# Large-scale data integration framework provides a comprehensive view on glioblastoma multiforme

**DOI:** 10.1186/gm186

**Published:** 2010-09-07

**Authors:** Kristian Ovaska, Marko Laakso, Saija Haapa-Paananen, Riku Louhimo, Ping Chen, Viljami Aittomäki, Erkka Valo, Javier Núñez-Fontarnau, Ville Rantanen, Sirkku Karinen, Kari Nousiainen, Anna-Maria Lahesmaa-Korpinen, Minna Miettinen, Lilli Saarinen, Pekka Kohonen, Jianmin Wu, Jukka Westermarck, Sampsa Hautaniemi

**Affiliations:** 1 Computational Systems Biology Laboratory, Institute of Biomedicine and Genome-Scale Biology Research Program, University of Helsinki, Haartmaninkatu 8, Helsinki, FIN-00014, Finland; 2 Medical Biotechnology, VTT Technical Research Centre and University of Turku, Itäinen Pitkäkatu 4C, Turku, FI-20521, Finland; 3 Turku Centre for Biotechnology, University of Turku and Åbo Akademi University, Tykistökatu 6A, Turku, FI-20520, Finland; 4 Department of Pathology, University of Turku and Turku University Hospital, Kiinamyllynkatu 4-8, Turku, FI-20521, Finland

## Abstract

**Background:**

Coordinated efforts to collect large-scale data sets provide a basis for systems level understanding of complex diseases. In order to translate these fragmented and heterogeneous data sets into knowledge and medical benefits, advanced computational methods for data analysis, integration and visualization are needed.

**Methods:**

We introduce a novel data integration framework, Anduril, for translating fragmented large-scale data into testable predictions. The Anduril framework allows rapid integration of heterogeneous data with state-of-the-art computational methods and existing knowledge in bio-databases. Anduril automatically generates thorough summary reports and a website that shows the most relevant features of each gene at a glance, allows sorting of data based on different parameters, and provides direct links to more detailed data on genes, transcripts or genomic regions. Anduril is open-source; all methods and documentation are freely available.

**Results:**

We have integrated multidimensional molecular and clinical data from 338 subjects having glioblastoma multiforme, one of the deadliest and most poorly understood cancers, using Anduril. The central objective of our approach is to identify genetic loci and genes that have significant survival effect. Our results suggest several novel genetic alterations linked to glioblastoma multiforme progression and, more specifically, reveal Moesin as a novel glioblastoma multiforme-associated gene that has a strong survival effect and whose depletion *in vitro *significantly inhibited cell proliferation. All analysis results are available as a comprehensive website.

**Conclusions:**

Our results demonstrate that integrated analysis and visualization of multidimensional and heterogeneous data by Anduril enables drawing conclusions on functional consequences of large-scale molecular data. Many of the identified genetic loci and genes having significant survival effect have not been reported earlier in the context of glioblastoma multiforme. Thus, in addition to generally applicable novel methodology, our results provide several glioblastoma multiforme candidate genes for further studies.

Anduril is available at http://csbi.ltdk.helsinki.fi/anduril/

The glioblastoma multiforme analysis results are available at http://csbi.ltdk.helsinki.fi/anduril/tcga-gbm/

## Background

Comprehensive characterization of complex diseases calls for coordinated efforts to collect and share genome-scale data from large patient cohorts. A prime example of such a coordinated effort is The Cancer Genome Atlas (TCGA), which currently provides more than five billion data points on glioblastoma multiforme (GBM) with the aim of improving diagnosis, treatment and prevention of GBM [1].

Translating genome-scale data into knowledge and further to effective diagnosis, treatment and prevention strategies requires computational tools that are designed for large-scale data analysis as well as for the integration of multidimensional data with clinical parameters and knowledge available in bio-databases. In addition, it is evident that until data integration tools are developed to the level that experimental scientists can independently interpret the vast amounts of data generated by genome-scale technologies, most of the potential of the generated data will be severely underexploited. In order to address these challenges, we have developed a data analysis and integration framework, Anduril, which facilitates the integration of various data formats, bio-databases and analysis techniques. Anduril manages and automates analysis workflows from importing raw data to reporting and visualizing the results. In order to facilitate interpretation of the large-scale data analysis results, Anduril generates a website that shows the most relevant features of each gene at a glance, allows sorting of data based on different parameters, and provides direct links to more detailed views of genes, transcripts, genomic regions, protein-protein interactions and pathways.

We demonstrate the utility of the Anduril framework by analyzing heterogeneous and multidimensional data from 338 GBM patients [1]. GBM is an aggressive brain cancer having a median survival of one year and is remarkably resistant to all current anti-cancer therapeutic regimens [2]. In order to understand the complex molecular mechanisms behind GBM, earlier efforts have analyzed data from one or two platforms, such as mutations, copy number and gene expression profiles and methylation patterns [3-7]. In contrast, we have analyzed all TCGA provided GBM data sets and collected the results into a comprehensive website that facilitates the interpretation of the data and allows an advanced view of genes and genomic regions crucial to GBM progression. Most importantly, Anduril can be applied to data from any accessible source.

## Materials and methods

Documentation for algorithms, their parameters and usage in the analysis together with all results are available in Additional file 1.

### Glioblastoma multiforme data set

The glioblastoma data set was originally released in 2008 [1] and has been updated online since then. An updated revision was used in the present work: comparative genomic hybridization array (aCGH), single nucleotide polymorphism (SNP), exon, gene expression and microRNA (miRNA) data were accessed May to August 2009, while methylation and clinical data were accessed October to November 2009. The data set consists of 338 primary glioblastoma patients with clinical annotations. Data were analyzed from the following microarray platforms: Affymetrix HU133A (269 GBM samples, 10 control samples), Affymetrix Human Exon 1.0 (298 GBM samples, 10 control samples), Agilent 244 k aCGH (238 GBM samples), Affymetrix SNP Array 6.0 (214 GBM blood samples), Illumina GoldenGate methylation array (243 GBM samples) and Agilent miRNA array (251 GBM samples, 10 control samples). Pre-normalized data (level 2) were used for gene, exon and miRNA expression and methylation arrays. Raw data (level 1) were used for aCGH and SNP platforms. Clinical annotations were used to compute the duration of patient survival in months from the initial diagnosis to death or to the last follow-up. The publicly available results in the present work do not reveal protected patient information.

### Gene expression analyses

The gene and exon expression platforms include ten control samples from brain tissue extracted from non-cancer patients in addition to the glioblastoma samples. Transcript level expressions are calculated from the exon level expression data by considering the problem of transforming the exon-level data to transcripts as a least squares problem. For *i*th gene having *m *exons and *n *transcripts in Ensembl (v.58) we define a vector e_*i *_of length *m *that denotes the measured exon expressions, and an *m *times *n *matrix **A**_*i*_, where the values in each column denote if the exon belongs to the transcript (1) or not (0). Transcript expression values **t**_*i *_are solved from the equation **A**_*i*_**t**_*i *_= **e**_*i *_using the QR decomposition to ensure numerical stability. The gene level expression values for the exon array platform were computed by taking a median of the intensity of all the exons linked with the gene in Ensembl.

Differential expression is determined by computing fold changes and applying a *t*-test between glioblastoma and control groups, followed by multiple hypotheses correction [8]. Fold changes are computed by dividing the mean of glioblastoma expression values by the mean of control expression values.

### Transcriptome survival analysis

Differentially expressed splice variants were selected as the basis of expression survival analysis. There were 8,887 splice variants (out of a total 75,083) that were differentially expressed having absolute fold change >2 and a multiple hypothesis corrected *P*-value < 0.05. For these splice variants we computed sample-specific fold changes by dividing the sample expression value by the mean of control expression values. These fold changes (FC) were discretized into classes denoted by '-1' (underexpression, FC < 0.5), '1' (overexpression, FC >2) and '0' (stable expression), and the samples were divided into three groups accordingly. This grouping was used in Kaplan-Meier survival analysis and groups with <20 patients were excluded. A log-rank test was computed for each differentially expressed splice variant.

### SNP survival analysis

Affymetrix SNP 6.0 genotypes were called with the CRLMM algorithm [9]. Samples with a signal-to-noise ratio below five and markers with call probabilities below 0.95 were discarded. We restricted our analysis to a genetically homogeneous pool of samples by using only ethnically similar samples. Markers with a relative minor allele frequency below 0.1 were excluded from the survival analysis. The study time in the survival analysis was 36 months. If the size of the patient group with the rare homozygote genotype in a marker was less than 15, or its frequency was less than 0.1, then the rare homozygote group was combined with the heterozygote group. The uncorrected *P*-value limit was set to 0.0001.

### Copy number and expression integration

Normalized aCGH data from tumor samples were segmented using circular binary segmentation [10]. A segment was called aberrated if its mean was over 0.632 or below -0.632. These thresholds were estimated from the 64 blood versus blood controls as two standard deviations from the mean of normalized probe intensities.

Based on gain and loss frequencies for each splice variant, aCGH and splice variant expression data were integrated with the statistical method originally applied to breast cancer [11,12]. Briefly, the samples are first divided into amplified and non-amplified groups. The difference of the expression means in these groups is divided by the sum of their standard deviation, resulting in a weight value. Then statistical significance for the weight value is computed by randomly permuting the samples into amplified and non-amplified groups and comparing the permuted weight value to the original.

### miRNA expression analysis

Differentially expressed miRNA genes were determined using the same procedure as for gene expression platforms. Annotations for target sites of miRNAs were obtained from the miRBase::Targets database [13]. Only target sites with a *P*-value < 10^-5 ^were included. MiRBase::Targets version 4 was used to match the annotations used in constructing the Agilent human miRNA array (G4470A).

### DNA methylation arrays

Illumina DNA Methylation Cancer Panel I (808 gene promoters) and a custom Illumina GoldenGate array (1,498 gene promoters) were used in the methylation analysis. Processed beta values were used as provided by the TCGA. The beta value is defined as *M*/(*M *+ *U*), where *M *and *U *are signal levels of methylation and unmethylation, respectively. The range of beta is 0 to 1, with 0 indicating hypomethylation and 1 indicating hypermethylation. Probes that target the same gene promoter were combined by taking the median of beta values so that each gene has a unique combined beta.

### Small interfering RNA assays

Cell lines A172 and U87MG were obtained from the European Collection of Cell Cultures (ECACC, Salisbury, UK), LN405 from Deutsche Sammlung von Microorganismen und Zellkulturen GmbH (DSMZ, Braunschweig, Germany) and SVGp12 from American Type Culture Collection (ATCC, Manassas, VA, USA). Cells were cultured in medium conditions recommended by the providers.

The small interfering RNAs (siRNAs) were purchased from Qiagen (Qiagen GmbH, Germany) and include AllStars Hs Cell Death Control siRNA and AllStars Negative Control siRNA; siRNA sequences for the other 11 genes are given in Additional file 2. Each siRNA was assayed as three replicate wells, and for each gene four siRNAs were used in reverse transfection. Briefly, the siRNAs were printed robotically to 384-well white, clear-bottom assay plates (Greiner Bio-One GmbH, Frickenhausen, Germany). SilentFect transfection agent (Bio-Rad Laboratories, Hercules, CA, USA) or Lipofectamine RNAiMax (Invitrogen, Carlsbad, CA, USA) diluted into OptiMEM (Gibco Invitrogen, Carlsbad, CA, USA) was aliquoted into each 384-plate well using a Multidrop 384 Microplate Dispenser (Thermo Fisher Scientific Inc, Waltham, MA, USA), and the plates were incubated for 1 h at room temperature. Subsequently, 35 μl of cell suspension (1,500 cells of A172, U87MG and SVGp12 or 1,200 LN405 cells) was added on top of the siRNA-lipid complexes (13 nM final siRNA concentration) and the plates were incubated for 48 h or 72 h at +37°C with 5% CO_2_.

### Proliferation assay and analysis of caspase-3 and -7 activities

Cell proliferation was assayed 72 h after transfection with CellTiter-Glo Cell Viability assay (Promega, Madison, WI, USA) and induction of caspase-3 and -7 activities was detected 48 h after transfection either with homogeneous Caspase-Glo 3/7 assay or Apo-ONE assay (Promega). All assays were performed according to the manufacturer's instructions. The signals were quantified by using an Envision Multilabel Plate Reader (Perkin-Elmer, Massachusetts, MA, USA). Both assays were repeated twice from independent transfections. Signals from the proliferation and caspase-3/7 assays were calculated and presented as relative signal to the mean of negative control siRNA replicate wells that was given a value of one. The values for each siRNA were then transformed into robust z-scores using median of the replicates and the median absolute deviation (MAD). A *t*-test (two-tailed, unequal variances) was calculated for each siRNA treatment and *P*-values < 0.05, < 0.01 and < 0.001 were taken as significant.

Data for *CDKN2A *and *MSN *are from an earlier siRNA screen and the values have been normalized to the background signal of each plate. The values were normalized using a LOESS method similar to the one implemented in the cellHTS2 R-package [14]. Briefly, the statistical outliers were down-weighted when a polynomial surface was fitted to the intensities within each assay plate using local regression [15]. This ensured a robust fit even if plates differ in hit-rate. The fit, representing a systematic background signal, was then subtracted from the values. A span of 0.35 and a degree of two for polynomial kernel were used. Robust z-scores were then calculated from the corrected data.

## Results

### Anduril framework

**Figure 1:**
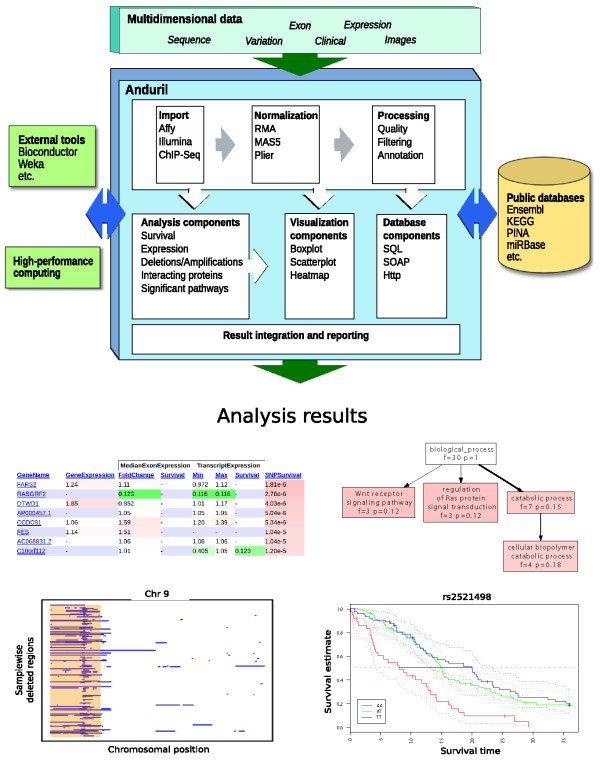
**Schematic of the Anduril platform**. Anduril is an extensible framework for analyzing large-scale data sets using workflows. Elementary analysis and reporting methods, as well as connections to external databases, are implemented as reusable Anduril components. Components can utilize libraries such as Bioconductor and Weka and are not limited to a particular programming language. Components are then wired into custom workflows, which implement complete analyses that take complex high-throughput data as input and automatically produce comprehensive final reports as result. Reports include generated web sites that show the most relevant features of genes at a glance, and detailed figures and tables produced by analysis methods such as Kaplan-Meier analysis, Gene Ontology enrichment, and so on. Analysis workflows and their parameters are also documented in reports.

Anduril is a flexible framework for processing large-scale data sets and integrating knowledge from bio-databases (Figure 1). Anduril architecture is based on the concept of workflows. A workflow consists of a series of interconnected processing steps, each of which executes a well-defined part of an analysis, such as data import or the generation of summary reports. Anduril can be invoked from Eclipse [16], a multipurpose graphical user interface, or from the command line. Anduril is available under an open source license and is actively maintained; new versions are released at least every three months. Anduril source code, component repository, extensive documentation, an installation guide and VirtualBox image for convenient testing are downloadable from the Anduril website [17]. Full technical details of the framework together with worked examples are available in the Anduril User Guide [18].

Workflows are constructed using a custom workflow language called AndurilScript that resembles traditional programming languages and is designed to enable rapid construction of complex workflows. The elementary processing steps in a workflow are implemented by Anduril components, which are reusable software packages written in various programming languages, for instance, R, Java, MATLAB, Octave, Python and Perl. Components are executable processes that communicate with the workflow through files. The component model is programming language independent since the only requirement is the ability to read and write files. At the AndurilScript level, components are accessed using their external interfaces, which hides implementation details. The components can use software libraries, such as Bioconductor [19] and Weka [20], to bring well-tested libraries to the workflow environment. It is also possible to invoke command-line programs from workflows. Currently, the Anduril core repository consists of more than a hundred components, and new components are added regularly. For instance, we designed a computational platform to generate networks from a list of genes by integrating pathway and protein-protein interaction data in Anduril [21]. This represents a component bundle that uses the Anduril framework but is distributed independently from the Anduril core.

Anduril includes advanced features for working with complex workflows. Large workflows can be divided into nested subworkflows, so that each hierarchical level is simple to maintain. When a workflow is executed several times, Anduril caches results of components and only executes the components whose configuration has changed since the last run, which reduces execution time significantly. Selected parts of workflows can be enabled based on dynamic conditions, which increases the flexibility of the workflows.

Compared to traditional programming environments, for instance, R coupled with Bioconductor, the advantages of Anduril are the use of workflows and the support for several programming languages. Workflows have a higher level of abstraction than R code, which increases productivity and enables visualization of analysis configuration. Compared to workflow frameworks GenePattern [22], Ergatis [23] and Taverna [24], Anduril provides several novel features, such as efficient programming-like workflow construction with an advanced workflow engine, algorithms specifically designed for large-scale data analysis and automated result website construction, that enable efficient analysis and visualization of large-scale data sets (see [18] for details).

### Anduril-generated result report and website for GBM data interpretation

We used Anduril to analyze high-throughput SNP, copy number, transcriptomics, miRNA, methylation and clinical data for 338 GBM patients (Table 1). Anduril reports the analysis results in two formats. Firstly, Anduril provides a comprehensive PDF document consisting of analysis workflow configurations, method parameters, tables and figures produced by individual components. This report is intended primarily for bioinformaticians as it contains all the necessary details to reproduce the results. The report file for the GBM analyses conducted herein is available in Additional file 1. Secondly, Anduril automatically generates a website that contains the results computed with the analysis pipelines without the technical details. The website is designed primarily for experimental scientists as it gives a comprehensive view of the data at a glance. The website for GBM analyses executed herein is available at [25].

**Table 1:** Analyses performed and corresponding TCGA glioblastoma data sets

Primary analysis	TCGA dataset(s)
Differentially expressed genes	Gene expression
Differentially expressed transcripts (DETs)	Exon expression
Differentially expressed miRNAs	miRNA expression
Survival affecting germline SNPs	Blood SNP arrays, clinical data
Survival affecting DETs	Exon expression, clinical data
Survival affecting differentially expressed miRNAs	miRNA expression, clinical data
Chromosomal aberrations (amplification and deletion)	aCGH
Integration of differential expression and chromosomal aberrations	aCGH, exon expression
DNA methylation	Methylation arrays

**Figure 2:**
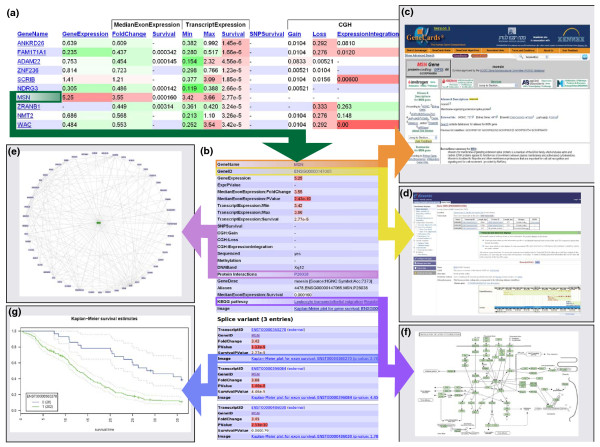
**Example of Anduril-generated result website and links to external sources**. Anduril generates a browsable website based on analysis results.** (a) **A screenshot of the gene level view of the data. The genes are sorted according to the survival *P*-value on the exon platform. The data are divided into 13 fields corresponding to analysis results and data sources. For example, the field 'GeneExpression' illustrates fold changes between GBM and control samples using data from gene expression microarrays. Exon array values are computed at the gene ('MedianExonExpression') and transcript levels ('TranscriptExpression'). For the transcript data the minimum and maximum transcript expression values show GBM-specific alternative splice variant candidates. The fields 'TranscriptExpression:Survival' and 'MedianExonExpression:Survival' show survival analysis *P*-values for the best transcript and gene in the exon arrays, whereas 'SNPSurvival' contains *P*-values for the survival associated SNPs. The green color for 'GeneExpression', 'FoldChange', 'Min', 'Max', 'Gain', 'Loss' and 'Methylation' denote downregulation and red denotes upregulation. The red color for *P*-values for the fields 'Survival', 'SNPSurvival' and 'ExonIntegration' denotes low *P*-values.** (b) **A web page that opens after clicking the gene *MSN*. This page contains detailed results and external links.** (c, d) **Clicking 'GeneName' opens a website in Genecards [28] (c), and 'GeneID' connects to Ensembl [29] (d).** (e) **Clicking 'Protein Interactions' opens a page listing known protein-protein interactions in PINA [27].** (f) **Clicking an entry in 'KEGG pathway' allows accessing pathways at the KEGG [26] website.** (g) **Each splice variant is listed separately and if the survival *P*-value is < 0.01, the users can view the Kaplan-Meier curves. The groups '1', '-1' and '0' denote overexpression, underexpression (not shown for *MSN*) and stable expression, respectively ('-1' is not present in the figure). The dotted lines are 95% confidence intervals.

An example of the Anduril generated web page is given in Figure 2. The genes are sorted according to survival effect in exon array data. Anduril provides hyperlinks to several important databases, such as the pathway database KEGG [26], the protein-protein interaction database PINA [27], the miRNA database miRBASE [13], and the gene annotation databases GeneCards [28] and Ensembl [29]. These links enable users to easily obtain more information on the function and structure of interesting genes.

### Integration of copy number and transcript expression GBM data

We identified genes that are frequently amplified or deleted in GBM samples and integrated these results with expression data in order to identify genes whose altered expression activity can potentially be explained by chromosomal aberrations. Genomic regions with significant amplifications include 7p11.2 (amplified in up to 54% of patients, housing *EGFR*), 12q13-12q15 (14%) and 4q12 (14%).

Integration of aCGH and exon expression data reveals 16 genes for which amplification is an explanatory factor for overexpression (*P *< 0.01 and gain frequency >5%). Of these, *EGFR *is amplified on the aCGH platform and overexpressed on both gene expression platforms (fold change 2.8 to 6.2; Additional file 3, panel A). *EGFR *is also hypomethylated (beta = 0.03), which may be an additional explanatory mechanism for its overexpression. However, not all genes located in the amplified region 7p11.2 show marked overexpression in the total patient population (Additional file 3, panel A). For example, *LANCL2 *(the closest annotated gene to *EGFR *in the 7p11.2 region) is amplified in 24% of patients but shows underexpression in the exon platform and only slight over-expression in the gene expression platform. Similar differential expression is seen also between *METTL1 *(overexpressed) and *AGAP2 *(underexpressed) in the amplified chromosomal location 12q14.1 (Additional file 3, panel B).

Gene deletions are generally thought to result in downregulation of the expression of genes coded by the deleted genomic region. Interestingly, Anduril-based analysis of the two most frequently deleted genes at 9p21.3, *MTAP *and *CDKN2A*, shows that even though the gene deletion is an explanatory factor for lower expression of these genes in patients with deletion, in total GBM patient material the *MTAP *expression is not inhibited and *CDKN2A *is overexpressed compared to normal tissue (Additional file 4). The seemingly contradictory correlation between gene deletion and overexpression suggests activation of *MTAP *and *CDKN2 *promoters, and thereby increased gene expression levels in patients who have not yet lost one or two copies of these genes. This hypothesis is supported by the observation that in patients with remaining *MTAP *and *CDKN2 *alleles, both *MTAP *and *CDKN2A *are hypomethylated. On the other hand, another gene at 9p21.3 (*ELAVL2*) shows classical behavior of a deleted gene; its expression correlates with deletion, and it is also significantly downregulated in both expression platforms.

These examples illustrate that Anduril allows researchers to detect critical parameters affecting expression levels of the gene of interest at a glance. Our results demonstrate that integrated data analysis combining amplification, expression, and methylation status is integral in order to draw conclusions about functional consequences of gene amplifications or deletions detected by aCGH microarrays.

### Survival analysis of GBM data

Probably the most important feature of the Anduril analysis of the GBM data is the integration of patient survival information with both expression and SNP data, thereby allowing the user to sort the genomic alterations according to their clinical relevance.

In order to examine the relevance of gene expression levels to patient survival in GBM, we first searched for genes whose overexpression correlated significantly with poor survival (*P *< 0.01). Among the 100 most upregulated genes, only 15 genes showed significant correlation with poor survival. On the other hand, out of the top ten survival affecting genes, only one gene (*MSN*, encoding Moesin) showed consistent overexpression in the gene and exon expression platforms (Figure 2a). All the other genes affecting survival in this group were underexpressed. Three of the top ten genes affecting survival (*ADAM22*, *SCRIB*, *WAC*) had at least one transcript that was overexpressed when analyzed on the exon array platform. However, survival effects of these genes are related to underexpressed splice variants instead of the overexpressed variants. Together these results show that gene repression is a common mode for gene regulation among the genes that have the most significant survival effect in GBM. These results challenge the general assumption that the level of gene overexpression is the major determinant to separate between clinically relevant and non-relevant genes.

In order to test the association between genetic alterations in GBM and their relevance to patient survival, we linked gene amplifications, expression profiles and survival data. Among the 300 most amplified genes, only filamin C gamma (*FLNC*; 7q32.1) is amplified (9% of the patients) with consistent overexpression in the gene and exon arrays and significant survival effect (*P *< 0.01). Together these results indicate that there is unexpectedly poor concordance between gene amplification, overexpression of the genes from the amplicons, and patient survival in GBM.

In general, individual miRNA survival effects in GBM were much smaller than expression survival effects, which may be explained by their indirect mechanism of action. The highest expressed miRNA in the GBM data was *hsa-miR-21 *(fold change 15.5), which has been shown to increase apoptotic activity and reduce tumor size *in vivo *[30-32]. Some of the most downregulated miRNAs according to our analysis were *hsa-miR-124a*, *hsa-miR-137*, *hsa-miR-7*, *hsa-miR-128a *and *hsa-miR-128b*. All of these have been connected functionally to glioblastoma, either via neuronal differentiation or growth regulation [33].

Finally, we correlated 550,000 SNPs on the SNP arrays to survival using Kaplan-Meier and log-rank methods. This analysis identified 50 genes that contain survival-associated SNPs. Of these genes, *KIAA0040 *is also overexpressed (fold change 1.7 to 2.6) and associated with poor survival in exon array data (*P *< 8.7 × 10^-4^). The role of *KIAA0040 *in cancer progression is also supported by a recent study where *KIAA0040 *overexpression was shown to correlate with poor prognosis in breast cancer [34]. Another example of a gene showing a significant survival-affecting SNP is rs17258085 of *ODZ3*. In contrast to *KIAA0040*, this gene is significantly underexpressed in the GBM samples.

### Functional analysis of survival-affecting genes in vitro

We chose 11 genes having overexpression and a survival effect on the GBM for functional analysis with three glioma cell lines (A172, LN405, U87MG) and one control cell line (SVG p12; SV40 transformed fetal astrocyte). Each gene was targeted with four siRNA constructs. The phenotypes were cell proliferation and induction of apoptosis via caspase-3 and -7 activities assayed 48 to 72 h after transfection in a 384-well format. Positive control siRNAs against *KIF11 *and *PLK1 *as well as AllStars Hs Cell Death Control siRNA gave clear anti-proliferative effects in all four cell lines (Additional file 5). Cell Death Control and *KIF11 *siRNAs also showed a clear induction of apoptosis in all four cell lines (Additional file 6). The results for the A172 cell line are presented in Table 2, and all functional analysis results are given in Additional file 2.

**Table 2:** Functional siRNA screening data for 11 GBM survival-associated genes in the A172 glioblastoma cell line

Symbol	Description	siRNA name	CTG	Caspase	Survival	Expression
	AllStars Cell Death Vontrol siRNA (Cell death ctrl)		**-13.80**	**19.62**	NA	NA
	AllStars Negative Control siRNA (siNEG)		0.00	0.00	NA	NA
*KIF11*	Kinesin family member 11	KIF11_7	**-9.56**	**7.39**	NA	NA
*PLK1*	Polo-like kinase 1	PLK1_7	**-5.92**	0.31	NA	NA
*FLNC*	Filamin C, gamma	FLNC_2	**2.56**	-0.89	0.000189	2.51
		FLNC_5	-0.37	0.29		
		FLNC_6	-0.57	-0.94		
		FLNC_7	**-5.39**	0.49		
*H19*	H19, imprinted	H19_1	0.38	-0.14	0.000588	3.54
	maternally expressed	H19_2	**-5.08**	1.17		
	transcript (non-protein	H19_3	**5.20**	**-2.71**		
	coding)	H19_4	**-2.97**	**2.71**		
*HIST1H4L*	Histone cluster 1, H4l	HIST1H4L_1	1.63	-0.60	0.001560	5.01
		HIST1H4L_2	**2.61**	-2.00		
		HIST1H4L_5	-0.36	-1.19		
		HIST1H4L_7	**-5.93**	-0.97		
*KIAA0040*	KIAA0040	KIAA0040_11	**-2.94**	-0.64	0.000867	2.63
		KIAA0040_12	0.63	-0.83		
		LOC100129443_3	0.69	-0.71		
		LOC100129443_4	-0.31	-0.30		
*LTF*	Lactotransferrin	LTF_1	-1.74	0.22	0.001570	3.95
		LTF_2	-0.03	0.07		
		LTF_5	-1.04	0.44		
		LTF_6	-1.18	-0.79		
*NNMT*	Nicotinamide N-	NNMT_5	-0.91	-1.29	0.000074	7.41
	methyltransferase	NNMT_6	-0.48	-1.87		
		NNMT_7	-1.26	-0.32		
		NNMT_8	-0.30	-0.10		
*POSTN*	Periostin, osteoblast	POSTN_1	0.24	-1.16	0.001950	14.9
	specific factor	POSTN_2	0.28	**-2.11**		
		POSTN_6	**-2.17**	0.56		
		POSTN_7	0.52	0.12		
*TAGLN2*	Transgelin 2	TAGLN2_10	**4.53**	-1.74	0.001010	4.21
		TAGLN2_11	**-3.78**	0.48		
		TAGLN2_8	-0.43	0.45		
		TAGLN2_9	**4.67**	-1.94		
*TIMP1*	TIMP metallopeptidase	TIMP1_2	-0.09	-0.96	0.000109	3.21
	inhibitor 1	TIMP1_4	1.07	0.88		
		TIMP1_5	0.56	-1.87		
		TIMP1_6	-0.08	0.41		
*MSN*	Moesin	MSN_8	-1.68	1.61	0.000028	3.42
		MSN_9	**-4.49**	-1.05		
		MSN_5	-0.36	-1.57		
		MSN_1	**-2.57**	0.80		
*CDKN2A*	Cyclin-dependent kinase inhibitor 2A	NA (gene deleted in A172)				

Cell proliferation (CTG) and induction of caspase-3 and -7 activities (Caspase) were assayed after transfection of A172 cells with four siRNAs against each gene. Z-scores from the proliferation and caspase-3/7 assays are presented, centered on the scramble siRNA. Values in bold diverge by more than two standard deviation units from the median of scramble negative control siRNA and are considered significant. For each gene, the best survival *P*-value (Survival) and the corresponding fold change in the exon array (Expression) are given.

**Figure 3:**
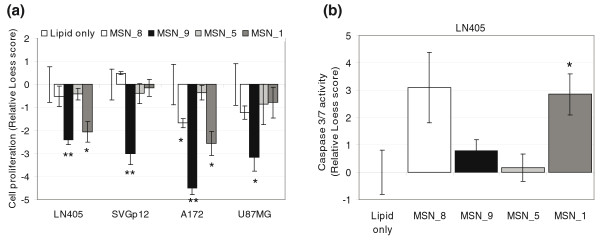
**Functional effects of knocking out *MSN *in three glioblastoma and one control cell line**. Four *MSN *targeting siRNAs at a final concentration of 13 nM were transfected with Silenfect (BioRad) transfection reagent to A172, LN405 and U87MG glioma cell lines and the SVGp12 control cell line.** (a) **Cell proliferation was assayed 72 h after transfection using CellTiter-Glo Cell Viability assay. **(b) **Induction of caspase-3 and -7 activities was detected 48 h after transfection with homogeneous Apo-ONE assay (Promega). Loess normalized signals from the proliferation and caspase-3/7 assays are presented as relative scores to the mean of lipid-containing wells. Significant *P*-values < 0.05*, < 0.01** and < 0.001*** calculated by *t*-test are shown. Error bars indicate standard error of the mean (SEM).

Of the tested genes, only the silencing of *MSN *caused consistent inhibition of cell proliferation in all four cell lines. In addition, it caused an increase in caspase-3/7 activity in LN405 (Figure 3). The silencing of *CDKN2A *caused inhibition of cell proliferation with two siRNAs and an increase in caspase-3/7 activity in the LN405 and SVGp12 cell lines that do not have the *CDKN2A *deletion (Additional file 7). The silencing of the other genes did not result in consistent effects on cell proliferation or induction of apoptosis in the tested glioblastoma cell lines.

## Discussion

Large-scale data gathering efforts require software and computational tools to facilitate interpretation of the data. We have developed Anduril, an efficient and systematic data integration framework, to conduct large-scale data analysis that necessarily requires a number of processing steps before the data can be interpreted. In the GBM analysis here, the workflow contained approximately 350 processing steps, demonstrating the efficiency of workflows - more code would be needed when working with traditional programming languages - as well as highlighting the need for complexity management in workflow software. The structure of the analysis is automatically documented together with all execution parameters of the participating components, which enables reproduction of the results. Anduril supports modular and programming-like workflow construction, which together with automated component testing and a version control system allows a team of bioinformaticians to work on the project simultaneously and to seamlessly integrate the analysis results.

We have demonstrated the utility of the Anduril framework with the GBM data from TCGA, one of the largest multidimensional cancer data sets currently available. We focused on the integration of mRNA expression, SNPs and copy number data to clinical parameters as these results can provide evidence of potential molecular markers with impact on GBM progression. This also facilitates the sorting of the genomic alterations according to their clinical relevance and further helps to focus future mechanistic studies on genetic alterations that have evidence of clinical relevance. While TCGA GBM data sources, such as The Cancer Genome Atlas Portal and the Cancer Molecular Analysis Portal, provide box-plots for single genes and genome-wide heatmaps, Anduril offers a significant step forward. It enables a comprehensive view of the most critical parameters influencing expression, miRNA, SNP and copy number levels, as well as correlation of these data to survival at a glance. In addition, Anduril provides a number of direct links to external databases, and is thus an easy access point for interpreting the vast amounts of heterogeneous data from multiple sources. These characteristics of Anduril facilitate scientists without bioinformatics training to interpret complex data sets, such as TCGA.

Analysis of the GBM data demonstrates the utility of Anduril in translating fragmented data to testable predictions. For example, detection of amplified genomic regions has traditionally been used to identify genes with potential causal roles in oncogenesis [35]. However, whether genomic amplification generally results in clinically relevant changes in gene expression from the amplicon has been difficult to assess because of the lack of Anduril-type websites combining gene expression, patient survival and aCGH amplification data. Our results show surprisingly poor concordance between gene amplification, overexpression of the genes in the amplicons, and patient survival. For example, even though *EGFR *is the most often amplified gene in GBM (54% of patients), and this amplification has been considered as a hallmark of the disease, *EGFR *overexpression does not correlate well with overall patient survival (*P *< 0.122). This result is supported by a recent study demonstrating that *EGFR *amplification does not determine patient survival in primary GBM [36]. Instead, our results demonstrate that gene repression, rather than activation, is a common mode for gene regulation among the genes that have the most significant effect on survival in GBM.

Interestingly, many of the most survival-affecting genes have not been previously implicated in GBM pathogenesis. An example of such a gene is *ZRANB1 *(encoding ubiquitin thioesterase), which is downregulated in exon arrays and has a strong survival effect (*P *< 3.2 × 10^-5^). It has been shown in *Drosophila *and in human cancer cell lines to function as a positive regulator of Wnt-signaling [37]. Another interesting survival-affecting gene revealed by our analysis is *MSN *(encoding Moesin). We have functionally demonstrated that Moesin depletion by siRNA significantly inhibited cell proliferation and induced apoptosis. Moesin is functionally involved in regulation of actin cytoskeleton and cell migration, which indicates that in GBM it may promote, in addition to proliferation, the highly invasive behavior of GBM cells.

## Conclusions

The different analysis approaches described herein demonstrate the ability of Anduril to integrate several types of genomic information and above all its capacity to determine which of the observed genetic alterations have an impact on patient survival. In this regard, Anduril clearly facilitates scientists to focus future functional analysis on those cancer-related genes that have already been verified to have clinical significance. Interestingly, each of the survival analyses described above (SNP, expression level, copy number changes) identified clinically relevant genomic alterations in genes for which cancer relevance is not presently established. It is anticipated that further studies of genes (for example, *MSN *and *ZRANB1*) and clinically relevant SNPs (for example, rs2285218 in *KIAA0040*) will produce interesting novel mechanistic insights into GBM progression and oncogenesis.

## Abbreviations

aCGH: comparative genomic hybridization array; GBM: glioblastoma multiforme; miRNA: microRNA; siRNA: small interfering RNA; SNP: single nucleotide polymorphism; TCGA: The Cancer Genome Atlas.

## Competing interests

The authors declare that they have no competing interests.

## Authors' contributions

KO designed and implemented the Anduril framework and contributed to writing the manuscript. ML contributed to overall design and Anduril component implementation. SHP performed siRNA experiments and contributed to writing the manuscript. GBM informatics analyses were conducted by RL, PC, VA, KO, ML and EV. Data analysis tools were designed and implemented by KO, ML, RL, PC, VA, EV, JNF, VR, SK, KN, AMLK, MM, LS and JWu. PK analyzed the siRNA data. SK and RL made Figures 1 and 2. JNF contributed to implementation of the Anduril core. JWe contributed to designing the case studies, interpreting the results from the informatics analyses and writing the manuscript. SH initiated and supervised the project and contributed to writing the manuscript. All authors read and approved the final manuscript.

## Supplementary Material

Additional file 1 **Automatically generated result file**. The report contains analysis configurations, parameter settings, result lists, tables and figures. The document also includes some analyses not reported in the manuscript, such as Gene Ontology analysis. Click here for file

Additional file 2 **Information and data for siRNA screens**. The information includes identifiers, siRNA target sequences, normalized mean intensities, standard errors of the mean and *P*-values for four cell lines used. Click here for file

Additional file 3 **Screenshot from the Anduril-generated web site**. Genes are sorted in decreasing order according to the fraction of amplification ('Gain') in the GBM samples. The strongest amplified region in the GBM samples is 7p11.2. Interestingly, the expression values of the genes in the same genomic region vary significantly. For example, *EGFR *is amplified and has high fold change whereas *LANCL2 *is amplified and downregulated (panel A). The same phenomenon is seen in another amplified region 12q14.1 (panel B). Click here for file

Additional file 4 **Screenshot from the Anduril-generated web site**. Genes are sorted in decreasing order according to the fraction of deletion ('Loss') in the GBM samples. The strongest deleted region in the GBM samples is 9p21.3. The fraction of deletion varies from 35% to 69%. Click here for file

Additional file 5 **The effect of gene silencing on cell proliferation**. Control siRNAs (13 nM final concentration) were transfected with Silenfect (BioRad) transfection reagent to A172, LN405 and U87MG glioma cell lines and the SVGp12 control cell line. Cell proliferation was assayed 72 h after transfection using CellTiter-Glo Cell Viability assay. The proliferation data are presented as relative score to the mean of scramble siRNA-containing wells. Error bars indicate median absolute deviation. Click here for file

Additional file 6 **The effect of gene silencing on caspase-3 and -7 activities**. Control siRNAs (13 nM final concentration) were transfected with Silenfect (BioRad) transfection reagent to A172, LN405 and U87MG glioma cell lines and the SVGp12 control cell line. Induction of caspase-3 and -7 activities was detected 48 h after transfection with homogeneous Caspase-Glo 3/7 assay (Promega). The caspase activity is presented as relative median score to the mean of scramble siRNA containing wells. Error bars indicate median absolute deviation. Click here for file

Additional file 7 **The effects of silencing *CDKN2A *in LN405 and SVGp12 cell lines on cell proliferation and apoptosis**. Click here for file
