# Supplementary material for: Large-scale data integration framework provides a comprehensive view on glioblastoma multiforme
Source: Genome Med. 2010 Sep 7;2(9):65. doi: 10.1186/gm186 (PMC3092116; doi:10.1186/gm186)
Supplement: Additional file 3 — Screenshot from the Anduril-generated web site. Genes are sorted in decreasing order according to the fraction of amplification ('Gain') in the GBM samples. The strongest amplified region in the GBM samples is 7p11.2. Interestingly, the expression values of the genes in the same genomic region vary significantly. For example, EGFR is amplified and has high fold change whereas LANCL2 is amplified and downregulated (panel A). The same phenomenon is seen in another amplified region 12q14.1 (panel B). [file gm186-S3.PDF]

A

		MedianExonExpression		TranscriptExpression				CGH				
GeneName	GeneExpression	FoldChange	Survival	Min	Max	Survival	SNPSurvival	Gain	Loss	ExpressionIntegration	Methylation	DNABand
EGFR	4.04	5.16	0.780	2.80	6.18	0.122	-	0.542	0.00521	0.00	0.0300	7p11.2
RP11-745C15.2	-	0.987	-	1.23	1.23	-	-	0.474	0.00521	0.00100	-	7p11.2
RP11-745C15.1	-	3.08	0.431	3.32	3.32	0.312	-	0.474	0.00521	-	-	7p11.2
RP4-791C19.1	-	1.14	-	1.00	1.31	-	-	0.458	0.00521	-	-	7p11.2
GS1-18A18.3	-	0.941	-	0.941	0.941	-	-	0.443	0.00521	-	-	7p11.2
AC011228.1	-	0.954	-	0.954	0.954	-	-	0.432	0.00521	-	-	7p11.2
GS1-18A18.1	-	0.257	-	0.103	0.103	-	-	0.375	0.00521	0.136	-	7p11.2
GS1-18A18.2	-	1.30	-	1.38	1.38	-	-	0.339	0.00521	-	-	7p11.2
RP11-436F9.2	-	1.37	-	1.24	1.39	-	-	0.307	0.00521	-	-	7p11.2
RP11-436F9.1	-	1.06	-	0.931	0.985	-	-	0.292	0.00521	0.747	-	7p11.2
RP5-1091E12.1	-	1.31	-	1.32	1.32	-	-	0.292	0.00521	0.0590	-	7p11.2
AC073269.1	-	1.08	-	1.08	1.08	-	-	0.276	0.00521	-	-	7p11.2
RP11-339F13.2	-	1.09	-	1.09	1.09	-	-	0.271	0.00521	-	-	7p11.2
RP11-339F13.1	-	0.809	-	0.809	0.809	-	-	0.260	0.00521	-	-	7p11.2
VOPP1	1.98	1.47	-	0.595	1.87	-	-	0.250	0.00521	0.00200	-	7p11.2
LANCL2	1.60	0.750	-	0.734	0.865	-	-	0.240	0.00521	0.00200	-	7p11.2

B

TSPAN31	2.09	1.49	-	1.51	1.69	-	-	0.141	-	0.0110	-	12q14.1
CDK4	6.41	5.04	-	4.46	4.56	-	-	0.141	-	0.0830	-	12q14.1
AC138607.1	-	0.762	-	0.762	0.762	-	-	0.135	-	-	-	4q12
TSFM	1.22	1.22	-	0.848	5.19	-	-	0.135	0.00521	0.0140	-	12q14.1
GSX2	-	1.26	-	1.28	1.28	-	-	0.135	-	-	-	4q12
MARCH9	-	1.29	-	1.30	1.30	-	-	0.135	-	-	-	12q14.1
CYP27B1	1.55	1.59	-	1.51	1.51	-	-	0.135	-	0.00200	-	12q14.1
CHIC2	3.40	1.93	-	1.66	1.66	-	-	0.135	-	0.0610	-	4q12
FAM119B	3.71	2.46	0.451	2.19	2.48	0.275	-	0.135	0.00521	0.00700	-	12q14.1
METTL1	2.17	2.61	0.0546	2.45	2.59	0.0482	-	0.135	-	-	-	12q14.1
AGAP2	0.493	0.153	-	0.141	0.171	-	-	0.130	-	0.0280	-	12q14.1
AC138779.1	-	0.808	-	0.808	0.808	-	-	0.130	-	-	-	4q12
