# Supplementary material for: Large-scale data integration framework provides a comprehensive view on glioblastoma multiforme
Source: Genome Med. 2010 Sep 7;2(9):65. doi: 10.1186/gm186 (PMC3092116; doi:10.1186/gm186)
Supplement: Additional file 4 — Screenshot from the Anduril-generated web site. Genes are sorted in decreasing order according to the fraction of deletion ('Loss') in the GBM samples. The strongest deleted region in the GBM samples is 9p21.3. The fraction of deletion varies from 35% to 69%. [file gm186-S4.PDF]

| | | MedianExonExpression | | TranscriptExpression | | | | CGH | | | | |
|---|---|---|---|---|---|---|---|---|---|---|---|---|
| GeneName | GeneExpression | FoldChange | Survival | Min | Max | Survival | SNPSurvival | Gain | Loss | ExpressionIntegration | Methylation | DNABand |
| MTAP | 0.949 | 1.17 | - | 0.827 | 1.42 | - | - | - | 0.693 | 0.00600 | 0.00 | 9p21.3 |
| CDKN2BAS | - | 1.39 | - | 1.43 | 3.40 | - | - | - | 0.630 | 0.0630 | - | 9p21.3 |
| CDKN2A | 1.93 | 2.36 | 0.603 | 1.95 | 8.57 | 0.0716 | - | - | 0.620 | 0.0100 | 0.0700 | 9p21.3 |
| CDKN2B | 0.920 | 1.40 | - | 1.42 | 1.54 | - | - | - | 0.599 | 0.0670 | 0.0500 | 9p21.3 |
| C9orf53 | 0.977 | 1.18 | - | 1.18 | 1.18 | - | - | - | 0.589 | 0.769 | - | 9p21.3 |
| UBA52P6 | - | 1.82 | - | 1.82 | 1.82 | - | - | - | 0.578 | - | - | 9p21.3 |
| RP11-408N14.1 | - | 1.01 | - | 0.966 | 0.966 | - | - | - | 0.469 | 0.131 | - | 9p21.3 |
| RP11-70L8.3 | - | 1.12 | - | 1.22 | 1.22 | - | - | - | 0.458 | - | - | 9p21.3 |
| RP11-70L8.1 | - | 1.15 | - | 1.15 | 1.15 | - | - | - | 0.458 | - | - | 9p21.3 |
| RP11-354P17.9 | - | 0.626 | - | 1.04 | 1.04 | - | - | - | 0.448 | 0.531 | - | 9p21.3 |
| AL355679.1 | - | 0.847 | - | 0.847 | 0.847 | - | - | - | 0.427 | - | - | 9p21.3 |
| IFNE | - | 1.04 | - | 1.03 | 1.05 | - | - | - | 0.427 | - | - | 9p21.3 |
| RP11-473O3.1 | - | 1.21 | - | 1.21 | 1.21 | - | - | - | 0.427 | - | - | 9p21.3 |
| MIR31 | - | 0.903 | - | 0.903 | 0.903 | - | - | - | 0.422 | - | - | 9p21.3 |
| IFNWP19 | - | 0.917 | - | 0.917 | 0.917 | - | - | - | 0.422 | - | - | 9p21.3 |
| IFNA8 | 0.845 | 1.09 | - | 1.09 | 1.09 | - | - | - | 0.417 | - | - | 9p21.3 |
| DMRTA1 | - | 1.27 | - | 1.36 | 1.36 | - | - | - | 0.417 | 0.576 | 0.0200 | 9p21.3 |
| IFNWP2 | - | 1.42 | - | 1.42 | 1.42 | - | - | - | 0.417 | - | - | 9p21.3 |
| IFNA2 | 0.997 | 0.887 | - | 0.887 | 0.887 | - | - | - | 0.406 | - | - | 9p21.3 |
| RP11-399D6.2 | - | 1.13 | - | 1.14 | 1.18 | - | - | - | 0.406 | 0.690 | - | 9p21.3 |
| KLHL9 | 0.749 | 0.598 | - | 0.598 | 0.598 | - | - | - | 0.391 | 0.00 | - | 9p21.3 |
| IFNA6 | 0.857 | 1.36 | - | 1.36 | 1.36 | - | - | - | 0.391 | - | - | 9p21.3 |
| PTEN | - | 0.494 | 0.352 | 0.500 | 0.930 | - | - | - | 0.385 | 0.286 | 0.0200 | 10q23.31 |
| ATAD1 | - | 0.452 | 0.0485 | 0.388 | 0.642 | 0.00581 | - | - | 0.380 | 0.459 | - | 10q23.31 |
| IFNA5 | 0.990 | 0.938 | - | 0.938 | 0.938 | - | - | - | 0.380 | - | - | 9p21.3 |
| KIAA1797 | 0.312 | 0.303 | 0.155 | 0.218 | 0.591 | - | - | - | 0.375 | 0.00 | - | 9p21.3 |
| IFNA22P | - | 0.927 | - | 0.927 | 0.927 | - | - | - | 0.375 | - | - | 9p21.3 |
| RP11-370B11.3 | - | 1.04 | - | 1.01 | 1.01 | - | - | - | 0.370 | - | - | 9p21.3 |
| RP11-370B11.1 | - | 1.18 | - | 1.11 | 1.26 | - | - | - | 0.370 | - | - | 9p21.3 |
| RNLS | 0.937 | 0.624 | - | 0.425 | 0.906 | 0.0558 | - | - | 0.365 | 0.00700 | 0.120 | 10q23.31 |
| CFLP1 | - | 0.955 | - | 0.735 | 1.09 | - | - | - | 0.365 | 0.0180 | - | 10q23.31 |
| AC063965.1 | - | 1.10 | - | 1.10 | 1.10 | - | - | - | 0.365 | - | - | 10q23.31 |
| CTNNA3 | 0.793 | 0.315 | 0.253 | 0.262 | 0.766 | 0.0328 | - | - | 0.354 | - | - | 10q21.3 |
| AC022016.1 | - | 0.891 | - | 0.891 | 0.891 | - | - | - | 0.354 | - | - | 10q23.31 |
| RP11-380G5.3 | - | 0.924 | - | 0.924 | 0.924 | - | - | - | 0.354 | - | - | 10q23.31 |
| AC022016.2 | - | 1.26 | - | 1.26 | 1.26 | - | - | - | 0.354 | - | - | 10q23.31 |
| ELAVL2 | 0.285 | 0.0712 | - | 0.0700 | 0.0993 | - | - | - | 0.349 | 0.0320 | - | 9p21.3 |
