# Supplementary figures and images for: Large-scale data integration framework provides a comprehensive view on glioblastoma multiforme
Source: Genome Med. 2010 Sep 7;2(9):65. doi: 10.1186/gm186 (PMC3092116; doi:10.1186/gm186)

### Control siRNA transfections - CTG assay

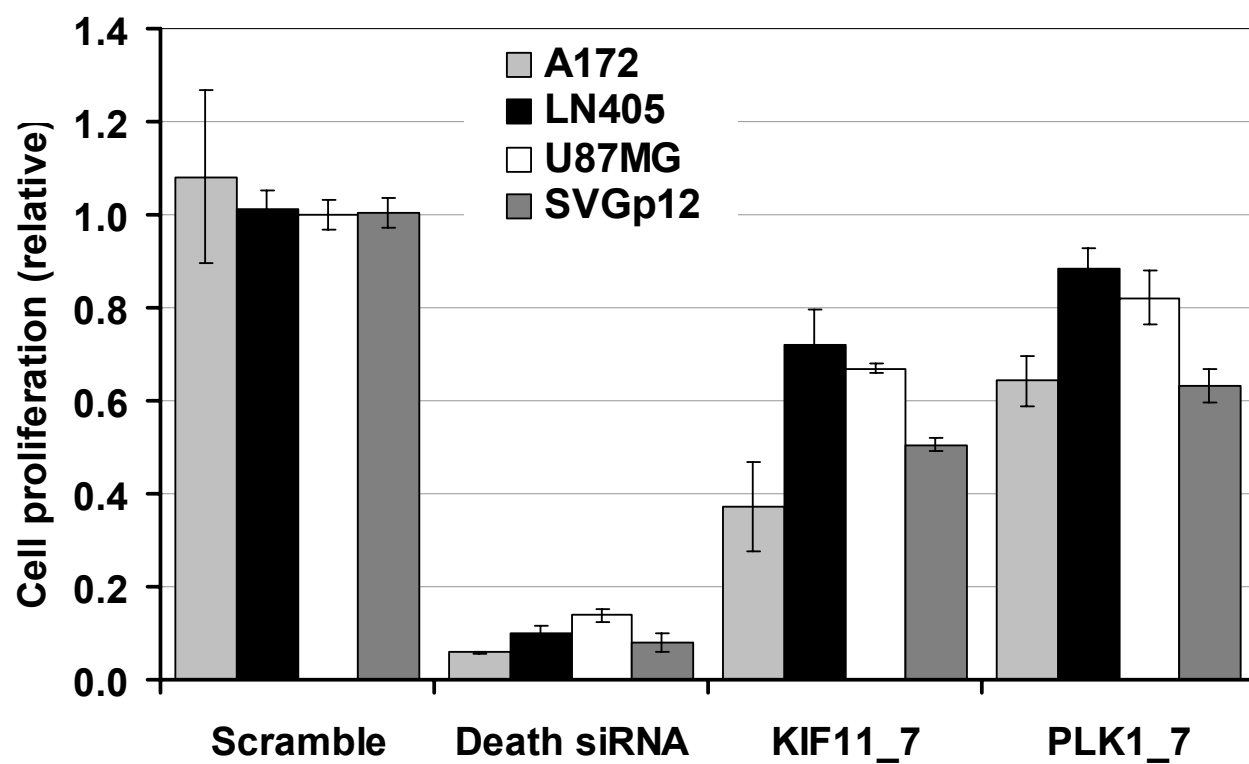

Supplement: Additional file 5 — The effect of gene silencing on cell proliferation. Control siRNAs (13 nM final concentration) were transfected with Silenfect (BioRad) transfection reagent to A172, LN405 and U87MG glioma cell lines and the SVGp12 control cell line. Cell proliferation was assayed 72 h after transfection using CellTiter-Glo Cell Viability assay. The proliferation data are presented as relative score to the mean of scramble siRNA-containing wells. Error bars indicate median absolute deviation. [file gm186-S5.PDF]

### Control siRNA transfections - Caspase 3/7 assay

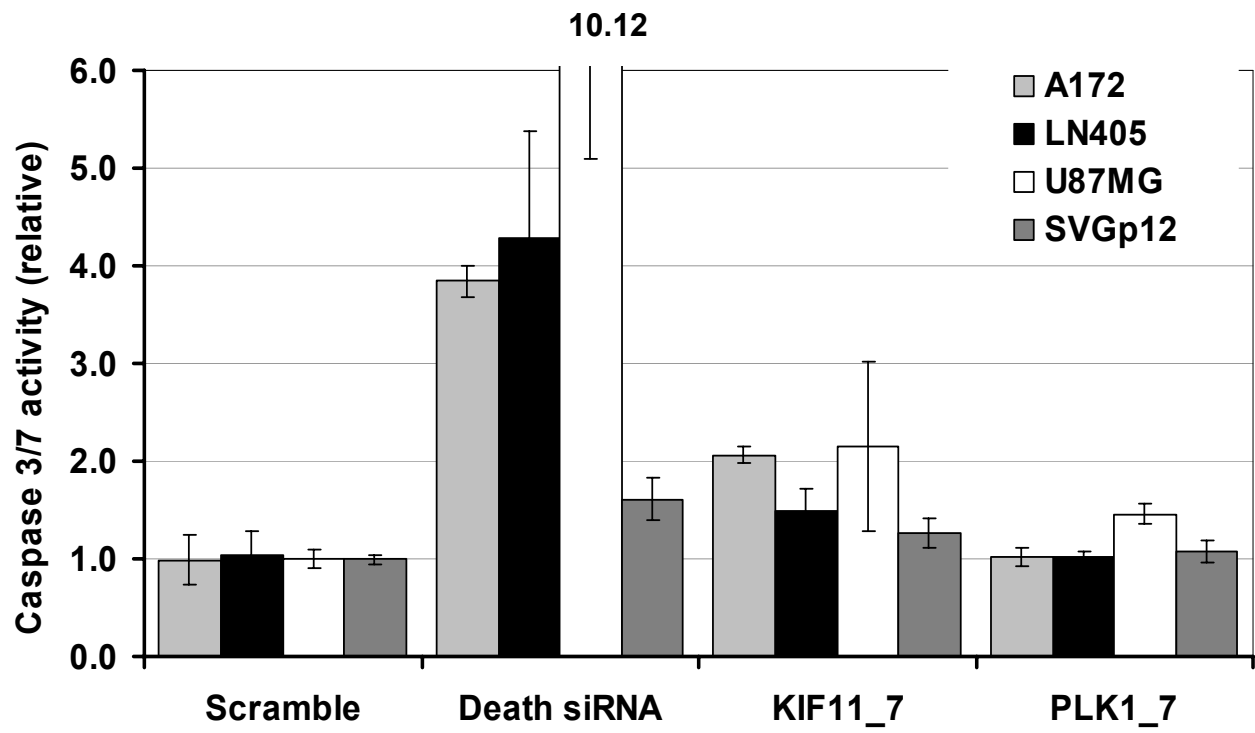

Supplement: Additional file 6 — The effect of gene silencing on caspase-3 and -7 activities. Control siRNAs (13 nM final concentration) were transfected with Silenfect (BioRad) transfection reagent to A172, LN405 and U87MG glioma cell lines and the SVGp12 control cell line. Induction of caspase-3 and -7 activities was detected 48 h after transfection with homogeneous Caspase-Glo 3/7 assay (Promega). The caspase activity is presented as relative median score to the mean of scramble siRNA containing wells. Error bars indicate median absolute deviation. [file gm186-S6.PDF]

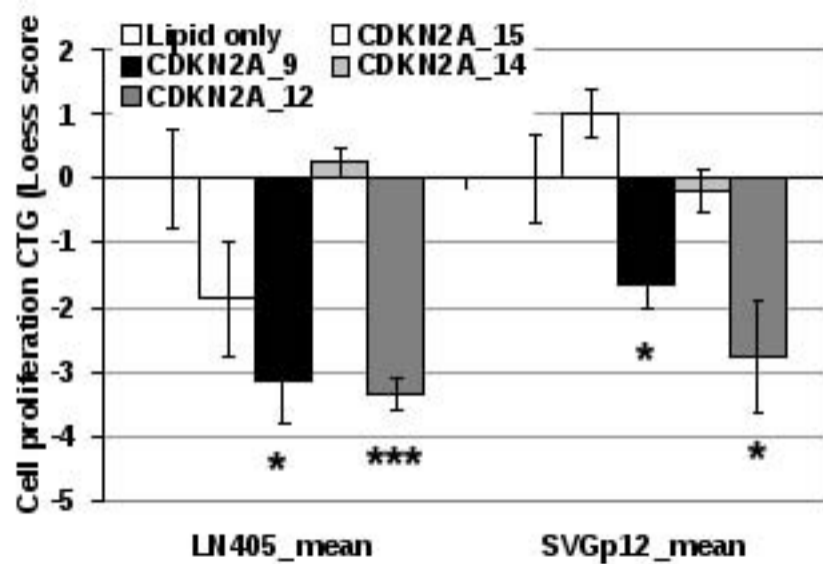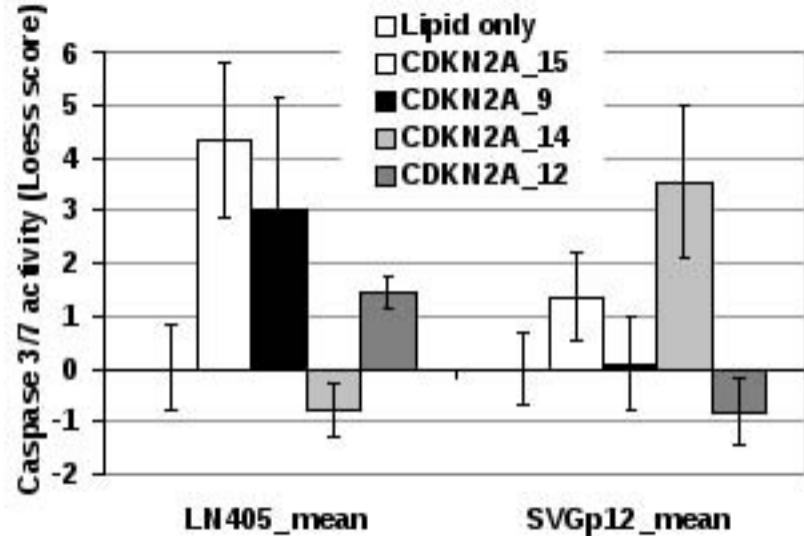

Supplement: Additional file 7 — The effects of silencing CDKN2A in LN405 and SVGp12 cell lines on cell proliferation and apoptosis. [file gm186-S7.PDF]
