# Supplementary material for: Large-scale data integration framework provides a comprehensive view on glioblastoma multiforme
Source: Genome Med. 2010 Sep 7;2(9):65. doi: 10.1186/gm186 (PMC3092116; doi:10.1186/gm186)
Supplement: Additional file 1 — Automatically generated result file. The report contains analysis configurations, parameter settings, result lists, tables and figures. The document also includes some analyses not reported in the manuscript, such as Gene Ontology analysis. [file SummaryReport.pdf › SummaryReport.pdf]

Analysis of Glioblastoma Samples

Kristian Ovaska Marko Laakso Riku Louhimo Erkka Valo Viljami Aittomäki
Sampsa Hautaniemi

July 16, 2010

Abstract

This analysis of The Cancer Genome Atlas (TCGA) provided data on glioblastoma multiforme patients [14] demonstrates the capabilities of Anduril in processing large and heterogenous data sets. The following links may be used to access:

1. gene expression results,
2. transcript specific expression results,
3. results of copy number analysis,
4. miRNA results, and
5. results of genotype associated survivals.

This document is generated by Anduril (Engine 1.0.4) and describes the main outputs of the analysis pipeline. The actual structure and the parameters of the analysis are listed in Section 19.

Contents

1 Gene expression analysis **29**

1.1 Summary of differentially expressed genes . . . 29

2 List of differentially expressed genes **29**

2.1 Gene set: over . . . 29

2.2 Gene set: under . . . 30

2.3 GO clustering . . . 31

3 Survival analysis for DEGs **34**

4 Summary of differentially expressed transcripts: fc > 2.0 and corrected p-value < 0.05 **185**

4.1 Ordered transcript expression values . . . 185

4.2 Multidimensional scaling of the samples . . . 186

4.3 GO clustering . . . 186

5 Survival analysis for transcript expression **190**

6 Signalling pathway impact analysis for differentially expressed transcripts with survival p < 0.01 **745**

7 Summary of differentially expressed genes: fc > 2.0 and corrected p-value < 0.05 **746**

7.1 Ordered transcript expression values . . . 746

7.2 Multidimensional scaling of the samples . . . 747

7.3 GO clustering . . . 747

8 Survival analysis for gene expression **750**

9 Signalling pathway impact analysis for differentially expressed genes evaluated from median exon expression with survival p < 0.01 **982**

10 ArrayCGH: Segmentation Results **984**

10.1 Interesting copy number 1 segments . . . 1008

10.2 Interesting copy number -1.0 segments . . . 1011

10.3 Copy number abberations . . . 1036

11 Quality Control **1067**

11.1 Box plots . . . 1067

12 Array CGH: integration results for CN gain regions and expression **1067**

13 Array CGH: integration results for CN loss regions and expression **1068**

14 Summary of differentially expressed miRNAs **1069**

14.1 Venn diagram of differentially expressed and statistically significant miRNAs . . . 1069

14.2 Expression quality control . . . 1070

14.2.1 Ordered miRNA expression values . . . 1070

14.2.2 Multidimensional scaling of the samples . . . 1070

15 Survival analysis for miRNA expression 1071

16 List of differentially expressed genes 1076
16.1 Gene set: over 1076
16.2 Gene set: under 1076

17 Genotype Specific Survival Estimates 1077
17.1 Survival analysis of rs7536561 1077
17.2 Survival analysis of rs10506216 1078
17.3 Survival analysis of rs1919793 1079
17.4 Survival analysis of rs2881513 1080
17.5 Survival analysis of rs555206 1081
17.6 Survival analysis of rs1479852 1082
17.7 Survival analysis of rs4382455 1083
17.8 Survival analysis of rs9395780 1084
17.9 Survival analysis of rs1349345 1085
17.10 Survival analysis of rs7892835 1086
17.11 Survival analysis of rs7572149 1087
17.12 Survival analysis of rs2326613 1088
17.13 Survival analysis of rs826911 1089
17.14 Survival analysis of rs17258085 1090
17.15 Survival analysis of rs12642867 1091
17.16 Survival analysis of rs1159766 1092
17.17 Survival analysis of rs1159764 1093
17.18 Survival analysis of rs17022010 1094
17.19 Survival analysis of rs4378238 1095
17.20 Survival analysis of rs1376946 1096
17.21 Survival analysis of rs10810961 1097
17.22 Survival analysis of rs11664633 1098
17.23 Survival analysis of rs9920861 1099
17.24 Survival analysis of rs5928288 1100
17.25 Survival analysis of rs11664762 1101
17.26 Survival analysis of rs11102233 1102
17.27 Survival analysis of rs4644918 1103
17.28 Survival analysis of rs12964779 1104
17.29 Survival analysis of rs8034783 1105
17.30 Survival analysis of rs12101080 1106
17.31 Survival analysis of rs11708378 1107
17.32 Survival analysis of rs6004869 1108
17.33 Survival analysis of rs4906048 1109
17.34 Survival analysis of rs10803885 1110
17.35 Survival analysis of rs7902084 1111

17.36 Survival analysis of rs306220 1112
17.37 Survival analysis of rs4489589 1113
17.38 Survival analysis of rs10221769 1114
17.39 Survival analysis of rs12891139 1115
17.40 Survival analysis of rs10827154 1116
17.41 Survival analysis of rs13168015 1117
17.42 Survival analysis of rs11692586 1118
17.43 Survival analysis of rs10793491 1119
17.44 Survival analysis of rs11007253 1120
17.45 Survival analysis of rs4498833 1121
17.46 Survival analysis of rs1436094 1122
17.47 Survival analysis of rs9403788 1123
17.48 Survival analysis of rs6077364 1124
17.49 Survival analysis of rs182174 1125
17.50 Survival analysis of rs10516167 1126
17.51 Survival analysis of rs701405 1127
17.52 Survival analysis of rs10107890 1128
17.53 Survival analysis of rs6728529 1129
17.54 Survival analysis of rs2521498 1130
17.55 Survival analysis of rs17403255 1131
17.56 Survival analysis of rs6671217 1132
17.57 Survival analysis of rs1506390 1133
17.58 Survival analysis of rs563460 1134
17.59 Survival analysis of rs11599561 1135
17.60 Survival analysis of rs7863546 1136
17.61 Survival analysis of rs2784761 1137
17.62 Survival analysis of rs12468464 1138
17.63 Survival analysis of rs13091723 1139
17.64 Survival analysis of rs6846040 1140
17.65 Survival analysis of rs2600077 1141
17.66 Survival analysis of rs10932321 1142
17.67 Survival analysis of rs1011127 1143
17.68 Survival analysis of rs464420 1144
17.69 Survival analysis of rs8093433 1145
17.70 Survival analysis of rs4921056 1146
17.71 Survival analysis of rs6427839 1147
17.72 Survival analysis of rs7069891 1148
17.73 Survival analysis of rs9635999 1149
17.74 Survival analysis of rs7571480 1150
17.75 Survival analysis of rs13423908 1151
17.76 Survival analysis of rs10749145 1152

17.77 Survival analysis of rs2581221 . . . 1153
17.78 Survival analysis of rs11897287 . . . 1154
17.79 Survival analysis of rs6756452 . . . 1155
17.80 Survival analysis of rs10169218 . . . 1156
17.81 Survival analysis of rs4962722 . . . 1157
17.82 Survival analysis of rs588115 . . . 1158
17.83 Survival analysis of rs2285218 . . . 1159
17.84 Survival analysis of rs9925903 . . . 1160
17.85 Survival analysis of rs3779936 . . . 1161
17.86 Survival analysis of rs7833024 . . . 1162
17.87 Survival analysis of rs1379149 . . . 1163
17.88 Survival analysis of rs7903448 . . . 1164
17.89 Survival analysis of rs1554336 . . . 1165
17.90 Survival analysis of rs13041611 . . . 1166
17.91 Survival analysis of rs12010722 . . . 1167
17.92 Survival analysis of rs17676096 . . . 1168
17.93 Survival analysis of rs7064020 . . . 1169
17.94 Gene rank . . . 1171
17.95 SNP database configuration . . . 1171

18 Gene set information 1172

18.1 GO clustering . . . 1172

19 Pipeline configuration 1175

19.1 ACGHabberGainFreqs (ACGHfrequencies) . . . 1215
19.2 ACGHabberGainFreqs-chrAnnot (KorvasieniAnnotator) . . . 1215
19.3 ACGHabberGainFreqs-filteredChrAnnot (TableQuery) . . . 1215
19.4 ACGHabberGainFreqs-frequencies (TableQuery) . . . 1216
19.5 ACGHabberGainFreqs-geneFreqs (TableQuery) . . . 1216
19.6 ACGHabberGainFreqs-geneList (SearchReplace) . . . 1217
19.7 ACGHabberGainFreqs-occurances (TableQuery) . . . 1217
19.8 ACGHabberGainFreqs-rules (INPUT) . . . 1218
19.9 ACGHabberLossFreqs (ACGHfrequencies) . . . 1218
19.10 ACGHabberLossFreqs-chrAnnot (KorvasieniAnnotator) . . . 1218
19.11 ACGHabberLossFreqs-filteredChrAnnot (TableQuery) . . . 1219
19.12 ACGHabberLossFreqs-frequencies (TableQuery) . . . 1220
19.13 ACGHabberLossFreqs-geneFreqs (TableQuery) . . . 1220
19.14 ACGHabberLossFreqs-geneList (SearchReplace) . . . 1220
19.15 ACGHabberLossFreqs-occurances (TableQuery) . . . 1221
19.16 ACGHabberLossFreqs-rules (INPUT) . . . 1222
19.17 ACGHmiRNAgainFreqs (ACGHfrequencies) . . . 1222
19.18 ACGHmiRNAgainFreqs-chrAnnot (CSVCleaner) . . . 1222

19.19 ACGHmiRNAgainFreqs-chrAnnotPre (KorvasieniAnnotator) . . . 1222
19.20 ACGHmiRNAgainFreqs-duplicateMirnas (TableQuery) . . . 1223
19.21 ACGHmiRNAgainFreqs-filteredChrAnnot (TableQuery) . . . 1224
19.22 ACGHmiRNAgainFreqs-frequencies (TableQuery) . . . 1224
19.23 ACGHmiRNAgainFreqs-geneFreqs (TableQuery) . . . 1224
19.24 ACGHmiRNAgainFreqs-geneList (SearchReplace) . . . 1225
19.25 ACGHmiRNAgainFreqs-mirnaIDs (SearchReplace) . . . 1226
19.26 ACGHmiRNAgainFreqs-occurances (TableQuery) . . . 1226
19.27 ACGHmiRNAgainFreqs-rules (INPUT) . . . 1227
19.28 ACGHmiRNAlossFreqs (ACGHfrequencies) . . . 1227
19.29 ACGHmiRNAlossFreqs-chrAnnot (CSVCleaner) . . . 1227
19.30 ACGHmiRNAlossFreqs-chrAnnotPre (KorvasieniAnnotator) . . . 1228
19.31 ACGHmiRNAlossFreqs-duplicateMirnas (TableQuery) . . . 1228
19.32 ACGHmiRNAlossFreqs-filteredChrAnnot (TableQuery) . . . 1229
19.33 ACGHmiRNAlossFreqs-frequencies (TableQuery) . . . 1229
19.34 ACGHmiRNAlossFreqs-geneFreqs (TableQuery) . . . 1230
19.35 ACGHmiRNAlossFreqs-geneList (SearchReplace) . . . 1230
19.36 ACGHmiRNAlossFreqs-mirnaIDs (SearchReplace) . . . 1231
19.37 ACGHmiRNAlossFreqs-occurances (TableQuery) . . . 1231
19.38 ACGHmiRNAlossFreqs-rules (INPUT) . . . 1232
19.39 OUTPUT1 (OUTPUT) . . . 1232
19.40 OUTPUT2 (OUTPUT) . . . 1232
19.41 OUTPUT3 (OUTPUT) . . . 1232
19.42 abstract (INPUT) . . . 1233
19.43 arrayCGHanalysis (ACGHAnalysis) . . . 1233
19.44 arrayCGHanalysis-aCGH (AgilentReader) . . . 1233
19.45 arrayCGHanalysis-abberSQL (INPUT) . . . 1234
19.46 arrayCGHanalysis-aberrations (QueryReport) . . . 1234
19.47 arrayCGHanalysis-aberrations-eqData (TableQuery) . . . 1234
19.48 arrayCGHanalysis-aberrations-eqTable (CSV2Latex) . . . 1235
19.49 arrayCGHanalysis-agil36to37map (INPUT) . . . 1235
19.50 arrayCGHanalysis-analysis (LatexCombiner) . . . 1236
19.51 arrayCGHanalysis-both (ACGHSegmentAnalysis) . . . 1236
19.52 arrayCGHanalysis-both-ArrayCGHgenes (TableQuery) . . . 1236
19.53 arrayCGHanalysis-both-associationLysis (associations) . . . 1237
19.54 arrayCGHanalysis-both-associationLysis-asc (INPUT) . . . 1237
19.55 arrayCGHanalysis-both-associationLysis-association (REvaluate) . . . 1237
19.56 arrayCGHanalysis-both-associationLysis-association1 (TableQuery) . . . 1238
19.57 arrayCGHanalysis-both-associationLysis-rBand (TableQuery) . . . 1238
19.58 arrayCGHanalysis-both-associationLysis-rev (INPUT) . . . 1238
19.59 arrayCGHanalysis-both-gAndLoss (AnnotateSegs) . . . 1239

19.60 arrayCGHanalysis-both-gAndLoss-annot (KorvasieniAnnotator) . . . 1239
19.61 arrayCGHanalysis-both-gAndLoss-annotExpand (ExpandCollapse) . . . 1239
19.62 arrayCGHanalysis-both-gAndLoss-annotGenes (KorvasieniAnnotator) . . . 1240
19.63 arrayCGHanalysis-both-gAndLoss-filtSegs (TableQuery) . . . 1241
19.64 arrayCGHanalysis-both-segplot (SegmentPlot) . . . 1241
19.65 arrayCGHanalysis-both-summaryReport (LatexCombiner) . . . 1242
19.66 arrayCGHanalysis-boxPlot (BoxPlot) . . . 1242
19.67 arrayCGHanalysis-chr1 (Segmentation) . . . 1242
19.68 arrayCGHanalysis-chr1-chrFilter (CSVFilter) . . . 1243
19.69 arrayCGHanalysis-chr1-filtNormCaseChan (CSVFilter) . . . 1243
19.70 arrayCGHanalysis-chr1-segmented (ACGHsegment) . . . 1244
19.71 arrayCGHanalysis-chr10 (Segmentation) . . . 1245
19.72 arrayCGHanalysis-chr10-chrFilter (CSVFilter) . . . 1245
19.73 arrayCGHanalysis-chr10-filtNormCaseChan (CSVFilter) . . . 1246
19.74 arrayCGHanalysis-chr10-segmented (ACGHsegment) . . . 1247
19.75 arrayCGHanalysis-chr11 (Segmentation) . . . 1248
19.76 arrayCGHanalysis-chr11-chrFilter (CSVFilter) . . . 1248
19.77 arrayCGHanalysis-chr11-filtNormCaseChan (CSVFilter) . . . 1249
19.78 arrayCGHanalysis-chr11-segmented (ACGHsegment) . . . 1250
19.79 arrayCGHanalysis-chr12 (Segmentation) . . . 1251
19.80 arrayCGHanalysis-chr12-chrFilter (CSVFilter) . . . 1251
19.81 arrayCGHanalysis-chr12-filtNormCaseChan (CSVFilter) . . . 1252
19.82 arrayCGHanalysis-chr12-segmented (ACGHsegment) . . . 1253
19.83 arrayCGHanalysis-chr13 (Segmentation) . . . 1254
19.84 arrayCGHanalysis-chr13-chrFilter (CSVFilter) . . . 1254
19.85 arrayCGHanalysis-chr13-filtNormCaseChan (CSVFilter) . . . 1255
19.86 arrayCGHanalysis-chr13-segmented (ACGHsegment) . . . 1255
19.87 arrayCGHanalysis-chr14 (Segmentation) . . . 1256
19.88 arrayCGHanalysis-chr14-chrFilter (CSVFilter) . . . 1256
19.89 arrayCGHanalysis-chr14-filtNormCaseChan (CSVFilter) . . . 1257
19.90 arrayCGHanalysis-chr14-segmented (ACGHsegment) . . . 1258
19.91 arrayCGHanalysis-chr15 (Segmentation) . . . 1259
19.92 arrayCGHanalysis-chr15-chrFilter (CSVFilter) . . . 1259
19.93 arrayCGHanalysis-chr15-filtNormCaseChan (CSVFilter) . . . 1260
19.94 arrayCGHanalysis-chr15-segmented (ACGHsegment) . . . 1261
19.95 arrayCGHanalysis-chr16 (Segmentation) . . . 1262
19.96 arrayCGHanalysis-chr16-chrFilter (CSVFilter) . . . 1262
19.97 arrayCGHanalysis-chr16-filtNormCaseChan (CSVFilter) . . . 1263
19.98 arrayCGHanalysis-chr16-segmented (ACGHsegment) . . . 1264
19.99 arrayCGHanalysis-chr17 (Segmentation) . . . 1265
19.100 arrayCGHanalysis-chr17-chrFilter (CSVFilter) . . . 1265

19.101 arrayCGHanalysis-chr17-filtNormCaseChan (CSVFilter) 1266
19.102 arrayCGHanalysis-chr17-segmented (ACGHsegment) 1266
19.103 arrayCGHanalysis-chr18 (Segmentation) 1267
19.104 arrayCGHanalysis-chr18-chrFilter (CSVFilter) 1267
19.105 arrayCGHanalysis-chr18-filtNormCaseChan (CSVFilter) 1268
19.106 arrayCGHanalysis-chr18-segmented (ACGHsegment) 1269
19.107 arrayCGHanalysis-chr19 (Segmentation) 1270
19.108 arrayCGHanalysis-chr19-chrFilter (CSVFilter) 1270
19.109 arrayCGHanalysis-chr19-filtNormCaseChan (CSVFilter) 1271
19.110 arrayCGHanalysis-chr19-segmented (ACGHsegment) 1272
19.111 arrayCGHanalysis-chr2 (Segmentation) 1273
19.112 arrayCGHanalysis-chr2-chrFilter (CSVFilter) 1273
19.113 arrayCGHanalysis-chr2-filtNormCaseChan (CSVFilter) 1274
19.114 arrayCGHanalysis-chr2-segmented (ACGHsegment) 1275
19.115 arrayCGHanalysis-chr20 (Segmentation) 1276
19.116 arrayCGHanalysis-chr20-chrFilter (CSVFilter) 1276
19.117 arrayCGHanalysis-chr20-filtNormCaseChan (CSVFilter) 1277
19.118 arrayCGHanalysis-chr20-segmented (ACGHsegment) 1277
19.119 arrayCGHanalysis-chr21 (Segmentation) 1278
19.120 arrayCGHanalysis-chr21-chrFilter (CSVFilter) 1278
19.121 arrayCGHanalysis-chr21-filtNormCaseChan (CSVFilter) 1279
19.122 arrayCGHanalysis-chr21-segmented (ACGHsegment) 1280
19.123 arrayCGHanalysis-chr22 (Segmentation) 1281
19.124 arrayCGHanalysis-chr22-chrFilter (CSVFilter) 1281
19.125 arrayCGHanalysis-chr22-filtNormCaseChan (CSVFilter) 1282
19.126 arrayCGHanalysis-chr22-segmented (ACGHsegment) 1283
19.127 arrayCGHanalysis-chr3 (Segmentation) 1284
19.128 arrayCGHanalysis-chr3-chrFilter (CSVFilter) 1284
19.129 arrayCGHanalysis-chr3-filtNormCaseChan (CSVFilter) 1285
19.130 arrayCGHanalysis-chr3-segmented (ACGHsegment) 1286
19.131 arrayCGHanalysis-chr4 (Segmentation) 1287
19.132 arrayCGHanalysis-chr4-chrFilter (CSVFilter) 1287
19.133 arrayCGHanalysis-chr4-filtNormCaseChan (CSVFilter) 1288
19.134 arrayCGHanalysis-chr4-segmented (ACGHsegment) 1288
19.135 arrayCGHanalysis-chr5 (Segmentation) 1289
19.136 arrayCGHanalysis-chr5-chrFilter (CSVFilter) 1289
19.137 arrayCGHanalysis-chr5-filtNormCaseChan (CSVFilter) 1290
19.138 arrayCGHanalysis-chr5-segmented (ACGHsegment) 1291
19.139 arrayCGHanalysis-chr6 (Segmentation) 1292
19.140 arrayCGHanalysis-chr6-chrFilter (CSVFilter) 1292
19.141 arrayCGHanalysis-chr6-filtNormCaseChan (CSVFilter) 1293

19.142 arrayCGHanalysis-chr6-segmented (ACGHsegment) . . . 1294
19.143 arrayCGHanalysis-chr7 (Segmentation) . . . 1295
19.144 arrayCGHanalysis-chr7-chrFilter (CSVFilter) . . . 1295
19.145 arrayCGHanalysis-chr7-filtNormCaseChan (CSVFilter) . . . 1296
19.146 arrayCGHanalysis-chr7-segmented (ACGHsegment) . . . 1297
19.147 arrayCGHanalysis-chr8 (Segmentation) . . . 1298
19.148 arrayCGHanalysis-chr8-chrFilter (CSVFilter) . . . 1298
19.149 arrayCGHanalysis-chr8-filtNormCaseChan (CSVFilter) . . . 1299
19.150 arrayCGHanalysis-chr8-segmented (ACGHsegment) . . . 1299
19.151 arrayCGHanalysis-chr9 (Segmentation) . . . 1300
19.152 arrayCGHanalysis-chr9-chrFilter (CSVFilter) . . . 1300
19.153 arrayCGHanalysis-chr9-filtNormCaseChan (CSVFilter) . . . 1301
19.154 arrayCGHanalysis-chr9-segmented (ACGHsegment) . . . 1302
19.155 arrayCGHanalysis-dataLoc (INPUT) . . . 1303
19.156 arrayCGHanalysis-fAnnot (CSVFilter) . . . 1303
19.157 arrayCGHanalysis-fChannels (QuantileFilter) . . . 1304
19.158 arrayCGHanalysis-gains (ACGHSegmentAnalysis) . . . 1304
19.159 arrayCGHanalysis-gains-ArrayCGHgenes (TableQuery) . . . 1305
19.160 arrayCGHanalysis-gains-abb (TableQuery) . . . 1305
19.161 arrayCGHanalysis-gains-abbers (CSV2Latex) . . . 1306
19.162 arrayCGHanalysis-gains-associationLysis (associations) . . . 1306
19.163 arrayCGHanalysis-gains-associationLysis-asc (INPUT) . . . 1306
19.164 arrayCGHanalysis-gains-associationLysis-association (REvaluate) . . . 1307
19.165 arrayCGHanalysis-gains-associationLysis-association1 (TableQuery) . . . 1307
19.166 arrayCGHanalysis-gains-associationLysis-rBand (TableQuery) . . . 1308
19.167 arrayCGHanalysis-gains-associationLysis-rev (INPUT) . . . 1308
19.168 arrayCGHanalysis-gains-gAndLoss (AnnotateSegs) . . . 1308
19.169 arrayCGHanalysis-gains-gAndLoss-annot (KorvasieniAnnotator) . . . 1308
19.170 arrayCGHanalysis-gains-gAndLoss-annotExpand (ExpandCollapse) . . . 1309
19.171 arrayCGHanalysis-gains-gAndLoss-annotGenes (KorvasieniAnnotator) . . . 1309
19.172 arrayCGHanalysis-gains-gAndLoss-filtSegs (TableQuery) . . . 1310
19.173 arrayCGHanalysis-gains-refs (INPUT) . . . 1311
19.174 arrayCGHanalysis-gains-segplot (SegmentPlot) . . . 1311
19.175 arrayCGHanalysis-gains-summaryReport (LatexCombiner) . . . 1311
19.176 arrayCGHanalysis-loclist (CSVFilter) . . . 1312
19.177 arrayCGHanalysis-losses (ACGHSegmentAnalysis) . . . 1313
19.178 arrayCGHanalysis-losses-ArrayCGHgenes (TableQuery) . . . 1313
19.179 arrayCGHanalysis-losses-abb (TableQuery) . . . 1313
19.180 arrayCGHanalysis-losses-abbers (CSV2Latex) . . . 1314
19.181 arrayCGHanalysis-losses-associationLysis (associations) . . . 1314
19.182 arrayCGHanalysis-losses-associationLysis-asc (INPUT) . . . 1315

19.183 arrayCGHanalysis-losses-associationLysis-association (REvaluate) . . . 1315
19.184 arrayCGHanalysis-losses-associationLysis-association1 (TableQuery) . . . 1315
19.185 arrayCGHanalysis-losses-associationLysis-rBand (TableQuery) . . . 1316
19.186 arrayCGHanalysis-losses-associationLysis-rev (INPUT) . . . 1316
19.187 arrayCGHanalysis-losses-gAndLoss (AnnotateSegs) . . . 1316
19.188 arrayCGHanalysis-losses-gAndLoss-annot (KorvasieniAnnotator) . . . 1317
19.189 arrayCGHanalysis-losses-gAndLoss-annotExpand (ExpandCollapse) . . . 1317
19.190 arrayCGHanalysis-losses-gAndLoss-annotGenes (KorvasieniAnnotator) . . . 1318
19.191 arrayCGHanalysis-losses-gAndLoss-filtSegs (TableQuery) . . . 1318
19.192 arrayCGHanalysis-losses-refs (INPUT) . . . 1319
19.193 arrayCGHanalysis-losses-segplot (SegmentPlot) . . . 1319
19.194 arrayCGHanalysis-losses-summaryReport (LatexCombiner) . . . 1320
19.195 arrayCGHanalysis-maPlot (Plot2D) . . . 1320
19.196 arrayCGHanalysis-normalized (ACGHnorm) . . . 1321
19.197 arrayCGHanalysis-preSegmentedGTSgain (CSVListJoin) . . . 1322
19.198 arrayCGHanalysis-probeIDs (CSV2IDList) . . . 1322
19.199 arrayCGHanalysis-qc (LatexCombiner) . . . 1323
19.200 arrayCGHanalysis-reMappedAnnot (TableQuery) . . . 1323
19.201 arrayCGHanalysis-rules (INPUT) . . . 1323
19.202 arrayCGHanalysis-sampFileMap (INPUT) . . . 1324
19.203 arrayCGHanalysis-samples (INPUT) . . . 1324
19.204 arrayCGHanalysis-segmented (TableQuery) . . . 1324
19.205 arrayCGHanalysis-segmentedGTSgain (CSVFilter) . . . 1325
19.206 arrayCGHanalysis-segmentedGTSloss (TableQuery) . . . 1325
19.207 arrayCGHanalysis-segmentedPart1 (LatexCombiner) . . . 1326
19.208 arrayCGHanalysis-segmentedPart2 (LatexCombiner) . . . 1326
19.209 arrayCGHanalysis-segmentedPart3 (LatexCombiner) . . . 1327
19.210 arrayCGHanalysis-segmentedReport (LatexCombiner) . . . 1327
19.211 arrayCGHanalysis-segmentedVals (StandardProcess) . . . 1328
19.212 arrayCGHanalysis-segplot (SegmentPlot) . . . 1328
19.213 arrayCGHanalysis-srFAnnot (SearchReplace) . . . 1329
19.214 arrayCGHanalysis-summaryReport (LatexPDF) . . . 1329
19.215 arrayCGHanalysis-template (LatexTemplate) . . . 1330
19.216 arrayCGHanalysis-unionSegs (TableQuery) . . . 1330
19.217 arrayCGHanalysis-unionSegs2 (TableQuery) . . . 1331
19.218 arrayCGHanalysis-unionSegsG (TableQuery) . . . 1332
19.219 arrayCGHanalysis-unionSegsG2 (TableQuery) . . . 1332
19.220 arrayCGHanalysis-unionSegsG3 (TableQuery) . . . 1333
19.221 arrayCGHanalysis-unionSegsL (TableQuery) . . . 1334
19.222 arrayCGHanalysis-unionSegsL2 (TableQuery) . . . 1335
19.223 arrayCGHexonIntGain (ACGHexonIntegration) . . . 1335

19.224 arrayCGHexonIntGain-AnnotProbeGenes (NextGene) . . . 1336
19.225 arrayCGHexonIntGain-ExonAnnot (INPUT) . . . 1336
19.226 arrayCGHexonIntGain-GTSoutputG (INPUT) . . . 1336
19.227 arrayCGHexonIntGain-ProbeIDs (REvaluate) . . . 1337
19.228 arrayCGHexonIntGain-assocExpand (ExpandCollapse) . . . 1337
19.229 arrayCGHexonIntGain-expr (INPUT) . . . 1338
19.230 arrayCGHexonIntGain-exprAnnot (INPUT) . . . 1338
19.231 arrayCGHexonIntGain-exprMat (REvaluate) . . . 1338
19.232 arrayCGHexonIntGain-gainsList (TableQuery) . . . 1339
19.233 arrayCGHexonIntGain-intGreat (ExpExpIntegration) . . . 1339
19.234 arrayCGHexonIntGain-integrated (CSVCleaner) . . . 1340
19.235 arrayCGHexonIntGain-integratedExonACGH (KorvasieniAnnotator) . . . 1340
19.236 arrayCGHexonIntGain-labelMat (REvaluate) . . . 1341
19.237 arrayCGHexonIntGain-matchAnnot (TableQuery) . . . 1342
19.238 arrayCGHexonIntGain-outputList (TableQuery) . . . 1342
19.239 arrayCGHexonIntGain-refs (INPUT) . . . 1342
19.240 arrayCGHexonIntGain-report (CSV2Latex) . . . 1343
19.241 arrayCGHexonIntGain-script (INPUT) . . . 1343
19.242 arrayCGHexonIntGain-script2 (INPUT) . . . 1344
19.243 arrayCGHexonIntGain-sortedIntegrated (TableQuery) . . . 1344
19.244 arrayCGHexonIntGain-transAnnot (TableQuery) . . . 1344
19.245 arrayCGHexonIntGain-unDuplic (RowJoin) . . . 1345
19.246 arrayCGHexonIntLoss (ACGHexonIntegration) . . . 1345
19.247 arrayCGHexonIntLoss-AnnotProbeGenes (NextGene) . . . 1345
19.248 arrayCGHexonIntLoss-ExonAnnot (INPUT) . . . 1346
19.249 arrayCGHexonIntLoss-GTSoutputL (INPUT) . . . 1346
19.250 arrayCGHexonIntLoss-ProbeIDs (REvaluate) . . . 1346
19.251 arrayCGHexonIntLoss-assocExpand (ExpandCollapse) . . . 1347
19.252 arrayCGHexonIntLoss-expr (INPUT) . . . 1347
19.253 arrayCGHexonIntLoss-exprAnnot (INPUT) . . . 1348
19.254 arrayCGHexonIntLoss-exprMat (REvaluate) . . . 1348
19.255 arrayCGHexonIntLoss-gainsList (TableQuery) . . . 1349
19.256 arrayCGHexonIntLoss-intGreat (ExpExpIntegration) . . . 1349
19.257 arrayCGHexonIntLoss-integrated (CSVCleaner) . . . 1350
19.258 arrayCGHexonIntLoss-integratedExonACGH (KorvasieniAnnotator) . . . 1350
19.259 arrayCGHexonIntLoss-labelMat (REvaluate) . . . 1351
19.260 arrayCGHexonIntLoss-matchAnnot (TableQuery) . . . 1351
19.261 arrayCGHexonIntLoss-outputList (TableQuery) . . . 1352
19.262 arrayCGHexonIntLoss-refs (INPUT) . . . 1352
19.263 arrayCGHexonIntLoss-report (CSV2Latex) . . . 1352
19.264 arrayCGHexonIntLoss-script (INPUT) . . . 1353

19.265 arrayCGHexonIntLoss-script2 (INPUT) 1353
19.266 arrayCGHexonIntLoss-sortedIntegrated (TableQuery) 1354
19.267 arrayCGHexonIntLoss-transAnnot (TableQuery) 1354
19.268 arrayCGHexonIntLoss-unDuplic (RowJoin) 1354
19.269 arrayCGHsummary (LatexCombiner) 1355
19.270 cfgDescMicroarray (INPUT) 1355
19.271 cfgDoc (LatexCombiner) 1356
19.272 cfgReport (ConfigurationReport) 1356
19.273 ensembl (INPUT) 1356
19.274 expressionSummary (LatexCombiner) 1357
19.275 geneExpression (GeneExpressionAnalysis) 1357
19.276 geneExpression-allLogratio (SampleCombiner) 1357
19.277 geneExpression-allMeans (SampleCombiner) 1358
19.278 geneExpression-allProbes (IDConvert) 1358
19.279 geneExpression-allProbesDup (TableQuery) 1359
19.280 geneExpression-degAnnot (REvaluate) 1360
19.281 geneExpression-degAnnotCols (INPUT) 1360
19.282 geneExpression-degAnnotK (KorvasieniAnnotator) 1360
19.283 geneExpression-degProbes (CSV2IDList) 1361
19.284 geneExpression-degReport (DEGReport) 1362
19.285 geneExpression-degSet (SetTransformer) 1362
19.286 geneExpression-degStat (TableQuery) 1363
19.287 geneExpression-degStatSQL (INPUT) 1363
19.288 geneExpression-degTransformation (INPUT) 1364
19.289 geneExpression-filterSamplesRScript (INPUT) 1364
19.290 geneExpression-foldChange (FoldChange) 1364
19.291 geneExpression-geneStatisticsQuery (INPUT) 1365
19.292 geneExpression-groupMeans (SampleCombiner) 1365
19.293 geneExpression-heatmap (GOClustering) 1365
19.294 geneExpression-logratio (SampleCombiner) 1366
19.295 geneExpression-metadata (INPUT) 1367
19.296 geneExpression-probeAnnot (BiomartAnnotator) 1367
19.297 geneExpression-probeAnnotExp (ExpandCollapse) 1368
19.298 geneExpression-probeFilter (CSVFilter) 1368
19.299 geneExpression-probeJoin (IDConvert) 1369
19.300 geneExpression-probeSelect (TableQuery) 1370
19.301 geneExpression-report (LatexCombiner) 1370
19.302 geneExpression-sampleFilter (REvaluate) 1371
19.303 geneExpression-sampleGroups (SampleGroupCreator) 1371
19.304 geneExpression-samplesWithoutClinicalAnnotation (INPUT) 1372
19.305 geneExpression-survival (TranscriptSurvival) 1372

19.306 geneExpression-survival-km (KaplanMeier) 1373
19.307 geneExpression-survival-kmReport (LatexCombiner) 1373
19.308 geneExpression-survival-sampleExpression (SampleExpression) 1374
19.309 geneExpression-survival-sampleExpressionTrans (MatrixTranspose) 1374
19.310 geneExpression-survival-sampleNames (ExpandCollapse) 1375
19.311 geneExpression-survival-survival (TableQuery) 1375
19.312 geneExpression-survival-survivalQuery (INPUT) 1375
19.313 geneExpression-survival-survivalTable (CSVJoin) 1376
19.314 geneExpression-survival-survivalTableConv (IDConvert) 1376
19.315 geneExpression-survivalQuery (INPUT) 1377
19.316 geneExpression-tTest (StatisticalTest) 1377
19.317 geneExpression-uniqProbesSQL (INPUT) 1379
19.318 geneExpression-varFilter (VariationFilter) 1379
19.319 geneListFreqs (CSV2IDList) 1379
19.320 genotypeDB (INPUT) 1380
19.321 mainTemplate (LatexTemplate) 1380
19.322 medianExonExpression (MedianExonExpressionAnalysis) 1381
19.323 medianExonExpression-allEntrez (KorvasieniAnnotator) 1381
19.324 medianExonExpression-allProbes (TableQuery) 1382
19.325 medianExonExpression-cumDistReport (Plot2D) 1382
19.326 medianExonExpression-deeTableQuery (INPUT) 1383
19.327 medianExonExpression-det (SetTransformer) 1383
19.328 medianExonExpression-detAnnot (KorvasieniAnnotator) 1384
19.329 medianExonExpression-detFoldChange (FoldChange) 1385
19.330 medianExonExpression-detIDList (CSV2IDList) 1385
19.331 medianExonExpression-detReport (DEGReport) 1386
19.332 medianExonExpression-detTTest (StatisticalTest) 1386
19.333 medianExonExpression-detTable (TableQuery) 1387
19.334 medianExonExpression-detVennDiagram (VennDiagram) 1388
19.335 medianExonExpression-exonSPIArefs (INPUT) 1388
19.336 medianExonExpression-filterSamplesRScript (INPUT) 1389
19.337 medianExonExpression-goClusters (GOClustering) 1389
19.338 medianExonExpression-groupMeans (SampleCombiner) 1390
19.339 medianExonExpression-intersectionTransformation (INPUT) 1390
19.340 medianExonExpression-km (TranscriptSurvival) 1390
19.341 medianExonExpression-km-km (KaplanMeier) 1391
19.342 medianExonExpression-km-kmReport (LatexCombiner) 1391
19.343 medianExonExpression-km-sampleExpression (SampleExpression) 1392
19.344 medianExonExpression-km-sampleExpressionFilt (CSVFilter) 1392
19.345 medianExonExpression-km-sampleExpressionTrans (MatrixTranspose) 1393
19.346 medianExonExpression-km-sampleNames (ExpandCollapse) 1394

19.347 medianExonExpression-km-survival (TableQuery) . . . 1394
19.348 medianExonExpression-km-survivalQuery (INPUT) . . . 1394
19.349 medianExonExpression-km-survivalTable (CSVJoin) . . . 1395
19.350 medianExonExpression-km-survivalTableConv (IDConvert) . . . 1395
19.351 medianExonExpression-logratio (SampleCombiner) . . . 1396
19.352 medianExonExpression-mdsReport (MDSPlot) . . . 1397
19.353 medianExonExpression-metadata (INPUT) . . . 1397
19.354 medianExonExpression-qcReport (LatexCombiner) . . . 1398
19.355 medianExonExpression-reference (CSV2IDList) . . . 1398
19.356 medianExonExpression-report (LatexCombiner) . . . 1399
19.357 medianExonExpression-sampleGroups (SampleGroupCreator) . . . 1399
19.358 medianExonExpression-samplesWithoutClinicalAnnotation (INPUT) . . . 1400
19.359 medianExonExpression-spia (SPIA) . . . 1400
19.360 medianExonExpression-spiaReport (CSV2Latex) . . . 1401
19.361 medianExonExpression-survGenes (CSVTransformer) . . . 1402
19.362 medianExonExpression-survGenesEntrez (KorvasieniAnnotator) . . . 1402
19.363 medianExonExpression-survLogratio (CSVFilter) . . . 1403
19.364 medianExonExpression-survLogratioEntrez (IDConvert) . . . 1404
19.365 medianExonExpression-survLogratioEntrezFilt (CSVFilter) . . . 1405
19.366 medianExonExpression-tExpr (REvaluate) . . . 1406
19.367 medianExonExpression-transcriptStatisticsQuery (INPUT) . . . 1406
19.368 medianExonExpressionMatrix (INPUT) . . . 1407
19.369 methylation (Methylation) . . . 1407
19.370 methylation-annotation002 (INPUT) . . . 1407
19.371 methylation-annotation003 (INPUT) . . . 1407
19.372 methylation-beta002raw (INPUT) . . . 1407
19.373 methylation-beta003raw (INPUT) . . . 1408
19.374 methylation-combined (CSVJoin) . . . 1408
19.375 methylation-results002 (MethylationAnalysis) . . . 1409
19.376 methylation-results002-beta (SampleCombiner) . . . 1409
19.377 methylation-results002-betaFilt (SearchReplace) . . . 1409
19.378 methylation-results002-betaFilt1 (CSVFilter) . . . 1410
19.379 methylation-results002-betaNames (CSVTransformer) . . . 1411
19.380 methylation-results002-betaResults (CSVTransformer) . . . 1411
19.381 methylation-results002-betaUnique (IDConvert) . . . 1412
19.382 methylation-results002-groups (SampleGroupCreator) . . . 1413
19.383 methylation-results003 (MethylationAnalysis) . . . 1414
19.384 methylation-results003-beta (SampleCombiner) . . . 1414
19.385 methylation-results003-betaFilt (SearchReplace) . . . 1414
19.386 methylation-results003-betaFilt1 (CSVFilter) . . . 1415
19.387 methylation-results003-betaNames (CSVTransformer) . . . 1416

19.388 methylation-results003-betaResults (CSVTransformer) 1416
19.389 methylation-results003-betaUnique (IDConvert) 1417
19.390 methylation-results003-groups (SampleGroupCreator) 1418
19.391 miRNAExprNormalized (INPUT) 1419
19.392 miRNANames (INPUT) 1419
19.393 miRNAProbeAnnot (INPUT) 1419
19.394 miRNAResults (MiRNAAnalysis) 1419
19.395 miRNAResults-collapsedMiRNANames (ExpandCollapse) 1420
19.396 miRNAResults-collapsedMiRNATargets (ExpandCollapse) 1420
19.397 miRNAResults-cumDistReport (Plot2D) 1421
19.398 miRNAResults-demFoldChange (FoldChange) 1422
19.399 miRNAResults-demReport (DEGReport) 1422
19.400 miRNAResults-demSet (SetTransformer) 1423
19.401 miRNAResults-demTransformation (INPUT) 1423
19.402 miRNAResults-demVennDiagram (VennDiagram) 1423
19.403 miRNAResults-dems (CSV2IDList) 1424
19.404 miRNAResults-filterSamplesRScript (INPUT) 1424
19.405 miRNAResults-filteredMiRNATargets (CSVFilter) 1425
19.406 miRNAResults-groupMeans (SampleCombiner) 1426
19.407 miRNAResults-km (KaplanMeier) 1426
19.408 miRNAResults-kmReport (LatexCombiner) 1427
19.409 miRNAResults-logratio (SampleCombiner) 1427
19.410 miRNAResults-mExpr (VariationFilter) 1428
19.411 miRNAResults-mds (MDSPlot) 1428
19.412 miRNAResults-miRNAAnnot (CSVJoin) 1429
19.413 miRNAResults-miRNAStat (TableQuery) 1429
19.414 miRNAResults-miRNAStatBand (KorvasieniAnnotator) 1430
19.415 miRNAResults-miRNAStatConverted (IDConvert) 1430
19.416 miRNAResults-miRNAStatFiltered (CSVFilter) 1431
19.417 miRNAResults-miRNAStatGenes (KorvasieniAnnotator) 1432
19.418 miRNAResults-miRNAStatQuery (INPUT) 1433
19.419 miRNAResults-patientMetadata (INPUT) 1433
19.420 miRNAResults-probeJoin (IDConvert) 1433
19.421 miRNAResults-qcReport (LatexCombiner) 1434
19.422 miRNAResults-report (LatexCombiner) 1435
19.423 miRNAResults-sampleExpression (SampleExpression) 1435
19.424 miRNAResults-sampleExpressionFilt (CSVFilter) 1436
19.425 miRNAResults-sampleExpressionTrans (MatrixTranspose) 1437
19.426 miRNAResults-sampleFilter (REvaluate) 1437
19.427 miRNAResults-sampleGroups (SampleGroupCreator) 1437
19.428 miRNAResults-sampleNames (ExpandCollapse) 1438

19.429 miRNAResults-samplesWithoutClinicalAnnotation (INPUT) . . . 1439
19.430 miRNAResults-survival (TableQuery) . . . 1439
19.431 miRNAResults-survivalAnnot (CSVFilter) . . . 1439
19.432 miRNAResults-survivalQuery (INPUT) . . . 1440
19.433 miRNAResults-survivalTable (CSVJoin) . . . 1440
19.434 miRNAResults-survivalTableConv (IDConvert) . . . 1441
19.435 miRNAResults-tTest (StatisticalTest) . . . 1442
19.436 miRNATargets (INPUT) . . . 1443
19.437 propertiesDoc (Properties2Latex) . . . 1443
19.438 RConfig (RConfigurationReport) . . . 1443
19.439 rawGeneExpression (INPUT) . . . 1444
19.440 snpSurvival (SNPSurvival) . . . 1444
19.441 snpSurvival-combinedSurvival (CSVListJoin) . . . 1444
19.442 snpSurvival-combinedSurvivalpost (SearchReplace) . . . 1445
19.443 snpSurvival-combinedSurvivalpre (CSVListJoin) . . . 1445
19.444 snpSurvival-finalMarkerSet (CSV2IDList) . . . 1446
19.445 snpSurvival-finalMarkerSetPre (AffySNPFetch) . . . 1446
19.446 snpSurvival-finalMarkerSetPre-markers (CSV2IDList) . . . 1447
19.447 snpSurvival-finalMarkerSetPre-rsNumbers (SQLSelect) . . . 1447
19.448 snpSurvival-markers1 (INPUT) . . . 1448
19.449 snpSurvival-markers10 (INPUT) . . . 1448
19.450 snpSurvival-markers2 (INPUT) . . . 1448
19.451 snpSurvival-markers3 (INPUT) . . . 1448
19.452 snpSurvival-markers4 (INPUT) . . . 1449
19.453 snpSurvival-markers5 (INPUT) . . . 1449
19.454 snpSurvival-markers6 (INPUT) . . . 1449
19.455 snpSurvival-markers7 (INPUT) . . . 1449
19.456 snpSurvival-markers8 (INPUT) . . . 1450
19.457 snpSurvival-markers9 (INPUT) . . . 1450
19.458 snpSurvival-survivalAll (SurvivalAnalysis) . . . 1450
19.459 snpSurvival-survivalAll-annotSQL (INPUT) . . . 1450
19.460 snpSurvival-survivalAll-caseGenotypes (GetGenotypes) . . . 1450
19.461 snpSurvival-survivalAll-caseGenotypes-freqCheck (AlleleCounter) . . . 1450
19.462 snpSurvival-survivalAll-caseGenotypes-ftMarkers (CSV2IDList) . . . 1451
19.463 snpSurvival-survivalAll-caseGenotypes-genotypes (SNPHelistinReader) . . . 1452
19.464 snpSurvival-survivalAll-caseGenotypes-gtypes (SNPHelistinReader) . . . 1452
19.465 snpSurvival-survivalAll-caseNames (CSV2IDList) . . . 1452
19.466 snpSurvival-survivalAll-clinicalData (INPUT) . . . 1453
19.467 snpSurvival-survivalAll-clinicalSelect (SourceCode2Latex) . . . 1453
19.468 snpSurvival-survivalAll-clinicalTypes (INPUT) . . . 1454
19.469 snpSurvival-survivalAll-geneRank (QueryReport) . . . 1454

19.470 snpSurvival-survivalAll-geneRank-eqData (TableQuery) 1454
19.471 snpSurvival-survivalAll-geneRank-eqTable (CSV2Latex) 1455
19.472 snpSurvival-survivalAll-geneRankQuery (INPUT) 1455
19.473 snpSurvival-survivalAll-missingDistC (CSV2Latex) 1456
19.474 snpSurvival-survivalAll-propertiesDoc (Properties2Latex) 1456
19.475 snpSurvival-survivalAll-refsGeneRank (INPUT) 1457
19.476 snpSurvival-survivalAll-refsHits (INPUT) 1457
19.477 snpSurvival-survivalAll-reportBody (LatexCombiner) 1457
19.478 snpSurvival-survivalAll-reportTemplate (LatexTemplate) 1458
19.479 snpSurvival-survivalAll-snpAnnotations (INPUT) 1458
19.480 snpSurvival-survivalAll-summaryReport (LatexPDF) 1458
19.481 snpSurvival-survivalAll-survSNPAnnot (AnnotateSNPs) 1459
19.482 snpSurvival-survivalAll-survSNPAnnot-annotData (TableQuery) 1459
19.483 snpSurvival-survivalAll-survSNPAnnot-annotTable (CSV2Latex) 1459
19.484 snpSurvival-survivalAll-survSNPAnnot-genes (KorvasieniAnnotator) 1460
19.485 snpSurvival-survivalAll-survSNPAnnot-snpLoci (RefSNPAnnotator) 1461
19.486 snpSurvival-survivalAll-survSNPAnnot-snpRel (ExpandCollapse) 1461
19.487 snpSurvival-survivalAll-survival (SNPKaplanMeier) 1461
19.488 snpSurvival-survivalAll-survivalClinical (TableQuery) 1462
19.489 snpSurvival-survivalAll-survivalParamsQuery (SearchReplace) 1463
19.490 snpSurvival-survivalAll-survivalParamsTemplate (INPUT) 1463
19.491 snpSurvival-survivalAll-survivalReport (LatexCombiner) 1463
19.492 snpSurvival-survivalSet1 (SurvivalAnalysis) 1464
19.493 snpSurvival-survivalSet1-annotSQL (INPUT) 1464
19.494 snpSurvival-survivalSet1-caseGenotypes (GetGenotypes) 1464
19.495 snpSurvival-survivalSet1-caseGenotypes-freqCheck (AlleleCounter) 1464
19.496 snpSurvival-survivalSet1-caseGenotypes-ftMarkers (CSV2IDList) 1465
19.497 snpSurvival-survivalSet1-caseGenotypes-genotypes (SNPHelistinReader) 1466
19.498 snpSurvival-survivalSet1-caseGenotypes-gtypes (SNPHelistinReader) 1466
19.499 snpSurvival-survivalSet1-caseNames (CSV2IDList) 1466
19.500 snpSurvival-survivalSet1-clinicalData (INPUT) 1467
19.501 snpSurvival-survivalSet1-clinicalSelect (SourceCode2Latex) 1467
19.502 snpSurvival-survivalSet1-clinicalTypes (INPUT) 1468
19.503 snpSurvival-survivalSet1-geneRank (QueryReport) 1468
19.504 snpSurvival-survivalSet1-geneRank-eqData (TableQuery) 1468
19.505 snpSurvival-survivalSet1-geneRank-eqTable (CSV2Latex) 1469
19.506 snpSurvival-survivalSet1-geneRankQuery (INPUT) 1469
19.507 snpSurvival-survivalSet1-missingDistC (CSV2Latex) 1470
19.508 snpSurvival-survivalSet1-propertiesDoc (Properties2Latex) 1470
19.509 snpSurvival-survivalSet1-refsGeneRank (INPUT) 1471
19.510 snpSurvival-survivalSet1-refsHits (INPUT) 1471

19.511 snpSurvival-survivalSet1-reportBody (LatexCombiner) . . . 1471
19.512 snpSurvival-survivalSet1-reportTemplate (LatexTemplate) . . . 1472
19.513 snpSurvival-survivalSet1-snpAnnotations (INPUT) . . . 1472
19.514 snpSurvival-survivalSet1-summaryReport (LatexPDF) . . . 1472
19.515 snpSurvival-survivalSet1-survSNPAnnot (AnnotateSNPs) . . . 1473
19.516 snpSurvival-survivalSet1-survSNPAnnot-annotData (TableQuery) . . . 1473
19.517 snpSurvival-survivalSet1-survSNPAnnot-annotTable (CSV2Latex) . . . 1473
19.518 snpSurvival-survivalSet1-survSNPAnnot-genes (KorvasieniAnnotator) . . . 1474
19.519 snpSurvival-survivalSet1-survSNPAnnot-snpLoci (RefSNPAnnotator) . . . 1475
19.520 snpSurvival-survivalSet1-survSNPAnnot-snpRel (ExpandCollapse) . . . 1475
19.521 snpSurvival-survivalSet1-survival (SNPKaplanMeier) . . . 1476
19.522 snpSurvival-survivalSet1-survivalClinical (TableQuery) . . . 1476
19.523 snpSurvival-survivalSet1-survivalParamsQuery (SearchReplace) . . . 1477
19.524 snpSurvival-survivalSet1-survivalParamsTemplate (INPUT) . . . 1477
19.525 snpSurvival-survivalSet1-survivalReport (LatexCombiner) . . . 1477
19.526 snpSurvival-survivalSet10 (SurvivalAnalysis) . . . 1478
19.527 snpSurvival-survivalSet10-annotSQL (INPUT) . . . 1478
19.528 snpSurvival-survivalSet10-caseGenotypes (GetGenotypes) . . . 1478
19.529 snpSurvival-survivalSet10-caseGenotypes-freqCheck (AlleleCounter) . . . 1478
19.530 snpSurvival-survivalSet10-caseGenotypes-ftMarkers (CSV2IDList) . . . 1479
19.531 snpSurvival-survivalSet10-caseGenotypes-genotypes (SNPHelistinReader) . . . 1480
19.532 snpSurvival-survivalSet10-caseGenotypes-gtypes (SNPHelistinReader) . . . 1480
19.533 snpSurvival-survivalSet10-caseNames (CSV2IDList) . . . 1480
19.534 snpSurvival-survivalSet10-clinicalData (INPUT) . . . 1481
19.535 snpSurvival-survivalSet10-clinicalSelect (SourceCode2Latex) . . . 1481
19.536 snpSurvival-survivalSet10-clinicalTypes (INPUT) . . . 1482
19.537 snpSurvival-survivalSet10-geneRank (QueryReport) . . . 1482
19.538 snpSurvival-survivalSet10-geneRank-eqData (TableQuery) . . . 1482
19.539 snpSurvival-survivalSet10-geneRank-eqTable (CSV2Latex) . . . 1483
19.540 snpSurvival-survivalSet10-geneRankQuery (INPUT) . . . 1483
19.541 snpSurvival-survivalSet10-missingDistC (CSV2Latex) . . . 1484
19.542 snpSurvival-survivalSet10-propertiesDoc (Properties2Latex) . . . 1484
19.543 snpSurvival-survivalSet10-refsGeneRank (INPUT) . . . 1485
19.544 snpSurvival-survivalSet10-refsHits (INPUT) . . . 1485
19.545 snpSurvival-survivalSet10-reportBody (LatexCombiner) . . . 1485
19.546 snpSurvival-survivalSet10-reportTemplate (LatexTemplate) . . . 1486
19.547 snpSurvival-survivalSet10-snpAnnotations (INPUT) . . . 1486
19.548 snpSurvival-survivalSet10-summaryReport (LatexPDF) . . . 1486
19.549 snpSurvival-survivalSet10-survSNPAnnot (AnnotateSNPs) . . . 1487
19.550 snpSurvival-survivalSet10-survSNPAnnot-annotData (TableQuery) . . . 1487
19.551 snpSurvival-survivalSet10-survSNPAnnot-annotTable (CSV2Latex) . . . 1487

19.552 snpSurvival-survivalSet10-survSNPAnnot-genes (KorvasieniAnnotator) . . . 1488
19.553 snpSurvival-survivalSet10-survSNPAnnot-snpLoci (RefSNPAnnotator) . . . 1489
19.554 snpSurvival-survivalSet10-survSNPAnnot-snpRel (ExpandCollapse) . . . 1489
19.555 snpSurvival-survivalSet10-survival (SNPKaplanMeier) . . . 1490
19.556 snpSurvival-survivalSet10-survivalClinical (TableQuery) . . . 1490
19.557 snpSurvival-survivalSet10-survivalParamsQuery (SearchReplace) . . . 1491
19.558 snpSurvival-survivalSet10-survivalParamsTemplate (INPUT) . . . 1491
19.559 snpSurvival-survivalSet10-survivalReport (LatexCombiner) . . . 1491
19.560 snpSurvival-survivalSet2 (SurvivalAnalysis) . . . 1492
19.561 snpSurvival-survivalSet2-annotSQL (INPUT) . . . 1492
19.562 snpSurvival-survivalSet2-caseGenotypes (GetGenotypes) . . . 1492
19.563 snpSurvival-survivalSet2-caseGenotypes-freqCheck (AlleleCounter) . . . 1492
19.564 snpSurvival-survivalSet2-caseGenotypes-ftMarkers (CSV2IDList) . . . 1493
19.565 snpSurvival-survivalSet2-caseGenotypes-genotypes (SNPHelistinReader) . . . 1494
19.566 snpSurvival-survivalSet2-caseGenotypes-gtypes (SNPHelistinReader) . . . 1494
19.567 snpSurvival-survivalSet2-caseNames (CSV2IDList) . . . 1494
19.568 snpSurvival-survivalSet2-clinicalData (INPUT) . . . 1495
19.569 snpSurvival-survivalSet2-clinicalSelect (SourceCode2Latex) . . . 1495
19.570 snpSurvival-survivalSet2-clinicalTypes (INPUT) . . . 1496
19.571 snpSurvival-survivalSet2-geneRank (QueryReport) . . . 1496
19.572 snpSurvival-survivalSet2-geneRank-eqData (TableQuery) . . . 1496
19.573 snpSurvival-survivalSet2-geneRank-eqTable (CSV2Latex) . . . 1497
19.574 snpSurvival-survivalSet2-geneRankQuery (INPUT) . . . 1497
19.575 snpSurvival-survivalSet2-missingDistC (CSV2Latex) . . . 1498
19.576 snpSurvival-survivalSet2-propertiesDoc (Properties2Latex) . . . 1498
19.577 snpSurvival-survivalSet2-refsGeneRank (INPUT) . . . 1499
19.578 snpSurvival-survivalSet2-refsHits (INPUT) . . . 1499
19.579 snpSurvival-survivalSet2-reportBody (LatexCombiner) . . . 1499
19.580 snpSurvival-survivalSet2-reportTemplate (LatexTemplate) . . . 1500
19.581 snpSurvival-survivalSet2-snpAnnotations (INPUT) . . . 1500
19.582 snpSurvival-survivalSet2-summaryReport (LatexPDF) . . . 1500
19.583 snpSurvival-survivalSet2-survSNPAnnot (AnnotateSNPs) . . . 1501
19.584 snpSurvival-survivalSet2-survSNPAnnot-annotData (TableQuery) . . . 1501
19.585 snpSurvival-survivalSet2-survSNPAnnot-annotTable (CSV2Latex) . . . 1501
19.586 snpSurvival-survivalSet2-survSNPAnnot-genes (KorvasieniAnnotator) . . . 1502
19.587 snpSurvival-survivalSet2-survSNPAnnot-snpLoci (RefSNPAnnotator) . . . 1503
19.588 snpSurvival-survivalSet2-survSNPAnnot-snpRel (ExpandCollapse) . . . 1503
19.589 snpSurvival-survivalSet2-survival (SNPKaplanMeier) . . . 1504
19.590 snpSurvival-survivalSet2-survivalClinical (TableQuery) . . . 1504
19.591 snpSurvival-survivalSet2-survivalParamsQuery (SearchReplace) . . . 1505
19.592 snpSurvival-survivalSet2-survivalParamsTemplate (INPUT) . . . 1505

19.593 snpSurvival-survivalSet2-survivalReport (LatexCombiner) . . . 1505
19.594 snpSurvival-survivalSet3 (SurvivalAnalysis) . . . 1506
19.595 snpSurvival-survivalSet3-annotSQL (INPUT) . . . 1506
19.596 snpSurvival-survivalSet3-caseGenotypes (GetGenotypes) . . . 1506
19.597 snpSurvival-survivalSet3-caseGenotypes-freqCheck (AlleleCounter) . . . 1506
19.598 snpSurvival-survivalSet3-caseGenotypes-ftMarkers (CSV2IDList) . . . 1507
19.599 snpSurvival-survivalSet3-caseGenotypes-genotypes (SNPHelistinReader) . . . 1508
19.600 snpSurvival-survivalSet3-caseGenotypes-gtypes (SNPHelistinReader) . . . 1508
19.601 snpSurvival-survivalSet3-caseNames (CSV2IDList) . . . 1508
19.602 snpSurvival-survivalSet3-clinicalData (INPUT) . . . 1509
19.603 snpSurvival-survivalSet3-clinicalSelect (SourceCode2Latex) . . . 1509
19.604 snpSurvival-survivalSet3-clinicalTypes (INPUT) . . . 1510
19.605 snpSurvival-survivalSet3-geneRank (QueryReport) . . . 1510
19.606 snpSurvival-survivalSet3-geneRank-eqData (TableQuery) . . . 1510
19.607 snpSurvival-survivalSet3-geneRank-eqTable (CSV2Latex) . . . 1511
19.608 snpSurvival-survivalSet3-geneRankQuery (INPUT) . . . 1511
19.609 snpSurvival-survivalSet3-missingDistC (CSV2Latex) . . . 1512
19.610 snpSurvival-survivalSet3-propertiesDoc (Properties2Latex) . . . 1512
19.611 snpSurvival-survivalSet3-refsGeneRank (INPUT) . . . 1513
19.612 snpSurvival-survivalSet3-refsHits (INPUT) . . . 1513
19.613 snpSurvival-survivalSet3-reportBody (LatexCombiner) . . . 1513
19.614 snpSurvival-survivalSet3-reportTemplate (LatexTemplate) . . . 1514
19.615 snpSurvival-survivalSet3-snpAnnotations (INPUT) . . . 1514
19.616 snpSurvival-survivalSet3-summaryReport (LatexPDF) . . . 1514
19.617 snpSurvival-survivalSet3-survSNPAnnot (AnnotateSNPs) . . . 1515
19.618 snpSurvival-survivalSet3-survSNPAnnot-annotData (TableQuery) . . . 1515
19.619 snpSurvival-survivalSet3-survSNPAnnot-annotTable (CSV2Latex) . . . 1515
19.620 snpSurvival-survivalSet3-survSNPAnnot-genes (KorvasieniAnnotator) . . . 1516
19.621 snpSurvival-survivalSet3-survSNPAnnot-snpLoci (RefSNPAnnotator) . . . 1517
19.622 snpSurvival-survivalSet3-survSNPAnnot-snpRel (ExpandCollapse) . . . 1517
19.623 snpSurvival-survivalSet3-survival (SNPKaplanMeier) . . . 1518
19.624 snpSurvival-survivalSet3-survivalClinical (TableQuery) . . . 1518
19.625 snpSurvival-survivalSet3-survivalParamsQuery (SearchReplace) . . . 1519
19.626 snpSurvival-survivalSet3-survivalParamsTemplate (INPUT) . . . 1519
19.627 snpSurvival-survivalSet3-survivalReport (LatexCombiner) . . . 1519
19.628 snpSurvival-survivalSet4 (SurvivalAnalysis) . . . 1520
19.629 snpSurvival-survivalSet4-annotSQL (INPUT) . . . 1520
19.630 snpSurvival-survivalSet4-caseGenotypes (GetGenotypes) . . . 1520
19.631 snpSurvival-survivalSet4-caseGenotypes-freqCheck (AlleleCounter) . . . 1520
19.632 snpSurvival-survivalSet4-caseGenotypes-ftMarkers (CSV2IDList) . . . 1521
19.633 snpSurvival-survivalSet4-caseGenotypes-genotypes (SNPHelistinReader) . . . 1522

19.634 snpSurvival-survivalSet4-caseGenotypes-gtypes (SNPHelistinReader) . . . 1522
19.635 snpSurvival-survivalSet4-caseNames (CSV2IDList) . . . 1522
19.636 snpSurvival-survivalSet4-clinicalData (INPUT) . . . 1523
19.637 snpSurvival-survivalSet4-clinicalSelect (SourceCode2Latex) . . . 1523
19.638 snpSurvival-survivalSet4-clinicalTypes (INPUT) . . . 1524
19.639 snpSurvival-survivalSet4-geneRank (QueryReport) . . . 1524
19.640 snpSurvival-survivalSet4-geneRank-eqData (TableQuery) . . . 1524
19.641 snpSurvival-survivalSet4-geneRank-eqTable (CSV2Latex) . . . 1525
19.642 snpSurvival-survivalSet4-geneRankQuery (INPUT) . . . 1525
19.643 snpSurvival-survivalSet4-missingDistC (CSV2Latex) . . . 1526
19.644 snpSurvival-survivalSet4-propertiesDoc (Properties2Latex) . . . 1526
19.645 snpSurvival-survivalSet4-refsGeneRank (INPUT) . . . 1527
19.646 snpSurvival-survivalSet4-refsHits (INPUT) . . . 1527
19.647 snpSurvival-survivalSet4-reportBody (LatexCombiner) . . . 1527
19.648 snpSurvival-survivalSet4-reportTemplate (LatexTemplate) . . . 1528
19.649 snpSurvival-survivalSet4-snpAnnotations (INPUT) . . . 1528
19.650 snpSurvival-survivalSet4-summaryReport (LatexPDF) . . . 1528
19.651 snpSurvival-survivalSet4-survSNPAnnot (AnnotateSNPs) . . . 1529
19.652 snpSurvival-survivalSet4-survSNPAnnot-annotData (TableQuery) . . . 1529
19.653 snpSurvival-survivalSet4-survSNPAnnot-annotTable (CSV2Latex) . . . 1529
19.654 snpSurvival-survivalSet4-survSNPAnnot-genes (KorvasieniAnnotator) . . . 1530
19.655 snpSurvival-survivalSet4-survSNPAnnot-snpLoci (RefSNPAnnotator) . . . 1531
19.656 snpSurvival-survivalSet4-survSNPAnnot-snpRel (ExpandCollapse) . . . 1531
19.657 snpSurvival-survivalSet4-survival (SNPKaplanMeier) . . . 1532
19.658 snpSurvival-survivalSet4-survivalClinical (TableQuery) . . . 1532
19.659 snpSurvival-survivalSet4-survivalParamsQuery (SearchReplace) . . . 1533
19.660 snpSurvival-survivalSet4-survivalParamsTemplate (INPUT) . . . 1533
19.661 snpSurvival-survivalSet4-survivalReport (LatexCombiner) . . . 1533
19.662 snpSurvival-survivalSet5 (SurvivalAnalysis) . . . 1534
19.663 snpSurvival-survivalSet5-annotSQL (INPUT) . . . 1534
19.664 snpSurvival-survivalSet5-caseGenotypes (GetGenotypes) . . . 1534
19.665 snpSurvival-survivalSet5-caseGenotypes-freqCheck (AlleleCounter) . . . 1534
19.666 snpSurvival-survivalSet5-caseGenotypes-ftMarkers (CSV2IDList) . . . 1535
19.667 snpSurvival-survivalSet5-caseGenotypes-genotypes (SNPHelistinReader) . . . 1536
19.668 snpSurvival-survivalSet5-caseGenotypes-gtypes (SNPHelistinReader) . . . 1536
19.669 snpSurvival-survivalSet5-caseNames (CSV2IDList) . . . 1536
19.670 snpSurvival-survivalSet5-clinicalData (INPUT) . . . 1537
19.671 snpSurvival-survivalSet5-clinicalSelect (SourceCode2Latex) . . . 1537
19.672 snpSurvival-survivalSet5-clinicalTypes (INPUT) . . . 1538
19.673 snpSurvival-survivalSet5-geneRank (QueryReport) . . . 1538
19.674 snpSurvival-survivalSet5-geneRank-eqData (TableQuery) . . . 1538

19.675 snpSurvival-survivalSet5-geneRank-eqTable (CSV2Latex) 1539
19.676 snpSurvival-survivalSet5-geneRankQuery (INPUT) 1539
19.677 snpSurvival-survivalSet5-missingDistC (CSV2Latex) 1540
19.678 snpSurvival-survivalSet5-propertiesDoc (Properties2Latex) 1540
19.679 snpSurvival-survivalSet5-refsGeneRank (INPUT) 1541
19.680 snpSurvival-survivalSet5-refsHits (INPUT) 1541
19.681 snpSurvival-survivalSet5-reportBody (LatexCombiner) 1541
19.682 snpSurvival-survivalSet5-reportTemplate (LatexTemplate) 1542
19.683 snpSurvival-survivalSet5-snpAnnotations (INPUT) 1542
19.684 snpSurvival-survivalSet5-summaryReport (LatexPDF) 1542
19.685 snpSurvival-survivalSet5-survSNPAnnot (AnnotateSNPs) 1543
19.686 snpSurvival-survivalSet5-survSNPAnnot-annotData (TableQuery) 1543
19.687 snpSurvival-survivalSet5-survSNPAnnot-annotTable (CSV2Latex) 1543
19.688 snpSurvival-survivalSet5-survSNPAnnot-genes (KorvasieniAnnotator) 1544
19.689 snpSurvival-survivalSet5-survSNPAnnot-snpLoci (RefSNPAnnotator) 1545
19.690 snpSurvival-survivalSet5-survSNPAnnot-snpRel (ExpandCollapse) 1545
19.691 snpSurvival-survivalSet5-survival (SNPKaplanMeier) 1546
19.692 snpSurvival-survivalSet5-survivalClinical (TableQuery) 1546
19.693 snpSurvival-survivalSet5-survivalParamsQuery (SearchReplace) 1547
19.694 snpSurvival-survivalSet5-survivalParamsTemplate (INPUT) 1547
19.695 snpSurvival-survivalSet5-survivalReport (LatexCombiner) 1547
19.696 snpSurvival-survivalSet6 (SurvivalAnalysis) 1548
19.697 snpSurvival-survivalSet6-annotSQL (INPUT) 1548
19.698 snpSurvival-survivalSet6-caseGenotypes (GetGenotypes) 1548
19.699 snpSurvival-survivalSet6-caseGenotypes-freqCheck (AlleleCounter) 1548
19.700 snpSurvival-survivalSet6-caseGenotypes-ftMarkers (CSV2IDList) 1549
19.701 snpSurvival-survivalSet6-caseGenotypes-genotypes (SNPHelistinReader) 1550
19.702 snpSurvival-survivalSet6-caseGenotypes-gtypes (SNPHelistinReader) 1550
19.703 snpSurvival-survivalSet6-caseNames (CSV2IDList) 1550
19.704 snpSurvival-survivalSet6-clinicalData (INPUT) 1551
19.705 snpSurvival-survivalSet6-clinicalSelect (SourceCode2Latex) 1551
19.706 snpSurvival-survivalSet6-clinicalTypes (INPUT) 1552
19.707 snpSurvival-survivalSet6-geneRank (QueryReport) 1552
19.708 snpSurvival-survivalSet6-geneRank-eqData (TableQuery) 1552
19.709 snpSurvival-survivalSet6-geneRank-eqTable (CSV2Latex) 1553
19.710 snpSurvival-survivalSet6-geneRankQuery (INPUT) 1553
19.711 snpSurvival-survivalSet6-missingDistC (CSV2Latex) 1554
19.712 snpSurvival-survivalSet6-propertiesDoc (Properties2Latex) 1554
19.713 snpSurvival-survivalSet6-refsGeneRank (INPUT) 1555
19.714 snpSurvival-survivalSet6-refsHits (INPUT) 1555
19.715 snpSurvival-survivalSet6-reportBody (LatexCombiner) 1555

19.716 snpSurvival-survivalSet6-reportTemplate (LatexTemplate) . . . 1556
19.717 snpSurvival-survivalSet6-snpAnnotations (INPUT) . . . 1556
19.718 snpSurvival-survivalSet6-summaryReport (LatexPDF) . . . 1556
19.719 snpSurvival-survivalSet6-survSNPAnnot (AnnotateSNPs) . . . 1557
19.720 snpSurvival-survivalSet6-survSNPAnnot-annotData (TableQuery) . . . 1557
19.721 snpSurvival-survivalSet6-survSNPAnnot-annotTable (CSV2Latex) . . . 1557
19.722 snpSurvival-survivalSet6-survSNPAnnot-genes (KorvasieniAnnotator) . . . 1558
19.723 snpSurvival-survivalSet6-survSNPAnnot-snpLoci (RefSNPAnnotator) . . . 1559
19.724 snpSurvival-survivalSet6-survSNPAnnot-snpRel (ExpandCollapse) . . . 1559
19.725 snpSurvival-survivalSet6-survival (SNPKaplanMeier) . . . 1560
19.726 snpSurvival-survivalSet6-survivalClinical (TableQuery) . . . 1560
19.727 snpSurvival-survivalSet6-survivalParamsQuery (SearchReplace) . . . 1561
19.728 snpSurvival-survivalSet6-survivalParamsTemplate (INPUT) . . . 1561
19.729 snpSurvival-survivalSet6-survivalReport (LatexCombiner) . . . 1561
19.730 snpSurvival-survivalSet7 (SurvivalAnalysis) . . . 1562
19.731 snpSurvival-survivalSet7-annotSQL (INPUT) . . . 1562
19.732 snpSurvival-survivalSet7-caseGenotypes (GetGenotypes) . . . 1562
19.733 snpSurvival-survivalSet7-caseGenotypes-freqCheck (AlleleCounter) . . . 1562
19.734 snpSurvival-survivalSet7-caseGenotypes-ftMarkers (CSV2IDList) . . . 1563
19.735 snpSurvival-survivalSet7-caseGenotypes-genotypes (SNPHelistinReader) . . . 1564
19.736 snpSurvival-survivalSet7-caseGenotypes-gtypes (SNPHelistinReader) . . . 1564
19.737 snpSurvival-survivalSet7-caseNames (CSV2IDList) . . . 1564
19.738 snpSurvival-survivalSet7-clinicalData (INPUT) . . . 1565
19.739 snpSurvival-survivalSet7-clinicalSelect (SourceCode2Latex) . . . 1565
19.740 snpSurvival-survivalSet7-clinicalTypes (INPUT) . . . 1566
19.741 snpSurvival-survivalSet7-geneRank (QueryReport) . . . 1566
19.742 snpSurvival-survivalSet7-geneRank-eqData (TableQuery) . . . 1566
19.743 snpSurvival-survivalSet7-geneRank-eqTable (CSV2Latex) . . . 1567
19.744 snpSurvival-survivalSet7-geneRankQuery (INPUT) . . . 1567
19.745 snpSurvival-survivalSet7-missingDistC (CSV2Latex) . . . 1568
19.746 snpSurvival-survivalSet7-propertiesDoc (Properties2Latex) . . . 1568
19.747 snpSurvival-survivalSet7-refsGeneRank (INPUT) . . . 1569
19.748 snpSurvival-survivalSet7-refsHits (INPUT) . . . 1569
19.749 snpSurvival-survivalSet7-reportBody (LatexCombiner) . . . 1569
19.750 snpSurvival-survivalSet7-reportTemplate (LatexTemplate) . . . 1570
19.751 snpSurvival-survivalSet7-snpAnnotations (INPUT) . . . 1570
19.752 snpSurvival-survivalSet7-summaryReport (LatexPDF) . . . 1570
19.753 snpSurvival-survivalSet7-survSNPAnnot (AnnotateSNPs) . . . 1571
19.754 snpSurvival-survivalSet7-survSNPAnnot-annotData (TableQuery) . . . 1571
19.755 snpSurvival-survivalSet7-survSNPAnnot-annotTable (CSV2Latex) . . . 1571
19.756 snpSurvival-survivalSet7-survSNPAnnot-genes (KorvasieniAnnotator) . . . 1572

19.757 snpSurvival-survivalSet7-survSNPAnnot-snpLoci (RefSNPAnnotator) 1573
19.758 snpSurvival-survivalSet7-survSNPAnnot-snpRel (ExpandCollapse) 1573
19.759 snpSurvival-survivalSet7-survival (SNPKaplanMeier) 1574
19.760 snpSurvival-survivalSet7-survivalClinical (TableQuery) 1574
19.761 snpSurvival-survivalSet7-survivalParamsQuery (SearchReplace) 1575
19.762 snpSurvival-survivalSet7-survivalParamsTemplate (INPUT) 1575
19.763 snpSurvival-survivalSet7-survivalReport (LatexCombiner) 1575
19.764 snpSurvival-survivalSet8 (SurvivalAnalysis) 1576
19.765 snpSurvival-survivalSet8-annotSQL (INPUT) 1576
19.766 snpSurvival-survivalSet8-caseGenotypes (GetGenotypes) 1576
19.767 snpSurvival-survivalSet8-caseGenotypes-freqCheck (AlleleCounter) 1576
19.768 snpSurvival-survivalSet8-caseGenotypes-ftMarkers (CSV2IDList) 1577
19.769 snpSurvival-survivalSet8-caseGenotypes-genotypes (SNPHelistinReader) 1578
19.770 snpSurvival-survivalSet8-caseGenotypes-gtypes (SNPHelistinReader) 1578
19.771 snpSurvival-survivalSet8-caseNames (CSV2IDList) 1578
19.772 snpSurvival-survivalSet8-clinicalData (INPUT) 1579
19.773 snpSurvival-survivalSet8-clinicalSelect (SourceCode2Latex) 1579
19.774 snpSurvival-survivalSet8-clinicalTypes (INPUT) 1580
19.775 snpSurvival-survivalSet8-geneRank (QueryReport) 1580
19.776 snpSurvival-survivalSet8-geneRank-eqData (TableQuery) 1580
19.777 snpSurvival-survivalSet8-geneRank-eqTable (CSV2Latex) 1581
19.778 snpSurvival-survivalSet8-geneRankQuery (INPUT) 1581
19.779 snpSurvival-survivalSet8-missingDistC (CSV2Latex) 1582
19.780 snpSurvival-survivalSet8-propertiesDoc (Properties2Latex) 1582
19.781 snpSurvival-survivalSet8-refsGeneRank (INPUT) 1583
19.782 snpSurvival-survivalSet8-refsHits (INPUT) 1583
19.783 snpSurvival-survivalSet8-reportBody (LatexCombiner) 1583
19.784 snpSurvival-survivalSet8-reportTemplate (LatexTemplate) 1584
19.785 snpSurvival-survivalSet8-snpAnnotations (INPUT) 1584
19.786 snpSurvival-survivalSet8-summaryReport (LatexPDF) 1584
19.787 snpSurvival-survivalSet8-survSNPAnnot (AnnotateSNPs) 1585
19.788 snpSurvival-survivalSet8-survSNPAnnot-annotData (TableQuery) 1585
19.789 snpSurvival-survivalSet8-survSNPAnnot-annotTable (CSV2Latex) 1585
19.790 snpSurvival-survivalSet8-survSNPAnnot-genes (KorvasieniAnnotator) 1586
19.791 snpSurvival-survivalSet8-survSNPAnnot-snpLoci (RefSNPAnnotator) 1587
19.792 snpSurvival-survivalSet8-survSNPAnnot-snpRel (ExpandCollapse) 1587
19.793 snpSurvival-survivalSet8-survival (SNPKaplanMeier) 1588
19.794 snpSurvival-survivalSet8-survivalClinical (TableQuery) 1588
19.795 snpSurvival-survivalSet8-survivalParamsQuery (SearchReplace) 1589
19.796 snpSurvival-survivalSet8-survivalParamsTemplate (INPUT) 1589
19.797 snpSurvival-survivalSet8-survivalReport (LatexCombiner) 1589

19.798 snpSurvival-survivalSet9 (SurvivalAnalysis) . . . 1590
19.799 snpSurvival-survivalSet9-annotSQL (INPUT) . . . 1590
19.800 snpSurvival-survivalSet9-caseGenotypes (GetGenotypes) . . . 1590
19.801 snpSurvival-survivalSet9-caseGenotypes-freqCheck (AlleleCounter) . . . 1590
19.802 snpSurvival-survivalSet9-caseGenotypes-ftMarkers (CSV2IDList) . . . 1591
19.803 snpSurvival-survivalSet9-caseGenotypes-genotypes (SNPHelistinReader) . . . 1592
19.804 snpSurvival-survivalSet9-caseGenotypes-gtypes (SNPHelistinReader) . . . 1592
19.805 snpSurvival-survivalSet9-caseNames (CSV2IDList) . . . 1592
19.806 snpSurvival-survivalSet9-clinicalData (INPUT) . . . 1593
19.807 snpSurvival-survivalSet9-clinicalSelect (SourceCode2Latex) . . . 1593
19.808 snpSurvival-survivalSet9-clinicalTypes (INPUT) . . . 1594
19.809 snpSurvival-survivalSet9-geneRank (QueryReport) . . . 1594
19.810 snpSurvival-survivalSet9-geneRank-eqData (TableQuery) . . . 1594
19.811 snpSurvival-survivalSet9-geneRank-eqTable (CSV2Latex) . . . 1595
19.812 snpSurvival-survivalSet9-geneRankQuery (INPUT) . . . 1595
19.813 snpSurvival-survivalSet9-missingDistC (CSV2Latex) . . . 1596
19.814 snpSurvival-survivalSet9-propertiesDoc (Properties2Latex) . . . 1596
19.815 snpSurvival-survivalSet9-refsGeneRank (INPUT) . . . 1597
19.816 snpSurvival-survivalSet9-refsHits (INPUT) . . . 1597
19.817 snpSurvival-survivalSet9-reportBody (LatexCombiner) . . . 1597
19.818 snpSurvival-survivalSet9-reportTemplate (LatexTemplate) . . . 1598
19.819 snpSurvival-survivalSet9-snpAnnotations (INPUT) . . . 1598
19.820 snpSurvival-survivalSet9-summaryReport (LatexPDF) . . . 1598
19.821 snpSurvival-survivalSet9-survSNPAnnot (AnnotateSNPs) . . . 1599
19.822 snpSurvival-survivalSet9-survSNPAnnot-annotData (TableQuery) . . . 1599
19.823 snpSurvival-survivalSet9-survSNPAnnot-annotTable (CSV2Latex) . . . 1599
19.824 snpSurvival-survivalSet9-survSNPAnnot-genes (KorvasieniAnnotator) . . . 1600
19.825 snpSurvival-survivalSet9-survSNPAnnot-snpLoci (RefSNPAnnotator) . . . 1601
19.826 snpSurvival-survivalSet9-survSNPAnnot-snpRel (ExpandCollapse) . . . 1601
19.827 snpSurvival-survivalSet9-survival (SNPKaplanMeier) . . . 1602
19.828 snpSurvival-survivalSet9-survivalClinical (TableQuery) . . . 1602
19.829 snpSurvival-survivalSet9-survivalParamsQuery (SearchReplace) . . . 1603
19.830 snpSurvival-survivalSet9-survivalParamsTemplate (INPUT) . . . 1603
19.831 snpSurvival-survivalSet9-survivalReport (LatexCombiner) . . . 1603
19.832 snpdb (INPUT) . . . 1604
19.833 summaryDoc (LatexCombiner) . . . 1604
19.834 summaryReport (LatexPDF) . . . 1605
19.835 summaryTable (SummaryTable) . . . 1605
19.836 summaryTable-acghExonCorrGainConv (CSVFilter) . . . 1606
19.837 summaryTable-acghExonCorrLossConv (CSVFilter) . . . 1606
19.838 summaryTable-acghExonCorrSummary (CSVJoin) . . . 1607

19.839 summaryTable-acghGainConv (CSVFilter) 1608
19.840 summaryTable-acghLossConv (CSVFilter) 1609
19.841 summaryTable-acghSummary (CSVJoin) 1610
19.842 summaryTable-annotation (KorvasieniAnnotator) 1610
19.843 summaryTable-correlation (INPUT) 1611
19.844 summaryTable-excel (CSV2Excel) 1611
19.845 summaryTable-exonAnnotation (CSVJoin) 1612
19.846 summaryTable-exonAnnotationFilt (CSVFilter) 1612
19.847 summaryTable-geneAlias (INPUT) 1613
19.848 summaryTable-geneInfo (GeneSetInfo) 1613
19.849 summaryTable-geneInfo-annotation (KorvasieniAnnotator) 1613
19.850 summaryTable-geneInfo-goEnrichment (GOEnrichment) 1614
19.851 summaryTable-geneInfo-goHeatmap (GOClustering) 1615
19.852 summaryTable-geneInfo-graphBP (GraphVisualizer) 1616
19.853 summaryTable-geneInfo-graphCC (GraphVisualizer) 1617
19.854 summaryTable-geneInfo-graphMF (GraphVisualizer) 1619
19.855 summaryTable-geneInfo-kegg (KEGGPathway) 1620
19.856 summaryTable-geneInfo-keggReport (CSV2Latex) 1621
19.857 summaryTable-geneInfo-report (LatexCombiner) 1621
19.858 summaryTable-geneInfo-uniprotExpanded (ExpandCollapse) 1622
19.859 summaryTable-geneSet (CSVTransformer) 1622
19.860 summaryTable-genes (CSV2IDList) 1623
19.861 summaryTable-imageMapping (TableQuery) 1624
19.862 summaryTable-imageMappingQuery (INPUT) 1624
19.863 summaryTable-images (LatexCombiner) 1624
19.864 summaryTable-miRNAstat (SearchReplace) 1625
19.865 summaryTable-miRNAtable (TableQuery) 1625
19.866 summaryTable-pathways (KEGGPathway) 1626
19.867 summaryTable-pathwaysEnsembl (CSVFilter) 1627
19.868 summaryTable-pathwaysEnsemblPre (IDConvert) 1627
19.869 summaryTable-refs (INPUT) 1628
19.870 summaryTable-sequenced (INPUT) 1629
19.871 summaryTable-site (HTMLReport) 1629
19.872 summaryTable-siteLabels (INPUT) 1631
19.873 summaryTable-siteMapping (INPUT) 1631
19.874 summaryTable-siteRefs (INPUT) 1631
19.875 summaryTable-style (INPUT) 1631
19.876 summaryTable-summaryQuery (INPUT) 1631
19.877 summaryTable-summaryTable (IDConvert) 1632
19.878 summaryTable-summaryTablePre (TableQuery) 1633
19.879 summaryTable-summaryTablePre2 (TableQuery) 1633

19.880 summaryTable-uniprot (CSVFilter) 1634
19.881 summaryTable-uniprotExp (ExpandCollapse) 1635
19.882 summaryTable-vttHits (INPUT) 1635
19.883 summaryTable-vttHits2 (INPUT) 1635
19.884 summaryTable-vttScreen (INPUT) 1636
19.885 transcriptAnnotation (INPUT) 1636
19.886 transcriptExpression (INPUT) 1636
19.887 transcriptExpressionResults (TranscriptExpressionAnalysis) 1636
19.888 transcriptExpressionResults-allEntrez (KorvasieniAnnotator) 1637
19.889 transcriptExpressionResults-allProbes (TableQuery) 1637
19.890 transcriptExpressionResults-cumDistReport (Plot2D) 1638
19.891 transcriptExpressionResults-det (SetTransformer) 1639
19.892 transcriptExpressionResults-detAnnot (KorvasieniAnnotator) 1639
19.893 transcriptExpressionResults-detFoldChange (FoldChange) 1640
19.894 transcriptExpressionResults-detIDList (CSV2IDList) 1641
19.895 transcriptExpressionResults-detReport (DEGReport) 1641
19.896 transcriptExpressionResults-detTTest (StatisticalTest) 1642
19.897 transcriptExpressionResults-detTable (TableQuery) 1643
19.898 transcriptExpressionResults-detTableQuery (INPUT) 1643
19.899 transcriptExpressionResults-detVennDiagram (VennDiagram) 1644
19.900 transcriptExpressionResults-exonSPIArefs (INPUT) 1644
19.901 transcriptExpressionResults-filterSamplesRScript (INPUT) 1644
19.902 transcriptExpressionResults-geneMapping (CSVFilter) 1644
19.903 transcriptExpressionResults-goClusters (GOClustering) 1645
19.904 transcriptExpressionResults-groupMeans (SampleCombiner) 1646
19.905 transcriptExpressionResults-intersectionTransformation (INPUT) 1647
19.906 transcriptExpressionResults-km (TranscriptSurvival) 1647
19.907 transcriptExpressionResults-km-km (KaplanMeier) 1647
19.908 transcriptExpressionResults-km-kmReport (LatexCombiner) 1648
19.909 transcriptExpressionResults-km-sampleExpression (SampleExpression) 1648
19.910 transcriptExpressionResults-km-sampleExpressionFilt (CSVFilter) 1649
19.911 transcriptExpressionResults-km-sampleExpressionTrans (MatrixTranspose) 1650
19.912 transcriptExpressionResults-km-sampleNames (ExpandCollapse) 1650
19.913 transcriptExpressionResults-km-survival (TableQuery) 1651
19.914 transcriptExpressionResults-km-survivalQuery (INPUT) 1651
19.915 transcriptExpressionResults-km-survivalTable (CSVJoin) 1651
19.916 transcriptExpressionResults-km-survivalTableConv (IDConvert) 1652
19.917 transcriptExpressionResults-logratio (SampleCombiner) 1653
19.918 transcriptExpressionResults-mdsReport (MDSPlot) 1653
19.919 transcriptExpressionResults-metadata (INPUT) 1654
19.920 transcriptExpressionResults-qcReport (LatexCombiner) 1654

19.921 transcriptExpressionResults-reference (CSV2IDList) . . . 1655
19.922 transcriptExpressionResults-report (LatexCombiner) . . . 1655
19.923 transcriptExpressionResults-sampleGroups (SampleGroupCreator) . . . 1656
19.924 transcriptExpressionResults-samplesWithoutClinicalAnnotation (INPUT) . . . 1657
19.925 transcriptExpressionResults-spia (SPIA) . . . 1657
19.926 transcriptExpressionResults-spiaReport (CSV2Latex) . . . 1657
19.927 transcriptExpressionResults-survGenes (CSVTransformer) . . . 1658
19.928 transcriptExpressionResults-survGenesEntrez (KorvasieniAnnotator) . . . 1659
19.929 transcriptExpressionResults-survLogratio (CSVFilter) . . . 1660
19.930 transcriptExpressionResults-survLogratioEntrez (IDConvert) . . . 1660
19.931 transcriptExpressionResults-survLogratioEntrezFilt (CSVFilter) . . . 1661
19.932 transcriptExpressionResults-tExpr (REvaluate) . . . 1662
19.933 transcriptExpressionResults-tExprTemp (IDConvert) . . . 1663
19.934 transcriptExpressionResults-transcriptAnnotation (INPUT) . . . 1664
19.935 transcriptExpressionResults-transcriptStatisticsQuery (INPUT) . . . 1664
19.936 R configuration . . . 1664
19.937 System configurations . . . 1665

1 Gene expression analysis

1.1 Summary of differentially expressed genes

Gene set	Size
over	668
under	740

2 List of differentially expressed genes

Overexpressed genes are sorted with the most overexpressed first and underexpressed genes with the most underexpressed first. Genes go by the column first and then by row.

2.1 Gene set: over

Number of genes: 668

RRM2	JAG1	PLAT	NUP107	PARP12	HIP1	MNDA	GALR1
FCGBP	IL10RB	CXCL10	SERPING1	CCDC102B	ETV1	DDR2	EFS
SERPINA3	STON1	FBXO5	SHC1	TMED2	LYN	ISG20	C13orf18
TOP2A	ASPM	NID1	RARRES3	EVI2B	KCNE4	CLEC7A	CD97
IGFBP2	ANGPT2	TRIP6	AP000654.1	PYCARD	CHRNA9	SCUBE2	SLC25A15
MLF1IP	HMOX1	NT5DC2	CD58	EFHC1	WHSC1	SLC39A8	IGSF6
COL4A1	FAM70A	STAB1	DLEU1	CASP4	ASPN	TRAF4	COL18A1
PTX3	LOX	TSPAN12	ITGB2	CCL2	RBM47	GINS3	ANXA4
PBK	CCNB1	AEBP1	IGFBP4	ITGA5	BCAT1	TGFB2	STIL
F2R	SOAT1	SOX4	ADORA3	NCAPG2	COPZ2	PDLIM3	METTL1
NUSAP1	ANGPTL2	C1S	LYPD1	ANKRD57	ARSJ	CEP55	SLC27A3
LTF	TUBB6	SERPINE1	ZYX	VAMP5	DDIT4	GMFG	CD37
AQP1	S100A11	NPL	MXRA5	IL1RAP	DONSON	FZD6	MSTN
TGFBI	VEGFA	CXCR7	VAMP8	IRF1	ATAD2	HELLS	KCNMB1
CHI3L2	KIF4A	PRC1	TYROBP	HOXC4	PLAC8	ZBTB10	C1QL1
TNC	IFI16	RNASE2	KIF11	PROM1	YAP1	HOXB7	MAT2A
NES	MDK	MGP	ANKRD10	HJURP	PMP2	SMC2	WIPI1
CD44	TTK	HK2	OIP5	KANK2	MYBL1	ANGPTL4	MGST2
CHI3L1	VCAM1	KIF20A	POLE2	TP53	RAD54B	CD48	TMEM185B
TMEM45A	IFI35	SAMSN1	CA3	CAV2	MEX3D	CTBS	CD300A
ANXA1	CD14	S100A8	IGFBP5	PCOLCE2	APOBEC3B	MALT1	LCP2
SRPX	PLOD2	ZFP36L2	SQRDL	TWSG1	PIPOX	BST2	OAS1
ID3	CD93	WNT5A	HAMP	IGF2BP2	EN1	STAC	NCKAP1L
CD163	COL1A2	DBF4	BLM	DAB2	ACTA2	CCNE2	LEFTY2
MS4A4A	MMP2	GEM	KLHL7	CARHSP1	TLR1	LY75	TFEC
LIMA1	WEE1	FAM60A	SLC26A2	PAXIP1	NAT1	GAS1	RND2
PDPN	SRPX2	PDIA5	IFI44	CD151	FOS	SLC25A24	ANGPT1
TIMP4	CDKN2C	EDNRA	IL13RA1	PLA2G4A	NRP1	MREG	NEDD4
DPYD	ADM	DTYMK	DIRAS3	BGN	SPRY4	DNALI1	DNMBP
NNMT	PLA2G2A	NID2	E2F5	LGALS3	TMSB15B	RFC5	LEPRE1
COL3A1	LY96	KIF15	SALL1	ANG	TMEM100	FAS	PI3
TGFB1I1	C1QA	PLOD1	LMNB1	CALCRL	ZNF83	HOXB2	C9orf82
MS4A6A	CTSC	BIRC5	UPP1	HNMT	CYBB	BBOX1	RASL12
SERPINH1	TAGLN2	RNASE6	MYC	RAI14	RTP4	C21orf45	CCNL2
GBP1	GBP2	HOXC6	RNASEH2A	FLNA	IL33	SELL	PION
CSRP2	BCHE	FNDC3B	SLC38A6	LOXL1	EHD2	BMP2	HOXC10
TYMS	FZD7	MAD2L1	MCM5	SNCAIP	AASS	GYPC	ZWILCH
FAM129A	SLC2A10	STK17A	HLA-DQB1	ADAM12	TMEM149	KIF14	SEMA5A
ACTL6A	DLGAP5	MCM2	GLIPR1	NCAPD2	PRIM1	FERMT1	CTSK
MELK	C1QB	TNFRSF12A	ABCC3	ALOX5AP	NMD3	TRAM2	SYNC
CFI	CA12	ALG6	KIF2C	ZFP36L1	LAMA2	MNS1	SLC25A20
C21orf62	FAM82B	PPIC	CHIC2	HCLS1	RHBDF1	FLNC	TSPAN31
EZH2	GPX7	DSE	SEC61G	IL10RA	TNFAIP8	ITGA7	TLR3
TMSB15A	CALD1	ELTD1	PLAU	CHEK1	FKBP10	LCAT	HOXA1
CPVL	APOC1	TLR2	KDELC1	ZBTB20	C21orf7	TCIRG1	NKX2-2
IGF2BP3	MATN2	CCDC109B	GPNMB	SFRP4	GPX3	CD53	GOLT1B
DTL	C7orf68	AURKA	ECT2	CLEC2B	SPON2	WDR5B	CENPF
IGFBP3	RAD51AP1	TMEM176B	ACP6	OAS3	OXTR	RP4-564F22.2	TIMP3
NMB	SERPINA1	BTN3A2	COL6A3	TM9SF1	KIF23	GYS1	KIAA0040
KIAA0101	PXDN	PLSCR1	GTSE1	MYCBP	S100A9	ELOVL2	VAMP4
TGIF1	FAM64A	TACC3	FAM114A1	ID4	HLA-DRB6	DCHS1	AC006276.1
CAV1	CASP1	PLIN2	C5AR1	MARCH3	COL6A2	OLFML2B	SYNPO
ABCA1	BUB1B	ZNF217	CLEC5A	TMEM140	APOC2	AHR	CDC45L
SMC4	FOXD1	SP100	CPM	ASCL1	ADAMDEC1	SLAMF8	CCL20
SOX11	IQGAP2	PRRX1	FPR1	AIM1	EFEMP2	CCR1	CSPG4
EMP3	SOD2	PLTP	PDLIM1	FABP7	SLC35F2	UCP2	RND3

UBE2C	TNFAIP6	HOXA5	DRAM1	BTN3A3	ID1	CASP7	C1orf38
COL5A2	S100A4	CP	ARAP3	SERPINB1	PTRF	ACSS3	MXRA8
LPL	CAPG	IFI44L	PSPH	MAFB	CARD8	ZNF227	CD86
CCNB2	TREM2	S100A11P1	PTPRC	FCGR2B	HAS2	LTBP1	UST
MEOX2	WWTR1	NCAPG	BCL2A1	POU3F2	RAB27A	GATM	TDO2
RGS1	TP53I3	EGFR	CENPA	HCK	KNTC1	RGS16	EDNRB
COL4A2	HERC5	CALU	C1RL	TRIB2	NOX4	SHMT2	CXCL9
EMP1	CXCR4	HMMR	RNASE4	IQCG	ERAP2	HP	AURKB
VCAN	POSTN	NUPR1	TFPI	PLVAP	NCF2	ASF1B	CDCP1
CKS2	LAMB1	ZNF107	SYNE2	MYOF	MVP	FMOD	C3AR1
MTHFD2	KLHDC8A	GINS1	FXYD5	FZD2	PDLIM4	RPE65	LBH
EFEMP1	RCAN1	TREM1	RAB20	DENND2A	IL8	PDGFD	SPC25
PYGL	TRIM22	GPR65	TRIM5	IL13RA2	TP53TG1	ATM	SMAGP
FAM46A	RIT1	PHLDA1	PLAUR	GRB10	CD302	C10orf10	MDFIC
CDC20	CYBRD1	NRAS	PNP	ZNF167	AZGP1	PLK1	PAN2
IFI30	FCER1G	F13A1	ENTPD1	SLC43A3	MGAT4A	CCDC99	COL15A1
CDK1	GINS2	GDF15	INSM1	HPSE	BAX	HS3ST3A1	TMEM39A
LYZ	HIST1H1C	CCNA2	PTGS1	TCF7L1	ACOX2	CDC7	PRR5L
VSIG4	FANCI	ECM2	RAB32	TRIB3	CYTIP	CREB5	AC023105.1
PLP2	ZWINT	FOXM1	BACE2	TMEM176A	CENPE	NOTCH1	TNFRSF1B
TIMP1	NDC80	ATF3	GALNT10	SOCS2	CDCA3	CENPN	TAPBPL
TPX2	CNGA3	CYTL1	HLA-DQA1	SP140L	SHOX2	LMNB2	FAM176B
ANXA2P2	PBEF1	TMEM38B	CYR61	TRIM21	DKK1	DDX60	THBS1
C8orf4	SLC16A4	SPRY1	GNS	COL5A1	AMMECR1	PCDHGC3	GTF2H2C
LUM	SLC7A7	CTSS	CDKN3	SPATA6	HPGDS	CCND1	
C1R	STEAP3	ZIC1	SNAI2	CDKN1A	AE000661.10	ADAMTS5	
LRRC17	CSTA	DHFR	PCOLCE	CLN5	HIST1H2AC	FNBP1L	
CDK4	TAGLN	FAM119B	SHCBP1	LEPREL1	DDB2	SLC2A5	

2.2 Gene set: under

Number of genes: 740

SST	PDE2A	PAK6	CCNA1	KIAA0284	OPCML	ABLIM3	CXCL14
VSNL1	DLG2	NPTX2	KIF3C	NOL4	CDH12	SEMA4D	FOSB
SLC17A7	PAK3	FEZF2	DUSP26	C10orf116	MYO16	RHOBTB2	RP11-404K5.1
SV2B	CA11	ITPKA	SYT13	RAPGEFL1	PAK1	RAI2	B4GALT6
CACNG3	ST6GALNAC5	ANK3	RGS20	RAB33A	KCNAB1	SH3BP5	AHNAK2
CCK	CAMKV	RTN1	SCAMP5	TLN2	NDRG2	NRN1	SOX10
GABRA1	GNAO1	DNM3	SLC25A22	ARHGAP32	NBL1	SLCO1C1	FSD1
NEFL	MAST3	RASL10A	WNT10B	RASGRP1	SIDT1	CTNNA2	SEMA3G
SYT1	PPP1R16B	CLSTN2	CACNB3	LDOC1	B3GAT1	SLC2A6	DZIP3
AC104308.1	GABBR2	GFOD1	NRXN3	FGF14	OPTN	RAB3B	ANKRD46
NEFM	RAB3A	VIPR1	DCLK1	KLK6	ACSL6	SOSTDC1	MAP6D1
SYN1	GPR22	MEG3	SSX2IP	ACTR3B	PPEF1	IL1RAPL1	PRKAG2
MYT1L	CAMK2B	TRHDE	FAM155A	STYK1	EPCAM	FLRT3	SORCS3
SLC12A5	TAC3	KCNJ6	PPP3R1	PK4P	JPH3	TRIM36	DPF1
SNAP91	GABRB1	AC011479.3	RAB6B	RUNX1T1	EFHD1	NUDT11	SIGIRR
SCN3B	PDYN	NRXN1	SV2C	NDN	NR3C2	WASF3	NME5
CRYM	CHGB	MYRIP	DUSP8	BCL11A	EGR4	ULK2	FGFR3
AK5	MAL	CHST1	ELOVL4	BAI3	AC079061.1	ZNF248	TF
NRGN	EPB49	CPNE6	COX7A1	PRMT8	LRRC8B	AC087284.2	PTH2R
GNG3	STXBP1	C1orf95	BSCL2	NPY1R	LRRTM2	ERBB4	ASPHD1
SYNGR3	CACNA2D3	CBX7	TUBA4A	CBFA2T3	C14orf132	TRIM23	ADRA2A
SNAP25	ACTL6B	GOT1	RAB15	GRIA2	ARHGEF9	PRKCQ	NLK
RIMBP2	RYR2	CDK5R2	SPOCK1	ZMAT4	CAMSAP1	NEDD4L	PIP4K2C
PCLO	CUX2	DDX25	SH3GLB2	MTUS2	TYRP1	DAAM2	CRLF1
INA	CHRM3	BASP1	GRM5	PLK2	CHRDL1	BAIAP3	CELSR3
SH3GL2	MICAL2	ITPR1	PALM	DHCR24	VAMP1	SCGB2A1	ETS2
HTR2A	FXYD7	GPC5	REEP1	GABRB3	ADCY1	KRT17	PDZRN4
ANO3	NPTXR	PGBD5	ATP2B2	PAIP2B	AC010336.3	AJAP1	SLC1A4
SH3GL3	CALB2	PCP4	P2RX5	BAI2	GALNT14	MFSD6	LRP8
WIF1	CNTNAP2	GRIN2A	MEF2C	RIMS3	SCG3	C3orf14	CDH7
SYN2	PTPRT	CABP1	ACOT7	NSG1	REPS2	ACSBG1	CXorf1
SNCB	AL691479.2	MCTP1	RUNDC3B	KIAA1467	COBL	PLP1	RNFT2
RGS4	EEF1A2	SYP	DLGAP2	FABP3	LIN7B	BIN1	BDH1
AC005774.2	ATRNL1	KCNV1	RAB26	S100A1	CACNA2D2	ZFPM2	LRRN2
STMN2	CALB1	NUAK1	CYFIP2	MAP7	FAM5B	SRGAP3	FHL2
PRKCB	PCSK2	EPB41L1	GABRA4	PRKAR2B	UROS	SPOCK3	PLEKHB1
KCNK1	CDH18	FGF9	EMX2	RAPGEF2	PCSK1	NRXN2	WWC1
FGF13	AC005277.1	PTPRN	CYP26B1	PTPRD	LDB3	SYT17	OPN3
DDN	NEFH	LDB2	FLRT2	SHC3	RASL11B	APBA2	PLA2G4C
CALY	SLC17A6	WFDC1	TMEM35	CDH10	YPEL1	SEPT4	PLCB4
DNM1	RCAN2	CHD5	KCNJ4	ME1	NOV	ODZ3	CRABP1
C1orf115	NAV3	NBEA	ATP8A1	SCG5	IQSEC2	ALDH5A1	RPP25
NELL1	KIF5A	ACCN1	KCNK3	HPRT1	CMAS	FAM184A	CKB
PHYHIP	DOCK3	ZNF204P	WFDC2	CDKN2D	ASS1	FAM169A	NR1D2

GABRG2	ZNF365	PTPRR	KBTBD11	POPDC3	ELAVL2	GRIA1	APOD
NPTX1	LHX6	FBXW7	CAMK2G	KIAA1462	TSPAN7	RPS6KA5	PKNOX2
AGXT2L1	KIAA1045	BEGAIN	CRHBP	AVPI1	NR4A2	C9orf126	TACC2
HS3ST2	RAP1GAP2	GNAL	IQSEC3	FAM171A1	AKAP6	DPP6	PID1
SERPINI1	DIRAS2	FRMPD4	ICAM5	GPR88	PPFIA4	FAM131B	PDE8B
LY6H	ENTPD3	MBP	ROGDI	CADPS2	KLHL26	ADAM23	HMGCS1
NPY	LGI1	TSPYL2	OGDHL	DUSP2	ATP1A2	SLC4A3	CACNA1A
AMPH	RAPGEF4	GLS2	MAPRE3	FA2H	EPHA4	ASTN1	CAMK1
GABRA2	SLC6A15	SHANK2	CSRNP3	C17orf108	EPHA5	SLC1A1	PTPRO
KIAA0513	HPCAL4	FAIM2	OMG	KLC2	RIMS1	BCR	GPHN
NMNAT2	NTSR2	FXYD1	DNAJC12	B3GALT2	DSP	KCNK10	TANC2
HSPA12A	PHACTR1	PTGDS	STXBP5L	SLIT2	THY1	CACNG2	THRA
SNCA	PPP1R1A	NDRG4	KCNK12	ABCC8	FHOD3	GRIA3	QPCT
CDC42	C20orf103	CAMK1G	KCNB1	FAM149A	MAP2	ADARB2	SCN1A
OLFM1	NELL2	EGR3	GLS	HTR1E	PCSK1N	BCL11B	SLITRK3
HPCA	SLITRK5	PAK7	CDS1	GABRA3	FLRT1	RP11-35N6.1	TMEM183A
CYP46A1	CAMK2A	STMN4	PRKAR1B	MAG	EDIL3	NCAM1	COCH
IPCEF1	PNOC	FBXO41	CAMK1D	ALDOC	LHX2	SYNJ2	GDAP1L1
RBP4	KIF1A	RAB40B	ZNF536	TNNT1	PPP1R12B	STXBP6	PKIA
KIAA1107	MOBP	KLHL3	PPL	HOMER1	HSPB3	RPRM	ITIH2
RUNDC3A	ENC1	PDP1	C1orf21	NTRK3	SELENBP1	RPS6KA2	SLC23A2
CHGA	RAP1GAP	FBXO2	CRH	RIT2	GNAI1	RALGPS1	TRPM3
DYNC1I1	ADCY2	DGKB	ELMO1	NCALD	PAQR6	CSGALNACT1	DTX4
CNKSR2	ATP1A3	KIF5C	TUSC3	SUSD4	SLC22A17	MBNL2	MLLT3
SCN2A	SULT4A1	FAM189A1	WASF1	UNC13A	CADM3	SLC38A1	ENPP4
RGS7	PPFIA2	CACNB4	KCNAB2	ARHGDIG	PCDH9	LHPP	RNF128
BSN	GPRASP1	KALRN	FABP6	CNTN1	FGF12	PYGM	PPP1R3C
ERC2	SEC14L5	NNAT	KLK7	PLLP	ASPA	FAM65B	SNX10
TAC1	LMO3	DOC2A	MAPK10	APLP1	ANXA3	EFNB3	INPP4B
CCKBR	AP3B2	ZIM2	CPEB1	LARGE	ARHGEF4	LY6E	MPHOSPH8
GRM3	SEMA4A	PEX5L	CA4	GNAZ	SLIT3	LRP1B	SMARCA2
RALYL	INPP5F	PPM1H	C2CD2L	BEX1	PPP1R9A	SLIT1	PPARGC1A
KIAA1644	PDE1A	CA10	SEZ6L2	SEC61A2	TESC	KCNMB4	TPBG
STX1A	AP003115.1	RP4-788L13.1	TBC1D30	SSBP3	AGAP1	OVGP1	CORO1A
DBC1	ELAVL4	NEUROD6	PIP5K1B	CNTNAP1	DHRS9	USP2	AGAP2
SLC8A2	KIAA0319	CD200	ENPP2	CDK5R1	MCF2L	GPR6	GPR17
NRIP3	CACNB2	PARM1	EPB41L3	PENK	NDST3	STARD13	LSAMP
ATP8A2	XK	SORBS2	TPD52L1	DIO2	ARHGEF12	FAM50B	SERPINF1
C9orf125	RPH3A	KCNMA1	KIF21B	SATB1	ZNF804A	PRODH	ALDH1A1
PRKCZ	CAMTA1	NAP1L3	LPCAT4	GLRB	KIAA1797	DGKI	RERGL
HLF	PRSS3	RAPGEF5	DGCR5	RND1	C11orf41	HSPA2	TUFT1
TAGLN3	SLC1A2	VIP	CIT	KCNS1	NT5DC3	SLC13A4	PDZD2
NAP1L2	BZRAP1	KCNC1	KIAA1598	CEND1	HSD17B6	UGT8	ZFYVE21
SLC30A3	BDNF	CAP2	PIGZ	KIF3A	KIT	BCAS1	CRMP1
RFPL1S	RASAL1	GREM2	PVALB	SYN3	TMEM90B	WFS1	ST18
AC093310.1	GUCY1B3	TCEAL2	RP5-1187M17.10	L1CAM	FAM49A	NINJ2	
EPHB6	EHD3	CLDN10	SMPX	TPPP	SLC3A1	CNTN2	
TUBB4	DNAJC6	FBXL2	GSTM5	GUCY1A3	PCDH8	FAM107A	
NECAB2	RELN	STAT4	CDH22	SLC6A1	HRK	PLEKHA5	

2.3 GO clustering

Gene products are clustered (grouped) based on their Gene Ontology (GO) annotations. Genes that have similar annotations belong to the same cluster. The distance between gene products is defined using information-theoretical semantic similarity measures.

In the table below, the most informative (most specific) common GO terms of each cluster are displayed. All gene products in the cluster are annotated with the GO term mentioned. A GO term is more informative if it occurs rarely in the GO annotations of the whole genome. The priori column contains the a priori probability p that a random gene product is annotated with the given GO term. Information is defined as $-\log_2 p$.

Generally, clusters with (1) a large number of gene products and (2) common GO terms with high information content are the most interesting.

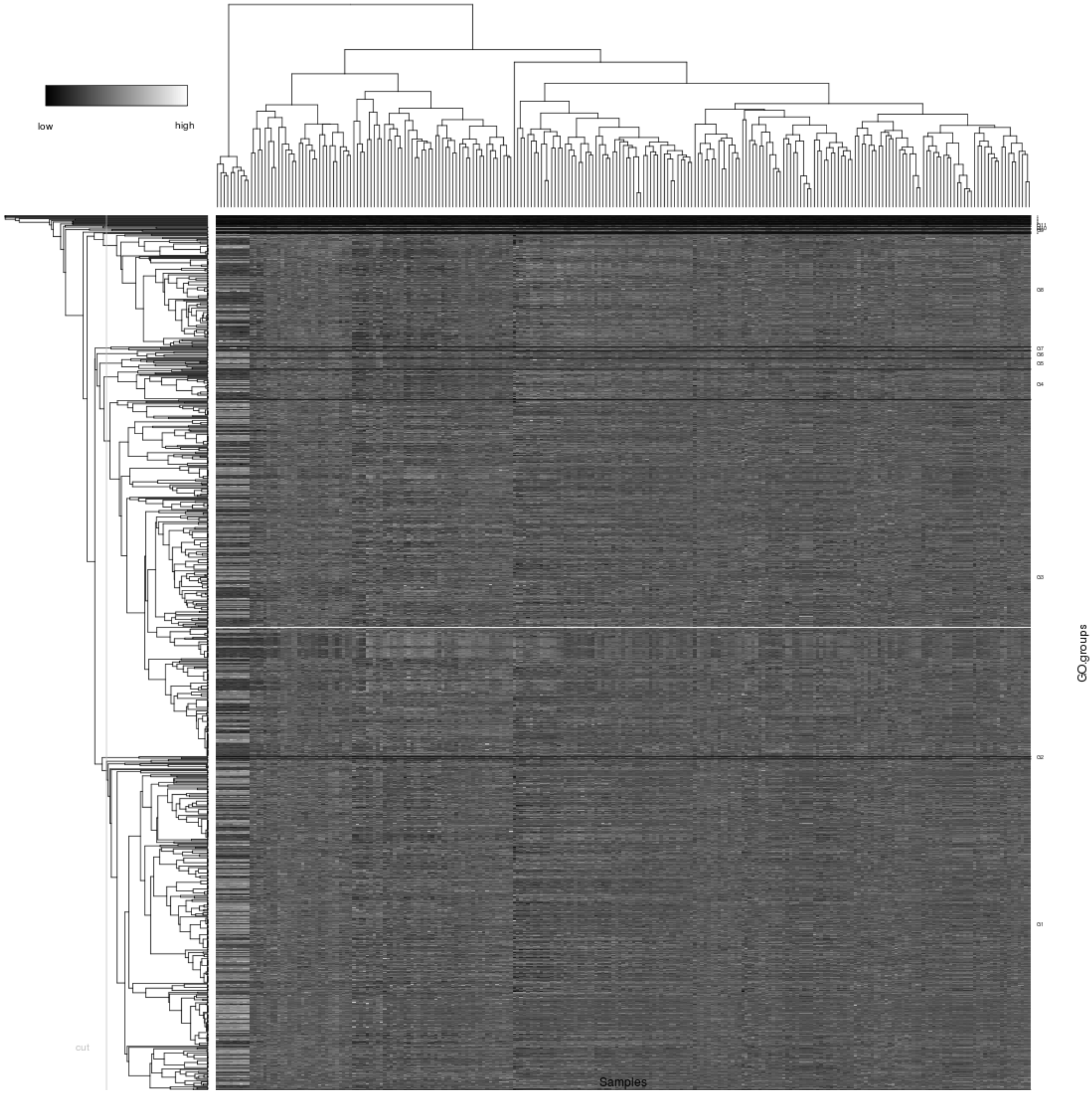

Figure 1: GO clustering dendrogram and expression heatmap. The analysis is based on 1363 gene products. The median number of GO annotations per gene product is 10. In addition, 45 gene products had no GO annotation and were excluded from the analysis. The colors represent expression values of the gene products. The lower expression values are indicated with black and the higher expressions with white. The left dendrogram corresponds to GO based clustering, while the top dendrogram uses expression values for clustering.

CLUSTER	SIZE	GOID	PRIORI	INFO	DESCRIPTION
G1	516				*[no interesting GO terms]*
G2	4	GO:0002376	0.042	4.57	immune system process
		GO:0044464	0.909	0.137	cell part
G3	556				*[no interesting GO terms]*
G4	47				*[no interesting GO terms]*
G5	17	GO:0046872	0.117	3.1	metal ion binding
G6	12	GO:0007165	0.114	3.13	signal transduction
G7	6	GO:0008152	0.286	1.805	metabolic process
		GO:0005737	0.295	1.763	cytoplasm
G8	176				*[no interesting GO terms]*
G9	4	GO:0003676	0.092	3.443	nucleic acid binding
G10	3	GO:0048731	0.1	3.316	system development
G11	2	GO:0005634	0.157	2.675	nucleus
		GO:0032502	0.174	2.522	developmental process

3 Survival analysis for DEGs

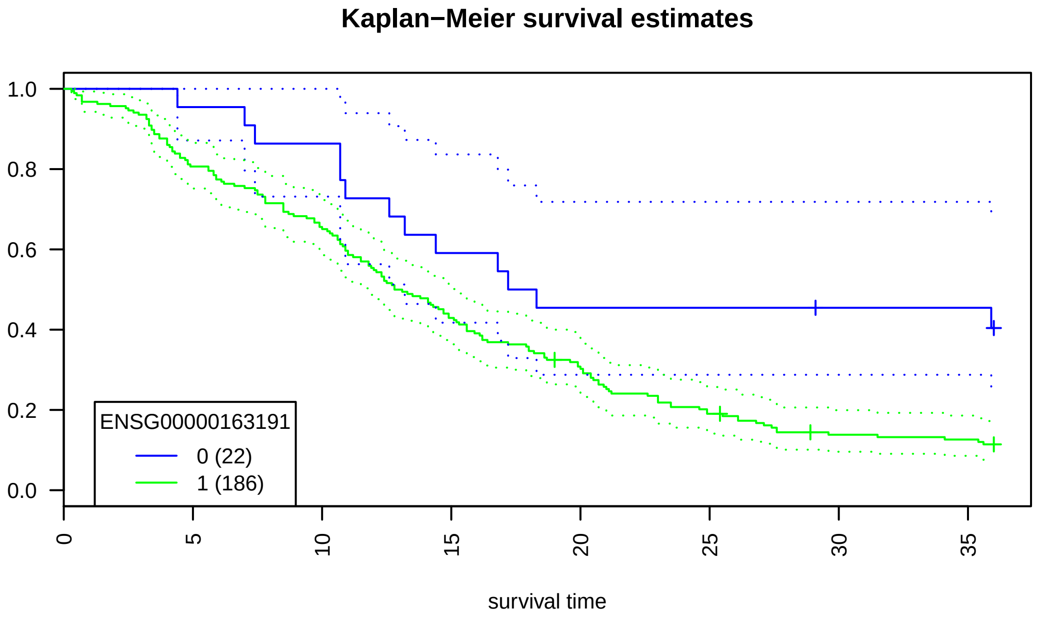

Figure 2: Each curve represents the relative number of ENSG00000163191 survived to the corresponding point of time. Dashed lines represent 0.95 confidence intervals for the curves with the corresponding color. Log-rank probability measure for the equality of these curves is 5.440705e-03. Additional annotations: *.GeneName*=S100A11, *.GeneDesc*=S100 calcium binding protein A11 [Source:HGNC Symbol;Acc:10488], *Uniprot/SWISSPROT*=P31949.

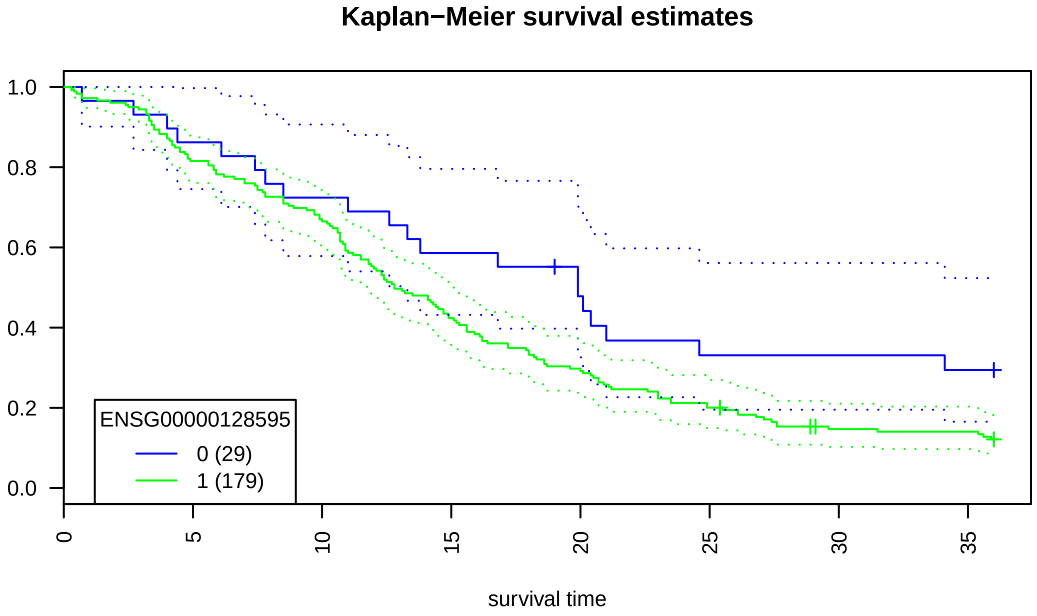

Figure 3: Each curve represents the relative number of ENSG00000128595 survived to the corresponding point of time. Dashed lines represent 0.95 confidence intervals for the curves with the corresponding color. Log-rank probability measure for the equality of these curves is 3.605709e-02. Additional annotations: *.GeneName*=CALU, *.GeneDesc*=calumenin [Source:HGNC Symbol;Acc:1458], *Uniprot/SWISSPROT*=O43852.

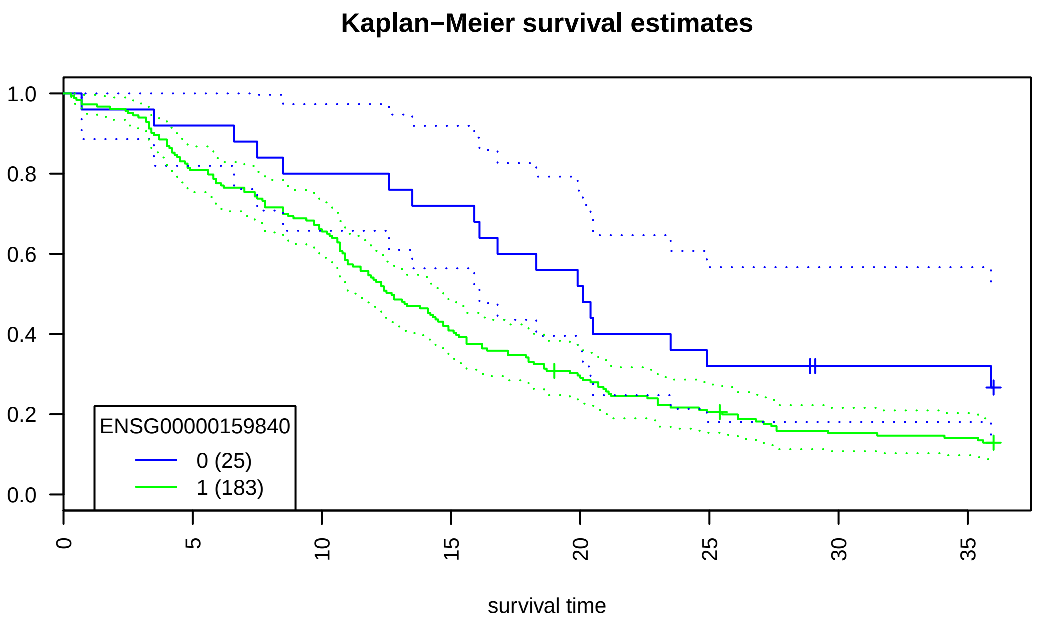

Figure 4: Each curve represents the relative number of ENSG00000159840 survived to the corresponding point of time. Dashed lines represent 0.95 confidence intervals for the curves with the corresponding color. Log-rank probability measure for the equality of these curves is 2.991130e-02. Additional annotations: *.GeneName*=ZYX, *.GeneDesc*=zyxin [Source:HGNC Symbol;Acc:13200], *Uniprot/SWISSPROT*=Q15942.

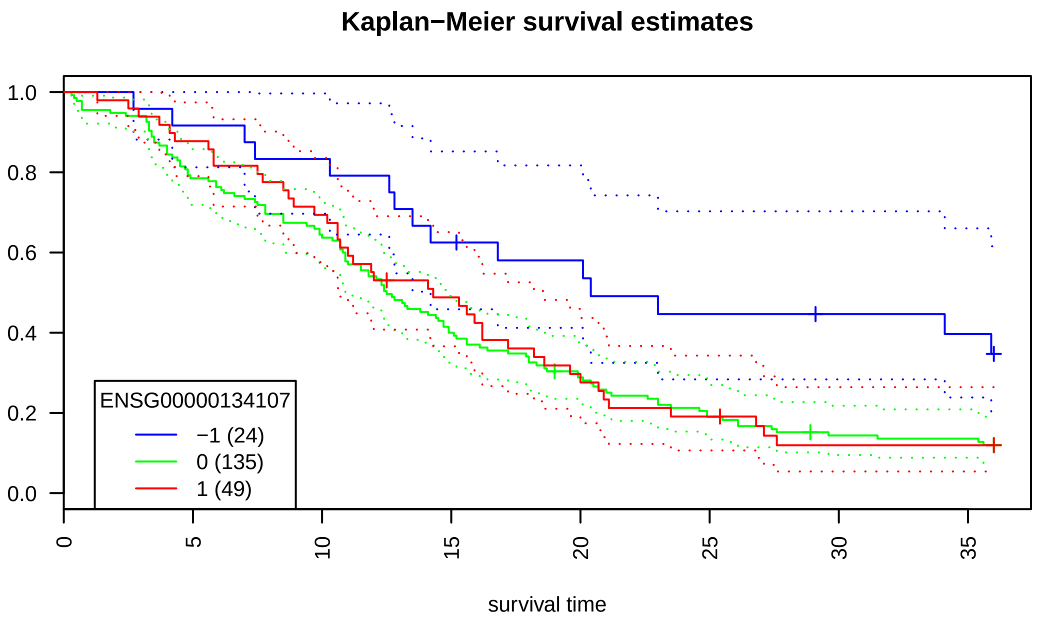

Figure 5: Each curve represents the relative number of ENSG00000134107 survived to the corresponding point of time. Dashed lines represent 0.95 confidence intervals for the curves with the corresponding color. Log-rank probability measure for the equality of these curves is 2.359757e-02.

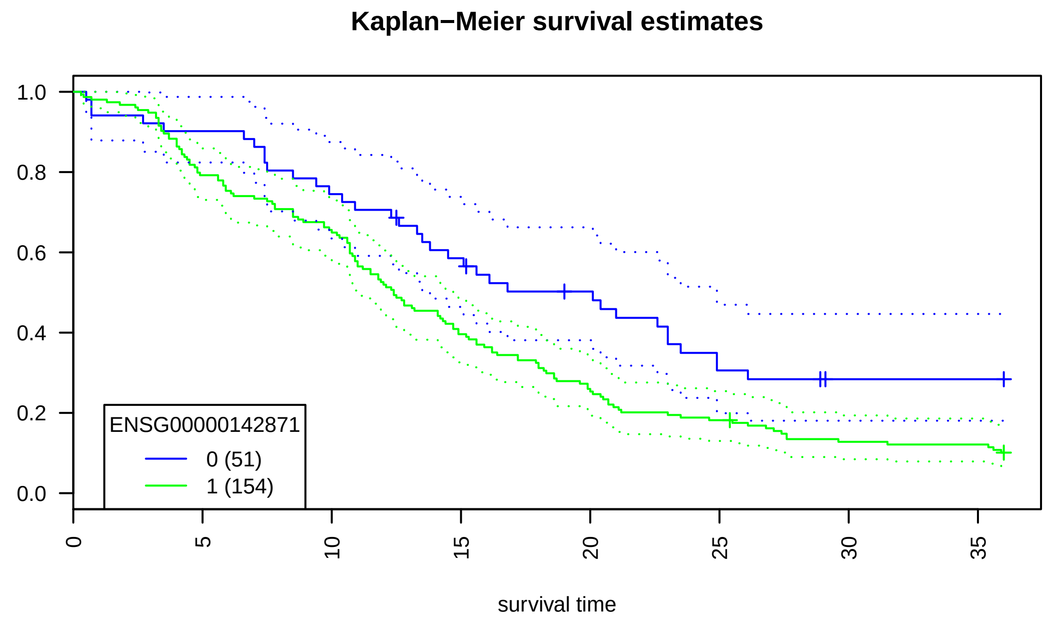

Figure 6: Each curve represents the relative number of ENSG00000142871 survived to the corresponding point of time. Dashed lines represent 0.95 confidence intervals for the curves with the corresponding color. Total of 3 observations deleted due to missingness. Log-rank probability measure for the equality of these curves is 3.694604e-03. Additional annotations: *.GeneName*=CYR61, *.GeneDesc*=cysteine-rich, angiogenic inducer, 61 [Source:HGNC Symbol;Acc:2654], *Uniprot/SWISSPROT*=O00622.

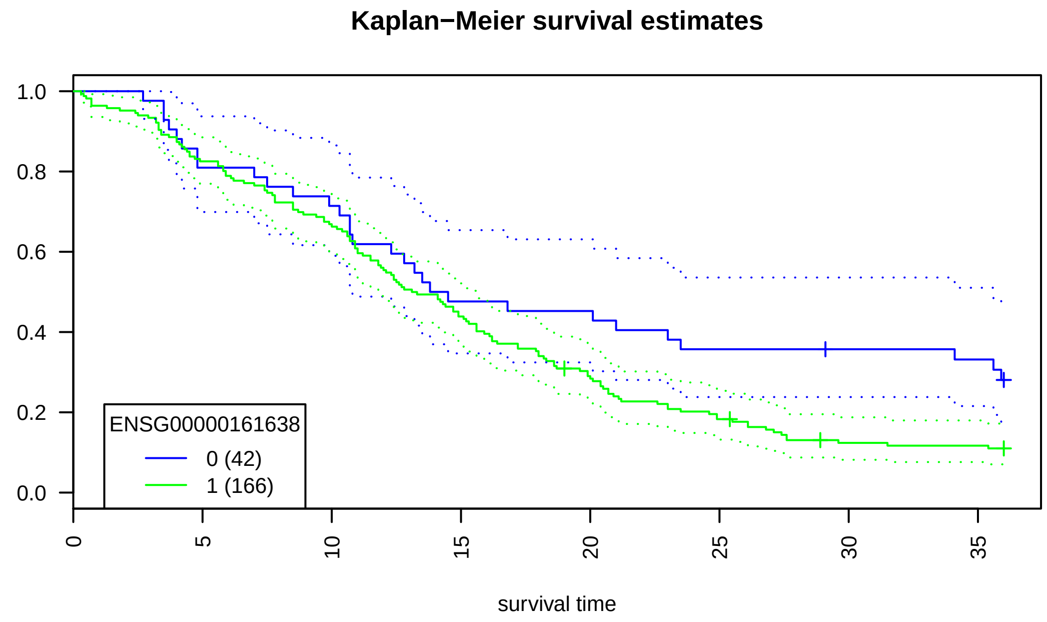

Figure 7: Each curve represents the relative number of ENSG00000161638 survived to the corresponding point of time. Dashed lines represent 0.95 confidence intervals for the curves with the corresponding color. Log-rank probability measure for the equality of these curves is 2.451210e-02. Additional annotations: *.GeneName*=ITGA5, *.GeneDesc*=integrin, alpha 5 (fibronectin receptor, alpha polypeptide) [Source:HGNC Symbol;Acc:6141], *Uniprot/SWISSPROT*=P08648.

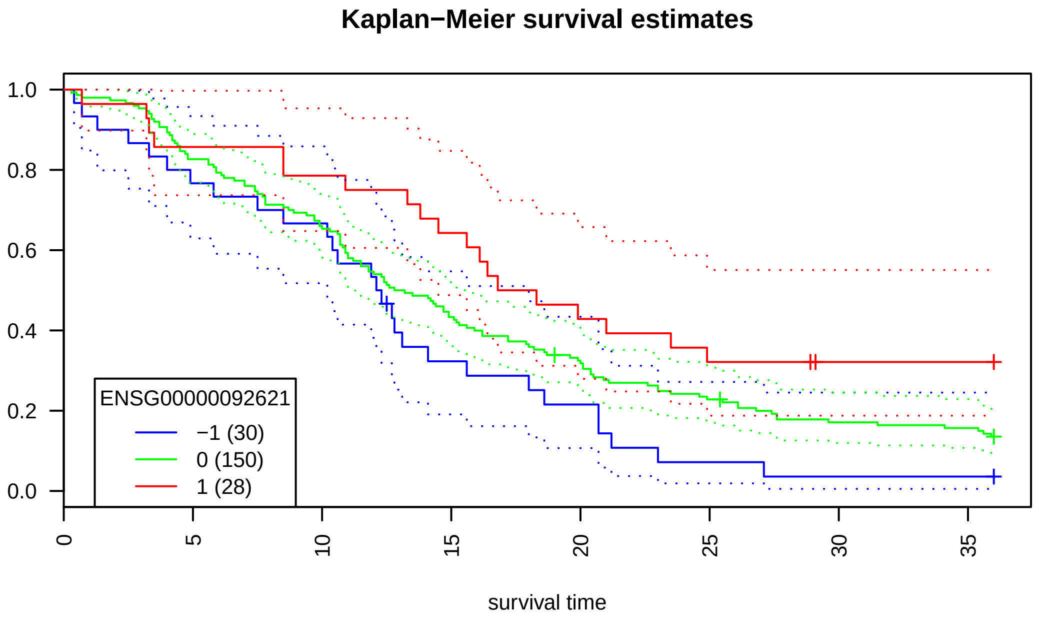

Figure 8: Each curve represents the relative number of ENSG00000092621 survived to the corresponding point of time. Dashed lines represent 0.95 confidence intervals for the curves with the corresponding color. Log-rank probability measure for the equality of these curves is 1.506444e-02.

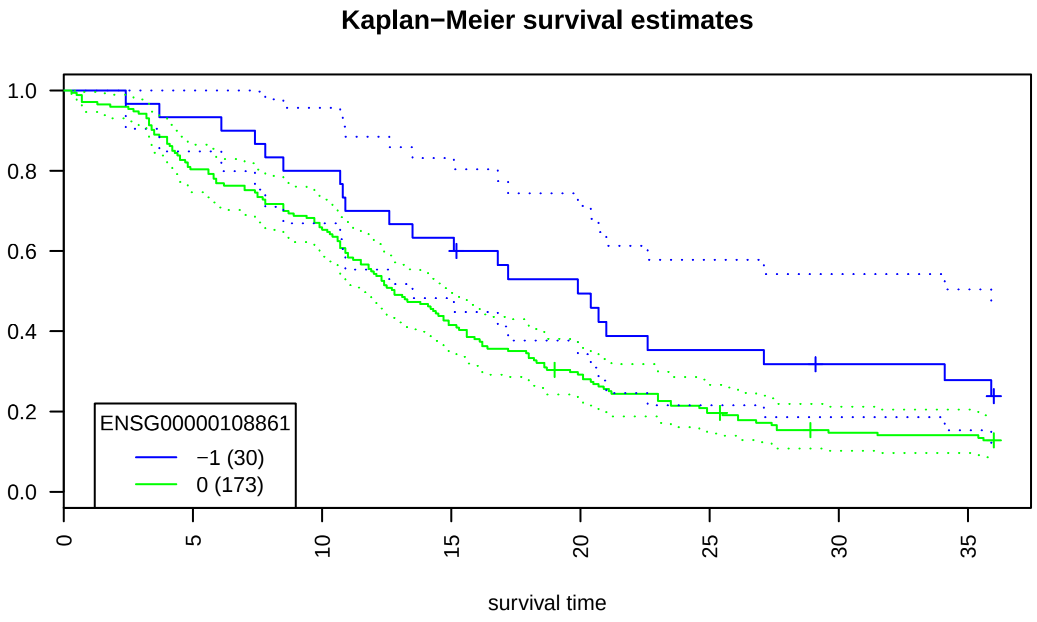

Figure 9: Each curve represents the relative number of ENSG00000108861 survived to the corresponding point of time. Dashed lines represent 0.95 confidence intervals for the curves with the corresponding color. Total of 5 observations deleted due to missingness. Log-rank probability measure for the equality of these curves is 4.343542e-02.

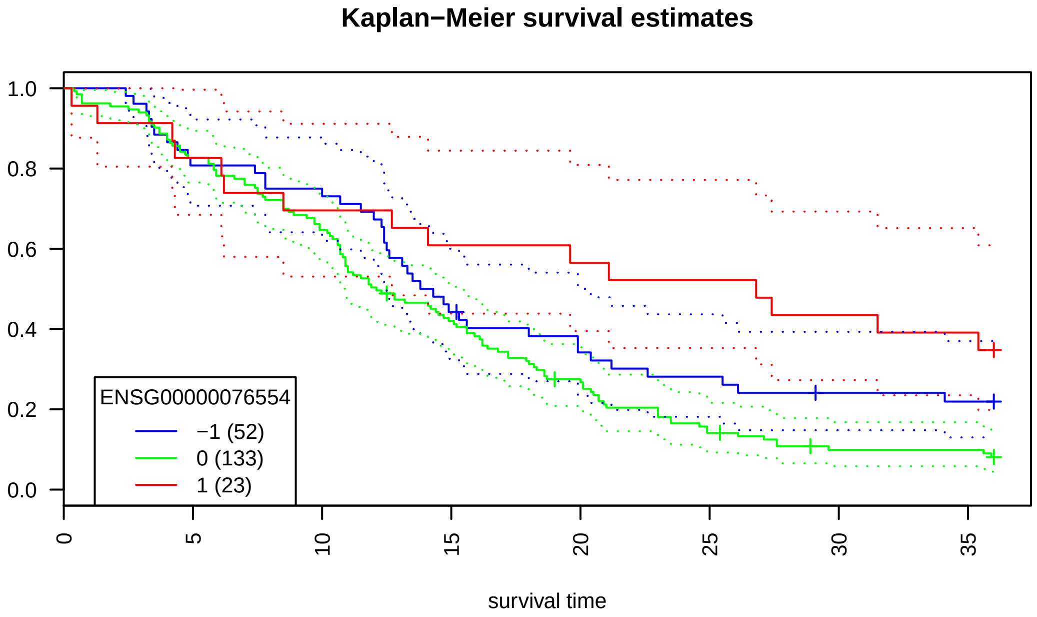

Figure 10: Each curve represents the relative number of ENSG00000076554 survived to the corresponding point of time. Dashed lines represent 0.95 confidence intervals for the curves with the corresponding color. Log-rank probability measure for the equality of these curves is 5.142641e-03.

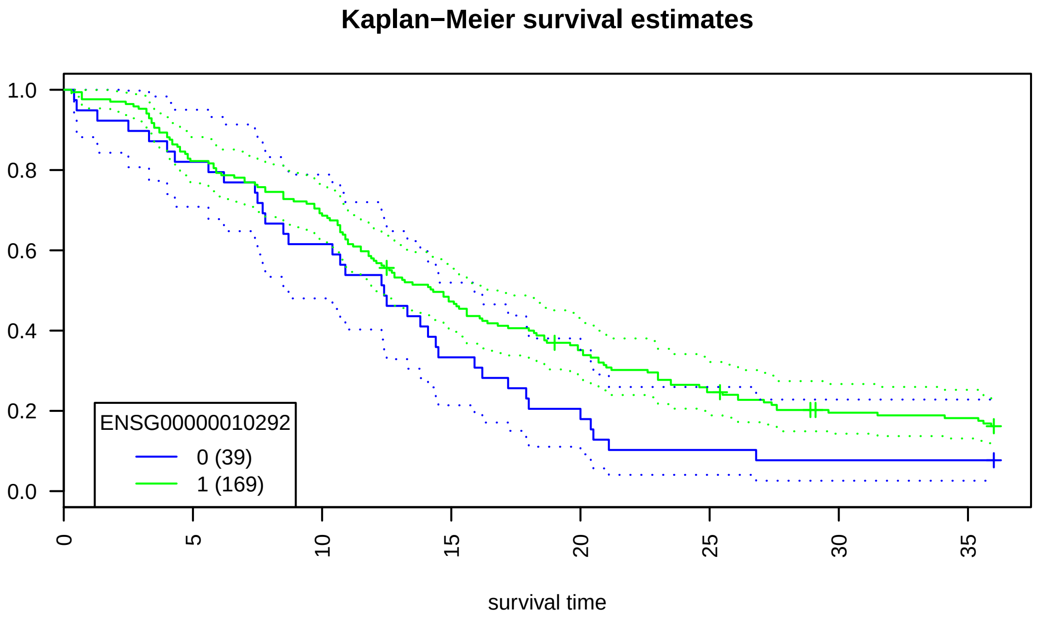

Figure 11: Each curve represents the relative number of ENSG00000010292 survived to the corresponding point of time. Dashed lines represent 0.95 confidence intervals for the curves with the corresponding color. Log-rank probability measure for the equality of these curves is 4.241218e-02. Additional annotations: *.GeneName*=NCAPD2, *.GeneDesc*=non-SMC condensin I complex, subunit D2 [Source:HGNC Symbol;Acc:24305], *Uniprot/SWISSPROT*=Q15021.

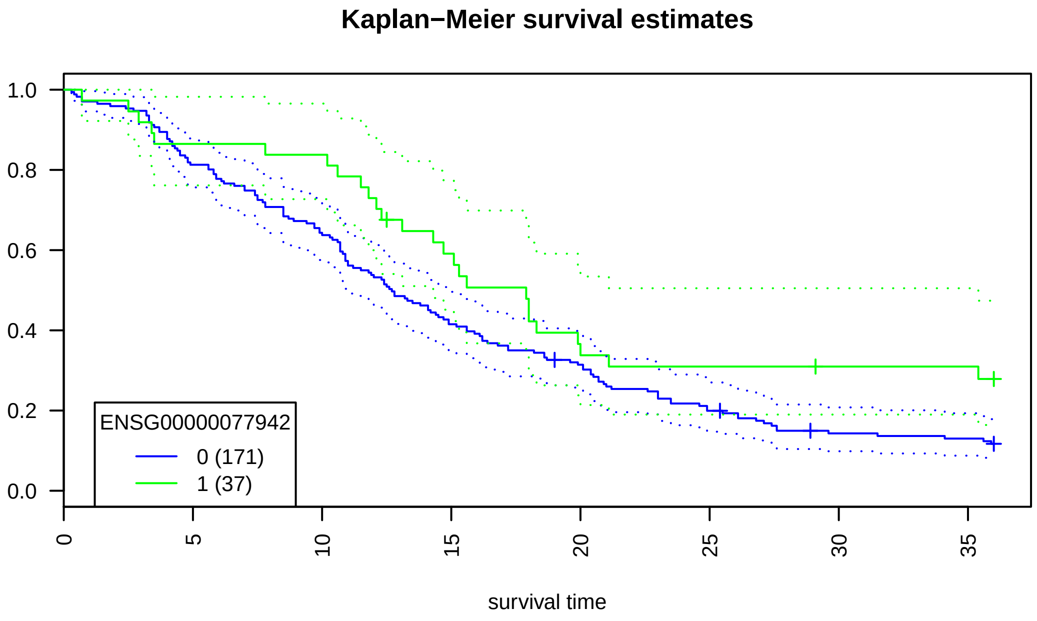

Figure 12: Each curve represents the relative number of ENSG00000077942 survived to the corresponding point of time. Dashed lines represent 0.95 confidence intervals for the curves with the corresponding color. Log-rank probability measure for the equality of these curves is 3.622037e-02.

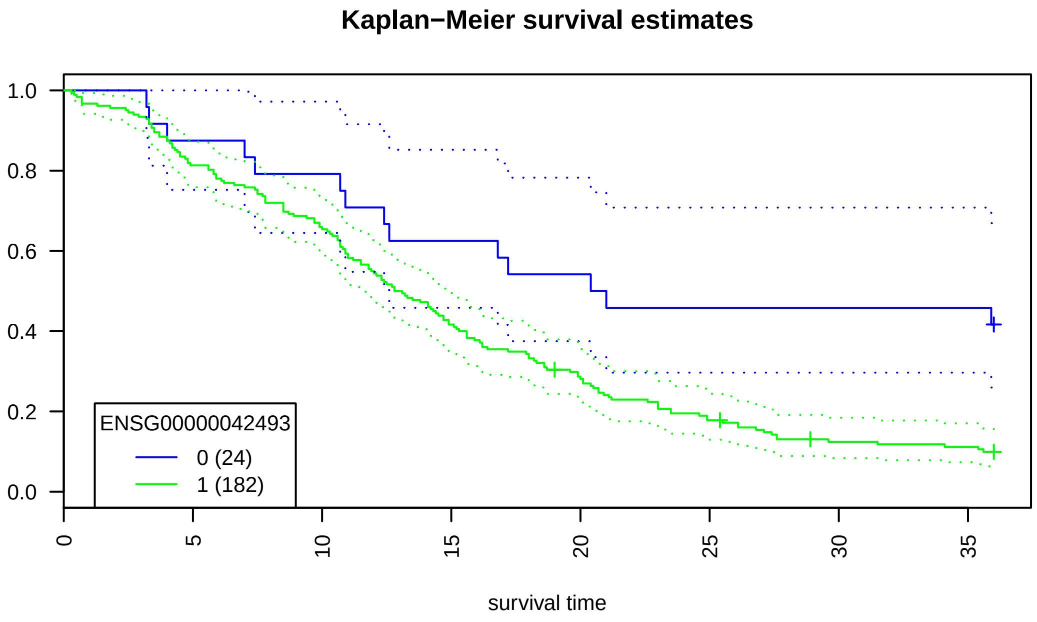

Figure 13: Each curve represents the relative number of ENSG00000042493 survived to the corresponding point of time. Dashed lines represent 0.95 confidence intervals for the curves with the corresponding color. Total of 2 observations deleted due to missingness. Log-rank probability measure for the equality of these curves is 1.908445e-03. Additional annotations: *.GeneName*=CAPG, *.GeneDesc*=capping protein (actin filament), gelsolin-like [Source:HGNC Symbol;Acc:1474], *Uniprot/SWISSPROT*=P40121.

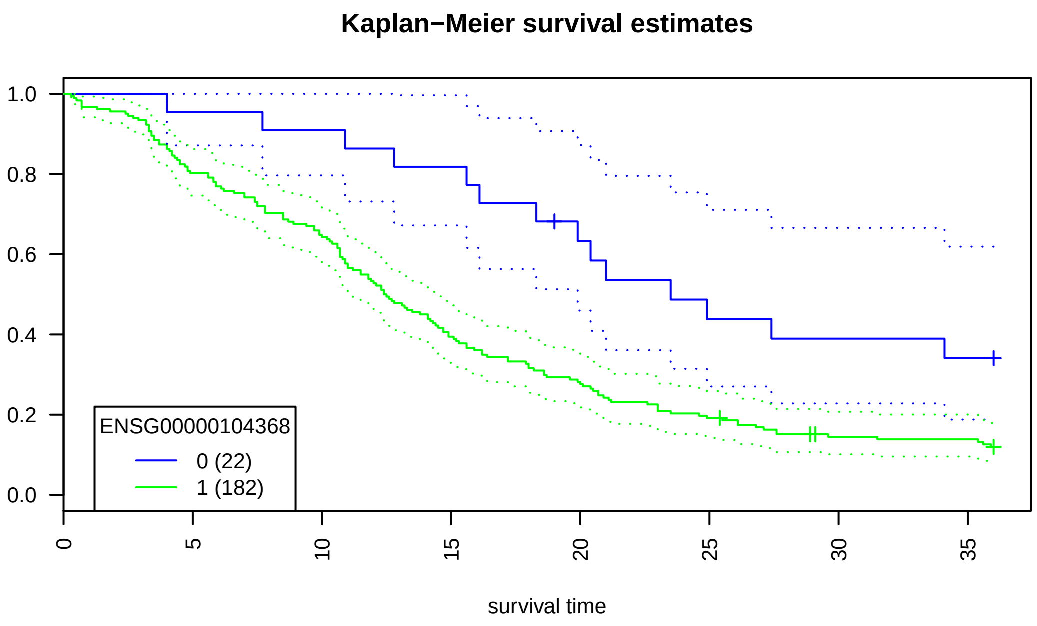

Figure 14: Each curve represents the relative number of ENSG00000104368 survived to the corresponding point of time. Dashed lines represent 0.95 confidence intervals for the curves with the corresponding color. Total of 4 observations deleted due to missingness. Log-rank probability measure for the equality of these curves is 2.088509e-03. Additional annotations: *.GeneName*=PLAT, *.GeneDesc*=plasminogen activator, tissue [Source:HGNC Symbol;Acc:9051], *Uniprot/SWISSPROT*=P00750.

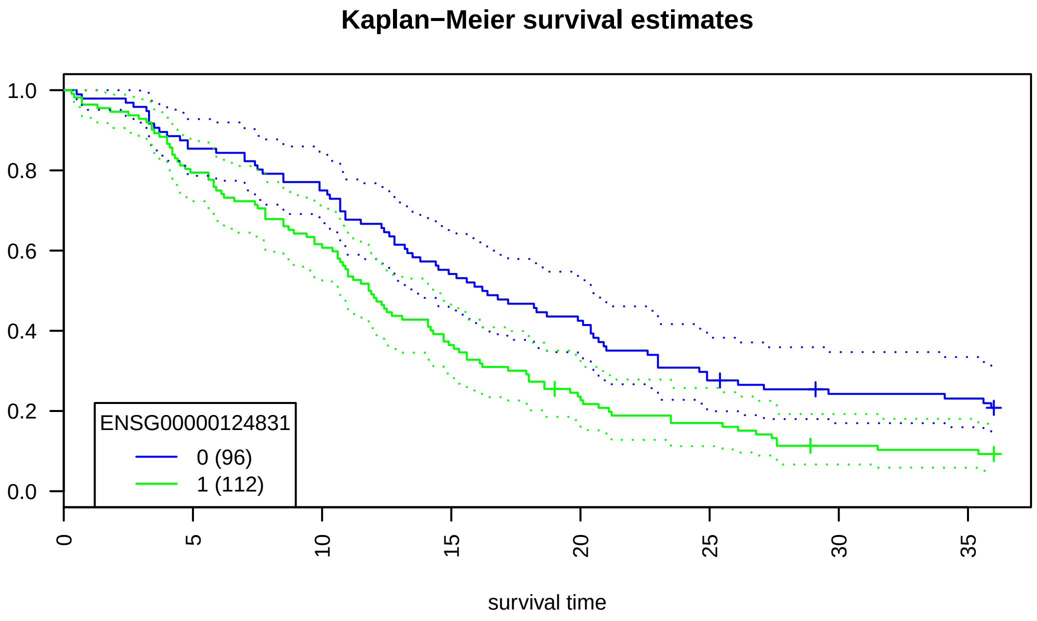

Figure 15: Each curve represents the relative number of ENSG00000124831 survived to the corresponding point of time. Dashed lines represent 0.95 confidence intervals for the curves with the corresponding color. Log-rank probability measure for the equality of these curves is 3.235318e-03.

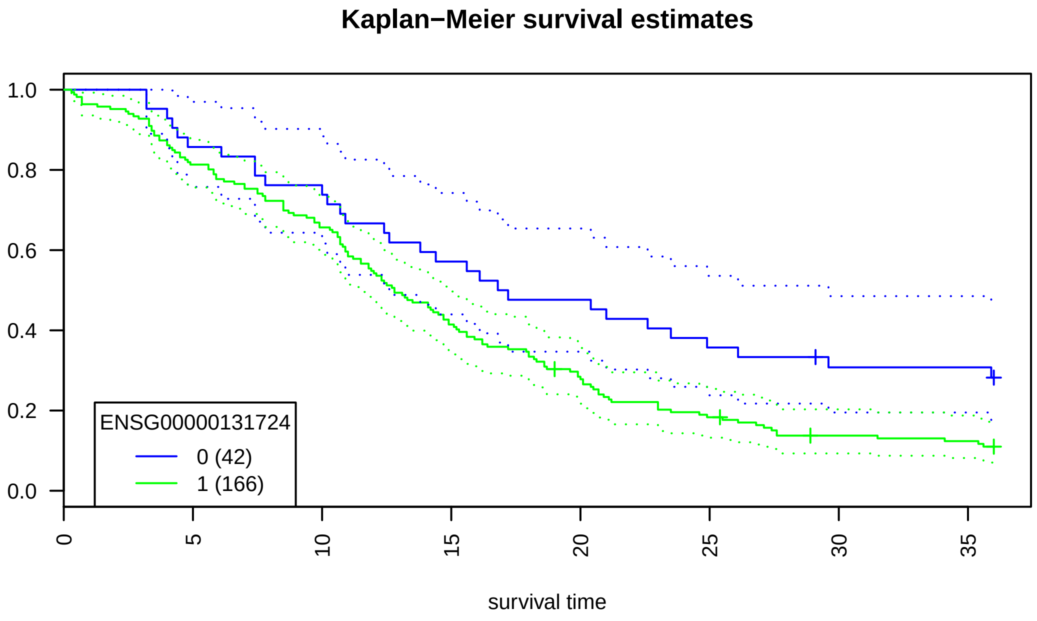

Figure 16: Each curve represents the relative number of ENSG00000131724 survived to the corresponding point of time. Dashed lines represent 0.95 confidence intervals for the curves with the corresponding color. Log-rank probability measure for the equality of these curves is 1.057691e-02. Additional annotations: *.GeneName*=IL13RA1, *.GeneDesc*=interleukin 13 receptor, alpha 1 [Source:HGNC Symbol;Acc:5974], *Uniprot/SWISSPROT*=P78552.

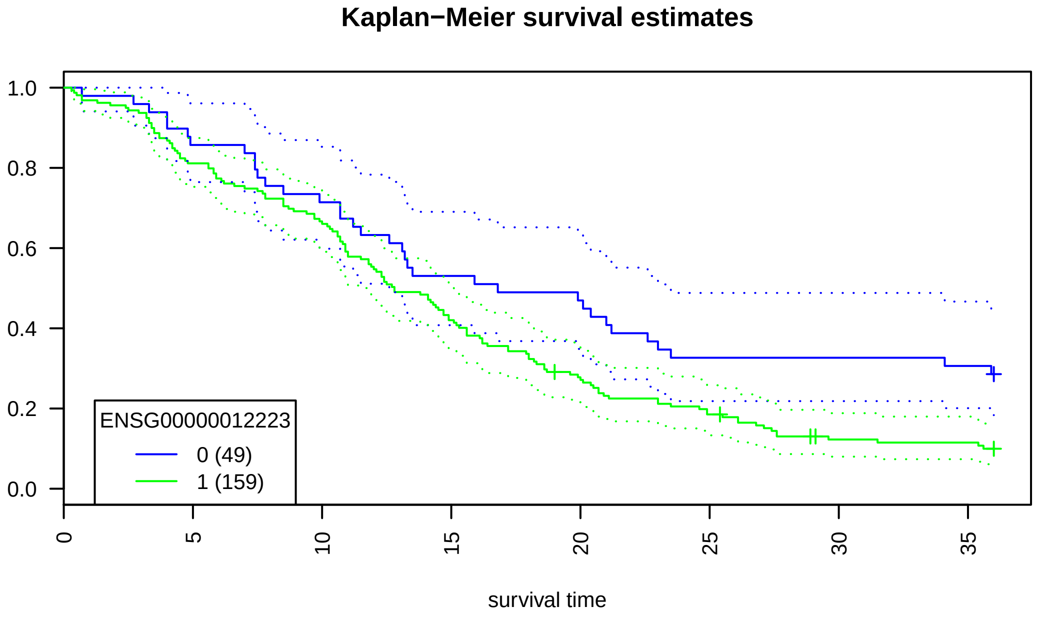

Figure 17: Each curve represents the relative number of ENSG00000012223 survived to the corresponding point of time. Dashed lines represent 0.95 confidence intervals for the curves with the corresponding color. Log-rank probability measure for the equality of these curves is 8.15989e-03. Additional annotations: *.GeneName*=LTF, *.GeneDesc*=lactotransferrin [Source:HGNC Symbol;Acc:6720], *Uniprot/SWISSPROT*=P02788.

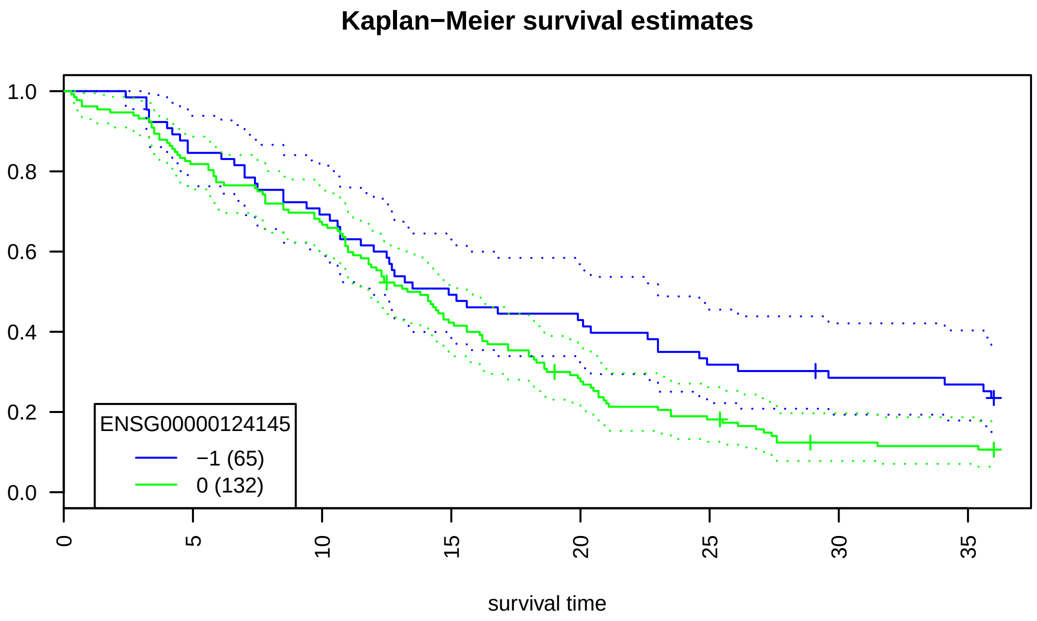

Figure 18: Each curve represents the relative number of ENSG00000124145 survived to the corresponding point of time. Dashed lines represent 0.95 confidence intervals for the curves with the corresponding color. Total of 11 observations deleted due to missingness. Log-rank probability measure for the equality of these curves is 3.380315e-02.

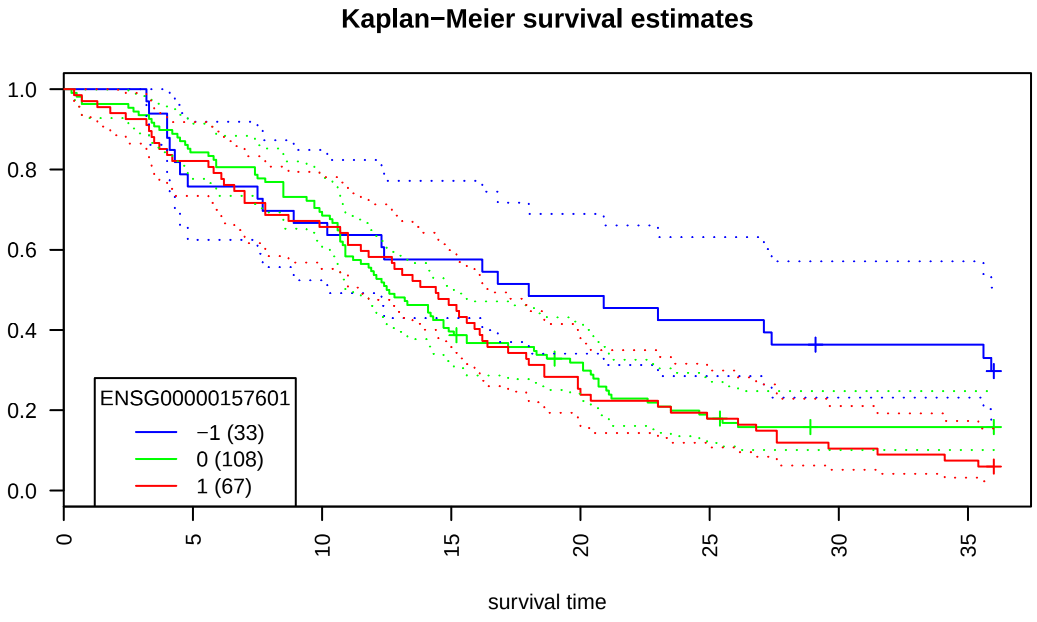

Figure 19: Each curve represents the relative number of ENSG00000157601 survived to the corresponding point of time. Dashed lines represent 0.95 confidence intervals for the curves with the corresponding color. Log-rank probability measure for the equality of these curves is 4.94741e-02.

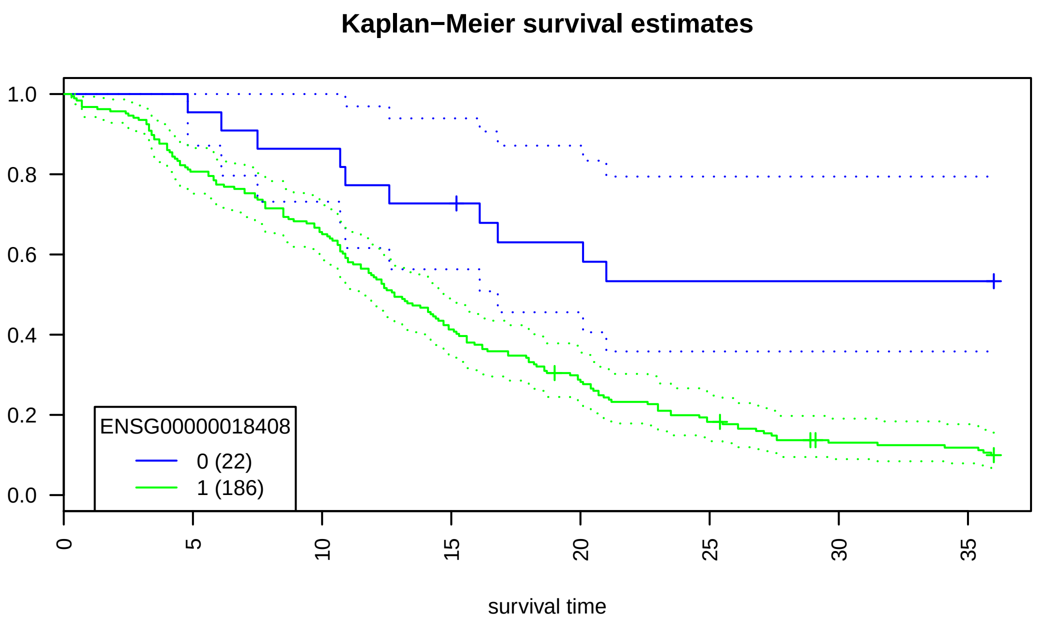

Figure 20: Each curve represents the relative number of ENSG00000018408 survived to the corresponding point of time. Dashed lines represent 0.95 confidence intervals for the curves with the corresponding color. Log-rank probability measure for the equality of these curves is 1.338237e-04. Additional annotations: *.GeneName*=WWTR1, *.GeneDesc*=WW domain containing transcription regulator 1 [Source:HGNC Symbol;Acc:24042], *Uniprot/SWISSPROT*=Q9GZV5.

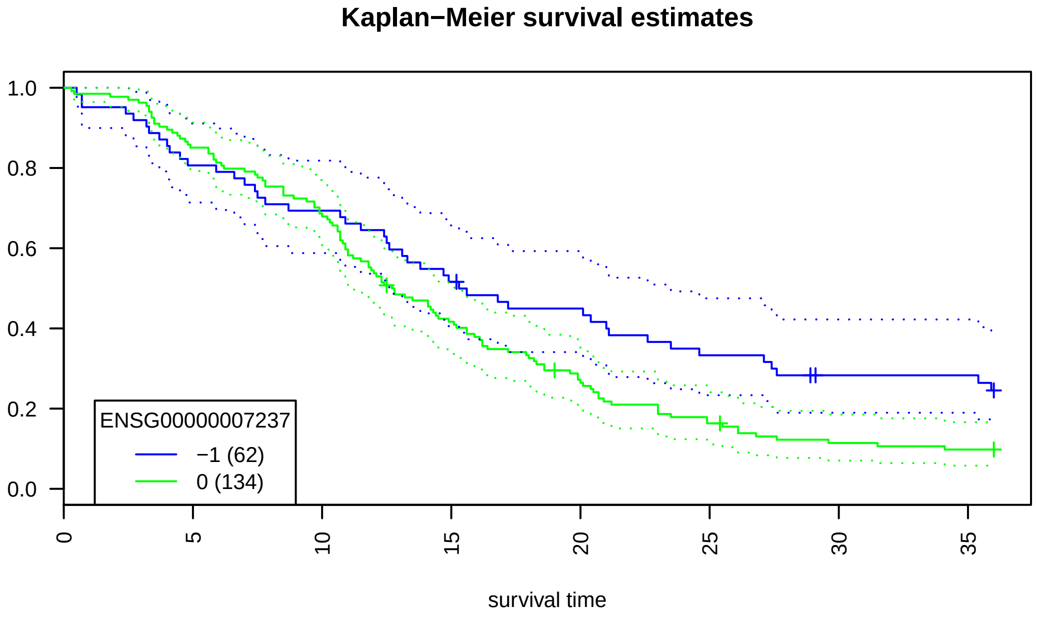

Figure 21: Each curve represents the relative number of ENSG00000007237 survived to the corresponding point of time. Dashed lines represent 0.95 confidence intervals for the curves with the corresponding color. Total of 12 observations deleted due to missingness. Log-rank probability measure for the equality of these curves is 1.799976e-02.

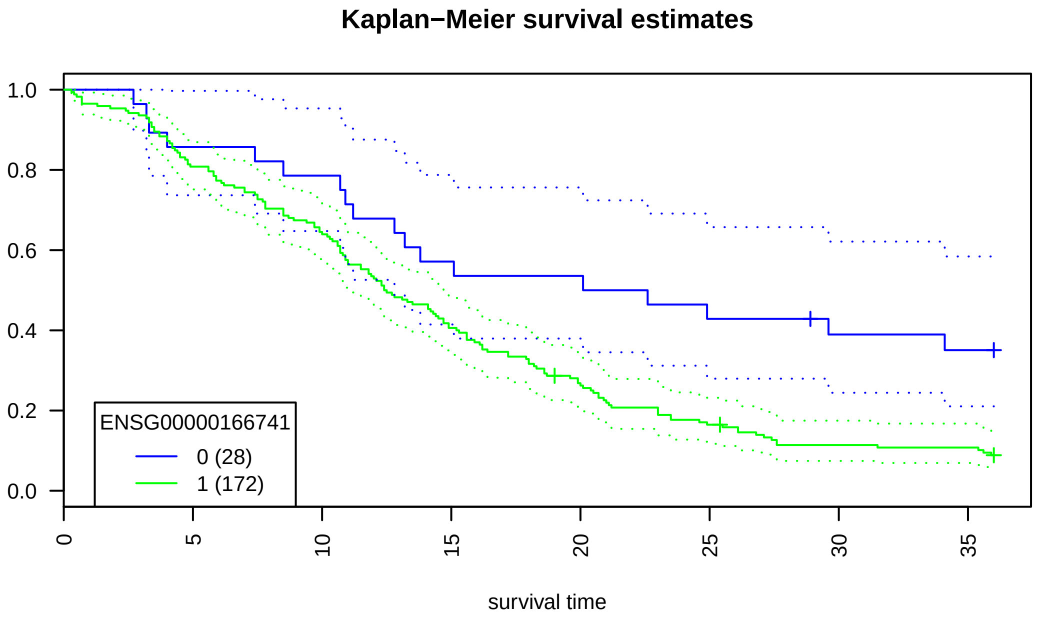

Figure 22: Each curve represents the relative number of ENSG00000166741 survived to the corresponding point of time. Dashed lines represent 0.95 confidence intervals for the curves with the corresponding color. Total of 8 observations deleted due to missingness. Log-rank probability measure for the equality of these curves is 2.255287e-03. Additional annotations: *.GeneName*=NNMT, *.GeneDesc*=nicotinamide N-methyltransferase [Source:HGNC Symbol;Acc:7861], *Uniprot/SWISSPROT*=P40261.

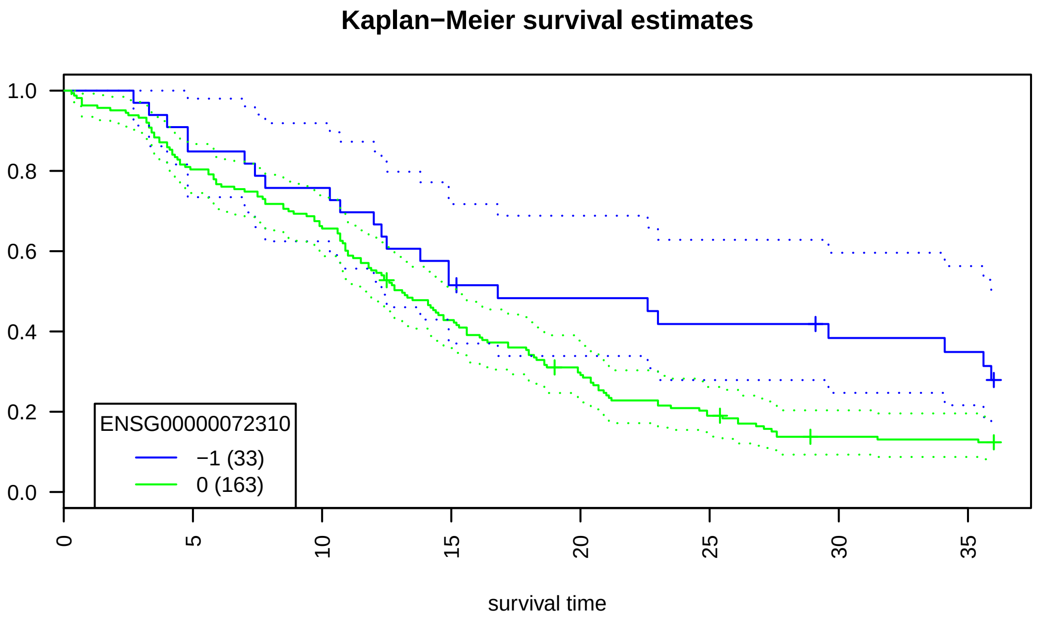

Figure 23: Each curve represents the relative number of ENSG00000072310 survived to the corresponding point of time. Dashed lines represent 0.95 confidence intervals for the curves with the corresponding color. Total of 12 observations deleted due to missingness. Log-rank probability measure for the equality of these curves is 2.255200e-02.

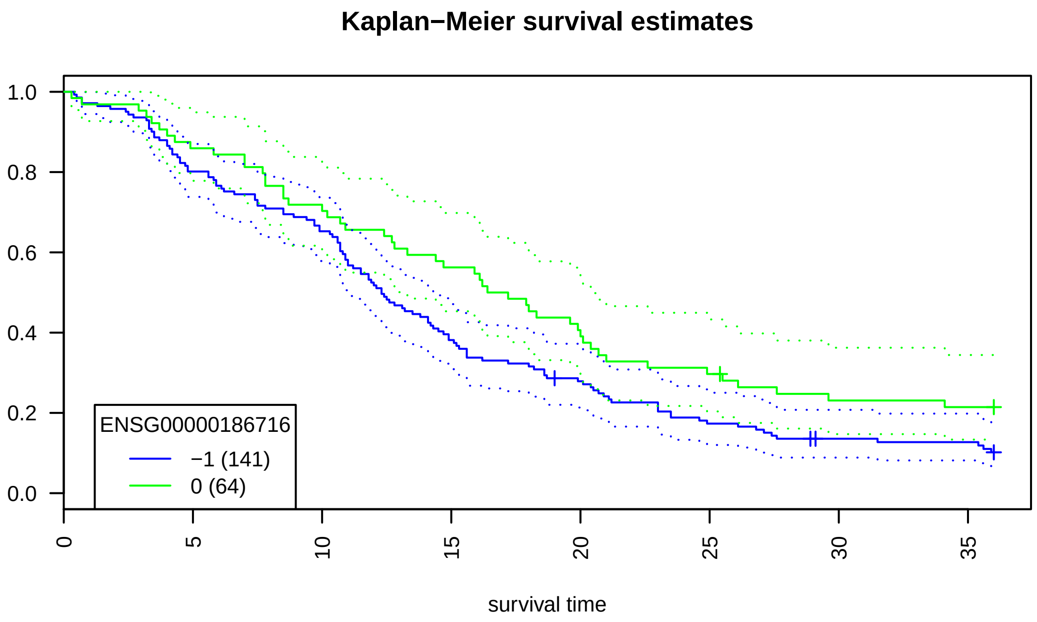

Figure 24: Each curve represents the relative number of ENSG00000186716 survived to the corresponding point of time. Dashed lines represent 0.95 confidence intervals for the curves with the corresponding color. Total of 3 observations deleted due to missingness. Log-rank probability measure for the equality of these curves is 2.575334e-02. Additional annotations: *.GeneName*=BCR, *.GeneDesc*=breakpoint cluster region [Source:HGNC Symbol;Acc:1014], *Uniprot/SWISSPROT*=P11274.

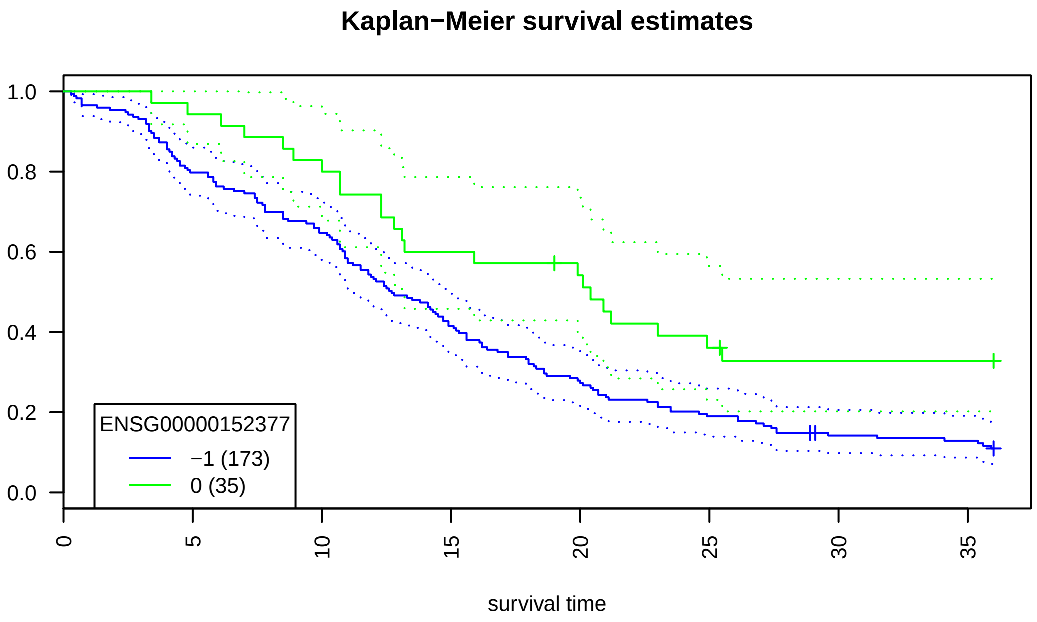

Figure 25: Each curve represents the relative number of ENSG00000152377 survived to the corresponding point of time. Dashed lines represent 0.95 confidence intervals for the curves with the corresponding color. Log-rank probability measure for the equality of these curves is 3.513647e-03. Additional annotations: *.GeneName*=SPOCK1, *.GeneDesc*=sparc/osteonectin, cwcv and kazal-like domains proteoglycan (testican) 1 [Source:HGNC Symbol;Acc:11251], *Uniprot/SWISSPROT*=Q08629.

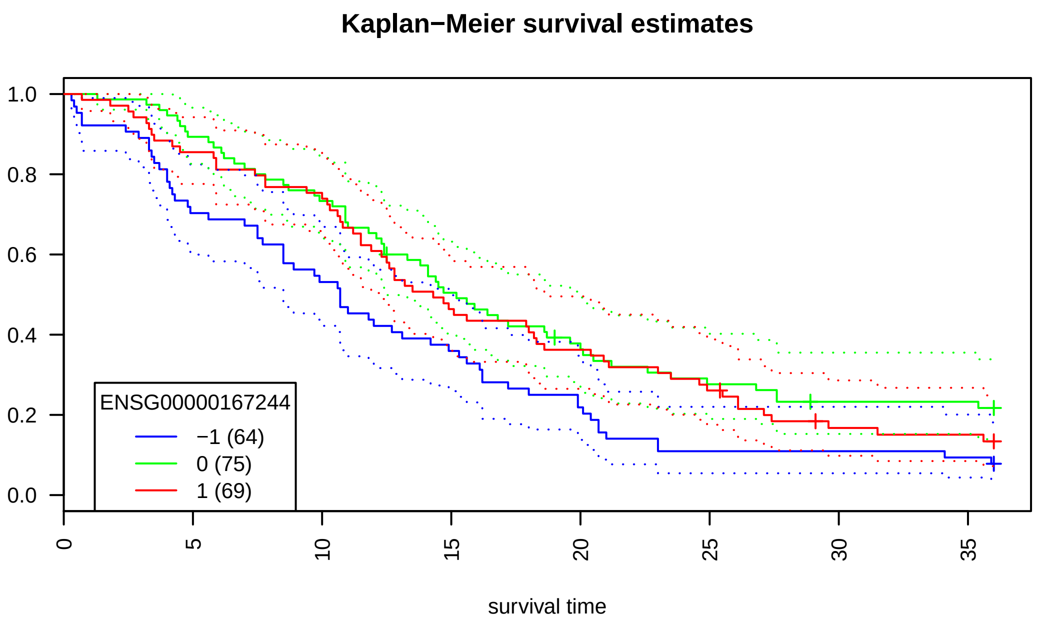

Figure 26: Each curve represents the relative number of ENSG00000167244 survived to the corresponding point of time. Dashed lines represent 0.95 confidence intervals for the curves with the corresponding color. Log-rank probability measure for the equality of these curves is 8.787045e-03.

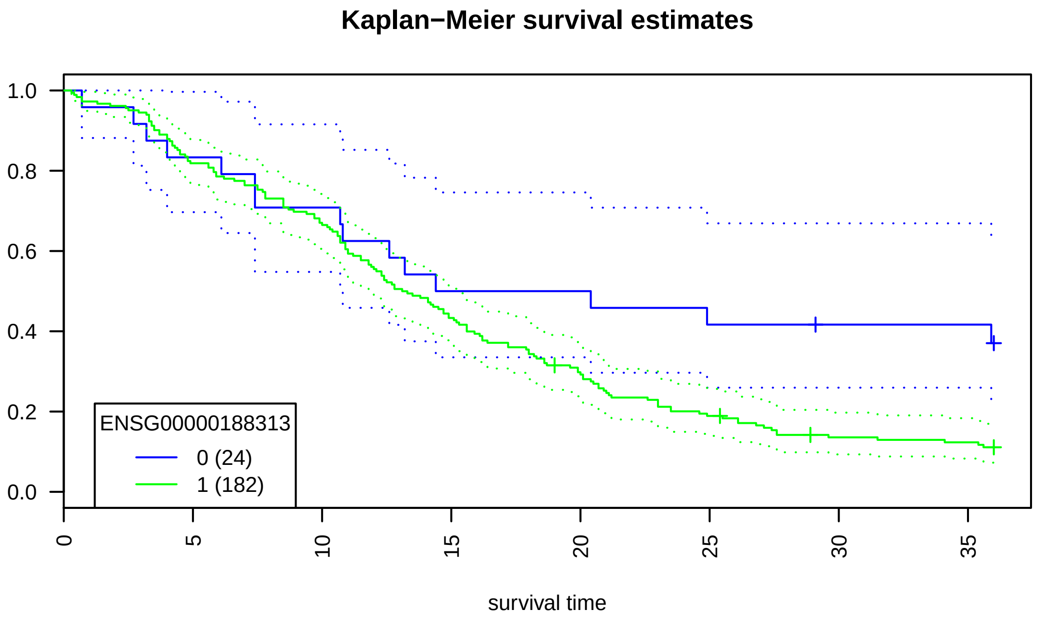

Figure 27: Each curve represents the relative number of ENSG00000188313 survived to the corresponding point of time. Dashed lines represent 0.95 confidence intervals for the curves with the corresponding color. Total of 2 observations deleted due to missingness. Log-rank probability measure for the equality of these curves is 1.879271e-02. Additional annotations: *.GeneName*=PLSCR1, *.GeneDesc*=phospholipid scramblase 1 [Source:HGNC Symbol;Acc:9092], *Uniprot/SWISSPROT*=O15162.

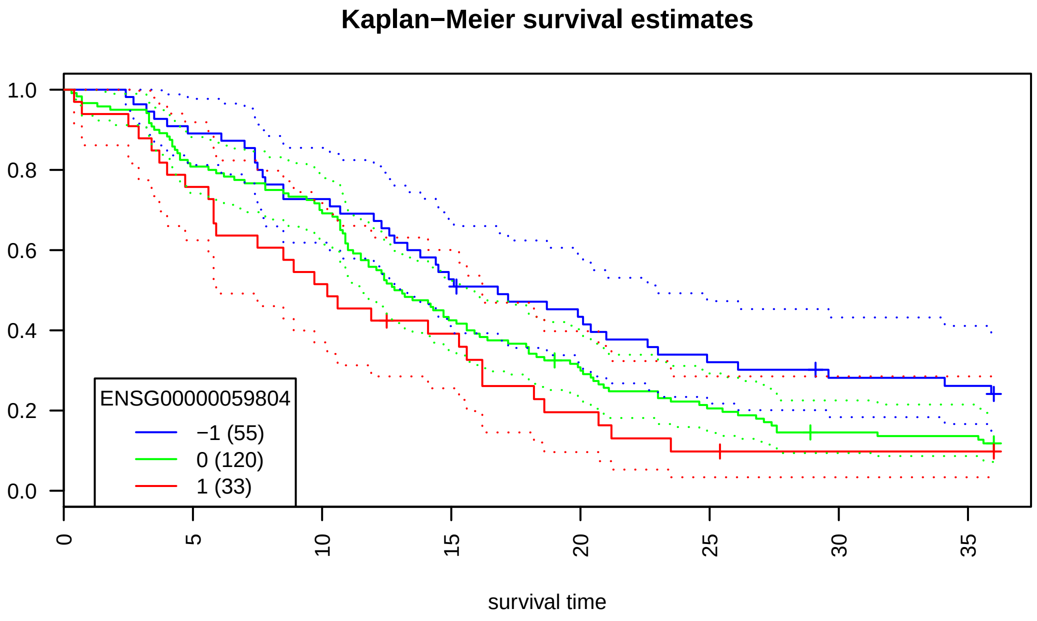

Figure 28: Each curve represents the relative number of ENSG00000059804 survived to the corresponding point of time. Dashed lines represent 0.95 confidence intervals for the curves with the corresponding color. Log-rank probability measure for the equality of these curves is 2.111483e-02.

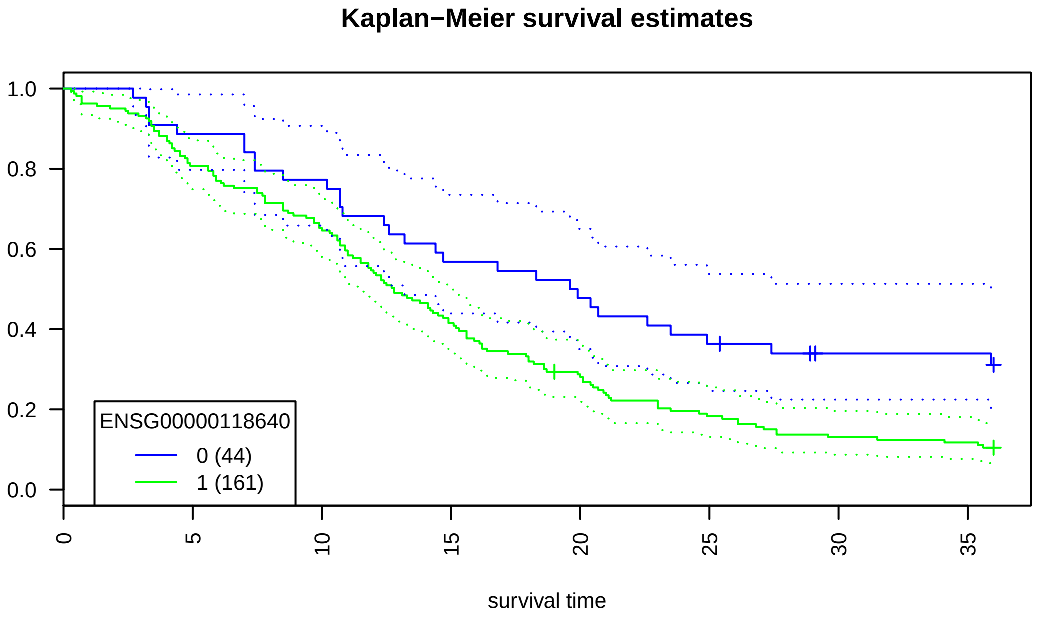

Figure 29: Each curve represents the relative number of ENSG00000118640 survived to the corresponding point of time. Dashed lines represent 0.95 confidence intervals for the curves with the corresponding color. Total of 3 observations deleted due to missingness. Log-rank probability measure for the equality of these curves is 3.260262e-03. Additional annotations: *.GeneName*=VAMP8, *.GeneDesc*=vesicle-associated membrane protein 8 (endobrevin) [Source:HGNC Symbol;Acc:12647], *Uniprot/SWISSPROT*=Q9BV40.

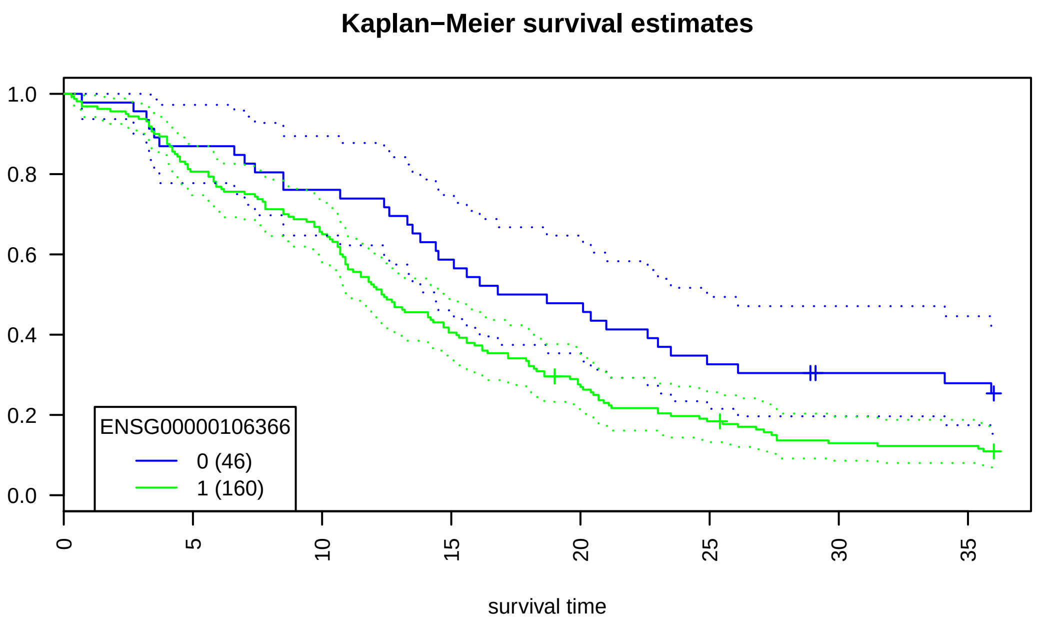

Figure 30: Each curve represents the relative number of ENSG00000106366 survived to the corresponding point of time. Dashed lines represent 0.95 confidence intervals for the curves with the corresponding color. Total of 2 observations deleted due to missingness. Log-rank probability measure for the equality of these curves is 9.589352e-03. Additional annotations: *.GeneName*=SERPINE1, *.GeneDesc*=serpin peptidase inhibitor, clade E (nexin, plasminogen activator inhibitor type 1), member 1 [Source:HGNC Symbol;Acc:8583], *Uniprot/SWISSPROT*=P05121.

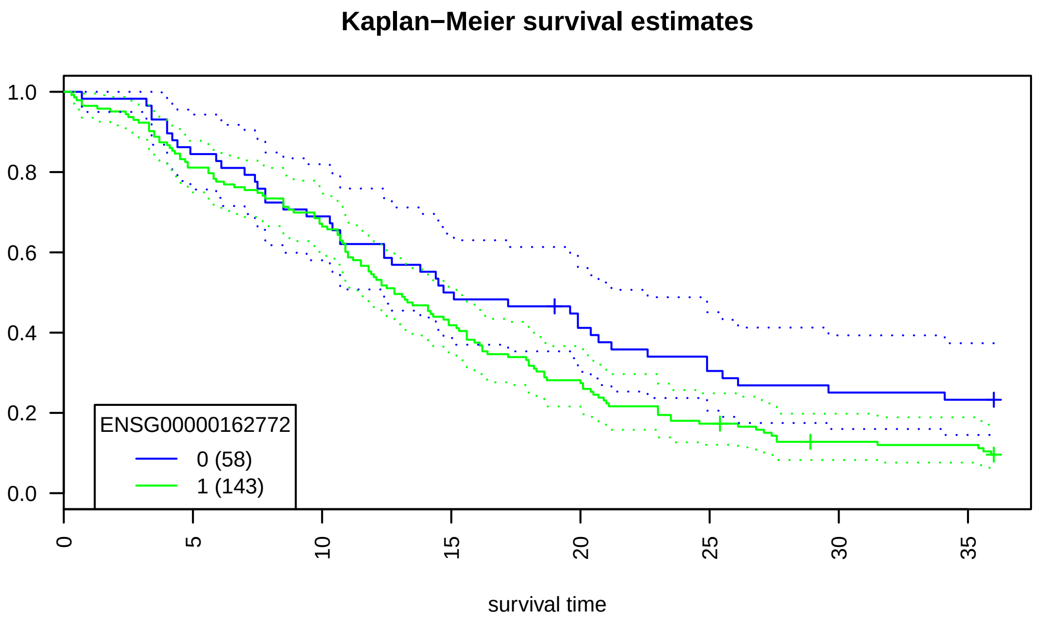

Figure 31: Each curve represents the relative number of ENSG00000162772 survived to the corresponding point of time. Dashed lines represent 0.95 confidence intervals for the curves with the corresponding color. Total of 7 observations deleted due to missingness. Log-rank probability measure for the equality of these curves is 3.083892e-02. Additional annotations: *.GeneName*=ATF3, *.GeneDesc*=activating transcription factor 3 [Source:HGNC Symbol;Acc:785], *Uniprot/SWISSPROT*=P18847.

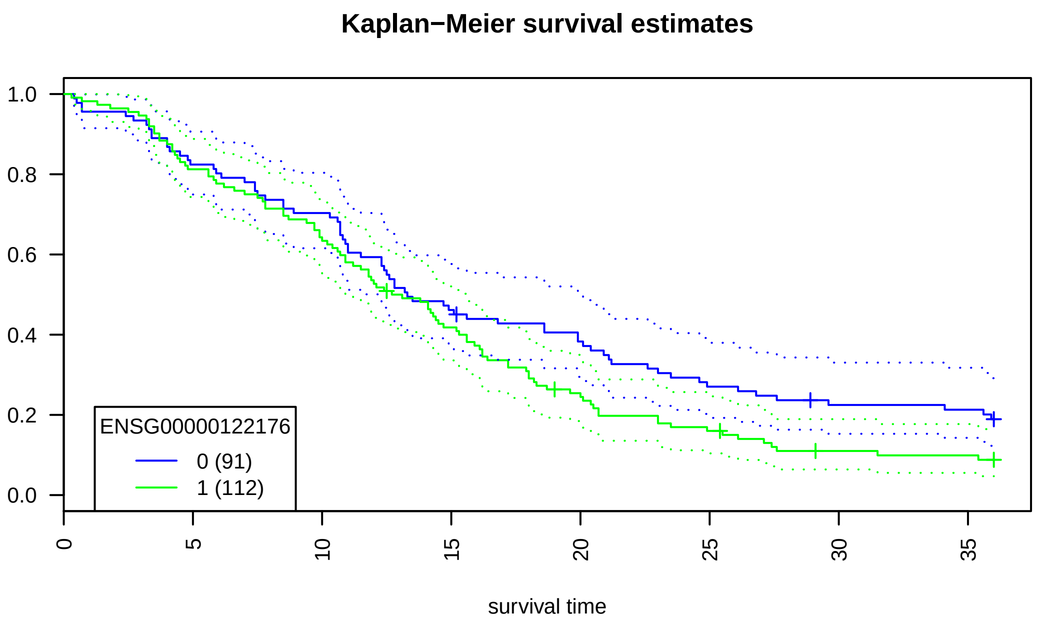

Figure 32: Each curve represents the relative number of ENSG00000122176 survived to the corresponding point of time. Dashed lines represent 0.95 confidence intervals for the curves with the corresponding color. Total of 5 observations deleted due to missingness. Log-rank probability measure for the equality of these curves is 4.962492e-02. Additional annotations: *.GeneName*=FMOD, *.GeneDesc*=fibromodulin [Source:HGNC Symbol;Acc:3774], *Uniprot/SWISSPROT*=Q06828.

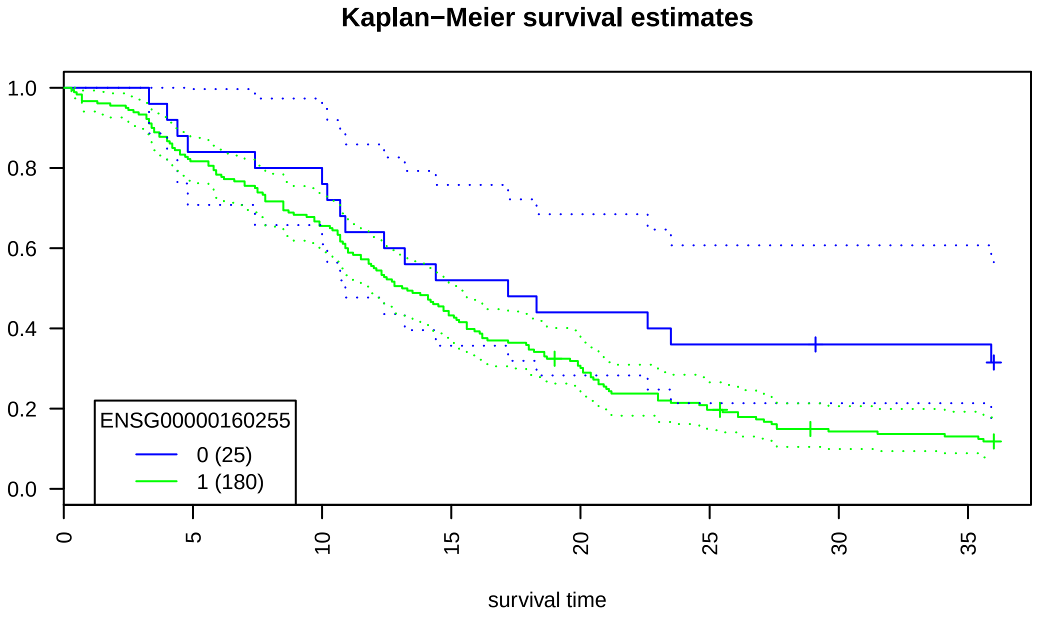

Figure 33: Each curve represents the relative number of ENSG00000160255 survived to the corresponding point of time. Dashed lines represent 0.95 confidence intervals for the curves with the corresponding color. Total of 3 observations deleted due to missingness. Log-rank probability measure for the equality of these curves is 4.20083e-02. Additional annotations: *.GeneName*=ITGB2, *.GeneDesc*=integrin, beta 2 (complement component 3 receptor 3 and 4 subunit) [Source:HGNC Symbol;Acc:6155], *Uniprot/SWISSPROT*=P05107.

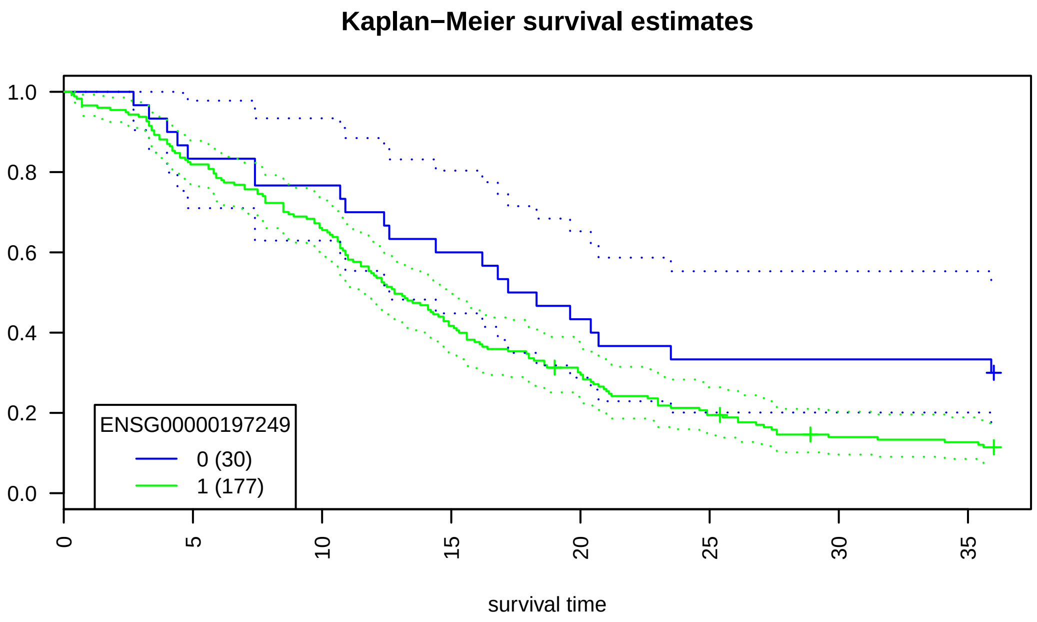

Figure 34: Each curve represents the relative number of ENSG00000197249 survived to the corresponding point of time. Dashed lines represent 0.95 confidence intervals for the curves with the corresponding color. Total of 1 observation deleted due to missingness. Log-rank probability measure for the equality of these curves is 2.640578e-02. Additional annotations: *.GeneName*=SERPINA1, *.GeneDesc*=serpin peptidase inhibitor, clade A (alpha-1 antiproteinase, antitrypsin), member 1 [Source:HGNC Symbol;Acc:8941], *Uniprot/SWISSPROT*=P01009.

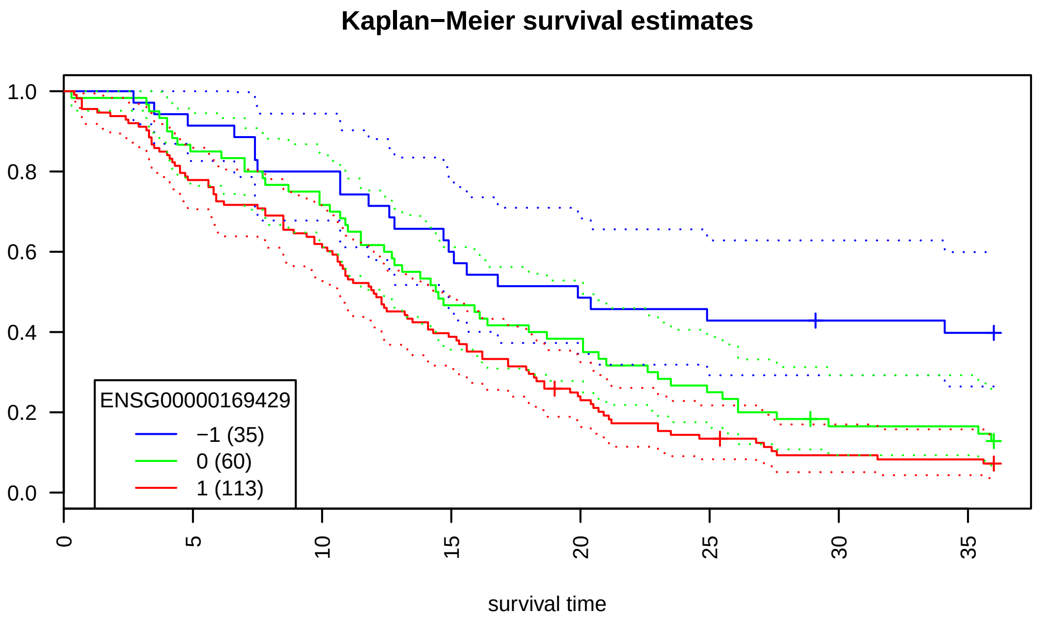

Figure 35: Each curve represents the relative number of ENSG00000169429 survived to the corresponding point of time. Dashed lines represent 0.95 confidence intervals for the curves with the corresponding color. Log-rank probability measure for the equality of these curves is 2.777298e-04. Additional annotations: *.GeneName*=IL8, *.GeneDesc*=interleukin 8 [Source:HGNC Symbol;Acc:6025], *Uniprot/SWISSPROT*=P10145.

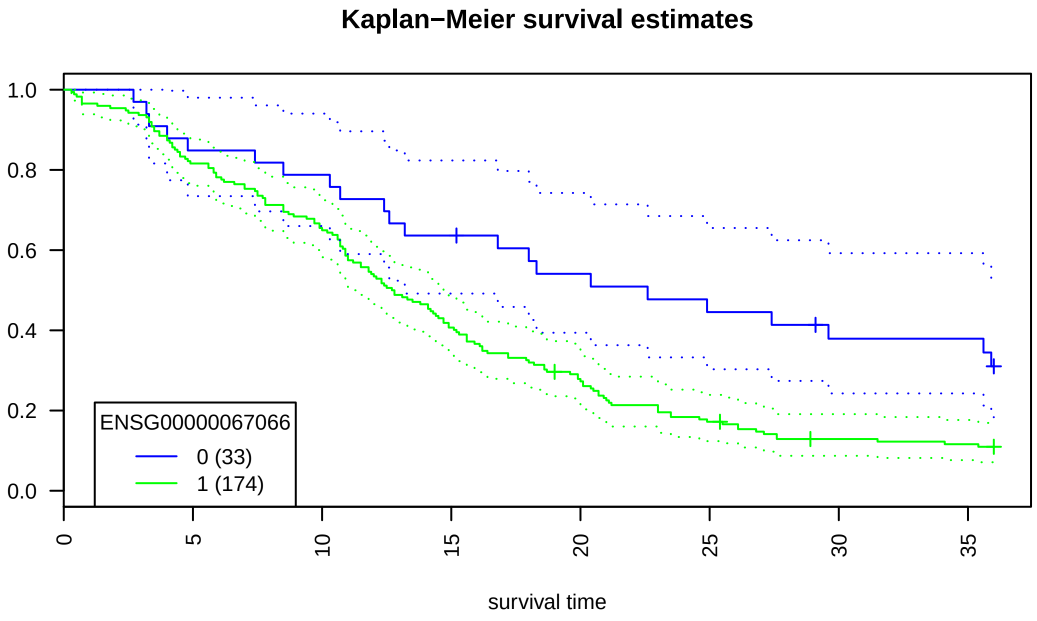

Figure 36: Each curve represents the relative number of ENSG00000067066 survived to the corresponding point of time. Dashed lines represent 0.95 confidence intervals for the curves with the corresponding color. Total of 1 observation deleted due to missingness. Log-rank probability measure for the equality of these curves is 2.592053e-03. Additional annotations: *.GeneName*=SP100, *.GeneDesc*=SP100 nuclear antigen [Source:HGNC Symbol;Acc:11206], *Uniprot/SWISSPROT*=P23497.

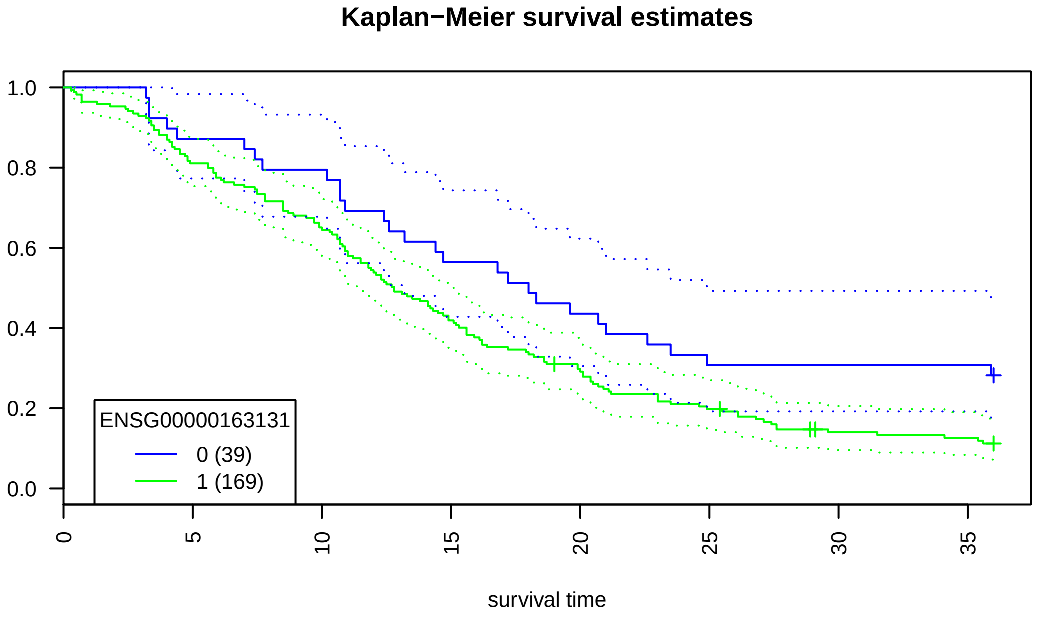

Figure 37: Each curve represents the relative number of ENSG00000163131 survived to the corresponding point of time. Dashed lines represent 0.95 confidence intervals for the curves with the corresponding color. Log-rank probability measure for the equality of these curves is 1.700268e-02. Additional annotations: *.GeneName*=CTSS, *.GeneDesc*=cathepsin S [Source:HGNC Symbol;Acc:2545], *Uniprot/SWISSPROT*=P25774.

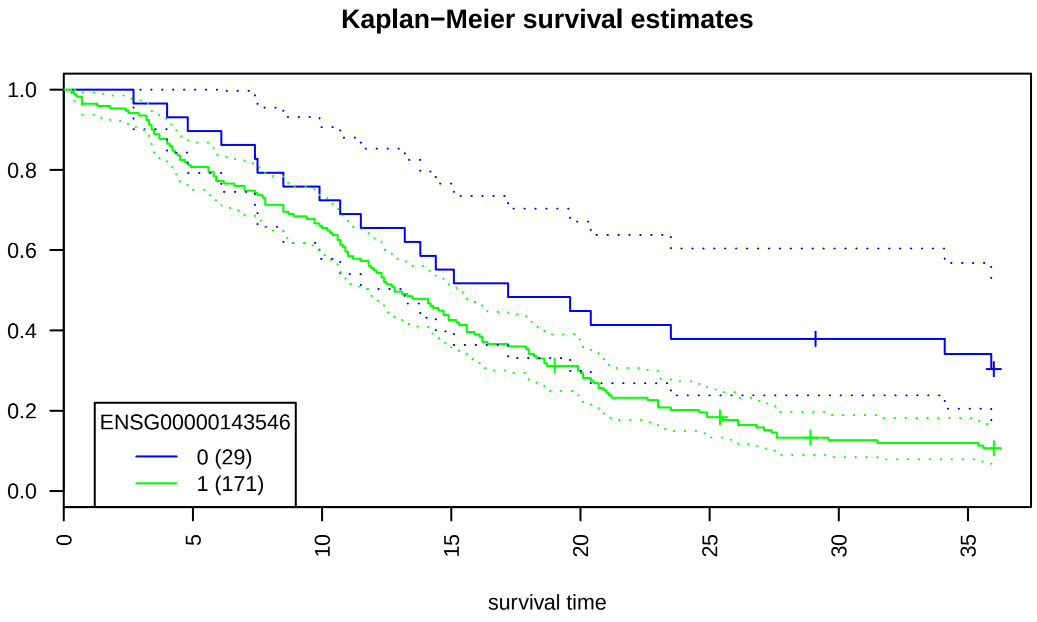

Figure 38: Each curve represents the relative number of ENSG00000143546 survived to the corresponding point of time. Dashed lines represent 0.95 confidence intervals for the curves with the corresponding color. Total of 8 observations deleted due to missingness. Log-rank probability measure for the equality of these curves is 1.771979e-02. Additional annotations: *.GeneName*=S100A8, *.GeneDesc*=S100 calcium binding protein A8 [Source:HGNC Symbol;Acc:10498], *Uniprot/SWISSPROT*=P05109.

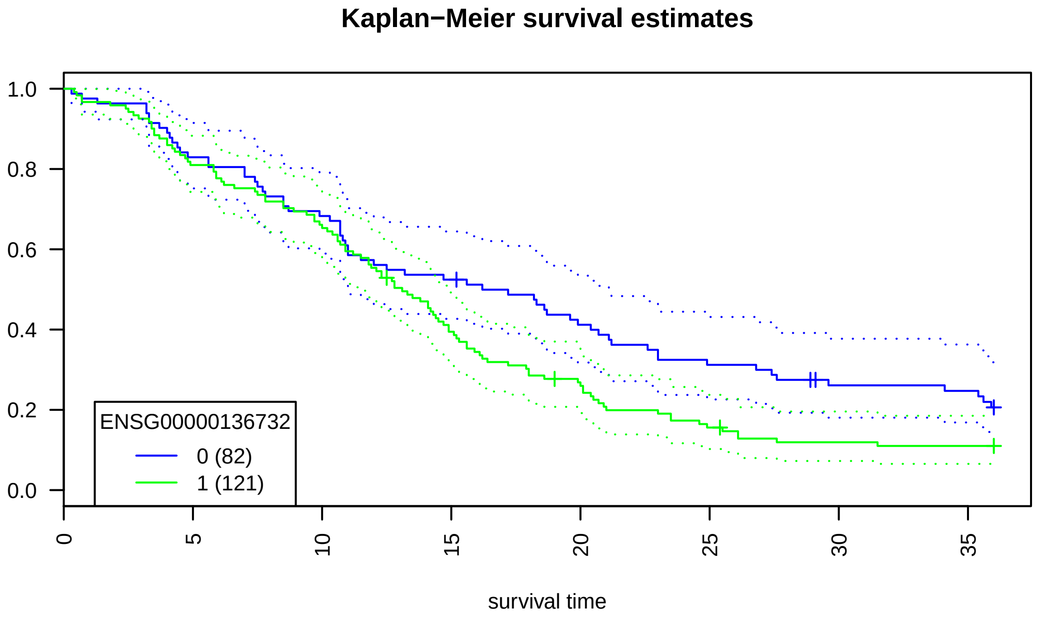

Figure 39: Each curve represents the relative number of ENSG00000136732 survived to the corresponding point of time. Dashed lines represent 0.95 confidence intervals for the curves with the corresponding color. Total of 5 observations deleted due to missingness. Log-rank probability measure for the equality of these curves is 2.767673e-02. Additional annotations: *.GeneName*=GYPC, *.GeneDesc*=glycophorin C (Gerbich blood group) [Source:HGNC Symbol;Acc:4704], *Uniprot/SWISSPROT*=P04921.

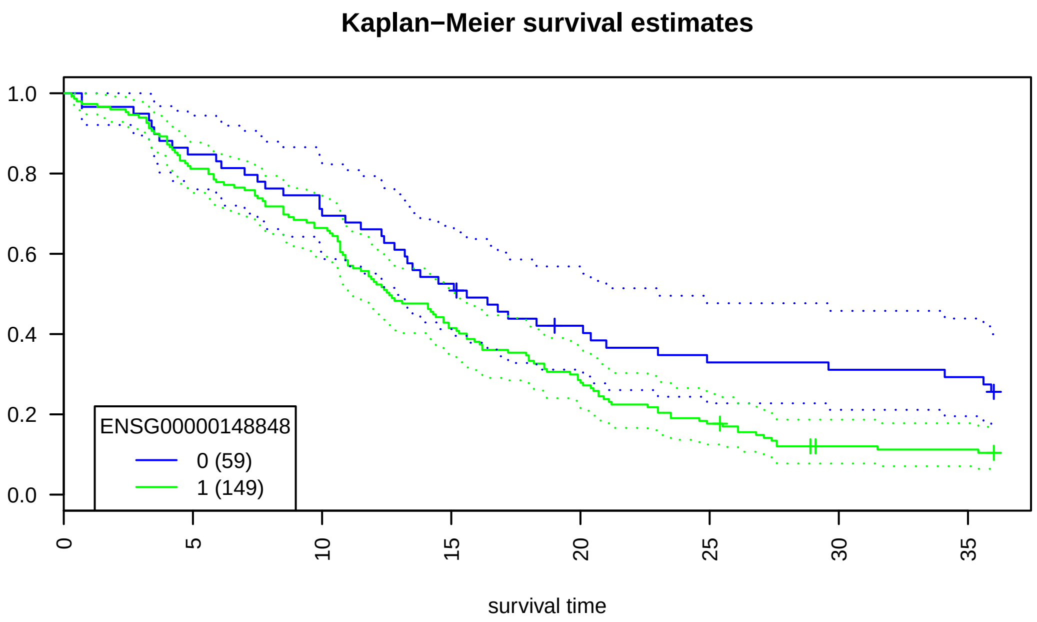

Figure 40: Each curve represents the relative number of ENSG00000148848 survived to the corresponding point of time. Dashed lines represent 0.95 confidence intervals for the curves with the corresponding color. Log-rank probability measure for the equality of these curves is 1.495896e-02. Additional annotations: *.GeneName*=ADAM12, *.GeneDesc*=ADAM metallopeptidase domain 12 [Source:HGNC Symbol;Acc:190], *Uniprot/SWISSPROT*=O43184.

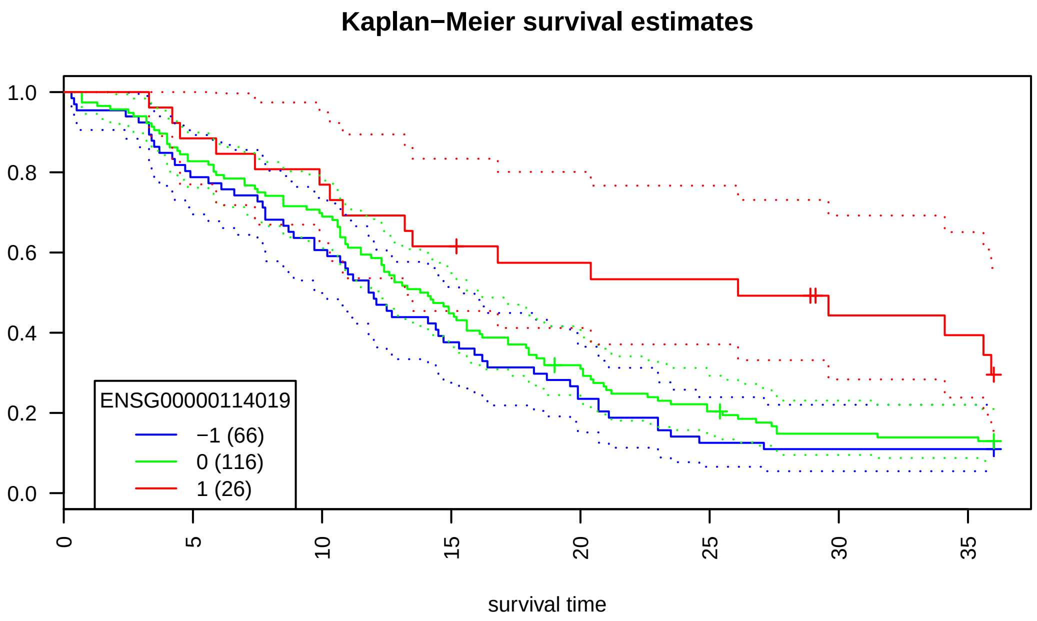

Figure 41: Each curve represents the relative number of ENSG00000114019 survived to the corresponding point of time. Dashed lines represent 0.95 confidence intervals for the curves with the corresponding color. Log-rank probability measure for the equality of these curves is 1.625997e-02.

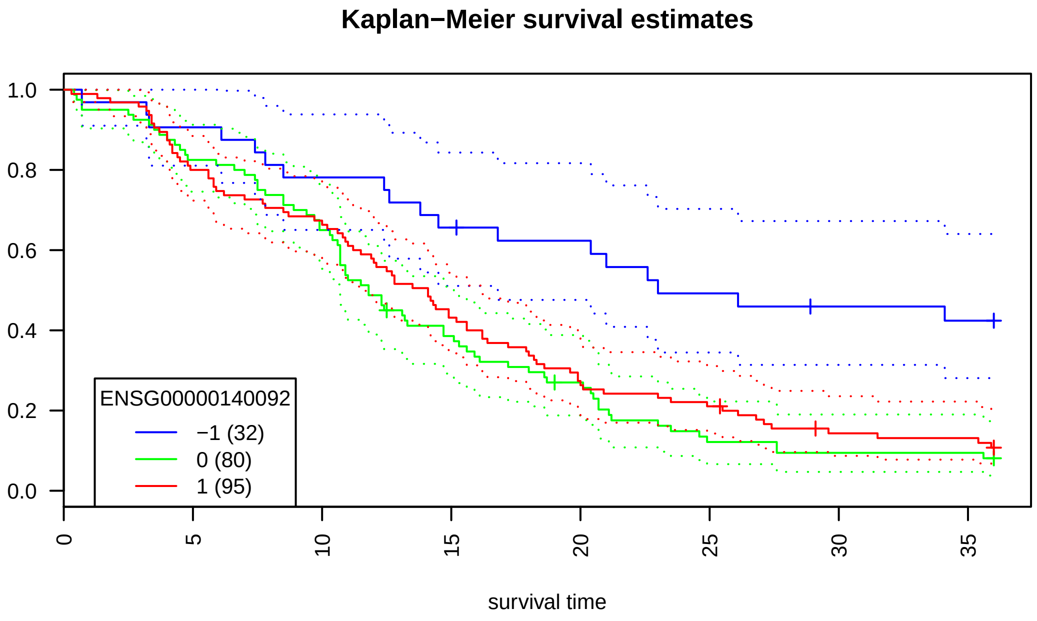

Figure 42: Each curve represents the relative number of ENSG00000140092 survived to the corresponding point of time. Dashed lines represent 0.95 confidence intervals for the curves with the corresponding color. Total of 1 observation deleted due to missingness. Log-rank probability measure for the equality of these curves is 4.096935e-04.

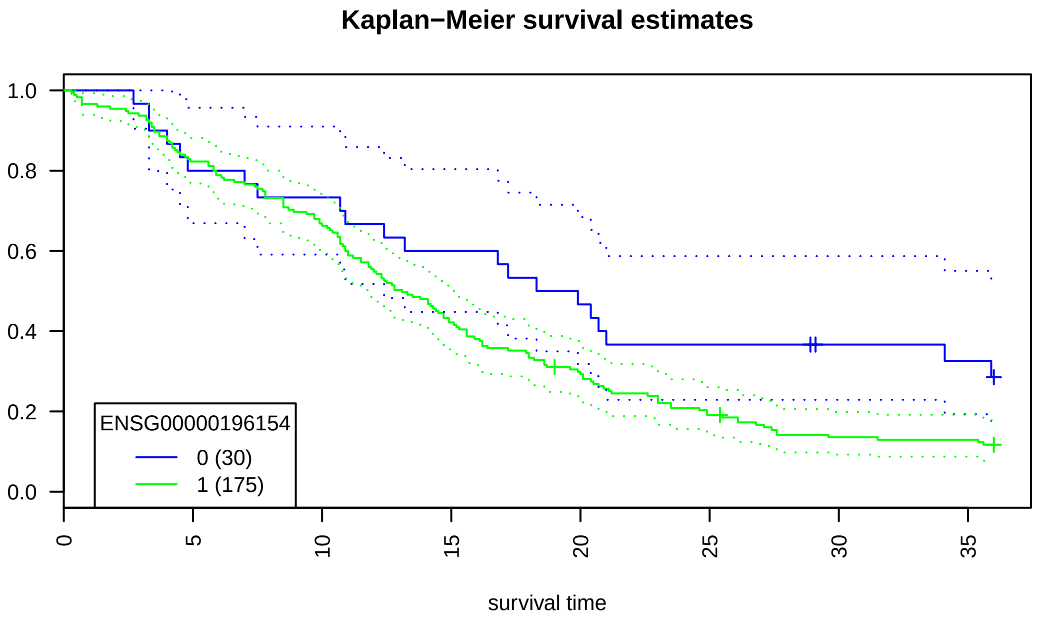

Figure 43: Each curve represents the relative number of ENSG00000196154 survived to the corresponding point of time. Dashed lines represent 0.95 confidence intervals for the curves with the corresponding color. Total of 3 observations deleted due to missingness. Log-rank probability measure for the equality of these curves is 3.056831e-02. Additional annotations: *.GeneName*=S100A4, *.GeneDesc*=S100 calcium binding protein A4 [Source:HGNC Symbol;Acc:10494], *Uniprot/SWISSPROT*=P26447.

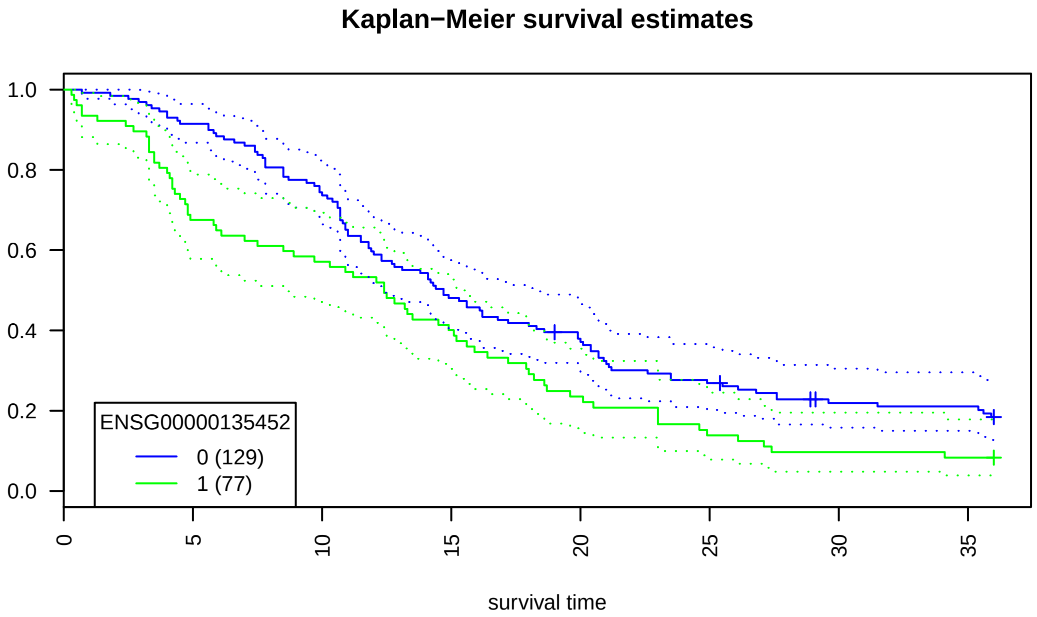

Figure 44: Each curve represents the relative number of ENSG00000135452 survived to the corresponding point of time. Dashed lines represent 0.95 confidence intervals for the curves with the corresponding color. Total of 2 observations deleted due to missingness. Log-rank probability measure for the equality of these curves is 7.820589e-03. Additional annotations: *.GeneName*=TSPAN31, *.GeneDesc*=tetraspanin 31 [Source:HGNC Symbol;Acc:10539], *Uniprot/SWISSPROT*=Q12999.

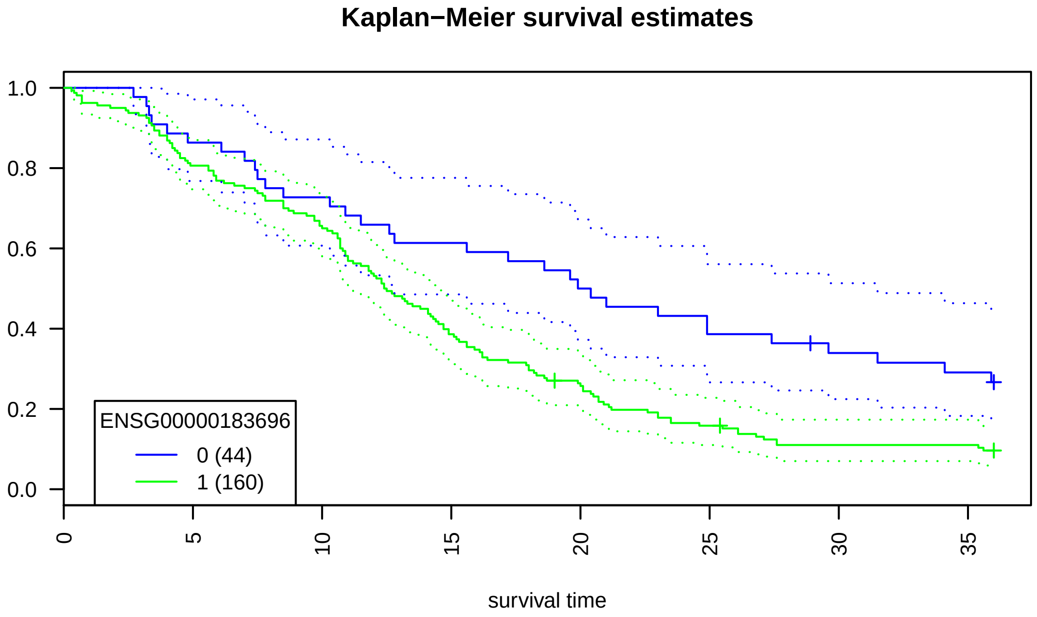

Figure 45: Each curve represents the relative number of ENSG00000183696 survived to the corresponding point of time. Dashed lines represent 0.95 confidence intervals for the curves with the corresponding color. Total of 4 observations deleted due to missingness. Log-rank probability measure for the equality of these curves is 1.873803e-03. Additional annotations: *.GeneName*=UPP1, *.GeneDesc*=uridine phosphorylase 1 [Source:HGNC Symbol;Acc:12576], *Uniprot/SWISSPROT*=Q16831.

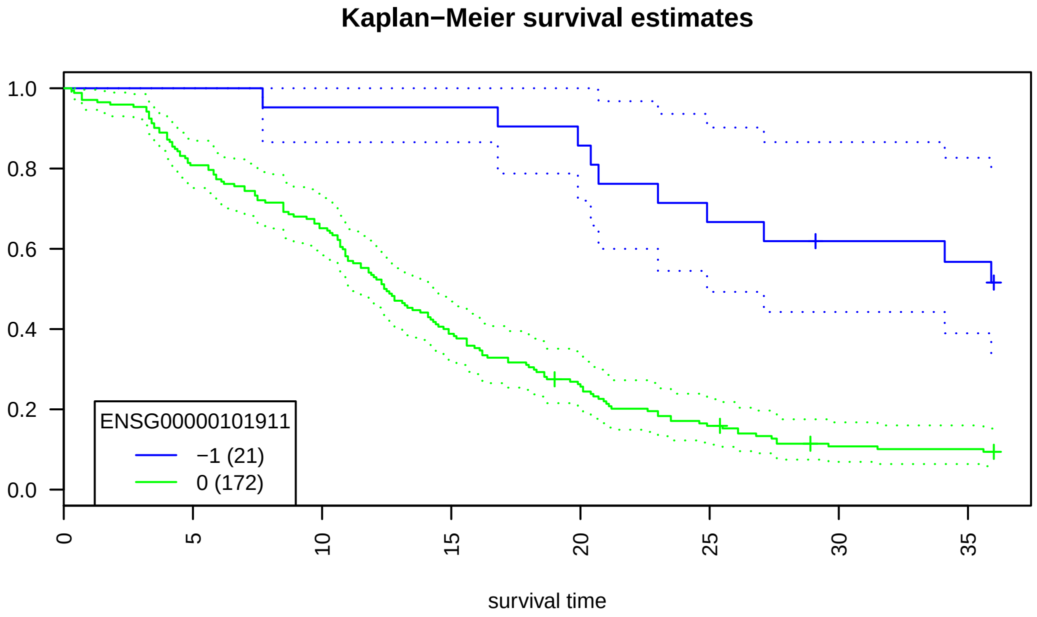

Figure 46: Each curve represents the relative number of ENSG00000101911 survived to the corresponding point of time. Dashed lines represent 0.95 confidence intervals for the curves with the corresponding color. Total of 15 observations deleted due to missingness. Log-rank probability measure for the equality of these curves is 1.695418e-06.

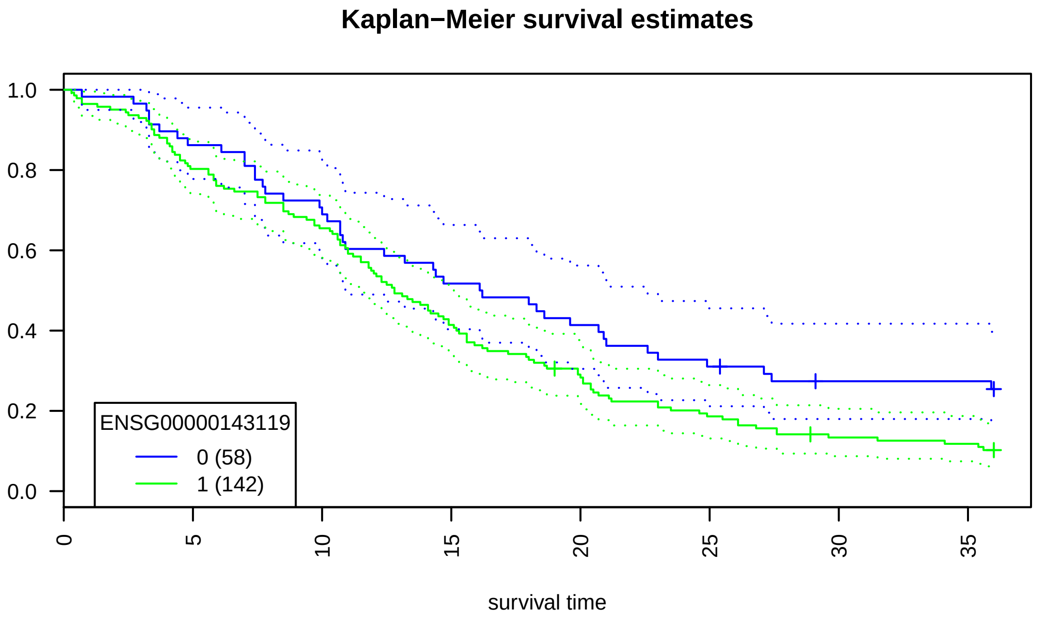

Figure 47: Each curve represents the relative number of ENSG00000143119 survived to the corresponding point of time. Dashed lines represent 0.95 confidence intervals for the curves with the corresponding color. Total of 8 observations deleted due to missingness. Log-rank probability measure for the equality of these curves is 2.326040e-02. Additional annotations: *.GeneName*=CD53, *.GeneDesc*=CD53 molecule [Source:HGNC Symbol;Acc:1686], *Uniprot/SWISSPROT*=P19397.

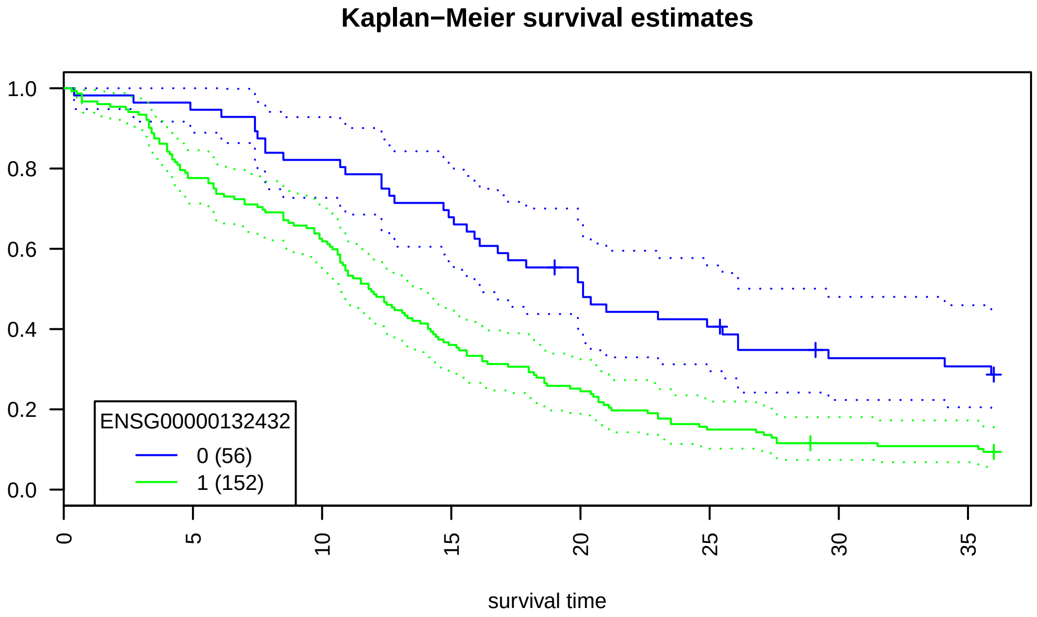

Figure 48: Each curve represents the relative number of ENSG00000132432 survived to the corresponding point of time. Dashed lines represent 0.95 confidence intervals for the curves with the corresponding color. Log-rank probability measure for the equality of these curves is 4.77105e-05. Additional annotations: *.GeneName*=SEC61G, *.GeneDesc*=Sec61 gamma subunit [Source:HGNC Symbol;Acc:18277], *Uniprot/SWISSPROT*=P60059.

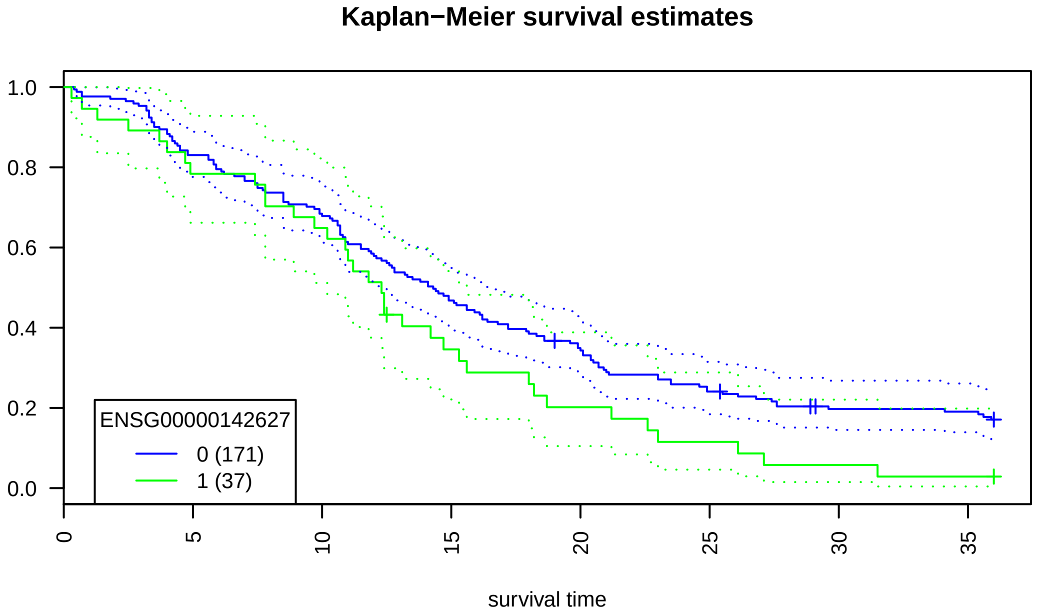

Figure 49: Each curve represents the relative number of ENSG00000142627 survived to the corresponding point of time. Dashed lines represent 0.95 confidence intervals for the curves with the corresponding color. Log-rank probability measure for the equality of these curves is 3.186032e-02.

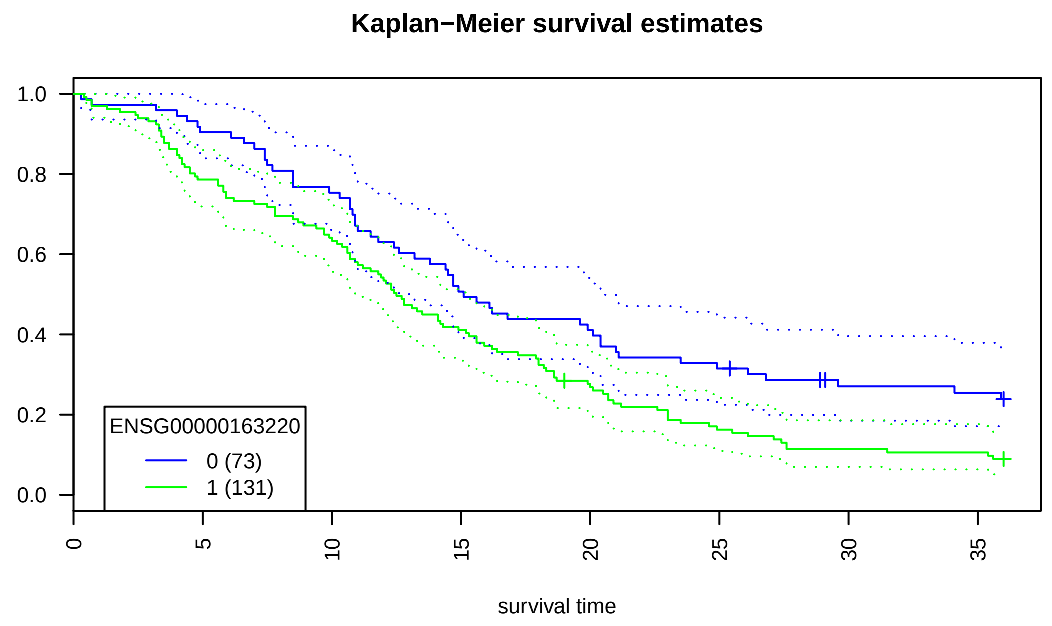

Figure 50: Each curve represents the relative number of ENSG00000163220 survived to the corresponding point of time. Dashed lines represent 0.95 confidence intervals for the curves with the corresponding color. Total of 4 observations deleted due to missingness. Log-rank probability measure for the equality of these curves is 5.482557e-03. Additional annotations: *.GeneName*=S100A9, *.GeneDesc*=S100 calcium binding protein A9 [Source:HGNC Symbol;Acc:10499], *Uniprot/SWISSPROT*=P06702.

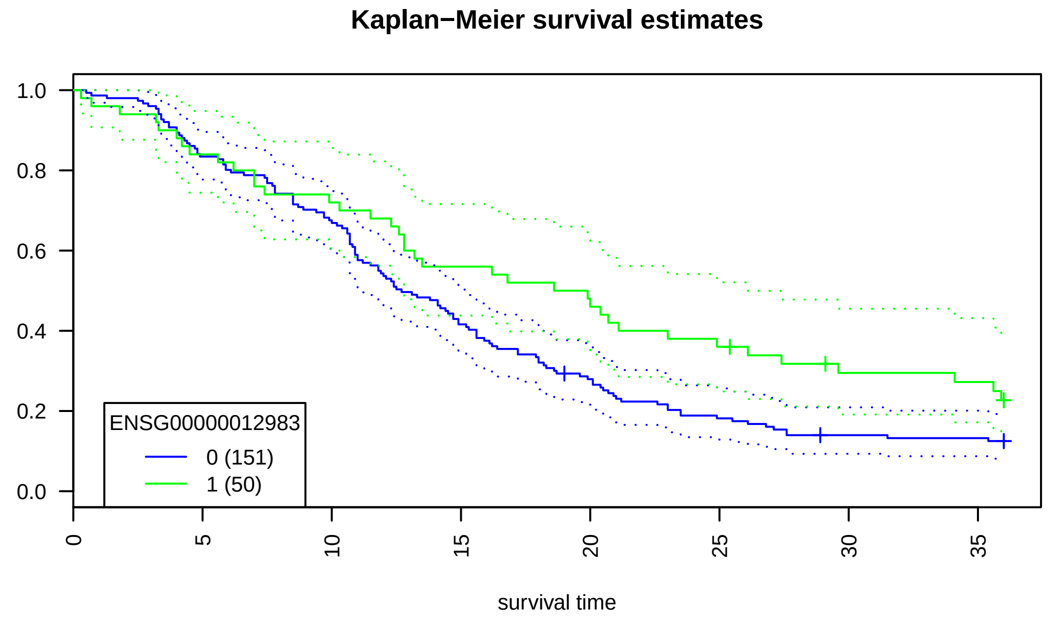

Figure 51: Each curve represents the relative number of ENSG00000012983 survived to the corresponding point of time. Dashed lines represent 0.95 confidence intervals for the curves with the corresponding color. Total of 7 observations deleted due to missingness. Log-rank probability measure for the equality of these curves is 3.155827e-02.

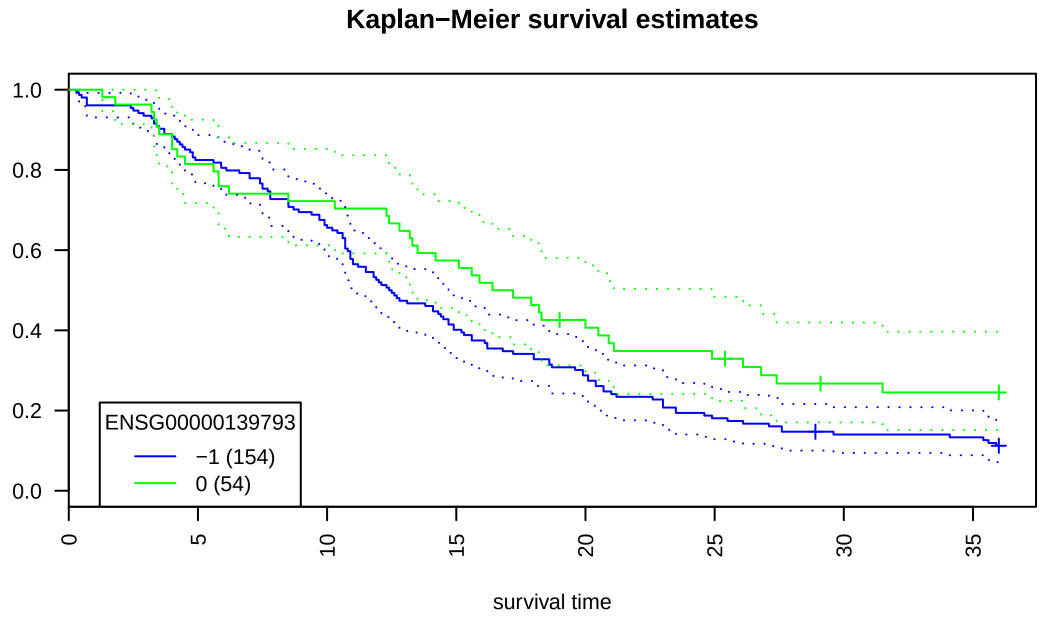

Figure 52: Each curve represents the relative number of ENSG00000139793 survived to the corresponding point of time. Dashed lines represent 0.95 confidence intervals for the curves with the corresponding color. Log-rank probability measure for the equality of these curves is 3.144083e-02. Additional annotations: *.GeneName*=MBNL2, *.GeneDesc*=muscleblind-like 2 (Drosophila) [Source:HGNC Symbol;Acc:16746], *Uniprot/SWISSPROT*=Q5VZF2.

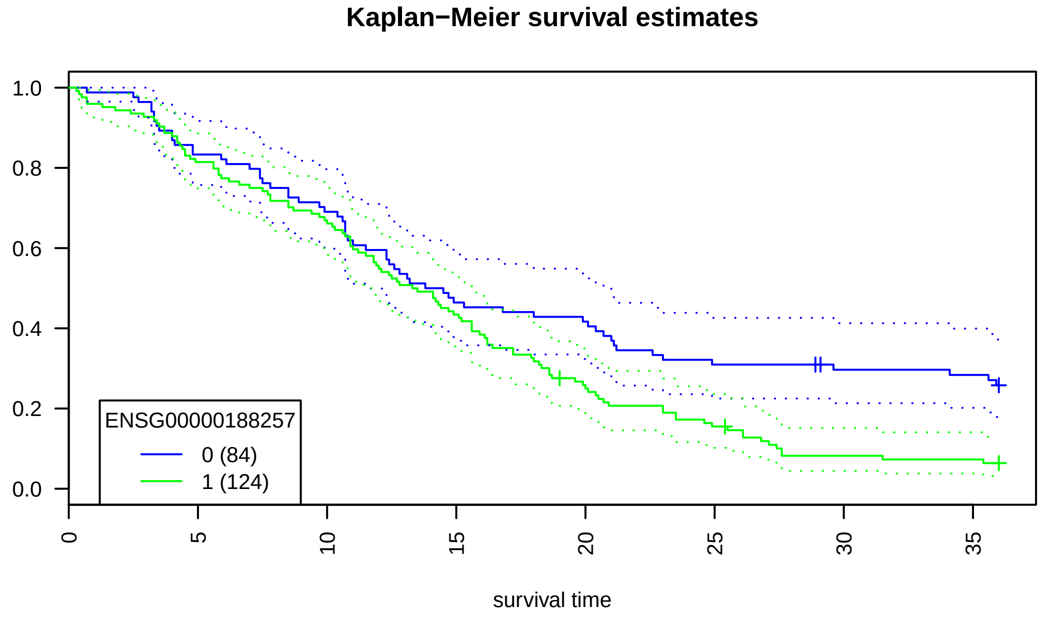

Figure 53: Each curve represents the relative number of ENSG00000188257 survived to the corresponding point of time. Dashed lines represent 0.95 confidence intervals for the curves with the corresponding color. Log-rank probability measure for the equality of these curves is 3.925003e-03. Additional annotations: *.GeneName*=PLA2G2A, *.GeneDesc*=phospholipase A2, group IIA (platelets, synovial fluid) [Source:HGNC Symbol;Acc:9031], *Uniprot/SWISSPROT*=P14555.

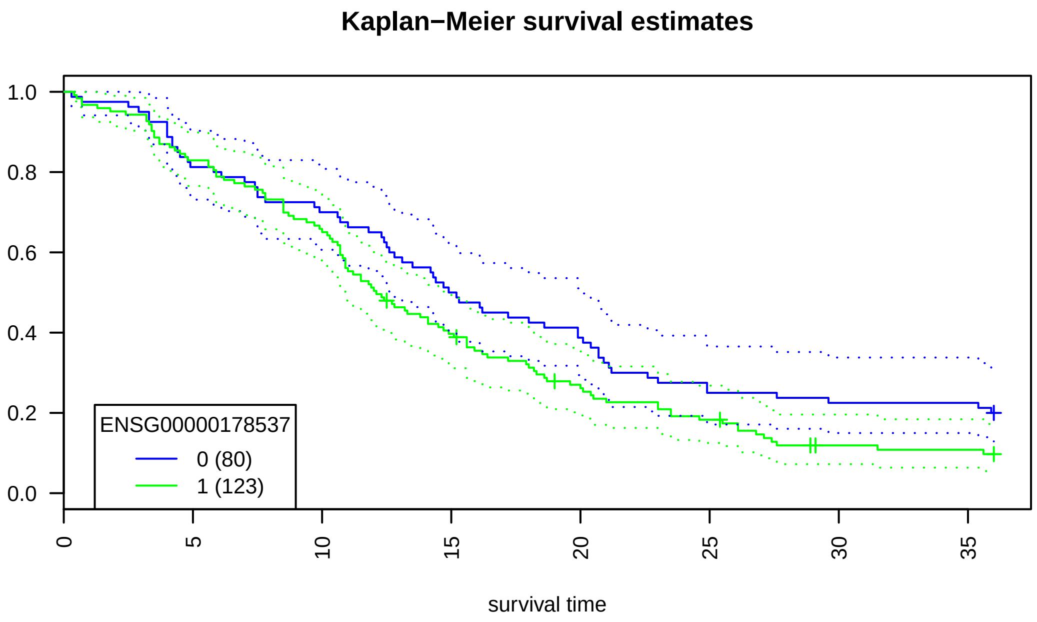

Figure 54: Each curve represents the relative number of ENSG00000178537 survived to the corresponding point of time. Dashed lines represent 0.95 confidence intervals for the curves with the corresponding color. Total of 5 observations deleted due to missingness. Log-rank probability measure for the equality of these curves is 4.434828e-02. Additional annotations: *.GeneName*=SLC25A20, *.GeneDesc*=solute carrier family 25 (carnitine/acylcarnitine translocase), member 20 [Source:HGNC Symbol;Acc:1421], *Uniprot/SWISSPROT*=O43772.

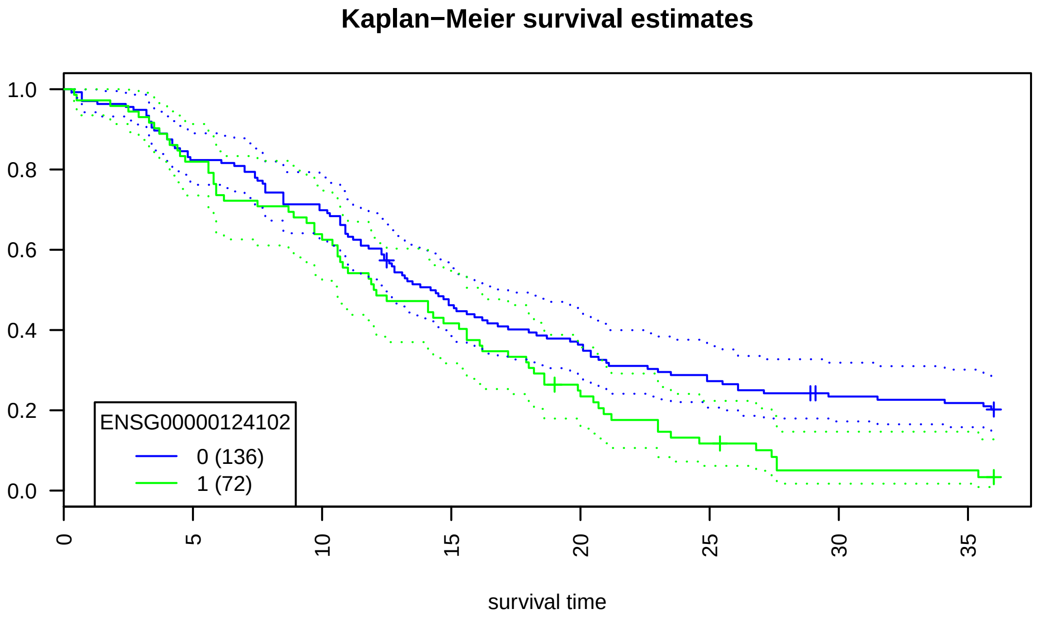

Figure 55: Each curve represents the relative number of ENSG00000124102 survived to the corresponding point of time. Dashed lines represent 0.95 confidence intervals for the curves with the corresponding color. Log-rank probability measure for the equality of these curves is 7.975771e-03. Additional annotations: *.GeneName*=PI3, *.GeneDesc*=peptidase inhibitor 3, skin-derived [Source:HGNC Symbol;Acc:8947], *Uniprot/SWISSPROT*=P19957.

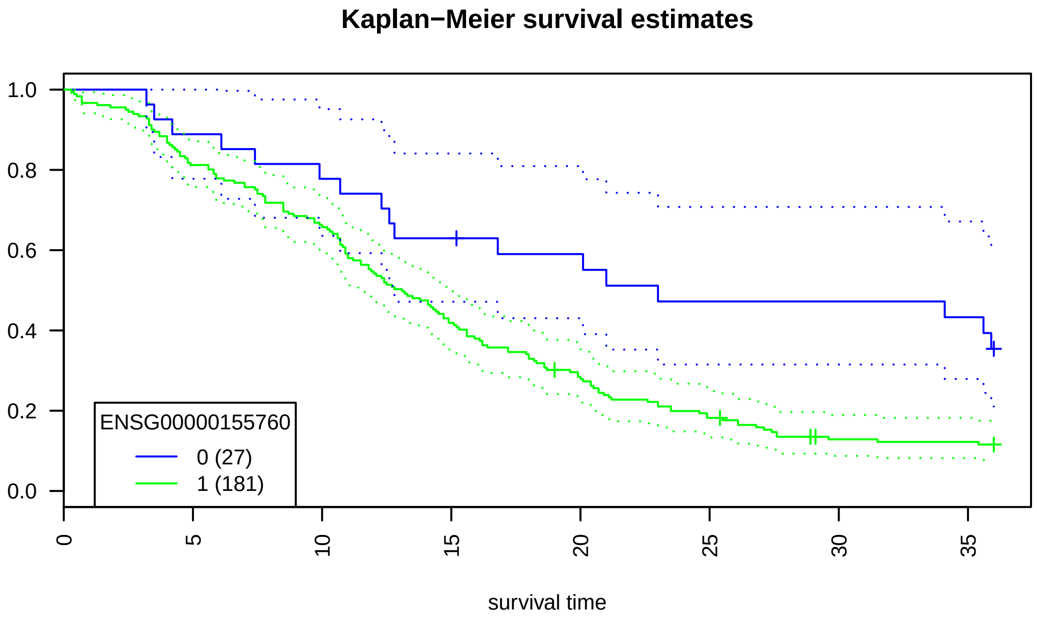

Figure 56: Each curve represents the relative number of ENSG00000155760 survived to the corresponding point of time. Dashed lines represent 0.95 confidence intervals for the curves with the corresponding color. Log-rank probability measure for the equality of these curves is 3.148372e-03. Additional annotations: *.GeneName*=FZD7, *.GeneDesc*=frizzled homolog 7 (Drosophila) [Source:HGNC Symbol;Acc:4045], *Uniprot/SWISSPROT*=O75084.

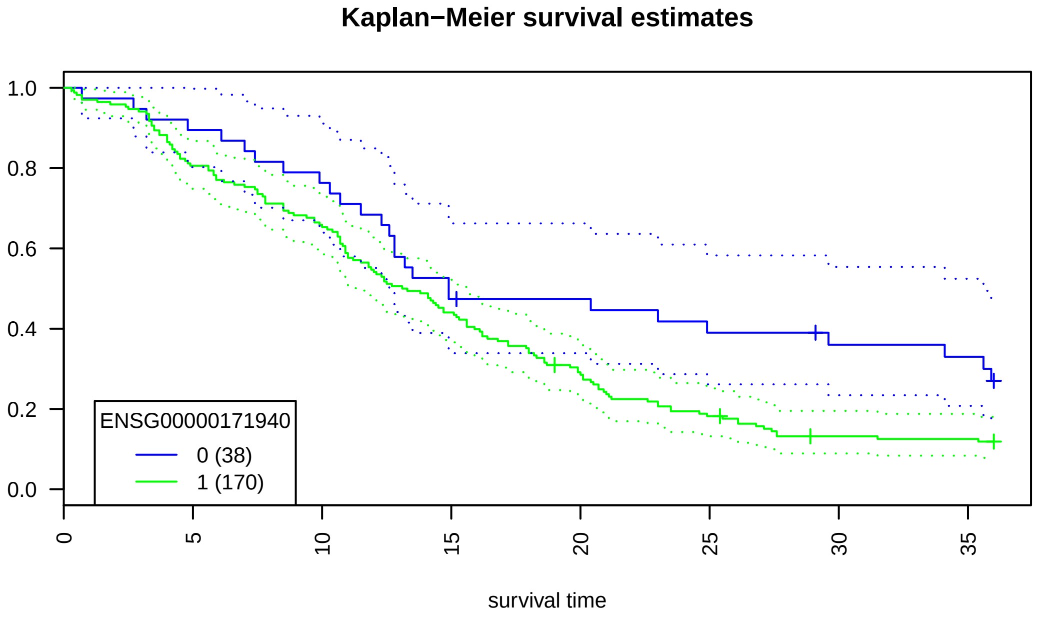

Figure 57: Each curve represents the relative number of ENSG00000171940 survived to the corresponding point of time. Dashed lines represent 0.95 confidence intervals for the curves with the corresponding color. Log-rank probability measure for the equality of these curves is 1.982926e-02. Additional annotations: *.GeneName*=ZNF217, *.GeneDesc*=zinc finger protein 217 [Source:HGNC Symbol;Acc:13009], *Uniprot/SWISSPROT*=O75362.

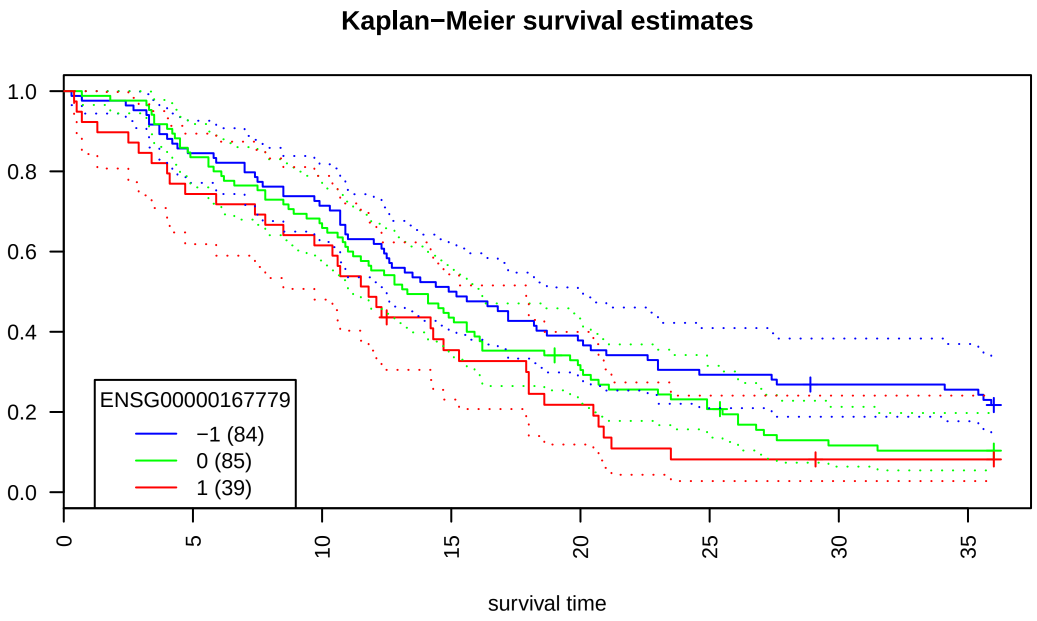

Figure 58: Each curve represents the relative number of ENSG00000167779 survived to the corresponding point of time. Dashed lines represent 0.95 confidence intervals for the curves with the corresponding color. Log-rank probability measure for the equality of these curves is 3.479069e-02.

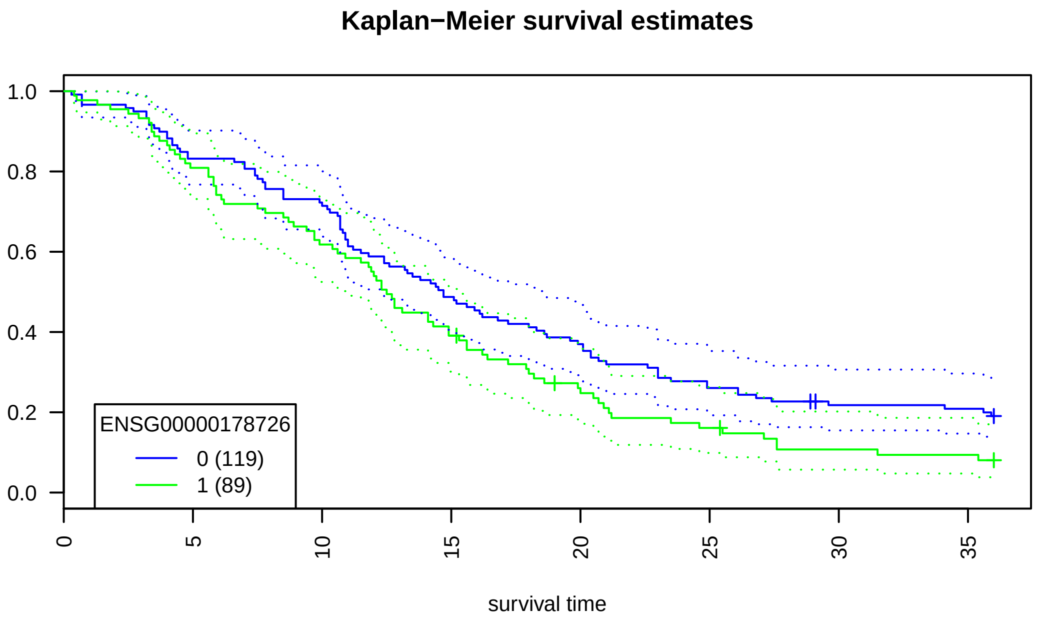

Figure 59: Each curve represents the relative number of ENSG00000178726 survived to the corresponding point of time. Dashed lines represent 0.95 confidence intervals for the curves with the corresponding color. Log-rank probability measure for the equality of these curves is 3.191867e-02.

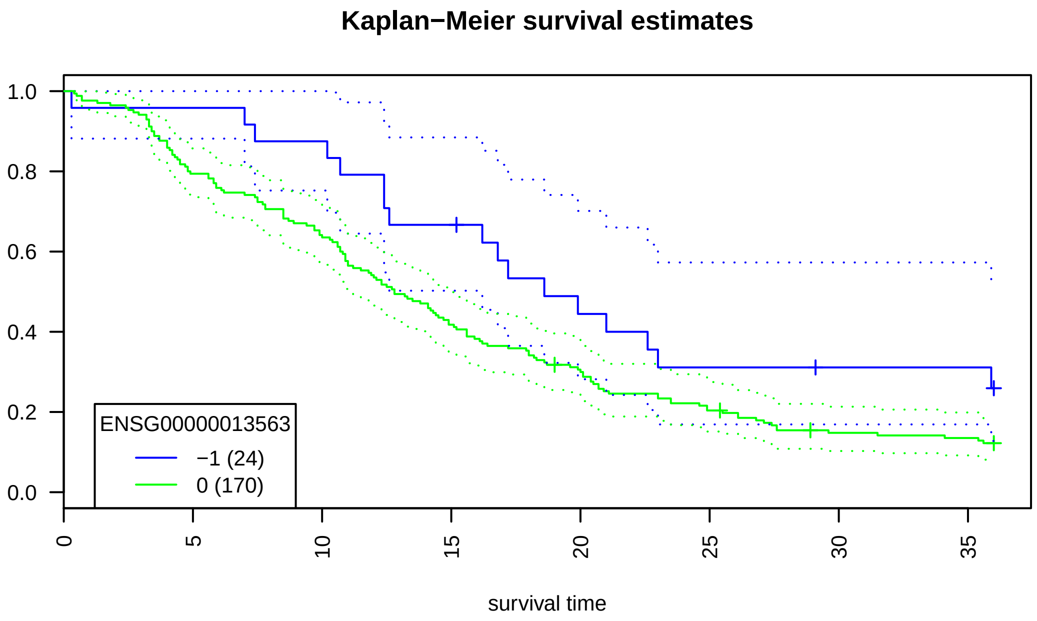

Figure 60: Each curve represents the relative number of ENSG00000013563 survived to the corresponding point of time. Dashed lines represent 0.95 confidence intervals for the curves with the corresponding color. Total of 14 observations deleted due to missingness. Log-rank probability measure for the equality of these curves is 4.219608e-02.

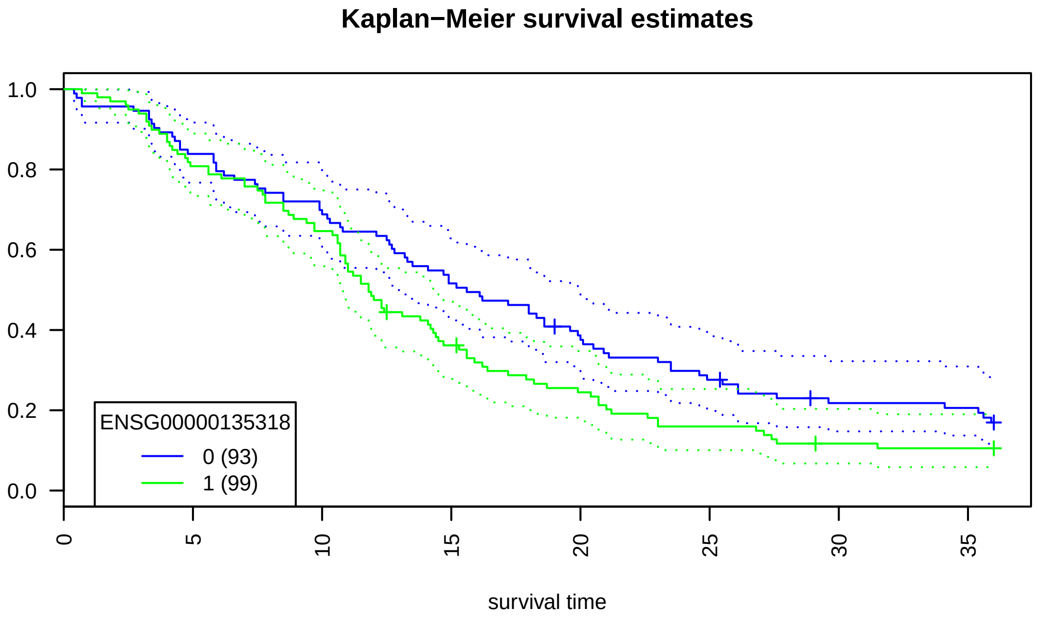

Figure 61: Each curve represents the relative number of ENSG00000135318 survived to the corresponding point of time. Dashed lines represent 0.95 confidence intervals for the curves with the corresponding color. Total of 16 observations deleted due to missingness. Log-rank probability measure for the equality of these curves is 3.709674e-02.

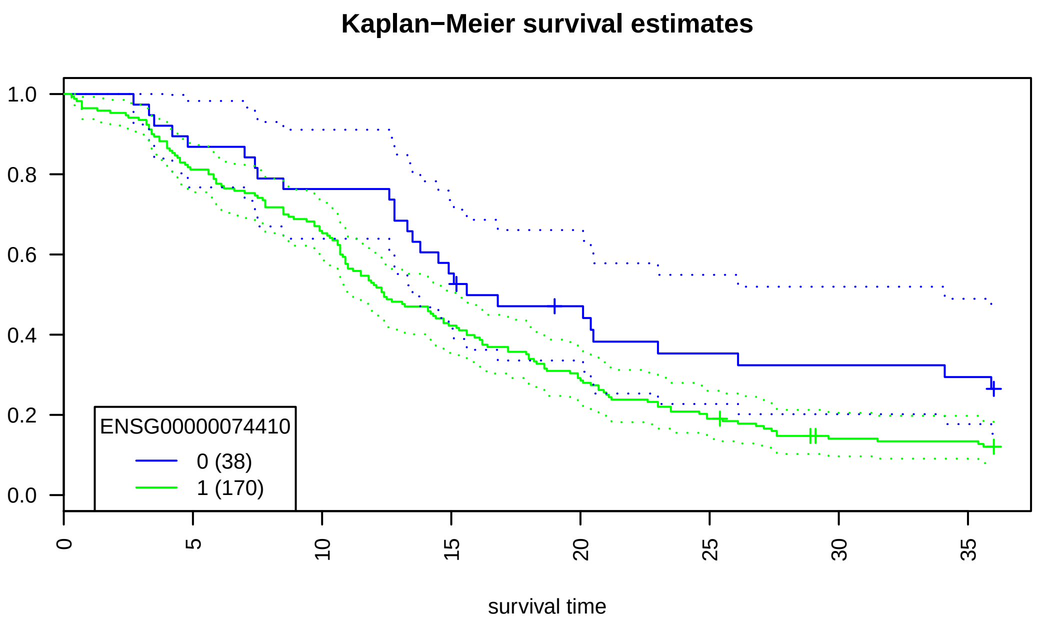

Figure 62: Each curve represents the relative number of ENSG00000074410 survived to the corresponding point of time. Dashed lines represent 0.95 confidence intervals for the curves with the corresponding color. Log-rank probability measure for the equality of these curves is 2.594344e-02. Additional annotations: *.GeneName*=CA12, *.GeneDesc*=carbonic anhydrase XII [Source:HGNC Symbol;Acc:1371], *Uniprot/SWISSPROT*=O43570.

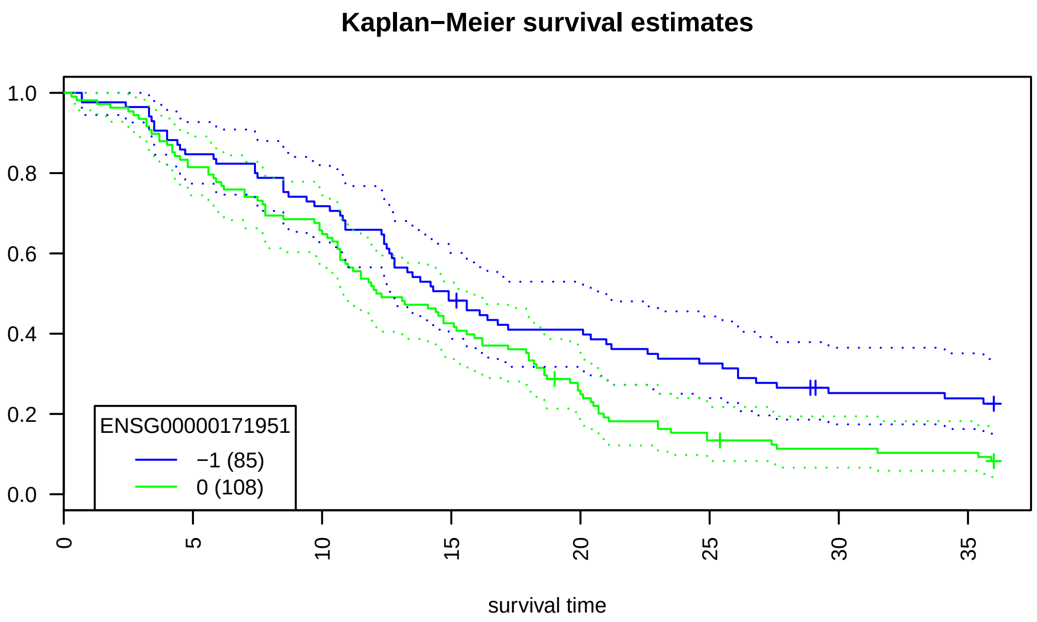

Figure 63: Each curve represents the relative number of ENSG00000171951 survived to the corresponding point of time. Dashed lines represent 0.95 confidence intervals for the curves with the corresponding color. Total of 15 observations deleted due to missingness. Log-rank probability measure for the equality of these curves is 9.255637e-03.

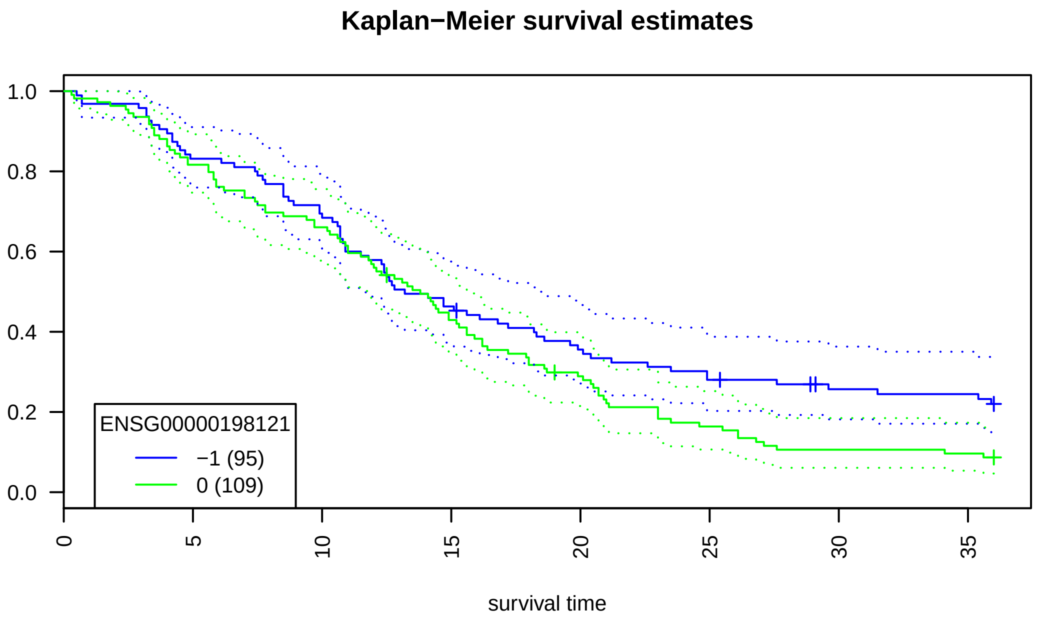

Figure 64: Each curve represents the relative number of ENSG00000198121 survived to the corresponding point of time. Dashed lines represent 0.95 confidence intervals for the curves with the corresponding color. Total of 4 observations deleted due to missingness. Log-rank probability measure for the equality of these curves is 4.511837e-02.

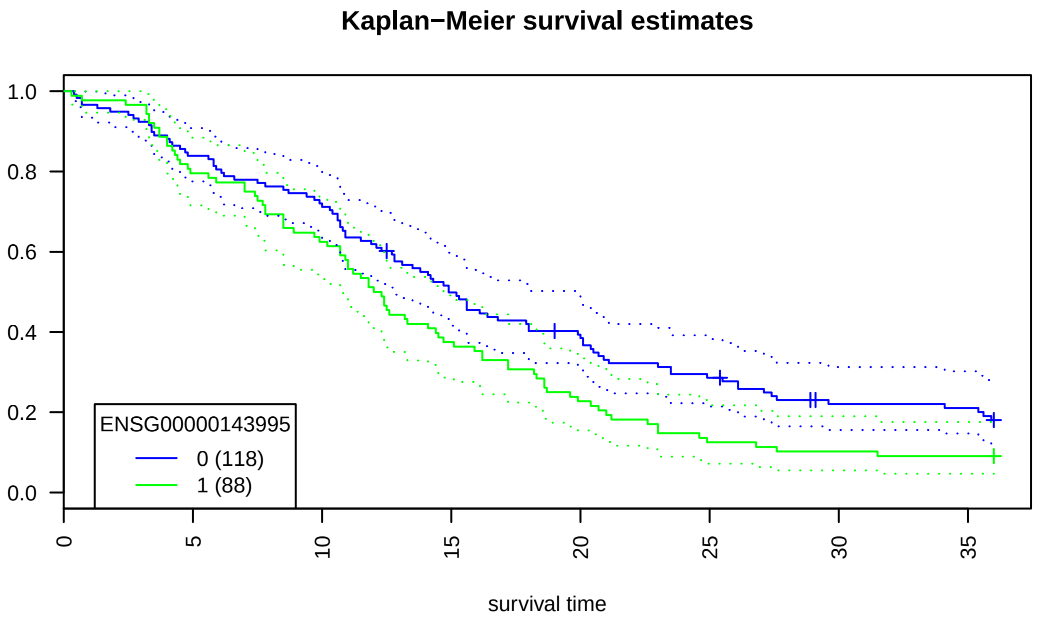

Figure 65: Each curve represents the relative number of ENSG00000143995 survived to the corresponding point of time. Dashed lines represent 0.95 confidence intervals for the curves with the corresponding color. Total of 2 observations deleted due to missingness. Log-rank probability measure for the equality of these curves is 1.940991e-02.

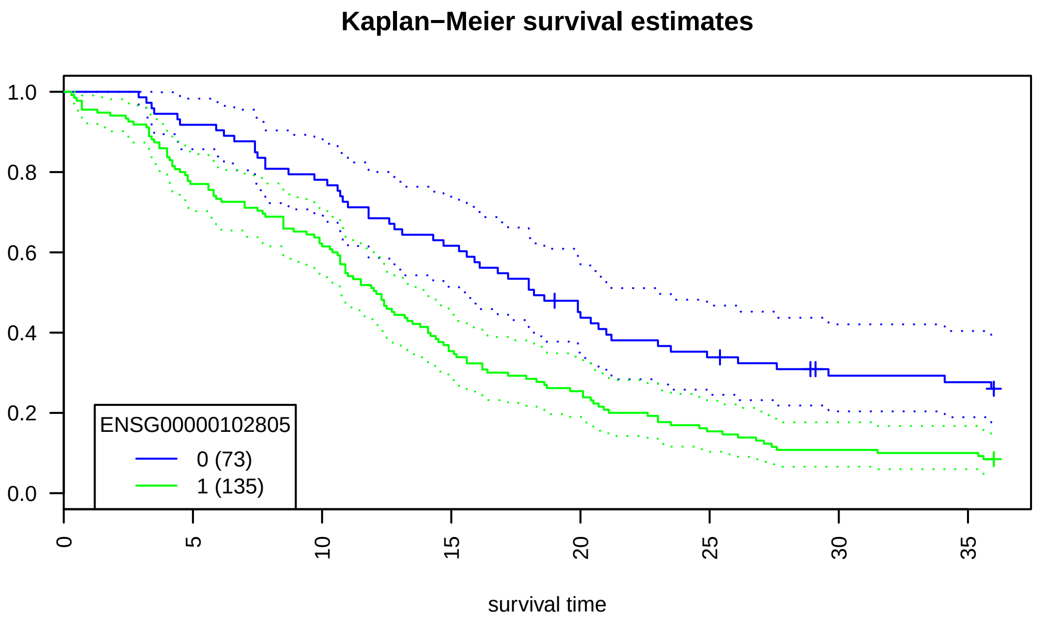

Figure 66: Each curve represents the relative number of ENSG00000102805 survived to the corresponding point of time. Dashed lines represent 0.95 confidence intervals for the curves with the corresponding color. Log-rank probability measure for the equality of these curves is 1.374999e-04. Additional annotations: *.GeneName*=CLN5, *.GeneDesc*=ceroid-lipofuscinosis, neuronal 5 [Source:HGNC Symbol;Acc:2076], *Uniprot/SWISSPROT*=O75503.

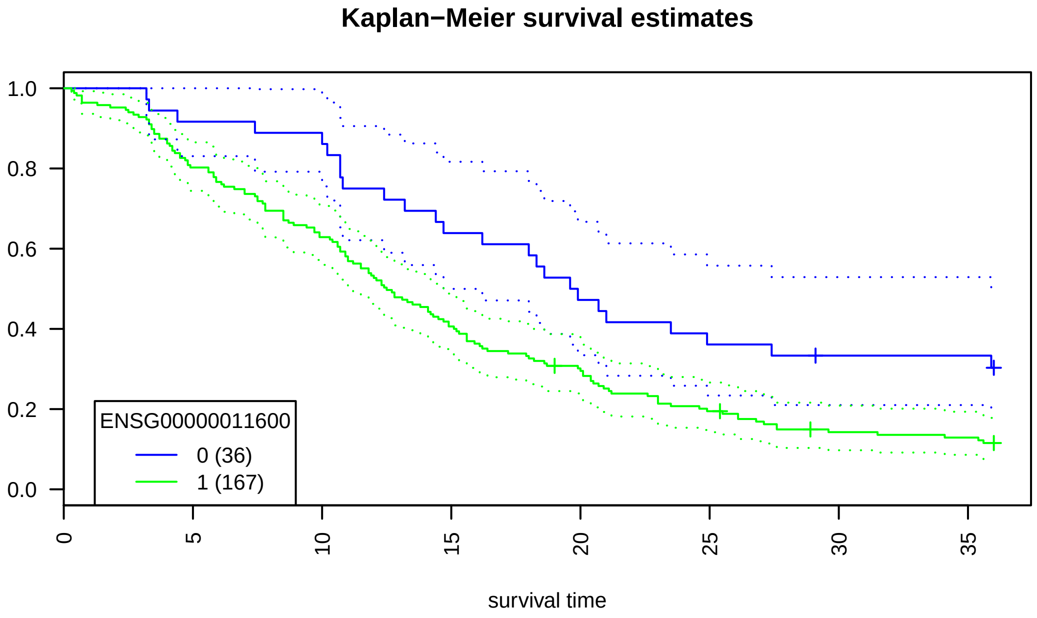

Figure 67: Each curve represents the relative number of ENSG00000011600 survived to the corresponding point of time. Dashed lines represent 0.95 confidence intervals for the curves with the corresponding color. Total of 5 observations deleted due to missingness. Log-rank probability measure for the equality of these curves is 3.700832e-03. Additional annotations: *.GeneName*=TYROBP, *.GeneDesc*=TYRO protein tyrosine kinase binding protein [Source:HGNC Symbol;Acc:12449], *Uniprot/SWISSPROT*=O43914.

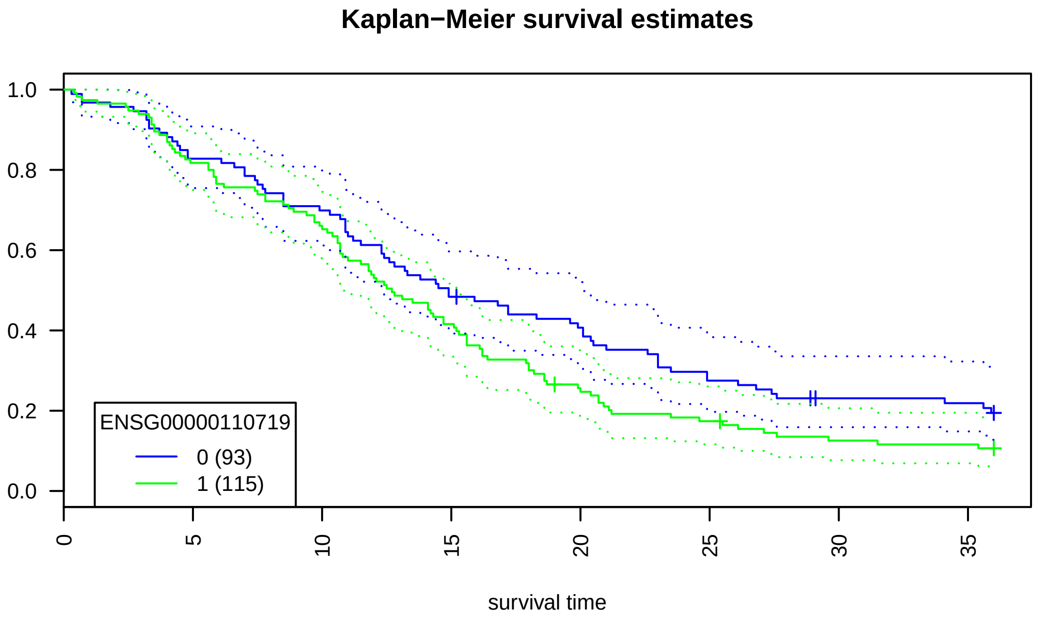

Figure 68: Each curve represents the relative number of ENSG00000110719 survived to the corresponding point of time. Dashed lines represent 0.95 confidence intervals for the curves with the corresponding color. Log-rank probability measure for the equality of these curves is 4.6785e-02. Additional annotations: *.GeneName*=TCIRG1, *.GeneDesc*=T-cell, immune regulator 1, ATPase, H+ transporting, lysosomal V0 subunit A3 [Source:HGNC Symbol;Acc:11647], *Uniprot/SWISSPROT*=Q13488.

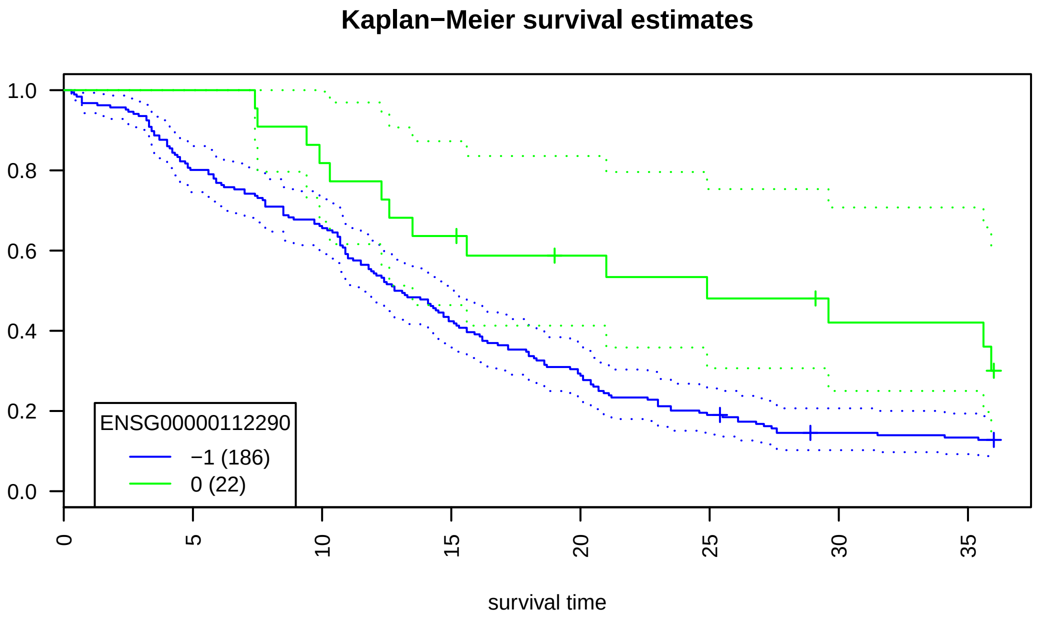

Figure 69: Each curve represents the relative number of ENSG00000112290 survived to the corresponding point of time. Dashed lines represent 0.95 confidence intervals for the curves with the corresponding color. Log-rank probability measure for the equality of these curves is 1.068366e-02. Additional annotations: *.GeneName*=WASF1, *.GeneDesc*=WAS protein family, member 1 [Source:HGNC Symbol;Acc:12732], *Uniprot/SWISSPROT*=Q92558.

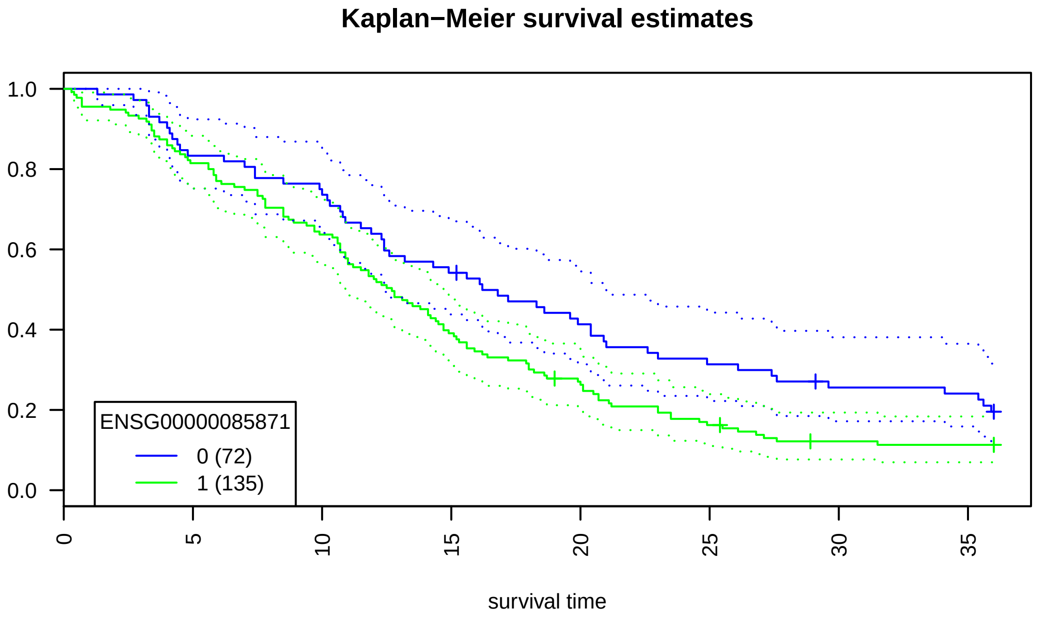

Figure 70: Each curve represents the relative number of ENSG00000085871 survived to the corresponding point of time. Dashed lines represent 0.95 confidence intervals for the curves with the corresponding color. Total of 1 observation deleted due to missingness. Log-rank probability measure for the equality of these curves is 2.194785e-02. Additional annotations: *.GeneName*=MGST2, *.GeneDesc*=microsomal glutathione S-transferase 2 [Source:HGNC Symbol;Acc:7063], *Uniprot/SWISSPROT*=Q99735.

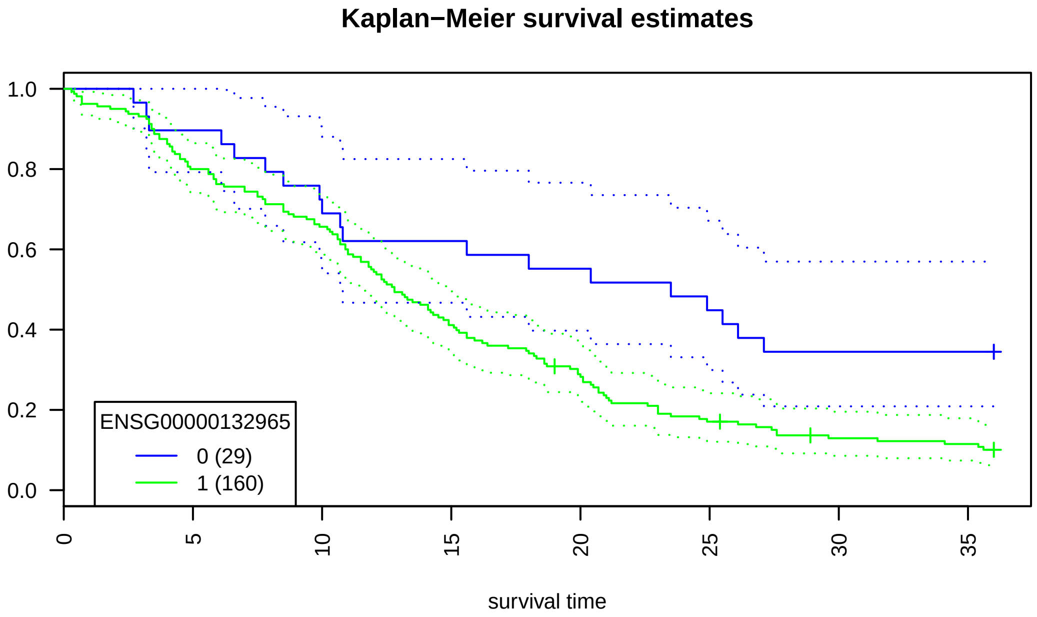

Figure 71: Each curve represents the relative number of ENSG00000132965 survived to the corresponding point of time. Dashed lines represent 0.95 confidence intervals for the curves with the corresponding color. Total of 19 observations deleted due to missingness. Log-rank probability measure for the equality of these curves is 4.362415e-03. Additional annotations: *.GeneName*=ALOX5AP, *.GeneDesc*=arachidonate 5-lipoxygenase-activating protein [Source:HGNC Symbol;Acc:436], *Uniprot/SWISSPROT*=P20292.

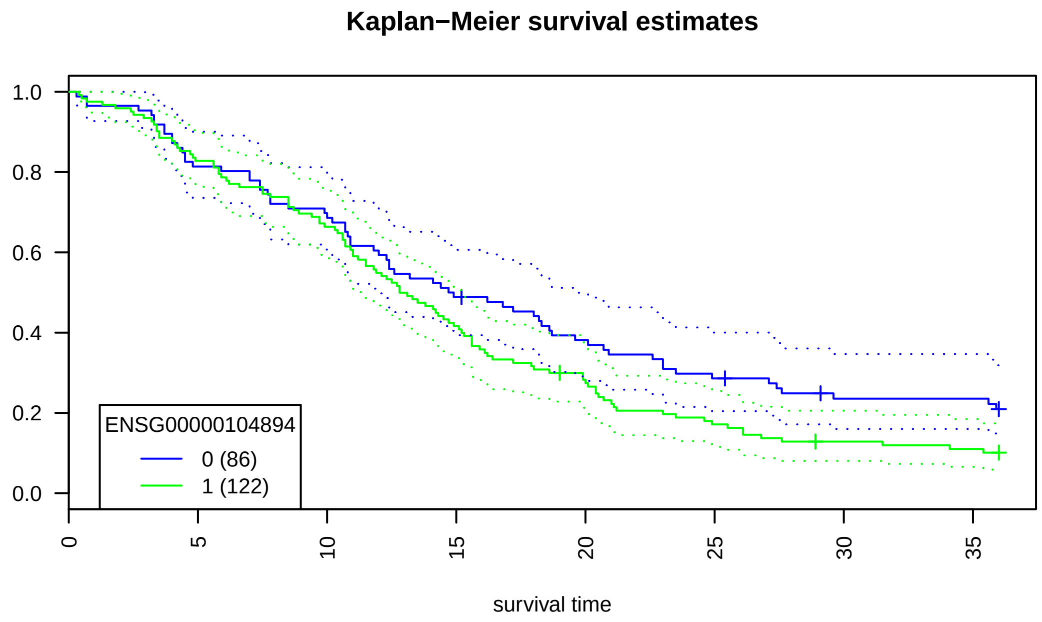

Figure 72: Each curve represents the relative number of ENSG00000104894 survived to the corresponding point of time. Dashed lines represent 0.95 confidence intervals for the curves with the corresponding color. Log-rank probability measure for the equality of these curves is 4.857582e-02. Additional annotations: *.GeneName*=CD37, *.GeneDesc*=CD37 molecule [Source:HGNC Symbol;Acc:1666], *Uniprot/SWISSPROT*=P11049.

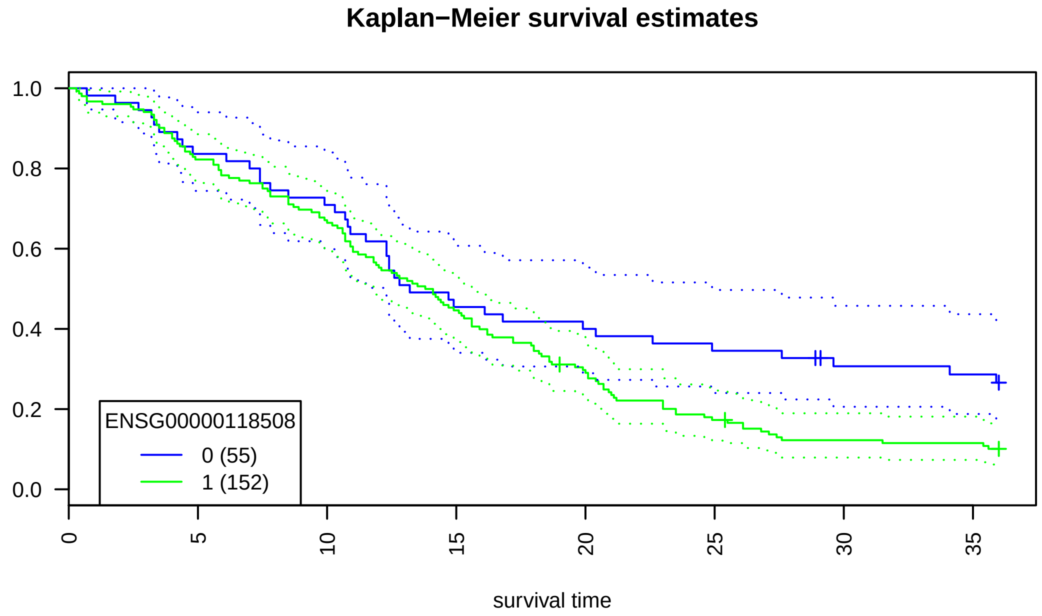

Figure 73: Each curve represents the relative number of ENSG00000118508 survived to the corresponding point of time. Dashed lines represent 0.95 confidence intervals for the curves with the corresponding color. Total of 1 observation deleted due to missingness. Log-rank probability measure for the equality of these curves is 2.681371e-02. Additional annotations: *.GeneName*=RAB32, *.GeneDesc*=RAB32, member RAS oncogene family [Source:HGNC Symbol;Acc:9772], *Uniprot/SWISSPROT*=Q13637.

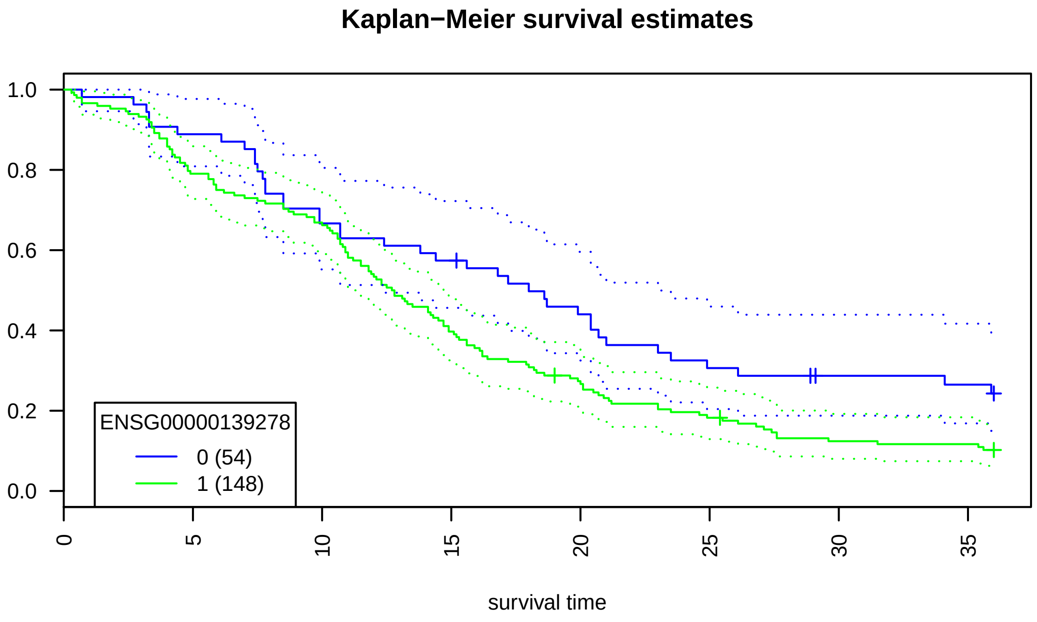

Figure 74: Each curve represents the relative number of ENSG00000139278 survived to the corresponding point of time. Dashed lines represent 0.95 confidence intervals for the curves with the corresponding color. Total of 6 observations deleted due to missingness. Log-rank probability measure for the equality of these curves is 1.389329e-02. Additional annotations: *.GeneName*=GLIPR1, *.GeneDesc*=GLI pathogenesis-related 1 [Source:HGNC Symbol;Acc:17001], *Uniprot/SWISSPROT*=P48060.

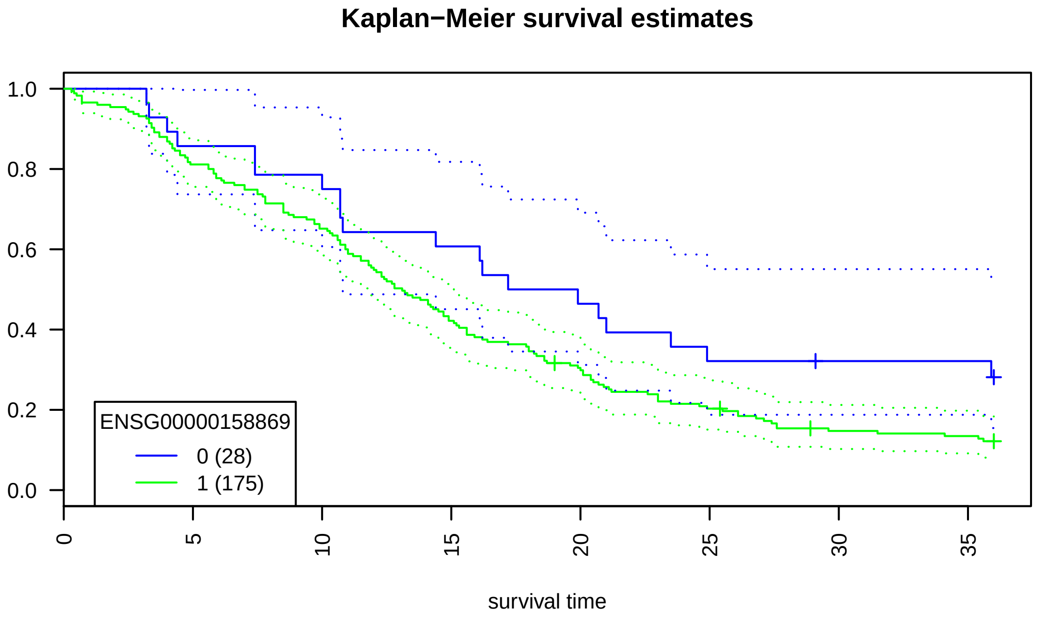

Figure 75: Each curve represents the relative number of ENSG00000158869 survived to the corresponding point of time. Dashed lines represent 0.95 confidence intervals for the curves with the corresponding color. Total of 5 observations deleted due to missingness. Log-rank probability measure for the equality of these curves is 4.385908e-02. Additional annotations: *.GeneName*=FCER1G, *.GeneDesc*=Fc fragment of IgE, high affinity I, receptor for; gamma polypeptide [Source:HGNC Symbol;Acc:3611], *Uniprot/SWISSPROT*=P30273.

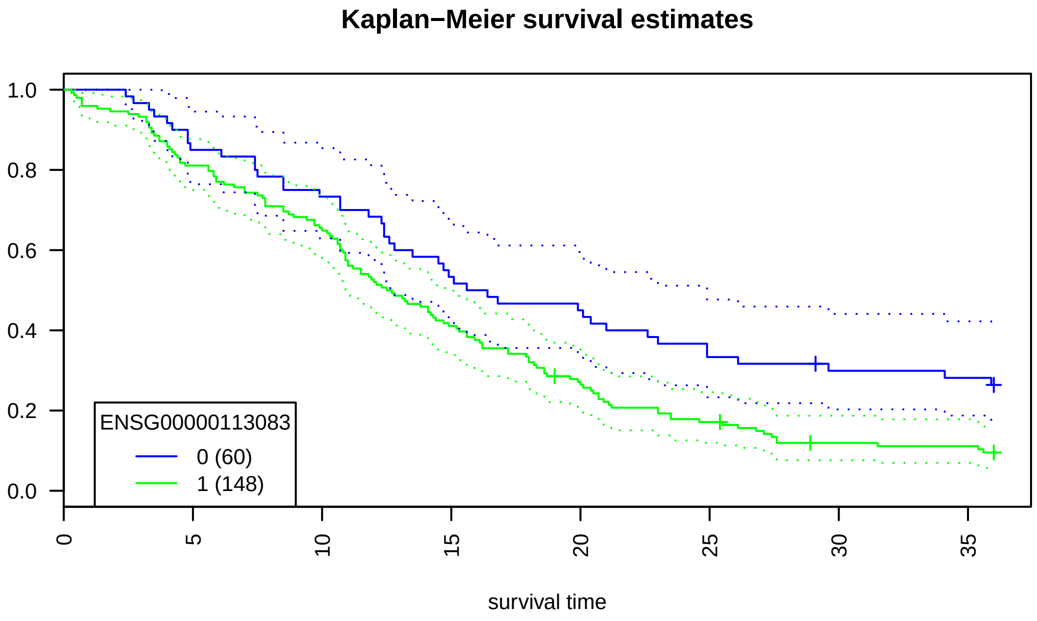

Figure 76: Each curve represents the relative number of ENSG00000113083 survived to the corresponding point of time. Dashed lines represent 0.95 confidence intervals for the curves with the corresponding color. Log-rank probability measure for the equality of these curves is 3.643139e-03. Additional annotations: *.GeneName*=LOX, *.GeneDesc*=lysyl oxidase [Source:HGNC Symbol;Acc:6664], *Uniprot/SWISSPROT*=P28300.

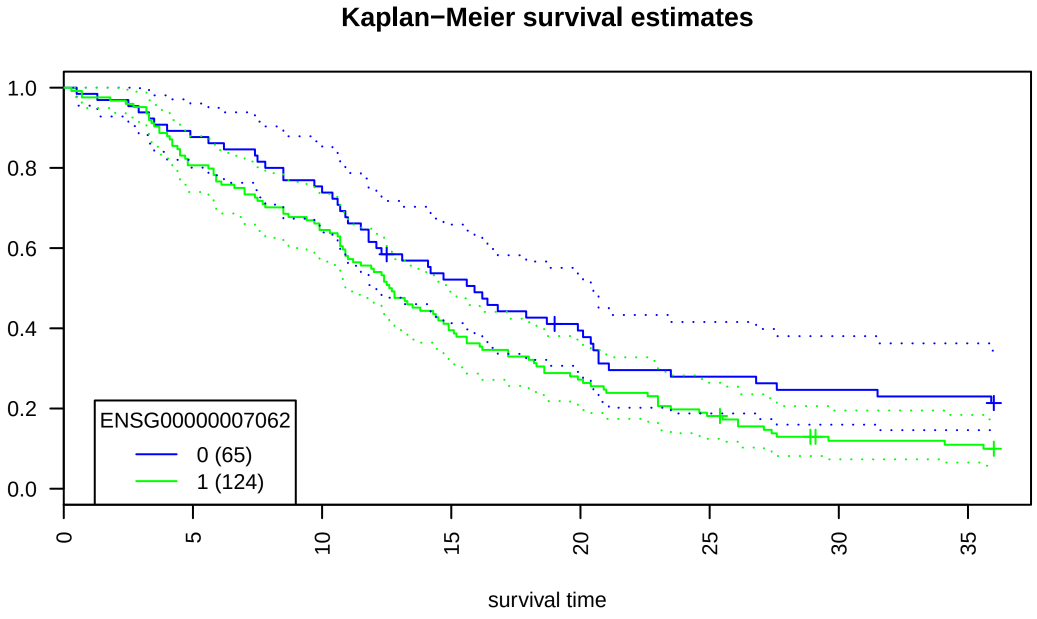

Figure 77: Each curve represents the relative number of ENSG00000007062 survived to the corresponding point of time. Dashed lines represent 0.95 confidence intervals for the curves with the corresponding color. Total of 19 observations deleted due to missingness. Log-rank probability measure for the equality of these curves is 4.286095e-02. Additional annotations: *.GeneName*=PROM1, *.GeneDesc*=prominin 1 [Source:HGNC Symbol;Acc:9454], *Uniprot/SWISSPROT*=O43490.

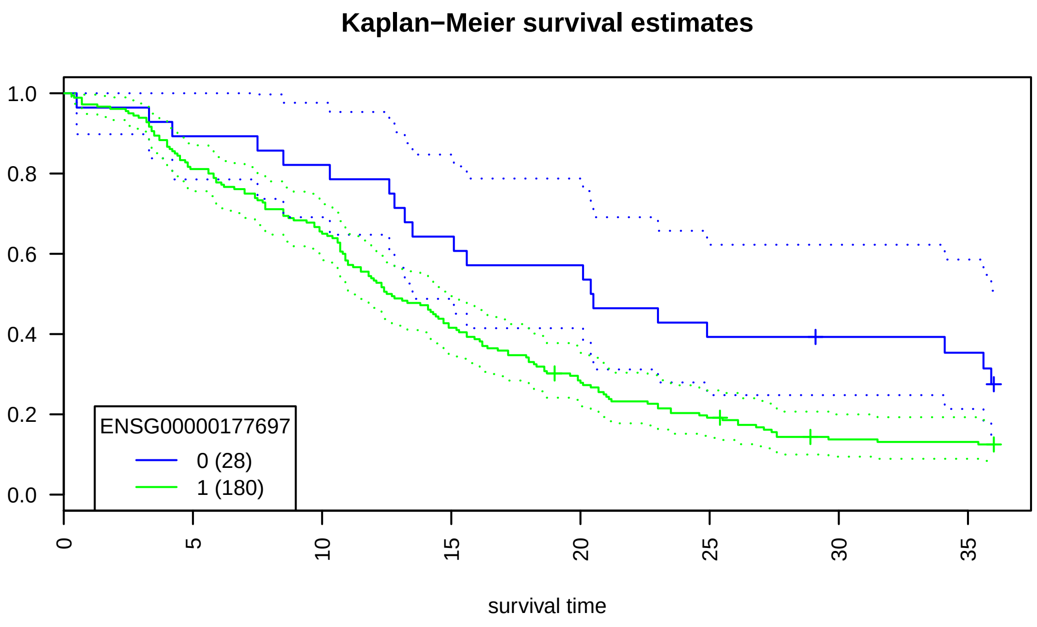

Figure 78: Each curve represents the relative number of ENSG00000177697 survived to the corresponding point of time. Dashed lines represent 0.95 confidence intervals for the curves with the corresponding color. Log-rank probability measure for the equality of these curves is 1.297343e-02. Additional annotations: *.GeneName*=CD151, *.GeneDesc*=CD151 molecule (Raph blood group) [Source:HGNC Symbol;Acc:1630], *Uniprot/SWISSPROT*=P48509.

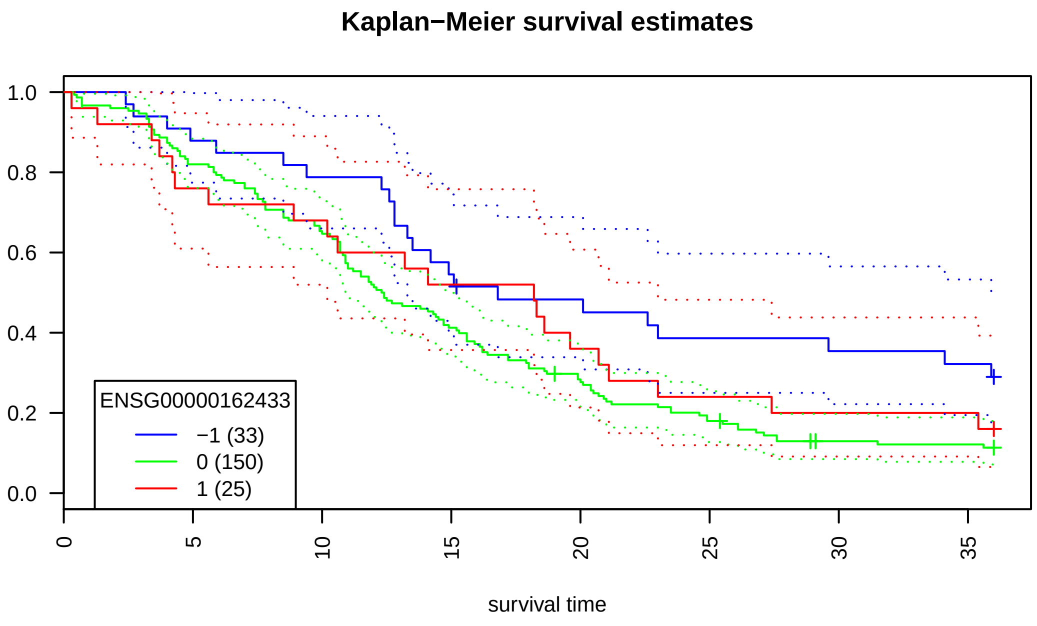

Figure 79: Each curve represents the relative number of ENSG00000162433 survived to the corresponding point of time. Dashed lines represent 0.95 confidence intervals for the curves with the corresponding color. Log-rank probability measure for the equality of these curves is 3.66864e-02.

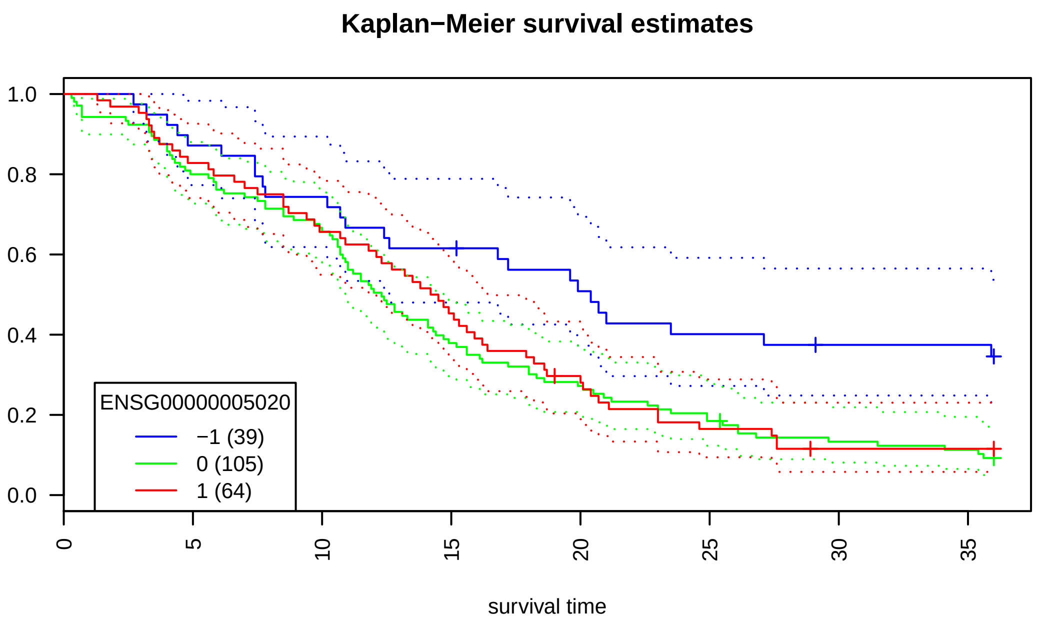

Figure 80: Each curve represents the relative number of ENSG00000005020 survived to the corresponding point of time. Dashed lines represent 0.95 confidence intervals for the curves with the corresponding color. Log-rank probability measure for the equality of these curves is 5.894795e-03.

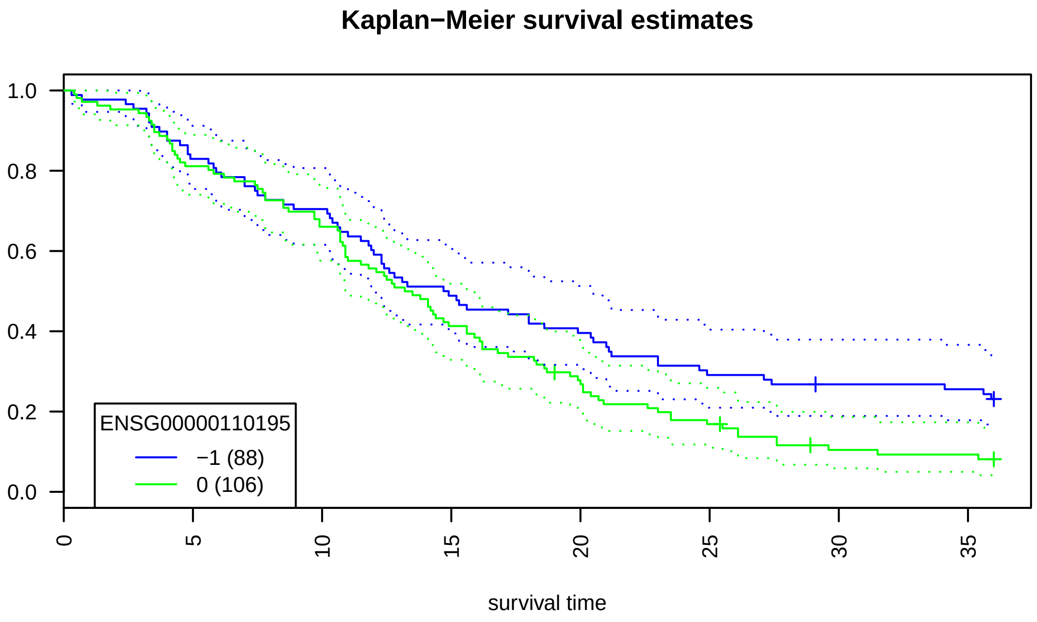

Figure 81: Each curve represents the relative number of ENSG00000110195 survived to the corresponding point of time. Dashed lines represent 0.95 confidence intervals for the curves with the corresponding color. Total of 14 observations deleted due to missingness. Log-rank probability measure for the equality of these curves is 2.463035e-02.

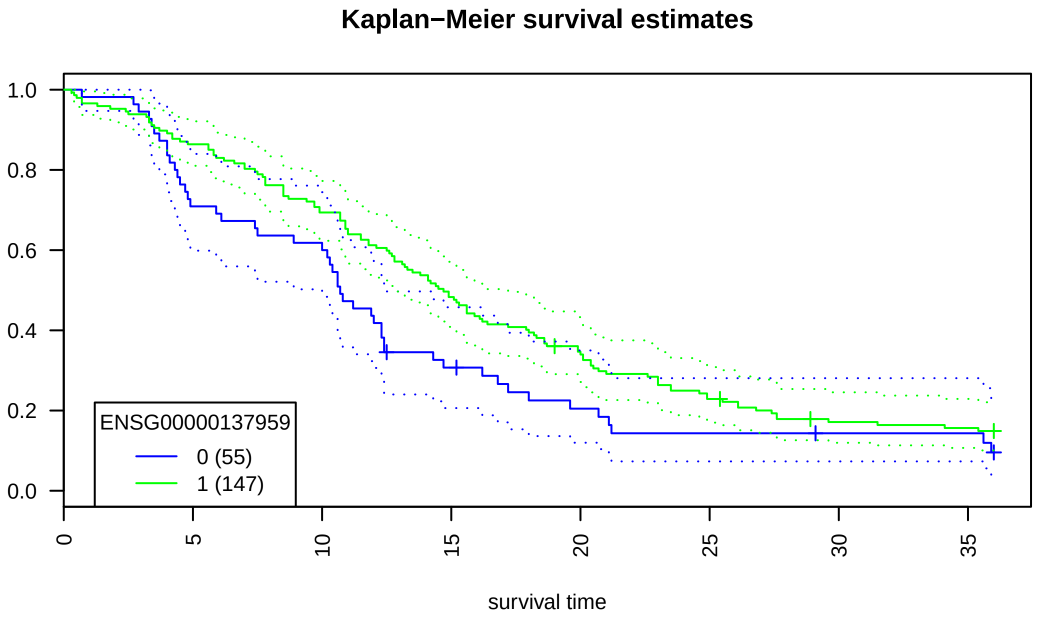

Figure 82: Each curve represents the relative number of ENSG00000137959 survived to the corresponding point of time. Dashed lines represent 0.95 confidence intervals for the curves with the corresponding color. Total of 6 observations deleted due to missingness. Log-rank probability measure for the equality of these curves is 3.454322e-02. Additional annotations: *.GeneName*=IFI44L, *.GeneDesc*=interferon-induced protein 44-like [Source:HGNC Symbol;Acc:17817], *Uniprot/SWISSPROT*=Q53G44.

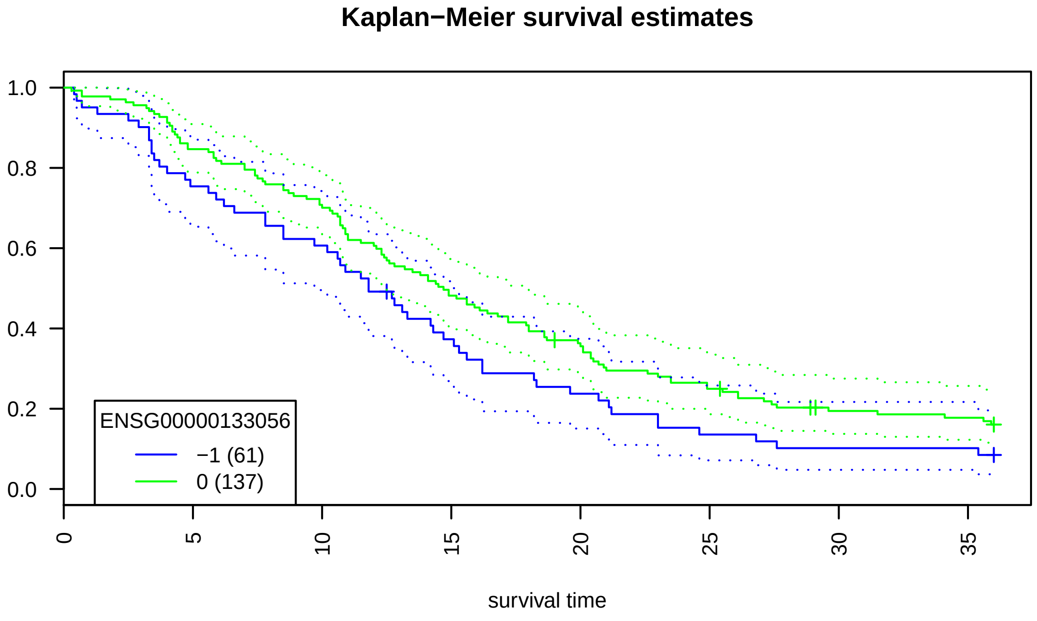

Figure 83: Each curve represents the relative number of ENSG00000133056 survived to the corresponding point of time. Dashed lines represent 0.95 confidence intervals for the curves with the corresponding color. Total of 10 observations deleted due to missingness. Log-rank probability measure for the equality of these curves is 4.176922e-02.

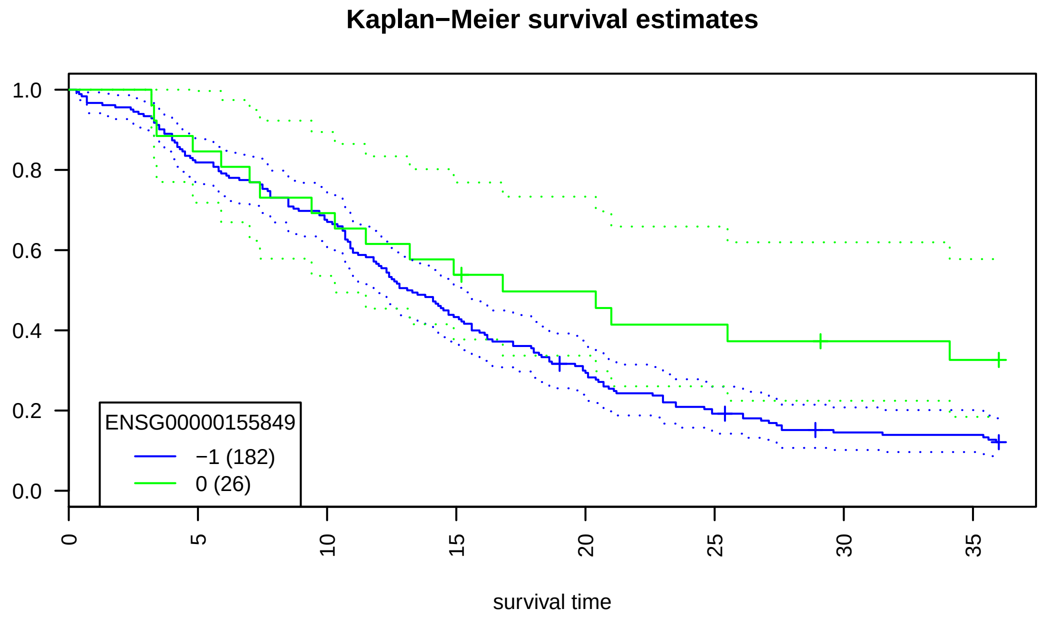

Figure 84: Each curve represents the relative number of ENSG00000155849 survived to the corresponding point of time. Dashed lines represent 0.95 confidence intervals for the curves with the corresponding color. Log-rank probability measure for the equality of these curves is 4.211765e-02. Additional annotations: *.GeneName*=ELMO1, *.GeneDesc*=engulfment and cell motility 1 [Source:HGNC Symbol;Acc:16286], *Uniprot/SWISSPROT*=Q92556.

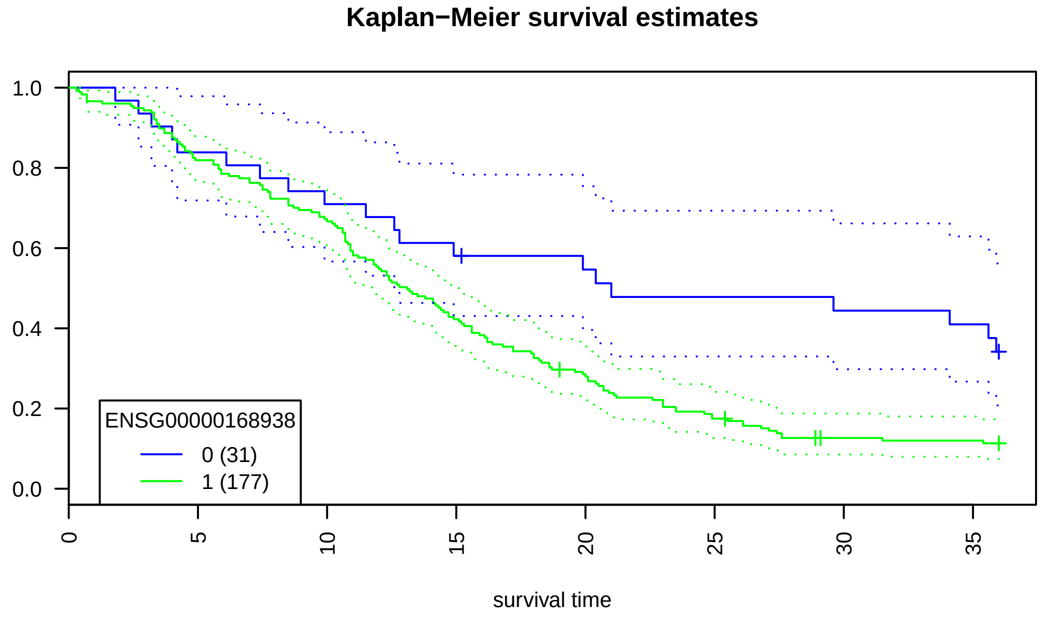

Figure 85: Each curve represents the relative number of ENSG00000168938 survived to the corresponding point of time. Dashed lines represent 0.95 confidence intervals for the curves with the corresponding color. Log-rank probability measure for the equality of these curves is 3.829558e-03. Additional annotations: *.GeneName*=PPIC, *.GeneDesc*=peptidylprolyl isomerase C (cyclophilin C) [Source:HGNC Symbol;Acc:9256], *Uniprot/SWISSPROT*=P45877.

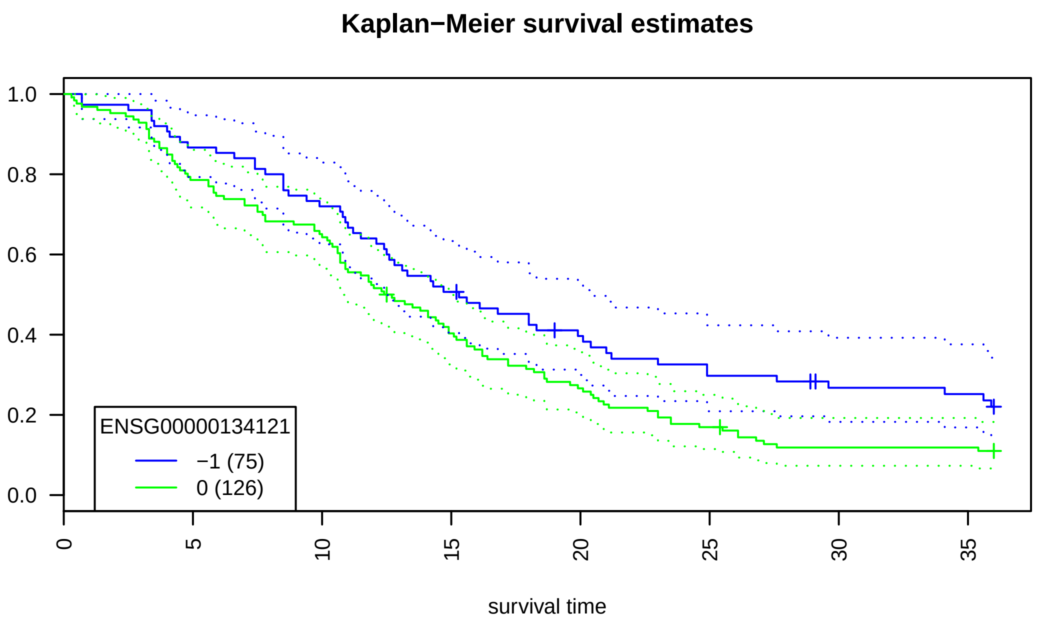

Figure 86: Each curve represents the relative number of ENSG00000134121 survived to the corresponding point of time. Dashed lines represent 0.95 confidence intervals for the curves with the corresponding color. Total of 7 observations deleted due to missingness. Log-rank probability measure for the equality of these curves is 1.977875e-02.

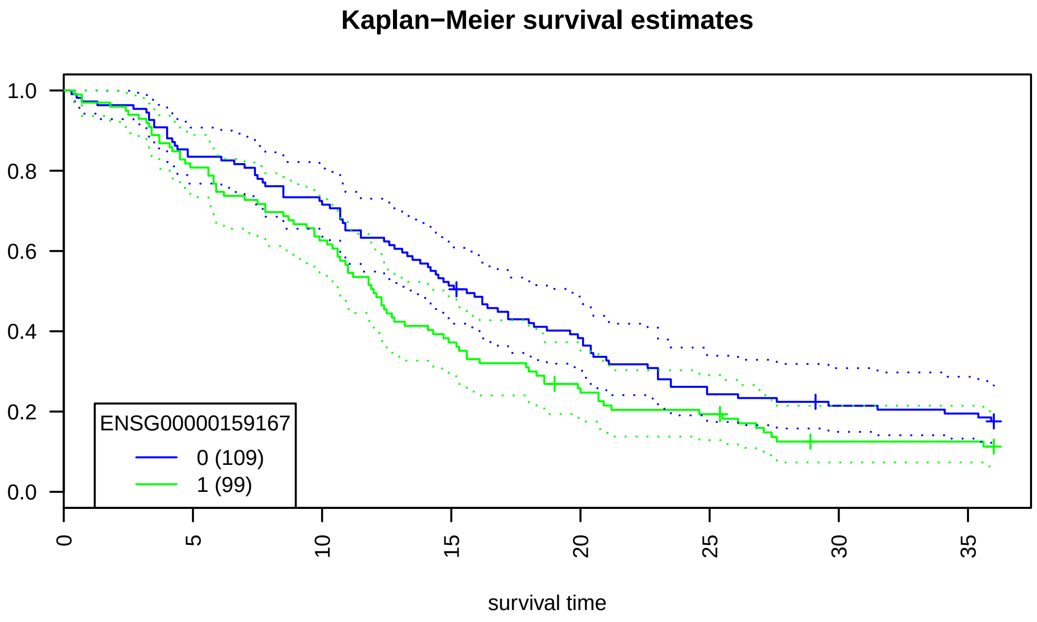

Figure 87: Each curve represents the relative number of ENSG00000159167 survived to the corresponding point of time. Dashed lines represent 0.95 confidence intervals for the curves with the corresponding color. Log-rank probability measure for the equality of these curves is 4.827371e-02.

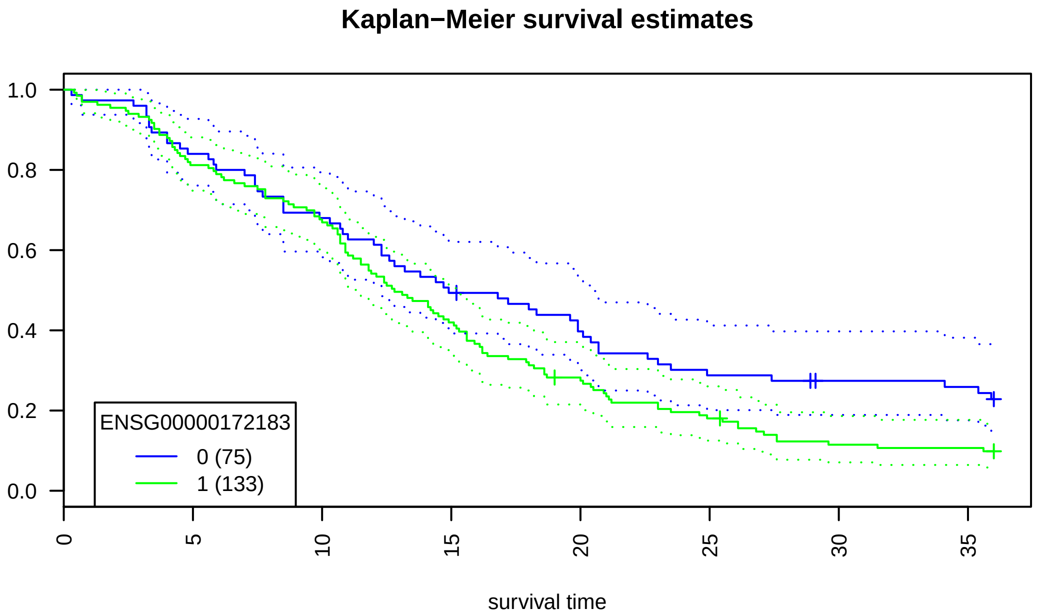

Figure 88: Each curve represents the relative number of ENSG00000172183 survived to the corresponding point of time. Dashed lines represent 0.95 confidence intervals for the curves with the corresponding color. Log-rank probability measure for the equality of these curves is 3.074586e-02. Additional annotations: *.GeneName*=ISG20, *.GeneDesc*=interferon stimulated exonuclease gene 20kDa [Source:HGNC Symbol;Acc:6130], *Uniprot/SWISSPROT*=Q96AZ6.

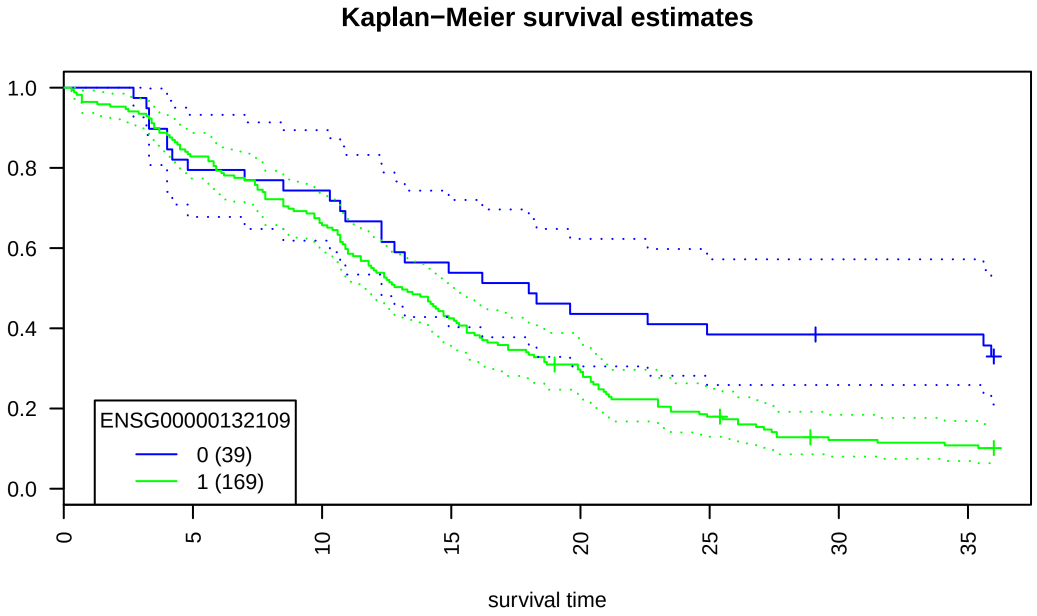

Figure 89: Each curve represents the relative number of ENSG00000132109 survived to the corresponding point of time. Dashed lines represent 0.95 confidence intervals for the curves with the corresponding color. Log-rank probability measure for the equality of these curves is 5.529078e-03. Additional annotations: *.GeneName*=TRIM21, *.GeneDesc*=tripartite motif-containing 21 [Source:HGNC Symbol;Acc:11312], *Uniprot/SWISSPROT*=P19474.

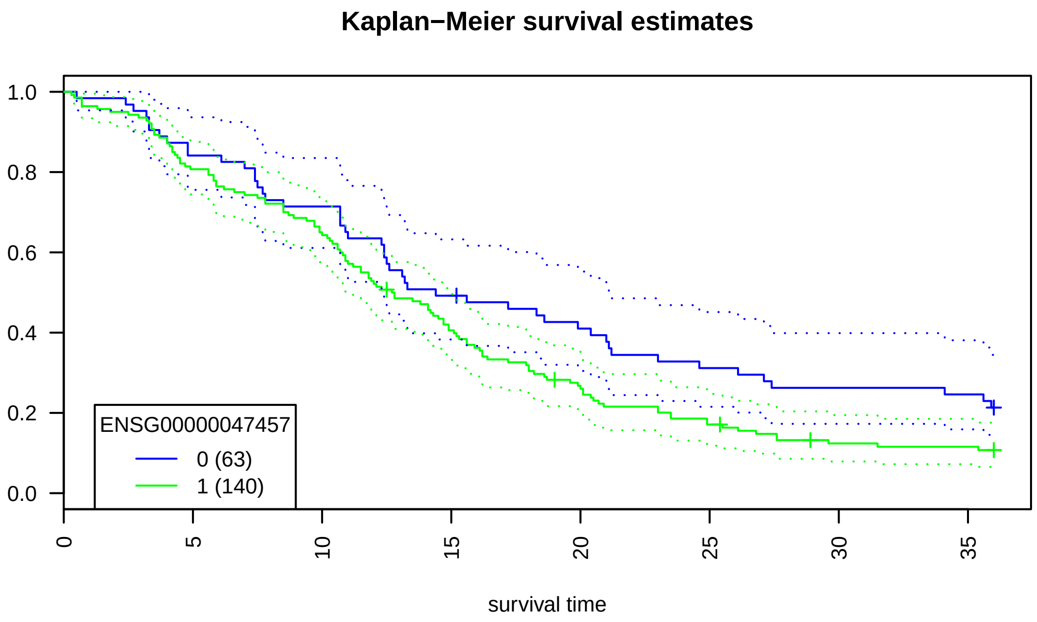

Figure 90: Each curve represents the relative number of ENSG00000047457 survived to the corresponding point of time. Dashed lines represent 0.95 confidence intervals for the curves with the corresponding color. Total of 5 observations deleted due to missingness. Log-rank probability measure for the equality of these curves is 3.980593e-02. Additional annotations: *.GeneName*=CP, *.GeneDesc*=ceruloplasmin (ferroxidase) [Source:HGNC Symbol;Acc:2295], *Uniprot/SWISSPROT*=P00450.

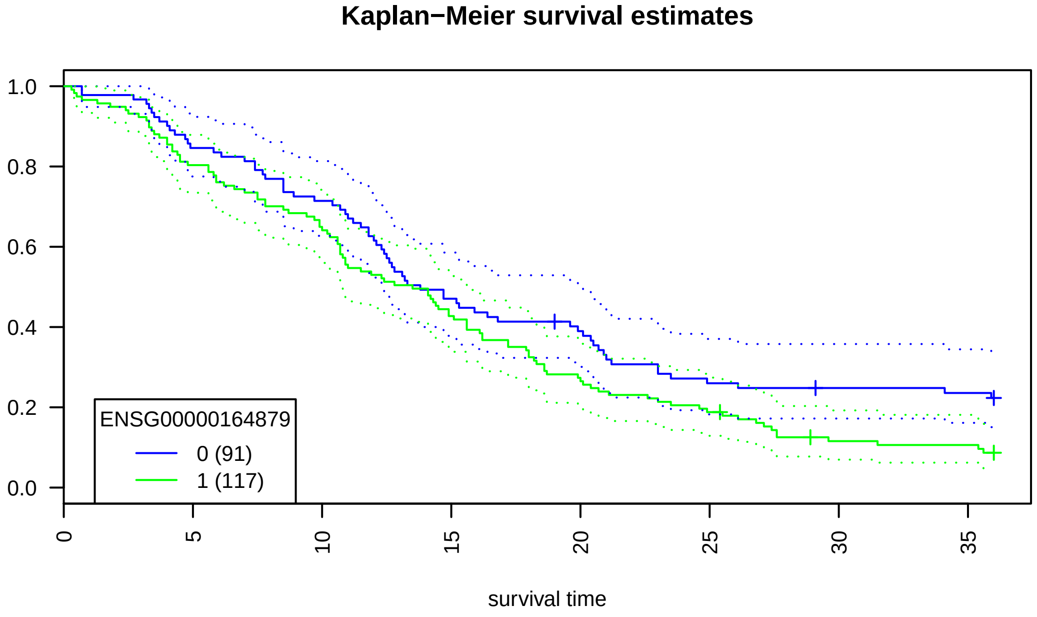

Figure 91: Each curve represents the relative number of ENSG00000164879 survived to the corresponding point of time. Dashed lines represent 0.95 confidence intervals for the curves with the corresponding color. Log-rank probability measure for the equality of these curves is 3.155501e-02. Additional annotations: *.GeneName*=CA3, *.GeneDesc*=carbonic anhydrase III, muscle specific [Source:HGNC Symbol;Acc:1374], *Uniprot/SWISSPROT*=P07451.

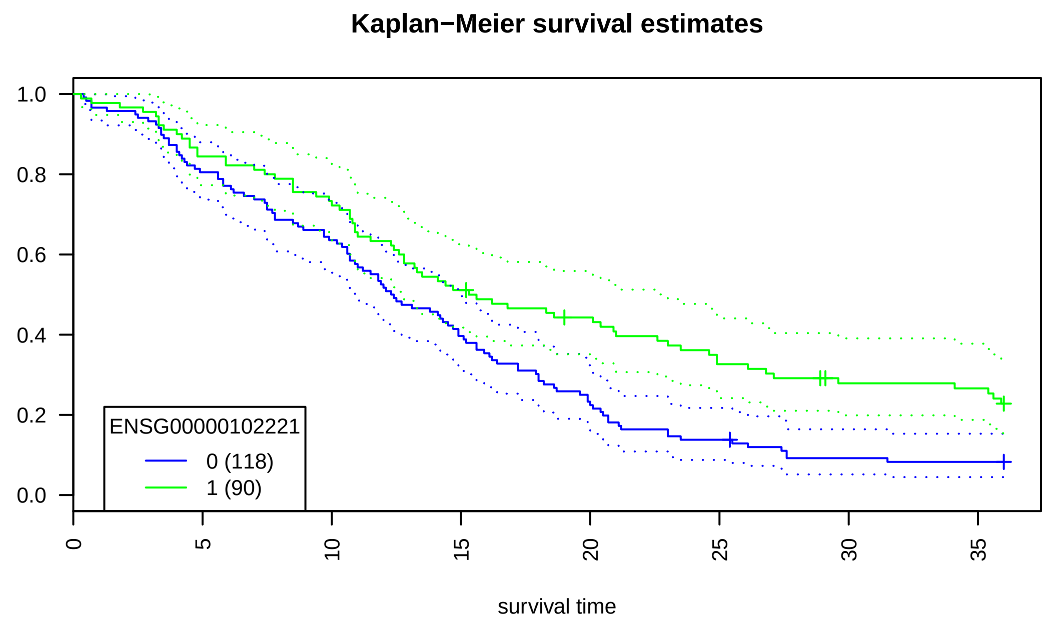

Figure 92: Each curve represents the relative number of ENSG00000102221 survived to the corresponding point of time. Dashed lines represent 0.95 confidence intervals for the curves with the corresponding color. Log-rank probability measure for the equality of these curves is 1.361260e-03.

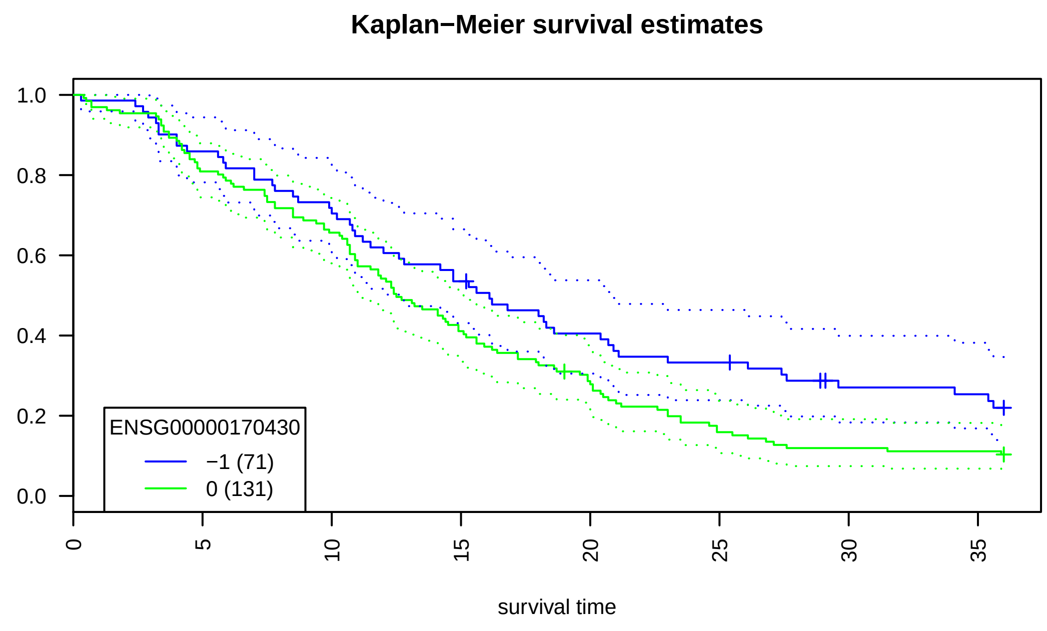

Figure 93: Each curve represents the relative number of ENSG00000170430 survived to the corresponding point of time. Dashed lines represent 0.95 confidence intervals for the curves with the corresponding color. Total of 6 observations deleted due to missingness. Log-rank probability measure for the equality of these curves is 2.323975e-02.

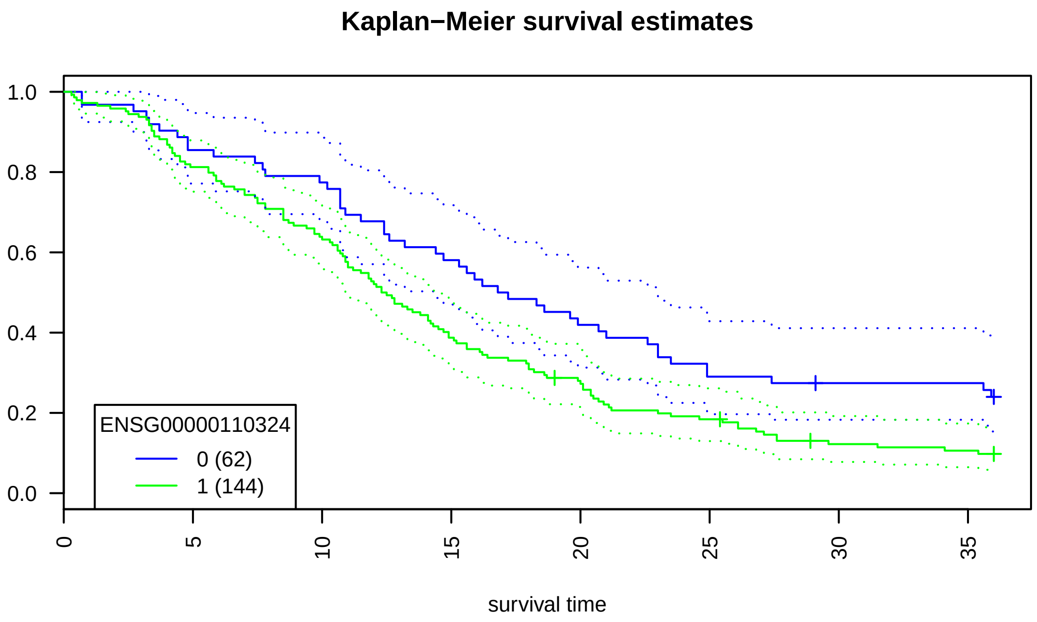

Figure 94: Each curve represents the relative number of ENSG00000110324 survived to the corresponding point of time. Dashed lines represent 0.95 confidence intervals for the curves with the corresponding color. Total of 2 observations deleted due to missingness. Log-rank probability measure for the equality of these curves is 5.922739e-03. Additional annotations: *.GeneName*=IL10RA, *.GeneDesc*=interleukin 10 receptor, alpha [Source:HGNC Symbol;Acc:5964], *Uniprot/SWISSPROT*=Q13651.

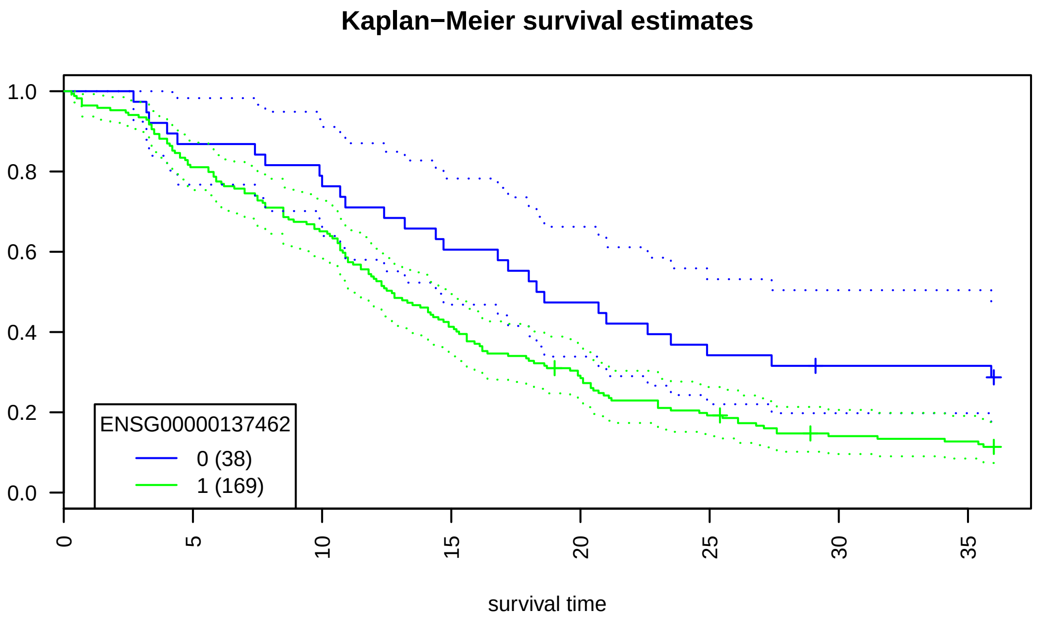

Figure 95: Each curve represents the relative number of ENSG00000137462 survived to the corresponding point of time. Dashed lines represent 0.95 confidence intervals for the curves with the corresponding color. Total of 1 observation deleted due to missingness. Log-rank probability measure for the equality of these curves is 8.683046e-03. Additional annotations: *.GeneName*=TLR2, *.GeneDesc*=toll-like receptor 2 [Source:HGNC Symbol;Acc:11848], *Uniprot/SWISSPROT*=O60603.

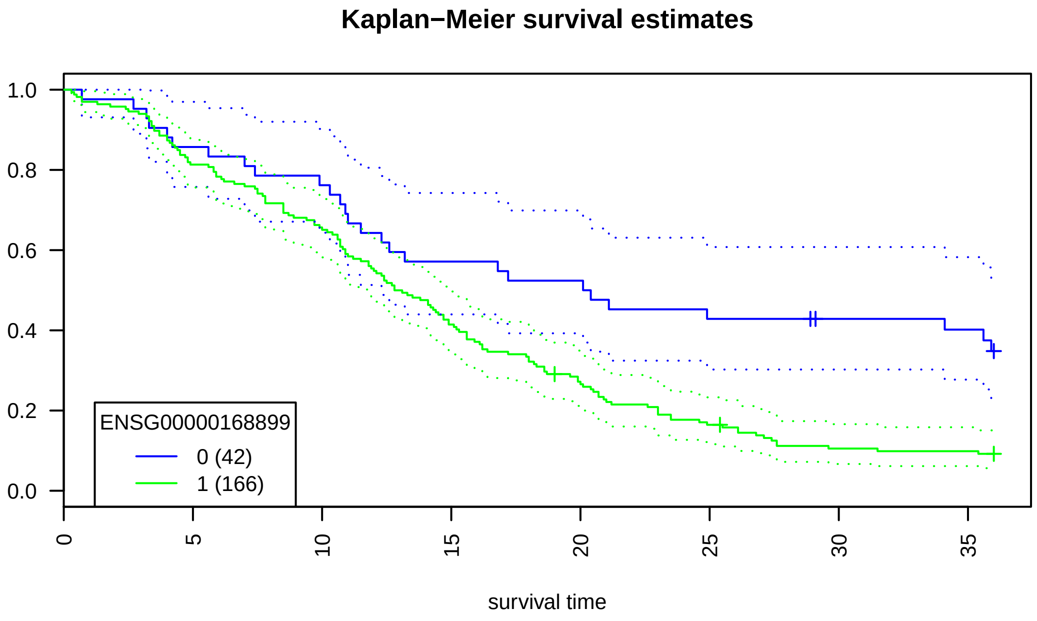

Figure 96: Each curve represents the relative number of ENSG00000168899 survived to the corresponding point of time. Dashed lines represent 0.95 confidence intervals for the curves with the corresponding color. Log-rank probability measure for the equality of these curves is 5.83041e-04. Additional annotations: *.GeneName*=VAMP5, *.GeneDesc*=vesicle-associated membrane protein 5 (myobrevin) [Source:HGNC Symbol;Acc:12646], *Uniprot/SWISSPROT*=O95183.

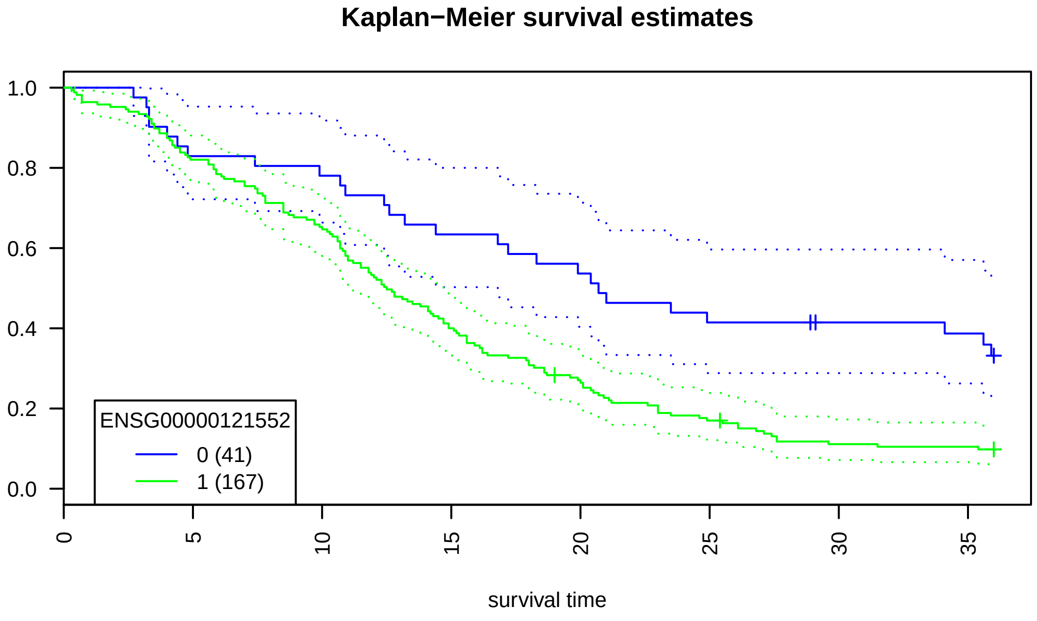

Figure 97: Each curve represents the relative number of ENSG00000121552 survived to the corresponding point of time. Dashed lines represent 0.95 confidence intervals for the curves with the corresponding color. Log-rank probability measure for the equality of these curves is 3.693856e-04. Additional annotations: *.GeneName*=CSTA, *.GeneDesc*=cystatin A (stefin A) [Source:HGNC Symbol;Acc:2481], *Uniprot/SWISSPROT*=P01040.

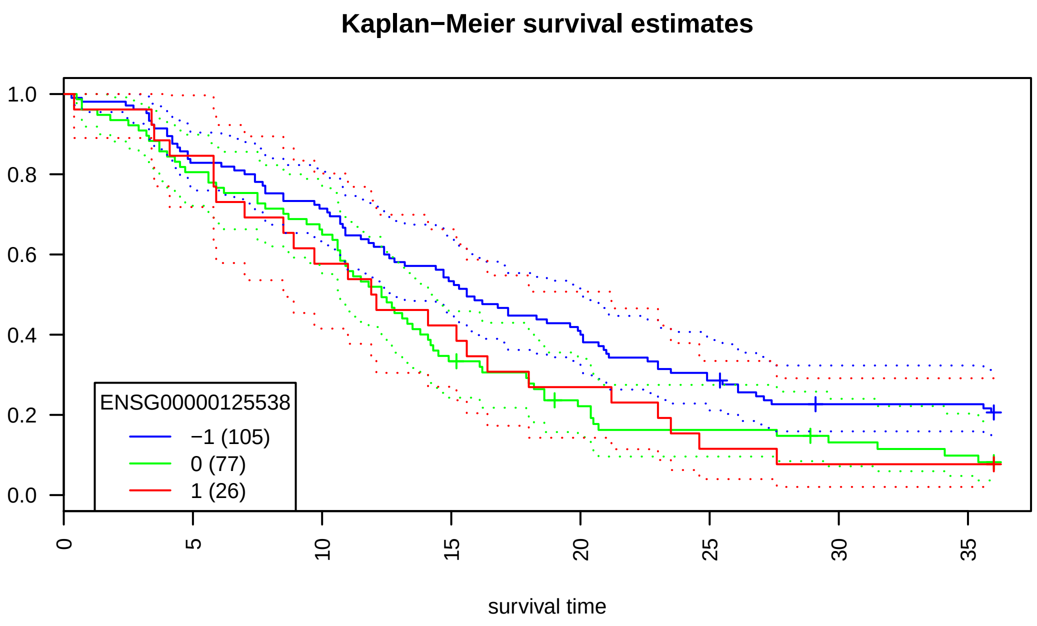

Figure 98: Each curve represents the relative number of ENSG00000125538 survived to the corresponding point of time. Dashed lines represent 0.95 confidence intervals for the curves with the corresponding color. Log-rank probability measure for the equality of these curves is 2.173727e-02.

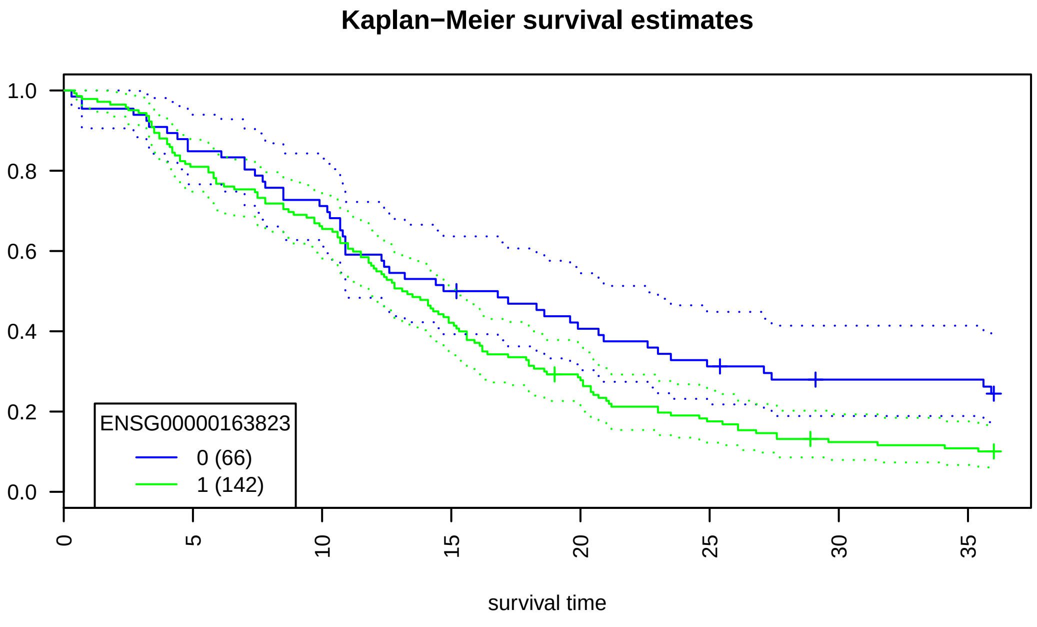

Figure 99: Each curve represents the relative number of ENSG00000163823 survived to the corresponding point of time. Dashed lines represent 0.95 confidence intervals for the curves with the corresponding color. Log-rank probability measure for the equality of these curves is 2.175933e-02. Additional annotations: *.GeneName*=CCR1, *.GeneDesc*=chemokine (C-C motif) receptor 1 [Source:HGNC Symbol;Acc:1602], *Uniprot/SWISSPROT*=P32246.

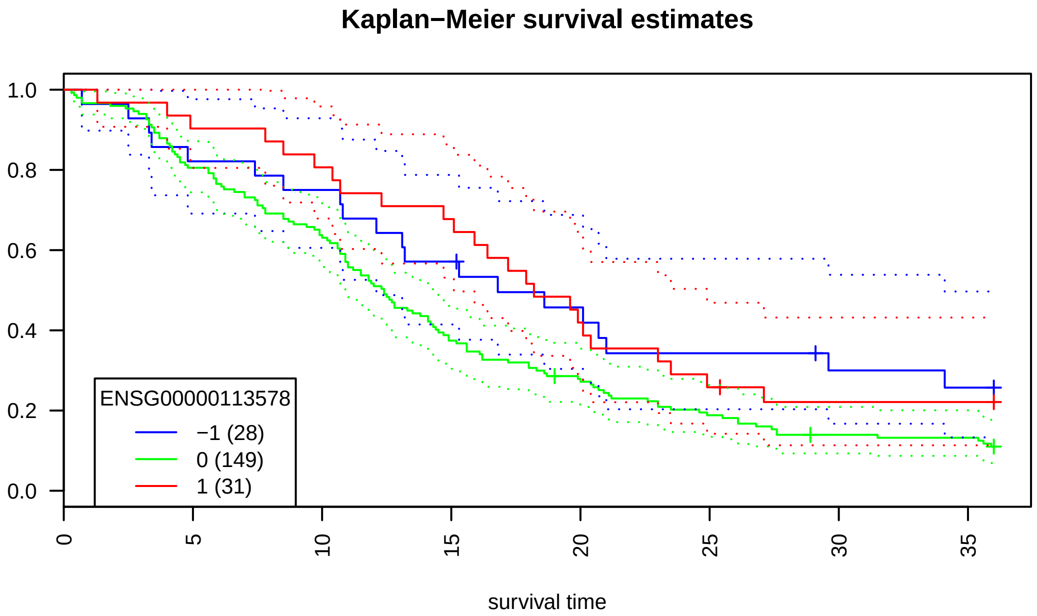

Figure 100: Each curve represents the relative number of ENSG00000113578 survived to the corresponding point of time. Dashed lines represent 0.95 confidence intervals for the curves with the corresponding color. Log-rank probability measure for the equality of these curves is 2.951934e-02.

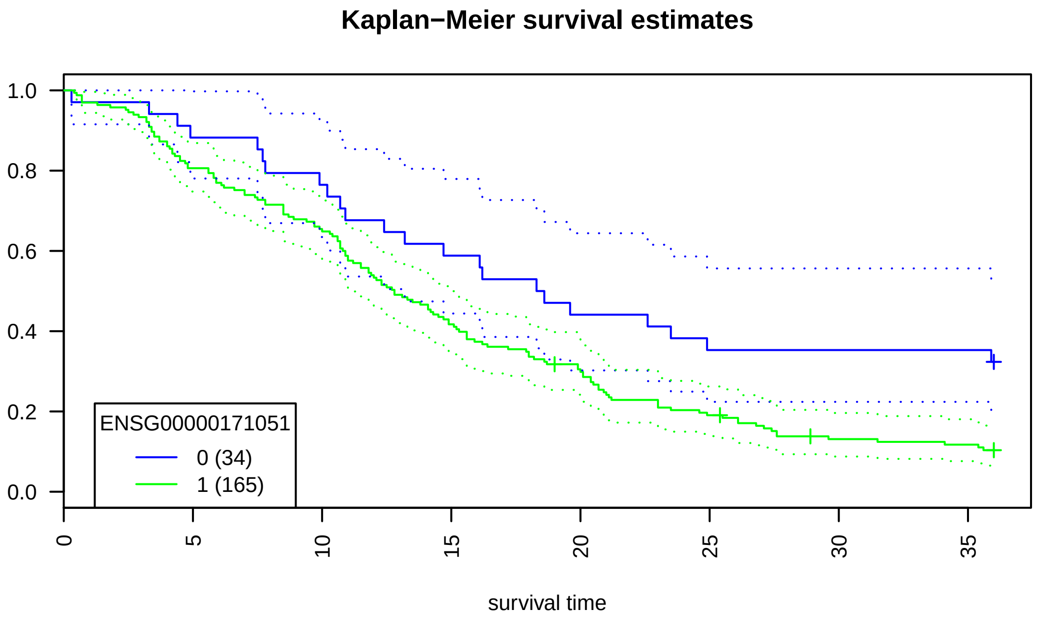

Figure 101: Each curve represents the relative number of ENSG00000171051 survived to the corresponding point of time. Dashed lines represent 0.95 confidence intervals for the curves with the corresponding color. Total of 9 observations deleted due to missingness. Log-rank probability measure for the equality of these curves is 6.660541e-03. Additional annotations: *.GeneName*=FPR1, *.GeneDesc*=formyl peptide receptor 1 [Source:HGNC Symbol;Acc:3826], *Uniprot/SWISSPROT*=P21462.

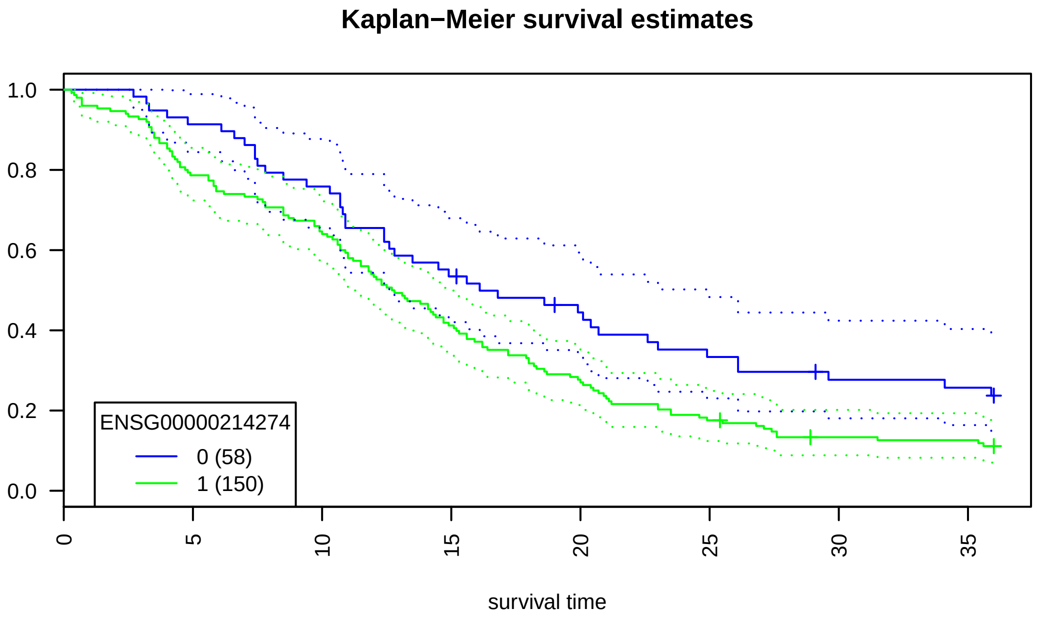

Figure 102: Each curve represents the relative number of ENSG00000214274 survived to the corresponding point of time. Dashed lines represent 0.95 confidence intervals for the curves with the corresponding color. Log-rank probability measure for the equality of these curves is 1.292568e-02. Additional annotations: *.GeneName*=ANG, *.GeneDesc*=angiogenin, ribonuclease, RNase A family, 5 [Source:HGNC Symbol;Acc:483], *Uniprot/SWISSPROT*=P03950.

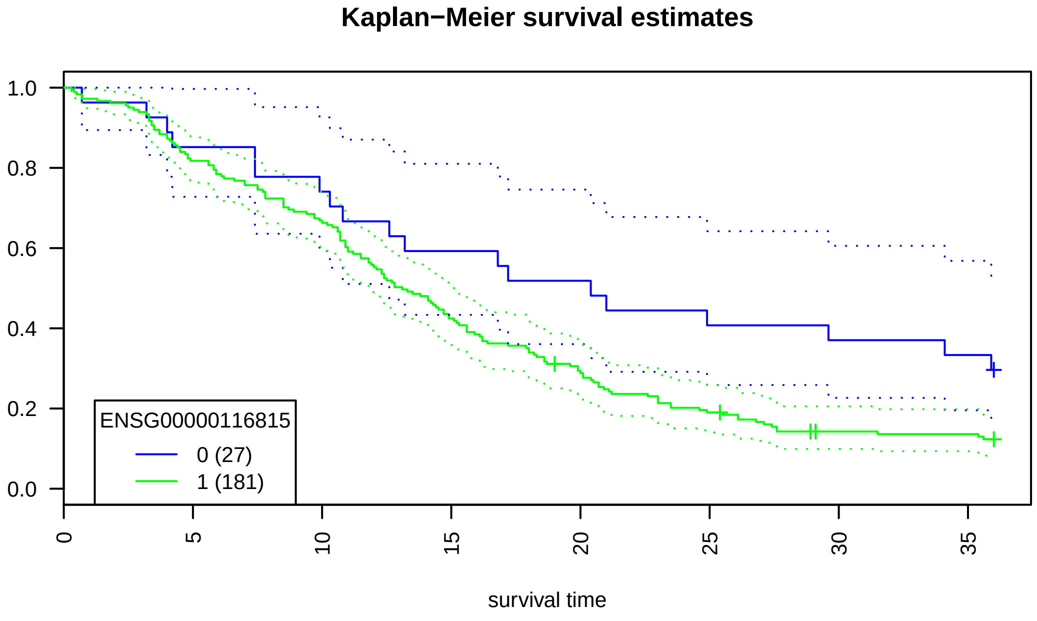

Figure 103: Each curve represents the relative number of ENSG00000116815 survived to the corresponding point of time. Dashed lines represent 0.95 confidence intervals for the curves with the corresponding color. Log-rank probability measure for the equality of these curves is 2.50925e-02. Additional annotations: *.GeneName*=CD58, *.GeneDesc*=CD58 molecule [Source:HGNC Symbol;Acc:1688], *Uniprot/SWISSPROT*=P19256.

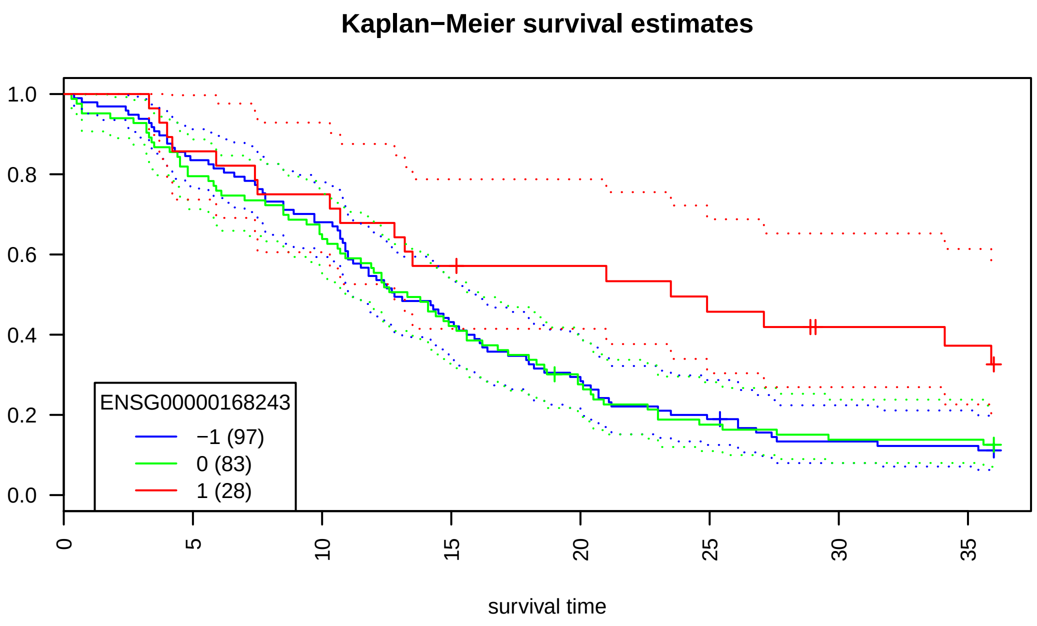

Figure 104: Each curve represents the relative number of ENSG00000168243 survived to the corresponding point of time. Dashed lines represent 0.95 confidence intervals for the curves with the corresponding color. Log-rank probability measure for the equality of these curves is 2.987092e-02.

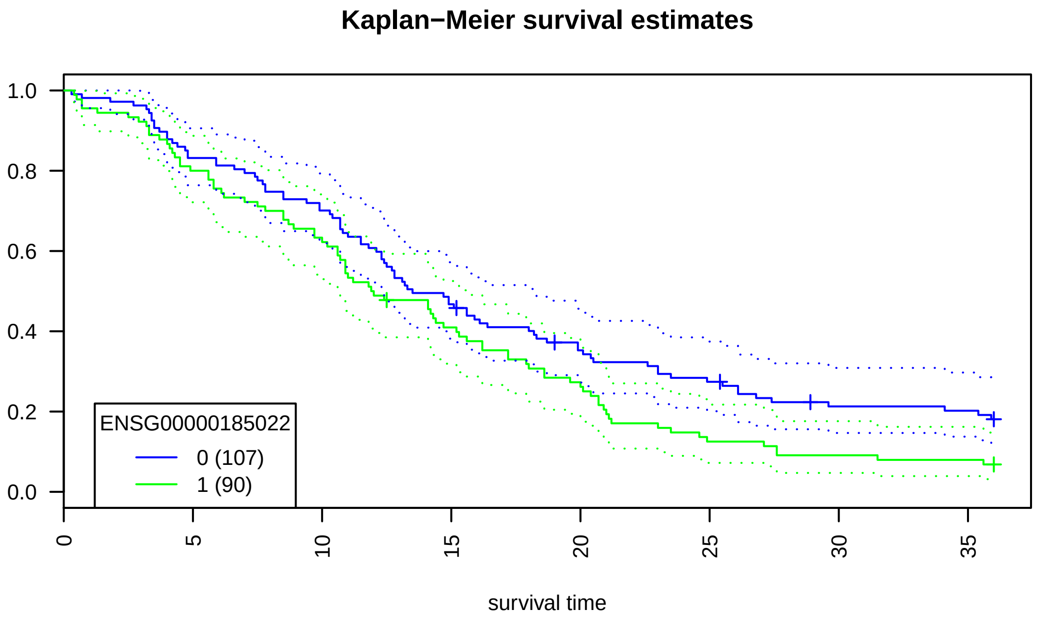

Figure 105: Each curve represents the relative number of ENSG00000185022 survived to the corresponding point of time. Dashed lines represent 0.95 confidence intervals for the curves with the corresponding color. Total of 11 observations deleted due to missingness. Log-rank probability measure for the equality of these curves is 2.595836e-02.

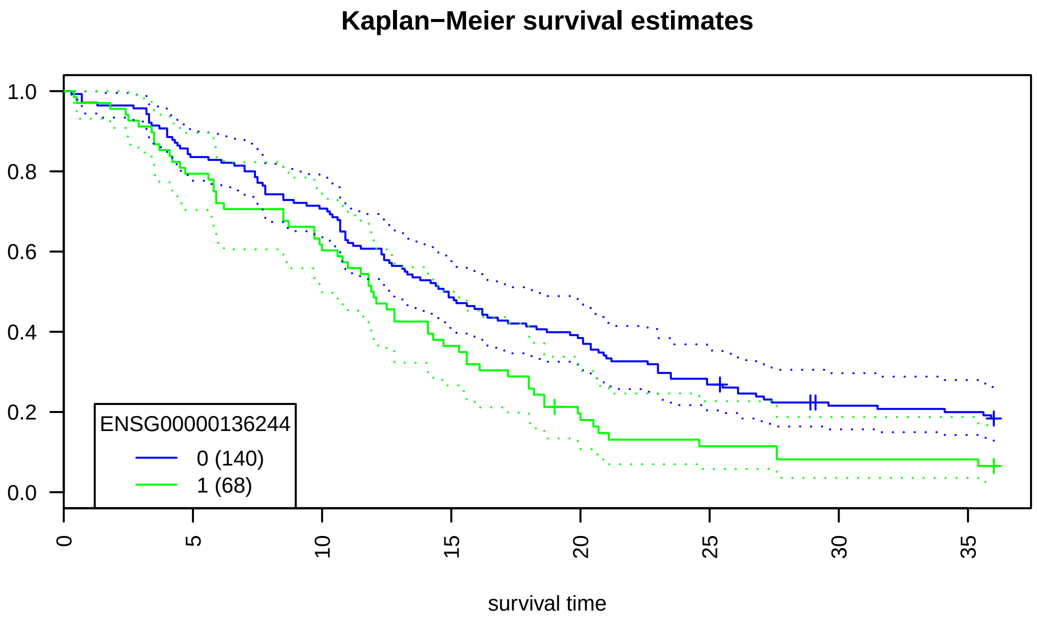

Figure 106: Each curve represents the relative number of ENSG00000136244 survived to the corresponding point of time. Dashed lines represent 0.95 confidence intervals for the curves with the corresponding color. Log-rank probability measure for the equality of these curves is 6.704588e-03.

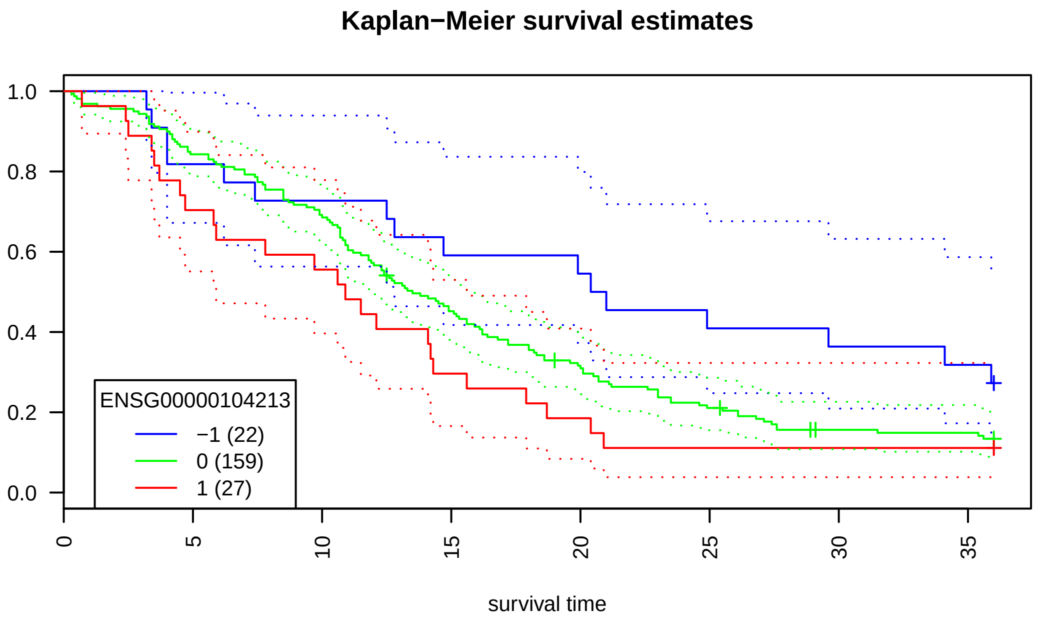

Figure 107: Each curve represents the relative number of ENSG00000104213 survived to the corresponding point of time. Dashed lines represent 0.95 confidence intervals for the curves with the corresponding color. Log-rank probability measure for the equality of these curves is 4.883469e-02.

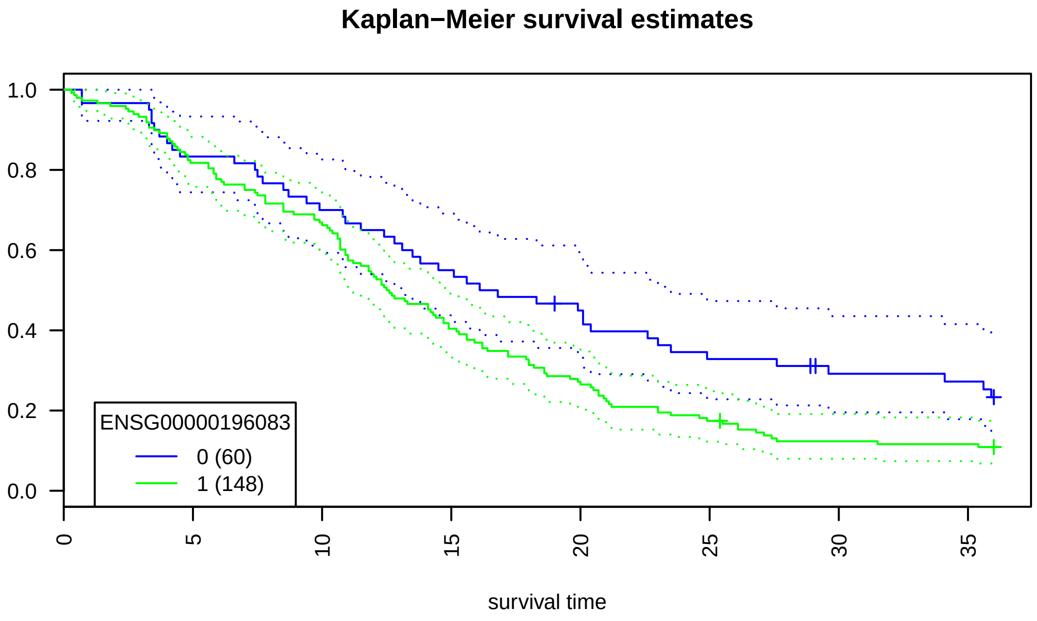

Figure 108: Each curve represents the relative number of ENSG00000196083 survived to the corresponding point of time. Dashed lines represent 0.95 confidence intervals for the curves with the corresponding color. Log-rank probability measure for the equality of these curves is 1.38863e-02. Additional annotations: *.GeneName*=IL1RAP, *.GeneDesc*=interleukin 1 receptor accessory protein [Source:HGNC Symbol;Acc:5995], *Uniprot/SWISSPROT*=Q9NPH3.

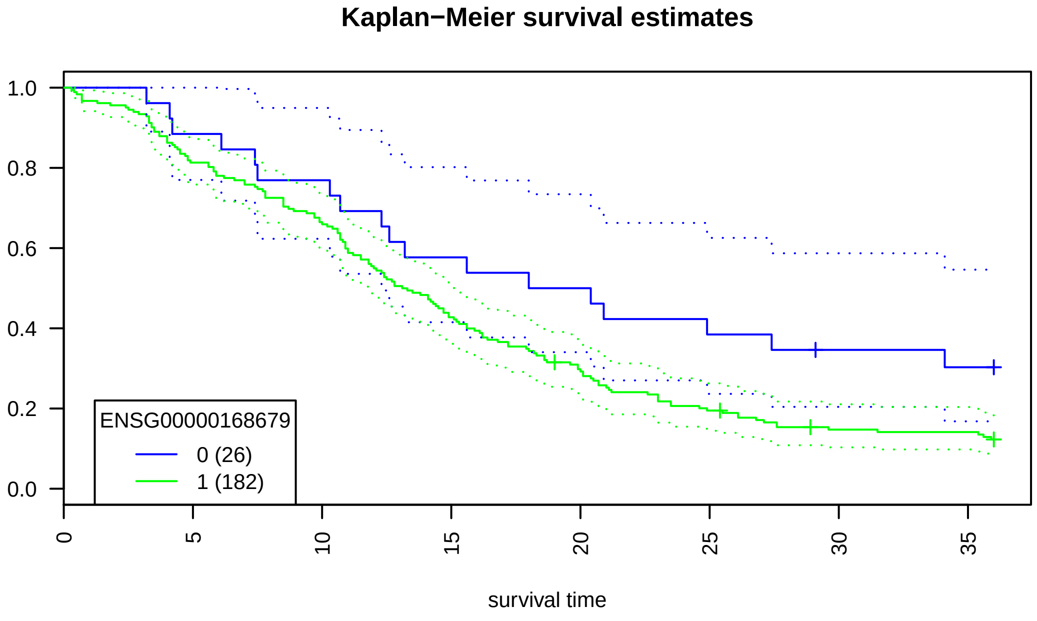

Figure 109: Each curve represents the relative number of ENSG00000168679 survived to the corresponding point of time. Dashed lines represent 0.95 confidence intervals for the curves with the corresponding color. Log-rank probability measure for the equality of these curves is 3.407635e-02. Additional annotations: *.GeneName*=SLC16A4, *.GeneDesc*=solute carrier family 16, member 4 (monocarboxylic acid transporter 5) [Source:HGNC Symbol;Acc:10925], *Uniprot/SWISSPROT*=O15374.

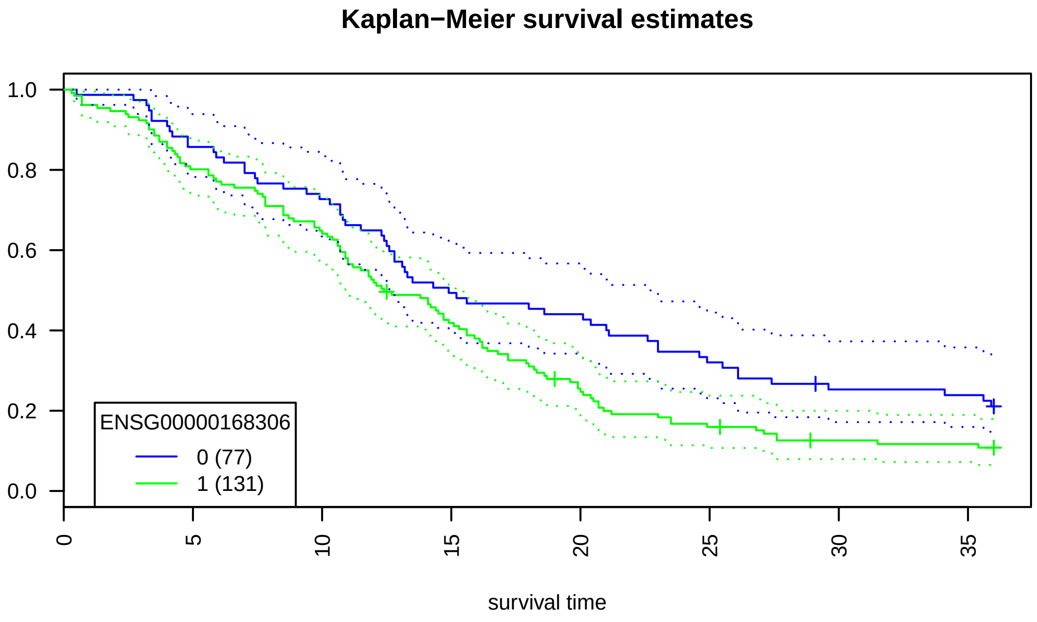

Figure 110: Each curve represents the relative number of ENSG00000168306 survived to the corresponding point of time. Dashed lines represent 0.95 confidence intervals for the curves with the corresponding color. Log-rank probability measure for the equality of these curves is 1.768102e-02. Additional annotations: *.GeneName*=ACOX2, *.GeneDesc*=acyl-Coenzyme A oxidase 2, branched chain [Source:HGNC Symbol;Acc:120], *Uniprot/SWISSPROT*=Q99424.

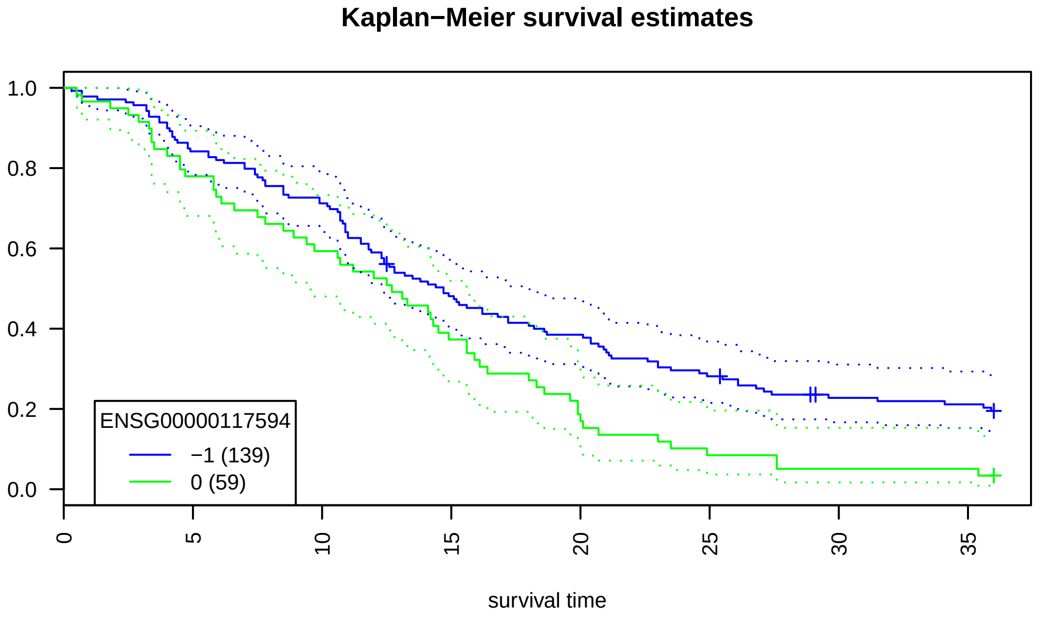

Figure 111: Each curve represents the relative number of ENSG00000117594 survived to the corresponding point of time. Dashed lines represent 0.95 confidence intervals for the curves with the corresponding color. Total of 10 observations deleted due to missingness. Log-rank probability measure for the equality of these curves is 2.120825e-03.

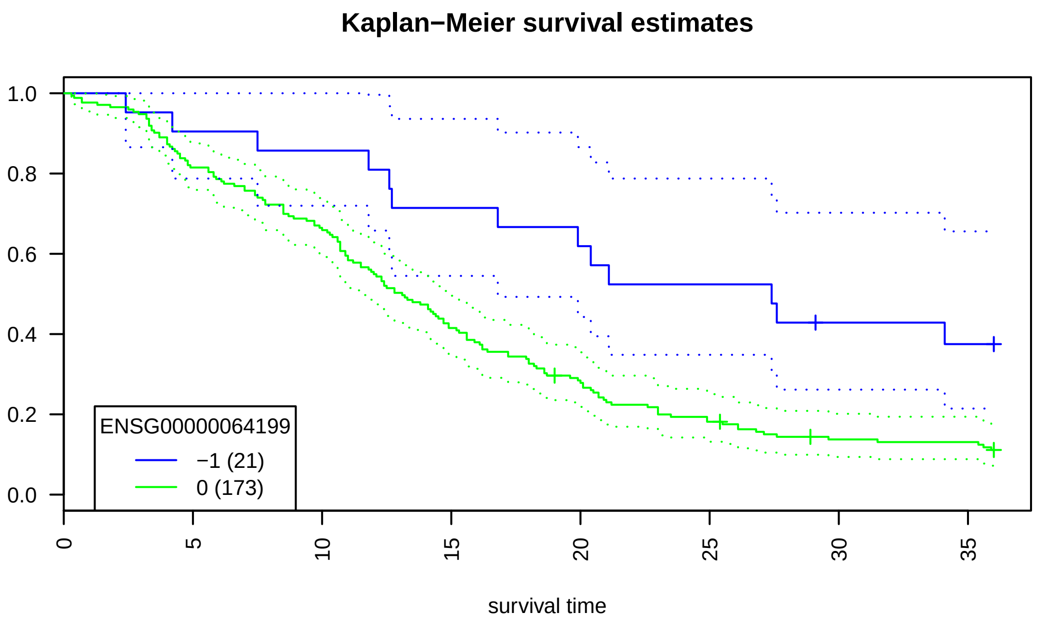

Figure 112: Each curve represents the relative number of ENSG00000064199 survived to the corresponding point of time. Dashed lines represent 0.95 confidence intervals for the curves with the corresponding color. Total of 14 observations deleted due to missingness. Log-rank probability measure for the equality of these curves is 2.334755e-03.

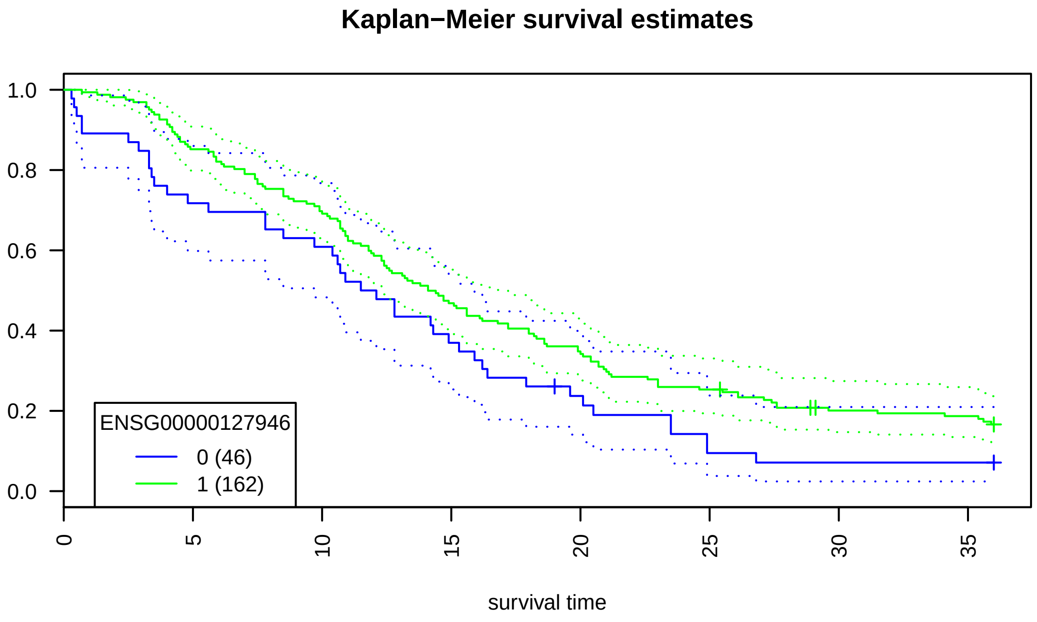

Figure 113: Each curve represents the relative number of ENSG00000127946 survived to the corresponding point of time. Dashed lines represent 0.95 confidence intervals for the curves with the corresponding color. Log-rank probability measure for the equality of these curves is 3.121217e-02. Additional annotations: *.GeneName*=HIP1, *.GeneDesc*=huntingtin interacting protein 1 [Source:HGNC Symbol;Acc:4913], *Uniprot/SWISSPROT*=O00291.

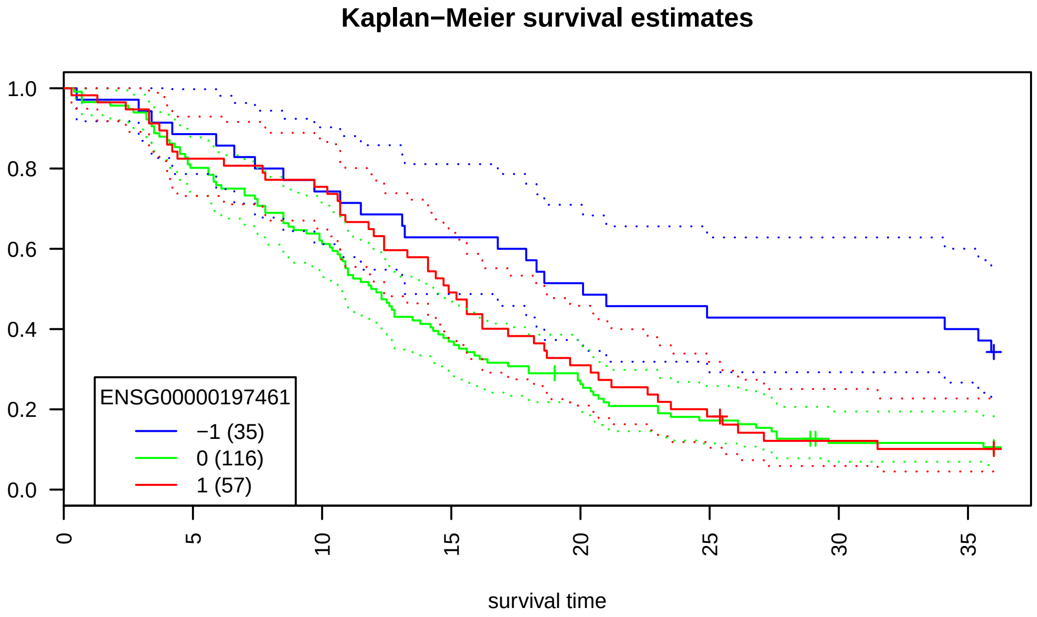

Figure 114: Each curve represents the relative number of ENSG00000197461 survived to the corresponding point of time. Dashed lines represent 0.95 confidence intervals for the curves with the corresponding color. Log-rank probability measure for the equality of these curves is 4.288501e-03.

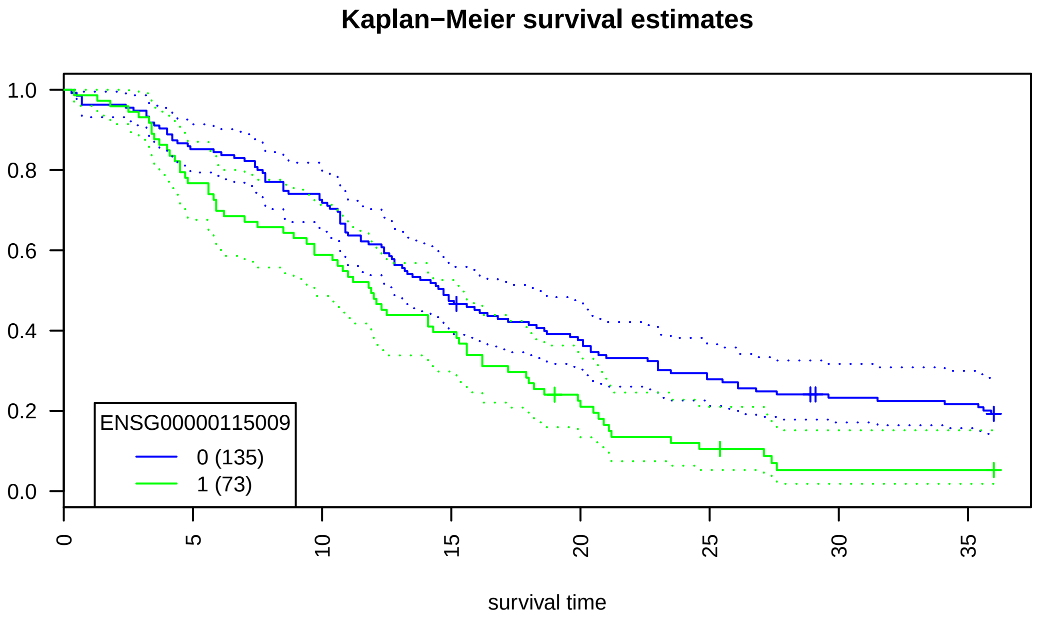

Figure 115: Each curve represents the relative number of ENSG00000115009 survived to the corresponding point of time. Dashed lines represent 0.95 confidence intervals for the curves with the corresponding color. Log-rank probability measure for the equality of these curves is 2.241243e-03. Additional annotations: *.GeneName*=CCL20, *.GeneDesc*=chemokine (C-C motif) ligand 20 [Source:HGNC Symbol;Acc:10619], *Uniprot/SWISSPROT*=P78556.

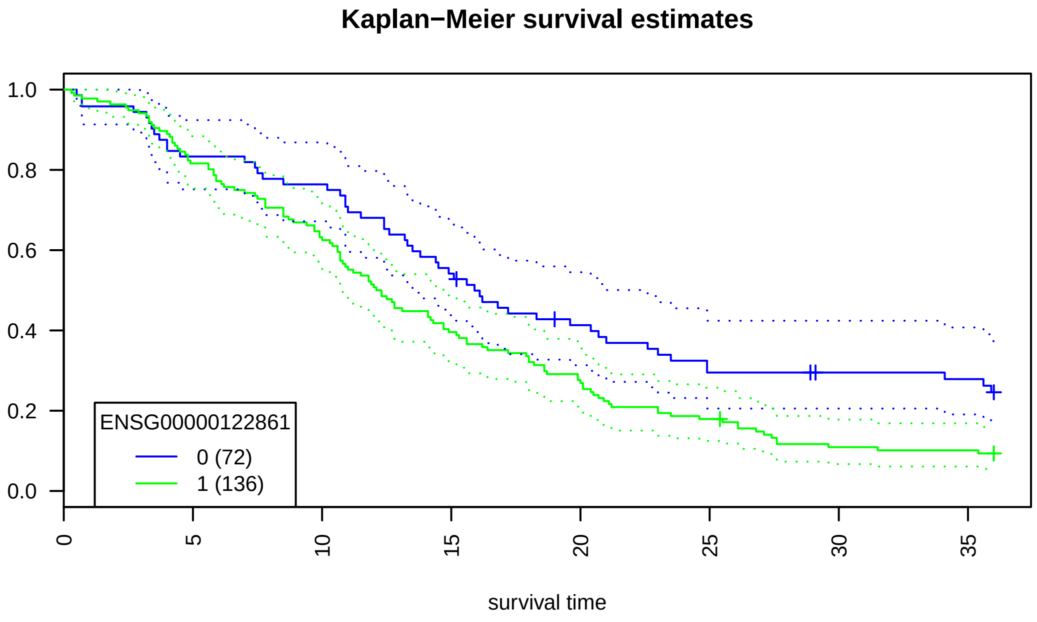

Figure 116: Each curve represents the relative number of ENSG00000122861 survived to the corresponding point of time. Dashed lines represent 0.95 confidence intervals for the curves with the corresponding color. Log-rank probability measure for the equality of these curves is 5.190632e-03. Additional annotations: *.GeneName*=PLAU, *.GeneDesc*=plasminogen activator, urokinase [Source:HGNC Symbol;Acc:9052], *Uniprot/SWISSPROT*=P00749.

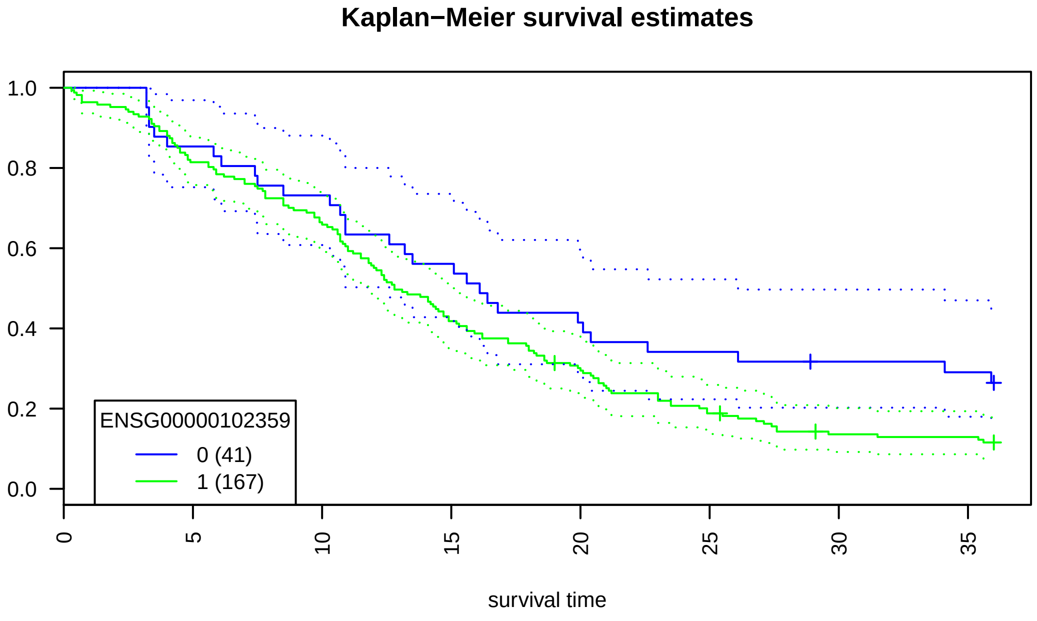

Figure 117: Each curve represents the relative number of ENSG00000102359 survived to the corresponding point of time. Dashed lines represent 0.95 confidence intervals for the curves with the corresponding color. Log-rank probability measure for the equality of these curves is 4.747793e-02. Additional annotations: *.GeneName*=SRPX2, *.GeneDesc*=sushi-repeat-containing protein, X-linked 2 [Source:HGNC Symbol;Acc:30668], *Uniprot/SWISSPROT*=O60687.

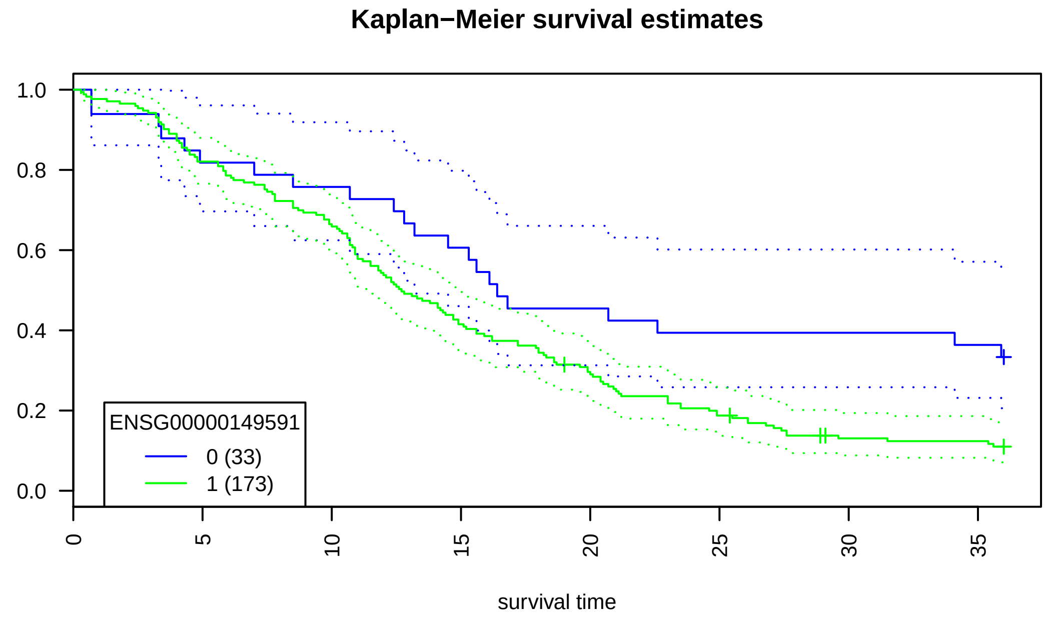

Figure 118: Each curve represents the relative number of ENSG00000149591 survived to the corresponding point of time. Dashed lines represent 0.95 confidence intervals for the curves with the corresponding color. Total of 2 observations deleted due to missingness. Log-rank probability measure for the equality of these curves is 8.307745e-03. Additional annotations: *.GeneName*=TAGLN, *.GeneDesc*=transgelin [Source:HGNC Symbol;Acc:11553], *Uniprot/SWISSPROT*=Q01995.

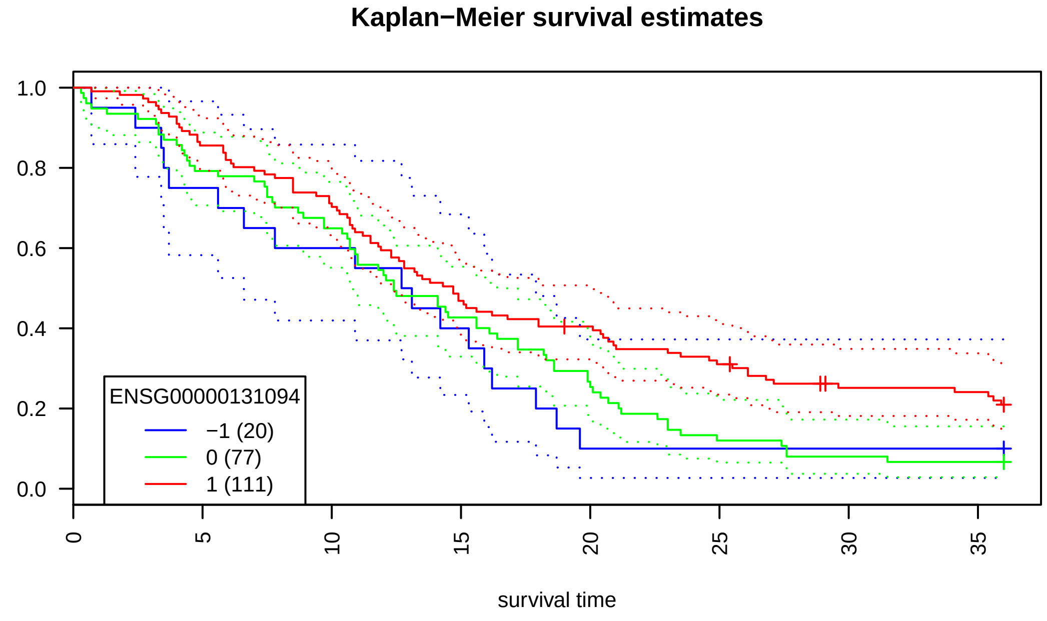

Figure 119: Each curve represents the relative number of ENSG00000131094 survived to the corresponding point of time. Dashed lines represent 0.95 confidence intervals for the curves with the corresponding color. Log-rank probability measure for the equality of these curves is 1.834348e-02. Additional annotations: *.GeneName*=C1QL1, *.GeneDesc*=complement component 1, q subcomponent-like 1 [Source:HGNC Symbol;Acc:24182], *Uniprot/SWISSPROT*=O75973.

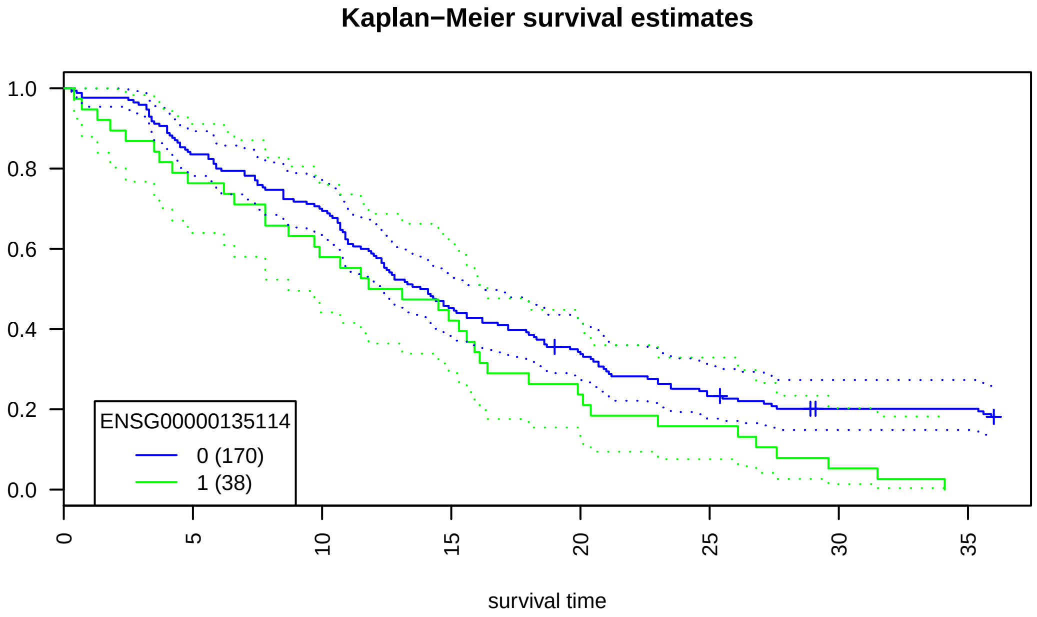

Figure 120: Each curve represents the relative number of ENSG00000135114 survived to the corresponding point of time. Dashed lines represent 0.95 confidence intervals for the curves with the corresponding color. Log-rank probability measure for the equality of these curves is 2.269761e-02.

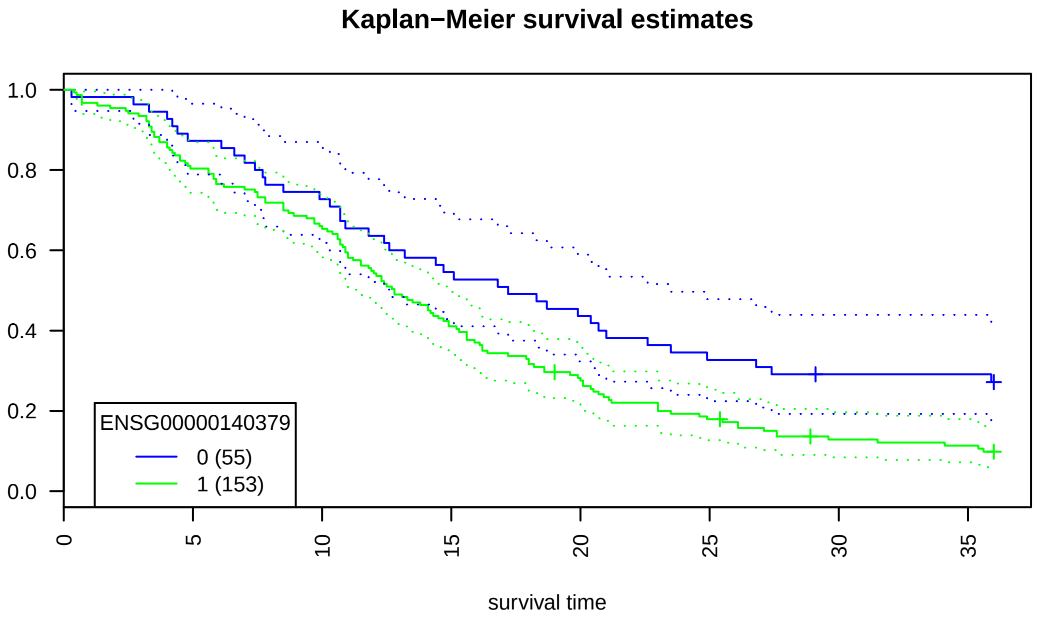

Figure 121: Each curve represents the relative number of ENSG00000140379 survived to the corresponding point of time. Dashed lines represent 0.95 confidence intervals for the curves with the corresponding color. Log-rank probability measure for the equality of these curves is 7.386227e-03. Additional annotations: *.GeneName*=BCL2A1, *.GeneDesc*=BCL2-related protein A1 [Source:HGNC Symbol;Acc:991], *Uniprot/SWISSPROT*=Q16548.

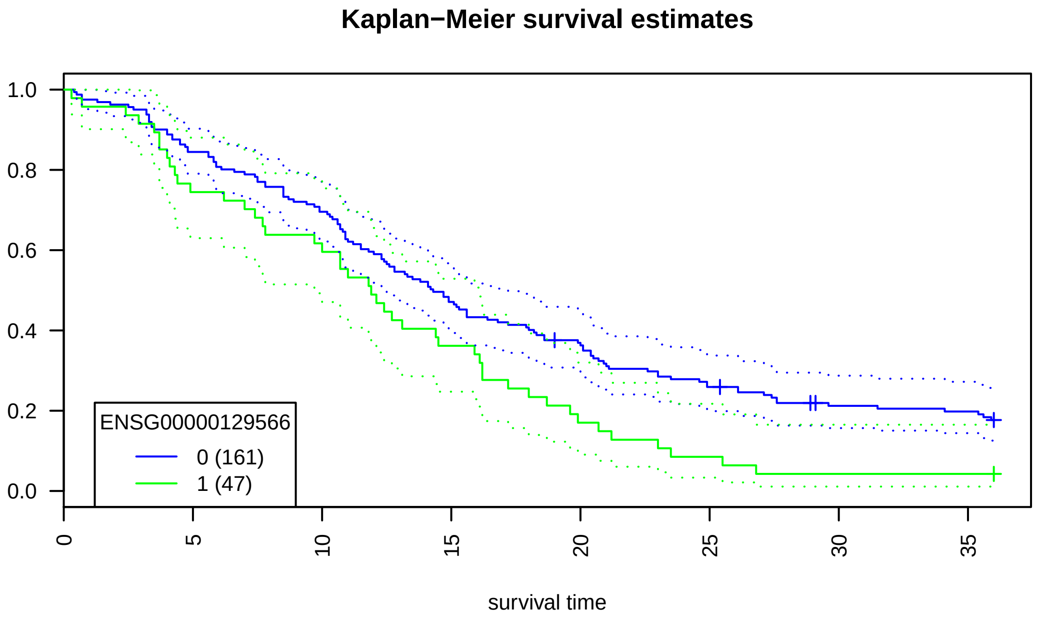

Figure 122: Each curve represents the relative number of ENSG00000129566 survived to the corresponding point of time. Dashed lines represent 0.95 confidence intervals for the curves with the corresponding color. Log-rank probability measure for the equality of these curves is 5.491567e-03.

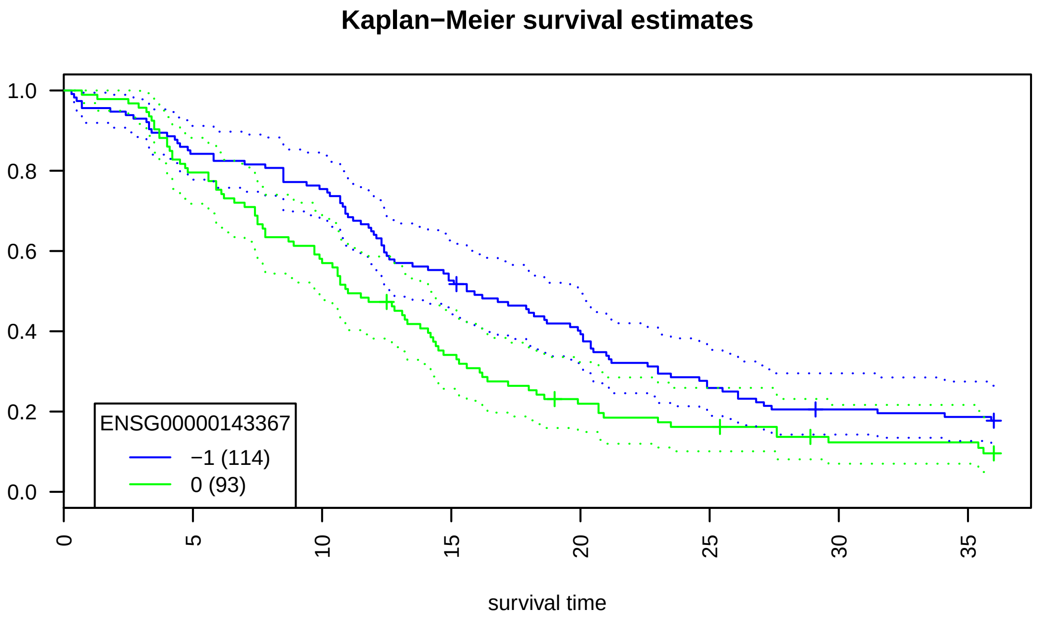

Figure 123: Each curve represents the relative number of ENSG00000143367 survived to the corresponding point of time. Dashed lines represent 0.95 confidence intervals for the curves with the corresponding color. Total of 1 observation deleted due to missingness. Log-rank probability measure for the equality of these curves is 1.037701e-02. Additional annotations: *.GeneName*=TUFT1, *.GeneDesc*=tuftelin 1 [Source:HGNC Symbol;Acc:12422], *Uniprot/SWISSPROT*=Q9NNX1.

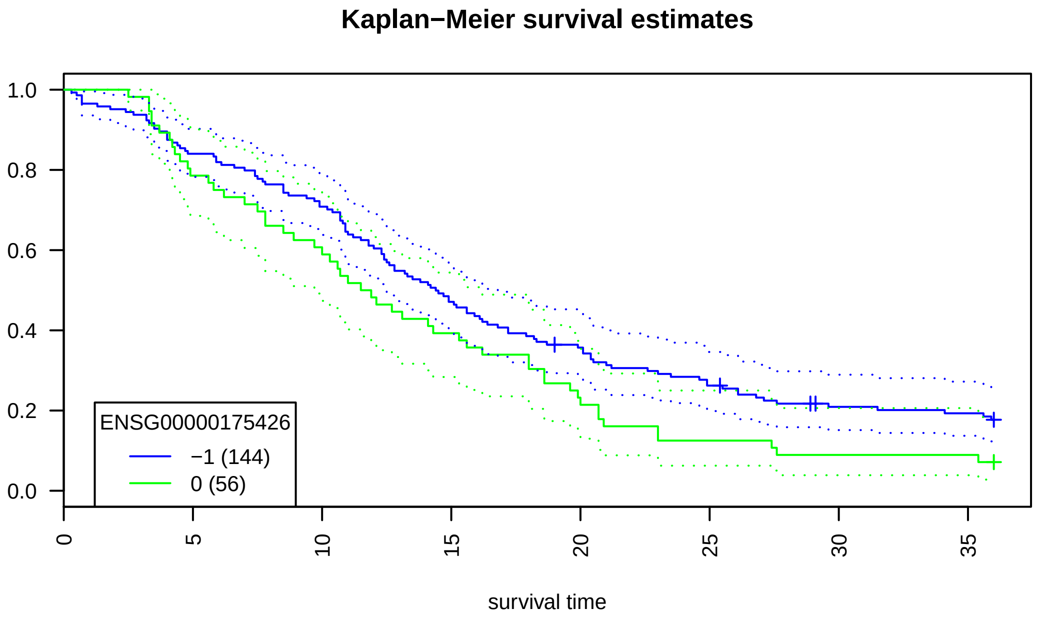

Figure 124: Each curve represents the relative number of ENSG00000175426 survived to the corresponding point of time. Dashed lines represent 0.95 confidence intervals for the curves with the corresponding color. Total of 8 observations deleted due to missingness. Log-rank probability measure for the equality of these curves is 3.336588e-02. Additional annotations: *.GeneName*=PCSK1, *.GeneDesc*=proprotein convertase subtilisin/kexin type 1 [Source:HGNC Symbol;Acc:8743], *Uniprot/SWISSPROT*=P29120.

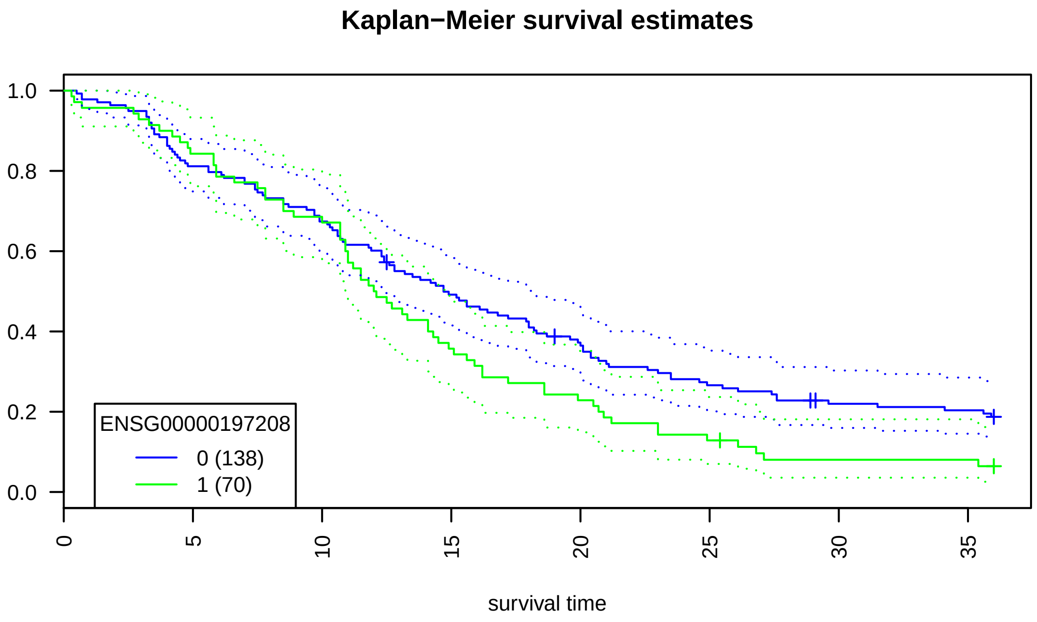

Figure 125: Each curve represents the relative number of ENSG00000197208 survived to the corresponding point of time. Dashed lines represent 0.95 confidence intervals for the curves with the corresponding color. Log-rank probability measure for the equality of these curves is 2.025889e-02.

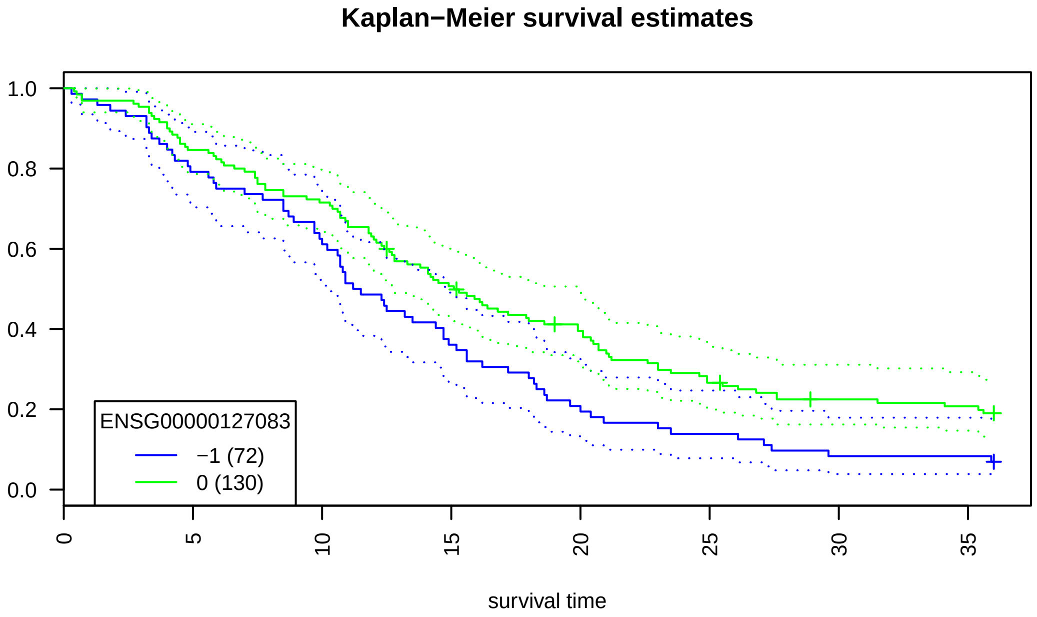

Figure 126: Each curve represents the relative number of ENSG00000127083 survived to the corresponding point of time. Dashed lines represent 0.95 confidence intervals for the curves with the corresponding color. Total of 6 observations deleted due to missingness. Log-rank probability measure for the equality of these curves is 5.484835e-03.

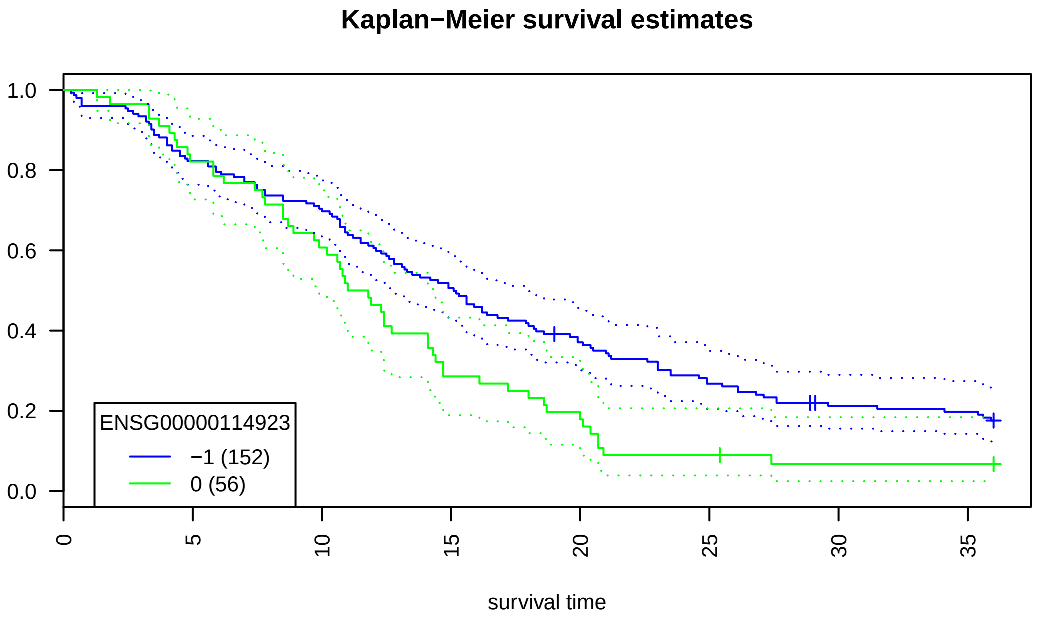

Figure 127: Each curve represents the relative number of ENSG00000114923 survived to the corresponding point of time. Dashed lines represent 0.95 confidence intervals for the curves with the corresponding color. Log-rank probability measure for the equality of these curves is 4.168374e-03. Additional annotations: *.GeneName*=SLC4A3, *.GeneDesc*=solute carrier family 4, anion exchanger, member 3 [Source:HGNC Symbol;Acc:11029], *Uniprot/SWISSPROT*=P48751.

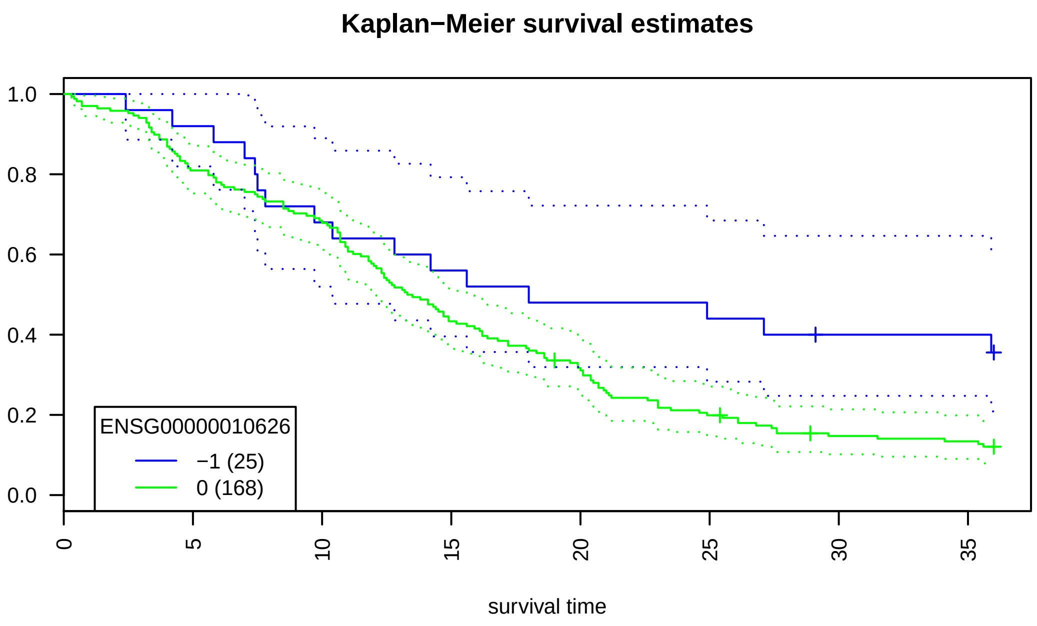

Figure 128: Each curve represents the relative number of ENSG00000010626 survived to the corresponding point of time. Dashed lines represent 0.95 confidence intervals for the curves with the corresponding color. Total of 15 observations deleted due to missingness. Log-rank probability measure for the equality of these curves is 1.958754e-02.

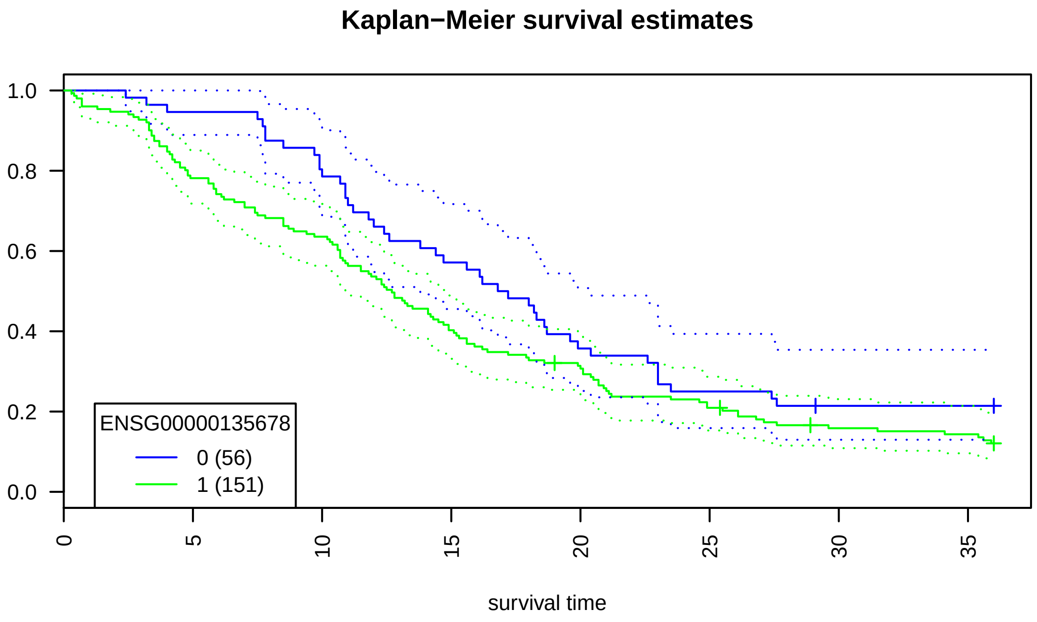

Figure 129: Each curve represents the relative number of ENSG00000135678 survived to the corresponding point of time. Dashed lines represent 0.95 confidence intervals for the curves with the corresponding color. Total of 1 observation deleted due to missingness. Log-rank probability measure for the equality of these curves is 4.602595e-02. Additional annotations: *.GeneName*=CPM, *.GeneDesc*=carboxypeptidase M [Source:HGNC Symbol;Acc:2311], *Uniprot/SWISSPROT*=P14384.

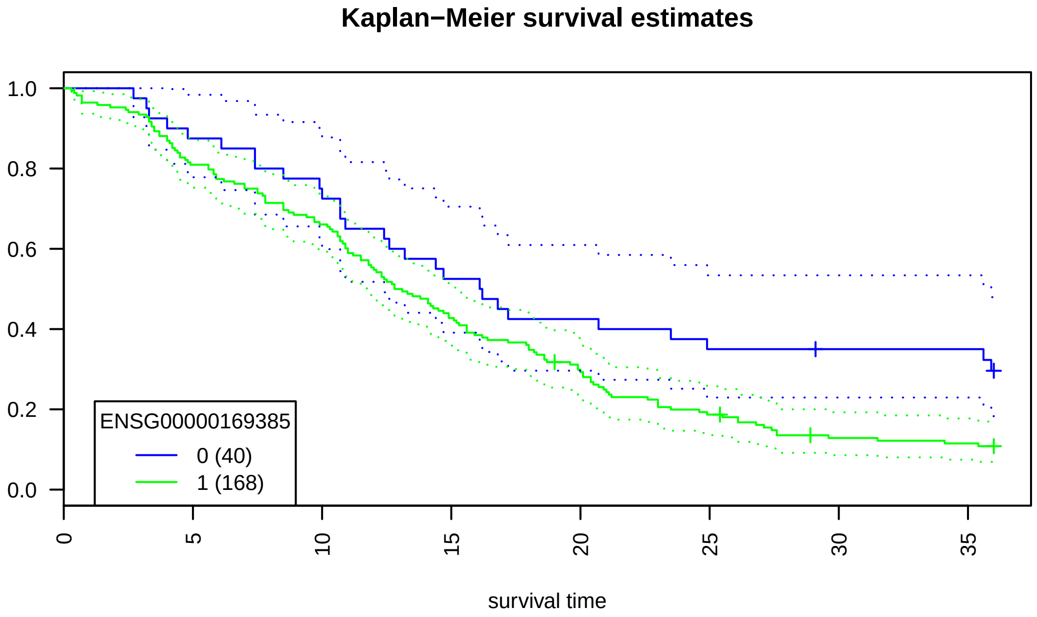

Figure 130: Each curve represents the relative number of ENSG00000169385 survived to the corresponding point of time. Dashed lines represent 0.95 confidence intervals for the curves with the corresponding color. Log-rank probability measure for the equality of these curves is 1.467089e-02. Additional annotations: *.GeneName*=RNASE2, *.GeneDesc*=ribonuclease, RNase A family, 2 (liver, eosinophil-derived neurotoxin) [Source:HGNC Symbol;Acc:10045], *Uniprot/SWISSPROT*=P10153.

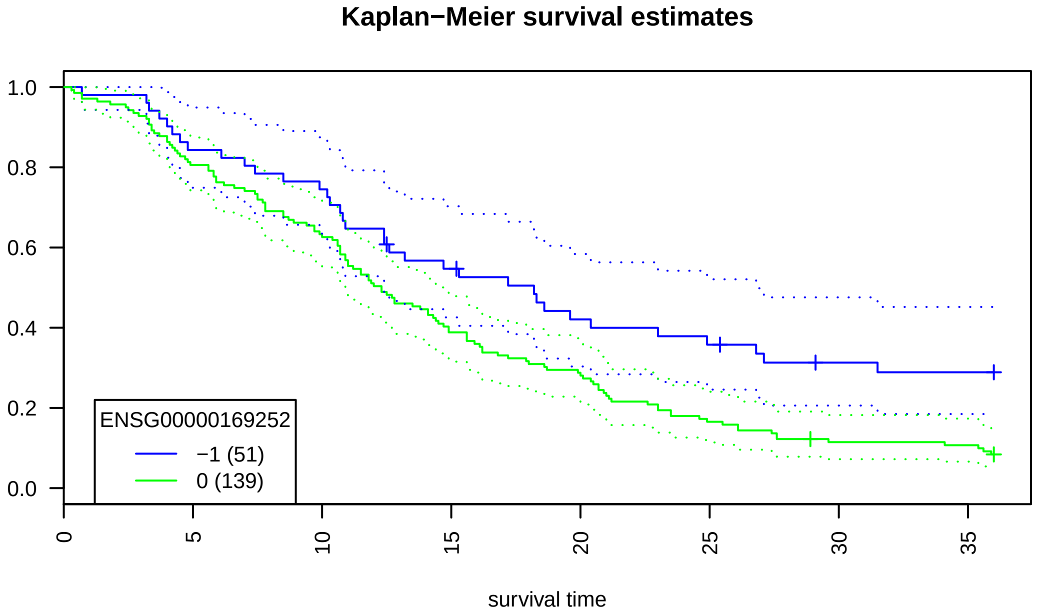

Figure 131: Each curve represents the relative number of ENSG00000169252 survived to the corresponding point of time. Dashed lines represent 0.95 confidence intervals for the curves with the corresponding color. Total of 18 observations deleted due to missingness. Log-rank probability measure for the equality of these curves is 3.725144e-03.

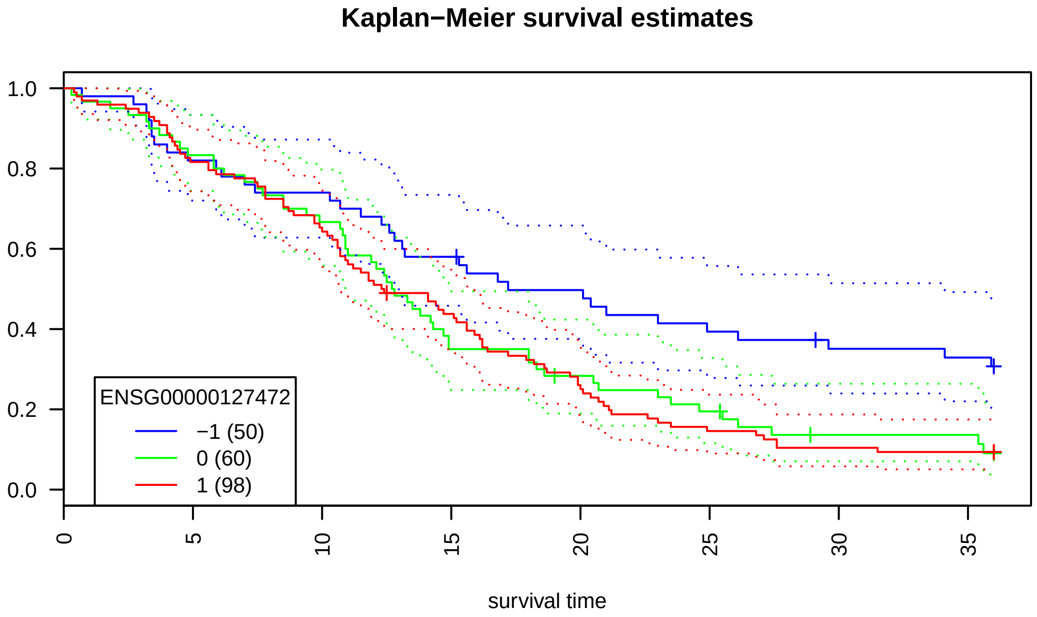

Figure 132: Each curve represents the relative number of ENSG00000127472 survived to the corresponding point of time. Dashed lines represent 0.95 confidence intervals for the curves with the corresponding color. Log-rank probability measure for the equality of these curves is 6.878145e-03.

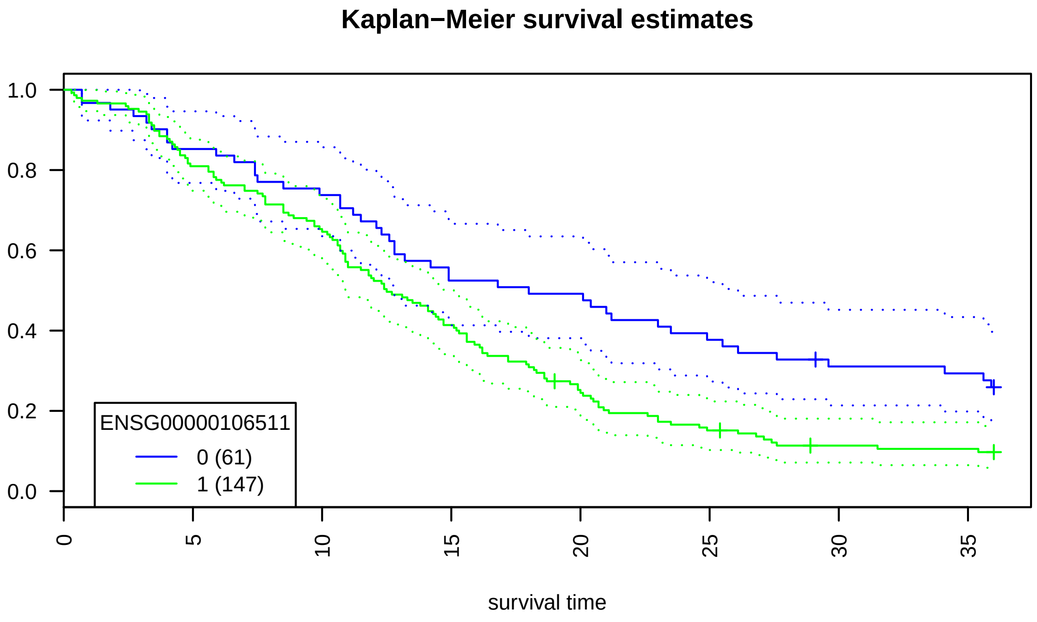

Figure 133: Each curve represents the relative number of ENSG00000106511 survived to the corresponding point of time. Dashed lines represent 0.95 confidence intervals for the curves with the corresponding color. Log-rank probability measure for the equality of these curves is 1.914883e-03. Additional annotations: *.GeneName*=MEOX2, *.GeneDesc*=mesenchyme homeobox 2 [Source:HGNC Symbol;Acc:7014], *Uniprot/SWISSPROT*=P50222.

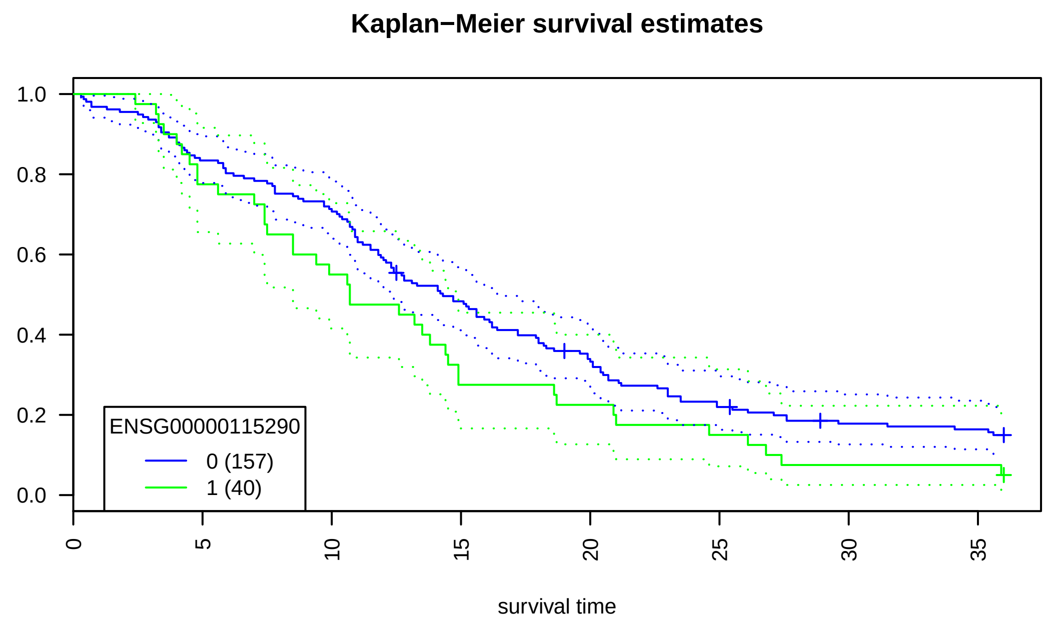

Figure 134: Each curve represents the relative number of ENSG00000115290 survived to the corresponding point of time. Dashed lines represent 0.95 confidence intervals for the curves with the corresponding color. Total of 11 observations deleted due to missingness. Log-rank probability measure for the equality of these curves is 4.812722e-02.

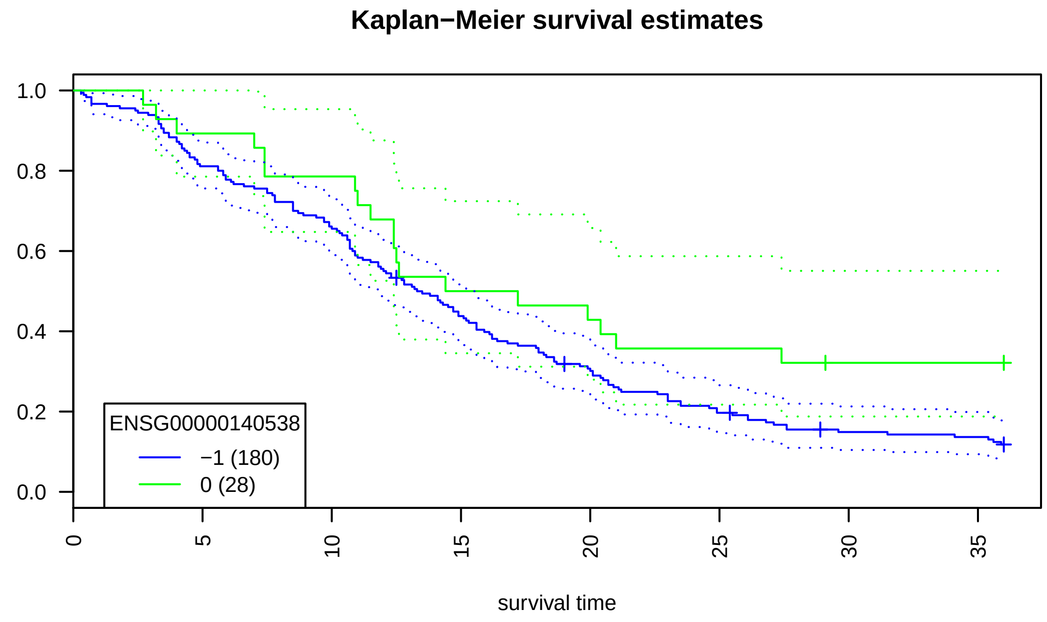

Figure 135: Each curve represents the relative number of ENSG00000140538 survived to the corresponding point of time. Dashed lines represent 0.95 confidence intervals for the curves with the corresponding color. Log-rank probability measure for the equality of these curves is 4.162891e-02. Additional annotations: *.GeneName*=NTRK3, *.GeneDesc*=neurotrophic tyrosine kinase, receptor, type 3 [Source:HGNC Symbol;Acc:8033], *Uniprot/SWISSPROT*=Q16288.

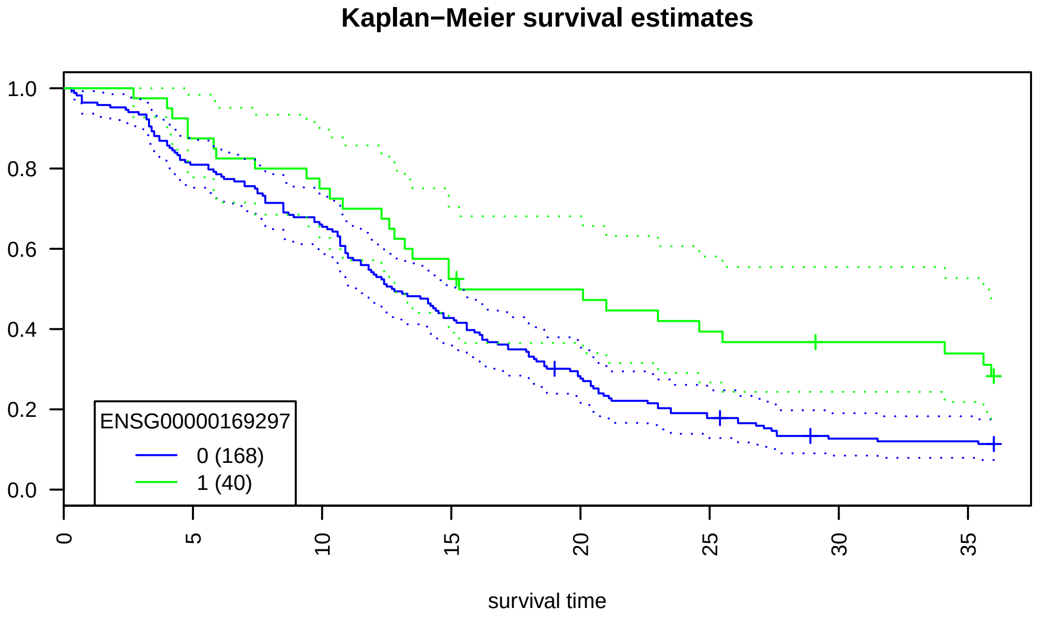

Figure 136: Each curve represents the relative number of ENSG00000169297 survived to the corresponding point of time. Dashed lines represent 0.95 confidence intervals for the curves with the corresponding color. Log-rank probability measure for the equality of these curves is 8.968465e-03.

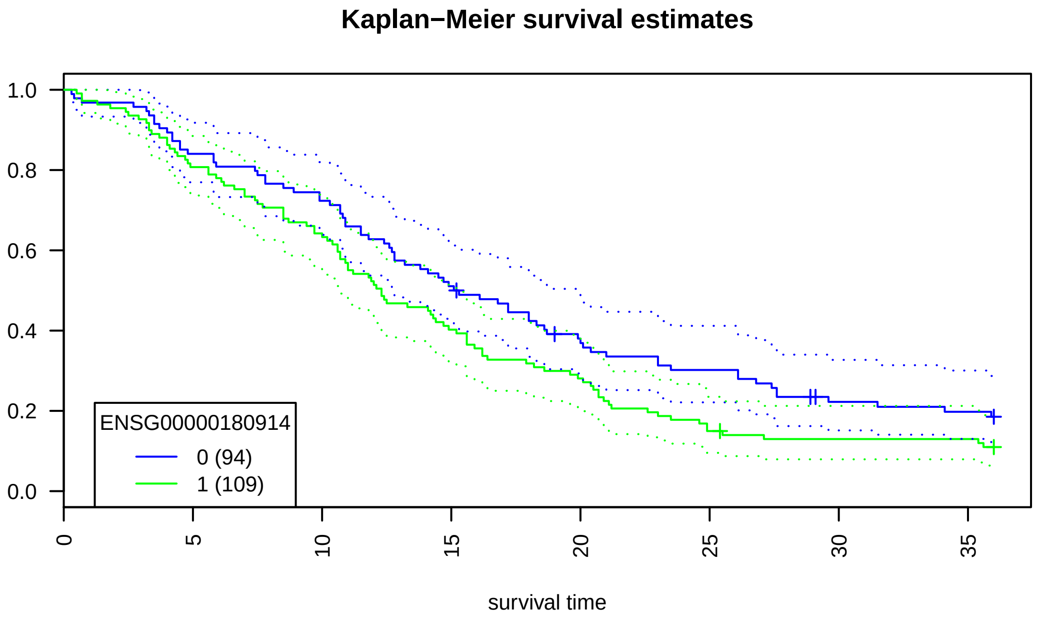

Figure 137: Each curve represents the relative number of ENSG00000180914 survived to the corresponding point of time. Dashed lines represent 0.95 confidence intervals for the curves with the corresponding color. Total of 5 observations deleted due to missingness. Log-rank probability measure for the equality of these curves is 4.017741e-02. Additional annotations: *.GeneName*=OXTR, *.GeneDesc*=oxytocin receptor [Source:HGNC Symbol;Acc:8529], *Uniprot/SWISSPROT*=P30559.

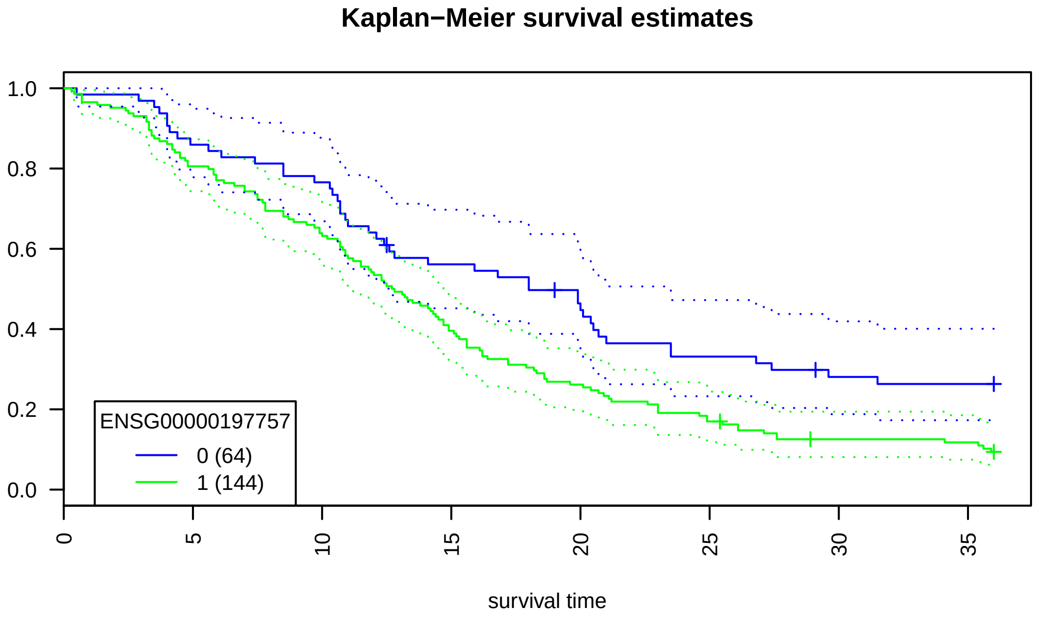

Figure 138: Each curve represents the relative number of ENSG00000197757 survived to the corresponding point of time. Dashed lines represent 0.95 confidence intervals for the curves with the corresponding color. Log-rank probability measure for the equality of these curves is 3.260395e-03. Additional annotations: *.GeneName*=HOXC6, *.GeneDesc*=homeobox C6 [Source:HGNC Symbol;Acc:5128], *Uniprot/SWISSPROT*=P09630.

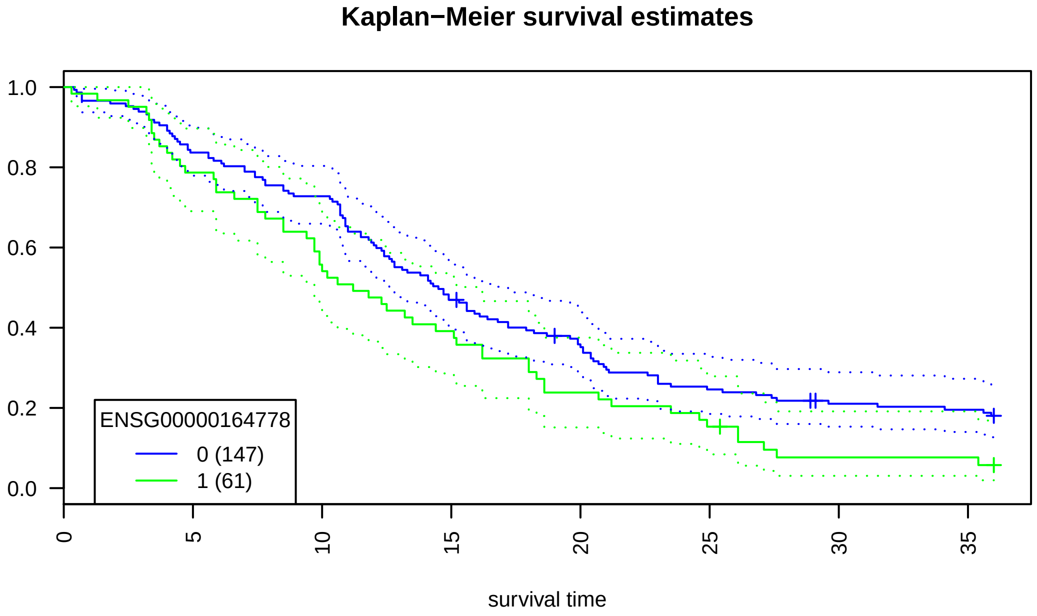

Figure 139: Each curve represents the relative number of ENSG00000164778 survived to the corresponding point of time. Dashed lines represent 0.95 confidence intervals for the curves with the corresponding color. Log-rank probability measure for the equality of these curves is 2.130232e-02.

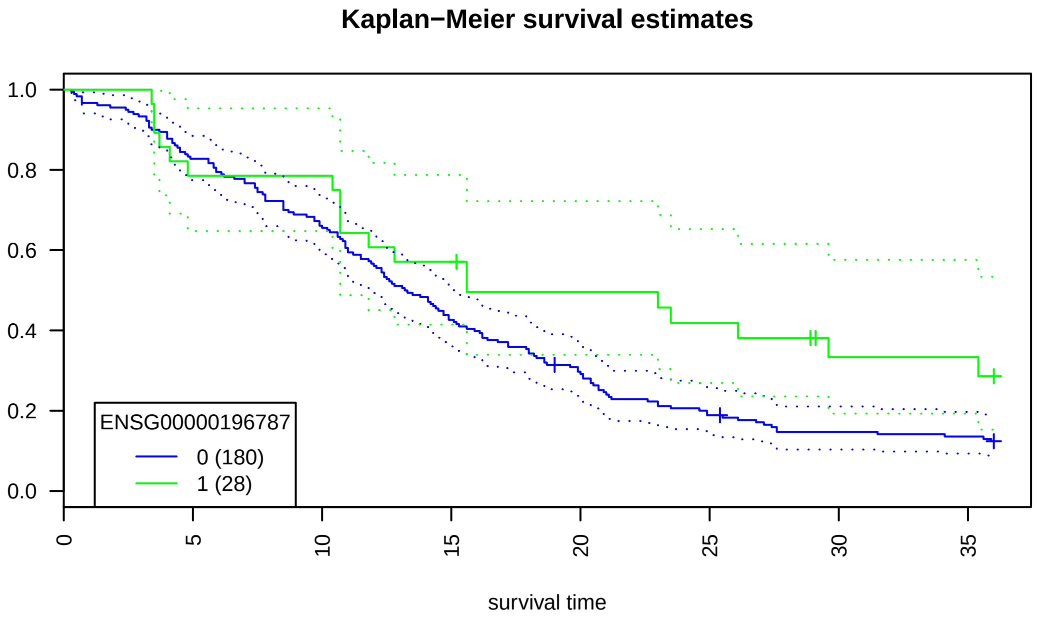

Figure 140: Each curve represents the relative number of ENSG00000196787 survived to the corresponding point of time. Dashed lines represent 0.95 confidence intervals for the curves with the corresponding color. Log-rank probability measure for the equality of these curves is 3.838757e-02.

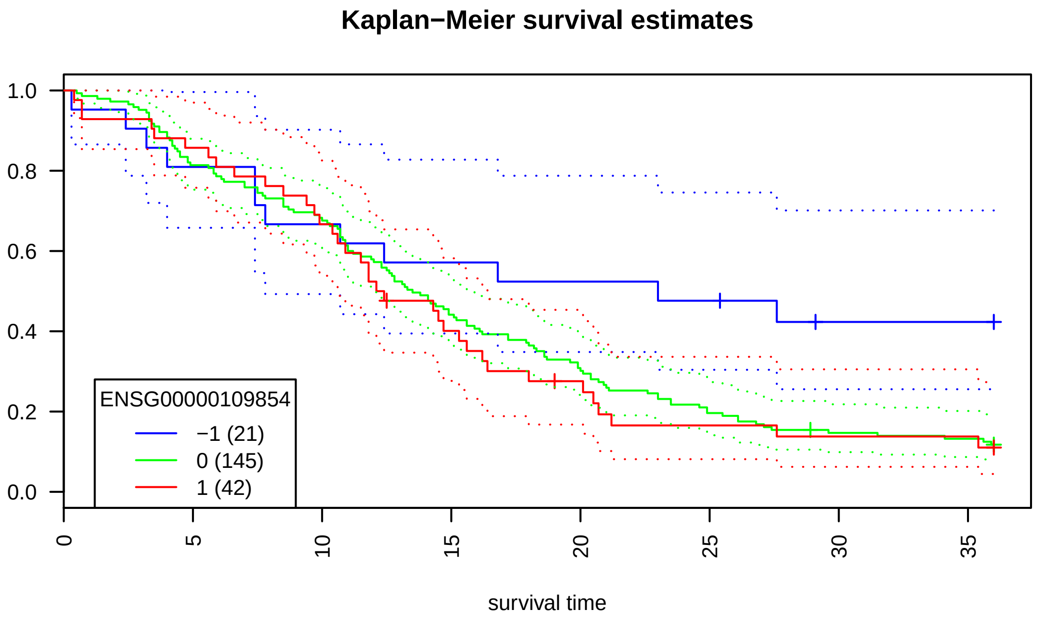

Figure 141: Each curve represents the relative number of ENSG00000109854 survived to the corresponding point of time. Dashed lines represent 0.95 confidence intervals for the curves with the corresponding color. Log-rank probability measure for the equality of these curves is 4.56366e-02.

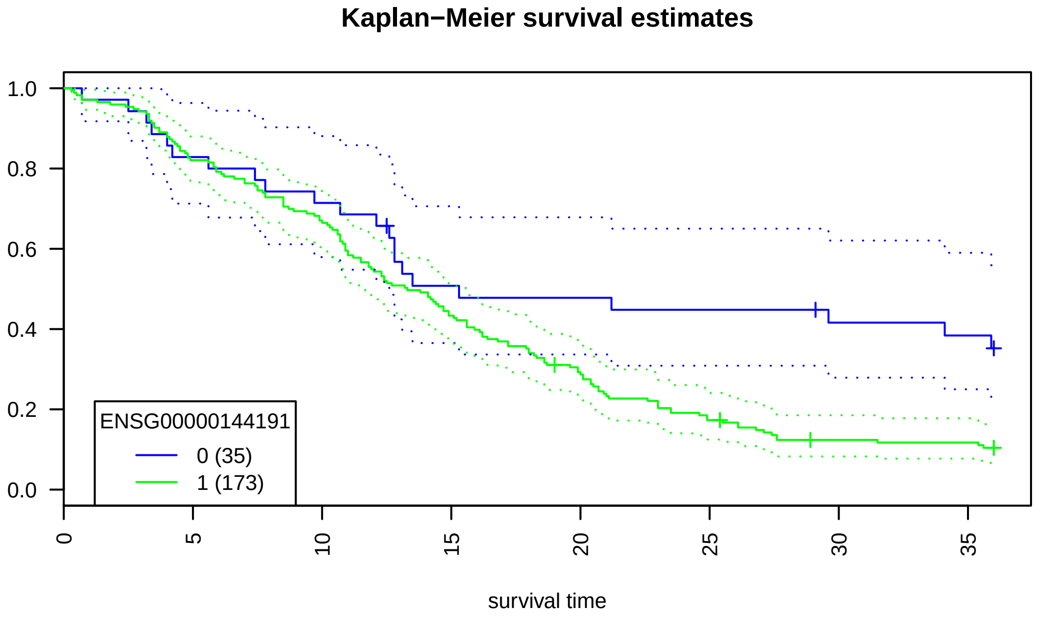

Figure 142: Each curve represents the relative number of ENSG00000144191 survived to the corresponding point of time. Dashed lines represent 0.95 confidence intervals for the curves with the corresponding color. Log-rank probability measure for the equality of these curves is 5.123026e-03. Additional annotations: *.GeneName*=CNGA3, *.GeneDesc*=cyclic nucleotide gated channel alpha 3 [Source:HGNC Symbol;Acc:2150], *Uniprot/SWISSPROT*=Q16281.

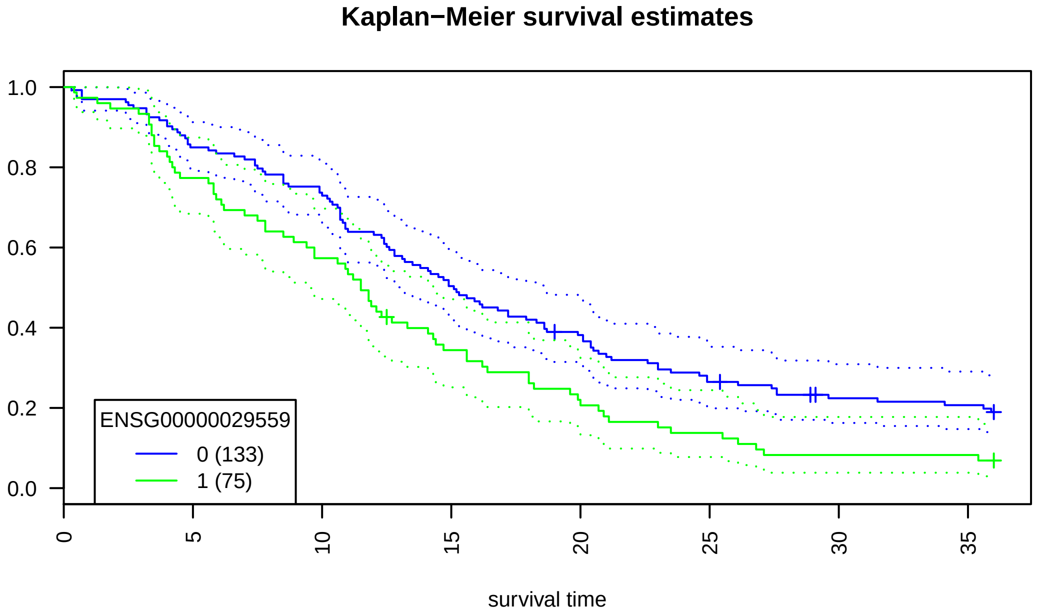

Figure 143: Each curve represents the relative number of ENSG00000029559 survived to the corresponding point of time. Dashed lines represent 0.95 confidence intervals for the curves with the corresponding color. Log-rank probability measure for the equality of these curves is 2.372866e-03.

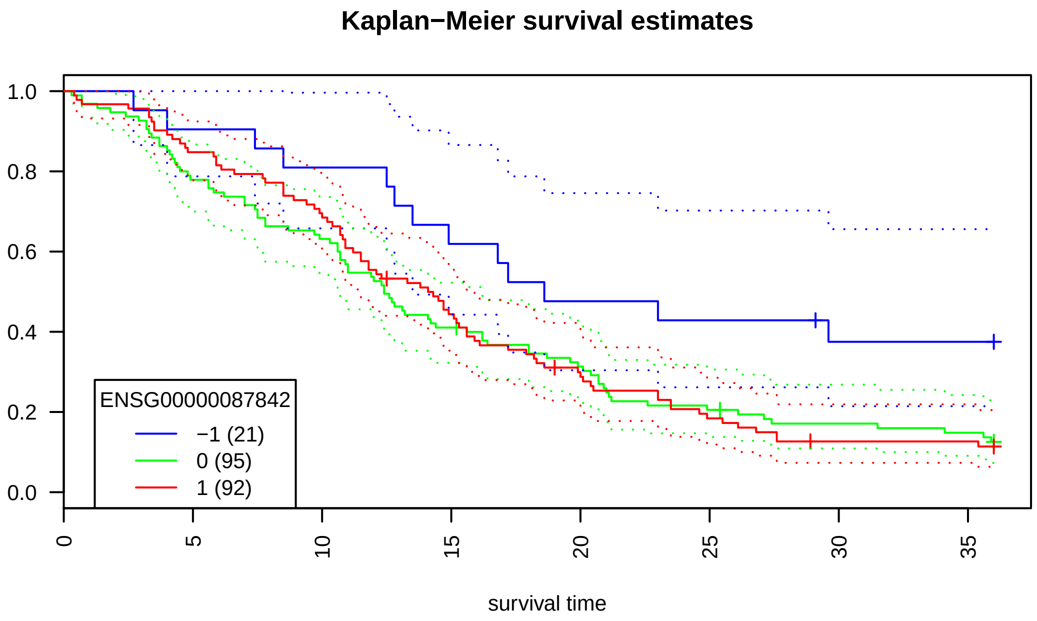

Figure 144: Each curve represents the relative number of ENSG00000087842 survived to the corresponding point of time. Dashed lines represent 0.95 confidence intervals for the curves with the corresponding color. Log-rank probability measure for the equality of these curves is 3.745253e-02.

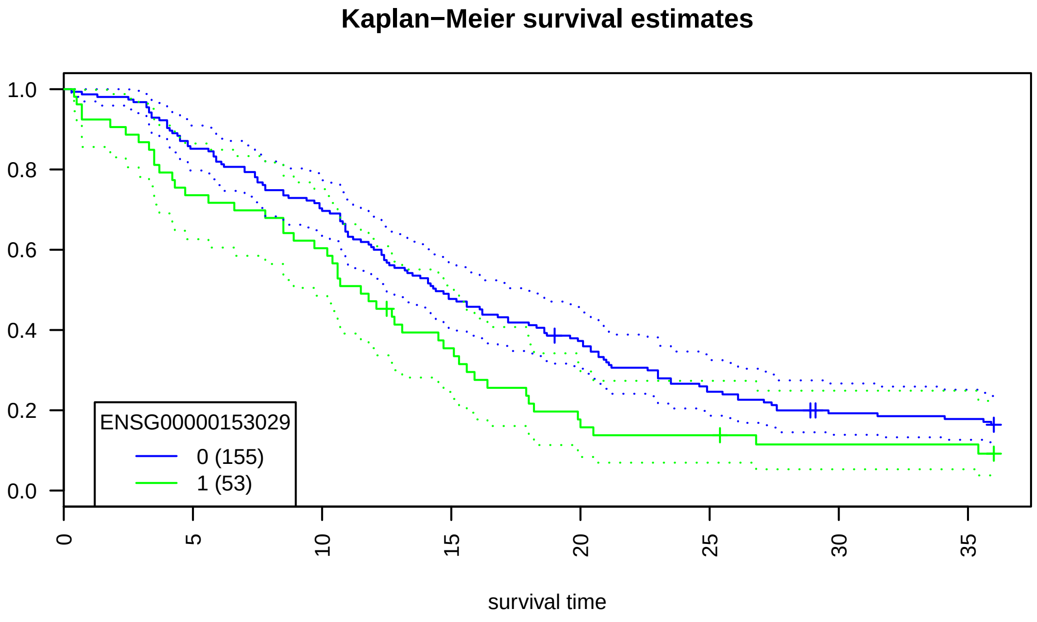

Figure 145: Each curve represents the relative number of ENSG00000153029 survived to the corresponding point of time. Dashed lines represent 0.95 confidence intervals for the curves with the corresponding color. Log-rank probability measure for the equality of these curves is 1.673967e-02.

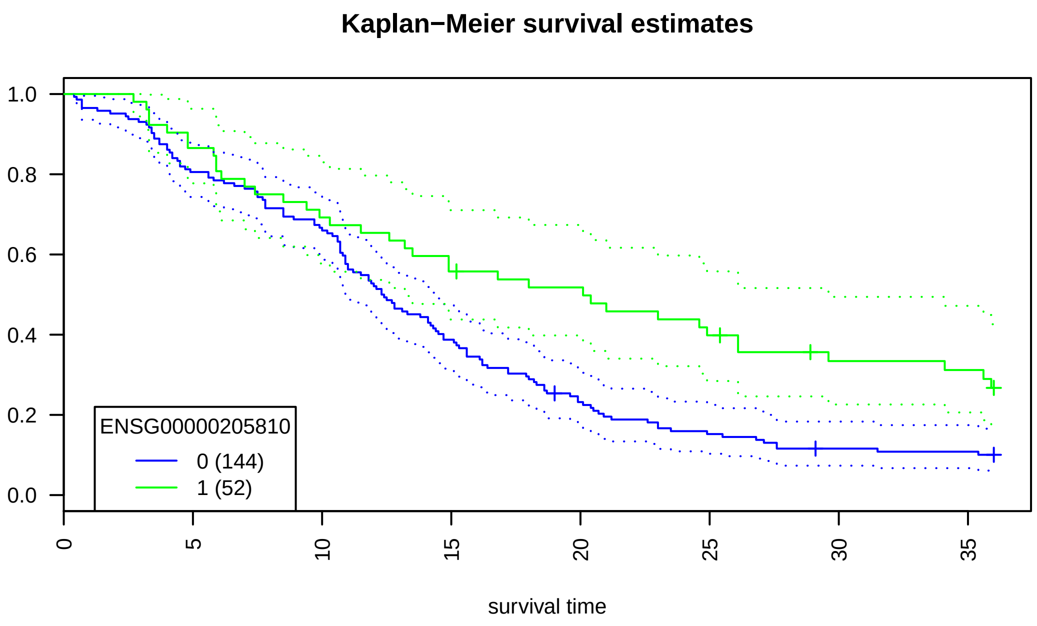

Figure 146: Each curve represents the relative number of ENSG00000205810 survived to the corresponding point of time. Dashed lines represent 0.95 confidence intervals for the curves with the corresponding color. Total of 12 observations deleted due to missingness. Log-rank probability measure for the equality of these curves is 1.749381e-03.

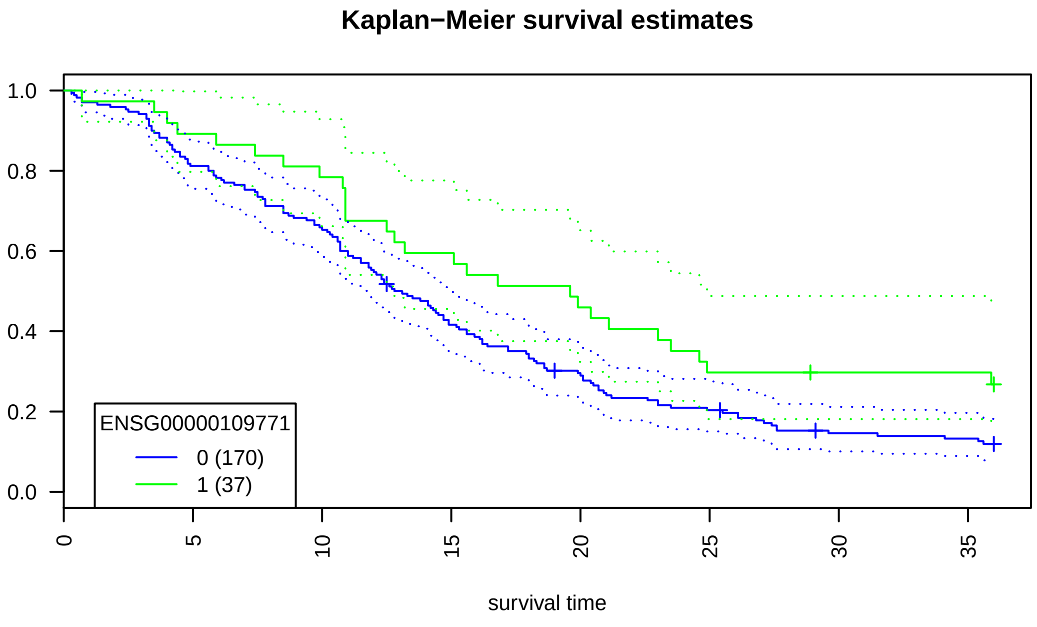

Figure 147: Each curve represents the relative number of ENSG00000109771 survived to the corresponding point of time. Dashed lines represent 0.95 confidence intervals for the curves with the corresponding color. Total of 1 observation deleted due to missingness. Log-rank probability measure for the equality of these curves is 2.542412e-02.

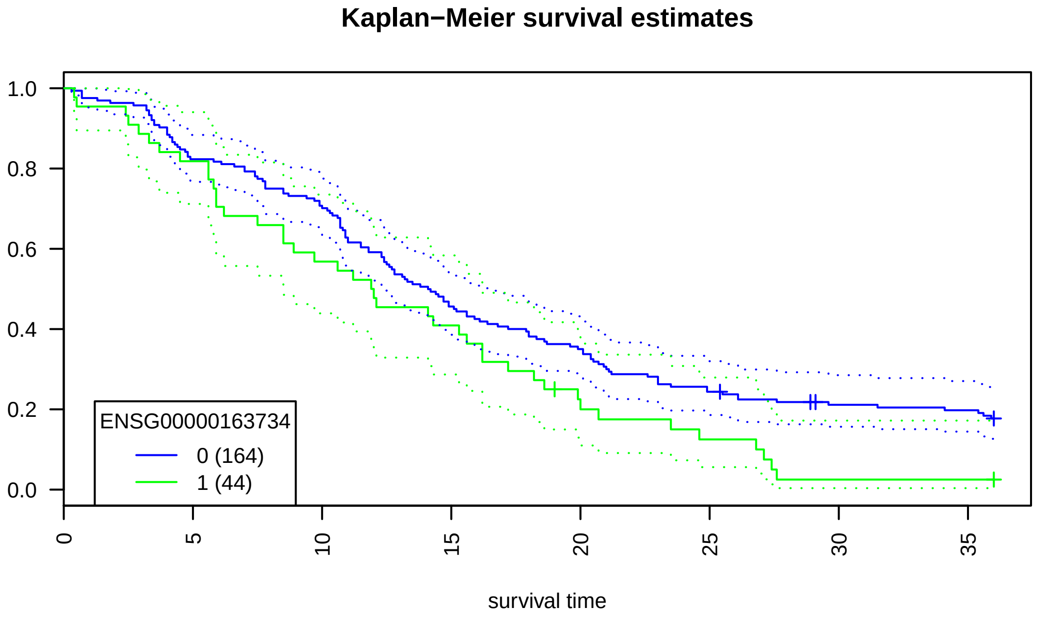

Figure 148: Each curve represents the relative number of ENSG00000163734 survived to the corresponding point of time. Dashed lines represent 0.95 confidence intervals for the curves with the corresponding color. Log-rank probability measure for the equality of these curves is 1.724340e-02.

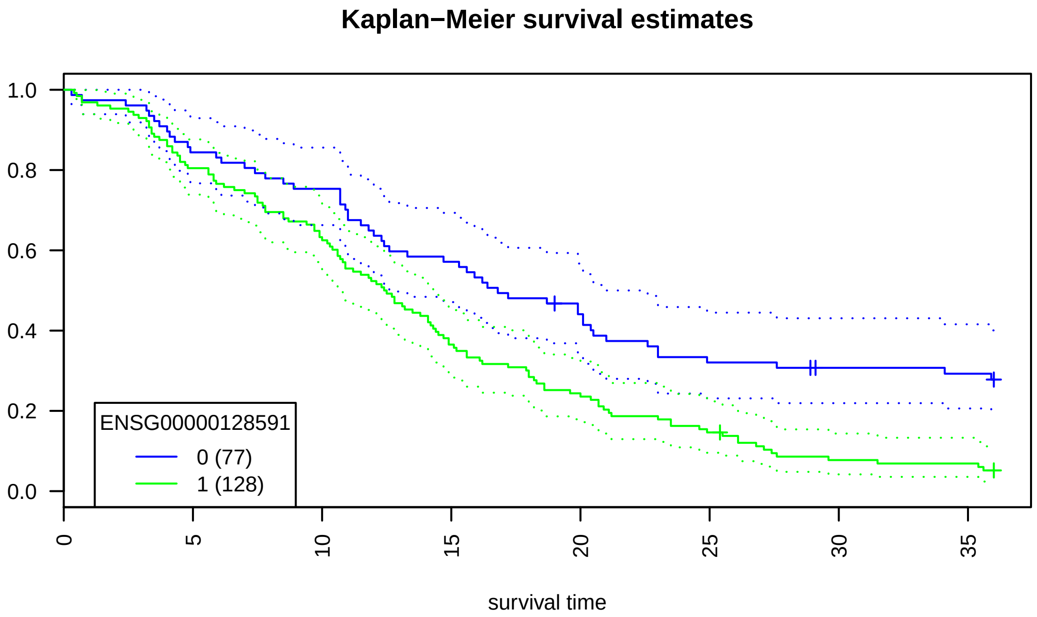

Figure 149: Each curve represents the relative number of ENSG00000128591 survived to the corresponding point of time. Dashed lines represent 0.95 confidence intervals for the curves with the corresponding color. Total of 3 observations deleted due to missingness. Log-rank probability measure for the equality of these curves is 1.009895e-04. Additional annotations: *.GeneName*=FLNC, *.GeneDesc*=filamin C, gamma [Source:HGNC Symbol;Acc:3756], *Uniprot/SWISSPROT*=Q14315.

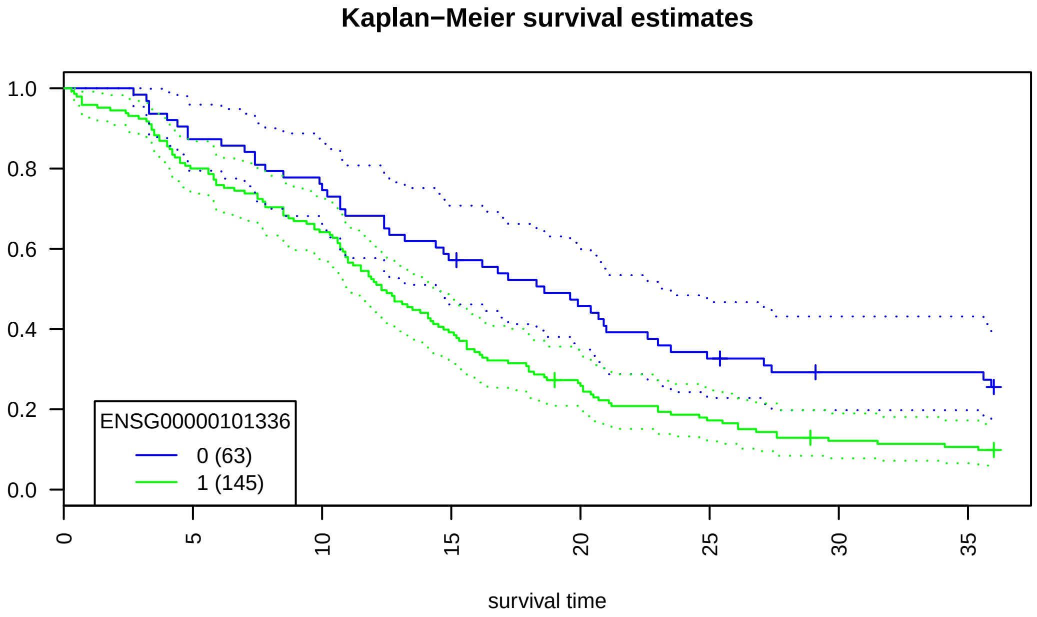

Figure 150: Each curve represents the relative number of ENSG00000101336 survived to the corresponding point of time. Dashed lines represent 0.95 confidence intervals for the curves with the corresponding color. Log-rank probability measure for the equality of these curves is 1.971287e-03. Additional annotations: *.GeneName*=HCK, *.GeneDesc*=hemopoietic cell kinase [Source:HGNC Symbol;Acc:4840], *Uniprot/SWISSPROT*=P08631.

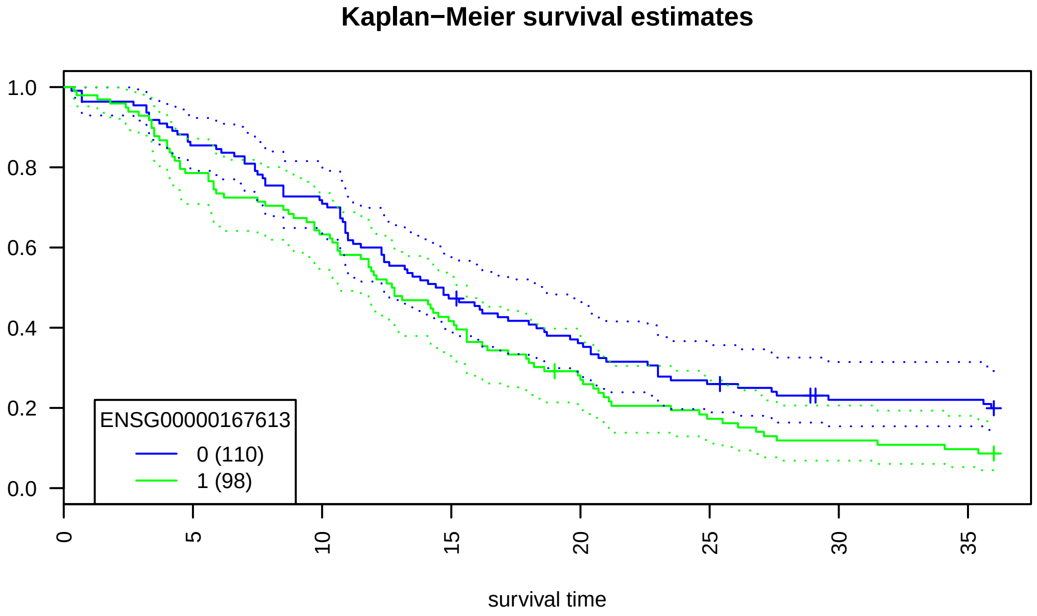

Figure 151: Each curve represents the relative number of ENSG00000167613 survived to the corresponding point of time. Dashed lines represent 0.95 confidence intervals for the curves with the corresponding color. Log-rank probability measure for the equality of these curves is 3.875319e-02.

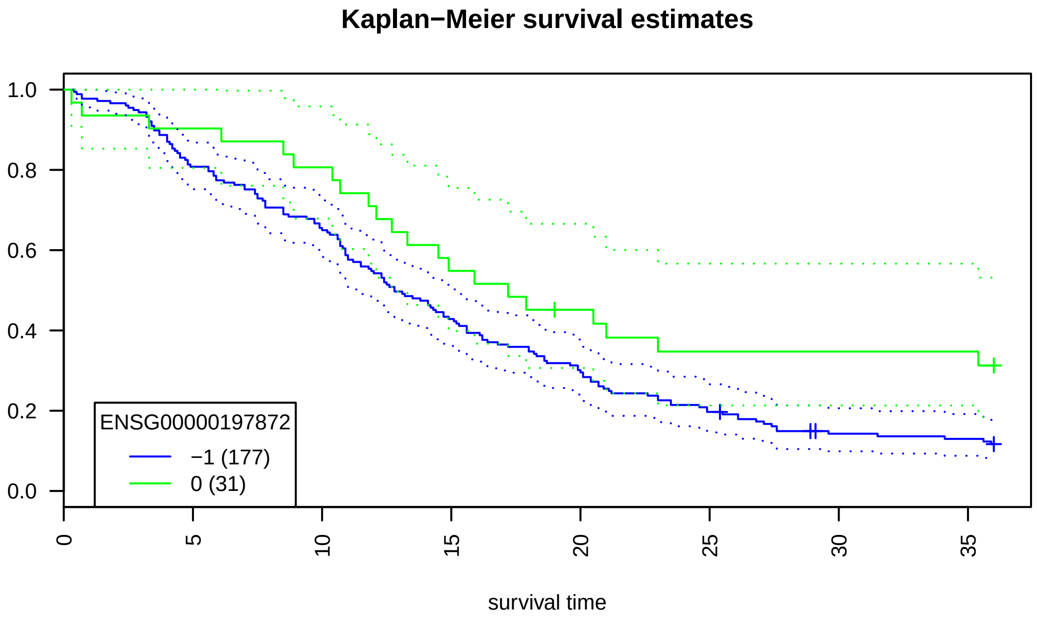

Figure 152: Each curve represents the relative number of ENSG00000197872 survived to the corresponding point of time. Dashed lines represent 0.95 confidence intervals for the curves with the corresponding color. Log-rank probability measure for the equality of these curves is 2.256624e-02. Additional annotations: *.GeneName*=FAM49A, *.GeneDesc*=family with sequence similarity 49, member A [Source:HGNC Symbol;Acc:25373], *Uniprot/SWISSPROT*=Q9H0Q0.

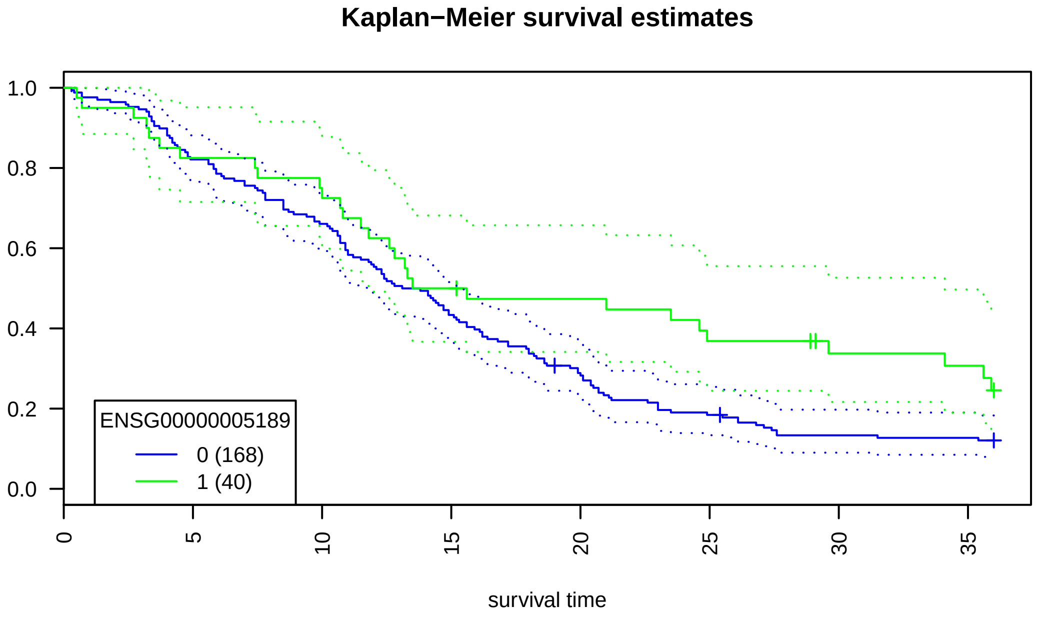

Figure 153: Each curve represents the relative number of ENSG00000005189 survived to the corresponding point of time. Dashed lines represent 0.95 confidence intervals for the curves with the corresponding color. Log-rank probability measure for the equality of these curves is 3.692041e-02.

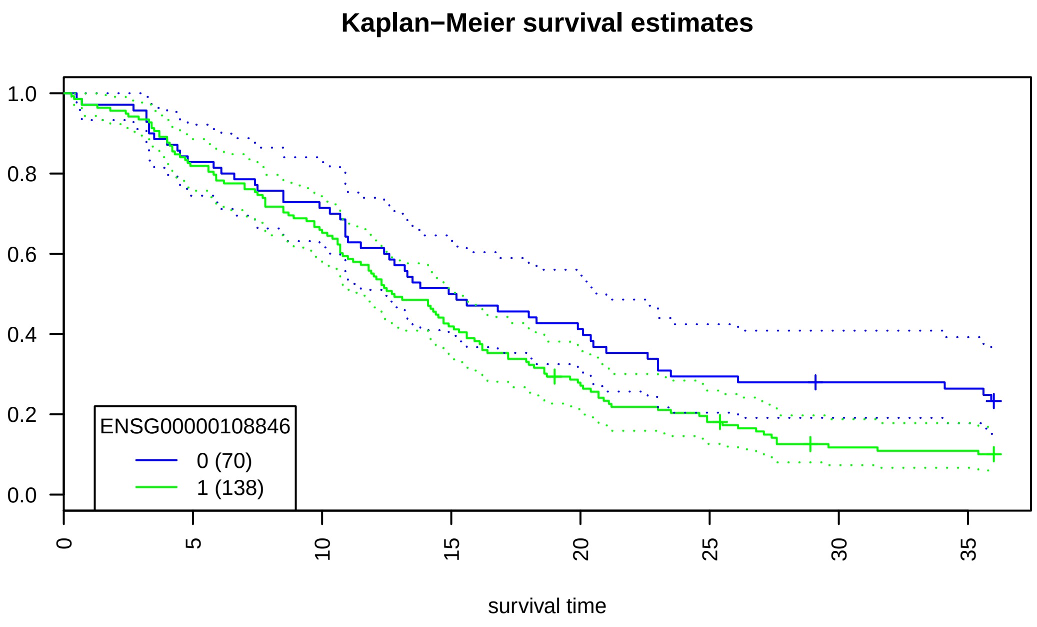

Figure 154: Each curve represents the relative number of ENSG00000108846 survived to the corresponding point of time. Dashed lines represent 0.95 confidence intervals for the curves with the corresponding color. Log-rank probability measure for the equality of these curves is 3.095684e-02. Additional annotations: *.GeneName*=ABCC3, *.GeneDesc*=ATP-binding cassette, sub-family C (CFTR/MRP), member 3 [Source:HGNC Symbol;Acc:54], *Uniprot/SWISSPROT*=O15438.

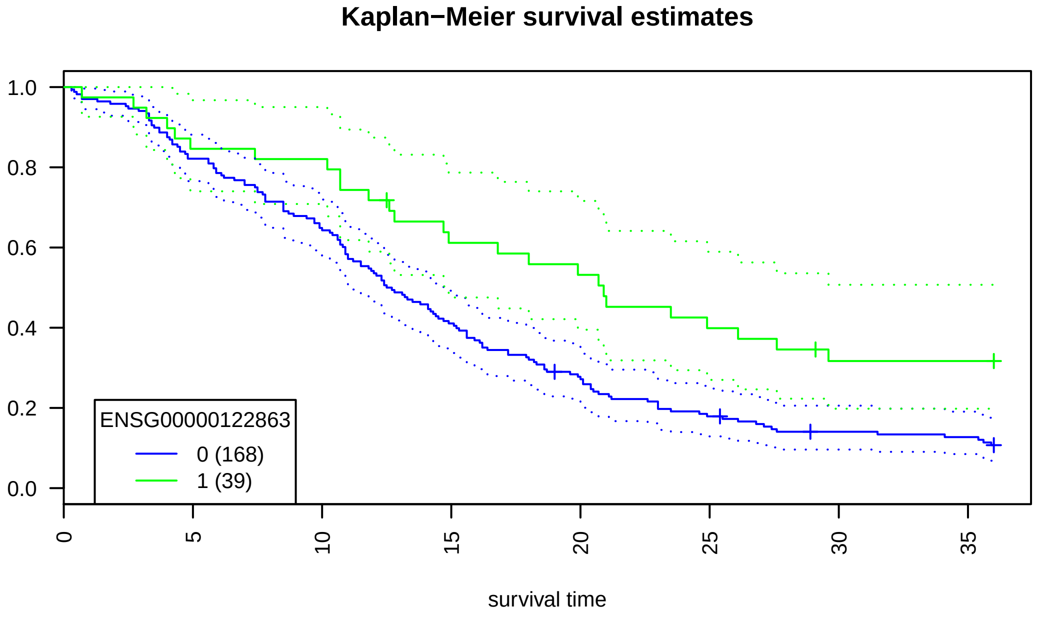

Figure 155: Each curve represents the relative number of ENSG00000122863 survived to the corresponding point of time. Dashed lines represent 0.95 confidence intervals for the curves with the corresponding color. Total of 1 observation deleted due to missingness. Log-rank probability measure for the equality of these curves is 1.893678e-03.

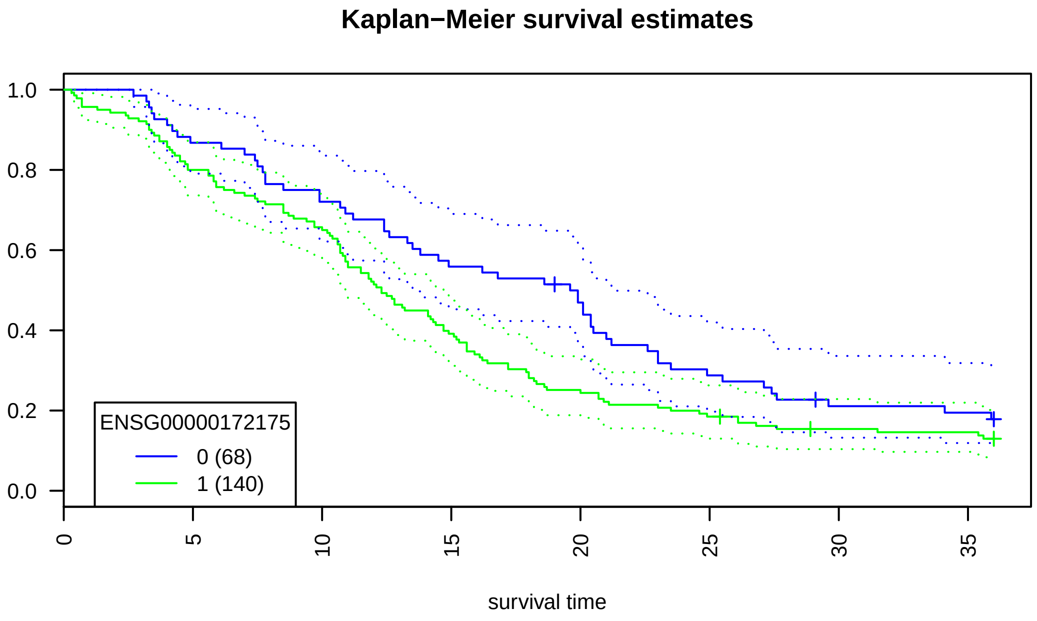

Figure 156: Each curve represents the relative number of ENSG00000172175 survived to the corresponding point of time. Dashed lines represent 0.95 confidence intervals for the curves with the corresponding color. Log-rank probability measure for the equality of these curves is 3.378065e-02. Additional annotations: *.GeneName*=MALT1, *.GeneDesc*=mucosa associated lymphoid tissue lymphoma translocation gene 1 [Source:HGNC Symbol;Acc:6819], *Uniprot/SWISSPROT*=Q9UDY8.

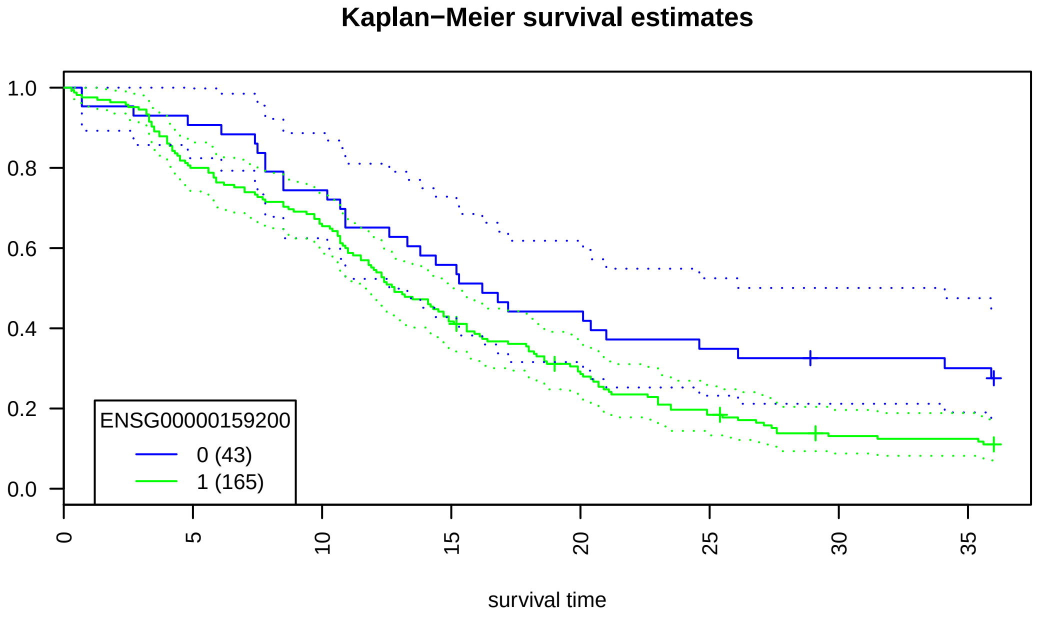

Figure 157: Each curve represents the relative number of ENSG00000159200 survived to the corresponding point of time. Dashed lines represent 0.95 confidence intervals for the curves with the corresponding color. Log-rank probability measure for the equality of these curves is 1.802075e-02. Additional annotations: *.GeneName*=RCAN1, *.GeneDesc*=regulator of calcineurin 1 [Source:HGNC Symbol;Acc:3040], *Uniprot/SWISSPROT*=P53805.

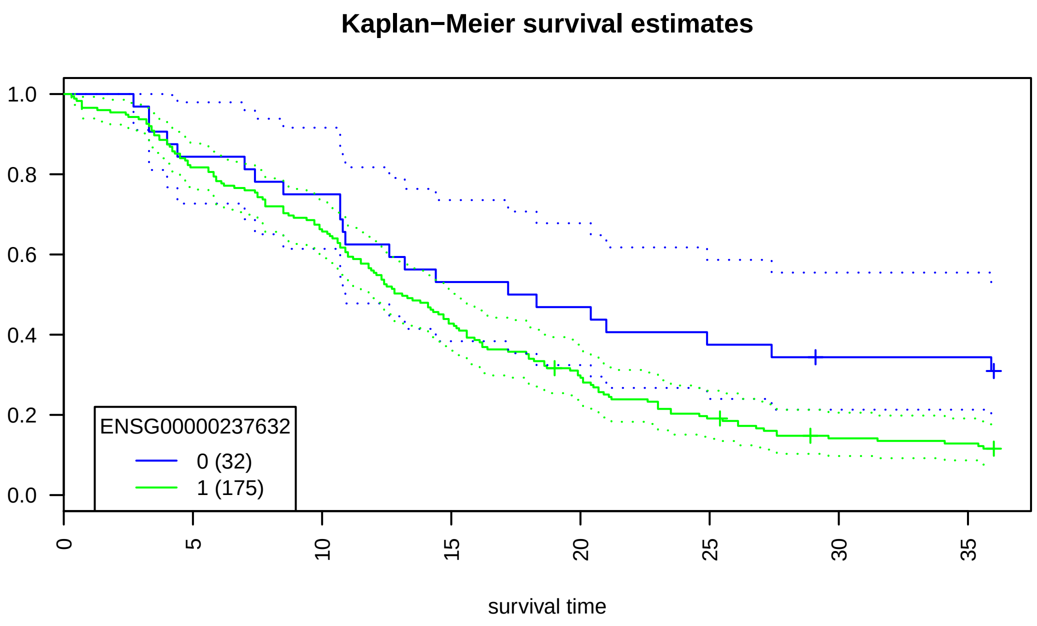

Figure 158: Each curve represents the relative number of ENSG00000237632 survived to the corresponding point of time. Dashed lines represent 0.95 confidence intervals for the curves with the corresponding color. Total of 1 observation deleted due to missingness. Log-rank probability measure for the equality of these curves is 2.407174e-02. Additional annotations: *.GeneName*=S100A11P1, *.GeneDesc*=S100 calcium binding protein A11 pseudogene 1 [Source:HGNC Symbol;Acc:10491].

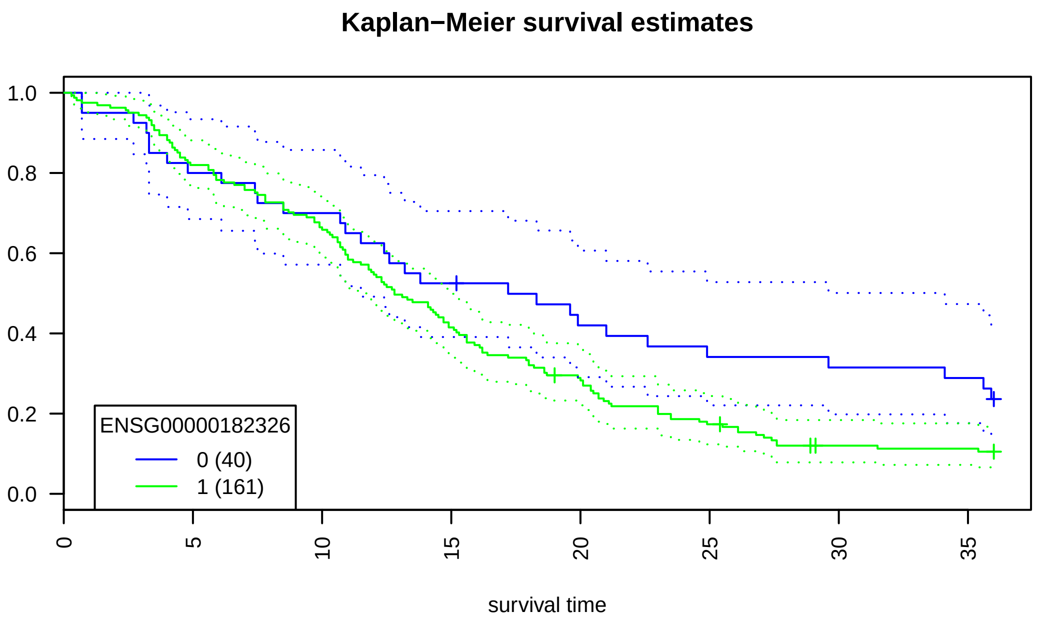

Figure 159: Each curve represents the relative number of ENSG00000182326 survived to the corresponding point of time. Dashed lines represent 0.95 confidence intervals for the curves with the corresponding color. Total of 7 observations deleted due to missingness. Log-rank probability measure for the equality of these curves is 4.185778e-02. Additional annotations: *.GeneName*=C1S, *.GeneDesc*=complement component 1, s subcomponent [Source:HGNC Symbol;Acc:1247], *Uniprot/SWISSPROT*=P09871.

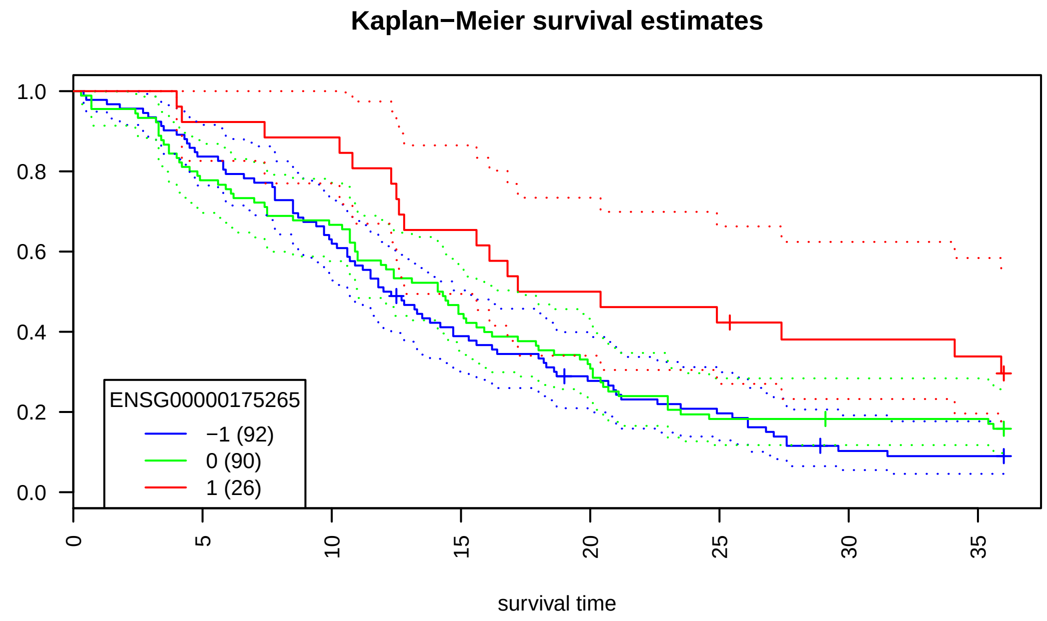

Figure 160: Each curve represents the relative number of ENSG00000175265 survived to the corresponding point of time. Dashed lines represent 0.95 confidence intervals for the curves with the corresponding color. Log-rank probability measure for the equality of these curves is 2.986685e-02.

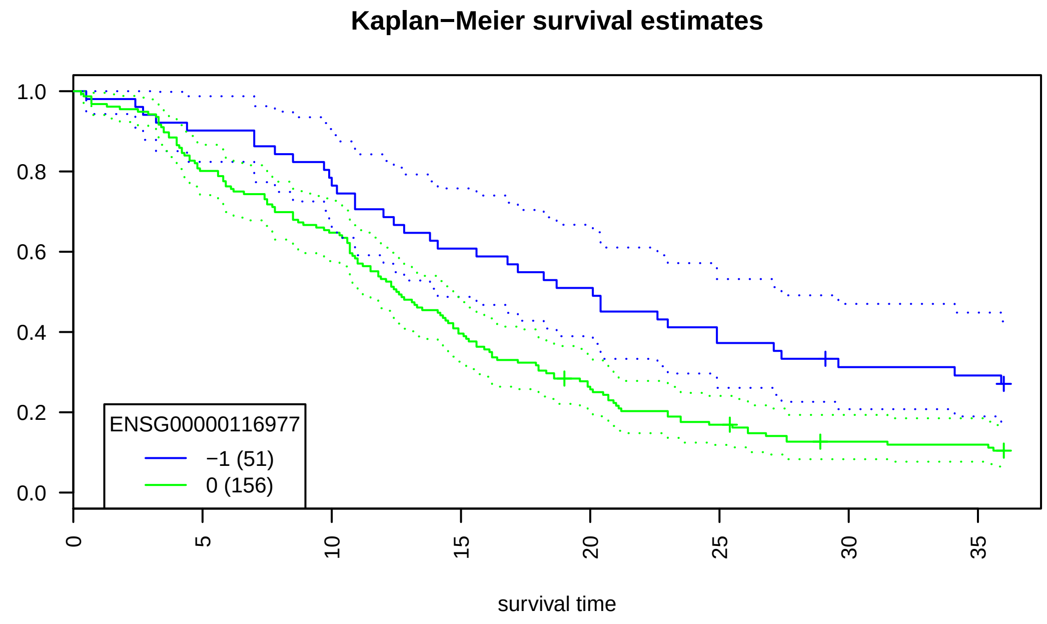

Figure 161: Each curve represents the relative number of ENSG00000116977 survived to the corresponding point of time. Dashed lines represent 0.95 confidence intervals for the curves with the corresponding color. Total of 1 observation deleted due to missingness. Log-rank probability measure for the equality of these curves is 1.434217e-03.

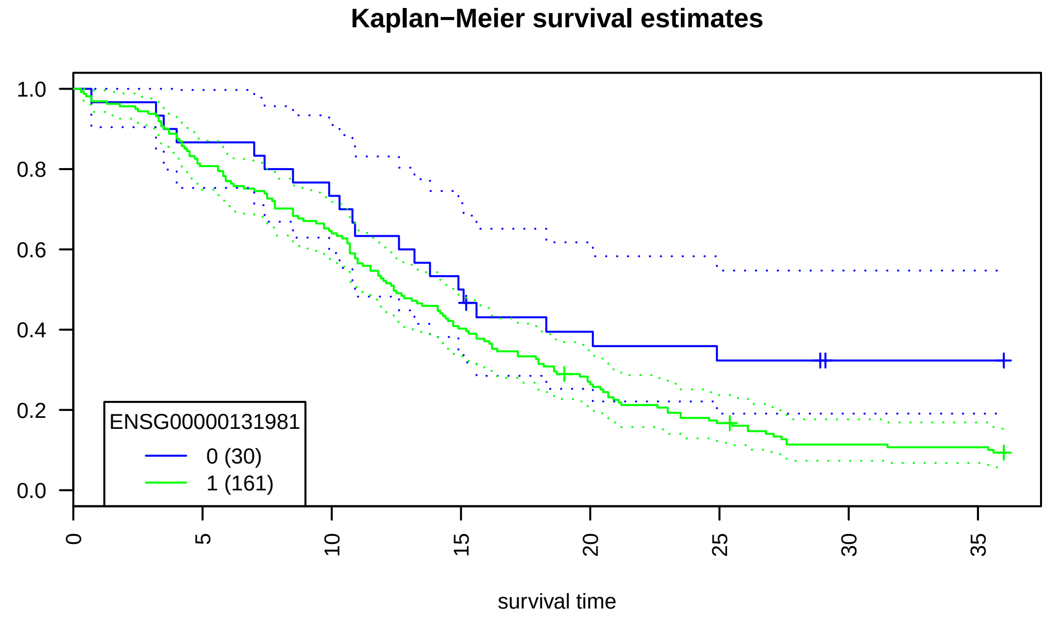

Figure 162: Each curve represents the relative number of ENSG00000131981 survived to the corresponding point of time. Dashed lines represent 0.95 confidence intervals for the curves with the corresponding color. Total of 17 observations deleted due to missingness. Log-rank probability measure for the equality of these curves is 2.796087e-02. Additional annotations: *.GeneName*=LGALS3, *.GeneDesc*=lectin, galactoside-binding, soluble, 3 [Source:HGNC Symbol;Acc:6563], *Uniprot/SWISSPROT*=P17931.

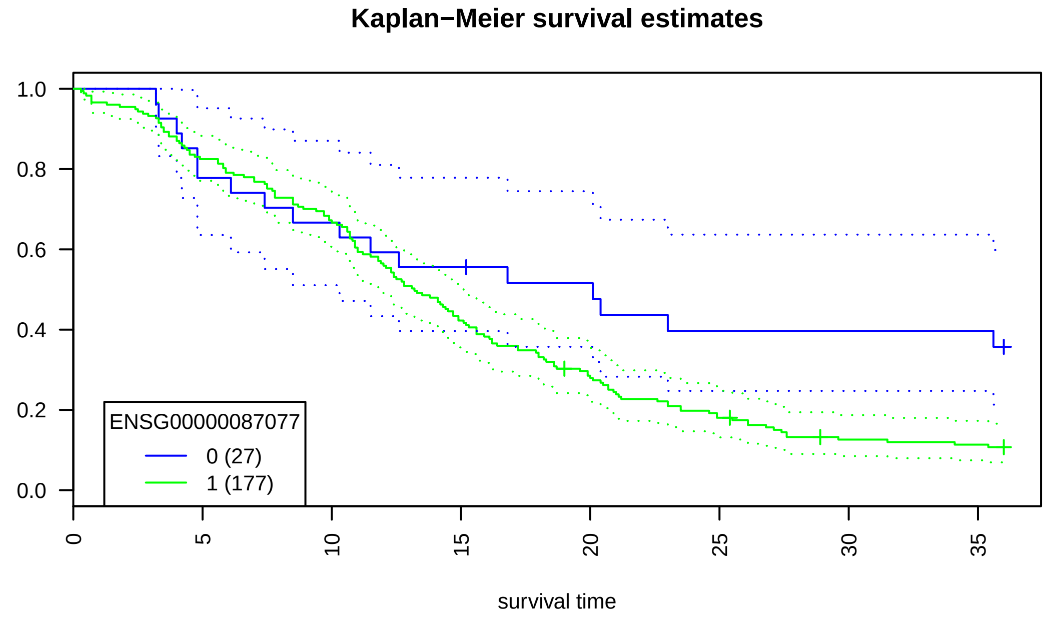

Figure 163: Each curve represents the relative number of ENSG00000087077 survived to the corresponding point of time. Dashed lines represent 0.95 confidence intervals for the curves with the corresponding color. Total of 4 observations deleted due to missingness. Log-rank probability measure for the equality of these curves is 1.795084e-02. Additional annotations: *.GeneName*=TRIP6, *.GeneDesc*=thyroid hormone receptor interactor 6 [Source:HGNC Symbol;Acc:12311], *Uniprot/SWISSPROT*=Q15654.

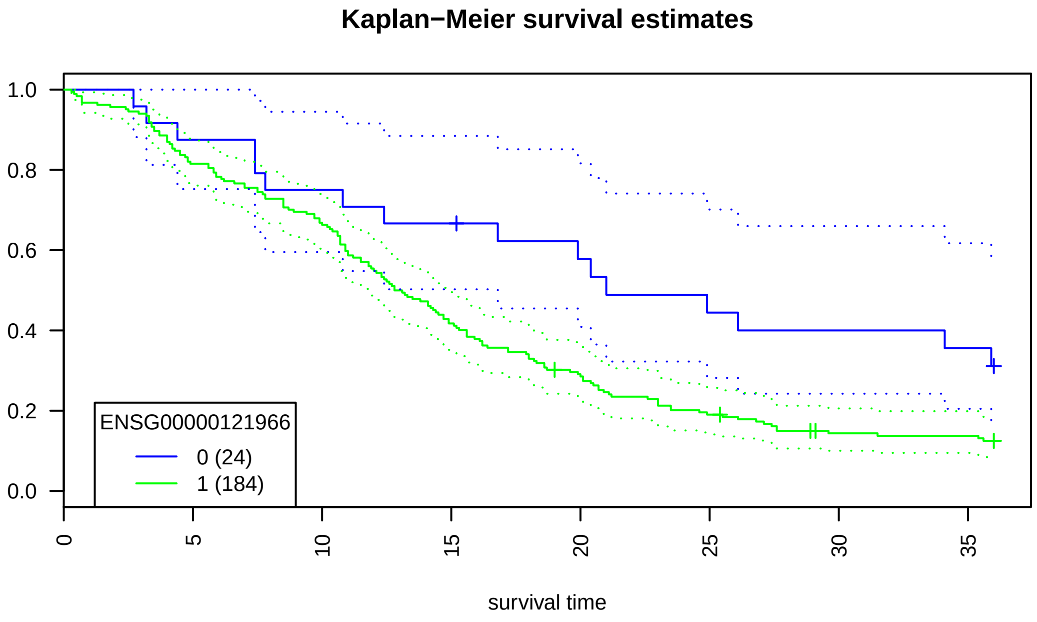

Figure 164: Each curve represents the relative number of ENSG00000121966 survived to the corresponding point of time. Dashed lines represent 0.95 confidence intervals for the curves with the corresponding color. Log-rank probability measure for the equality of these curves is 1.442131e-02. Additional annotations: *.GeneName*=CXCR4, *.GeneDesc*=chemokine (C-X-C motif) receptor 4 [Source:HGNC Symbol;Acc:2561], *Uniprot/SWISSPROT*=P61073.

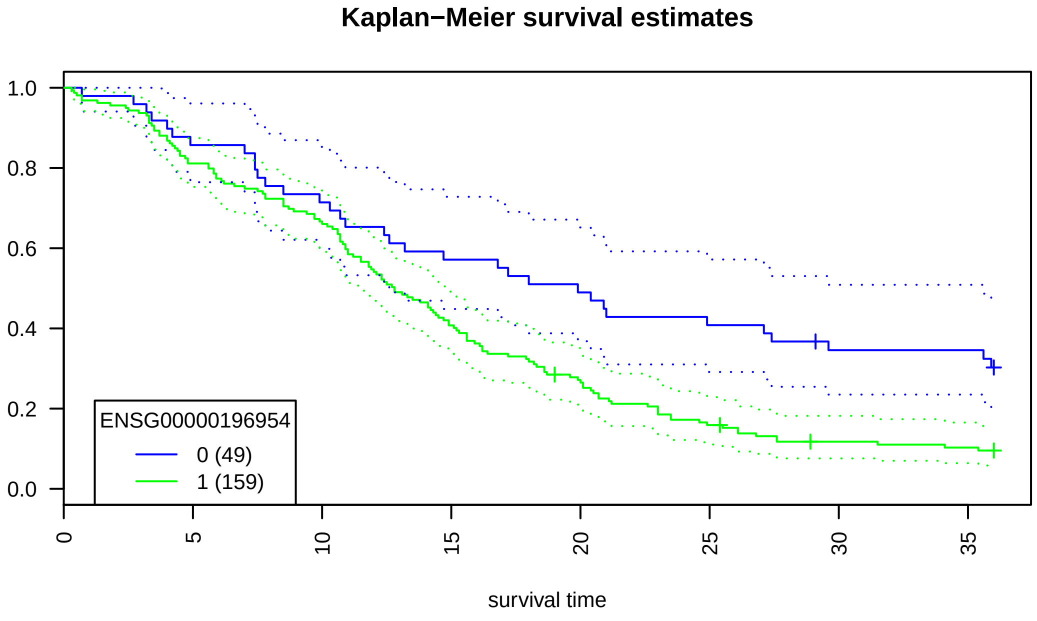

Figure 165: Each curve represents the relative number of ENSG00000196954 survived to the corresponding point of time. Dashed lines represent 0.95 confidence intervals for the curves with the corresponding color. Log-rank probability measure for the equality of these curves is 1.228843e-03. Additional annotations: *.GeneName*=CASP4, *.GeneDesc*=caspase 4, apoptosis-related cysteine peptidase [Source:HGNC Symbol;Acc:1505], *Uniprot/SWISSPROT*=P49662.

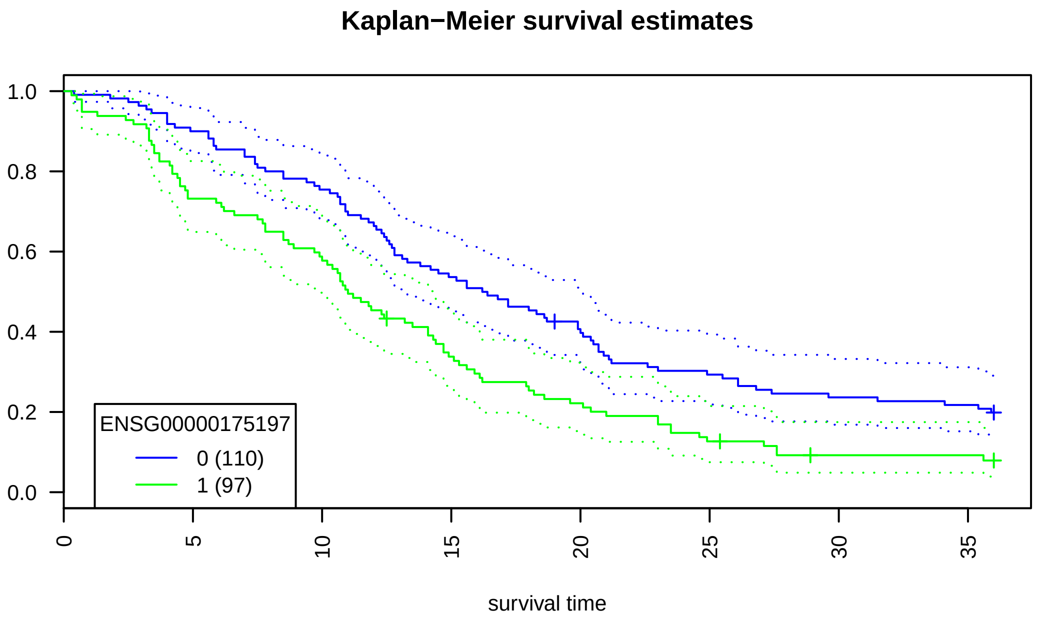

Figure 166: Each curve represents the relative number of ENSG00000175197 survived to the corresponding point of time. Dashed lines represent 0.95 confidence intervals for the curves with the corresponding color. Total of 1 observation deleted due to missingness. Log-rank probability measure for the equality of these curves is 6.289214e-04.

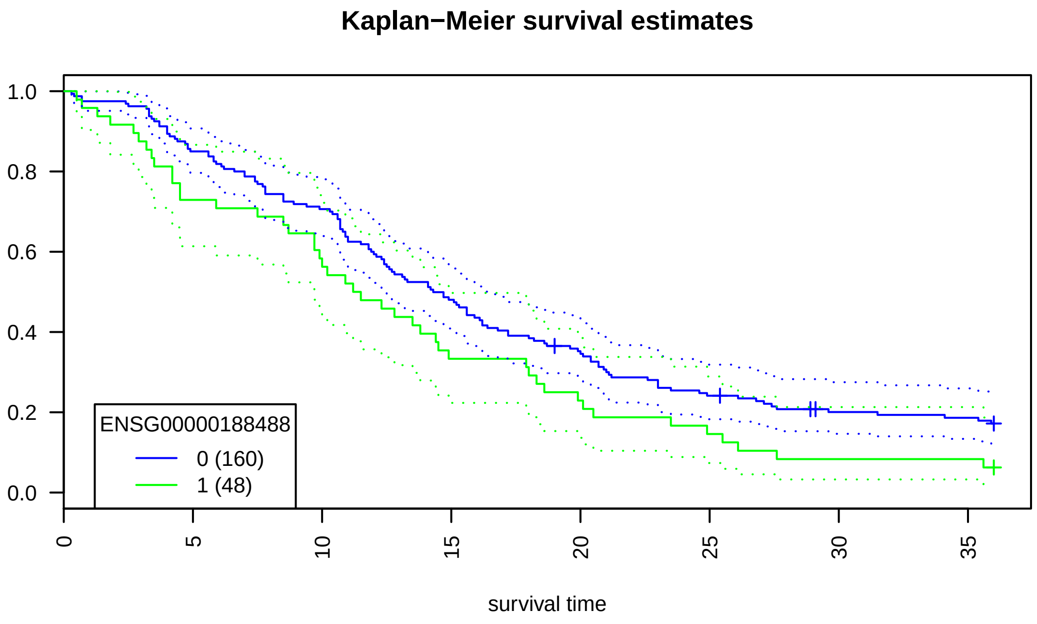

Figure 167: Each curve represents the relative number of ENSG00000188488 survived to the corresponding point of time. Dashed lines represent 0.95 confidence intervals for the curves with the corresponding color. Log-rank probability measure for the equality of these curves is 2.844514e-02.

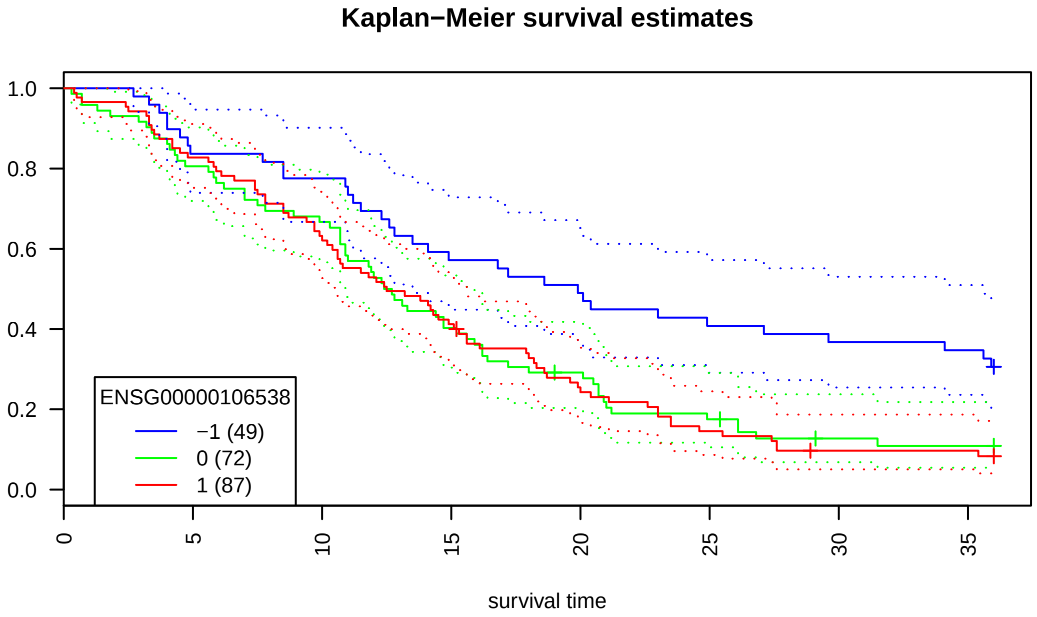

Figure 168: Each curve represents the relative number of ENSG00000106538 survived to the corresponding point of time. Dashed lines represent 0.95 confidence intervals for the curves with the corresponding color. Log-rank probability measure for the equality of these curves is 2.222543e-03.

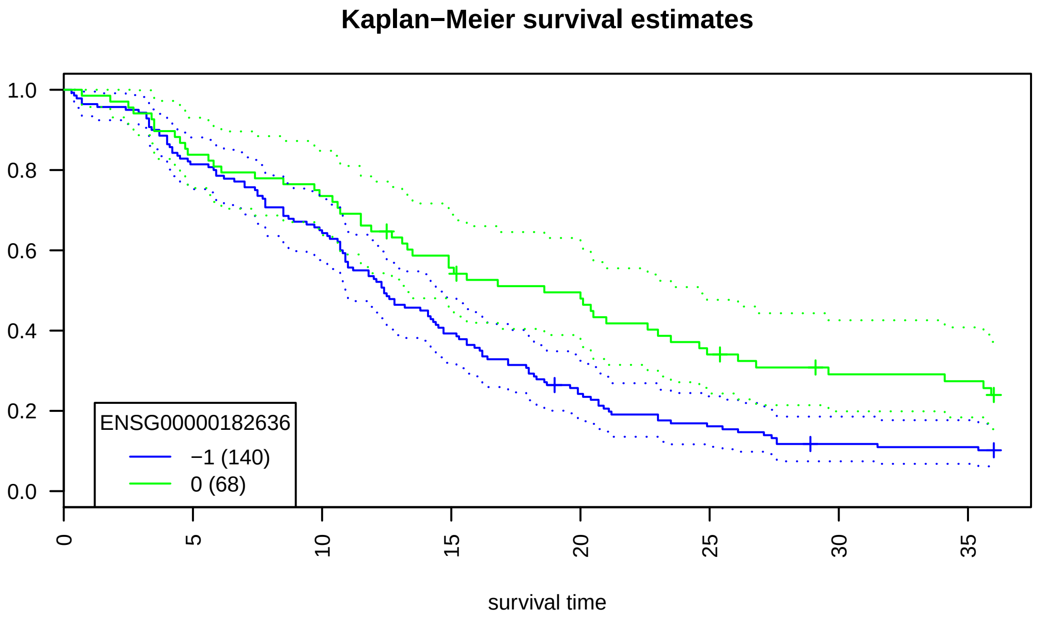

Figure 169: Each curve represents the relative number of ENSG00000182636 survived to the corresponding point of time. Dashed lines represent 0.95 confidence intervals for the curves with the corresponding color. Log-rank probability measure for the equality of these curves is 2.961199e-03. Additional annotations: *.GeneName*=NDN, *.GeneDesc*=necdin homolog (mouse) [Source:HGNC Symbol;Acc:7675], *Uniprot/SWISSPROT*=Q99608.

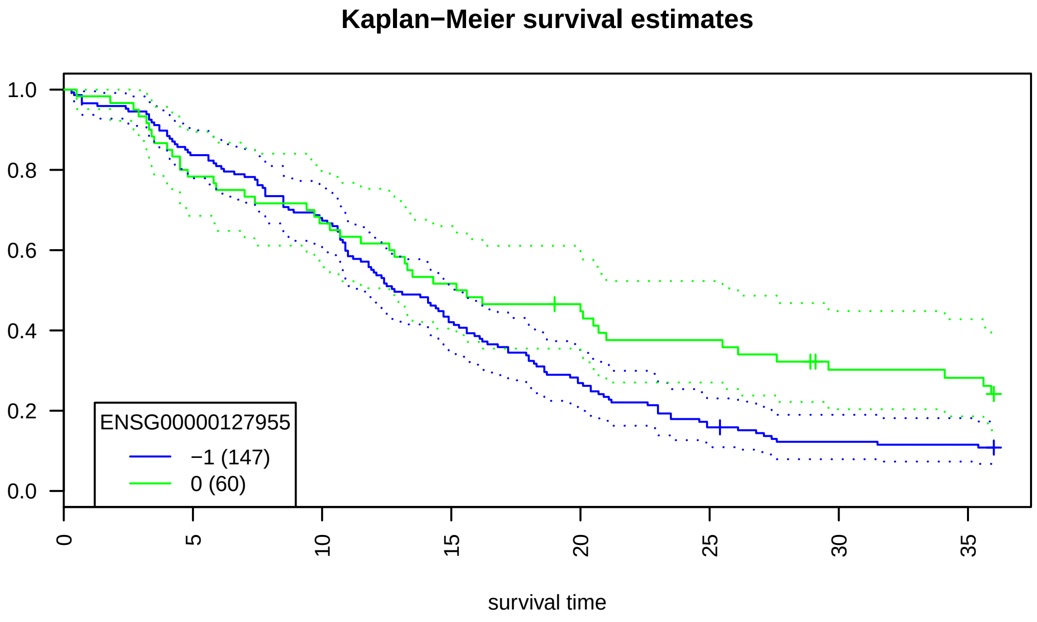

Figure 170: Each curve represents the relative number of ENSG00000127955 survived to the corresponding point of time. Dashed lines represent 0.95 confidence intervals for the curves with the corresponding color. Total of 1 observation deleted due to missingness. Log-rank probability measure for the equality of these curves is 2.604887e-02. Additional annotations: *.GeneName*=GNAI1, *.GeneDesc*=guanine nucleotide binding protein (G protein), alpha inhibiting activity polypeptide 1 [Source:HGNC Symbol;Acc:4384], *Uniprot/SWISSPROT*=P63096.

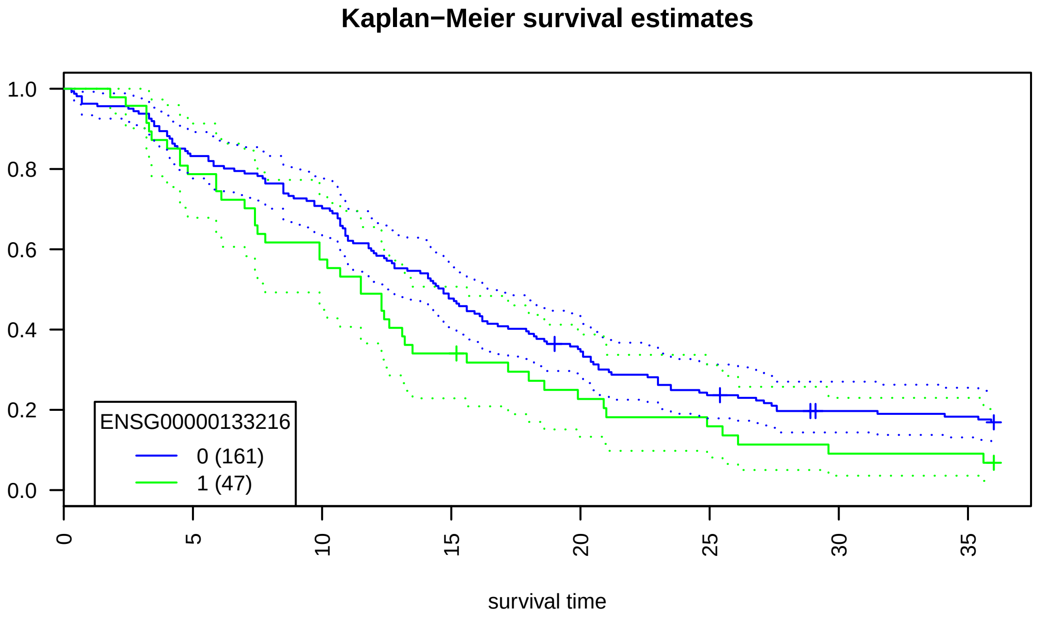

Figure 171: Each curve represents the relative number of ENSG00000133216 survived to the corresponding point of time. Dashed lines represent 0.95 confidence intervals for the curves with the corresponding color. Log-rank probability measure for the equality of these curves is 4.217110e-02.

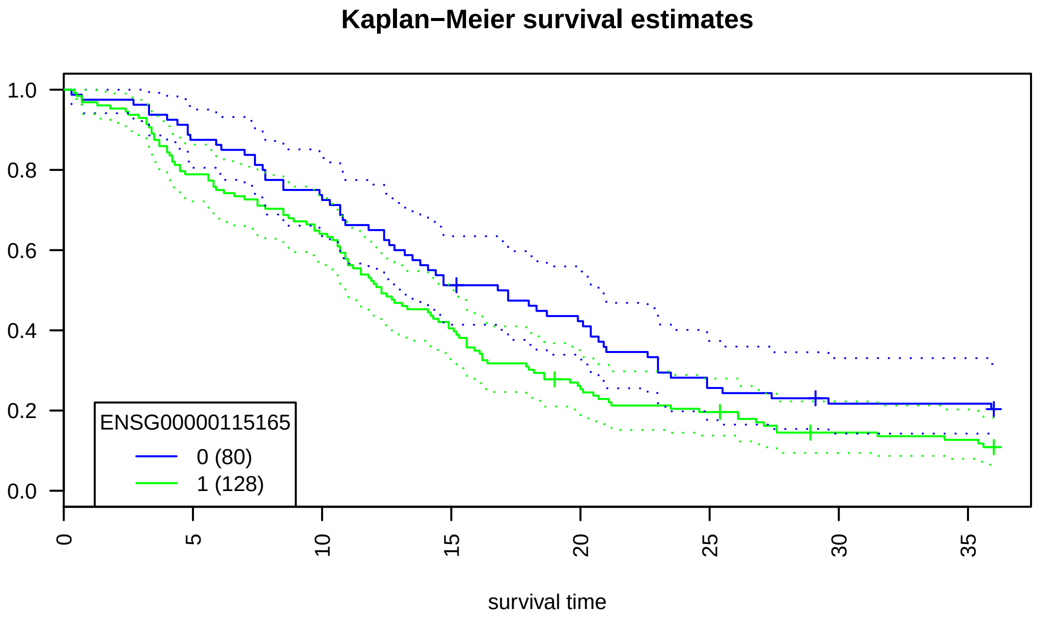

Figure 172: Each curve represents the relative number of ENSG00000115165 survived to the corresponding point of time. Dashed lines represent 0.95 confidence intervals for the curves with the corresponding color. Log-rank probability measure for the equality of these curves is 2.619382e-02. Additional annotations: *.GeneName*=CYTIP, *.GeneDesc*=cytohesin 1 interacting protein [Source:HGNC Symbol;Acc:9506], *Uniprot/SWISSPROT*=O60759.

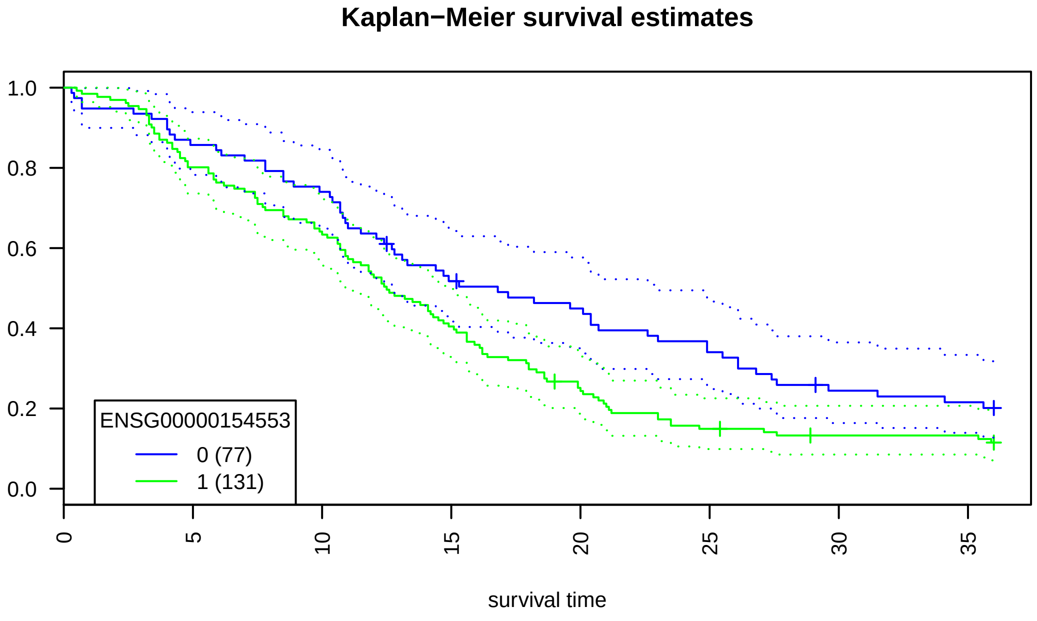

Figure 173: Each curve represents the relative number of ENSG00000154553 survived to the corresponding point of time. Dashed lines represent 0.95 confidence intervals for the curves with the corresponding color. Log-rank probability measure for the equality of these curves is 1.613108e-02. Additional annotations: *.GeneName*=PDLIM3, *.GeneDesc*=PDZ and LIM domain 3 [Source:HGNC Symbol;Acc:20767], *Uniprot/SWISSPROT*=Q53GG5.

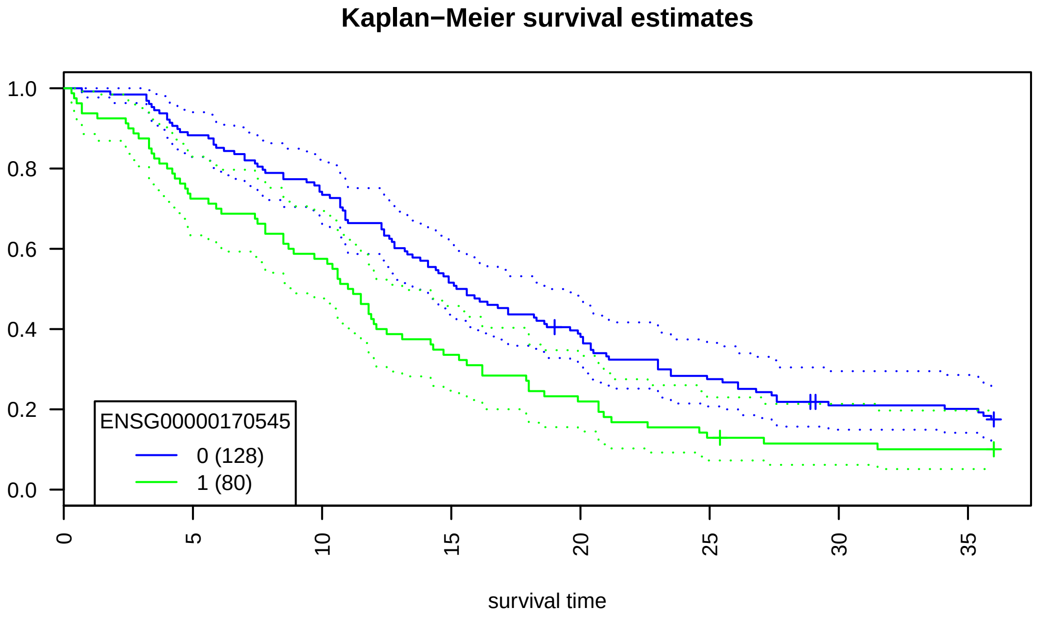

Figure 174: Each curve represents the relative number of ENSG00000170545 survived to the corresponding point of time. Dashed lines represent 0.95 confidence intervals for the curves with the corresponding color. Log-rank probability measure for the equality of these curves is 2.882896e-03. Additional annotations: *.GeneName*=SMAGP, *.GeneDesc*=small cell adhesion glycoprotein [Source:HGNC Symbol;Acc:26918], *Uniprot/SWISSPROT*=Q0VAQ4.

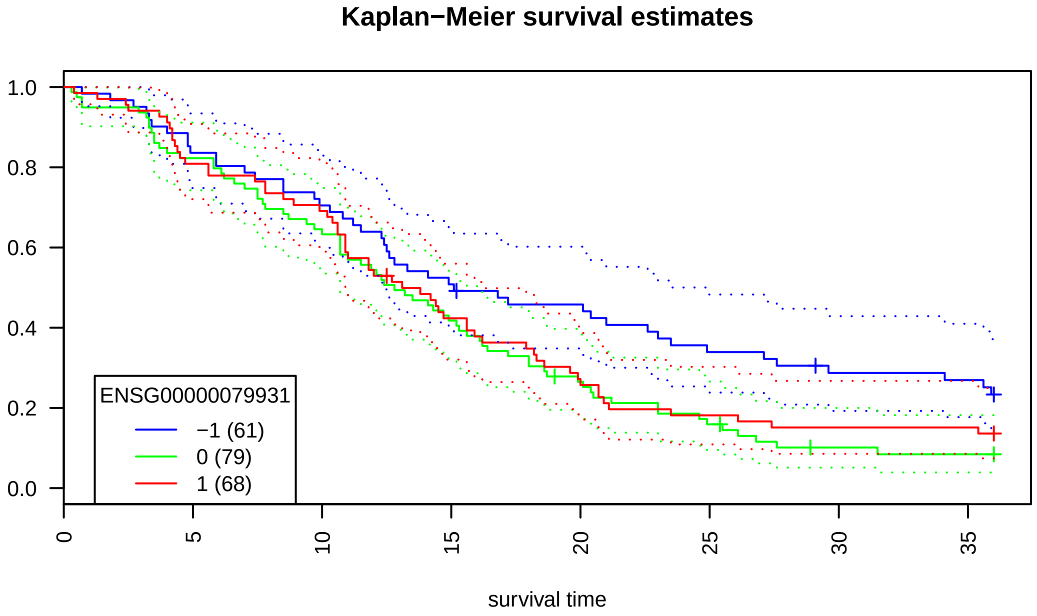

Figure 175: Each curve represents the relative number of ENSG00000079931 survived to the corresponding point of time. Dashed lines represent 0.95 confidence intervals for the curves with the corresponding color. Log-rank probability measure for the equality of these curves is 4.99971e-02.

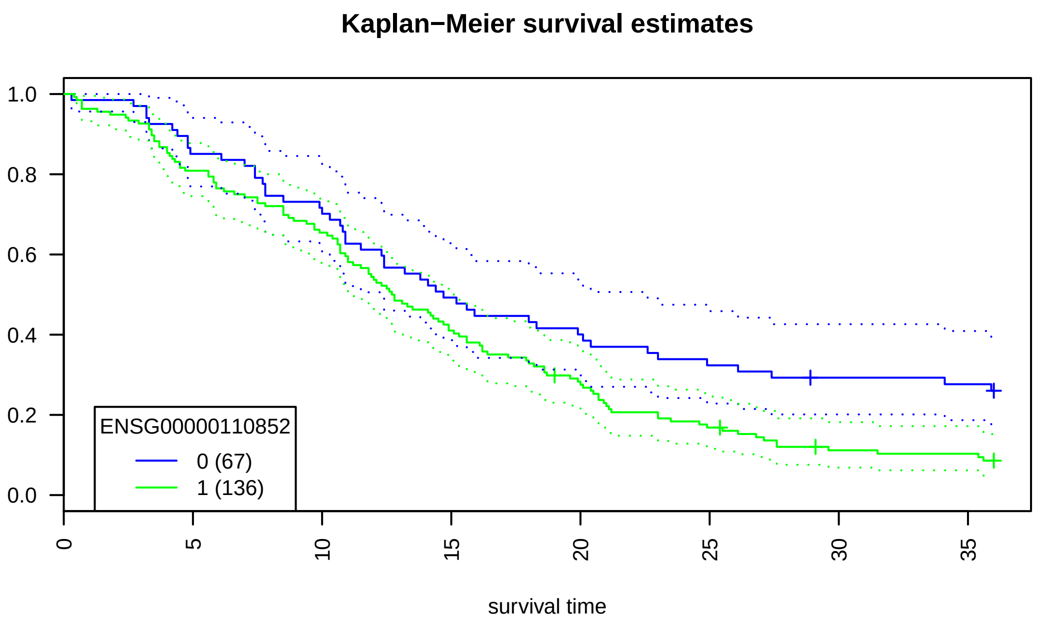

Figure 176: Each curve represents the relative number of ENSG00000110852 survived to the corresponding point of time. Dashed lines represent 0.95 confidence intervals for the curves with the corresponding color. Total of 5 observations deleted due to missingness. Log-rank probability measure for the equality of these curves is 9.724197e-03. Additional annotations: *.GeneName*=CLEC2B, *.GeneDesc*=C-type lectin domain family 2, member B [Source:HGNC Symbol;Acc:2053], *Uniprot/SWISSPROT*=Q92478.

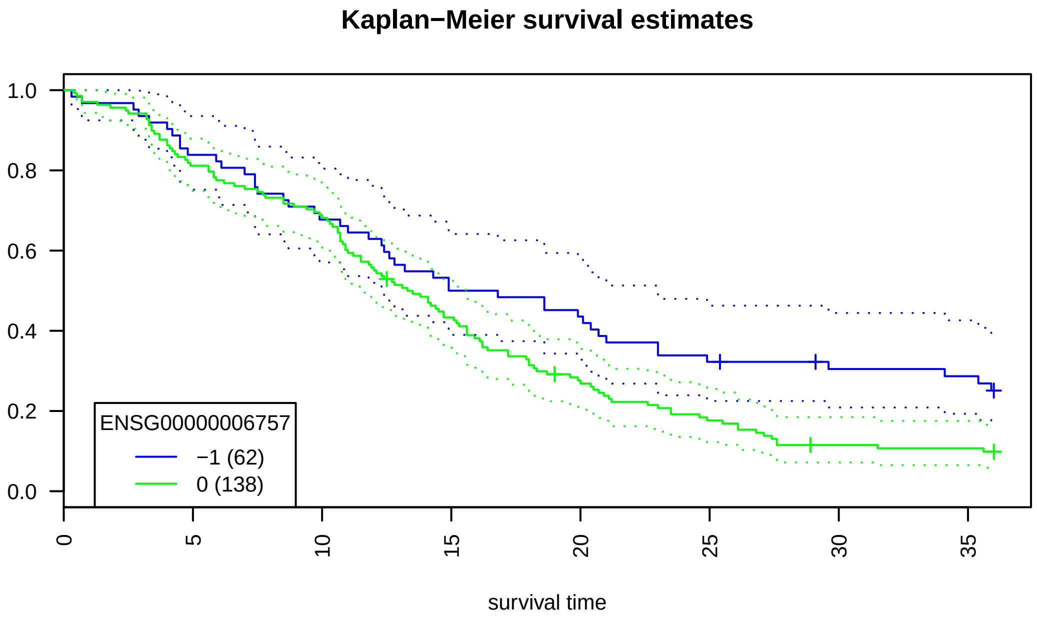

Figure 177: Each curve represents the relative number of ENSG00000006757 survived to the corresponding point of time. Dashed lines represent 0.95 confidence intervals for the curves with the corresponding color. Total of 8 observations deleted due to missingness. Log-rank probability measure for the equality of these curves is 1.448589e-02.

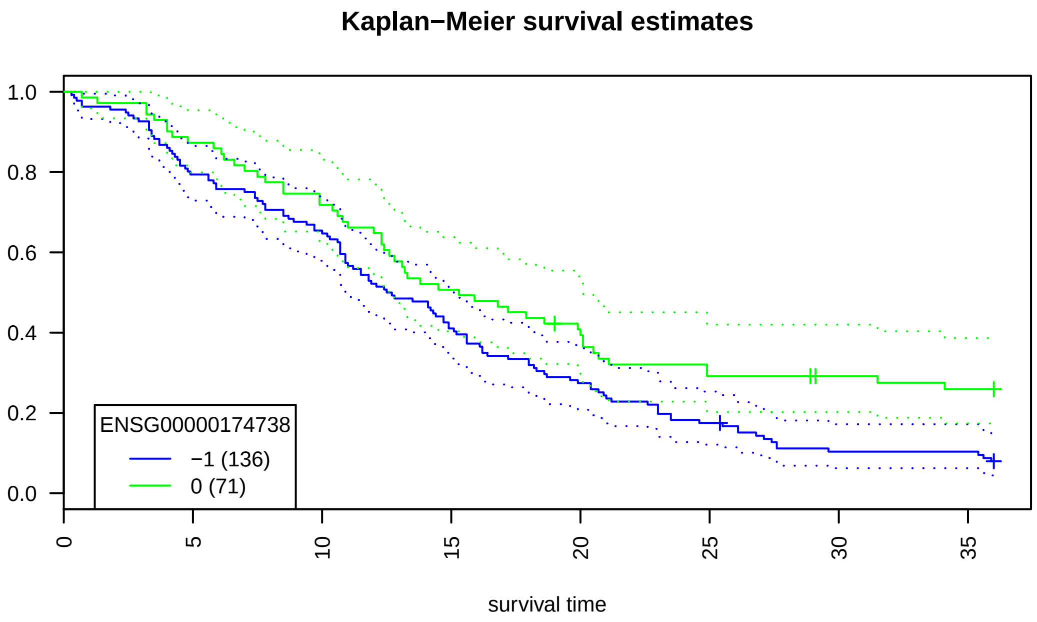

Figure 178: Each curve represents the relative number of ENSG00000174738 survived to the corresponding point of time. Dashed lines represent 0.95 confidence intervals for the curves with the corresponding color. Total of 1 observation deleted due to missingness. Log-rank probability measure for the equality of these curves is 6.614613e-03. Additional annotations: *.GeneName*=NR1D2, *.GeneDesc*=nuclear receptor subfamily 1, group D, member 2 [Source:HGNC Symbol;Acc:7963], *Uniprot/SWISSPROT*=Q14995.

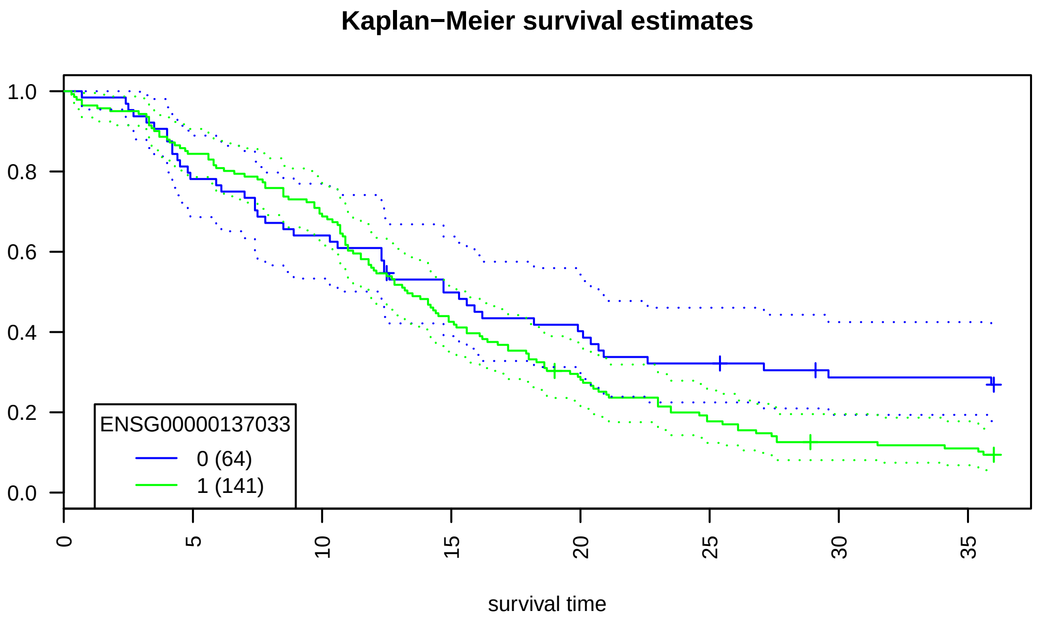

Figure 179: Each curve represents the relative number of ENSG00000137033 survived to the corresponding point of time. Dashed lines represent 0.95 confidence intervals for the curves with the corresponding color. Total of 3 observations deleted due to missingness. Log-rank probability measure for the equality of these curves is 3.892563e-02. Additional annotations: *.GeneName*=IL33, *.GeneDesc*=interleukin 33 [Source:HGNC Symbol;Acc:16028], *Uniprot/SWISSPROT*=O95760.

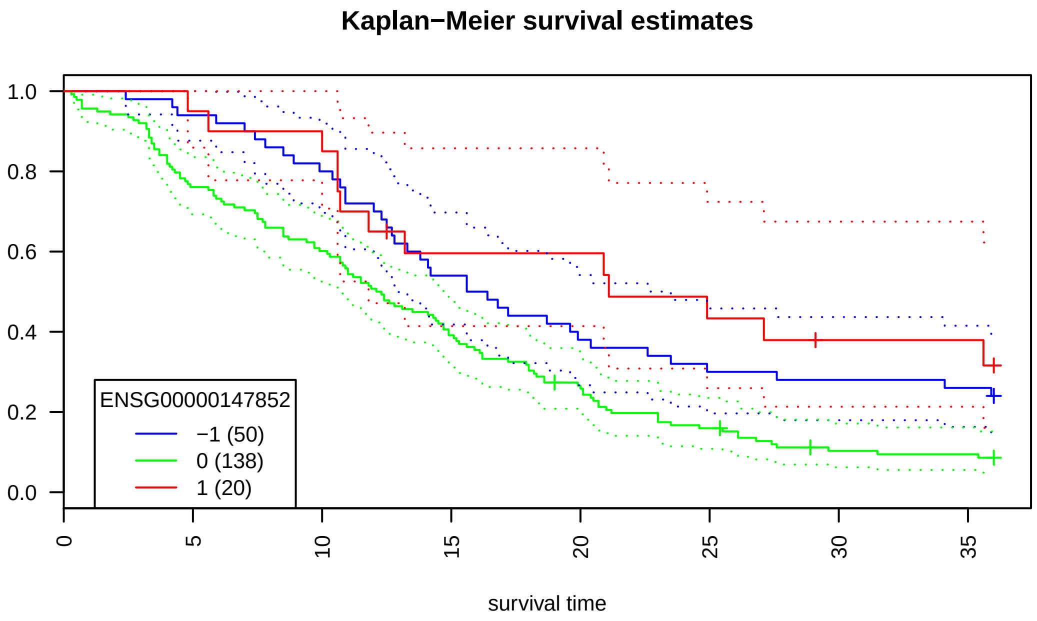

Figure 180: Each curve represents the relative number of ENSG00000147852 survived to the corresponding point of time. Dashed lines represent 0.95 confidence intervals for the curves with the corresponding color. Log-rank probability measure for the equality of these curves is 1.047859e-03.

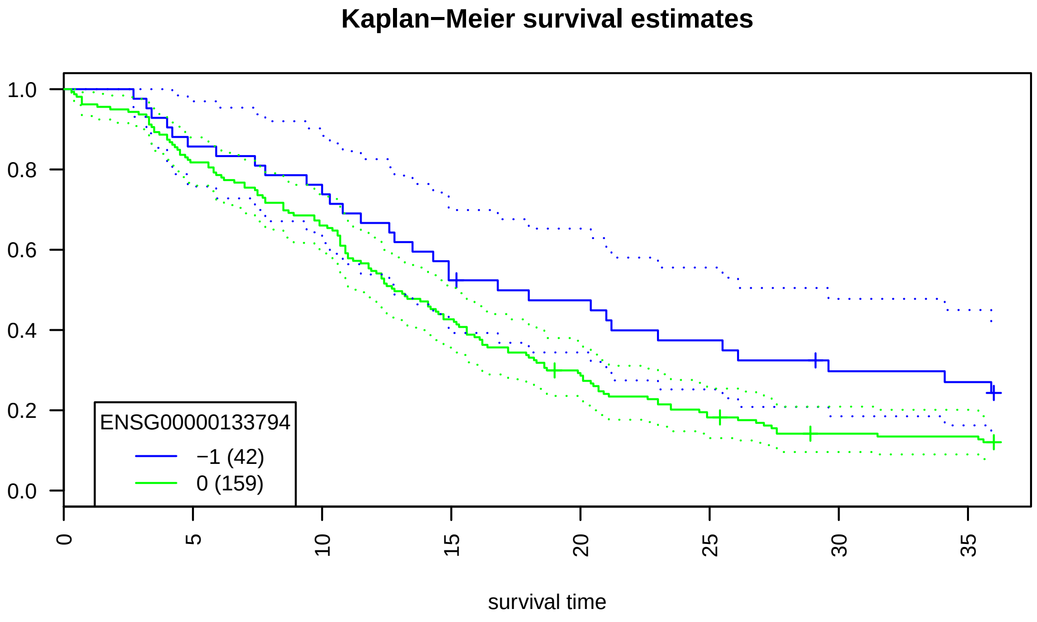

Figure 181: Each curve represents the relative number of ENSG00000133794 survived to the corresponding point of time. Dashed lines represent 0.95 confidence intervals for the curves with the corresponding color. Total of 7 observations deleted due to missingness. Log-rank probability measure for the equality of these curves is 3.090772e-02.

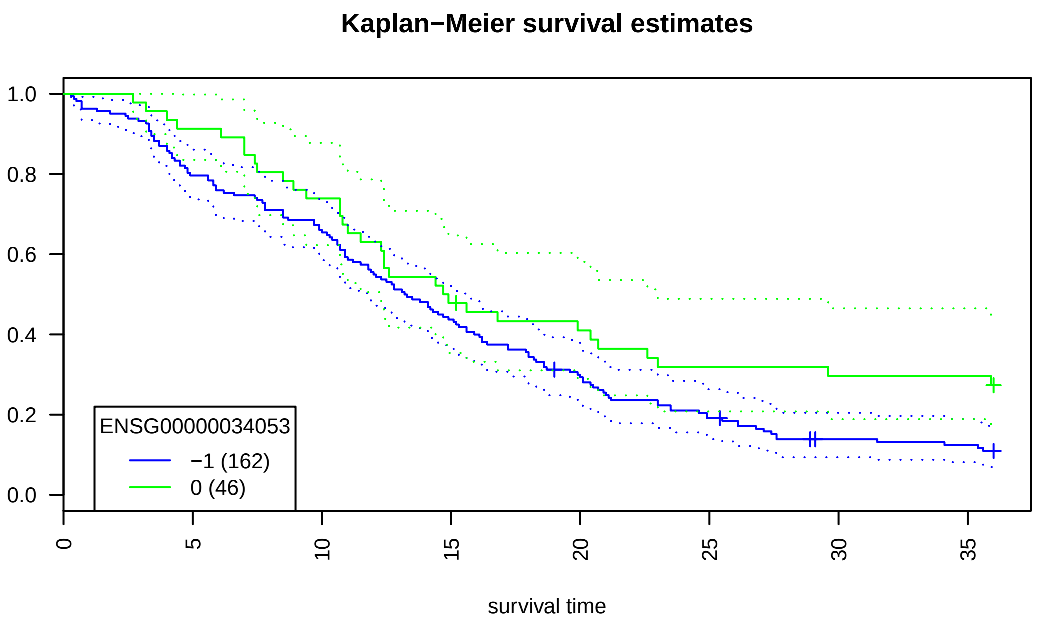

Figure 182: Each curve represents the relative number of ENSG00000034053 survived to the corresponding point of time. Dashed lines represent 0.95 confidence intervals for the curves with the corresponding color. Log-rank probability measure for the equality of these curves is 3.139202e-02. Additional annotations: *.GeneName*=APBA2, *.GeneDesc*=amyloid beta (A4) precursor protein-binding, family A, member 2 [Source:HGNC Symbol;Acc:579], *Uniprot/SWISSPROT*=Q99767.

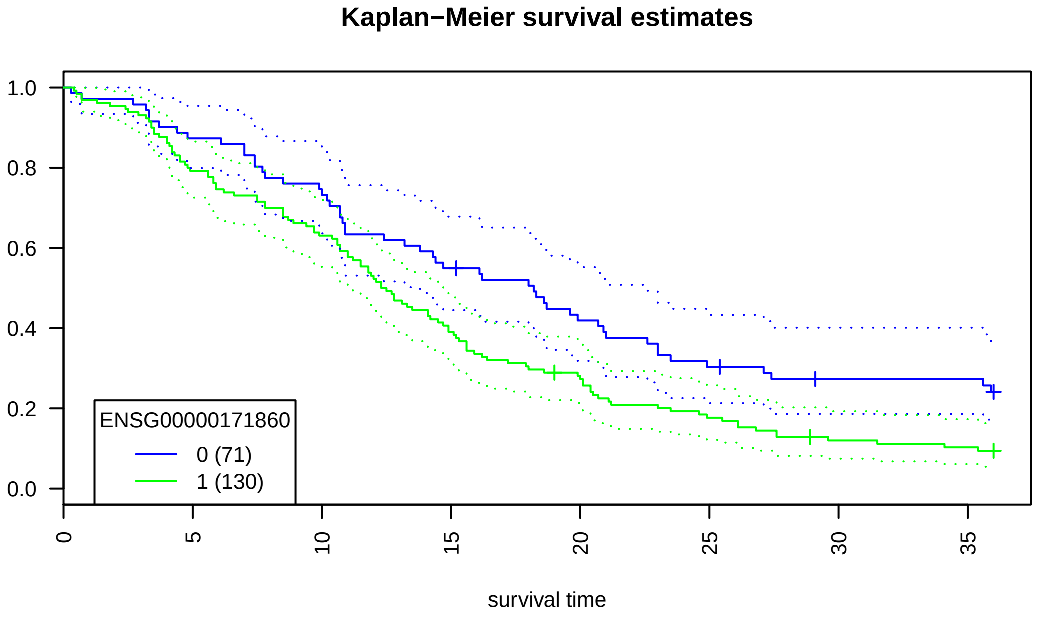

Figure 183: Each curve represents the relative number of ENSG00000171860 survived to the corresponding point of time. Dashed lines represent 0.95 confidence intervals for the curves with the corresponding color. Total of 7 observations deleted due to missingness. Log-rank probability measure for the equality of these curves is 5.135078e-03. Additional annotations: *.GeneName*=C3AR1, *.GeneDesc*=complement component 3a receptor 1 [Source:HGNC Symbol;Acc:1319], *Uniprot/SWISSPROT*=Q16581.

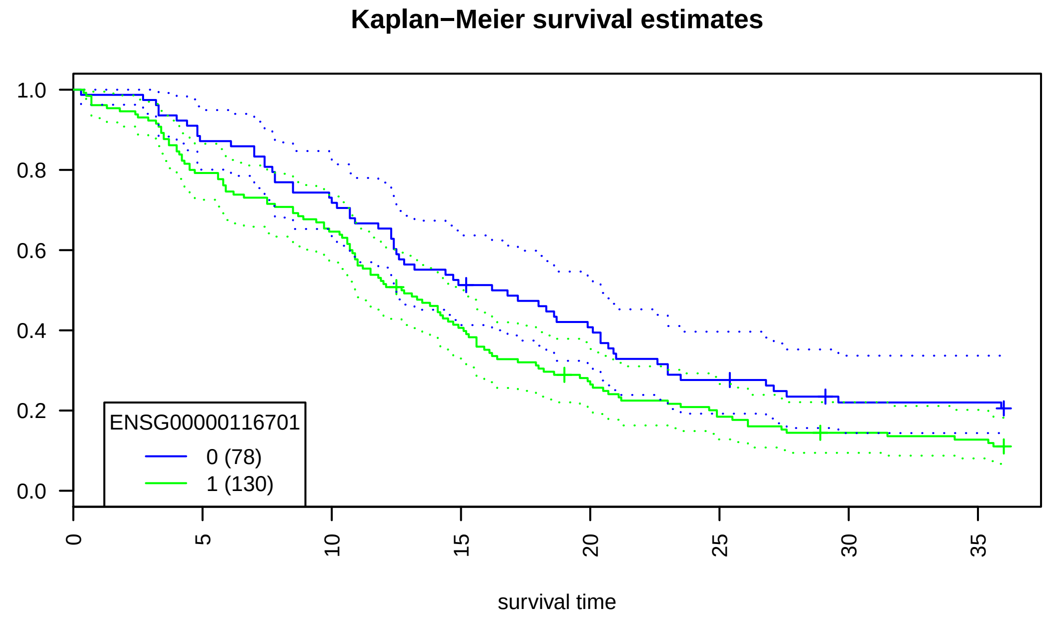

Figure 184: Each curve represents the relative number of ENSG00000116701 survived to the corresponding point of time. Dashed lines represent 0.95 confidence intervals for the curves with the corresponding color. Log-rank probability measure for the equality of these curves is 3.423657e-02. Additional annotations: *.GeneName*=NCF2, *.GeneDesc*=neutrophil cytosolic factor 2 [Source:HGNC Symbol;Acc:7661], *Uniprot/SWISSPROT*=P19878.

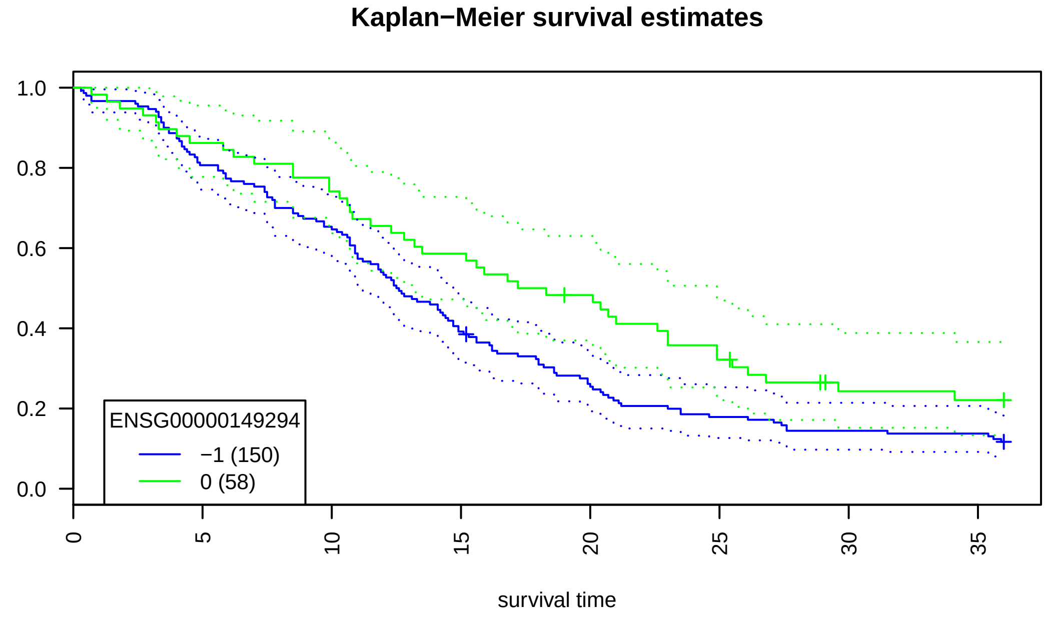

Figure 185: Each curve represents the relative number of ENSG00000149294 survived to the corresponding point of time. Dashed lines represent 0.95 confidence intervals for the curves with the corresponding color. Log-rank probability measure for the equality of these curves is 1.918981e-02. Additional annotations: *.GeneName*=NCAM1, *.GeneDesc*=neural cell adhesion molecule 1 [Source:HGNC Symbol;Acc:7656], *Uniprot/SWISSPROT*=P13591.

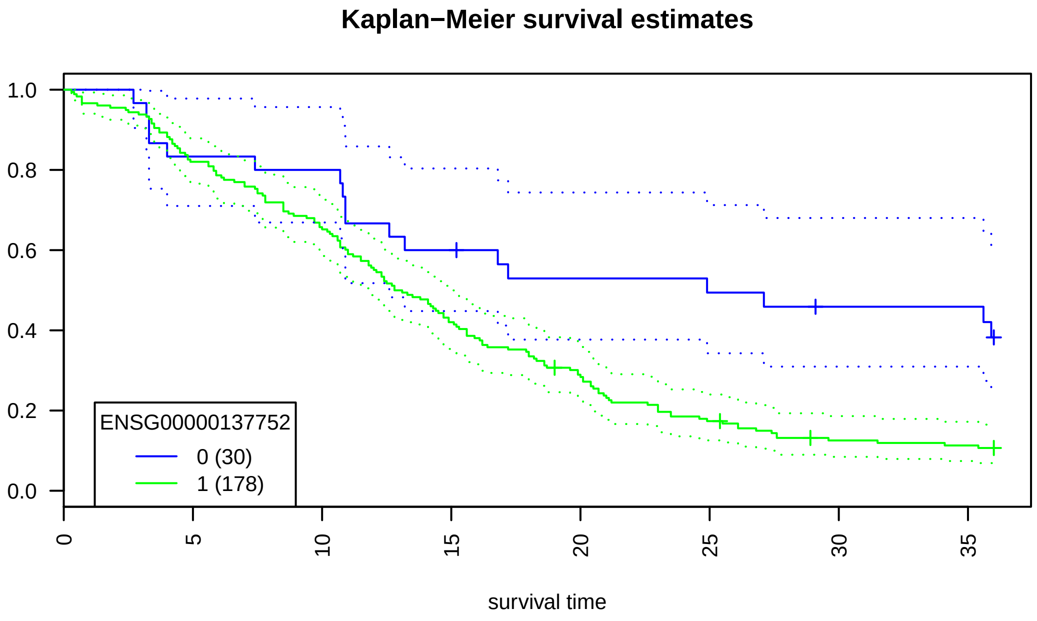

Figure 186: Each curve represents the relative number of ENSG00000137752 survived to the corresponding point of time. Dashed lines represent 0.95 confidence intervals for the curves with the corresponding color. Log-rank probability measure for the equality of these curves is 1.623007e-03. Additional annotations: *.GeneName*=CASP1, *.GeneDesc*=caspase 1, apoptosis-related cysteine peptidase (interleukin 1, beta, convertase) [Source:HGNC Symbol;Acc:1499], *Uniprot/SWISSPROT*=P29466,Q5XLA6.

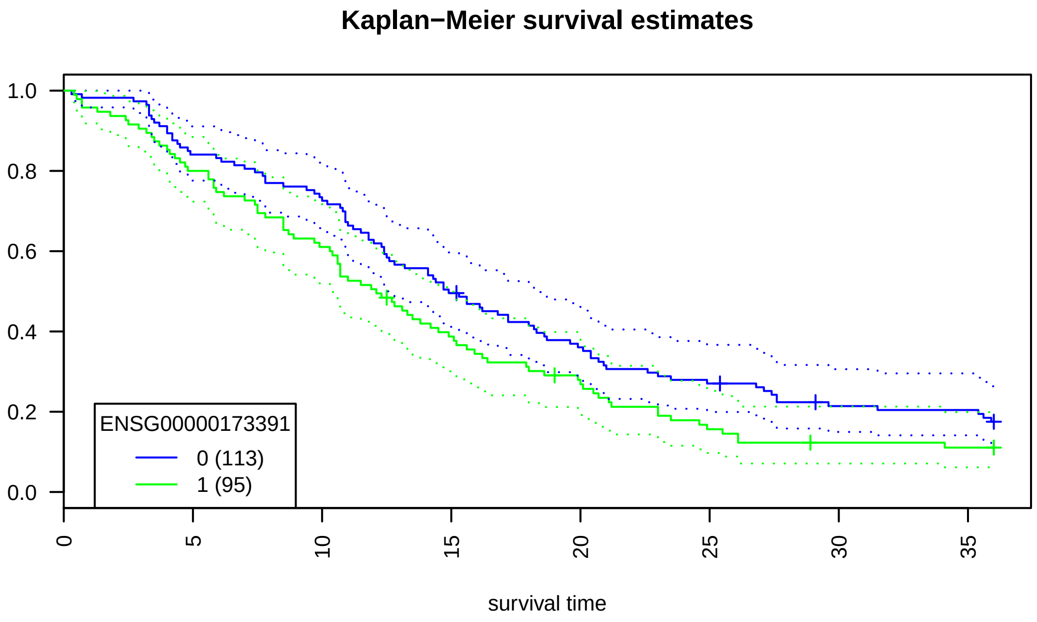

Figure 187: Each curve represents the relative number of ENSG00000173391 survived to the corresponding point of time. Dashed lines represent 0.95 confidence intervals for the curves with the corresponding color. Log-rank probability measure for the equality of these curves is 4.26762e-02.

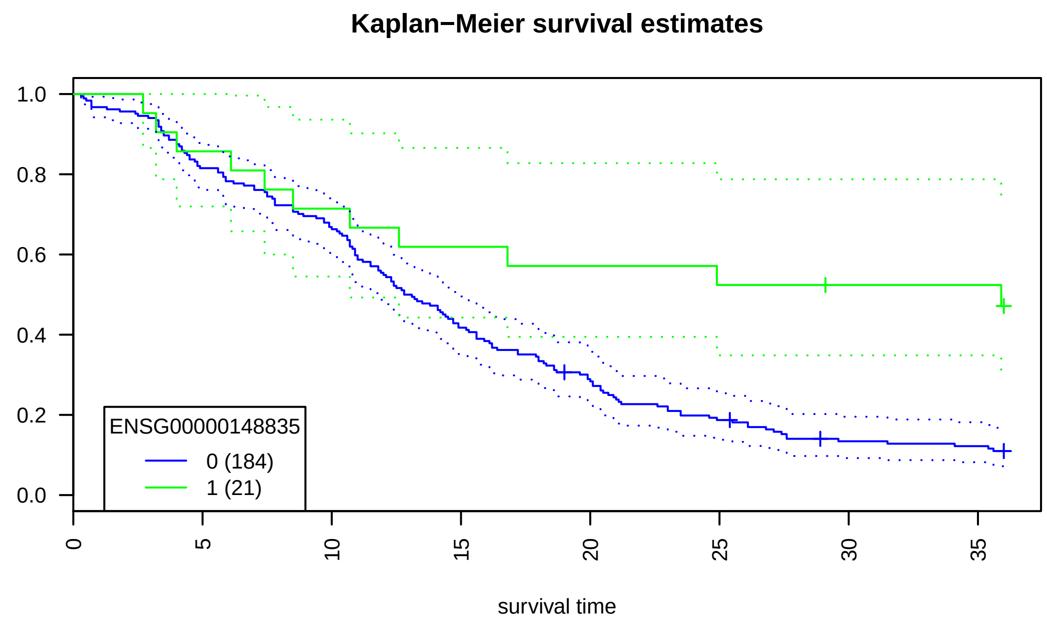

Figure 188: Each curve represents the relative number of ENSG00000148835 survived to the corresponding point of time. Dashed lines represent 0.95 confidence intervals for the curves with the corresponding color. Total of 3 observations deleted due to missingness. Log-rank probability measure for the equality of these curves is 1.778314e-03.

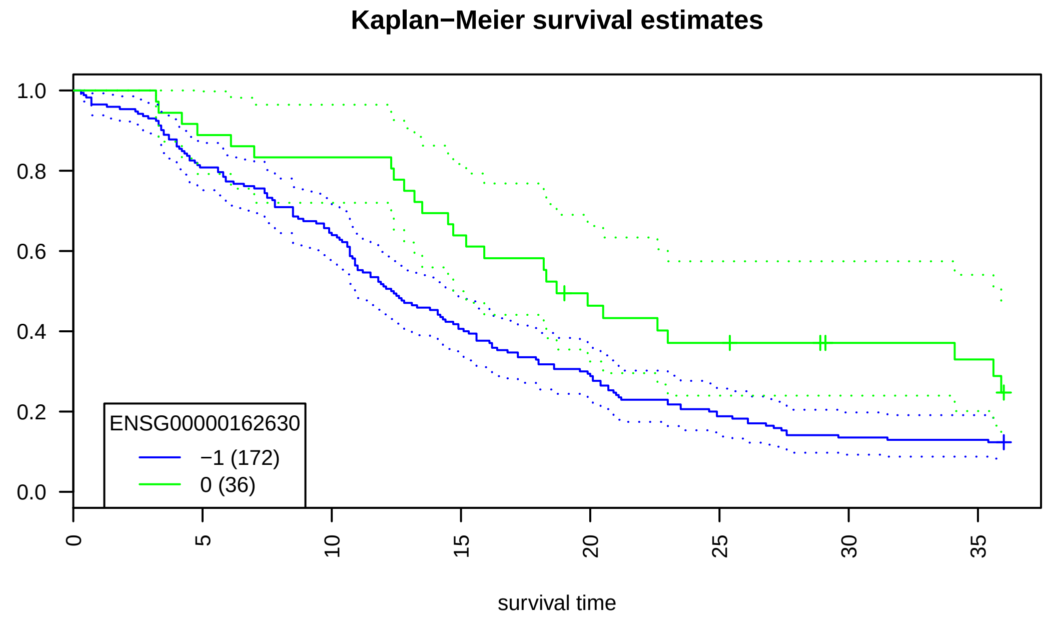

Figure 189: Each curve represents the relative number of ENSG00000162630 survived to the corresponding point of time. Dashed lines represent 0.95 confidence intervals for the curves with the corresponding color. Log-rank probability measure for the equality of these curves is 8.552792e-03. Additional annotations: *.GeneName*=B3GALT2, *.GeneDesc*=UDP-Gal:betaGlcNAc beta 1,3-galactosyltransferase, polypeptide 2 [Source:HGNC Symbol;Acc:917], *Uniprot/SWISSPROT*=O43825.

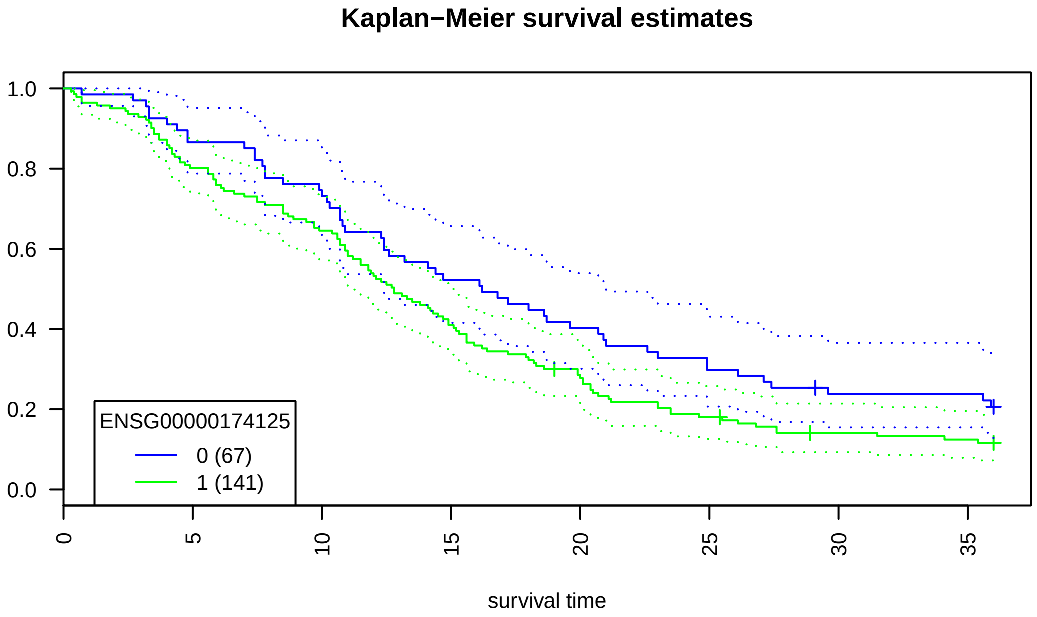

Figure 190: Each curve represents the relative number of ENSG00000174125 survived to the corresponding point of time. Dashed lines represent 0.95 confidence intervals for the curves with the corresponding color. Log-rank probability measure for the equality of these curves is 3.865896e-02. Additional annotations: *.GeneName*=TLR1, *.GeneDesc*=toll-like receptor 1 [Source:HGNC Symbol;Acc:11847], *Uniprot/SWISSPROT*=Q15399.

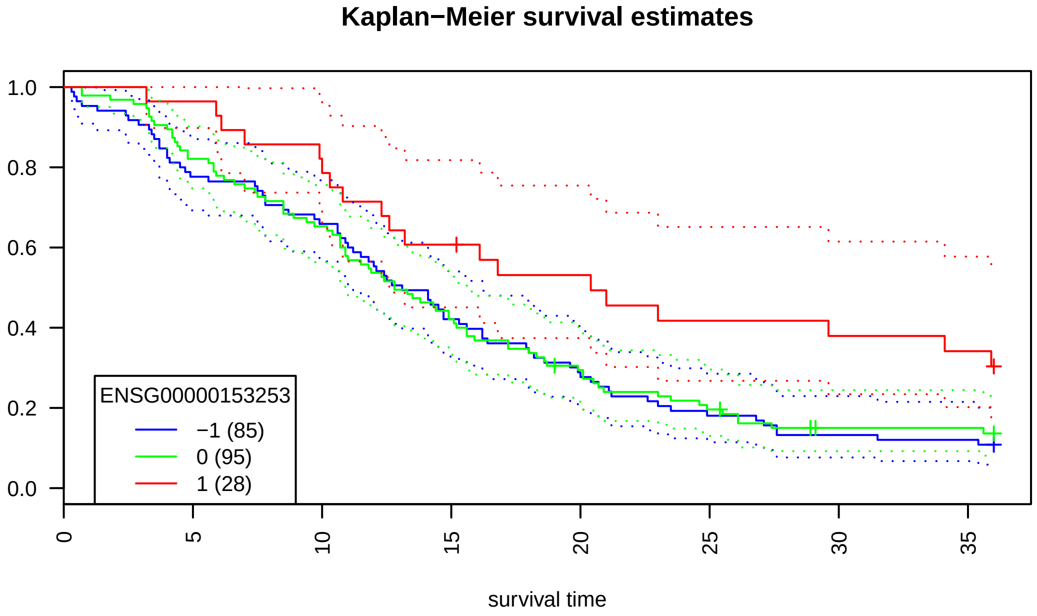

Figure 191: Each curve represents the relative number of ENSG00000153253 survived to the corresponding point of time. Dashed lines represent 0.95 confidence intervals for the curves with the corresponding color. Log-rank probability measure for the equality of these curves is 4.347141e-02.

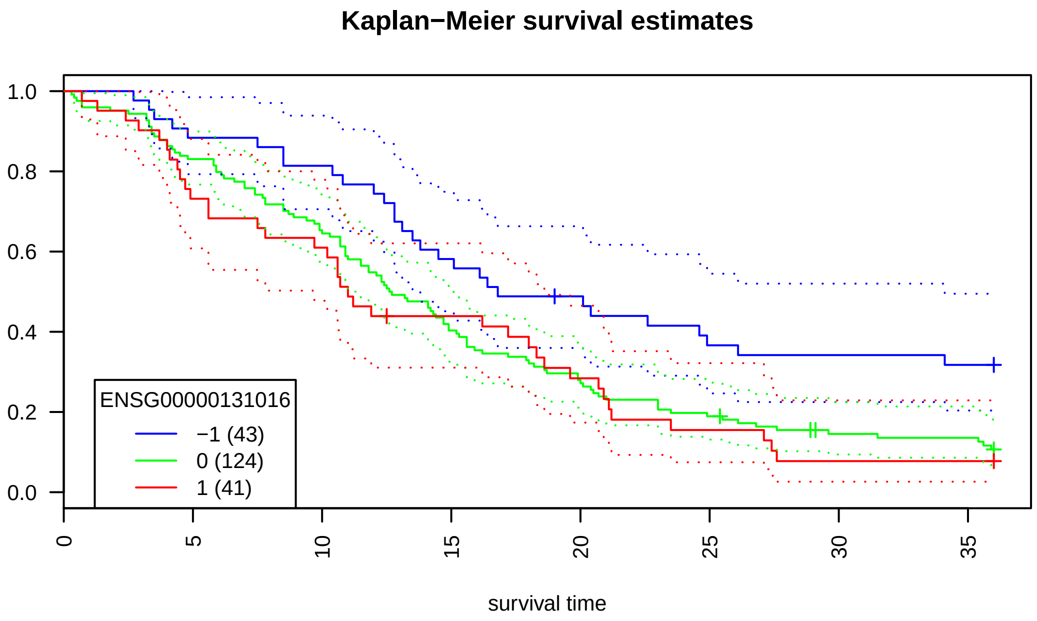

Figure 192: Each curve represents the relative number of ENSG00000131016 survived to the corresponding point of time. Dashed lines represent 0.95 confidence intervals for the curves with the corresponding color. Log-rank probability measure for the equality of these curves is 6.327992e-03.

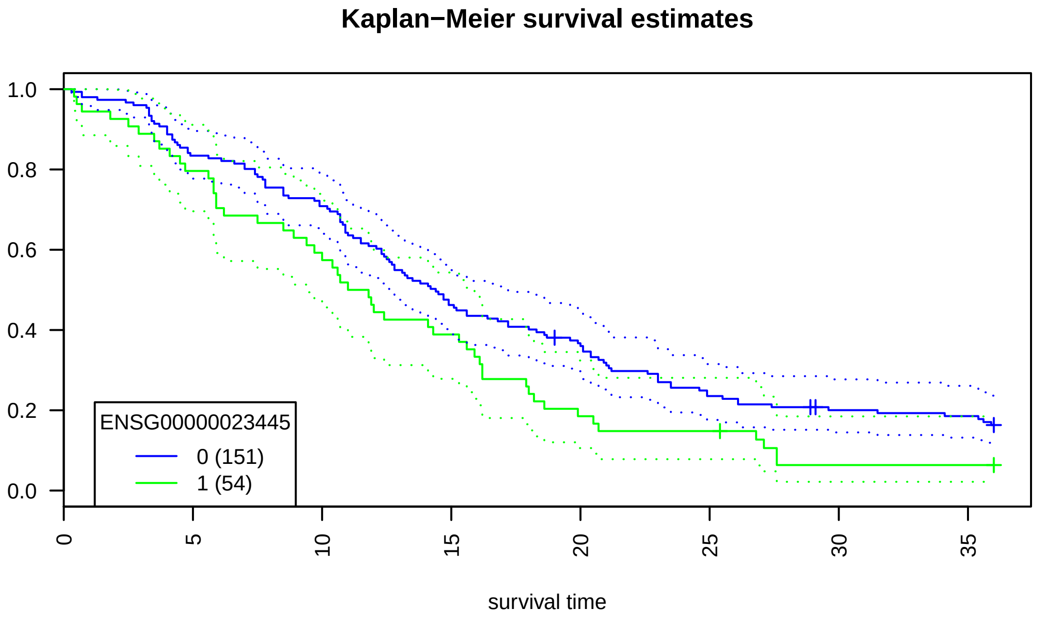

Figure 193: Each curve represents the relative number of ENSG00000023445 survived to the corresponding point of time. Dashed lines represent 0.95 confidence intervals for the curves with the corresponding color. Total of 3 observations deleted due to missingness. Log-rank probability measure for the equality of these curves is 1.700216e-02.

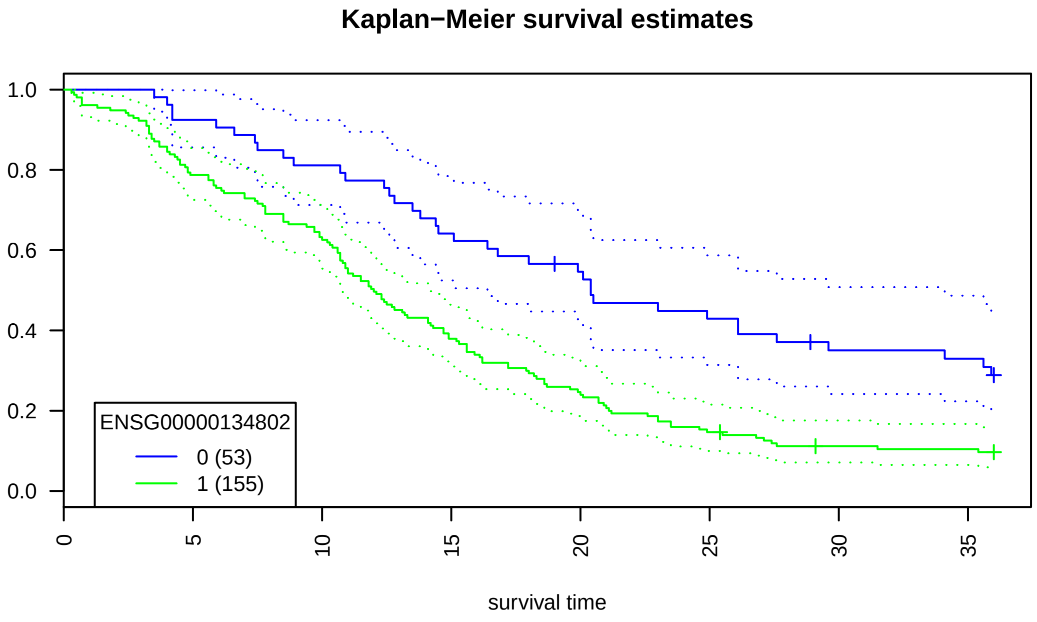

Figure 194: Each curve represents the relative number of ENSG00000134802 survived to the corresponding point of time. Dashed lines represent 0.95 confidence intervals for the curves with the corresponding color. Log-rank probability measure for the equality of these curves is 5.604875e-05. Additional annotations: *.GeneName*=SLC43A3, *.GeneDesc*=solute carrier family 43, member 3 [Source:HGNC Symbol;Acc:17466], *Uniprot/SWISSPROT*=Q8NBI5.

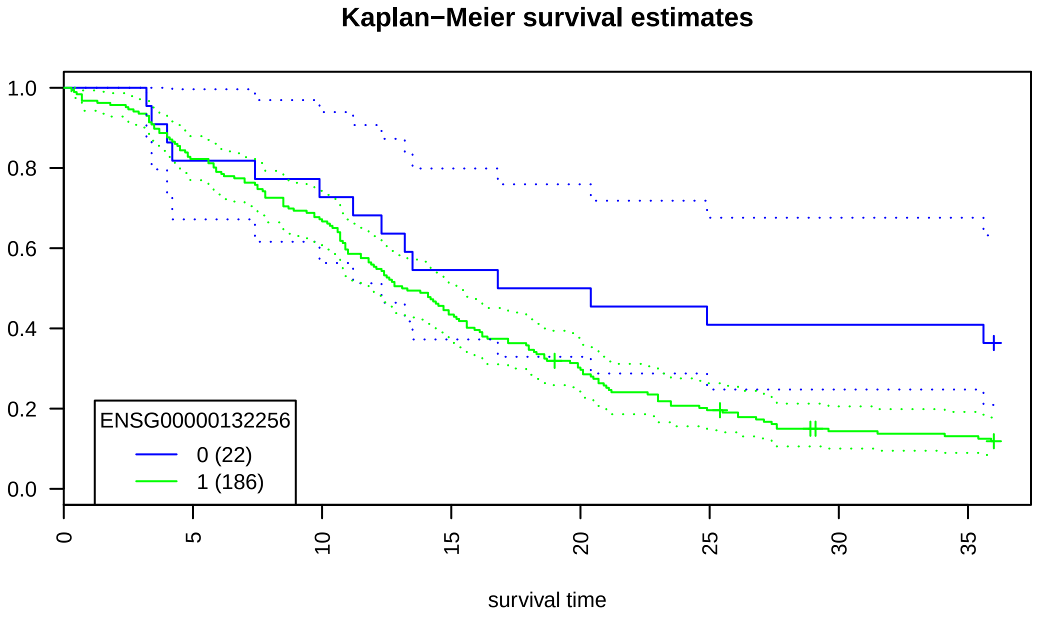

Figure 195: Each curve represents the relative number of ENSG00000132256 survived to the corresponding point of time. Dashed lines represent 0.95 confidence intervals for the curves with the corresponding color. Log-rank probability measure for the equality of these curves is 2.269909e-02. Additional annotations: *.GeneName*=TRIM5, *.GeneDesc*=tripartite motif-containing 5 [Source:HGNC Symbol;Acc:16276], *Uniprot/SWISSPROT*=Q9C035.

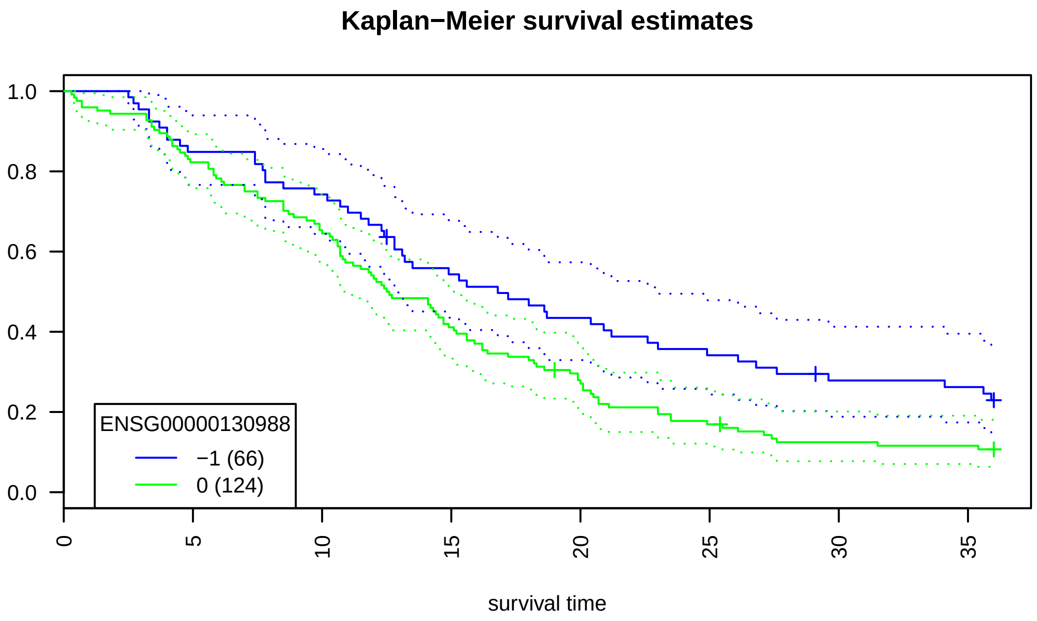

Figure 196: Each curve represents the relative number of ENSG00000130988 survived to the corresponding point of time. Dashed lines represent 0.95 confidence intervals for the curves with the corresponding color. Total of 18 observations deleted due to missingness. Log-rank probability measure for the equality of these curves is 1.193359e-02.

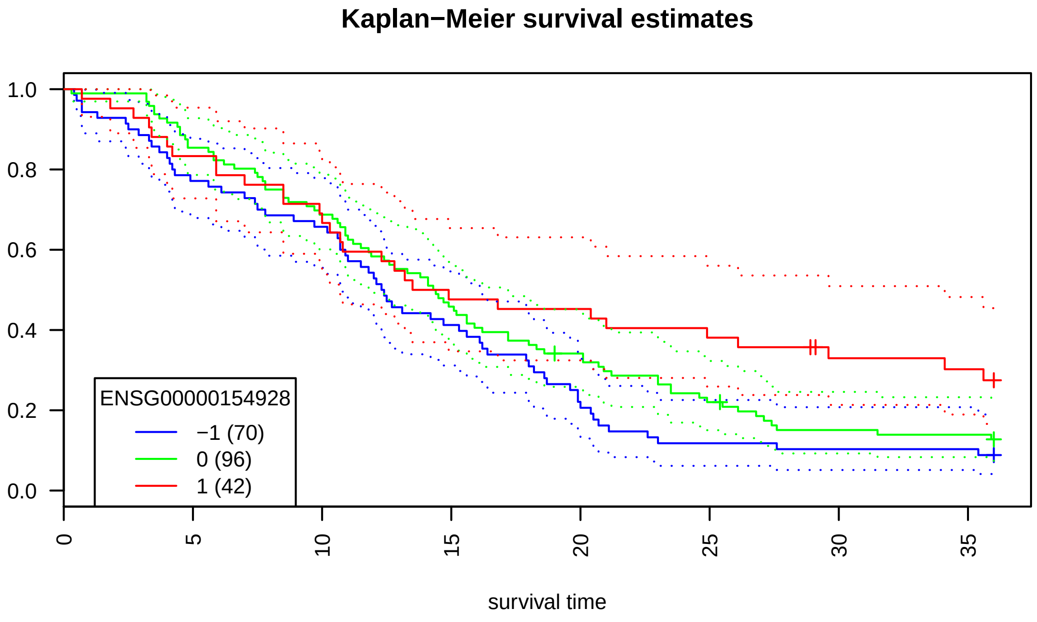

Figure 197: Each curve represents the relative number of ENSG00000154928 survived to the corresponding point of time. Dashed lines represent 0.95 confidence intervals for the curves with the corresponding color. Log-rank probability measure for the equality of these curves is 3.642803e-02.

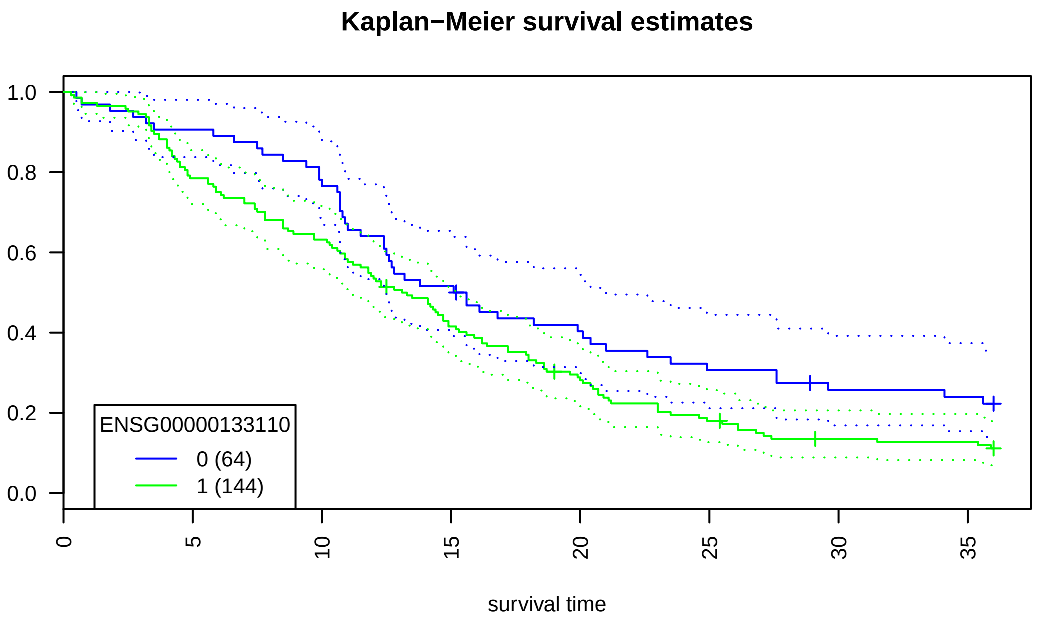

Figure 198: Each curve represents the relative number of ENSG00000133110 survived to the corresponding point of time. Dashed lines represent 0.95 confidence intervals for the curves with the corresponding color. Log-rank probability measure for the equality of these curves is 2.997424e-02. Additional annotations: *.GeneName*=POSTN, *.GeneDesc*=periostin, osteoblast specific factor [Source:HGNC Symbol;Acc:16953], *Uniprot/SWISSPROT*=Q15063.

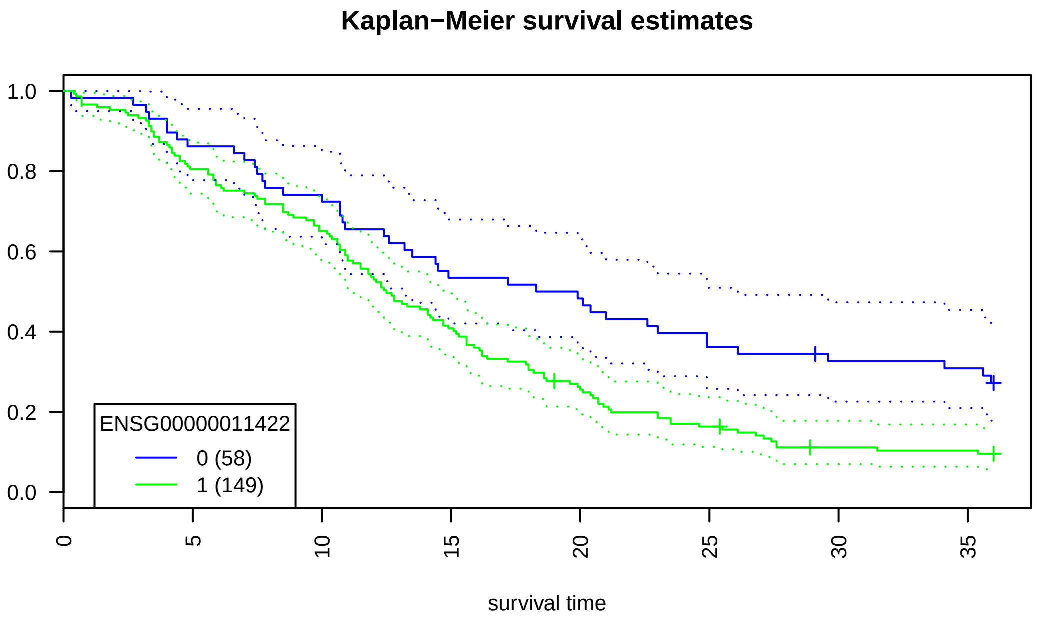

Figure 199: Each curve represents the relative number of ENSG00000011422 survived to the corresponding point of time. Dashed lines represent 0.95 confidence intervals for the curves with the corresponding color. Total of 1 observation deleted due to missingness. Log-rank probability measure for the equality of these curves is 1.604042e-03. Additional annotations: *.GeneName*=PLAUR, *.GeneDesc*=plasminogen activator, urokinase receptor [Source:HGNC Symbol;Acc:9053], *Uniprot/SWISSPROT*=Q03405.

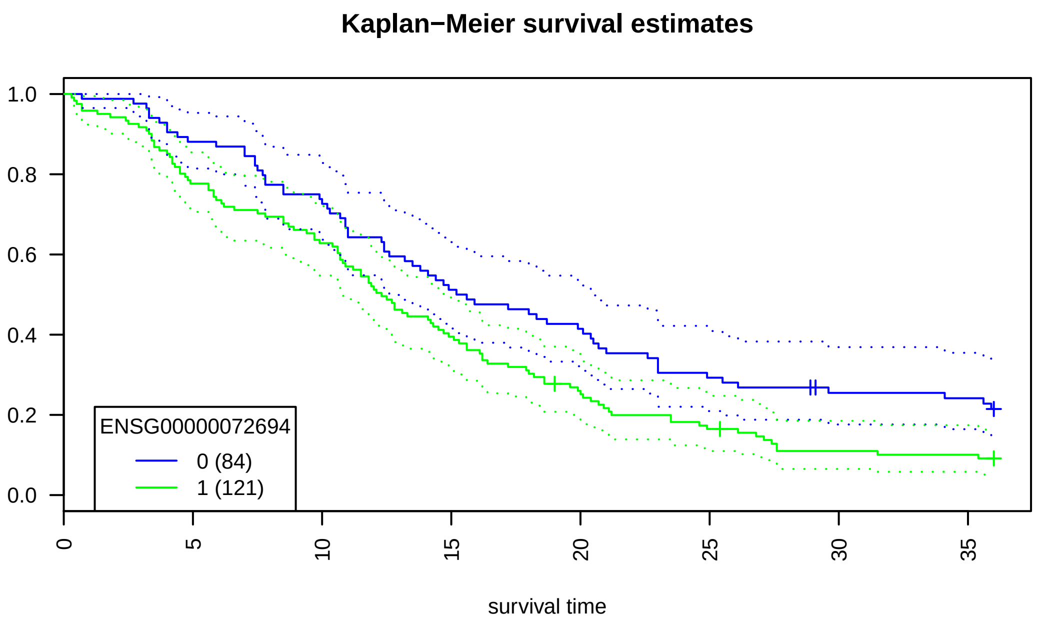

Figure 200: Each curve represents the relative number of ENSG00000072694 survived to the corresponding point of time. Dashed lines represent 0.95 confidence intervals for the curves with the corresponding color. Total of 3 observations deleted due to missingness. Log-rank probability measure for the equality of these curves is 7.319123e-03. Additional annotations: *.GeneName*=FCGR2B, *.GeneDesc*=Fc fragment of IgG, low affinity IIb, receptor (CD32) [Source:HGNC Symbol;Acc:3618], *Uniprot/SWISSPROT*=P31994.

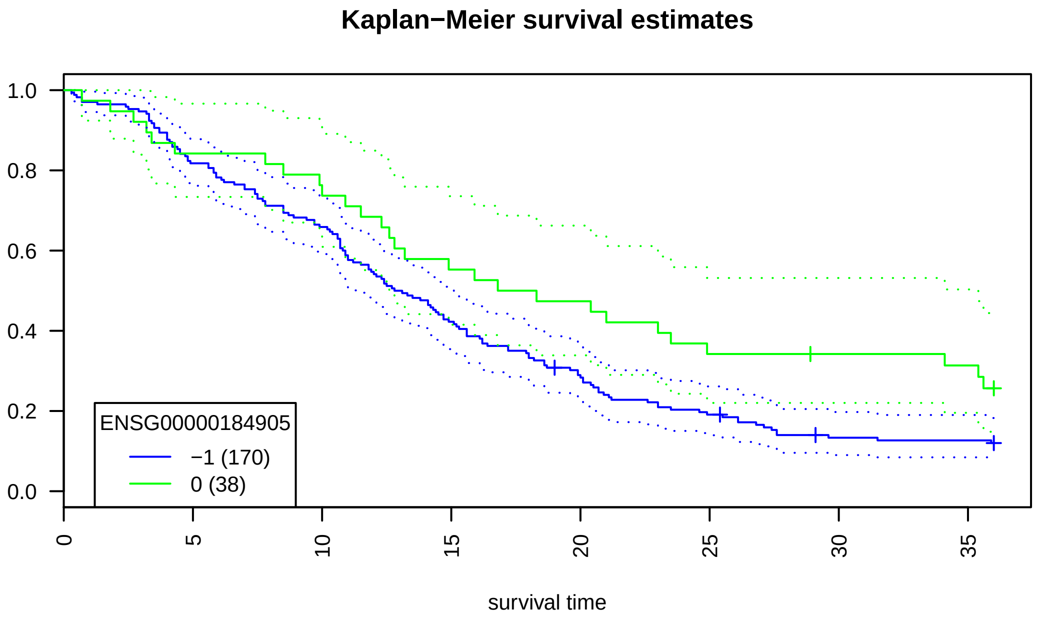

Figure 201: Each curve represents the relative number of ENSG00000184905 survived to the corresponding point of time. Dashed lines represent 0.95 confidence intervals for the curves with the corresponding color. Log-rank probability measure for the equality of these curves is 2.636018e-02. Additional annotations: *.GeneName*=TCEAL2, *.GeneDesc*=transcription elongation factor A (SII)-like 2 [Source:HGNC Symbol;Acc:29818], *Uniprot/SWISSPROT*=Q9H3H9.

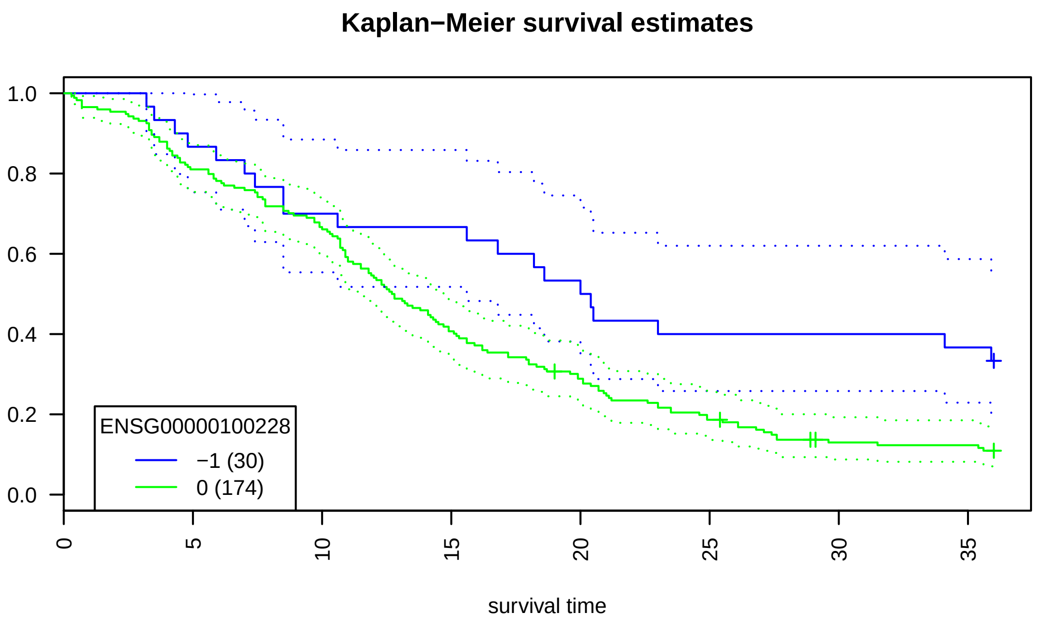

Figure 202: Each curve represents the relative number of ENSG00000100228 survived to the corresponding point of time. Dashed lines represent 0.95 confidence intervals for the curves with the corresponding color. Total of 4 observations deleted due to missingness. Log-rank probability measure for the equality of these curves is 6.052052e-03.

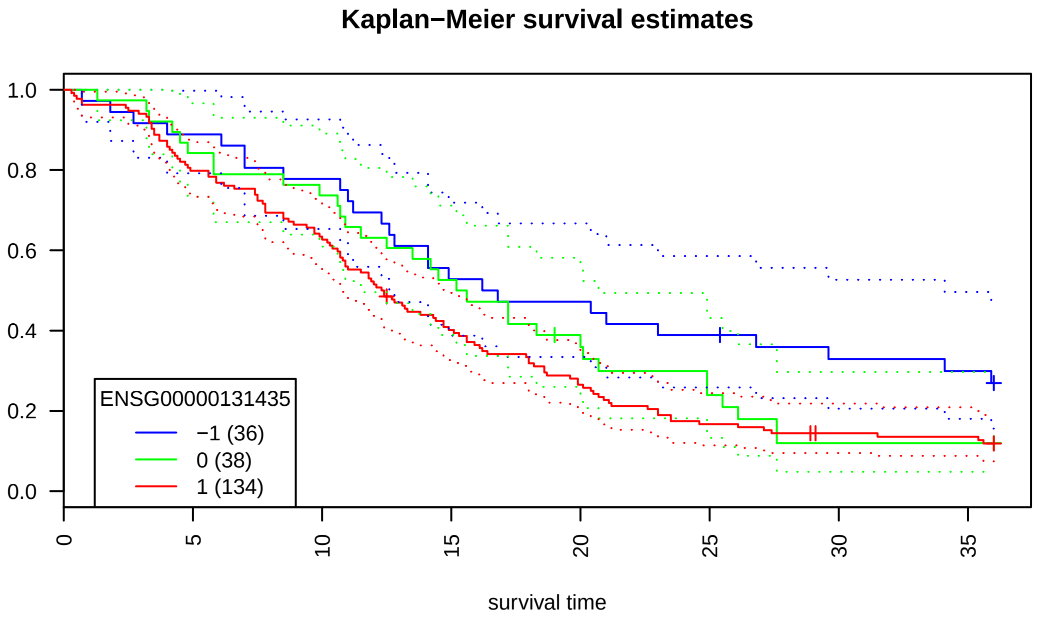

Figure 203: Each curve represents the relative number of ENSG00000131435 survived to the corresponding point of time. Dashed lines represent 0.95 confidence intervals for the curves with the corresponding color. Log-rank probability measure for the equality of these curves is 4.689361e-02. Additional annotations: *.GeneName*=PDLIM4, *.GeneDesc*=PDZ and LIM domain 4 [Source:HGNC Symbol;Acc:16501], *Uniprot/SWISSPROT*=P50479.

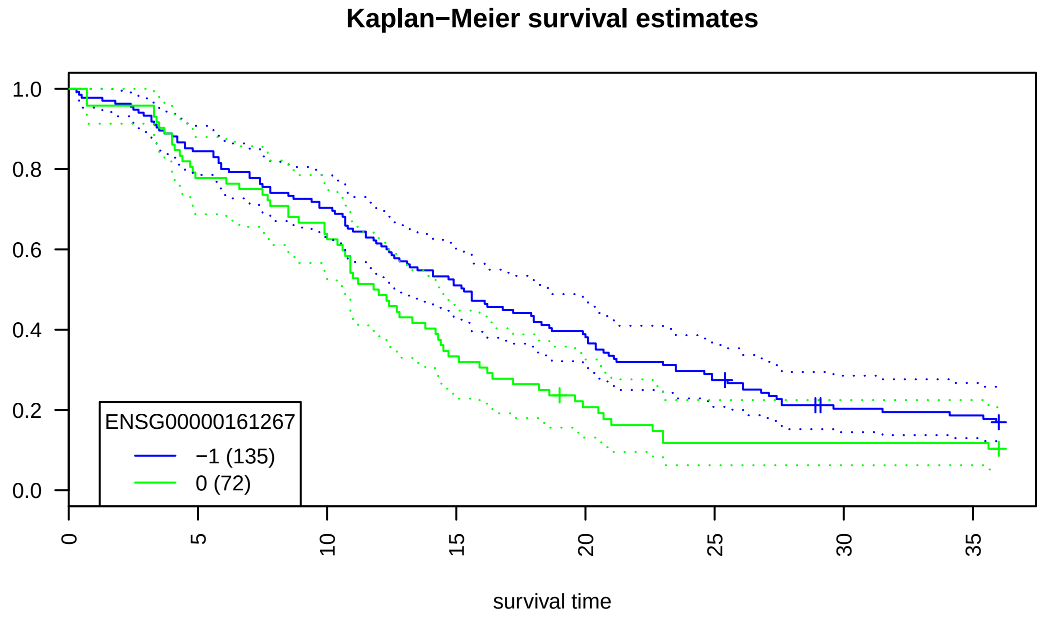

Figure 204: Each curve represents the relative number of ENSG00000161267 survived to the corresponding point of time. Dashed lines represent 0.95 confidence intervals for the curves with the corresponding color. Total of 1 observation deleted due to missingness. Log-rank probability measure for the equality of these curves is 2.447722e-02. Additional annotations: *.GeneName*=BDH1, *.GeneDesc*=3-hydroxybutyrate dehydrogenase, type 1 [Source:HGNC Symbol;Acc:1027], *Uniprot/SWISSPROT*=Q02338.

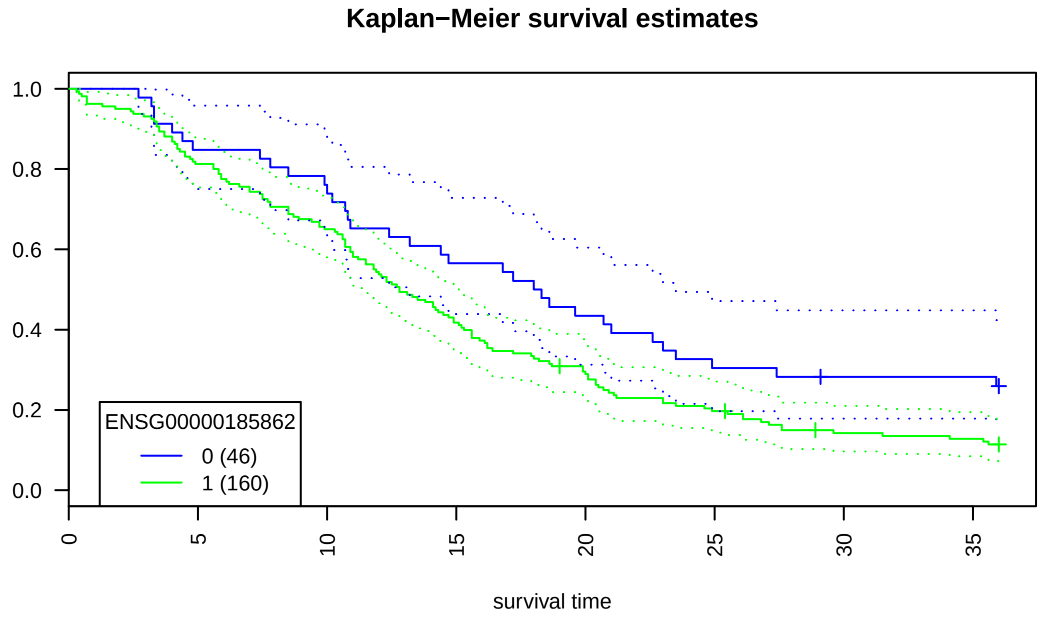

Figure 205: Each curve represents the relative number of ENSG00000185862 survived to the corresponding point of time. Dashed lines represent 0.95 confidence intervals for the curves with the corresponding color. Total of 2 observations deleted due to missingness. Log-rank probability measure for the equality of these curves is 2.148569e-02. Additional annotations: *.GeneName*=EVI2B, *.GeneDesc*=ecotropic viral integration site 2B [Source:HGNC Symbol;Acc:3500], *Uniprot/SWISSPROT*=P34910.

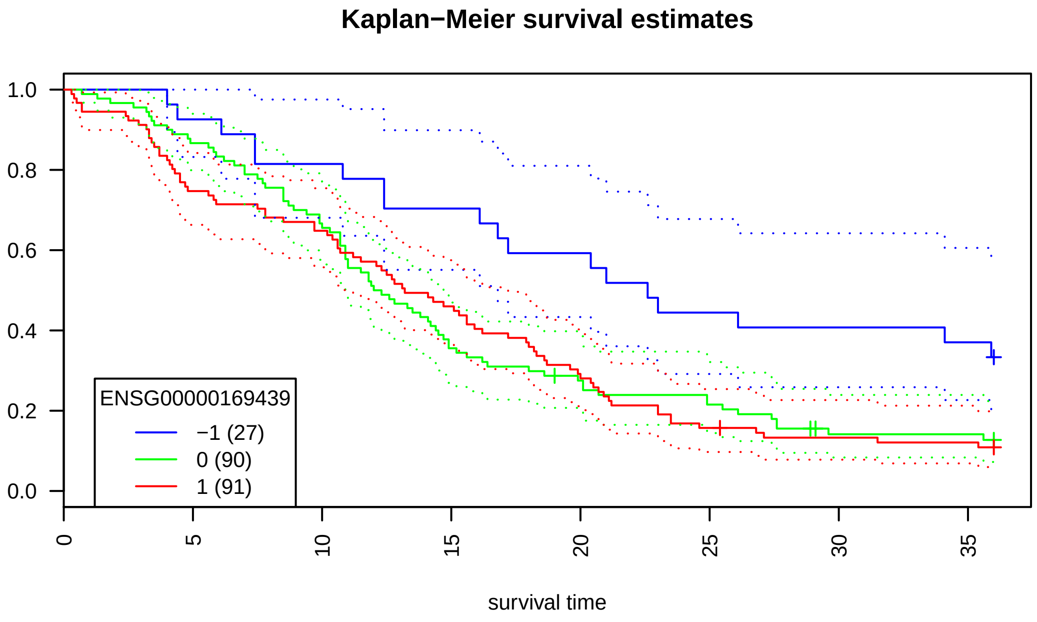

Figure 206: Each curve represents the relative number of ENSG00000169439 survived to the corresponding point of time. Dashed lines represent 0.95 confidence intervals for the curves with the corresponding color. Log-rank probability measure for the equality of these curves is 1.200666e-02.

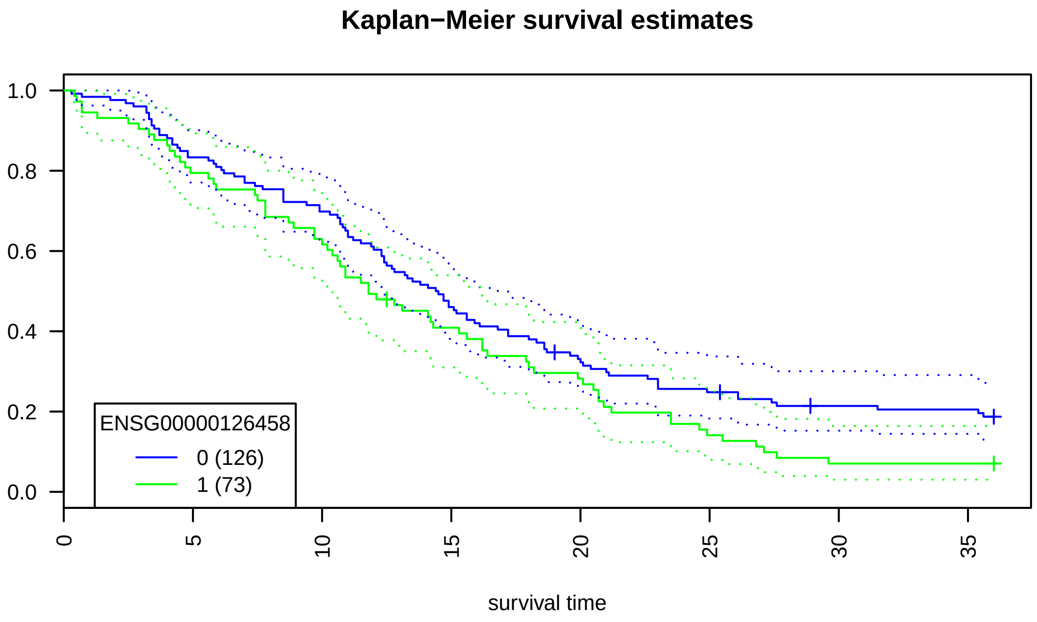

Figure 207: Each curve represents the relative number of ENSG00000126458 survived to the corresponding point of time. Dashed lines represent 0.95 confidence intervals for the curves with the corresponding color. Total of 9 observations deleted due to missingness. Log-rank probability measure for the equality of these curves is 4.823388e-02.

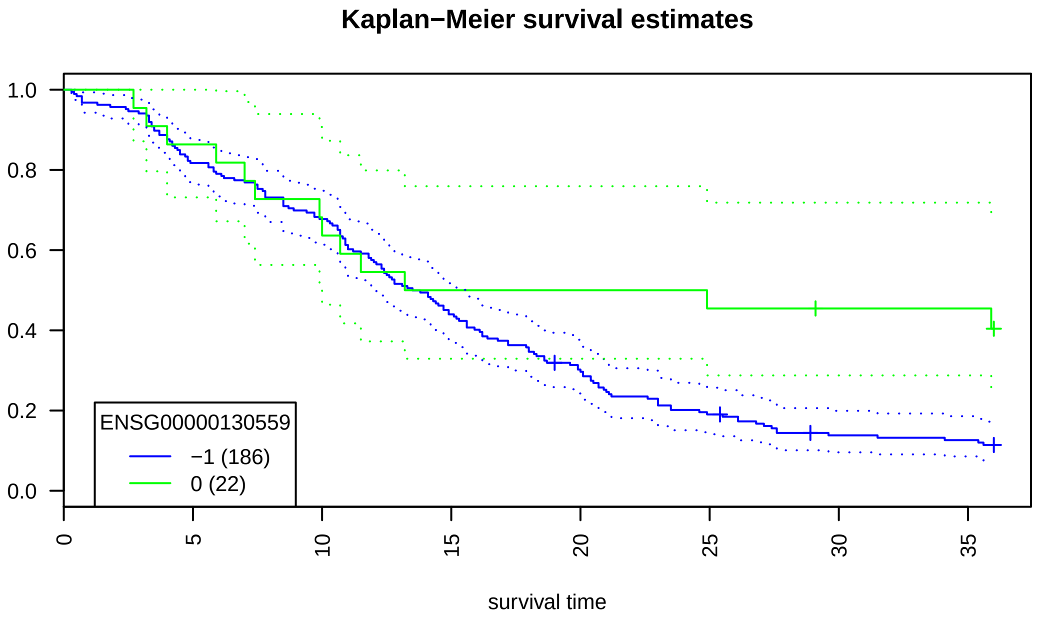

Figure 208: Each curve represents the relative number of ENSG00000130559 survived to the corresponding point of time. Dashed lines represent 0.95 confidence intervals for the curves with the corresponding color. Log-rank probability measure for the equality of these curves is 1.562771e-02. Additional annotations: *.GeneName*=CAMSAP1, *.GeneDesc*=calmodulin regulated spectrin-associated protein 1 [Source:HGNC Symbol;Acc:19946], *Uniprot/SWISSPROT*=Q5T5Y3.

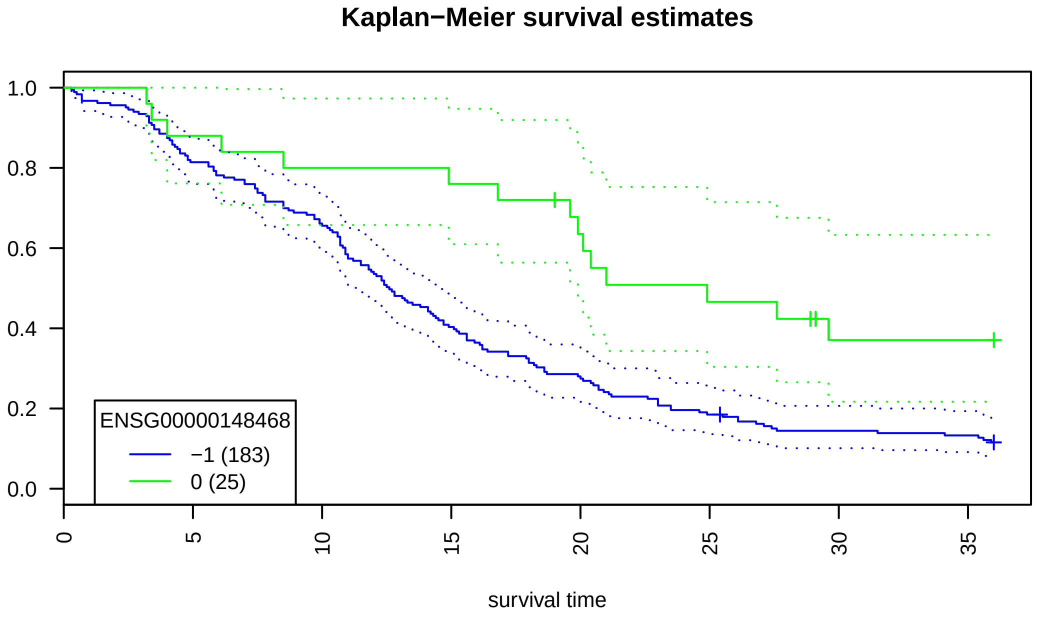

Figure 209: Each curve represents the relative number of ENSG00000148468 survived to the corresponding point of time. Dashed lines represent 0.95 confidence intervals for the curves with the corresponding color. Log-rank probability measure for the equality of these curves is 1.182729e-03. Additional annotations: *.GeneName*=FAM171A1, *.GeneDesc*=family with sequence similarity 171, member A1 [Source:HGNC Symbol;Acc:23522], *Uniprot/SWISSPROT*=Q5VUB5.

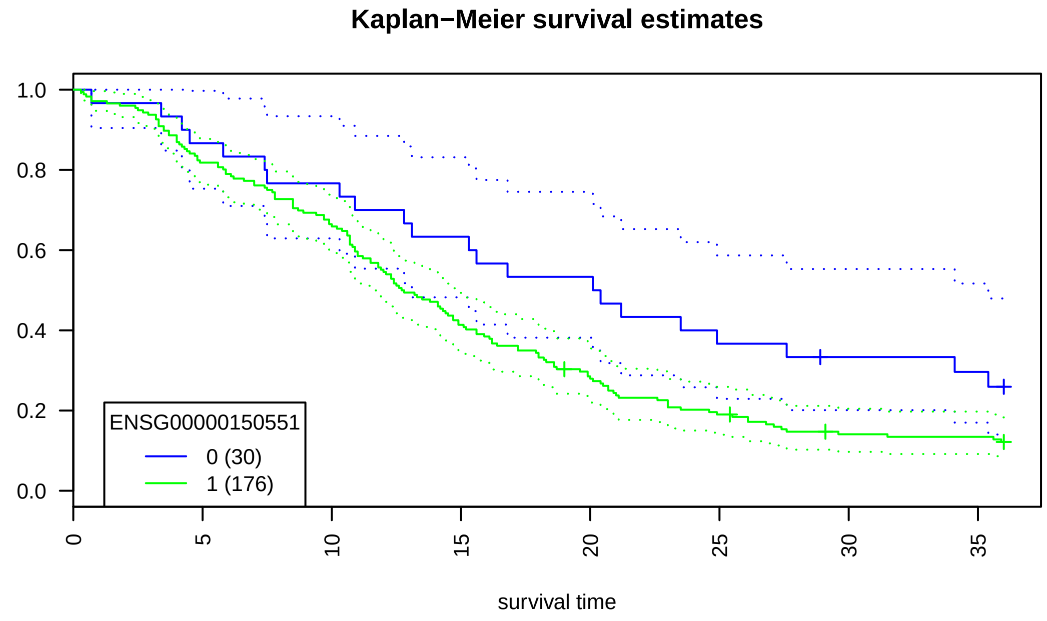

Figure 210: Each curve represents the relative number of ENSG00000150551 survived to the corresponding point of time. Dashed lines represent 0.95 confidence intervals for the curves with the corresponding color. Total of 2 observations deleted due to missingness. Log-rank probability measure for the equality of these curves is 2.733495e-02. Additional annotations: *.GeneName*=LYPD1, *.GeneDesc*=LY6/PLAUR domain containing 1 [Source:HGNC Symbol;Acc:28431], *Uniprot/SWISSPROT*=Q8N2G4.

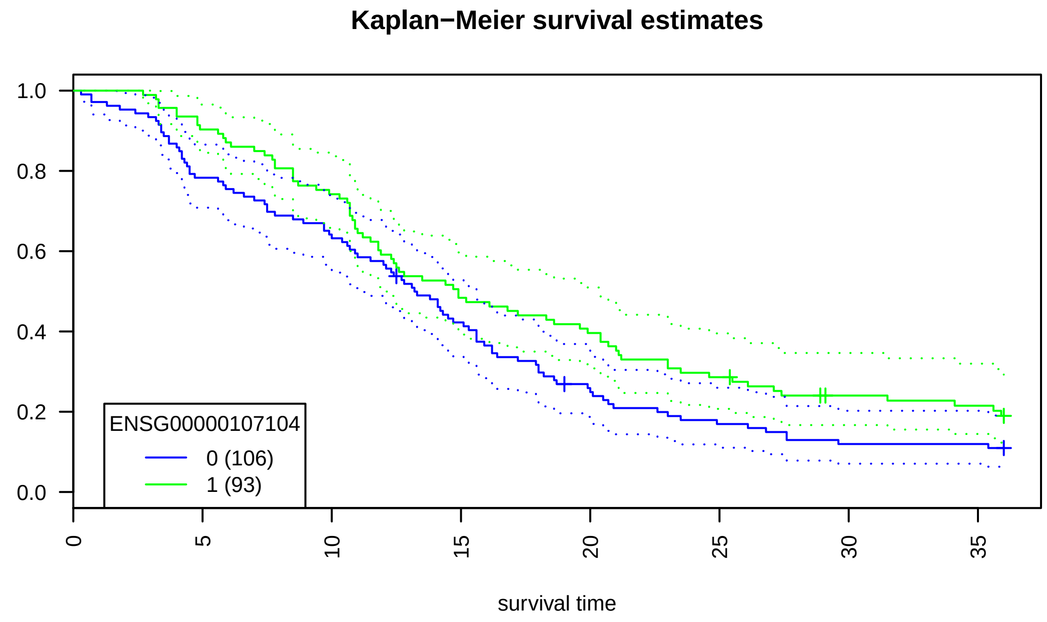

Figure 211: Each curve represents the relative number of ENSG00000107104 survived to the corresponding point of time. Dashed lines represent 0.95 confidence intervals for the curves with the corresponding color. Total of 9 observations deleted due to missingness. Log-rank probability measure for the equality of these curves is 3.984149e-02.

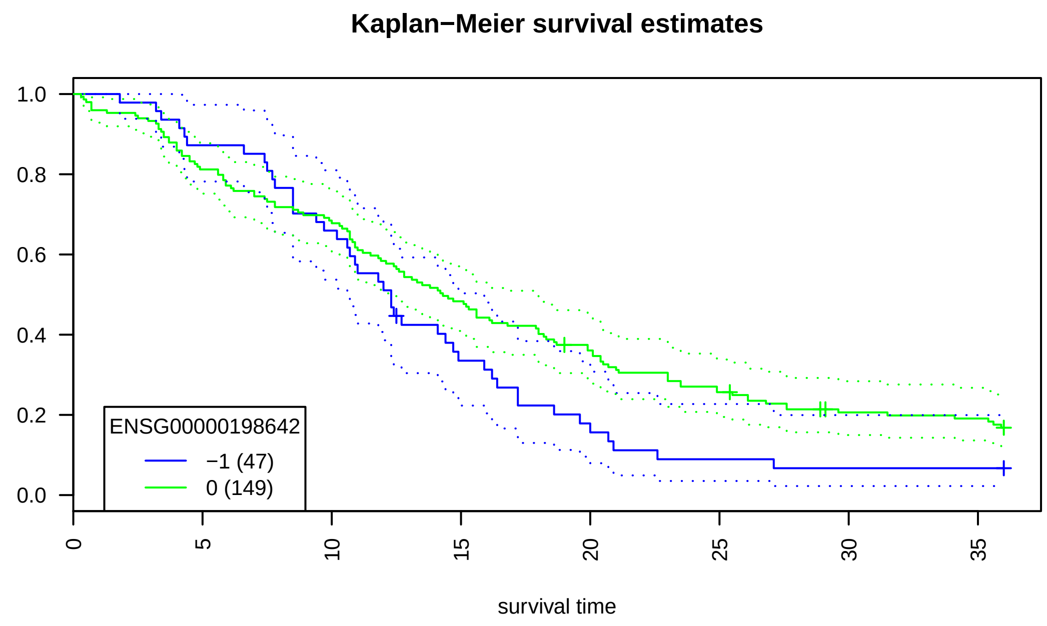

Figure 212: Each curve represents the relative number of ENSG00000198642 survived to the corresponding point of time. Dashed lines represent 0.95 confidence intervals for the curves with the corresponding color. Total of 12 observations deleted due to missingness. Log-rank probability measure for the equality of these curves is 4.059941e-02.

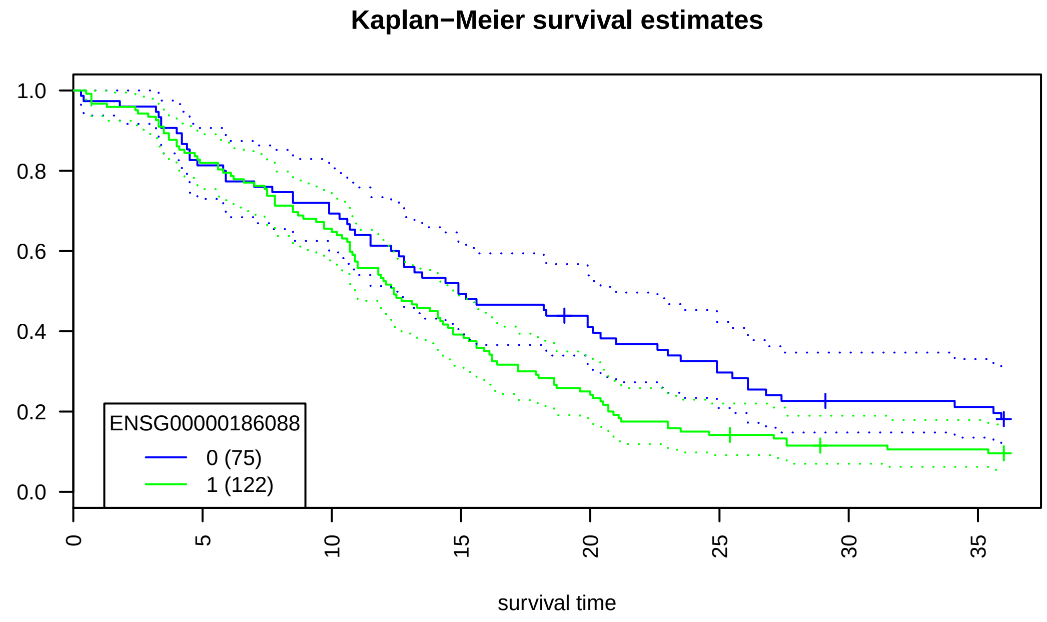

Figure 213: Each curve represents the relative number of ENSG00000186088 survived to the corresponding point of time. Dashed lines represent 0.95 confidence intervals for the curves with the corresponding color. Total of 11 observations deleted due to missingness. Log-rank probability measure for the equality of these curves is 3.10244e-02. Additional annotations: *.GeneName*=PION, *.GeneDesc*=pigeon homolog (Drosophila) [Source:HGNC Symbol;Acc:28042], *Uniprot/SWISSPROT*=A4D1B5.

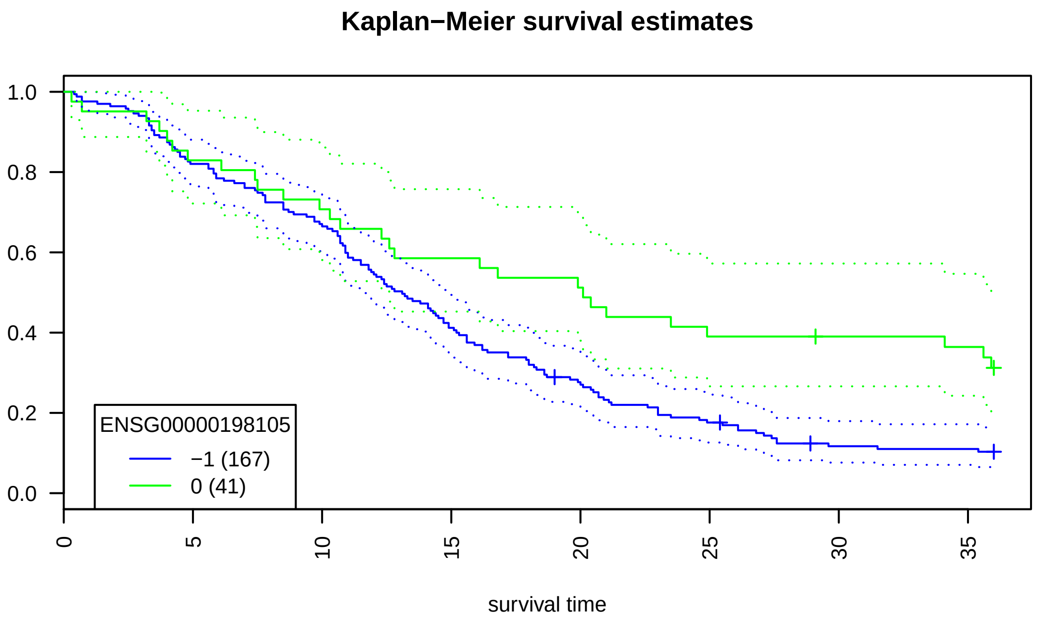

Figure 214: Each curve represents the relative number of ENSG00000198105 survived to the corresponding point of time. Dashed lines represent 0.95 confidence intervals for the curves with the corresponding color. Log-rank probability measure for the equality of these curves is 3.391210e-03. Additional annotations: *.GeneName*=ZNF248, *.GeneDesc*=zinc finger protein 248 [Source:HGNC Symbol;Acc:13041], *Uniprot/SWISSPROT*=Q8NDW4.

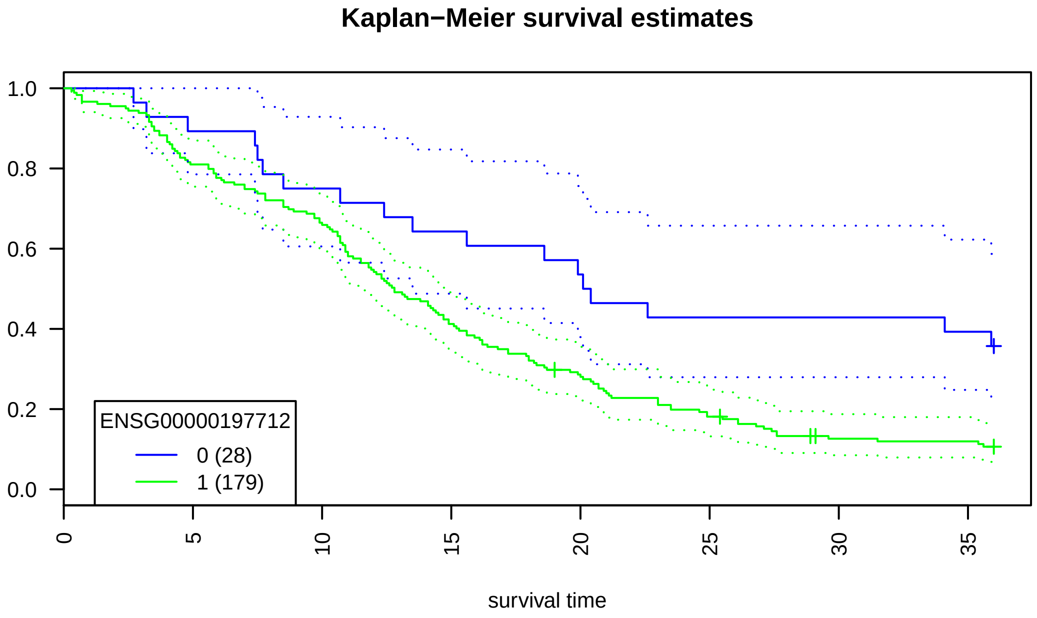

Figure 215: Each curve represents the relative number of ENSG00000197712 survived to the corresponding point of time. Dashed lines represent 0.95 confidence intervals for the curves with the corresponding color. Total of 1 observation deleted due to missingness. Log-rank probability measure for the equality of these curves is 2.966935e-03. Additional annotations: *.GeneName*=FAM114A1, *.GeneDesc*=family with sequence similarity 114, member A1 [Source:HGNC Symbol;Acc:25087], *Uniprot/SWISSPROT*=Q8IWE2.

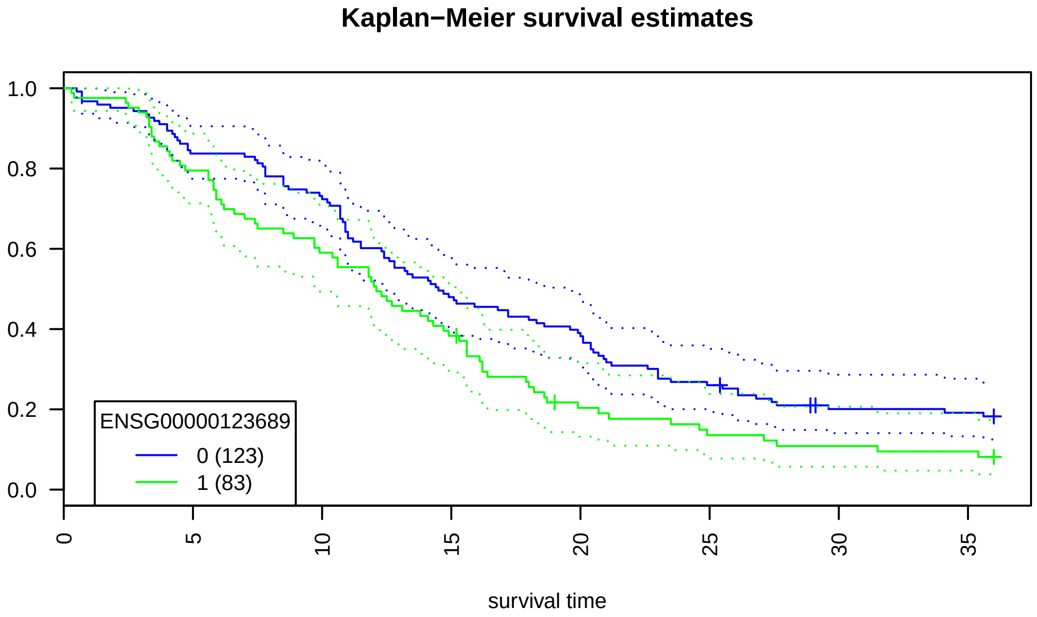

Figure 216: Each curve represents the relative number of ENSG00000123689 survived to the corresponding point of time. Dashed lines represent 0.95 confidence intervals for the curves with the corresponding color. Total of 2 observations deleted due to missingness. Log-rank probability measure for the equality of these curves is 1.300513e-02.

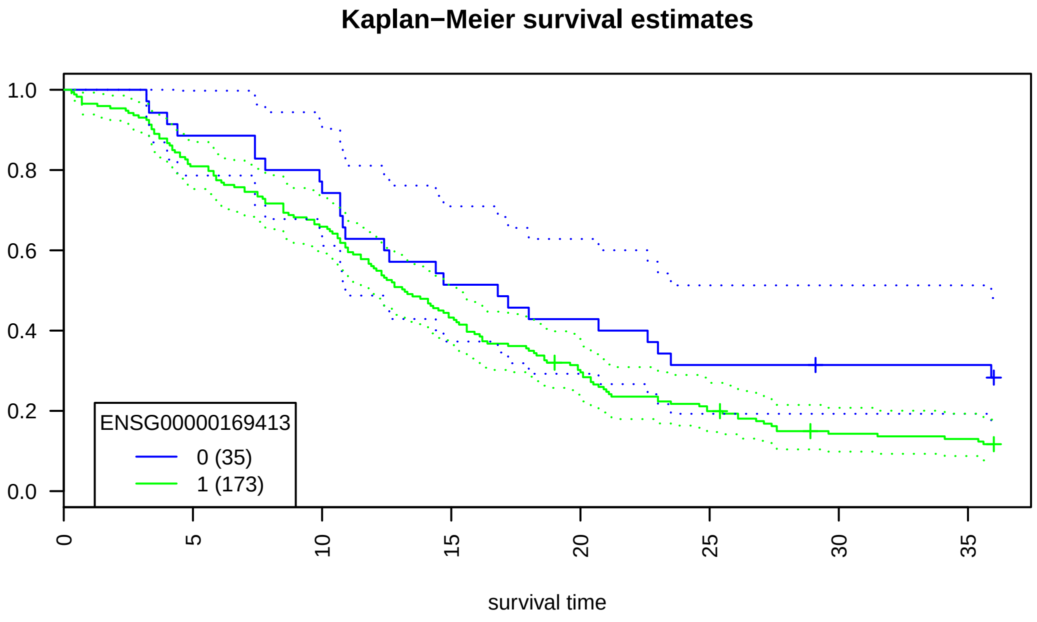

Figure 217: Each curve represents the relative number of ENSG00000169413 survived to the corresponding point of time. Dashed lines represent 0.95 confidence intervals for the curves with the corresponding color. Log-rank probability measure for the equality of these curves is 4.14972e-02. Additional annotations: *.GeneName*=RNASE6, *.GeneDesc*=ribonuclease, RNase A family, k6 [Source:HGNC Symbol;Acc:10048], *Uniprot/SWISSPROT*=Q93091.

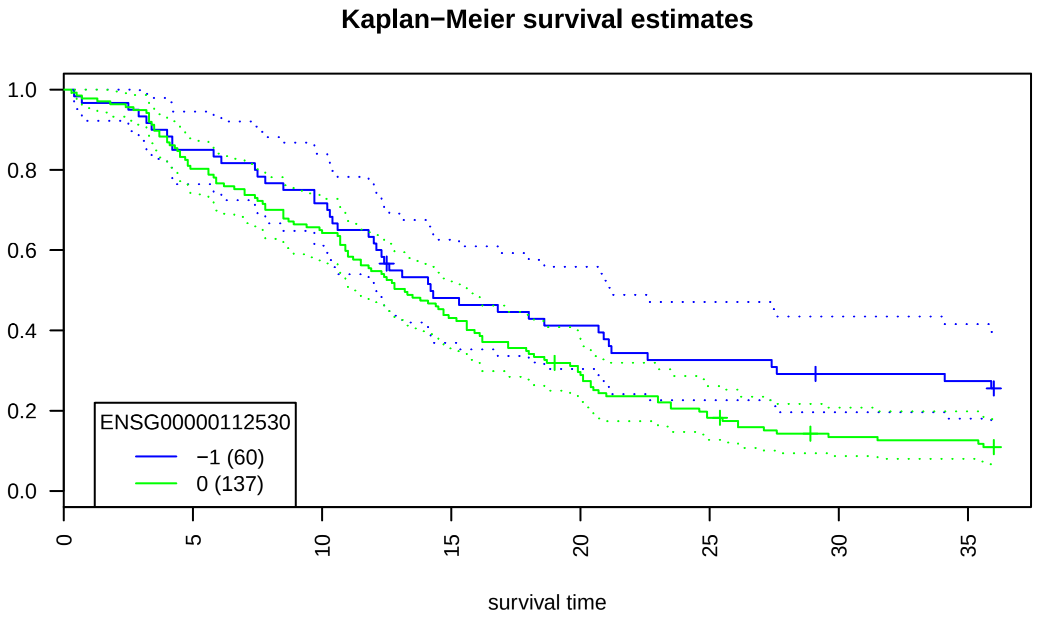

Figure 218: Each curve represents the relative number of ENSG00000112530 survived to the corresponding point of time. Dashed lines represent 0.95 confidence intervals for the curves with the corresponding color. Total of 11 observations deleted due to missingness. Log-rank probability measure for the equality of these curves is 3.870647e-02.

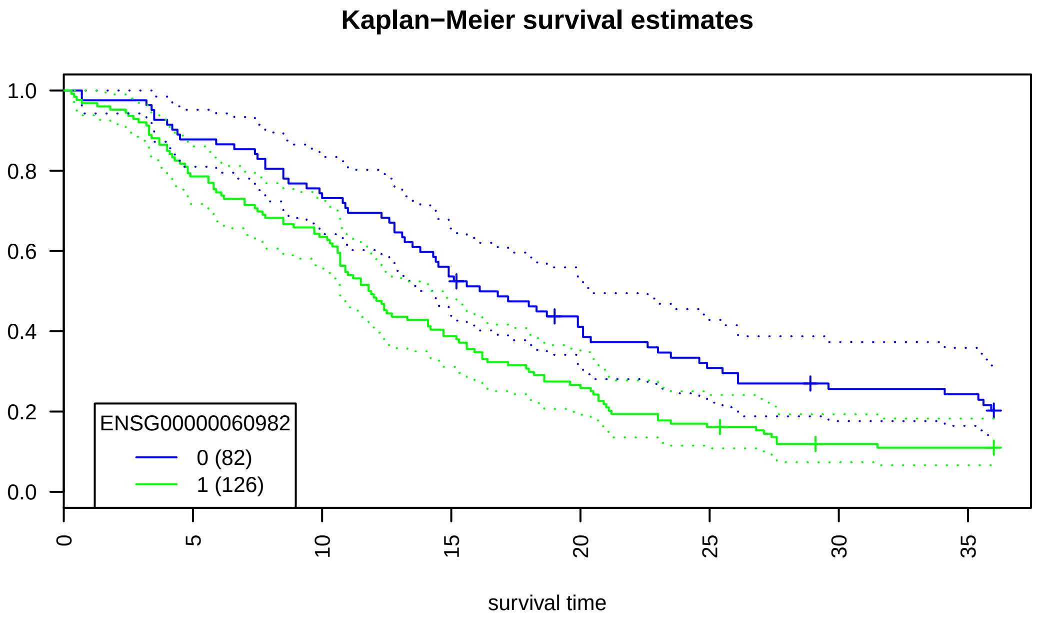

Figure 219: Each curve represents the relative number of ENSG00000060982 survived to the corresponding point of time. Dashed lines represent 0.95 confidence intervals for the curves with the corresponding color. Log-rank probability measure for the equality of these curves is 6.293597e-03. Additional annotations: *.GeneName*=BCAT1, *.GeneDesc*=branched chain aminotransferase 1, cytosolic [Source:HGNC Symbol;Acc:976], *Uniprot/SWISSPROT*=P54687.

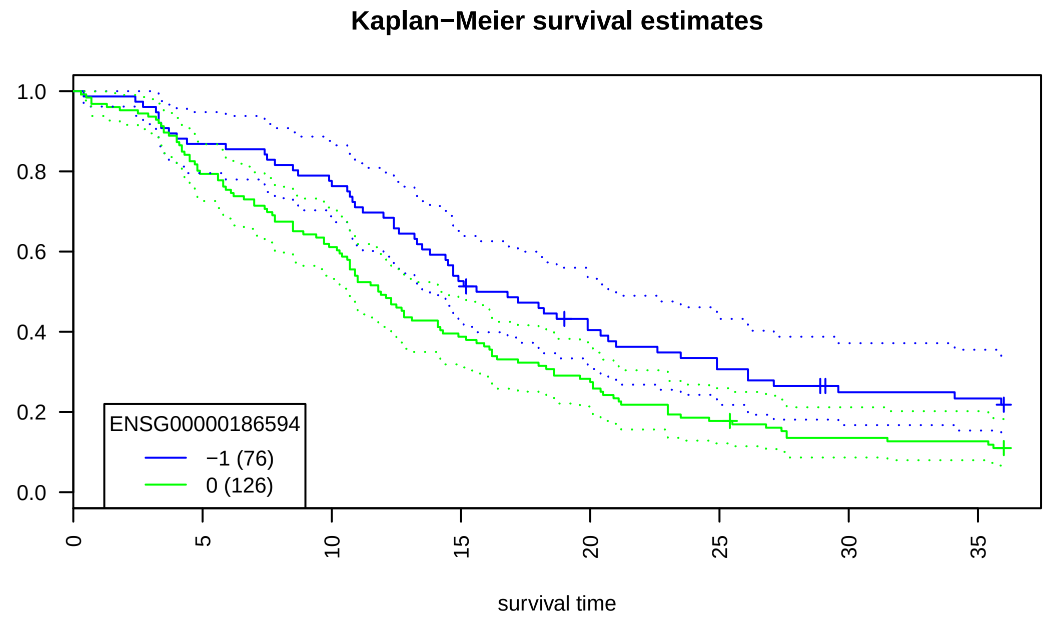

Figure 220: Each curve represents the relative number of ENSG00000186594 survived to the corresponding point of time. Dashed lines represent 0.95 confidence intervals for the curves with the corresponding color. Total of 6 observations deleted due to missingness. Log-rank probability measure for the equality of these curves is 9.36516e-03.

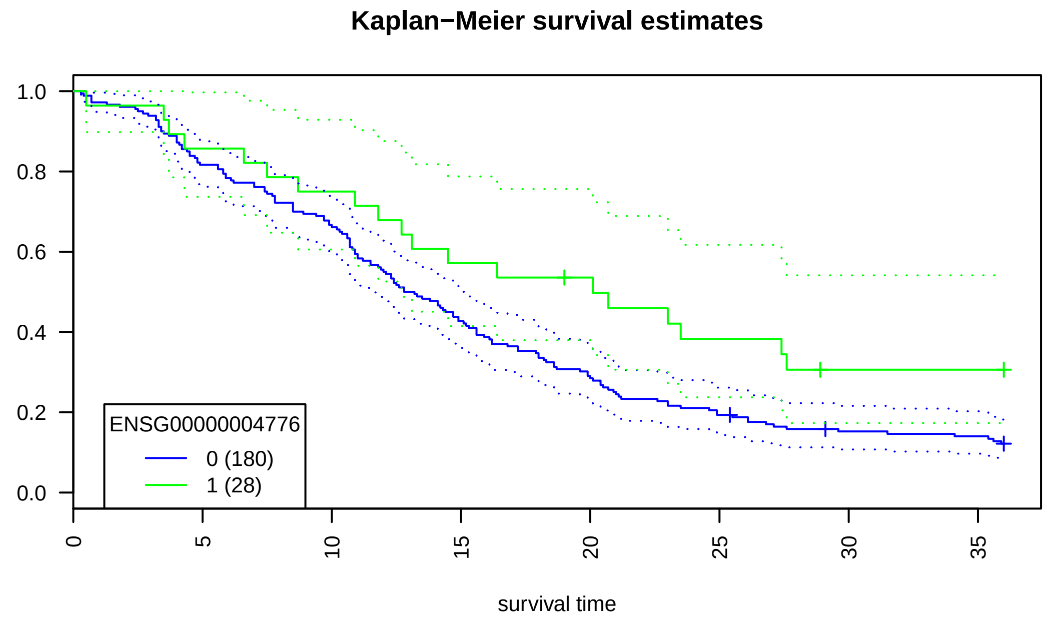

Figure 221: Each curve represents the relative number of ENSG00000004776 survived to the corresponding point of time. Dashed lines represent 0.95 confidence intervals for the curves with the corresponding color. Log-rank probability measure for the equality of these curves is 2.619156e-02.

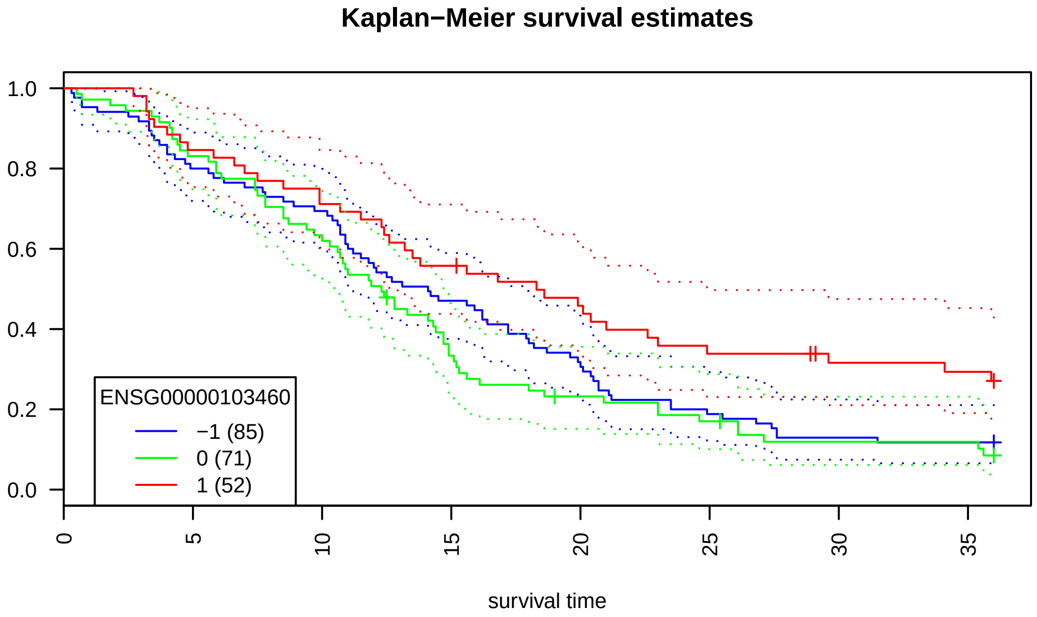

Figure 222: Each curve represents the relative number of ENSG00000103460 survived to the corresponding point of time. Dashed lines represent 0.95 confidence intervals for the curves with the corresponding color. Log-rank probability measure for the equality of these curves is 1.676424e-02.

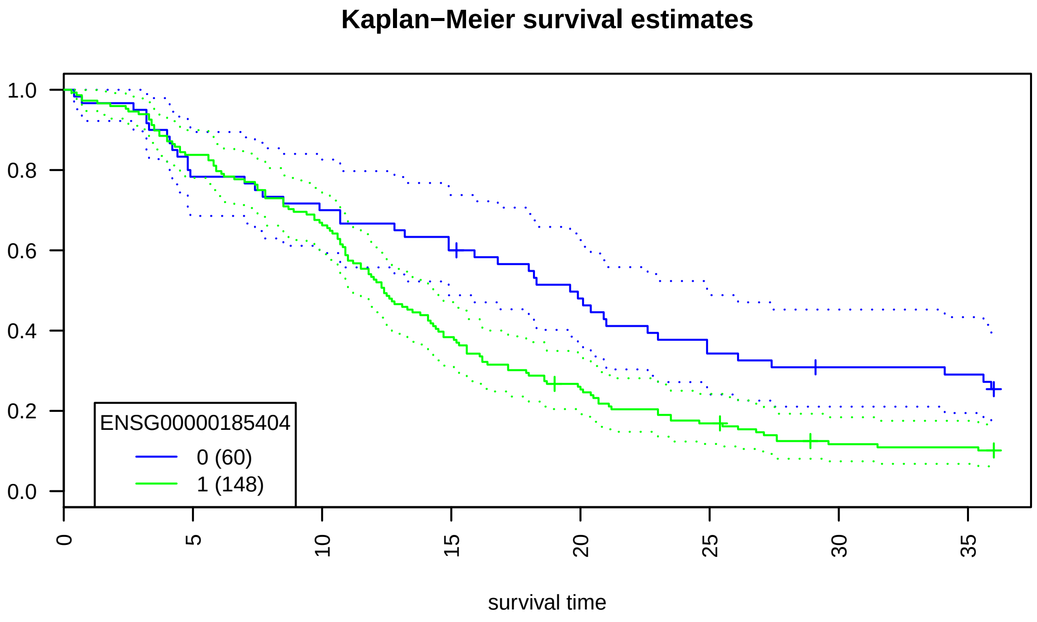

Figure 223: Each curve represents the relative number of ENSG00000185404 survived to the corresponding point of time. Dashed lines represent 0.95 confidence intervals for the curves with the corresponding color. Log-rank probability measure for the equality of these curves is 2.916788e-03. Additional annotations: *.GeneName*=SP140L, *.GeneDesc*=SP140 nuclear body protein-like [Source:HGNC Symbol;Acc:25105], *Uniprot/SWISSPROT*=Q9H930.

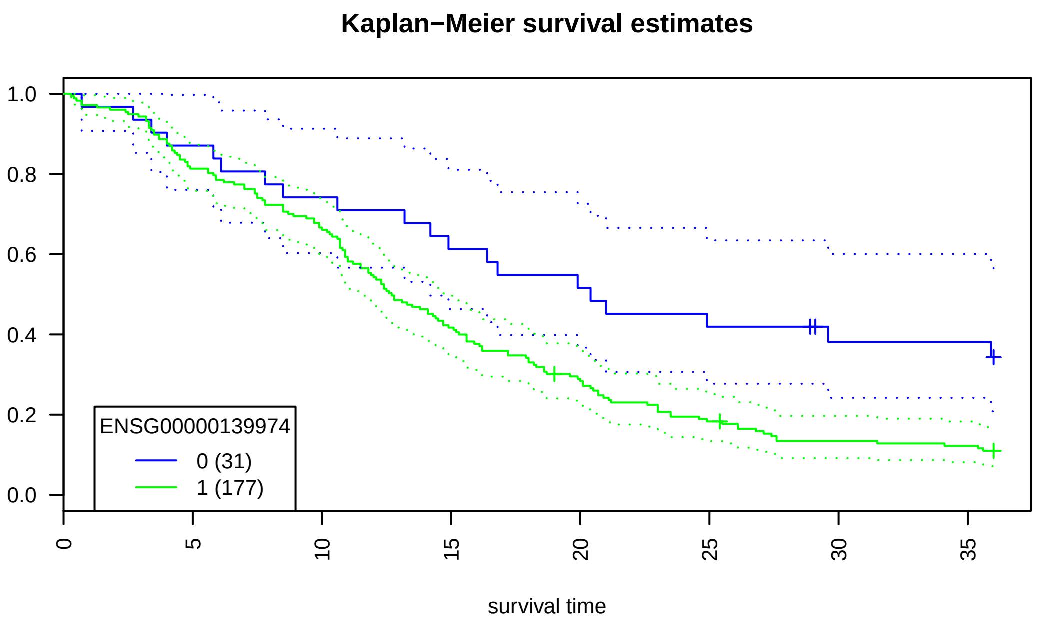

Figure 224: Each curve represents the relative number of ENSG00000139974 survived to the corresponding point of time. Dashed lines represent 0.95 confidence intervals for the curves with the corresponding color. Log-rank probability measure for the equality of these curves is 3.682199e-03. Additional annotations: *.GeneName*=SLC38A6, *.GeneDesc*=solute carrier family 38, member 6 [Source:HGNC Symbol;Acc:19863], *Uniprot/SWISSPROT*=Q8IZM9.

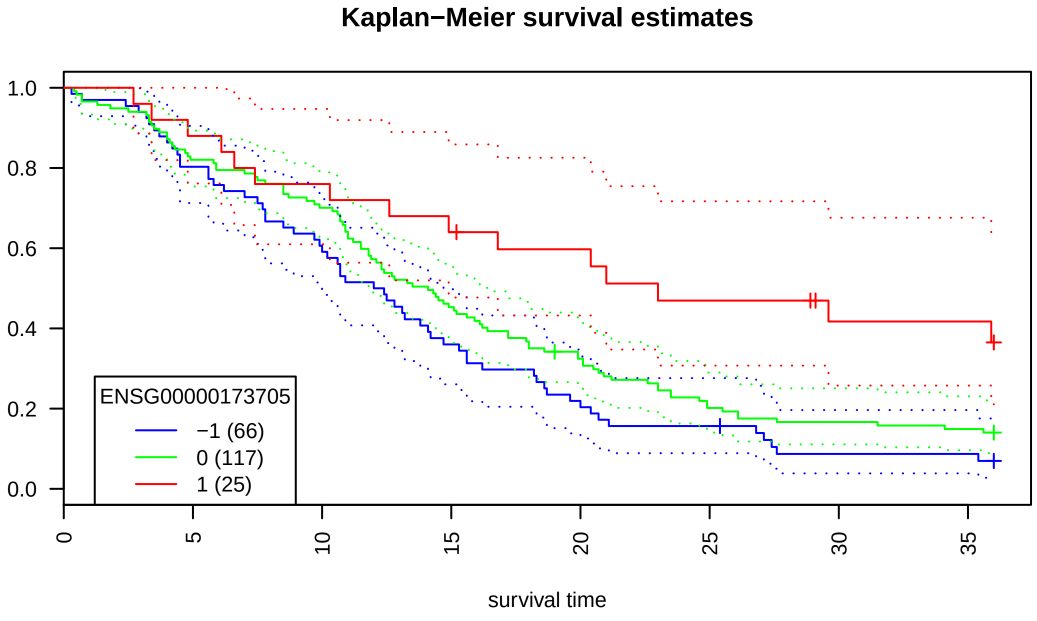

Figure 225: Each curve represents the relative number of ENSG00000173705 survived to the corresponding point of time. Dashed lines represent 0.95 confidence intervals for the curves with the corresponding color. Log-rank probability measure for the equality of these curves is 3.801605e-03.

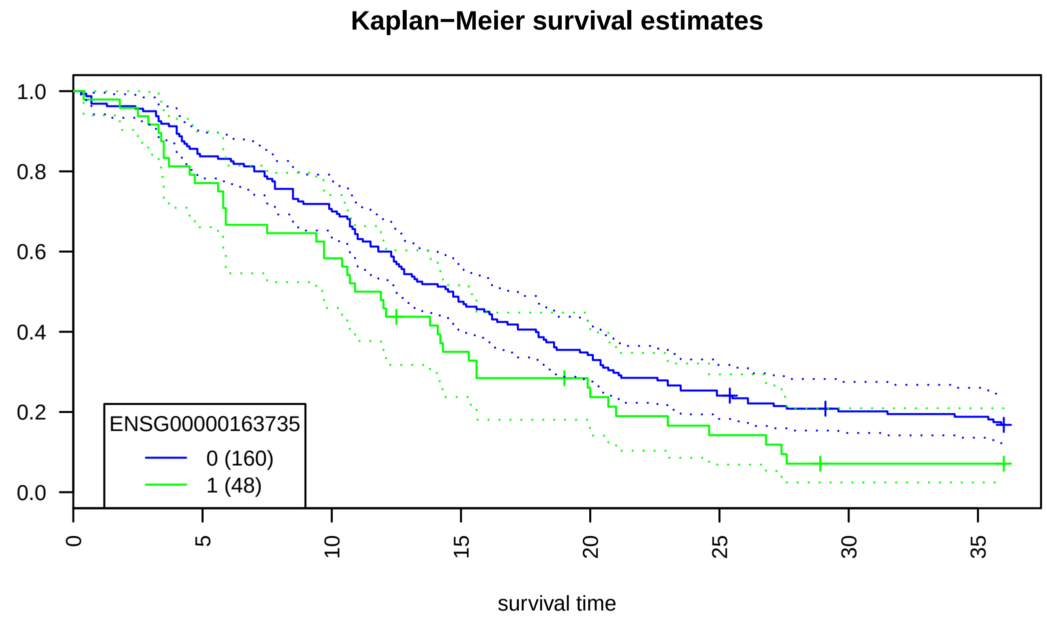

Figure 226: Each curve represents the relative number of ENSG00000163735 survived to the corresponding point of time. Dashed lines represent 0.95 confidence intervals for the curves with the corresponding color. Log-rank probability measure for the equality of these curves is 3.468096e-02.

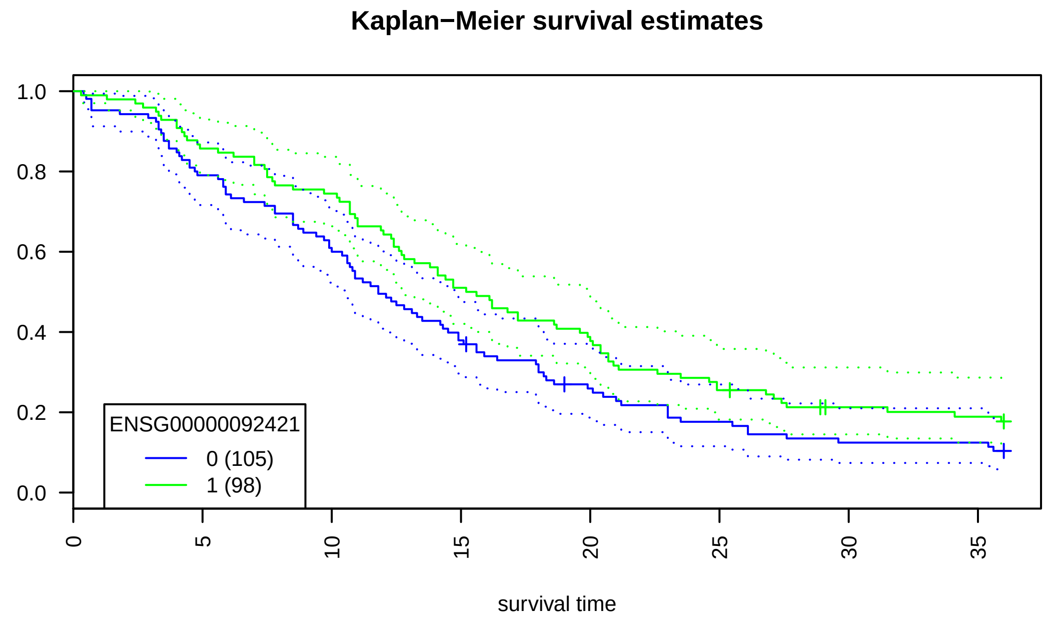

Figure 227: Each curve represents the relative number of ENSG00000092421 survived to the corresponding point of time. Dashed lines represent 0.95 confidence intervals for the curves with the corresponding color. Total of 5 observations deleted due to missingness. Log-rank probability measure for the equality of these curves is 3.473019e-02.

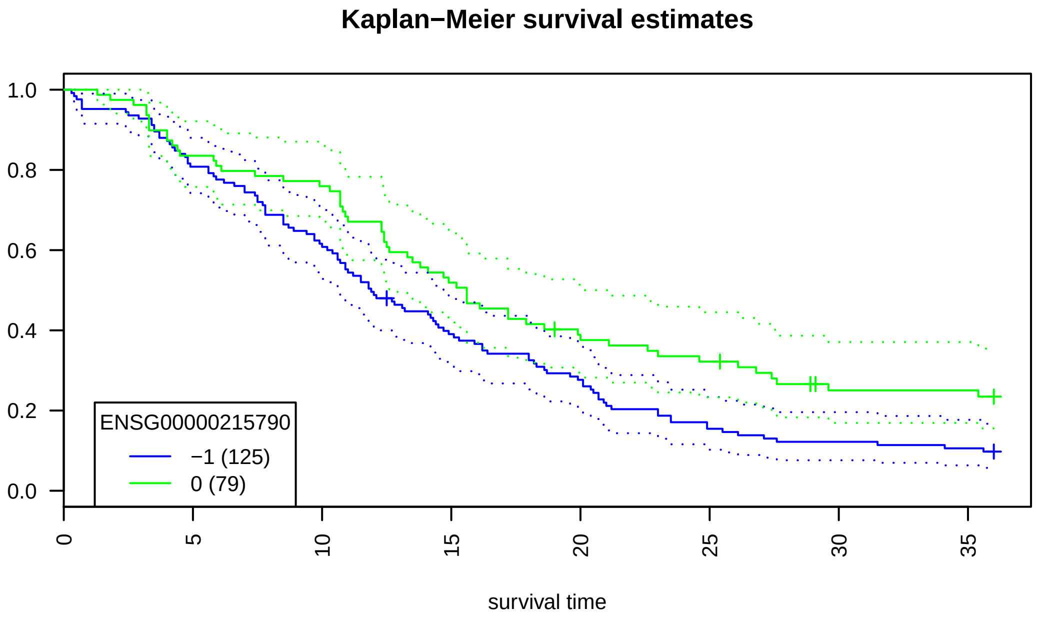

Figure 228: Each curve represents the relative number of ENSG00000215790 survived to the corresponding point of time. Dashed lines represent 0.95 confidence intervals for the curves with the corresponding color. Total of 4 observations deleted due to missingness. Log-rank probability measure for the equality of these curves is 8.858018e-03.

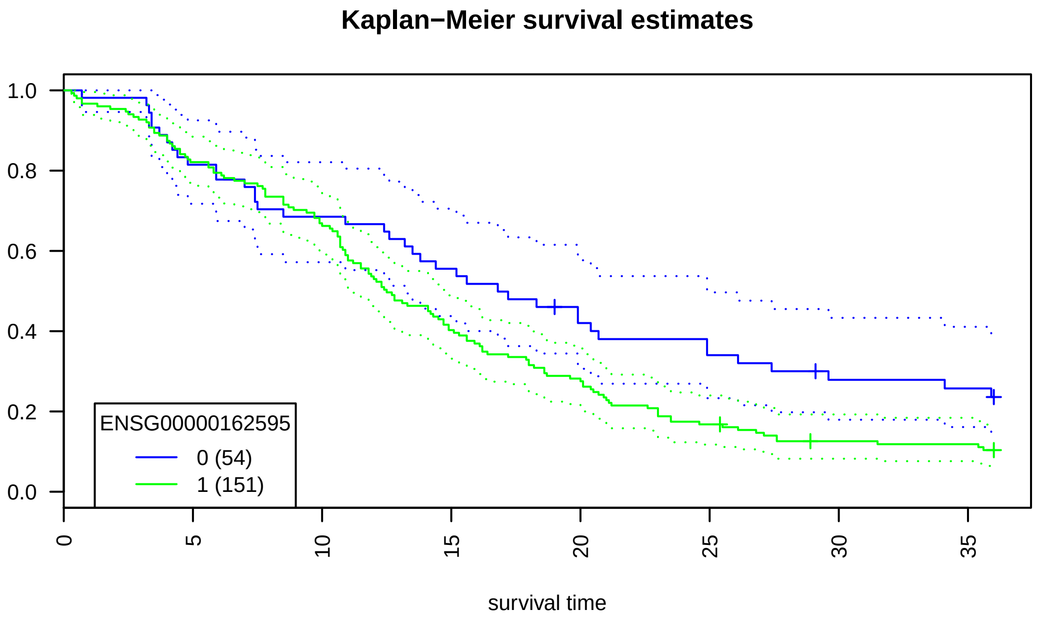

Figure 229: Each curve represents the relative number of ENSG00000162595 survived to the corresponding point of time. Dashed lines represent 0.95 confidence intervals for the curves with the corresponding color. Total of 3 observations deleted due to missingness. Log-rank probability measure for the equality of these curves is 1.875622e-02. Additional annotations: *.GeneName*=DIRAS3, *.GeneDesc*=DIRAS family, GTP-binding RAS-like 3 [Source:HGNC Symbol;Acc:687], *Uniprot/SWISSPROT*=O95661.

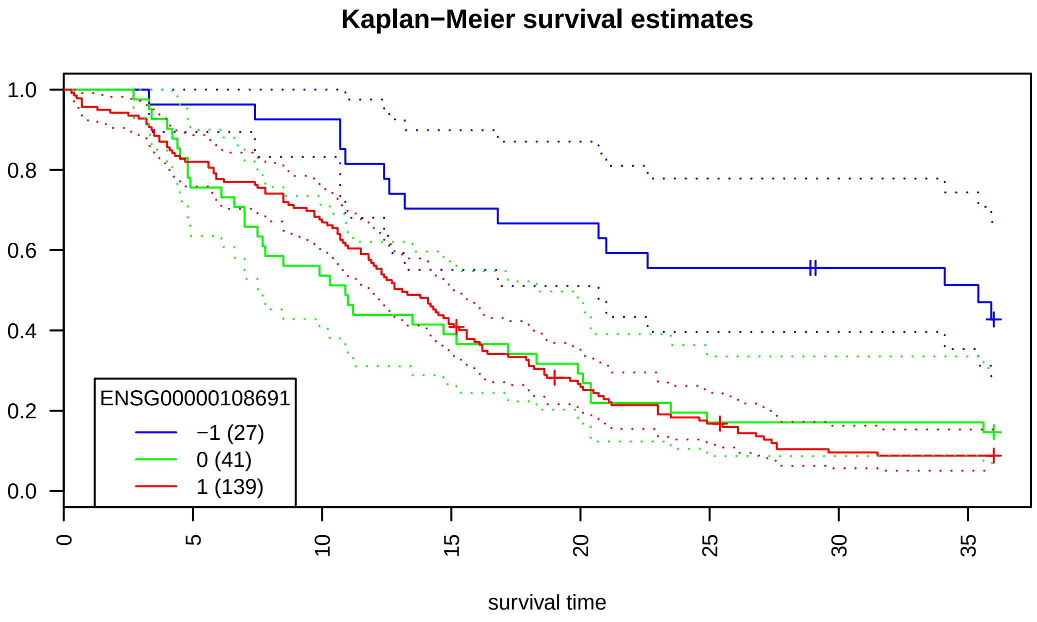

Figure 230: Each curve represents the relative number of ENSG00000108691 survived to the corresponding point of time. Dashed lines represent 0.95 confidence intervals for the curves with the corresponding color. Total of 1 observation deleted due to missingness. Log-rank probability measure for the equality of these curves is 2.602334e-04. Additional annotations: *.GeneName*=CCL2, *.GeneDesc*=chemokine (C-C motif) ligand 2 [Source:HGNC Symbol;Acc:10618], *Uniprot/SWISSPROT*=P13500.

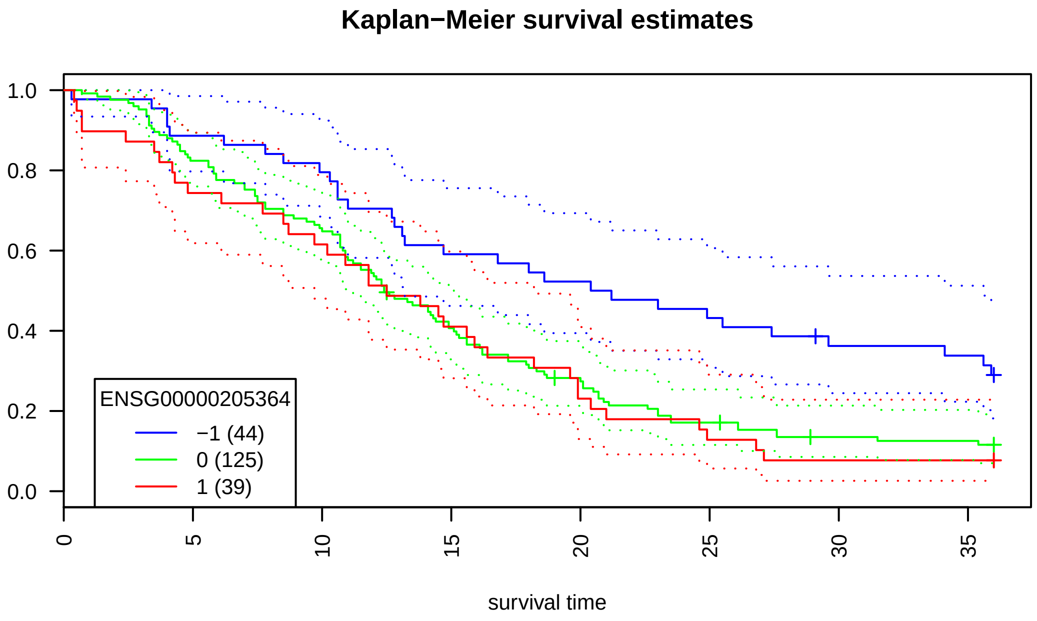

Figure 231: Each curve represents the relative number of ENSG00000205364 survived to the corresponding point of time. Dashed lines represent 0.95 confidence intervals for the curves with the corresponding color. Log-rank probability measure for the equality of these curves is 3.611837e-03.

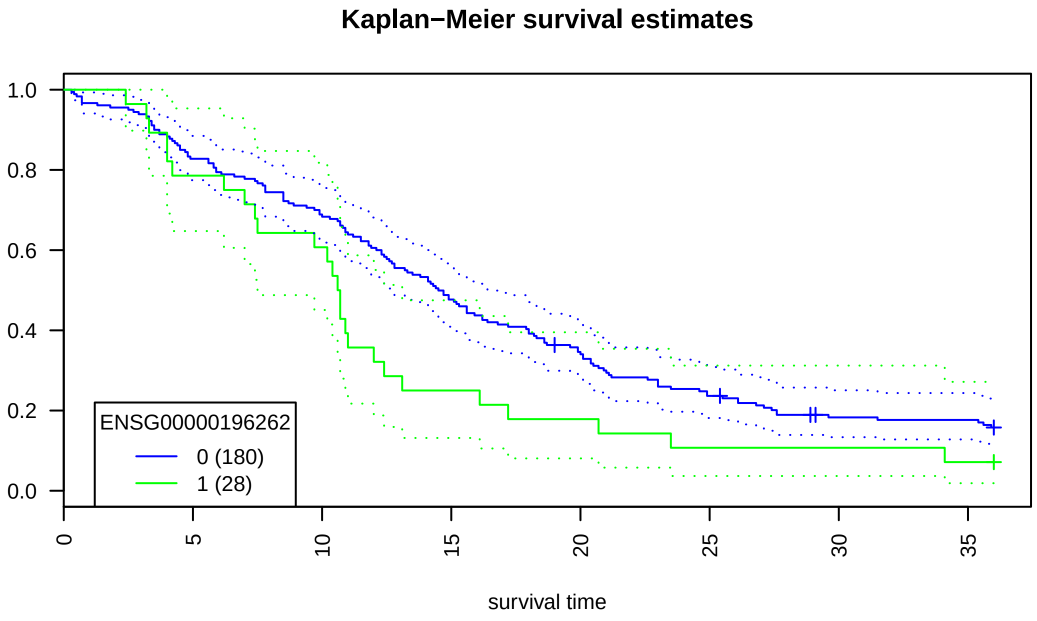

Figure 232: Each curve represents the relative number of ENSG00000196262 survived to the corresponding point of time. Dashed lines represent 0.95 confidence intervals for the curves with the corresponding color. Log-rank probability measure for the equality of these curves is 2.415542e-02.

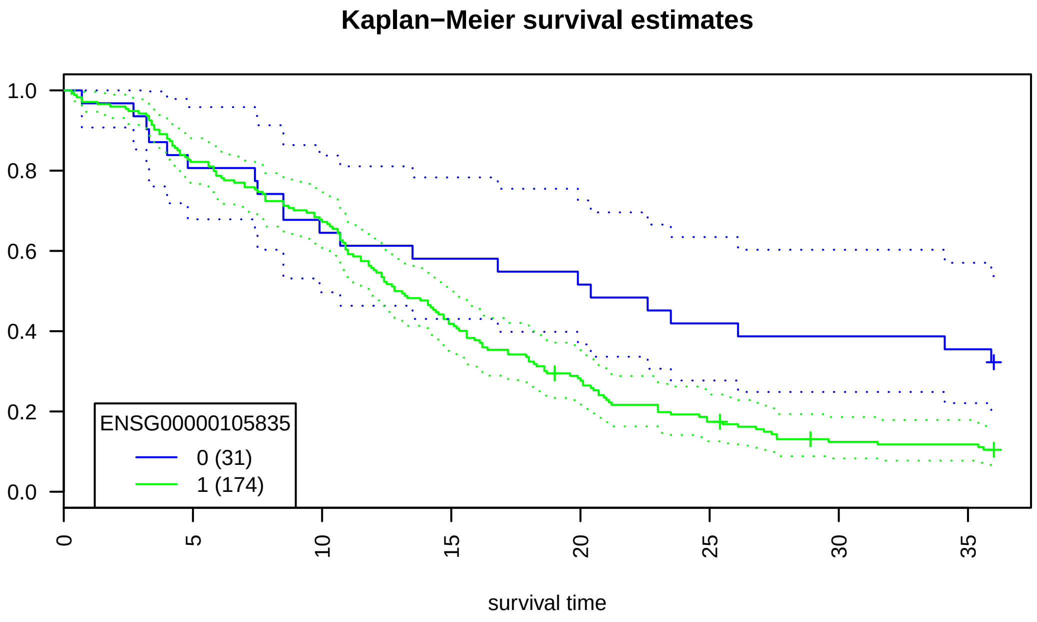

Figure 233: Each curve represents the relative number of ENSG00000105835 survived to the corresponding point of time. Dashed lines represent 0.95 confidence intervals for the curves with the corresponding color. Total of 3 observations deleted due to missingness. Log-rank probability measure for the equality of these curves is 9.367011e-03. Additional annotations: *.GeneName*=PBEF1, *.GeneDesc*=nicotinamide phosphoribosyltransferase [Source:HGNC Symbol;Acc:30092], *Uniprot/SWISSPROT*=P43490.

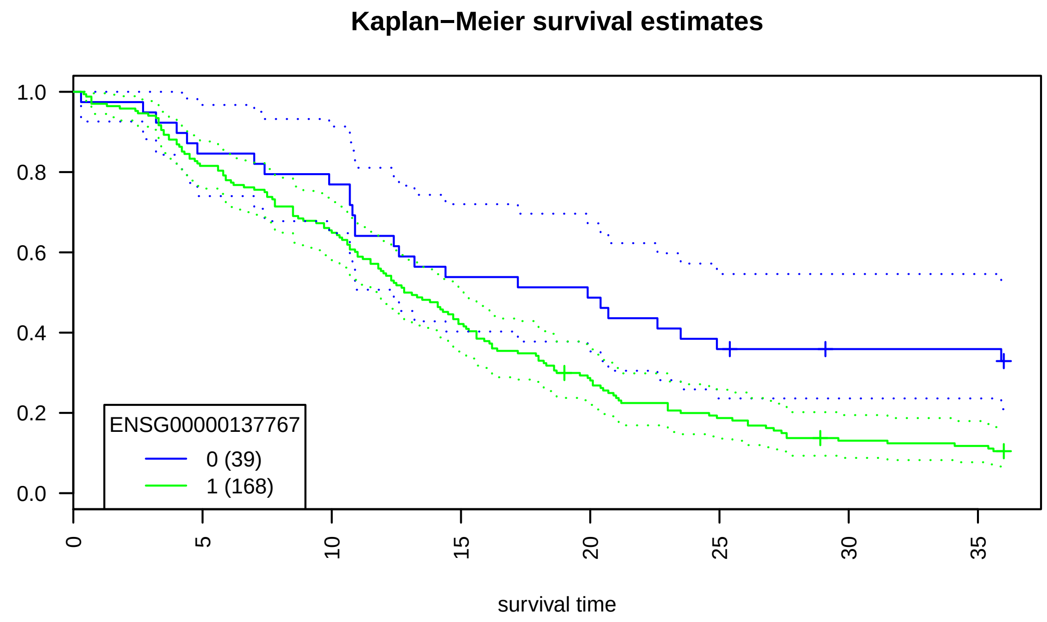

Figure 234: Each curve represents the relative number of ENSG00000137767 survived to the corresponding point of time. Dashed lines represent 0.95 confidence intervals for the curves with the corresponding color. Total of 1 observation deleted due to missingness. Log-rank probability measure for the equality of these curves is 5.705298e-03. Additional annotations: *.GeneName*=SQRDL, *.GeneDesc*=sulfide quinone reductase-like (yeast) [Source:HGNC Symbol;Acc:20390], *Uniprot/SWISSPROT*=Q9Y6N5.

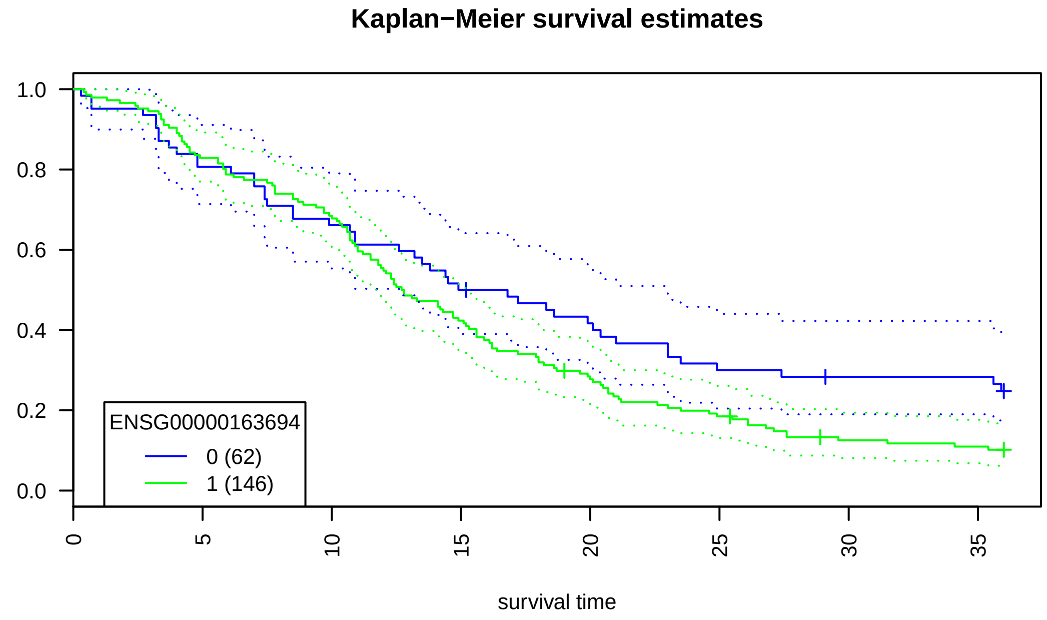

Figure 235: Each curve represents the relative number of ENSG00000163694 survived to the corresponding point of time. Dashed lines represent 0.95 confidence intervals for the curves with the corresponding color. Log-rank probability measure for the equality of these curves is 3.253382e-02. Additional annotations: *.GeneName*=RBM47, *.GeneDesc*=RNA binding motif protein 47 [Source:HGNC Symbol;Acc:30358], *Uniprot/SWISSPROT*=A0AV96.

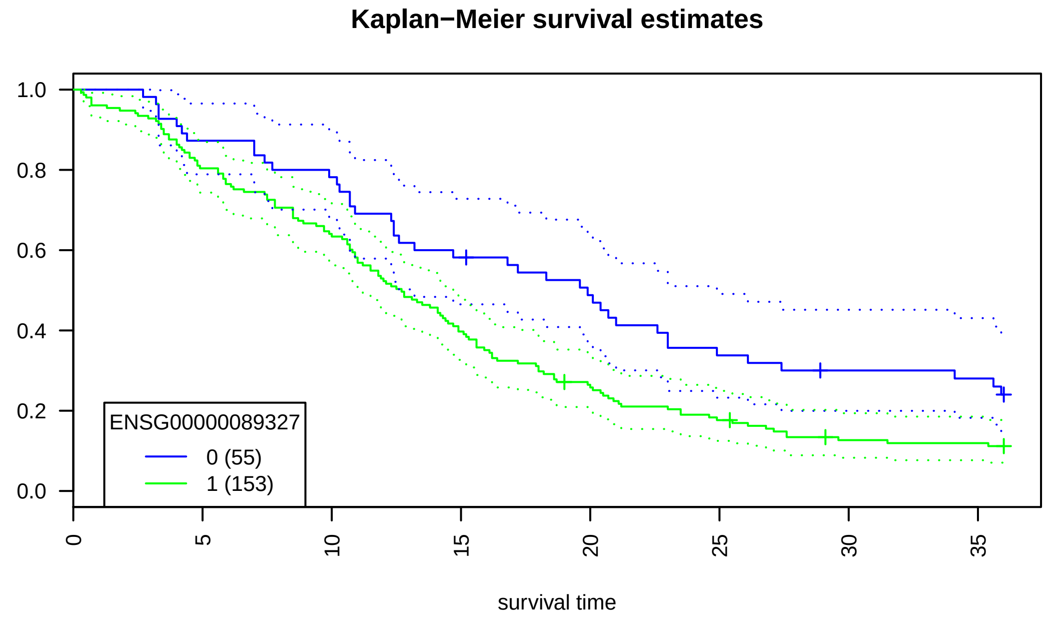

Figure 236: Each curve represents the relative number of ENSG00000089327 survived to the corresponding point of time. Dashed lines represent 0.95 confidence intervals for the curves with the corresponding color. Log-rank probability measure for the equality of these curves is 5.401432e-03. Additional annotations: *.GeneName*=FXYD5, *.GeneDesc*=FXYD domain containing ion transport regulator 5 [Source:HGNC Symbol;Acc:4029], *Uniprot/SWISSPROT*=Q96DB9.

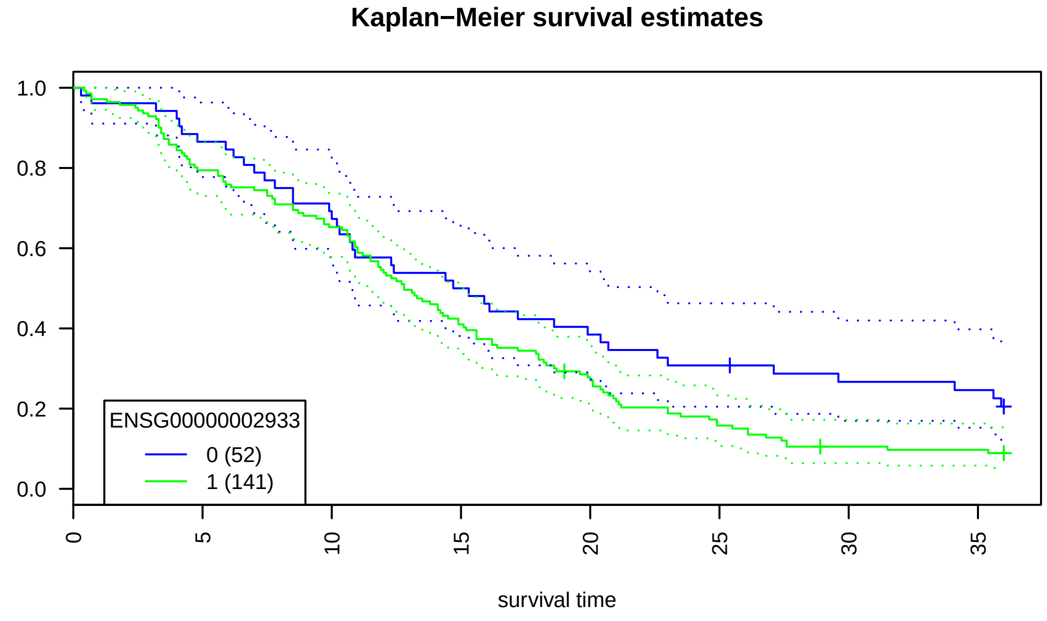

Figure 237: Each curve represents the relative number of ENSG00000002933 survived to the corresponding point of time. Dashed lines represent 0.95 confidence intervals for the curves with the corresponding color. Total of 15 observations deleted due to missingness. Log-rank probability measure for the equality of these curves is 4.702661e-02. Additional annotations: *.GeneName*=TMEM176A, *.GeneDesc*=transmembrane protein 176A [Source:HGNC Symbol;Acc:24930], *Uniprot/SWISSPROT*=Q96HP8.

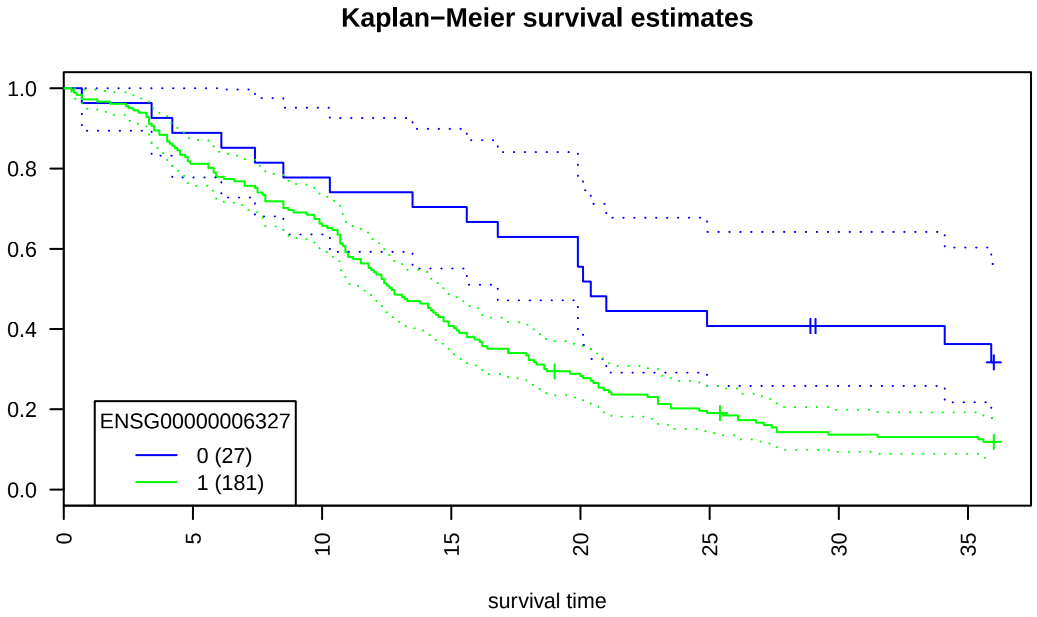

Figure 238: Each curve represents the relative number of ENSG00000006327 survived to the corresponding point of time. Dashed lines represent 0.95 confidence intervals for the curves with the corresponding color. Log-rank probability measure for the equality of these curves is 6.05377e-03. Additional annotations: *.GeneName*=TNFRSF12A, *.GeneDesc*=tumor necrosis factor receptor superfamily, member 12A [Source:HGNC Symbol;Acc:18152], *Uniprot/SWISSPROT*=Q9NP84.

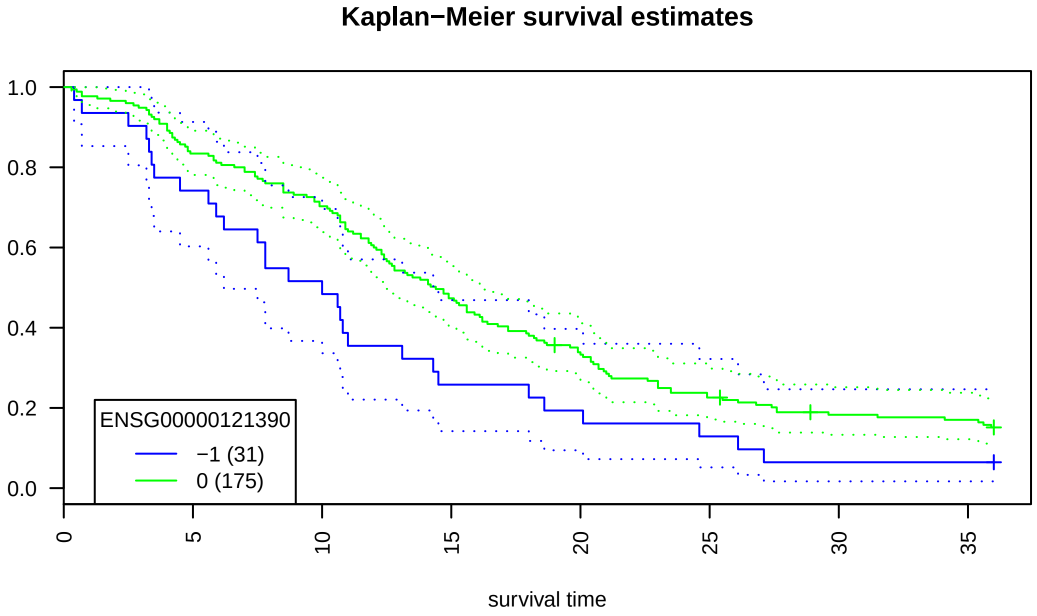

Figure 239: Each curve represents the relative number of ENSG00000121390 survived to the corresponding point of time. Dashed lines represent 0.95 confidence intervals for the curves with the corresponding color. Total of 2 observations deleted due to missingness. Log-rank probability measure for the equality of these curves is 1.477253e-02.

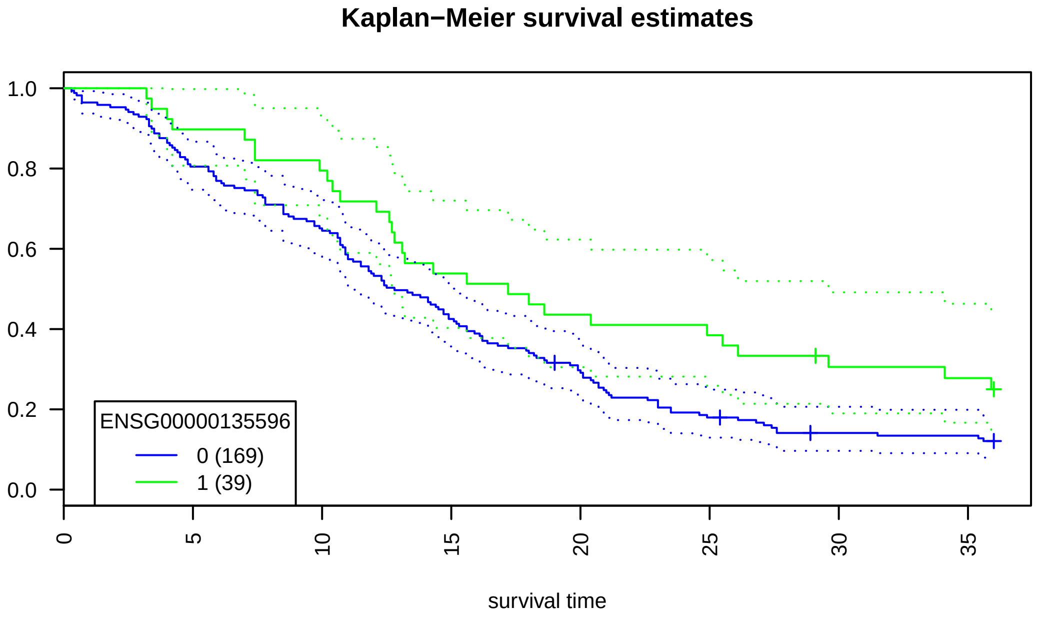

Figure 240: Each curve represents the relative number of ENSG00000135596 survived to the corresponding point of time. Dashed lines represent 0.95 confidence intervals for the curves with the corresponding color. Log-rank probability measure for the equality of these curves is 2.560625e-02.

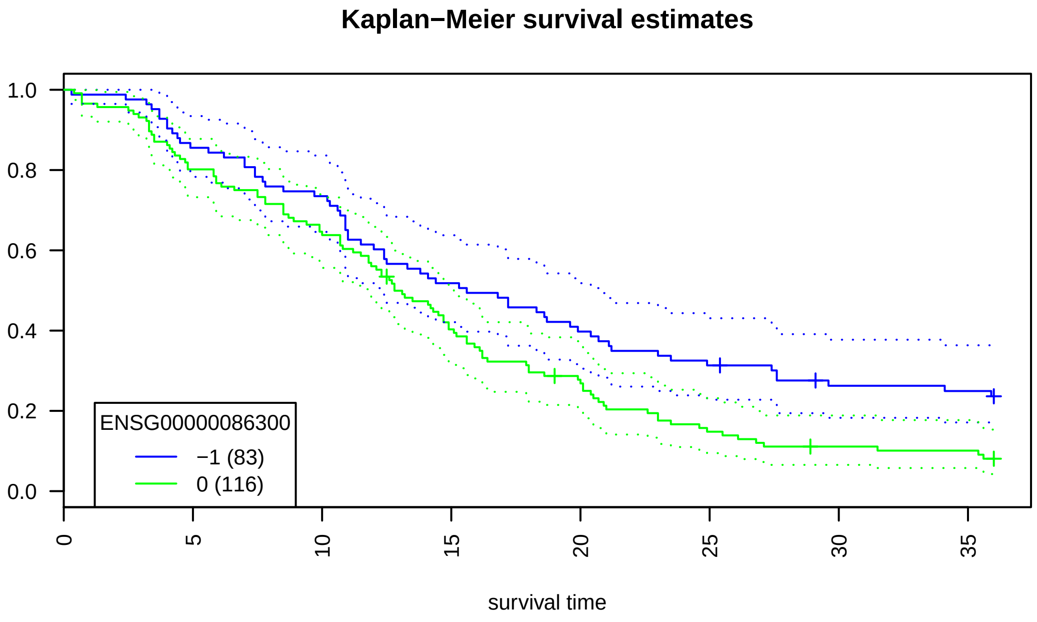

Figure 241: Each curve represents the relative number of ENSG00000086300 survived to the corresponding point of time. Dashed lines represent 0.95 confidence intervals for the curves with the corresponding color. Total of 9 observations deleted due to missingness. Log-rank probability measure for the equality of these curves is 5.706826e-03. Additional annotations: *.GeneName*=SNX10, *.GeneDesc*=sorting nexin 10 [Source:HGNC Symbol;Acc:14974], *Uniprot/SWISSPROT*=Q9Y5X0.

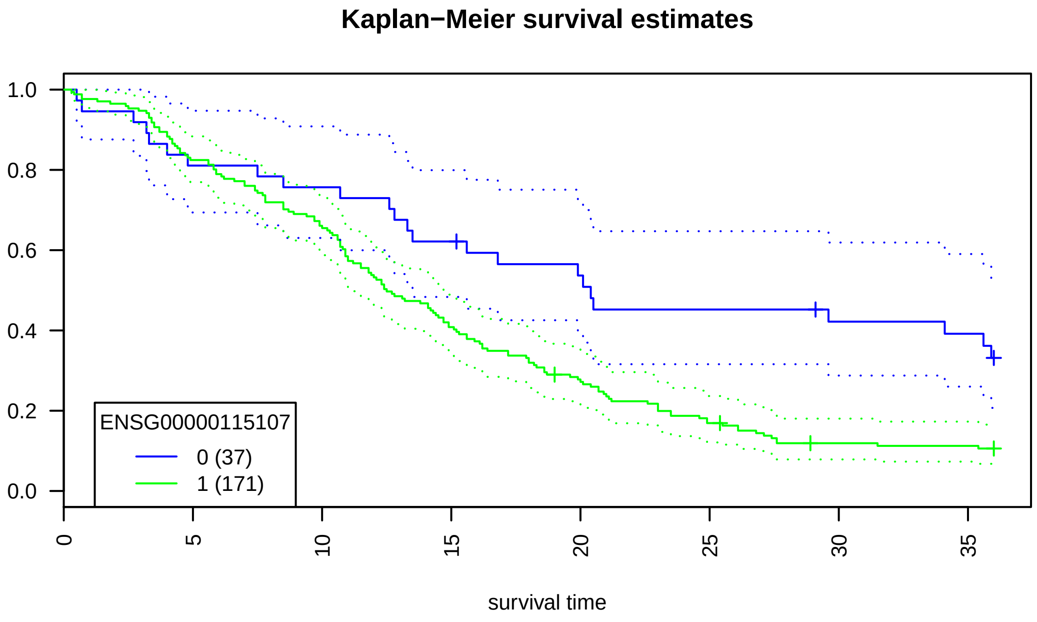

Figure 242: Each curve represents the relative number of ENSG00000115107 survived to the corresponding point of time. Dashed lines represent 0.95 confidence intervals for the curves with the corresponding color. Log-rank probability measure for the equality of these curves is 1.492763e-03. Additional annotations: *.GeneName*=STEAP3, *.GeneDesc*=STEAP family member 3 [Source:HGNC Symbol;Acc:24592], *Uniprot/SWISSPROT*=Q658P3.

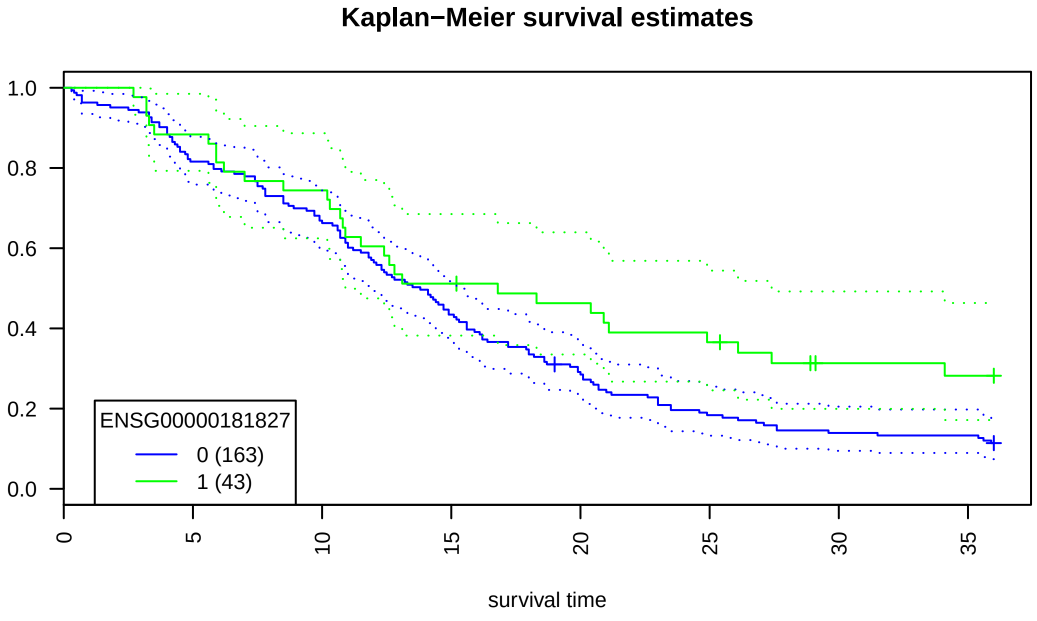

Figure 243: Each curve represents the relative number of ENSG00000181827 survived to the corresponding point of time. Dashed lines represent 0.95 confidence intervals for the curves with the corresponding color. Total of 2 observations deleted due to missingness. Log-rank probability measure for the equality of these curves is 3.284067e-02.

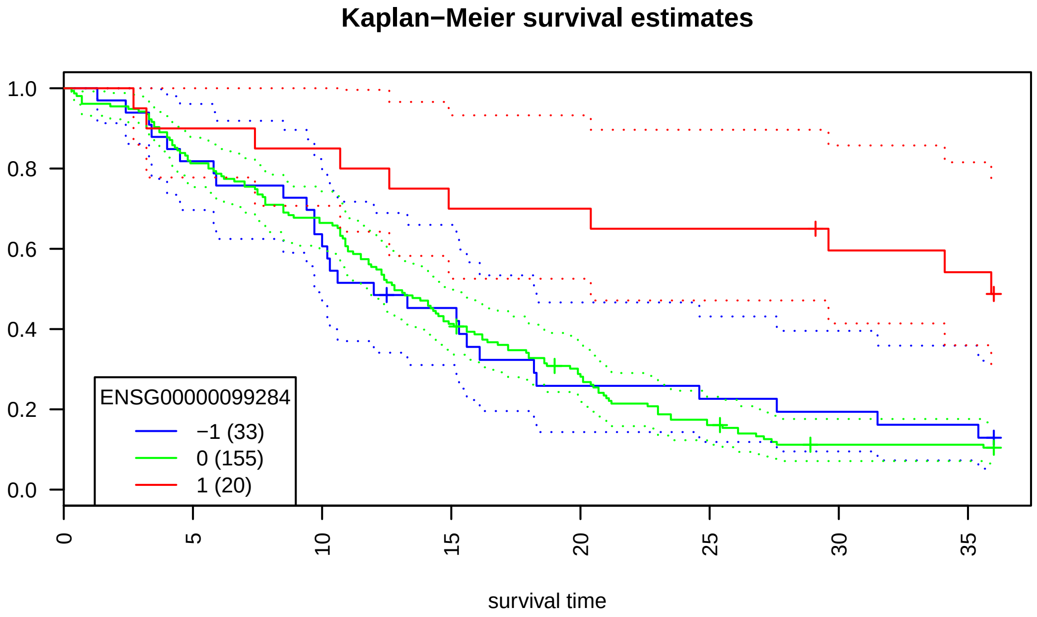

Figure 244: Each curve represents the relative number of ENSG00000099284 survived to the corresponding point of time. Dashed lines represent 0.95 confidence intervals for the curves with the corresponding color. Log-rank probability measure for the equality of these curves is 8.395068e-04.

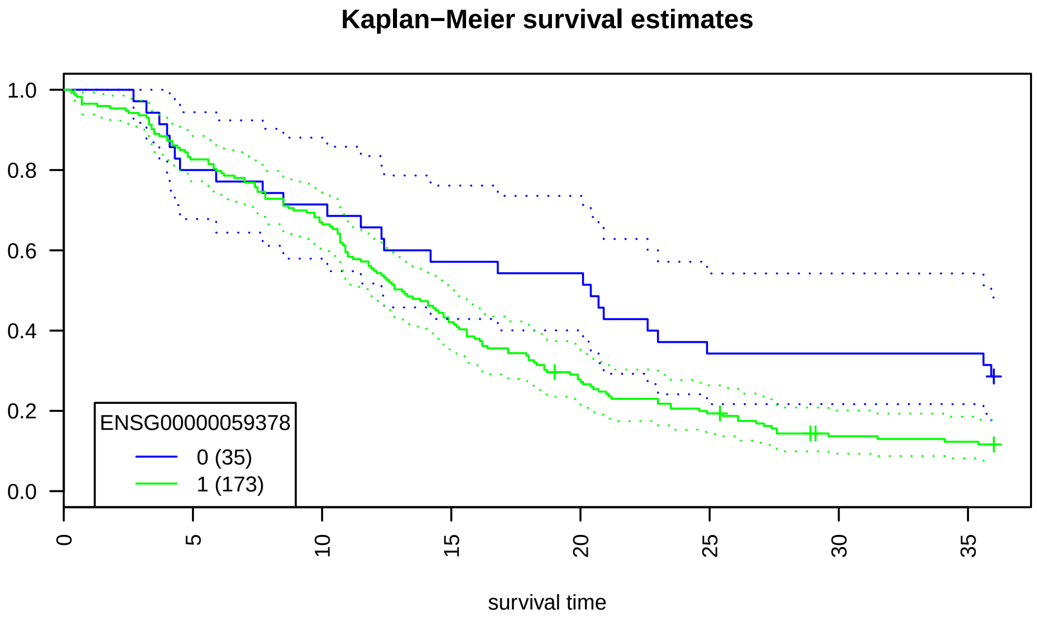

Figure 245: Each curve represents the relative number of ENSG00000059378 survived to the corresponding point of time. Dashed lines represent 0.95 confidence intervals for the curves with the corresponding color. Log-rank probability measure for the equality of these curves is 2.041065e-02. Additional annotations: *.GeneName*=PARP12, *.GeneDesc*=poly (ADP-ribose) polymerase family, member 12 [Source:HGNC Symbol;Acc:21919], *Uniprot/SWISSPROT*=Q9H0J9.

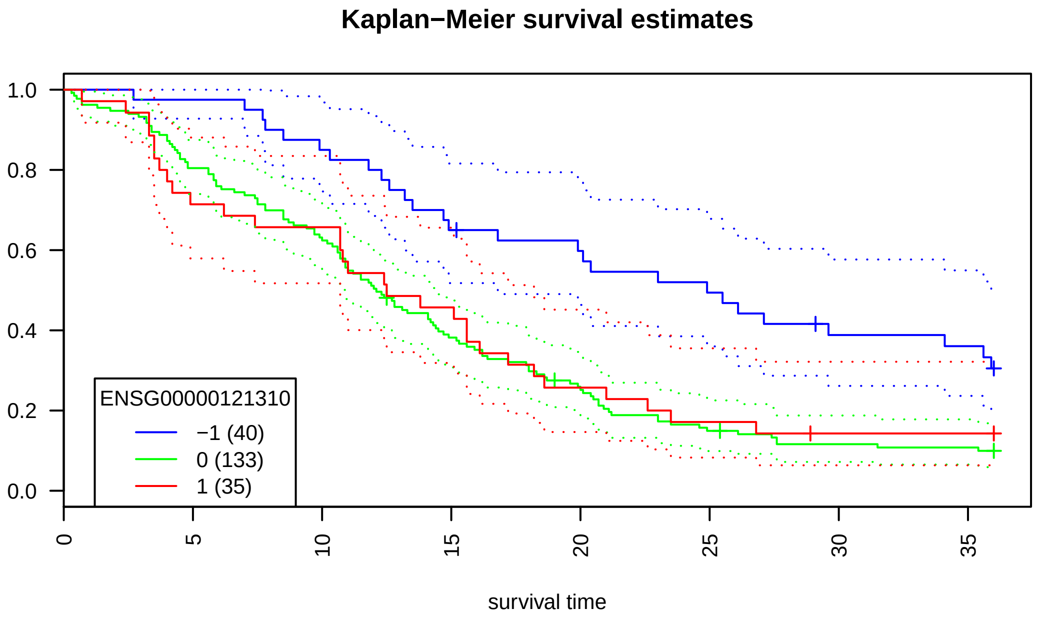

Figure 246: Each curve represents the relative number of ENSG00000121310 survived to the corresponding point of time. Dashed lines represent 0.95 confidence intervals for the curves with the corresponding color. Log-rank probability measure for the equality of these curves is 5.639072e-04.

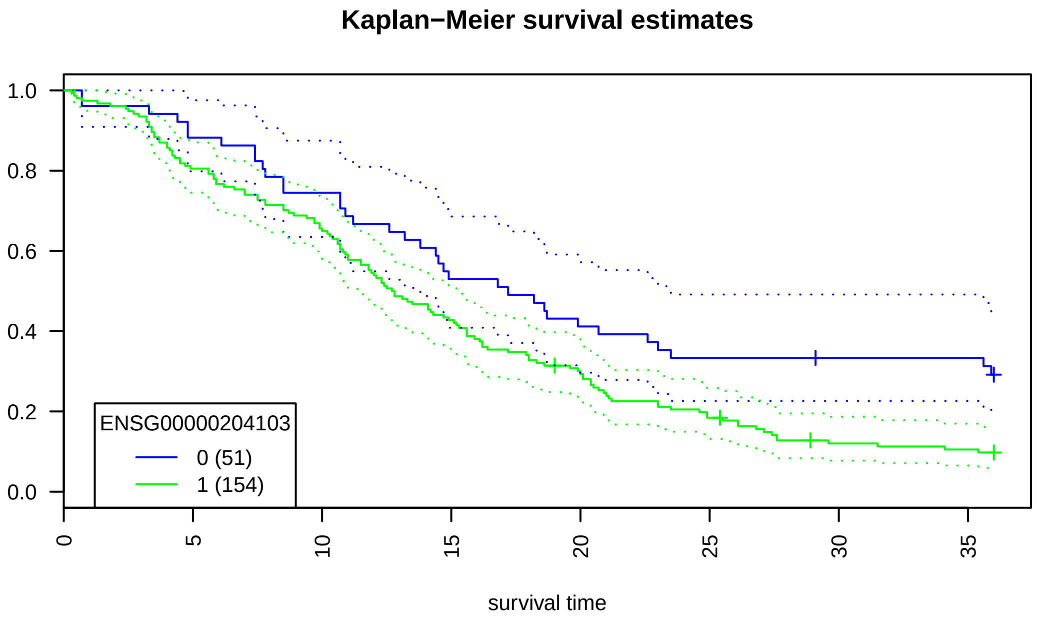

Figure 247: Each curve represents the relative number of ENSG00000204103 survived to the corresponding point of time. Dashed lines represent 0.95 confidence intervals for the curves with the corresponding color. Total of 3 observations deleted due to missingness. Log-rank probability measure for the equality of these curves is 4.587298e-03. Additional annotations: *.GeneName*=MAFB, *.GeneDesc*=v-maf musculoaponeurotic fibrosarcoma oncogene homolog B (avian) [Source:HGNC Symbol;Acc:6408], *Uniprot/SWISSPROT*=Q9Y5Q3.

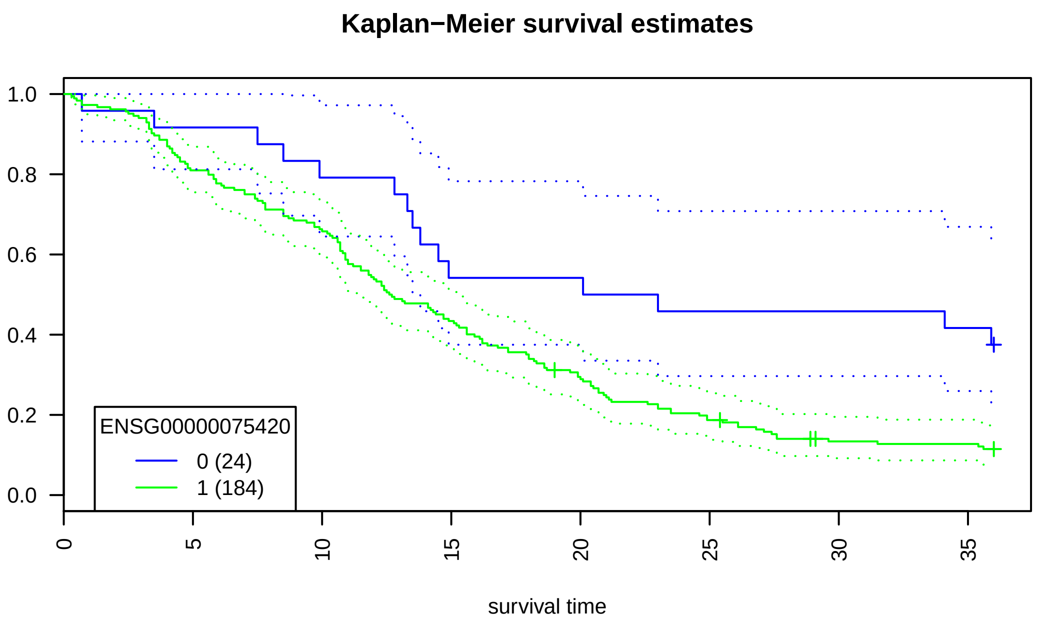

Figure 248: Each curve represents the relative number of ENSG00000075420 survived to the corresponding point of time. Dashed lines represent 0.95 confidence intervals for the curves with the corresponding color. Log-rank probability measure for the equality of these curves is 4.299502e-03. Additional annotations: *.GeneName*=FNDC3B, *.GeneDesc*=fibronectin type III domain containing 3B [Source:HGNC Symbol;Acc:24670], *Uniprot/SWISSPROT*=Q53EP0.

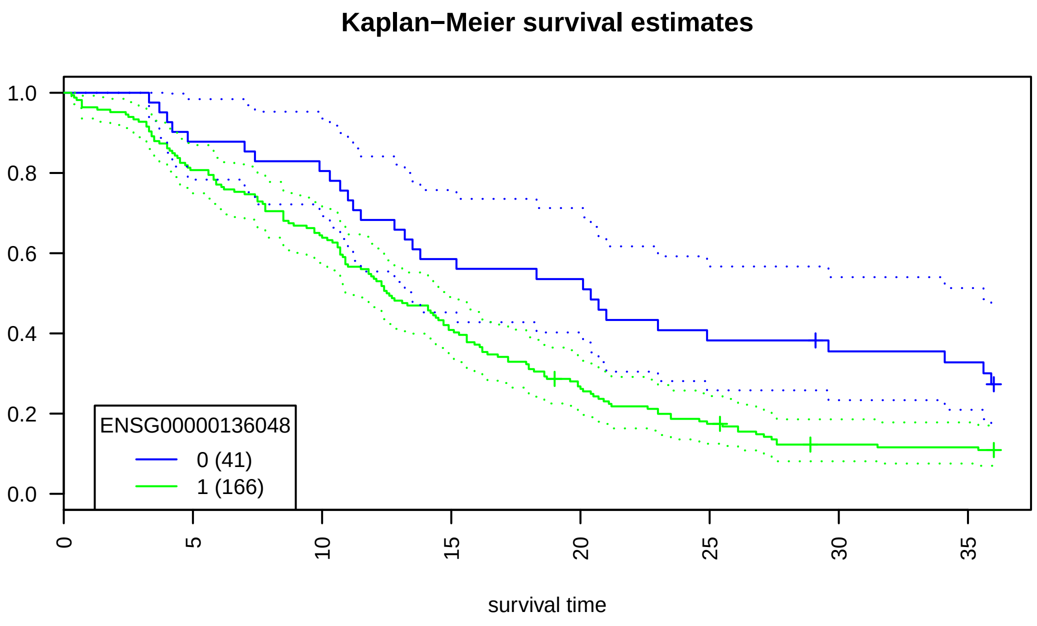

Figure 249: Each curve represents the relative number of ENSG00000136048 survived to the corresponding point of time. Dashed lines represent 0.95 confidence intervals for the curves with the corresponding color. Total of 1 observation deleted due to missingness. Log-rank probability measure for the equality of these curves is 3.438643e-03. Additional annotations: *.GeneName*=DRAM1, *.GeneDesc*=DNA-damage regulated autophagy modulator 1 [Source:HGNC Symbol;Acc:25645], *Uniprot/SWISSPROT*=Q8N682.

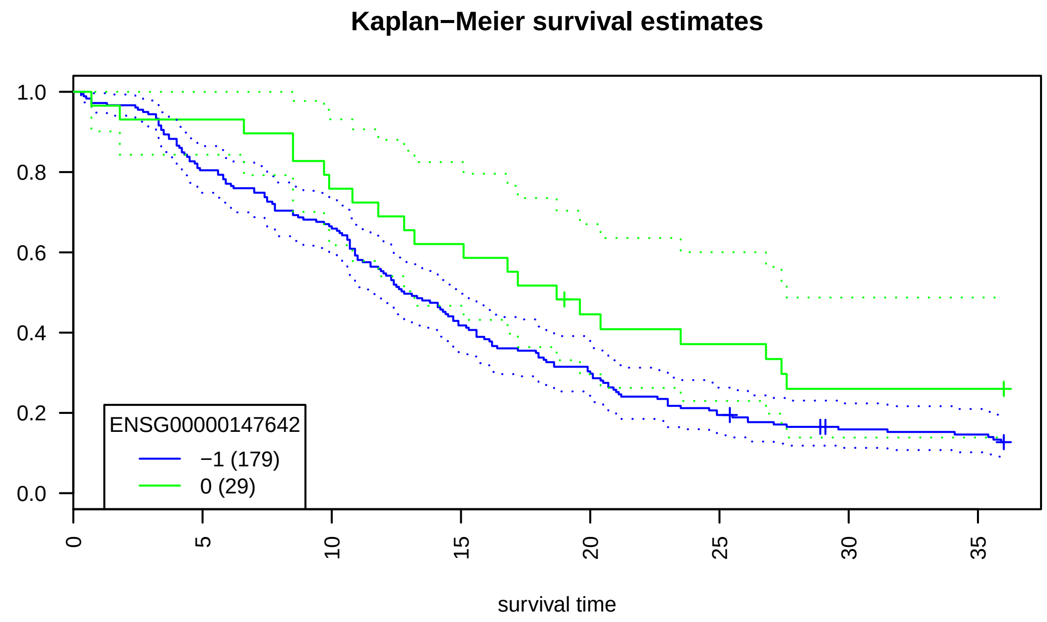

Figure 250: Each curve represents the relative number of ENSG00000147642 survived to the corresponding point of time. Dashed lines represent 0.95 confidence intervals for the curves with the corresponding color. Log-rank probability measure for the equality of these curves is 4.714119e-02. Additional annotations: *.GeneName*=AC079061.1, *.GeneDesc*=Syntabulin (Syntaxin-1-binding protein)(Golgi-localized syntaphilin-related protein) [Source:UniProtKB/Swiss-Prot;Acc:Q9NX95], *Uniprot/SWISSPROT*=Q9NX95.

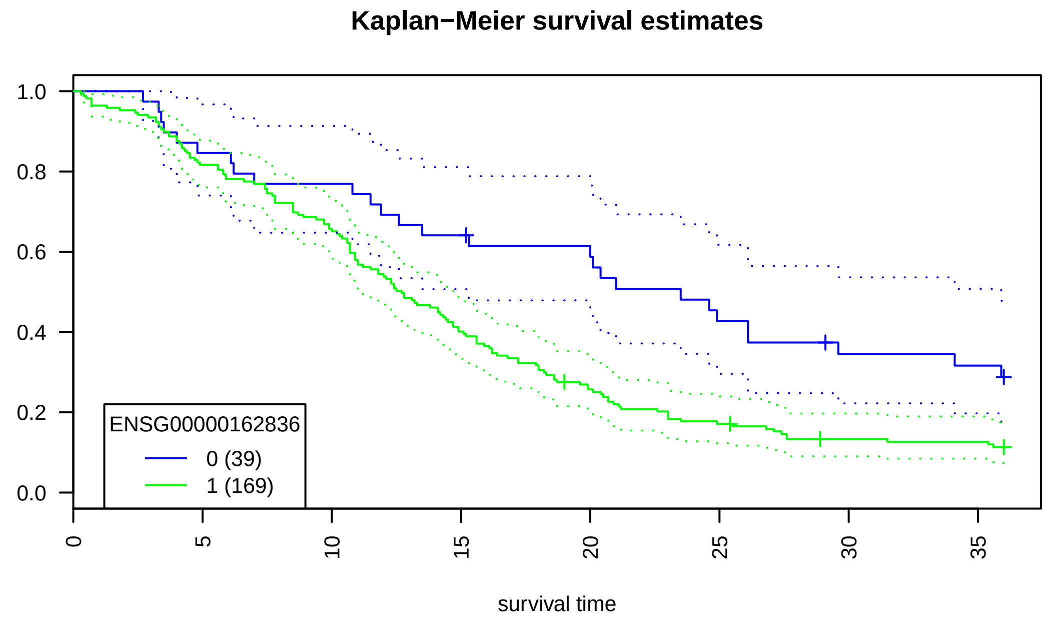

Figure 251: Each curve represents the relative number of ENSG00000162836 survived to the corresponding point of time. Dashed lines represent 0.95 confidence intervals for the curves with the corresponding color. Log-rank probability measure for the equality of these curves is 2.110883e-03. Additional annotations: *.GeneName*=ACP6, *.GeneDesc*=acid phosphatase 6, lysophosphatidic [Source:HGNC Symbol;Acc:29609], *Uniprot/SWISSPROT*=Q9NPH0.

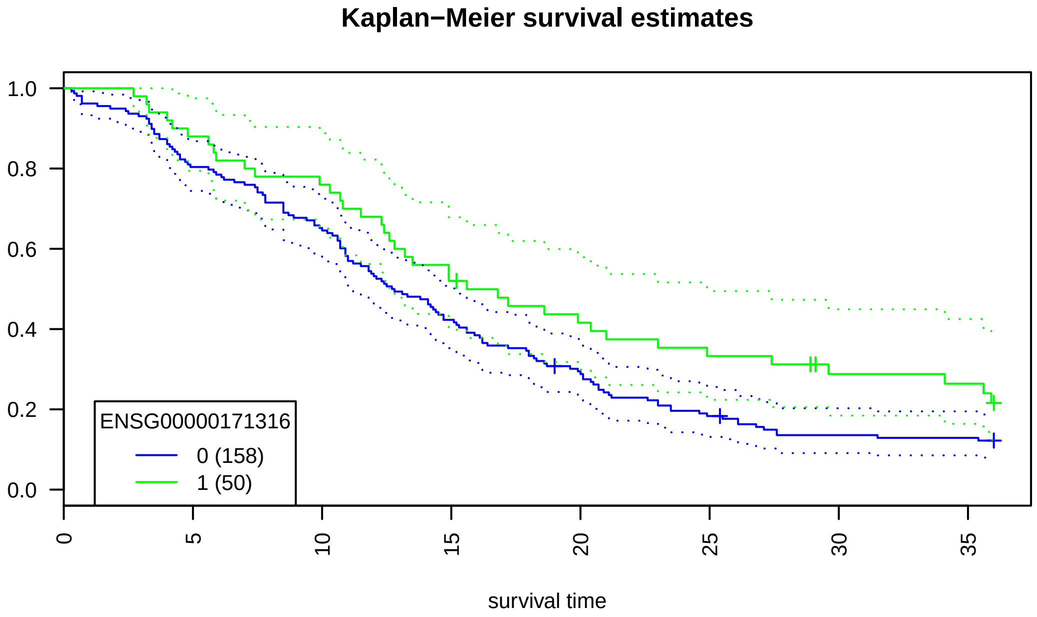

Figure 252: Each curve represents the relative number of ENSG00000171316 survived to the corresponding point of time. Dashed lines represent 0.95 confidence intervals for the curves with the corresponding color. Log-rank probability measure for the equality of these curves is 3.979804e-02.

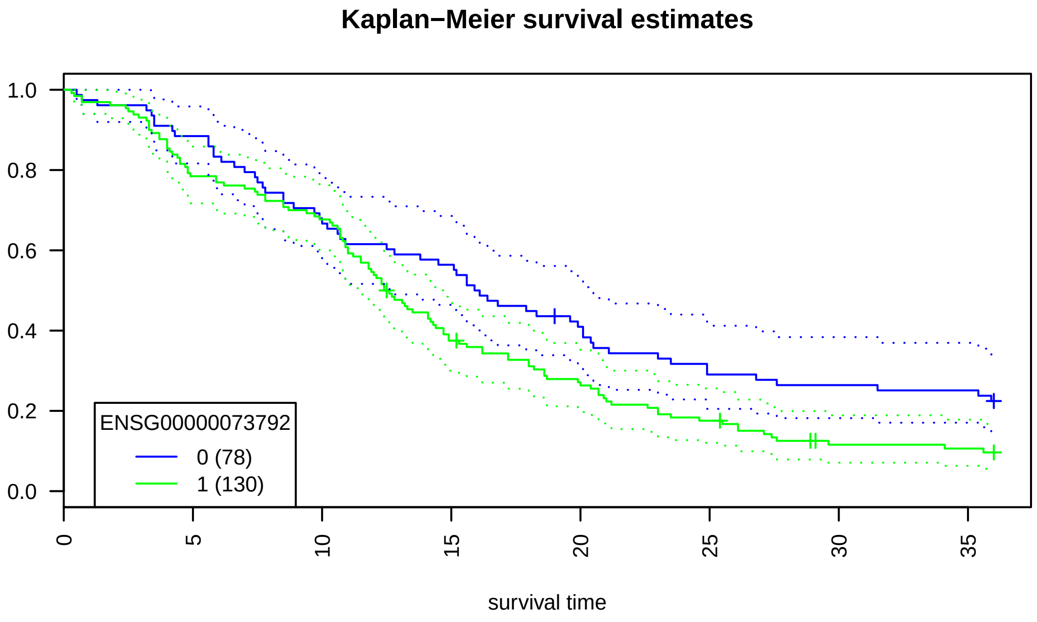

Figure 253: Each curve represents the relative number of ENSG00000073792 survived to the corresponding point of time. Dashed lines represent 0.95 confidence intervals for the curves with the corresponding color. Log-rank probability measure for the equality of these curves is 1.632523e-02. Additional annotations: *.GeneName*=IGF2BP2, *.GeneDesc*=insulin-like growth factor 2 mRNA binding protein 2 [Source:HGNC Symbol;Acc:28867], *Uniprot/SWISSPROT*=Q9Y6M1.

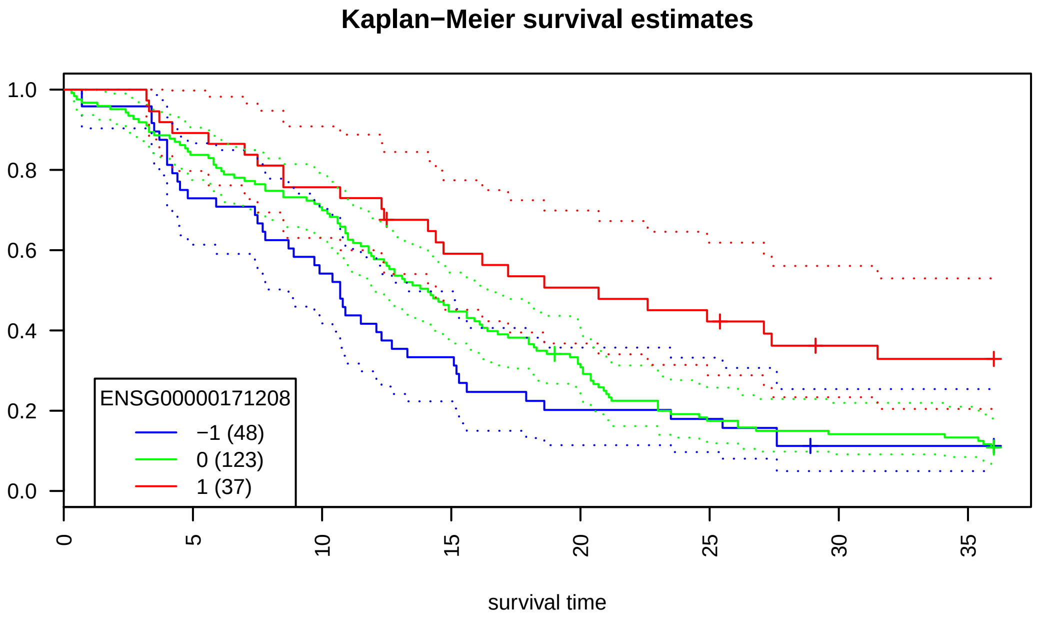

Figure 254: Each curve represents the relative number of ENSG00000171208 survived to the corresponding point of time. Dashed lines represent 0.95 confidence intervals for the curves with the corresponding color. Log-rank probability measure for the equality of these curves is 4.289499e-03.

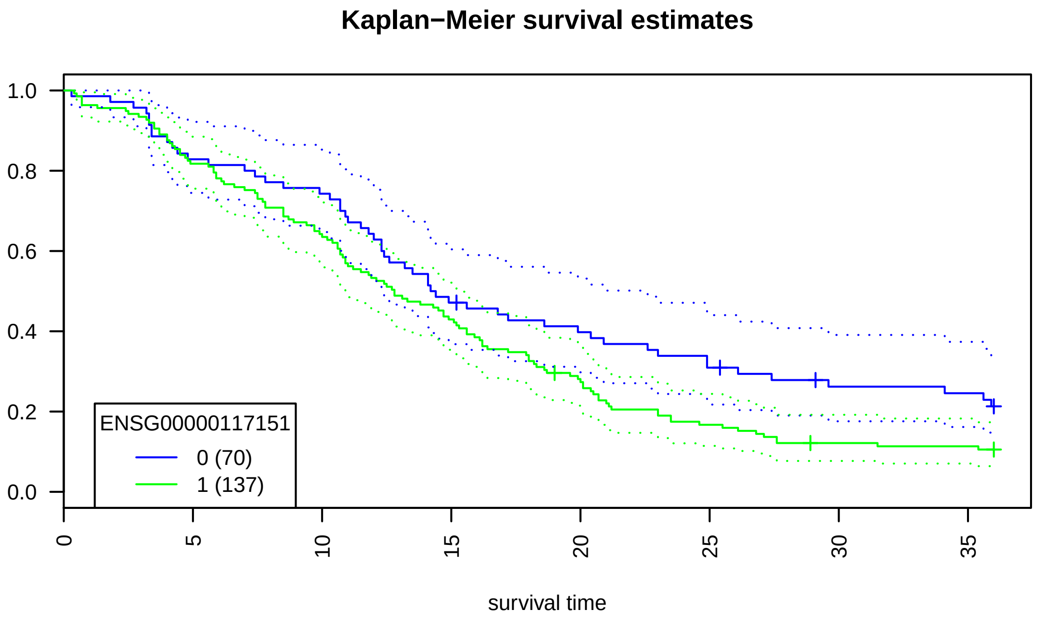

Figure 255: Each curve represents the relative number of ENSG00000117151 survived to the corresponding point of time. Dashed lines represent 0.95 confidence intervals for the curves with the corresponding color. Total of 1 observation deleted due to missingness. Log-rank probability measure for the equality of these curves is 3.255694e-02. Additional annotations: *.GeneName*=CTBS, *.GeneDesc*=chitobiase, di-N-acetyl- [Source:HGNC Symbol;Acc:2496], *Uniprot/SWISSPROT*=Q01459.

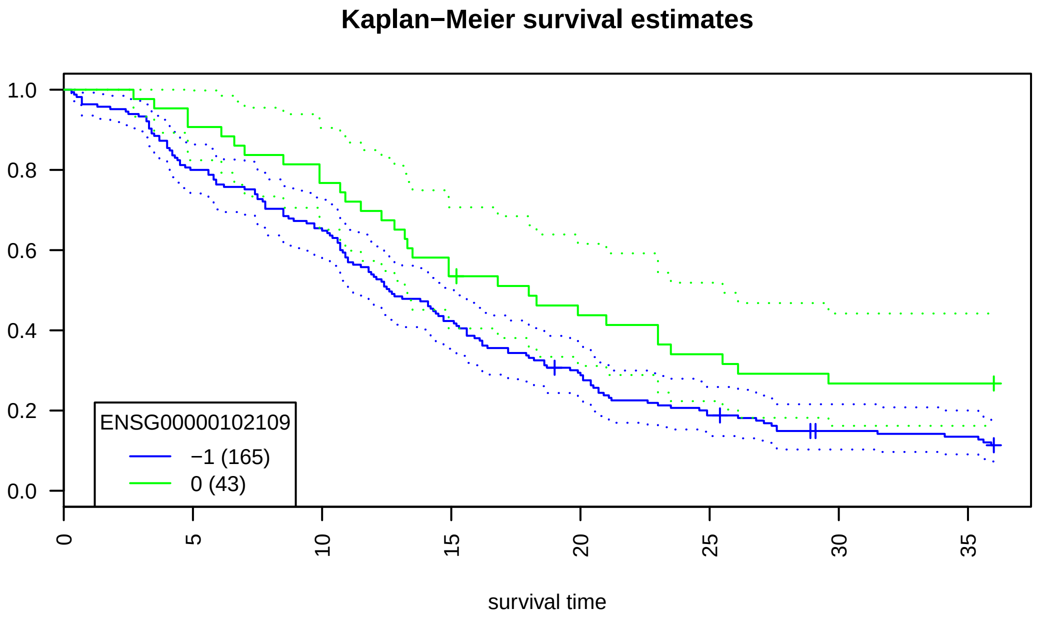

Figure 256: Each curve represents the relative number of ENSG00000102109 survived to the corresponding point of time. Dashed lines represent 0.95 confidence intervals for the curves with the corresponding color. Log-rank probability measure for the equality of these curves is 1.496281e-02. Additional annotations: *.GeneName*=PCSK1N, *.GeneDesc*=proprotein convertase subtilisin/kexin type 1 inhibitor [Source:HGNC Symbol;Acc:17301], *Uniprot/SWISSPROT*=Q9UHG2.

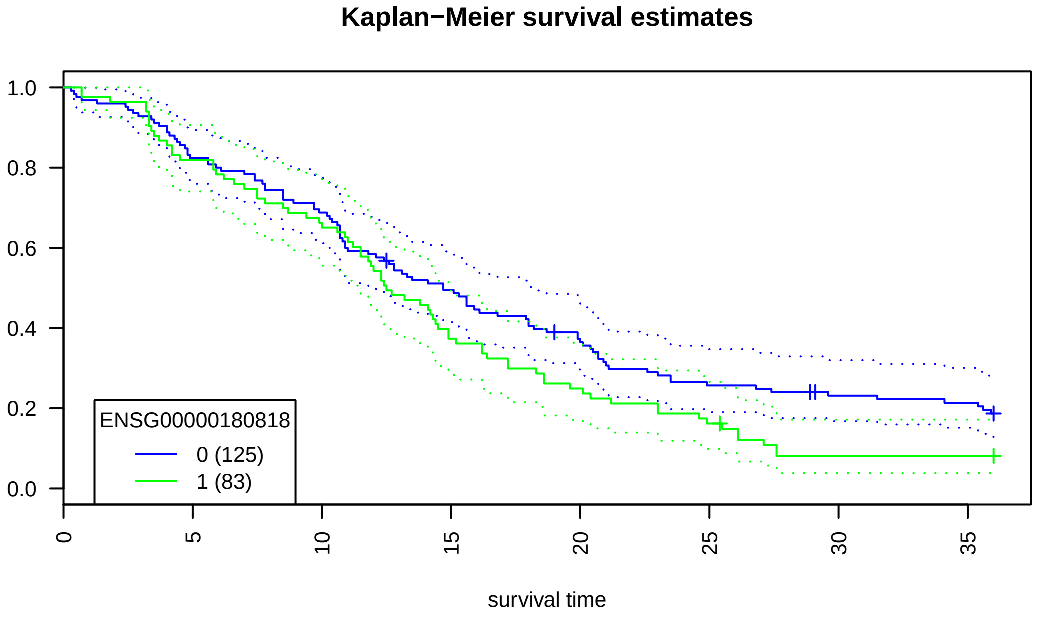

Figure 257: Each curve represents the relative number of ENSG00000180818 survived to the corresponding point of time. Dashed lines represent 0.95 confidence intervals for the curves with the corresponding color. Log-rank probability measure for the equality of these curves is 4.74607e-02. Additional annotations: *.GeneName*=HOXC10, *.GeneDesc*=homeobox C10 [Source:HGNC Symbol;Acc:5122], *Uniprot/SWISSPROT*=Q9NYD6.

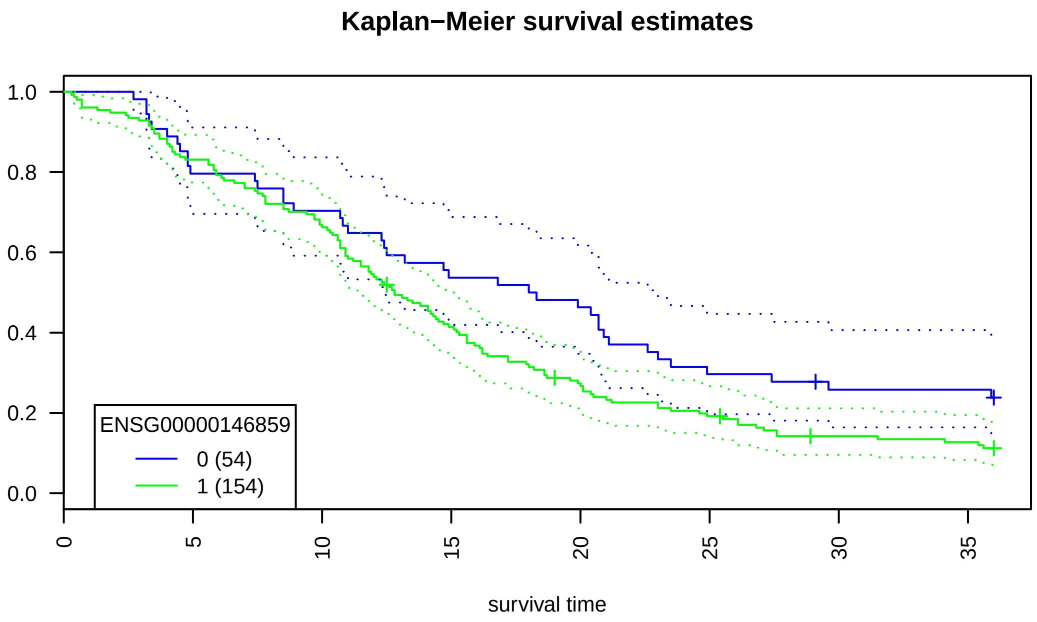

Figure 258: Each curve represents the relative number of ENSG00000146859 survived to the corresponding point of time. Dashed lines represent 0.95 confidence intervals for the curves with the corresponding color. Log-rank probability measure for the equality of these curves is 2.74095e-02. Additional annotations: *.GeneName*=TMEM140, *.GeneDesc*=transmembrane protein 140 [Source:HGNC Symbol;Acc:21870], *Uniprot/SWISSPROT*=Q9NV12.

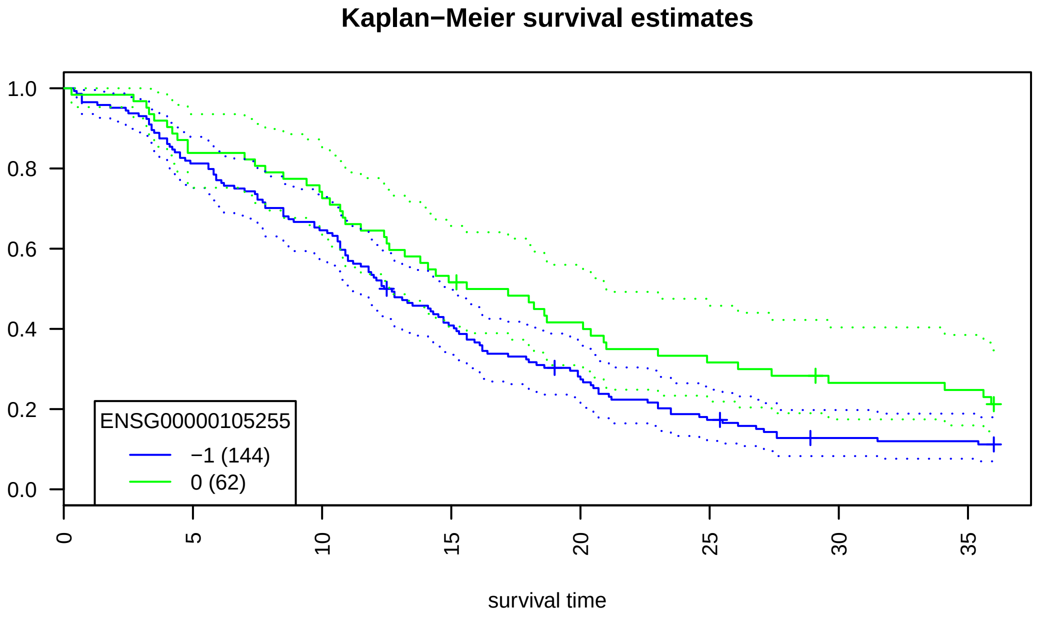

Figure 259: Each curve represents the relative number of ENSG00000105255 survived to the corresponding point of time. Dashed lines represent 0.95 confidence intervals for the curves with the corresponding color. Total of 2 observations deleted due to missingness. Log-rank probability measure for the equality of these curves is 2.886701e-02. Additional annotations: *.GeneName*=FSD1, *.GeneDesc*=fibronectin type III and SPRY domain containing 1 [Source:HGNC Symbol;Acc:13745], *Uniprot/SWISSPROT*=Q9BTV5.

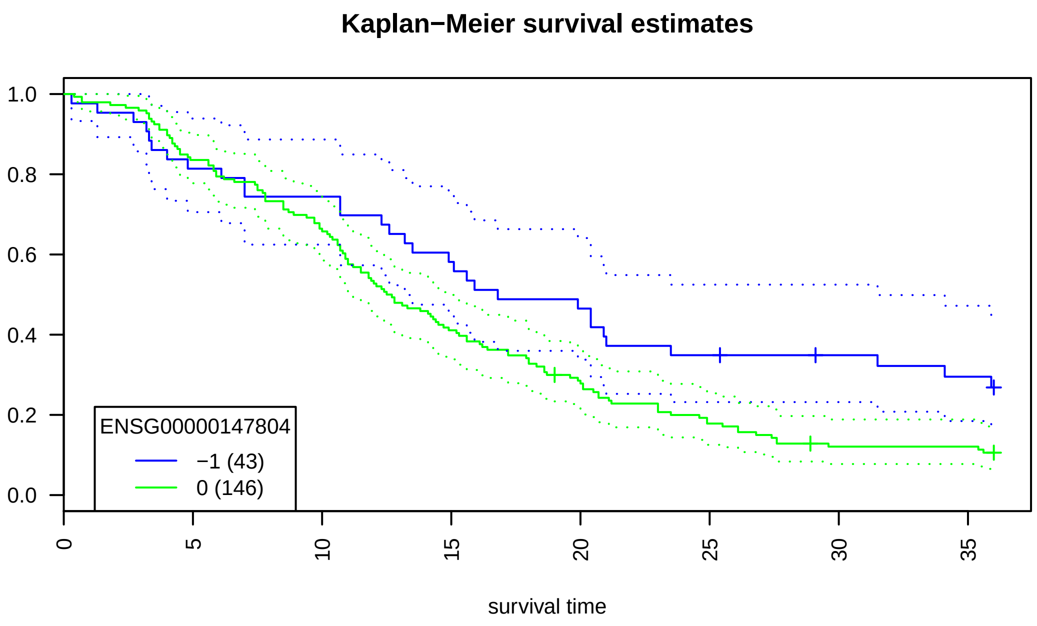

Figure 260: Each curve represents the relative number of ENSG00000147804 survived to the corresponding point of time. Dashed lines represent 0.95 confidence intervals for the curves with the corresponding color. Total of 19 observations deleted due to missingness. Log-rank probability measure for the equality of these curves is 1.565091e-02.

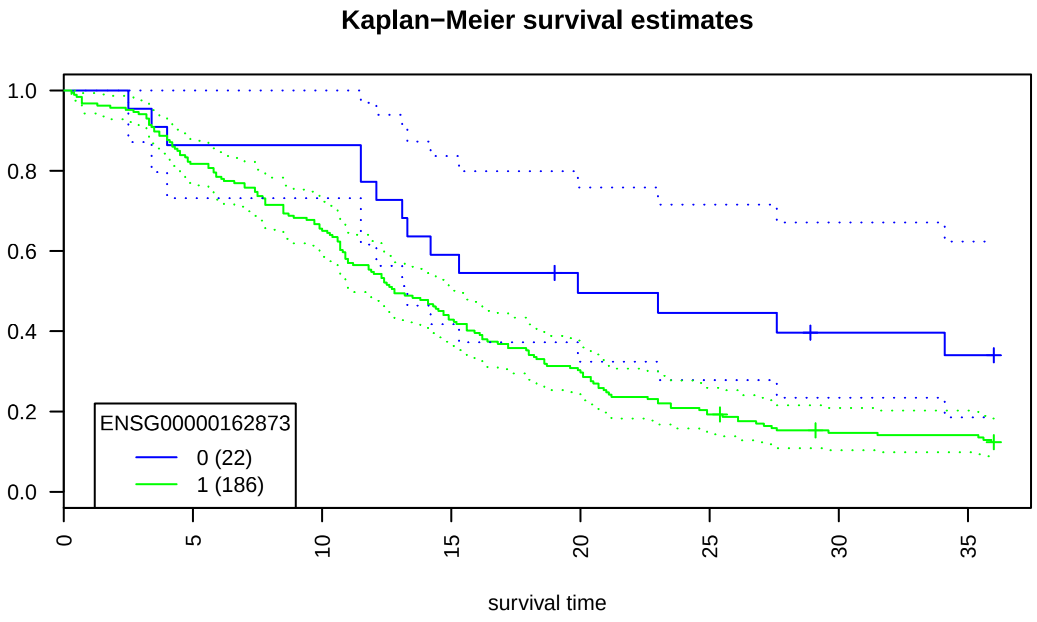

Figure 261: Each curve represents the relative number of ENSG00000162873 survived to the corresponding point of time. Dashed lines represent 0.95 confidence intervals for the curves with the corresponding color. Log-rank probability measure for the equality of these curves is 1.634323e-02. Additional annotations: *.GeneName*=KLHDC8A, *.GeneDesc*=kelch domain containing 8A [Source:HGNC Symbol;Acc:25573], *Uniprot/SWISSPROT*=Q8IYD2.

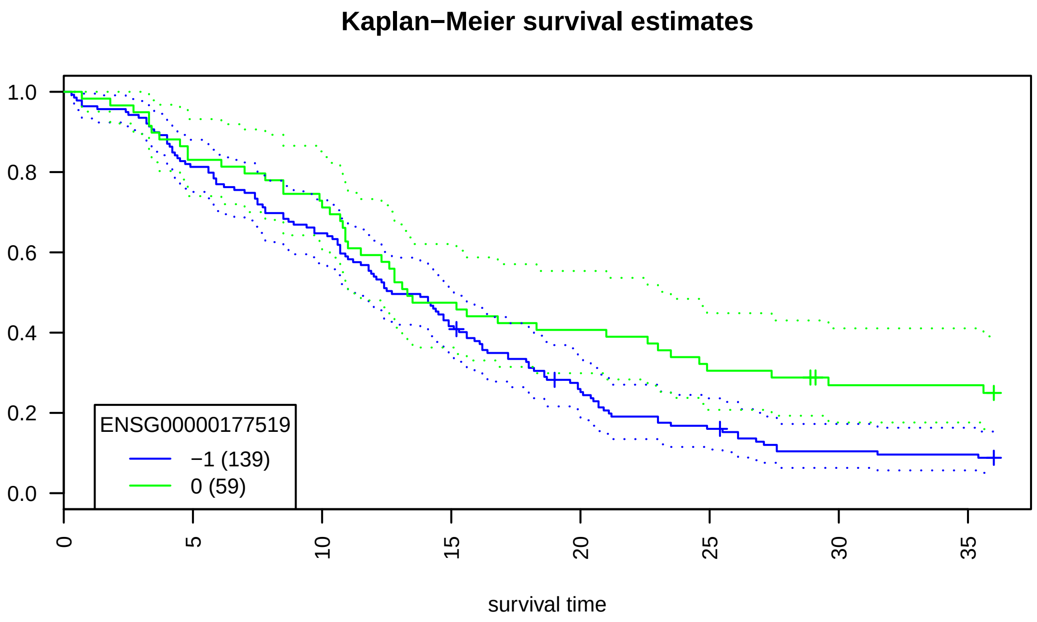

Figure 262: Each curve represents the relative number of ENSG00000177519 survived to the corresponding point of time. Dashed lines represent 0.95 confidence intervals for the curves with the corresponding color. Total of 10 observations deleted due to missingness. Log-rank probability measure for the equality of these curves is 1.601964e-02. Additional annotations: *.GeneName*=RPRM, *.GeneDesc*=reprimo, TP53 dependent G2 arrest mediator candidate [Source:HGNC Symbol;Acc:24201], *Uniprot/SWISSPROT*=Q9NS64.

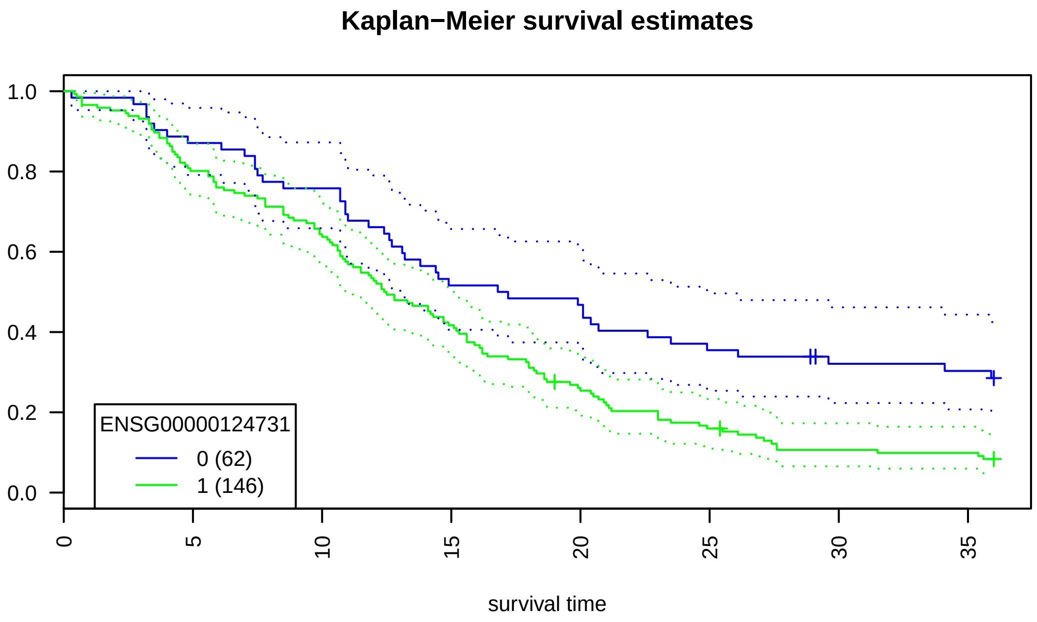

Figure 263: Each curve represents the relative number of ENSG00000124731 survived to the corresponding point of time. Dashed lines represent 0.95 confidence intervals for the curves with the corresponding color. Log-rank probability measure for the equality of these curves is 9.49878e-04. Additional annotations: *.GeneName*=TREM1, *.GeneDesc*=triggering receptor expressed on myeloid cells 1 [Source:HGNC Symbol;Acc:17760], *Uniprot/SWISSPROT*=Q9NP99.

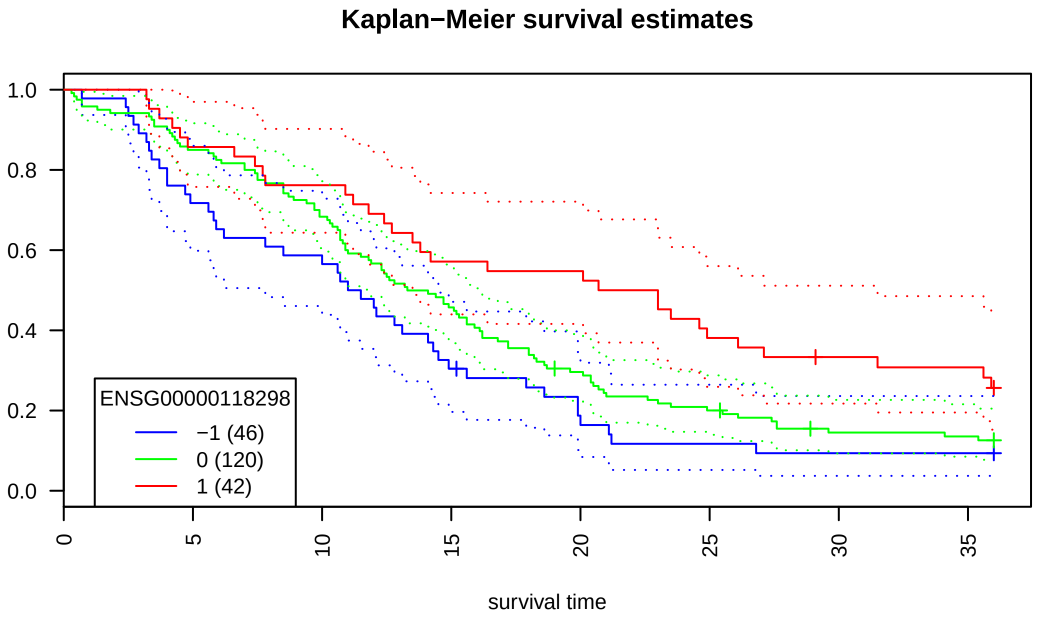

Figure 264: Each curve represents the relative number of ENSG00000118298 survived to the corresponding point of time. Dashed lines represent 0.95 confidence intervals for the curves with the corresponding color. Log-rank probability measure for the equality of these curves is 5.764107e-03.

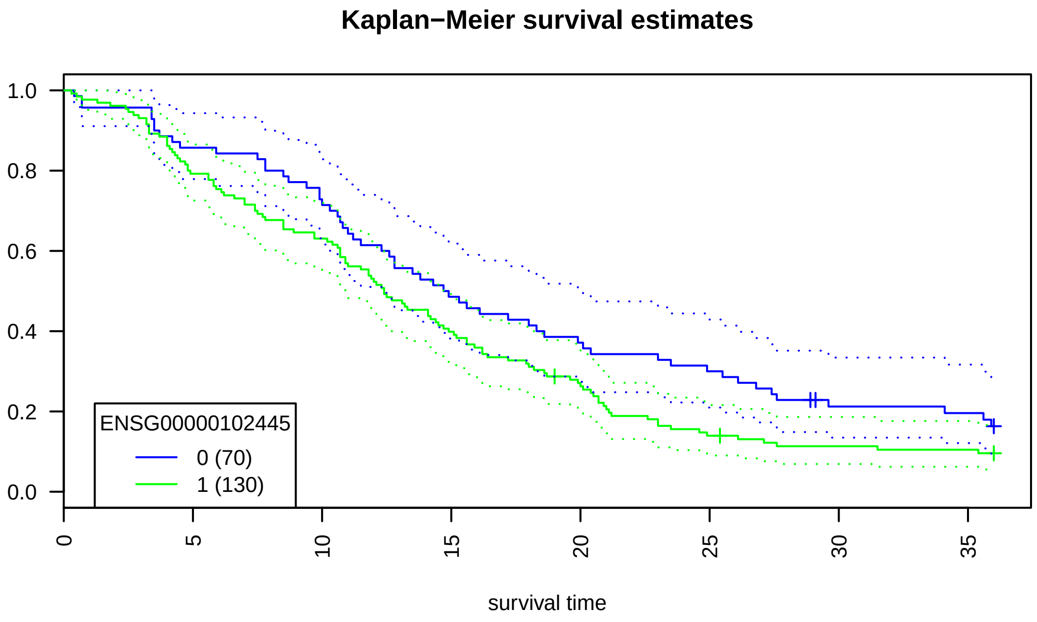

Figure 265: Each curve represents the relative number of ENSG00000102445 survived to the corresponding point of time. Dashed lines represent 0.95 confidence intervals for the curves with the corresponding color. Total of 8 observations deleted due to missingness. Log-rank probability measure for the equality of these curves is 4.43564e-02. Additional annotations: *.GeneName*=C13orf18, *.GeneDesc*=Uncharacterized protein C13orf18 [Source:UniProtKB/Swiss-Prot;Acc:Q9H714], *Uniprot/SWISSPROT*=Q9H714.

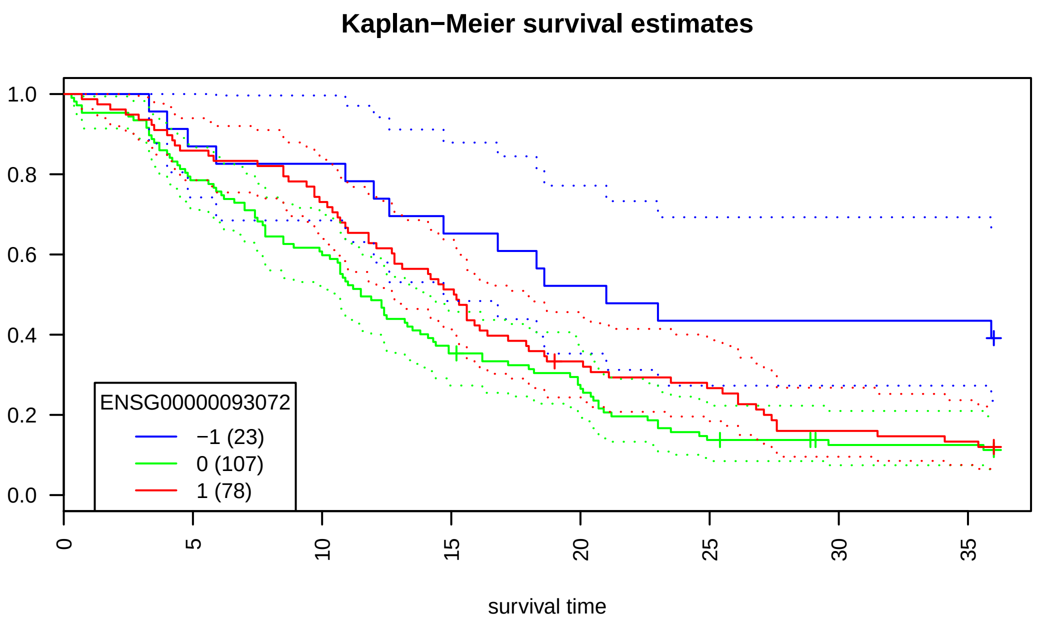

Figure 266: Each curve represents the relative number of ENSG00000093072 survived to the corresponding point of time. Dashed lines represent 0.95 confidence intervals for the curves with the corresponding color. Log-rank probability measure for the equality of these curves is 5.019474e-03.

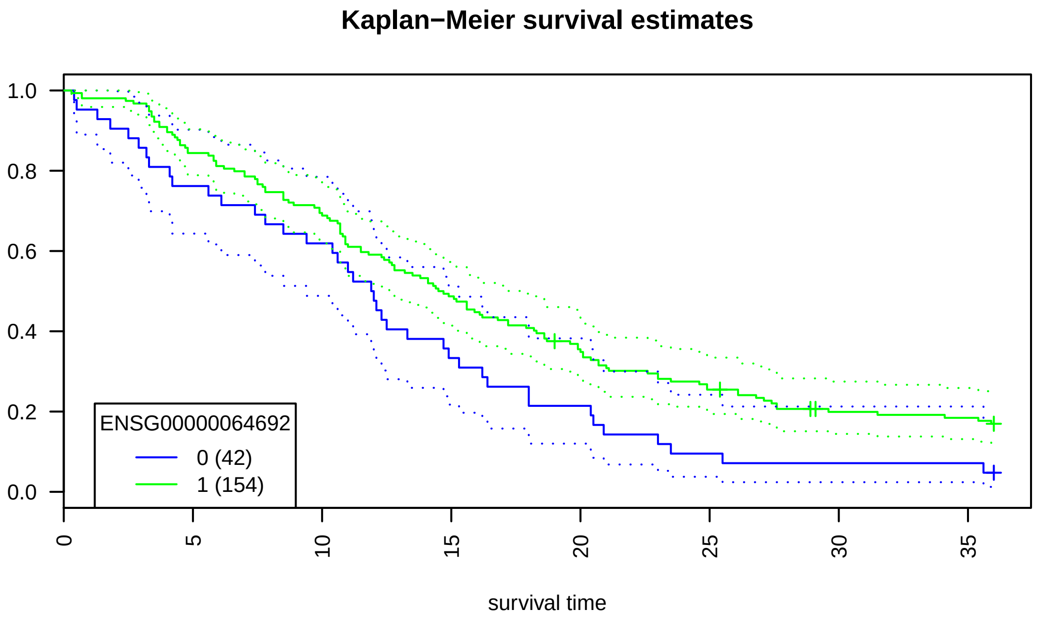

Figure 267: Each curve represents the relative number of ENSG00000064692 survived to the corresponding point of time. Dashed lines represent 0.95 confidence intervals for the curves with the corresponding color. Total of 12 observations deleted due to missingness. Log-rank probability measure for the equality of these curves is 1.002267e-02. Additional annotations: *.GeneName*=SNCAIP, *.GeneDesc*=synuclein, alpha interacting protein [Source:HGNC Symbol;Acc:11139], *Uniprot/SWISSPROT*=Q9Y6H5.

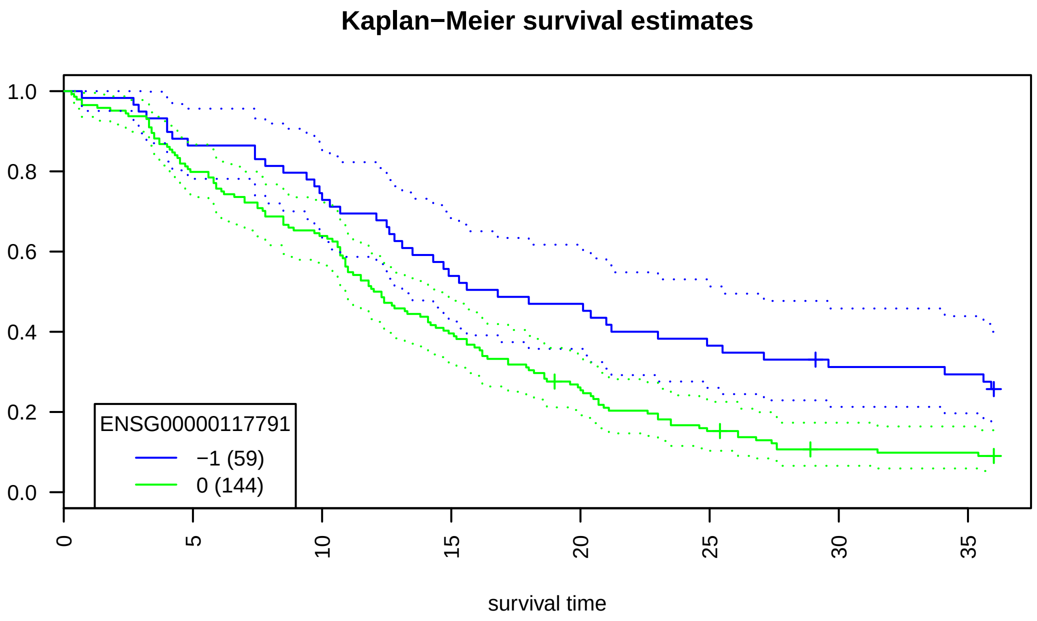

Figure 268: Each curve represents the relative number of ENSG00000117791 survived to the corresponding point of time. Dashed lines represent 0.95 confidence intervals for the curves with the corresponding color. Total of 5 observations deleted due to missingness. Log-rank probability measure for the equality of these curves is 1.425184e-03.

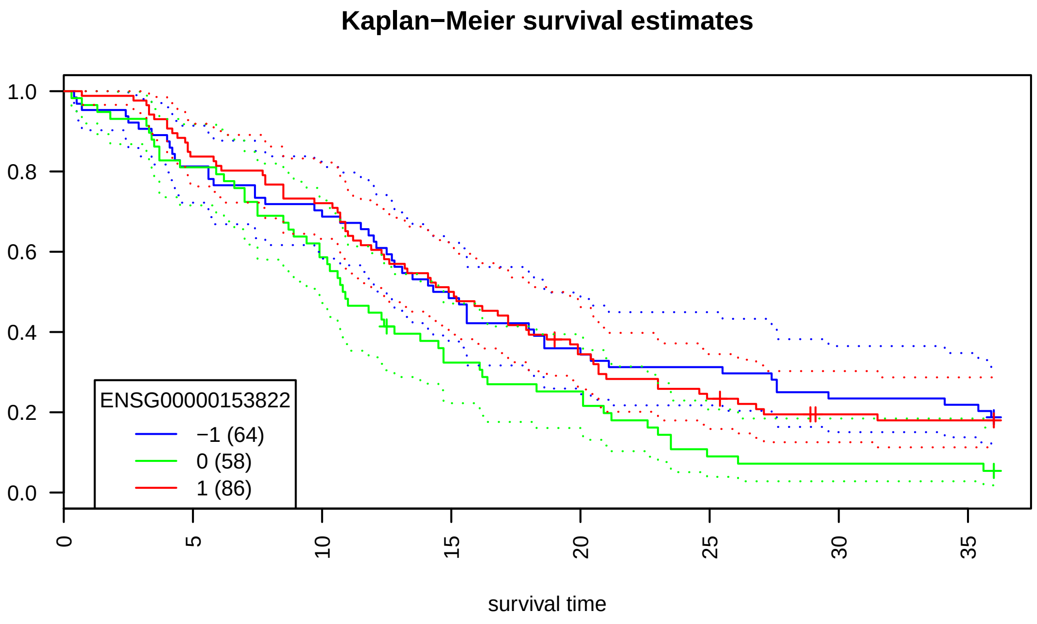

Figure 269: Each curve represents the relative number of ENSG00000153822 survived to the corresponding point of time. Dashed lines represent 0.95 confidence intervals for the curves with the corresponding color. Log-rank probability measure for the equality of these curves is 2.231879e-02.

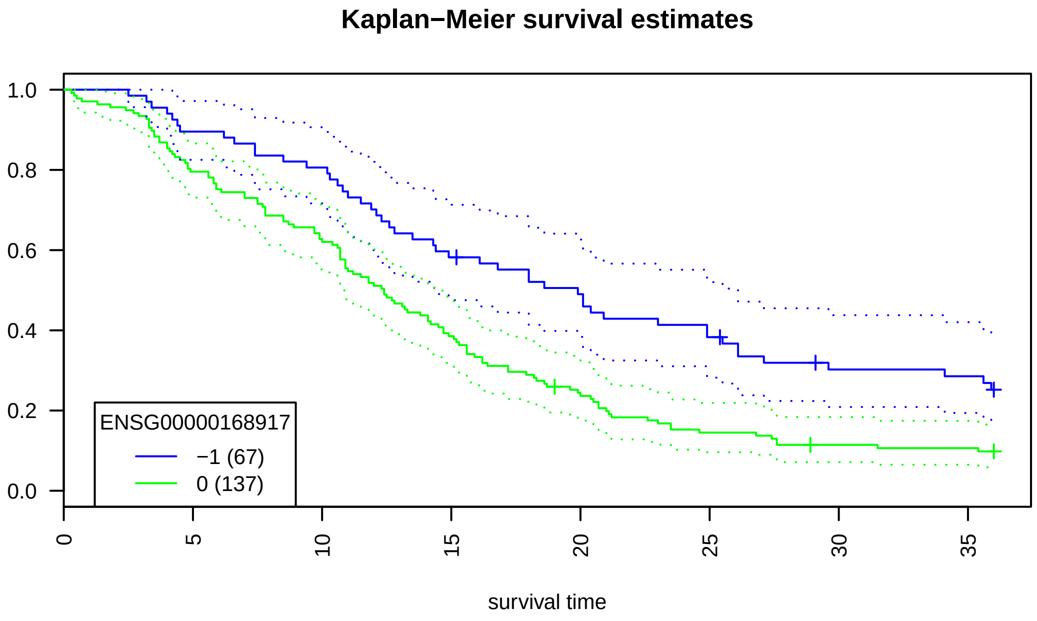

Figure 270: Each curve represents the relative number of ENSG00000168917 survived to the corresponding point of time. Dashed lines represent 0.95 confidence intervals for the curves with the corresponding color. Total of 4 observations deleted due to missingness. Log-rank probability measure for the equality of these curves is 3.357792e-04.

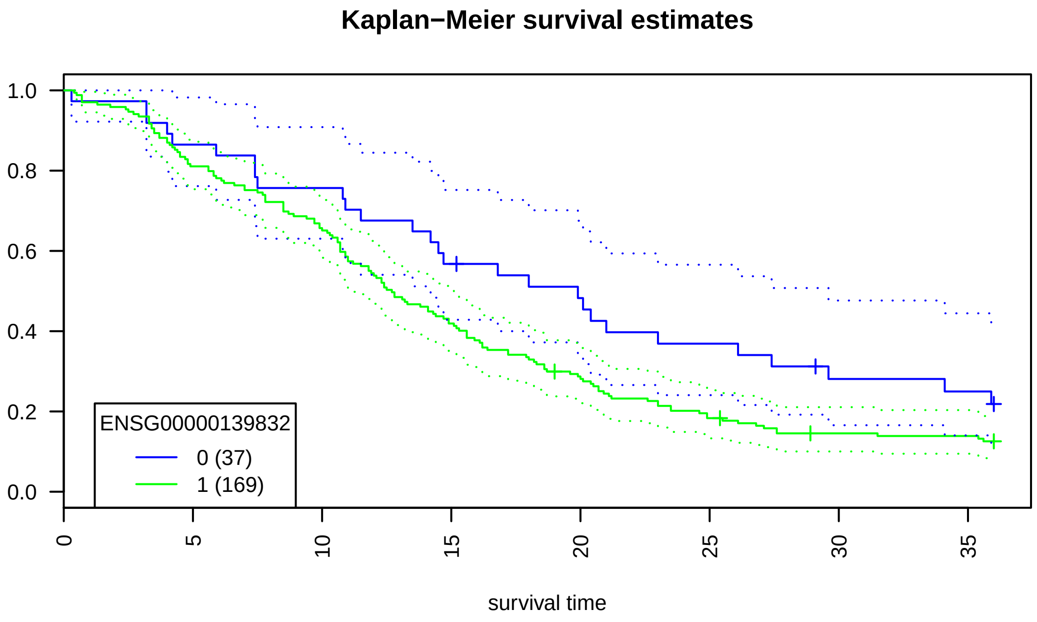

Figure 271: Each curve represents the relative number of ENSG00000139832 survived to the corresponding point of time. Dashed lines represent 0.95 confidence intervals for the curves with the corresponding color. Total of 2 observations deleted due to missingness. Log-rank probability measure for the equality of these curves is 4.522553e-02. Additional annotations: *.GeneName*=RAB20, *.GeneDesc*=RAB20, member RAS oncogene family [Source:HGNC Symbol;Acc:18260], *Uniprot/SWISSPROT*=Q9NX57.

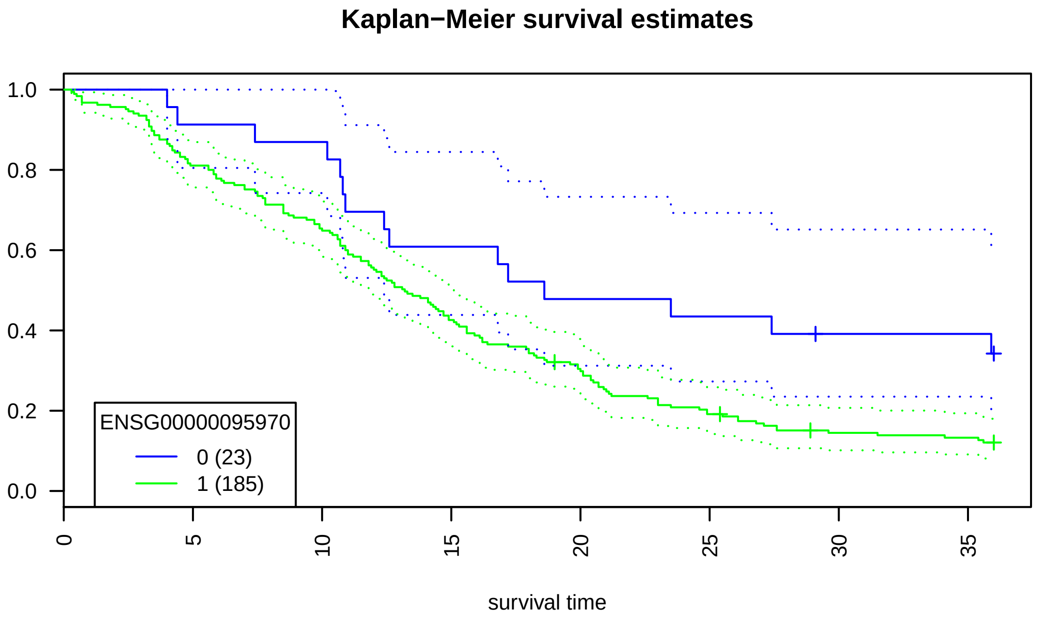

Figure 272: Each curve represents the relative number of ENSG00000095970 survived to the corresponding point of time. Dashed lines represent 0.95 confidence intervals for the curves with the corresponding color. Log-rank probability measure for the equality of these curves is 1.364541e-02. Additional annotations: *.GeneName*=TREM2, *.GeneDesc*=triggering receptor expressed on myeloid cells 2 [Source:HGNC Symbol;Acc:17761], *Uniprot/SWISSPROT*=Q9NZC2.

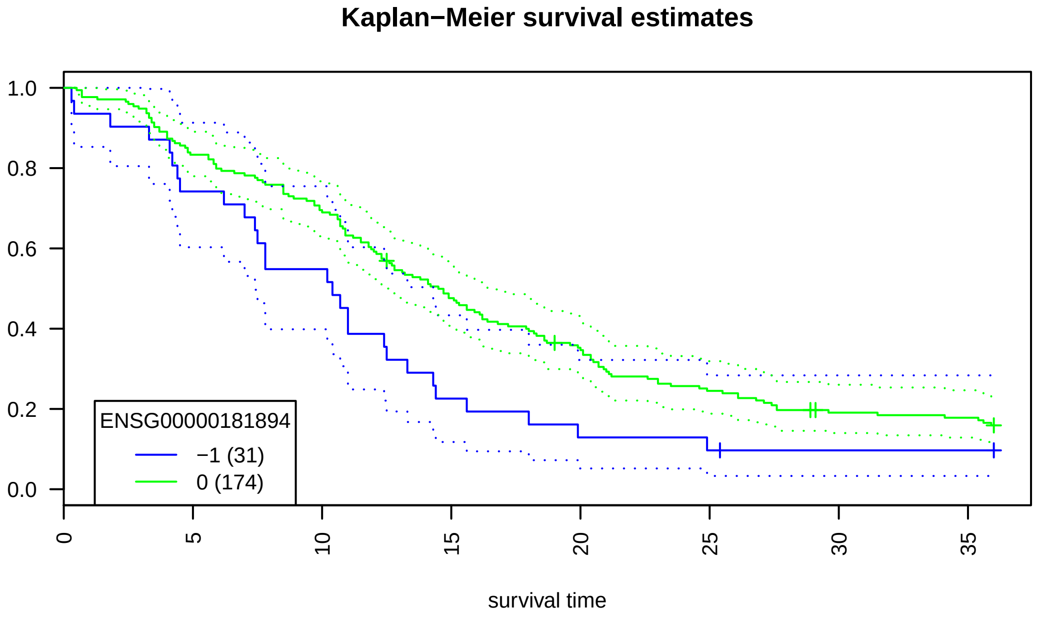

Figure 273: Each curve represents the relative number of ENSG00000181894 survived to the corresponding point of time. Dashed lines represent 0.95 confidence intervals for the curves with the corresponding color. Total of 3 observations deleted due to missingness. Log-rank probability measure for the equality of these curves is 1.279859e-02.

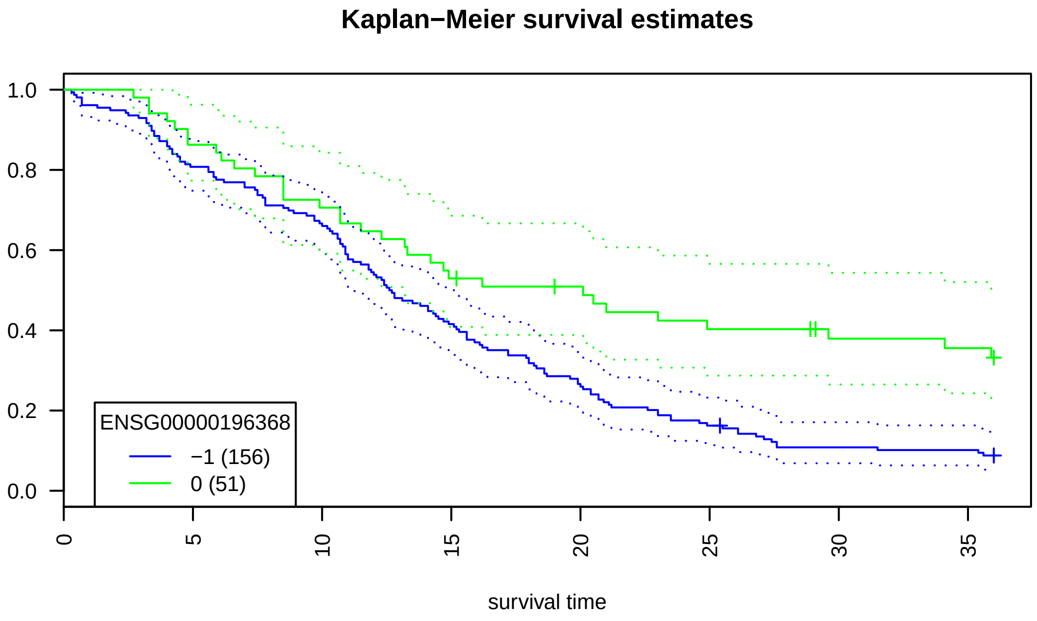

Figure 274: Each curve represents the relative number of ENSG00000196368 survived to the corresponding point of time. Dashed lines represent 0.95 confidence intervals for the curves with the corresponding color. Total of 1 observation deleted due to missingness. Log-rank probability measure for the equality of these curves is 5.311659e-04. Additional annotations: *.GeneName*=NUDT11, *.GeneDesc*=nudix (nucleoside diphosphate linked moiety X)-type motif 11 [Source:HGNC Symbol;Acc:18011], *Uniprot/SWISSPROT*=Q96G61.

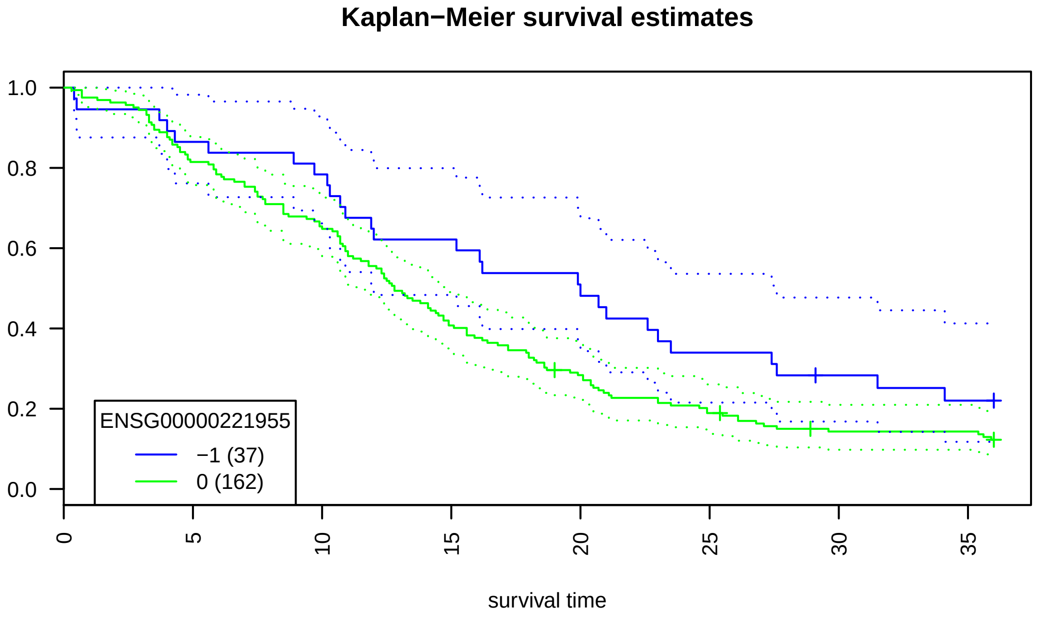

Figure 275: Each curve represents the relative number of ENSG00000221955 survived to the corresponding point of time. Dashed lines represent 0.95 confidence intervals for the curves with the corresponding color. Total of 9 observations deleted due to missingness. Log-rank probability measure for the equality of these curves is 4.437391e-02.

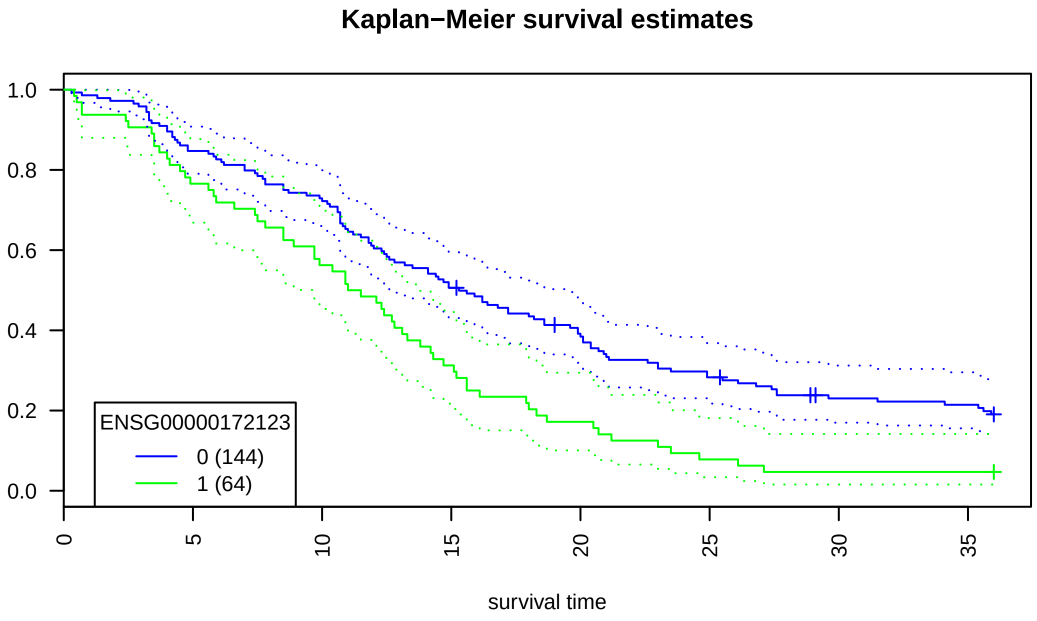

Figure 276: Each curve represents the relative number of ENSG00000172123 survived to the corresponding point of time. Dashed lines represent 0.95 confidence intervals for the curves with the corresponding color. Log-rank probability measure for the equality of these curves is 1.869911e-04.

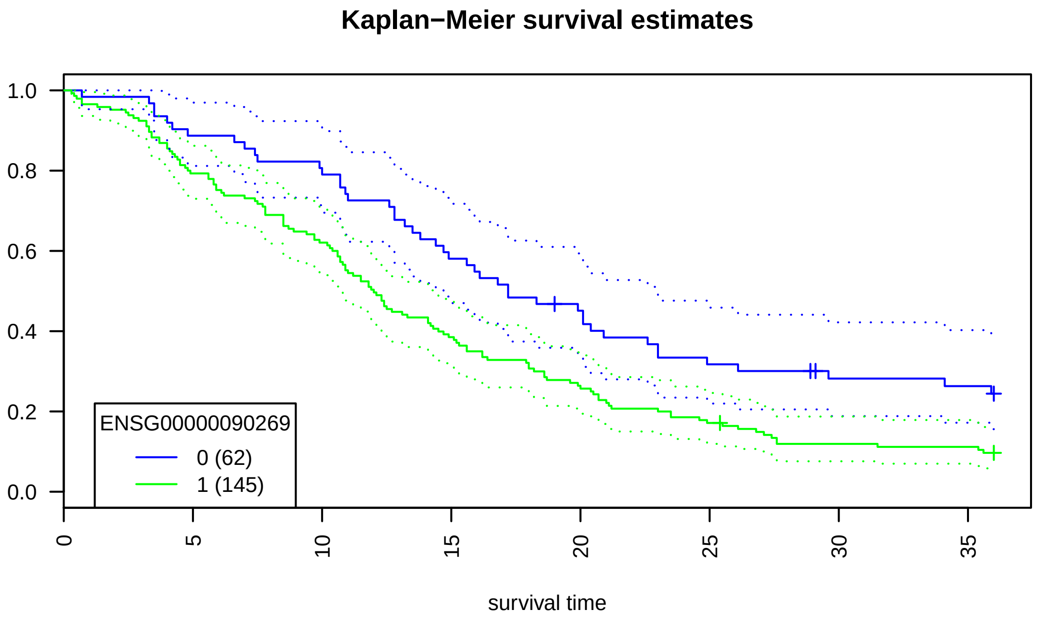

Figure 277: Each curve represents the relative number of ENSG00000090269 survived to the corresponding point of time. Dashed lines represent 0.95 confidence intervals for the curves with the corresponding color. Total of 1 observation deleted due to missingness. Log-rank probability measure for the equality of these curves is 1.651155e-03. Additional annotations: *.GeneName*=CLEC5A, *.GeneDesc*=C-type lectin domain family 5, member A [Source:HGNC Symbol;Acc:2054], *Uniprot/SWISSPROT*=Q9NY25.

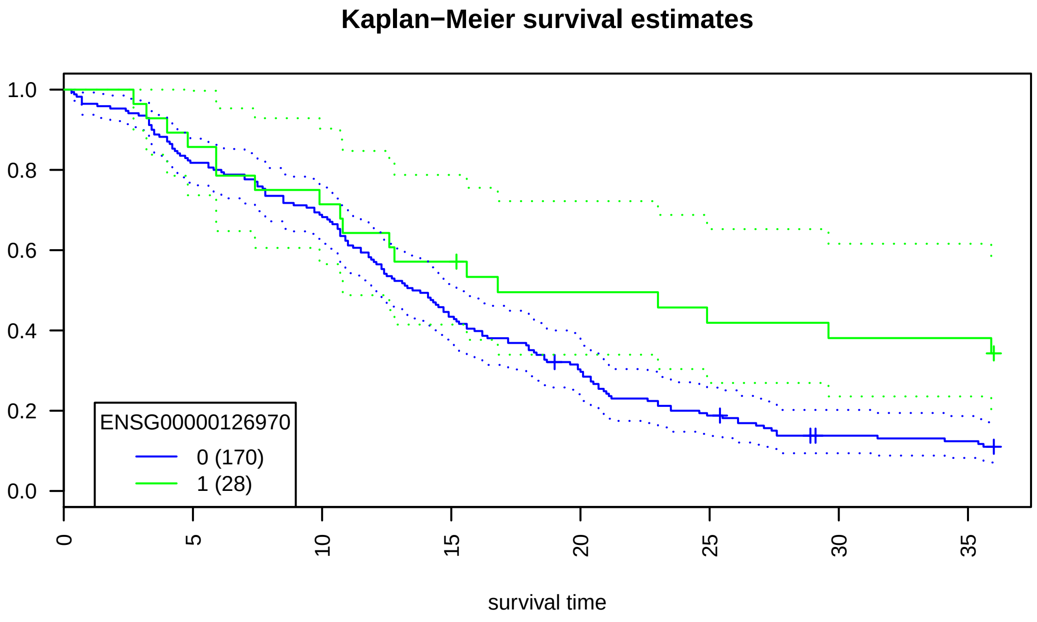

Figure 278: Each curve represents the relative number of ENSG00000126970 survived to the corresponding point of time. Dashed lines represent 0.95 confidence intervals for the curves with the corresponding color. Total of 10 observations deleted due to missingness. Log-rank probability measure for the equality of these curves is 1.355477e-02.

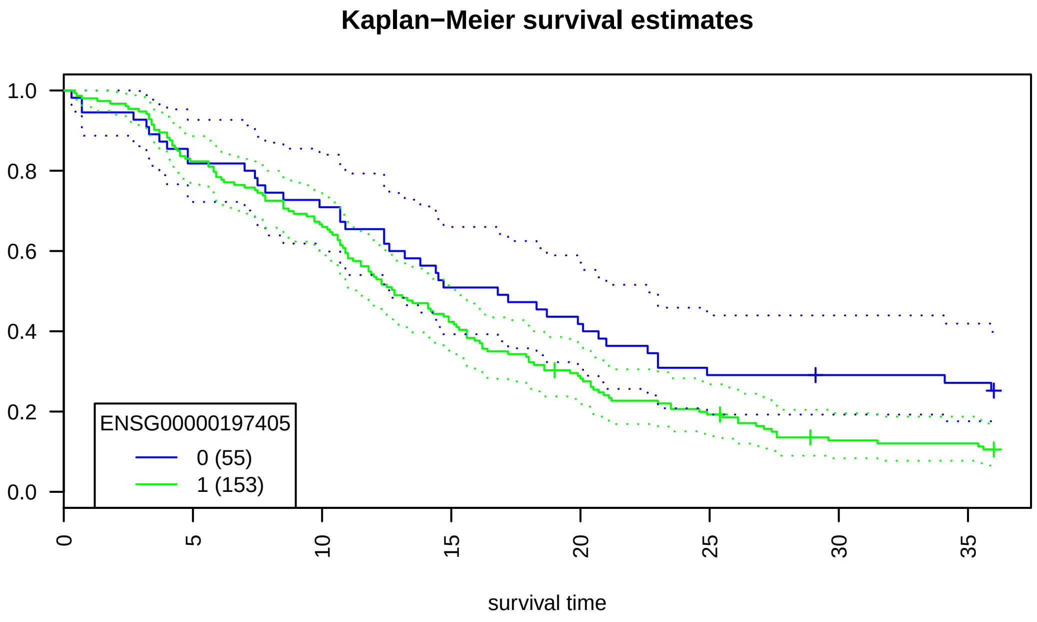

Figure 279: Each curve represents the relative number of ENSG00000197405 survived to the corresponding point of time. Dashed lines represent 0.95 confidence intervals for the curves with the corresponding color. Log-rank probability measure for the equality of these curves is 2.844111e-02. Additional annotations: *.GeneName*=C5AR1, *.GeneDesc*=complement component 5a receptor 1 [Source:HGNC Symbol;Acc:1338], *Uniprot/SWISSPROT*=P21730.

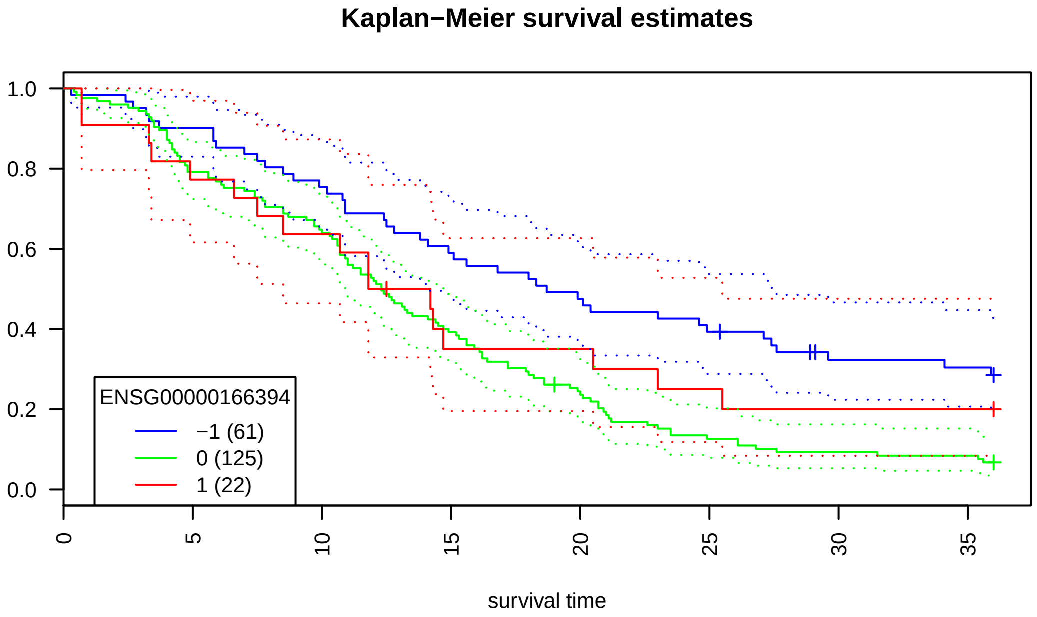

Figure 280: Each curve represents the relative number of ENSG00000166394 survived to the corresponding point of time. Dashed lines represent 0.95 confidence intervals for the curves with the corresponding color. Log-rank probability measure for the equality of these curves is 5.368334e-04.

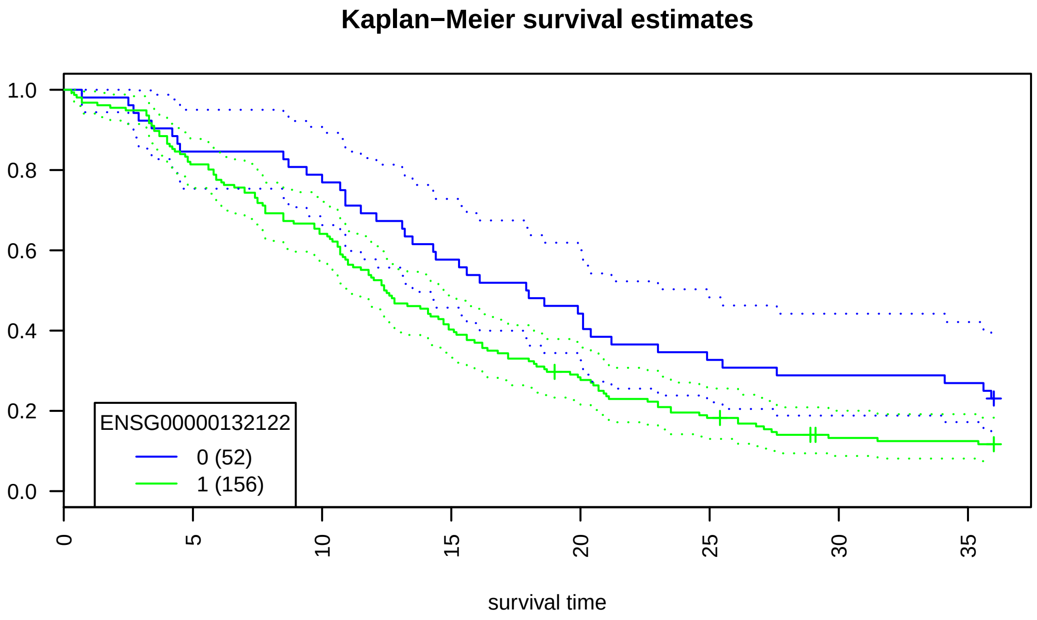

Figure 281: Each curve represents the relative number of ENSG00000132122 survived to the corresponding point of time. Dashed lines represent 0.95 confidence intervals for the curves with the corresponding color. Log-rank probability measure for the equality of these curves is 1.752196e-02. Additional annotations: *.GeneName*=SPATA6, *.GeneDesc*=spermatogenesis associated 6 [Source:HGNC Symbol;Acc:18309], *Uniprot/SWISSPROT*=Q9NWH7.

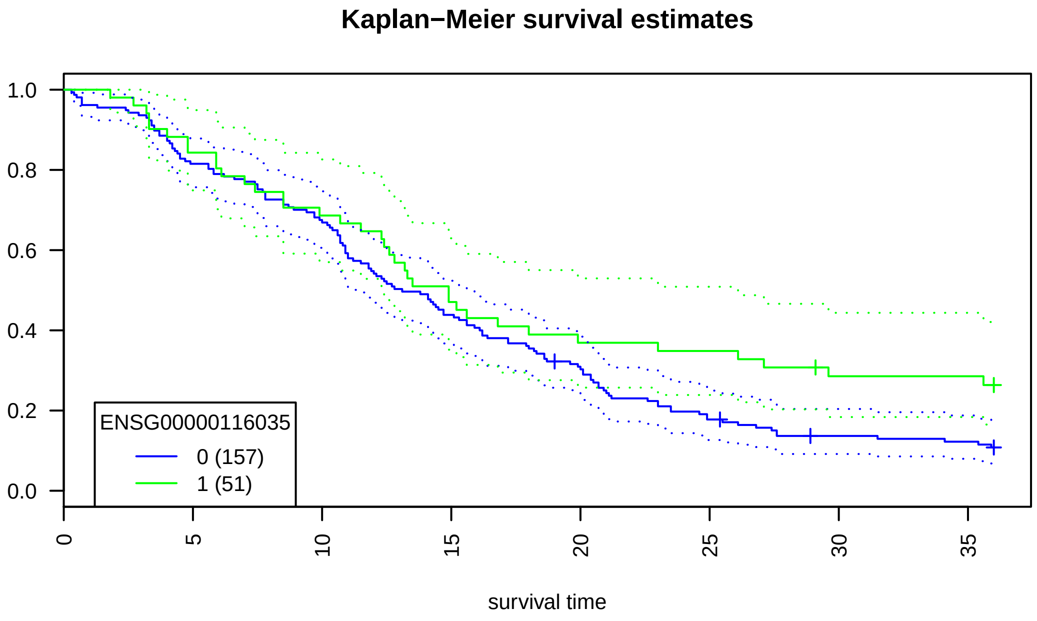

Figure 282: Each curve represents the relative number of ENSG00000116035 survived to the corresponding point of time. Dashed lines represent 0.95 confidence intervals for the curves with the corresponding color. Log-rank probability measure for the equality of these curves is 4.889437e-02.

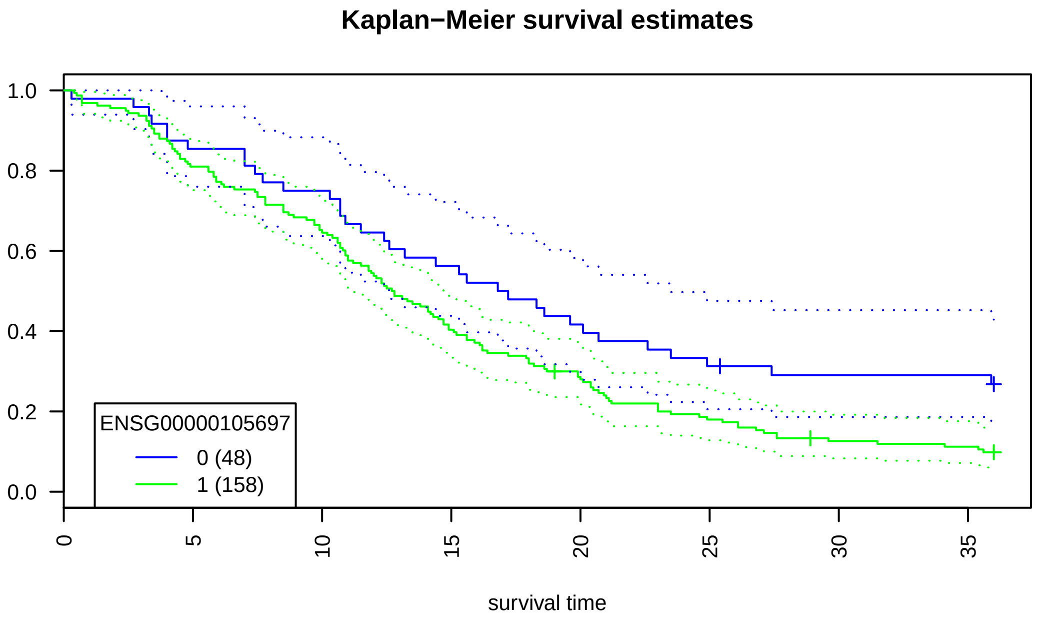

Figure 283: Each curve represents the relative number of ENSG00000105697 survived to the corresponding point of time. Dashed lines represent 0.95 confidence intervals for the curves with the corresponding color. Total of 2 observations deleted due to missingness. Log-rank probability measure for the equality of these curves is 1.337244e-02. Additional annotations: *.GeneName*=HAMP, *.GeneDesc*=hepcidin antimicrobial peptide [Source:HGNC Symbol;Acc:15598], *Uniprot/SWISSPROT*=P81172.

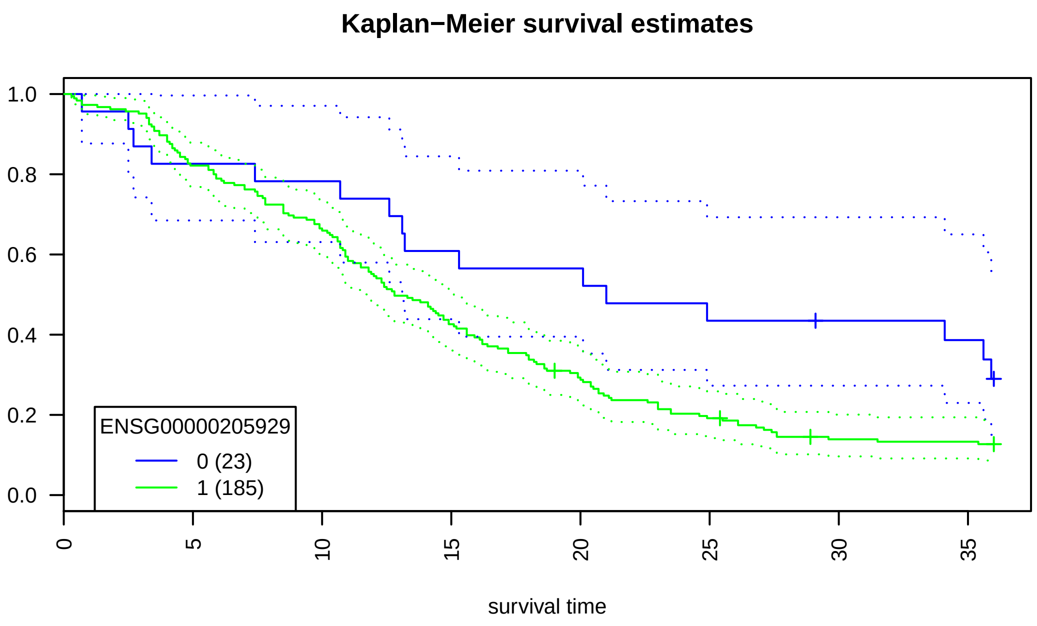

Figure 284: Each curve represents the relative number of ENSG00000205929 survived to the corresponding point of time. Dashed lines represent 0.95 confidence intervals for the curves with the corresponding color. Log-rank probability measure for the equality of these curves is 2.621968e-02. Additional annotations: *.GeneName*=C21orf62, *.GeneDesc*=Uncharacterized protein C21orf62 (B37) [Source:UniProtKB/Swiss-Prot;Acc:Q9NYP8], *Uniprot/SWISSPROT*=Q9NYP8.

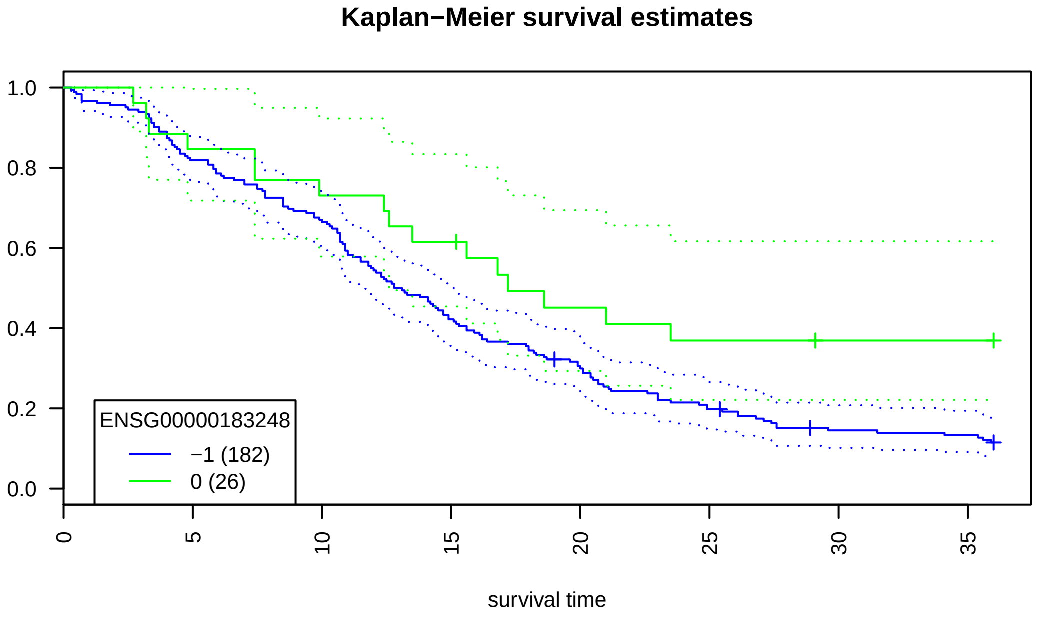

Figure 285: Each curve represents the relative number of ENSG00000183248 survived to the corresponding point of time. Dashed lines represent 0.95 confidence intervals for the curves with the corresponding color. Log-rank probability measure for the equality of these curves is 1.560712e-02. Additional annotations: *.GeneName*=AC010336.3, *.GeneDesc*=Putative uncharacterized protein FLJ22184 [Source:UniProtKB/Swiss-Prot;Acc:Q9H6K5], *Uniprot/SWISSPROT*=Q9H6K5.

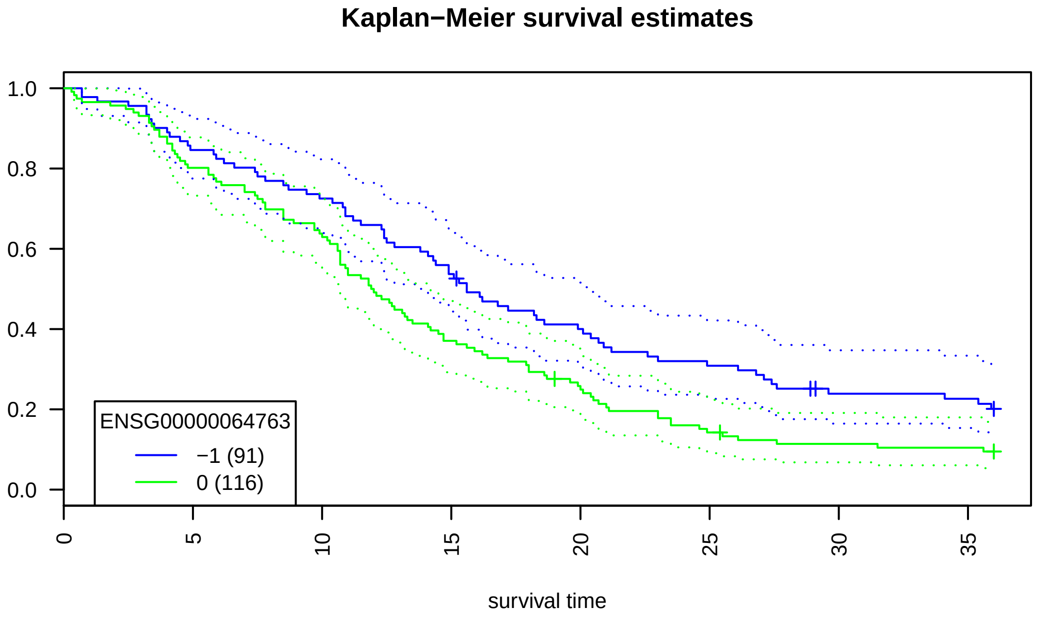

Figure 286: Each curve represents the relative number of ENSG00000064763 survived to the corresponding point of time. Dashed lines represent 0.95 confidence intervals for the curves with the corresponding color. Total of 1 observation deleted due to missingness. Log-rank probability measure for the equality of these curves is 7.072713e-03.

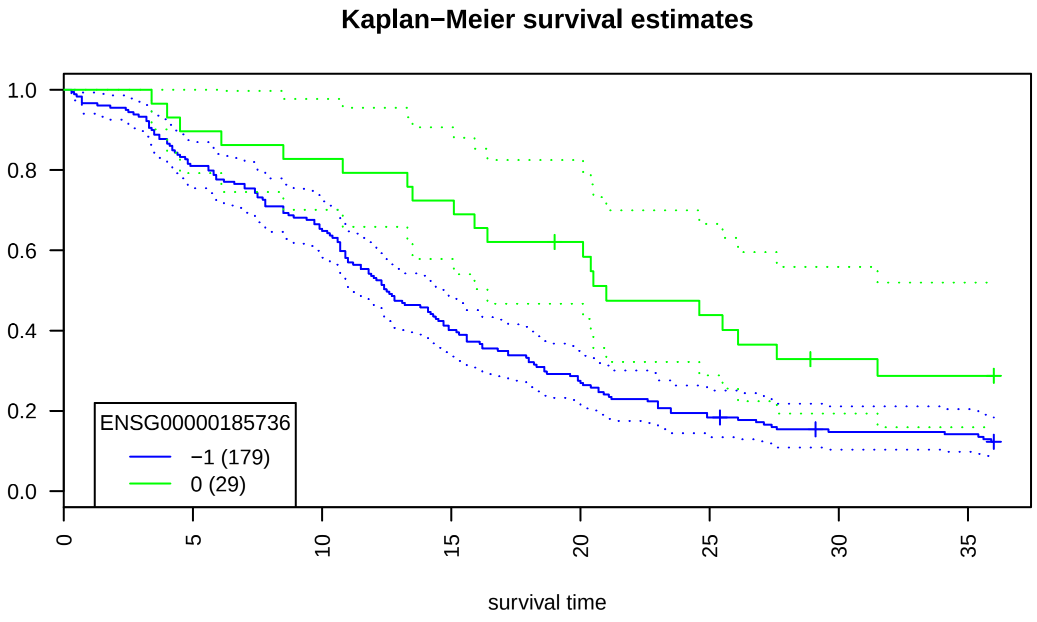

Figure 287: Each curve represents the relative number of ENSG00000185736 survived to the corresponding point of time. Dashed lines represent 0.95 confidence intervals for the curves with the corresponding color. Log-rank probability measure for the equality of these curves is 5.615161e-03. Additional annotations: *.GeneName*=ADARB2, *.GeneDesc*=adenosine deaminase, RNA-specific, B2 (RED2 homolog rat) [Source:HGNC Symbol;Acc:227], *Uniprot/SWISSPROT*=Q9NS39.

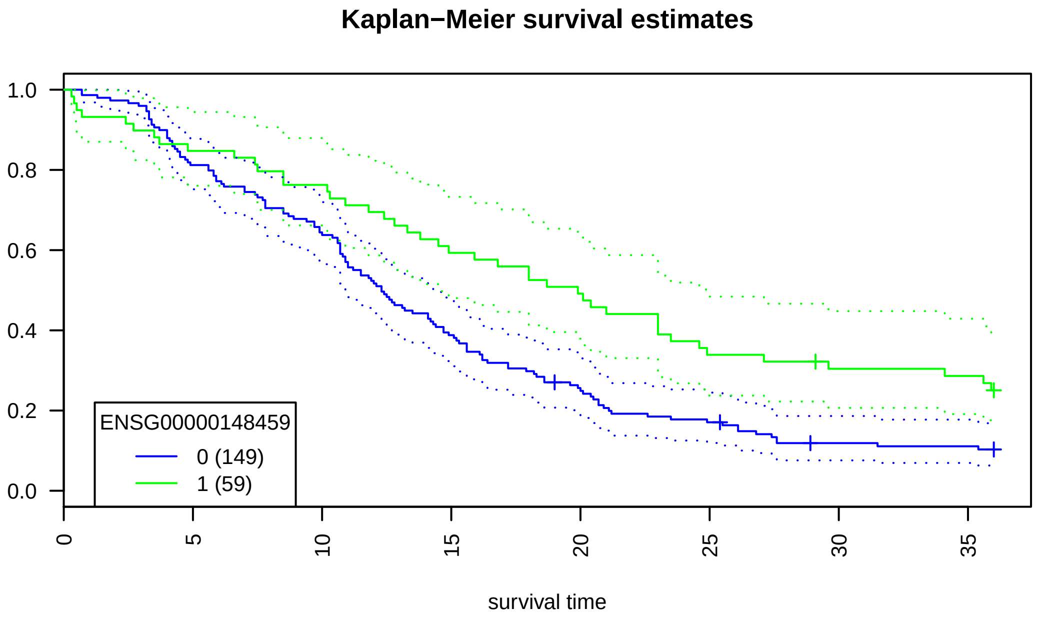

Figure 288: Each curve represents the relative number of ENSG00000148459 survived to the corresponding point of time. Dashed lines represent 0.95 confidence intervals for the curves with the corresponding color. Log-rank probability measure for the equality of these curves is 1.697726e-03.

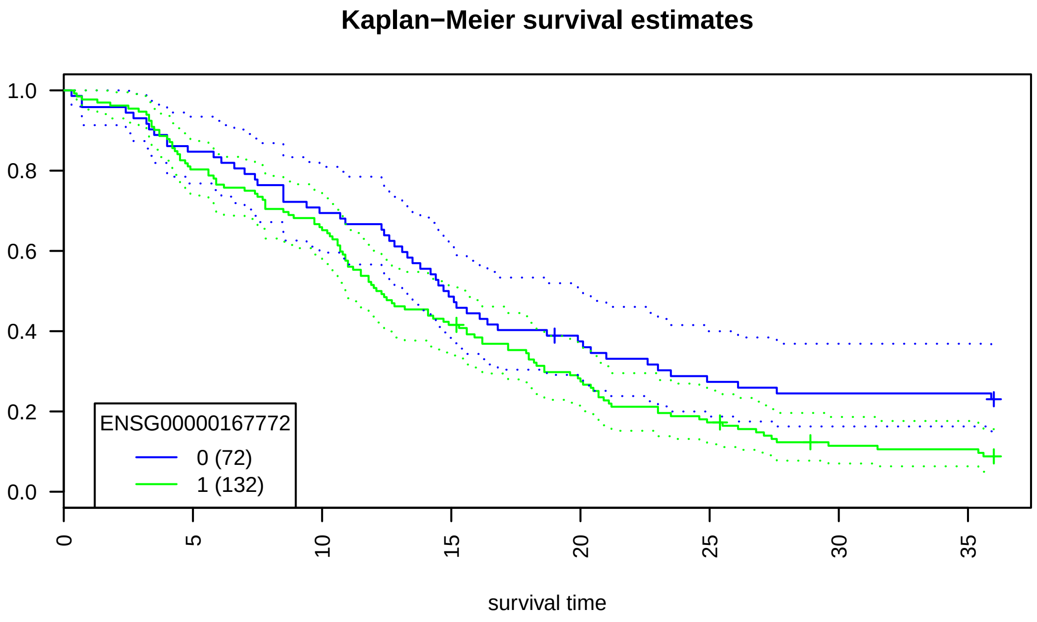

Figure 289: Each curve represents the relative number of ENSG00000167772 survived to the corresponding point of time. Dashed lines represent 0.95 confidence intervals for the curves with the corresponding color. Total of 4 observations deleted due to missingness. Log-rank probability measure for the equality of these curves is 2.722234e-02. Additional annotations: *.GeneName*=ANGPTL4, *.GeneDesc*=angiopoietin-like 4 [Source:HGNC Symbol;Acc:16039], *Uniprot/SWISSPROT*=Q9BY76.

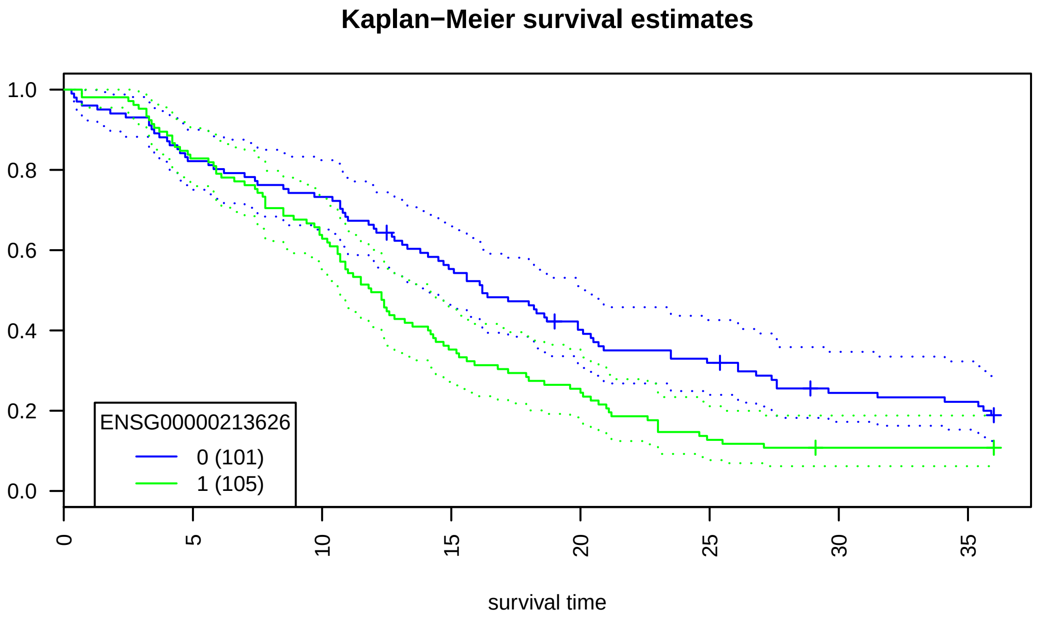

Figure 290: Each curve represents the relative number of ENSG00000213626 survived to the corresponding point of time. Dashed lines represent 0.95 confidence intervals for the curves with the corresponding color. Total of 2 observations deleted due to missingness. Log-rank probability measure for the equality of these curves is 7.229362e-03. Additional annotations: *.GeneName*=LBH, *.GeneDesc*=limb bud and heart development homolog (mouse) [Source:HGNC Symbol;Acc:29532], *Uniprot/SWISSPROT*=Q53QV2.

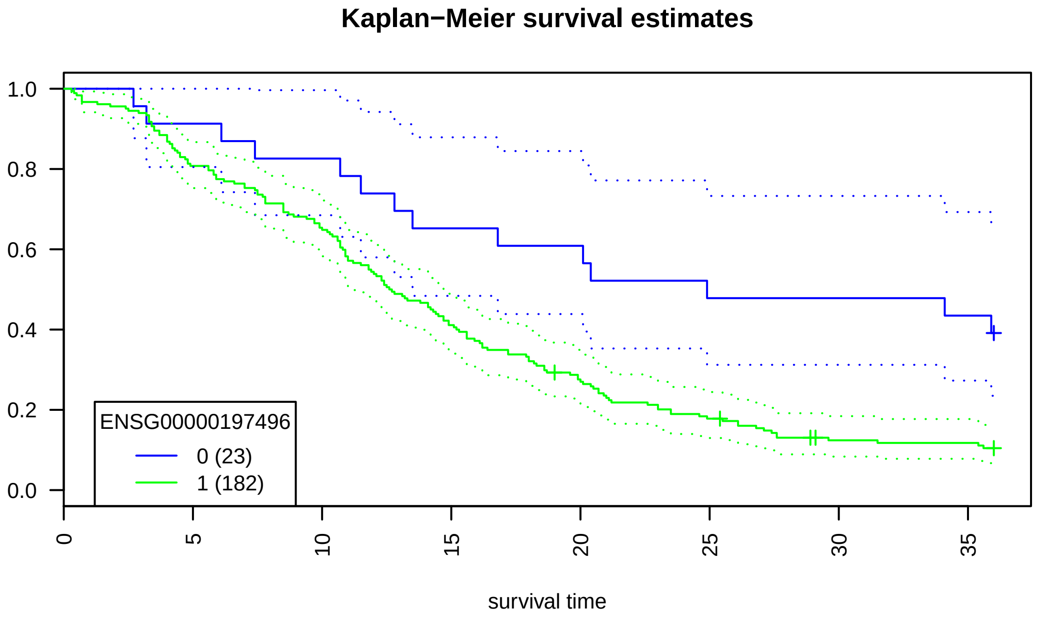

Figure 291: Each curve represents the relative number of ENSG00000197496 survived to the corresponding point of time. Dashed lines represent 0.95 confidence intervals for the curves with the corresponding color. Total of 3 observations deleted due to missingness. Log-rank probability measure for the equality of these curves is 1.542875e-03. Additional annotations: *.GeneName*=SLC2A10, *.GeneDesc*=solute carrier family 2 (facilitated glucose transporter), member 10 [Source:HGNC Symbol;Acc:13444], *Uniprot/SWISSPROT*=O95528.

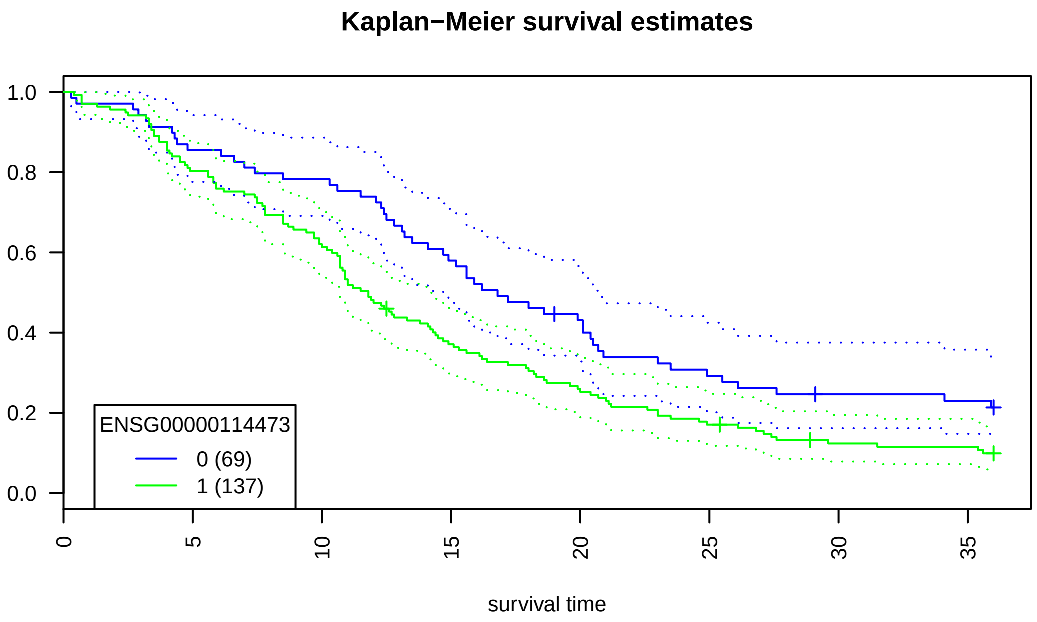

Figure 292: Each curve represents the relative number of ENSG00000114473 survived to the corresponding point of time. Dashed lines represent 0.95 confidence intervals for the curves with the corresponding color. Total of 2 observations deleted due to missingness. Log-rank probability measure for the equality of these curves is 6.406202e-03. Additional annotations: *.GeneName*=IQCG, *.GeneDesc*=IQ motif containing G [Source:HGNC Symbol;Acc:25251], *Uniprot/SWISSPROT*=Q9H095.

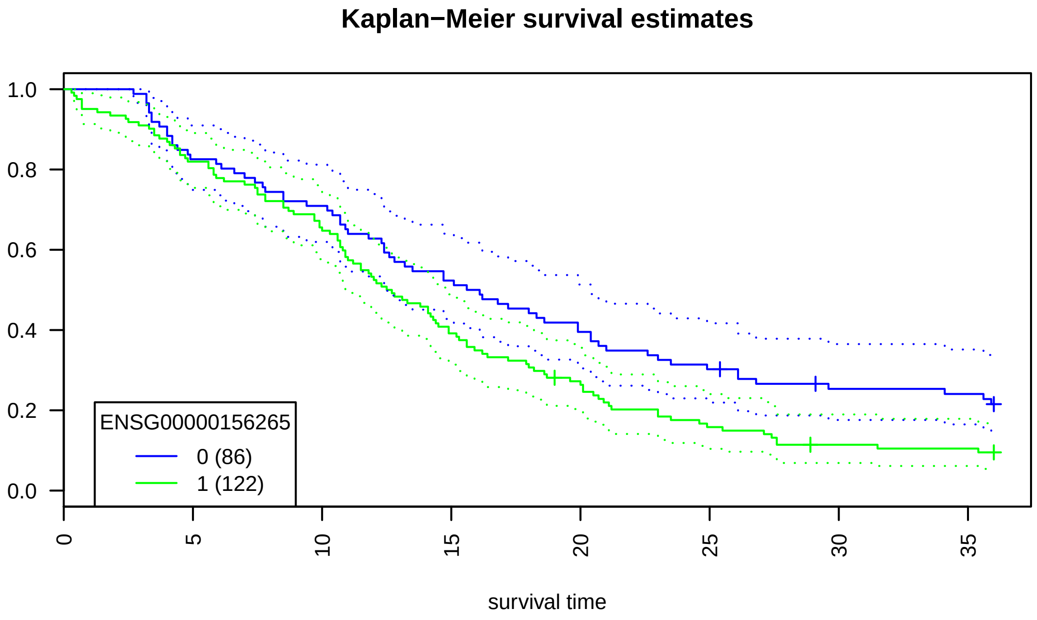

Figure 293: Each curve represents the relative number of ENSG00000156265 survived to the corresponding point of time. Dashed lines represent 0.95 confidence intervals for the curves with the corresponding color. Log-rank probability measure for the equality of these curves is 1.497465e-02. Additional annotations: *.GeneName*=C21orf7, *.GeneDesc*=TAK1-like protein [Source:UniProtKB/Swiss-Prot;Acc:P57077], *Uniprot/SWISSPROT*=P57077.

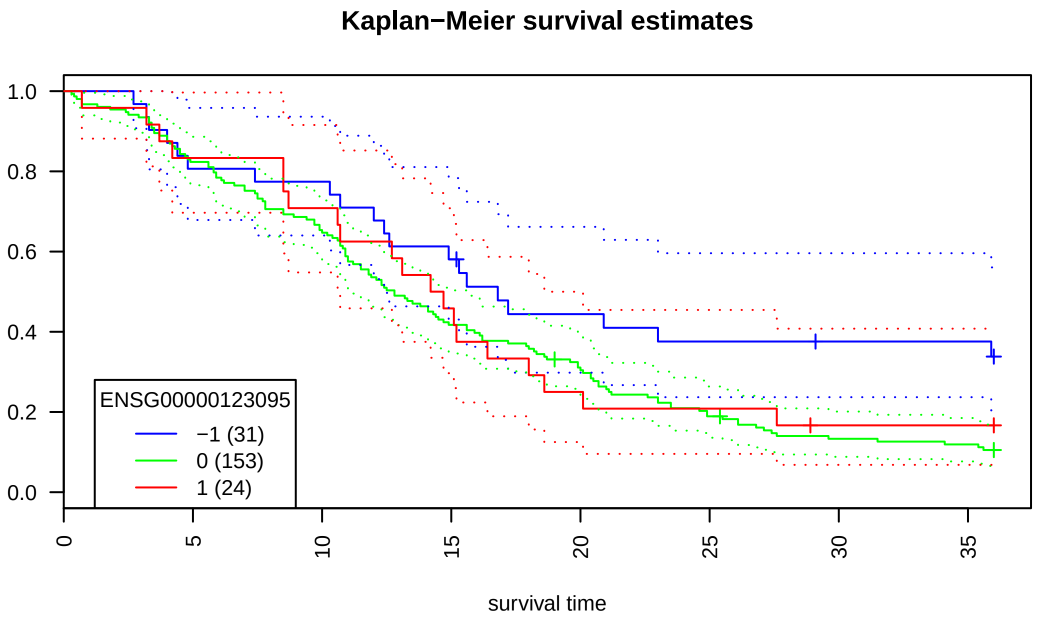

Figure 294: Each curve represents the relative number of ENSG00000123095 survived to the corresponding point of time. Dashed lines represent 0.95 confidence intervals for the curves with the corresponding color. Log-rank probability measure for the equality of these curves is 4.99821e-02.

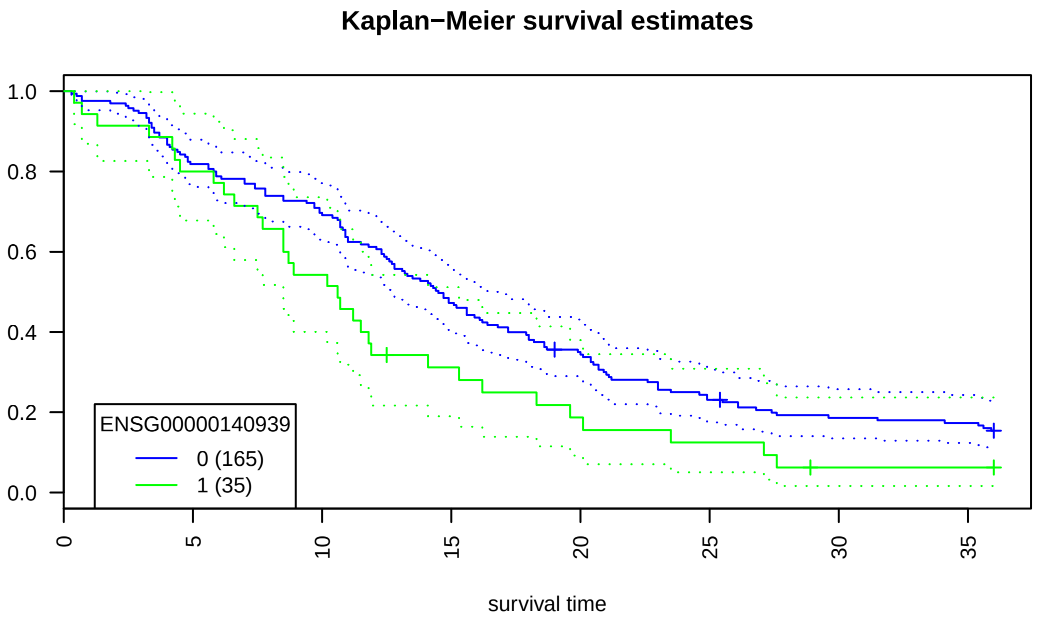

Figure 295: Each curve represents the relative number of ENSG00000140939 survived to the corresponding point of time. Dashed lines represent 0.95 confidence intervals for the curves with the corresponding color. Total of 8 observations deleted due to missingness. Log-rank probability measure for the equality of these curves is 2.007605e-02.

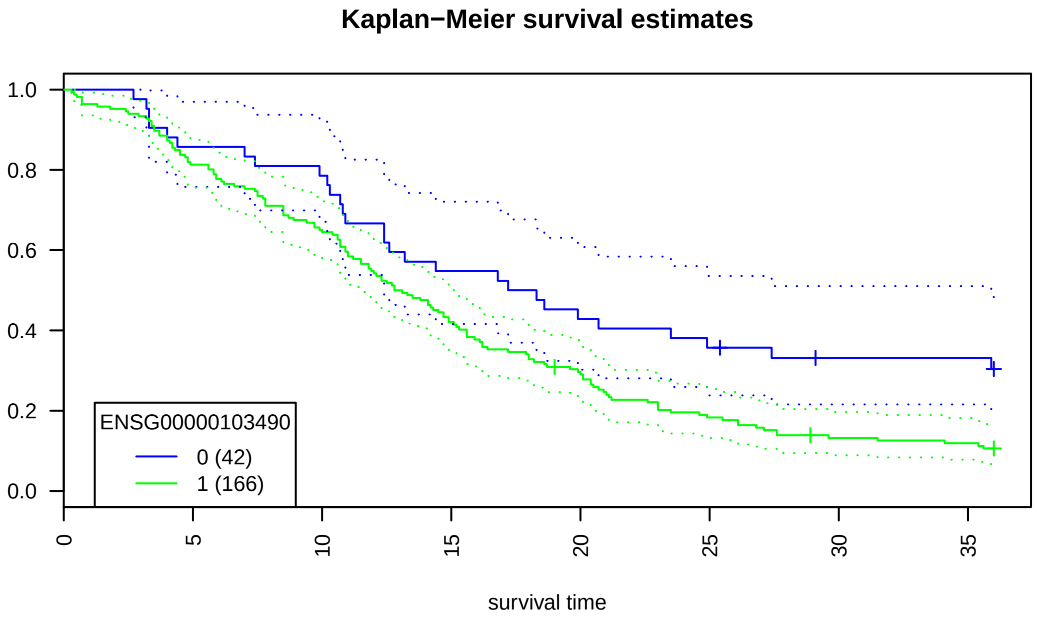

Figure 296: Each curve represents the relative number of ENSG00000103490 survived to the corresponding point of time. Dashed lines represent 0.95 confidence intervals for the curves with the corresponding color. Log-rank probability measure for the equality of these curves is 8.302423e-03. Additional annotations: *.GeneName*=PYCARD, *.GeneDesc*=PYD and CARD domain containing [Source:HGNC Symbol;Acc:16608], *Uniprot/SWISSPROT*=Q9ULZ3.

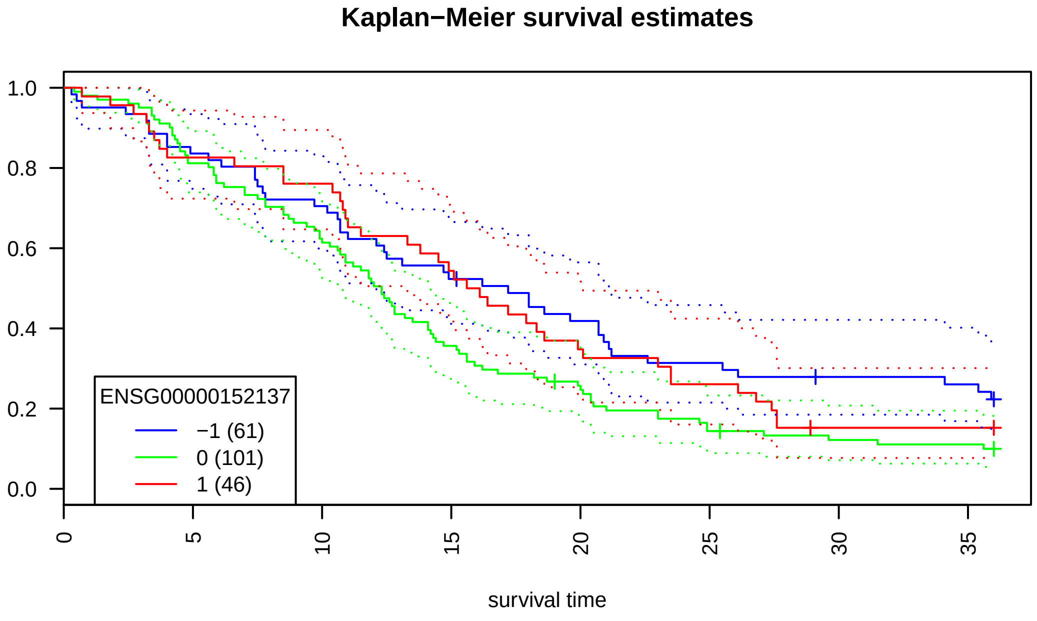

Figure 297: Each curve represents the relative number of ENSG00000152137 survived to the corresponding point of time. Dashed lines represent 0.95 confidence intervals for the curves with the corresponding color. Log-rank probability measure for the equality of these curves is 4.769681e-02.

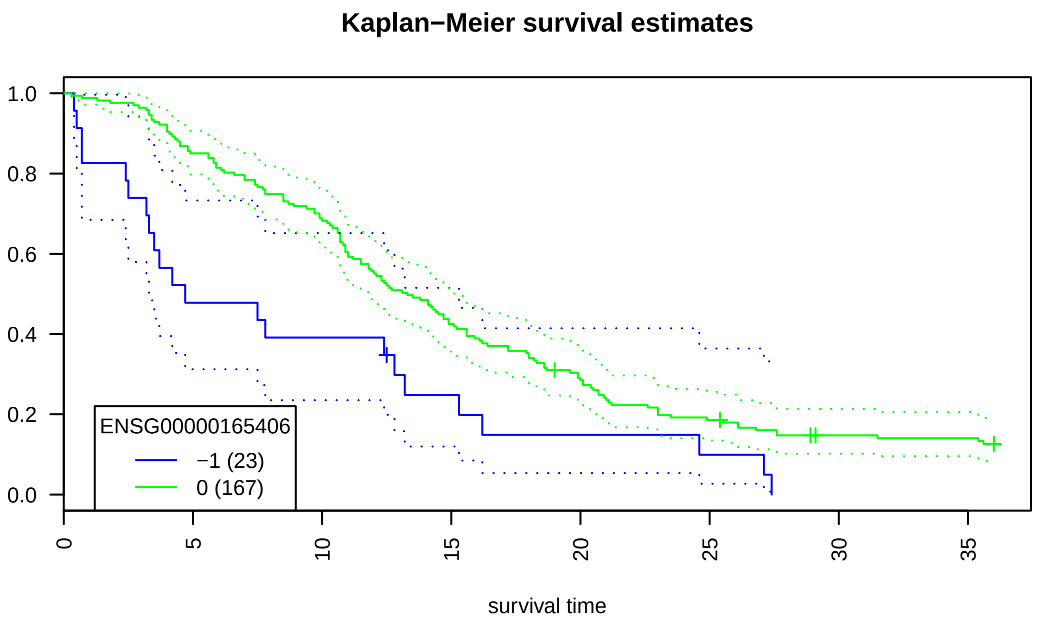

Figure 298: Each curve represents the relative number of ENSG00000165406 survived to the corresponding point of time. Dashed lines represent 0.95 confidence intervals for the curves with the corresponding color. Total of 18 observations deleted due to missingness. Log-rank probability measure for the equality of these curves is 2.358877e-03.

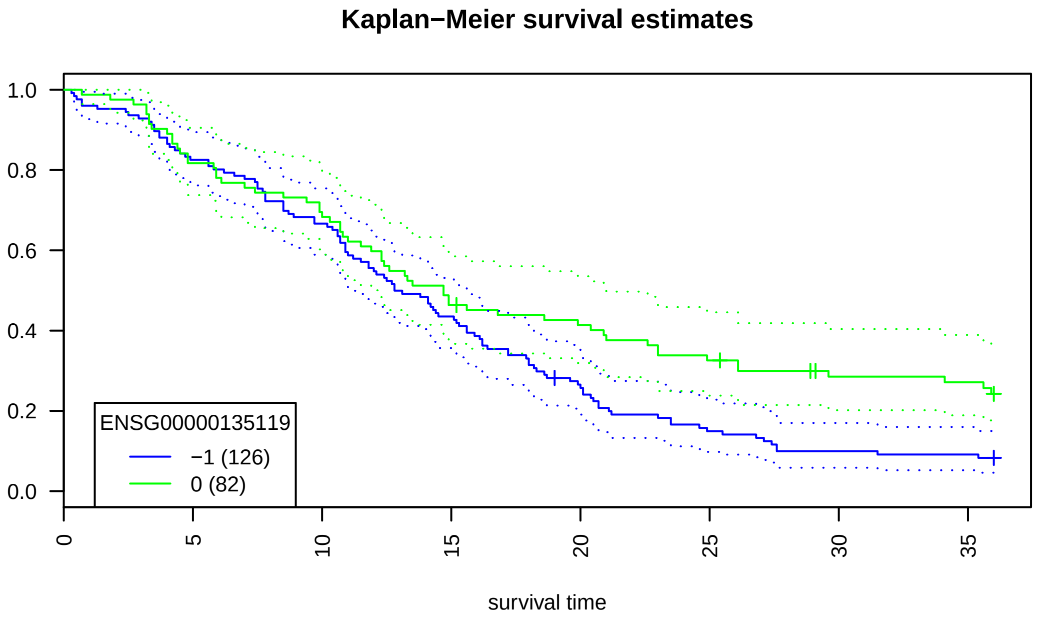

Figure 299: Each curve represents the relative number of ENSG00000135119 survived to the corresponding point of time. Dashed lines represent 0.95 confidence intervals for the curves with the corresponding color. Log-rank probability measure for the equality of these curves is 9.190749e-03. Additional annotations: *.GeneName*=RNFT2, *.GeneDesc*=ring finger protein, transmembrane 2 [Source:HGNC Symbol;Acc:25905], *Uniprot/SWISSPROT*=Q96EX2.

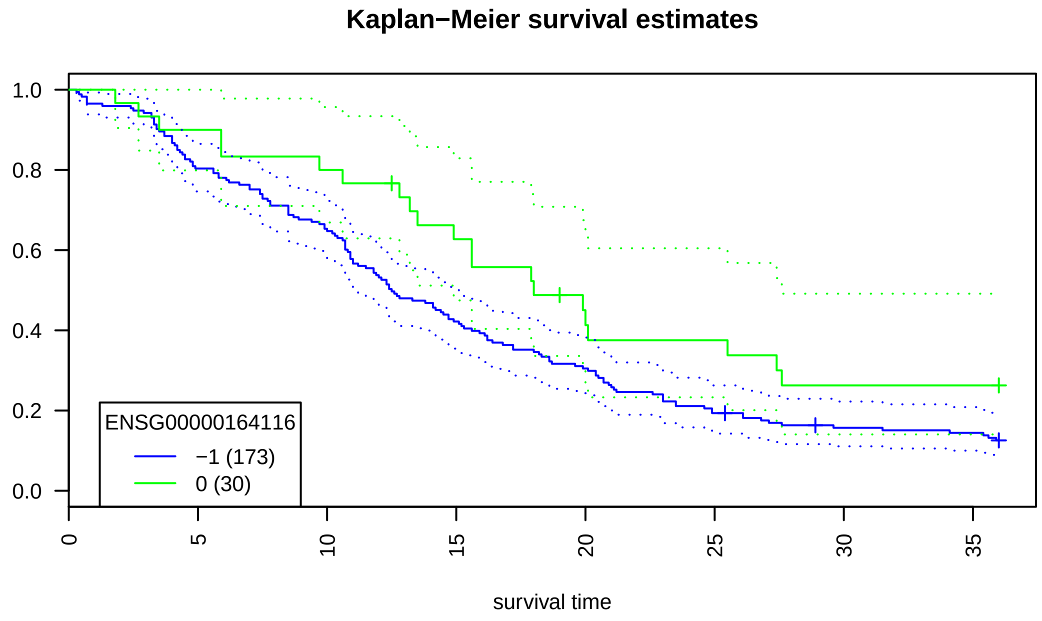

Figure 300: Each curve represents the relative number of ENSG00000164116 survived to the corresponding point of time. Dashed lines represent 0.95 confidence intervals for the curves with the corresponding color. Total of 5 observations deleted due to missingness. Log-rank probability measure for the equality of these curves is 4.124004e-02. Additional annotations: *.GeneName*=GUCY1A3, *.GeneDesc*=guanylate cyclase 1, soluble, alpha 3 [Source:HGNC Symbol;Acc:4685], *Uniprot/SWISSPROT*=Q02108.

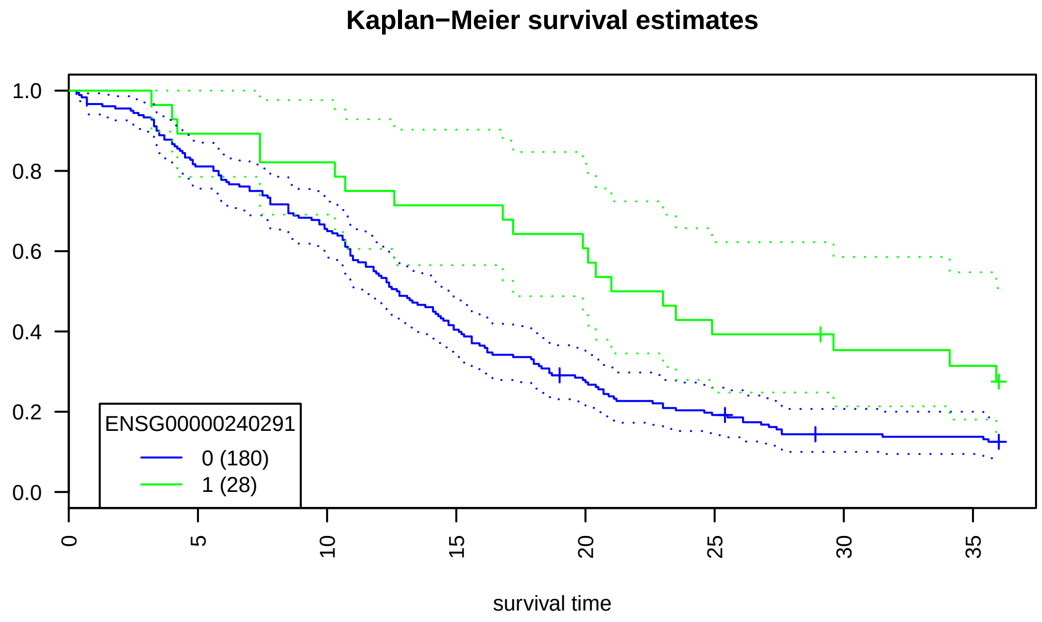

Figure 301: Each curve represents the relative number of ENSG00000240291 survived to the corresponding point of time. Dashed lines represent 0.95 confidence intervals for the curves with the corresponding color. Log-rank probability measure for the equality of these curves is 7.243605e-03.

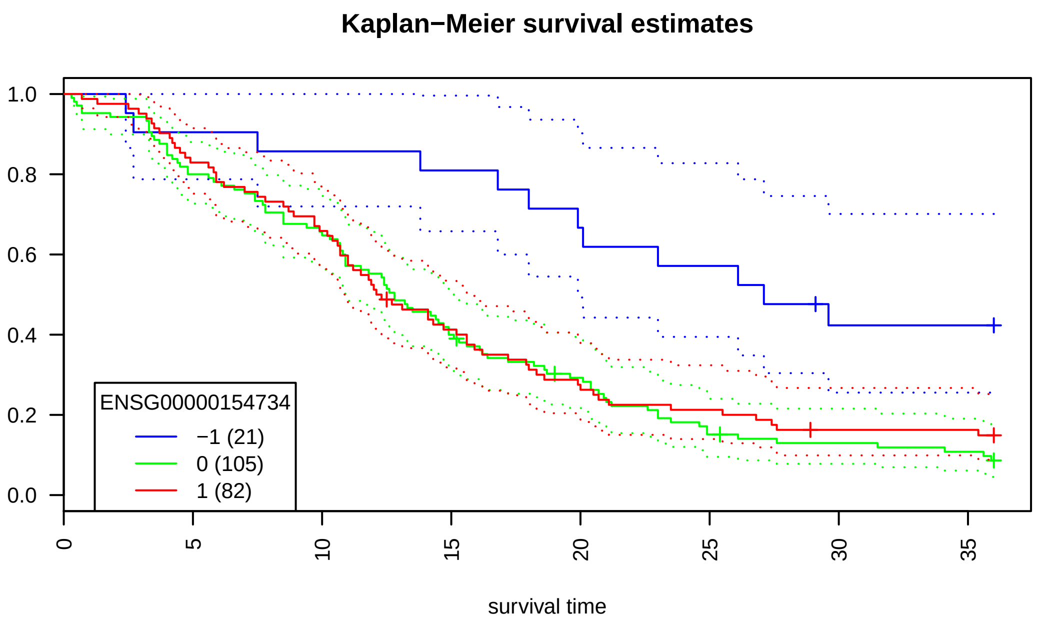

Figure 302: Each curve represents the relative number of ENSG00000154734 survived to the corresponding point of time. Dashed lines represent 0.95 confidence intervals for the curves with the corresponding color. Log-rank probability measure for the equality of these curves is 2.160889e-03.

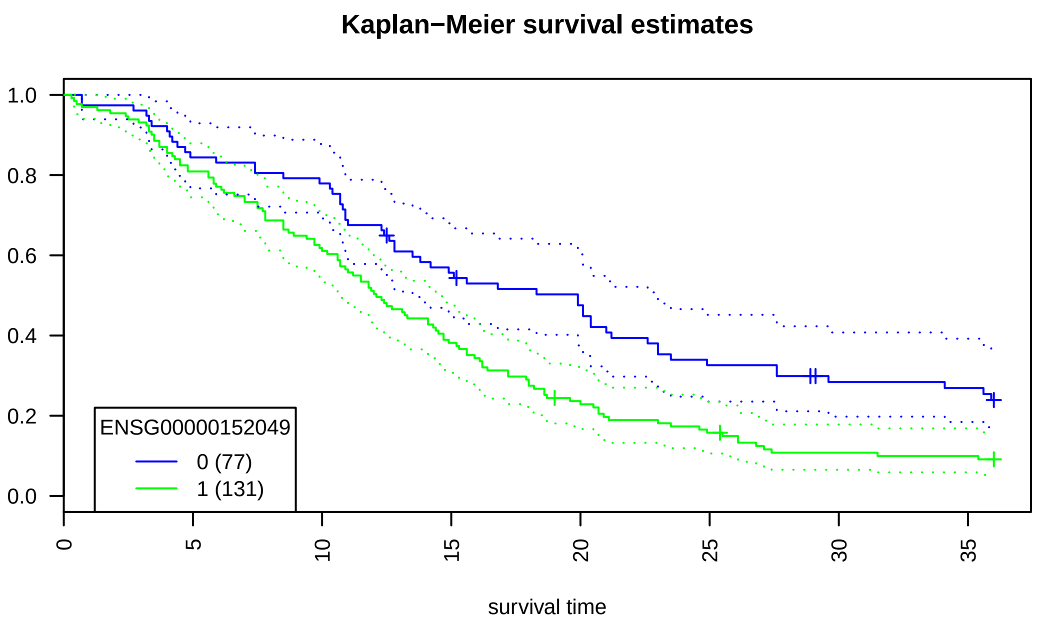

Figure 303: Each curve represents the relative number of ENSG00000152049 survived to the corresponding point of time. Dashed lines represent 0.95 confidence intervals for the curves with the corresponding color. Log-rank probability measure for the equality of these curves is 7.576815e-04. Additional annotations: *.GeneName*=KCNE4, *.GeneDesc*=potassium voltage-gated channel, Isk-related family, member 4 [Source:HGNC Symbol;Acc:6244], *Uniprot/SWISSPROT*=Q8WWG9.

4 Summary of differentially expressed transcripts: fc > 2.0 and corrected p-value < 0.05

Gene set	Size
over	3507
under	5380

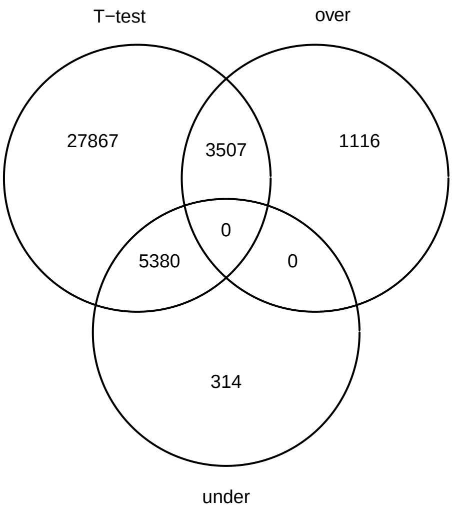

(a)

Figure 304: Intersections of sets: (a) T-test, over and under.

4.1 Ordered transcript expression values

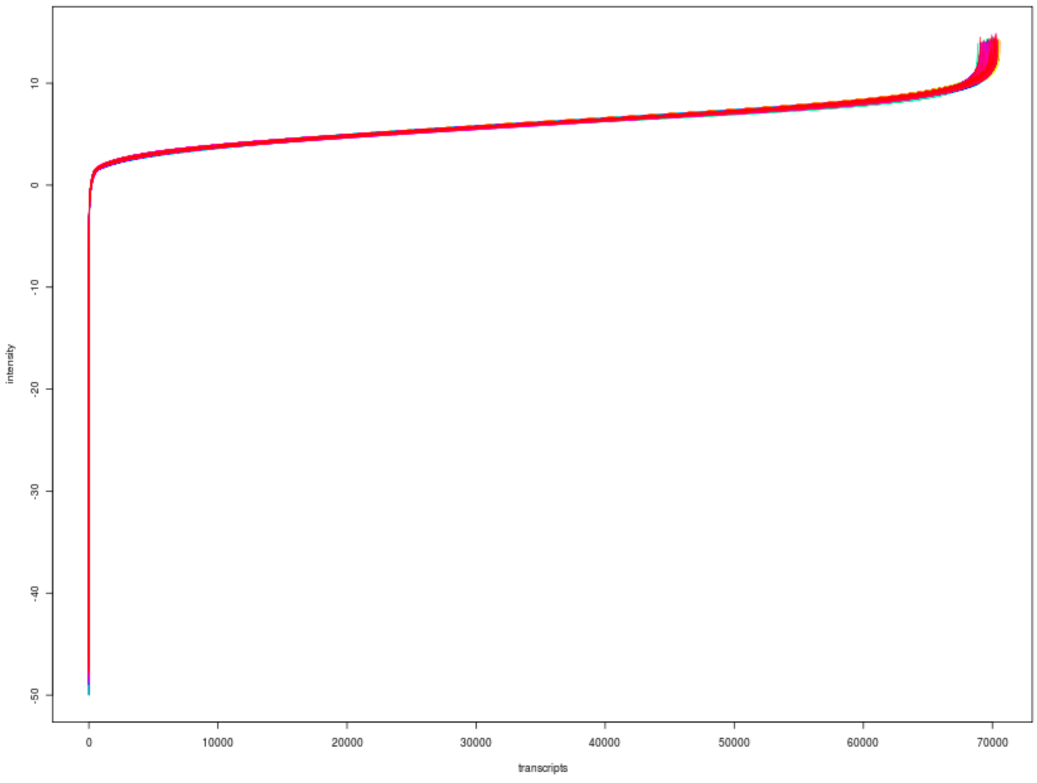

Figure 305: Distribution of transcript expression values (log2) for the samples.

4.2 Multidimensional scaling of the samples

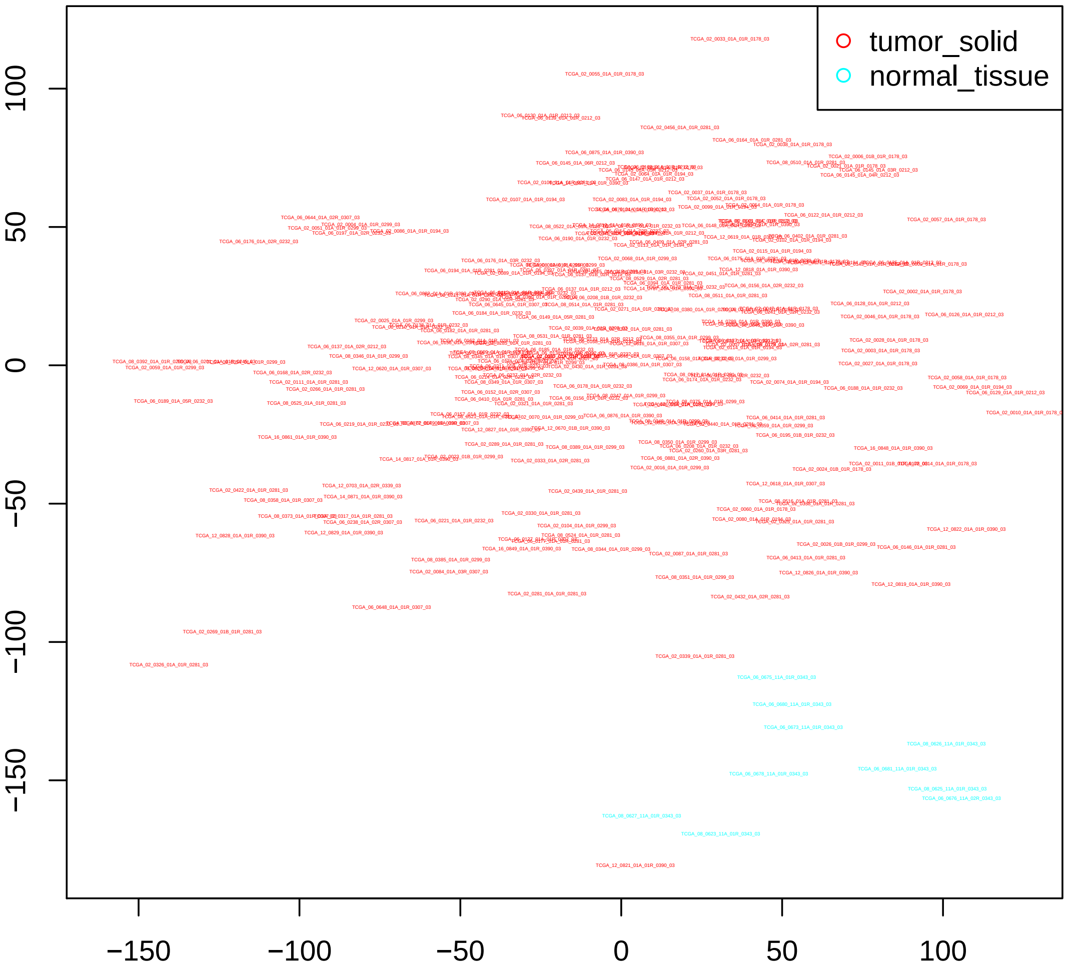

Figure 306: Multidimensional Scaling

4.3 GO clustering

Gene products are clustered (grouped) based on their Gene Ontology (GO) annotations. Genes that have similar annotations belong to the same cluster. The distance between gene products is defined using information-theoretical semantic similarity measures.

In the table below, the most informative (most specific) common GO terms of each cluster are displayed. All gene products in the cluster are annotated with the GO term mentioned. A GO term is more informative if it occurs rarely in the GO annotations of the whole genome. The priori column contains the a priori probability p that a random gene product is annotated with the given GO term. Information is defined as $-\log_2 p$.

Generally, clusters with (1) a large number of gene products and (2) common GO terms with high information content are the most interesting.

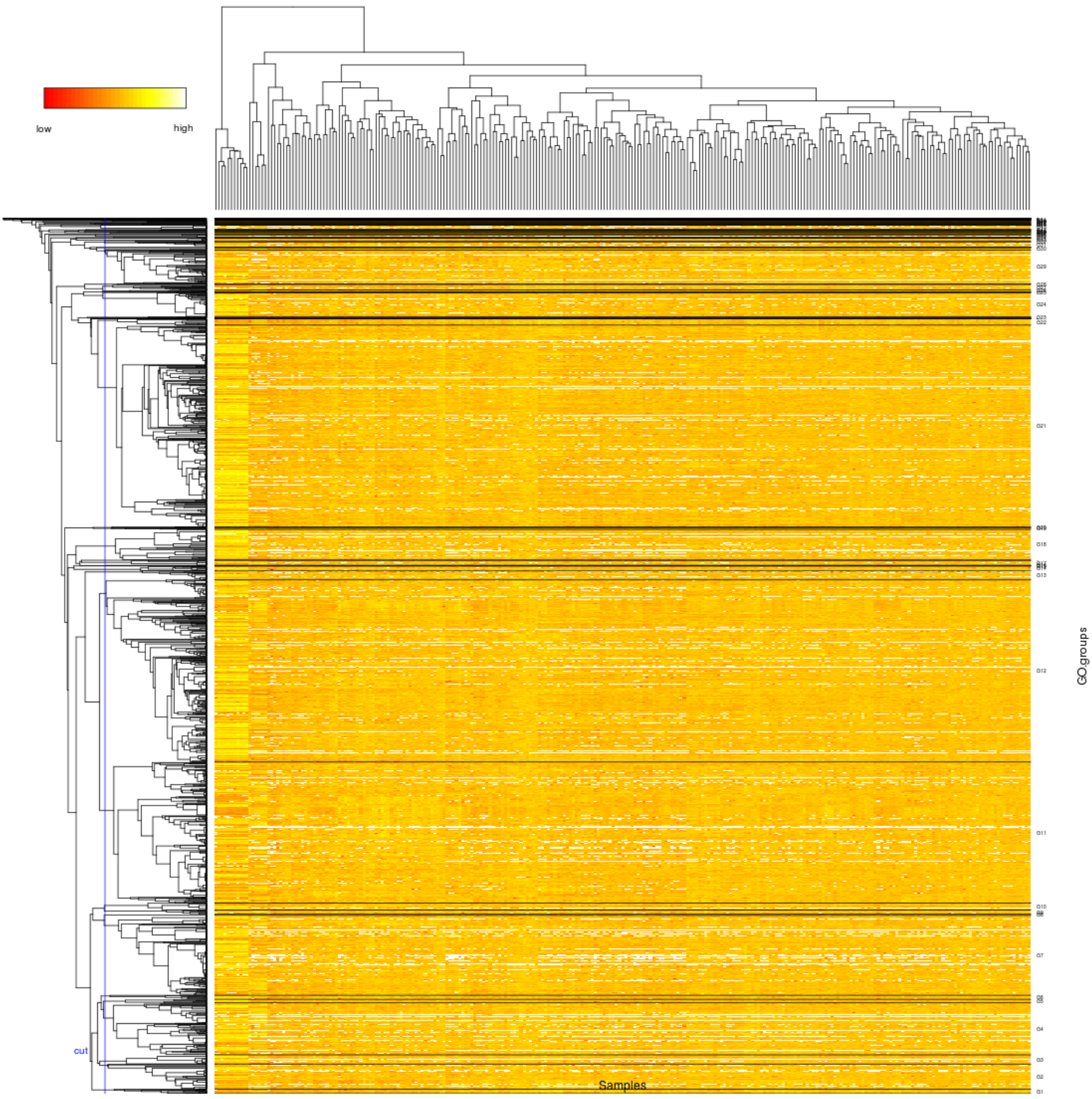

Figure 307: GO clustering dendrogram and expression heatmap. The analysis is based on 4529 gene products. The median number of GO annotations per gene product is 5. In addition, 4358 gene products had no GO annotation and were excluded from the analysis. The colors represent expression values of the gene products. The lower expression values are indicated with red and higher expressions with white. The left dendrogram corresponds to GO based clustering, while the top dendrogram uses expression values for clustering.

CLUSTER	SIZE	GOID	PRIORI	INFO	DESCRIPTION
G1	23	GO:0005975	0.015	6.053	carbohydrate metabolic process
G2	130				*[no interesting GO terms]*
G3	48	GO:0003824	0.228	2.13	catalytic activity
G4	269				*[no interesting GO terms]*
G5	18	GO:0016740	0.081	3.625	transferase activity
G6	21	GO:0005739	0.038	4.706	mitochondrion
G7	413				*[no interesting GO terms]*
G8	6	GO:0004143	0	11.646	diacylglycerol kinase activity
		GO:0007205	0.001	10.809	activation of protein kinase C activity by G-protein coupled receptor protein signaling pathway
G9	21	GO:0006457	0.003	8.342	protein folding
G10	36				*[no interesting GO terms]*
G11	730				*[no interesting GO terms]*
G12	942				*[no interesting GO terms]*
G13	46	GO:0008092	0.012	6.342	cytoskeletal protein binding
G14	25	GO:0005783	0.034	4.867	endoplasmic reticulum
G15	2	GO:0008021	0.002	9.137	synaptic vesicle
G16	2	GO:0033554	0.014	6.114	cellular response to stress
		GO:0050794	0.277	1.85	regulation of cellular process
		GO:0005737	0.295	1.763	cytoplasm
G17	26	GO:0005622	0.554	0.852	intracellular
G18	159				*[no interesting GO terms]*
G19	7	GO:0016830	0.001	10.092	carbon-carbon lyase activity
		GO:0044238	0.234	2.093	primary metabolic process
		GO:0044237	0.235	2.086	cellular metabolic process
G20	6	GO:0055086	0.011	6.458	nucleobase, nucleoside and nucleotide metabolic process
G21	1043				*[no interesting GO terms]*
G22	33	GO:0050896	0.127	2.982	response to stimulus
G23	7	GO:0044464	0.909	0.137	cell part
G24	122	GO:0005509	0.012	6.354	calcium ion binding
G25	5	GO:0010038	0.003	8.566	response to metal ion
G26	10				*[no interesting GO terms]*
G27	28				*[no interesting GO terms]*
G28	3	GO:0019842	0.003	8.486	vitamin binding
G29	175				*[no interesting GO terms]*
G30	12				*[no interesting GO terms]*
G31	26	GO:0005102	0.026	5.24	receptor binding
G32	7	GO:0007275	0.13	2.946	multicellular organismal development
G33	14	GO:0004867	0.002	9.092	serine-type endopeptidase inhibitor activity
G34	3	GO:0042254	0.003	8.462	ribosome biogenesis
G35	12	GO:0045454	0.001	9.852	cell redox homeostasis
G36	3	GO:0016853	0.005	7.698	isomerase activity
G37	8	GO:0031072	0.001	9.398	heat shock protein binding
G38	2	GO:0016746	0.007	7.127	transferase activity, transferring acyl groups
G39	4	GO:0044271	0.004	7.996	cellular nitrogen compound biosynthetic process
		GO:0016740	0.081	3.625	transferase activity
G40	4	GO:0003950	0	11.187	NAD+ ADP-ribosyltransferase activity
G41	4	GO:0001727	0.001	10.398	lipid kinase activity
		GO:0004428	0.001	9.646	inositol or phosphatidylinositol kinase activity
		GO:0016773	0.022	5.489	phosphotransferase activity, alcohol group as acceptor
G42	4	GO:0004359	0	14.646	glutaminase activity
		GO:0006541	0	11.727	glutamine metabolic process
G43	20				*[no interesting GO terms]*
G44	2	GO:0030704	0	15.897	vitelline membrane formation
		GO:0008316	0	15.646	structural constituent of vitelline membrane
G45	2	GO:0008537	0	14.011	proteasome activator complex
		GO:0008538	0	13.324	proteasome activator activity
G46	4	GO:0006810	0.096	3.38	transport
G47	5	GO:0000145	0	12.426	exocyst
		GO:0006904	0	11.253	vesicle docking during exocytosis
G48	3	GO:0006357	0.015	6.089	regulation of transcription from RNA polymerase II promoter
		GO:0030528	0.044	4.514	transcription regulator activity

G49	3	GO:0007217	0	12.897	tachykinin receptor signaling pathway
G50	4	GO:0007417	0.011	6.464	central nervous system development
G51	4	GO:0016667	0.001	10.031	oxidoreductase activity, acting on sulfur group of donors

5 Survival analysis for transcript expression

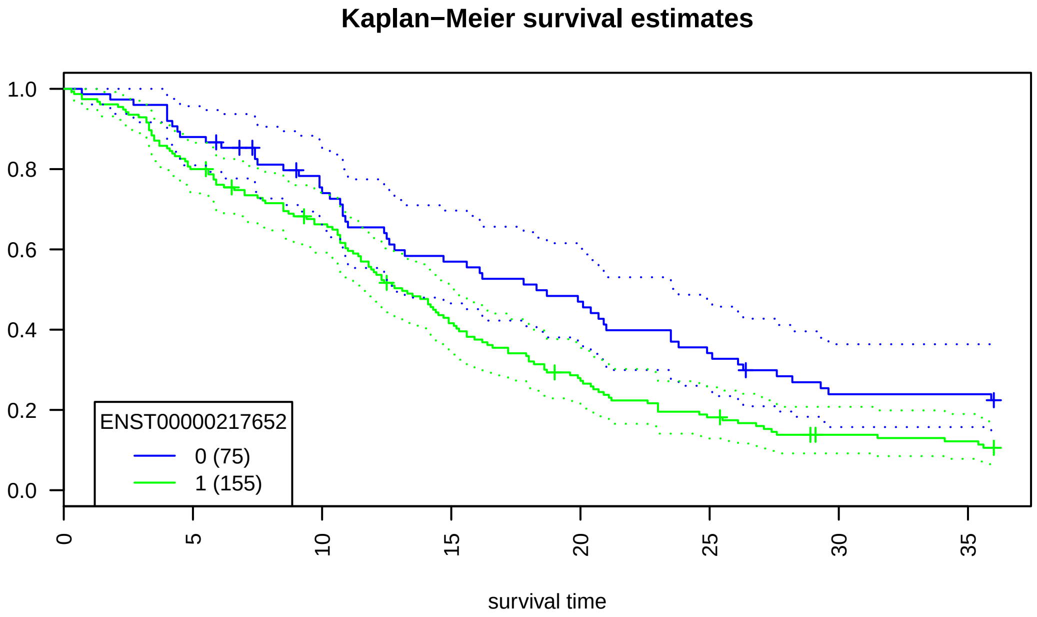

Figure 308: Each curve represents the relative number of ENST00000217652 survived to the corresponding point of time. Dashed lines represent 0.95 confidence intervals for the curves with the corresponding color. Log-rank probability measure for the equality of these curves is 6.588337e-03. Additional annotations: *.GeneId*=ENSG00000101608, *.GeneName*=MYL12A, *.GeneDesc*=myosin, light chain 12A, regulatory, non-sarcomeric [Source:HGNC Symbol;Acc:16701].

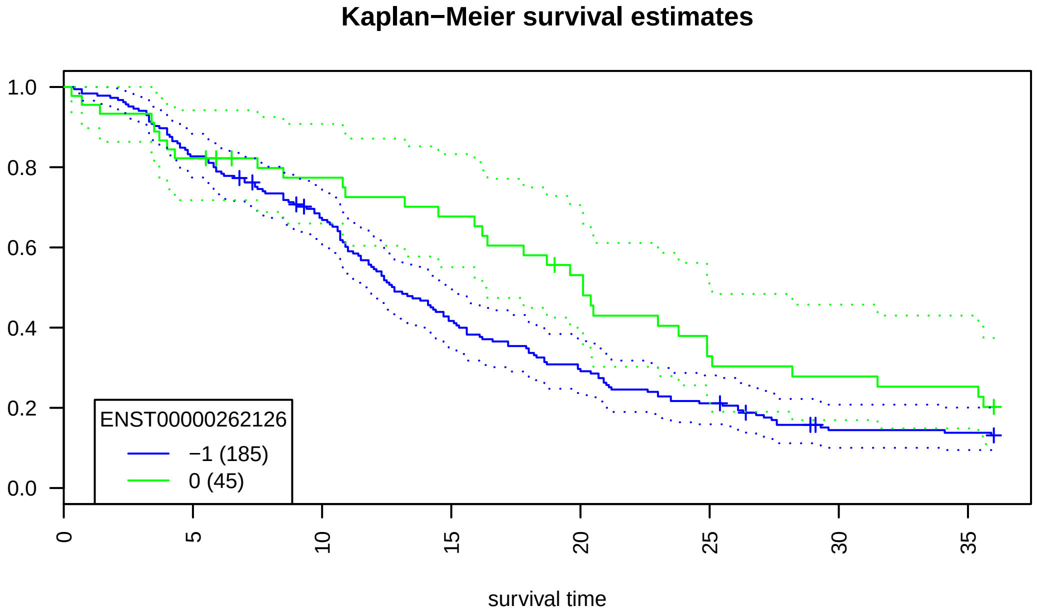

Figure 309: Each curve represents the relative number of ENST00000262126 survived to the corresponding point of time. Dashed lines represent 0.95 confidence intervals for the curves with the corresponding color. Log-rank probability measure for the equality of these curves is 4.076213e-02. Additional annotations: *.GeneId*=ENSG00000101745, *.GeneName*=ANKRD12, *.GeneDesc*=ankyrin repeat domain 12 [Source:HGNC Symbol;Acc:29135].

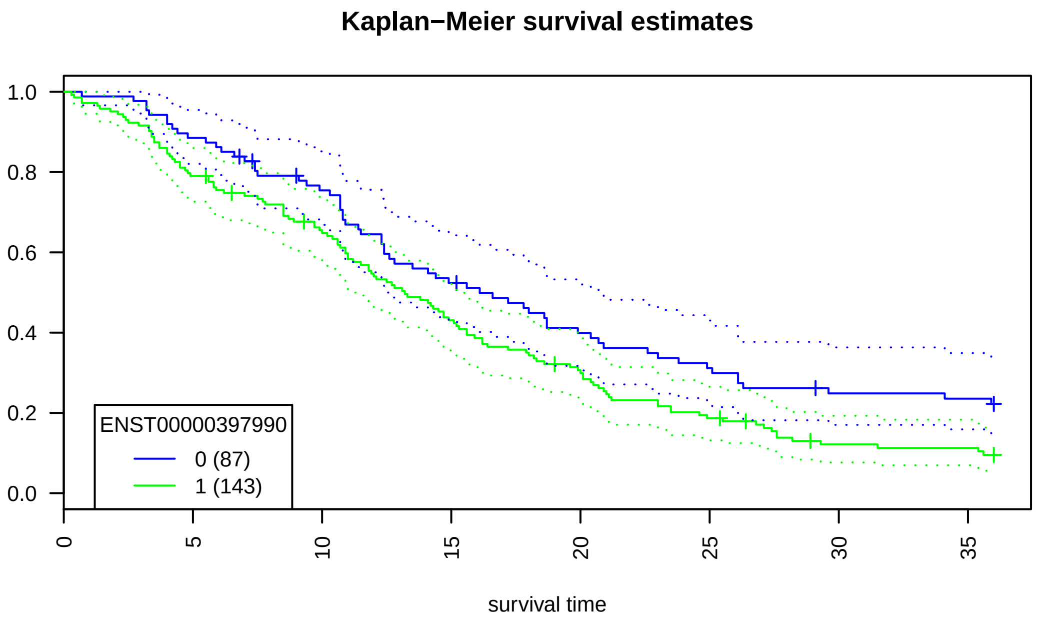

Figure 310: Each curve represents the relative number of ENST00000397990 survived to the corresponding point of time. Dashed lines represent 0.95 confidence intervals for the curves with the corresponding color. Log-rank probability measure for the equality of these curves is 1.428984e-02. Additional annotations: *.GeneId*=ENSG00000214274, *.GeneName*=ANG, *.GeneDesc*=angiogenin, ribonuclease, RNase A family, 5 [Source:HGNC Symbol;Acc:483].

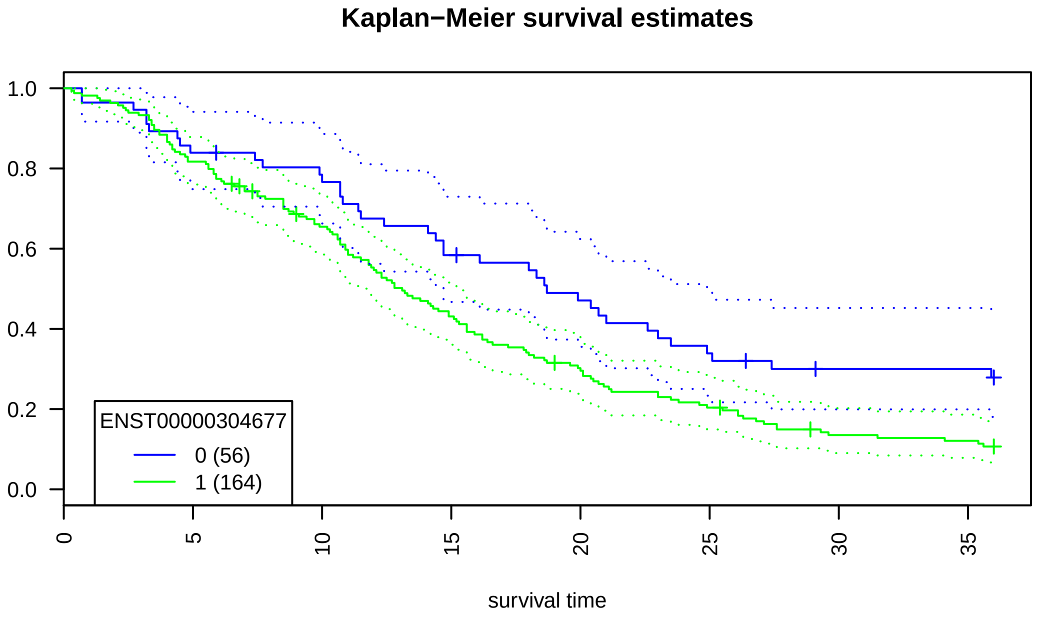

Figure 311: Each curve represents the relative number of ENST00000304677 survived to the corresponding point of time. Dashed lines represent 0.95 confidence intervals for the curves with the corresponding color. Total of 10 observations deleted due to missingness. Log-rank probability measure for the equality of these curves is 5.689881e-03. Additional annotations: *.GeneId*=ENSG00000169413, *.GeneName*=RNASE6, *.GeneDesc*=ribonuclease, RNase A family, k6 [Source:HGNC Symbol;Acc:10048].

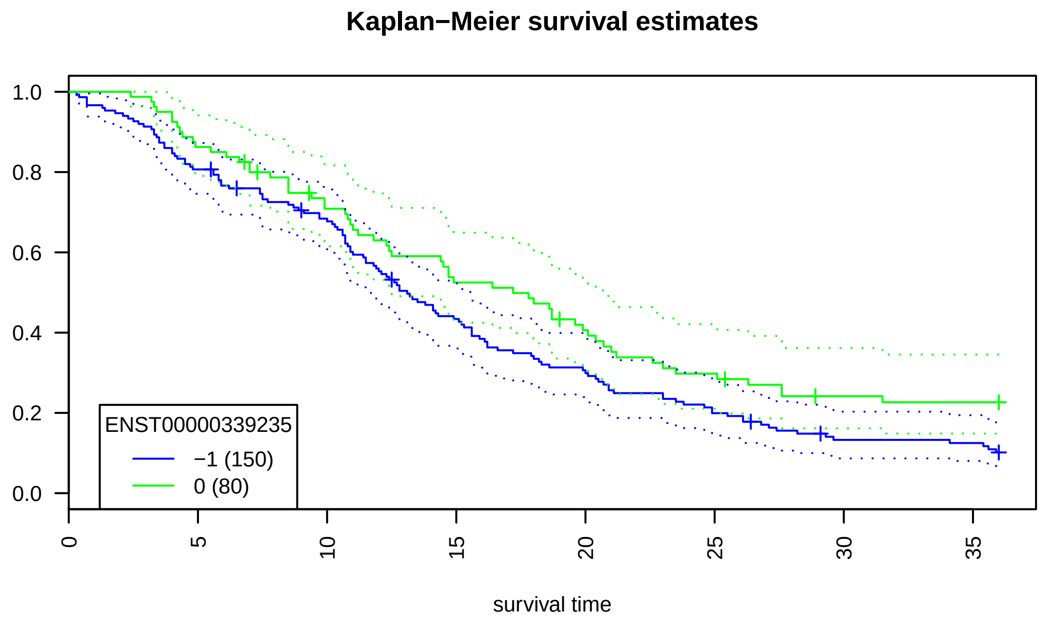

Figure 312: Each curve represents the relative number of ENST00000339235 survived to the corresponding point of time. Dashed lines represent 0.95 confidence intervals for the curves with the corresponding color. Log-rank probability measure for the equality of these curves is 2.685937e-02. Additional annotations: *.GeneId*=ENSG00000171823, *.GeneName*=FBXL14, *.GeneDesc*=F-box and leucine-rich repeat protein 14 [Source:HGNC Symbol;Acc:28624].

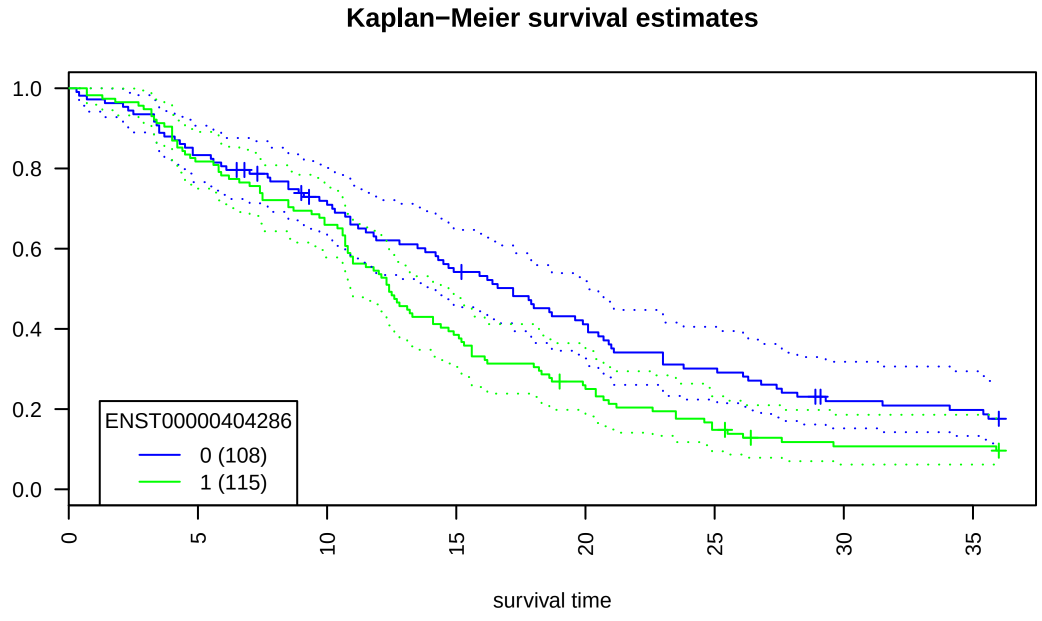

Figure 313: Each curve represents the relative number of ENST00000404286 survived to the corresponding point of time. Dashed lines represent 0.95 confidence intervals for the curves with the corresponding color. Total of 7 observations deleted due to missingness. Log-rank probability measure for the equality of these curves is 1.602200e-02. Additional annotations: *.GeneId*=ENSG00000169026, *.GeneName*=MFSD7, *.GeneDesc*=major facilitator superfamily domain containing 7 [Source:HGNC Symbol;Acc:26177].

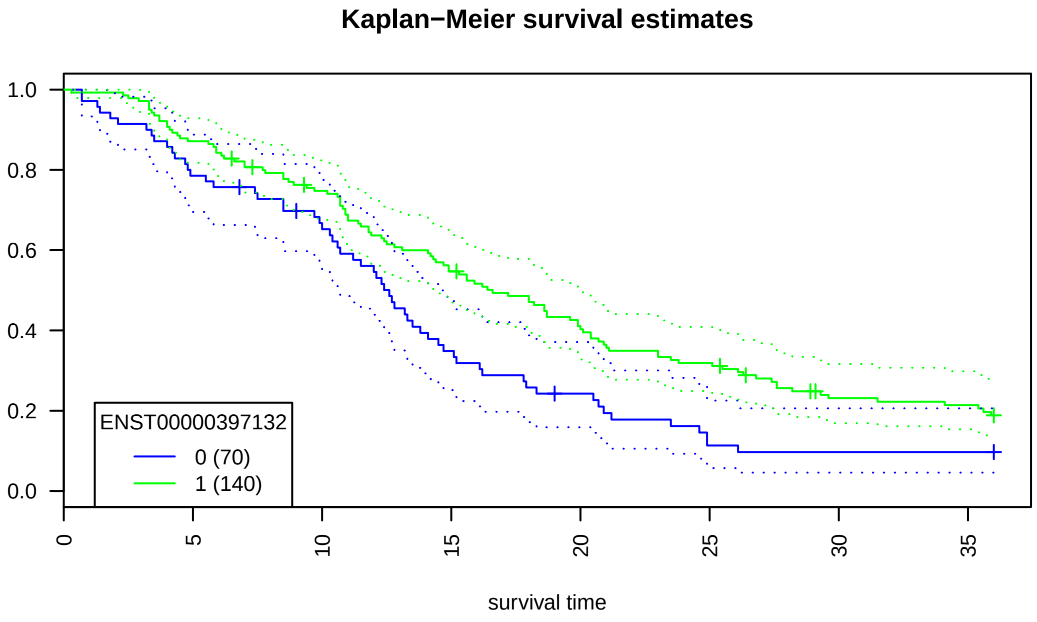

Figure 314: Each curve represents the relative number of ENST00000397132 survived to the corresponding point of time. Dashed lines represent 0.95 confidence intervals for the curves with the corresponding color. Total of 20 observations deleted due to missingness. Log-rank probability measure for the equality of these curves is 4.817129e-03. Additional annotations: *.GeneId*=ENSG00000078246, *.GeneName*=TULP3, *.GeneDesc*=tubby like protein 3 [Source:HGNC Symbol;Acc:12425].

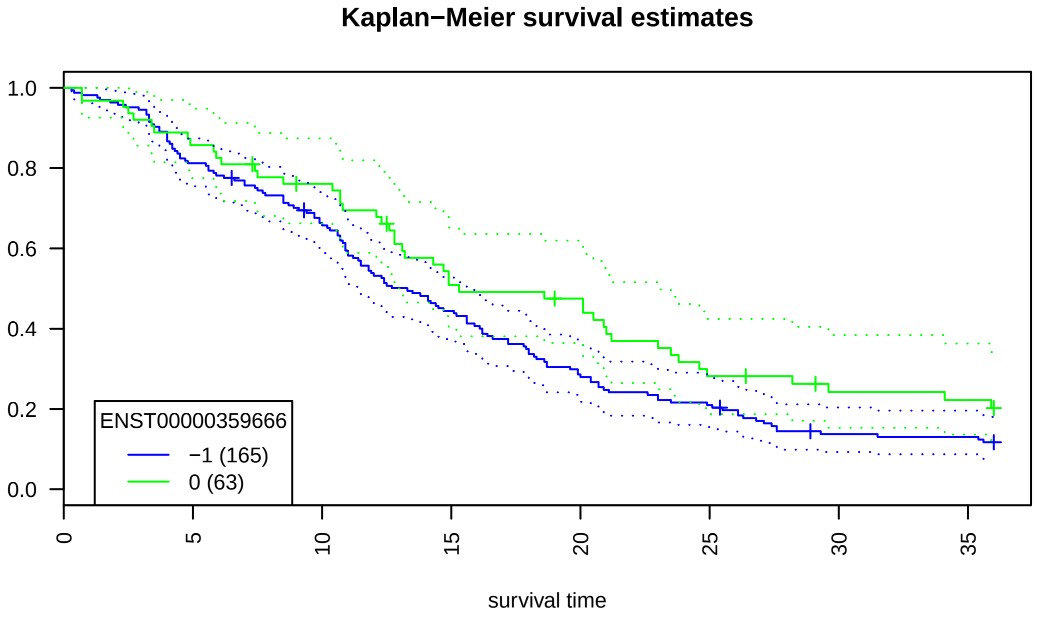

Figure 315: Each curve represents the relative number of ENST00000359666 survived to the corresponding point of time. Dashed lines represent 0.95 confidence intervals for the curves with the corresponding color. Total of 2 observations deleted due to missingness. Log-rank probability measure for the equality of these curves is 4.901269e-02. Additional annotations: *.GeneId*=ENSG00000104723, *.GeneName*=TUSC3, *.GeneDesc*=tumor suppressor candidate 3 [Source:HGNC Symbol;Acc:30242].

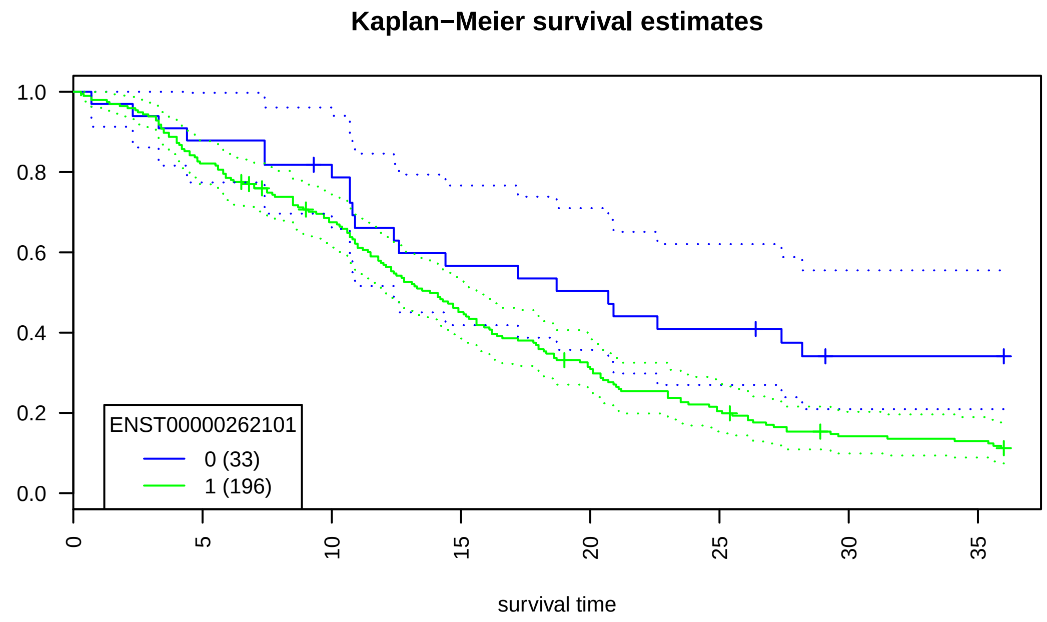

Figure 316: Each curve represents the relative number of ENST00000262101 survived to the corresponding point of time. Dashed lines represent 0.95 confidence intervals for the curves with the corresponding color. Total of 1 observation deleted due to missingness. Log-rank probability measure for the equality of these curves is 1.027462e-02. Additional annotations: *.GeneId*=ENSG00000038945, *.GeneName*=MSR1, *.GeneDesc*=macrophage scavenger receptor 1 [Source:HGNC Symbol;Acc:7376].

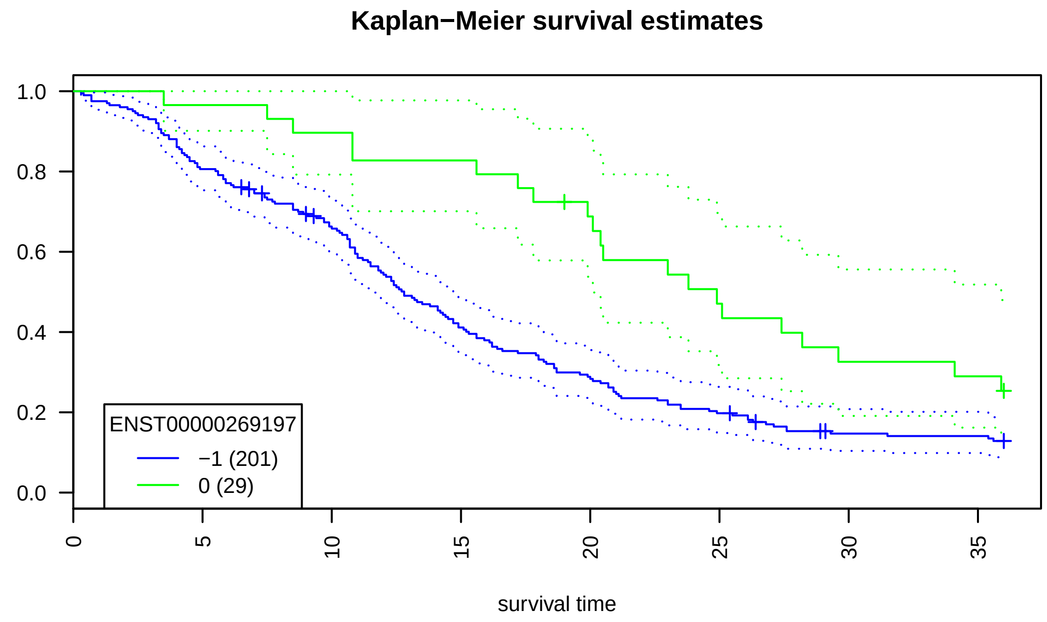

Figure 317: Each curve represents the relative number of ENST00000269197 survived to the corresponding point of time. Dashed lines represent 0.95 confidence intervals for the curves with the corresponding color. Log-rank probability measure for the equality of these curves is 2.257697e-03. Additional annotations: *.GeneId*=ENSG00000141431, *.GeneName*=ASXL3, *.GeneDesc*=additional sex combs like 3 (Drosophila) [Source:HGNC Symbol;Acc:29357].

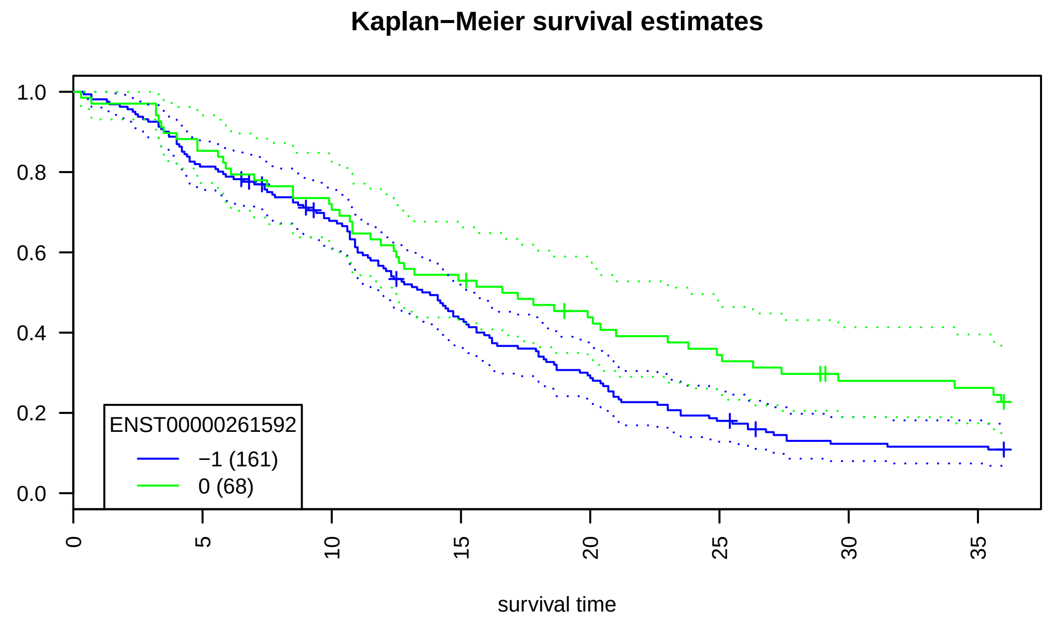

Figure 318: Each curve represents the relative number of ENST00000261592 survived to the corresponding point of time. Dashed lines represent 0.95 confidence intervals for the curves with the corresponding color. Total of 1 observation deleted due to missingness. Log-rank probability measure for the equality of these curves is 2.271807e-02. Additional annotations: *.GeneId*=ENSG00000101746, *.GeneName*=NOL4, *.GeneDesc*=nucleolar protein 4 [Source:HGNC Symbol;Acc:7870].

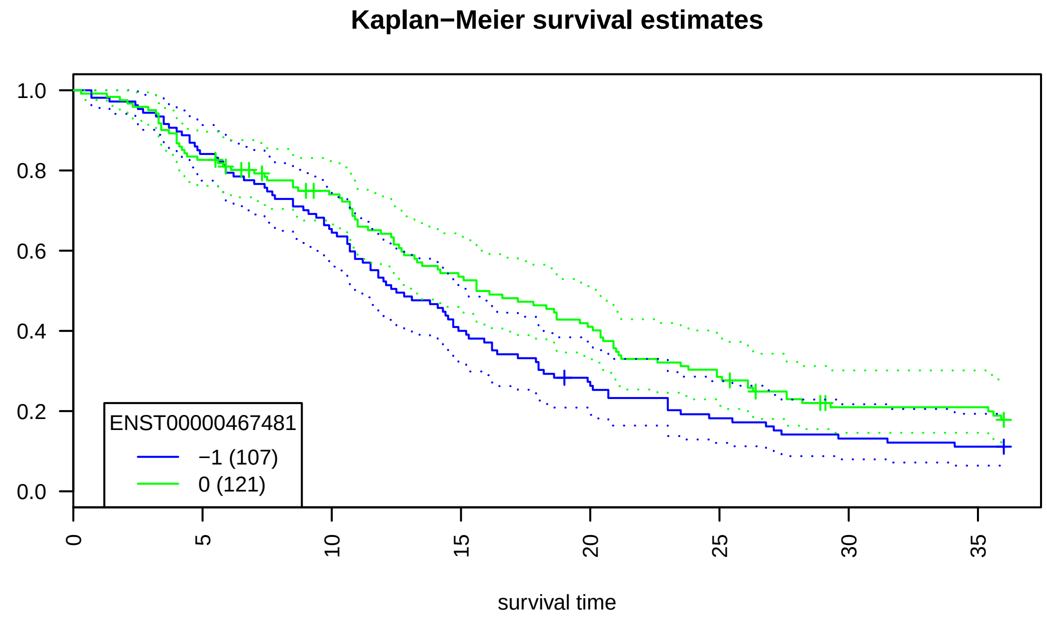

Figure 319: Each curve represents the relative number of ENST00000467481 survived to the corresponding point of time. Dashed lines represent 0.95 confidence intervals for the curves with the corresponding color. Total of 2 observations deleted due to missingness. Log-rank probability measure for the equality of these curves is 4.950403e-02. Additional annotations: *.GeneId*=ENSG00000196199, *.GeneName*=MPHOSPH8, *.GeneDesc*=M-phase phosphoprotein 8 [Source:HGNC Symbol;Acc:29810].

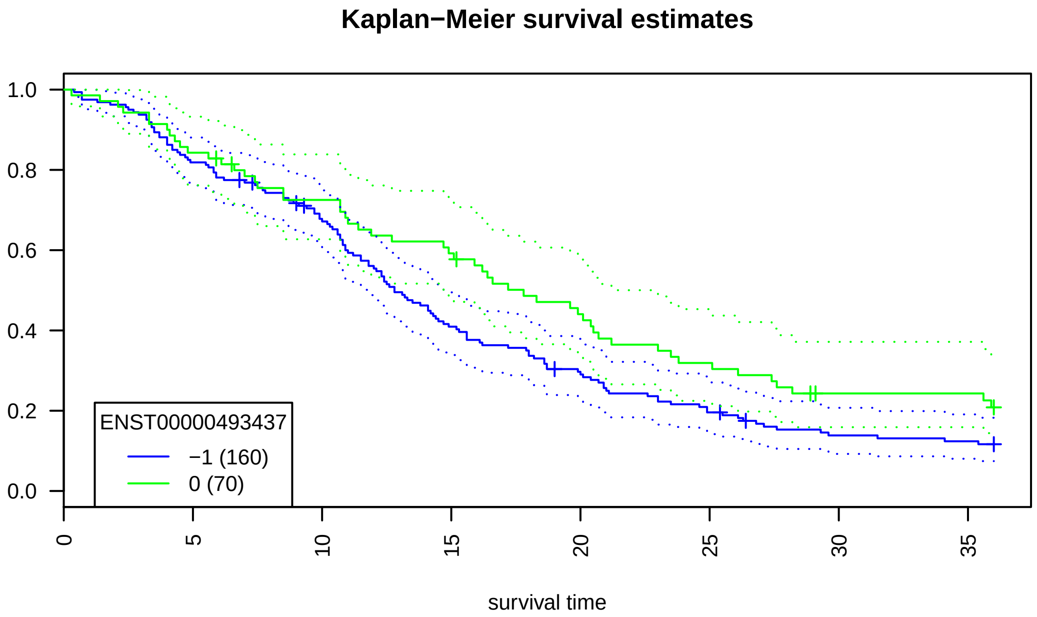

Figure 320: Each curve represents the relative number of ENST00000493437 survived to the corresponding point of time. Dashed lines represent 0.95 confidence intervals for the curves with the corresponding color. Log-rank probability measure for the equality of these curves is 3.703613e-02. Additional annotations: *.GeneId*=ENSG00000196199, *.GeneName*=MPHOSPH8, *.GeneDesc*=M-phase phosphoprotein 8 [Source:HGNC Symbol;Acc:29810].

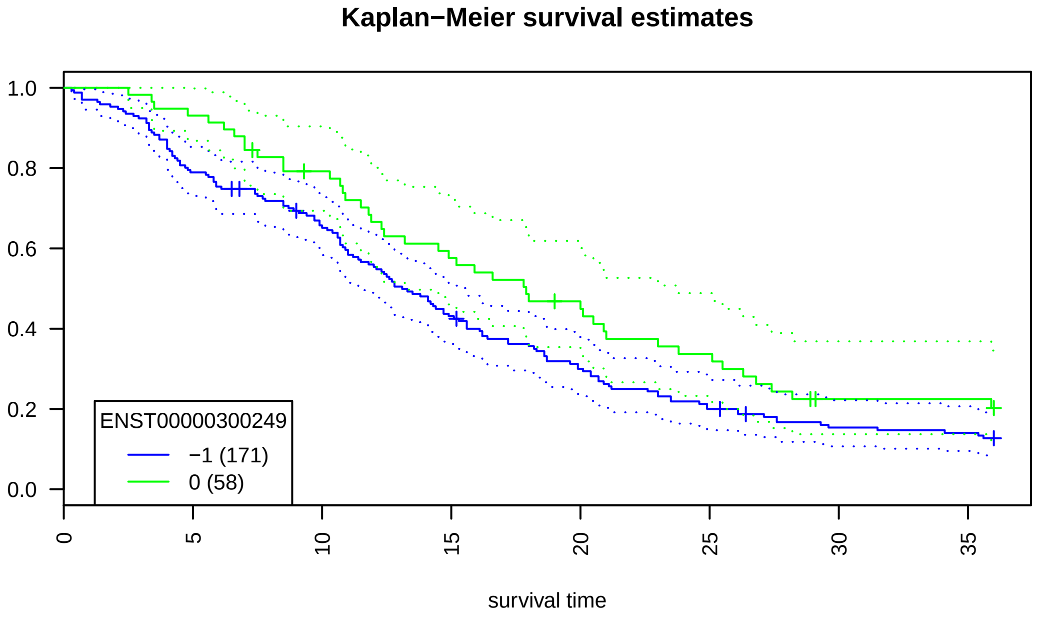

Figure 321: Each curve represents the relative number of ENST00000300249 survived to the corresponding point of time. Dashed lines represent 0.95 confidence intervals for the curves with the corresponding color. Total of 1 observation deleted due to missingness. Log-rank probability measure for the equality of these curves is 4.91182e-02. Additional annotations: *.GeneId*=ENSG00000166974, *.GeneName*=MAPRE2, *.GeneDesc*=microtubule-associated protein, RP/EB family, member 2 [Source:HGNC Symbol;Acc:6891].

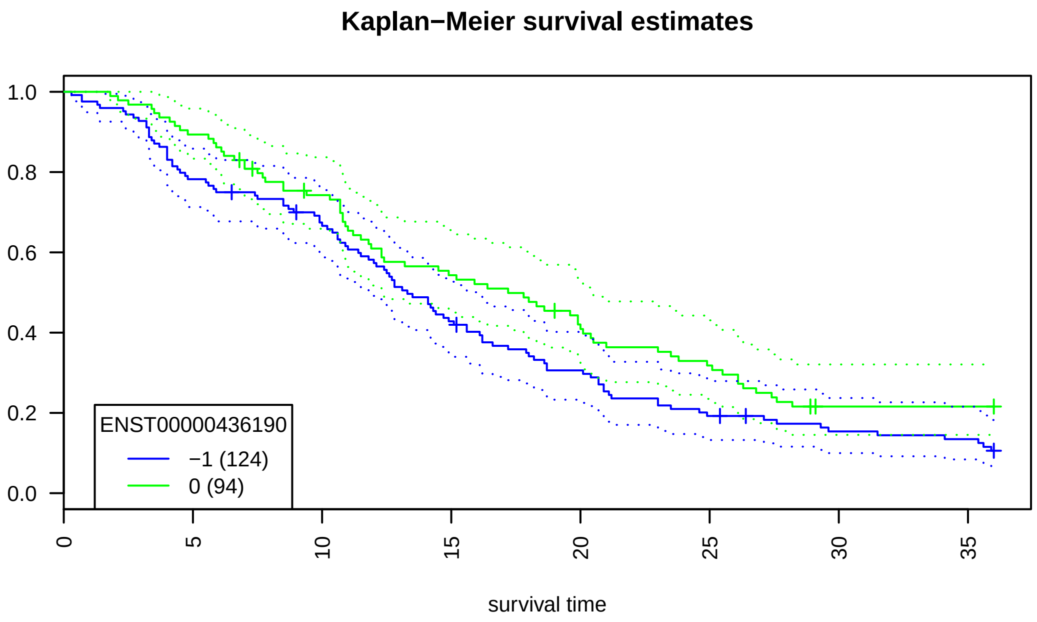

Figure 322: Each curve represents the relative number of ENST00000436190 survived to the corresponding point of time. Dashed lines represent 0.95 confidence intervals for the curves with the corresponding color. Total of 12 observations deleted due to missingness. Log-rank probability measure for the equality of these curves is 3.548902e-02. Additional annotations: *.GeneId*=ENSG00000166974, *.GeneName*=MAPRE2, *.GeneDesc*=microtubule-associated protein, RP/EB family, member 2 [Source:HGNC Symbol;Acc:6891].

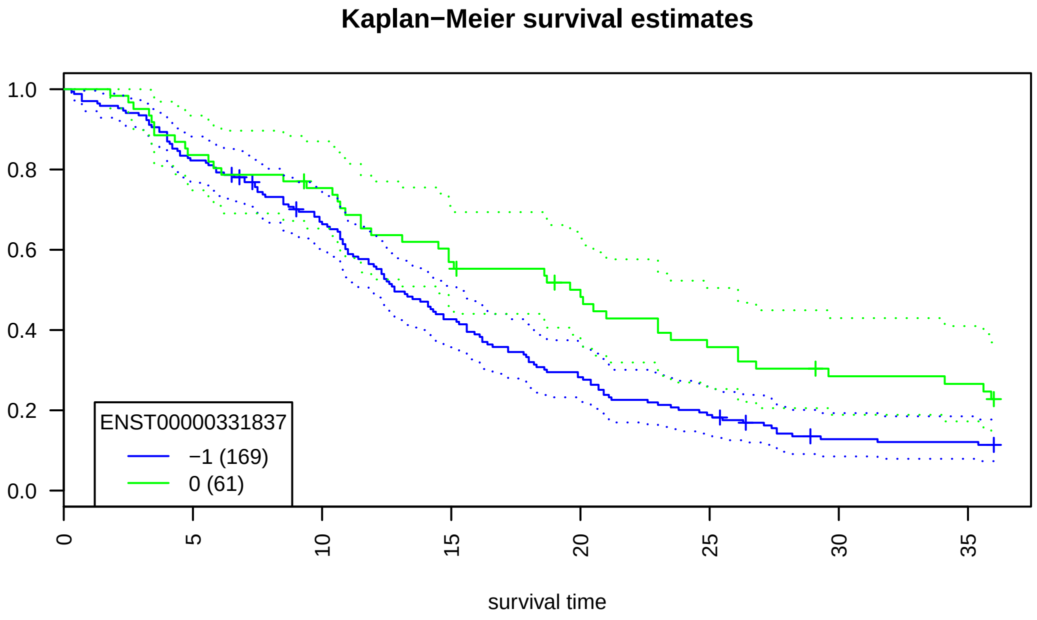

Figure 323: Each curve represents the relative number of ENST00000331837 survived to the corresponding point of time. Dashed lines represent 0.95 confidence intervals for the curves with the corresponding color. Log-rank probability measure for the equality of these curves is 1.10241e-02. Additional annotations: *.GeneId*=ENSG00000182636, *.GeneName*=NDN, *.GeneDesc*=necdin homolog (mouse) [Source:HGNC Symbol;Acc:7675].

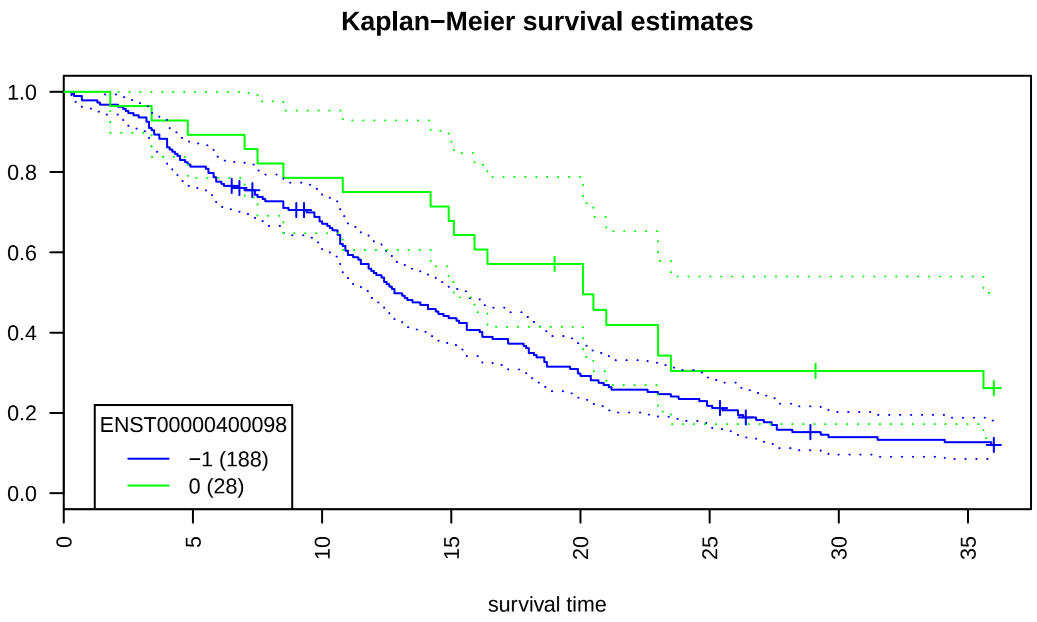

Figure 324: Each curve represents the relative number of ENST00000400098 survived to the corresponding point of time. Dashed lines represent 0.95 confidence intervals for the curves with the corresponding color. Total of 14 observations deleted due to missingness. Log-rank probability measure for the equality of these curves is 3.316628e-02. Additional annotations: *.GeneId*=ENSG00000128739, *.GeneName*=SNRPN, *.GeneDesc*=small nuclear ribonucleoprotein polypeptide N [Source:HGNC Symbol;Acc:11164].

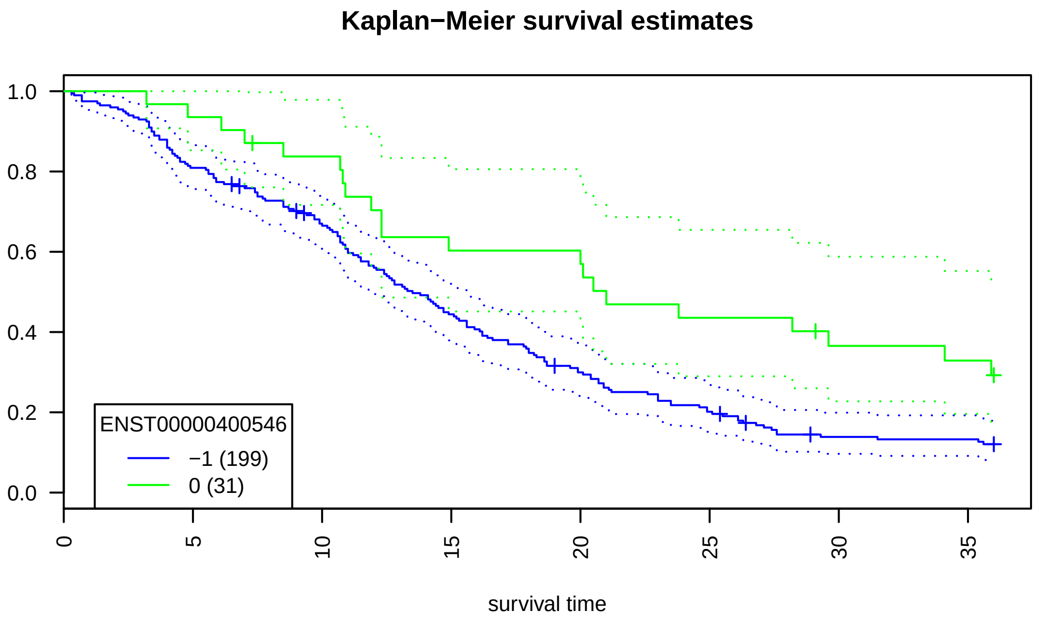

Figure 325: Each curve represents the relative number of ENST00000400546 survived to the corresponding point of time. Dashed lines represent 0.95 confidence intervals for the curves with the corresponding color. Log-rank probability measure for the equality of these curves is 7.142827e-03. Additional annotations: *.GeneId*=ENSG00000154654, *.GeneName*=NCAM2, *.GeneDesc*=neural cell adhesion molecule 2 [Source:HGNC Symbol;Acc:7657].

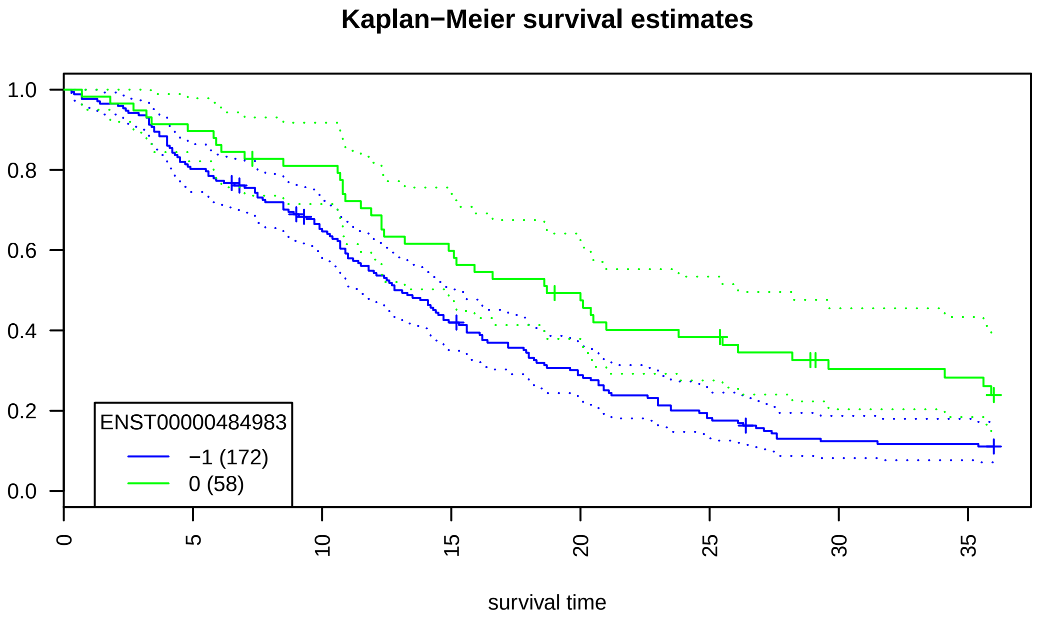

Figure 326: Each curve represents the relative number of ENST00000484983 survived to the corresponding point of time. Dashed lines represent 0.95 confidence intervals for the curves with the corresponding color. Log-rank probability measure for the equality of these curves is 5.067612e-03. Additional annotations: *.GeneId*=ENSG00000154654, *.GeneName*=NCAM2, *.GeneDesc*=neural cell adhesion molecule 2 [Source:HGNC Symbol;Acc:7657].

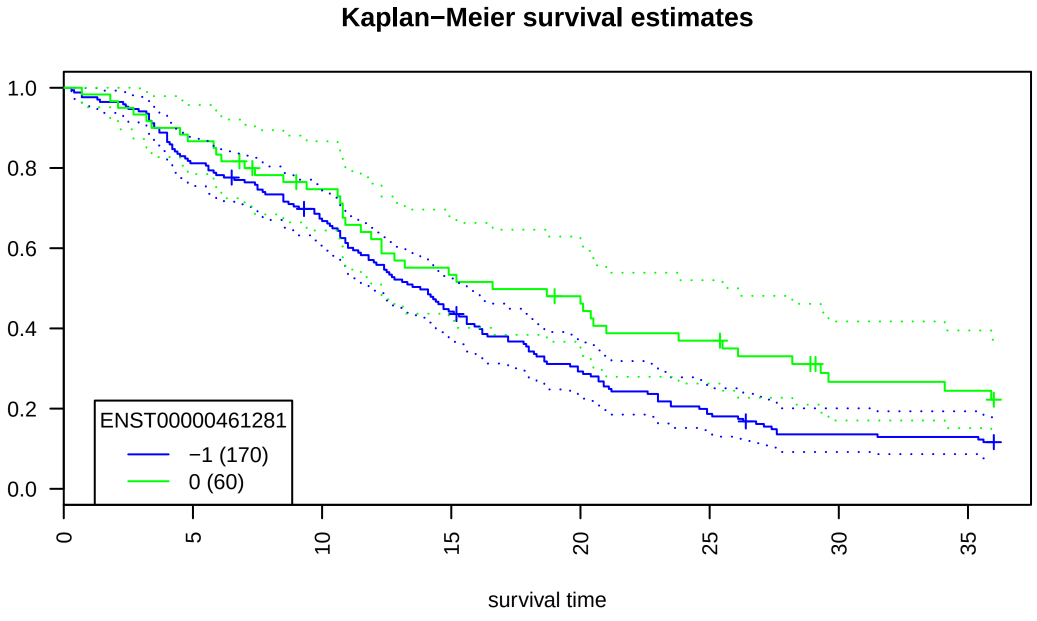

Figure 327: Each curve represents the relative number of ENST00000461281 survived to the corresponding point of time. Dashed lines represent 0.95 confidence intervals for the curves with the corresponding color. Log-rank probability measure for the equality of these curves is 3.071453e-02. Additional annotations: *.GeneId*=ENSG00000154654, *.GeneName*=NCAM2, *.GeneDesc*=neural cell adhesion molecule 2 [Source:HGNC Symbol;Acc:7657].

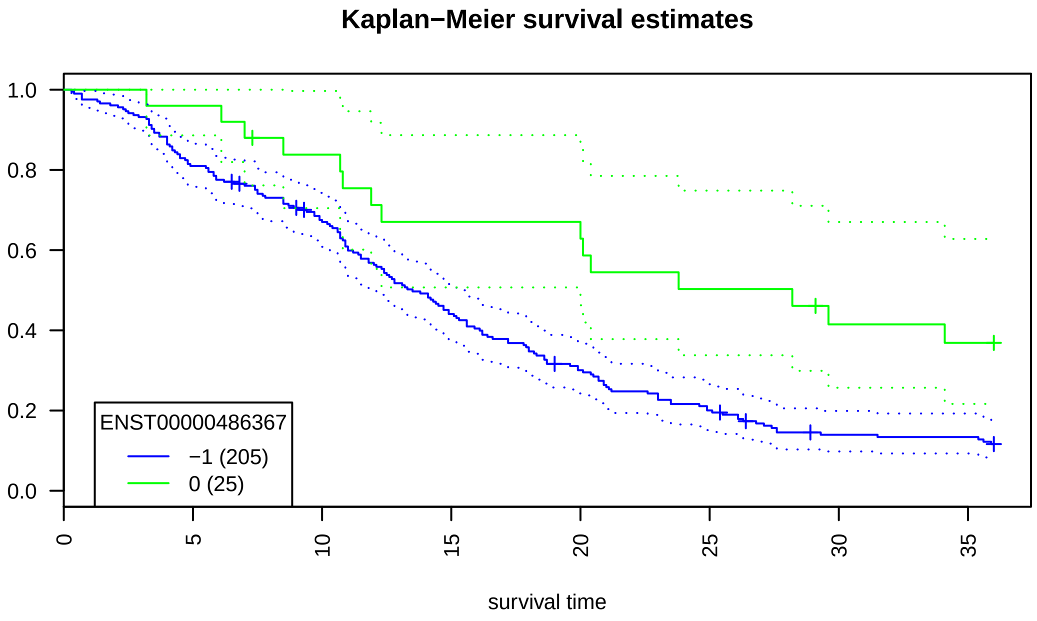

Figure 328: Each curve represents the relative number of ENST00000486367 survived to the corresponding point of time. Dashed lines represent 0.95 confidence intervals for the curves with the corresponding color. Log-rank probability measure for the equality of these curves is 2.351626e-03. Additional annotations: *.GeneId*=ENSG00000154654, *.GeneName*=NCAM2, *.GeneDesc*=neural cell adhesion molecule 2 [Source:HGNC Symbol;Acc:7657].

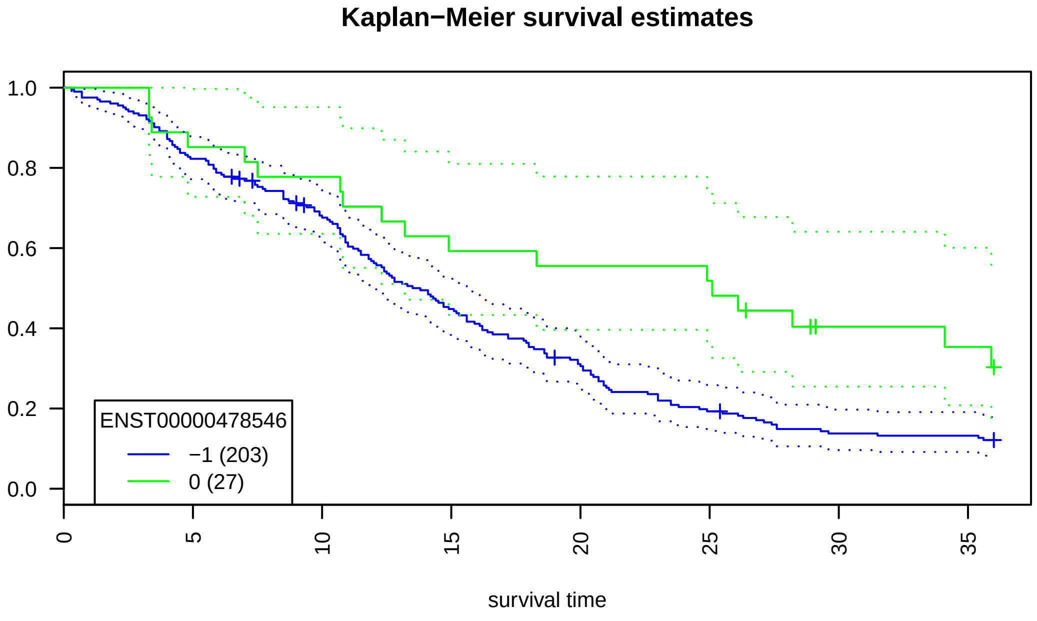

Figure 329: Each curve represents the relative number of ENST00000478546 survived to the corresponding point of time. Dashed lines represent 0.95 confidence intervals for the curves with the corresponding color. Log-rank probability measure for the equality of these curves is 9.300007e-03. Additional annotations: *.GeneId*=ENSG00000102678, *.GeneName*=FGF9, *.GeneDesc*=fibroblast growth factor 9 (glia-activating factor) [Source:HGNC Symbol;Acc:3687].

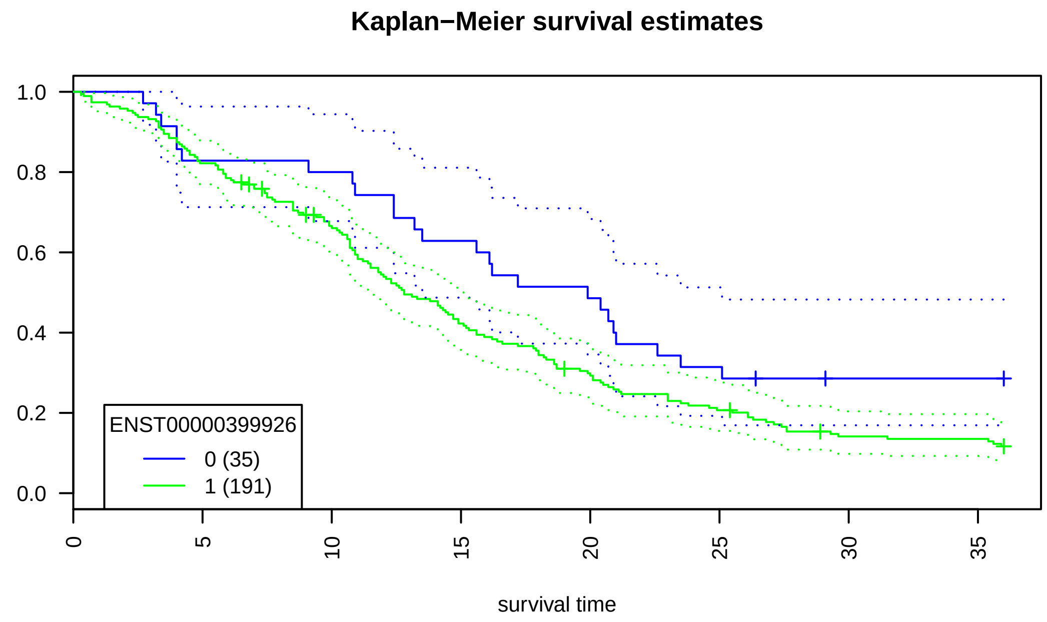

Figure 330: Each curve represents the relative number of ENST00000399926 survived to the corresponding point of time. Dashed lines represent 0.95 confidence intervals for the curves with the corresponding color. Total of 4 observations deleted due to missingness. Log-rank probability measure for the equality of these curves is 2.644754e-02. Additional annotations: *.GeneId*=ENSG00000156265, *.GeneName*=C21orf7, *.GeneDesc*=TAK1-like protein [Source:UniProtKB/Swiss-Prot;Acc:P57077].

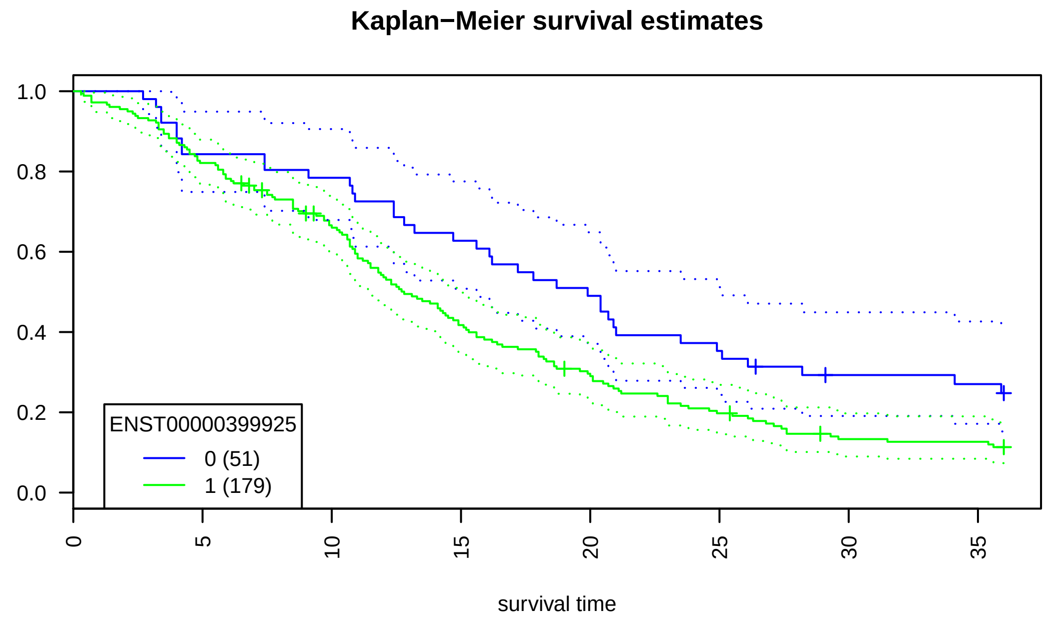

Figure 331: Each curve represents the relative number of ENST00000399925 survived to the corresponding point of time. Dashed lines represent 0.95 confidence intervals for the curves with the corresponding color. Log-rank probability measure for the equality of these curves is 8.694838e-03. Additional annotations: *.GeneId*=ENSG00000156265, *.GeneName*=C21orf7, *.GeneDesc*=TAK1-like protein [Source:UniProtKB/Swiss-Prot;Acc:P57077].

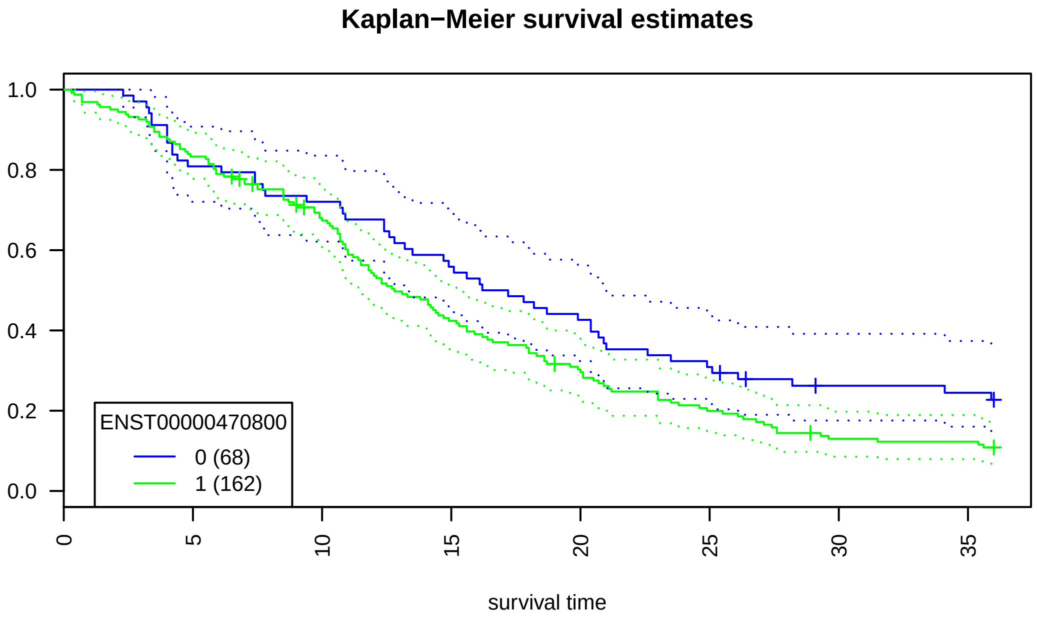

Figure 332: Each curve represents the relative number of ENST00000470800 survived to the corresponding point of time. Dashed lines represent 0.95 confidence intervals for the curves with the corresponding color. Log-rank probability measure for the equality of these curves is 3.249679e-02. Additional annotations: *.GeneId*=ENSG00000156265, *.GeneName*=C21orf7, *.GeneDesc*=TAK1-like protein [Source:UniProtKB/Swiss-Prot;Acc:P57077].

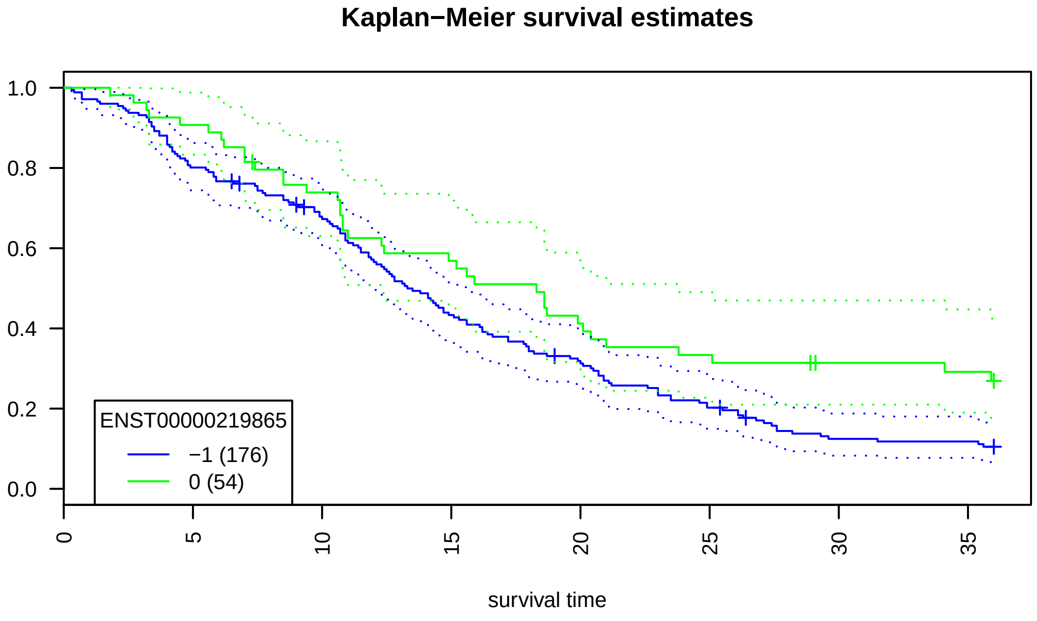

Figure 333: Each curve represents the relative number of ENST00000219865 survived to the corresponding point of time. Dashed lines represent 0.95 confidence intervals for the curves with the corresponding color. Log-rank probability measure for the equality of these curves is 1.851717e-02. Additional annotations: *.GeneId*=ENSG00000034053, *.GeneName*=APBA2, *.GeneDesc*=amyloid beta (A4) precursor protein-binding, family A, member 2 [Source:HGNC Symbol;Acc:579].

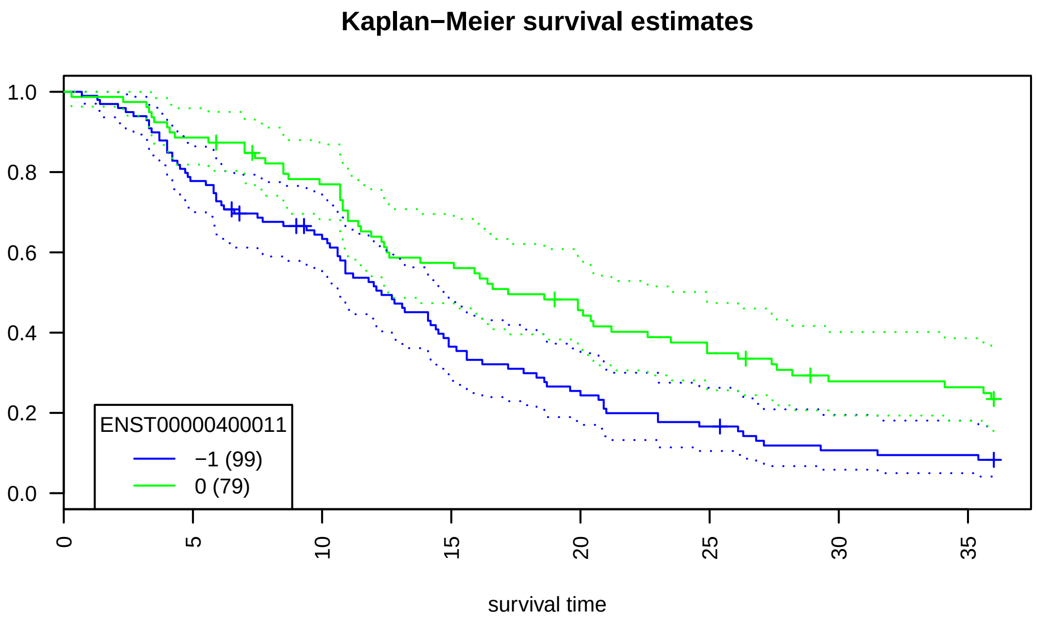

Figure 334: Each curve represents the relative number of ENST00000400011 survived to the corresponding point of time. Dashed lines represent 0.95 confidence intervals for the curves with the corresponding color. Total of 52 observations deleted due to missingness. Log-rank probability measure for the equality of these curves is 1.224502e-03. Additional annotations: *.GeneId*=ENSG00000104067, *.GeneName*=TJP1, *.GeneDesc*=tight junction protein 1 (zona occludens 1) [Source:HGNC Symbol;Acc:11827].

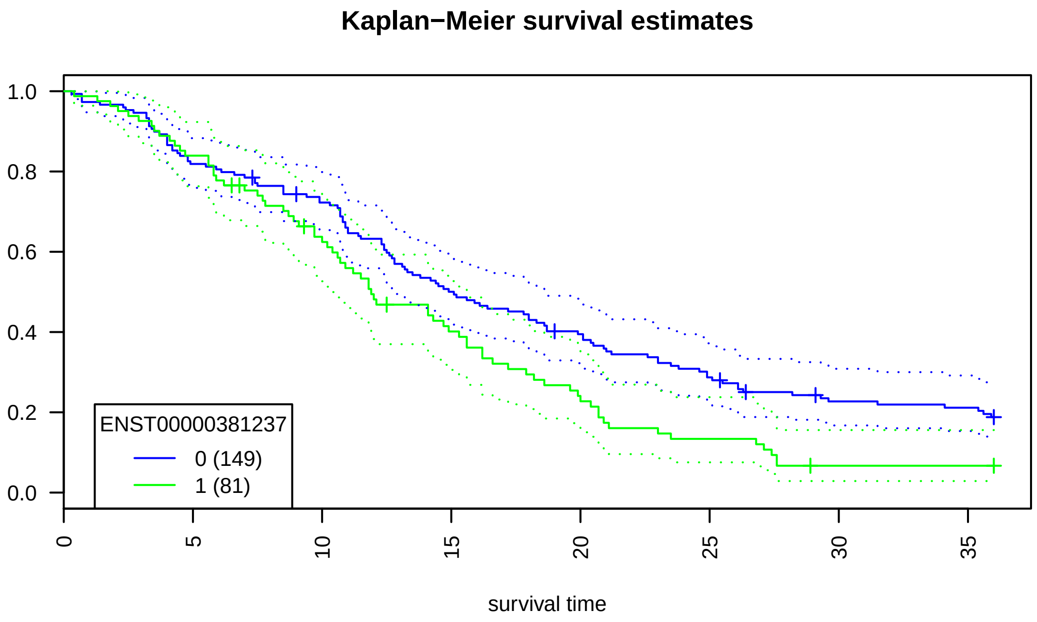

Figure 335: Each curve represents the relative number of ENST00000381237 survived to the corresponding point of time. Dashed lines represent 0.95 confidence intervals for the curves with the corresponding color. Log-rank probability measure for the equality of these curves is 5.425101e-03. Additional annotations: *.GeneId*=ENSG00000104635, *.GeneName*=SLC39A14, *.GeneDesc*=solute carrier family 39 (zinc transporter), member 14 [Source:HGNC Symbol;Acc:20858].

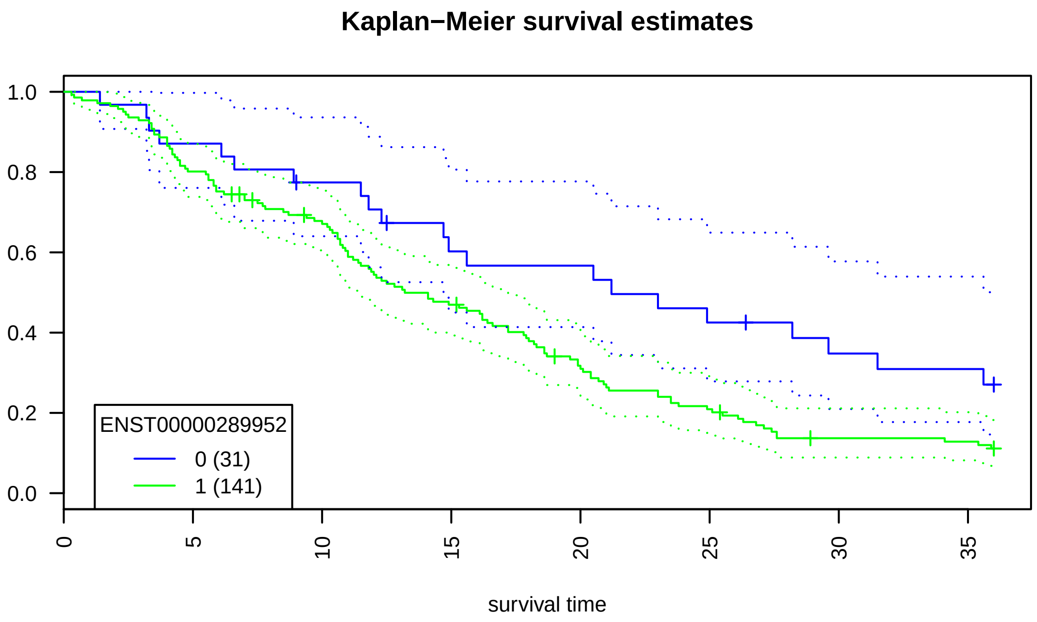

Figure 336: Each curve represents the relative number of ENST00000289952 survived to the corresponding point of time. Dashed lines represent 0.95 confidence intervals for the curves with the corresponding color. Total of 58 observations deleted due to missingness. Log-rank probability measure for the equality of these curves is 1.581052e-02. Additional annotations: *.GeneId*=ENSG00000104635, *.GeneName*=SLC39A14, *.GeneDesc*=solute carrier family 39 (zinc transporter), member 14 [Source:HGNC Symbol;Acc:20858].

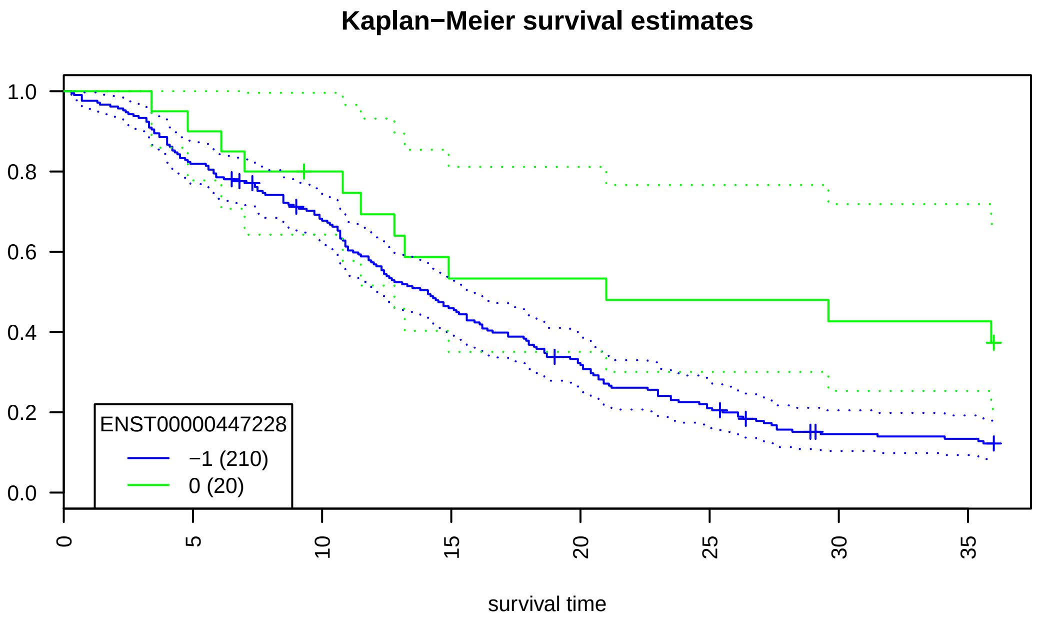

Figure 337: Each curve represents the relative number of ENST00000447228 survived to the corresponding point of time. Dashed lines represent 0.95 confidence intervals for the curves with the corresponding color. Log-rank probability measure for the equality of these curves is 1.803689e-02. Additional annotations: *.GeneId*=ENSG00000176788, *.GeneName*=BASP1, *.GeneDesc*=brain abundant, membrane attached signal protein 1 [Source:HGNC Symbol;Acc:957].

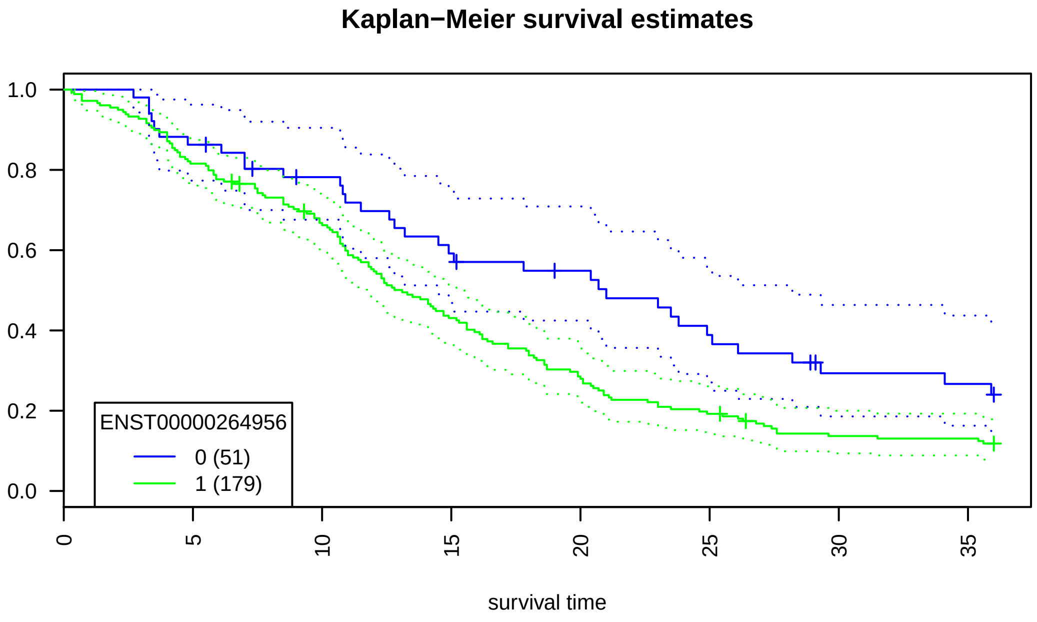

Figure 338: Each curve represents the relative number of ENST00000264956 survived to the corresponding point of time. Dashed lines represent 0.95 confidence intervals for the curves with the corresponding color. Log-rank probability measure for the equality of these curves is 7.81745e-03. Additional annotations: *.GeneId*=ENSG00000072840, *.GeneName*=EVC, *.GeneDesc*=Ellis van Creveld syndrome [Source:HGNC Symbol;Acc:3497].

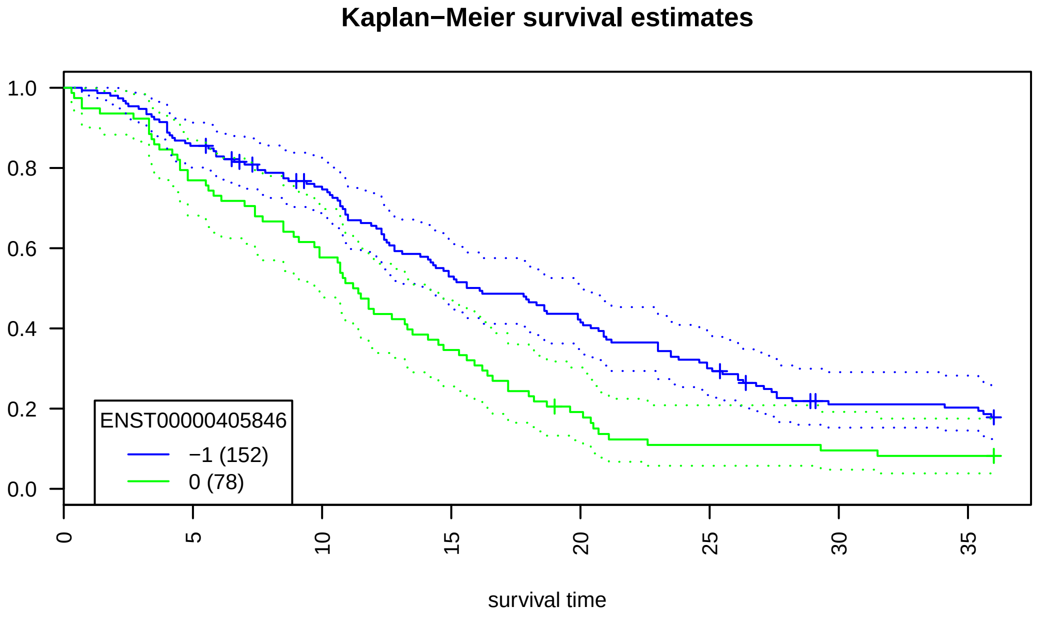

Figure 339: Each curve represents the relative number of ENST00000405846 survived to the corresponding point of time. Dashed lines represent 0.95 confidence intervals for the curves with the corresponding color. Log-rank probability measure for the equality of these curves is 4.535576e-04. Additional annotations: *.GeneId*=ENSG00000132975, *.GeneName*=GPR12, *.GeneDesc*=G protein-coupled receptor 12 [Source:HGNC Symbol;Acc:4466].

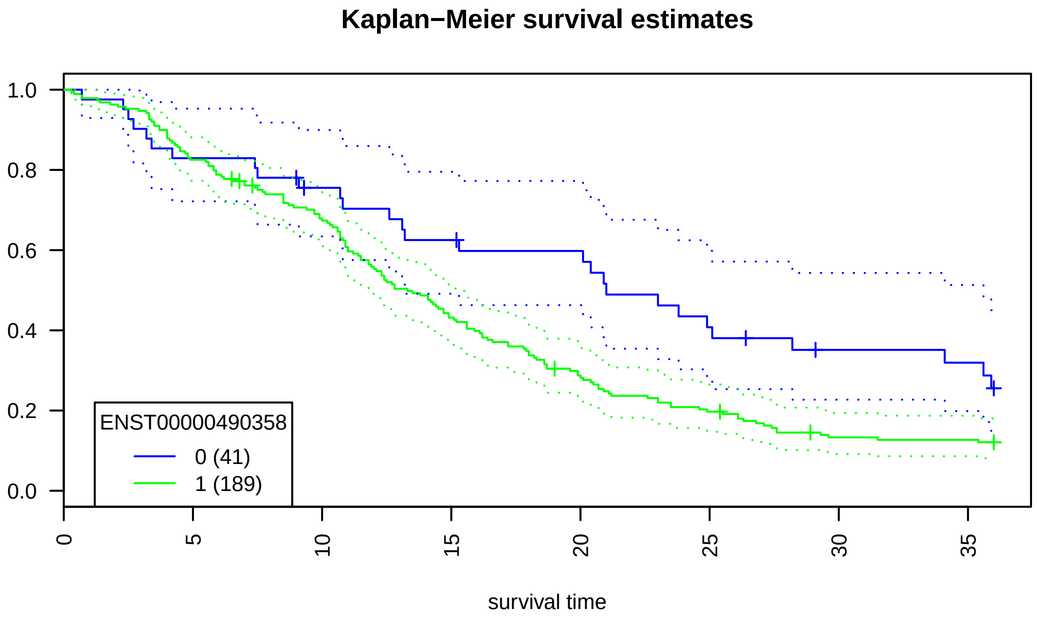

Figure 340: Each curve represents the relative number of ENST00000490358 survived to the corresponding point of time. Dashed lines represent 0.95 confidence intervals for the curves with the corresponding color. Log-rank probability measure for the equality of these curves is 1.050803e-02. Additional annotations: *.GeneId*=ENSG00000205929, *.GeneName*=C21orf62, *.GeneDesc*=Uncharacterized protein C21orf62 (B37) [Source:UniProtKB/Swiss-Prot;Acc:Q9NYP8].

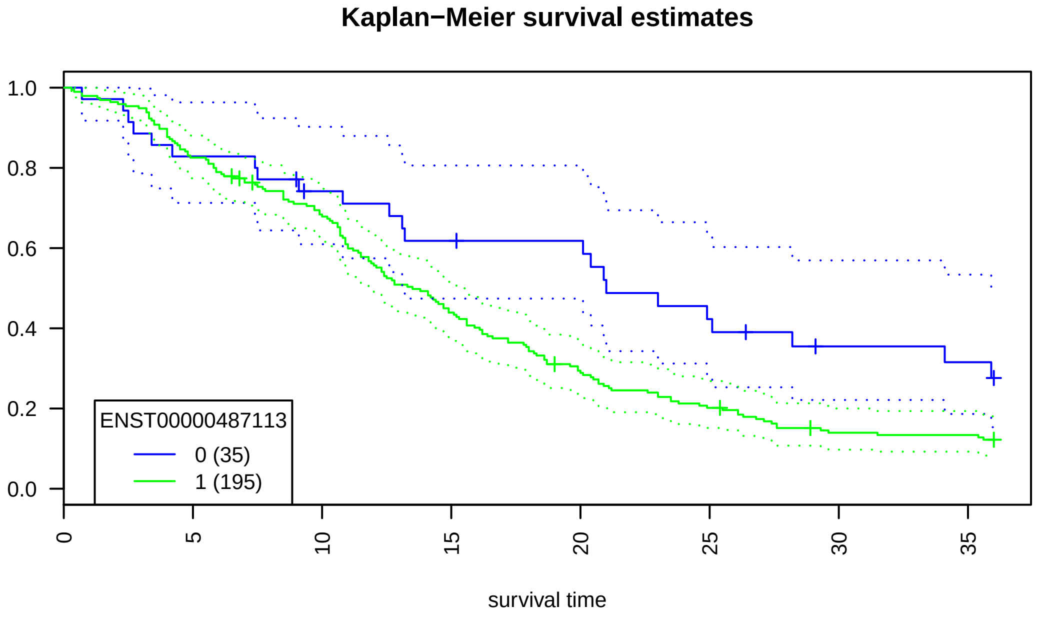

Figure 341: Each curve represents the relative number of ENST00000487113 survived to the corresponding point of time. Dashed lines represent 0.95 confidence intervals for the curves with the corresponding color. Log-rank probability measure for the equality of these curves is 1.469182e-02. Additional annotations: *.GeneId*=ENSG00000205929, *.GeneName*=C21orf62, *.GeneDesc*=Uncharacterized protein C21orf62 (B37) [Source:UniProtKB/Swiss-Prot;Acc:Q9NYP8].

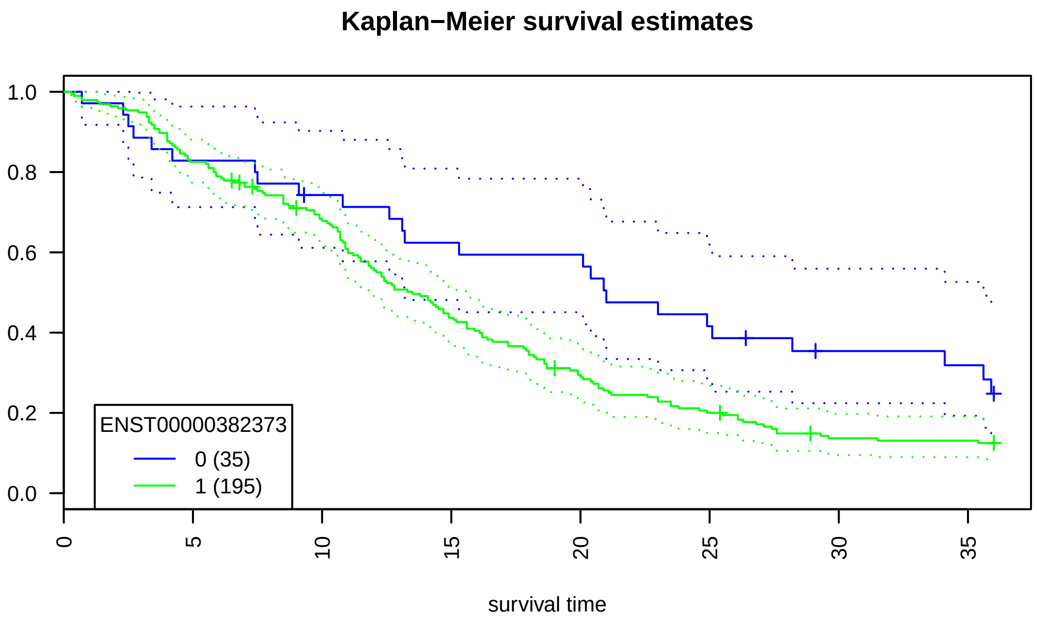

Figure 342: Each curve represents the relative number of ENST00000382373 survived to the corresponding point of time. Dashed lines represent 0.95 confidence intervals for the curves with the corresponding color. Log-rank probability measure for the equality of these curves is 2.006789e-02. Additional annotations: *.GeneId*=ENSG00000205929, *.GeneName*=C21orf62, *.GeneDesc*=Uncharacterized protein C21orf62 (B37) [Source:UniProtKB/Swiss-Prot;Acc:Q9NYP8].

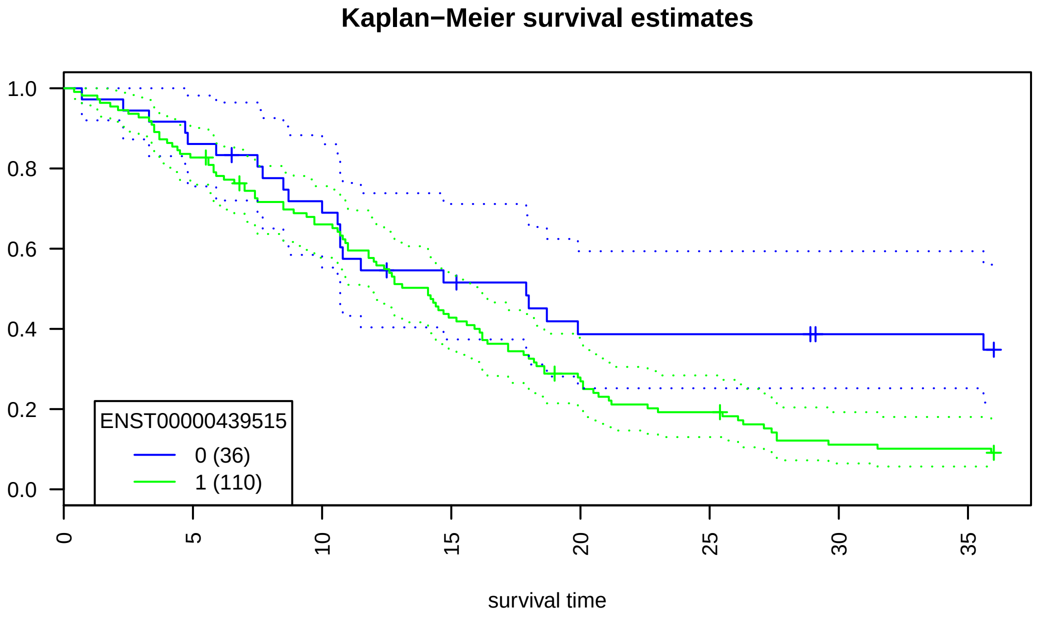

Figure 343: Each curve represents the relative number of ENST00000439515 survived to the corresponding point of time. Dashed lines represent 0.95 confidence intervals for the curves with the corresponding color. Total of 84 observations deleted due to missingness. Log-rank probability measure for the equality of these curves is 1.73163e-02. Additional annotations: *.GeneId*=ENSG00000132405, *.GeneName*=TBC1D14, *.GeneDesc*=TBC1 domain family, member 14 [Source:HGNC Symbol;Acc:29246].

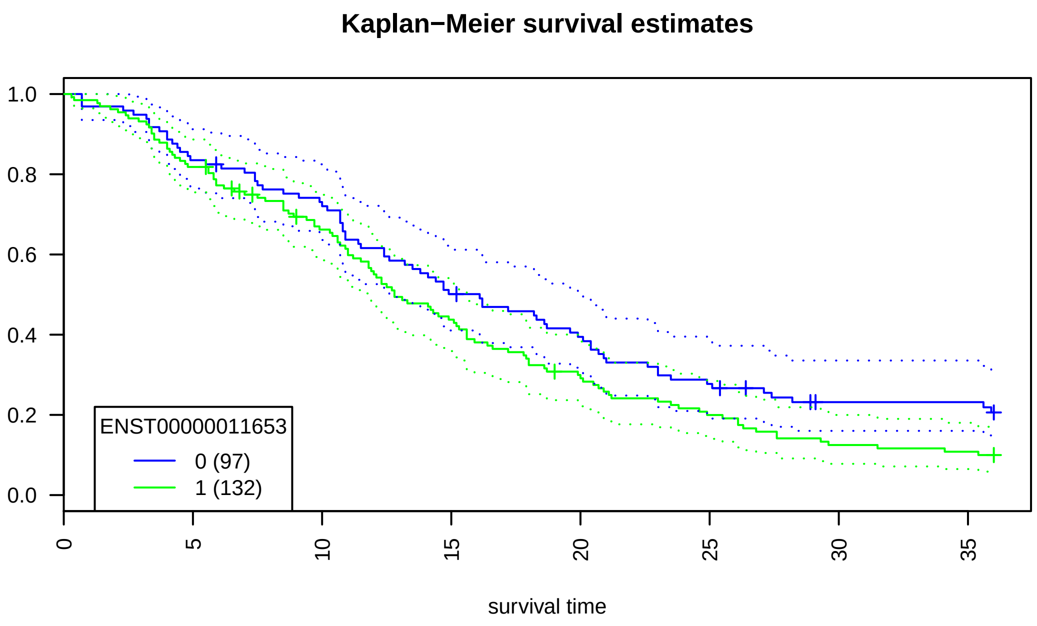

Figure 344: Each curve represents the relative number of ENST00000011653 survived to the corresponding point of time. Dashed lines represent 0.95 confidence intervals for the curves with the corresponding color. Total of 1 observation deleted due to missingness. Log-rank probability measure for the equality of these curves is 4.973438e-02. Additional annotations: *.GeneId*=ENSG00000010610, *.GeneName*=CD4, *.GeneDesc*=CD4 molecule [Source:HGNC Symbol;Acc:1678].

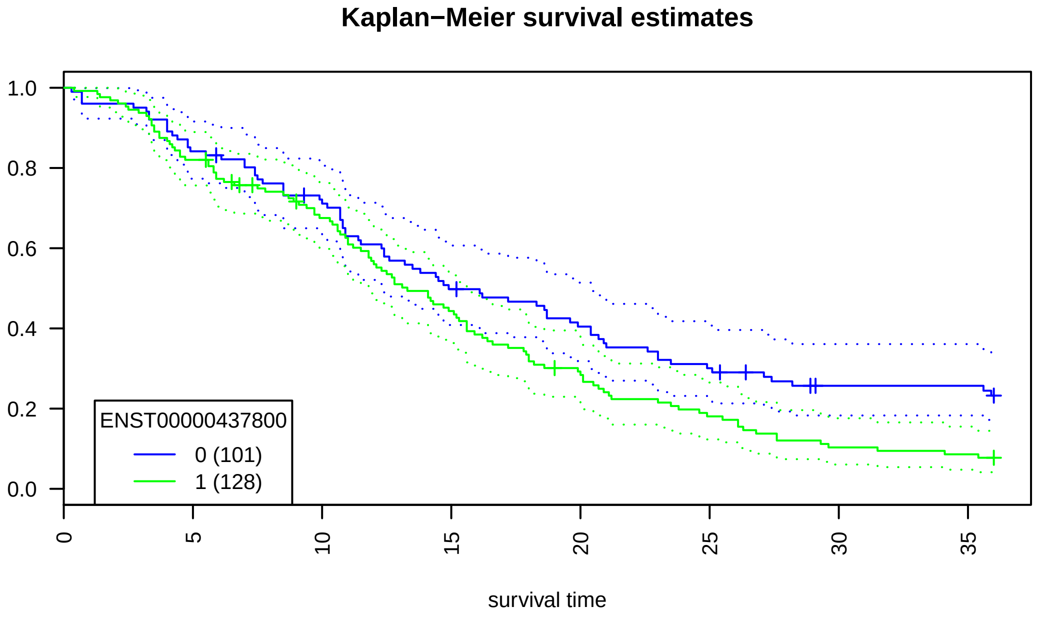

Figure 345: Each curve represents the relative number of ENST00000437800 survived to the corresponding point of time. Dashed lines represent 0.95 confidence intervals for the curves with the corresponding color. Total of 1 observation deleted due to missingness. Log-rank probability measure for the equality of these curves is 1.036891e-02. Additional annotations: *.GeneId*=ENSG00000010610, *.GeneName*=CD4, *.GeneDesc*=CD4 molecule [Source:HGNC Symbol;Acc:1678].

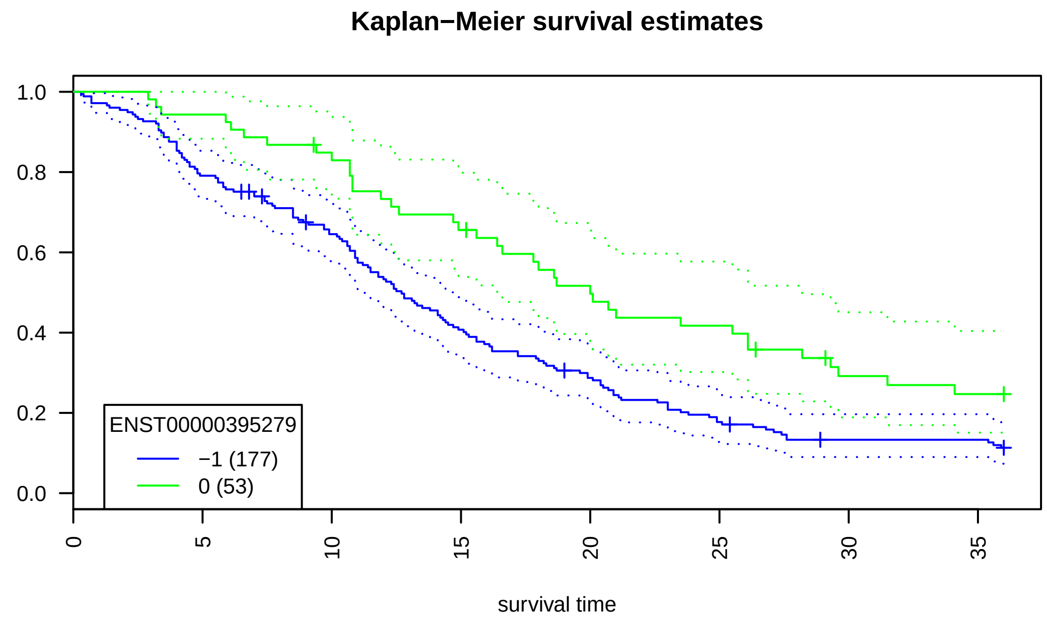

Figure 346: Each curve represents the relative number of ENST00000395279 survived to the corresponding point of time. Dashed lines represent 0.95 confidence intervals for the curves with the corresponding color. Log-rank probability measure for the equality of these curves is 1.393053e-03. Additional annotations: *.GeneId*=ENSG00000008853, *.GeneName*=RHOBTB2, *.GeneDesc*=Rho-related BTB domain containing 2 [Source:HGNC Symbol;Acc:18756].

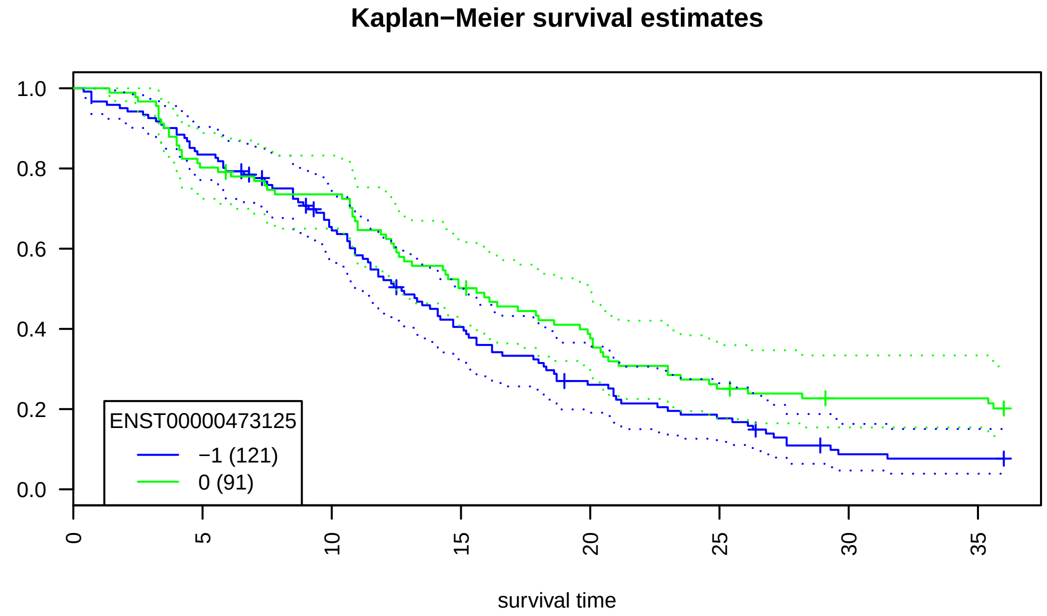

Figure 347: Each curve represents the relative number of ENST00000473125 survived to the corresponding point of time. Dashed lines represent 0.95 confidence intervals for the curves with the corresponding color. Total of 18 observations deleted due to missingness. Log-rank probability measure for the equality of these curves is 2.973444e-02. Additional annotations: *.GeneId*=ENSG00000175265, *.GeneName*=GOLGA8A, *.GeneDesc*=golgin A8 family, member A [Source:HGNC Symbol;Acc:31972].

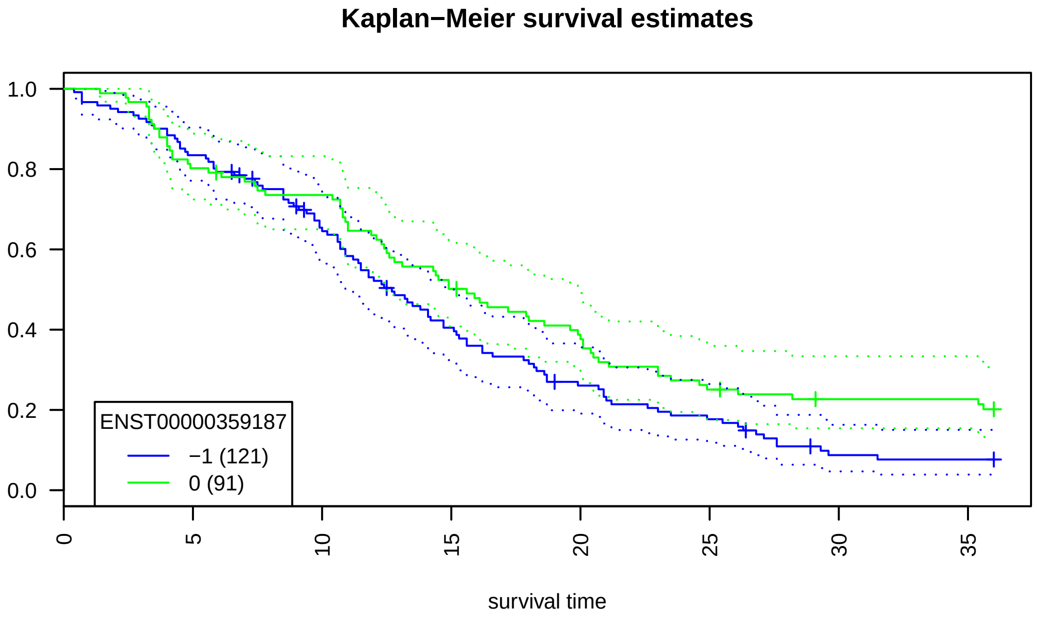

Figure 348: Each curve represents the relative number of ENST00000359187 survived to the corresponding point of time. Dashed lines represent 0.95 confidence intervals for the curves with the corresponding color. Total of 18 observations deleted due to missingness. Log-rank probability measure for the equality of these curves is 2.973444e-02. Additional annotations: *.GeneId*=ENSG00000175265, *.GeneName*=GOLGA8A, *.GeneDesc*=golgin A8 family, member A [Source:HGNC Symbol;Acc:31972].

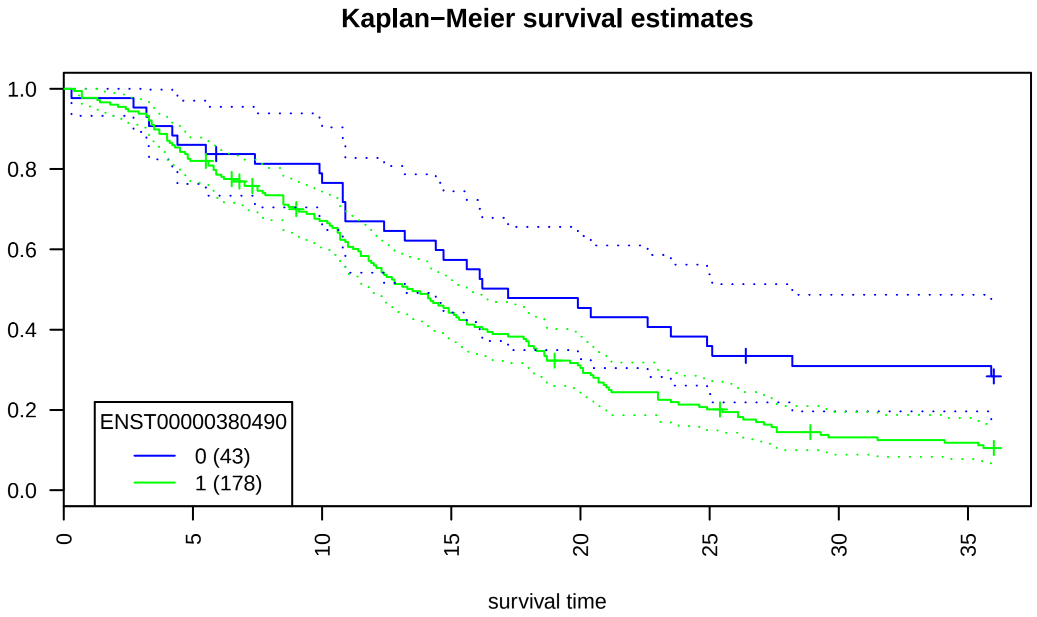

Figure 349: Each curve represents the relative number of ENST00000380490 survived to the corresponding point of time. Dashed lines represent 0.95 confidence intervals for the curves with the corresponding color. Total of 9 observations deleted due to missingness. Log-rank probability measure for the equality of these curves is 1.368409e-02. Additional annotations: *.GeneId*=ENSG00000132965, *.GeneName*=ALOX5AP, *.GeneDesc*=arachidonate 5-lipoxygenase-activating protein [Source:HGNC Symbol;Acc:436].

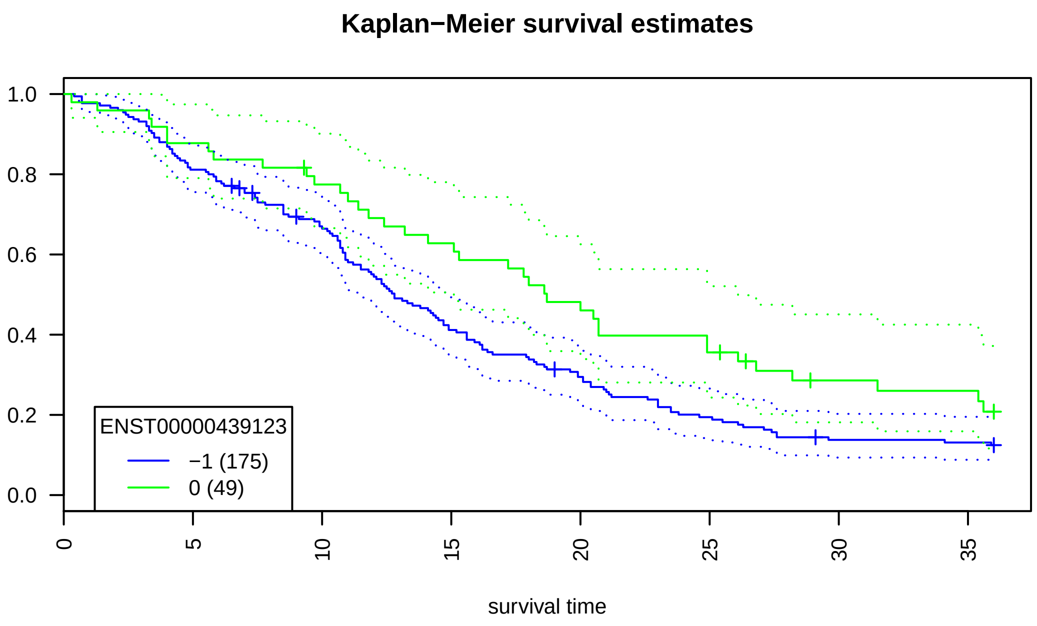

Figure 350: Each curve represents the relative number of ENST00000439123 survived to the corresponding point of time. Dashed lines represent 0.95 confidence intervals for the curves with the corresponding color. Total of 6 observations deleted due to missingness. Log-rank probability measure for the equality of these curves is 2.389675e-02. Additional annotations: *.GeneId*=ENSG00000120694, *.GeneName*=HSPH1, *.GeneDesc*=heat shock 105kDa/110kDa protein 1 [Source:HGNC Symbol;Acc:16969].

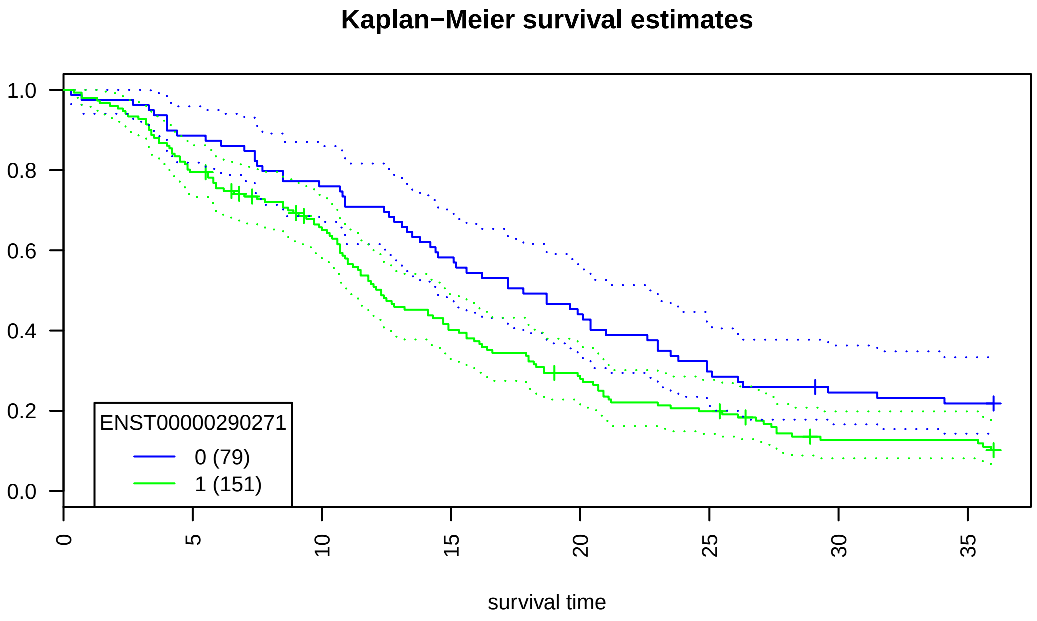

Figure 351: Each curve represents the relative number of ENST00000290271 survived to the corresponding point of time. Dashed lines represent 0.95 confidence intervals for the curves with the corresponding color. Log-rank probability measure for the equality of these curves is 6.490555e-03. Additional annotations: *.GeneId*=ENSG00000159167, *.GeneName*=STC1, *.GeneDesc*=stanniocalcin 1 [Source:HGNC Symbol;Acc:11373].

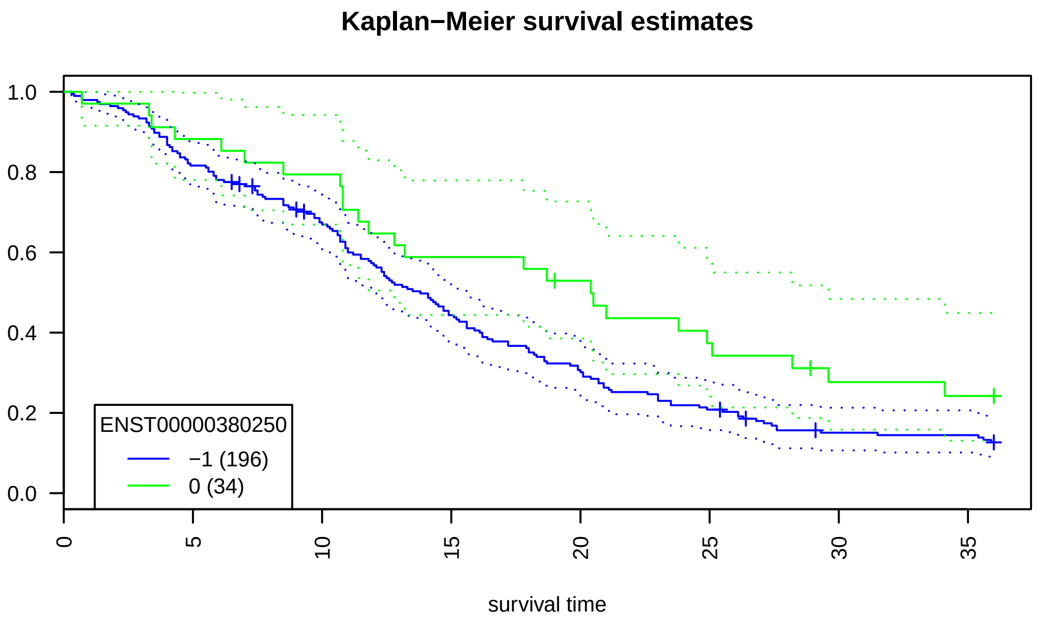

Figure 352: Each curve represents the relative number of ENST00000380250 survived to the corresponding point of time. Dashed lines represent 0.95 confidence intervals for the curves with the corresponding color. Log-rank probability measure for the equality of these curves is 4.137257e-02. Additional annotations: *.GeneId*=ENSG00000073910, *.GeneName*=FRY, *.GeneDesc*=furry homolog (Drosophila) [Source:HGNC Symbol;Acc:20367].

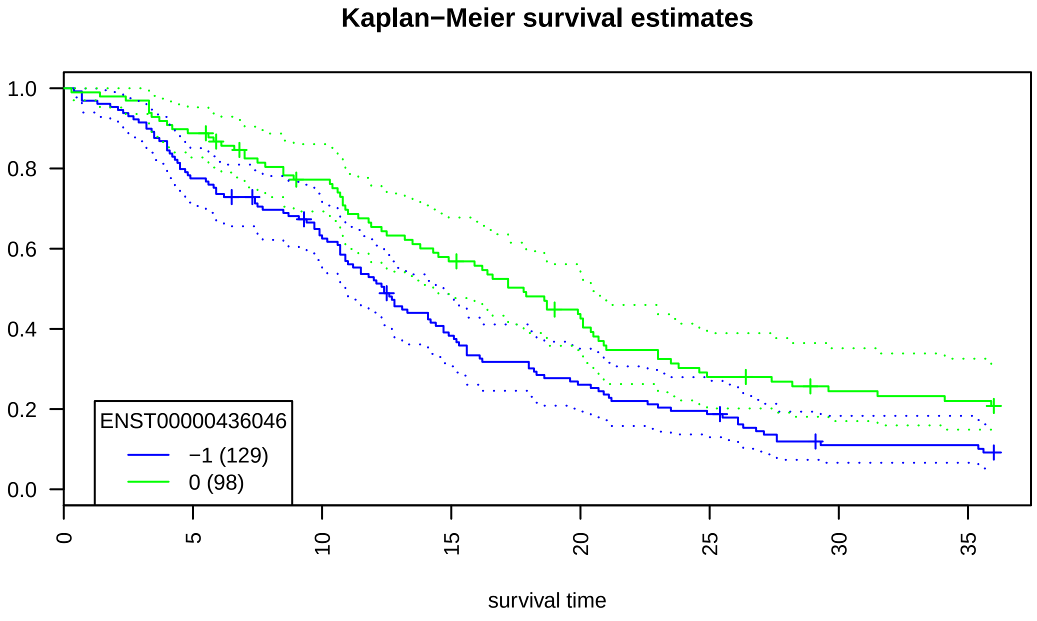

Figure 353: Each curve represents the relative number of ENST00000436046 survived to the corresponding point of time. Dashed lines represent 0.95 confidence intervals for the curves with the corresponding color. Total of 3 observations deleted due to missingness. Log-rank probability measure for the equality of these curves is 3.018222e-03. Additional annotations: *.GeneId*=ENSG00000073910, *.GeneName*=FRY, *.GeneDesc*=furry homolog (Drosophila) [Source:HGNC Symbol;Acc:20367].

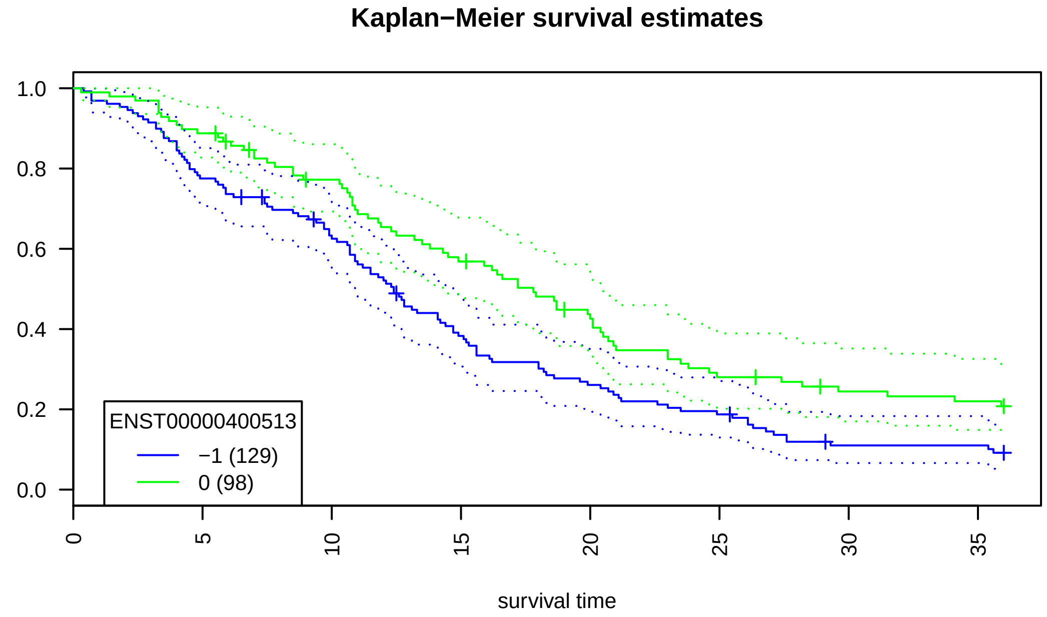

Figure 354: Each curve represents the relative number of ENST00000400513 survived to the corresponding point of time. Dashed lines represent 0.95 confidence intervals for the curves with the corresponding color. Total of 3 observations deleted due to missingness. Log-rank probability measure for the equality of these curves is 3.018222e-03.

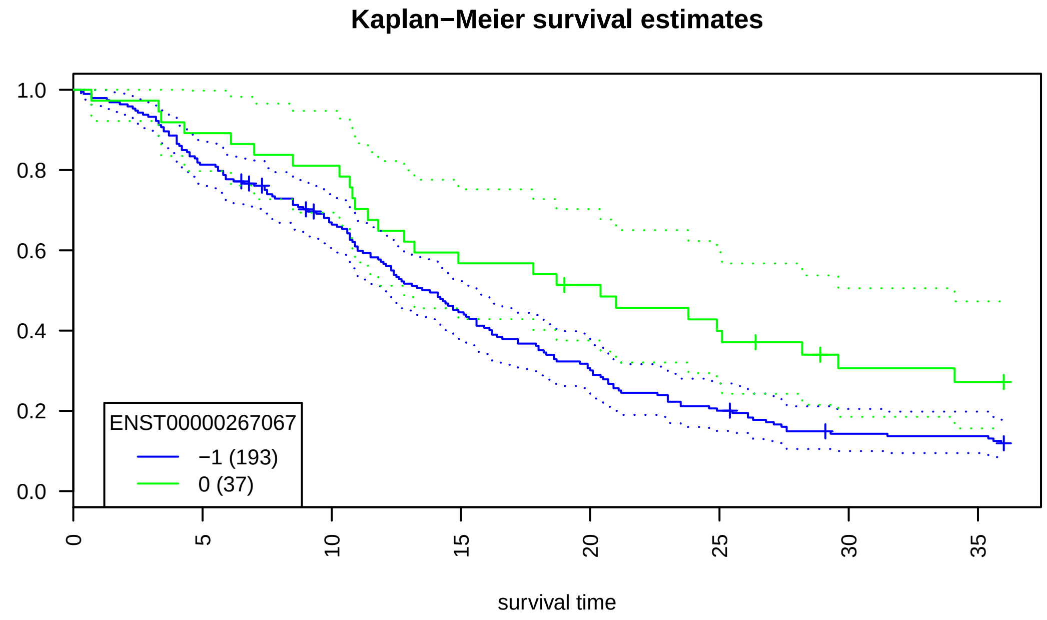

Figure 355: Each curve represents the relative number of ENST00000267067 survived to the corresponding point of time. Dashed lines represent 0.95 confidence intervals for the curves with the corresponding color. Log-rank probability measure for the equality of these curves is 1.555738e-02. Additional annotations: *.GeneId*=ENSG00000073910, *.GeneName*=FRY, *.GeneDesc*=furry homolog (Drosophila) [Source:HGNC Symbol;Acc:20367].

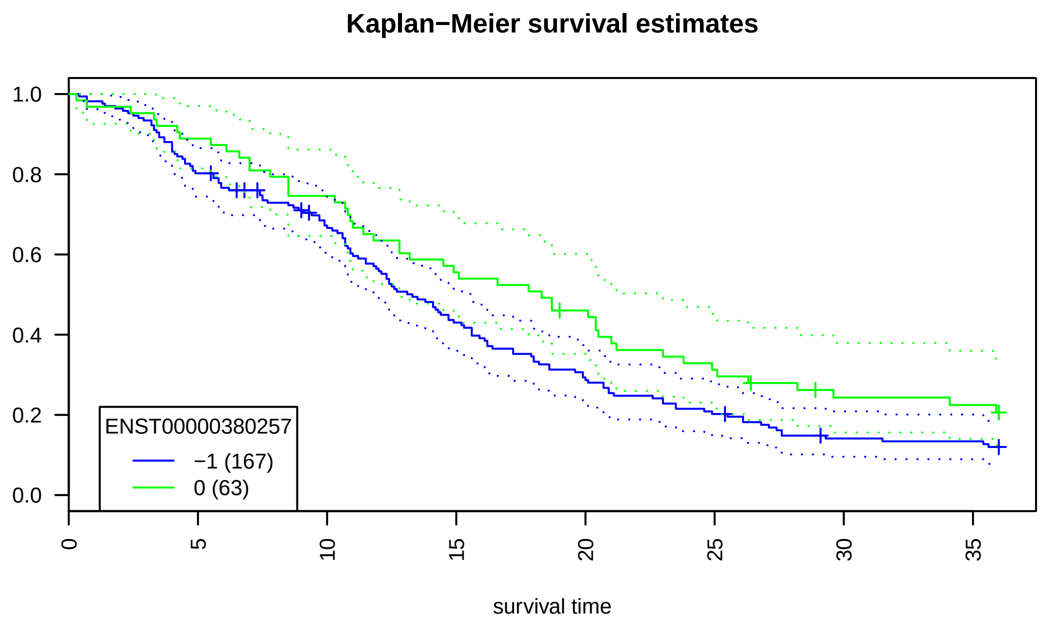

Figure 356: Each curve represents the relative number of ENST00000380257 survived to the corresponding point of time. Dashed lines represent 0.95 confidence intervals for the curves with the corresponding color. Log-rank probability measure for the equality of these curves is 4.394367e-02. Additional annotations: *.GeneId*=ENSG00000073910, *.GeneName*=FRY, *.GeneDesc*=furry homolog (Drosophila) [Source:HGNC Symbol;Acc:20367].

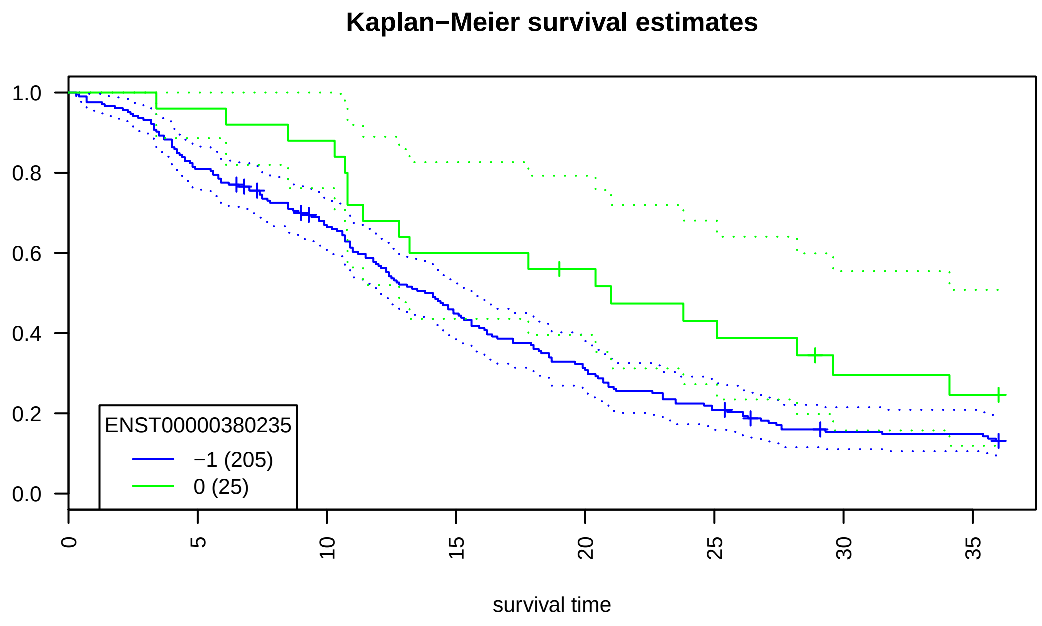

Figure 357: Each curve represents the relative number of ENST00000380235 survived to the corresponding point of time. Dashed lines represent 0.95 confidence intervals for the curves with the corresponding color. Log-rank probability measure for the equality of these curves is 4.265322e-02. Additional annotations: *.GeneId*=ENSG00000073910, *.GeneName*=FRY, *.GeneDesc*=furry homolog (Drosophila) [Source:HGNC Symbol;Acc:20367].

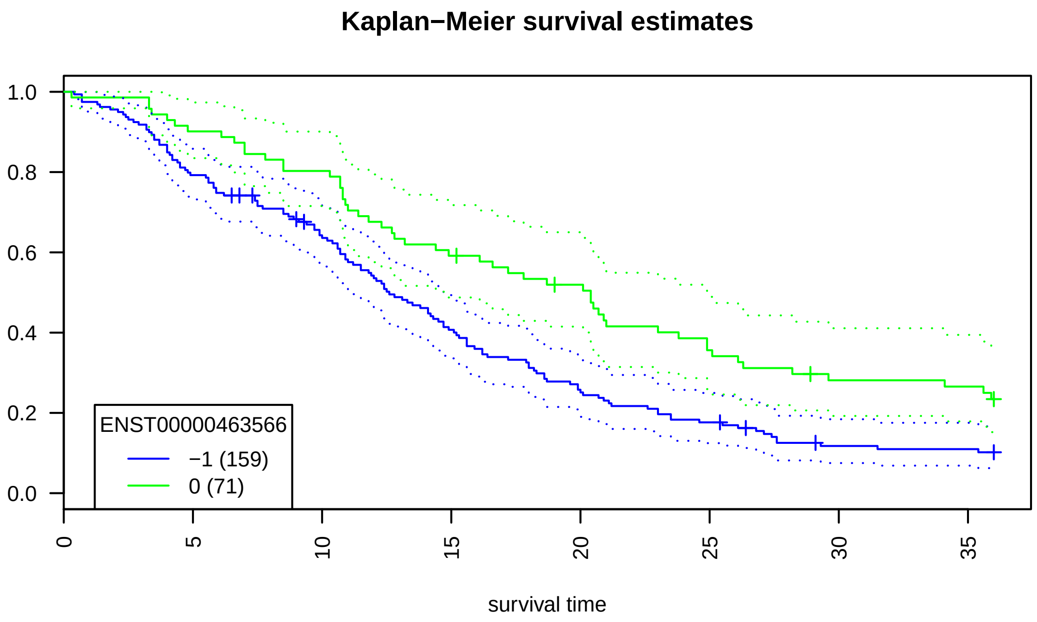

Figure 358: Each curve represents the relative number of ENST00000463566 survived to the corresponding point of time. Dashed lines represent 0.95 confidence intervals for the curves with the corresponding color. Log-rank probability measure for the equality of these curves is 9.942376e-04. Additional annotations: *.GeneId*=ENSG00000073910, *.GeneName*=FRY, *.GeneDesc*=furry homolog (Drosophila) [Source:HGNC Symbol;Acc:20367].

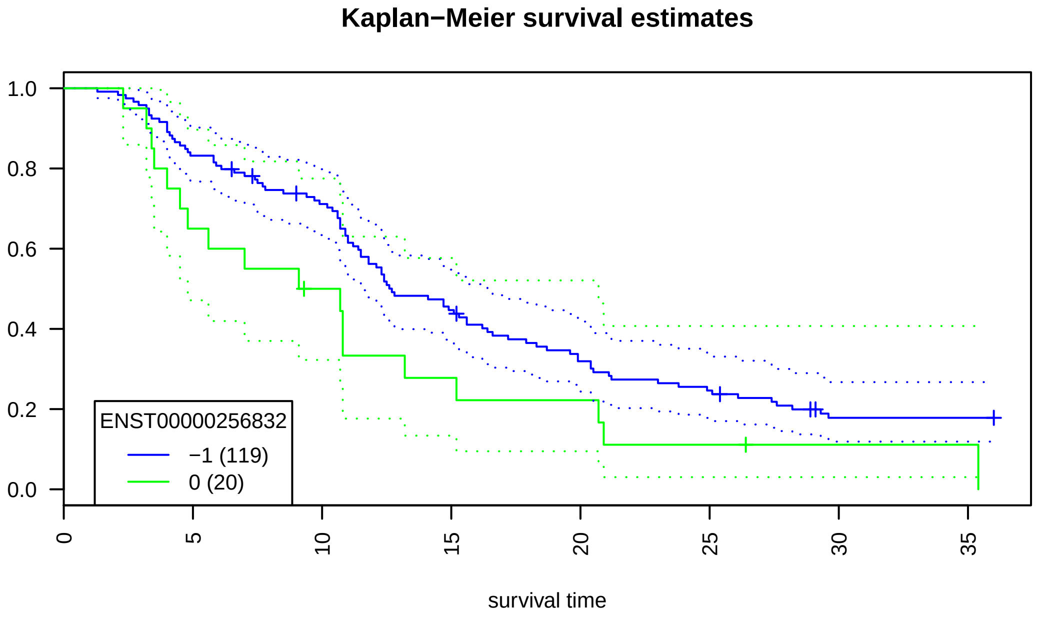

Figure 359: Each curve represents the relative number of ENST00000256832 survived to the corresponding point of time. Dashed lines represent 0.95 confidence intervals for the curves with the corresponding color. Total of 91 observations deleted due to missingness. Log-rank probability measure for the equality of these curves is 2.760272e-02. Additional annotations: *.GeneId*=ENSG00000049759, *.GeneName*=NEDD4L, *.GeneDesc*=neural precursor cell expressed, developmentally down-regulated 4-like [Source:HGNC Symbol;Acc:7728].

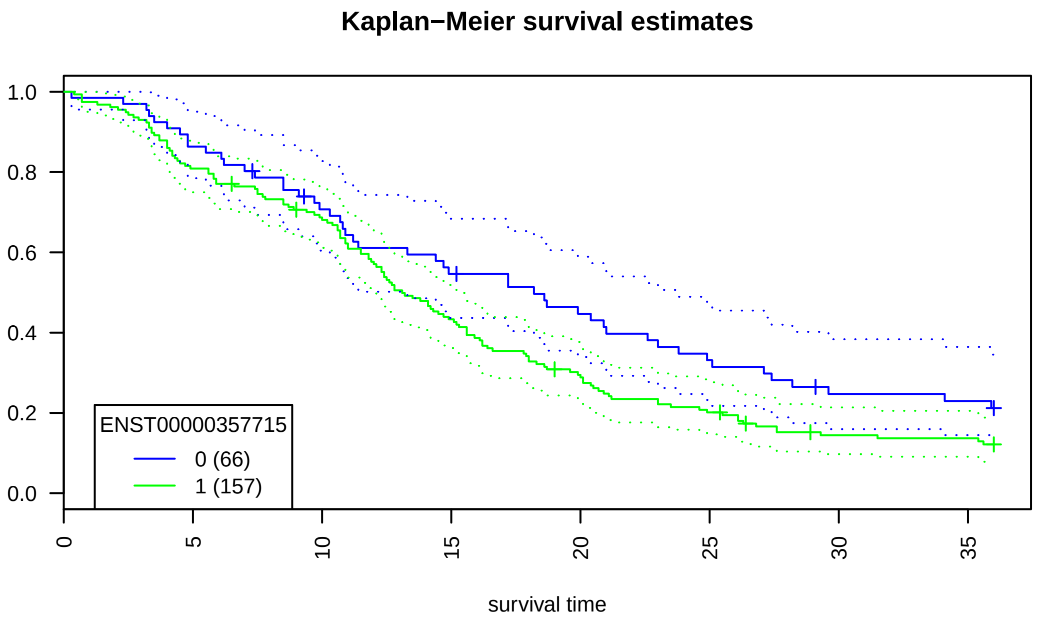

Figure 360: Each curve represents the relative number of ENST00000357715 survived to the corresponding point of time. Dashed lines represent 0.95 confidence intervals for the curves with the corresponding color. Total of 7 observations deleted due to missingness. Log-rank probability measure for the equality of these curves is 4.180143e-02. Additional annotations: *.GeneId*=ENSG00000129465, *.GeneName*=RIPK3, *.GeneDesc*=receptor-interacting serine-threonine kinase 3 [Source:HGNC Symbol;Acc:10021].

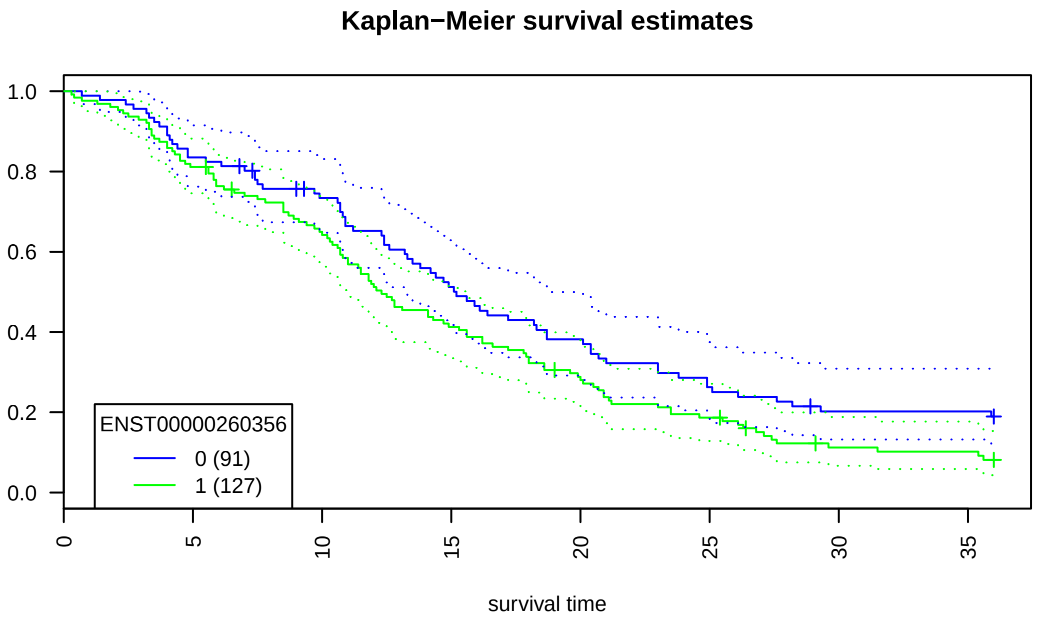

Figure 361: Each curve represents the relative number of ENST00000260356 survived to the corresponding point of time. Dashed lines represent 0.95 confidence intervals for the curves with the corresponding color. Total of 12 observations deleted due to missingness. Log-rank probability measure for the equality of these curves is 3.628751e-02. Additional annotations: *.GeneId*=ENSG00000137801, *.GeneName*=THBS1, *.GeneDesc*=thrombospondin 1 [Source:HGNC Symbol;Acc:11785].

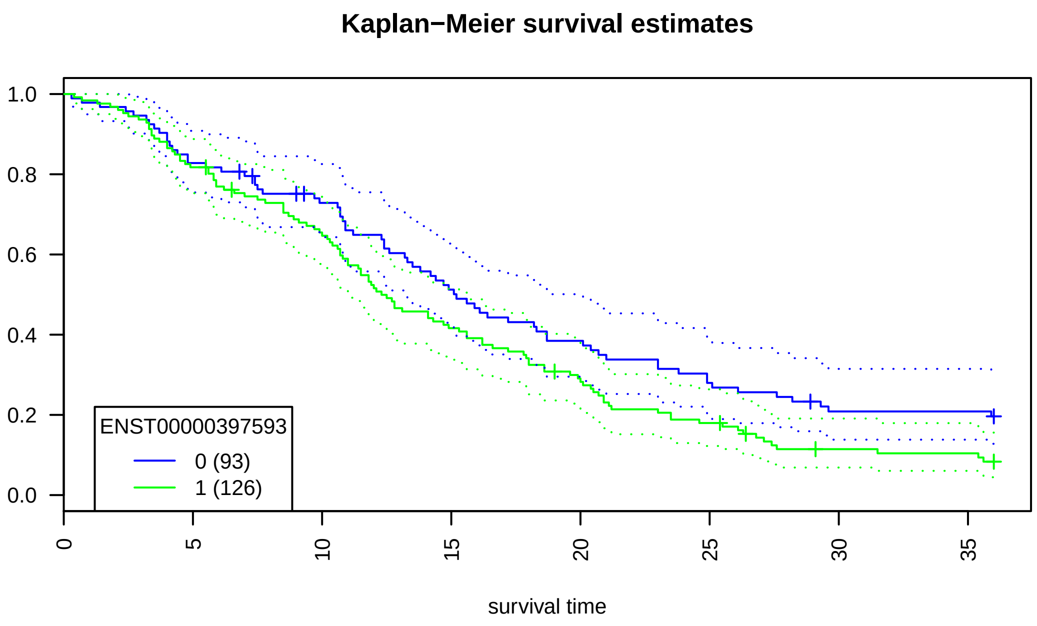

Figure 362: Each curve represents the relative number of ENST00000397593 survived to the corresponding point of time. Dashed lines represent 0.95 confidence intervals for the curves with the corresponding color. Total of 11 observations deleted due to missingness. Log-rank probability measure for the equality of these curves is 2.795249e-02. Additional annotations: *.GeneId*=ENSG00000137801, *.GeneName*=THBS1, *.GeneDesc*=thrombospondin 1 [Source:HGNC Symbol;Acc:11785].

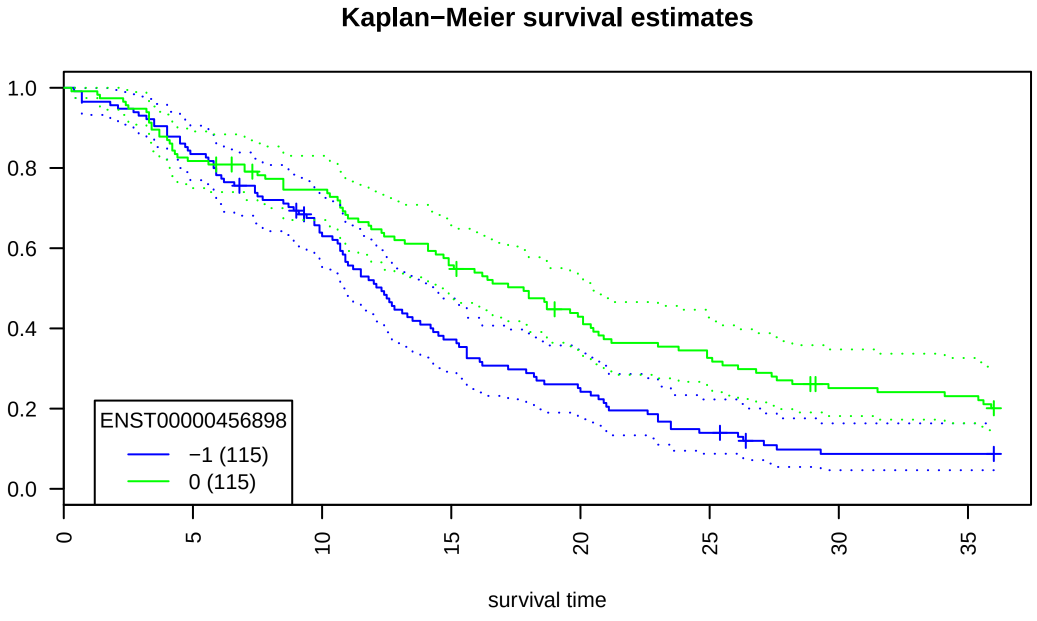

Figure 363: Each curve represents the relative number of ENST00000456898 survived to the corresponding point of time. Dashed lines represent 0.95 confidence intervals for the curves with the corresponding color. Log-rank probability measure for the equality of these curves is 1.540100e-03. Additional annotations: *.GeneId*=ENSG00000083642, *.GeneName*=PDS5B, *.GeneDesc*=PDS5, regulator of cohesion maintenance, homolog B (S. cerevisiae) [Source:HGNC Symbol;Acc:20418].

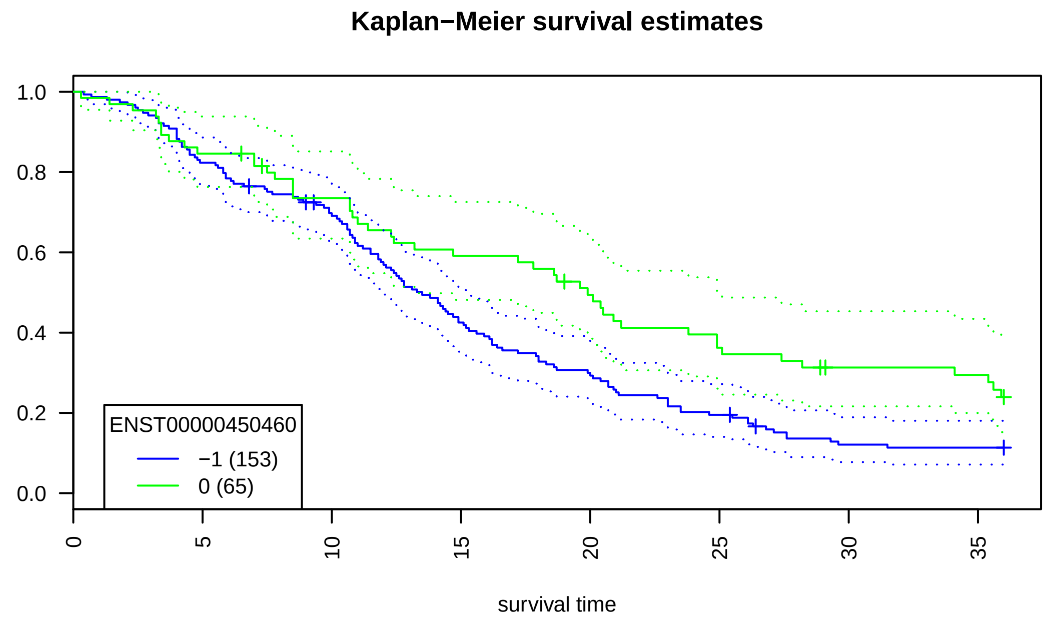

Figure 364: Each curve represents the relative number of ENST00000450460 survived to the corresponding point of time. Dashed lines represent 0.95 confidence intervals for the curves with the corresponding color. Total of 12 observations deleted due to missingness. Log-rank probability measure for the equality of these curves is 8.120661e-03. Additional annotations: *.GeneId*=ENSG00000083642, *.GeneName*=PDS5B, *.GeneDesc*=PDS5, regulator of cohesion maintenance, homolog B (S. cerevisiae) [Source:HGNC Symbol;Acc:20418].

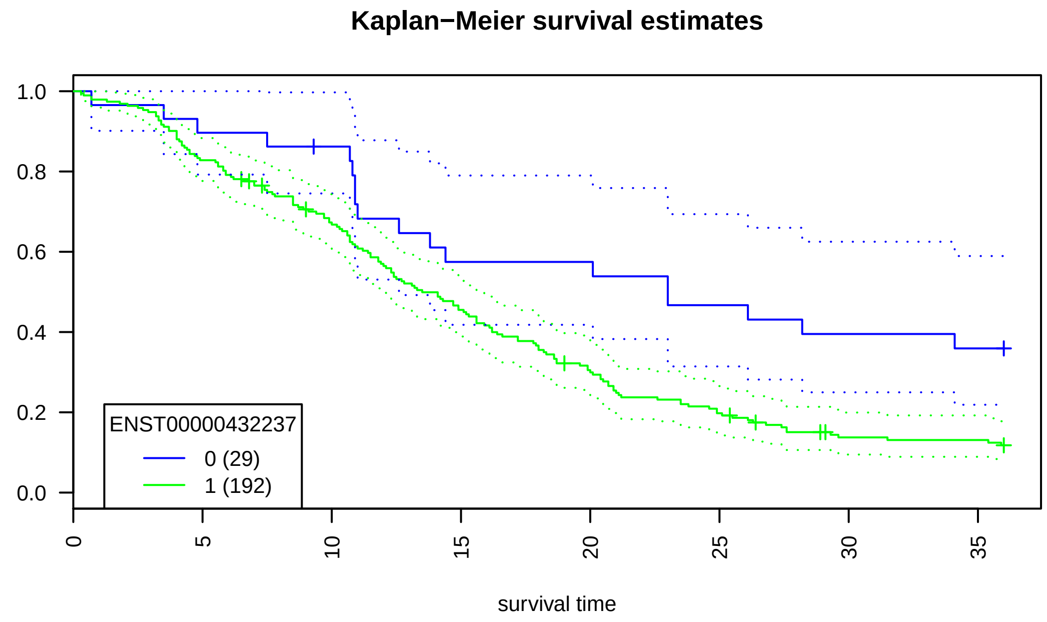

Figure 365: Each curve represents the relative number of ENST00000432237 survived to the corresponding point of time. Dashed lines represent 0.95 confidence intervals for the curves with the corresponding color. Total of 9 observations deleted due to missingness. Log-rank probability measure for the equality of these curves is 4.23847e-03. Additional annotations: *.GeneId*=ENSG00000177575, *.GeneName*=CD163, *.GeneDesc*=CD163 molecule [Source:HGNC Symbol;Acc:1631].

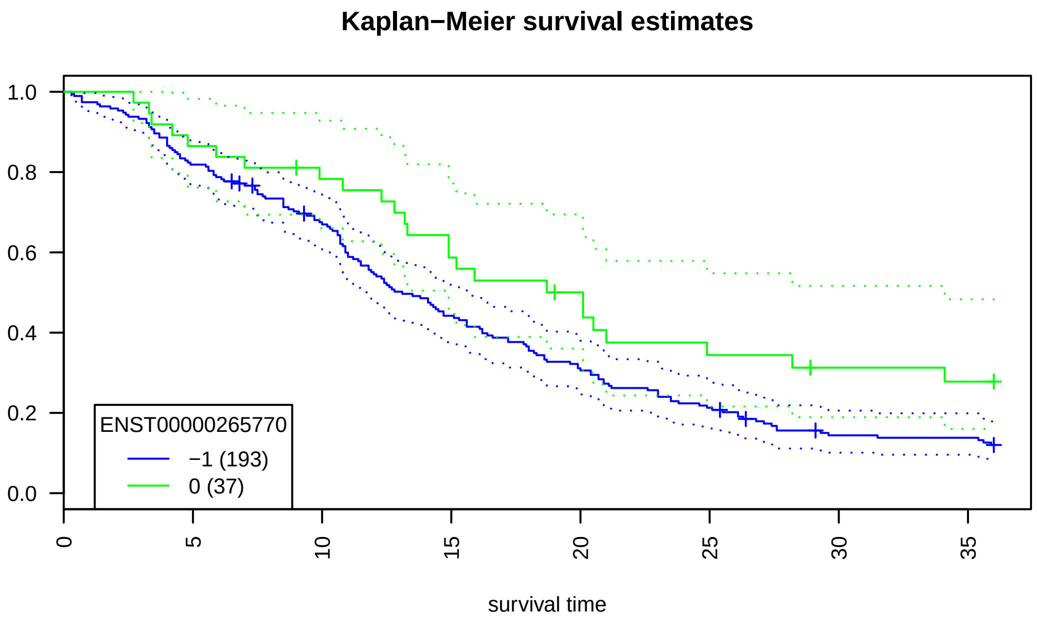

Figure 366: Each curve represents the relative number of ENST00000265770 survived to the corresponding point of time. Dashed lines represent 0.95 confidence intervals for the curves with the corresponding color. Log-rank probability measure for the equality of these curves is 2.467909e-02. Additional annotations: *.GeneId*=ENSG00000015592, *.GeneName*=STMN4, *.GeneDesc*=stathmin-like 4 [Source:HGNC Symbol;Acc:16078].

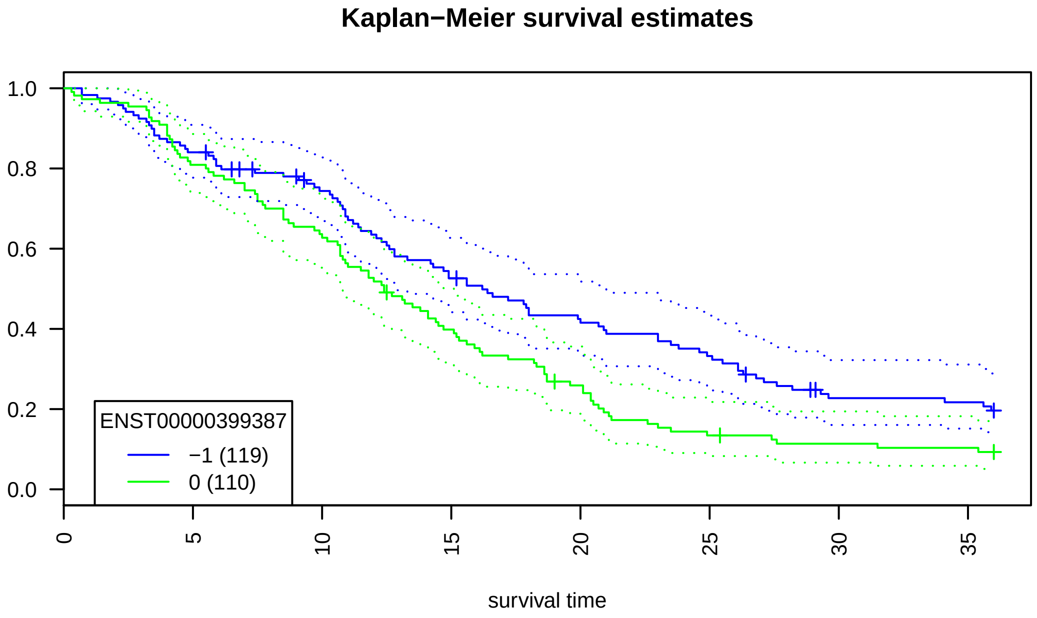

Figure 367: Each curve represents the relative number of ENST00000399387 survived to the corresponding point of time. Dashed lines represent 0.95 confidence intervals for the curves with the corresponding color. Total of 1 observation deleted due to missingness. Log-rank probability measure for the equality of these curves is 4.368096e-03. Additional annotations: *.GeneId*=ENSG00000186960, *.GeneName*=C14orf23, *.GeneDesc*=Uncharacterized protein C14orf23 [Source:UniProtKB/Swiss-Prot;Acc:Q86U37].

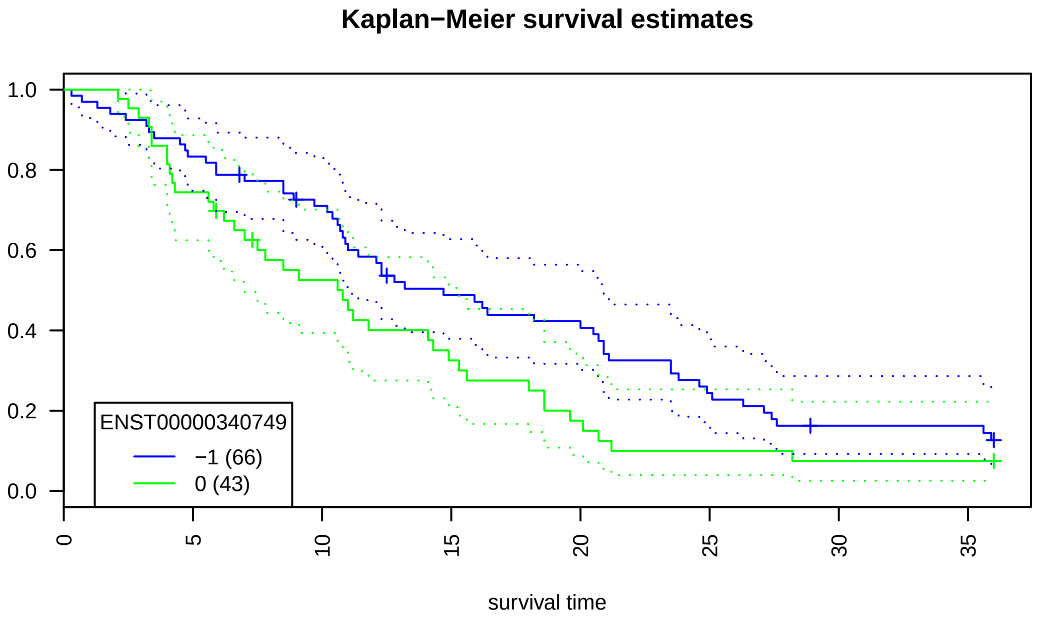

Figure 368: Each curve represents the relative number of ENST00000340749 survived to the corresponding point of time. Dashed lines represent 0.95 confidence intervals for the curves with the corresponding color. Total of 121 observations deleted due to missingness. Log-rank probability measure for the equality of these curves is 3.752605e-02. Additional annotations: *.GeneId*=ENSG00000173262, *.GeneName*=SLC2A14, *.GeneDesc*=solute carrier family 2 (facilitated glucose transporter), member 14 [Source:HGNC Symbol;Acc:18301].

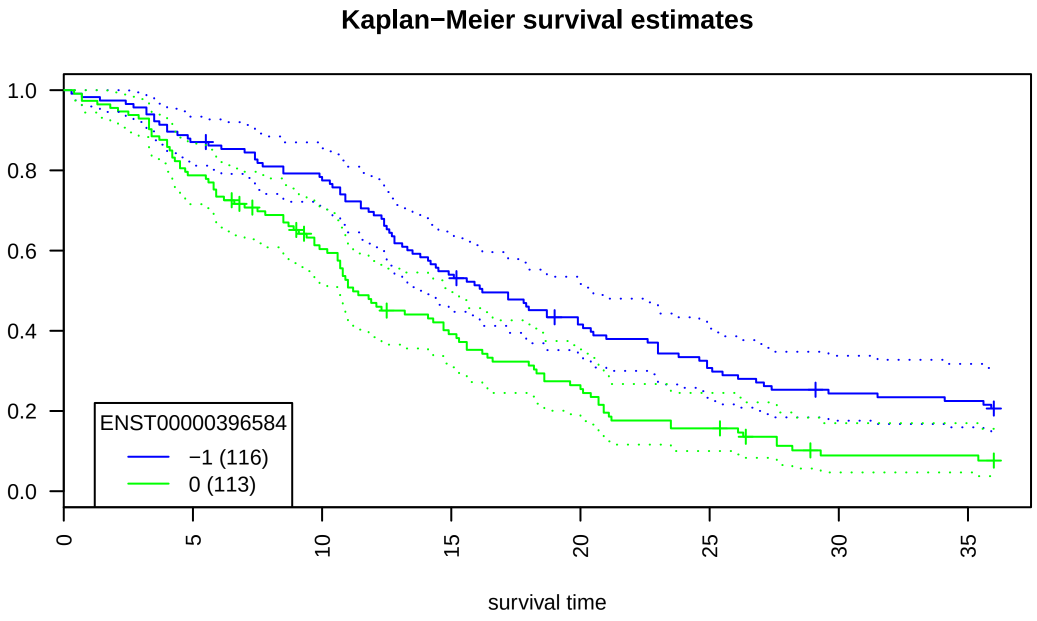

Figure 369: Each curve represents the relative number of ENST00000396584 survived to the corresponding point of time. Dashed lines represent 0.95 confidence intervals for the curves with the corresponding color. Total of 1 observation deleted due to missingness. Log-rank probability measure for the equality of these curves is 6.914245e-04.

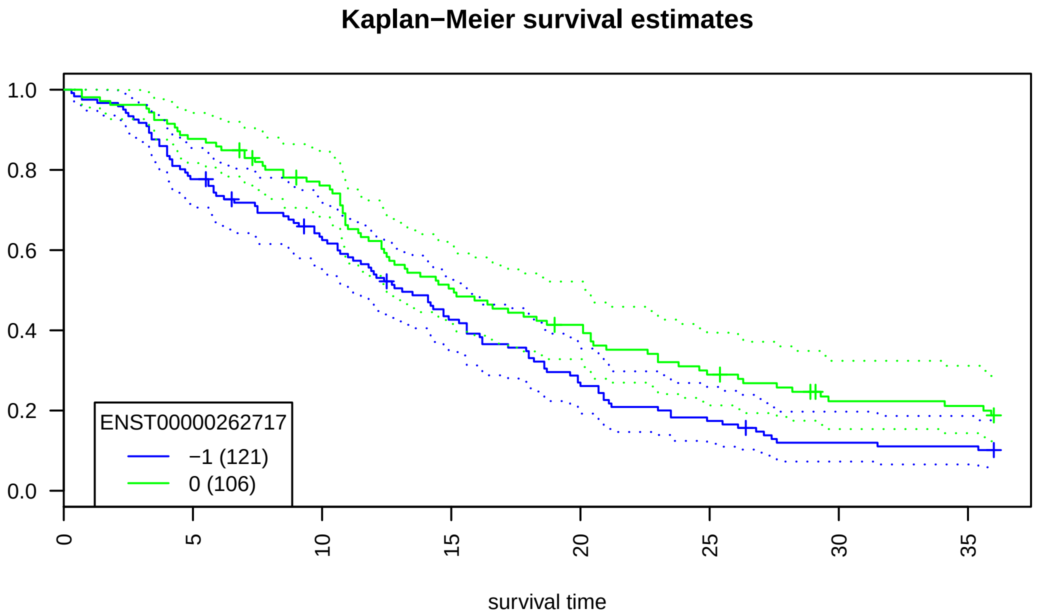

Figure 370: Each curve represents the relative number of ENST00000262717 survived to the corresponding point of time. Dashed lines represent 0.95 confidence intervals for the curves with the corresponding color. Total of 3 observations deleted due to missingness. Log-rank probability measure for the equality of these curves is 2.262387e-02. Additional annotations: .*GeneId*=ENSG00000101542, .*GeneName*=CDH20, .*GeneDesc*=cadherin 20, type 2 [Source:HGNC Symbol;Acc:1760].

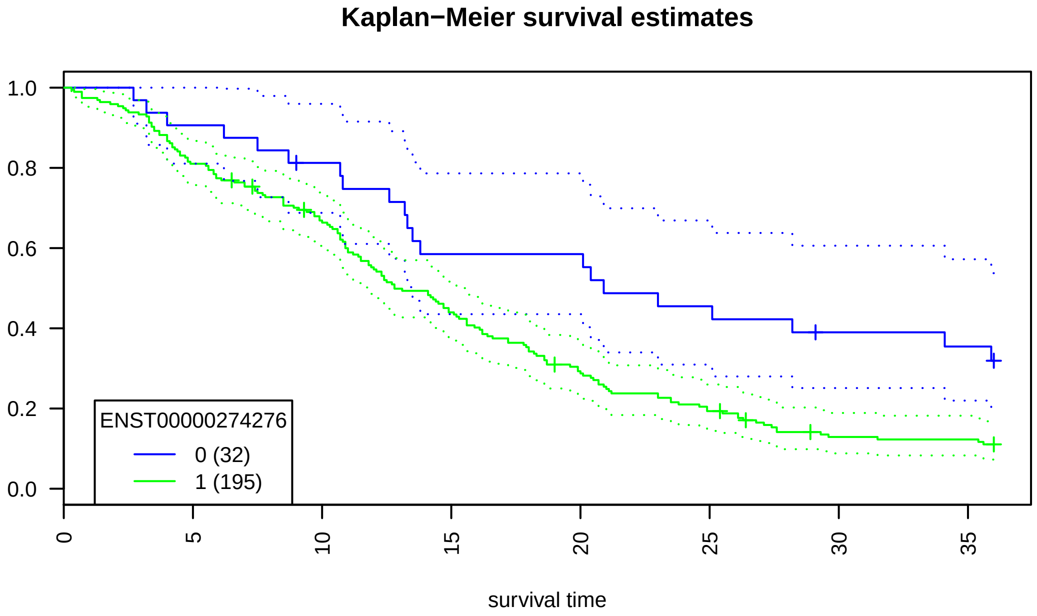

Figure 371: Each curve represents the relative number of ENST00000274276 survived to the corresponding point of time. Dashed lines represent 0.95 confidence intervals for the curves with the corresponding color. Total of 3 observations deleted due to missingness. Log-rank probability measure for the equality of these curves is 3.617339e-03. Additional annotations: .*GeneId*=ENSG00000145623, .*GeneName*=OSMR, .*GeneDesc*=oncostatin M receptor [Source:HGNC Symbol;Acc:8507].

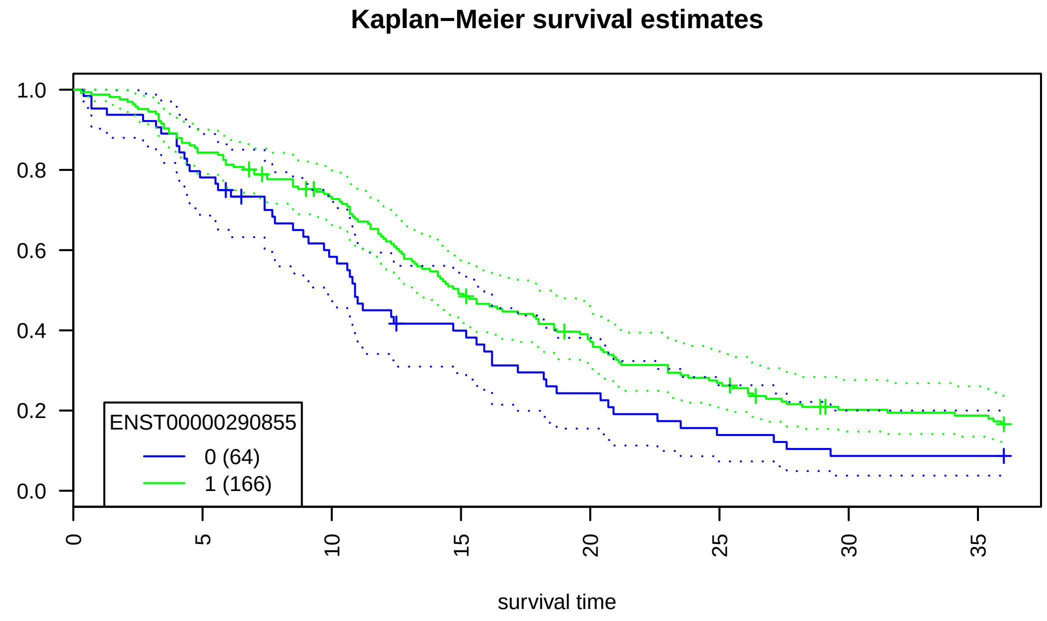

Figure 372: Each curve represents the relative number of ENST00000290855 survived to the corresponding point of time. Dashed lines represent 0.95 confidence intervals for the curves with the corresponding color. Log-rank probability measure for the equality of these curves is 1.916832e-02. Additional annotations: *.GeneId*=ENSG00000069493, *.GeneName*=CLEC2D, *.GeneDesc*=C-type lectin domain family 2, member D [Source:HGNC Symbol;Acc:14351].

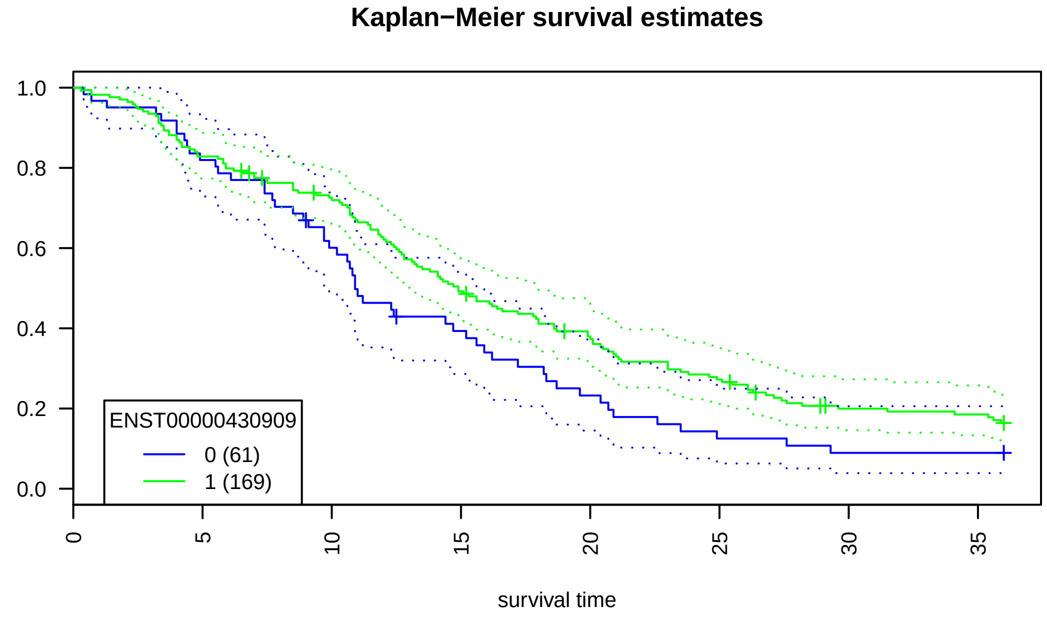

Figure 373: Each curve represents the relative number of ENST00000430909 survived to the corresponding point of time. Dashed lines represent 0.95 confidence intervals for the curves with the corresponding color. Log-rank probability measure for the equality of these curves is 3.328293e-02. Additional annotations: *.GeneId*=ENSG00000069493, *.GeneName*=CLEC2D, *.GeneDesc*=C-type lectin domain family 2, member D [Source:HGNC Symbol;Acc:14351].

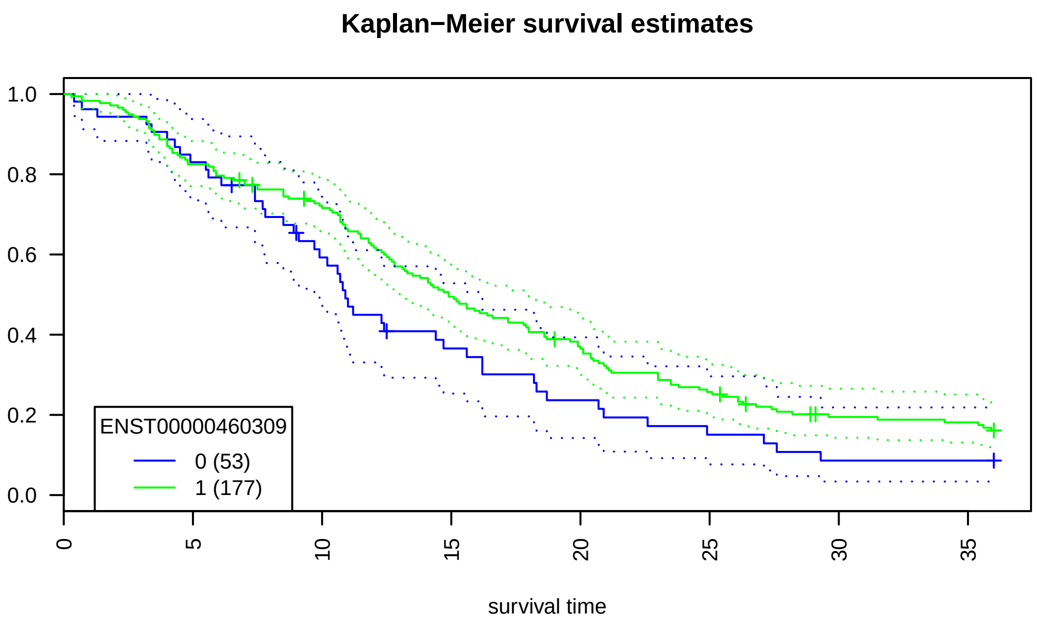

Figure 374: Each curve represents the relative number of ENST00000460309 survived to the corresponding point of time. Dashed lines represent 0.95 confidence intervals for the curves with the corresponding color. Log-rank probability measure for the equality of these curves is 4.97146e-02. Additional annotations: *.GeneId*=ENSG00000069493, *.GeneName*=CLEC2D, *.GeneDesc*=C-type lectin domain family 2, member D [Source:HGNC Symbol;Acc:14351].

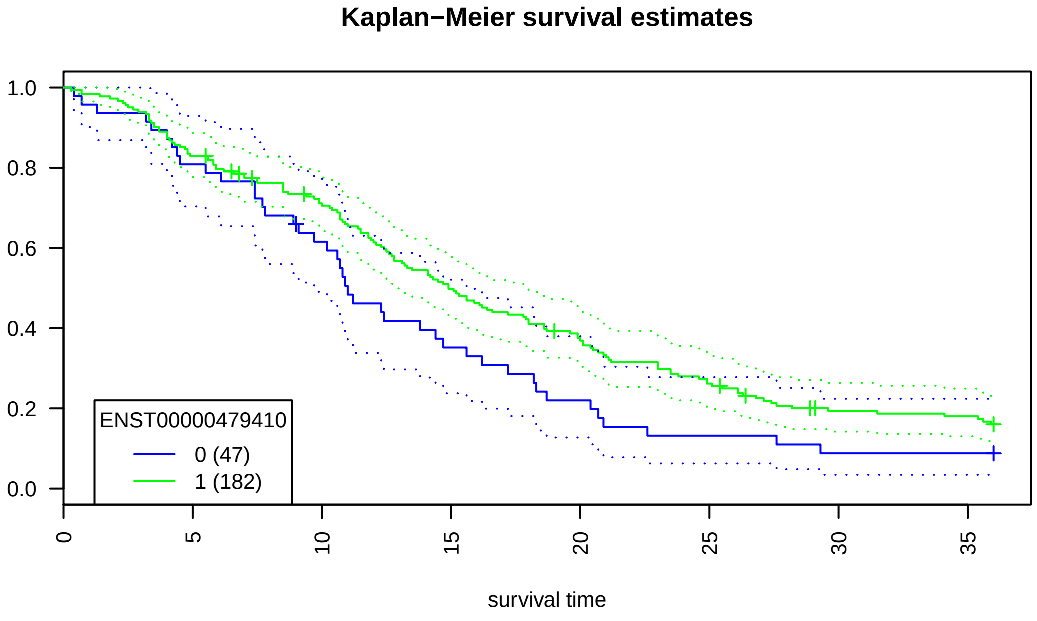

Figure 375: Each curve represents the relative number of ENST00000479410 survived to the corresponding point of time. Dashed lines represent 0.95 confidence intervals for the curves with the corresponding color. Total of 1 observation deleted due to missingness. Log-rank probability measure for the equality of these curves is 3.747357e-02. Additional annotations: *.GeneId*=ENSG00000069493, *.GeneName*=CLEC2D, *.GeneDesc*=C-type lectin domain family 2, member D [Source:HGNC Symbol;Acc:14351].

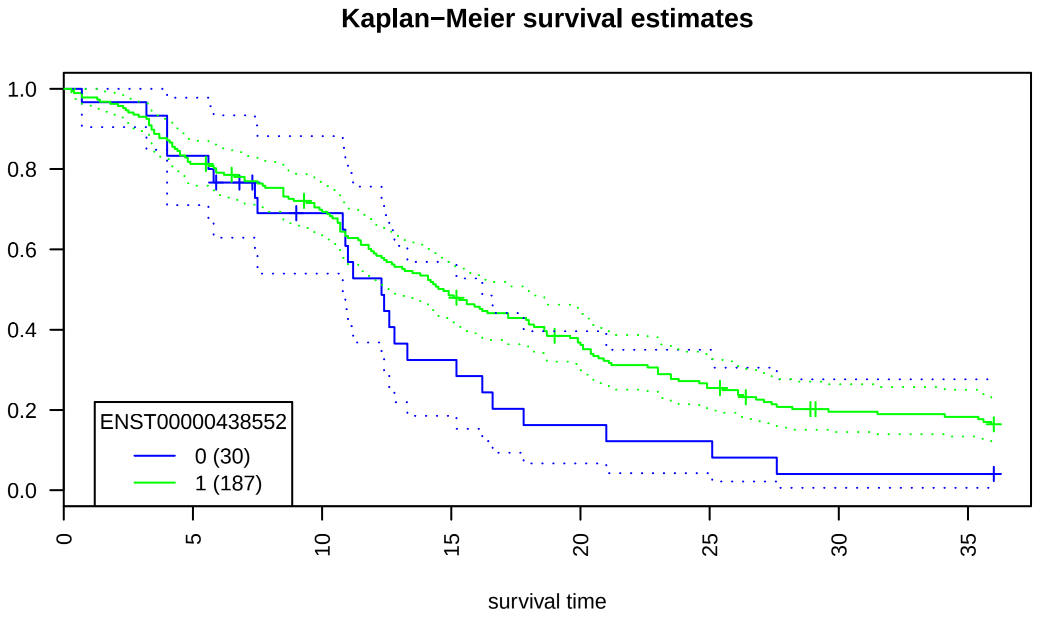

Figure 376: Each curve represents the relative number of ENST00000438552 survived to the corresponding point of time. Dashed lines represent 0.95 confidence intervals for the curves with the corresponding color. Total of 13 observations deleted due to missingness. Log-rank probability measure for the equality of these curves is 4.132925e-02. Additional annotations: *.GeneId*=ENSG00000125835, *.GeneName*=SNRPB, *.GeneDesc*=small nuclear ribonucleoprotein polypeptides B and B1 [Source:HGNC Symbol;Acc:11153].

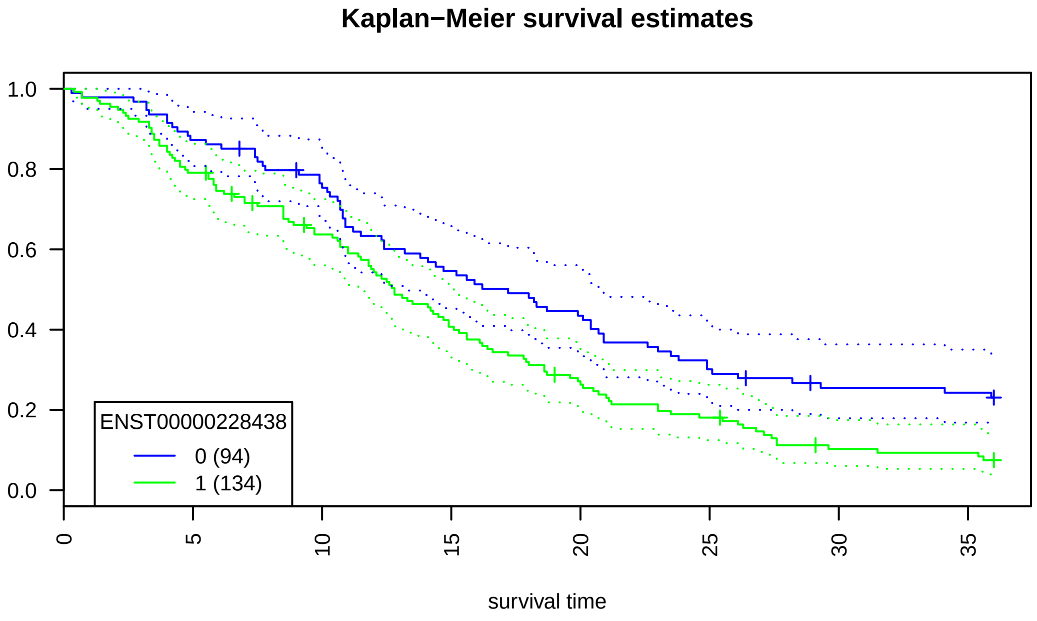

Figure 377: Each curve represents the relative number of ENST00000228438 survived to the corresponding point of time. Dashed lines represent 0.95 confidence intervals for the curves with the corresponding color. Total of 2 observations deleted due to missingness. Log-rank probability measure for the equality of these curves is 2.046411e-03. Additional annotations: *.GeneId*=ENSG00000110852, *.GeneName*=CLEC2B, *.GeneDesc*=C-type lectin domain family 2, member B [Source:HGNC Symbol;Acc:2053].

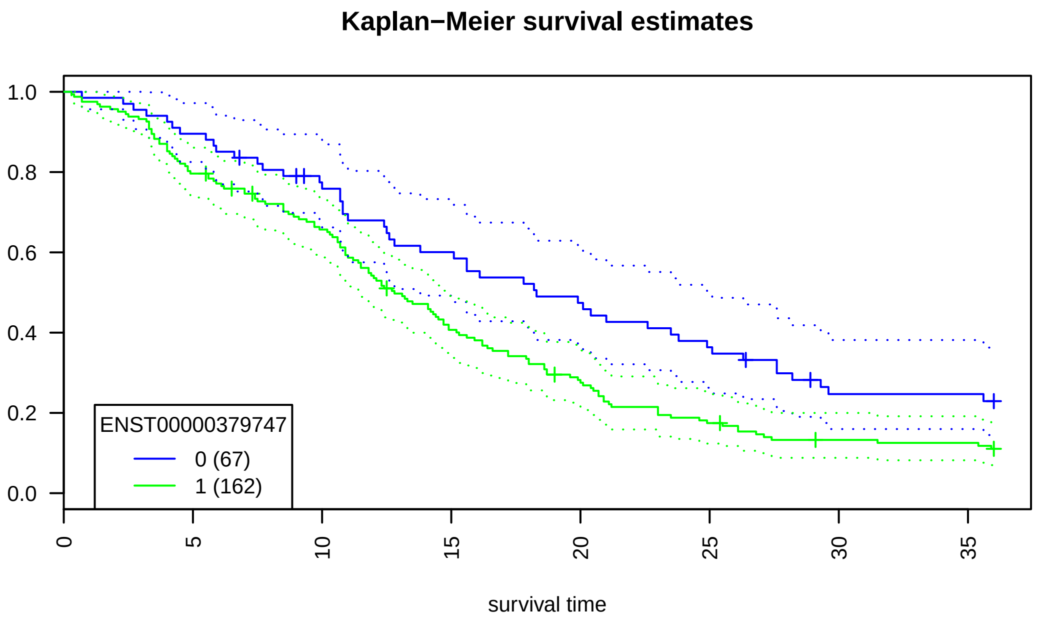

Figure 378: Each curve represents the relative number of ENST00000379747 survived to the corresponding point of time. Dashed lines represent 0.95 confidence intervals for the curves with the corresponding color. Total of 1 observation deleted due to missingness. Log-rank probability measure for the equality of these curves is 4.897593e-03. Additional annotations: *.GeneId*=ENSG00000133110, *.GeneName*=POSTN, *.GeneDesc*=periostin, osteoblast specific factor [Source:HGNC Symbol;Acc:16953].

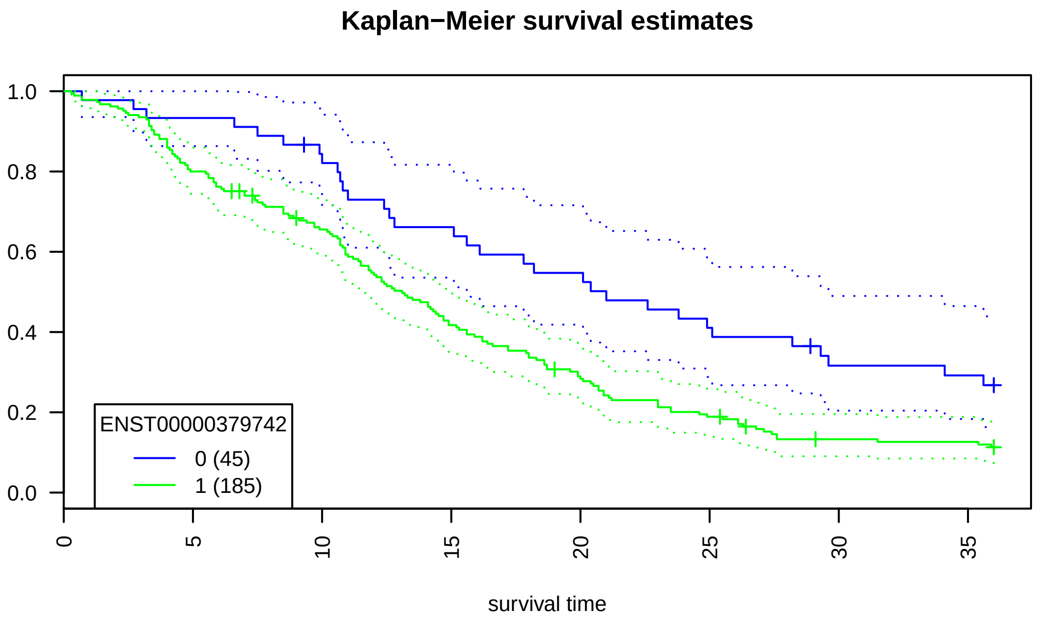

Figure 379: Each curve represents the relative number of ENST00000379742 survived to the corresponding point of time. Dashed lines represent 0.95 confidence intervals for the curves with the corresponding color. Log-rank probability measure for the equality of these curves is 1.946112e-03. Additional annotations: *.GeneId*=ENSG00000133110, *.GeneName*=POSTN, *.GeneDesc*=periostin, osteoblast specific factor [Source:HGNC Symbol;Acc:16953].

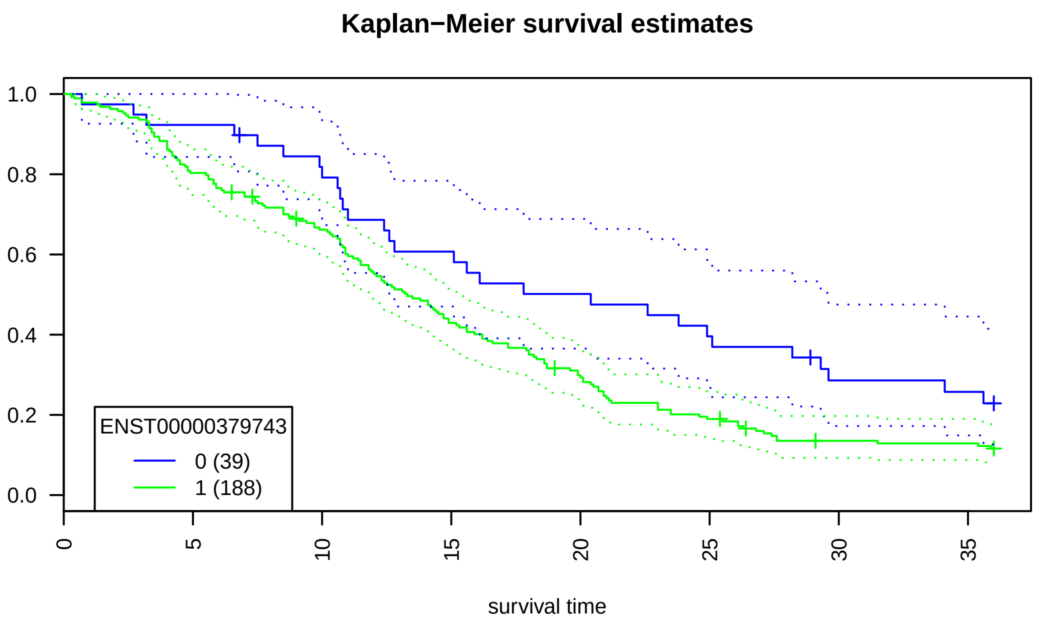

Figure 380: Each curve represents the relative number of ENST00000379743 survived to the corresponding point of time. Dashed lines represent 0.95 confidence intervals for the curves with the corresponding color. Total of 3 observations deleted due to missingness. Log-rank probability measure for the equality of these curves is 1.723576e-02. Additional annotations: *.GeneId*=ENSG00000133110, *.GeneName*=POSTN, *.GeneDesc*=periostin, osteoblast specific factor [Source:HGNC Symbol;Acc:16953].

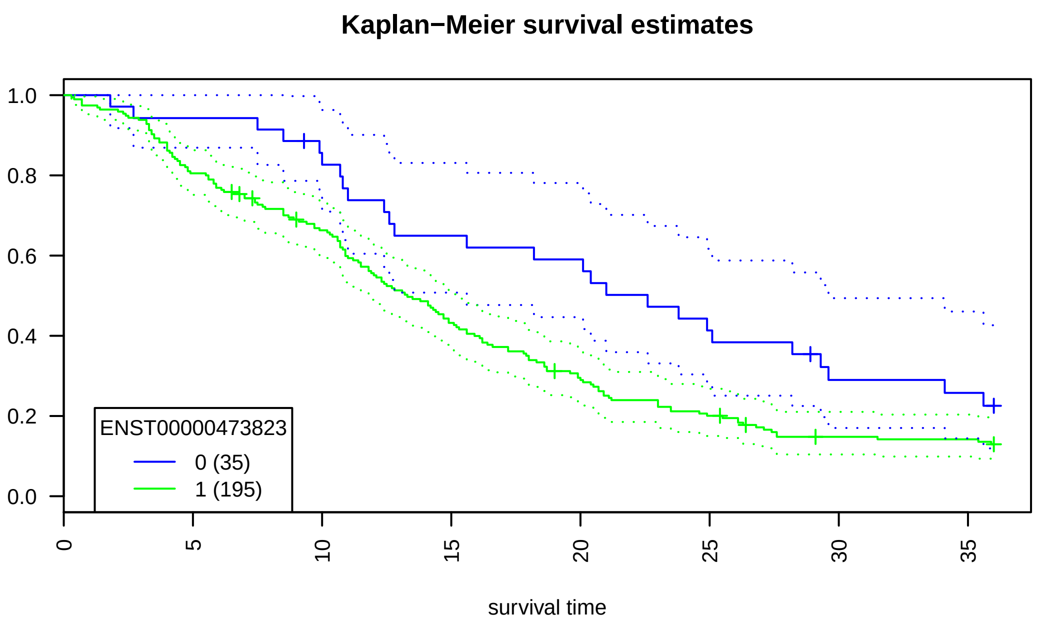

Figure 381: Each curve represents the relative number of ENST00000473823 survived to the corresponding point of time. Dashed lines represent 0.95 confidence intervals for the curves with the corresponding color. Log-rank probability measure for the equality of these curves is 1.336865e-02. Additional annotations: *.GeneId*=ENSG00000133110, *.GeneName*=POSTN, *.GeneDesc*=periostin, osteoblast specific factor [Source:HGNC Symbol;Acc:16953].

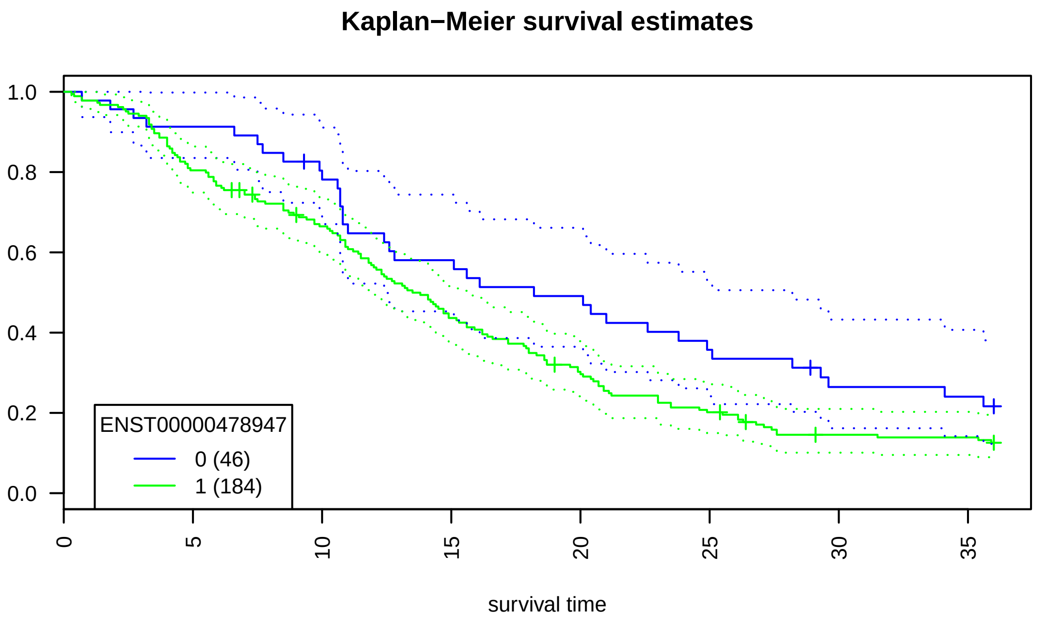

Figure 382: Each curve represents the relative number of ENST00000478947 survived to the corresponding point of time. Dashed lines represent 0.95 confidence intervals for the curves with the corresponding color. Log-rank probability measure for the equality of these curves is 4.0595e-02. Additional annotations: *.GeneId*=ENSG00000133110, *.GeneName*=POSTN, *.GeneDesc*=periostin, osteoblast specific factor [Source:HGNC Symbol;Acc:16953].

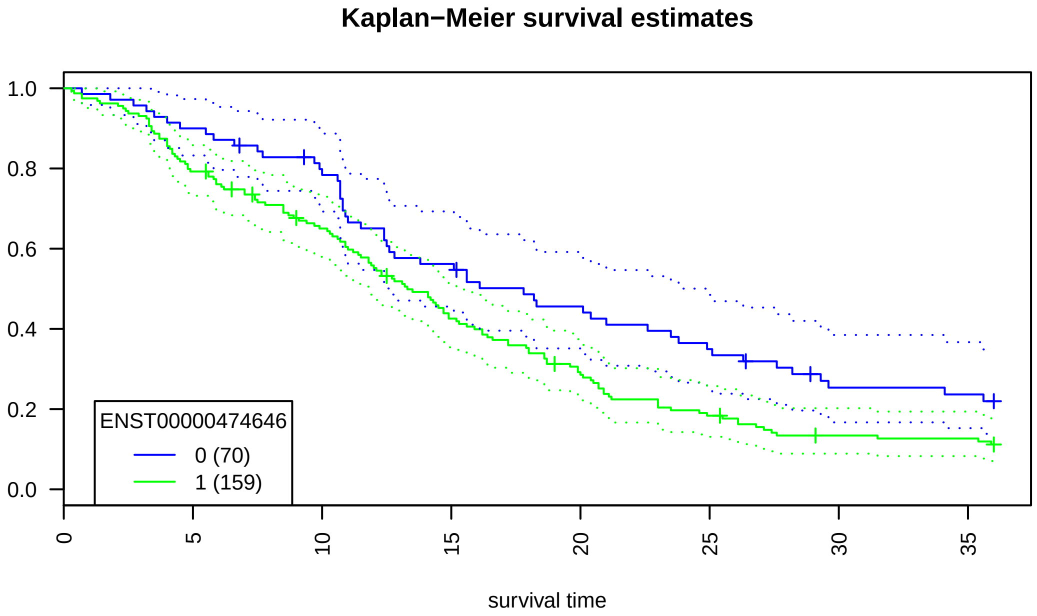

Figure 383: Each curve represents the relative number of ENST00000474646 survived to the corresponding point of time. Dashed lines represent 0.95 confidence intervals for the curves with the corresponding color. Total of 1 observation deleted due to missingness. Log-rank probability measure for the equality of these curves is 1.176622e-02. Additional annotations: *.GeneId*=ENSG00000133110, *.GeneName*=POSTN, *.GeneDesc*=periostin, osteoblast specific factor [Source:HGNC Symbol;Acc:16953].

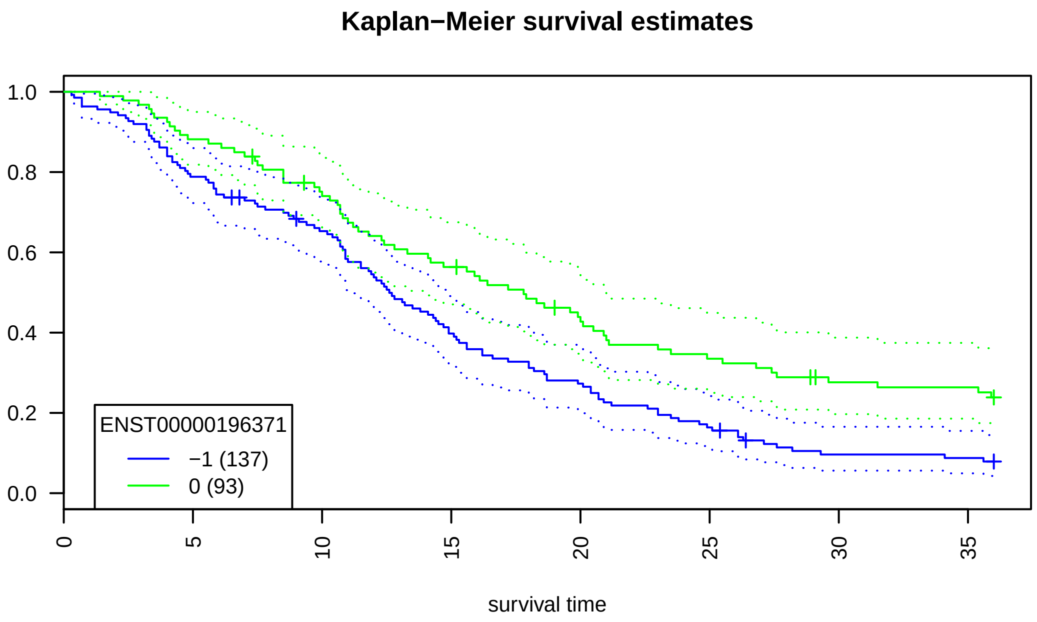

Figure 384: Each curve represents the relative number of ENST00000196371 survived to the corresponding point of time. Dashed lines represent 0.95 confidence intervals for the curves with the corresponding color. Log-rank probability measure for the equality of these curves is 7.464405e-04. Additional annotations: *.GeneId*=ENSG00000083720, *.GeneName*=OXCT1, *.GeneDesc*=3-oxoacid CoA transferase 1 [Source:HGNC Symbol;Acc:8527].

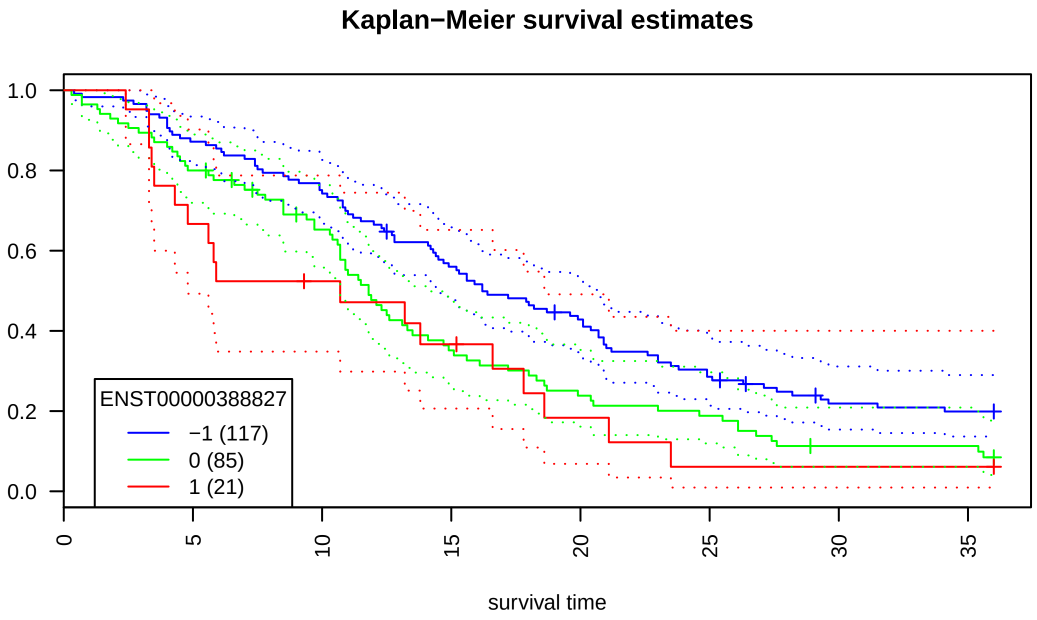

Figure 385: Each curve represents the relative number of ENST00000388827 survived to the corresponding point of time. Dashed lines represent 0.95 confidence intervals for the curves with the corresponding color. Total of 7 observations deleted due to missingness. Log-rank probability measure for the equality of these curves is 3.144377e-03. Additional annotations: *.GeneId*=ENSG00000198865, *.GeneName*=CCDC152, *.GeneDesc*=coiled-coil domain containing 152 [Source:HGNC Symbol;Acc:34438].

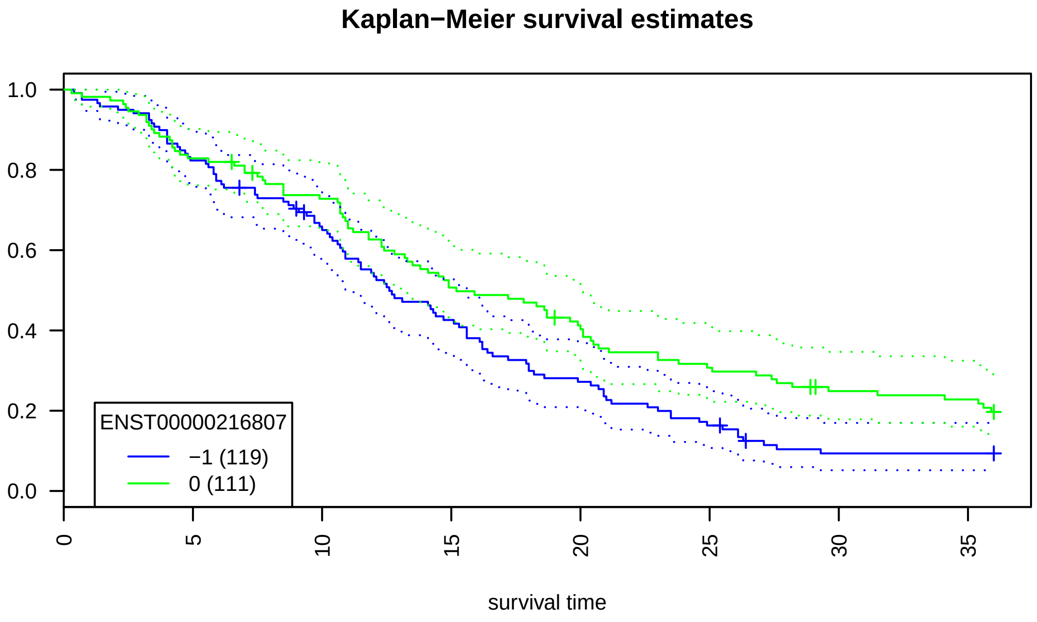

Figure 386: Each curve represents the relative number of ENST00000216807 survived to the corresponding point of time. Dashed lines represent 0.95 confidence intervals for the curves with the corresponding color. Log-rank probability measure for the equality of these curves is 1.283744e-02. Additional annotations: *.GeneId*=ENSG00000100916, *.GeneName*=BRMS1L, *.GeneDesc*=breast cancer metastasis-suppressor 1-like [Source:HGNC Symbol;Acc:20512].

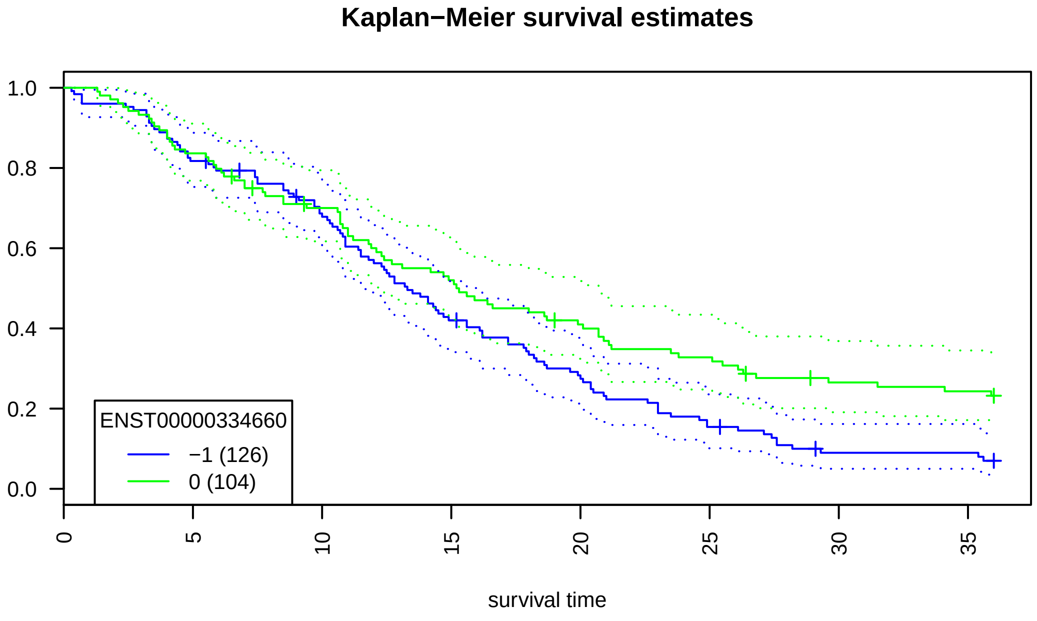

Figure 387: Each curve represents the relative number of ENST00000334660 survived to the corresponding point of time. Dashed lines represent 0.95 confidence intervals for the curves with the corresponding color. Log-rank probability measure for the equality of these curves is 8.178639e-03. Additional annotations: *.GeneId*=ENSG00000187446, *.GeneName*=AC012652.1, *.GeneDesc*=Calcium-binding protein p22 (Calcium-binding protein CHP)(Calcineurin homologous protein)(Calcineurin B homolog) [Source:UniProtKB/Swiss-Prot;Acc:Q99653].

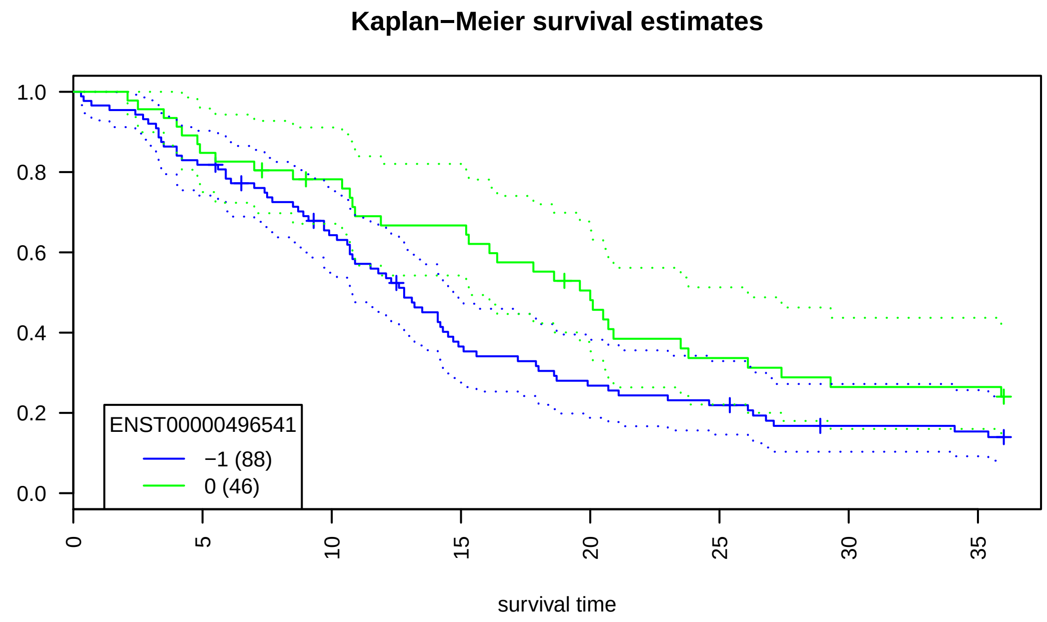

Figure 388: Each curve represents the relative number of ENST00000496541 survived to the corresponding point of time. Dashed lines represent 0.95 confidence intervals for the curves with the corresponding color. Total of 96 observations deleted due to missingness. Log-rank probability measure for the equality of these curves is 3.936969e-02. Additional annotations: *.GeneId*=ENSG00000162039, *.GeneName*=C16orf73, *.GeneDesc*=Uncharacterized protein C16orf73 [Source:UniProtKB/Swiss-Prot;Acc:Q8N635].

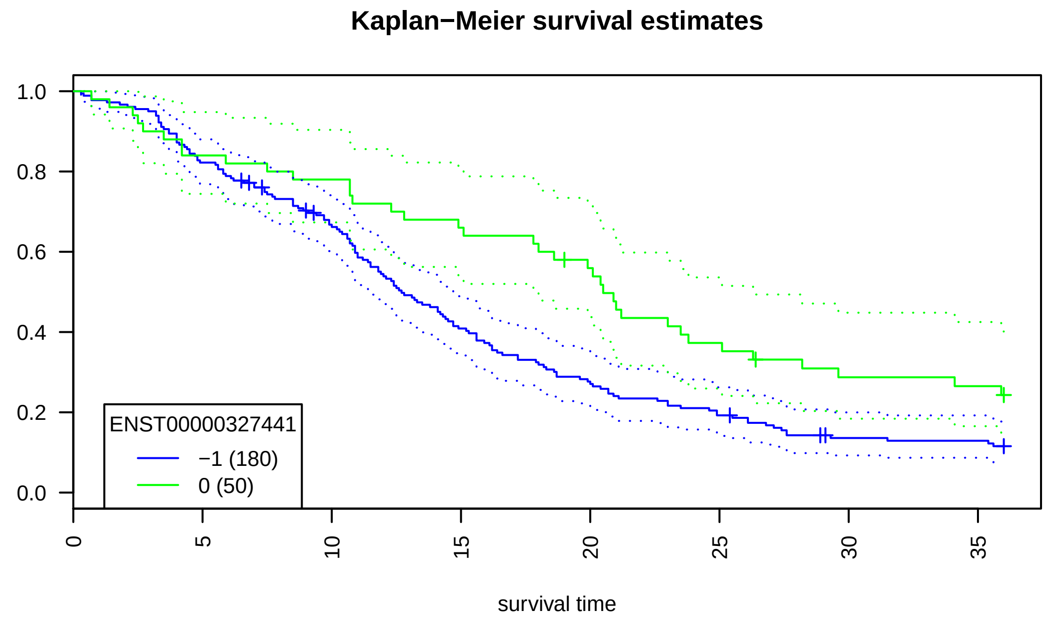

Figure 389: Each curve represents the relative number of ENST00000327441 survived to the corresponding point of time. Dashed lines represent 0.95 confidence intervals for the curves with the corresponding color. Log-rank probability measure for the equality of these curves is 4.576582e-03. Additional annotations: *.GeneId*=ENSG00000151338, *.GeneName*=MIPOL1, *.GeneDesc*=mirror-image polydactyly 1 [Source:HGNC Symbol;Acc:21460].

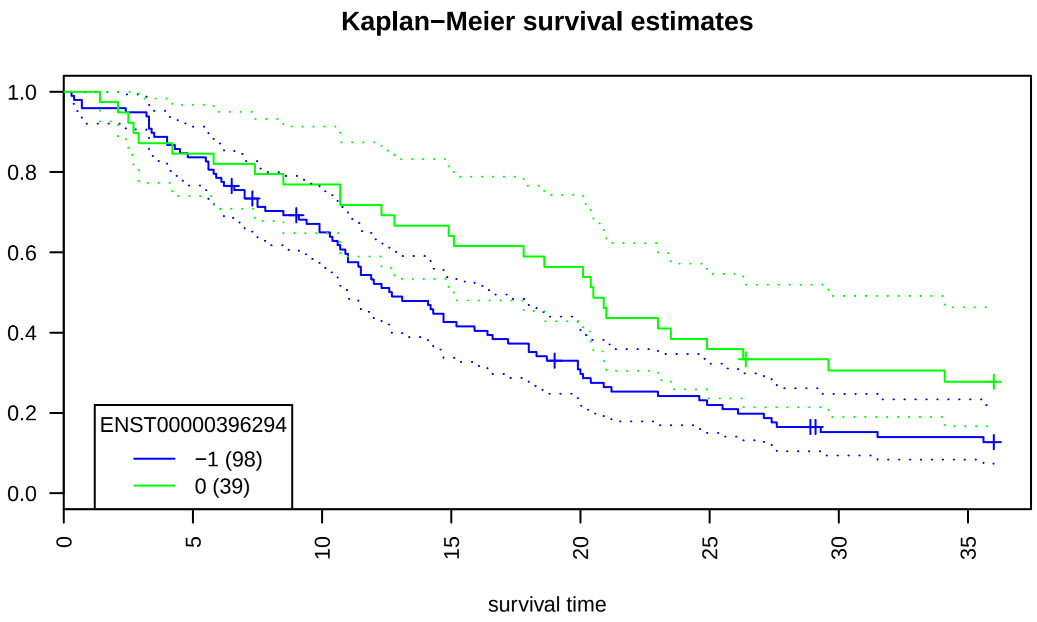

Figure 390: Each curve represents the relative number of ENST00000396294 survived to the corresponding point of time. Dashed lines represent 0.95 confidence intervals for the curves with the corresponding color. Total of 93 observations deleted due to missingness. Log-rank probability measure for the equality of these curves is 2.355003e-02. Additional annotations: *.GeneId*=ENSG00000151338, *.GeneName*=MIPOL1, *.GeneDesc*=mirror-image polydactyly 1 [Source:HGNC Symbol;Acc:21460].

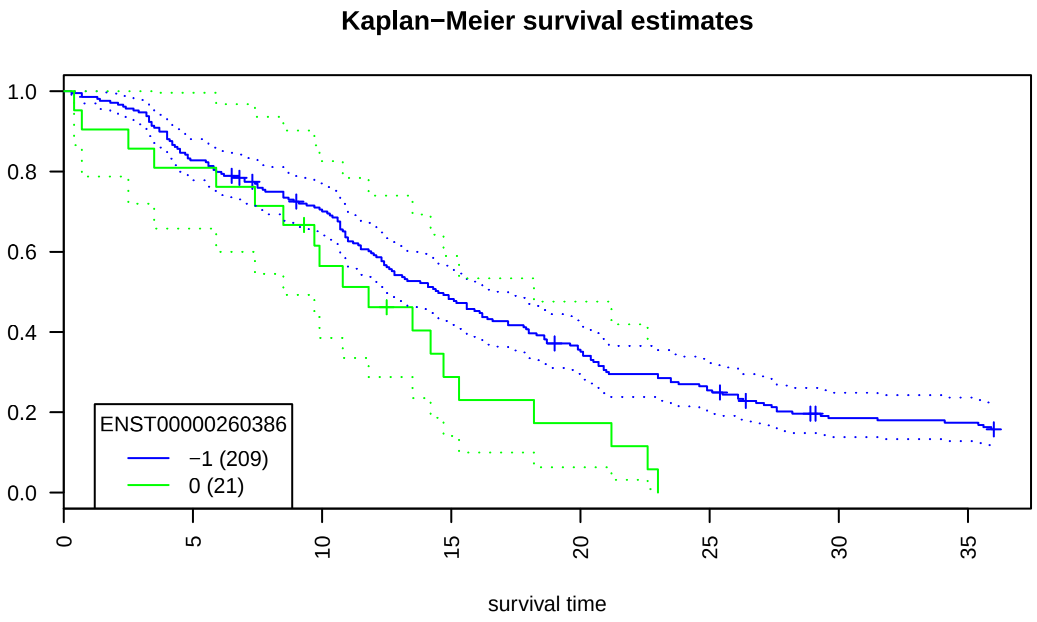

Figure 391: Each curve represents the relative number of ENST00000260386 survived to the corresponding point of time. Dashed lines represent 0.95 confidence intervals for the curves with the corresponding color. Log-rank probability measure for the equality of these curves is 1.756051e-02. Additional annotations: *.GeneId*=ENSG00000137825, *.GeneName*=ITPKA, *.GeneDesc*=inositol 1,4,5-trisphosphate 3-kinase A [Source:HGNC Symbol;Acc:6178].

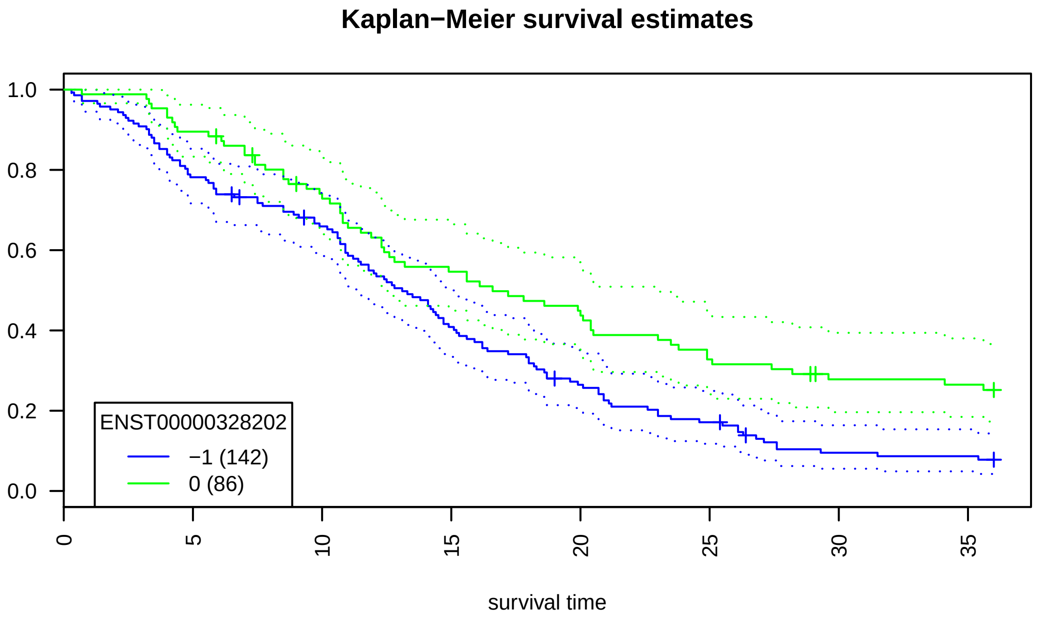

Figure 392: Each curve represents the relative number of ENST00000328202 survived to the corresponding point of time. Dashed lines represent 0.95 confidence intervals for the curves with the corresponding color. Total of 2 observations deleted due to missingness. Log-rank probability measure for the equality of these curves is 1.011498e-03. Additional annotations: *.GeneId*=ENSG00000182139, *.GeneName*=AC123788.1, *.GeneDesc*=Putative uncharacterized protein [Source:UniProtKB/TrEMBL;Acc:Q96FU4].

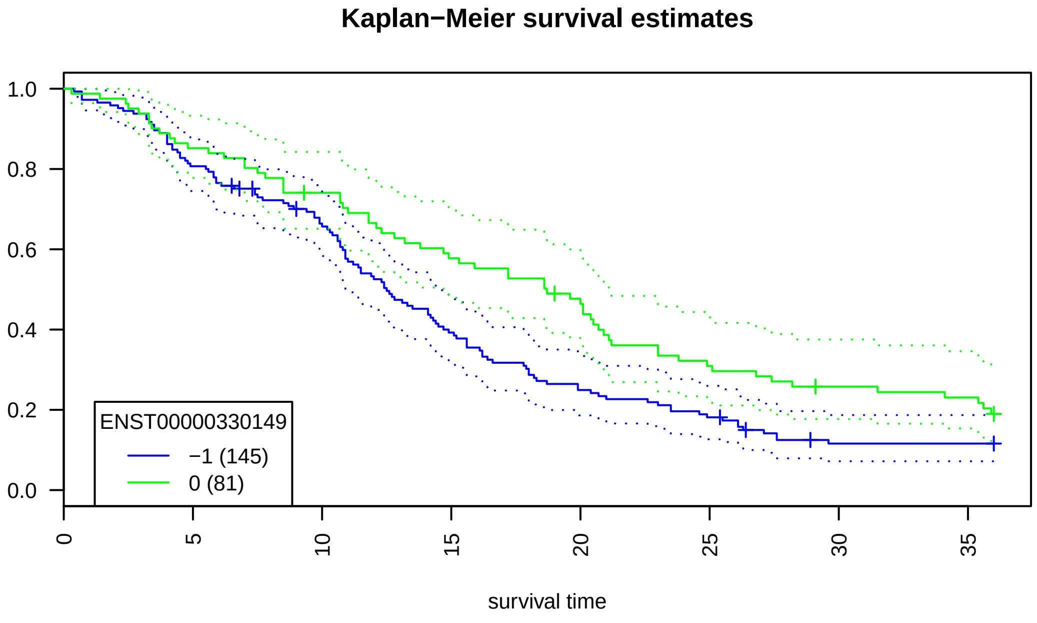

Figure 393: Each curve represents the relative number of ENST00000330149 survived to the corresponding point of time. Dashed lines represent 0.95 confidence intervals for the curves with the corresponding color. Total of 4 observations deleted due to missingness. Log-rank probability measure for the equality of these curves is 1.192687e-02. Additional annotations: *.GeneId*=ENSG00000182400, *.GeneName*=TRAPPC6B, *.GeneDesc*=trafficking protein particle complex 6B [Source:HGNC Symbol;Acc:23066].

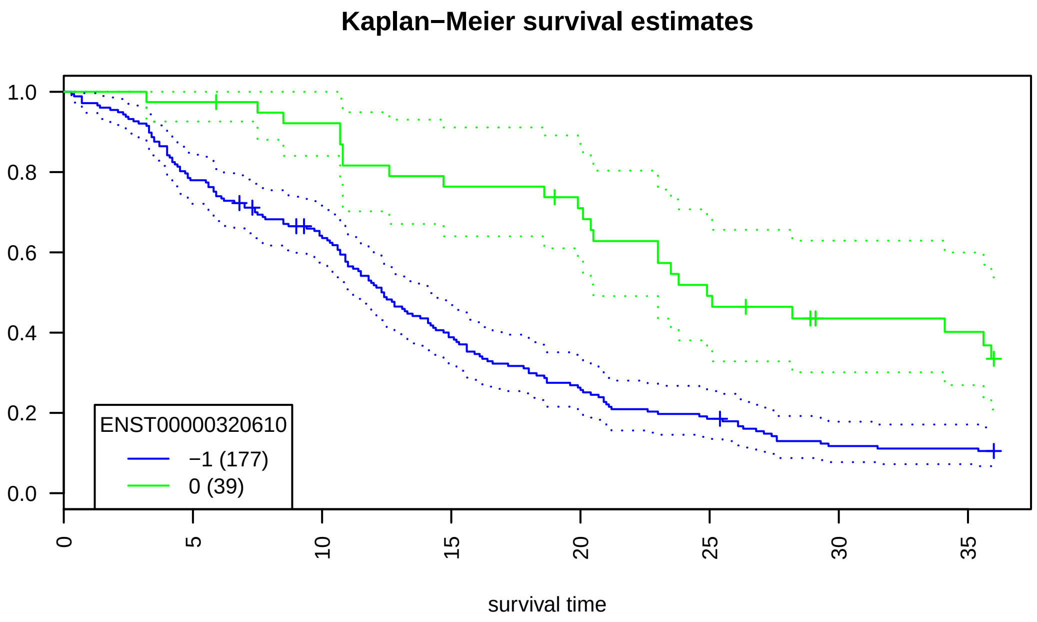

Figure 394: Each curve represents the relative number of ENST00000320610 survived to the corresponding point of time. Dashed lines represent 0.95 confidence intervals for the curves with the corresponding color. Total of 14 observations deleted due to missingness. Log-rank probability measure for the equality of these curves is 1.229427e-05. Additional annotations: *.GeneId*=ENSG00000130856, *.GeneName*=ZNF236, *.GeneDesc*=zinc finger protein 236 [Source:HGNC Symbol;Acc:13028].

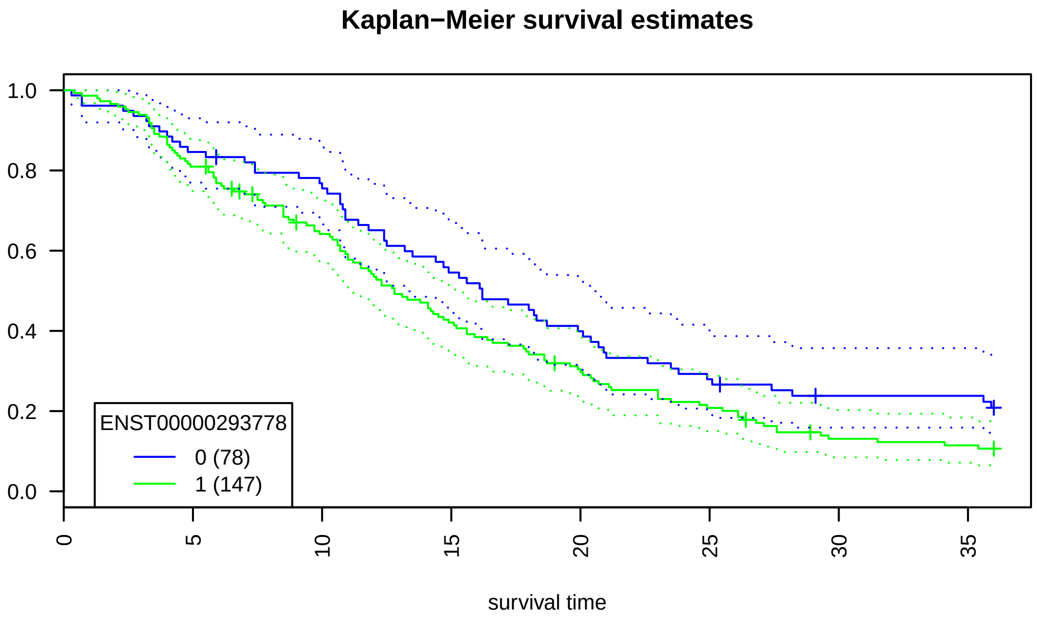

Figure 395: Each curve represents the relative number of ENST00000293778 survived to the corresponding point of time. Dashed lines represent 0.95 confidence intervals for the curves with the corresponding color. Total of 5 observations deleted due to missingness. Log-rank probability measure for the equality of these curves is 4.316051e-02. Additional annotations: *.GeneId*=ENSG00000161921, *.GeneName*=CXCL16, *.GeneDesc*=chemokine (C-X-C motif) ligand 16 [Source:HGNC Symbol;Acc:16642].

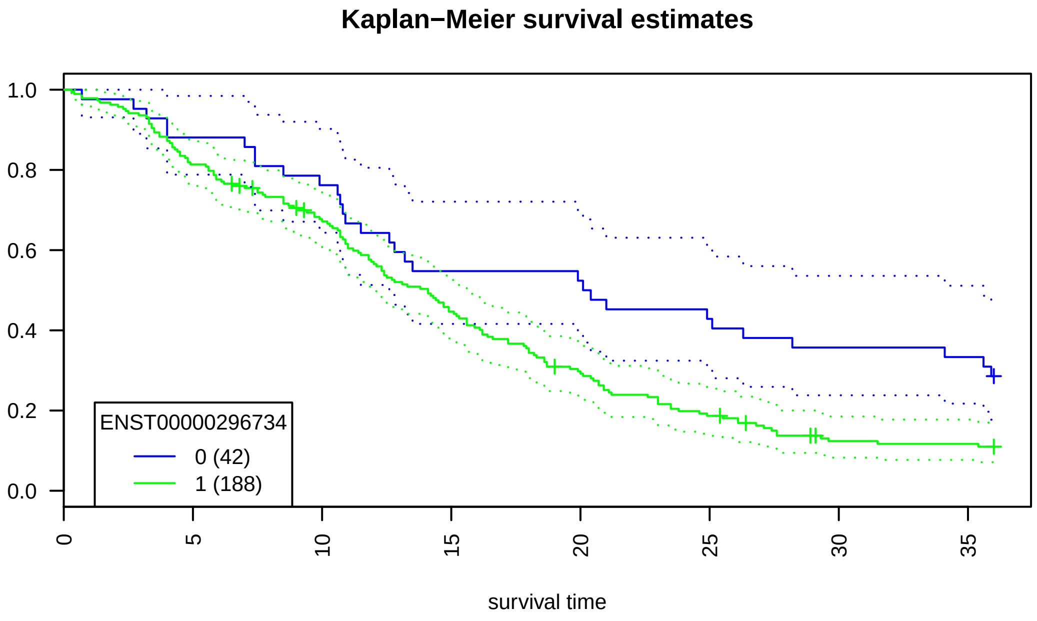

Figure 396: Each curve represents the relative number of ENST00000296734 survived to the corresponding point of time. Dashed lines represent 0.95 confidence intervals for the curves with the corresponding color. Log-rank probability measure for the equality of these curves is 5.936744e-03. Additional annotations: *.GeneId*=ENSG00000164294, *.GeneName*=GPX8, *.GeneDesc*=glutathione peroxidase 8 (putative) [Source:HGNC Symbol;Acc:33100].

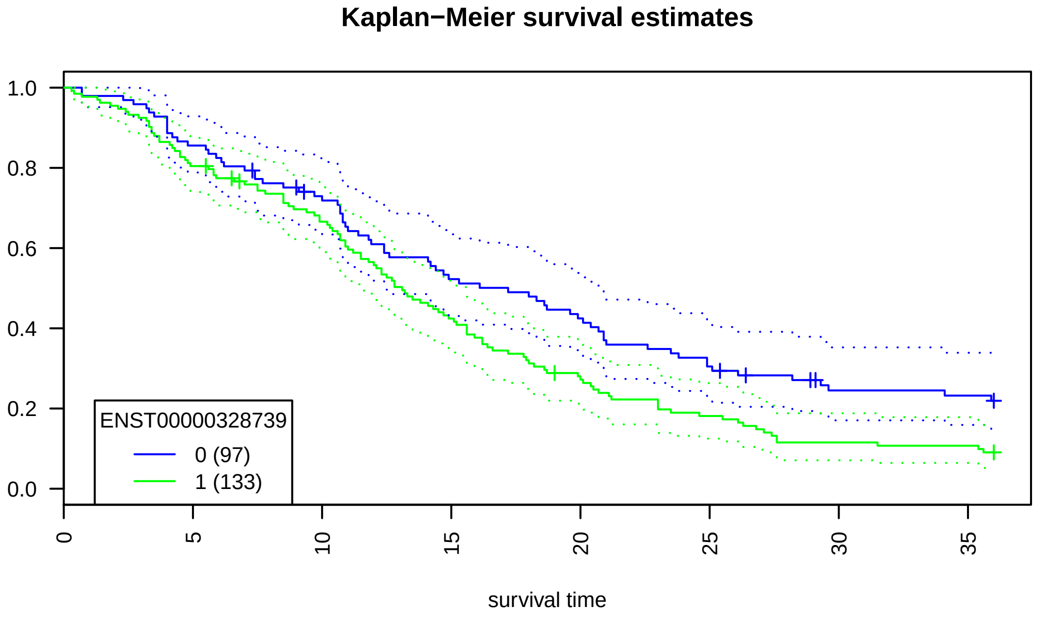

Figure 397: Each curve represents the relative number of ENST00000328739 survived to the corresponding point of time. Dashed lines represent 0.95 confidence intervals for the curves with the corresponding color. Log-rank probability measure for the equality of these curves is 9.52681e-03. Additional annotations: *.GeneId*=ENSG00000182853, *.GeneName*=VMO1, *.GeneDesc*=vitelline membrane outer layer 1 homolog (chicken) [Source:HGNC Symbol;Acc:30387].

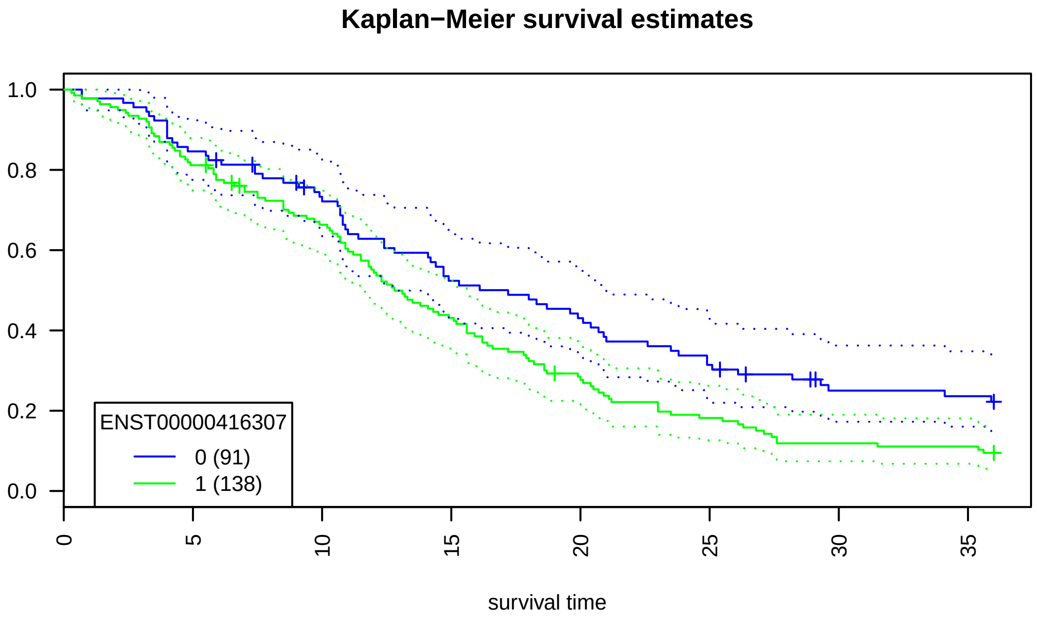

Figure 398: Each curve represents the relative number of ENST00000416307 survived to the corresponding point of time. Dashed lines represent 0.95 confidence intervals for the curves with the corresponding color. Total of 1 observation deleted due to missingness. Log-rank probability measure for the equality of these curves is 8.99011e-03. Additional annotations: *.GeneId*=ENSG00000182853, *.GeneName*=VMO1, *.GeneDesc*=vitelline membrane outer layer 1 homolog (chicken) [Source:HGNC Symbol;Acc:30387].

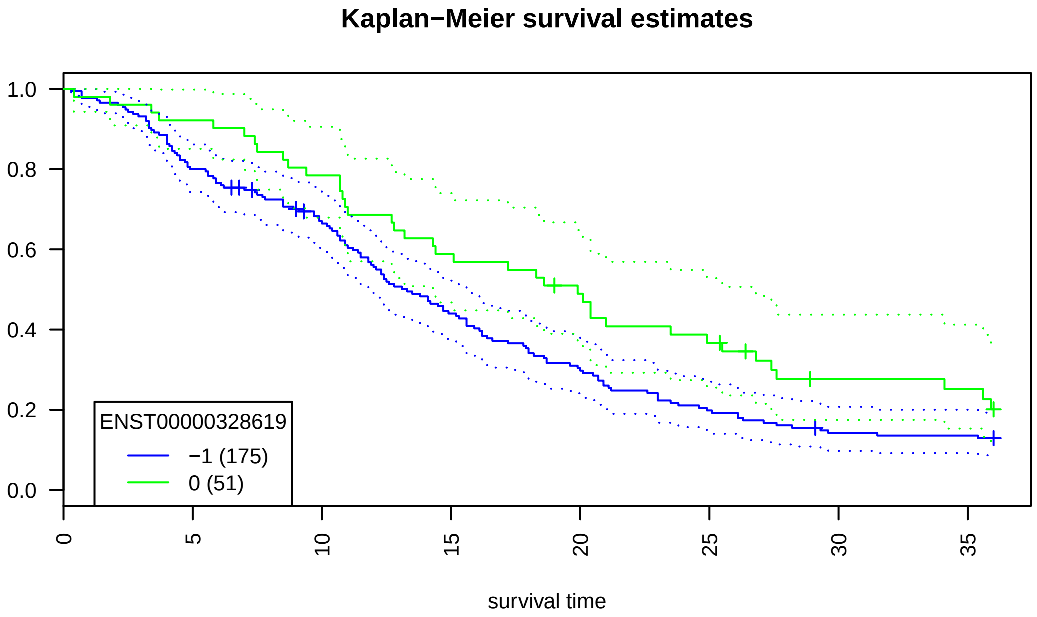

Figure 399: Each curve represents the relative number of ENST00000328619 survived to the corresponding point of time. Dashed lines represent 0.95 confidence intervals for the curves with the corresponding color. Total of 4 observations deleted due to missingness. Log-rank probability measure for the equality of these curves is 2.890229e-02. Additional annotations: *.GeneId*=ENSG00000183036, *.GeneName*=PCP4, *.GeneDesc*=Purkinje cell protein 4 [Source:HGNC Symbol;Acc:8742].

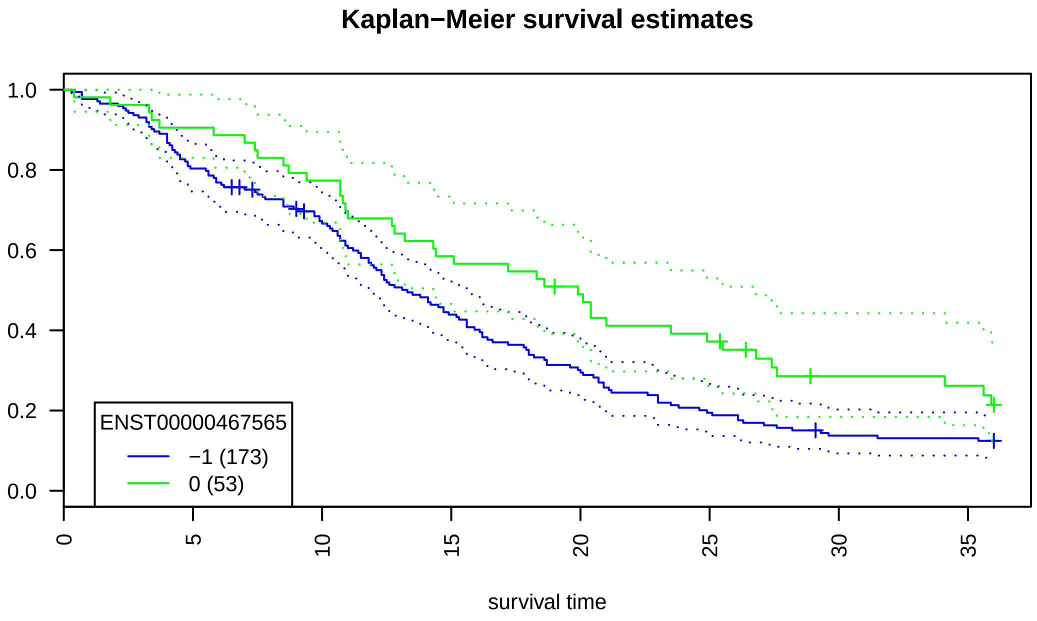

Figure 400: Each curve represents the relative number of ENST00000467565 survived to the corresponding point of time. Dashed lines represent 0.95 confidence intervals for the curves with the corresponding color. Total of 4 observations deleted due to missingness. Log-rank probability measure for the equality of these curves is 2.017722e-02. Additional annotations: *.GeneId*=ENSG00000183036, *.GeneName*=PCP4, *.GeneDesc*=Purkinje cell protein 4 [Source:HGNC Symbol;Acc:8742].

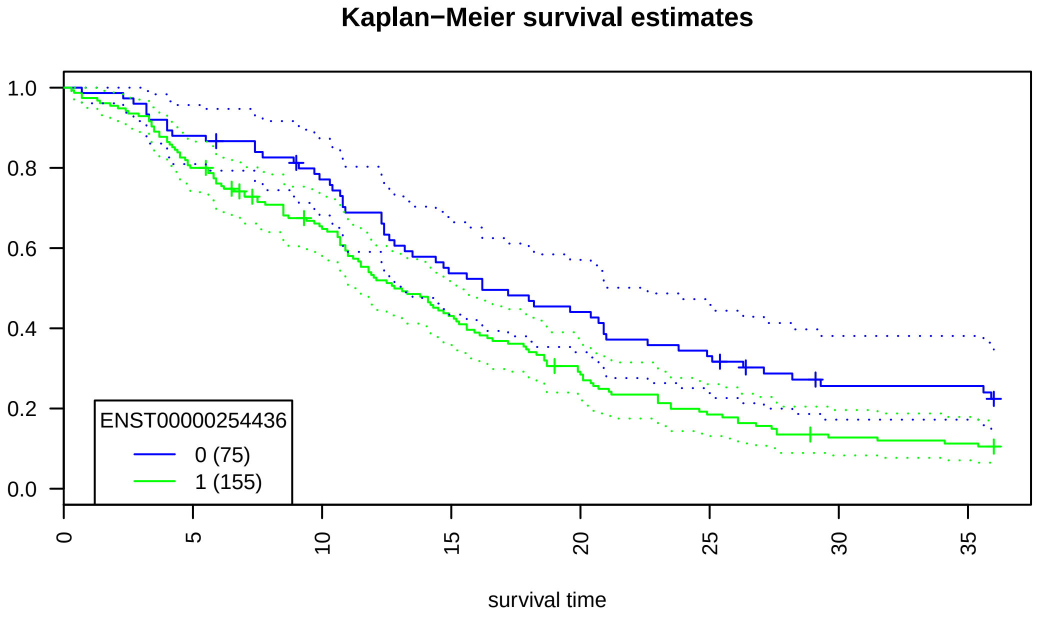

Figure 401: Each curve represents the relative number of ENST00000254436 survived to the corresponding point of time. Dashed lines represent 0.95 confidence intervals for the curves with the corresponding color. Log-rank probability measure for the equality of these curves is 9.76697e-03. Additional annotations: *.GeneId*=ENSG00000132109, *.GeneName*=TRIM21, *.GeneDesc*=tripartite motif-containing 21 [Source:HGNC Symbol;Acc:11312].

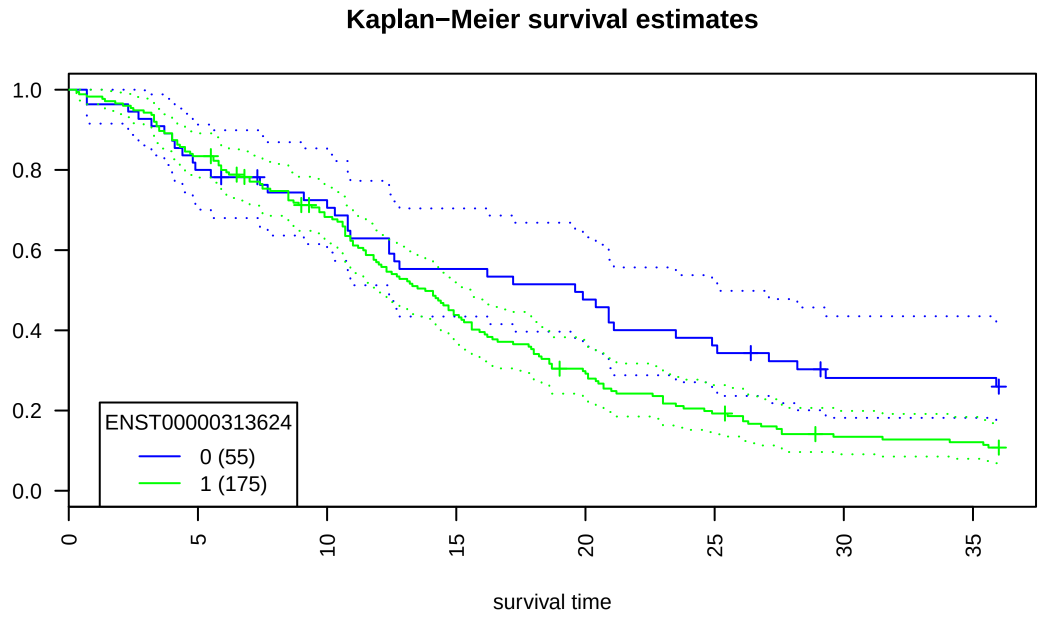

Figure 402: Each curve represents the relative number of ENST00000313624 survived to the corresponding point of time. Dashed lines represent 0.95 confidence intervals for the curves with the corresponding color. Log-rank probability measure for the equality of these curves is 1.91909e-02. Additional annotations: *.GeneId*=ENSG00000133106, *.GeneName*=EPSTI1, *.GeneDesc*=epithelial stromal interaction 1 (breast) [Source:HGNC Symbol;Acc:16465].

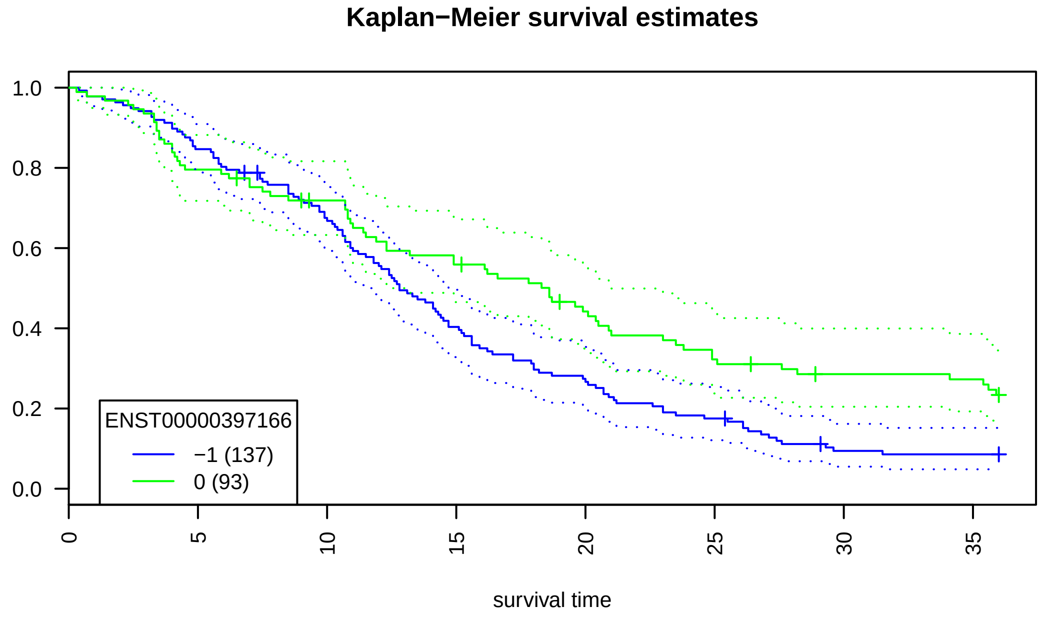

Figure 403: Each curve represents the relative number of ENST00000397166 survived to the corresponding point of time. Dashed lines represent 0.95 confidence intervals for the curves with the corresponding color. Log-rank probability measure for the equality of these curves is 3.746542e-03. Additional annotations: *.GeneId*=ENSG00000085788, *.GeneName*=DDHD2, *.GeneDesc*=DDHD domain containing 2 [Source:HGNC Symbol;Acc:29106].

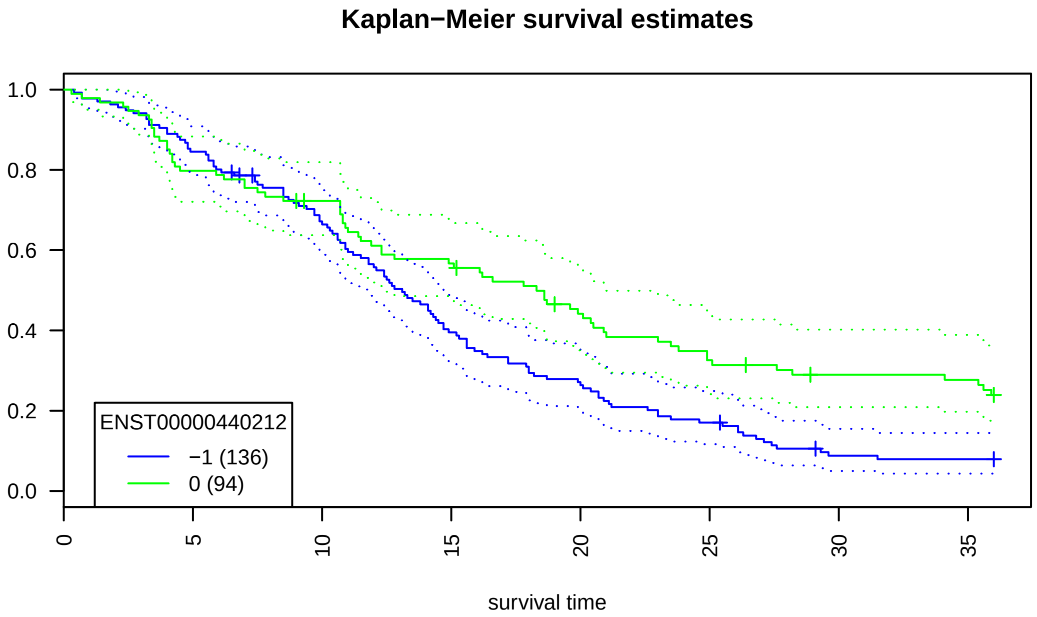

Figure 404: Each curve represents the relative number of ENST00000440212 survived to the corresponding point of time. Dashed lines represent 0.95 confidence intervals for the curves with the corresponding color. Log-rank probability measure for the equality of these curves is 2.129356e-03. Additional annotations: *.GeneId*=ENSG00000085788, *.GeneName*=DDHD2, *.GeneDesc*=DDHD domain containing 2 [Source:HGNC Symbol;Acc:29106].

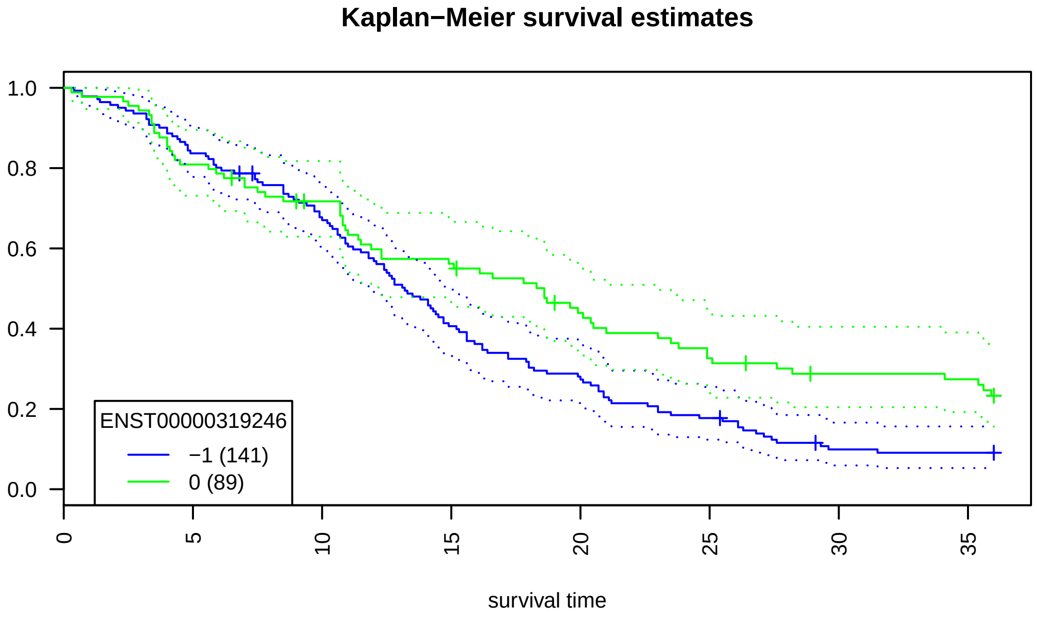

Figure 405: Each curve represents the relative number of ENST00000319246 survived to the corresponding point of time. Dashed lines represent 0.95 confidence intervals for the curves with the corresponding color. Log-rank probability measure for the equality of these curves is 6.042226e-03. Additional annotations: *.GeneId*=ENSG00000085788, *.GeneName*=DDHD2, *.GeneDesc*=DDHD domain containing 2 [Source:HGNC Symbol;Acc:29106].

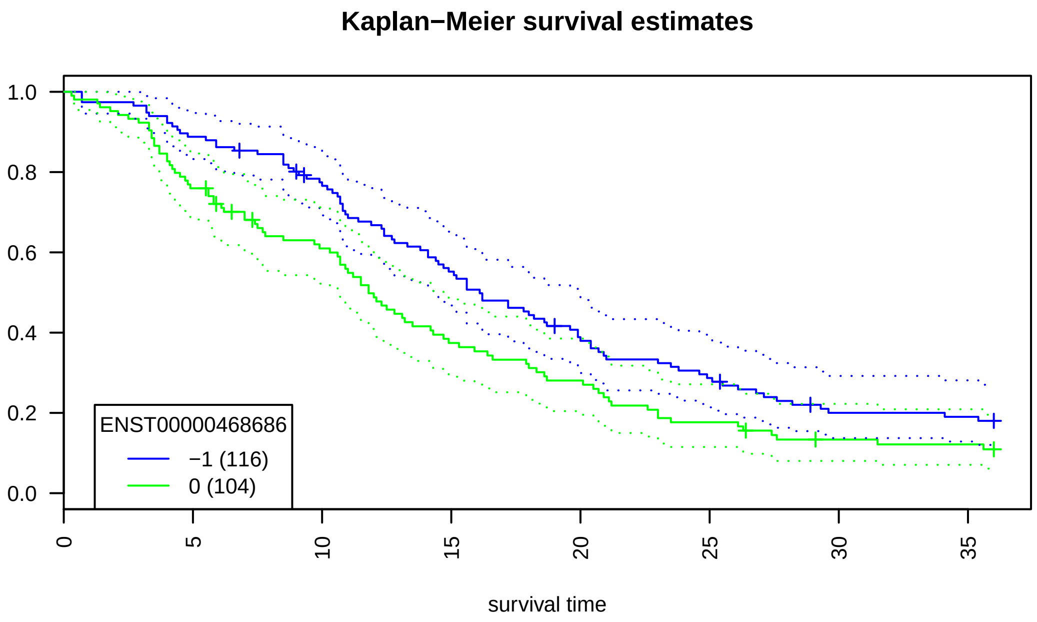

Figure 406: Each curve represents the relative number of ENST00000468686 survived to the corresponding point of time. Dashed lines represent 0.95 confidence intervals for the curves with the corresponding color. Total of 10 observations deleted due to missingness. Log-rank probability measure for the equality of these curves is 1.246880e-02. Additional annotations: *.GeneId*=ENSG00000161179, *.GeneName*=YDJC, *.GeneDesc*=YdjC homolog (bacterial) [Source:HGNC Symbol;Acc:27158].

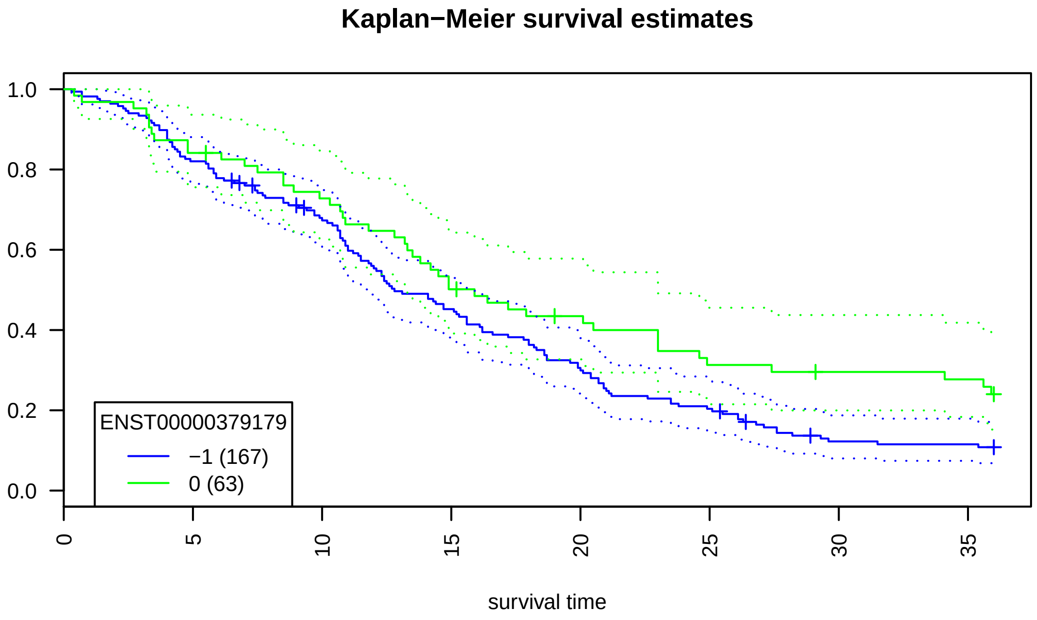

Figure 407: Each curve represents the relative number of ENST00000379179 survived to the corresponding point of time. Dashed lines represent 0.95 confidence intervals for the curves with the corresponding color. Log-rank probability measure for the equality of these curves is 2.846706e-02. Additional annotations: *.GeneId*=ENSG00000151778, *.GeneName*=SERP2, *.GeneDesc*=stress-associated endoplasmic reticulum protein family member 2 [Source:HGNC Symbol;Acc:20607].

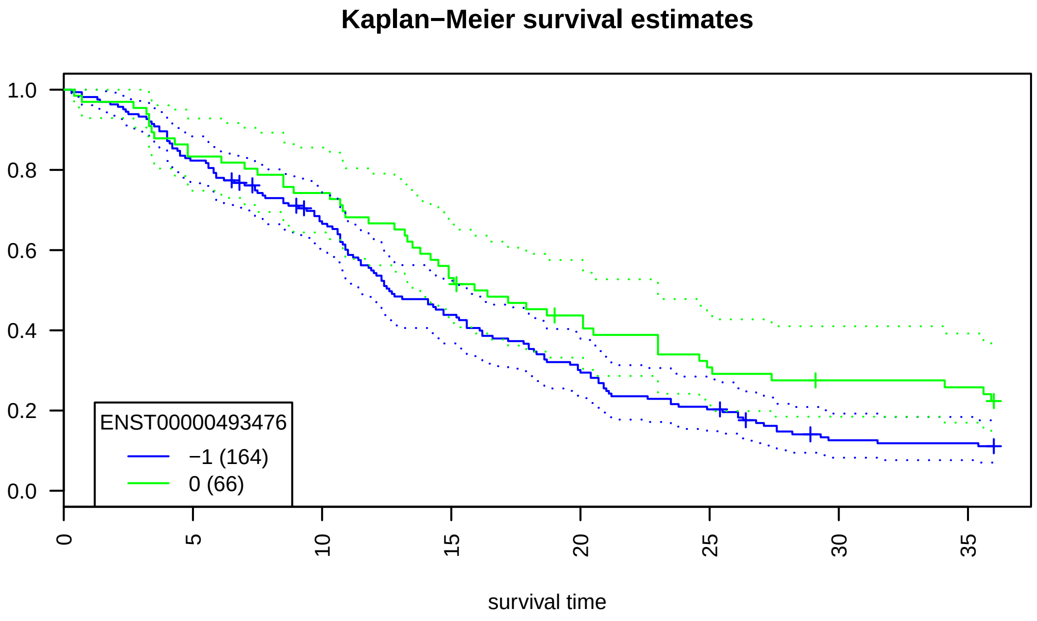

Figure 408: Each curve represents the relative number of ENST00000493476 survived to the corresponding point of time. Dashed lines represent 0.95 confidence intervals for the curves with the corresponding color. Log-rank probability measure for the equality of these curves is 3.172853e-02. Additional annotations: *.GeneId*=ENSG00000151778, *.GeneName*=SERP2, *.GeneDesc*=stress-associated endoplasmic reticulum protein family member 2 [Source:HGNC Symbol;Acc:20607].

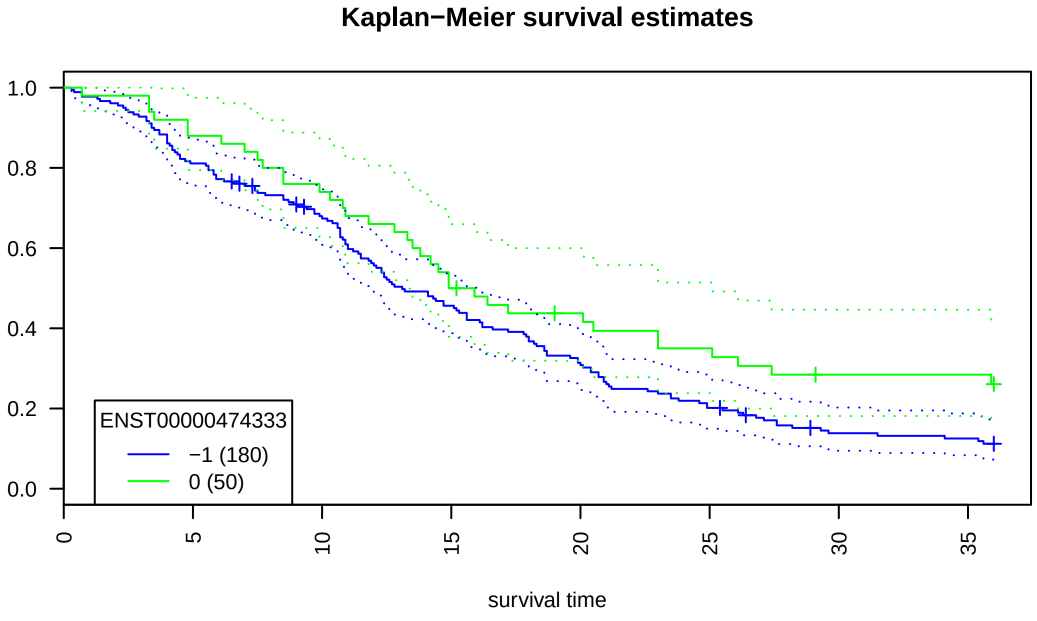

Figure 409: Each curve represents the relative number of ENST00000474333 survived to the corresponding point of time. Dashed lines represent 0.95 confidence intervals for the curves with the corresponding color. Log-rank probability measure for the equality of these curves is 3.350642e-02. Additional annotations: *.GeneId*=ENSG00000151778, *.GeneName*=SERP2, *.GeneDesc*=stress-associated endoplasmic reticulum protein family member 2 [Source:HGNC Symbol;Acc:20607].

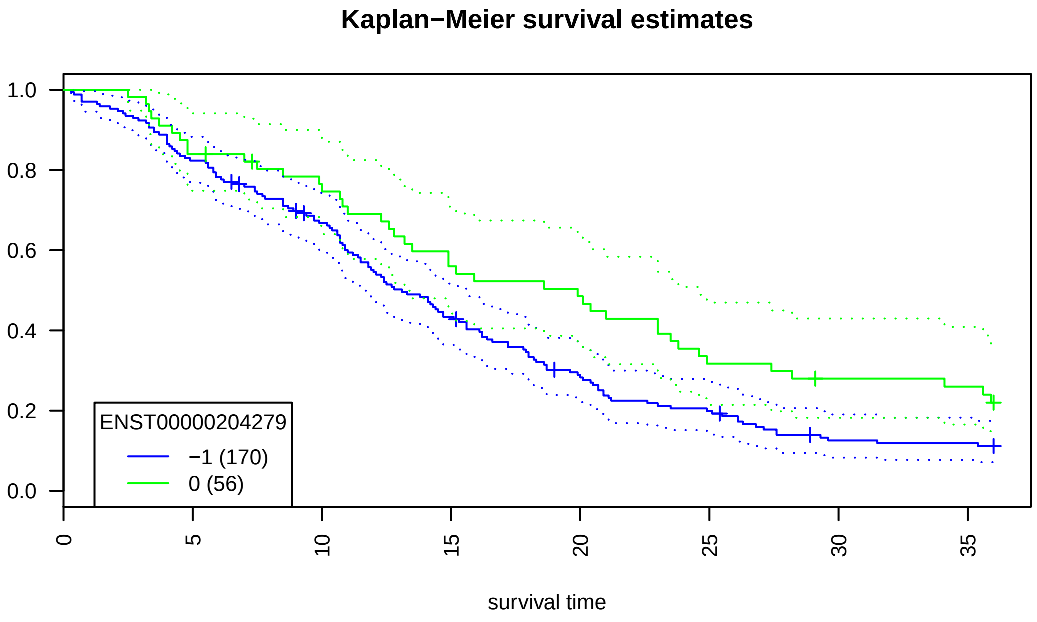

Figure 410: Each curve represents the relative number of ENST00000204279 survived to the corresponding point of time. Dashed lines represent 0.95 confidence intervals for the curves with the corresponding color. Total of 4 observations deleted due to missingness. Log-rank probability measure for the equality of these curves is 1.623553e-02. Additional annotations: *.GeneId*=ENSG00000100027, *.GeneName*=YPEL1, *.GeneDesc*=yippee-like 1 (Drosophila) [Source:HGNC Symbol;Acc:12845].

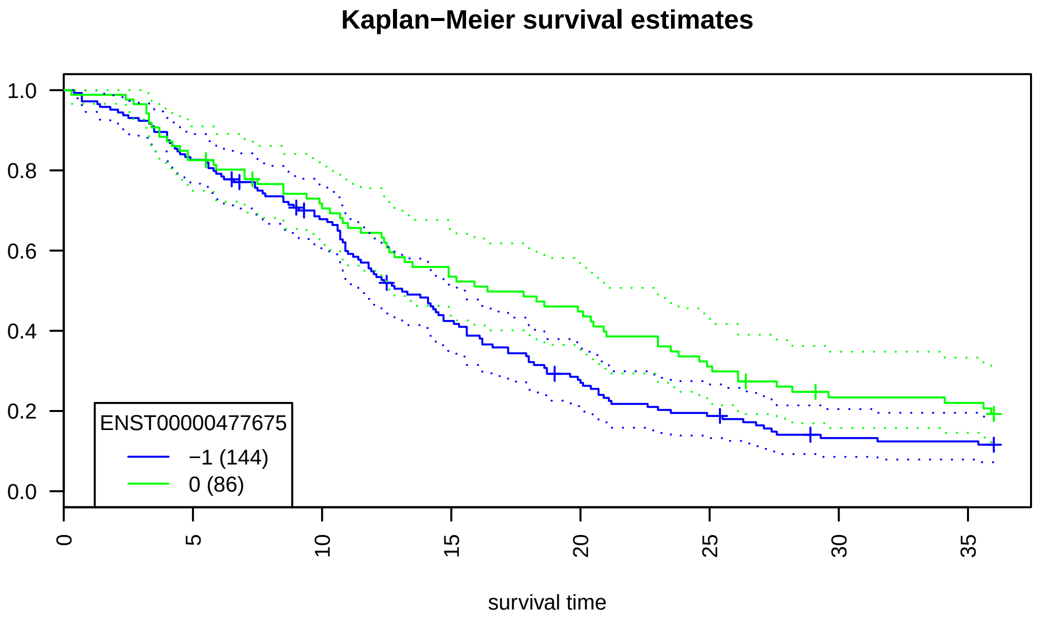

Figure 411: Each curve represents the relative number of ENST00000477675 survived to the corresponding point of time. Dashed lines represent 0.95 confidence intervals for the curves with the corresponding color. Log-rank probability measure for the equality of these curves is 3.946665e-02. Additional annotations: *.GeneId*=ENSG00000100027, *.GeneName*=YPEL1, *.GeneDesc*=yippee-like 1 (Drosophila) [Source:HGNC Symbol;Acc:12845].

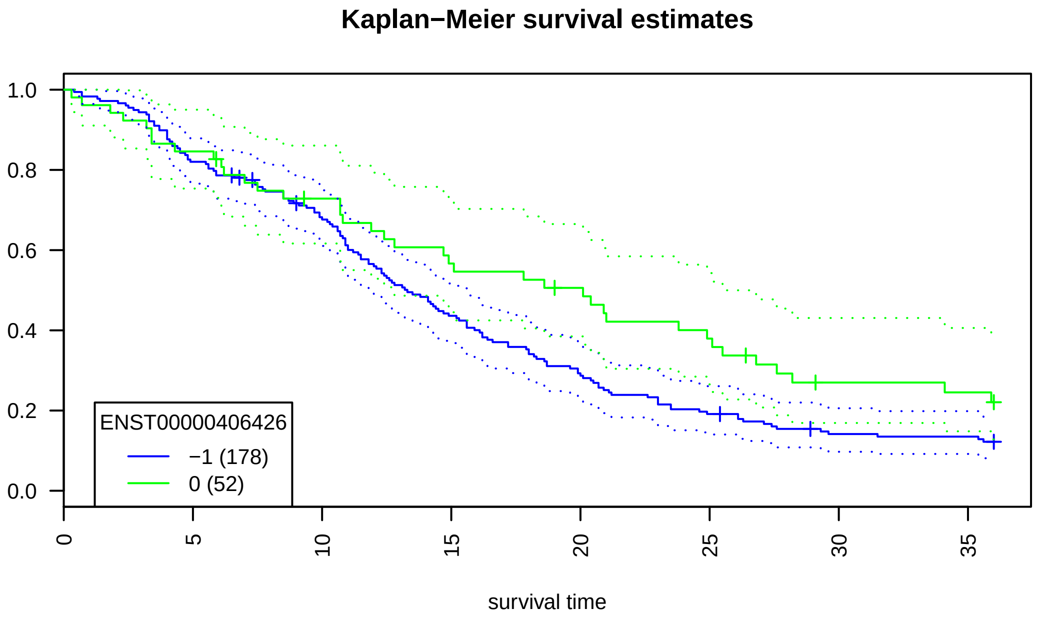

Figure 412: Each curve represents the relative number of ENST00000406426 survived to the corresponding point of time. Dashed lines represent 0.95 confidence intervals for the curves with the corresponding color. Log-rank probability measure for the equality of these curves is 3.823339e-02. Additional annotations: .*GeneId*=ENSG00000198477, .*GeneName*=ZNF280B, .*GeneDesc*=zinc finger protein 280B [Source:HGNC Symbol;Acc:23022].

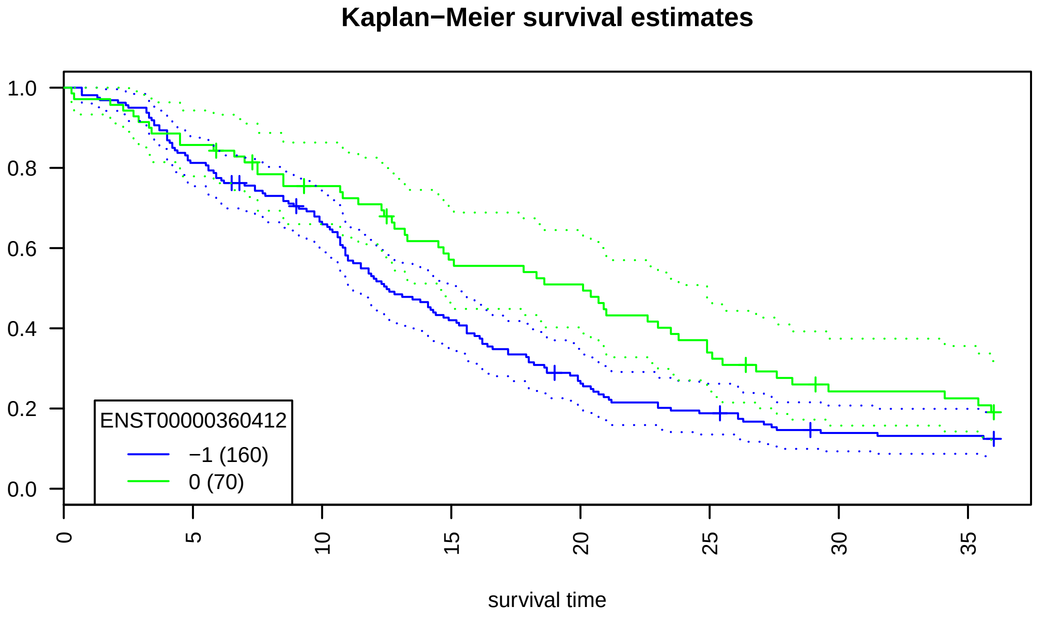

Figure 413: Each curve represents the relative number of ENST00000360412 survived to the corresponding point of time. Dashed lines represent 0.95 confidence intervals for the curves with the corresponding color. Log-rank probability measure for the equality of these curves is 1.703387e-02. Additional annotations: .*GeneId*=ENSG00000198477, .*GeneName*=ZNF280B, .*GeneDesc*=zinc finger protein 280B [Source:HGNC Symbol;Acc:23022].

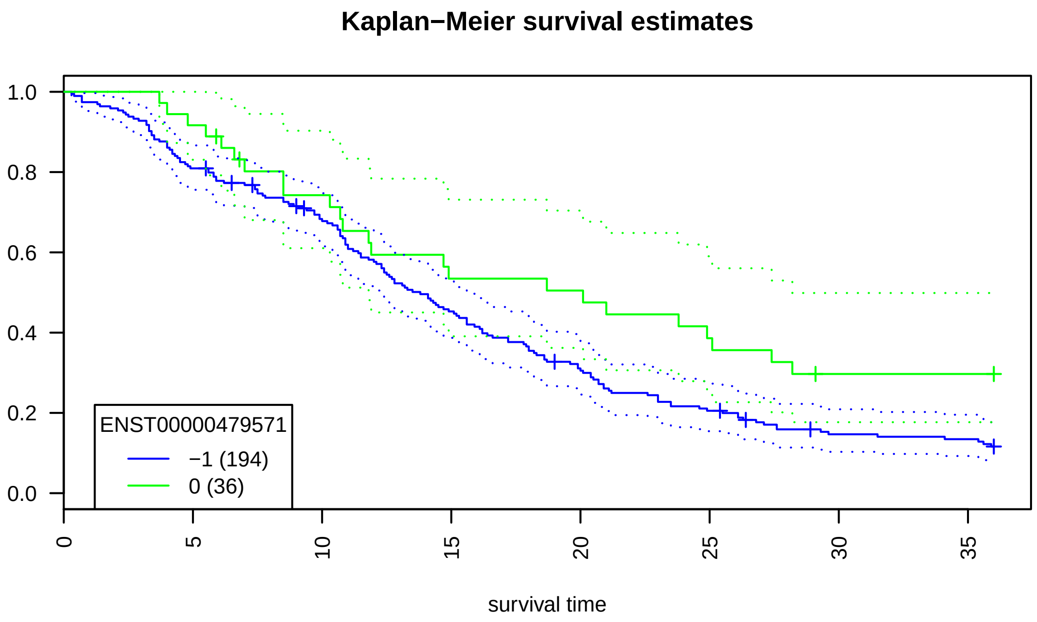

Figure 414: Each curve represents the relative number of ENST00000479571 survived to the corresponding point of time. Dashed lines represent 0.95 confidence intervals for the curves with the corresponding color. Log-rank probability measure for the equality of these curves is 2.304514e-02. Additional annotations: *.GeneId*=ENSG00000128266, *.GeneName*=GNAZ, *.GeneDesc*=guanine nucleotide binding protein (G protein), alpha z polypeptide [Source:HGNC Symbol;Acc:4395].

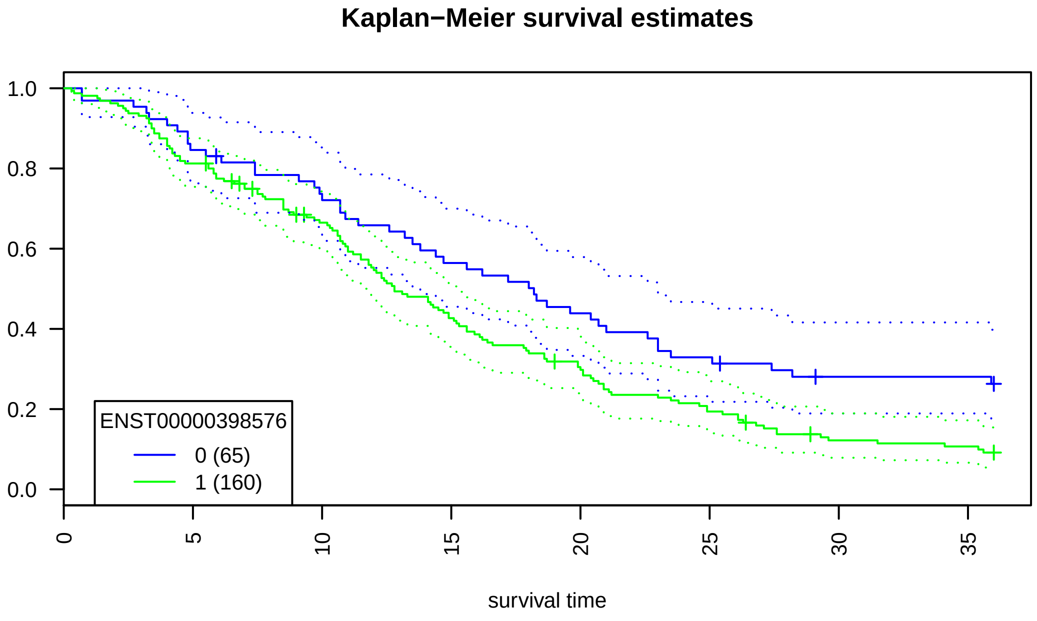

Figure 415: Each curve represents the relative number of ENST00000398576 survived to the corresponding point of time. Dashed lines represent 0.95 confidence intervals for the curves with the corresponding color. Total of 5 observations deleted due to missingness. Log-rank probability measure for the equality of these curves is 5.836214e-03. Additional annotations: *.GeneId*=ENSG00000136167, *.GeneName*=LCP1, *.GeneDesc*=lymphocyte cytosolic protein 1 (L-plastin) [Source:HGNC Symbol;Acc:6528].

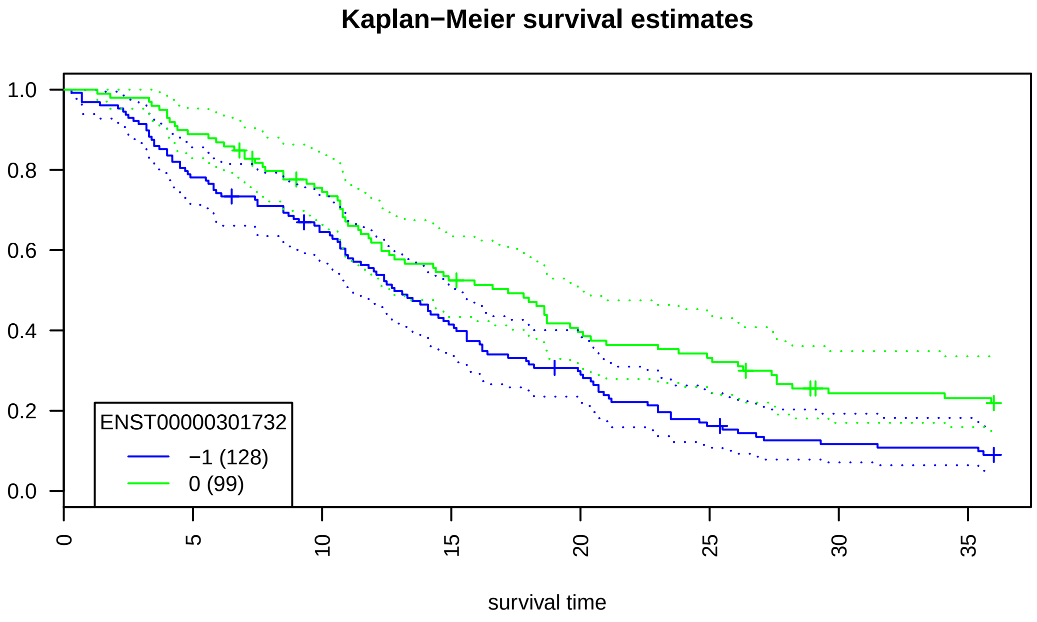

Figure 416: Each curve represents the relative number of ENST00000301732 survived to the corresponding point of time. Dashed lines represent 0.95 confidence intervals for the curves with the corresponding color. Total of 3 observations deleted due to missingness. Log-rank probability measure for the equality of these curves is 4.530704e-03. Additional annotations: *.GeneId*=ENSG00000167972, *.GeneName*=ABCA3, *.GeneDesc*=ATP-binding cassette, sub-family A (ABC1), member 3 [Source:HGNC Symbol;Acc:33].

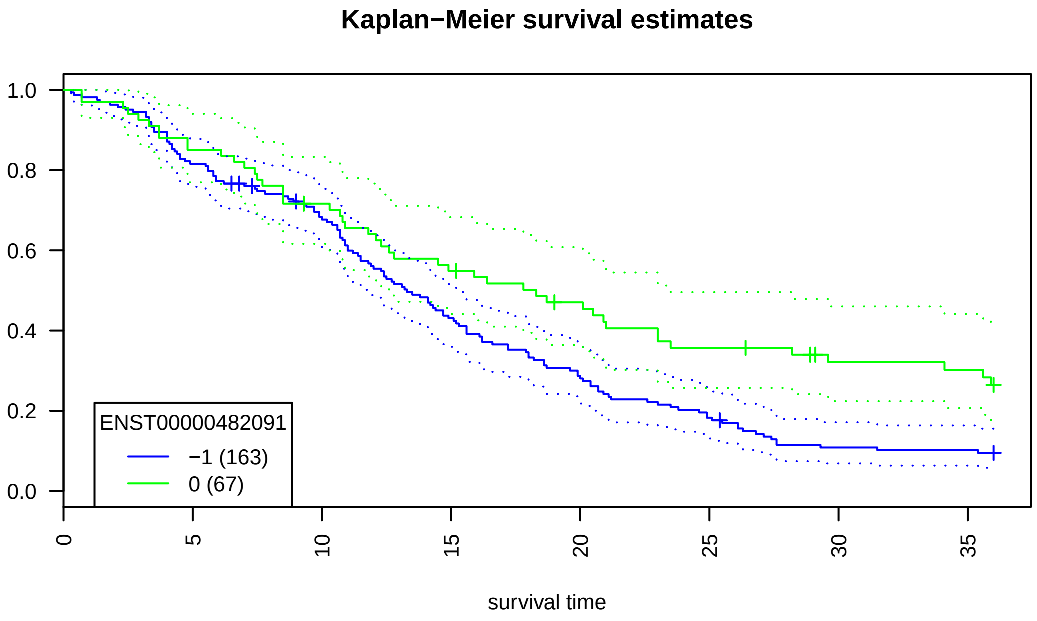

Figure 417: Each curve represents the relative number of ENST00000482091 survived to the corresponding point of time. Dashed lines represent 0.95 confidence intervals for the curves with the corresponding color. Log-rank probability measure for the equality of these curves is 3.660216e-03. Additional annotations: *.GeneId*=ENSG00000067064, *.GeneName*=IDI1, *.GeneDesc*=isopentenyl-diphosphate delta isomerase 1 [Source:HGNC Symbol;Acc:5387].

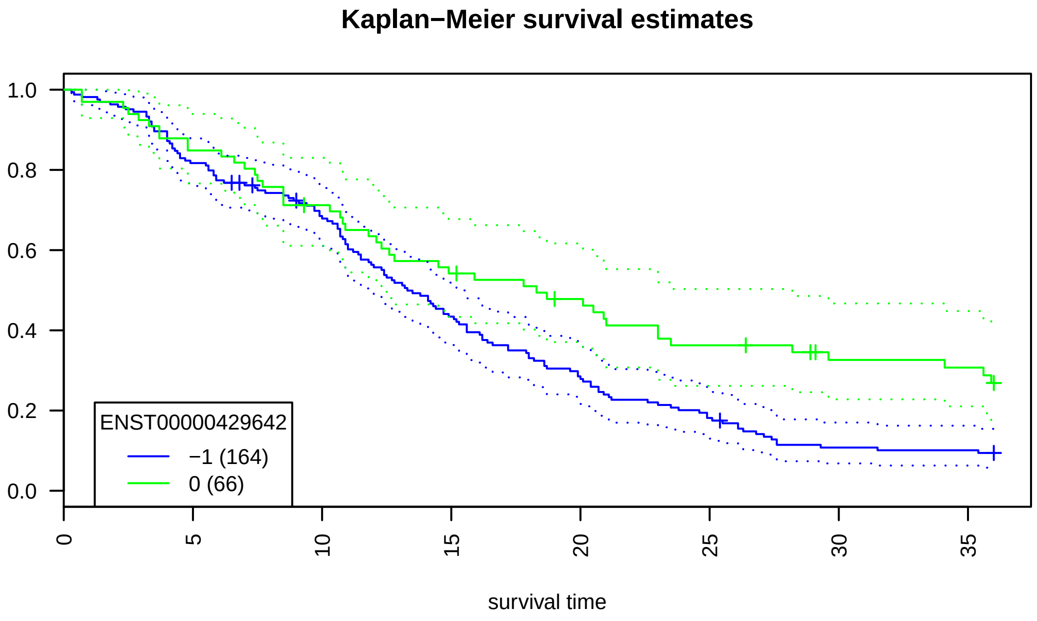

Figure 418: Each curve represents the relative number of ENST00000429642 survived to the corresponding point of time. Dashed lines represent 0.95 confidence intervals for the curves with the corresponding color. Log-rank probability measure for the equality of these curves is 3.315636e-03. Additional annotations: *.GeneId*=ENSG00000067064, *.GeneName*=IDI1, *.GeneDesc*=isopentenyl-diphosphate delta isomerase 1 [Source:HGNC Symbol;Acc:5387].

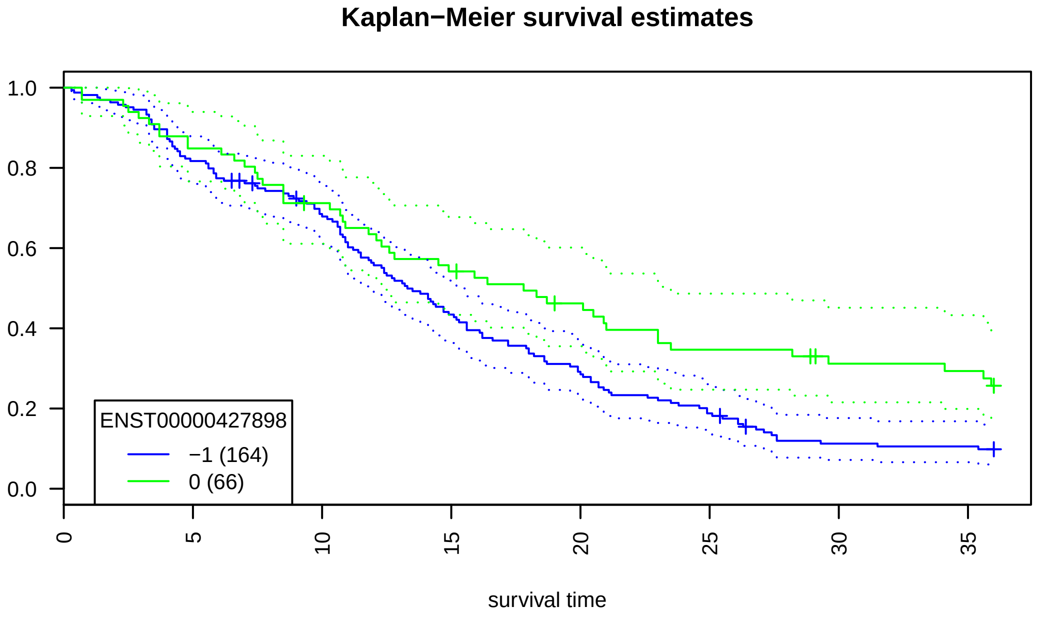

Figure 419: Each curve represents the relative number of ENST00000427898 survived to the corresponding point of time. Dashed lines represent 0.95 confidence intervals for the curves with the corresponding color. Log-rank probability measure for the equality of these curves is 7.340914e-03. Additional annotations: *.GeneId*=ENSG00000067064, *.GeneName*=IDI1, *.GeneDesc*=isopentenyl-diphosphate delta isomerase 1 [Source:HGNC Symbol;Acc:5387].

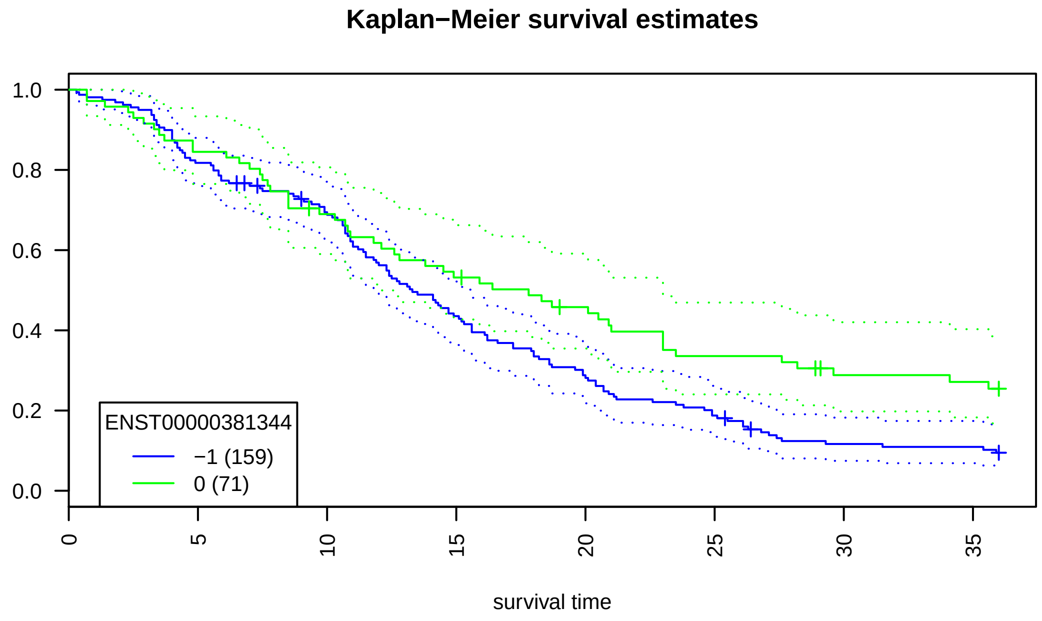

Figure 420: Each curve represents the relative number of ENST00000381344 survived to the corresponding point of time. Dashed lines represent 0.95 confidence intervals for the curves with the corresponding color. Log-rank probability measure for the equality of these curves is 9.083752e-03. Additional annotations: *.GeneId*=ENSG00000067064, *.GeneName*=IDI1, *.GeneDesc*=isopentenyl-diphosphate delta isomerase 1 [Source:HGNC Symbol;Acc:5387].

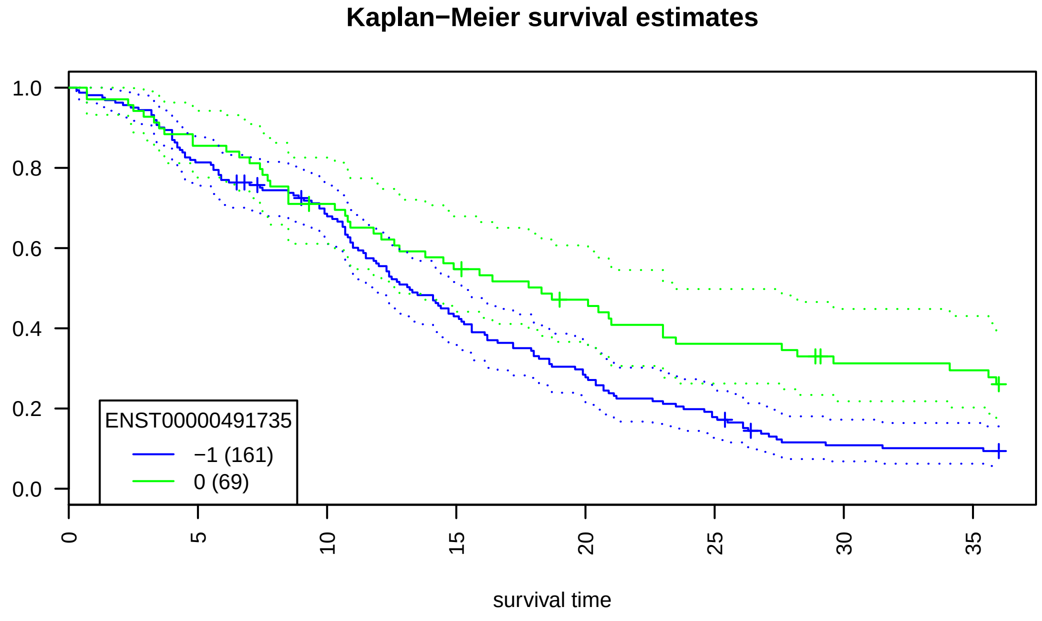

Figure 421: Each curve represents the relative number of ENST00000491735 survived to the corresponding point of time. Dashed lines represent 0.95 confidence intervals for the curves with the corresponding color. Log-rank probability measure for the equality of these curves is 3.152560e-03. Additional annotations: *.GeneId*=ENSG00000067064, *.GeneName*=IDI1, *.GeneDesc*=isopentenyl-diphosphate delta isomerase 1 [Source:HGNC Symbol;Acc:5387].

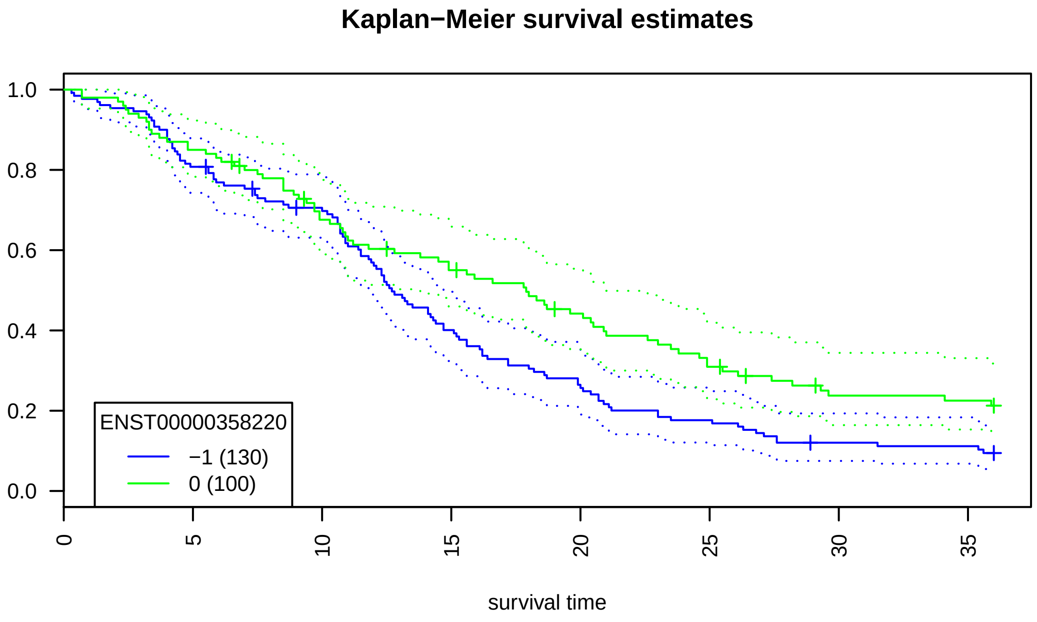

Figure 422: Each curve represents the relative number of ENST00000358220 survived to the corresponding point of time. Dashed lines represent 0.95 confidence intervals for the curves with the corresponding color. Log-rank probability measure for the equality of these curves is 8.11164e-03. Additional annotations: *.GeneId*=ENSG00000047056, *.GeneName*=WDR37, *.GeneDesc*=WD repeat domain 37 [Source:HGNC Symbol;Acc:31406].

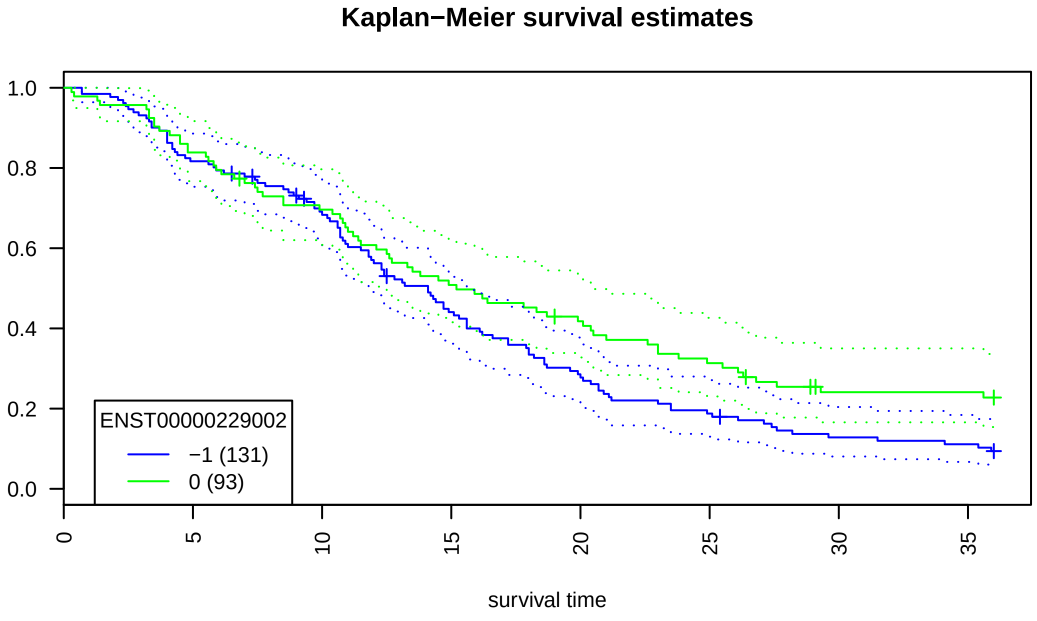

Figure 423: Each curve represents the relative number of ENST00000229002 survived to the corresponding point of time. Dashed lines represent 0.95 confidence intervals for the curves with the corresponding color. Total of 6 observations deleted due to missingness. Log-rank probability measure for the equality of these curves is 3.194778e-02. Additional annotations: *.GeneId*=ENSG00000111404, *.GeneName*=RERGL, *.GeneDesc*=RERG/RAS-like [Source:HGNC Symbol;Acc:26213].

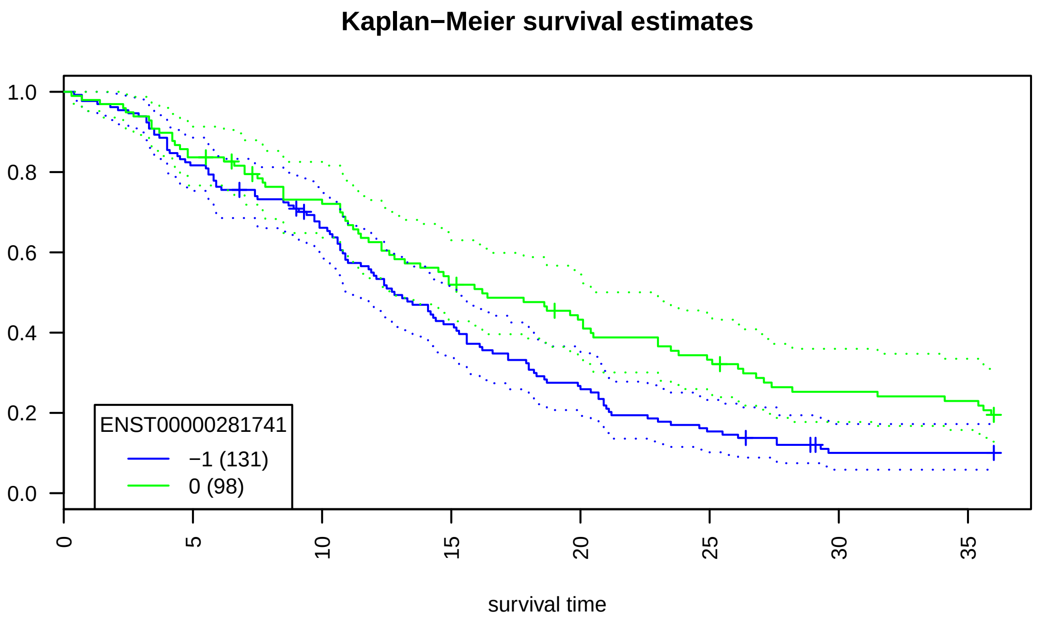

Figure 424: Each curve represents the relative number of ENST00000281741 survived to the corresponding point of time. Dashed lines represent 0.95 confidence intervals for the curves with the corresponding color. Total of 1 observation deleted due to missingness. Log-rank probability measure for the equality of these curves is 1.003457e-02. Additional annotations: *.GeneId*=ENSG00000087301, *.GeneName*=TXNDC16, *.GeneDesc*=thioredoxin domain containing 16 [Source:HGNC Symbol;Acc:19965].

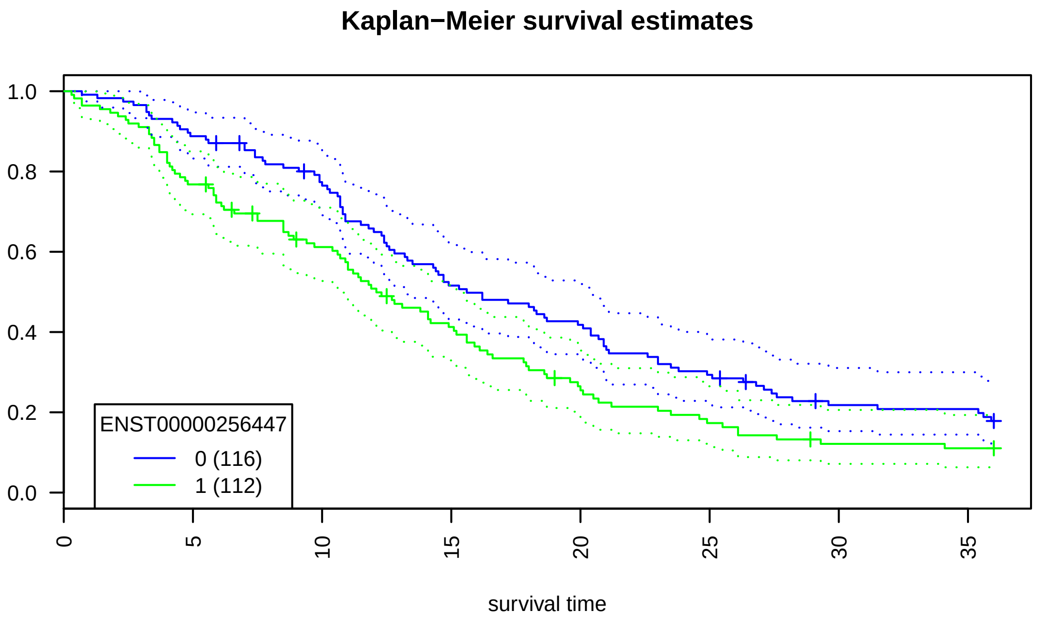

Figure 425: Each curve represents the relative number of ENST00000256447 survived to the corresponding point of time. Dashed lines represent 0.95 confidence intervals for the curves with the corresponding color. Total of 2 observations deleted due to missingness. Log-rank probability measure for the equality of these curves is 1.194485e-02. Additional annotations: *.GeneId*=ENSG00000134061, *.GeneName*=CD180, *.GeneDesc*=CD180 molecule [Source:HGNC Symbol;Acc:6726].

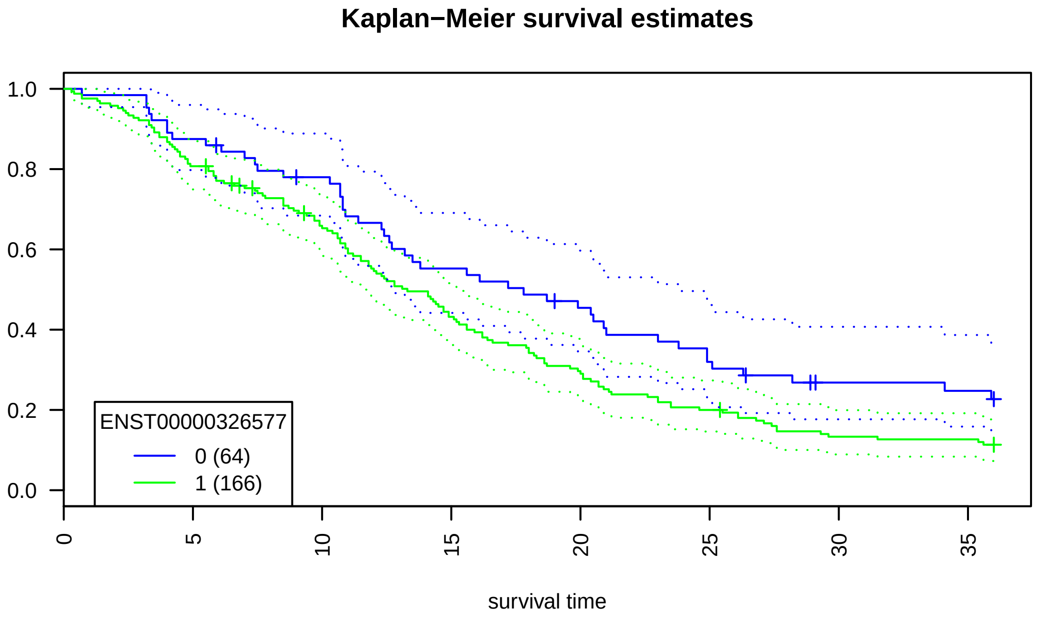

Figure 426: Each curve represents the relative number of ENST00000326577 survived to the corresponding point of time. Dashed lines represent 0.95 confidence intervals for the curves with the corresponding color. Log-rank probability measure for the equality of these curves is 2.189089e-02. Additional annotations: *.GeneId*=ENSG00000006327, *.GeneName*=TNFRSF12A, *.GeneDesc*=tumor necrosis factor receptor superfamily, member 12A [Source:HGNC Symbol;Acc:18152].

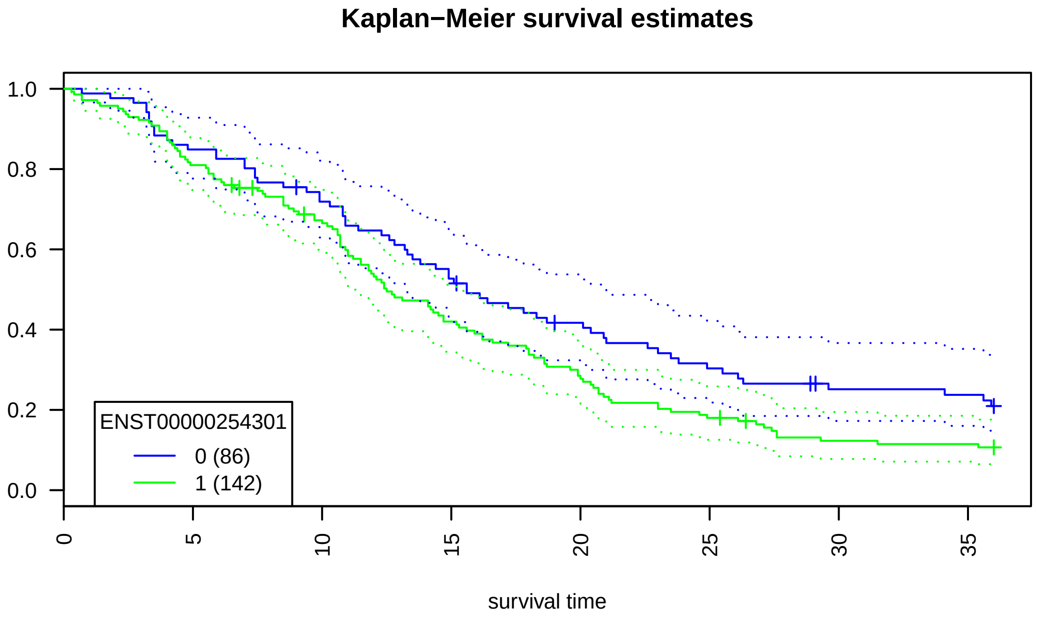

Figure 427: Each curve represents the relative number of ENST00000254301 survived to the corresponding point of time. Dashed lines represent 0.95 confidence intervals for the curves with the corresponding color. Total of 2 observations deleted due to missingness. Log-rank probability measure for the equality of these curves is 2.644250e-02. Additional annotations: *.GeneId*=ENSG00000131981, *.GeneName*=LGALS3, *.GeneDesc*=lectin, galactoside-binding, soluble, 3 [Source:HGNC Symbol;Acc:6563].

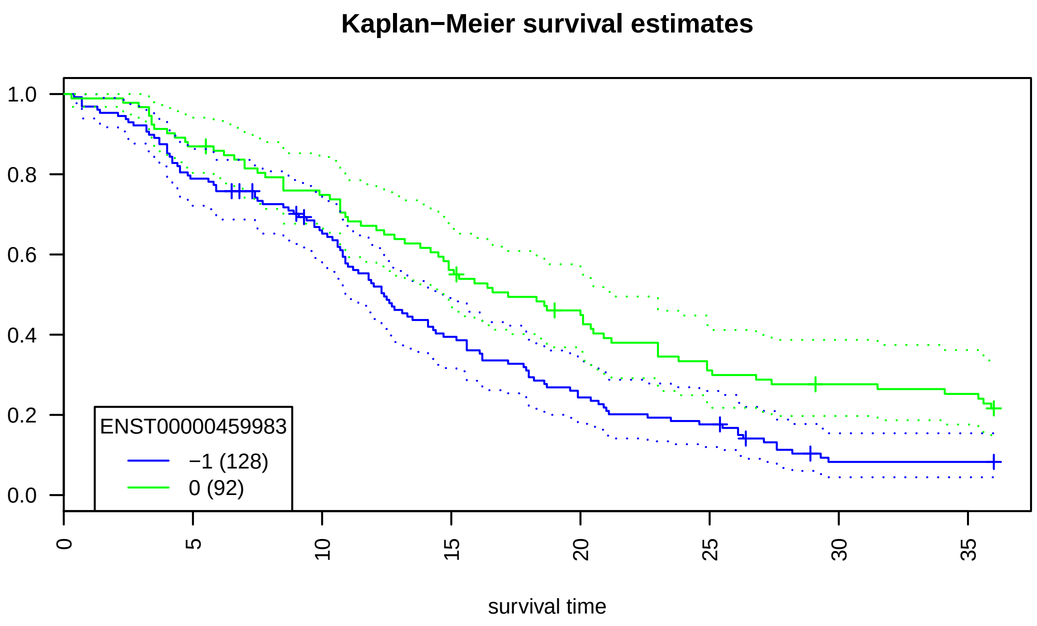

Figure 428: Each curve represents the relative number of ENST00000459983 survived to the corresponding point of time. Dashed lines represent 0.95 confidence intervals for the curves with the corresponding color. Total of 10 observations deleted due to missingness. Log-rank probability measure for the equality of these curves is 1.349813e-03. Additional annotations: *.GeneId*=ENSG00000136104, *.GeneName*=RNASEH2B, *.GeneDesc*=ribonuclease H2, subunit B [Source:HGNC Symbol;Acc:25671].

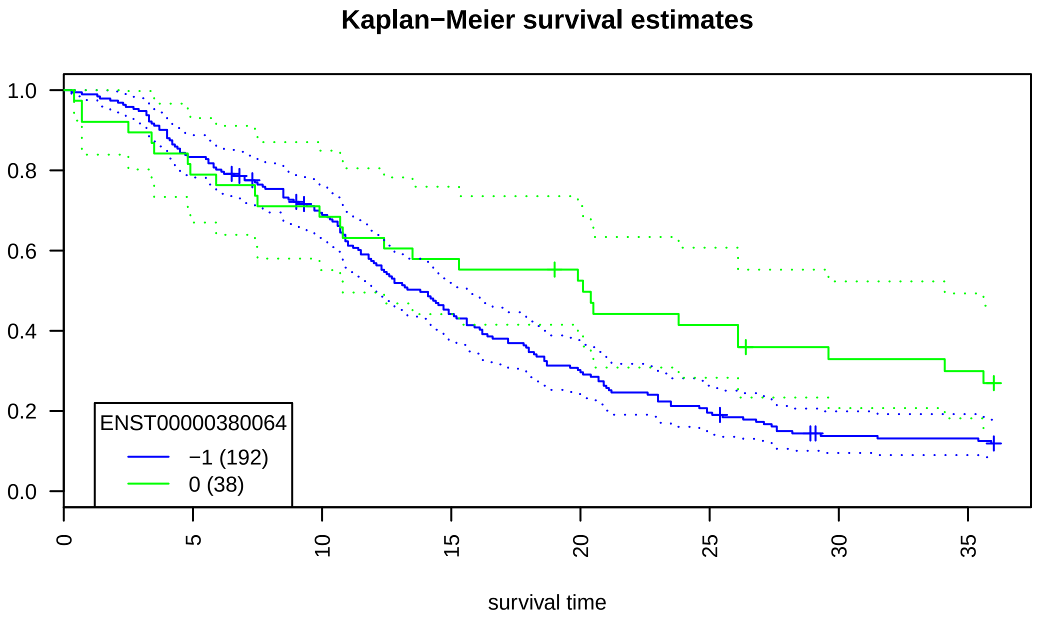

Figure 429: Each curve represents the relative number of ENST00000380064 survived to the corresponding point of time. Dashed lines represent 0.95 confidence intervals for the curves with the corresponding color. Log-rank probability measure for the equality of these curves is 3.043572e-02. Additional annotations: *.GeneId*=ENSG00000169891, *.GeneName*=REPS2, *.GeneDesc*=RALBP1 associated Eps domain containing 2 [Source:HGNC Symbol;Acc:9963].

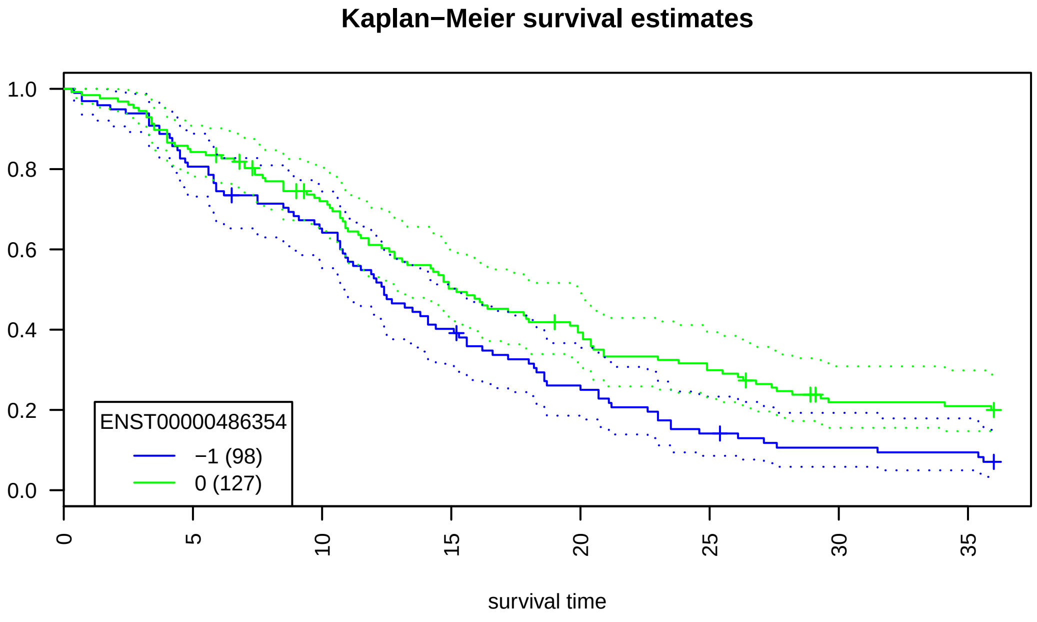

Figure 430: Each curve represents the relative number of ENST00000486354 survived to the corresponding point of time. Dashed lines represent 0.95 confidence intervals for the curves with the corresponding color. Total of 5 observations deleted due to missingness. Log-rank probability measure for the equality of these curves is 7.380788e-03. Additional annotations: *.GeneId*=ENSG00000173848, *.GeneName*=NET1, *.GeneDesc*=neuroepithelial cell transforming 1 [Source:HGNC Symbol;Acc:14592].

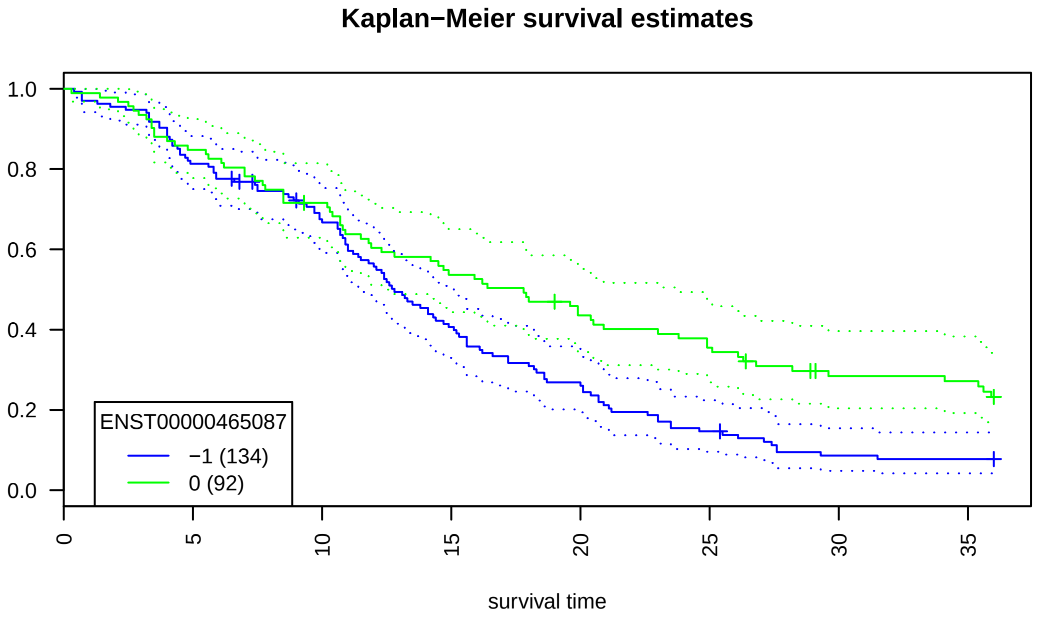

Figure 431: Each curve represents the relative number of ENST00000465087 survived to the corresponding point of time. Dashed lines represent 0.95 confidence intervals for the curves with the corresponding color. Total of 4 observations deleted due to missingness. Log-rank probability measure for the equality of these curves is 1.530081e-03. Additional annotations: *.GeneId*=ENSG00000173848, *.GeneName*=NET1, *.GeneDesc*=neuroepithelial cell transforming 1 [Source:HGNC Symbol;Acc:14592].

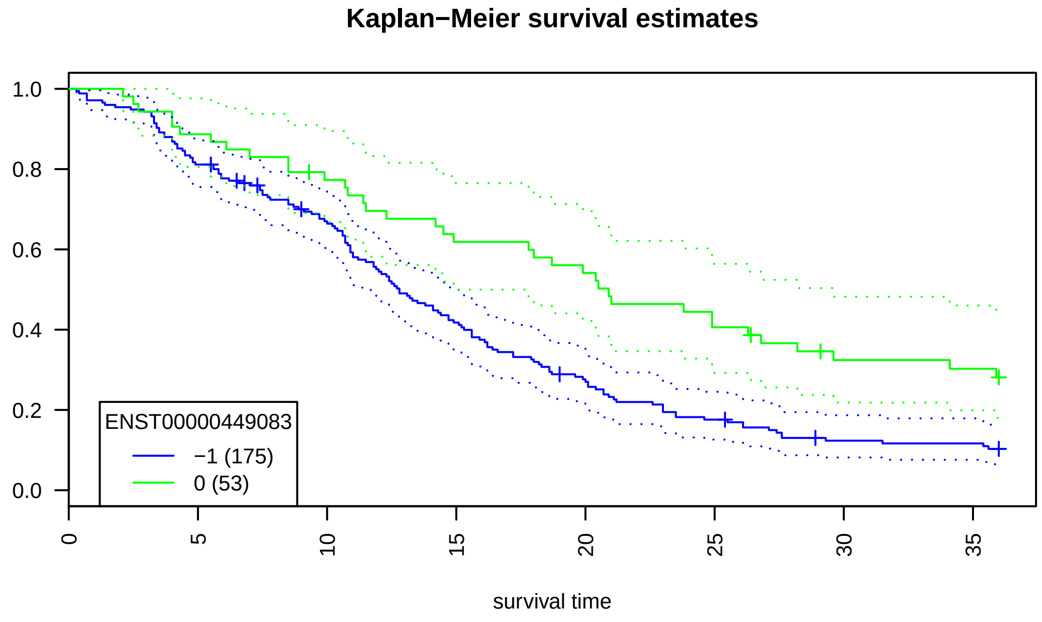

Figure 432: Each curve represents the relative number of ENST00000449083 survived to the corresponding point of time. Dashed lines represent 0.95 confidence intervals for the curves with the corresponding color. Total of 2 observations deleted due to missingness. Log-rank probability measure for the equality of these curves is 5.819943e-04. Additional annotations: *.GeneId*=ENSG00000173848, *.GeneName*=NET1, *.GeneDesc*=neuroepithelial cell transforming 1 [Source:HGNC Symbol;Acc:14592].

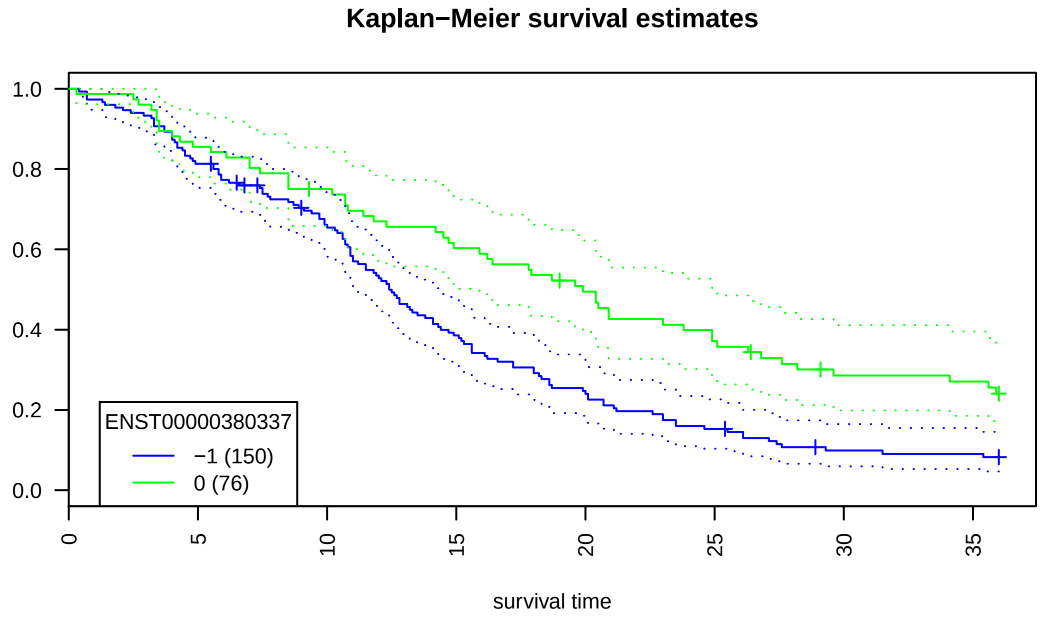

Figure 433: Each curve represents the relative number of ENST00000380337 survived to the corresponding point of time. Dashed lines represent 0.95 confidence intervals for the curves with the corresponding color. Total of 4 observations deleted due to missingness. Log-rank probability measure for the equality of these curves is 2.189335e-04. Additional annotations: *.GeneId*=ENSG00000173848, *.GeneName*=NET1, *.GeneDesc*=neuroepithelial cell transforming 1 [Source:HGNC Symbol;Acc:14592].

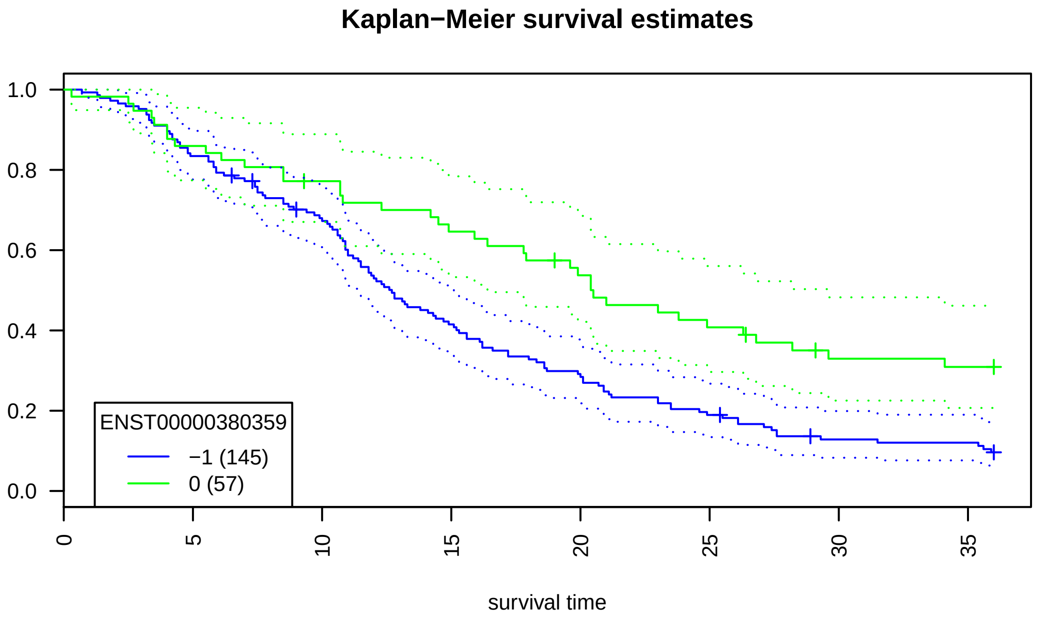

Figure 434: Each curve represents the relative number of ENST00000380359 survived to the corresponding point of time. Dashed lines represent 0.95 confidence intervals for the curves with the corresponding color. Total of 28 observations deleted due to missingness. Log-rank probability measure for the equality of these curves is 4.340759e-04. Additional annotations: *.GeneId*=ENSG00000173848, *.GeneName*=NET1, *.GeneDesc*=neuroepithelial cell transforming 1 [Source:HGNC Symbol;Acc:14592].

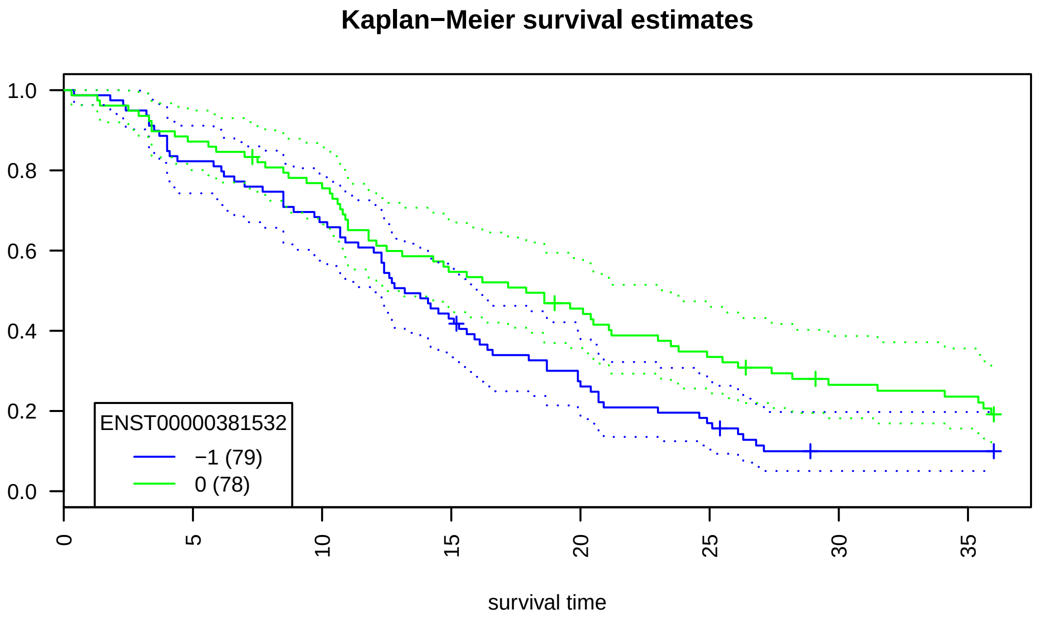

Figure 435: Each curve represents the relative number of ENST00000381532 survived to the corresponding point of time. Dashed lines represent 0.95 confidence intervals for the curves with the corresponding color. Total of 73 observations deleted due to missingness. Log-rank probability measure for the equality of these curves is 2.105641e-02. Additional annotations: *.GeneId*=ENSG00000107036, *.GeneName*=KIAA1432, *.GeneDesc*=KIAA1432 [Source:HGNC Symbol;Acc:17686].

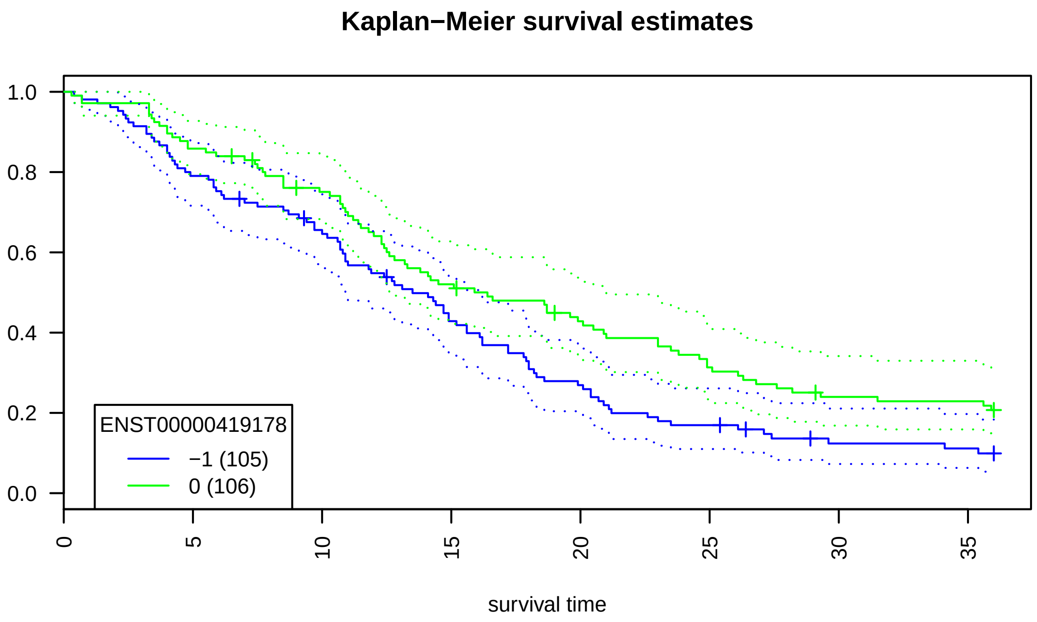

Figure 436: Each curve represents the relative number of ENST00000419178 survived to the corresponding point of time. Dashed lines represent 0.95 confidence intervals for the curves with the corresponding color. Total of 19 observations deleted due to missingness. Log-rank probability measure for the equality of these curves is 1.143357e-02. Additional annotations: *.GeneId*=ENSG00000125863, *.GeneName*=MKKS, *.GeneDesc*=McKusick-Kaufman syndrome [Source:HGNC Symbol;Acc:7108].

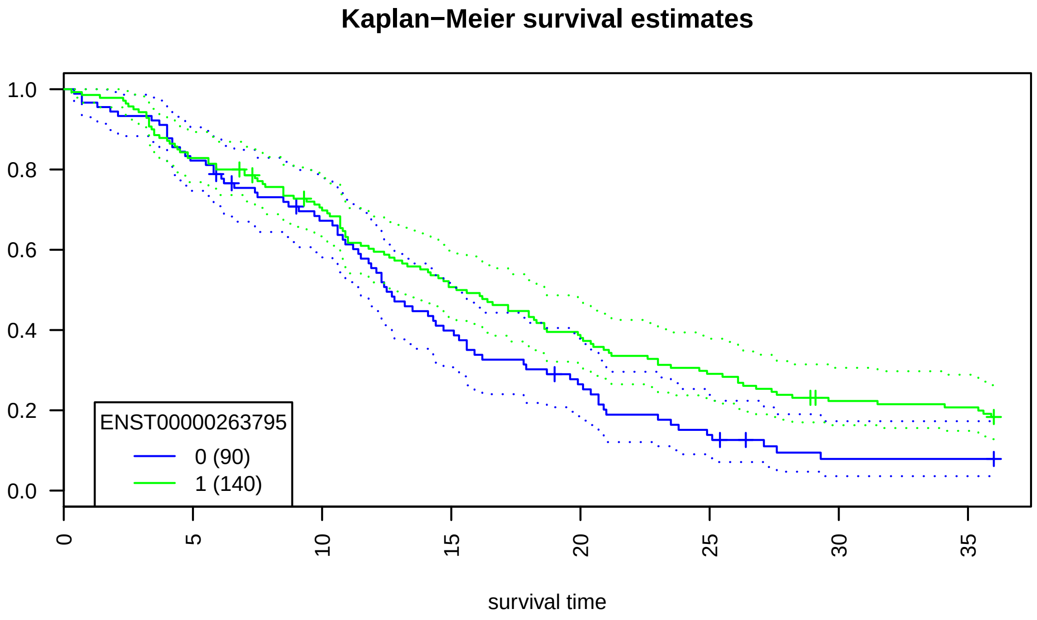

Figure 437: Each curve represents the relative number of ENST00000263795 survived to the corresponding point of time. Dashed lines represent 0.95 confidence intervals for the curves with the corresponding color. Log-rank probability measure for the equality of these curves is 1.975583e-02. Additional annotations: *.GeneId*=ENSG00000092470, *.GeneName*=WDR76, *.GeneDesc*=WD repeat domain 76 [Source:HGNC Symbol;Acc:25773].

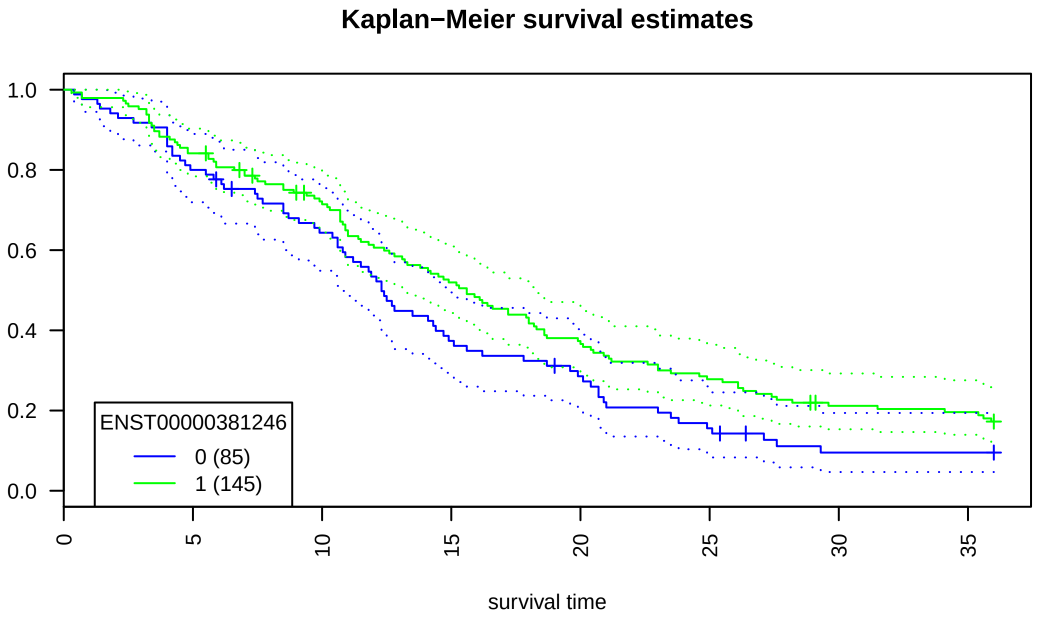

Figure 438: Each curve represents the relative number of ENST00000381246 survived to the corresponding point of time. Dashed lines represent 0.95 confidence intervals for the curves with the corresponding color. Log-rank probability measure for the equality of these curves is 4.070817e-02. Additional annotations: *.GeneId*=ENSG00000092470, *.GeneName*=WDR76, *.GeneDesc*=WD repeat domain 76 [Source:HGNC Symbol;Acc:25773].

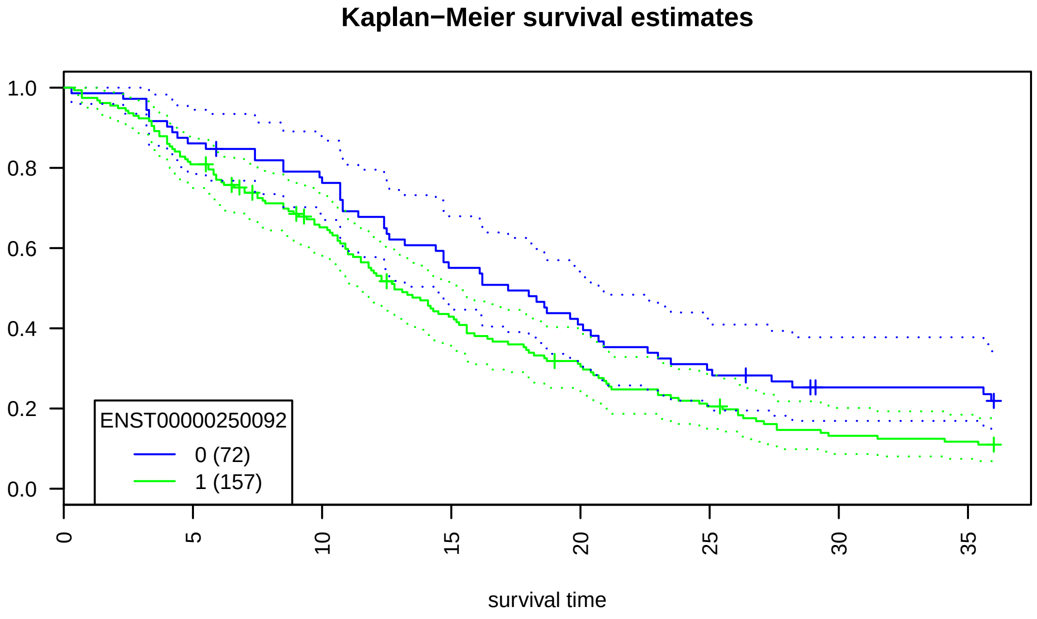

Figure 439: Each curve represents the relative number of ENST00000250092 survived to the corresponding point of time. Dashed lines represent 0.95 confidence intervals for the curves with the corresponding color. Total of 1 observation deleted due to missingness. Log-rank probability measure for the equality of these curves is 2.469507e-02. Additional annotations: *.GeneId*=ENSG00000129226, *.GeneName*=CD68, *.GeneDesc*=CD68 molecule [Source:HGNC Symbol;Acc:1693].

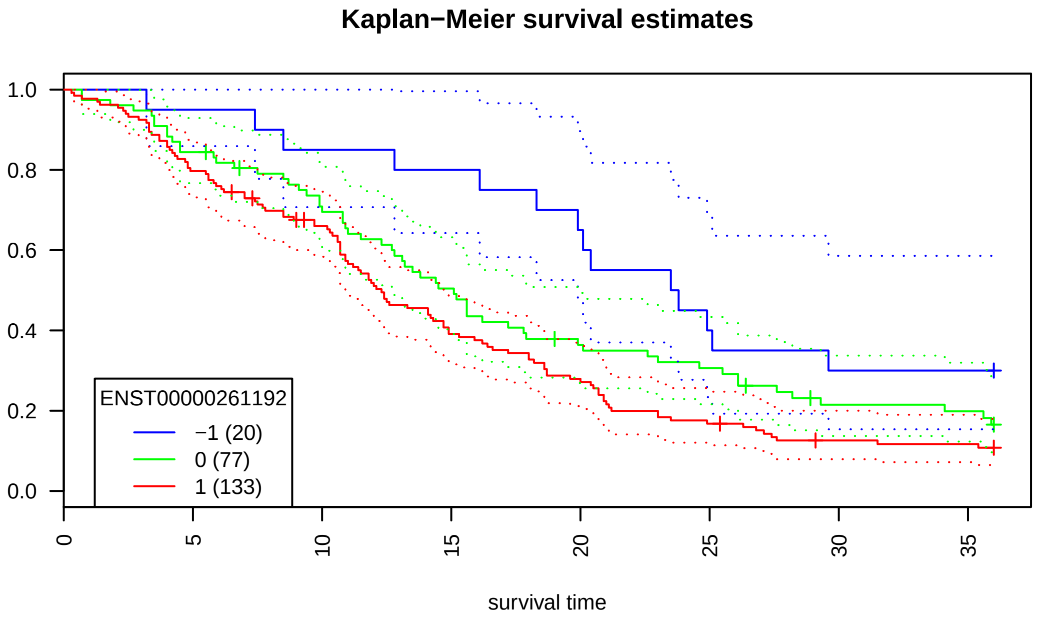

Figure 440: Each curve represents the relative number of ENST00000261192 survived to the corresponding point of time. Dashed lines represent 0.95 confidence intervals for the curves with the corresponding color. Log-rank probability measure for the equality of these curves is 9.721105e-03. Additional annotations: *.GeneId*=ENSG00000060982, *.GeneName*=BCAT1, *.GeneDesc*=branched chain aminotransferase 1, cytosolic [Source:HGNC Symbol;Acc:976].

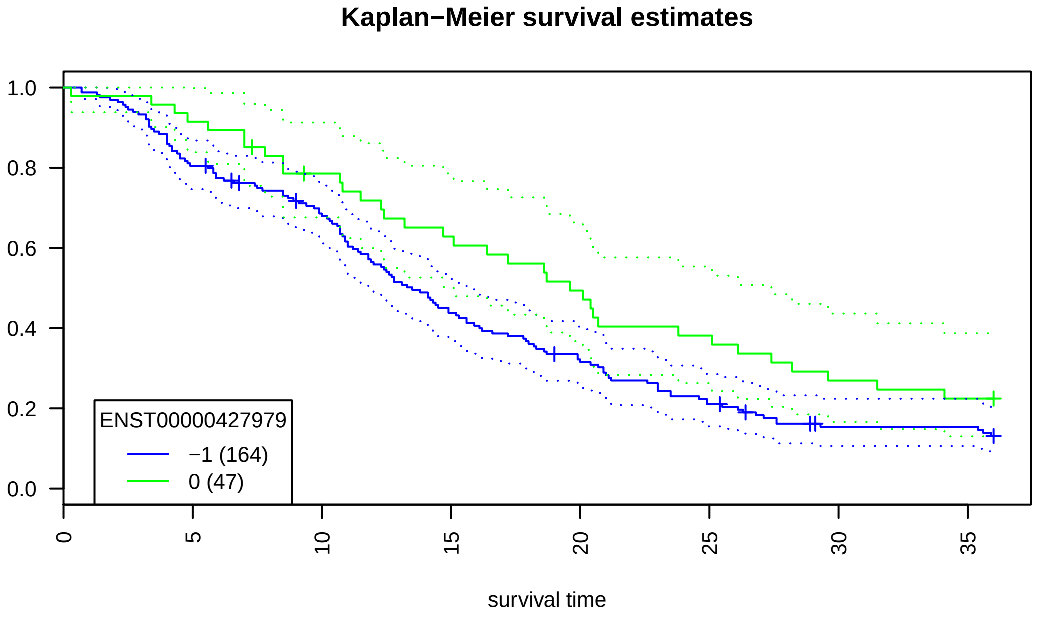

Figure 441: Each curve represents the relative number of ENST00000427979 survived to the corresponding point of time. Dashed lines represent 0.95 confidence intervals for the curves with the corresponding color. Total of 19 observations deleted due to missingness. Log-rank probability measure for the equality of these curves is 3.612566e-02. Additional annotations: *.GeneId*=ENSG00000032219, *.GeneName*=ARID4A, *.GeneDesc*=AT rich interactive domain 4A (RBP1-like) [Source:HGNC Symbol;Acc:9885].

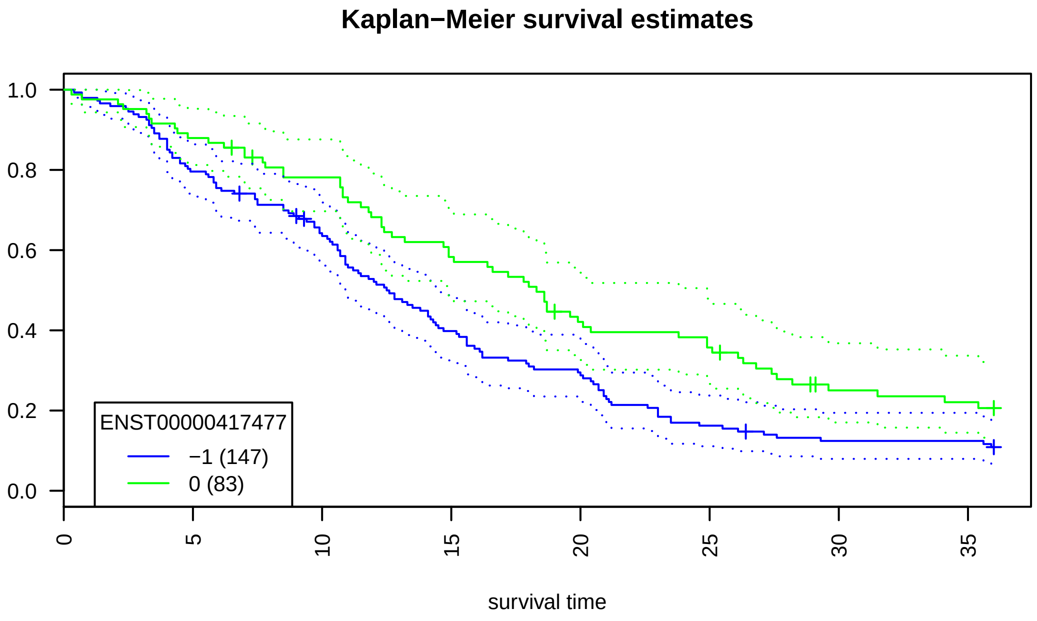

Figure 442: Each curve represents the relative number of ENST00000417477 survived to the corresponding point of time. Dashed lines represent 0.95 confidence intervals for the curves with the corresponding color. Log-rank probability measure for the equality of these curves is 3.813981e-03. Additional annotations: *.GeneId*=ENSG00000032219, *.GeneName*=ARID4A, *.GeneDesc*=AT rich interactive domain 4A (RBP1-like) [Source:HGNC Symbol;Acc:9885].

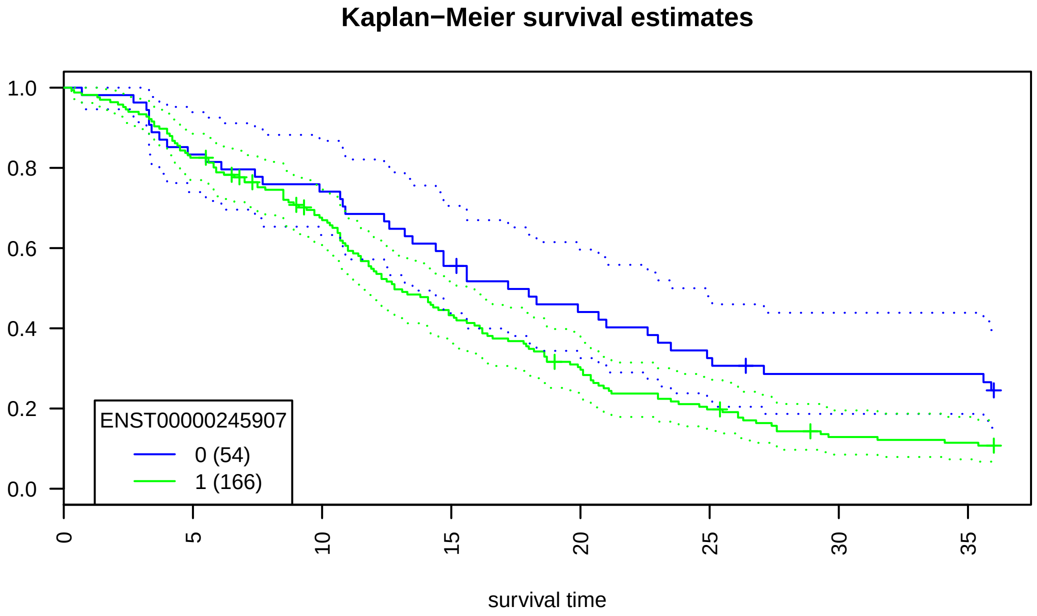

Figure 443: Each curve represents the relative number of ENST00000245907 survived to the corresponding point of time. Dashed lines represent 0.95 confidence intervals for the curves with the corresponding color. Total of 10 observations deleted due to missingness. Log-rank probability measure for the equality of these curves is 2.161419e-02. Additional annotations: *.GeneId*=ENSG00000125730, *.GeneName*=C3, *.GeneDesc*=complement component 3 [Source:HGNC Symbol;Acc:1318].

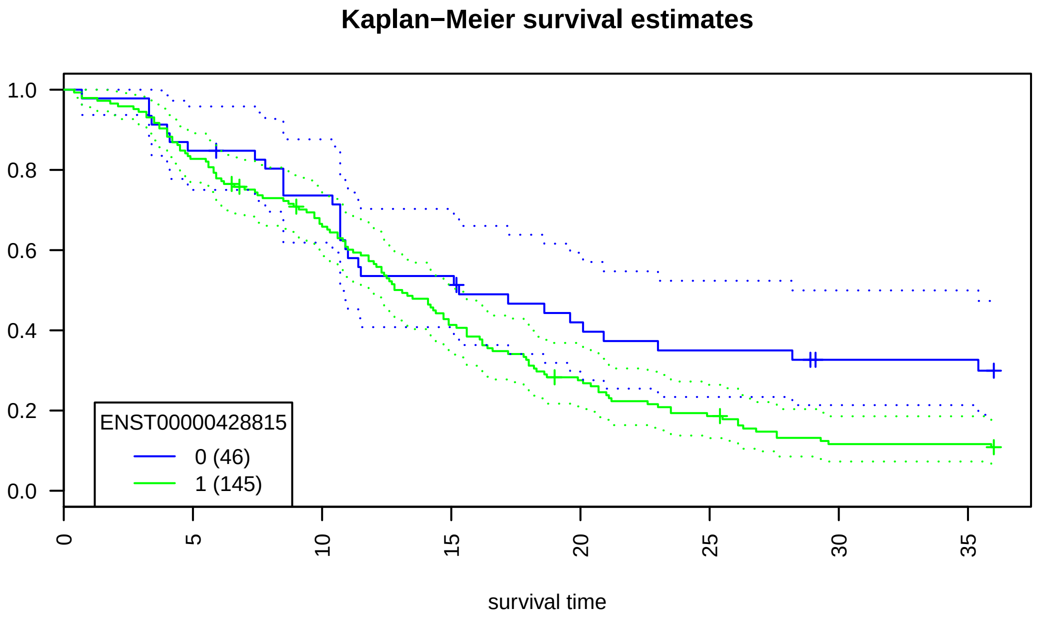

Figure 444: Each curve represents the relative number of ENST00000428815 survived to the corresponding point of time. Dashed lines represent 0.95 confidence intervals for the curves with the corresponding color. Total of 39 observations deleted due to missingness. Log-rank probability measure for the equality of these curves is 2.353013e-02. Additional annotations: *.GeneId*=ENSG00000125730, *.GeneName*=C3, *.GeneDesc*=complement component 3 [Source:HGNC Symbol;Acc:1318].

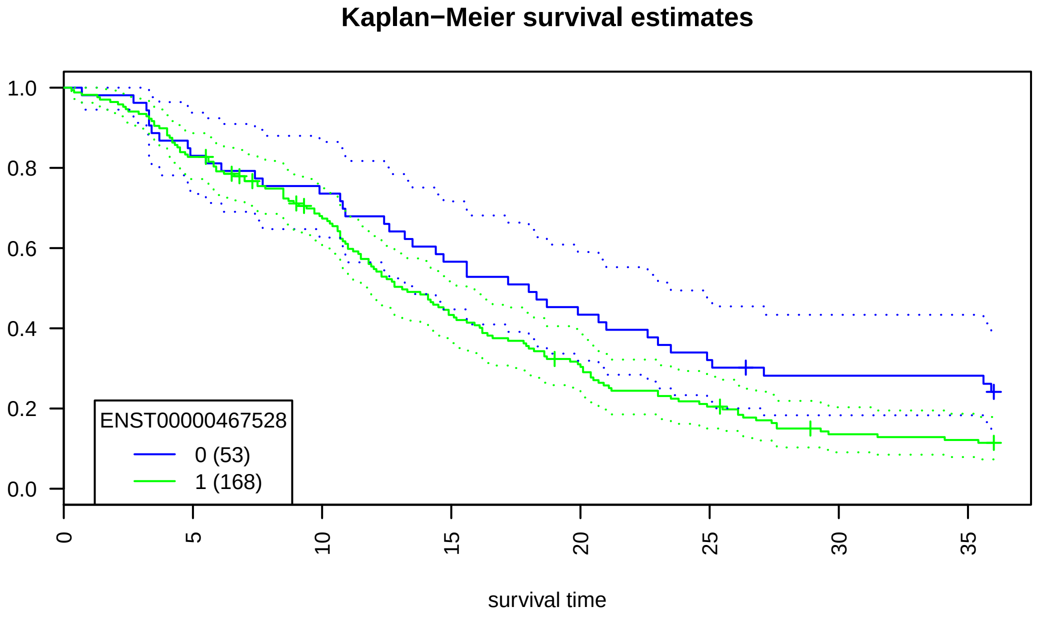

Figure 445: Each curve represents the relative number of ENST00000467528 survived to the corresponding point of time. Dashed lines represent 0.95 confidence intervals for the curves with the corresponding color. Total of 9 observations deleted due to missingness. Log-rank probability measure for the equality of these curves is 3.276115e-02. Additional annotations: *.GeneId*=ENSG00000125730, *.GeneName*=C3, *.GeneDesc*=complement component 3 [Source:HGNC Symbol;Acc:1318].

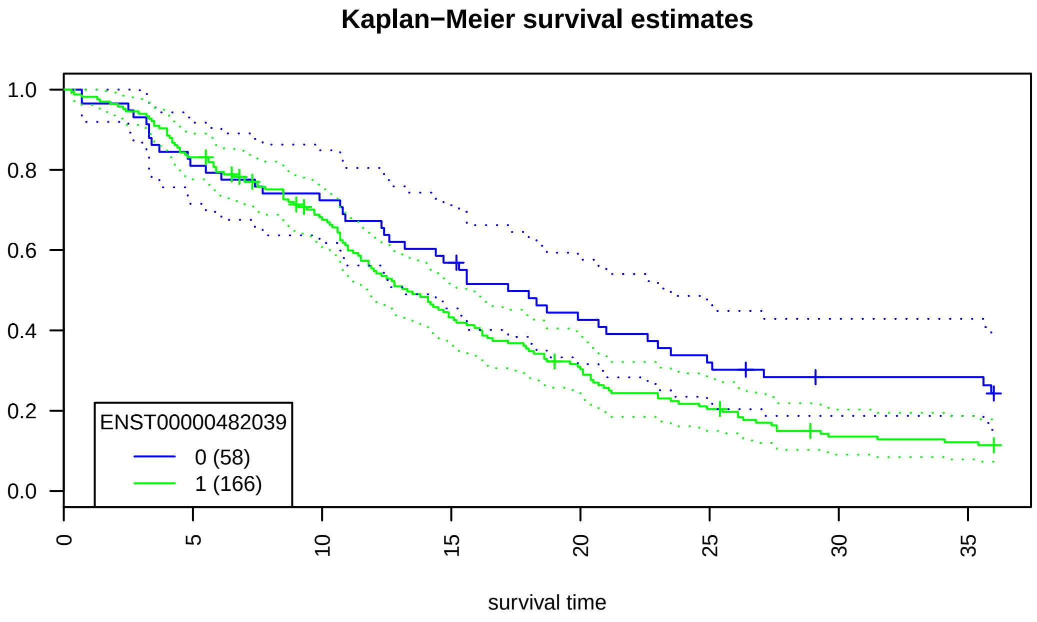

Figure 446: Each curve represents the relative number of ENST00000482039 survived to the corresponding point of time. Dashed lines represent 0.95 confidence intervals for the curves with the corresponding color. Total of 6 observations deleted due to missingness. Log-rank probability measure for the equality of these curves is 3.565453e-02. Additional annotations: *.GeneId*=ENSG00000125730, *.GeneName*=C3, *.GeneDesc*=complement component 3 [Source:HGNC Symbol;Acc:1318].

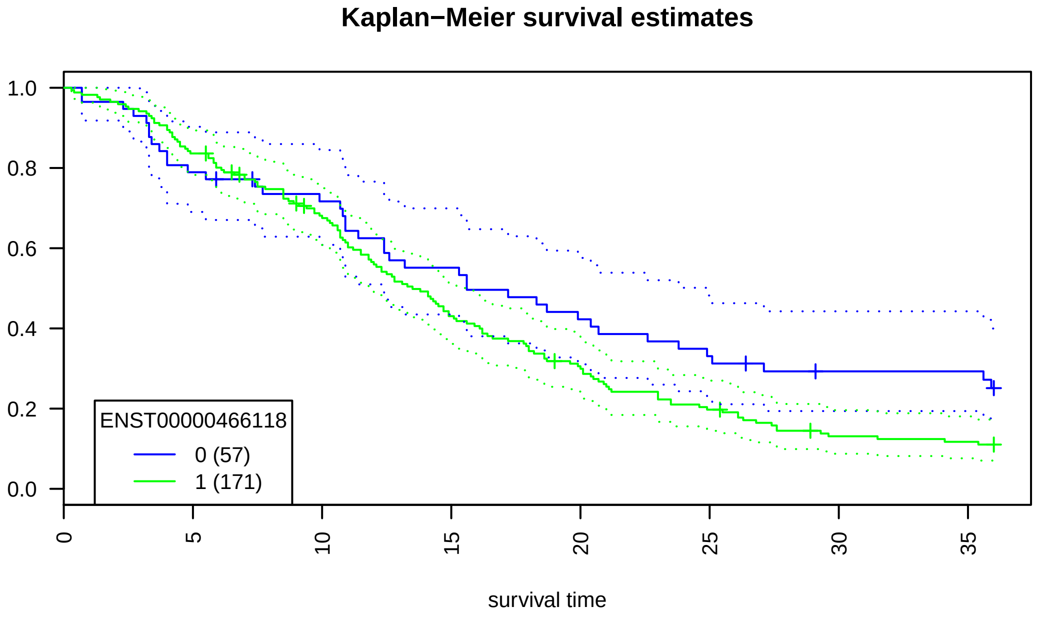

Figure 447: Each curve represents the relative number of ENST00000466118 survived to the corresponding point of time. Dashed lines represent 0.95 confidence intervals for the curves with the corresponding color. Total of 2 observations deleted due to missingness. Log-rank probability measure for the equality of these curves is 4.271932e-02. Additional annotations: *.GeneId*=ENSG00000125730, *.GeneName*=C3, *.GeneDesc*=complement component 3 [Source:HGNC Symbol;Acc:1318].

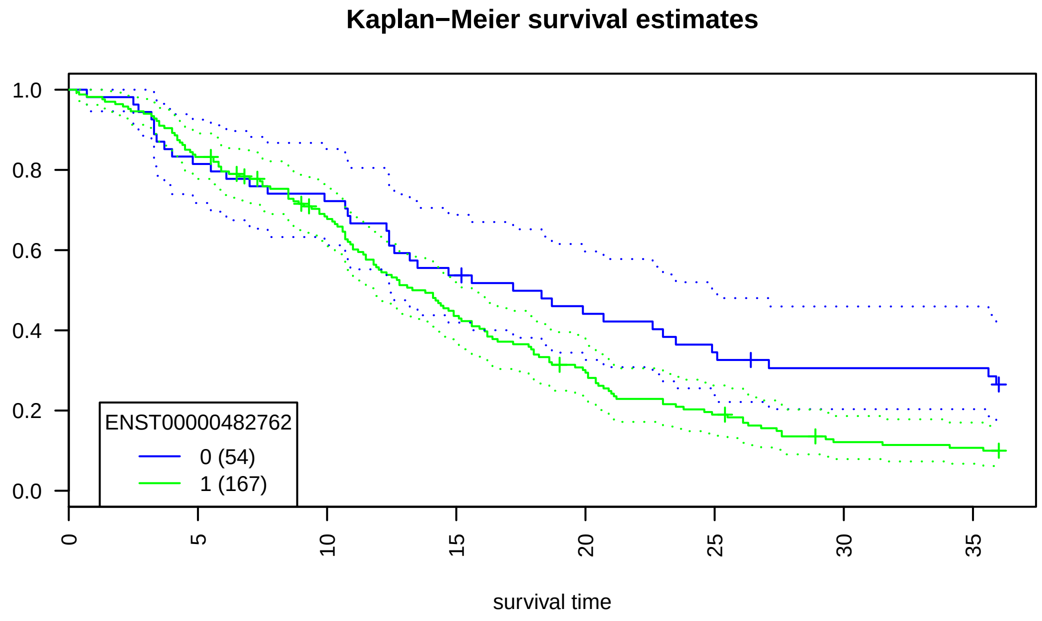

Figure 448: Each curve represents the relative number of ENST00000482762 survived to the corresponding point of time. Dashed lines represent 0.95 confidence intervals for the curves with the corresponding color. Total of 9 observations deleted due to missingness. Log-rank probability measure for the equality of these curves is 1.469062e-02. Additional annotations: *.GeneId*=ENSG00000125730, *.GeneName*=C3, *.GeneDesc*=complement component 3 [Source:HGNC Symbol;Acc:1318].

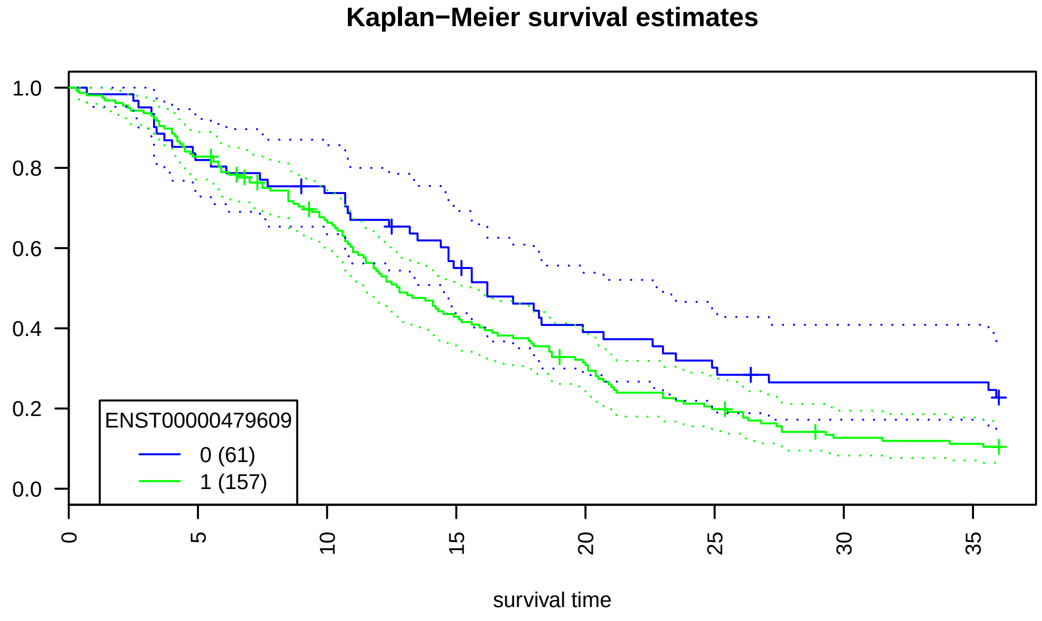

Figure 449: Each curve represents the relative number of ENST00000479609 survived to the corresponding point of time. Dashed lines represent 0.95 confidence intervals for the curves with the corresponding color. Total of 12 observations deleted due to missingness. Log-rank probability measure for the equality of these curves is 4.046509e-02. Additional annotations: *.GeneId*=ENSG00000125730, *.GeneName*=C3, *.GeneDesc*=complement component 3 [Source:HGNC Symbol;Acc:1318].

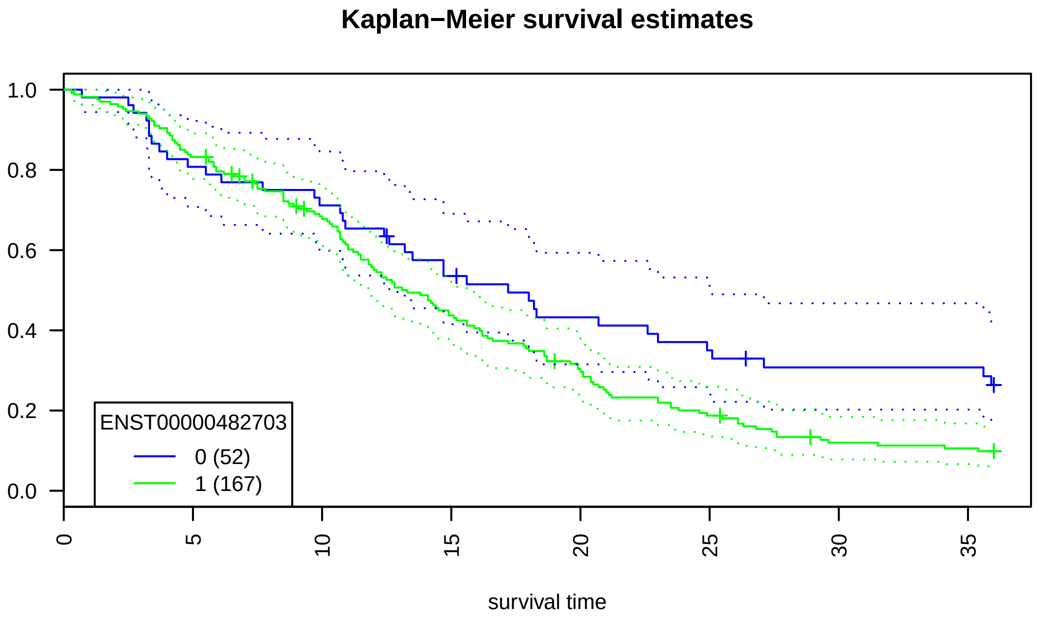

Figure 450: Each curve represents the relative number of ENST00000482703 survived to the corresponding point of time. Dashed lines represent 0.95 confidence intervals for the curves with the corresponding color. Total of 11 observations deleted due to missingness. Log-rank probability measure for the equality of these curves is 1.852674e-02. Additional annotations: *.GeneId*=ENSG00000125730, *.GeneName*=C3, *.GeneDesc*=complement component 3 [Source:HGNC Symbol;Acc:1318].

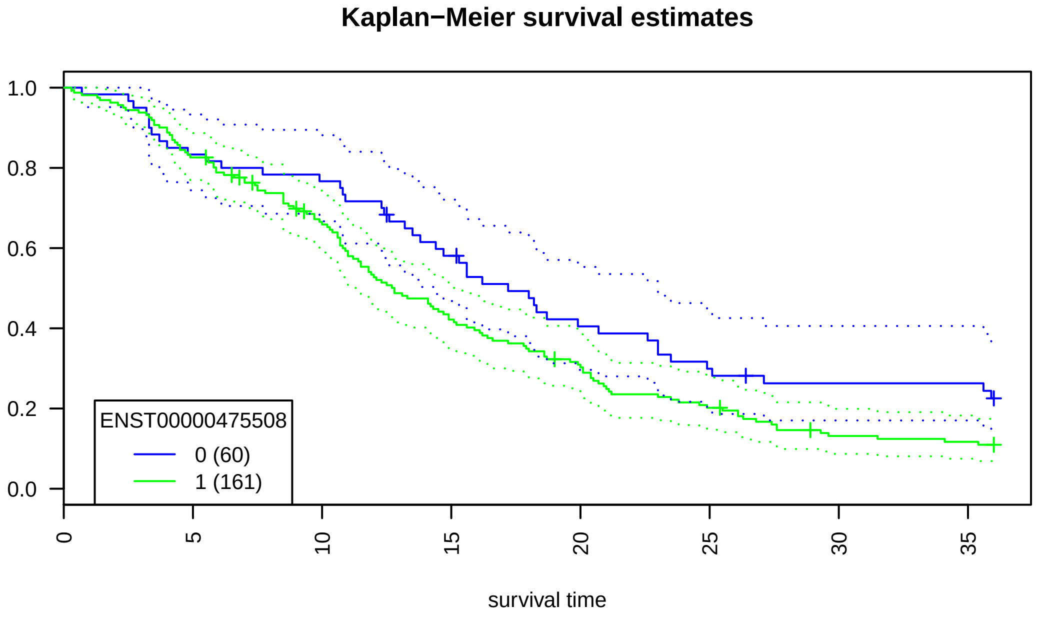

Figure 451: Each curve represents the relative number of ENST00000475508 survived to the corresponding point of time. Dashed lines represent 0.95 confidence intervals for the curves with the corresponding color. Total of 9 observations deleted due to missingness. Log-rank probability measure for the equality of these curves is 2.828386e-02. Additional annotations: *.GeneId*=ENSG00000125730, *.GeneName*=C3, *.GeneDesc*=complement component 3 [Source:HGNC Symbol;Acc:1318].

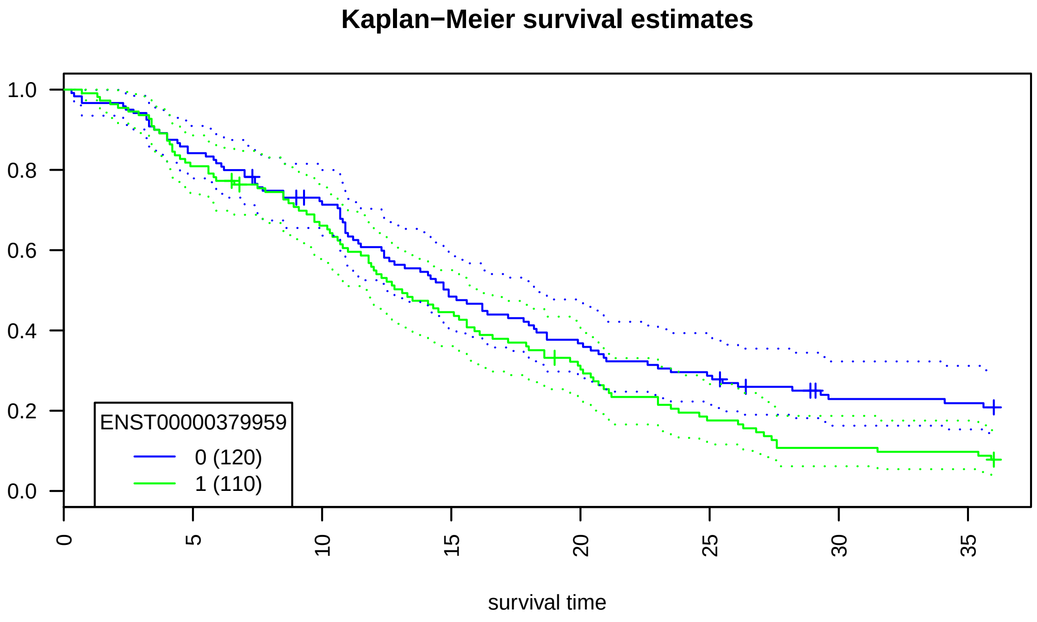

Figure 452: Each curve represents the relative number of ENST00000379959 survived to the corresponding point of time. Dashed lines represent 0.95 confidence intervals for the curves with the corresponding color. Log-rank probability measure for the equality of these curves is 3.658407e-02. Additional annotations: *.GeneId*=ENSG00000134460, *.GeneName*=IL2RA, *.GeneDesc*=interleukin 2 receptor, alpha [Source:HGNC Symbol;Acc:6008].

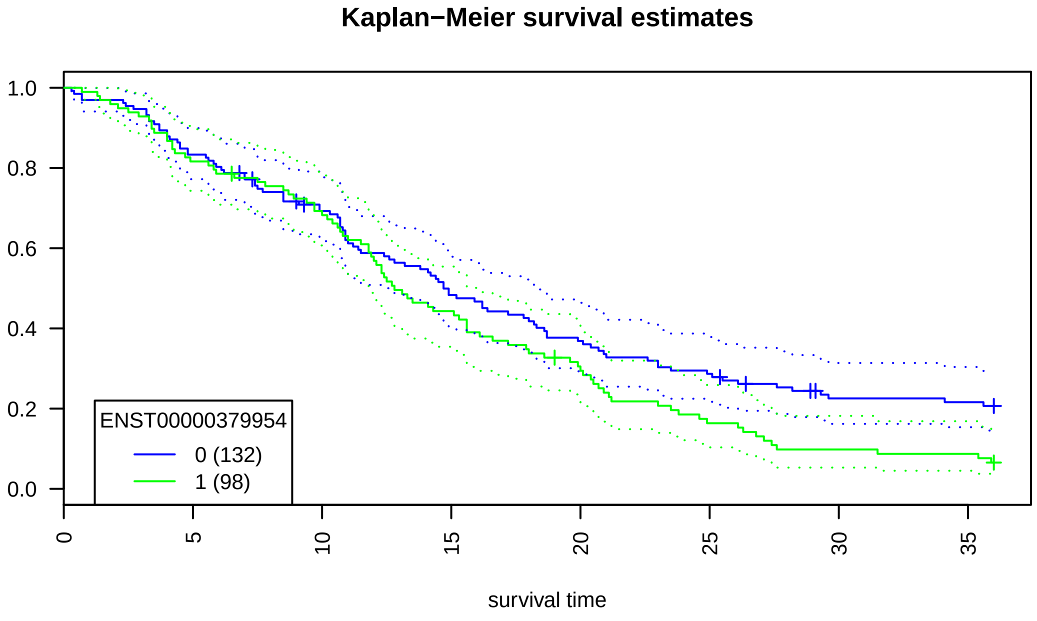

Figure 453: Each curve represents the relative number of ENST00000379954 survived to the corresponding point of time. Dashed lines represent 0.95 confidence intervals for the curves with the corresponding color. Log-rank probability measure for the equality of these curves is 2.966396e-02. Additional annotations: *.GeneId*=ENSG00000134460, *.GeneName*=IL2RA, *.GeneDesc*=interleukin 2 receptor, alpha [Source:HGNC Symbol;Acc:6008].

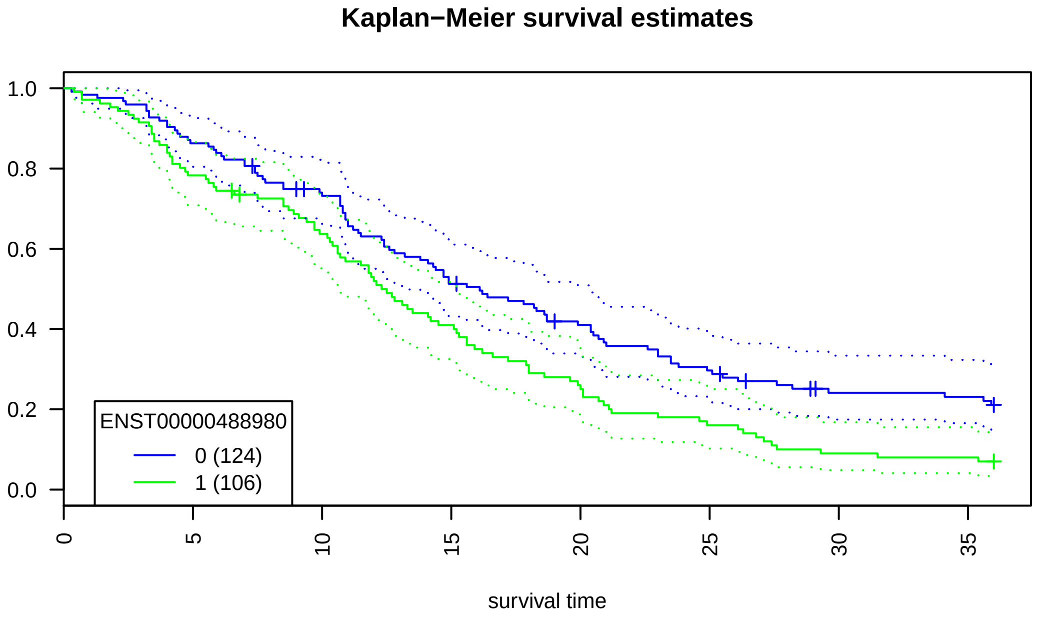

Figure 454: Each curve represents the relative number of ENST00000488980 survived to the corresponding point of time. Dashed lines represent 0.95 confidence intervals for the curves with the corresponding color. Log-rank probability measure for the equality of these curves is 1.911385e-03. Additional annotations: .*GeneId*=ENSG00000134460, .*GeneName*=IL2RA, .*GeneDesc*=interleukin 2 receptor, alpha [Source:HGNC Symbol;Acc:6008].

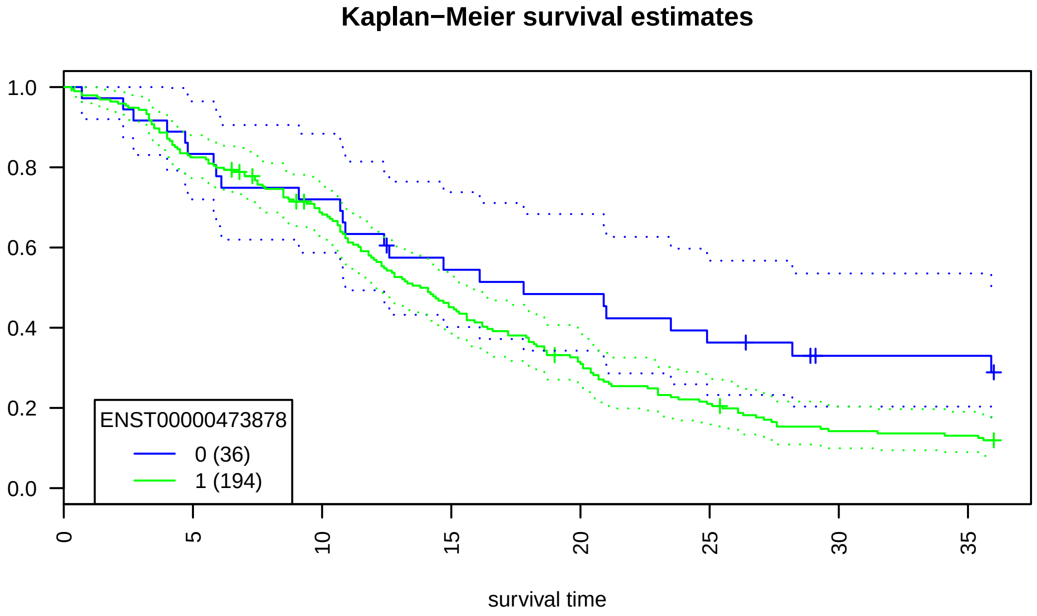

Figure 455: Each curve represents the relative number of ENST00000473878 survived to the corresponding point of time. Dashed lines represent 0.95 confidence intervals for the curves with the corresponding color. Log-rank probability measure for the equality of these curves is 4.322426e-02.

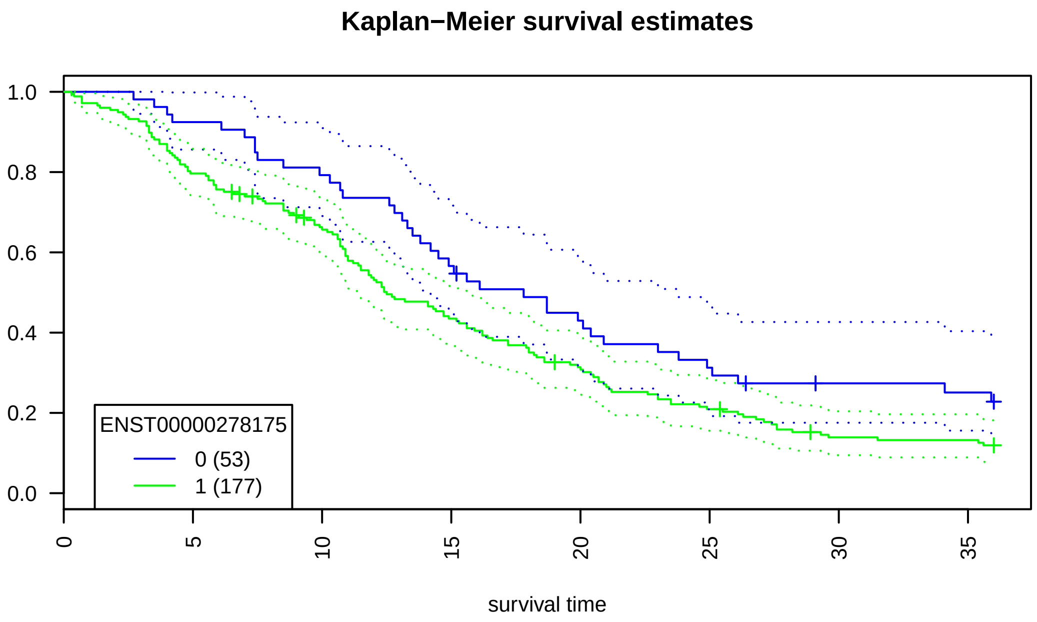

Figure 456: Each curve represents the relative number of ENST00000278175 survived to the corresponding point of time. Dashed lines represent 0.95 confidence intervals for the curves with the corresponding color. Log-rank probability measure for the equality of these curves is 2.649243e-02. Additional annotations: *.GeneId*=ENSG00000148926, *.GeneName*=ADM, *.GeneDesc*=adrenomedullin [Source:HGNC Symbol;Acc:259].

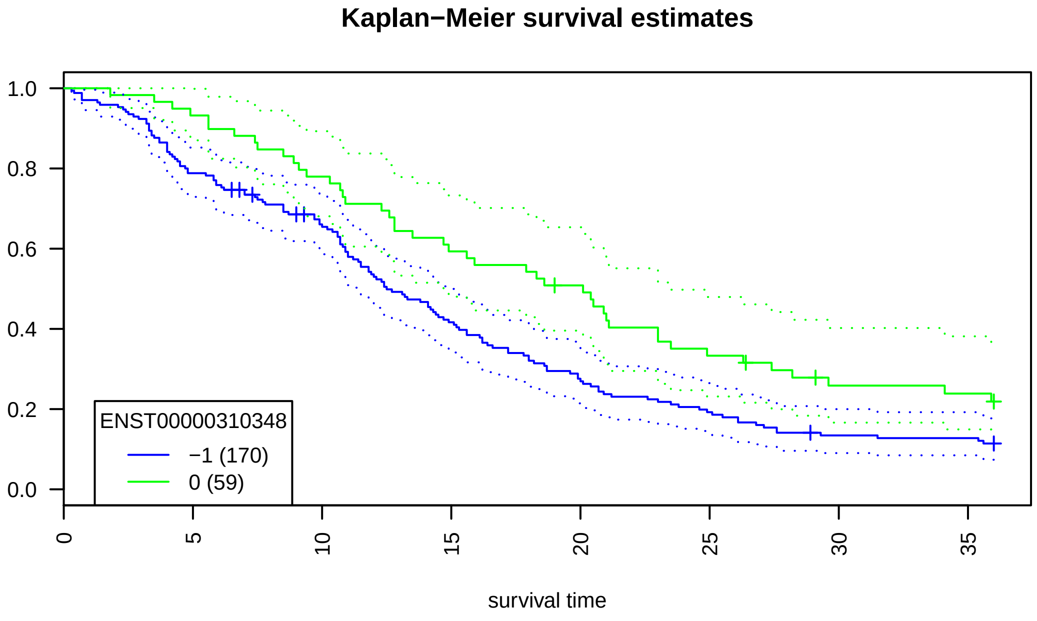

Figure 457: Each curve represents the relative number of ENST00000310348 survived to the corresponding point of time. Dashed lines represent 0.95 confidence intervals for the curves with the corresponding color. Total of 1 observation deleted due to missingness. Log-rank probability measure for the equality of these curves is 6.05198e-03. Additional annotations: *.GeneId*=ENSG00000172264, *.GeneName*=MACROD2, *.GeneDesc*=MACRO domain containing 2 [Source:HGNC Symbol;Acc:16126].

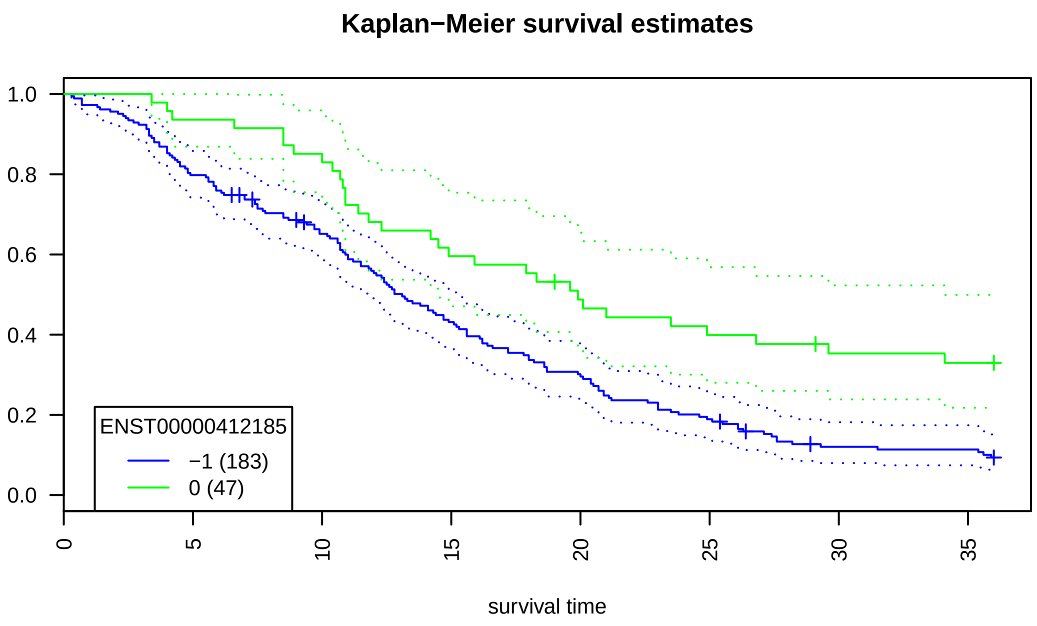

Figure 458: Each curve represents the relative number of ENST00000412185 survived to the corresponding point of time. Dashed lines represent 0.95 confidence intervals for the curves with the corresponding color. Log-rank probability measure for the equality of these curves is 3.838808e-04. Additional annotations: .*GeneId*=ENSG00000089486, .*GeneName*=C16orf5, .*GeneDesc*=LITAF-like protein (Transmembrane protein I1)(Cell death-inducing protein) [Source:UniProtKB/Swiss-Prot;Acc:Q9H305].

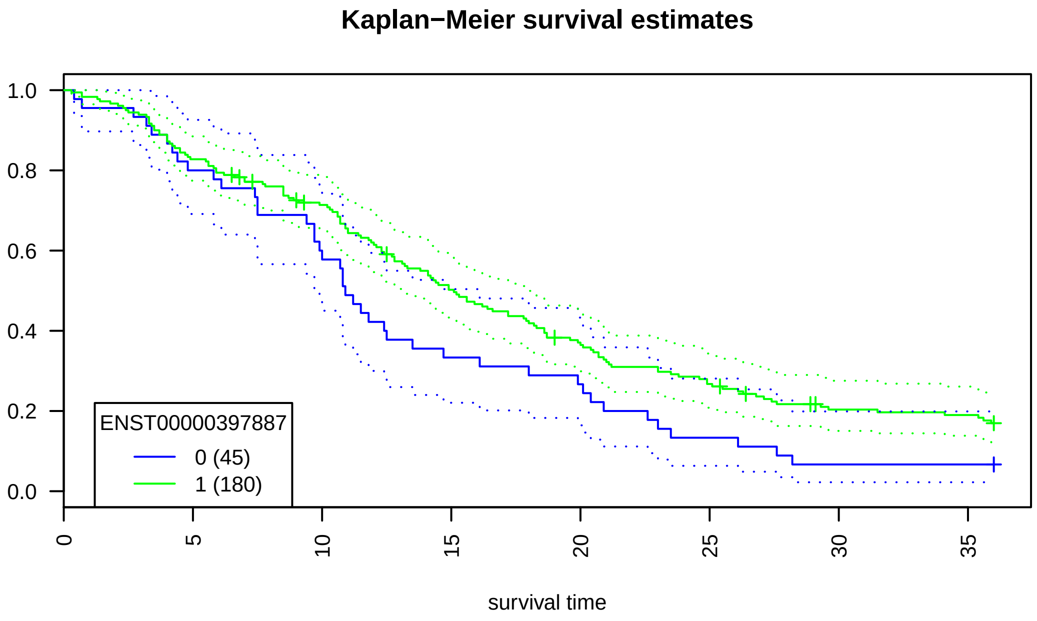

Figure 459: Each curve represents the relative number of ENST00000397887 survived to the corresponding point of time. Dashed lines represent 0.95 confidence intervals for the curves with the corresponding color. Total of 5 observations deleted due to missingness. Log-rank probability measure for the equality of these curves is 2.221958e-02. Additional annotations: .*GeneId*=ENSG00000183255, .*GeneName*=PTTG1IP, .*GeneDesc*=pituitary tumor-transforming 1 interacting protein [Source:HGNC Symbol;Acc:13524].

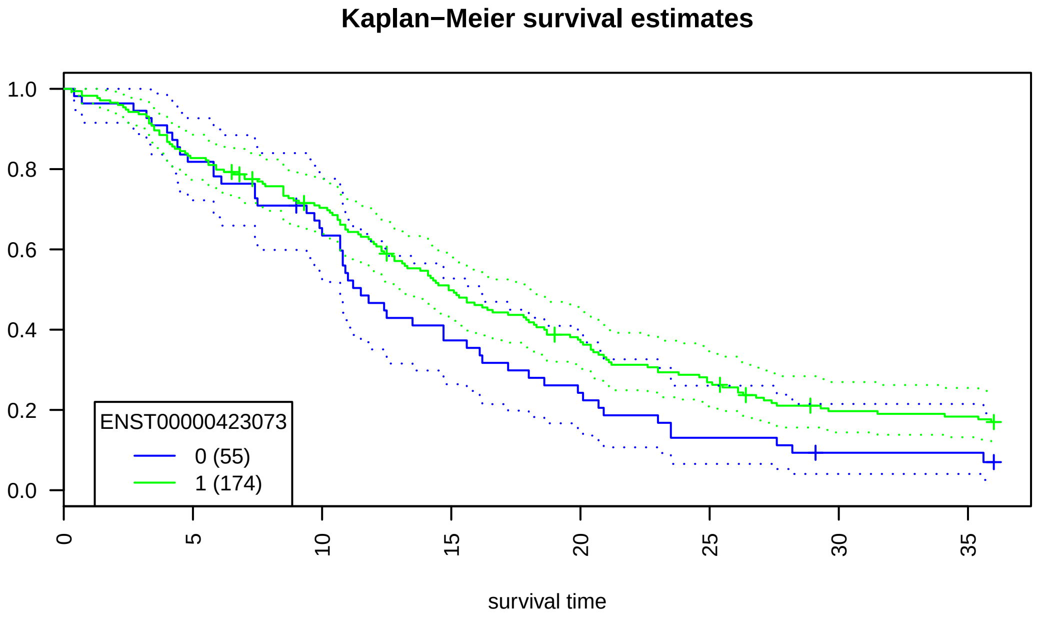

Figure 460: Each curve represents the relative number of ENST00000423073 survived to the corresponding point of time. Dashed lines represent 0.95 confidence intervals for the curves with the corresponding color. Total of 1 observation deleted due to missingness. Log-rank probability measure for the equality of these curves is 3.594331e-02. Additional annotations: *.GeneId*=ENSG00000183255, *.GeneName*=PTTG1IP, *.GeneDesc*=pituitary tumor-transforming 1 interacting protein [Source:HGNC Symbol;Acc:13524].

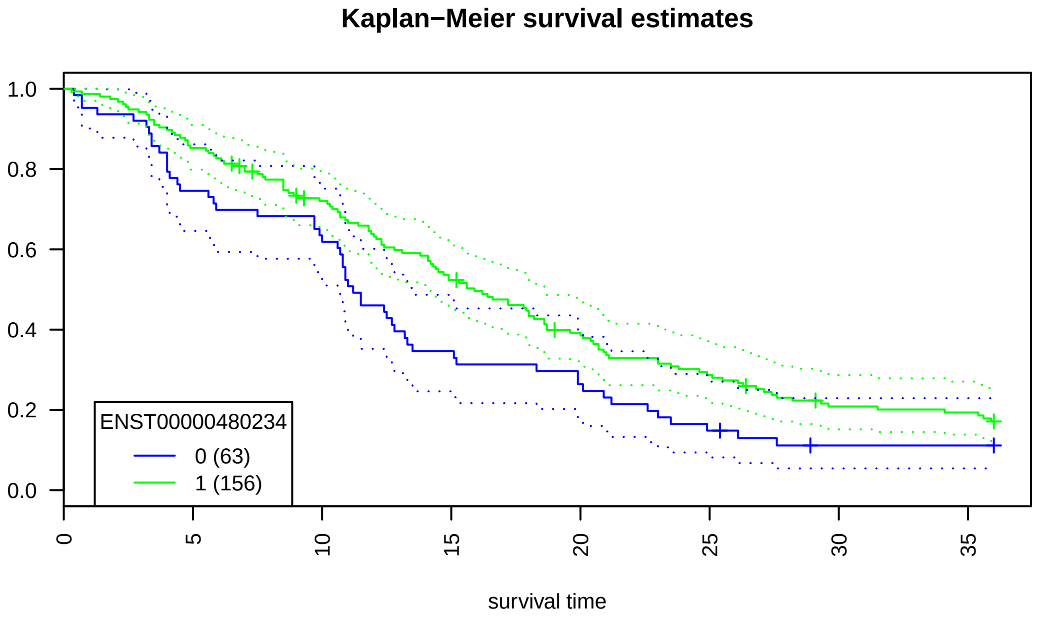

Figure 461: Each curve represents the relative number of ENST00000480234 survived to the corresponding point of time. Dashed lines represent 0.95 confidence intervals for the curves with the corresponding color. Total of 11 observations deleted due to missingness. Log-rank probability measure for the equality of these curves is 2.115179e-02. Additional annotations: *.GeneId*=ENSG00000183255, *.GeneName*=PTTG1IP, *.GeneDesc*=pituitary tumor-transforming 1 interacting protein [Source:HGNC Symbol;Acc:13524].

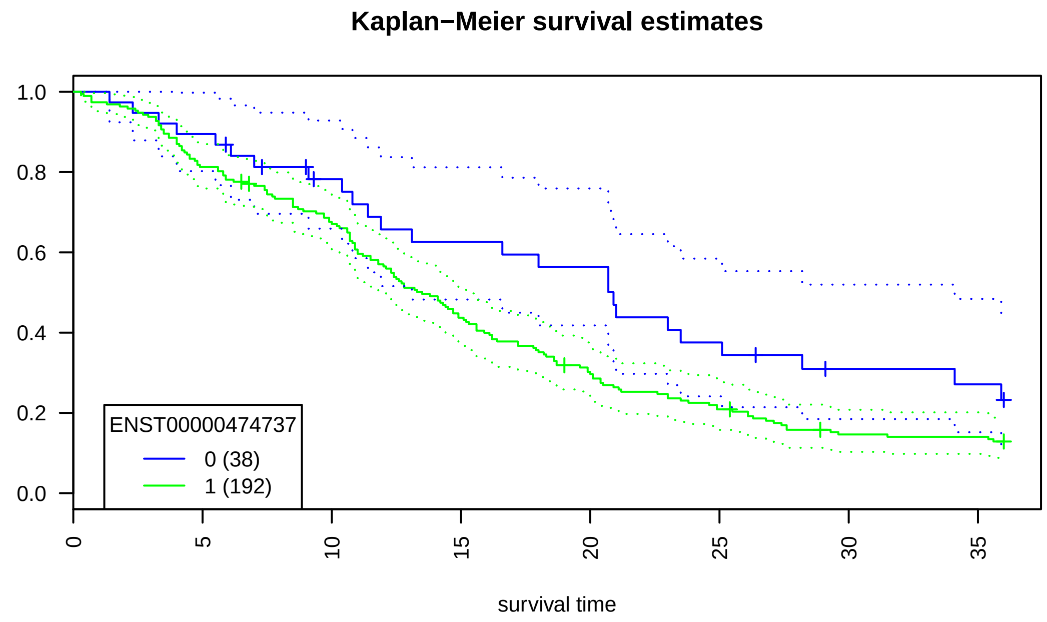

Figure 462: Each curve represents the relative number of ENST00000474737 survived to the corresponding point of time. Dashed lines represent 0.95 confidence intervals for the curves with the corresponding color. Log-rank probability measure for the equality of these curves is 3.844573e-02. Additional annotations: *.GeneId*=ENSG00000183255, *.GeneName*=PTTG1IP, *.GeneDesc*=pituitary tumor-transforming 1 interacting protein [Source:HGNC Symbol;Acc:13524].

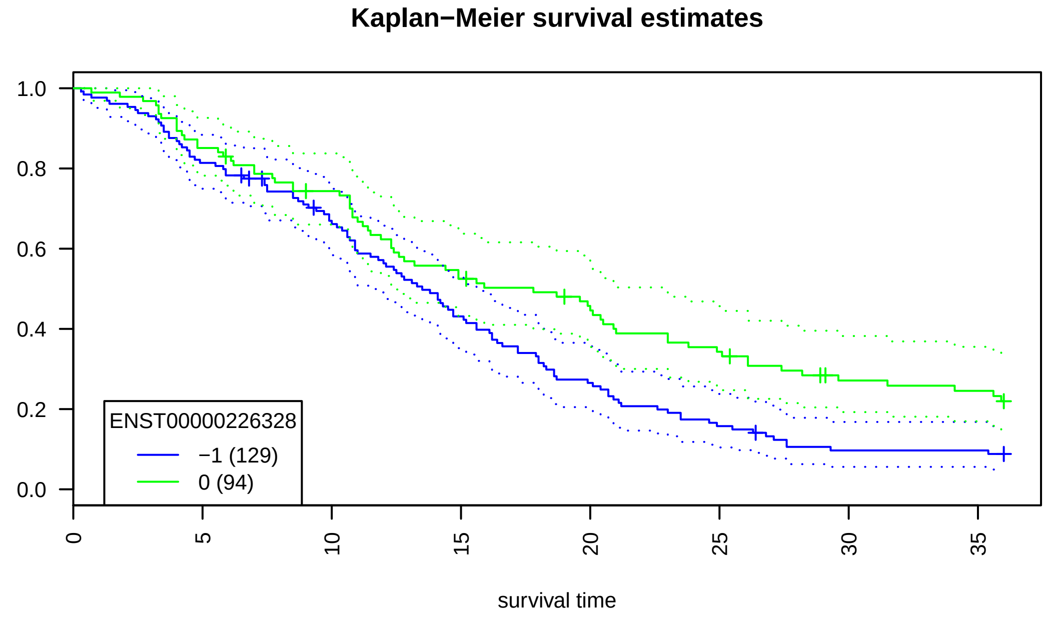

Figure 463: Each curve represents the relative number of ENST00000226328 survived to the corresponding point of time. Dashed lines represent 0.95 confidence intervals for the curves with the corresponding color. Total of 7 observations deleted due to missingness. Log-rank probability measure for the equality of these curves is 4.165303e-03. Additional annotations: *.GeneId*=ENSG00000018189, *.GeneName*=RUFY3, *.GeneDesc*=RUN and FYVE domain containing 3 [Source:HGNC Symbol;Acc:30285].

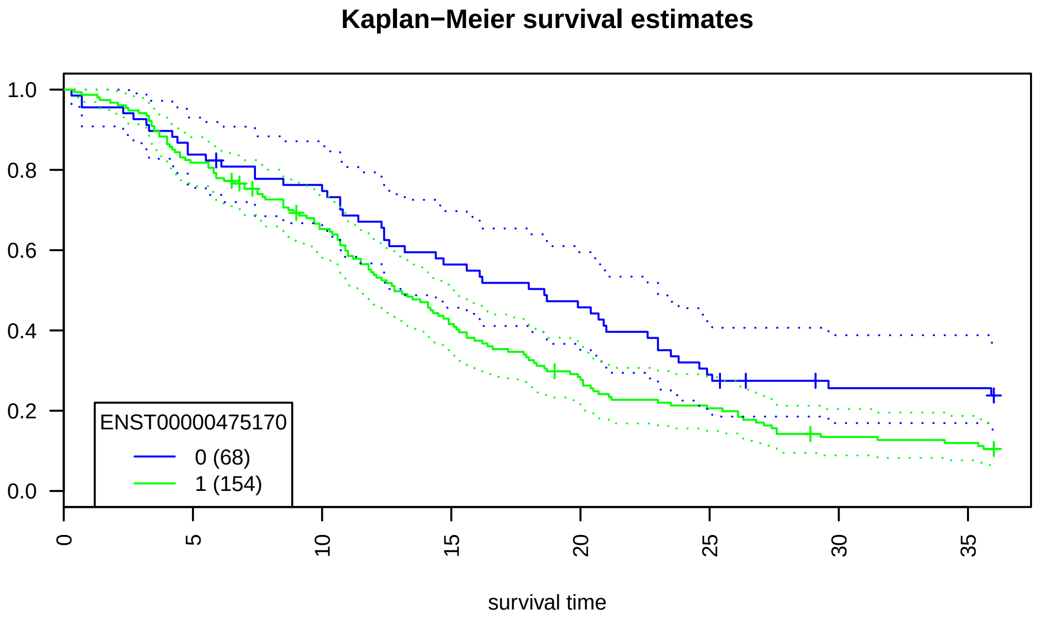

Figure 464: Each curve represents the relative number of ENST00000475170 survived to the corresponding point of time. Dashed lines represent 0.95 confidence intervals for the curves with the corresponding color. Total of 8 observations deleted due to missingness. Log-rank probability measure for the equality of these curves is 1.540883e-02. Additional annotations: *.GeneId*=ENSG00000160255, *.GeneName*=ITGB2, *.GeneDesc*=integrin, beta 2 (complement component 3 receptor 3 and 4 subunit) [Source:HGNC Symbol;Acc:6155].

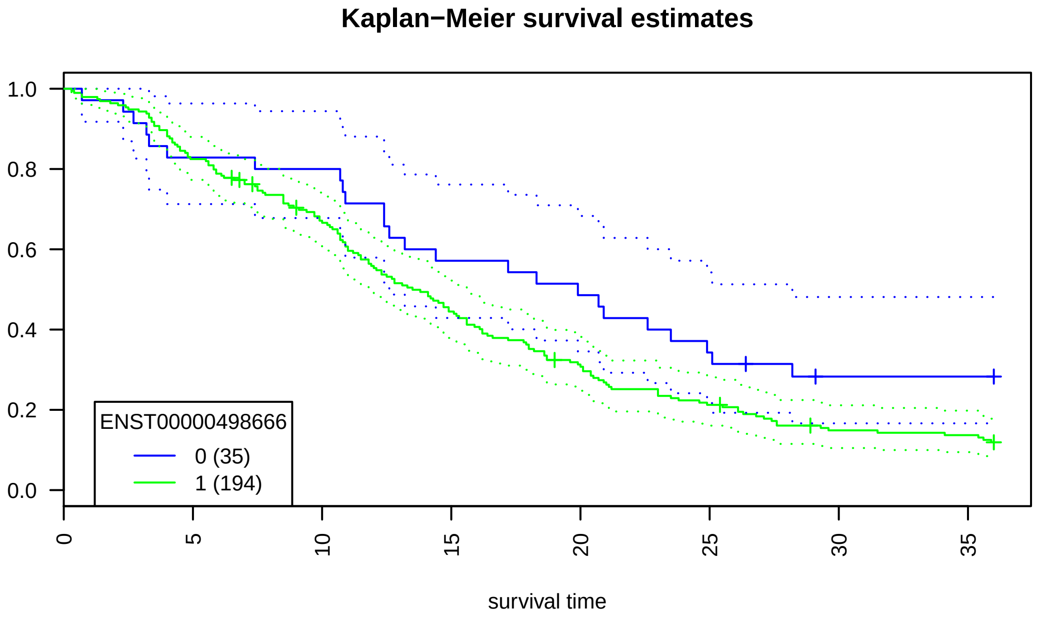

Figure 465: Each curve represents the relative number of ENST00000498666 survived to the corresponding point of time. Dashed lines represent 0.95 confidence intervals for the curves with the corresponding color. Total of 1 observation deleted due to missingness. Log-rank probability measure for the equality of these curves is 3.481259e-02. Additional annotations: *.GeneId*=ENSG00000160255, *.GeneName*=ITGB2, *.GeneDesc*=integrin, beta 2 (complement component 3 receptor 3 and 4 subunit) [Source:HGNC Symbol;Acc:6155].

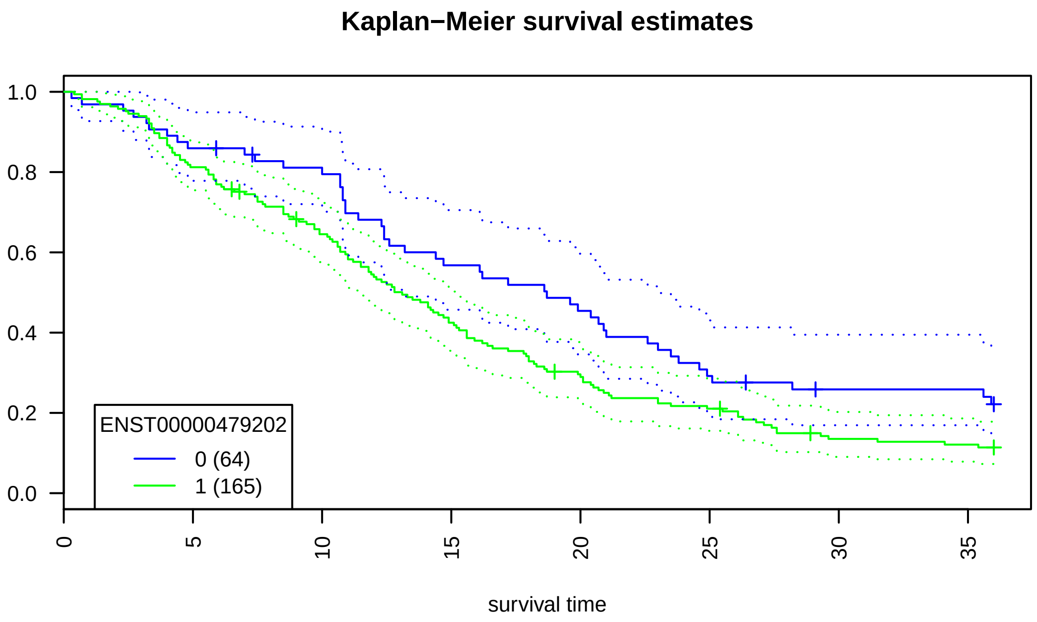

Figure 466: Each curve represents the relative number of ENST00000479202 survived to the corresponding point of time. Dashed lines represent 0.95 confidence intervals for the curves with the corresponding color. Total of 1 observation deleted due to missingness. Log-rank probability measure for the equality of these curves is 1.917378e-02. Additional annotations: *.GeneId*=ENSG00000160255, *.GeneName*=ITGB2, *.GeneDesc*=integrin, beta 2 (complement component 3 receptor 3 and 4 subunit) [Source:HGNC Symbol;Acc:6155].

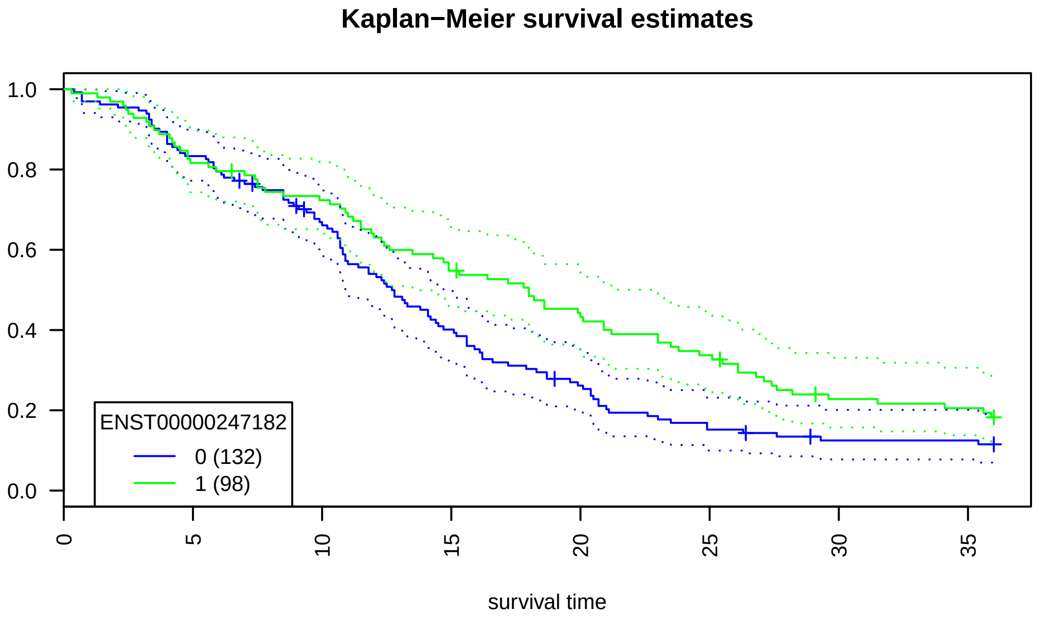

Figure 467: Each curve represents the relative number of ENST00000247182 survived to the corresponding point of time. Dashed lines represent 0.95 confidence intervals for the curves with the corresponding color. Log-rank probability measure for the equality of these curves is 1.637959e-02. Additional annotations: *.GeneId*=ENSG00000126778, *.GeneName*=SIX1, *.GeneDesc*=SIX homeobox 1 [Source:HGNC Symbol;Acc:10887].

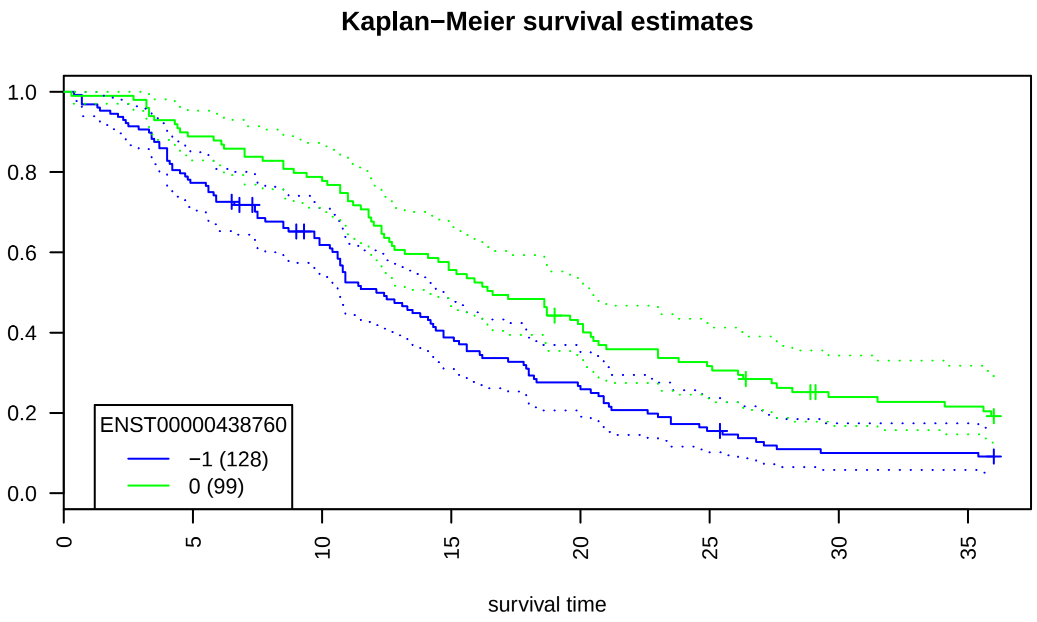

Figure 468: Each curve represents the relative number of ENST00000438760 survived to the corresponding point of time. Dashed lines represent 0.95 confidence intervals for the curves with the corresponding color. Total of 3 observations deleted due to missingness. Log-rank probability measure for the equality of these curves is 1.763644e-03. Additional annotations: *.GeneId*=ENSG00000138606, *.GeneName*=SHF, *.GeneDesc*=Src homology 2 domain containing F [Source:HGNC Symbol;Acc:25116].

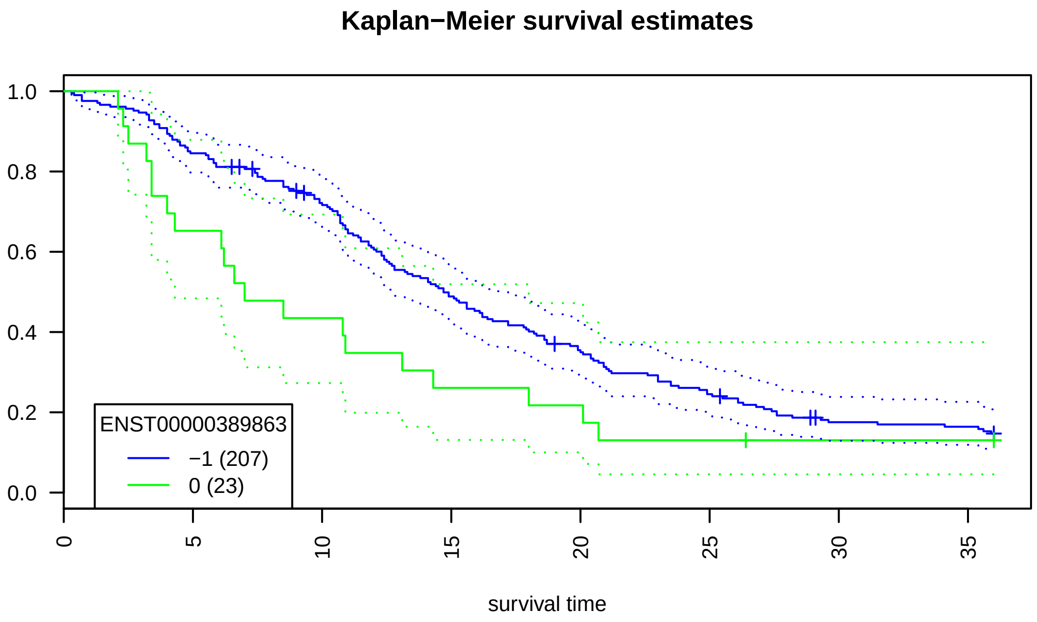

Figure 469: Each curve represents the relative number of ENST00000389863 survived to the corresponding point of time. Dashed lines represent 0.95 confidence intervals for the curves with the corresponding color. Log-rank probability measure for the equality of these curves is 3.777123e-02. Additional annotations: *.GeneId*=ENSG00000197381, *.GeneName*=ADARB1, *.GeneDesc*=adenosine deaminase, RNA-specific, B1 (RED1 homolog rat) [Source:HGNC Symbol;Acc:226].

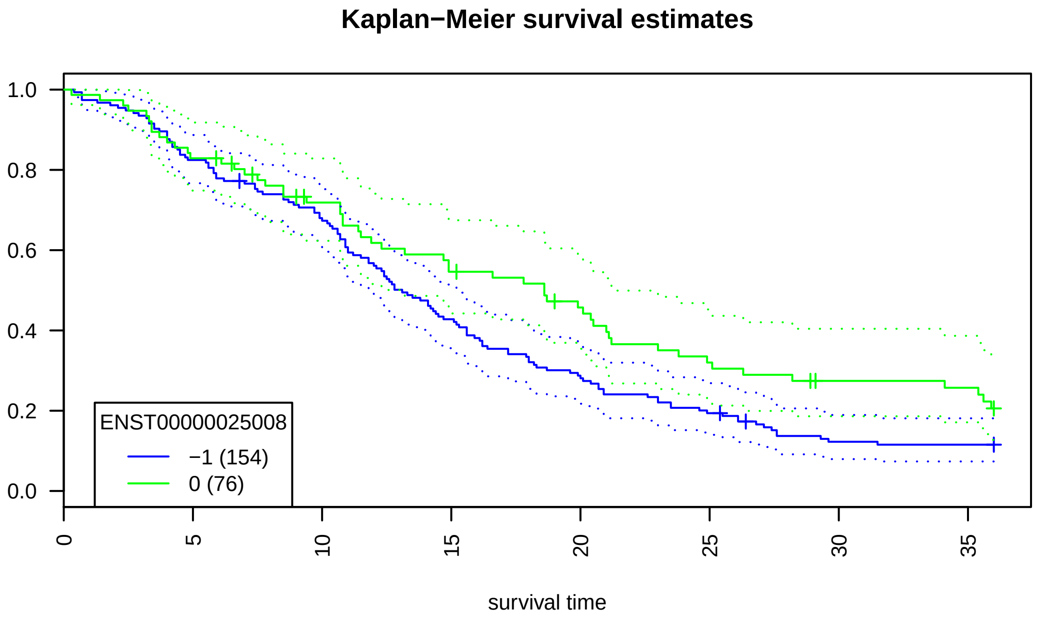

Figure 470: Each curve represents the relative number of ENST00000025008 survived to the corresponding point of time. Dashed lines represent 0.95 confidence intervals for the curves with the corresponding color. Log-rank probability measure for the equality of these curves is 3.042131e-02. Additional annotations: *.GeneId*=ENSG00000023287, *.GeneName*=RB1CC1, *.GeneDesc*=RB1-inducible coiled-coil 1 [Source:HGNC Symbol;Acc:15574].

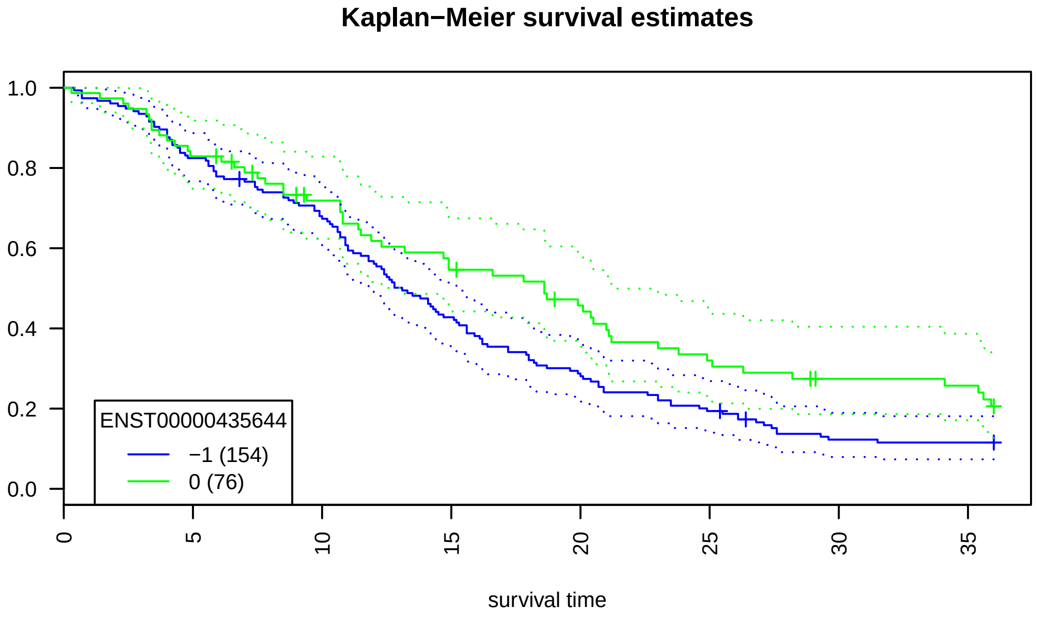

Figure 471: Each curve represents the relative number of ENST00000435644 survived to the corresponding point of time. Dashed lines represent 0.95 confidence intervals for the curves with the corresponding color. Log-rank probability measure for the equality of these curves is 3.042131e-02. Additional annotations: *.GeneId*=ENSG00000023287, *.GeneName*=RB1CC1, *.GeneDesc*=RB1-inducible coiled-coil 1 [Source:HGNC Symbol;Acc:15574].

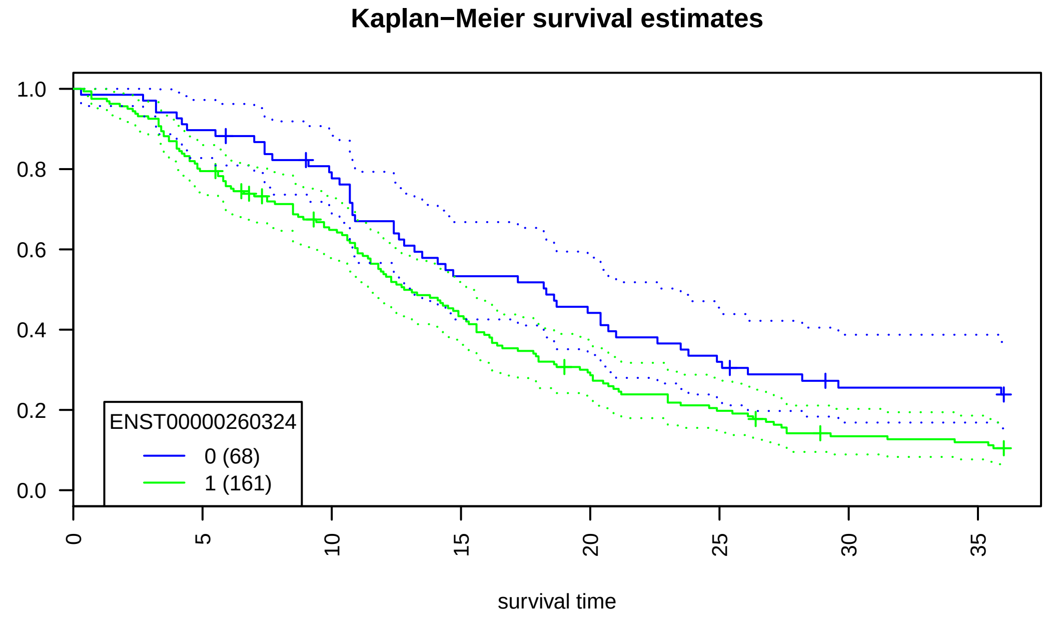

Figure 472: Each curve represents the relative number of ENST00000260324 survived to the corresponding point of time. Dashed lines represent 0.95 confidence intervals for the curves with the corresponding color. Total of 1 observation deleted due to missingness. Log-rank probability measure for the equality of these curves is 9.574336e-03. Additional annotations: *.GeneId*=ENSG00000137767, *.GeneName*=SQRDL, *.GeneDesc*=sulfide quinone reductase-like (yeast) [Source:HGNC Symbol;Acc:20390].

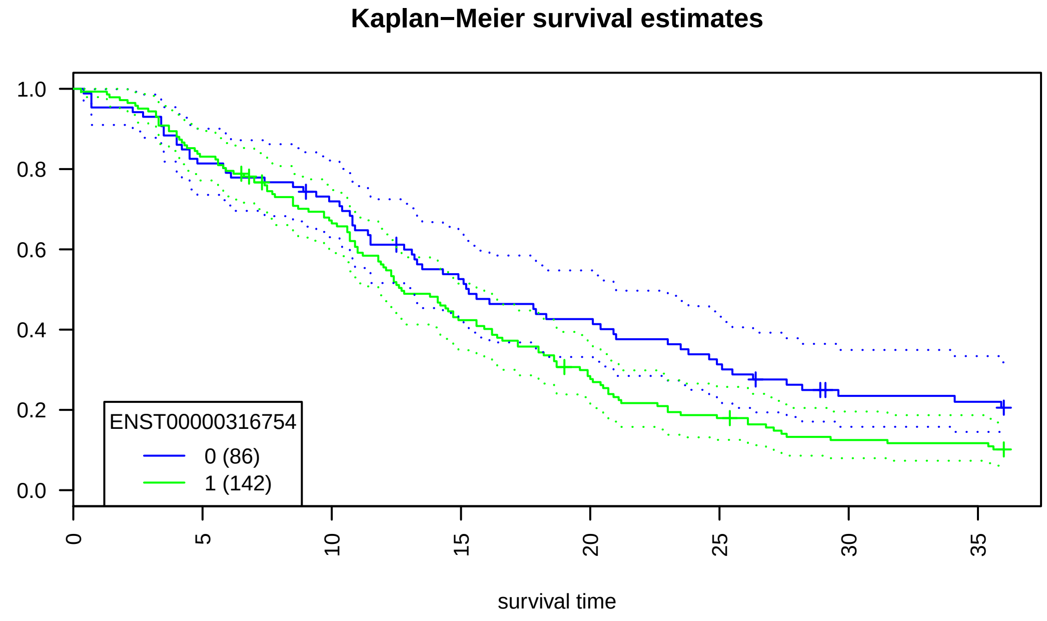

Figure 473: Each curve represents the relative number of ENST00000316754 survived to the corresponding point of time. Dashed lines represent 0.95 confidence intervals for the curves with the corresponding color. Total of 2 observations deleted due to missingness. Log-rank probability measure for the equality of these curves is 3.713448e-02. Additional annotations: *.GeneId*=ENSG00000126785, *.GeneName*=RHOJ, *.GeneDesc*=ras homolog gene family, member J [Source:HGNC Symbol;Acc:688].

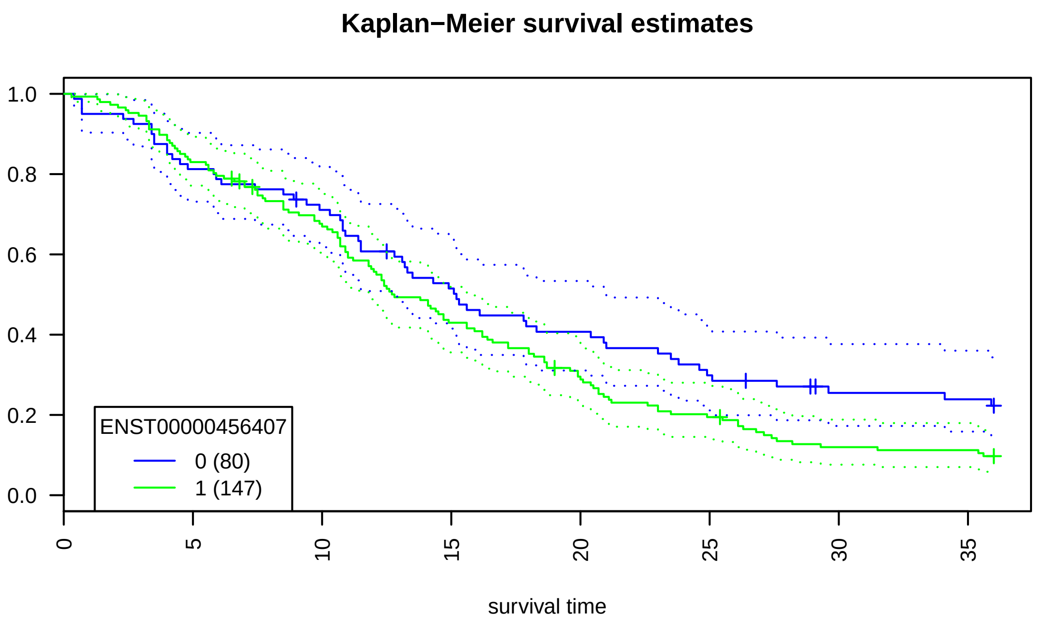

Figure 474: Each curve represents the relative number of ENST00000456407 survived to the corresponding point of time. Dashed lines represent 0.95 confidence intervals for the curves with the corresponding color. Total of 3 observations deleted due to missingness. Log-rank probability measure for the equality of these curves is 4.103781e-02. Additional annotations: *.GeneId*=ENSG00000126785, *.GeneName*=RHOJ, *.GeneDesc*=ras homolog gene family, member J [Source:HGNC Symbol;Acc:688].

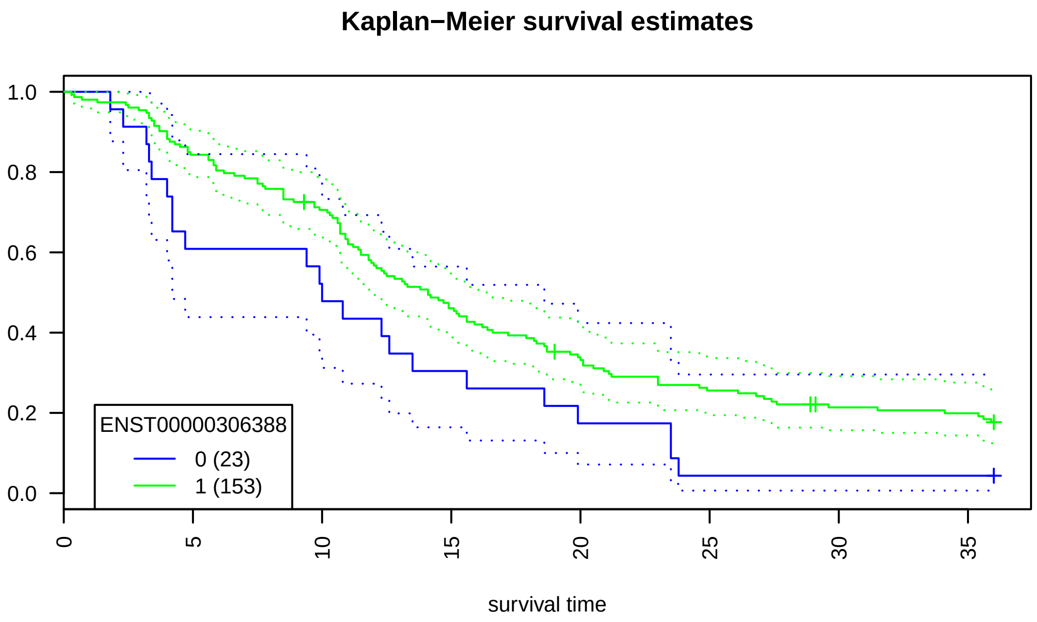

Figure 475: Each curve represents the relative number of ENST00000306388 survived to the corresponding point of time. Dashed lines represent 0.95 confidence intervals for the curves with the corresponding color. Total of 54 observations deleted due to missingness. Log-rank probability measure for the equality of these curves is 1.694739e-02. Additional annotations: *.GeneId*=ENSG00000171530, *.GeneName*=TBCA, *.GeneDesc*=tubulin folding cofactor A [Source:HGNC Symbol;Acc:11579].

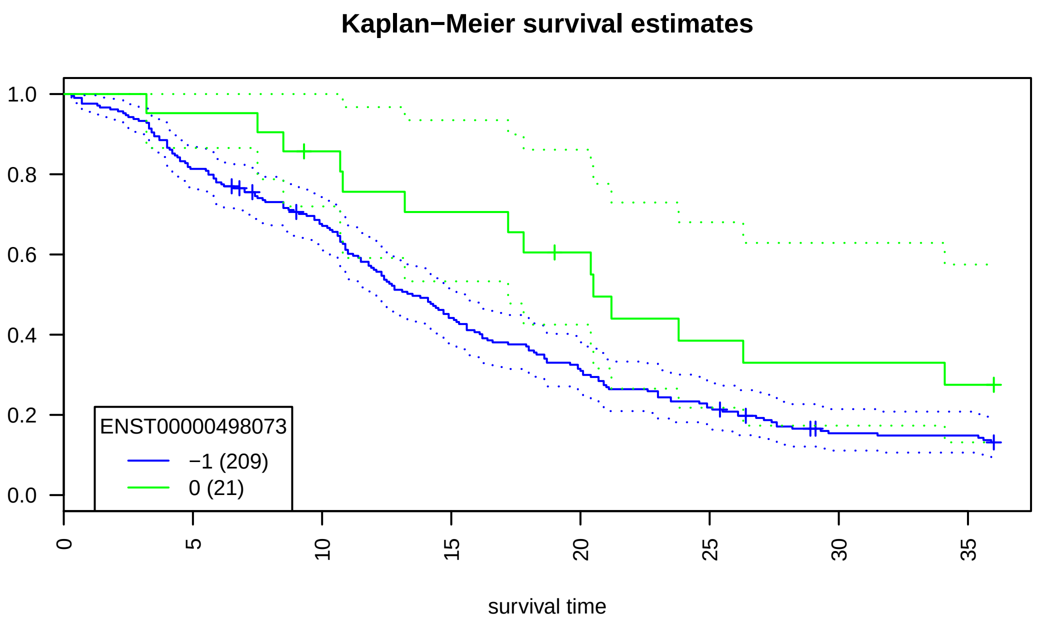

Figure 476: Each curve represents the relative number of ENST00000498073 survived to the corresponding point of time. Dashed lines represent 0.95 confidence intervals for the curves with the corresponding color. Log-rank probability measure for the equality of these curves is 3.948265e-02. Additional annotations: *.GeneId*=ENSG00000005810, *.GeneName*=MYCBP2, *.GeneDesc*=MYC binding protein 2 [Source:HGNC Symbol;Acc:23386].

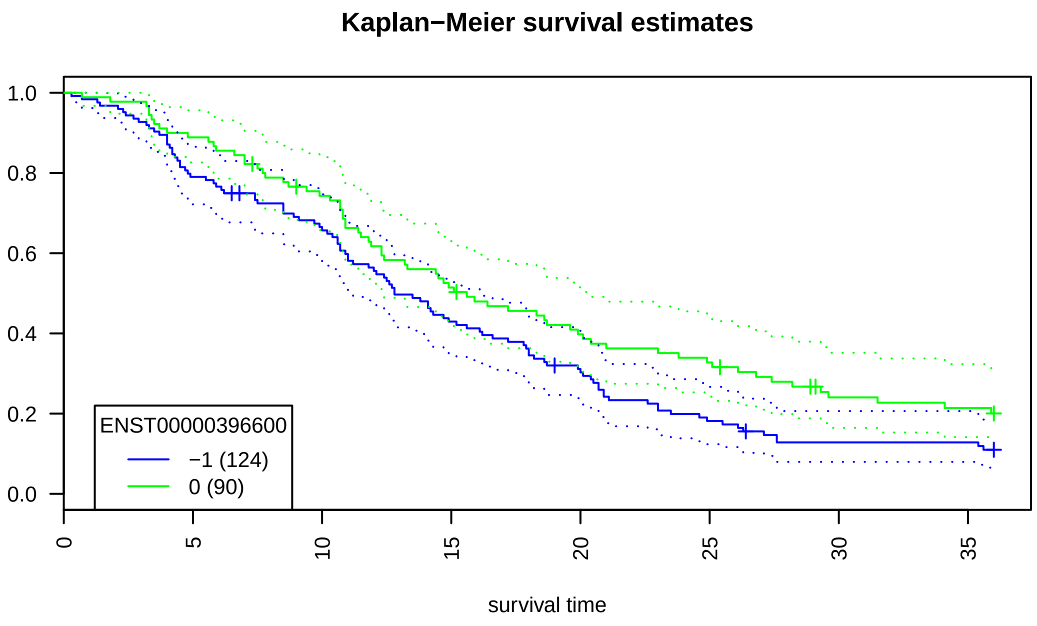

Figure 477: Each curve represents the relative number of ENST00000396600 survived to the corresponding point of time. Dashed lines represent 0.95 confidence intervals for the curves with the corresponding color. Total of 16 observations deleted due to missingness. Log-rank probability measure for the equality of these curves is 3.406394e-02. Additional annotations: *.GeneId*=ENSG00000183044, *.GeneName*=ABAT, *.GeneDesc*=4-aminobutyrate aminotransferase [Source:HGNC Symbol;Acc:23].

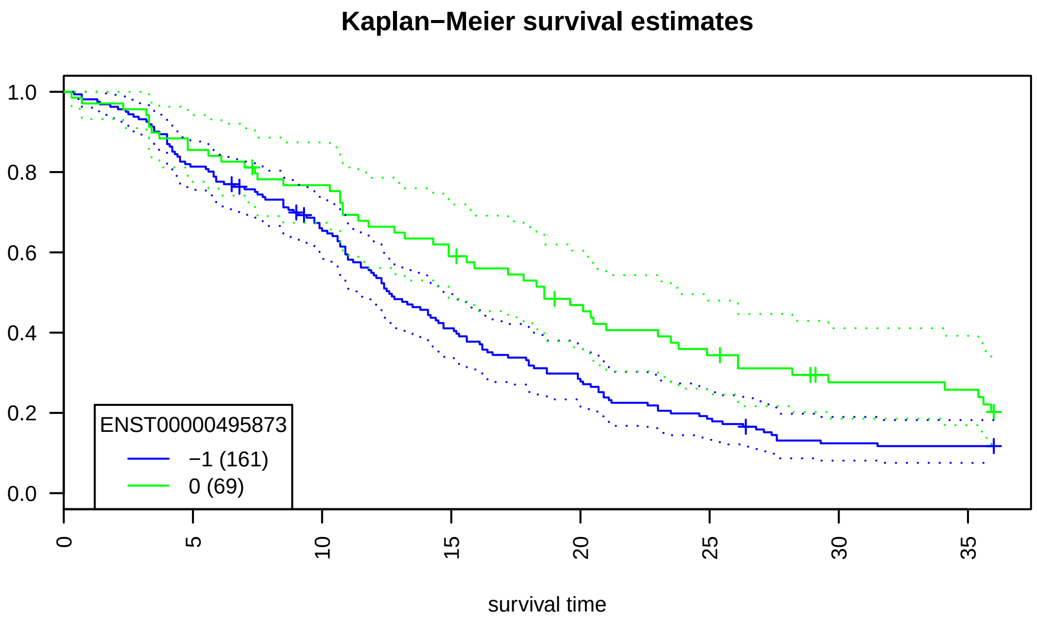

Figure 478: Each curve represents the relative number of ENST00000495873 survived to the corresponding point of time. Dashed lines represent 0.95 confidence intervals for the curves with the corresponding color. Log-rank probability measure for the equality of these curves is 1.078094e-02. Additional annotations: *.GeneId*=ENSG00000164985, *.GeneName*=PSIP1, *.GeneDesc*=PC4 and SFRS1 interacting protein 1 [Source:HGNC Symbol;Acc:9527].

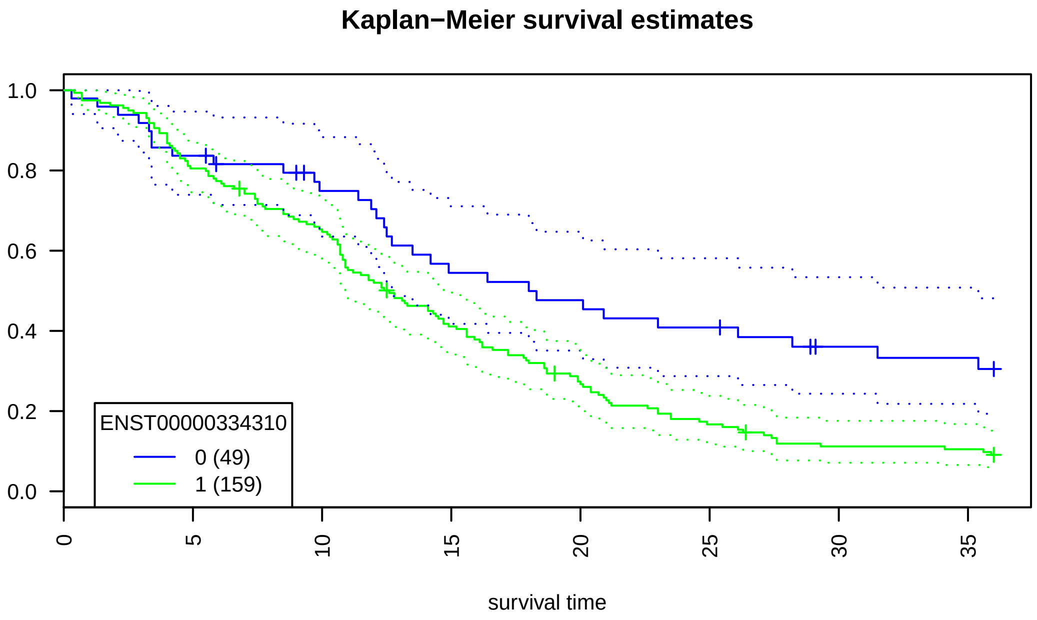

Figure 479: Each curve represents the relative number of ENST00000334310 survived to the corresponding point of time. Dashed lines represent 0.95 confidence intervals for the curves with the corresponding color. Total of 22 observations deleted due to missingness. Log-rank probability measure for the equality of these curves is 1.926536e-03. Additional annotations: *.GeneId*=ENSG00000187079, *.GeneName*=TEAD1, *.GeneDesc*=TEA domain family member 1 (SV40 transcriptional enhancer factor) [Source:HGNC Symbol;Acc:11714].

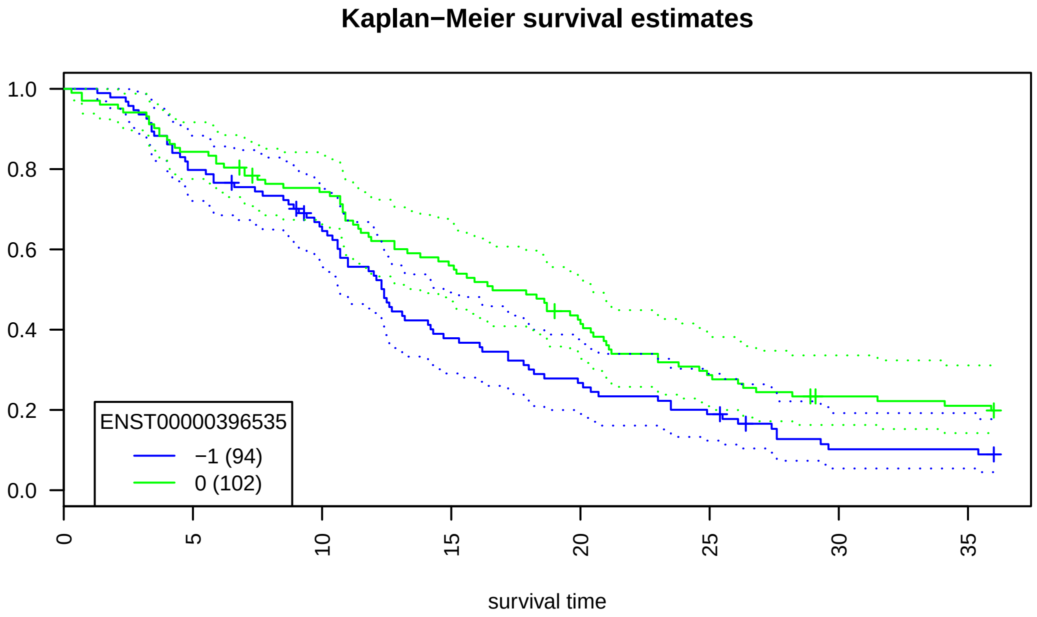

Figure 480: Each curve represents the relative number of ENST00000396535 survived to the corresponding point of time. Dashed lines represent 0.95 confidence intervals for the curves with the corresponding color. Total of 34 observations deleted due to missingness. Log-rank probability measure for the equality of these curves is 1.816794e-02. Additional annotations: *.GeneId*=ENSG00000185634, *.GeneName*=SHC4, *.GeneDesc*=SHC (Src homology 2 domain containing) family, member 4 [Source:HGNC Symbol;Acc:16743].

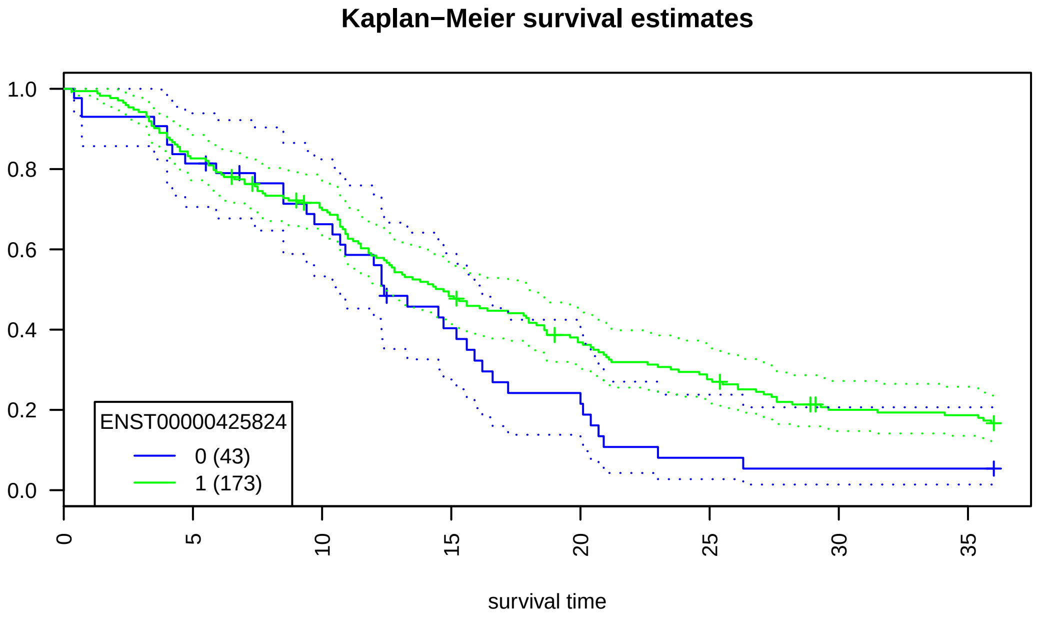

Figure 481: Each curve represents the relative number of ENST00000425824 survived to the corresponding point of time. Dashed lines represent 0.95 confidence intervals for the curves with the corresponding color. Total of 14 observations deleted due to missingness. Log-rank probability measure for the equality of these curves is 3.189532e-02. Additional annotations: *.GeneId*=ENSG00000044459, *.GeneName*=CNTLN, *.GeneDesc*=centlein, centrosomal protein [Source:HGNC Symbol;Acc:23432].

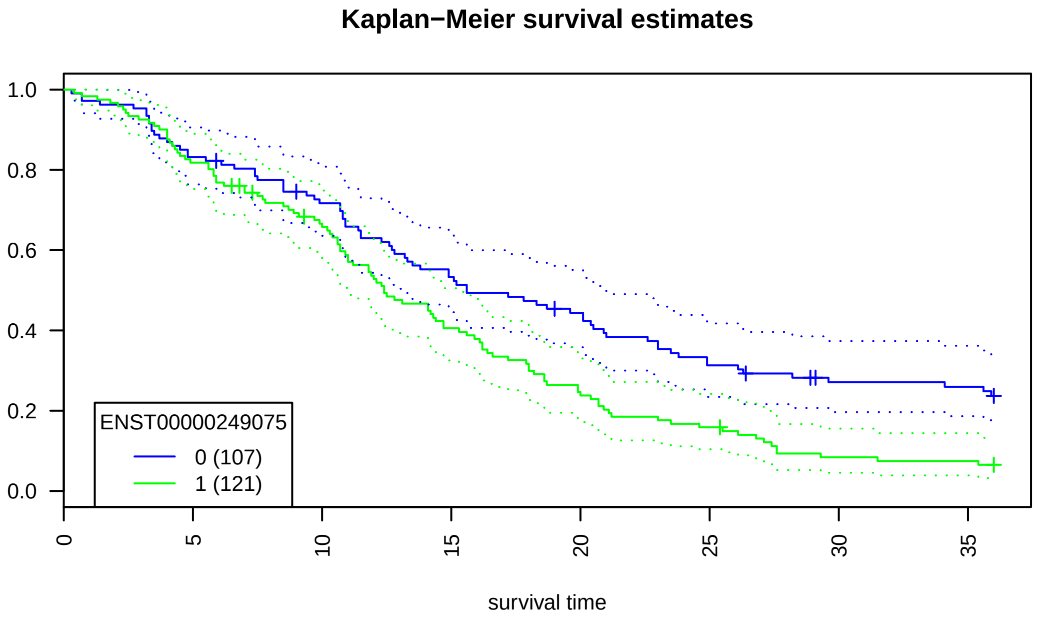

Figure 482: Each curve represents the relative number of ENST00000249075 survived to the corresponding point of time. Dashed lines represent 0.95 confidence intervals for the curves with the corresponding color. Total of 2 observations deleted due to missingness. Log-rank probability measure for the equality of these curves is 8.716821e-04. Additional annotations: *.GeneId*=ENSG00000128342, *.GeneName*=LIF, *.GeneDesc*=leukemia inhibitory factor (cholinergic differentiation factor) [Source:HGNC Symbol;Acc:6596].

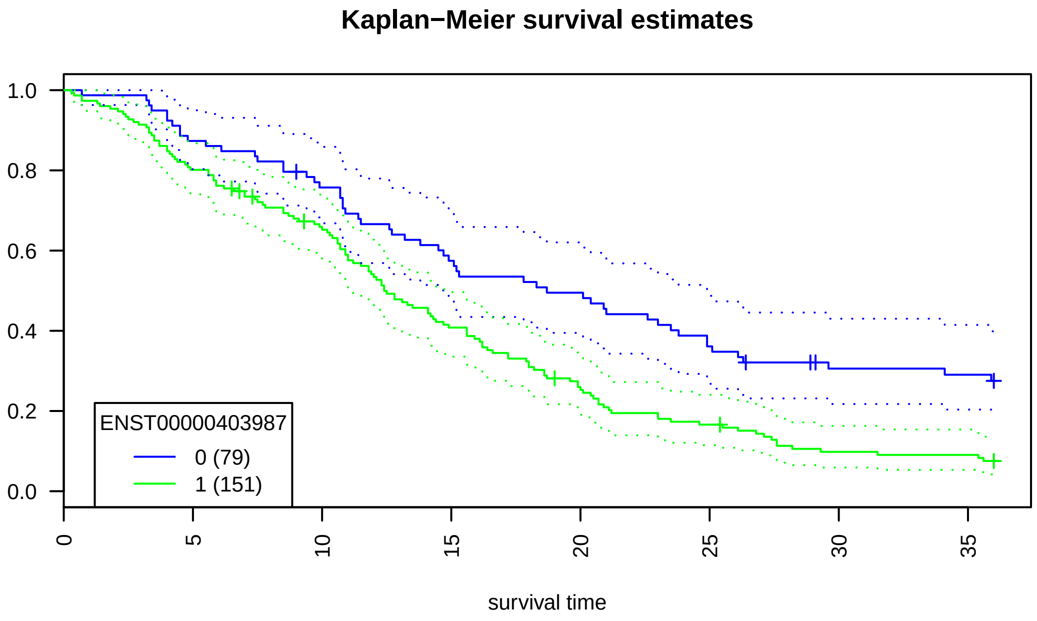

Figure 483: Each curve represents the relative number of ENST00000403987 survived to the corresponding point of time. Dashed lines represent 0.95 confidence intervals for the curves with the corresponding color. Log-rank probability measure for the equality of these curves is 1.173384e-04. Additional annotations: *.GeneId*=ENSG00000128342, *.GeneName*=LIF, *.GeneDesc*=leukemia inhibitory factor (cholinergic differentiation factor) [Source:HGNC Symbol;Acc:6596].

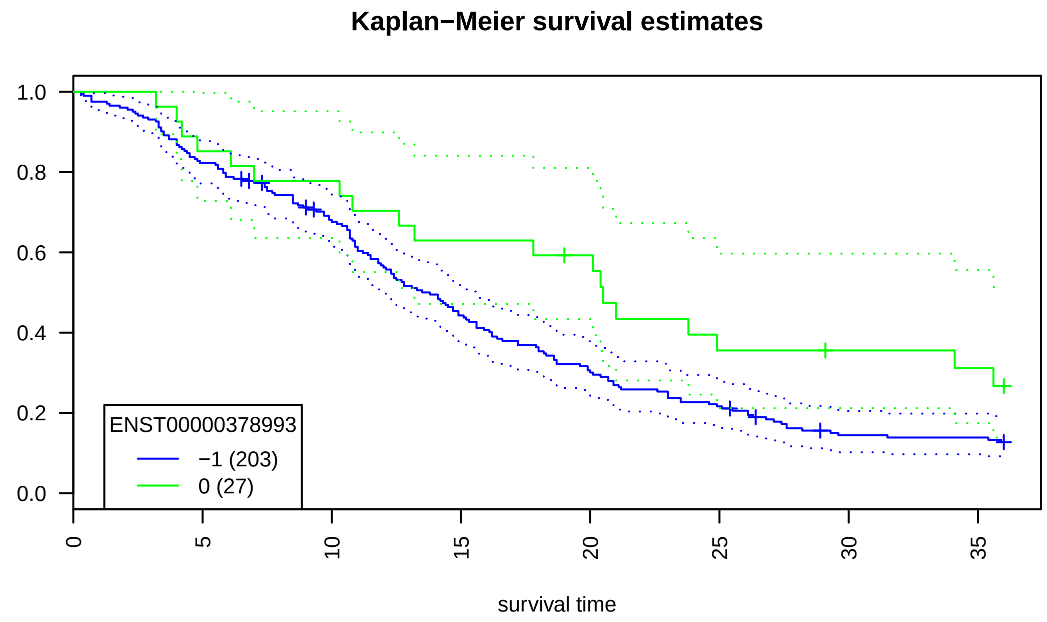

Figure 484: Each curve represents the relative number of ENST00000378993 survived to the corresponding point of time. Dashed lines represent 0.95 confidence intervals for the curves with the corresponding color. Log-rank probability measure for the equality of these curves is 3.571791e-02. Additional annotations: *.GeneId*=ENSG00000169306, *.GeneName*=IL1RAPL1, *.GeneDesc*=interleukin 1 receptor accessory protein-like 1 [Source:HGNC Symbol;Acc:5996].

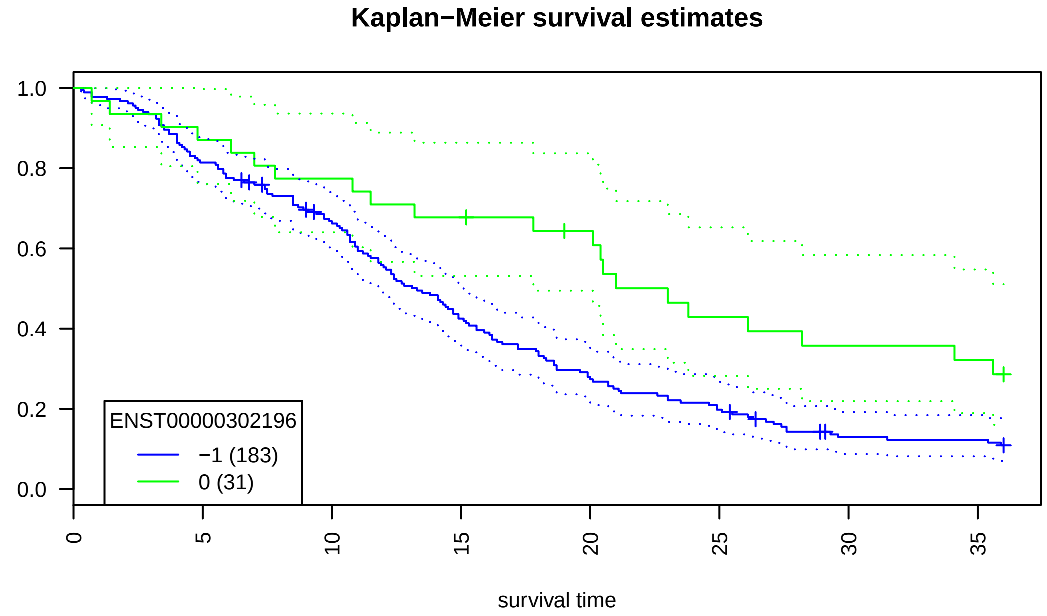

Figure 485: Each curve represents the relative number of ENST00000302196 survived to the corresponding point of time. Dashed lines represent 0.95 confidence intervals for the curves with the corresponding color. Total of 16 observations deleted due to missingness. Log-rank probability measure for the equality of these curves is 5.015856e-03. Additional annotations: *.GeneId*=ENSG00000169306, *.GeneName*=IL1RAPL1, *.GeneDesc*=interleukin 1 receptor accessory protein-like 1 [Source:HGNC Symbol;Acc:5996].

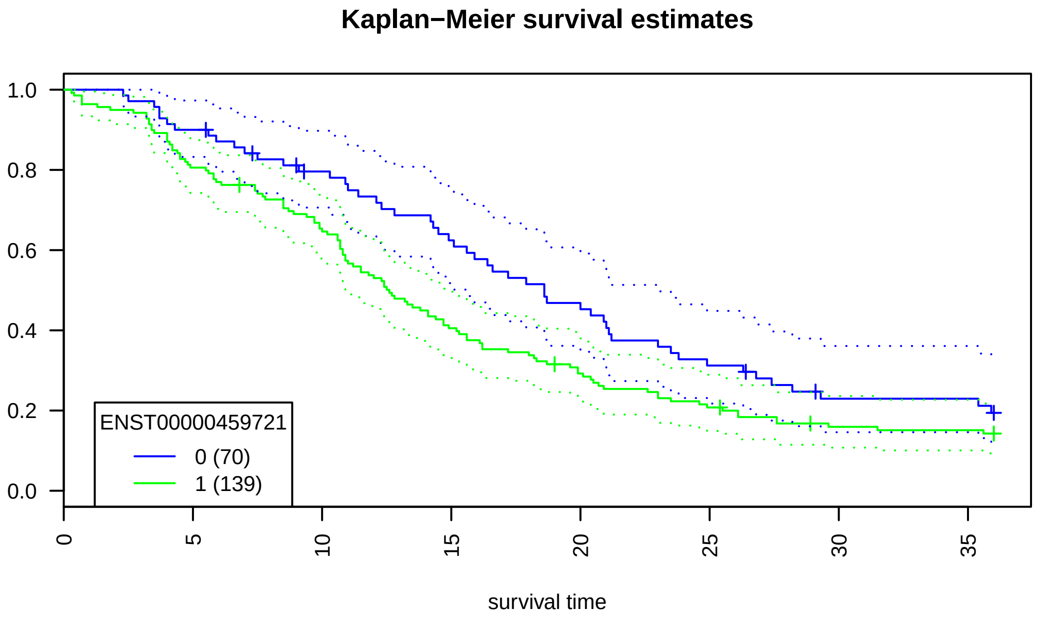

Figure 486: Each curve represents the relative number of ENST00000459721 survived to the corresponding point of time. Dashed lines represent 0.95 confidence intervals for the curves with the corresponding color. Total of 21 observations deleted due to missingness. Log-rank probability measure for the equality of these curves is 3.13378e-02. Additional annotations: *.GeneId*=ENSG00000132669, *.GeneName*=RIN2, *.GeneDesc*=Ras and Rab interactor 2 [Source:HGNC Symbol;Acc:18750].

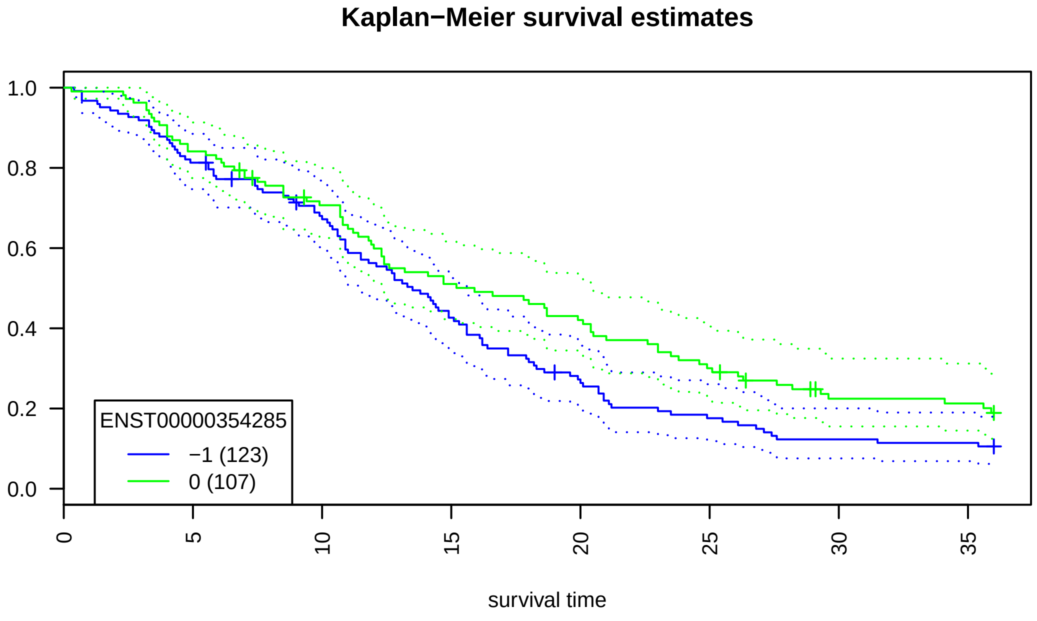

Figure 487: Each curve represents the relative number of ENST00000354285 survived to the corresponding point of time. Dashed lines represent 0.95 confidence intervals for the curves with the corresponding color. Log-rank probability measure for the equality of these curves is 3.583934e-02. Additional annotations: *.GeneId*=ENSG00000170456, *.GeneName*=DENND5B, *.GeneDesc*=DENN/MADD domain containing 5B [Source:HGNC Symbol;Acc:28338].

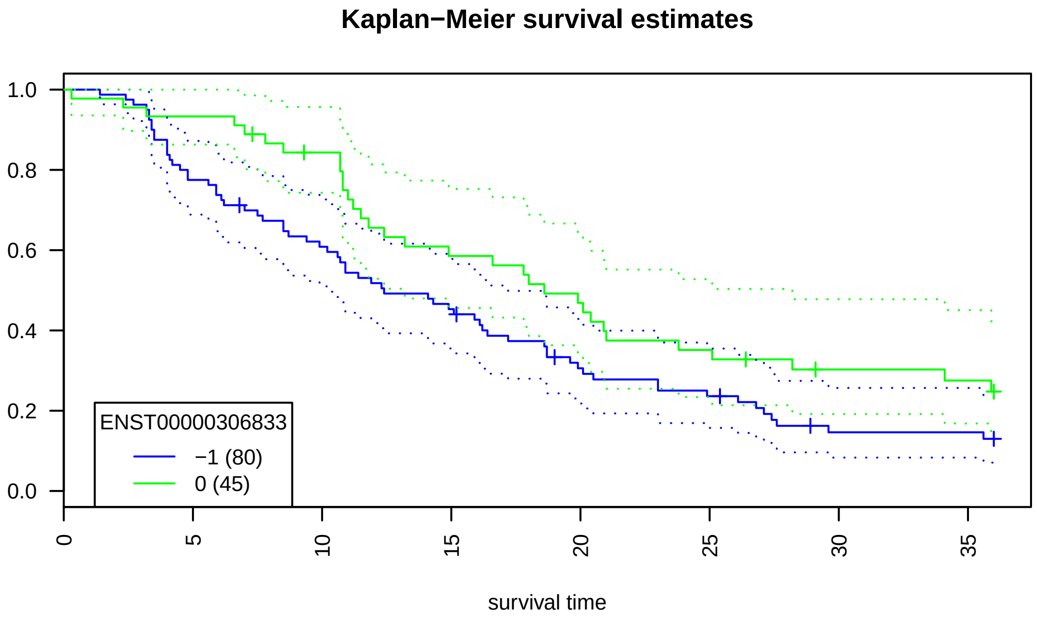

Figure 488: Each curve represents the relative number of ENST00000306833 survived to the corresponding point of time. Dashed lines represent 0.95 confidence intervals for the curves with the corresponding color. Total of 105 observations deleted due to missingness. Log-rank probability measure for the equality of these curves is 3.785241e-02. Additional annotations: *.GeneId*=ENSG00000170456, *.GeneName*=DENND5B, *.GeneDesc*=DENN/MADD domain containing 5B [Source:HGNC Symbol;Acc:28338].

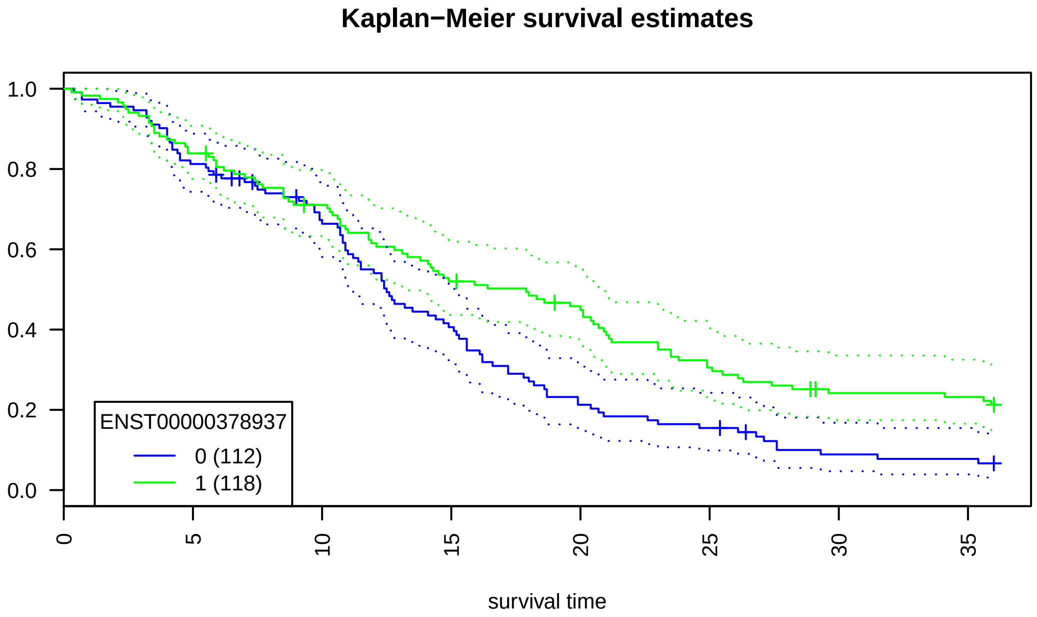

Figure 489: Each curve represents the relative number of ENST00000378937 survived to the corresponding point of time. Dashed lines represent 0.95 confidence intervals for the curves with the corresponding color. Log-rank probability measure for the equality of these curves is 1.841032e-03. Additional annotations: *.GeneId*=ENSG00000165609, *.GeneName*=NUDT5, *.GeneDesc*=nudix (nucleoside diphosphate linked moiety X)-type motif 5 [Source:HGNC Symbol;Acc:8052].

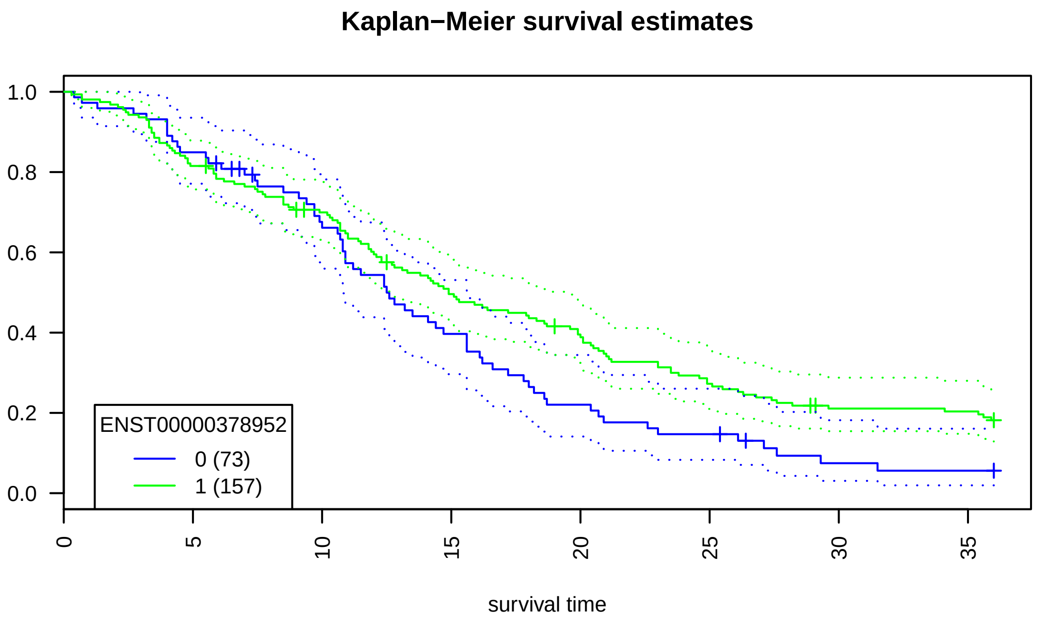

Figure 490: Each curve represents the relative number of ENST00000378952 survived to the corresponding point of time. Dashed lines represent 0.95 confidence intervals for the curves with the corresponding color. Log-rank probability measure for the equality of these curves is 1.923944e-02. Additional annotations: *.GeneId*=ENSG00000165609, *.GeneName*=NUDT5, *.GeneDesc*=nudix (nucleoside diphosphate linked moiety X)-type motif 5 [Source:HGNC Symbol;Acc:8052].

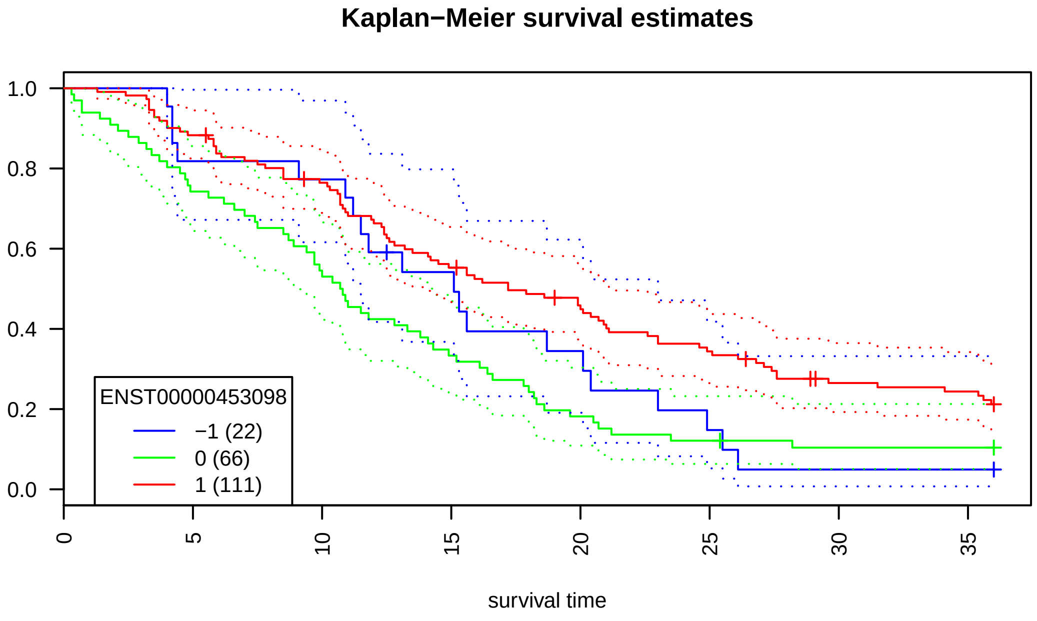

Figure 491: Each curve represents the relative number of ENST00000453098 survived to the corresponding point of time. Dashed lines represent 0.95 confidence intervals for the curves with the corresponding color. Total of 31 observations deleted due to missingness. Log-rank probability measure for the equality of these curves is 1.189705e-03. Additional annotations: *.GeneId*=ENSG00000171316, *.GeneName*=CHD7, *.GeneDesc*=chromodomain helicase DNA binding protein 7 [Source:HGNC Symbol;Acc:20626].

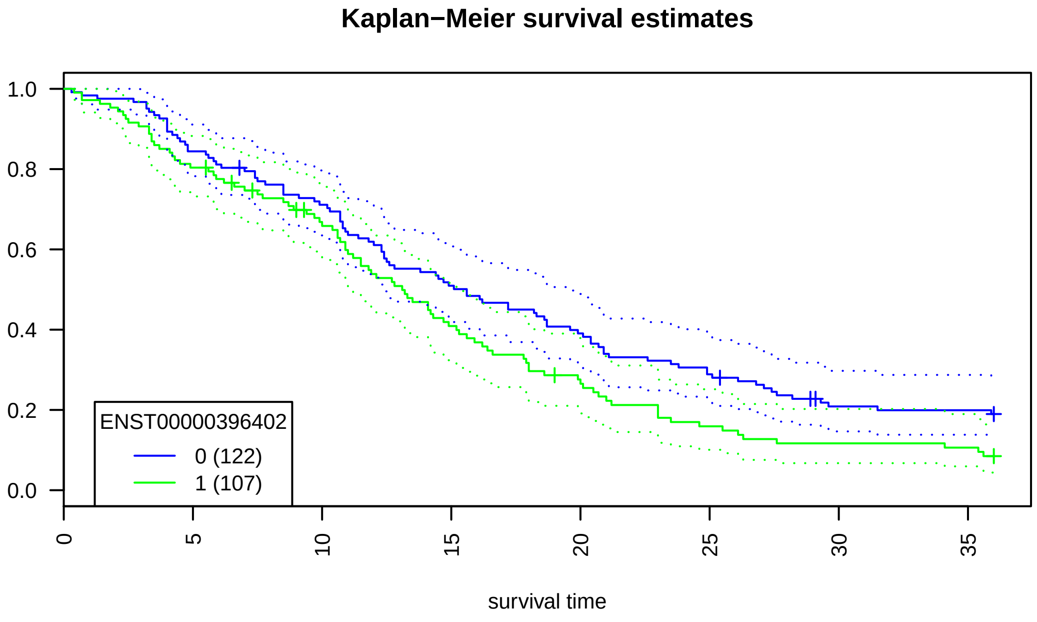

Figure 492: Each curve represents the relative number of ENST00000396402 survived to the corresponding point of time. Dashed lines represent 0.95 confidence intervals for the curves with the corresponding color. Total of 1 observation deleted due to missingness. Log-rank probability measure for the equality of these curves is 2.138595e-02. Additional annotations: *.GeneId*=ENSG00000137869, *.GeneName*=CYP19A1, *.GeneDesc*=cytochrome P450, family 19, subfamily A, polypeptide 1 [Source:HGNC Symbol;Acc:2594].

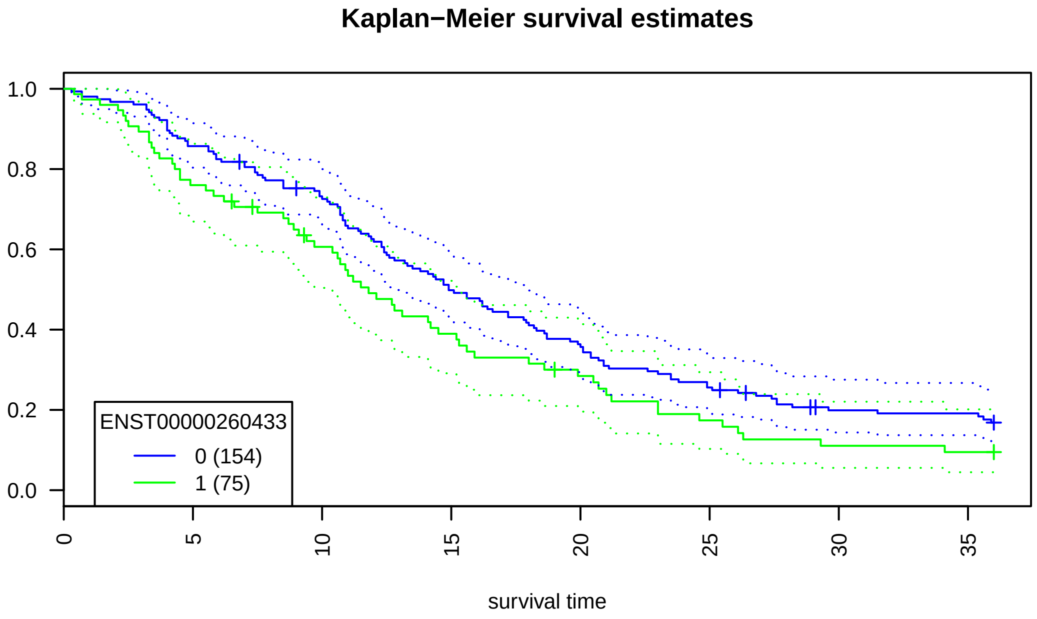

Figure 493: Each curve represents the relative number of ENST00000260433 survived to the corresponding point of time. Dashed lines represent 0.95 confidence intervals for the curves with the corresponding color. Total of 1 observation deleted due to missingness. Log-rank probability measure for the equality of these curves is 4.317483e-02. Additional annotations: *.GeneId*=ENSG00000137869, *.GeneName*=CYP19A1, *.GeneDesc*=cytochrome P450, family 19, subfamily A, polypeptide 1 [Source:HGNC Symbol;Acc:2594].

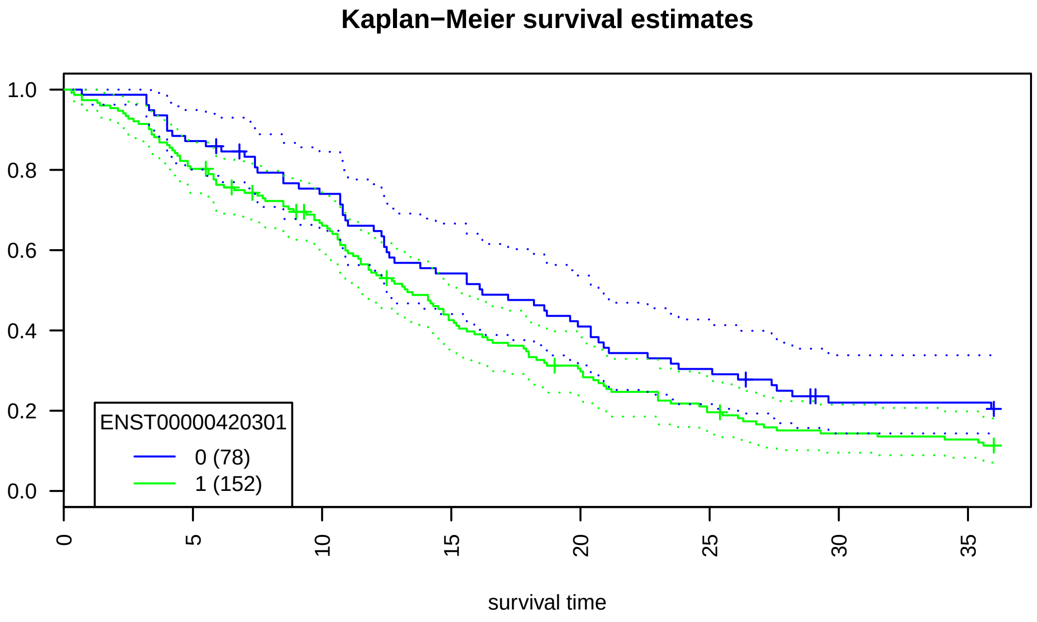

Figure 494: Each curve represents the relative number of ENST00000420301 survived to the corresponding point of time. Dashed lines represent 0.95 confidence intervals for the curves with the corresponding color. Log-rank probability measure for the equality of these curves is 4.519187e-02. Additional annotations: *.GeneId*=ENSG00000137869, *.GeneName*=CYP19A1, *.GeneDesc*=cytochrome P450, family 19, subfamily A, polypeptide 1 [Source:HGNC Symbol;Acc:2594].

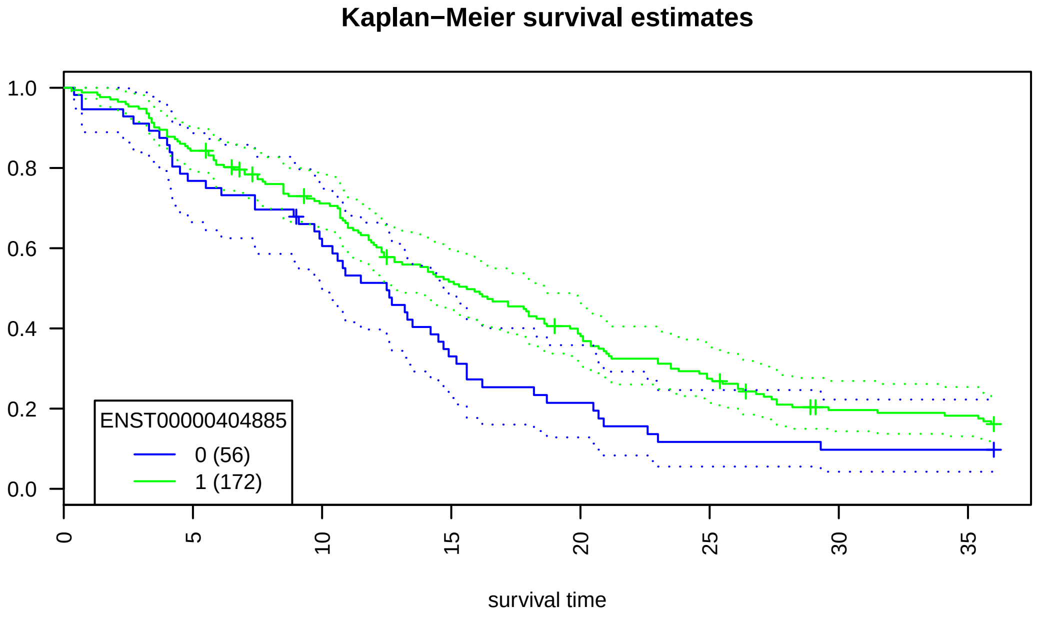

Figure 495: Each curve represents the relative number of ENST00000404885 survived to the corresponding point of time. Dashed lines represent 0.95 confidence intervals for the curves with the corresponding color. Total of 2 observations deleted due to missingness. Log-rank probability measure for the equality of these curves is 1.962447e-02. Additional annotations: *.GeneId*=ENSG00000167065, *.GeneName*=DUSP18, *.GeneDesc*=dual specificity phosphatase 18 [Source:HGNC Symbol;Acc:18484].

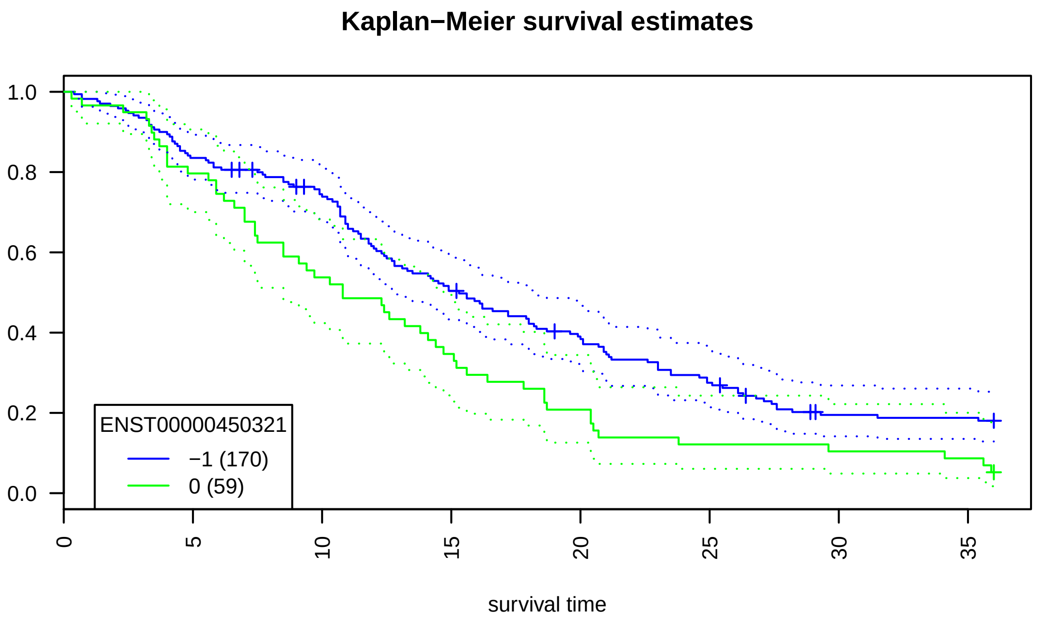

Figure 496: Each curve represents the relative number of ENST00000450321 survived to the corresponding point of time. Dashed lines represent 0.95 confidence intervals for the curves with the corresponding color. Total of 1 observation deleted due to missingness. Log-rank probability measure for the equality of these curves is 2.31515e-03. Additional annotations: *.GeneId*=ENSG00000164199, *.GeneName*=GPR98, *.GeneDesc*=G protein-coupled receptor 98 [Source:HGNC Symbol;Acc:17416].

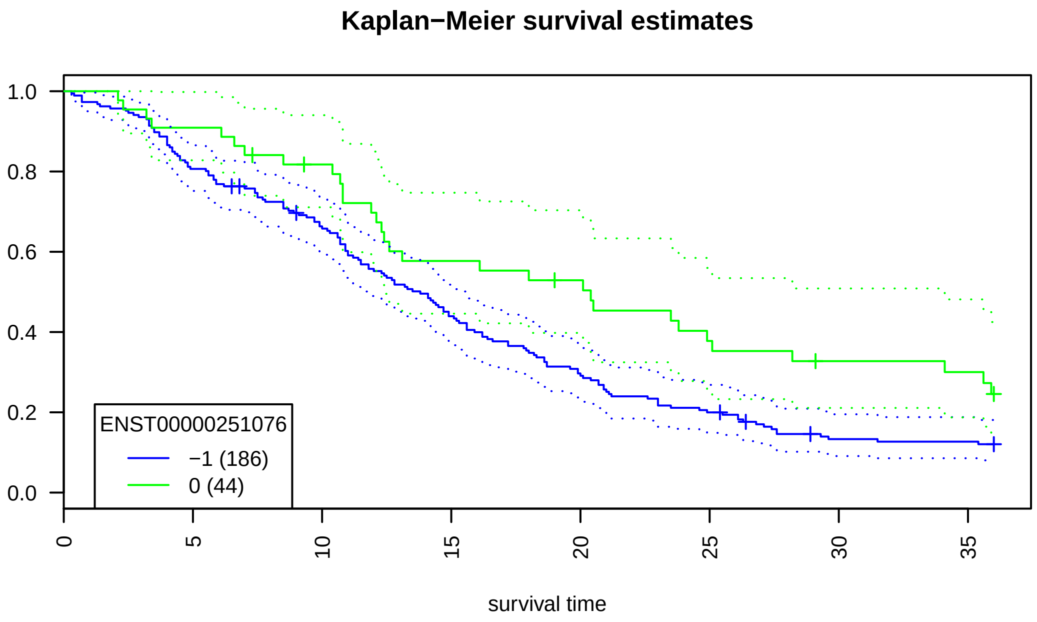

Figure 497: Each curve represents the relative number of ENST00000251076 survived to the corresponding point of time. Dashed lines represent 0.95 confidence intervals for the curves with the corresponding color. Log-rank probability measure for the equality of these curves is 1.465686e-02. Additional annotations: *.GeneId*=ENSG00000104093, *.GeneName*=DMXL2, *.GeneDesc*=Dmx-like 2 [Source:HGNC Symbol;Acc:2938].

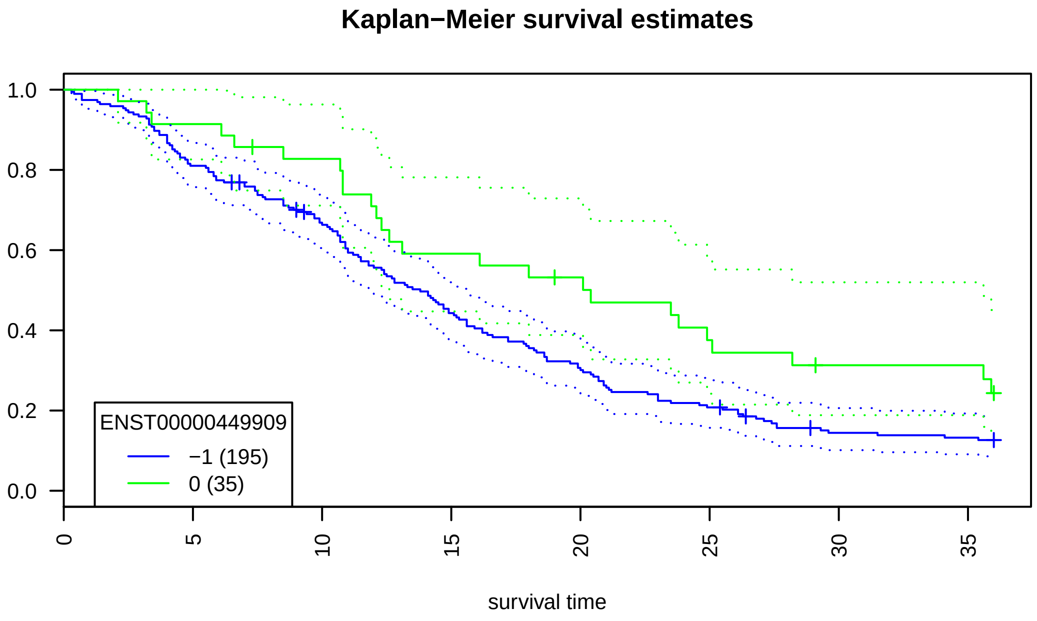

Figure 498: Each curve represents the relative number of ENST00000449909 survived to the corresponding point of time. Dashed lines represent 0.95 confidence intervals for the curves with the corresponding color. Log-rank probability measure for the equality of these curves is 2.926968e-02. Additional annotations: *.GeneId*=ENSG00000104093, *.GeneName*=DMXL2, *.GeneDesc*=Dmx-like 2 [Source:HGNC Symbol;Acc:2938].

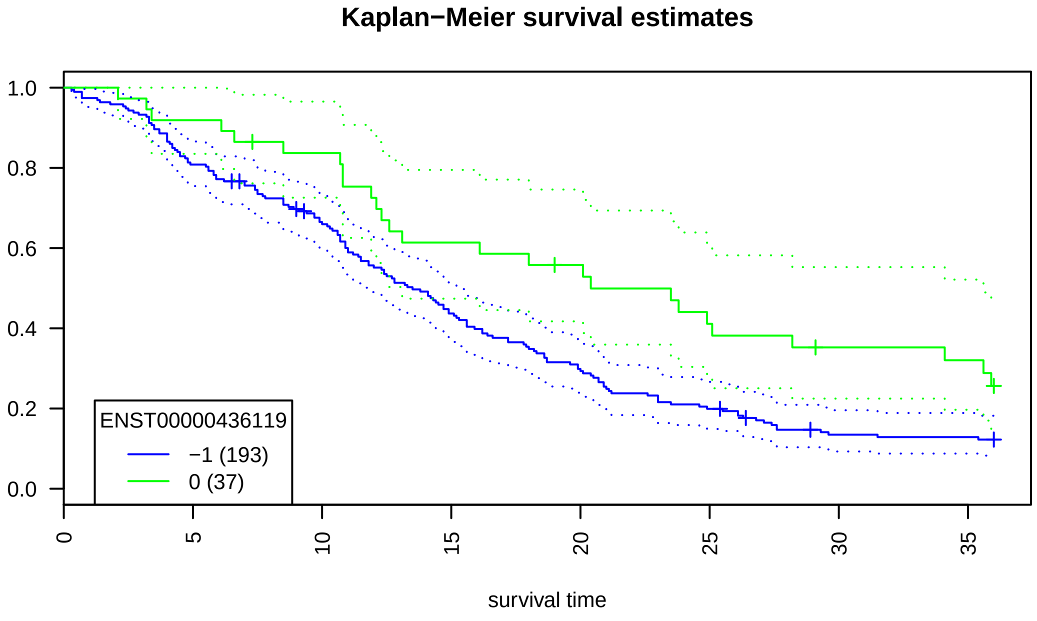

Figure 499: Each curve represents the relative number of ENST00000436119 survived to the corresponding point of time. Dashed lines represent 0.95 confidence intervals for the curves with the corresponding color. Log-rank probability measure for the equality of these curves is 9.397318e-03. Additional annotations: *.GeneId*=ENSG00000104093, *.GeneName*=DMXL2, *.GeneDesc*=Dmx-like 2 [Source:HGNC Symbol;Acc:2938].

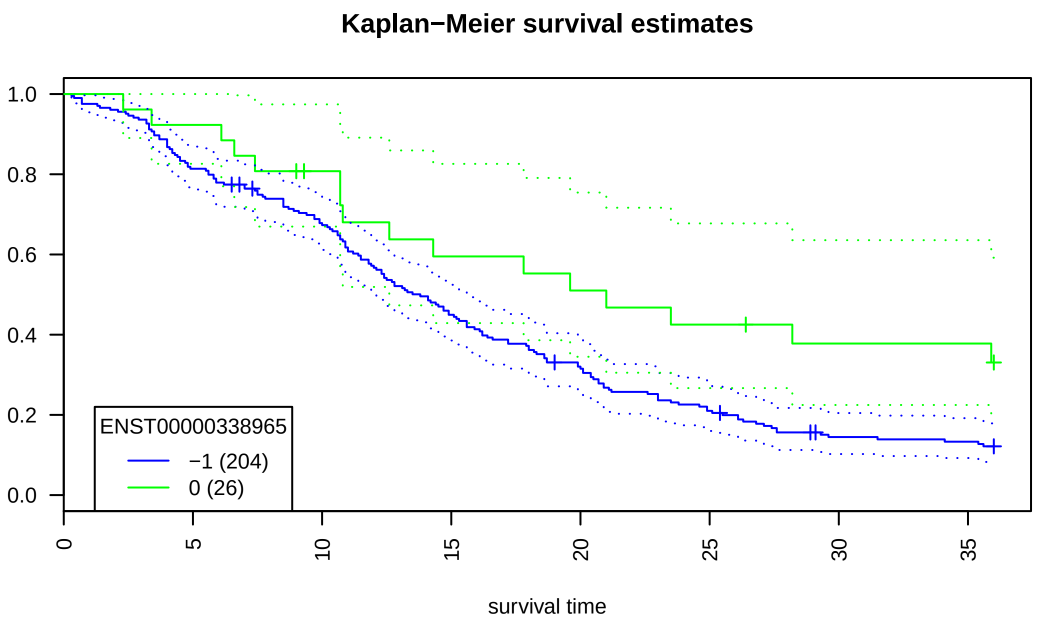

Figure 500: Each curve represents the relative number of ENST00000338965 survived to the corresponding point of time. Dashed lines represent 0.95 confidence intervals for the curves with the corresponding color. Log-rank probability measure for the equality of these curves is 1.859849e-02. Additional annotations: *.GeneId*=ENSG00000188211, *.GeneName*=AC124798.1, *.GeneDesc*=Putative Ig-like domain-containing protein DKFZp686O24166/DKFZp686I21167 Precursor [Source:UniProtKB/Swiss-Prot;Acc:Q68D85].

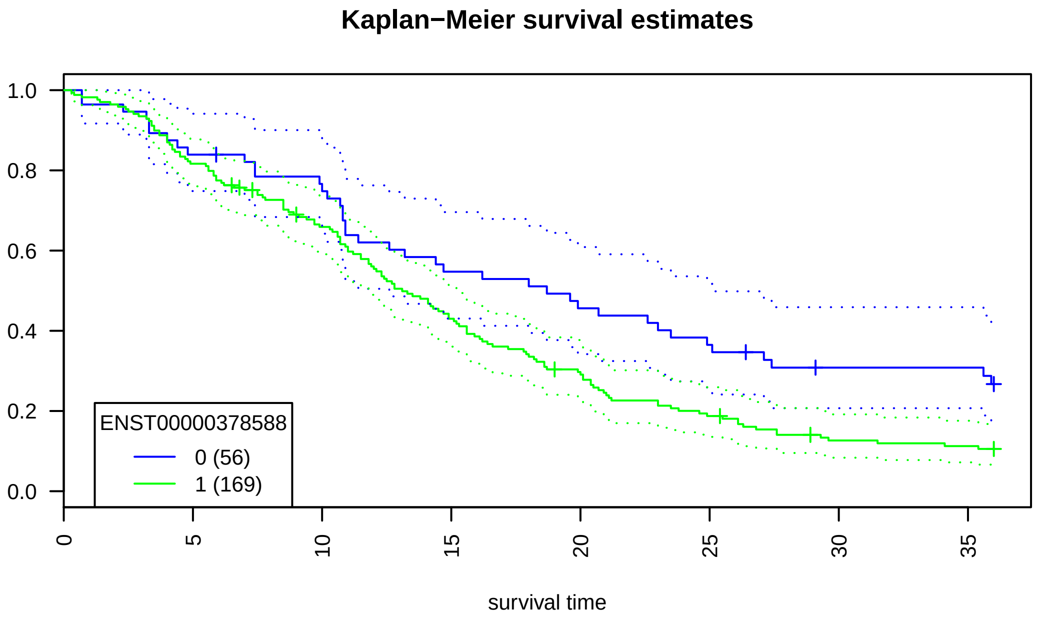

Figure 501: Each curve represents the relative number of ENST00000378588 survived to the corresponding point of time. Dashed lines represent 0.95 confidence intervals for the curves with the corresponding color. Total of 5 observations deleted due to missingness. Log-rank probability measure for the equality of these curves is 7.502318e-03. Additional annotations: *.GeneId*=ENSG00000165168, *.GeneName*=CYBB, *.GeneDesc*=cytochrome b-245, beta polypeptide [Source:HGNC Symbol;Acc:2578].

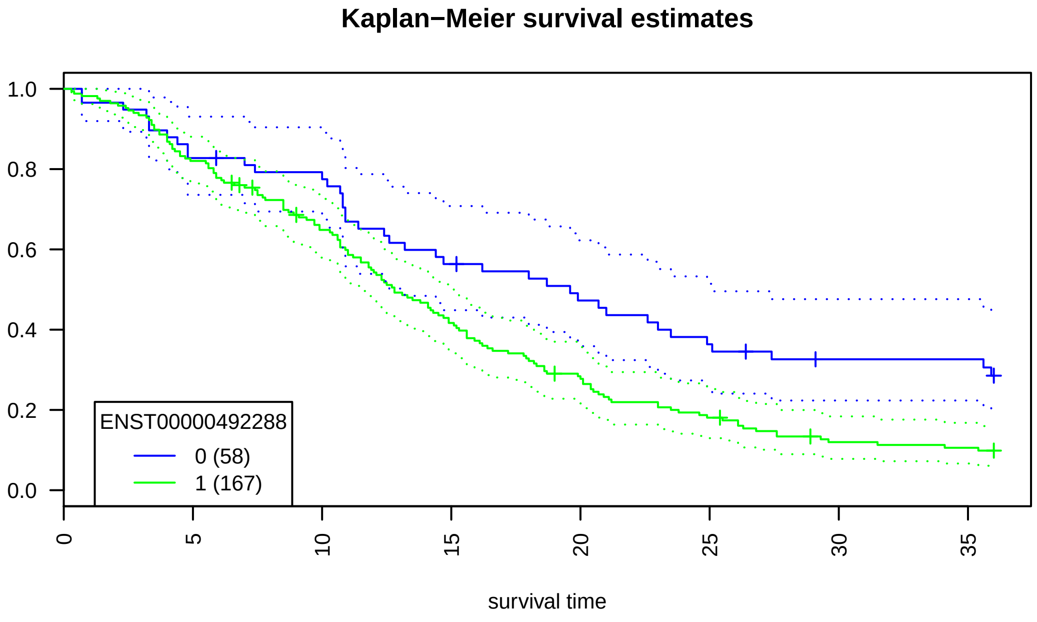

Figure 502: Each curve represents the relative number of ENST00000492288 survived to the corresponding point of time. Dashed lines represent 0.95 confidence intervals for the curves with the corresponding color. Total of 5 observations deleted due to missingness. Log-rank probability measure for the equality of these curves is 2.138172e-03. Additional annotations: *.GeneId*=ENSG00000165168, *.GeneName*=CYBB, *.GeneDesc*=cytochrome b-245, beta polypeptide [Source:HGNC Symbol;Acc:2578].

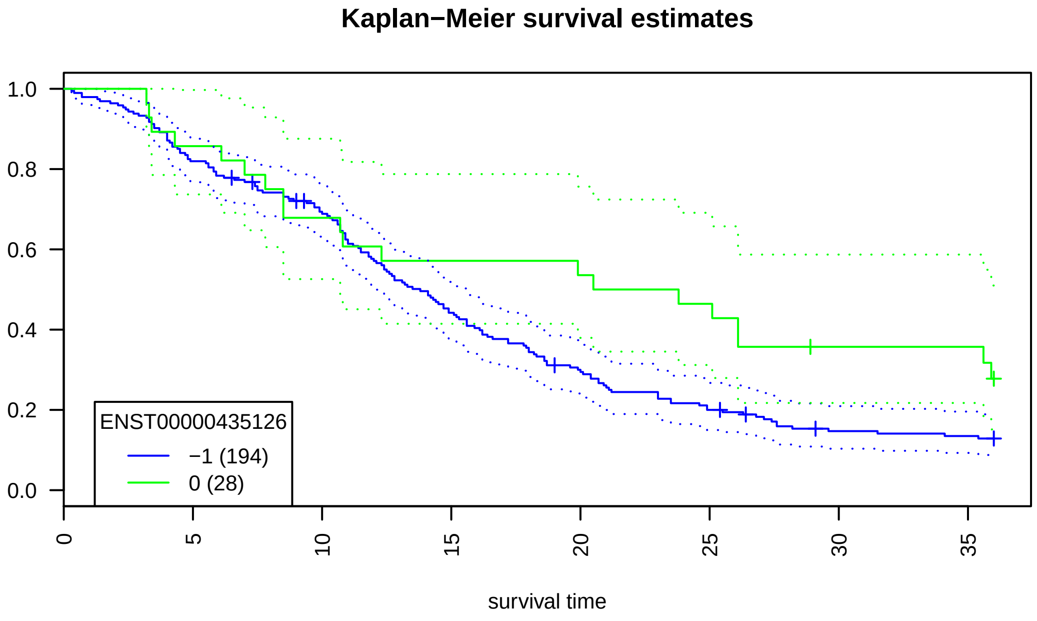

Figure 503: Each curve represents the relative number of ENST00000435126 survived to the corresponding point of time. Dashed lines represent 0.95 confidence intervals for the curves with the corresponding color. Total of 8 observations deleted due to missingness. Log-rank probability measure for the equality of these curves is 4.208581e-02. Additional annotations: *.GeneId*=ENSG00000128872, *.GeneName*=TMOD2, *.GeneDesc*=tropomodulin 2 (neuronal) [Source:HGNC Symbol;Acc:11872].

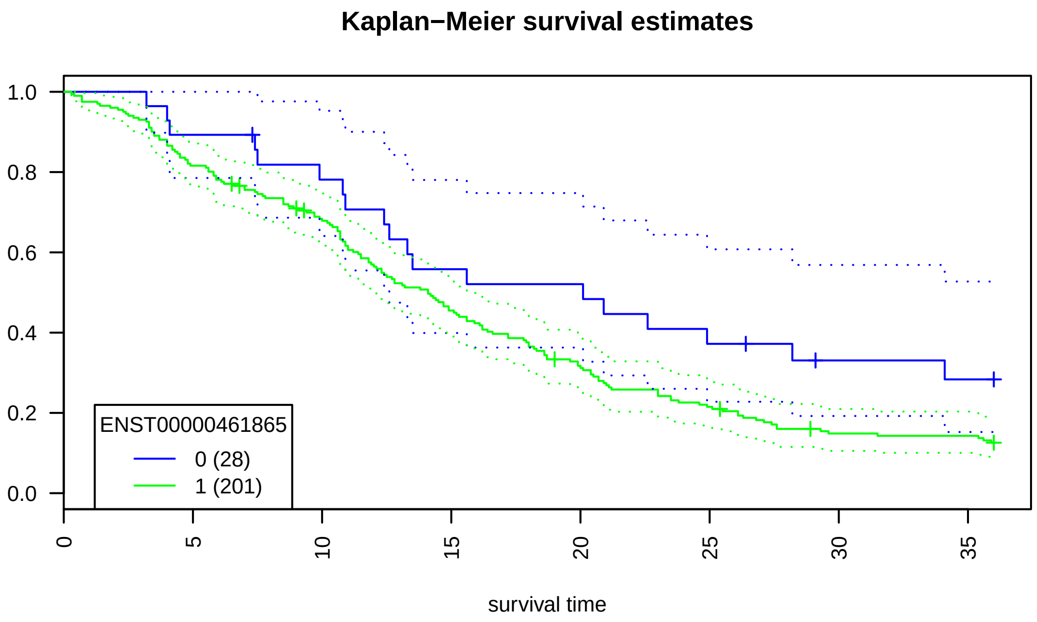

Figure 504: Each curve represents the relative number of ENST00000461865 survived to the corresponding point of time. Dashed lines represent 0.95 confidence intervals for the curves with the corresponding color. Total of 1 observation deleted due to missingness. Log-rank probability measure for the equality of these curves is 4.343112e-02. Additional annotations: *.GeneId*=ENSG00000101955, *.GeneName*=SRPX, *.GeneDesc*=sushi-repeat-containing protein, X-linked [Source:HGNC Symbol;Acc:11309].

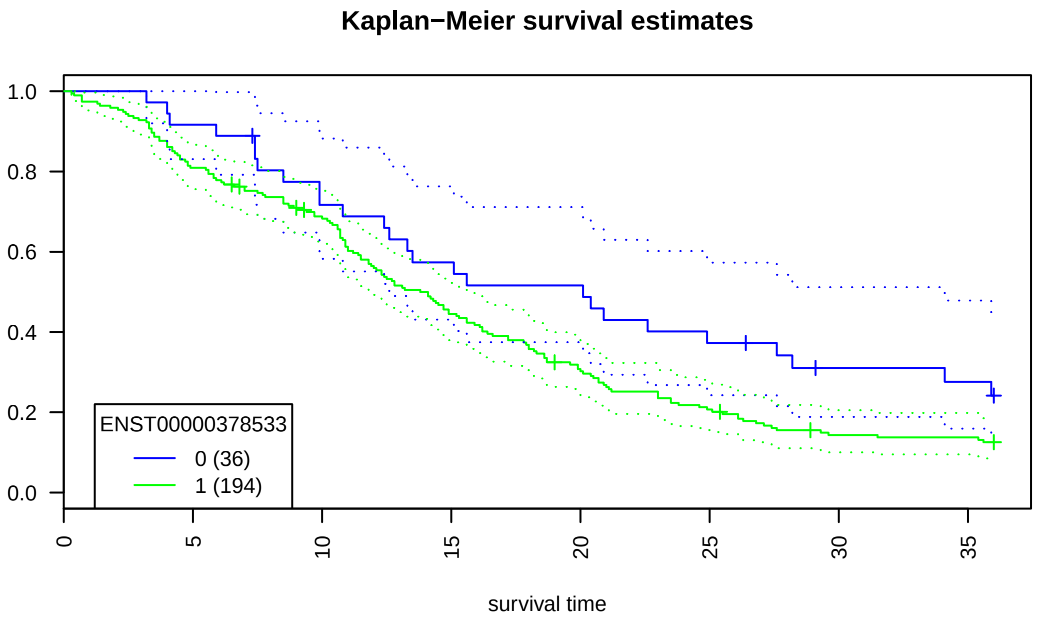

Figure 505: Each curve represents the relative number of ENST00000378533 survived to the corresponding point of time. Dashed lines represent 0.95 confidence intervals for the curves with the corresponding color. Log-rank probability measure for the equality of these curves is 3.996613e-02. Additional annotations: *.GeneId*=ENSG00000101955, *.GeneName*=SRPX, *.GeneDesc*=sushi-repeat-containing protein, X-linked [Source:HGNC Symbol;Acc:11309].

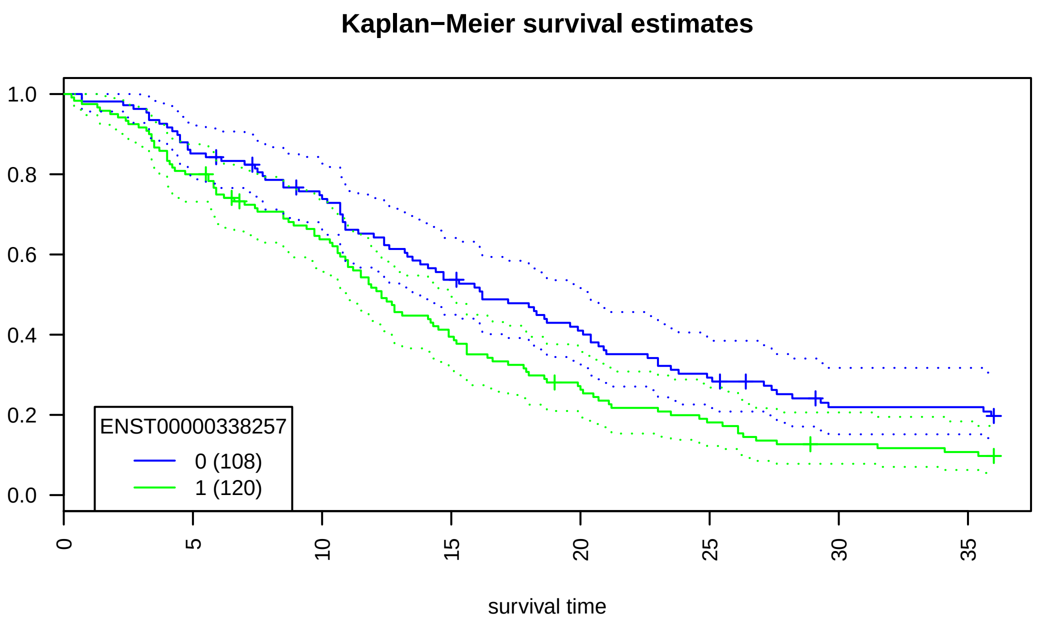

Figure 506: Each curve represents the relative number of ENST00000338257 survived to the corresponding point of time. Dashed lines represent 0.95 confidence intervals for the curves with the corresponding color. Total of 2 observations deleted due to missingness. Log-rank probability measure for the equality of these curves is 8.897653e-03. Additional annotations: *.GeneId*=ENSG00000142347, *.GeneName*=MYO1F, *.GeneDesc*=myosin IF [Source:HGNC Symbol;Acc:7600].

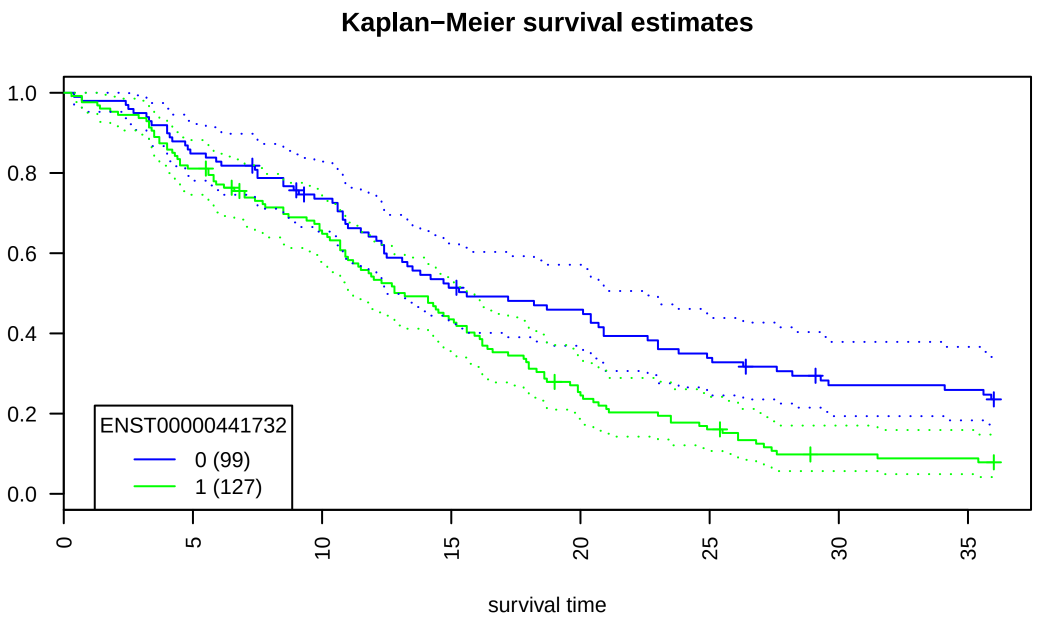

Figure 507: Each curve represents the relative number of ENST00000441732 survived to the corresponding point of time. Dashed lines represent 0.95 confidence intervals for the curves with the corresponding color. Total of 4 observations deleted due to missingness. Log-rank probability measure for the equality of these curves is 1.584841e-03. Additional annotations: *.GeneId*=ENSG00000233670, *.GeneName*=AC015908.1, *.GeneDesc*=Phosphoinositide-interacting protein [Source:UniProtKB/Swiss-Prot;Acc:P0C851].

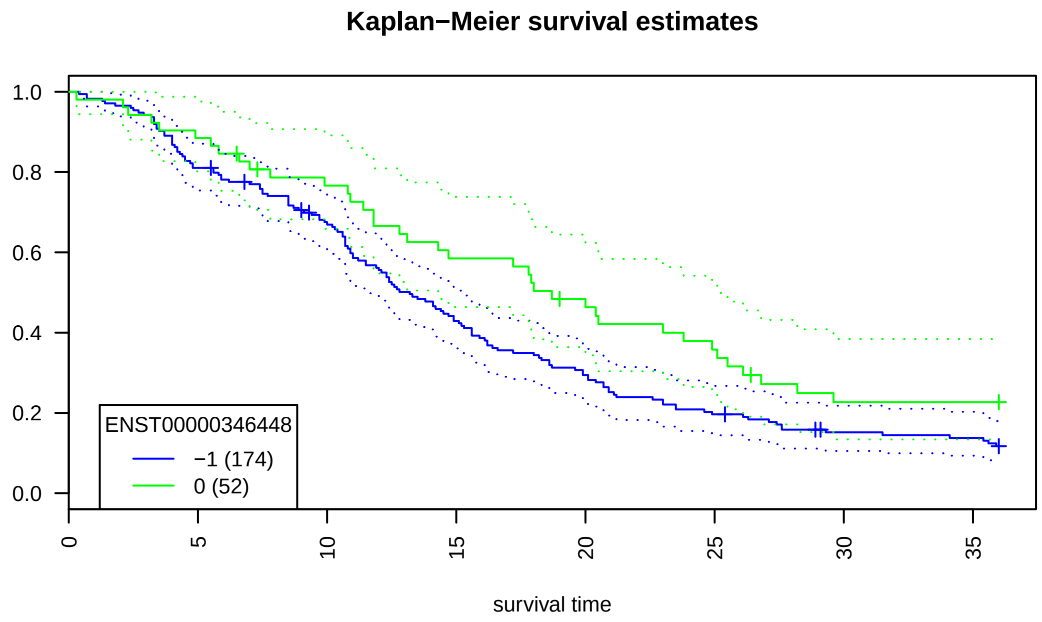

Figure 508: Each curve represents the relative number of ENST00000346448 survived to the corresponding point of time. Dashed lines represent 0.95 confidence intervals for the curves with the corresponding color. Total of 4 observations deleted due to missingness. Log-rank probability measure for the equality of these curves is 2.759728e-02. Additional annotations: *.GeneId*=ENSG00000120156, *.GeneName*=TEK, *.GeneDesc*=TEK tyrosine kinase, endothelial [Source:HGNC Symbol;Acc:11724].

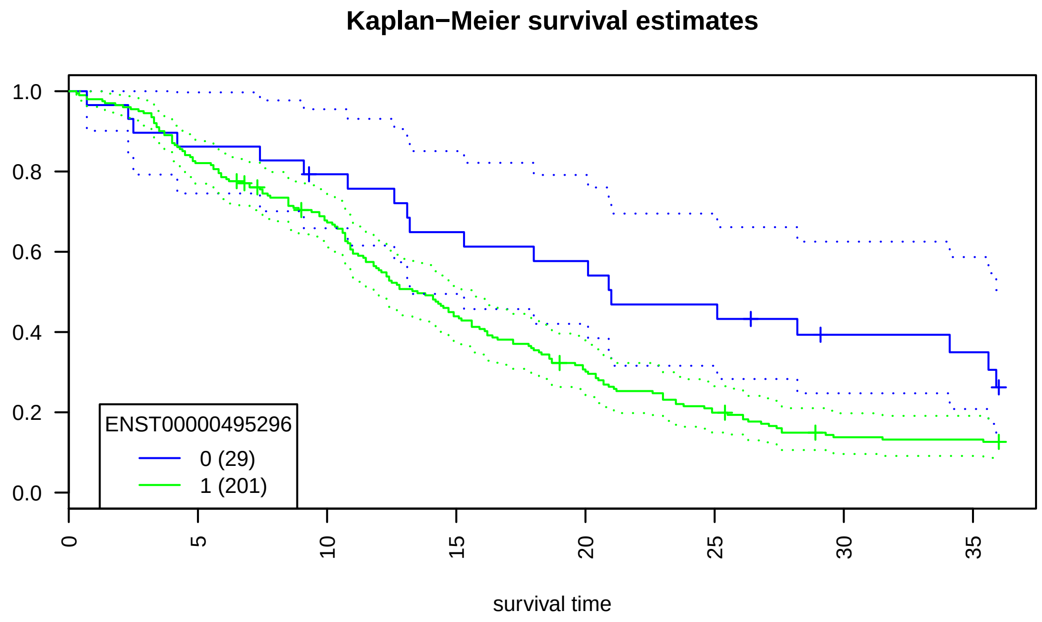

Figure 509: Each curve represents the relative number of ENST00000495296 survived to the corresponding point of time. Dashed lines represent 0.95 confidence intervals for the curves with the corresponding color. Log-rank probability measure for the equality of these curves is 1.622247e-02. Additional annotations: *.GeneId*=ENSG00000239565, *.GeneName*=AP000280.66, *.GeneDesc*=[undefined].

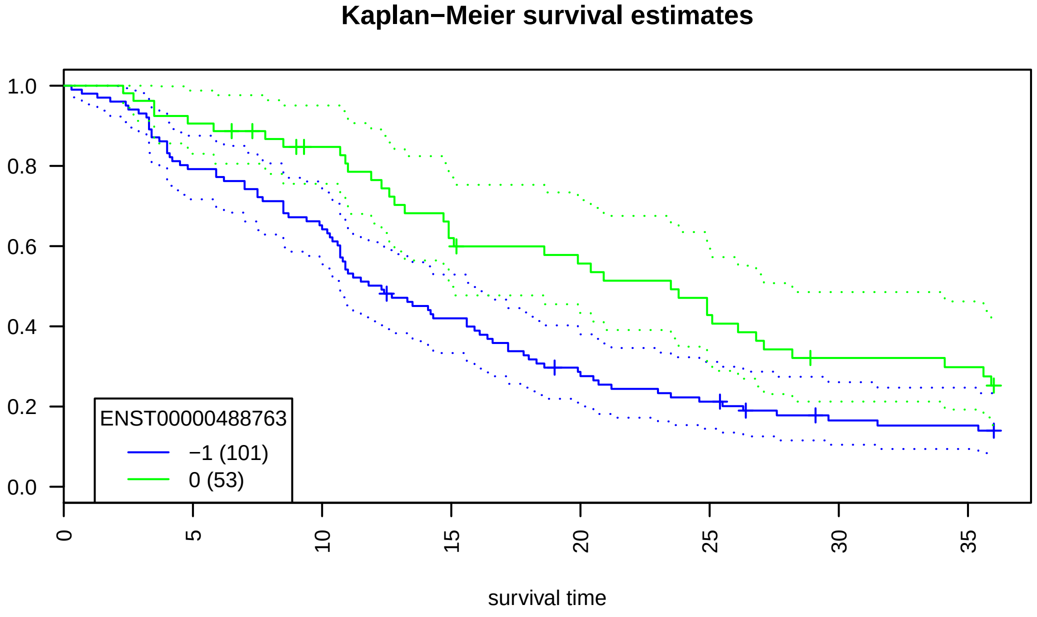

Figure 510: Each curve represents the relative number of ENST00000488763 survived to the corresponding point of time. Dashed lines represent 0.95 confidence intervals for the curves with the corresponding color. Total of 76 observations deleted due to missingness. Log-rank probability measure for the equality of these curves is 4.983249e-03. Additional annotations: *.GeneId*=ENSG00000180537, *.GeneName*=RNF182, *.GeneDesc*=ring finger protein 182 [Source:HGNC Symbol;Acc:28522].

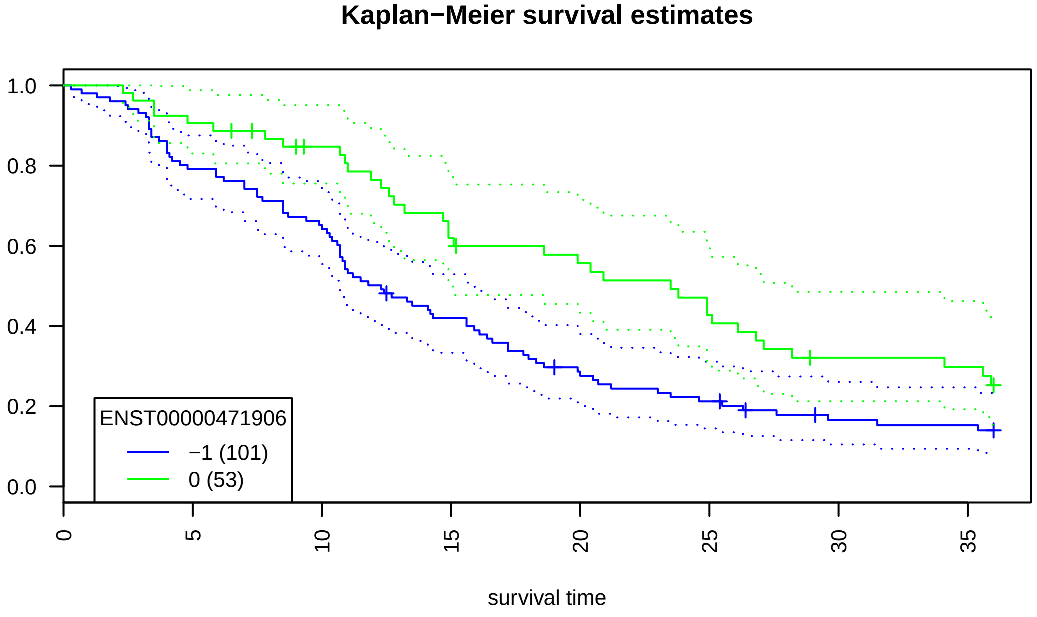

Figure 511: Each curve represents the relative number of ENST00000471906 survived to the corresponding point of time. Dashed lines represent 0.95 confidence intervals for the curves with the corresponding color. Total of 76 observations deleted due to missingness. Log-rank probability measure for the equality of these curves is 4.983249e-03. Additional annotations: *.GeneId*=ENSG00000180537, *.GeneName*=RNF182, *.GeneDesc*=ring finger protein 182 [Source:HGNC Symbol;Acc:28522].

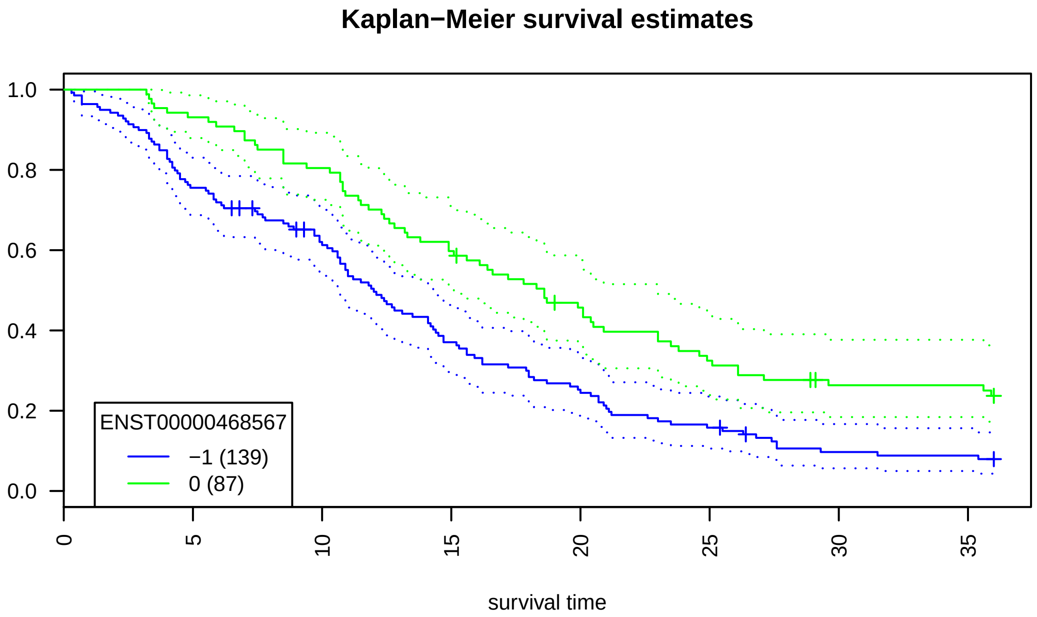

Figure 512: Each curve represents the relative number of ENST00000468567 survived to the corresponding point of time. Dashed lines represent 0.95 confidence intervals for the curves with the corresponding color. Total of 4 observations deleted due to missingness. Log-rank probability measure for the equality of these curves is 9.070578e-05. Additional annotations: *.GeneId*=ENSG00000219545, *.GeneName*=AC006465.3, *.GeneDesc*=cDNA FLJ54628HCG19535Hypothetical gene supported by AK027125 ;; [Source:UniProtKB/TrEMBL;Acc:A4D104].

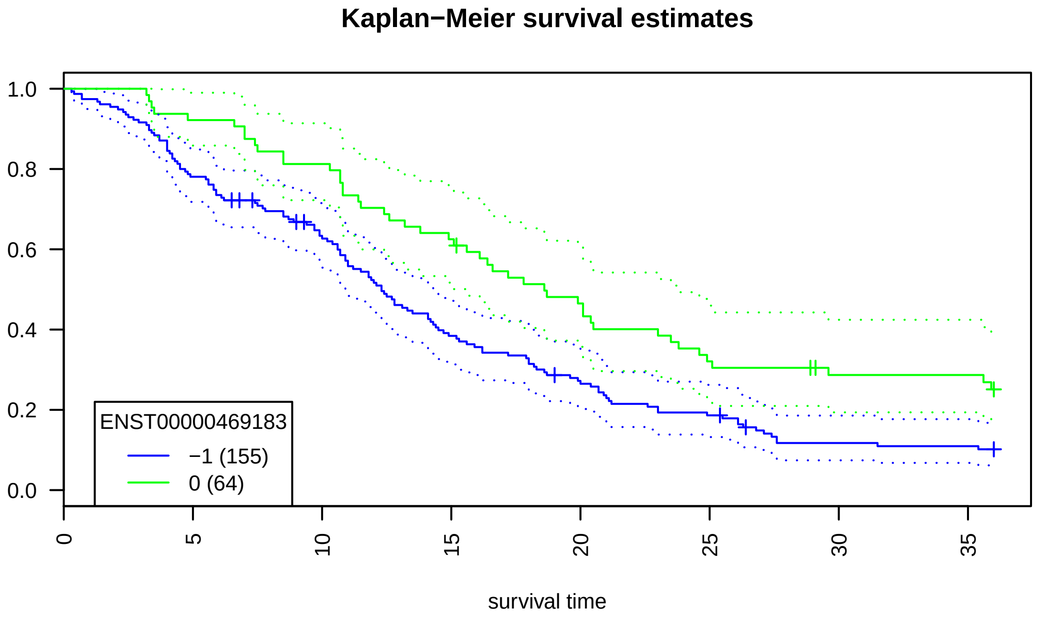

Figure 513: Each curve represents the relative number of ENST00000469183 survived to the corresponding point of time. Dashed lines represent 0.95 confidence intervals for the curves with the corresponding color. Total of 11 observations deleted due to missingness. Log-rank probability measure for the equality of these curves is 1.172827e-03. Additional annotations: *.GeneId*=ENSG00000219545, *.GeneName*=AC006465.3, *.GeneDesc*=cDNA FLJ54628HCG19535Hypothetical gene supported by AK027125 ;; [Source:UniProtKB/TrEMBL;Acc:A4D104].

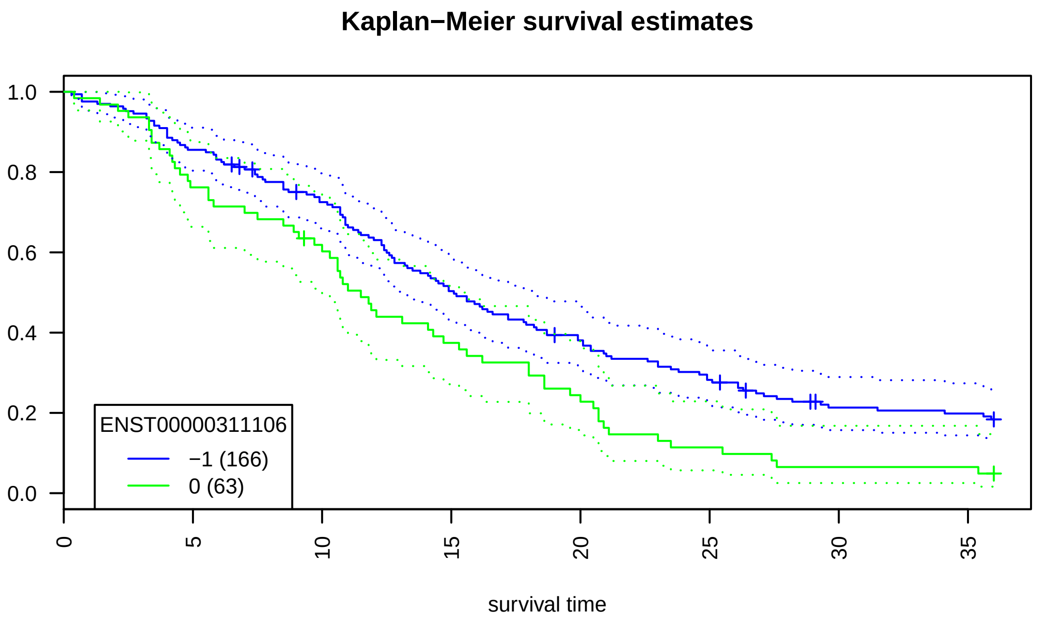

Figure 514: Each curve represents the relative number of ENST00000311106 survived to the corresponding point of time. Dashed lines represent 0.95 confidence intervals for the curves with the corresponding color. Total of 1 observation deleted due to missingness. Log-rank probability measure for the equality of these curves is 1.907356e-03. Additional annotations: *.GeneId*=ENSG00000175426, *.GeneName*=PCSK1, *.GeneDesc*=proprotein convertase subtilisin/kexin type 1 [Source:HGNC Symbol;Acc:8743].

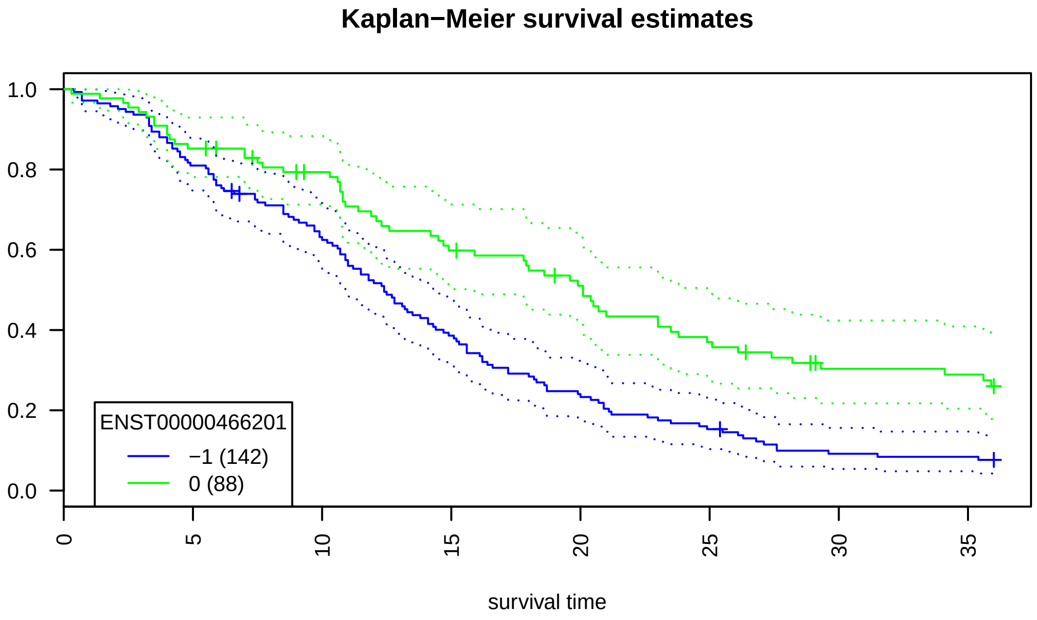

Figure 515: Each curve represents the relative number of ENST00000466201 survived to the corresponding point of time. Dashed lines represent 0.95 confidence intervals for the curves with the corresponding color. Log-rank probability measure for the equality of these curves is 3.261555e-05. Additional annotations: *.GeneId*=ENSG00000152465, *.GeneName*=NMT2, *.GeneDesc*=N-myristoyltransferase 2 [Source:HGNC Symbol;Acc:7858].

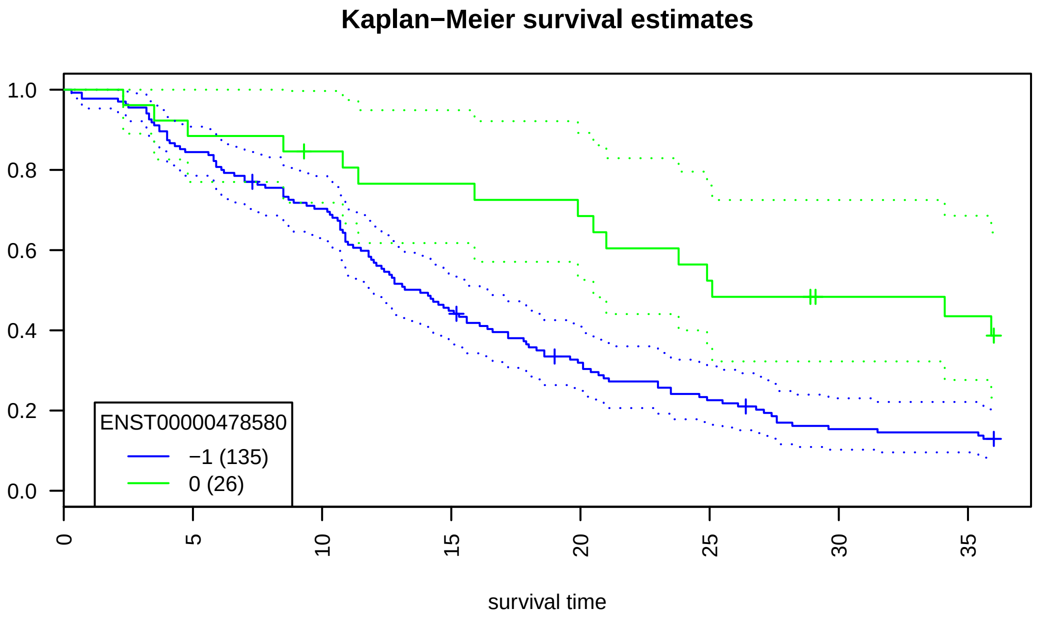

Figure 516: Each curve represents the relative number of ENST00000478580 survived to the corresponding point of time. Dashed lines represent 0.95 confidence intervals for the curves with the corresponding color. Total of 69 observations deleted due to missingness. Log-rank probability measure for the equality of these curves is 1.755666e-03. Additional annotations: *.GeneId*=ENSG00000152465, *.GeneName*=NMT2, *.GeneDesc*=N-myristoyltransferase 2 [Source:HGNC Symbol;Acc:7858].

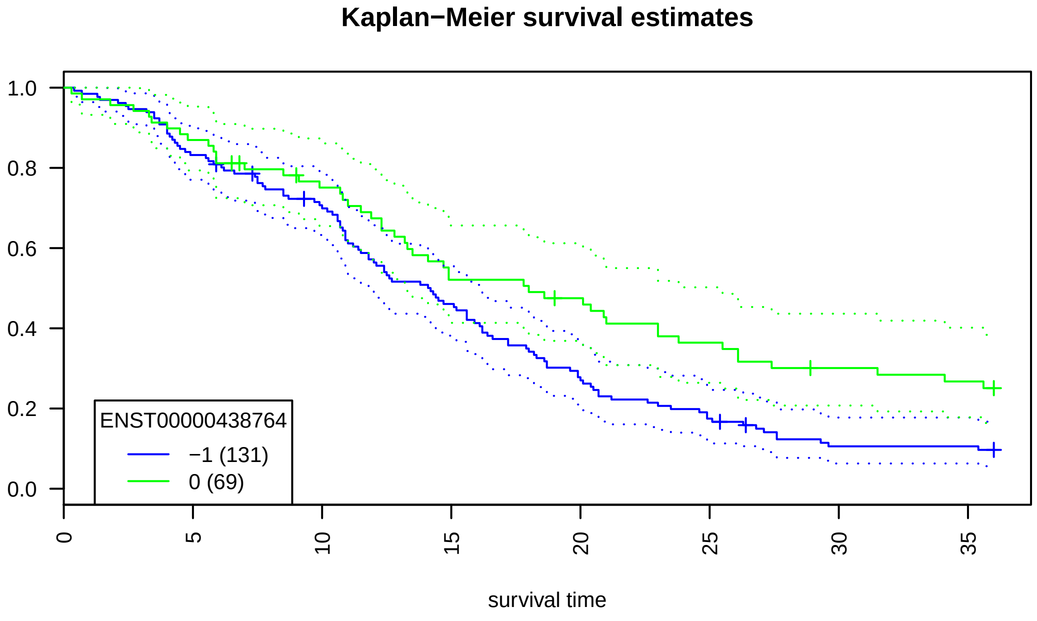

Figure 517: Each curve represents the relative number of ENST00000438764 survived to the corresponding point of time. Dashed lines represent 0.95 confidence intervals for the curves with the corresponding color. Total of 30 observations deleted due to missingness. Log-rank probability measure for the equality of these curves is 6.981234e-03. Additional annotations: *.GeneId*=ENSG00000122584, *.GeneName*=NXPH1, *.GeneDesc*=neurexophilin 1 [Source:HGNC Symbol;Acc:20693].

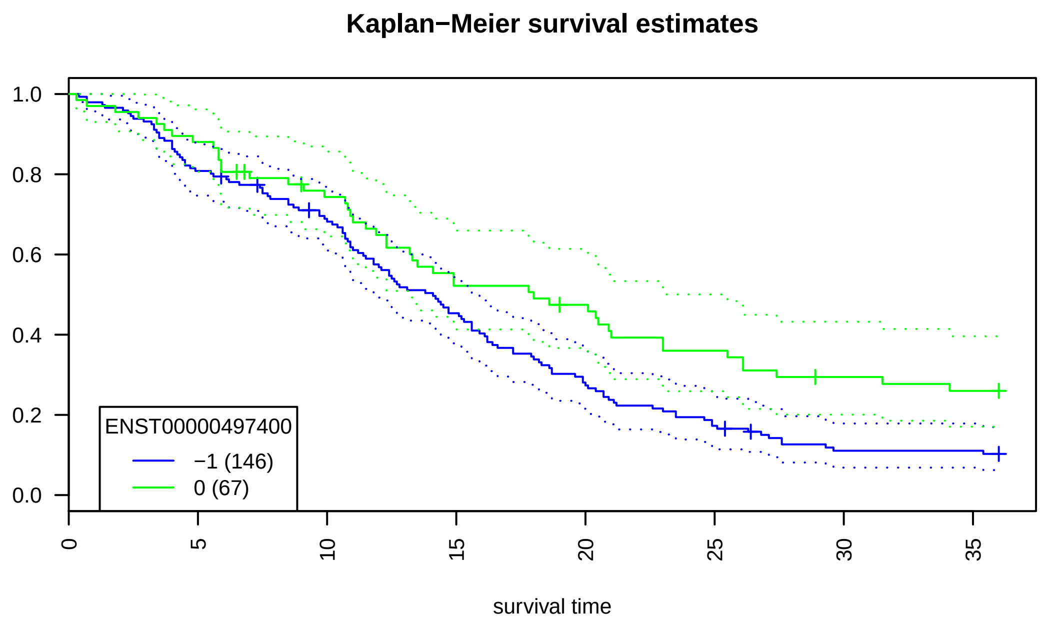

Figure 518: Each curve represents the relative number of ENST00000497400 survived to the corresponding point of time. Dashed lines represent 0.95 confidence intervals for the curves with the corresponding color. Total of 17 observations deleted due to missingness. Log-rank probability measure for the equality of these curves is 8.437234e-03. Additional annotations: *.GeneId*=ENSG00000122584, *.GeneName*=NXPH1, *.GeneDesc*=neurexophilin 1 [Source:HGNC Symbol;Acc:20693].

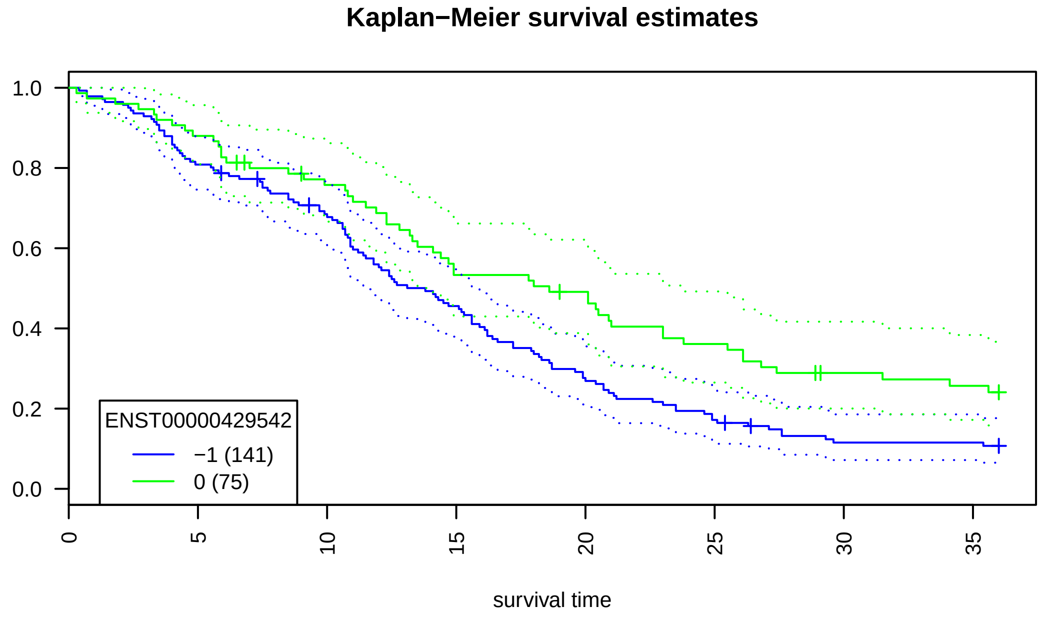

Figure 519: Each curve represents the relative number of ENST00000429542 survived to the corresponding point of time. Dashed lines represent 0.95 confidence intervals for the curves with the corresponding color. Total of 14 observations deleted due to missingness. Log-rank probability measure for the equality of these curves is 5.660342e-03. Additional annotations: *.GeneId*=ENSG00000122584, *.GeneName*=NXPH1, *.GeneDesc*=neurexophilin 1 [Source:HGNC Symbol;Acc:20693].

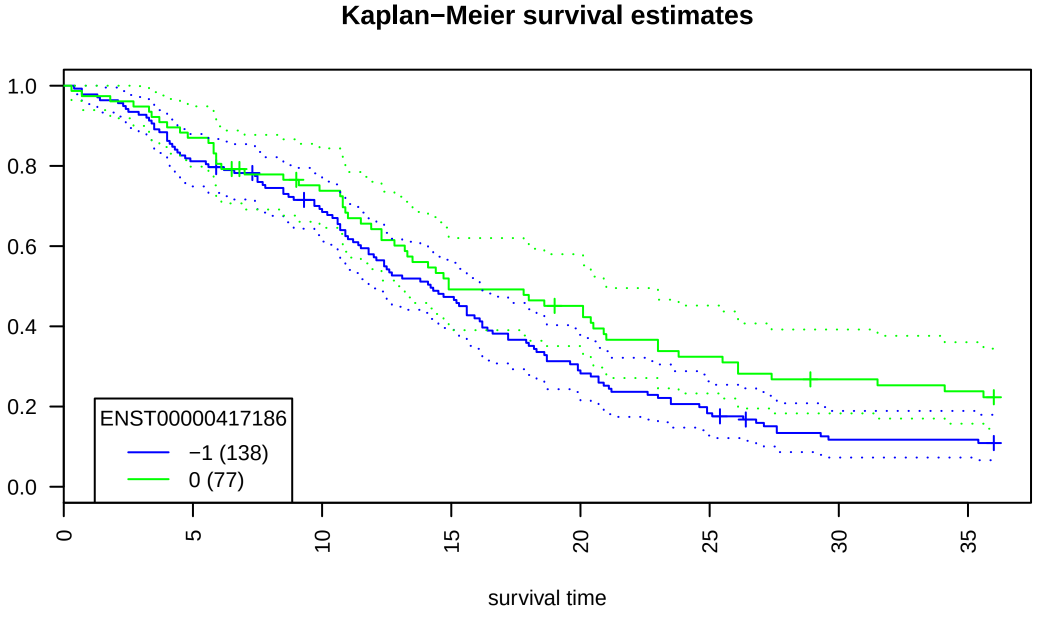

Figure 520: Each curve represents the relative number of ENST00000417186 survived to the corresponding point of time. Dashed lines represent 0.95 confidence intervals for the curves with the corresponding color. Total of 15 observations deleted due to missingness. Log-rank probability measure for the equality of these curves is 3.646988e-02. Additional annotations: *.GeneId*=ENSG00000122584, *.GeneName*=NXPH1, *.GeneDesc*=neurexophilin 1 [Source:HGNC Symbol;Acc:20693].

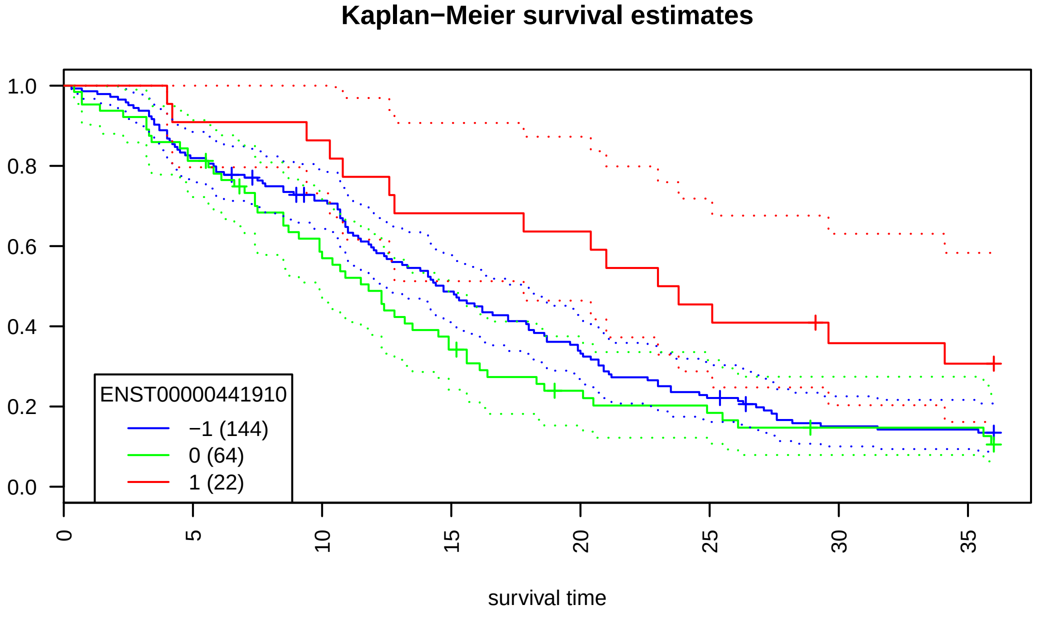

Figure 521: Each curve represents the relative number of ENST00000441910 survived to the corresponding point of time. Dashed lines represent 0.95 confidence intervals for the curves with the corresponding color. Log-rank probability measure for the equality of these curves is 1.577348e-02. Additional annotations: *.GeneId*=ENSG00000233756, *.GeneName*=AF165176.2, *.GeneDesc*=[undefined].

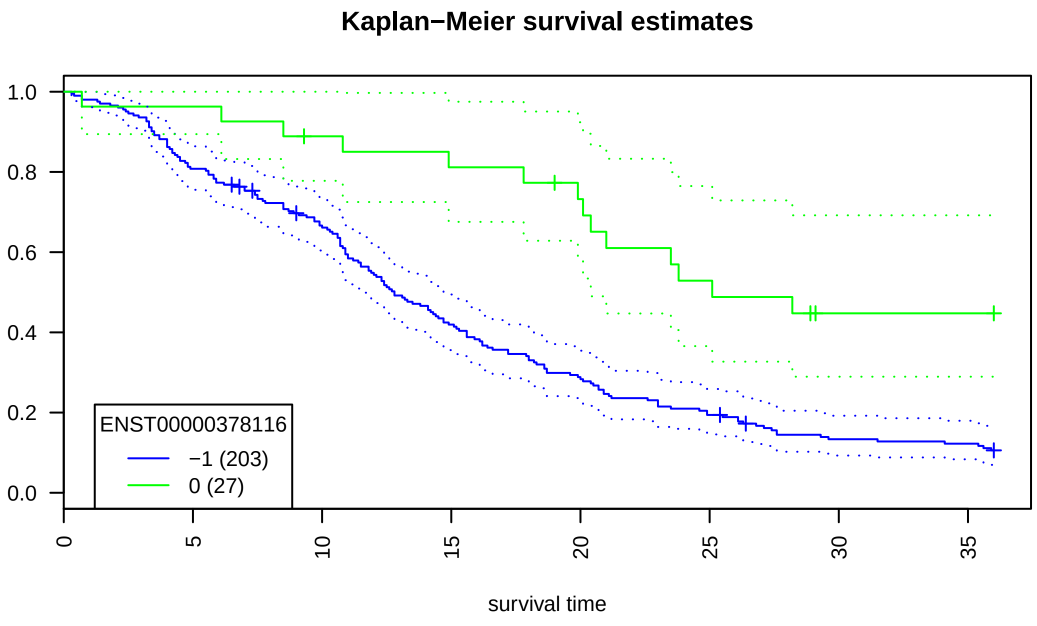

Figure 522: Each curve represents the relative number of ENST00000378116 survived to the corresponding point of time. Dashed lines represent 0.95 confidence intervals for the curves with the corresponding color. Log-rank probability measure for the equality of these curves is 6.194739e-05. Additional annotations: *.GeneId*=ENSG00000148468, *.GeneName*=FAM171A1, *.GeneDesc*=family with sequence similarity 171, member A1 [Source:HGNC Symbol;Acc:23522].

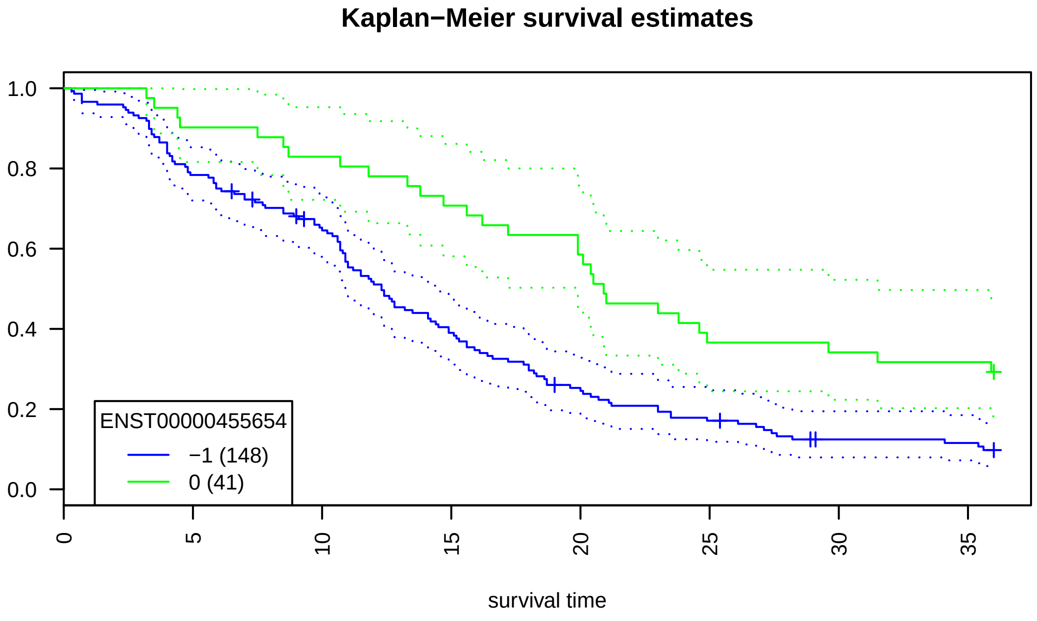

Figure 523: Each curve represents the relative number of ENST00000455654 survived to the corresponding point of time. Dashed lines represent 0.95 confidence intervals for the curves with the corresponding color. Total of 41 observations deleted due to missingness. Log-rank probability measure for the equality of these curves is 2.668121e-04. Additional annotations: *.GeneId*=ENSG00000148468, *.GeneName*=FAM171A1, *.GeneDesc*=family with sequence similarity 171, member A1 [Source:HGNC Symbol;Acc:23522].

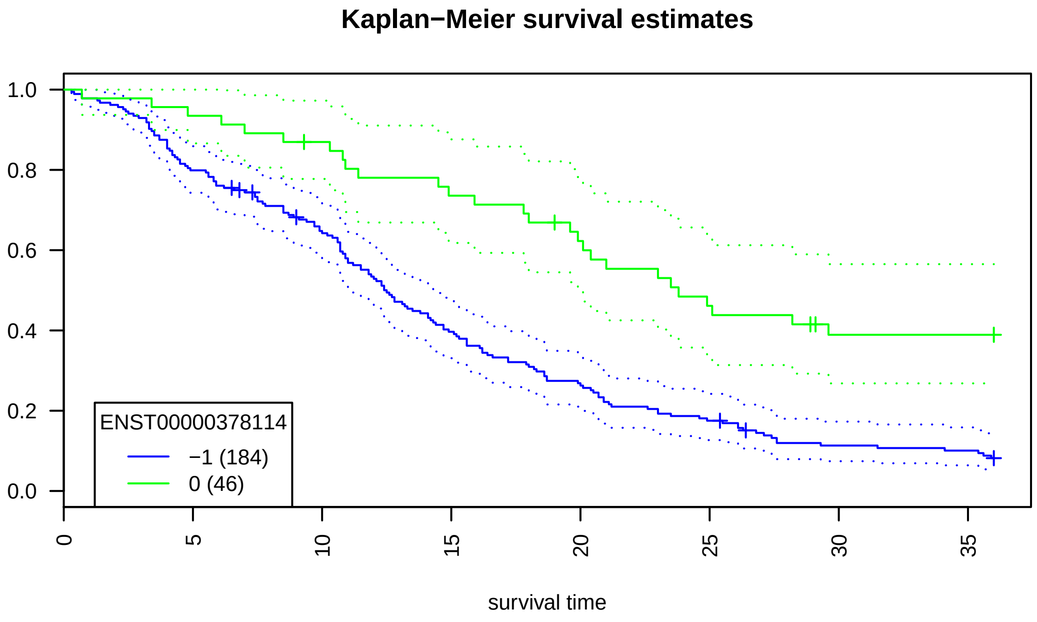

Figure 524: Each curve represents the relative number of ENST00000378114 survived to the corresponding point of time. Dashed lines represent 0.95 confidence intervals for the curves with the corresponding color. Log-rank probability measure for the equality of these curves is 1.658953e-06. Additional annotations: *.GeneId*=ENSG00000148468, *.GeneName*=FAM171A1, *.GeneDesc*=family with sequence similarity 171, member A1 [Source:HGNC Symbol;Acc:23522].

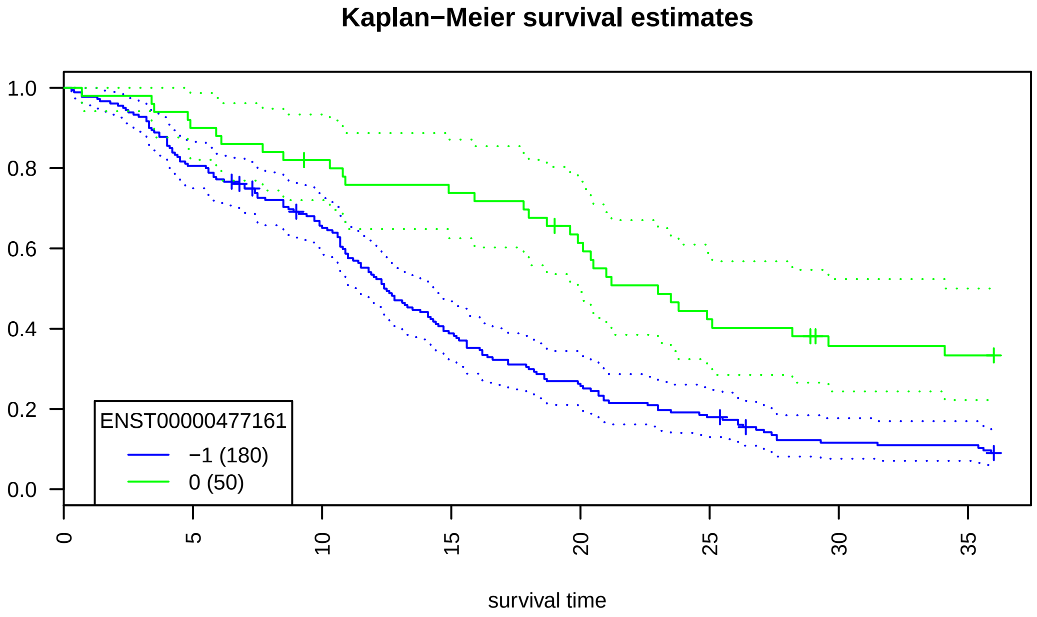

Figure 525: Each curve represents the relative number of ENST00000477161 survived to the corresponding point of time. Dashed lines represent 0.95 confidence intervals for the curves with the corresponding color. Log-rank probability measure for the equality of these curves is 1.333930e-05. Additional annotations: *.GeneId*=ENSG00000148468, *.GeneName*=FAM171A1, *.GeneDesc*=family with sequence similarity 171, member A1 [Source:HGNC Symbol;Acc:23522].

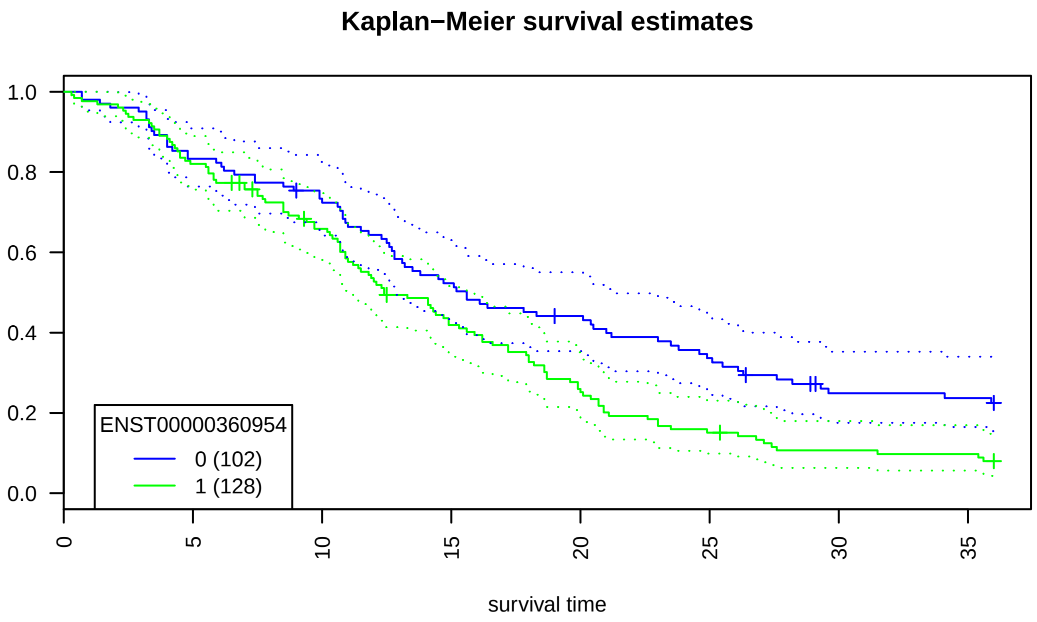

Figure 526: Each curve represents the relative number of ENST00000360954 survived to the corresponding point of time. Dashed lines represent 0.95 confidence intervals for the curves with the corresponding color. Log-rank probability measure for the equality of these curves is 2.993379e-03. Additional annotations: *.GeneId*=ENSG00000125430, *.GeneName*=HS3ST3B1, *.GeneDesc*=heparan sulfate (glucosamine) 3-O-sulfotransferase 3B1 [Source:HGNC Symbol;Acc:5198].

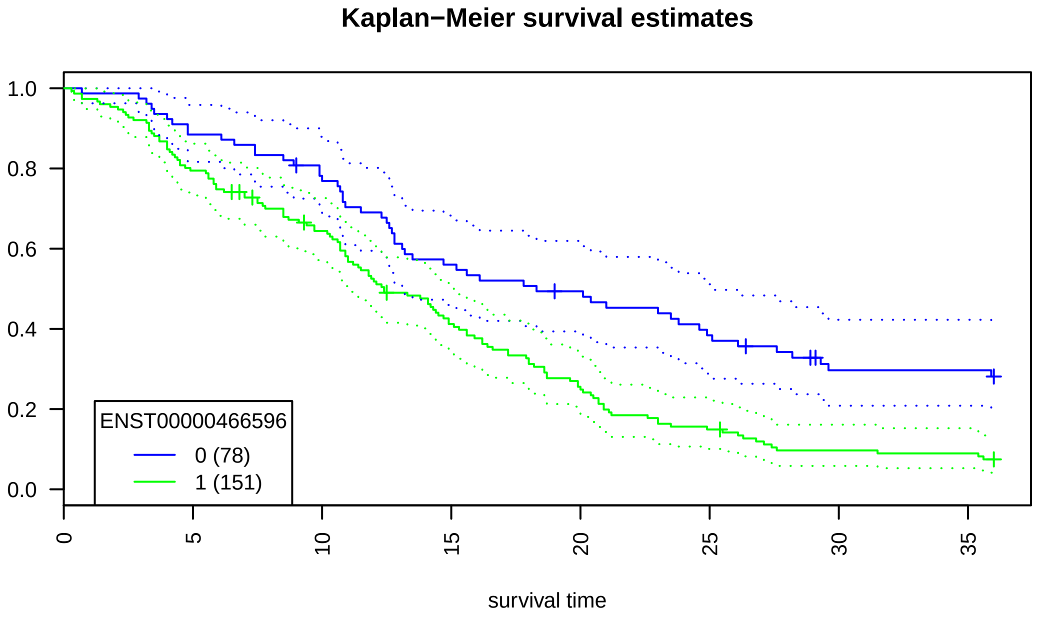

Figure 527: Each curve represents the relative number of ENST00000466596 survived to the corresponding point of time. Dashed lines represent 0.95 confidence intervals for the curves with the corresponding color. Total of 1 observation deleted due to missingness. Log-rank probability measure for the equality of these curves is 5.039389e-05. Additional annotations: *.GeneId*=ENSG00000125430, *.GeneName*=HS3ST3B1, *.GeneDesc*=heparan sulfate (glucosamine) 3-O-sulfotransferase 3B1 [Source:HGNC Symbol;Acc:5198].

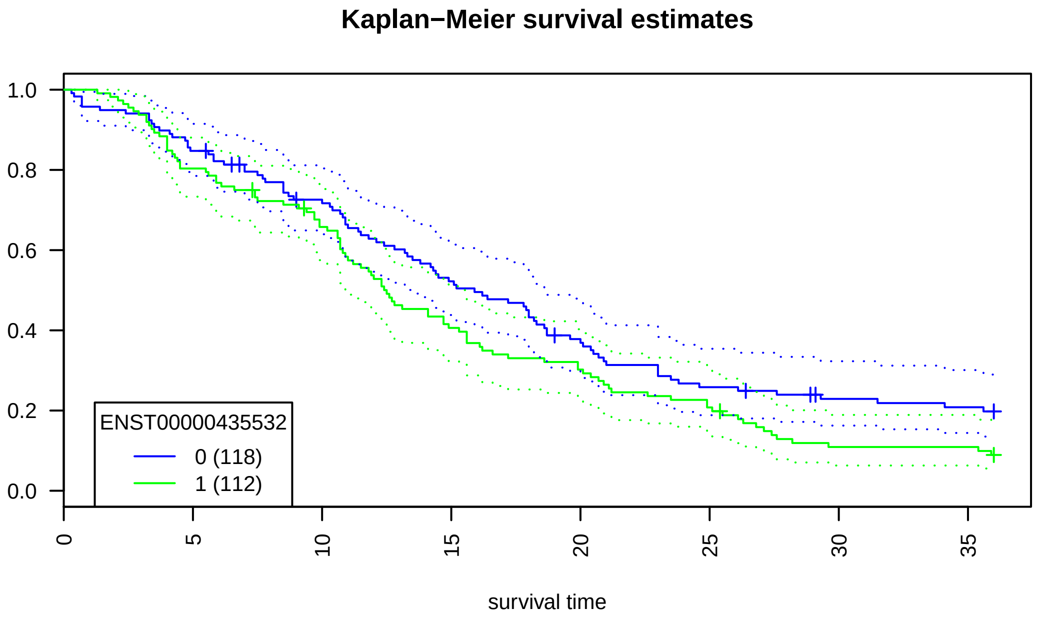

Figure 528: Each curve represents the relative number of ENST00000435532 survived to the corresponding point of time. Dashed lines represent 0.95 confidence intervals for the curves with the corresponding color. Log-rank probability measure for the equality of these curves is 3.836156e-02. Additional annotations: *.GeneId*=ENSG00000069869, *.GeneName*=NEDD4, *.GeneDesc*=neural precursor cell expressed, developmentally down-regulated 4 [Source:HGNC Symbol;Acc:7727].

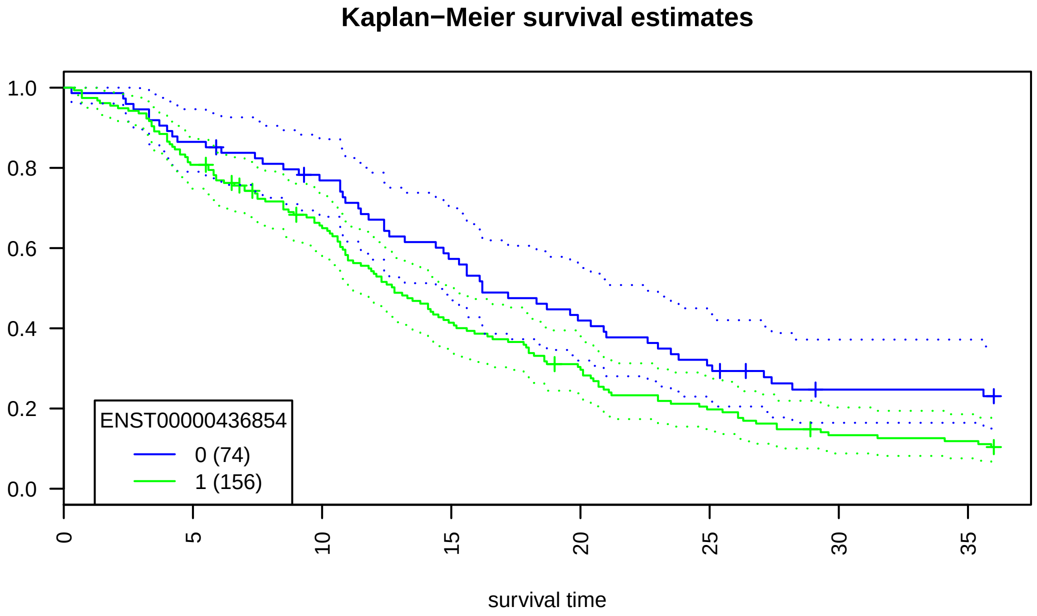

Figure 529: Each curve represents the relative number of ENST00000436854 survived to the corresponding point of time. Dashed lines represent 0.95 confidence intervals for the curves with the corresponding color. Log-rank probability measure for the equality of these curves is 1.244767e-02. Additional annotations: *.GeneId*=ENSG00000006747, *.GeneName*=SCIN, *.GeneDesc*=scinderin [Source:HGNC Symbol;Acc:21695].

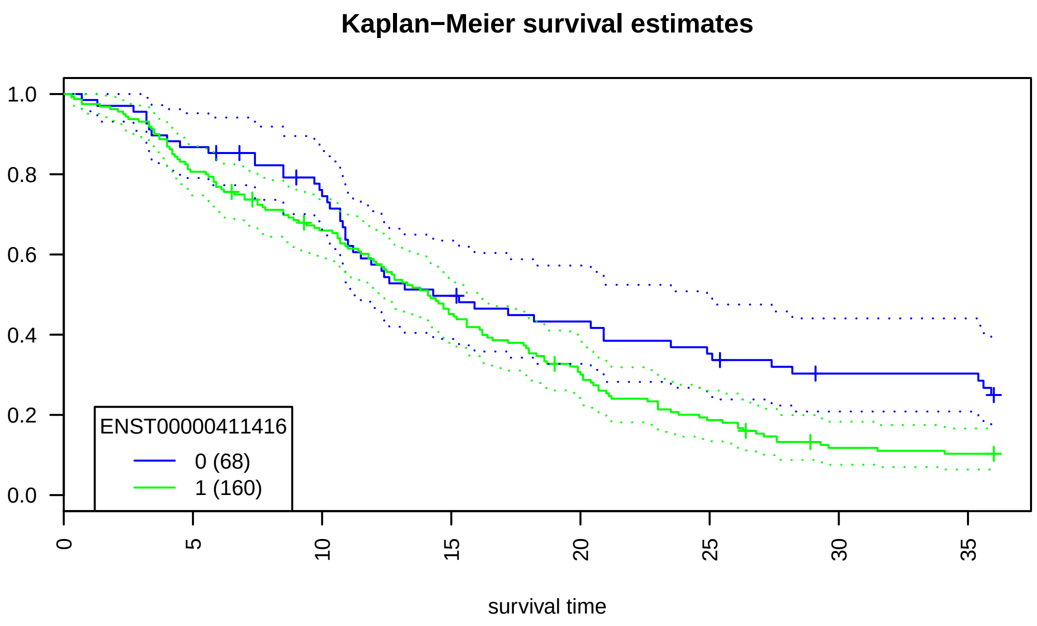

Figure 530: Each curve represents the relative number of ENST00000411416 survived to the corresponding point of time. Dashed lines represent 0.95 confidence intervals for the curves with the corresponding color. Total of 2 observations deleted due to missingness. Log-rank probability measure for the equality of these curves is 1.934451e-02. Additional annotations: *.GeneId*=ENSG00000145287, *.GeneName*=PLAC8, *.GeneDesc*=placenta-specific 8 [Source:HGNC Symbol;Acc:19254].

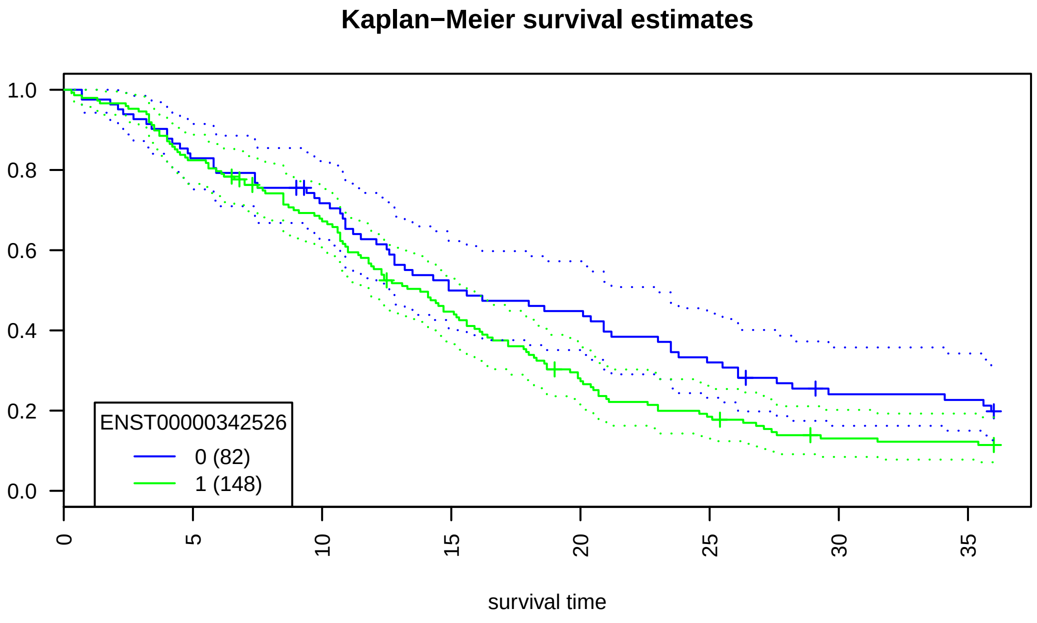

Figure 531: Each curve represents the relative number of ENST00000342526 survived to the corresponding point of time. Dashed lines represent 0.95 confidence intervals for the curves with the corresponding color. Log-rank probability measure for the equality of these curves is 4.703488e-02. Additional annotations: *.GeneId*=ENSG00000187546, *.GeneName*=TMEM195, *.GeneDesc*=transmembrane protein 195 [Source:HGNC Symbol;Acc:33784].

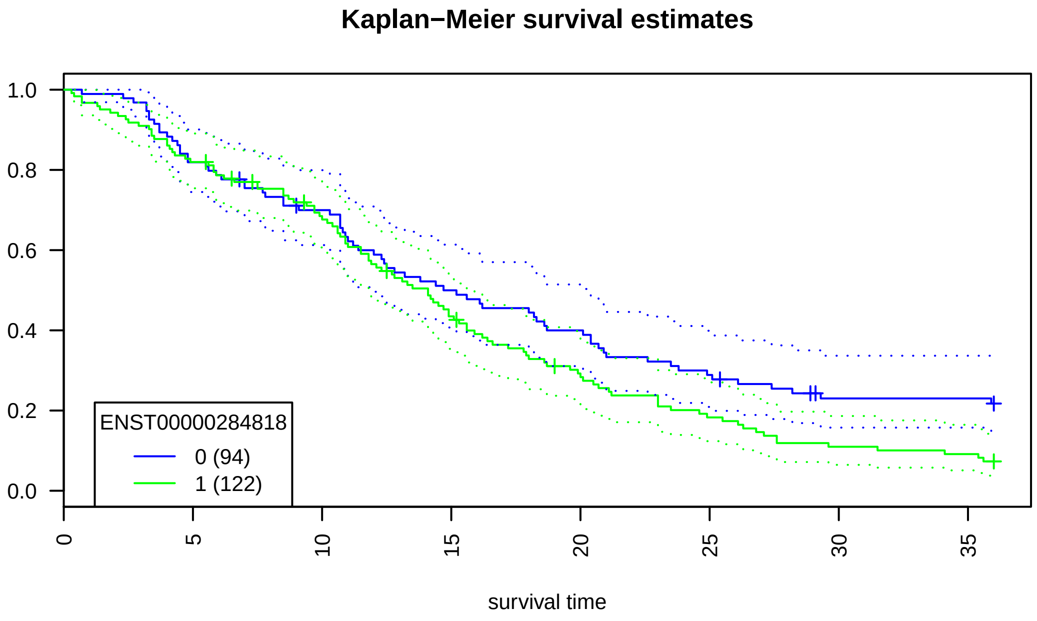

Figure 532: Each curve represents the relative number of ENST00000284818 survived to the corresponding point of time. Dashed lines represent 0.95 confidence intervals for the curves with the corresponding color. Total of 14 observations deleted due to missingness. Log-rank probability measure for the equality of these curves is 3.267666e-02. Additional annotations: *.GeneId*=ENSG00000154589, *.GeneName*=LY96, *.GeneDesc*=lymphocyte antigen 96 [Source:HGNC Symbol;Acc:17156].

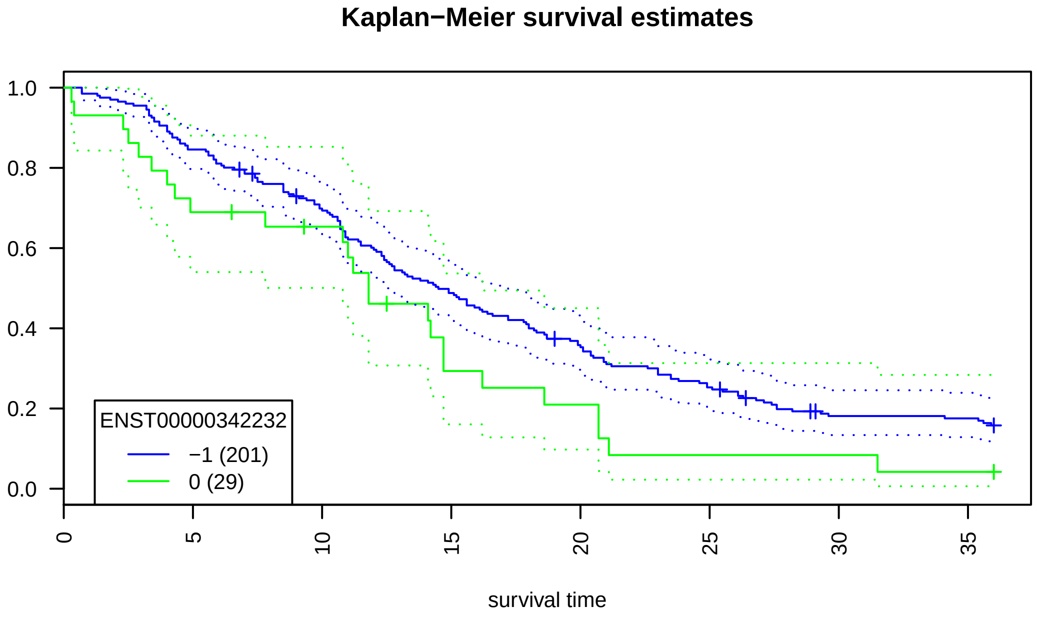

Figure 533: Each curve represents the relative number of ENST00000342232 survived to the corresponding point of time. Dashed lines represent 0.95 confidence intervals for the curves with the corresponding color. Log-rank probability measure for the equality of these curves is 3.217617e-02. Additional annotations: *.GeneId*=ENSG00000104369, *.GeneName*=JPH1, *.GeneDesc*=junctophilin 1 [Source:HGNC Symbol;Acc:14201].

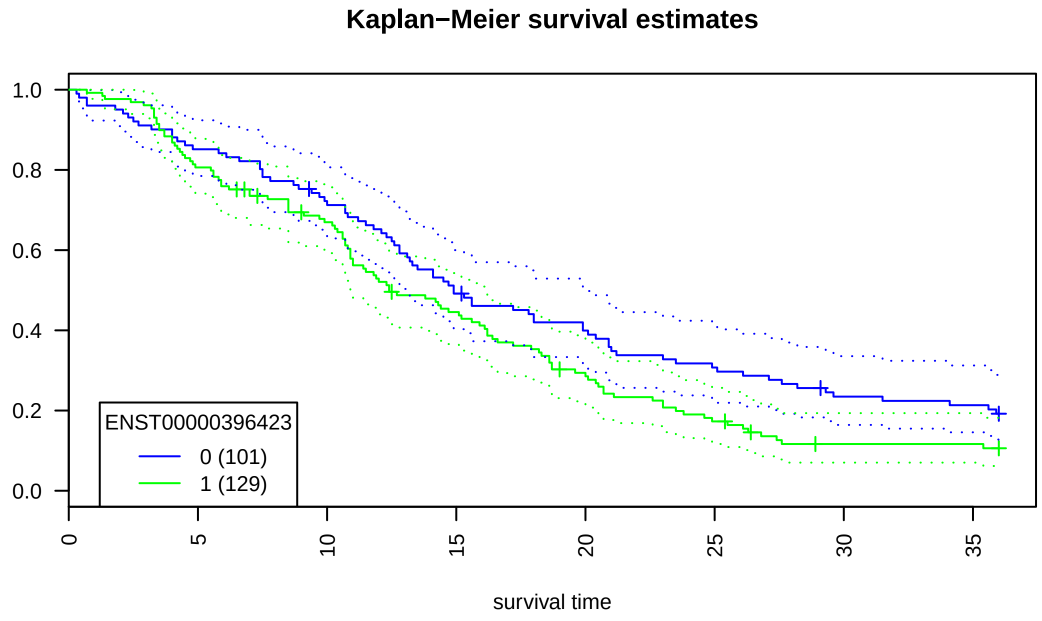

Figure 534: Each curve represents the relative number of ENST00000396423 survived to the corresponding point of time. Dashed lines represent 0.95 confidence intervals for the curves with the corresponding color. Log-rank probability measure for the equality of these curves is 3.516428e-02. Additional annotations: *.GeneId*=ENSG00000164749, *.GeneName*=HNF4G, *.GeneDesc*=hepatocyte nuclear factor 4, gamma [Source:HGNC Symbol;Acc:5026].

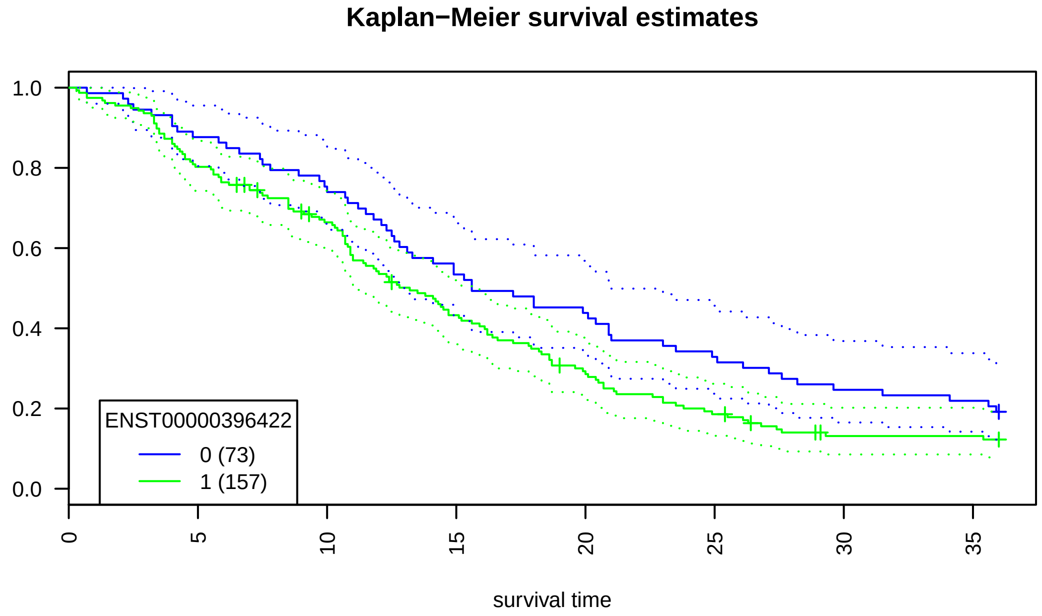

Figure 535: Each curve represents the relative number of ENST00000396422 survived to the corresponding point of time. Dashed lines represent 0.95 confidence intervals for the curves with the corresponding color. Log-rank probability measure for the equality of these curves is 3.626793e-02. Additional annotations: *.GeneId*=ENSG00000164749, *.GeneName*=HNF4G, *.GeneDesc*=hepatocyte nuclear factor 4, gamma [Source:HGNC Symbol;Acc:5026].

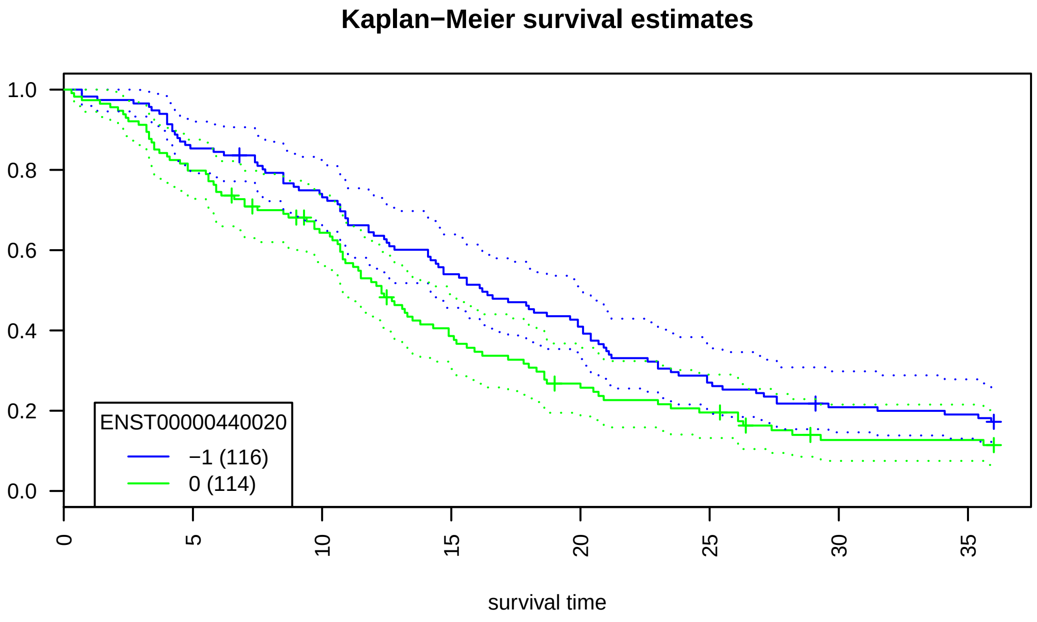

Figure 536: Each curve represents the relative number of ENST00000440020 survived to the corresponding point of time. Dashed lines represent 0.95 confidence intervals for the curves with the corresponding color. Log-rank probability measure for the equality of these curves is 2.946904e-02. Additional annotations: *.GeneId*=ENSG00000106537, *.GeneName*=TSPAN13, *.GeneDesc*=tetraspanin 13 [Source:HGNC Symbol;Acc:21643].

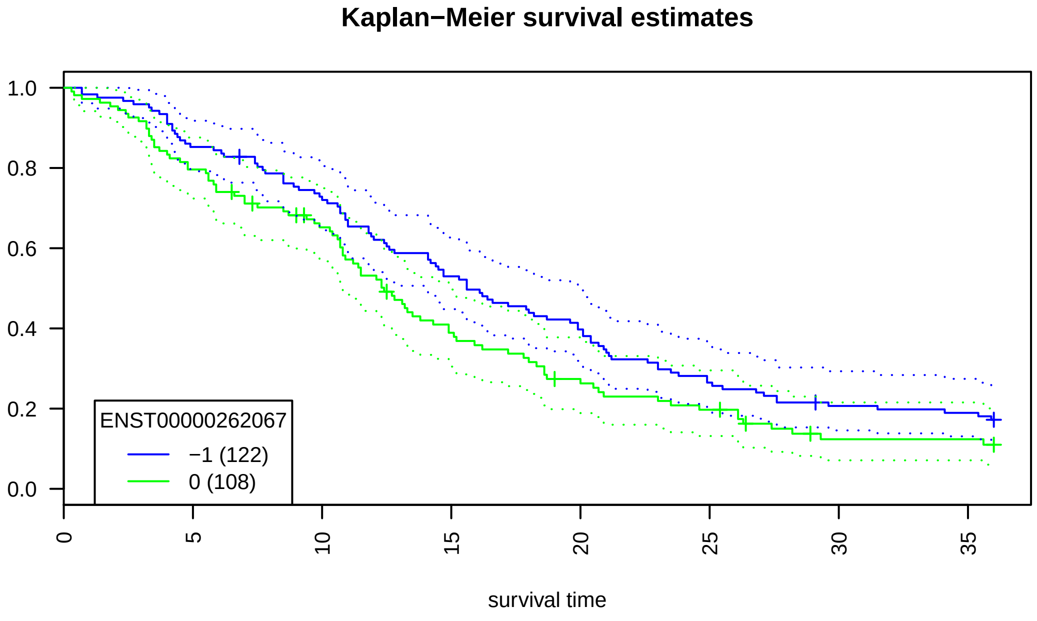

Figure 537: Each curve represents the relative number of ENST00000262067 survived to the corresponding point of time. Dashed lines represent 0.95 confidence intervals for the curves with the corresponding color. Log-rank probability measure for the equality of these curves is 4.251998e-02. Additional annotations: *.GeneId*=ENSG00000106537, *.GeneName*=TSPAN13, *.GeneDesc*=tetraspanin 13 [Source:HGNC Symbol;Acc:21643].

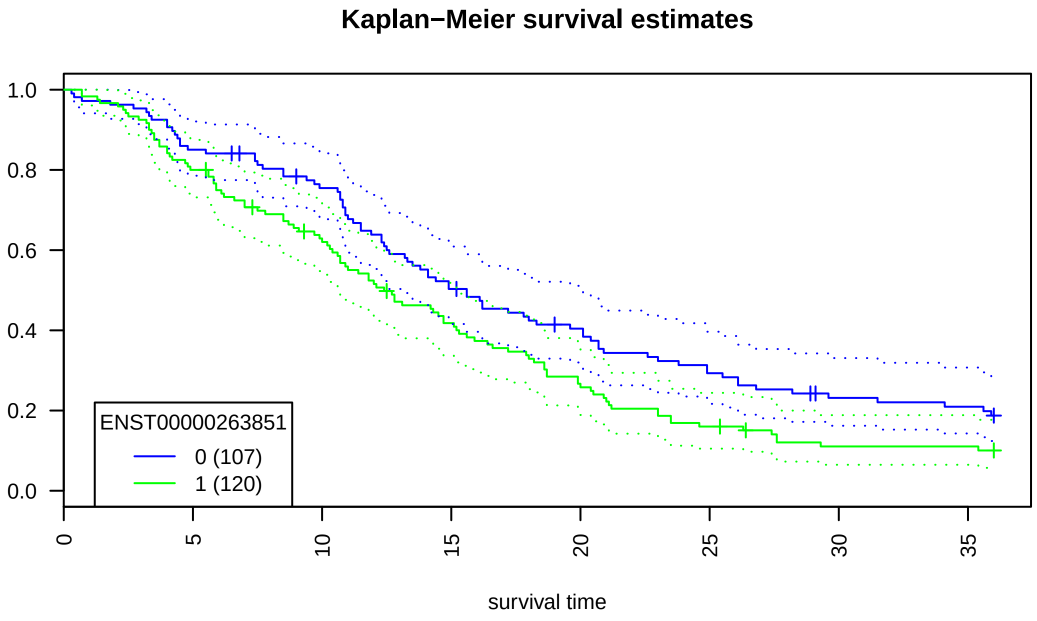

Figure 538: Each curve represents the relative number of ENST00000263851 survived to the corresponding point of time. Dashed lines represent 0.95 confidence intervals for the curves with the corresponding color. Total of 3 observations deleted due to missingness. Log-rank probability measure for the equality of these curves is 1.666306e-02. Additional annotations: *.GeneId*=ENSG00000104432, *.GeneName*=IL7, *.GeneDesc*=interleukin 7 [Source:HGNC Symbol;Acc:6023].

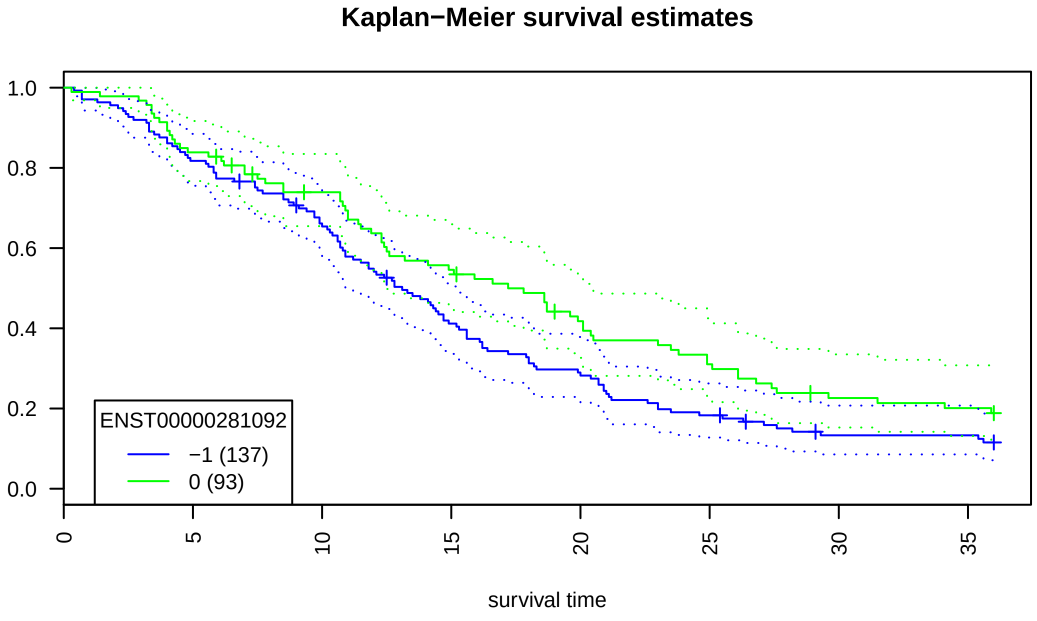

Figure 539: Each curve represents the relative number of ENST00000281092 survived to the corresponding point of time. Dashed lines represent 0.95 confidence intervals for the curves with the corresponding color. Log-rank probability measure for the equality of these curves is 3.661343e-02. Additional annotations: *.GeneId*=ENSG00000151422, *.GeneName*=FER, *.GeneDesc*=fer (fps/fes related) tyrosine kinase [Source:HGNC Symbol;Acc:3655].

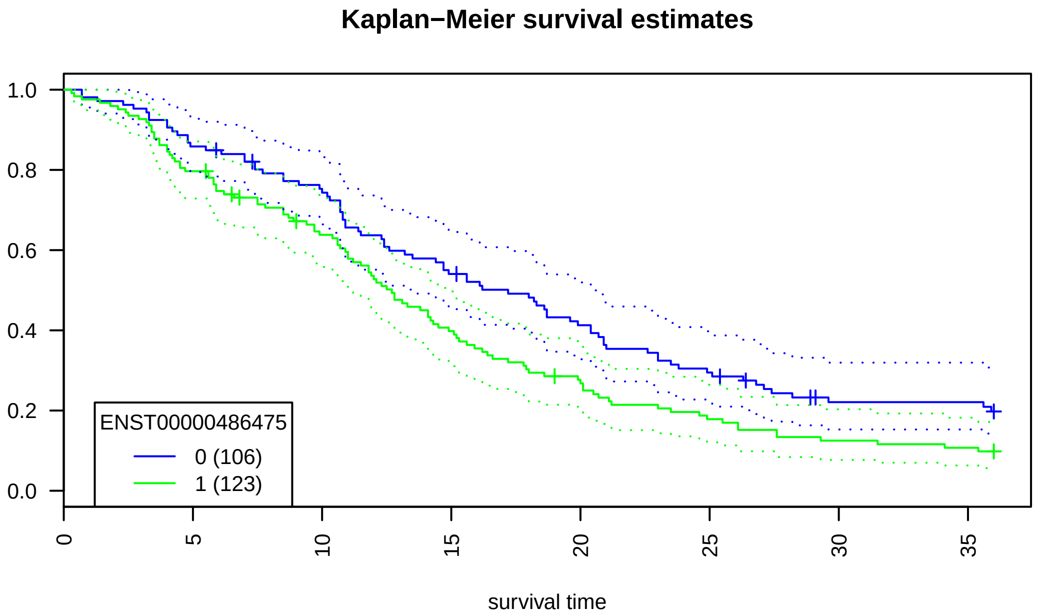

Figure 540: Each curve represents the relative number of ENST00000486475 survived to the corresponding point of time. Dashed lines represent 0.95 confidence intervals for the curves with the corresponding color. Total of 1 observation deleted due to missingness. Log-rank probability measure for the equality of these curves is 1.020724e-02. Additional annotations: *.GeneId*=ENSG00000101336, *.GeneName*=HCK, *.GeneDesc*=hemopoietic cell kinase [Source:HGNC Symbol;Acc:4840].

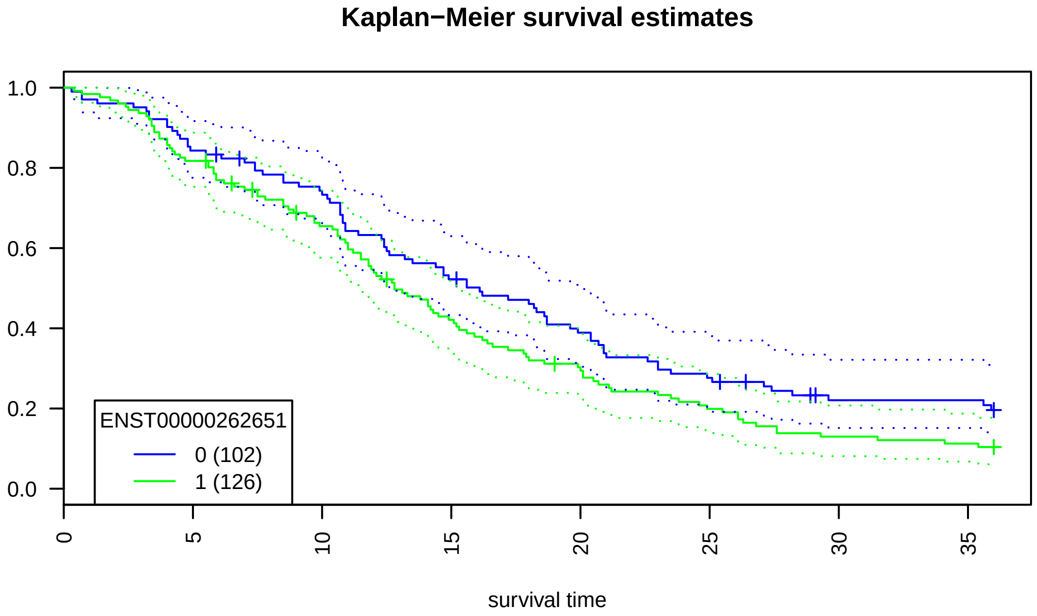

Figure 541: Each curve represents the relative number of ENST00000262651 survived to the corresponding point of time. Dashed lines represent 0.95 confidence intervals for the curves with the corresponding color. Total of 2 observations deleted due to missingness. Log-rank probability measure for the equality of these curves is 4.64337e-02. Additional annotations: *.GeneId*=ENSG00000101336, *.GeneName*=HCK, *.GeneDesc*=hemopoietic cell kinase [Source:HGNC Symbol;Acc:4840].

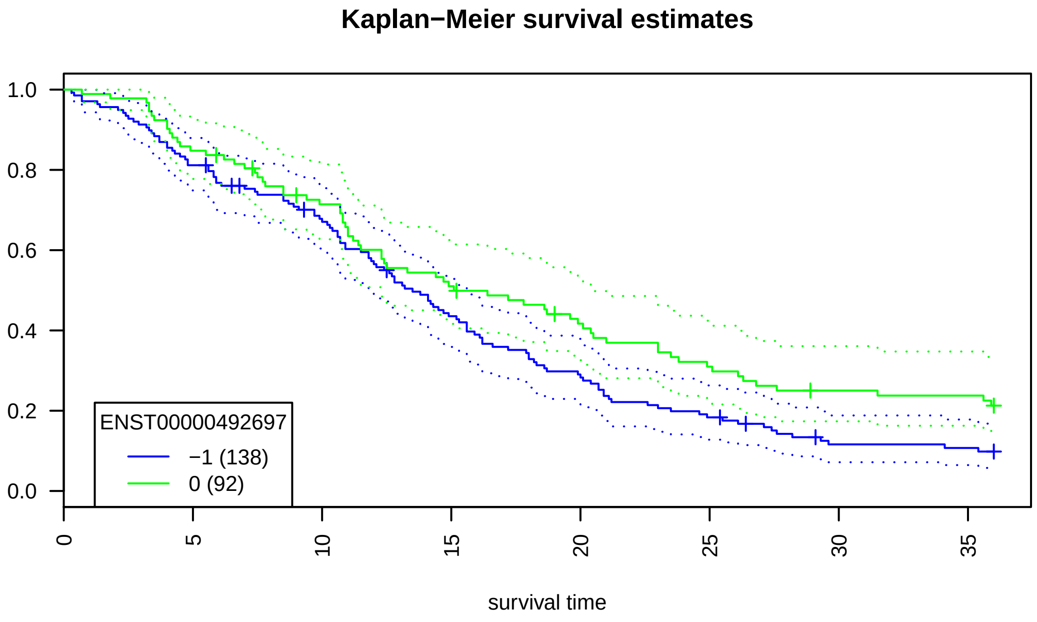

Figure 542: Each curve represents the relative number of ENST00000492697 survived to the corresponding point of time. Dashed lines represent 0.95 confidence intervals for the curves with the corresponding color. Log-rank probability measure for the equality of these curves is 2.536893e-02. Additional annotations: *.GeneId*=ENSG00000112294, *.GeneName*=ALDH5A1, *.GeneDesc*=aldehyde dehydrogenase 5 family, member A1 [Source:HGNC Symbol;Acc:408].

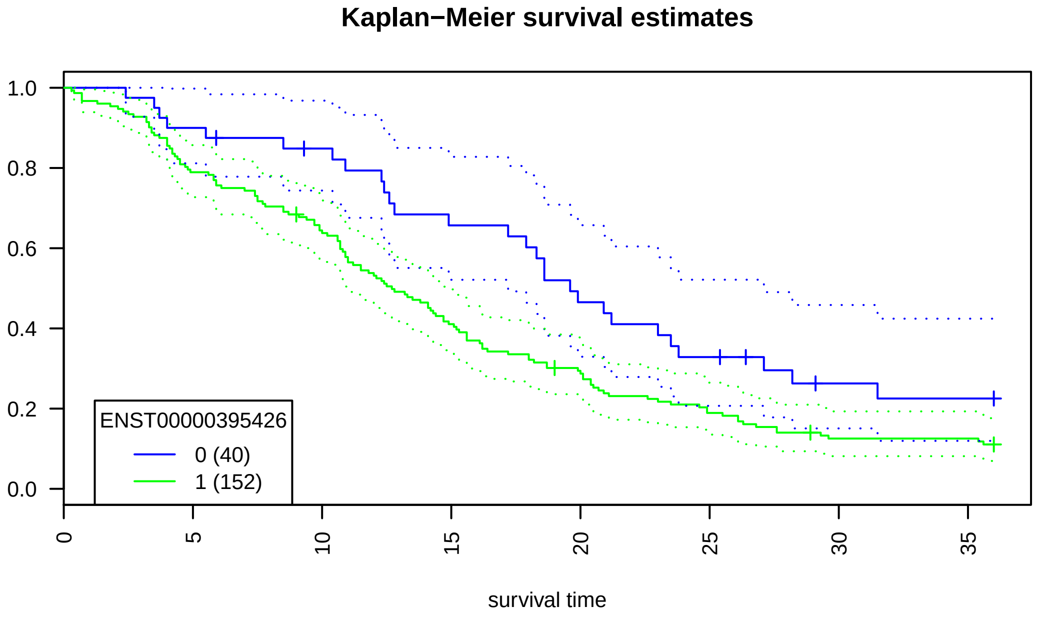

Figure 543: Each curve represents the relative number of ENST00000395426 survived to the corresponding point of time. Dashed lines represent 0.95 confidence intervals for the curves with the corresponding color. Total of 38 observations deleted due to missingness. Log-rank probability measure for the equality of these curves is 1.192523e-02. Additional annotations: *.GeneId*=ENSG00000139209, *.GeneName*=SLC38A4, *.GeneDesc*=solute carrier family 38, member 4 [Source:HGNC Symbol;Acc:14679].

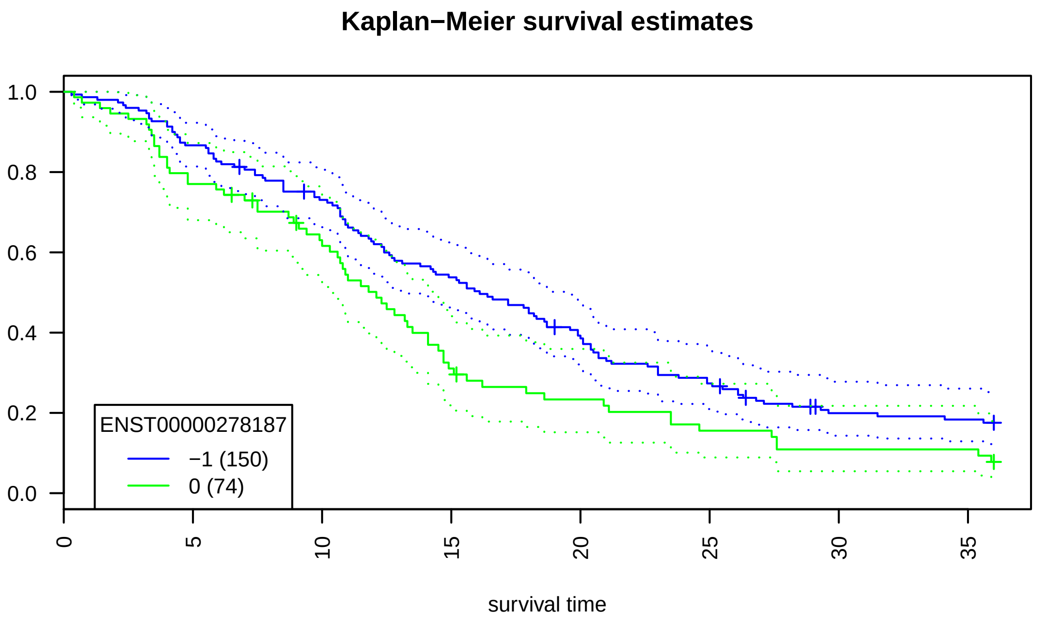

Figure 544: Each curve represents the relative number of ENST00000278187 survived to the corresponding point of time. Dashed lines represent 0.95 confidence intervals for the curves with the corresponding color. Total of 6 observations deleted due to missingness. Log-rank probability measure for the equality of these curves is 8.325907e-03. Additional annotations: *.GeneId*=ENSG00000148935, *.GeneName*=GAS2, *.GeneDesc*=growth arrest-specific 2 [Source:HGNC Symbol;Acc:4167].

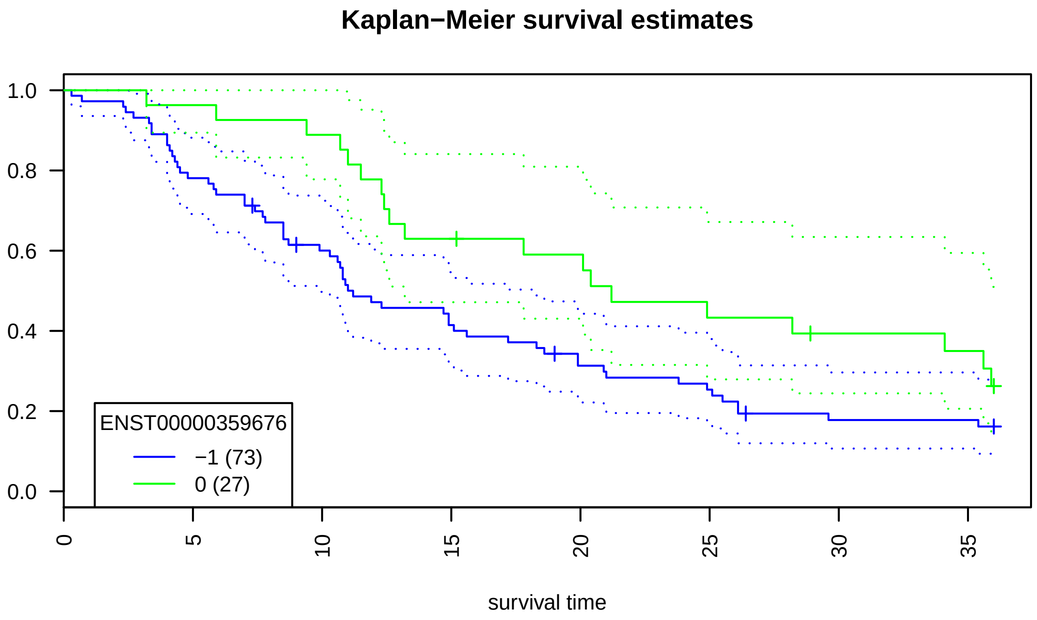

Figure 545: Each curve represents the relative number of ENST00000359676 survived to the corresponding point of time. Dashed lines represent 0.95 confidence intervals for the curves with the corresponding color. Total of 130 observations deleted due to missingness. Log-rank probability measure for the equality of these curves is 3.446485e-02. Additional annotations: *.GeneId*=ENSG00000197183, *.GeneName*=C20orf112, *.GeneDesc*=Uncharacterized protein C20orf112 [Source:UniProtKB/Swiss-Prot;Acc:Q96MY1].

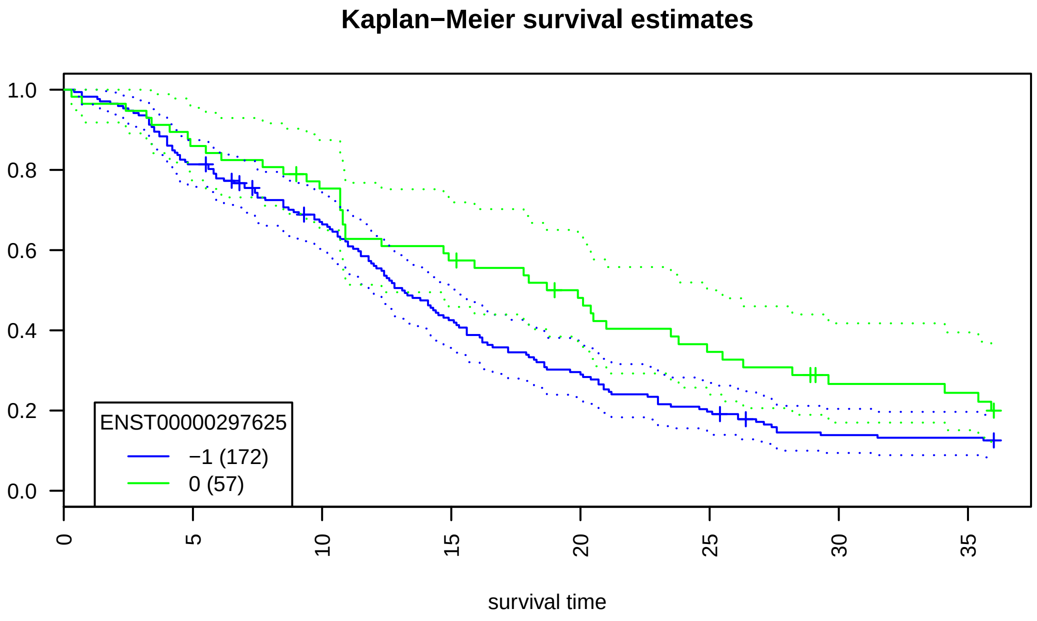

Figure 546: Each curve represents the relative number of ENST00000297625 survived to the corresponding point of time. Dashed lines represent 0.95 confidence intervals for the curves with the corresponding color. Total of 1 observation deleted due to missingness. Log-rank probability measure for the equality of these curves is 3.558239e-02. Additional annotations: *.GeneId*=ENSG00000164976, *.GeneName*=KIAA1161, *.GeneDesc*=KIAA1161 [Source:HGNC Symbol;Acc:19918].

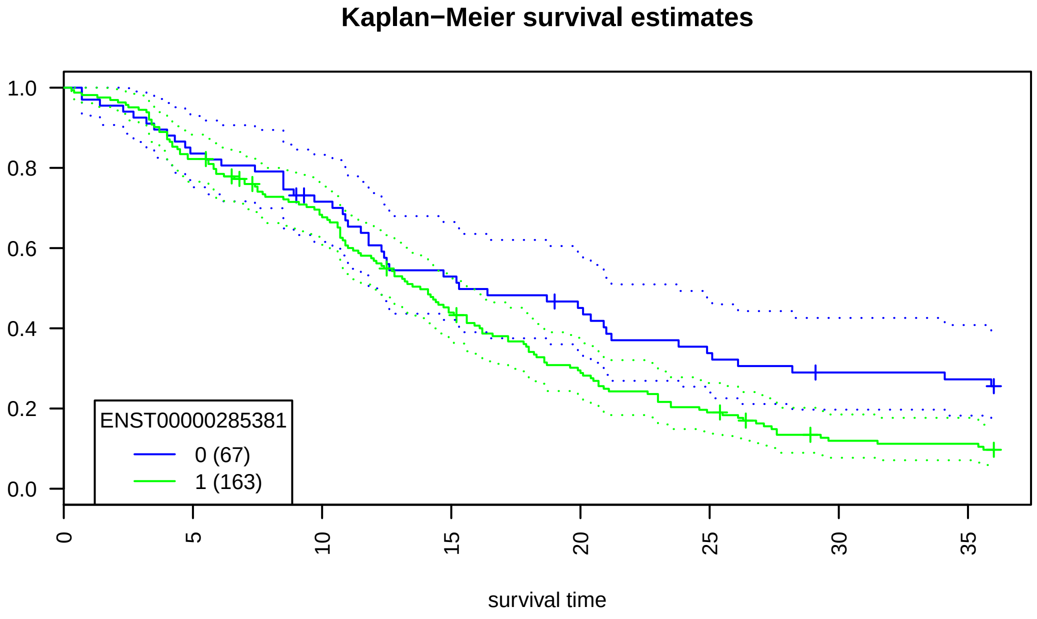

Figure 547: Each curve represents the relative number of ENST00000285381 survived to the corresponding point of time. Dashed lines represent 0.95 confidence intervals for the curves with the corresponding color. Log-rank probability measure for the equality of these curves is 1.516996e-02. Additional annotations: *.GeneId*=ENSG00000164879, *.GeneName*=CA3, *.GeneDesc*=carbonic anhydrase III, muscle specific [Source:HGNC Symbol;Acc:1374].

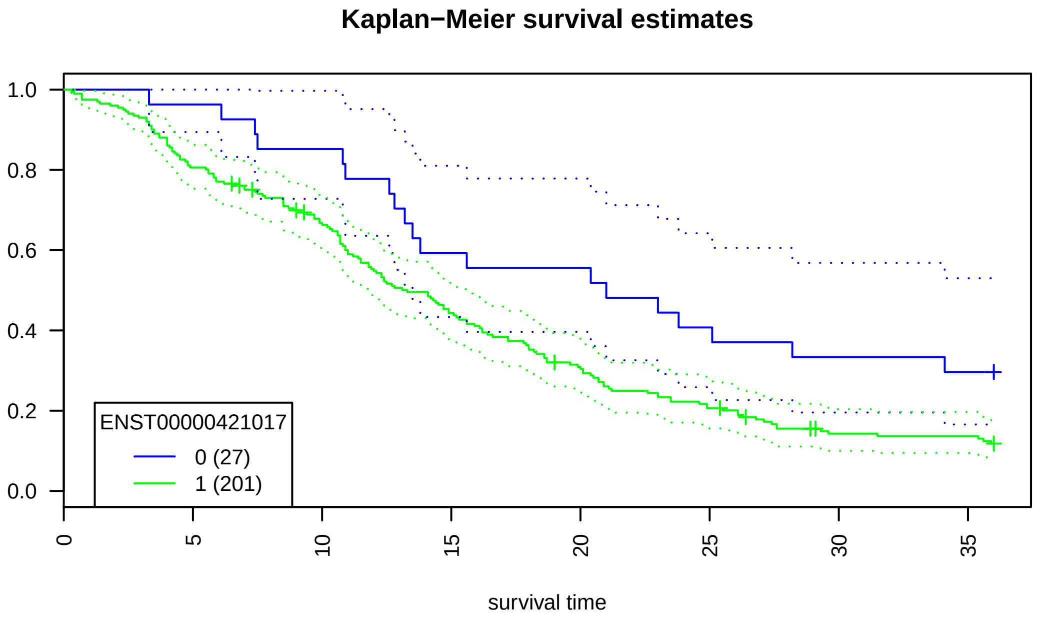

Figure 548: Each curve represents the relative number of ENST00000421017 survived to the corresponding point of time. Dashed lines represent 0.95 confidence intervals for the curves with the corresponding color. Total of 2 observations deleted due to missingness. Log-rank probability measure for the equality of these curves is 1.382311e-02. Additional annotations: *.GeneId*=ENSG00000182718, *.GeneName*=ANXA2, *.GeneDesc*=annexin A2 [Source:HGNC Symbol;Acc:537].

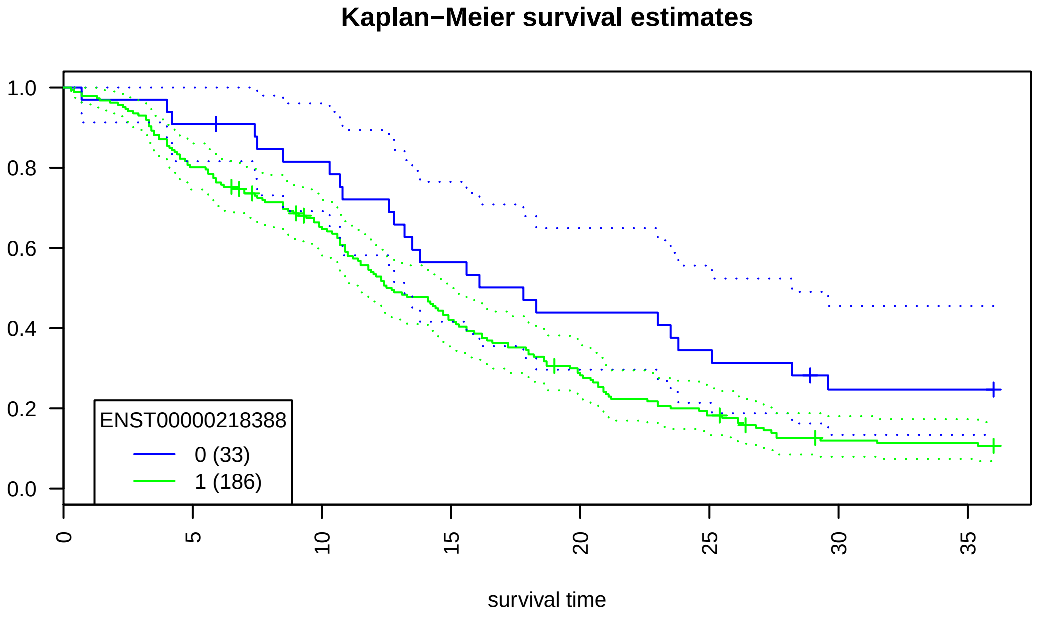

Figure 549: Each curve represents the relative number of ENST00000218388 survived to the corresponding point of time. Dashed lines represent 0.95 confidence intervals for the curves with the corresponding color. Total of 11 observations deleted due to missingness. Log-rank probability measure for the equality of these curves is 2.606388e-02. Additional annotations: *.GeneId*=ENSG00000102265, *.GeneName*=TIMP1, *.GeneDesc*=TIMP metallopeptidase inhibitor 1 [Source:HGNC Symbol;Acc:11820].

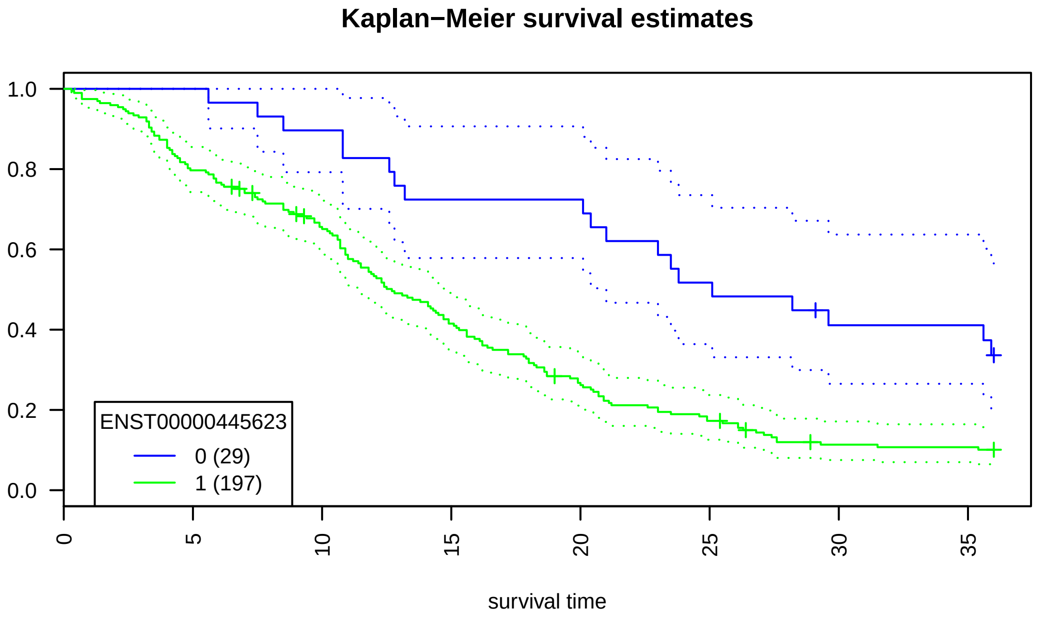

Figure 550: Each curve represents the relative number of ENST00000445623 survived to the corresponding point of time. Dashed lines represent 0.95 confidence intervals for the curves with the corresponding color. Total of 4 observations deleted due to missingness. Log-rank probability measure for the equality of these curves is 1.094017e-04. Additional annotations: *.GeneId*=ENSG00000102265, *.GeneName*=TIMP1, *.GeneDesc*=TIMP metallopeptidase inhibitor 1 [Source:HGNC Symbol;Acc:11820].

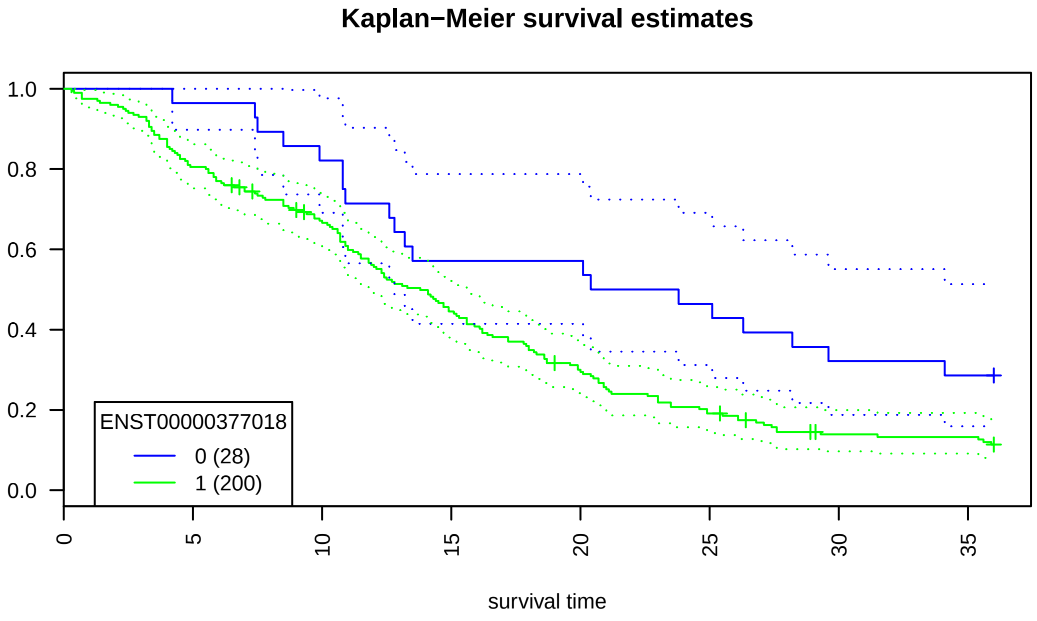

Figure 551: Each curve represents the relative number of ENST00000377018 survived to the corresponding point of time. Dashed lines represent 0.95 confidence intervals for the curves with the corresponding color. Total of 2 observations deleted due to missingness. Log-rank probability measure for the equality of these curves is 1.034906e-02. Additional annotations: *.GeneId*=ENSG00000102265, *.GeneName*=TIMP1, *.GeneDesc*=TIMP metallopeptidase inhibitor 1 [Source:HGNC Symbol;Acc:11820].

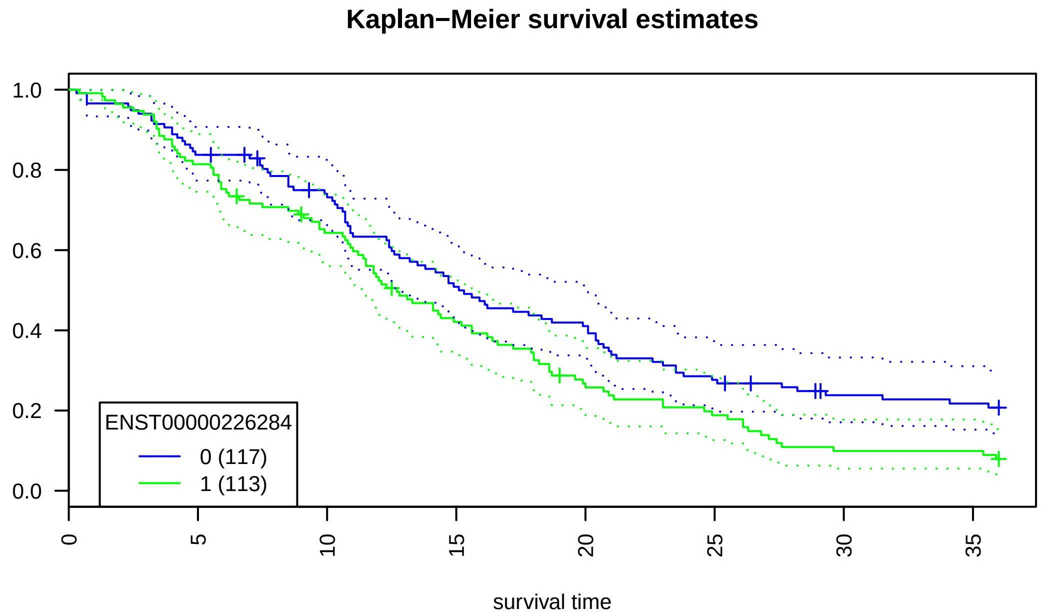

Figure 552: Each curve represents the relative number of ENST00000226284 survived to the corresponding point of time. Dashed lines represent 0.95 confidence intervals for the curves with the corresponding color. Log-rank probability measure for the equality of these curves is 1.362805e-02. Additional annotations: *.GeneId*=ENSG00000029559, *.GeneName*=IBSP, *.GeneDesc*=integrin-binding sialoprotein [Source:HGNC Symbol;Acc:5341].

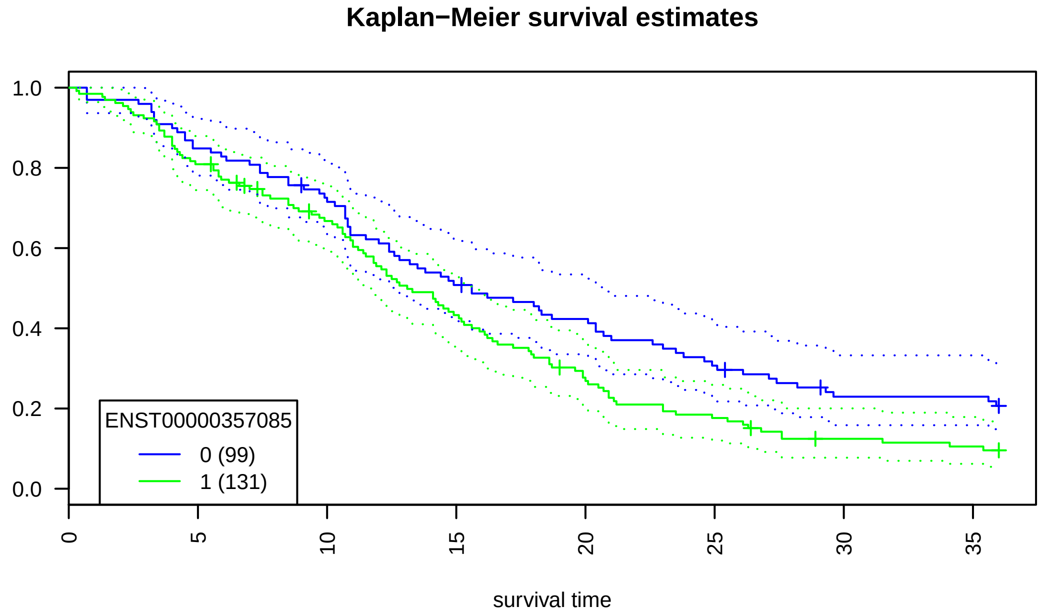

Figure 553: Each curve represents the relative number of ENST00000357085 survived to the corresponding point of time. Dashed lines represent 0.95 confidence intervals for the curves with the corresponding color. Log-rank probability measure for the equality of these curves is 1.925917e-02. Additional annotations: *.GeneId*=ENSG00000112343, *.GeneName*=TRIM38, *.GeneDesc*=tripartite motif-containing 38 [Source:HGNC Symbol;Acc:10059].

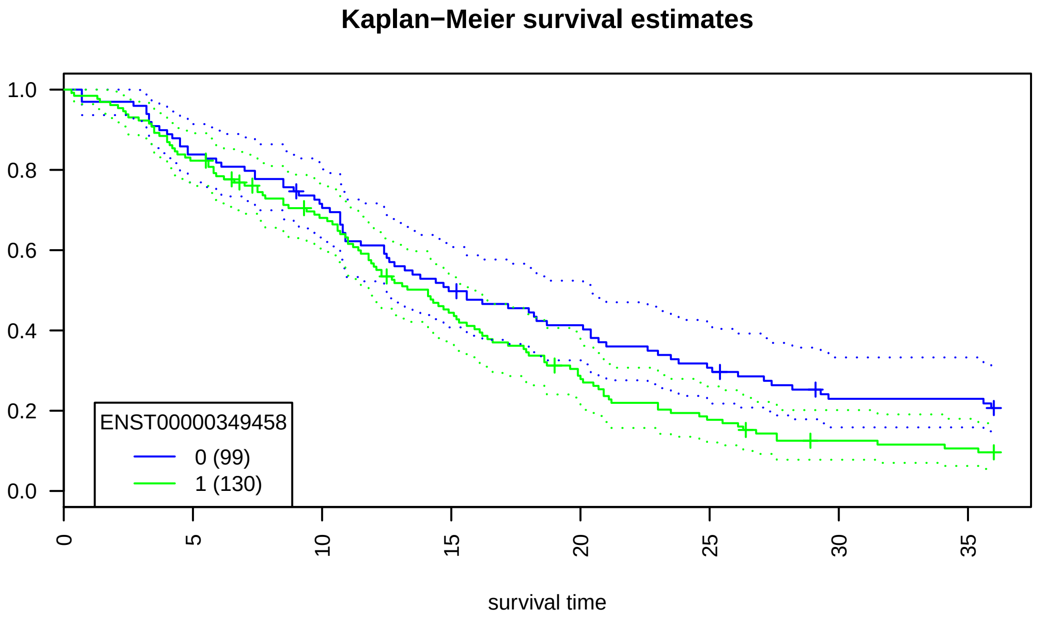

Figure 554: Each curve represents the relative number of ENST00000349458 survived to the corresponding point of time. Dashed lines represent 0.95 confidence intervals for the curves with the corresponding color. Total of 1 observation deleted due to missingness. Log-rank probability measure for the equality of these curves is 3.453434e-02. Additional annotations: *.GeneId*=ENSG00000112343, *.GeneName*=TRIM38, *.GeneDesc*=tripartite motif-containing 38 [Source:HGNC Symbol;Acc:10059].

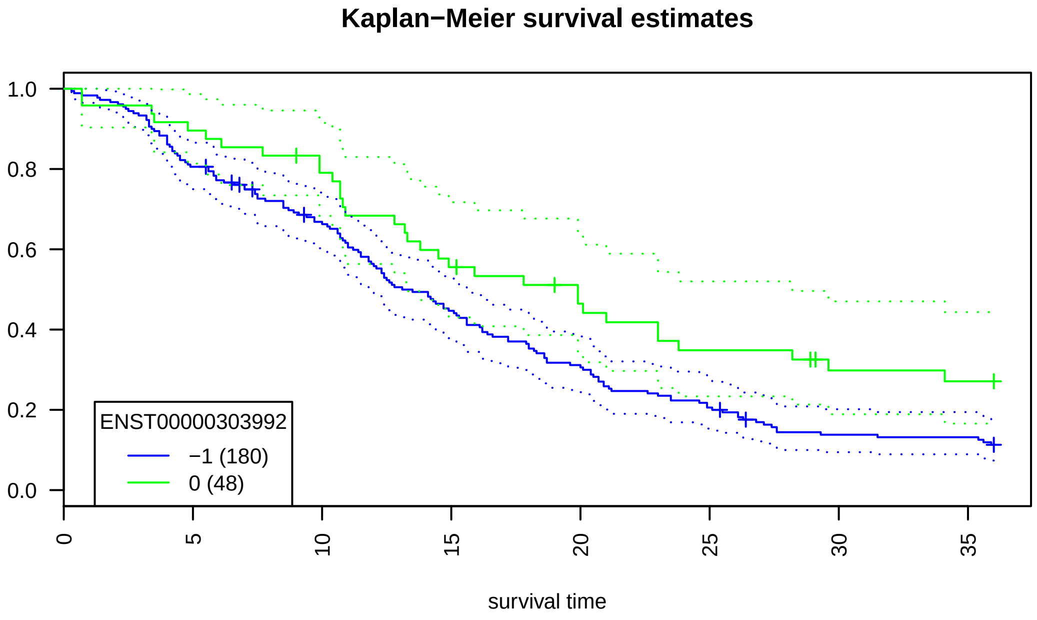

Figure 555: Each curve represents the relative number of ENST00000303992 survived to the corresponding point of time. Dashed lines represent 0.95 confidence intervals for the curves with the corresponding color. Total of 2 observations deleted due to missingness. Log-rank probability measure for the equality of these curves is 1.136903e-02. Additional annotations: *.GeneId*=ENSG00000168913, *.GeneName*=ENHO, *.GeneDesc*=energy homeostasis associated [Source:HGNC Symbol;Acc:24838].

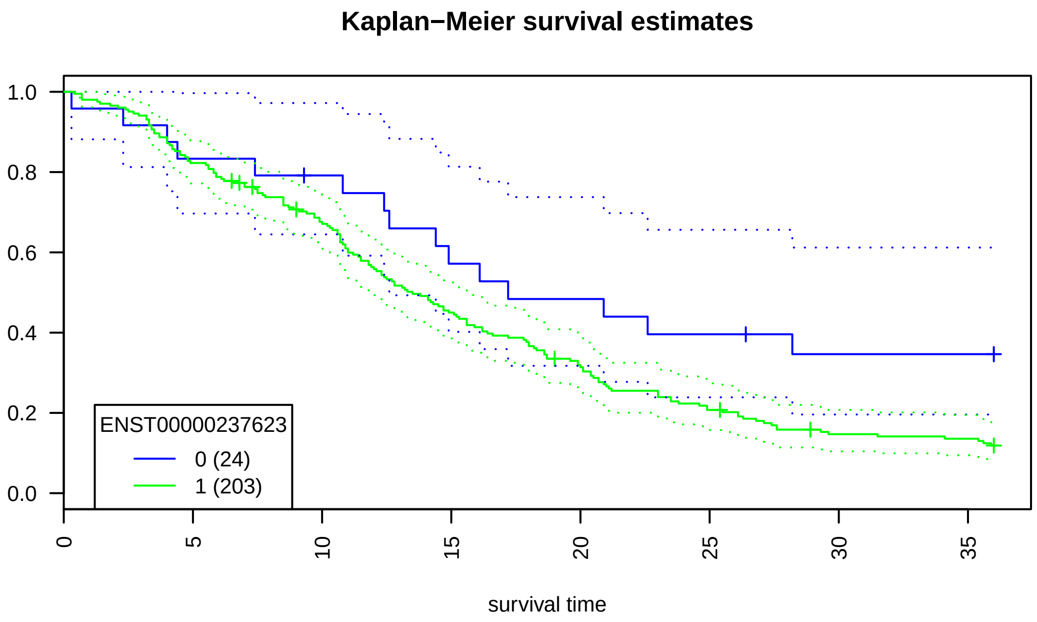

Figure 556: Each curve represents the relative number of ENST00000237623 survived to the corresponding point of time. Dashed lines represent 0.95 confidence intervals for the curves with the corresponding color. Total of 3 observations deleted due to missingness. Log-rank probability measure for the equality of these curves is 2.882977e-02. Additional annotations: *.GeneId*=ENSG00000118785, *.GeneName*=SPP1, *.GeneDesc*=secreted phosphoprotein 1 [Source:HGNC Symbol;Acc:11255].

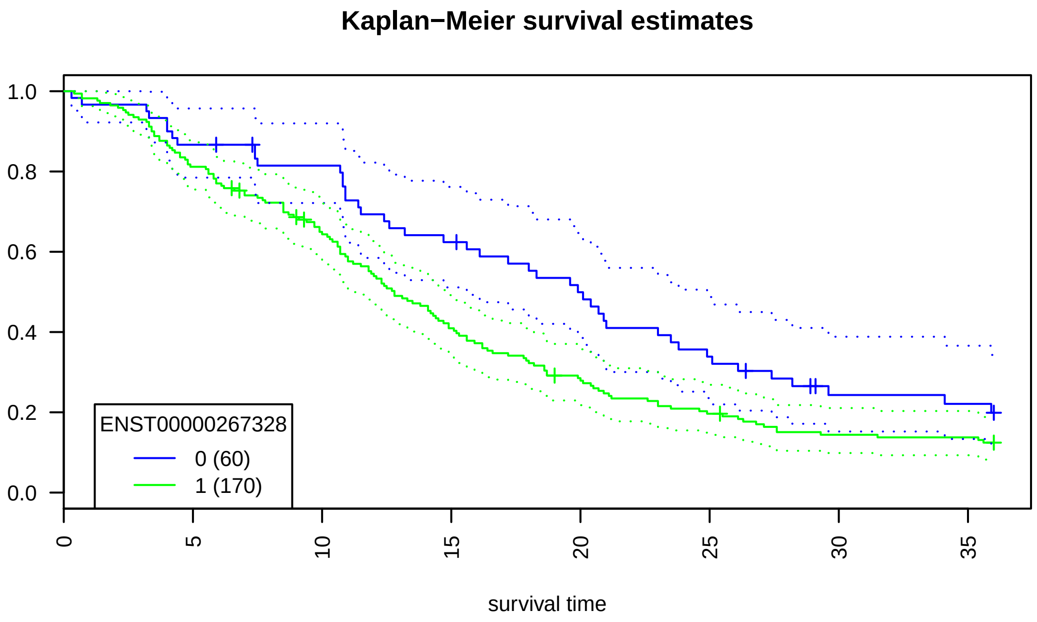

Figure 557: Each curve represents the relative number of ENST00000267328 survived to the corresponding point of time. Dashed lines represent 0.95 confidence intervals for the curves with the corresponding color. Log-rank probability measure for the equality of these curves is 1.406099e-02. Additional annotations: *.GeneId*=ENSG00000139832, *.GeneName*=RAB20, *.GeneDesc*=RAB20, member RAS oncogene family [Source:HGNC Symbol;Acc:18260].

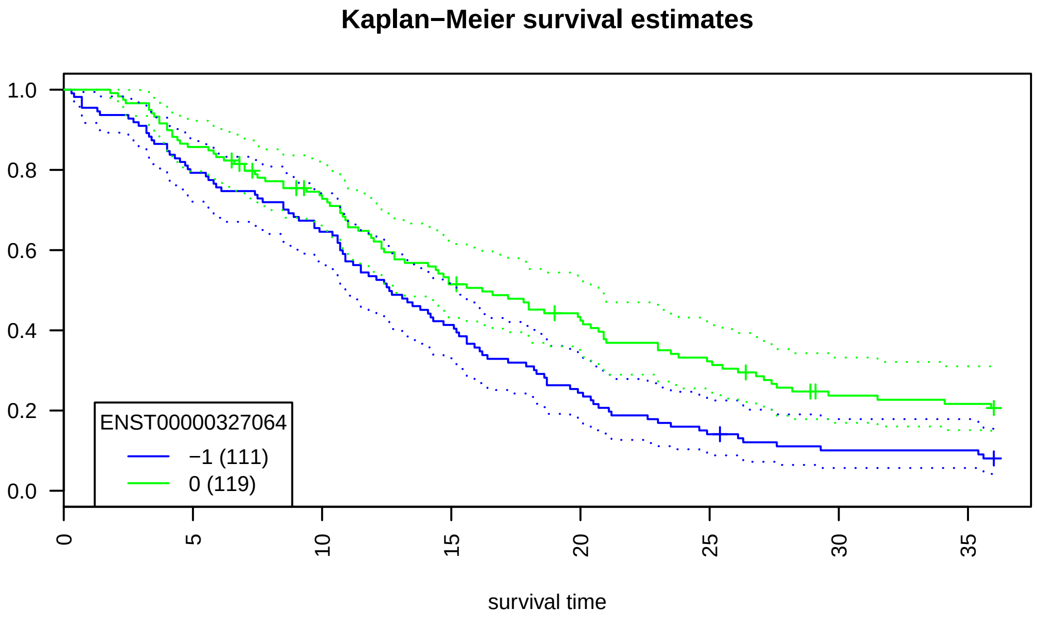

Figure 558: Each curve represents the relative number of ENST00000327064 survived to the corresponding point of time. Dashed lines represent 0.95 confidence intervals for the curves with the corresponding color. Log-rank probability measure for the equality of these curves is 2.399471e-03. Additional annotations: *.GeneId*=ENSG00000142453, *.GeneName*=CARM1, *.GeneDesc*=coactivator-associated arginine methyltransferase 1 [Source:HGNC Symbol;Acc:23393].

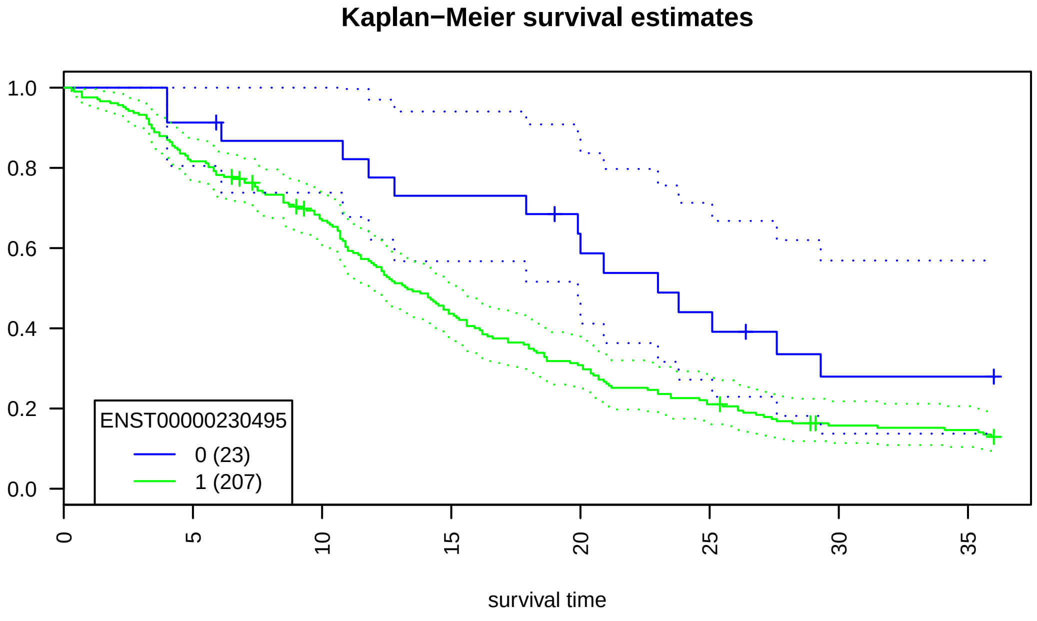

Figure 559: Each curve represents the relative number of ENST00000230495 survived to the corresponding point of time. Dashed lines represent 0.95 confidence intervals for the curves with the corresponding color. Log-rank probability measure for the equality of these curves is 1.648849e-02. Additional annotations: *.GeneId*=ENSG00000112727, *.GeneName*=HIST1H3F, *.GeneDesc*=histone cluster 1, H3f [Source:HGNC Symbol;Acc:4773].

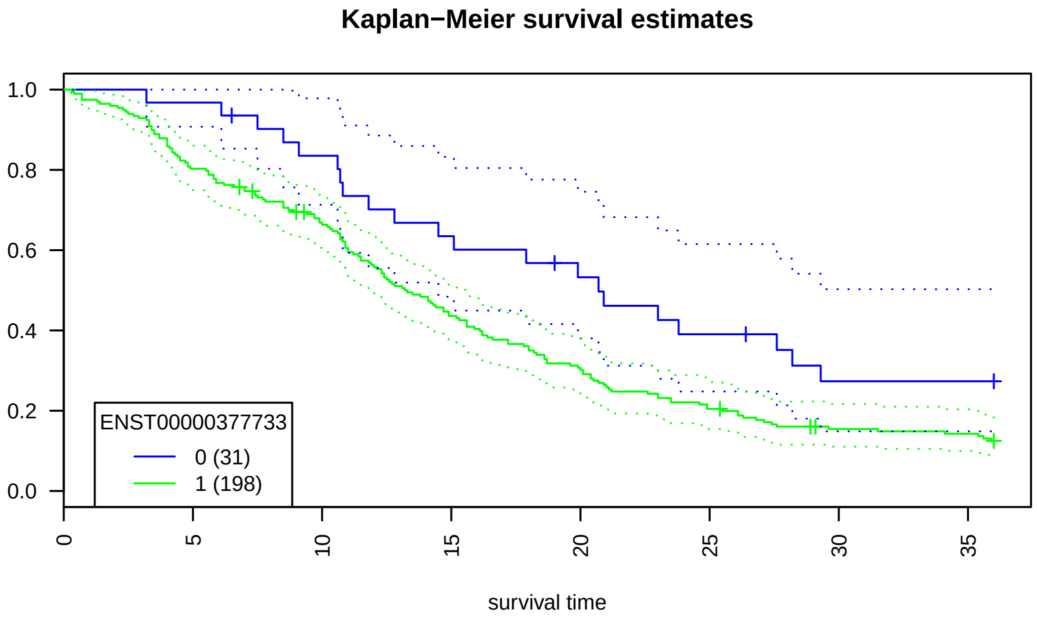

Figure 560: Each curve represents the relative number of ENST00000377733 survived to the corresponding point of time. Dashed lines represent 0.95 confidence intervals for the curves with the corresponding color. Total of 1 observation deleted due to missingness. Log-rank probability measure for the equality of these curves is 1.561564e-02. Additional annotations: *.GeneId*=ENSG00000168242, *.GeneName*=HIST1H2BI, *.GeneDesc*=histone cluster 1, H2bi [Source:HGNC Symbol;Acc:4756].

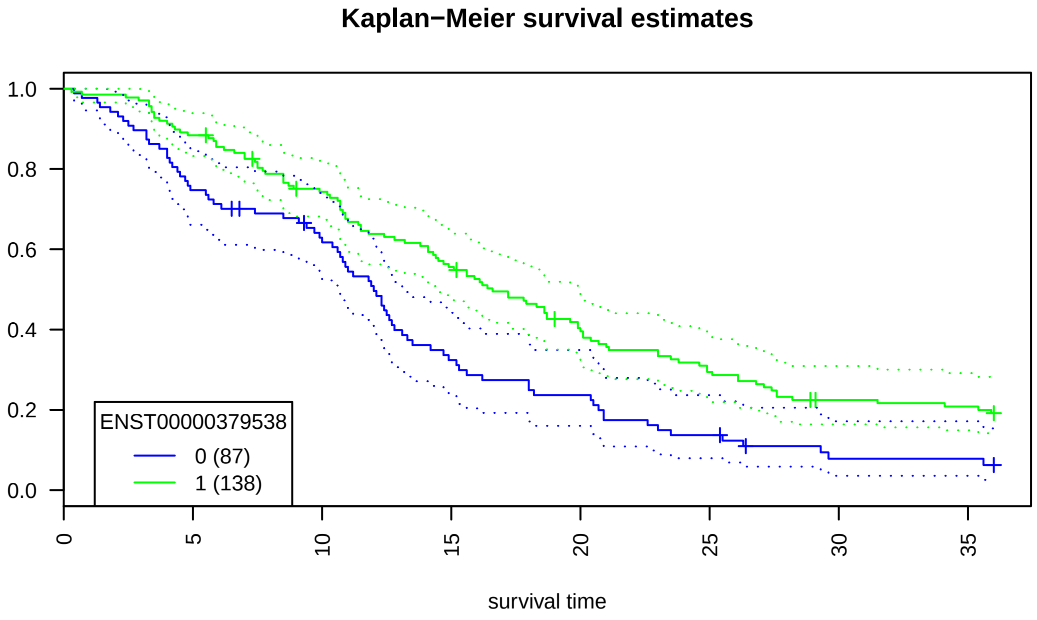

Figure 561: Each curve represents the relative number of ENST00000379538 survived to the corresponding point of time. Dashed lines represent 0.95 confidence intervals for the curves with the corresponding color. Total of 5 observations deleted due to missingness. Log-rank probability measure for the equality of these curves is 3.369827e-04. Additional annotations: *.GeneId*=ENSG00000064692, *.GeneName*=SNCAIP, *.GeneDesc*=synuclein, alpha interacting protein [Source:HGNC Symbol;Acc:11139].

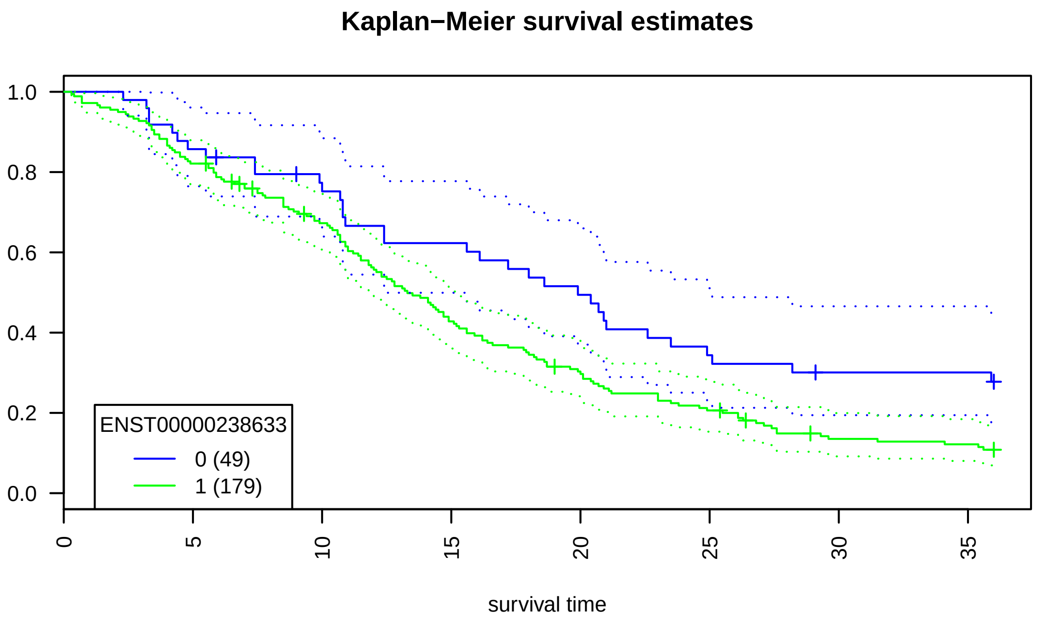

Figure 562: Each curve represents the relative number of ENST00000238633 survived to the corresponding point of time. Dashed lines represent 0.95 confidence intervals for the curves with the corresponding color. Total of 2 observations deleted due to missingness. Log-rank probability measure for the equality of these curves is 8.826111e-03. Additional annotations: *.GeneId*=ENSG00000119655, *.GeneName*=NPC2, *.GeneDesc*=Niemann-Pick disease, type C2 [Source:HGNC Symbol;Acc:14537].

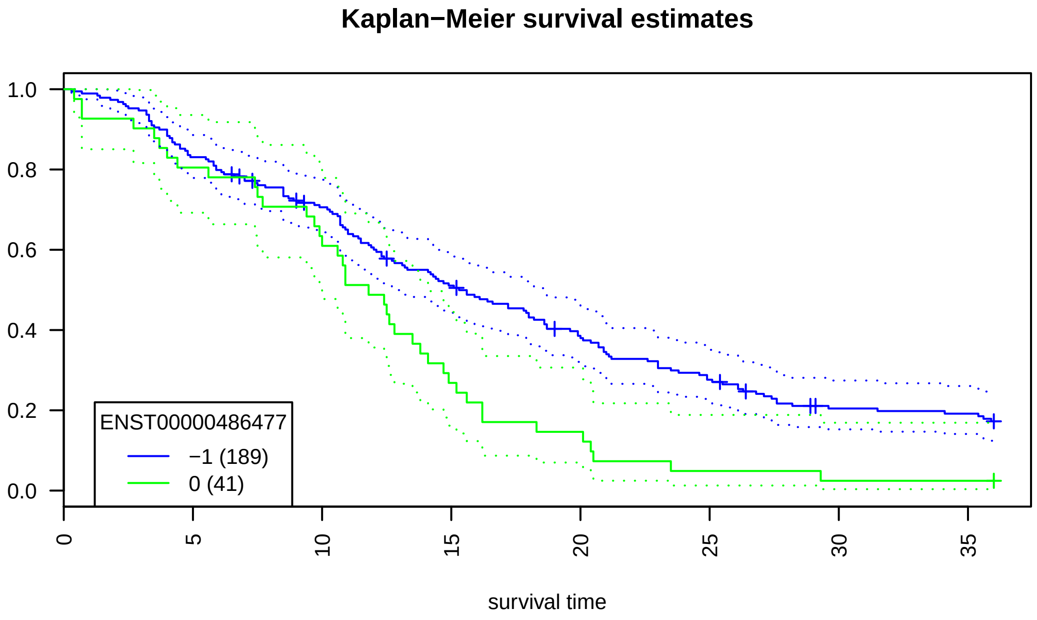

Figure 563: Each curve represents the relative number of ENST00000486477 survived to the corresponding point of time. Dashed lines represent 0.95 confidence intervals for the curves with the corresponding color. Log-rank probability measure for the equality of these curves is 3.912128e-04. Additional annotations: *.GeneId*=ENSG00000122733, *.GeneName*=KIAA1045, *.GeneDesc*=KIAA1045 [Source:HGNC Symbol;Acc:29180].

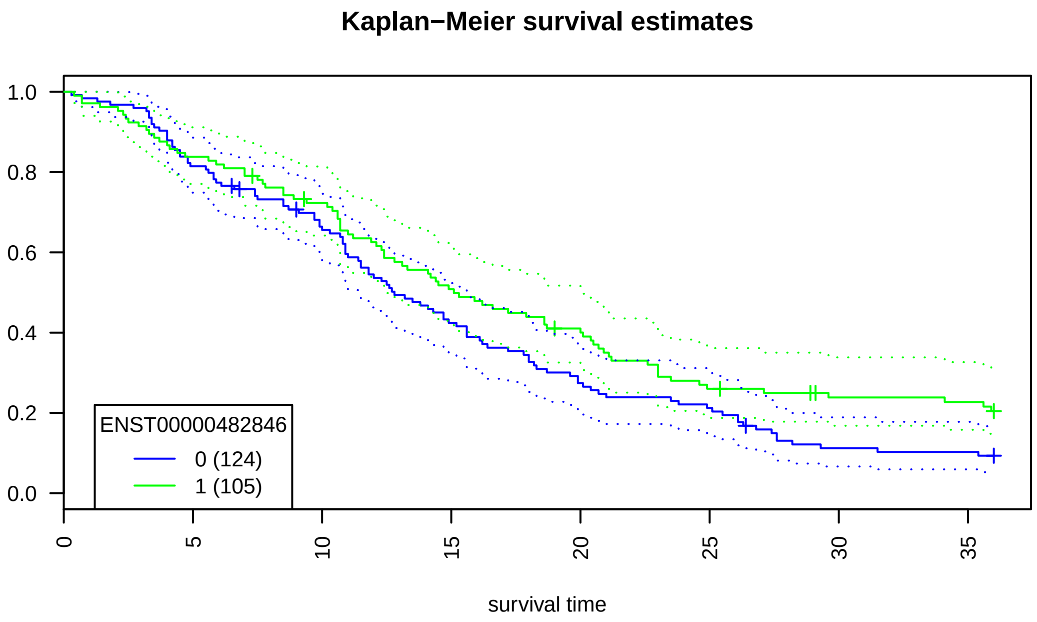

Figure 564: Each curve represents the relative number of ENST00000482846 survived to the corresponding point of time. Dashed lines represent 0.95 confidence intervals for the curves with the corresponding color. Total of 1 observation deleted due to missingness. Log-rank probability measure for the equality of these curves is 4.507656e-02. Additional annotations: *.GeneId*=ENSG00000185088, *.GeneName*=RPS27L, *.GeneDesc*=ribosomal protein S27-like [Source:HGNC Symbol;Acc:18476].

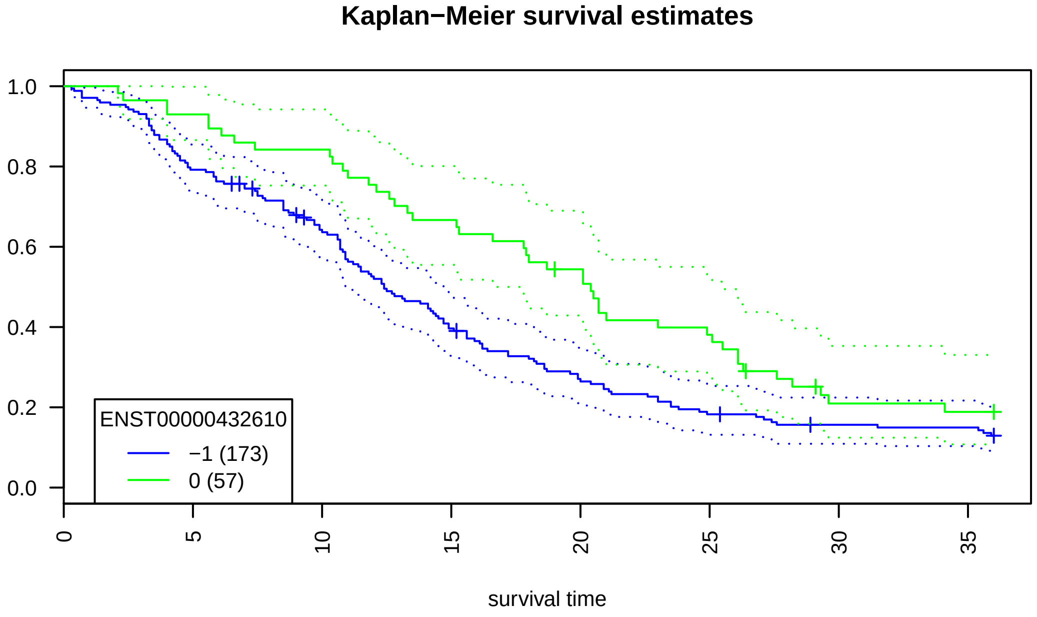

Figure 565: Each curve represents the relative number of ENST00000432610 survived to the corresponding point of time. Dashed lines represent 0.95 confidence intervals for the curves with the corresponding color. Log-rank probability measure for the equality of these curves is 8.936648e-03. Additional annotations: *.GeneId*=ENSG00000150867, *.GeneName*=PIP5K2A, *.GeneDesc*=phosphatidylinositol-5-phosphate 4-kinase, type II, alpha [Source:HGNC Symbol;Acc:8997].

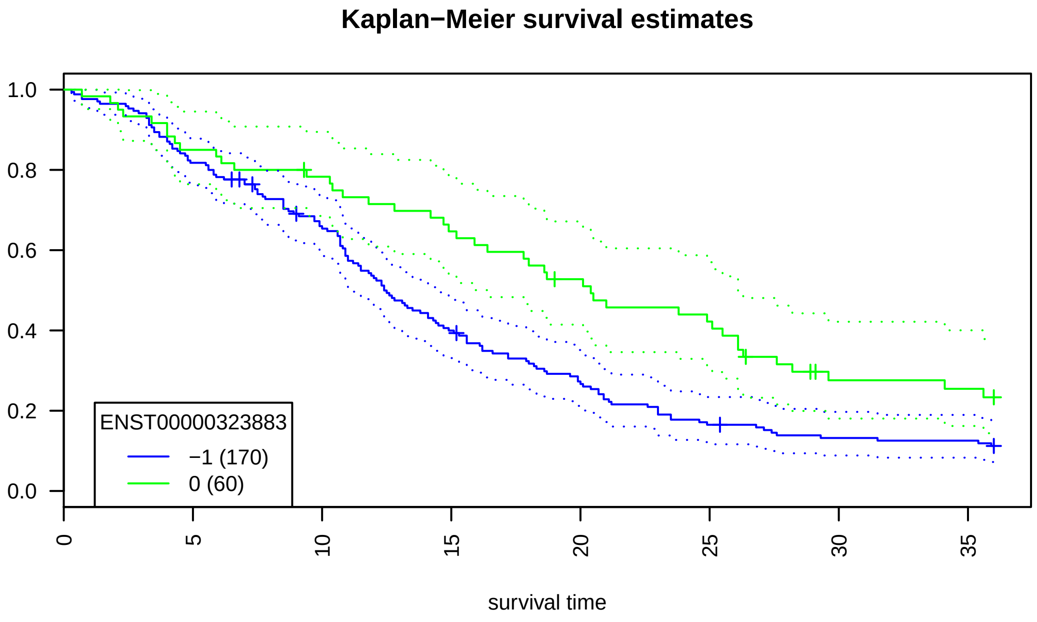

Figure 566: Each curve represents the relative number of ENST00000323883 survived to the corresponding point of time. Dashed lines represent 0.95 confidence intervals for the curves with the corresponding color. Log-rank probability measure for the equality of these curves is 1.822262e-03. Additional annotations: *.GeneId*=ENSG00000150867, *.GeneName*=PIP5K2A, *.GeneDesc*=phosphatidylinositol-5-phosphate 4-kinase, type II, alpha [Source:HGNC Symbol;Acc:8997].

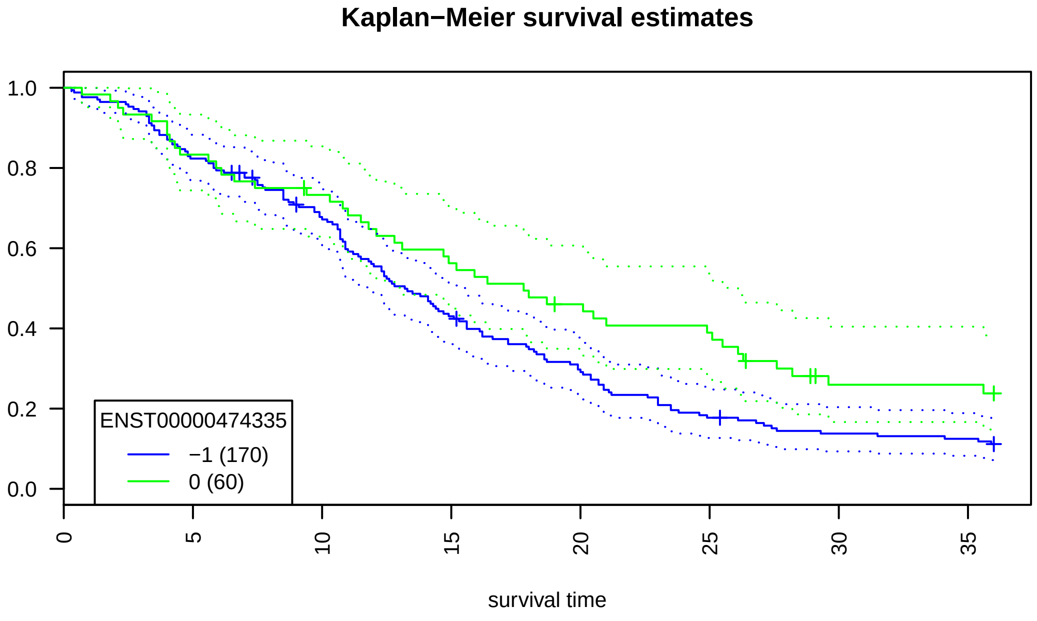

Figure 567: Each curve represents the relative number of ENST00000474335 survived to the corresponding point of time. Dashed lines represent 0.95 confidence intervals for the curves with the corresponding color. Log-rank probability measure for the equality of these curves is 1.855299e-02. Additional annotations: *.GeneId*=ENSG00000150867, *.GeneName*=PIP5K2A, *.GeneDesc*=phosphatidylinositol-5-phosphate 4-kinase, type II, alpha [Source:HGNC Symbol;Acc:8997].

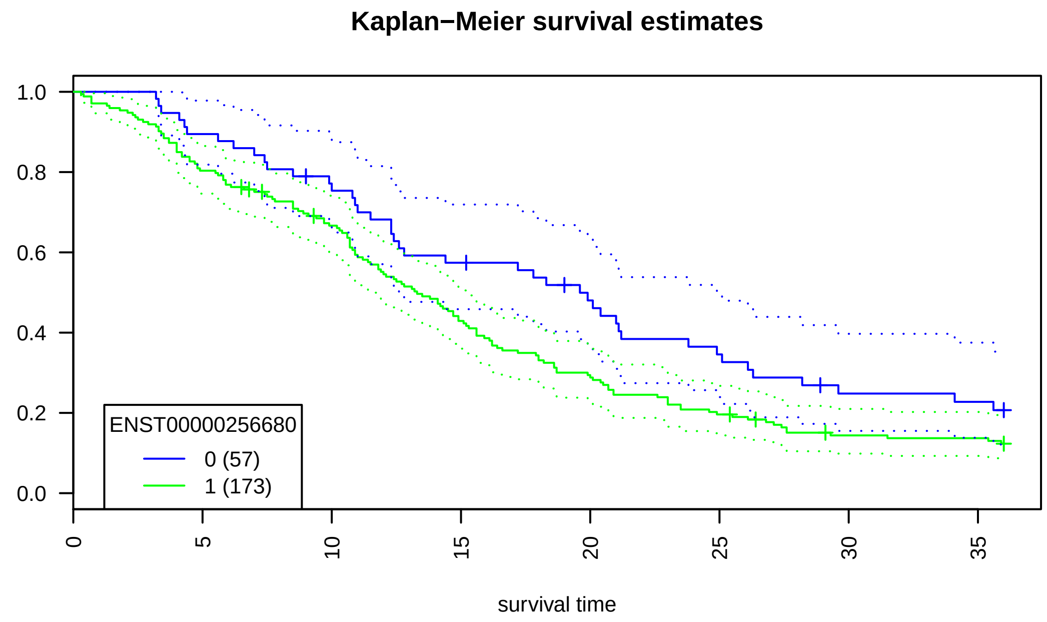

Figure 568: Each curve represents the relative number of ENST00000256680 survived to the corresponding point of time. Dashed lines represent 0.95 confidence intervals for the curves with the corresponding color. Log-rank probability measure for the equality of these curves is 2.602194e-02. Additional annotations: *.GeneId*=ENSG00000134285, *.GeneName*=FKBP11, *.GeneDesc*=FK506 binding protein 11, 19 kDa [Source:HGNC Symbol;Acc:18624].

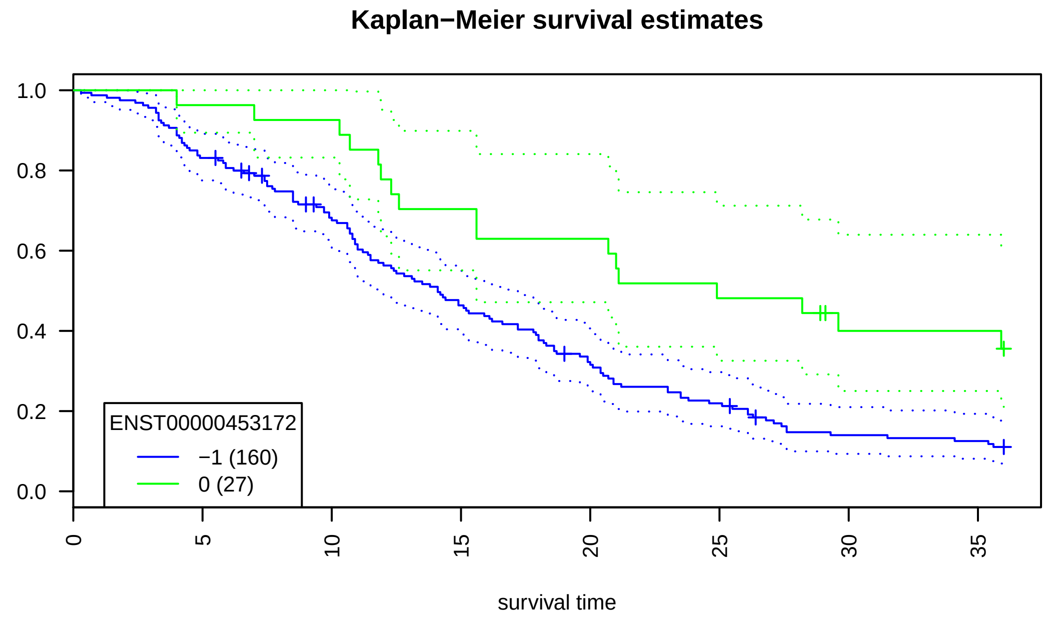

Figure 569: Each curve represents the relative number of ENST00000453172 survived to the corresponding point of time. Dashed lines represent 0.95 confidence intervals for the curves with the corresponding color. Total of 43 observations deleted due to missingness. Log-rank probability measure for the equality of these curves is 1.421899e-03. Additional annotations: *.GeneId*=ENSG00000134285, *.GeneName*=FKBP11, *.GeneDesc*=FK506 binding protein 11, 19 kDa [Source:HGNC Symbol;Acc:18624].

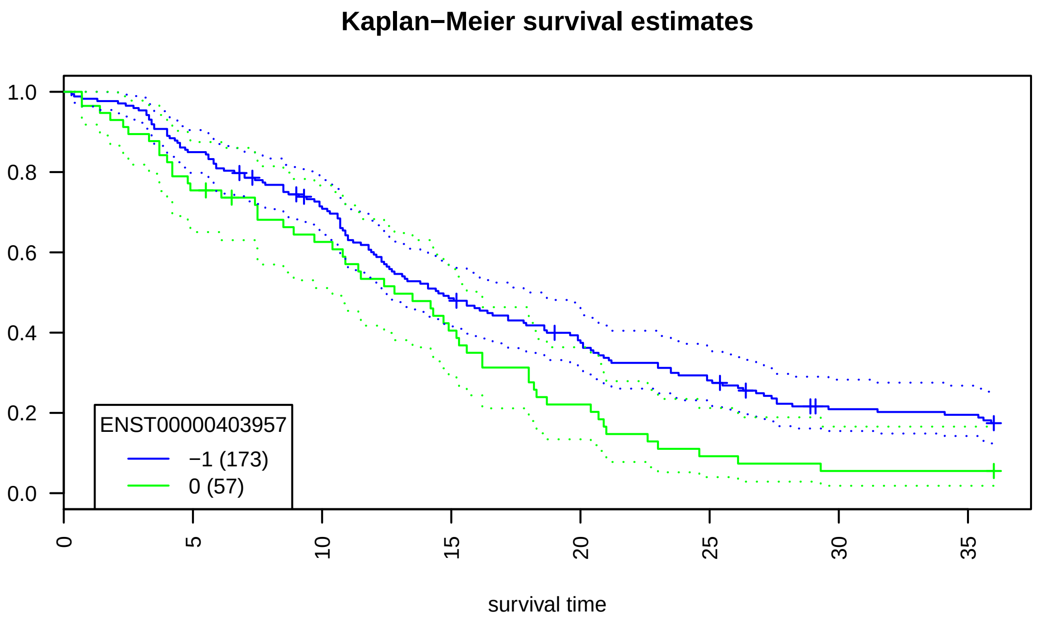

Figure 570: Each curve represents the relative number of ENST00000403957 survived to the corresponding point of time. Dashed lines represent 0.95 confidence intervals for the curves with the corresponding color. Log-rank probability measure for the equality of these curves is 8.264817e-03. Additional annotations: *.GeneId*=ENSG00000169884, *.GeneName*=WNT10B, *.GeneDesc*=wingless-type MMTV integration site family, member 10B [Source:HGNC Symbol;Acc:12775].

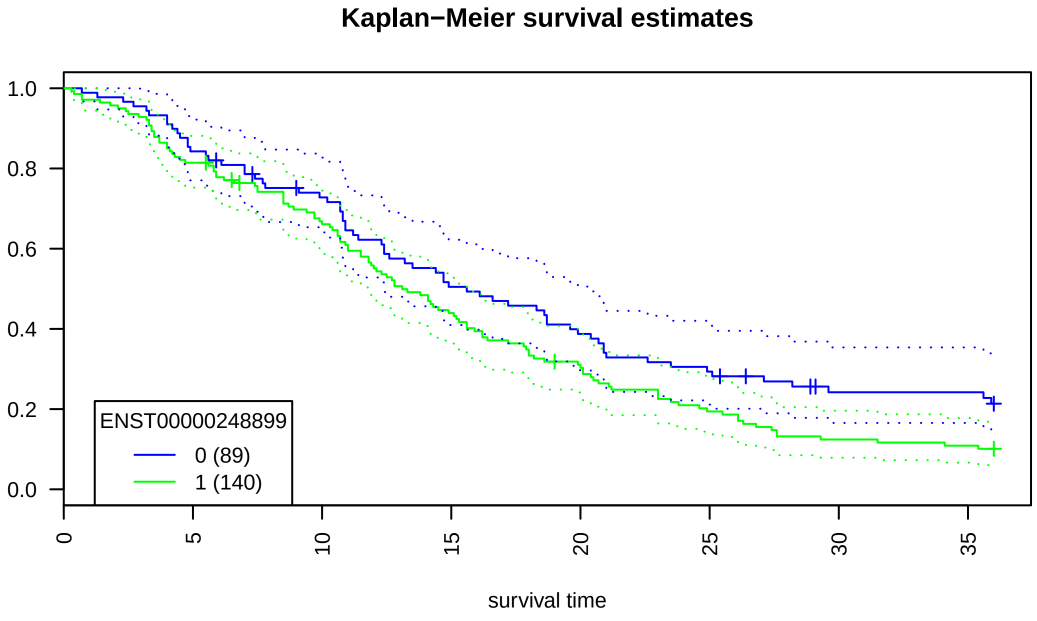

Figure 571: Each curve represents the relative number of ENST00000248899 survived to the corresponding point of time. Dashed lines represent 0.95 confidence intervals for the curves with the corresponding color. Total of 1 observation deleted due to missingness. Log-rank probability measure for the equality of these curves is 3.856396e-02. Additional annotations: *.GeneId*=ENSG00000100365, *.GeneName*=NCF4, *.GeneDesc*=neutrophil cytosolic factor 4, 40kDa [Source:HGNC Symbol;Acc:7662].

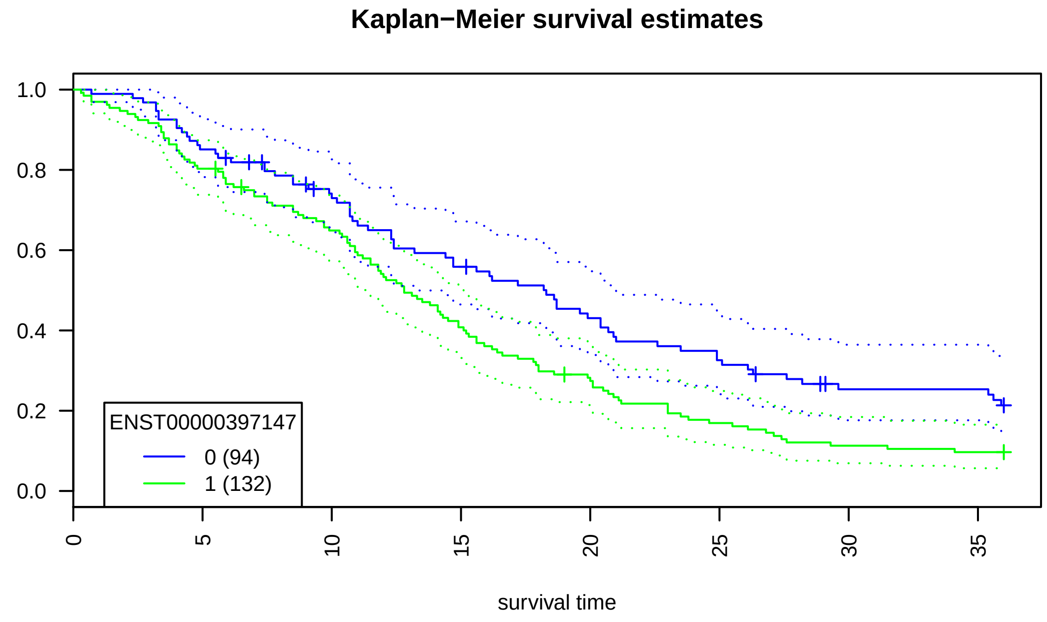

Figure 572: Each curve represents the relative number of ENST00000397147 survived to the corresponding point of time. Dashed lines represent 0.95 confidence intervals for the curves with the corresponding color. Total of 4 observations deleted due to missingness. Log-rank probability measure for the equality of these curves is 4.752384e-03. Additional annotations: *.GeneId*=ENSG00000100365, *.GeneName*=NCF4, *.GeneDesc*=neutrophil cytosolic factor 4, 40kDa [Source:HGNC Symbol;Acc:7662].

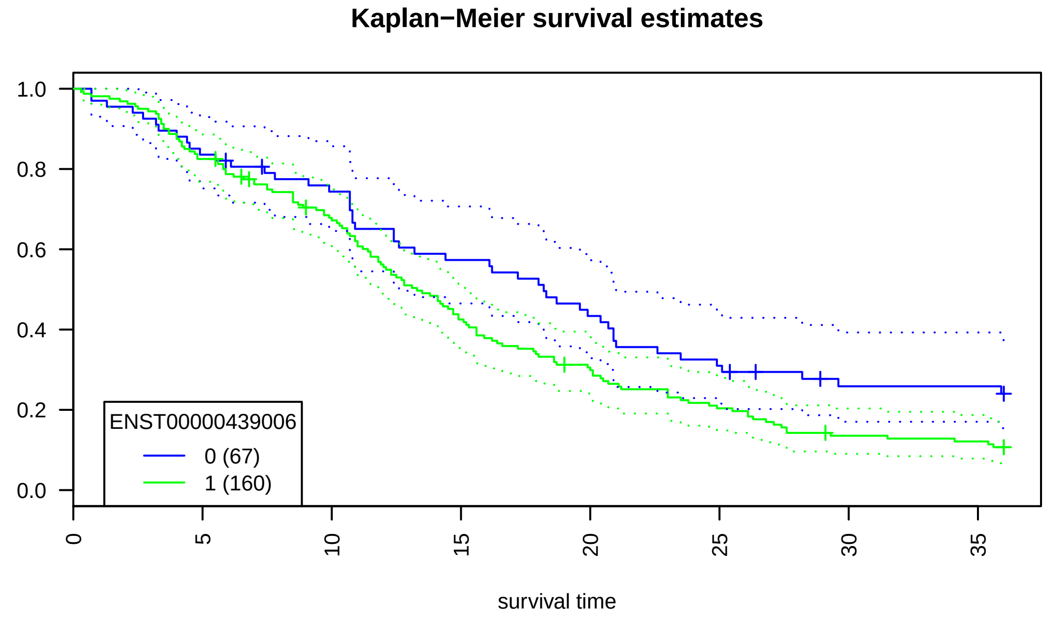

Figure 573: Each curve represents the relative number of ENST00000439006 survived to the corresponding point of time. Dashed lines represent 0.95 confidence intervals for the curves with the corresponding color. Total of 3 observations deleted due to missingness. Log-rank probability measure for the equality of these curves is 2.238076e-02. Additional annotations: *.GeneId*=ENSG00000100365, *.GeneName*=NCF4, *.GeneDesc*=neutrophil cytosolic factor 4, 40kDa [Source:HGNC Symbol;Acc:7662].

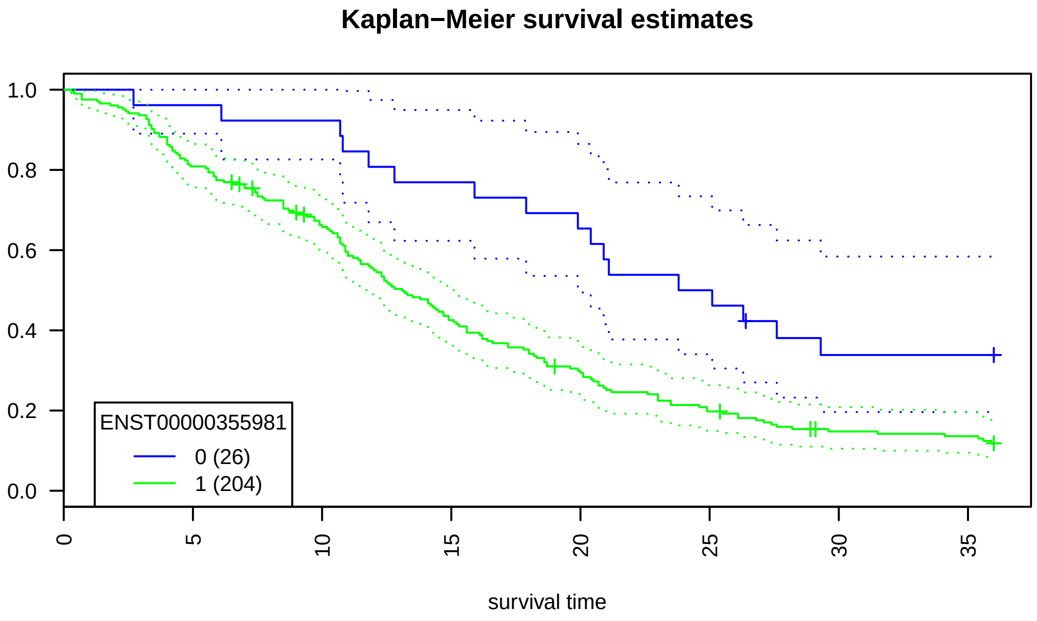

Figure 574: Each curve represents the relative number of ENST00000355981 survived to the corresponding point of time. Dashed lines represent 0.95 confidence intervals for the curves with the corresponding color. Log-rank probability measure for the equality of these curves is 1.562658e-03. Additional annotations: $.GeneId$=ENSG00000198558, $.GeneName$=HIST1H4L, $.GeneDesc$=histone cluster 1, H4l [Source:HGNC Symbol;Acc:4791].

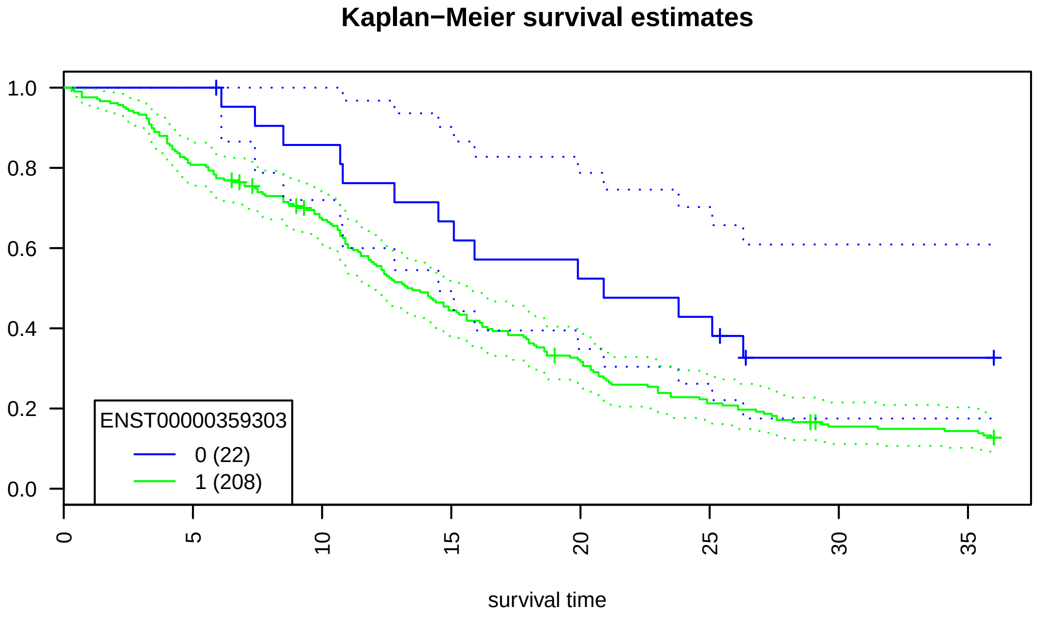

Figure 575: Each curve represents the relative number of ENST00000359303 survived to the corresponding point of time. Dashed lines represent 0.95 confidence intervals for the curves with the corresponding color. Log-rank probability measure for the equality of these curves is 2.649082e-02. Additional annotations: $.GeneId$=ENSG00000197153, $.GeneName$=HIST1H3J, $.GeneDesc$=histone cluster 1, H3j [Source:HGNC Symbol;Acc:4774].

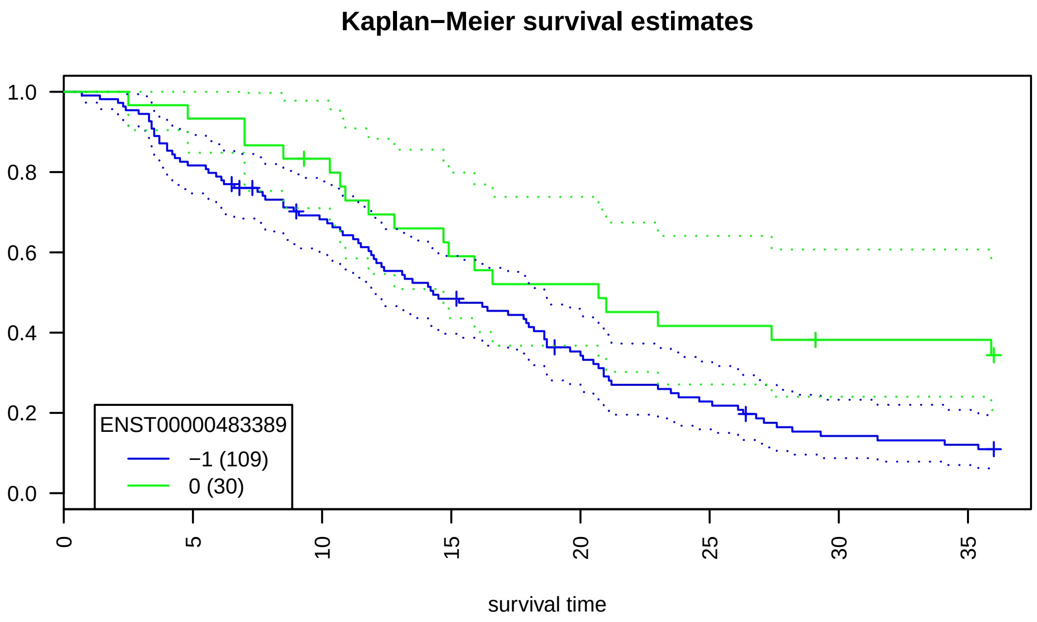

Figure 576: Each curve represents the relative number of ENST00000483389 survived to the corresponding point of time. Dashed lines represent 0.95 confidence intervals for the curves with the corresponding color. Total of 91 observations deleted due to missingness. Log-rank probability measure for the equality of these curves is 1.23884e-02. Additional annotations: *.GeneId*=ENSG00000100379, *.GeneName*=KCTD17, *.GeneDesc*=potassium channel tetramerisation domain containing 17 [Source:HGNC Symbol;Acc:25705].

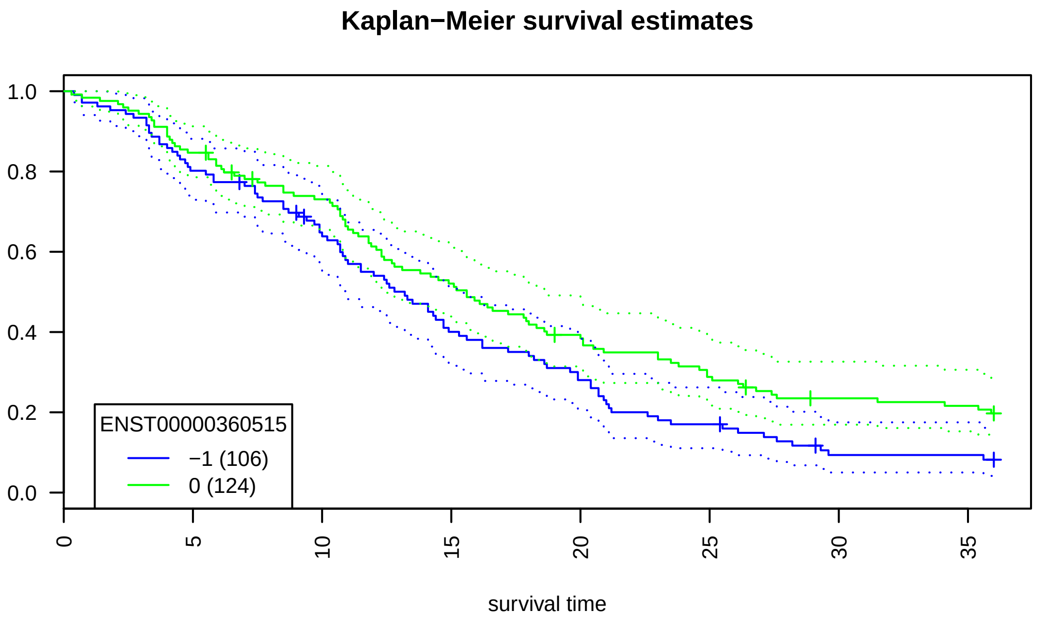

Figure 577: Each curve represents the relative number of ENST00000360515 survived to the corresponding point of time. Dashed lines represent 0.95 confidence intervals for the curves with the corresponding color. Log-rank probability measure for the equality of these curves is 1.945491e-02. Additional annotations: *.GeneId*=ENSG00000158985, *.GeneName*=CDC42SE2, *.GeneDesc*=CDC42 small effector 2 [Source:HGNC Symbol;Acc:18547].

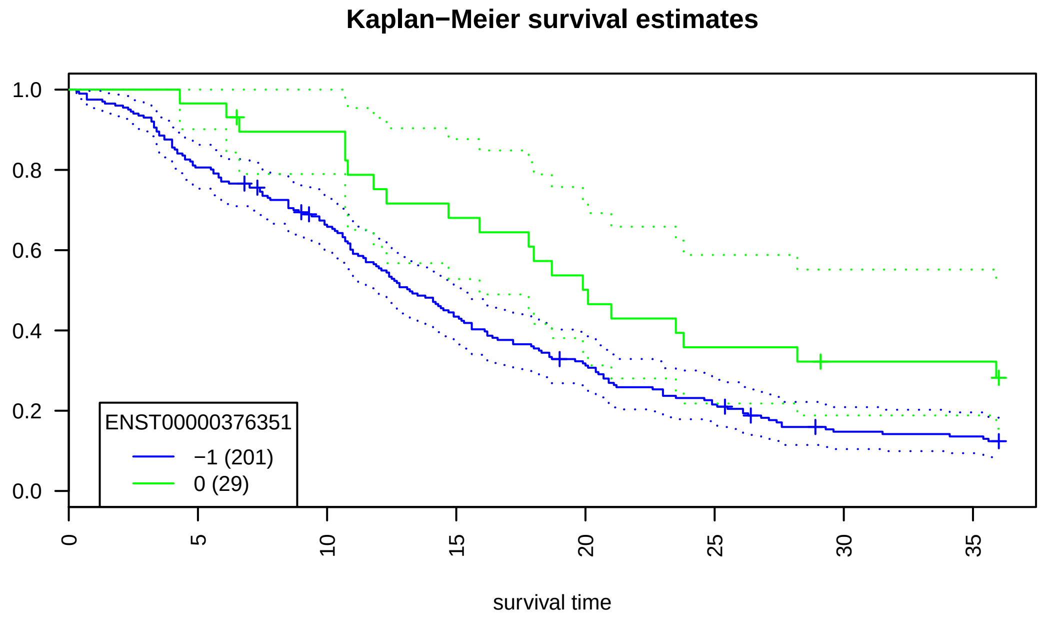

Figure 578: Each curve represents the relative number of ENST00000376351 survived to the corresponding point of time. Dashed lines represent 0.95 confidence intervals for the curves with the corresponding color. Log-rank probability measure for the equality of these curves is 1.448301e-02. Additional annotations: *.GeneId*=ENSG00000151025, *.GeneName*=GPR158, *.GeneDesc*=G protein-coupled receptor 158 [Source:HGNC Symbol;Acc:23689].

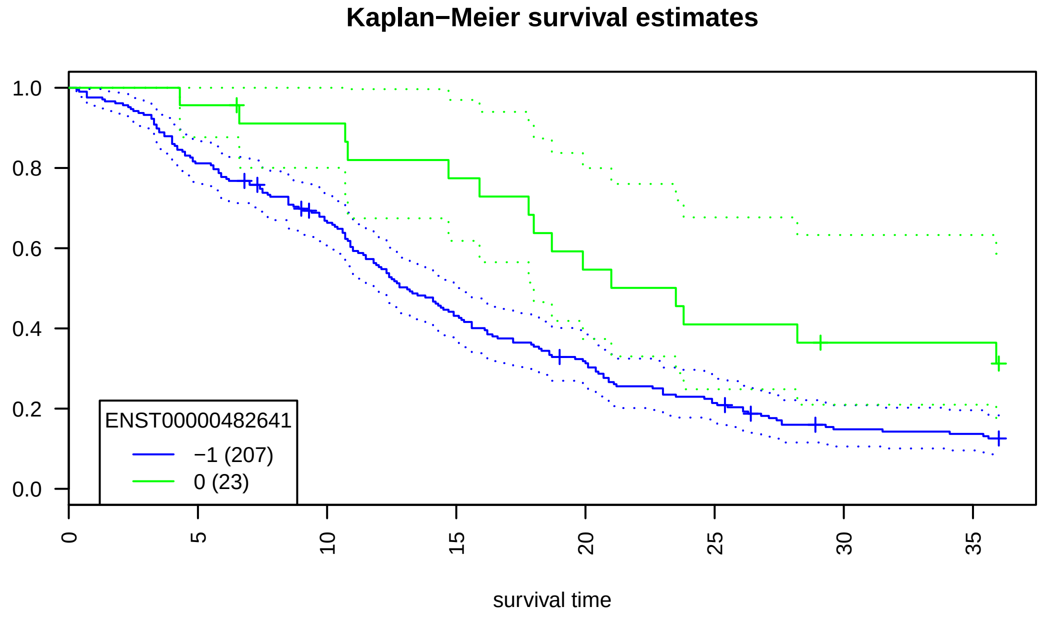

Figure 579: Each curve represents the relative number of ENST00000482641 survived to the corresponding point of time. Dashed lines represent 0.95 confidence intervals for the curves with the corresponding color. Log-rank probability measure for the equality of these curves is 7.92818e-03. Additional annotations: *.GeneId*=ENSG00000151025, *.GeneName*=GPR158, *.GeneDesc*=G protein-coupled receptor 158 [Source:HGNC Symbol;Acc:23689].

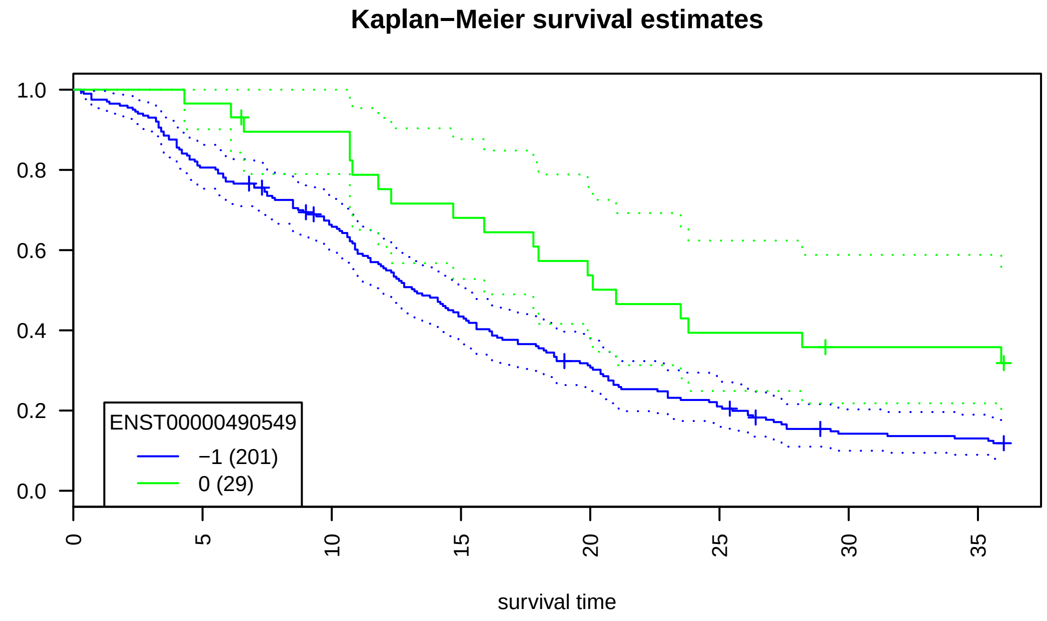

Figure 580: Each curve represents the relative number of ENST00000490549 survived to the corresponding point of time. Dashed lines represent 0.95 confidence intervals for the curves with the corresponding color. Log-rank probability measure for the equality of these curves is 5.331831e-03. Additional annotations: *.GeneId*=ENSG00000151025, *.GeneName*=GPR158, *.GeneDesc*=G protein-coupled receptor 158 [Source:HGNC Symbol;Acc:23689].

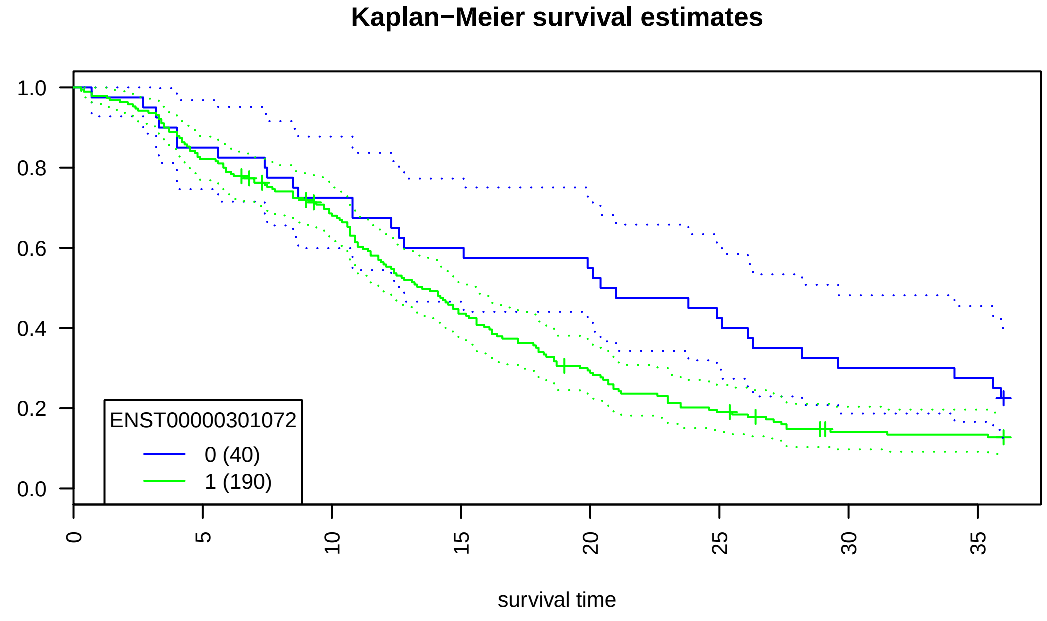

Figure 581: Each curve represents the relative number of ENST00000301072 survived to the corresponding point of time. Dashed lines represent 0.95 confidence intervals for the curves with the corresponding color. Log-rank probability measure for the equality of these curves is 2.799661e-02. Additional annotations: *.GeneId*=ENSG00000167553, *.GeneName*=TUBA1C, *.GeneDesc*=tubulin, alpha 1c [Source:HGNC Symbol;Acc:20768].

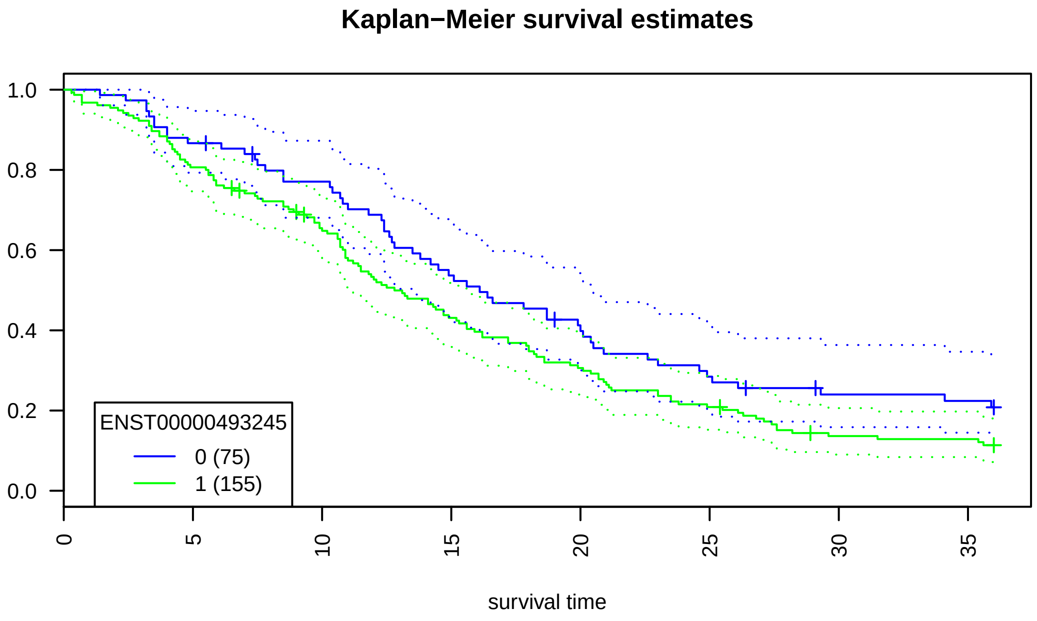

Figure 582: Each curve represents the relative number of ENST00000493245 survived to the corresponding point of time. Dashed lines represent 0.95 confidence intervals for the curves with the corresponding color. Log-rank probability measure for the equality of these curves is 4.739236e-02. Additional annotations: *.GeneId*=ENSG00000107159, *.GeneName*=CA9, *.GeneDesc*=carbonic anhydrase IX [Source:HGNC Symbol;Acc:1383].

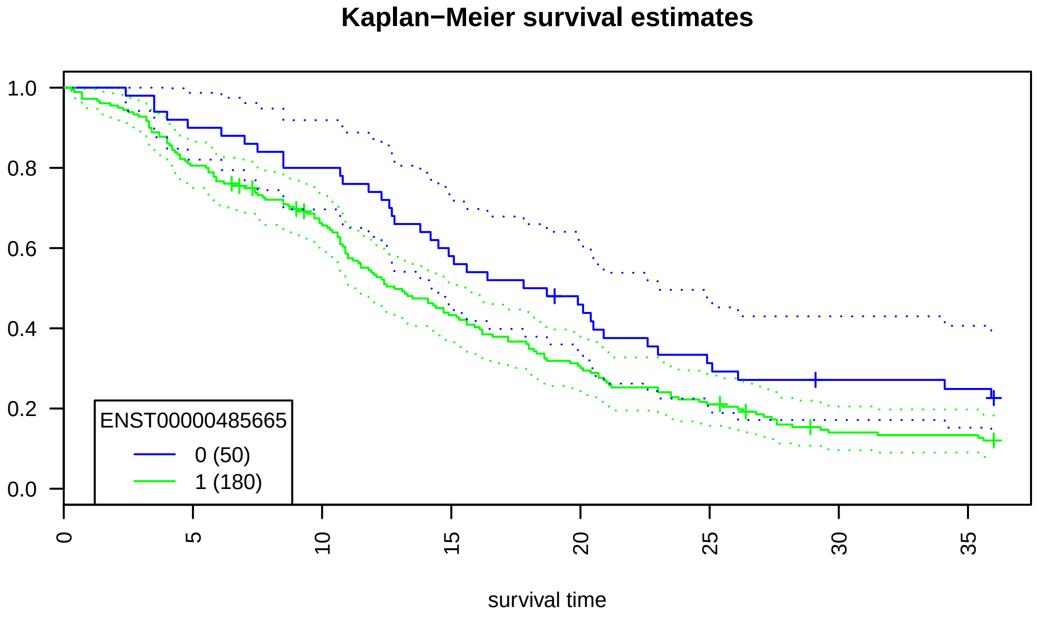

Figure 583: Each curve represents the relative number of ENST00000485665 survived to the corresponding point of time. Dashed lines represent 0.95 confidence intervals for the curves with the corresponding color. Log-rank probability measure for the equality of these curves is 2.802245e-02. Additional annotations: *.GeneId*=ENSG00000107159, *.GeneName*=CA9, *.GeneDesc*=carbonic anhydrase IX [Source:HGNC Symbol;Acc:1383].

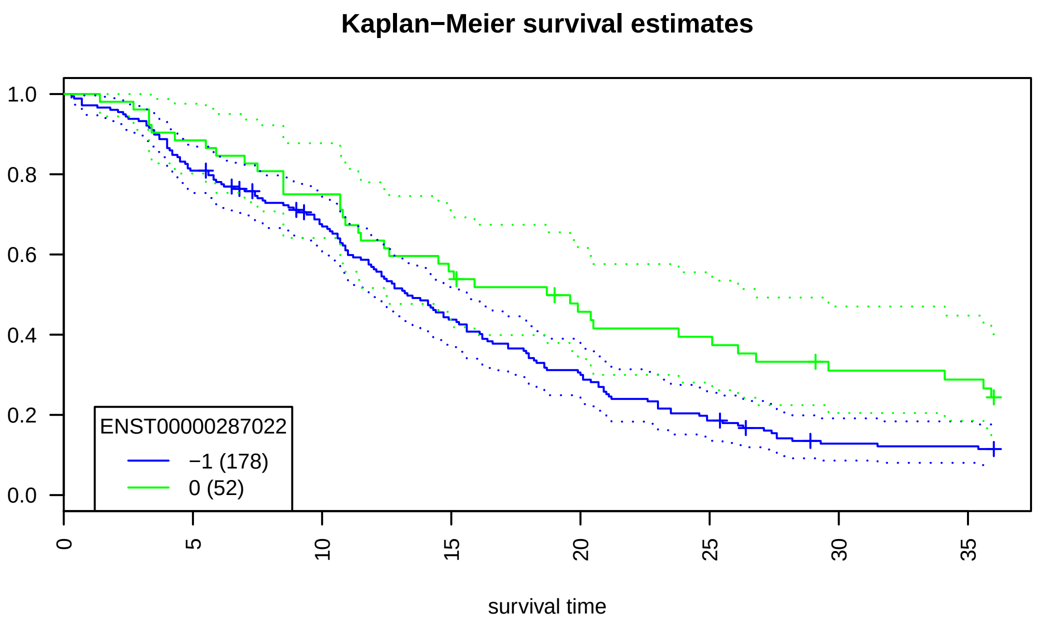

Figure 584: Each curve represents the relative number of ENST00000287022 survived to the corresponding point of time. Dashed lines represent 0.95 confidence intervals for the curves with the corresponding color. Log-rank probability measure for the equality of these curves is 1.557249e-02. Additional annotations: *.GeneId*=ENSG00000156467, *.GeneName*=UQCRB, *.GeneDesc*=ubiquinol-cytochrome c reductase binding protein [Source:HGNC Symbol;Acc:12582].

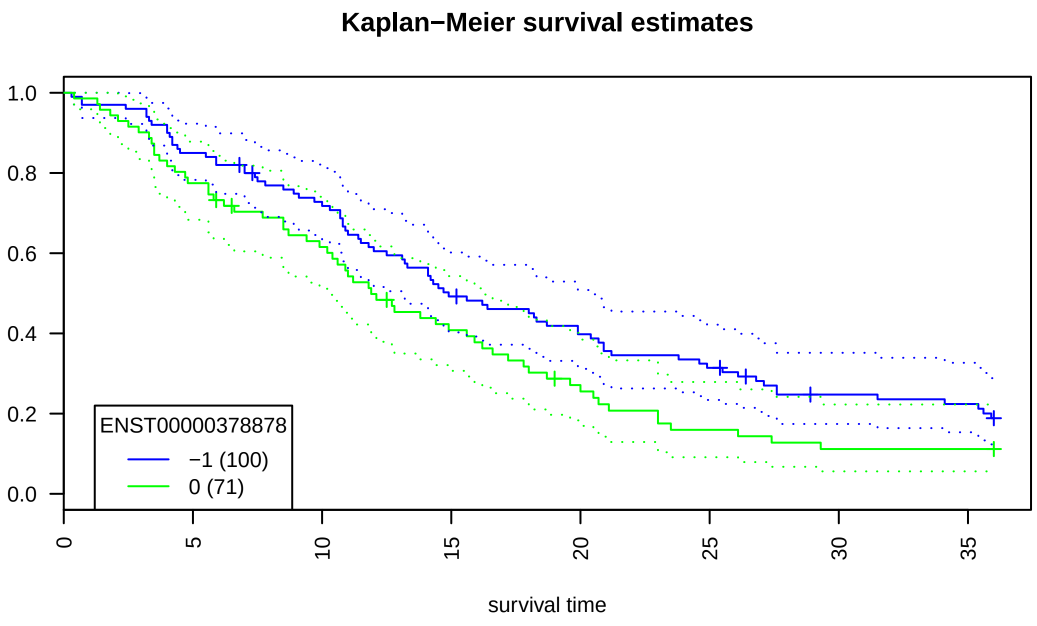

Figure 585: Each curve represents the relative number of ENST00000378878 survived to the corresponding point of time. Dashed lines represent 0.95 confidence intervals for the curves with the corresponding color. Total of 59 observations deleted due to missingness. Log-rank probability measure for the equality of these curves is 3.968371e-02. Additional annotations: *.GeneId*=ENSG00000149090, *.GeneName*=PAMR1, *.GeneDesc*=peptidase domain containing associated with muscle regeneration 1 [Source:HGNC Symbol;Acc:24554].

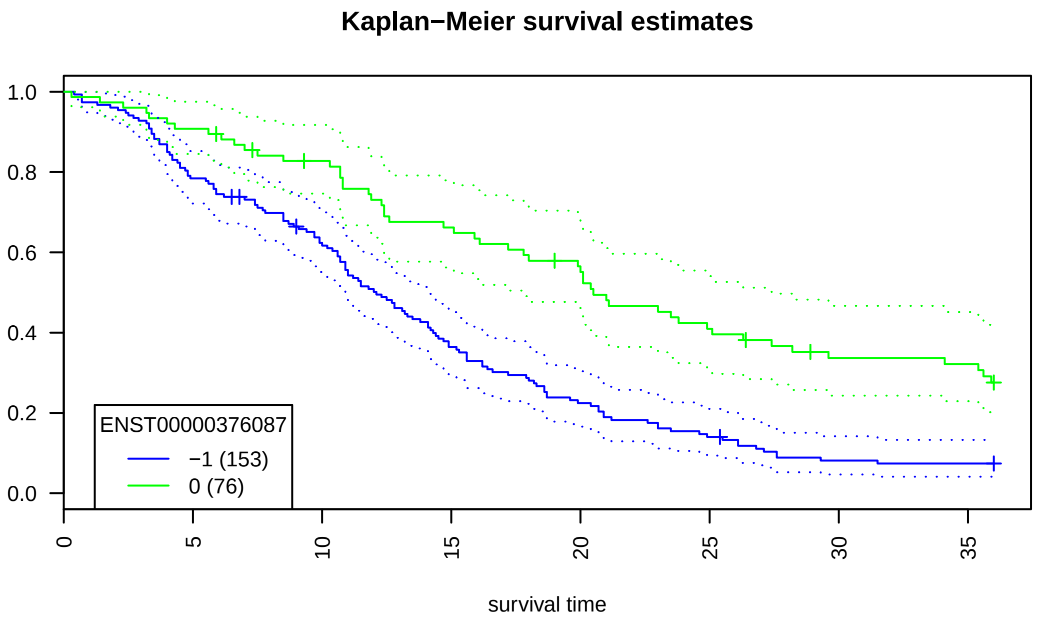

Figure 586: Each curve represents the relative number of ENST00000376087 survived to the corresponding point of time. Dashed lines represent 0.95 confidence intervals for the curves with the corresponding color. Total of 1 observation deleted due to missingness. Log-rank probability measure for the equality of these curves is 1.450819e-06. Additional annotations: *.GeneId*=ENSG00000107890, *.GeneName*=ANKRD26, *.GeneDesc*=ankyrin repeat domain 26 [Source:HGNC Symbol;Acc:29186].

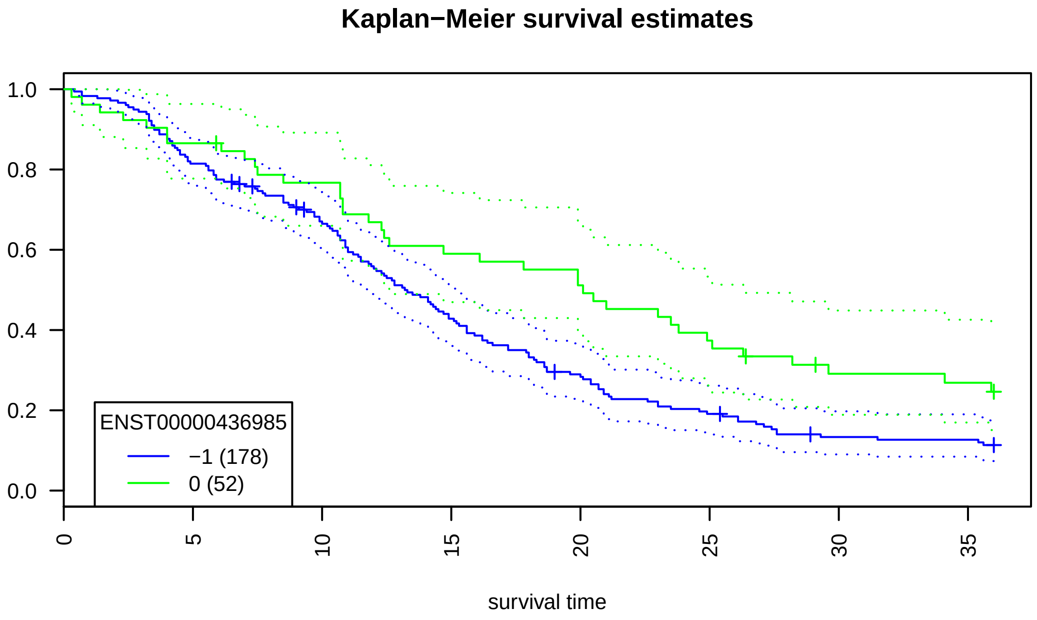

Figure 587: Each curve represents the relative number of ENST00000436985 survived to the corresponding point of time. Dashed lines represent 0.95 confidence intervals for the curves with the corresponding color. Log-rank probability measure for the equality of these curves is 8.342444e-03. Additional annotations: *.GeneId*=ENSG00000107890, *.GeneName*=ANKRD26, *.GeneDesc*=ankyrin repeat domain 26 [Source:HGNC Symbol;Acc:29186].

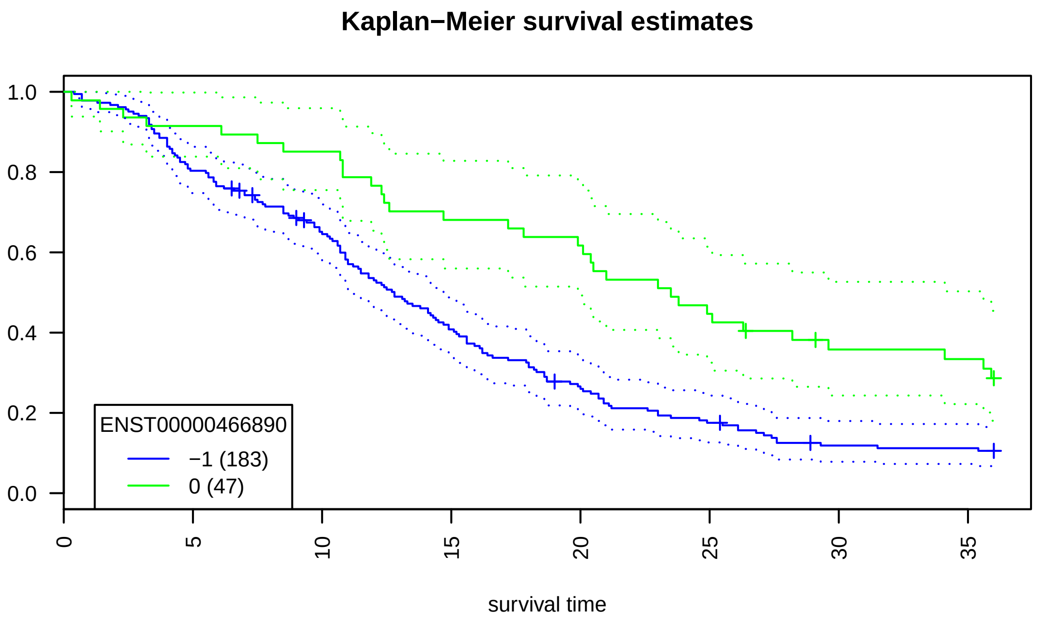

Figure 588: Each curve represents the relative number of ENST00000466890 survived to the corresponding point of time. Dashed lines represent 0.95 confidence intervals for the curves with the corresponding color. Log-rank probability measure for the equality of these curves is 1.275723e-04. Additional annotations: *.GeneId*=ENSG00000107890, *.GeneName*=ANKRD26, *.GeneDesc*=ankyrin repeat domain 26 [Source:HGNC Symbol;Acc:29186].

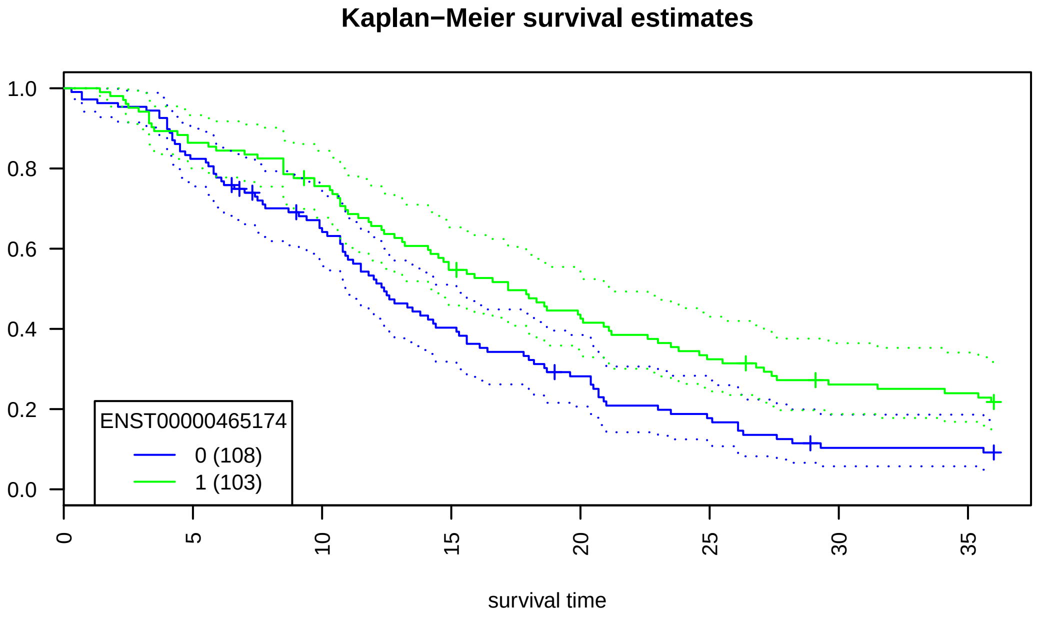

Figure 589: Each curve represents the relative number of ENST00000465174 survived to the corresponding point of time. Dashed lines represent 0.95 confidence intervals for the curves with the corresponding color. Total of 19 observations deleted due to missingness. Log-rank probability measure for the equality of these curves is 3.422011e-03. Additional annotations: *.GeneId*=ENSG00000198176, *.GeneName*=TFDP1, *.GeneDesc*=transcription factor Dp-1 [Source:HGNC Symbol;Acc:11749].

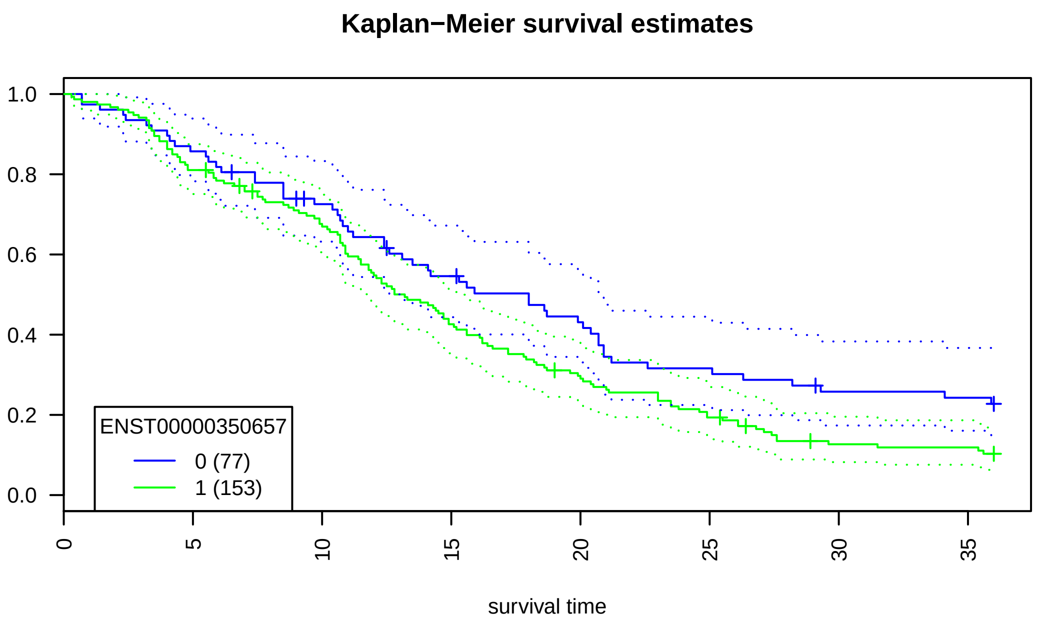

Figure 590: Each curve represents the relative number of ENST00000350657 survived to the corresponding point of time. Dashed lines represent 0.95 confidence intervals for the curves with the corresponding color. Log-rank probability measure for the equality of these curves is 2.683681e-02. Additional annotations: *.GeneId*=ENSG00000180638, *.GeneName*=SLC47A2, *.GeneDesc*=solute carrier family 47, member 2 [Source:HGNC Symbol;Acc:26439].

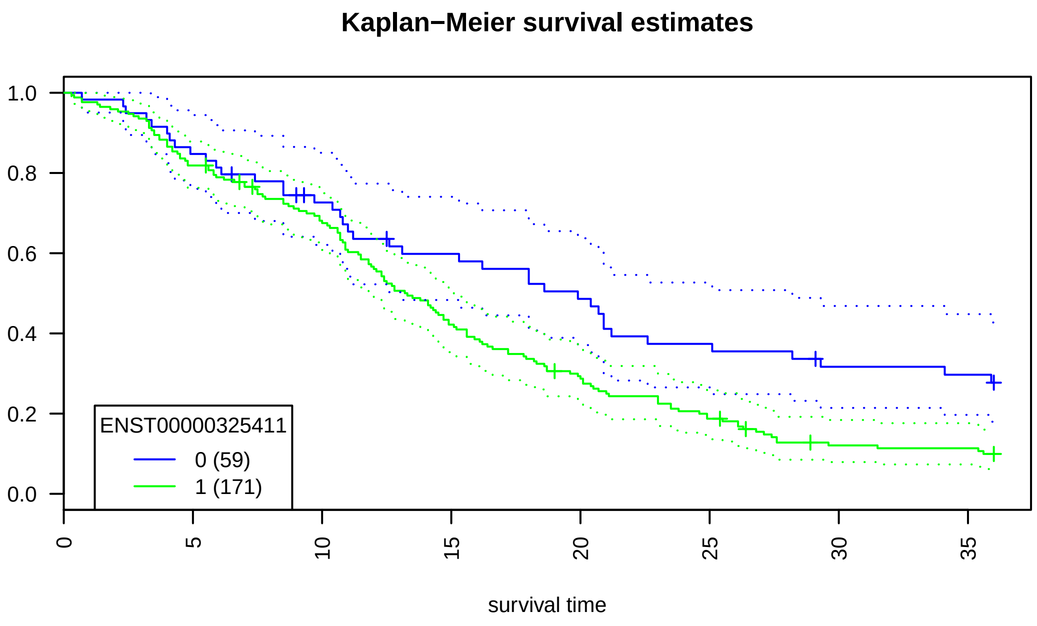

Figure 591: Each curve represents the relative number of ENST00000325411 survived to the corresponding point of time. Dashed lines represent 0.95 confidence intervals for the curves with the corresponding color. Log-rank probability measure for the equality of these curves is 4.400754e-03. Additional annotations: *.GeneId*=ENSG00000180638, *.GeneName*=SLC47A2, *.GeneDesc*=solute carrier family 47, member 2 [Source:HGNC Symbol;Acc:26439].

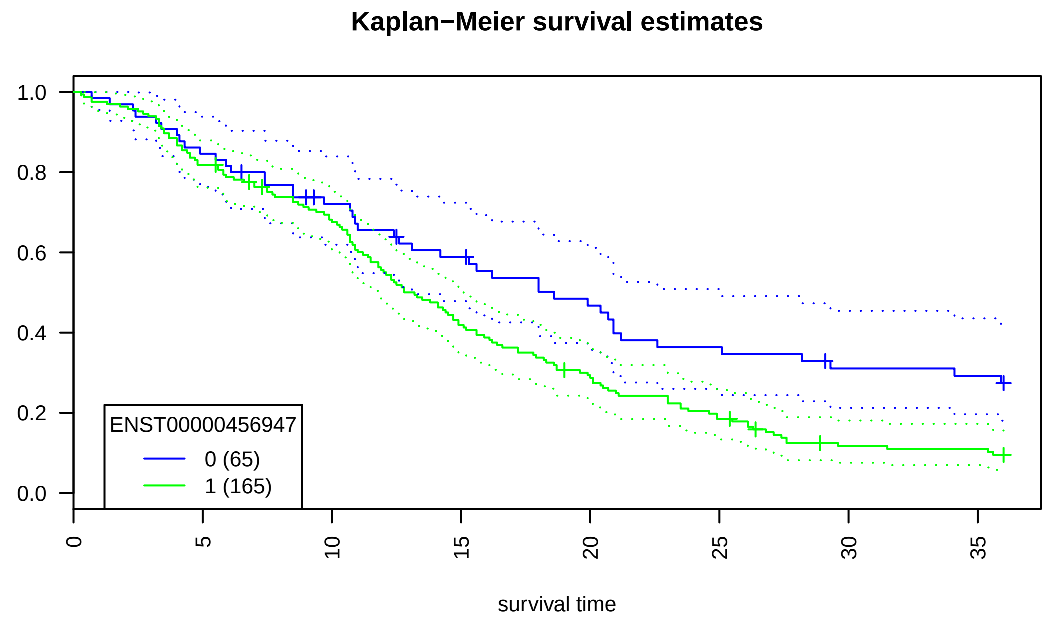

Figure 592: Each curve represents the relative number of ENST00000456947 survived to the corresponding point of time. Dashed lines represent 0.95 confidence intervals for the curves with the corresponding color. Log-rank probability measure for the equality of these curves is 3.819344e-03. Additional annotations: *.GeneId*=ENSG00000180638, *.GeneName*=SLC47A2, *.GeneDesc*=solute carrier family 47, member 2 [Source:HGNC Symbol;Acc:26439].

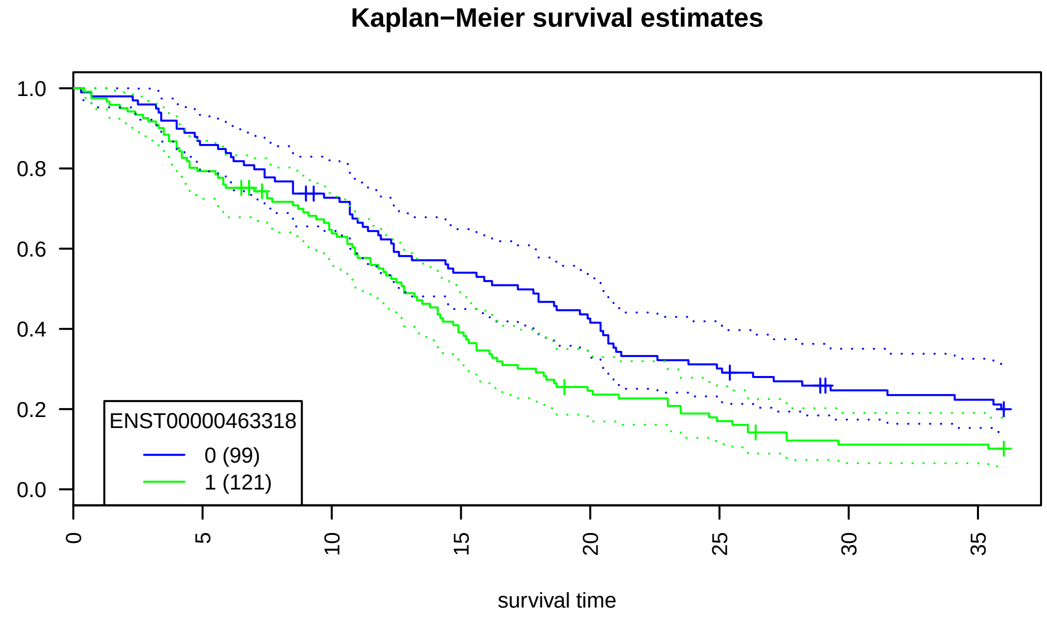

Figure 593: Each curve represents the relative number of ENST00000463318 survived to the corresponding point of time. Dashed lines represent 0.95 confidence intervals for the curves with the corresponding color. Total of 10 observations deleted due to missingness. Log-rank probability measure for the equality of these curves is 1.154647e-02. Additional annotations: *.GeneId*=ENSG00000180638, *.GeneName*=SLC47A2, *.GeneDesc*=solute carrier family 47, member 2 [Source:HGNC Symbol;Acc:26439].

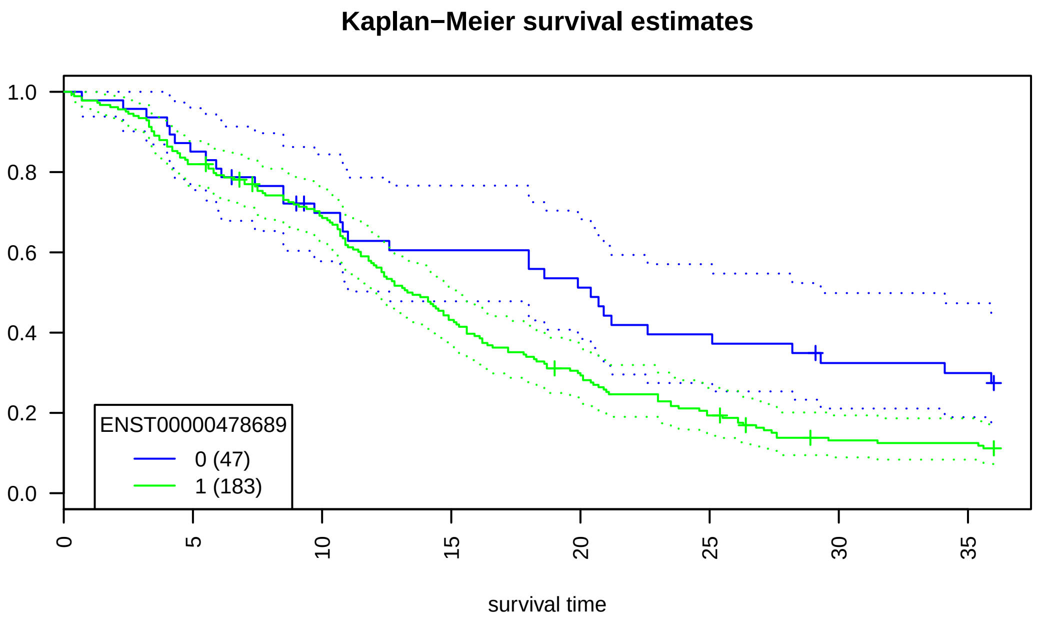

Figure 594: Each curve represents the relative number of ENST00000478689 survived to the corresponding point of time. Dashed lines represent 0.95 confidence intervals for the curves with the corresponding color. Log-rank probability measure for the equality of these curves is 1.083442e-02. Additional annotations: *.GeneId*=ENSG00000180638, *.GeneName*=SLC47A2, *.GeneDesc*=solute carrier family 47, member 2 [Source:HGNC Symbol;Acc:26439].

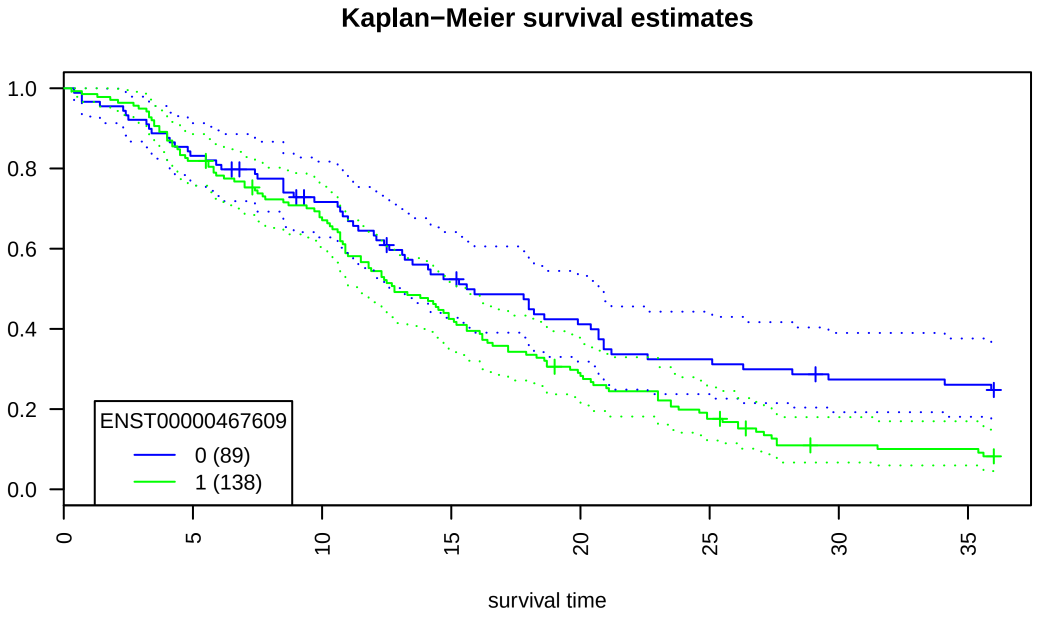

Figure 595: Each curve represents the relative number of ENST00000467609 survived to the corresponding point of time. Dashed lines represent 0.95 confidence intervals for the curves with the corresponding color. Total of 3 observations deleted due to missingness. Log-rank probability measure for the equality of these curves is 8.3293e-03. Additional annotations: *.GeneId*=ENSG00000180638, *.GeneName*=SLC47A2, *.GeneDesc*=solute carrier family 47, member 2 [Source:HGNC Symbol;Acc:26439].

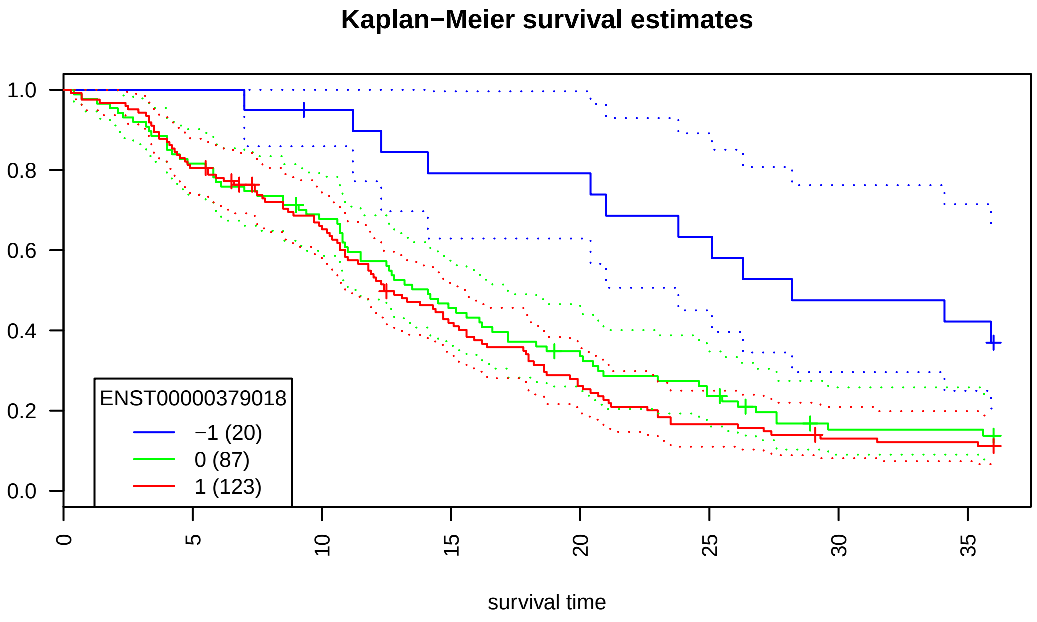

Figure 596: Each curve represents the relative number of ENST00000379018 survived to the corresponding point of time. Dashed lines represent 0.95 confidence intervals for the curves with the corresponding color. Log-rank probability measure for the equality of these curves is 2.842421e-03. Additional annotations: *.GeneId*=ENSG00000131435, *.GeneName*=PDLIM4, *.GeneDesc*=PDZ and LIM domain 4 [Source:HGNC Symbol;Acc:16501].

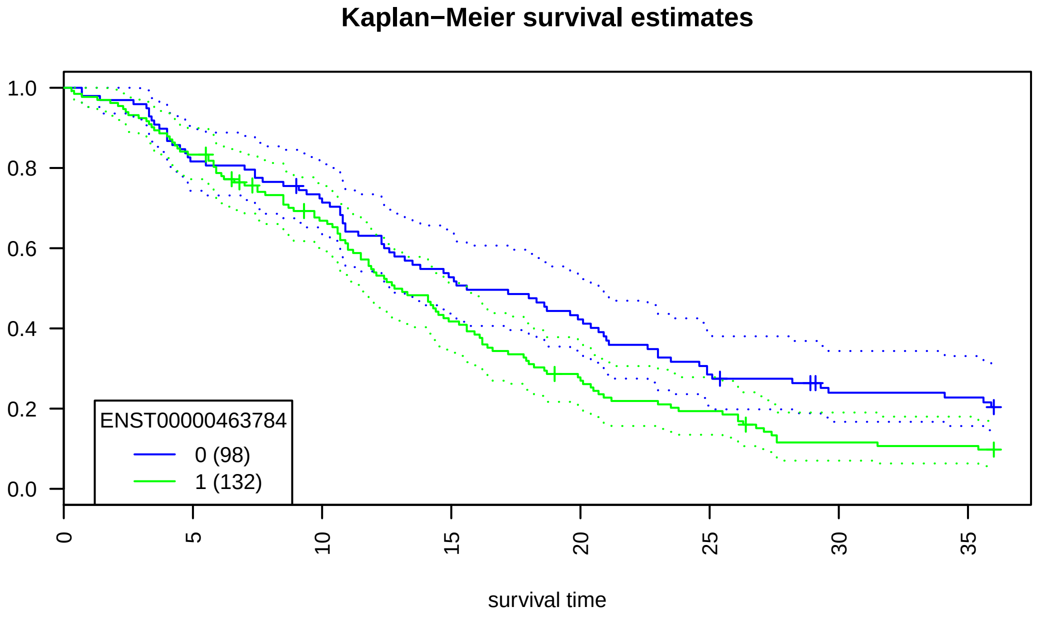

Figure 597: Each curve represents the relative number of ENST00000463784 survived to the corresponding point of time. Dashed lines represent 0.95 confidence intervals for the curves with the corresponding color. Log-rank probability measure for the equality of these curves is 2.04551e-02. Additional annotations: *.GeneId*=ENSG00000125347, *.GeneName*=IRF1, *.GeneDesc*=interferon regulatory factor 1 [Source:HGNC Symbol;Acc:6116].

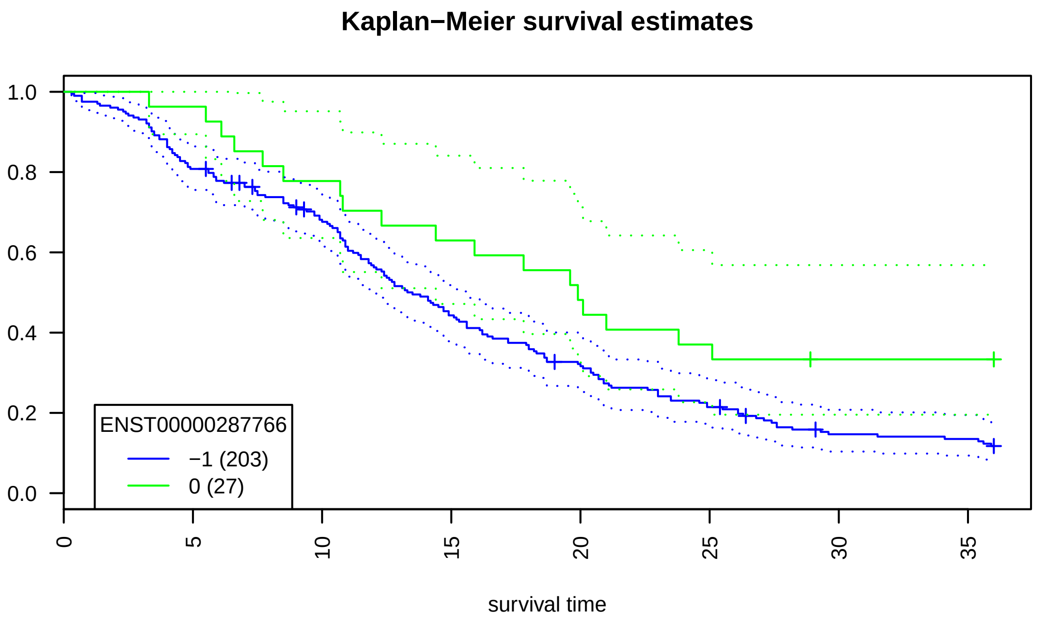

Figure 598: Each curve represents the relative number of ENST00000287766 survived to the corresponding point of time. Dashed lines represent 0.95 confidence intervals for the curves with the corresponding color. Log-rank probability measure for the equality of these curves is 1.958672e-02. Additional annotations: *.GeneId*=ENSG00000157103, *.GeneName*=SLC6A1, *.GeneDesc*=solute carrier family 6 (neurotransmitter transporter, GABA), member 1 [Source:HGNC Symbol;Acc:11042].

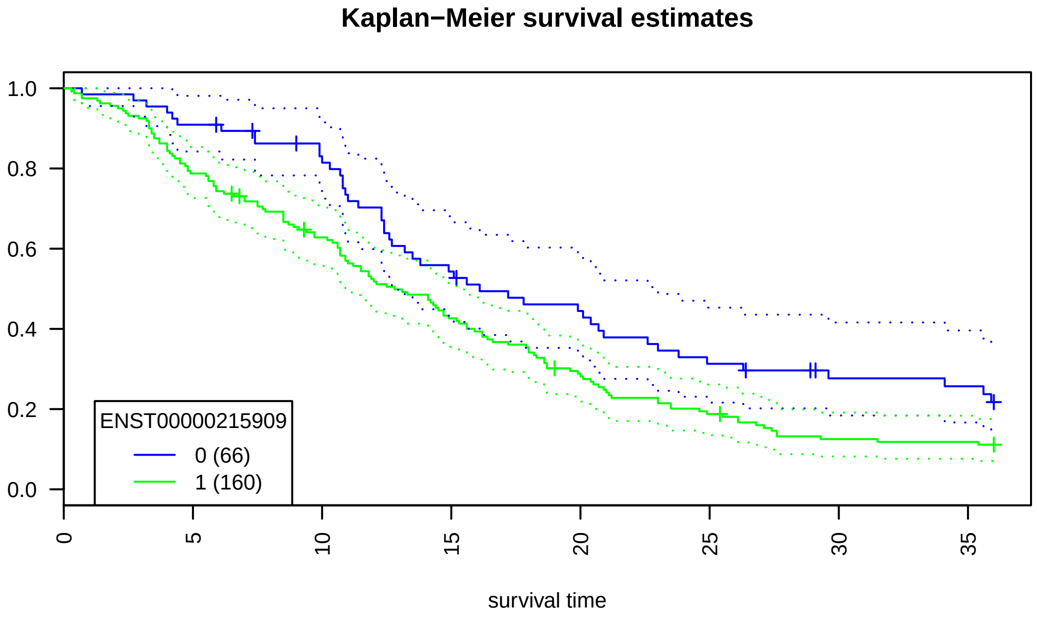

Figure 599: Each curve represents the relative number of ENST00000215909 survived to the corresponding point of time. Dashed lines represent 0.95 confidence intervals for the curves with the corresponding color. Total of 4 observations deleted due to missingness. Log-rank probability measure for the equality of these curves is 1.002088e-02. Additional annotations: *.GeneId*=ENSG00000100097, *.GeneName*=LGALS1, *.GeneDesc*=lectin, galactoside-binding, soluble, 1 [Source:HGNC Symbol;Acc:6561].

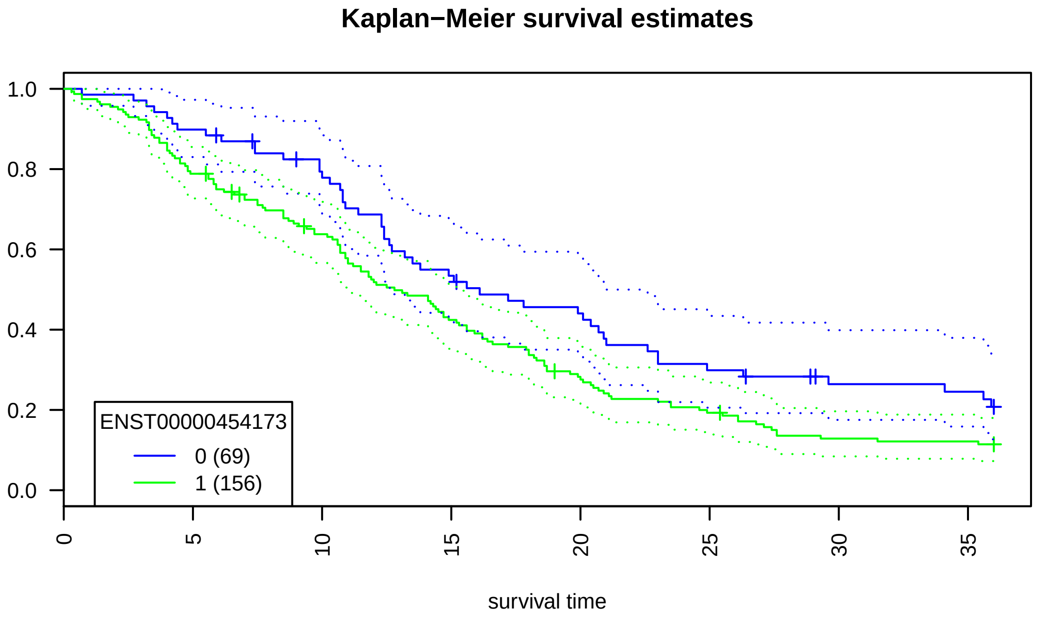

Figure 600: Each curve represents the relative number of ENST00000454173 survived to the corresponding point of time. Dashed lines represent 0.95 confidence intervals for the curves with the corresponding color. Total of 5 observations deleted due to missingness. Log-rank probability measure for the equality of these curves is 2.005665e-02. Additional annotations: *.GeneId*=ENSG00000100097, *.GeneName*=LGALS1, *.GeneDesc*=lectin, galactoside-binding, soluble, 1 [Source:HGNC Symbol;Acc:6561].

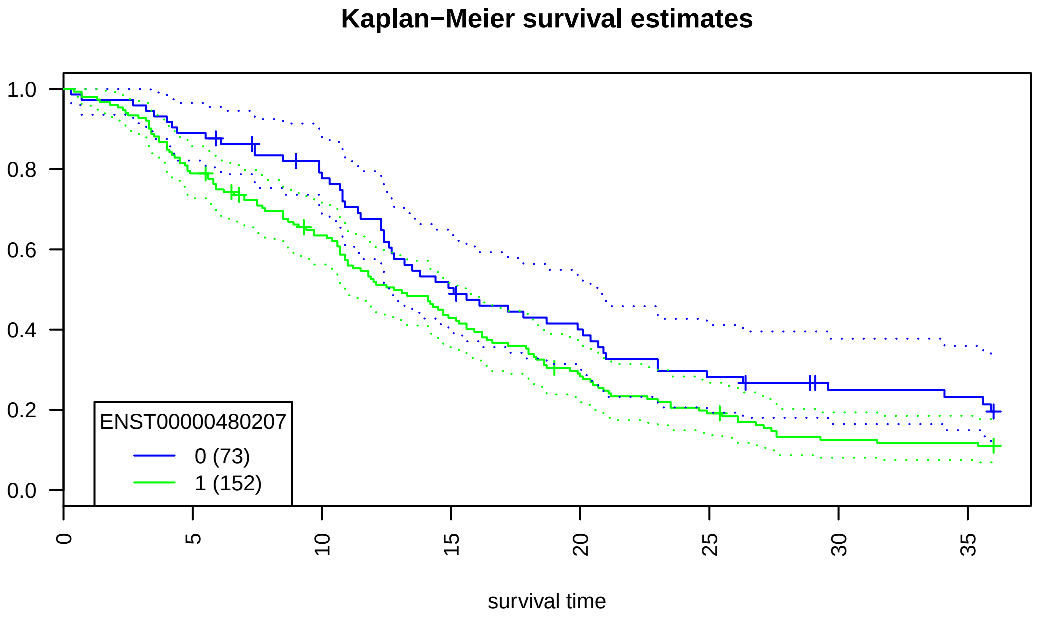

Figure 601: Each curve represents the relative number of ENST00000480207 survived to the corresponding point of time. Dashed lines represent 0.95 confidence intervals for the curves with the corresponding color. Total of 5 observations deleted due to missingness. Log-rank probability measure for the equality of these curves is 3.810649e-02. Additional annotations: *.GeneId*=ENSG00000100097, *.GeneName*=LGALS1, *.GeneDesc*=lectin, galactoside-binding, soluble, 1 [Source:HGNC Symbol;Acc:6561].

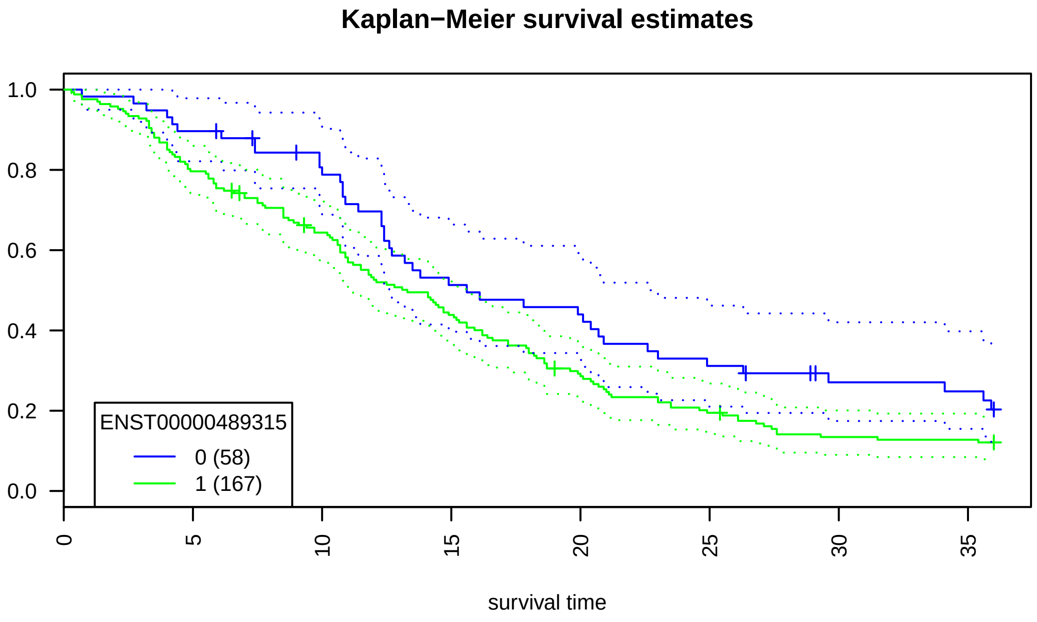

Figure 602: Each curve represents the relative number of ENST00000489315 survived to the corresponding point of time. Dashed lines represent 0.95 confidence intervals for the curves with the corresponding color. Total of 5 observations deleted due to missingness. Log-rank probability measure for the equality of these curves is 3.599486e-02. Additional annotations: *.GeneId*=ENSG00000100097, *.GeneName*=LGALS1, *.GeneDesc*=lectin, galactoside-binding, soluble, 1 [Source:HGNC Symbol;Acc:6561].

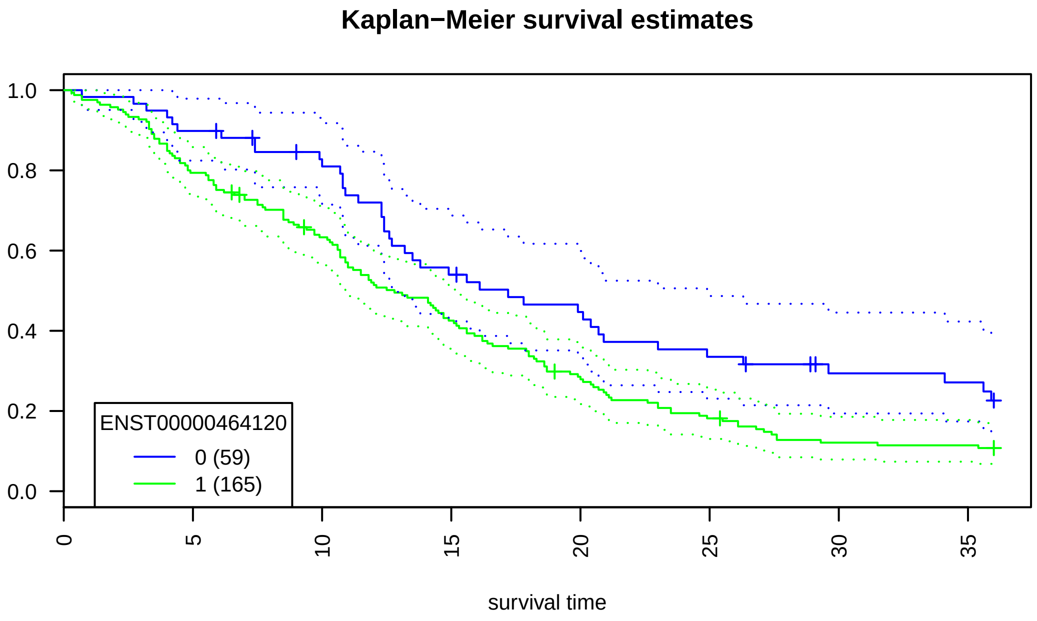

Figure 603: Each curve represents the relative number of ENST00000464120 survived to the corresponding point of time. Dashed lines represent 0.95 confidence intervals for the curves with the corresponding color. Total of 6 observations deleted due to missingness. Log-rank probability measure for the equality of these curves is 8.074061e-03. Additional annotations: *.GeneId*=ENSG00000100097, *.GeneName*=LGALS1, *.GeneDesc*=lectin, galactoside-binding, soluble, 1 [Source:HGNC Symbol;Acc:6561].

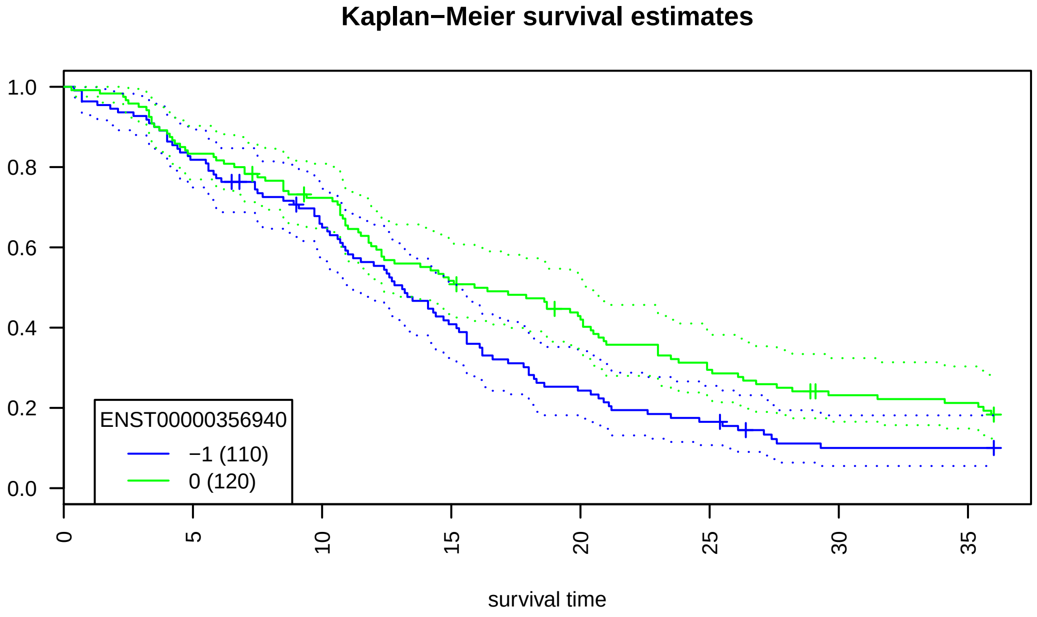

Figure 604: Each curve represents the relative number of ENST00000356940 survived to the corresponding point of time. Dashed lines represent 0.95 confidence intervals for the curves with the corresponding color. Log-rank probability measure for the equality of these curves is 1.808222e-02. Additional annotations: *.GeneId*=ENSG00000099246, *.GeneName*=RAB18, *.GeneDesc*=RAB18, member RAS oncogene family [Source:HGNC Symbol;Acc:14244].

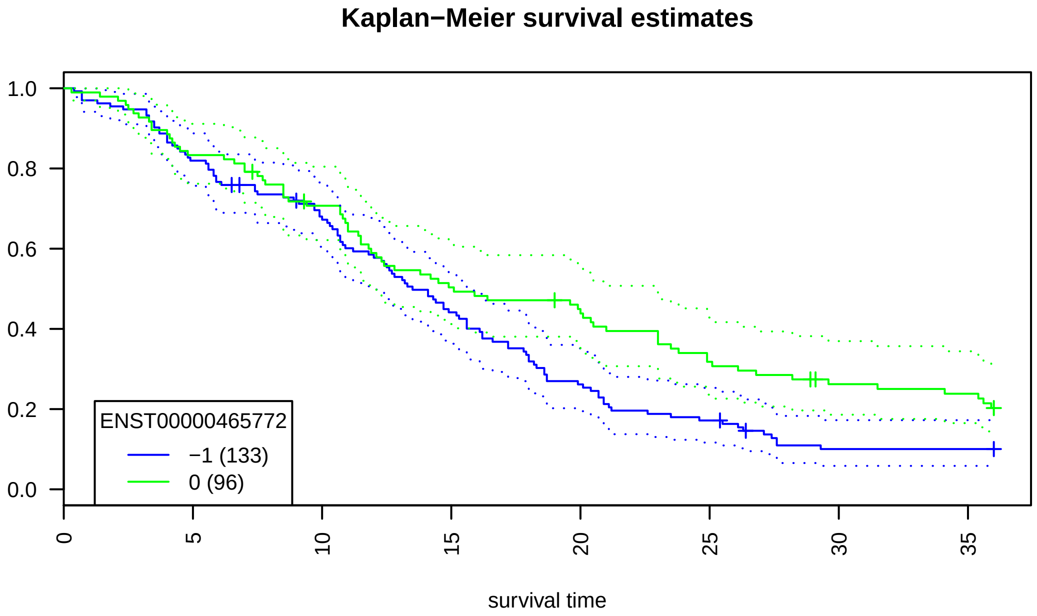

Figure 605: Each curve represents the relative number of ENST00000465772 survived to the corresponding point of time. Dashed lines represent 0.95 confidence intervals for the curves with the corresponding color. Total of 1 observation deleted due to missingness. Log-rank probability measure for the equality of these curves is 1.824673e-02. Additional annotations: *.GeneId*=ENSG00000099246, *.GeneName*=RAB18, *.GeneDesc*=RAB18, member RAS oncogene family [Source:HGNC Symbol;Acc:14244].

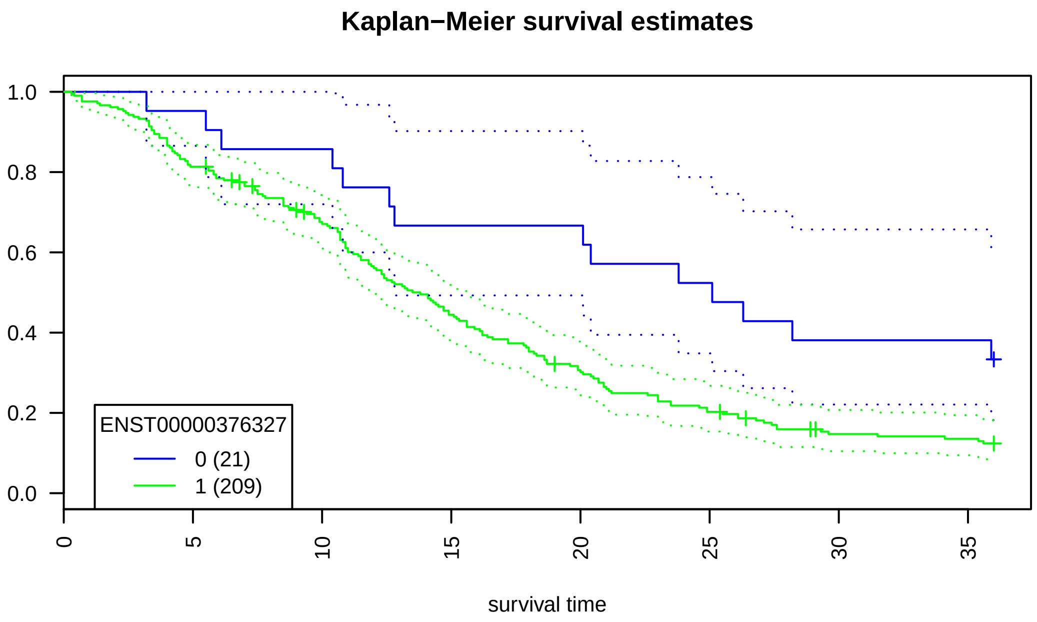

Figure 606: Each curve represents the relative number of ENST00000376327 survived to the corresponding point of time. Dashed lines represent 0.95 confidence intervals for the curves with the corresponding color. Log-rank probability measure for the equality of these curves is 9.287354e-03. Additional annotations: *.GeneId*=ENSG00000102007, *.GeneName*=PLP2, *.GeneDesc*=proteolipid protein 2 (colonic epithelium-enriched) [Source:HGNC Symbol;Acc:9087].

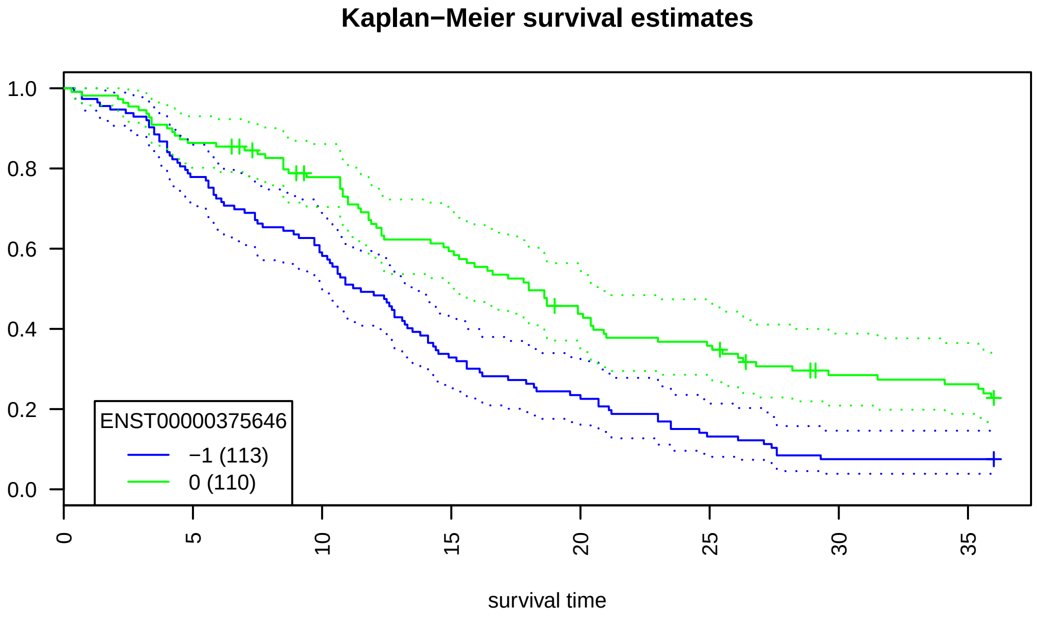

Figure 607: Each curve represents the relative number of ENST00000375646 survived to the corresponding point of time. Dashed lines represent 0.95 confidence intervals for the curves with the corresponding color. Total of 7 observations deleted due to missingness. Log-rank probability measure for the equality of these curves is 3.420700e-05. Additional annotations: *.GeneId*=ENSG00000095787, *.GeneName*=WAC, *.GeneDesc*=WW domain containing adaptor with coiled-coil [Source:HGNC Symbol;Acc:17327].

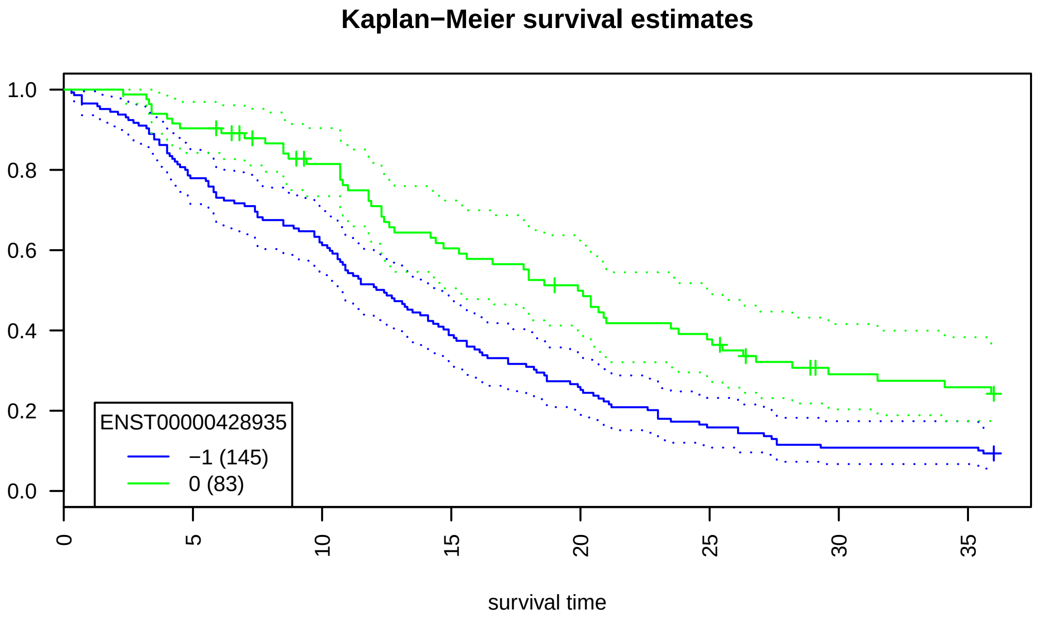

Figure 608: Each curve represents the relative number of ENST00000428935 survived to the corresponding point of time. Dashed lines represent 0.95 confidence intervals for the curves with the corresponding color. Total of 2 observations deleted due to missingness. Log-rank probability measure for the equality of these curves is 1.181688e-04. Additional annotations: *.GeneId*=ENSG00000095787, *.GeneName*=WAC, *.GeneDesc*=WW domain containing adaptor with coiled-coil [Source:HGNC Symbol;Acc:17327].

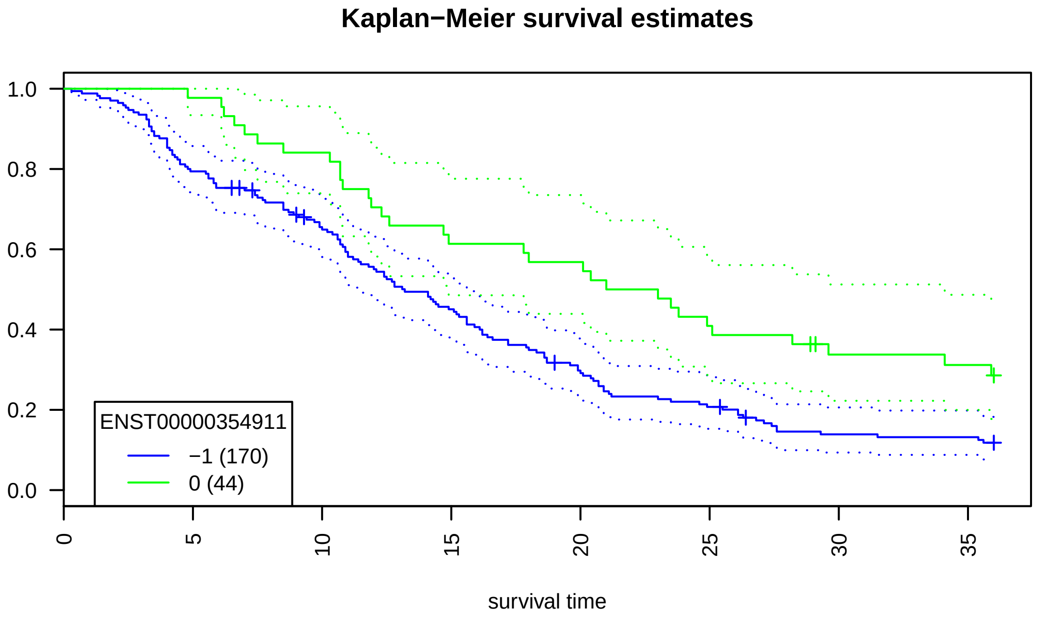

Figure 609: Each curve represents the relative number of ENST00000354911 survived to the corresponding point of time. Dashed lines represent 0.95 confidence intervals for the curves with the corresponding color. Total of 16 observations deleted due to missingness. Log-rank probability measure for the equality of these curves is 1.829499e-03. Additional annotations: *.GeneId*=ENSG00000095787, *.GeneName*=WAC, *.GeneDesc*=WW domain containing adaptor with coiled-coil [Source:HGNC Symbol;Acc:17327].

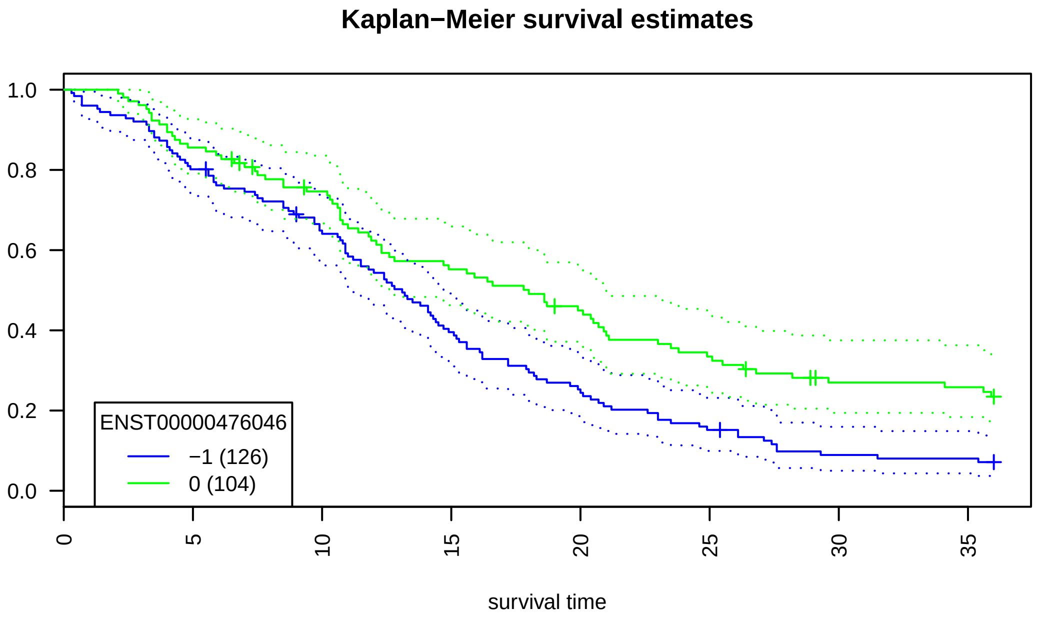

Figure 610: Each curve represents the relative number of ENST00000476046 survived to the corresponding point of time. Dashed lines represent 0.95 confidence intervals for the curves with the corresponding color. Log-rank probability measure for the equality of these curves is 6.095737e-04. Additional annotations: *.GeneId*=ENSG00000095787, *.GeneName*=WAC, *.GeneDesc*=WW domain containing adaptor with coiled-coil [Source:HGNC Symbol;Acc:17327].

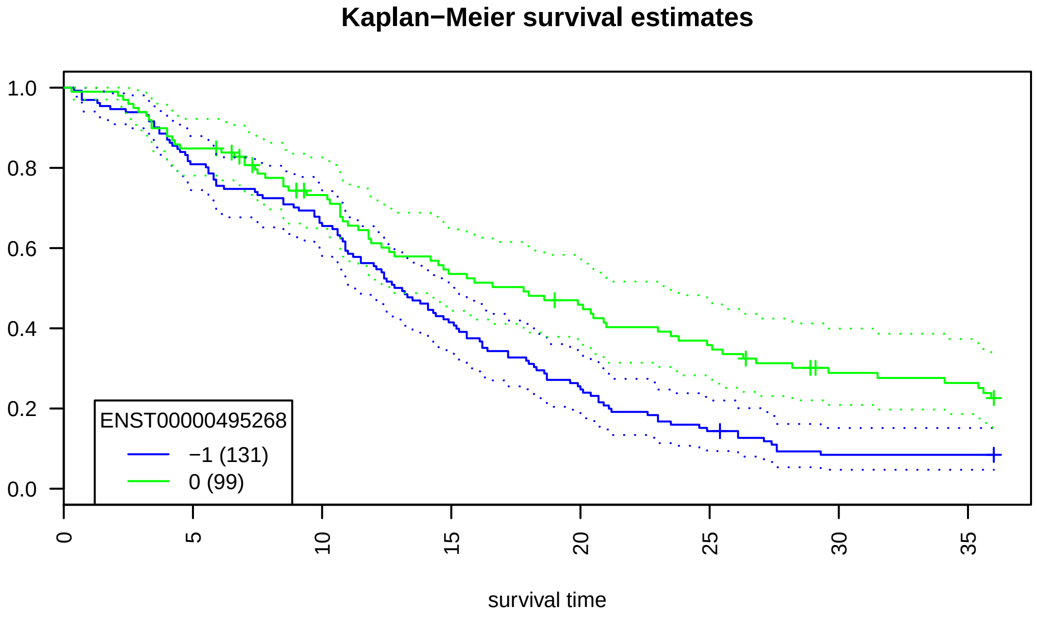

Figure 611: Each curve represents the relative number of ENST00000495268 survived to the corresponding point of time. Dashed lines represent 0.95 confidence intervals for the curves with the corresponding color. Log-rank probability measure for the equality of these curves is 1.183129e-03. Additional annotations: *.GeneId*=ENSG00000095787, *.GeneName*=WAC, *.GeneDesc*=WW domain containing adaptor with coiled-coil [Source:HGNC Symbol;Acc:17327].

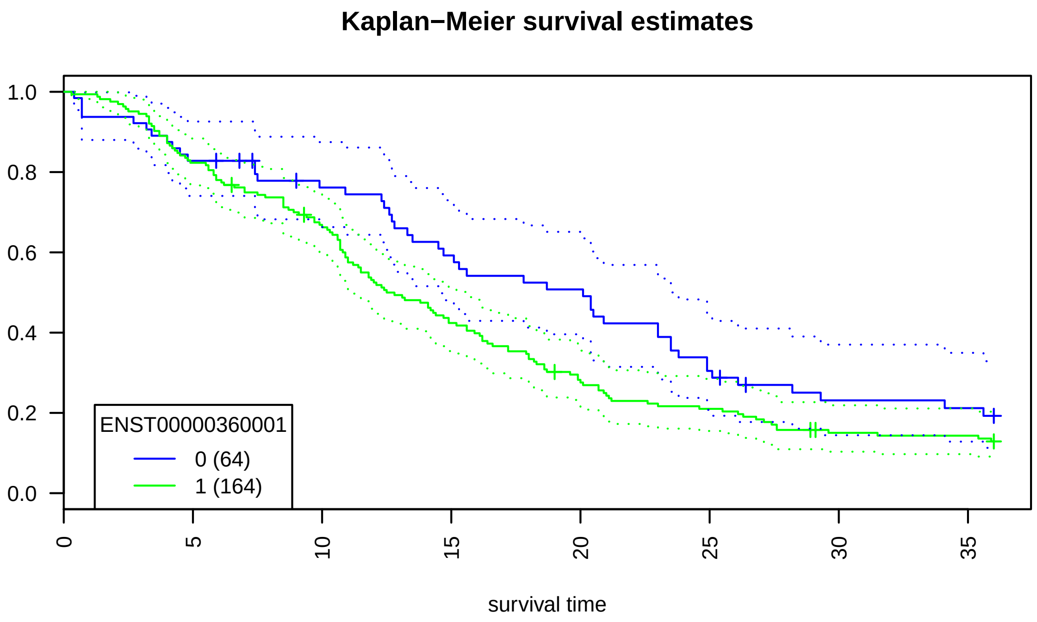

Figure 612: Each curve represents the relative number of ENST00000360001 survived to the corresponding point of time. Dashed lines represent 0.95 confidence intervals for the curves with the corresponding color. Total of 2 observations deleted due to missingness. Log-rank probability measure for the equality of these curves is 4.111133e-02. Additional annotations: *.GeneId*=ENSG00000078808, *.GeneName*=SDF4, *.GeneDesc*=stromal cell derived factor 4 [Source:HGNC Symbol;Acc:24188].

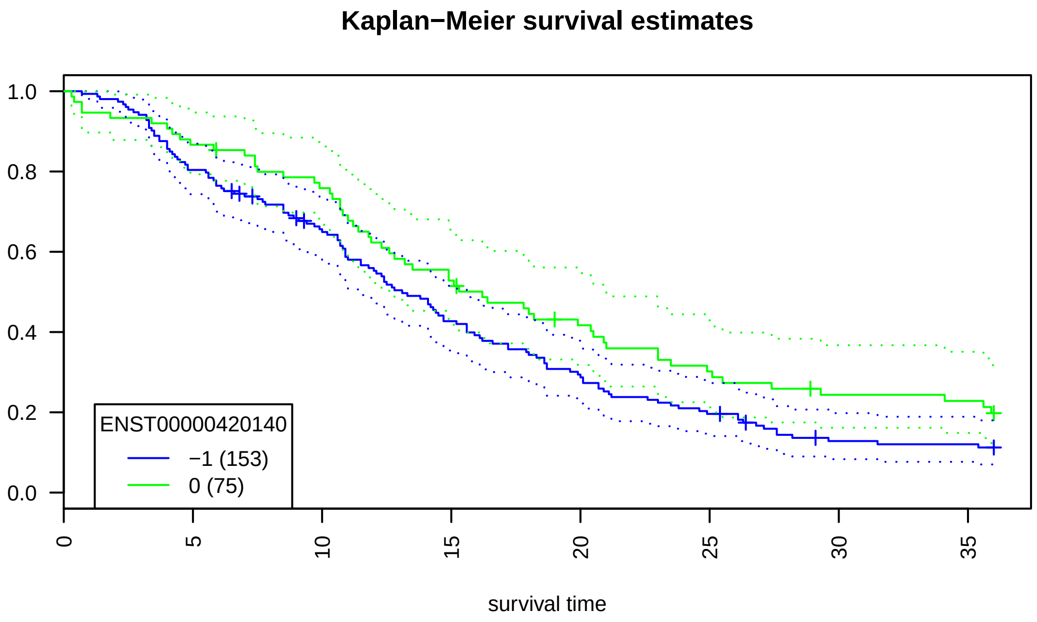

Figure 613: Each curve represents the relative number of ENST00000420140 survived to the corresponding point of time. Dashed lines represent 0.95 confidence intervals for the curves with the corresponding color. Total of 2 observations deleted due to missingness. Log-rank probability measure for the equality of these curves is 4.76141e-02. Additional annotations: *.GeneId*=ENSG00000228009, *.GeneName*=RP11-756A22.7, *.GeneDesc*=[undefined].

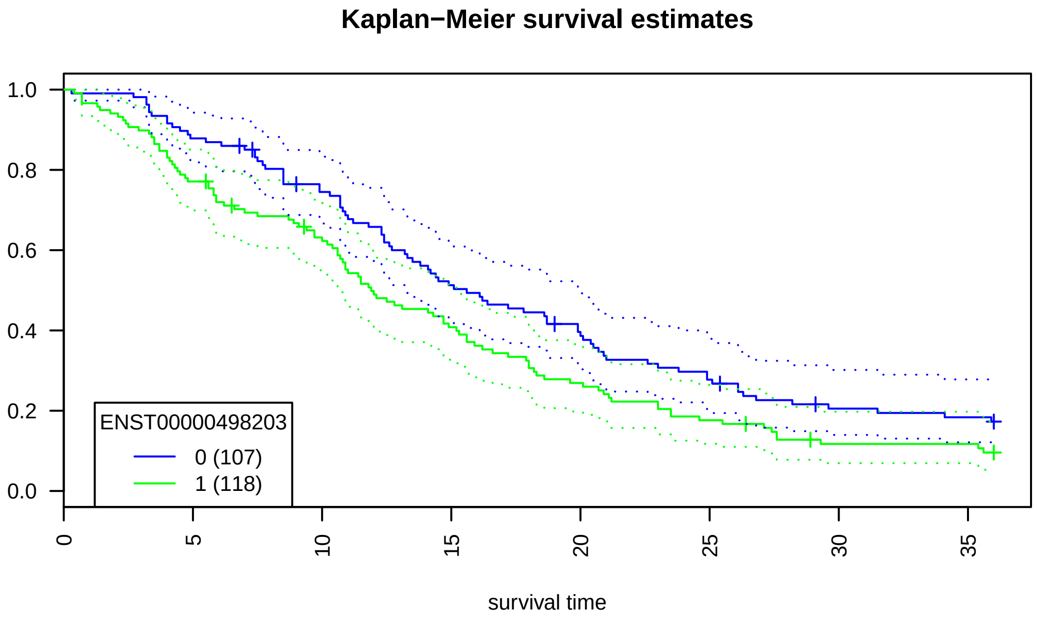

Figure 614: Each curve represents the relative number of ENST00000498203 survived to the corresponding point of time. Dashed lines represent 0.95 confidence intervals for the curves with the corresponding color. Total of 5 observations deleted due to missingness. Log-rank probability measure for the equality of these curves is 1.986477e-02. Additional annotations: *.GeneId*=ENSG00000061656, *.GeneName*=SPAG4, *.GeneDesc*=sperm associated antigen 4 [Source:HGNC Symbol;Acc:11214].

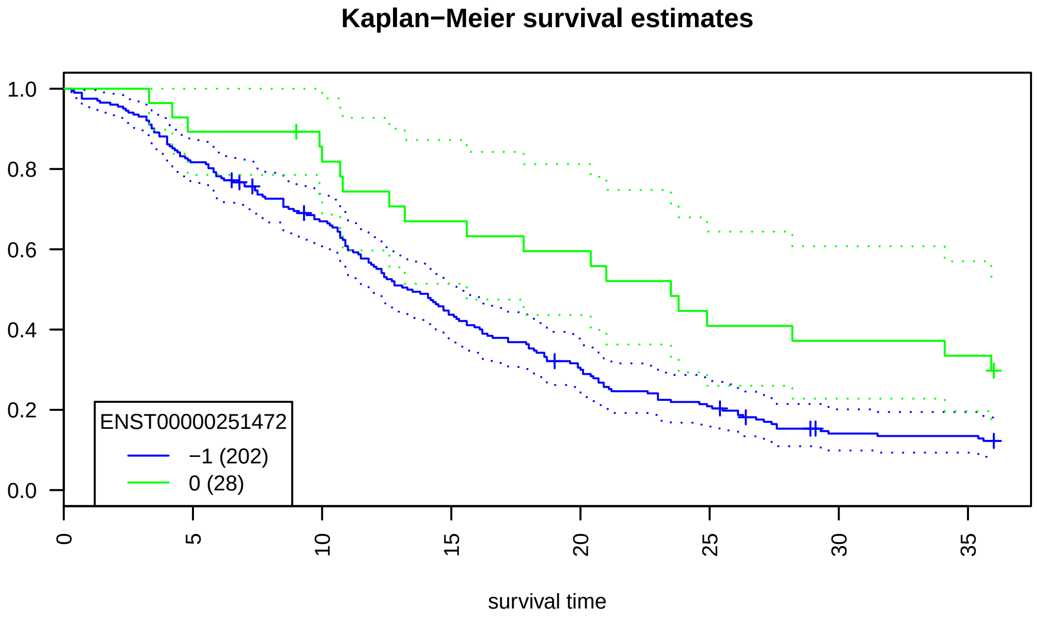

Figure 615: Each curve represents the relative number of ENST00000251472 survived to the corresponding point of time. Dashed lines represent 0.95 confidence intervals for the curves with the corresponding color. Log-rank probability measure for the equality of these curves is 7.946338e-03. Additional annotations: *.GeneId*=ENSG00000105613, *.GeneName*=MAST1, *.GeneDesc*=microtubule associated serine/threonine kinase 1 [Source:HGNC Symbol;Acc:19034].

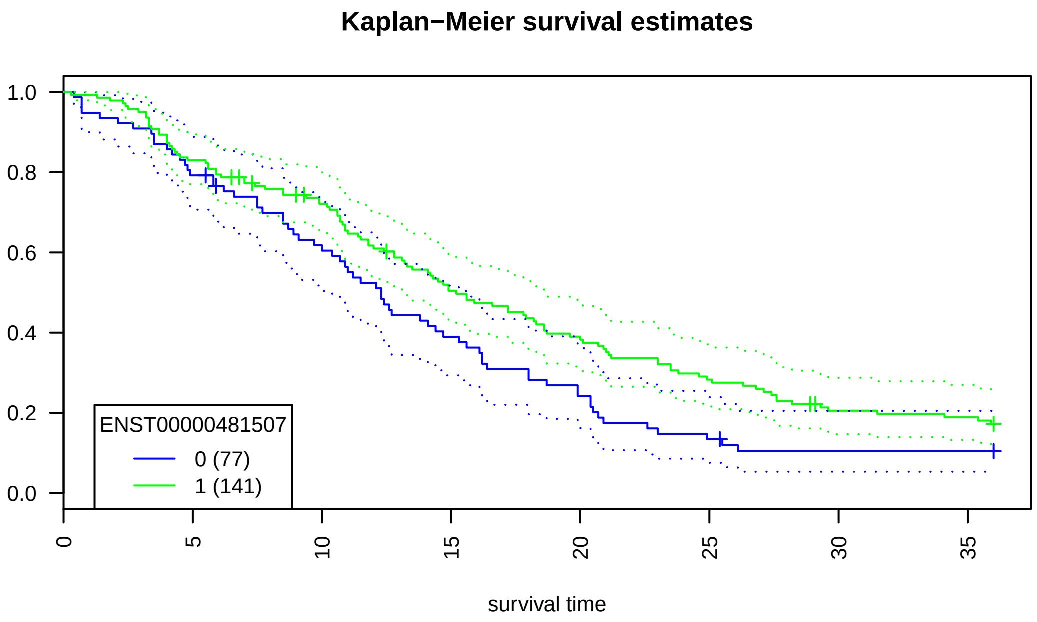

Figure 616: Each curve represents the relative number of ENST00000481507 survived to the corresponding point of time. Dashed lines represent 0.95 confidence intervals for the curves with the corresponding color. Total of 12 observations deleted due to missingness. Log-rank probability measure for the equality of these curves is 2.275884e-02. Additional annotations: *.GeneId*=ENSG00000147905, *.GeneName*=ZCCHC7, *.GeneDesc*=zinc finger, CCHC domain containing 7 [Source:HGNC Symbol;Acc:26209].

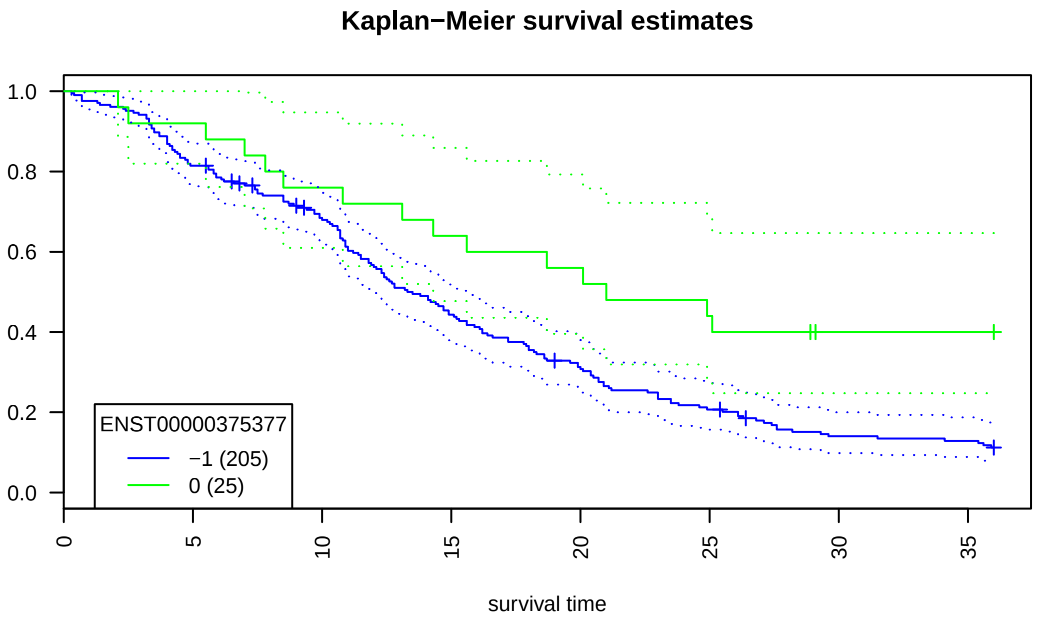

Figure 617: Each curve represents the relative number of ENST00000375377 survived to the corresponding point of time. Dashed lines represent 0.95 confidence intervals for the curves with the corresponding color. Log-rank probability measure for the equality of these curves is 4.943414e-03. Additional annotations: *.GeneId*=ENSG00000165757, *.GeneName*=KIAA1462, *.GeneDesc*=KIAA1462 [Source:HGNC Symbol;Acc:29283].

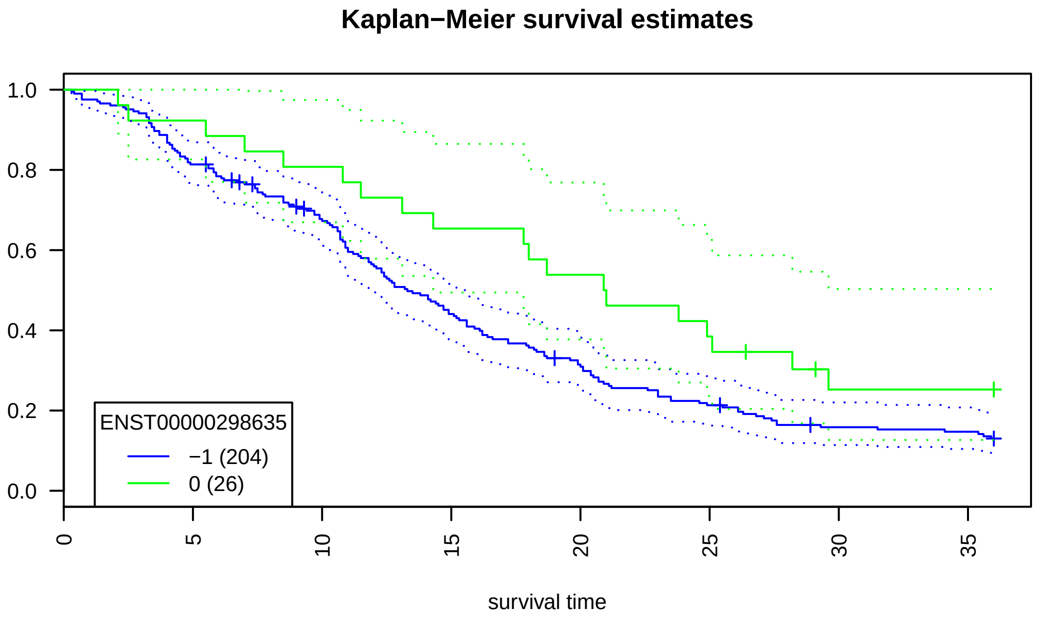

Figure 618: Each curve represents the relative number of ENST00000298635 survived to the corresponding point of time. Dashed lines represent 0.95 confidence intervals for the curves with the corresponding color. Log-rank probability measure for the equality of these curves is 4.122139e-02. Additional annotations: .*GeneId*=ENSG00000165757, .*GeneName*=KIAA1462, .*GeneDesc*=KIAA1462 [Source:HGNC Symbol;Acc:29283].

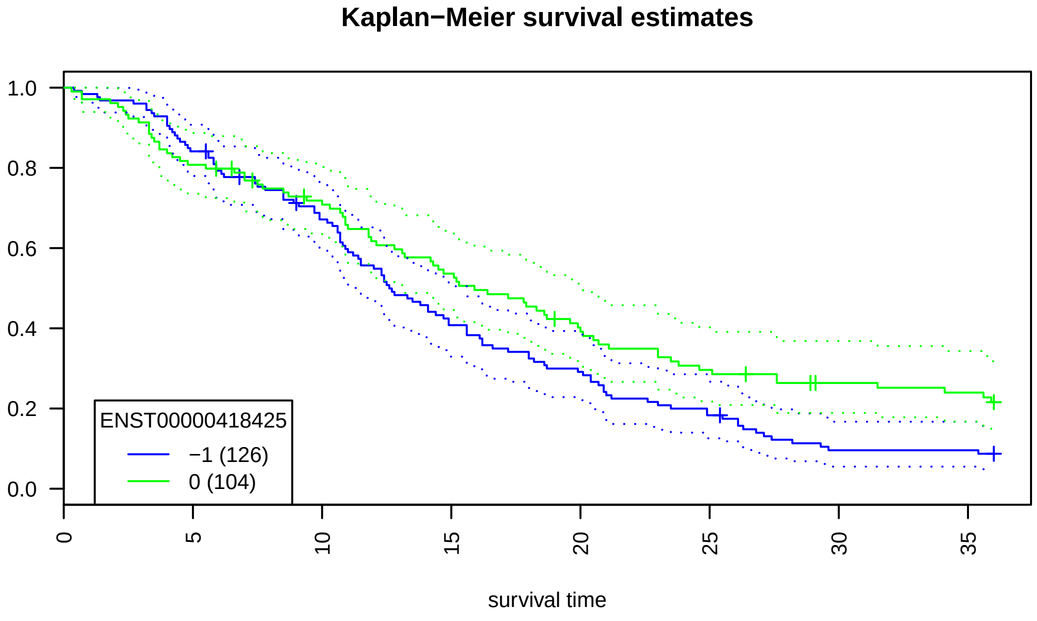

Figure 619: Each curve represents the relative number of ENST00000418425 survived to the corresponding point of time. Dashed lines represent 0.95 confidence intervals for the curves with the corresponding color. Log-rank probability measure for the equality of these curves is 1.816369e-02. Additional annotations: .*GeneId*=ENSG00000050426, .*GeneName*=LETMD1, .*GeneDesc*=LETM1 domain containing 1 [Source:HGNC Symbol;Acc:24241].

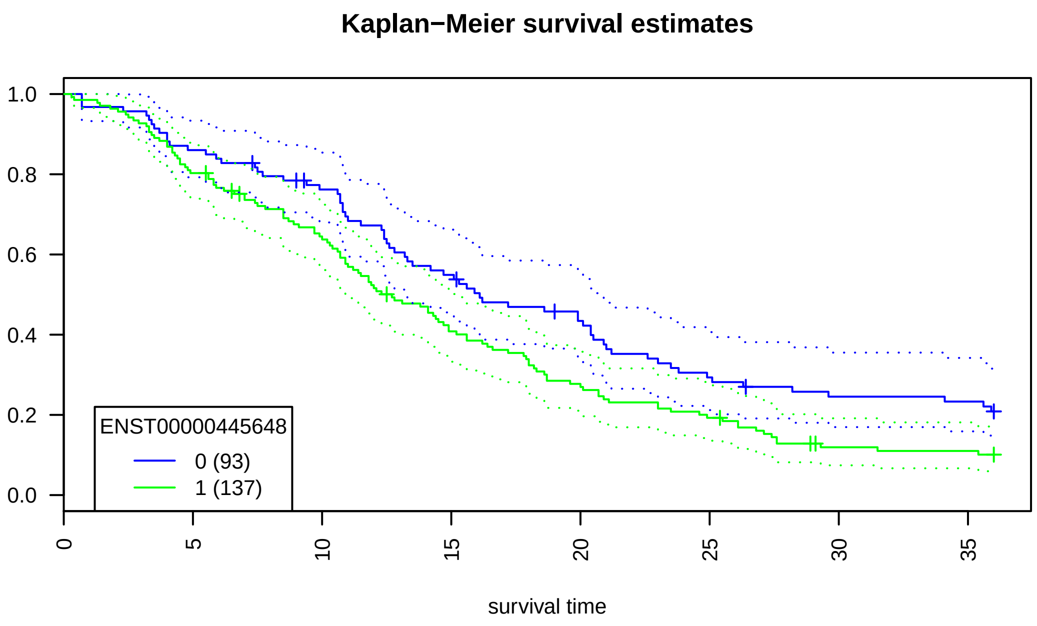

Figure 620: Each curve represents the relative number of ENST00000445648 survived to the corresponding point of time. Dashed lines represent 0.95 confidence intervals for the curves with the corresponding color. Log-rank probability measure for the equality of these curves is 1.167393e-02. Additional annotations: *.GeneId*=ENSG00000162576, *.GeneName*=MXRA8, *.GeneDesc*=matrix-remodelling associated 8 [Source:HGNC Symbol;Acc:7542].

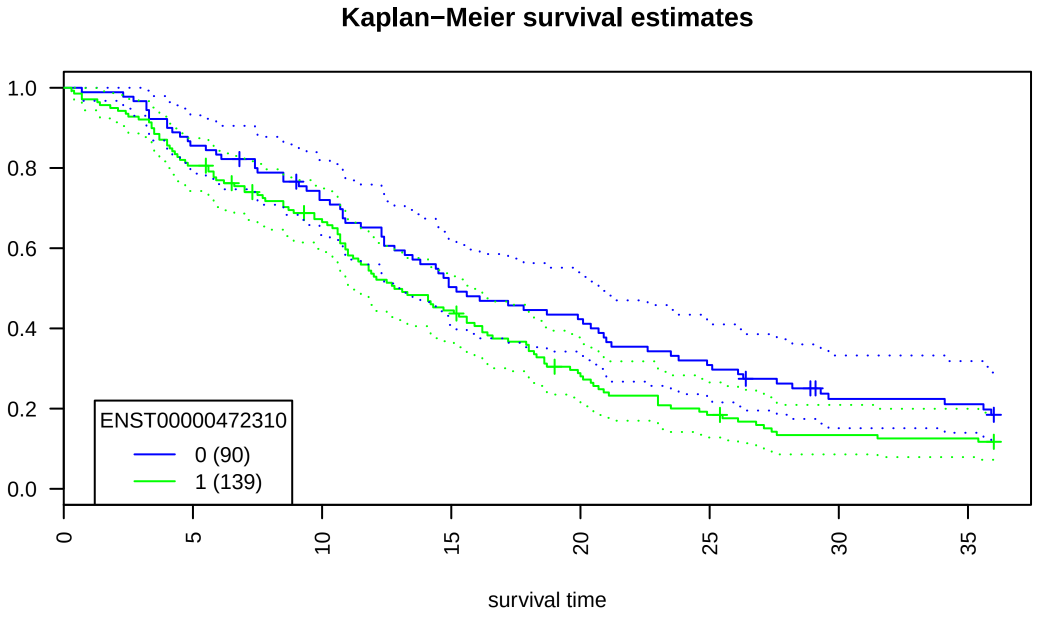

Figure 621: Each curve represents the relative number of ENST00000472310 survived to the corresponding point of time. Dashed lines represent 0.95 confidence intervals for the curves with the corresponding color. Total of 1 observation deleted due to missingness. Log-rank probability measure for the equality of these curves is 4.987711e-02. Additional annotations: *.GeneId*=ENSG00000005059, *.GeneName*=CCDC109B, *.GeneDesc*=coiled-coil domain containing 109B [Source:HGNC Symbol;Acc:26076].

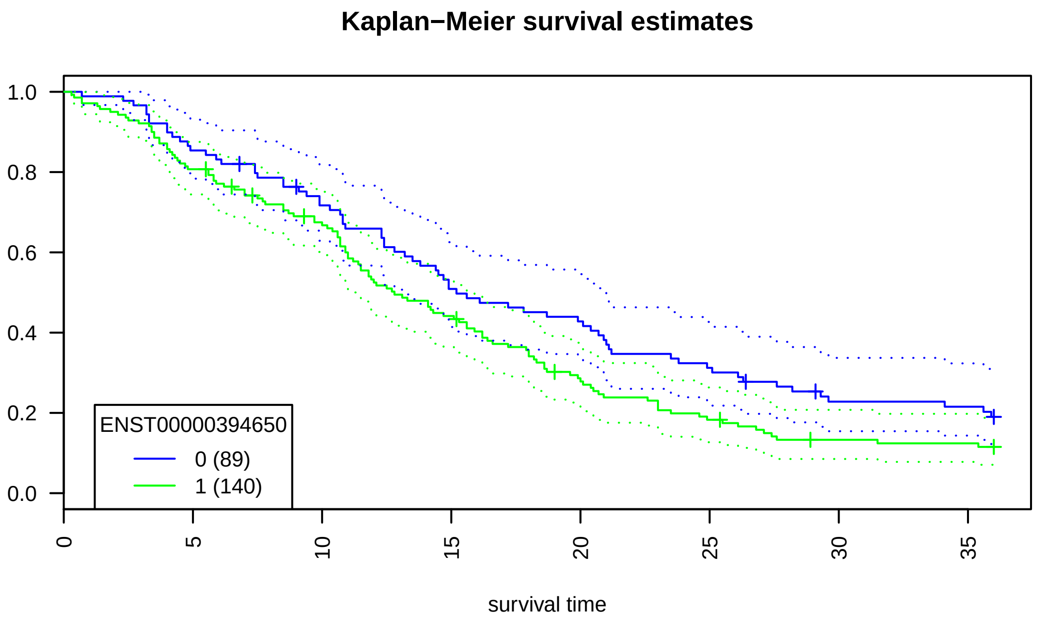

Figure 622: Each curve represents the relative number of ENST00000394650 survived to the corresponding point of time. Dashed lines represent 0.95 confidence intervals for the curves with the corresponding color. Total of 1 observation deleted due to missingness. Log-rank probability measure for the equality of these curves is 3.91113e-02. Additional annotations: *.GeneId*=ENSG00000005059, *.GeneName*=CCDC109B, *.GeneDesc*=coiled-coil domain containing 109B [Source:HGNC Symbol;Acc:26076].

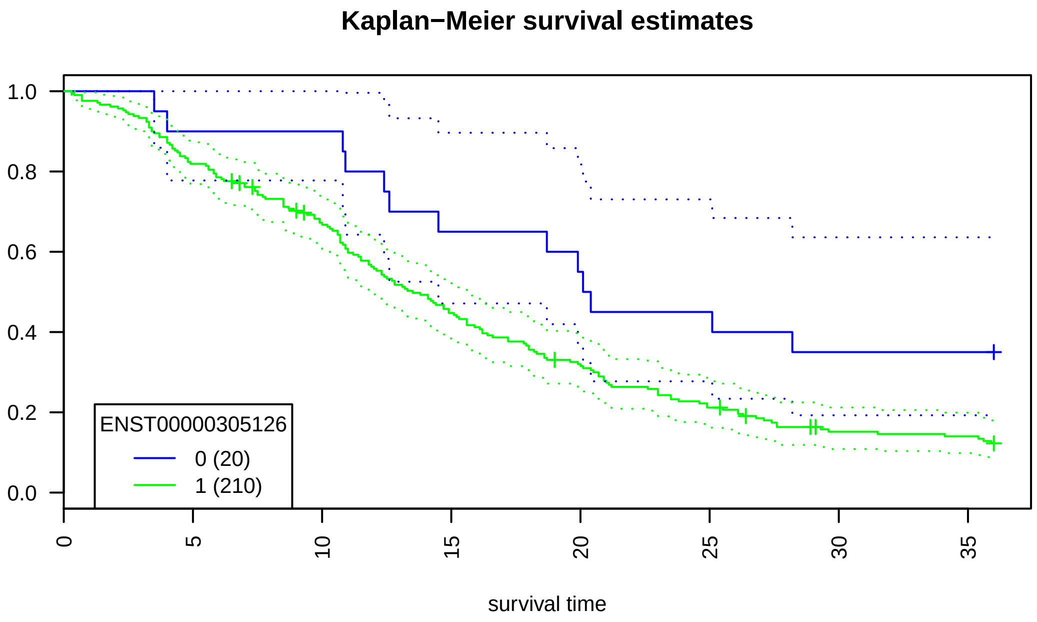

Figure 623: Each curve represents the relative number of ENST00000305126 survived to the corresponding point of time. Dashed lines represent 0.95 confidence intervals for the curves with the corresponding color. Log-rank probability measure for the equality of these curves is 1.570129e-02. Additional annotations: *.GeneId*=ENSG00000120708, *.GeneName*=TGFBI, *.GeneDesc*=transforming growth factor, beta-induced, 68kDa [Source:HGNC Symbol;Acc:11771].

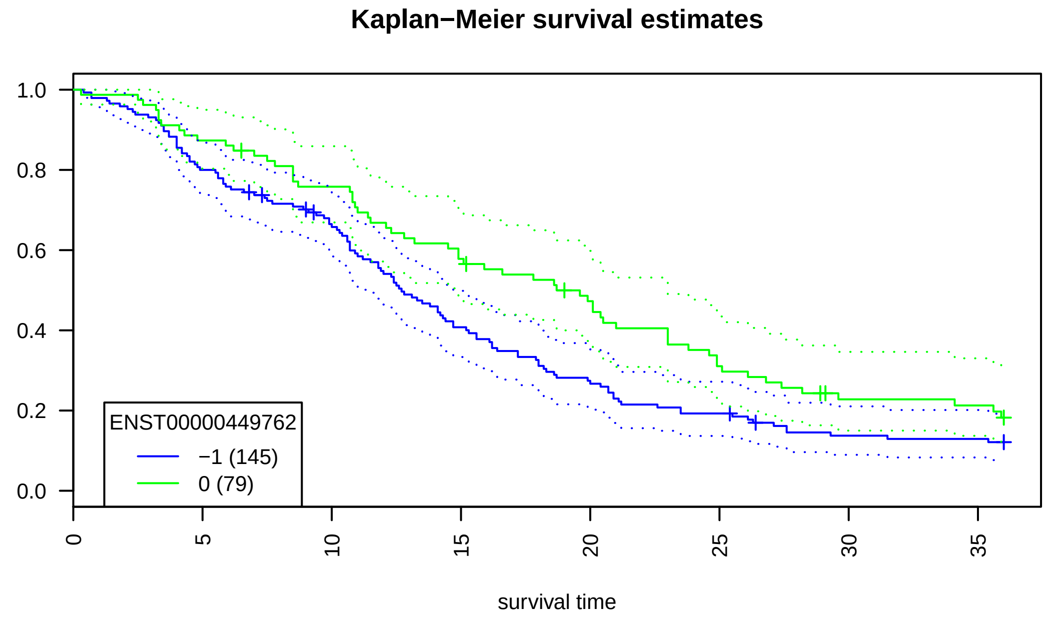

Figure 624: Each curve represents the relative number of ENST00000449762 survived to the corresponding point of time. Dashed lines represent 0.95 confidence intervals for the curves with the corresponding color. Total of 6 observations deleted due to missingness. Log-rank probability measure for the equality of these curves is 1.864106e-02. Additional annotations: *.GeneId*=ENSG00000164830, *.GeneName*=OXR1, *.GeneDesc*=oxidation resistance 1 [Source:HGNC Symbol;Acc:15822].

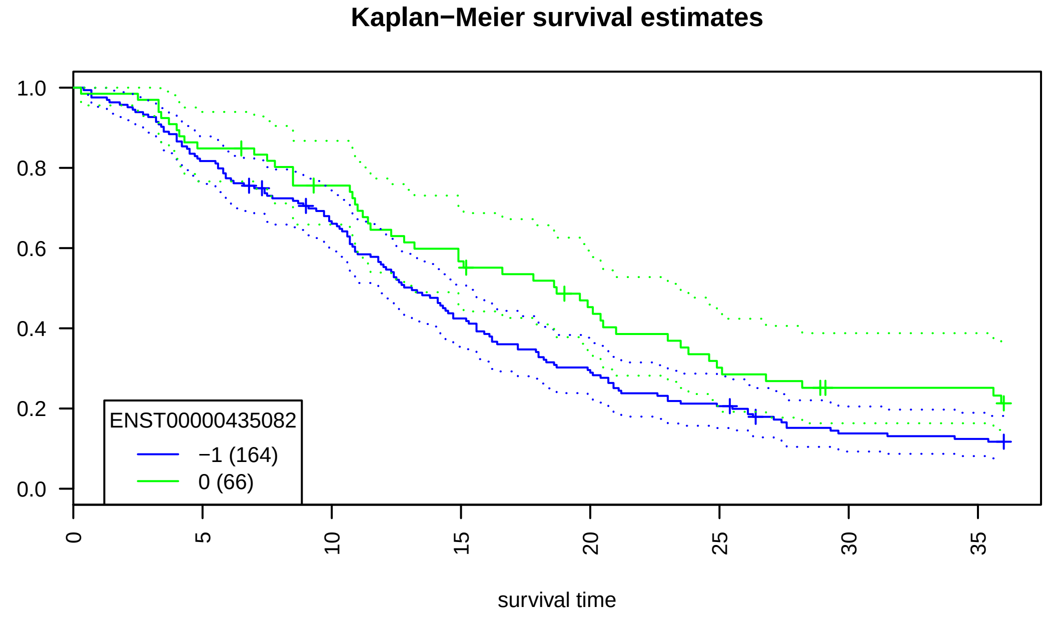

Figure 625: Each curve represents the relative number of ENST00000435082 survived to the corresponding point of time. Dashed lines represent 0.95 confidence intervals for the curves with the corresponding color. Log-rank probability measure for the equality of these curves is 2.830797e-02. Additional annotations: *.GeneId*=ENSG00000164830, *.GeneName*=OXR1, *.GeneDesc*=oxidation resistance 1 [Source:HGNC Symbol;Acc:15822].

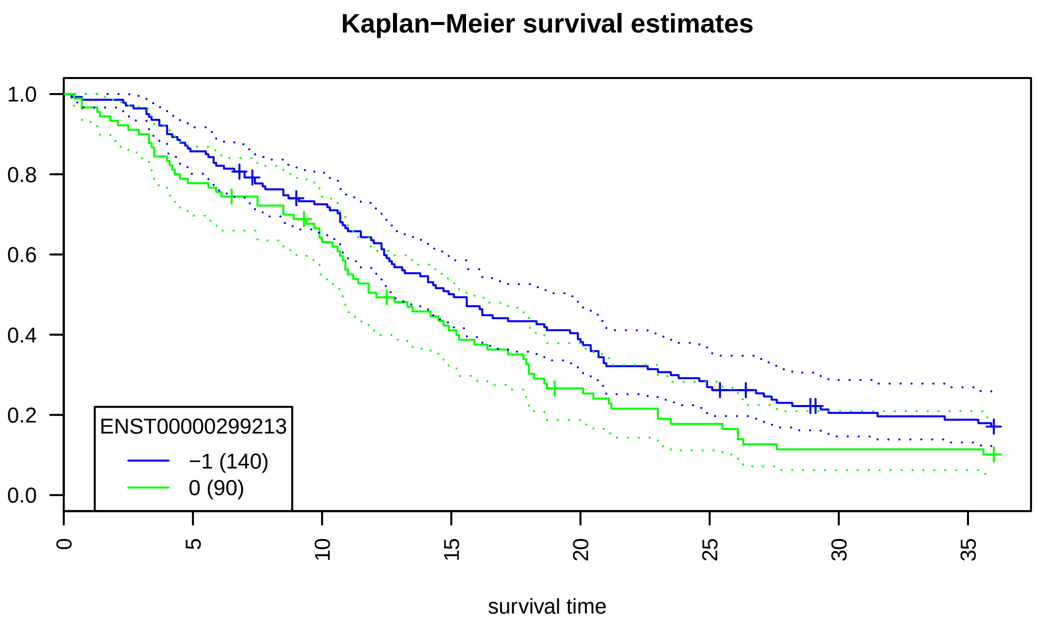

Figure 626: Each curve represents the relative number of ENST00000299213 survived to the corresponding point of time. Dashed lines represent 0.95 confidence intervals for the curves with the corresponding color. Log-rank probability measure for the equality of these curves is 4.148539e-02. Additional annotations: *.GeneId*=ENSG00000166173, *.GeneName*=LARP6, *.GeneDesc*=La ribonucleoprotein domain family, member 6 [Source:HGNC Symbol;Acc:24012].

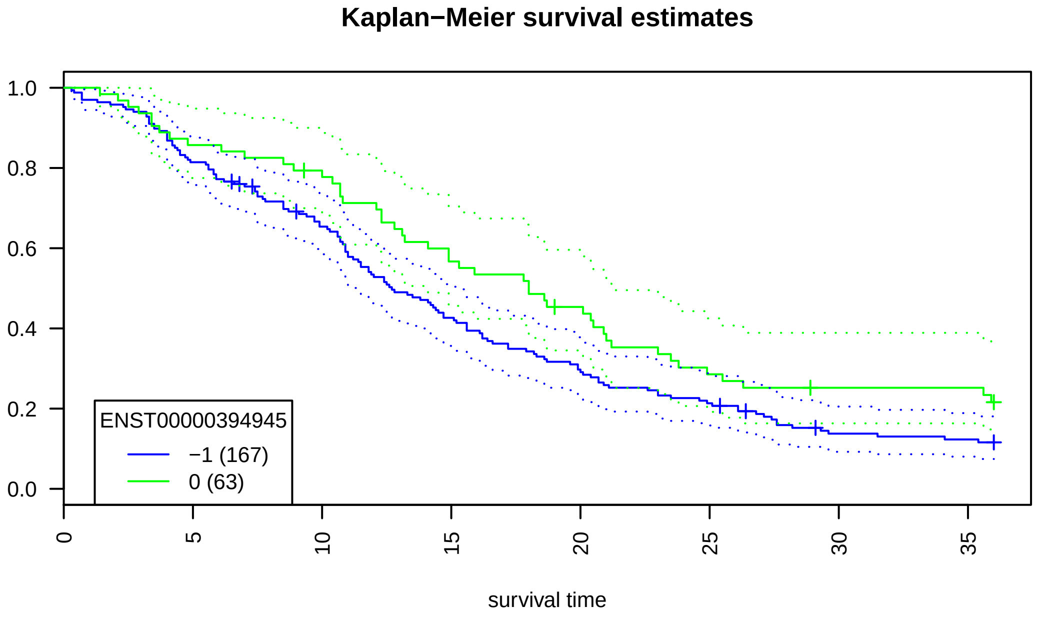

Figure 627: Each curve represents the relative number of ENST00000394945 survived to the corresponding point of time. Dashed lines represent 0.95 confidence intervals for the curves with the corresponding color. Log-rank probability measure for the equality of these curves is 3.148726e-02. Additional annotations: *.GeneId*=ENSG00000152377, *.GeneName*=SPOCK1, *.GeneDesc*=sparc/osteonectin, cwcv and kazal-like domains proteoglycan (testican) 1 [Source:HGNC Symbol;Acc:11251].

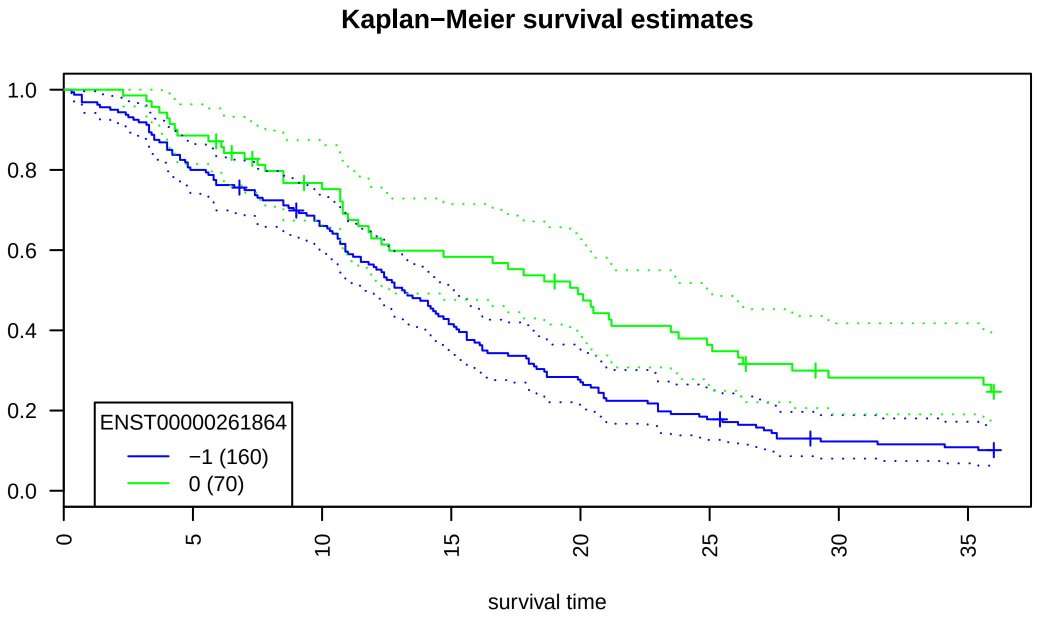

Figure 628: Each curve represents the relative number of ENST00000261864 survived to the corresponding point of time. Dashed lines represent 0.95 confidence intervals for the curves with the corresponding color. Log-rank probability measure for the equality of these curves is 2.280648e-03. Additional annotations: *.GeneId*=ENSG00000066933, *.GeneName*=MYO9A, *.GeneDesc*=myosin IXA [Source:HGNC Symbol;Acc:7608].

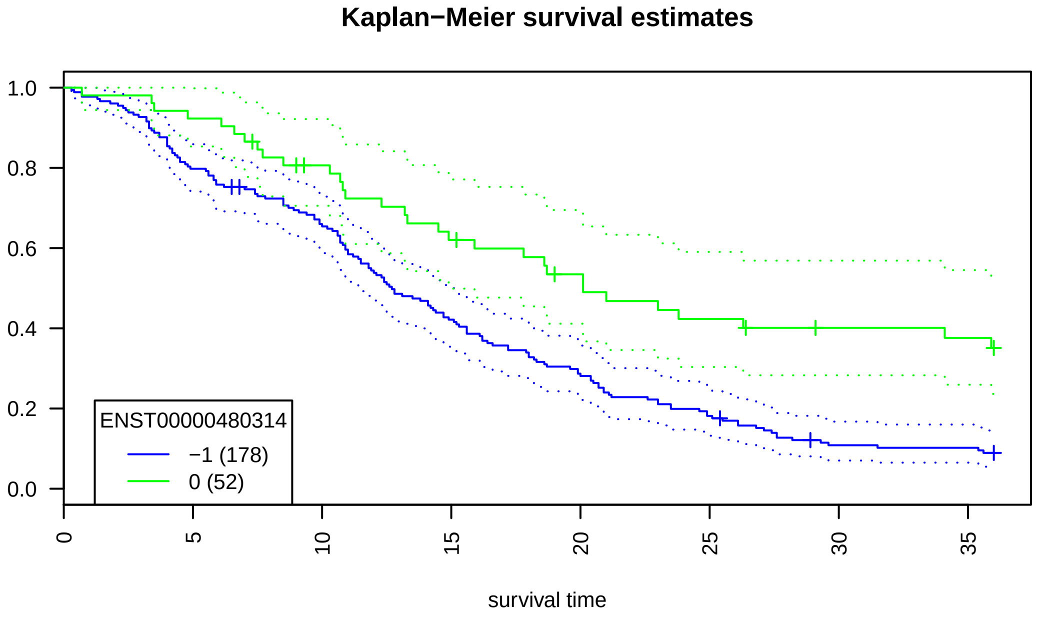

Figure 629: Each curve represents the relative number of ENST00000480314 survived to the corresponding point of time. Dashed lines represent 0.95 confidence intervals for the curves with the corresponding color. Log-rank probability measure for the equality of these curves is 9.398618e-05. Additional annotations: *.GeneId*=ENSG00000101079, *.GeneName*=NDRG3, *.GeneDesc*=NDRG family member 3 [Source:HGNC Symbol;Acc:14462].

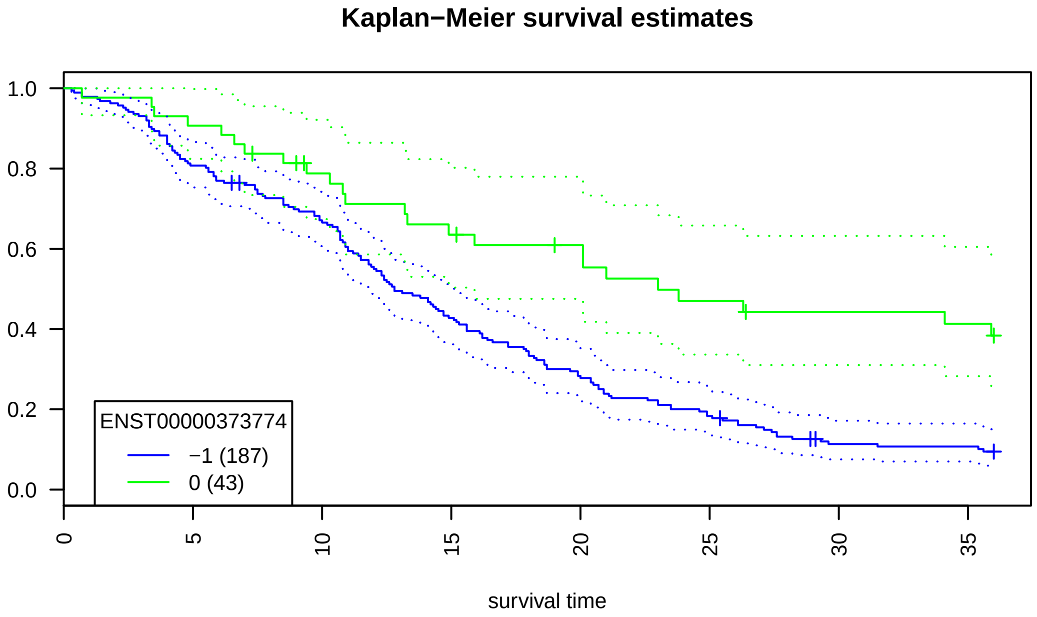

Figure 630: Each curve represents the relative number of ENST00000373774 survived to the corresponding point of time. Dashed lines represent 0.95 confidence intervals for the curves with the corresponding color. Log-rank probability measure for the equality of these curves is 1.09279e-04.

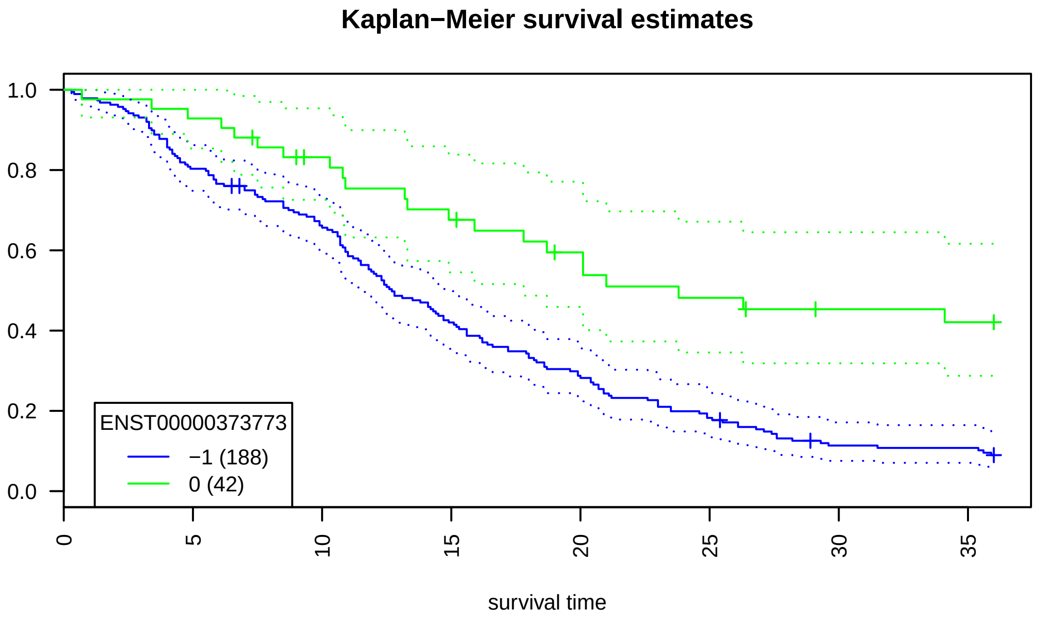

Figure 631: Each curve represents the relative number of ENST00000373773 survived to the corresponding point of time. Dashed lines represent 0.95 confidence intervals for the curves with the corresponding color. Log-rank probability measure for the equality of these curves is 2.655173e-05. Additional annotations: *.GeneId*=ENSG00000101079, *.GeneName*=NDRG3, *.GeneDesc*=NDRG family member 3 [Source:HGNC Symbol;Acc:14462].

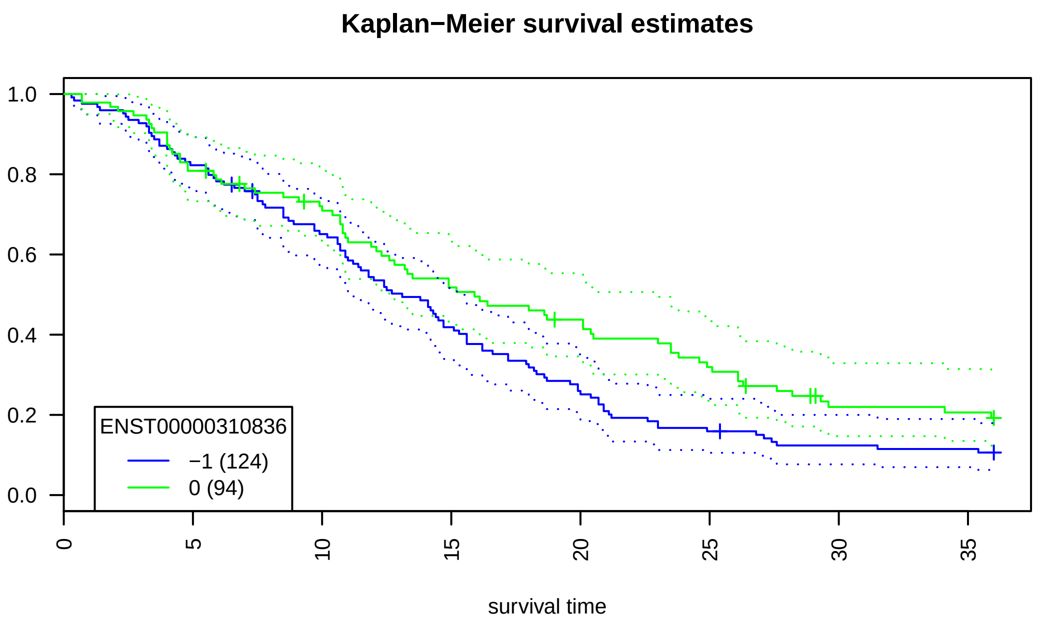

Figure 632: Each curve represents the relative number of ENST00000310836 survived to the corresponding point of time. Dashed lines represent 0.95 confidence intervals for the curves with the corresponding color. Total of 12 observations deleted due to missingness. Log-rank probability measure for the equality of these curves is 2.741559e-02. Additional annotations: *.GeneId*=ENSG00000174607, *.GeneName*=UGT8, *.GeneDesc*=UDP glycosyltransferase 8 [Source:HGNC Symbol;Acc:12555].

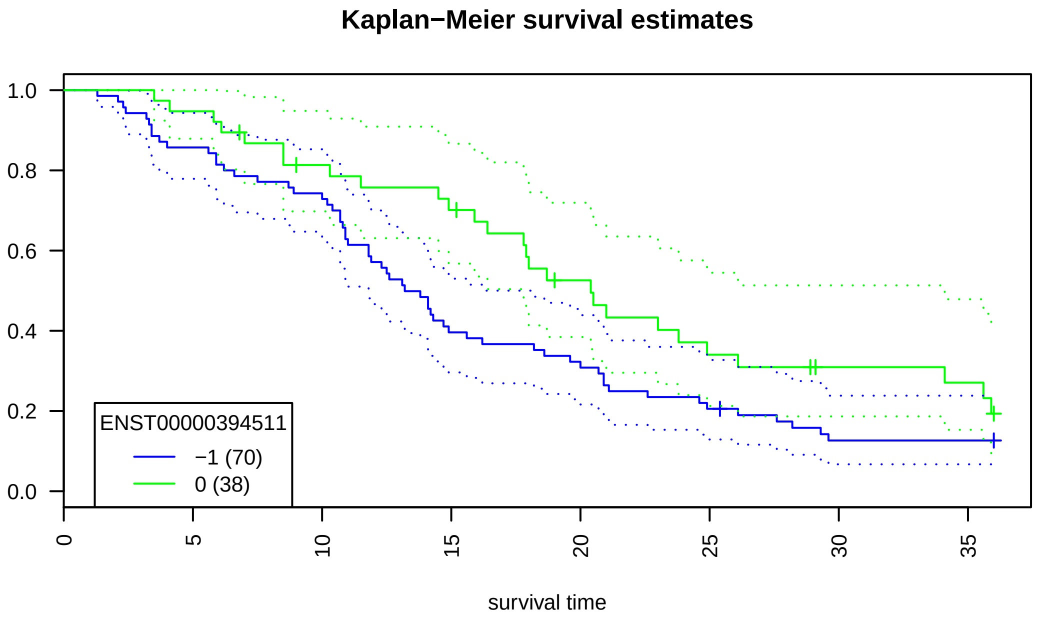

Figure 633: Each curve represents the relative number of ENST00000394511 survived to the corresponding point of time. Dashed lines represent 0.95 confidence intervals for the curves with the corresponding color. Total of 122 observations deleted due to missingness. Log-rank probability measure for the equality of these curves is 4.511531e-02. Additional annotations: *.GeneId*=ENSG00000174607, *.GeneName*=UGT8, *.GeneDesc*=UDP glycosyltransferase 8 [Source:HGNC Symbol;Acc:12555].

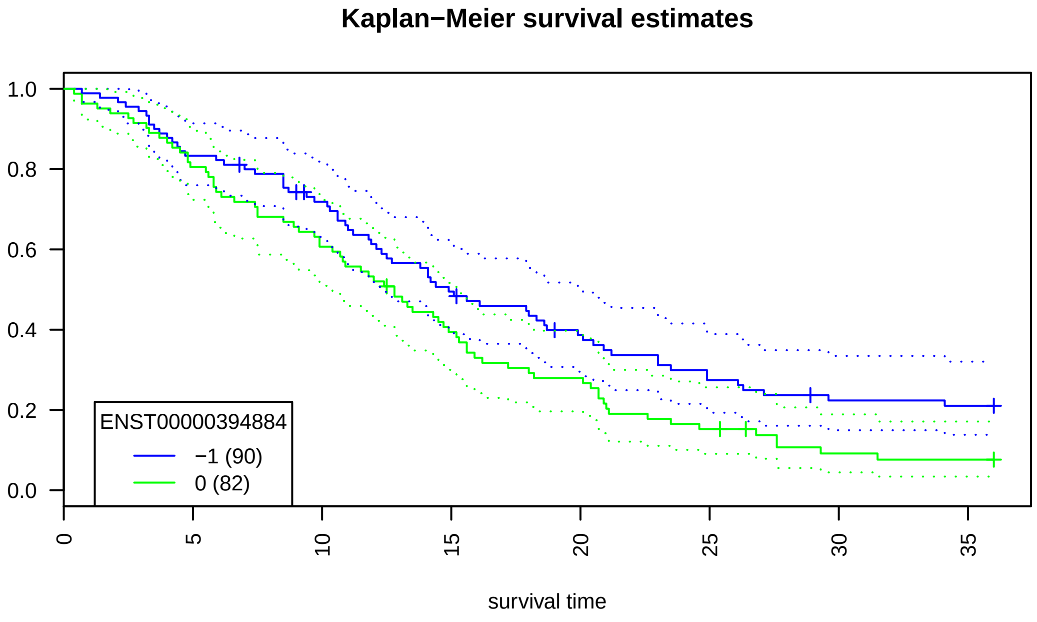

Figure 634: Each curve represents the relative number of ENST00000394884 survived to the corresponding point of time. Dashed lines represent 0.95 confidence intervals for the curves with the corresponding color. Total of 58 observations deleted due to missingness. Log-rank probability measure for the equality of these curves is 2.222927e-02. Additional annotations: *.GeneId*=ENSG00000094880, *.GeneName*=CDC23, *.GeneDesc*=cell division cycle 23 homolog (S. cerevisiae) [Source:HGNC Symbol;Acc:1724].

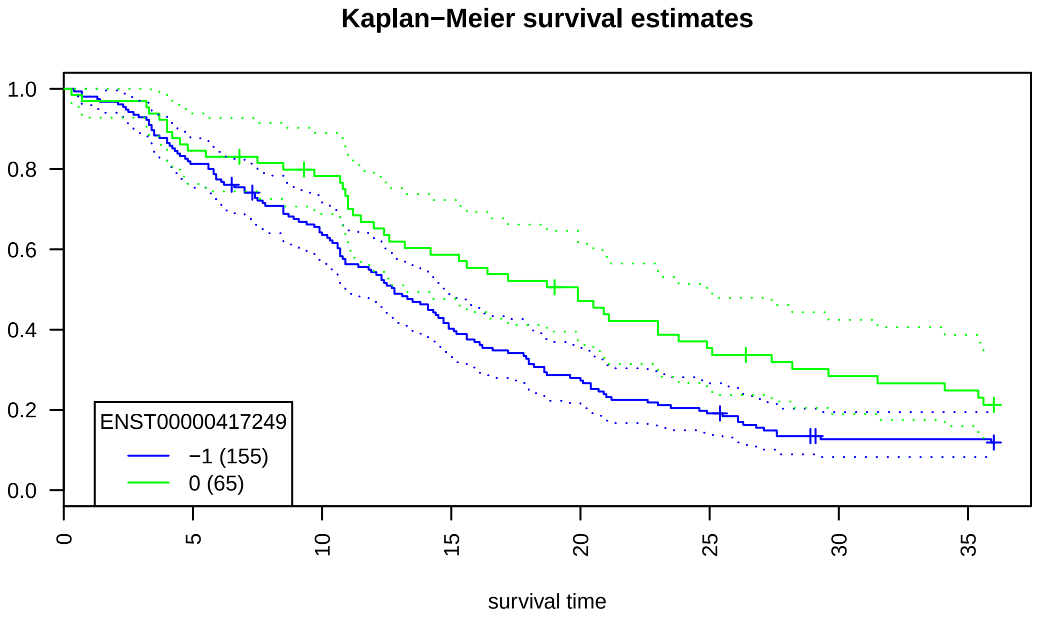

Figure 635: Each curve represents the relative number of ENST00000417249 survived to the corresponding point of time. Dashed lines represent 0.95 confidence intervals for the curves with the corresponding color. Total of 10 observations deleted due to missingness. Log-rank probability measure for the equality of these curves is 9.61398e-03. Additional annotations: *.GeneId*=ENSG00000100796, *.GeneName*=SMEK1, *.GeneDesc*=SMEK homolog 1, suppressor of mek1 (Dictyostelium) [Source:HGNC Symbol;Acc:20219].

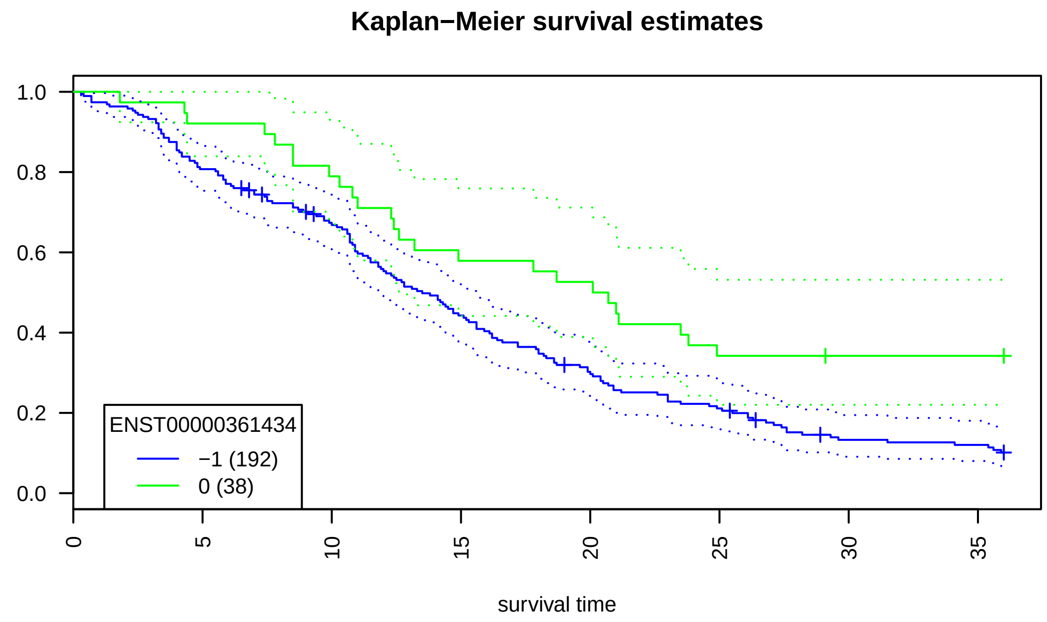

Figure 636: Each curve represents the relative number of ENST00000361434 survived to the corresponding point of time. Dashed lines represent 0.95 confidence intervals for the curves with the corresponding color. Log-rank probability measure for the equality of these curves is 2.721071e-03. Additional annotations: *.GeneId*=ENSG00000072071, *.GeneName*=LPHN1, *.GeneDesc*=latrophilin 1 [Source:HGNC Symbol;Acc:20973].

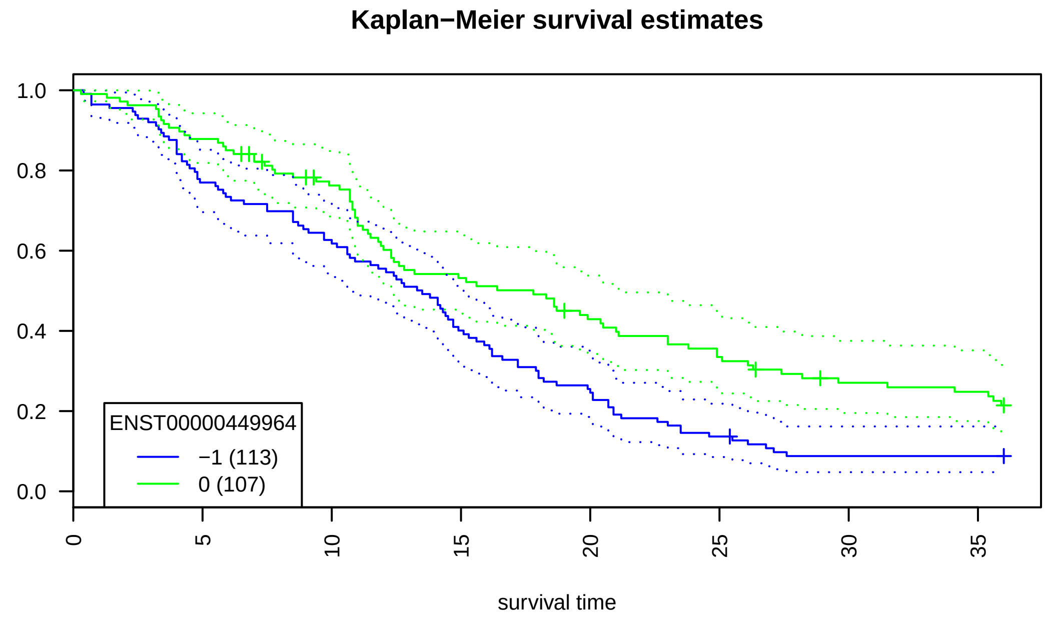

Figure 637: Each curve represents the relative number of ENST00000449964 survived to the corresponding point of time. Dashed lines represent 0.95 confidence intervals for the curves with the corresponding color. Total of 10 observations deleted due to missingness. Log-rank probability measure for the equality of these curves is 1.327436e-03. Additional annotations: *.GeneId*=ENSG00000131381, *.GeneName*=ZFYVE20, *.GeneDesc*=zinc finger, FYVE domain containing 20 [Source:HGNC Symbol;Acc:20759].

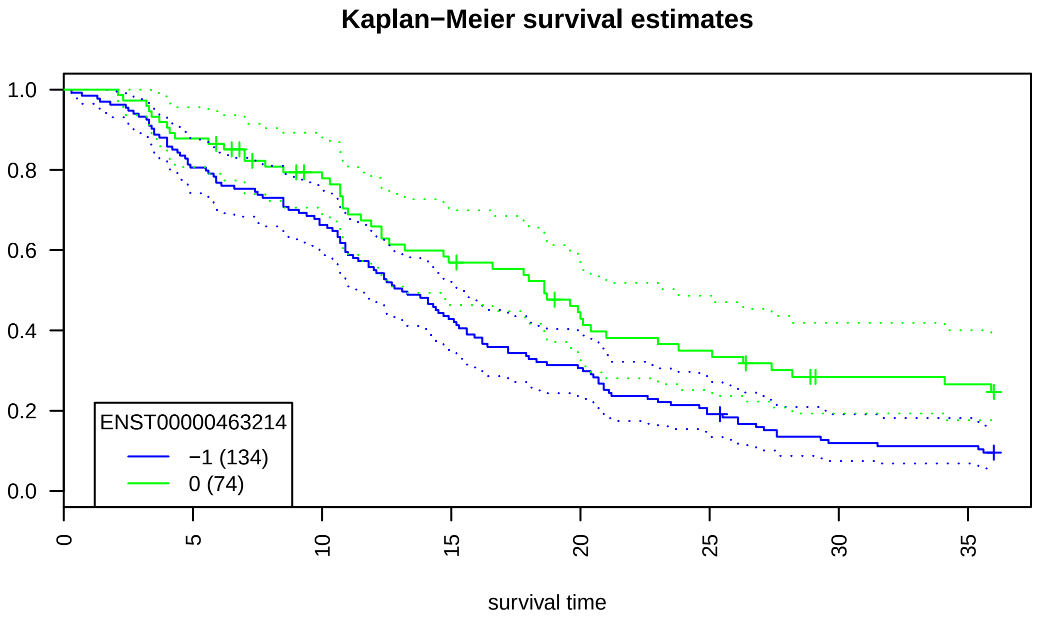

Figure 638: Each curve represents the relative number of ENST00000463214 survived to the corresponding point of time. Dashed lines represent 0.95 confidence intervals for the curves with the corresponding color. Total of 22 observations deleted due to missingness. Log-rank probability measure for the equality of these curves is 5.477117e-03. Additional annotations: *.GeneId*=ENSG00000131381, *.GeneName*=ZFYVE20, *.GeneDesc*=zinc finger, FYVE domain containing 20 [Source:HGNC Symbol;Acc:20759].

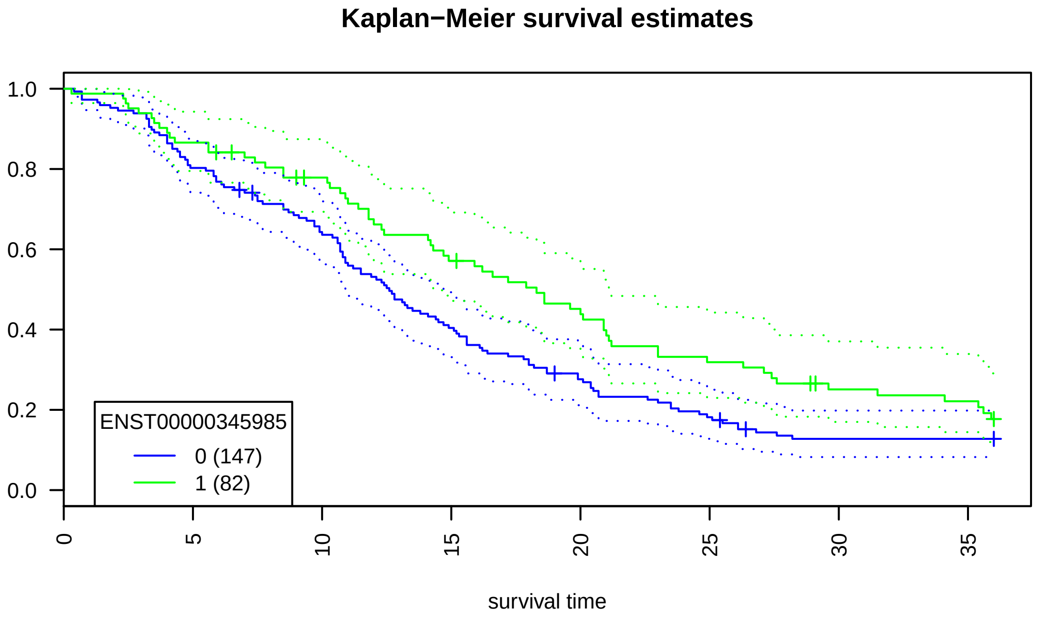

Figure 639: Each curve represents the relative number of ENST00000345985 survived to the corresponding point of time. Dashed lines represent 0.95 confidence intervals for the curves with the corresponding color. Total of 1 observation deleted due to missingness. Log-rank probability measure for the equality of these curves is 1.977140e-02. Additional annotations: *.GeneId*=ENSG00000115761, *.GeneName*=NOL10, *.GeneDesc*=nucleolar protein 10 [Source:HGNC Symbol;Acc:25862].

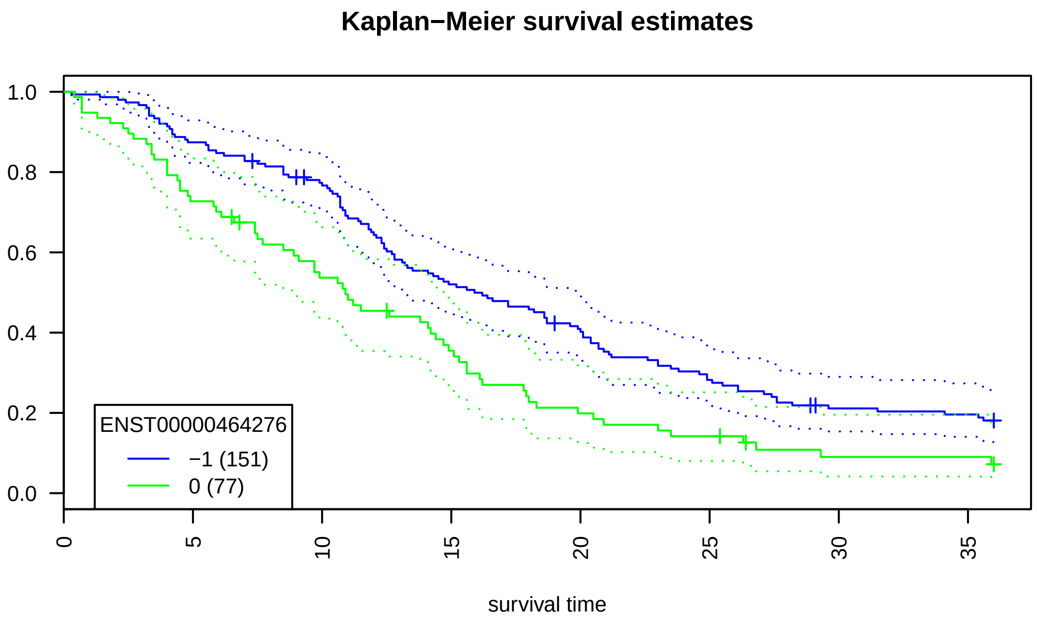

Figure 640: Each curve represents the relative number of ENST00000464276 survived to the corresponding point of time. Dashed lines represent 0.95 confidence intervals for the curves with the corresponding color. Total of 2 observations deleted due to missingness. Log-rank probability measure for the equality of these curves is 6.906127e-04. Additional annotations: *.GeneId*=ENSG00000106536, *.GeneName*=POU6F2, *.GeneDesc*=POU class 6 homeobox 2 [Source:HGNC Symbol;Acc:21694].

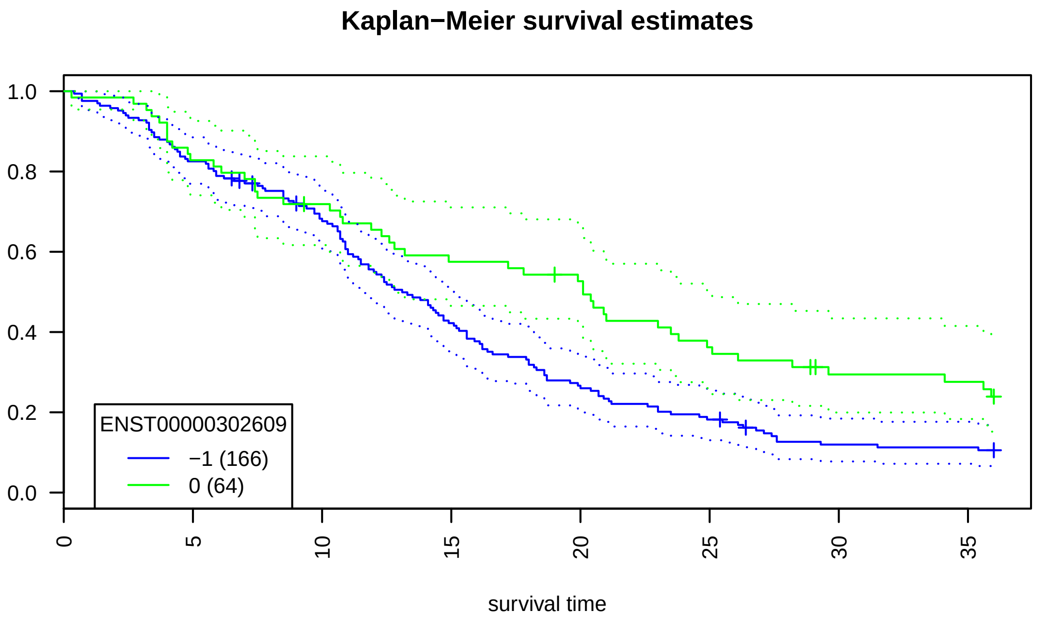

Figure 641: Each curve represents the relative number of ENST00000302609 survived to the corresponding point of time. Dashed lines represent 0.95 confidence intervals for the curves with the corresponding color. Log-rank probability measure for the equality of these curves is 4.80974e-03. Additional annotations: *.GeneId*=ENSG00000175395, *.GeneName*=ZNF25, *.GeneDesc*=zinc finger protein 25 [Source:HGNC Symbol;Acc:13043].

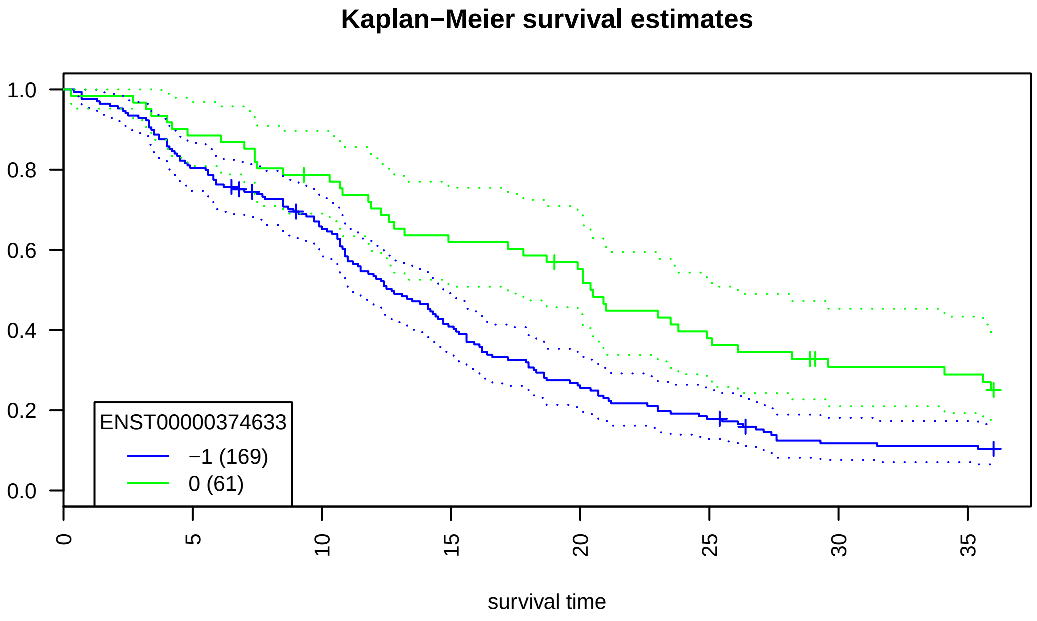

Figure 642: Each curve represents the relative number of ENST00000374633 survived to the corresponding point of time. Dashed lines represent 0.95 confidence intervals for the curves with the corresponding color. Log-rank probability measure for the equality of these curves is 6.834596e-04. Additional annotations: *.GeneId*=ENSG00000175395, *.GeneName*=ZNF25, *.GeneDesc*=zinc finger protein 25 [Source:HGNC Symbol;Acc:13043].

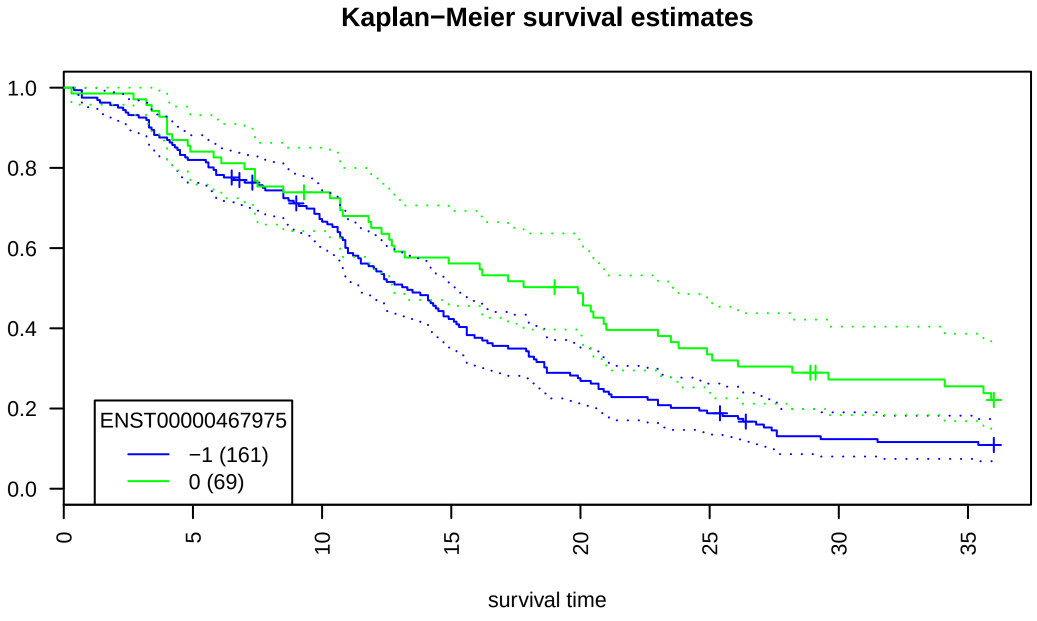

Figure 643: Each curve represents the relative number of ENST00000467975 survived to the corresponding point of time. Dashed lines represent 0.95 confidence intervals for the curves with the corresponding color. Log-rank probability measure for the equality of these curves is 1.193630e-02. Additional annotations: *.GeneId*=ENSG00000175395, *.GeneName*=ZNF25, *.GeneDesc*=zinc finger protein 25 [Source:HGNC Symbol;Acc:13043].

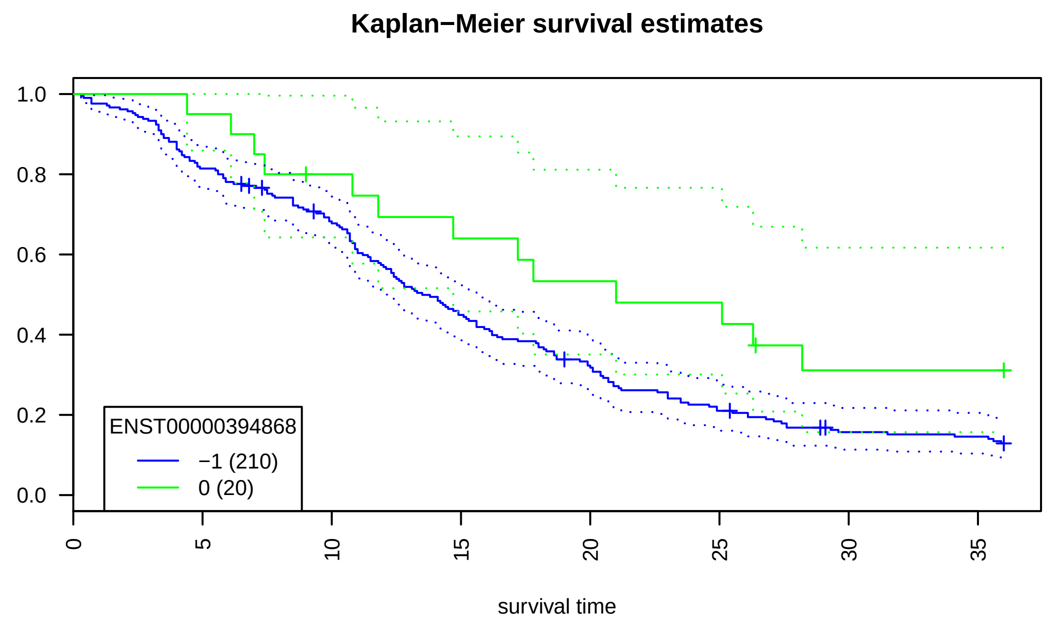

Figure 644: Each curve represents the relative number of ENST00000394868 survived to the corresponding point of time. Dashed lines represent 0.95 confidence intervals for the curves with the corresponding color. Log-rank probability measure for the equality of these curves is 4.30549e-02. Additional annotations: *.GeneId*=ENSG00000108262, *.GeneName*=GIT1, *.GeneDesc*=G protein-coupled receptor kinase interacting ArfGAP 1 [Source:HGNC Symbol;Acc:4272].

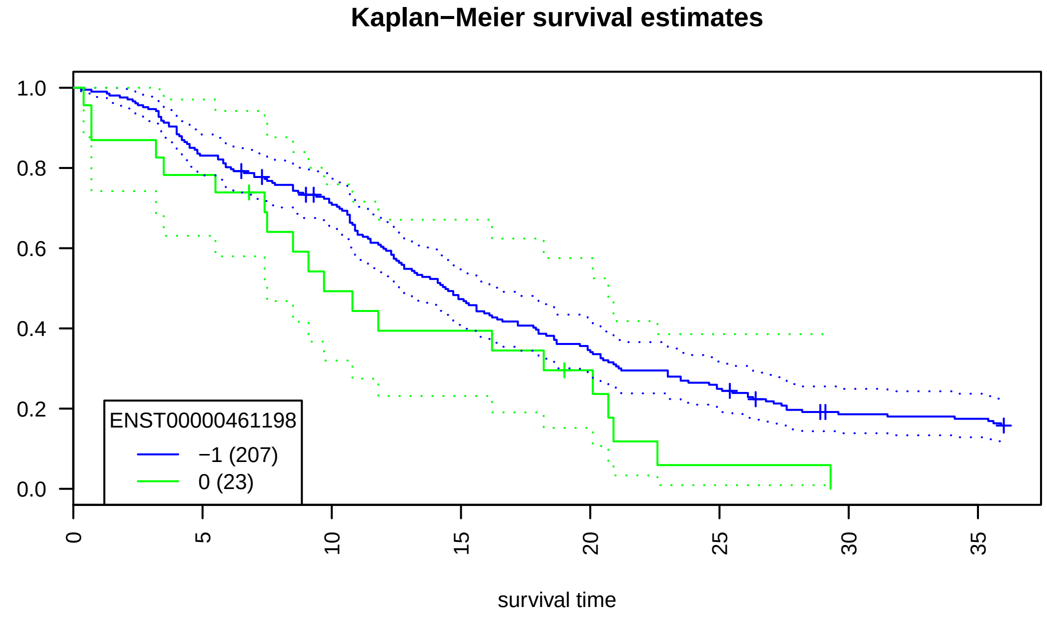

Figure 645: Each curve represents the relative number of ENST00000461198 survived to the corresponding point of time. Dashed lines represent 0.95 confidence intervals for the curves with the corresponding color. Log-rank probability measure for the equality of these curves is 3.403815e-02. Additional annotations: *.GeneId*=ENSG00000164112, *.GeneName*=TMEM155, *.GeneDesc*=transmembrane protein 155 [Source:HGNC Symbol;Acc:26418].

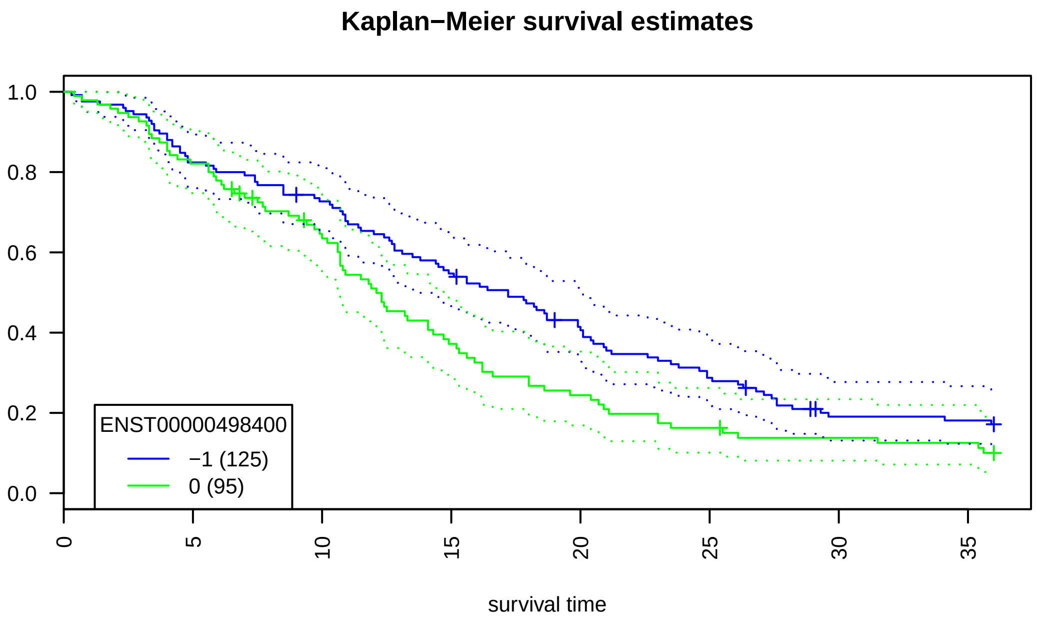

Figure 646: Each curve represents the relative number of ENST00000498400 survived to the corresponding point of time. Dashed lines represent 0.95 confidence intervals for the curves with the corresponding color. Total of 10 observations deleted due to missingness. Log-rank probability measure for the equality of these curves is 1.584620e-02. Additional annotations: *.GeneId*=ENSG00000128285, *.GeneName*=MCHR1, *.GeneDesc*=melanin-concentrating hormone receptor 1 [Source:HGNC Symbol;Acc:4479].

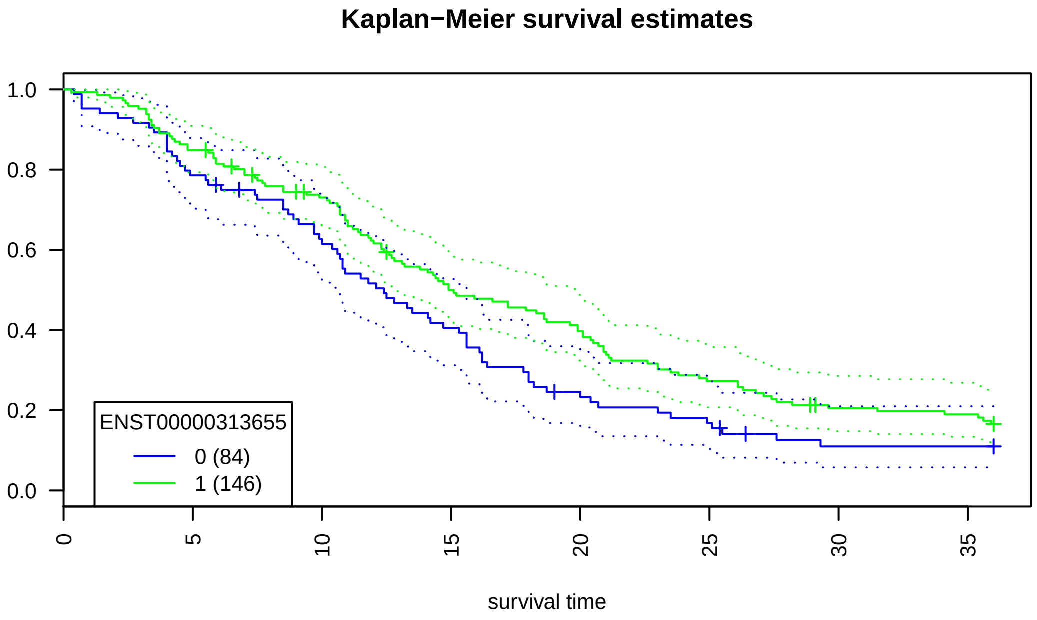

Figure 647: Each curve represents the relative number of ENST00000313655 survived to the corresponding point of time. Dashed lines represent 0.95 confidence intervals for the curves with the corresponding color. Log-rank probability measure for the equality of these curves is 3.487352e-02. Additional annotations: *.GeneId*=ENSG00000136982, *.GeneName*=DSCC1, *.GeneDesc*=defective in sister chromatid cohesion 1 homolog (S. cerevisiae) [Source:HGNC Symbol;Acc:24453].

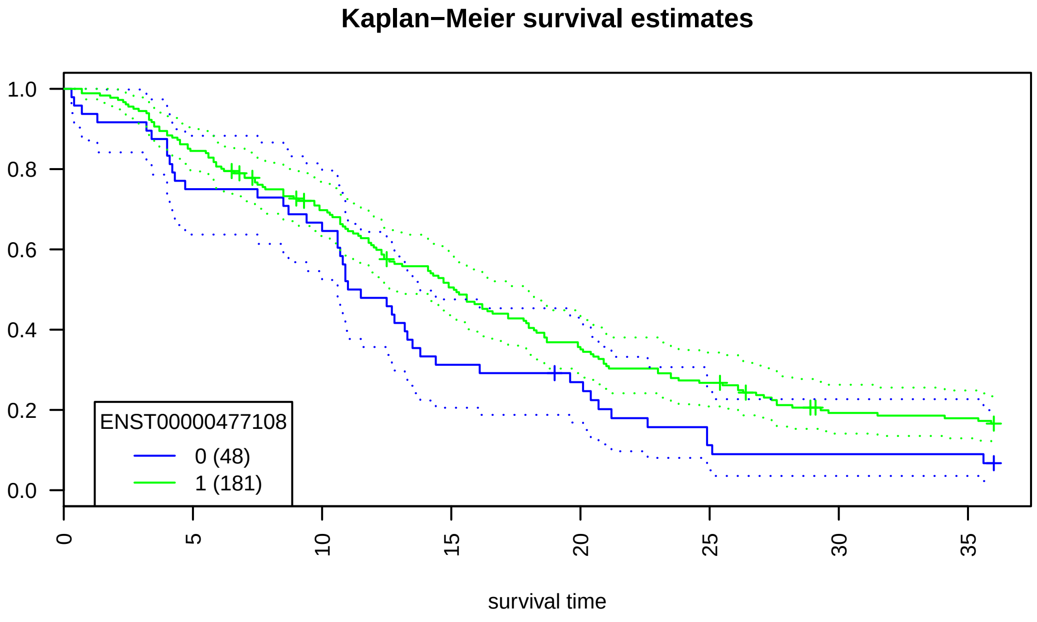

Figure 648: Each curve represents the relative number of ENST00000477108 survived to the corresponding point of time. Dashed lines represent 0.95 confidence intervals for the curves with the corresponding color. Total of 1 observation deleted due to missingness. Log-rank probability measure for the equality of these curves is 2.493249e-02. Additional annotations: *.GeneId*=ENSG00000169813, *.GeneName*=HNRNPF, *.GeneDesc*=heterogeneous nuclear ribonucleoprotein F [Source:HGNC Symbol;Acc:5039].

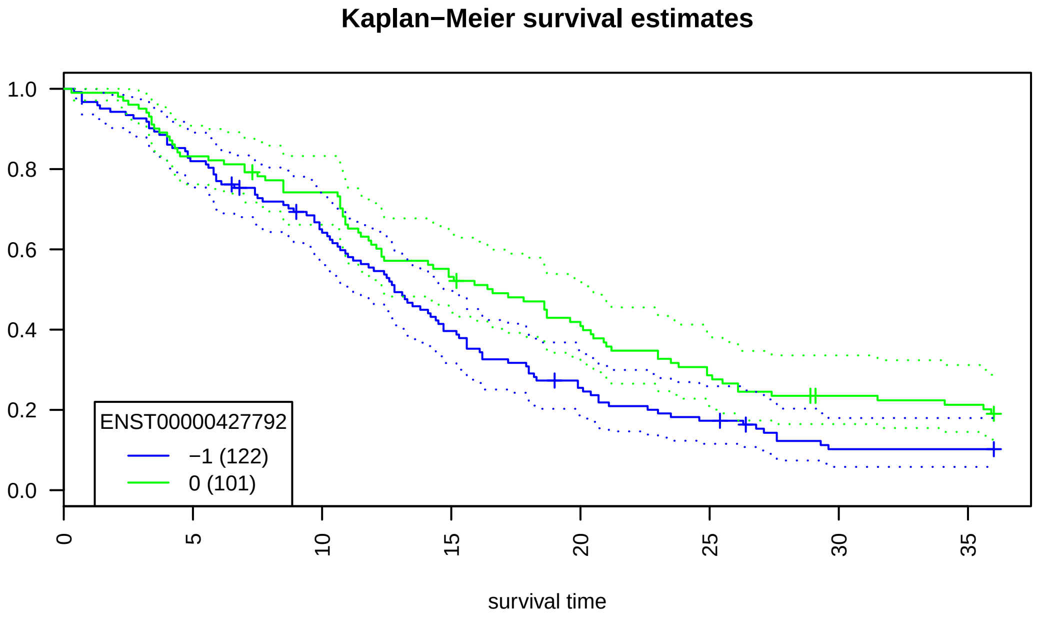

Figure 649: Each curve represents the relative number of ENST00000427792 survived to the corresponding point of time. Dashed lines represent 0.95 confidence intervals for the curves with the corresponding color. Total of 7 observations deleted due to missingness. Log-rank probability measure for the equality of these curves is 2.198440e-02. Additional annotations: *.GeneId*=ENSG00000151779, *.GeneName*=NBAS, *.GeneDesc*=neuroblastoma amplified sequence [Source:HGNC Symbol;Acc:15625].

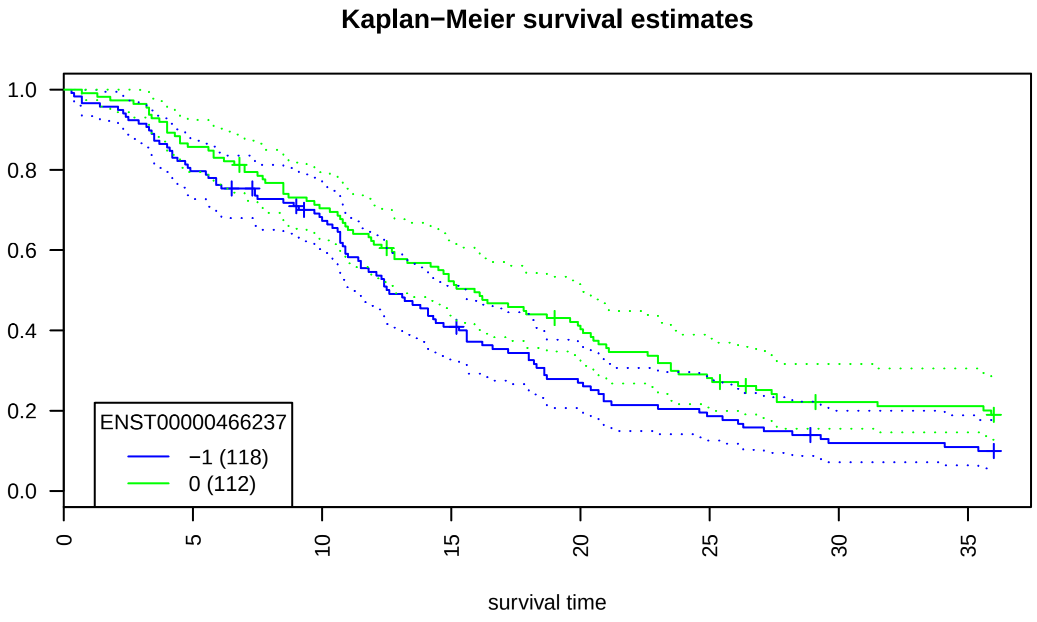

Figure 650: Each curve represents the relative number of ENST00000466237 survived to the corresponding point of time. Dashed lines represent 0.95 confidence intervals for the curves with the corresponding color. Log-rank probability measure for the equality of these curves is 3.201804e-02. Additional annotations: *.GeneId*=ENSG00000100412, *.GeneName*=ACO2, *.GeneDesc*=aconitase 2, mitochondrial [Source:HGNC Symbol;Acc:118].

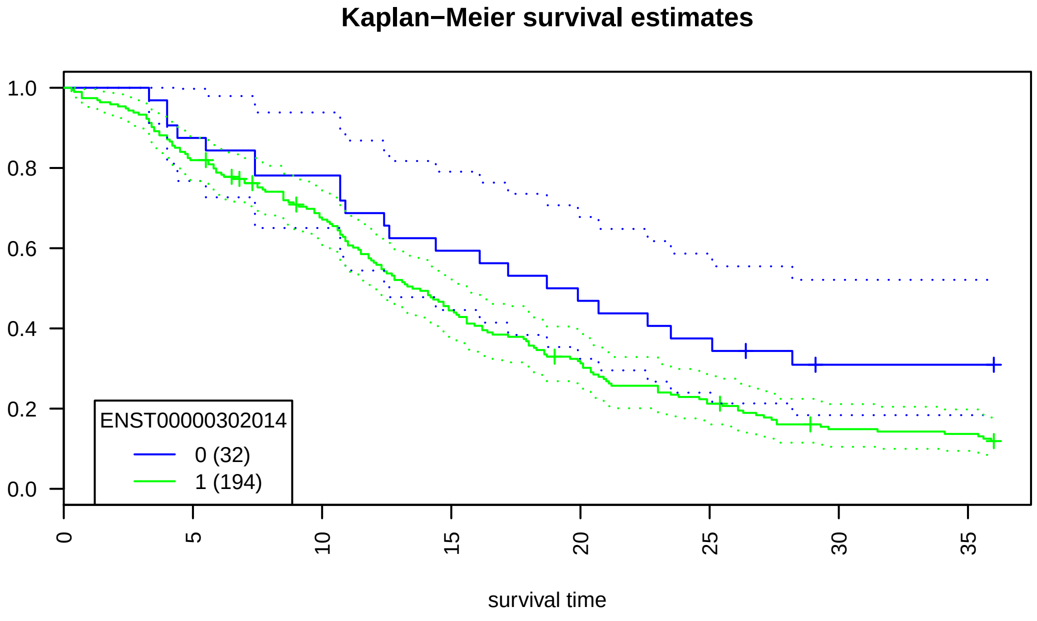

Figure 651: Each curve represents the relative number of ENST00000302014 survived to the corresponding point of time. Dashed lines represent 0.95 confidence intervals for the curves with the corresponding color. Total of 4 observations deleted due to missingness. Log-rank probability measure for the equality of these curves is 2.518466e-02. Additional annotations: *.GeneId*=ENSG00000170458, *.GeneName*=CD14, *.GeneDesc*=CD14 molecule [Source:HGNC Symbol;Acc:1628].

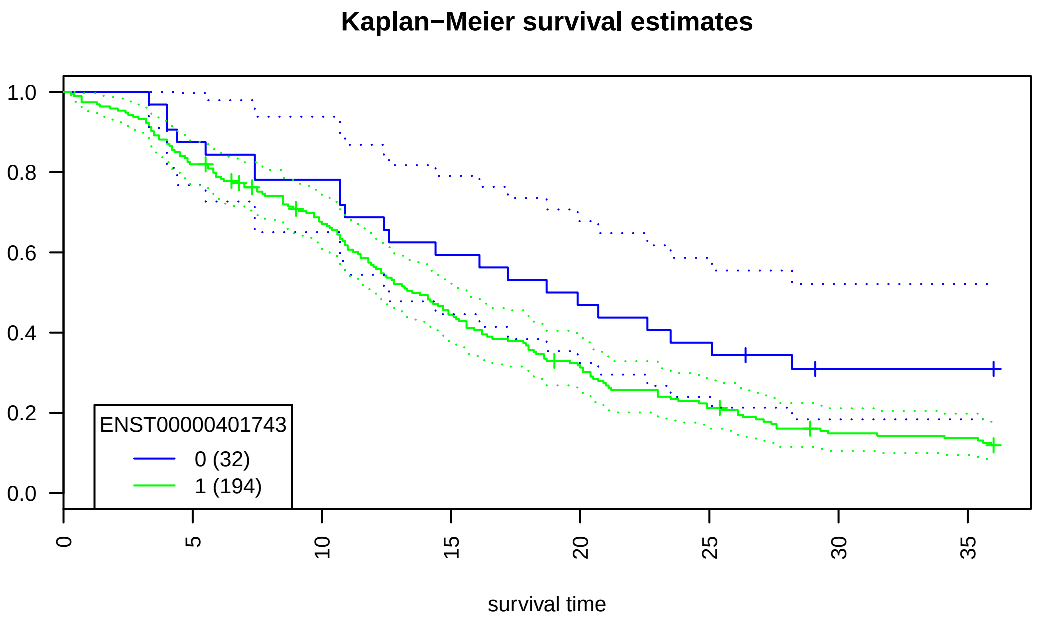

Figure 652: Each curve represents the relative number of ENST00000401743 survived to the corresponding point of time. Dashed lines represent 0.95 confidence intervals for the curves with the corresponding color. Total of 4 observations deleted due to missingness. Log-rank probability measure for the equality of these curves is 2.518466e-02. Additional annotations: *.GeneId*=ENSG00000170458, *.GeneName*=CD14, *.GeneDesc*=CD14 molecule [Source:HGNC Symbol;Acc:1628].

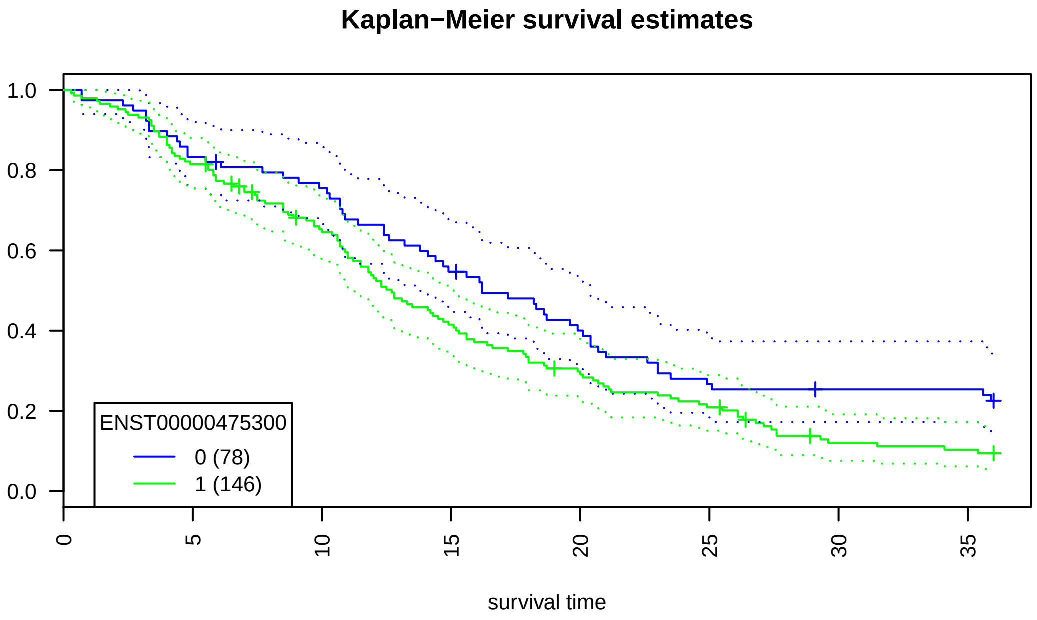

Figure 653: Each curve represents the relative number of ENST00000475300 survived to the corresponding point of time. Dashed lines represent 0.95 confidence intervals for the curves with the corresponding color. Total of 6 observations deleted due to missingness. Log-rank probability measure for the equality of these curves is 2.100069e-02. Additional annotations: *.GeneId*=ENSG00000012779, *.GeneName*=ALOX5, *.GeneDesc*=arachidonate 5-lipoxygenase [Source:HGNC Symbol;Acc:435].

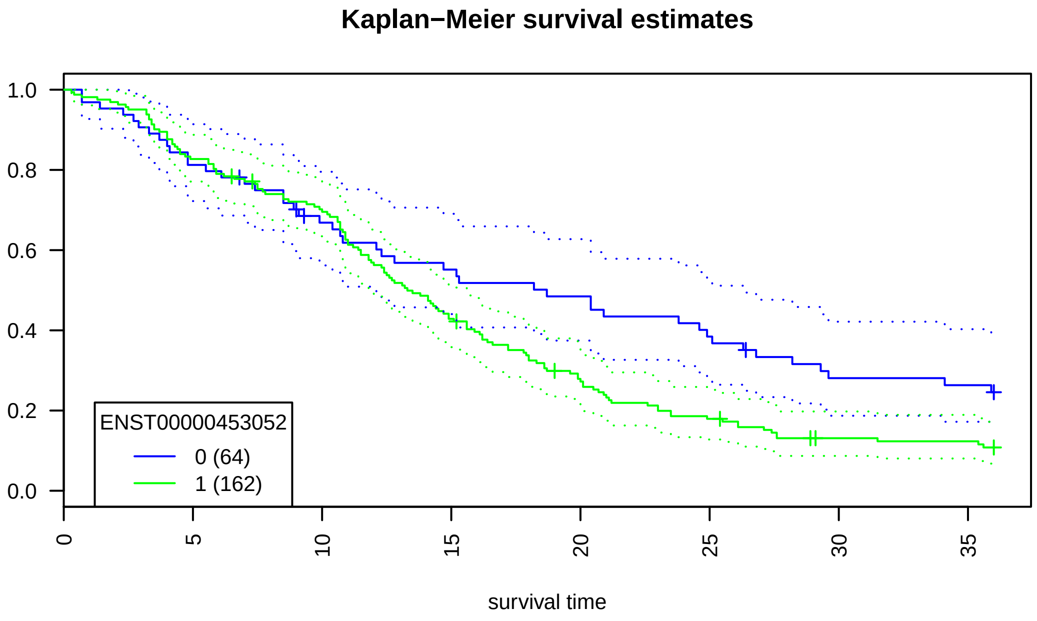

Figure 654: Each curve represents the relative number of ENST00000453052 survived to the corresponding point of time. Dashed lines represent 0.95 confidence intervals for the curves with the corresponding color. Total of 4 observations deleted due to missingness. Log-rank probability measure for the equality of these curves is 1.531777e-02. Additional annotations: *.GeneId*=ENSG00000106624, *.GeneName*=AEBP1, *.GeneDesc*=AE binding protein 1 [Source:HGNC Symbol;Acc:303].

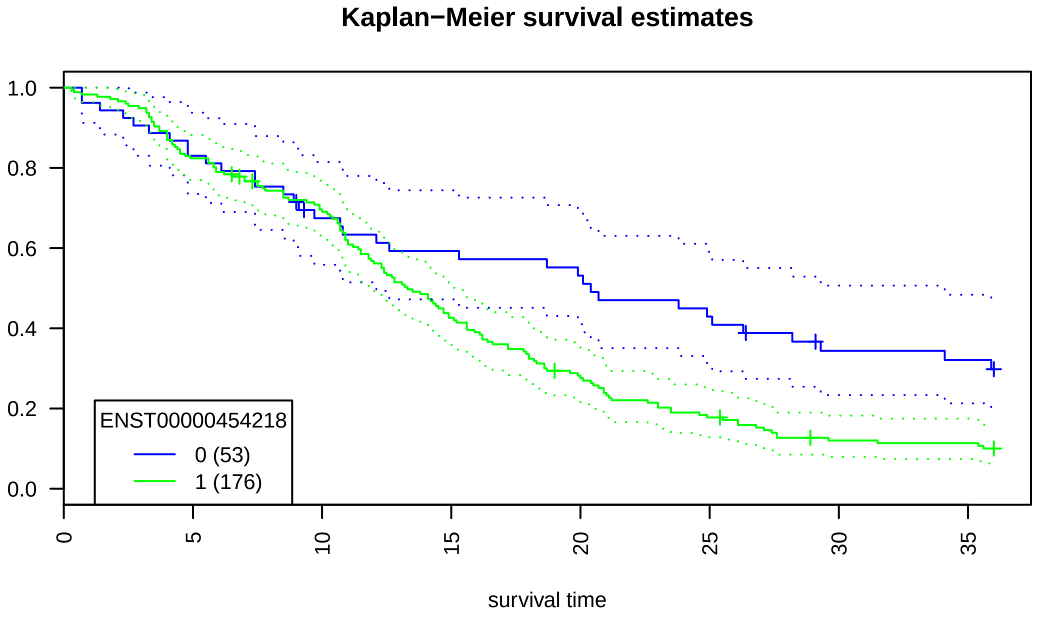

Figure 655: Each curve represents the relative number of ENST00000454218 survived to the corresponding point of time. Dashed lines represent 0.95 confidence intervals for the curves with the corresponding color. Total of 1 observation deleted due to missingness. Log-rank probability measure for the equality of these curves is 2.49332e-03. Additional annotations: *.GeneId*=ENSG00000106624, *.GeneName*=AEBP1, *.GeneDesc*=AE binding protein 1 [Source:HGNC Symbol;Acc:303].

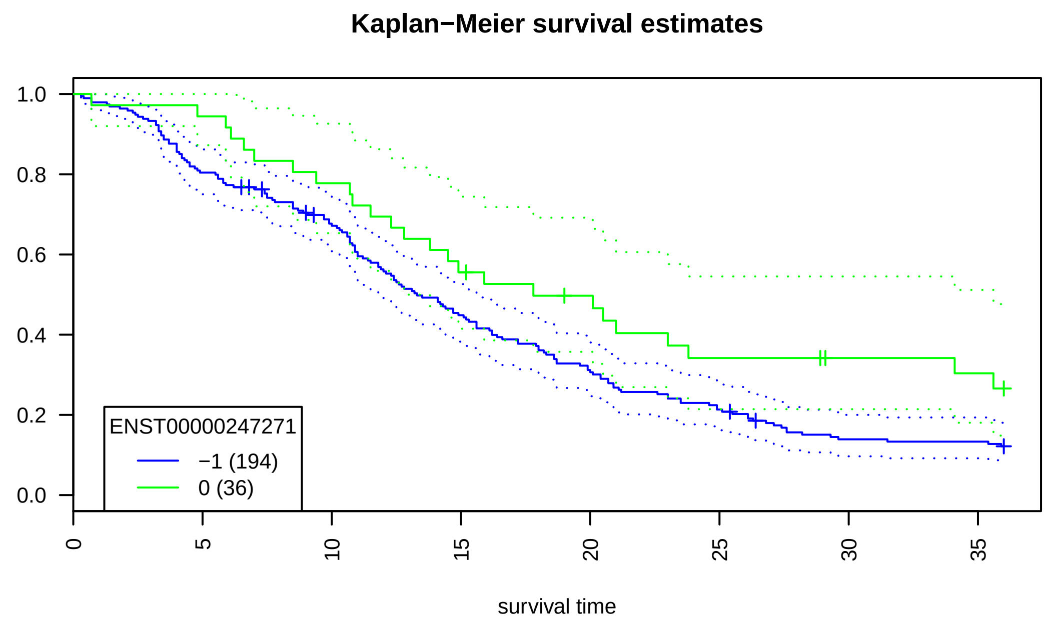

Figure 656: Each curve represents the relative number of ENST00000247271 survived to the corresponding point of time. Dashed lines represent 0.95 confidence intervals for the curves with the corresponding color. Log-rank probability measure for the equality of these curves is 2.922255e-02. Additional annotations: *.GeneId*=ENSG00000126861, *.GeneName*=OMG, *.GeneDesc*=oligodendrocyte myelin glycoprotein [Source:HGNC Symbol;Acc:8135].

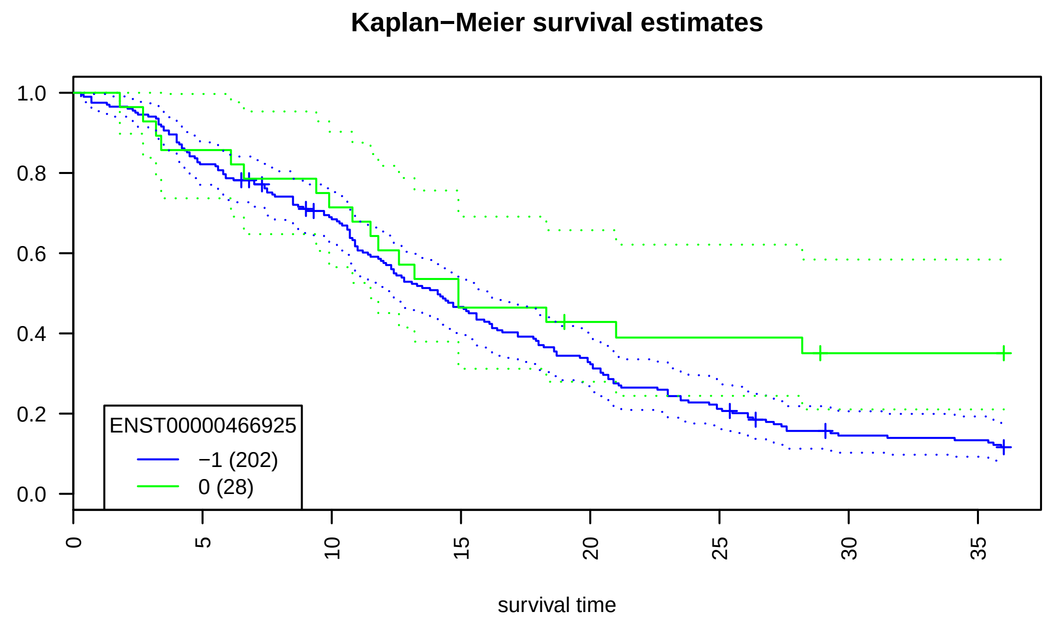

Figure 657: Each curve represents the relative number of ENST00000466925 survived to the corresponding point of time. Dashed lines represent 0.95 confidence intervals for the curves with the corresponding color. Log-rank probability measure for the equality of these curves is 4.564561e-02. Additional annotations: *.GeneId*=ENSG00000131089, *.GeneName*=ARHGEF9, *.GeneDesc*=Cdc42 guanine nucleotide exchange factor (GEF) 9 [Source:HGNC Symbol;Acc:14561].

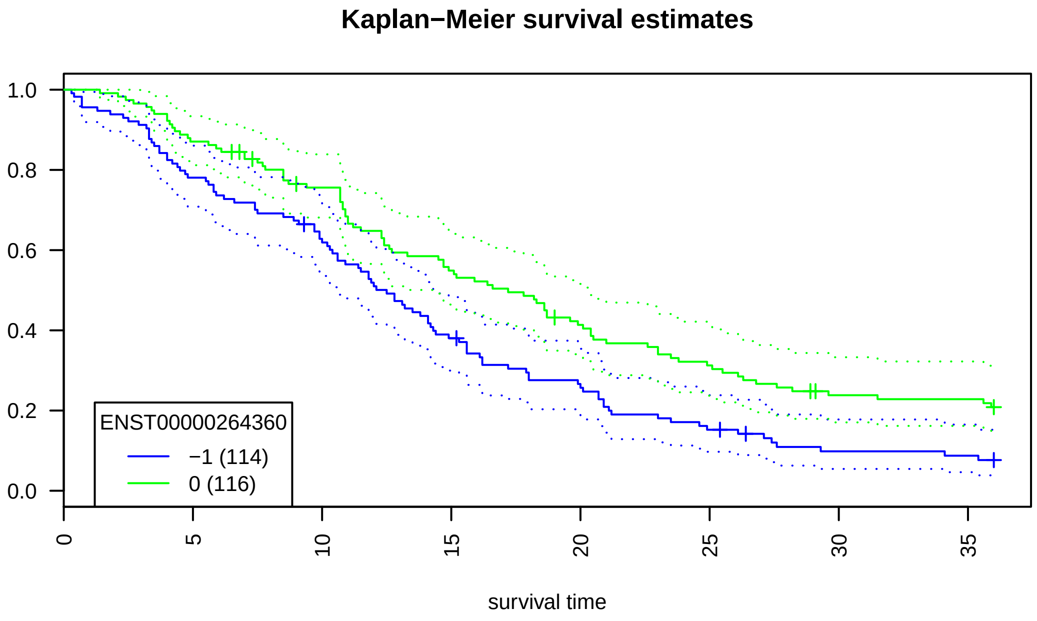

Figure 658: Each curve represents the relative number of ENST00000264360 survived to the corresponding point of time. Dashed lines represent 0.95 confidence intervals for the curves with the corresponding color. Log-rank probability measure for the equality of these curves is 1.040692e-03. Additional annotations: *.GeneId*=ENSG00000138650, *.GeneName*=PCDH10, *.GeneDesc*=protocadherin 10 [Source:HGNC Symbol;Acc:13404].

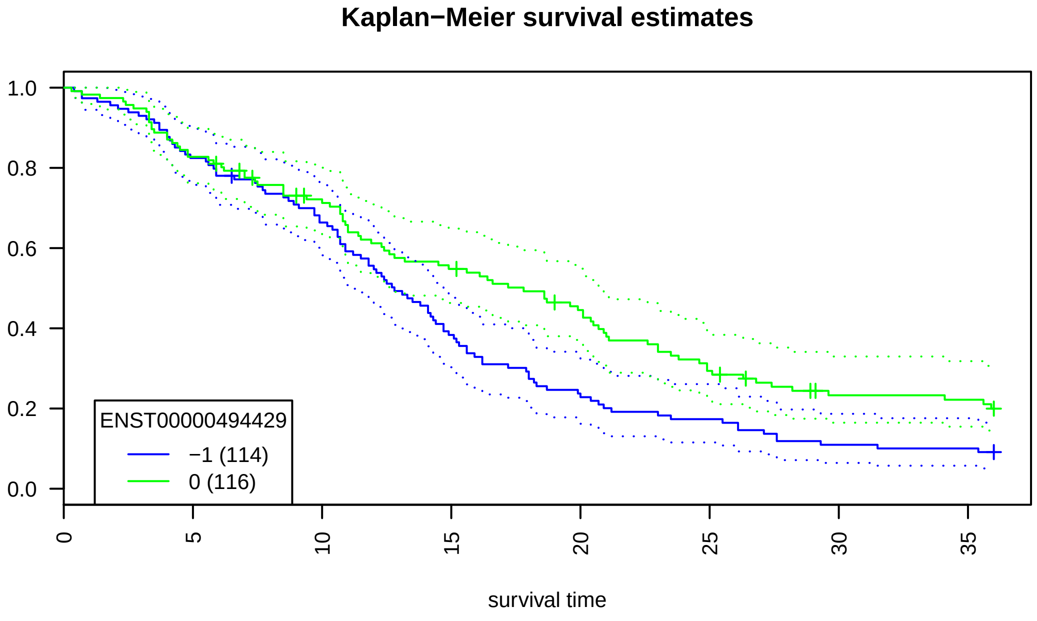

Figure 659: Each curve represents the relative number of ENST00000494429 survived to the corresponding point of time. Dashed lines represent 0.95 confidence intervals for the curves with the corresponding color. Log-rank probability measure for the equality of these curves is 6.873693e-03. Additional annotations: *.GeneId*=ENSG00000178691, *.GeneName*=SUZ12, *.GeneDesc*=suppressor of zeste 12 homolog pseudogene [Source:HGNC Symbol;Acc:32421].

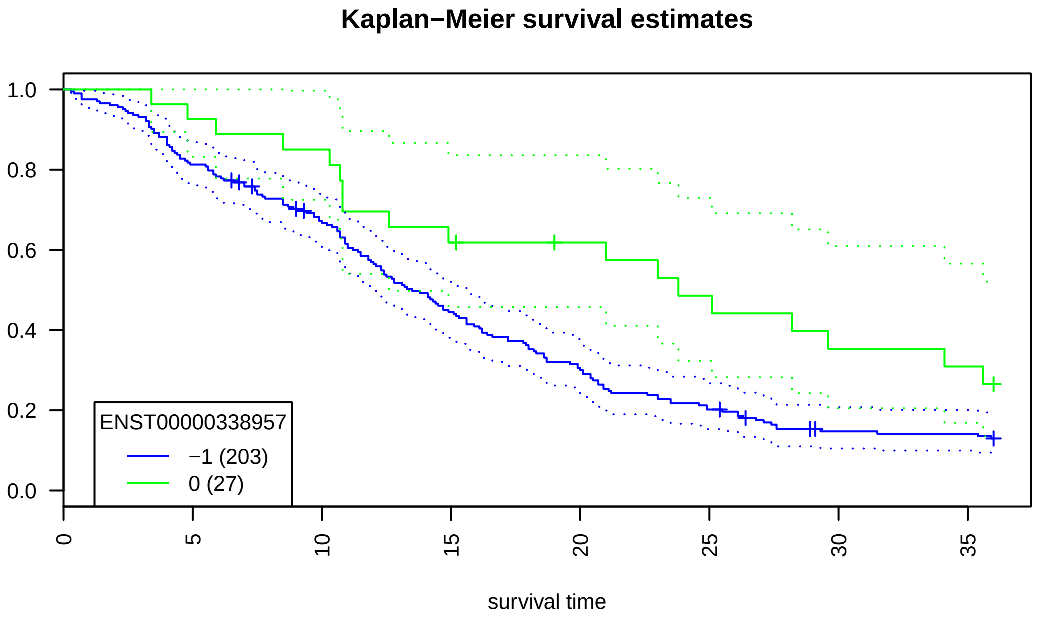

Figure 660: Each curve represents the relative number of ENST00000338957 survived to the corresponding point of time. Dashed lines represent 0.95 confidence intervals for the curves with the corresponding color. Log-rank probability measure for the equality of these curves is 1.625673e-02. Additional annotations: *.GeneId*=ENSG00000102053, *.GeneName*=ZC3H12B, *.GeneDesc*=zinc finger CCCH-type containing 12B [Source:HGNC Symbol;Acc:17407].

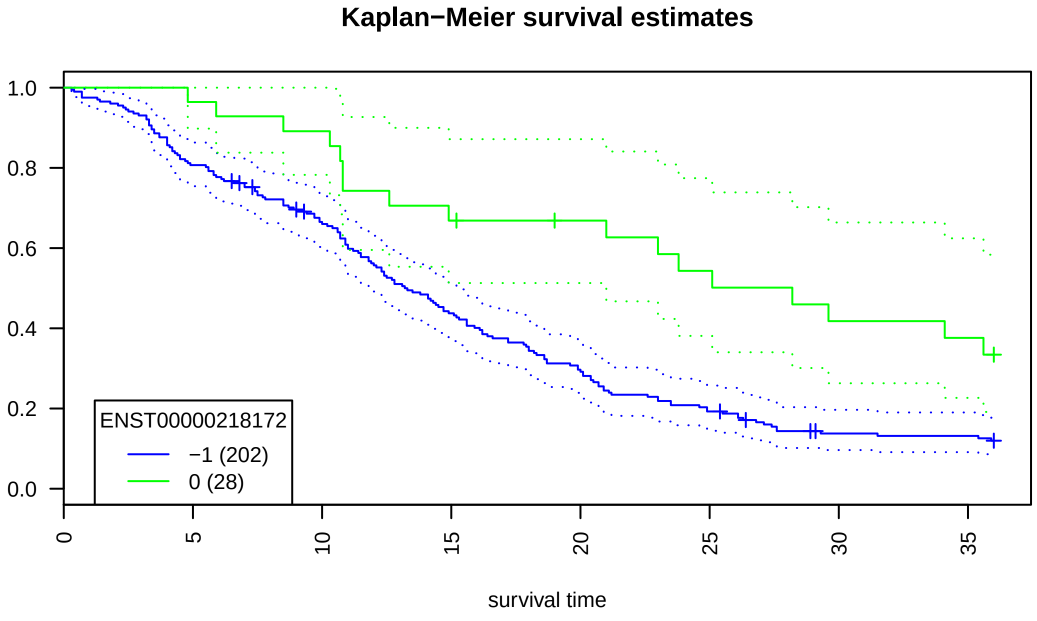

Figure 661: Each curve represents the relative number of ENST00000218172 survived to the corresponding point of time. Dashed lines represent 0.95 confidence intervals for the curves with the corresponding color. Log-rank probability measure for the equality of these curves is 1.316977e-03. Additional annotations: *.GeneId*=ENSG00000102053, *.GeneName*=ZC3H12B, *.GeneDesc*=zinc finger CCCH-type containing 12B [Source:HGNC Symbol;Acc:17407].

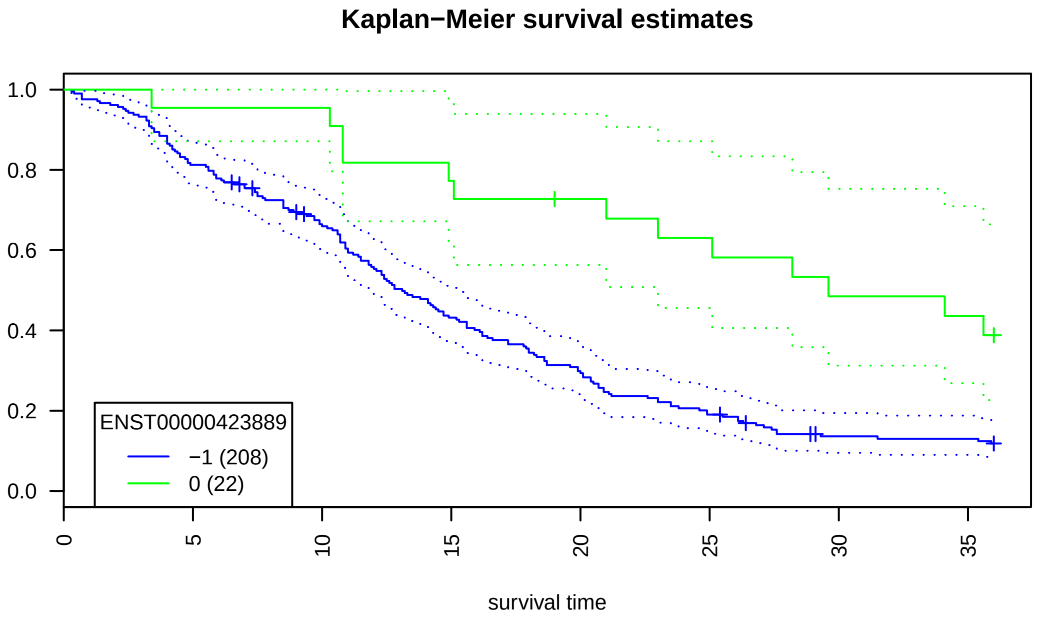

Figure 662: Each curve represents the relative number of ENST00000423889 survived to the corresponding point of time. Dashed lines represent 0.95 confidence intervals for the curves with the corresponding color. Log-rank probability measure for the equality of these curves is 4.597476e-04. Additional annotations: *.GeneId*=ENSG00000102053, *.GeneName*=ZC3H12B, *.GeneDesc*=zinc finger CCCH-type containing 12B [Source:HGNC Symbol;Acc:17407].

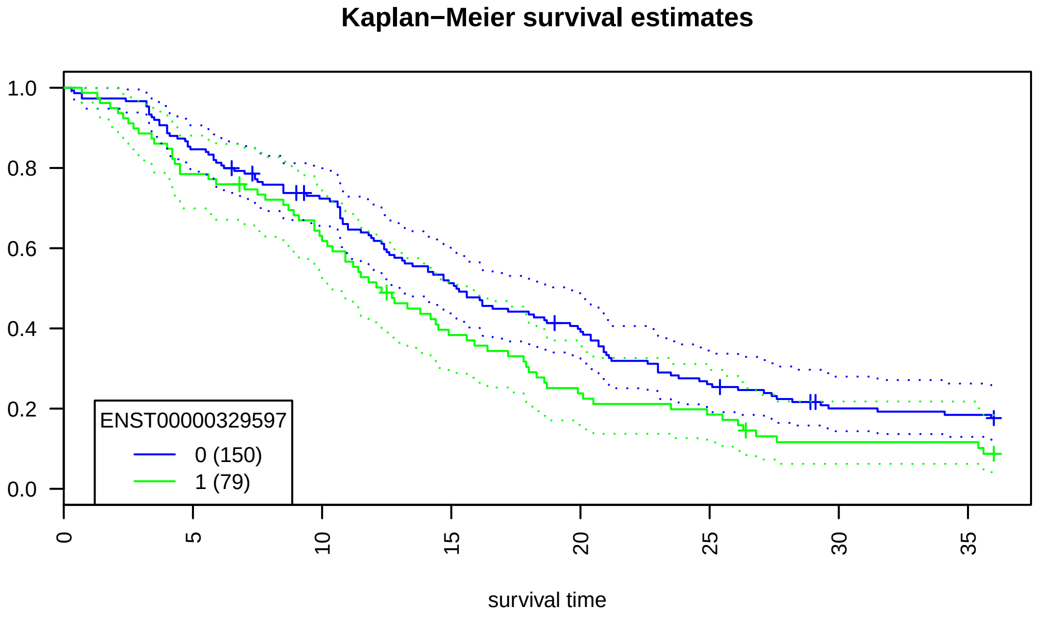

Figure 663: Each curve represents the relative number of ENST00000329597 survived to the corresponding point of time. Dashed lines represent 0.95 confidence intervals for the curves with the corresponding color. Total of 1 observation deleted due to missingness. Log-rank probability measure for the equality of these curves is 2.884961e-02. Additional annotations: *.GeneId*=ENSG00000188488, *.GeneName*=SERPINA5, *.GeneDesc*=serpin peptidase inhibitor, clade A (alpha-1 antiproteinase, antitrypsin), member 5 [Source:HGNC Symbol;Acc:8723].

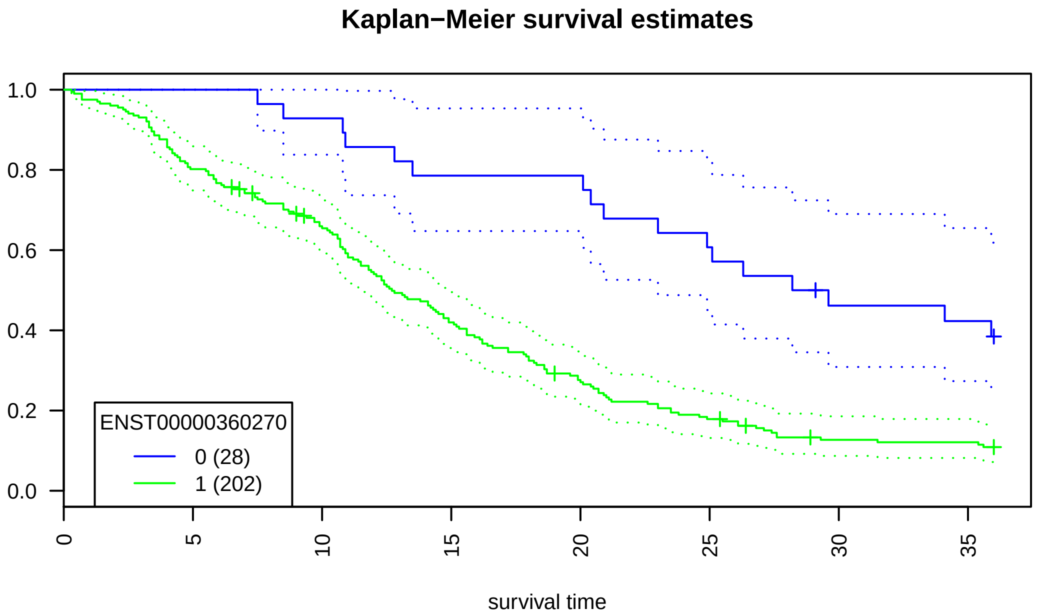

Figure 664: Each curve represents the relative number of ENST00000360270 survived to the corresponding point of time. Dashed lines represent 0.95 confidence intervals for the curves with the corresponding color. Log-rank probability measure for the equality of these curves is 2.769993e-05. Additional annotations: *.GeneId*=ENSG00000147065, *.GeneName*=MSN, *.GeneDesc*=moesin [Source:HGNC Symbol;Acc:7373].

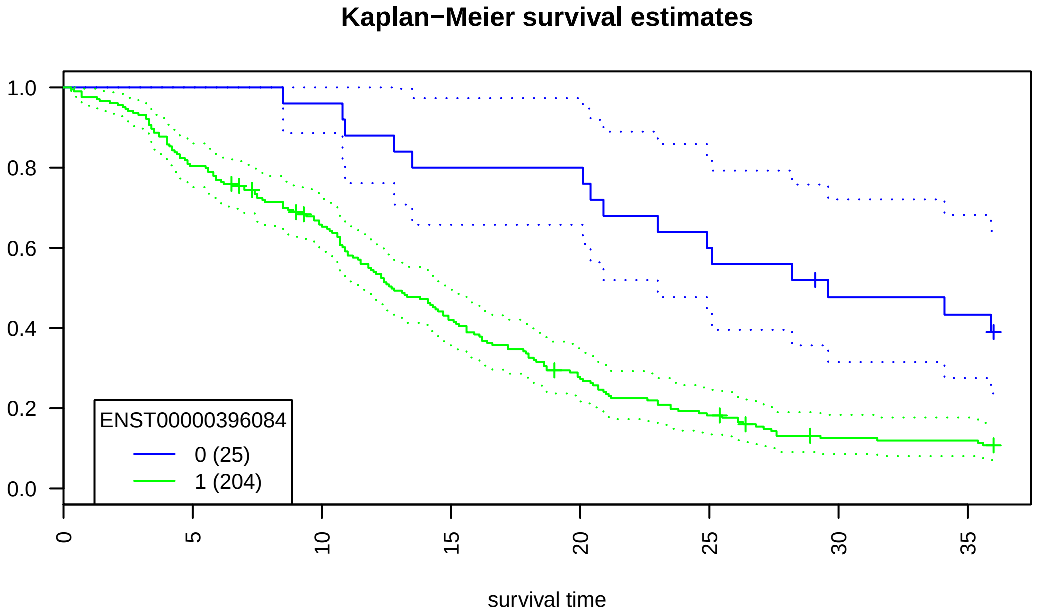

Figure 665: Each curve represents the relative number of ENST00000396084 survived to the corresponding point of time. Dashed lines represent 0.95 confidence intervals for the curves with the corresponding color. Total of 1 observation deleted due to missingness. Log-rank probability measure for the equality of these curves is 4.456713e-05. Additional annotations: *.GeneId*=ENSG00000147065, *.GeneName*=MSN, *.GeneDesc*=moesin [Source:HGNC Symbol;Acc:7373].

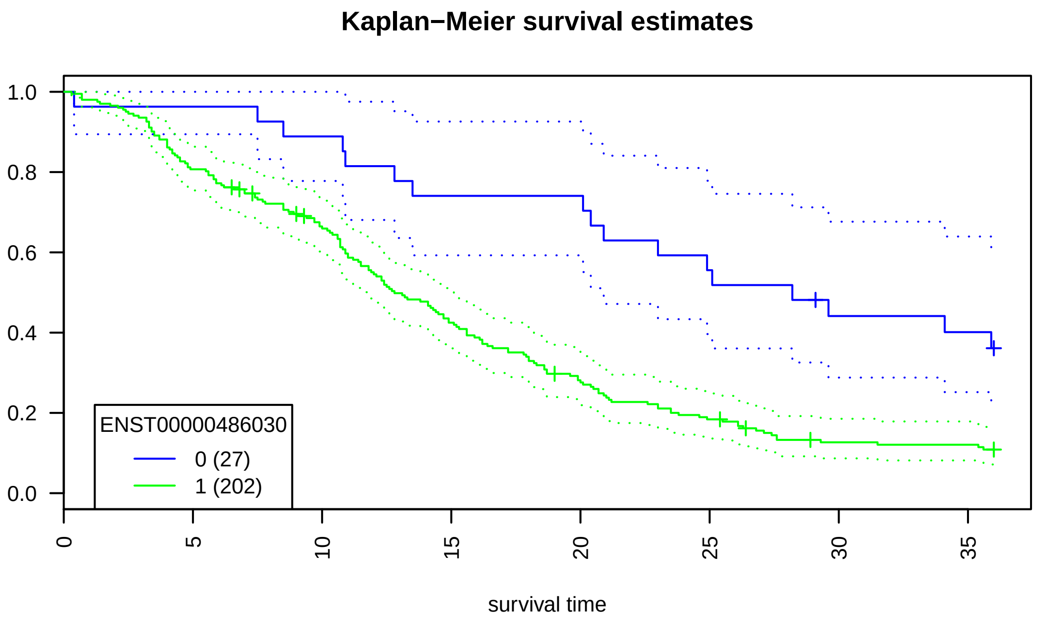

Figure 666: Each curve represents the relative number of ENST00000486030 survived to the corresponding point of time. Dashed lines represent 0.95 confidence intervals for the curves with the corresponding color. Total of 1 observation deleted due to missingness. Log-rank probability measure for the equality of these curves is 1.787260e-04. Additional annotations: *.GeneId*=ENSG00000147065, *.GeneName*=MSN, *.GeneDesc*=moesin [Source:HGNC Symbol;Acc:7373].

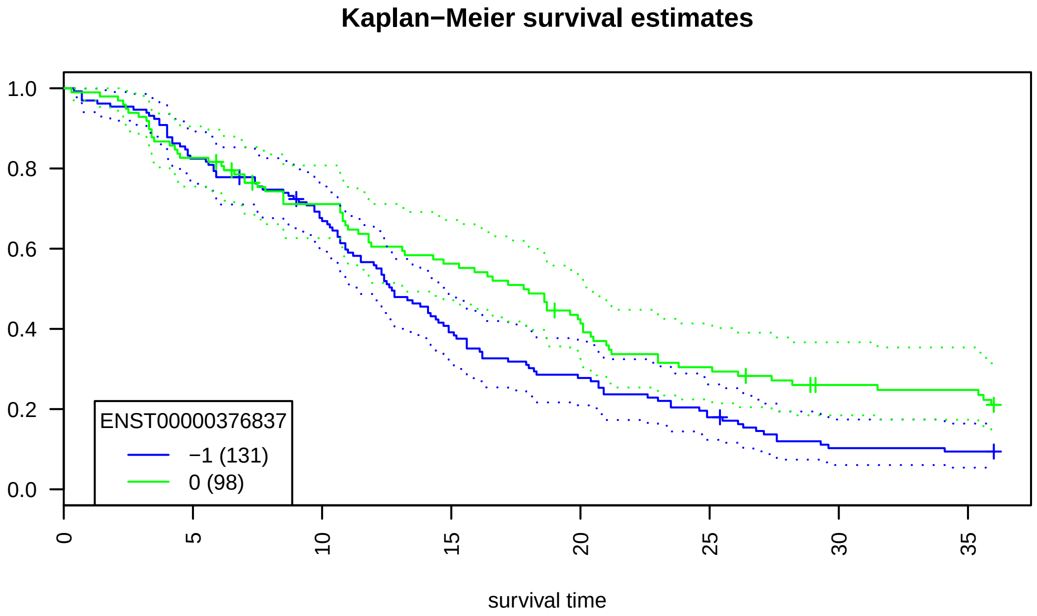

Figure 667: Each curve represents the relative number of ENST00000376837 survived to the corresponding point of time. Dashed lines represent 0.95 confidence intervals for the curves with the corresponding color. Total of 1 observation deleted due to missingness. Log-rank probability measure for the equality of these curves is 1.376097e-02. Additional annotations: *.GeneId*=ENSG00000156017, *.GeneName*=C9orf41, *.GeneDesc*=UPF0586 protein C9orf41 [Source:UniProtKB/Swiss-Prot;Acc:Q8N4J0].

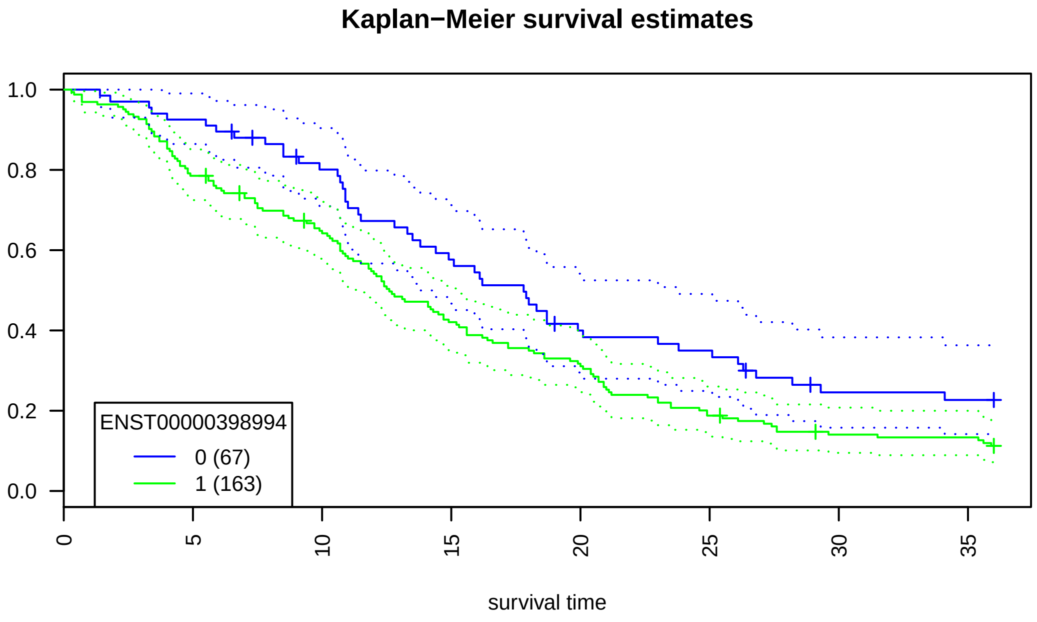

Figure 668: Each curve represents the relative number of ENST00000398994 survived to the corresponding point of time. Dashed lines represent 0.95 confidence intervals for the curves with the corresponding color. Log-rank probability measure for the equality of these curves is 1.271800e-02.

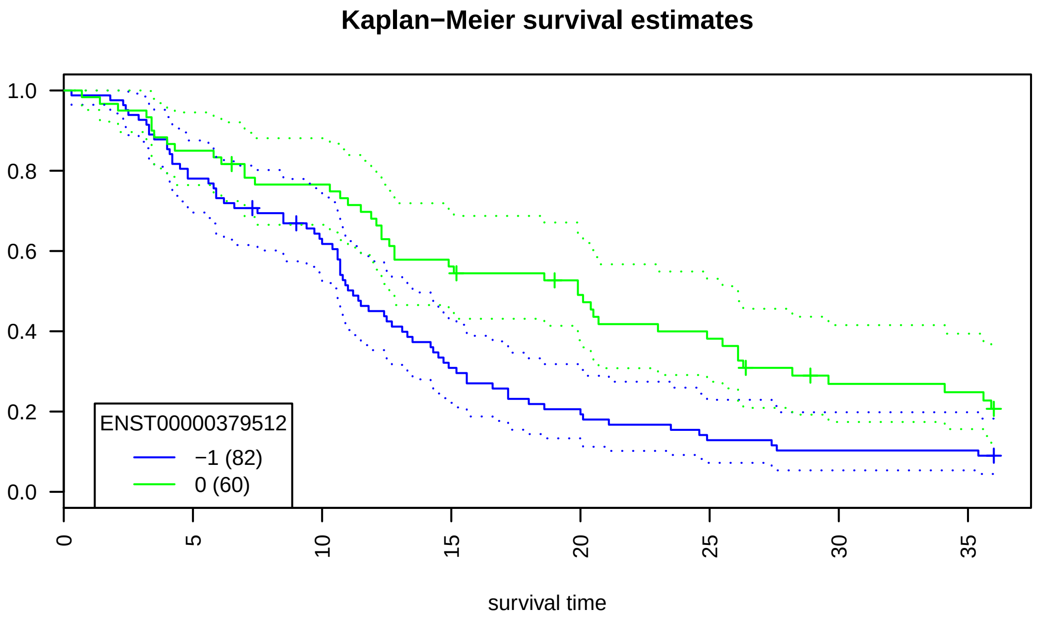

Figure 669: Each curve represents the relative number of ENST00000379512 survived to the corresponding point of time. Dashed lines represent 0.95 confidence intervals for the curves with the corresponding color. Total of 88 observations deleted due to missingness. Log-rank probability measure for the equality of these curves is 2.246317e-03. Additional annotations: *.GeneId*=ENSG00000170153, *.GeneName*=RNF150, *.GeneDesc*=ring finger protein 150 [Source:HGNC Symbol;Acc:23138].

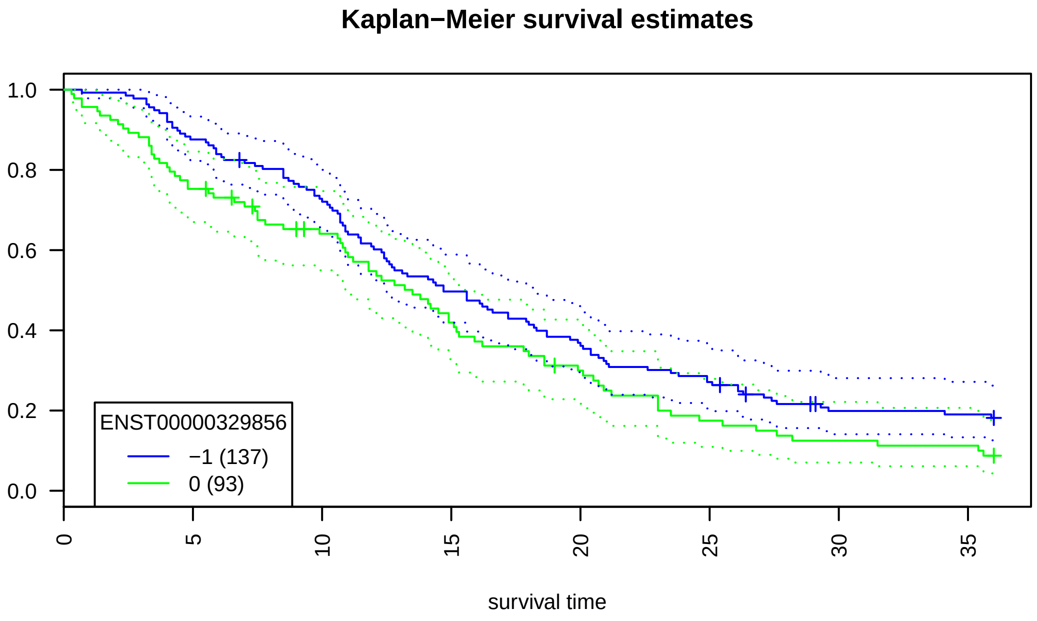

Figure 670: Each curve represents the relative number of ENST00000329856 survived to the corresponding point of time. Dashed lines represent 0.95 confidence intervals for the curves with the corresponding color. Log-rank probability measure for the equality of these curves is 4.335352e-02. Additional annotations: *.GeneId*=ENSG00000100744, *.GeneName*=C14orf129, *.GeneDesc*=GSK3-beta interaction protein (GSKIP) [Source:UniProtKB/Swiss-Prot;Acc:Q9P0R6].

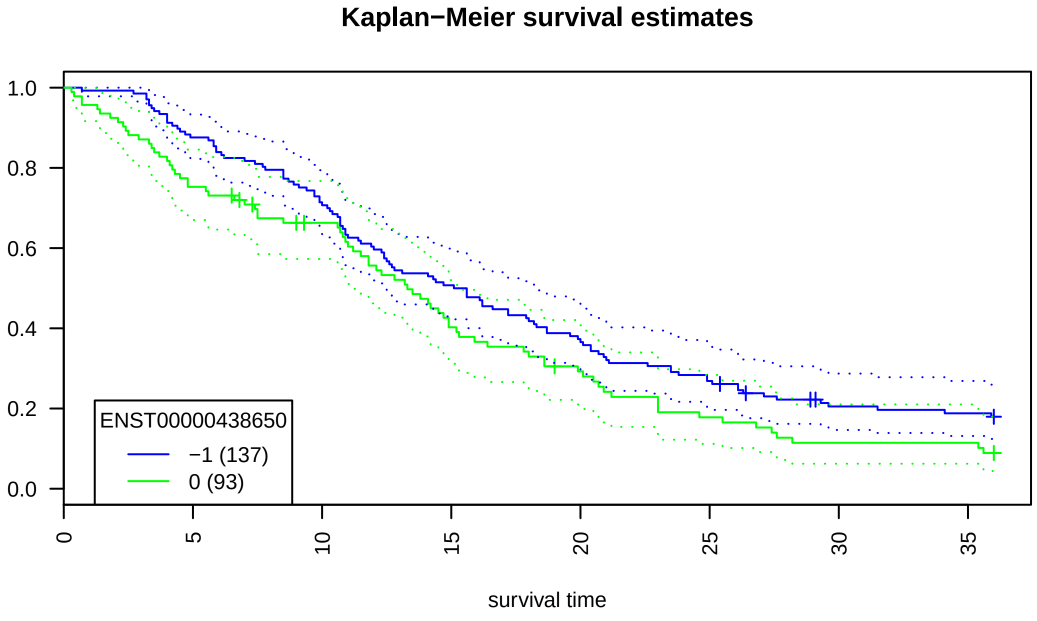

Figure 671: Each curve represents the relative number of ENST00000438650 survived to the corresponding point of time. Dashed lines represent 0.95 confidence intervals for the curves with the corresponding color. Log-rank probability measure for the equality of these curves is 4.581882e-02. Additional annotations: *.GeneId*=ENSG00000100744, *.GeneName*=C14orf129, *.GeneDesc*=GSK3-beta interaction protein (GSKIP) [Source:UniProtKB/Swiss-Prot;Acc:Q9P0R6].

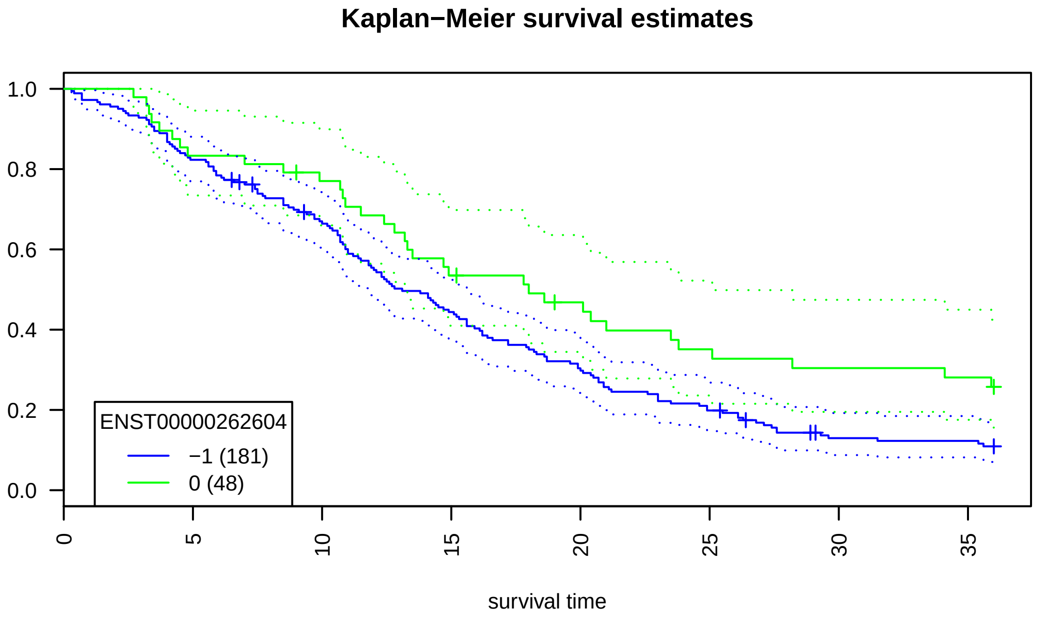

Figure 672: Each curve represents the relative number of ENST00000262604 survived to the corresponding point of time. Dashed lines represent 0.95 confidence intervals for the curves with the corresponding color. Total of 1 observation deleted due to missingness. Log-rank probability measure for the equality of these curves is 1.749676e-02. Additional annotations: *.GeneId*=ENSG00000124194, *.GeneName*=GDAP1L1, *.GeneDesc*=ganglioside-induced differentiation-associated protein 1-like 1 [Source:HGNC Symbol;Acc:4213].

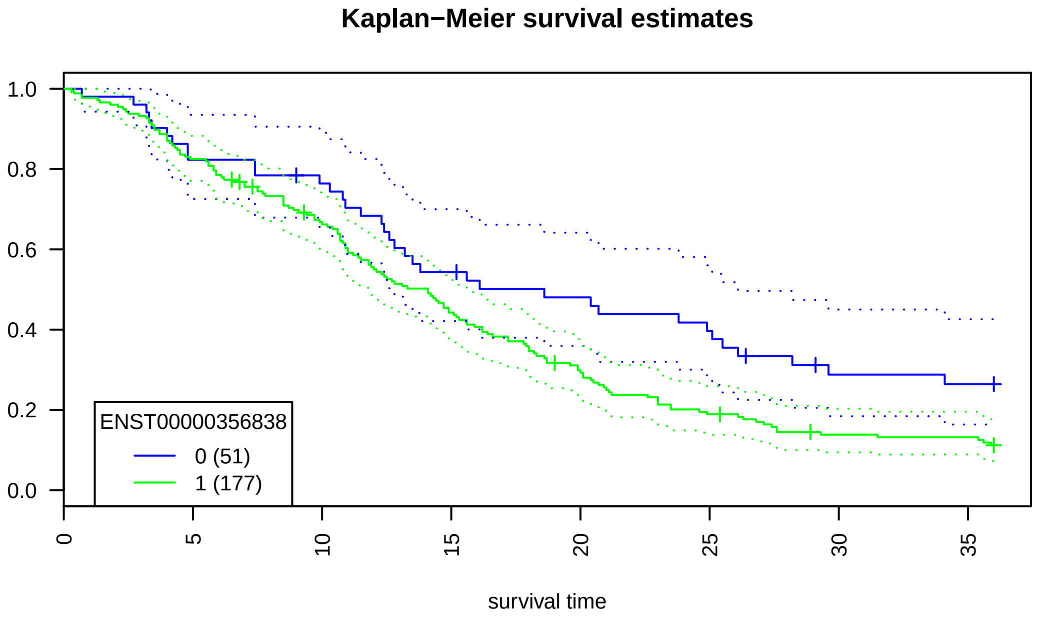

Figure 673: Each curve represents the relative number of ENST00000356838 survived to the corresponding point of time. Dashed lines represent 0.95 confidence intervals for the curves with the corresponding color. Total of 2 observations deleted due to missingness. Log-rank probability measure for the equality of these curves is 1.386294e-02. Additional annotations: *.GeneId*=ENSG00000165071, *.GeneName*=TMEM71, *.GeneDesc*=transmembrane protein 71 [Source:HGNC Symbol;Acc:26572].

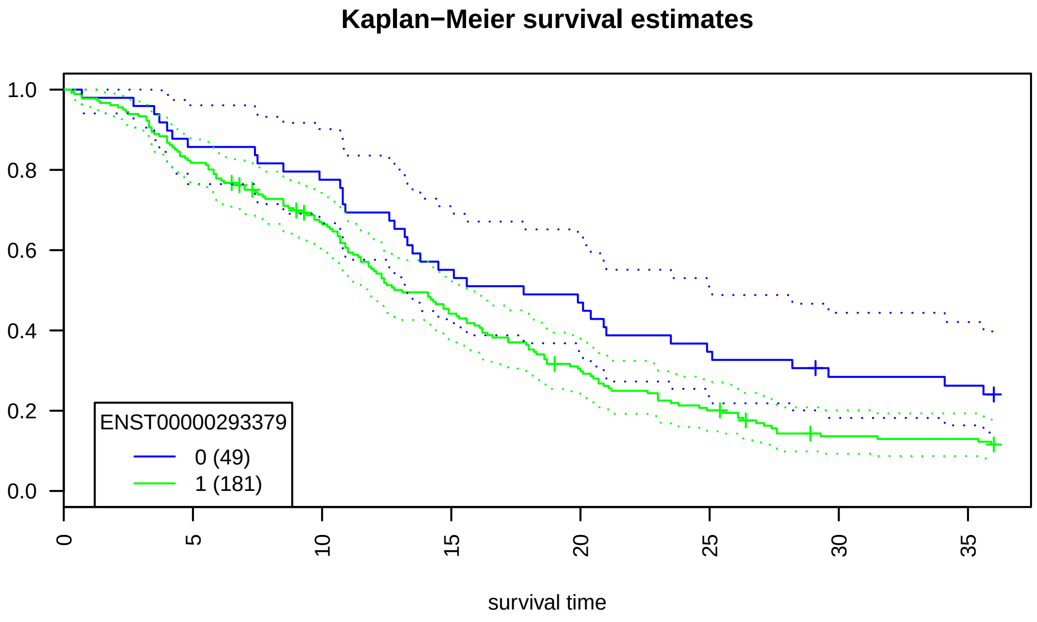

Figure 674: Each curve represents the relative number of ENST00000293379 survived to the corresponding point of time. Dashed lines represent 0.95 confidence intervals for the curves with the corresponding color. Log-rank probability measure for the equality of these curves is 2.236658e-02. Additional annotations: *.GeneId*=ENSG00000161638, *.GeneName*=ITGA5, *.GeneDesc*=integrin, alpha 5 (fibronectin receptor, alpha polypeptide) [Source:HGNC Symbol;Acc:6141].

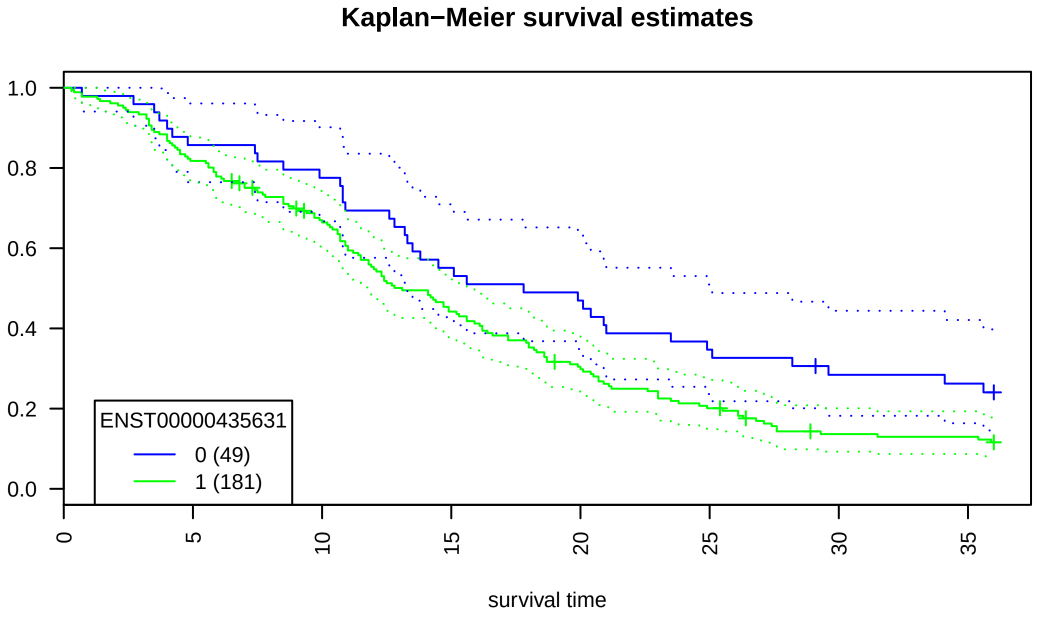

Figure 675: Each curve represents the relative number of ENST00000435631 survived to the corresponding point of time. Dashed lines represent 0.95 confidence intervals for the curves with the corresponding color. Log-rank probability measure for the equality of these curves is 2.236658e-02. Additional annotations: *.GeneId*=ENSG00000161638, *.GeneName*=ITGA5, *.GeneDesc*=integrin, alpha 5 (fibronectin receptor, alpha polypeptide) [Source:HGNC Symbol;Acc:6141].

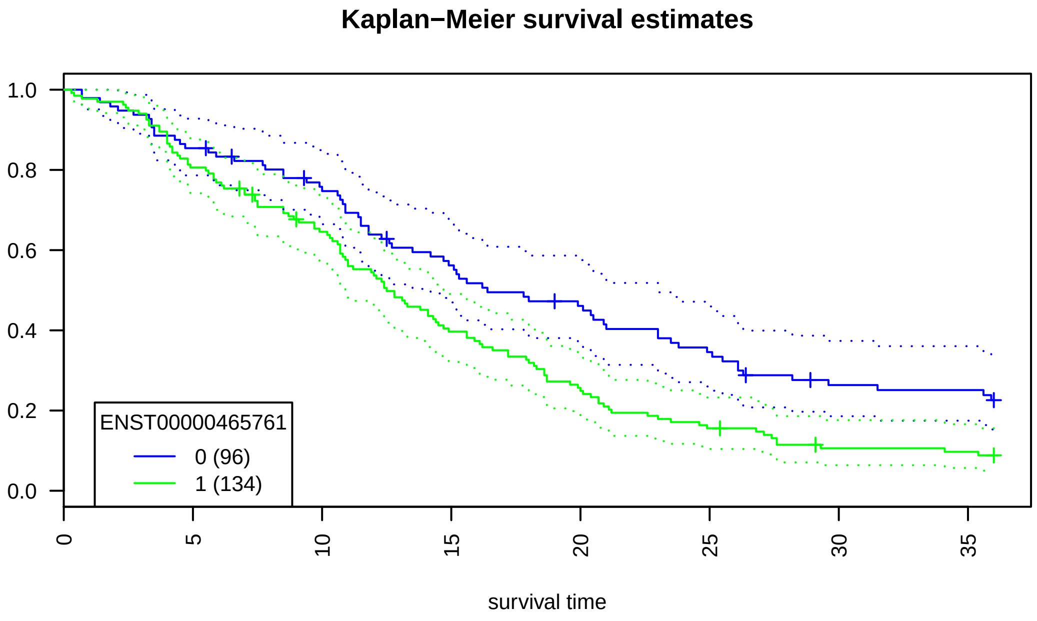

Figure 676: Each curve represents the relative number of ENST00000465761 survived to the corresponding point of time. Dashed lines represent 0.95 confidence intervals for the curves with the corresponding color. Log-rank probability measure for the equality of these curves is 1.490069e-03. Additional annotations: *.GeneId*=ENSG00000101104, *.GeneName*=PABPC1L, *.GeneDesc*=poly(A) binding protein, cytoplasmic 1-like [Source:HGNC Symbol;Acc:15797].

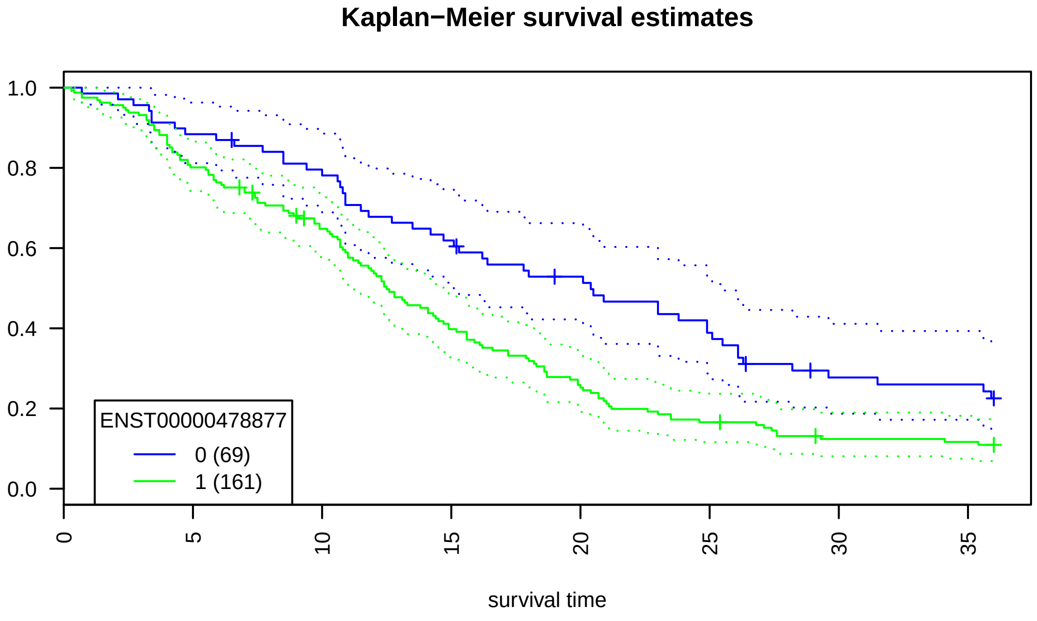

Figure 677: Each curve represents the relative number of ENST00000478877 survived to the corresponding point of time. Dashed lines represent 0.95 confidence intervals for the curves with the corresponding color. Log-rank probability measure for the equality of these curves is 1.271400e-03. Additional annotations: *.GeneId*=ENSG00000101104, *.GeneName*=PABPC1L, *.GeneDesc*=poly(A) binding protein, cytoplasmic 1-like [Source:HGNC Symbol;Acc:15797].

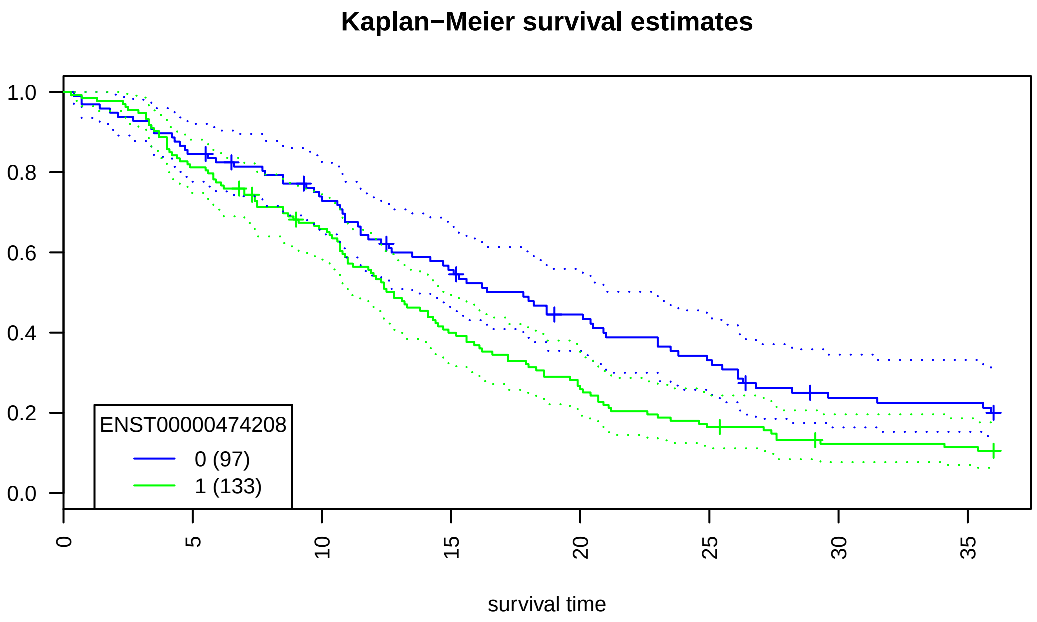

Figure 678: Each curve represents the relative number of ENST00000474208 survived to the corresponding point of time. Dashed lines represent 0.95 confidence intervals for the curves with the corresponding color. Log-rank probability measure for the equality of these curves is 1.037203e-02. Additional annotations: *.GeneId*=ENSG00000101104, *.GeneName*=PABPC1L, *.GeneDesc*=poly(A) binding protein, cytoplasmic 1-like [Source:HGNC Symbol;Acc:15797].

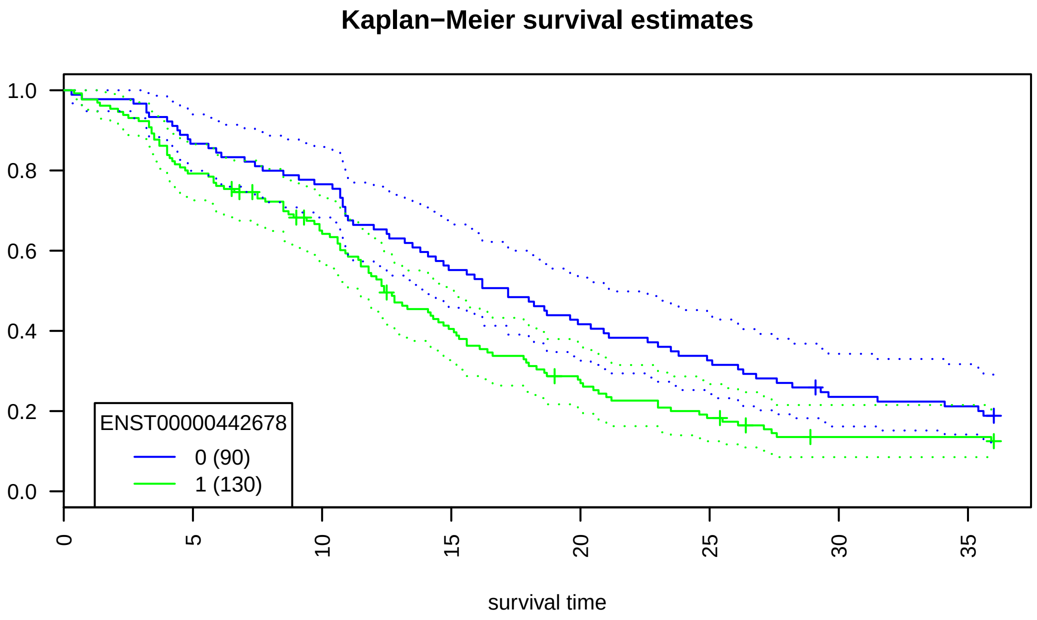

Figure 679: Each curve represents the relative number of ENST00000442678 survived to the corresponding point of time. Dashed lines represent 0.95 confidence intervals for the curves with the corresponding color. Total of 10 observations deleted due to missingness. Log-rank probability measure for the equality of these curves is 2.086075e-02. Additional annotations: *.GeneId*=ENSG00000172716, *.GeneName*=SLFN11, *.GeneDesc*=schlafen family member 11 [Source:HGNC Symbol;Acc:26633].

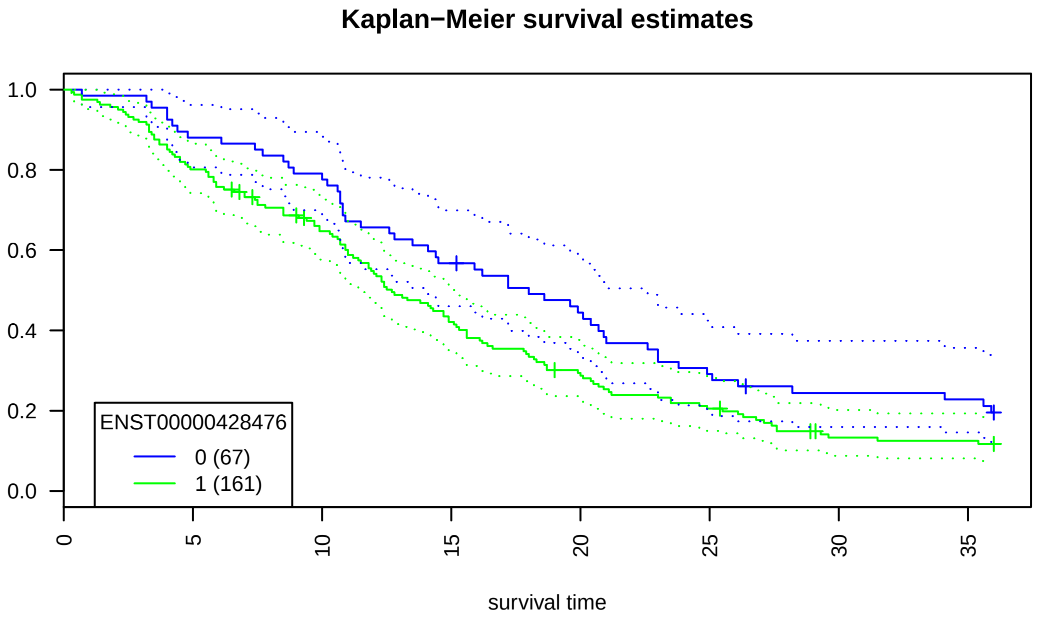

Figure 680: Each curve represents the relative number of ENST00000428476 survived to the corresponding point of time. Dashed lines represent 0.95 confidence intervals for the curves with the corresponding color. Total of 2 observations deleted due to missingness. Log-rank probability measure for the equality of these curves is 3.201043e-02. Additional annotations: *.GeneId*=ENSG00000172123, *.GeneName*=SLFN12, *.GeneDesc*=schlafen family member 12 [Source:HGNC Symbol;Acc:25500].

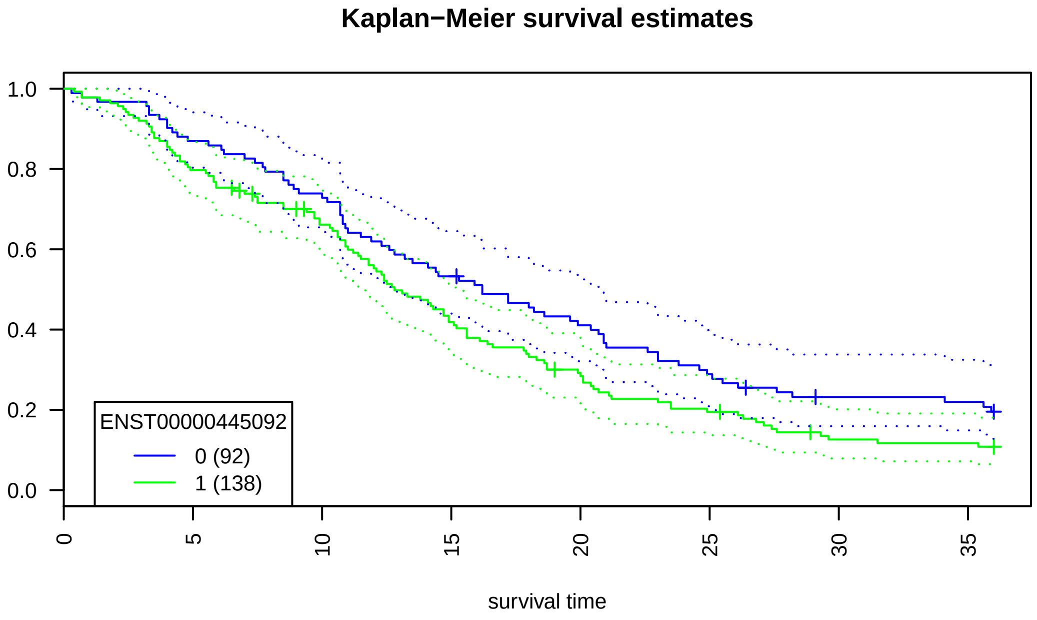

Figure 681: Each curve represents the relative number of ENST00000445092 survived to the corresponding point of time. Dashed lines represent 0.95 confidence intervals for the curves with the corresponding color. Log-rank probability measure for the equality of these curves is 3.736633e-02. Additional annotations: *.GeneId*=ENSG00000172123, *.GeneName*=SLFN12, *.GeneDesc*=schlafen family member 12 [Source:HGNC Symbol;Acc:25500].

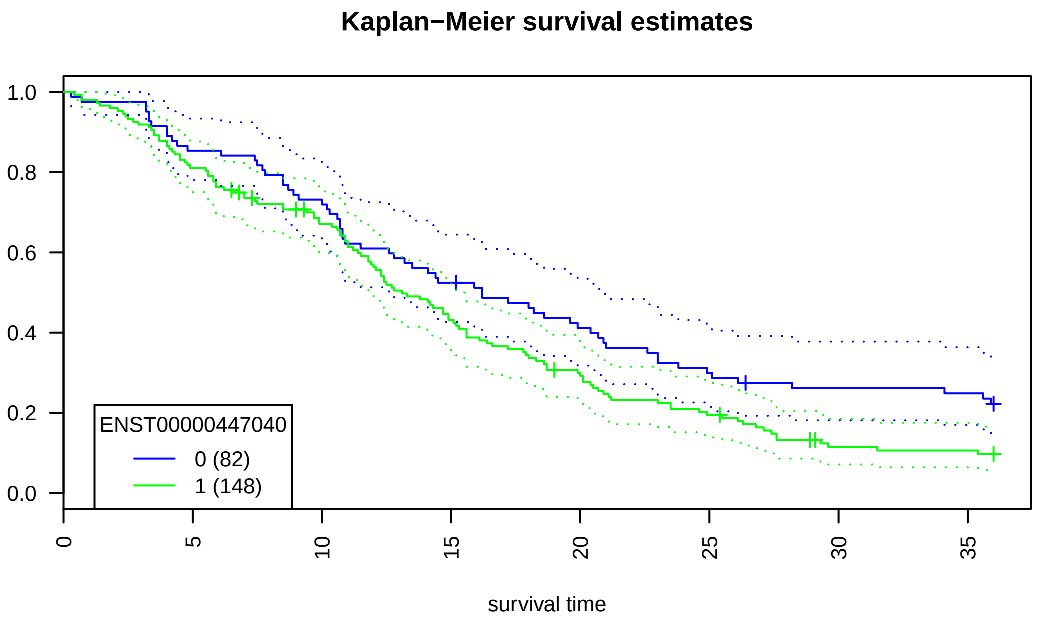

Figure 682: Each curve represents the relative number of ENST00000447040 survived to the corresponding point of time. Dashed lines represent 0.95 confidence intervals for the curves with the corresponding color. Log-rank probability measure for the equality of these curves is 2.255874e-02. Additional annotations: *.GeneId*=ENSG00000172123, *.GeneName*=SLFN12, *.GeneDesc*=schlafen family member 12 [Source:HGNC Symbol;Acc:25500].

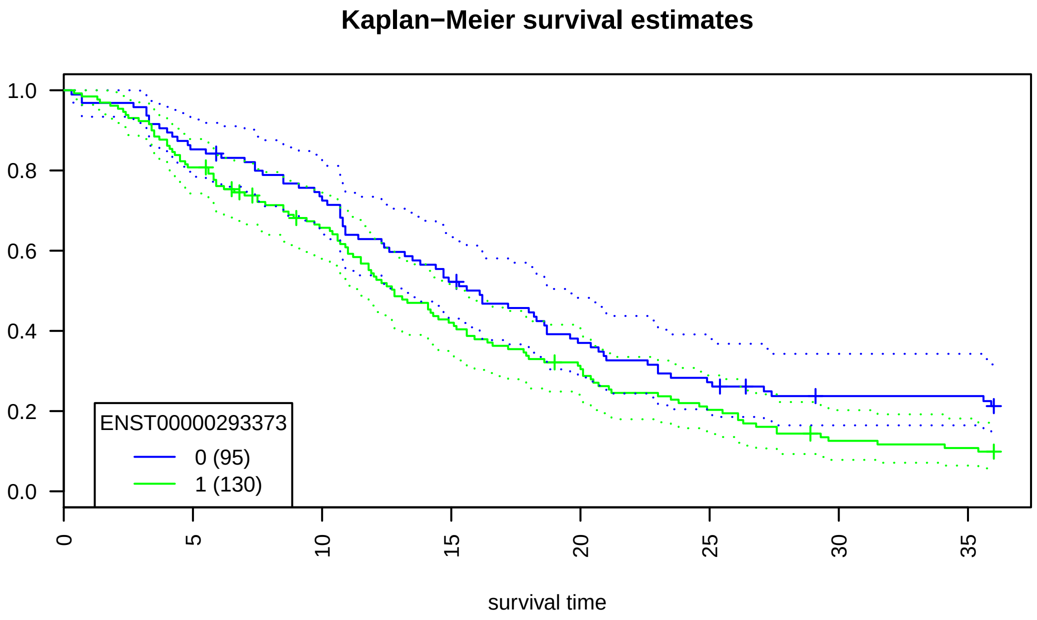

Figure 683: Each curve represents the relative number of ENST00000293373 survived to the corresponding point of time. Dashed lines represent 0.95 confidence intervals for the curves with the corresponding color. Total of 5 observations deleted due to missingness. Log-rank probability measure for the equality of these curves is 3.961106e-02. Additional annotations: *.GeneId*=ENSG00000123338, *.GeneName*=NCKAP1L, *.GeneDesc*=NCK-associated protein 1-like [Source:HGNC Symbol;Acc:4862].

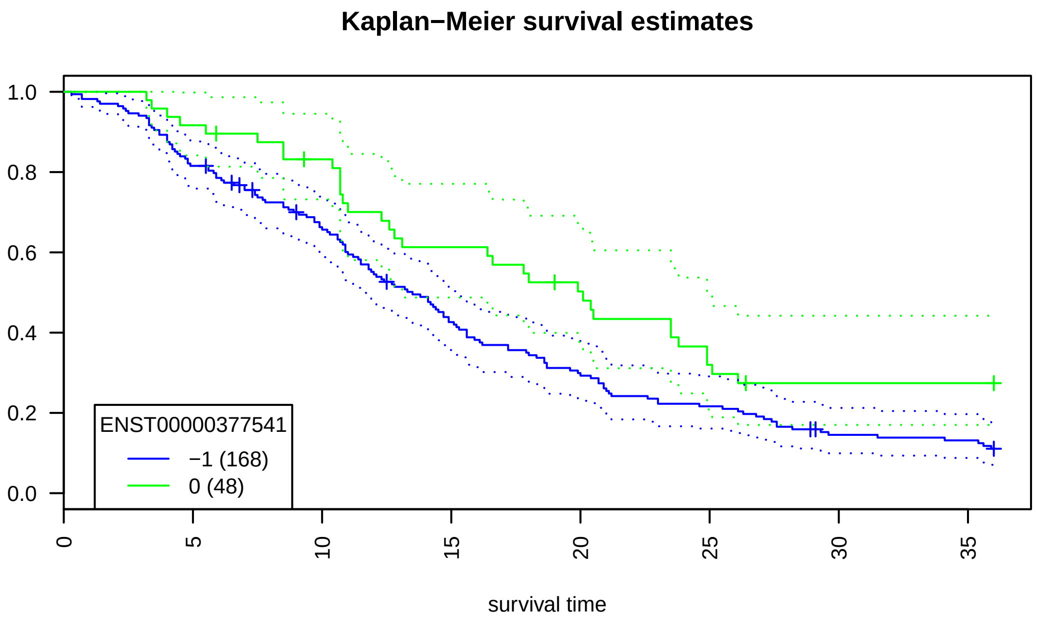

Figure 684: Each curve represents the relative number of ENST00000377541 survived to the corresponding point of time. Dashed lines represent 0.95 confidence intervals for the curves with the corresponding color. Total of 14 observations deleted due to missingness. Log-rank probability measure for the equality of these curves is 9.685867e-03. Additional annotations: *.GeneId*=ENSG00000049246, *.GeneName*=PER3, *.GeneDesc*=period homolog 3 (Drosophila) [Source:HGNC Symbol;Acc:8847].

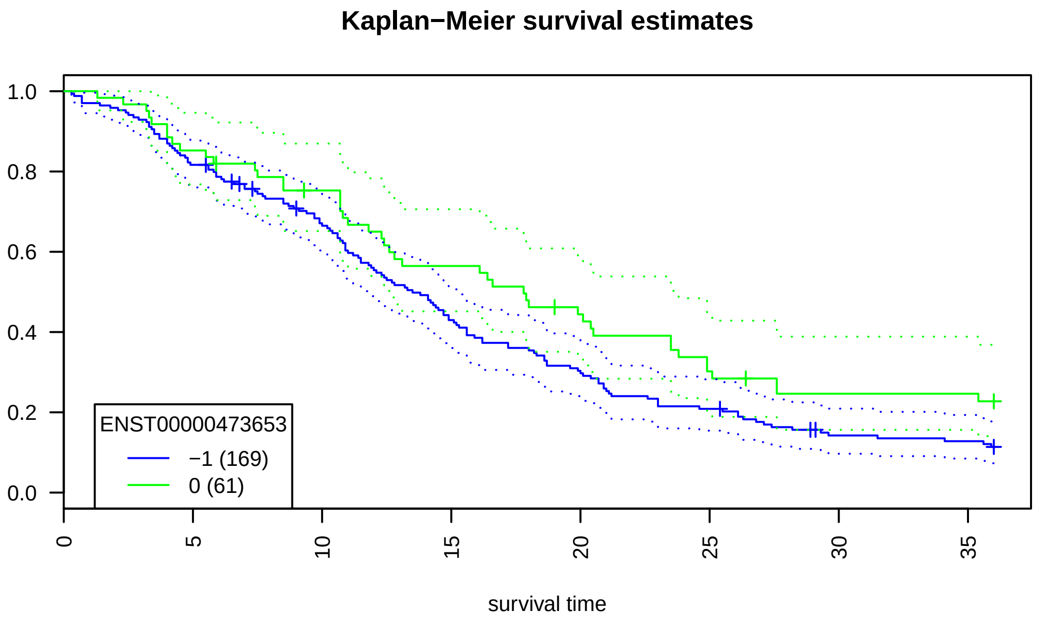

Figure 685: Each curve represents the relative number of ENST00000473653 survived to the corresponding point of time. Dashed lines represent 0.95 confidence intervals for the curves with the corresponding color. Log-rank probability measure for the equality of these curves is 4.061849e-02. Additional annotations: *.GeneId*=ENSG00000049246, *.GeneName*=PER3, *.GeneDesc*=period homolog 3 (Drosophila) [Source:HGNC Symbol;Acc:8847].

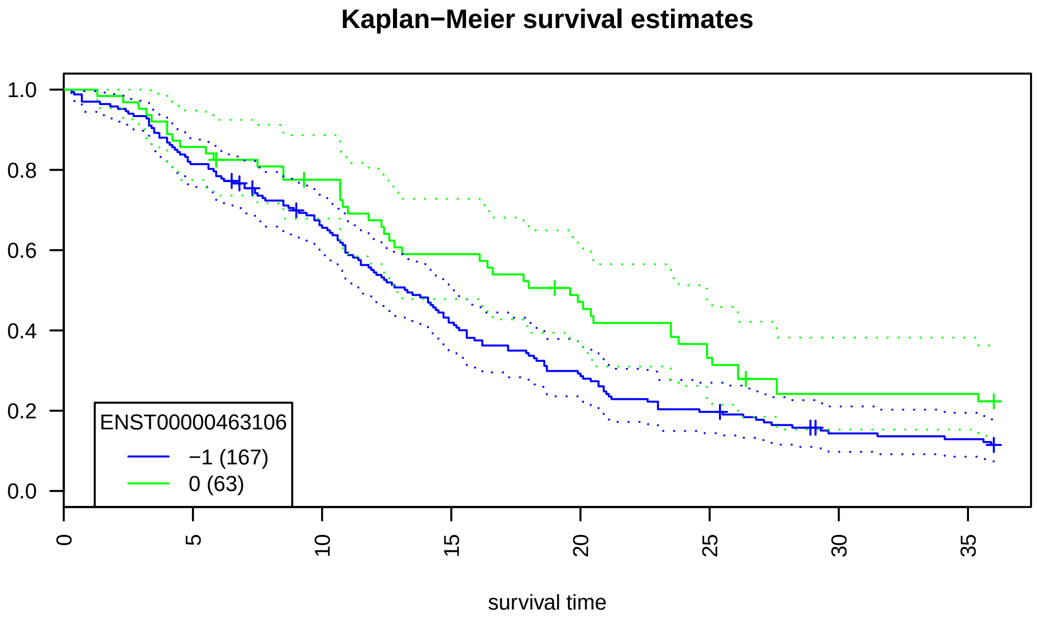

Figure 686: Each curve represents the relative number of ENST00000463106 survived to the corresponding point of time. Dashed lines represent 0.95 confidence intervals for the curves with the corresponding color. Log-rank probability measure for the equality of these curves is 1.90088e-02. Additional annotations: *.GeneId*=ENSG00000049246, *.GeneName*=PER3, *.GeneDesc*=period homolog 3 (Drosophila) [Source:HGNC Symbol;Acc:8847].

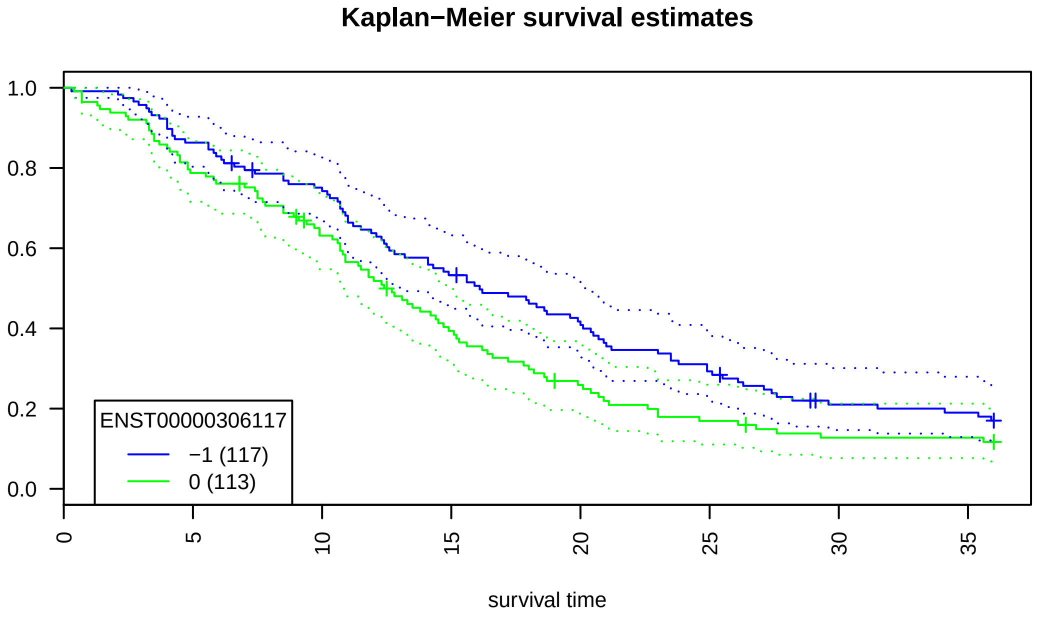

Figure 687: Each curve represents the relative number of ENST00000306117 survived to the corresponding point of time. Dashed lines represent 0.95 confidence intervals for the curves with the corresponding color. Log-rank probability measure for the equality of these curves is 2.865134e-02. Additional annotations: *.GeneId*=ENSG00000124134, *.GeneName*=KCNS1, *.GeneDesc*=potassium voltage-gated channel, delayed-rectifier, subfamily S, member 1 [Source:HGNC Symbol;Acc:6300].

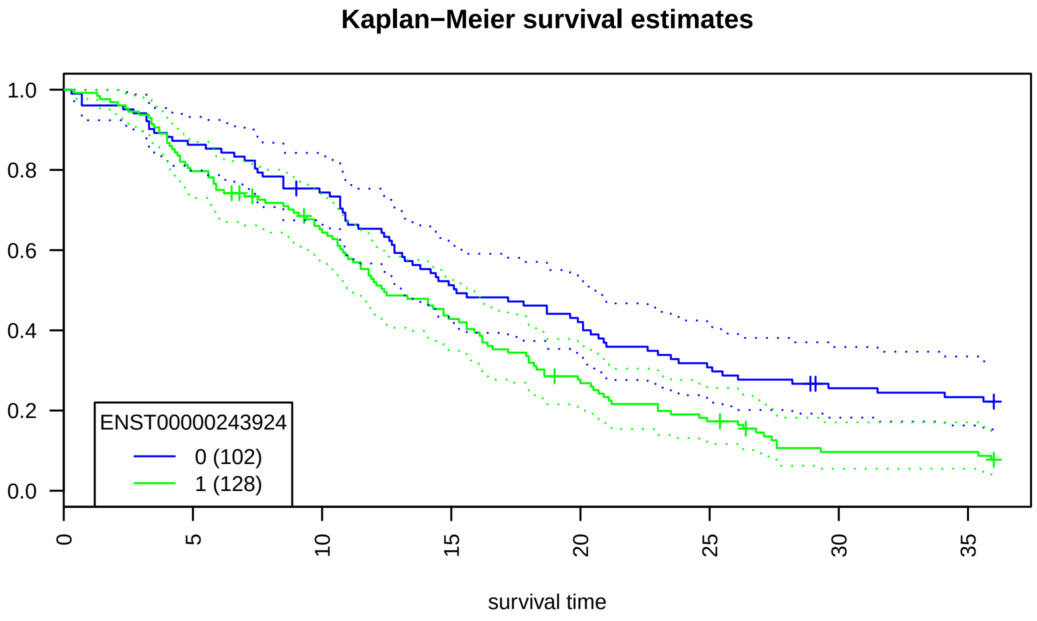

Figure 688: Each curve represents the relative number of ENST00000243924 survived to the corresponding point of time. Dashed lines represent 0.95 confidence intervals for the curves with the corresponding color. Log-rank probability measure for the equality of these curves is 4.960258e-03. Additional annotations: *.GeneId*=ENSG00000124102, *.GeneName*=PI3, *.GeneDesc*=peptidase inhibitor 3, skin-derived [Source:HGNC Symbol;Acc:8947].

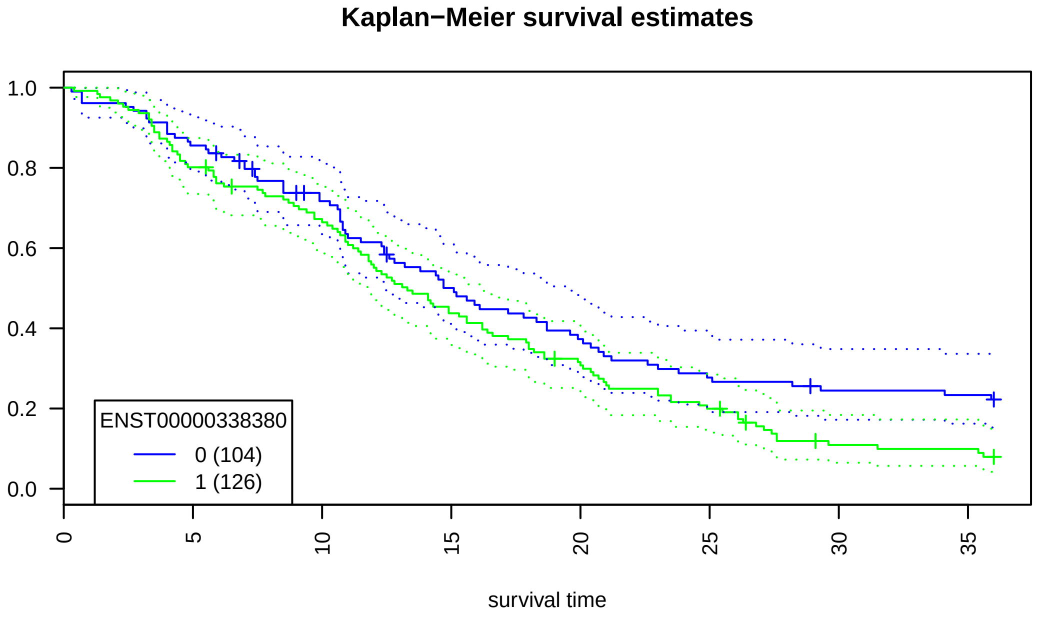

Figure 689: Each curve represents the relative number of ENST00000338380 survived to the corresponding point of time. Dashed lines represent 0.95 confidence intervals for the curves with the corresponding color. Log-rank probability measure for the equality of these curves is 3.247892e-02. Additional annotations: *.GeneId*=ENSG00000124107, *.GeneName*=SLPI, *.GeneDesc*=secretory leukocyte peptidase inhibitor [Source:HGNC Symbol;Acc:11092].

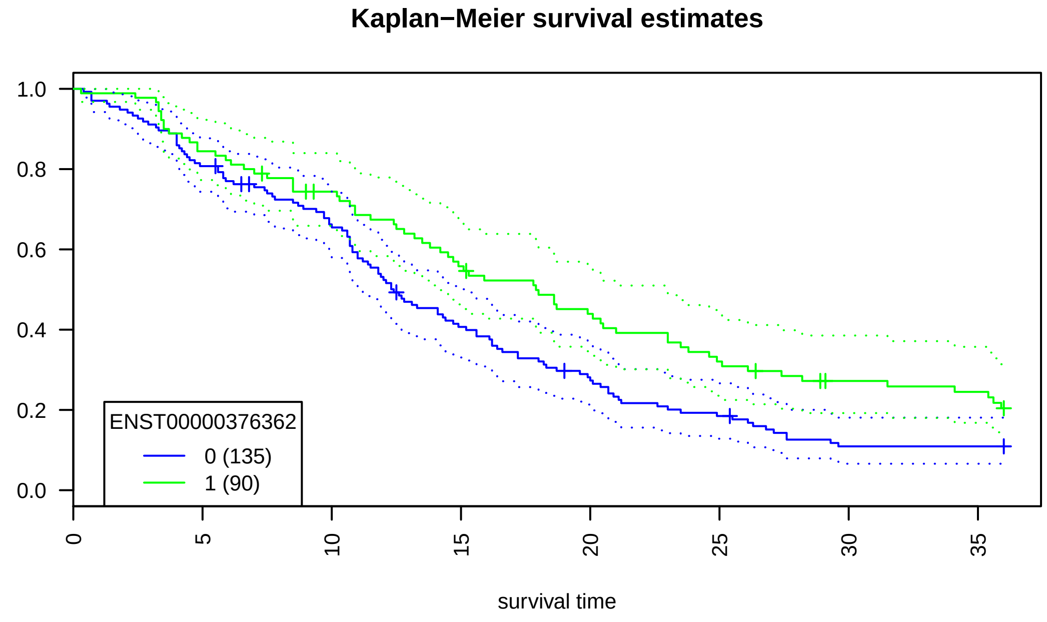

Figure 690: Each curve represents the relative number of ENST00000376362 survived to the corresponding point of time. Dashed lines represent 0.95 confidence intervals for the curves with the corresponding color. Total of 5 observations deleted due to missingness. Log-rank probability measure for the equality of these curves is 9.471538e-03. Additional annotations: *.GeneId*=ENSG00000165113, *.GeneName*=GKAP1, *.GeneDesc*=G kinase anchoring protein 1 [Source:HGNC Symbol;Acc:17496].

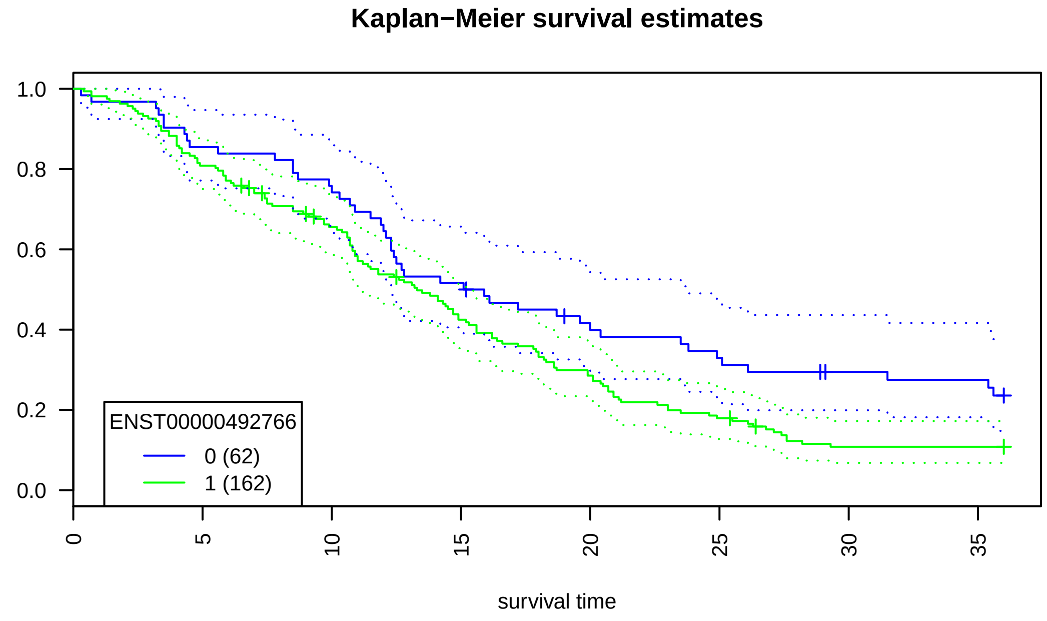

Figure 691: Each curve represents the relative number of ENST00000492766 survived to the corresponding point of time. Dashed lines represent 0.95 confidence intervals for the curves with the corresponding color. Total of 6 observations deleted due to missingness. Log-rank probability measure for the equality of these curves is 2.150478e-02. Additional annotations: *.GeneId*=ENSG00000142599, *.GeneName*=RERE, *.GeneDesc*=arginine-glutamic acid dipeptide (RE) repeats [Source:HGNC Symbol;Acc:9965].

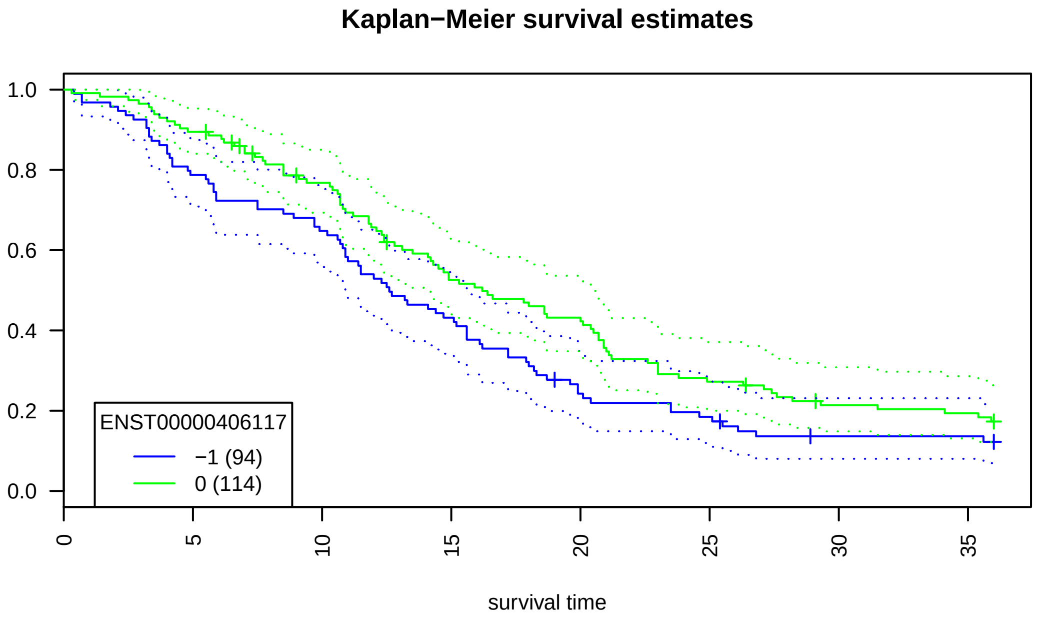

Figure 692: Each curve represents the relative number of ENST00000406117 survived to the corresponding point of time. Dashed lines represent 0.95 confidence intervals for the curves with the corresponding color. Total of 22 observations deleted due to missingness. Log-rank probability measure for the equality of these curves is 3.397931e-02. Additional annotations: *.GeneId*=ENSG00000100344, *.GeneName*=PNPLA3, *.GeneDesc*=patatin-like phospholipase domain containing 3 [Source:HGNC Symbol;Acc:18590].

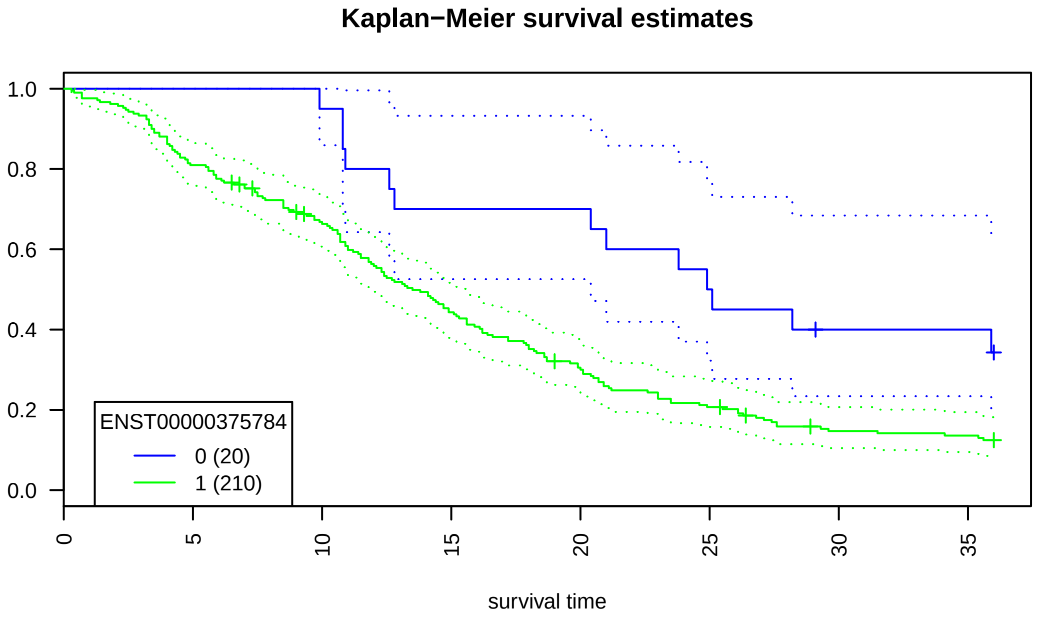

Figure 693: Each curve represents the relative number of ENST00000375784 survived to the corresponding point of time. Dashed lines represent 0.95 confidence intervals for the curves with the corresponding color. Log-rank probability measure for the equality of these curves is 4.49591e-03. Additional annotations: *.GeneId*=ENSG00000213719, *.GeneName*=CLIC1, *.GeneDesc*=Chloride intracellular channel protein 1 (Nuclear chloride ion channel 27)(NCC27)(Chloride channel ABP)(Regulatory nuclear chloride ion channel protein)(hRNCC) [Source:UniProtKB/Swiss-Prot;Acc:O00299].

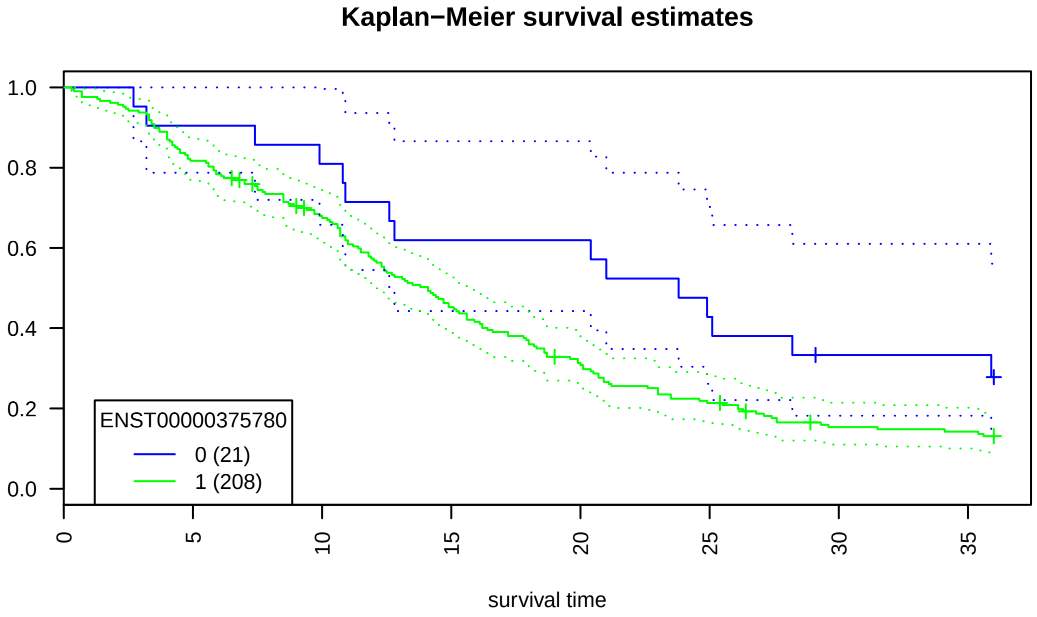

Figure 694: Each curve represents the relative number of ENST00000375780 survived to the corresponding point of time. Dashed lines represent 0.95 confidence intervals for the curves with the corresponding color. Total of 1 observation deleted due to missingness. Log-rank probability measure for the equality of these curves is 4.034975e-02. Additional annotations: *.GeneId*=ENSG00000213719, *.GeneName*=CLIC1, *.GeneDesc*=Chloride intracellular channel protein 1 (Nuclear chloride ion channel 27)(NCC27)(Chloride channel ABP)(Regulatory nuclear chloride ion channel protein)(hRNCC) [Source:UniProtKB/Swiss-Prot;Acc:O00299].

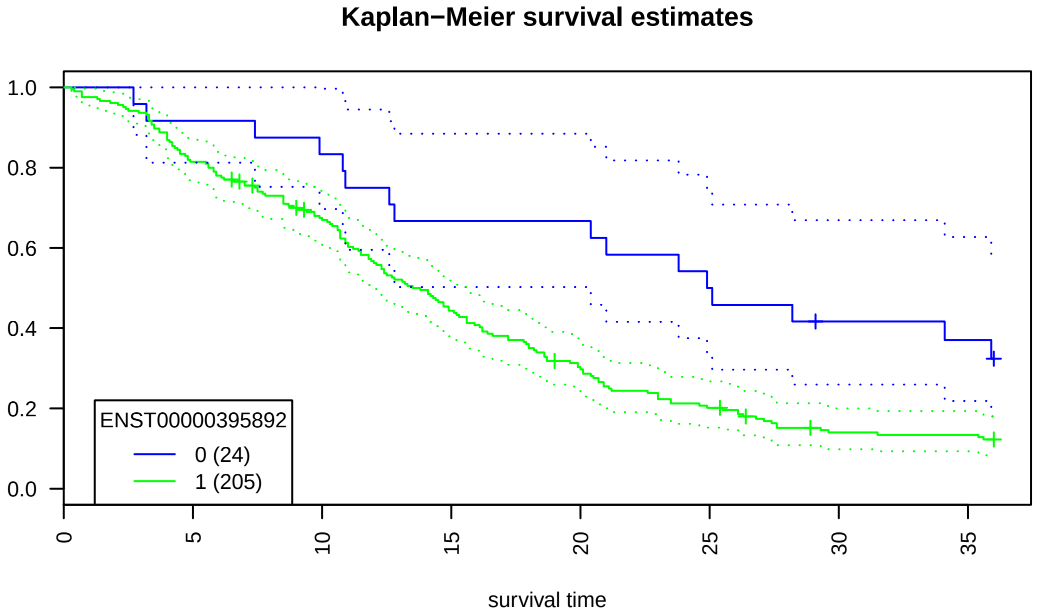

Figure 695: Each curve represents the relative number of ENST00000395892 survived to the corresponding point of time. Dashed lines represent 0.95 confidence intervals for the curves with the corresponding color. Total of 1 observation deleted due to missingness. Log-rank probability measure for the equality of these curves is 4.734071e-03. Additional annotations: *.GeneId*=ENSG00000213719, *.GeneName*=CLIC1, *.GeneDesc*=Chloride intracellular channel protein 1 (Nuclear chloride ion channel 27)(NCC27)(Chloride channel ABP)(Regulatory nuclear chloride ion channel protein)(hRNCC) [Source:UniProtKB/Swiss-Prot;Acc:O00299].

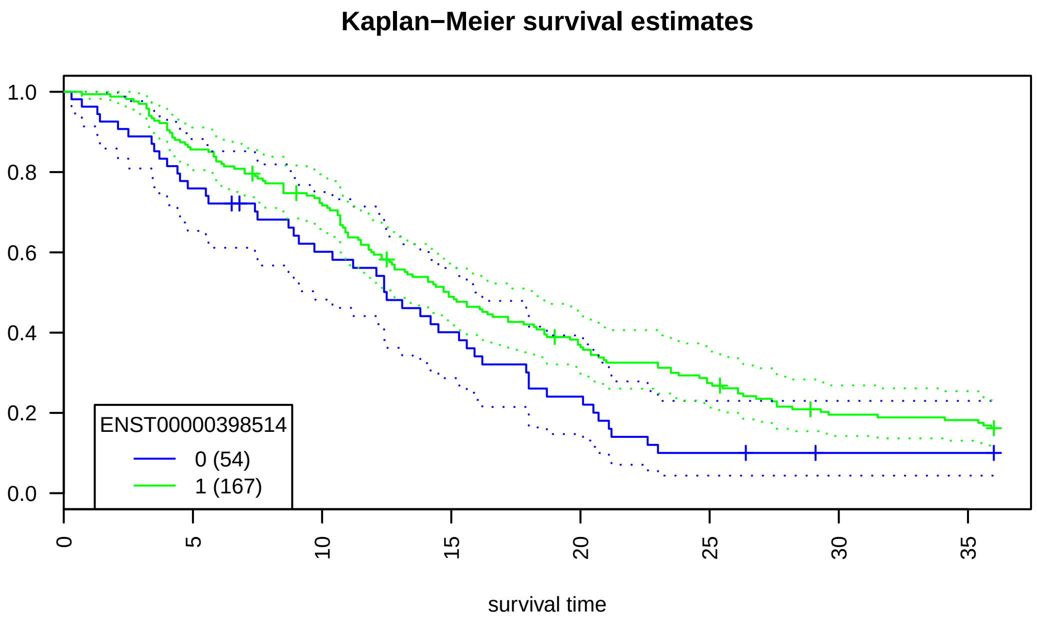

Figure 696: Each curve represents the relative number of ENST00000398514 survived to the corresponding point of time. Dashed lines represent 0.95 confidence intervals for the curves with the corresponding color. Total of 9 observations deleted due to missingness. Log-rank probability measure for the equality of these curves is 2.766526e-02. Additional annotations: *.GeneId*=ENSG00000113657, *.GeneName*=DPYSL3, *.GeneDesc*=dihydropyrimidinase-like 3 [Source:HGNC Symbol;Acc:3015].

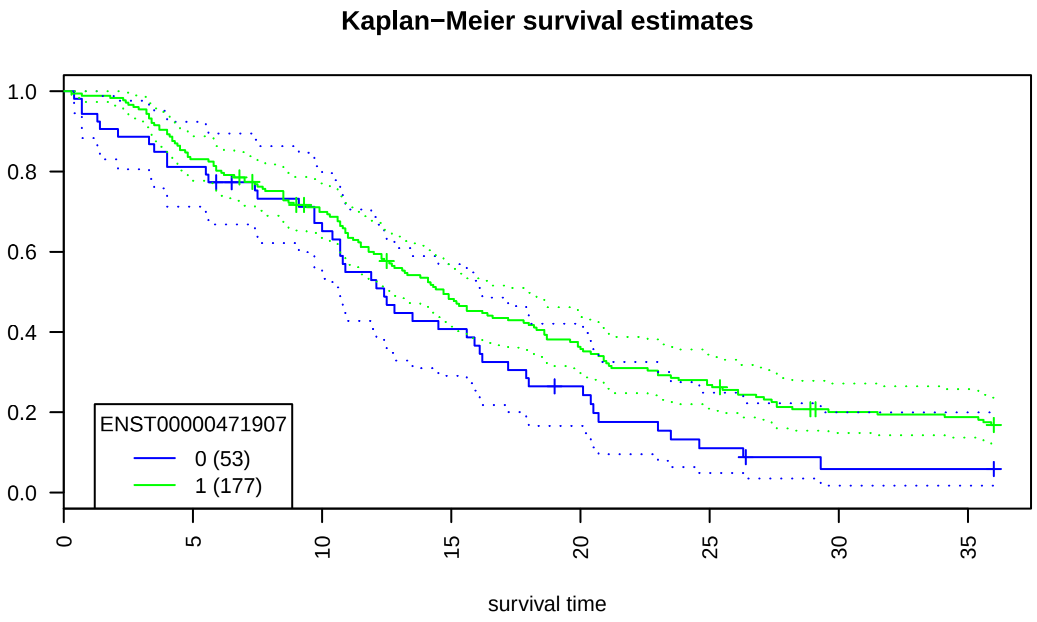

Figure 697: Each curve represents the relative number of ENST00000471907 survived to the corresponding point of time. Dashed lines represent 0.95 confidence intervals for the curves with the corresponding color. Log-rank probability measure for the equality of these curves is 2.959991e-02. Additional annotations: *.GeneId*=ENSG00000147140, *.GeneName*=NONO, *.GeneDesc*=non-POU domain containing, octamer-binding [Source:HGNC Symbol;Acc:7871].

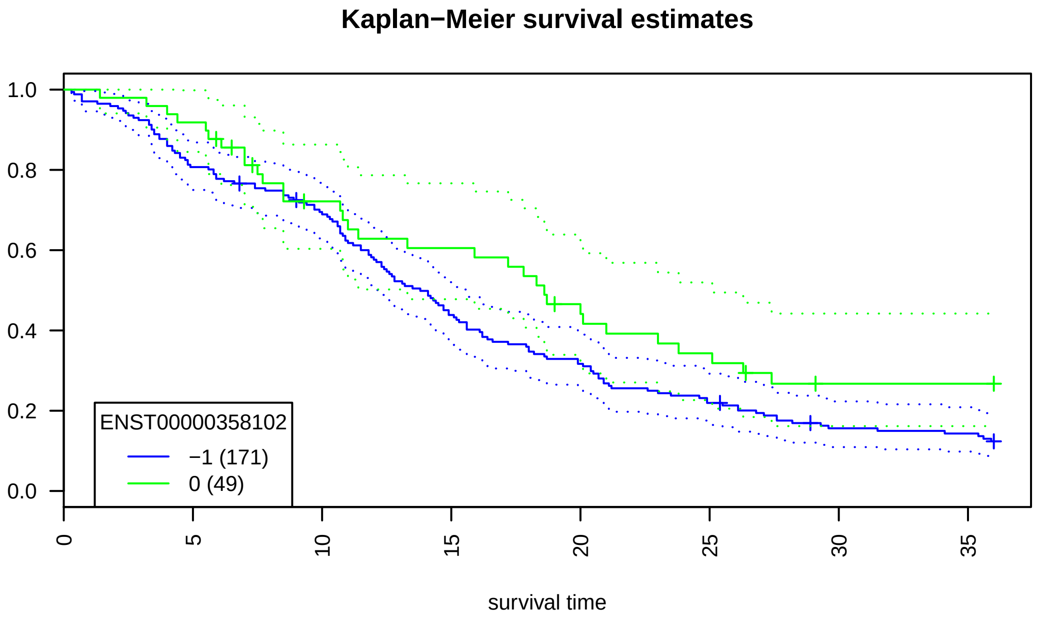

Figure 698: Each curve represents the relative number of ENST00000358102 survived to the corresponding point of time. Dashed lines represent 0.95 confidence intervals for the curves with the corresponding color. Total of 10 observations deleted due to missingness. Log-rank probability measure for the equality of these curves is 4.544041e-02. Additional annotations: *.GeneId*=ENSG00000151623, *.GeneName*=NR3C2, *.GeneDesc*=nuclear receptor subfamily 3, group C, member 2 [Source:HGNC Symbol;Acc:7979].

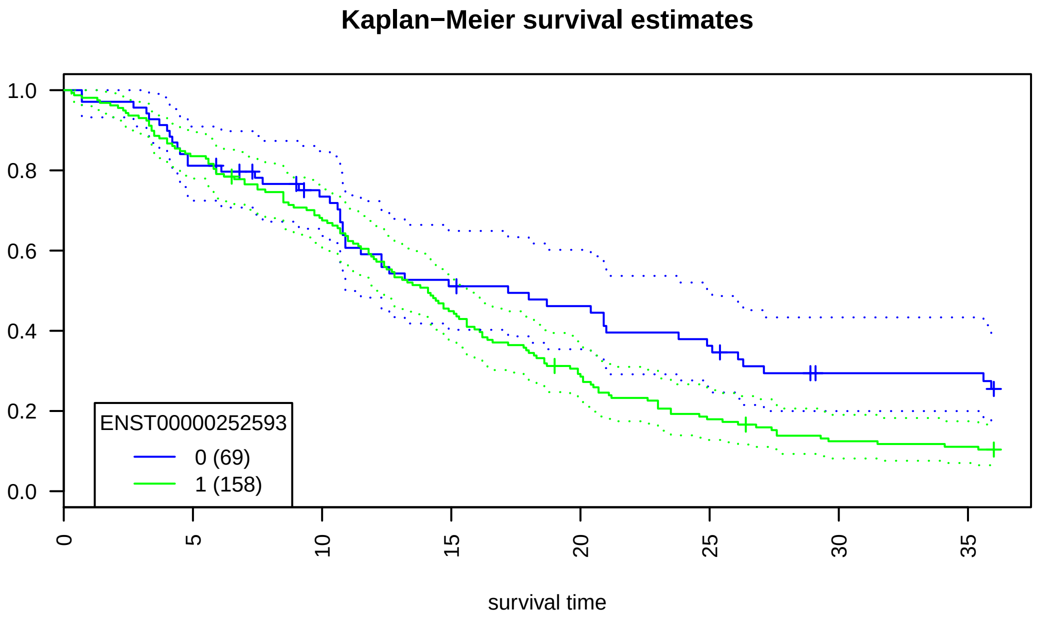

Figure 699: Each curve represents the relative number of ENST00000252593 survived to the corresponding point of time. Dashed lines represent 0.95 confidence intervals for the curves with the corresponding color. Total of 3 observations deleted due to missingness. Log-rank probability measure for the equality of these curves is 1.767899e-02. Additional annotations: *.GeneId*=ENSG00000130303, *.GeneName*=BST2, *.GeneDesc*=bone marrow stromal cell antigen 2 [Source:HGNC Symbol;Acc:1119].

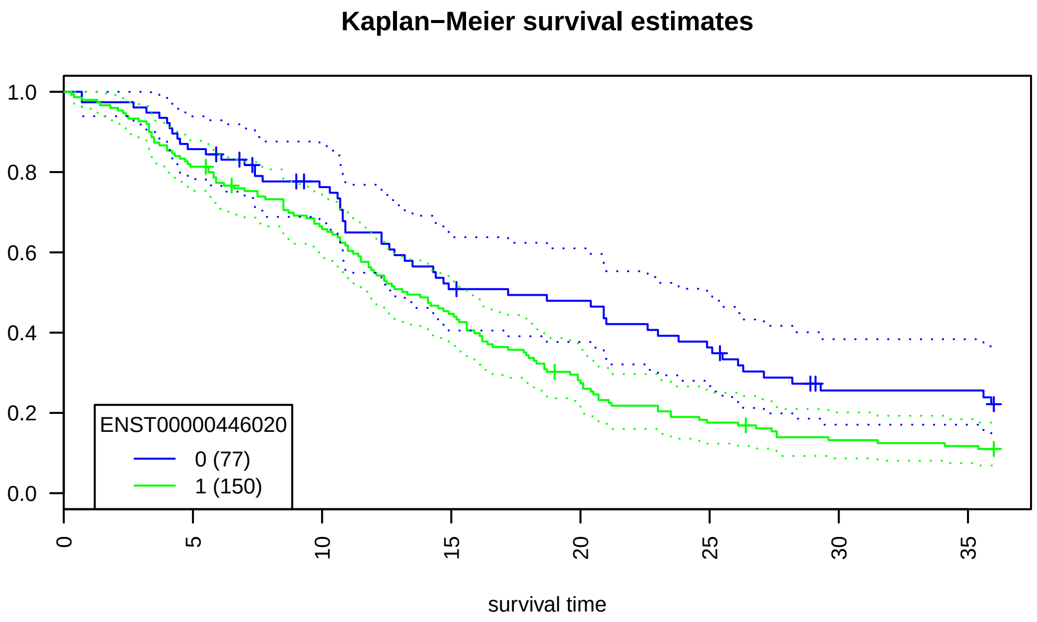

Figure 700: Each curve represents the relative number of ENST00000446020 survived to the corresponding point of time. Dashed lines represent 0.95 confidence intervals for the curves with the corresponding color. Total of 3 observations deleted due to missingness. Log-rank probability measure for the equality of these curves is 1.234586e-02. Additional annotations: *.GeneId*=ENSG00000130303, *.GeneName*=BST2, *.GeneDesc*=bone marrow stromal cell antigen 2 [Source:HGNC Symbol;Acc:1119].

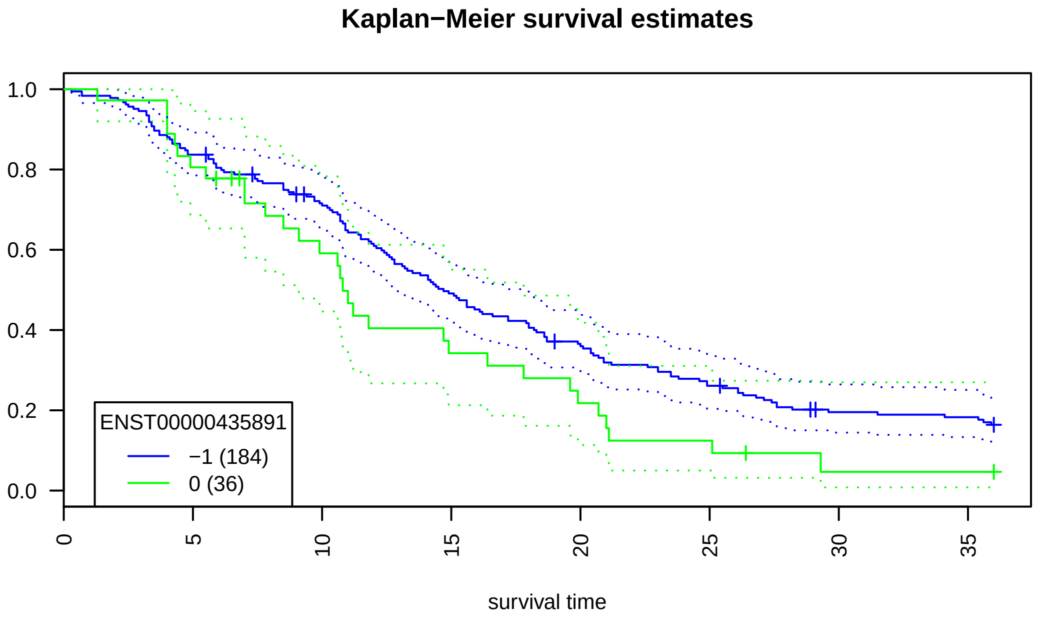

Figure 701: Each curve represents the relative number of ENST00000435891 survived to the corresponding point of time. Dashed lines represent 0.95 confidence intervals for the curves with the corresponding color. Total of 10 observations deleted due to missingness. Log-rank probability measure for the equality of these curves is 3.470486e-02. Additional annotations: *.GeneId*=ENSG00000171603, *.GeneName*=CLSTN1, *.GeneDesc*=calsyntenin 1 [Source:HGNC Symbol;Acc:17447].

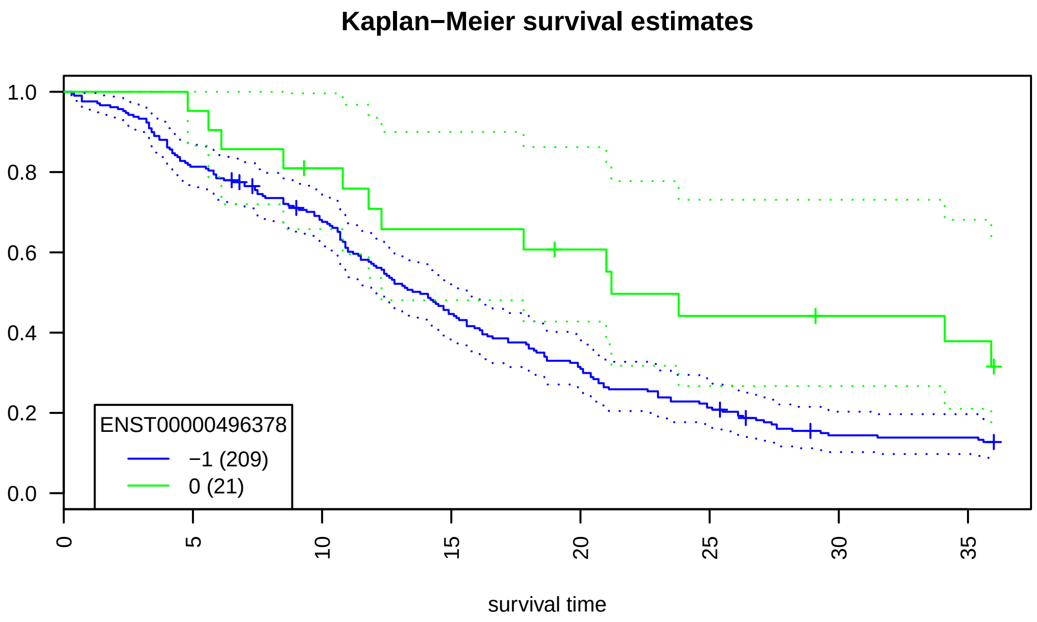

Figure 702: Each curve represents the relative number of ENST00000496378 survived to the corresponding point of time. Dashed lines represent 0.95 confidence intervals for the curves with the corresponding color. Log-rank probability measure for the equality of these curves is 1.846555e-02. Additional annotations: *.GeneId*=ENSG00000084731, *.GeneName*=KIF3C, *.GeneDesc*=kinesin family member 3C [Source:HGNC Symbol;Acc:6321].

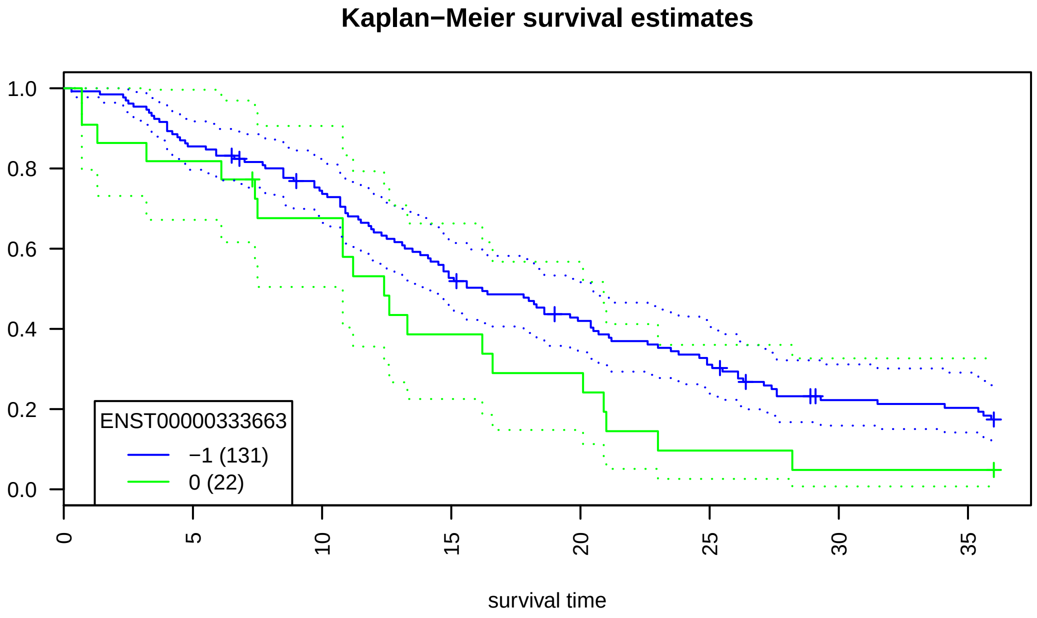

Figure 703: Each curve represents the relative number of ENST00000333663 survived to the corresponding point of time. Dashed lines represent 0.95 confidence intervals for the curves with the corresponding color. Total of 77 observations deleted due to missingness. Log-rank probability measure for the equality of these curves is 3.062098e-02. Additional annotations: *.GeneId*=ENSG00000106078, *.GeneName*=COBL, *.GeneDesc*=cordon-bleu homolog (mouse) [Source:HGNC Symbol;Acc:22199].

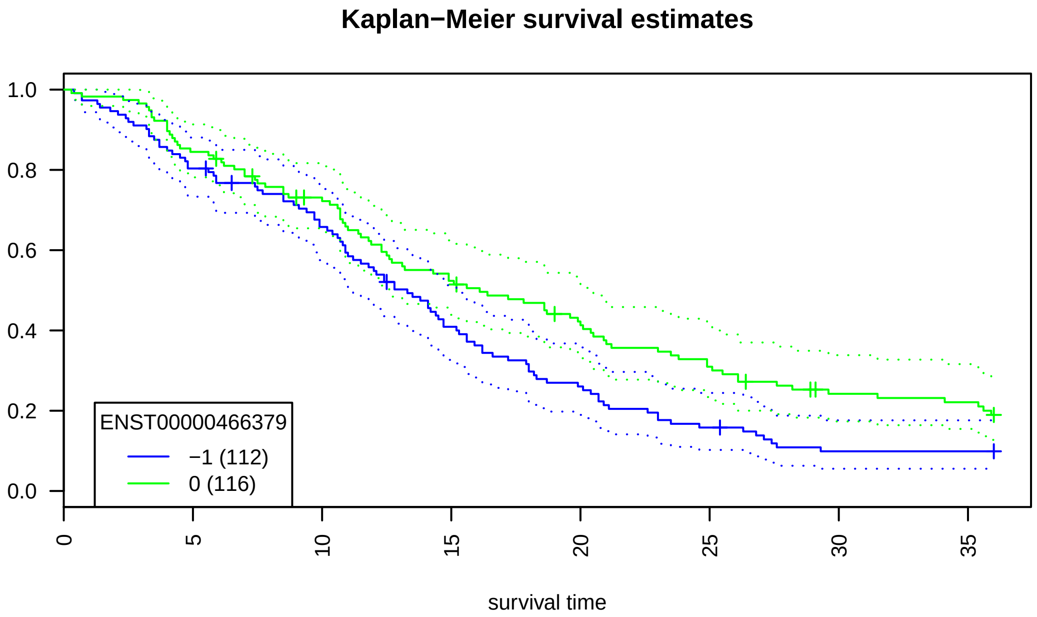

Figure 704: Each curve represents the relative number of ENST00000466379 survived to the corresponding point of time. Dashed lines represent 0.95 confidence intervals for the curves with the corresponding color. Total of 2 observations deleted due to missingness. Log-rank probability measure for the equality of these curves is 1.368796e-02. Additional annotations: *.GeneId*=ENSG00000130939, *.GeneName*=UBE4B, *.GeneDesc*=ubiquitination factor E4B (UFD2 homolog, yeast) [Source:HGNC Symbol;Acc:12500].

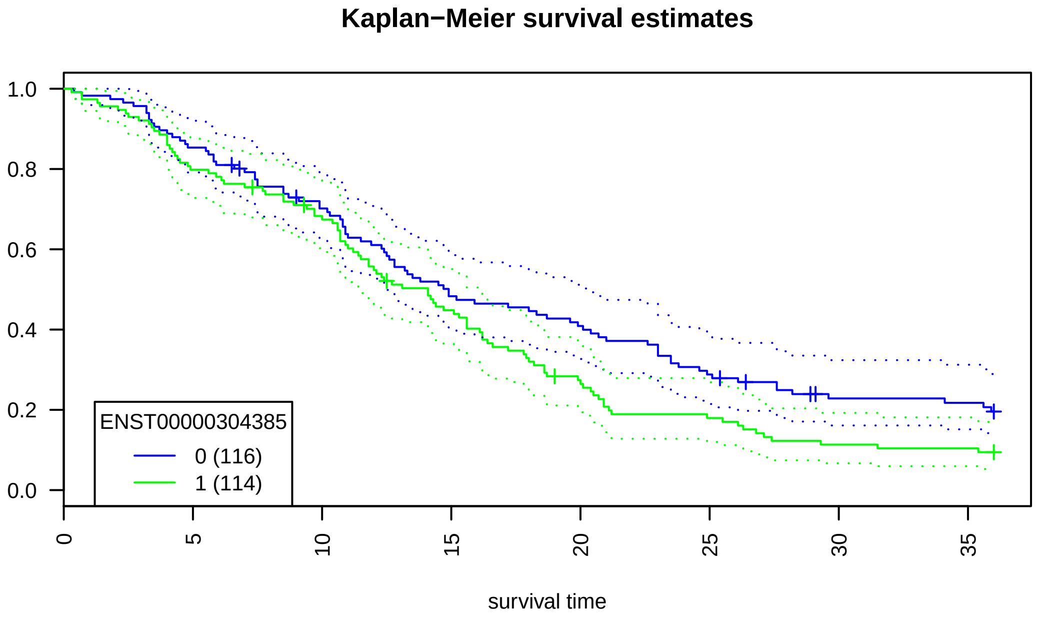

Figure 705: Each curve represents the relative number of ENST00000304385 survived to the corresponding point of time. Dashed lines represent 0.95 confidence intervals for the curves with the corresponding color. Log-rank probability measure for the equality of these curves is 2.948919e-02. Additional annotations: *.GeneId*=ENSG00000170006, *.GeneName*=TMEM154, *.GeneDesc*=transmembrane protein 154 [Source:HGNC Symbol;Acc:26489].

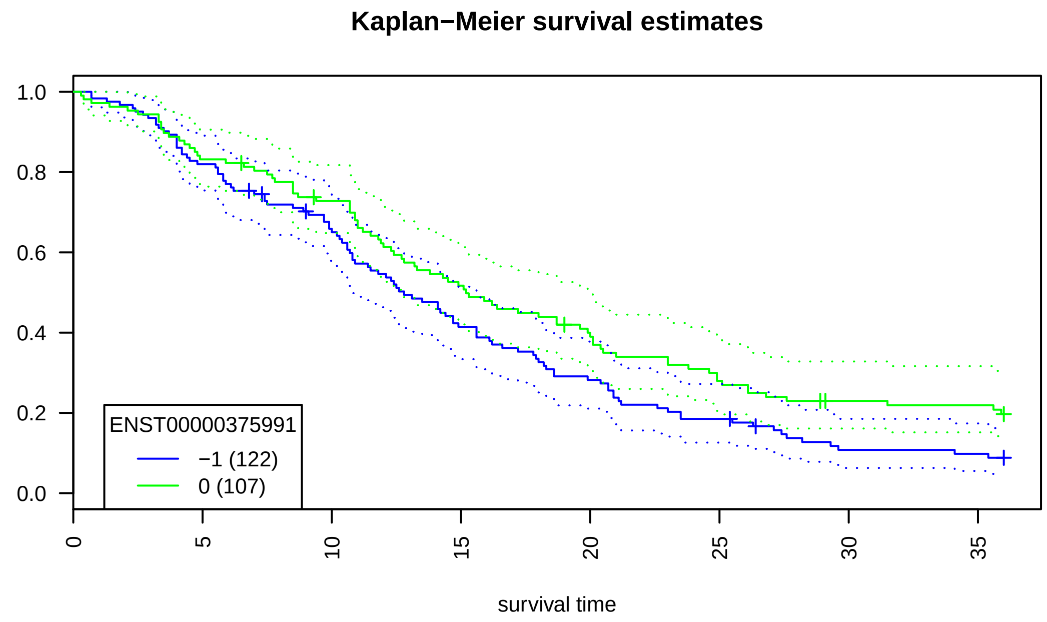

Figure 706: Each curve represents the relative number of ENST00000375991 survived to the corresponding point of time. Dashed lines represent 0.95 confidence intervals for the curves with the corresponding color. Total of 1 observation deleted due to missingness. Log-rank probability measure for the equality of these curves is 2.788116e-02. Additional annotations: *.GeneId*=ENSG00000135070, *.GeneName*=ISCA1, *.GeneDesc*=iron-sulfur cluster assembly 1 homolog (S. cerevisiae) [Source:HGNC Symbol;Acc:28660].

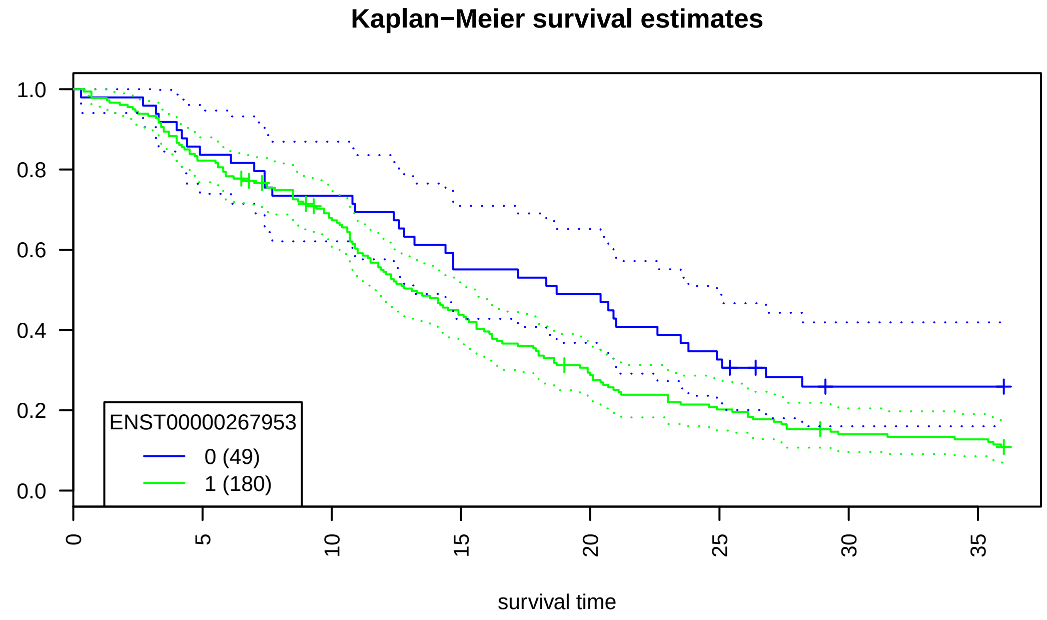

Figure 707: Each curve represents the relative number of ENST00000267953 survived to the corresponding point of time. Dashed lines represent 0.95 confidence intervals for the curves with the corresponding color. Total of 1 observation deleted due to missingness. Log-rank probability measure for the equality of these curves is 1.992042e-02. Additional annotations: *.GeneId*=ENSG00000140379, *.GeneName*=BCL2A1, *.GeneDesc*=BCL2-related protein A1 [Source:HGNC Symbol;Acc:991].

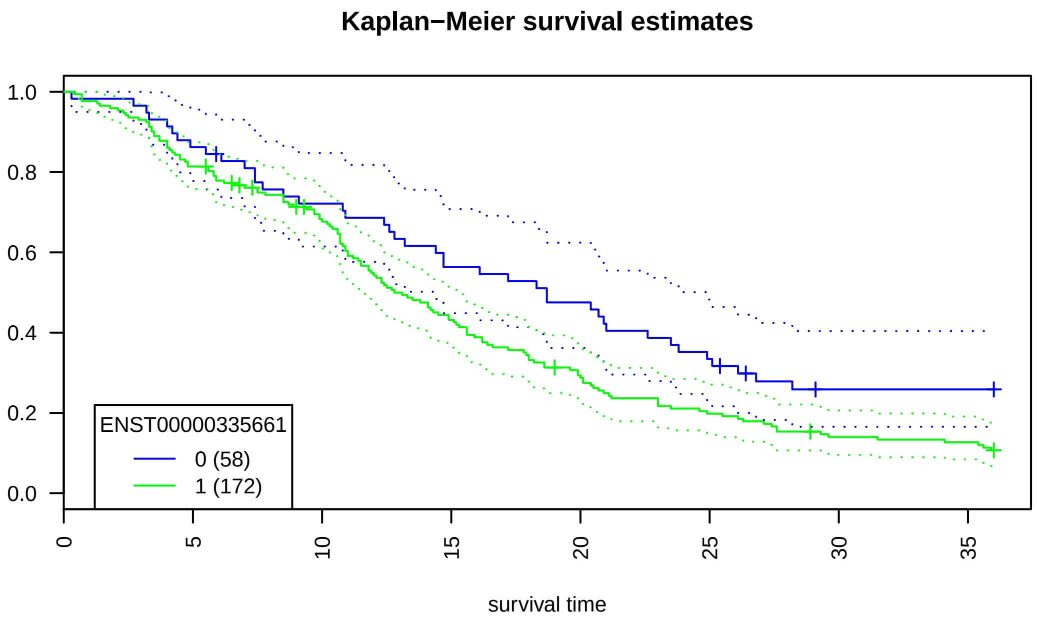

Figure 708: Each curve represents the relative number of ENST00000335661 survived to the corresponding point of time. Dashed lines represent 0.95 confidence intervals for the curves with the corresponding color. Log-rank probability measure for the equality of these curves is 1.216610e-02. Additional annotations: *.GeneId*=ENSG00000140379, *.GeneName*=BCL2A1, *.GeneDesc*=BCL2-related protein A1 [Source:HGNC Symbol;Acc:991].

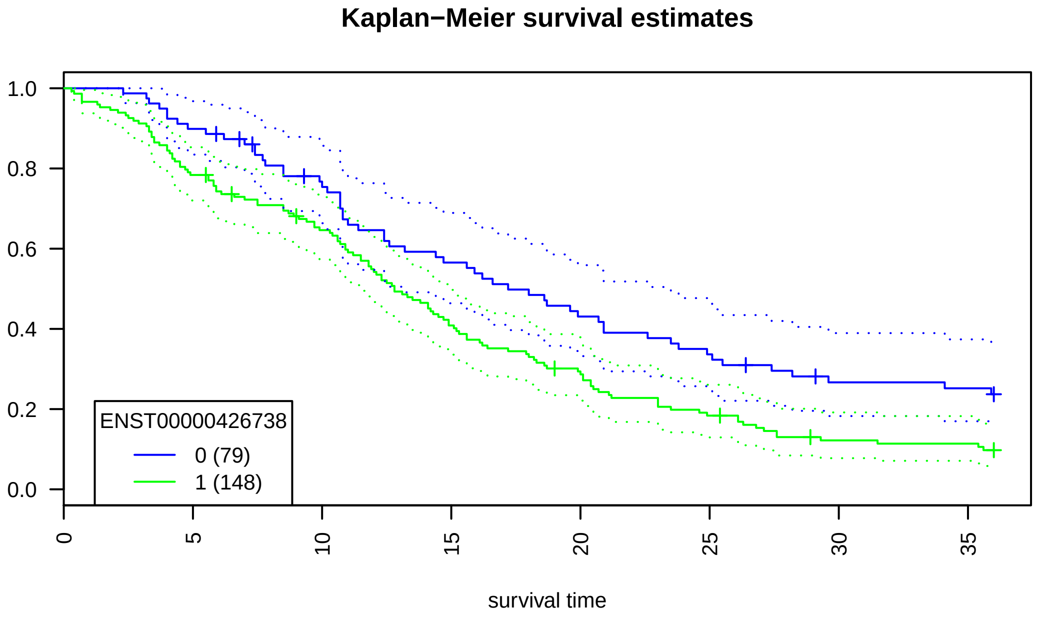

Figure 709: Each curve represents the relative number of ENST00000426738 survived to the corresponding point of time. Dashed lines represent 0.95 confidence intervals for the curves with the corresponding color. Total of 3 observations deleted due to missingness. Log-rank probability measure for the equality of these curves is 3.426234e-03. Additional annotations: *.GeneId*=ENSG00000110077, *.GeneName*=MS4A6A, *.GeneDesc*=membrane-spanning 4-domains, subfamily A, member 6A [Source:HGNC Symbol;Acc:13375].

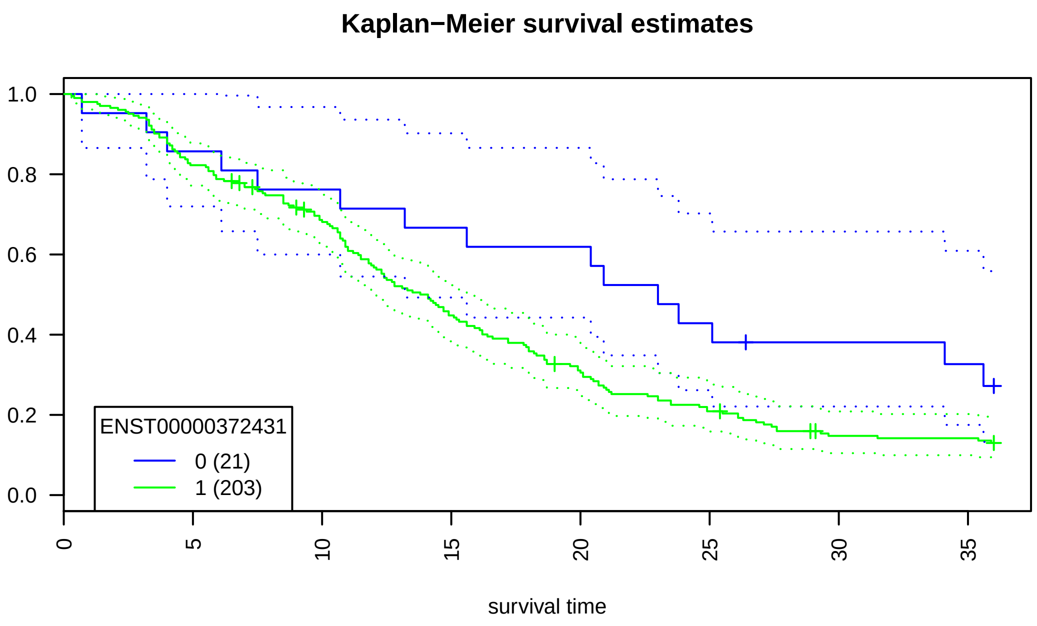

Figure 710: Each curve represents the relative number of ENST00000372431 survived to the corresponding point of time. Dashed lines represent 0.95 confidence intervals for the curves with the corresponding color. Total of 6 observations deleted due to missingness. Log-rank probability measure for the equality of these curves is 4.617764e-02. Additional annotations: *.GeneId*=ENSG00000100979, *.GeneName*=PLTP, *.GeneDesc*=phospholipid transfer protein [Source:HGNC Symbol;Acc:9093].

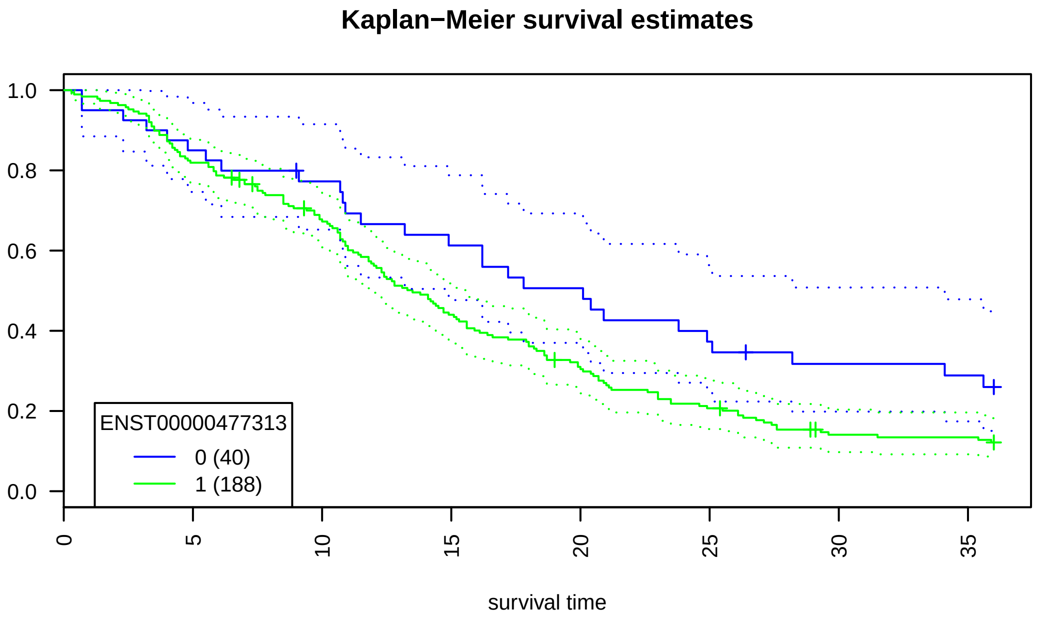

Figure 711: Each curve represents the relative number of ENST00000477313 survived to the corresponding point of time. Dashed lines represent 0.95 confidence intervals for the curves with the corresponding color. Total of 2 observations deleted due to missingness. Log-rank probability measure for the equality of these curves is 2.774260e-02. Additional annotations: *.GeneId*=ENSG00000100979, *.GeneName*=PLTP, *.GeneDesc*=phospholipid transfer protein [Source:HGNC Symbol;Acc:9093].

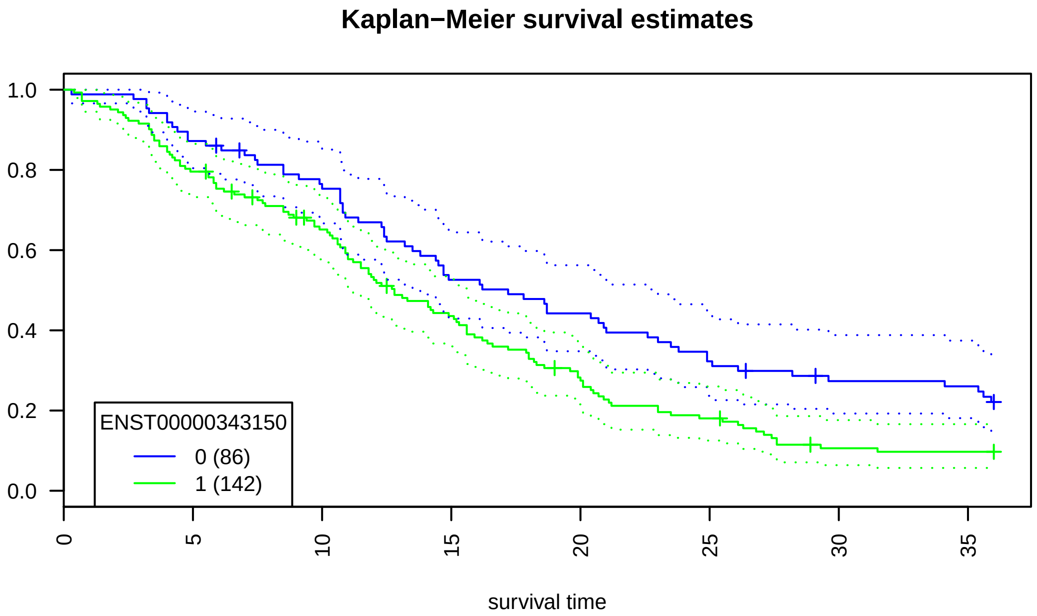

Figure 712: Each curve represents the relative number of ENST00000343150 survived to the corresponding point of time. Dashed lines represent 0.95 confidence intervals for the curves with the corresponding color. Total of 2 observations deleted due to missingness. Log-rank probability measure for the equality of these curves is 3.596069e-03. Additional annotations: *.GeneId*=ENSG00000135047, *.GeneName*=CTSL1, *.GeneDesc*=cathepsin L1 [Source:HGNC Symbol;Acc:2537].

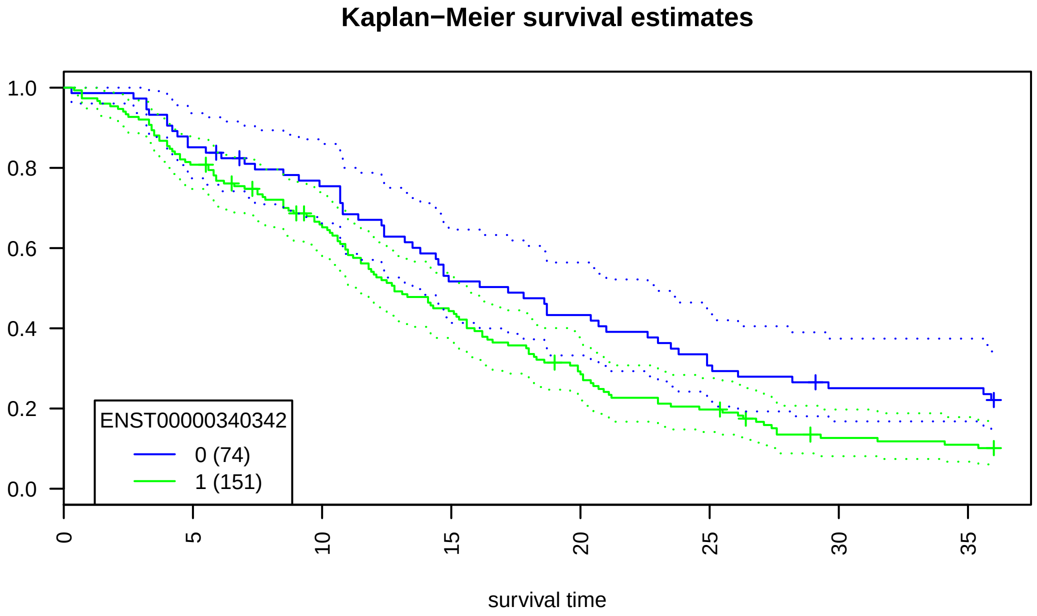

Figure 713: Each curve represents the relative number of ENST00000340342 survived to the corresponding point of time. Dashed lines represent 0.95 confidence intervals for the curves with the corresponding color. Total of 5 observations deleted due to missingness. Log-rank probability measure for the equality of these curves is 1.41091e-02. Additional annotations: *.GeneId*=ENSG00000135047, *.GeneName*=CTSL1, *.GeneDesc*=cathepsin L1 [Source:HGNC Symbol;Acc:2537].

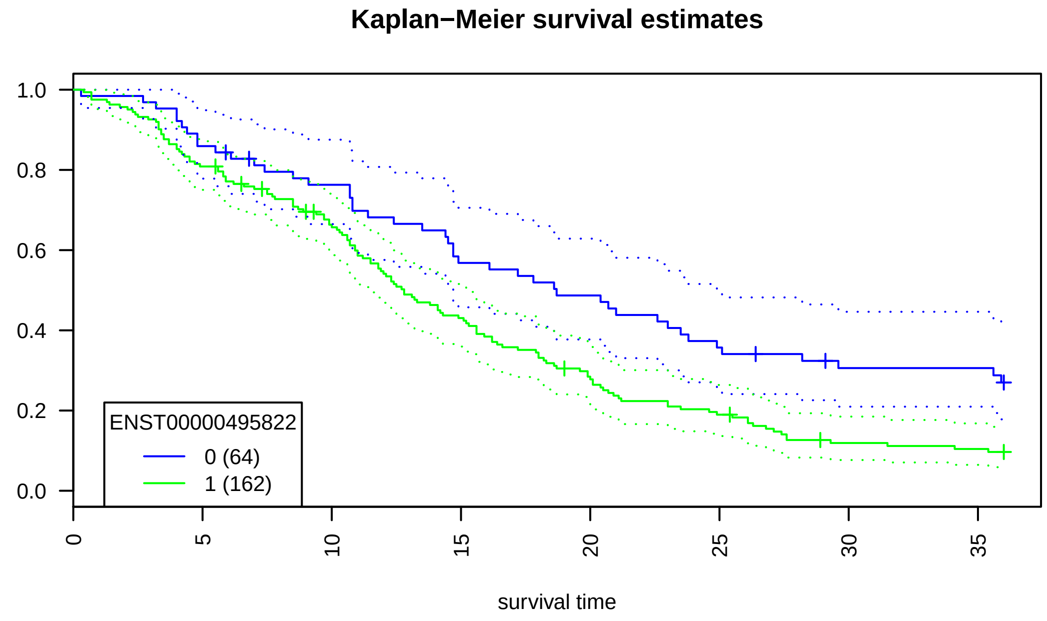

Figure 714: Each curve represents the relative number of ENST00000495822 survived to the corresponding point of time. Dashed lines represent 0.95 confidence intervals for the curves with the corresponding color. Total of 4 observations deleted due to missingness. Log-rank probability measure for the equality of these curves is 1.406596e-03. Additional annotations: *.GeneId*=ENSG00000135047, *.GeneName*=CTSL1, *.GeneDesc*=cathepsin L1 [Source:HGNC Symbol;Acc:2537].

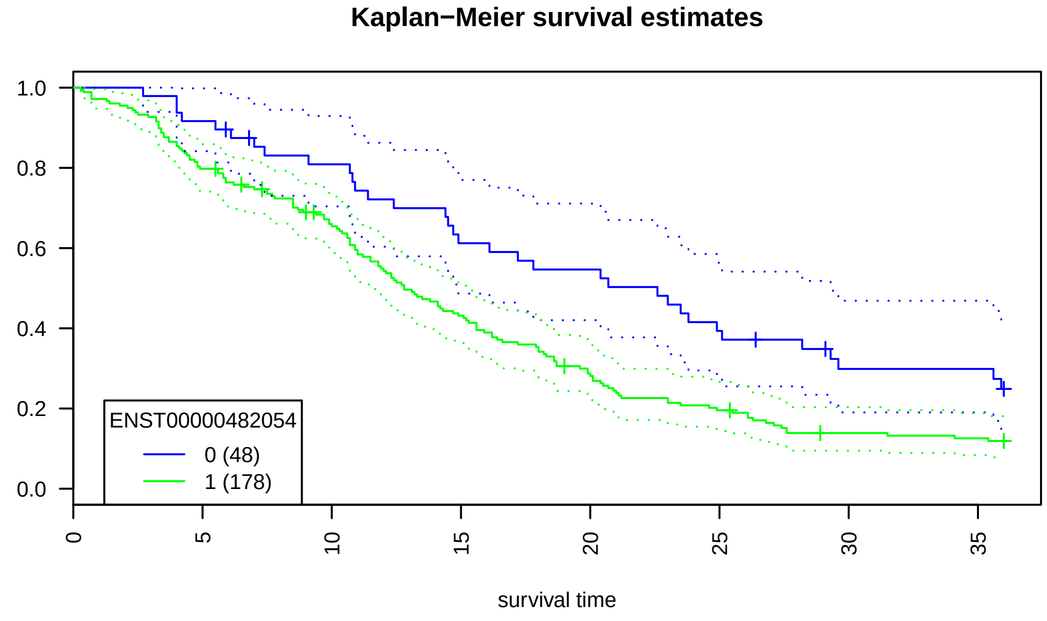

Figure 715: Each curve represents the relative number of ENST00000482054 survived to the corresponding point of time. Dashed lines represent 0.95 confidence intervals for the curves with the corresponding color. Total of 4 observations deleted due to missingness. Log-rank probability measure for the equality of these curves is 3.304049e-03. Additional annotations: *.GeneId*=ENSG00000135047, *.GeneName*=CTSL1, *.GeneDesc*=cathepsin L1 [Source:HGNC Symbol;Acc:2537].

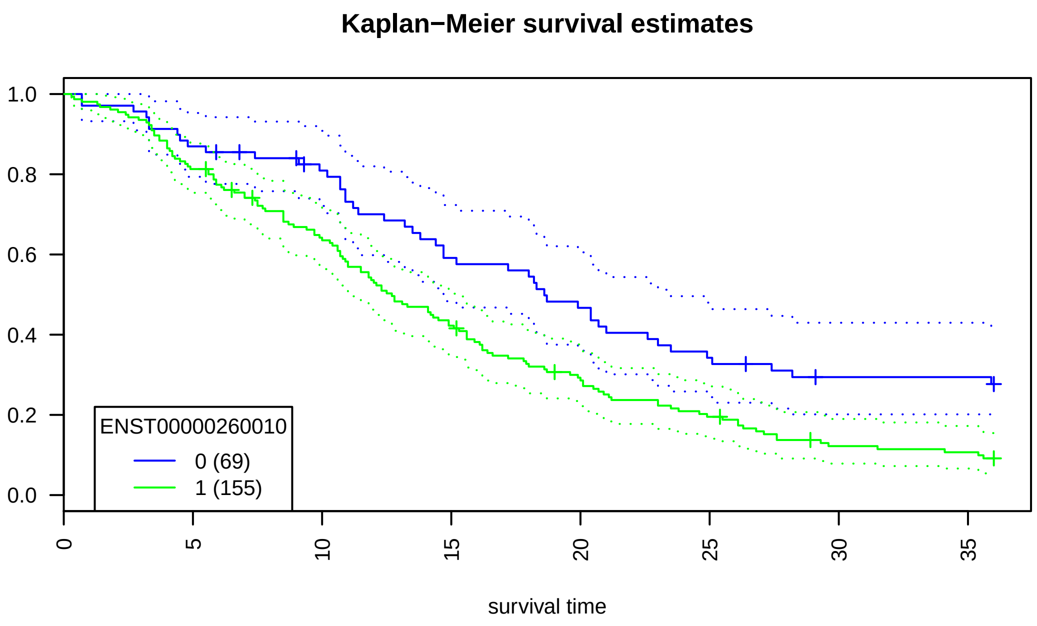

Figure 716: Each curve represents the relative number of ENST00000260010 survived to the corresponding point of time. Dashed lines represent 0.95 confidence intervals for the curves with the corresponding color. Total of 6 observations deleted due to missingness. Log-rank probability measure for the equality of these curves is 9.821117e-04. Additional annotations: *.GeneId*=ENSG00000137462, *.GeneName*=TLR2, *.GeneDesc*=toll-like receptor 2 [Source:HGNC Symbol;Acc:11848].

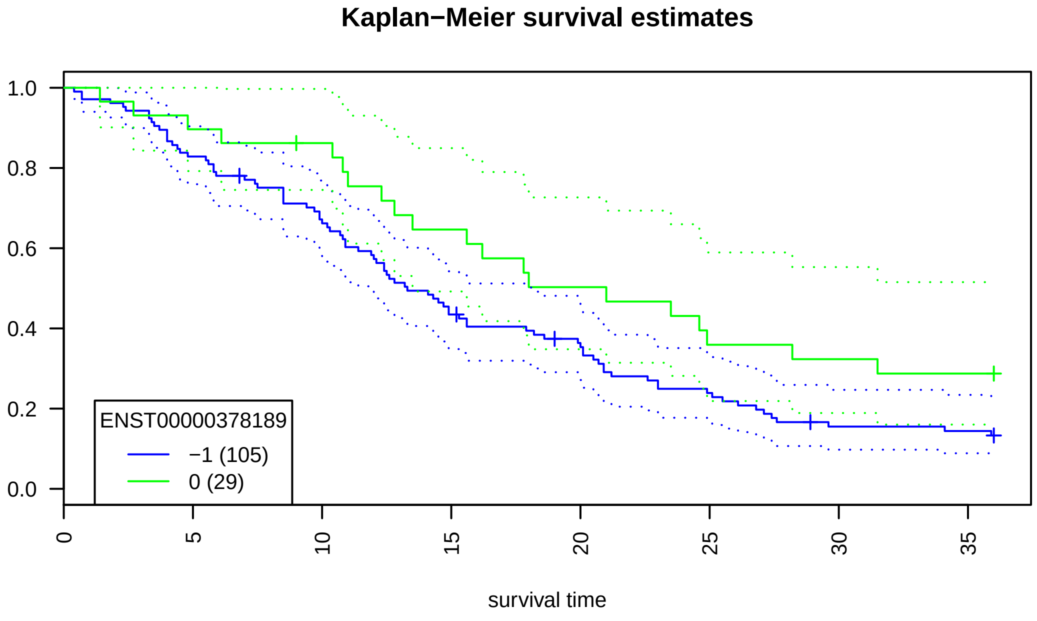

Figure 717: Each curve represents the relative number of ENST00000378189 survived to the corresponding point of time. Dashed lines represent 0.95 confidence intervals for the curves with the corresponding color. Total of 96 observations deleted due to missingness. Log-rank probability measure for the equality of these curves is 4.235964e-02. Additional annotations: *.GeneId*=ENSG00000006114, *.GeneName*=SYNRG, *.GeneDesc*=synergin, gamma [Source:HGNC Symbol;Acc:557].

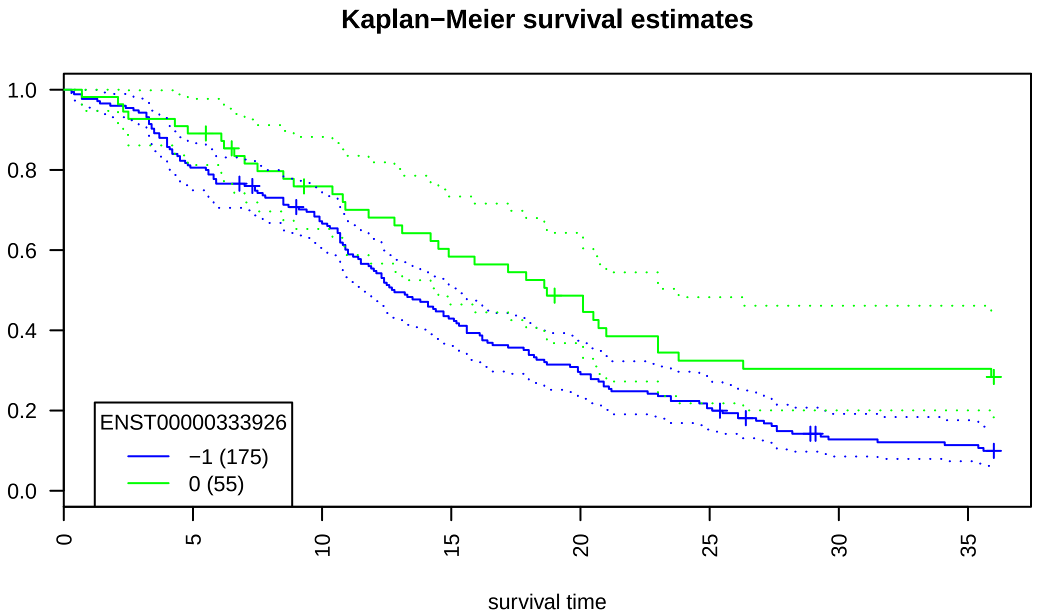

Figure 718: Each curve represents the relative number of ENST00000333926 survived to the corresponding point of time. Dashed lines represent 0.95 confidence intervals for the curves with the corresponding color. Log-rank probability measure for the equality of these curves is 5.092165e-03. Additional annotations: *.GeneId*=ENSG00000122873, *.GeneName*=CISD1, *.GeneDesc*=CDGSH iron sulfur domain 1 [Source:HGNC Symbol;Acc:30880].

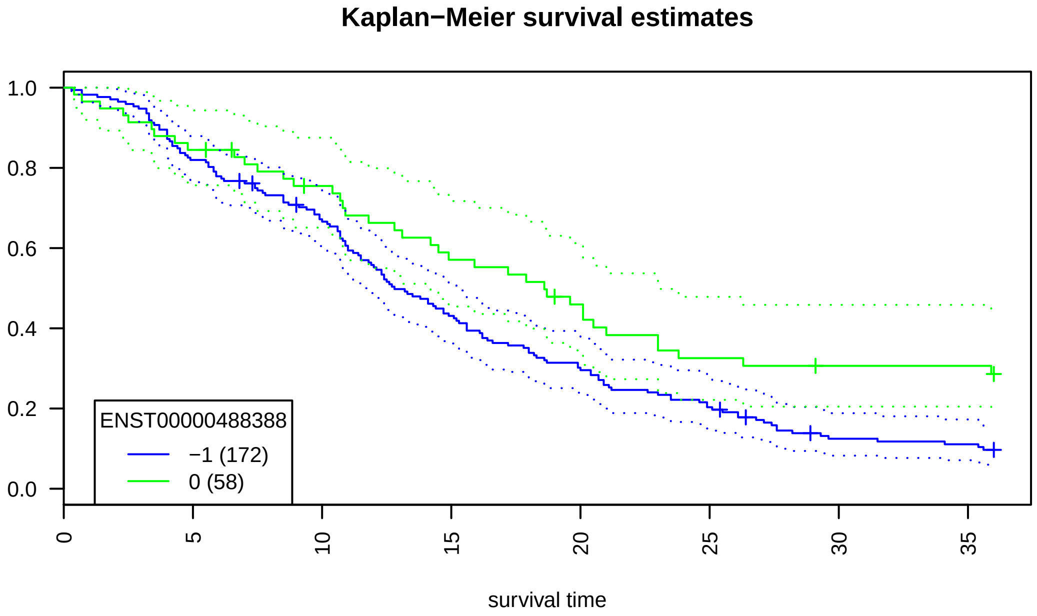

Figure 719: Each curve represents the relative number of ENST00000488388 survived to the corresponding point of time. Dashed lines represent 0.95 confidence intervals for the curves with the corresponding color. Log-rank probability measure for the equality of these curves is 6.333458e-03. Additional annotations: *.GeneId*=ENSG00000122873, *.GeneName*=CISD1, *.GeneDesc*=CDGSH iron sulfur domain 1 [Source:HGNC Symbol;Acc:30880].

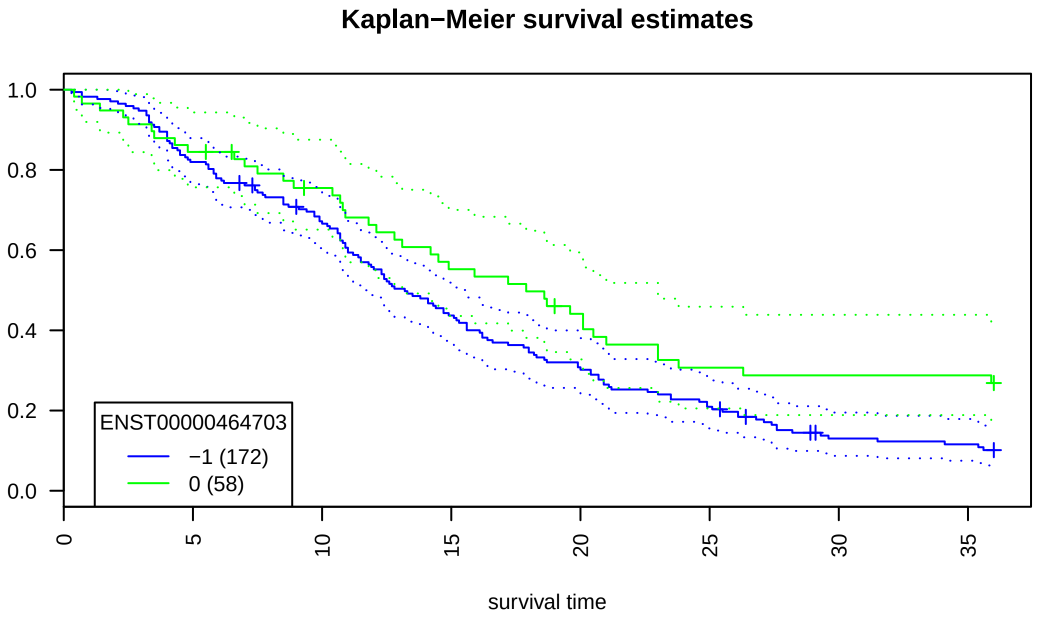

Figure 720: Each curve represents the relative number of ENST00000464703 survived to the corresponding point of time. Dashed lines represent 0.95 confidence intervals for the curves with the corresponding color. Log-rank probability measure for the equality of these curves is 1.653311e-02. Additional annotations: *.GeneId*=ENSG00000122873, *.GeneName*=CISD1, *.GeneDesc*=CDGSH iron sulfur domain 1 [Source:HGNC Symbol;Acc:30880].

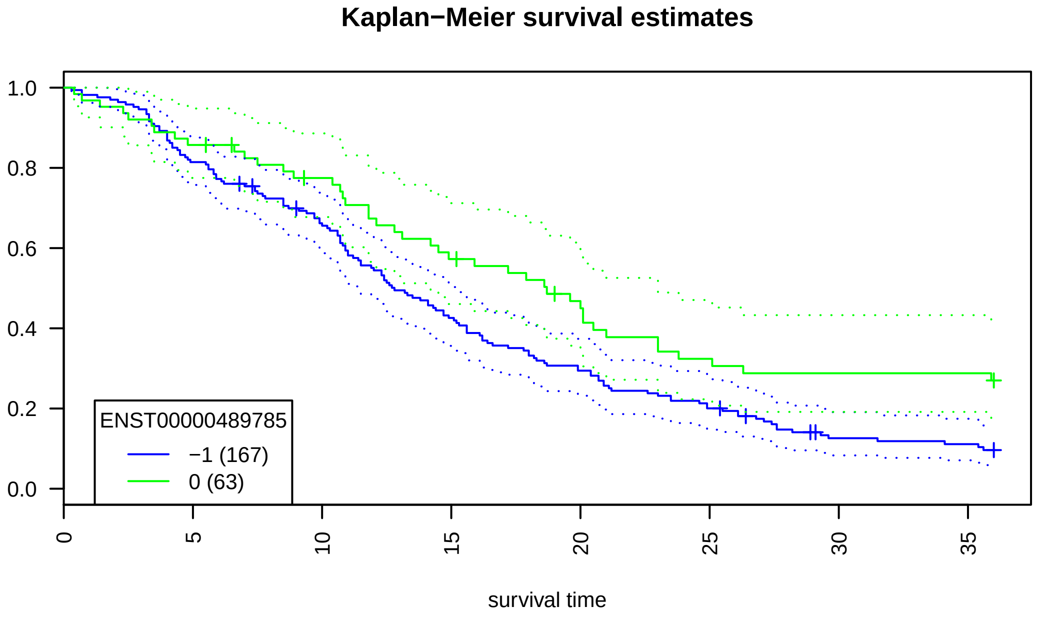

Figure 721: Each curve represents the relative number of ENST00000489785 survived to the corresponding point of time. Dashed lines represent 0.95 confidence intervals for the curves with the corresponding color. Log-rank probability measure for the equality of these curves is 5.66935e-03. Additional annotations: *.GeneId*=ENSG00000122873, *.GeneName*=CISD1, *.GeneDesc*=CDGSH iron sulfur domain 1 [Source:HGNC Symbol;Acc:30880].

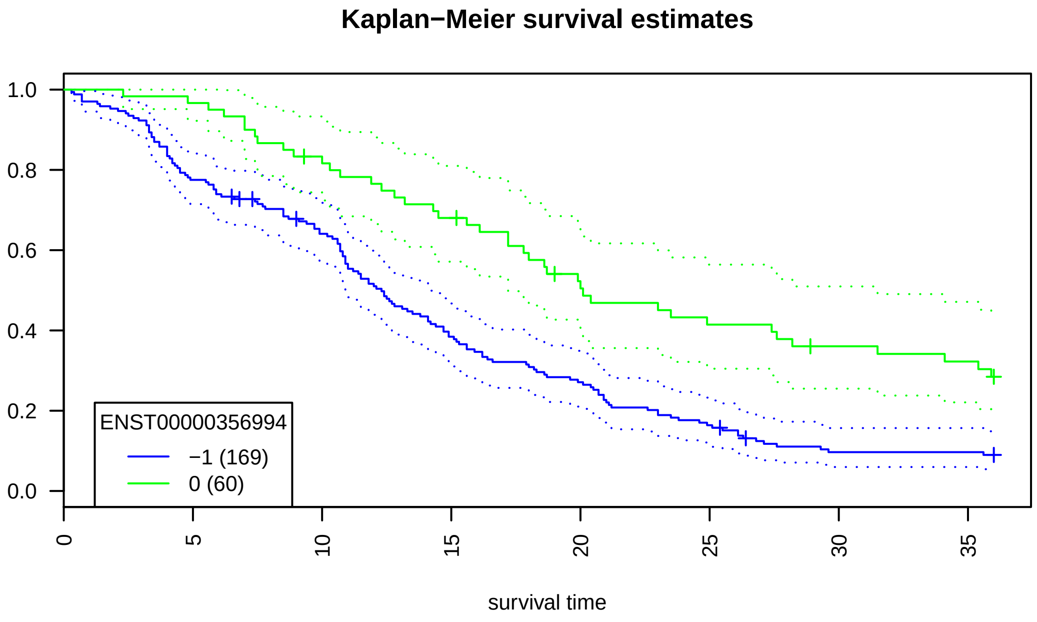

Figure 722: Each curve represents the relative number of ENST00000356994 survived to the corresponding point of time. Dashed lines represent 0.95 confidence intervals for the curves with the corresponding color. Total of 1 observation deleted due to missingness. Log-rank probability measure for the equality of these curves is 1.853222e-05. Additional annotations: *.GeneId*=ENSG00000180900, *.GeneName*=SCRIB, *.GeneDesc*=scribbled homolog (Drosophila) [Source:HGNC Symbol;Acc:30377].

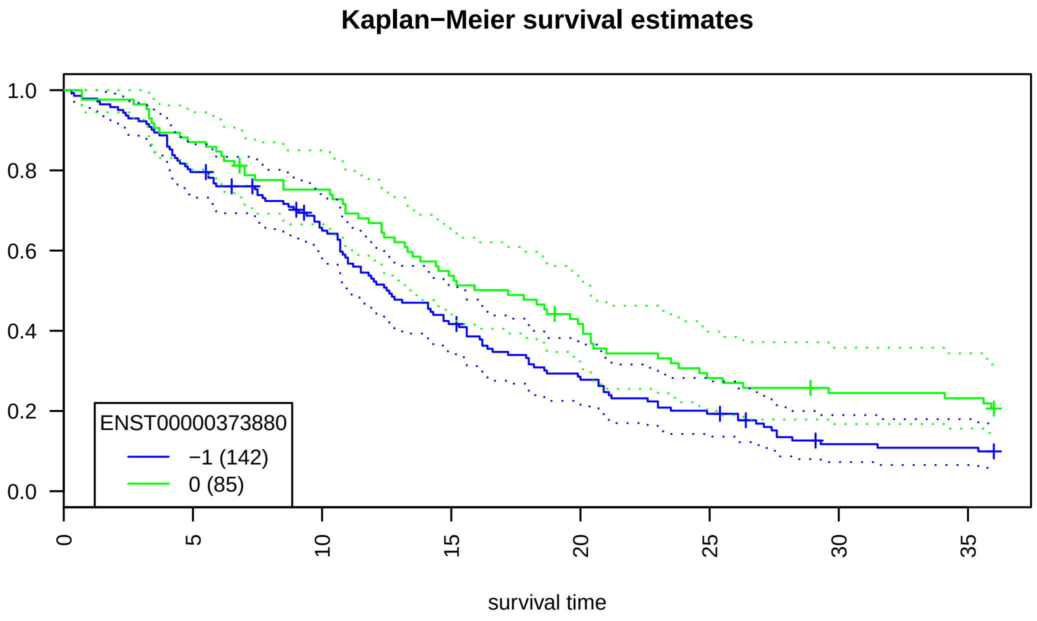

Figure 723: Each curve represents the relative number of ENST00000373880 survived to the corresponding point of time. Dashed lines represent 0.95 confidence intervals for the curves with the corresponding color. Total of 3 observations deleted due to missingness. Log-rank probability measure for the equality of these curves is 1.852511e-02. Additional annotations: *.GeneId*=ENSG00000165443, *.GeneName*=PHYHIPL, *.GeneDesc*=phytanoyl-CoA 2-hydroxylase interacting protein-like [Source:HGNC Symbol;Acc:29378].

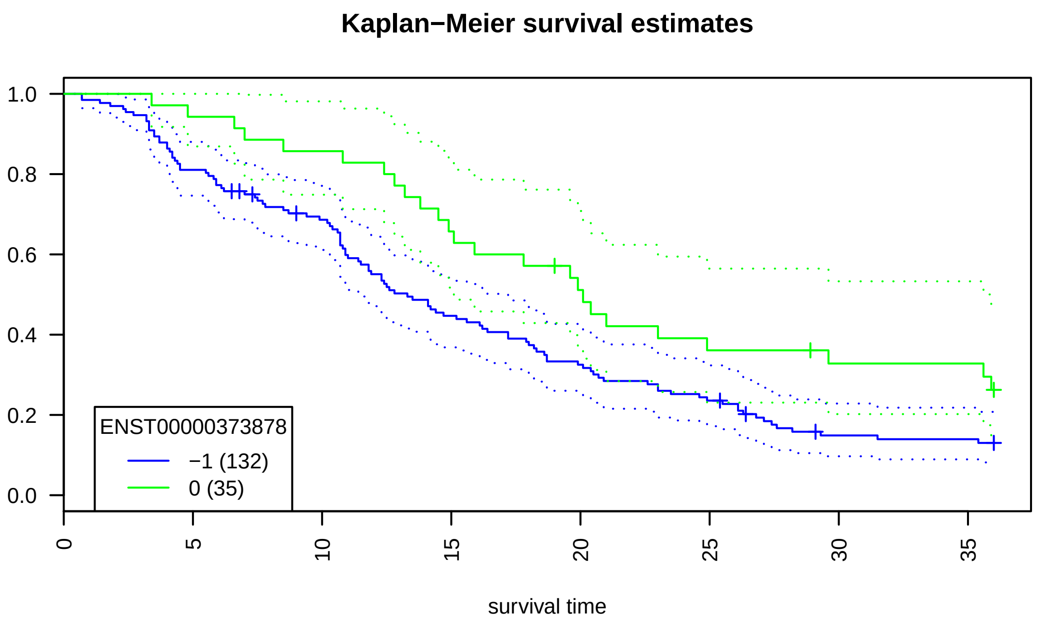

Figure 724: Each curve represents the relative number of ENST00000373878 survived to the corresponding point of time. Dashed lines represent 0.95 confidence intervals for the curves with the corresponding color. Total of 63 observations deleted due to missingness. Log-rank probability measure for the equality of these curves is 1.540825e-02. Additional annotations: *.GeneId*=ENSG00000165443, *.GeneName*=PHYHIPL, *.GeneDesc*=phytanoyl-CoA 2-hydroxylase interacting protein-like [Source:HGNC Symbol;Acc:29378].

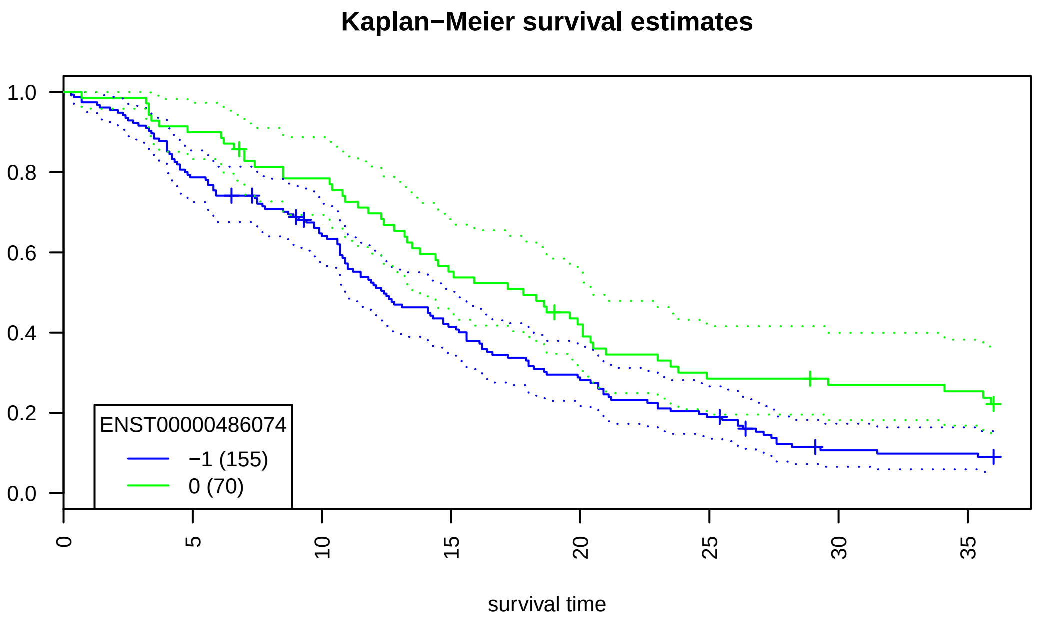

Figure 725: Each curve represents the relative number of ENST00000486074 survived to the corresponding point of time. Dashed lines represent 0.95 confidence intervals for the curves with the corresponding color. Total of 5 observations deleted due to missingness. Log-rank probability measure for the equality of these curves is 5.261301e-03. Additional annotations: *.GeneId*=ENSG00000165443, *.GeneName*=PHYHIPL, *.GeneDesc*=phytanoyl-CoA 2-hydroxylase interacting protein-like [Source:HGNC Symbol;Acc:29378].

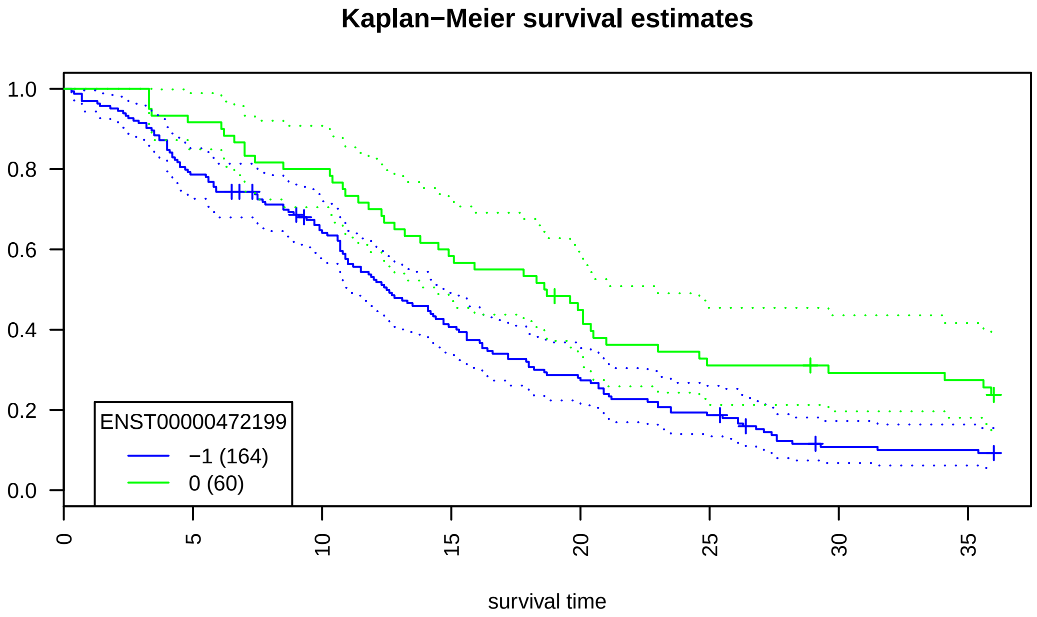

Figure 726: Each curve represents the relative number of ENST00000472199 survived to the corresponding point of time. Dashed lines represent 0.95 confidence intervals for the curves with the corresponding color. Total of 6 observations deleted due to missingness. Log-rank probability measure for the equality of these curves is 2.522276e-03. Additional annotations: *.GeneId*=ENSG00000165443, *.GeneName*=PHYHIPL, *.GeneDesc*=phytanoyl-CoA 2-hydroxylase interacting protein-like [Source:HGNC Symbol;Acc:29378].

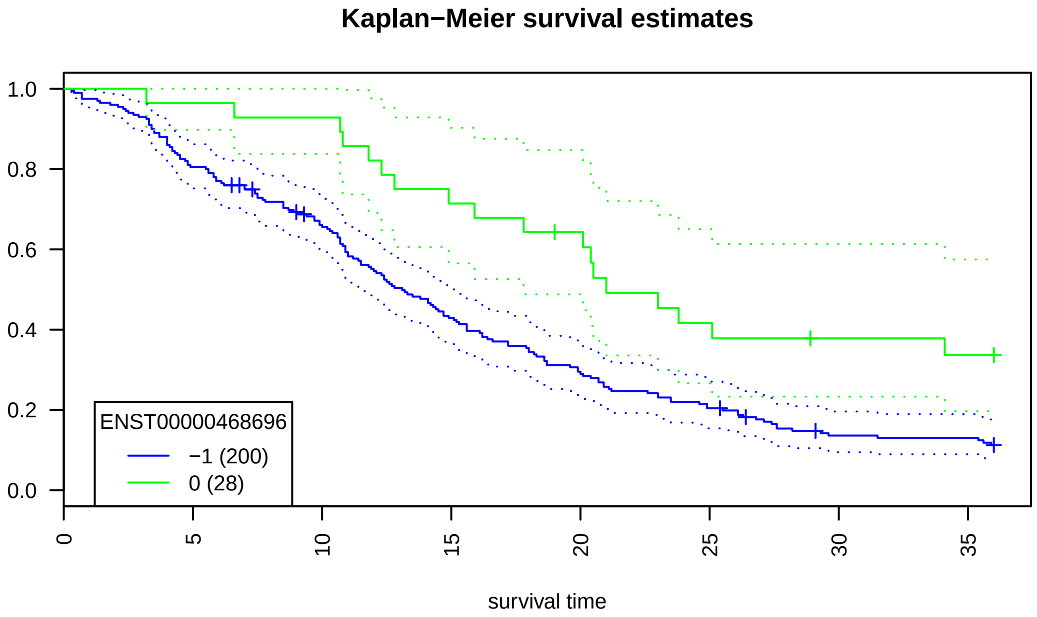

Figure 727: Each curve represents the relative number of ENST00000468696 survived to the corresponding point of time. Dashed lines represent 0.95 confidence intervals for the curves with the corresponding color. Total of 2 observations deleted due to missingness. Log-rank probability measure for the equality of these curves is 1.832498e-03. Additional annotations: *.GeneId*=ENSG00000148541, *.GeneName*=FAM13C, *.GeneDesc*=family with sequence similarity 13, member C [Source:HGNC Symbol;Acc:19371].

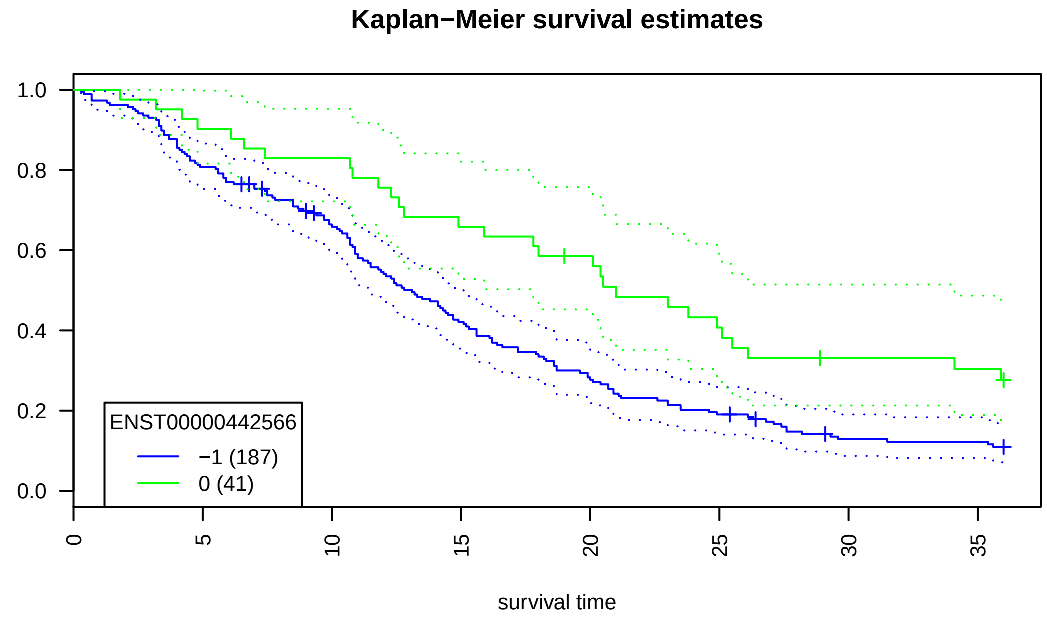

Figure 728: Each curve represents the relative number of ENST00000442566 survived to the corresponding point of time. Dashed lines represent 0.95 confidence intervals for the curves with the corresponding color. Total of 2 observations deleted due to missingness. Log-rank probability measure for the equality of these curves is 2.058904e-03. Additional annotations: *.GeneId*=ENSG00000148541, *.GeneName*=FAM13C, *.GeneDesc*=family with sequence similarity 13, member C [Source:HGNC Symbol;Acc:19371].

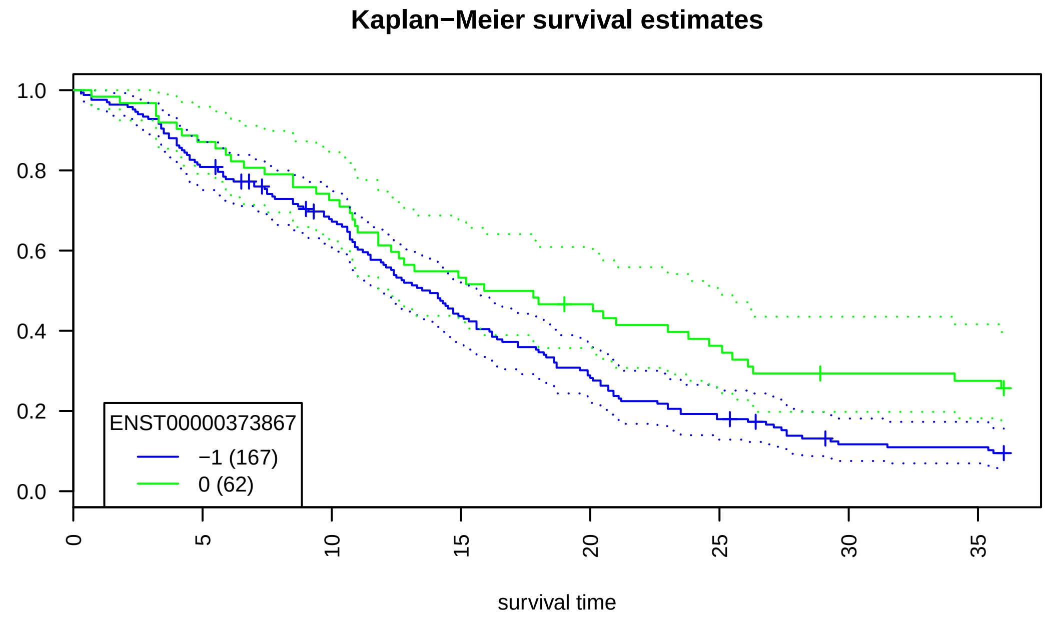

Figure 729: Each curve represents the relative number of ENST00000373867 survived to the corresponding point of time. Dashed lines represent 0.95 confidence intervals for the curves with the corresponding color. Total of 1 observation deleted due to missingness. Log-rank probability measure for the equality of these curves is 9.32061e-03. Additional annotations: *.GeneId*=ENSG00000148541, *.GeneName*=FAM13C, *.GeneDesc*=family with sequence similarity 13, member C [Source:HGNC Symbol;Acc:19371].

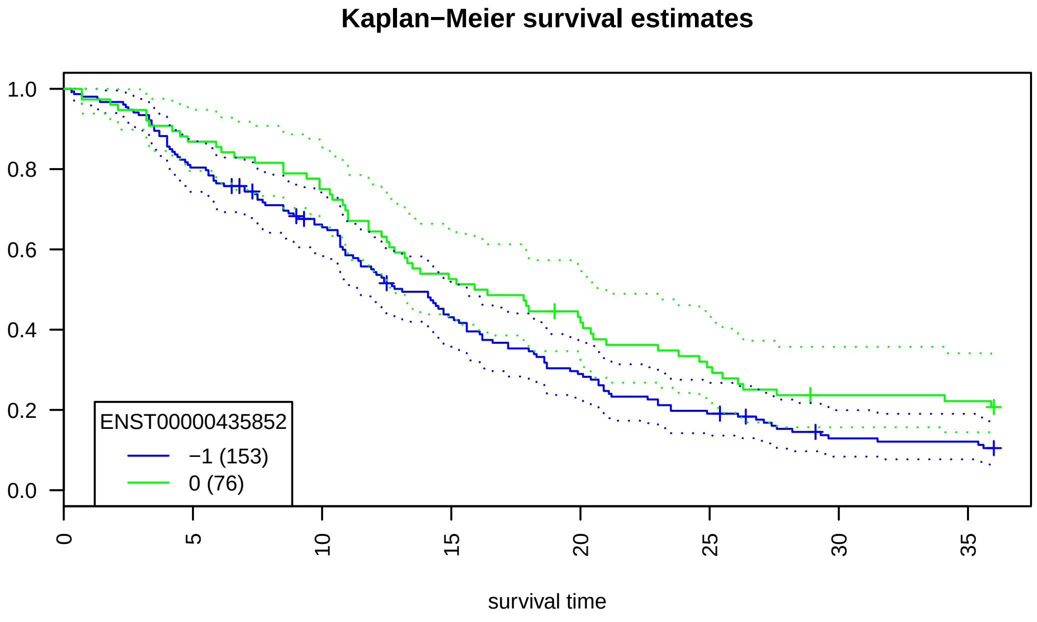

Figure 730: Each curve represents the relative number of ENST00000435852 survived to the corresponding point of time. Dashed lines represent 0.95 confidence intervals for the curves with the corresponding color. Total of 1 observation deleted due to missingness. Log-rank probability measure for the equality of these curves is 3.344162e-02. Additional annotations: *.GeneId*=ENSG00000148541, *.GeneName*=FAM13C, *.GeneDesc*=family with sequence similarity 13, member C [Source:HGNC Symbol;Acc:19371].

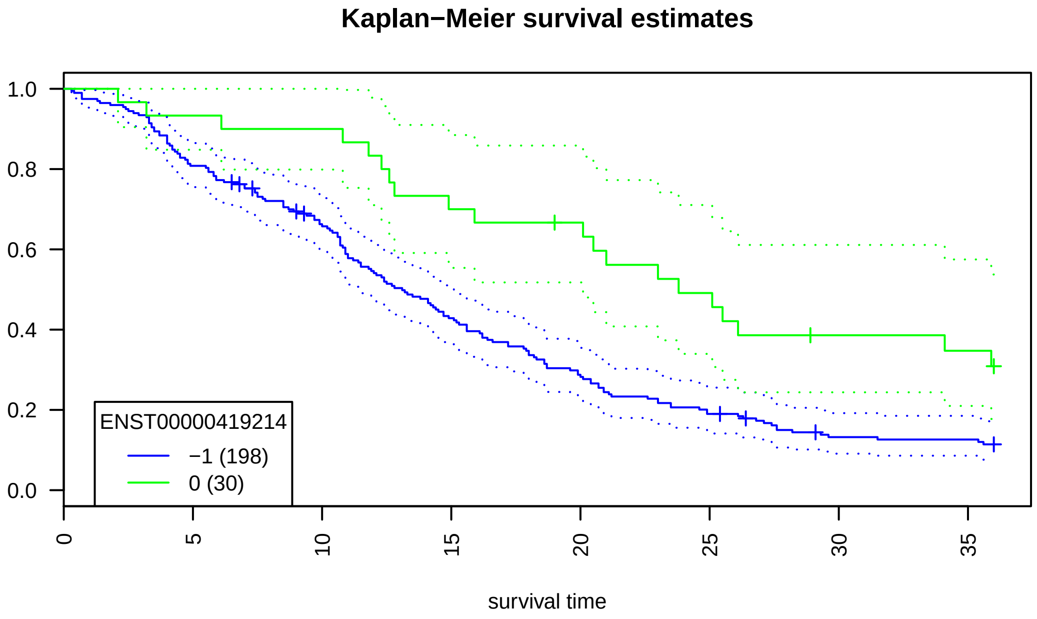

Figure 731: Each curve represents the relative number of ENST00000419214 survived to the corresponding point of time. Dashed lines represent 0.95 confidence intervals for the curves with the corresponding color. Total of 2 observations deleted due to missingness. Log-rank probability measure for the equality of these curves is 1.24827e-03. Additional annotations: *.GeneId*=ENSG00000148541, *.GeneName*=FAM13C, *.GeneDesc*=family with sequence similarity 13, member C [Source:HGNC Symbol;Acc:19371].

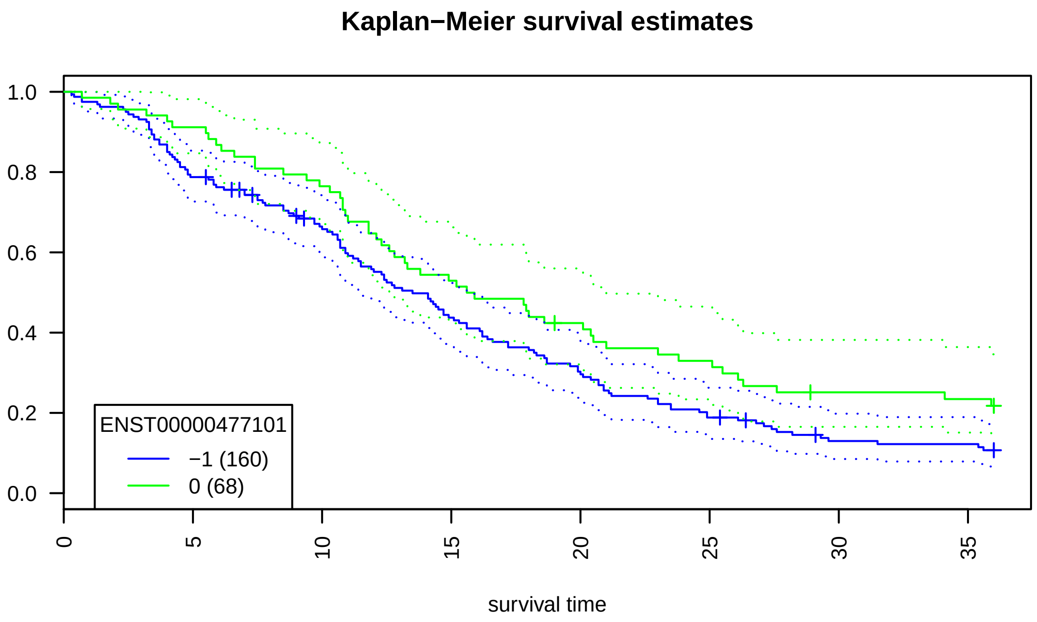

Figure 732: Each curve represents the relative number of ENST00000477101 survived to the corresponding point of time. Dashed lines represent 0.95 confidence intervals for the curves with the corresponding color. Total of 2 observations deleted due to missingness. Log-rank probability measure for the equality of these curves is 3.407293e-02. Additional annotations: *.GeneId*=ENSG00000148541, *.GeneName*=FAM13C, *.GeneDesc*=family with sequence similarity 13, member C [Source:HGNC Symbol;Acc:19371].

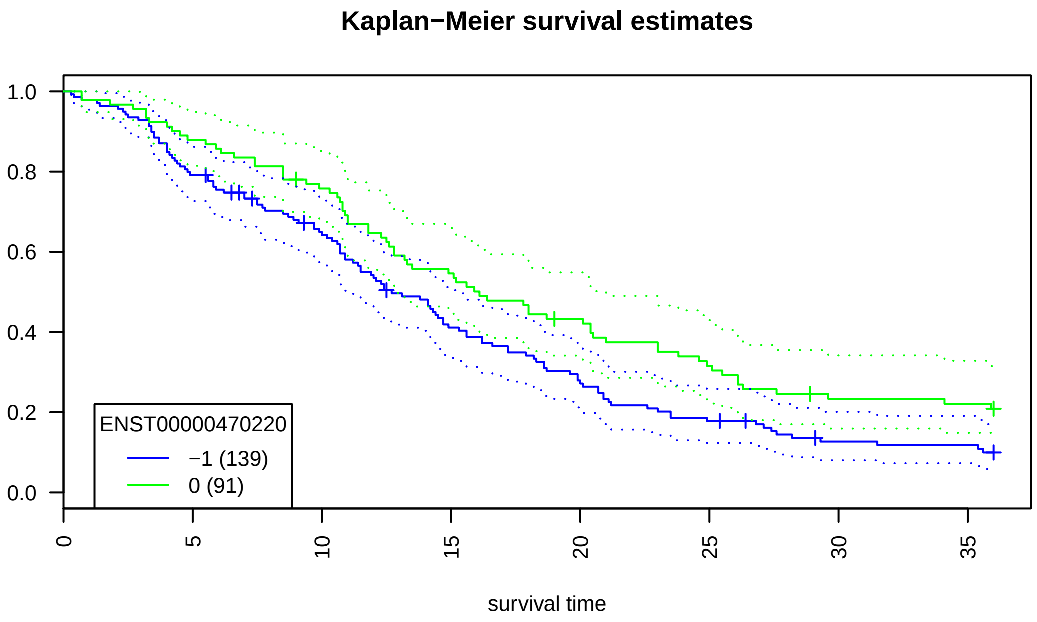

Figure 733: Each curve represents the relative number of ENST00000470220 survived to the corresponding point of time. Dashed lines represent 0.95 confidence intervals for the curves with the corresponding color. Log-rank probability measure for the equality of these curves is 1.278773e-02. Additional annotations: *.GeneId*=ENSG00000148541, *.GeneName*=FAM13C, *.GeneDesc*=family with sequence similarity 13, member C [Source:HGNC Symbol;Acc:19371].

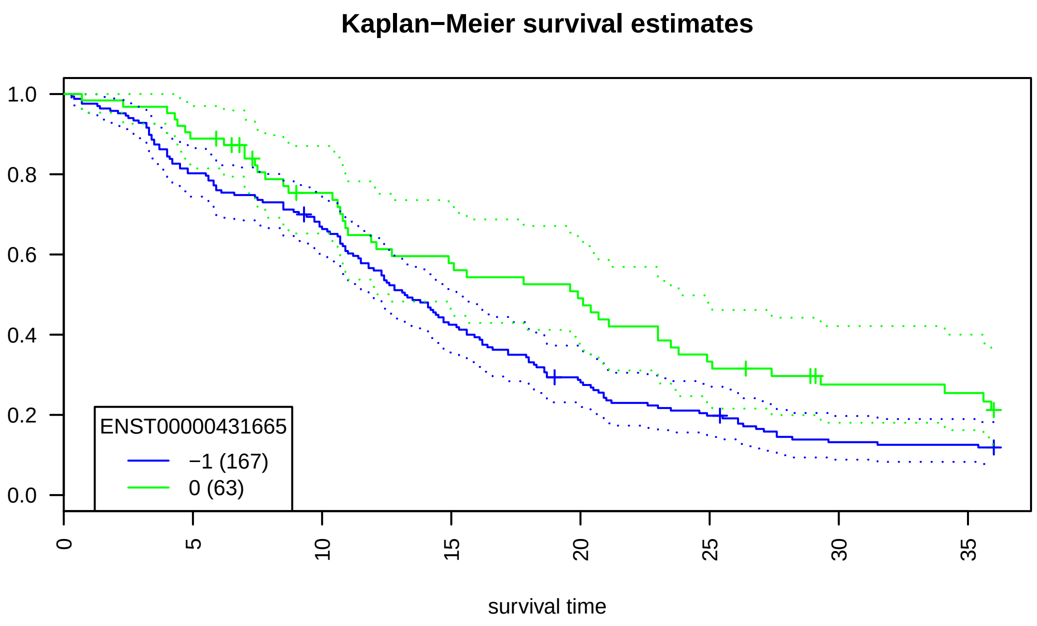

Figure 734: Each curve represents the relative number of ENST00000431665 survived to the corresponding point of time. Dashed lines represent 0.95 confidence intervals for the curves with the corresponding color. Log-rank probability measure for the equality of these curves is 1.723802e-02.

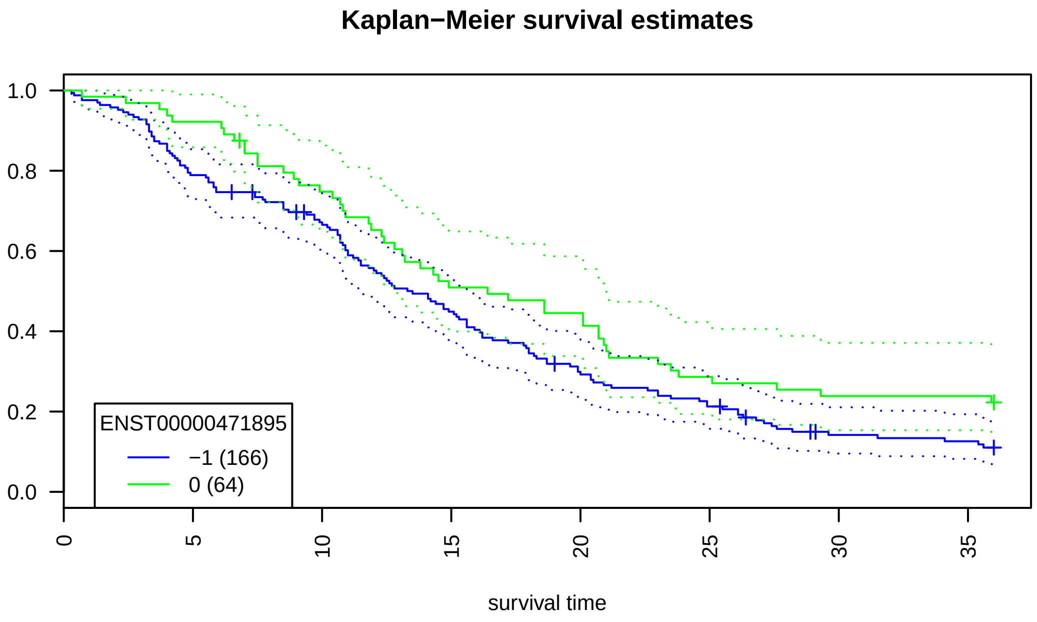

Figure 735: Each curve represents the relative number of ENST00000471895 survived to the corresponding point of time. Dashed lines represent 0.95 confidence intervals for the curves with the corresponding color. Log-rank probability measure for the equality of these curves is 3.875348e-02. Additional annotations: *.GeneId*=ENSG00000132879, *.GeneName*=FBXO44, *.GeneDesc*=F-box protein 44 [Source:HGNC Symbol;Acc:24847].

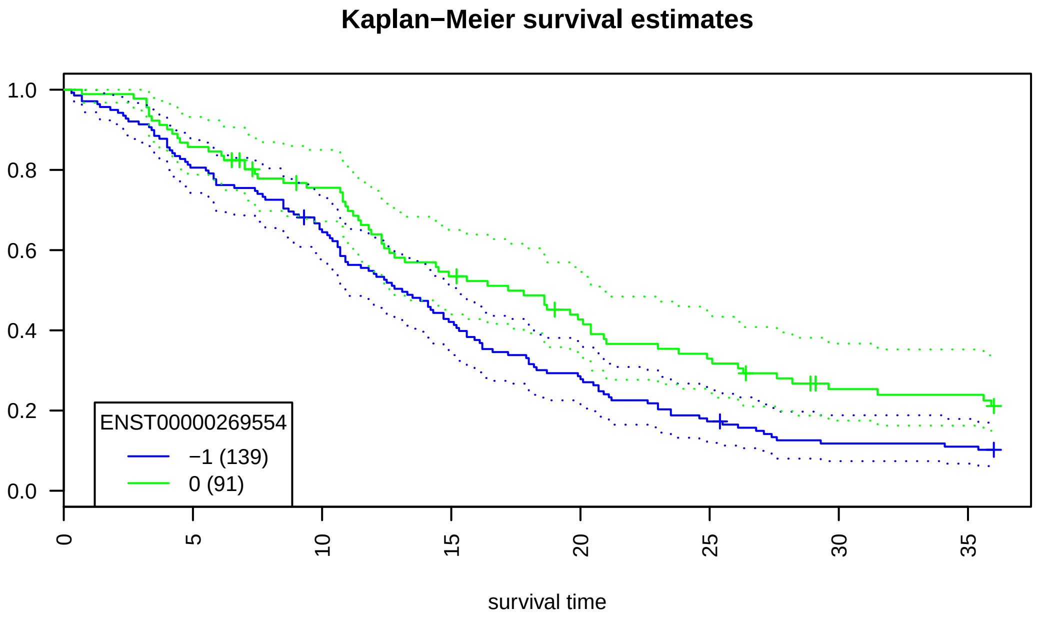

Figure 736: Each curve represents the relative number of ENST00000269554 survived to the corresponding point of time. Dashed lines represent 0.95 confidence intervals for the curves with the corresponding color. Log-rank probability measure for the equality of these curves is 8.580595e-03. Additional annotations: *.GeneId*=ENSG00000141720, *.GeneName*=PIP5K2B, *.GeneDesc*=phosphatidylinositol-5-phosphate 4-kinase, type II, beta [Source:HGNC Symbol;Acc:8998].

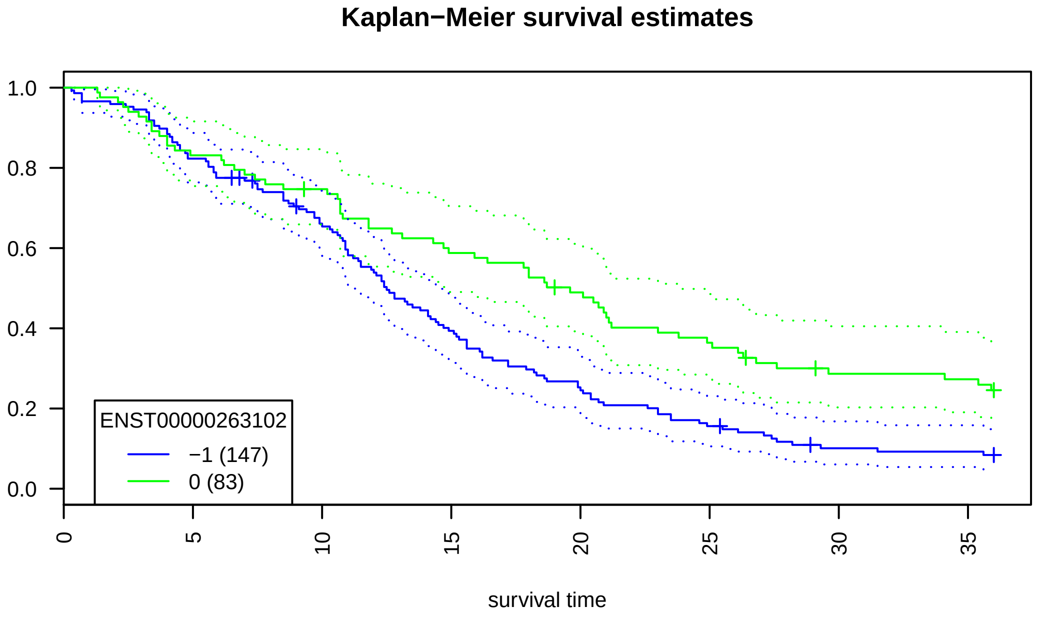

Figure 737: Each curve represents the relative number of ENST00000263102 survived to the corresponding point of time. Dashed lines represent 0.95 confidence intervals for the curves with the corresponding color. Log-rank probability measure for the equality of these curves is 4.674368e-04. Additional annotations: *.GeneId*=ENSG00000108091, *.GeneName*=CCDC6, *.GeneDesc*=coiled-coil domain containing 6 [Source:HGNC Symbol;Acc:18782].

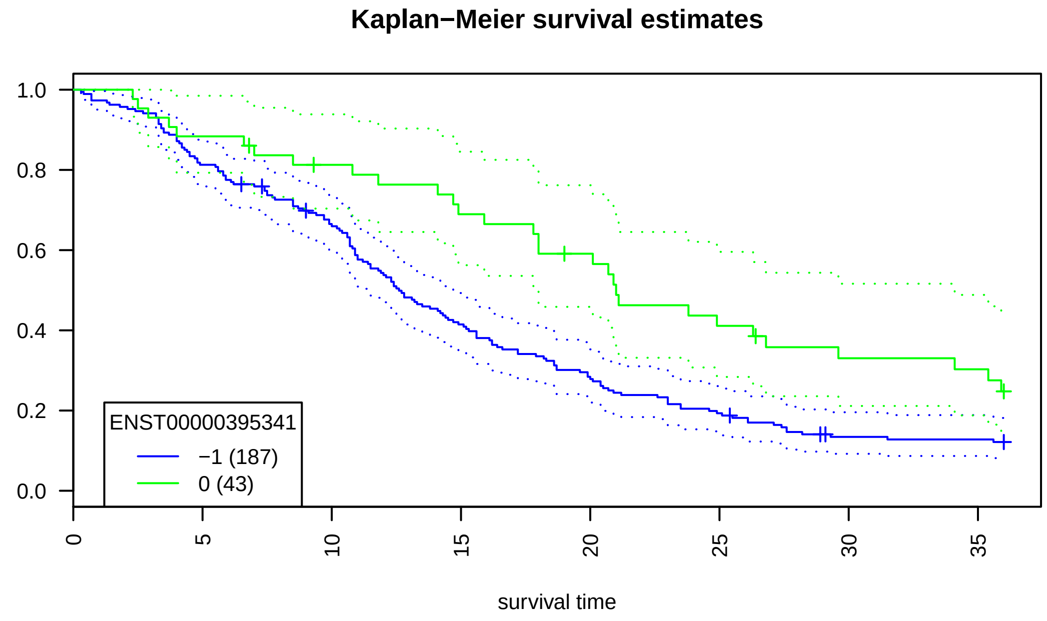

Figure 738: Each curve represents the relative number of ENST00000395341 survived to the corresponding point of time. Dashed lines represent 0.95 confidence intervals for the curves with the corresponding color. Log-rank probability measure for the equality of these curves is 2.743288e-03. Additional annotations: .*GeneId*=ENSG00000108091, .*GeneName*=CCDC6, .*GeneDesc*=coiled-coil domain containing 6 [Source:HGNC Symbol;Acc:18782].

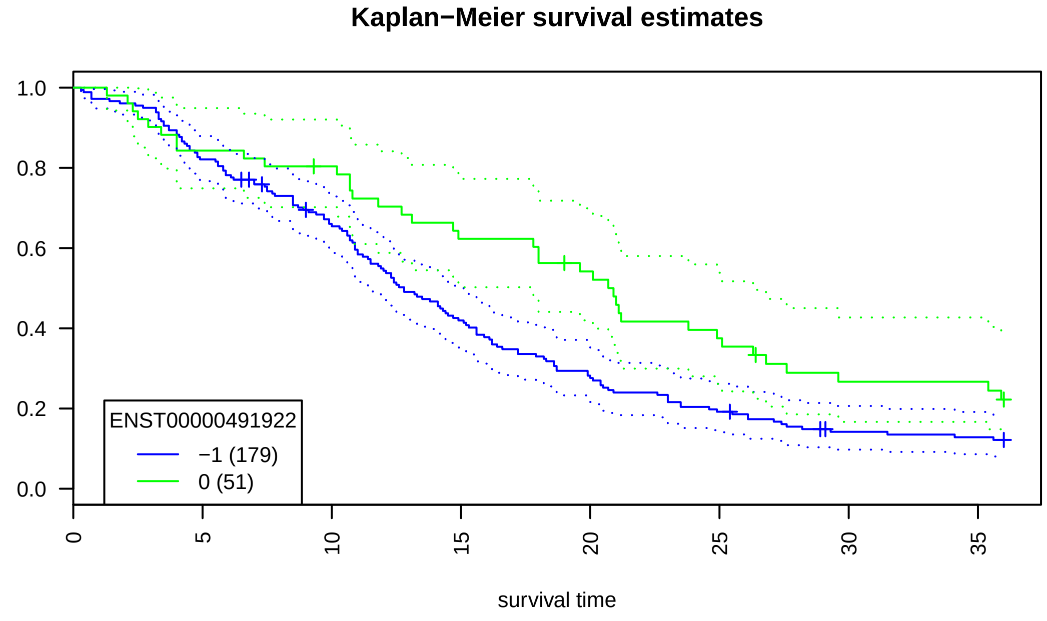

Figure 739: Each curve represents the relative number of ENST00000491922 survived to the corresponding point of time. Dashed lines represent 0.95 confidence intervals for the curves with the corresponding color. Log-rank probability measure for the equality of these curves is 9.950367e-03. Additional annotations: .*GeneId*=ENSG00000108091, .*GeneName*=CCDC6, .*GeneDesc*=coiled-coil domain containing 6 [Source:HGNC Symbol;Acc:18782].

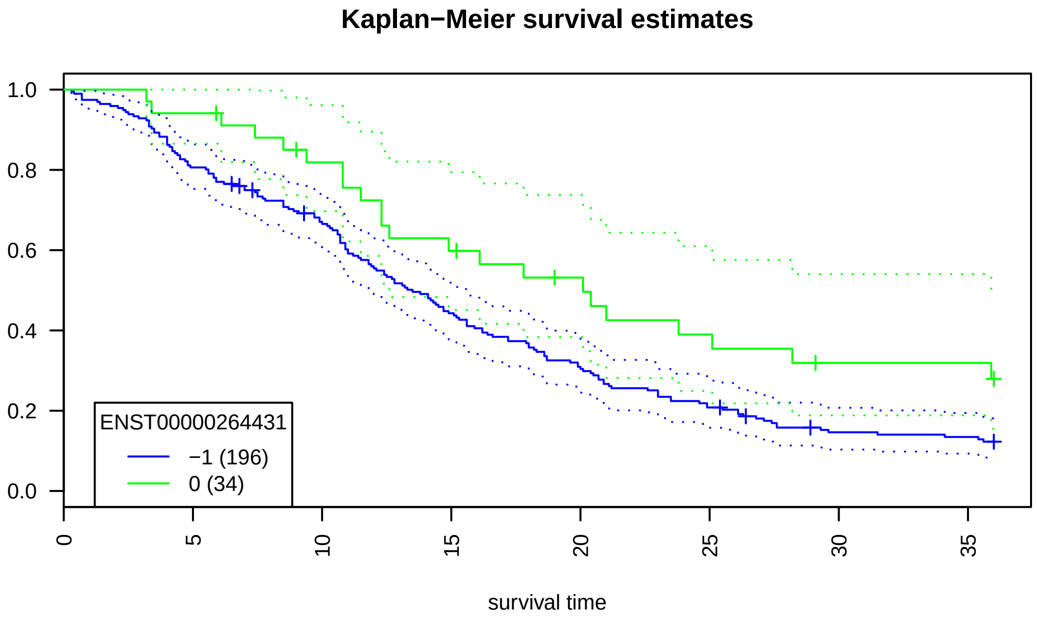

Figure 740: Each curve represents the relative number of ENST00000264431 survived to the corresponding point of time. Dashed lines represent 0.95 confidence intervals for the curves with the corresponding color. Log-rank probability measure for the equality of these curves is 1.898172e-02. Additional annotations: *.GeneId*=ENSG00000109756, *.GeneName*=RAPGEF2, *.GeneDesc*=Rap guanine nucleotide exchange factor (GEF) 2 [Source:HGNC Symbol;Acc:16854].

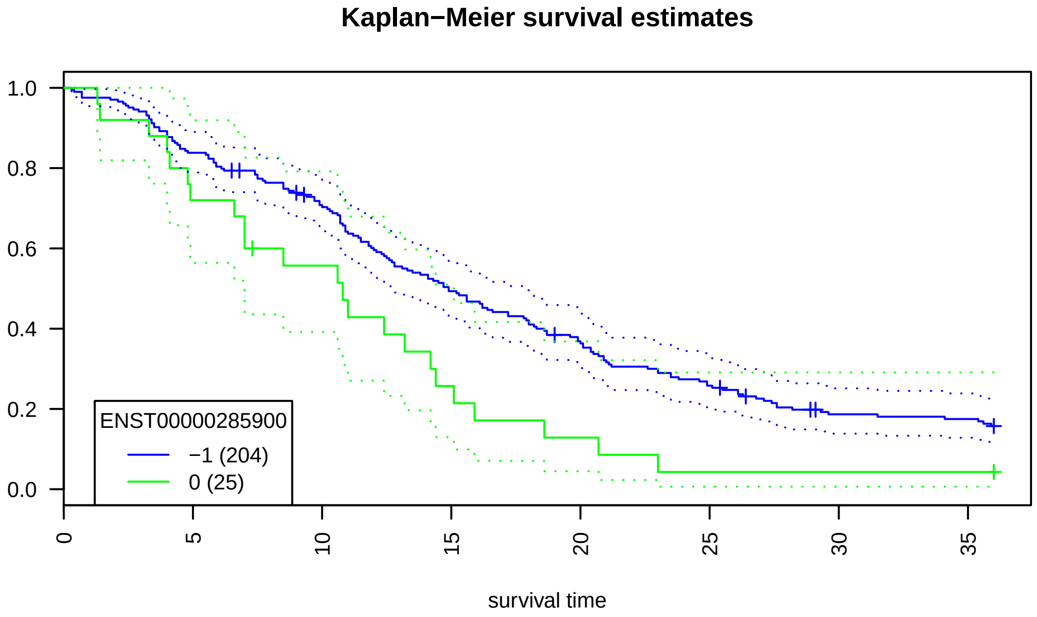

Figure 741: Each curve represents the relative number of ENST00000285900 survived to the corresponding point of time. Dashed lines represent 0.95 confidence intervals for the curves with the corresponding color. Total of 1 observation deleted due to missingness. Log-rank probability measure for the equality of these curves is 4.106547e-03. Additional annotations: *.GeneId*=ENSG00000155511, *.GeneName*=GRIA1, *.GeneDesc*=glutamate receptor, ionotropic, AMPA 1 [Source:HGNC Symbol;Acc:4571].

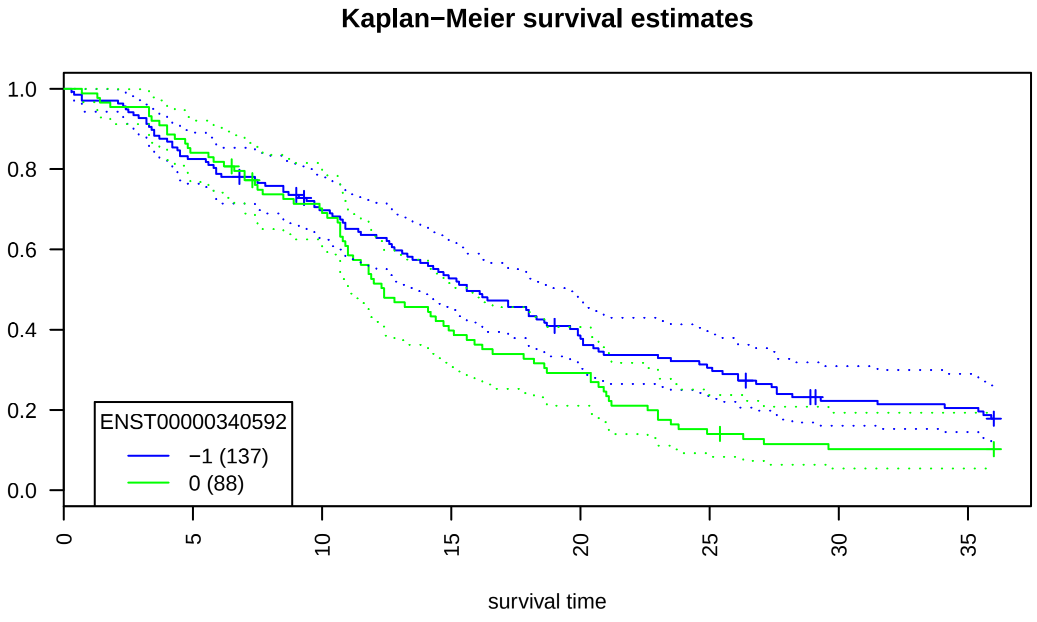

Figure 742: Each curve represents the relative number of ENST00000340592 survived to the corresponding point of time. Dashed lines represent 0.95 confidence intervals for the curves with the corresponding color. Total of 5 observations deleted due to missingness. Log-rank probability measure for the equality of these curves is 4.632422e-02. Additional annotations: *.GeneId*=ENSG00000155511, *.GeneName*=GRIA1, *.GeneDesc*=glutamate receptor, ionotropic, AMPA 1 [Source:HGNC Symbol;Acc:4571].

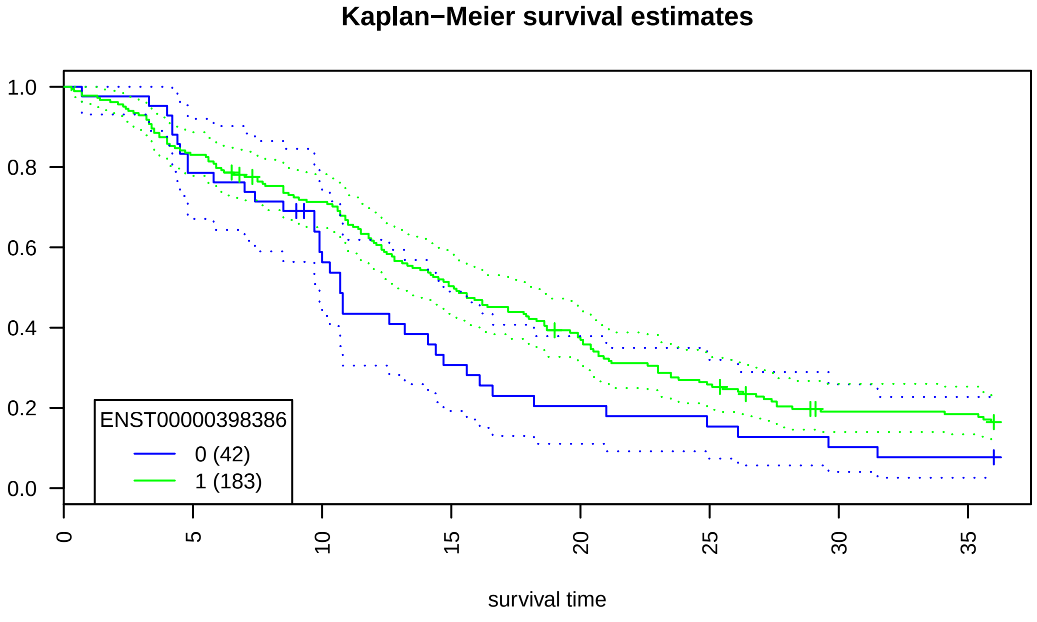

Figure 743: Each curve represents the relative number of ENST00000398386 survived to the corresponding point of time. Dashed lines represent 0.95 confidence intervals for the curves with the corresponding color. Total of 5 observations deleted due to missingness. Log-rank probability measure for the equality of these curves is 3.864969e-02. Additional annotations: *.GeneId*=ENSG00000140612, *.GeneName*=SEC11A, *.GeneDesc*=SEC11 homolog B (S. cerevisiae) [Source:HGNC Symbol;Acc:31884].

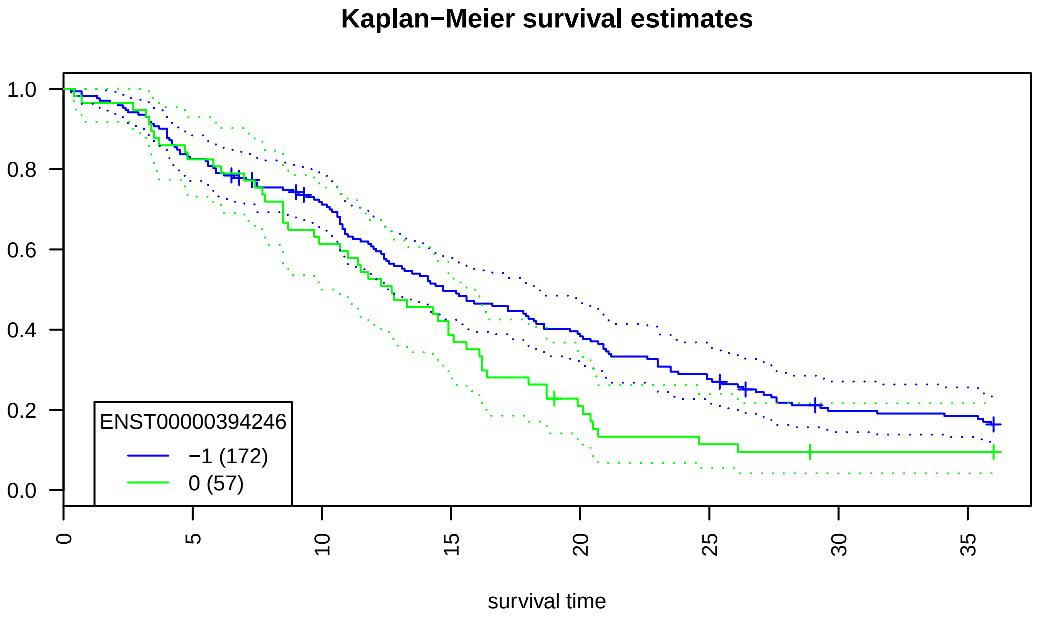

Figure 744: Each curve represents the relative number of ENST00000394246 survived to the corresponding point of time. Dashed lines represent 0.95 confidence intervals for the curves with the corresponding color. Total of 1 observation deleted due to missingness. Log-rank probability measure for the equality of these curves is 3.160242e-02. Additional annotations: *.GeneId*=ENSG00000141744, *.GeneName*=PNMT, *.GeneDesc*=phenylethanolamine N-methyltransferase [Source:HGNC Symbol;Acc:9160].

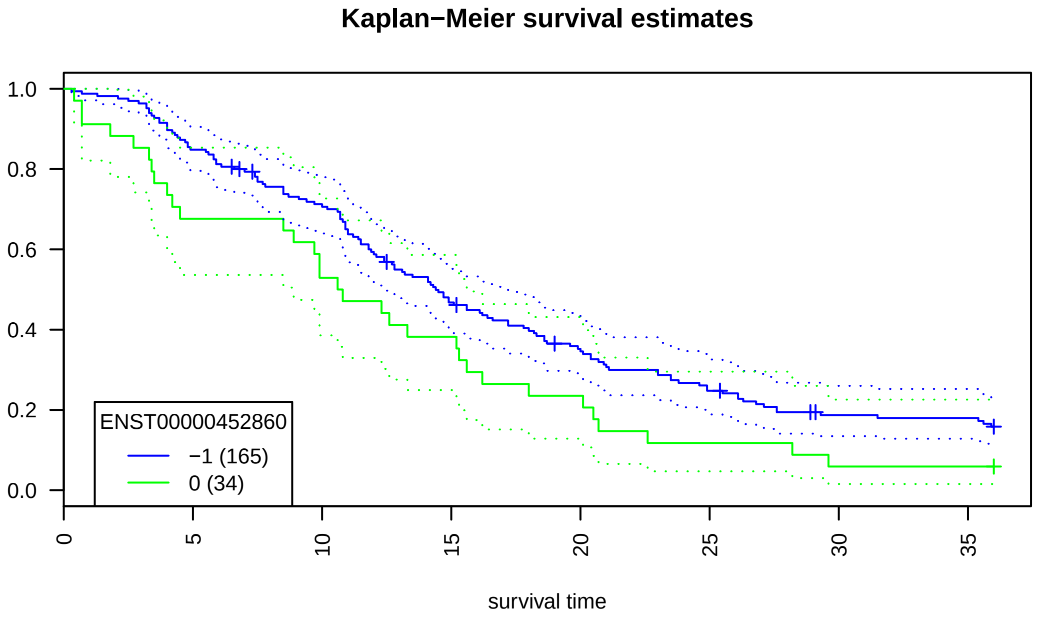

Figure 745: Each curve represents the relative number of ENST00000452860 survived to the corresponding point of time. Dashed lines represent 0.95 confidence intervals for the curves with the corresponding color. Total of 31 observations deleted due to missingness. Log-rank probability measure for the equality of these curves is 2.204529e-02. Additional annotations: *.GeneId*=ENSG00000163793, *.GeneName*=DNAJC5G, *.GeneDesc*=DnaJ (Hsp40) homolog, subfamily C, member 5 gamma [Source:HGNC Symbol;Acc:24844].

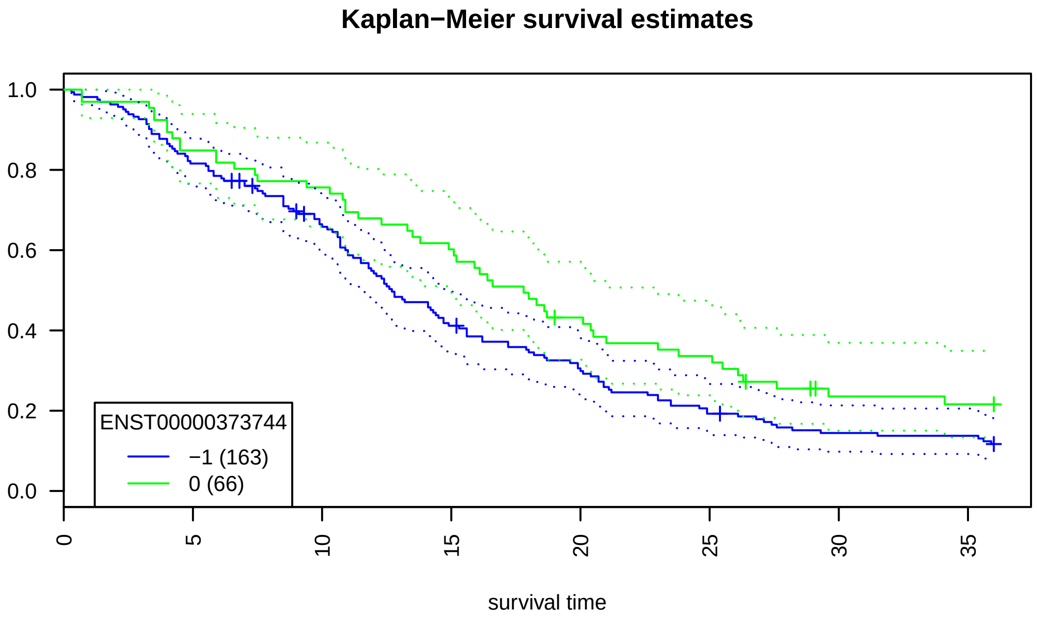

Figure 746: Each curve represents the relative number of ENST00000373744 survived to the corresponding point of time. Dashed lines represent 0.95 confidence intervals for the curves with the corresponding color. Total of 1 observation deleted due to missingness. Log-rank probability measure for the equality of these curves is 3.280763e-02. Additional annotations: *.GeneId*=ENSG00000183230, *.GeneName*=CTNNA3, *.GeneDesc*=catenin (cadherin-associated protein), alpha 3 [Source:HGNC Symbol;Acc:2511].

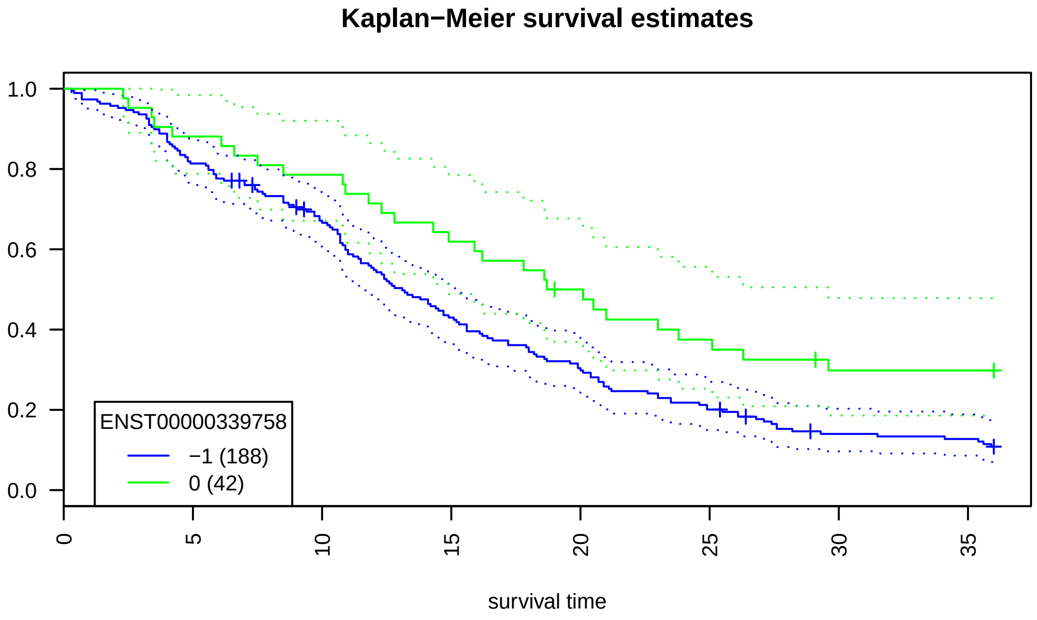

Figure 747: Each curve represents the relative number of ENST00000339758 survived to the corresponding point of time. Dashed lines represent 0.95 confidence intervals for the curves with the corresponding color. Log-rank probability measure for the equality of these curves is 5.794537e-03. Additional annotations: *.GeneId*=ENSG00000108176, *.GeneName*=DNAJC12, *.GeneDesc*=DnaJ (Hsp40) homolog, subfamily C, member 12 [Source:HGNC Symbol;Acc:28908].

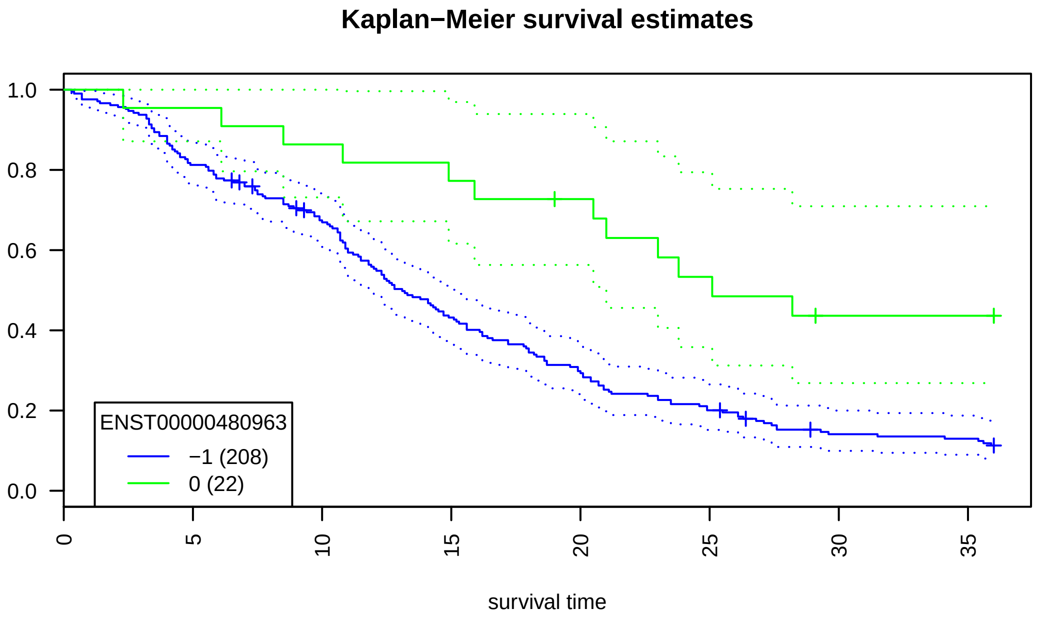

Figure 748: Each curve represents the relative number of ENST00000480963 survived to the corresponding point of time. Dashed lines represent 0.95 confidence intervals for the curves with the corresponding color. Log-rank probability measure for the equality of these curves is 5.366528e-04. Additional annotations: *.GeneId*=ENSG00000108176, *.GeneName*=DNAJC12, *.GeneDesc*=DnaJ (Hsp40) homolog, subfamily C, member 12 [Source:HGNC Symbol;Acc:28908].

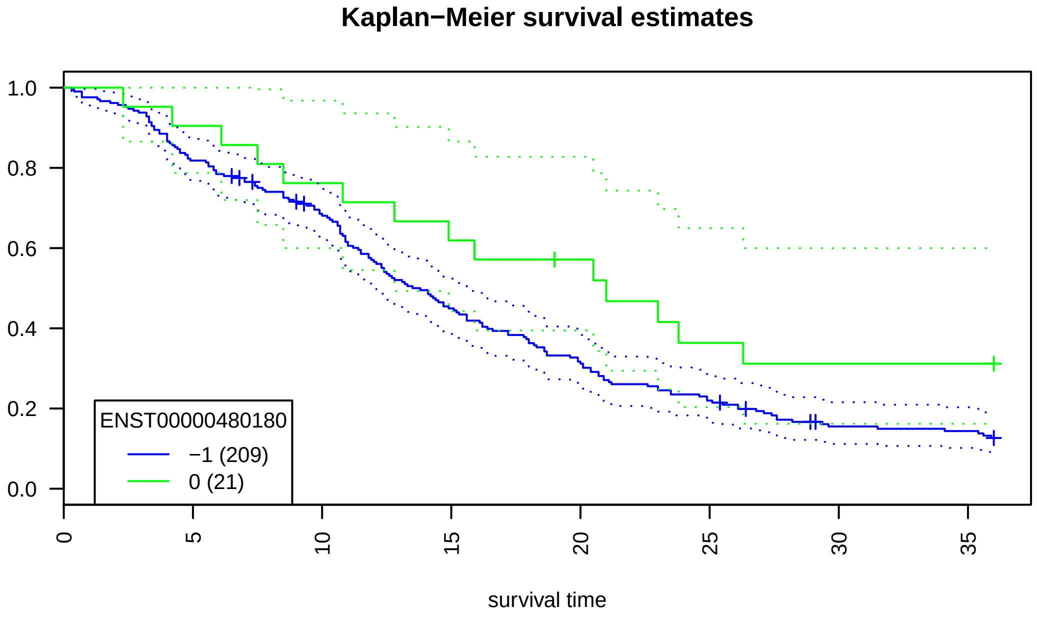

Figure 749: Each curve represents the relative number of ENST00000480180 survived to the corresponding point of time. Dashed lines represent 0.95 confidence intervals for the curves with the corresponding color. Log-rank probability measure for the equality of these curves is 4.713333e-02. Additional annotations: *.GeneId*=ENSG00000108176, *.GeneName*=DNAJC12, *.GeneDesc*=DnaJ (Hsp40) homolog, subfamily C, member 12 [Source:HGNC Symbol;Acc:28908].

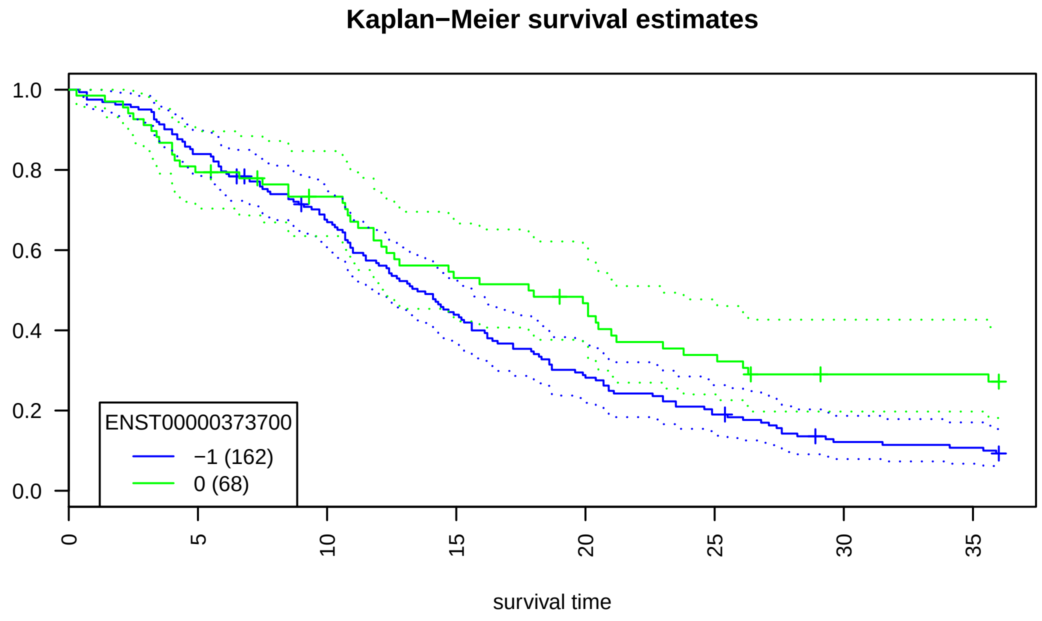

Figure 750: Each curve represents the relative number of ENST00000373700 survived to the corresponding point of time. Dashed lines represent 0.95 confidence intervals for the curves with the corresponding color. Log-rank probability measure for the equality of these curves is 1.148897e-02. Additional annotations: *.GeneId*=ENSG00000148634, *.GeneName*=HERC4, *.GeneDesc*=hect domain and RLD 4 [Source:HGNC Symbol;Acc:24521].

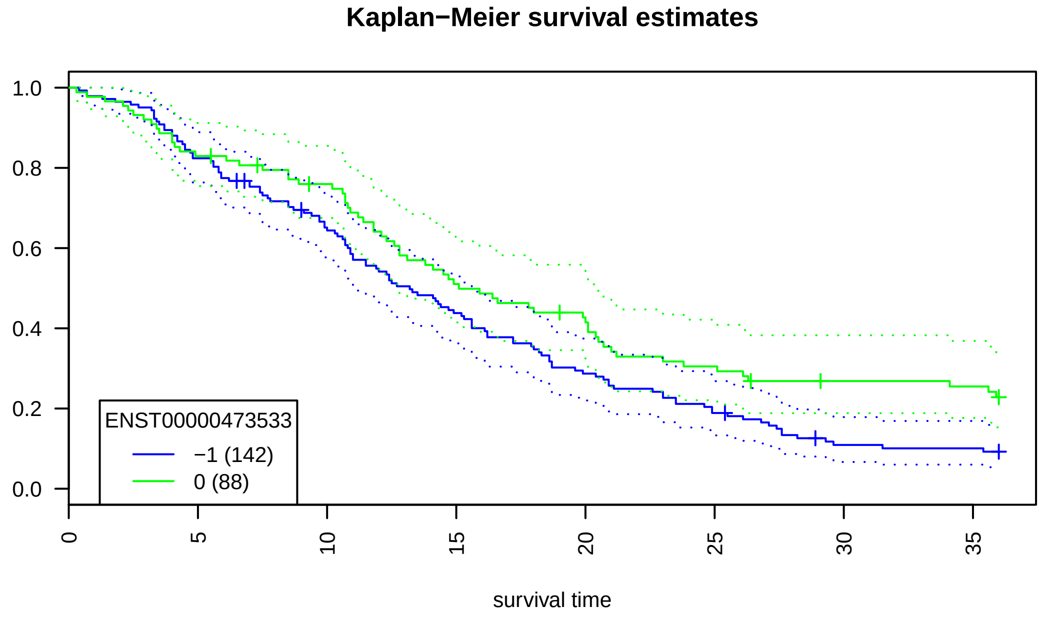

Figure 751: Each curve represents the relative number of ENST00000473533 survived to the corresponding point of time. Dashed lines represent 0.95 confidence intervals for the curves with the corresponding color. Log-rank probability measure for the equality of these curves is 1.904008e-02. Additional annotations: *.GeneId*=ENSG00000148634, *.GeneName*=HERC4, *.GeneDesc*=hect domain and RLD 4 [Source:HGNC Symbol;Acc:24521].

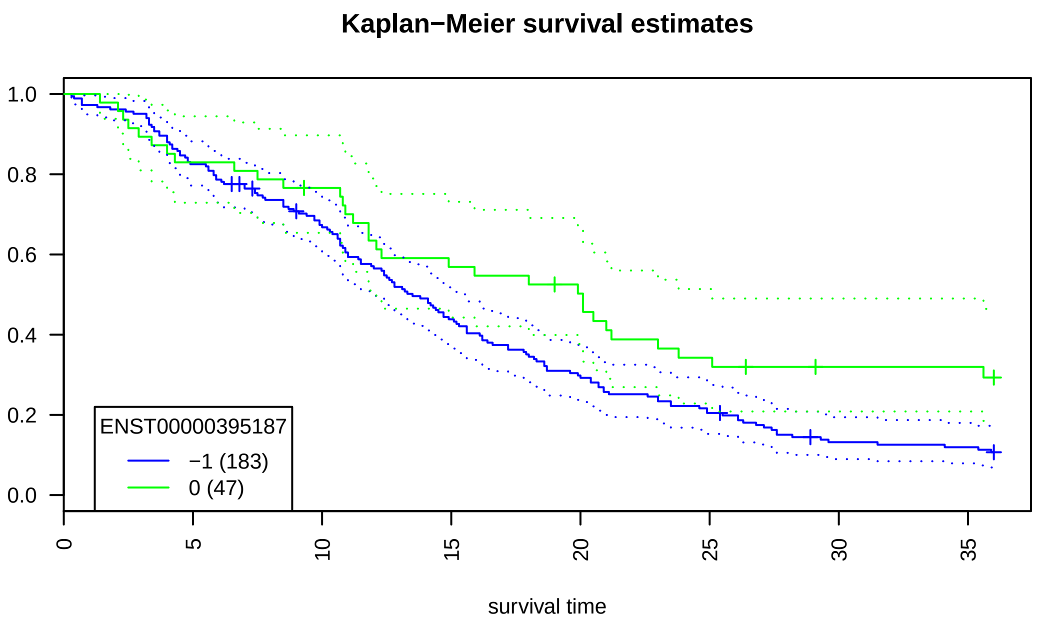

Figure 752: Each curve represents the relative number of ENST00000395187 survived to the corresponding point of time. Dashed lines represent 0.95 confidence intervals for the curves with the corresponding color. Log-rank probability measure for the equality of these curves is 1.260142e-02. Additional annotations: *.GeneId*=ENSG00000148634, *.GeneName*=HERC4, *.GeneDesc*=hect domain and RLD 4 [Source:HGNC Symbol;Acc:24521].

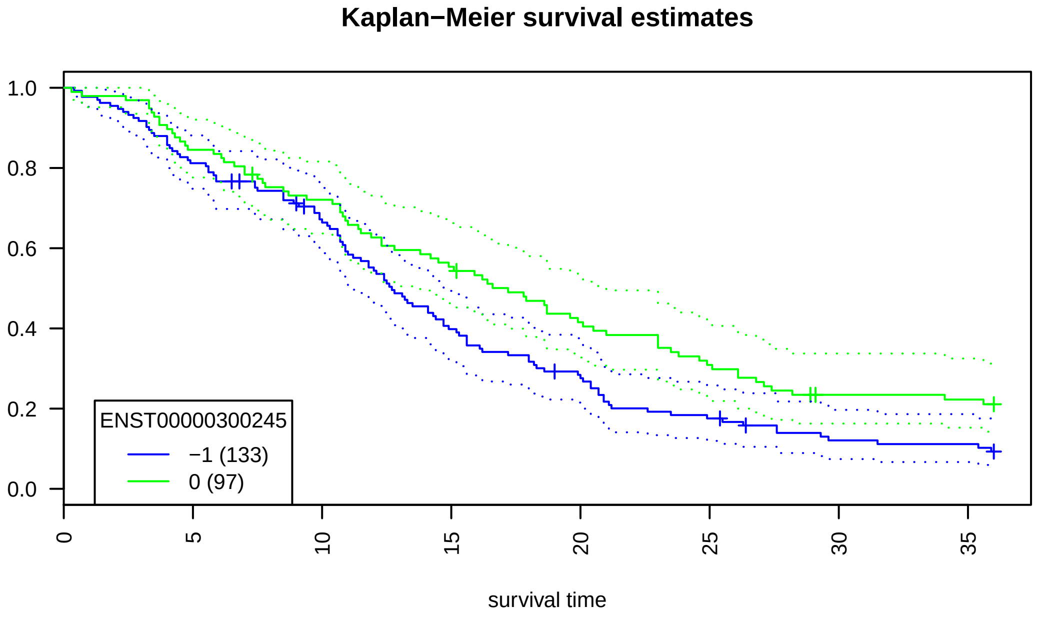

Figure 753: Each curve represents the relative number of ENST00000300245 survived to the corresponding point of time. Dashed lines represent 0.95 confidence intervals for the curves with the corresponding color. Log-rank probability measure for the equality of these curves is 9.911593e-03. Additional annotations: *.GeneId*=ENSG00000166971, *.GeneName*=AKTIP, *.GeneDesc*=AKT interacting protein [Source:HGNC Symbol;Acc:16710].

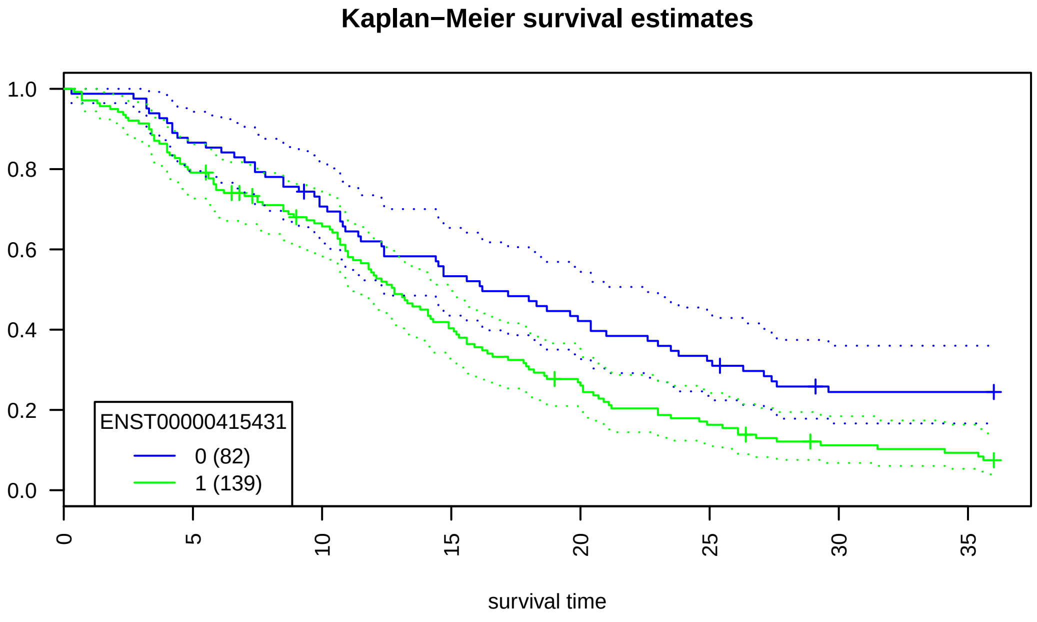

Figure 754: Each curve represents the relative number of ENST00000415431 survived to the corresponding point of time. Dashed lines represent 0.95 confidence intervals for the curves with the corresponding color. Total of 9 observations deleted due to missingness. Log-rank probability measure for the equality of these curves is 2.353557e-03. Additional annotations: *.GeneId*=ENSG00000165140, *.GeneName*=FBP1, *.GeneDesc*=fructose-1,6-bisphosphatase 1 [Source:HGNC Symbol;Acc:3606].

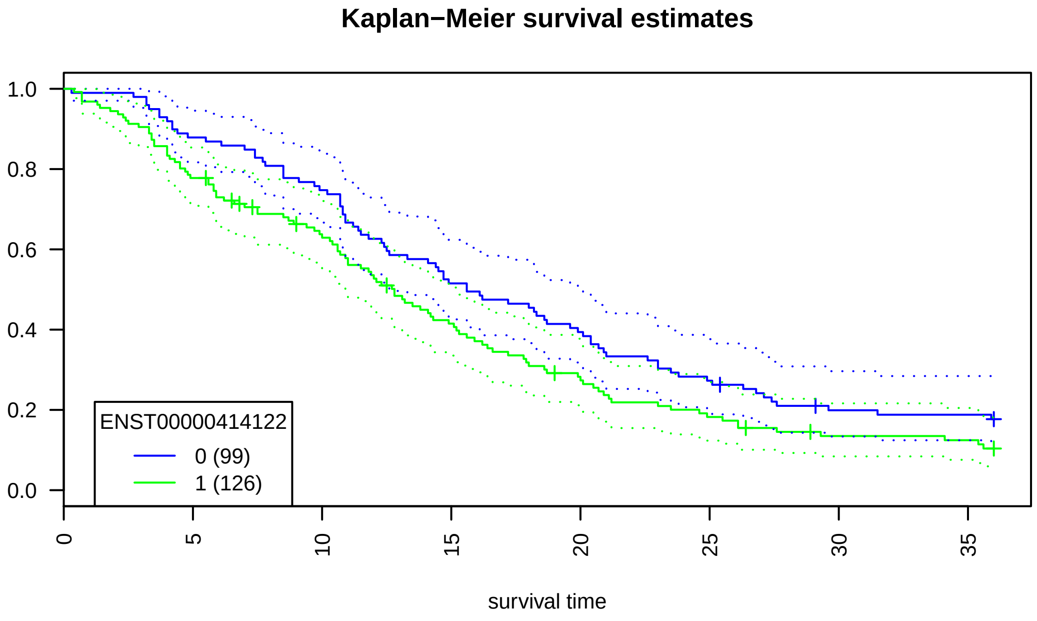

Figure 755: Each curve represents the relative number of ENST00000414122 survived to the corresponding point of time. Dashed lines represent 0.95 confidence intervals for the curves with the corresponding color. Total of 5 observations deleted due to missingness. Log-rank probability measure for the equality of these curves is 3.337546e-02. Additional annotations: *.GeneId*=ENSG00000165140, *.GeneName*=FBP1, *.GeneDesc*=fructose-1,6-bisphosphatase 1 [Source:HGNC Symbol;Acc:3606].

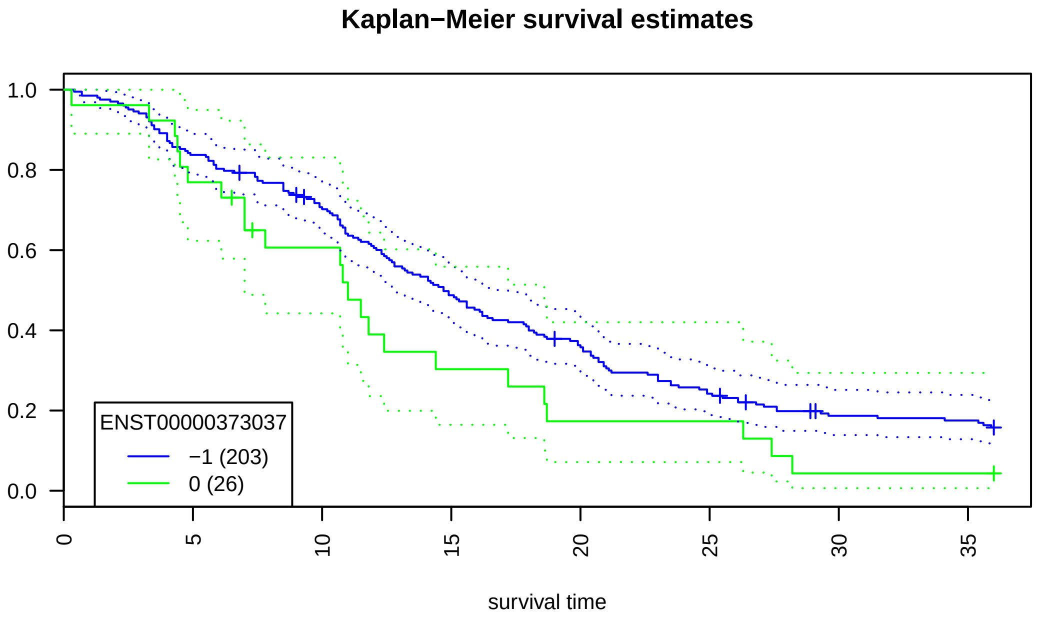

Figure 756: Each curve represents the relative number of ENST00000373037 survived to the corresponding point of time. Dashed lines represent 0.95 confidence intervals for the curves with the corresponding color. Total of 1 observation deleted due to missingness. Log-rank probability measure for the equality of these curves is 4.841468e-02.

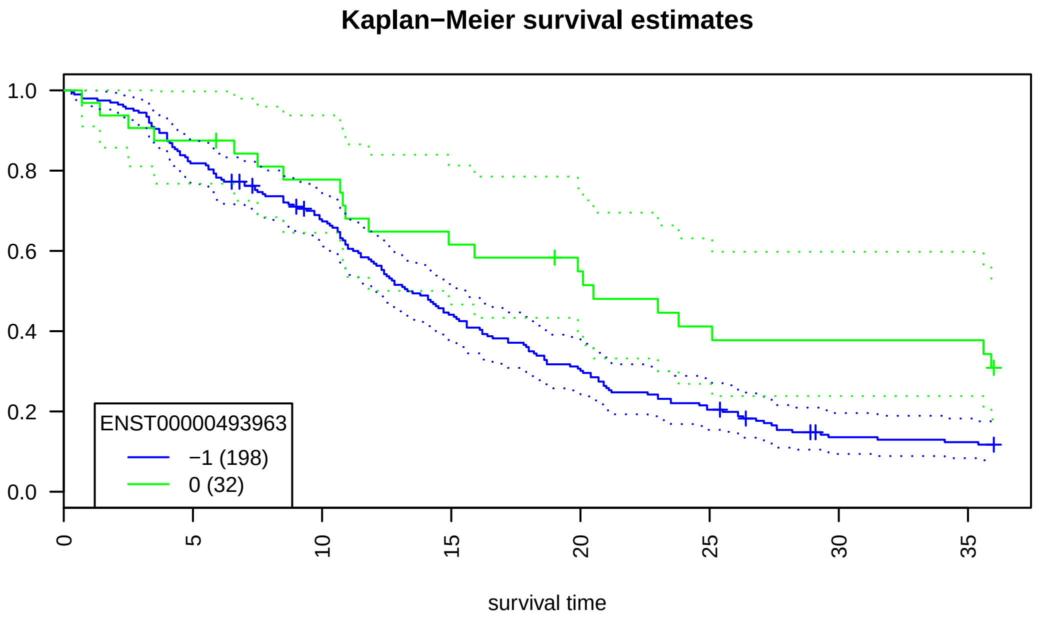

Figure 757: Each curve represents the relative number of ENST00000493963 survived to the corresponding point of time. Dashed lines represent 0.95 confidence intervals for the curves with the corresponding color. Log-rank probability measure for the equality of these curves is 1.111638e-02. Additional annotations: *.GeneId*=ENSG00000122912, *.GeneName*=SLC25A16, *.GeneDesc*=solute carrier family 25 (mitochondrial carrier; Graves disease autoantigen), member 16 [Source:HGNC Symbol;Acc:10986].

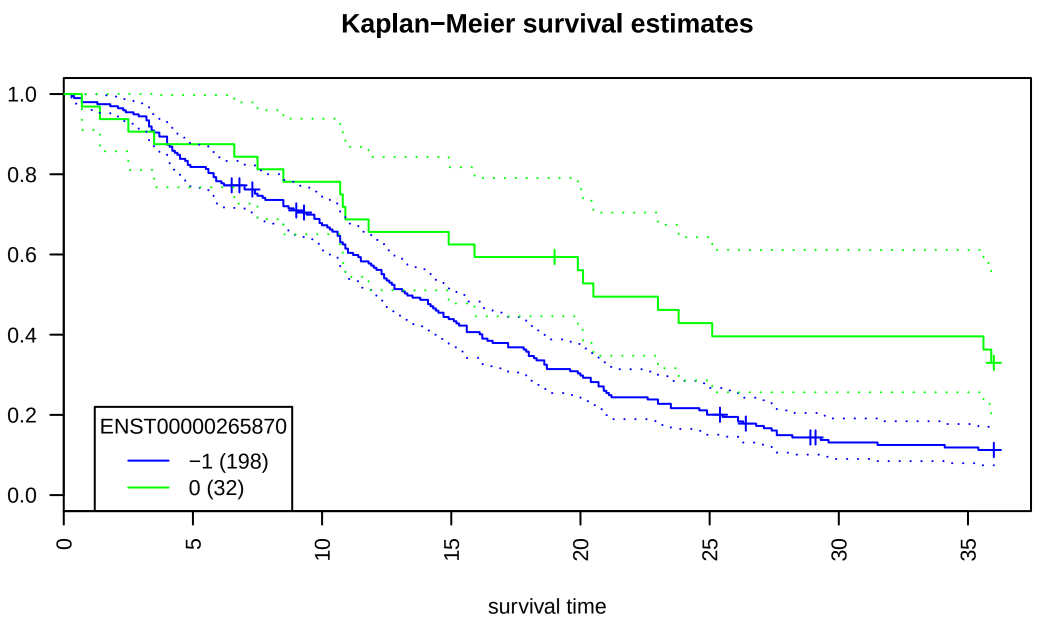

Figure 758: Each curve represents the relative number of ENST00000265870 survived to the corresponding point of time. Dashed lines represent 0.95 confidence intervals for the curves with the corresponding color. Log-rank probability measure for the equality of these curves is 4.876191e-03. Additional annotations: *.GeneId*=ENSG00000122912, *.GeneName*=SLC25A16, *.GeneDesc*=solute carrier family 25 (mitochondrial carrier; Graves disease autoantigen), member 16 [Source:HGNC Symbol;Acc:10986].

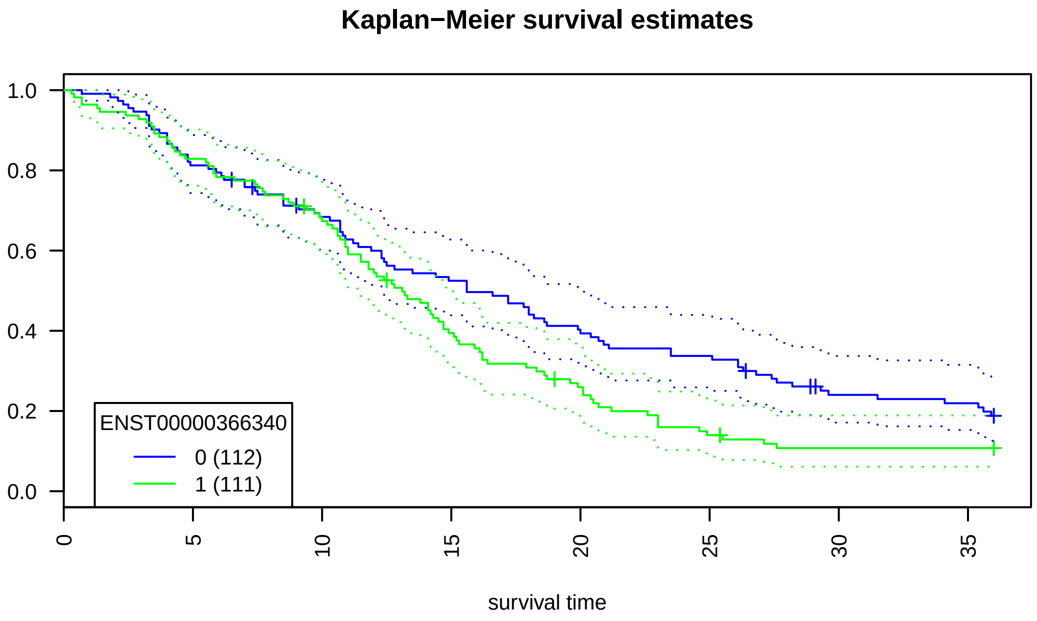

Figure 759: Each curve represents the relative number of ENST00000366340 survived to the corresponding point of time. Dashed lines represent 0.95 confidence intervals for the curves with the corresponding color. Total of 7 observations deleted due to missingness. Log-rank probability measure for the equality of these curves is 2.656161e-02. Additional annotations: *.GeneId*=ENSG00000106077, *.GeneName*=ABHD11, *.GeneDesc*=abhydrolase domain containing 11 [Source:HGNC Symbol;Acc:16407].

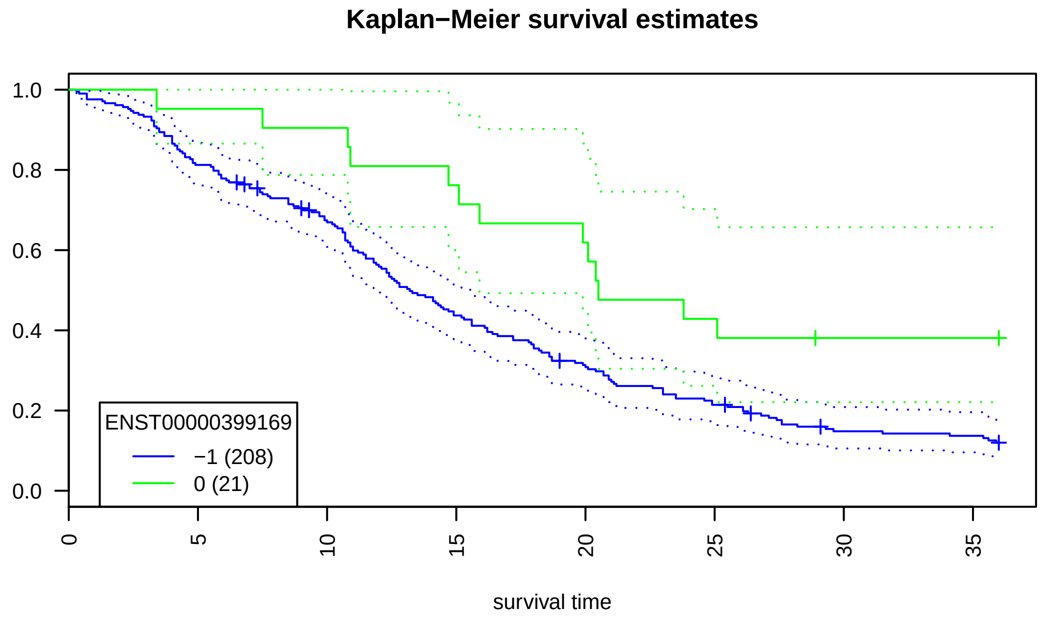

Figure 760: Each curve represents the relative number of ENST00000399169 survived to the corresponding point of time. Dashed lines represent 0.95 confidence intervals for the curves with the corresponding color. Total of 1 observation deleted due to missingness. Log-rank probability measure for the equality of these curves is 5.741752e-03. Additional annotations: *.GeneId*=ENSG00000165730, *.GeneName*=STOX1, *.GeneDesc*=storkhead box 1 [Source:HGNC Symbol;Acc:23508].

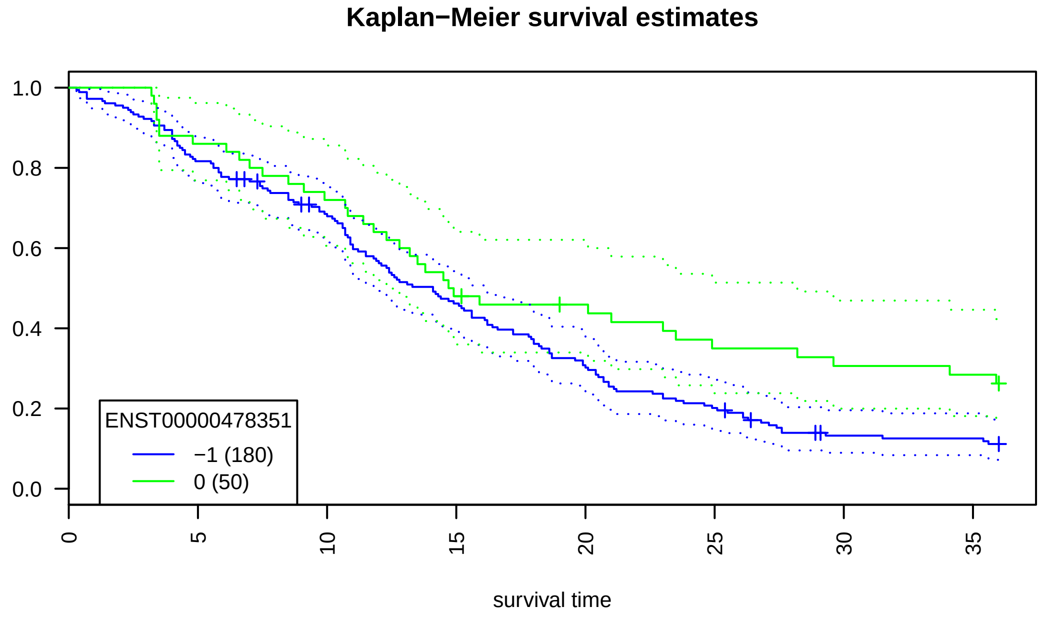

Figure 761: Each curve represents the relative number of ENST00000478351 survived to the corresponding point of time. Dashed lines represent 0.95 confidence intervals for the curves with the corresponding color. Log-rank probability measure for the equality of these curves is 2.579154e-02. Additional annotations: *.GeneId*=ENSG00000126950, *.GeneName*=TMEM35, *.GeneDesc*=transmembrane protein 35 [Source:HGNC Symbol;Acc:25864].

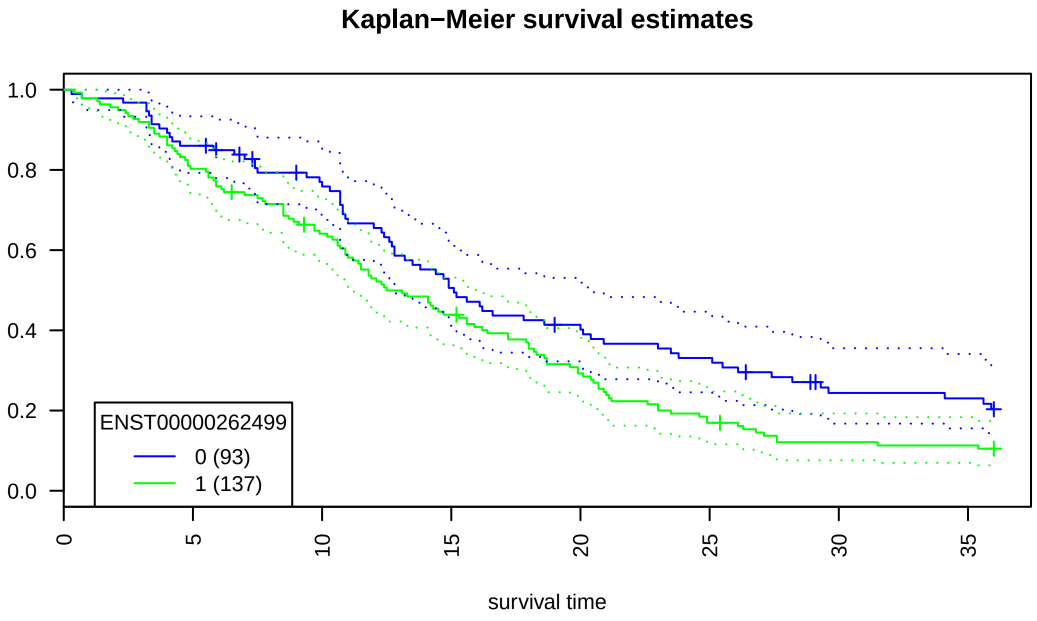

Figure 762: Each curve represents the relative number of ENST00000262499 survived to the corresponding point of time. Dashed lines represent 0.95 confidence intervals for the curves with the corresponding color. Log-rank probability measure for the equality of these curves is 2.034878e-02. Additional annotations: *.GeneId*=ENSG00000162840, *.GeneName*=RP11-4I17.1, *.GeneDesc*=[undefined].

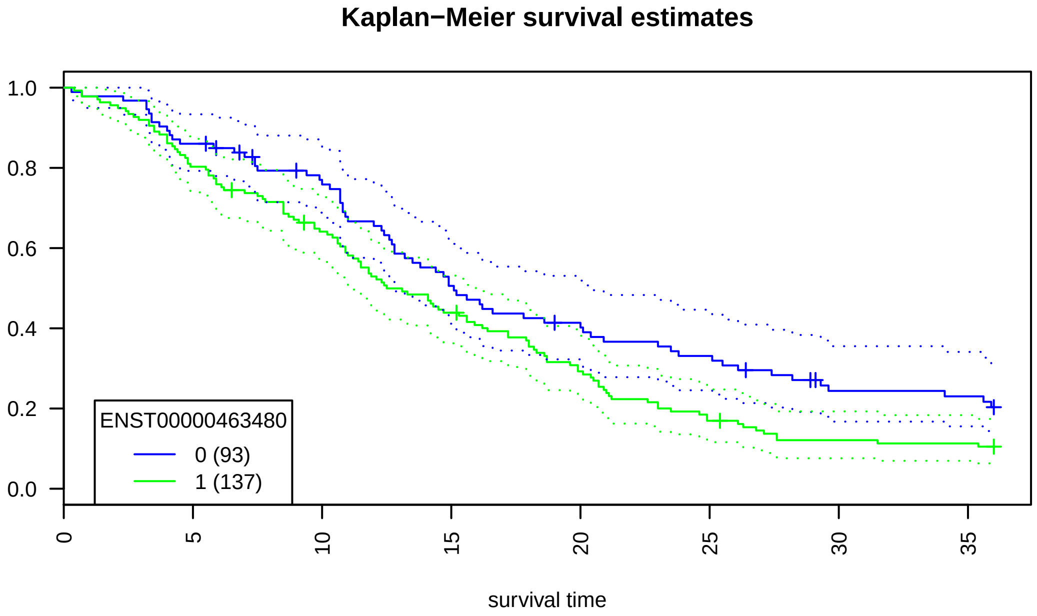

Figure 763: Each curve represents the relative number of ENST00000463480 survived to the corresponding point of time. Dashed lines represent 0.95 confidence intervals for the curves with the corresponding color. Log-rank probability measure for the equality of these curves is 2.034878e-02. Additional annotations: *.GeneId*=ENSG00000205361, *.GeneName*=MT1DP, *.GeneDesc*=metallothionein 1D (pseudogene) [Source:HGNC Symbol;Acc:7396].

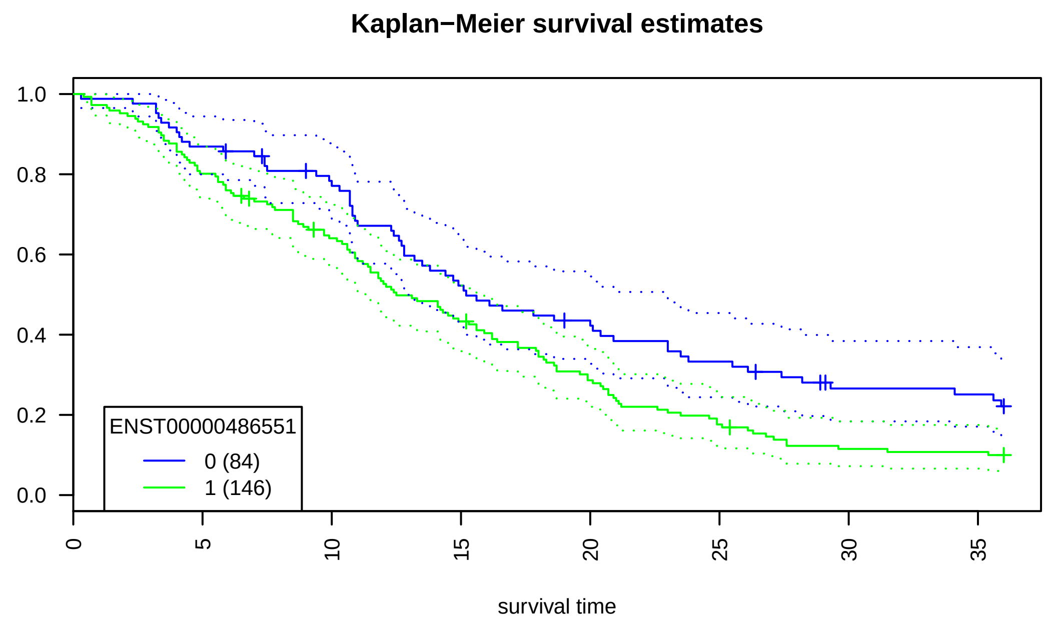

Figure 764: Each curve represents the relative number of ENST00000486551 survived to the corresponding point of time. Dashed lines represent 0.95 confidence intervals for the curves with the corresponding color. Log-rank probability measure for the equality of these curves is 7.879176e-03. Additional annotations: *.GeneId*=ENSG00000205361, *.GeneName*=MT1DP, *.GeneDesc*=metallothionein 1D (pseudogene) [Source:HGNC Symbol;Acc:7396].

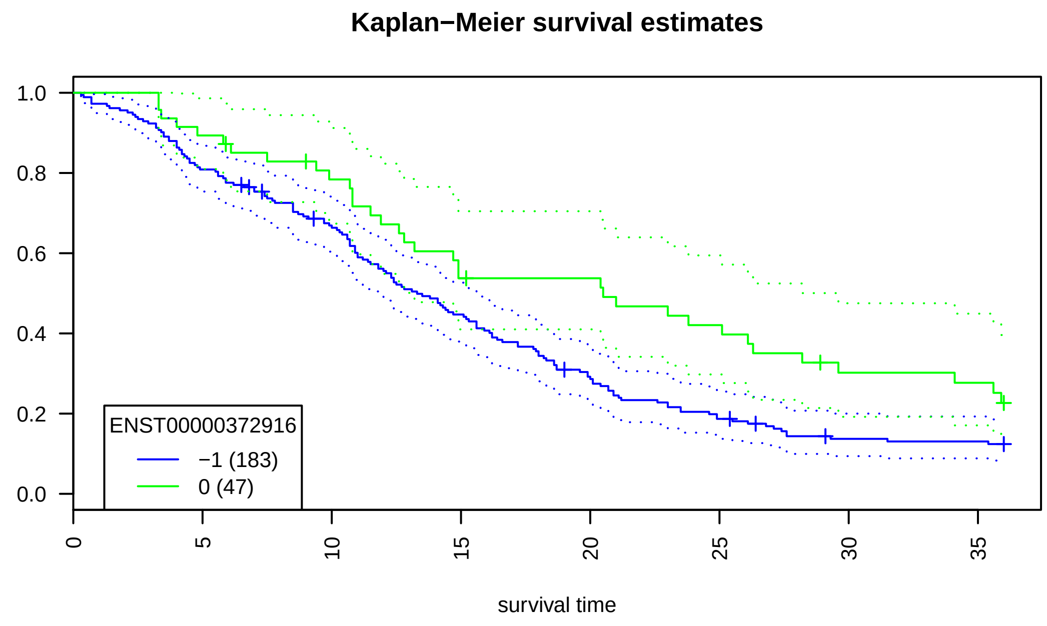

Figure 765: Each curve represents the relative number of ENST00000372916 survived to the corresponding point of time. Dashed lines represent 0.95 confidence intervals for the curves with the corresponding color. Log-rank probability measure for the equality of these curves is 1.407461e-02. Additional annotations: *.GeneId*=ENSG00000102385, *.GeneName*=DRP2, *.GeneDesc*=dystrophin related protein 2 [Source:HGNC Symbol;Acc:3032].

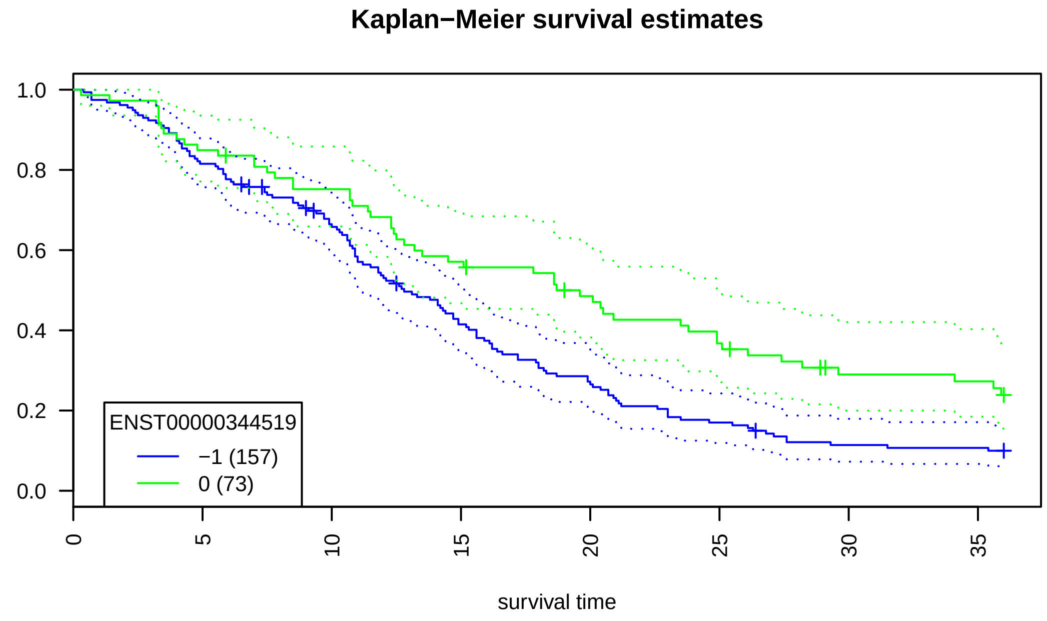

Figure 766: Each curve represents the relative number of ENST00000344519 survived to the corresponding point of time. Dashed lines represent 0.95 confidence intervals for the curves with the corresponding color. Log-rank probability measure for the equality of these curves is 1.841847e-03. Additional annotations: *.GeneId*=ENSG00000198153, *.GeneName*=ZNF737, *.GeneDesc*=zinc finger protein 737 [Source:HGNC Symbol;Acc:32468].

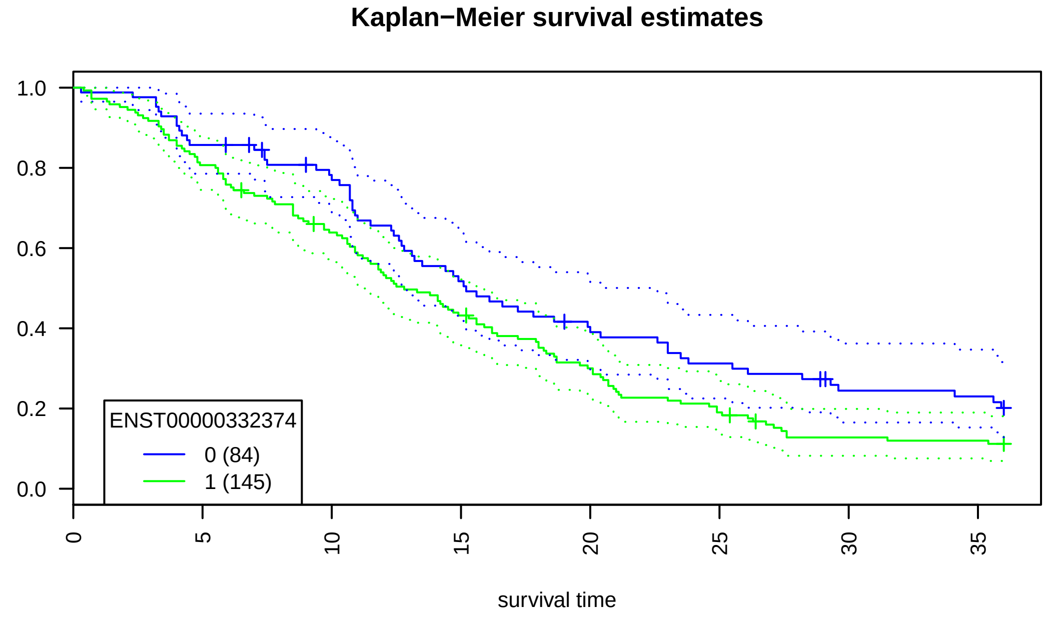

Figure 767: Each curve represents the relative number of ENST00000332374 survived to the corresponding point of time. Dashed lines represent 0.95 confidence intervals for the curves with the corresponding color. Total of 1 observation deleted due to missingness. Log-rank probability measure for the equality of these curves is 2.959298e-02. Additional annotations: *.GeneId*=ENSG00000205358, *.GeneName*=MT1H, *.GeneDesc*=metallothionein 1H [Source:HGNC Symbol;Acc:7400].

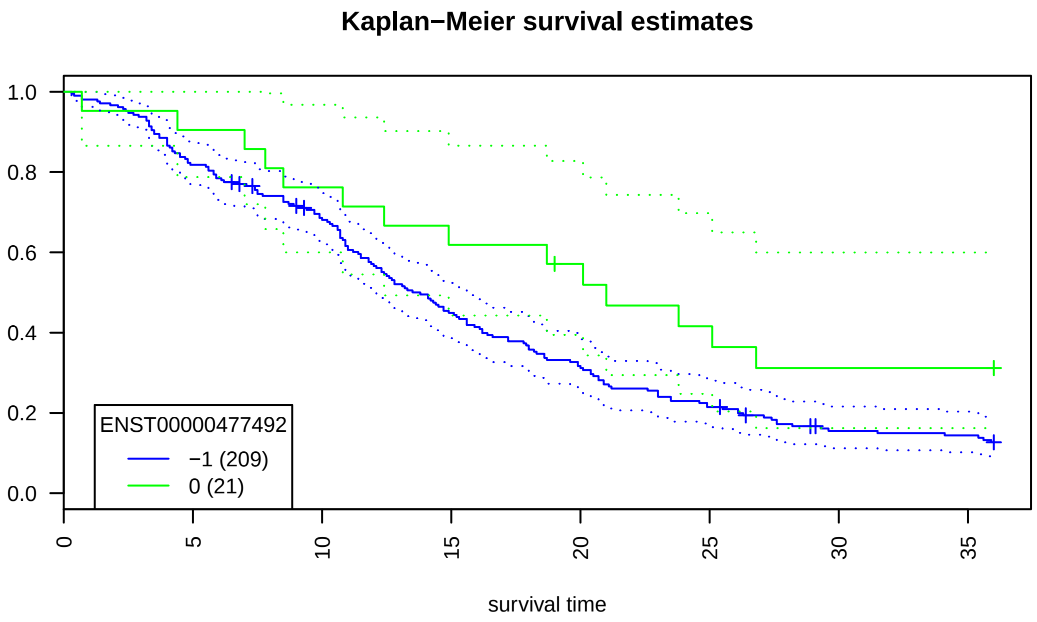

Figure 768: Each curve represents the relative number of ENST00000477492 survived to the corresponding point of time. Dashed lines represent 0.95 confidence intervals for the curves with the corresponding color. Log-rank probability measure for the equality of these curves is 4.137046e-02. Additional annotations: *.GeneId*=ENSG00000054793, *.GeneName*=ATP9A, *.GeneDesc*=ATPase, class II, type 9A [Source:HGNC Symbol;Acc:13540].

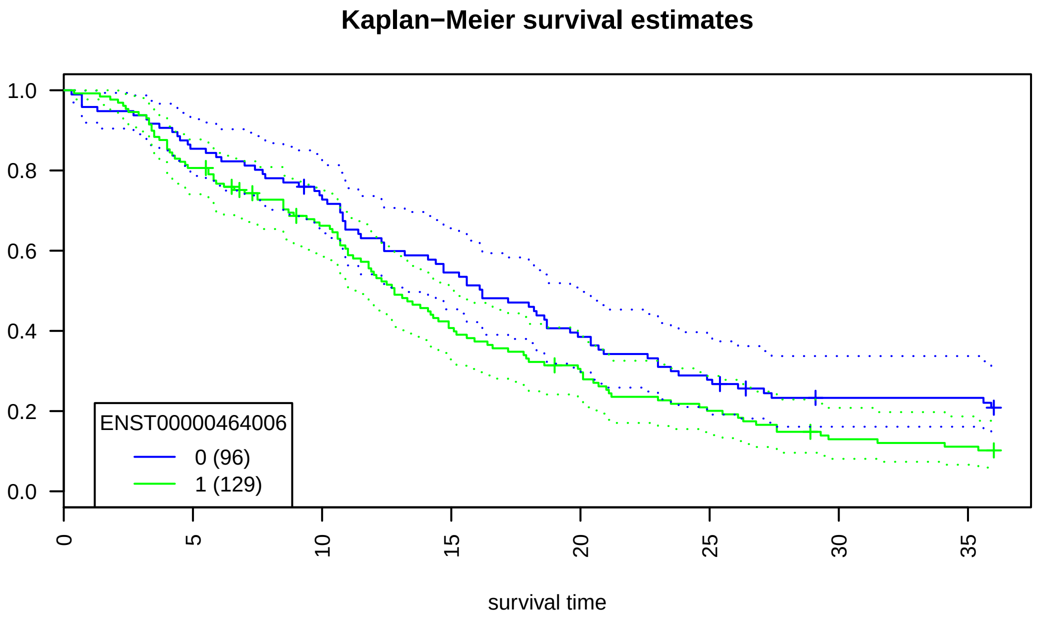

Figure 769: Each curve represents the relative number of ENST00000464006 survived to the corresponding point of time. Dashed lines represent 0.95 confidence intervals for the curves with the corresponding color. Total of 5 observations deleted due to missingness. Log-rank probability measure for the equality of these curves is 3.559544e-02. Additional annotations: *.GeneId*=ENSG00000010671, *.GeneName*=BTK, *.GeneDesc*=Bruton agammaglobulinemia tyrosine kinase [Source:HGNC Symbol;Acc:1133].

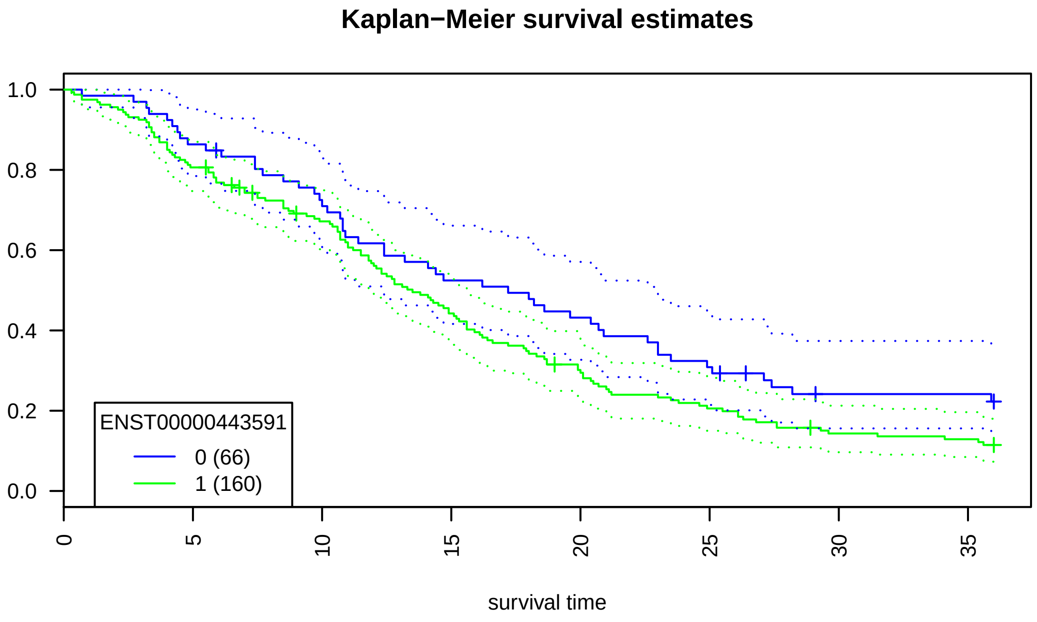

Figure 770: Each curve represents the relative number of ENST00000443591 survived to the corresponding point of time. Dashed lines represent 0.95 confidence intervals for the curves with the corresponding color. Total of 4 observations deleted due to missingness. Log-rank probability measure for the equality of these curves is 4.809036e-02. Additional annotations: *.GeneId*=ENSG00000010671, *.GeneName*=BTK, *.GeneDesc*=Bruton agammaglobulinemia tyrosine kinase [Source:HGNC Symbol;Acc:1133].

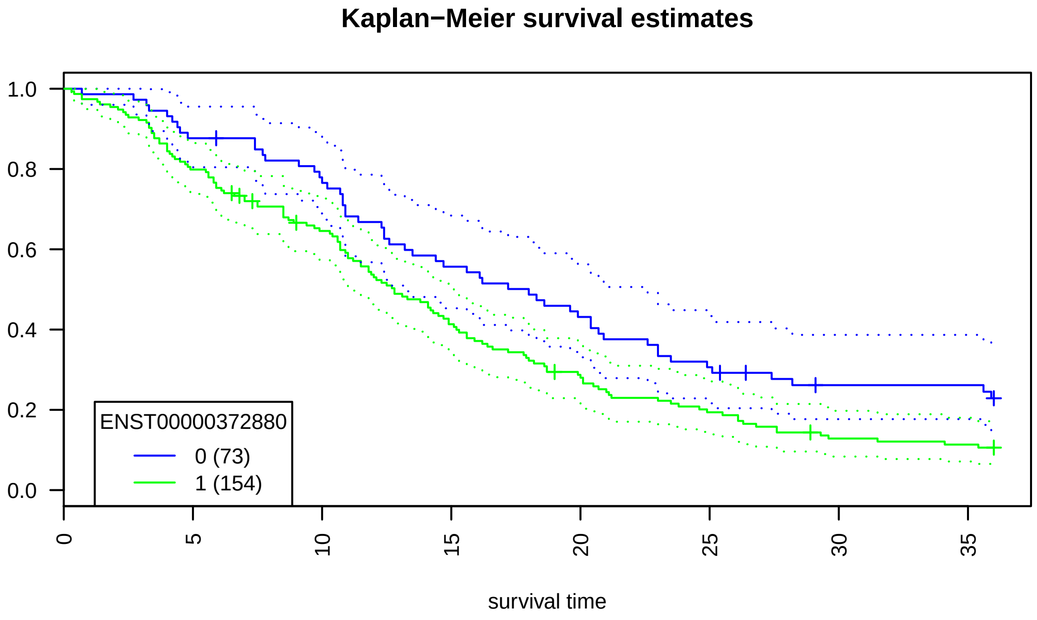

Figure 771: Each curve represents the relative number of ENST00000372880 survived to the corresponding point of time. Dashed lines represent 0.95 confidence intervals for the curves with the corresponding color. Total of 3 observations deleted due to missingness. Log-rank probability measure for the equality of these curves is 8.752437e-03. Additional annotations: *.GeneId*=ENSG00000010671, *.GeneName*=BTK, *.GeneDesc*=Bruton agammaglobulinemia tyrosine kinase [Source:HGNC Symbol;Acc:1133].

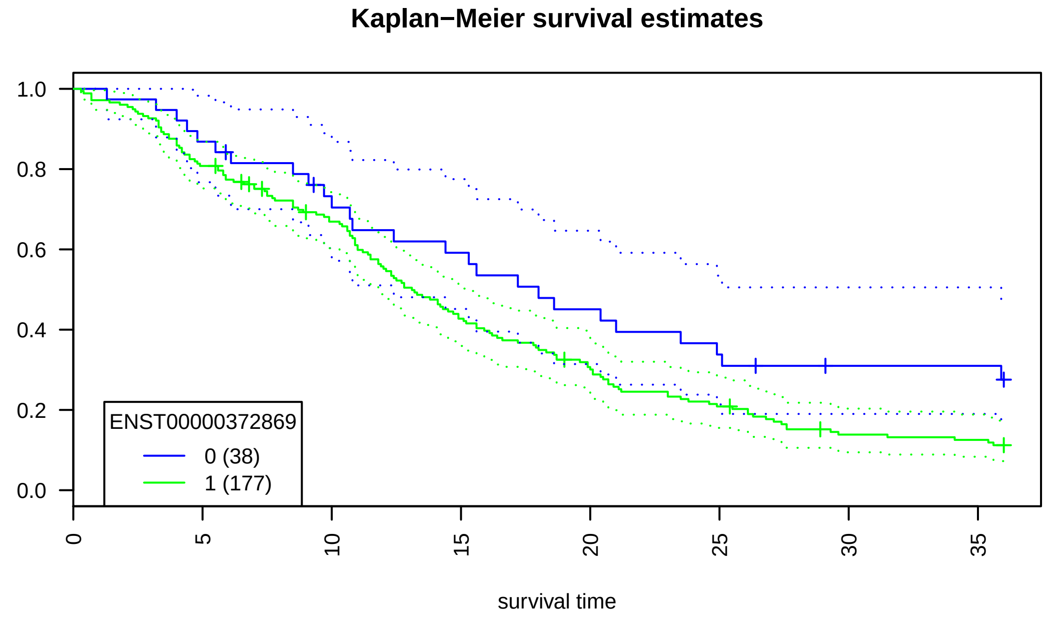

Figure 772: Each curve represents the relative number of ENST00000372869 survived to the corresponding point of time. Dashed lines represent 0.95 confidence intervals for the curves with the corresponding color. Total of 15 observations deleted due to missingness. Log-rank probability measure for the equality of these curves is 3.467511e-02. Additional annotations: *.GeneId*=ENSG00000010671, *.GeneName*=BTK, *.GeneDesc*=Bruton agammaglobulinemia tyrosine kinase [Source:HGNC Symbol;Acc:1133].

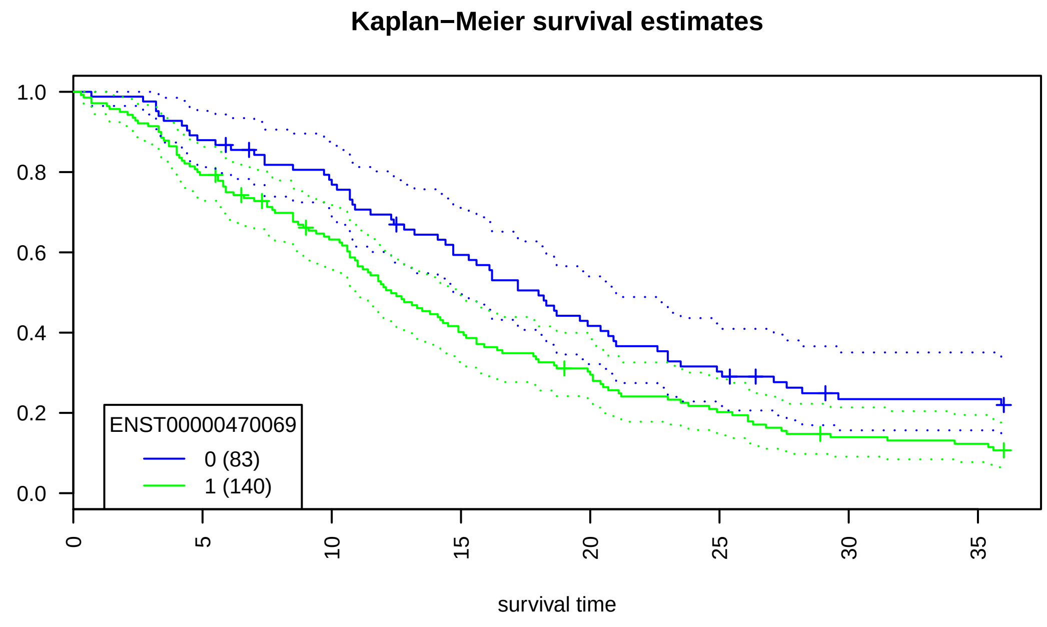

Figure 773: Each curve represents the relative number of ENST00000470069 survived to the corresponding point of time. Dashed lines represent 0.95 confidence intervals for the curves with the corresponding color. Total of 7 observations deleted due to missingness. Log-rank probability measure for the equality of these curves is 7.753651e-03. Additional annotations: *.GeneId*=ENSG00000010671, *.GeneName*=BTK, *.GeneDesc*=Bruton agammaglobulinemia tyrosine kinase [Source:HGNC Symbol;Acc:1133].

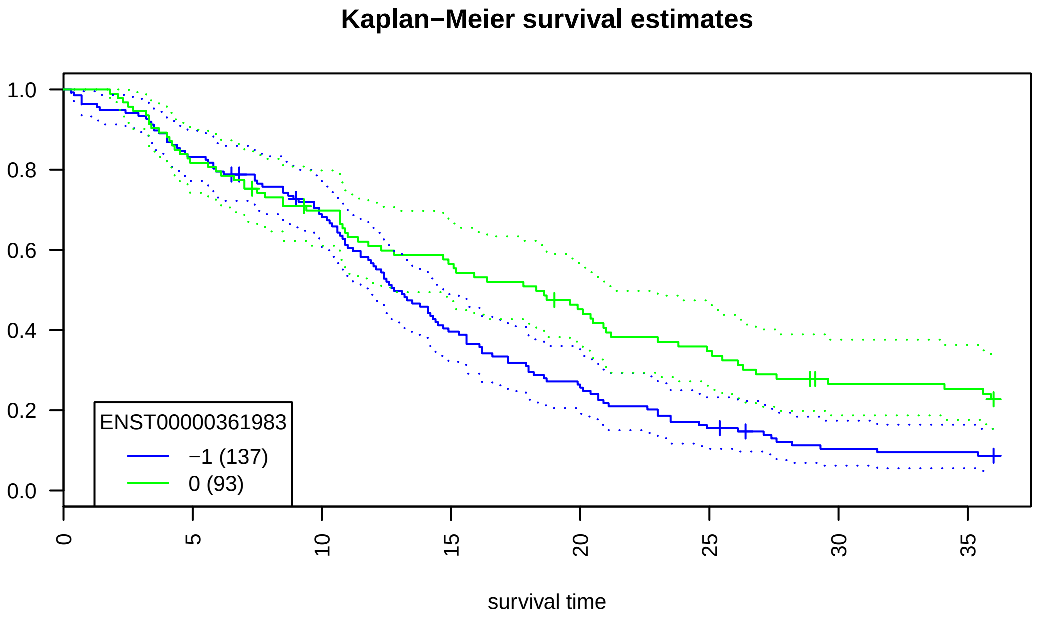

Figure 774: Each curve represents the relative number of ENST00000361983 survived to the corresponding point of time. Dashed lines represent 0.95 confidence intervals for the curves with the corresponding color. Log-rank probability measure for the equality of these curves is 3.692554e-03. Additional annotations: *.GeneId*=ENSG00000198954, *.GeneName*=KIAA1279, *.GeneDesc*=KIAA1279 [Source:HGNC Symbol;Acc:23419].

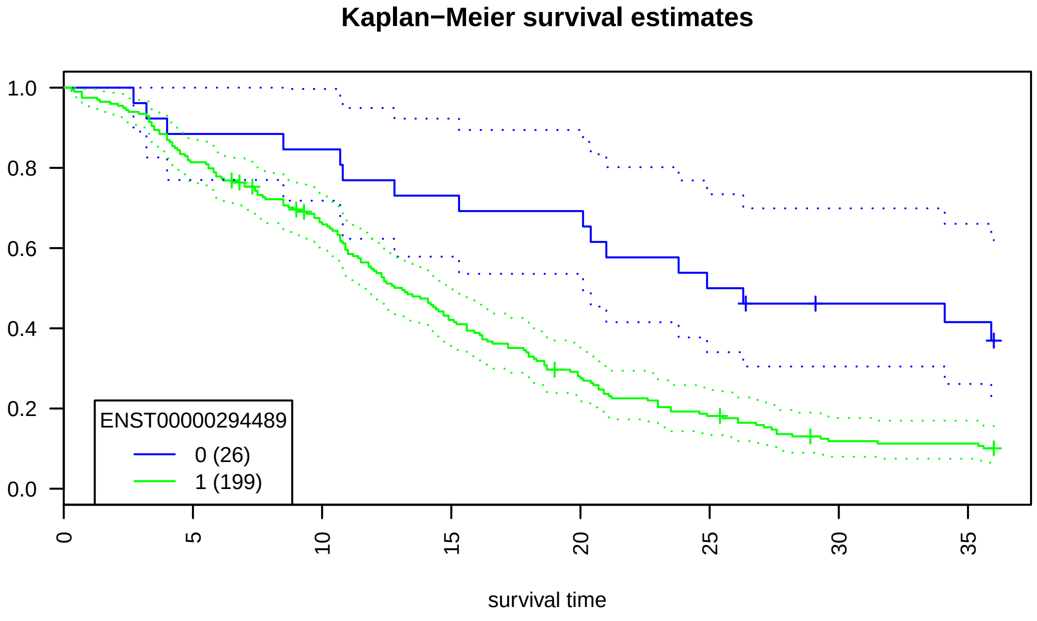

Figure 775: Each curve represents the relative number of ENST00000294489 survived to the corresponding point of time. Dashed lines represent 0.95 confidence intervals for the curves with the corresponding color. Total of 5 observations deleted due to missingness. Log-rank probability measure for the equality of these curves is 4.559118e-04. Additional annotations: *.GeneId*=ENSG00000162493, *.GeneName*=PDPN, *.GeneDesc*=podoplanin [Source:HGNC Symbol;Acc:29602].

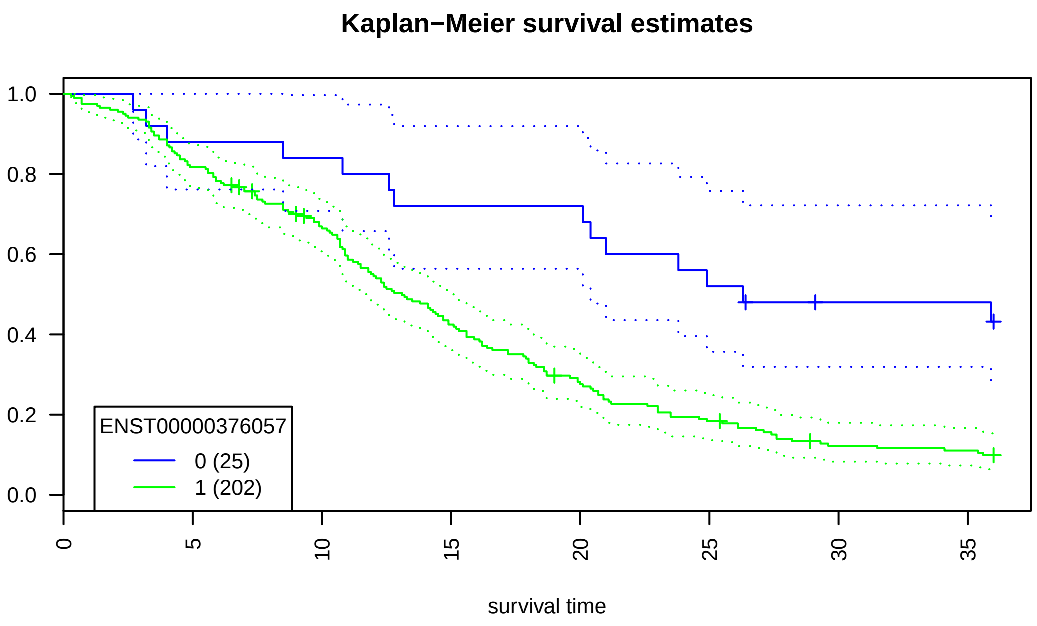

Figure 776: Each curve represents the relative number of ENST00000376057 survived to the corresponding point of time. Dashed lines represent 0.95 confidence intervals for the curves with the corresponding color. Total of 3 observations deleted due to missingness. Log-rank probability measure for the equality of these curves is 1.699631e-04. Additional annotations: *.GeneId*=ENSG00000162493, *.GeneName*=PDPN, *.GeneDesc*=podoplanin [Source:HGNC Symbol;Acc:29602].

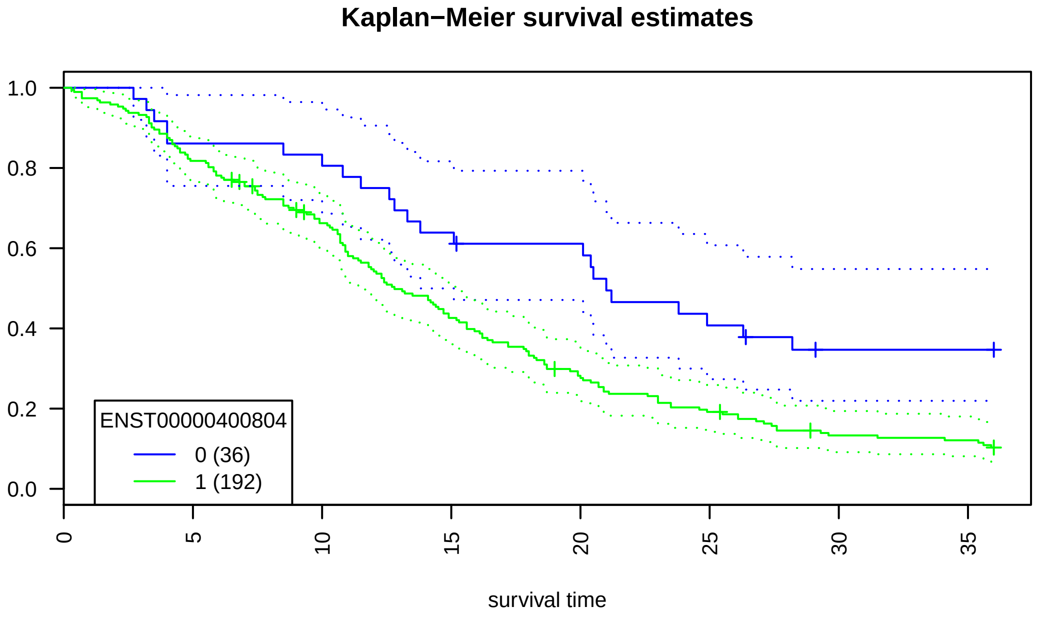

Figure 777: Each curve represents the relative number of ENST00000400804 survived to the corresponding point of time. Dashed lines represent 0.95 confidence intervals for the curves with the corresponding color. Total of 2 observations deleted due to missingness. Log-rank probability measure for the equality of these curves is 1.226660e-03. Additional annotations: *.GeneId*=ENSG00000162493, *.GeneName*=PDPN, *.GeneDesc*=podoplanin [Source:HGNC Symbol;Acc:29602].

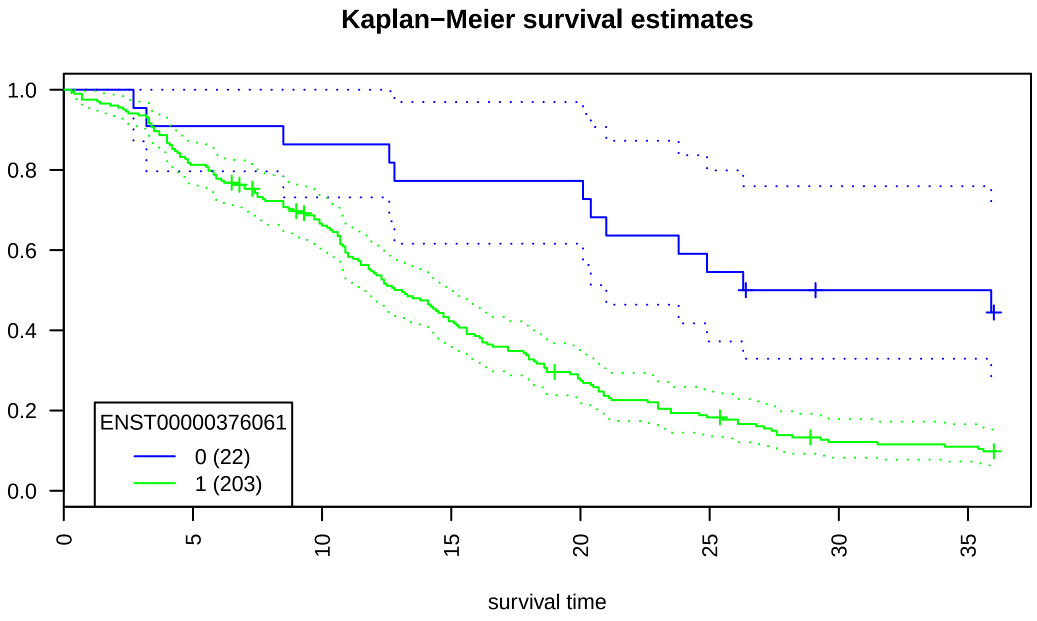

Figure 778: Each curve represents the relative number of ENST00000376061 survived to the corresponding point of time. Dashed lines represent 0.95 confidence intervals for the curves with the corresponding color. Total of 5 observations deleted due to missingness. Log-rank probability measure for the equality of these curves is 1.336665e-04. Additional annotations: *.GeneId*=ENSG00000162493, *.GeneName*=PDPN, *.GeneDesc*=podoplanin [Source:HGNC Symbol;Acc:29602].

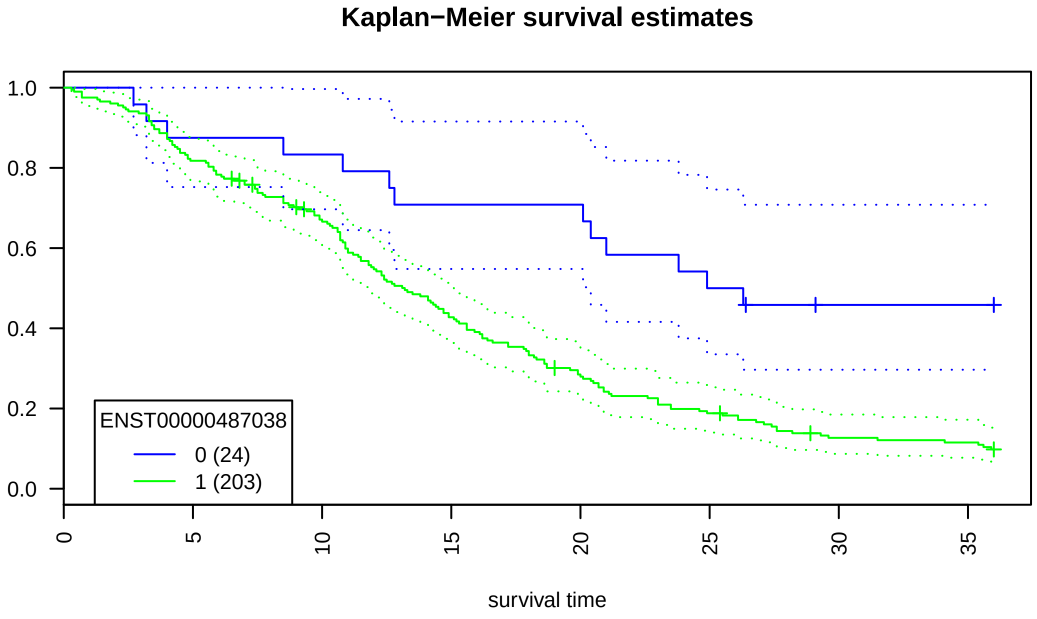

Figure 779: Each curve represents the relative number of ENST00000487038 survived to the corresponding point of time. Dashed lines represent 0.95 confidence intervals for the curves with the corresponding color. Total of 3 observations deleted due to missingness. Log-rank probability measure for the equality of these curves is 2.617393e-04. Additional annotations: *.GeneId*=ENSG00000162493, *.GeneName*=PDPN, *.GeneDesc*=podoplanin [Source:HGNC Symbol;Acc:29602].

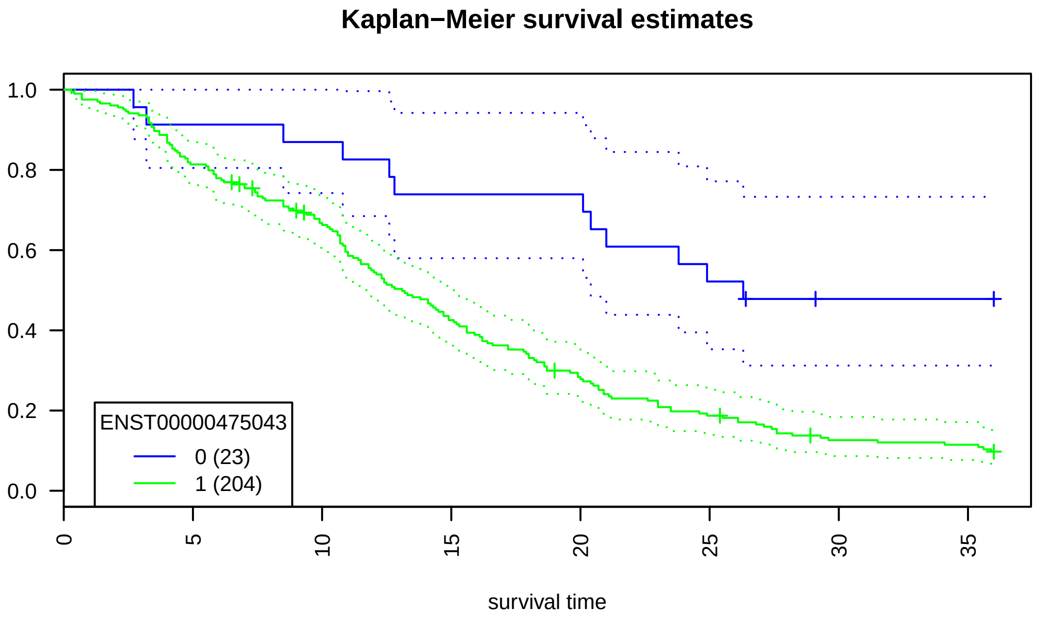

Figure 780: Each curve represents the relative number of ENST00000475043 survived to the corresponding point of time. Dashed lines represent 0.95 confidence intervals for the curves with the corresponding color. Total of 3 observations deleted due to missingness. Log-rank probability measure for the equality of these curves is 1.278976e-04. Additional annotations: *.GeneId*=ENSG00000162493, *.GeneName*=PDPN, *.GeneDesc*=podoplanin [Source:HGNC Symbol;Acc:29602].

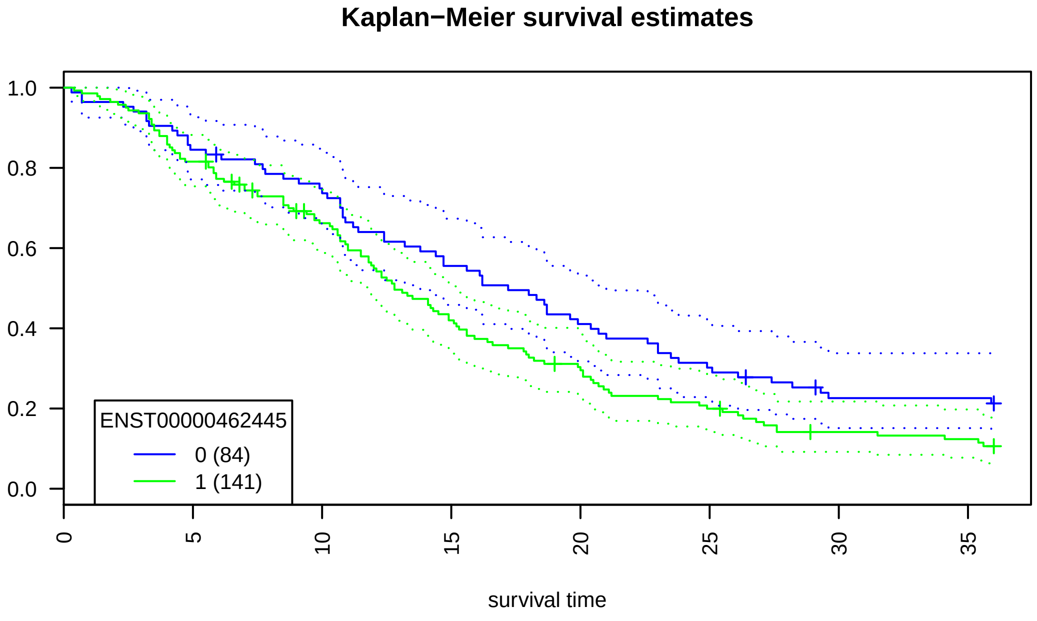

Figure 781: Each curve represents the relative number of ENST00000462445 survived to the corresponding point of time. Dashed lines represent 0.95 confidence intervals for the curves with the corresponding color. Total of 5 observations deleted due to missingness. Log-rank probability measure for the equality of these curves is 2.408614e-02. Additional annotations: *.GeneId*=ENSG00000122862, *.GeneName*=SRGN, *.GeneDesc*=serglycin [Source:HGNC Symbol;Acc:9361].

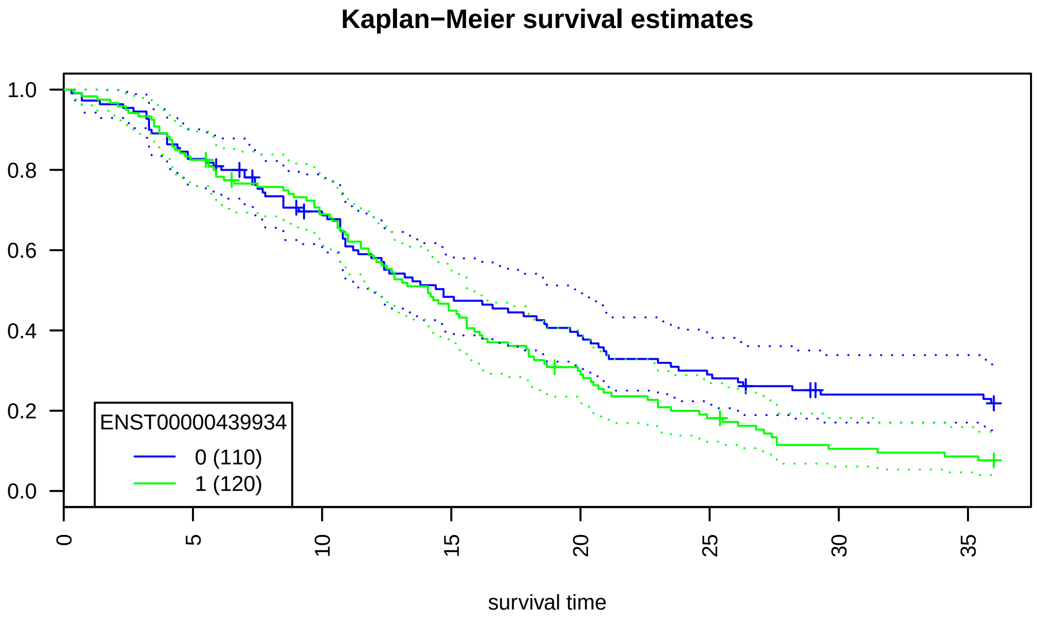

Figure 782: Each curve represents the relative number of ENST00000439934 survived to the corresponding point of time. Dashed lines represent 0.95 confidence intervals for the curves with the corresponding color. Log-rank probability measure for the equality of these curves is 3.988662e-02. Additional annotations: *.GeneId*=ENSG00000130489, *.GeneName*=SCO2, *.GeneDesc*=SCO cytochrome oxidase deficient homolog 2 (yeast) [Source:HGNC Symbol;Acc:10604].

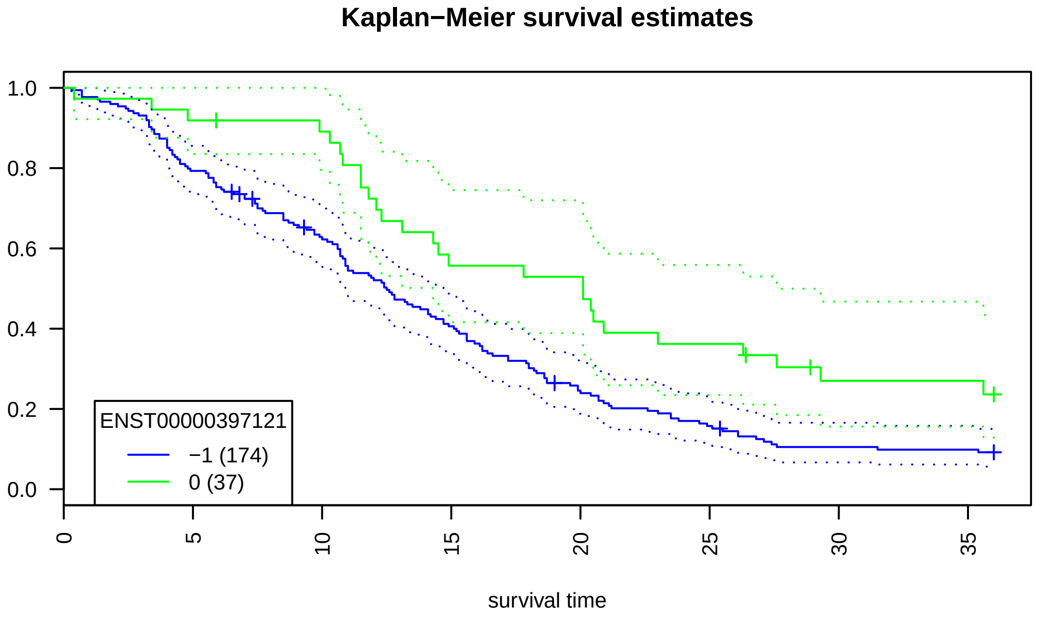

Figure 783: Each curve represents the relative number of ENST00000397121 survived to the corresponding point of time. Dashed lines represent 0.95 confidence intervals for the curves with the corresponding color. Total of 19 observations deleted due to missingness. Log-rank probability measure for the equality of these curves is 3.604590e-03. Additional annotations: *.GeneId*=ENSG00000196109, *.GeneName*=ZNF676, *.GeneDesc*=zinc finger protein 676 [Source:HGNC Symbol;Acc:20429].

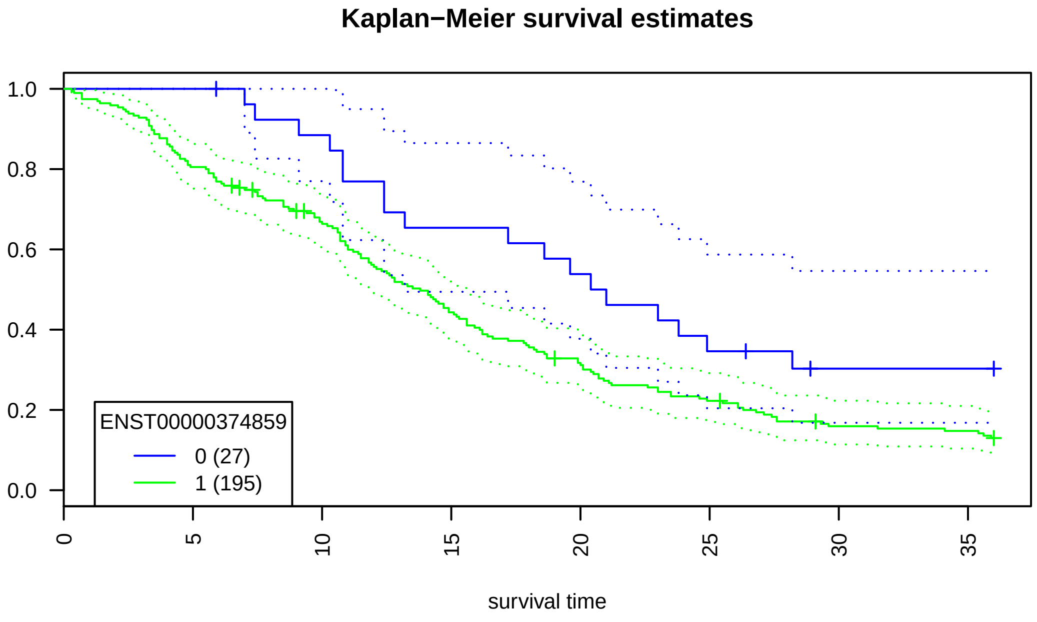

Figure 784: Each curve represents the relative number of ENST00000374859 survived to the corresponding point of time. Dashed lines represent 0.95 confidence intervals for the curves with the corresponding color. Total of 8 observations deleted due to missingness. Log-rank probability measure for the equality of these curves is 1.922561e-02. Additional annotations: *.GeneId*=ENSG00000240065, *.GeneName*=PSMB9, *.GeneDesc*=Proteasome subunit beta type-9 Precursor (EC 3.4.25.1)(Proteasome subunit beta-1i)(Proteasome chain 7)(Macropain chain 7)(Multicatalytic endopeptidase complex chain 7)(Really interesting new gene 12 protein)(Low molecular mass protein 2) [Source:UniProtKB/Swiss-Prot;Acc:P28065].

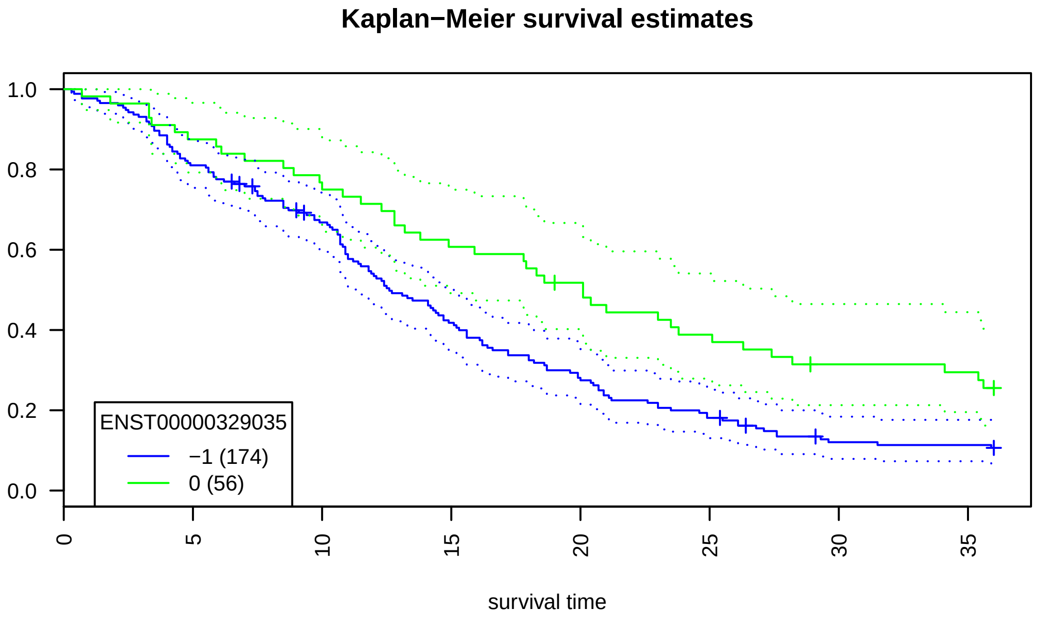

Figure 785: Each curve represents the relative number of ENST00000329035 survived to the corresponding point of time. Dashed lines represent 0.95 confidence intervals for the curves with the corresponding color. Log-rank probability measure for the equality of these curves is 2.145423e-03. Additional annotations: *.GeneId*=ENSG00000184905, *.GeneName*=TCEAL2, *.GeneDesc*=transcription elongation factor A (SII)-like 2 [Source:HGNC Symbol;Acc:29818].

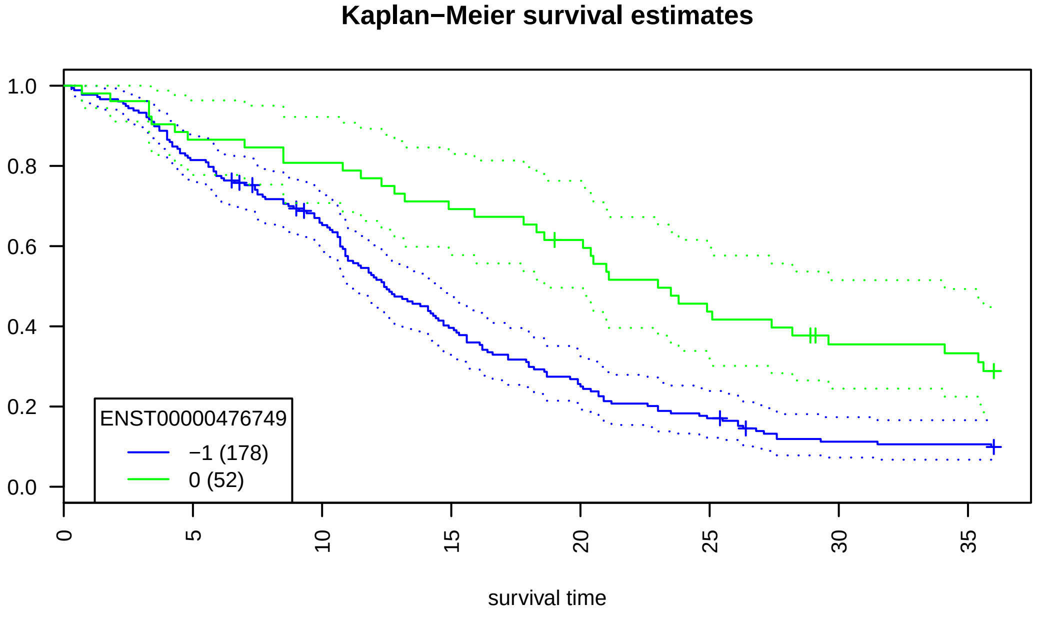

Figure 786: Each curve represents the relative number of ENST00000476749 survived to the corresponding point of time. Dashed lines represent 0.95 confidence intervals for the curves with the corresponding color. Log-rank probability measure for the equality of these curves is 4.944152e-05. Additional annotations: *.GeneId*=ENSG00000184905, *.GeneName*=TCEAL2, *.GeneDesc*=transcription elongation factor A (SII)-like 2 [Source:HGNC Symbol;Acc:29818].

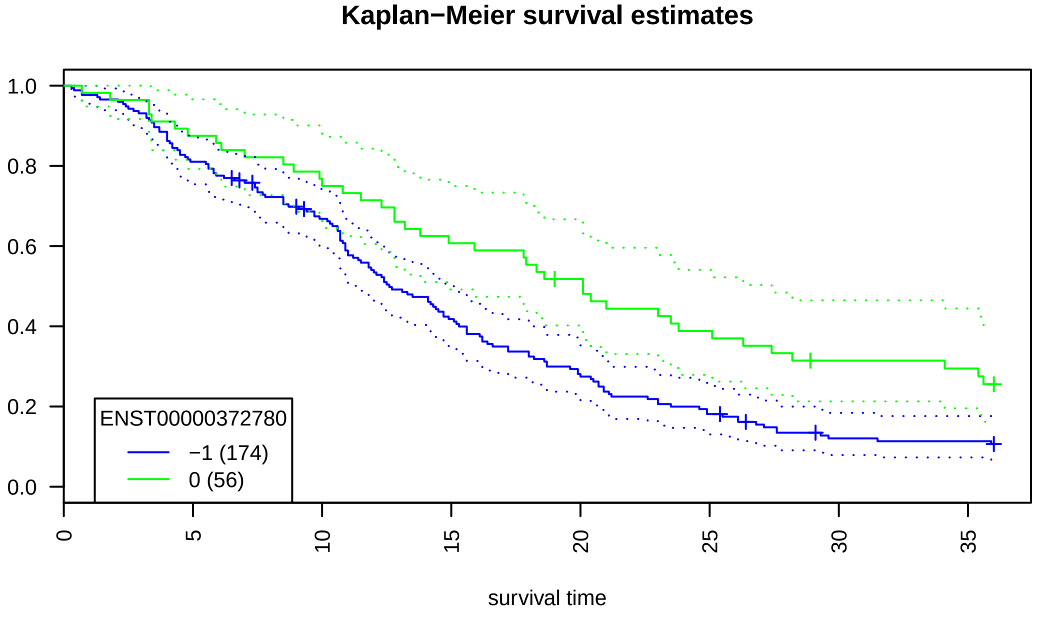

Figure 787: Each curve represents the relative number of ENST00000372780 survived to the corresponding point of time. Dashed lines represent 0.95 confidence intervals for the curves with the corresponding color. Log-rank probability measure for the equality of these curves is 2.145423e-03. Additional annotations: *.GeneId*=ENSG00000184905, *.GeneName*=TCEAL2, *.GeneDesc*=transcription elongation factor A (SII)-like 2 [Source:HGNC Symbol;Acc:29818].

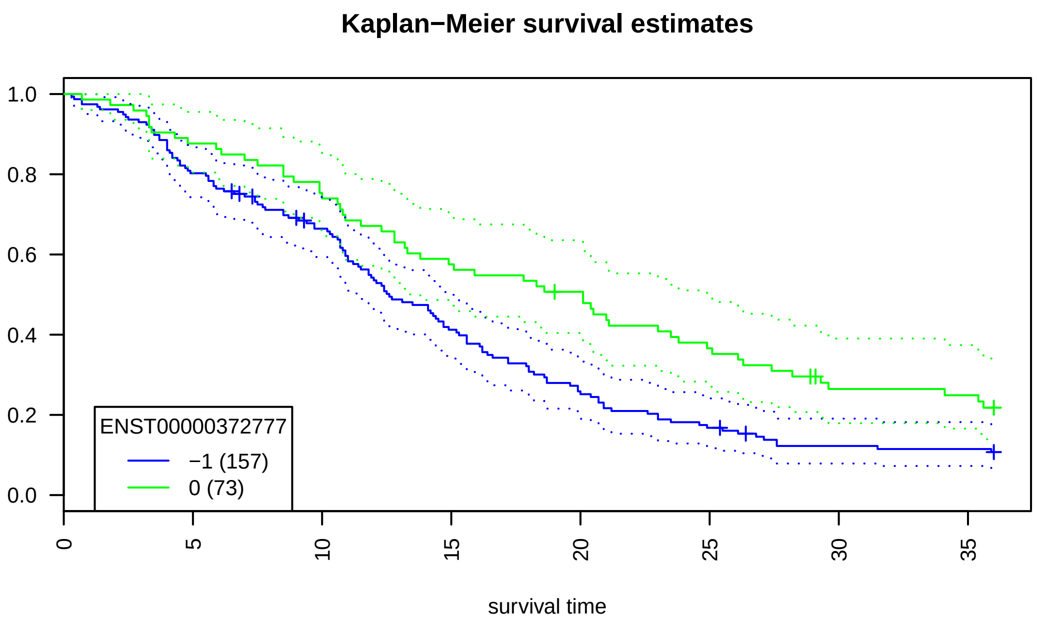

Figure 788: Each curve represents the relative number of ENST00000372777 survived to the corresponding point of time. Dashed lines represent 0.95 confidence intervals for the curves with the corresponding color. Log-rank probability measure for the equality of these curves is 2.934100e-03.

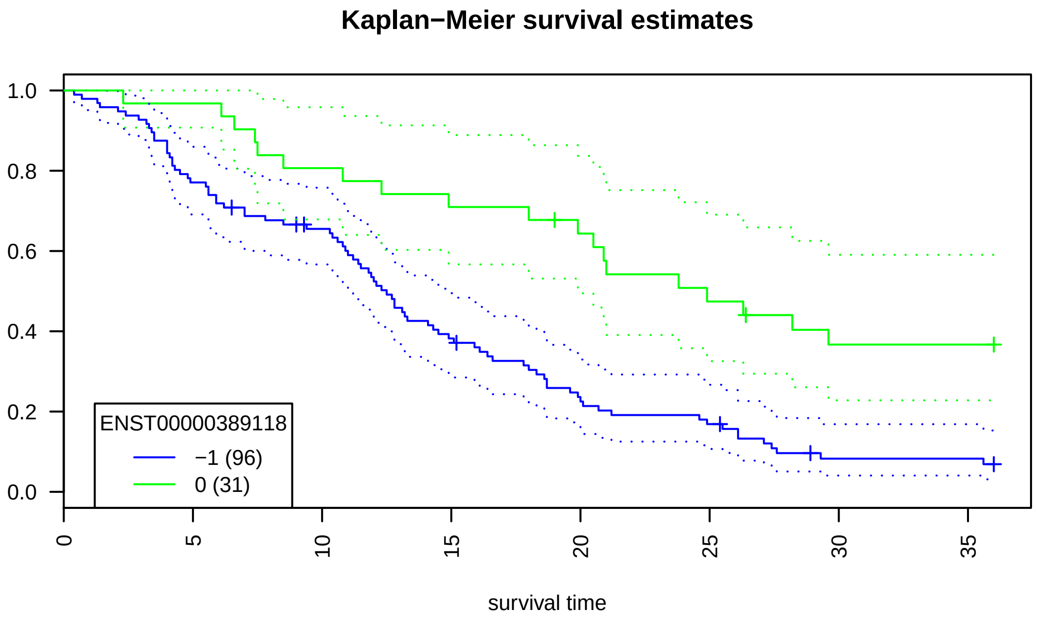

Figure 789: Each curve represents the relative number of ENST00000389118 survived to the corresponding point of time. Dashed lines represent 0.95 confidence intervals for the curves with the corresponding color. Total of 103 observations deleted due to missingness. Log-rank probability measure for the equality of these curves is 6.197774e-05. Additional annotations: *.GeneId*=ENSG00000204764, *.GeneName*=RANBP17, *.GeneDesc*=RAN binding protein 17 [Source:HGNC Symbol;Acc:14428].

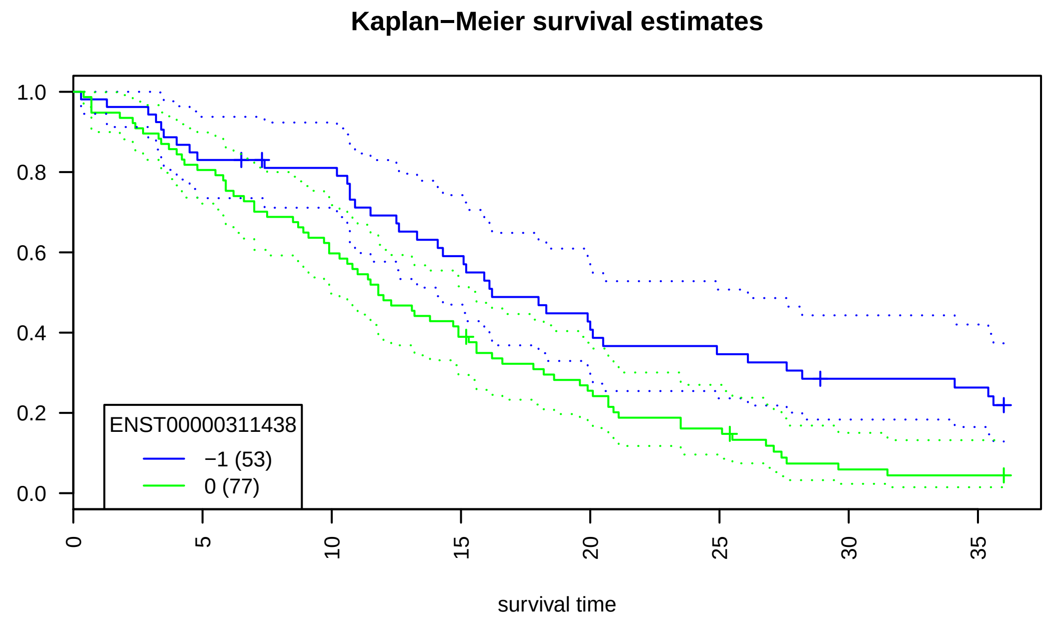

Figure 790: Each curve represents the relative number of ENST00000311438 survived to the corresponding point of time. Dashed lines represent 0.95 confidence intervals for the curves with the corresponding color. Total of 100 observations deleted due to missingness. Log-rank probability measure for the equality of these curves is 1.971315e-03. Additional annotations: *.GeneId*=ENSG00000149452, *.GeneName*=SLC22A8, *.GeneDesc*=solute carrier family 22 (organic anion transporter), member 8 [Source:HGNC Symbol;Acc:10972].

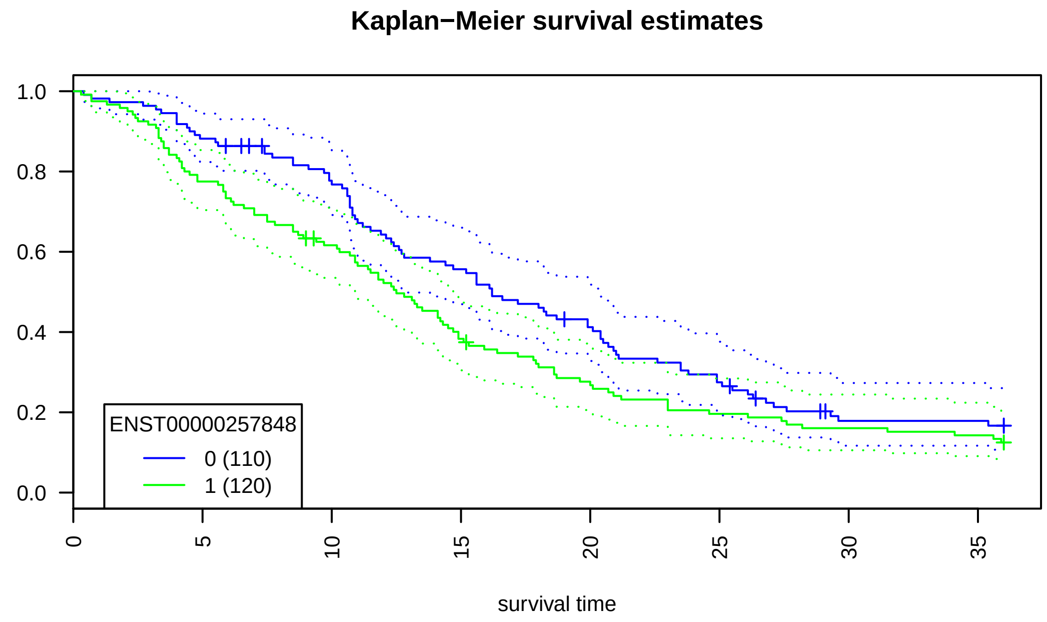

Figure 791: Each curve represents the relative number of ENST00000257848 survived to the corresponding point of time. Dashed lines represent 0.95 confidence intervals for the curves with the corresponding color. Log-rank probability measure for the equality of these curves is 4.822542e-02. Additional annotations: *.GeneId*=ENSG00000037897, *.GeneName*=METTL1, *.GeneDesc*=methyltransferase like 1 [Source:HGNC Symbol;Acc:7030].

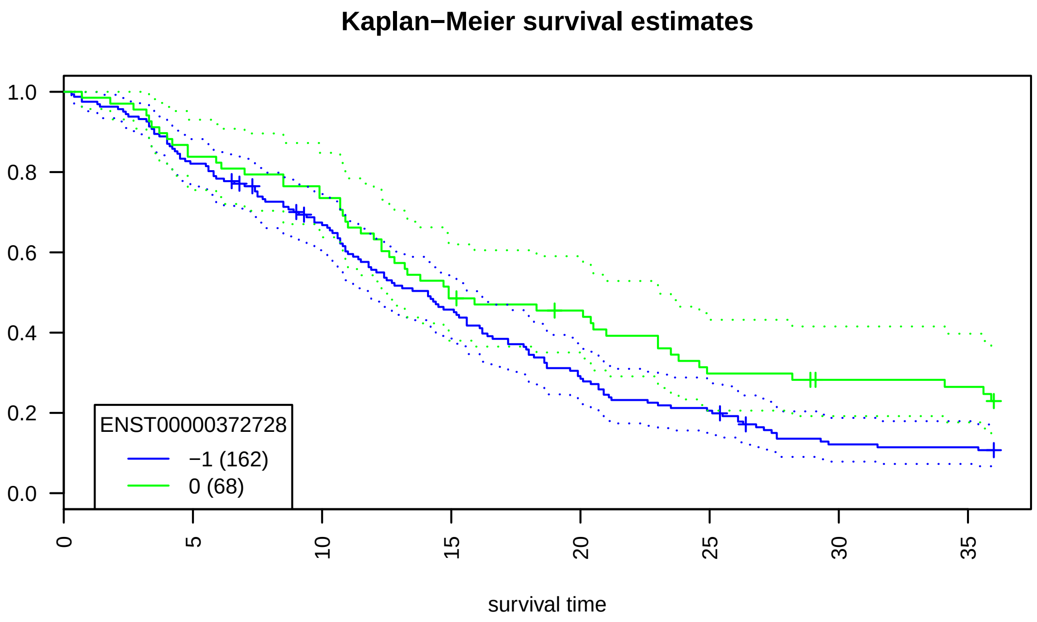

Figure 792: Each curve represents the relative number of ENST00000372728 survived to the corresponding point of time. Dashed lines represent 0.95 confidence intervals for the curves with the corresponding color. Log-rank probability measure for the equality of these curves is 2.844885e-02. Additional annotations: *.GeneId*=ENSG00000133169, *.GeneName*=BEX1, *.GeneDesc*=brain expressed, X-linked 1 [Source:HGNC Symbol;Acc:1036].

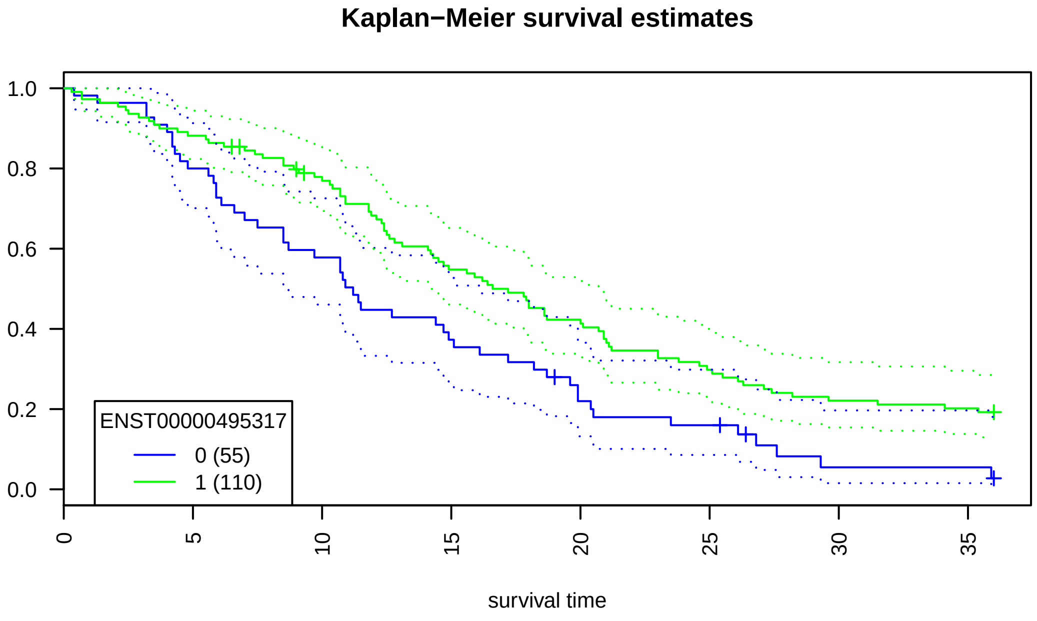

Figure 793: Each curve represents the relative number of ENST00000495317 survived to the corresponding point of time. Dashed lines represent 0.95 confidence intervals for the curves with the corresponding color. Total of 65 observations deleted due to missingness. Log-rank probability measure for the equality of these curves is 2.069361e-03. Additional annotations: *.GeneId*=ENSG00000141698, *.GeneName*=NT5C3L, *.GeneDesc*=5'-nucleotidase, cytosolic III-like [Source:HGNC Symbol;Acc:28300].

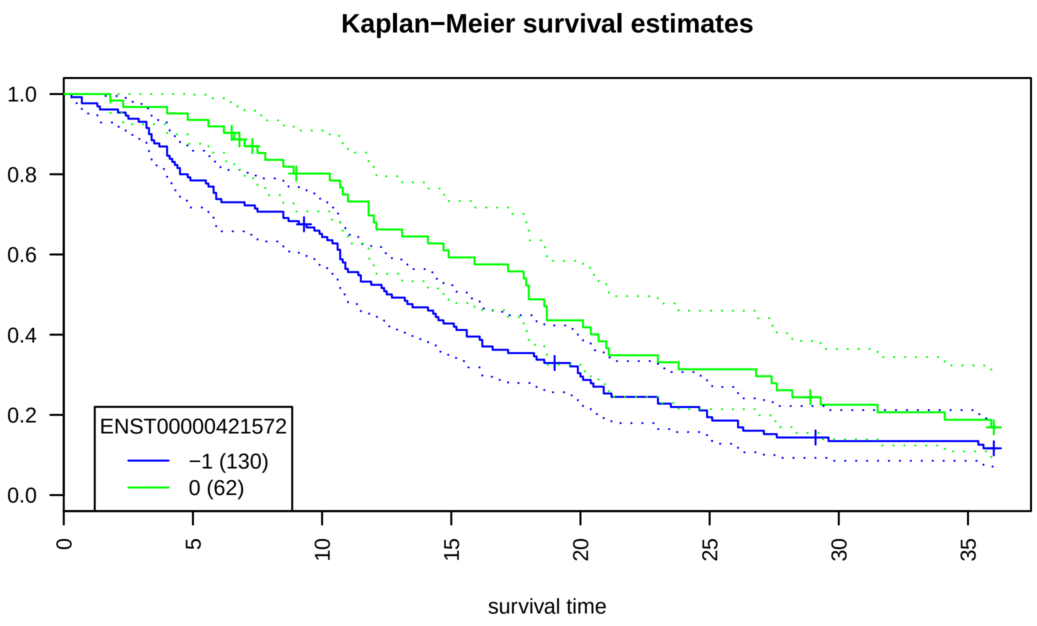

Figure 794: Each curve represents the relative number of ENST00000421572 survived to the corresponding point of time. Dashed lines represent 0.95 confidence intervals for the curves with the corresponding color. Total of 38 observations deleted due to missingness. Log-rank probability measure for the equality of these curves is 3.644187e-02. Additional annotations: *.GeneId*=ENSG00000237517, *.GeneName*=DGCR5, *.GeneDesc*=DiGeorge syndrome critical region gene 5 (non-protein coding) [Source:HGNC Symbol;Acc:16757].

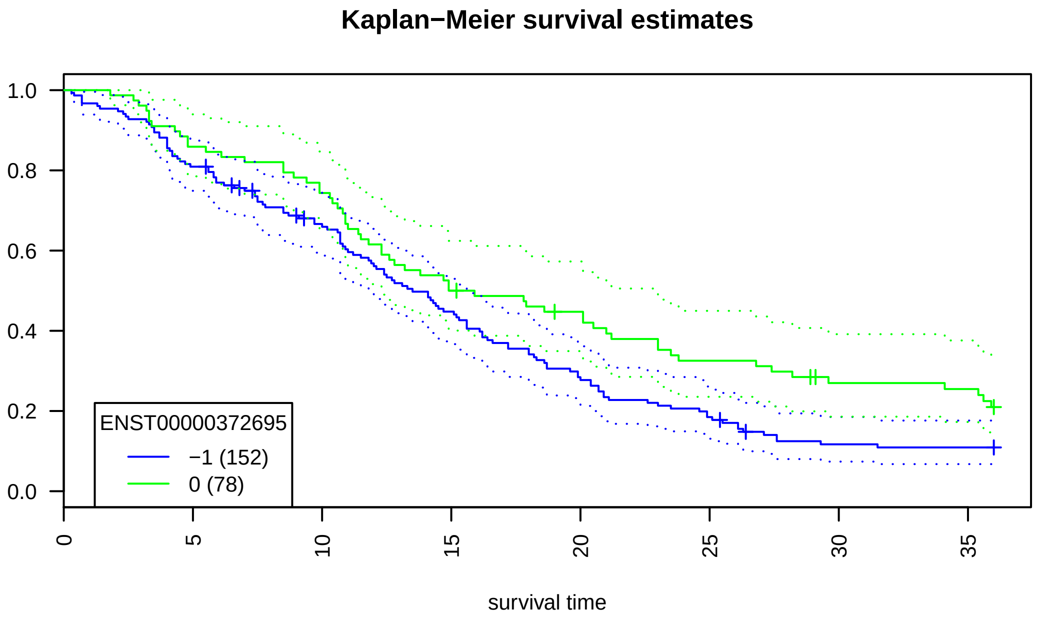

Figure 795: Each curve represents the relative number of ENST00000372695 survived to the corresponding point of time. Dashed lines represent 0.95 confidence intervals for the curves with the corresponding color. Log-rank probability measure for the equality of these curves is 2.027773e-02. Additional annotations: *.GeneId*=ENSG00000102409, *.GeneName*=BEX4, *.GeneDesc*=brain expressed, X-linked 4 [Source:HGNC Symbol;Acc:25475].

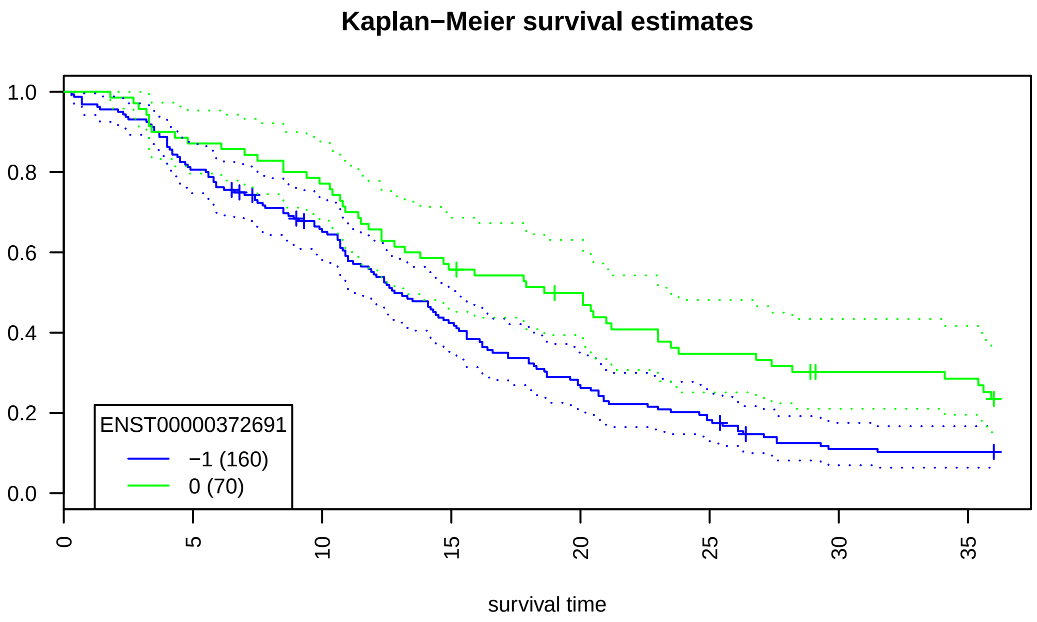

Figure 796: Each curve represents the relative number of ENST00000372691 survived to the corresponding point of time. Dashed lines represent 0.95 confidence intervals for the curves with the corresponding color. Log-rank probability measure for the equality of these curves is 2.907006e-03. Additional annotations: *.GeneId*=ENSG00000102409, *.GeneName*=BEX4, *.GeneDesc*=brain expressed, X-linked 4 [Source:HGNC Symbol;Acc:25475].

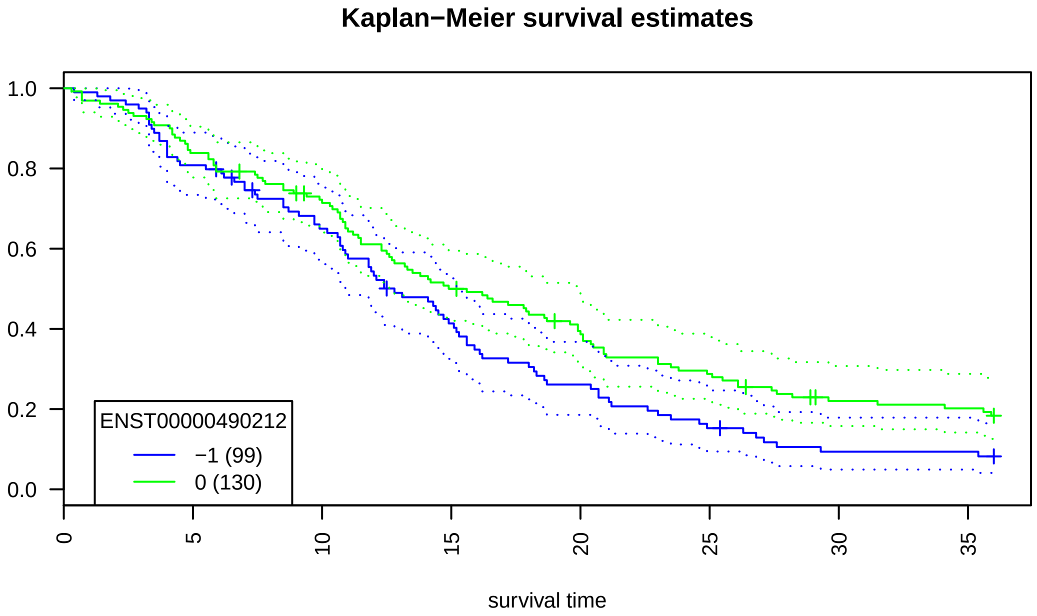

Figure 797: Each curve represents the relative number of ENST00000490212 survived to the corresponding point of time. Dashed lines represent 0.95 confidence intervals for the curves with the corresponding color. Total of 1 observation deleted due to missingness. Log-rank probability measure for the equality of these curves is 2.053039e-02. Additional annotations: *.GeneId*=ENSG00000158089, *.GeneName*=GALNT14, *.GeneDesc*=UDP-N-acetyl-alpha-D-galactosamine:polypeptide N-acetylgalactosaminyltransferase 14 (GalNAc-T14) [Source:HGNC Symbol;Acc:22946].

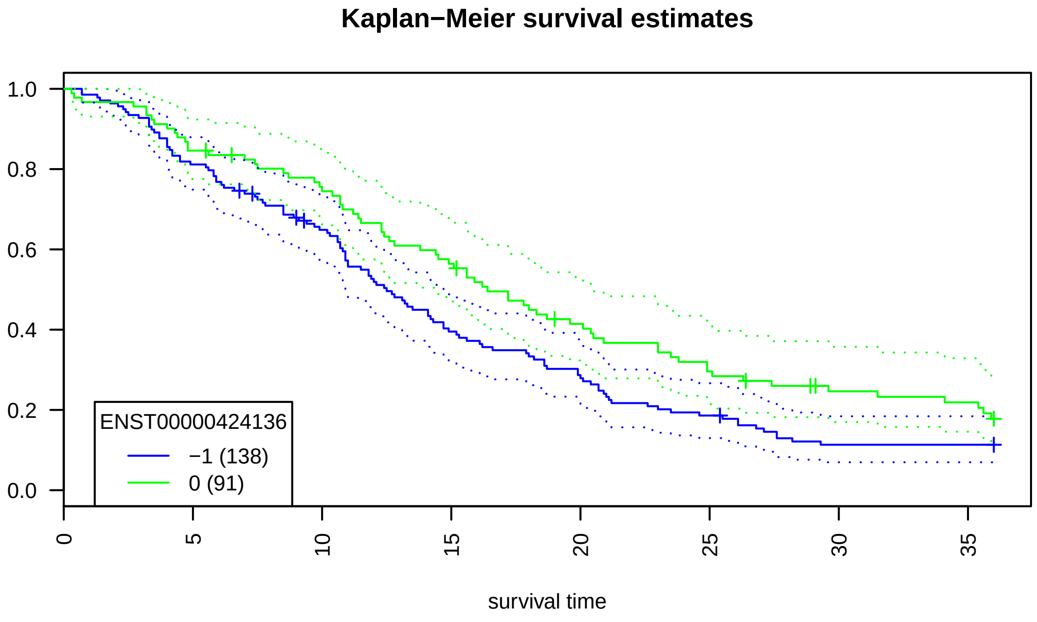

Figure 798: Each curve represents the relative number of ENST00000424136 survived to the corresponding point of time. Dashed lines represent 0.95 confidence intervals for the curves with the corresponding color. Total of 1 observation deleted due to missingness. Log-rank probability measure for the equality of these curves is 2.332001e-02. Additional annotations: *.GeneId*=ENSG00000158089, *.GeneName*=GALNT14, *.GeneDesc*=UDP-N-acetyl-alpha-D-galactosamine:polypeptide N-acetylgalactosaminyltransferase 14 (GalNAc-T14) [Source:HGNC Symbol;Acc:22946].

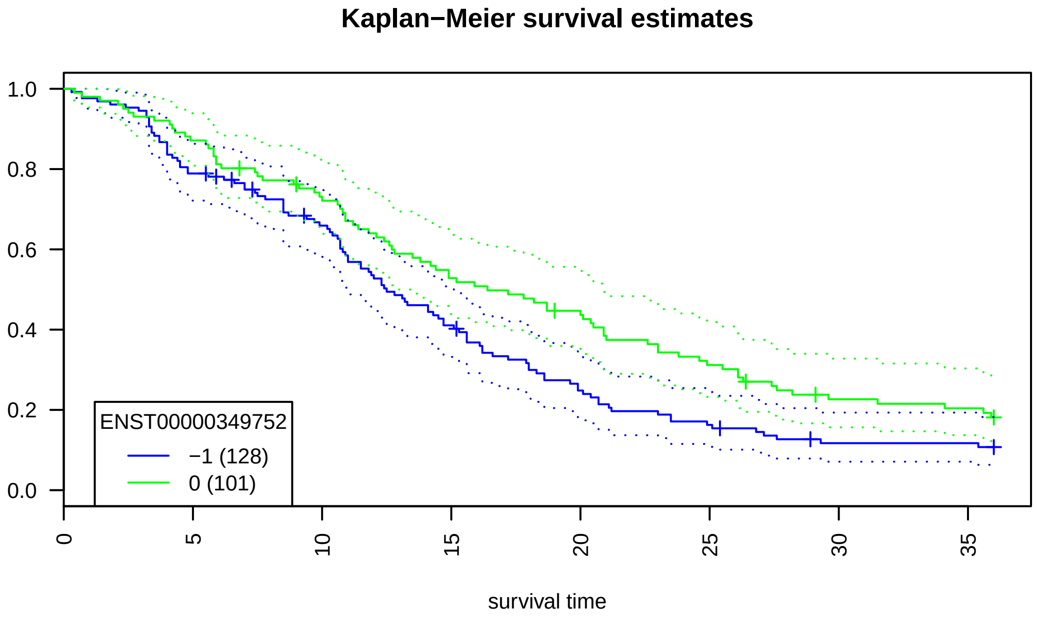

Figure 799: Each curve represents the relative number of ENST00000349752 survived to the corresponding point of time. Dashed lines represent 0.95 confidence intervals for the curves with the corresponding color. Total of 1 observation deleted due to missingness. Log-rank probability measure for the equality of these curves is 1.448422e-02. Additional annotations: *.GeneId*=ENSG00000158089, *.GeneName*=GALNT14, *.GeneDesc*=UDP-N-acetyl-alpha-D-galactosamine:polypeptide N-acetylgalactosaminyltransferase 14 (GalNAc-T14) [Source:HGNC Symbol;Acc:22946].

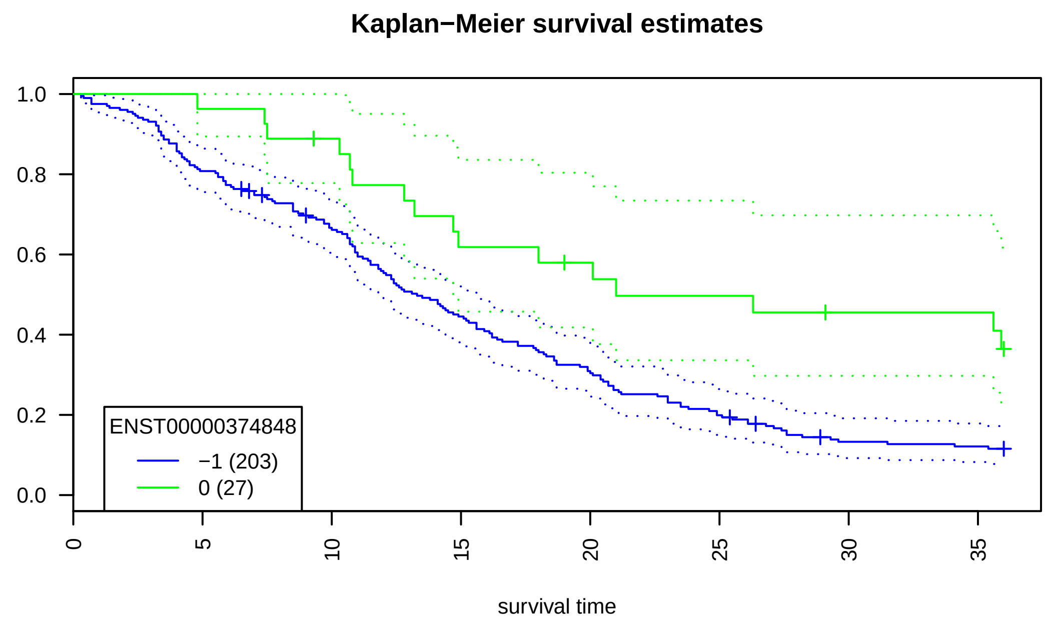

Figure 800: Each curve represents the relative number of ENST00000374848 survived to the corresponding point of time. Dashed lines represent 0.95 confidence intervals for the curves with the corresponding color. Log-rank probability measure for the equality of these curves is 1.932825e-03. Additional annotations: .*GeneId*=ENSG00000165152, .*GeneName*=C9orf125, .*GeneDesc*=Transmembrane protein C9orf125 [Source:UniProtKB/Swiss-Prot;Acc:Q9BRR3].

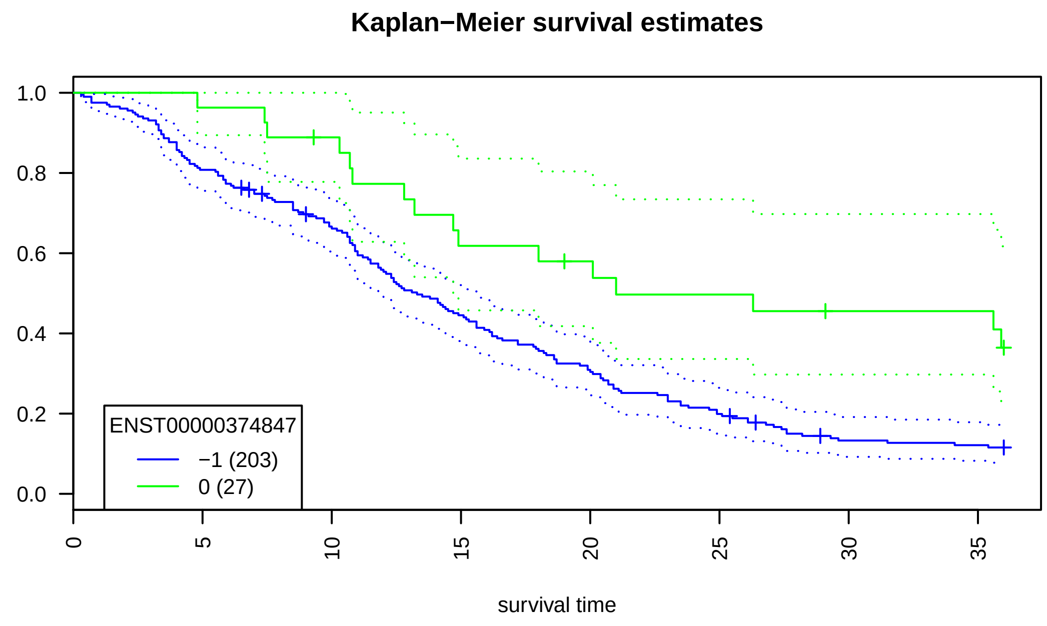

Figure 801: Each curve represents the relative number of ENST00000374847 survived to the corresponding point of time. Dashed lines represent 0.95 confidence intervals for the curves with the corresponding color. Log-rank probability measure for the equality of these curves is 1.932825e-03. Additional annotations: .*GeneId*=ENSG00000165152, .*GeneName*=C9orf125, .*GeneDesc*=Transmembrane protein C9orf125 [Source:UniProtKB/Swiss-Prot;Acc:Q9BRR3].

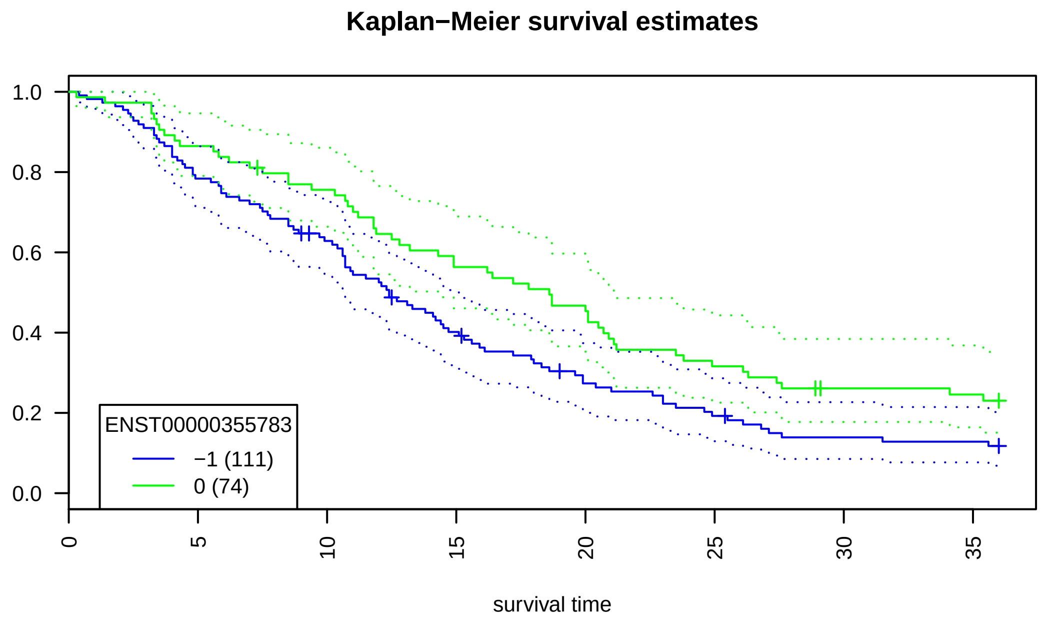

Figure 802: Each curve represents the relative number of ENST00000355783 survived to the corresponding point of time. Dashed lines represent 0.95 confidence intervals for the curves with the corresponding color. Total of 45 observations deleted due to missingness. Log-rank probability measure for the equality of these curves is 1.440495e-02. Additional annotations: *.GeneId*=ENSG00000165671, *.GeneName*=NSD1, *.GeneDesc*=nuclear receptor binding SET domain protein 1 [Source:HGNC Symbol;Acc:14234].

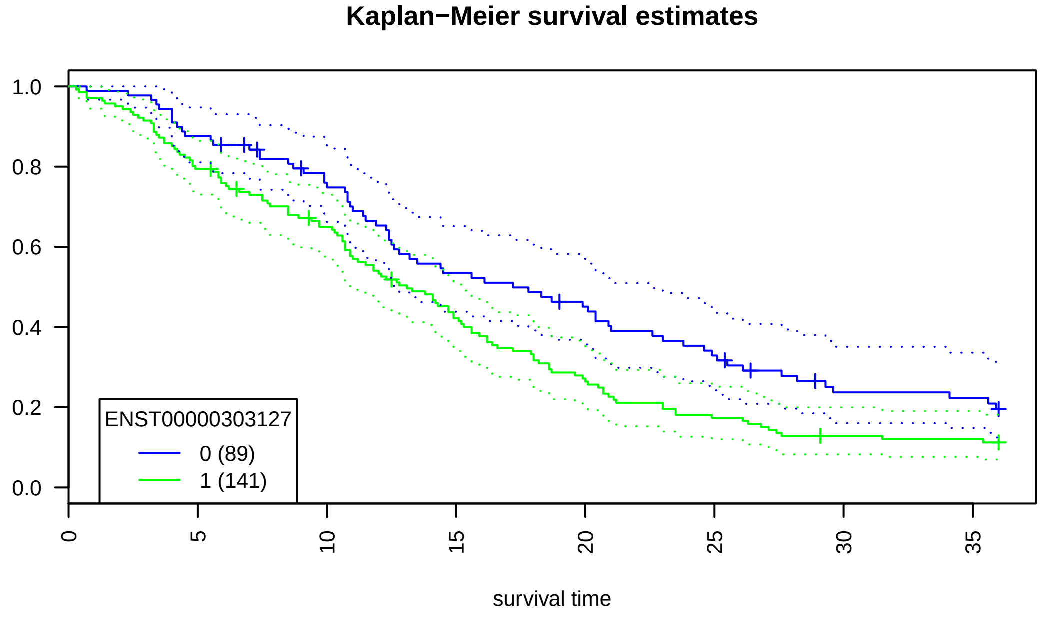

Figure 803: Each curve represents the relative number of ENST00000303127 survived to the corresponding point of time. Dashed lines represent 0.95 confidence intervals for the curves with the corresponding color. Log-rank probability measure for the equality of these curves is 1.054630e-02. Additional annotations: *.GeneId*=ENSG00000169223, *.GeneName*=LMAN2, *.GeneDesc*=lectin, mannose-binding 2 [Source:HGNC Symbol;Acc:16986].

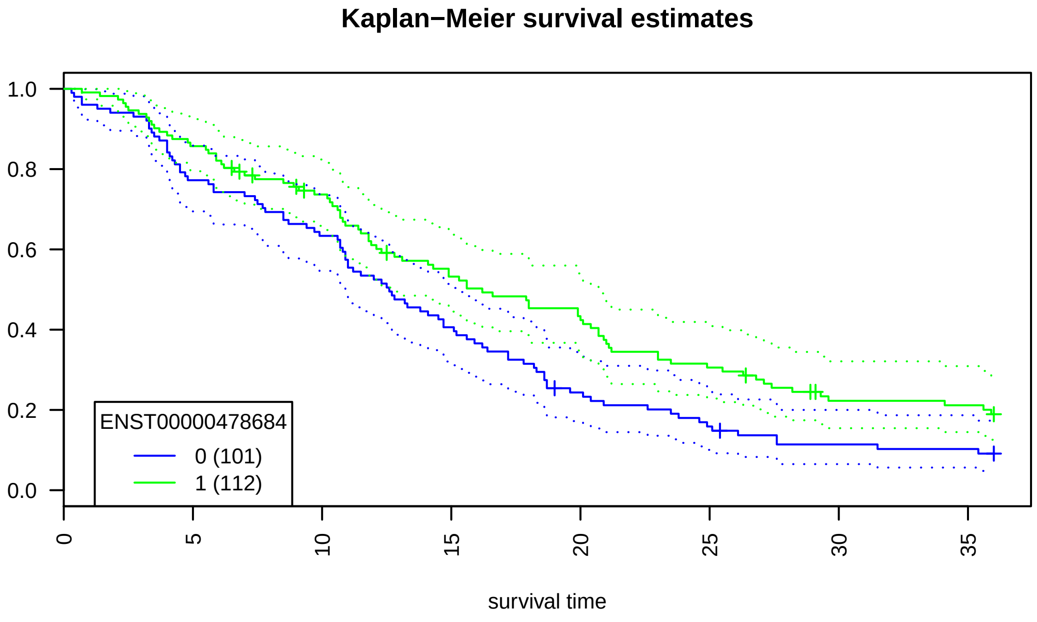

Figure 804: Each curve represents the relative number of ENST00000478684 survived to the corresponding point of time. Dashed lines represent 0.95 confidence intervals for the curves with the corresponding color. Total of 17 observations deleted due to missingness. Log-rank probability measure for the equality of these curves is 1.010906e-02. Additional annotations: *.GeneId*=ENSG00000117122, *.GeneName*=MFAP2, *.GeneDesc*=microfibrillar-associated protein 2 [Source:HGNC Symbol;Acc:7033].

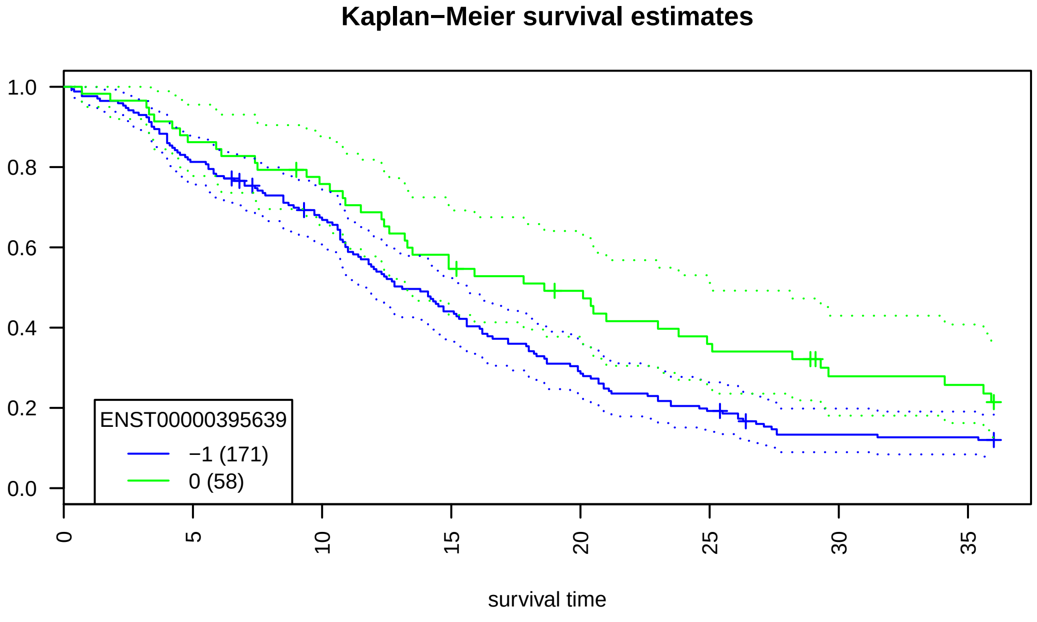

Figure 805: Each curve represents the relative number of ENST00000395639 survived to the corresponding point of time. Dashed lines represent 0.95 confidence intervals for the curves with the corresponding color. Total of 1 observation deleted due to missingness. Log-rank probability measure for the equality of these curves is 1.809159e-02. Additional annotations: *.GeneId*=ENSG00000087495, *.GeneName*=PHACTR3, *.GeneDesc*=phosphatase and actin regulator 3 [Source:HGNC Symbol;Acc:15833].

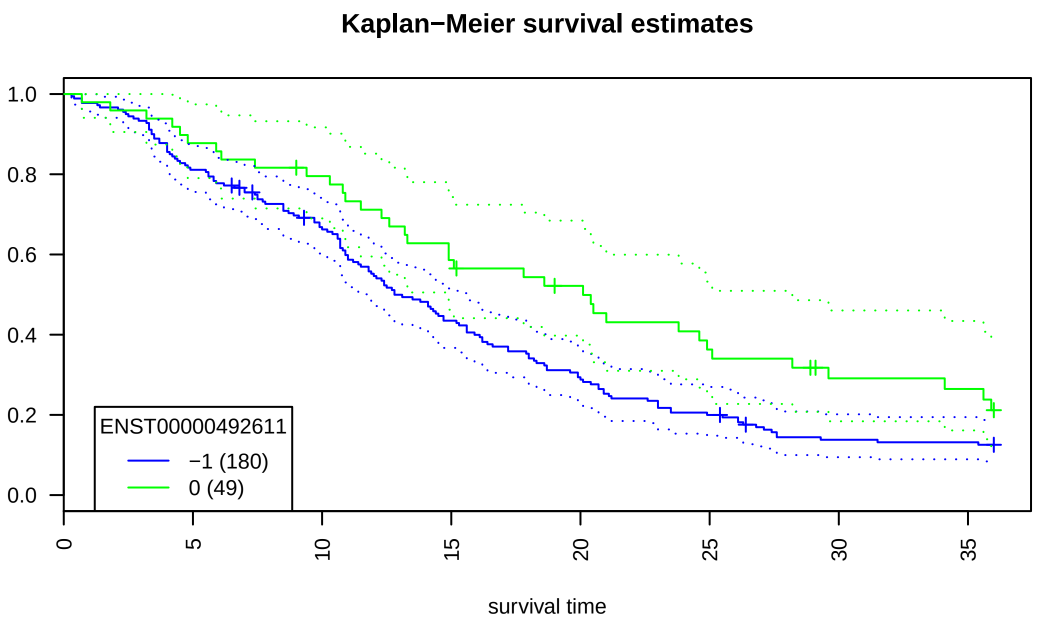

Figure 806: Each curve represents the relative number of ENST00000492611 survived to the corresponding point of time. Dashed lines represent 0.95 confidence intervals for the curves with the corresponding color. Total of 1 observation deleted due to missingness. Log-rank probability measure for the equality of these curves is 1.808671e-02. Additional annotations: *.GeneId*=ENSG00000087495, *.GeneName*=PHACTR3, *.GeneDesc*=phosphatase and actin regulator 3 [Source:HGNC Symbol;Acc:15833].

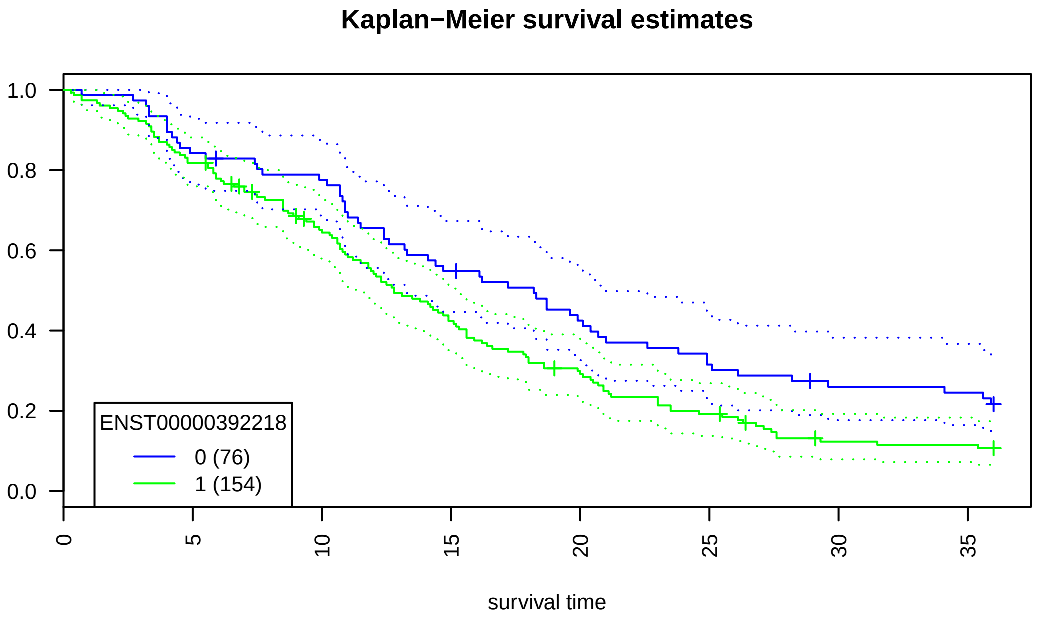

Figure 807: Each curve represents the relative number of ENST00000392218 survived to the corresponding point of time. Dashed lines represent 0.95 confidence intervals for the curves with the corresponding color. Log-rank probability measure for the equality of these curves is 1.312218e-02. Additional annotations: *.GeneId*=ENSG00000089327, *.GeneName*=FXYD5, *.GeneDesc*=FXYD domain containing ion transport regulator 5 [Source:HGNC Symbol;Acc:4029].

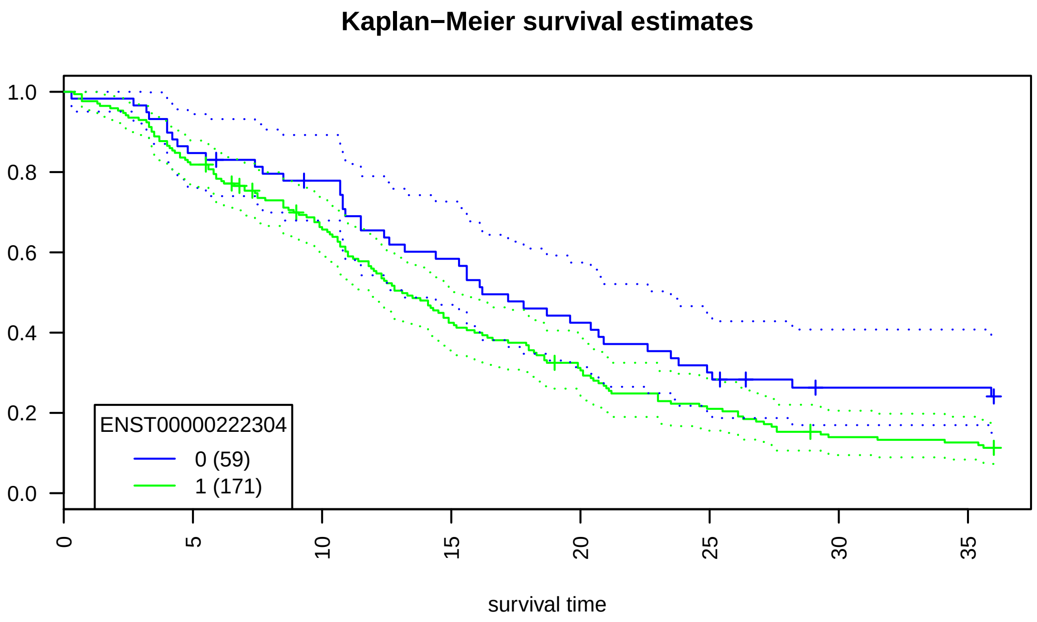

Figure 808: Each curve represents the relative number of ENST00000222304 survived to the corresponding point of time. Dashed lines represent 0.95 confidence intervals for the curves with the corresponding color. Log-rank probability measure for the equality of these curves is 3.378469e-02. Additional annotations: *.GeneId*=ENSG00000105697, *.GeneName*=HAMP, *.GeneDesc*=hepcidin antimicrobial peptide [Source:HGNC Symbol;Acc:15598].

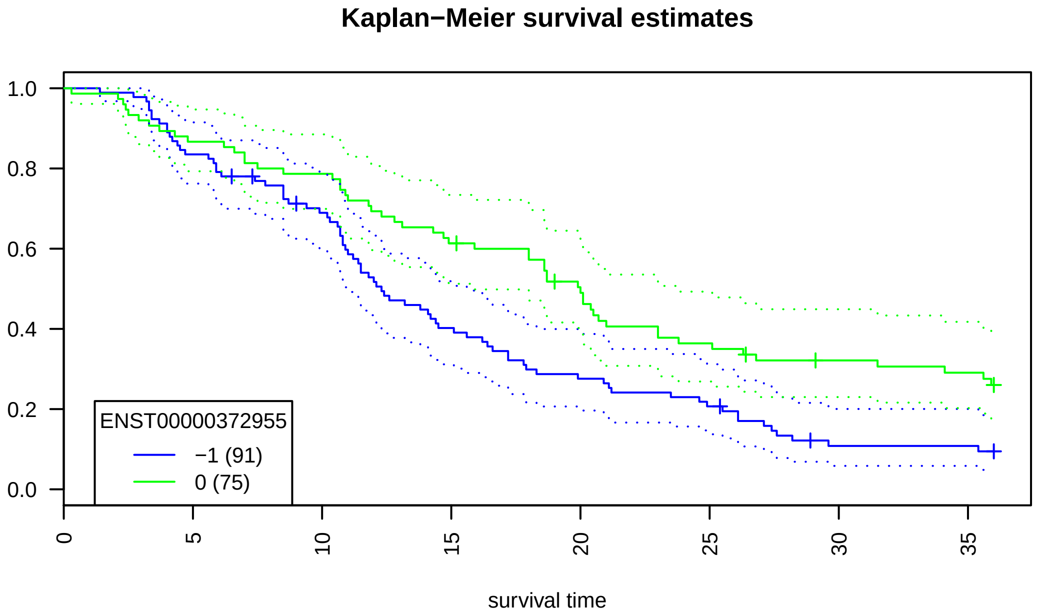

Figure 809: Each curve represents the relative number of ENST00000372955 survived to the corresponding point of time. Dashed lines represent 0.95 confidence intervals for the curves with the corresponding color. Total of 64 observations deleted due to missingness. Log-rank probability measure for the equality of these curves is 2.03691e-03. Additional annotations: *.GeneId*=ENSG00000138286, *.GeneName*=FAM149B1, *.GeneDesc*=family with sequence similarity 149, member B1 [Source:HGNC Symbol;Acc:29162].

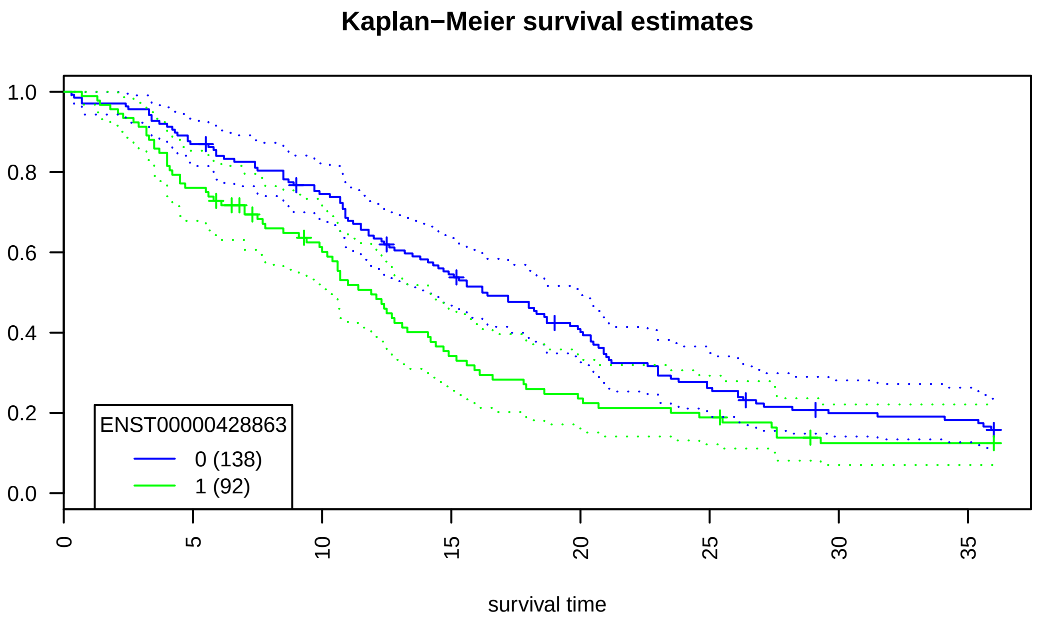

Figure 810: Each curve represents the relative number of ENST00000428863 survived to the corresponding point of time. Dashed lines represent 0.95 confidence intervals for the curves with the corresponding color. Log-rank probability measure for the equality of these curves is 2.174921e-02. Additional annotations: *.GeneId*=ENSG00000226959, *.GeneName*=AC025423.2, *.GeneDesc*=cDNA, FLJ92387 [Source:UniProtKB/TrEMBL;Acc:B2R594].

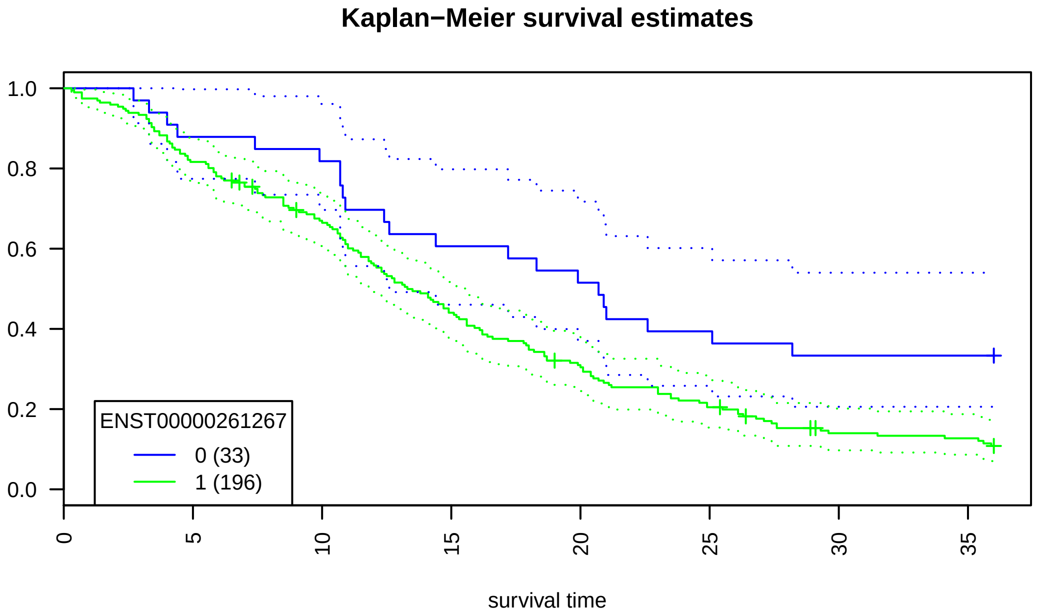

Figure 811: Each curve represents the relative number of ENST00000261267 survived to the corresponding point of time. Dashed lines represent 0.95 confidence intervals for the curves with the corresponding color. Total of 1 observation deleted due to missingness. Log-rank probability measure for the equality of these curves is 5.787865e-03. Additional annotations: *.GeneId*=ENSG00000090382, *.GeneName*=LYZ, *.GeneDesc*=lysozyme (renal amyloidosis) [Source:HGNC Symbol;Acc:6740].

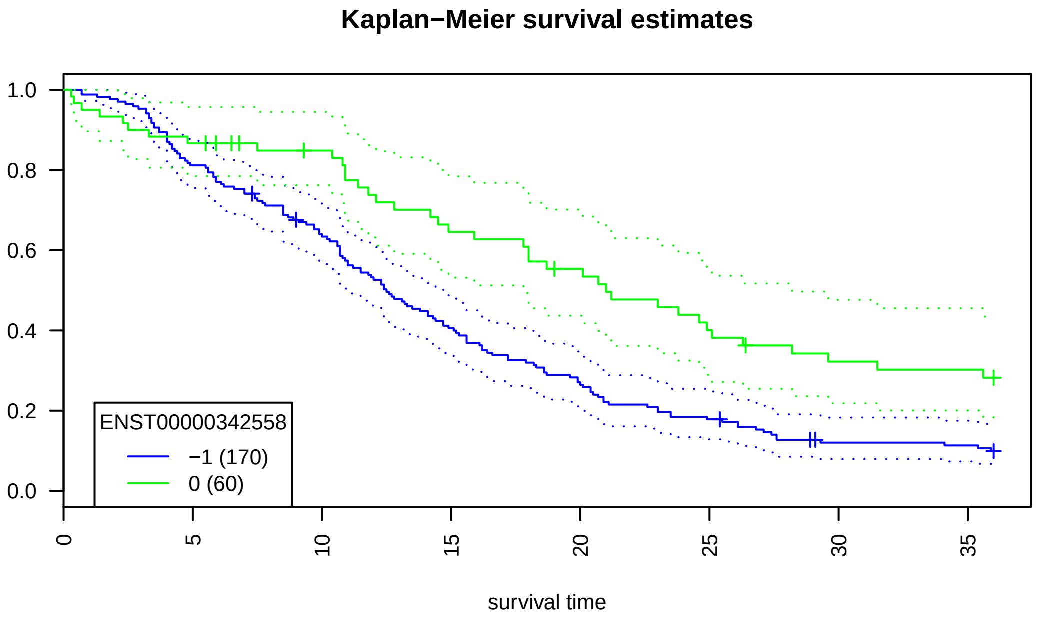

Figure 812: Each curve represents the relative number of ENST00000342558 survived to the corresponding point of time. Dashed lines represent 0.95 confidence intervals for the curves with the corresponding color. Log-rank probability measure for the equality of these curves is 2.120692e-04. Additional annotations: *.GeneId*=ENSG00000107758, *.GeneName*=PPP3CB, *.GeneDesc*=protein phosphatase 3, catalytic subunit, beta isozyme [Source:HGNC Symbol;Acc:9315].

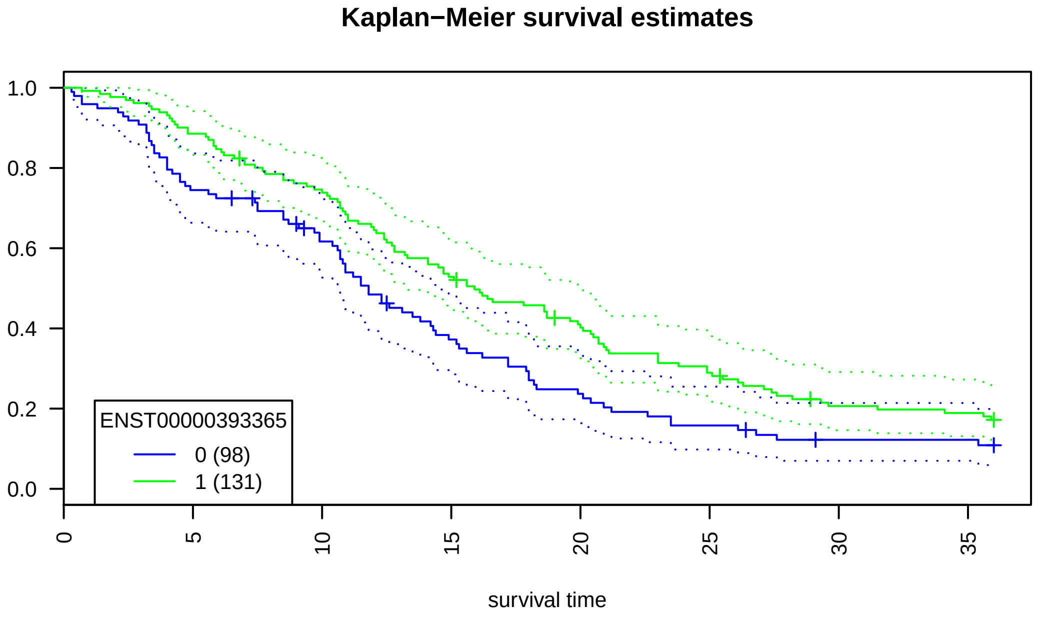

Figure 813: Each curve represents the relative number of ENST00000393365 survived to the corresponding point of time. Dashed lines represent 0.95 confidence intervals for the curves with the corresponding color. Total of 1 observation deleted due to missingness. Log-rank probability measure for the equality of these curves is 8.715445e-03. Additional annotations: *.GeneId*=ENSG00000127325, *.GeneName*=BEST3, *.GeneDesc*=bestrophin 3 [Source:HGNC Symbol;Acc:17105].

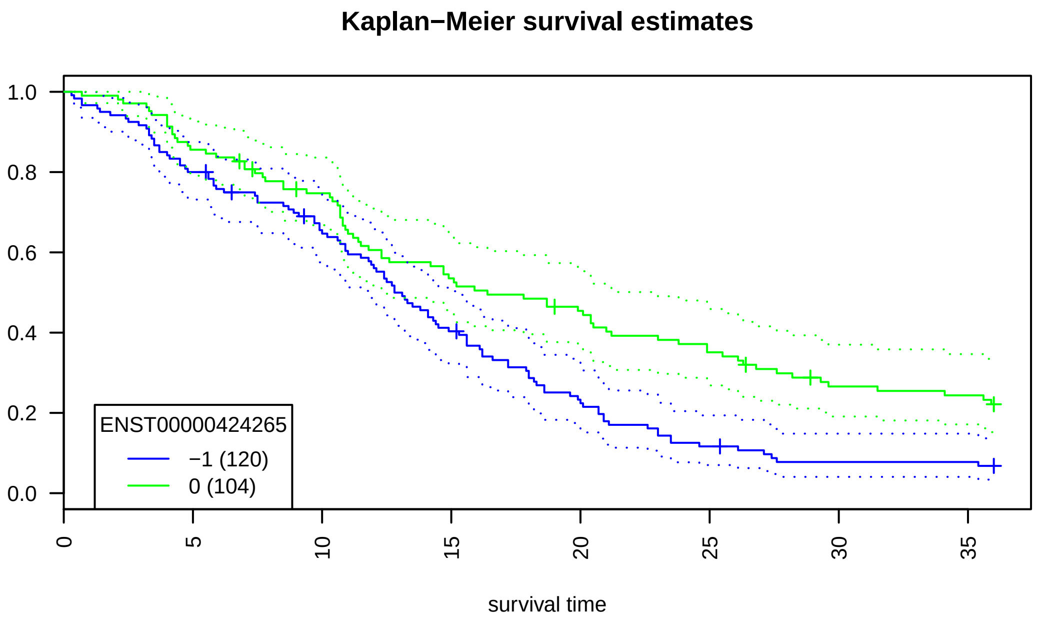

Figure 814: Each curve represents the relative number of ENST00000424265 survived to the corresponding point of time. Dashed lines represent 0.95 confidence intervals for the curves with the corresponding color. Total of 6 observations deleted due to missingness. Log-rank probability measure for the equality of these curves is 3.349629e-04. Additional annotations: *.GeneId*=ENSG00000166348, *.GeneName*=USP54, *.GeneDesc*=ubiquitin specific peptidase 54 [Source:HGNC Symbol;Acc:23513].

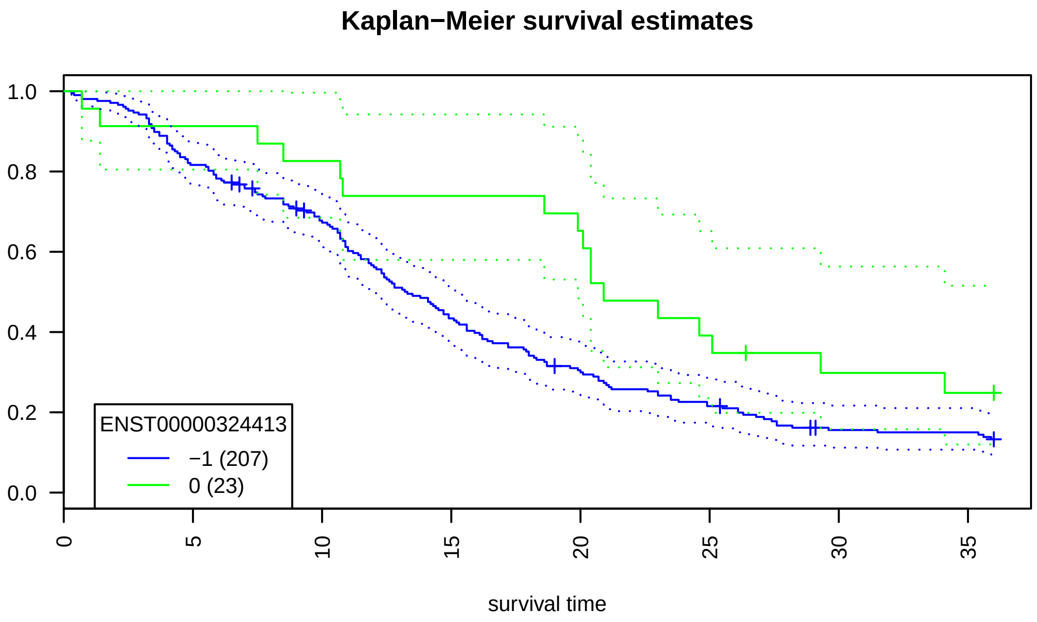

Figure 815: Each curve represents the relative number of ENST00000324413 survived to the corresponding point of time. Dashed lines represent 0.95 confidence intervals for the curves with the corresponding color. Log-rank probability measure for the equality of these curves is 3.526550e-02. Additional annotations: *.GeneId*=ENSG00000240746, *.GeneName*=AC026495.2, *.GeneDesc*=[undefined].

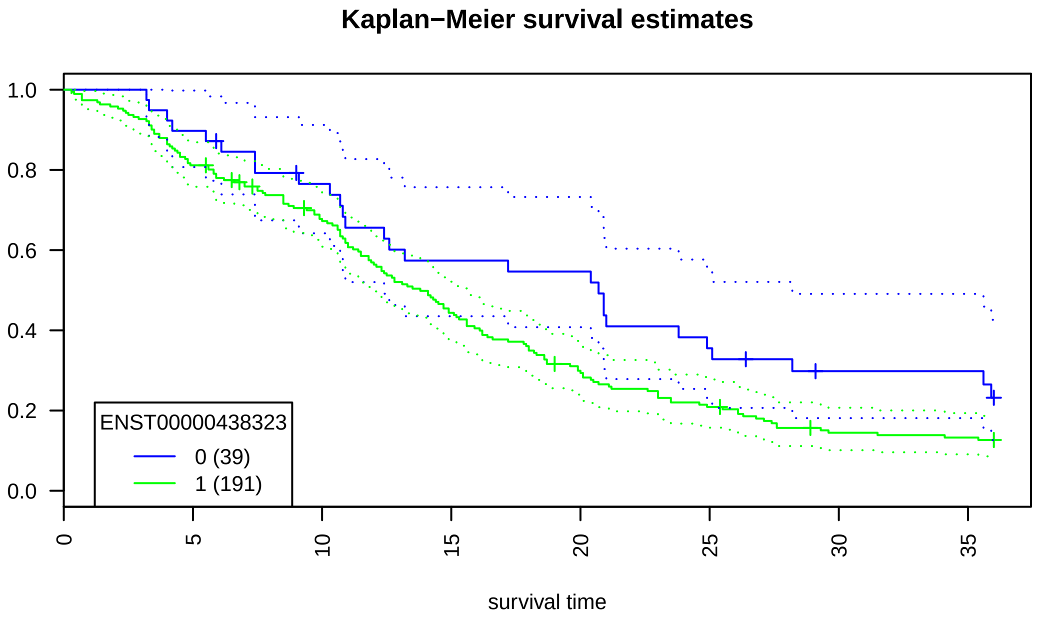

Figure 816: Each curve represents the relative number of ENST00000438323 survived to the corresponding point of time. Dashed lines represent 0.95 confidence intervals for the curves with the corresponding color. Log-rank probability measure for the equality of these curves is 4.364437e-02. Additional annotations: *.GeneId*=ENSG00000068079, *.GeneName*=IFI35, *.GeneDesc*=interferon-induced protein 35 [Source:HGNC Symbol;Acc:5399].

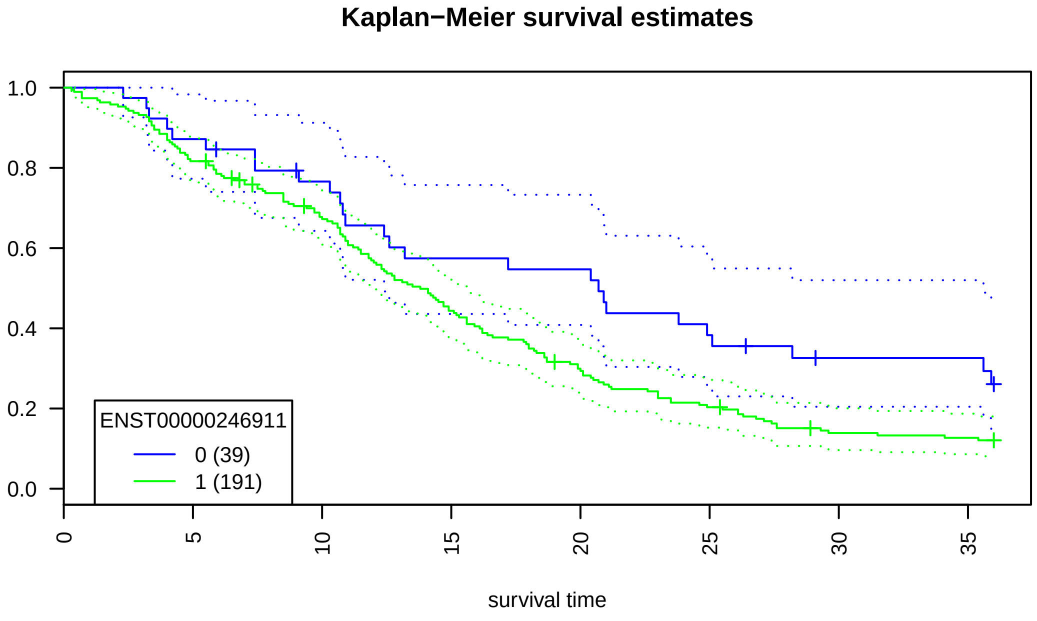

Figure 817: Each curve represents the relative number of ENST00000246911 survived to the corresponding point of time. Dashed lines represent 0.95 confidence intervals for the curves with the corresponding color. Log-rank probability measure for the equality of these curves is 2.235799e-02. Additional annotations: *.GeneId*=ENSG00000068079, *.GeneName*=IFI35, *.GeneDesc*=interferon-induced protein 35 [Source:HGNC Symbol;Acc:5399].

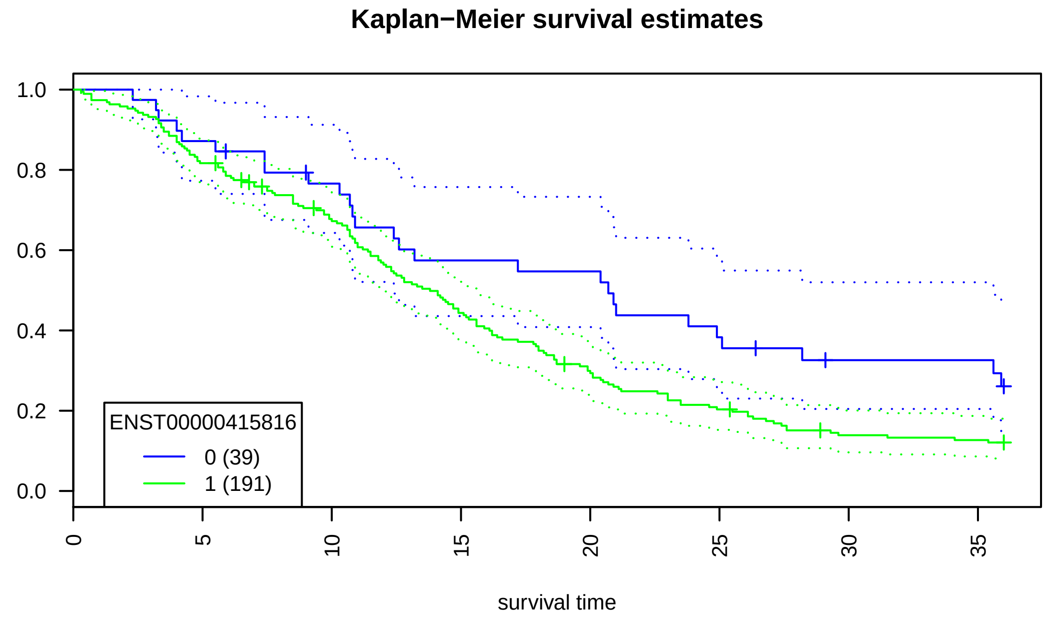

Figure 818: Each curve represents the relative number of ENST00000415816 survived to the corresponding point of time. Dashed lines represent 0.95 confidence intervals for the curves with the corresponding color. Log-rank probability measure for the equality of these curves is 2.235799e-02. Additional annotations: *.GeneId*=ENSG00000068079, *.GeneName*=IFI35, *.GeneDesc*=interferon-induced protein 35 [Source:HGNC Symbol;Acc:5399].

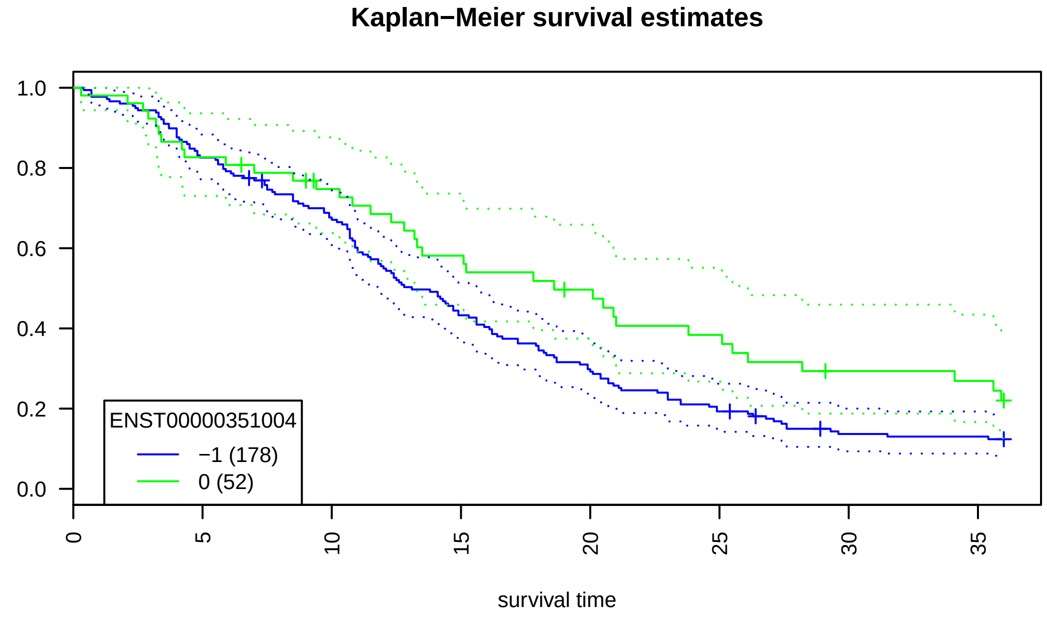

Figure 819: Each curve represents the relative number of ENST00000351004 survived to the corresponding point of time. Dashed lines represent 0.95 confidence intervals for the curves with the corresponding color. Log-rank probability measure for the equality of these curves is 3.994224e-02. Additional annotations: *.GeneId*=ENSG00000127955, *.GeneName*=GNAI1, *.GeneDesc*=guanine nucleotide binding protein (G protein), alpha inhibiting activity polypeptide 1 [Source:HGNC Symbol;Acc:4384].

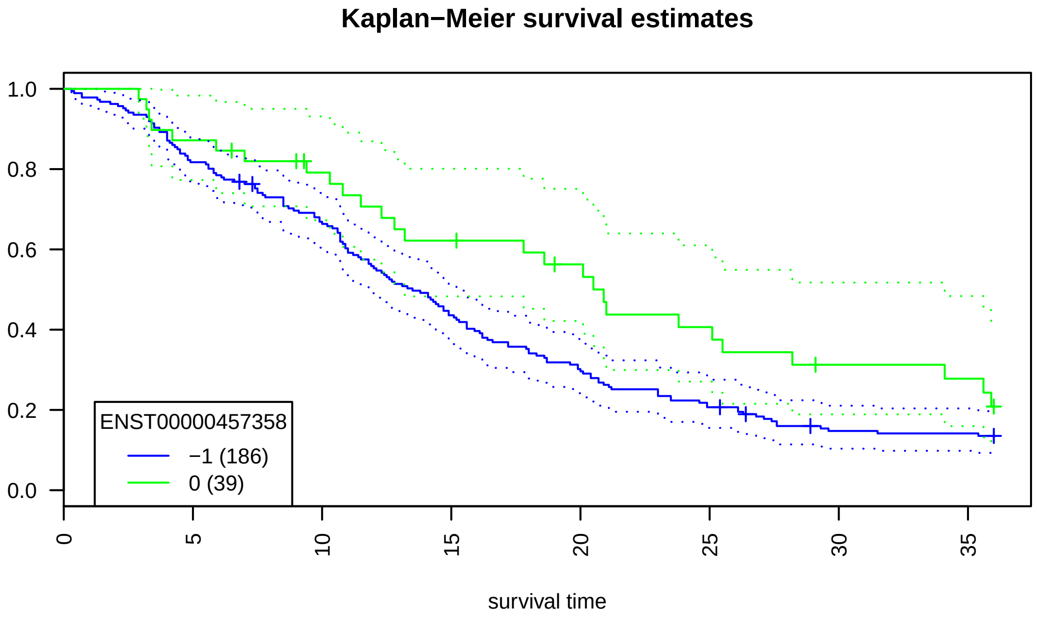

Figure 820: Each curve represents the relative number of ENST00000457358 survived to the corresponding point of time. Dashed lines represent 0.95 confidence intervals for the curves with the corresponding color. Total of 5 observations deleted due to missingness. Log-rank probability measure for the equality of these curves is 4.009749e-02. Additional annotations: *.GeneId*=ENSG00000127955, *.GeneName*=GNAI1, *.GeneDesc*=guanine nucleotide binding protein (G protein), alpha inhibiting activity polypeptide 1 [Source:HGNC Symbol;Acc:4384].

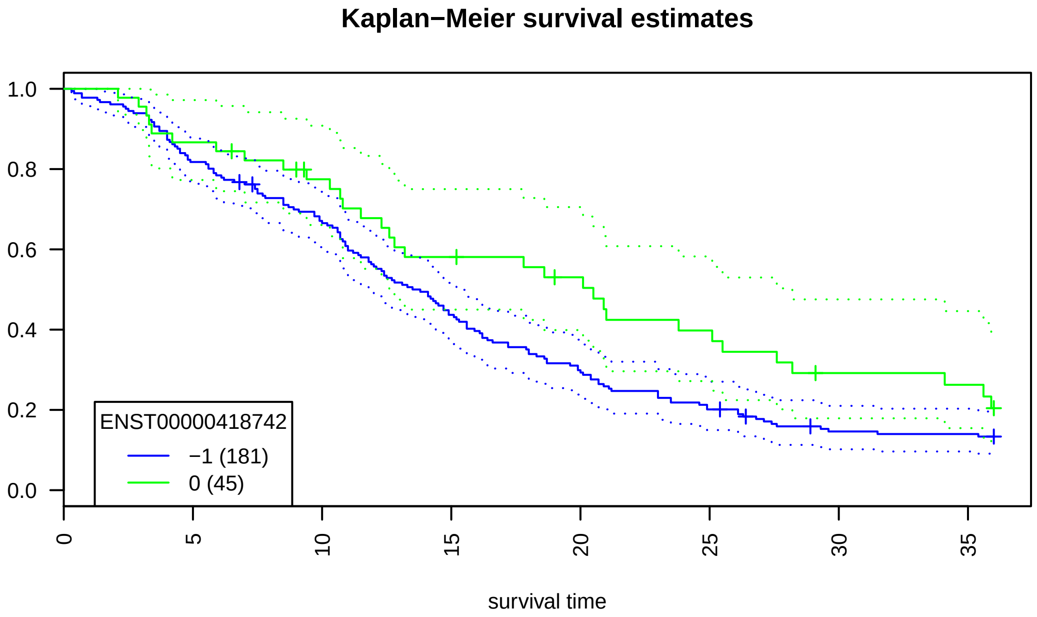

Figure 821: Each curve represents the relative number of ENST00000418742 survived to the corresponding point of time. Dashed lines represent 0.95 confidence intervals for the curves with the corresponding color. Total of 4 observations deleted due to missingness. Log-rank probability measure for the equality of these curves is 4.923584e-02. Additional annotations: *.GeneId*=ENSG00000127955, *.GeneName*=GNAI1, *.GeneDesc*=guanine nucleotide binding protein (G protein), alpha inhibiting activity polypeptide 1 [Source:HGNC Symbol;Acc:4384].

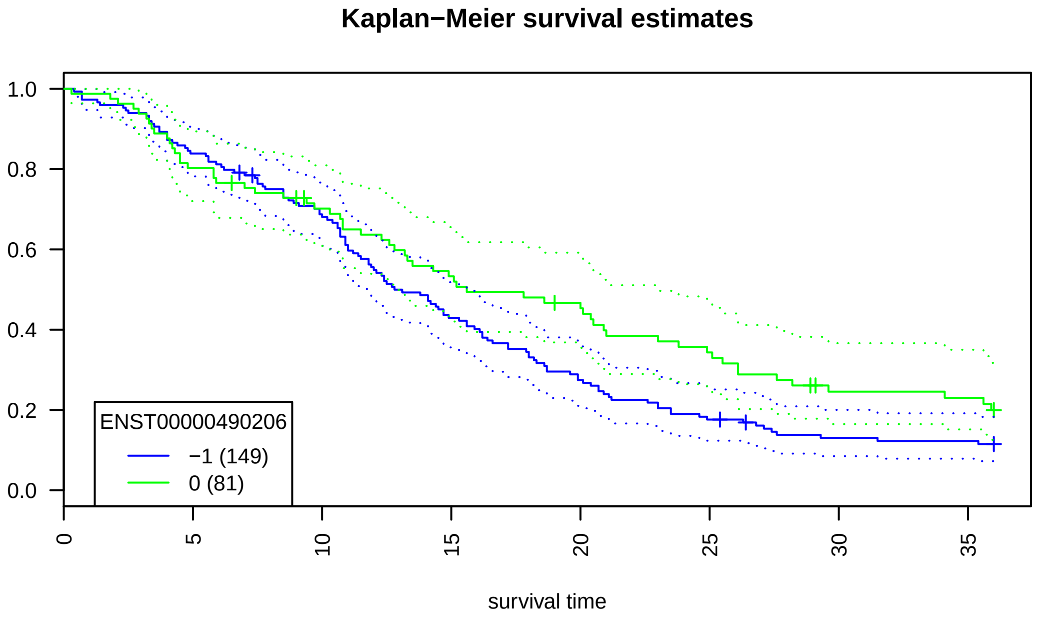

Figure 822: Each curve represents the relative number of ENST00000490206 survived to the corresponding point of time. Dashed lines represent 0.95 confidence intervals for the curves with the corresponding color. Log-rank probability measure for the equality of these curves is 4.466887e-02. Additional annotations: *.GeneId*=ENSG00000127955, *.GeneName*=GNAI1, *.GeneDesc*=guanine nucleotide binding protein (G protein), alpha inhibiting activity polypeptide 1 [Source:HGNC Symbol;Acc:4384].

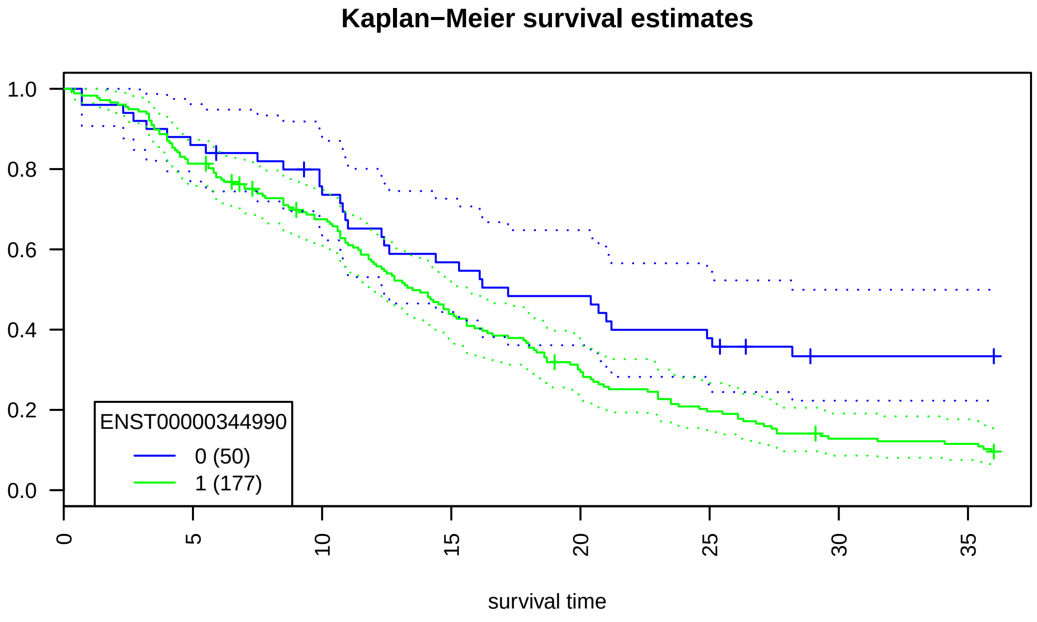

Figure 823: Each curve represents the relative number of ENST00000344990 survived to the corresponding point of time. Dashed lines represent 0.95 confidence intervals for the curves with the corresponding color. Total of 3 observations deleted due to missingness. Log-rank probability measure for the equality of these curves is 3.578693e-03. Additional annotations: *.GeneId*=ENSG00000126246, *.GeneName*=TMEM149, *.GeneDesc*=transmembrane protein 149 [Source:HGNC Symbol;Acc:23620].

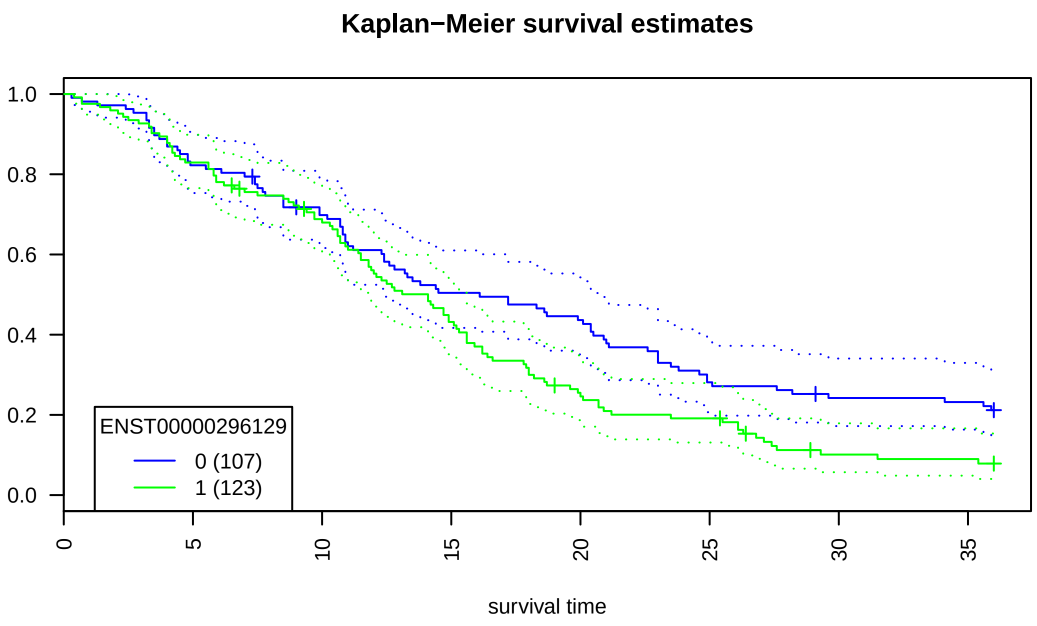

Figure 824: Each curve represents the relative number of ENST00000296129 survived to the corresponding point of time. Dashed lines represent 0.95 confidence intervals for the curves with the corresponding color. Log-rank probability measure for the equality of these curves is 1.238264e-02. Additional annotations: *.GeneId*=ENSG00000163814, *.GeneName*=CDCP1, *.GeneDesc*=CUB domain containing protein 1 [Source:HGNC Symbol;Acc:24357].

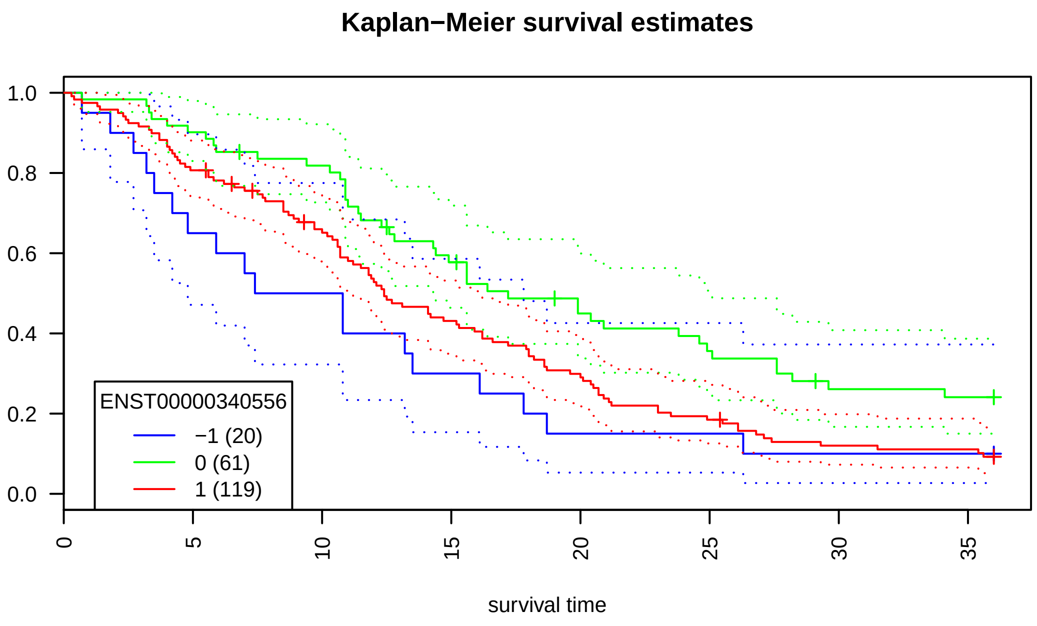

Figure 825: Each curve represents the relative number of ENST00000340556 survived to the corresponding point of time. Dashed lines represent 0.95 confidence intervals for the curves with the corresponding color. Total of 30 observations deleted due to missingness. Log-rank probability measure for the equality of these curves is 5.238196e-03. Additional annotations: *.GeneId*=ENSG00000188010, *.GeneName*=MORN2, *.GeneDesc*=MORN repeat containing 2 [Source:HGNC Symbol;Acc:30166].

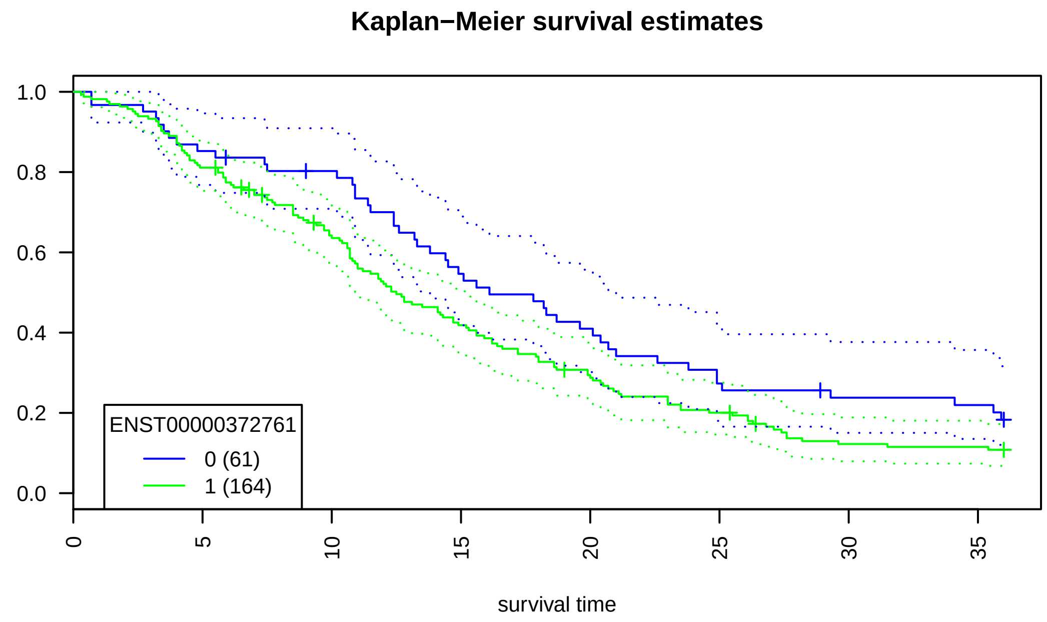

Figure 826: Each curve represents the relative number of ENST00000372761 survived to the corresponding point of time. Dashed lines represent 0.95 confidence intervals for the curves with the corresponding color. Total of 5 observations deleted due to missingness. Log-rank probability measure for the equality of these curves is 4.767973e-02. Additional annotations: *.GeneId*=ENSG00000122861, *.GeneName*=PLAU, *.GeneDesc*=plasminogen activator, urokinase [Source:HGNC Symbol;Acc:9052].

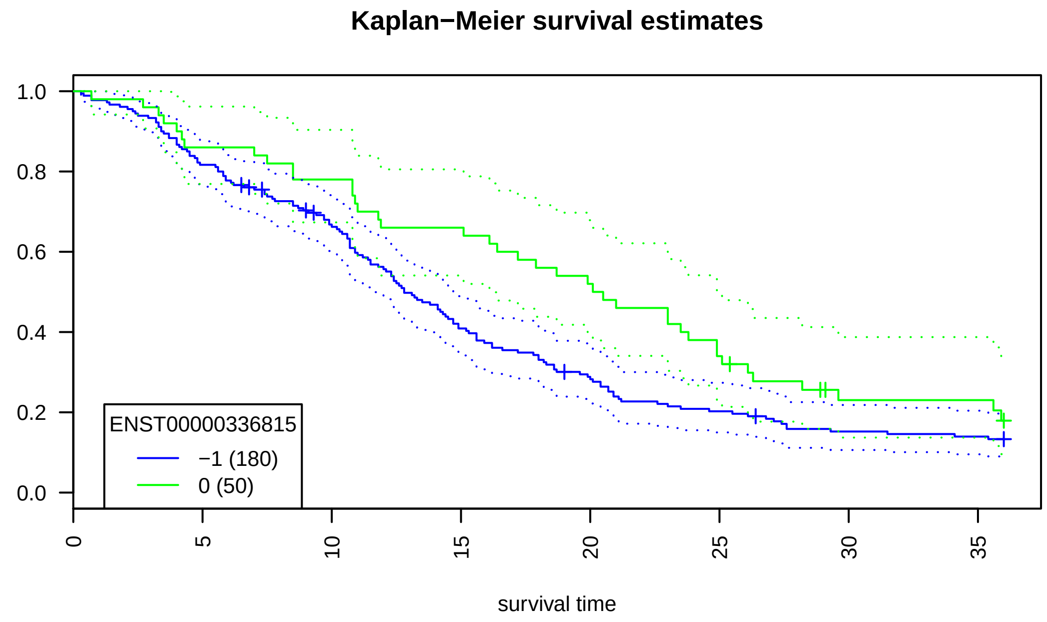

Figure 827: Each curve represents the relative number of ENST00000336815 survived to the corresponding point of time. Dashed lines represent 0.95 confidence intervals for the curves with the corresponding color. Log-rank probability measure for the equality of these curves is 3.329249e-02. Additional annotations: *.GeneId*=ENSG00000180881, *.GeneName*=CAPS2, *.GeneDesc*=calcyphosine 2 [Source:HGNC Symbol;Acc:16471].

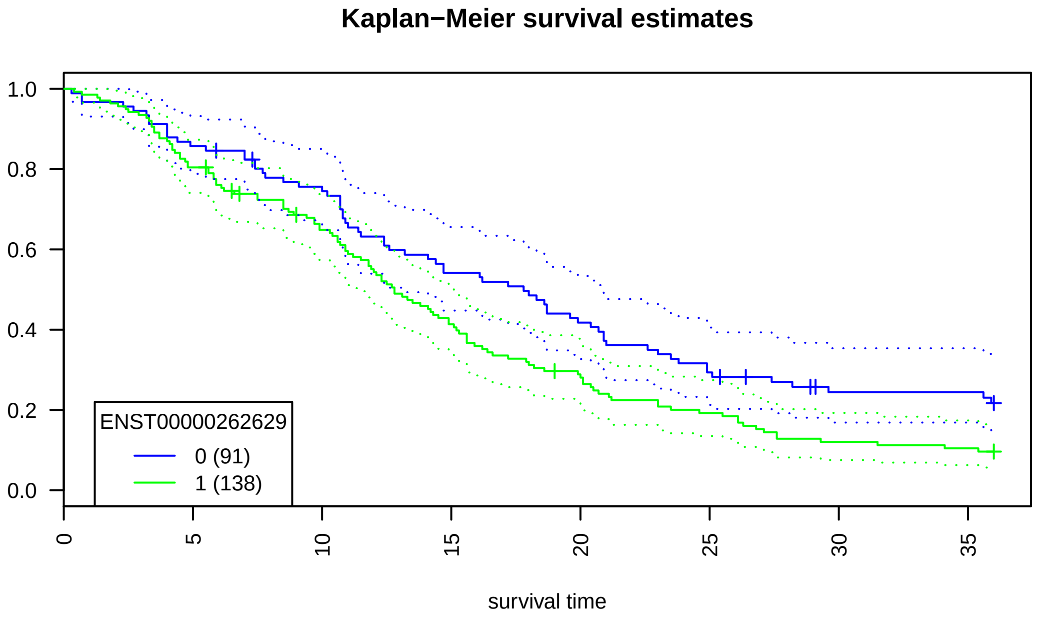

Figure 828: Each curve represents the relative number of ENST00000262629 survived to the corresponding point of time. Dashed lines represent 0.95 confidence intervals for the curves with the corresponding color. Total of 1 observation deleted due to missingness. Log-rank probability measure for the equality of these curves is 9.34209e-03. Additional annotations: *.GeneId*=ENSG00000011600, *.GeneName*=TYROBP, *.GeneDesc*=TYRO protein tyrosine kinase binding protein [Source:HGNC Symbol;Acc:12449].

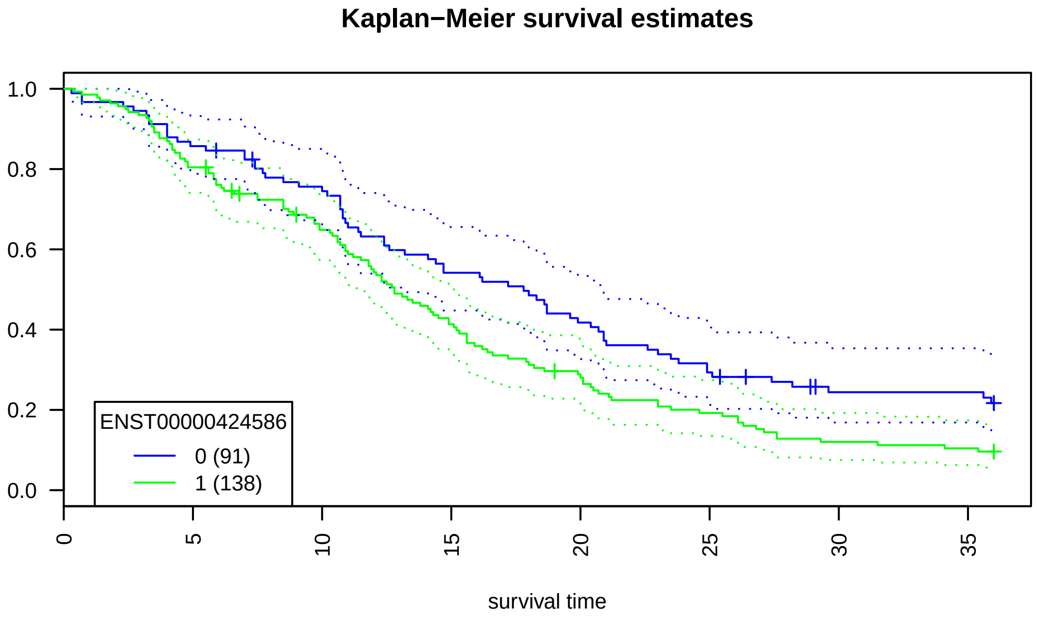

Figure 829: Each curve represents the relative number of ENST00000424586 survived to the corresponding point of time. Dashed lines represent 0.95 confidence intervals for the curves with the corresponding color. Total of 1 observation deleted due to missingness. Log-rank probability measure for the equality of these curves is 9.34209e-03. Additional annotations: *.GeneId*=ENSG00000011600, *.GeneName*=TYROBP, *.GeneDesc*=TYRO protein tyrosine kinase binding protein [Source:HGNC Symbol;Acc:12449].

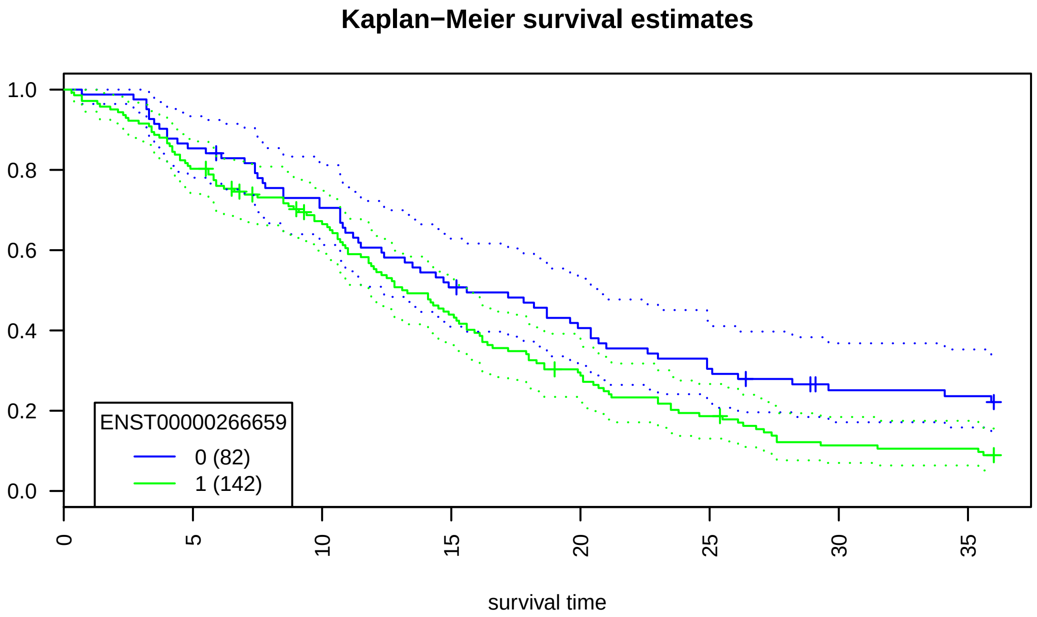

Figure 830: Each curve represents the relative number of ENST00000266659 survived to the corresponding point of time. Dashed lines represent 0.95 confidence intervals for the curves with the corresponding color. Total of 6 observations deleted due to missingness. Log-rank probability measure for the equality of these curves is 1.810272e-02. Additional annotations: *.GeneId*=ENSG00000139278, *.GeneName*=GLIPR1, *.GeneDesc*=GLI pathogenesis-related 1 [Source:HGNC Symbol;Acc:17001].

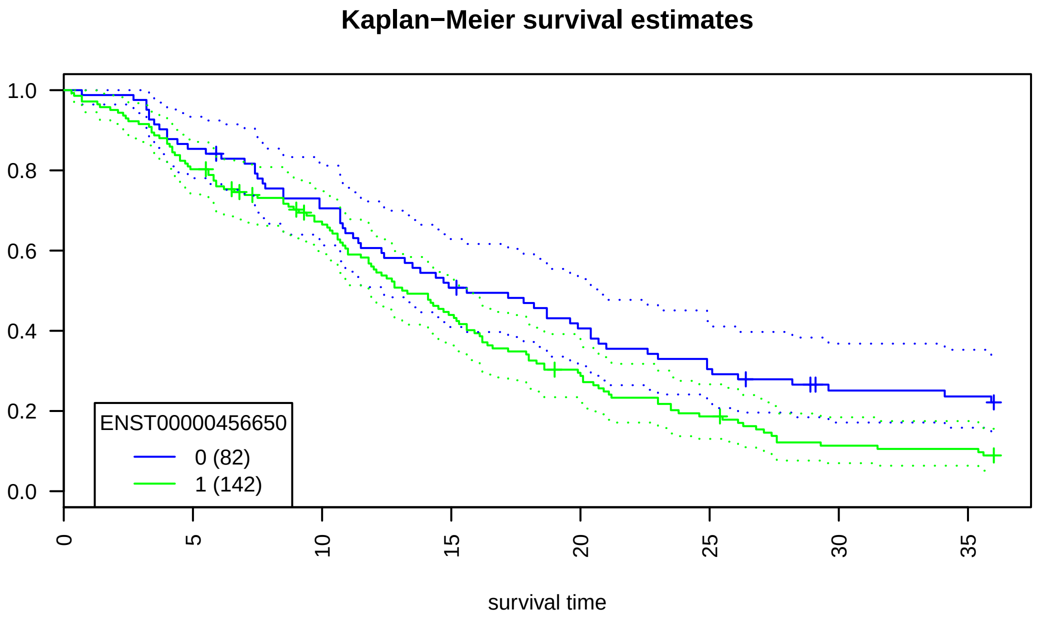

Figure 831: Each curve represents the relative number of ENST00000456650 survived to the corresponding point of time. Dashed lines represent 0.95 confidence intervals for the curves with the corresponding color. Total of 6 observations deleted due to missingness. Log-rank probability measure for the equality of these curves is 1.810272e-02. Additional annotations: *.GeneId*=ENSG00000139278, *.GeneName*=GLIPR1, *.GeneDesc*=GLI pathogenesis-related 1 [Source:HGNC Symbol;Acc:17001].

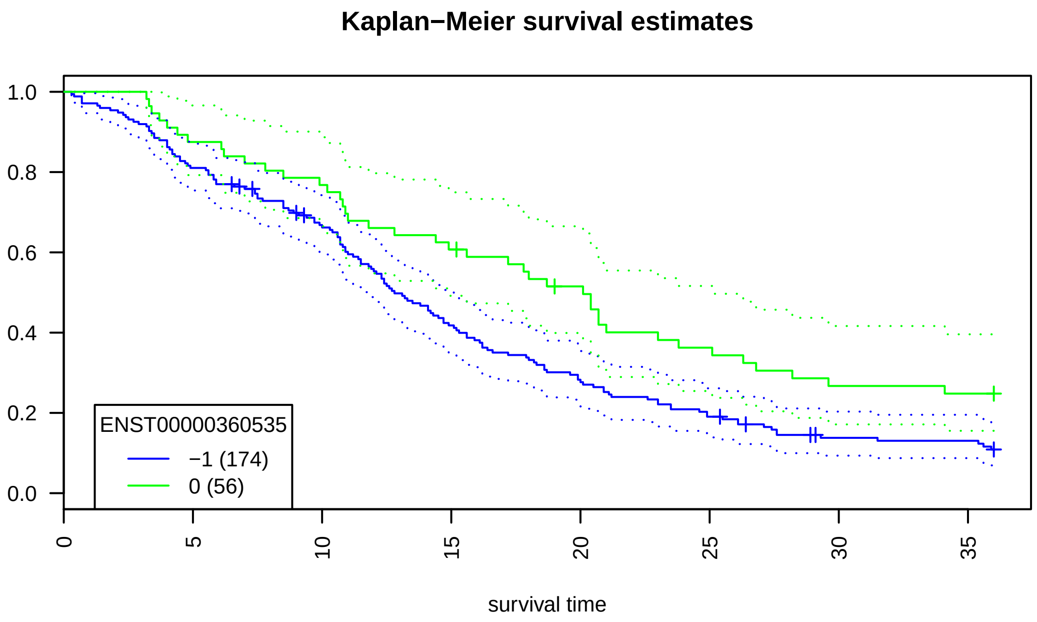

Figure 832: Each curve represents the relative number of ENST00000360535 survived to the corresponding point of time. Dashed lines represent 0.95 confidence intervals for the curves with the corresponding color. Log-rank probability measure for the equality of these curves is 6.086192e-03. Additional annotations: *.GeneId*=ENSG00000105270, *.GeneName*=CLIP3, *.GeneDesc*=CAP-GLY domain containing linker protein 3 [Source:HGNC Symbol;Acc:24314].

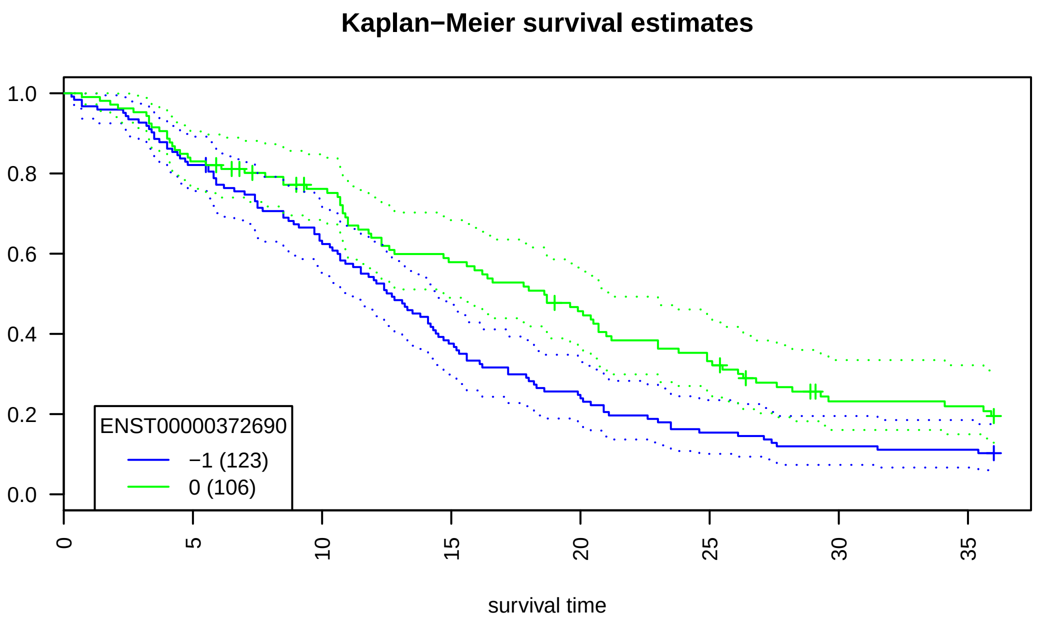

Figure 833: Each curve represents the relative number of ENST00000372690 survived to the corresponding point of time. Dashed lines represent 0.95 confidence intervals for the curves with the corresponding color. Total of 1 observation deleted due to missingness. Log-rank probability measure for the equality of these curves is 2.819953e-03. Additional annotations: *.GeneId*=ENSG00000156671, *.GeneName*=SAMD8, *.GeneDesc*=sterile alpha motif domain containing 8 [Source:HGNC Symbol;Acc:26320].

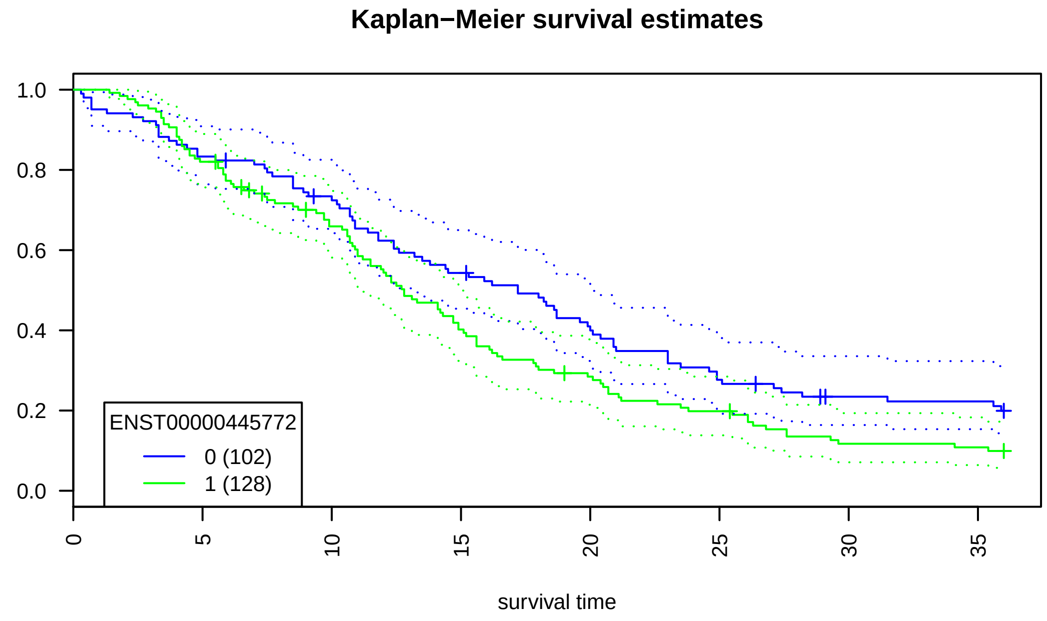

Figure 834: Each curve represents the relative number of ENST00000445772 survived to the corresponding point of time. Dashed lines represent 0.95 confidence intervals for the curves with the corresponding color. Log-rank probability measure for the equality of these curves is 2.681793e-02. Additional annotations: *.GeneId*=ENSG00000160791, *.GeneName*=CCR5, *.GeneDesc*=chemokine (C-C motif) receptor 5 [Source:HGNC Symbol;Acc:1606].

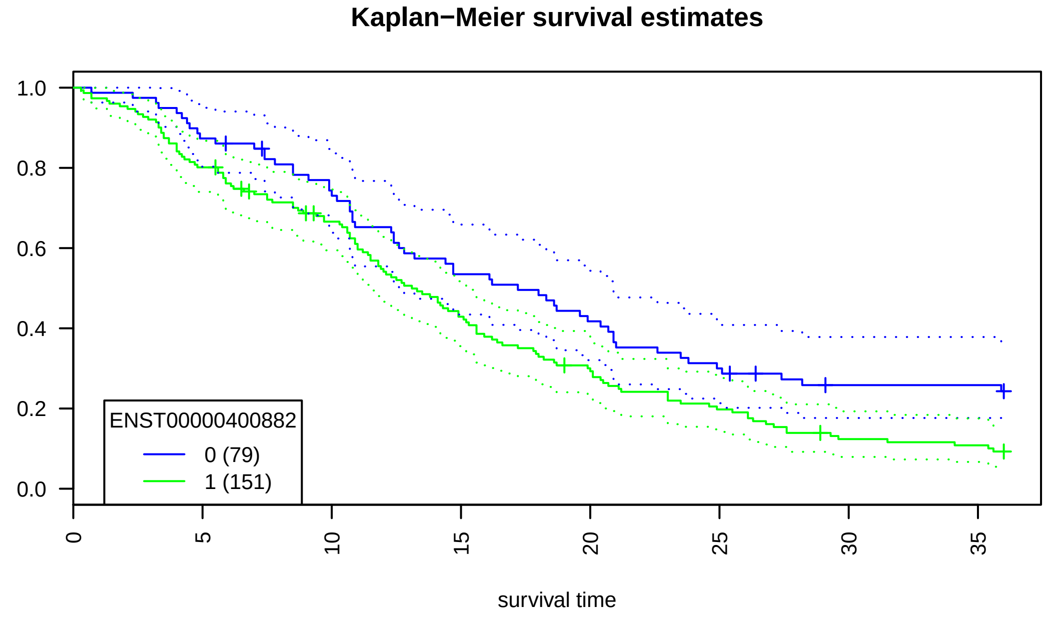

Figure 835: Each curve represents the relative number of ENST00000400882 survived to the corresponding point of time. Dashed lines represent 0.95 confidence intervals for the curves with the corresponding color. Log-rank probability measure for the equality of these curves is 8.420114e-03. Additional annotations: *.GeneId*=ENSG00000121797, *.GeneName*=CCRL2, *.GeneDesc*=chemokine (C-C motif) receptor-like 2 [Source:HGNC Symbol;Acc:1612].

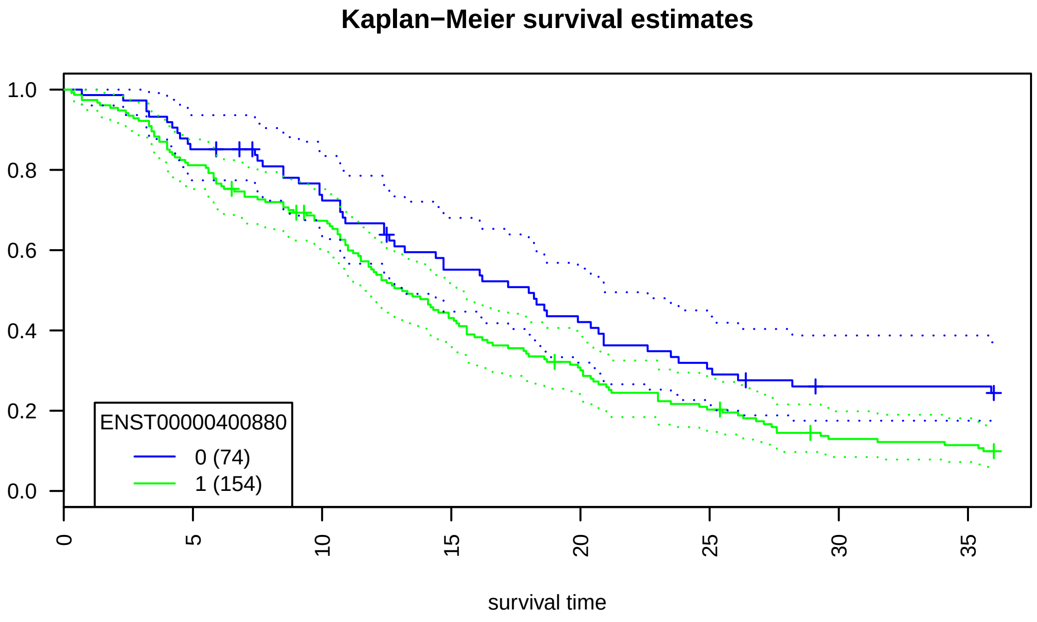

Figure 836: Each curve represents the relative number of ENST00000400880 survived to the corresponding point of time. Dashed lines represent 0.95 confidence intervals for the curves with the corresponding color. Total of 2 observations deleted due to missingness. Log-rank probability measure for the equality of these curves is 1.219415e-02. Additional annotations: *.GeneId*=ENSG00000121797, *.GeneName*=CCRL2, *.GeneDesc*=chemokine (C-C motif) receptor-like 2 [Source:HGNC Symbol;Acc:1612].

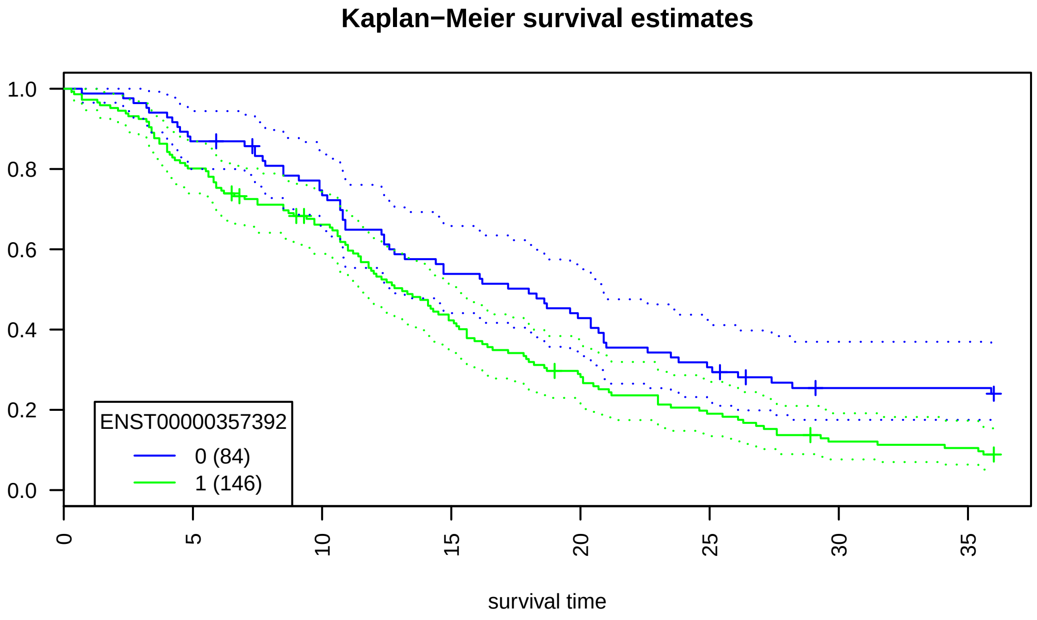

Figure 837: Each curve represents the relative number of ENST00000357392 survived to the corresponding point of time. Dashed lines represent 0.95 confidence intervals for the curves with the corresponding color. Log-rank probability measure for the equality of these curves is 5.290474e-03. Additional annotations: *.GeneId*=ENSG00000121797, *.GeneName*=CCRL2, *.GeneDesc*=chemokine (C-C motif) receptor-like 2 [Source:HGNC Symbol;Acc:1612].

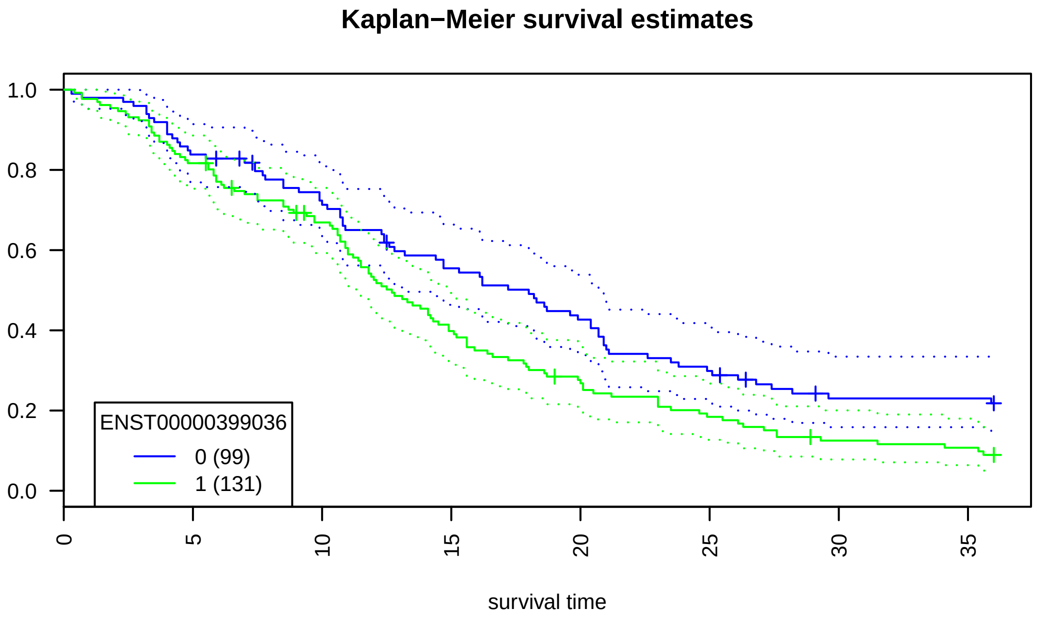

Figure 838: Each curve represents the relative number of ENST00000399036 survived to the corresponding point of time. Dashed lines represent 0.95 confidence intervals for the curves with the corresponding color. Log-rank probability measure for the equality of these curves is 7.862744e-03. Additional annotations: *.GeneId*=ENSG00000121797, *.GeneName*=CCRL2, *.GeneDesc*=chemokine (C-C motif) receptor-like 2 [Source:HGNC Symbol;Acc:1612].

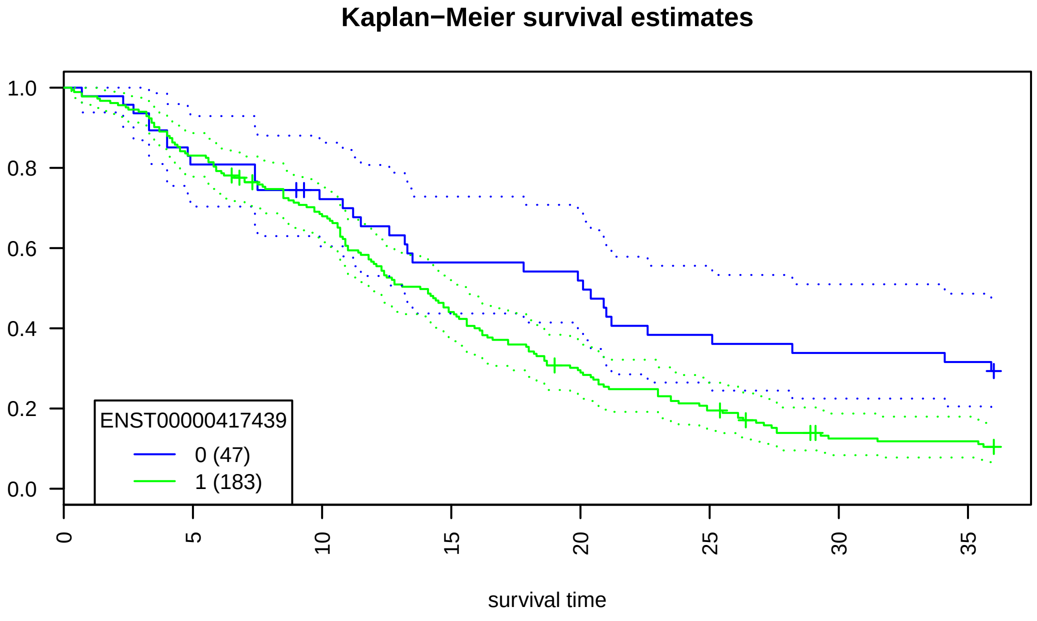

Figure 839: Each curve represents the relative number of ENST00000417439 survived to the corresponding point of time. Dashed lines represent 0.95 confidence intervals for the curves with the corresponding color. Log-rank probability measure for the equality of these curves is 7.412629e-03. Additional annotations: *.GeneId*=ENSG00000012223, *.GeneName*=LTF, *.GeneDesc*=lactotransferrin [Source:HGNC Symbol;Acc:6720].

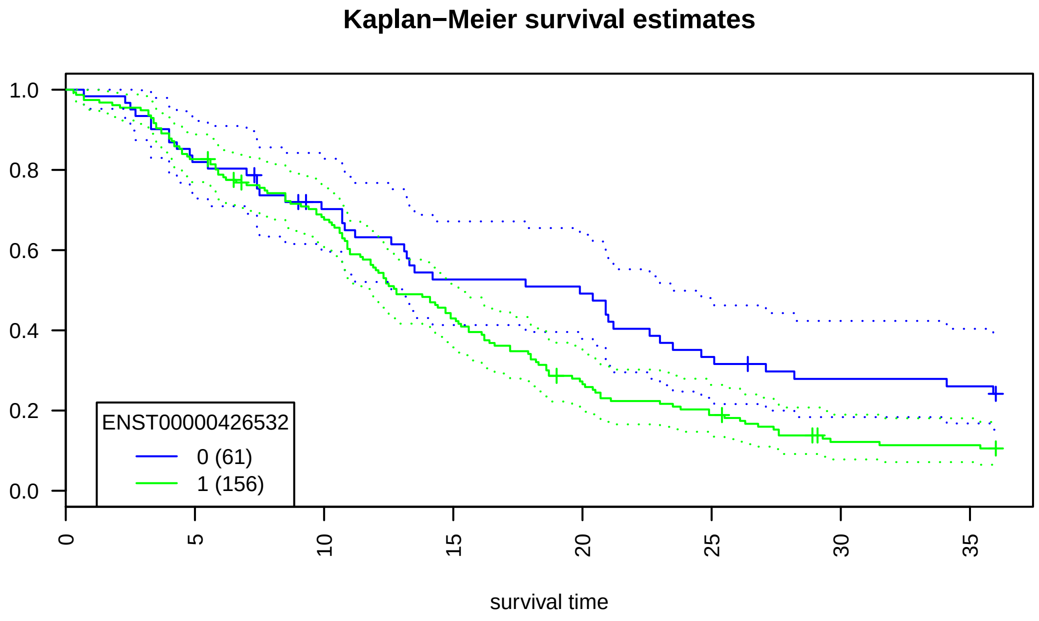

Figure 840: Each curve represents the relative number of ENST00000426532 survived to the corresponding point of time. Dashed lines represent 0.95 confidence intervals for the curves with the corresponding color. Total of 13 observations deleted due to missingness. Log-rank probability measure for the equality of these curves is 1.895262e-02. Additional annotations: *.GeneId*=ENSG00000012223, *.GeneName*=LTF, *.GeneDesc*=lactotransferrin [Source:HGNC Symbol;Acc:6720].

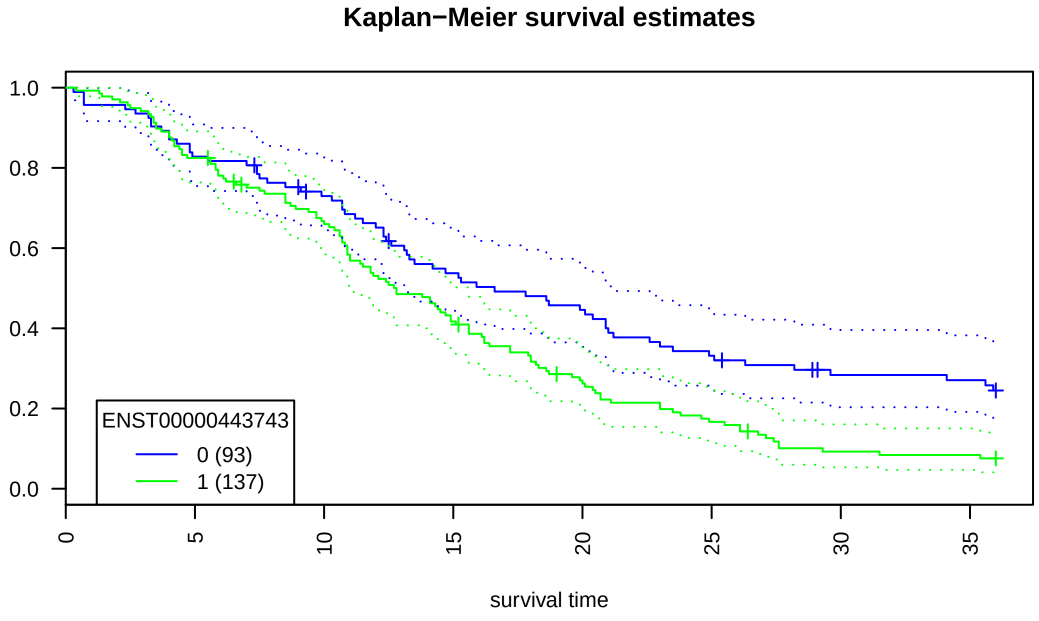

Figure 841: Each curve represents the relative number of ENST00000443743 survived to the corresponding point of time. Dashed lines represent 0.95 confidence intervals for the curves with the corresponding color. Log-rank probability measure for the equality of these curves is 1.573143e-03. Additional annotations: *.GeneId*=ENSG00000012223, *.GeneName*=LTF, *.GeneDesc*=lactotransferrin [Source:HGNC Symbol;Acc:6720].

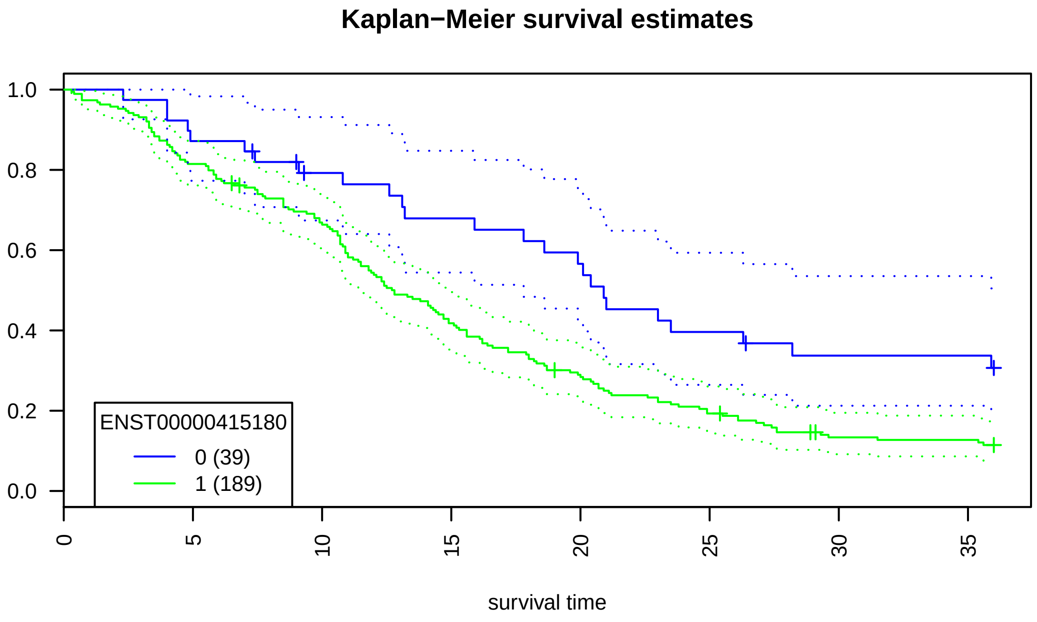

Figure 842: Each curve represents the relative number of ENST00000415180 survived to the corresponding point of time. Dashed lines represent 0.95 confidence intervals for the curves with the corresponding color. Total of 2 observations deleted due to missingness. Log-rank probability measure for the equality of these curves is 2.633544e-03. Additional annotations: *.GeneId*=ENSG00000012223, *.GeneName*=LTF, *.GeneDesc*=lactotransferrin [Source:HGNC Symbol;Acc:6720].

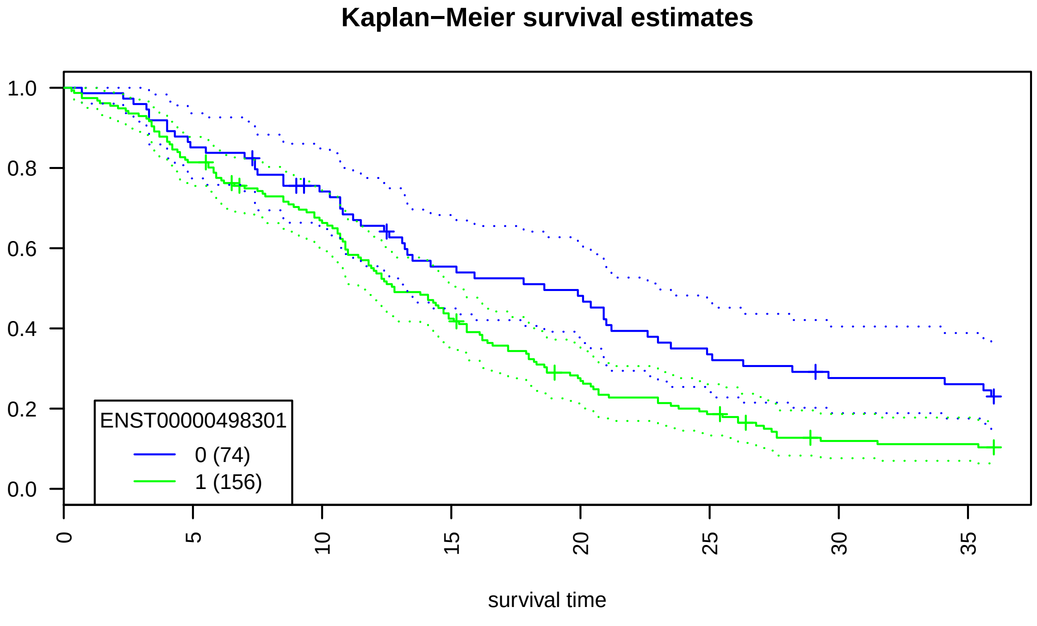

Figure 843: Each curve represents the relative number of ENST00000498301 survived to the corresponding point of time. Dashed lines represent 0.95 confidence intervals for the curves with the corresponding color. Log-rank probability measure for the equality of these curves is 7.065765e-03. Additional annotations: *.GeneId*=ENSG00000012223, *.GeneName*=LTF, *.GeneDesc*=lactotransferrin [Source:HGNC Symbol;Acc:6720].

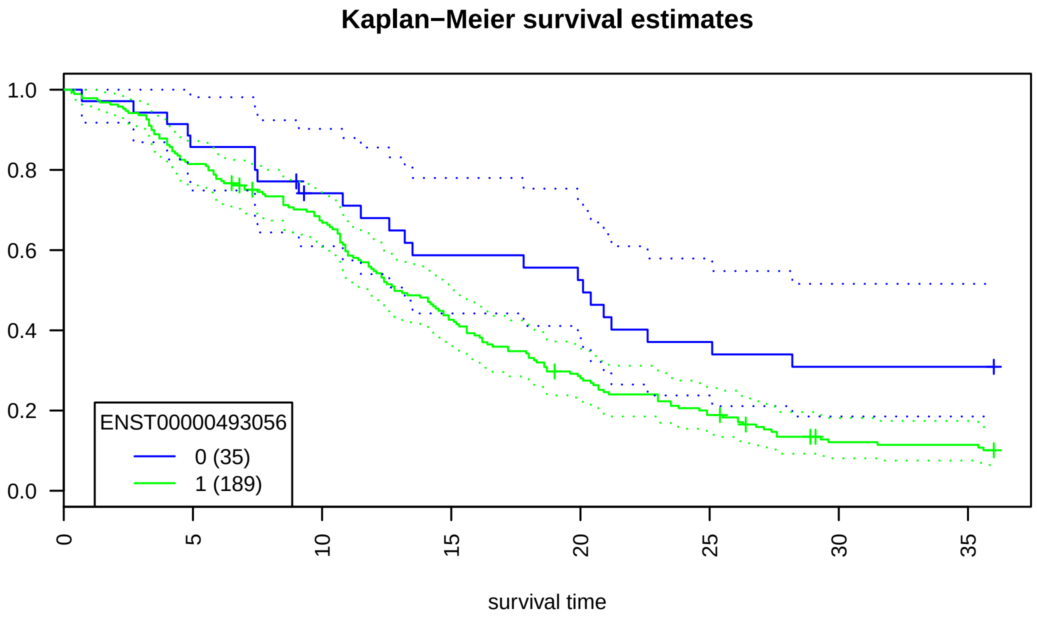

Figure 844: Each curve represents the relative number of ENST00000493056 survived to the corresponding point of time. Dashed lines represent 0.95 confidence intervals for the curves with the corresponding color. Total of 6 observations deleted due to missingness. Log-rank probability measure for the equality of these curves is 9.463864e-03. Additional annotations: *.GeneId*=ENSG00000012223, *.GeneName*=LTF, *.GeneDesc*=lactotransferrin [Source:HGNC Symbol;Acc:6720].

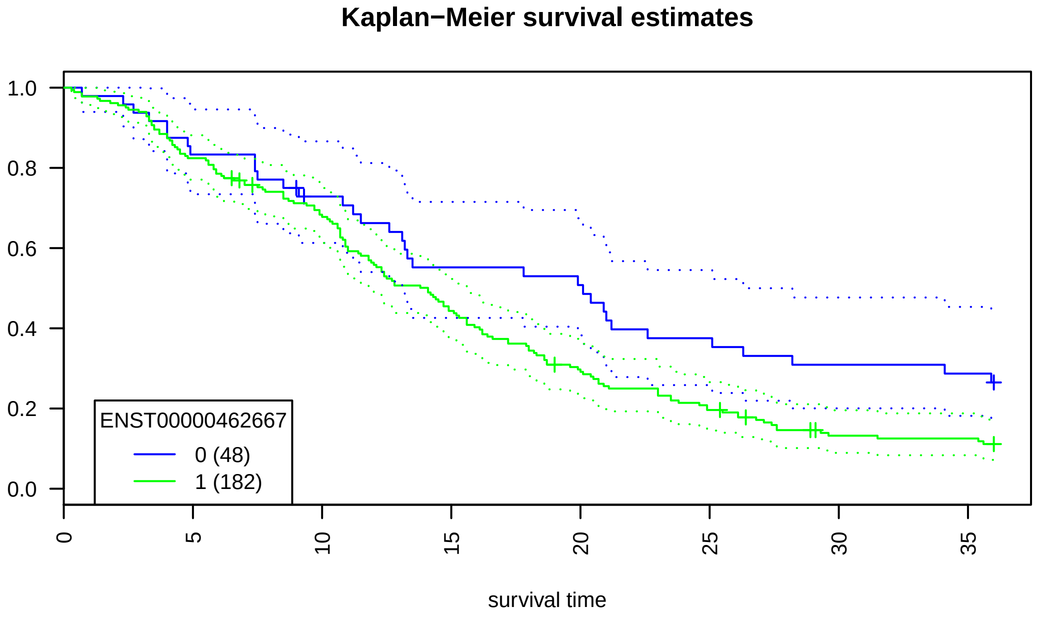

Figure 845: Each curve represents the relative number of ENST00000462667 survived to the corresponding point of time. Dashed lines represent 0.95 confidence intervals for the curves with the corresponding color. Log-rank probability measure for the equality of these curves is 1.629459e-02. Additional annotations: *.GeneId*=ENSG00000012223, *.GeneName*=LTF, *.GeneDesc*=lactotransferrin [Source:HGNC Symbol;Acc:6720].

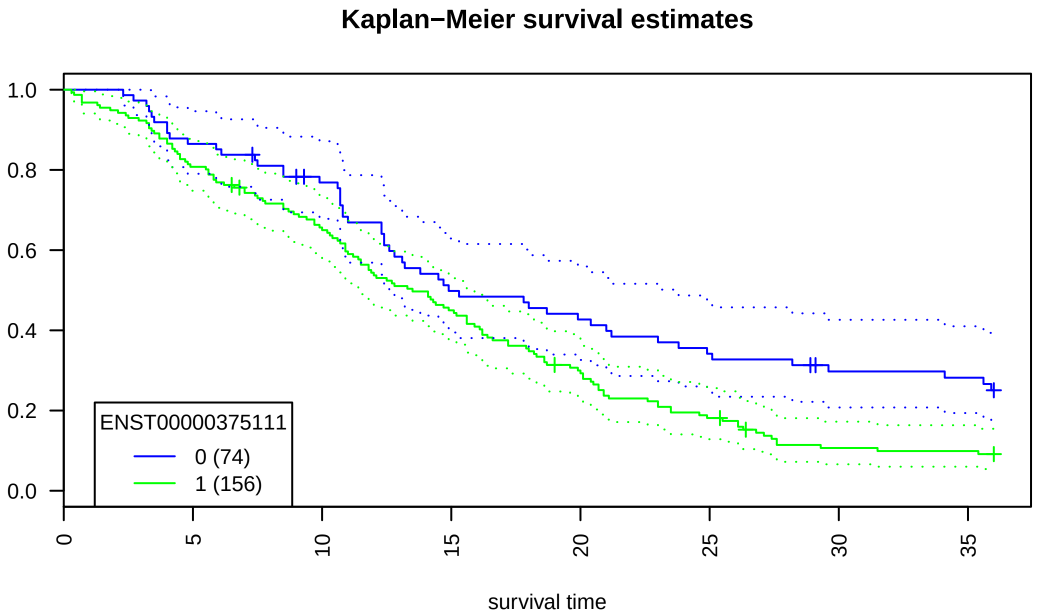

Figure 846: Each curve represents the relative number of ENST00000375111 survived to the corresponding point of time. Dashed lines represent 0.95 confidence intervals for the curves with the corresponding color. Log-rank probability measure for the equality of these curves is 4.238378e-03. Additional annotations: *.GeneId*=ENSG00000188257, *.GeneName*=PLA2G2A, *.GeneDesc*=phospholipase A2, group IIA (platelets, synovial fluid) [Source:HGNC Symbol;Acc:9031].

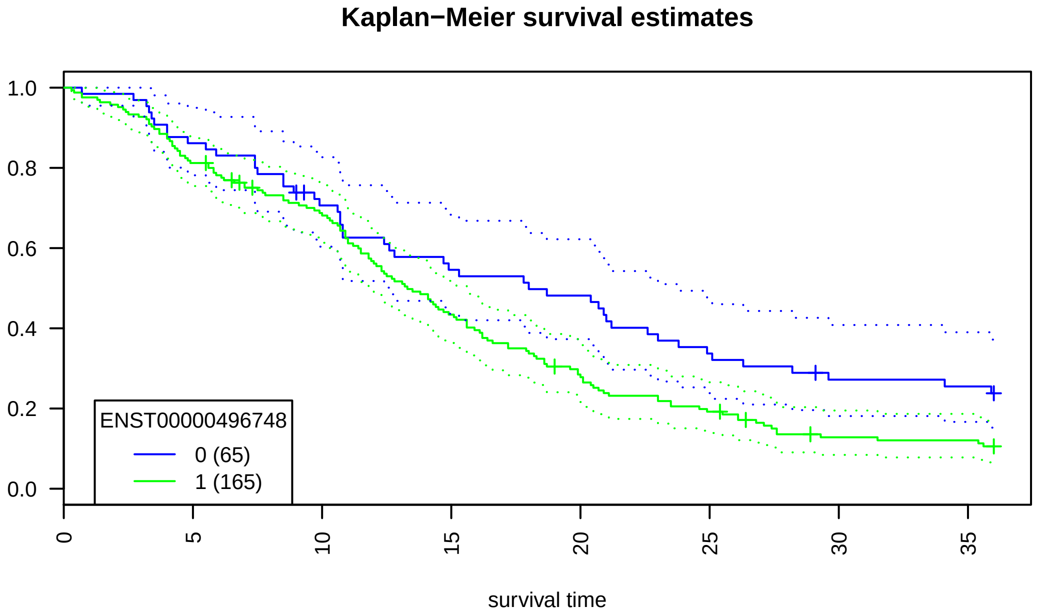

Figure 847: Each curve represents the relative number of ENST00000496748 survived to the corresponding point of time. Dashed lines represent 0.95 confidence intervals for the curves with the corresponding color. Log-rank probability measure for the equality of these curves is 1.402852e-02. Additional annotations: *.GeneId*=ENSG00000188257, *.GeneName*=PLA2G2A, *.GeneDesc*=phospholipase A2, group IIA (platelets, synovial fluid) [Source:HGNC Symbol;Acc:9031].

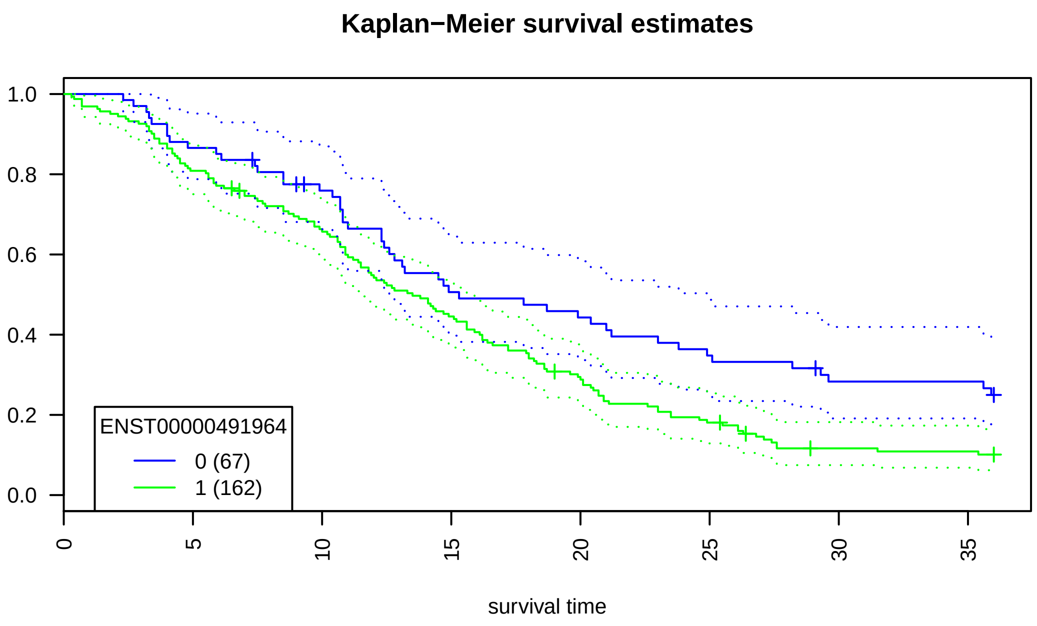

Figure 848: Each curve represents the relative number of ENST00000491964 survived to the corresponding point of time. Dashed lines represent 0.95 confidence intervals for the curves with the corresponding color. Total of 1 observation deleted due to missingness. Log-rank probability measure for the equality of these curves is 6.567946e-03. Additional annotations: *.GeneId*=ENSG00000188257, *.GeneName*=PLA2G2A, *.GeneDesc*=phospholipase A2, group IIA (platelets, synovial fluid) [Source:HGNC Symbol;Acc:9031].

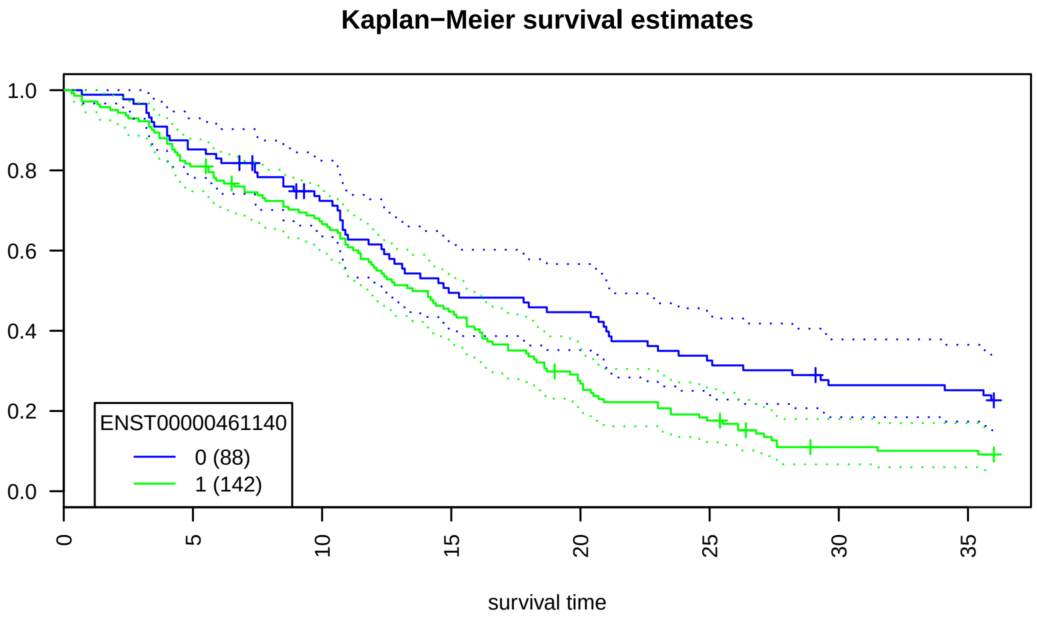

Figure 849: Each curve represents the relative number of ENST00000461140 survived to the corresponding point of time. Dashed lines represent 0.95 confidence intervals for the curves with the corresponding color. Log-rank probability measure for the equality of these curves is 8.962702e-03. Additional annotations: *.GeneId*=ENSG00000188257, *.GeneName*=PLA2G2A, *.GeneDesc*=phospholipase A2, group IIA (platelets, synovial fluid) [Source:HGNC Symbol;Acc:9031].

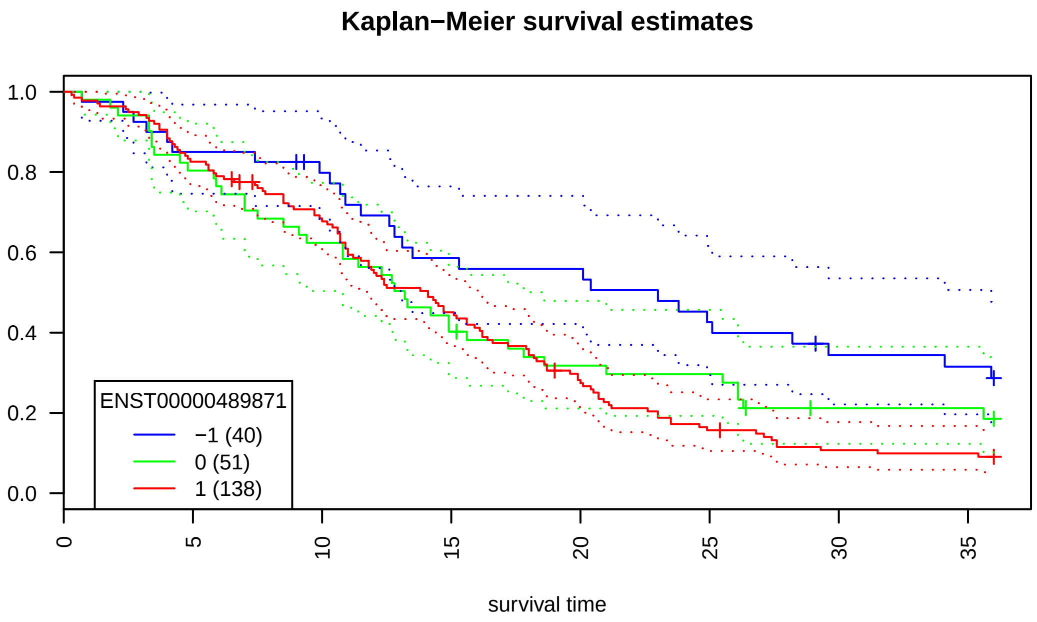

Figure 850: Each curve represents the relative number of ENST00000489871 survived to the corresponding point of time. Dashed lines represent 0.95 confidence intervals for the curves with the corresponding color. Total of 1 observation deleted due to missingness. Log-rank probability measure for the equality of these curves is 1.352089e-02. Additional annotations: *.GeneId*=ENSG00000127472, *.GeneName*=PLA2G5, *.GeneDesc*=phospholipase A2, group V [Source:HGNC Symbol;Acc:9038].

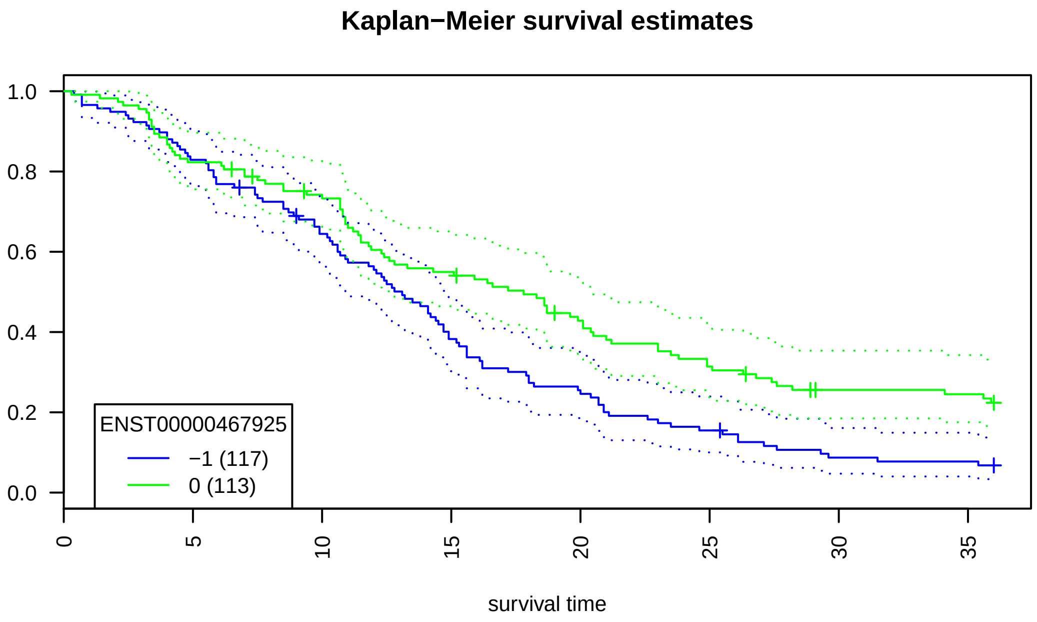

Figure 851: Each curve represents the relative number of ENST00000467925 survived to the corresponding point of time. Dashed lines represent 0.95 confidence intervals for the curves with the corresponding color. Log-rank probability measure for the equality of these curves is 1.114026e-03. Additional annotations: *.GeneId*=ENSG00000057935, *.GeneName*=MTA3, *.GeneDesc*=metastasis associated 1 family, member 3 [Source:HGNC Symbol;Acc:23784].

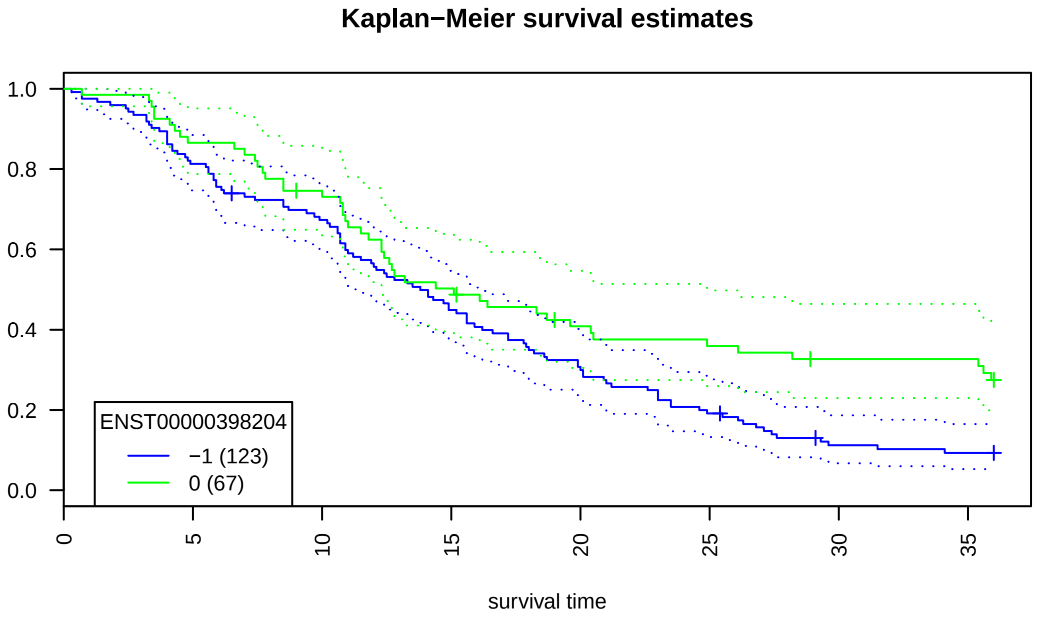

Figure 852: Each curve represents the relative number of ENST00000398204 survived to the corresponding point of time. Dashed lines represent 0.95 confidence intervals for the curves with the corresponding color. Total of 40 observations deleted due to missingness. Log-rank probability measure for the equality of these curves is 9.699394e-03. Additional annotations: *.GeneId*=ENSG00000008277, *.GeneName*=ADAM22, *.GeneDesc*=ADAM metallopeptidase domain 22 [Source:HGNC Symbol;Acc:201].

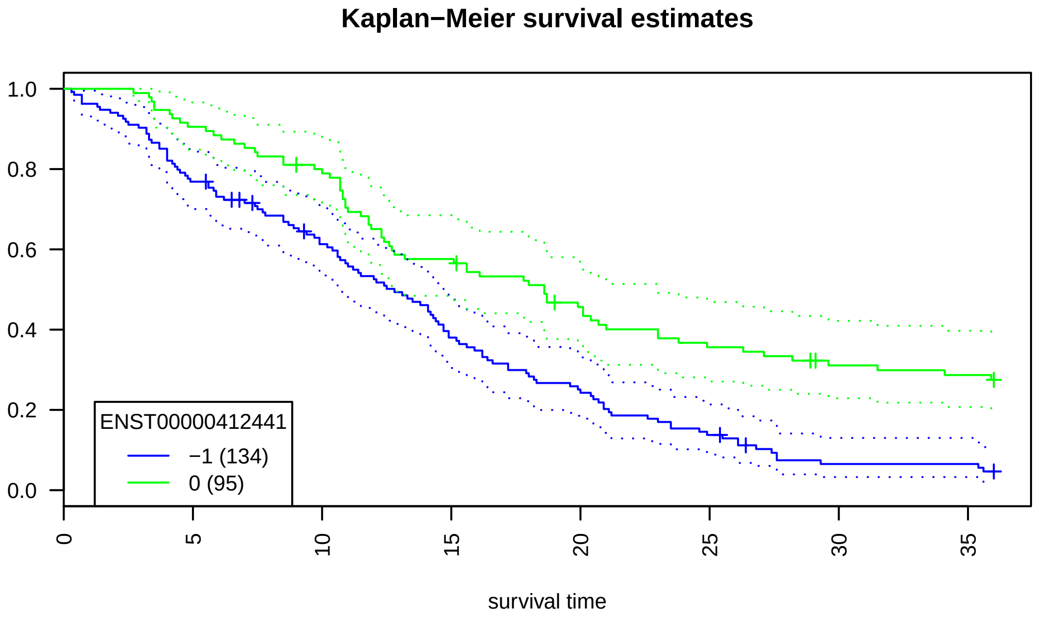

Figure 853: Each curve represents the relative number of ENST00000412441 survived to the corresponding point of time. Dashed lines represent 0.95 confidence intervals for the curves with the corresponding color. Total of 1 observation deleted due to missingness. Log-rank probability measure for the equality of these curves is 4.560019e-06. Additional annotations: *.GeneId*=ENSG00000008277, *.GeneName*=ADAM22, *.GeneDesc*=ADAM metallopeptidase domain 22 [Source:HGNC Symbol;Acc:201].

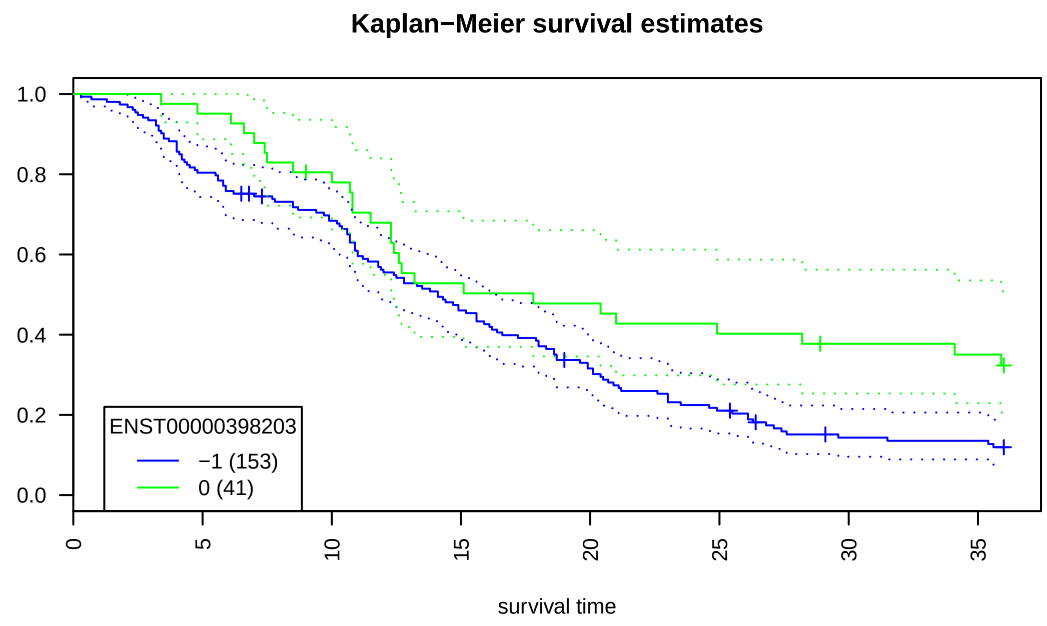

Figure 854: Each curve represents the relative number of ENST00000398203 survived to the corresponding point of time. Dashed lines represent 0.95 confidence intervals for the curves with the corresponding color. Total of 36 observations deleted due to missingness. Log-rank probability measure for the equality of these curves is 9.15915e-03. Additional annotations: *.GeneId*=ENSG00000008277, *.GeneName*=ADAM22, *.GeneDesc*=ADAM metallopeptidase domain 22 [Source:HGNC Symbol;Acc:201].

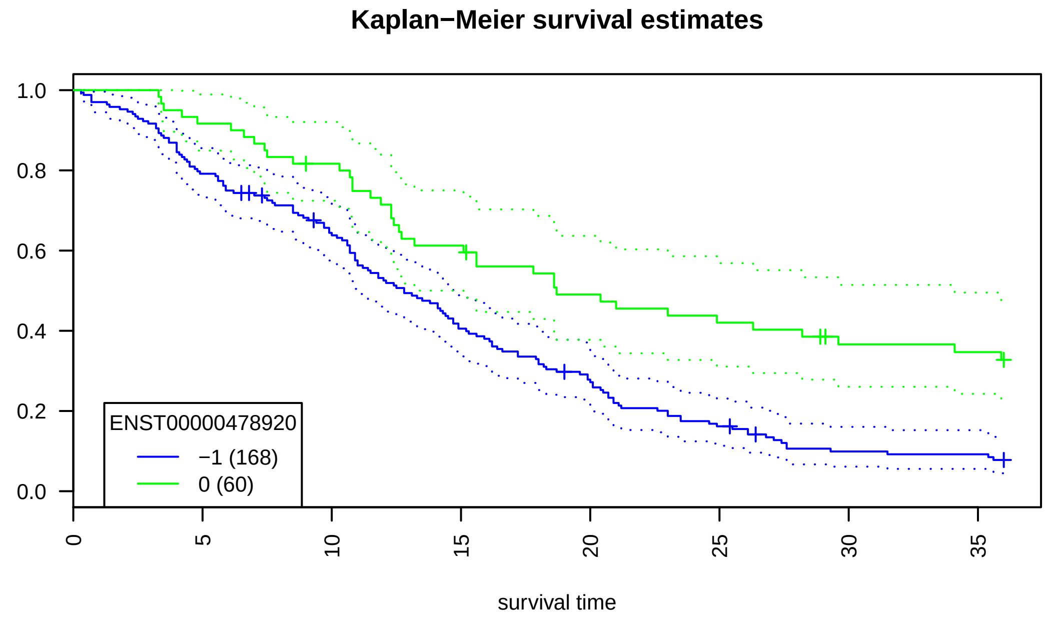

Figure 855: Each curve represents the relative number of ENST00000478920 survived to the corresponding point of time. Dashed lines represent 0.95 confidence intervals for the curves with the corresponding color. Total of 2 observations deleted due to missingness. Log-rank probability measure for the equality of these curves is 2.756643e-05. Additional annotations: *.GeneId*=ENSG00000008277, *.GeneName*=ADAM22, *.GeneDesc*=ADAM metallopeptidase domain 22 [Source:HGNC Symbol;Acc:201].

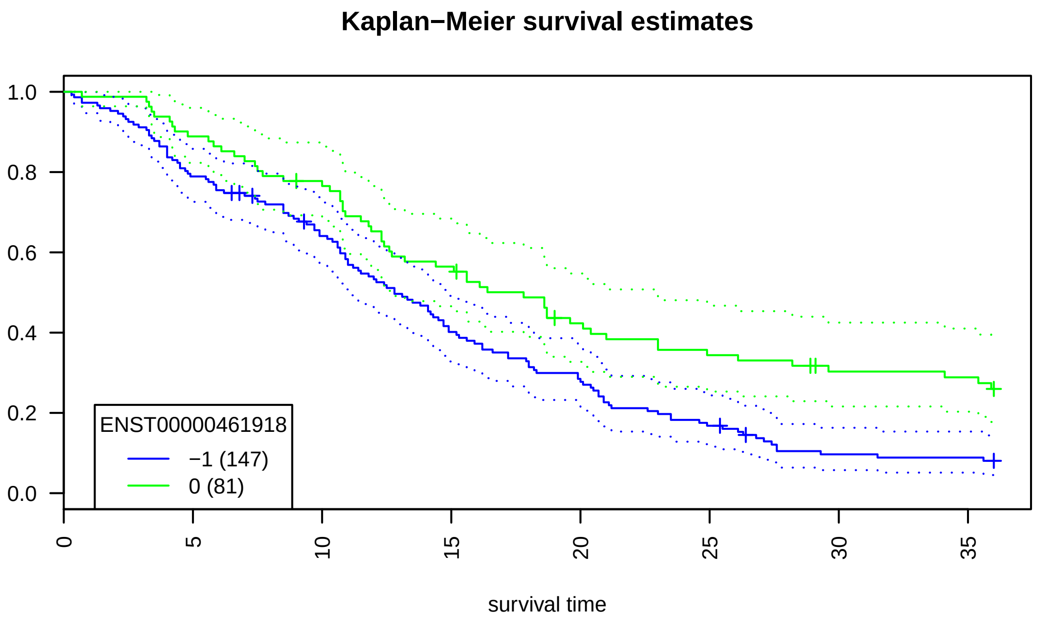

Figure 856: Each curve represents the relative number of ENST00000461918 survived to the corresponding point of time. Dashed lines represent 0.95 confidence intervals for the curves with the corresponding color. Total of 2 observations deleted due to missingness. Log-rank probability measure for the equality of these curves is 7.139474e-04. Additional annotations: *.GeneId*=ENSG00000008277, *.GeneName*=ADAM22, *.GeneDesc*=ADAM metallopeptidase domain 22 [Source:HGNC Symbol;Acc:201].

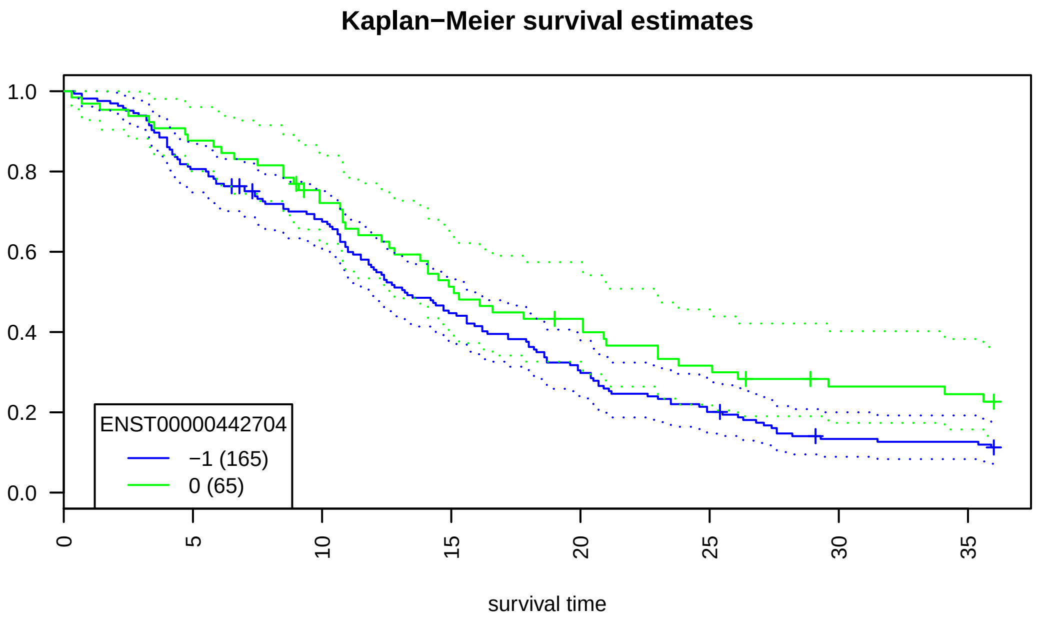

Figure 857: Each curve represents the relative number of ENST00000442704 survived to the corresponding point of time. Dashed lines represent 0.95 confidence intervals for the curves with the corresponding color. Log-rank probability measure for the equality of these curves is 4.971254e-02. Additional annotations: *.GeneId*=ENSG00000165899, *.GeneName*=C12orf64, *.GeneDesc*=Uncharacterized protein C12orf64 [Source:UniProtKB/Swiss-Prot;Acc:Q3ZCN5].

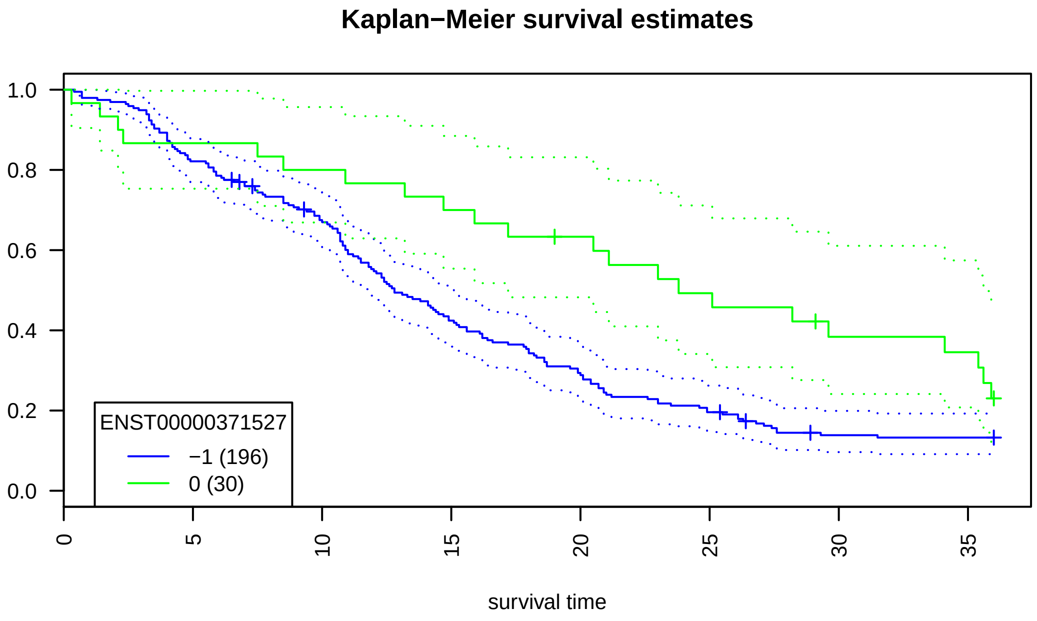

Figure 858: Each curve represents the relative number of ENST00000371527 survived to the corresponding point of time. Dashed lines represent 0.95 confidence intervals for the curves with the corresponding color. Total of 4 observations deleted due to missingness. Log-rank probability measure for the equality of these curves is 9.07949e-03. Additional annotations: *.GeneId*=ENSG00000186416, *.GeneName*=NKRF, *.GeneDesc*=NFKB repressing factor [Source:HGNC Symbol;Acc:19374].

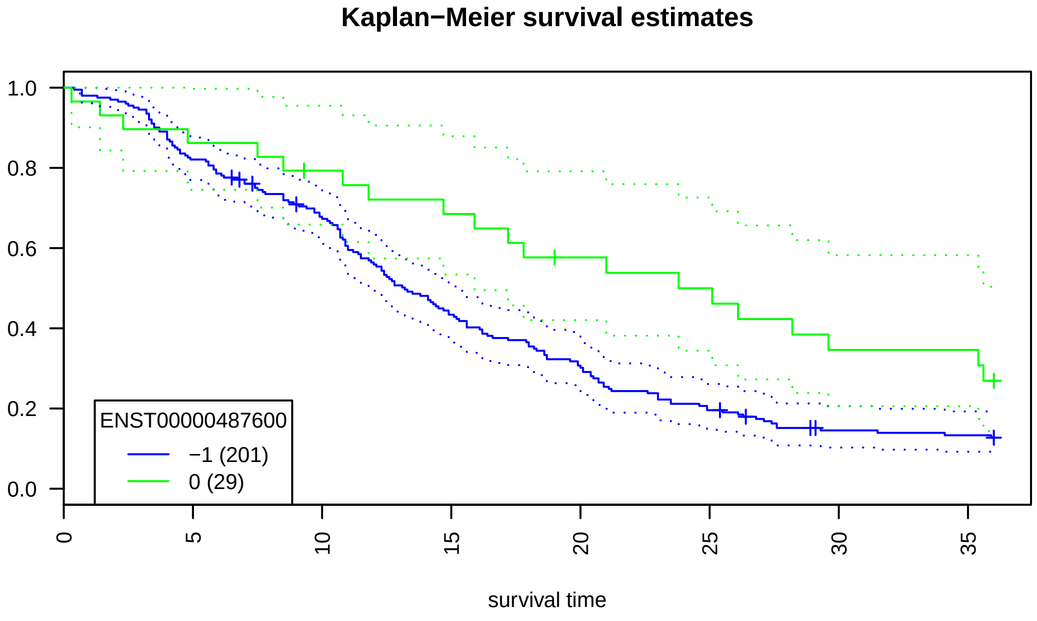

Figure 859: Each curve represents the relative number of ENST00000487600 survived to the corresponding point of time. Dashed lines represent 0.95 confidence intervals for the curves with the corresponding color. Log-rank probability measure for the equality of these curves is 1.311659e-02. Additional annotations: *.GeneId*=ENSG00000186416, *.GeneName*=NKRF, *.GeneDesc*=NFKB repressing factor [Source:HGNC Symbol;Acc:19374].

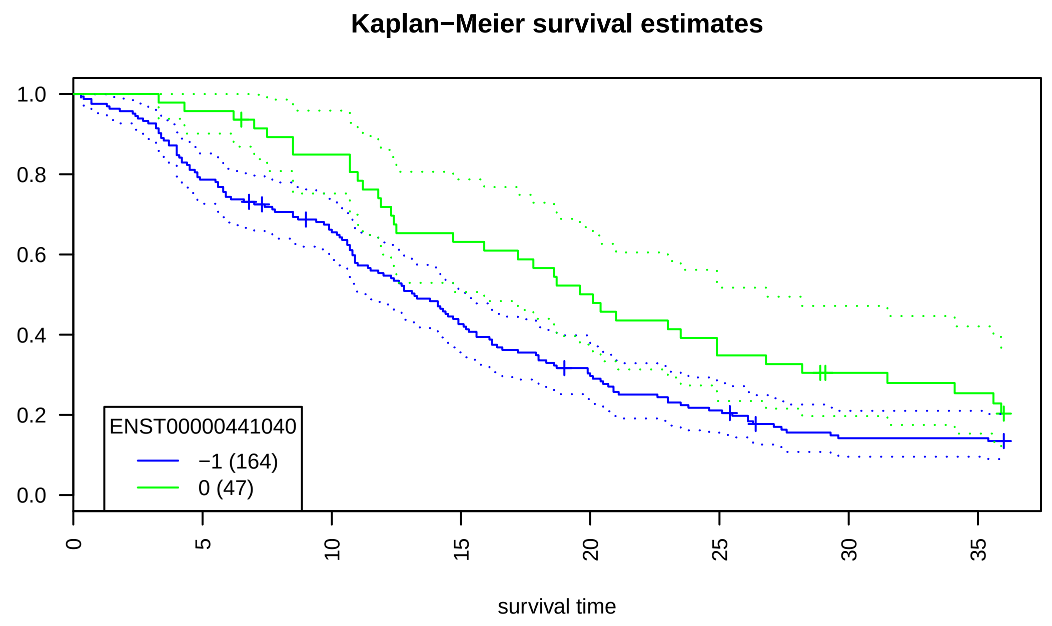

Figure 860: Each curve represents the relative number of ENST00000441040 survived to the corresponding point of time. Dashed lines represent 0.95 confidence intervals for the curves with the corresponding color. Total of 19 observations deleted due to missingness. Log-rank probability measure for the equality of these curves is 1.603515e-02. Additional annotations: *.GeneId*=ENSG00000161298, *.GeneName*=ZNF382, *.GeneDesc*=zinc finger protein 382 [Source:HGNC Symbol;Acc:17409].

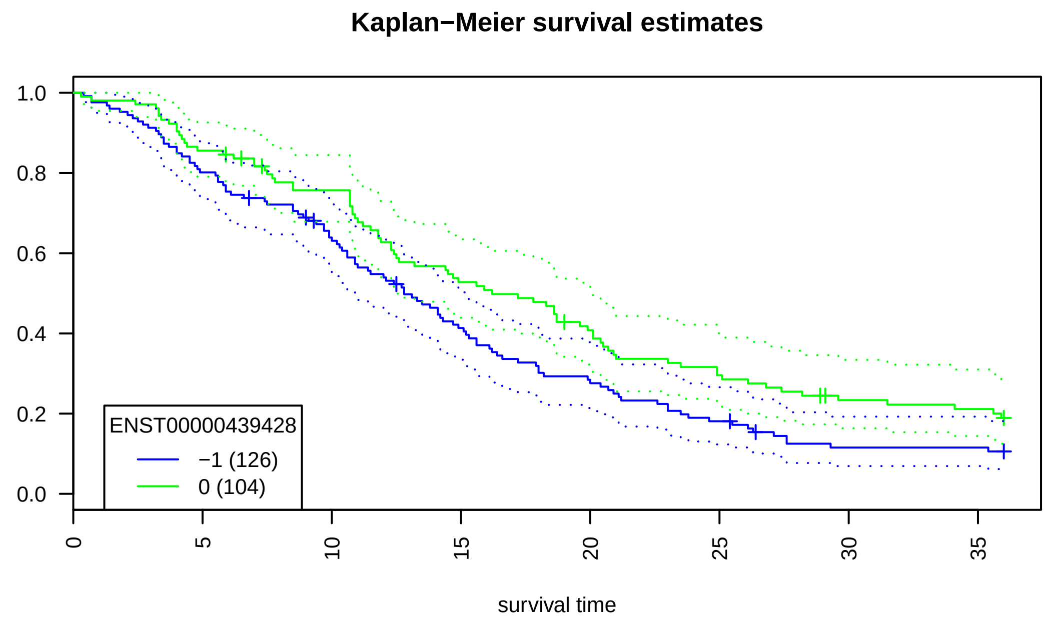

Figure 861: Each curve represents the relative number of ENST00000439428 survived to the corresponding point of time. Dashed lines represent 0.95 confidence intervals for the curves with the corresponding color. Log-rank probability measure for the equality of these curves is 1.978462e-02. Additional annotations: *.GeneId*=ENSG00000161298, *.GeneName*=ZNF382, *.GeneDesc*=zinc finger protein 382 [Source:HGNC Symbol;Acc:17409].

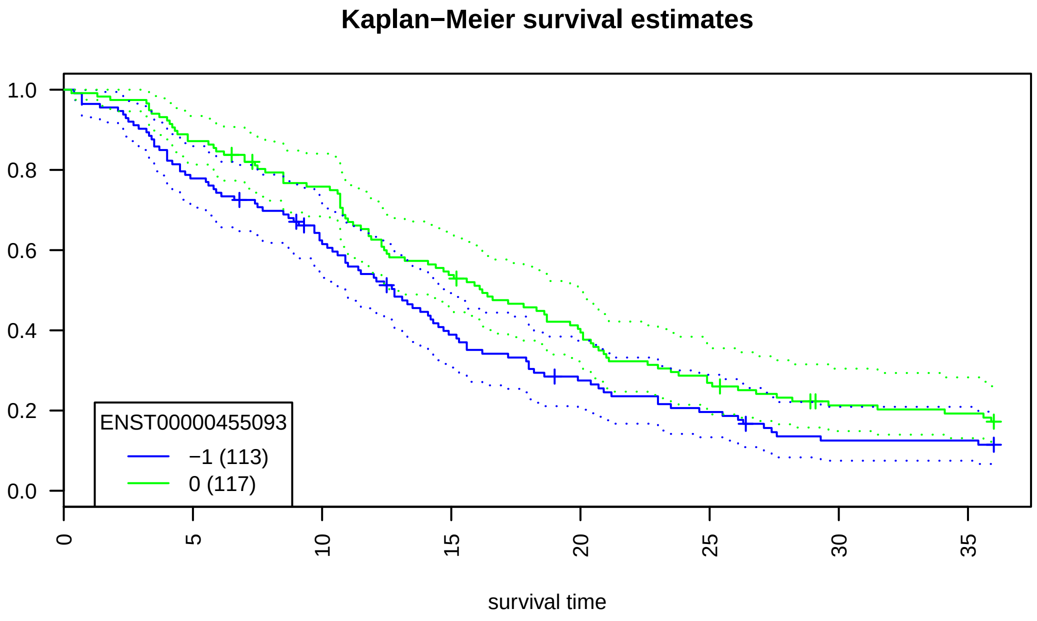

Figure 862: Each curve represents the relative number of ENST00000455093 survived to the corresponding point of time. Dashed lines represent 0.95 confidence intervals for the curves with the corresponding color. Log-rank probability measure for the equality of these curves is 3.731857e-02. Additional annotations: *.GeneId*=ENSG00000161298, *.GeneName*=ZNF382, *.GeneDesc*=zinc finger protein 382 [Source:HGNC Symbol;Acc:17409].

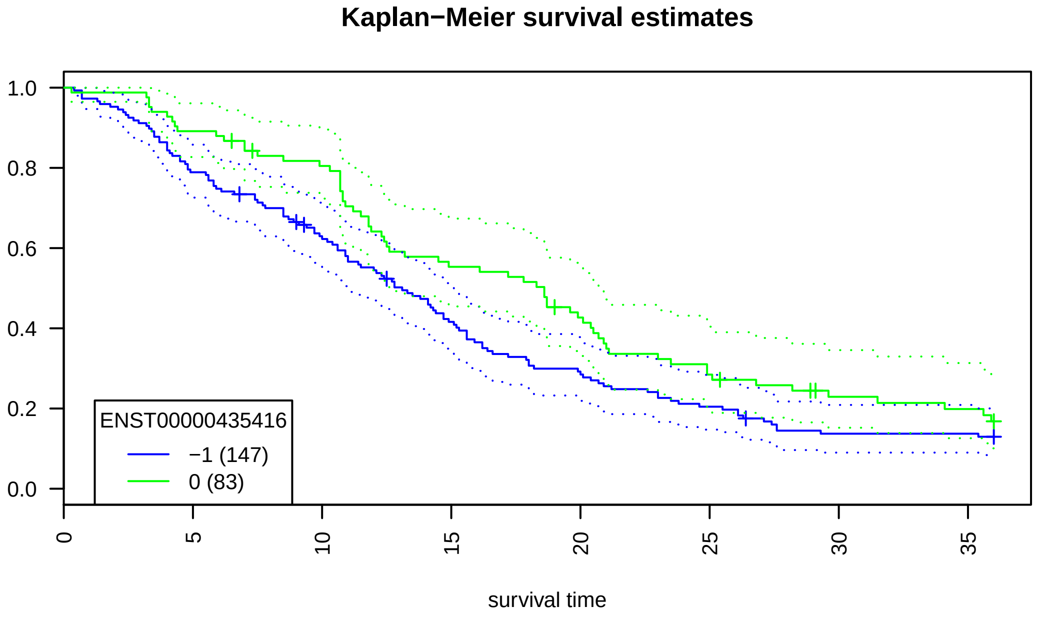

Figure 863: Each curve represents the relative number of ENST00000435416 survived to the corresponding point of time. Dashed lines represent 0.95 confidence intervals for the curves with the corresponding color. Log-rank probability measure for the equality of these curves is 4.32169e-02. Additional annotations: *.GeneId*=ENSG00000161298, *.GeneName*=ZNF382, *.GeneDesc*=zinc finger protein 382 [Source:HGNC Symbol;Acc:17409].

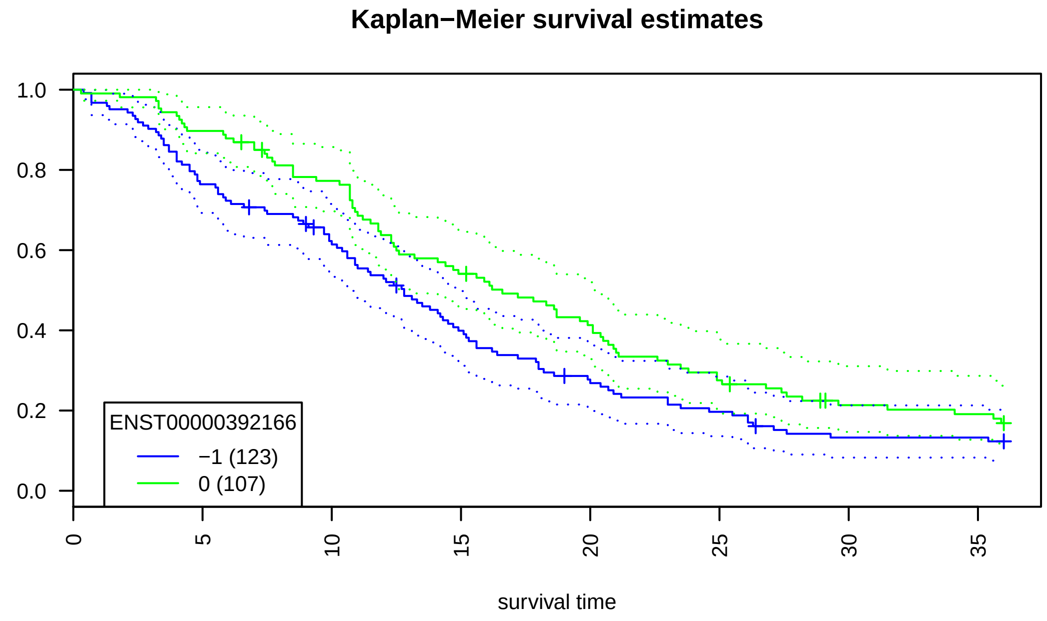

Figure 864: Each curve represents the relative number of ENST00000392166 survived to the corresponding point of time. Dashed lines represent 0.95 confidence intervals for the curves with the corresponding color. Log-rank probability measure for the equality of these curves is 3.007594e-02.

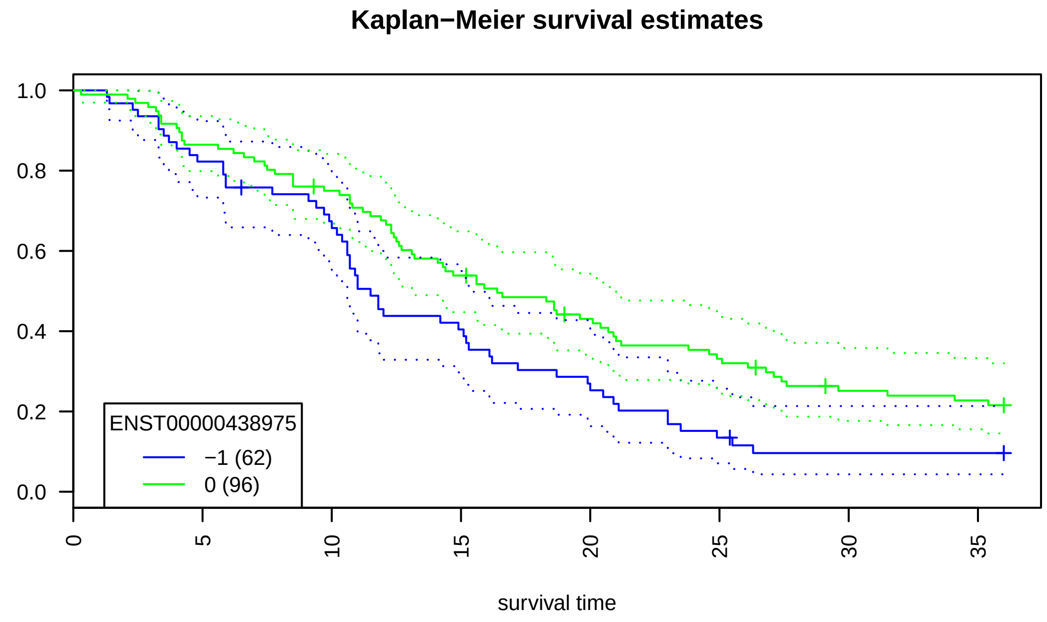

Figure 865: Each curve represents the relative number of ENST00000438975 survived to the corresponding point of time. Dashed lines represent 0.95 confidence intervals for the curves with the corresponding color. Total of 72 observations deleted due to missingness. Log-rank probability measure for the equality of these curves is 7.814067e-03. Additional annotations: *.GeneId*=ENSG00000075151, *.GeneName*=EIF4G3, *.GeneDesc*=eukaryotic translation initiation factor 4 gamma, 3 [Source:HGNC Symbol;Acc:3298].

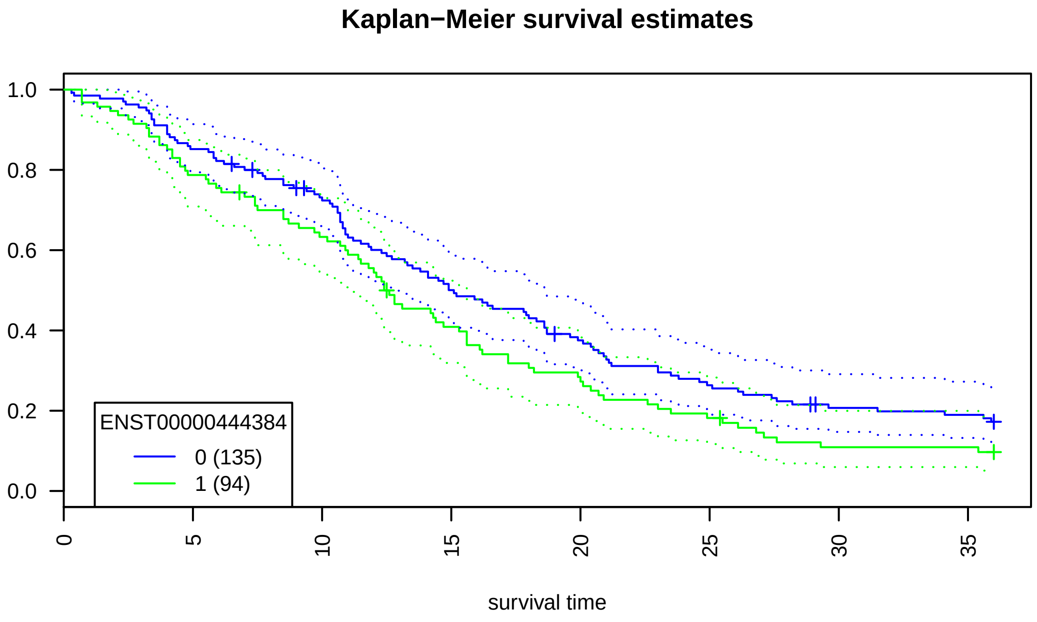

Figure 866: Each curve represents the relative number of ENST00000444384 survived to the corresponding point of time. Dashed lines represent 0.95 confidence intervals for the curves with the corresponding color. Total of 1 observation deleted due to missingness. Log-rank probability measure for the equality of these curves is 4.983082e-02. Additional annotations: *.GeneId*=ENSG00000133661, *.GeneName*=SFTPD, *.GeneDesc*=surfactant protein D [Source:HGNC Symbol;Acc:10803].

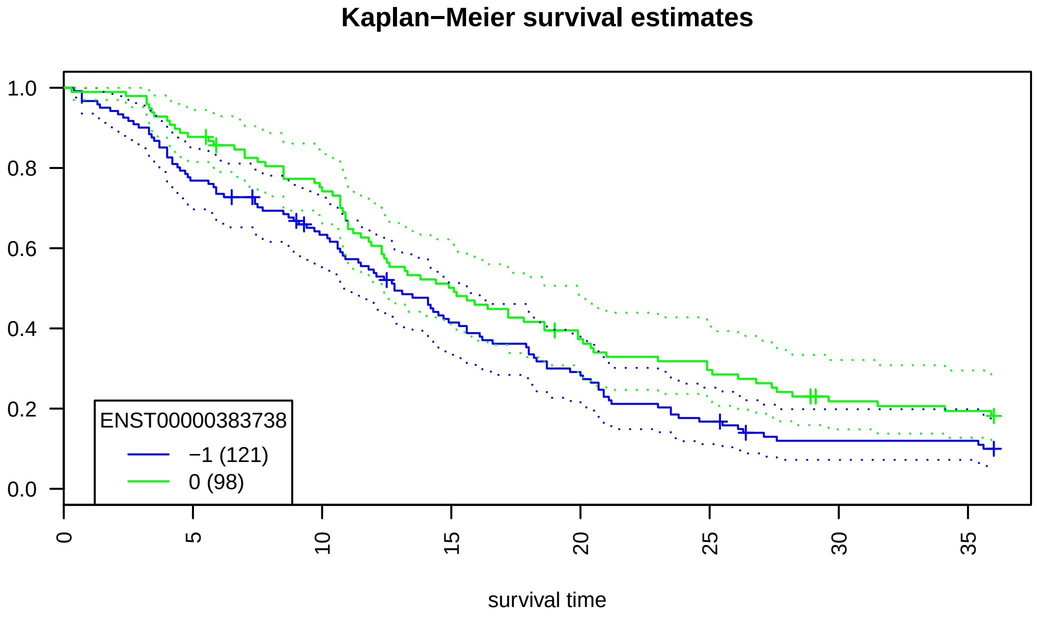

Figure 867: Each curve represents the relative number of ENST00000383738 survived to the corresponding point of time. Dashed lines represent 0.95 confidence intervals for the curves with the corresponding color. Total of 11 observations deleted due to missingness. Log-rank probability measure for the equality of these curves is 3.543912e-02. Additional annotations: *.GeneId*=ENSG00000114646, *.GeneName*=CSPG5, *.GeneDesc*=chondroitin sulfate proteoglycan 5 (neuroglycan C) [Source:HGNC Symbol;Acc:2467].

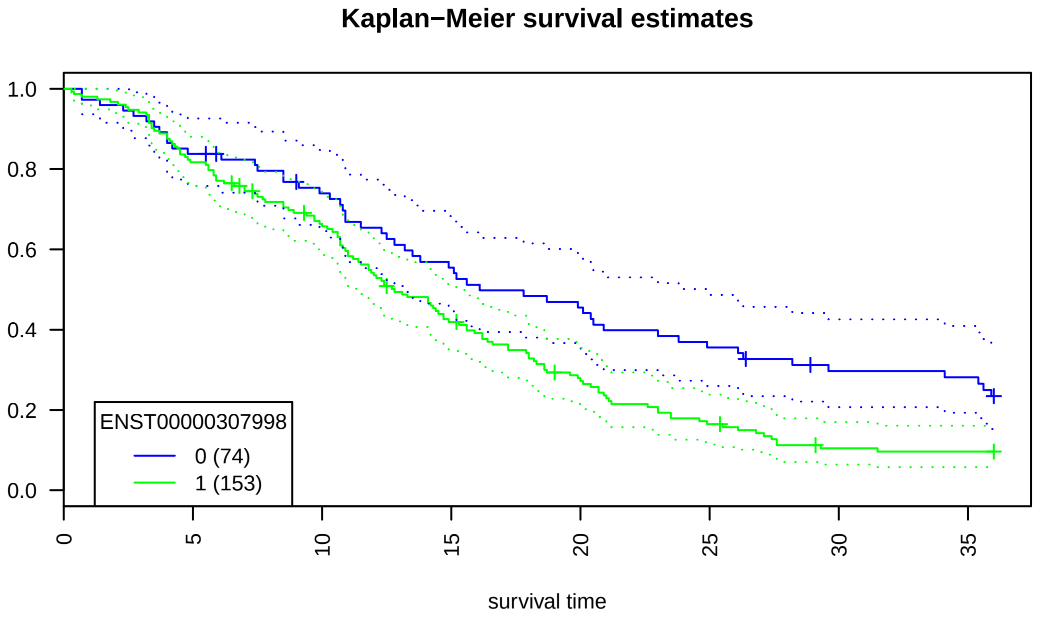

Figure 868: Each curve represents the relative number of ENST00000307998 survived to the corresponding point of time. Dashed lines represent 0.95 confidence intervals for the curves with the corresponding color. Total of 3 observations deleted due to missingness. Log-rank probability measure for the equality of these curves is 4.132291e-03. Additional annotations: *.GeneId*=ENSG00000172638, *.GeneName*=EFEMP2, *.GeneDesc*=EGF-containing fibulin-like extracellular matrix protein 2 [Source:HGNC Symbol;Acc:3219].

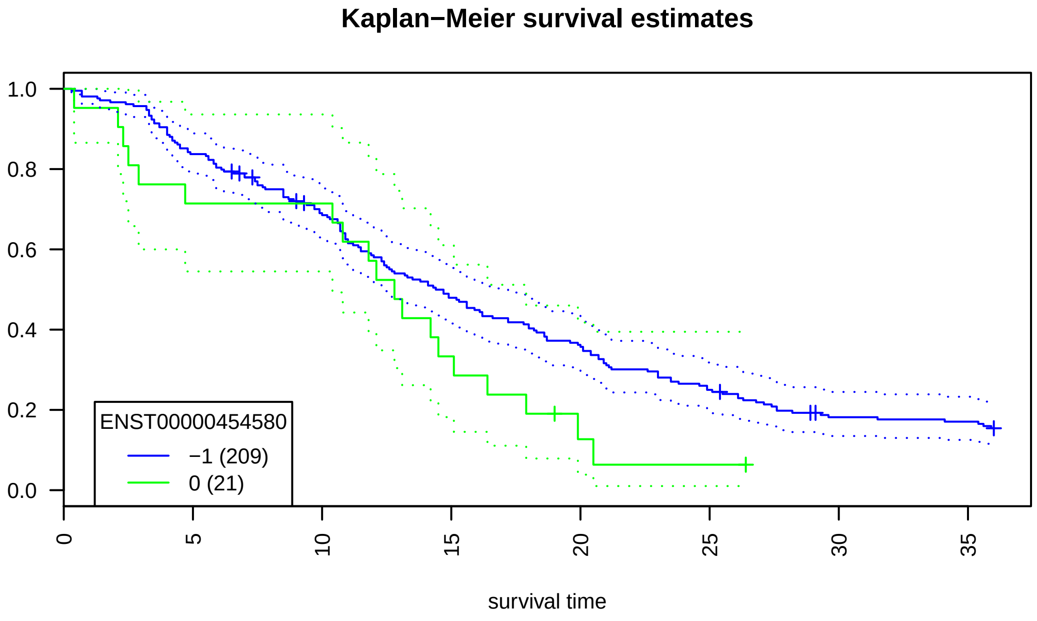

Figure 869: Each curve represents the relative number of ENST00000454580 survived to the corresponding point of time. Dashed lines represent 0.95 confidence intervals for the curves with the corresponding color. Log-rank probability measure for the equality of these curves is 4.744353e-02. Additional annotations: *.GeneId*=ENSG00000167642, *.GeneName*=AC011479.3, *.GeneDesc*=Kunitz-type protease inhibitor 2 Precursor (Hepatocyte growth factor activator inhibitor type 2)(HAI-2)(Placental bikunin) [Source:UniProtKB/Swiss-Prot;Acc:O43291].

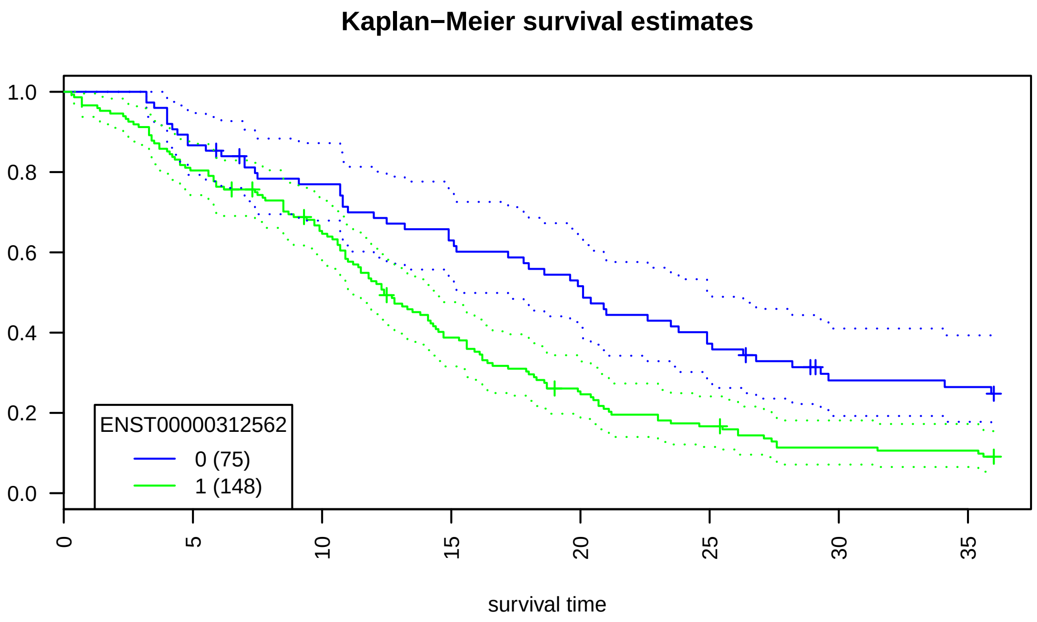

Figure 870: Each curve represents the relative number of ENST00000312562 survived to the corresponding point of time. Dashed lines represent 0.95 confidence intervals for the curves with the corresponding color. Total of 7 observations deleted due to missingness. Log-rank probability measure for the equality of these curves is 2.08165e-04. Additional annotations: *.GeneId*=ENSG00000175592, *.GeneName*=FOSL1, *.GeneDesc*=FOS-like antigen 1 [Source:HGNC Symbol;Acc:13718].

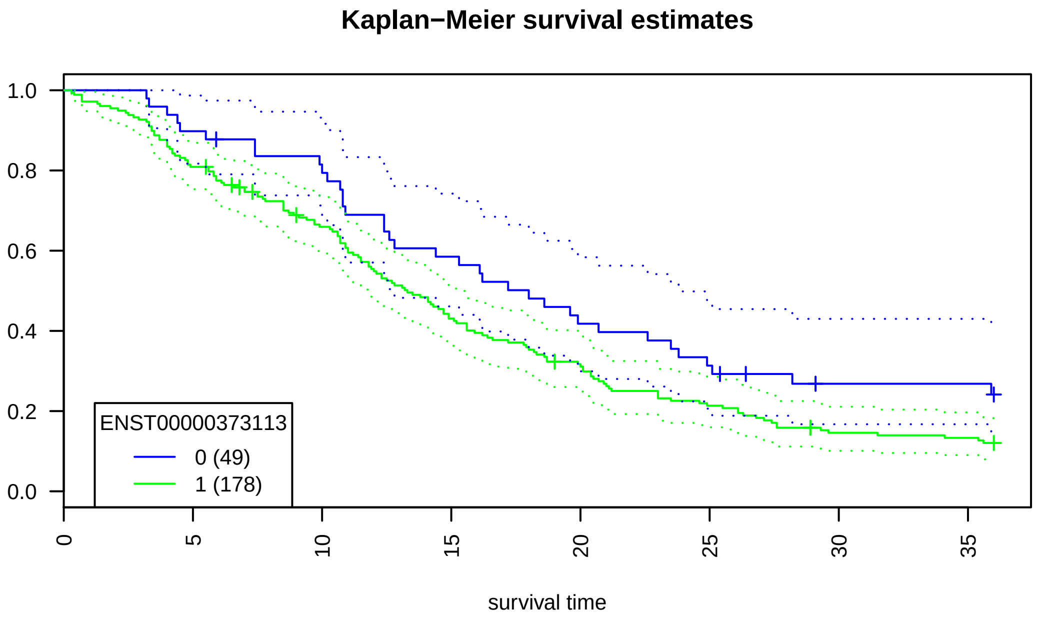

Figure 871: Each curve represents the relative number of ENST00000373113 survived to the corresponding point of time. Dashed lines represent 0.95 confidence intervals for the curves with the corresponding color. Total of 3 observations deleted due to missingness. Log-rank probability measure for the equality of these curves is 3.46705e-02. Additional annotations: *.GeneId*=ENSG00000095970, *.GeneName*=TREM2, *.GeneDesc*=triggering receptor expressed on myeloid cells 2 [Source:HGNC Symbol;Acc:17761].

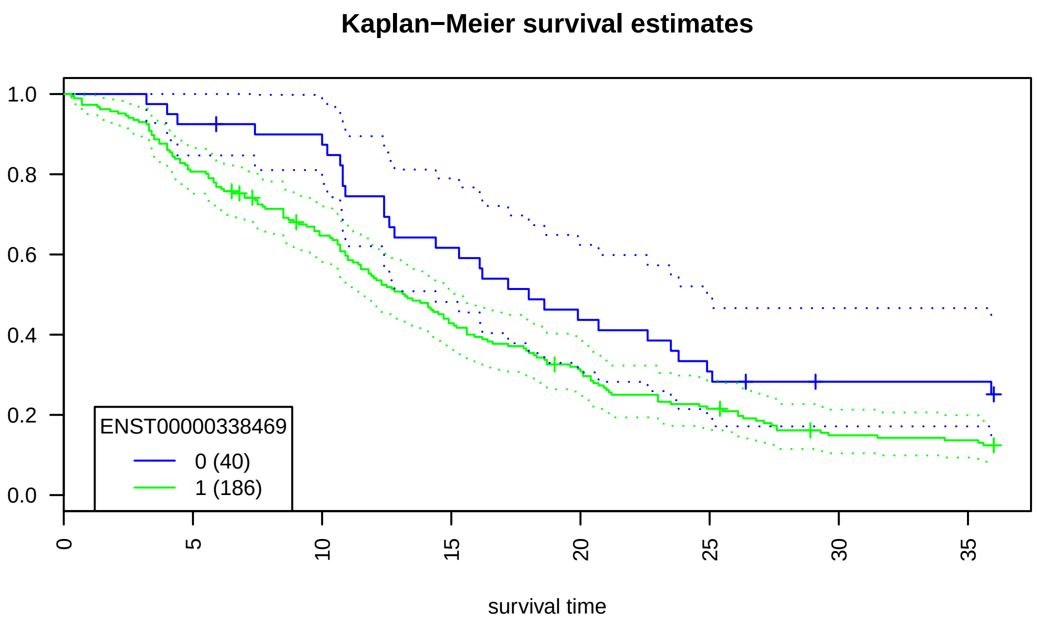

Figure 872: Each curve represents the relative number of ENST00000338469 survived to the corresponding point of time. Dashed lines represent 0.95 confidence intervals for the curves with the corresponding color. Total of 4 observations deleted due to missingness. Log-rank probability measure for the equality of these curves is 2.630478e-02. Additional annotations: *.GeneId*=ENSG00000095970, *.GeneName*=TREM2, *.GeneDesc*=triggering receptor expressed on myeloid cells 2 [Source:HGNC Symbol;Acc:17761].

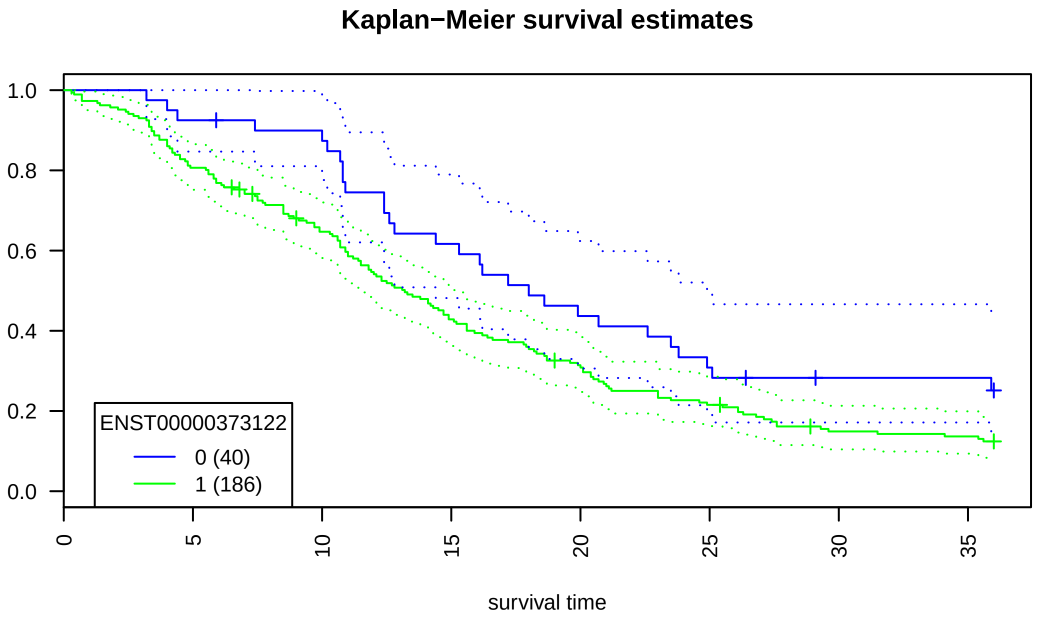

Figure 873: Each curve represents the relative number of ENST00000373122 survived to the corresponding point of time. Dashed lines represent 0.95 confidence intervals for the curves with the corresponding color. Total of 4 observations deleted due to missingness. Log-rank probability measure for the equality of these curves is 2.630478e-02. Additional annotations: *.GeneId*=ENSG00000095970, *.GeneName*=TREM2, *.GeneDesc*=triggering receptor expressed on myeloid cells 2 [Source:HGNC Symbol;Acc:17761].

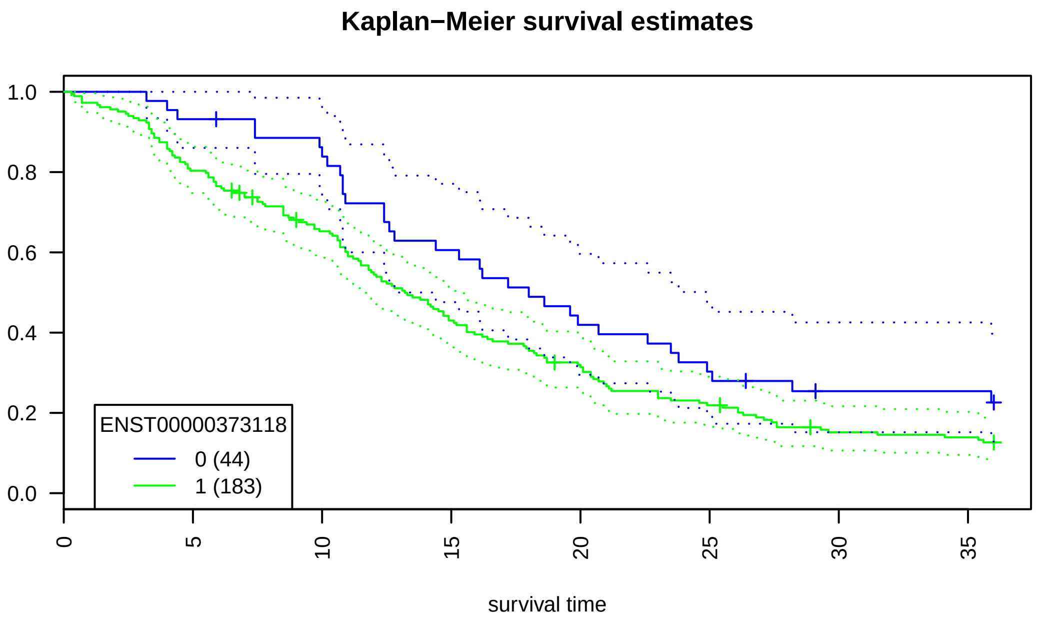

Figure 874: Each curve represents the relative number of ENST00000373118 survived to the corresponding point of time. Dashed lines represent 0.95 confidence intervals for the curves with the corresponding color. Total of 3 observations deleted due to missingness. Log-rank probability measure for the equality of these curves is 4.227017e-02. Additional annotations: *.GeneId*=ENSG00000095970, *.GeneName*=TREM2, *.GeneDesc*=triggering receptor expressed on myeloid cells 2 [Source:HGNC Symbol;Acc:17761].

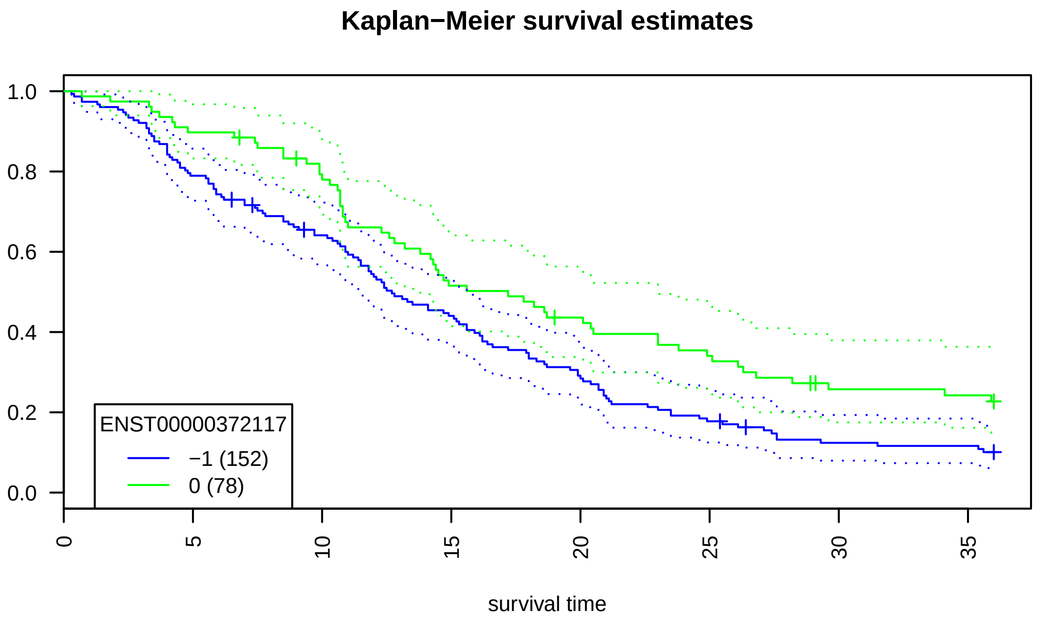

Figure 875: Each curve represents the relative number of ENST00000372117 survived to the corresponding point of time. Dashed lines represent 0.95 confidence intervals for the curves with the corresponding color. Log-rank probability measure for the equality of these curves is 5.431265e-03. Additional annotations: *.GeneId*=ENSG00000148600, *.GeneName*=PCDH21, *.GeneDesc*=cadherin-related family member 1 [Source:HGNC Symbol;Acc:14550].

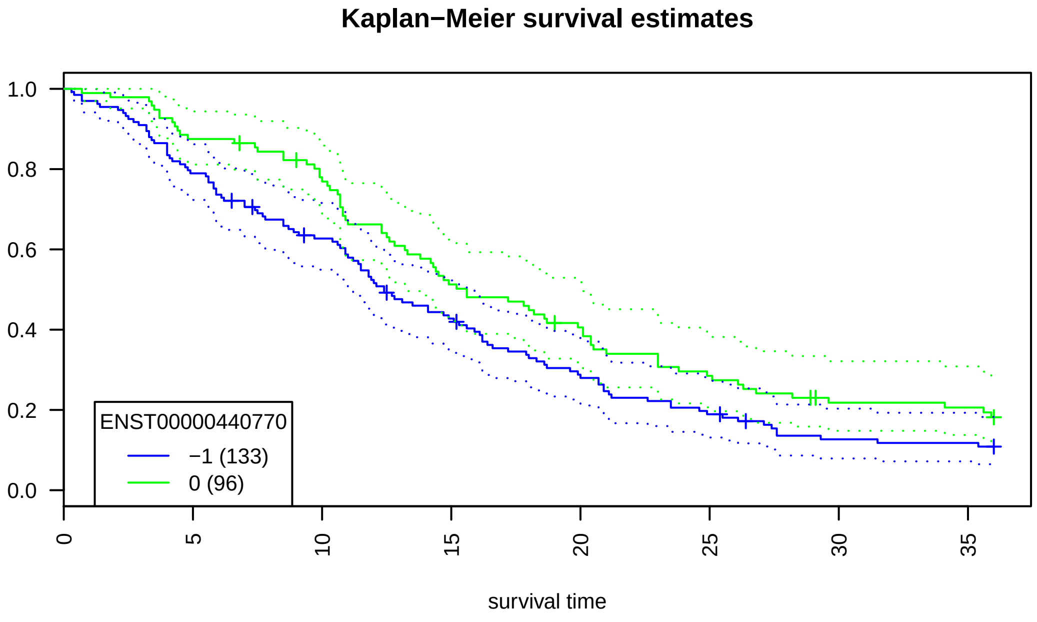

Figure 876: Each curve represents the relative number of ENST00000440770 survived to the corresponding point of time. Dashed lines represent 0.95 confidence intervals for the curves with the corresponding color. Total of 1 observation deleted due to missingness. Log-rank probability measure for the equality of these curves is 2.839221e-02. Additional annotations: *.GeneId*=ENSG00000148600, *.GeneName*=PCDH21, *.GeneDesc*=cadherin-related family member 1 [Source:HGNC Symbol;Acc:14550].

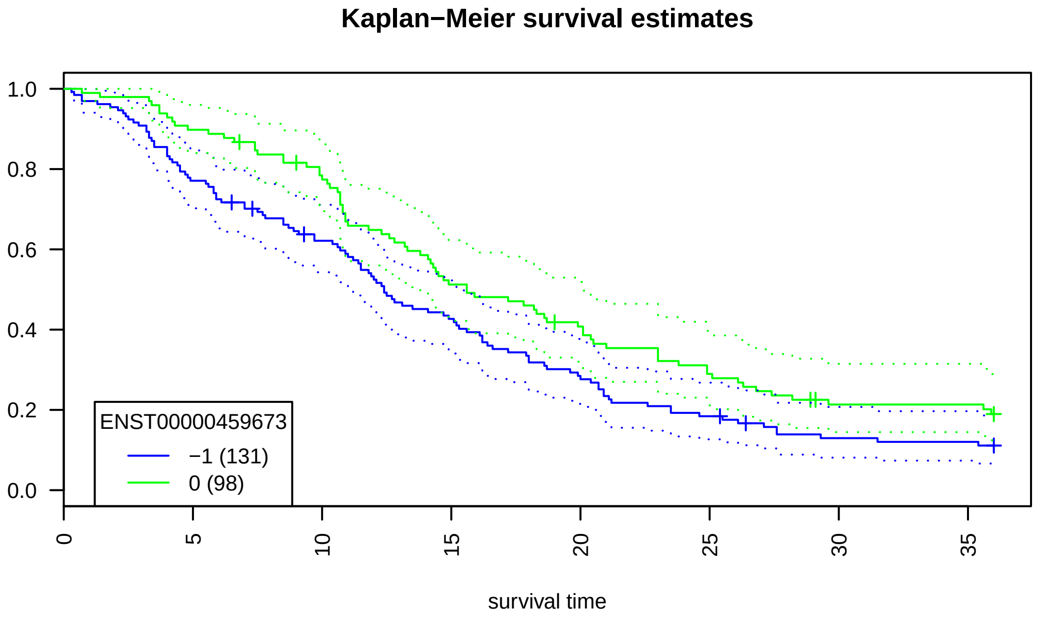

Figure 877: Each curve represents the relative number of ENST00000459673 survived to the corresponding point of time. Dashed lines represent 0.95 confidence intervals for the curves with the corresponding color. Total of 1 observation deleted due to missingness. Log-rank probability measure for the equality of these curves is 1.856027e-02. Additional annotations: *.GeneId*=ENSG00000148600, *.GeneName*=PCDH21, *.GeneDesc*=cadherin-related family member 1 [Source:HGNC Symbol;Acc:14550].

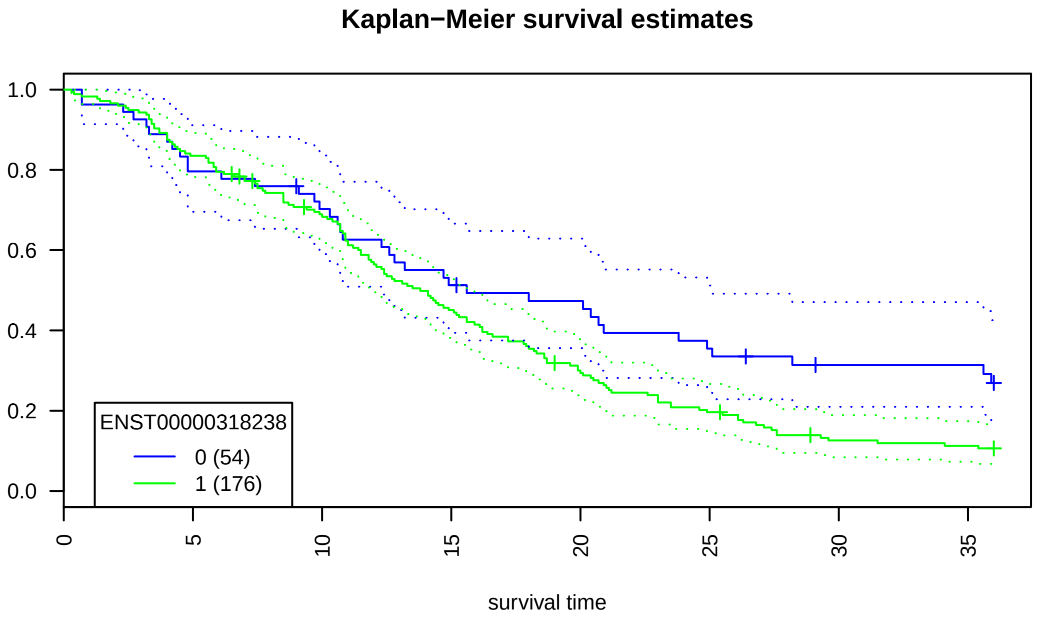

Figure 878: Each curve represents the relative number of ENST00000318238 survived to the corresponding point of time. Dashed lines represent 0.95 confidence intervals for the curves with the corresponding color. Log-rank probability measure for the equality of these curves is 2.290006e-02. Additional annotations: *.GeneId*=ENSG00000177409, *.GeneName*=SAMD9L, *.GeneDesc*=sterile alpha motif domain containing 9-like [Source:HGNC Symbol;Acc:1349].

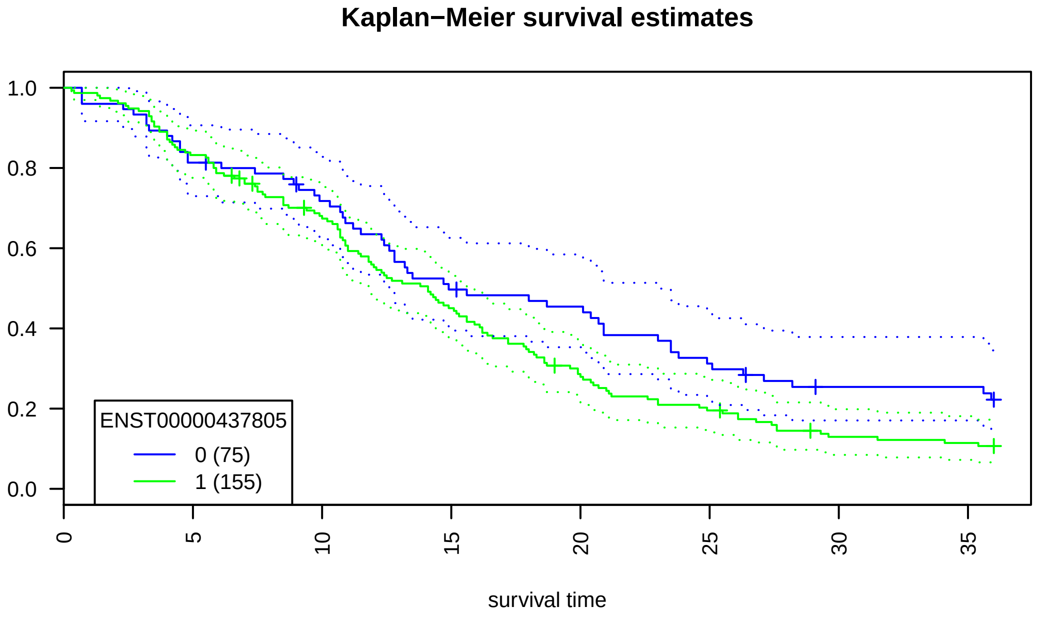

Figure 879: Each curve represents the relative number of ENST00000437805 survived to the corresponding point of time. Dashed lines represent 0.95 confidence intervals for the curves with the corresponding color. Log-rank probability measure for the equality of these curves is 3.642978e-02. Additional annotations: *.GeneId*=ENSG00000177409, *.GeneName*=SAMD9L, *.GeneDesc*=sterile alpha motif domain containing 9-like [Source:HGNC Symbol;Acc:1349].

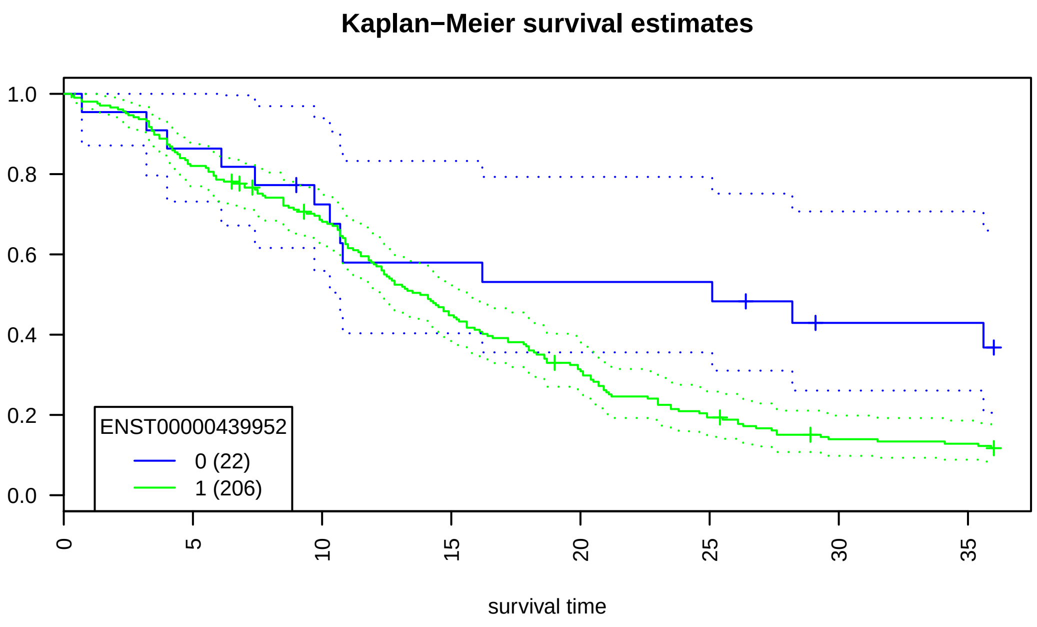

Figure 880: Each curve represents the relative number of ENST00000439952 survived to the corresponding point of time. Dashed lines represent 0.95 confidence intervals for the curves with the corresponding color. Total of 2 observations deleted due to missingness. Log-rank probability measure for the equality of these curves is 1.827590e-02. Additional annotations: *.GeneId*=ENSG00000177409, *.GeneName*=SAMD9L, *.GeneDesc*=sterile alpha motif domain containing 9-like [Source:HGNC Symbol;Acc:1349].

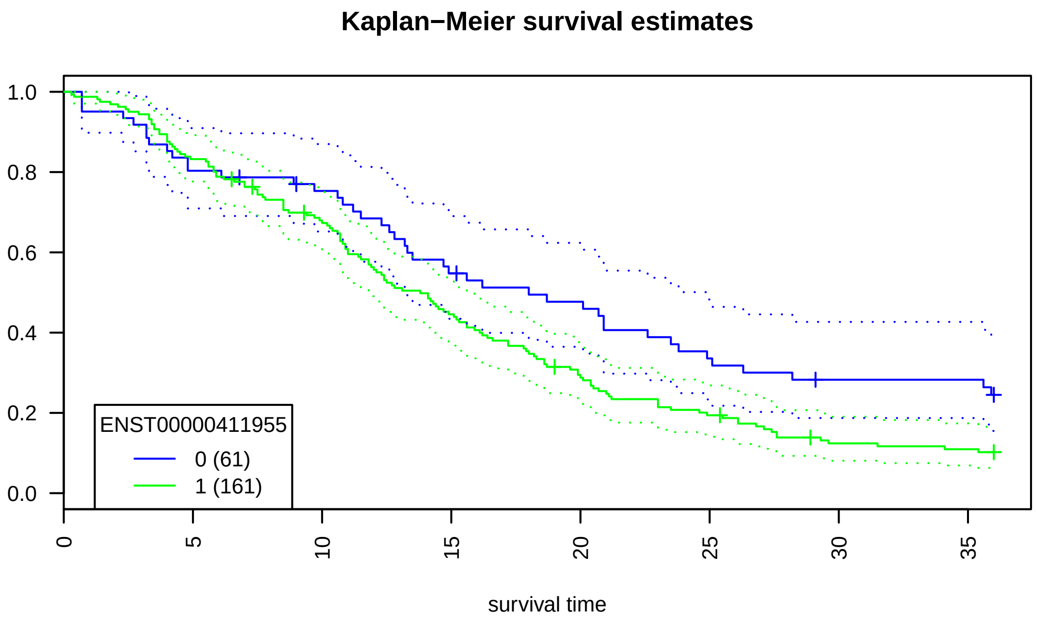

Figure 881: Each curve represents the relative number of ENST00000411955 survived to the corresponding point of time. Dashed lines represent 0.95 confidence intervals for the curves with the corresponding color. Total of 8 observations deleted due to missingness. Log-rank probability measure for the equality of these curves is 1.412874e-02. Additional annotations: *.GeneId*=ENSG00000177409, *.GeneName*=SAMD9L, *.GeneDesc*=sterile alpha motif domain containing 9-like [Source:HGNC Symbol;Acc:1349].

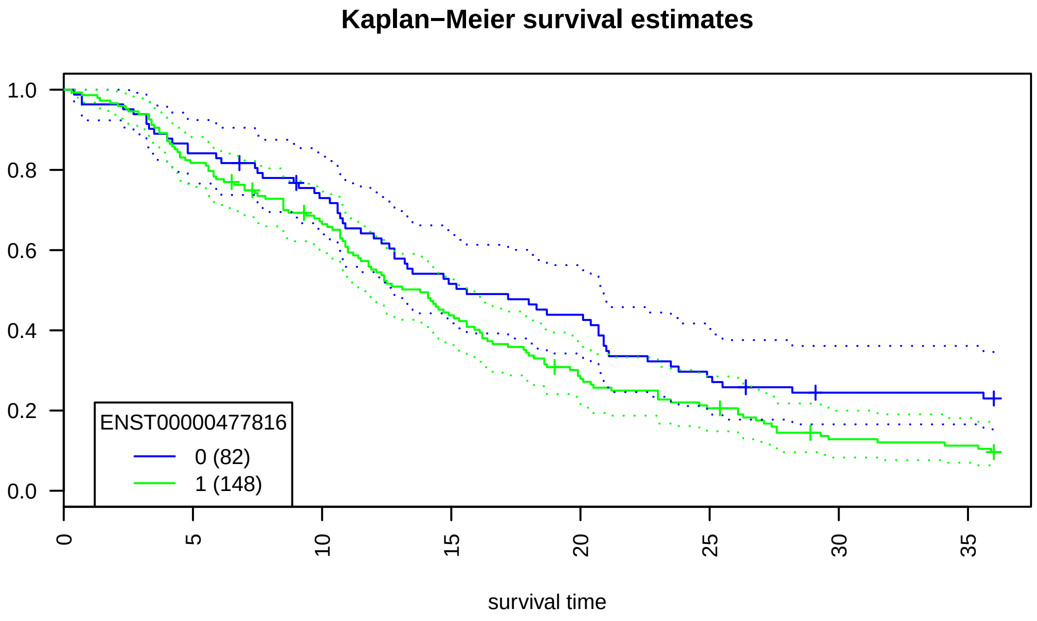

Figure 882: Each curve represents the relative number of ENST00000477816 survived to the corresponding point of time. Dashed lines represent 0.95 confidence intervals for the curves with the corresponding color. Log-rank probability measure for the equality of these curves is 3.026988e-02. Additional annotations: .*GeneId*=ENSG00000177409, .*GeneName*=SAMD9L, .*GeneDesc*=sterile alpha motif domain containing 9-like [Source:HGNC Symbol;Acc:1349].

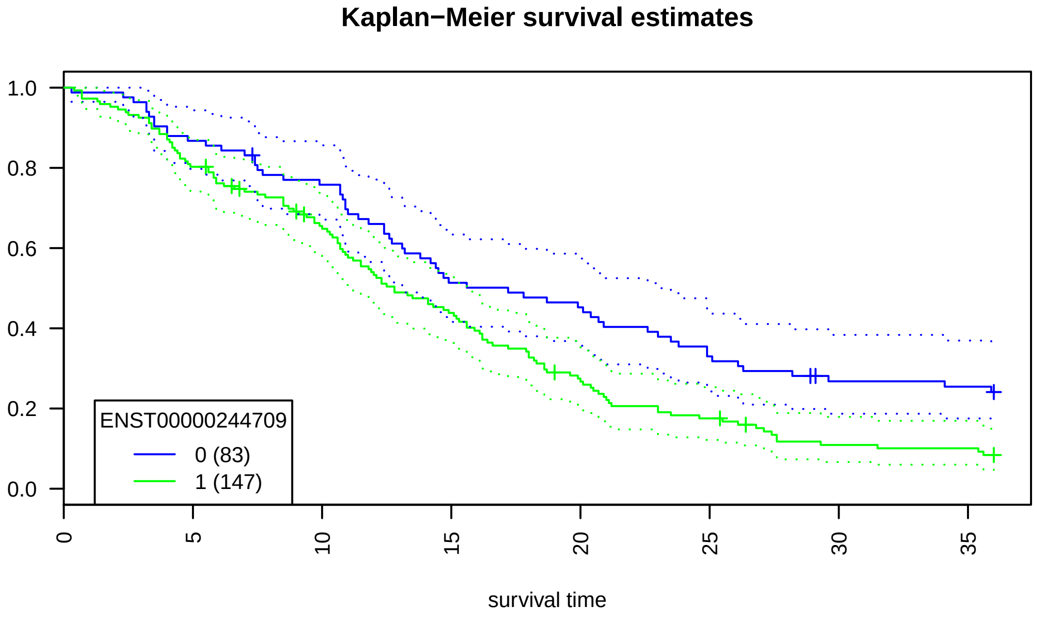

Figure 883: Each curve represents the relative number of ENST00000244709 survived to the corresponding point of time. Dashed lines represent 0.95 confidence intervals for the curves with the corresponding color. Log-rank probability measure for the equality of these curves is 2.156442e-03. Additional annotations: .*GeneId*=ENSG00000124731, .*GeneName*=TREM1, .*GeneDesc*=triggering receptor expressed on myeloid cells 1 [Source:HGNC Symbol;Acc:17760].

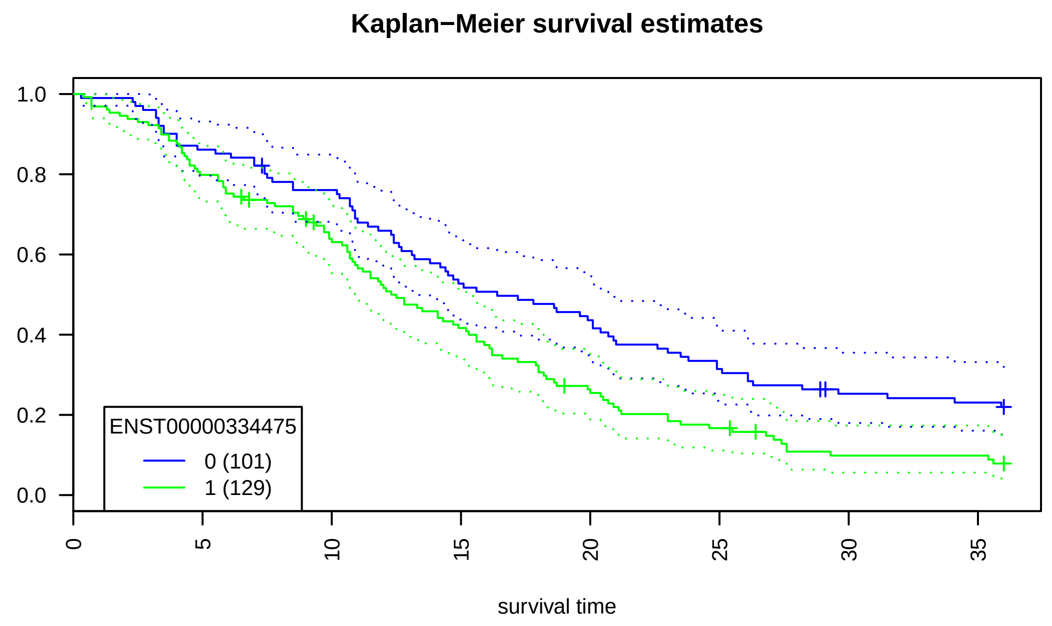

Figure 884: Each curve represents the relative number of ENST00000334475 survived to the corresponding point of time. Dashed lines represent 0.95 confidence intervals for the curves with the corresponding color. Log-rank probability measure for the equality of these curves is 2.138315e-03. Additional annotations: *.GeneId*=ENSG00000124731, *.GeneName*=TREM1, *.GeneDesc*=triggering receptor expressed on myeloid cells 1 [Source:HGNC Symbol;Acc:17760].

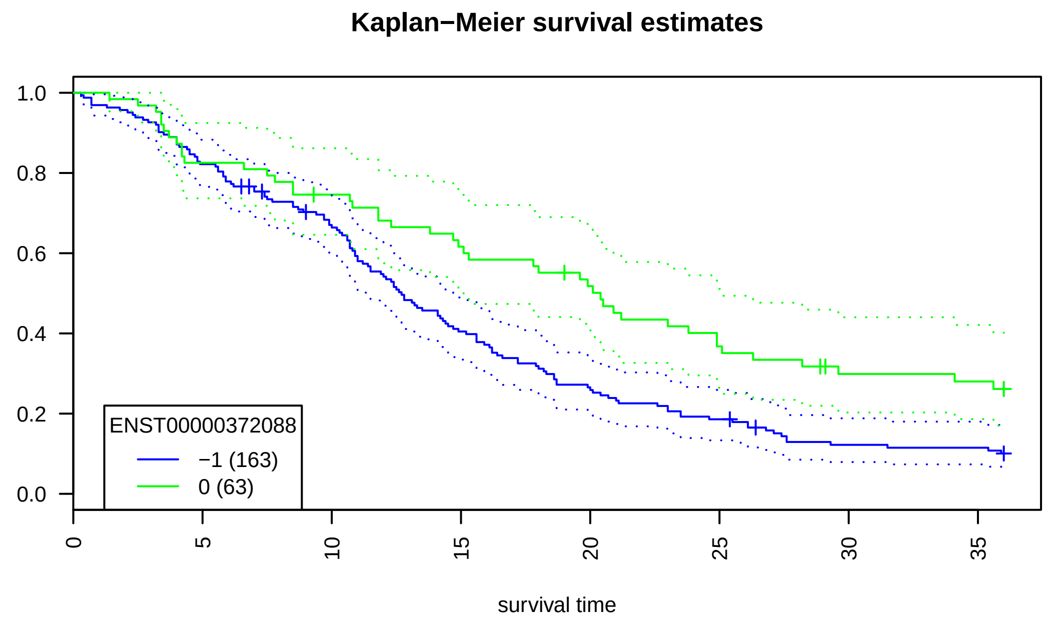

Figure 885: Each curve represents the relative number of ENST00000372088 survived to the corresponding point of time. Dashed lines represent 0.95 confidence intervals for the curves with the corresponding color. Total of 4 observations deleted due to missingness. Log-rank probability measure for the equality of these curves is 1.322831e-03. Additional annotations: *.GeneId*=ENSG00000107771, *.GeneName*=FAM190, *.GeneDesc*=family with sequence similarity 190, member B [Source:HGNC Symbol;Acc:29197].

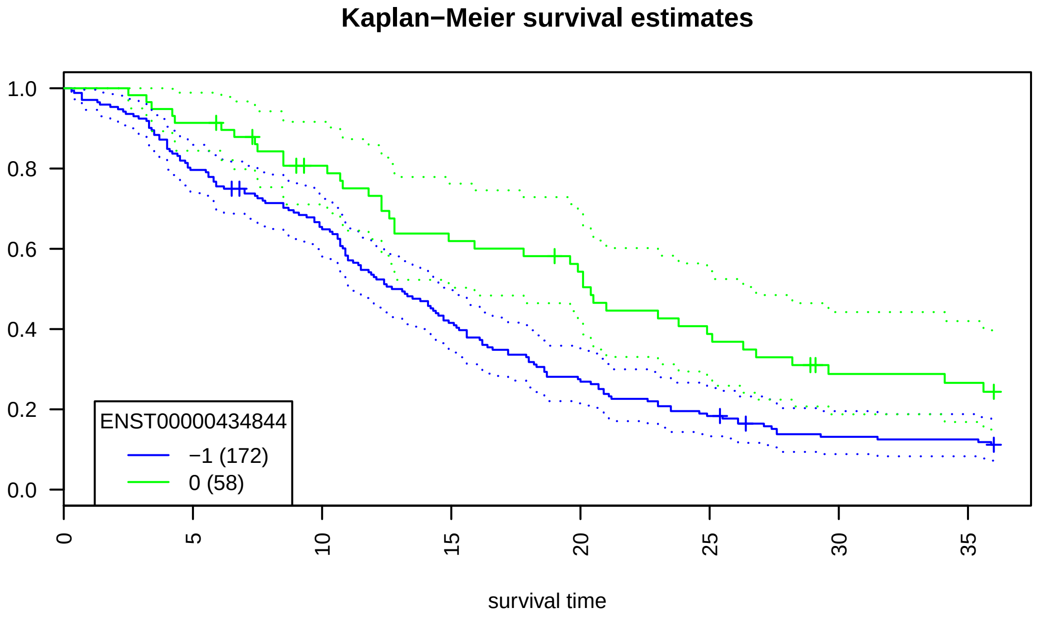

Figure 886: Each curve represents the relative number of ENST00000434844 survived to the corresponding point of time. Dashed lines represent 0.95 confidence intervals for the curves with the corresponding color. Log-rank probability measure for the equality of these curves is 1.757988e-03.

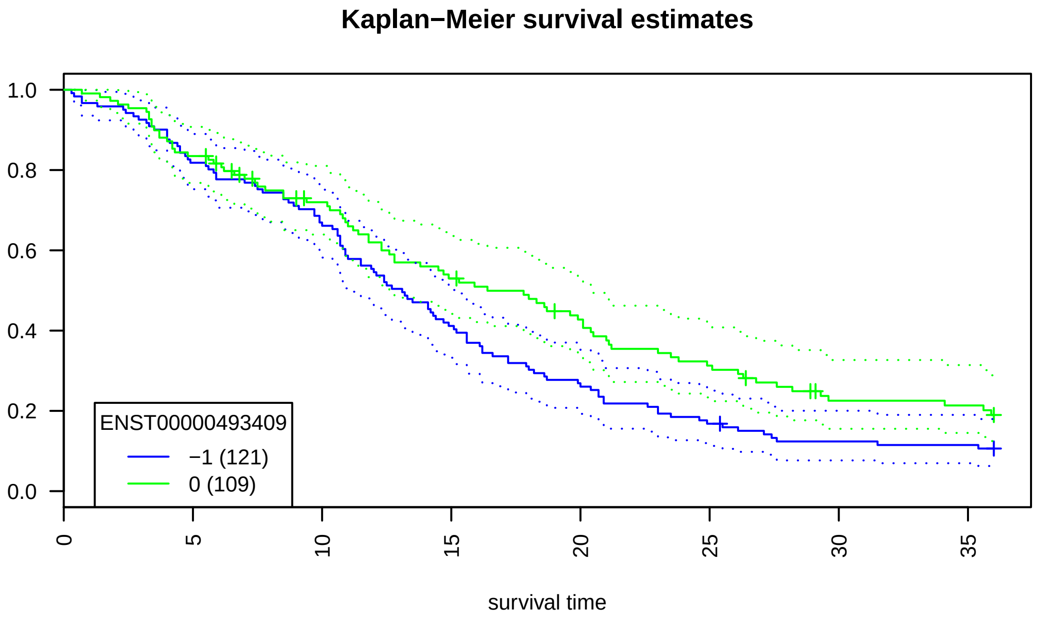

Figure 887: Each curve represents the relative number of ENST00000493409 survived to the corresponding point of time. Dashed lines represent 0.95 confidence intervals for the curves with the corresponding color. Log-rank probability measure for the equality of these curves is 2.335856e-02. Additional annotations: *.GeneId*=ENSG00000107771, *.GeneName*=FAM190, *.GeneDesc*=family with sequence similarity 190, member B [Source:HGNC Symbol;Acc:29197].

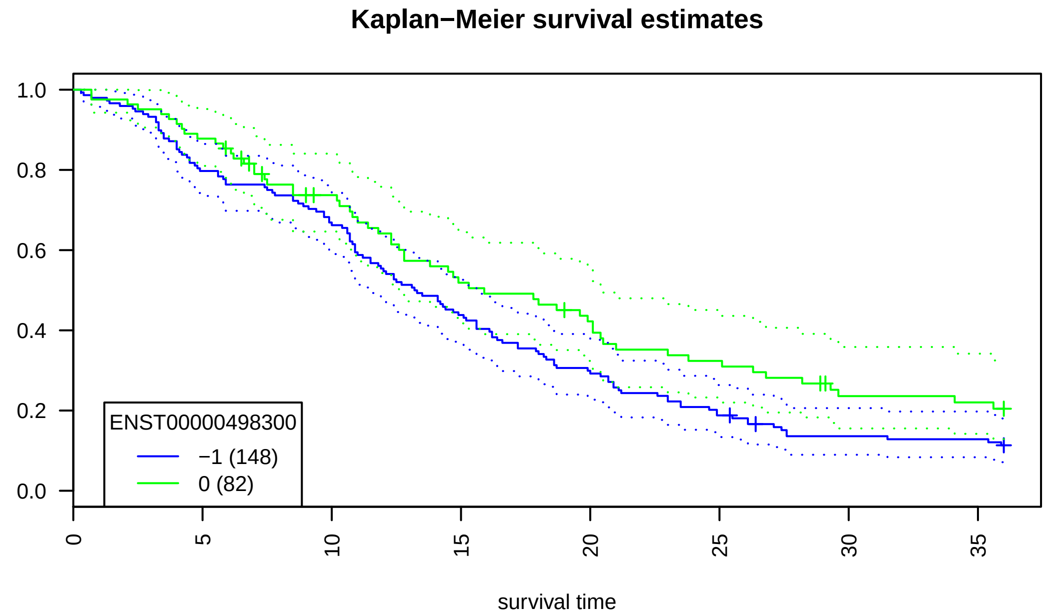

Figure 888: Each curve represents the relative number of ENST00000498300 survived to the corresponding point of time. Dashed lines represent 0.95 confidence intervals for the curves with the corresponding color. Log-rank probability measure for the equality of these curves is 4.252031e-02. Additional annotations: *.GeneId*=ENSG00000107771, *.GeneName*=FAM190, *.GeneDesc*=family with sequence similarity 190, member B [Source:HGNC Symbol;Acc:29197].

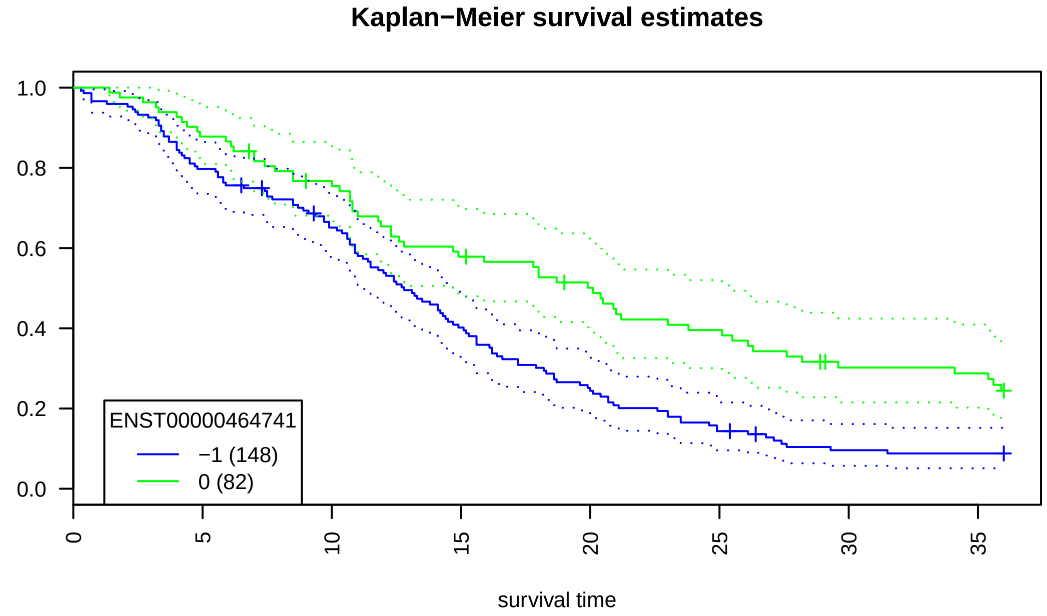

Figure 889: Each curve represents the relative number of ENST00000464741 survived to the corresponding point of time. Dashed lines represent 0.95 confidence intervals for the curves with the corresponding color. Log-rank probability measure for the equality of these curves is 2.038895e-04. Additional annotations: *.GeneId*=ENSG00000182771, *.GeneName*=GRID1, *.GeneDesc*=glutamate receptor, ionotropic, delta 1 [Source:HGNC Symbol;Acc:4575].

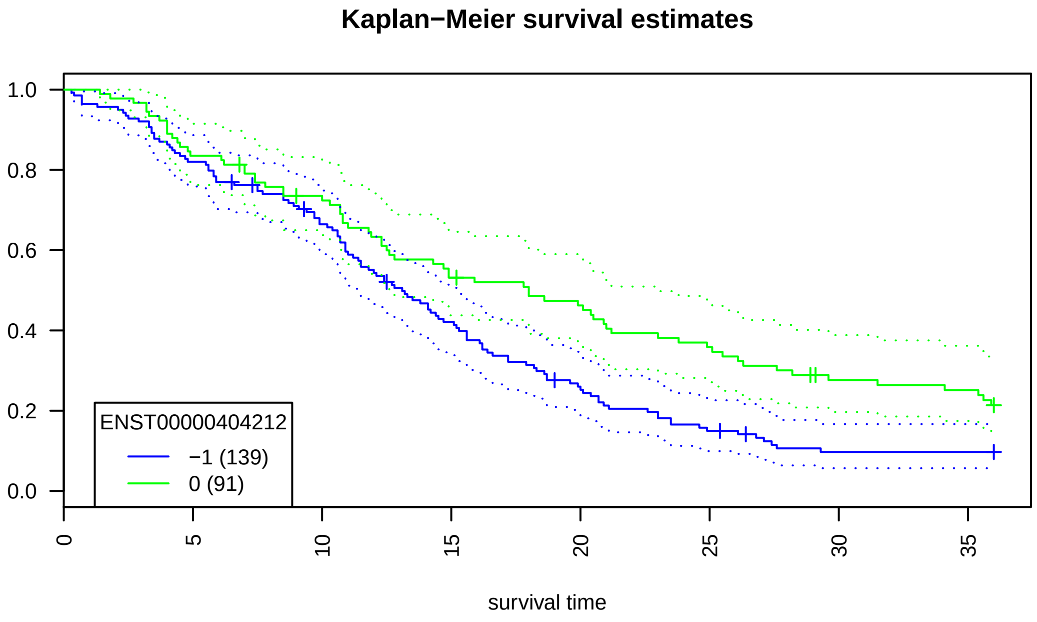

Figure 890: Each curve represents the relative number of ENST00000404212 survived to the corresponding point of time. Dashed lines represent 0.95 confidence intervals for the curves with the corresponding color. Log-rank probability measure for the equality of these curves is 3.337792e-03. Additional annotations: *.GeneId*=ENSG00000182771, *.GeneName*=GRID1, *.GeneDesc*=glutamate receptor, ionotropic, delta 1 [Source:HGNC Symbol;Acc:4575].

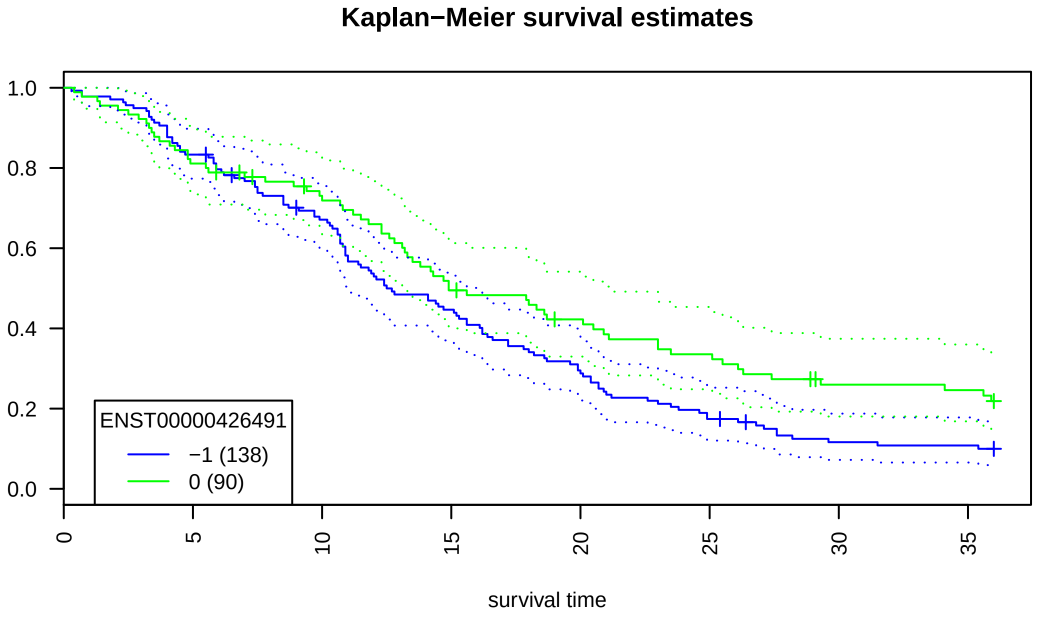

Figure 891: Each curve represents the relative number of ENST00000426491 survived to the corresponding point of time. Dashed lines represent 0.95 confidence intervals for the curves with the corresponding color. Total of 2 observations deleted due to missingness. Log-rank probability measure for the equality of these curves is 2.054734e-02. Additional annotations: *.GeneId*=ENSG00000227906, *.GeneName*=RP5-839B4.7, *.GeneDesc*=[undefined].

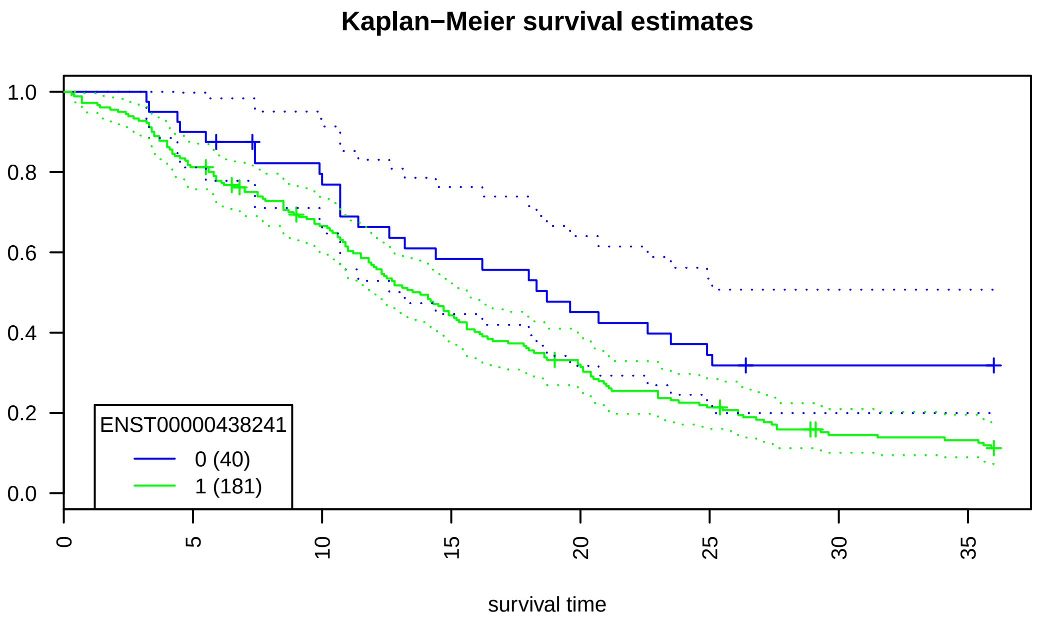

Figure 892: Each curve represents the relative number of ENST00000438241 survived to the corresponding point of time. Dashed lines represent 0.95 confidence intervals for the curves with the corresponding color. Total of 9 observations deleted due to missingness. Log-rank probability measure for the equality of these curves is 1.228493e-02. Additional annotations: *.GeneId*=ENSG00000173372, *.GeneName*=C1QA, *.GeneDesc*=complement component 1, q subcomponent, A chain [Source:HGNC Symbol;Acc:1241].

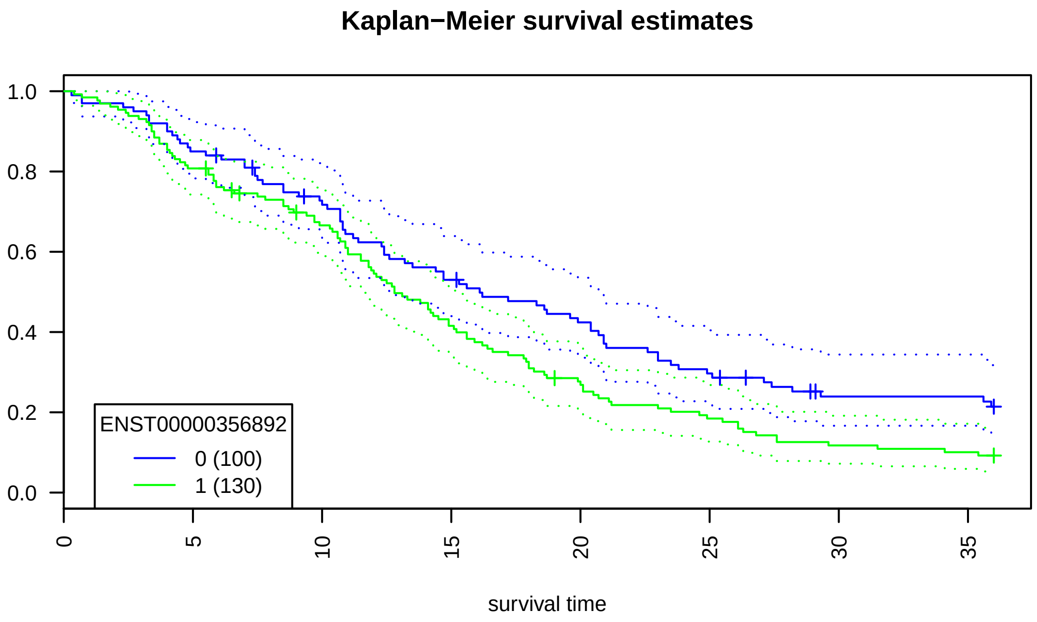

Figure 893: Each curve represents the relative number of ENST00000356892 survived to the corresponding point of time. Dashed lines represent 0.95 confidence intervals for the curves with the corresponding color. Log-rank probability measure for the equality of these curves is 1.187373e-02. Additional annotations: *.GeneId*=ENSG00000122122, *.GeneName*=SASH3, *.GeneDesc*=SAM and SH3 domain containing 3 [Source:HGNC Symbol;Acc:15975].

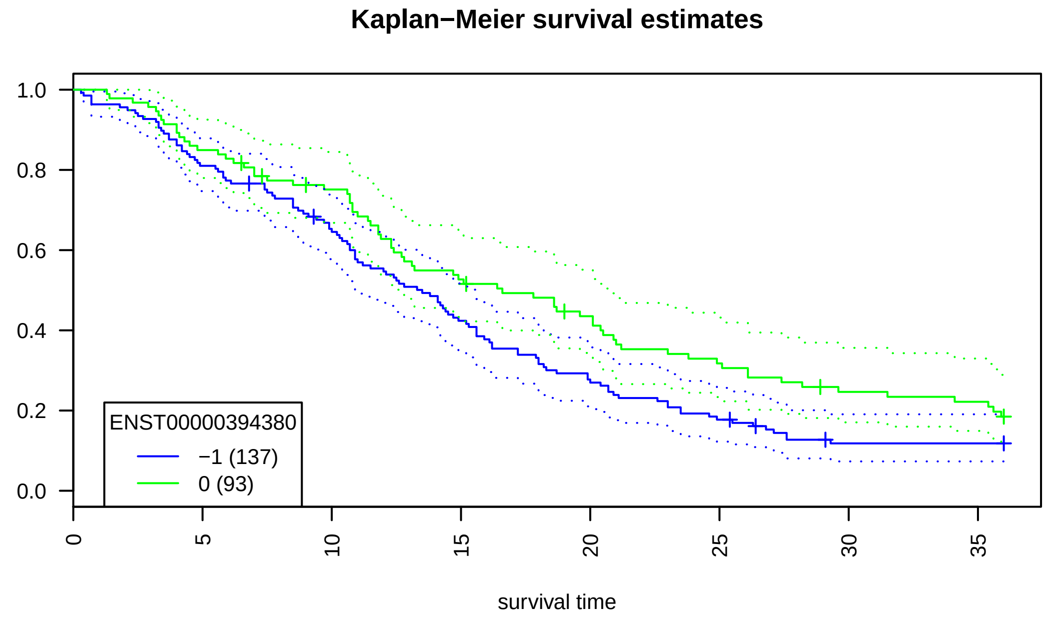

Figure 894: Each curve represents the relative number of ENST00000394380 survived to the corresponding point of time. Dashed lines represent 0.95 confidence intervals for the curves with the corresponding color. Log-rank probability measure for the equality of these curves is 3.219880e-02. Additional annotations: *.GeneId*=ENSG00000127995, *.GeneName*=CASD1, *.GeneDesc*=CAS1 domain containing 1 [Source:HGNC Symbol;Acc:16014].

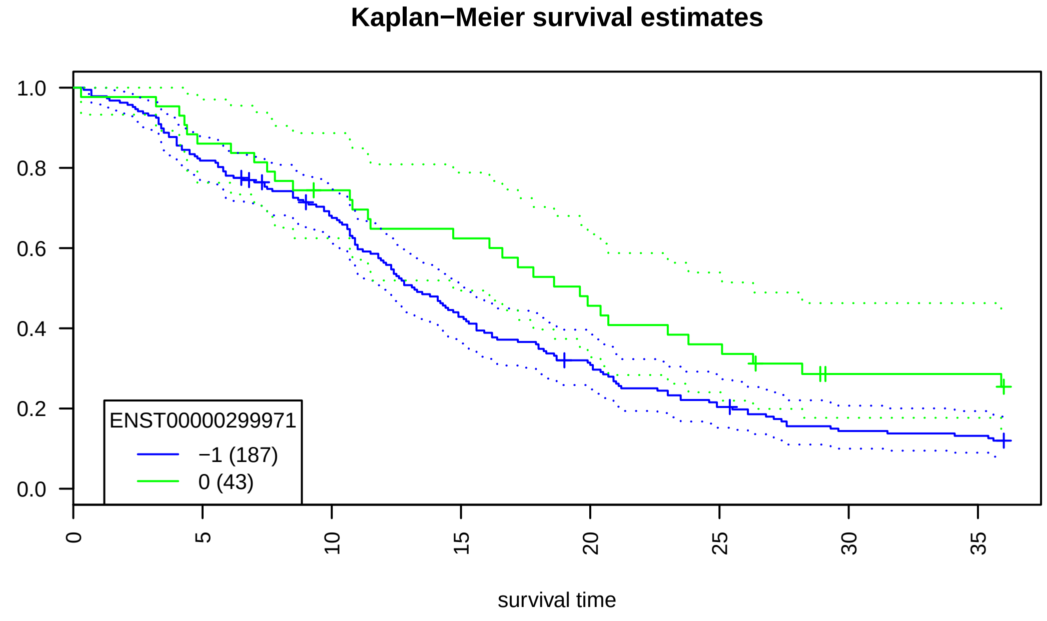

Figure 895: Each curve represents the relative number of ENST00000299971 survived to the corresponding point of time. Dashed lines represent 0.95 confidence intervals for the curves with the corresponding color. Log-rank probability measure for the equality of these curves is 2.813673e-02. Additional annotations: *.GeneId*=ENSG00000040199, *.GeneName*=PHLPP2, *.GeneDesc*=PH domain and leucine rich repeat protein phosphatase 2 [Source:HGNC Symbol;Acc:29149].

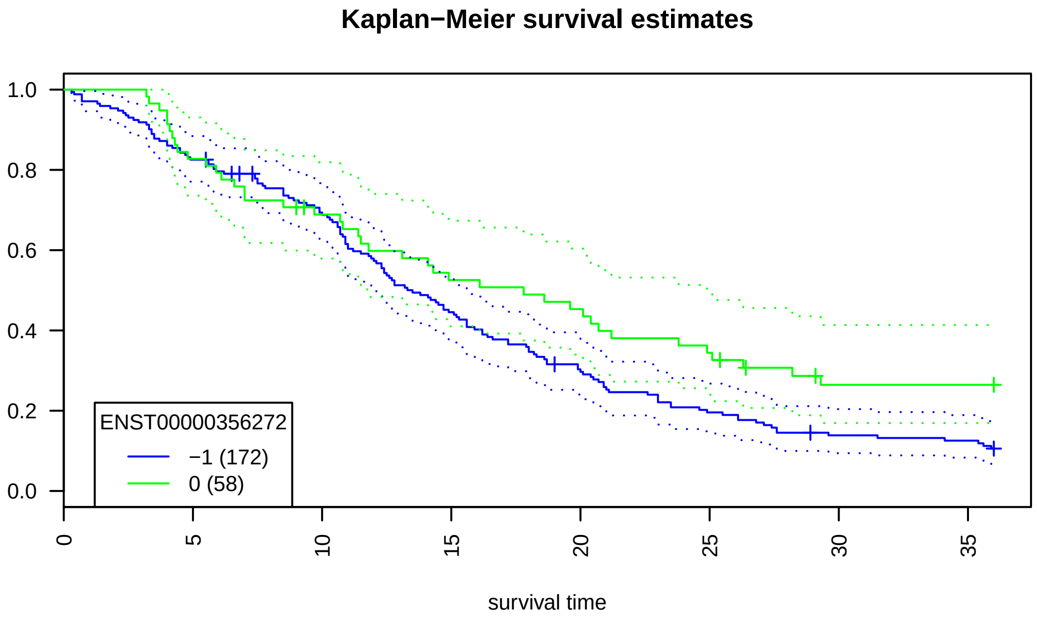

Figure 896: Each curve represents the relative number of ENST00000356272 survived to the corresponding point of time. Dashed lines represent 0.95 confidence intervals for the curves with the corresponding color. Log-rank probability measure for the equality of these curves is 2.675339e-02. Additional annotations: *.GeneId*=ENSG00000040199, *.GeneName*=PHLPP2, *.GeneDesc*=PH domain and leucine rich repeat protein phosphatase 2 [Source:HGNC Symbol;Acc:29149].

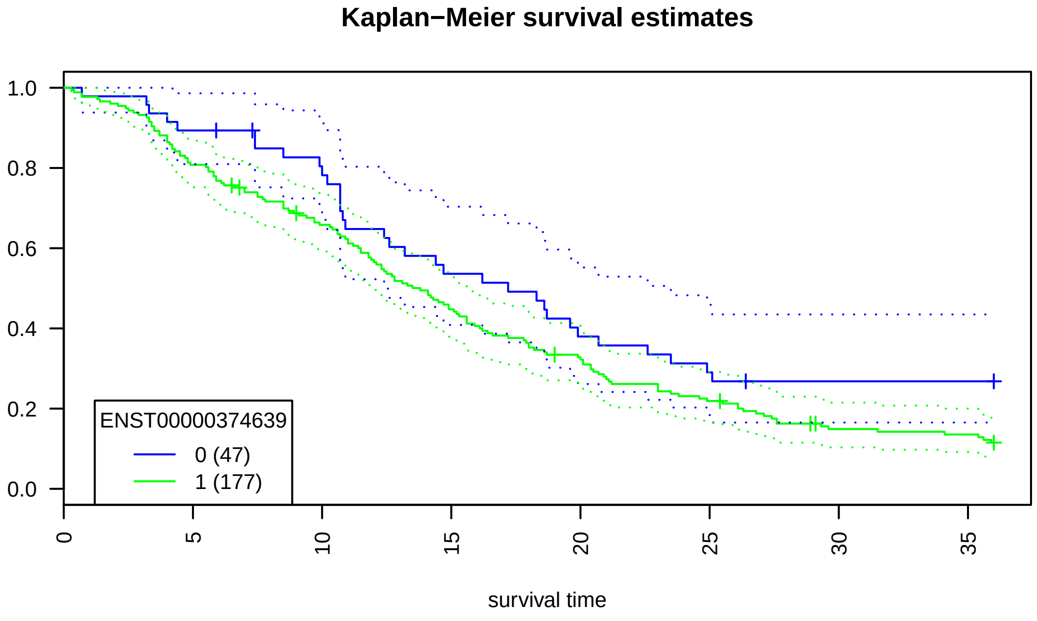

Figure 897: Each curve represents the relative number of ENST00000374639 survived to the corresponding point of time. Dashed lines represent 0.95 confidence intervals for the curves with the corresponding color. Total of 6 observations deleted due to missingness. Log-rank probability measure for the equality of these curves is 4.768416e-02. Additional annotations: *.GeneId*=ENSG00000159189, *.GeneName*=C1QC, *.GeneDesc*=complement component 1, q subcomponent, C chain [Source:HGNC Symbol;Acc:1245].

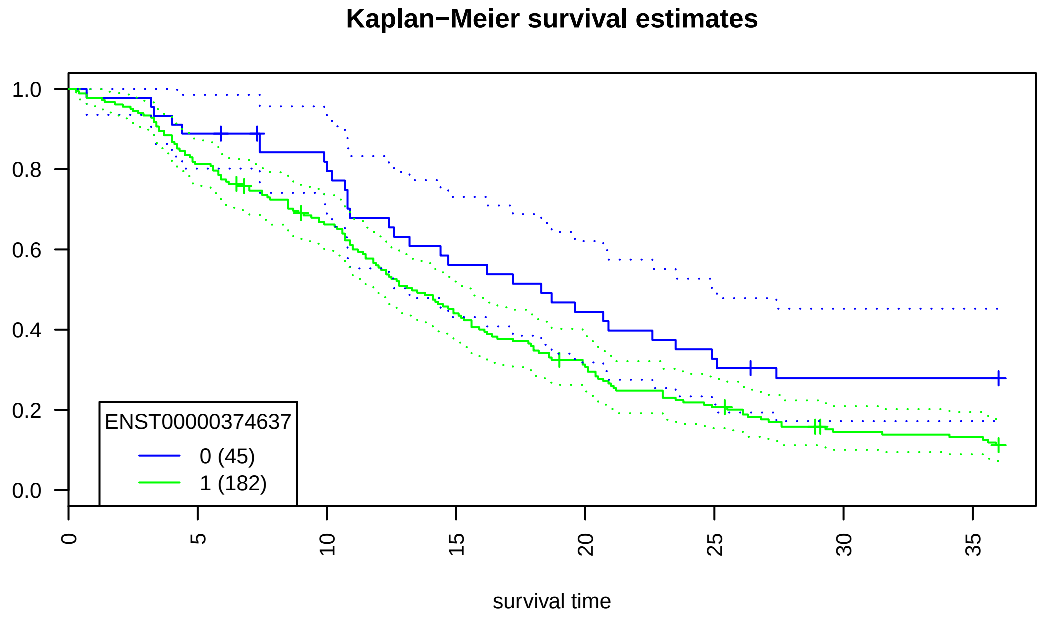

Figure 898: Each curve represents the relative number of ENST00000374637 survived to the corresponding point of time. Dashed lines represent 0.95 confidence intervals for the curves with the corresponding color. Total of 3 observations deleted due to missingness. Log-rank probability measure for the equality of these curves is 1.823568e-02. Additional annotations: *.GeneId*=ENSG00000159189, *.GeneName*=C1QC, *.GeneDesc*=complement component 1, q subcomponent, C chain [Source:HGNC Symbol;Acc:1245].

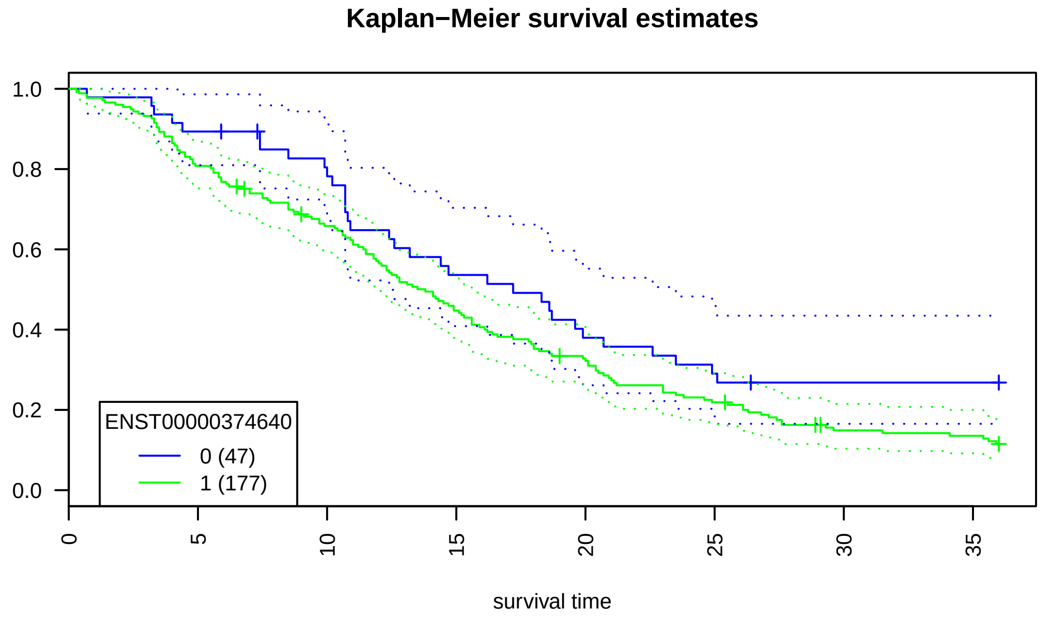

Figure 899: Each curve represents the relative number of ENST00000374640 survived to the corresponding point of time. Dashed lines represent 0.95 confidence intervals for the curves with the corresponding color. Total of 6 observations deleted due to missingness. Log-rank probability measure for the equality of these curves is 4.768416e-02. Additional annotations: *.GeneId*=ENSG00000159189, *.GeneName*=C1QC, *.GeneDesc*=complement component 1, q subcomponent, C chain [Source:HGNC Symbol;Acc:1245].

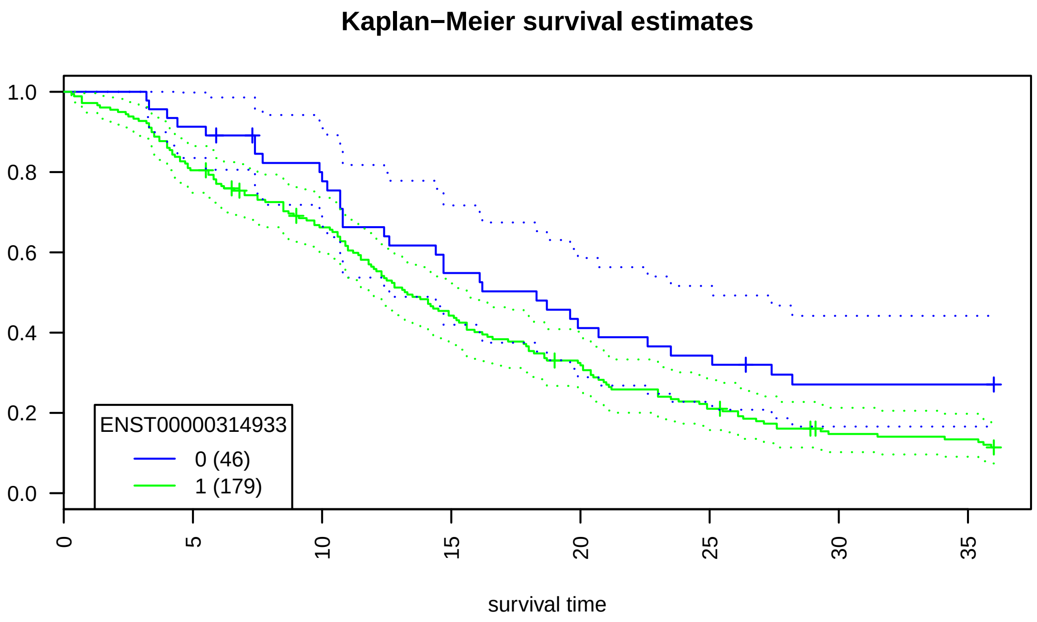

Figure 900: Each curve represents the relative number of ENST00000314933 survived to the corresponding point of time. Dashed lines represent 0.95 confidence intervals for the curves with the corresponding color. Total of 5 observations deleted due to missingness. Log-rank probability measure for the equality of these curves is 2.524263e-02. Additional annotations: *.GeneId*=ENSG00000173369, *.GeneName*=C1QB, *.GeneDesc*=complement component 1, q subcomponent, B chain [Source:HGNC Symbol;Acc:1242].

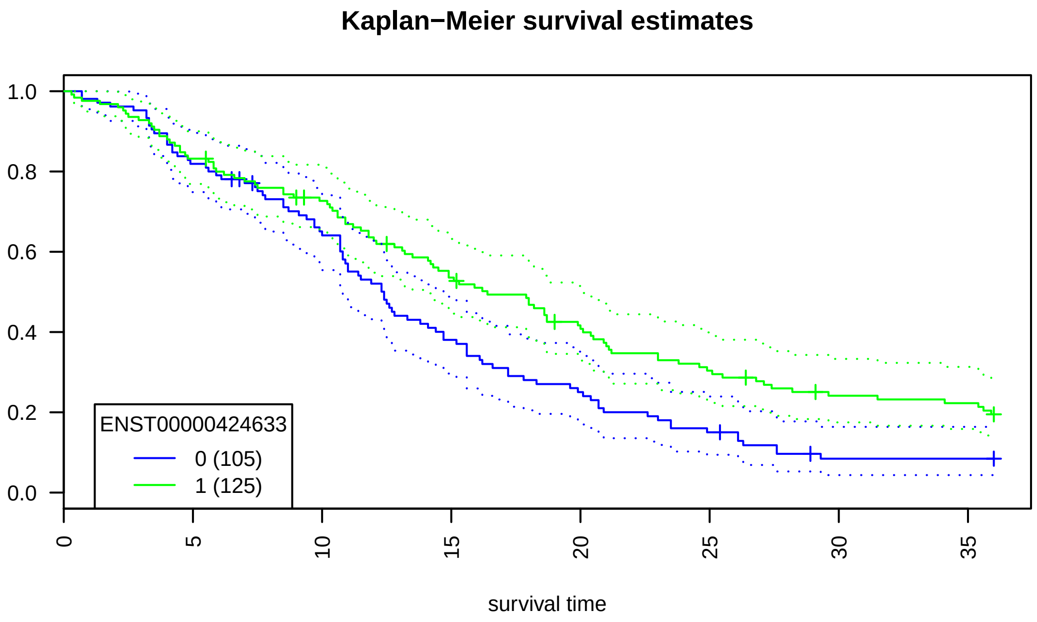

Figure 901: Each curve represents the relative number of ENST00000424633 survived to the corresponding point of time. Dashed lines represent 0.95 confidence intervals for the curves with the corresponding color. Log-rank probability measure for the equality of these curves is 4.338715e-03. Additional annotations: *.GeneId*=ENSG00000236992, *.GeneName*=RPL12L3, *.GeneDesc*=[undefined].

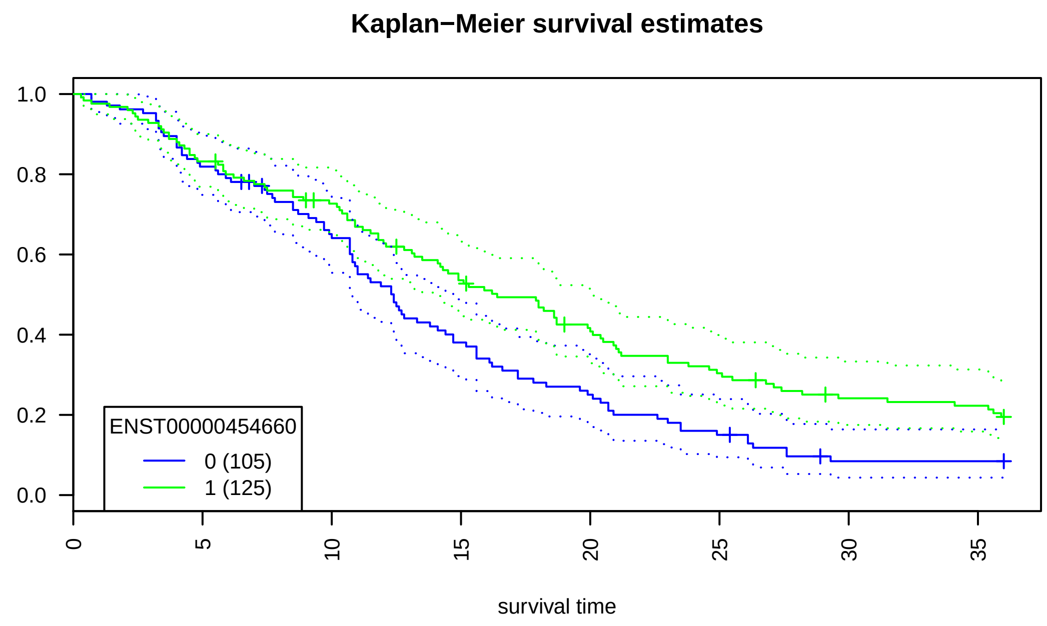

Figure 902: Each curve represents the relative number of ENST00000454660 survived to the corresponding point of time. Dashed lines represent 0.95 confidence intervals for the curves with the corresponding color. Log-rank probability measure for the equality of these curves is 4.338715e-03. Additional annotations: *.GeneId*=ENSG00000236992, *.GeneName*=RPL12L3, *.GeneDesc*=[undefined].

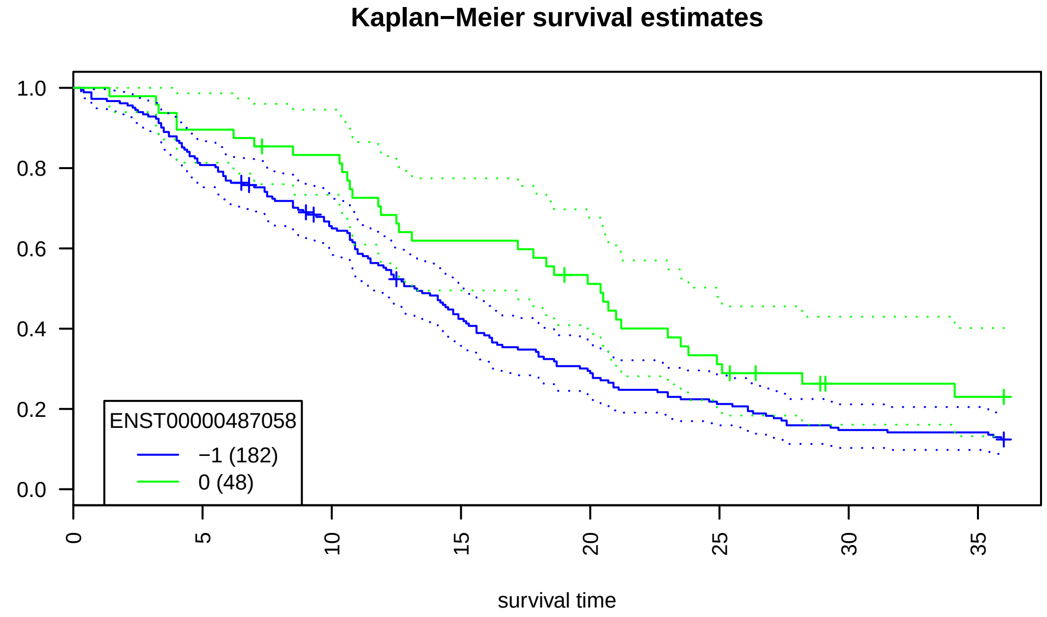

Figure 903: Each curve represents the relative number of ENST00000487058 survived to the corresponding point of time. Dashed lines represent 0.95 confidence intervals for the curves with the corresponding color. Log-rank probability measure for the equality of these curves is 2.238032e-02. Additional annotations: *.GeneId*=ENSG00000148672, *.GeneName*=GLUD1, *.GeneDesc*=glutamate dehydrogenase 1 [Source:HGNC Symbol;Acc:4335].

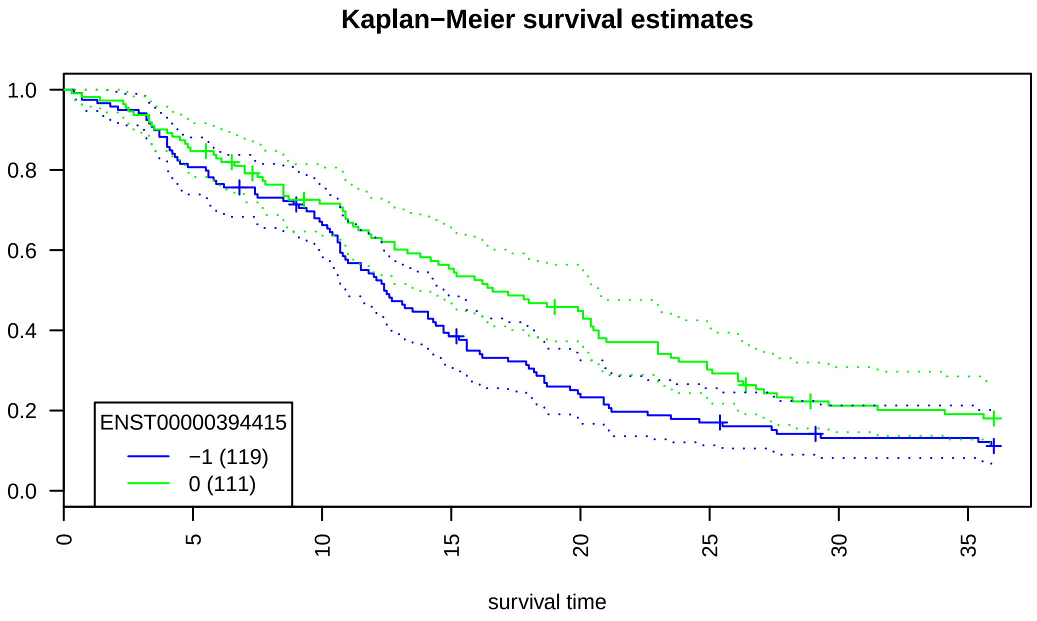

Figure 904: Each curve represents the relative number of ENST00000394415 survived to the corresponding point of time. Dashed lines represent 0.95 confidence intervals for the curves with the corresponding color. Log-rank probability measure for the equality of these curves is 1.944107e-02. Additional annotations: *.GeneId*=ENSG00000148672, *.GeneName*=GLUD1, *.GeneDesc*=glutamate dehydrogenase 1 [Source:HGNC Symbol;Acc:4335].

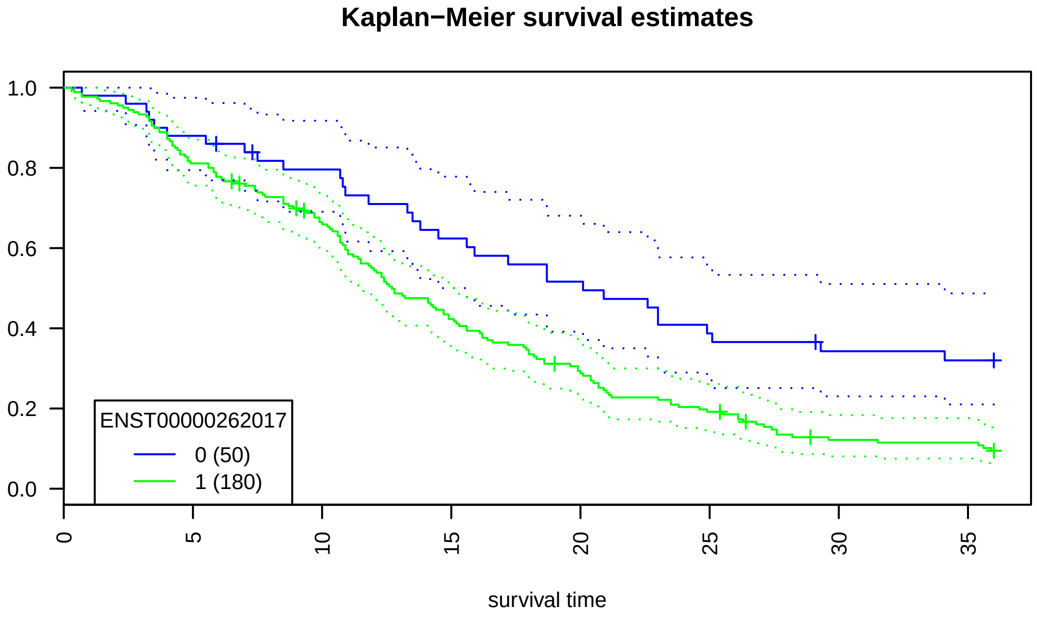

Figure 905: Each curve represents the relative number of ENST00000262017 survived to the corresponding point of time. Dashed lines represent 0.95 confidence intervals for the curves with the corresponding color. Log-rank probability measure for the equality of these curves is 7.049374e-04. Additional annotations: *.GeneId*=ENSG00000056345, *.GeneName*=ITGB3, *.GeneDesc*=integrin, beta 3 (platelet glycoprotein IIIa, antigen CD61) [Source:HGNC Symbol;Acc:6156].

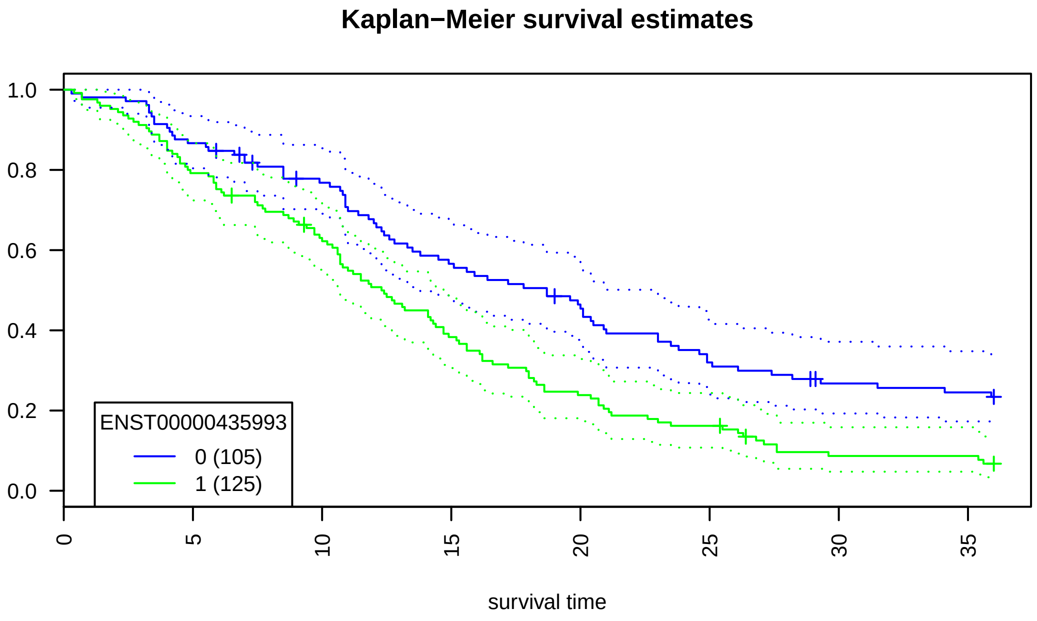

Figure 906: Each curve represents the relative number of ENST00000435993 survived to the corresponding point of time. Dashed lines represent 0.95 confidence intervals for the curves with the corresponding color. Log-rank probability measure for the equality of these curves is 1.411380e-04. Additional annotations: *.GeneId*=ENSG00000056345, *.GeneName*=ITGB3, *.GeneDesc*=integrin, beta 3 (platelet glycoprotein IIIa, antigen CD61) [Source:HGNC Symbol;Acc:6156].

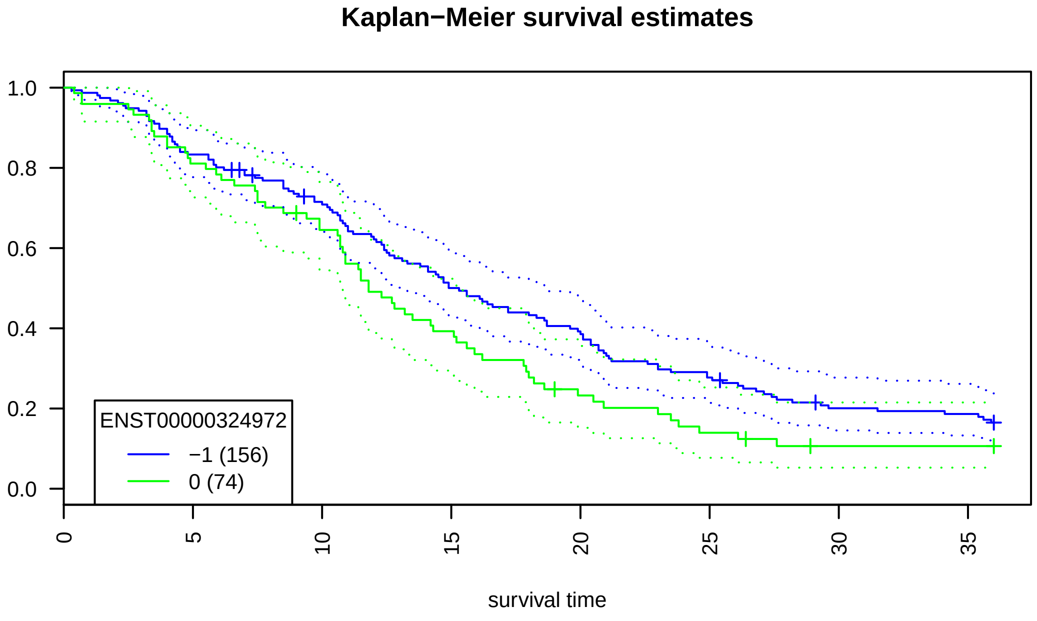

Figure 907: Each curve represents the relative number of ENST00000324972 survived to the corresponding point of time. Dashed lines represent 0.95 confidence intervals for the curves with the corresponding color. Log-rank probability measure for the equality of these curves is 3.896568e-02. Additional annotations: *.GeneId*=ENSG00000158560, *.GeneName*=DYNC1I1, *.GeneDesc*=dynein, cytoplasmic 1, intermediate chain 1 [Source:HGNC Symbol;Acc:2963].

Kaplan−Meier survival estimates

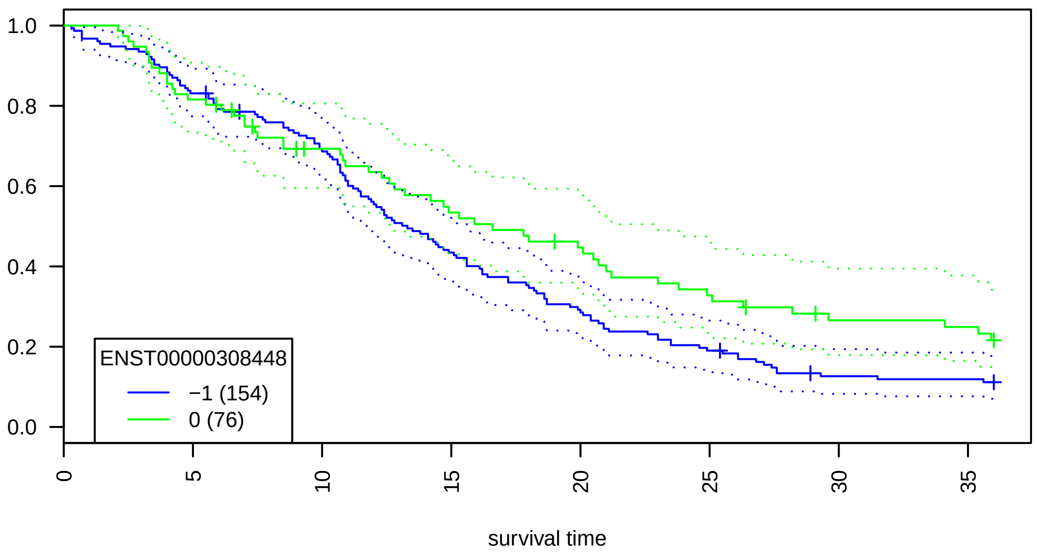

Figure 908: Each curve represents the relative number of ENST00000308448 survived to the corresponding point of time. Dashed lines represent 0.95 confidence intervals for the curves with the corresponding color. Log-rank probability measure for the equality of these curves is 4.095532e-02. Additional annotations: *.GeneId*=ENSG00000138138, *.GeneName*=ATAD1, *.GeneDesc*=ATPase family, AAA domain containing 1 [Source:HGNC Symbol;Acc:25903].

Kaplan−Meier survival estimates

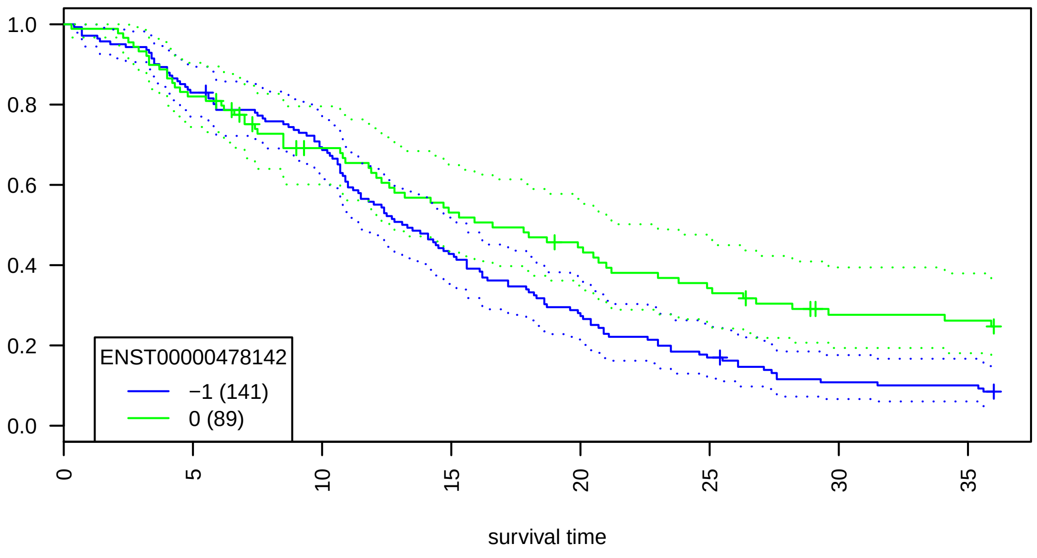

Figure 909: Each curve represents the relative number of ENST00000478142 survived to the corresponding point of time. Dashed lines represent 0.95 confidence intervals for the curves with the corresponding color. Log-rank probability measure for the equality of these curves is 5.805582e-03. Additional annotations: *.GeneId*=ENSG00000138138, *.GeneName*=ATAD1, *.GeneDesc*=ATPase family, AAA domain containing 1 [Source:HGNC Symbol;Acc:25903].

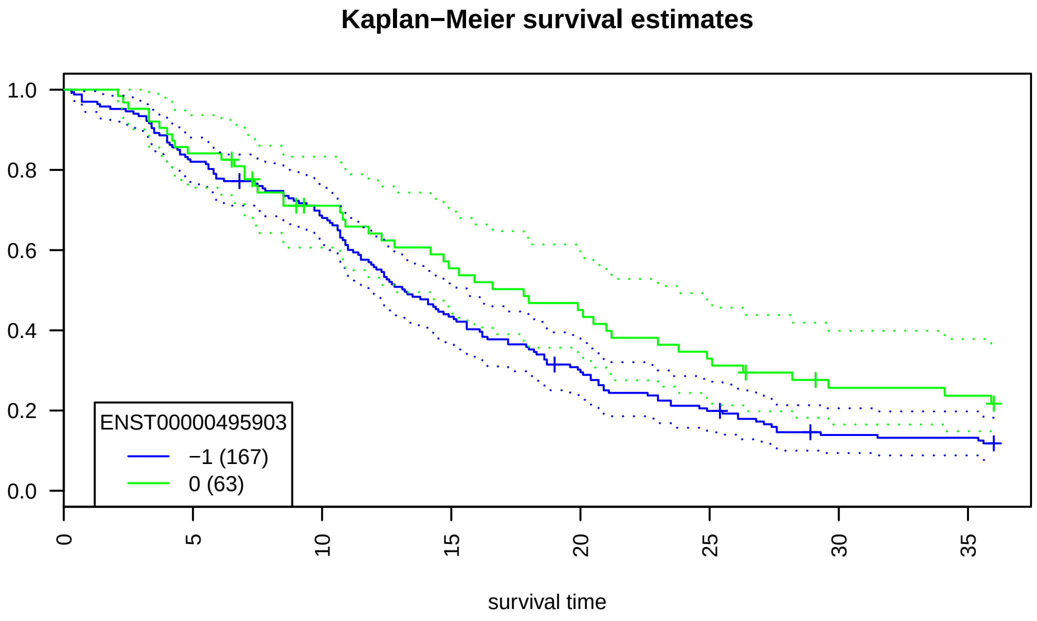

Figure 910: Each curve represents the relative number of ENST00000495903 survived to the corresponding point of time. Dashed lines represent 0.95 confidence intervals for the curves with the corresponding color. Log-rank probability measure for the equality of these curves is 4.794358e-02. Additional annotations: *.GeneId*=ENSG00000138138, *.GeneName*=ATAD1, *.GeneDesc*=ATPase family, AAA domain containing 1 [Source:HGNC Symbol;Acc:25903].

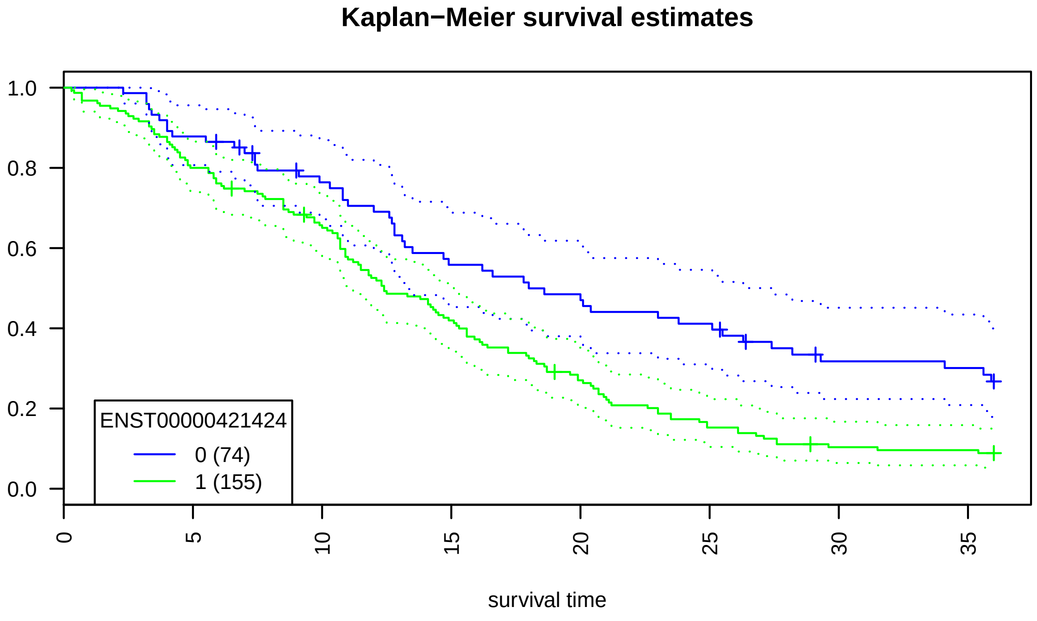

Figure 911: Each curve represents the relative number of ENST00000421424 survived to the corresponding point of time. Dashed lines represent 0.95 confidence intervals for the curves with the corresponding color. Total of 1 observation deleted due to missingness. Log-rank probability measure for the equality of these curves is 3.234496e-04. Additional annotations: *.GeneId*=ENSG00000229230, *.GeneName*=C20orf127, *.GeneDesc*=[undefined].

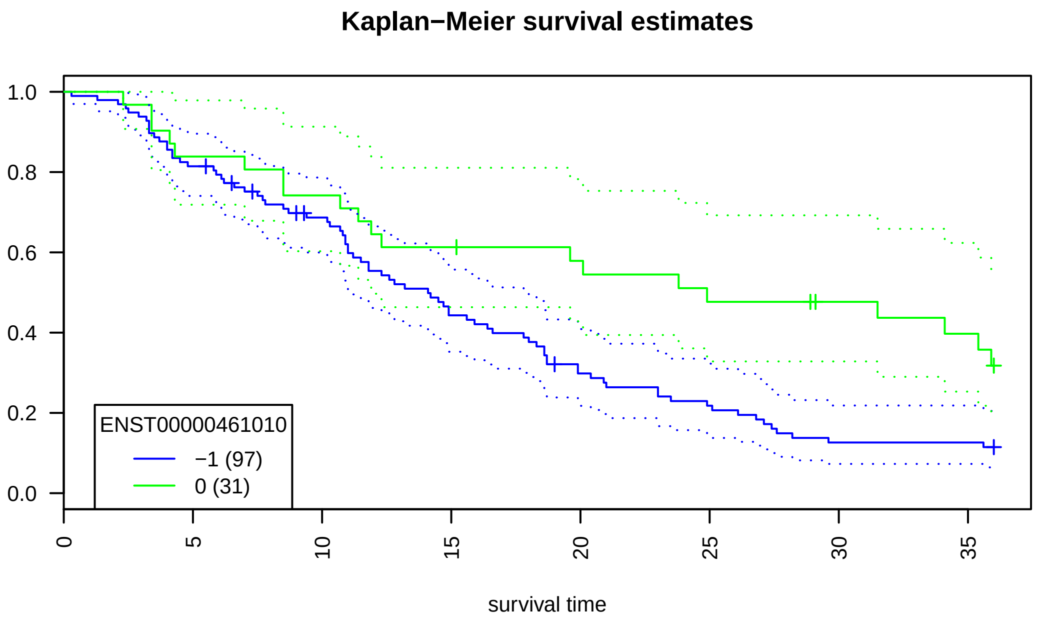

Figure 912: Each curve represents the relative number of ENST00000461010 survived to the corresponding point of time. Dashed lines represent 0.95 confidence intervals for the curves with the corresponding color. Total of 102 observations deleted due to missingness. Log-rank probability measure for the equality of these curves is 7.528528e-03. Additional annotations: *.GeneId*=ENSG00000112640, *.GeneName*=PPP2R5D, *.GeneDesc*=protein phosphatase 2, regulatory subunit B', delta isoform [Source:HGNC Symbol;Acc:9312].

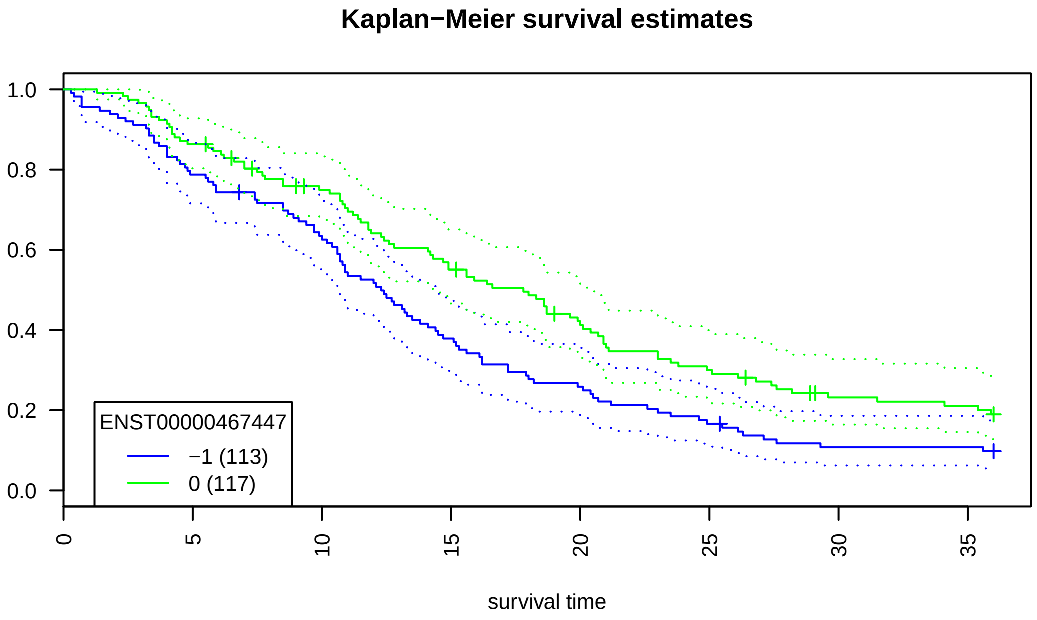

Figure 913: Each curve represents the relative number of ENST00000467447 survived to the corresponding point of time. Dashed lines represent 0.95 confidence intervals for the curves with the corresponding color. Log-rank probability measure for the equality of these curves is 3.917496e-03. Additional annotations: *.GeneId*=ENSG00000112640, *.GeneName*=PPP2R5D, *.GeneDesc*=protein phosphatase 2, regulatory subunit B', delta isoform [Source:HGNC Symbol;Acc:9312].

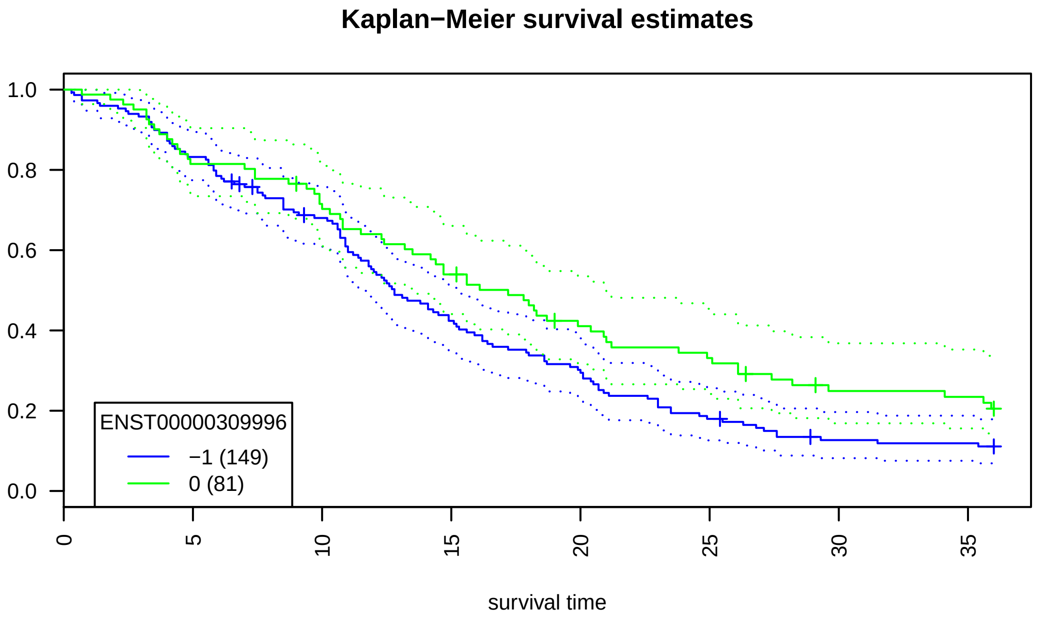

Figure 914: Each curve represents the relative number of ENST00000309996 survived to the corresponding point of time. Dashed lines represent 0.95 confidence intervals for the curves with the corresponding color. Log-rank probability measure for the equality of these curves is 3.119913e-02. Additional annotations: *.GeneId*=ENSG00000173898, *.GeneName*=SPTBN2, *.GeneDesc*=spectrin, beta, non-erythrocytic 2 [Source:HGNC Symbol;Acc:11276].

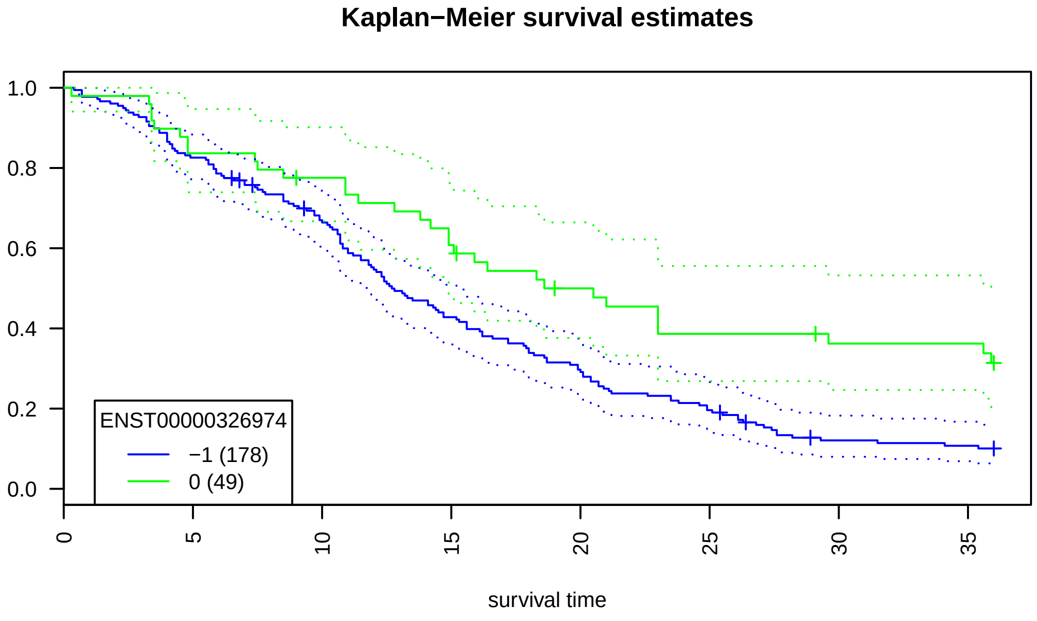

Figure 915: Each curve represents the relative number of ENST00000326974 survived to the corresponding point of time. Dashed lines represent 0.95 confidence intervals for the curves with the corresponding color. Total of 3 observations deleted due to missingness. Log-rank probability measure for the equality of these curves is 1.224886e-03. Additional annotations: *.GeneId*=ENSG00000124702, *.GeneName*=KLHDC3, *.GeneDesc*=kelch domain containing 3 [Source:HGNC Symbol;Acc:20704].

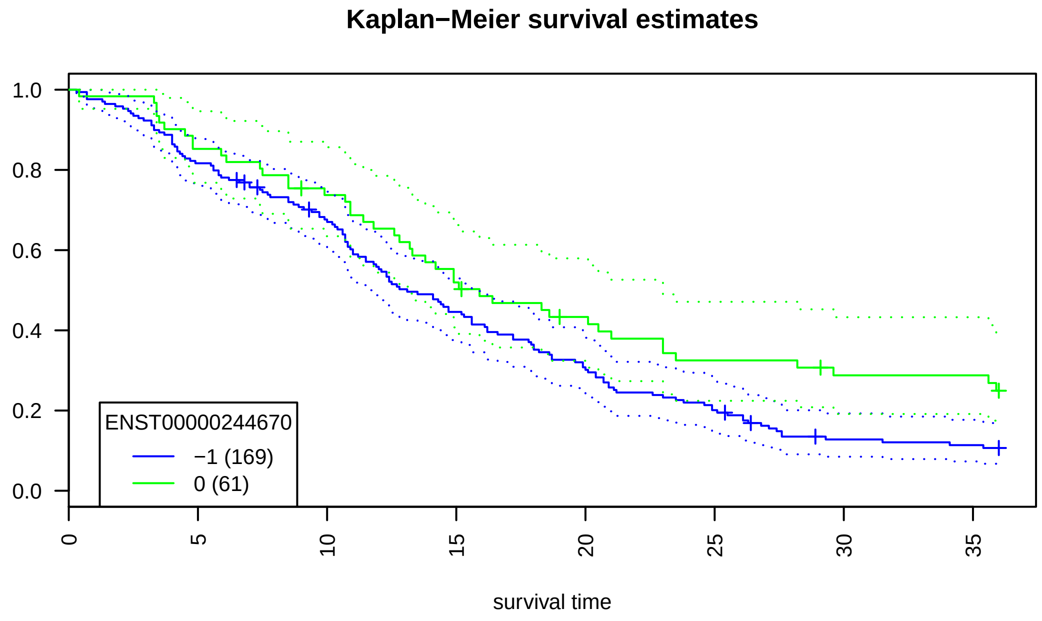

Figure 916: Each curve represents the relative number of ENST00000244670 survived to the corresponding point of time. Dashed lines represent 0.95 confidence intervals for the curves with the corresponding color. Log-rank probability measure for the equality of these curves is 1.918259e-02. Additional annotations: *.GeneId*=ENSG00000124702, *.GeneName*=KLHDC3, *.GeneDesc*=kelch domain containing 3 [Source:HGNC Symbol;Acc:20704].

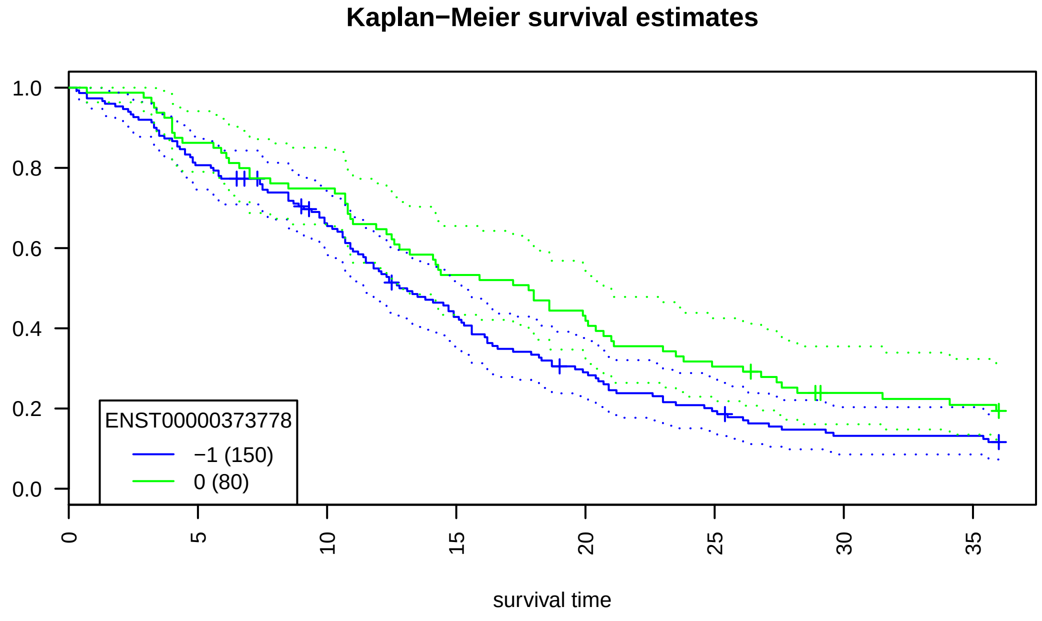

Figure 917: Each curve represents the relative number of ENST00000373778 survived to the corresponding point of time. Dashed lines represent 0.95 confidence intervals for the curves with the corresponding color. Log-rank probability measure for the equality of these curves is 4.12585e-02. Additional annotations: *.GeneId*=ENSG00000175764, *.GeneName*=TTLL11, *.GeneDesc*=tubulin tyrosine ligase-like family, member 11 [Source:HGNC Symbol;Acc:18113].

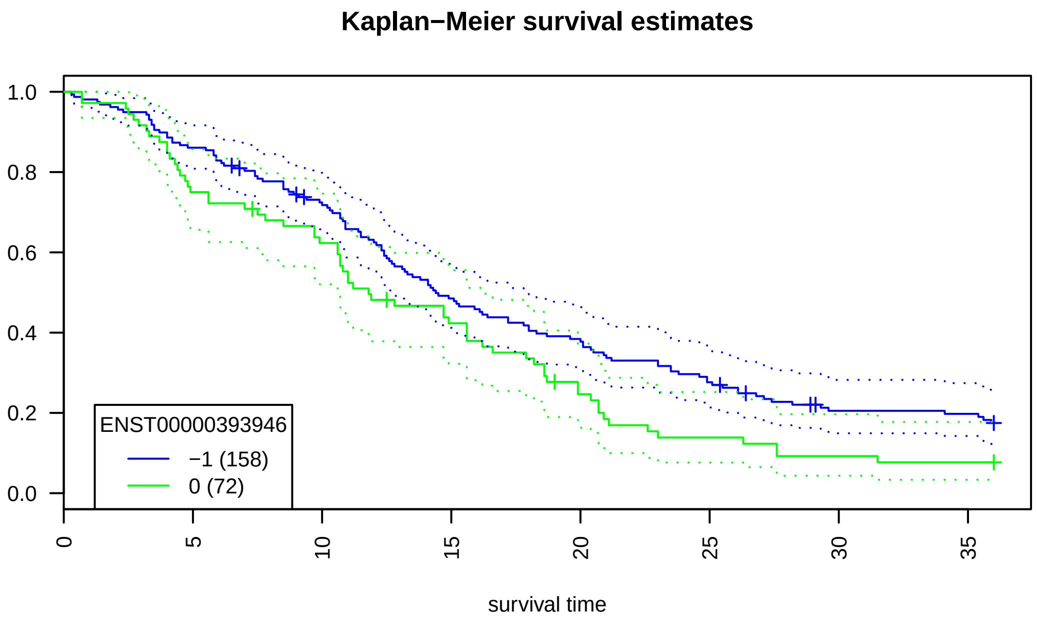

Figure 918: Each curve represents the relative number of ENST00000393946 survived to the corresponding point of time. Dashed lines represent 0.95 confidence intervals for the curves with the corresponding color. Log-rank probability measure for the equality of these curves is 2.037799e-02. Additional annotations: *.GeneId*=ENSG00000173227, *.GeneName*=SYT12, *.GeneDesc*=synaptotagmin XII [Source:HGNC Symbol;Acc:18381].

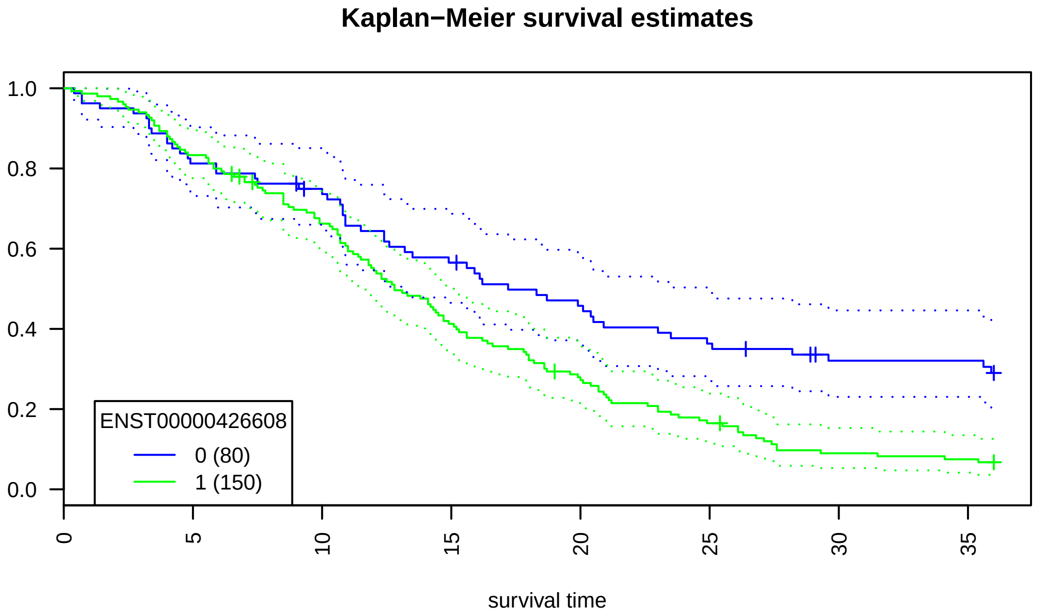

Figure 919: Each curve represents the relative number of ENST00000426608 survived to the corresponding point of time. Dashed lines represent 0.95 confidence intervals for the curves with the corresponding color. Log-rank probability measure for the equality of these curves is 3.185861e-04. Additional annotations: *.GeneId*=ENSG00000095303, *.GeneName*=PTGS1, *.GeneDesc*=prostaglandin-endoperoxide synthase 1 (prostaglandin G/H synthase and cyclooxygenase) [Source:HGNC Symbol;Acc:9604].

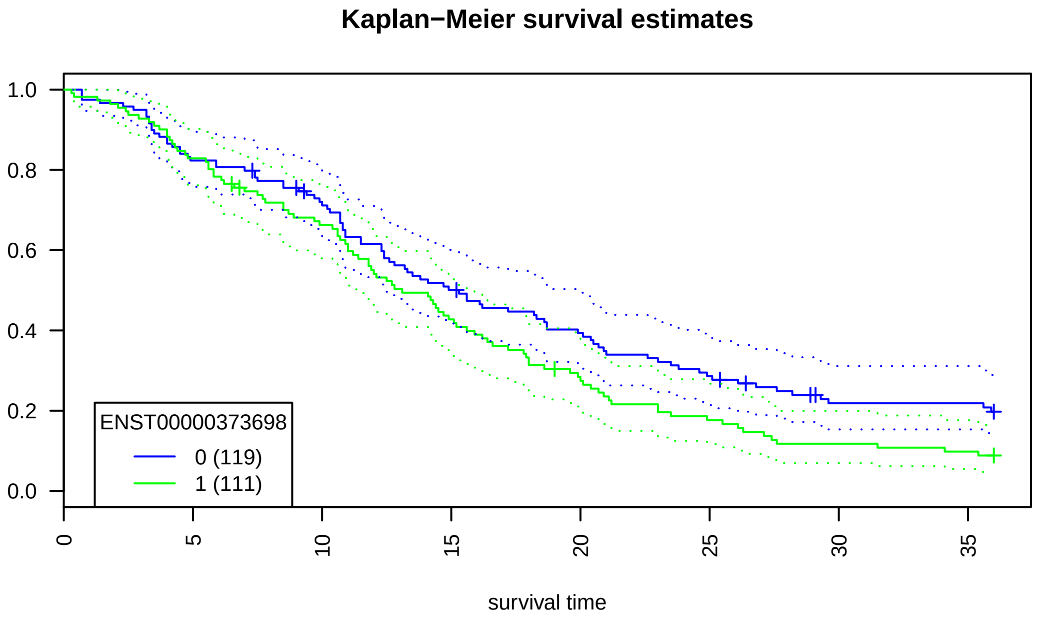

Figure 920: Each curve represents the relative number of ENST00000373698 survived to the corresponding point of time. Dashed lines represent 0.95 confidence intervals for the curves with the corresponding color. Log-rank probability measure for the equality of these curves is 3.270605e-02. Additional annotations: *.GeneId*=ENSG00000095303, *.GeneName*=PTGS1, *.GeneDesc*=prostaglandin-endoperoxide synthase 1 (prostaglandin G/H synthase and cyclooxygenase) [Source:HGNC Symbol;Acc:9604].

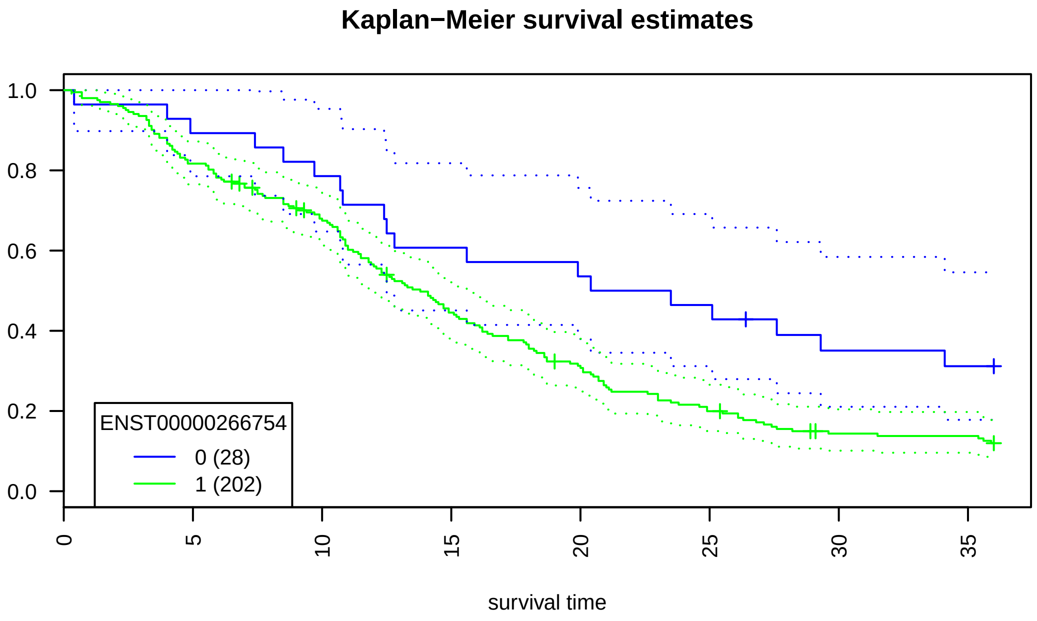

Figure 921: Each curve represents the relative number of ENST00000266754 survived to the corresponding point of time. Dashed lines represent 0.95 confidence intervals for the curves with the corresponding color. Log-rank probability measure for the equality of these curves is 1.150882e-02. Additional annotations: *.GeneId*=ENSG00000139354, *.GeneName*=GAS2L3, *.GeneDesc*=growth arrest-specific 2 like 3 [Source:HGNC Symbol;Acc:27475].

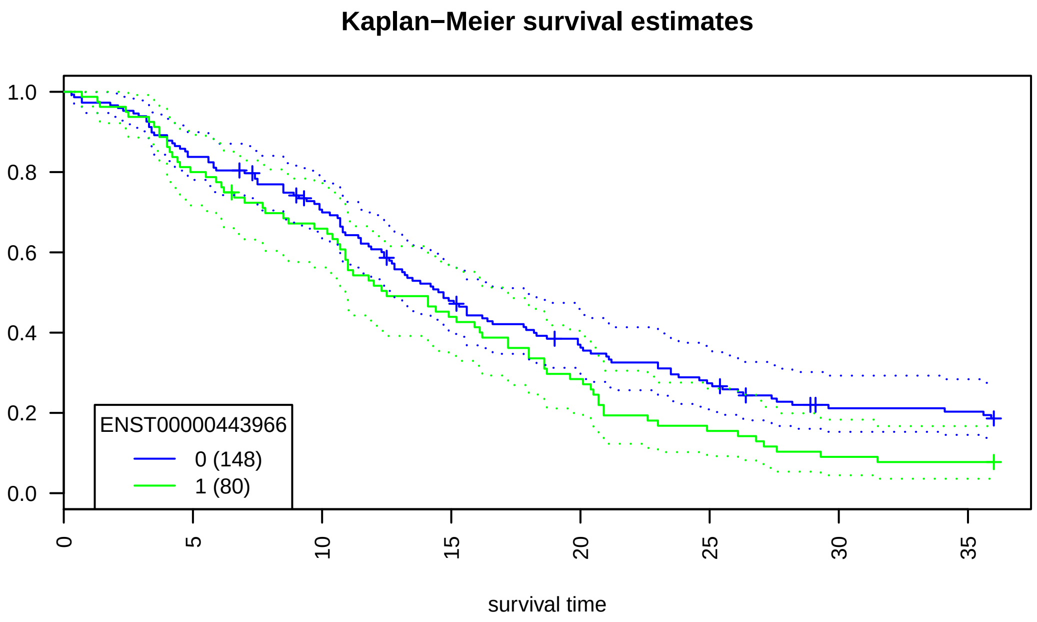

Figure 922: Each curve represents the relative number of ENST00000443966 survived to the corresponding point of time. Dashed lines represent 0.95 confidence intervals for the curves with the corresponding color. Total of 2 observations deleted due to missingness. Log-rank probability measure for the equality of these curves is 4.004961e-02. Additional annotations: *.GeneId*=ENSG00000235590, *.GeneName*=GNASAS, *.GeneDesc*=GNAS antisense RNA (non-protein coding) [Source:HGNC Symbol;Acc:24872].

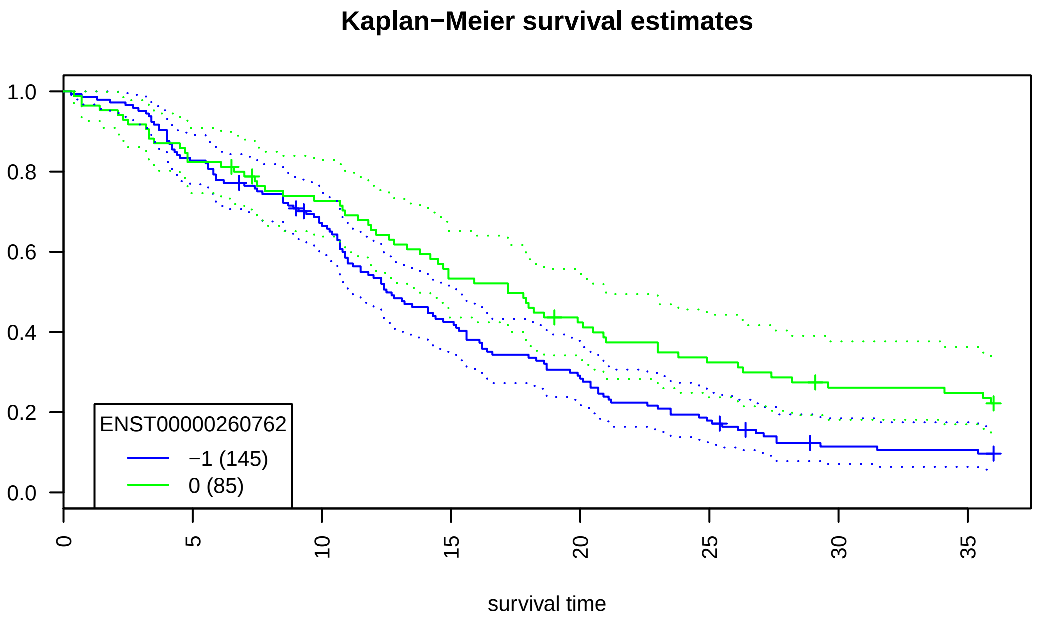

Figure 923: Each curve represents the relative number of ENST00000260762 survived to the corresponding point of time. Dashed lines represent 0.95 confidence intervals for the curves with the corresponding color. Log-rank probability measure for the equality of these curves is 9.637672e-03. Additional annotations: *.GeneId*=ENSG00000138190, *.GeneName*=EXOC6, *.GeneDesc*=exocyst complex component 6 [Source:HGNC Symbol;Acc:23196].

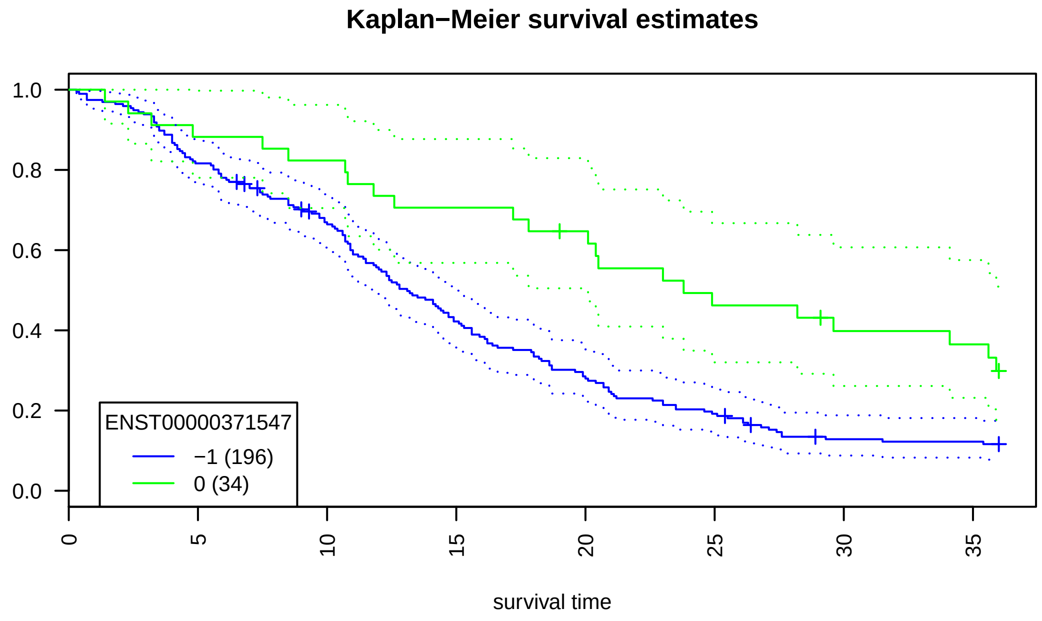

Figure 924: Each curve represents the relative number of ENST00000371547 survived to the corresponding point of time. Dashed lines represent 0.95 confidence intervals for the curves with the corresponding color. Log-rank probability measure for the equality of these curves is 1.044527e-03. Additional annotations: *.GeneId*=ENSG00000138190, *.GeneName*=EXOC6, *.GeneDesc*=exocyst complex component 6 [Source:HGNC Symbol;Acc:23196].

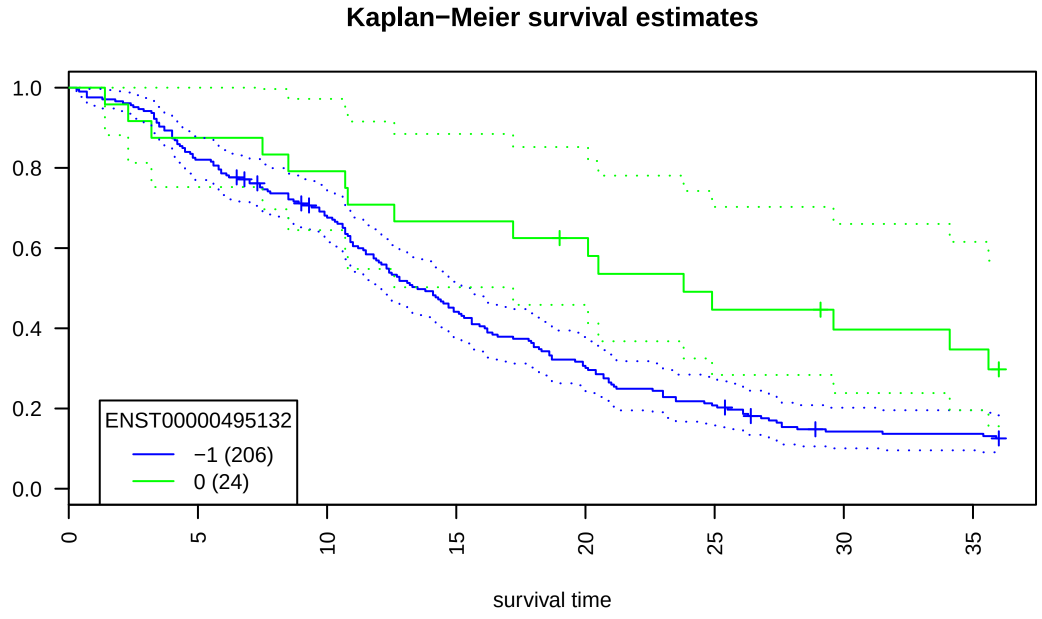

Figure 925: Each curve represents the relative number of ENST00000495132 survived to the corresponding point of time. Dashed lines represent 0.95 confidence intervals for the curves with the corresponding color. Log-rank probability measure for the equality of these curves is 1.381511e-02. Additional annotations: *.GeneId*=ENSG00000138190, *.GeneName*=EXOC6, *.GeneDesc*=exocyst complex component 6 [Source:HGNC Symbol;Acc:23196].

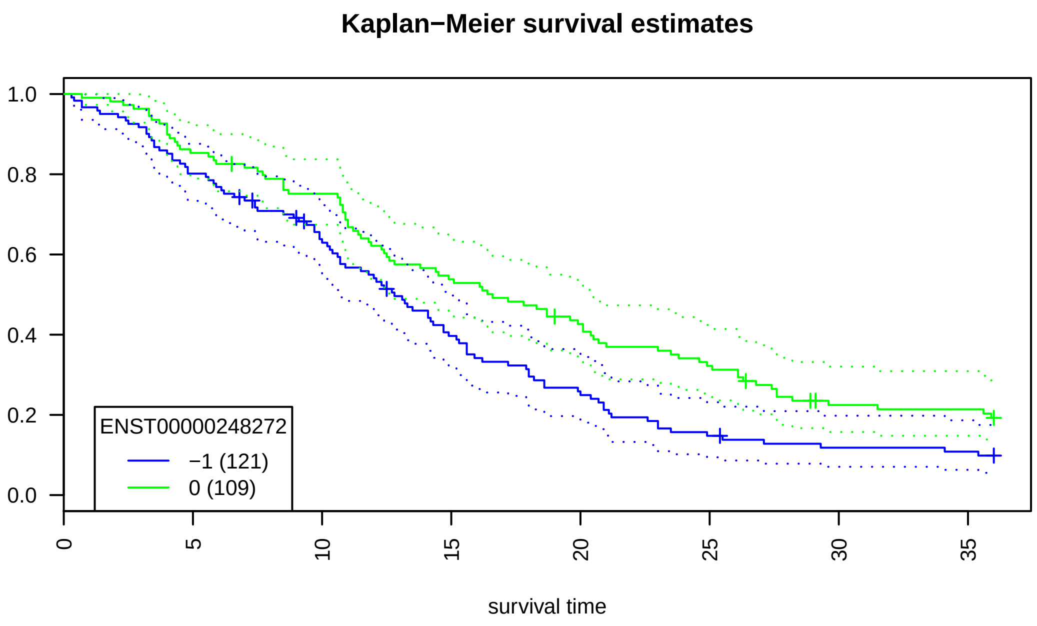

Figure 926: Each curve represents the relative number of ENST00000248272 survived to the corresponding point of time. Dashed lines represent 0.95 confidence intervals for the curves with the corresponding color. Log-rank probability measure for the equality of these curves is 5.908303e-03. Additional annotations: *.GeneId*=ENSG00000127688, *.GeneName*=GAN, *.GeneDesc*=gigaxonin [Source:HGNC Symbol;Acc:4137].

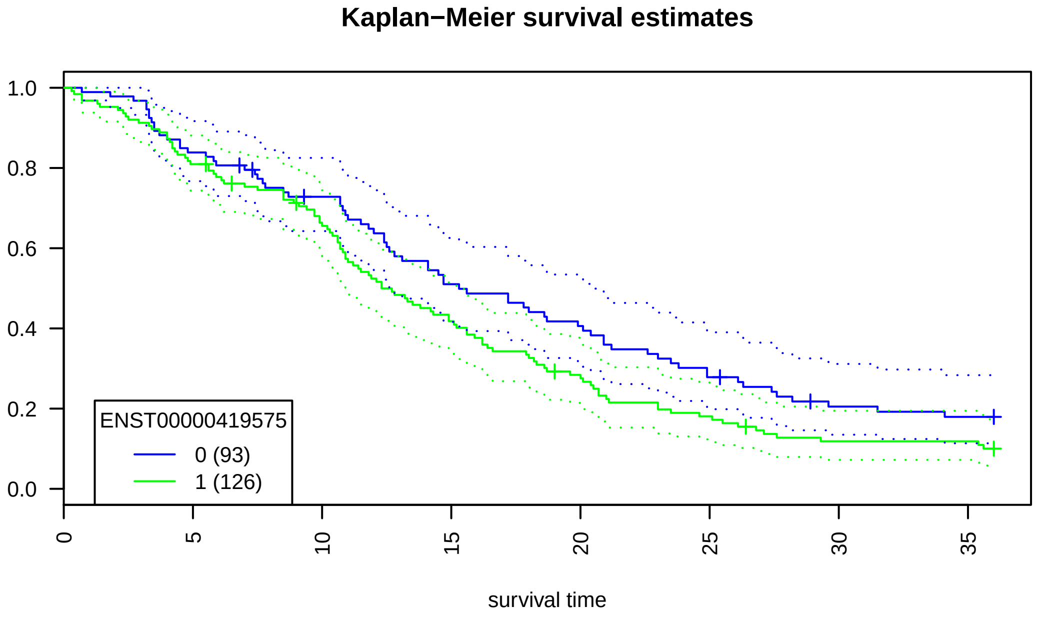

Figure 927: Each curve represents the relative number of ENST00000419575 survived to the corresponding point of time. Dashed lines represent 0.95 confidence intervals for the curves with the corresponding color. Total of 11 observations deleted due to missingness. Log-rank probability measure for the equality of these curves is 4.204941e-02. Additional annotations: *.GeneId*=ENSG00000160862, *.GeneName*=AZGP1, *.GeneDesc*=alpha-2-glycoprotein 1, zinc-binding [Source:HGNC Symbol;Acc:910].

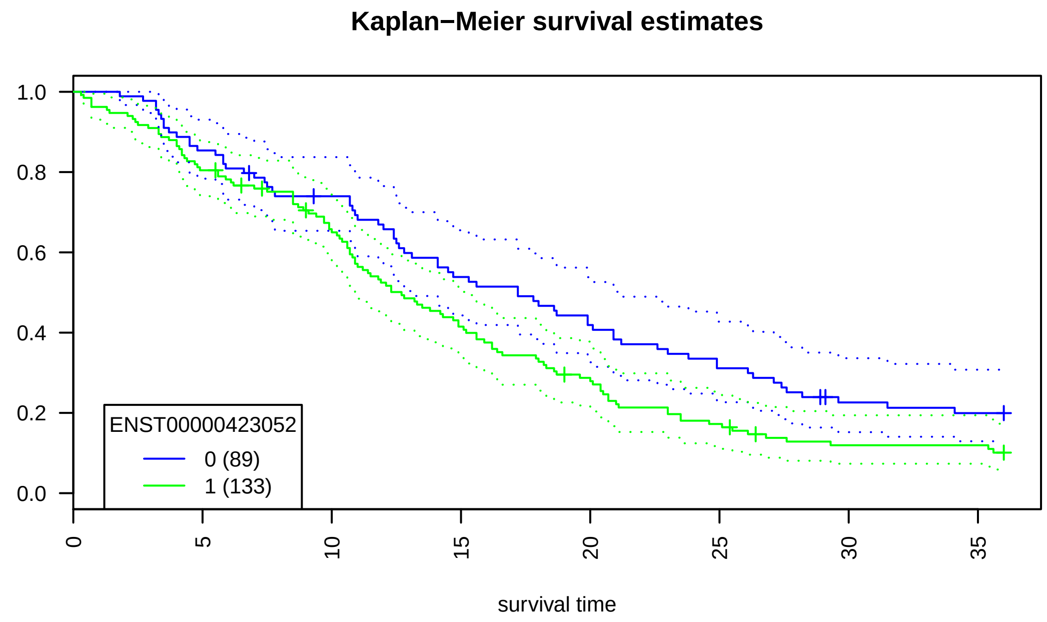

Figure 928: Each curve represents the relative number of ENST00000423052 survived to the corresponding point of time. Dashed lines represent 0.95 confidence intervals for the curves with the corresponding color. Total of 8 observations deleted due to missingness. Log-rank probability measure for the equality of these curves is 1.374069e-02. Additional annotations: *.GeneId*=ENSG00000160862, *.GeneName*=AZGP1, *.GeneDesc*=alpha-2-glycoprotein 1, zinc-binding [Source:HGNC Symbol;Acc:910].

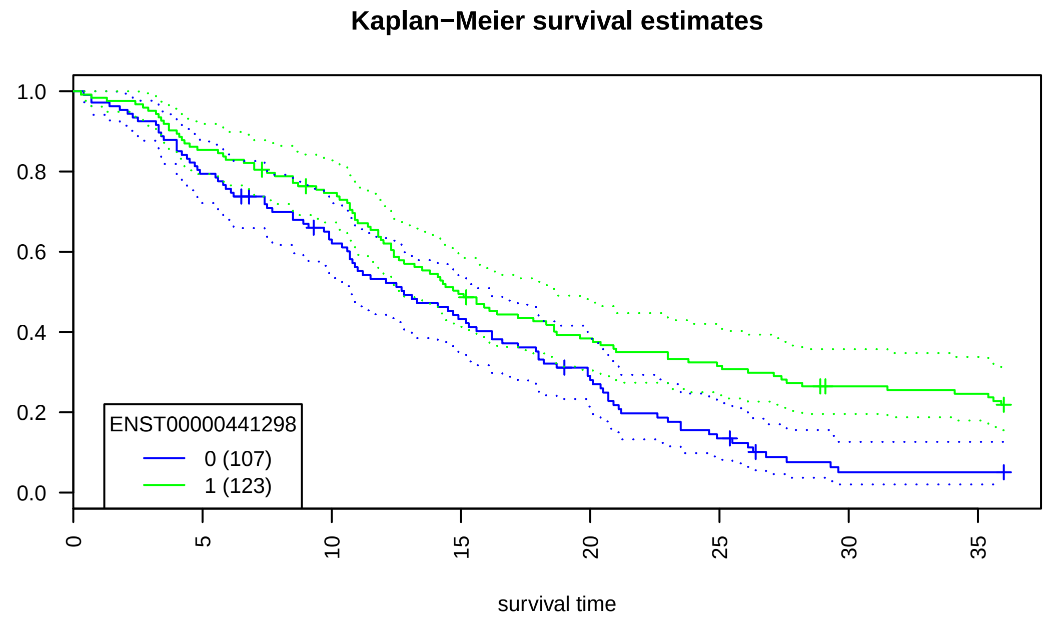

Figure 929: Each curve represents the relative number of ENST00000441298 survived to the corresponding point of time. Dashed lines represent 0.95 confidence intervals for the curves with the corresponding color. Log-rank probability measure for the equality of these curves is 1.501988e-03. Additional annotations: *.GeneId*=ENSG00000166526, *.GeneName*=ZNF3, *.GeneDesc*=zinc finger protein 3 [Source:HGNC Symbol;Acc:13089].

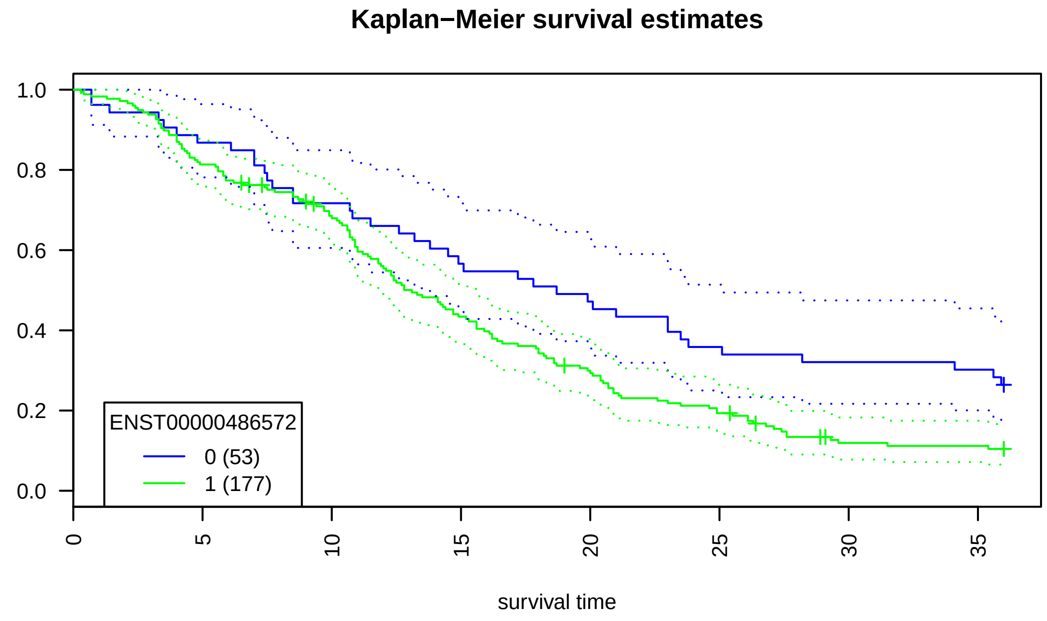

Figure 930: Each curve represents the relative number of ENST00000486572 survived to the corresponding point of time. Dashed lines represent 0.95 confidence intervals for the curves with the corresponding color. Log-rank probability measure for the equality of these curves is 7.165986e-03. Additional annotations: $.GeneId$=ENSG00000108821, $.GeneName$=COL1A1, $.GeneDesc$=collagen, type I, alpha 1 [Source:HGNC Symbol;Acc:2197].

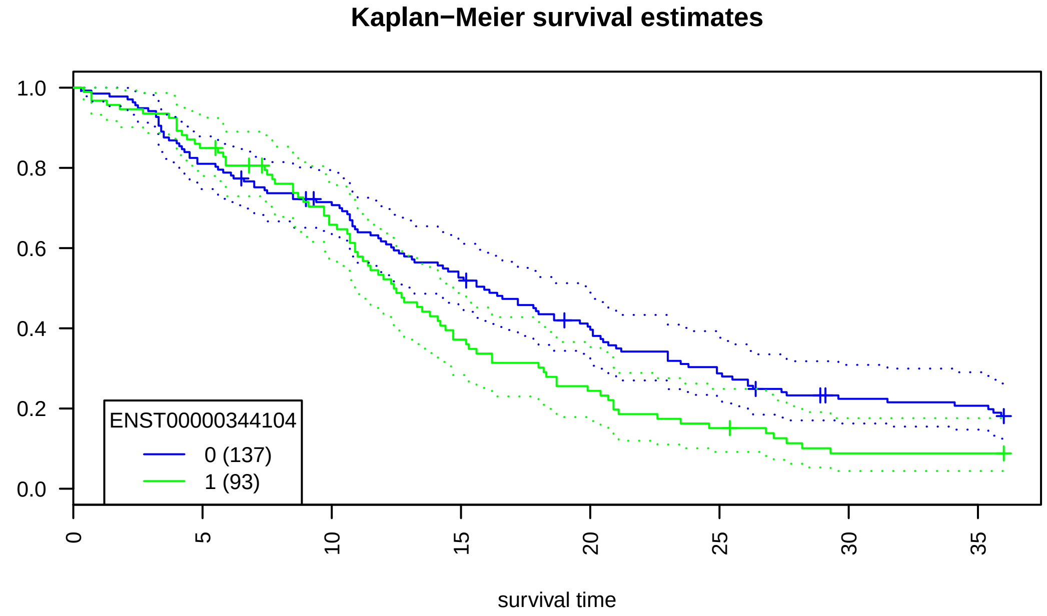

Figure 931: Each curve represents the relative number of ENST00000344104 survived to the corresponding point of time. Dashed lines represent 0.95 confidence intervals for the curves with the corresponding color. Log-rank probability measure for the equality of these curves is 2.337435e-02. Additional annotations: $.GeneId$=ENSG00000160460, $.GeneName$=SPTBN4, $.GeneDesc$=spectrin, beta, non-erythrocytic 4 [Source:HGNC Symbol;Acc:14896].

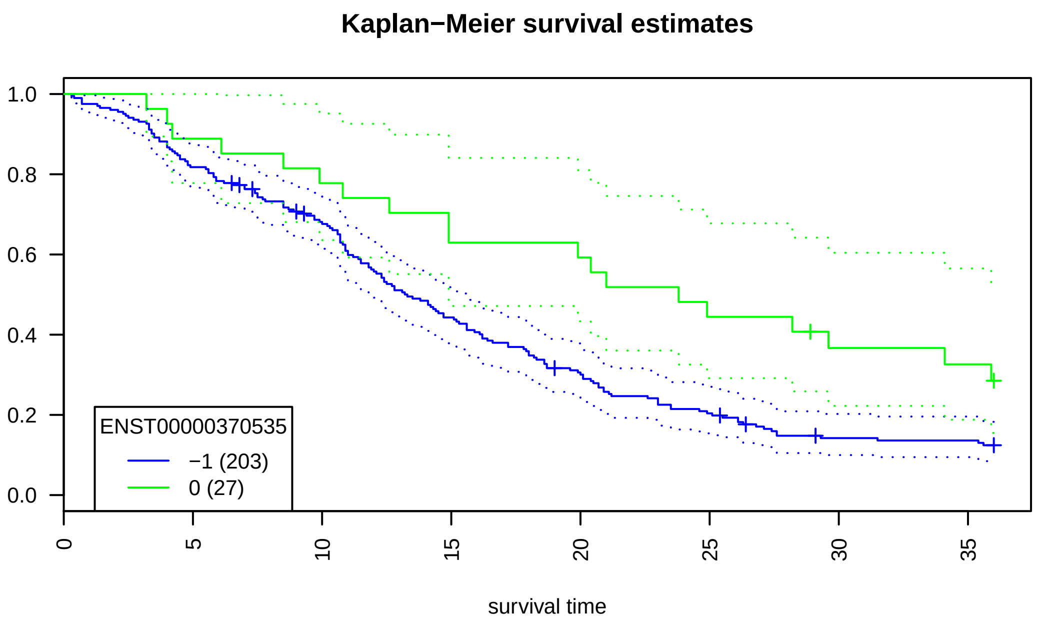

Figure 932: Each curve represents the relative number of ENST00000370535 survived to the corresponding point of time. Dashed lines represent 0.95 confidence intervals for the curves with the corresponding color. Log-rank probability measure for the equality of these curves is 8.950045e-03. Additional annotations: *.GeneId*=ENSG00000203930, *.GeneName*=RP1-177G6.2, *.GeneDesc*=Novel protein [Source:UniProtKB/TrEMBL;Acc:Q5JPA2].

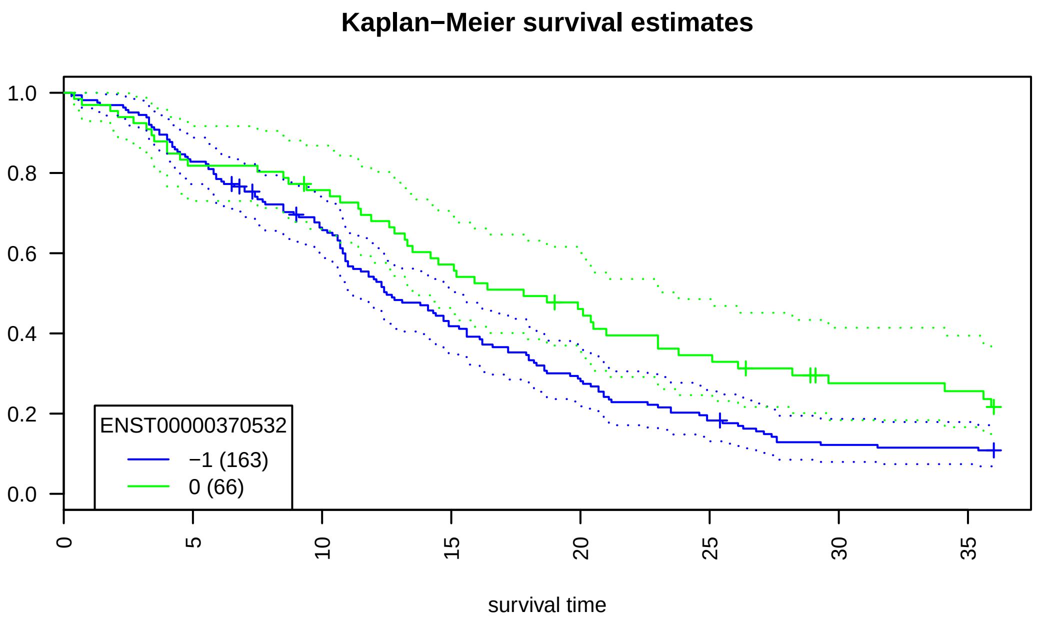

Figure 933: Each curve represents the relative number of ENST00000370532 survived to the corresponding point of time. Dashed lines represent 0.95 confidence intervals for the curves with the corresponding color. Total of 1 observation deleted due to missingness. Log-rank probability measure for the equality of these curves is 1.280911e-02. Additional annotations: *.GeneId*=ENSG00000184258, *.GeneName*=CDR1, *.GeneDesc*=cerebellar degeneration-related protein 1, 34kDa [Source:HGNC Symbol;Acc:1798].

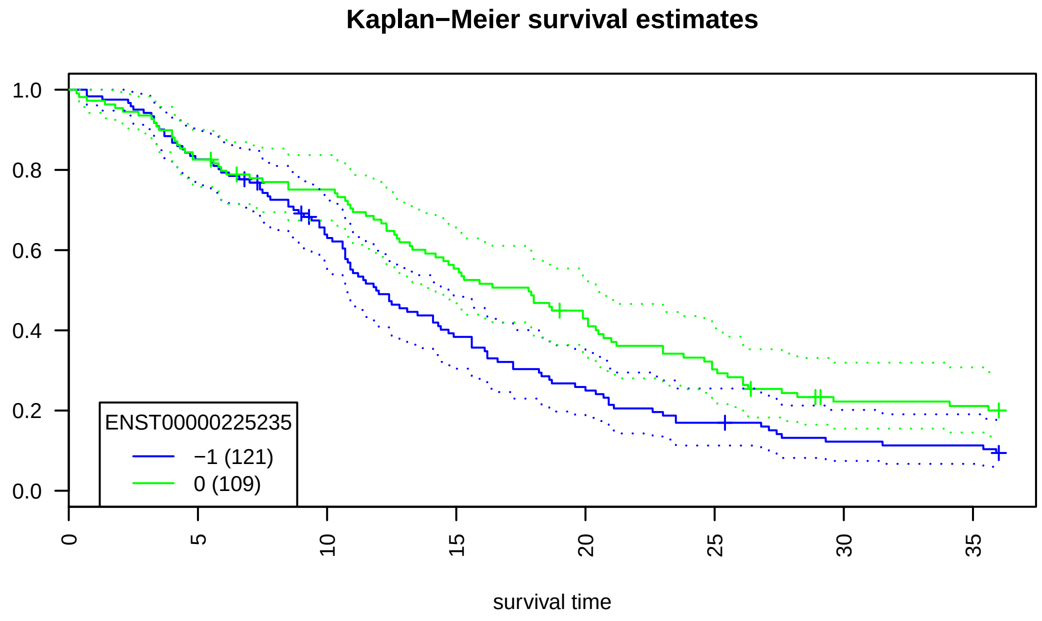

Figure 934: Each curve represents the relative number of ENST00000225235 survived to the corresponding point of time. Dashed lines represent 0.95 confidence intervals for the curves with the corresponding color. Log-rank probability measure for the equality of these curves is 6.670455e-03. Additional annotations: *.GeneId*=ENSG00000108239, *.GeneName*=TBC1D12, *.GeneDesc*=TBC1 domain family, member 12 [Source:HGNC Symbol;Acc:29082].

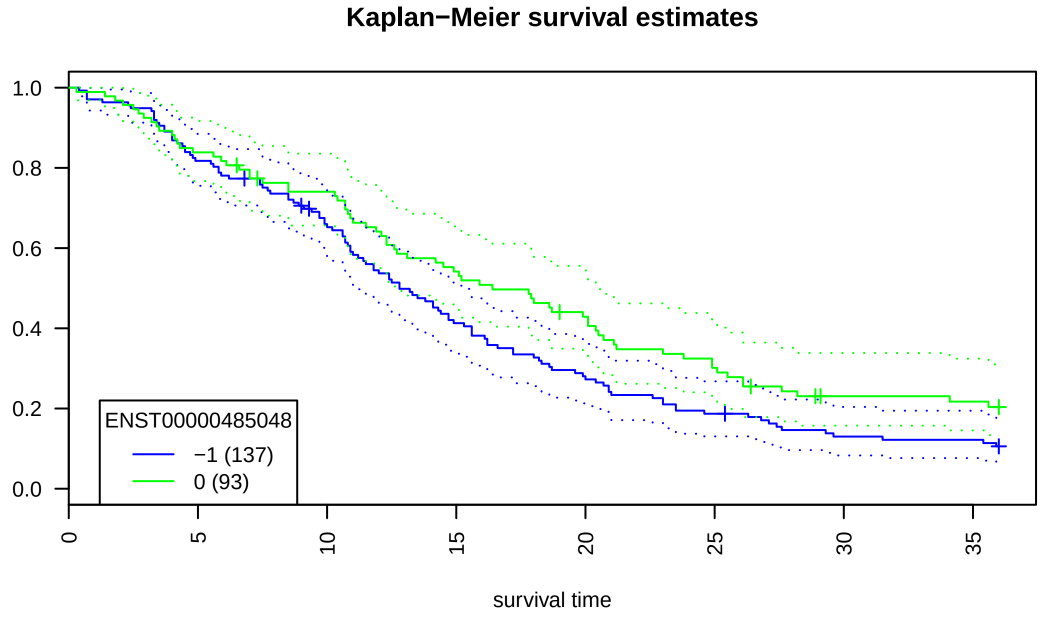

Figure 935: Each curve represents the relative number of ENST00000485048 survived to the corresponding point of time. Dashed lines represent 0.95 confidence intervals for the curves with the corresponding color. Log-rank probability measure for the equality of these curves is 3.391503e-02. Additional annotations: *.GeneId*=ENSG00000108239, *.GeneName*=TBC1D12, *.GeneDesc*=TBC1 domain family, member 12 [Source:HGNC Symbol;Acc:29082].

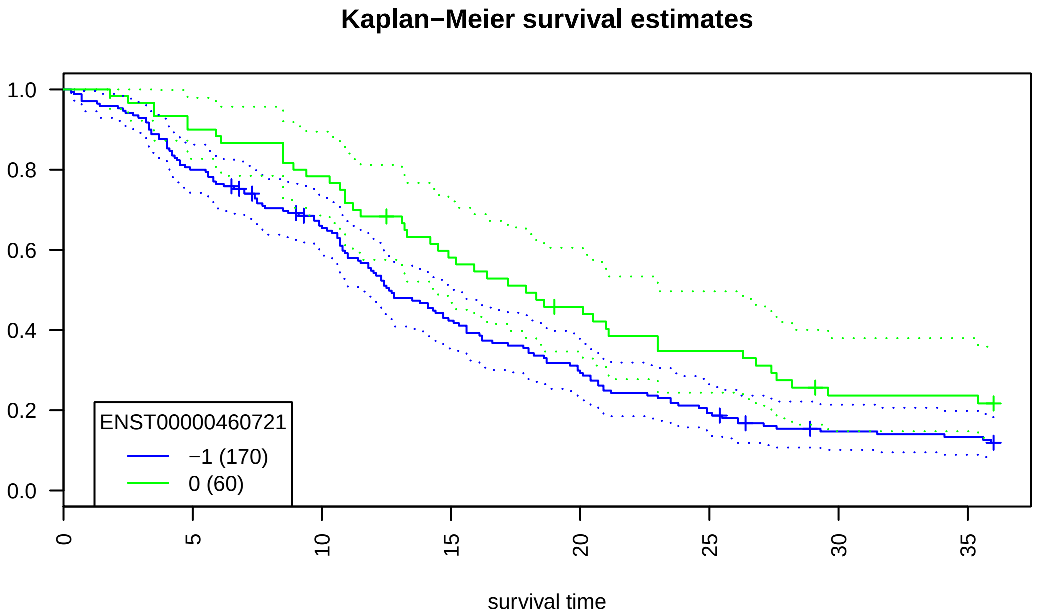

Figure 936: Each curve represents the relative number of ENST00000460721 survived to the corresponding point of time. Dashed lines represent 0.95 confidence intervals for the curves with the corresponding color. Log-rank probability measure for the equality of these curves is 1.723375e-02. Additional annotations: *.GeneId*=ENSG00000182195, *.GeneName*=LDOC1, *.GeneDesc*=leucine zipper, down-regulated in cancer 1 [Source:HGNC Symbol;Acc:6548].

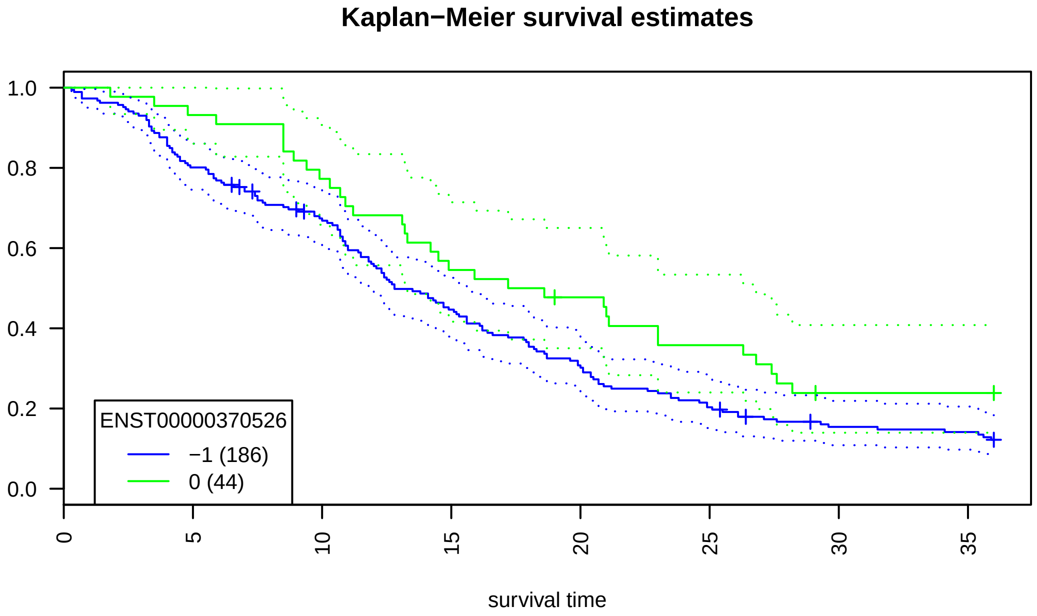

Figure 937: Each curve represents the relative number of ENST00000370526 survived to the corresponding point of time. Dashed lines represent 0.95 confidence intervals for the curves with the corresponding color. Log-rank probability measure for the equality of these curves is 3.171656e-02. Additional annotations: *.GeneId*=ENSG00000182195, *.GeneName*=LDOC1, *.GeneDesc*=leucine zipper, down-regulated in cancer 1 [Source:HGNC Symbol;Acc:6548].

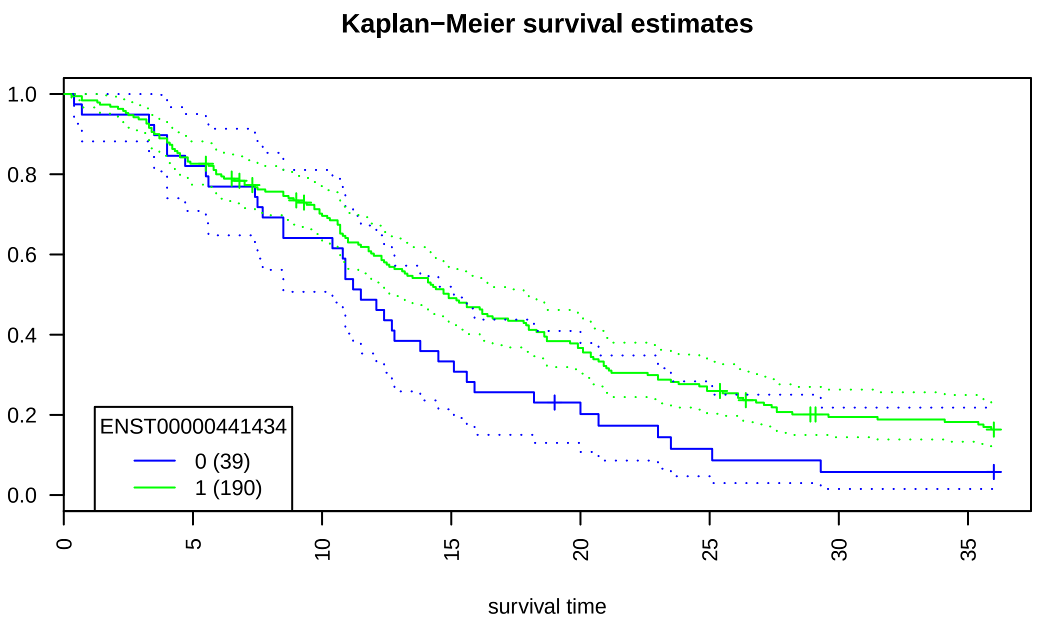

Figure 938: Each curve represents the relative number of ENST00000441434 survived to the corresponding point of time. Dashed lines represent 0.95 confidence intervals for the curves with the corresponding color. Total of 1 observation deleted due to missingness. Log-rank probability measure for the equality of these curves is 2.346288e-02. Additional annotations: *.GeneId*=ENSG00000119969, *.GeneName*=HELLS, *.GeneDesc*=helicase, lymphoid-specific [Source:HGNC Symbol;Acc:4861].

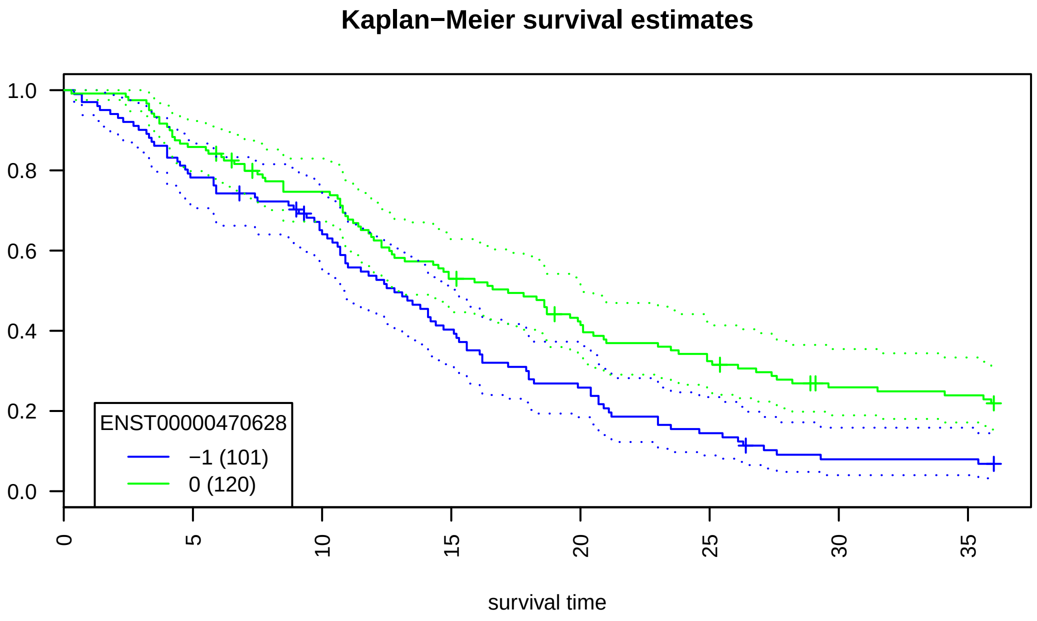

Figure 939: Each curve represents the relative number of ENST00000470628 survived to the corresponding point of time. Dashed lines represent 0.95 confidence intervals for the curves with the corresponding color. Total of 9 observations deleted due to missingness. Log-rank probability measure for the equality of these curves is 6.939529e-04. Additional annotations: *.GeneId*=ENSG00000111785, *.GeneName*=RIC8B, *.GeneDesc*=resistance to inhibitors of cholinesterase 8 homolog B (C. elegans) [Source:HGNC Symbol;Acc:25555].

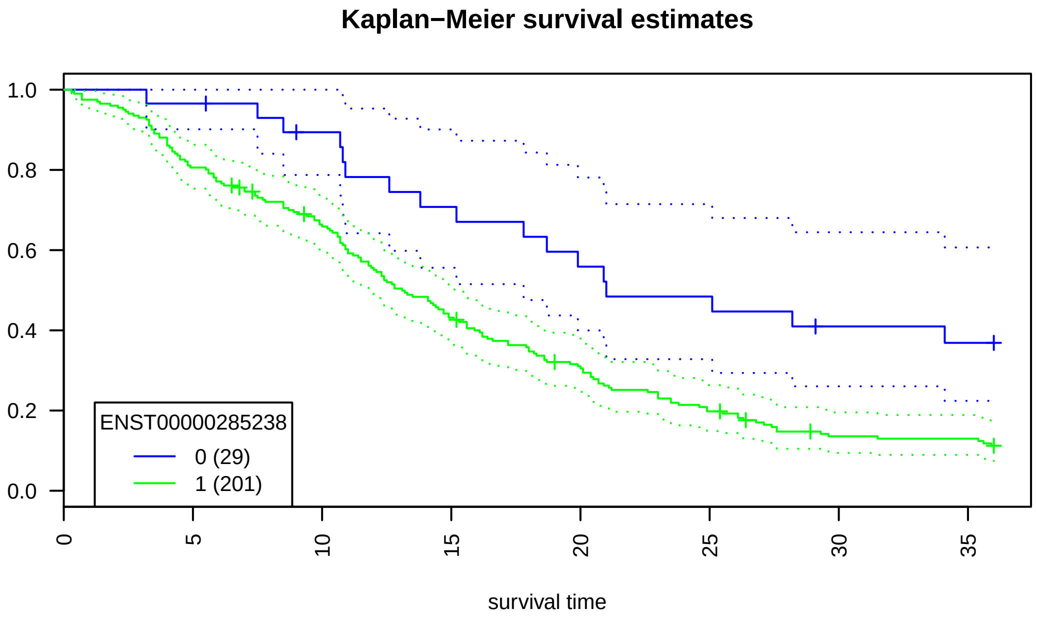

Figure 940: Each curve represents the relative number of ENST00000285238 survived to the corresponding point of time. Dashed lines represent 0.95 confidence intervals for the curves with the corresponding color. Log-rank probability measure for the equality of these curves is 1.176504e-03. Additional annotations: *.GeneId*=ENSG00000108846, *.GeneName*=ABCC3, *.GeneDesc*=ATP-binding cassette, sub-family C (CFTR/MRP), member 3 [Source:HGNC Symbol;Acc:54].

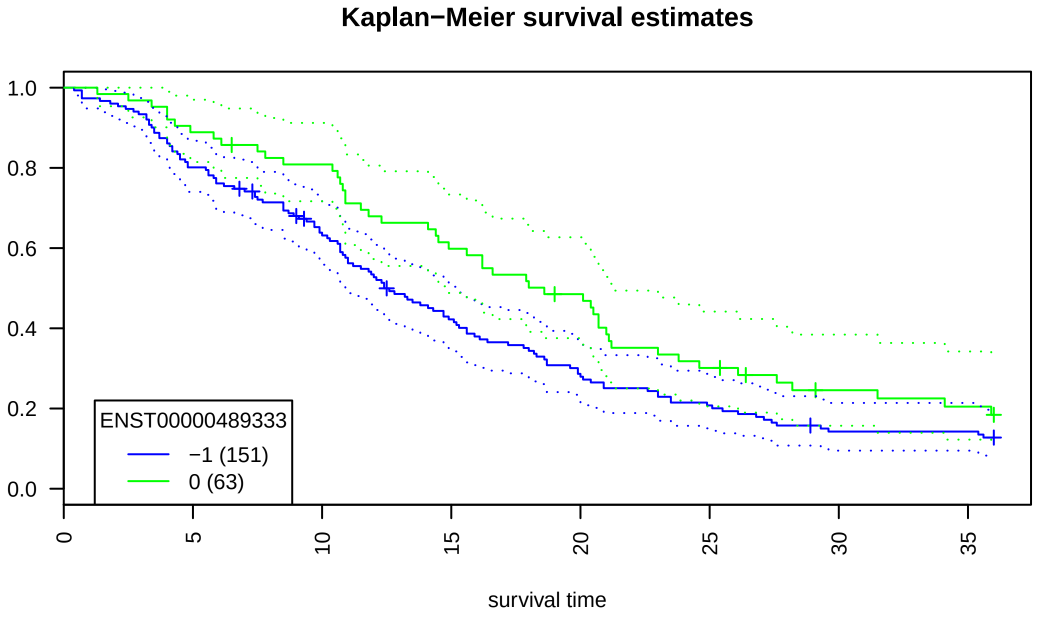

Figure 941: Each curve represents the relative number of ENST00000489333 survived to the corresponding point of time. Dashed lines represent 0.95 confidence intervals for the curves with the corresponding color. Total of 16 observations deleted due to missingness. Log-rank probability measure for the equality of these curves is 3.32488e-02. Additional annotations: *.GeneId*=ENSG00000102287, *.GeneName*=GABRE, *.GeneDesc*=microRNA 224 [Source:HGNC Symbol;Acc:31604].

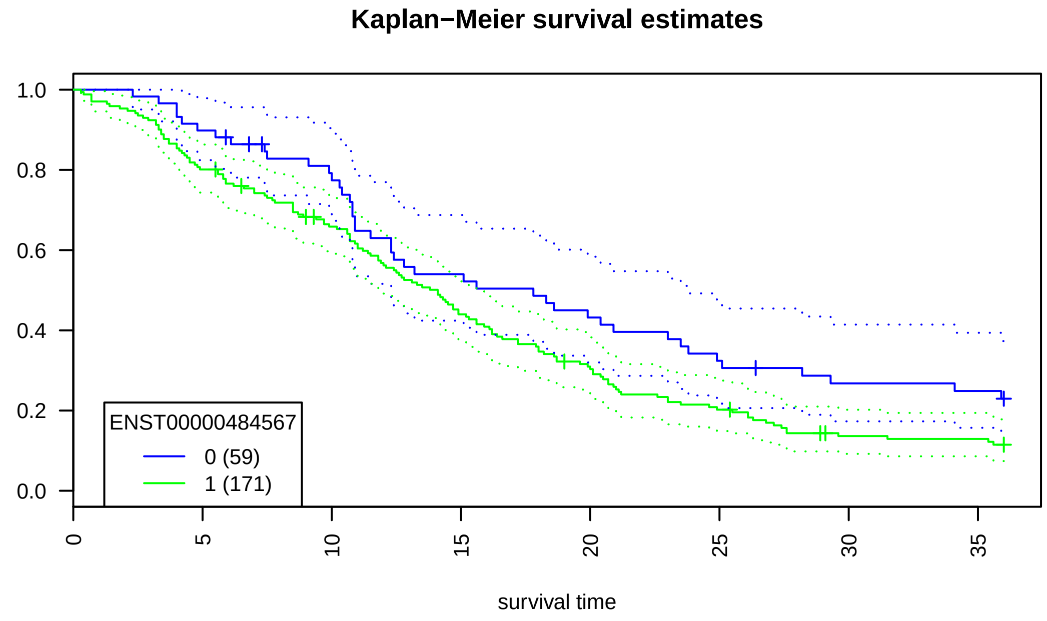

Figure 942: Each curve represents the relative number of ENST00000484567 survived to the corresponding point of time. Dashed lines represent 0.95 confidence intervals for the curves with the corresponding color. Log-rank probability measure for the equality of these curves is 2.944201e-02. Additional annotations: *.GeneId*=ENSG00000103335, *.GeneName*=FAM38A, *.GeneDesc*=family with sequence similarity 38, member A [Source:HGNC Symbol;Acc:28993].

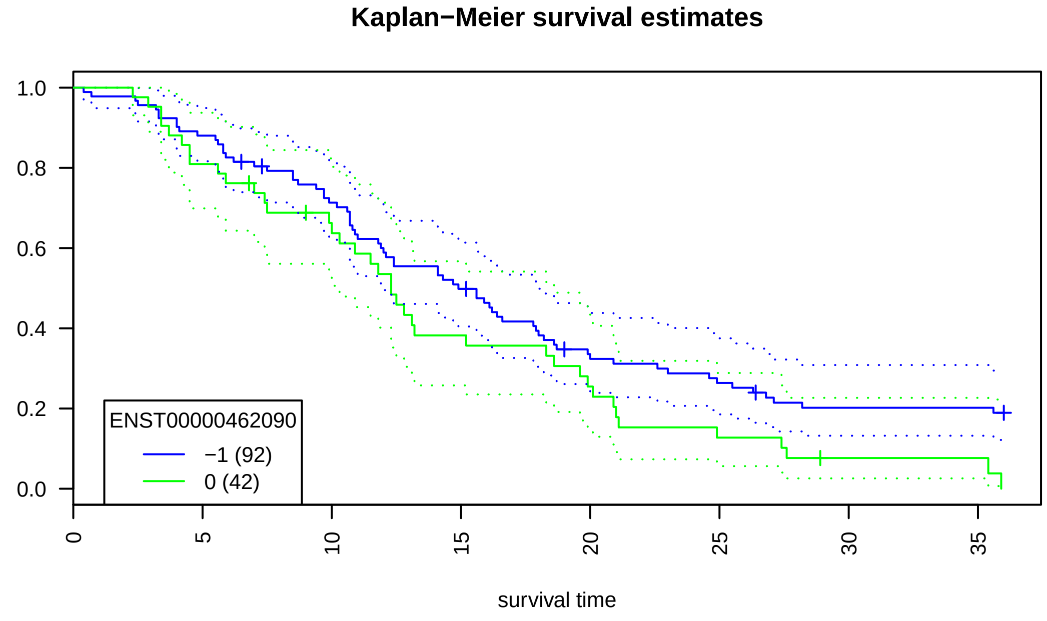

Figure 943: Each curve represents the relative number of ENST00000462090 survived to the corresponding point of time. Dashed lines represent 0.95 confidence intervals for the curves with the corresponding color. Total of 96 observations deleted due to missingness. Log-rank probability measure for the equality of these curves is 4.301958e-02. Additional annotations: *.GeneId*=ENSG00000106327, *.GeneName*=TFR2, *.GeneDesc*=transferrin receptor 2 [Source:HGNC Symbol;Acc:11762].

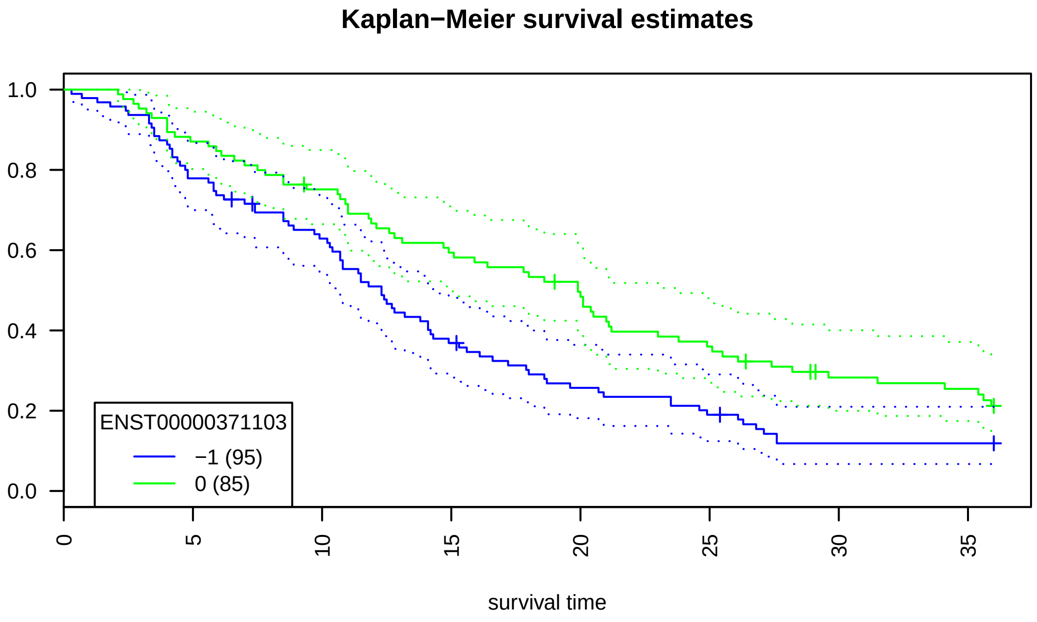

Figure 944: Each curve represents the relative number of ENST00000371103 survived to the corresponding point of time. Dashed lines represent 0.95 confidence intervals for the curves with the corresponding color. Total of 50 observations deleted due to missingness. Log-rank probability measure for the equality of these curves is 5.268097e-03. Additional annotations: .*GeneId*=ENSG00000196233, .*GeneName*=LCOR, .*GeneDesc*=ligand dependent nuclear receptor corepressor [Source:HGNC Symbol;Acc:29503].

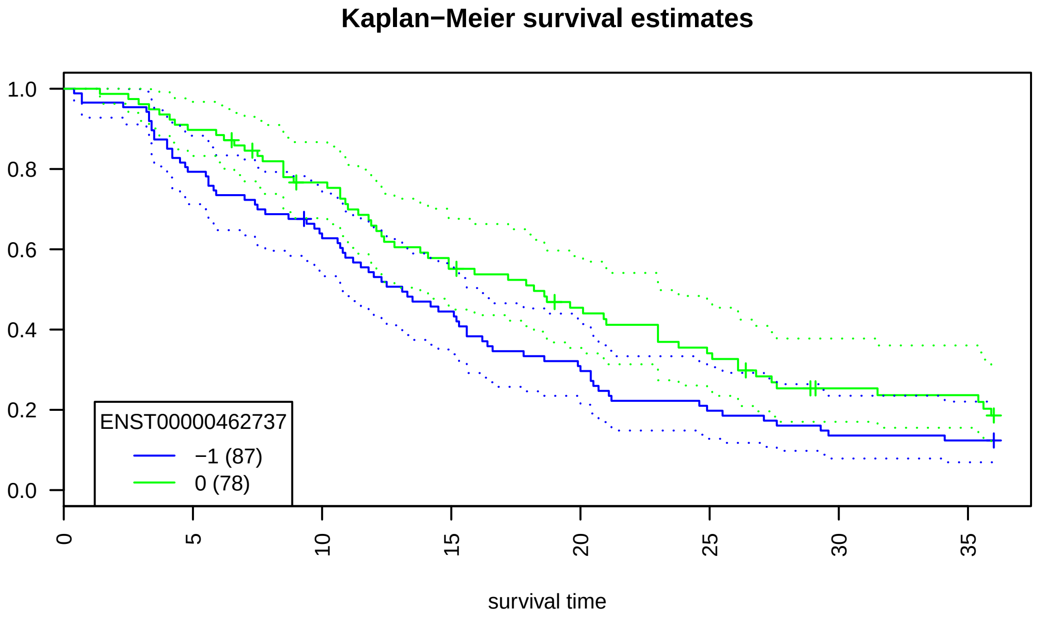

Figure 945: Each curve represents the relative number of ENST00000462737 survived to the corresponding point of time. Dashed lines represent 0.95 confidence intervals for the curves with the corresponding color. Total of 65 observations deleted due to missingness. Log-rank probability measure for the equality of these curves is 4.085402e-02. Additional annotations: .*GeneId*=ENSG00000119862, .*GeneName*=AC008074.1, .*GeneDesc*=Galectin-related protein [Source:UniProtKB/Swiss-Prot;Acc:Q3ZCW2].

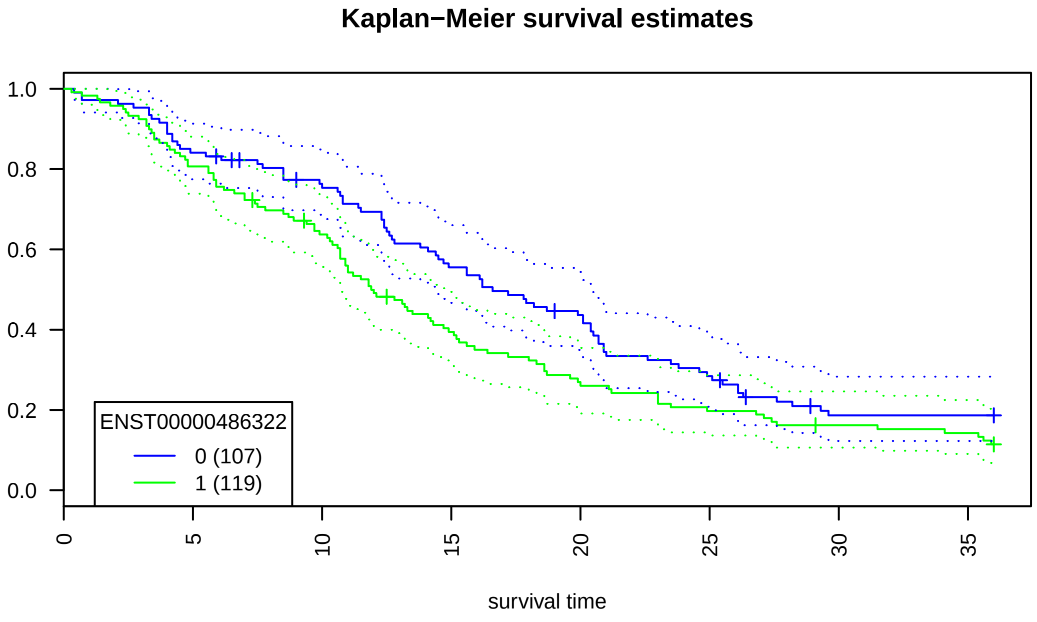

Figure 946: Each curve represents the relative number of ENST00000486322 survived to the corresponding point of time. Dashed lines represent 0.95 confidence intervals for the curves with the corresponding color. Total of 4 observations deleted due to missingness. Log-rank probability measure for the equality of these curves is 3.087789e-02. Additional annotations: *.GeneId*=ENSG00000214706, *.GeneName*=IFRD2, *.GeneDesc*=interferon-related developmental regulator 2 [Source:HGNC Symbol;Acc:5457].

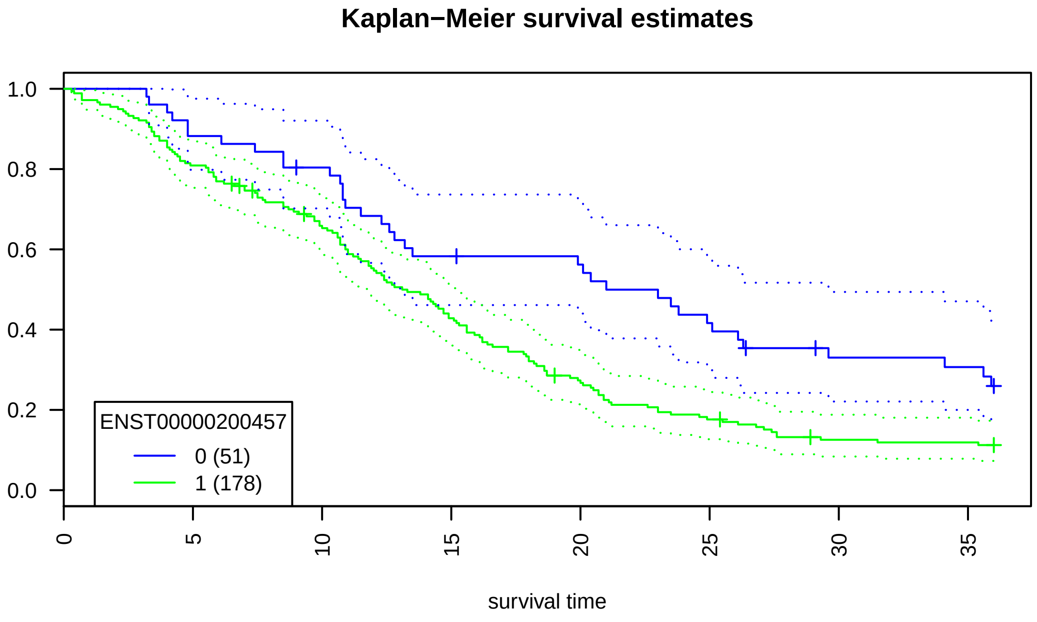

Figure 947: Each curve represents the relative number of ENST00000200457 survived to the corresponding point of time. Dashed lines represent 0.95 confidence intervals for the curves with the corresponding color. Total of 1 observation deleted due to missingness. Log-rank probability measure for the equality of these curves is 1.813824e-03. Additional annotations: *.GeneId*=ENSG00000087077, *.GeneName*=TRIP6, *.GeneDesc*=thyroid hormone receptor interactor 6 [Source:HGNC Symbol;Acc:12311].

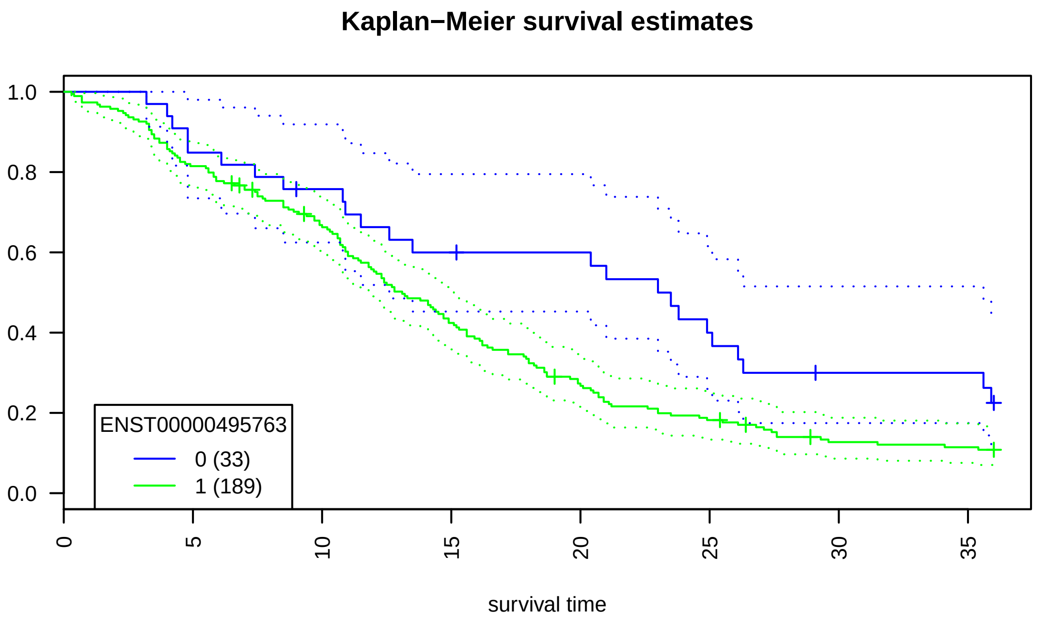

Figure 948: Each curve represents the relative number of ENST00000495763 survived to the corresponding point of time. Dashed lines represent 0.95 confidence intervals for the curves with the corresponding color. Total of 8 observations deleted due to missingness. Log-rank probability measure for the equality of these curves is 1.694435e-02. Additional annotations: *.GeneId*=ENSG00000087077, *.GeneName*=TRIP6, *.GeneDesc*=thyroid hormone receptor interactor 6 [Source:HGNC Symbol;Acc:12311].

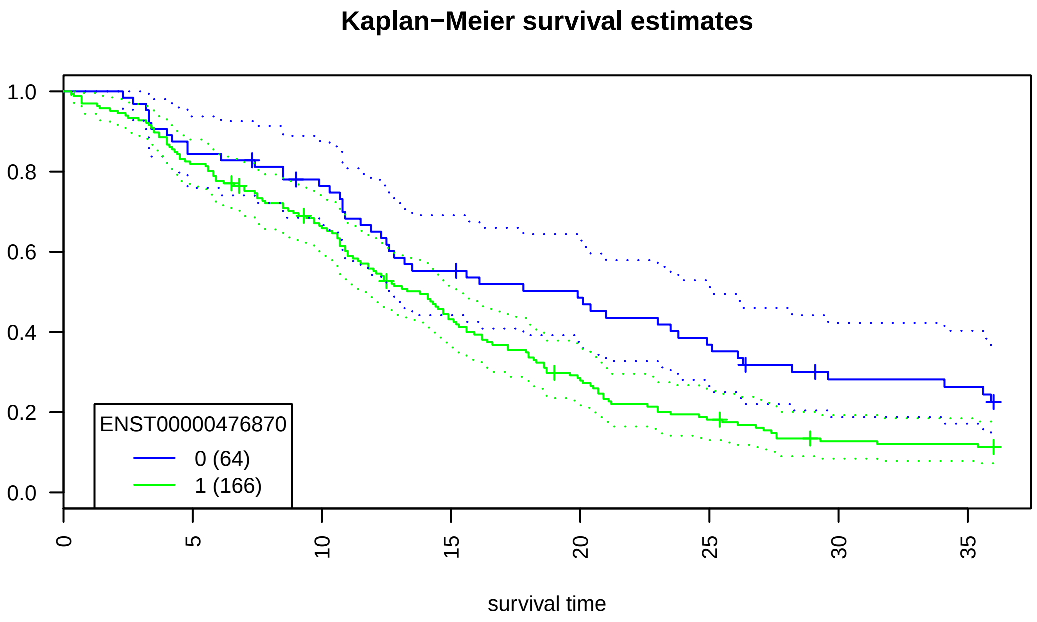

Figure 949: Each curve represents the relative number of ENST00000476870 survived to the corresponding point of time. Dashed lines represent 0.95 confidence intervals for the curves with the corresponding color. Log-rank probability measure for the equality of these curves is 1.101144e-02. Additional annotations: *.GeneId*=ENSG00000087077, *.GeneName*=TRIP6, *.GeneDesc*=thyroid hormone receptor interactor 6 [Source:HGNC Symbol;Acc:12311].

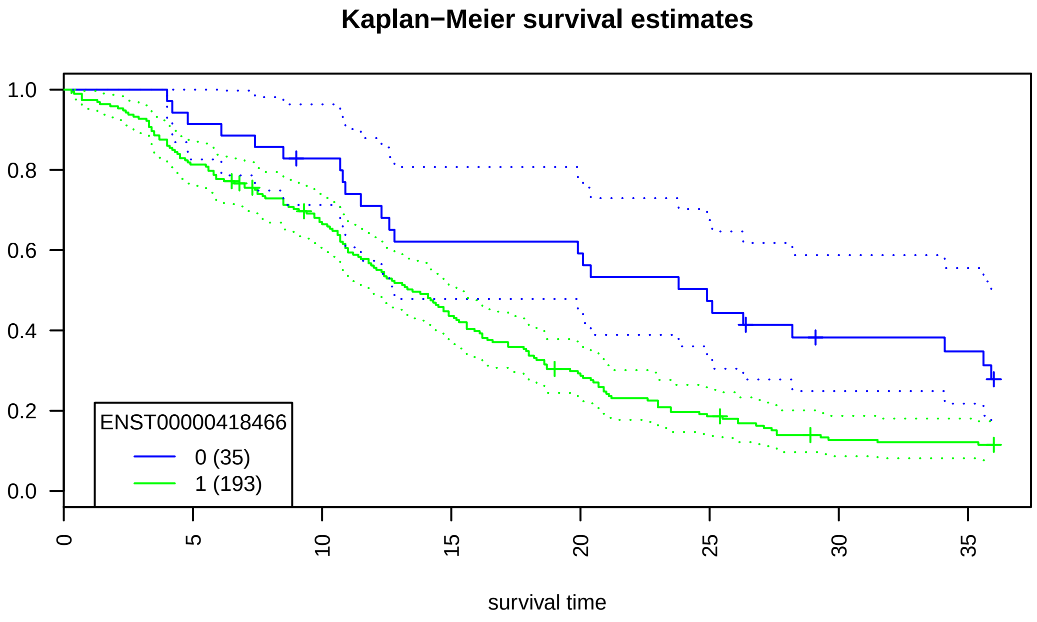

Figure 950: Each curve represents the relative number of ENST00000418466 survived to the corresponding point of time. Dashed lines represent 0.95 confidence intervals for the curves with the corresponding color. Total of 2 observations deleted due to missingness. Log-rank probability measure for the equality of these curves is 2.851924e-03. Additional annotations: *.GeneId*=ENSG00000087077, *.GeneName*=TRIP6, *.GeneDesc*=thyroid hormone receptor interactor 6 [Source:HGNC Symbol;Acc:12311].

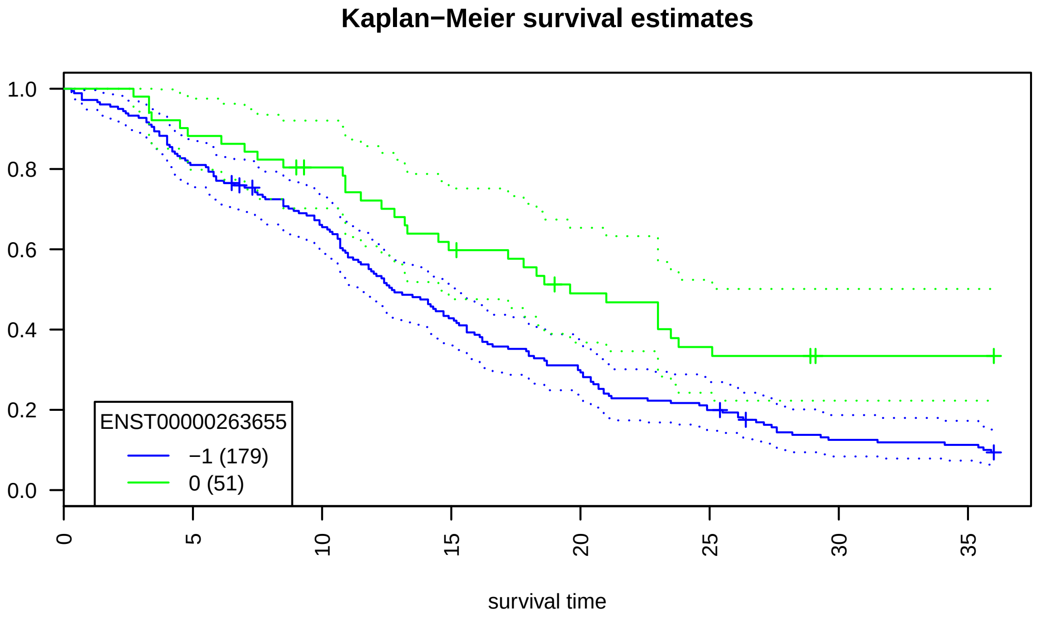

Figure 951: Each curve represents the relative number of ENST00000263655 survived to the corresponding point of time. Dashed lines represent 0.95 confidence intervals for the curves with the corresponding color. Log-rank probability measure for the equality of these curves is 7.235113e-04. Additional annotations: *.GeneId*=ENSG00000119865, *.GeneName*=CNRIP1, *.GeneDesc*=cannabinoid receptor interacting protein 1 [Source:HGNC Symbol;Acc:24546].

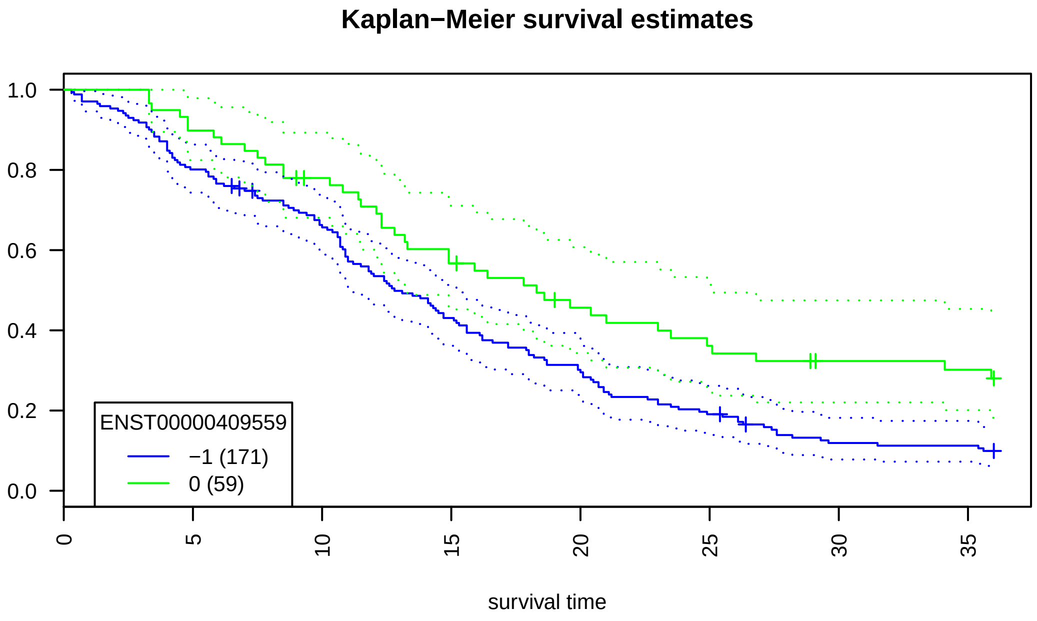

Figure 952: Each curve represents the relative number of ENST00000409559 survived to the corresponding point of time. Dashed lines represent 0.95 confidence intervals for the curves with the corresponding color. Log-rank probability measure for the equality of these curves is 2.235074e-03. Additional annotations: $.GeneId$=ENSG00000119865, $.GeneName$=CNRIP1, $.GeneDesc$=cannabinoid receptor interacting protein 1 [Source:HGNC Symbol;Acc:24546].

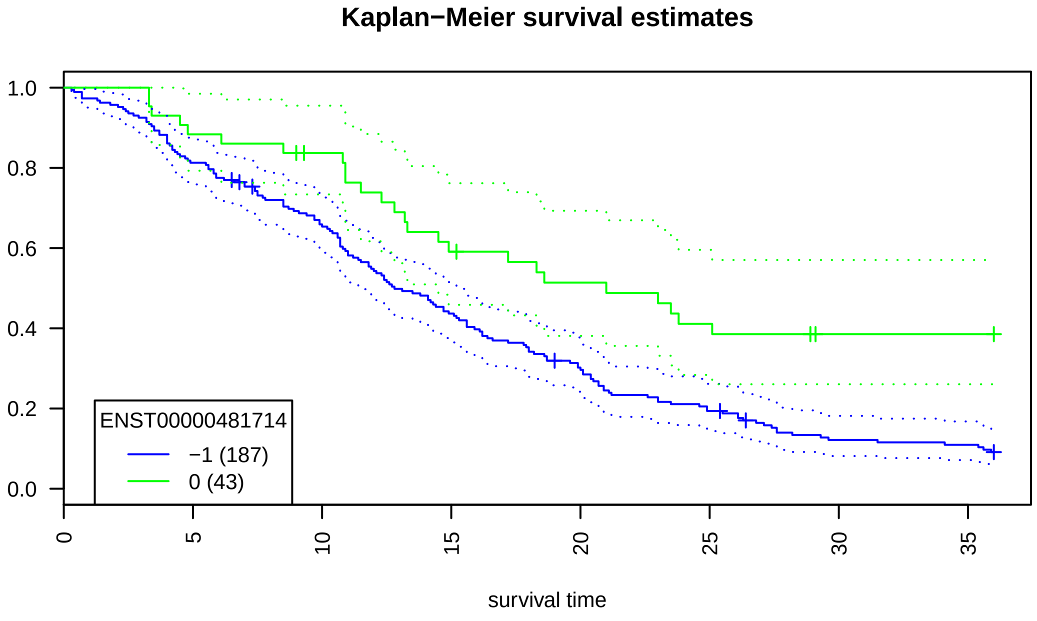

Figure 953: Each curve represents the relative number of ENST00000481714 survived to the corresponding point of time. Dashed lines represent 0.95 confidence intervals for the curves with the corresponding color. Log-rank probability measure for the equality of these curves is 2.523811e-04. Additional annotations: $.GeneId$=ENSG00000119865, $.GeneName$=CNRIP1, $.GeneDesc$=cannabinoid receptor interacting protein 1 [Source:HGNC Symbol;Acc:24546].

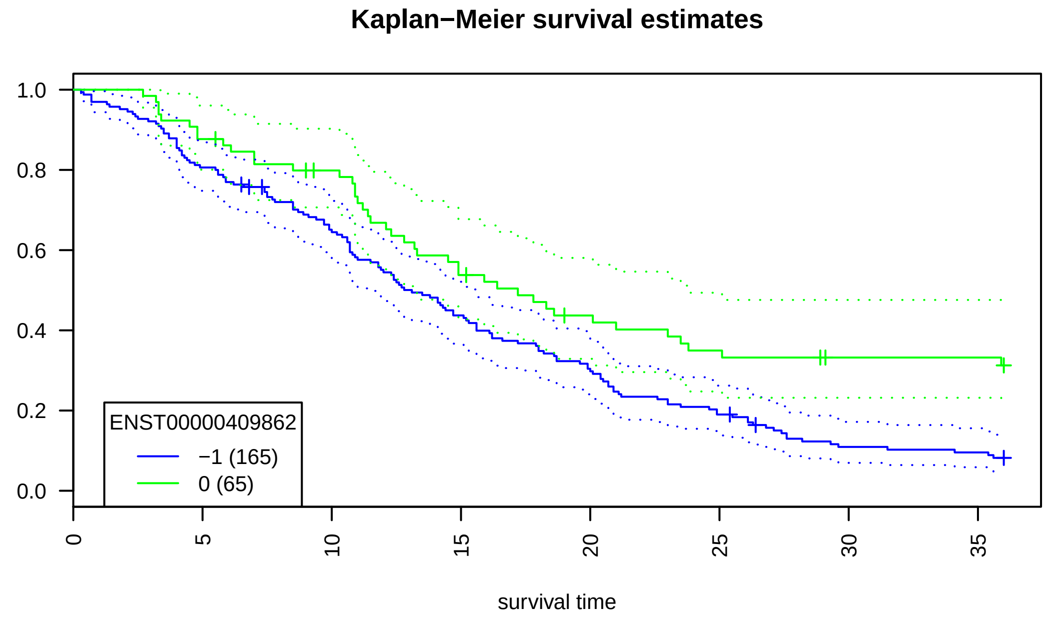

Figure 954: Each curve represents the relative number of ENST00000409862 survived to the corresponding point of time. Dashed lines represent 0.95 confidence intervals for the curves with the corresponding color. Log-rank probability measure for the equality of these curves is 9.393646e-04. Additional annotations: *.GeneId*=ENSG00000119865, *.GeneName*=CNRIP1, *.GeneDesc*=cannabinoid receptor interacting protein 1 [Source:HGNC Symbol;Acc:24546].

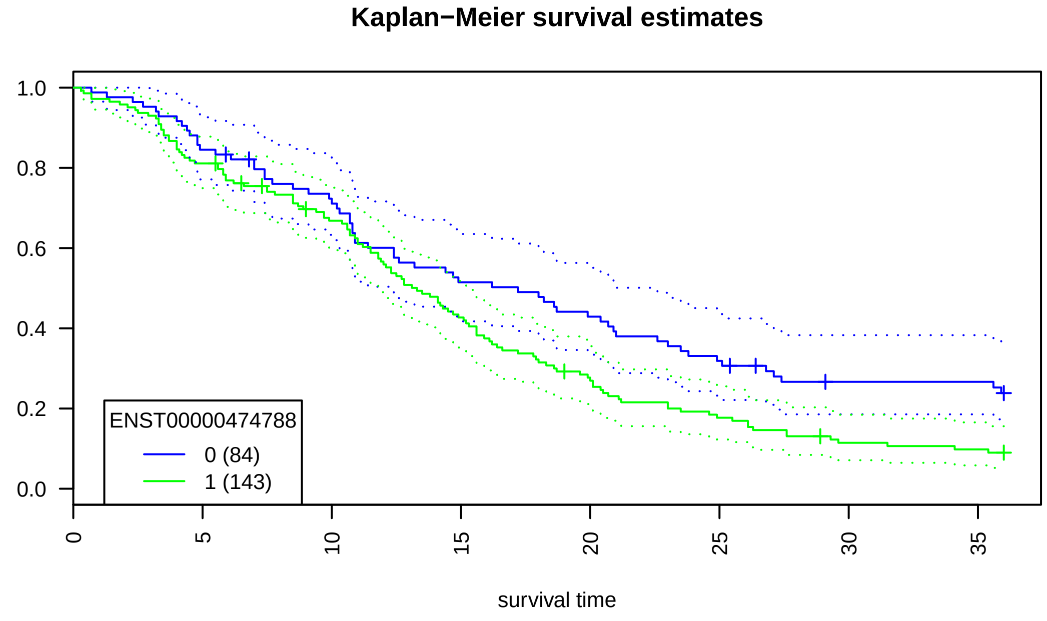

Figure 955: Each curve represents the relative number of ENST00000474788 survived to the corresponding point of time. Dashed lines represent 0.95 confidence intervals for the curves with the corresponding color. Total of 3 observations deleted due to missingness. Log-rank probability measure for the equality of these curves is 8.180176e-03. Additional annotations: *.GeneId*=ENSG00000115956, *.GeneName*=PLEK, *.GeneDesc*=pleckstrin [Source:HGNC Symbol;Acc:9070].

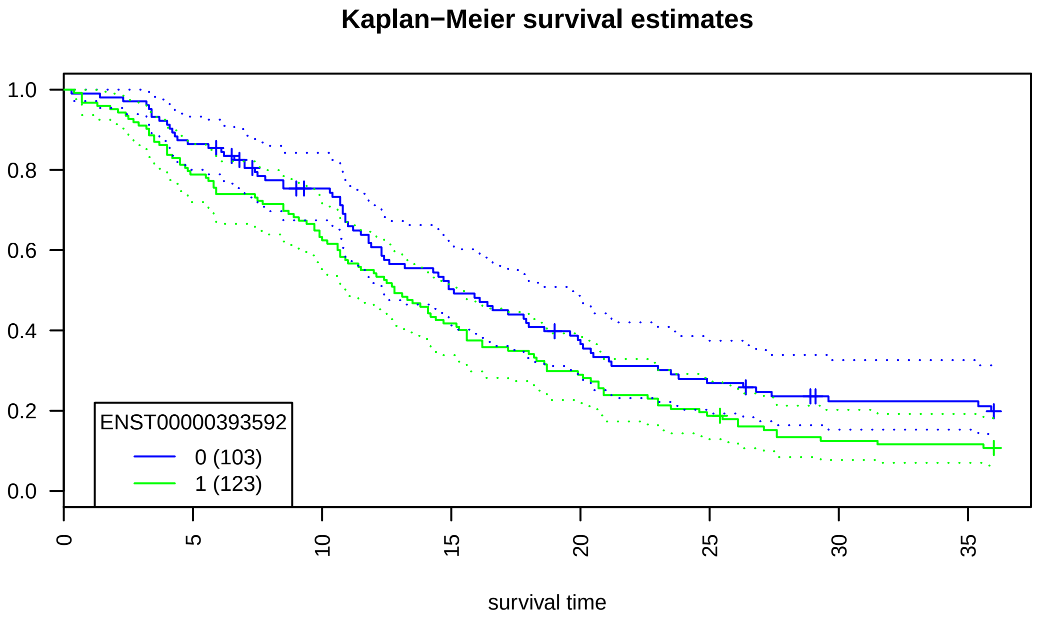

Figure 956: Each curve represents the relative number of ENST00000393592 survived to the corresponding point of time. Dashed lines represent 0.95 confidence intervals for the curves with the corresponding color. Total of 4 observations deleted due to missingness. Log-rank probability measure for the equality of these curves is 4.68463e-02. Additional annotations: *.GeneId*=ENSG00000171631, *.GeneName*=P2RY6, *.GeneDesc*=pyrimidinergic receptor P2Y, G-protein coupled, 6 [Source:HGNC Symbol;Acc:8543].

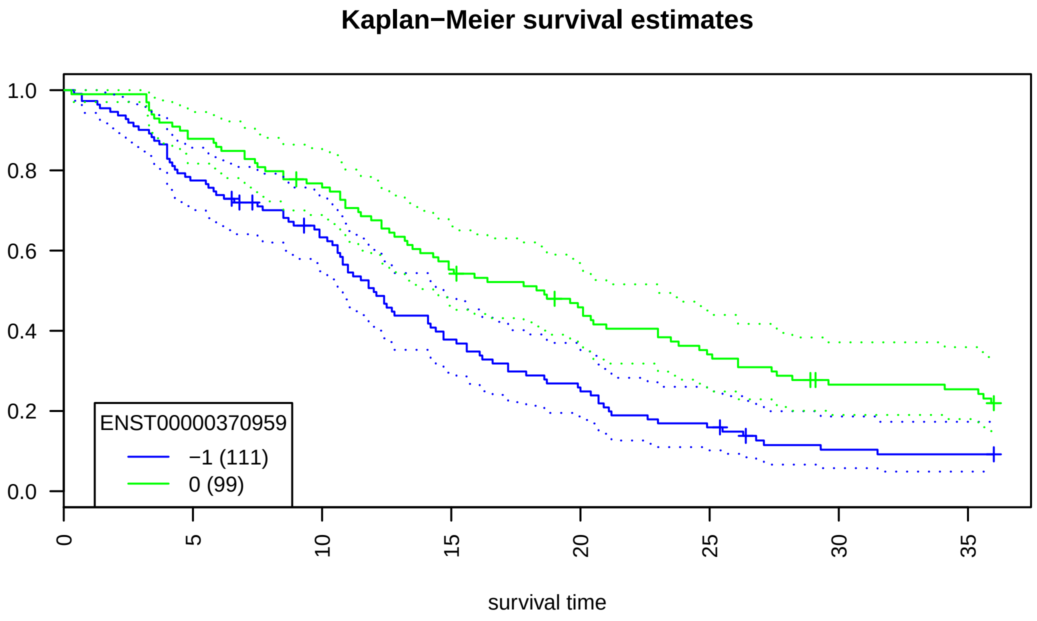

Figure 957: Each curve represents the relative number of ENST00000370959 survived to the corresponding point of time. Dashed lines represent 0.95 confidence intervals for the curves with the corresponding color. Total of 20 observations deleted due to missingness. Log-rank probability measure for the equality of these curves is 6.902709e-04. Additional annotations: *.GeneId*=ENSG00000170899, *.GeneName*=GSTA4, *.GeneDesc*=glutathione S-transferase alpha 4 [Source:HGNC Symbol;Acc:4629].

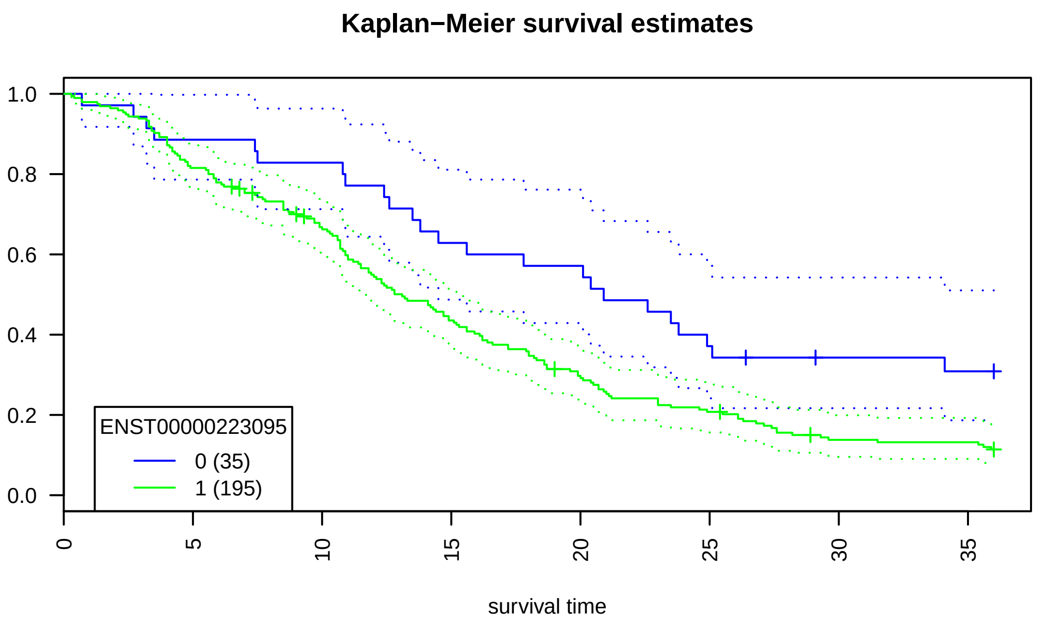

Figure 958: Each curve represents the relative number of ENST00000223095 survived to the corresponding point of time. Dashed lines represent 0.95 confidence intervals for the curves with the corresponding color. Log-rank probability measure for the equality of these curves is 5.445564e-03. Additional annotations: *.GeneId*=ENSG00000106366, *.GeneName*=SERPINE1, *.GeneDesc*=serpin peptidase inhibitor, clade E (nexin, plasminogen activator inhibitor type 1), member 1 [Source:HGNC Symbol;Acc:8583].

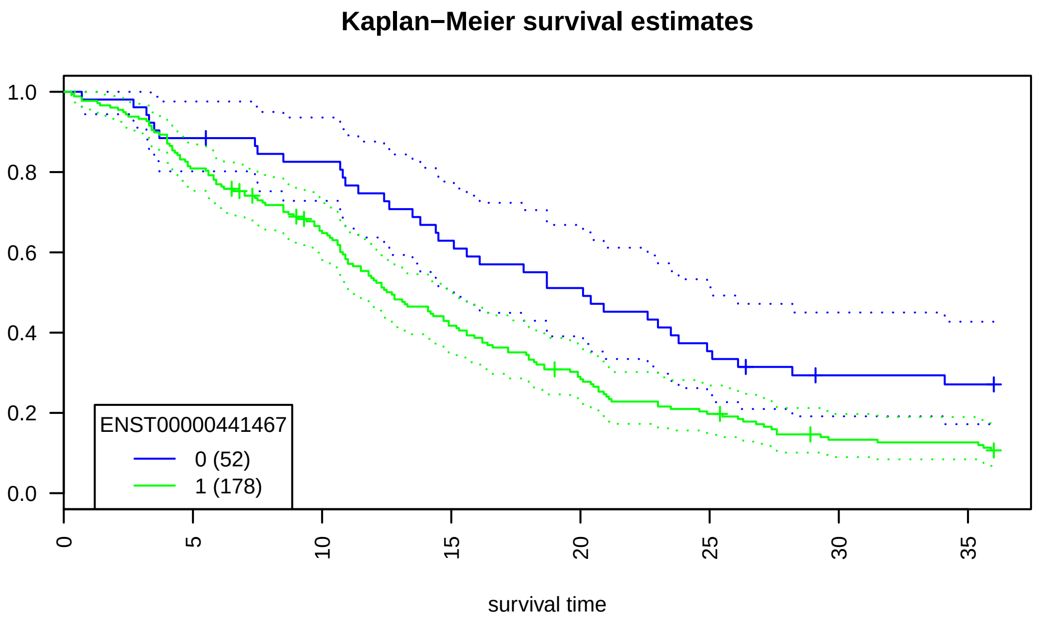

Figure 959: Each curve represents the relative number of ENST00000441467 survived to the corresponding point of time. Dashed lines represent 0.95 confidence intervals for the curves with the corresponding color. Log-rank probability measure for the equality of these curves is 2.597464e-03. Additional annotations: *.GeneId*=ENSG00000106366, *.GeneName*=SERPINE1, *.GeneDesc*=serpin peptidase inhibitor, clade E (nexin, plasminogen activator inhibitor type 1), member 1 [Source:HGNC Symbol;Acc:8583].

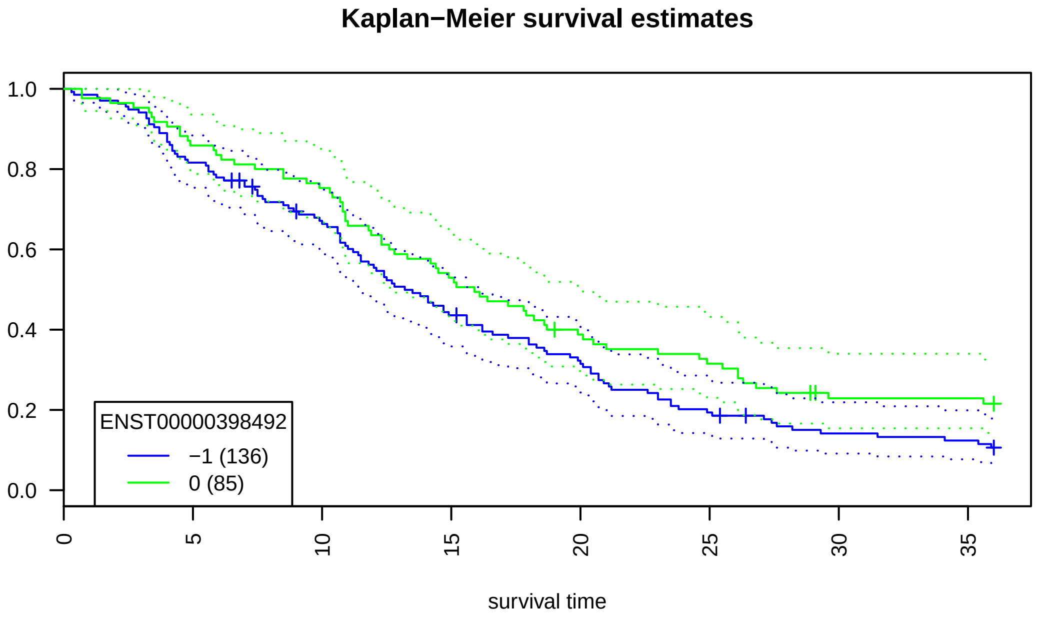

Figure 960: Each curve represents the relative number of ENST00000398492 survived to the corresponding point of time. Dashed lines represent 0.95 confidence intervals for the curves with the corresponding color. Total of 9 observations deleted due to missingness. Log-rank probability measure for the equality of these curves is 4.832353e-02. Additional annotations: *.GeneId*=ENSG00000021300, *.GeneName*=PLEKHB1, *.GeneDesc*=pleckstrin homology domain containing, family B (evectins) member 1 [Source:HGNC Symbol;Acc:19079].

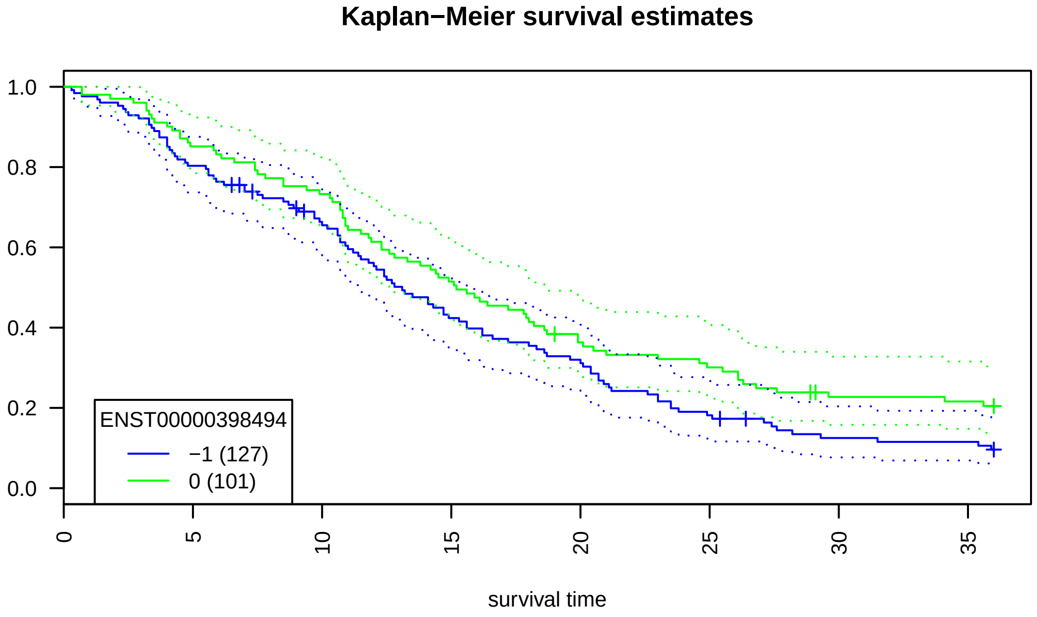

Figure 961: Each curve represents the relative number of ENST00000398494 survived to the corresponding point of time. Dashed lines represent 0.95 confidence intervals for the curves with the corresponding color. Total of 2 observations deleted due to missingness. Log-rank probability measure for the equality of these curves is 4.467546e-02. Additional annotations: *.GeneId*=ENSG00000021300, *.GeneName*=PLEKHB1, *.GeneDesc*=pleckstrin homology domain containing, family B (evectins) member 1 [Source:HGNC Symbol;Acc:19079].

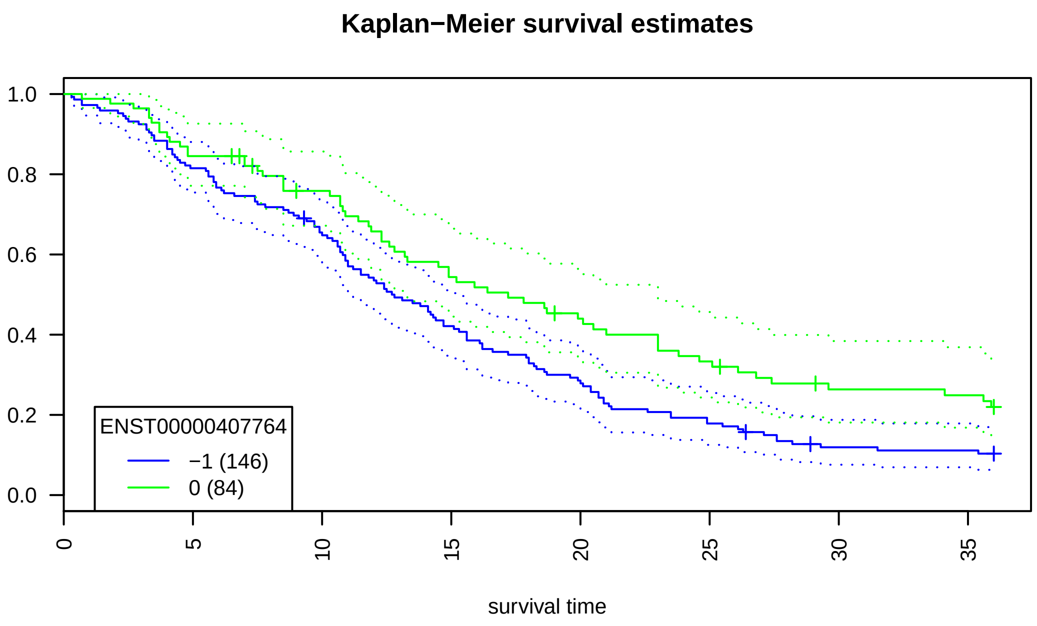

Figure 962: Each curve represents the relative number of ENST00000407764 survived to the corresponding point of time. Dashed lines represent 0.95 confidence intervals for the curves with the corresponding color. Log-rank probability measure for the equality of these curves is 7.317895e-03. Additional annotations: *.GeneId*=ENSG00000174456, *.GeneName*=C12orf76, *.GeneDesc*=Uncharacterized protein C12orf76 [Source:UniProtKB/Swiss-Prot;Acc:Q8N812].

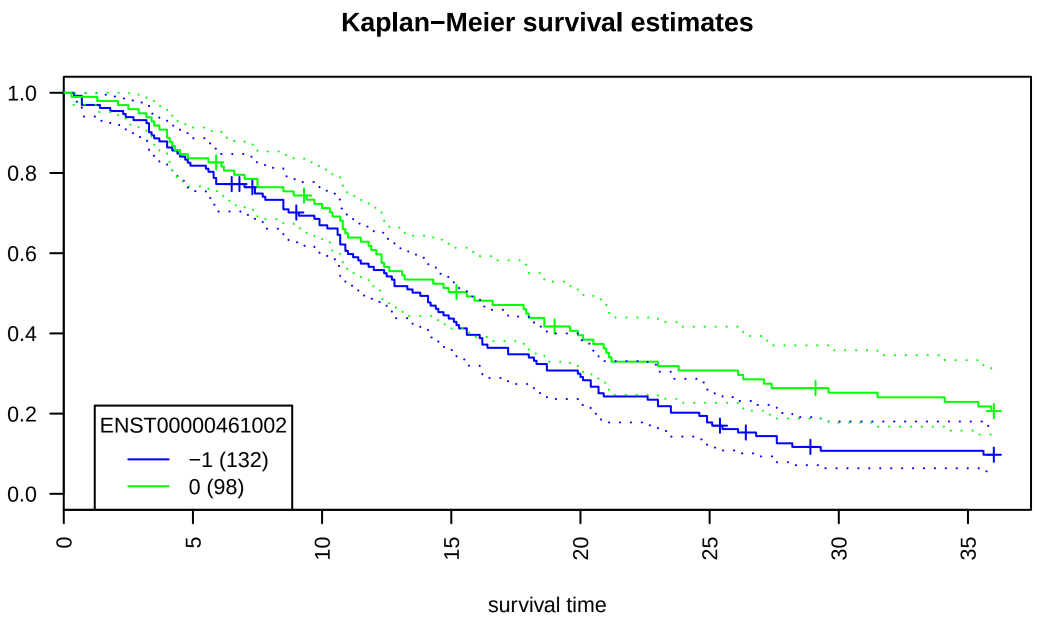

Figure 963: Each curve represents the relative number of ENST00000461002 survived to the corresponding point of time. Dashed lines represent 0.95 confidence intervals for the curves with the corresponding color. Log-rank probability measure for the equality of these curves is 3.450118e-02. Additional annotations: *.GeneId*=ENSG00000115977, *.GeneName*=AAK1, *.GeneDesc*=AP2 associated kinase 1 [Source:HGNC Symbol;Acc:19679].

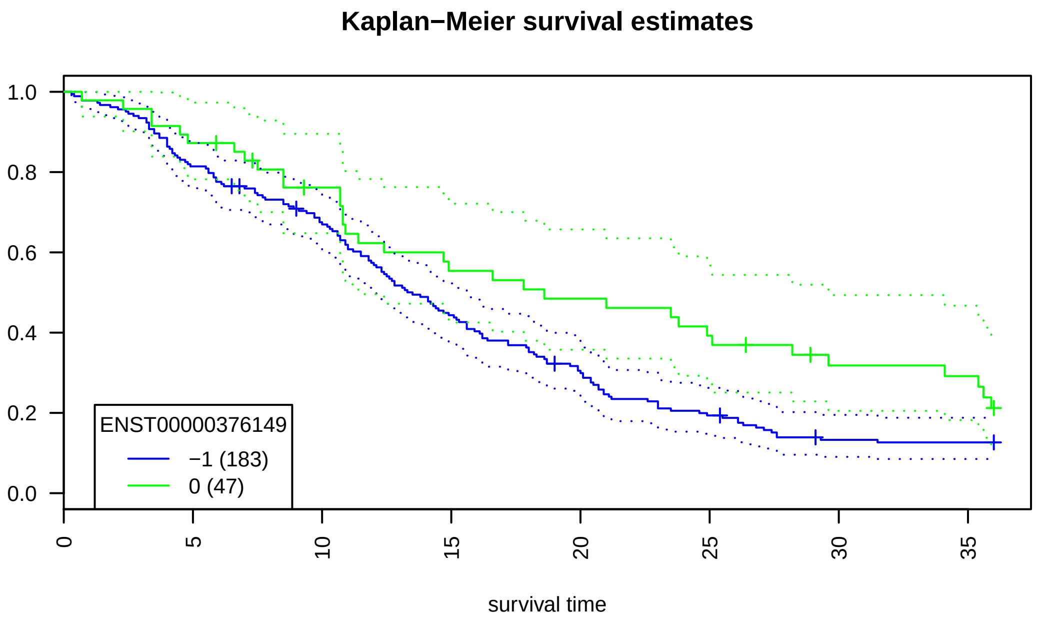

Figure 964: Each curve represents the relative number of ENST00000376149 survived to the corresponding point of time. Dashed lines represent 0.95 confidence intervals for the curves with the corresponding color. Log-rank probability measure for the equality of these curves is 2.927404e-02. Additional annotations: *.GeneId*=ENSG00000108395, *.GeneName*=TRIM37, *.GeneDesc*=tripartite motif-containing 37 [Source:HGNC Symbol;Acc:7523].

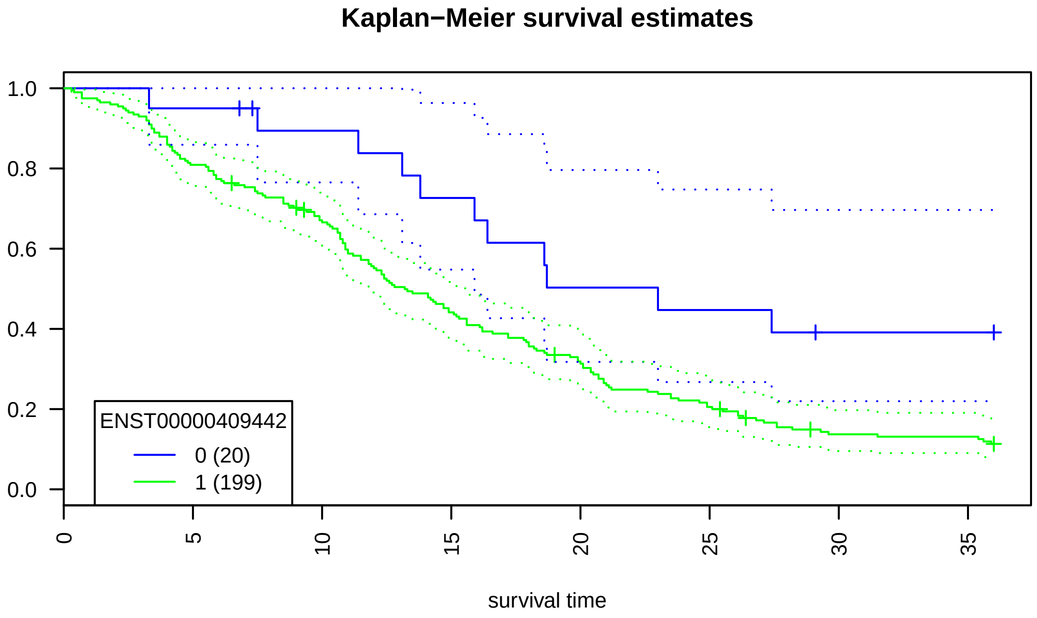

Figure 965: Each curve represents the relative number of ENST00000409442 survived to the corresponding point of time. Dashed lines represent 0.95 confidence intervals for the curves with the corresponding color. Total of 11 observations deleted due to missingness. Log-rank probability measure for the equality of these curves is 6.978677e-03. Additional annotations: *.GeneId*=ENSG00000059728, *.GeneName*=MXD1, *.GeneDesc*=MAX dimerization protein 1 [Source:HGNC Symbol;Acc:6761].

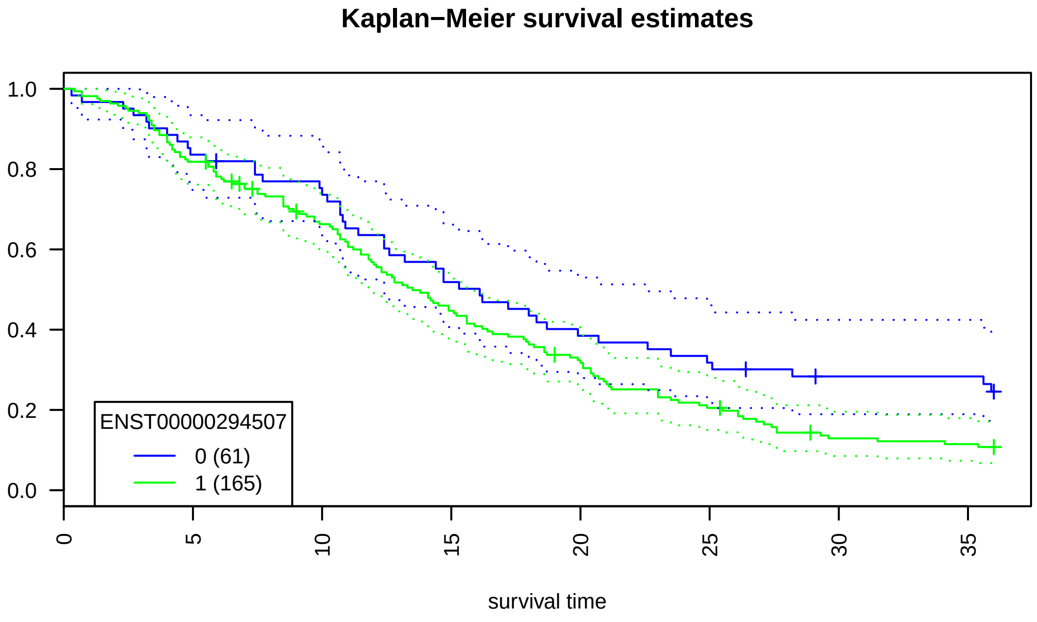

Figure 966: Each curve represents the relative number of ENST00000294507 survived to the corresponding point of time. Dashed lines represent 0.95 confidence intervals for the curves with the corresponding color. Total of 4 observations deleted due to missingness. Log-rank probability measure for the equality of these curves is 4.13508e-02. Additional annotations: *.GeneId*=ENSG00000162511, *.GeneName*=LAPTM5, *.GeneDesc*=lysosomal protein transmembrane 5 [Source:HGNC Symbol;Acc:29612].

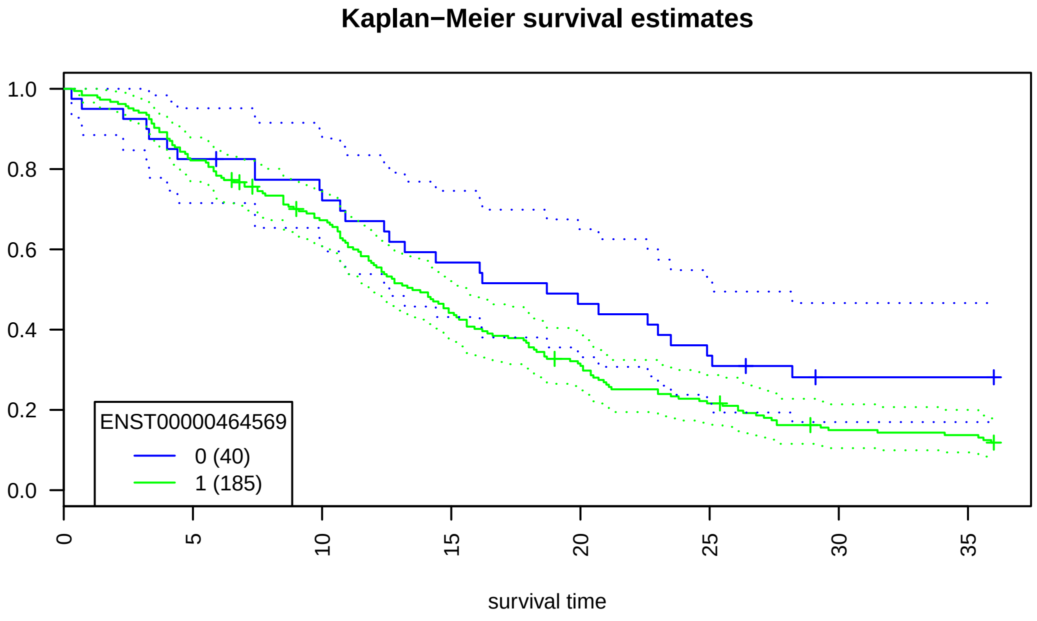

Figure 967: Each curve represents the relative number of ENST00000464569 survived to the corresponding point of time. Dashed lines represent 0.95 confidence intervals for the curves with the corresponding color. Total of 5 observations deleted due to missingness. Log-rank probability measure for the equality of these curves is 4.133774e-02. Additional annotations: *.GeneId*=ENSG00000162511, *.GeneName*=LAPTM5, *.GeneDesc*=lysosomal protein transmembrane 5 [Source:HGNC Symbol;Acc:29612].

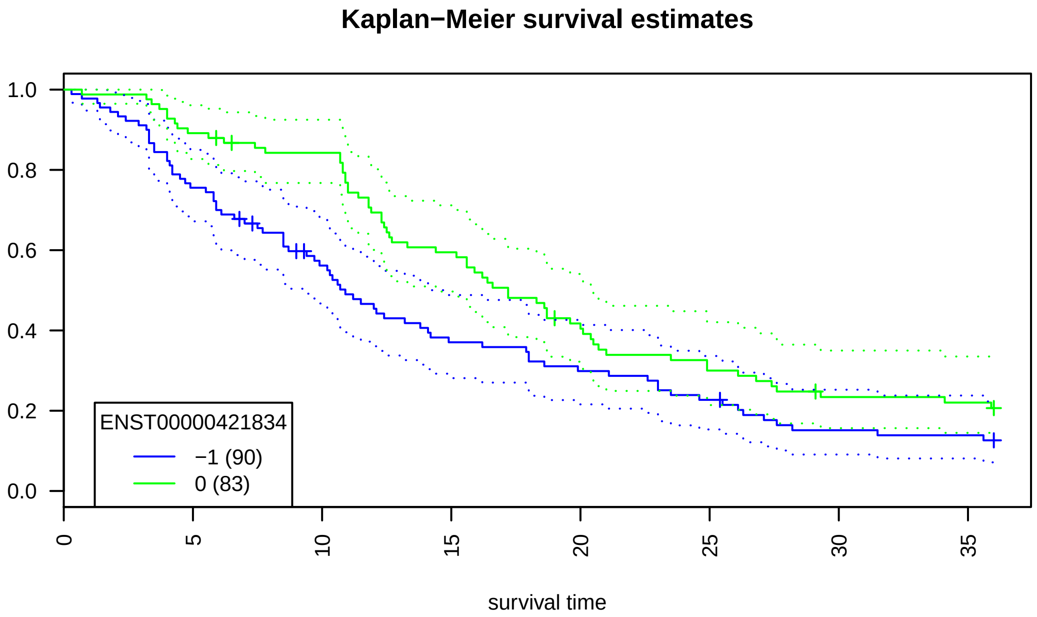

Figure 968: Each curve represents the relative number of ENST00000421834 survived to the corresponding point of time. Dashed lines represent 0.95 confidence intervals for the curves with the corresponding color. Total of 57 observations deleted due to missingness. Log-rank probability measure for the equality of these curves is 1.488327e-02. Additional annotations: *.GeneId*=ENSG00000151914, *.GeneName*=DST, *.GeneDesc*=dystonin [Source:HGNC Symbol;Acc:1090].

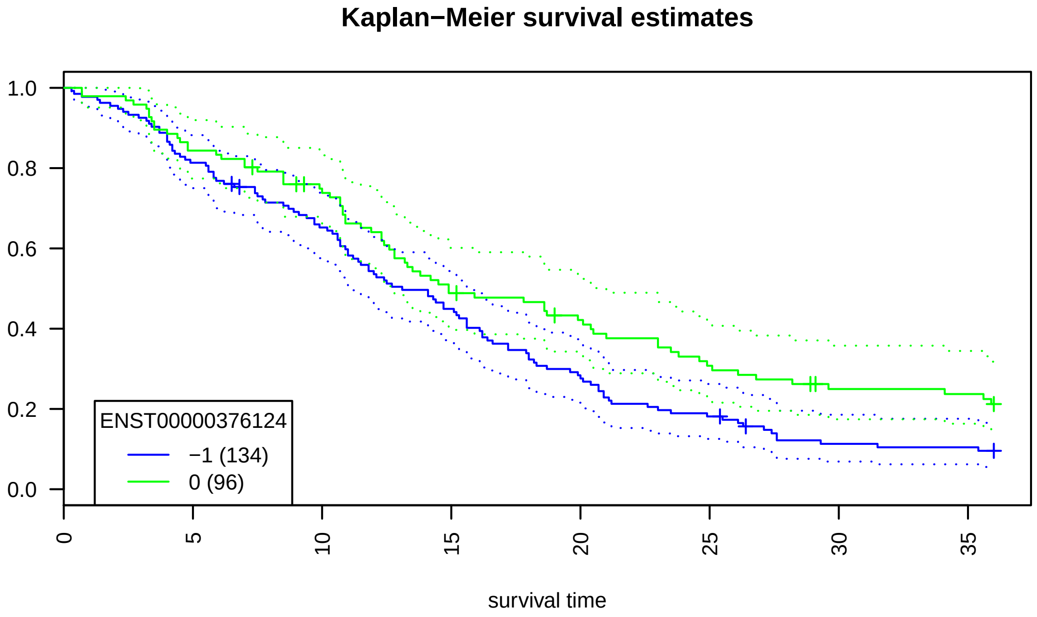

Figure 969: Each curve represents the relative number of ENST00000376124 survived to the corresponding point of time. Dashed lines represent 0.95 confidence intervals for the curves with the corresponding color. Log-rank probability measure for the equality of these curves is 1.384118e-02. Additional annotations: *.GeneId*=ENSG00000153982, *.GeneName*=GDPD1, *.GeneDesc*=glycerophosphodiester phosphodiesterase domain containing 1 [Source:HGNC Symbol;Acc:20883].

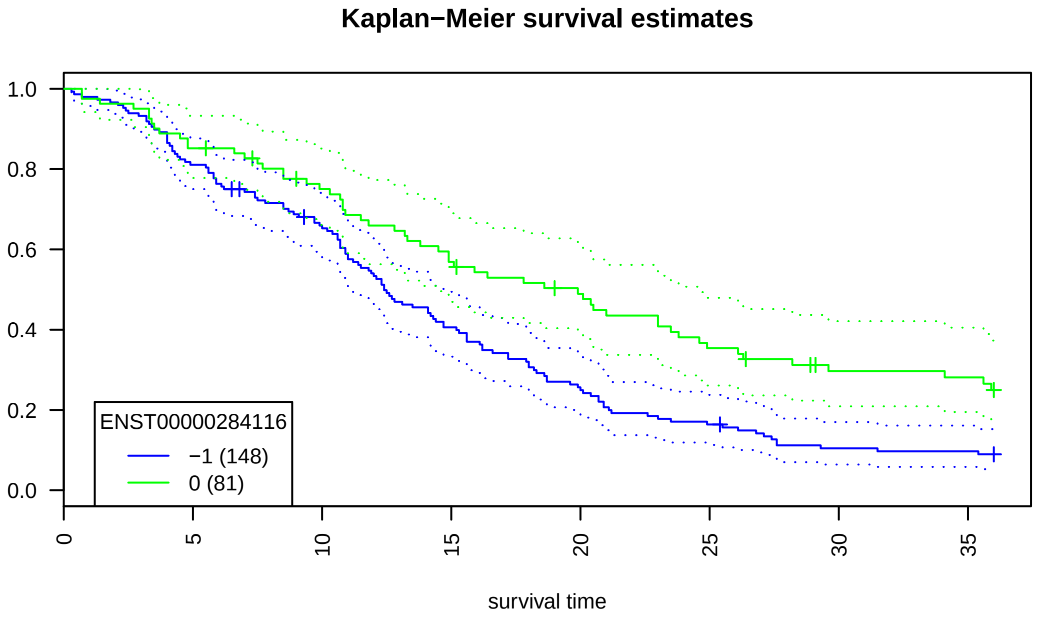

Figure 970: Each curve represents the relative number of ENST00000284116 survived to the corresponding point of time. Dashed lines represent 0.95 confidence intervals for the curves with the corresponding color. Total of 1 observation deleted due to missingness. Log-rank probability measure for the equality of these curves is 4.755757e-04. Additional annotations: *.GeneId*=ENSG00000153982, *.GeneName*=GDPD1, *.GeneDesc*=glycerophosphodiester phosphodiesterase domain containing 1 [Source:HGNC Symbol;Acc:20883].

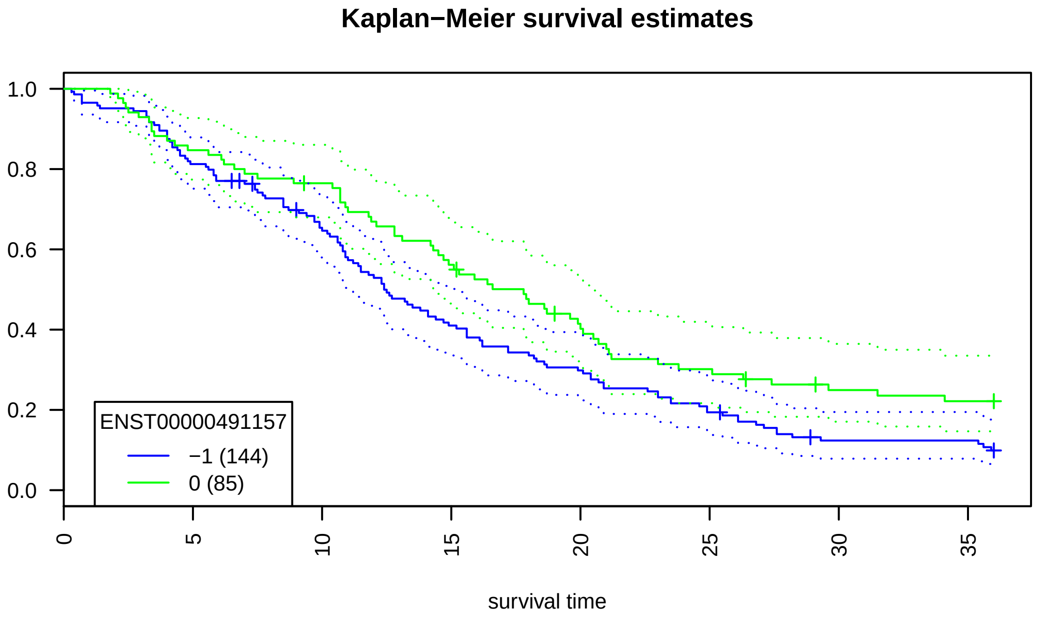

Figure 971: Each curve represents the relative number of ENST00000491157 survived to the corresponding point of time. Dashed lines represent 0.95 confidence intervals for the curves with the corresponding color. Total of 1 observation deleted due to missingness. Log-rank probability measure for the equality of these curves is 1.439878e-02. Additional annotations: *.GeneId*=ENSG00000155287, *.GeneName*=SLC25A28, *.GeneDesc*=solute carrier family 25, member 28 [Source:HGNC Symbol;Acc:23472].

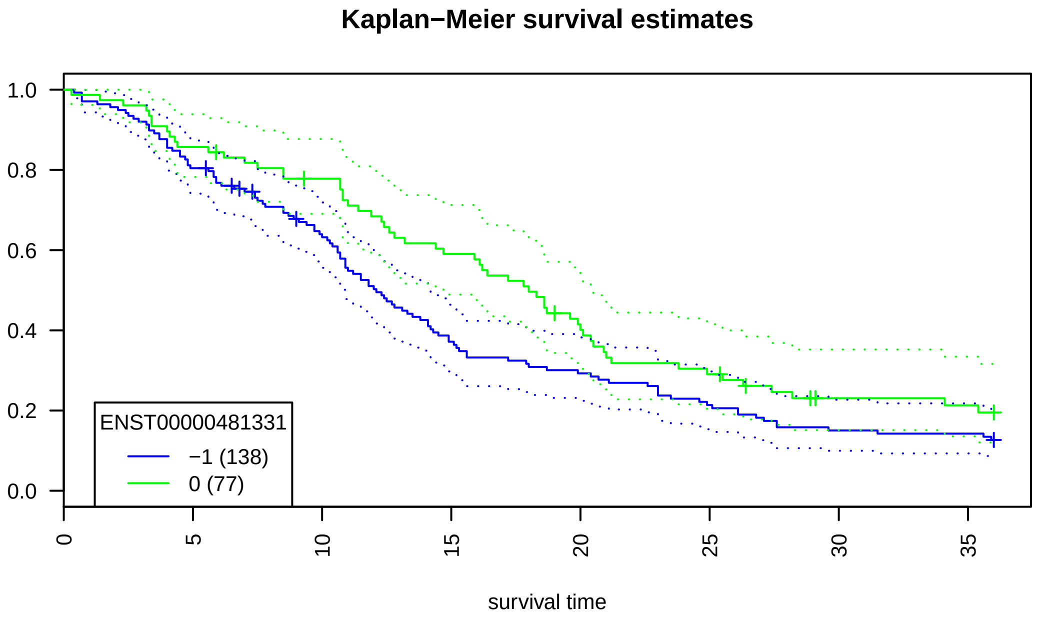

Figure 972: Each curve represents the relative number of ENST00000481331 survived to the corresponding point of time. Dashed lines represent 0.95 confidence intervals for the curves with the corresponding color. Total of 15 observations deleted due to missingness. Log-rank probability measure for the equality of these curves is 3.806166e-02. Additional annotations: *.GeneId*=ENSG00000204842, *.GeneName*=ATXN2, *.GeneDesc*=ataxin 2 [Source:HGNC Symbol;Acc:10555].

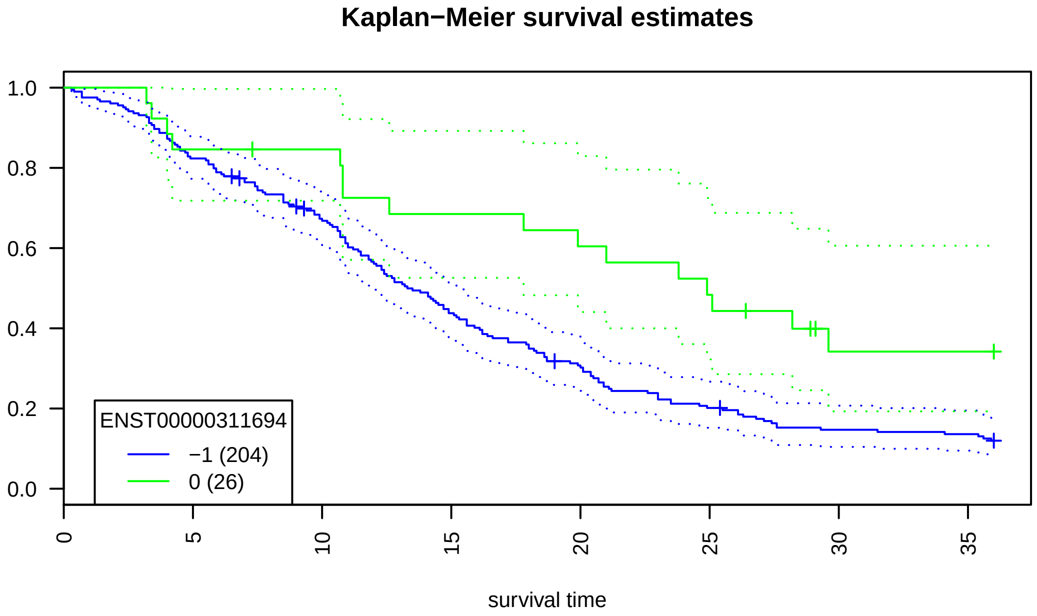

Figure 973: Each curve represents the relative number of ENST00000311694 survived to the corresponding point of time. Dashed lines represent 0.95 confidence intervals for the curves with the corresponding color. Log-rank probability measure for the equality of these curves is 4.823673e-03. Additional annotations: *.GeneId*=ENSG00000173064, *.GeneName*=C12orf51, *.GeneDesc*=Probable E3 ubiquitin-protein ligase KIAA0614 (EC 6.3.2.-) [Source:UniProtKB/Swiss-Prot;Acc:Q9Y4D8].

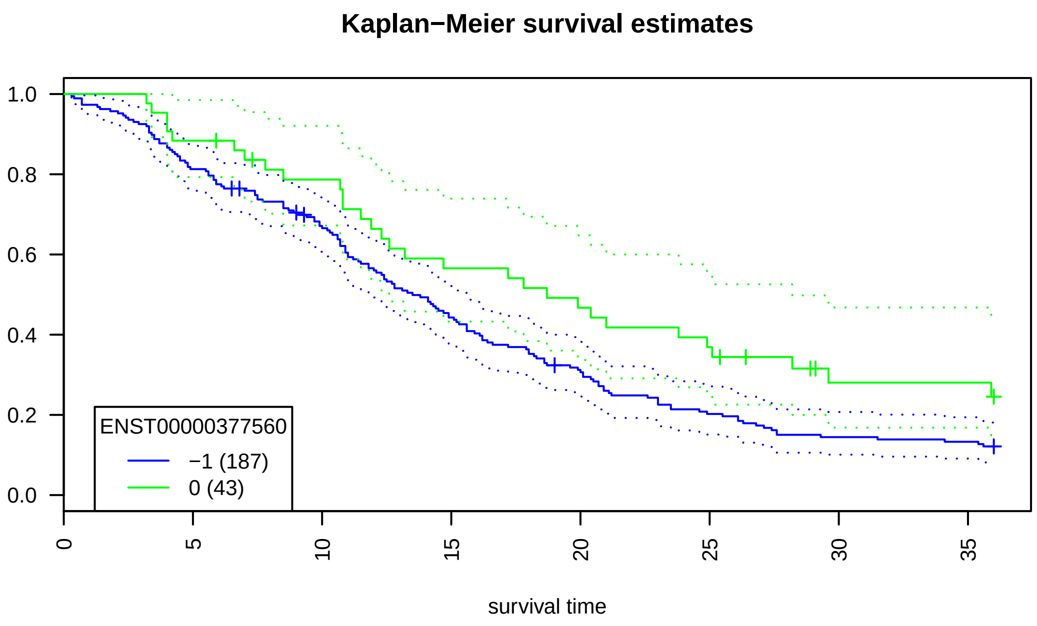

Figure 974: Each curve represents the relative number of ENST00000377560 survived to the corresponding point of time. Dashed lines represent 0.95 confidence intervals for the curves with the corresponding color. Log-rank probability measure for the equality of these curves is 2.475894e-02. Additional annotations: *.GeneId*=ENSG00000173064, *.GeneName*=C12orf51, *.GeneDesc*=Probable E3 ubiquitin-protein ligase KIAA0614 (EC 6.3.2.-) [Source:UniProtKB/Swiss-Prot;Acc:Q9Y4D8].

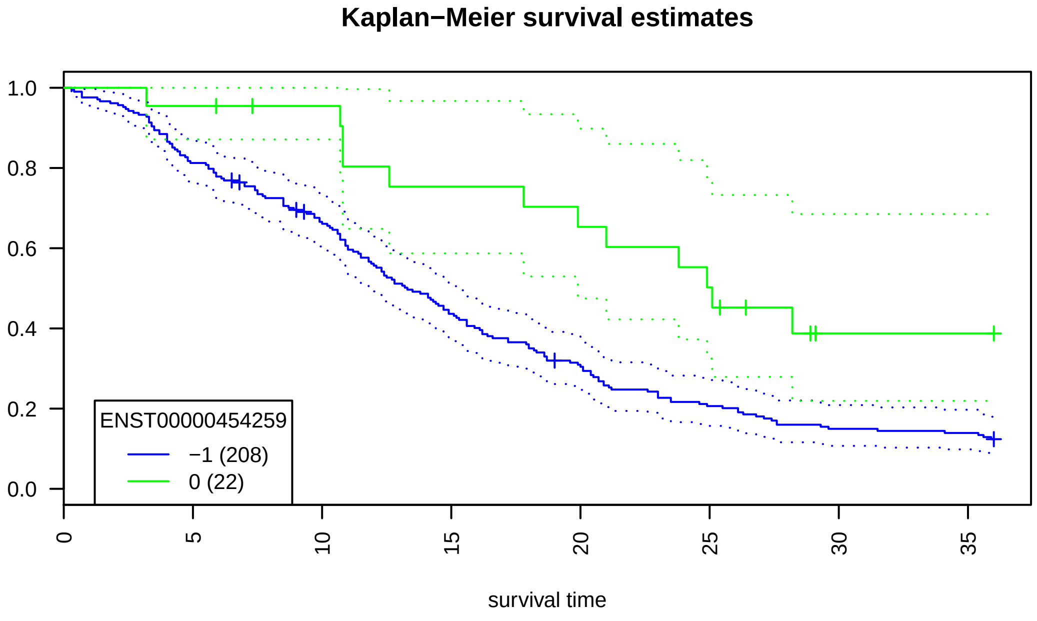

Figure 975: Each curve represents the relative number of ENST00000454259 survived to the corresponding point of time. Dashed lines represent 0.95 confidence intervals for the curves with the corresponding color. Log-rank probability measure for the equality of these curves is 3.308164e-03. Additional annotations: *.GeneId*=ENSG00000173064, *.GeneName*=C12orf51, *.GeneDesc*=Probable E3 ubiquitin-protein ligase KIAA0614 (EC 6.3.2.-) [Source:UniProtKB/Swiss-Prot;Acc:Q9Y4D8].

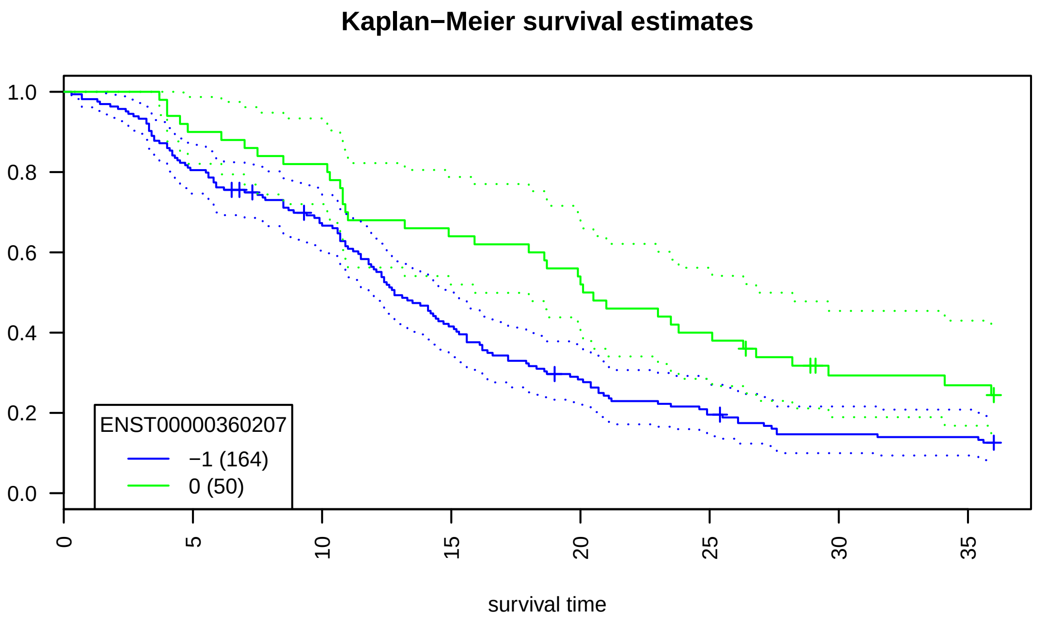

Figure 976: Each curve represents the relative number of ENST00000360207 survived to the corresponding point of time. Dashed lines represent 0.95 confidence intervals for the curves with the corresponding color. Total of 16 observations deleted due to missingness. Log-rank probability measure for the equality of these curves is 4.981428e-03. Additional annotations: *.GeneId*=ENSG00000141376, *.GeneName*=BCAS3, *.GeneDesc*=breast carcinoma amplified sequence 3 [Source:HGNC Symbol;Acc:14347].

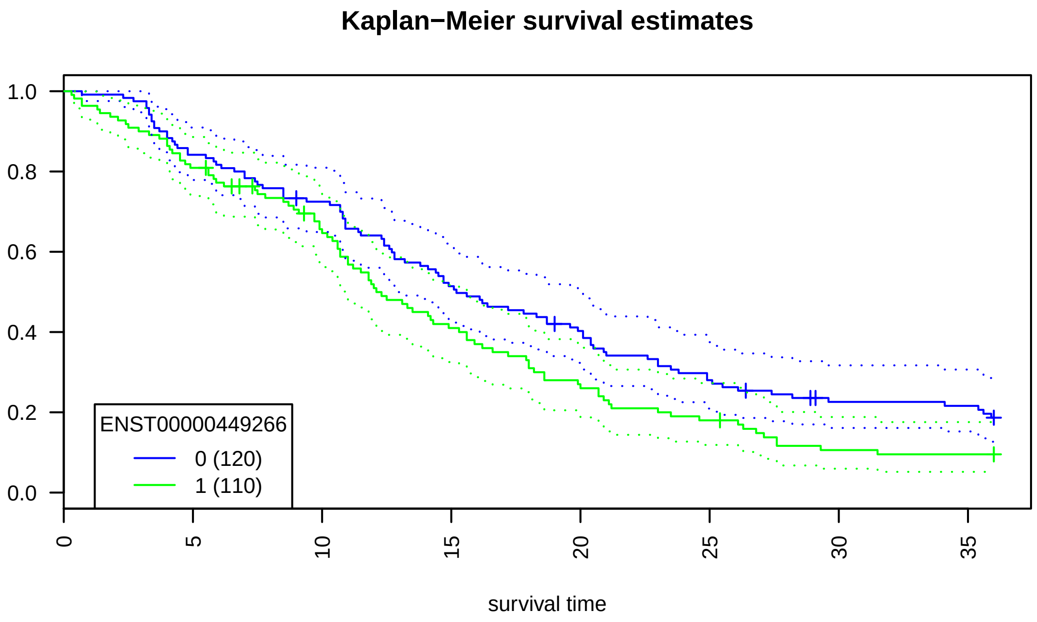

Figure 977: Each curve represents the relative number of ENST00000449266 survived to the corresponding point of time. Dashed lines represent 0.95 confidence intervals for the curves with the corresponding color. Log-rank probability measure for the equality of these curves is 2.318917e-02. Additional annotations: *.GeneId*=ENSG00000134668, *.GeneName*=SPOCD1, *.GeneDesc*=SPOC domain containing 1 [Source:HGNC Symbol;Acc:26338].

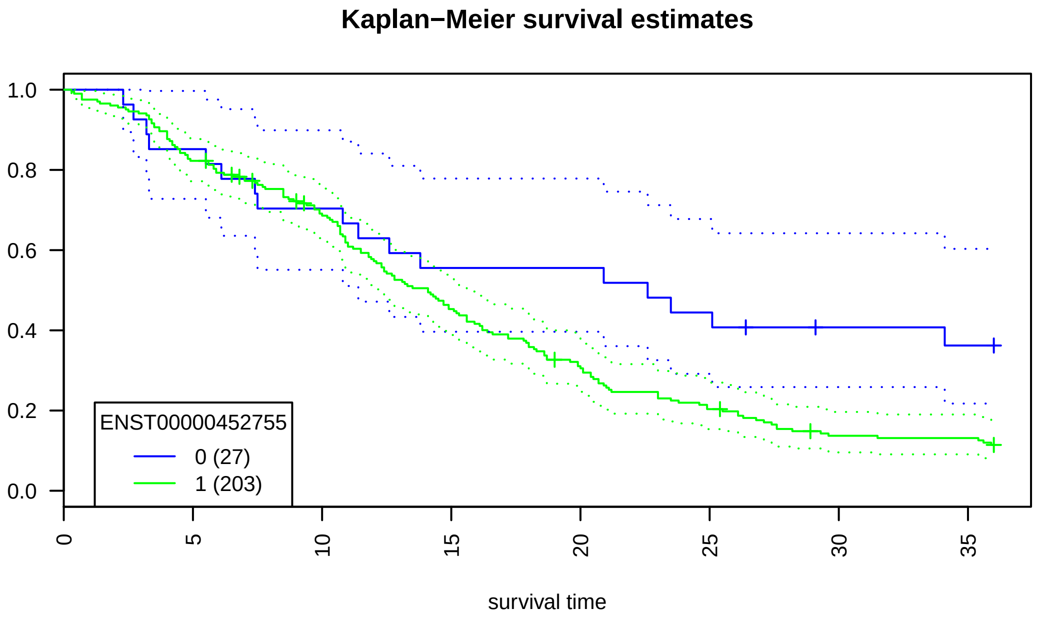

Figure 978: Each curve represents the relative number of ENST00000452755 survived to the corresponding point of time. Dashed lines represent 0.95 confidence intervals for the curves with the corresponding color. Log-rank probability measure for the equality of these curves is 1.33337e-02. Additional annotations: *.GeneId*=ENSG00000134668, *.GeneName*=SPOCD1, *.GeneDesc*=SPOC domain containing 1 [Source:HGNC Symbol;Acc:26338].

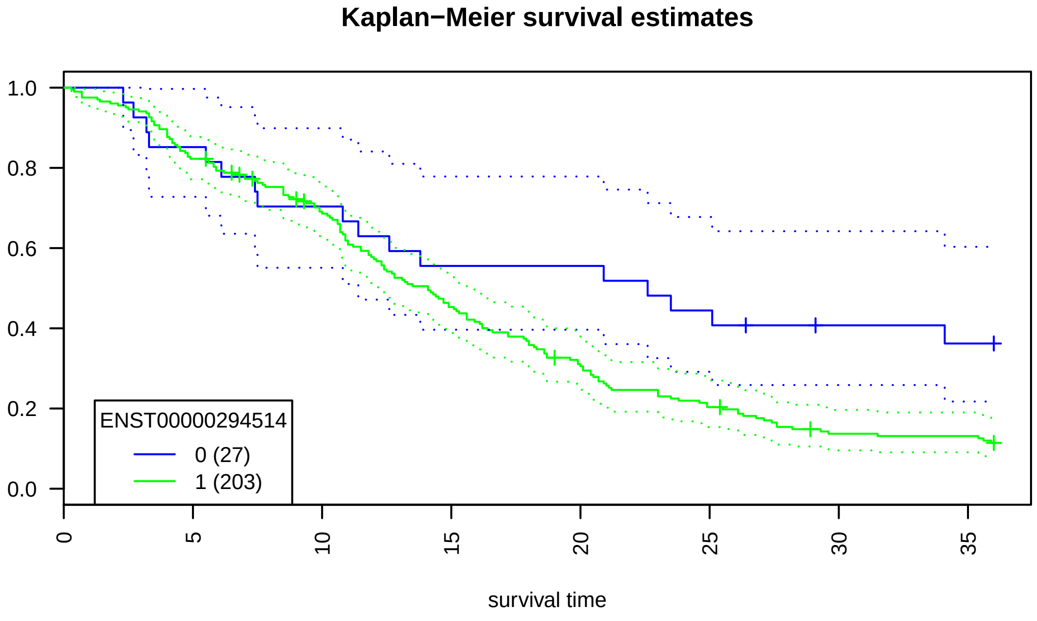

Figure 979: Each curve represents the relative number of ENST00000294514 survived to the corresponding point of time. Dashed lines represent 0.95 confidence intervals for the curves with the corresponding color. Log-rank probability measure for the equality of these curves is 1.33337e-02. Additional annotations: *.GeneId*=ENSG00000134668, *.GeneName*=SPOCD1, *.GeneDesc*=SPOC domain containing 1 [Source:HGNC Symbol;Acc:26338].

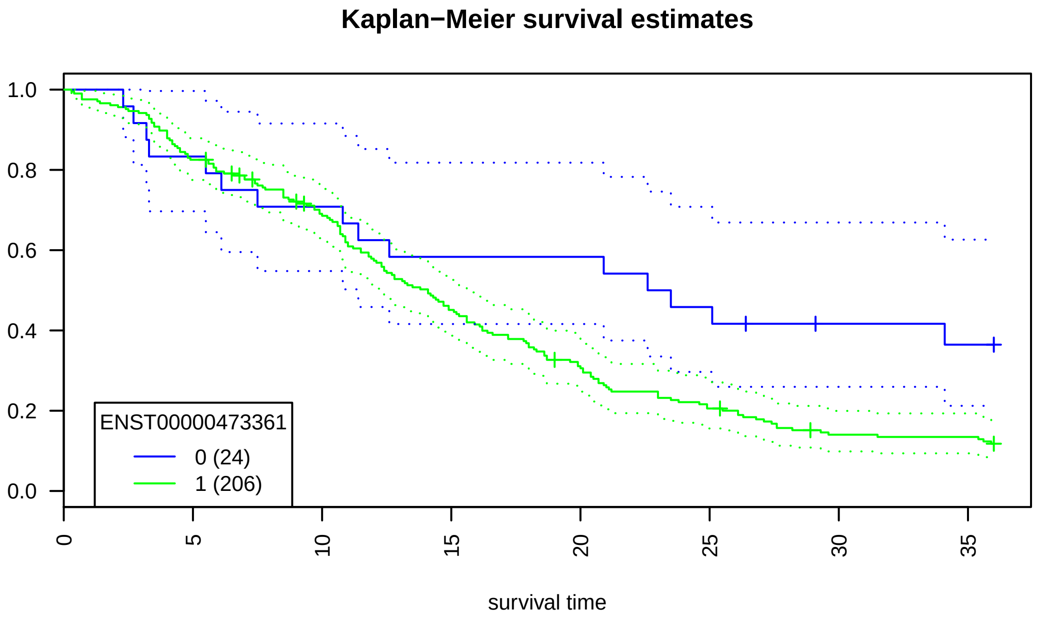

Figure 980: Each curve represents the relative number of ENST00000473361 survived to the corresponding point of time. Dashed lines represent 0.95 confidence intervals for the curves with the corresponding color. Log-rank probability measure for the equality of these curves is 1.634271e-02. Additional annotations: *.GeneId*=ENSG00000134668, *.GeneName*=SPOCD1, *.GeneDesc*=SPOC domain containing 1 [Source:HGNC Symbol;Acc:26338].

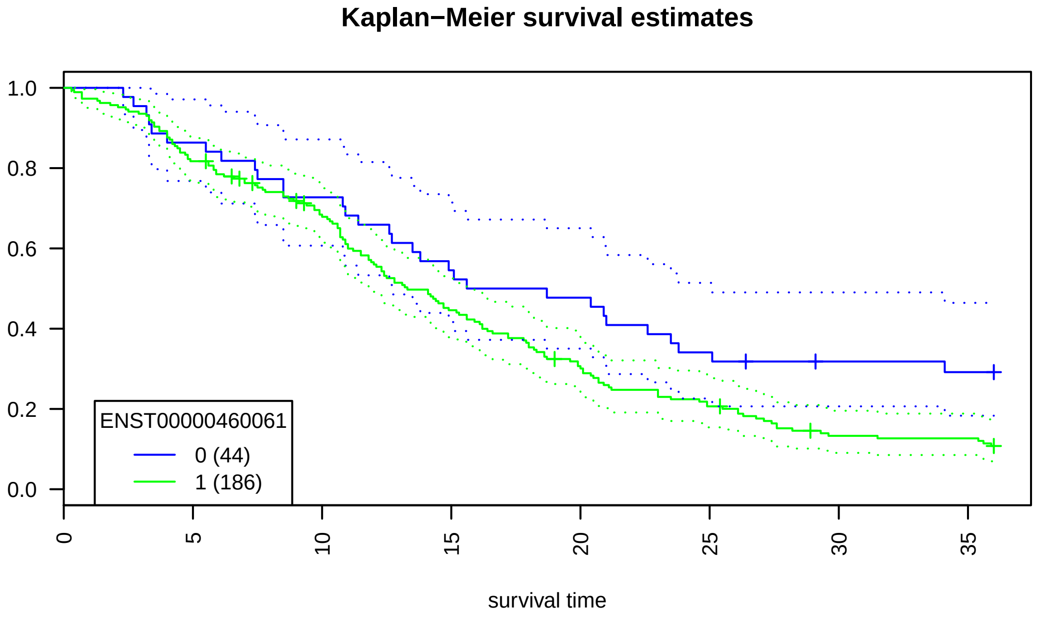

Figure 981: Each curve represents the relative number of ENST00000460061 survived to the corresponding point of time. Dashed lines represent 0.95 confidence intervals for the curves with the corresponding color. Log-rank probability measure for the equality of these curves is 1.865977e-02. Additional annotations: *.GeneId*=ENSG00000134668, *.GeneName*=SPOCD1, *.GeneDesc*=SPOC domain containing 1 [Source:HGNC Symbol;Acc:26338].

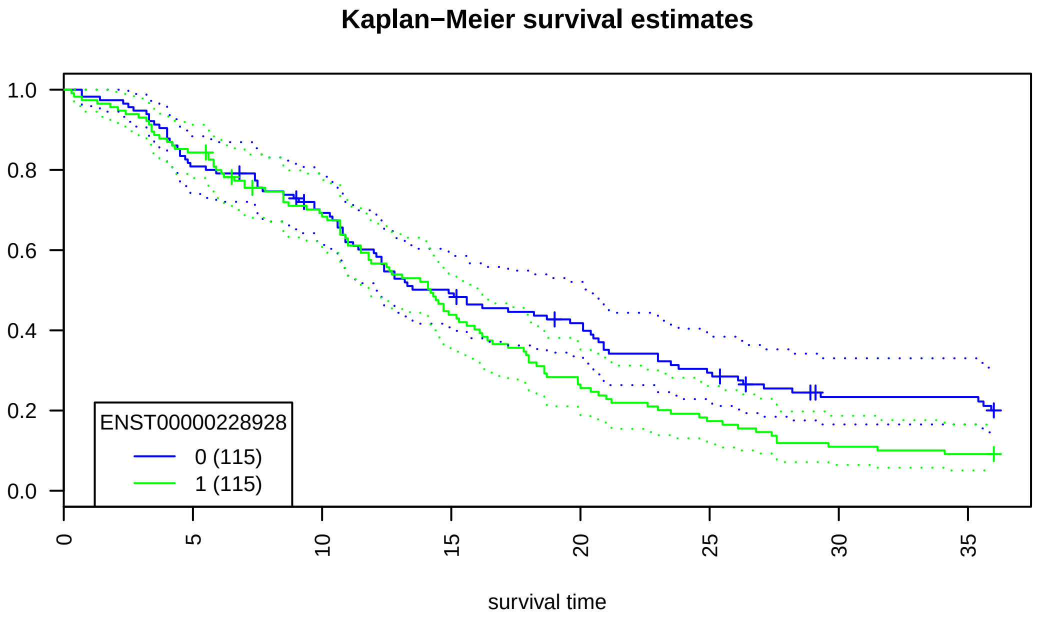

Figure 982: Each curve represents the relative number of ENST00000228928 survived to the corresponding point of time. Dashed lines represent 0.95 confidence intervals for the curves with the corresponding color. Log-rank probability measure for the equality of these curves is 4.776084e-02. Additional annotations: *.GeneId*=ENSG00000111331, *.GeneName*=OAS3, *.GeneDesc*=2'-5'-oligoadenylate synthetase 3, 100kDa [Source:HGNC Symbol;Acc:8088].

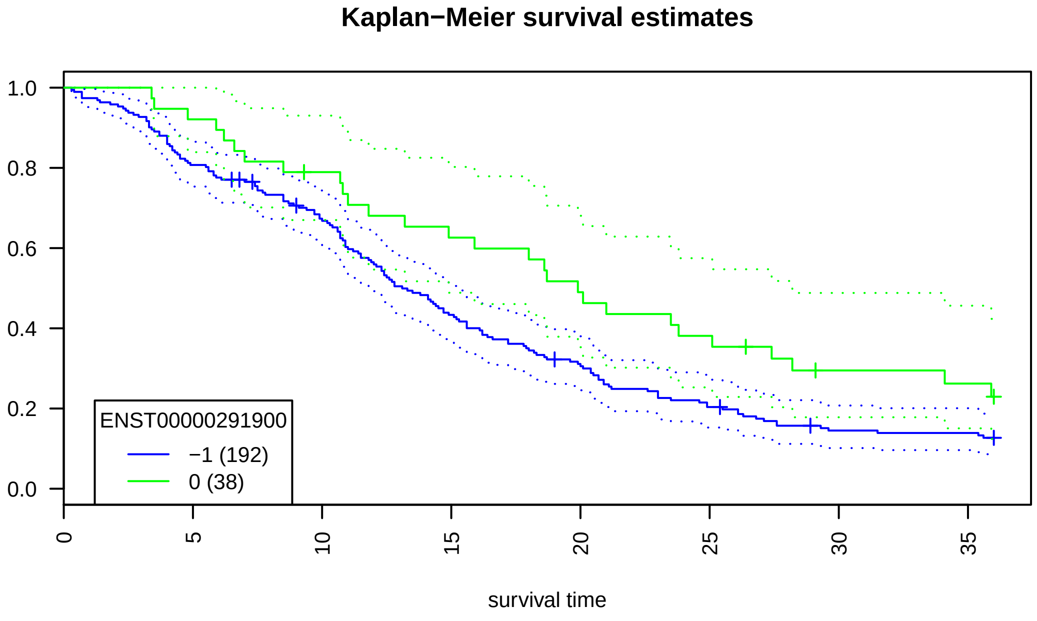

Figure 983: Each curve represents the relative number of ENST00000291900 survived to the corresponding point of time. Dashed lines represent 0.95 confidence intervals for the curves with the corresponding color. Log-rank probability measure for the equality of these curves is 2.651128e-02. Additional annotations: *.GeneId*=ENSG00000160445, *.GeneName*=ZER1, *.GeneDesc*=zer-1 homolog (C. elegans) [Source:HGNC Symbol;Acc:30960].

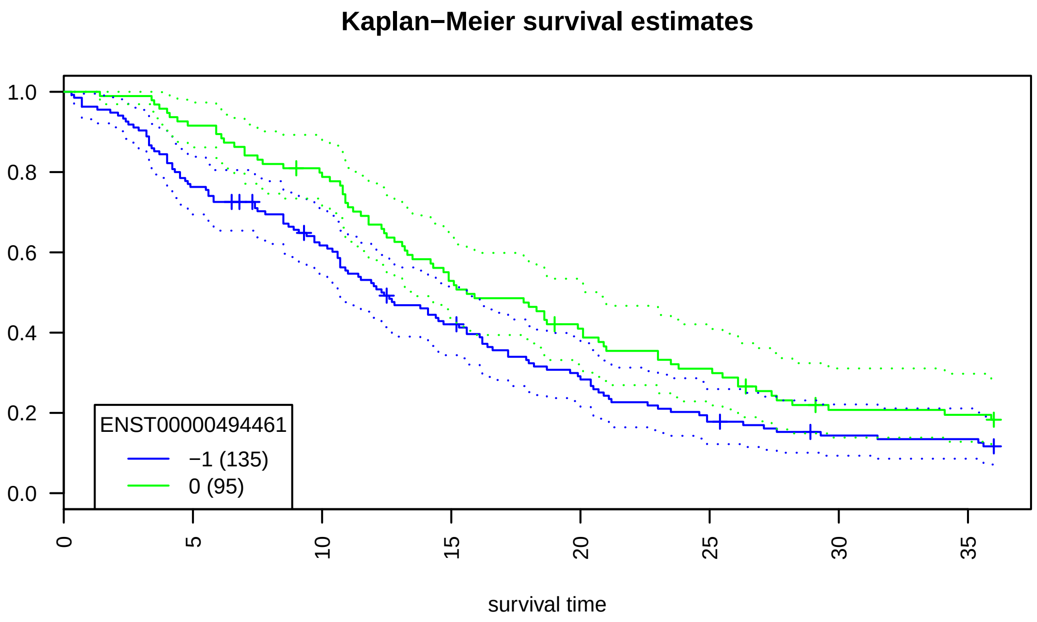

Figure 984: Each curve represents the relative number of ENST00000494461 survived to the corresponding point of time. Dashed lines represent 0.95 confidence intervals for the curves with the corresponding color. Log-rank probability measure for the equality of these curves is 1.879053e-02. Additional annotations: *.GeneId*=ENSG00000160445, *.GeneName*=ZER1, *.GeneDesc*=zer-1 homolog (C. elegans) [Source:HGNC Symbol;Acc:30960].

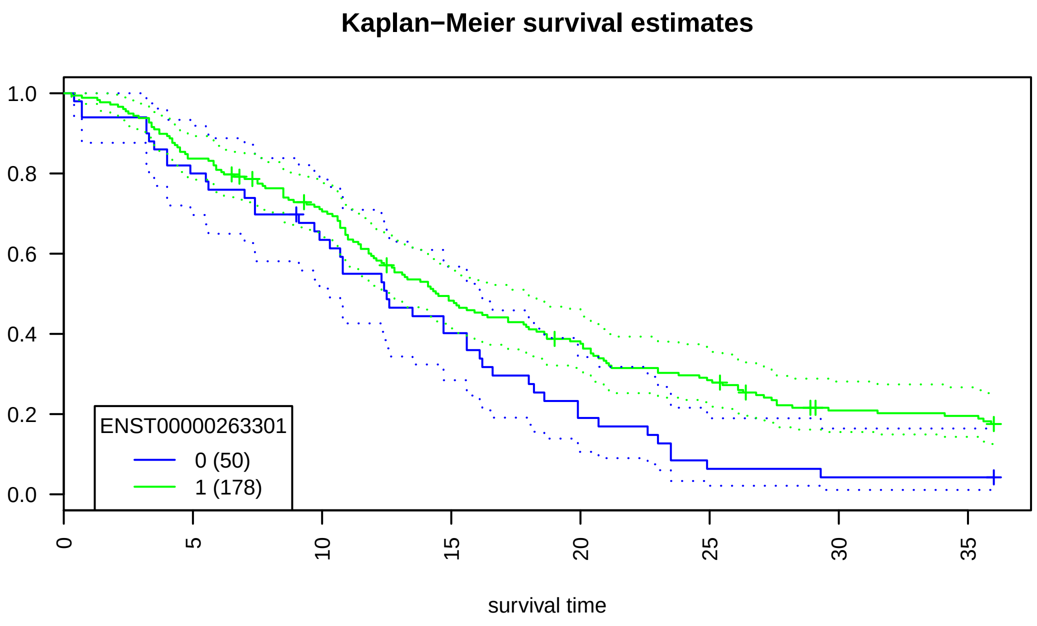

Figure 985: Each curve represents the relative number of ENST00000263301 survived to the corresponding point of time. Dashed lines represent 0.95 confidence intervals for the curves with the corresponding color. Total of 2 observations deleted due to missingness. Log-rank probability measure for the equality of these curves is 8.505022e-03. Additional annotations: *.GeneId*=ENSG00000087884, *.GeneName*=C11orf67, *.GeneDesc*=UPF0366 protein C11orf67 [Source:UniProtKB/Swiss-Prot;Acc:Q9H7C9].

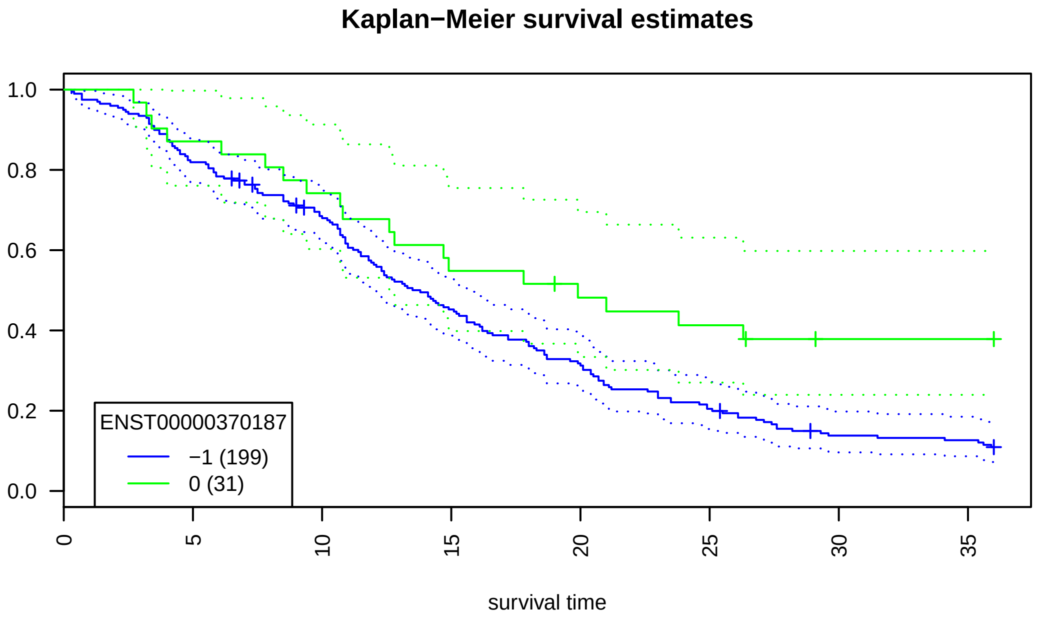

Figure 986: Each curve represents the relative number of ENST00000370187 survived to the corresponding point of time. Dashed lines represent 0.95 confidence intervals for the curves with the corresponding color. Log-rank probability measure for the equality of these curves is 7.53295e-03. Additional annotations: *.GeneId*=ENSG00000166167, *.GeneName*=BTRC, *.GeneDesc*=beta-transducin repeat containing [Source:HGNC Symbol;Acc:1144].

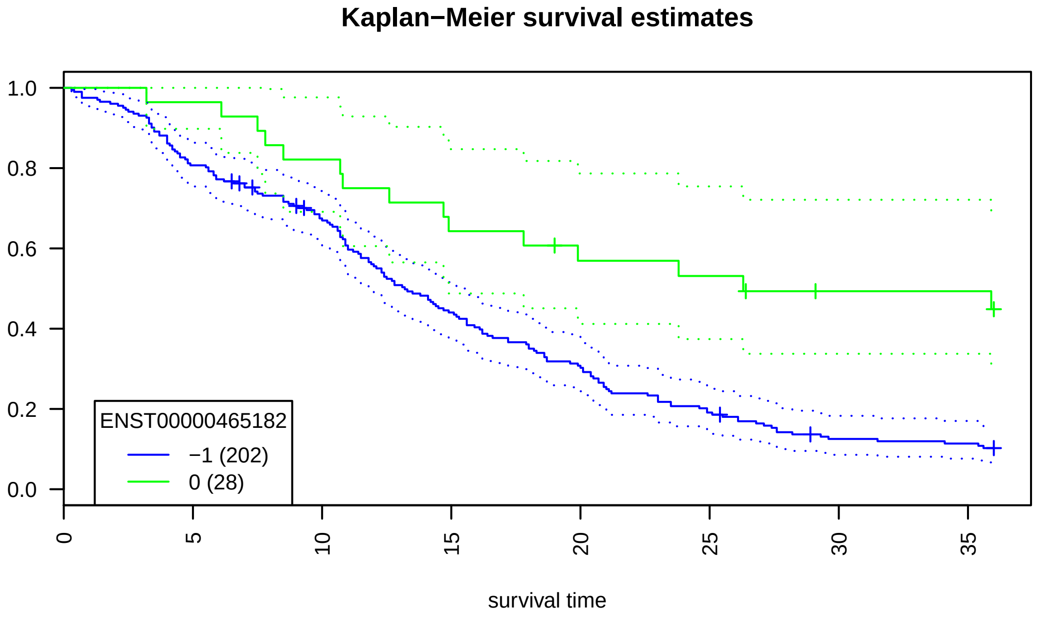

Figure 987: Each curve represents the relative number of ENST00000465182 survived to the corresponding point of time. Dashed lines represent 0.95 confidence intervals for the curves with the corresponding color. Log-rank probability measure for the equality of these curves is 2.04414e-04. Additional annotations: *.GeneId*=ENSG00000166167, *.GeneName*=BTRC, *.GeneDesc*=beta-transducin repeat containing [Source:HGNC Symbol;Acc:1144].

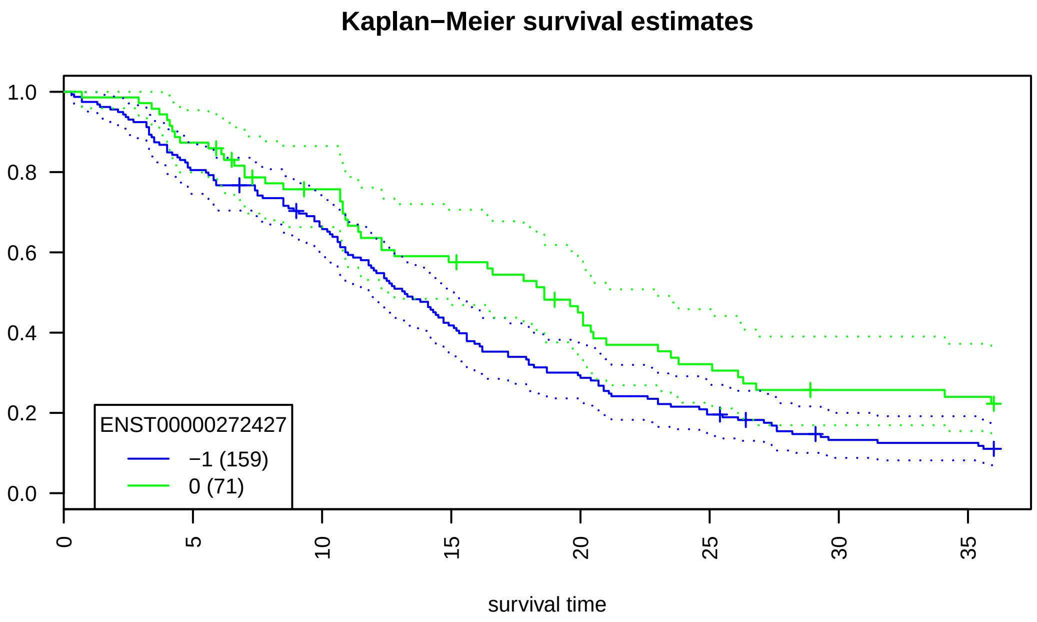

Figure 988: Each curve represents the relative number of ENST00000272427 survived to the corresponding point of time. Dashed lines represent 0.95 confidence intervals for the curves with the corresponding color. Log-rank probability measure for the equality of these curves is 1.968872e-02. Additional annotations: *.GeneId*=ENSG00000144036, *.GeneName*=EXOC6B, *.GeneDesc*=exocyst complex component 6B [Source:HGNC Symbol;Acc:17085].

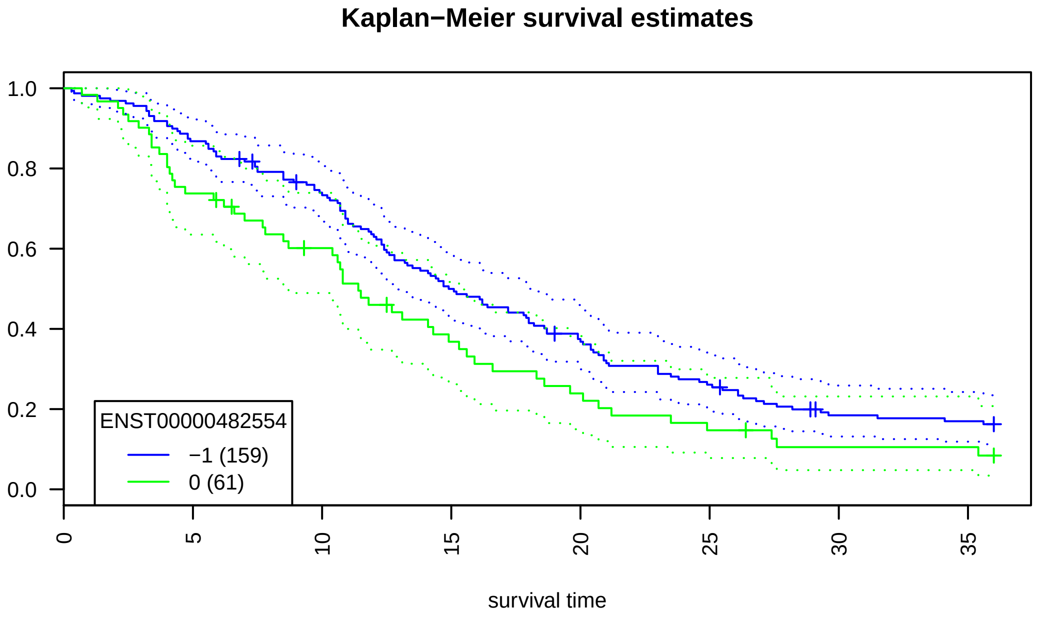

Figure 989: Each curve represents the relative number of ENST00000482554 survived to the corresponding point of time. Dashed lines represent 0.95 confidence intervals for the curves with the corresponding color. Total of 10 observations deleted due to missingness. Log-rank probability measure for the equality of these curves is 2.077322e-02. Additional annotations: *.GeneId*=ENSG00000135631, *.GeneName*=RAB11FIP5, *.GeneDesc*=RAB11 family interacting protein 5 (class I) [Source:HGNC Symbol;Acc:24845].

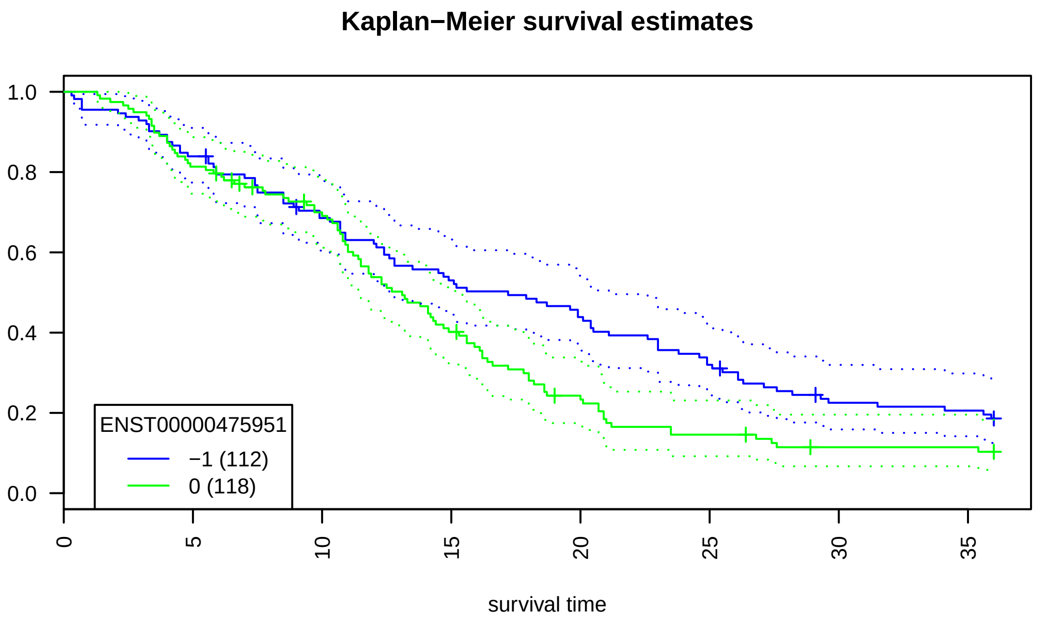

Figure 990: Each curve represents the relative number of ENST00000475951 survived to the corresponding point of time. Dashed lines represent 0.95 confidence intervals for the curves with the corresponding color. Log-rank probability measure for the equality of these curves is 1.411389e-02. Additional annotations: *.GeneId*=ENSG00000135631, *.GeneName*=RAB11FIP5, *.GeneDesc*=RAB11 family interacting protein 5 (class I) [Source:HGNC Symbol;Acc:24845].

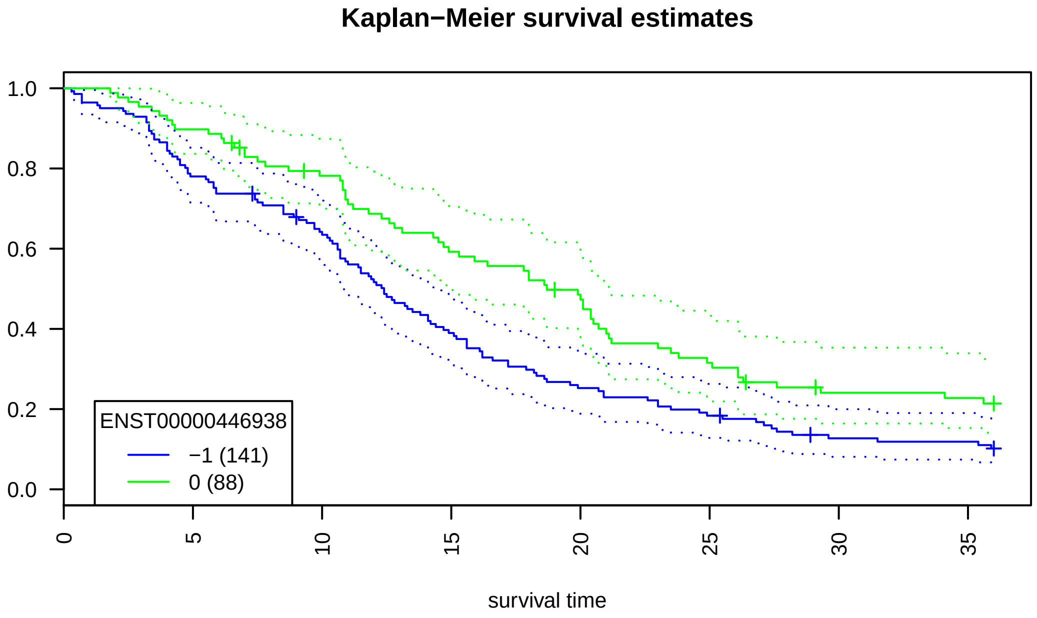

Figure 991: Each curve represents the relative number of ENST00000446938 survived to the corresponding point of time. Dashed lines represent 0.95 confidence intervals for the curves with the corresponding color. Total of 1 observation deleted due to missingness. Log-rank probability measure for the equality of these curves is 2.102811e-03. Additional annotations: *.GeneId*=ENSG00000107829, *.GeneName*=FBXW4, *.GeneDesc*=F-box and WD repeat domain containing 4 [Source:HGNC Symbol;Acc:10847].

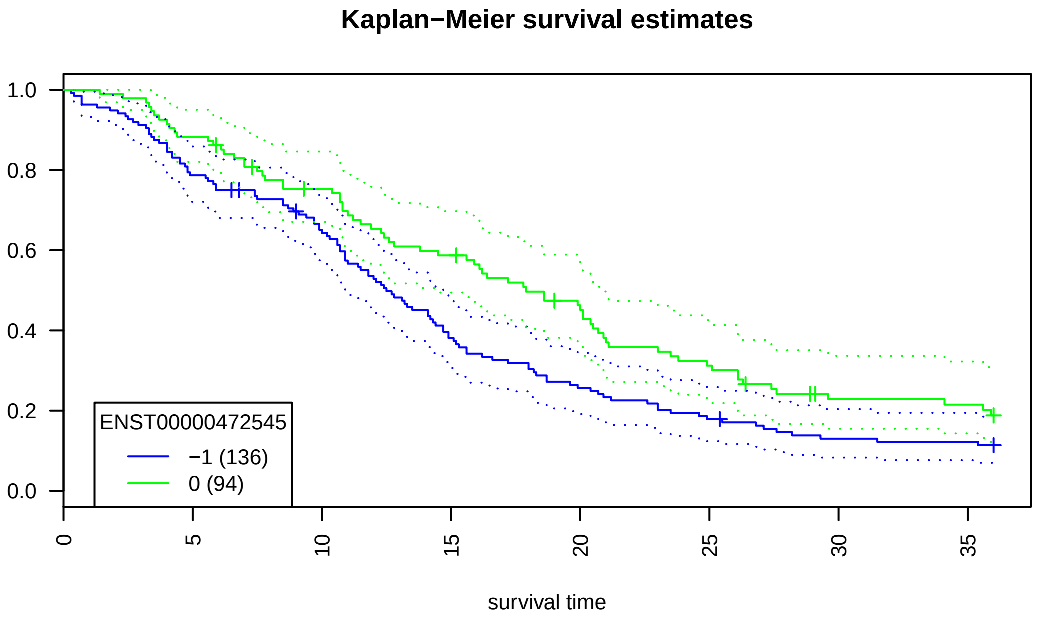

Figure 992: Each curve represents the relative number of ENST00000472545 survived to the corresponding point of time. Dashed lines represent 0.95 confidence intervals for the curves with the corresponding color. Log-rank probability measure for the equality of these curves is 1.090582e-02. Additional annotations: *.GeneId*=ENSG00000150672, *.GeneName*=DLG2, *.GeneDesc*=discs, large homolog 2 (Drosophila) [Source:HGNC Symbol;Acc:2901].

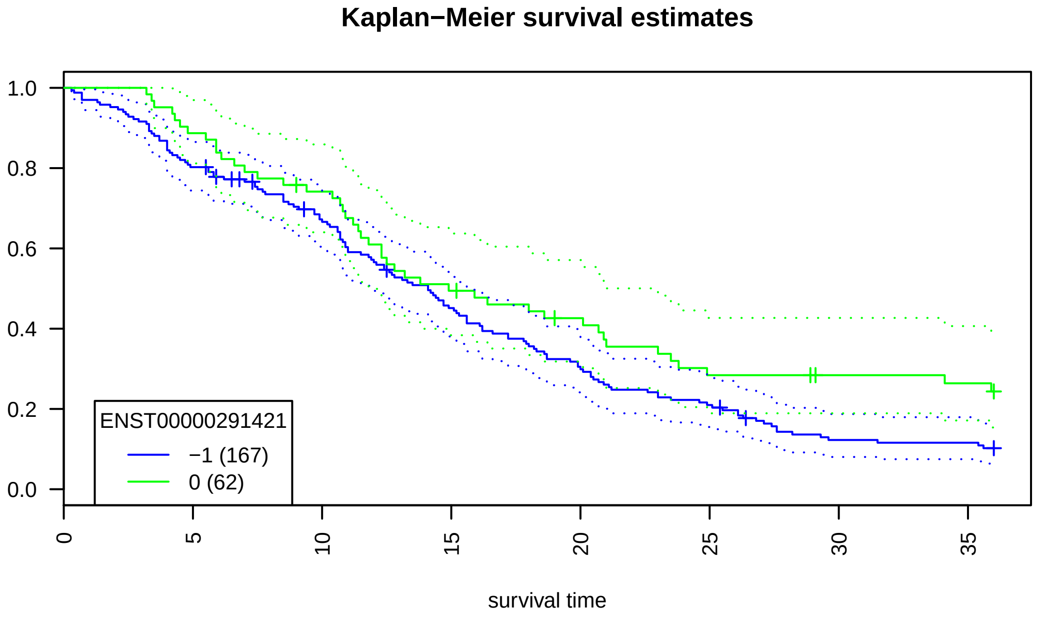

Figure 993: Each curve represents the relative number of ENST00000291421 survived to the corresponding point of time. Dashed lines represent 0.95 confidence intervals for the curves with the corresponding color. Total of 1 observation deleted due to missingness. Log-rank probability measure for the equality of these curves is 3.609446e-02. Additional annotations: *.GeneId*=ENSG00000160097, *.GeneName*=FNDC5, *.GeneDesc*=fibronectin type III domain containing 5 [Source:HGNC Symbol;Acc:20240].

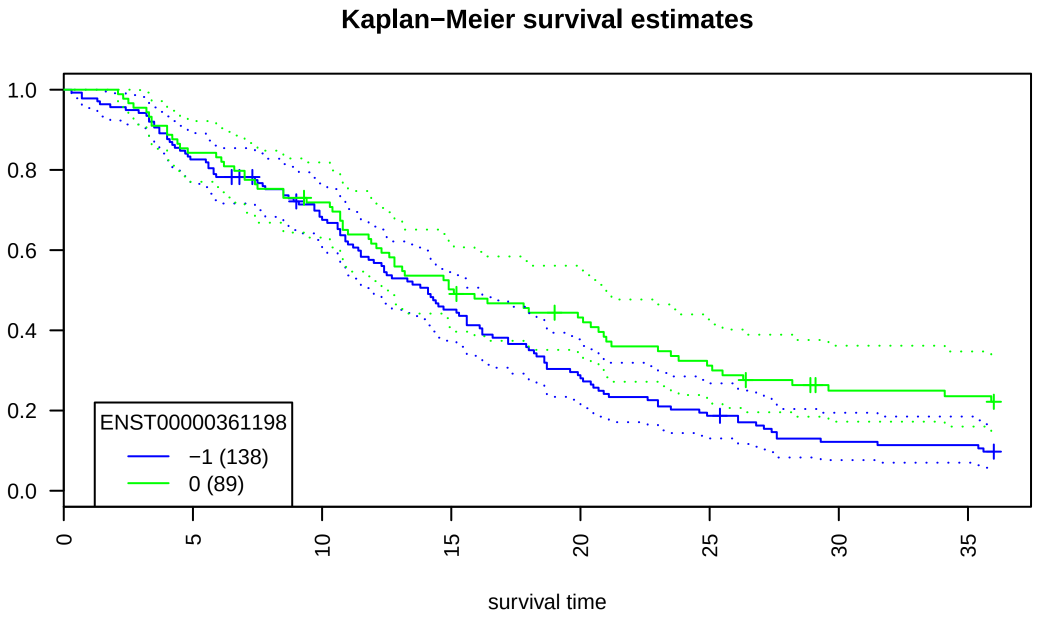

Figure 994: Each curve represents the relative number of ENST00000361198 survived to the corresponding point of time. Dashed lines represent 0.95 confidence intervals for the curves with the corresponding color. Total of 3 observations deleted due to missingness. Log-rank probability measure for the equality of these curves is 3.079783e-02. Additional annotations: *.GeneId*=ENSG00000198728, *.GeneName*=LDB1, *.GeneDesc*=LIM domain binding 1 [Source:HGNC Symbol;Acc:6532].

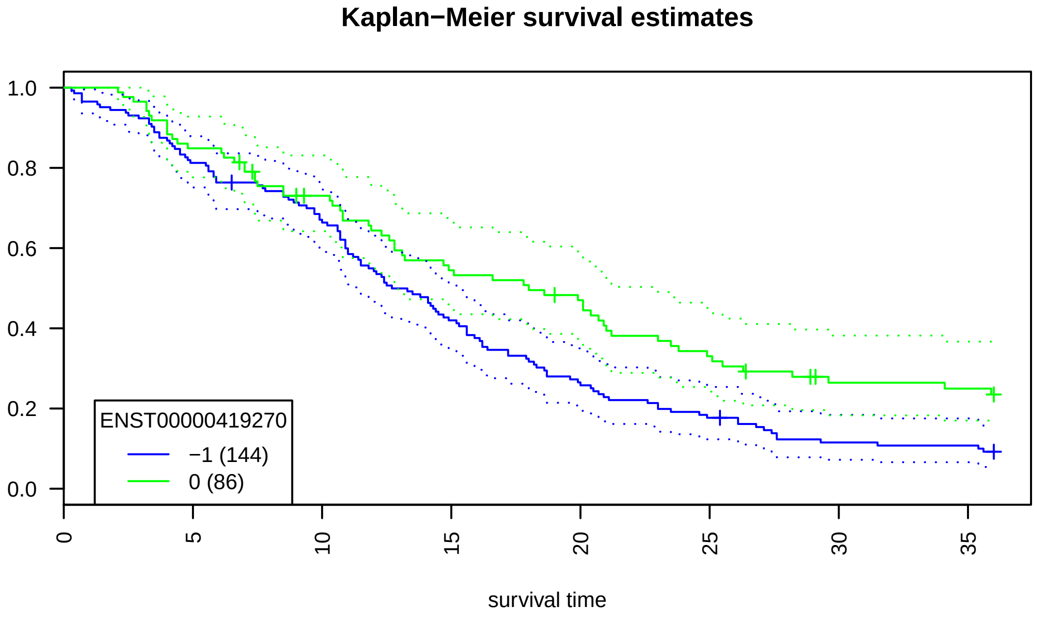

Figure 995: Each curve represents the relative number of ENST00000419270 survived to the corresponding point of time. Dashed lines represent 0.95 confidence intervals for the curves with the corresponding color. Log-rank probability measure for the equality of these curves is 4.360745e-03. Additional annotations: *.GeneId*=ENSG00000198728, *.GeneName*=LDB1, *.GeneDesc*=LIM domain binding 1 [Source:HGNC Symbol;Acc:6532].

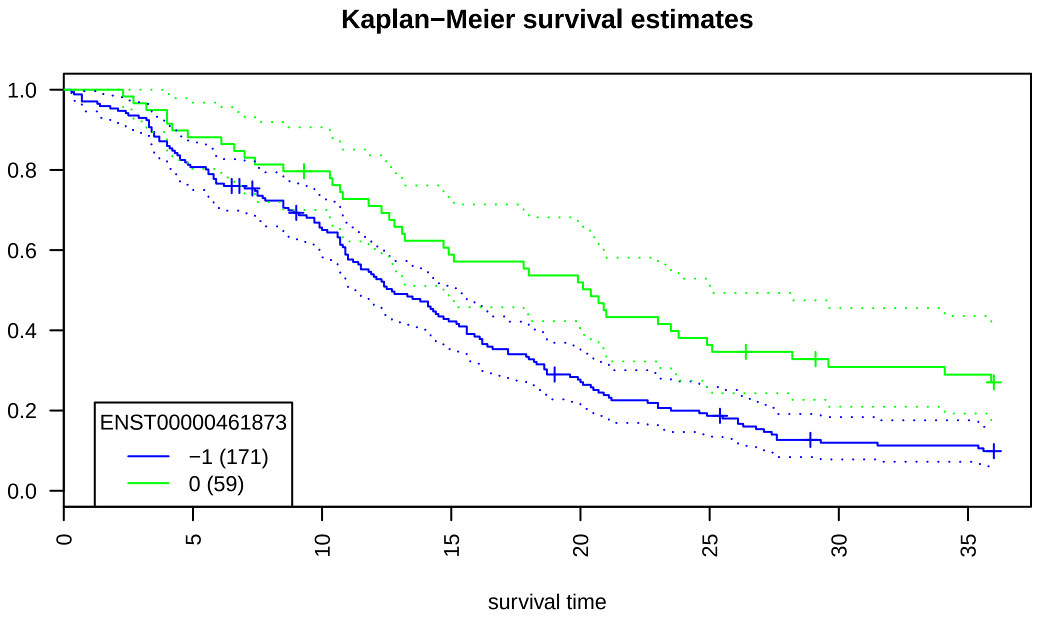

Figure 996: Each curve represents the relative number of ENST00000461873 survived to the corresponding point of time. Dashed lines represent 0.95 confidence intervals for the curves with the corresponding color. Log-rank probability measure for the equality of these curves is 1.035294e-03. Additional annotations: *.GeneId*=ENSG00000198728, *.GeneName*=LDB1, *.GeneDesc*=LIM domain binding 1 [Source:HGNC Symbol;Acc:6532].

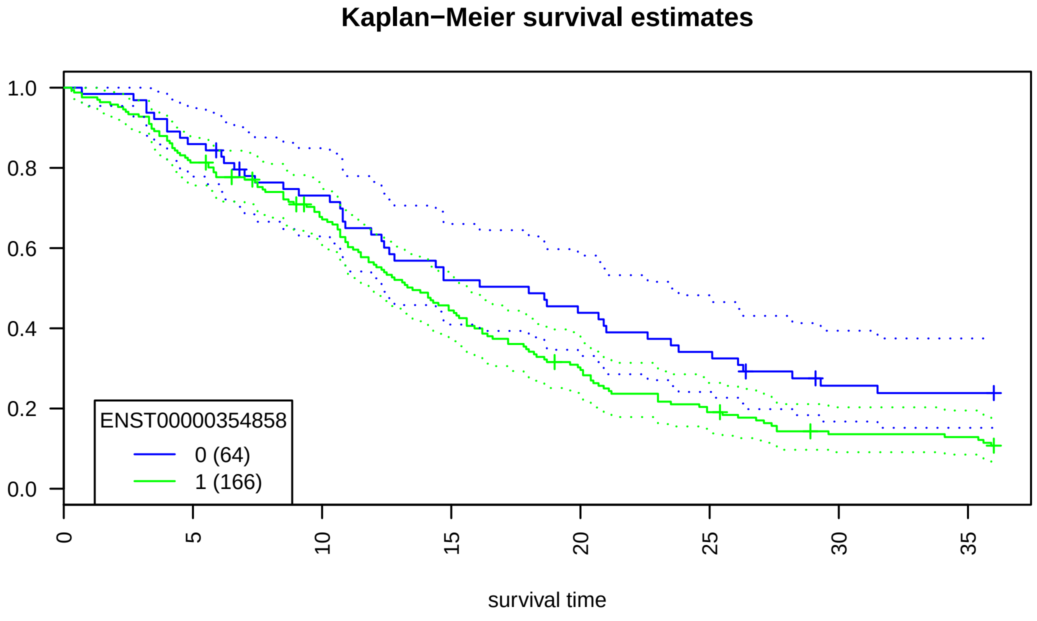

Figure 997: Each curve represents the relative number of ENST00000354858 survived to the corresponding point of time. Dashed lines represent 0.95 confidence intervals for the curves with the corresponding color. Log-rank probability measure for the equality of these curves is 2.587483e-02. Additional annotations: *.GeneId*=ENSG00000004455, *.GeneName*=AK2, *.GeneDesc*=adenylate kinase 2 [Source:HGNC Symbol;Acc:362].

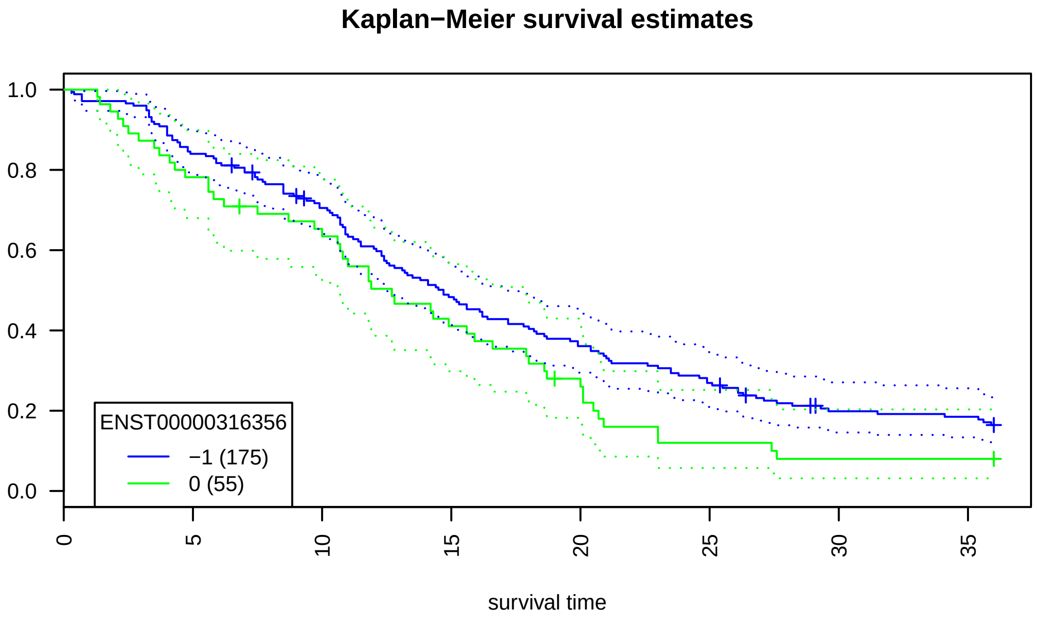

Figure 998: Each curve represents the relative number of ENST00000316356 survived to the corresponding point of time. Dashed lines represent 0.95 confidence intervals for the curves with the corresponding color. Log-rank probability measure for the equality of these curves is 4.60627e-02. Additional annotations: *.GeneId*=ENSG00000137501, *.GeneName*=SYTL2, *.GeneDesc*=synaptotagmin-like 2 [Source:HGNC Symbol;Acc:15585].

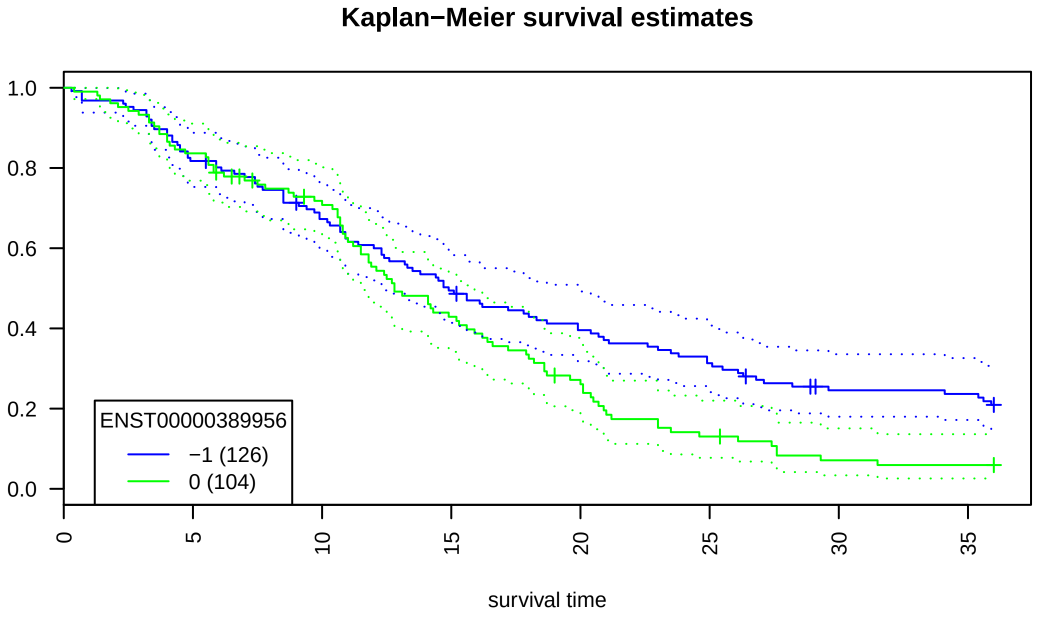

Figure 999: Each curve represents the relative number of ENST00000389956 survived to the corresponding point of time. Dashed lines represent 0.95 confidence intervals for the curves with the corresponding color. Log-rank probability measure for the equality of these curves is 6.868827e-03. Additional annotations: *.GeneId*=ENSG00000137501, *.GeneName*=SYTL2, *.GeneDesc*=synaptotagmin-like 2 [Source:HGNC Symbol;Acc:15585].

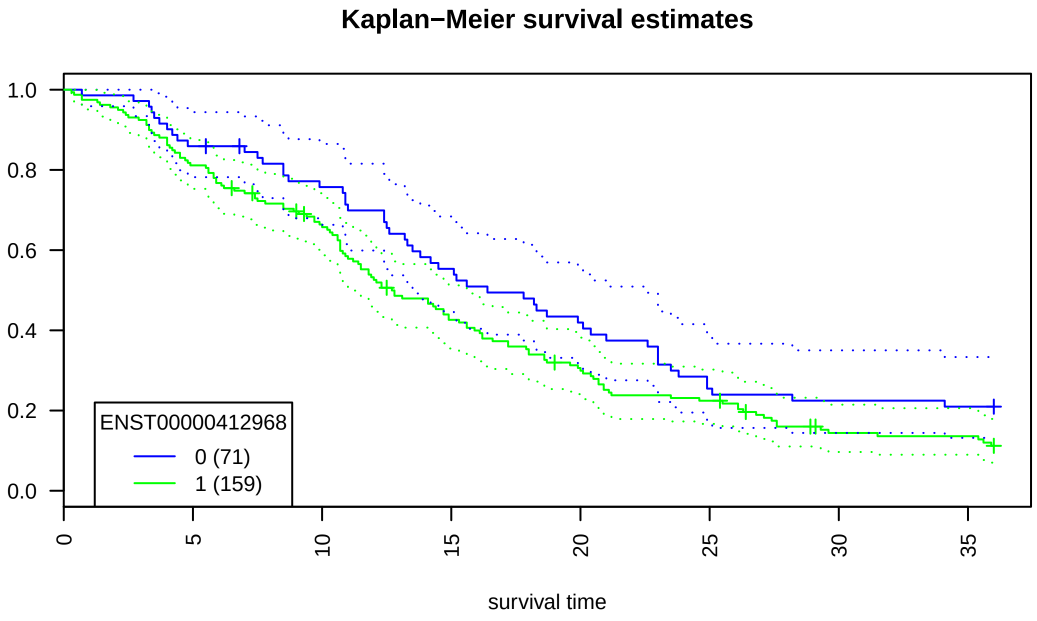

Figure 1000: Each curve represents the relative number of ENST00000412968 survived to the corresponding point of time. Dashed lines represent 0.95 confidence intervals for the curves with the corresponding color. Log-rank probability measure for the equality of these curves is 4.557375e-02. Additional annotations: *.GeneId*=ENSG00000091136, *.GeneName*=LAMB1, *.GeneDesc*=laminin, beta 1 [Source:HGNC Symbol;Acc:6486].

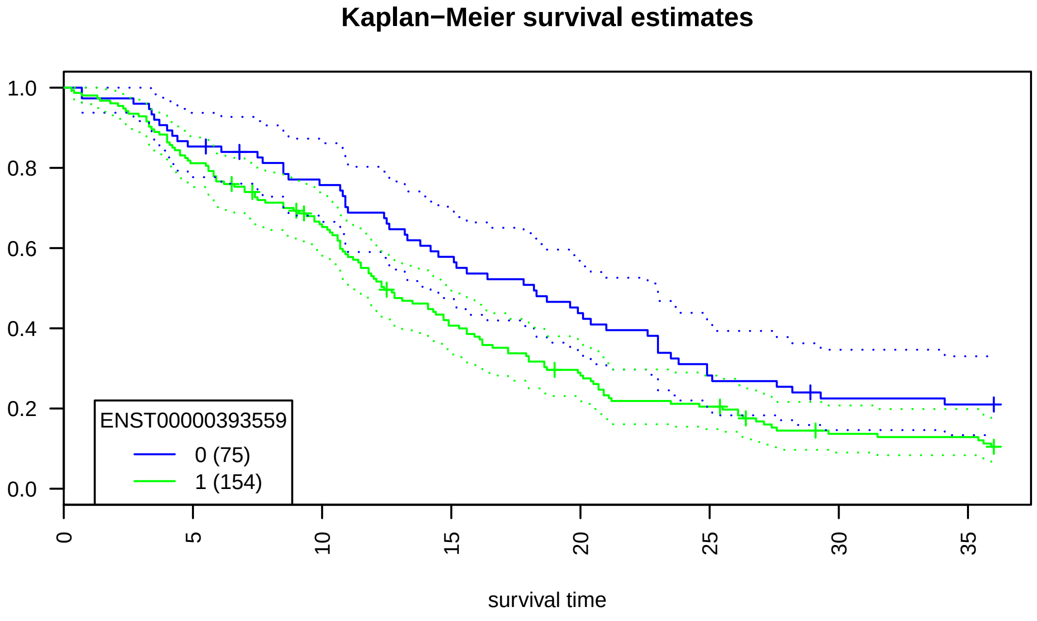

Figure 1001: Each curve represents the relative number of ENST00000393559 survived to the corresponding point of time. Dashed lines represent 0.95 confidence intervals for the curves with the corresponding color. Total of 1 observation deleted due to missingness. Log-rank probability measure for the equality of these curves is 1.479559e-02. Additional annotations: *.GeneId*=ENSG00000091136, *.GeneName*=LAMB1, *.GeneDesc*=laminin, beta 1 [Source:HGNC Symbol;Acc:6486].

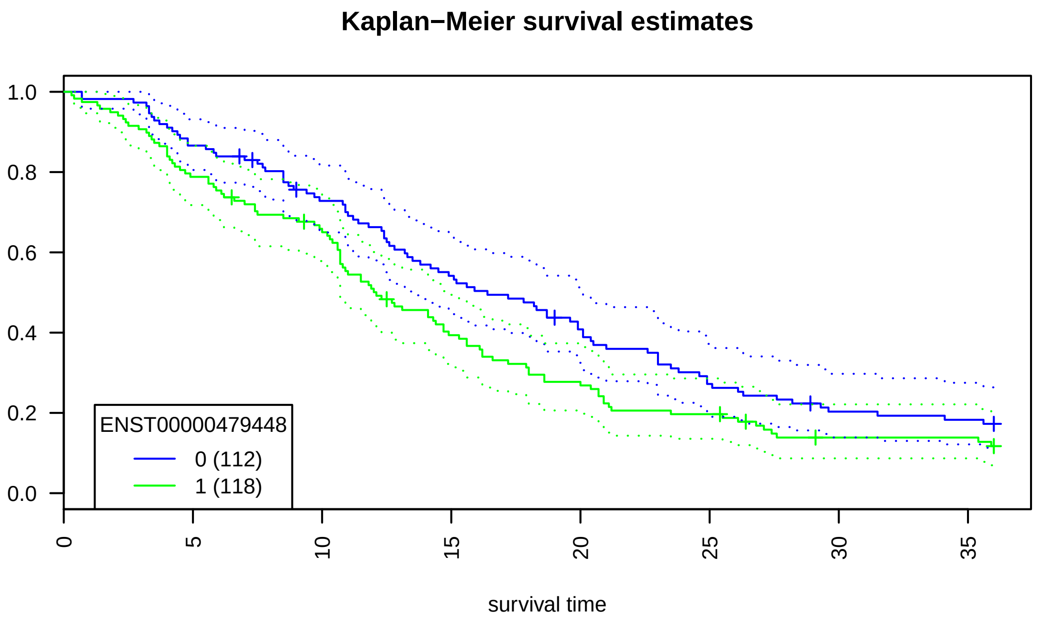

Figure 1002: Each curve represents the relative number of ENST00000479448 survived to the corresponding point of time. Dashed lines represent 0.95 confidence intervals for the curves with the corresponding color. Log-rank probability measure for the equality of these curves is 2.932227e-02. Additional annotations: *.GeneId*=ENSG00000091136, *.GeneName*=LAMB1, *.GeneDesc*=laminin, beta 1 [Source:HGNC Symbol;Acc:6486].

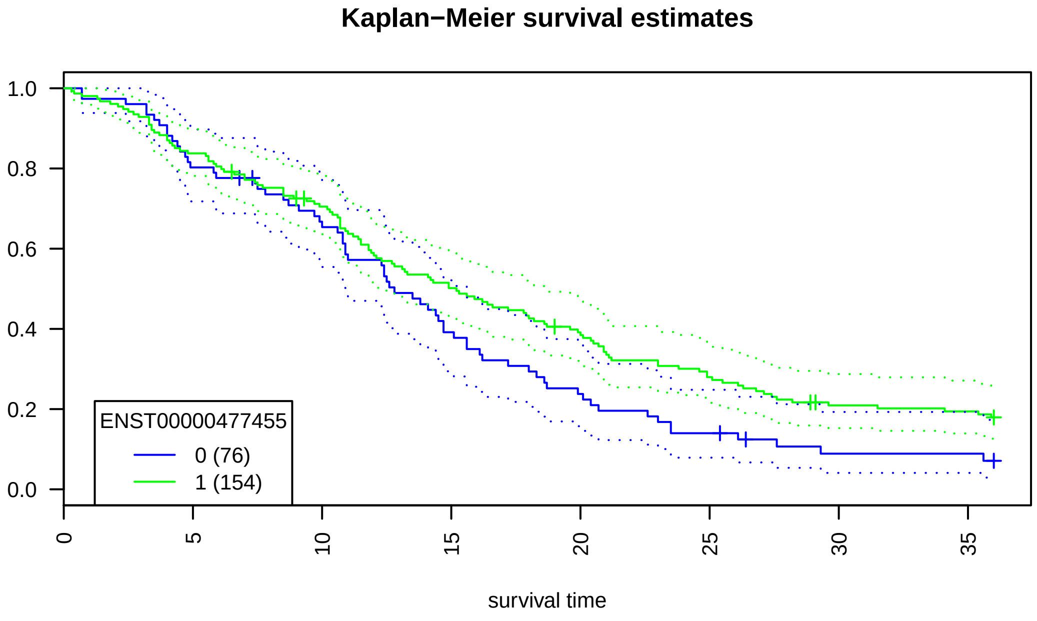

Figure 1003: Each curve represents the relative number of ENST00000477455 survived to the corresponding point of time. Dashed lines represent 0.95 confidence intervals for the curves with the corresponding color. Log-rank probability measure for the equality of these curves is 2.92815e-02. Additional annotations: *.GeneId*=ENSG00000065911, *.GeneName*=MTHFD2, *.GeneDesc*=methylenetetrahydrofolate dehydrogenase (NADP+ dependent) 2, methenyltetrahydrofolate cyclohydrolase [Source:HGNC Symbol;Acc:7434].

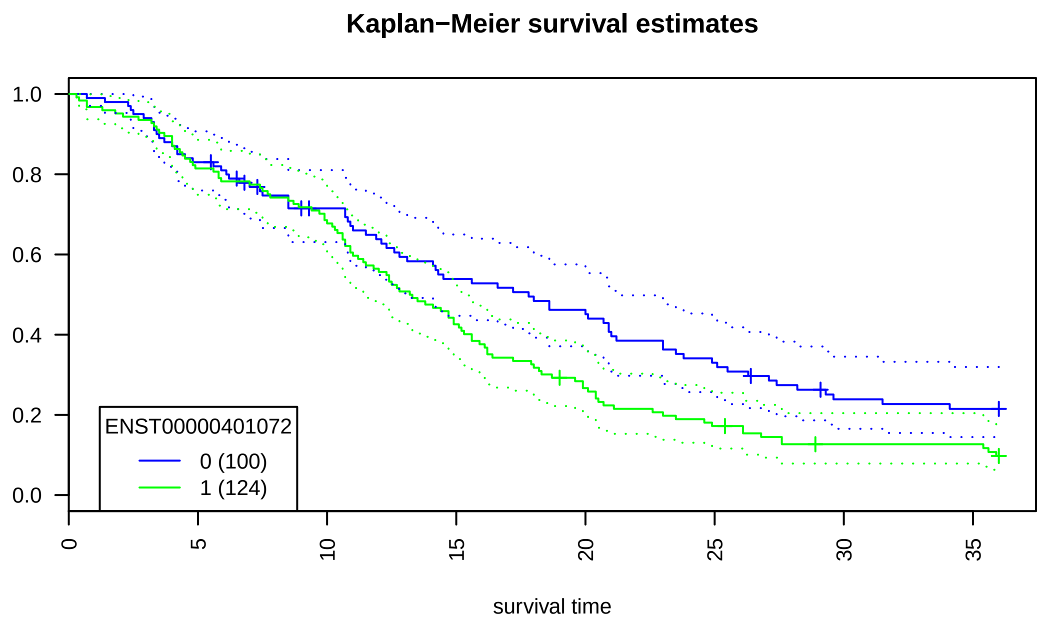

Figure 1004: Each curve represents the relative number of ENST00000401072 survived to the corresponding point of time. Dashed lines represent 0.95 confidence intervals for the curves with the corresponding color. Total of 6 observations deleted due to missingness. Log-rank probability measure for the equality of these curves is 1.193382e-02. Additional annotations: *.GeneId*=ENSG00000121903, *.GeneName*=ZSCAN20, *.GeneDesc*=zinc finger and SCAN domain containing 20 [Source:HGNC Symbol;Acc:13093].

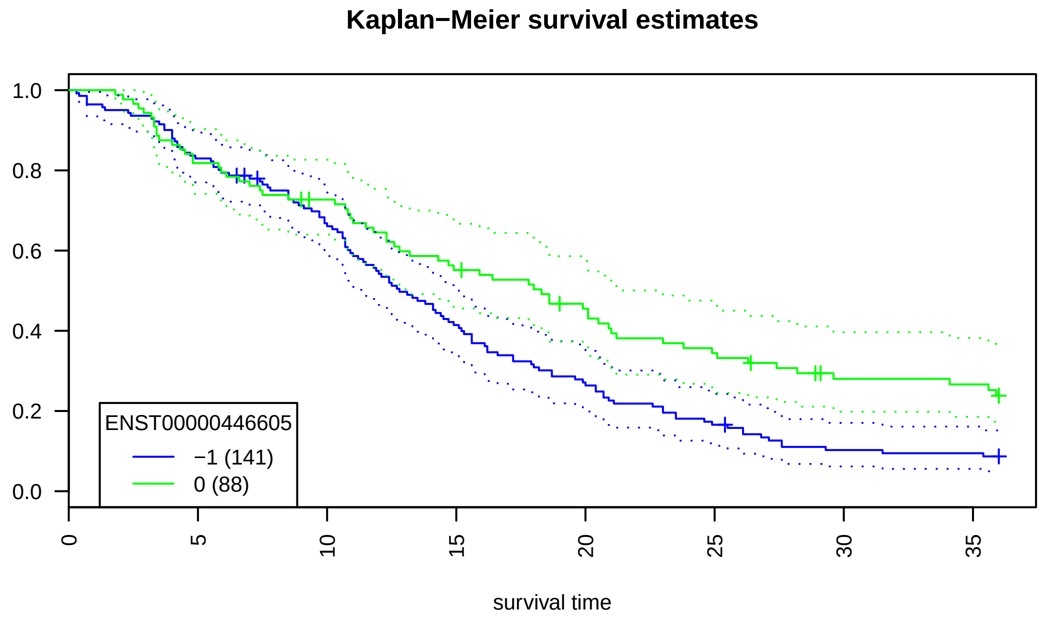

Figure 1005: Each curve represents the relative number of ENST00000446605 survived to the corresponding point of time. Dashed lines represent 0.95 confidence intervals for the curves with the corresponding color. Total of 1 observation deleted due to missingness. Log-rank probability measure for the equality of these curves is 2.438135e-03. Additional annotations: *.GeneId*=ENSG00000138107, *.GeneName*=ACTR1A, *.GeneDesc*=ARP1 actin-related protein 1 homolog A, centractin alpha (yeast) [Source:HGNC Symbol;Acc:167].

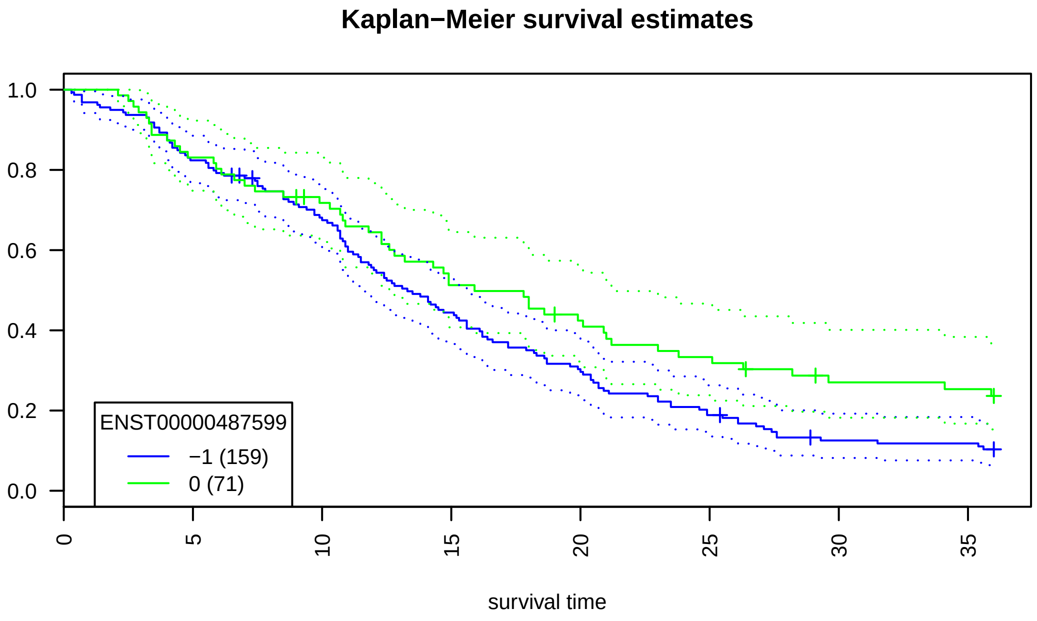

Figure 1006: Each curve represents the relative number of ENST00000487599 survived to the corresponding point of time. Dashed lines represent 0.95 confidence intervals for the curves with the corresponding color. Log-rank probability measure for the equality of these curves is 2.363003e-02. Additional annotations: *.GeneId*=ENSG00000138107, *.GeneName*=ACTR1A, *.GeneDesc*=ARP1 actin-related protein 1 homolog A, centractin alpha (yeast) [Source:HGNC Symbol;Acc:167].

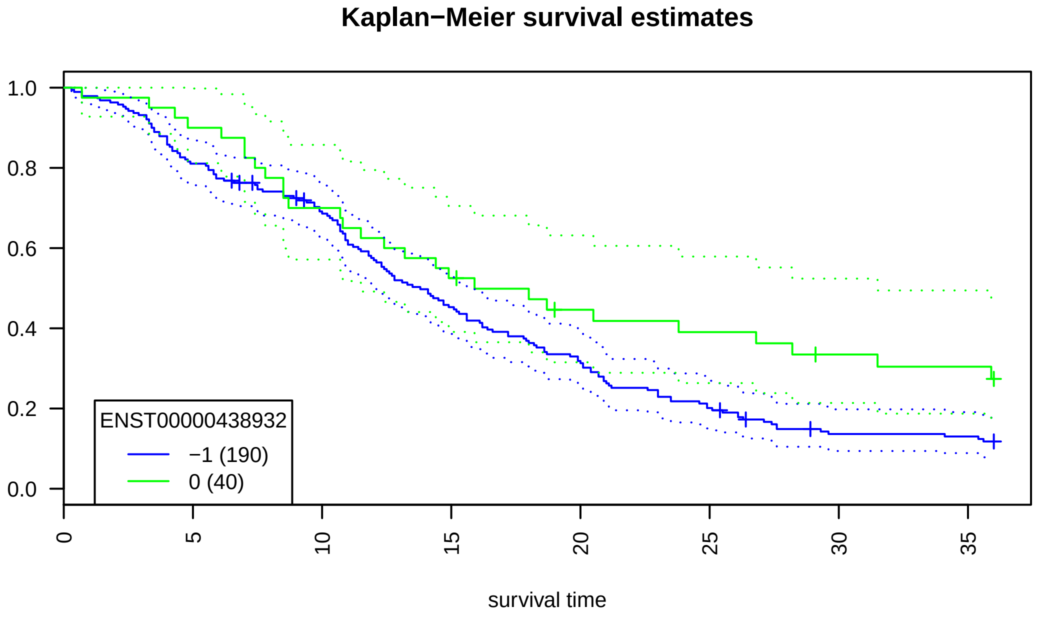

Figure 1007: Each curve represents the relative number of ENST00000438932 survived to the corresponding point of time. Dashed lines represent 0.95 confidence intervals for the curves with the corresponding color. Log-rank probability measure for the equality of these curves is 3.32982e-02. Additional annotations: *.GeneId*=ENSG00000182013, *.GeneName*=PNMAL1, *.GeneDesc*=PNMA-like 1 [Source:HGNC Symbol;Acc:25578].

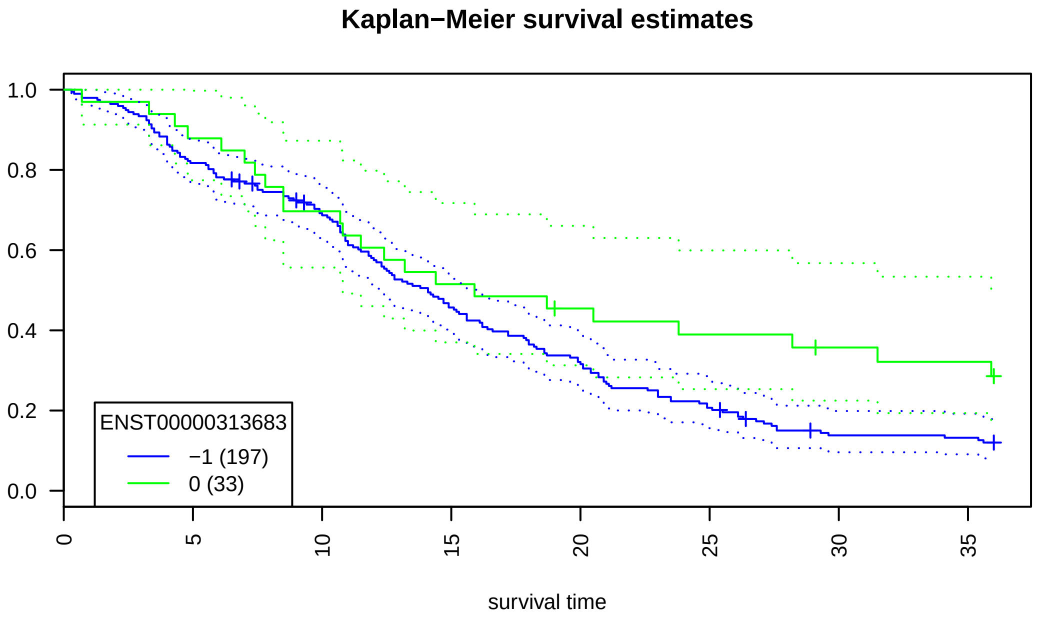

Figure 1008: Each curve represents the relative number of ENST00000313683 survived to the corresponding point of time. Dashed lines represent 0.95 confidence intervals for the curves with the corresponding color. Log-rank probability measure for the equality of these curves is 4.755469e-02. Additional annotations: *.GeneId*=ENSG00000182013, *.GeneName*=PNMAL1, *.GeneDesc*=PNMA-like 1 [Source:HGNC Symbol;Acc:25578].

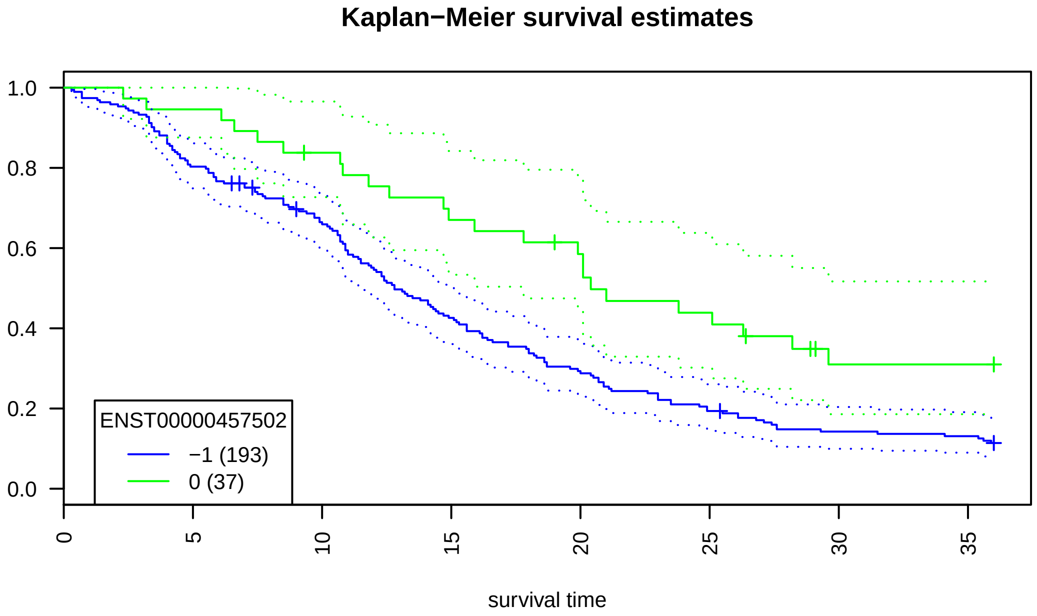

Figure 1009: Each curve represents the relative number of ENST00000457502 survived to the corresponding point of time. Dashed lines represent 0.95 confidence intervals for the curves with the corresponding color. Log-rank probability measure for the equality of these curves is 1.867177e-03. Additional annotations: *.GeneId*=ENSG00000148842, *.GeneName*=CNNM2, *.GeneDesc*=cyclin M2 [Source:HGNC Symbol;Acc:103].

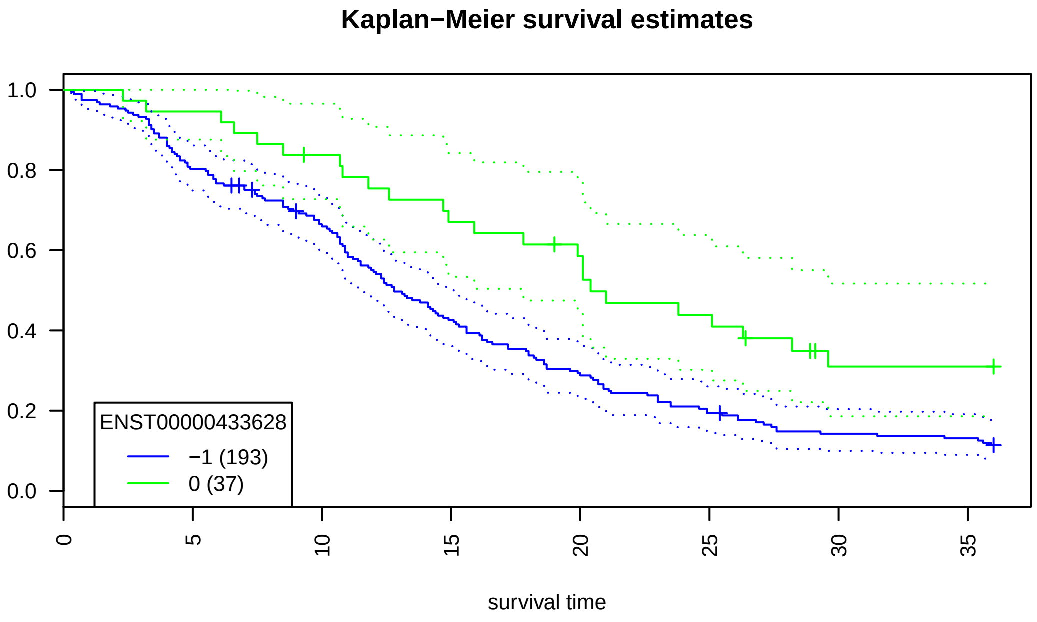

Figure 1010: Each curve represents the relative number of ENST00000433628 survived to the corresponding point of time. Dashed lines represent 0.95 confidence intervals for the curves with the corresponding color. Log-rank probability measure for the equality of these curves is 1.867177e-03. Additional annotations: *.GeneId*=ENSG00000148842, *.GeneName*=CNNM2, *.GeneDesc*=cyclin M2 [Source:HGNC Symbol;Acc:103].

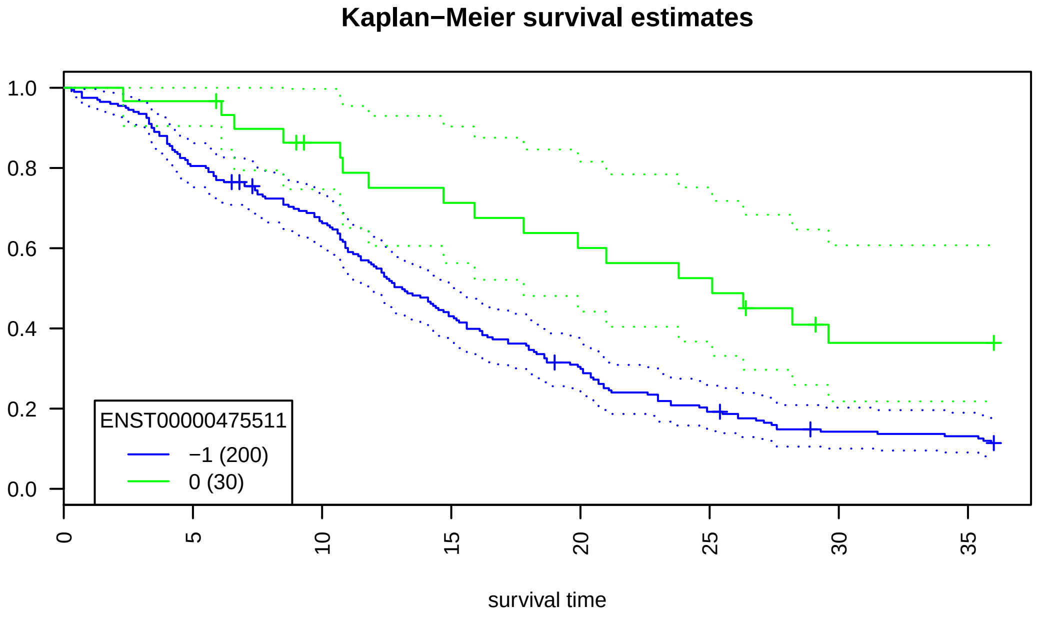

Figure 1011: Each curve represents the relative number of ENST00000475511 survived to the corresponding point of time. Dashed lines represent 0.95 confidence intervals for the curves with the corresponding color. Log-rank probability measure for the equality of these curves is 9.575278e-04. Additional annotations: *.GeneId*=ENSG00000148842, *.GeneName*=CNNM2, *.GeneDesc*=cyclin M2 [Source:HGNC Symbol;Acc:103].

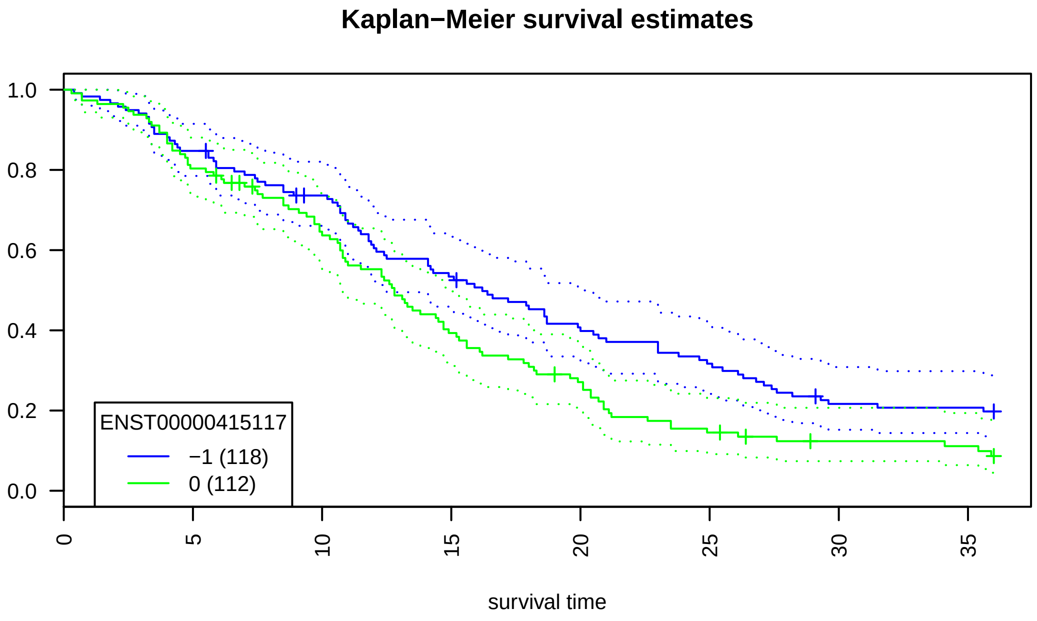

Figure 1012: Each curve represents the relative number of ENST00000415117 survived to the corresponding point of time. Dashed lines represent 0.95 confidence intervals for the curves with the corresponding color. Log-rank probability measure for the equality of these curves is 8.914662e-03. Additional annotations: .*GeneId*=ENSG00000236187, .*GeneName*=RP1-245G19.2, .*GeneDesc*=[undefined].

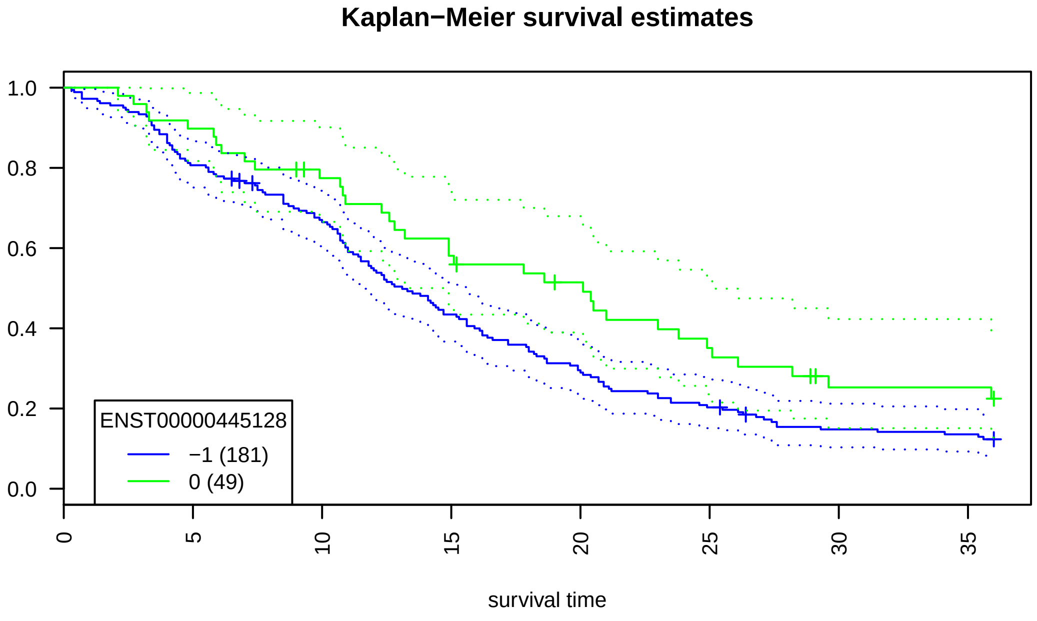

Figure 1013: Each curve represents the relative number of ENST00000445128 survived to the corresponding point of time. Dashed lines represent 0.95 confidence intervals for the curves with the corresponding color. Log-rank probability measure for the equality of these curves is 2.559491e-02. Additional annotations: .*GeneId*=ENSG00000164604, .*GeneName*=GPR85, .*GeneDesc*=G protein-coupled receptor 85 [Source:HGNC Symbol;Acc:4536].

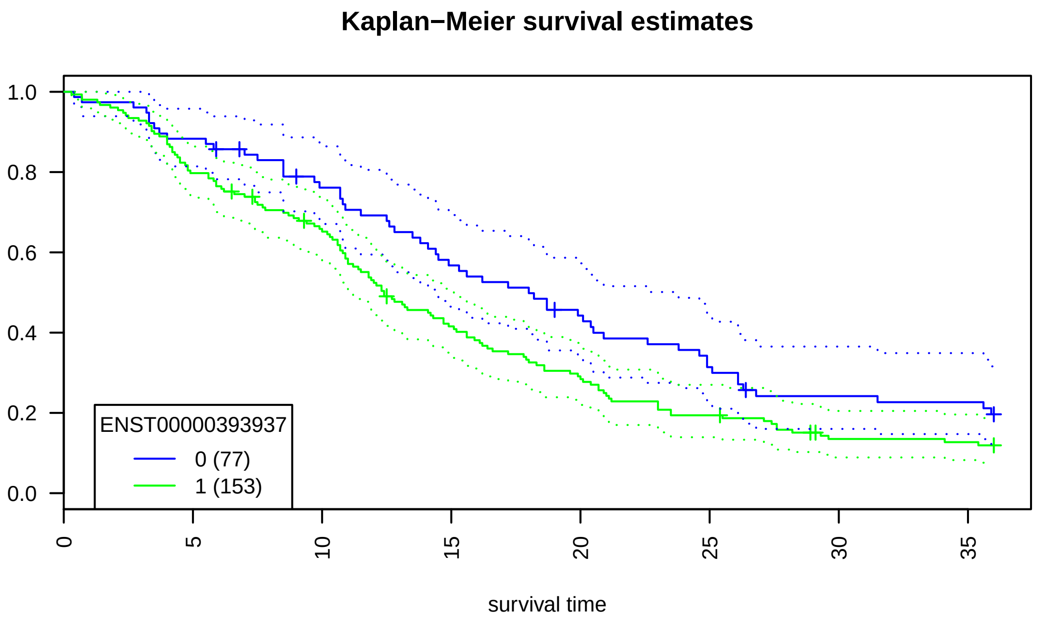

Figure 1014: Each curve represents the relative number of ENST00000393937 survived to the corresponding point of time. Dashed lines represent 0.95 confidence intervals for the curves with the corresponding color. Log-rank probability measure for the equality of these curves is 2.169942e-02. Additional annotations: *.GeneId*=ENSG00000115318, *.GeneName*=LOXL3, *.GeneDesc*=lysyl oxidase-like 3 [Source:HGNC Symbol;Acc:13869].

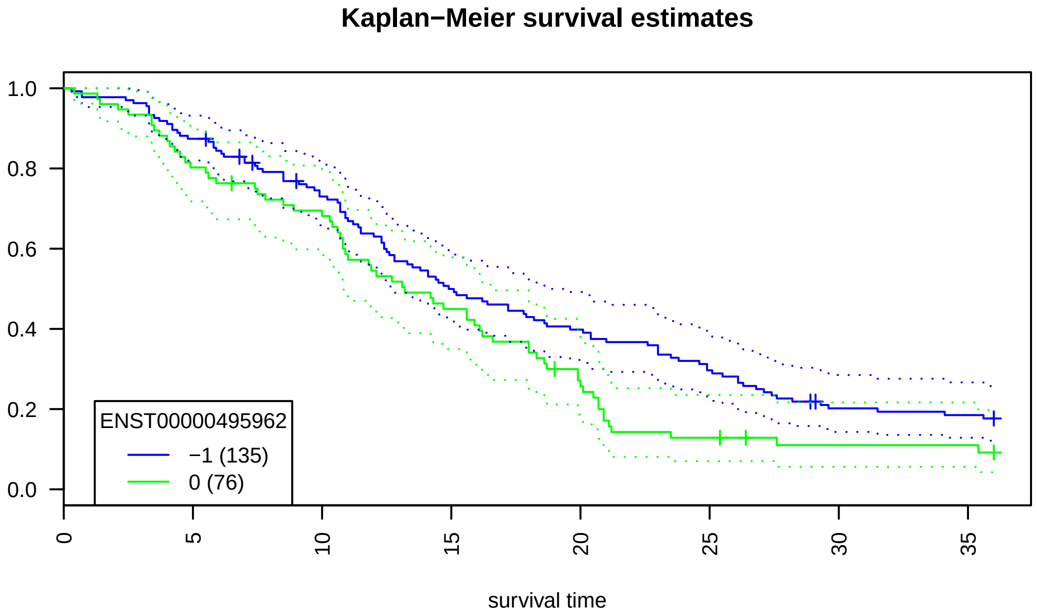

Figure 1015: Each curve represents the relative number of ENST00000495962 survived to the corresponding point of time. Dashed lines represent 0.95 confidence intervals for the curves with the corresponding color. Total of 19 observations deleted due to missingness. Log-rank probability measure for the equality of these curves is 2.908972e-02. Additional annotations: *.GeneId*=ENSG00000105976, *.GeneName*=MET, *.GeneDesc*=met proto-oncogene (hepatocyte growth factor receptor) [Source:HGNC Symbol;Acc:7029].

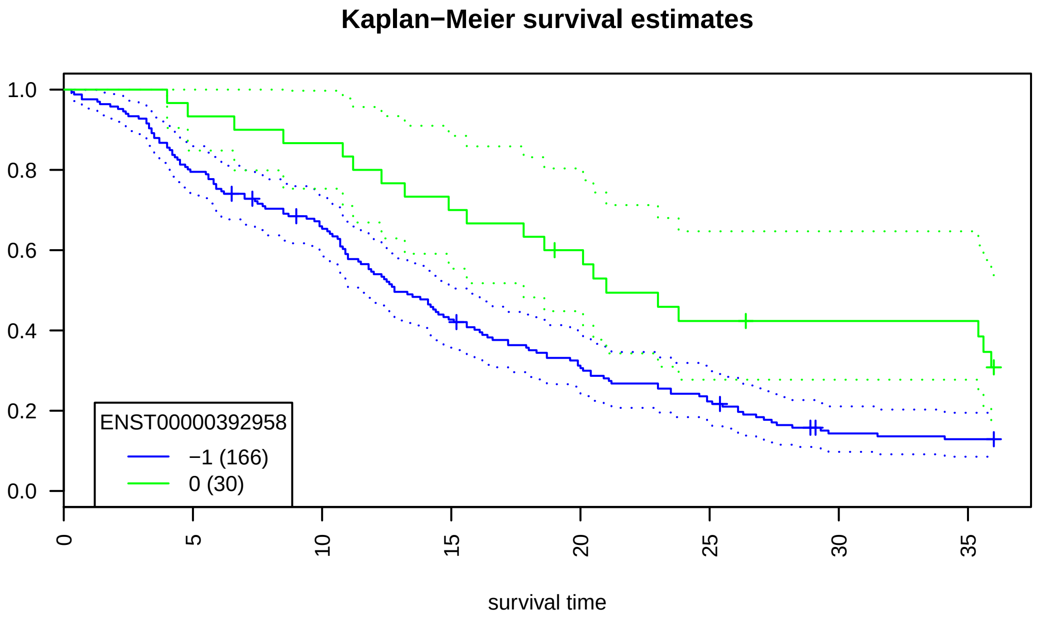

Figure 1016: Each curve represents the relative number of ENST00000392958 survived to the corresponding point of time. Dashed lines represent 0.95 confidence intervals for the curves with the corresponding color. Total of 34 observations deleted due to missingness. Log-rank probability measure for the equality of these curves is 4.136008e-03. Additional annotations: *.GeneId*=ENSG00000198478, *.GeneName*=SH3BGRL2, *.GeneDesc*=SH3 domain binding glutamic acid-rich protein like 2 [Source:HGNC Symbol;Acc:15567].

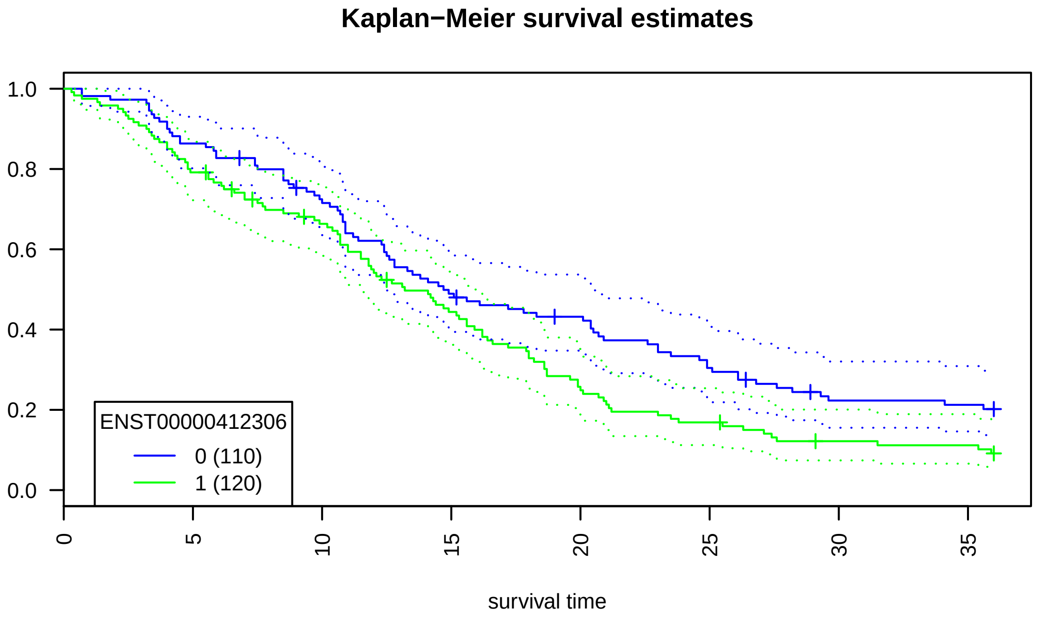

Figure 1017: Each curve represents the relative number of ENST00000412306 survived to the corresponding point of time. Dashed lines represent 0.95 confidence intervals for the curves with the corresponding color. Log-rank probability measure for the equality of these curves is 1.570287e-02. Additional annotations: *.GeneId*=ENSG00000112773, *.GeneName*=FAM46A, *.GeneDesc*=family with sequence similarity 46, member A [Source:HGNC Symbol;Acc:18345].

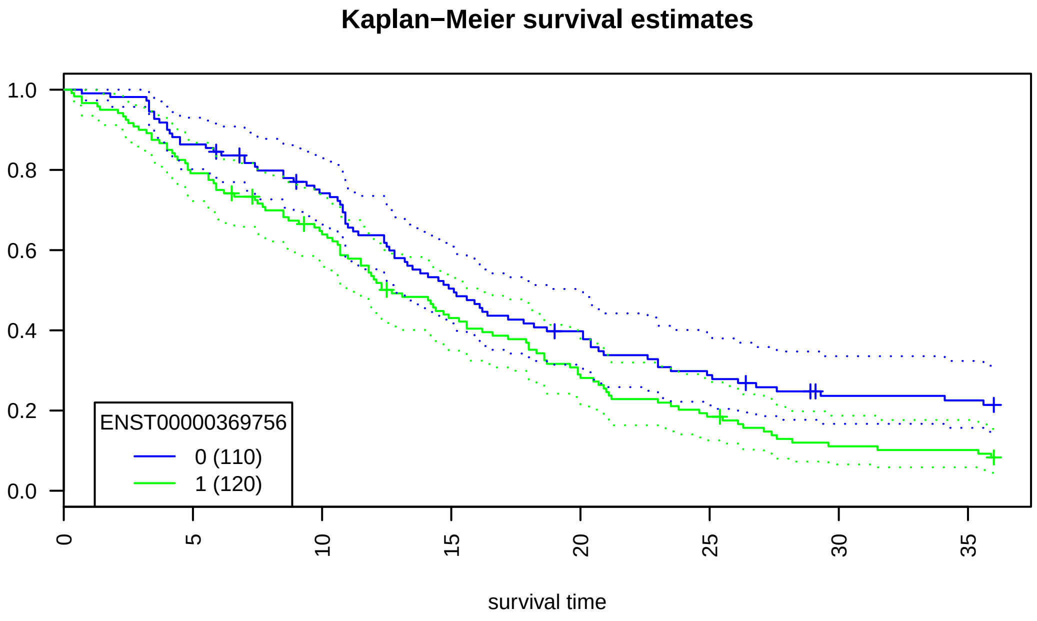

Figure 1018: Each curve represents the relative number of ENST00000369756 survived to the corresponding point of time. Dashed lines represent 0.95 confidence intervals for the curves with the corresponding color. Log-rank probability measure for the equality of these curves is 1.588135e-02. Additional annotations: *.GeneId*=ENSG00000112773, *.GeneName*=FAM46A, *.GeneDesc*=family with sequence similarity 46, member A [Source:HGNC Symbol;Acc:18345].

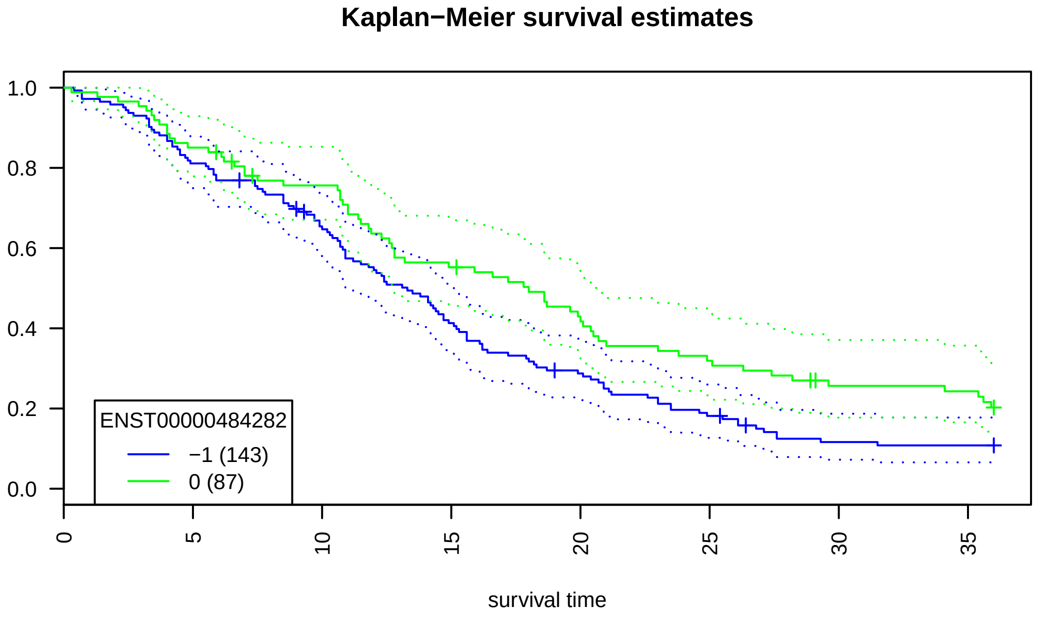

Figure 1019: Each curve represents the relative number of ENST00000484282 survived to the corresponding point of time. Dashed lines represent 0.95 confidence intervals for the curves with the corresponding color. Log-rank probability measure for the equality of these curves is 1.562435e-02. Additional annotations: *.GeneId*=ENSG00000083097, *.GeneName*=DOPEY1, *.GeneDesc*=dopey family member 1 [Source:HGNC Symbol;Acc:21194].

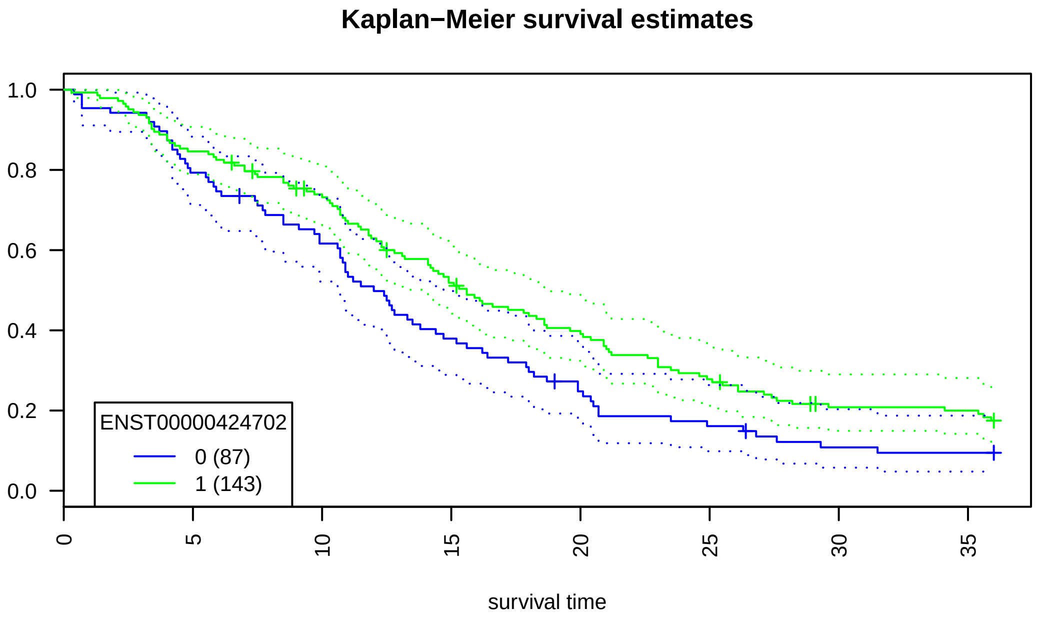

Figure 1020: Each curve represents the relative number of ENST00000424702 survived to the corresponding point of time. Dashed lines represent 0.95 confidence intervals for the curves with the corresponding color. Log-rank probability measure for the equality of these curves is 1.709652e-02. Additional annotations: *.GeneId*=ENSG00000128534, *.GeneName*=LSM8, *.GeneDesc*=N(alpha)-acetyltransferase 38, NatC auxiliary subunit [Source:HGNC Symbol;Acc:20471].

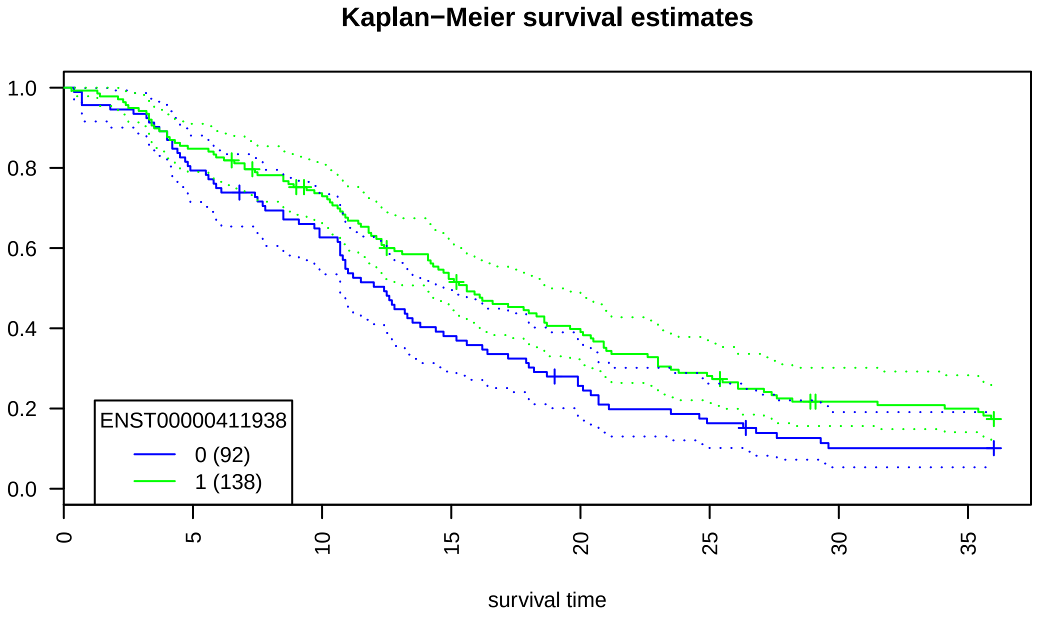

Figure 1021: Each curve represents the relative number of ENST00000411938 survived to the corresponding point of time. Dashed lines represent 0.95 confidence intervals for the curves with the corresponding color. Log-rank probability measure for the equality of these curves is 2.366955e-02. Additional annotations: *.GeneId*=ENSG00000128534, *.GeneName*=LSM8, *.GeneDesc*=N(alpha)-acetyltransferase 38, NatC auxiliary subunit [Source:HGNC Symbol;Acc:20471].

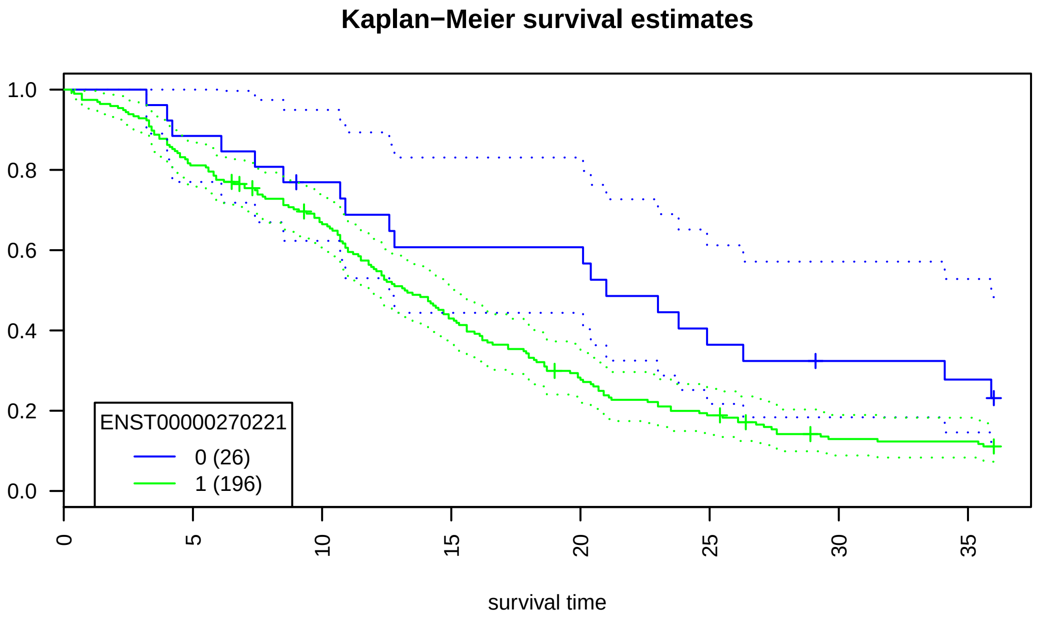

Figure 1022: Each curve represents the relative number of ENST00000270221 survived to the corresponding point of time. Dashed lines represent 0.95 confidence intervals for the curves with the corresponding color. Total of 8 observations deleted due to missingness. Log-rank probability measure for the equality of these curves is 3.210082e-02. Additional annotations: *.GeneId*=ENSG00000142227, *.GeneName*=EMP3, *.GeneDesc*=epithelial membrane protein 3 [Source:HGNC Symbol;Acc:3335].

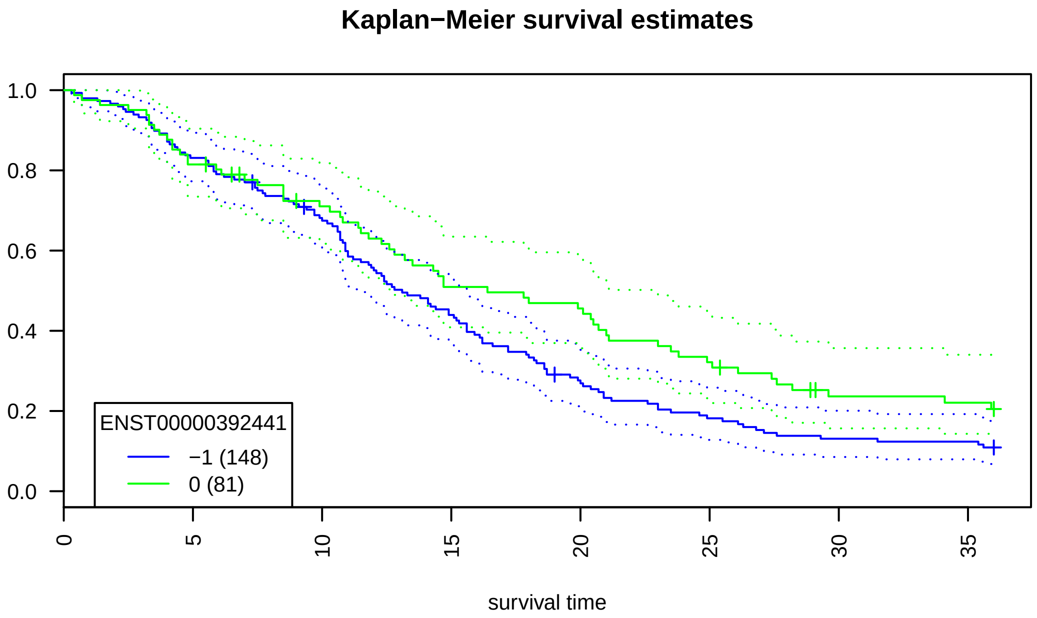

Figure 1023: Each curve represents the relative number of ENST00000392441 survived to the corresponding point of time. Dashed lines represent 0.95 confidence intervals for the curves with the corresponding color. Total of 1 observation deleted due to missingness. Log-rank probability measure for the equality of these curves is 3.383395e-02. Additional annotations: *.GeneId*=ENSG00000130783, *.GeneName*=CCDC62, *.GeneDesc*=coiled-coil domain containing 62 [Source:HGNC Symbol;Acc:30723].

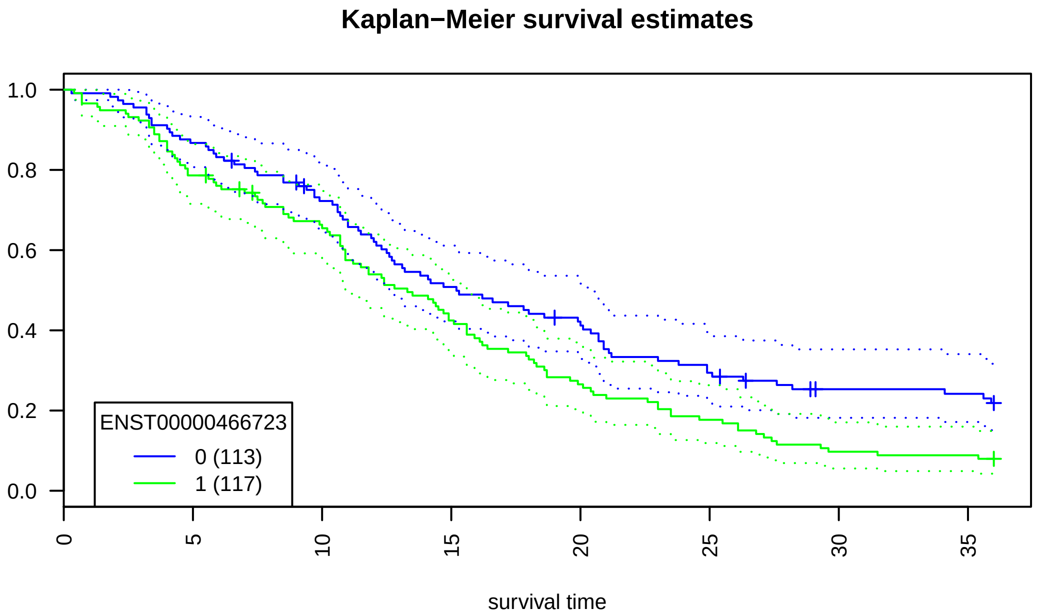

Figure 1024: Each curve represents the relative number of ENST00000466723 survived to the corresponding point of time. Dashed lines represent 0.95 confidence intervals for the curves with the corresponding color. Log-rank probability measure for the equality of these curves is 7.069909e-03. Additional annotations: *.GeneId*=ENSG00000163879, *.GeneName*=DNALI1, *.GeneDesc*=dynein, axonemal, light intermediate chain 1 [Source:HGNC Symbol;Acc:14353].

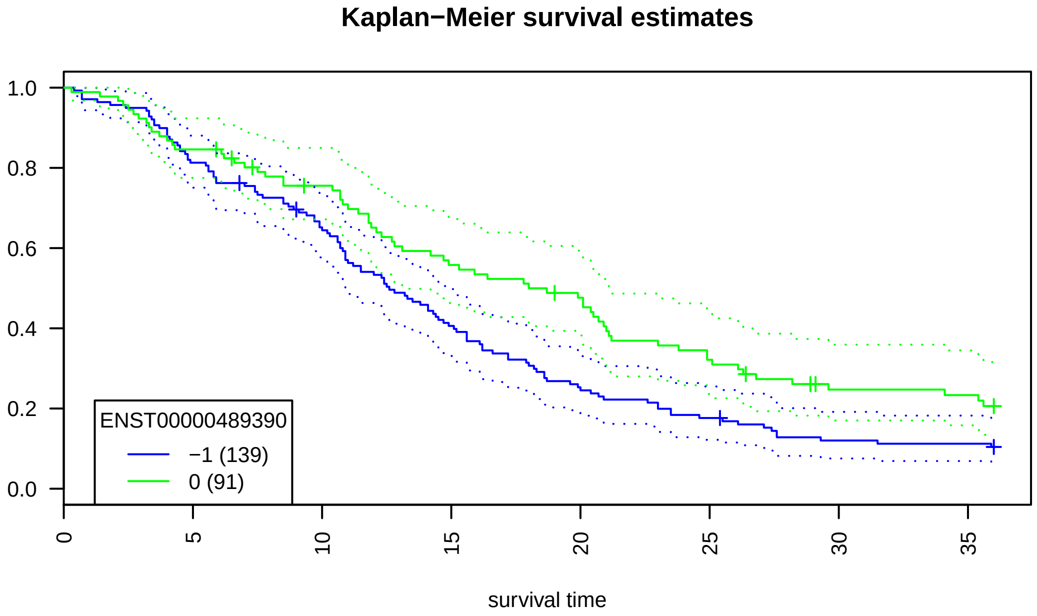

Figure 1025: Each curve represents the relative number of ENST00000489390 survived to the corresponding point of time. Dashed lines represent 0.95 confidence intervals for the curves with the corresponding color. Log-rank probability measure for the equality of these curves is 7.005428e-03. Additional annotations: *.GeneId*=ENSG00000108061, *.GeneName*=SHOC2, *.GeneDesc*=soc-2 suppressor of clear homolog (C. elegans) [Source:HGNC Symbol;Acc:15454].

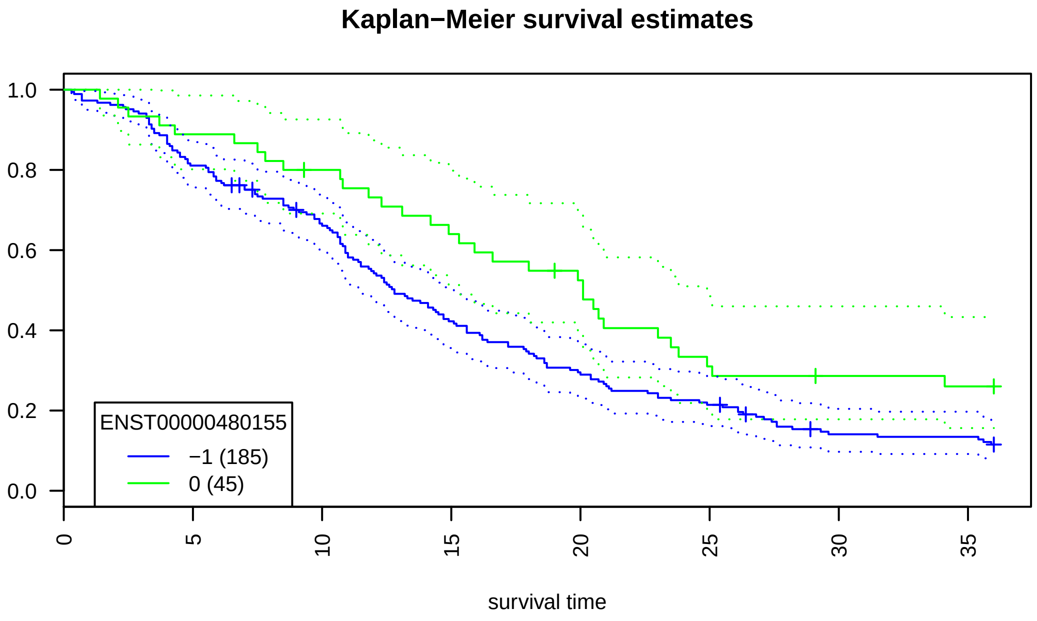

Figure 1026: Each curve represents the relative number of ENST00000480155 survived to the corresponding point of time. Dashed lines represent 0.95 confidence intervals for the curves with the corresponding color. Log-rank probability measure for the equality of these curves is 1.158311e-02. Additional annotations: *.GeneId*=ENSG00000108061, *.GeneName*=SHOC2, *.GeneDesc*=soc-2 suppressor of clear homolog (C. elegans) [Source:HGNC Symbol;Acc:15454].

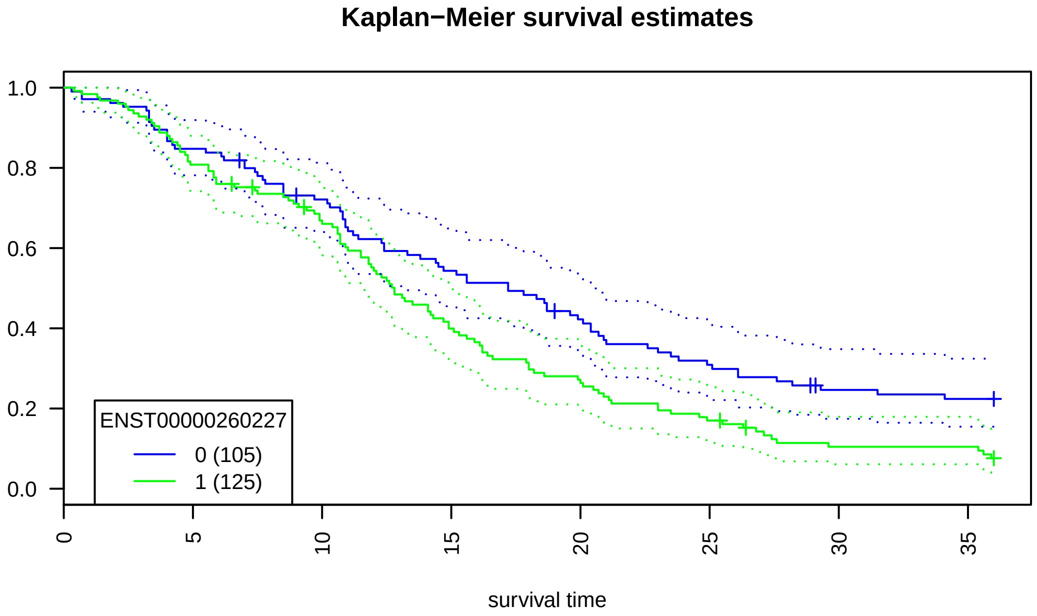

Figure 1027: Each curve represents the relative number of ENST00000260227 survived to the corresponding point of time. Dashed lines represent 0.95 confidence intervals for the curves with the corresponding color. Log-rank probability measure for the equality of these curves is 4.561144e-03. Additional annotations: *.GeneId*=ENSG00000137673, *.GeneName*=MMP7, *.GeneDesc*=matrix metallopeptidase 7 (matrilysin, uterine) [Source:HGNC Symbol;Acc:7174].

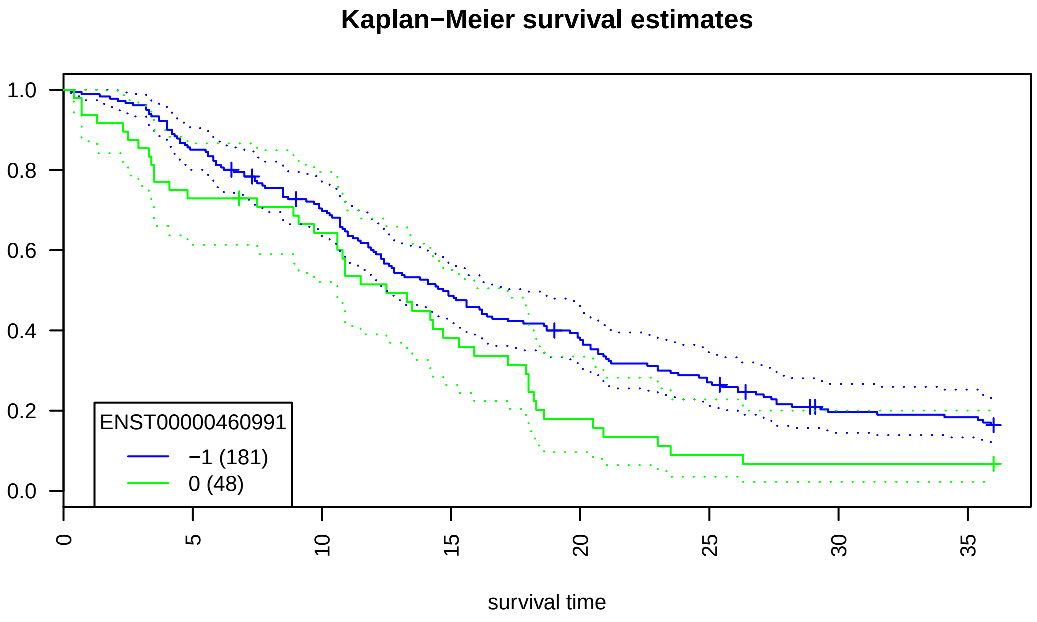

Figure 1028: Each curve represents the relative number of ENST00000460991 survived to the corresponding point of time. Dashed lines represent 0.95 confidence intervals for the curves with the corresponding color. Total of 1 observation deleted due to missingness. Log-rank probability measure for the equality of these curves is 1.293299e-02. Additional annotations: *.GeneId*=ENSG00000186854, *.GeneName*=C2orf89, *.GeneDesc*=UPF0632 protein B Precursor [Source:UniProtKB/Swiss-Prot;Acc:Q86V40].

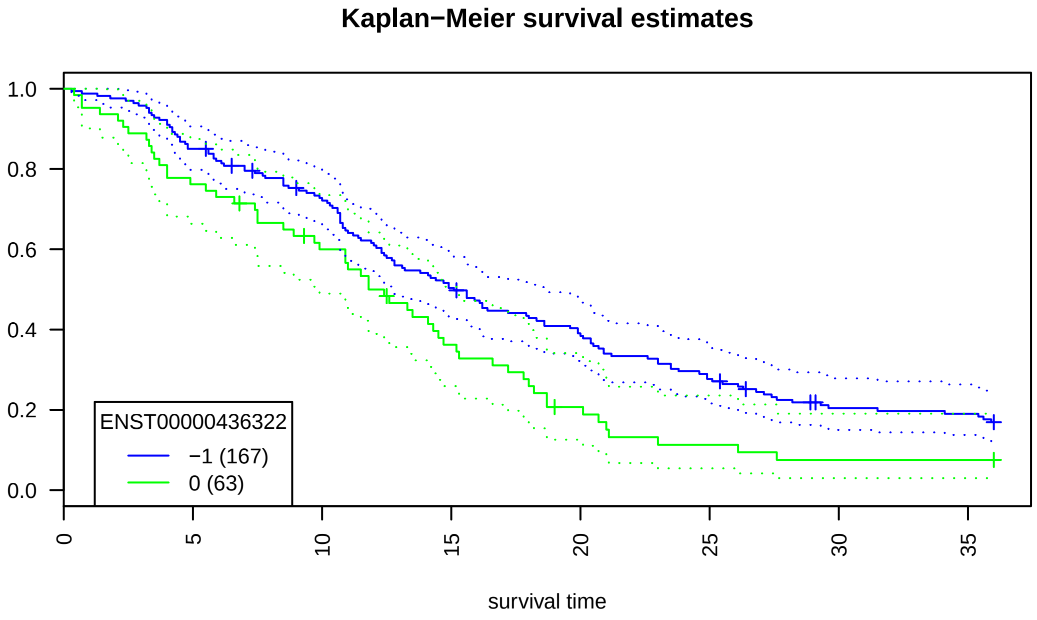

Figure 1029: Each curve represents the relative number of ENST00000436322 survived to the corresponding point of time. Dashed lines represent 0.95 confidence intervals for the curves with the corresponding color. Log-rank probability measure for the equality of these curves is 5.796418e-03. Additional annotations: *.GeneId*=ENSG00000186854, *.GeneName*=C2orf89, *.GeneDesc*=UPF0632 protein B Precursor [Source:UniProtKB/Swiss-Prot;Acc:Q86V40].

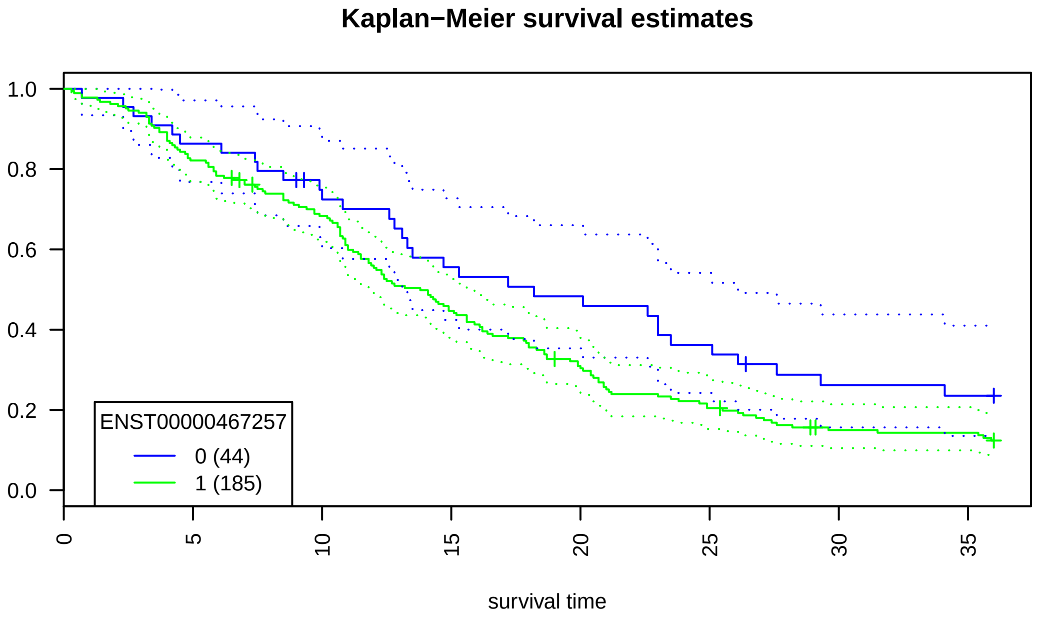

Figure 1030: Each curve represents the relative number of ENST00000467257 survived to the corresponding point of time. Dashed lines represent 0.95 confidence intervals for the curves with the corresponding color. Total of 1 observation deleted due to missingness. Log-rank probability measure for the equality of these curves is 4.944365e-02. Additional annotations: *.GeneId*=ENSG00000163638, *.GeneName*=ADAMTS9, *.GeneDesc*=ADAM metallopeptidase with thrombospondin type 1 motif, 9 [Source:HGNC Symbol;Acc:13202].

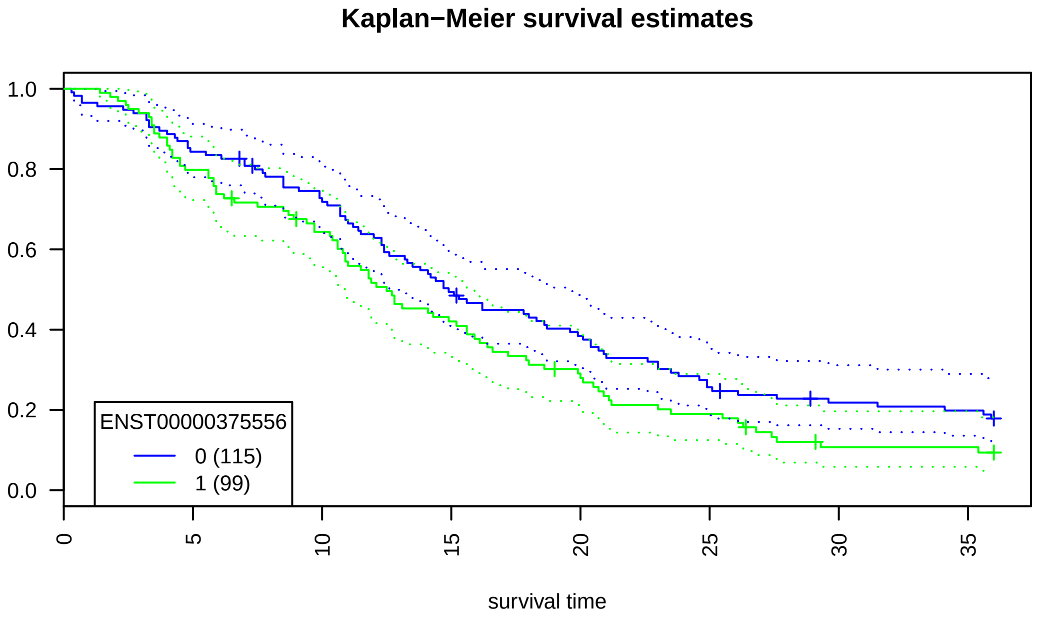

Figure 1031: Each curve represents the relative number of ENST00000375556 survived to the corresponding point of time. Dashed lines represent 0.95 confidence intervals for the curves with the corresponding color. Total of 16 observations deleted due to missingness. Log-rank probability measure for the equality of these curves is 4.914773e-02. Additional annotations: *.GeneId*=ENSG00000108950, *.GeneName*=FAM20A, *.GeneDesc*=family with sequence similarity 20, member A [Source:HGNC Symbol;Acc:23015].

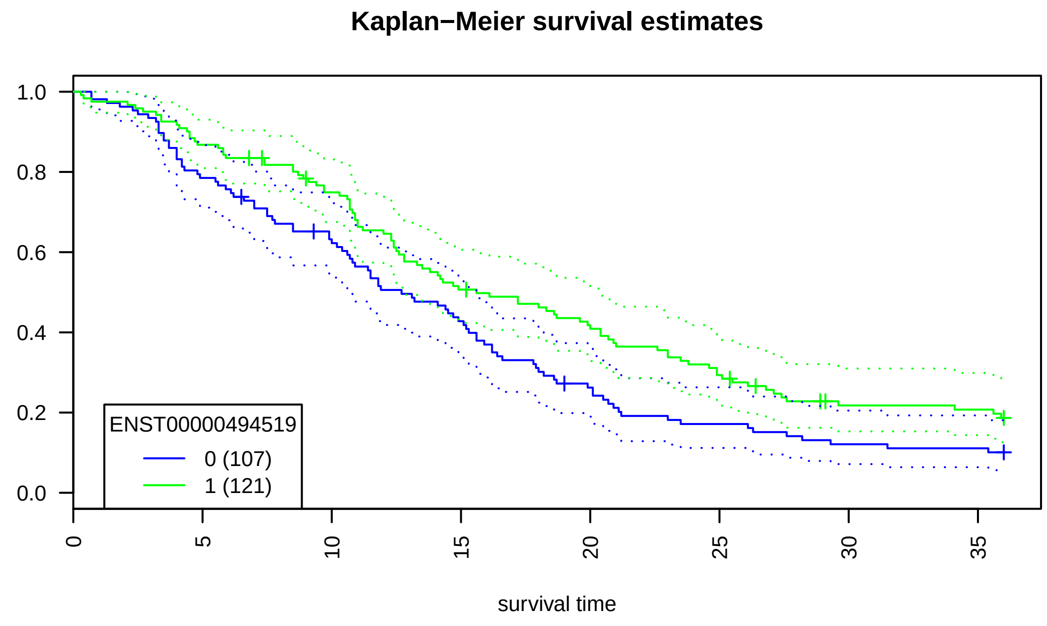

Figure 1032: Each curve represents the relative number of ENST00000494519 survived to the corresponding point of time. Dashed lines represent 0.95 confidence intervals for the curves with the corresponding color. Total of 2 observations deleted due to missingness. Log-rank probability measure for the equality of these curves is 1.244602e-02. Additional annotations: *.GeneId*=ENSG00000152284, *.GeneName*=TCF7L1, *.GeneDesc*=transcription factor 7-like 1 (T-cell specific, HMG-box) [Source:HGNC Symbol;Acc:11640].

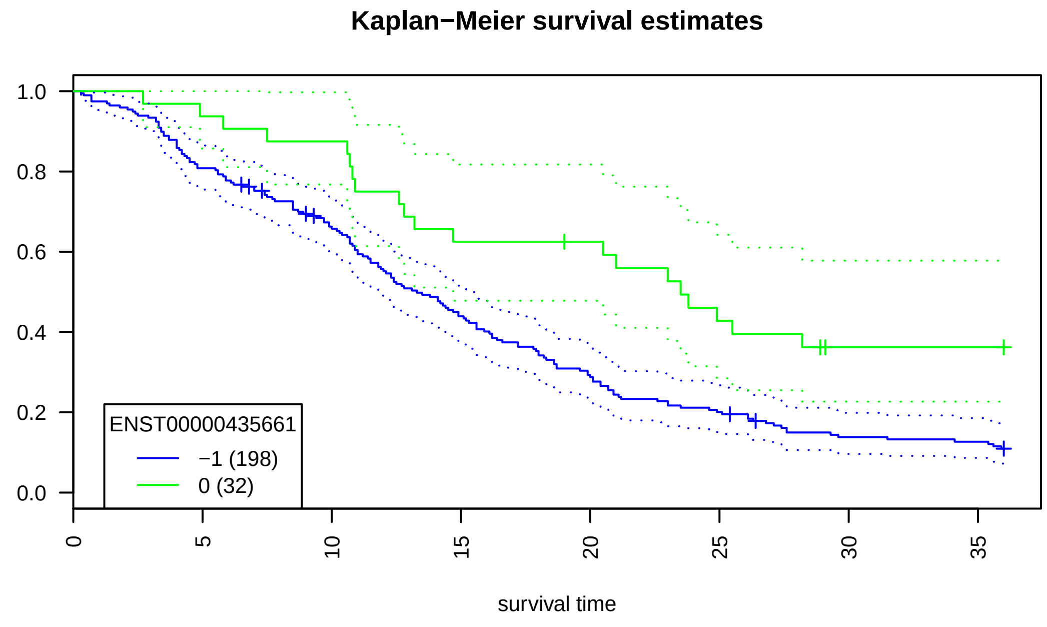

Figure 1033: Each curve represents the relative number of ENST00000435661 survived to the corresponding point of time. Dashed lines represent 0.95 confidence intervals for the curves with the corresponding color. Log-rank probability measure for the equality of these curves is 1.047822e-03. Additional annotations: *.GeneId*=ENSG00000179603, *.GeneName*=GRM8, *.GeneDesc*=glutamate receptor, metabotropic 8 [Source:HGNC Symbol;Acc:4600].

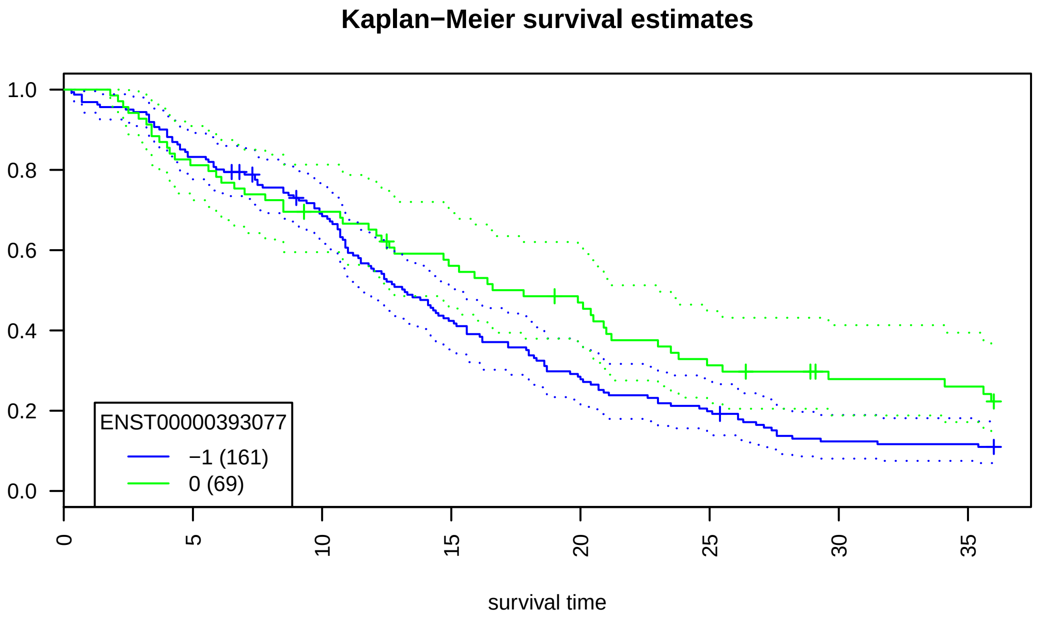

Figure 1034: Each curve represents the relative number of ENST00000393077 survived to the corresponding point of time. Dashed lines represent 0.95 confidence intervals for the curves with the corresponding color. Log-rank probability measure for the equality of these curves is 2.755647e-02. Additional annotations: *.GeneId*=ENSG00000151532, *.GeneName*=VTI1A, *.GeneDesc*=vesicle transport through interaction with t-SNAREs homolog 1A (yeast) [Source:HGNC Symbol;Acc:17792].

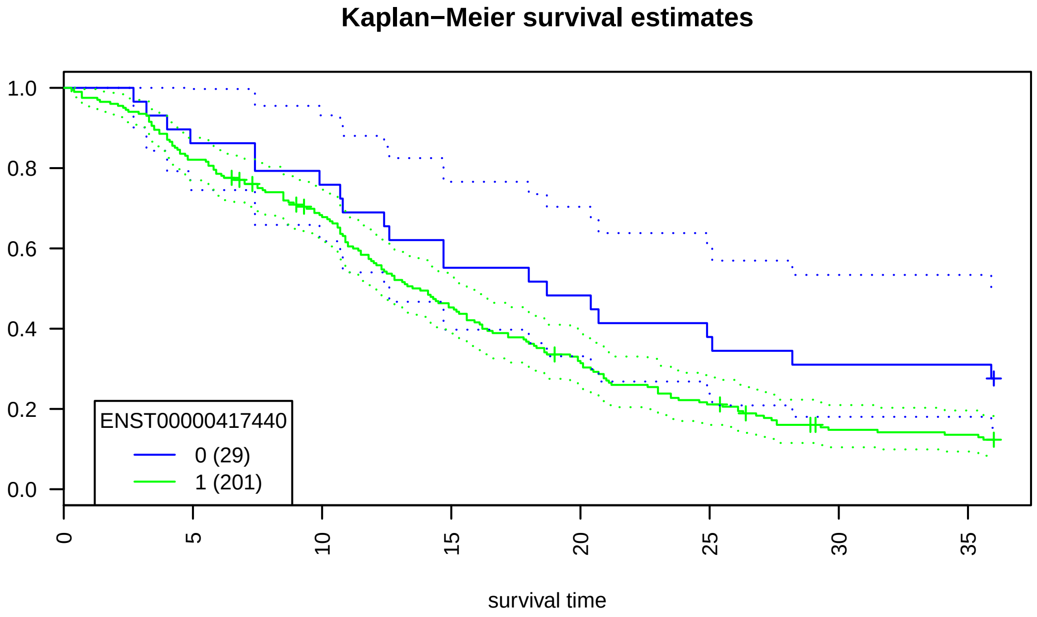

Figure 1035: Each curve represents the relative number of ENST00000417440 survived to the corresponding point of time. Dashed lines represent 0.95 confidence intervals for the curves with the corresponding color. Log-rank probability measure for the equality of these curves is 4.700178e-02. Additional annotations: *.GeneId*=ENSG00000196954, *.GeneName*=CASP4, *.GeneDesc*=caspase 4, apoptosis-related cysteine peptidase [Source:HGNC Symbol;Acc:1505].

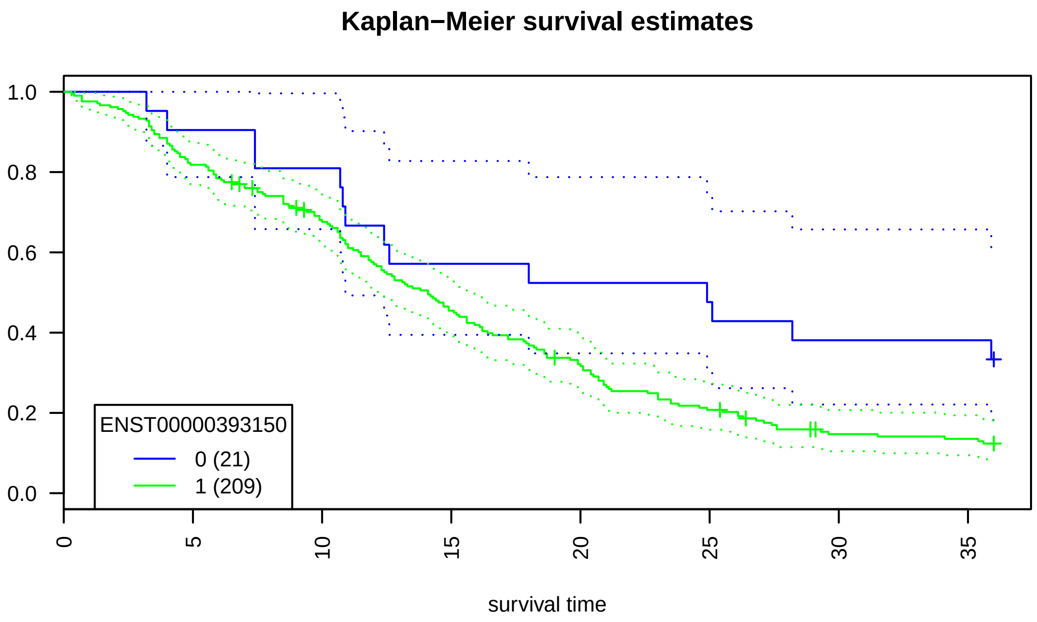

Figure 1036: Each curve represents the relative number of ENST00000393150 survived to the corresponding point of time. Dashed lines represent 0.95 confidence intervals for the curves with the corresponding color. Log-rank probability measure for the equality of these curves is 2.546173e-02. Additional annotations: *.GeneId*=ENSG00000196954, *.GeneName*=CASP4, *.GeneDesc*=caspase 4, apoptosis-related cysteine peptidase [Source:HGNC Symbol;Acc:1505].

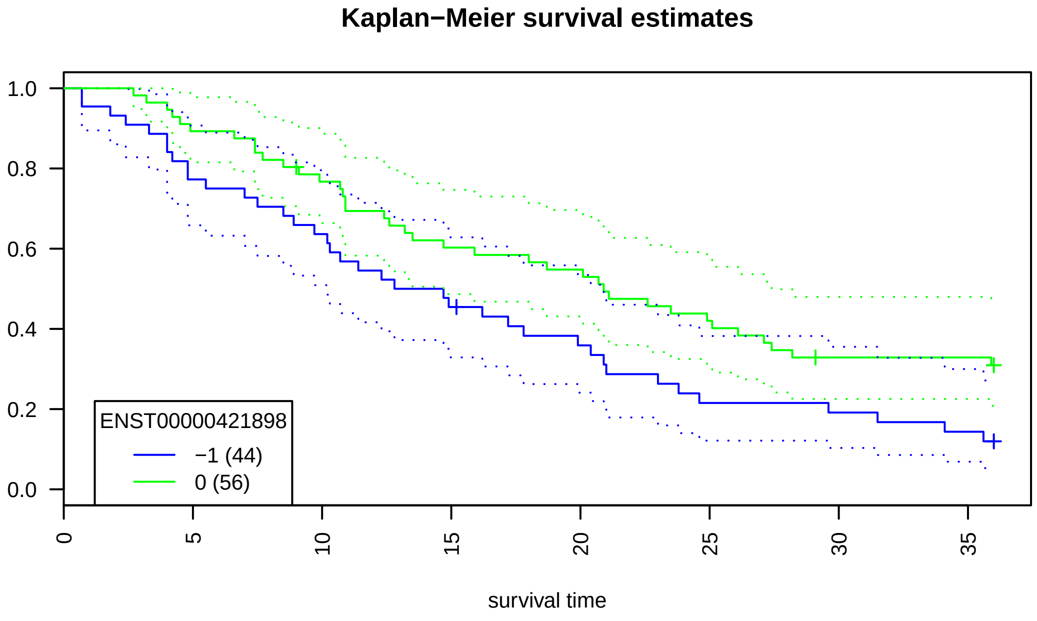

Figure 1037: Each curve represents the relative number of ENST00000421898 survived to the corresponding point of time. Dashed lines represent 0.95 confidence intervals for the curves with the corresponding color. Total of 130 observations deleted due to missingness. Log-rank probability measure for the equality of these curves is 1.696656e-02. Additional annotations: *.GeneId*=ENSG00000196954, *.GeneName*=CASP4, *.GeneDesc*=caspase 4, apoptosis-related cysteine peptidase [Source:HGNC Symbol;Acc:1505].

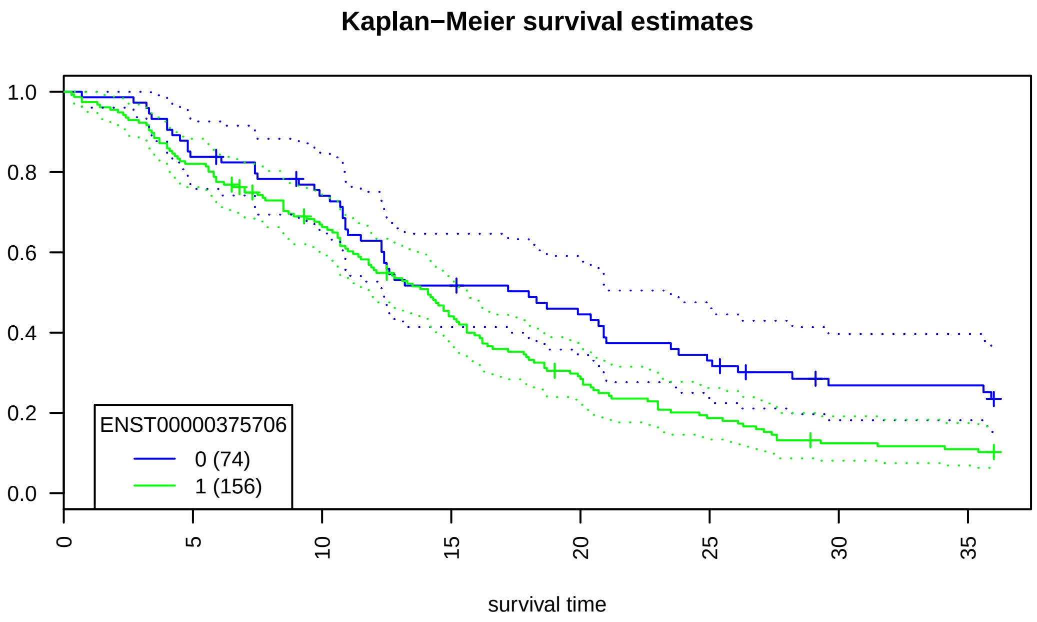

Figure 1038: Each curve represents the relative number of ENST00000375706 survived to the corresponding point of time. Dashed lines represent 0.95 confidence intervals for the curves with the corresponding color. Log-rank probability measure for the equality of these curves is 1.409366e-02. Additional annotations: *.GeneId*=ENSG00000204397, *.GeneName*=CARD16, *.GeneDesc*=caspase recruitment domain family, member 16 [Source:HGNC Symbol;Acc:33701].

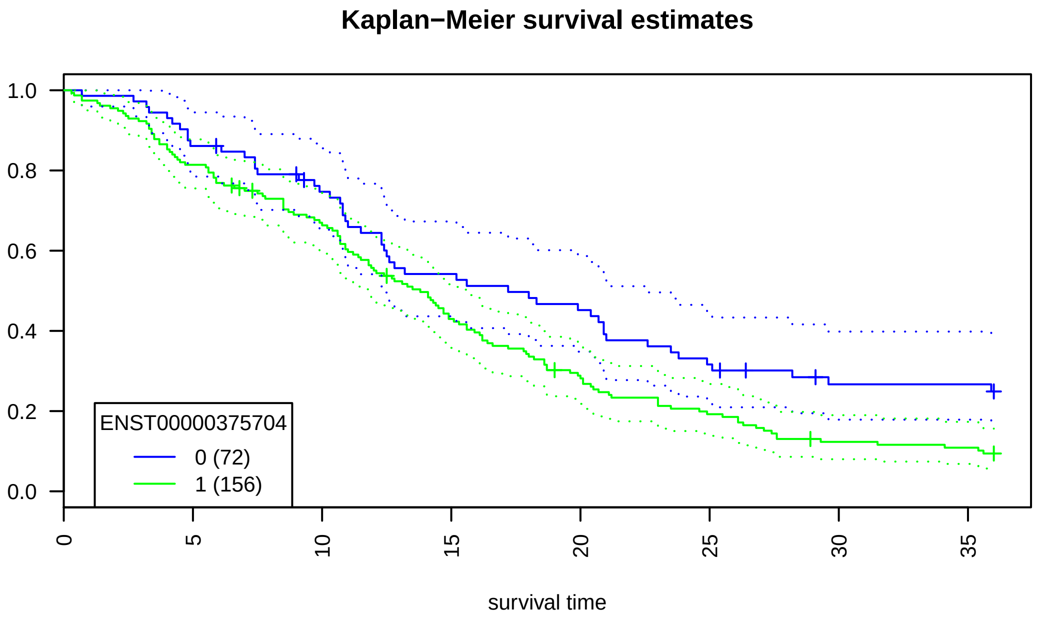

Figure 1039: Each curve represents the relative number of ENST00000375704 survived to the corresponding point of time. Dashed lines represent 0.95 confidence intervals for the curves with the corresponding color. Total of 2 observations deleted due to missingness. Log-rank probability measure for the equality of these curves is 7.915266e-03. Additional annotations: *.GeneId*=ENSG00000204397, *.GeneName*=CARD16, *.GeneDesc*=caspase recruitment domain family, member 16 [Source:HGNC Symbol;Acc:33701].

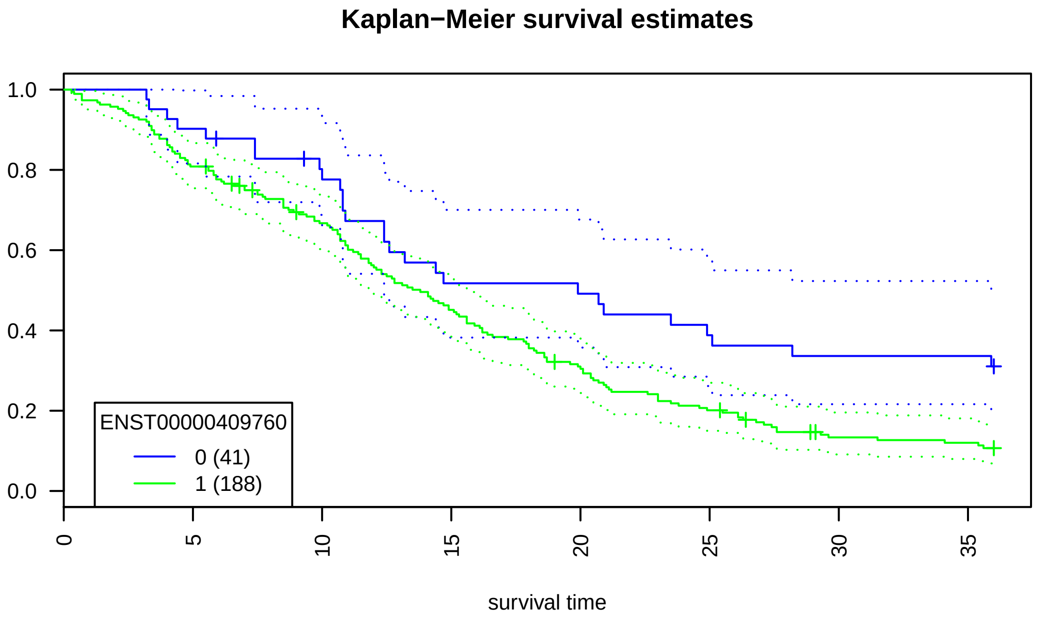

Figure 1040: Each curve represents the relative number of ENST00000409760 survived to the corresponding point of time. Dashed lines represent 0.95 confidence intervals for the curves with the corresponding color. Total of 1 observation deleted due to missingness. Log-rank probability measure for the equality of these curves is 6.516997e-03. Additional annotations: *.GeneId*=ENSG00000118640, *.GeneName*=VAMP8, *.GeneDesc*=vesicle-associated membrane protein 8 (endobrevin) [Source:HGNC Symbol;Acc:12647].

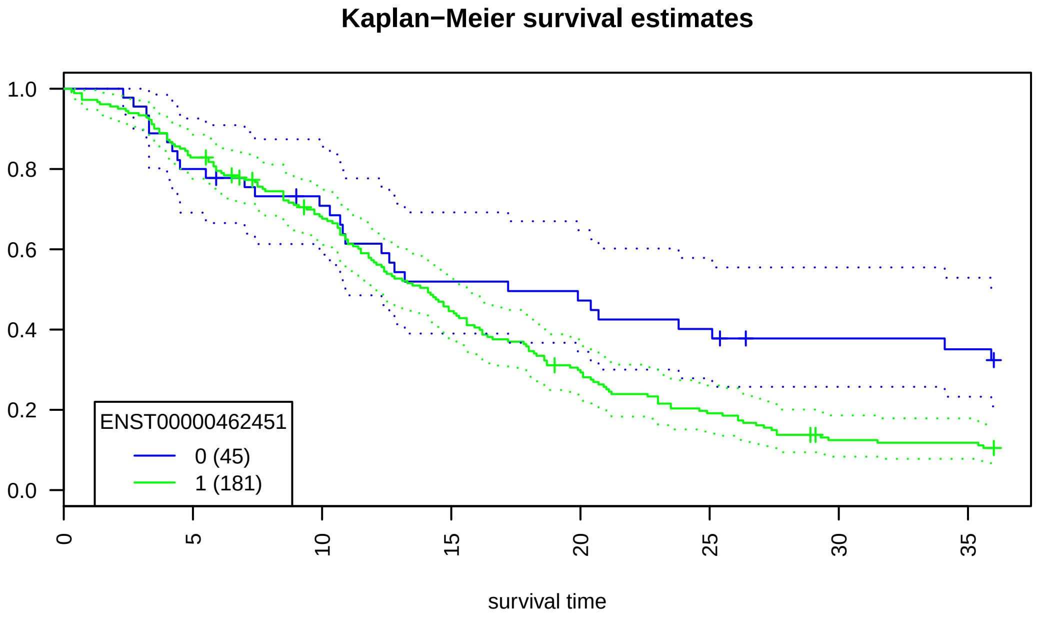

Figure 1041: Each curve represents the relative number of ENST00000462451 survived to the corresponding point of time. Dashed lines represent 0.95 confidence intervals for the curves with the corresponding color. Total of 4 observations deleted due to missingness. Log-rank probability measure for the equality of these curves is 1.060381e-02. Additional annotations: *.GeneId*=ENSG00000168899, *.GeneName*=VAMP5, *.GeneDesc*=vesicle-associated membrane protein 5 (myobrevin) [Source:HGNC Symbol;Acc:12646].

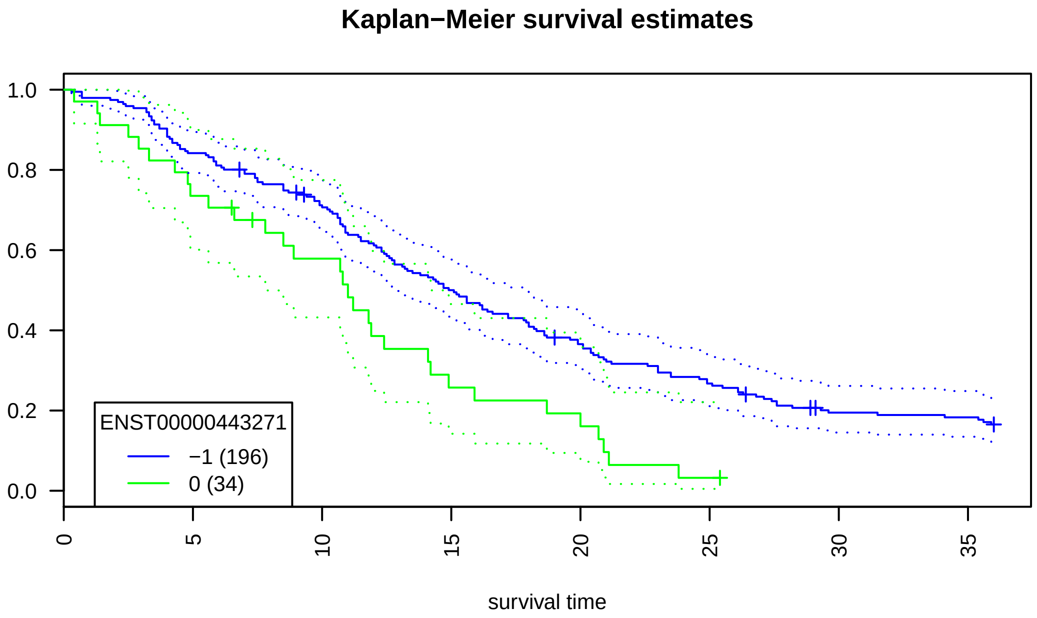

Figure 1042: Each curve represents the relative number of ENST00000443271 survived to the corresponding point of time. Dashed lines represent 0.95 confidence intervals for the curves with the corresponding color. Log-rank probability measure for the equality of these curves is 7.929753e-04. Additional annotations: *.GeneId*=ENSG00000110675, *.GeneName*=ELMOD1, *.GeneDesc*=ELMO/CED-12 domain containing 1 [Source:HGNC Symbol;Acc:25334].

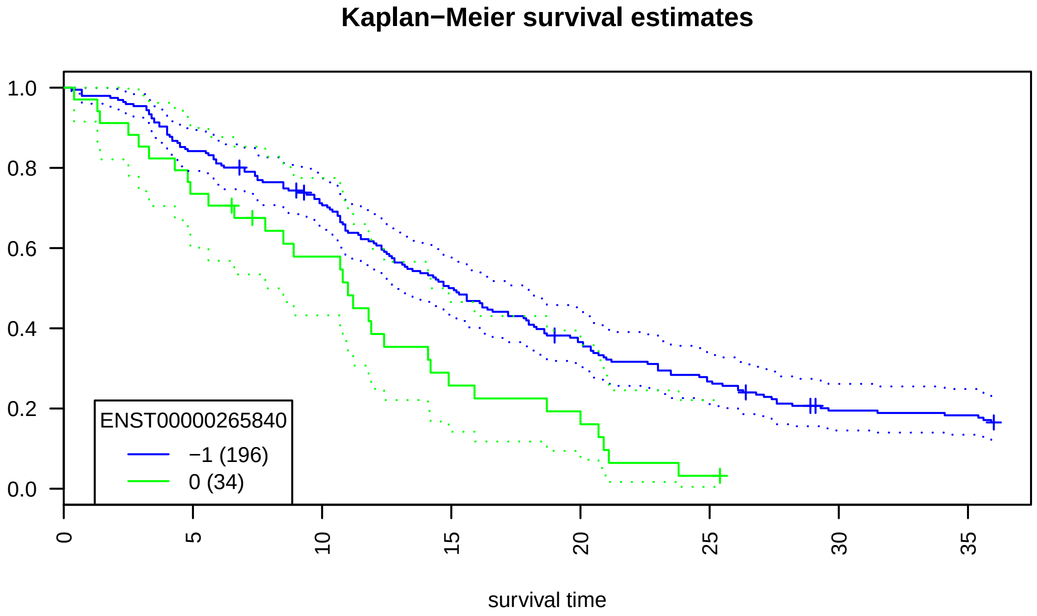

Figure 1043: Each curve represents the relative number of ENST00000265840 survived to the corresponding point of time. Dashed lines represent 0.95 confidence intervals for the curves with the corresponding color. Log-rank probability measure for the equality of these curves is 7.929753e-04. Additional annotations: *.GeneId*=ENSG00000110675, *.GeneName*=ELMOD1, *.GeneDesc*=ELMO/CED-12 domain containing 1 [Source:HGNC Symbol;Acc:25334].

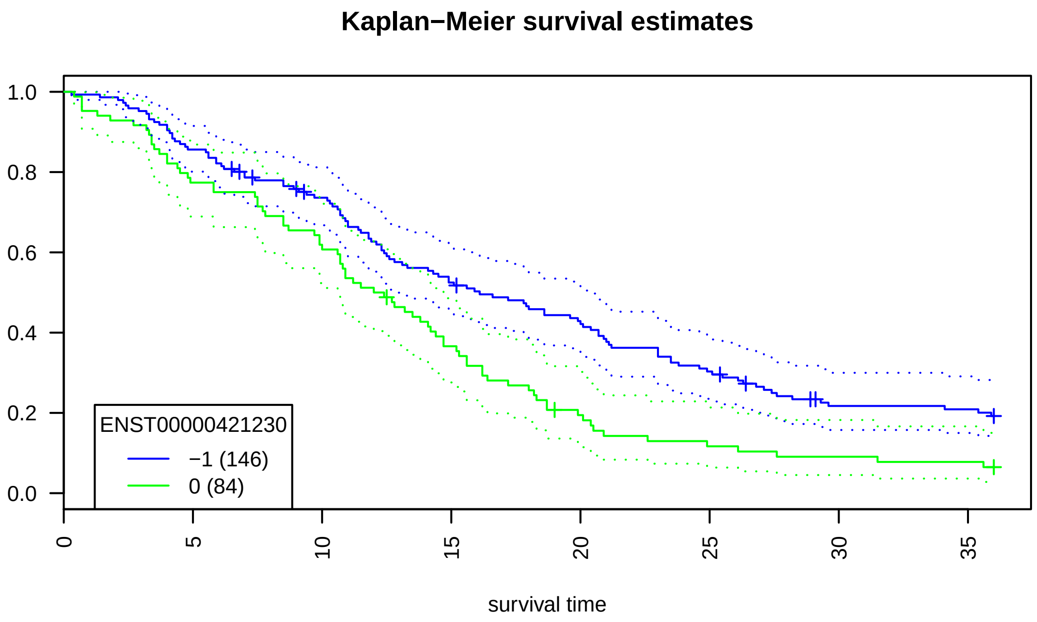

Figure 1044: Each curve represents the relative number of ENST00000421230 survived to the corresponding point of time. Dashed lines represent 0.95 confidence intervals for the curves with the corresponding color. Log-rank probability measure for the equality of these curves is 5.410284e-04. Additional annotations: *.GeneId*=ENSG00000177380, *.GeneName*=PPFIA3, *.GeneDesc*=protein tyrosine phosphatase, receptor type, f polypeptide (PTPRF), interacting protein (liprin), alpha 3 [Source:HGNC Symbol;Acc:9247].

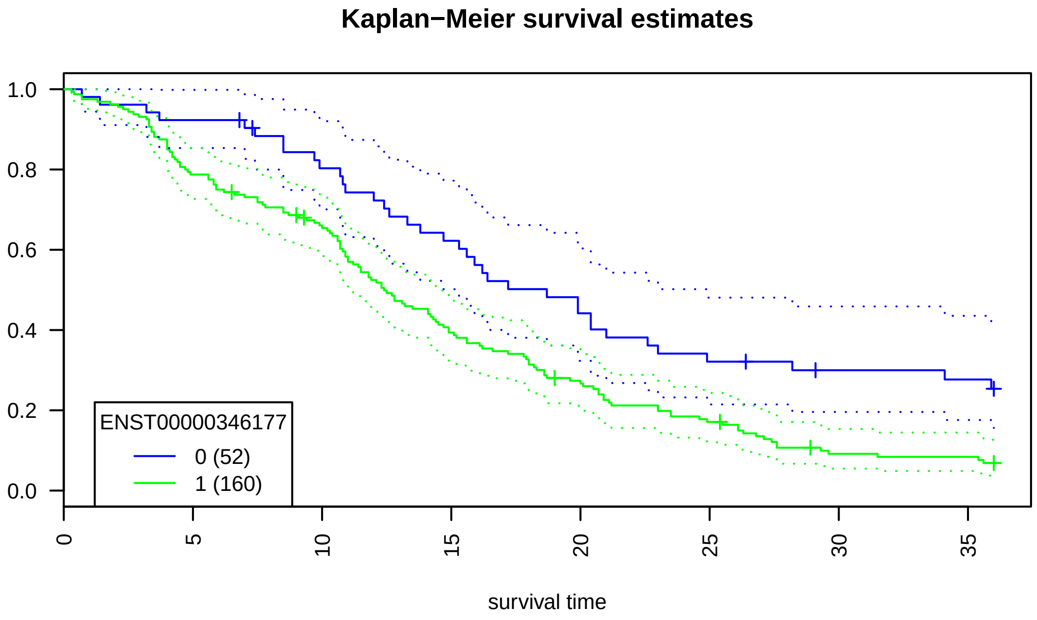

Figure 1045: Each curve represents the relative number of ENST00000346177 survived to the corresponding point of time. Dashed lines represent 0.95 confidence intervals for the curves with the corresponding color. Total of 18 observations deleted due to missingness. Log-rank probability measure for the equality of these curves is 8.091029e-04. Additional annotations: *.GeneId*=ENSG00000128591, *.GeneName*=FLNC, *.GeneDesc*=filamin C, gamma [Source:HGNC Symbol;Acc:3756].

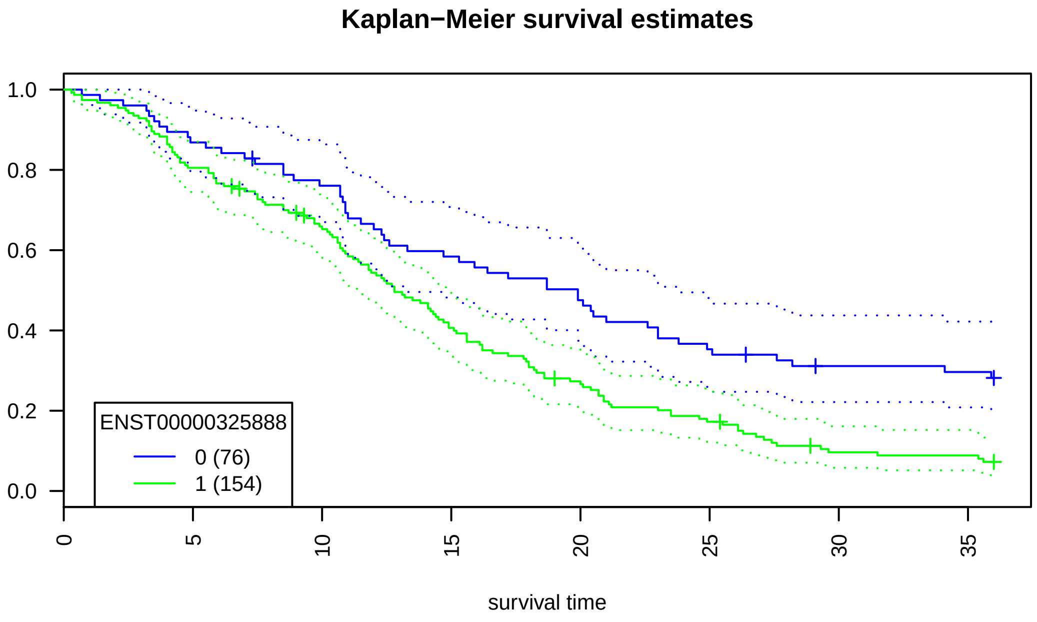

Figure 1046: Each curve represents the relative number of ENST00000325888 survived to the corresponding point of time. Dashed lines represent 0.95 confidence intervals for the curves with the corresponding color. Log-rank probability measure for the equality of these curves is 1.890704e-04. Additional annotations: *.GeneId*=ENSG00000128591, *.GeneName*=FLNC, *.GeneDesc*=filamin C, gamma [Source:HGNC Symbol;Acc:3756].

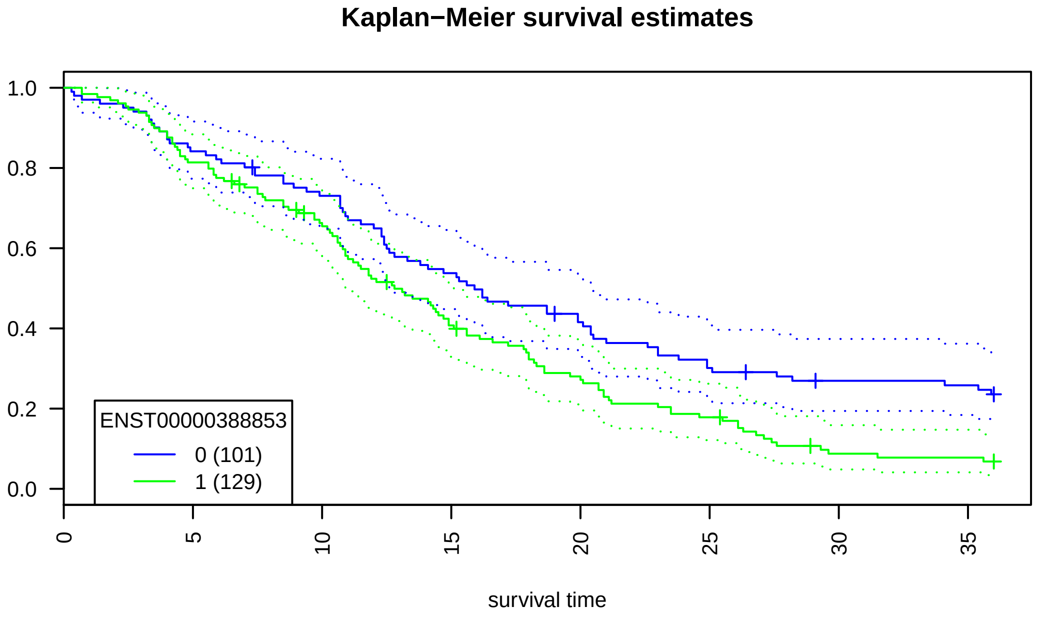

Figure 1047: Each curve represents the relative number of ENST00000388853 survived to the corresponding point of time. Dashed lines represent 0.95 confidence intervals for the curves with the corresponding color. Log-rank probability measure for the equality of these curves is 2.644541e-03. Additional annotations: *.GeneId*=ENSG00000128591, *.GeneName*=FLNC, *.GeneDesc*=filamin C, gamma [Source:HGNC Symbol;Acc:3756].

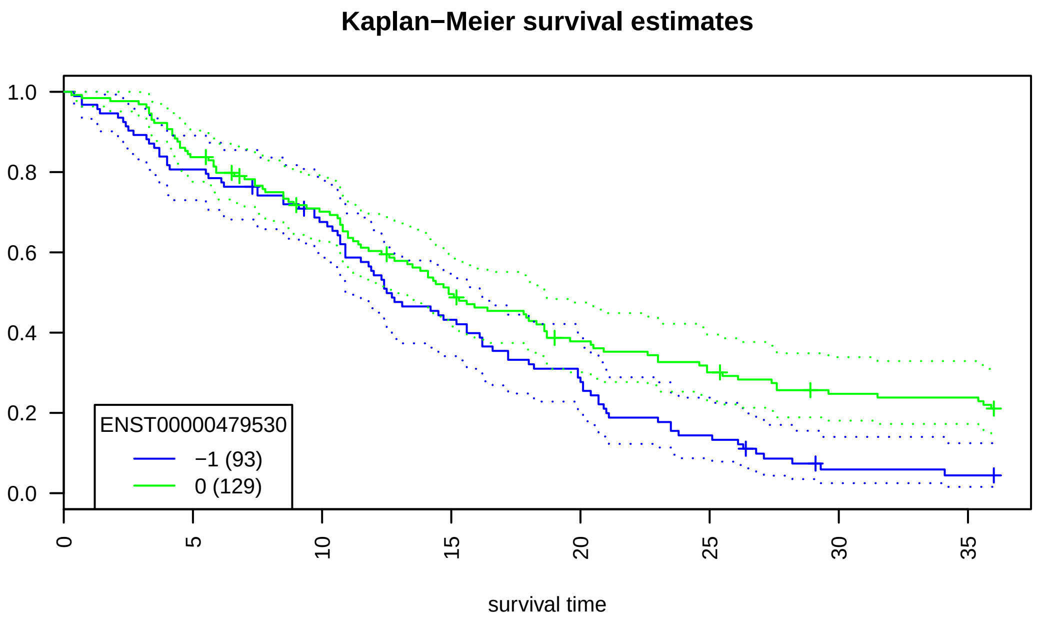

Figure 1048: Each curve represents the relative number of ENST00000479530 survived to the corresponding point of time. Dashed lines represent 0.95 confidence intervals for the curves with the corresponding color. Total of 8 observations deleted due to missingness. Log-rank probability measure for the equality of these curves is 3.106535e-03. Additional annotations: *.GeneId*=ENSG00000121440, *.GeneName*=PDZRN3, *.GeneDesc*=PDZ domain containing ring finger 3 [Source:HGNC Symbol;Acc:17704].

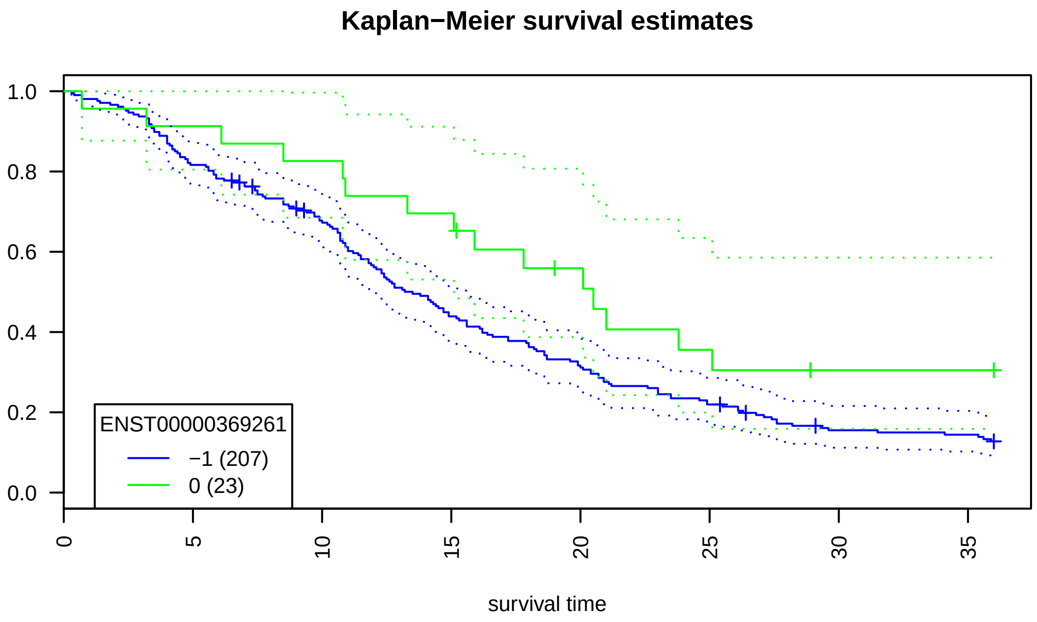

Figure 1049: Each curve represents the relative number of ENST00000369261 survived to the corresponding point of time. Dashed lines represent 0.95 confidence intervals for the curves with the corresponding color. Log-rank probability measure for the equality of these curves is 4.537338e-02. Additional annotations: *.GeneId*=ENSG00000186231, *.GeneName*=KLHL32, *.GeneDesc*=kelch-like 32 (Drosophila) [Source:HGNC Symbol;Acc:21221].

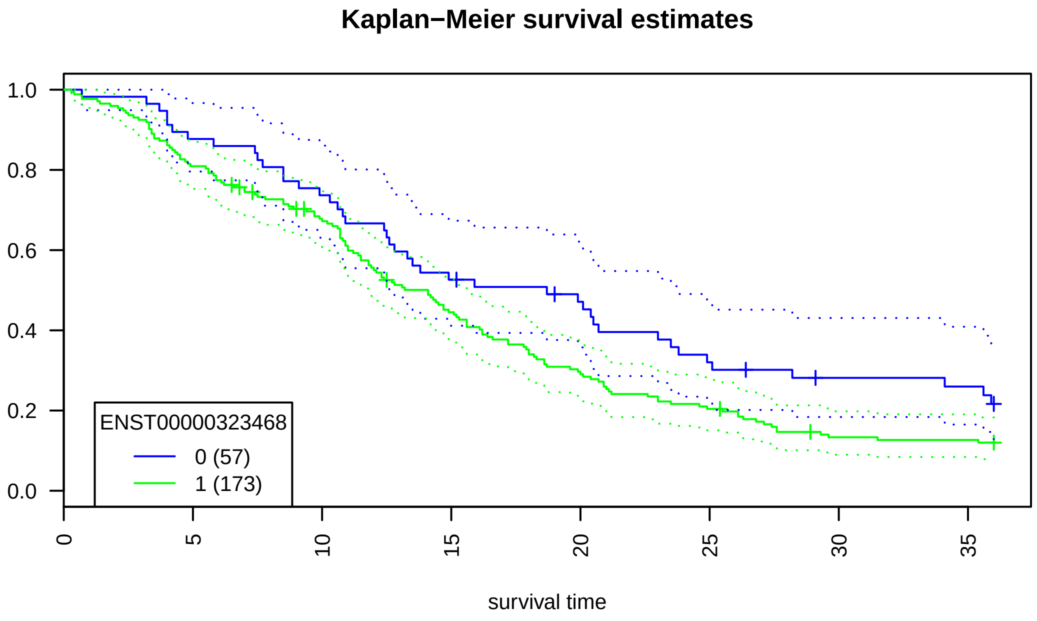

Figure 1050: Each curve represents the relative number of ENST00000323468 survived to the corresponding point of time. Dashed lines represent 0.95 confidence intervals for the curves with the corresponding color. Log-rank probability measure for the equality of these curves is 3.680543e-02. Additional annotations: *.GeneId*=ENSG00000178202, *.GeneName*=KDELC2, *.GeneDesc*=KDEL (Lys-Asp-Glu-Leu) containing 2 [Source:HGNC Symbol;Acc:28496].

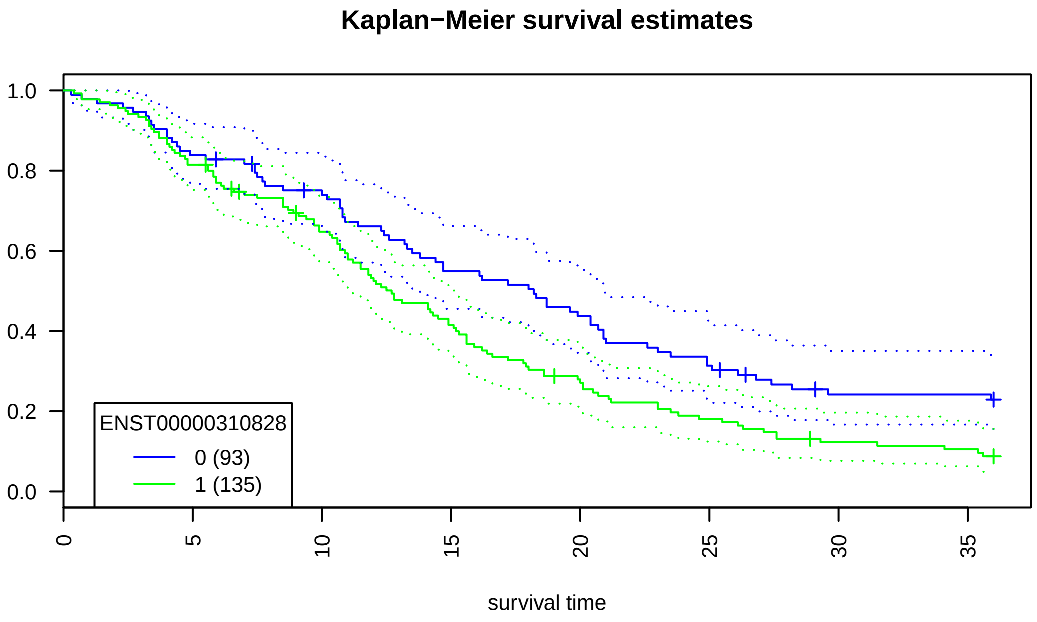

Figure 1051: Each curve represents the relative number of ENST00000310828 survived to the corresponding point of time. Dashed lines represent 0.95 confidence intervals for the curves with the corresponding color. Total of 2 observations deleted due to missingness. Log-rank probability measure for the equality of these curves is 4.131736e-03. Additional annotations: *.GeneId*=ENSG00000167851, *.GeneName*=CD300A, *.GeneDesc*=CD300a molecule [Source:HGNC Symbol;Acc:19319].

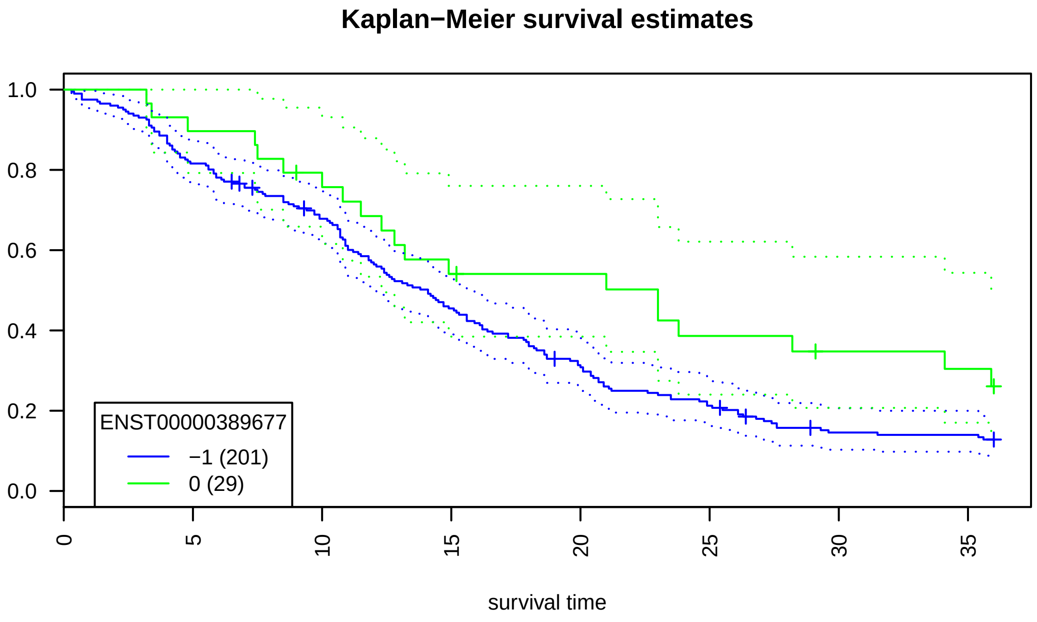

Figure 1052: Each curve represents the relative number of ENST00000389677 survived to the corresponding point of time. Dashed lines represent 0.95 confidence intervals for the curves with the corresponding color. Log-rank probability measure for the equality of these curves is 4.272724e-02. Additional annotations: *.GeneId*=ENSG00000146267, *.GeneName*=C6orf168, *.GeneDesc*=Uncharacterized protein C6orf168 [Source:UniProtKB/Swiss-Prot;Acc:Q5TGI0].

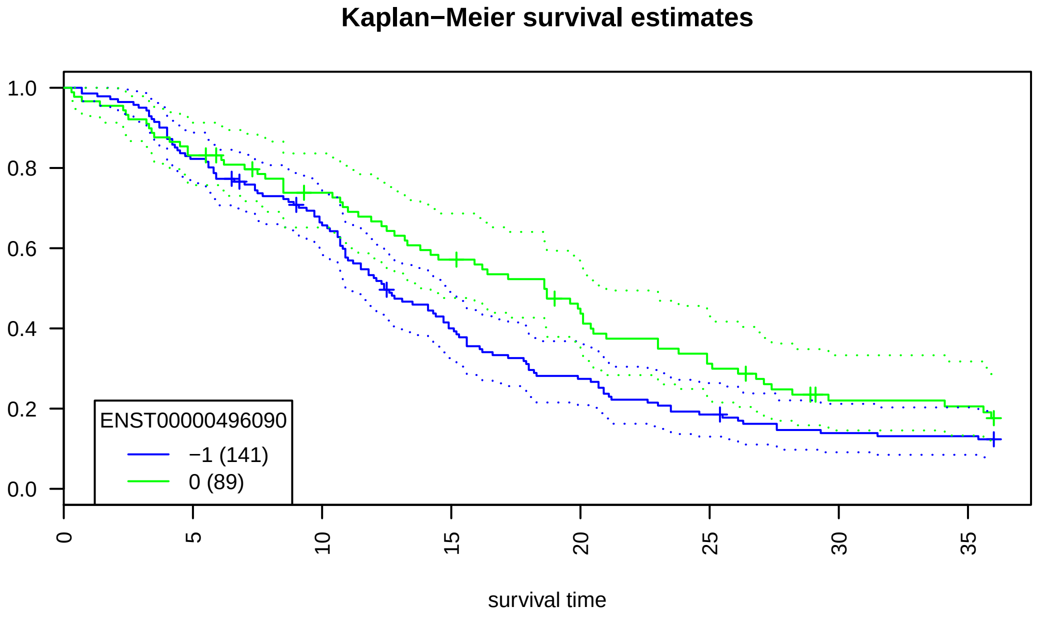

Figure 1053: Each curve represents the relative number of ENST00000496090 survived to the corresponding point of time. Dashed lines represent 0.95 confidence intervals for the curves with the corresponding color. Log-rank probability measure for the equality of these curves is 3.327139e-02. Additional annotations: *.GeneId*=ENSG00000123552, *.GeneName*=USP45, *.GeneDesc*=ubiquitin specific peptidase 45 [Source:HGNC Symbol;Acc:20080].

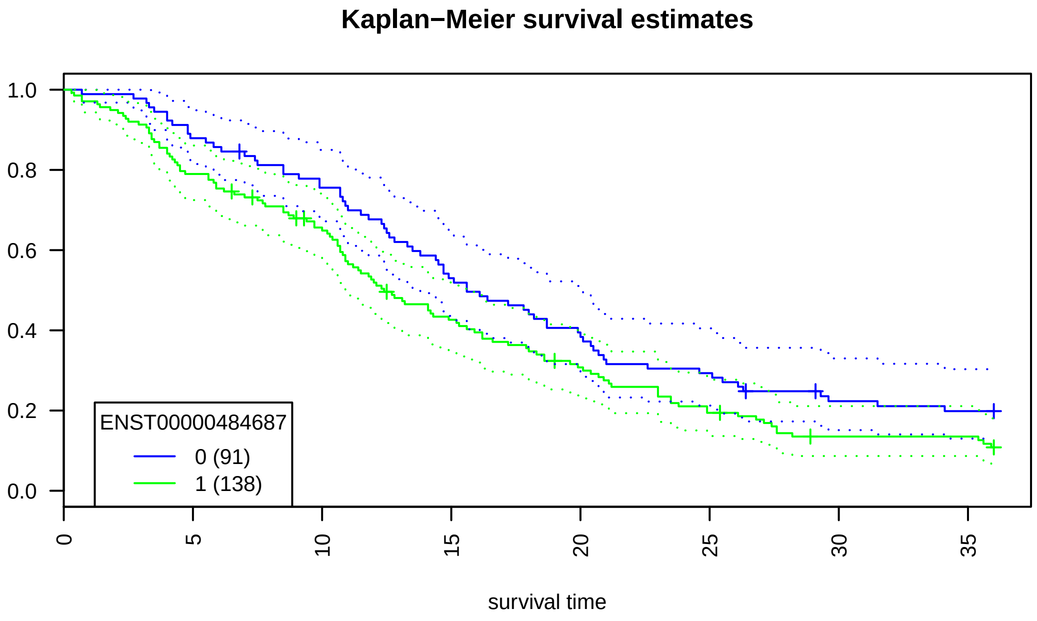

Figure 1054: Each curve represents the relative number of ENST00000484687 survived to the corresponding point of time. Dashed lines represent 0.95 confidence intervals for the curves with the corresponding color. Total of 1 observation deleted due to missingness. Log-rank probability measure for the equality of these curves is 3.559685e-02. Additional annotations: *.GeneId*=ENSG00000114480, *.GeneName*=GBE1, *.GeneDesc*=glucan (1,4-alpha-), branching enzyme 1 [Source:HGNC Symbol;Acc:4180].

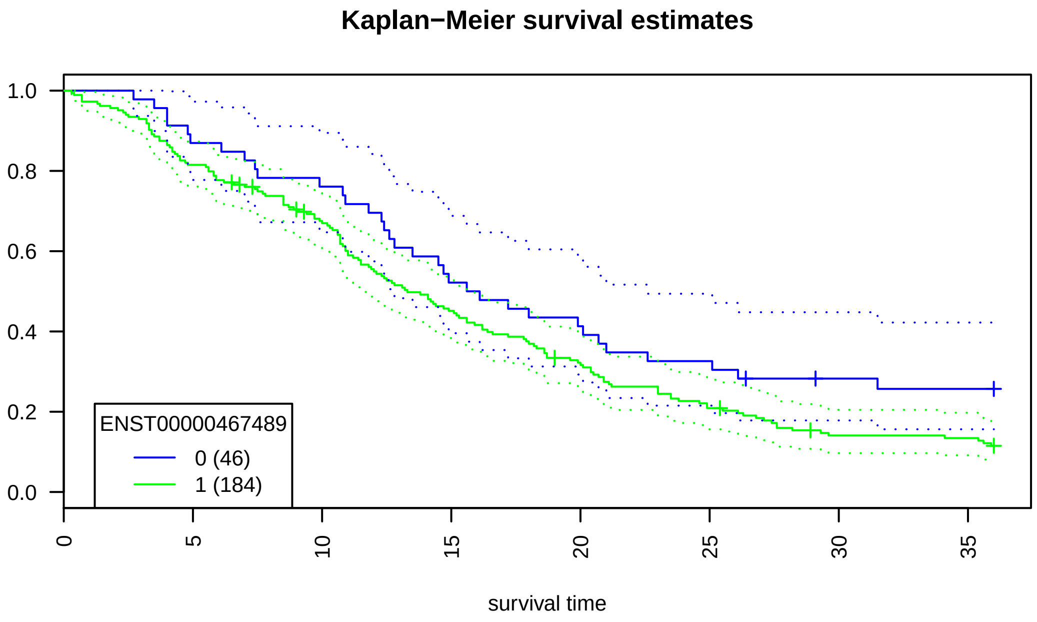

Figure 1055: Each curve represents the relative number of ENST00000467489 survived to the corresponding point of time. Dashed lines represent 0.95 confidence intervals for the curves with the corresponding color. Log-rank probability measure for the equality of these curves is 4.998935e-02.

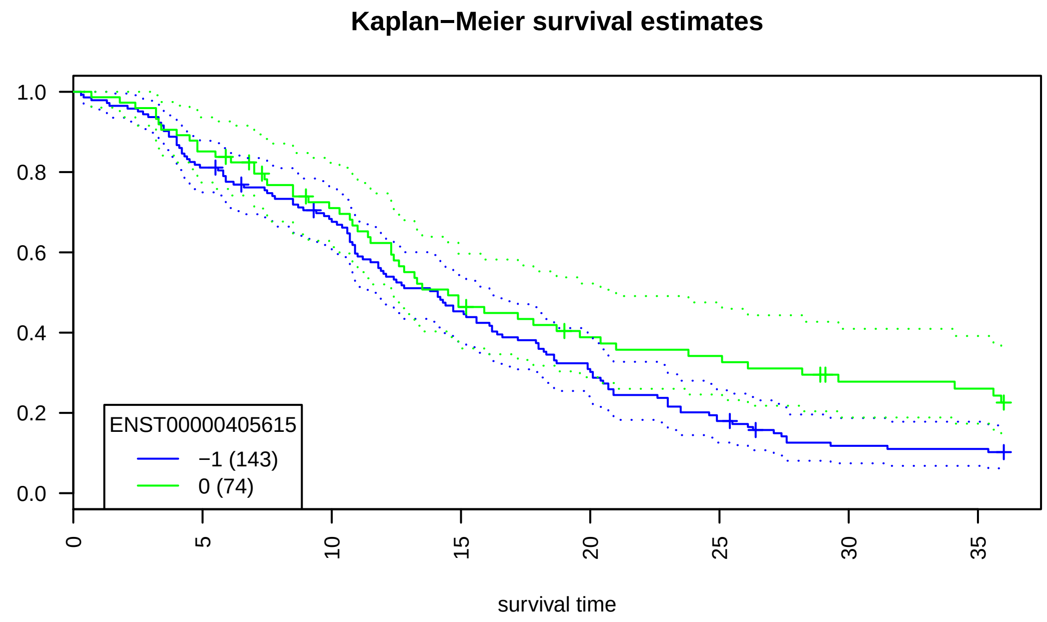

Figure 1056: Each curve represents the relative number of ENST00000405615 survived to the corresponding point of time. Dashed lines represent 0.95 confidence intervals for the curves with the corresponding color. Total of 13 observations deleted due to missingness. Log-rank probability measure for the equality of these curves is 4.245462e-02. Additional annotations: .*GeneId*=ENSG00000175161, .*GeneName*=CADM2, .*GeneDesc*=cell adhesion molecule 2 [Source:HGNC Symbol;Acc:29849].

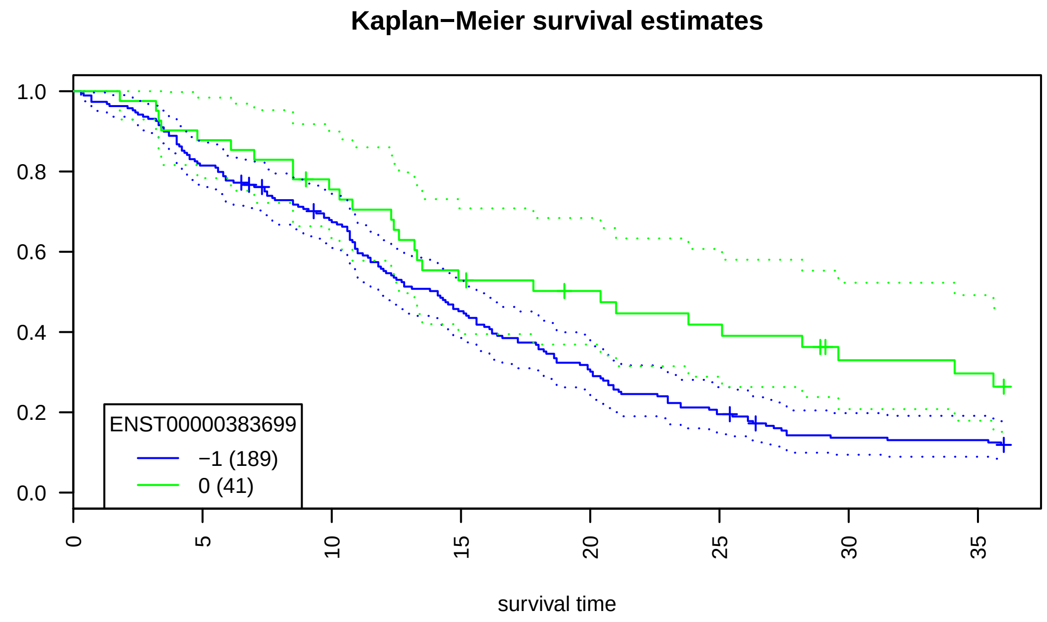

Figure 1057: Each curve represents the relative number of ENST00000383699 survived to the corresponding point of time. Dashed lines represent 0.95 confidence intervals for the curves with the corresponding color. Log-rank probability measure for the equality of these curves is 1.775390e-02. Additional annotations: .*GeneId*=ENSG00000175161, .*GeneName*=CADM2, .*GeneDesc*=cell adhesion molecule 2 [Source:HGNC Symbol;Acc:29849].

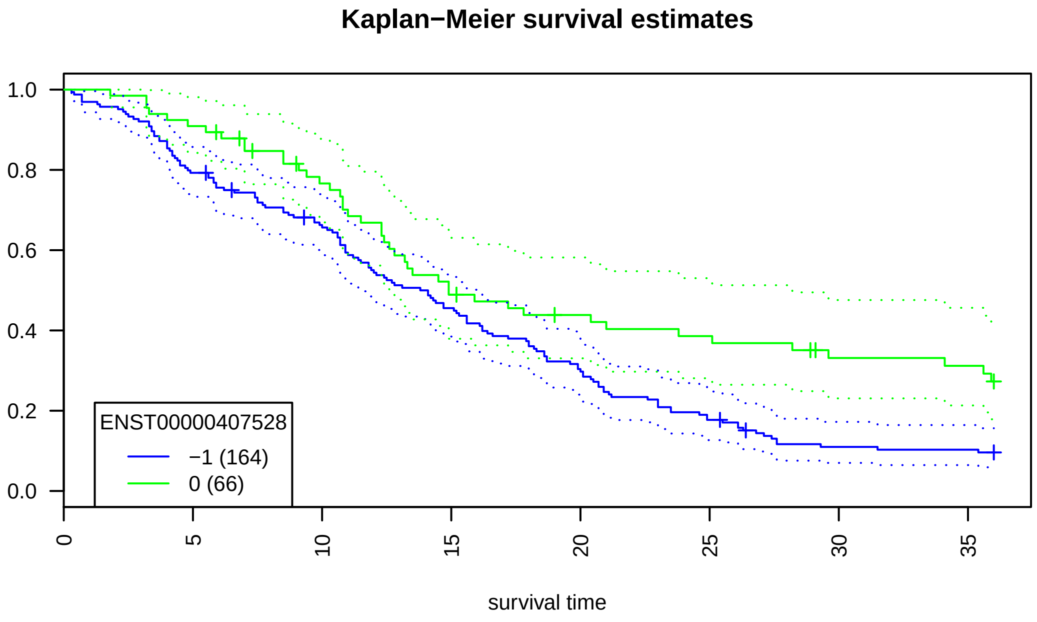

Figure 1058: Each curve represents the relative number of ENST00000407528 survived to the corresponding point of time. Dashed lines represent 0.95 confidence intervals for the curves with the corresponding color. Log-rank probability measure for the equality of these curves is 2.981332e-03. Additional annotations: .*GeneId*=ENSG00000175161, .*GeneName*=CADM2, .*GeneDesc*=cell adhesion molecule 2 [Source:HGNC Symbol;Acc:29849].

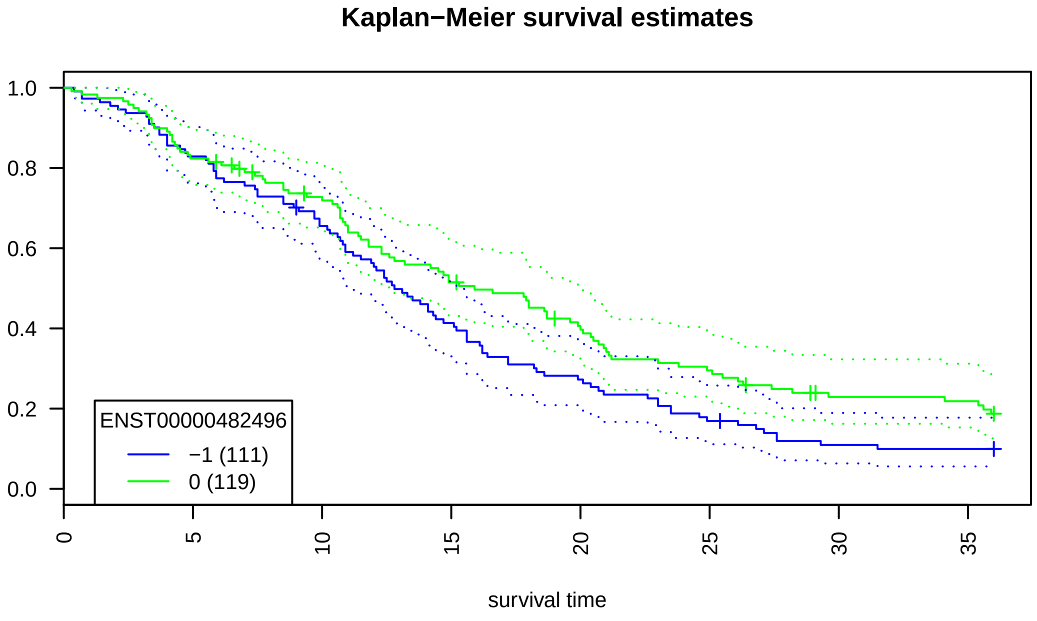

Figure 1059: Each curve represents the relative number of ENST00000482496 survived to the corresponding point of time. Dashed lines represent 0.95 confidence intervals for the curves with the corresponding color. Log-rank probability measure for the equality of these curves is 3.131683e-02. Additional annotations: .*GeneId*=ENSG00000165650, .*GeneName*=PDZD8, .*GeneDesc*=PDZ domain containing 8 [Source:HGNC Symbol;Acc:26974].

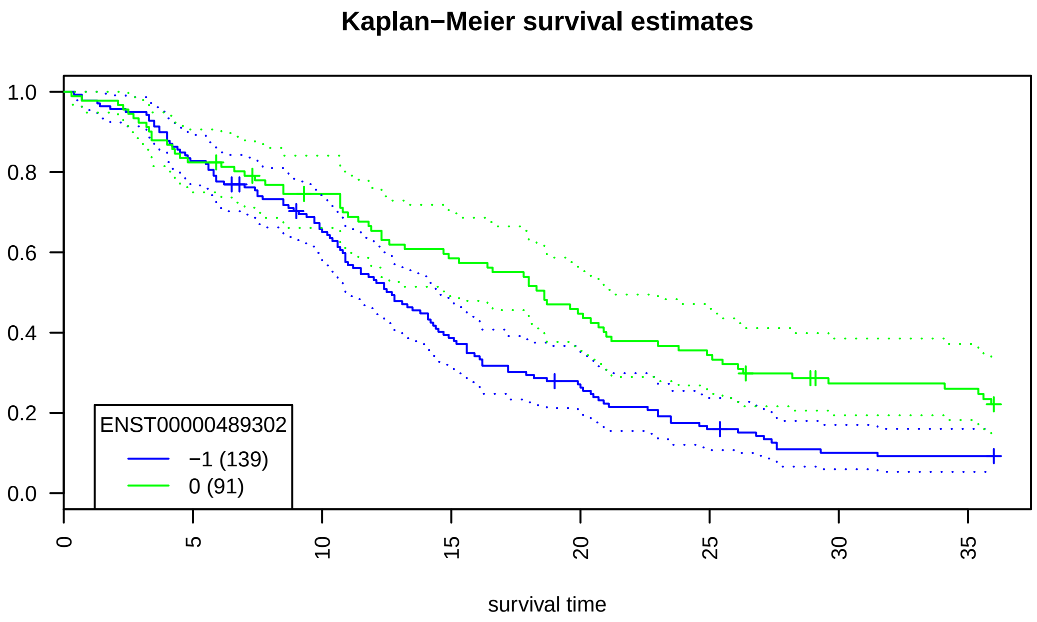

Figure 1060: Each curve represents the relative number of ENST00000489302 survived to the corresponding point of time. Dashed lines represent 0.95 confidence intervals for the curves with the corresponding color. Log-rank probability measure for the equality of these curves is 1.842449e-03. Additional annotations: *.GeneId*=ENSG00000165650, *.GeneName*=PDZD8, *.GeneDesc*=PDZ domain containing 8 [Source:HGNC Symbol;Acc:26974].

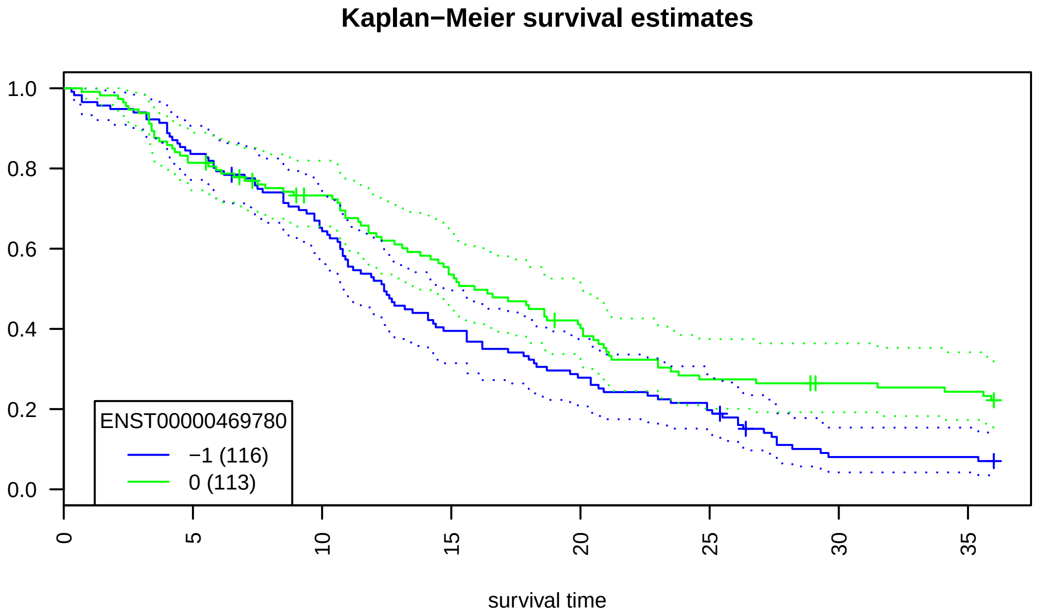

Figure 1061: Each curve represents the relative number of ENST00000469780 survived to the corresponding point of time. Dashed lines represent 0.95 confidence intervals for the curves with the corresponding color. Total of 1 observation deleted due to missingness. Log-rank probability measure for the equality of these curves is 6.770127e-03. Additional annotations: *.GeneId*=ENSG00000165669, *.GeneName*=C10orf84, *.GeneDesc*=Uncharacterized protein C10orf84 [Source:UniProtKB/Swiss-Prot;Acc:Q9H8W3].

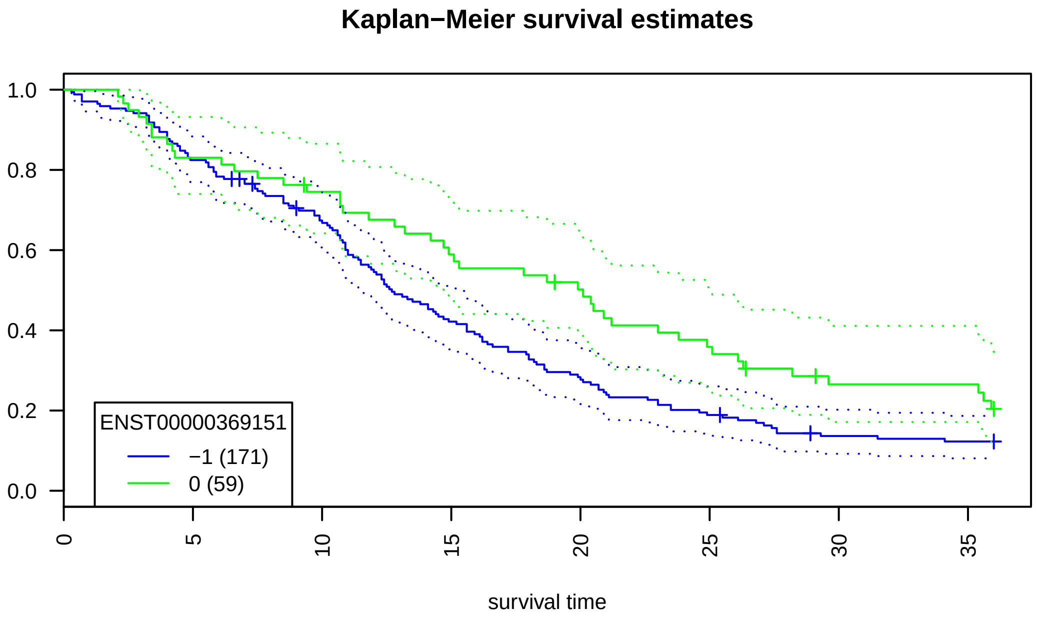

Figure 1062: Each curve represents the relative number of ENST00000369151 survived to the corresponding point of time. Dashed lines represent 0.95 confidence intervals for the curves with the corresponding color. Log-rank probability measure for the equality of these curves is 2.099681e-02. Additional annotations: *.GeneId*=ENSG00000151893, *.GeneName*=C10orf46, *.GeneDesc*=Uncharacterized protein C10orf46 [Source:UniProtKB/Swiss-Prot;Acc:Q86Y37].

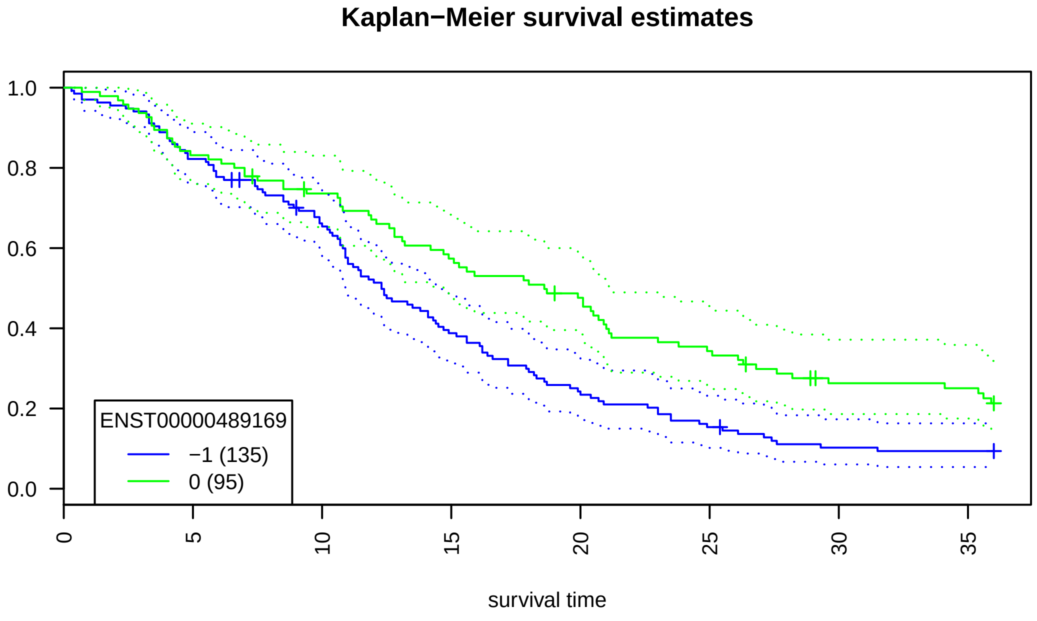

Figure 1063: Each curve represents the relative number of ENST00000489169 survived to the corresponding point of time. Dashed lines represent 0.95 confidence intervals for the curves with the corresponding color. Log-rank probability measure for the equality of these curves is 1.543092e-03. Additional annotations: *.GeneId*=ENSG00000151893, *.GeneName*=C10orf46, *.GeneDesc*=Uncharacterized protein C10orf46 [Source:UniProtKB/Swiss-Prot;Acc:Q86Y37].

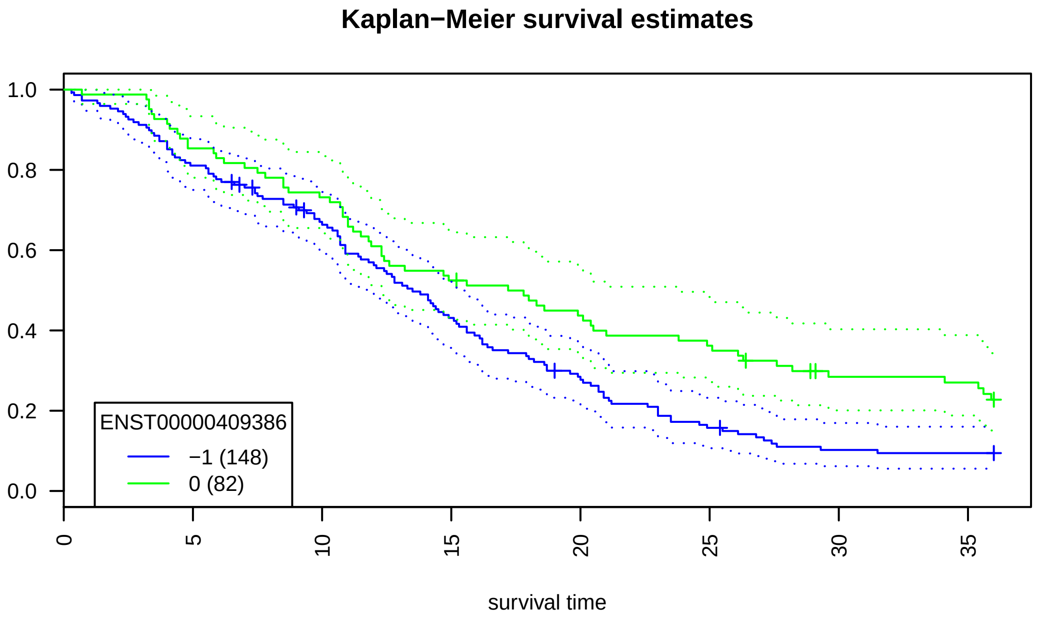

Figure 1064: Each curve represents the relative number of ENST00000409386 survived to the corresponding point of time. Dashed lines represent 0.95 confidence intervals for the curves with the corresponding color. Log-rank probability measure for the equality of these curves is 4.511268e-03. Additional annotations: *.GeneId*=ENSG00000130559, *.GeneName*=CAMSAP1, *.GeneDesc*=calmodulin regulated spectrin-associated protein 1 [Source:HGNC Symbol;Acc:19946].

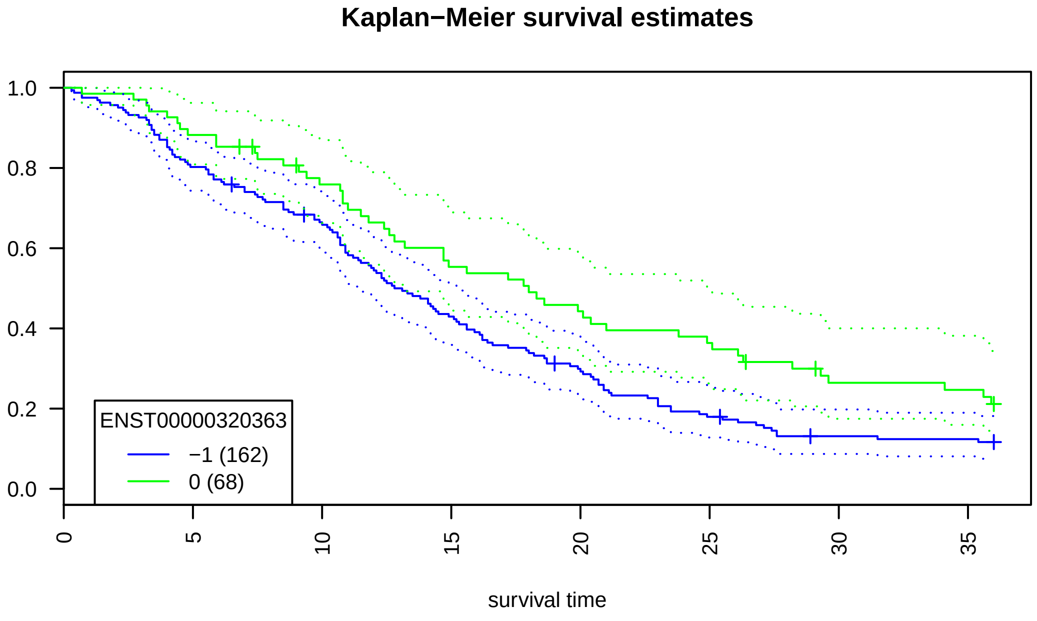

Figure 1065: Each curve represents the relative number of ENST00000320363 survived to the corresponding point of time. Dashed lines represent 0.95 confidence intervals for the curves with the corresponding color. Log-rank probability measure for the equality of these curves is 1.204050e-02. Additional annotations: *.GeneId*=ENSG00000130559, *.GeneName*=CAMSAP1, *.GeneDesc*=calmodulin regulated spectrin-associated protein 1 [Source:HGNC Symbol;Acc:19946].

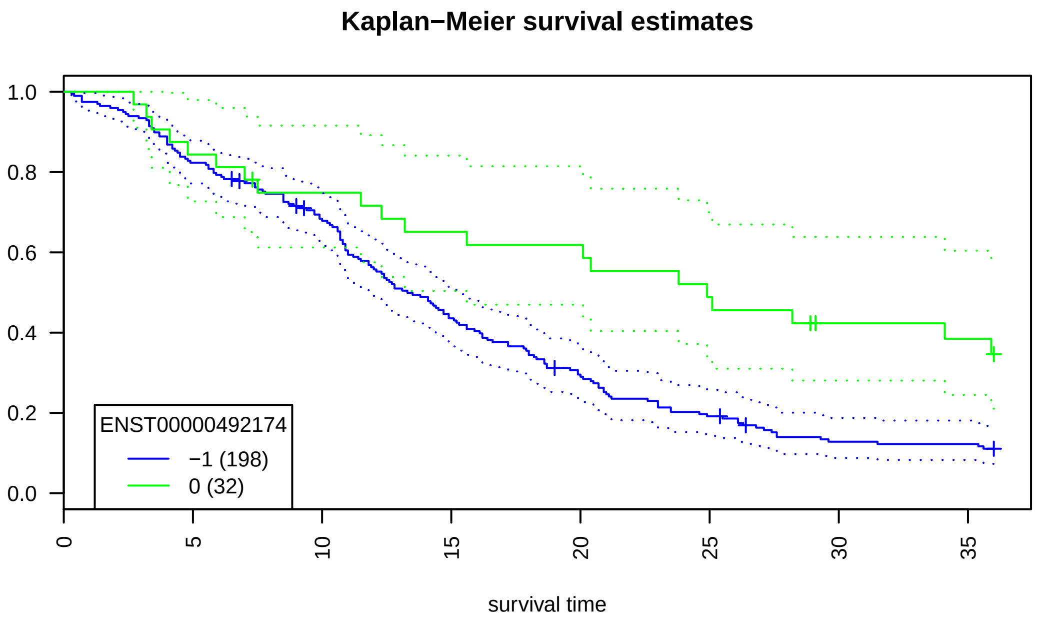

Figure 1066: Each curve represents the relative number of ENST00000492174 survived to the corresponding point of time. Dashed lines represent 0.95 confidence intervals for the curves with the corresponding color. Log-rank probability measure for the equality of these curves is 1.809767e-03. Additional annotations: *.GeneId*=ENSG00000130559, *.GeneName*=CAMSAP1, *.GeneDesc*=calmodulin regulated spectrin-associated protein 1 [Source:HGNC Symbol;Acc:19946].

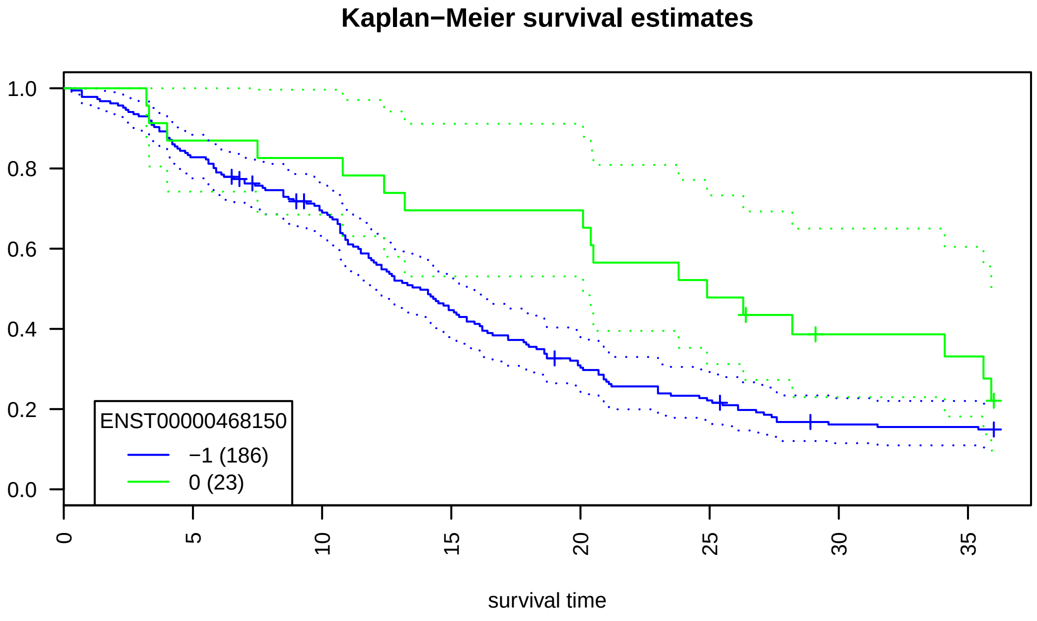

Figure 1067: Each curve represents the relative number of ENST00000468150 survived to the corresponding point of time. Dashed lines represent 0.95 confidence intervals for the curves with the corresponding color. Total of 21 observations deleted due to missingness. Log-rank probability measure for the equality of these curves is 3.485158e-02. Additional annotations: *.GeneId*=ENSG00000130559, *.GeneName*=CAMSAP1, *.GeneDesc*=calmodulin regulated spectrin-associated protein 1 [Source:HGNC Symbol;Acc:19946].

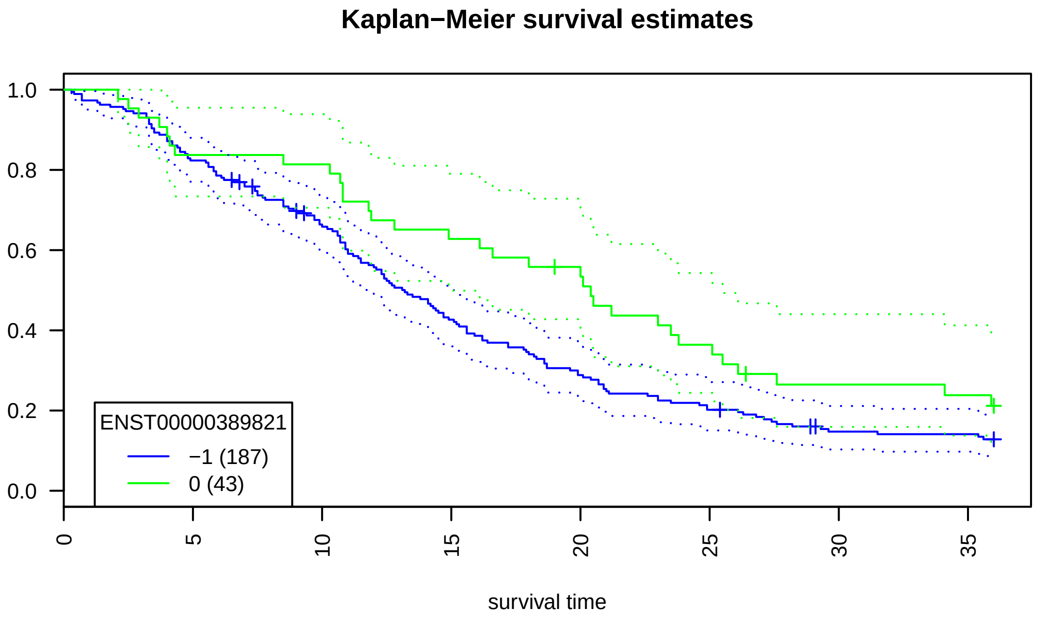

Figure 1068: Each curve represents the relative number of ENST00000389821 survived to the corresponding point of time. Dashed lines represent 0.95 confidence intervals for the curves with the corresponding color. Log-rank probability measure for the equality of these curves is 3.282549e-02. Additional annotations: .*GeneId*=ENSG00000150764, .*GeneName*=DIXDC1, .*GeneDesc*=DIX domain containing 1 [Source:HGNC Symbol;Acc:23695].

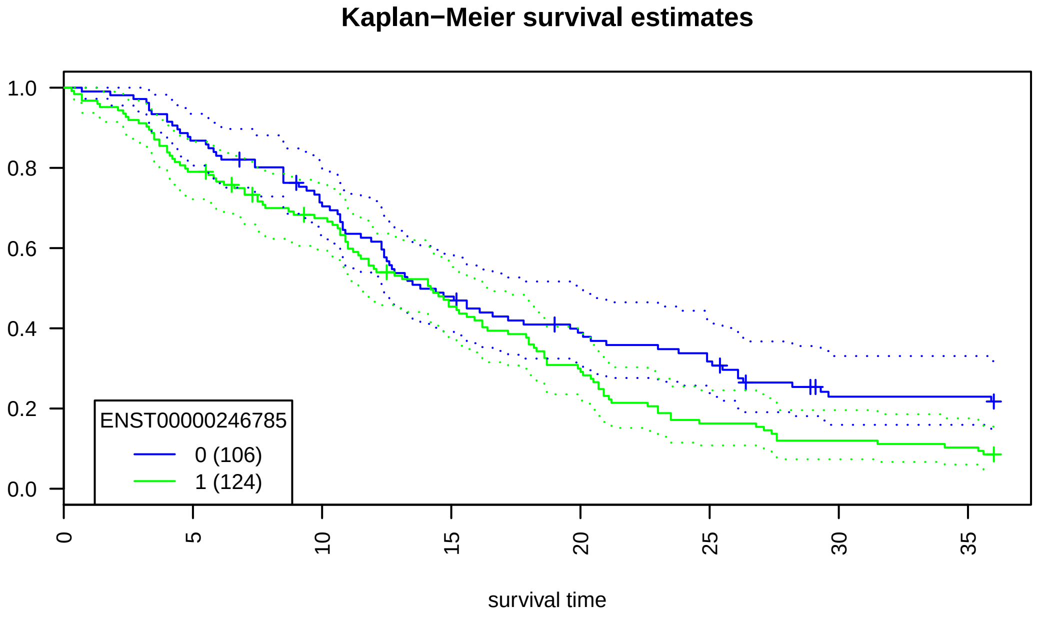

Figure 1069: Each curve represents the relative number of ENST00000246785 survived to the corresponding point of time. Dashed lines represent 0.95 confidence intervals for the curves with the corresponding color. Log-rank probability measure for the equality of these curves is 2.144576e-02. Additional annotations: .*GeneId*=ENSG00000126453, .*GeneName*=BCL2L12, .*GeneDesc*=BCL2-like 12 (proline rich) [Source:HGNC Symbol;Acc:13787].

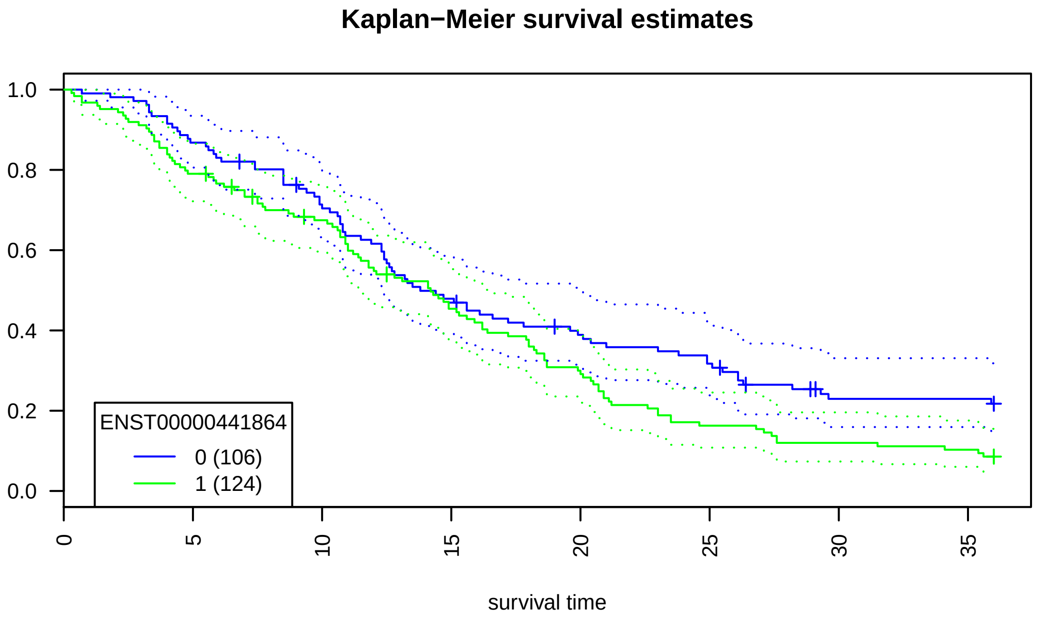

Figure 1070: Each curve represents the relative number of ENST00000441864 survived to the corresponding point of time. Dashed lines represent 0.95 confidence intervals for the curves with the corresponding color. Log-rank probability measure for the equality of these curves is 2.144576e-02. Additional annotations: *.GeneId*=ENSG00000126453, *.GeneName*=BCL2L12, *.GeneDesc*=BCL2-like 12 (proline rich) [Source:HGNC Symbol;Acc:13787].

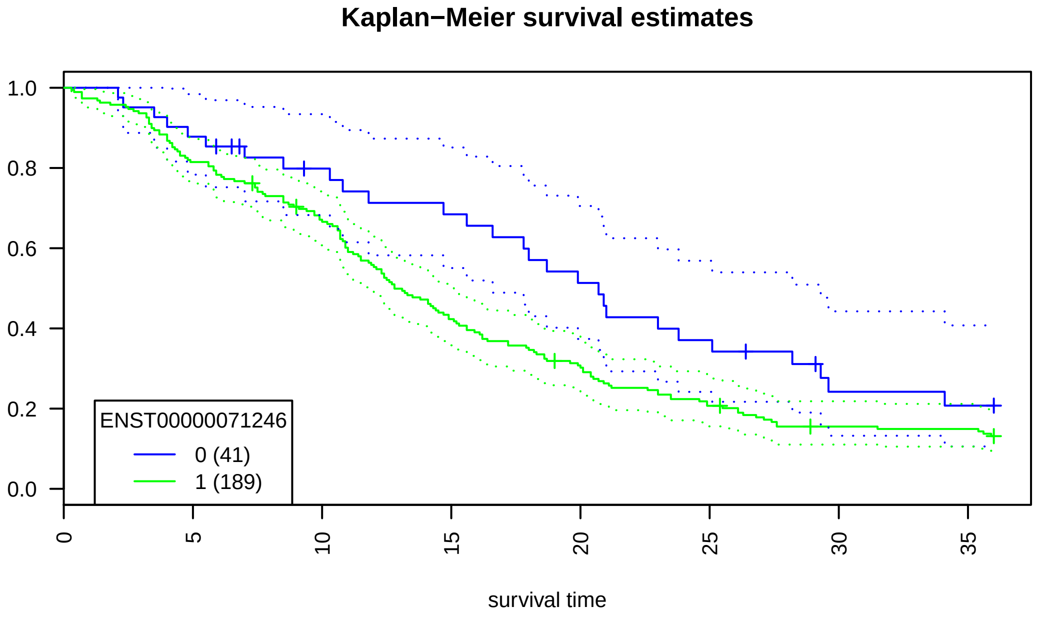

Figure 1071: Each curve represents the relative number of ENST00000071246 survived to the corresponding point of time. Dashed lines represent 0.95 confidence intervals for the curves with the corresponding color. Log-rank probability measure for the equality of these curves is 3.399923e-02. Additional annotations: *.GeneId*=ENSG00000057657, *.GeneName*=PRDM1, *.GeneDesc*=PR domain containing 1, with ZNF domain [Source:HGNC Symbol;Acc:9346].

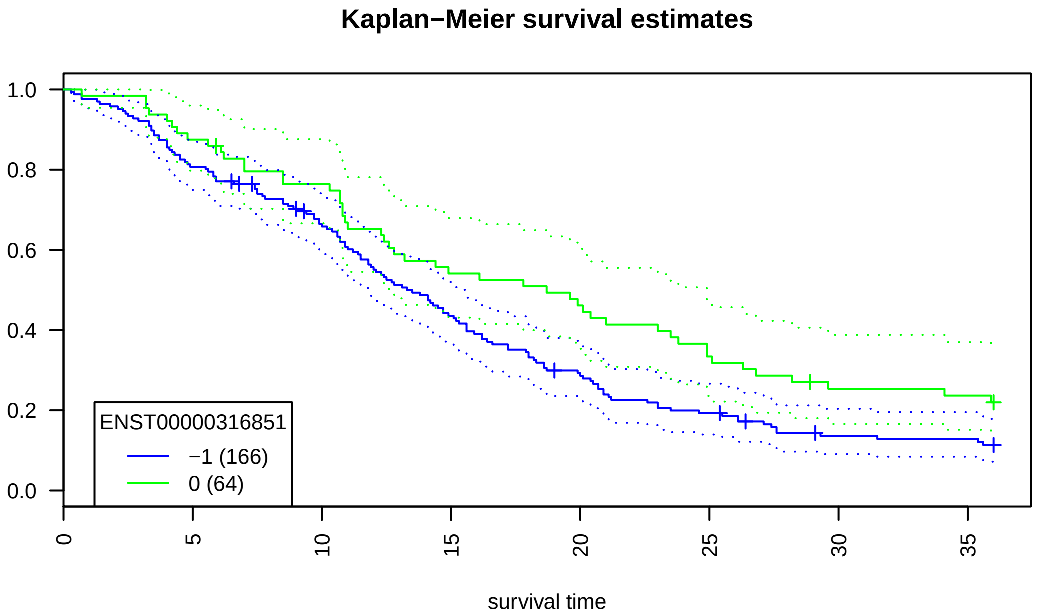

Figure 1072: Each curve represents the relative number of ENST00000316851 survived to the corresponding point of time. Dashed lines represent 0.95 confidence intervals for the curves with the corresponding color. Log-rank probability measure for the equality of these curves is 1.836731e-02. Additional annotations: *.GeneId*=ENSG00000149294, *.GeneName*=NCAM1, *.GeneDesc*=neural cell adhesion molecule 1 [Source:HGNC Symbol;Acc:7656].

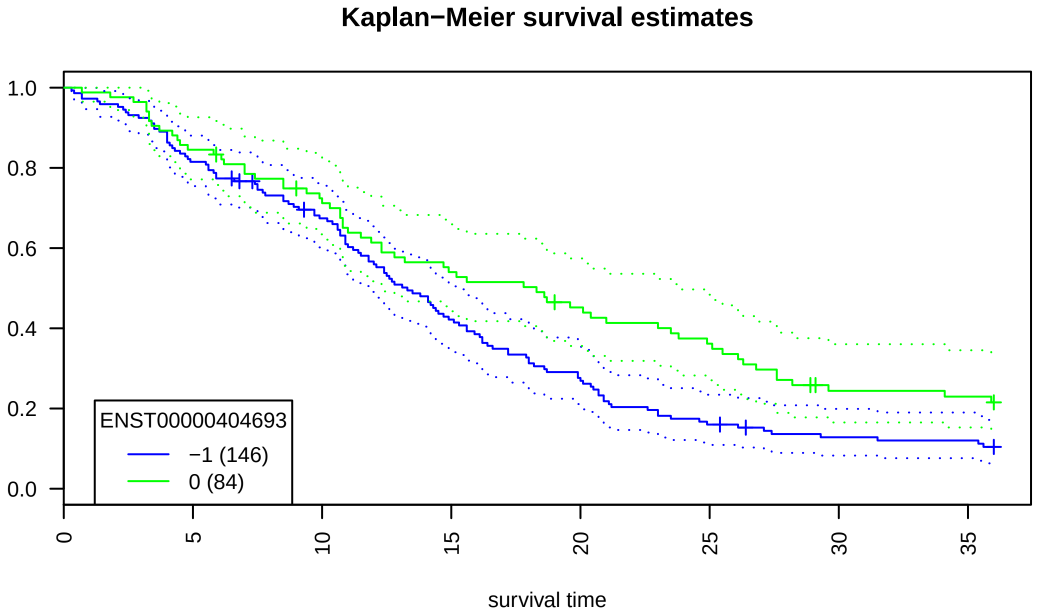

Figure 1073: Each curve represents the relative number of ENST00000404693 survived to the corresponding point of time. Dashed lines represent 0.95 confidence intervals for the curves with the corresponding color. Log-rank probability measure for the equality of these curves is 1.039980e-02. Additional annotations: *.GeneId*=ENSG00000149294, *.GeneName*=NCAM1, *.GeneDesc*=neural cell adhesion molecule 1 [Source:HGNC Symbol;Acc:7656].

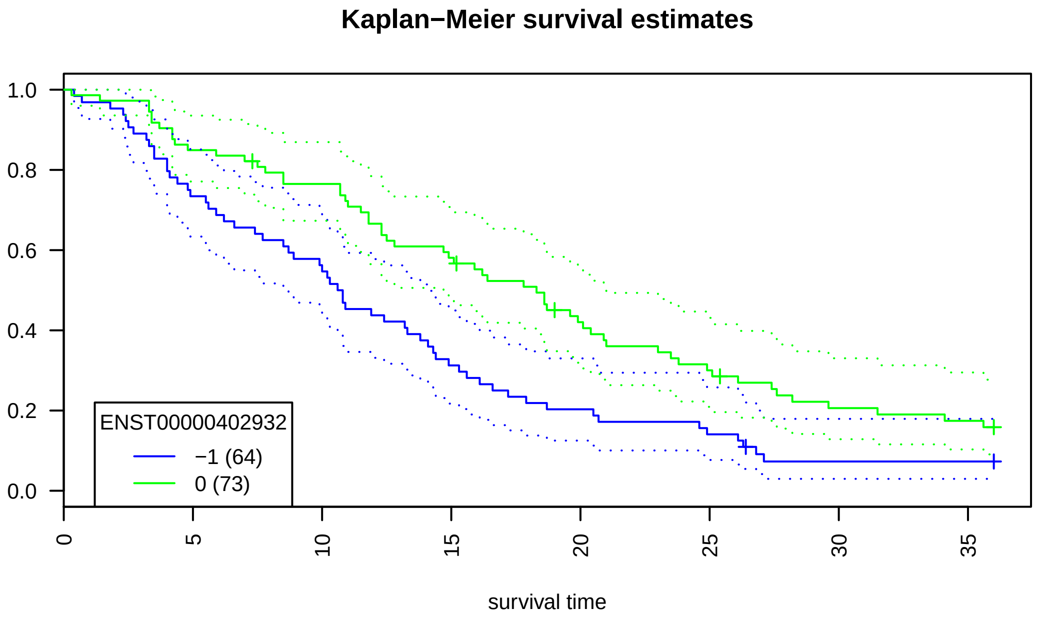

Figure 1074: Each curve represents the relative number of ENST00000402932 survived to the corresponding point of time. Dashed lines represent 0.95 confidence intervals for the curves with the corresponding color. Total of 93 observations deleted due to missingness. Log-rank probability measure for the equality of these curves is 2.082039e-03. Additional annotations: *.GeneId*=ENSG00000090612, *.GeneName*=ZNF10, *.GeneDesc*=zinc finger protein 268 [Source:HGNC Symbol;Acc:13061].

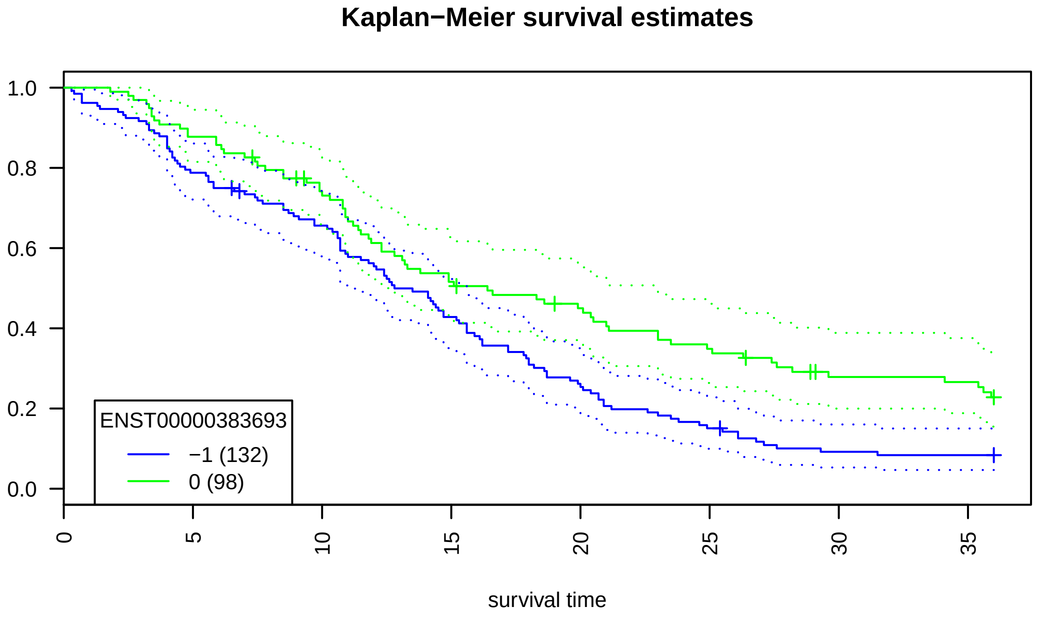

Figure 1075: Each curve represents the relative number of ENST00000383693 survived to the corresponding point of time. Dashed lines represent 0.95 confidence intervals for the curves with the corresponding color. Log-rank probability measure for the equality of these curves is 1.190815e-03. Additional annotations: *.GeneId*=ENSG00000206535, *.GeneName*=LNP1, *.GeneDesc*=leukemia NUP98 fusion partner 1 [Source:HGNC Symbol;Acc:28014].

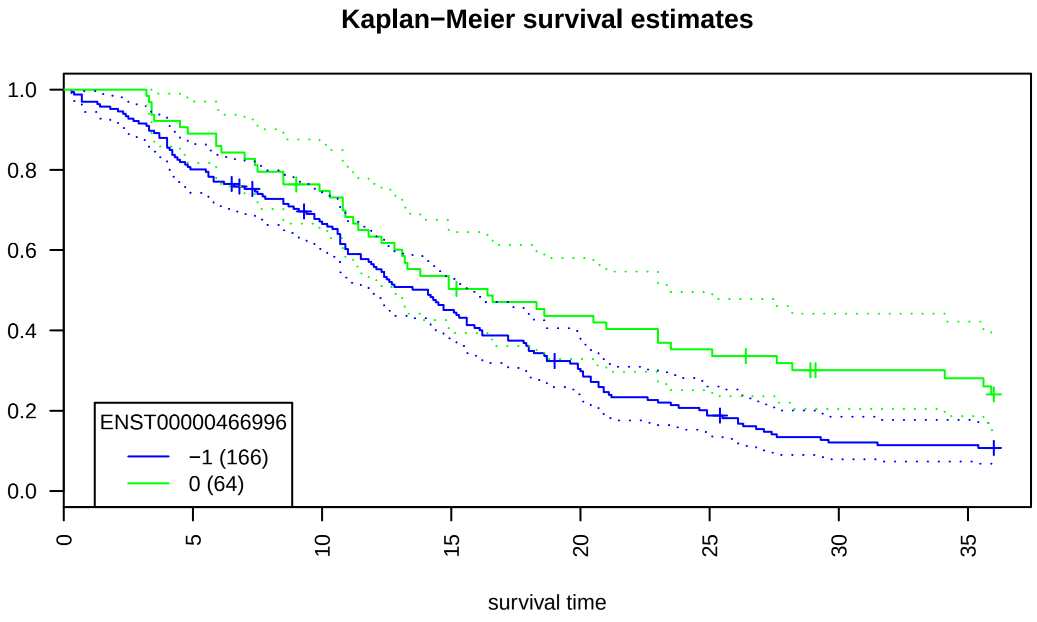

Figure 1076: Each curve represents the relative number of ENST00000466996 survived to the corresponding point of time. Dashed lines represent 0.95 confidence intervals for the curves with the corresponding color. Log-rank probability measure for the equality of these curves is 1.405479e-02. Additional annotations: *.GeneId*=ENSG00000206535, *.GeneName*=LNP1, *.GeneDesc*=leukemia NUP98 fusion partner 1 [Source:HGNC Symbol;Acc:28014].

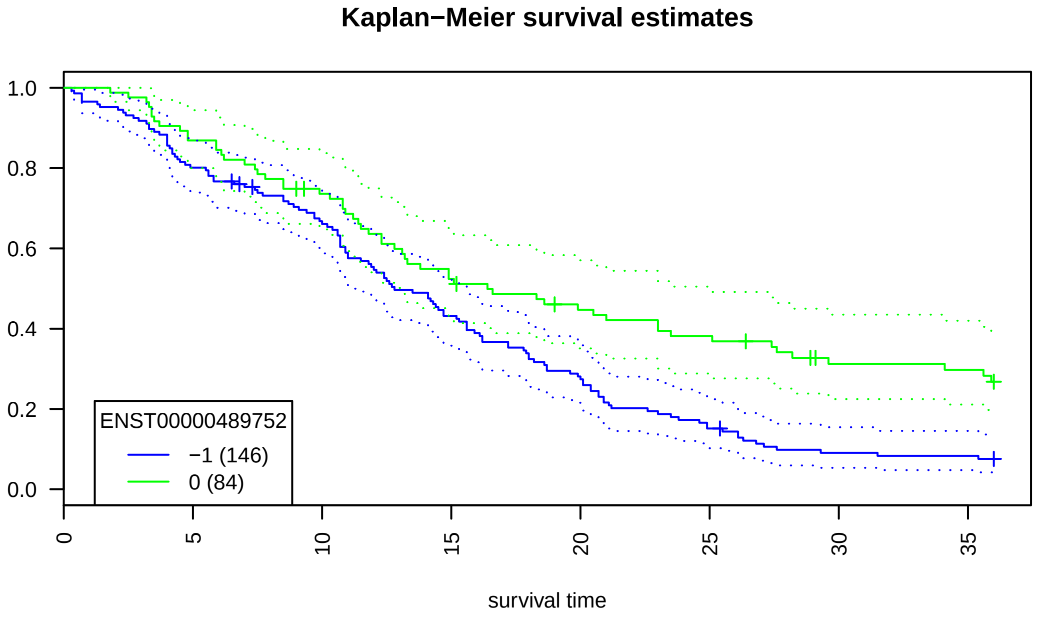

Figure 1077: Each curve represents the relative number of ENST00000489752 survived to the corresponding point of time. Dashed lines represent 0.95 confidence intervals for the curves with the corresponding color. Log-rank probability measure for the equality of these curves is 3.900548e-04. Additional annotations: *.GeneId*=ENSG00000206535, *.GeneName*=LNP1, *.GeneDesc*=leukemia NUP98 fusion partner 1 [Source:HGNC Symbol;Acc:28014].

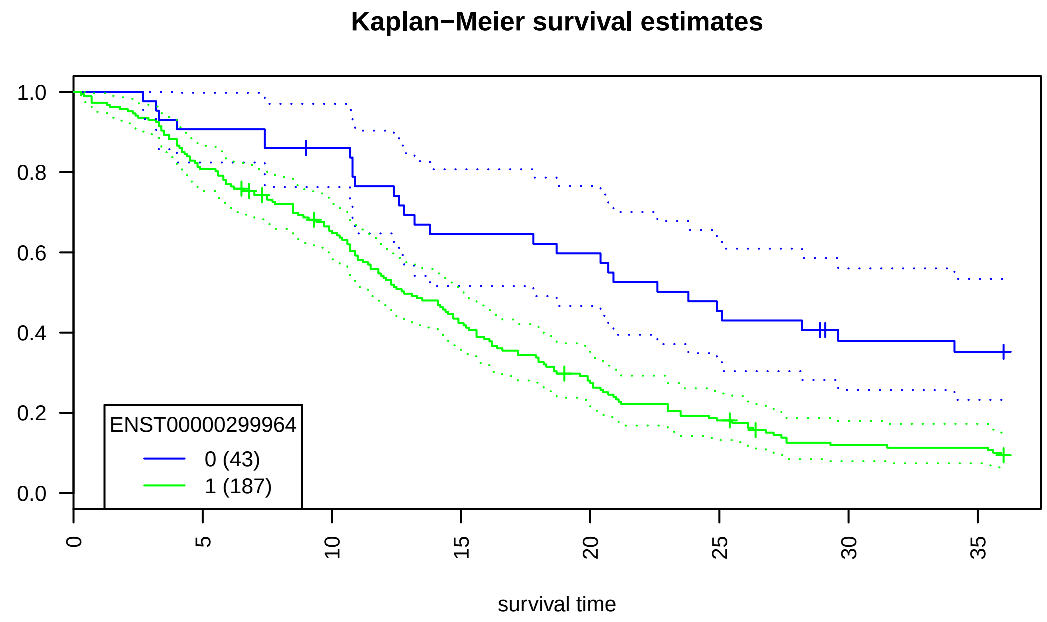

Figure 1078: Each curve represents the relative number of ENST00000299964 survived to the corresponding point of time. Dashed lines represent 0.95 confidence intervals for the curves with the corresponding color. Log-rank probability measure for the equality of these curves is 7.431457e-05. Additional annotations: *.GeneId*=ENSG00000166741, *.GeneName*=NNMT, *.GeneDesc*=nicotinamide N-methyltransferase [Source:HGNC Symbol;Acc:7861].

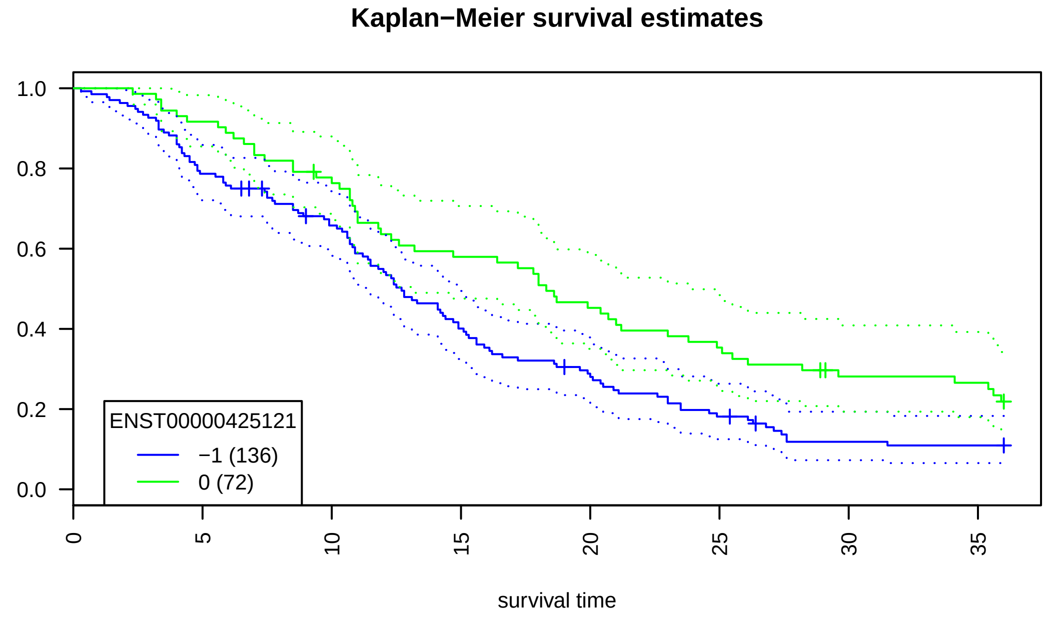

Figure 1079: Each curve represents the relative number of ENST00000425121 survived to the corresponding point of time. Dashed lines represent 0.95 confidence intervals for the curves with the corresponding color. Total of 22 observations deleted due to missingness. Log-rank probability measure for the equality of these curves is 5.814547e-03. Additional annotations: *.GeneId*=ENSG00000196642, *.GeneName*=C9orf86, *.GeneDesc*=Putative GTP-binding protein Parf (Partner of ARF) [Source:UniProtKB/Swiss-Prot;Acc:Q3YEC7].

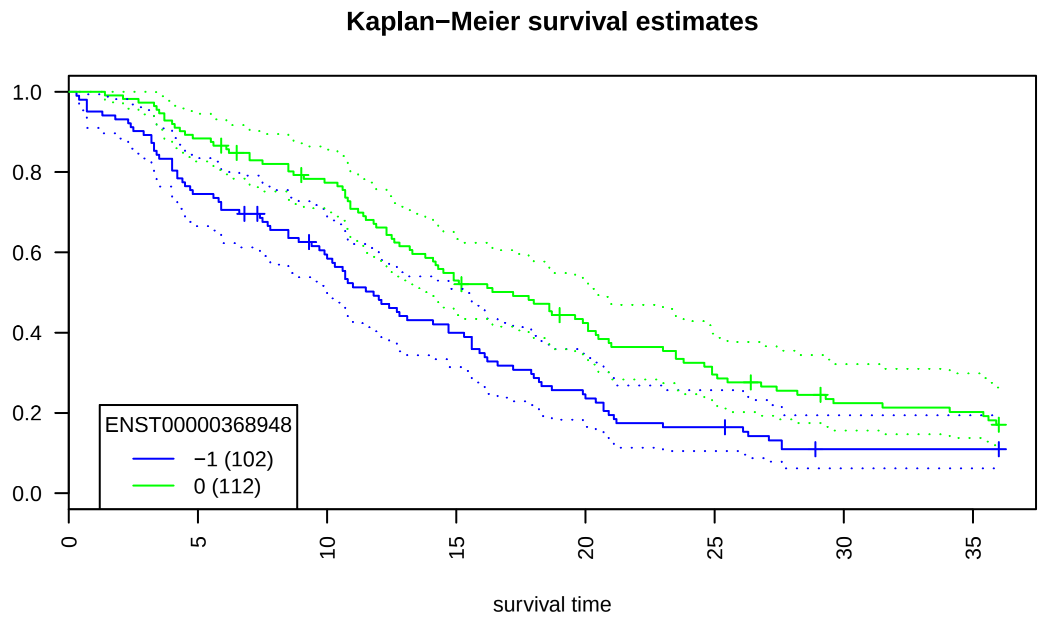

Figure 1080: Each curve represents the relative number of ENST00000368948 survived to the corresponding point of time. Dashed lines represent 0.95 confidence intervals for the curves with the corresponding color. Total of 16 observations deleted due to missingness. Log-rank probability measure for the equality of these curves is 4.18366e-03. Additional annotations: *.GeneId*=ENSG00000155085, *.GeneName*=AKD1, *.GeneDesc*=adenylate kinase domain containing 1 [Source:HGNC Symbol;Acc:33814].

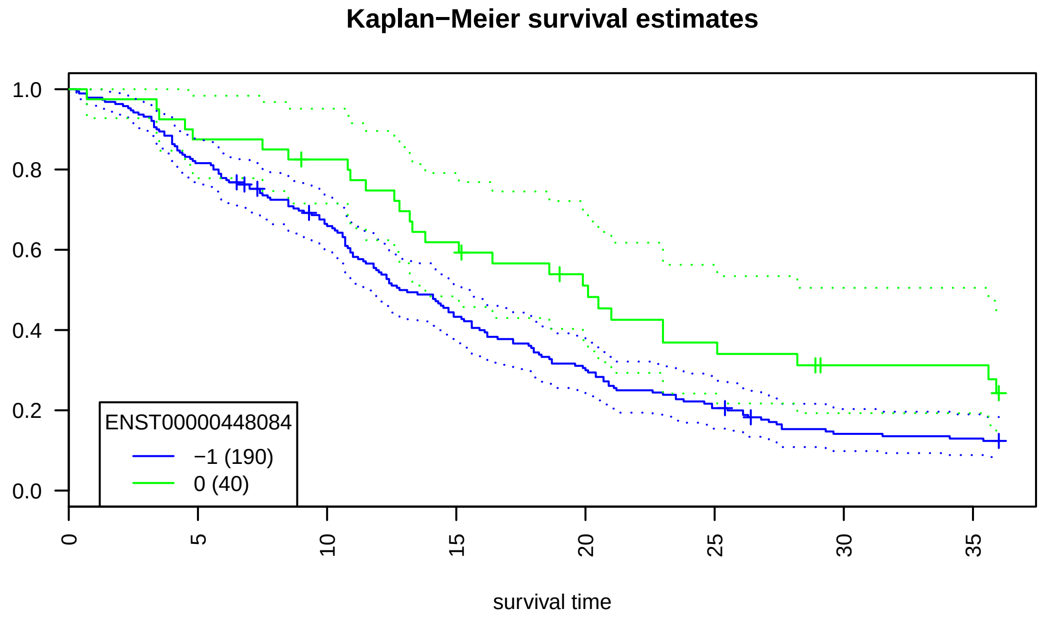

Figure 1081: Each curve represents the relative number of ENST00000448084 survived to the corresponding point of time. Dashed lines represent 0.95 confidence intervals for the curves with the corresponding color. Log-rank probability measure for the equality of these curves is 1.681903e-02. Additional annotations: *.GeneId*=ENSG00000155085, *.GeneName*=AKD1, *.GeneDesc*=adenylate kinase domain containing 1 [Source:HGNC Symbol;Acc:33814].

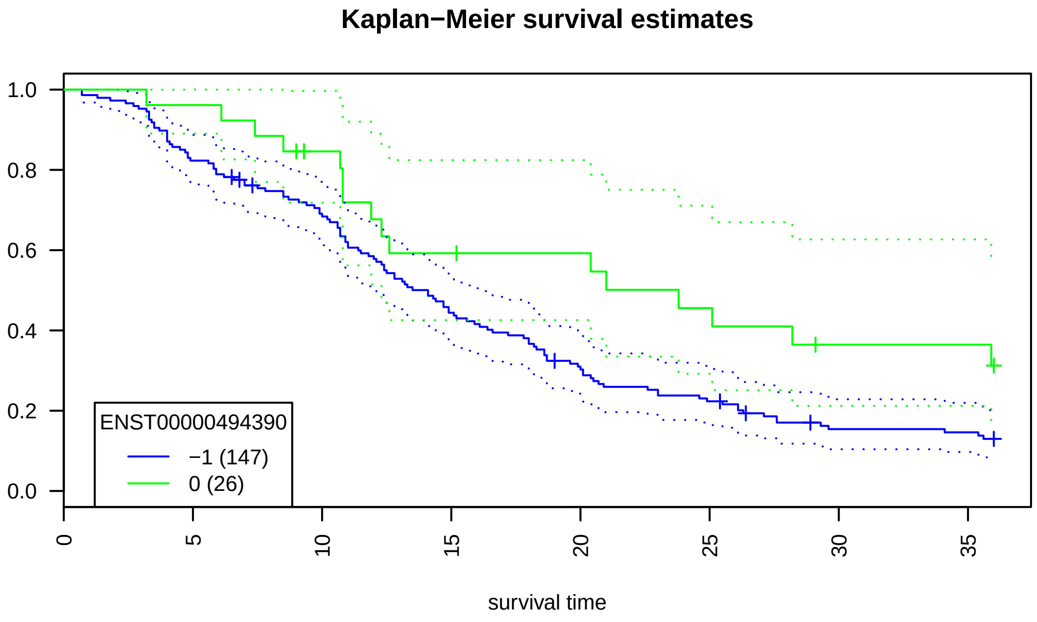

Figure 1082: Each curve represents the relative number of ENST00000494390 survived to the corresponding point of time. Dashed lines represent 0.95 confidence intervals for the curves with the corresponding color. Total of 57 observations deleted due to missingness. Log-rank probability measure for the equality of these curves is 2.558293e-02. Additional annotations: *.GeneId*=ENSG00000157680, *.GeneName*=DGKI, *.GeneDesc*=diacylglycerol kinase, iota [Source:HGNC Symbol;Acc:2855].

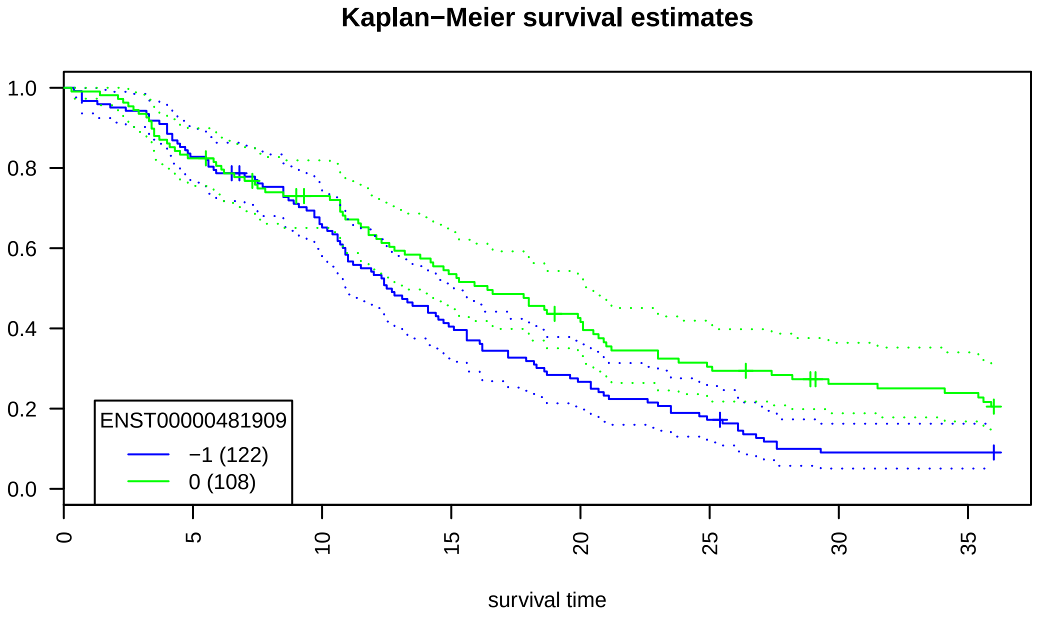

Figure 1083: Each curve represents the relative number of ENST00000481909 survived to the corresponding point of time. Dashed lines represent 0.95 confidence intervals for the curves with the corresponding color. Log-rank probability measure for the equality of these curves is 9.787729e-03. Additional annotations: *.GeneId*=ENSG00000119965, *.GeneName*=C10orf88, *.GeneDesc*=Uncharacterized protein C10orf88 [Source:UniProtKB/Swiss-Prot;Acc:Q9H8K7].

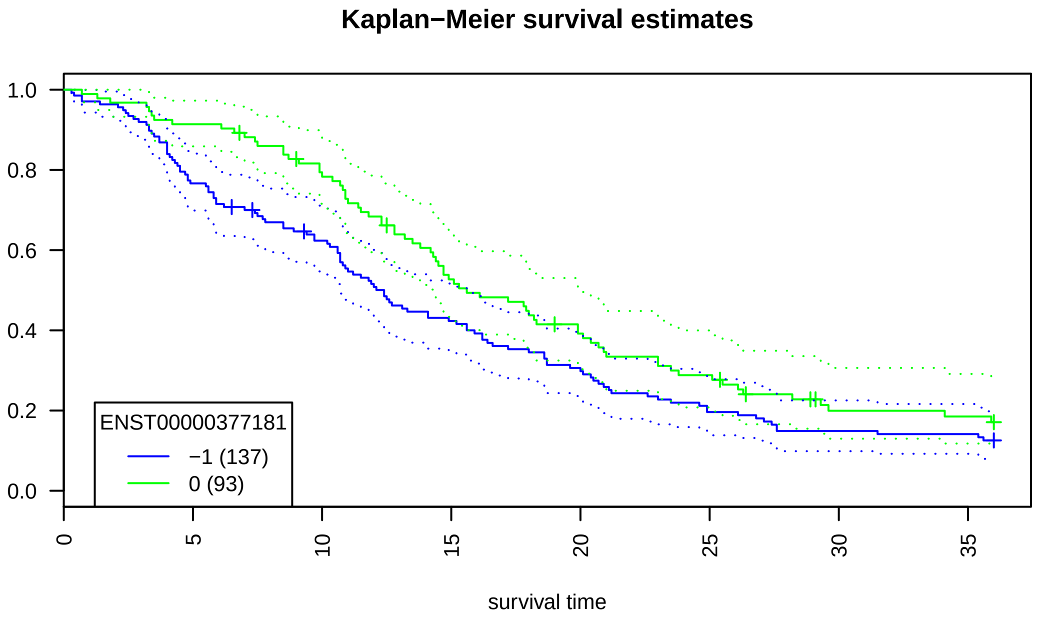

Figure 1084: Each curve represents the relative number of ENST00000377181 survived to the corresponding point of time. Dashed lines represent 0.95 confidence intervals for the curves with the corresponding color. Log-rank probability measure for the equality of these curves is 4.465175e-02. Additional annotations: *.GeneId*=ENSG00000115041, *.GeneName*=KCNIP3, *.GeneDesc*=Kv channel interacting protein 3, calsenilin [Source:HGNC Symbol;Acc:15523].

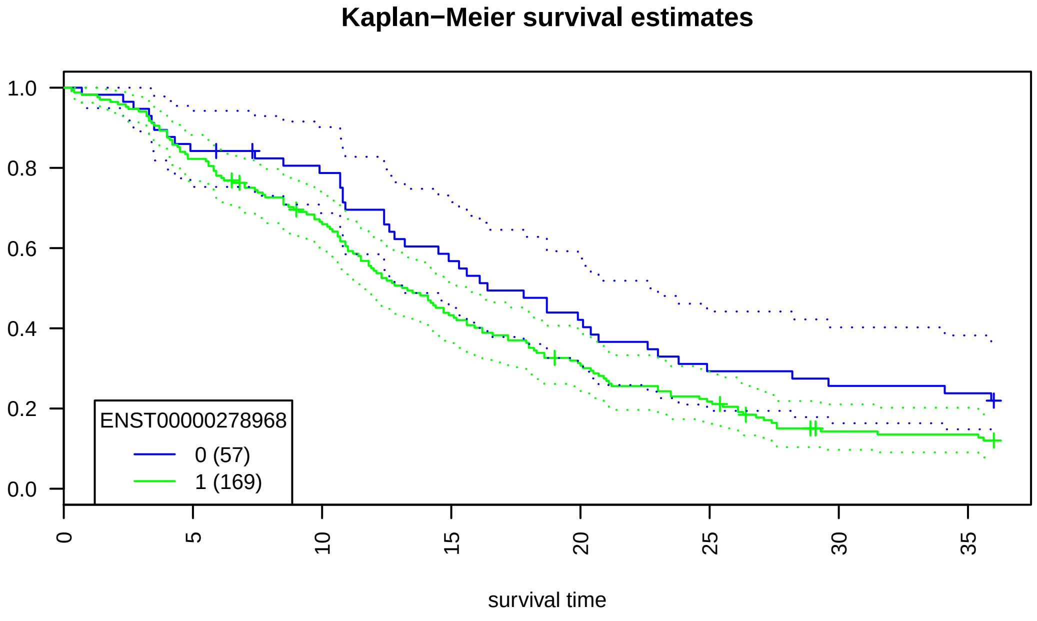

Figure 1085: Each curve represents the relative number of ENST00000278968 survived to the corresponding point of time. Dashed lines represent 0.95 confidence intervals for the curves with the corresponding color. Total of 4 observations deleted due to missingness. Log-rank probability measure for the equality of these curves is 4.976019e-02. Additional annotations: *.GeneId*=ENSG00000149591, *.GeneName*=TAGLN, *.GeneDesc*=transgelin [Source:HGNC Symbol;Acc:11553].

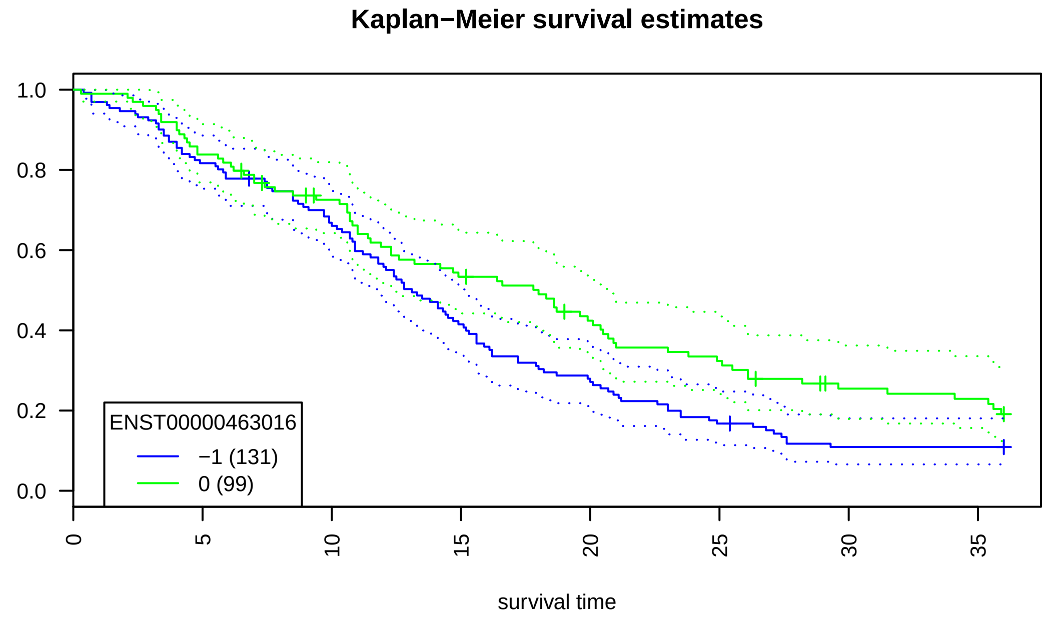

Figure 1086: Each curve represents the relative number of ENST00000463016 survived to the corresponding point of time. Dashed lines represent 0.95 confidence intervals for the curves with the corresponding color. Log-rank probability measure for the equality of these curves is 2.314512e-02. Additional annotations: .*GeneId*=ENSG00000155111, .*GeneName*=CDC2L6, .*GeneDesc*=cyclin-dependent kinase 19 [Source:HGNC Symbol;Acc:19338].

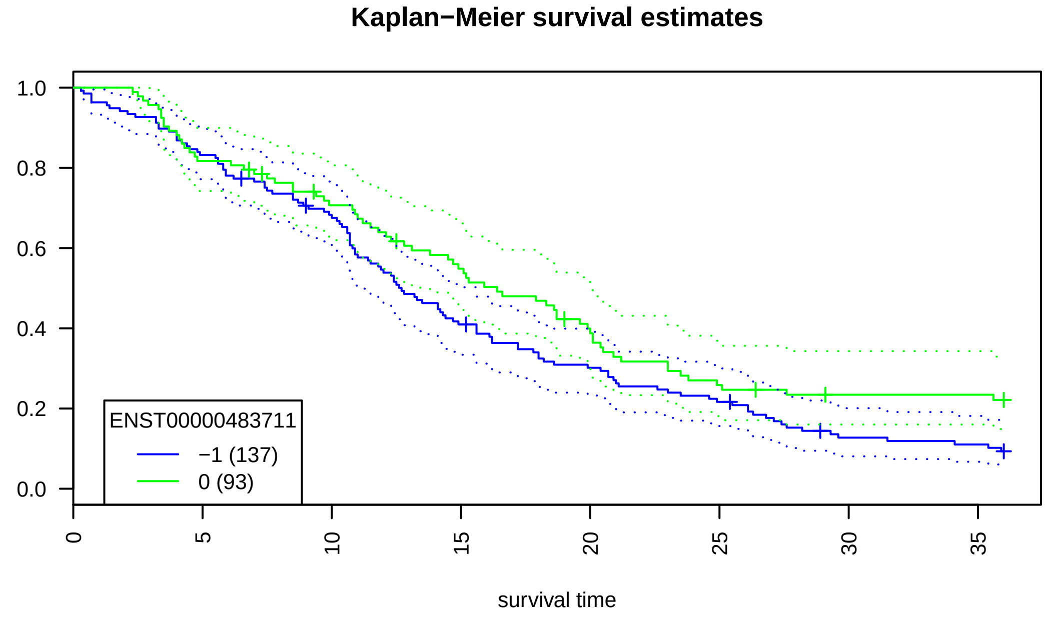

Figure 1087: Each curve represents the relative number of ENST00000483711 survived to the corresponding point of time. Dashed lines represent 0.95 confidence intervals for the curves with the corresponding color. Log-rank probability measure for the equality of these curves is 3.511262e-02. Additional annotations: .*GeneId*=ENSG00000065154, .*GeneName*=OAT, .*GeneDesc*=ornithine aminotransferase [Source:HGNC Symbol;Acc:8091].

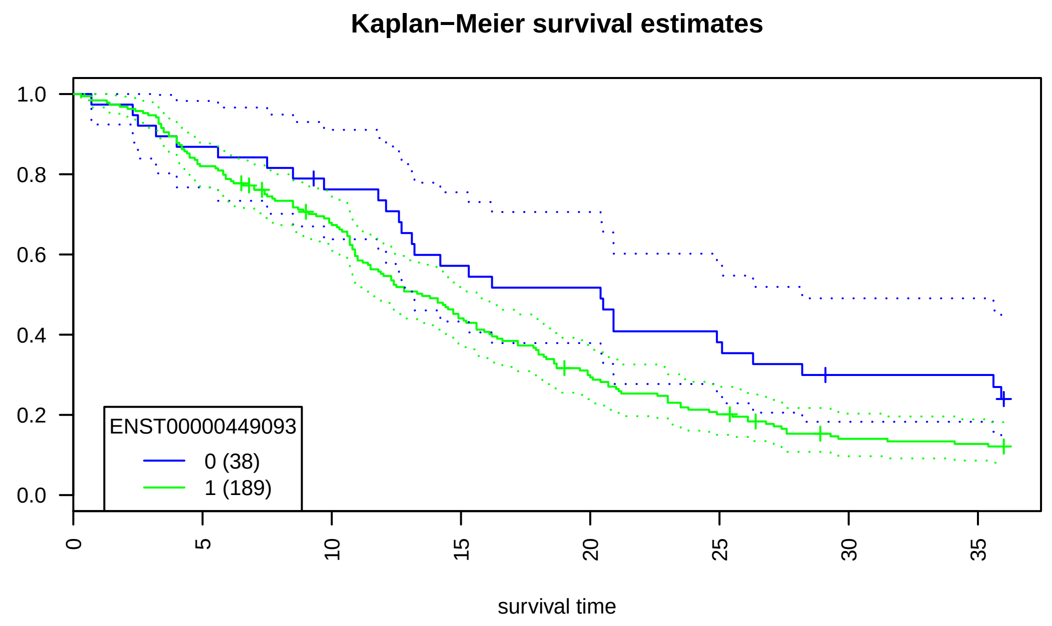

Figure 1088: Each curve represents the relative number of ENST00000449093 survived to the corresponding point of time. Dashed lines represent 0.95 confidence intervals for the curves with the corresponding color. Total of 3 observations deleted due to missingness. Log-rank probability measure for the equality of these curves is 3.568067e-02. Additional annotations: *.GeneId*=ENSG00000129654, *.GeneName*=FOXJ1, *.GeneDesc*=forkhead box J1 [Source:HGNC Symbol;Acc:3816].

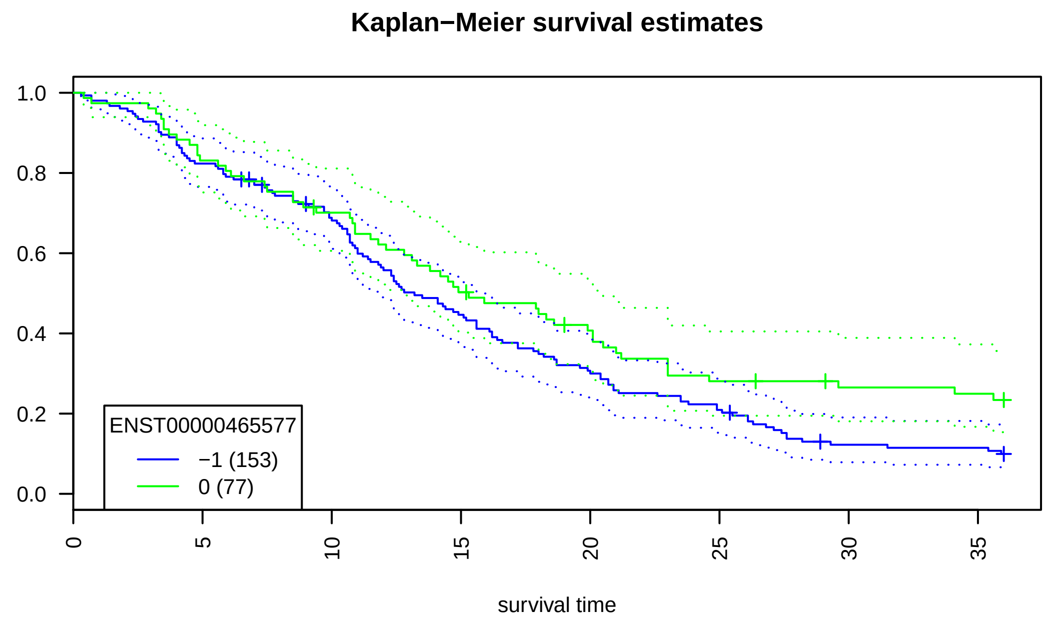

Figure 1089: Each curve represents the relative number of ENST00000465577 survived to the corresponding point of time. Dashed lines represent 0.95 confidence intervals for the curves with the corresponding color. Log-rank probability measure for the equality of these curves is 4.136171e-02. Additional annotations: *.GeneId*=ENSG00000188690, *.GeneName*=UROS, *.GeneDesc*=uroporphyrinogen III synthase [Source:HGNC Symbol;Acc:12592].

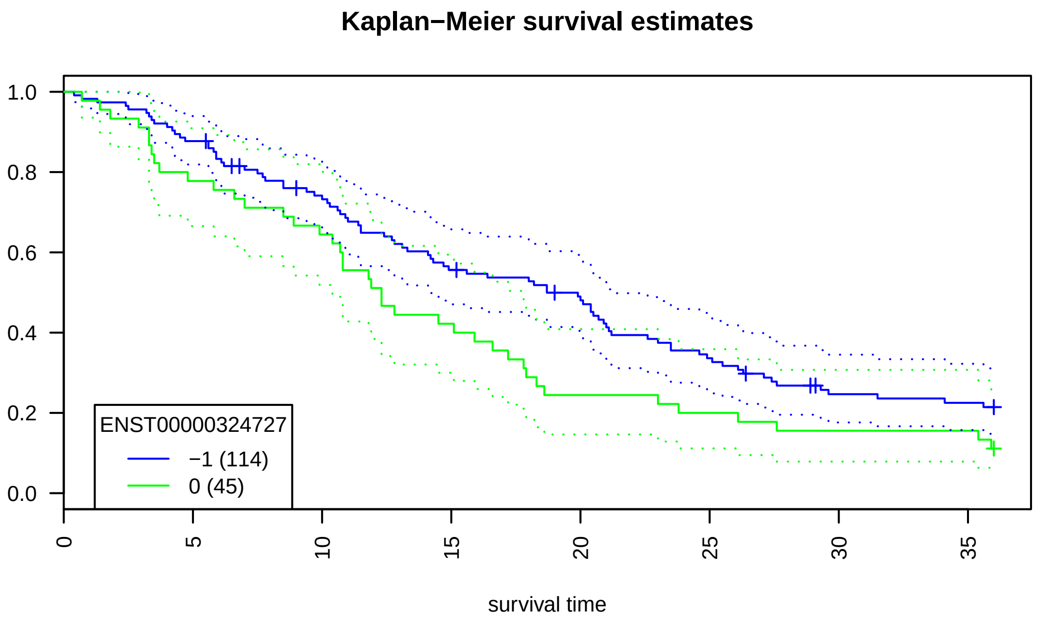

Figure 1090: Each curve represents the relative number of ENST00000324727 survived to the corresponding point of time. Dashed lines represent 0.95 confidence intervals for the curves with the corresponding color. Total of 71 observations deleted due to missingness. Log-rank probability measure for the equality of these curves is 3.176230e-02. Additional annotations: *.GeneId*=ENSG00000177098, *.GeneName*=SCN4B, *.GeneDesc*=sodium channel, voltage-gated, type IV, beta [Source:HGNC Symbol;Acc:10592].

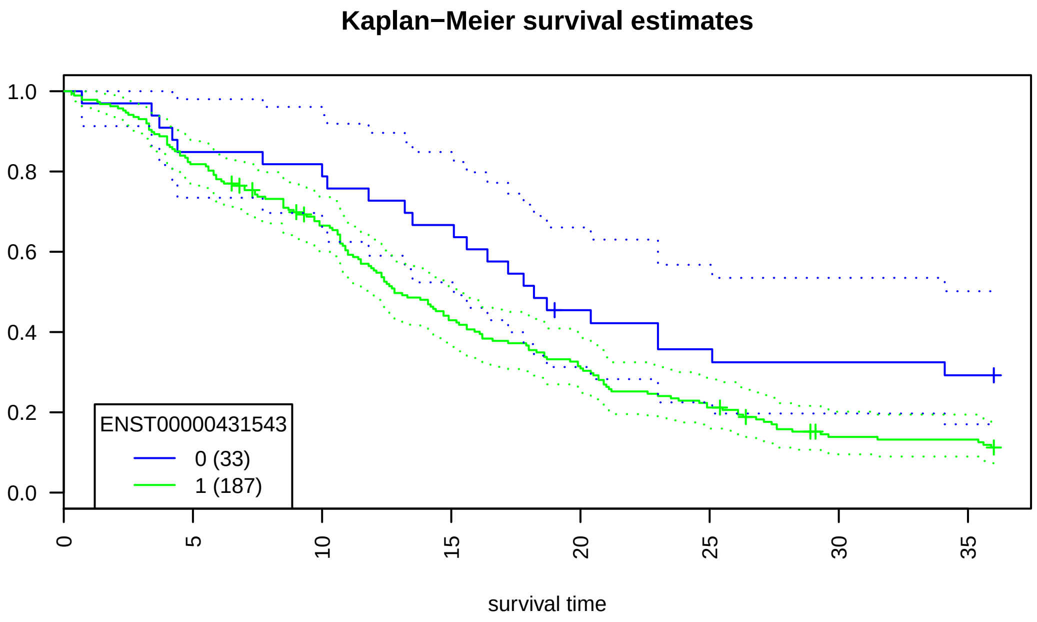

Figure 1091: Each curve represents the relative number of ENST00000431543 survived to the corresponding point of time. Dashed lines represent 0.95 confidence intervals for the curves with the corresponding color. Total of 10 observations deleted due to missingness. Log-rank probability measure for the equality of these curves is 1.945194e-02. Additional annotations: *.GeneId*=ENSG00000112769, *.GeneName*=LAMA4, *.GeneDesc*=laminin, alpha 4 [Source:HGNC Symbol;Acc:6484].

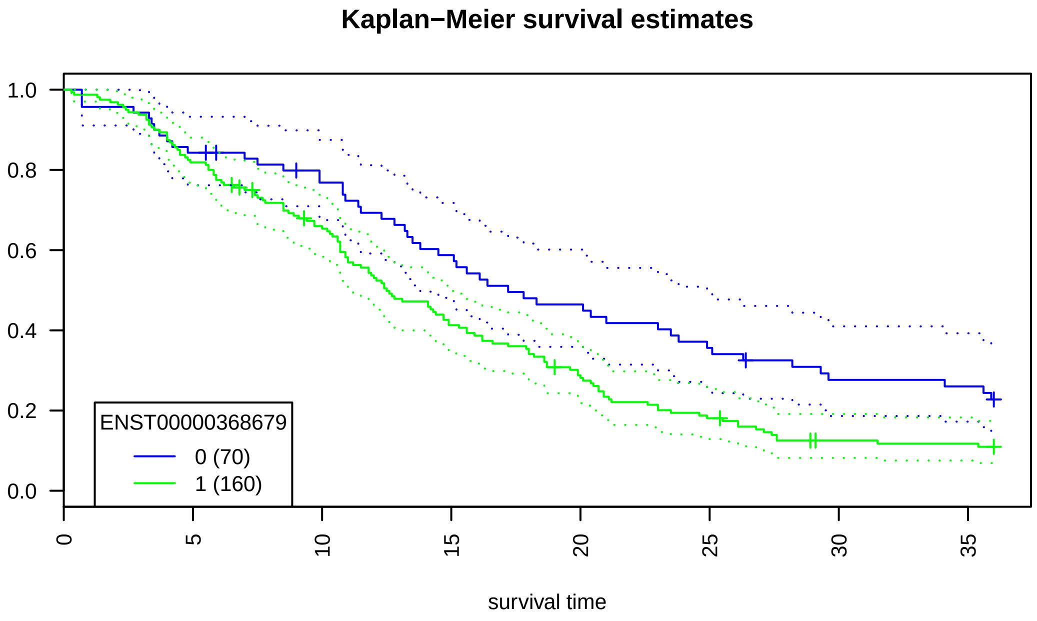

Figure 1092: Each curve represents the relative number of ENST00000368679 survived to the corresponding point of time. Dashed lines represent 0.95 confidence intervals for the curves with the corresponding color. Log-rank probability measure for the equality of these curves is 5.84444e-03. Additional annotations: *.GeneId*=ENSG00000148848, *.GeneName*=ADAM12, *.GeneDesc*=ADAM metallopeptidase domain 12 [Source:HGNC Symbol;Acc:190].

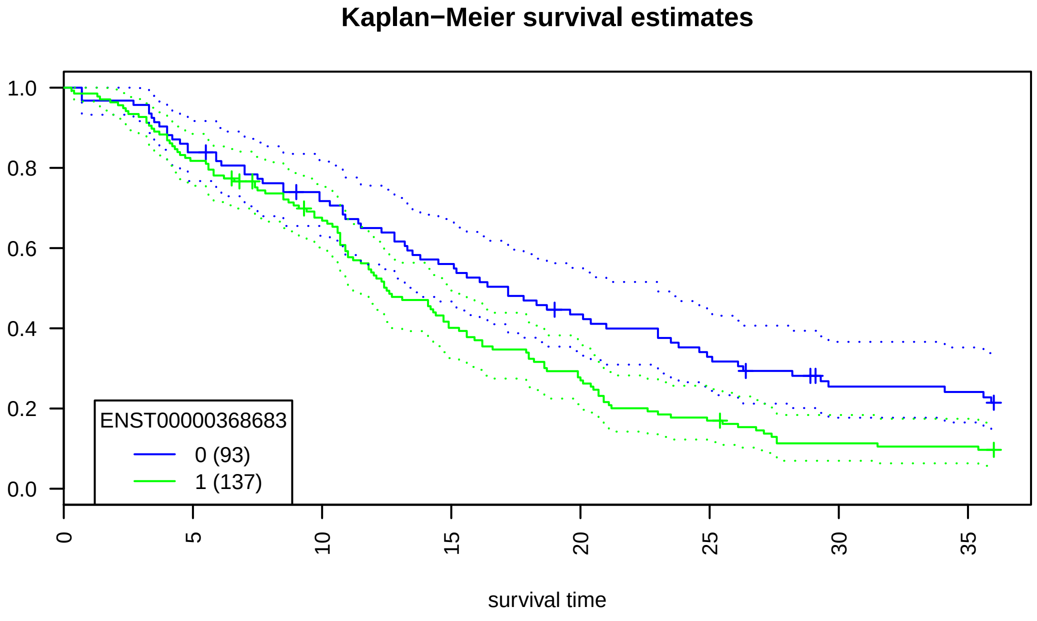

Figure 1093: Each curve represents the relative number of ENST00000368683 survived to the corresponding point of time. Dashed lines represent 0.95 confidence intervals for the curves with the corresponding color. Log-rank probability measure for the equality of these curves is 5.936725e-03. Additional annotations: *.GeneId*=ENSG00000148848, *.GeneName*=ADAM12, *.GeneDesc*=ADAM metallopeptidase domain 12 [Source:HGNC Symbol;Acc:190].

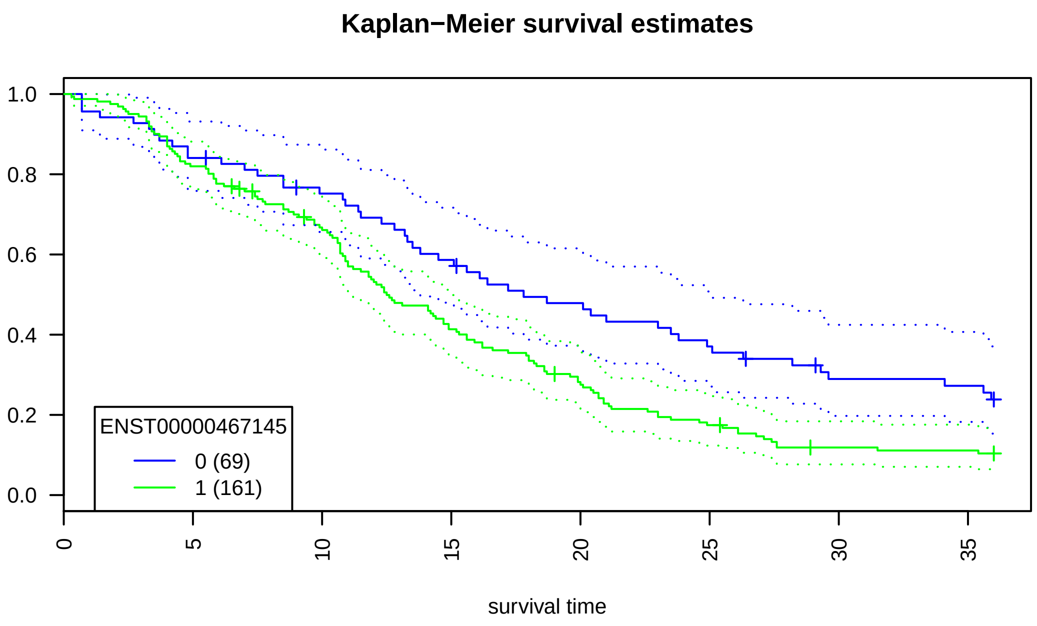

Figure 1094: Each curve represents the relative number of ENST00000467145 survived to the corresponding point of time. Dashed lines represent 0.95 confidence intervals for the curves with the corresponding color. Log-rank probability measure for the equality of these curves is 2.863905e-03. Additional annotations: *.GeneId*=ENSG00000148848, *.GeneName*=ADAM12, *.GeneDesc*=ADAM metallopeptidase domain 12 [Source:HGNC Symbol;Acc:190].

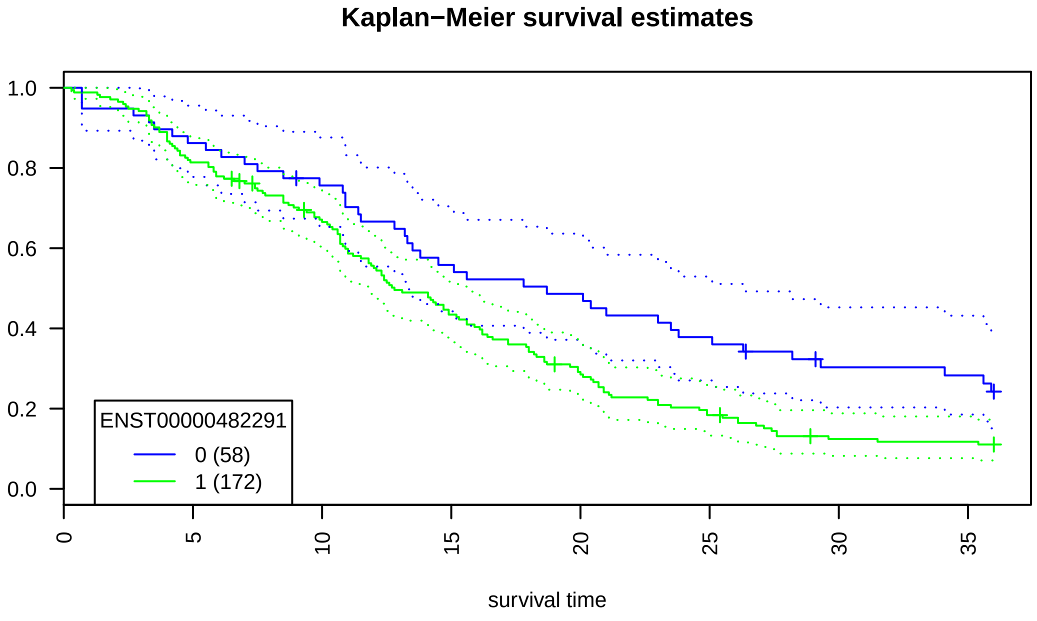

Figure 1095: Each curve represents the relative number of ENST00000482291 survived to the corresponding point of time. Dashed lines represent 0.95 confidence intervals for the curves with the corresponding color. Log-rank probability measure for the equality of these curves is 8.504107e-03. Additional annotations: *.GeneId*=ENSG00000148848, *.GeneName*=ADAM12, *.GeneDesc*=ADAM metallopeptidase domain 12 [Source:HGNC Symbol;Acc:190].

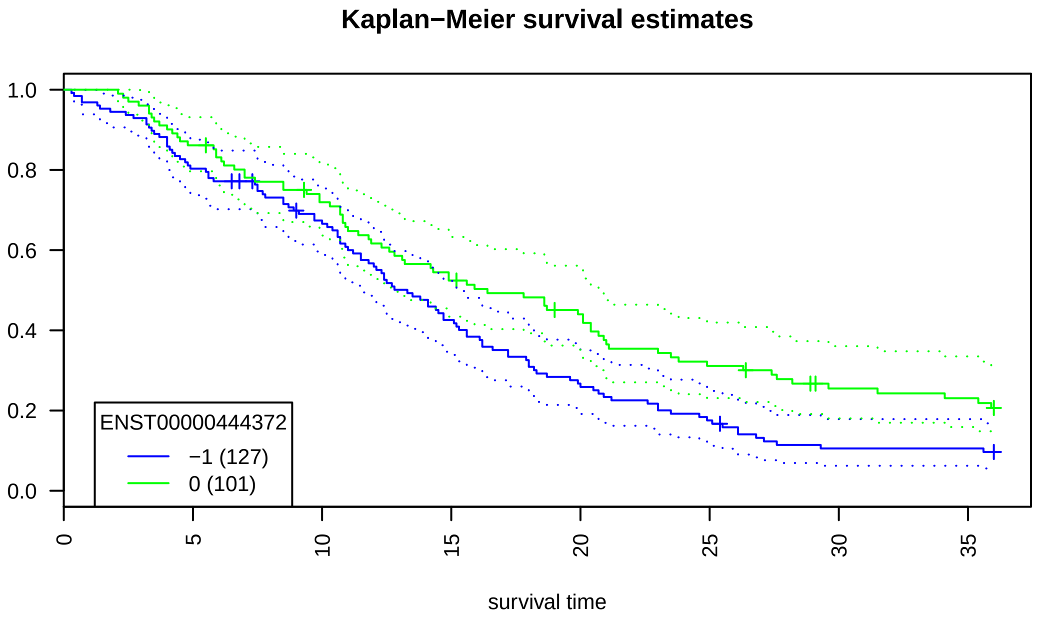

Figure 1096: Each curve represents the relative number of ENST00000444372 survived to the corresponding point of time. Dashed lines represent 0.95 confidence intervals for the curves with the corresponding color. Total of 2 observations deleted due to missingness. Log-rank probability measure for the equality of these curves is 9.9623e-03. Additional annotations: *.GeneId*=ENSG00000187147, *.GeneName*=RNF220, *.GeneDesc*=ring finger protein 220 [Source:HGNC Symbol;Acc:25552].

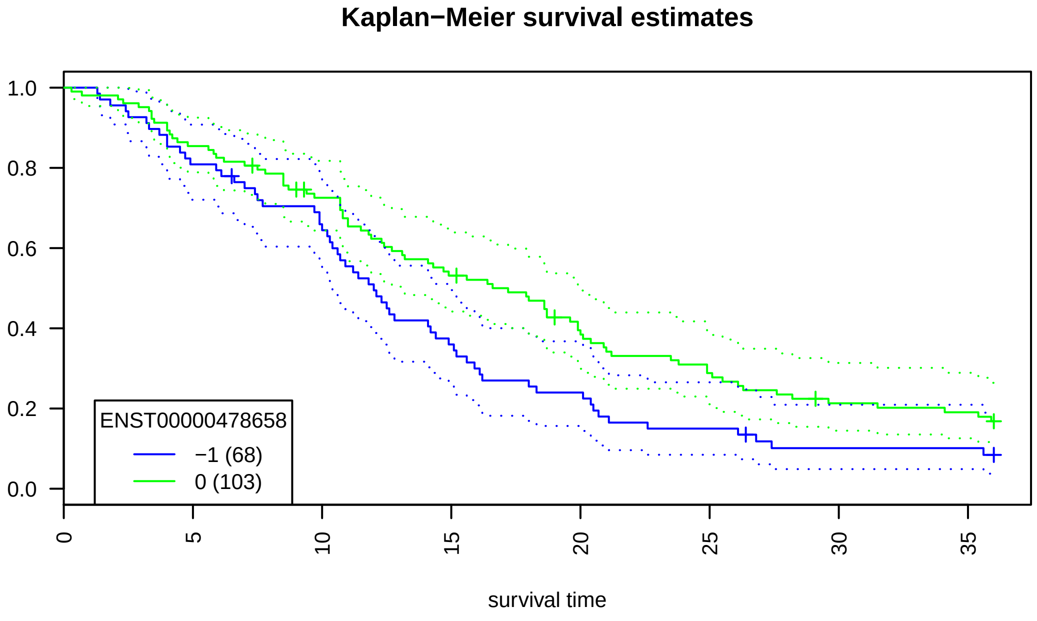

Figure 1097: Each curve represents the relative number of ENST00000478658 survived to the corresponding point of time. Dashed lines represent 0.95 confidence intervals for the curves with the corresponding color. Total of 59 observations deleted due to missingness. Log-rank probability measure for the equality of these curves is 1.536126e-02. Additional annotations: *.GeneId*=ENSG00000176542, *.GeneName*=KIAA2018, *.GeneDesc*=KIAA2018 [Source:HGNC Symbol;Acc:30494].

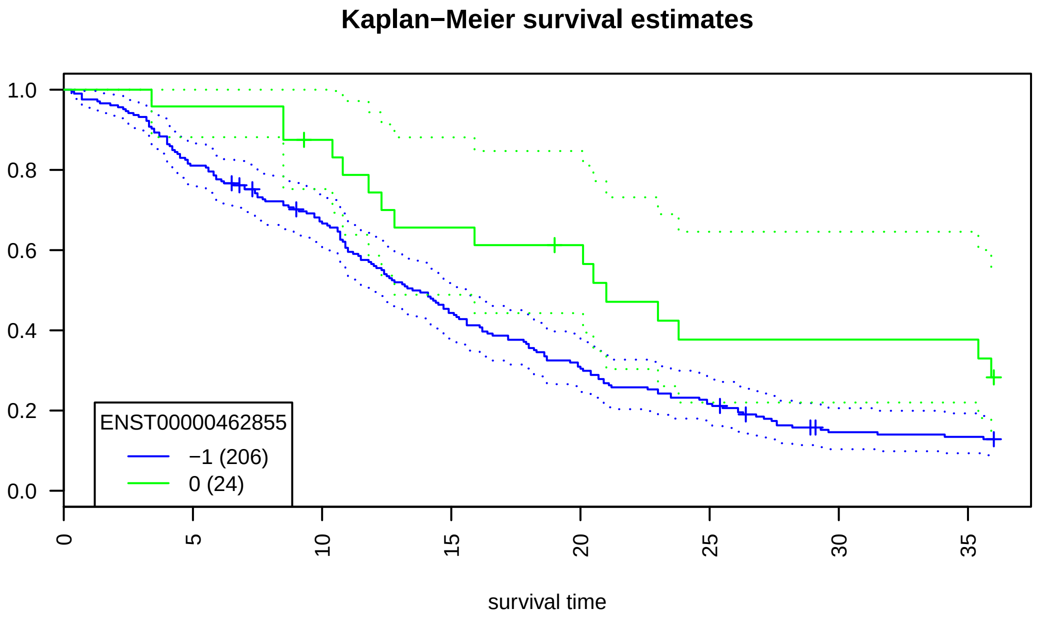

Figure 1098: Each curve represents the relative number of ENST00000462855 survived to the corresponding point of time. Dashed lines represent 0.95 confidence intervals for the curves with the corresponding color. Log-rank probability measure for the equality of these curves is 2.20062e-02. Additional annotations: *.GeneId*=ENSG00000114573, *.GeneName*=ATP6V1A, *.GeneDesc*=ATPase, H+ transporting, lysosomal 70kDa, V1 subunit A [Source:HGNC Symbol;Acc:851].

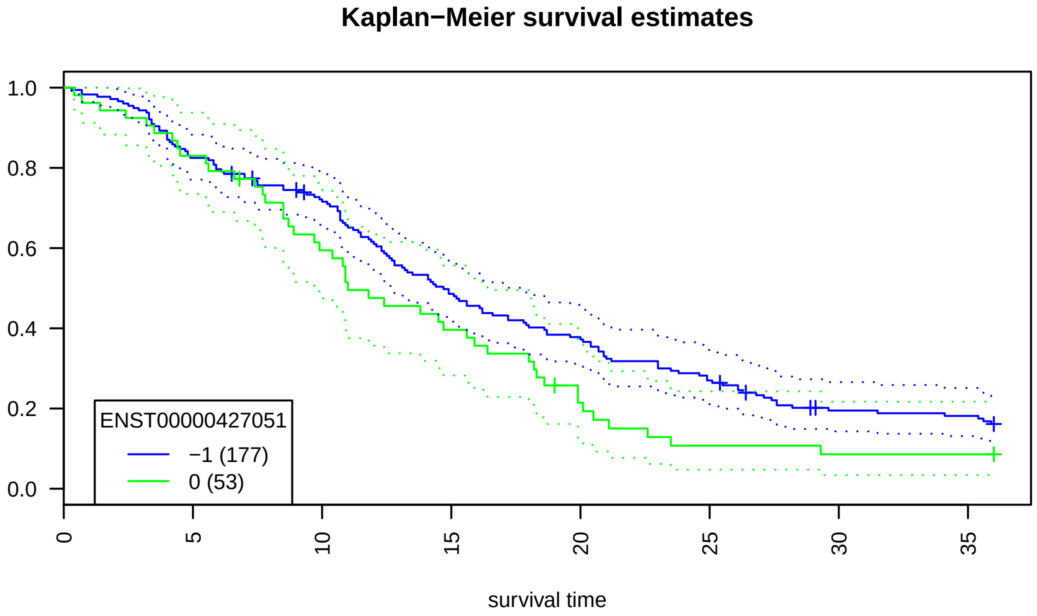

Figure 1099: Each curve represents the relative number of ENST00000427051 survived to the corresponding point of time. Dashed lines represent 0.95 confidence intervals for the curves with the corresponding color. Log-rank probability measure for the equality of these curves is 4.397904e-02. Additional annotations: *.GeneId*=ENSG00000171798, *.GeneName*=KNDC1, *.GeneDesc*=kinase non-catalytic C-lobe domain (KIND) containing 1 [Source:HGNC Symbol;Acc:29374].

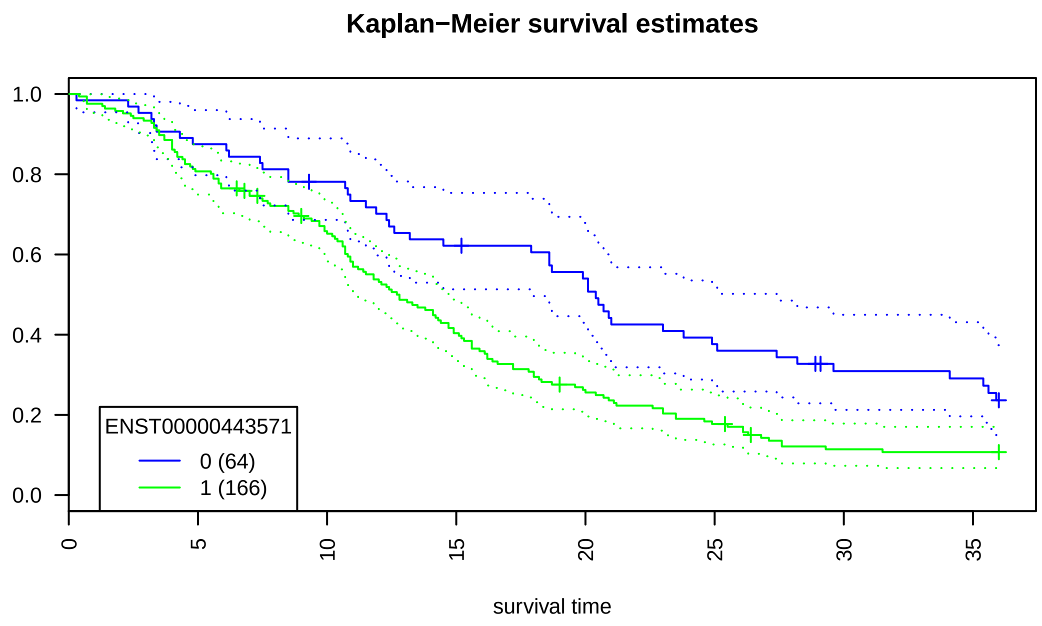

Figure 1100: Each curve represents the relative number of ENST00000443571 survived to the corresponding point of time. Dashed lines represent 0.95 confidence intervals for the curves with the corresponding color. Log-rank probability measure for the equality of these curves is 9.210141e-04. Additional annotations: *.GeneId*=ENSG00000197448, *.GeneName*=GSTK1, *.GeneDesc*=glutathione S-transferase kappa 1 [Source:HGNC Symbol;Acc:16906].

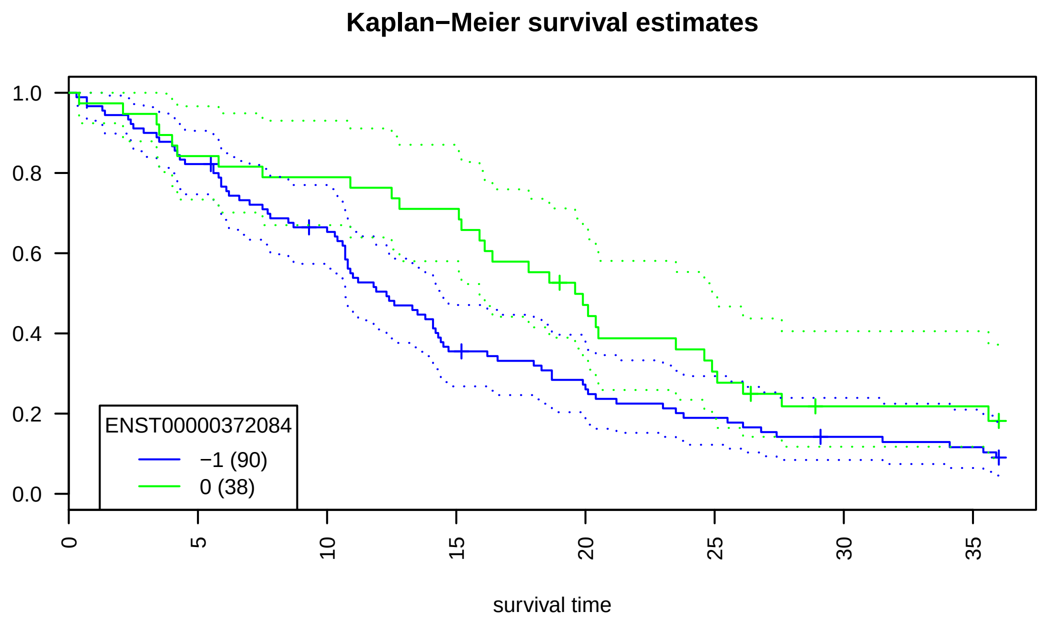

Figure 1101: Each curve represents the relative number of ENST00000372084 survived to the corresponding point of time. Dashed lines represent 0.95 confidence intervals for the curves with the corresponding color. Total of 102 observations deleted due to missingness. Log-rank probability measure for the equality of these curves is 3.098361e-02. Additional annotations: *.GeneId*=ENSG00000070759, *.GeneName*=TESK2, *.GeneDesc*=testis-specific kinase 2 [Source:HGNC Symbol;Acc:11732].

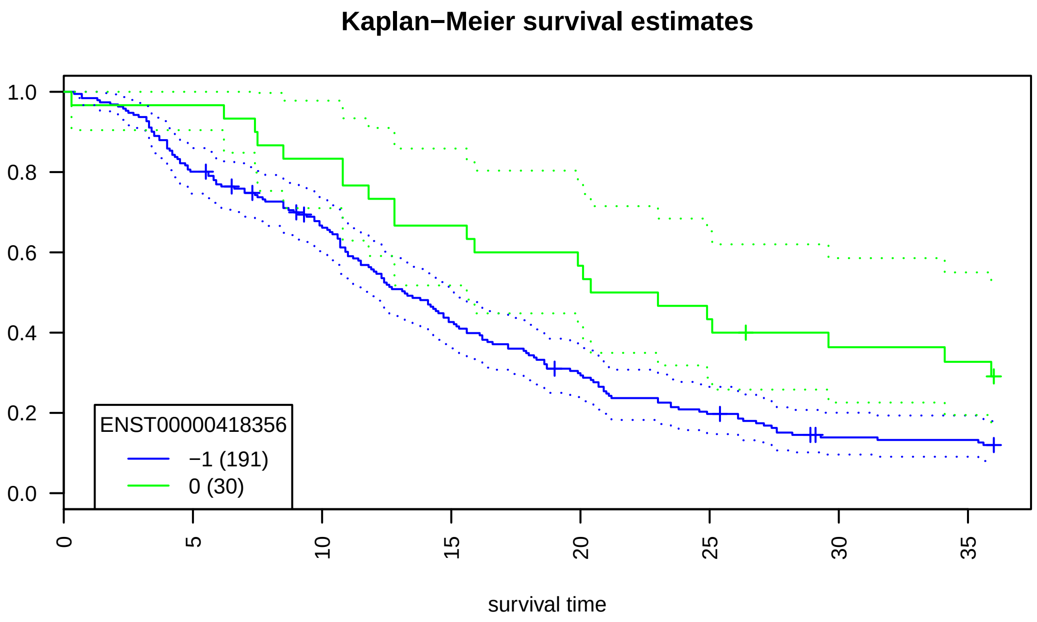

Figure 1102: Each curve represents the relative number of ENST00000418356 survived to the corresponding point of time. Dashed lines represent 0.95 confidence intervals for the curves with the corresponding color. Total of 9 observations deleted due to missingness. Log-rank probability measure for the equality of these curves is 6.14581e-03. Additional annotations: *.GeneId*=ENSG00000130649, *.GeneName*=CYP2E1, *.GeneDesc*=cytochrome P450, family 2, subfamily E, polypeptide 1 [Source:HGNC Symbol;Acc:2631].

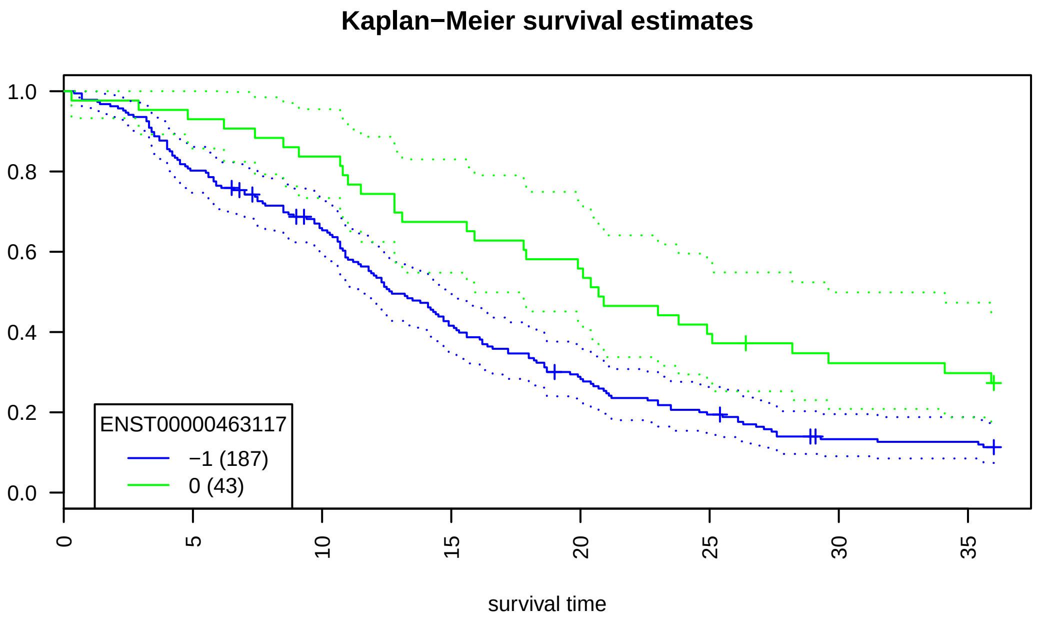

Figure 1103: Each curve represents the relative number of ENST00000463117 survived to the corresponding point of time. Dashed lines represent 0.95 confidence intervals for the curves with the corresponding color. Log-rank probability measure for the equality of these curves is 1.689632e-03. Additional annotations: *.GeneId*=ENSG00000130649, *.GeneName*=CYP2E1, *.GeneDesc*=cytochrome P450, family 2, subfamily E, polypeptide 1 [Source:HGNC Symbol;Acc:2631].

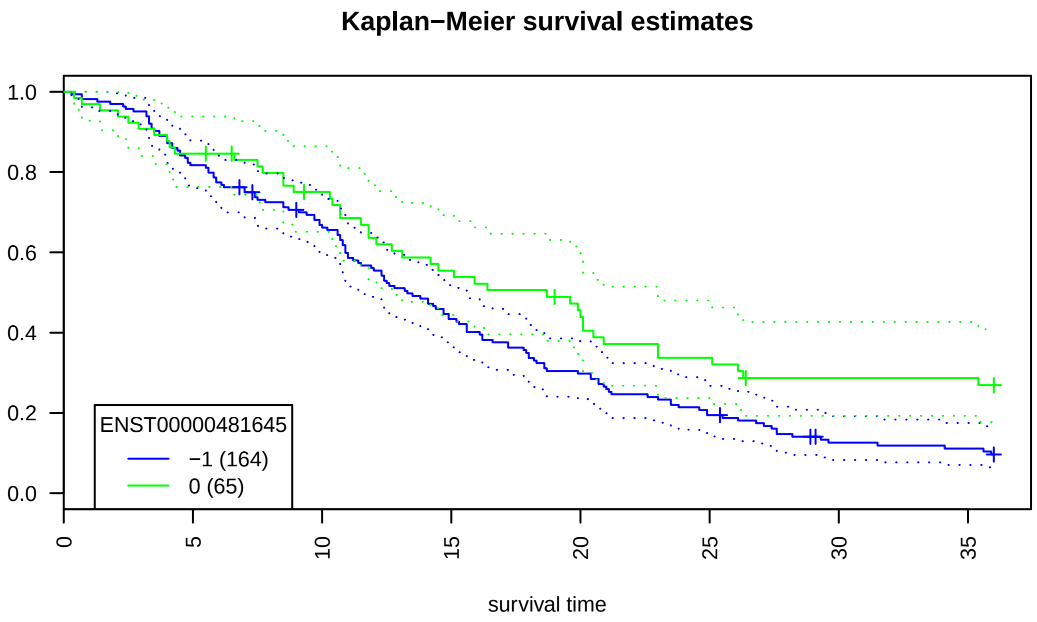

Figure 1104: Each curve represents the relative number of ENST00000481645 survived to the corresponding point of time. Dashed lines represent 0.95 confidence intervals for the curves with the corresponding color. Total of 1 observation deleted due to missingness. Log-rank probability measure for the equality of these curves is 1.100716e-02. Additional annotations: *.GeneId*=ENSG00000196511, *.GeneName*=TPK1, *.GeneDesc*=thiamin pyrophosphokinase 1 [Source:HGNC Symbol;Acc:17358].

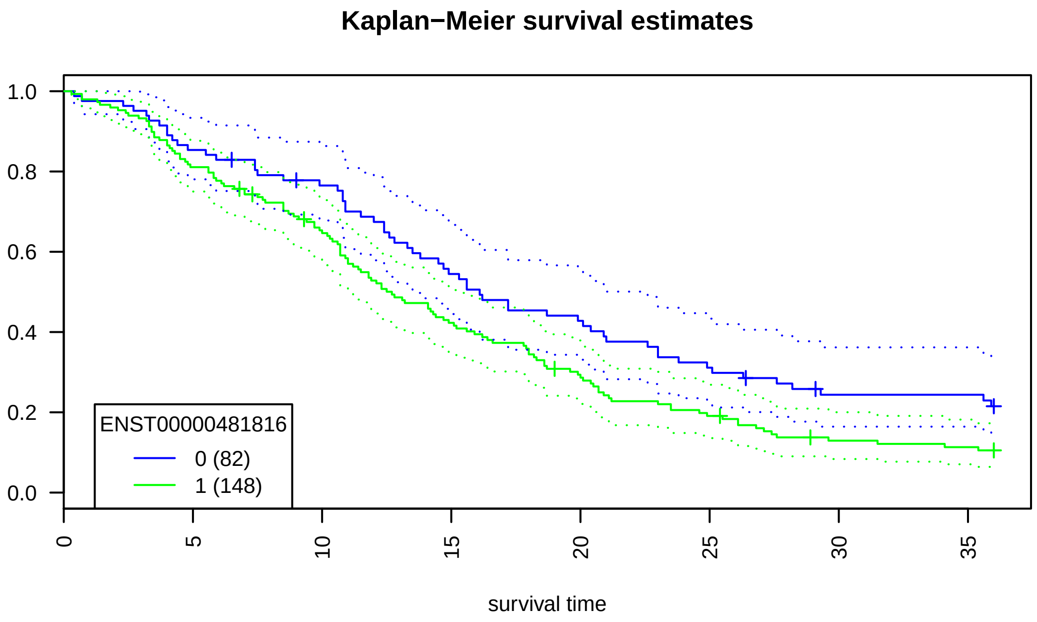

Figure 1105: Each curve represents the relative number of ENST00000481816 survived to the corresponding point of time. Dashed lines represent 0.95 confidence intervals for the curves with the corresponding color. Log-rank probability measure for the equality of these curves is 1.496282e-02. Additional annotations: *.GeneId*=ENSG00000144843, *.GeneName*=ADPRH, *.GeneDesc*=ADP-ribosylarginine hydrolase [Source:HGNC Symbol;Acc:269].

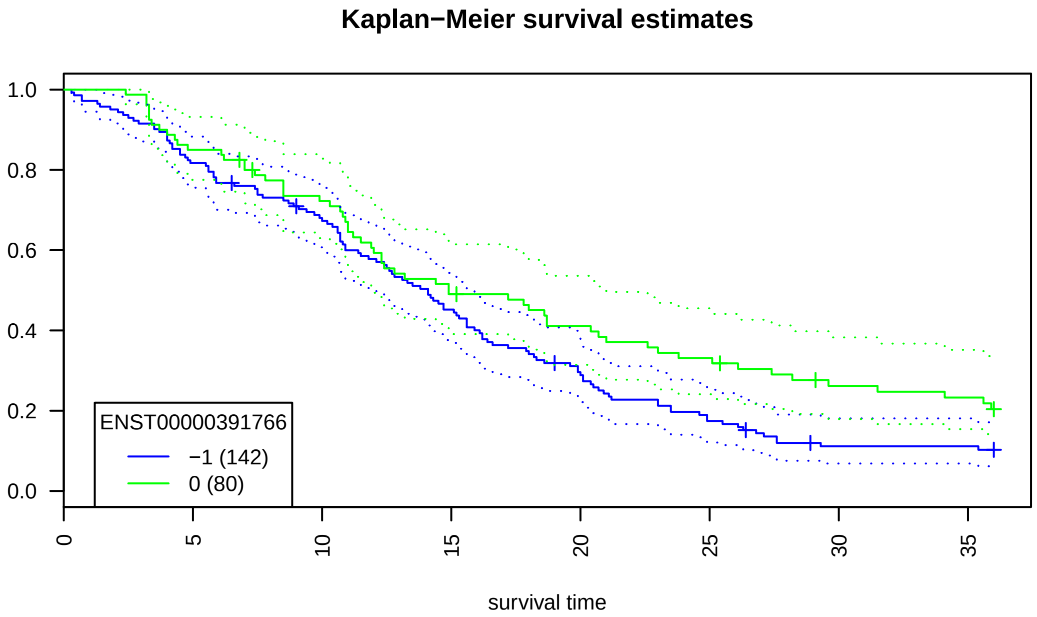

Figure 1106: Each curve represents the relative number of ENST00000391766 survived to the corresponding point of time. Dashed lines represent 0.95 confidence intervals for the curves with the corresponding color. Total of 8 observations deleted due to missingness. Log-rank probability measure for the equality of these curves is 3.447246e-02. Additional annotations: *.GeneId*=ENSG00000105605, *.GeneName*=CACNG7, *.GeneDesc*=calcium channel, voltage-dependent, gamma subunit 7 [Source:HGNC Symbol;Acc:13626].

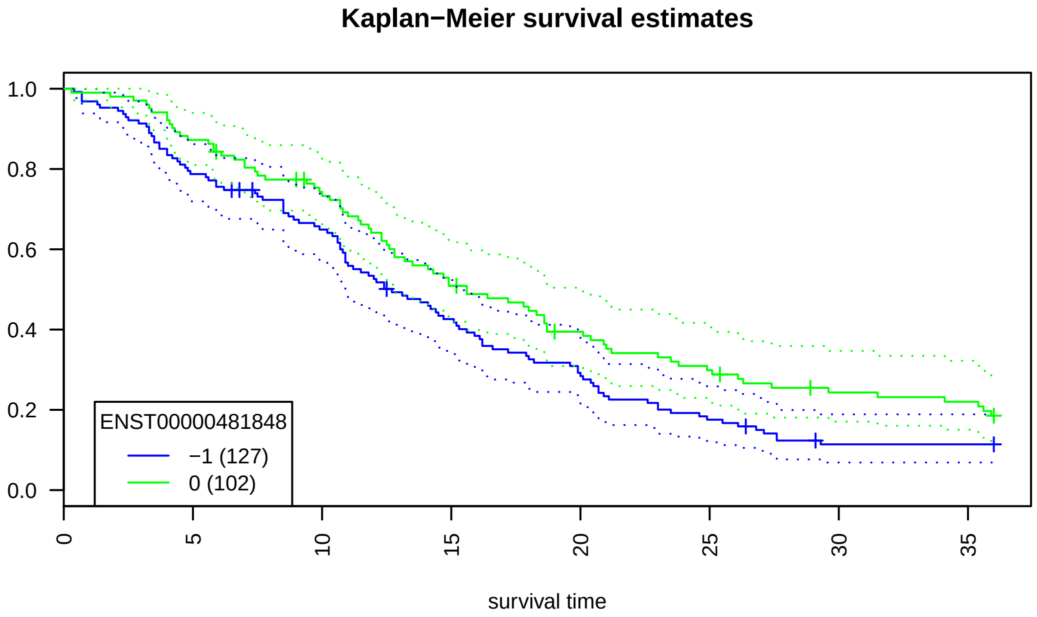

Figure 1107: Each curve represents the relative number of ENST00000481848 survived to the corresponding point of time. Dashed lines represent 0.95 confidence intervals for the curves with the corresponding color. Total of 1 observation deleted due to missingness. Log-rank probability measure for the equality of these curves is 2.984948e-02. Additional annotations: *.GeneId*=ENSG00000189367, *.GeneName*=C6orf174, *.GeneDesc*=KIAA0408 [Source:HGNC Symbol;Acc:21636].

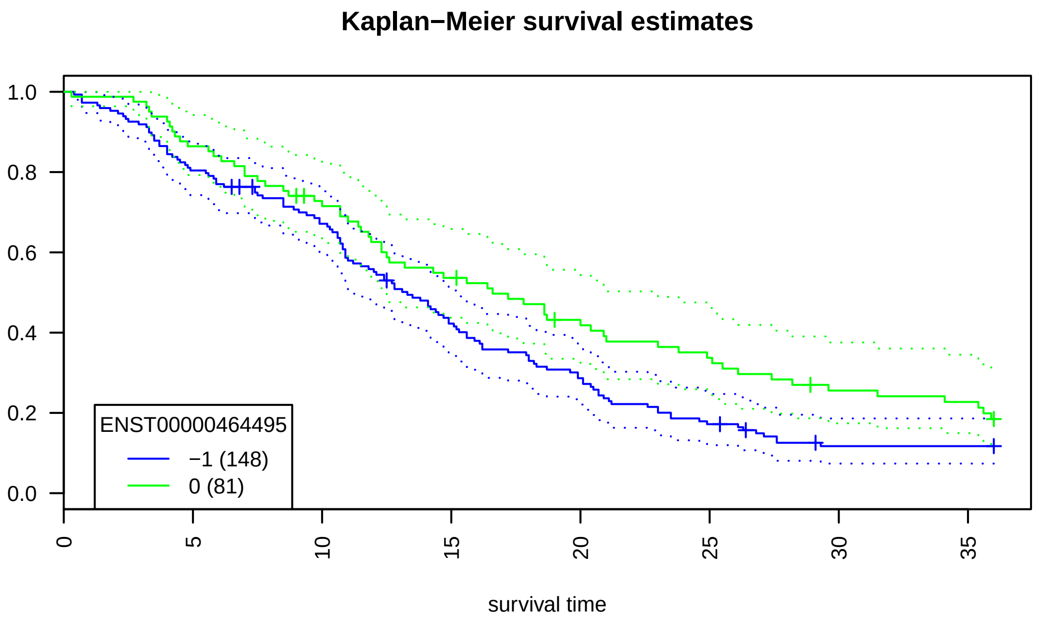

Figure 1108: Each curve represents the relative number of ENST00000464495 survived to the corresponding point of time. Dashed lines represent 0.95 confidence intervals for the curves with the corresponding color. Total of 1 observation deleted due to missingness. Log-rank probability measure for the equality of these curves is 3.200373e-02. Additional annotations: *.GeneId*=ENSG00000189367, *.GeneName*=C6orf174, *.GeneDesc*=KIAA0408 [Source:HGNC Symbol;Acc:21636].

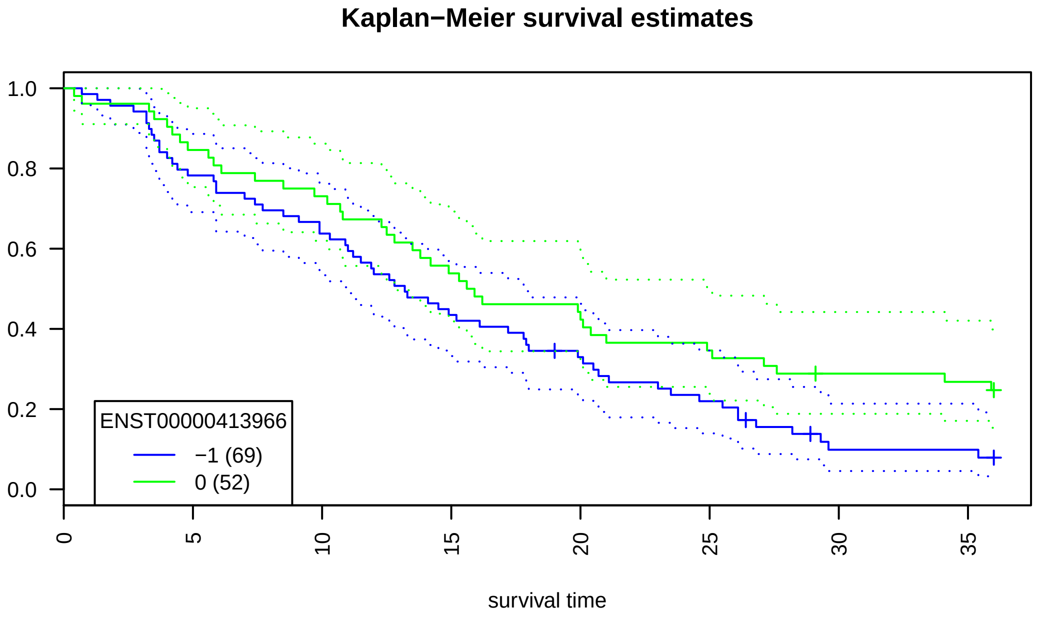

Figure 1109: Each curve represents the relative number of ENST00000413966 survived to the corresponding point of time. Dashed lines represent 0.95 confidence intervals for the curves with the corresponding color. Total of 109 observations deleted due to missingness. Log-rank probability measure for the equality of these curves is 3.711683e-02. Additional annotations: *.GeneId*=ENSG00000155660, *.GeneName*=PDIA4, *.GeneDesc*=protein disulfide isomerase family A, member 4 [Source:HGNC Symbol;Acc:30167].

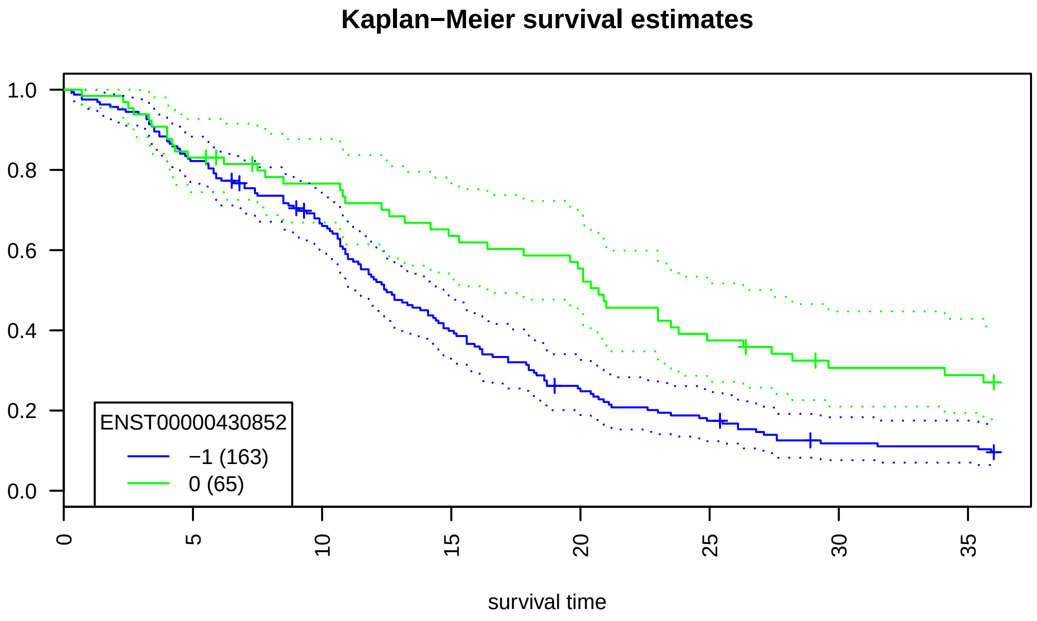

Figure 1110: Each curve represents the relative number of ENST00000430852 survived to the corresponding point of time. Dashed lines represent 0.95 confidence intervals for the curves with the corresponding color. Total of 2 observations deleted due to missingness. Log-rank probability measure for the equality of these curves is 3.314046e-04. Additional annotations: *.GeneId*=ENSG00000240180, *.GeneName*=RP11-318E3.4, *.GeneDesc*=[undefined].

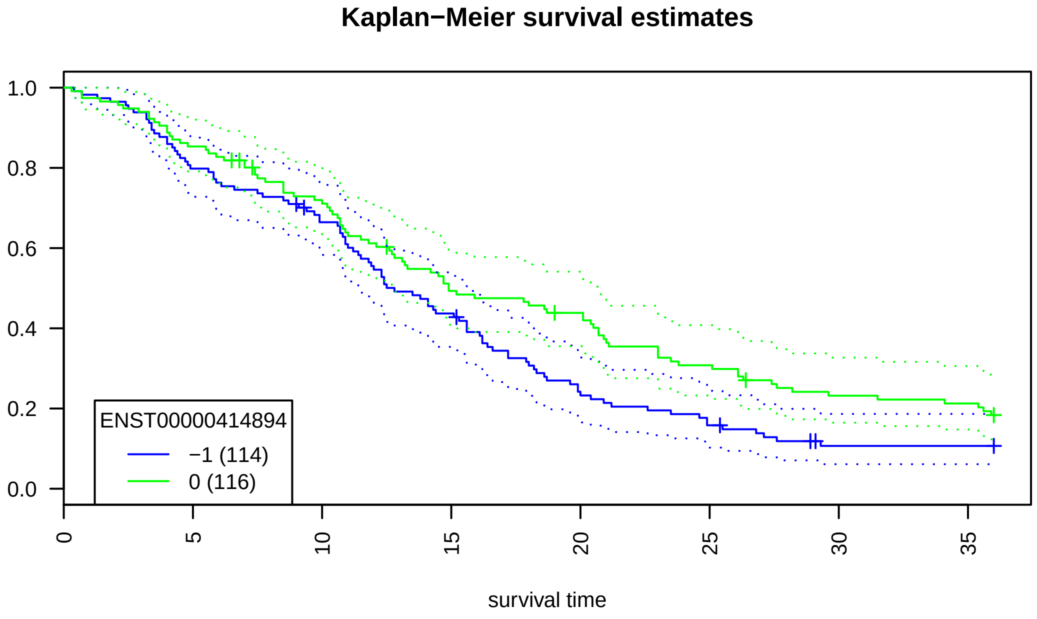

Figure 1111: Each curve represents the relative number of ENST00000414894 survived to the corresponding point of time. Dashed lines represent 0.95 confidence intervals for the curves with the corresponding color. Log-rank probability measure for the equality of these curves is 2.697483e-02. Additional annotations: *.GeneId*=ENSG00000237943, *.GeneName*=RP11-5N23.2, *.GeneDesc*=[undefined].

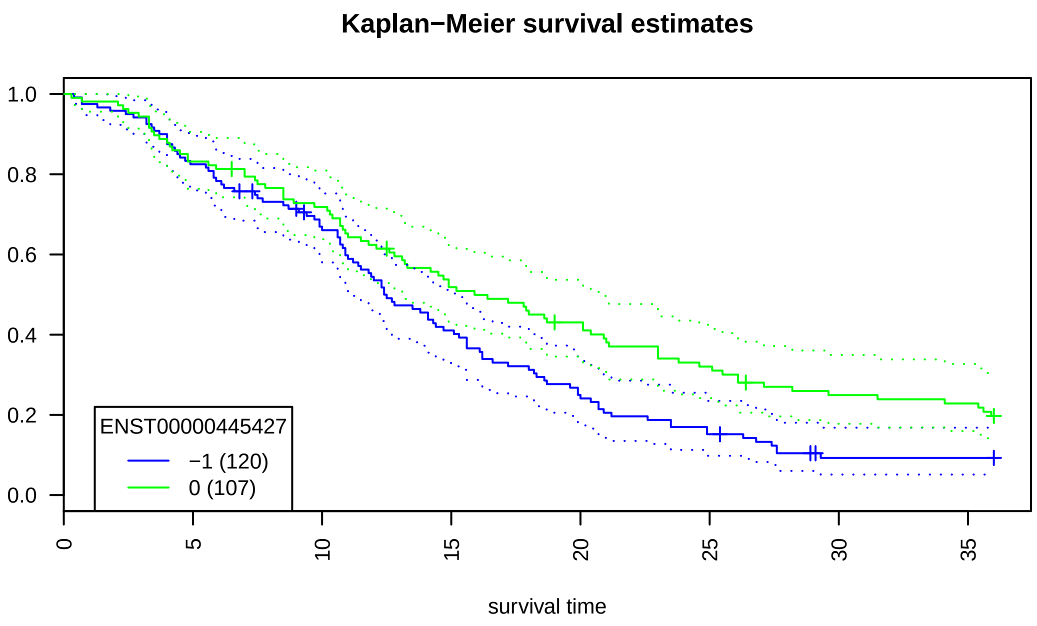

Figure 1112: Each curve represents the relative number of ENST00000445427 survived to the corresponding point of time. Dashed lines represent 0.95 confidence intervals for the curves with the corresponding color. Total of 3 observations deleted due to missingness. Log-rank probability measure for the equality of these curves is 9.002304e-03. Additional annotations: *.GeneId*=ENSG00000237943, *.GeneName*=RP11-5N23.2, *.GeneDesc*=[undefined].

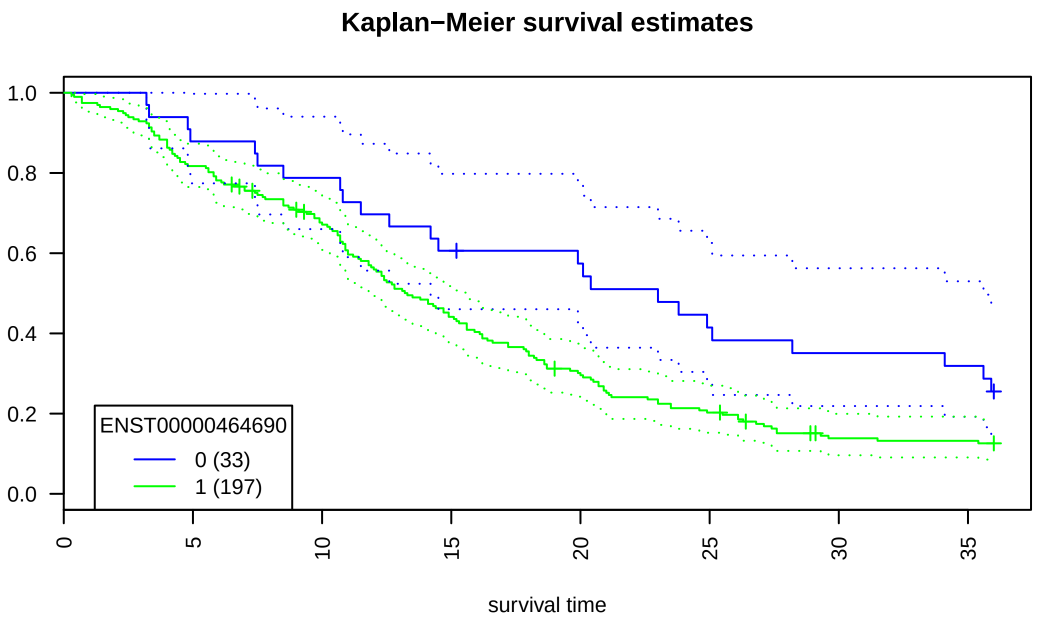

Figure 1113: Each curve represents the relative number of ENST00000464690 survived to the corresponding point of time. Dashed lines represent 0.95 confidence intervals for the curves with the corresponding color. Log-rank probability measure for the equality of these curves is 1.506583e-02. Additional annotations: *.GeneId*=ENSG00000163430, *.GeneName*=FSTL1, *.GeneDesc*=microRNA 198 [Source:HGNC Symbol;Acc:31570].

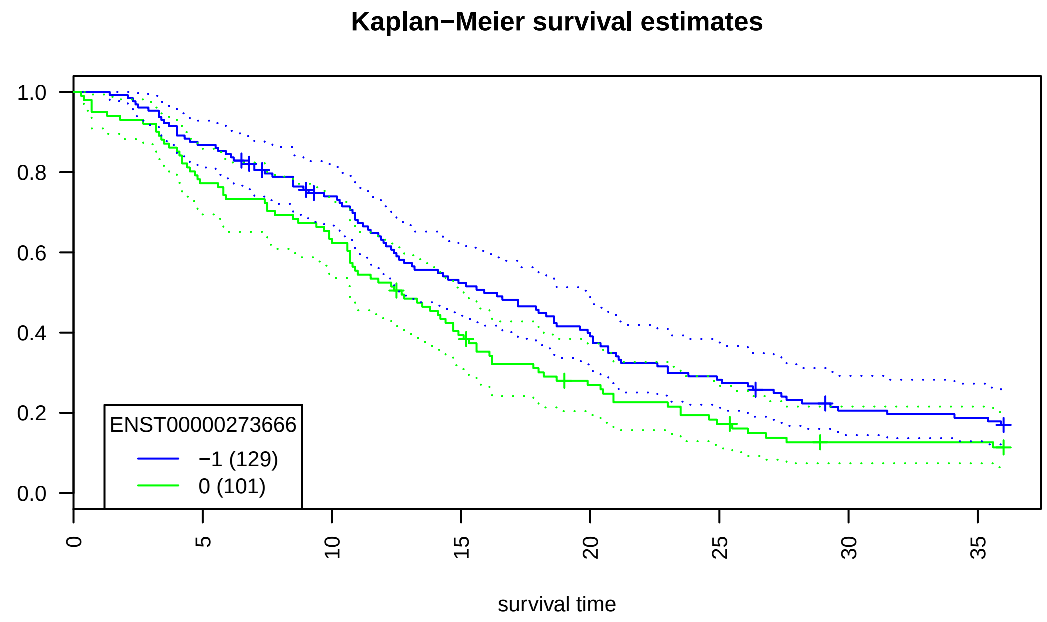

Figure 1114: Each curve represents the relative number of ENST00000273666 survived to the corresponding point of time. Dashed lines represent 0.95 confidence intervals for the curves with the corresponding color. Log-rank probability measure for the equality of these curves is 3.574523e-02. Additional annotations: *.GeneId*=ENSG00000145087, *.GeneName*=STXBP5L, *.GeneDesc*=syntaxin binding protein 5-like [Source:HGNC Symbol;Acc:30757].

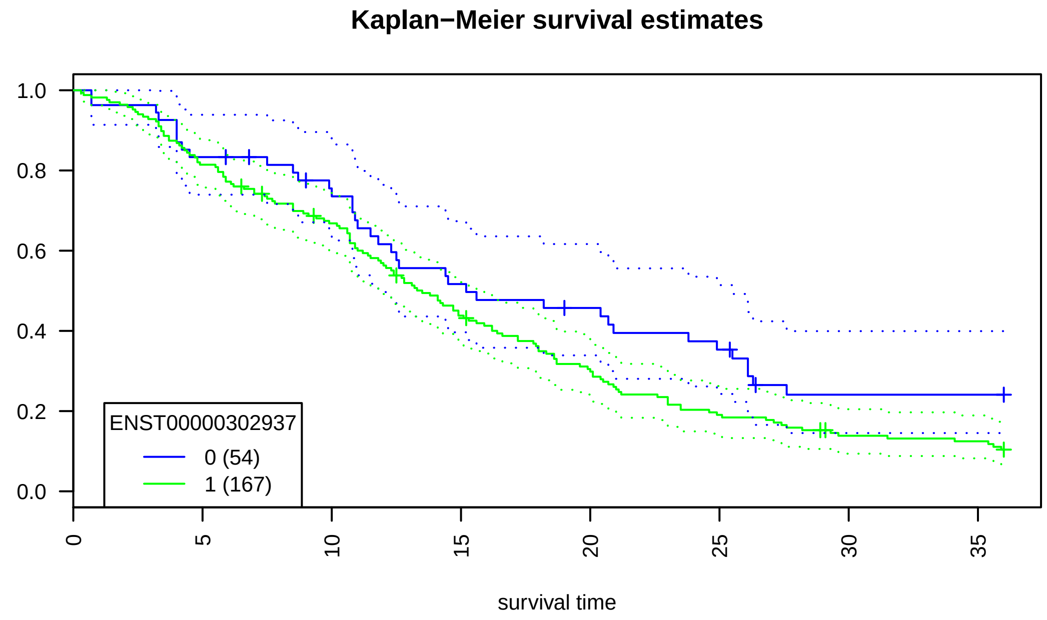

Figure 1115: Each curve represents the relative number of ENST00000302937 survived to the corresponding point of time. Dashed lines represent 0.95 confidence intervals for the curves with the corresponding color. Total of 9 observations deleted due to missingness. Log-rank probability measure for the equality of these curves is 3.991242e-02. Additional annotations: *.GeneId*=ENSG00000170892, *.GeneName*=TSEN34, *.GeneDesc*=tRNA splicing endonuclease 34 homolog (S. cerevisiae) [Source:HGNC Symbol;Acc:15506].

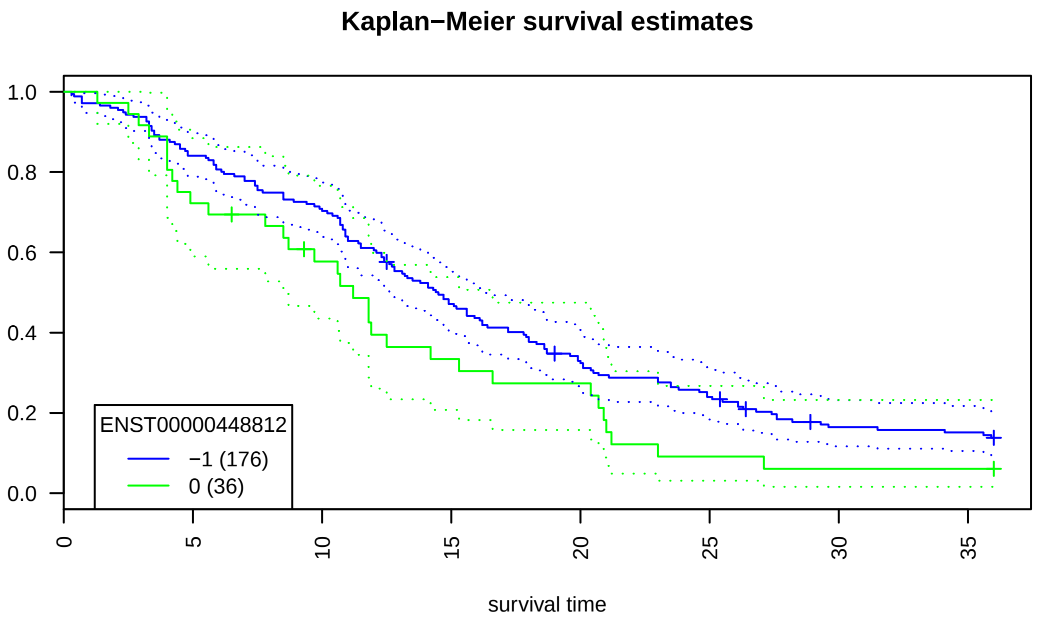

Figure 1116: Each curve represents the relative number of ENST00000448812 survived to the corresponding point of time. Dashed lines represent 0.95 confidence intervals for the curves with the corresponding color. Total of 18 observations deleted due to missingness. Log-rank probability measure for the equality of these curves is 4.240485e-02. Additional annotations: *.GeneId*=ENSG00000170485, *.GeneName*=NPAS2, *.GeneDesc*=neuronal PAS domain protein 2 [Source:HGNC Symbol;Acc:7895].

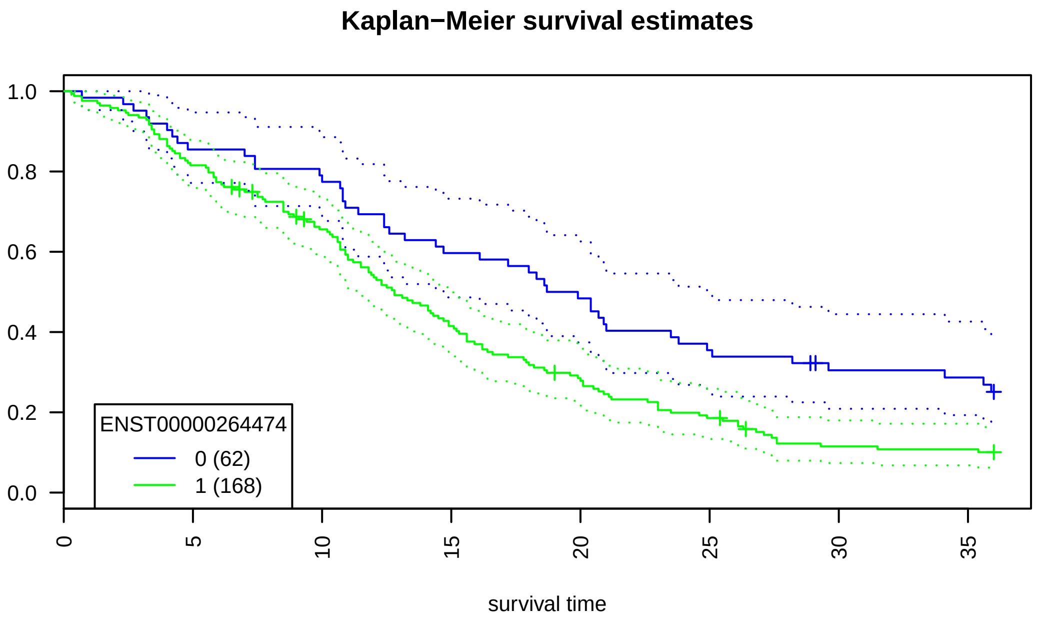

Figure 1117: Each curve represents the relative number of ENST00000264474 survived to the corresponding point of time. Dashed lines represent 0.95 confidence intervals for the curves with the corresponding color. Log-rank probability measure for the equality of these curves is 1.785975e-03. Additional annotations: *.GeneId*=ENSG00000121552, *.GeneName*=CSTA, *.GeneDesc*=cystatin A (stefin A) [Source:HGNC Symbol;Acc:2481].

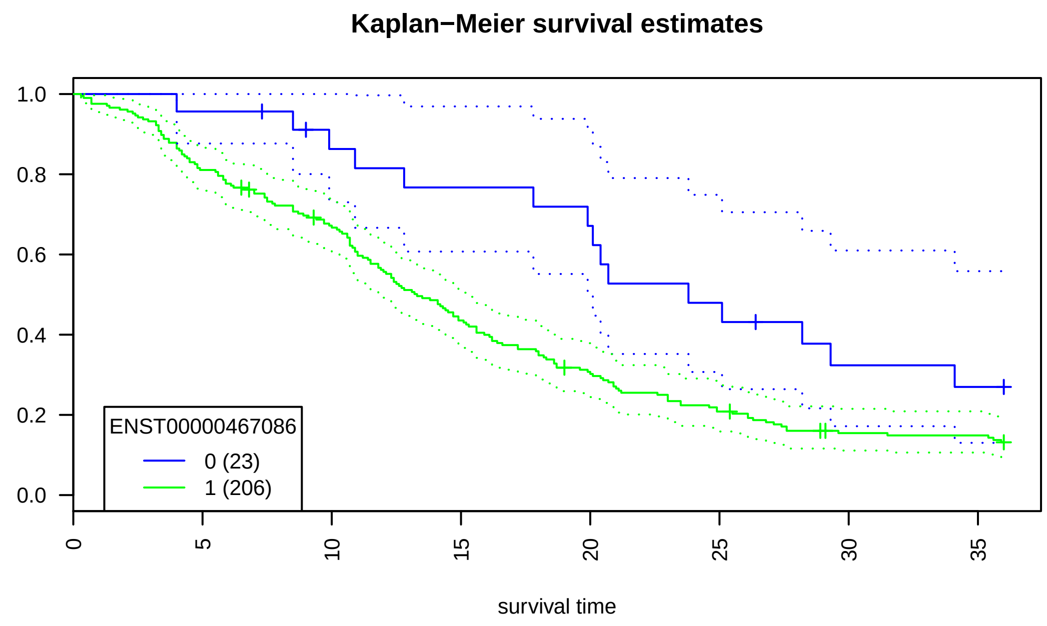

Figure 1118: Each curve represents the relative number of ENST00000467086 survived to the corresponding point of time. Dashed lines represent 0.95 confidence intervals for the curves with the corresponding color. Total of 1 observation deleted due to missingness. Log-rank probability measure for the equality of these curves is 1.173580e-02. Additional annotations: *.GeneId*=ENSG00000185624, *.GeneName*=P4HB, *.GeneDesc*=prolyl 4-hydroxylase, beta polypeptide [Source:HGNC Symbol;Acc:8548].

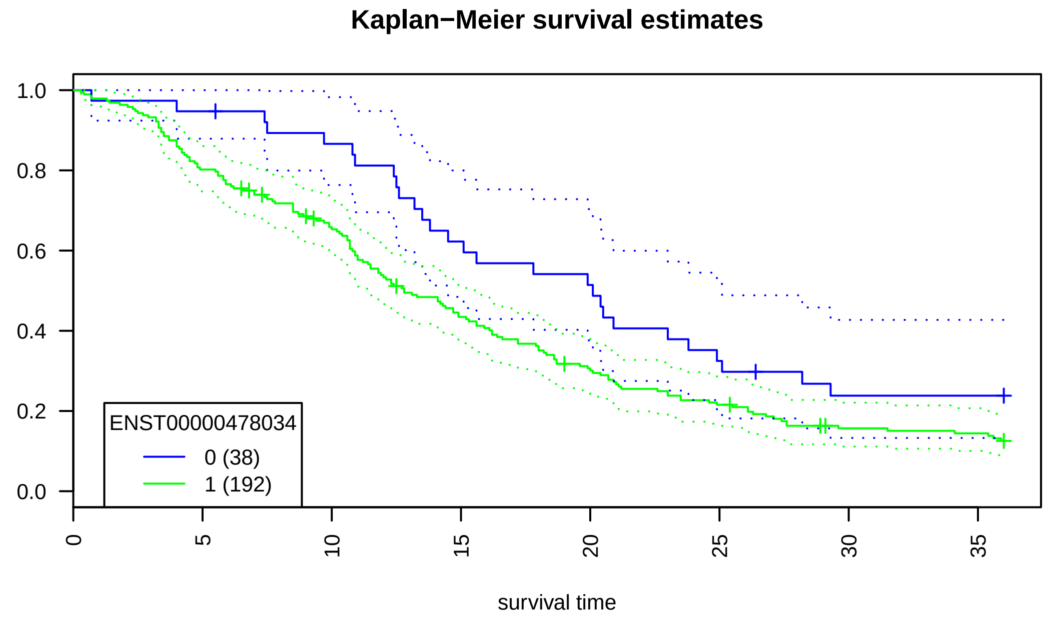

Figure 1119: Each curve represents the relative number of ENST00000478034 survived to the corresponding point of time. Dashed lines represent 0.95 confidence intervals for the curves with the corresponding color. Log-rank probability measure for the equality of these curves is 2.363613e-02. Additional annotations: *.GeneId*=ENSG00000185624, *.GeneName*=P4HB, *.GeneDesc*=prolyl 4-hydroxylase, beta polypeptide [Source:HGNC Symbol;Acc:8548].

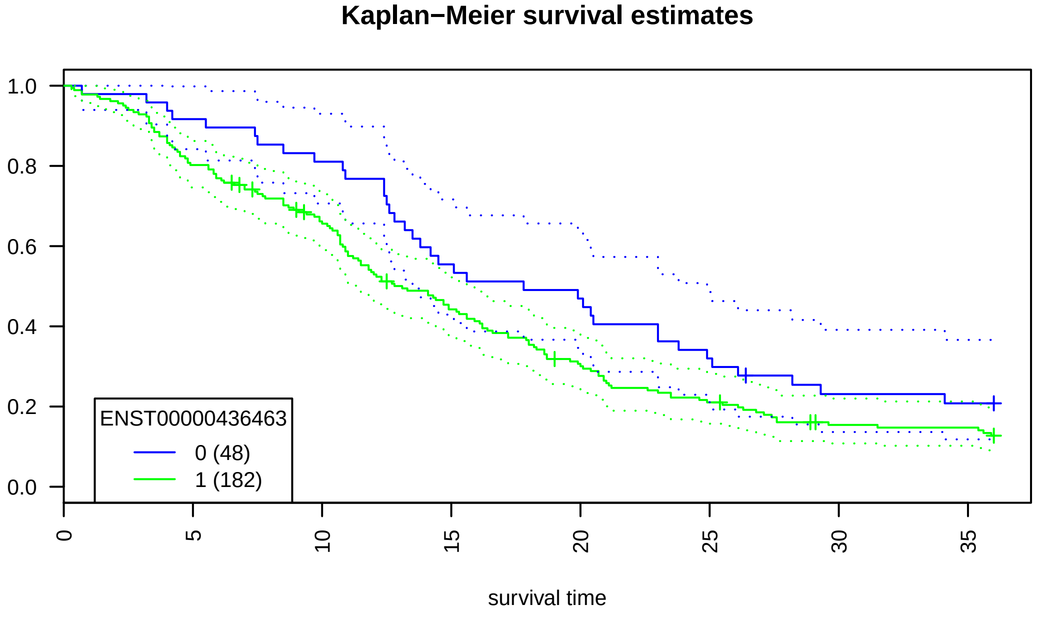

Figure 1120: Each curve represents the relative number of ENST00000436463 survived to the corresponding point of time. Dashed lines represent 0.95 confidence intervals for the curves with the corresponding color. Log-rank probability measure for the equality of these curves is 4.998912e-02. Additional annotations: *.GeneId*=ENSG00000185624, *.GeneName*=P4HB, *.GeneDesc*=prolyl 4-hydroxylase, beta polypeptide [Source:HGNC Symbol;Acc:8548].

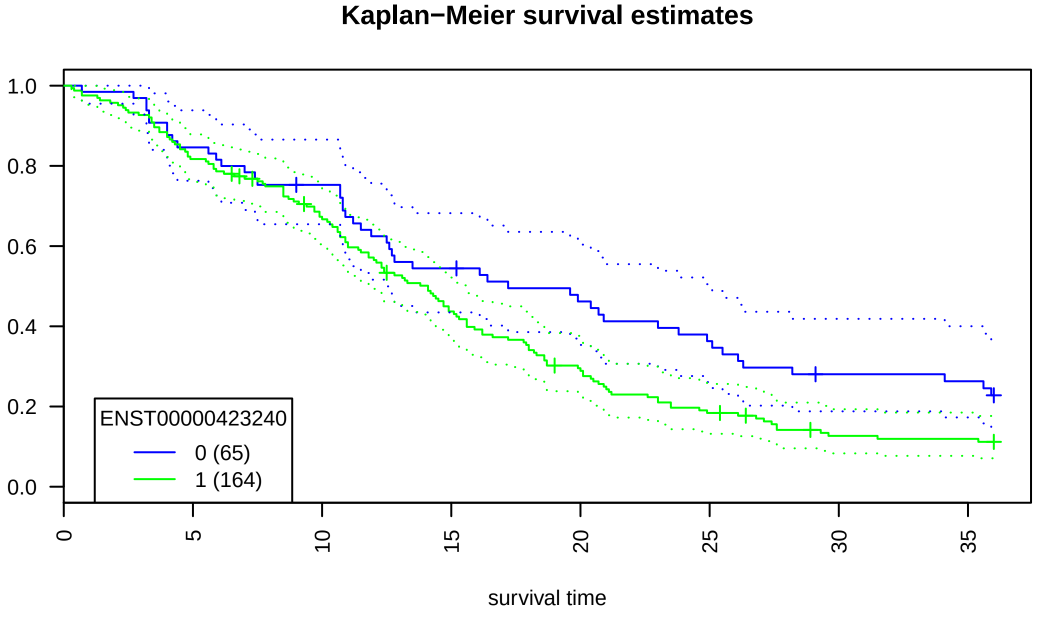

Figure 1121: Each curve represents the relative number of ENST00000423240 survived to the corresponding point of time. Dashed lines represent 0.95 confidence intervals for the curves with the corresponding color. Total of 1 observation deleted due to missingness. Log-rank probability measure for the equality of these curves is 2.122252e-02. Additional annotations: *.GeneId*=ENSG00000236060, *.GeneName*=RP11-346E17.4, *.GeneDesc*=[undefined].

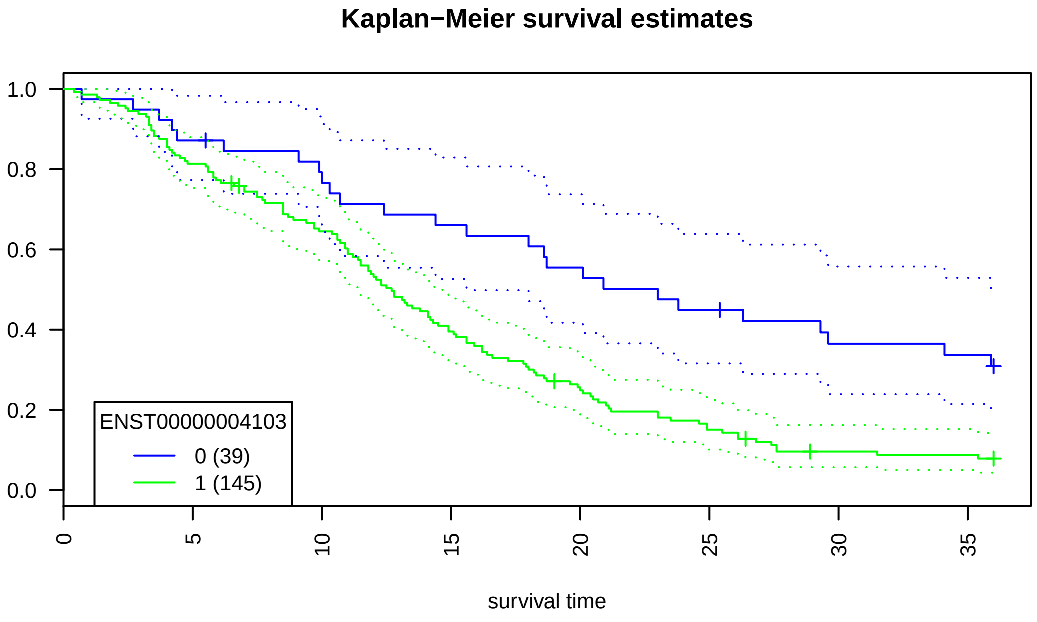

Figure 1122: Each curve represents the relative number of ENST00000004103 survived to the corresponding point of time. Dashed lines represent 0.95 confidence intervals for the curves with the corresponding color. Total of 46 observations deleted due to missingness. Log-rank probability measure for the equality of these curves is 2.059854e-04. Additional annotations: *.GeneId*=ENSG00000002933, *.GeneName*=TMEM176A, *.GeneDesc*=transmembrane protein 176A [Source:HGNC Symbol;Acc:24930].

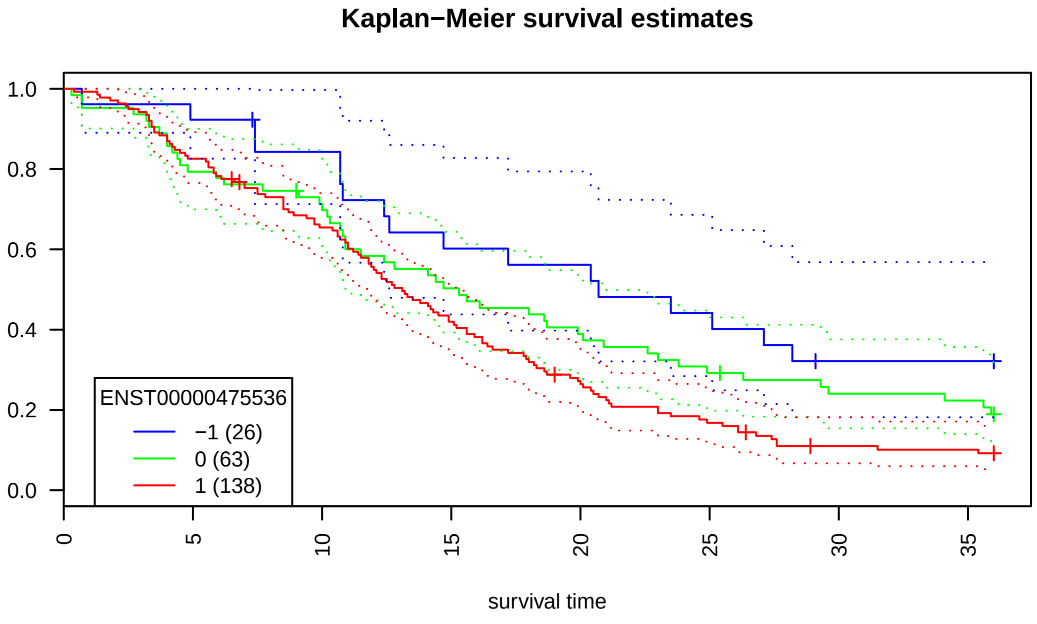

Figure 1123: Each curve represents the relative number of ENST00000475536 survived to the corresponding point of time. Dashed lines represent 0.95 confidence intervals for the curves with the corresponding color. Total of 3 observations deleted due to missingness. Log-rank probability measure for the equality of these curves is 9.296938e-03. Additional annotations: *.GeneId*=ENSG00000002933, *.GeneName*=TMEM176A, *.GeneDesc*=transmembrane protein 176A [Source:HGNC Symbol;Acc:24930].

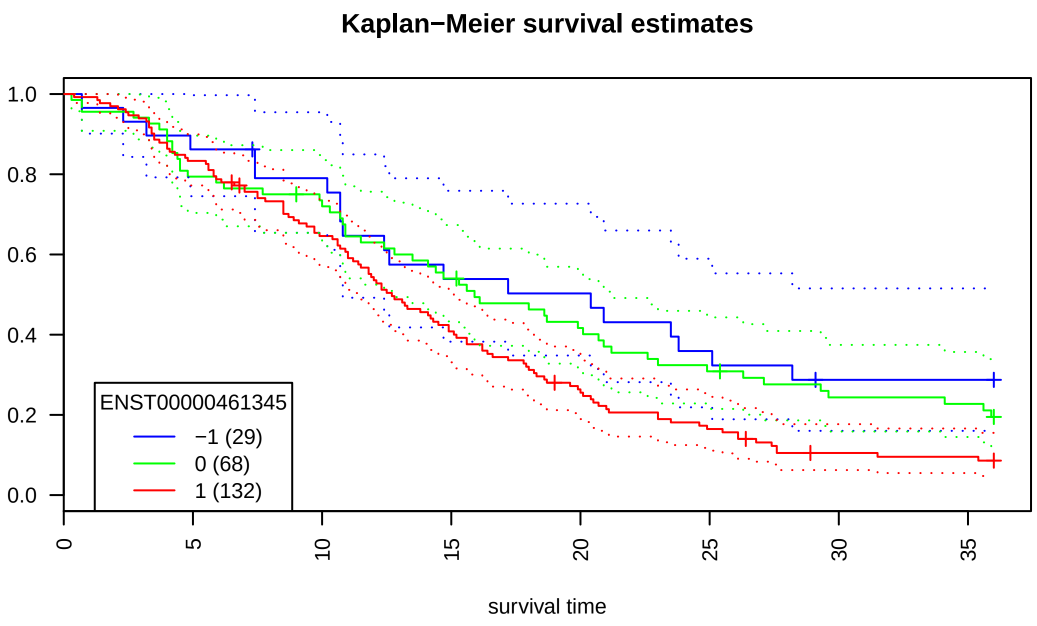

Figure 1124: Each curve represents the relative number of ENST00000461345 survived to the corresponding point of time. Dashed lines represent 0.95 confidence intervals for the curves with the corresponding color. Total of 1 observation deleted due to missingness. Log-rank probability measure for the equality of these curves is 1.113378e-02. Additional annotations: *.GeneId*=ENSG00000002933, *.GeneName*=TMEM176A, *.GeneDesc*=transmembrane protein 176A [Source:HGNC Symbol;Acc:24930].

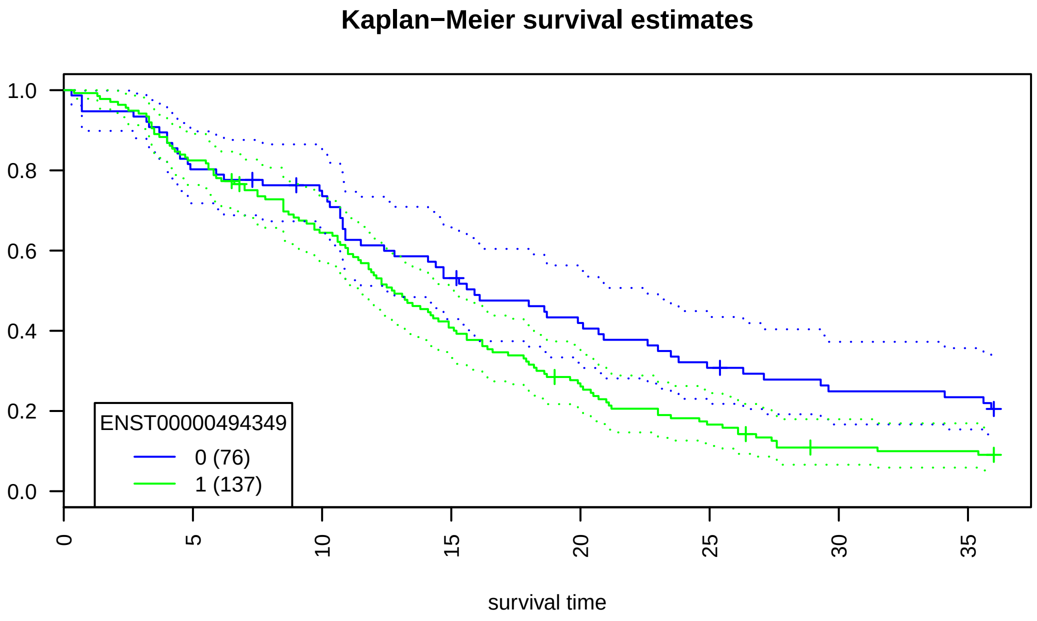

Figure 1125: Each curve represents the relative number of ENST00000494349 survived to the corresponding point of time. Dashed lines represent 0.95 confidence intervals for the curves with the corresponding color. Total of 17 observations deleted due to missingness. Log-rank probability measure for the equality of these curves is 1.587155e-02. Additional annotations: *.GeneId*=ENSG00000002933, *.GeneName*=TMEM176A, *.GeneDesc*=transmembrane protein 176A [Source:HGNC Symbol;Acc:24930].

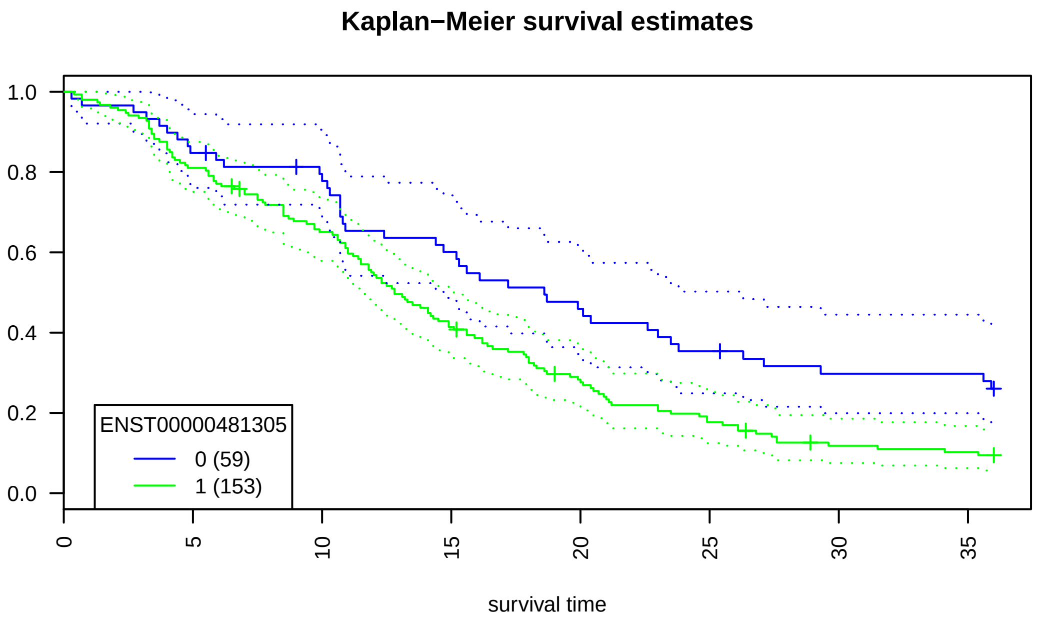

Figure 1126: Each curve represents the relative number of ENST00000481305 survived to the corresponding point of time. Dashed lines represent 0.95 confidence intervals for the curves with the corresponding color. Total of 18 observations deleted due to missingness. Log-rank probability measure for the equality of these curves is 3.464752e-03. Additional annotations: *.GeneId*=ENSG00000002933, *.GeneName*=TMEM176A, *.GeneDesc*=transmembrane protein 176A [Source:HGNC Symbol;Acc:24930].

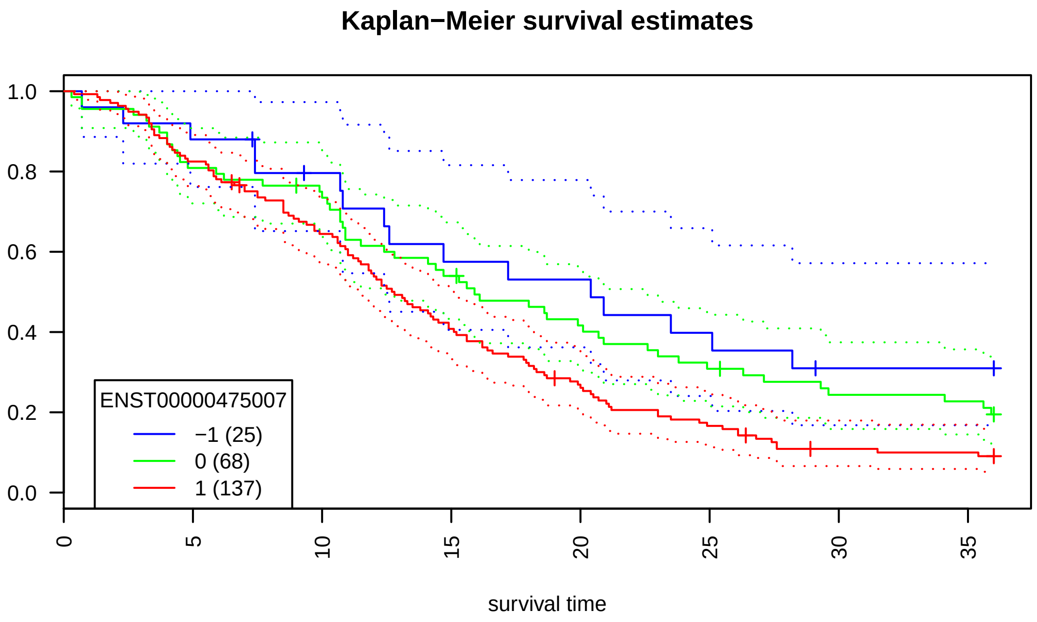

Figure 1127: Each curve represents the relative number of ENST00000475007 survived to the corresponding point of time. Dashed lines represent 0.95 confidence intervals for the curves with the corresponding color. Log-rank probability measure for the equality of these curves is 9.42895e-03. Additional annotations: *.GeneId*=ENSG00000002933, *.GeneName*=TMEM176A, *.GeneDesc*=transmembrane protein 176A [Source:HGNC Symbol;Acc:24930].

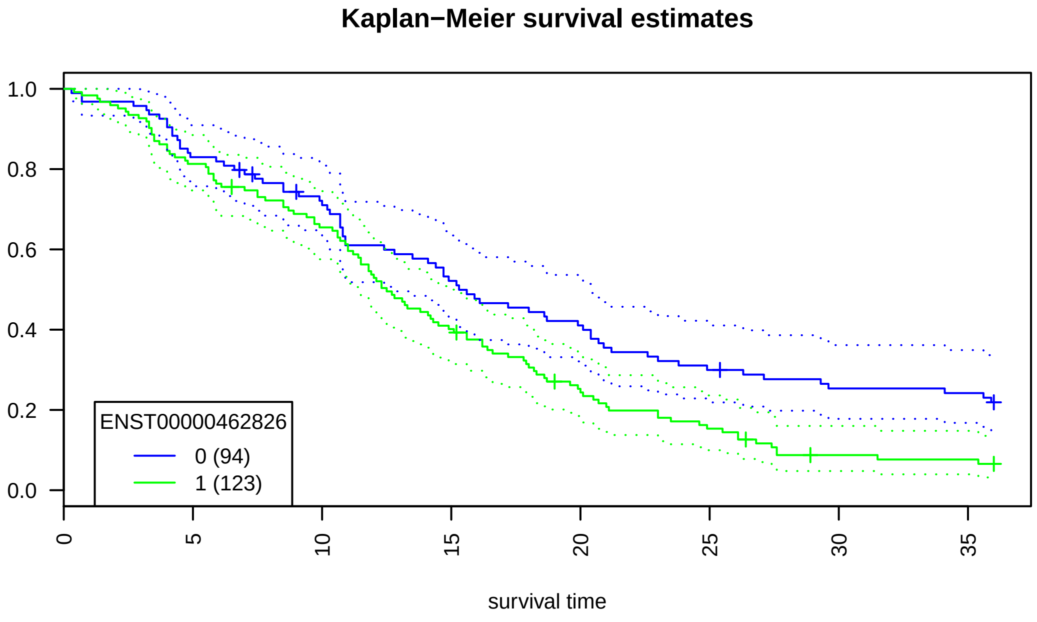

Figure 1128: Each curve represents the relative number of ENST00000462826 survived to the corresponding point of time. Dashed lines represent 0.95 confidence intervals for the curves with the corresponding color. Total of 13 observations deleted due to missingness. Log-rank probability measure for the equality of these curves is 3.521188e-03. Additional annotations: *.GeneId*=ENSG00000002933, *.GeneName*=TMEM176A, *.GeneDesc*=transmembrane protein 176A [Source:HGNC Symbol;Acc:24930].

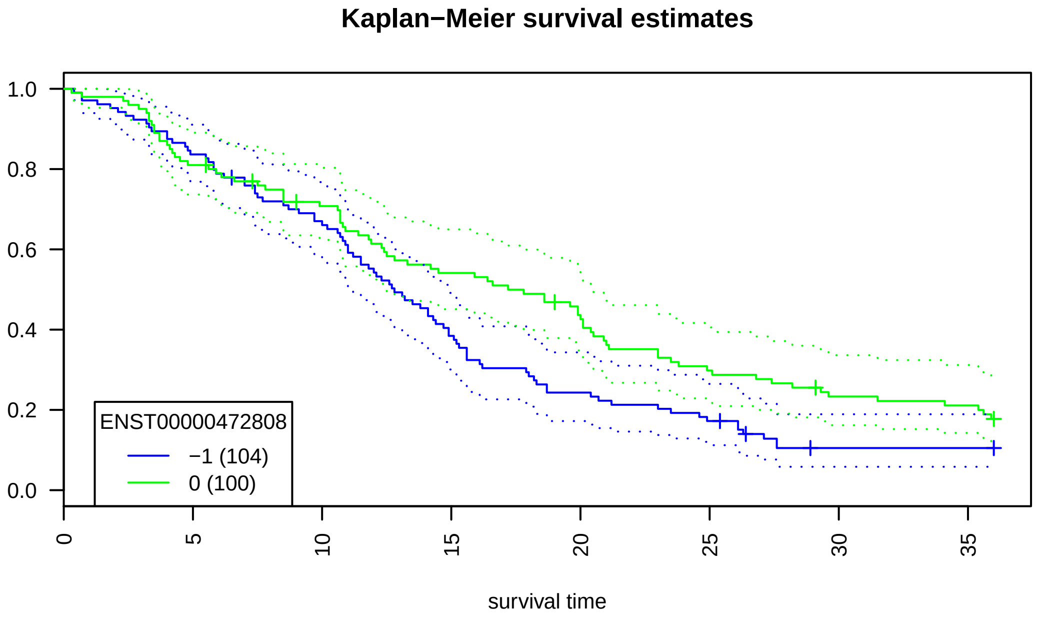

Figure 1129: Each curve represents the relative number of ENST00000472808 survived to the corresponding point of time. Dashed lines represent 0.95 confidence intervals for the curves with the corresponding color. Total of 26 observations deleted due to missingness. Log-rank probability measure for the equality of these curves is 2.53717e-02. Additional annotations: *.GeneId*=ENSG00000185104, *.GeneName*=FAF1, *.GeneDesc*=Fas (TNFRSF6) associated factor 1 [Source:HGNC Symbol;Acc:3578].

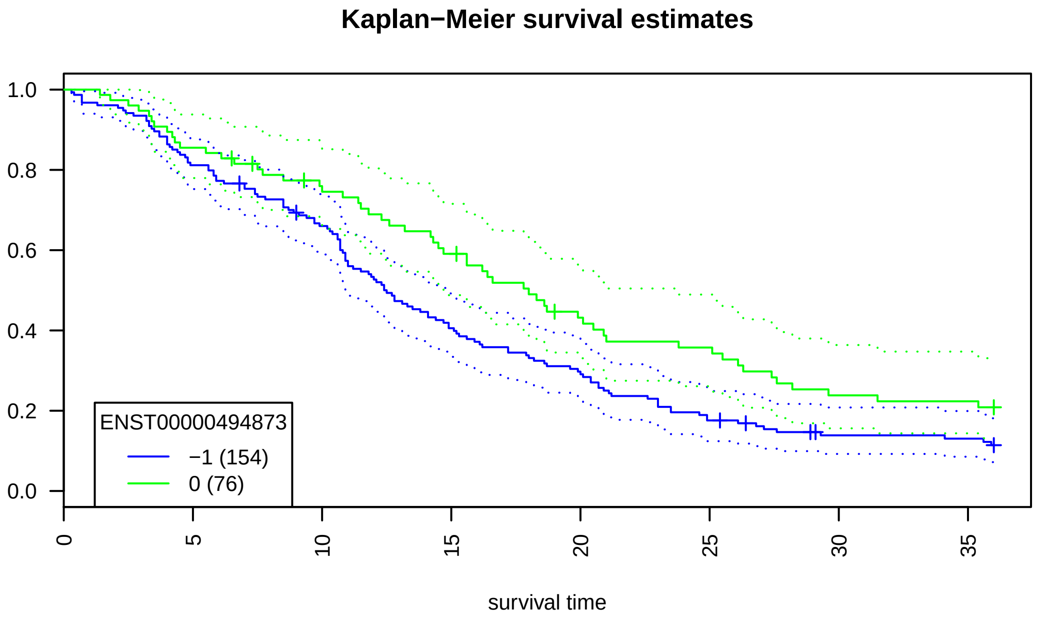

Figure 1130: Each curve represents the relative number of ENST00000494873 survived to the corresponding point of time. Dashed lines represent 0.95 confidence intervals for the curves with the corresponding color. Log-rank probability measure for the equality of these curves is 1.370147e-02. Additional annotations: *.GeneId*=ENSG00000123091, *.GeneName*=RNF11, *.GeneDesc*=ring finger protein 11 [Source:HGNC Symbol;Acc:10056].

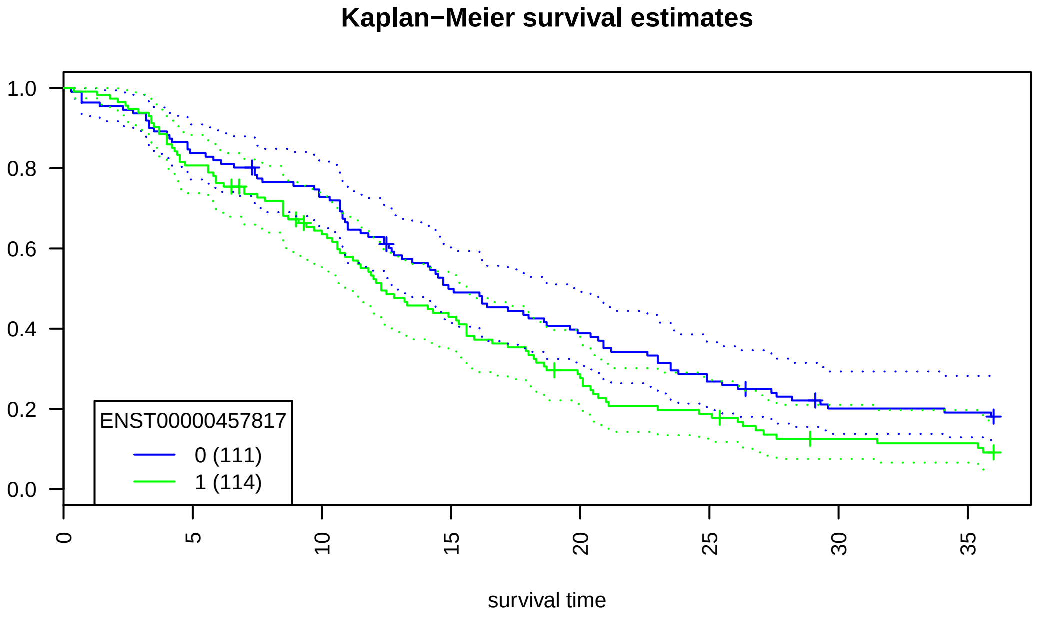

Figure 1131: Each curve represents the relative number of ENST00000457817 survived to the corresponding point of time. Dashed lines represent 0.95 confidence intervals for the curves with the corresponding color. Total of 5 observations deleted due to missingness. Log-rank probability measure for the equality of these curves is 3.688109e-02. Additional annotations: *.GeneId*=ENSG00000115590, *.GeneName*=IL1R2, *.GeneDesc*=interleukin 1 receptor, type II [Source:HGNC Symbol;Acc:5994].

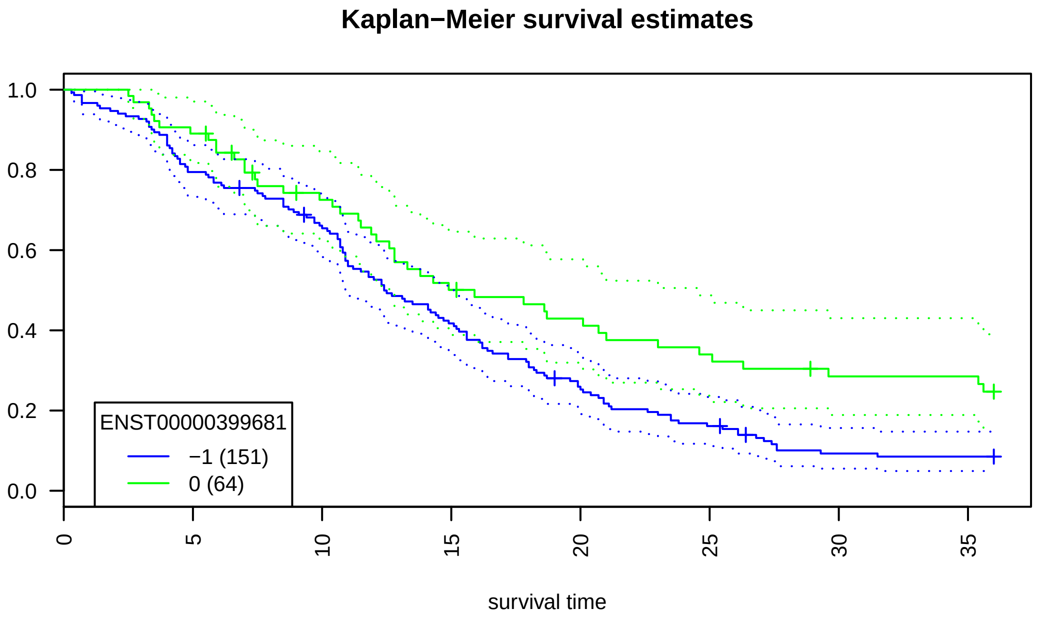

Figure 1132: Each curve represents the relative number of ENST00000399681 survived to the corresponding point of time. Dashed lines represent 0.95 confidence intervals for the curves with the corresponding color. Total of 15 observations deleted due to missingness. Log-rank probability measure for the equality of these curves is 5.484532e-03. Additional annotations: *.GeneId*=ENSG00000215146, *.GeneName*=RP11-313J2.1, *.GeneDesc*=[undefined].

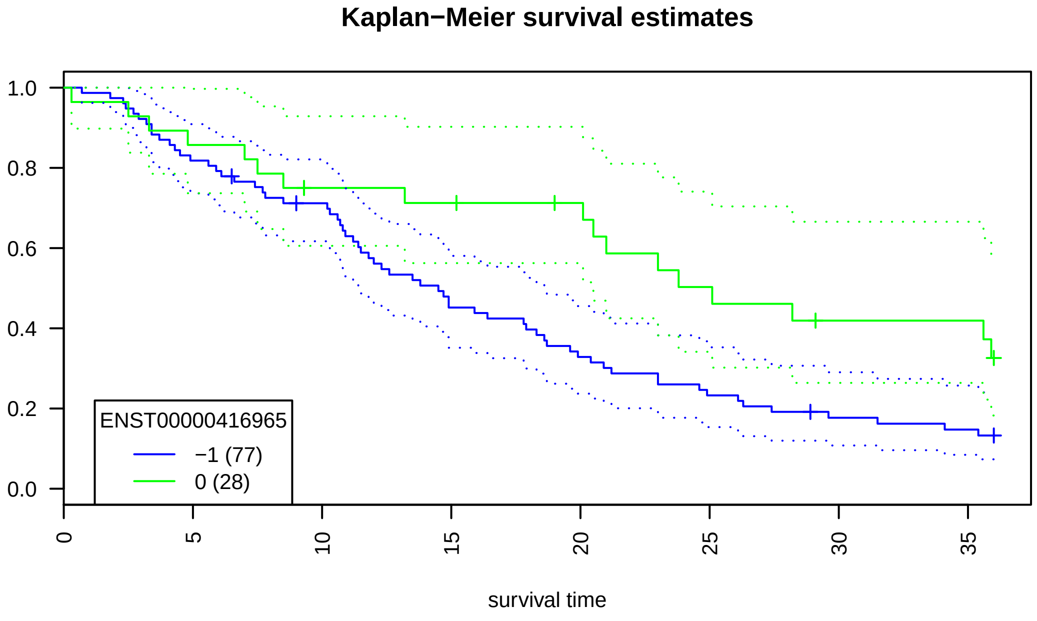

Figure 1133: Each curve represents the relative number of ENST00000416965 survived to the corresponding point of time. Dashed lines represent 0.95 confidence intervals for the curves with the corresponding color. Total of 125 observations deleted due to missingness. Log-rank probability measure for the equality of these curves is 1.379419e-02. Additional annotations: *.GeneId*=ENSG00000028839, *.GeneName*=TBPL1, *.GeneDesc*=TBP-like 1 [Source:HGNC Symbol;Acc:11589].

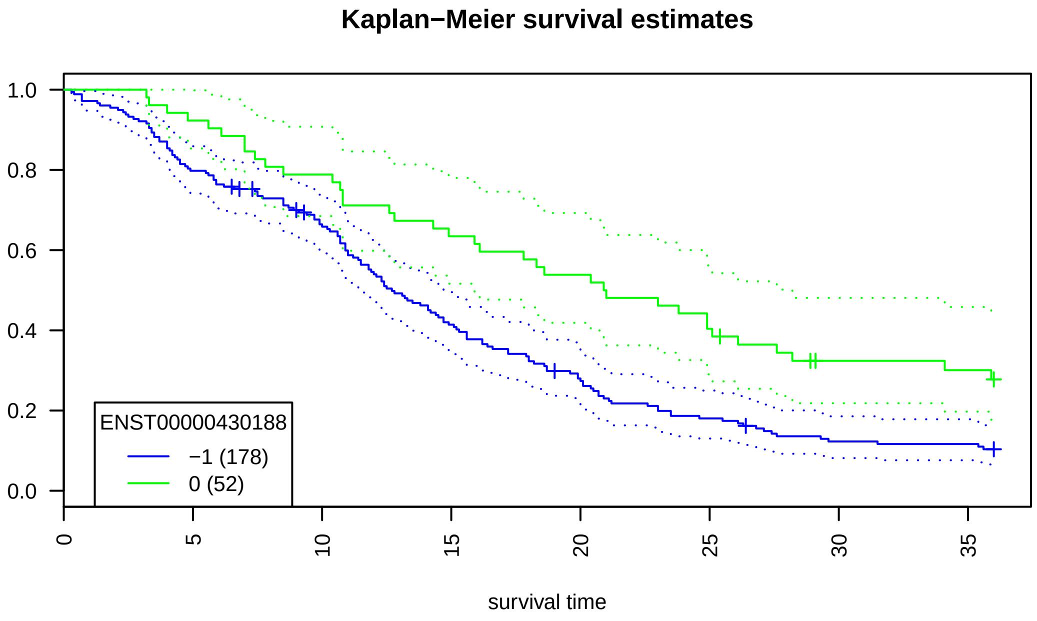

Figure 1134: Each curve represents the relative number of ENST00000430188 survived to the corresponding point of time. Dashed lines represent 0.95 confidence intervals for the curves with the corresponding color. Log-rank probability measure for the equality of these curves is 6.408545e-04. Additional annotations: *.GeneId*=ENSG00000231187, *.GeneName*=RP11-38L15.3, *.GeneDesc*=[undefined].

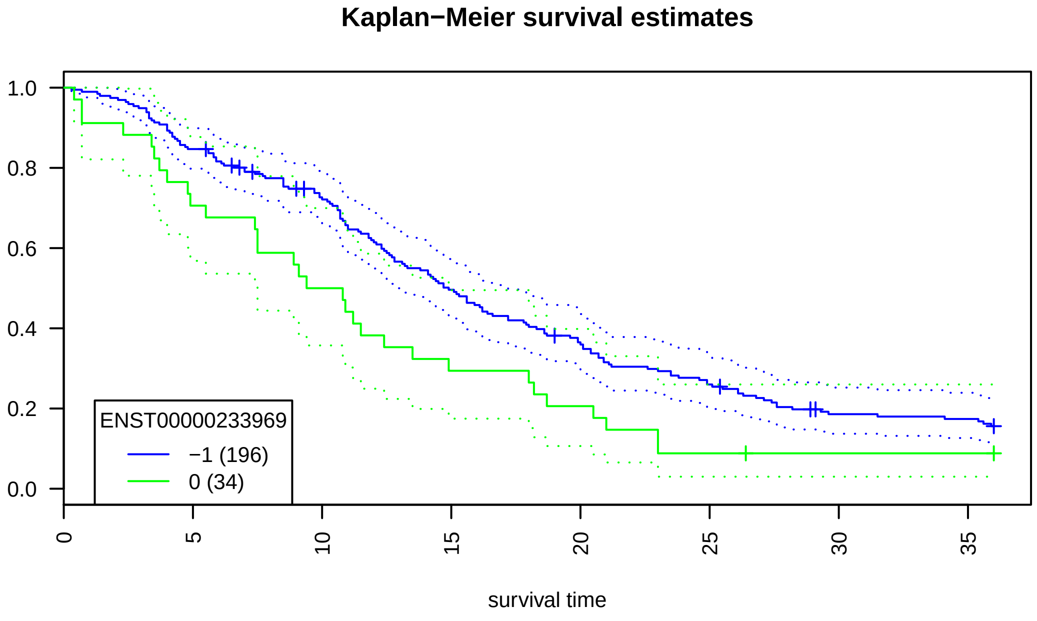

Figure 1135: Each curve represents the relative number of ENST00000233969 survived to the corresponding point of time. Dashed lines represent 0.95 confidence intervals for the curves with the corresponding color. Log-rank probability measure for the equality of these curves is 1.067151e-02. Additional annotations: *.GeneId*=ENSG00000115616, *.GeneName*=SLC9A2, *.GeneDesc*=solute carrier family 9 (sodium/hydrogen exchanger), member 2 [Source:HGNC Symbol;Acc:11072].

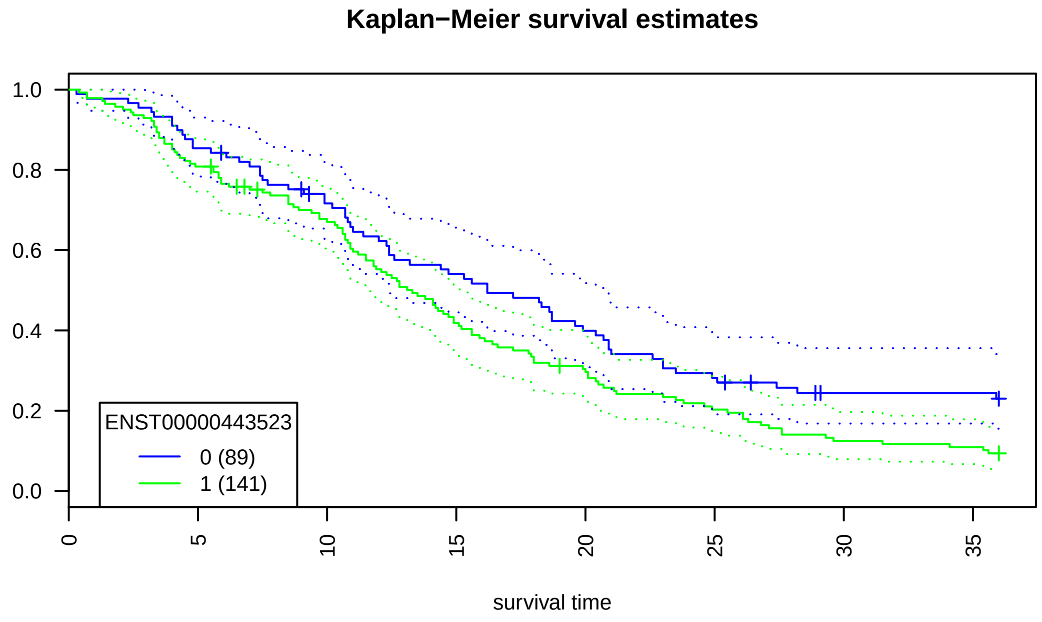

Figure 1136: Each curve represents the relative number of ENST00000443523 survived to the corresponding point of time. Dashed lines represent 0.95 confidence intervals for the curves with the corresponding color. Log-rank probability measure for the equality of these curves is 2.094019e-02. Additional annotations: *.GeneId*=ENSG00000228651, *.GeneName*=RP11-556E13.1, *.GeneDesc*=[undefined].

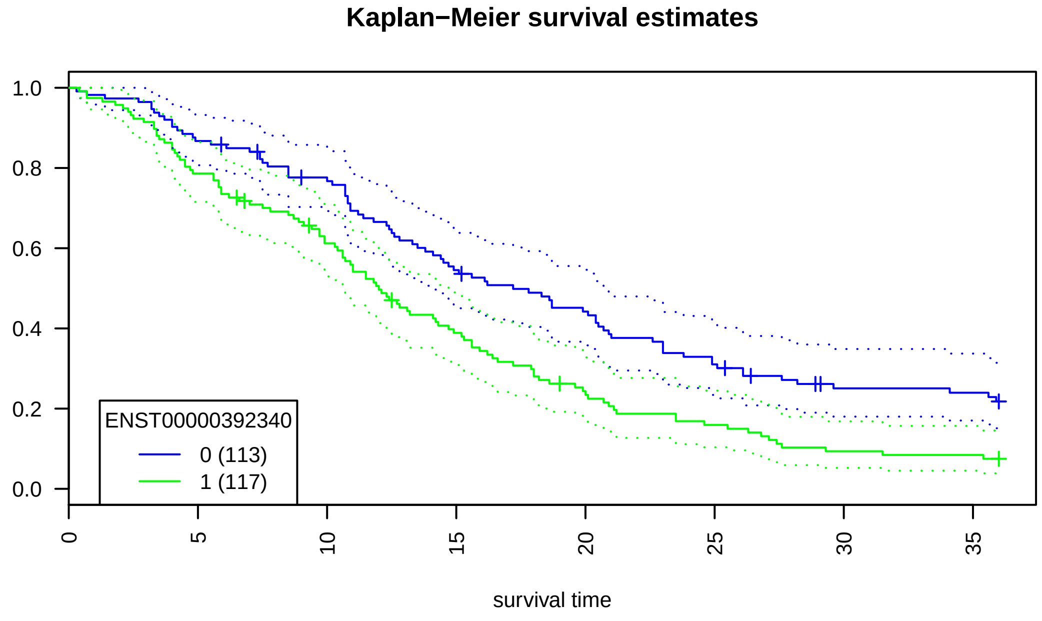

Figure 1137: Each curve represents the relative number of ENST00000392340 survived to the corresponding point of time. Dashed lines represent 0.95 confidence intervals for the curves with the corresponding color. Log-rank probability measure for the equality of these curves is 3.849447e-04. Additional annotations: *.GeneId*=ENSG00000141526, *.GeneName*=SLC16A3, *.GeneDesc*=solute carrier family 16, member 3 (monocarboxylic acid transporter 4) [Source:HGNC Symbol;Acc:10924].

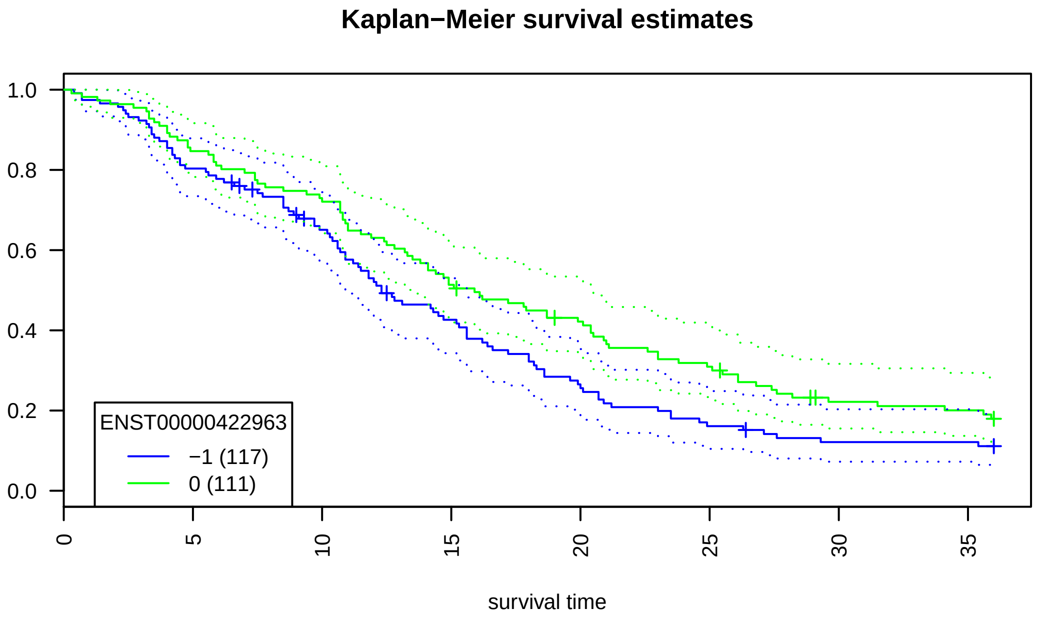

Figure 1138: Each curve represents the relative number of ENST00000422963 survived to the corresponding point of time. Dashed lines represent 0.95 confidence intervals for the curves with the corresponding color. Total of 2 observations deleted due to missingness. Log-rank probability measure for the equality of these curves is 3.028061e-02. Additional annotations: *.GeneId*=ENSG00000225299, *.GeneName*=RP11-344A5.1, *.GeneDesc*=[undefined].

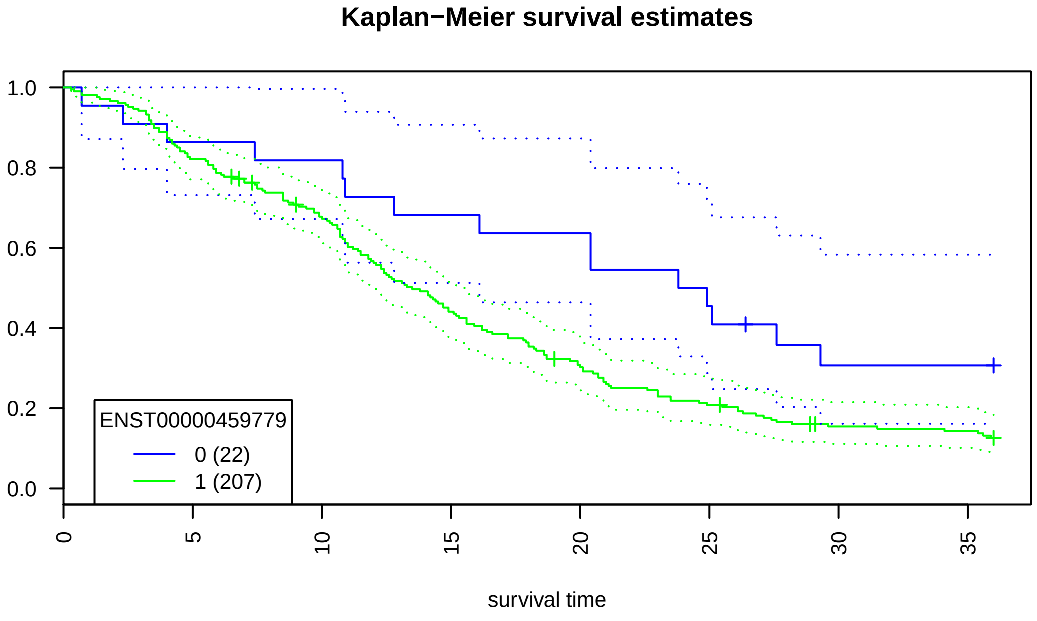

Figure 1139: Each curve represents the relative number of ENST00000459779 survived to the corresponding point of time. Dashed lines represent 0.95 confidence intervals for the curves with the corresponding color. Total of 1 observation deleted due to missingness. Log-rank probability measure for the equality of these curves is 2.019475e-02. Additional annotations: *.GeneId*=ENSG00000116157, *.GeneName*=GPX7, *.GeneDesc*=glutathione peroxidase 7 [Source:HGNC Symbol;Acc:4559].

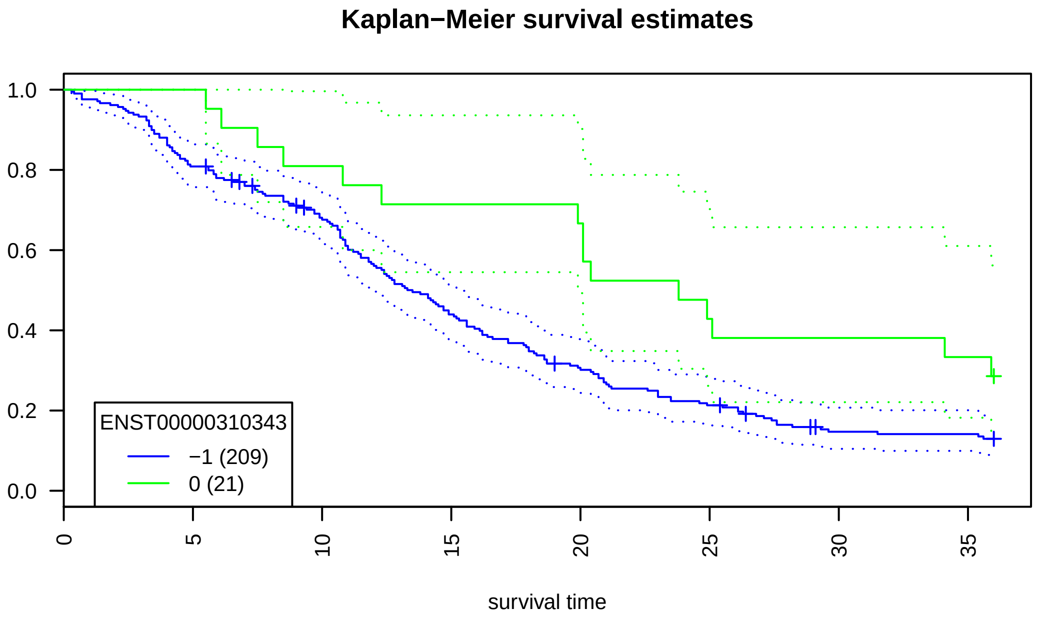

Figure 1140: Each curve represents the relative number of ENST00000310343 survived to the corresponding point of time. Dashed lines represent 0.95 confidence intervals for the curves with the corresponding color. Log-rank probability measure for the equality of these curves is 1.929801e-02. Additional annotations: *.GeneId*=ENSG00000134909, *.GeneName*=ARHGAP32, *.GeneDesc*=Rho GTPase activating protein 32 [Source:HGNC Symbol;Acc:17399].

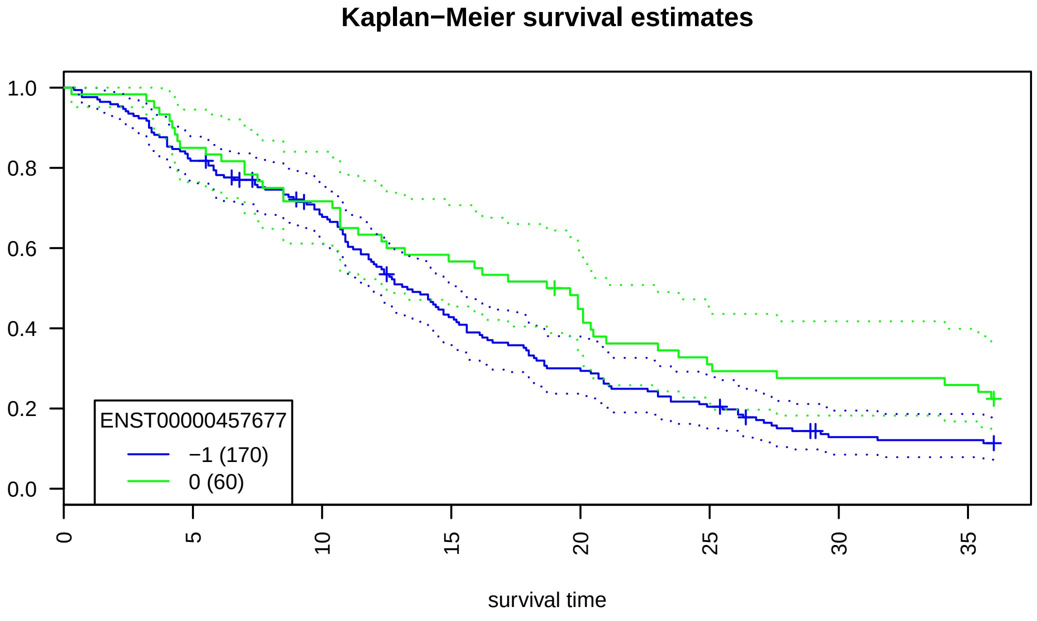

Figure 1141: Each curve represents the relative number of ENST00000457677 survived to the corresponding point of time. Dashed lines represent 0.95 confidence intervals for the curves with the corresponding color. Log-rank probability measure for the equality of these curves is 3.552867e-02. Additional annotations: *.GeneId*=ENSG00000134909, *.GeneName*=ARHGAP32, *.GeneDesc*=Rho GTPase activating protein 32 [Source:HGNC Symbol;Acc:17399].

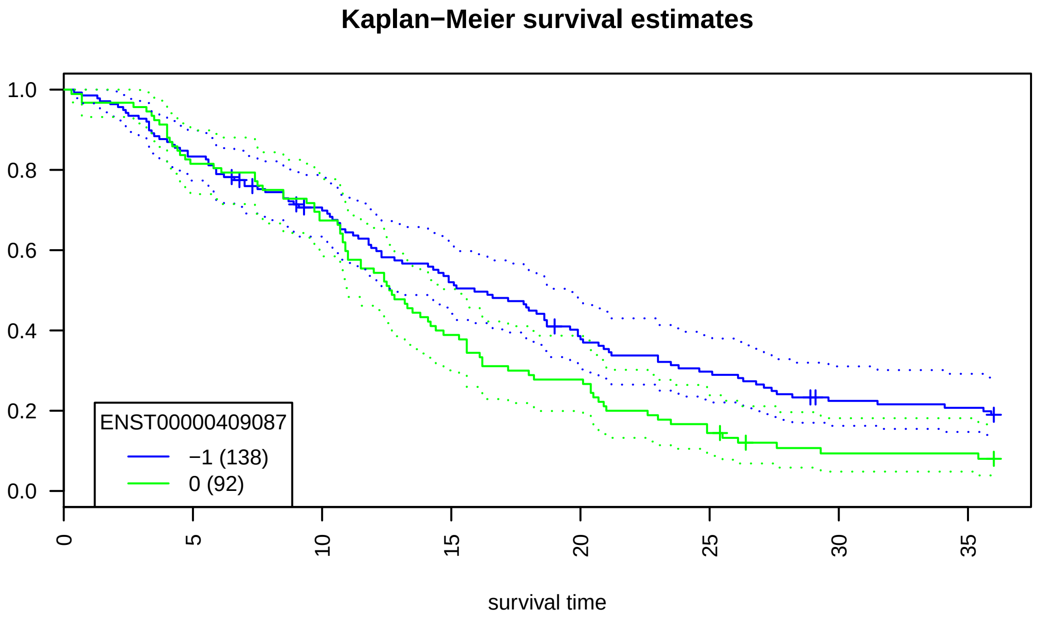

Figure 1142: Each curve represents the relative number of ENST00000409087 survived to the corresponding point of time. Dashed lines represent 0.95 confidence intervals for the curves with the corresponding color. Log-rank probability measure for the equality of these curves is 1.701562e-02. Additional annotations: *.GeneId*=ENSG00000144057, *.GeneName*=ST6GAL2, *.GeneDesc*=ST6 beta-galactosamide alpha-2,6-sialyltranferase 2 [Source:HGNC Symbol;Acc:10861].

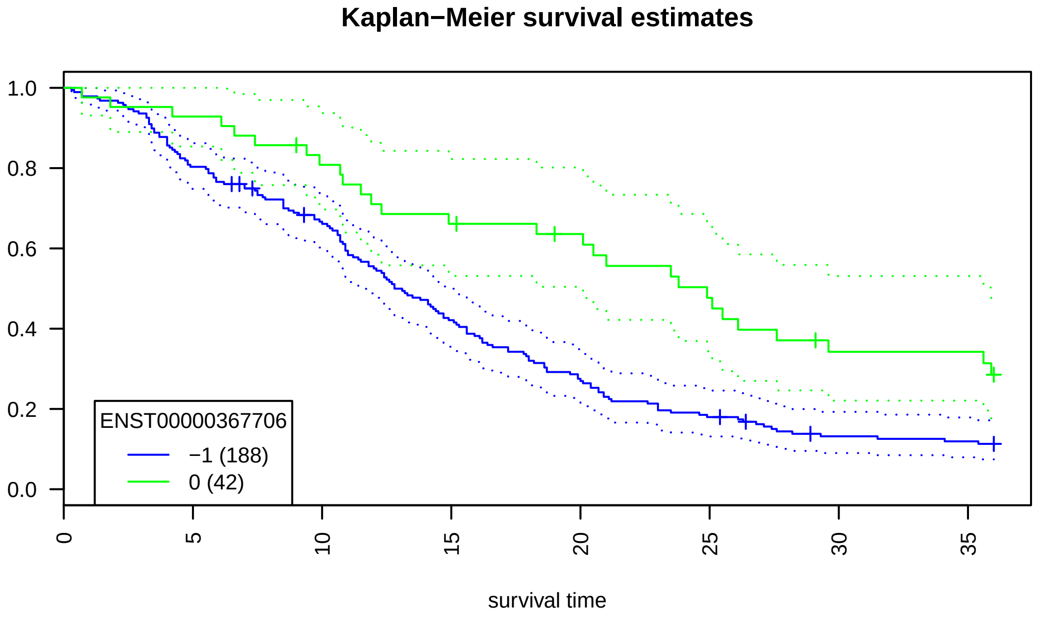

Figure 1143: Each curve represents the relative number of ENST00000367706 survived to the corresponding point of time. Dashed lines represent 0.95 confidence intervals for the curves with the corresponding color. Log-rank probability measure for the equality of these curves is 6.182931e-04. Additional annotations: *.GeneId*=ENSG00000112379, *.GeneName*=KIAA1244, *.GeneDesc*=KIAA1244 [Source:HGNC Symbol;Acc:21213].

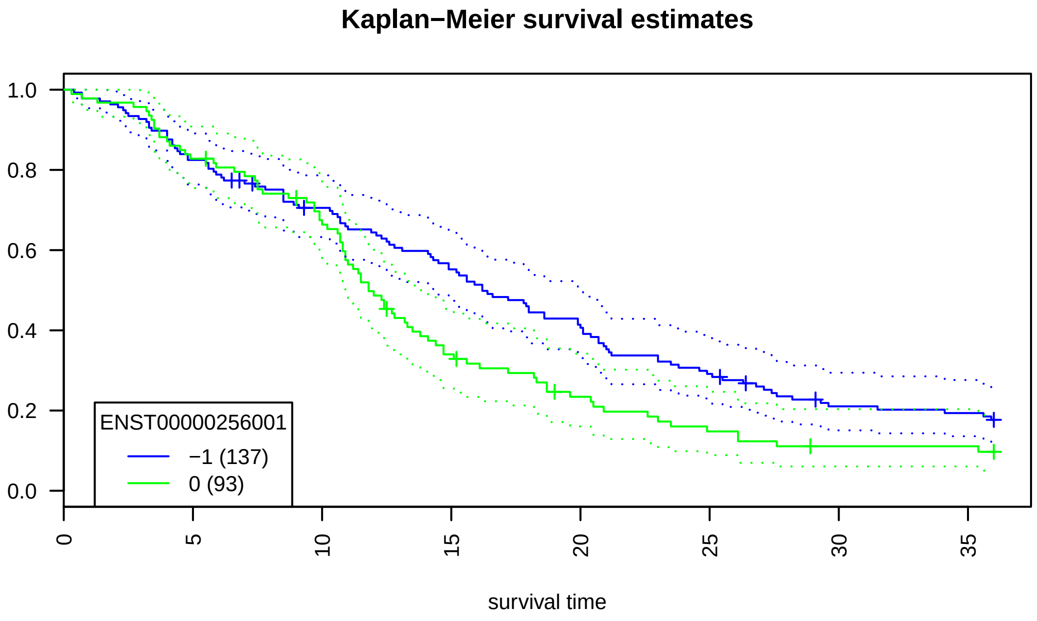

Figure 1144: Each curve represents the relative number of ENST00000256001 survived to the corresponding point of time. Dashed lines represent 0.95 confidence intervals for the curves with the corresponding color. Log-rank probability measure for the equality of these curves is 1.277938e-02. Additional annotations: *.GeneId*=ENSG00000133627, *.GeneName*=ACTR3B, *.GeneDesc*=ARP3 actin-related protein 3 homolog B (yeast) [Source:HGNC Symbol;Acc:17256].

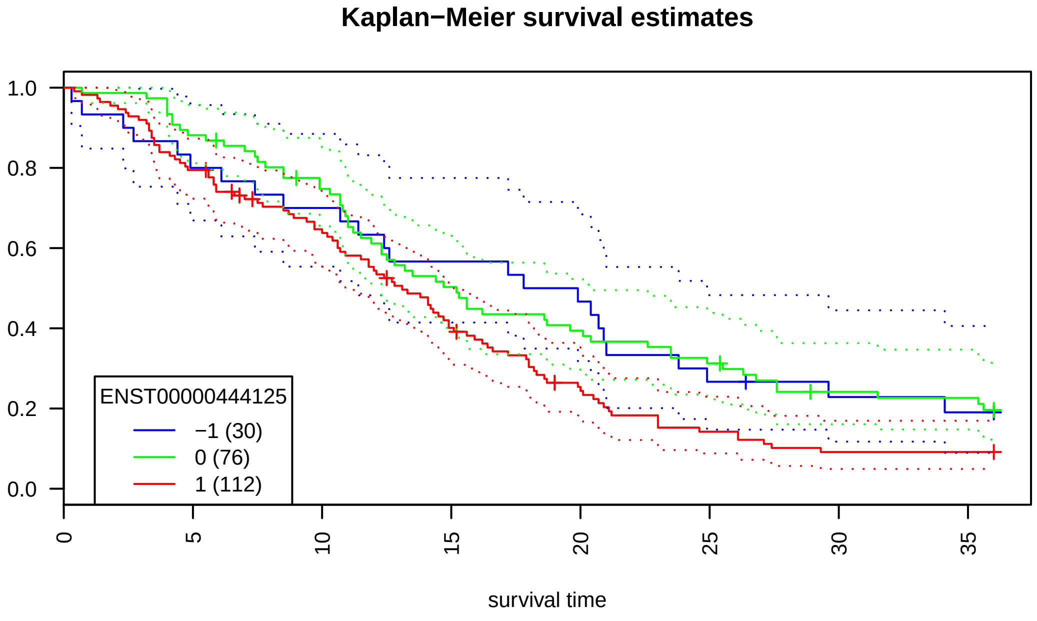

Figure 1145: Each curve represents the relative number of ENST00000444125 survived to the corresponding point of time. Dashed lines represent 0.95 confidence intervals for the curves with the corresponding color. Total of 12 observations deleted due to missingness. Log-rank probability measure for the equality of these curves is 2.784386e-02. Additional annotations: *.GeneId*=ENSG00000233901, *.GeneName*=RP11-65J3.1, *.GeneDesc*=[undefined].

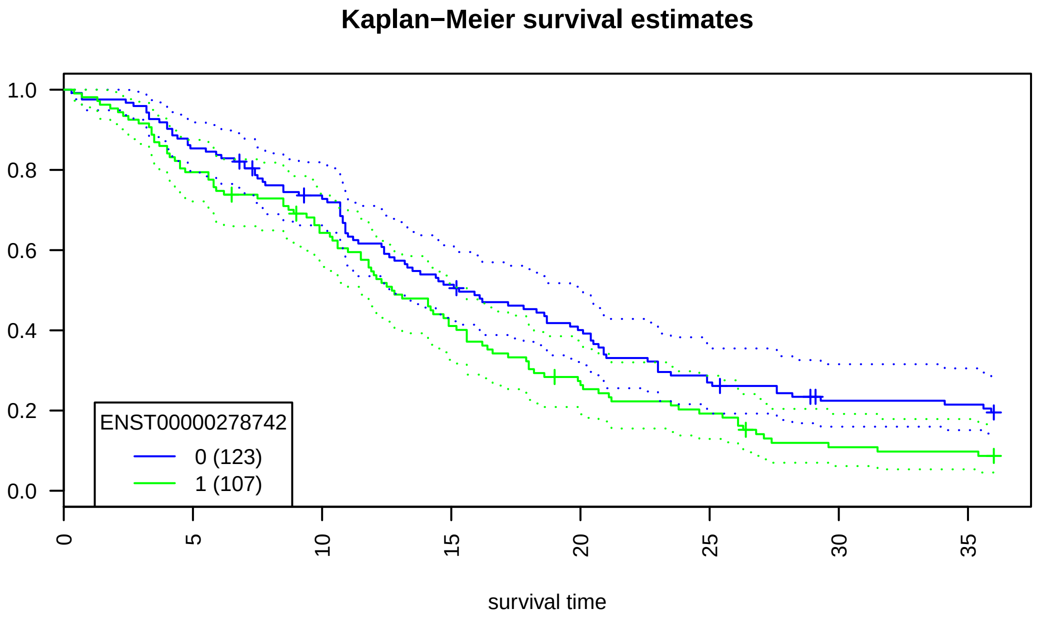

Figure 1146: Each curve represents the relative number of ENST00000278742 survived to the corresponding point of time. Dashed lines represent 0.95 confidence intervals for the curves with the corresponding color. Log-rank probability measure for the equality of these curves is 2.027188e-02. Additional annotations: .*GeneId*=ENSG00000149418, .*GeneName*=ST14, .*GeneDesc*=suppression of tumorigenicity 14 (colon carcinoma) [Source:HGNC Symbol;Acc:11344].

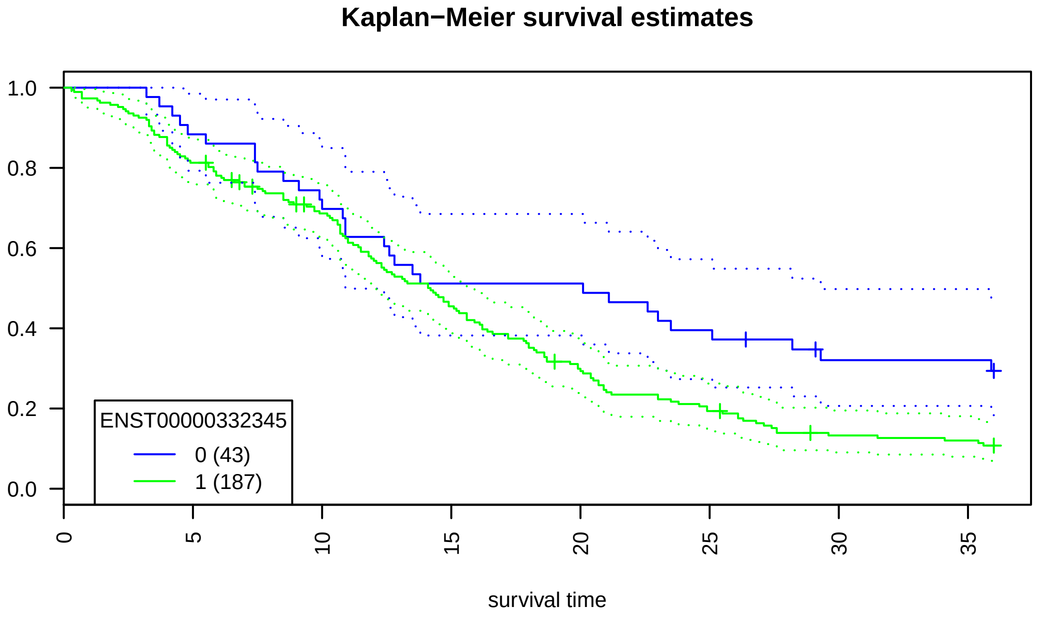

Figure 1147: Each curve represents the relative number of ENST00000332345 survived to the corresponding point of time. Dashed lines represent 0.95 confidence intervals for the curves with the corresponding color. Log-rank probability measure for the equality of these curves is 9.361196e-03. Additional annotations: .*GeneId*=ENSG00000169756, .*GeneName*=LIMS1, .*GeneDesc*=LIM and senescent cell antigen-like domains 1 [Source:HGNC Symbol;Acc:6616].

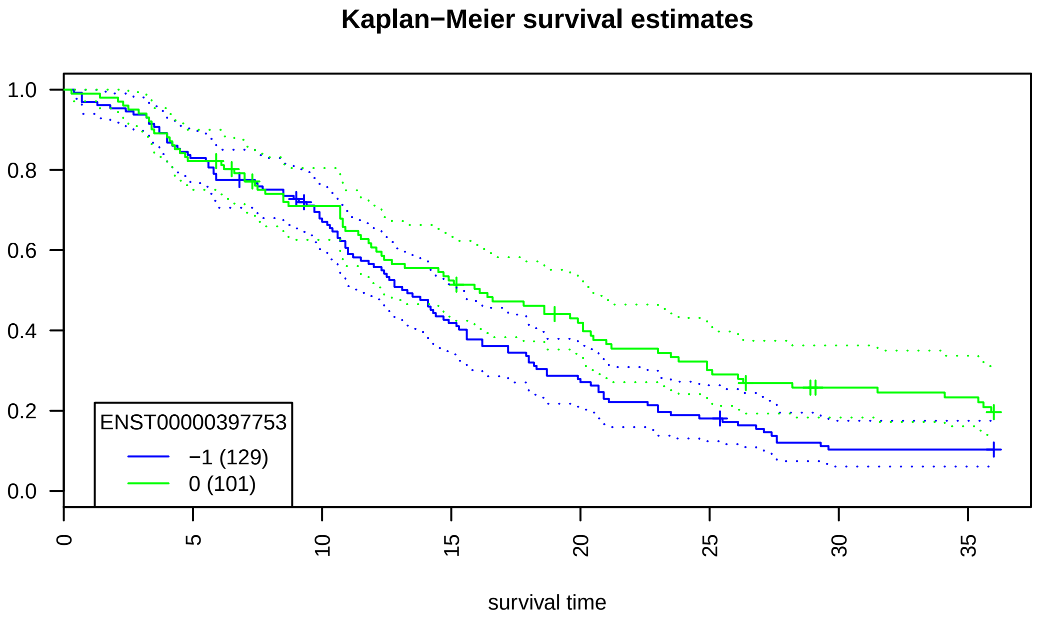

Figure 1148: Each curve represents the relative number of ENST00000397753 survived to the corresponding point of time. Dashed lines represent 0.95 confidence intervals for the curves with the corresponding color. Log-rank probability measure for the equality of these curves is 3.327439e-02. Additional annotations: *.GeneId*=ENSG00000196323, *.GeneName*=ZBTB44, *.GeneDesc*=zinc finger and BTB domain containing 44 [Source:HGNC Symbol;Acc:25001].

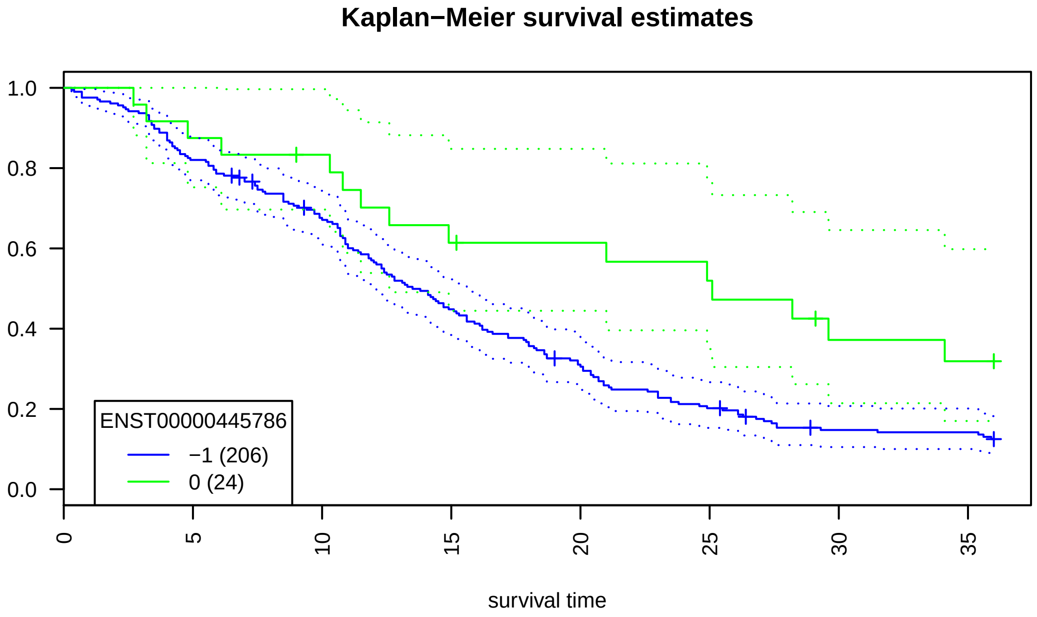

Figure 1149: Each curve represents the relative number of ENST00000445786 survived to the corresponding point of time. Dashed lines represent 0.95 confidence intervals for the curves with the corresponding color. Log-rank probability measure for the equality of these curves is 1.212771e-02.

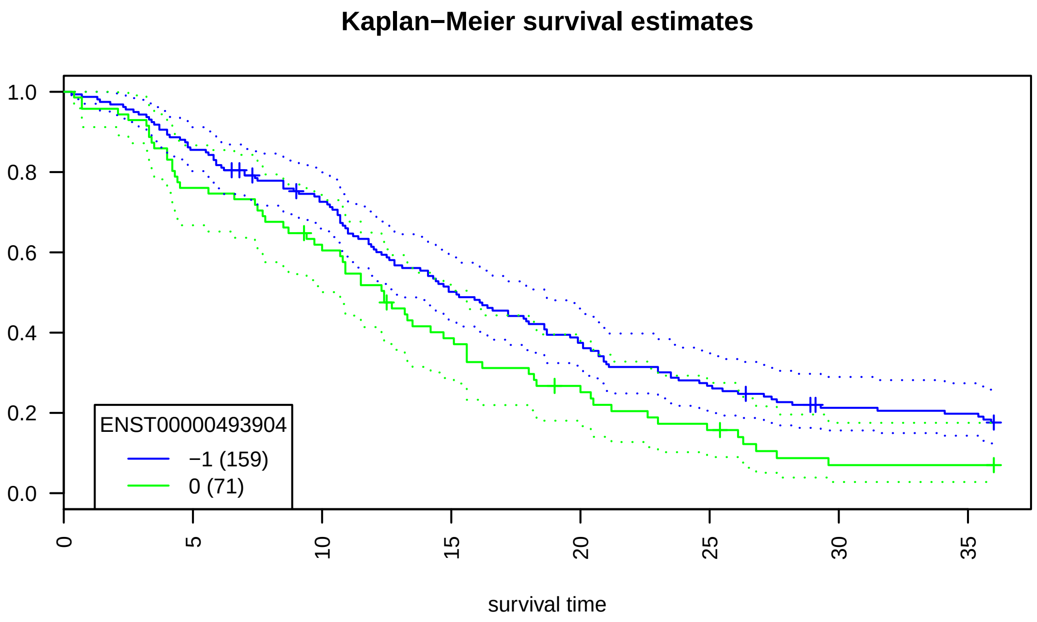

Figure 1150: Each curve represents the relative number of ENST00000493904 survived to the corresponding point of time. Dashed lines represent 0.95 confidence intervals for the curves with the corresponding color. Log-rank probability measure for the equality of these curves is 1.315732e-02. Additional annotations: *.GeneId*=ENSG00000220575, *.GeneName*=AC093726.4, *.GeneDesc*=cDNA FLJ50038 [Source:UniProtKB/TrEMBL;Acc:B7Z8E6].

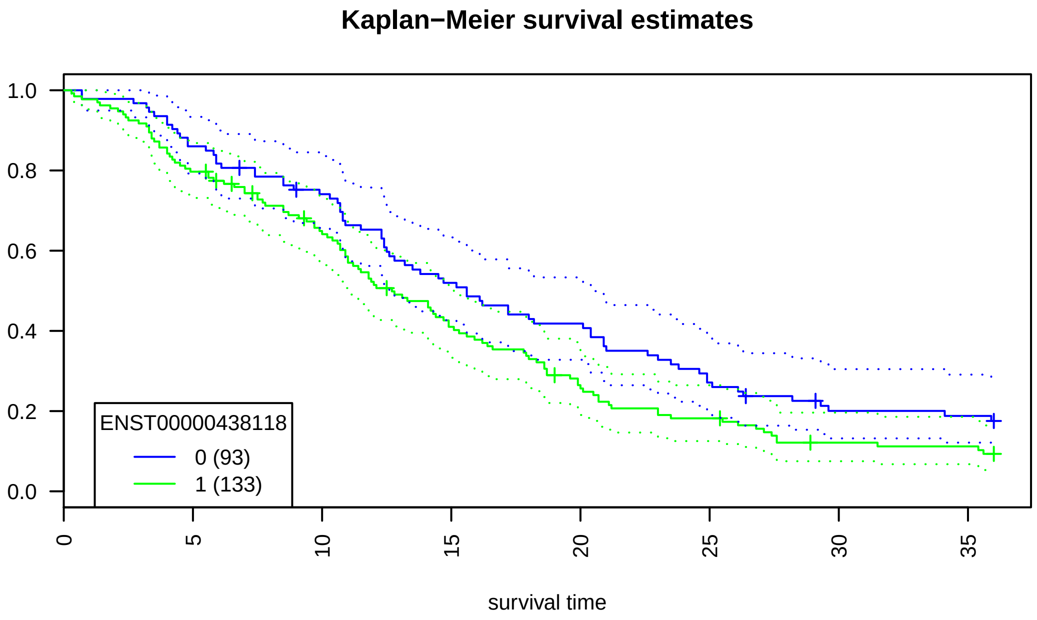

Figure 1151: Each curve represents the relative number of ENST00000438118 survived to the corresponding point of time. Dashed lines represent 0.95 confidence intervals for the curves with the corresponding color. Total of 4 observations deleted due to missingness. Log-rank probability measure for the equality of these curves is 3.059960e-02. Additional annotations: *.GeneId*=ENSG00000001036, *.GeneName*=FUCA2, *.GeneDesc*=fucosidase, alpha-L- 2, plasma [Source:HGNC Symbol;Acc:4008].

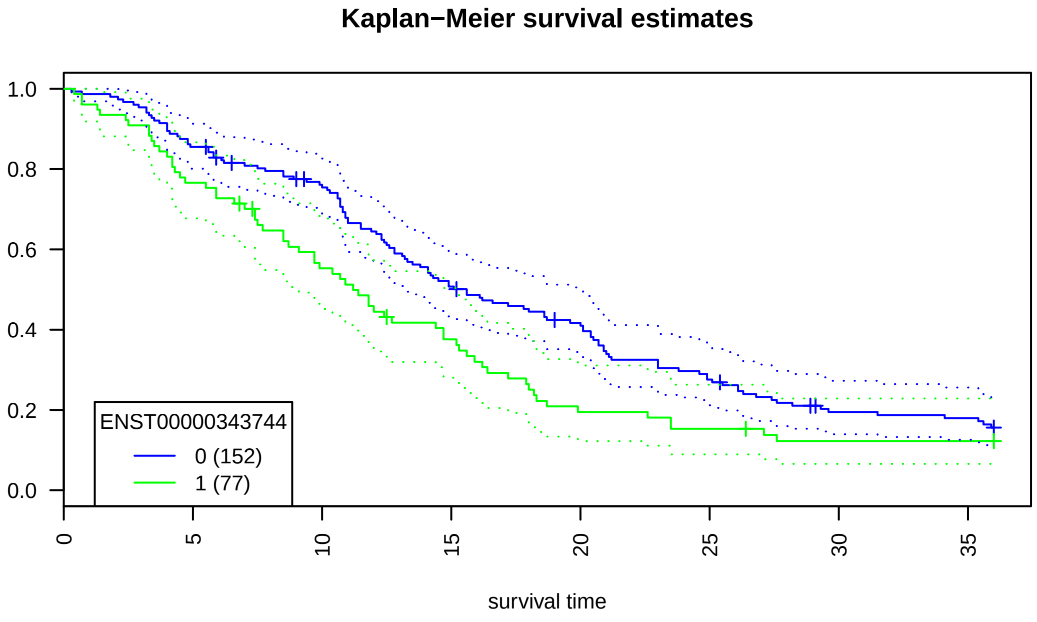

Figure 1152: Each curve represents the relative number of ENST00000343744 survived to the corresponding point of time. Dashed lines represent 0.95 confidence intervals for the curves with the corresponding color. Total of 1 observation deleted due to missingness. Log-rank probability measure for the equality of these curves is 1.687280e-02. Additional annotations: *.GeneId*=ENSG00000162390, *.GeneName*=ACOT11, *.GeneDesc*=acyl-CoA thioesterase 11 [Source:HGNC Symbol;Acc:18156].

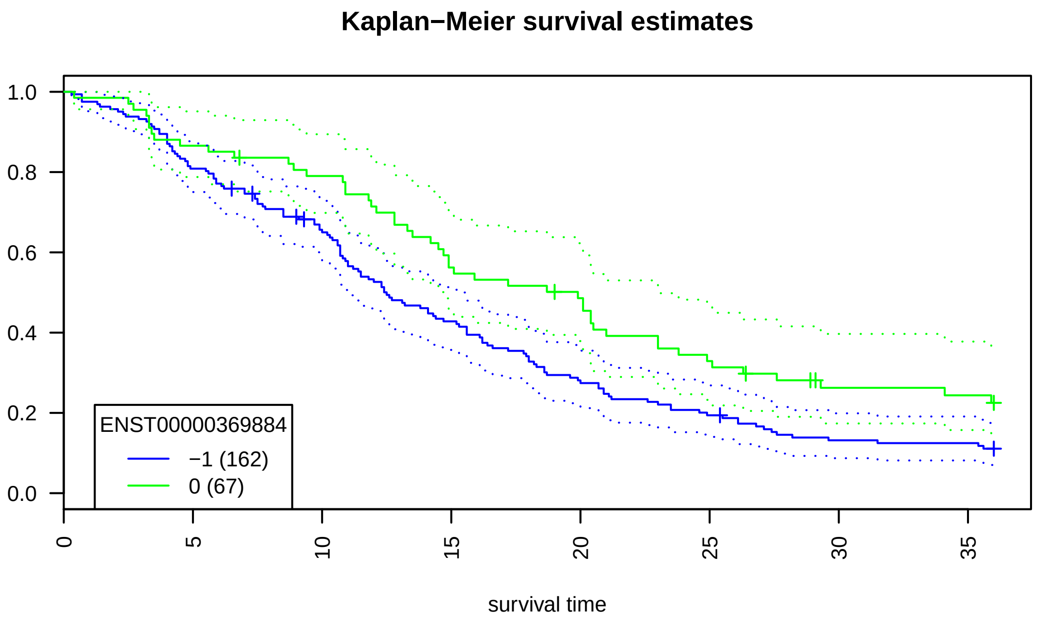

Figure 1153: Each curve represents the relative number of ENST00000369884 survived to the corresponding point of time. Dashed lines represent 0.95 confidence intervals for the curves with the corresponding color. Total of 1 observation deleted due to missingness. Log-rank probability measure for the equality of these curves is 7.432132e-03. Additional annotations: *.GeneId*=ENSG00000203886, *.GeneName*=CYP17A1OS, *.GeneDesc*=cytochrome P450, family 17, subfamily A, polypeptide 1 opposite strand [Source:HGNC Symbol;Acc:31671].

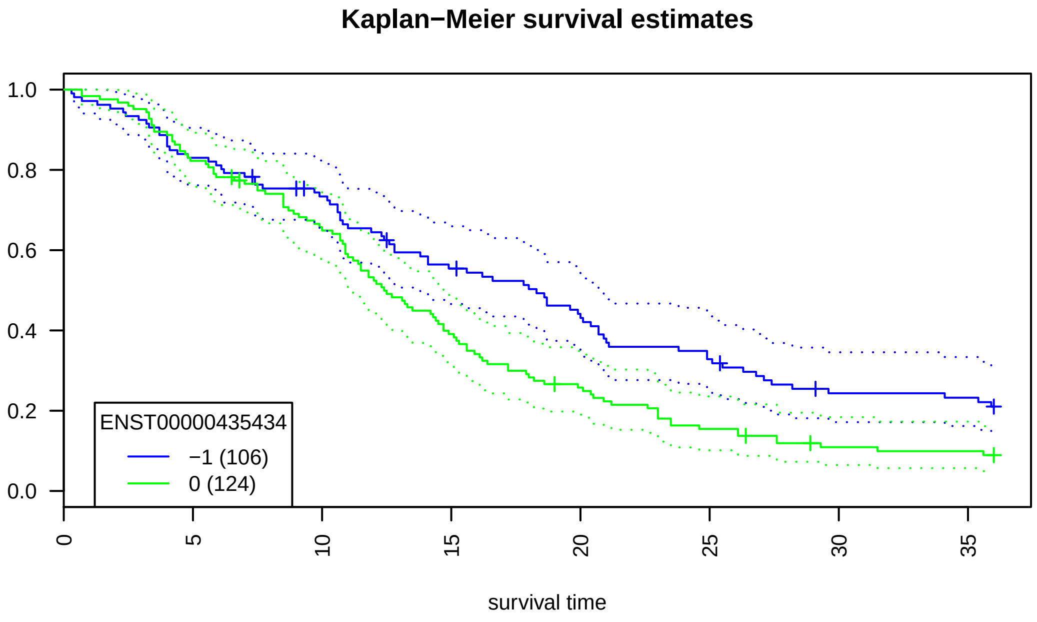

Figure 1154: Each curve represents the relative number of ENST00000435434 survived to the corresponding point of time. Dashed lines represent 0.95 confidence intervals for the curves with the corresponding color. Log-rank probability measure for the equality of these curves is 3.970294e-03. Additional annotations: *.GeneId*=ENSG00000231233, *.GeneName*=RP11-127L20.6, *.GeneDesc*=[undefined].

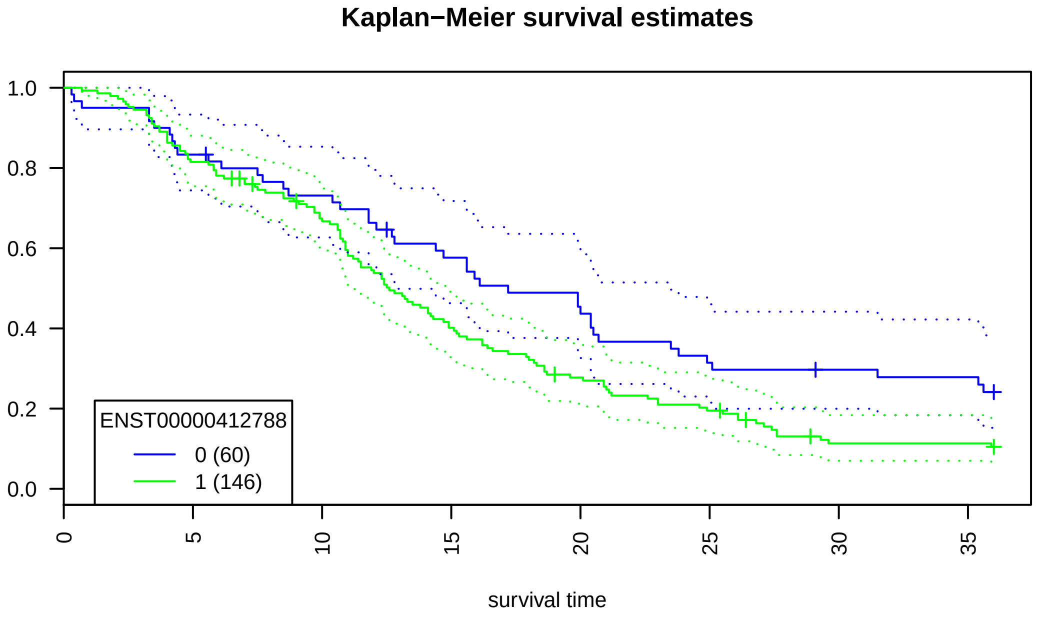

Figure 1155: Each curve represents the relative number of ENST00000412788 survived to the corresponding point of time. Dashed lines represent 0.95 confidence intervals for the curves with the corresponding color. Total of 24 observations deleted due to missingness. Log-rank probability measure for the equality of these curves is 1.687227e-02. Additional annotations: *.GeneId*=ENSG00000130600, *.GeneName*=H19, *.GeneDesc*=microRNA 675 [Source:HGNC Symbol;Acc:33351].

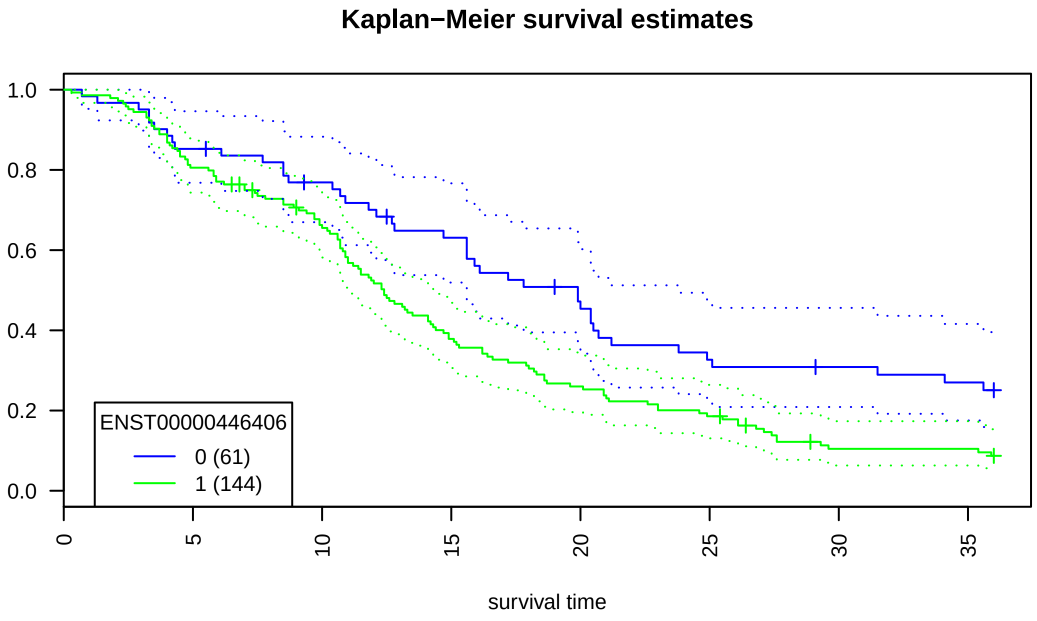

Figure 1156: Each curve represents the relative number of ENST00000446406 survived to the corresponding point of time. Dashed lines represent 0.95 confidence intervals for the curves with the corresponding color. Total of 25 observations deleted due to missingness. Log-rank probability measure for the equality of these curves is 2.291312e-03. Additional annotations: *.GeneId*=ENSG00000130600, *.GeneName*=H19, *.GeneDesc*=microRNA 675 [Source:HGNC Symbol;Acc:33351].

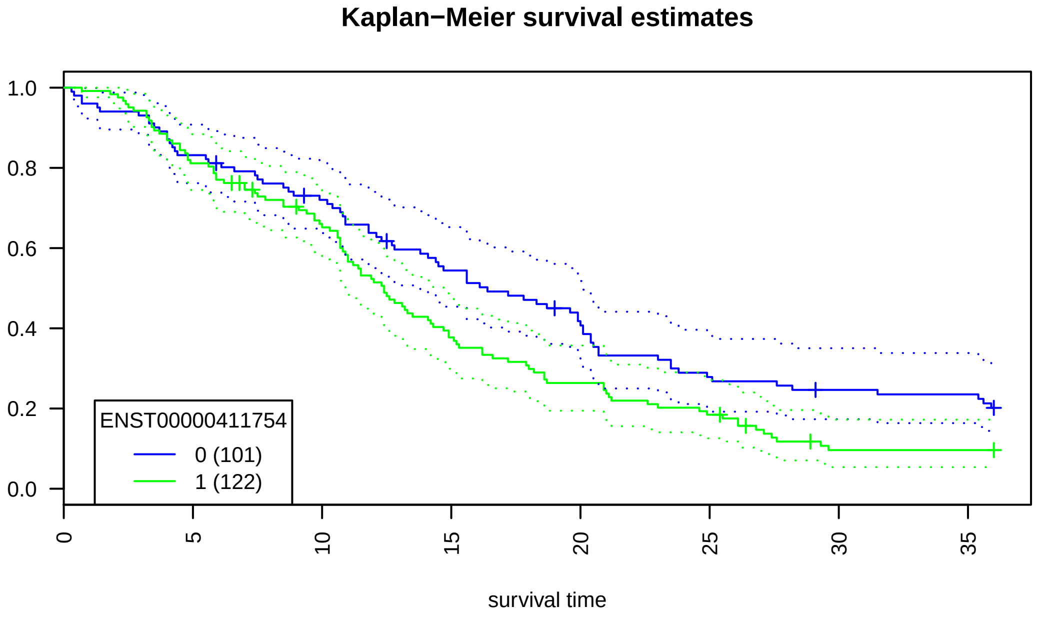

Figure 1157: Each curve represents the relative number of ENST00000411754 survived to the corresponding point of time. Dashed lines represent 0.95 confidence intervals for the curves with the corresponding color. Total of 7 observations deleted due to missingness. Log-rank probability measure for the equality of these curves is 1.630720e-02. Additional annotations: *.GeneId*=ENSG00000130600, *.GeneName*=H19, *.GeneDesc*=microRNA 675 [Source:HGNC Symbol;Acc:33351].

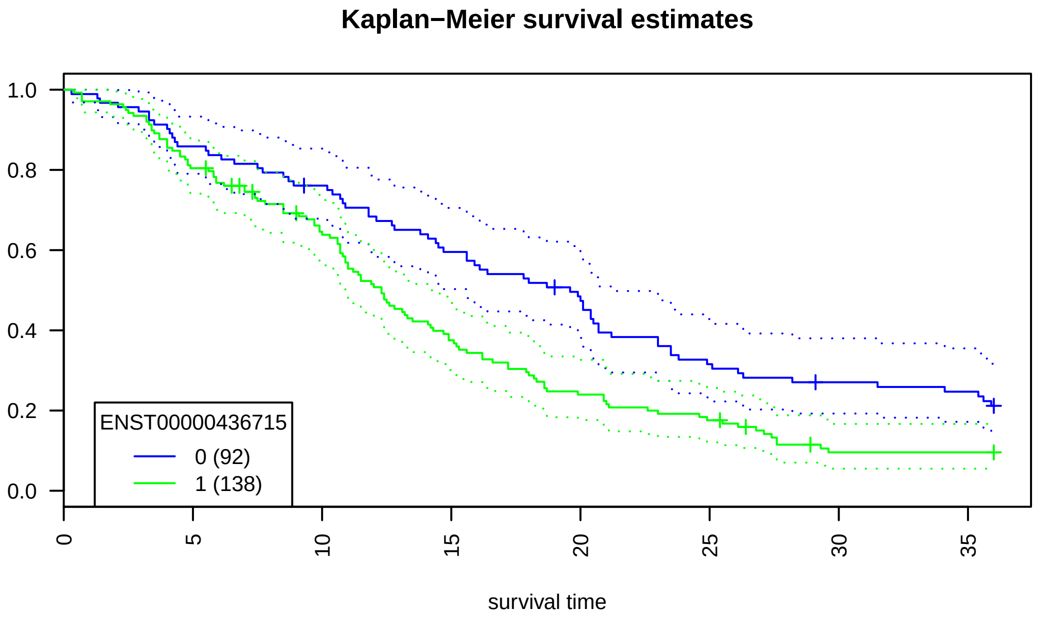

Figure 1158: Each curve represents the relative number of ENST00000436715 survived to the corresponding point of time. Dashed lines represent 0.95 confidence intervals for the curves with the corresponding color. Log-rank probability measure for the equality of these curves is 1.245201e-03. Additional annotations: *.GeneId*=ENSG00000130600, *.GeneName*=H19, *.GeneDesc*=microRNA 675 [Source:HGNC Symbol;Acc:33351].

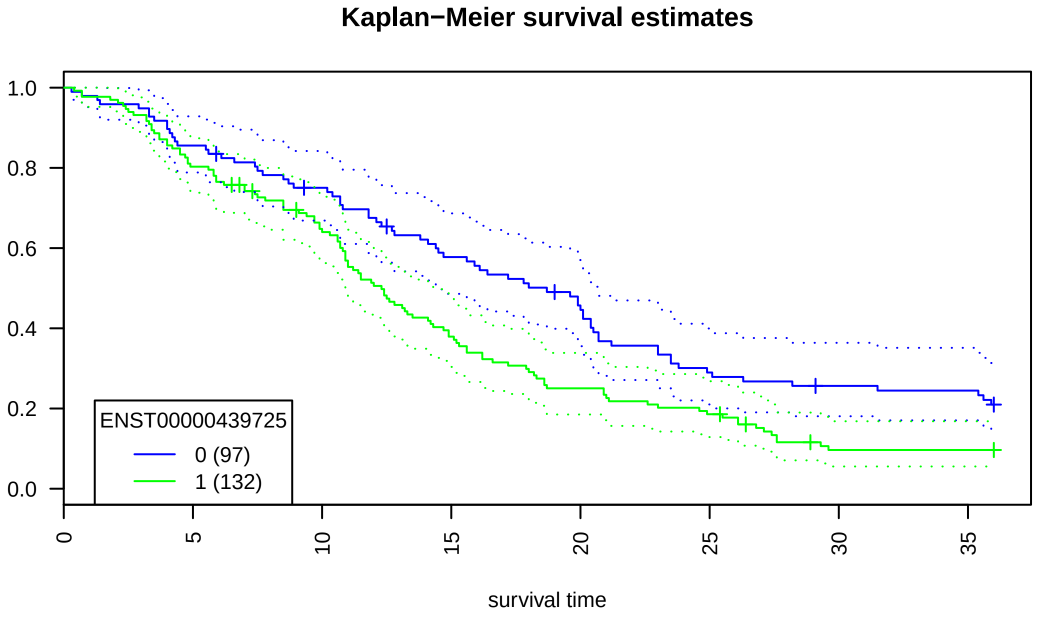

Figure 1159: Each curve represents the relative number of ENST00000439725 survived to the corresponding point of time. Dashed lines represent 0.95 confidence intervals for the curves with the corresponding color. Total of 1 observation deleted due to missingness. Log-rank probability measure for the equality of these curves is 3.367551e-03. Additional annotations: *.GeneId*=ENSG00000130600, *.GeneName*=H19, *.GeneDesc*=microRNA 675 [Source:HGNC Symbol;Acc:33351].

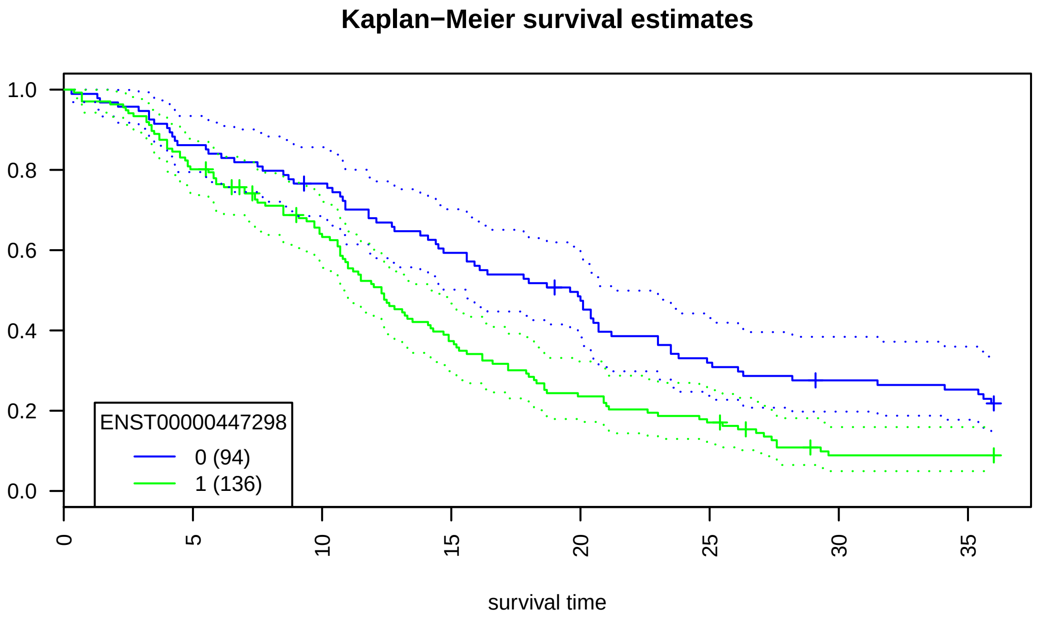

Figure 1160: Each curve represents the relative number of ENST00000447298 survived to the corresponding point of time. Dashed lines represent 0.95 confidence intervals for the curves with the corresponding color. Log-rank probability measure for the equality of these curves is 5.882097e-04. Additional annotations: *.GeneId*=ENSG00000130600, *.GeneName*=H19, *.GeneDesc*=microRNA 675 [Source:HGNC Symbol;Acc:33351].

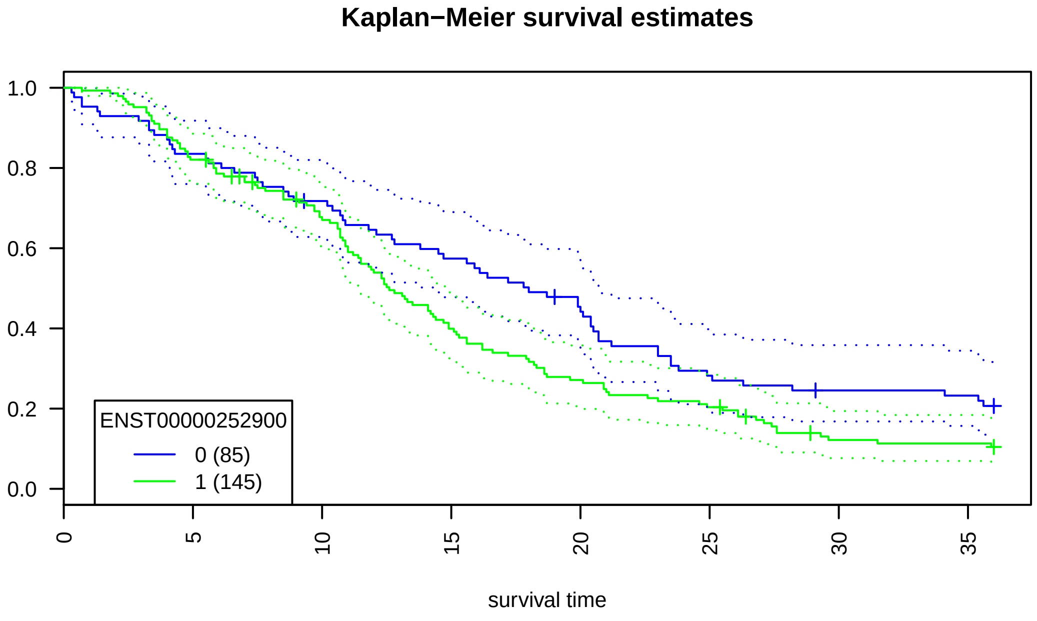

Figure 1161: Each curve represents the relative number of ENST00000252900 survived to the corresponding point of time. Dashed lines represent 0.95 confidence intervals for the curves with the corresponding color. Log-rank probability measure for the equality of these curves is 2.600394e-02.

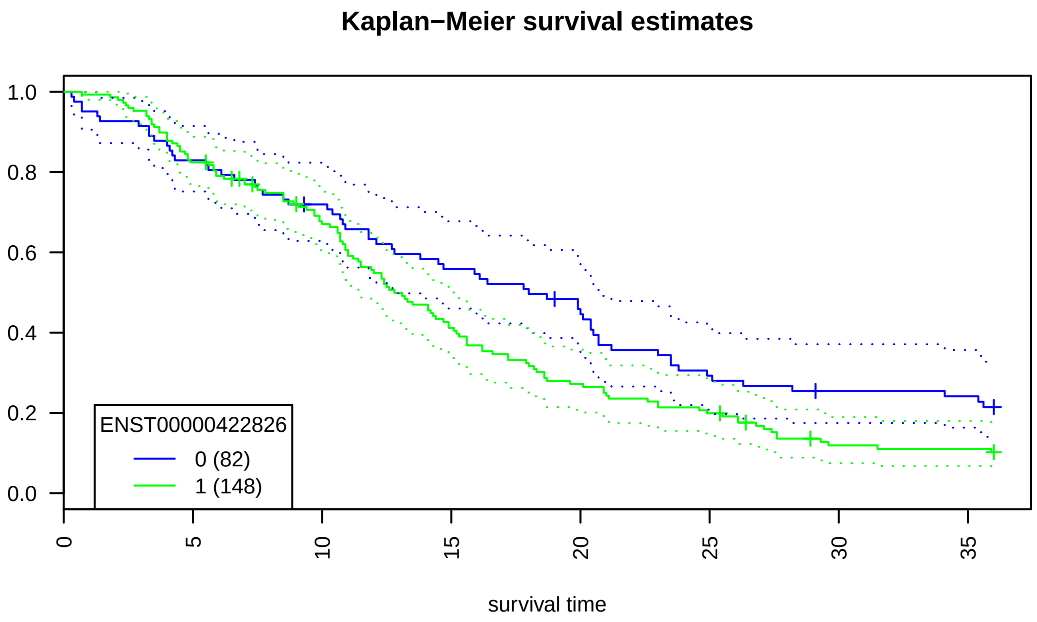

Figure 1162: Each curve represents the relative number of ENST00000422826 survived to the corresponding point of time. Dashed lines represent 0.95 confidence intervals for the curves with the corresponding color. Log-rank probability measure for the equality of these curves is 2.385224e-02. Additional annotations: *.GeneId*=ENSG00000130600, *.GeneName*=H19, *.GeneDesc*=microRNA 675 [Source:HGNC Symbol;Acc:33351].

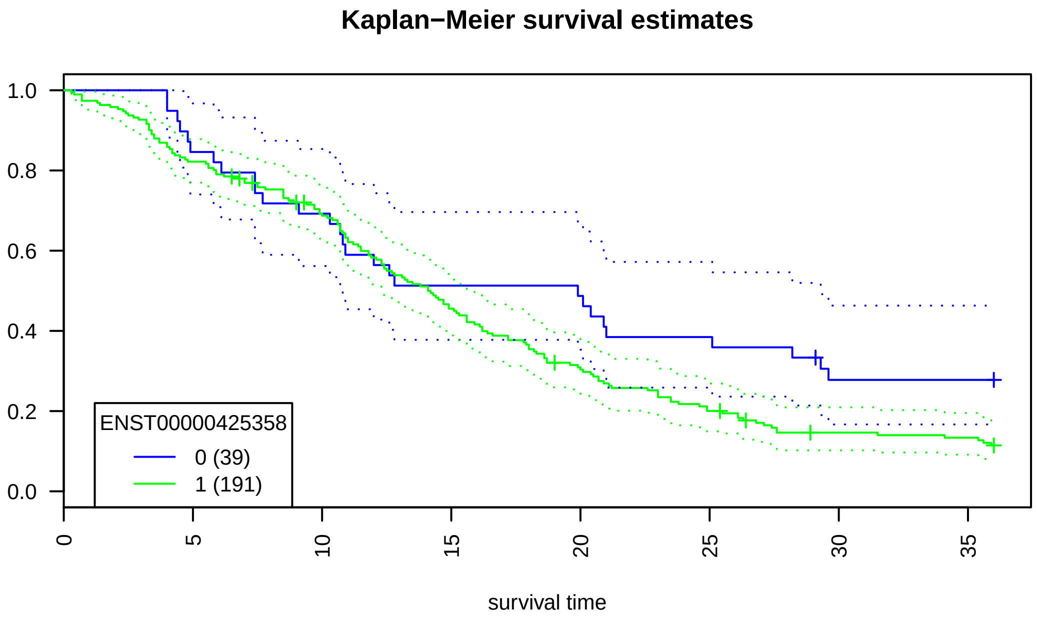

Figure 1163: Each curve represents the relative number of ENST00000425358 survived to the corresponding point of time. Dashed lines represent 0.95 confidence intervals for the curves with the corresponding color. Log-rank probability measure for the equality of these curves is 4.051174e-02. Additional annotations: *.GeneId*=ENSG00000233429, *.GeneName*=HOTAIRM1, *.GeneDesc*=hox transcript antisense RNA, myeloid-specific 1 (non-protein coding) [Source:HGNC Symbol;Acc:37117].

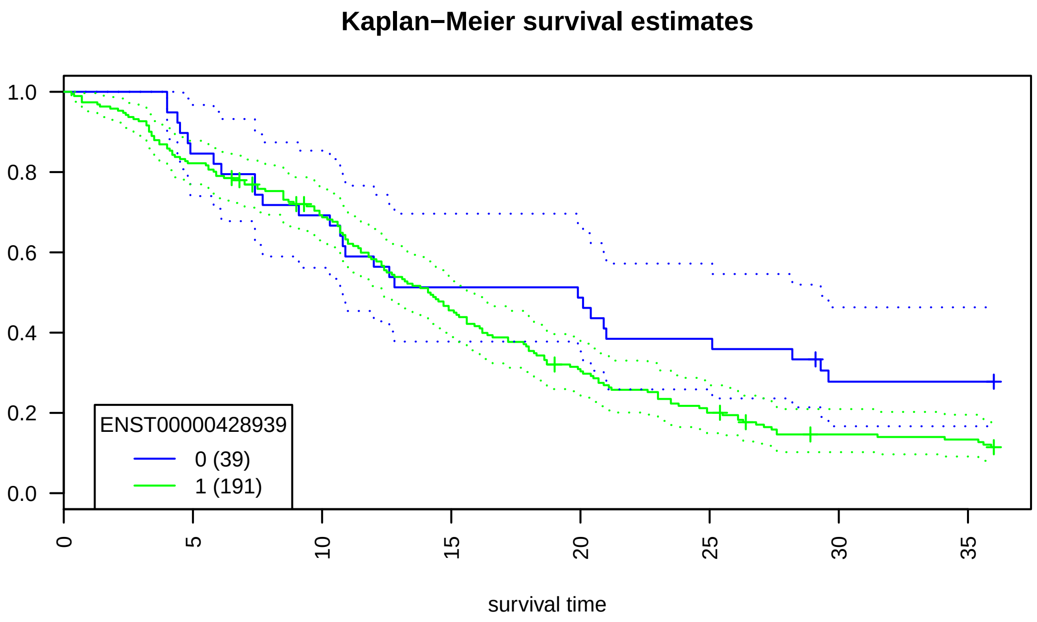

Figure 1164: Each curve represents the relative number of ENST00000428939 survived to the corresponding point of time. Dashed lines represent 0.95 confidence intervals for the curves with the corresponding color. Log-rank probability measure for the equality of these curves is 4.051174e-02. Additional annotations: *.GeneId*=ENSG00000233429, *.GeneName*=HOTAIRM1, *.GeneDesc*=hox transcript antisense RNA, myeloid-specific 1 (non-protein coding) [Source:HGNC Symbol;Acc:37117].

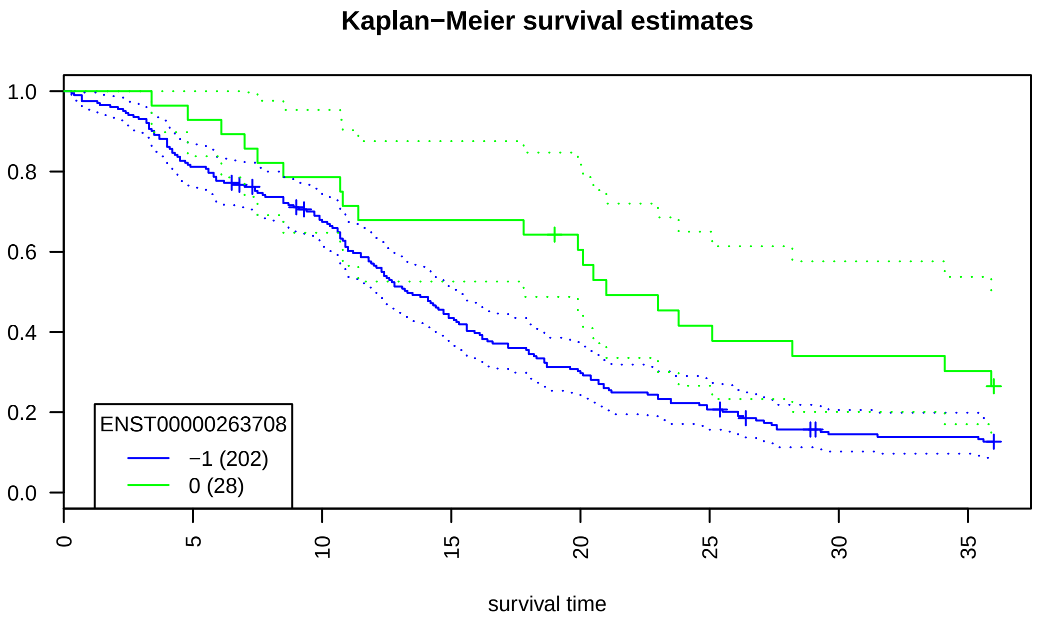

Figure 1165: Each curve represents the relative number of ENST00000263708 survived to the corresponding point of time. Dashed lines represent 0.95 confidence intervals for the curves with the corresponding color. Log-rank probability measure for the equality of these curves is 1.679715e-02. Additional annotations: *.GeneId*=ENSG00000088179, *.GeneName*=PTPN4, *.GeneDesc*=protein tyrosine phosphatase, non-receptor type 4 (megakaryocyte) [Source:HGNC Symbol;Acc:9656].

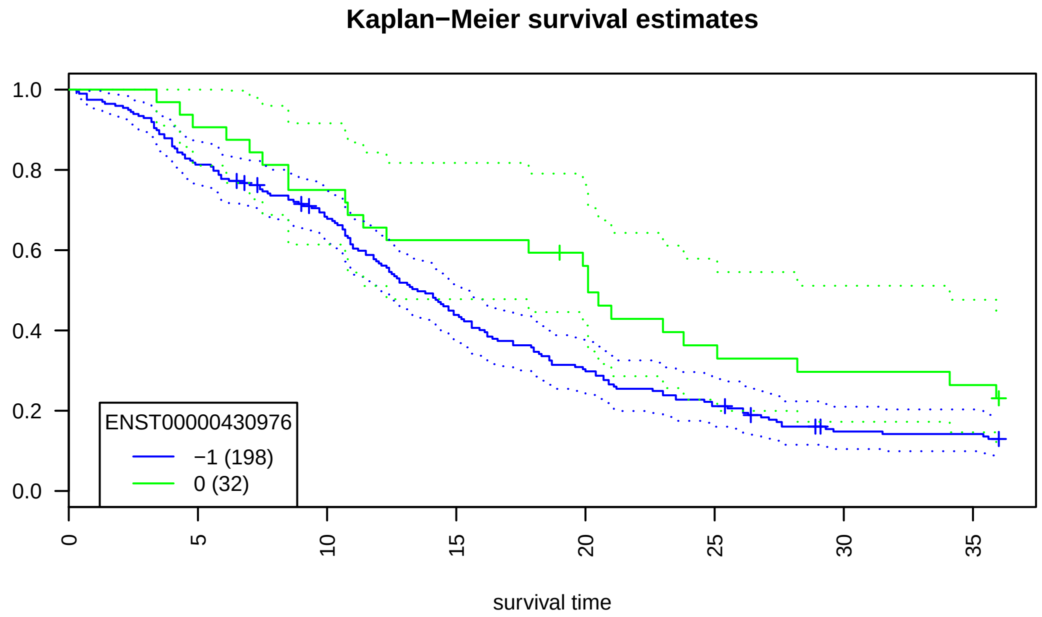

Figure 1166: Each curve represents the relative number of ENST00000430976 survived to the corresponding point of time. Dashed lines represent 0.95 confidence intervals for the curves with the corresponding color. Log-rank probability measure for the equality of these curves is 4.829254e-02. Additional annotations: *.GeneId*=ENSG00000088179, *.GeneName*=PTPN4, *.GeneDesc*=protein tyrosine phosphatase, non-receptor type 4 (megakaryocyte) [Source:HGNC Symbol;Acc:9656].

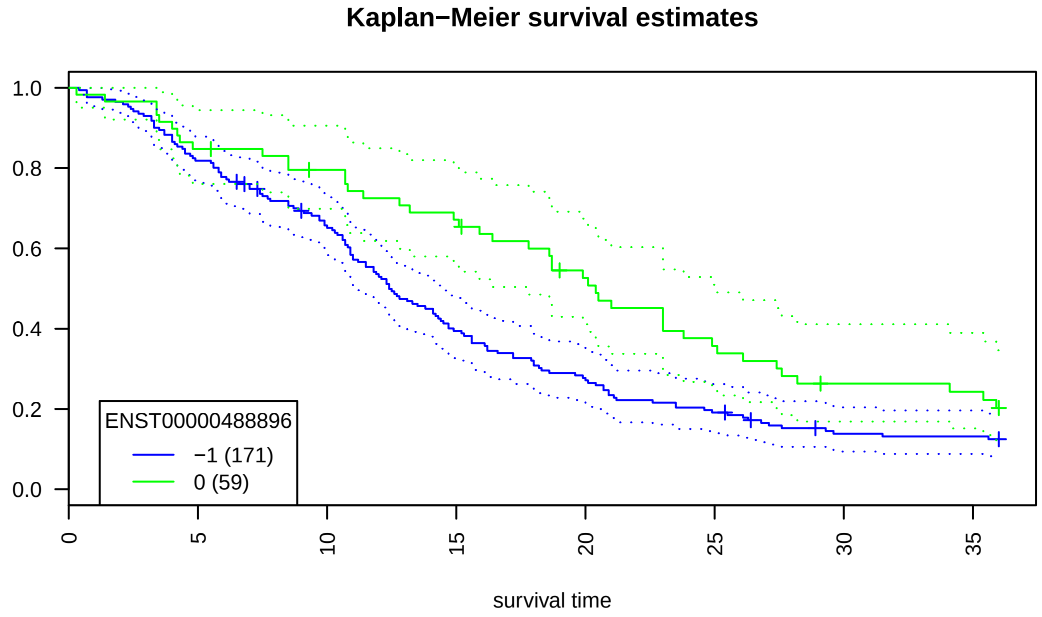

Figure 1167: Each curve represents the relative number of ENST00000488896 survived to the corresponding point of time. Dashed lines represent 0.95 confidence intervals for the curves with the corresponding color. Log-rank probability measure for the equality of these curves is 6.36446e-03. Additional annotations: *.GeneId*=ENSG00000088179, *.GeneName*=PTPN4, *.GeneDesc*=protein tyrosine phosphatase, non-receptor type 4 (megakaryocyte) [Source:HGNC Symbol;Acc:9656].

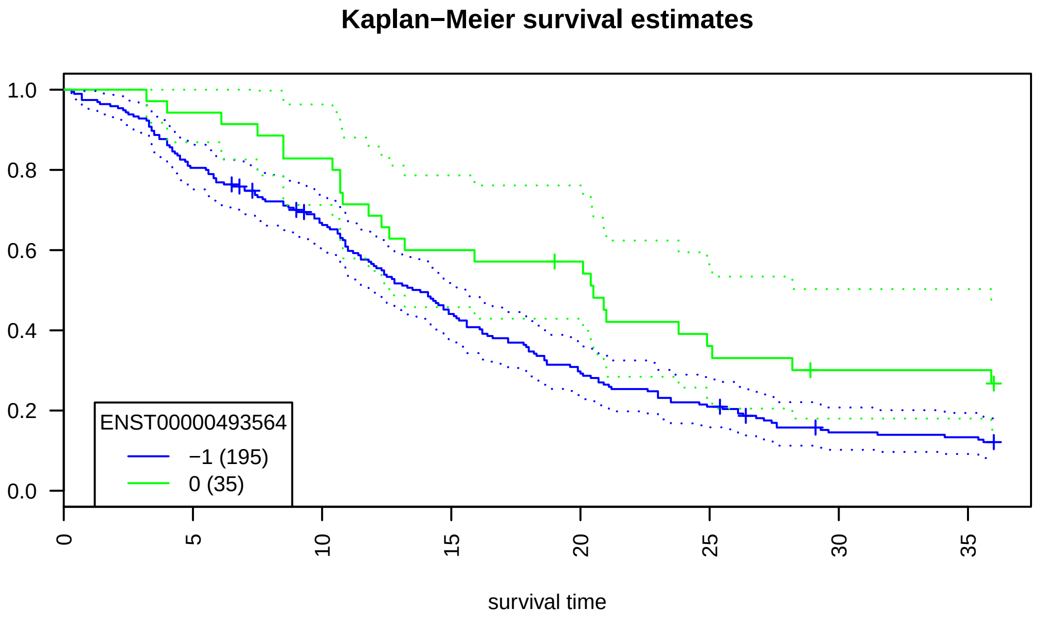

Figure 1168: Each curve represents the relative number of ENST00000493564 survived to the corresponding point of time. Dashed lines represent 0.95 confidence intervals for the curves with the corresponding color. Log-rank probability measure for the equality of these curves is 1.746700e-02. Additional annotations: *.GeneId*=ENSG00000118473, *.GeneName*=SGIP1, *.GeneDesc*=SH3-domain GRB2-like (endophilin) interacting protein 1 [Source:HGNC Symbol;Acc:25412].

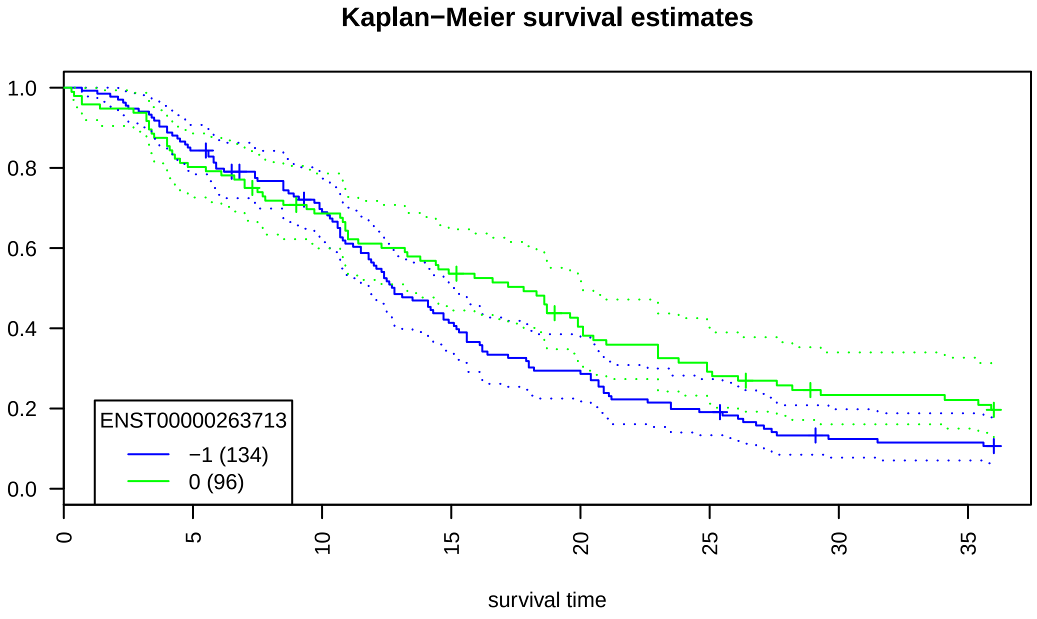

Figure 1169: Each curve represents the relative number of ENST00000263713 survived to the corresponding point of time. Dashed lines represent 0.95 confidence intervals for the curves with the corresponding color. Log-rank probability measure for the equality of these curves is 4.986017e-02. Additional annotations: *.GeneId*=ENSG00000115109, *.GeneName*=EPB41L5, *.GeneDesc*=erythrocyte membrane protein band 4.1 like 5 [Source:HGNC Symbol;Acc:19819].

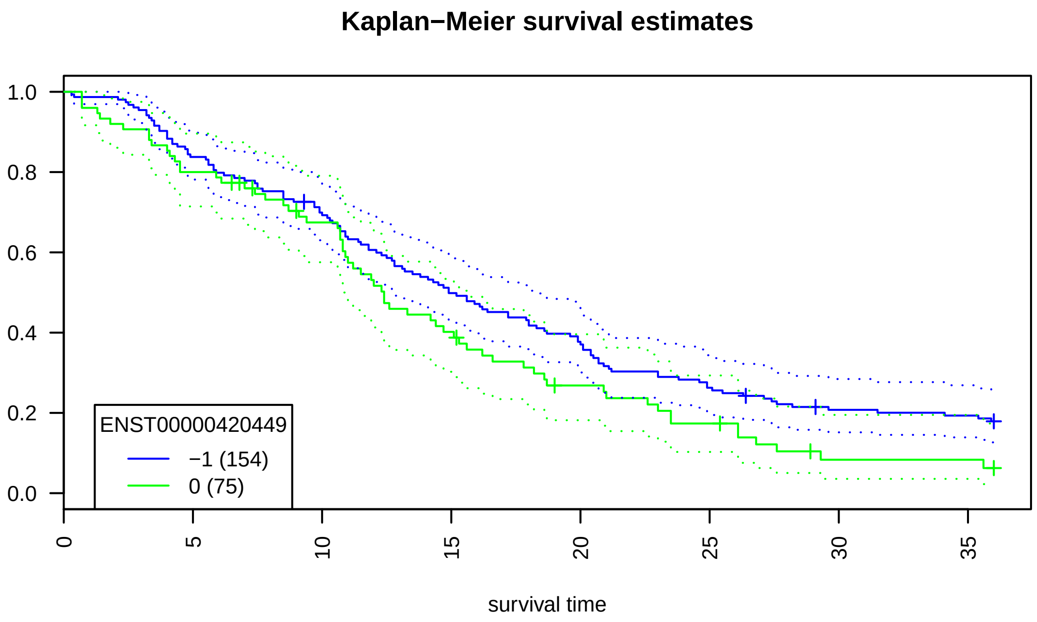

Figure 1170: Each curve represents the relative number of ENST00000420449 survived to the corresponding point of time. Dashed lines represent 0.95 confidence intervals for the curves with the corresponding color. Total of 1 observation deleted due to missingness. Log-rank probability measure for the equality of these curves is 3.973413e-02. Additional annotations: *.GeneId*=ENSG00000228204, *.GeneName*=RP4-724E13.2, *.GeneDesc*=[undefined].

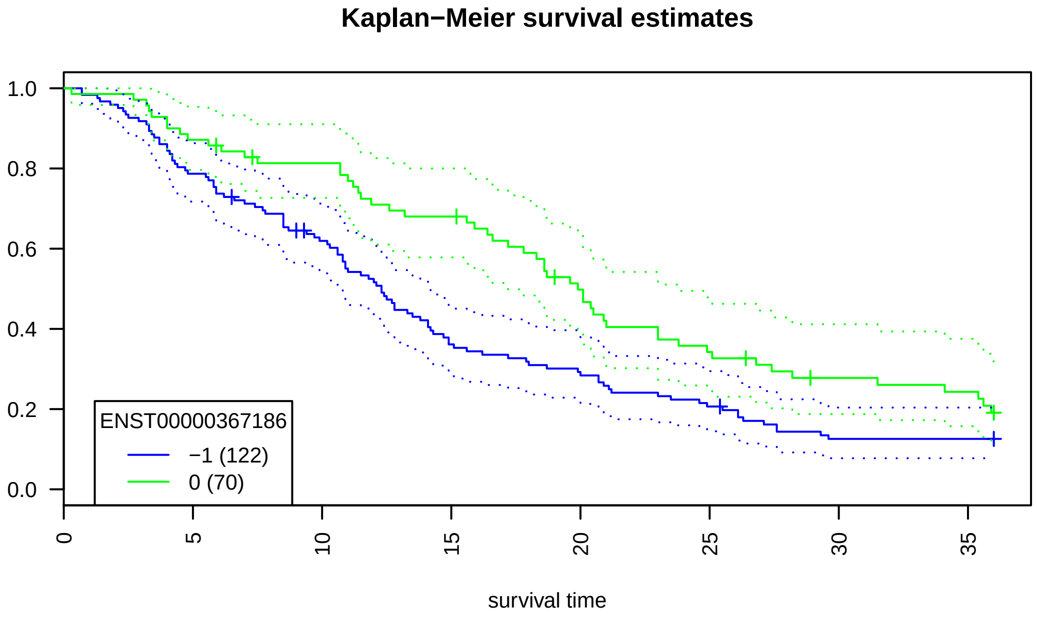

Figure 1171: Each curve represents the relative number of ENST00000367186 survived to the corresponding point of time. Dashed lines represent 0.95 confidence intervals for the curves with the corresponding color. Total of 38 observations deleted due to missingness. Log-rank probability measure for the equality of these curves is 7.208916e-03. Additional annotations: *.GeneId*=ENSG00000213079, *.GeneName*=RBM16, *.GeneDesc*=RNA binding motif protein 16 [Source:HGNC Symbol;Acc:20959].

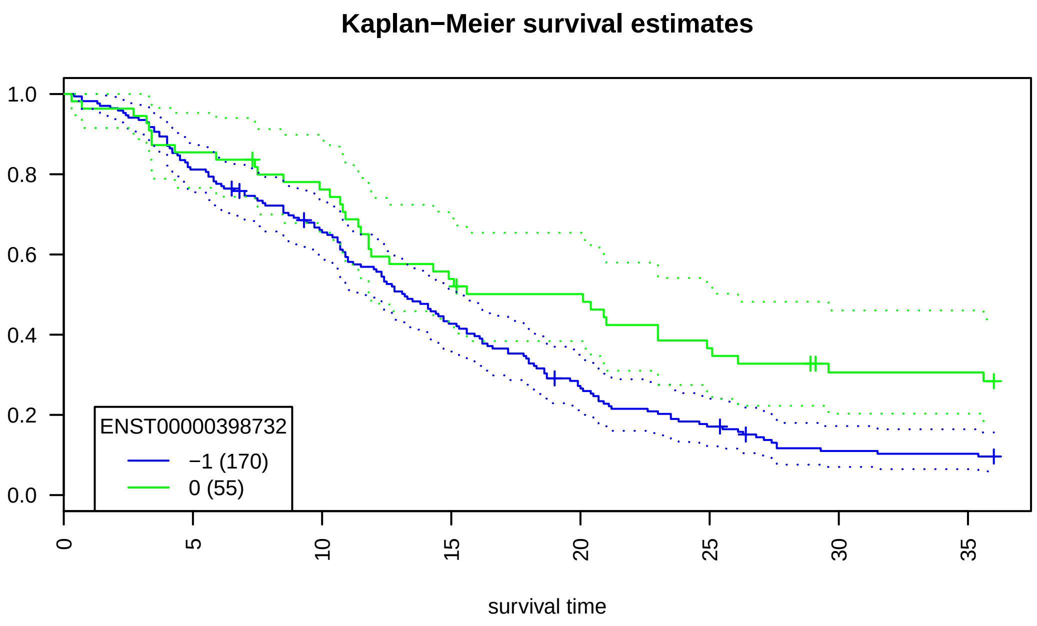

Figure 1172: Each curve represents the relative number of ENST00000398732 survived to the corresponding point of time. Dashed lines represent 0.95 confidence intervals for the curves with the corresponding color. Total of 5 observations deleted due to missingness. Log-rank probability measure for the equality of these curves is 3.742420e-03. Additional annotations: *.GeneId*=ENSG00000198155, *.GeneName*=ZNF732, *.GeneDesc*=zinc finger protein 876 (pseudogene) [Source:HGNC Symbol;Acc:32472].

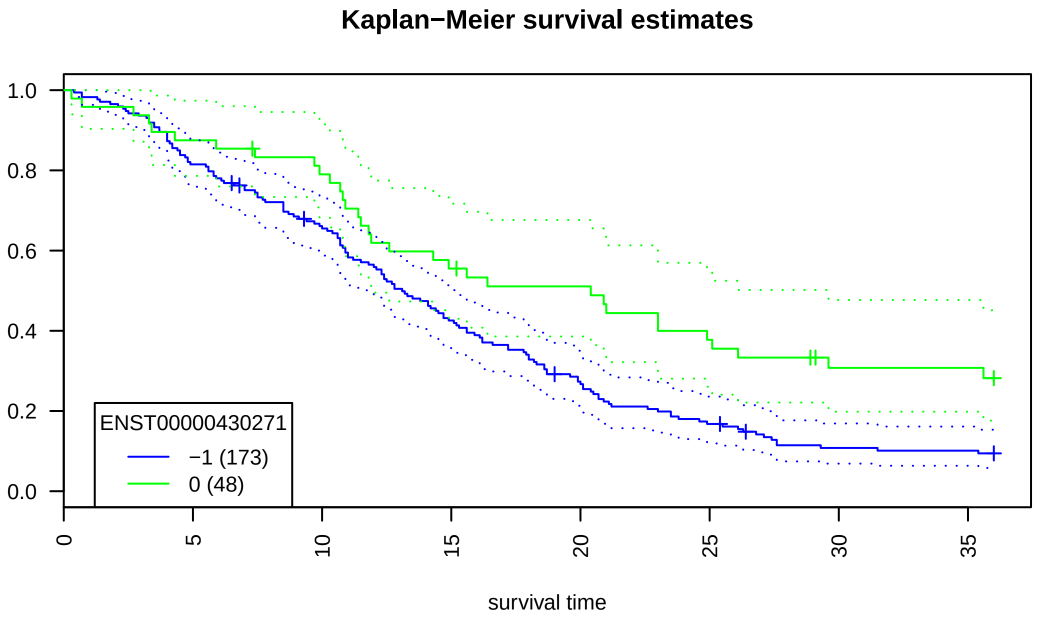

Figure 1173: Each curve represents the relative number of ENST00000430271 survived to the corresponding point of time. Dashed lines represent 0.95 confidence intervals for the curves with the corresponding color. Total of 9 observations deleted due to missingness. Log-rank probability measure for the equality of these curves is 2.849718e-03. Additional annotations: *.GeneId*=ENSG00000244117, *.GeneName*=RP11-3N2.1, *.GeneDesc*=[undefined].

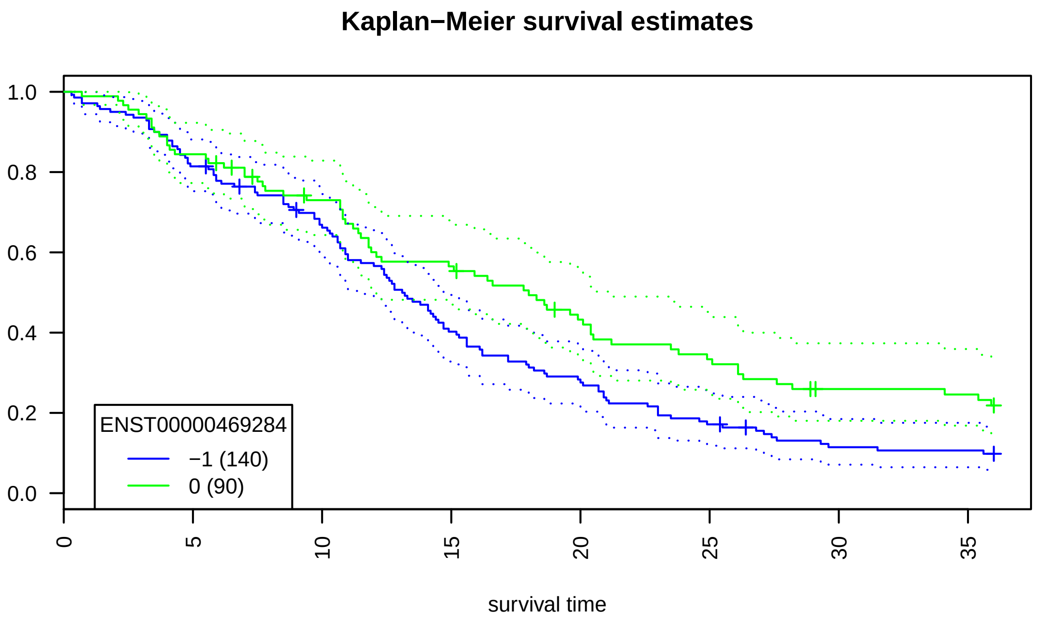

Figure 1174: Each curve represents the relative number of ENST00000469284 survived to the corresponding point of time. Dashed lines represent 0.95 confidence intervals for the curves with the corresponding color. Log-rank probability measure for the equality of these curves is 1.018382e-02. Additional annotations: *.GeneId*=ENSG00000051382, *.GeneName*=PIK3CB, *.GeneDesc*=phosphoinositide-3-kinase, catalytic, beta polypeptide [Source:HGNC Symbol;Acc:8976].

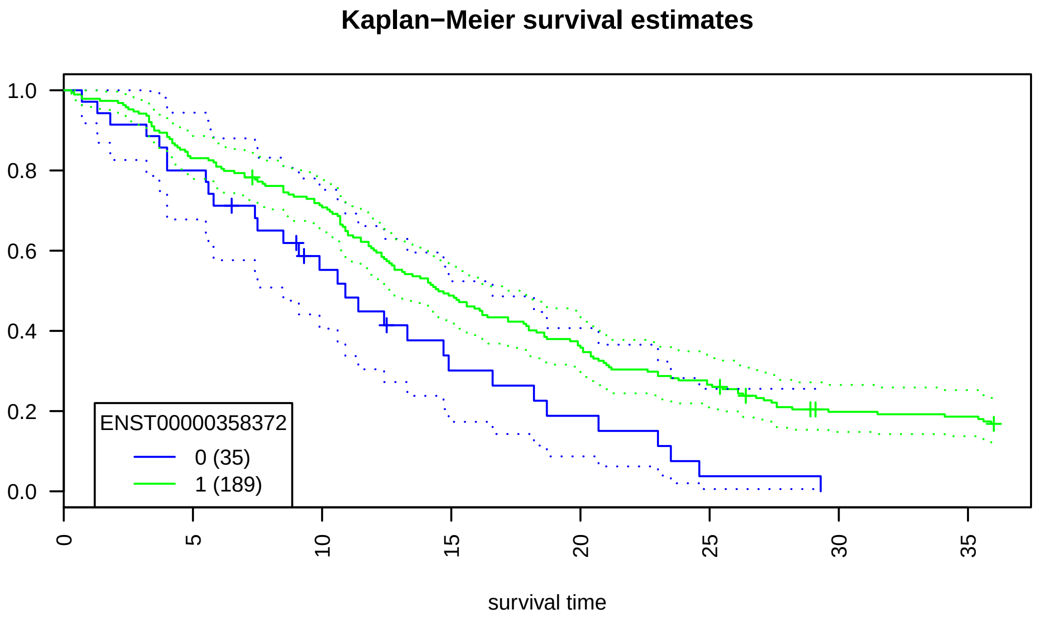

Figure 1175: Each curve represents the relative number of ENST00000358372 survived to the corresponding point of time. Dashed lines represent 0.95 confidence intervals for the curves with the corresponding color. Total of 6 observations deleted due to missingness. Log-rank probability measure for the equality of these curves is 4.054729e-03. Additional annotations: *.GeneId*=ENSG00000146457, *.GeneName*=WTAP, *.GeneDesc*=Wilms tumor 1 associated protein [Source:HGNC Symbol;Acc:16846].

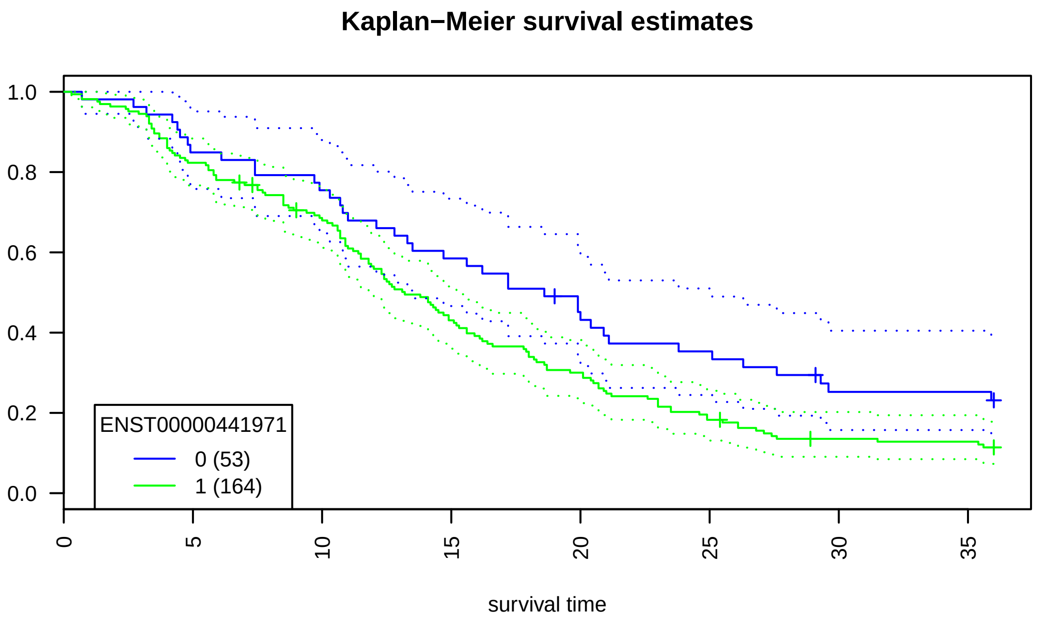

Figure 1176: Each curve represents the relative number of ENST00000441971 survived to the corresponding point of time. Dashed lines represent 0.95 confidence intervals for the curves with the corresponding color. Total of 13 observations deleted due to missingness. Log-rank probability measure for the equality of these curves is 2.038863e-02. Additional annotations: *.GeneId*=ENSG00000223969, *.GeneName*=AC002456.2, *.GeneDesc*=[undefined].

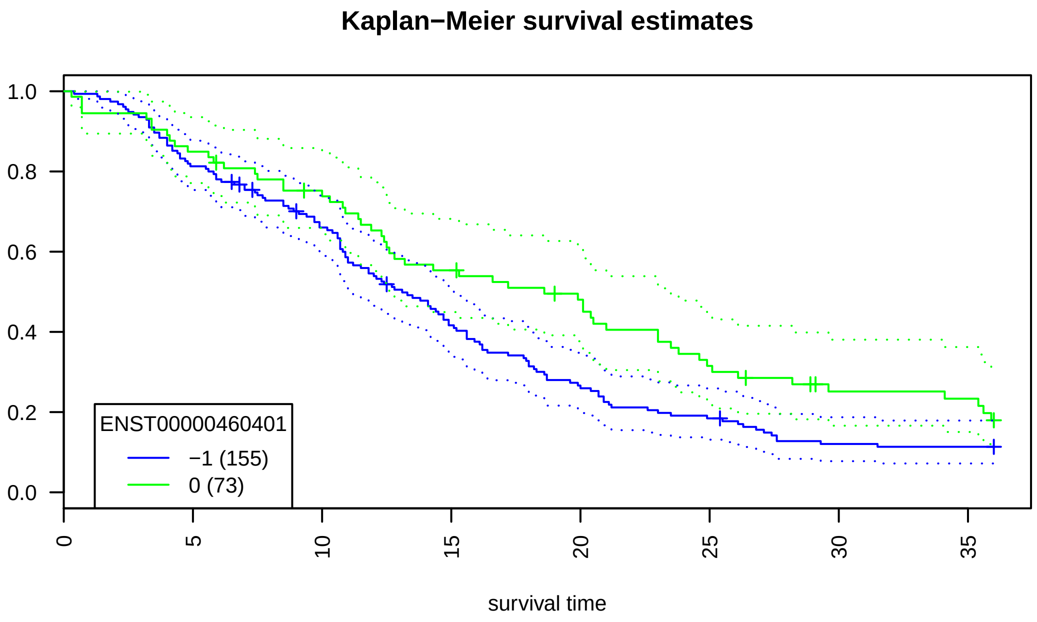

Figure 1177: Each curve represents the relative number of ENST00000460401 survived to the corresponding point of time. Dashed lines represent 0.95 confidence intervals for the curves with the corresponding color. Total of 2 observations deleted due to missingness. Log-rank probability measure for the equality of these curves is 2.433581e-02. Additional annotations: *.GeneId*=ENSG00000144935, *.GeneName*=TRPC1, *.GeneDesc*=transient receptor potential cation channel, subfamily C, member 1 [Source:HGNC Symbol;Acc:12333].

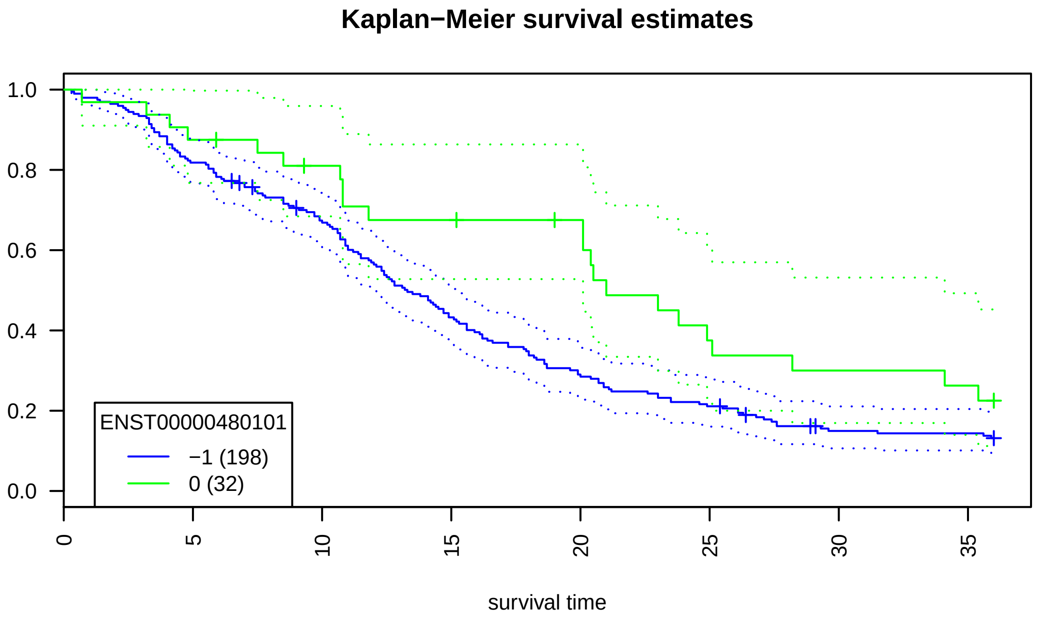

Figure 1178: Each curve represents the relative number of ENST00000480101 survived to the corresponding point of time. Dashed lines represent 0.95 confidence intervals for the curves with the corresponding color. Log-rank probability measure for the equality of these curves is 2.812696e-02. Additional annotations: *.GeneId*=ENSG00000144935, *.GeneName*=TRPC1, *.GeneDesc*=transient receptor potential cation channel, subfamily C, member 1 [Source:HGNC Symbol;Acc:12333].

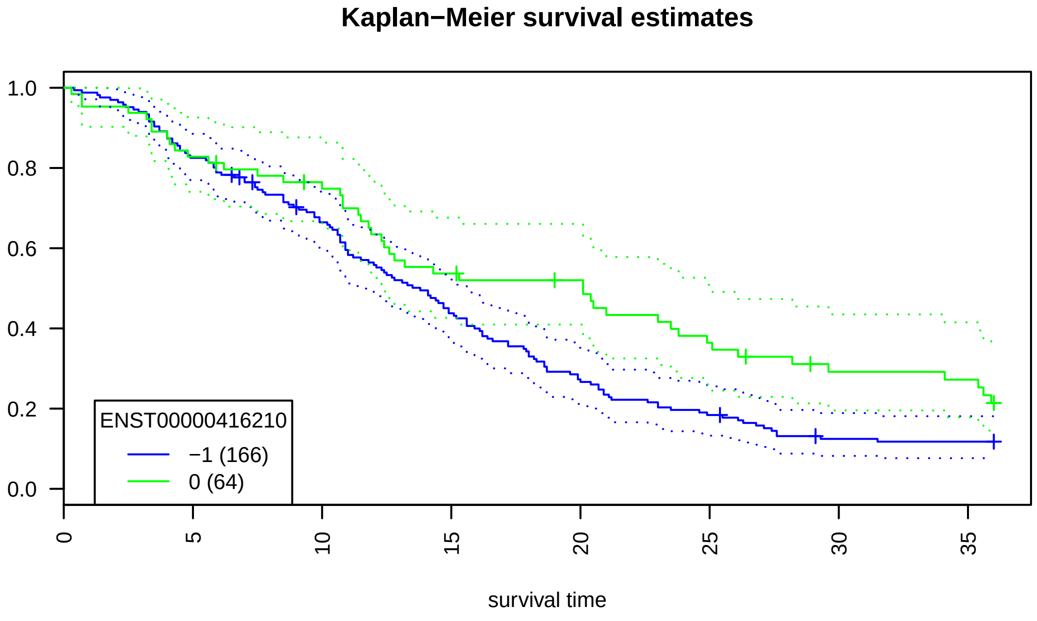

Figure 1179: Each curve represents the relative number of ENST00000416210 survived to the corresponding point of time. Dashed lines represent 0.95 confidence intervals for the curves with the corresponding color. Log-rank probability measure for the equality of these curves is 1.795947e-02. Additional annotations: *.GeneId*=ENSG00000144935, *.GeneName*=TRPC1, *.GeneDesc*=transient receptor potential cation channel, subfamily C, member 1 [Source:HGNC Symbol;Acc:12333].

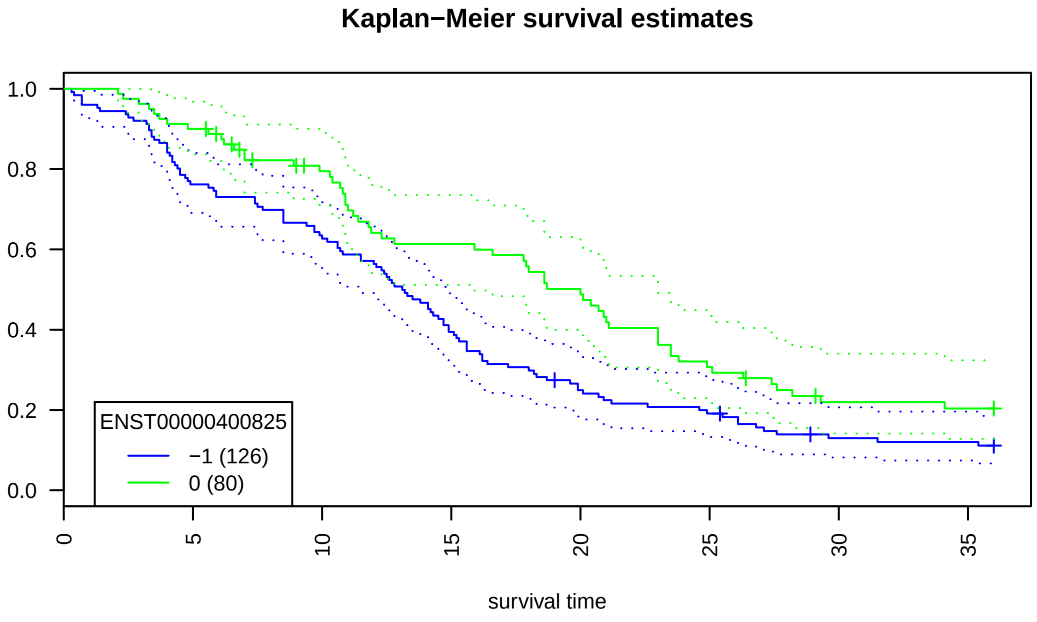

Figure 1180: Each curve represents the relative number of ENST00000400825 survived to the corresponding point of time. Dashed lines represent 0.95 confidence intervals for the curves with the corresponding color. Total of 24 observations deleted due to missingness. Log-rank probability measure for the equality of these curves is 5.885786e-03. Additional annotations: *.GeneId*=ENSG00000130396, *.GeneName*=MLLT4, *.GeneDesc*=myeloid/lymphoid or mixed-lineage leukemia (trithorax homolog, Drosophila); translocated to, 4 [Source:HGNC Symbol;Acc:7137].

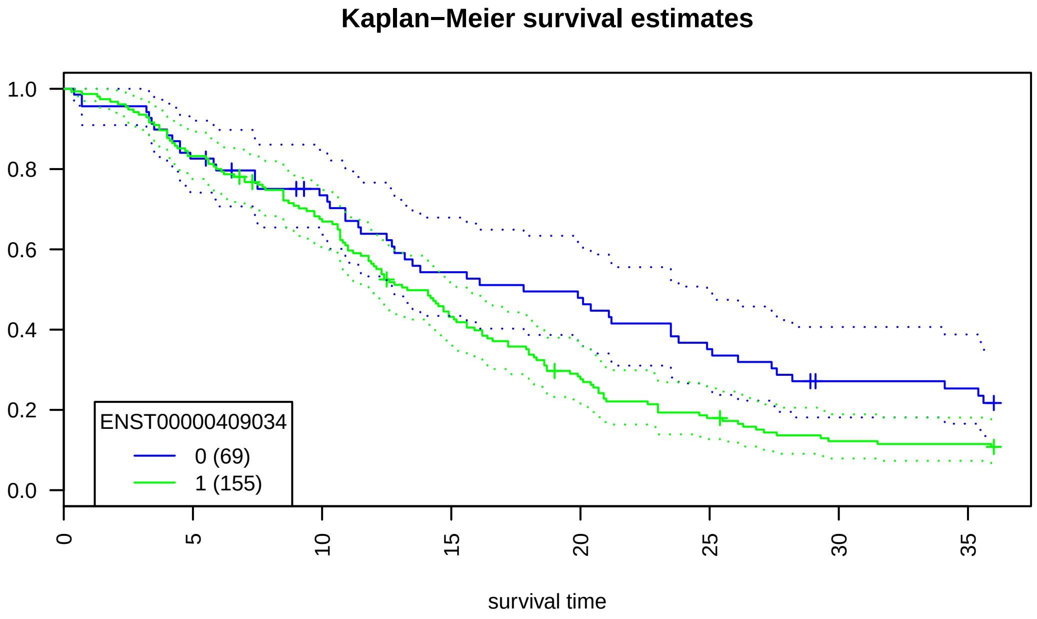

Figure 1181: Each curve represents the relative number of ENST00000409034 survived to the corresponding point of time. Dashed lines represent 0.95 confidence intervals for the curves with the corresponding color. Total of 6 observations deleted due to missingness. Log-rank probability measure for the equality of these curves is 1.970096e-02. Additional annotations: *.GeneId*=ENSG00000150551, *.GeneName*=LYPD1, *.GeneDesc*=LY6/PLAUR domain containing 1 [Source:HGNC Symbol;Acc:28431].

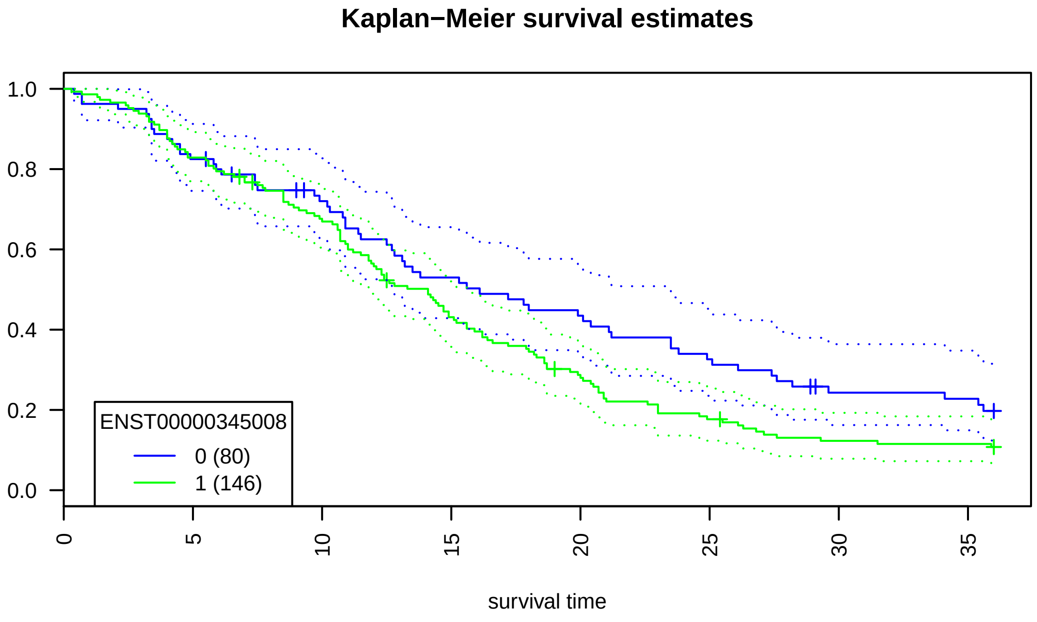

Figure 1182: Each curve represents the relative number of ENST00000345008 survived to the corresponding point of time. Dashed lines represent 0.95 confidence intervals for the curves with the corresponding color. Total of 4 observations deleted due to missingness. Log-rank probability measure for the equality of these curves is 4.047717e-02. Additional annotations: *.GeneId*=ENSG00000150551, *.GeneName*=LYPD1, *.GeneDesc*=LY6/PLAUR domain containing 1 [Source:HGNC Symbol;Acc:28431].

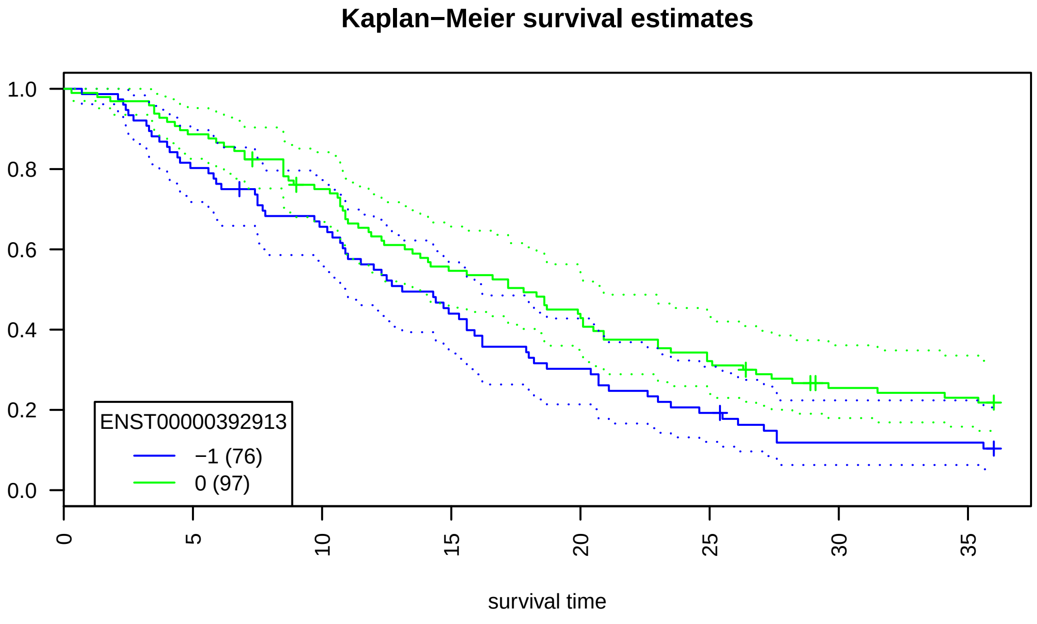

Figure 1183: Each curve represents the relative number of ENST00000392913 survived to the corresponding point of time. Dashed lines represent 0.95 confidence intervals for the curves with the corresponding color. Total of 57 observations deleted due to missingness. Log-rank probability measure for the equality of these curves is 2.156890e-02. Additional annotations: *.GeneId*=ENSG00000071794, *.GeneName*=HLTF, *.GeneDesc*=helicase-like transcription factor [Source:HGNC Symbol;Acc:11099].

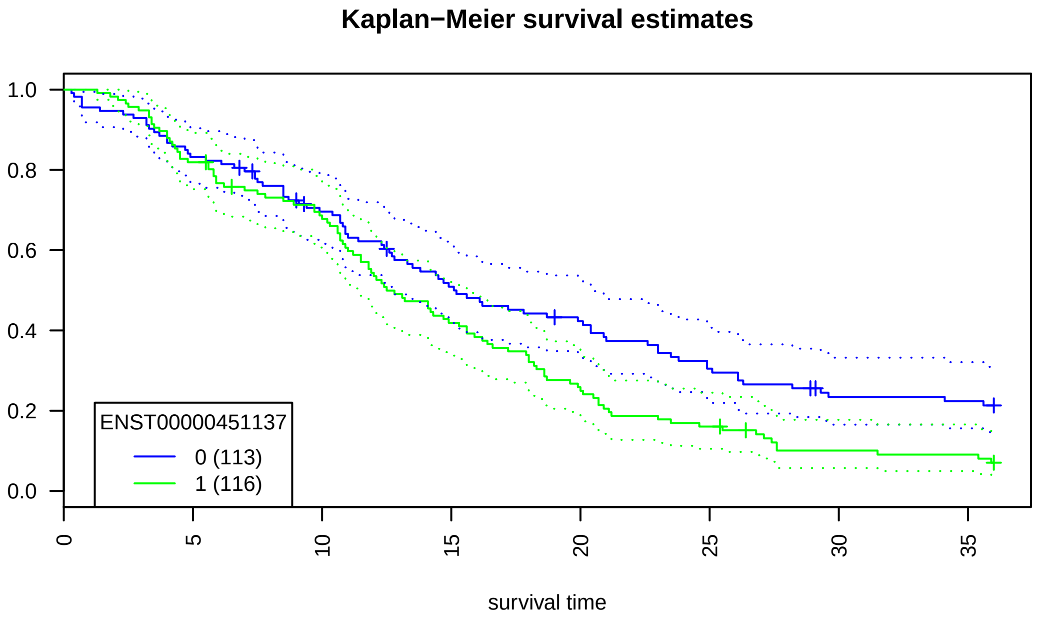

Figure 1184: Each curve represents the relative number of ENST00000451137 survived to the corresponding point of time. Dashed lines represent 0.95 confidence intervals for the curves with the corresponding color. Total of 1 observation deleted due to missingness. Log-rank probability measure for the equality of these curves is 7.29325e-03. Additional annotations: *.GeneId*=ENSG00000142871, *.GeneName*=CYR61, *.GeneDesc*=cysteine-rich, angiogenic inducer, 61 [Source:HGNC Symbol;Acc:2654].

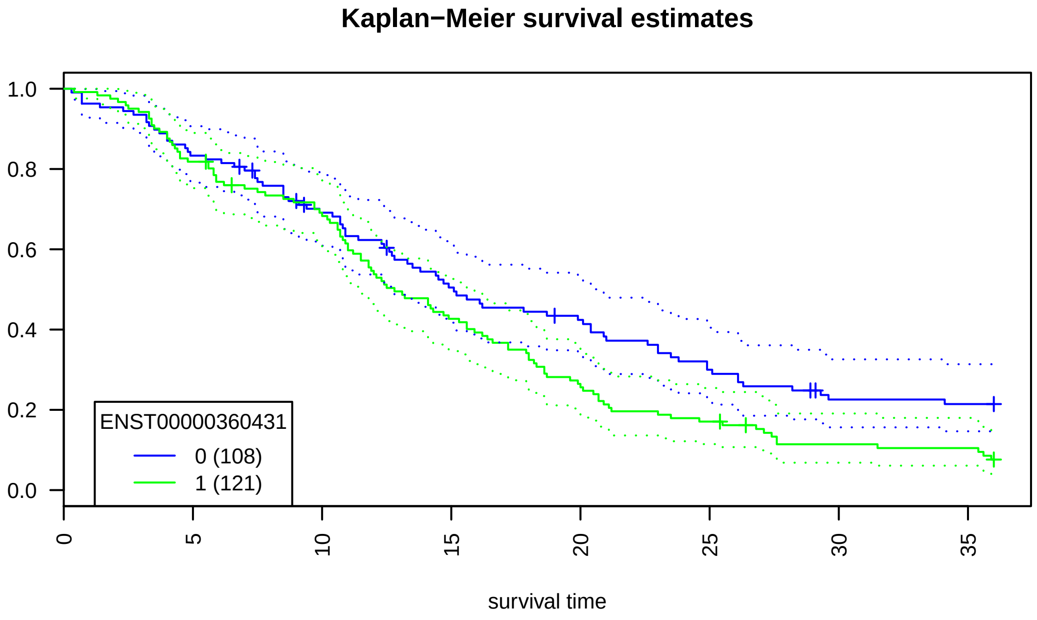

Figure 1185: Each curve represents the relative number of ENST00000360431 survived to the corresponding point of time. Dashed lines represent 0.95 confidence intervals for the curves with the corresponding color. Total of 1 observation deleted due to missingness. Log-rank probability measure for the equality of these curves is 1.299071e-02. Additional annotations: *.GeneId*=ENSG00000142871, *.GeneName*=CYR61, *.GeneDesc*=cysteine-rich, angiogenic inducer, 61 [Source:HGNC Symbol;Acc:2654].

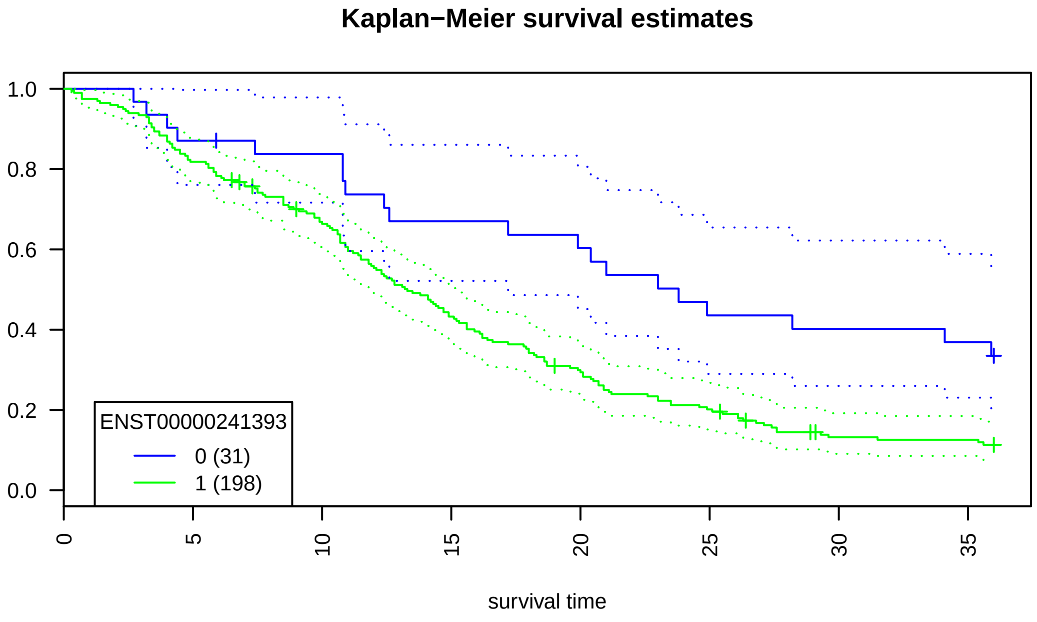

Figure 1186: Each curve represents the relative number of ENST00000241393 survived to the corresponding point of time. Dashed lines represent 0.95 confidence intervals for the curves with the corresponding color. Total of 1 observation deleted due to missingness. Log-rank probability measure for the equality of these curves is 1.943428e-03. Additional annotations: *.GeneId*=ENSG00000121966, *.GeneName*=CXCR4, *.GeneDesc*=chemokine (C-X-C motif) receptor 4 [Source:HGNC Symbol;Acc:2561].

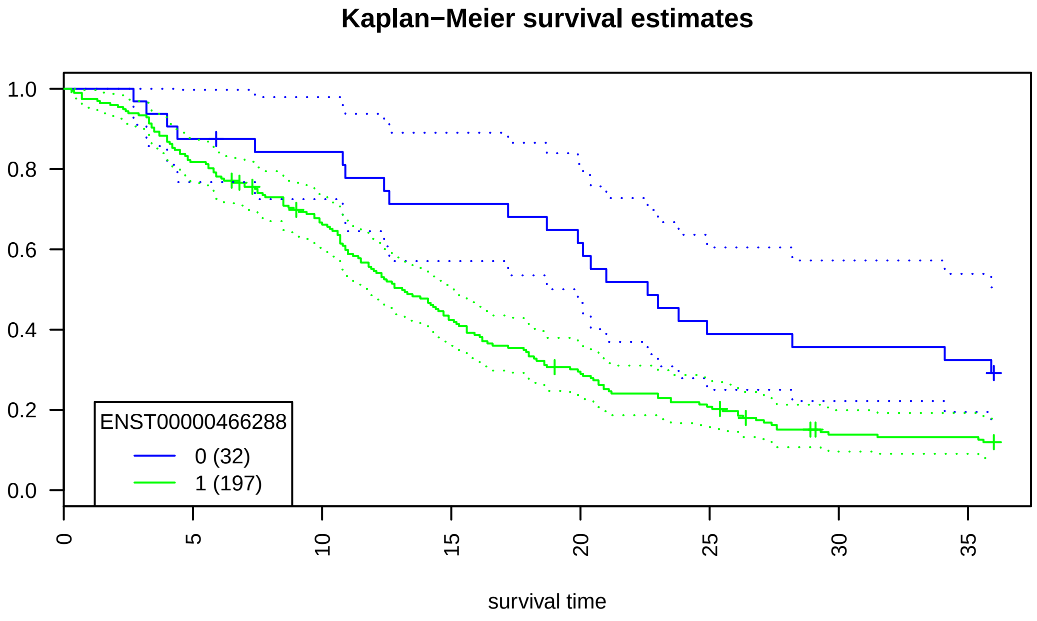

Figure 1187: Each curve represents the relative number of ENST00000466288 survived to the corresponding point of time. Dashed lines represent 0.95 confidence intervals for the curves with the corresponding color. Total of 1 observation deleted due to missingness. Log-rank probability measure for the equality of these curves is 3.935059e-03. Additional annotations: *.GeneId*=ENSG00000121966, *.GeneName*=CXCR4, *.GeneDesc*=chemokine (C-X-C motif) receptor 4 [Source:HGNC Symbol;Acc:2561].

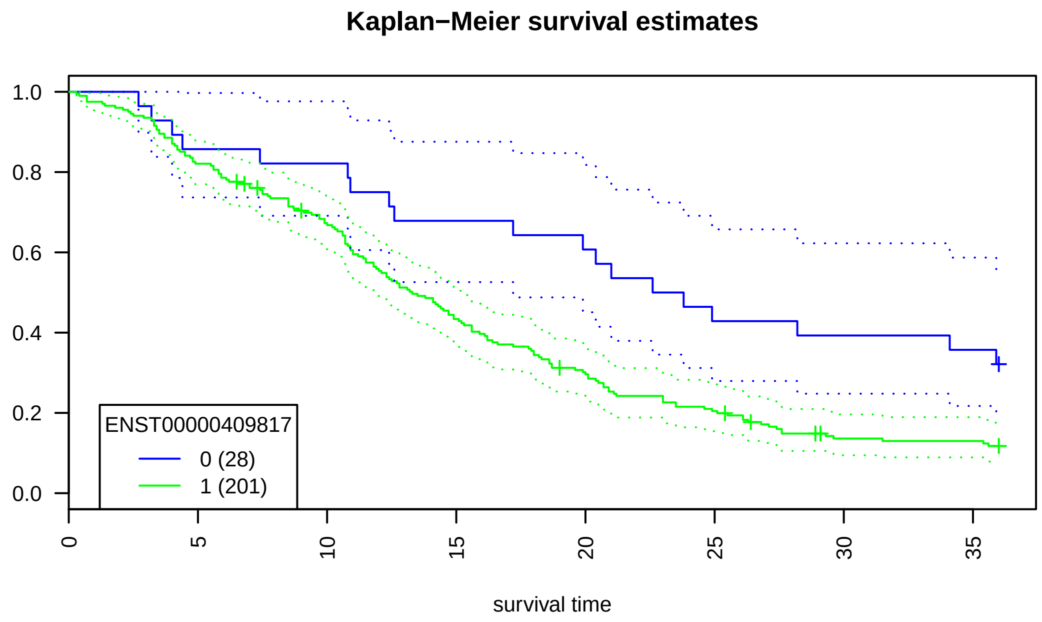

Figure 1188: Each curve represents the relative number of ENST00000409817 survived to the corresponding point of time. Dashed lines represent 0.95 confidence intervals for the curves with the corresponding color. Total of 1 observation deleted due to missingness. Log-rank probability measure for the equality of these curves is 4.094105e-03. Additional annotations: *.GeneId*=ENSG00000121966, *.GeneName*=CXCR4, *.GeneDesc*=chemokine (C-X-C motif) receptor 4 [Source:HGNC Symbol;Acc:2561].

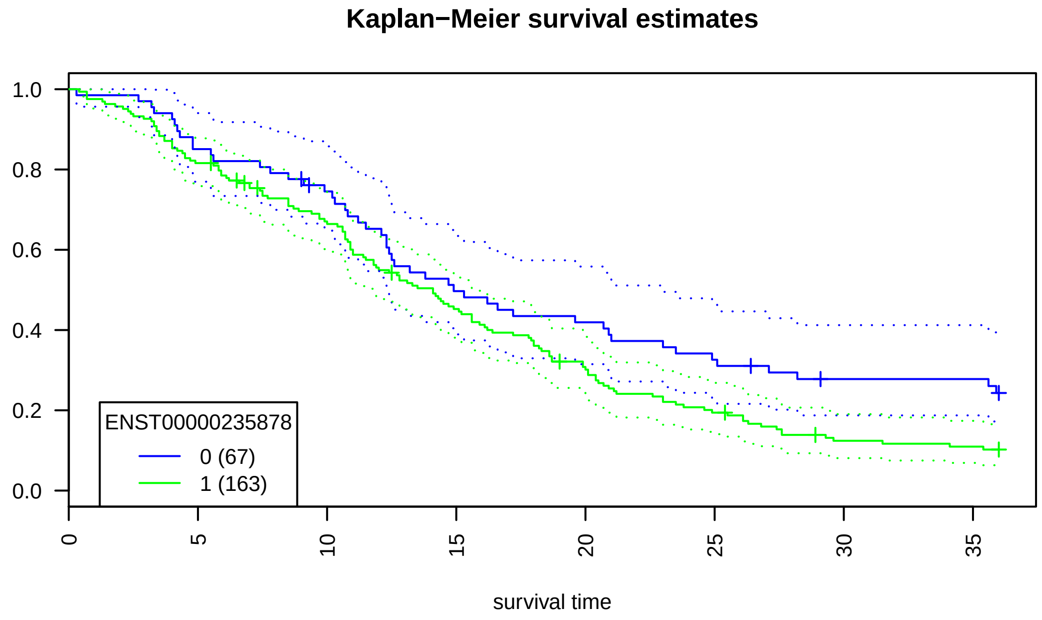

Figure 1189: Each curve represents the relative number of ENST00000235878 survived to the corresponding point of time. Dashed lines represent 0.95 confidence intervals for the curves with the corresponding color. Log-rank probability measure for the equality of these curves is 2.36801e-02. Additional annotations: *.GeneId*=ENSG00000117226, *.GeneName*=GBP3, *.GeneDesc*=guanylate binding protein 3 [Source:HGNC Symbol;Acc:4184].

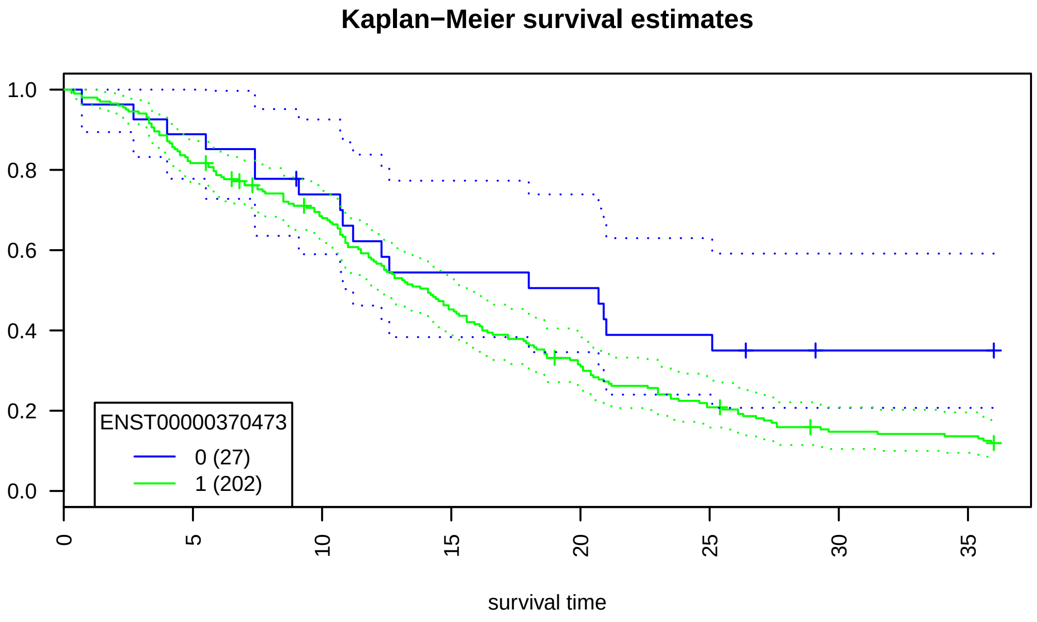

Figure 1190: Each curve represents the relative number of ENST00000370473 survived to the corresponding point of time. Dashed lines represent 0.95 confidence intervals for the curves with the corresponding color. Total of 1 observation deleted due to missingness. Log-rank probability measure for the equality of these curves is 3.831523e-02. Additional annotations: *.GeneId*=ENSG00000117228, *.GeneName*=GBP1, *.GeneDesc*=guanylate binding protein 1, interferon-inducible, 67kDa [Source:HGNC Symbol;Acc:4182].

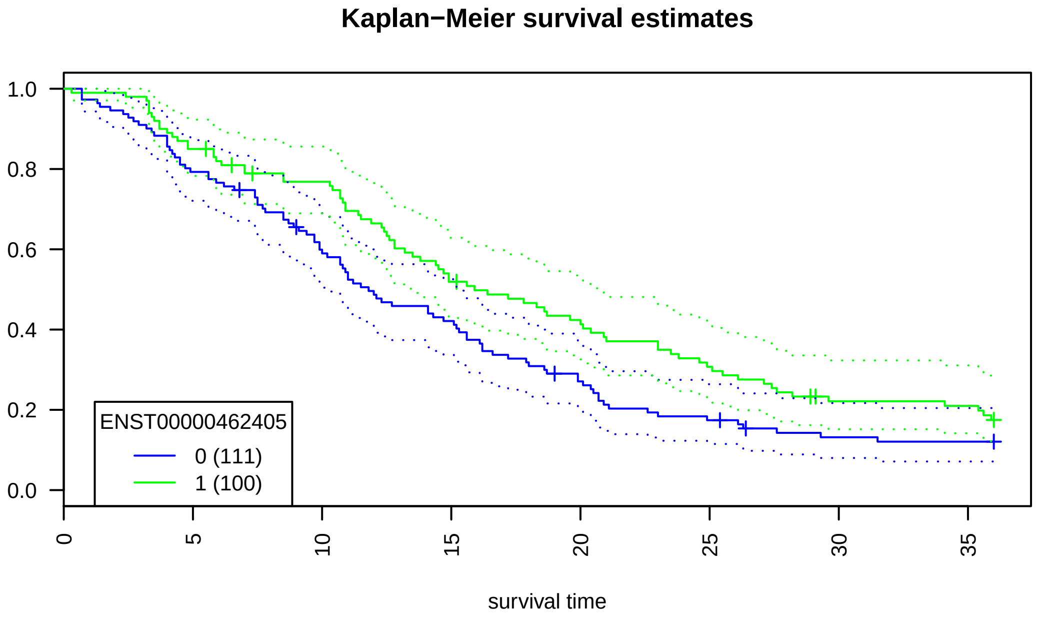

Figure 1191: Each curve represents the relative number of ENST00000462405 survived to the corresponding point of time. Dashed lines represent 0.95 confidence intervals for the curves with the corresponding color. Total of 19 observations deleted due to missingness. Log-rank probability measure for the equality of these curves is 2.589796e-02. Additional annotations: *.GeneId*=ENSG00000162669, *.GeneName*=HFM1, *.GeneDesc*=HFM1, ATP-dependent DNA helicase homolog (S. cerevisiae) [Source:HGNC Symbol;Acc:20193].

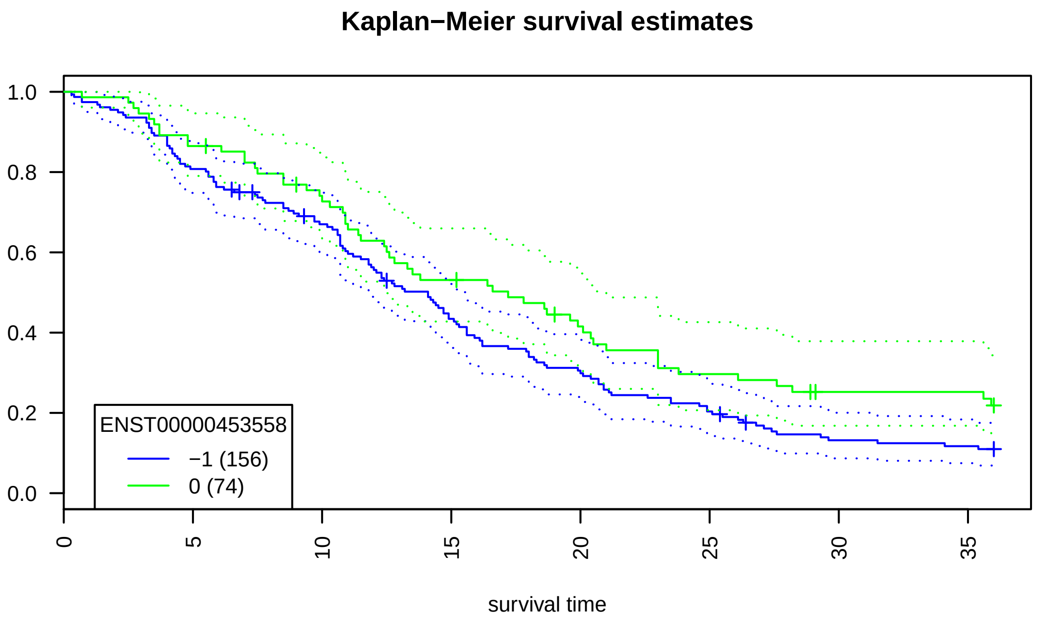

Figure 1192: Each curve represents the relative number of ENST00000453558 survived to the corresponding point of time. Dashed lines represent 0.95 confidence intervals for the curves with the corresponding color. Log-rank probability measure for the equality of these curves is 3.781923e-02. Additional annotations: *.GeneId*=ENSG00000242996, *.GeneName*=HCG17, *.GeneDesc*=[undefined].

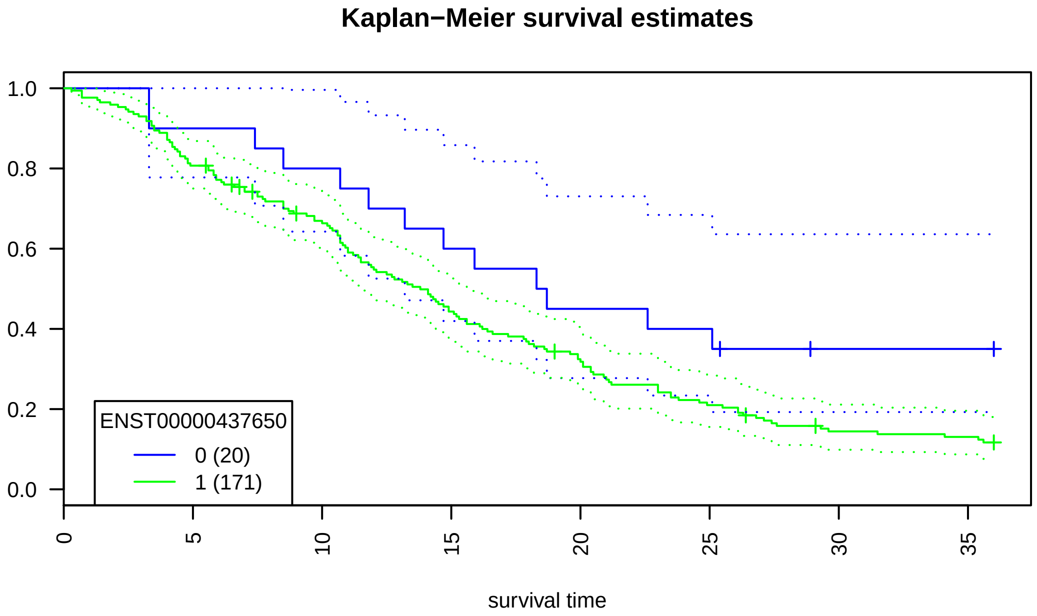

Figure 1193: Each curve represents the relative number of ENST00000437650 survived to the corresponding point of time. Dashed lines represent 0.95 confidence intervals for the curves with the corresponding color. Total of 39 observations deleted due to missingness. Log-rank probability measure for the equality of these curves is 4.339106e-02. Additional annotations: *.GeneId*=ENSG00000229391, *.GeneName*=HLA-DRB6, *.GeneDesc*=major histocompatibility complex, class II, DR beta 6 (pseudogene) [Source:HGNC Symbol;Acc:4954].

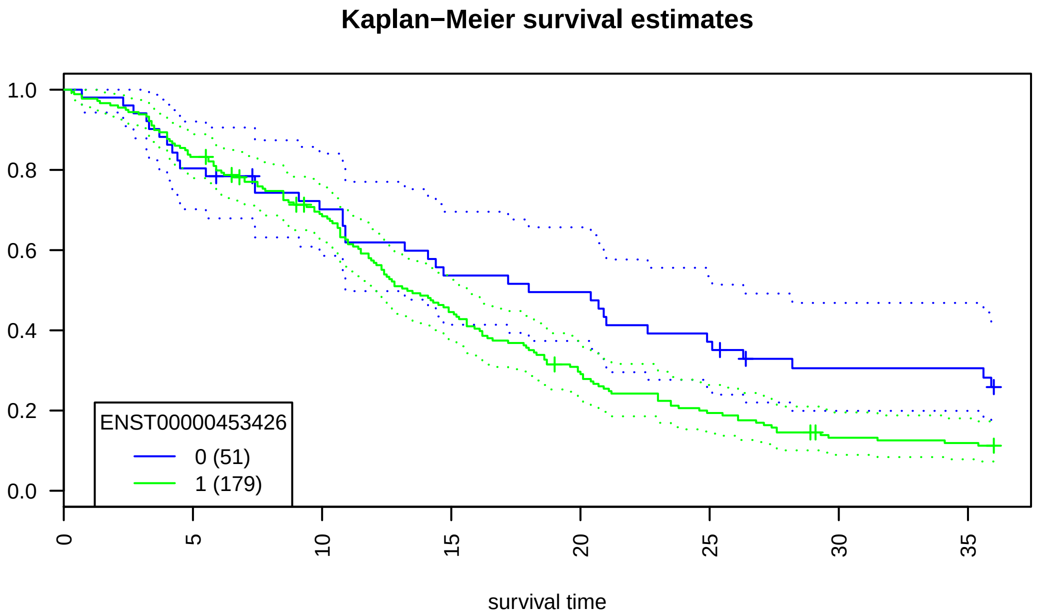

Figure 1194: Each curve represents the relative number of ENST00000453426 survived to the corresponding point of time. Dashed lines represent 0.95 confidence intervals for the curves with the corresponding color. Log-rank probability measure for the equality of these curves is 2.446814e-02. Additional annotations: *.GeneId*=ENSG00000204261, *.GeneName*=XXbac-BPG246D15.8, *.GeneDesc*=[undefined].

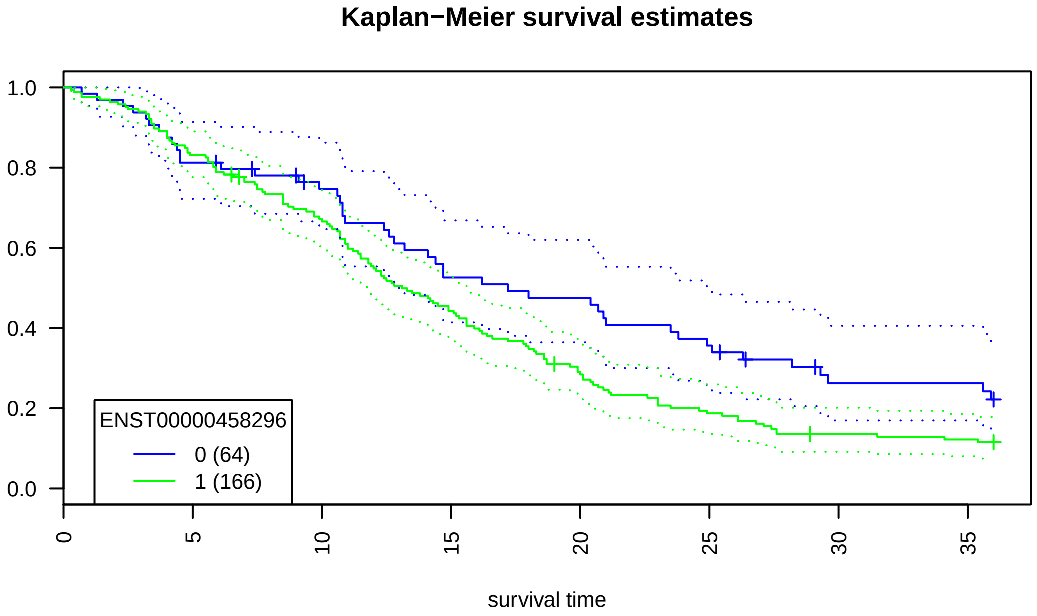

Figure 1195: Each curve represents the relative number of ENST00000458296 survived to the corresponding point of time. Dashed lines represent 0.95 confidence intervals for the curves with the corresponding color. Log-rank probability measure for the equality of these curves is 2.253103e-02. Additional annotations: *.GeneId*=ENSG00000204261, *.GeneName*=XXbac-BPG246D15.8, *.GeneDesc*=[undefined].

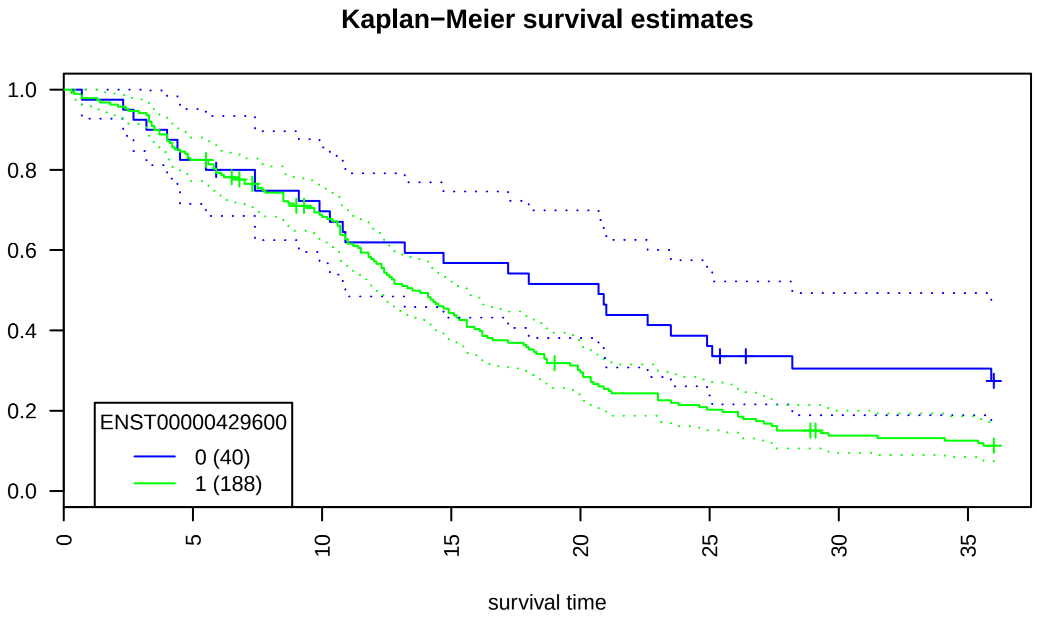

Figure 1196: Each curve represents the relative number of ENST00000429600 survived to the corresponding point of time. Dashed lines represent 0.95 confidence intervals for the curves with the corresponding color. Total of 2 observations deleted due to missingness. Log-rank probability measure for the equality of these curves is 2.788688e-02. Additional annotations: *.GeneId*=ENSG00000204261, *.GeneName*=XXbac-BPG246D15.8, *.GeneDesc*=[undefined].

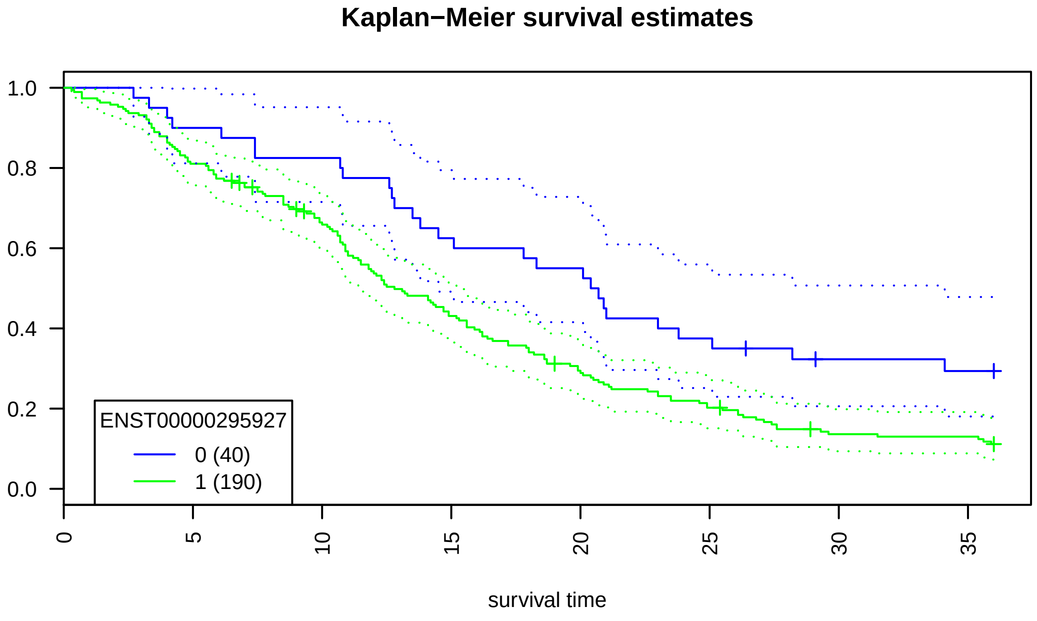

Figure 1197: Each curve represents the relative number of ENST00000295927 survived to the corresponding point of time. Dashed lines represent 0.95 confidence intervals for the curves with the corresponding color. Log-rank probability measure for the equality of these curves is 4.160777e-03. Additional annotations: *.GeneId*=ENSG00000163661, *.GeneName*=PTX3, *.GeneDesc*=pentraxin-related gene, rapidly induced by IL-1 beta [Source:HGNC Symbol;Acc:9692].

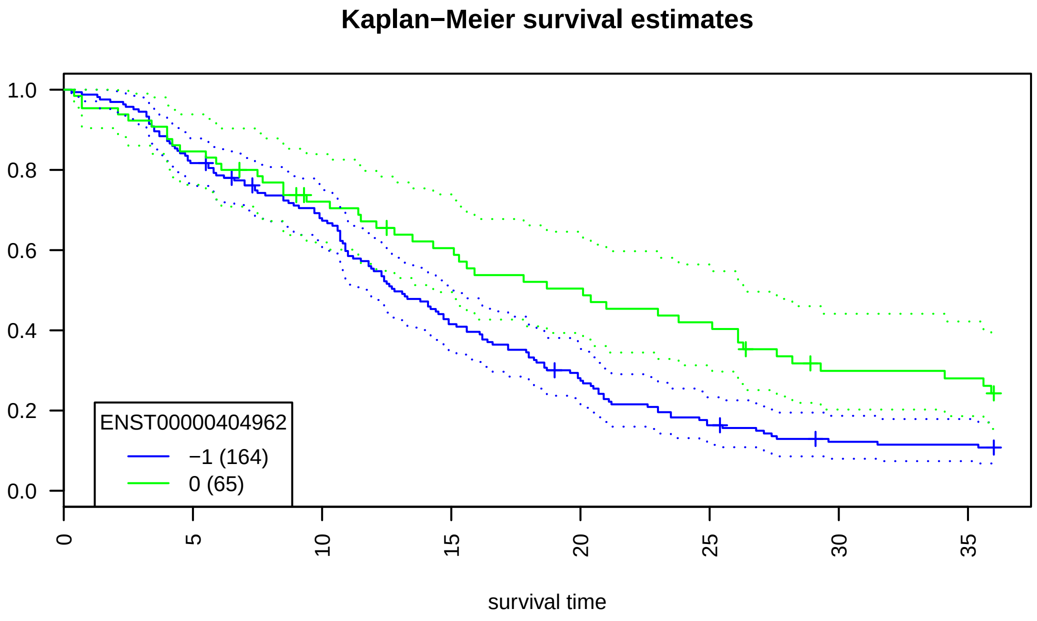

Figure 1198: Each curve represents the relative number of ENST00000404962 survived to the corresponding point of time. Dashed lines represent 0.95 confidence intervals for the curves with the corresponding color. Total of 1 observation deleted due to missingness. Log-rank probability measure for the equality of these curves is 3.90062e-03. Additional annotations: *.GeneId*=ENSG00000218337, *.GeneName*=RP4-724P12.1, *.GeneDesc*=[undefined].

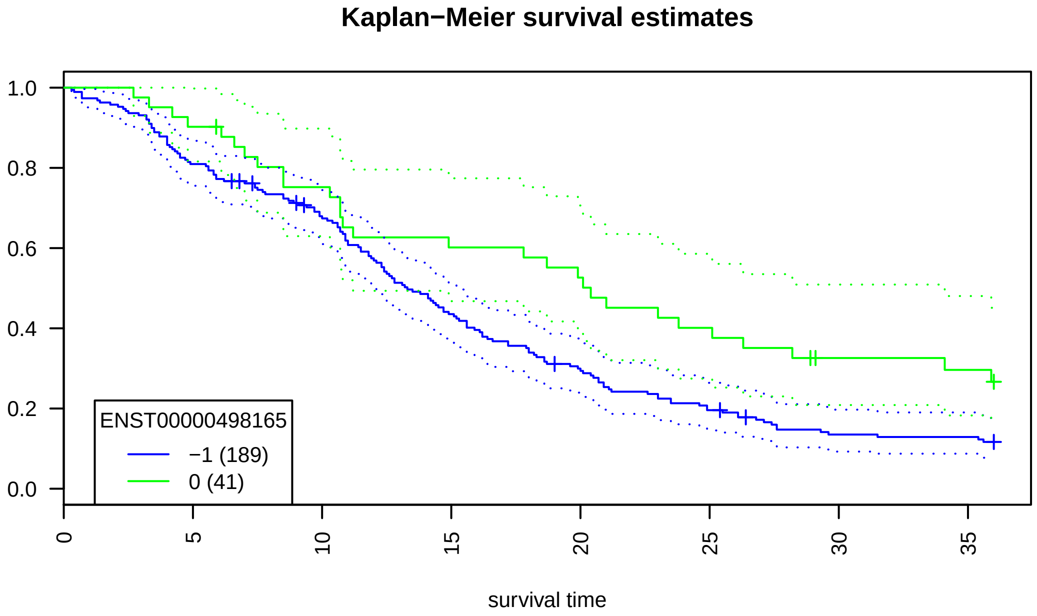

Figure 1199: Each curve represents the relative number of ENST00000498165 survived to the corresponding point of time. Dashed lines represent 0.95 confidence intervals for the curves with the corresponding color. Log-rank probability measure for the equality of these curves is 1.101030e-02. Additional annotations: *.GeneId*=ENSG00000163590, *.GeneName*=PPM1L, *.GeneDesc*=protein phosphatase, Mg2+/Mn2+ dependent, 1L [Source:HGNC Symbol;Acc:16381].

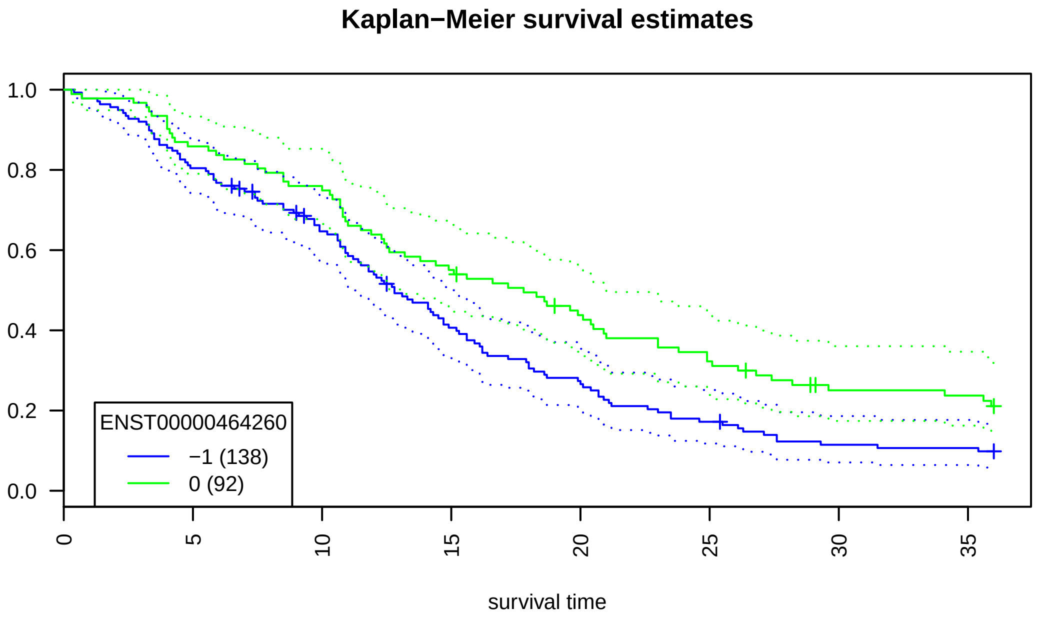

Figure 1200: Each curve represents the relative number of ENST00000464260 survived to the corresponding point of time. Dashed lines represent 0.95 confidence intervals for the curves with the corresponding color. Log-rank probability measure for the equality of these curves is 5.412405e-03. Additional annotations: .*GeneId*=ENSG00000163590, .*GeneName*=PPM1L, .*GeneDesc*=protein phosphatase, Mg2+/Mn2+ dependent, 1L [Source:HGNC Symbol;Acc:16381].

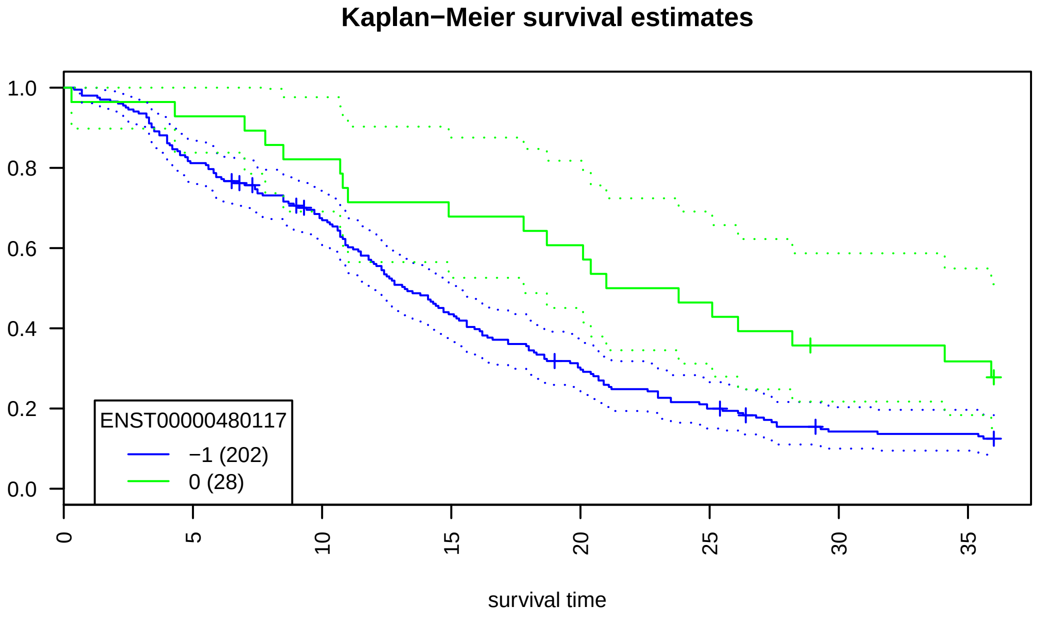

Figure 1201: Each curve represents the relative number of ENST00000480117 survived to the corresponding point of time. Dashed lines represent 0.95 confidence intervals for the curves with the corresponding color. Log-rank probability measure for the equality of these curves is 9.50785e-03. Additional annotations: .*GeneId*=ENSG00000163590, .*GeneName*=PPM1L, .*GeneDesc*=protein phosphatase, Mg2+/Mn2+ dependent, 1L [Source:HGNC Symbol;Acc:16381].

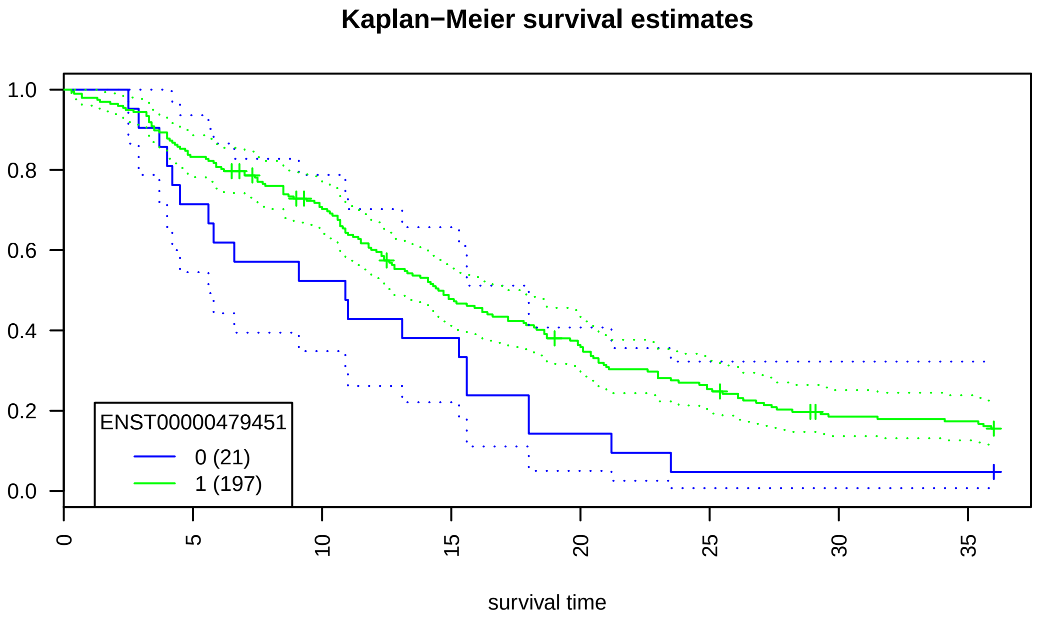

Figure 1202: Each curve represents the relative number of ENST00000479451 survived to the corresponding point of time. Dashed lines represent 0.95 confidence intervals for the curves with the corresponding color. Total of 12 observations deleted due to missingness. Log-rank probability measure for the equality of these curves is 1.844914e-02. Additional annotations: *.GeneId*=ENSG00000114200, *.GeneName*=BCHE, *.GeneDesc*=butyrylcholinesterase [Source:HGNC Symbol;Acc:983].

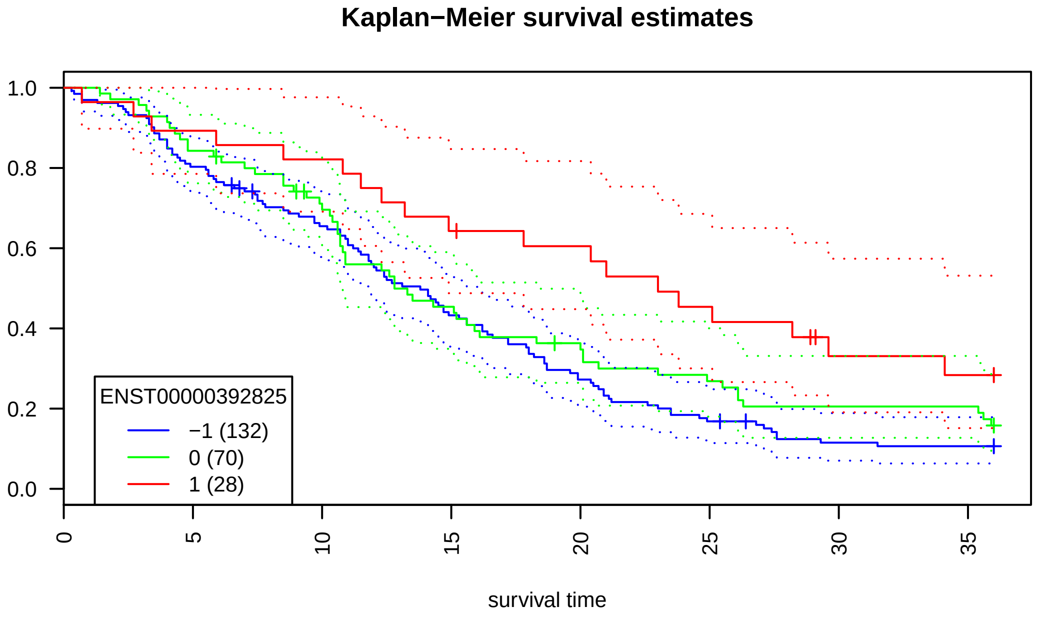

Figure 1203: Each curve represents the relative number of ENST00000392825 survived to the corresponding point of time. Dashed lines represent 0.95 confidence intervals for the curves with the corresponding color. Log-rank probability measure for the equality of these curves is 2.173693e-02. Additional annotations: *.GeneId*=ENSG00000144278, *.GeneName*=GALNT13, *.GeneDesc*=UDP-N-acetyl-alpha-D-galactosamine:polypeptide N-acetylgalactosaminyltransferase 13 (GalNAc-T13) [Source:HGNC Symbol;Acc:23242].

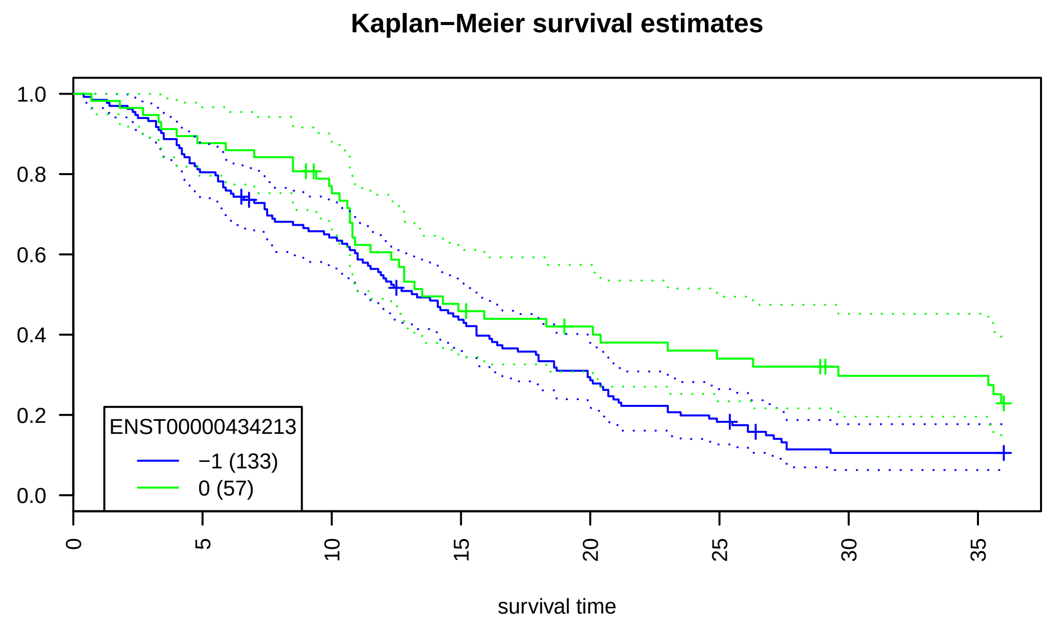

Figure 1204: Each curve represents the relative number of ENST00000434213 survived to the corresponding point of time. Dashed lines represent 0.95 confidence intervals for the curves with the corresponding color. Total of 40 observations deleted due to missingness. Log-rank probability measure for the equality of these curves is 3.343211e-02. Additional annotations: *.GeneId*=ENSG00000144278, *.GeneName*=GALNT13, *.GeneDesc*=UDP-N-acetyl-alpha-D-galactosamine:polypeptide N-acetylgalactosaminyltransferase 13 (GalNAc-T13) [Source:HGNC Symbol;Acc:23242].

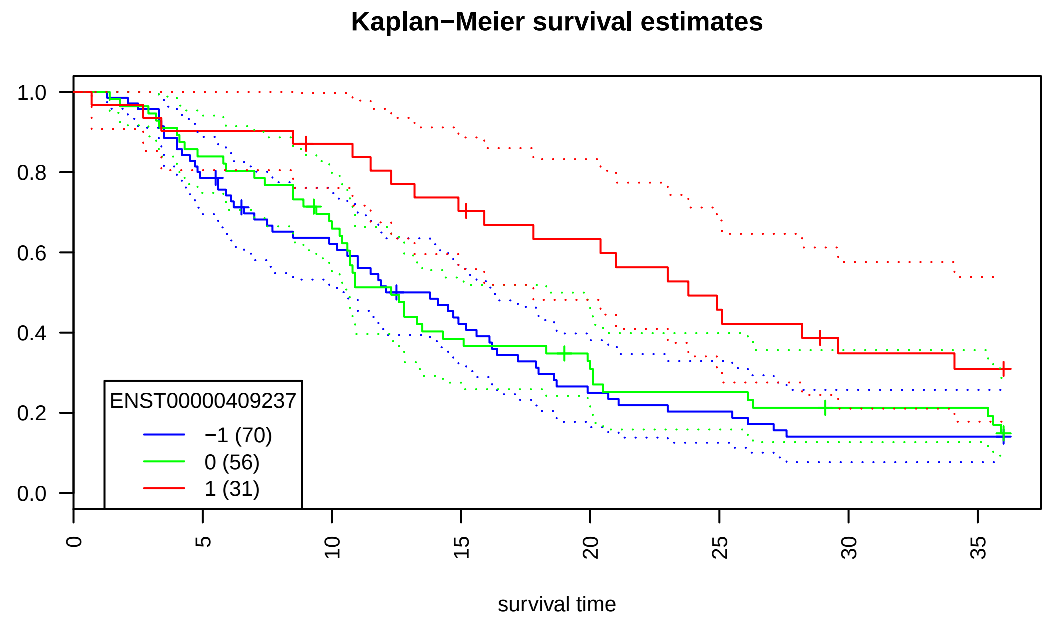

Figure 1205: Each curve represents the relative number of ENST00000409237 survived to the corresponding point of time. Dashed lines represent 0.95 confidence intervals for the curves with the corresponding color. Total of 73 observations deleted due to missingness. Log-rank probability measure for the equality of these curves is 2.014890e-02. Additional annotations: *.GeneId*=ENSG00000144278, *.GeneName*=GALNT13, *.GeneDesc*=UDP-N-acetyl-alpha-D-galactosamine:polypeptide N-acetylgalactosaminyltransferase 13 (GalNAc-T13) [Source:HGNC Symbol;Acc:23242].

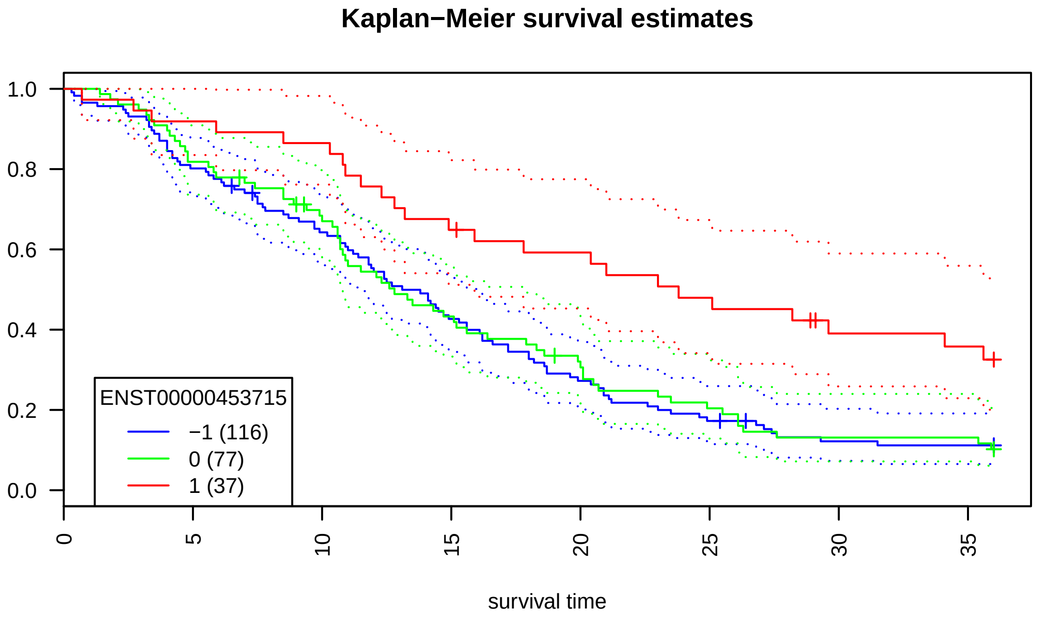

Figure 1206: Each curve represents the relative number of ENST00000453715 survived to the corresponding point of time. Dashed lines represent 0.95 confidence intervals for the curves with the corresponding color. Log-rank probability measure for the equality of these curves is 2.357006e-03. Additional annotations: *.GeneId*=ENSG00000144278, *.GeneName*=GALNT13, *.GeneDesc*=UDP-N-acetyl-alpha-D-galactosamine:polypeptide N-acetylgalactosaminyltransferase 13 (GalNAc-T13) [Source:HGNC Symbol;Acc:23242].

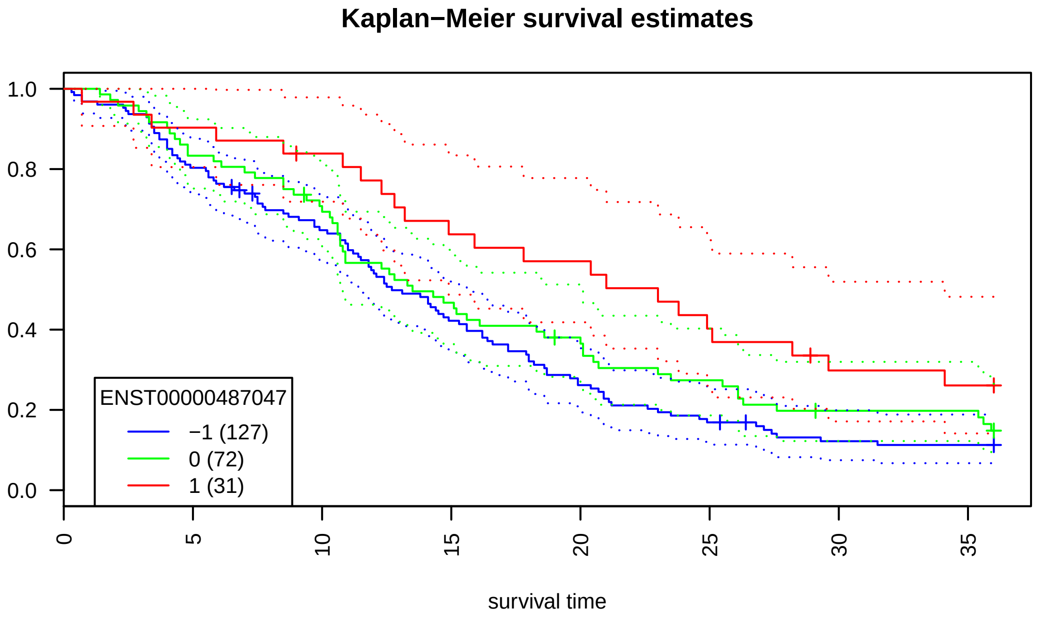

Figure 1207: Each curve represents the relative number of ENST00000487047 survived to the corresponding point of time. Dashed lines represent 0.95 confidence intervals for the curves with the corresponding color. Log-rank probability measure for the equality of these curves is 2.961819e-02. Additional annotations: *.GeneId*=ENSG00000144278, *.GeneName*=GALNT13, *.GeneDesc*=UDP-N-acetyl-alpha-D-galactosamine:polypeptide N-acetylgalactosaminyltransferase 13 (GalNAc-T13) [Source:HGNC Symbol;Acc:23242].

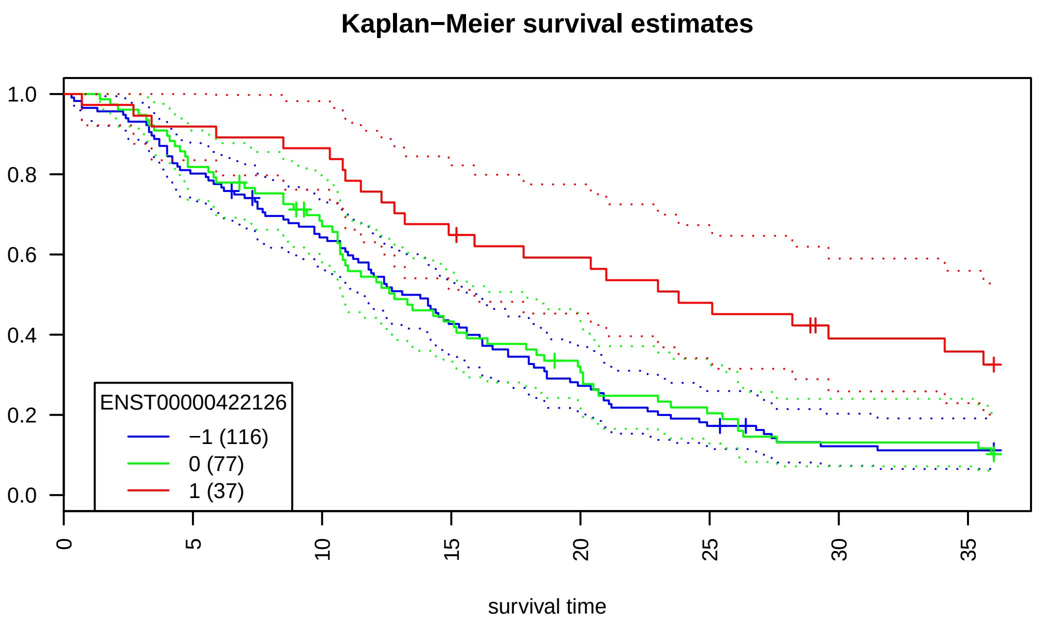

Figure 1208: Each curve represents the relative number of ENST00000422126 survived to the corresponding point of time. Dashed lines represent 0.95 confidence intervals for the curves with the corresponding color. Log-rank probability measure for the equality of these curves is 2.357006e-03. Additional annotations: *.GeneId*=ENSG00000144278, *.GeneName*=GALNT13, *.GeneDesc*=UDP-N-acetyl-alpha-D-galactosamine:polypeptide N-acetylgalactosaminyltransferase 13 (GalNAc-T13) [Source:HGNC Symbol;Acc:23242].

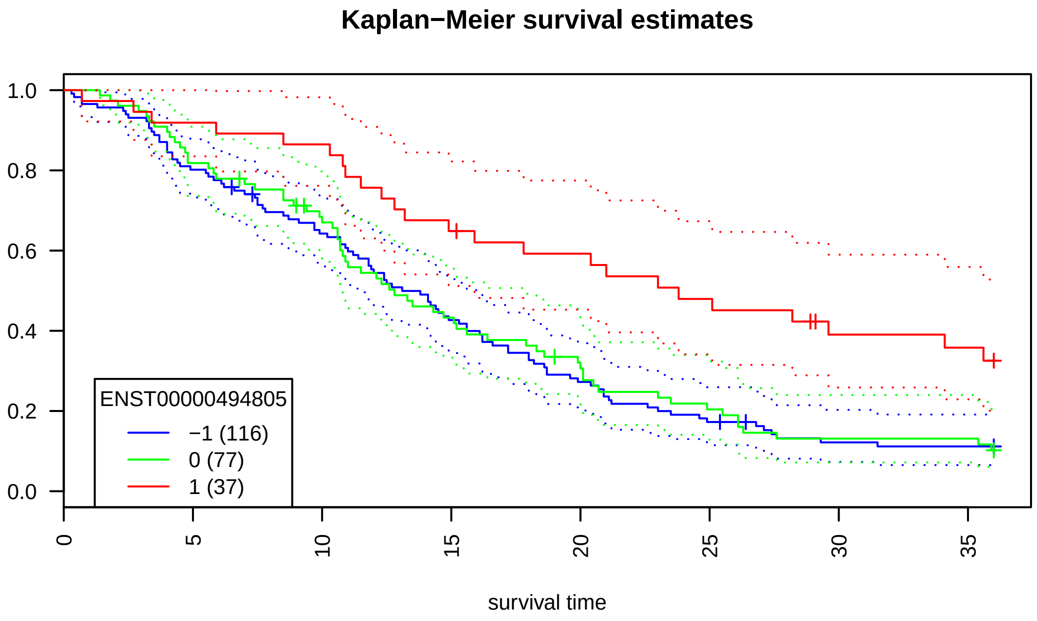

Figure 1209: Each curve represents the relative number of ENST00000494805 survived to the corresponding point of time. Dashed lines represent 0.95 confidence intervals for the curves with the corresponding color. Log-rank probability measure for the equality of these curves is 2.357006e-03. Additional annotations: *.GeneId*=ENSG00000144278, *.GeneName*=GALNT13, *.GeneDesc*=UDP-N-acetyl-alpha-D-galactosamine:polypeptide N-acetylgalactosaminyltransferase 13 (GalNAc-T13) [Source:HGNC Symbol;Acc:23242].

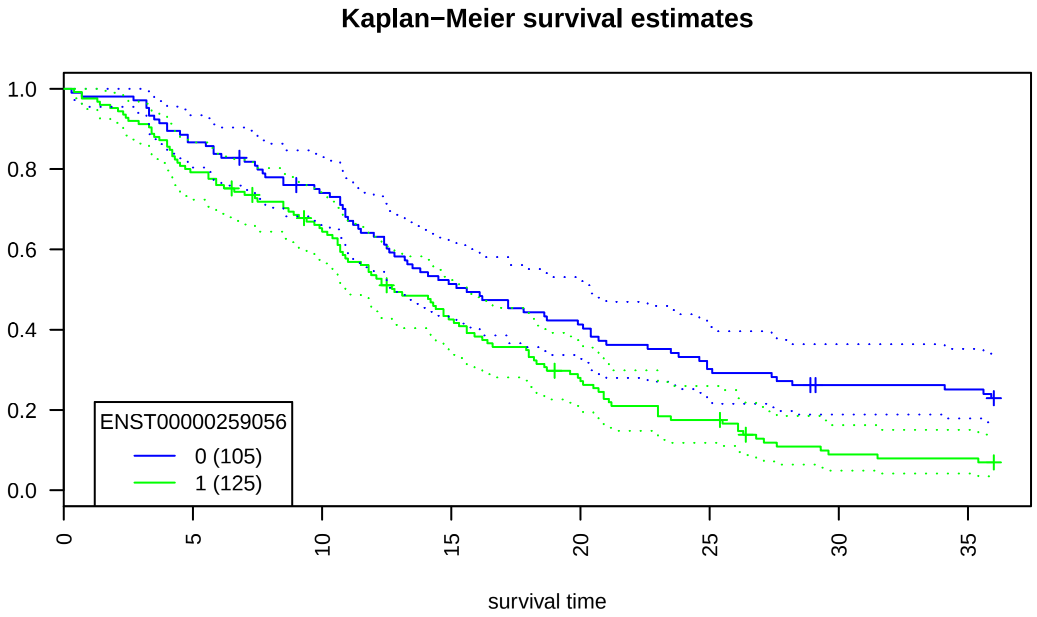

Figure 1210: Each curve represents the relative number of ENST00000259056 survived to the corresponding point of time. Dashed lines represent 0.95 confidence intervals for the curves with the corresponding color. Log-rank probability measure for the equality of these curves is 2.611869e-03. Additional annotations: *.GeneId*=ENSG00000136542, *.GeneName*=GALNT5, *.GeneDesc*=UDP-N-acetyl-alpha-D-galactosamine:polypeptide N-acetylgalactosaminyltransferase 5 (GalNAc-T5) [Source:HGNC Symbol;Acc:4127].

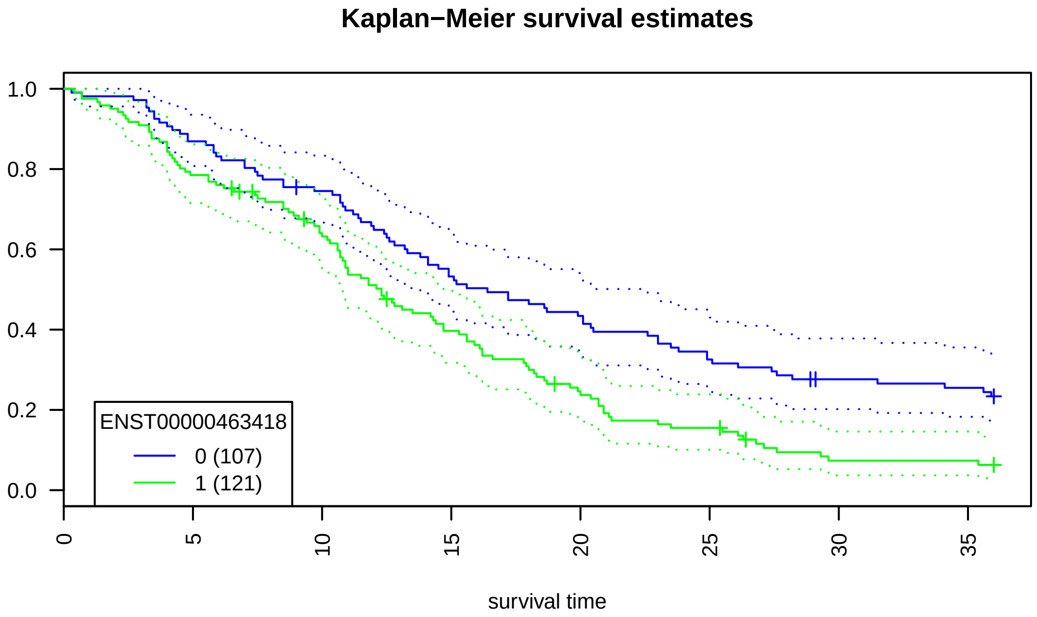

Figure 1211: Each curve represents the relative number of ENST00000463418 survived to the corresponding point of time. Dashed lines represent 0.95 confidence intervals for the curves with the corresponding color. Total of 2 observations deleted due to missingness. Log-rank probability measure for the equality of these curves is 2.685131e-04. Additional annotations: *.GeneId*=ENSG00000136542, *.GeneName*=GALNT5, *.GeneDesc*=UDP-N-acetyl-alpha-D-galactosamine:polypeptide N-acetylgalactosaminyltransferase 5 (GalNAc-T5) [Source:HGNC Symbol;Acc:4127].

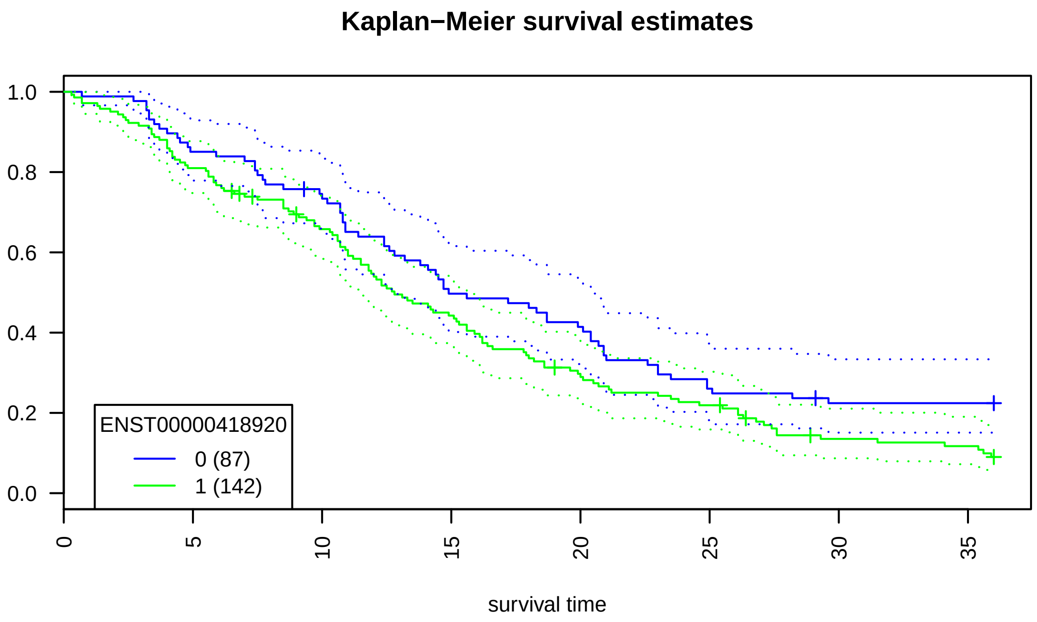

Figure 1212: Each curve represents the relative number of ENST00000418920 survived to the corresponding point of time. Dashed lines represent 0.95 confidence intervals for the curves with the corresponding color. Total of 1 observation deleted due to missingness. Log-rank probability measure for the equality of these curves is 2.856494e-02. Additional annotations: *.GeneId*=ENSG00000115165, *.GeneName*=CYTIP, *.GeneDesc*=cytohesin 1 interacting protein [Source:HGNC Symbol;Acc:9506].

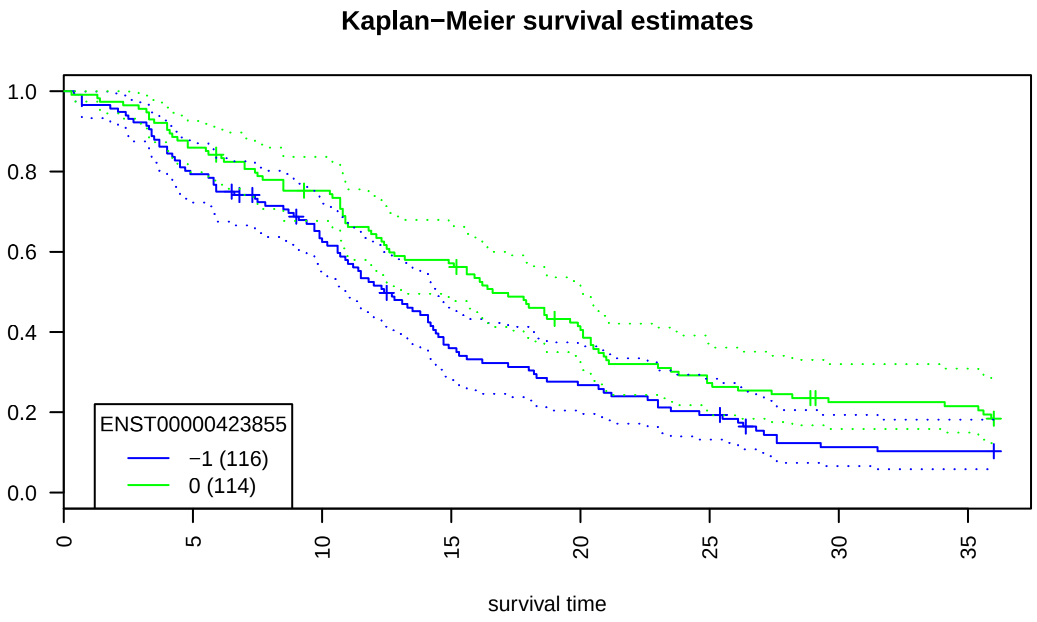

Figure 1213: Each curve represents the relative number of ENST00000423855 survived to the corresponding point of time. Dashed lines represent 0.95 confidence intervals for the curves with the corresponding color. Log-rank probability measure for the equality of these curves is 1.677432e-02. Additional annotations: *.GeneId*=ENSG00000185946, *.GeneName*=RNPC3, *.GeneDesc*=RNA-binding region (RNP1, RRM) containing 3 [Source:HGNC Symbol;Acc:18666].

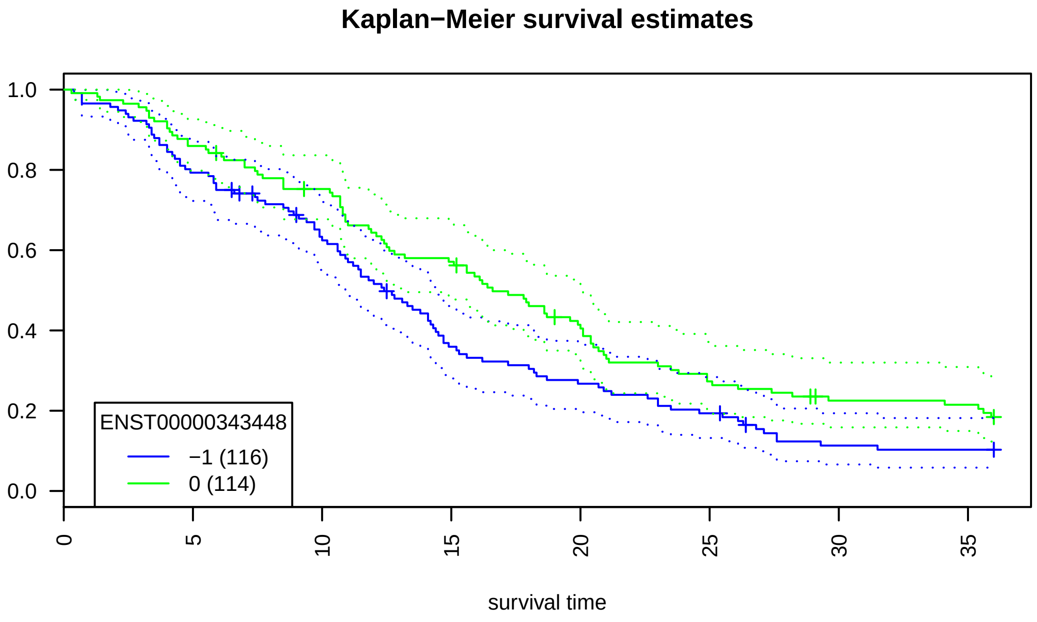

Figure 1214: Each curve represents the relative number of ENST00000343448 survived to the corresponding point of time. Dashed lines represent 0.95 confidence intervals for the curves with the corresponding color. Log-rank probability measure for the equality of these curves is 1.677432e-02. Additional annotations: *.GeneId*=ENSG00000185946, *.GeneName*=RNPC3, *.GeneDesc*=RNA-binding region (RNP1, RRM) containing 3 [Source:HGNC Symbol;Acc:18666].

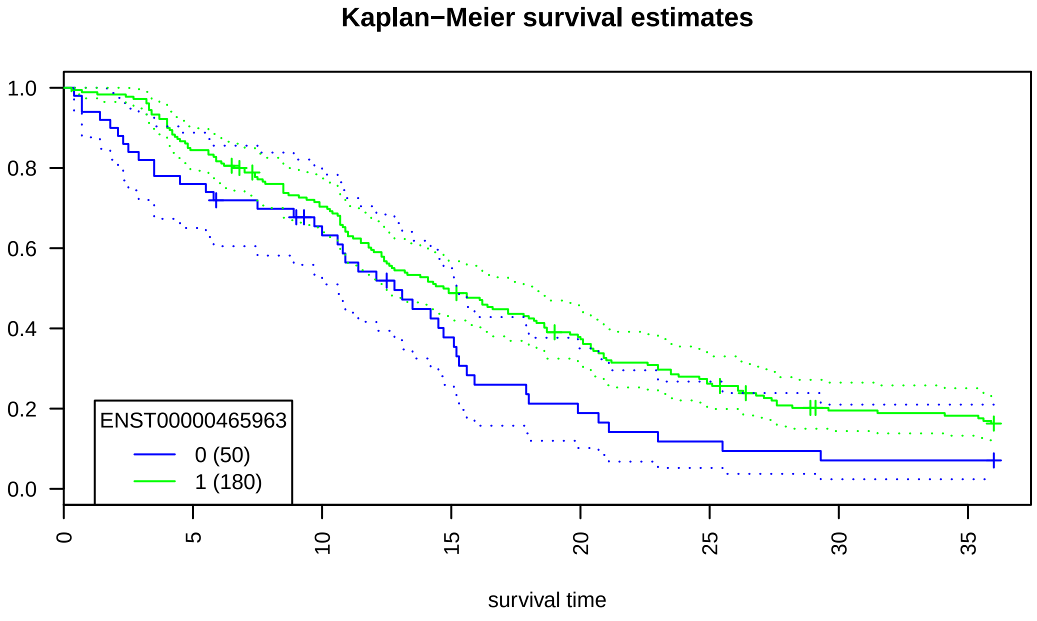

Figure 1215: Each curve represents the relative number of ENST00000465963 survived to the corresponding point of time. Dashed lines represent 0.95 confidence intervals for the curves with the corresponding color. Log-rank probability measure for the equality of these curves is 1.800107e-02. Additional annotations: *.GeneId*=ENSG00000115183, *.GeneName*=TANC1, *.GeneDesc*=tetratricopeptide repeat, ankyrin repeat and coiled-coil containing 1 [Source:HGNC Symbol;Acc:29364].

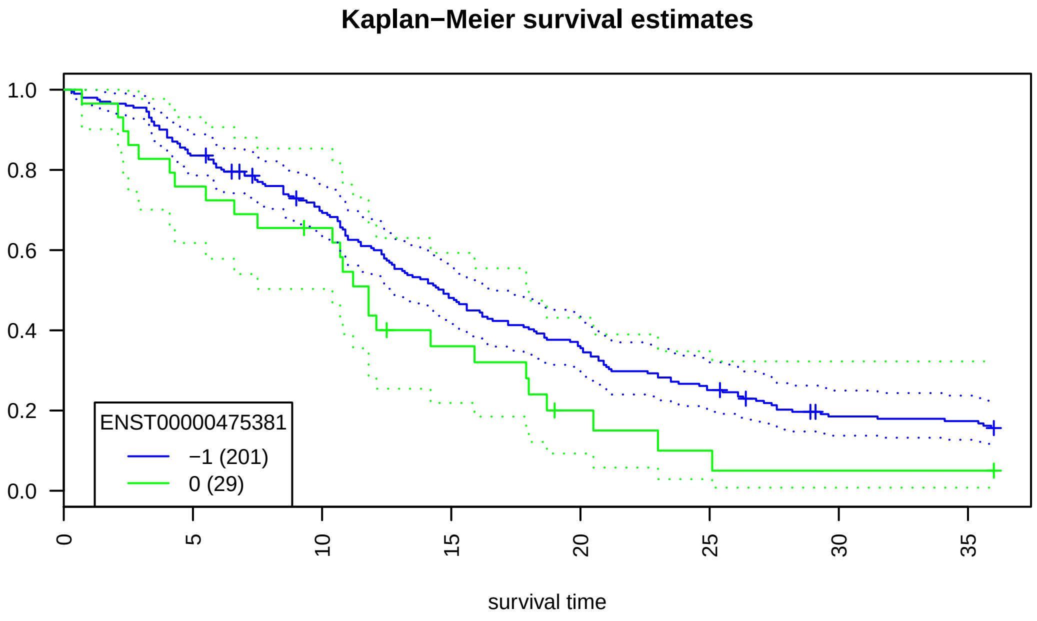

Figure 1216: Each curve represents the relative number of ENST00000475381 survived to the corresponding point of time. Dashed lines represent 0.95 confidence intervals for the curves with the corresponding color. Log-rank probability measure for the equality of these curves is 3.562269e-02. Additional annotations: .*GeneId*=ENSG00000144959, .*GeneName*=AADACL1, .*GeneDesc*=neutral cholesterol ester hydrolase 1 [Source:HGNC Symbol;Acc:29260].

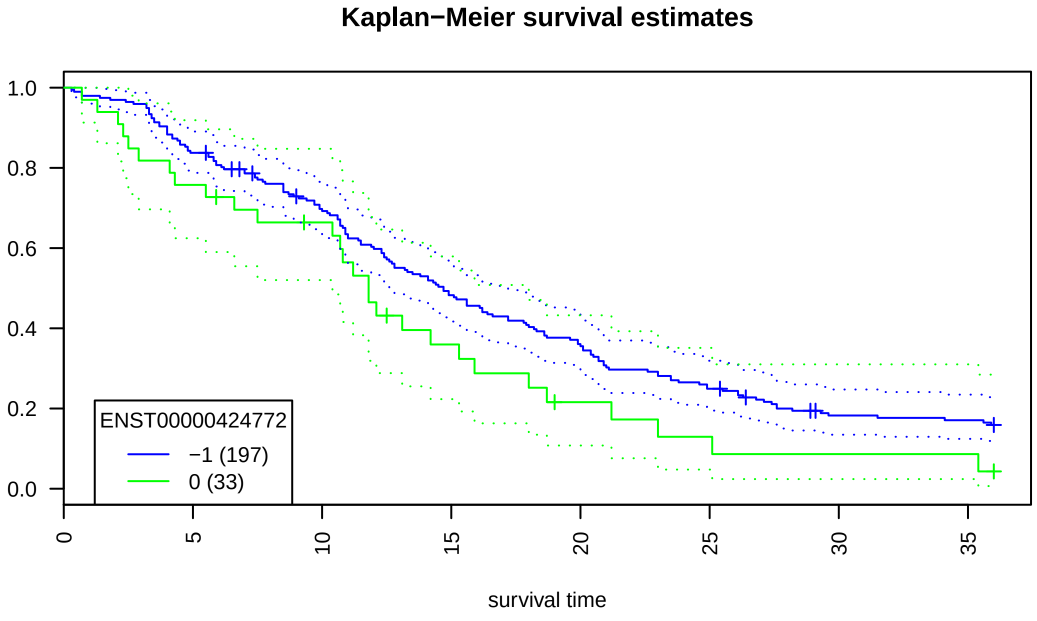

Figure 1217: Each curve represents the relative number of ENST00000424772 survived to the corresponding point of time. Dashed lines represent 0.95 confidence intervals for the curves with the corresponding color. Log-rank probability measure for the equality of these curves is 4.178598e-02. Additional annotations: .*GeneId*=ENSG00000144959, .*GeneName*=AADACL1, .*GeneDesc*=neutral cholesterol ester hydrolase 1 [Source:HGNC Symbol;Acc:29260].

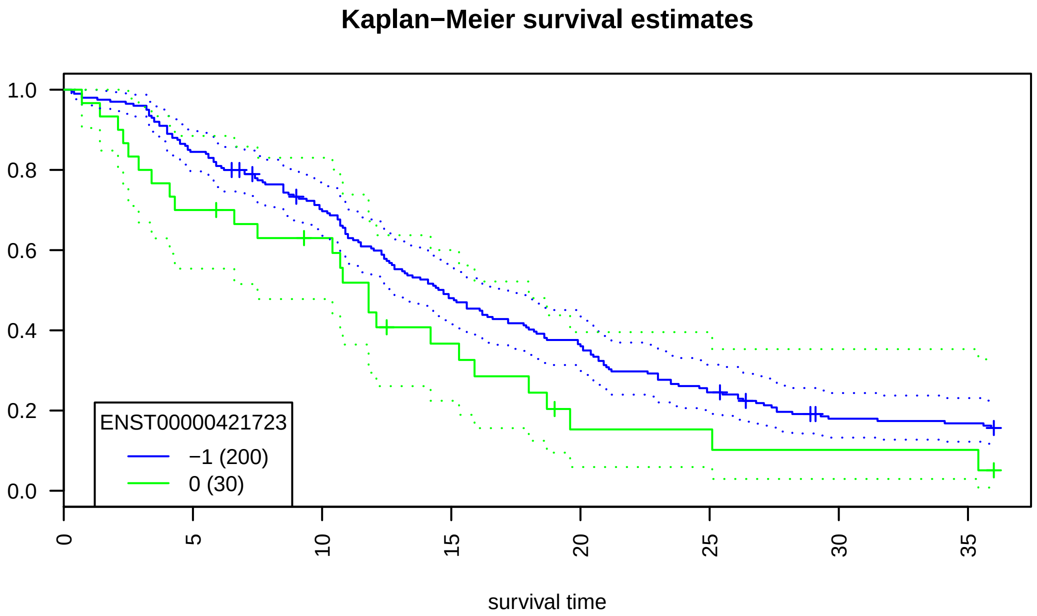

Figure 1218: Each curve represents the relative number of ENST00000421723 survived to the corresponding point of time. Dashed lines represent 0.95 confidence intervals for the curves with the corresponding color. Log-rank probability measure for the equality of these curves is 3.413165e-02. Additional annotations: $.GeneId$=ENSG00000144959, $.GeneName$=AADACL1, $.GeneDesc$=neutral cholesterol ester hydrolase 1 [Source:HGNC Symbol;Acc:29260].

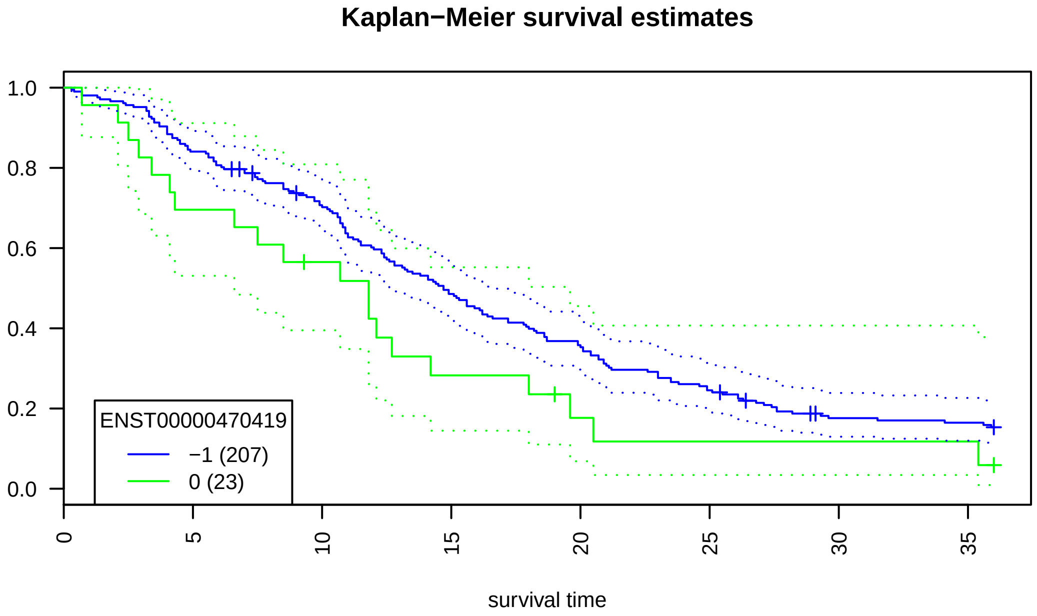

Figure 1219: Each curve represents the relative number of ENST00000470419 survived to the corresponding point of time. Dashed lines represent 0.95 confidence intervals for the curves with the corresponding color. Log-rank probability measure for the equality of these curves is 4.341107e-02. Additional annotations: $.GeneId$=ENSG00000144959, $.GeneName$=AADACL1, $.GeneDesc$=neutral cholesterol ester hydrolase 1 [Source:HGNC Symbol;Acc:29260].

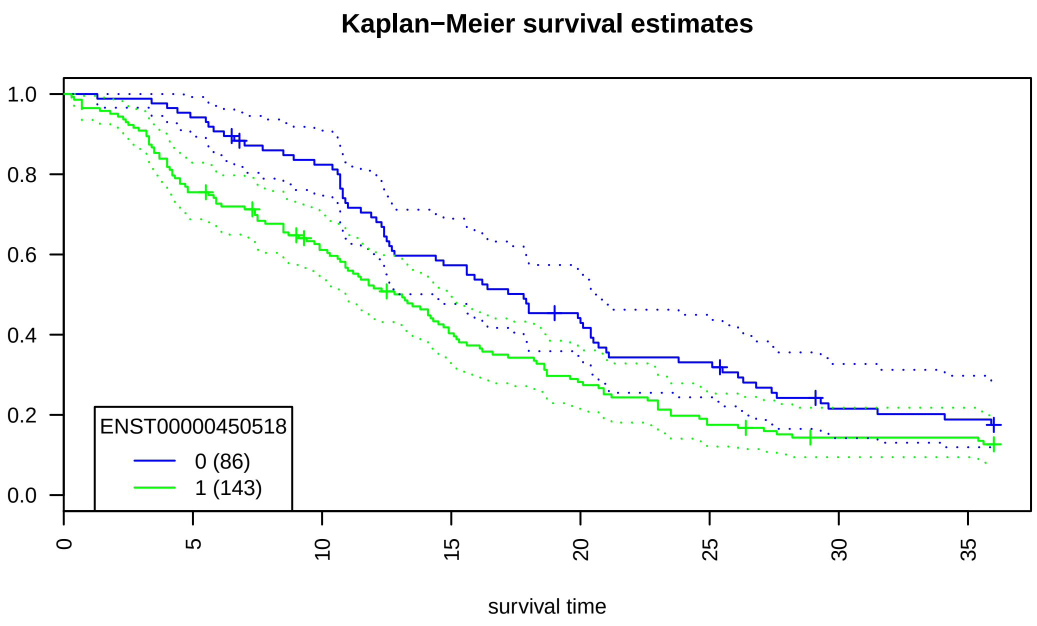

Figure 1220: Each curve represents the relative number of ENST00000450518 survived to the corresponding point of time. Dashed lines represent 0.95 confidence intervals for the curves with the corresponding color. Total of 1 observation deleted due to missingness. Log-rank probability measure for the equality of these curves is 1.735895e-02. Additional annotations: *.GeneId*=ENSG00000136518, *.GeneName*=ACTL6A, *.GeneDesc*=actin-like 6A [Source:HGNC Symbol;Acc:24124].

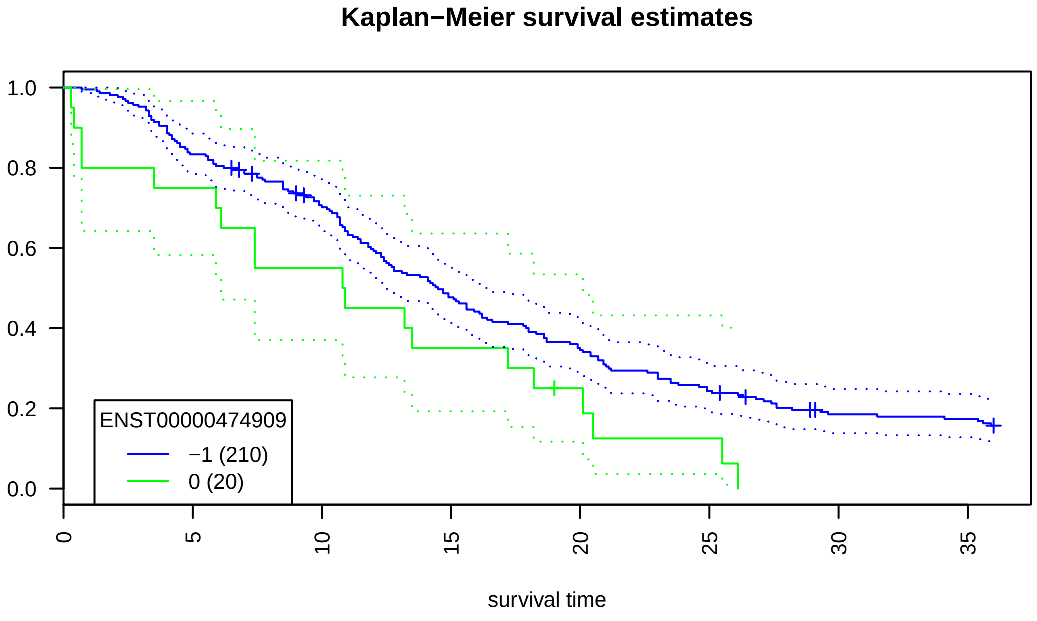

Figure 1221: Each curve represents the relative number of ENST00000474909 survived to the corresponding point of time. Dashed lines represent 0.95 confidence intervals for the curves with the corresponding color. Log-rank probability measure for the equality of these curves is 2.24269e-02. Additional annotations: *.GeneId*=ENSG00000114757, *.GeneName*=PEX5L, *.GeneDesc*=peroxisomal biogenesis factor 5-like [Source:HGNC Symbol;Acc:30024].

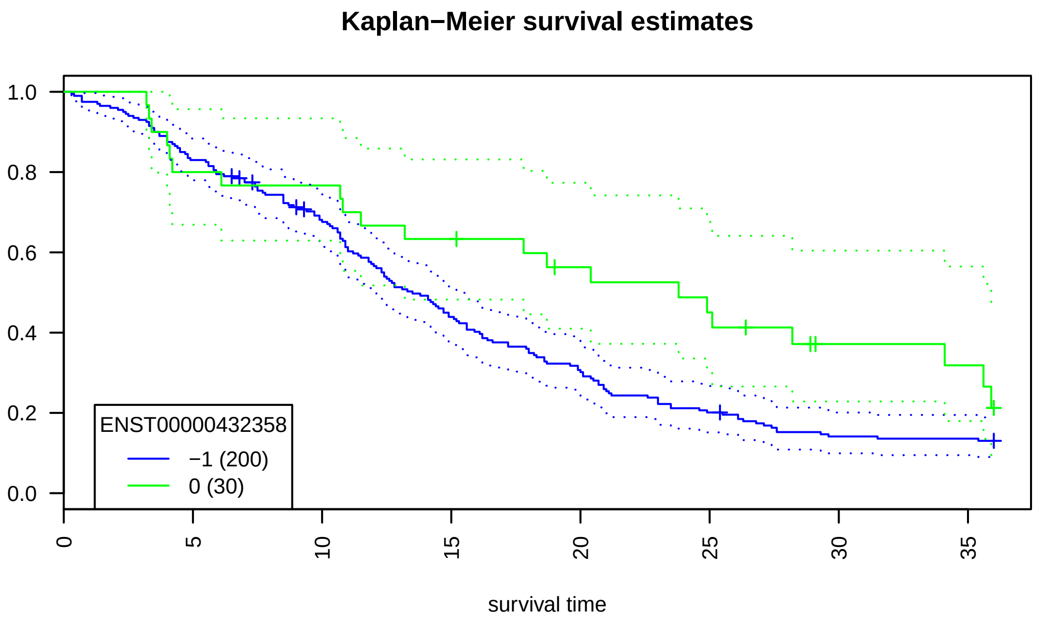

Figure 1222: Each curve represents the relative number of ENST00000432358 survived to the corresponding point of time. Dashed lines represent 0.95 confidence intervals for the curves with the corresponding color. Log-rank probability measure for the equality of these curves is 3.110375e-02. Additional annotations: *.GeneId*=ENSG00000236537, *.GeneName*=RP11-732M18.3, *.GeneDesc*=[undefined].

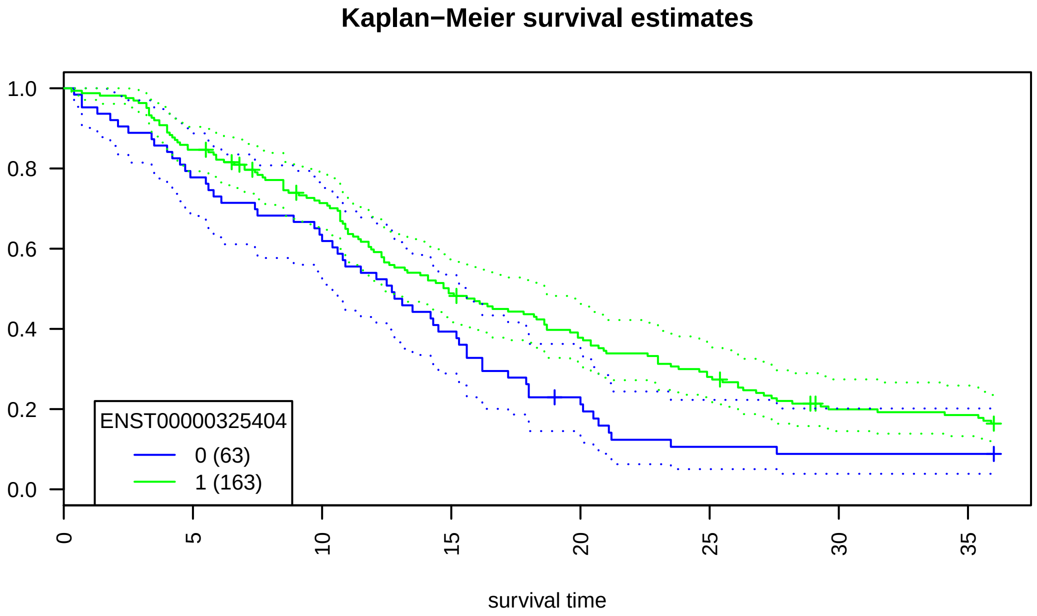

Figure 1223: Each curve represents the relative number of ENST00000325404 survived to the corresponding point of time. Dashed lines represent 0.95 confidence intervals for the curves with the corresponding color. Total of 4 observations deleted due to missingness. Log-rank probability measure for the equality of these curves is 1.529828e-02. Additional annotations: *.GeneId*=ENSG00000181449, *.GeneName*=SOX2, *.GeneDesc*=SRY (sex determining region Y)-box 2 [Source:HGNC Symbol;Acc:11195].

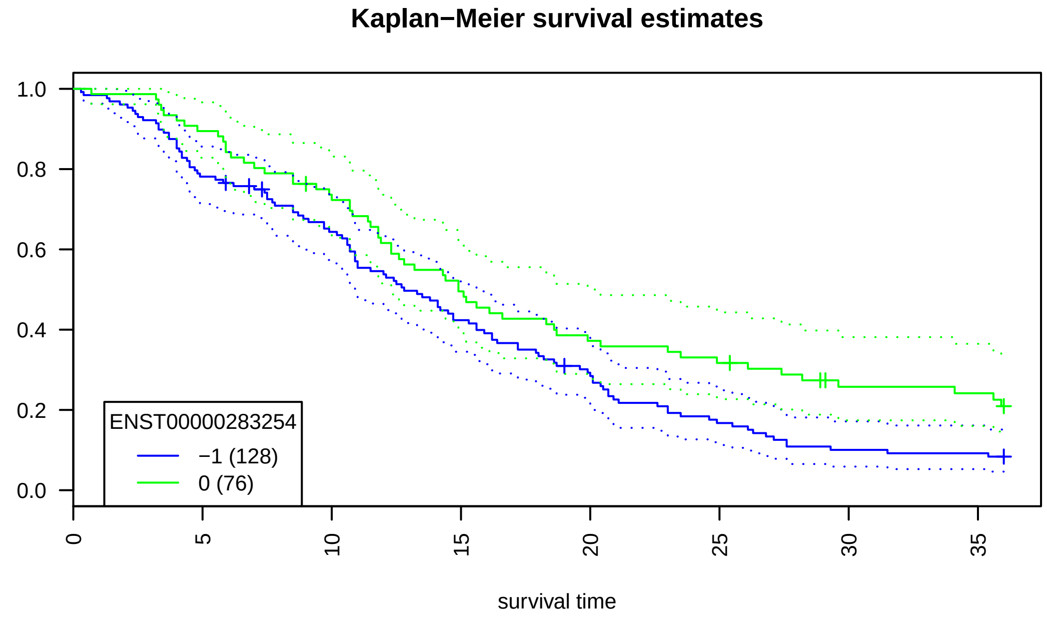

Figure 1224: Each curve represents the relative number of ENST00000283254 survived to the corresponding point of time. Dashed lines represent 0.95 confidence intervals for the curves with the corresponding color. Total of 26 observations deleted due to missingness. Log-rank probability measure for the equality of these curves is 1.449045e-02. Additional annotations: *.GeneId*=ENSG00000153253, *.GeneName*=SCN3A, *.GeneDesc*=sodium channel, voltage-gated, type III, alpha subunit [Source:HGNC Symbol;Acc:10590].

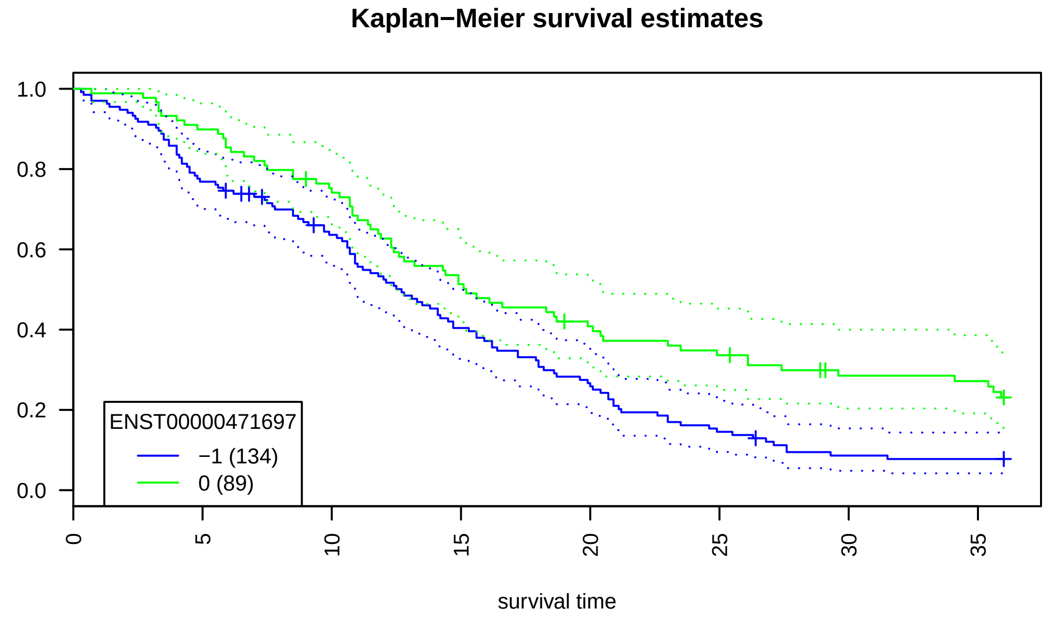

Figure 1225: Each curve represents the relative number of ENST00000471697 survived to the corresponding point of time. Dashed lines represent 0.95 confidence intervals for the curves with the corresponding color. Total of 7 observations deleted due to missingness. Log-rank probability measure for the equality of these curves is 1.356073e-03. Additional annotations: *.GeneId*=ENSG00000153253, *.GeneName*=SCN3A, *.GeneDesc*=sodium channel, voltage-gated, type III, alpha subunit [Source:HGNC Symbol;Acc:10590].

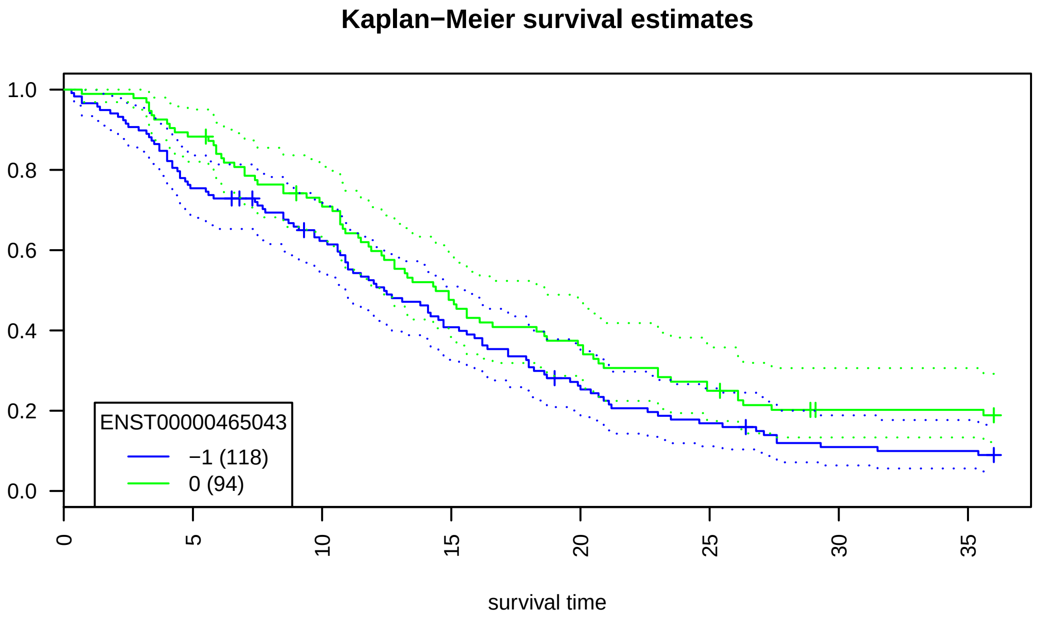

Figure 1226: Each curve represents the relative number of ENST00000465043 survived to the corresponding point of time. Dashed lines represent 0.95 confidence intervals for the curves with the corresponding color. Total of 18 observations deleted due to missingness. Log-rank probability measure for the equality of these curves is 4.839499e-02. Additional annotations: *.GeneId*=ENSG00000153253, *.GeneName*=SCN3A, *.GeneDesc*=sodium channel, voltage-gated, type III, alpha subunit [Source:HGNC Symbol;Acc:10590].

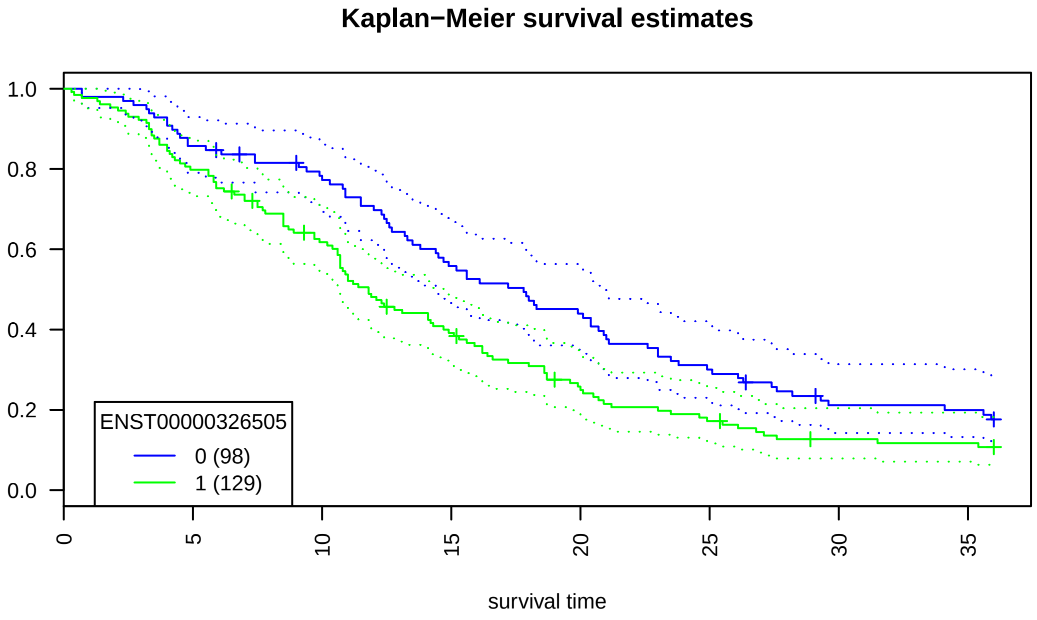

Figure 1227: Each curve represents the relative number of ENST00000326505 survived to the corresponding point of time. Dashed lines represent 0.95 confidence intervals for the curves with the corresponding color. Total of 3 observations deleted due to missingness. Log-rank probability measure for the equality of these curves is 6.341923e-03. Additional annotations: *.GeneId*=ENSG00000176597, *.GeneName*=B3GNT5, *.GeneDesc*=UDP-GlcNAc:betaGal beta-1,3-N-acetylglucosaminyltransferase 5 [Source:HGNC Symbol;Acc:15684].

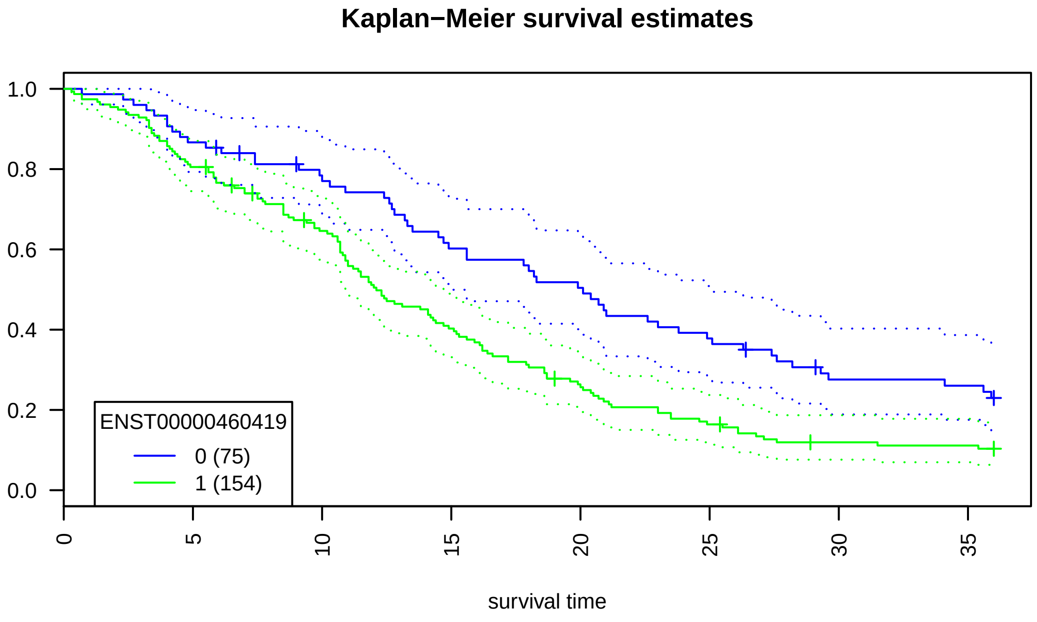

Figure 1228: Each curve represents the relative number of ENST00000460419 survived to the corresponding point of time. Dashed lines represent 0.95 confidence intervals for the curves with the corresponding color. Total of 1 observation deleted due to missingness. Log-rank probability measure for the equality of these curves is 5.426188e-04. Additional annotations: *.GeneId*=ENSG00000176597, *.GeneName*=B3GNT5, *.GeneDesc*=UDP-GlcNAc:betaGal beta-1,3-N-acetylglucosaminyltransferase 5 [Source:HGNC Symbol;Acc:15684].

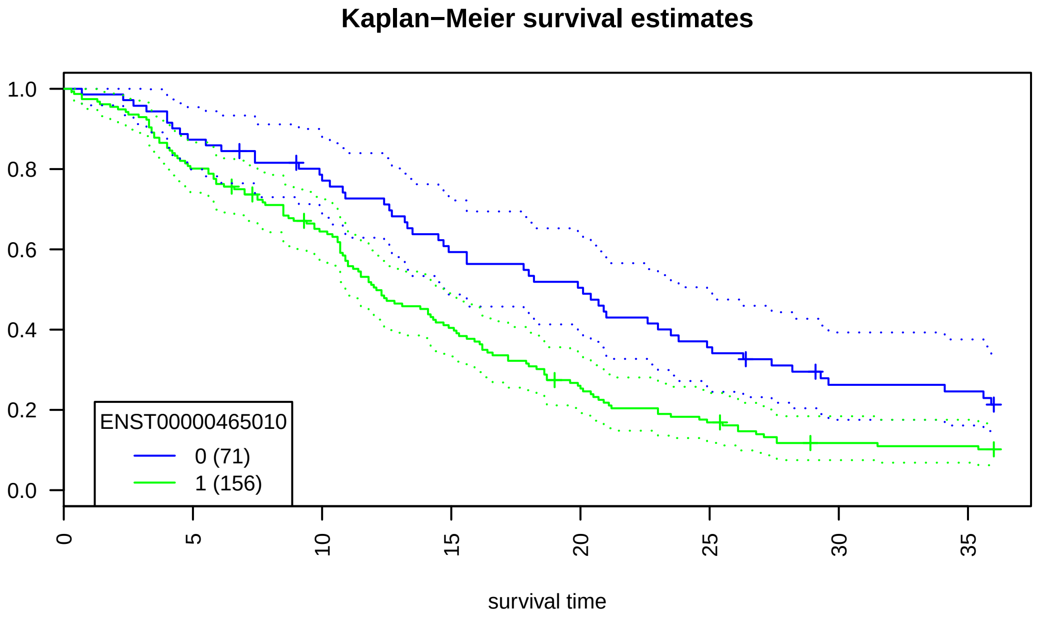

Figure 1229: Each curve represents the relative number of ENST00000465010 survived to the corresponding point of time. Dashed lines represent 0.95 confidence intervals for the curves with the corresponding color. Total of 3 observations deleted due to missingness. Log-rank probability measure for the equality of these curves is 1.269672e-03. Additional annotations: *.GeneId*=ENSG00000176597, *.GeneName*=B3GNT5, *.GeneDesc*=UDP-GlcNAc:betaGal beta-1,3-N-acetylglucosaminyltransferase 5 [Source:HGNC Symbol;Acc:15684].

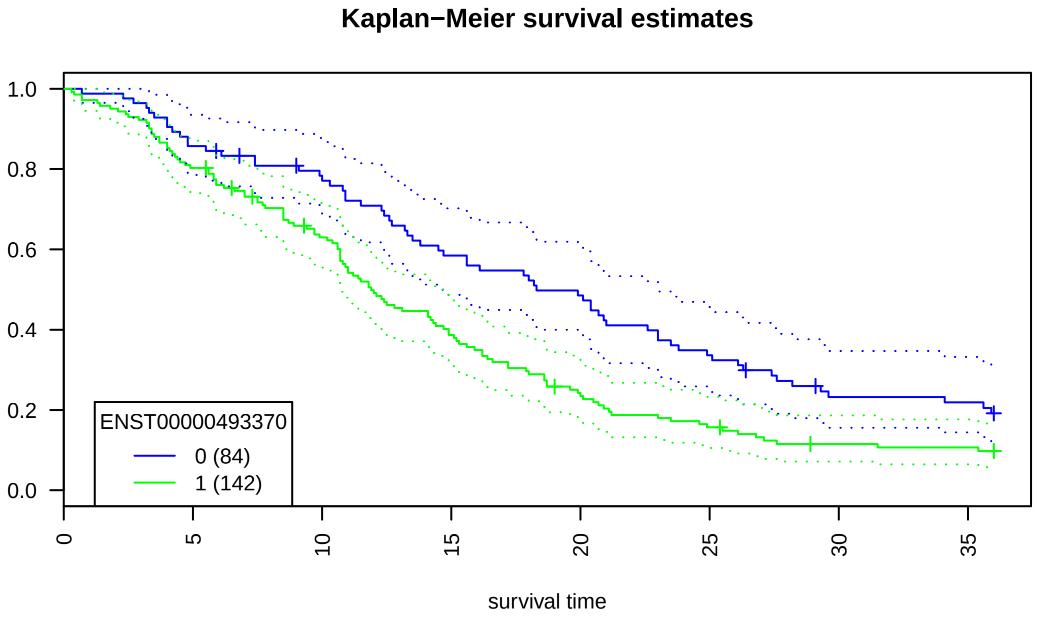

Figure 1230: Each curve represents the relative number of ENST00000493370 survived to the corresponding point of time. Dashed lines represent 0.95 confidence intervals for the curves with the corresponding color. Total of 4 observations deleted due to missingness. Log-rank probability measure for the equality of these curves is 1.230806e-03. Additional annotations: *.GeneId*=ENSG00000176597, *.GeneName*=B3GNT5, *.GeneDesc*=UDP-GlcNAc:betaGal beta-1,3-N-acetylglucosaminyltransferase 5 [Source:HGNC Symbol;Acc:15684].

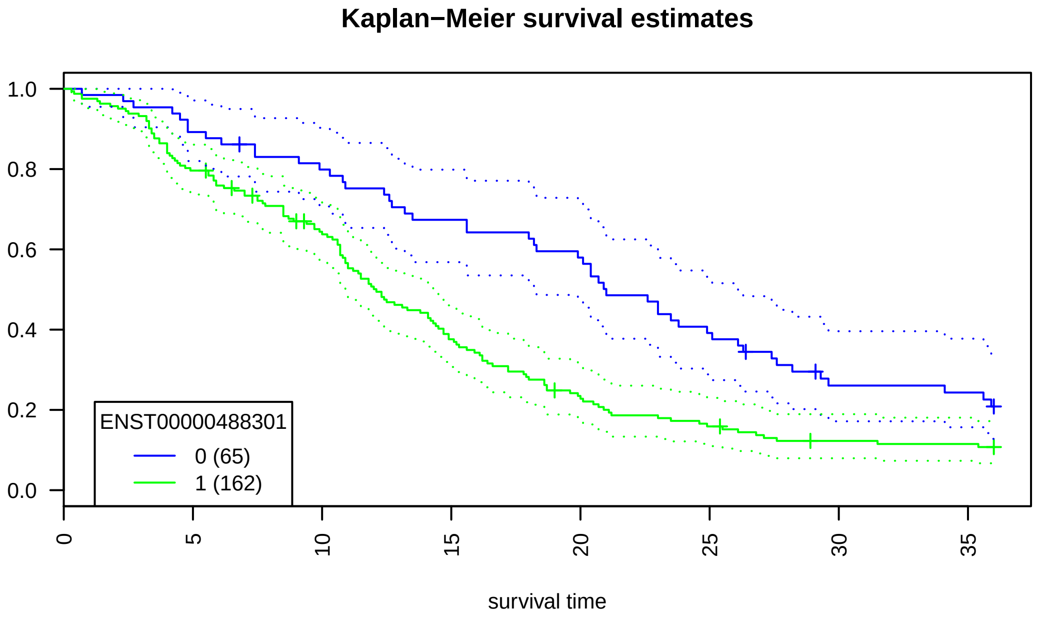

Figure 1231: Each curve represents the relative number of ENST00000488301 survived to the corresponding point of time. Dashed lines represent 0.95 confidence intervals for the curves with the corresponding color. Total of 3 observations deleted due to missingness. Log-rank probability measure for the equality of these curves is 2.302435e-04. Additional annotations: *.GeneId*=ENSG00000176597, *.GeneName*=B3GNT5, *.GeneDesc*=UDP-GlcNAc:betaGal beta-1,3-N-acetylglucosaminyltransferase 5 [Source:HGNC Symbol;Acc:15684].

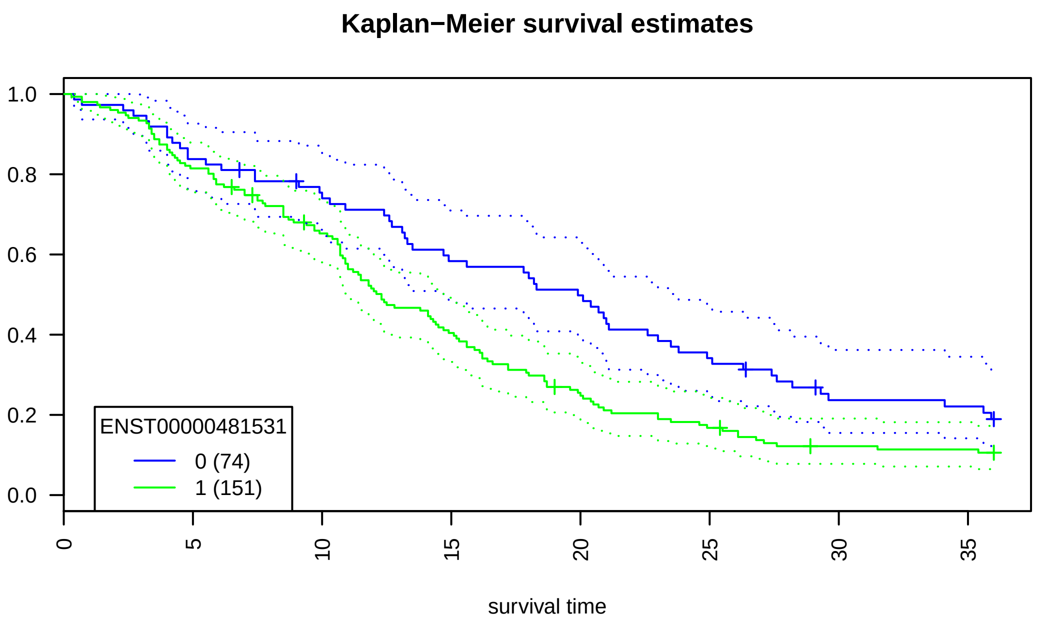

Figure 1232: Each curve represents the relative number of ENST00000481531 survived to the corresponding point of time. Dashed lines represent 0.95 confidence intervals for the curves with the corresponding color. Total of 5 observations deleted due to missingness. Log-rank probability measure for the equality of these curves is 4.352366e-03. Additional annotations: *.GeneId*=ENSG00000176597, *.GeneName*=B3GNT5, *.GeneDesc*=UDP-GlcNAc:betaGal beta-1,3-N-acetylglucosaminyltransferase 5 [Source:HGNC Symbol;Acc:15684].

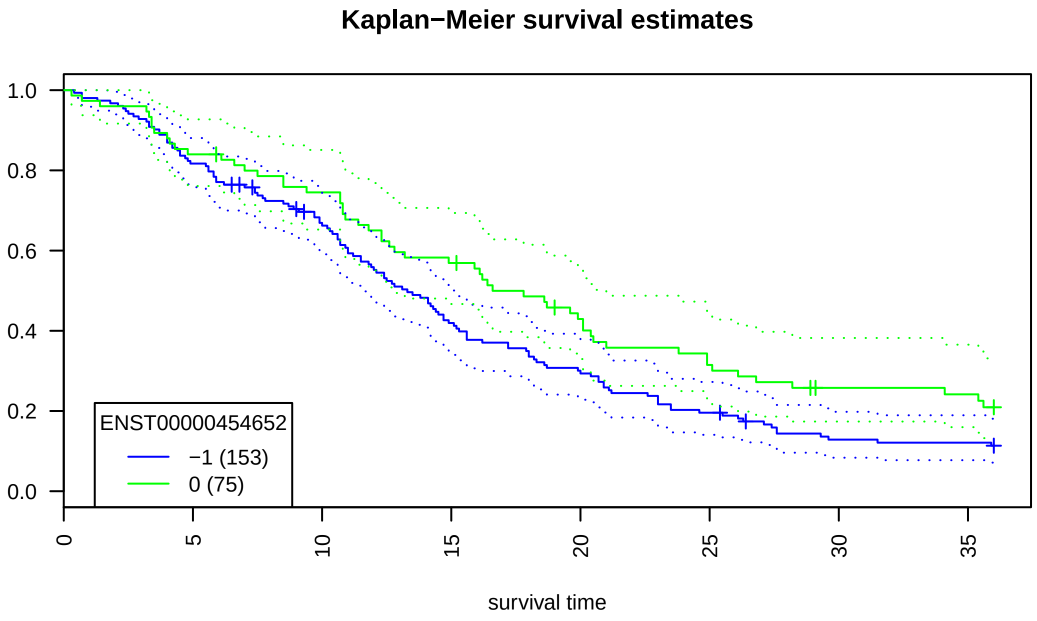

Figure 1233: Each curve represents the relative number of ENST00000454652 survived to the corresponding point of time. Dashed lines represent 0.95 confidence intervals for the curves with the corresponding color. Total of 2 observations deleted due to missingness. Log-rank probability measure for the equality of these curves is 3.188866e-02. Additional annotations: *.GeneId*=ENSG00000114796, *.GeneName*=KLHL24, *.GeneDesc*=kelch-like 24 (Drosophila) [Source:HGNC Symbol;Acc:25947].

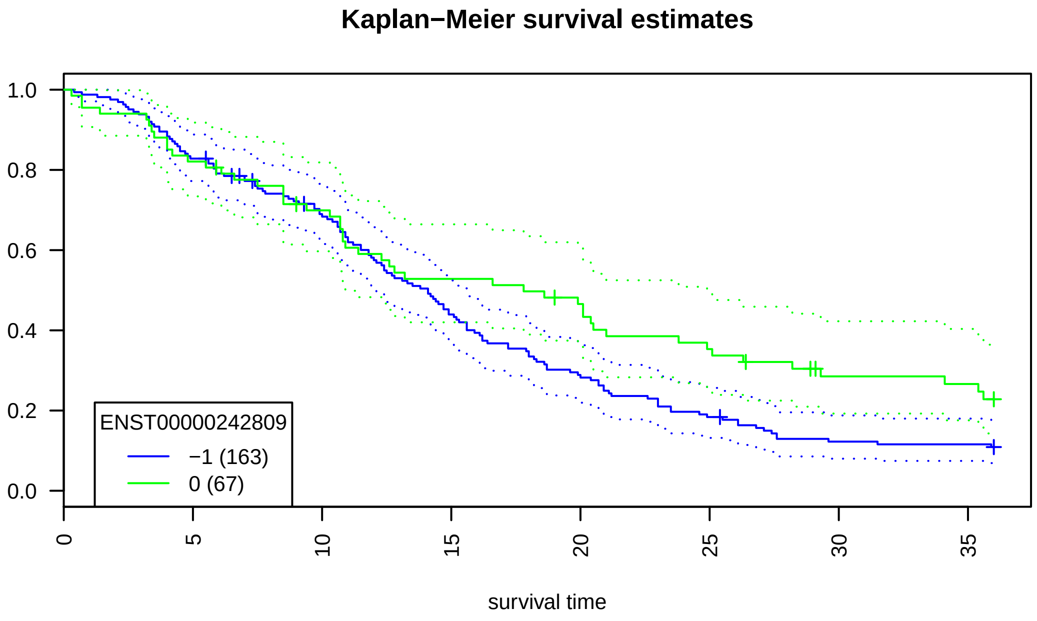

Figure 1234: Each curve represents the relative number of ENST00000242809 survived to the corresponding point of time. Dashed lines represent 0.95 confidence intervals for the curves with the corresponding color. Log-rank probability measure for the equality of these curves is 3.205853e-02. Additional annotations: *.GeneId*=ENSG00000114796, *.GeneName*=KLHL24, *.GeneDesc*=kelch-like 24 (Drosophila) [Source:HGNC Symbol;Acc:25947].

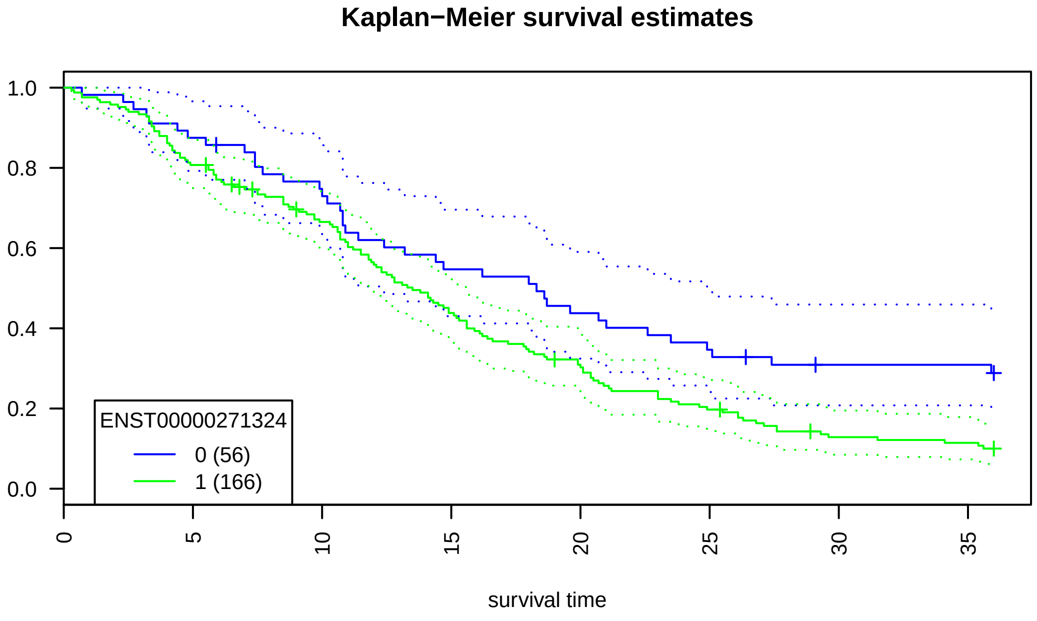

Figure 1235: Each curve represents the relative number of ENST00000271324 survived to the corresponding point of time. Dashed lines represent 0.95 confidence intervals for the curves with the corresponding color. Total of 8 observations deleted due to missingness. Log-rank probability measure for the equality of these curves is 7.432822e-03. Additional annotations: *.GeneId*=ENSG00000143119, *.GeneName*=CD53, *.GeneDesc*=CD53 molecule [Source:HGNC Symbol;Acc:1686].

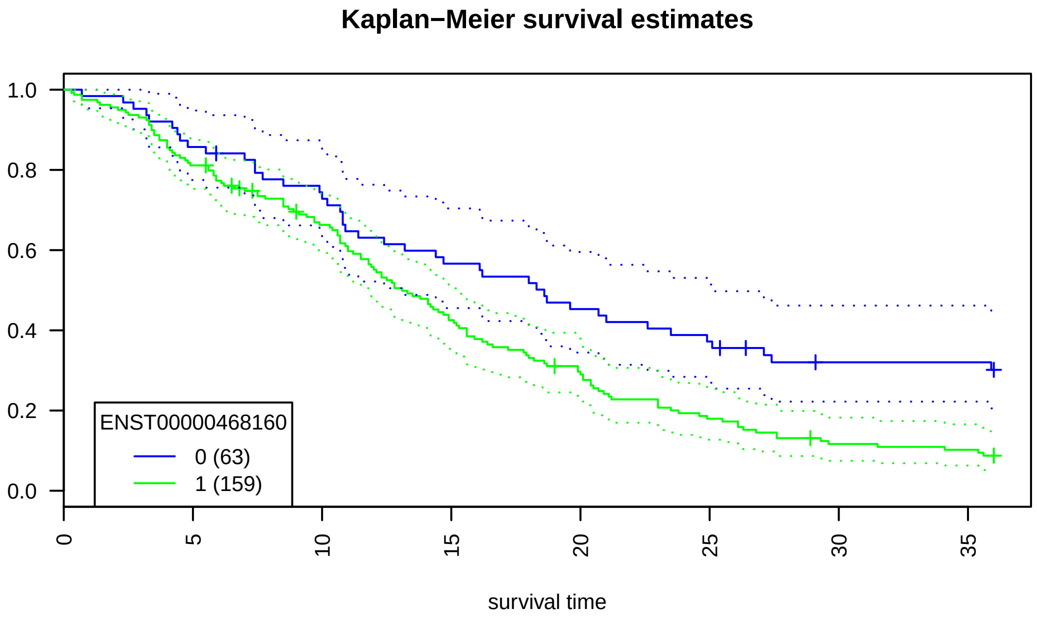

Figure 1236: Each curve represents the relative number of ENST00000468160 survived to the corresponding point of time. Dashed lines represent 0.95 confidence intervals for the curves with the corresponding color. Total of 8 observations deleted due to missingness. Log-rank probability measure for the equality of these curves is 1.283358e-03. Additional annotations: *.GeneId*=ENSG00000143119, *.GeneName*=CD53, *.GeneDesc*=CD53 molecule [Source:HGNC Symbol;Acc:1686].

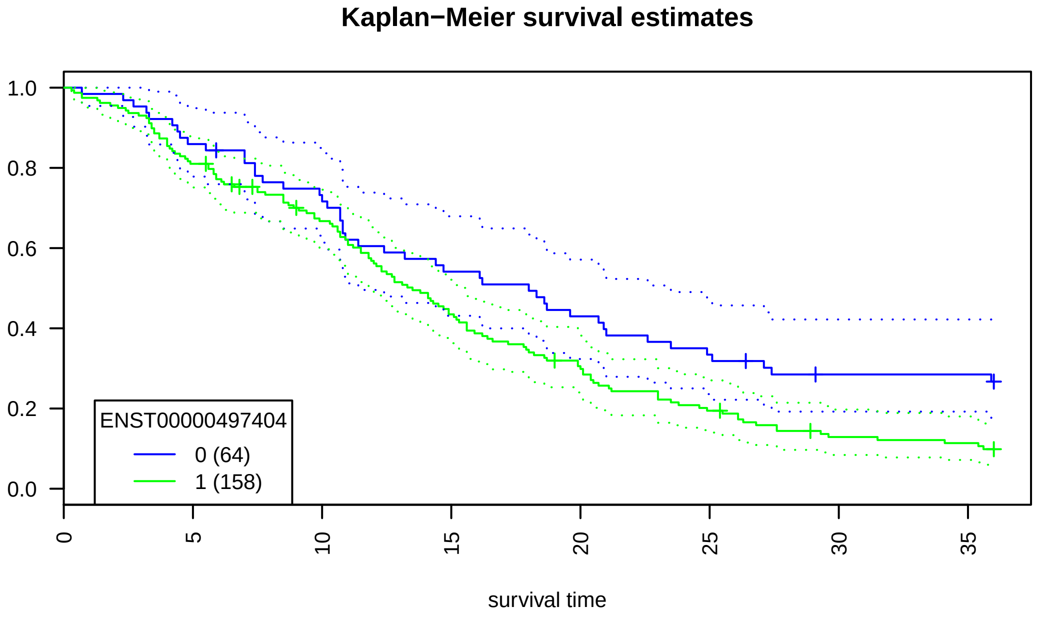

Figure 1237: Each curve represents the relative number of ENST00000497404 survived to the corresponding point of time. Dashed lines represent 0.95 confidence intervals for the curves with the corresponding color. Total of 8 observations deleted due to missingness. Log-rank probability measure for the equality of these curves is 1.153471e-02. Additional annotations: *.GeneId*=ENSG00000143119, *.GeneName*=CD53, *.GeneDesc*=CD53 molecule [Source:HGNC Symbol;Acc:1686].

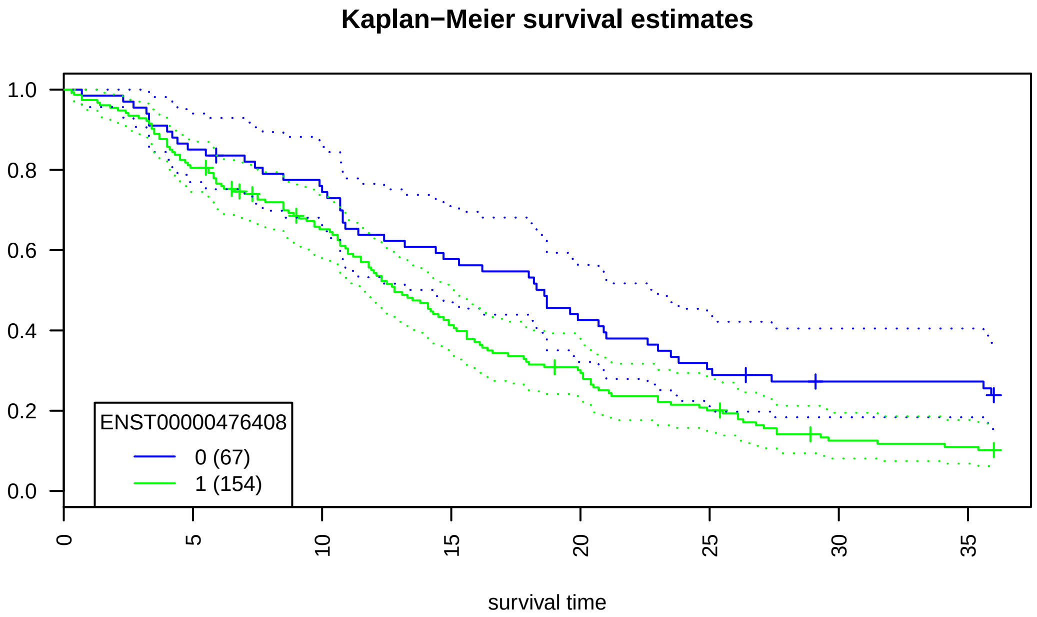

Figure 1238: Each curve represents the relative number of ENST00000476408 survived to the corresponding point of time. Dashed lines represent 0.95 confidence intervals for the curves with the corresponding color. Total of 9 observations deleted due to missingness. Log-rank probability measure for the equality of these curves is 1.101583e-02. Additional annotations: *.GeneId*=ENSG00000143119, *.GeneName*=CD53, *.GeneDesc*=CD53 molecule [Source:HGNC Symbol;Acc:1686].

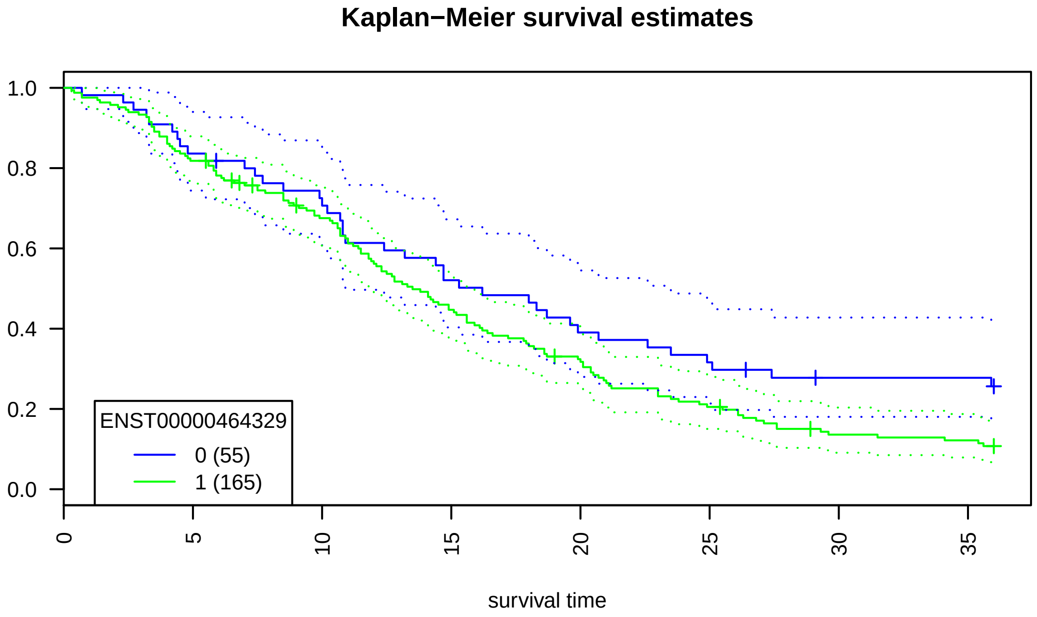

Figure 1239: Each curve represents the relative number of ENST00000464329 survived to the corresponding point of time. Dashed lines represent 0.95 confidence intervals for the curves with the corresponding color. Total of 10 observations deleted due to missingness. Log-rank probability measure for the equality of these curves is 4.695173e-02. Additional annotations: *.GeneId*=ENSG00000143119, *.GeneName*=CD53, *.GeneDesc*=CD53 molecule [Source:HGNC Symbol;Acc:1686].

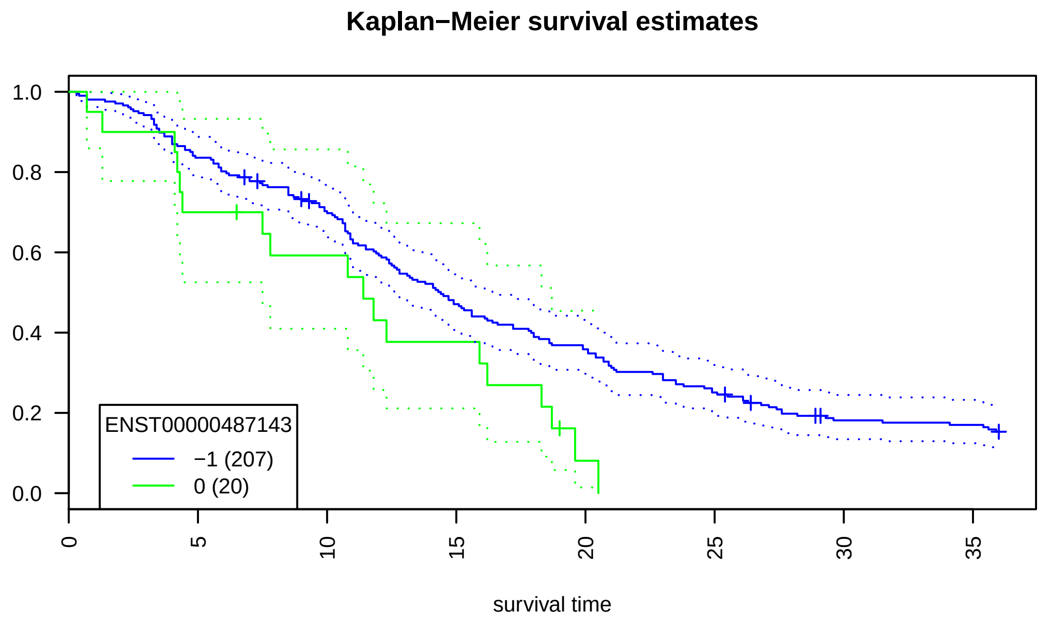

Figure 1240: Each curve represents the relative number of ENST00000487143 survived to the corresponding point of time. Dashed lines represent 0.95 confidence intervals for the curves with the corresponding color. Total of 3 observations deleted due to missingness. Log-rank probability measure for the equality of these curves is 1.582451e-02. Additional annotations: *.GeneId*=ENSG00000198648, *.GeneName*=STK39, *.GeneDesc*=serine threonine kinase 39 (STE20/SPS1 homolog, yeast) [Source:HGNC Symbol;Acc:17717].

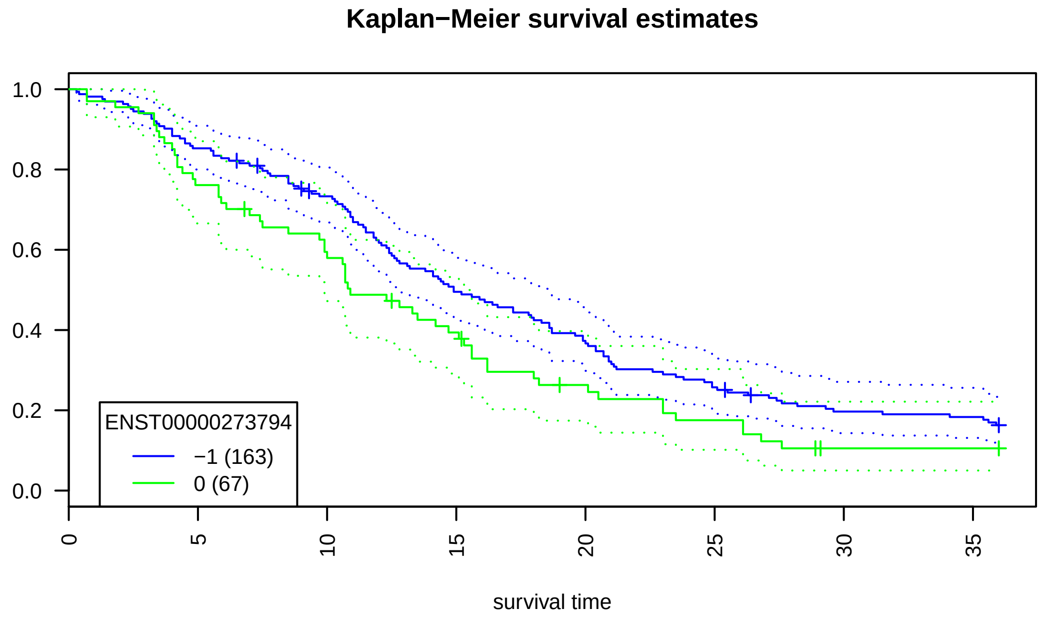

Figure 1241: Each curve represents the relative number of ENST00000273794 survived to the corresponding point of time. Dashed lines represent 0.95 confidence intervals for the curves with the corresponding color. Log-rank probability measure for the equality of these curves is 3.618219e-02. Additional annotations: *.GeneId*=ENSG00000145198, *.GeneName*=VWA5B2, *.GeneDesc*=von Willebrand factor A domain containing 5B2 [Source:HGNC Symbol;Acc:25144].

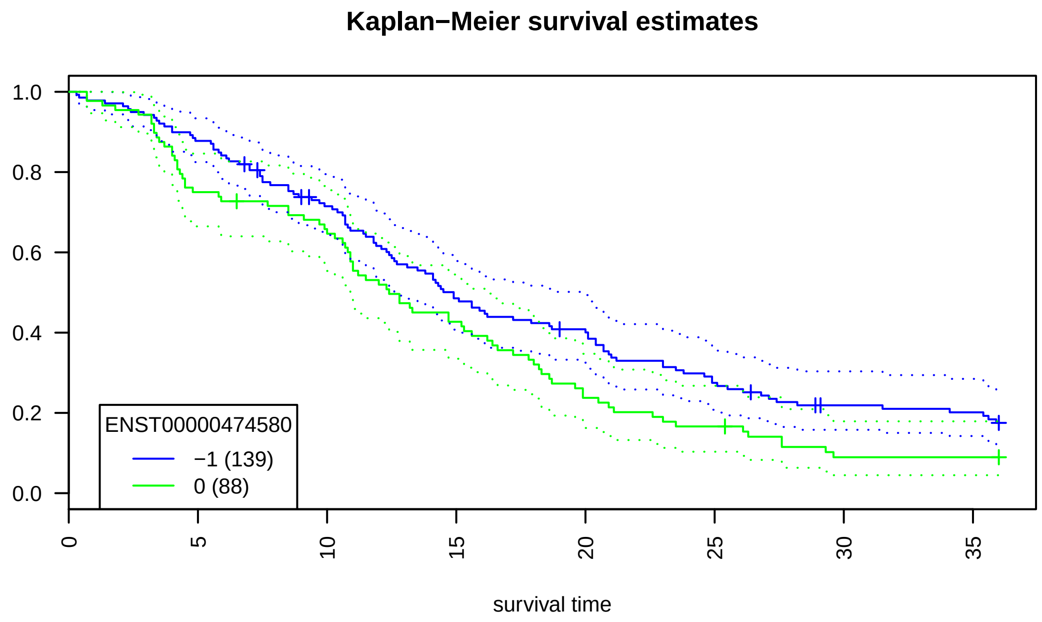

Figure 1242: Each curve represents the relative number of ENST00000474580 survived to the corresponding point of time. Dashed lines represent 0.95 confidence intervals for the curves with the corresponding color. Total of 3 observations deleted due to missingness. Log-rank probability measure for the equality of these curves is 2.881966e-02. Additional annotations: *.GeneId*=ENSG00000145198, *.GeneName*=VWA5B2, *.GeneDesc*=von Willebrand factor A domain containing 5B2 [Source:HGNC Symbol;Acc:25144].

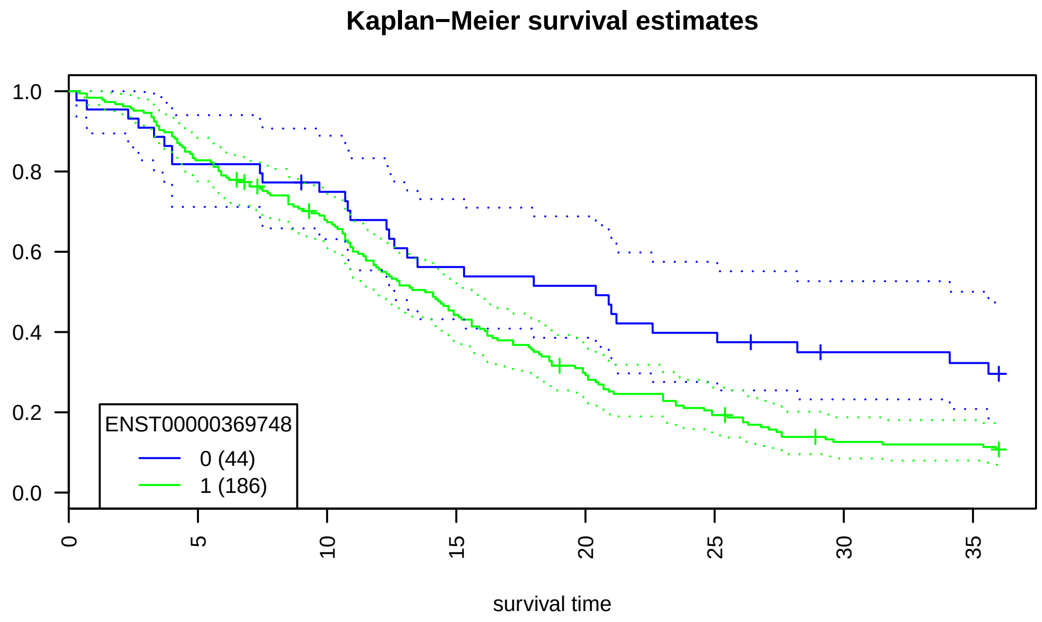

Figure 1243: Each curve represents the relative number of ENST00000369748 survived to the corresponding point of time. Dashed lines represent 0.95 confidence intervals for the curves with the corresponding color. Log-rank probability measure for the equality of these curves is 8.545916e-03. Additional annotations: *.GeneId*=ENSG00000064886, *.GeneName*=CHI3L2, *.GeneDesc*=chitinase 3-like 2 [Source:HGNC Symbol;Acc:1933].

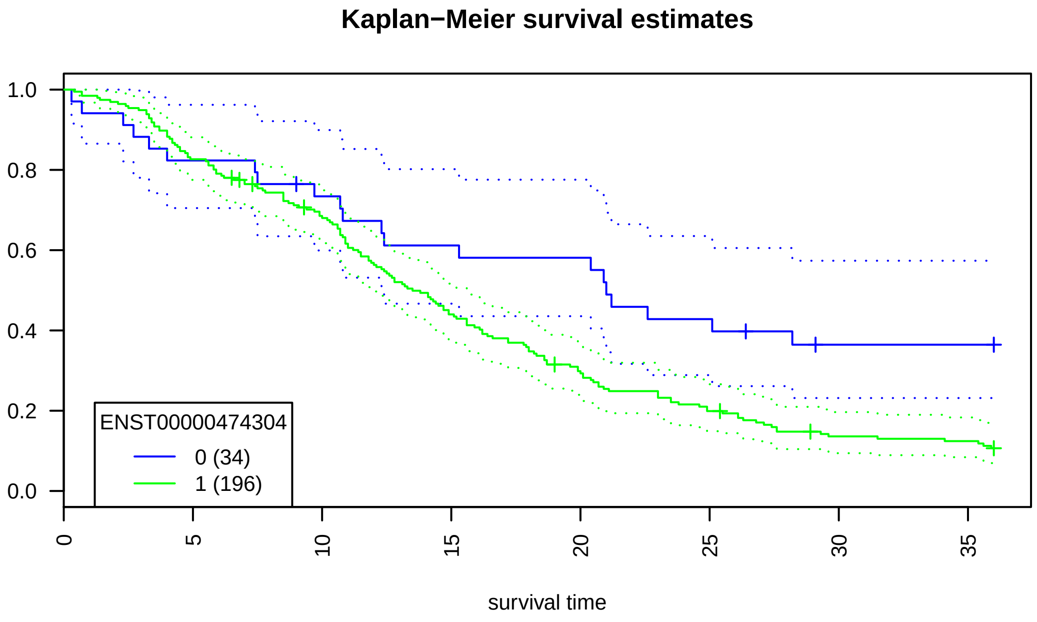

Figure 1244: Each curve represents the relative number of ENST00000474304 survived to the corresponding point of time. Dashed lines represent 0.95 confidence intervals for the curves with the corresponding color. Log-rank probability measure for the equality of these curves is 4.754889e-03. Additional annotations: *.GeneId*=ENSG00000064886, *.GeneName*=CHI3L2, *.GeneDesc*=chitinase 3-like 2 [Source:HGNC Symbol;Acc:1933].

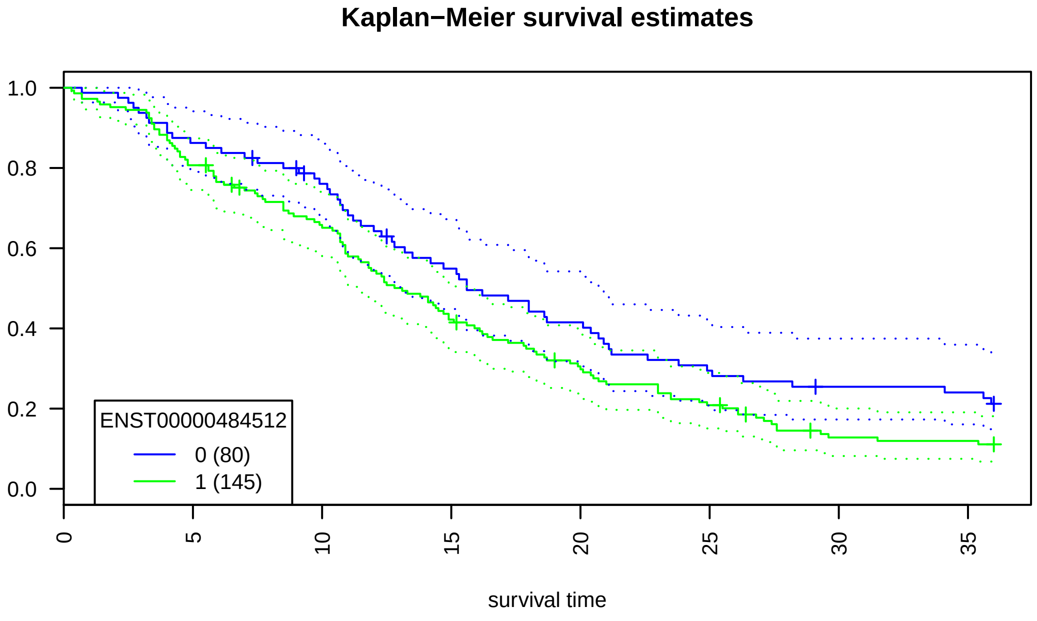

Figure 1245: Each curve represents the relative number of ENST00000484512 survived to the corresponding point of time. Dashed lines represent 0.95 confidence intervals for the curves with the corresponding color. Total of 5 observations deleted due to missingness. Log-rank probability measure for the equality of these curves is 3.947353e-02. Additional annotations: *.GeneId*=ENSG00000173947, *.GeneName*=C1orf88, *.GeneDesc*=Uncharacterized protein C1orf88 [Source:UniProtKB/Swiss-Prot;Acc:Q8TCI5].

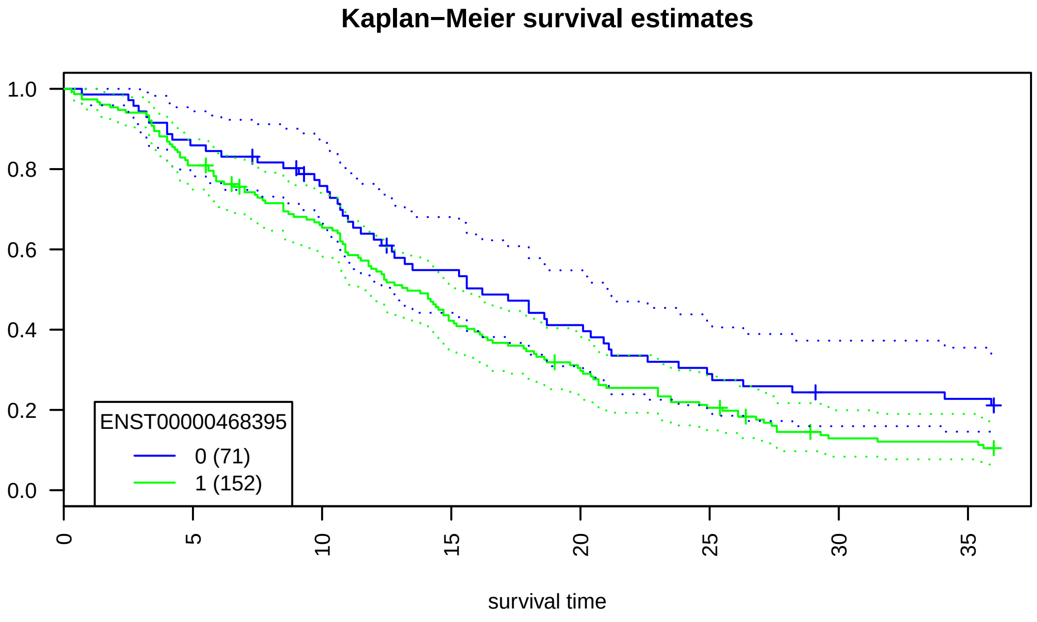

Figure 1246: Each curve represents the relative number of ENST00000468395 survived to the corresponding point of time. Dashed lines represent 0.95 confidence intervals for the curves with the corresponding color. Total of 7 observations deleted due to missingness. Log-rank probability measure for the equality of these curves is 4.888507e-02. Additional annotations: *.GeneId*=ENSG00000173947, *.GeneName*=C1orf88, *.GeneDesc*=Uncharacterized protein C1orf88 [Source:UniProtKB/Swiss-Prot;Acc:Q8TCI5].

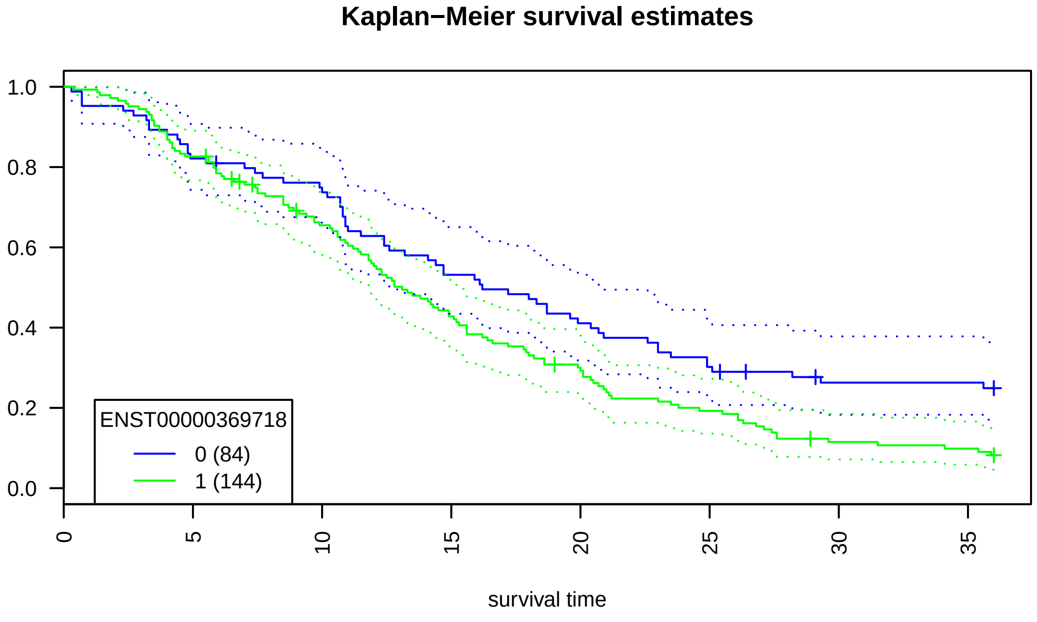

Figure 1247: Each curve represents the relative number of ENST00000369718 survived to the corresponding point of time. Dashed lines represent 0.95 confidence intervals for the curves with the corresponding color. Total of 2 observations deleted due to missingness. Log-rank probability measure for the equality of these curves is 6.791367e-03. Additional annotations: *.GeneId*=ENSG00000143110, *.GeneName*=C1orf162, *.GeneDesc*=Transmembrane protein C1orf162 [Source:UniProtKB/Swiss-Prot;Acc:Q8NEQ5].

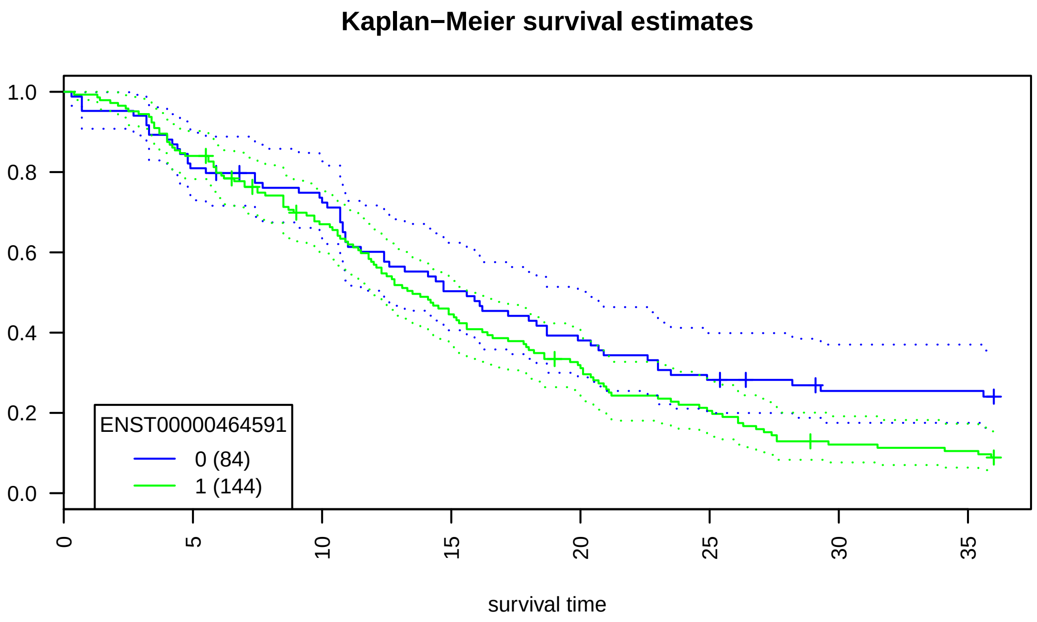

Figure 1248: Each curve represents the relative number of ENST00000464591 survived to the corresponding point of time. Dashed lines represent 0.95 confidence intervals for the curves with the corresponding color. Total of 2 observations deleted due to missingness. Log-rank probability measure for the equality of these curves is 3.735562e-02. Additional annotations: *.GeneId*=ENSG00000143110, *.GeneName*=C1orf162, *.GeneDesc*=Transmembrane protein C1orf162 [Source:UniProtKB/Swiss-Prot;Acc:Q8NEQ5].

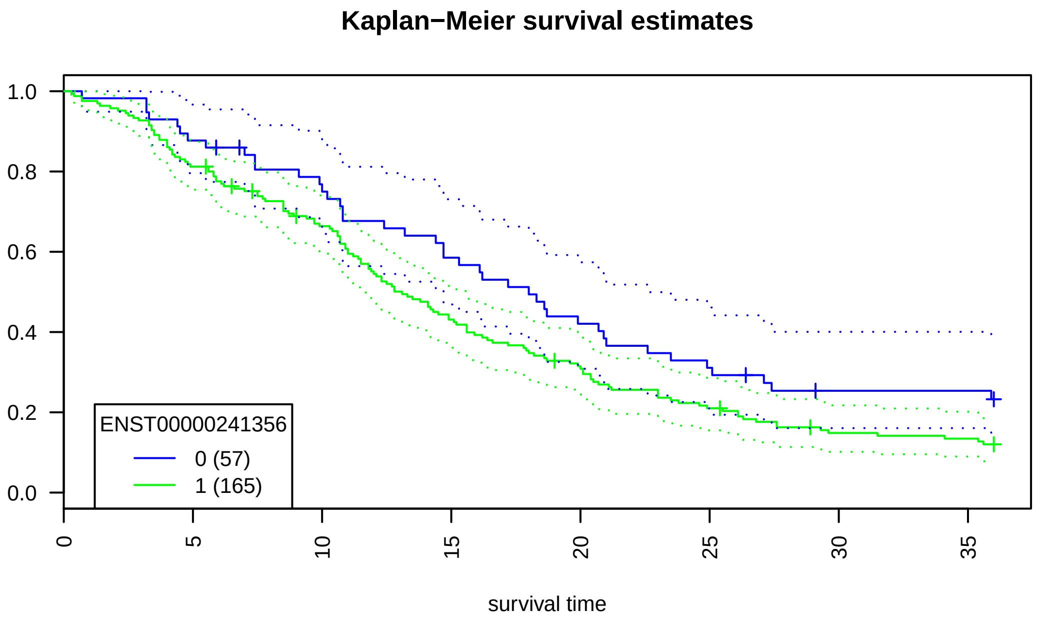

Figure 1249: Each curve represents the relative number of ENST00000241356 survived to the corresponding point of time. Dashed lines represent 0.95 confidence intervals for the curves with the corresponding color. Total of 8 observations deleted due to missingness. Log-rank probability measure for the equality of these curves is 3.696694e-02. Additional annotations: *.GeneId*=ENSG00000121933, *.GeneName*=ADORA3, *.GeneDesc*=adenosine A3 receptor [Source:HGNC Symbol;Acc:268].

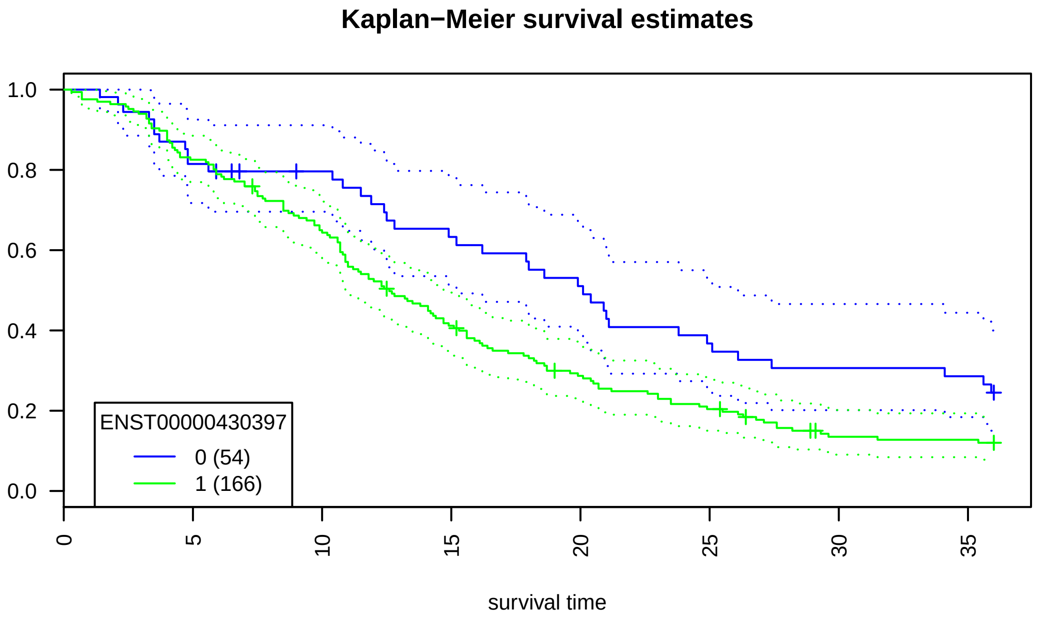

Figure 1250: Each curve represents the relative number of ENST00000430397 survived to the corresponding point of time. Dashed lines represent 0.95 confidence intervals for the curves with the corresponding color. Total of 10 observations deleted due to missingness. Log-rank probability measure for the equality of these curves is 9.710465e-03. Additional annotations: *.GeneId*=ENSG00000114859, *.GeneName*=CLCN2, *.GeneDesc*=chloride channel 2 [Source:HGNC Symbol;Acc:2020].

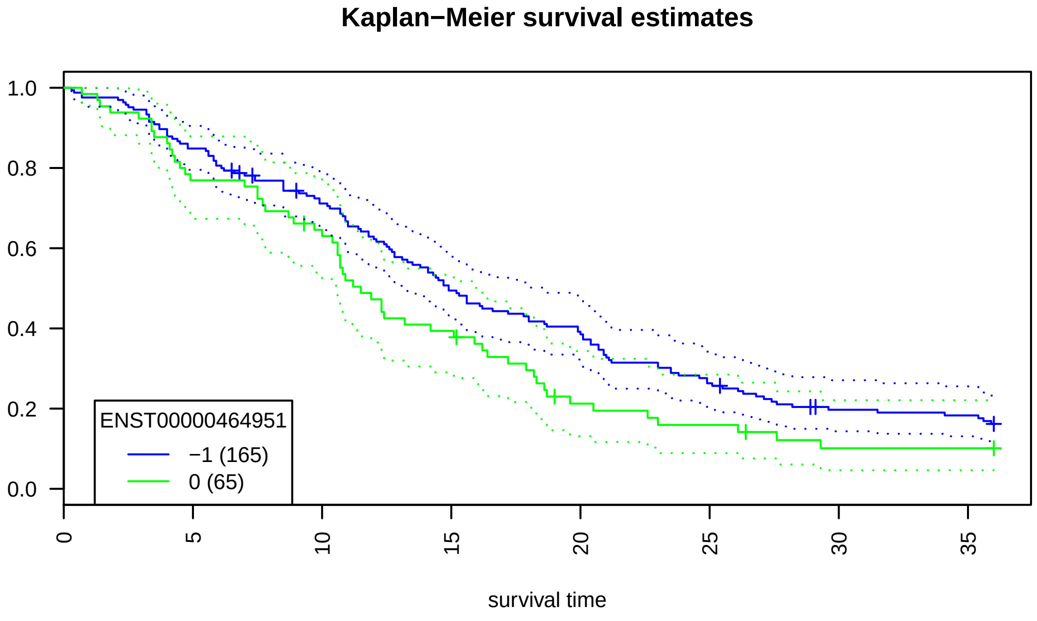

Figure 1251: Each curve represents the relative number of ENST00000464951 survived to the corresponding point of time. Dashed lines represent 0.95 confidence intervals for the curves with the corresponding color. Log-rank probability measure for the equality of these curves is 3.784229e-02. Additional annotations: *.GeneId*=ENSG00000155367, *.GeneName*=PPM1J, *.GeneDesc*=protein phosphatase, Mg2+/Mn2+ dependent, 1J [Source:HGNC Symbol;Acc:20785].

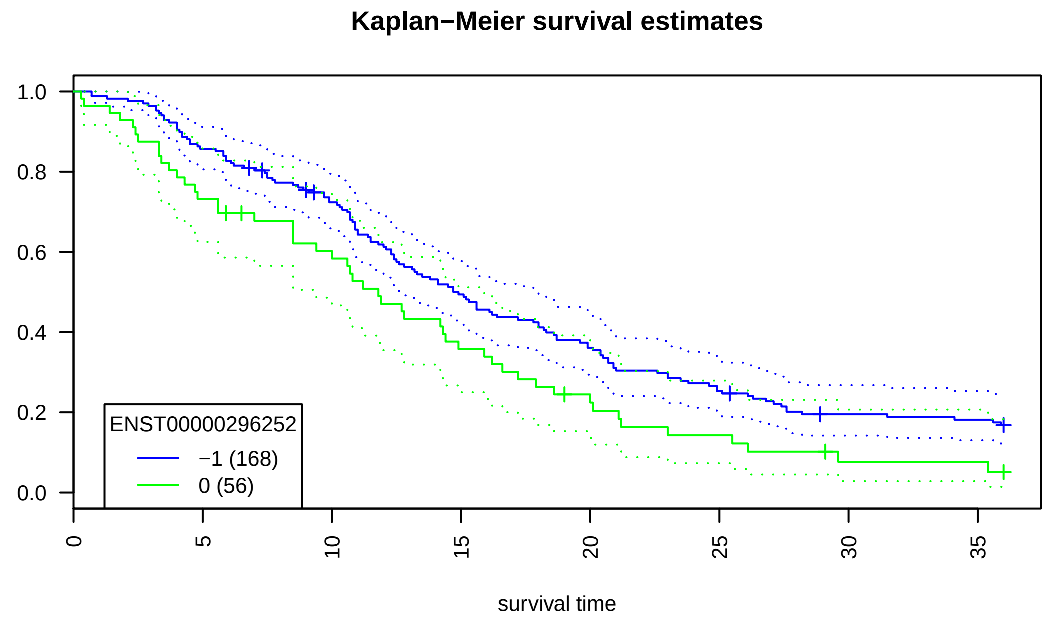

Figure 1252: Each curve represents the relative number of ENST00000296252 survived to the corresponding point of time. Dashed lines represent 0.95 confidence intervals for the curves with the corresponding color. Total of 6 observations deleted due to missingness. Log-rank probability measure for the equality of these curves is 9.604985e-03. Additional annotations: *.GeneId*=ENSG00000163898, *.GeneName*=LIPH, *.GeneDesc*=lipase, member H [Source:HGNC Symbol;Acc:18483].

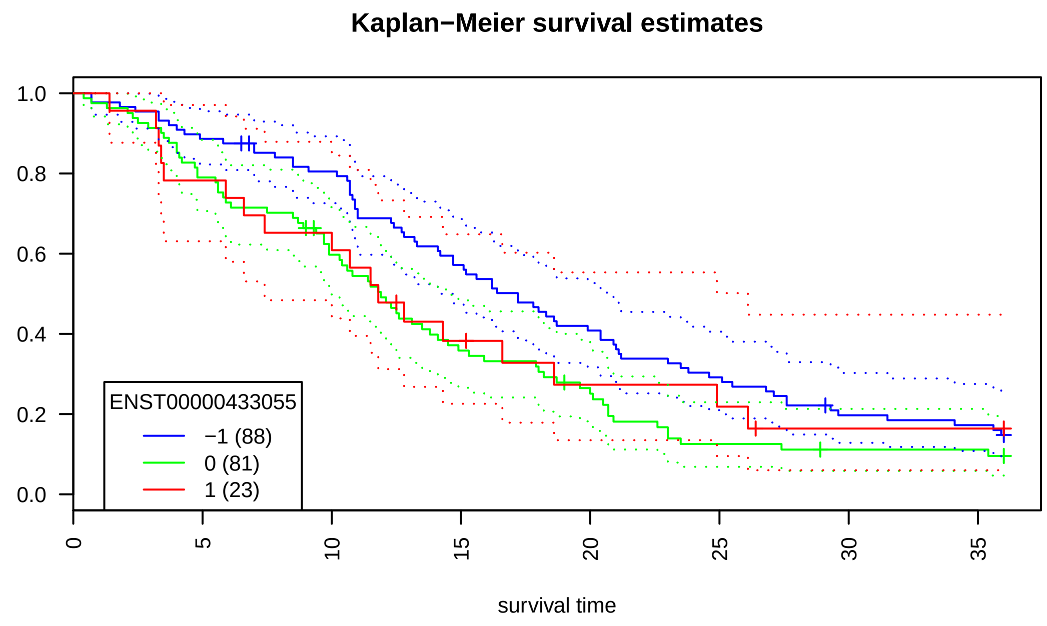

Figure 1253: Each curve represents the relative number of ENST00000433055 survived to the corresponding point of time. Dashed lines represent 0.95 confidence intervals for the curves with the corresponding color. Total of 38 observations deleted due to missingness. Log-rank probability measure for the equality of these curves is 4.102695e-02. Additional annotations: *.GeneId*=ENSG00000163923, *.GeneName*=RPL39L, *.GeneDesc*=ribosomal protein L39-like [Source:HGNC Symbol;Acc:17094].

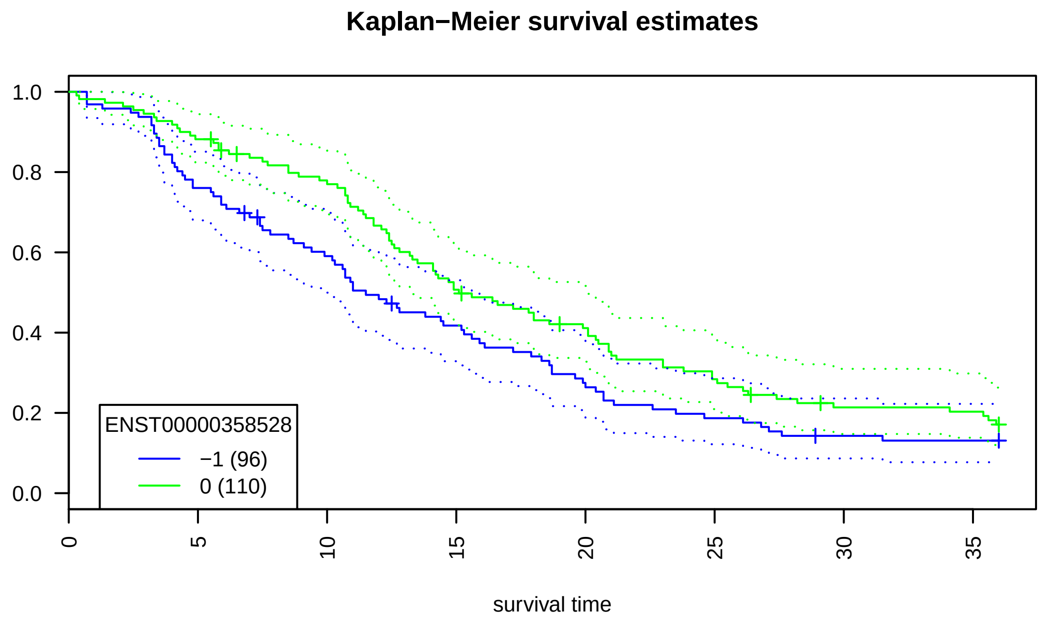

Figure 1254: Each curve represents the relative number of ENST00000358528 survived to the corresponding point of time. Dashed lines represent 0.95 confidence intervals for the curves with the corresponding color. Total of 24 observations deleted due to missingness. Log-rank probability measure for the equality of these curves is 4.359679e-02. Additional annotations: *.GeneId*=ENSG00000009307, *.GeneName*=CSDE1, *.GeneDesc*=cold shock domain containing E1, RNA-binding [Source:HGNC Symbol;Acc:29905].

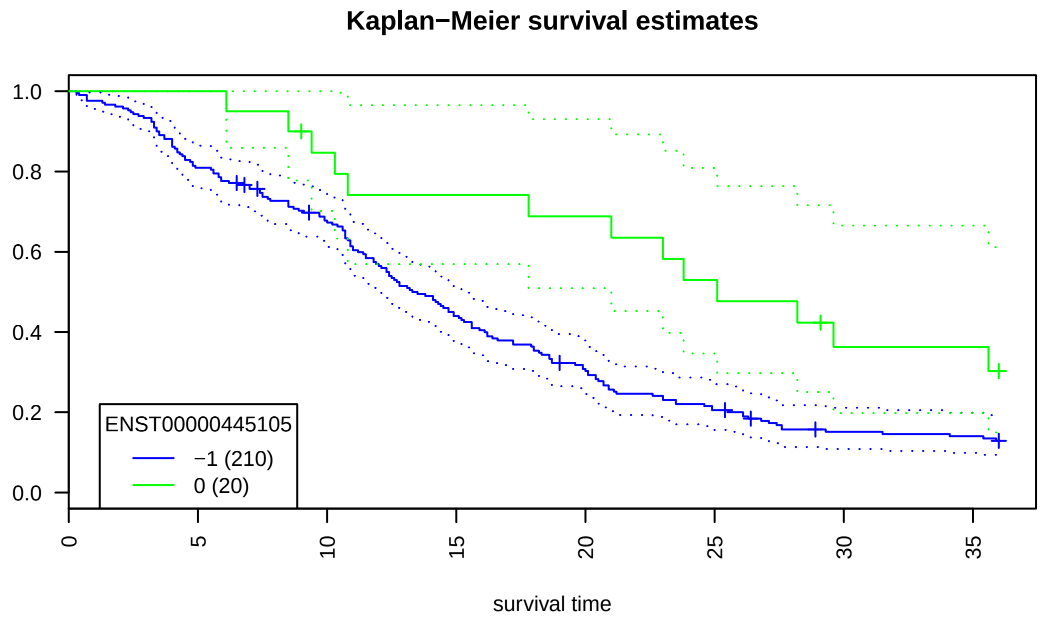

Figure 1255: Each curve represents the relative number of ENST00000445105 survived to the corresponding point of time. Dashed lines represent 0.95 confidence intervals for the curves with the corresponding color. Log-rank probability measure for the equality of these curves is 9.383247e-03. Additional annotations: *.GeneId*=ENSG00000114279, *.GeneName*=FGF12, *.GeneDesc*=fibroblast growth factor 12 [Source:HGNC Symbol;Acc:3668].

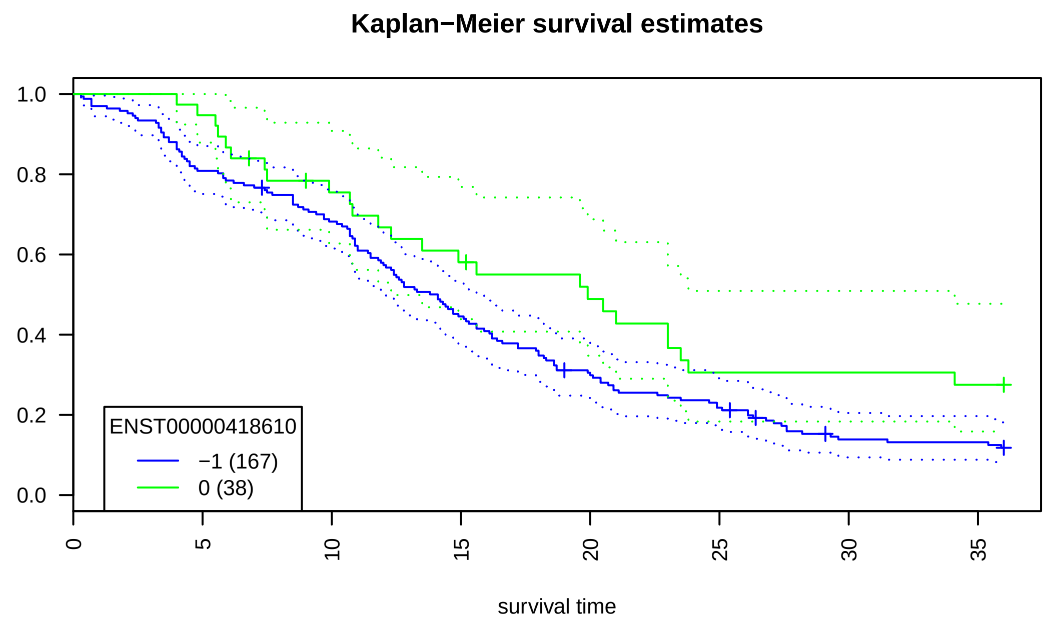

Figure 1256: Each curve represents the relative number of ENST00000418610 survived to the corresponding point of time. Dashed lines represent 0.95 confidence intervals for the curves with the corresponding color. Total of 25 observations deleted due to missingness. Log-rank probability measure for the equality of these curves is 3.040907e-02. Additional annotations: *.GeneId*=ENSG00000114279, *.GeneName*=FGF12, *.GeneDesc*=fibroblast growth factor 12 [Source:HGNC Symbol;Acc:3668].

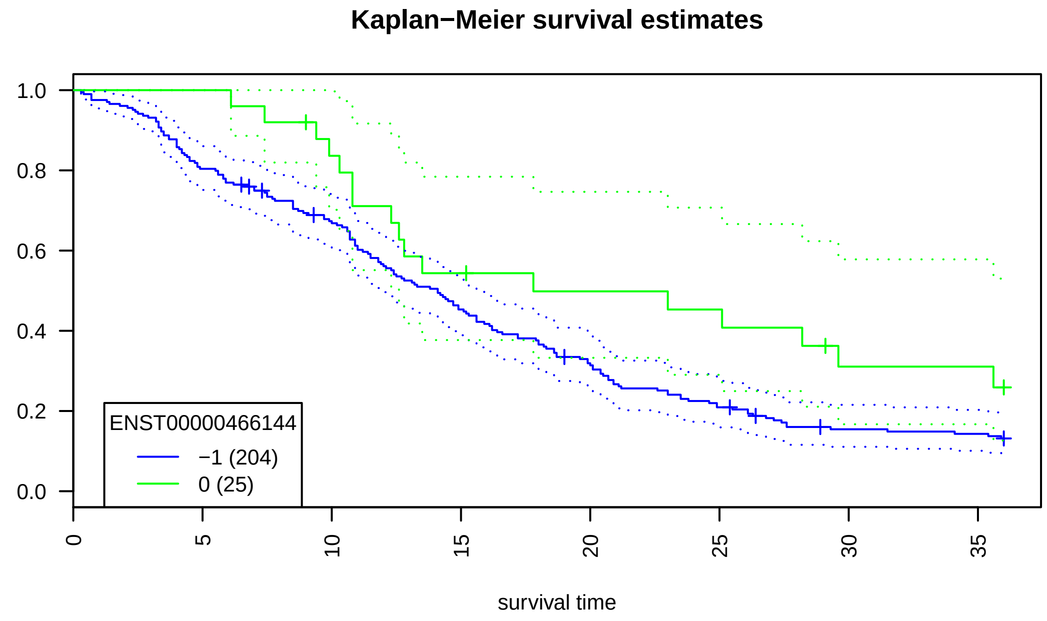

Figure 1257: Each curve represents the relative number of ENST00000466144 survived to the corresponding point of time. Dashed lines represent 0.95 confidence intervals for the curves with the corresponding color. Total of 1 observation deleted due to missingness. Log-rank probability measure for the equality of these curves is 4.845872e-02. Additional annotations: *.GeneId*=ENSG00000114279, *.GeneName*=FGF12, *.GeneDesc*=fibroblast growth factor 12 [Source:HGNC Symbol;Acc:3668].

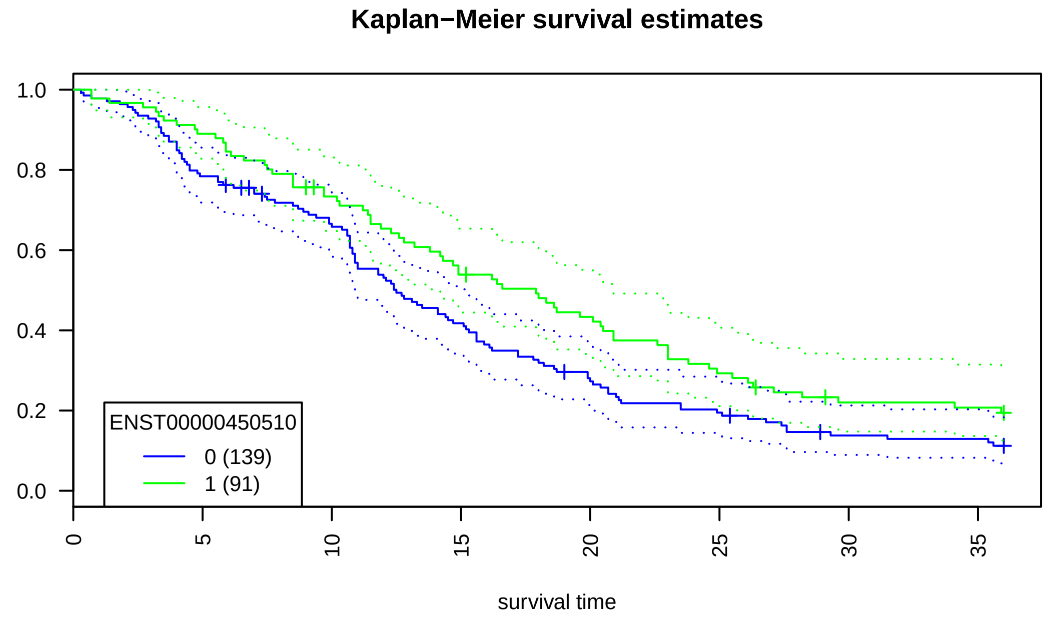

Figure 1258: Each curve represents the relative number of ENST00000450510 survived to the corresponding point of time. Dashed lines represent 0.95 confidence intervals for the curves with the corresponding color. Log-rank probability measure for the equality of these curves is 2.170979e-02. Additional annotations: *.GeneId*=ENSG00000175879, *.GeneName*=HOXD8, *.GeneDesc*=homeobox D8 [Source:HGNC Symbol;Acc:5139].

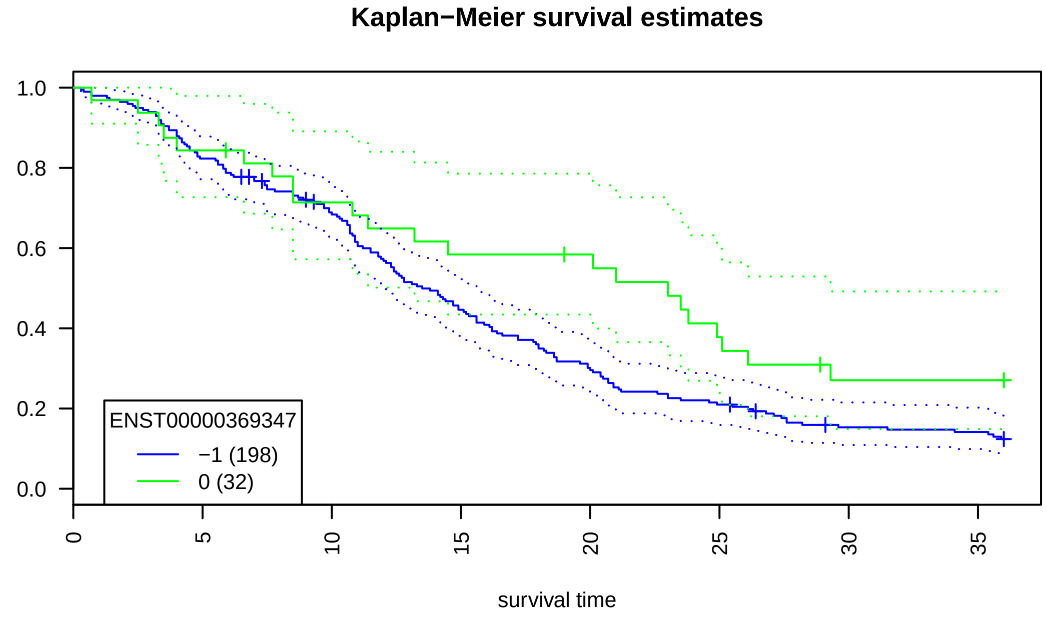

Figure 1259: Each curve represents the relative number of ENST00000369347 survived to the corresponding point of time. Dashed lines represent 0.95 confidence intervals for the curves with the corresponding color. Log-rank probability measure for the equality of these curves is 3.660169e-02. Additional annotations: *.GeneId*=ENSG00000178104, *.GeneName*=PDE4DIP, *.GeneDesc*=phosphodiesterase 4D interacting protein [Source:HGNC Symbol;Acc:15580].

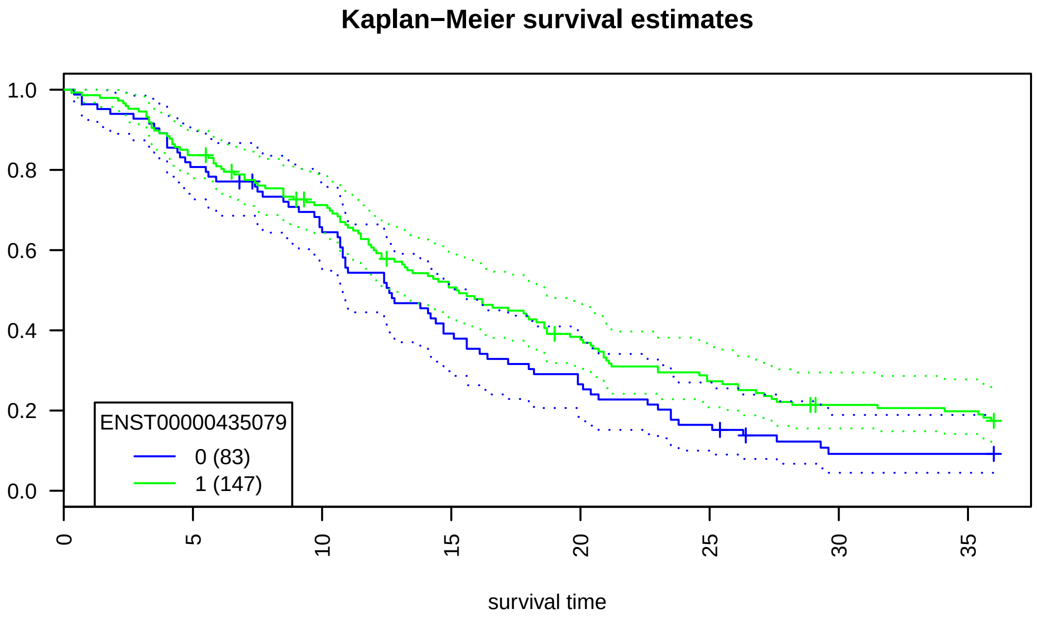

Figure 1260: Each curve represents the relative number of ENST00000435079 survived to the corresponding point of time. Dashed lines represent 0.95 confidence intervals for the curves with the corresponding color. Log-rank probability measure for the equality of these curves is 4.777294e-02. Additional annotations: *.GeneId*=ENSG00000079150, *.GeneName*=FKBP7, *.GeneDesc*=FK506 binding protein 7 [Source:HGNC Symbol;Acc:3723].

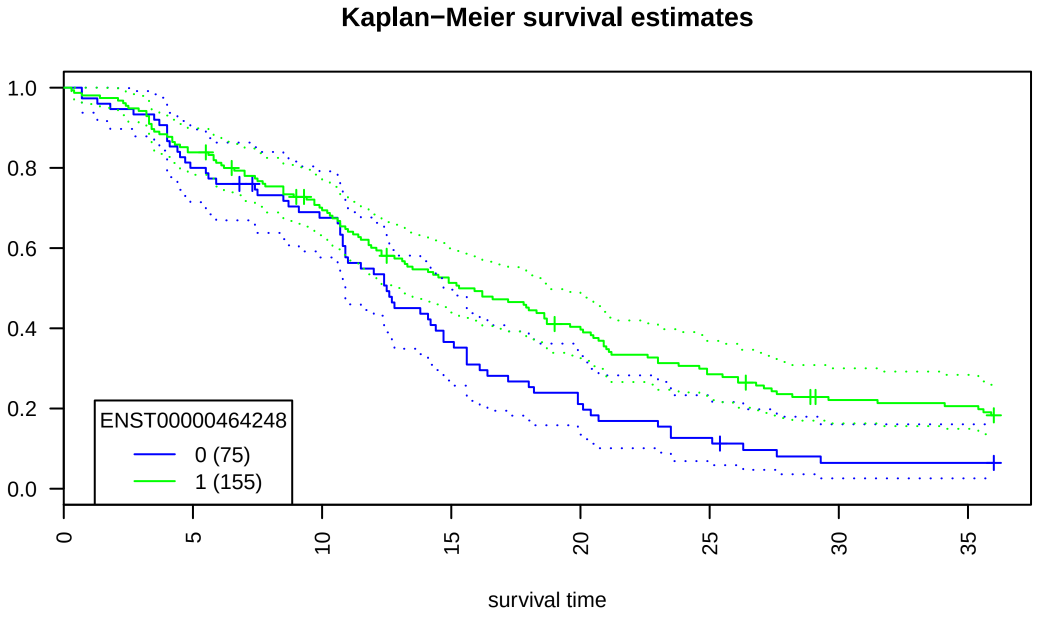

Figure 1261: Each curve represents the relative number of ENST00000464248 survived to the corresponding point of time. Dashed lines represent 0.95 confidence intervals for the curves with the corresponding color. Log-rank probability measure for the equality of these curves is 5.098906e-03. Additional annotations: *.GeneId*=ENSG00000079150, *.GeneName*=FKBP7, *.GeneDesc*=FK506 binding protein 7 [Source:HGNC Symbol;Acc:3723].

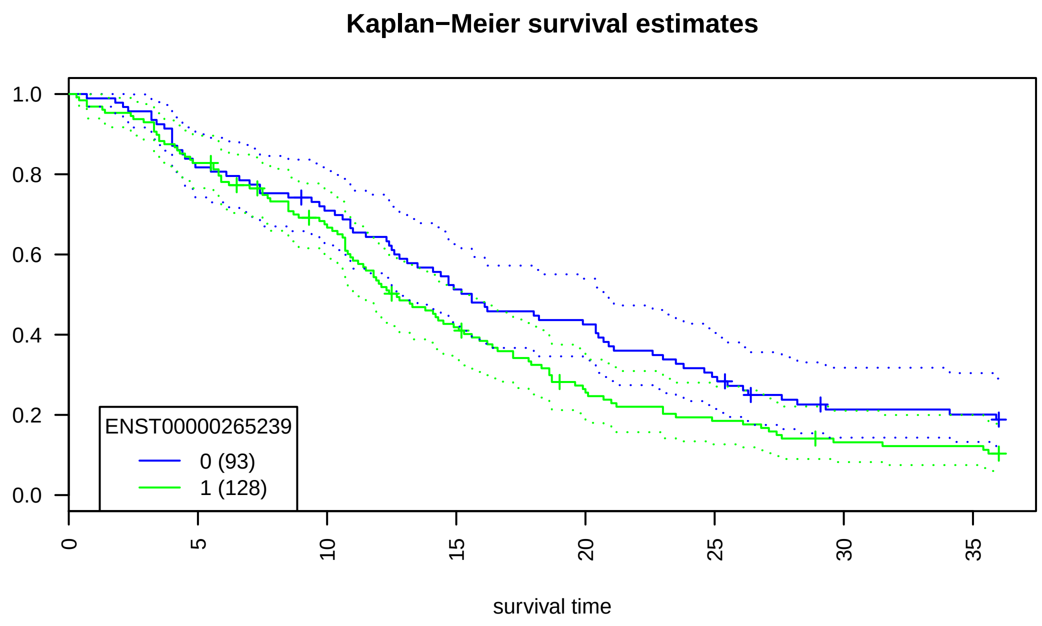

Figure 1262: Each curve represents the relative number of ENST00000265239 survived to the corresponding point of time. Dashed lines represent 0.95 confidence intervals for the curves with the corresponding color. Total of 9 observations deleted due to missingness. Log-rank probability measure for the equality of these curves is 4.739559e-02. Additional annotations: *.GeneId*=ENSG00000114473, *.GeneName*=IQCG, *.GeneDesc*=IQ motif containing G [Source:HGNC Symbol;Acc:25251].

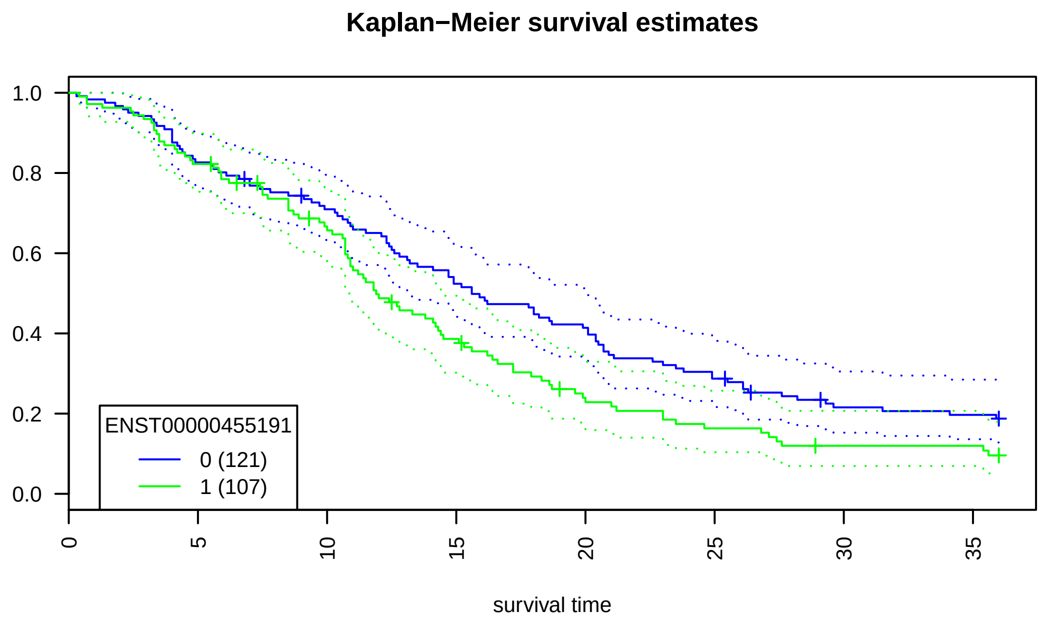

Figure 1263: Each curve represents the relative number of ENST00000455191 survived to the corresponding point of time. Dashed lines represent 0.95 confidence intervals for the curves with the corresponding color. Total of 2 observations deleted due to missingness. Log-rank probability measure for the equality of these curves is 1.814426e-02. Additional annotations: *.GeneId*=ENSG00000114473, *.GeneName*=IQCG, *.GeneDesc*=IQ motif containing G [Source:HGNC Symbol;Acc:25251].

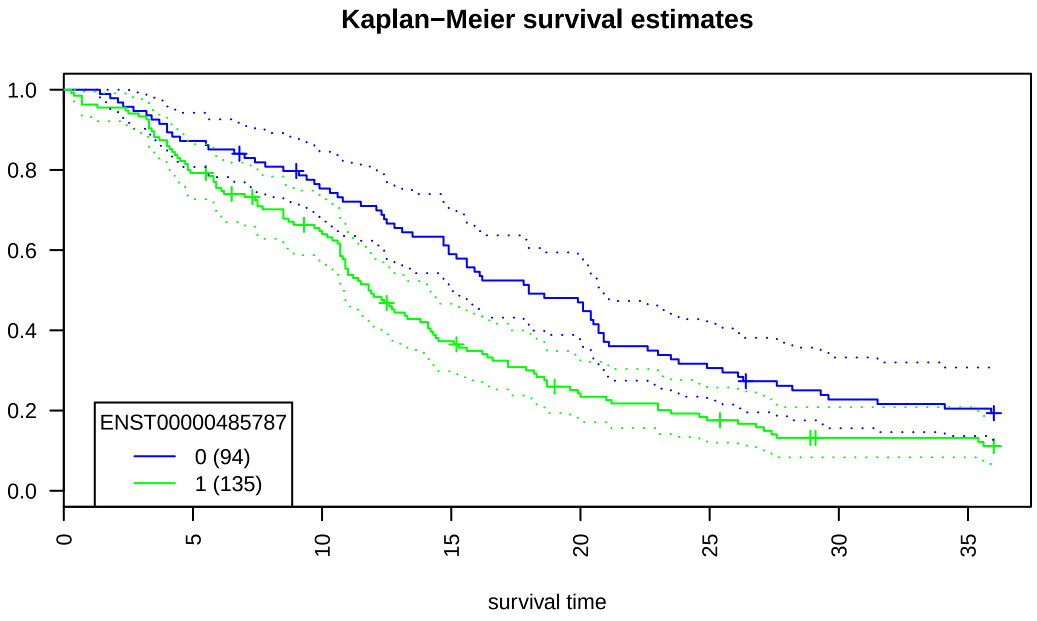

Figure 1264: Each curve represents the relative number of ENST00000485787 survived to the corresponding point of time. Dashed lines represent 0.95 confidence intervals for the curves with the corresponding color. Total of 1 observation deleted due to missingness. Log-rank probability measure for the equality of these curves is 4.358341e-03. Additional annotations: *.GeneId*=ENSG00000114473, *.GeneName*=IQCG, *.GeneDesc*=IQ motif containing G [Source:HGNC Symbol;Acc:25251].

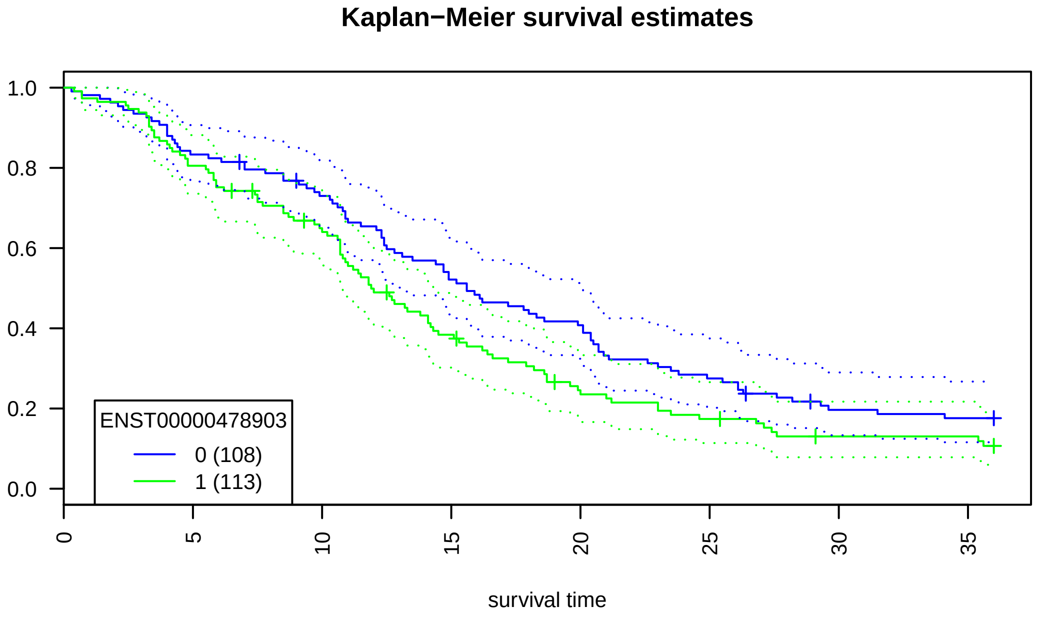

Figure 1265: Each curve represents the relative number of ENST00000478903 survived to the corresponding point of time. Dashed lines represent 0.95 confidence intervals for the curves with the corresponding color. Total of 9 observations deleted due to missingness. Log-rank probability measure for the equality of these curves is 3.644978e-02. Additional annotations: *.GeneId*=ENSG00000114473, *.GeneName*=IQCG, *.GeneDesc*=IQ motif containing G [Source:HGNC Symbol;Acc:25251].

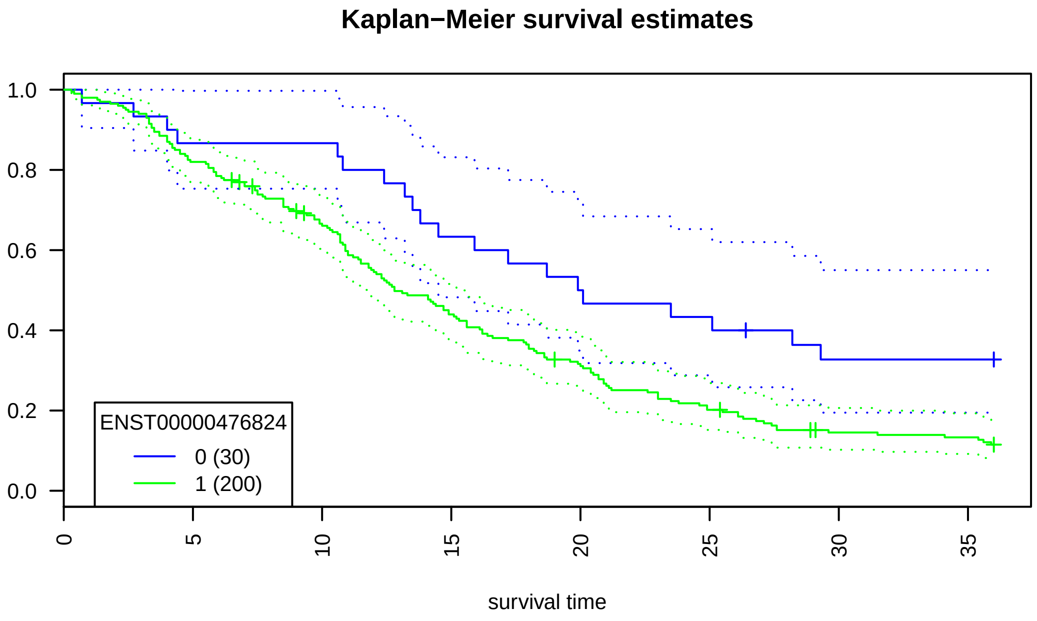

Figure 1266: Each curve represents the relative number of ENST00000476824 survived to the corresponding point of time. Dashed lines represent 0.95 confidence intervals for the curves with the corresponding color. Log-rank probability measure for the equality of these curves is 5.933426e-03. Additional annotations: .*GeneId*=ENSG00000115232, .*GeneName*=ITGA4, .*GeneDesc*=integrin, alpha 4 (antigen CD49D, alpha 4 subunit of VLA-4 receptor) [Source:HGNC Symbol;Acc:6140].

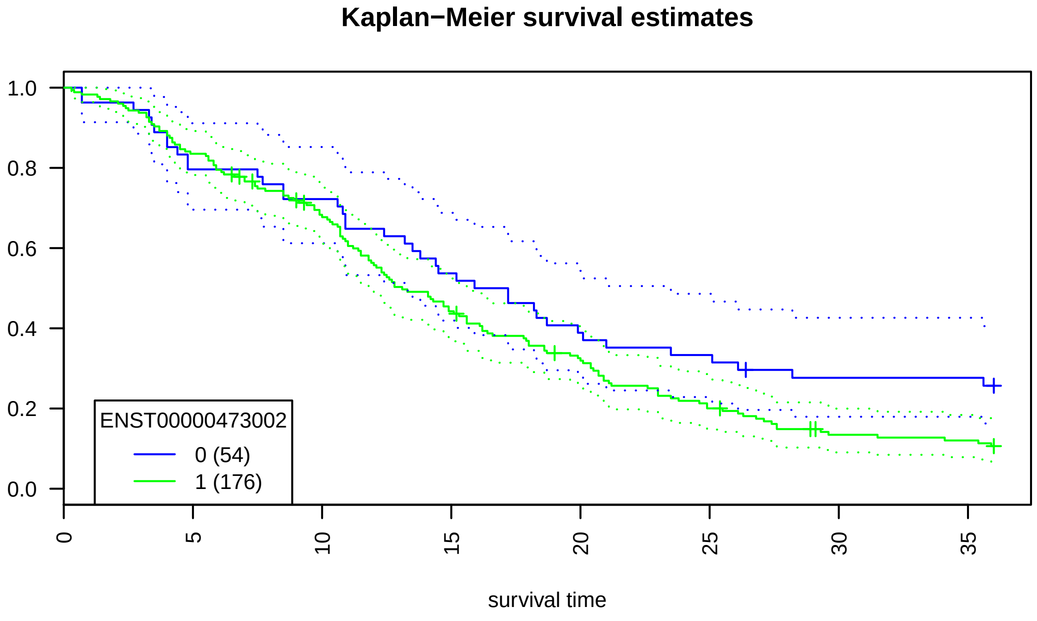

Figure 1267: Each curve represents the relative number of ENST00000473002 survived to the corresponding point of time. Dashed lines represent 0.95 confidence intervals for the curves with the corresponding color. Log-rank probability measure for the equality of these curves is 4.324670e-02. Additional annotations: .*GeneId*=ENSG00000115232, .*GeneName*=ITGA4, .*GeneDesc*=integrin, alpha 4 (antigen CD49D, alpha 4 subunit of VLA-4 receptor) [Source:HGNC Symbol;Acc:6140].

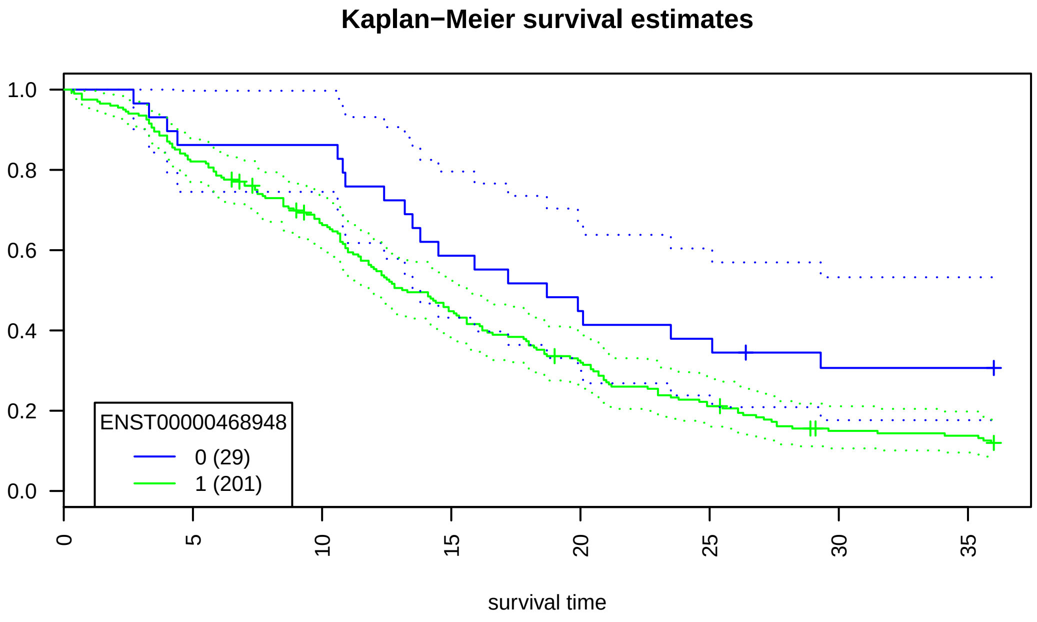

Figure 1268: Each curve represents the relative number of ENST00000468948 survived to the corresponding point of time. Dashed lines represent 0.95 confidence intervals for the curves with the corresponding color. Log-rank probability measure for the equality of these curves is 2.502401e-02. Additional annotations: *.GeneId*=ENSG00000115232, *.GeneName*=ITGA4, *.GeneDesc*=integrin, alpha 4 (antigen CD49D, alpha 4 subunit of VLA-4 receptor) [Source:HGNC Symbol;Acc:6140].

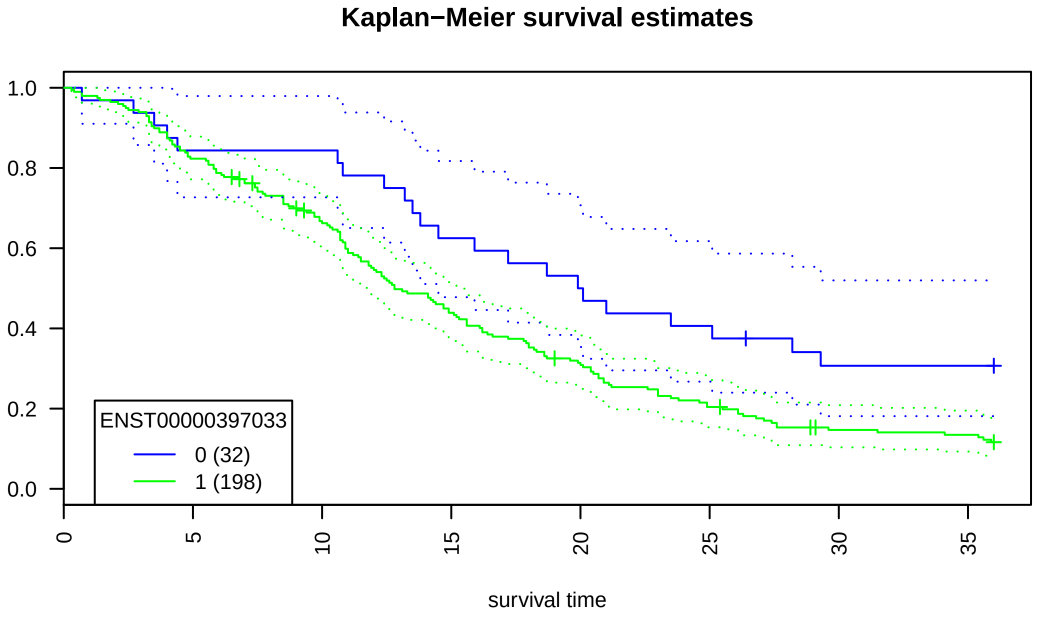

Figure 1269: Each curve represents the relative number of ENST00000397033 survived to the corresponding point of time. Dashed lines represent 0.95 confidence intervals for the curves with the corresponding color. Log-rank probability measure for the equality of these curves is 9.730589e-03. Additional annotations: *.GeneId*=ENSG00000115232, *.GeneName*=ITGA4, *.GeneDesc*=integrin, alpha 4 (antigen CD49D, alpha 4 subunit of VLA-4 receptor) [Source:HGNC Symbol;Acc:6140].

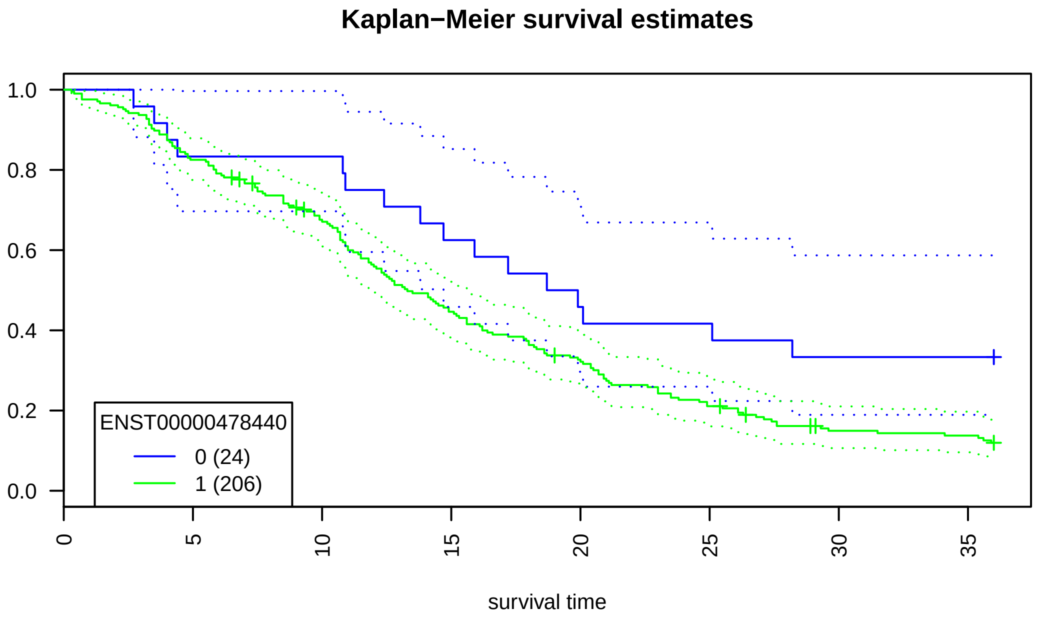

Figure 1270: Each curve represents the relative number of ENST00000478440 survived to the corresponding point of time. Dashed lines represent 0.95 confidence intervals for the curves with the corresponding color. Log-rank probability measure for the equality of these curves is 2.336840e-02. Additional annotations: *.GeneId*=ENSG00000115232, *.GeneName*=ITGA4, *.GeneDesc*=integrin, alpha 4 (antigen CD49D, alpha 4 subunit of VLA-4 receptor) [Source:HGNC Symbol;Acc:6140].

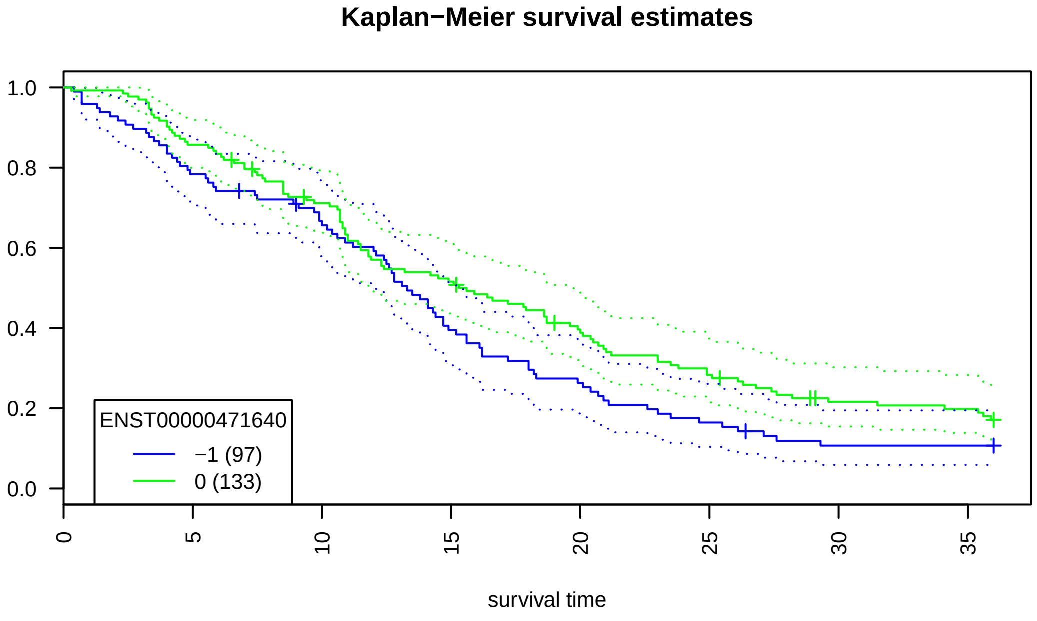

Figure 1271: Each curve represents the relative number of ENST00000471640 survived to the corresponding point of time. Dashed lines represent 0.95 confidence intervals for the curves with the corresponding color. Log-rank probability measure for the equality of these curves is 4.815092e-02. Additional annotations: *.GeneId*=ENSG00000061676, *.GeneName*=NCKAP1, *.GeneDesc*=NCK-associated protein 1 [Source:HGNC Symbol;Acc:7666].

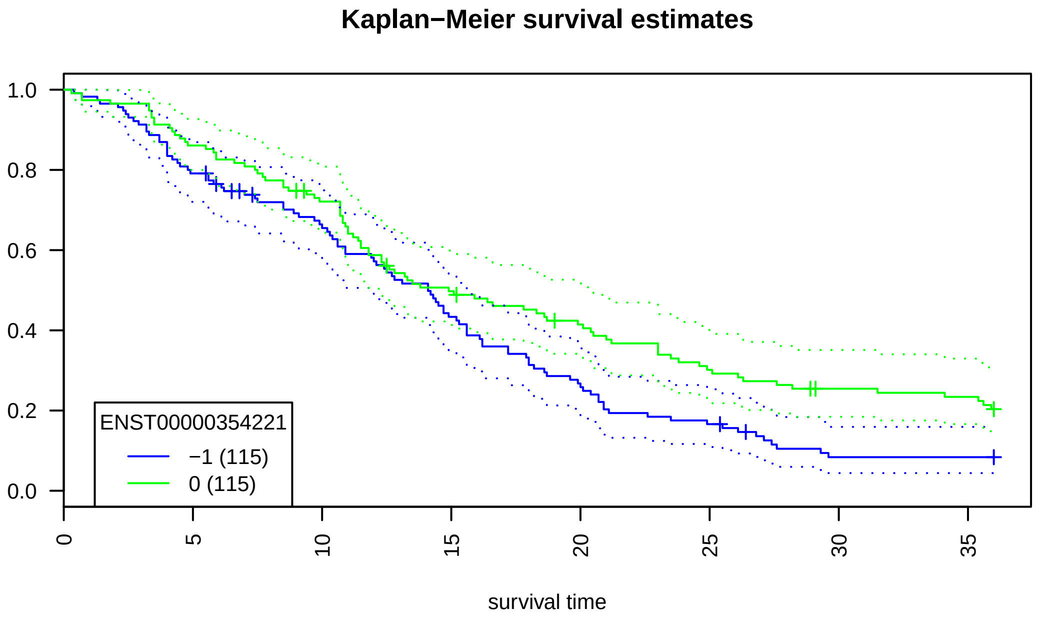

Figure 1272: Each curve represents the relative number of ENST00000354221 survived to the corresponding point of time. Dashed lines represent 0.95 confidence intervals for the curves with the corresponding color. Log-rank probability measure for the equality of these curves is 1.038603e-02. Additional annotations: .*GeneId*=ENSG00000162999, .*GeneName*=DUSP19, .*GeneDesc*=dual specificity phosphatase 19 [Source:HGNC Symbol;Acc:18894].

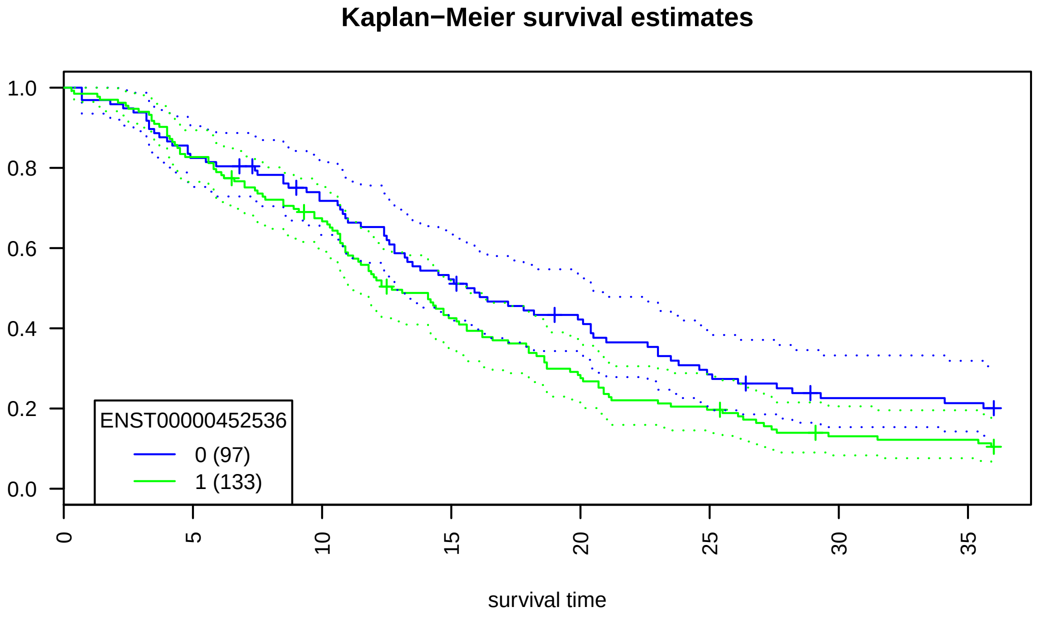

Figure 1273: Each curve represents the relative number of ENST00000452536 survived to the corresponding point of time. Dashed lines represent 0.95 confidence intervals for the curves with the corresponding color. Log-rank probability measure for the equality of these curves is 4.259957e-02. Additional annotations: .*GeneId*=ENSG00000204262, .*GeneName*=COL5A2, .*GeneDesc*=collagen, type V, alpha 2 [Source:HGNC Symbol;Acc:2210].

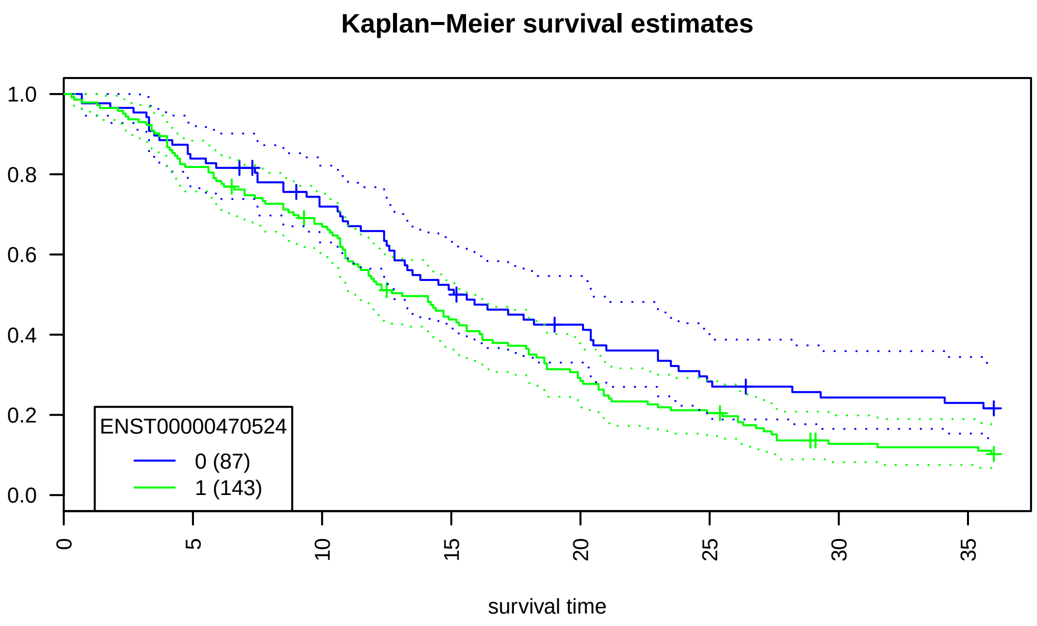

Figure 1274: Each curve represents the relative number of ENST00000470524 survived to the corresponding point of time. Dashed lines represent 0.95 confidence intervals for the curves with the corresponding color. Log-rank probability measure for the equality of these curves is 3.698099e-02. Additional annotations: *.GeneId*=ENSG00000204262, *.GeneName*=COL5A2, *.GeneDesc*=collagen, type V, alpha 2 [Source:HGNC Symbol;Acc:2210].

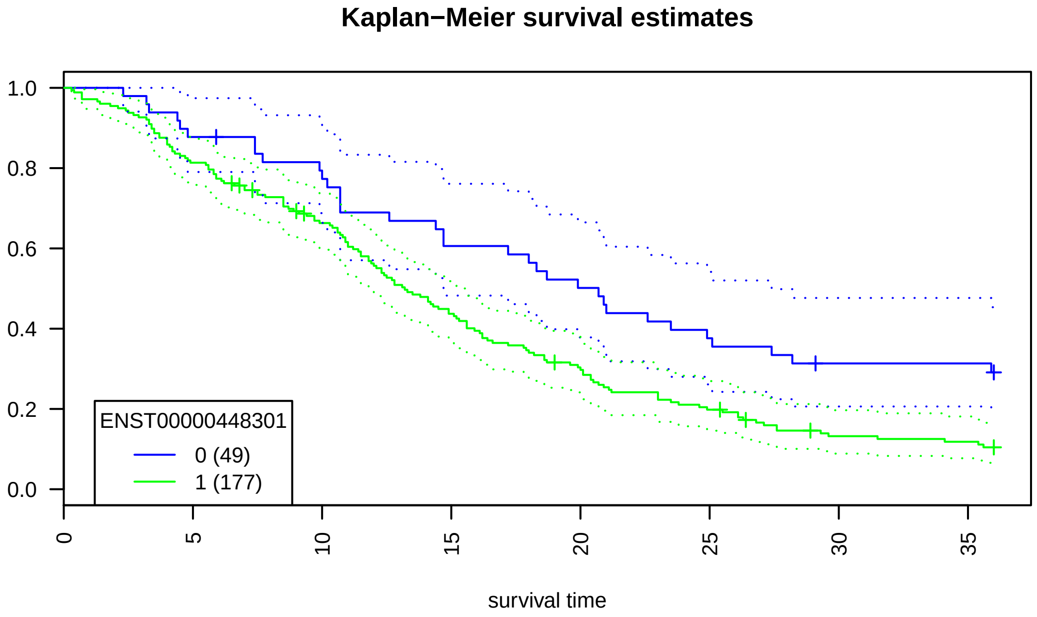

Figure 1275: Each curve represents the relative number of ENST00000448301 survived to the corresponding point of time. Dashed lines represent 0.95 confidence intervals for the curves with the corresponding color. Total of 4 observations deleted due to missingness. Log-rank probability measure for the equality of these curves is 2.471562e-03. Additional annotations: *.GeneId*=ENSG00000163131, *.GeneName*=CTSS, *.GeneDesc*=cathepsin S [Source:HGNC Symbol;Acc:2545].

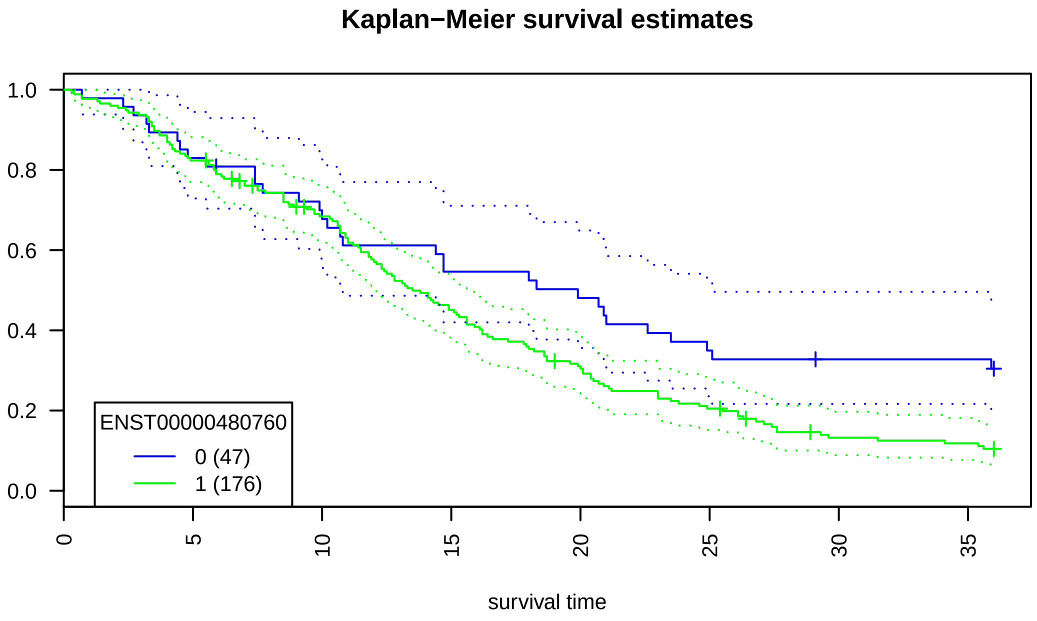

Figure 1276: Each curve represents the relative number of ENST00000480760 survived to the corresponding point of time. Dashed lines represent 0.95 confidence intervals for the curves with the corresponding color. Total of 7 observations deleted due to missingness. Log-rank probability measure for the equality of these curves is 1.203362e-02. Additional annotations: *.GeneId*=ENSG00000163131, *.GeneName*=CTSS, *.GeneDesc*=cathepsin S [Source:HGNC Symbol;Acc:2545].

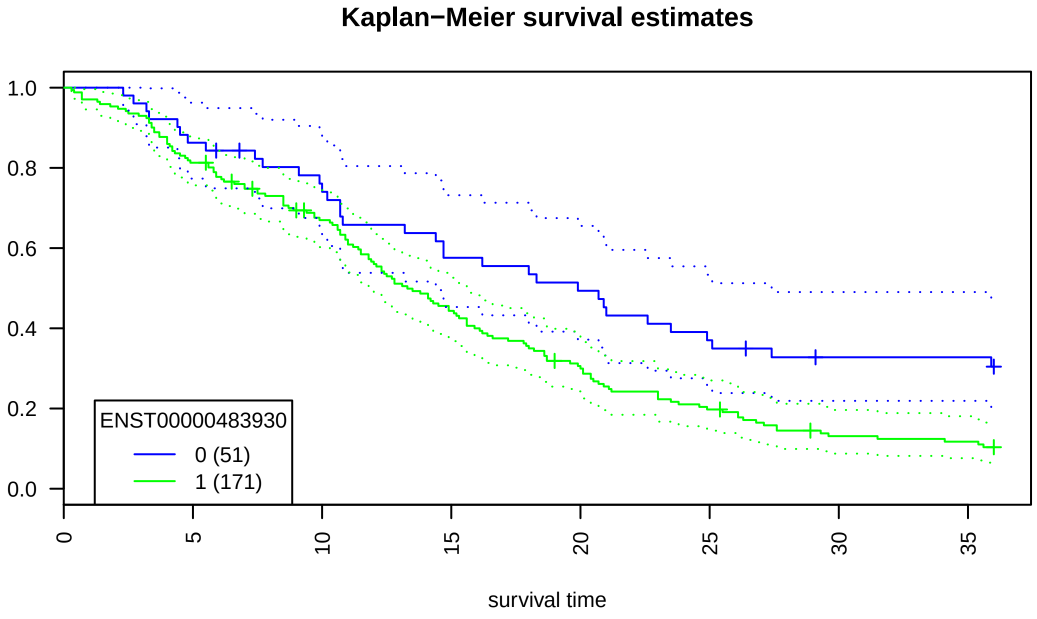

Figure 1277: Each curve represents the relative number of ENST00000483930 survived to the corresponding point of time. Dashed lines represent 0.95 confidence intervals for the curves with the corresponding color. Total of 8 observations deleted due to missingness. Log-rank probability measure for the equality of these curves is 3.647569e-03. Additional annotations: *.GeneId*=ENSG00000163131, *.GeneName*=CTSS, *.GeneDesc*=cathepsin S [Source:HGNC Symbol;Acc:2545].

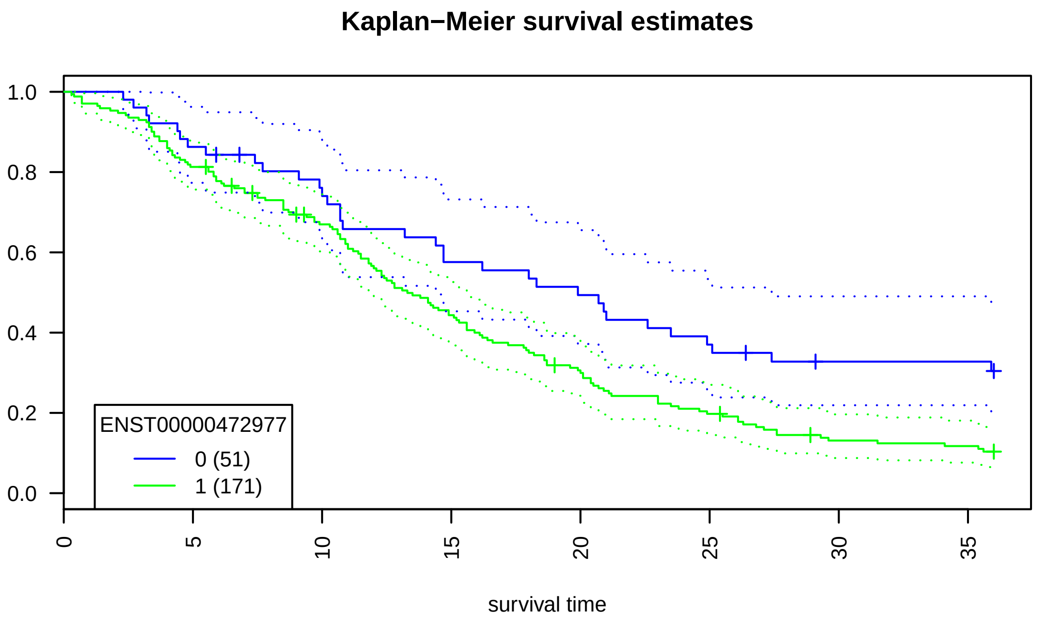

Figure 1278: Each curve represents the relative number of ENST00000472977 survived to the corresponding point of time. Dashed lines represent 0.95 confidence intervals for the curves with the corresponding color. Total of 8 observations deleted due to missingness. Log-rank probability measure for the equality of these curves is 3.647569e-03. Additional annotations: *.GeneId*=ENSG00000163131, *.GeneName*=CTSS, *.GeneDesc*=cathepsin S [Source:HGNC Symbol;Acc:2545].

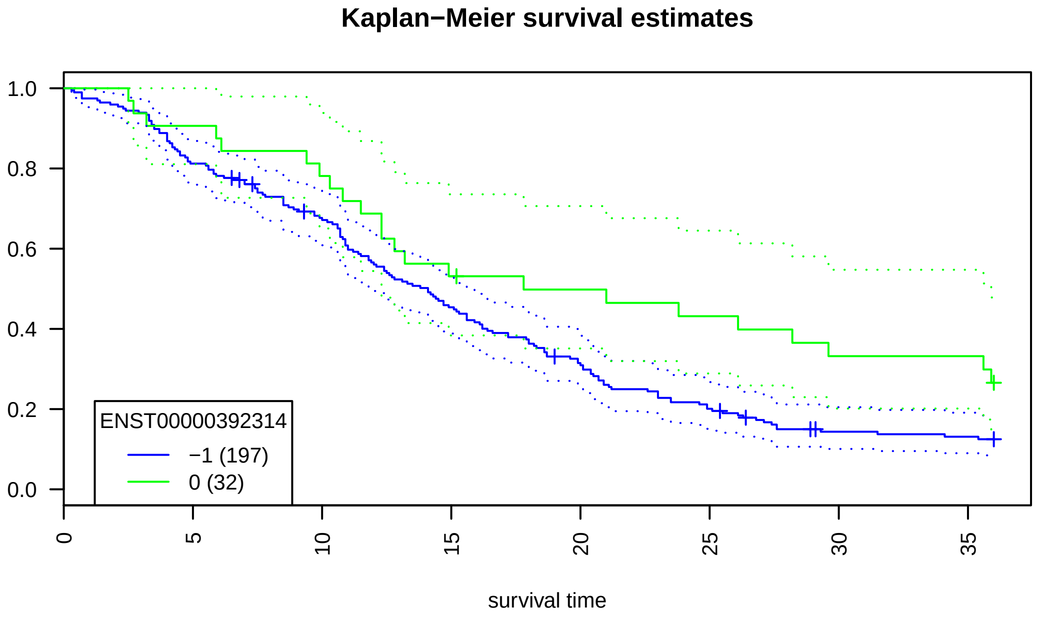

Figure 1279: Each curve represents the relative number of ENST00000392314 survived to the corresponding point of time. Dashed lines represent 0.95 confidence intervals for the curves with the corresponding color. Total of 1 observation deleted due to missingness. Log-rank probability measure for the equality of these curves is 3.119914e-02. Additional annotations: *.GeneId*=ENSG00000144339, *.GeneName*=TMEFF2, *.GeneDesc*=transmembrane protein with EGF-like and two follistatin-like domains 2 [Source:HGNC Symbol;Acc:11867].

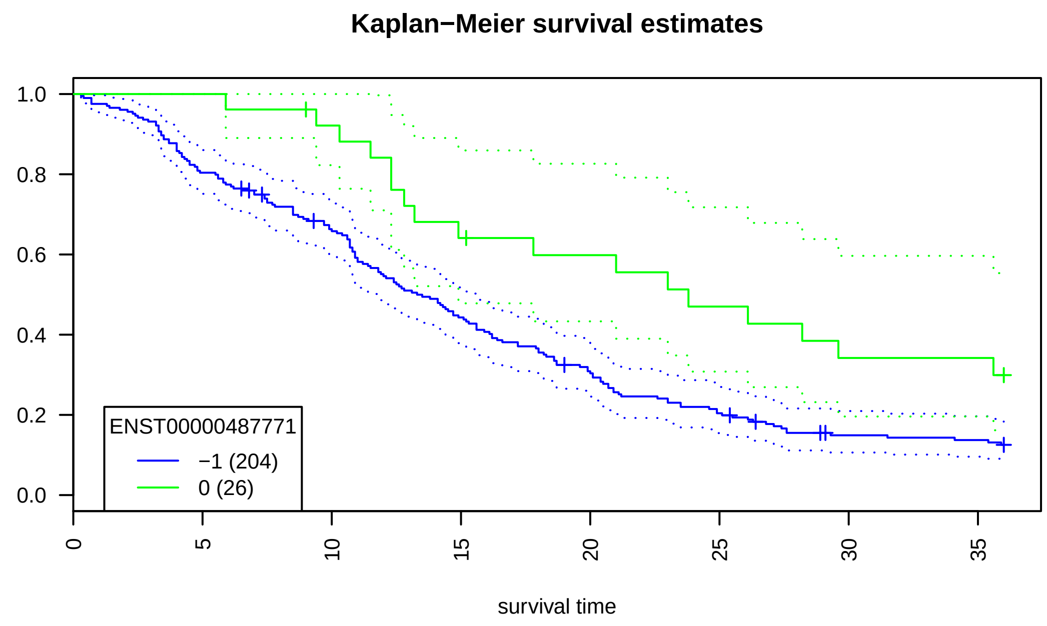

Figure 1280: Each curve represents the relative number of ENST00000487771 survived to the corresponding point of time. Dashed lines represent 0.95 confidence intervals for the curves with the corresponding color. Log-rank probability measure for the equality of these curves is 5.984658e-03. Additional annotations: *.GeneId*=ENSG00000144339, *.GeneName*=TMEFF2, *.GeneDesc*=transmembrane protein with EGF-like and two follistatin-like domains 2 [Source:HGNC Symbol;Acc:11867].

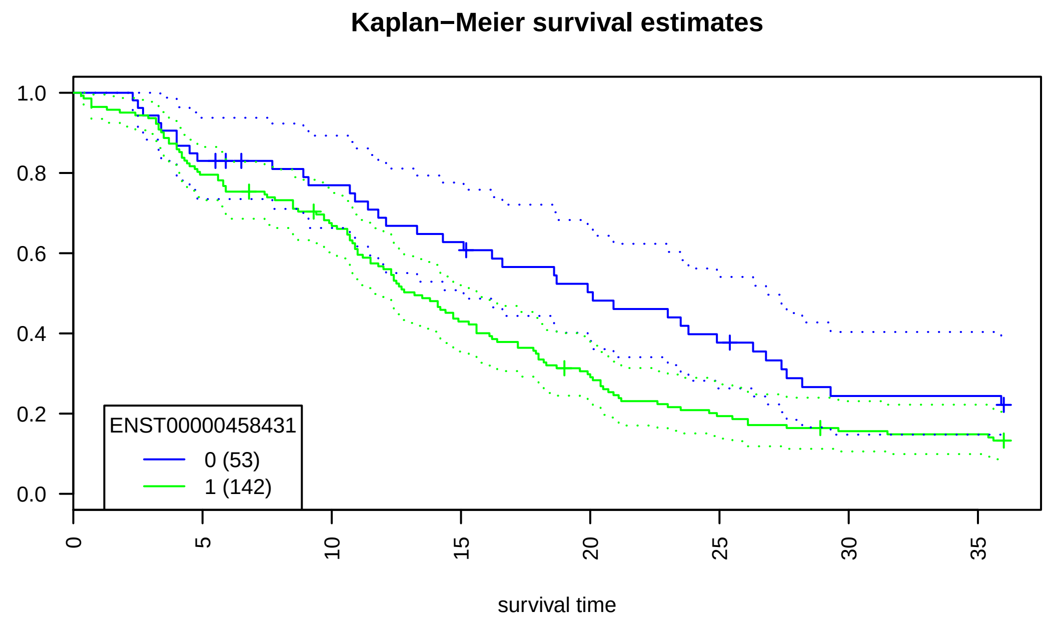

Figure 1281: Each curve represents the relative number of ENST00000458431 survived to the corresponding point of time. Dashed lines represent 0.95 confidence intervals for the curves with the corresponding color. Total of 35 observations deleted due to missingness. Log-rank probability measure for the equality of these curves is 2.419435e-02. Additional annotations: *.GeneId*=ENSG00000182134, *.GeneName*=TDRKH, *.GeneDesc*=tudor and KH domain containing [Source:HGNC Symbol;Acc:11713].

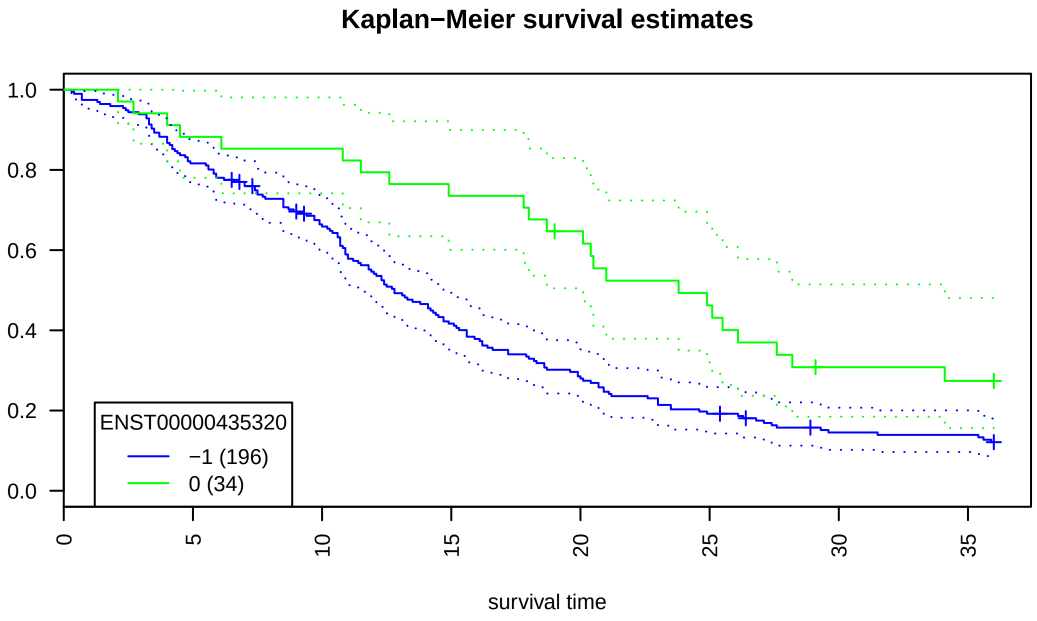

Figure 1282: Each curve represents the relative number of ENST00000435320 survived to the corresponding point of time. Dashed lines represent 0.95 confidence intervals for the curves with the corresponding color. Log-rank probability measure for the equality of these curves is 2.395211e-03. Additional annotations: *.GeneId*=ENSG00000115896, *.GeneName*=PLCL1, *.GeneDesc*=phospholipase C-like 1 [Source:HGNC Symbol;Acc:9063].

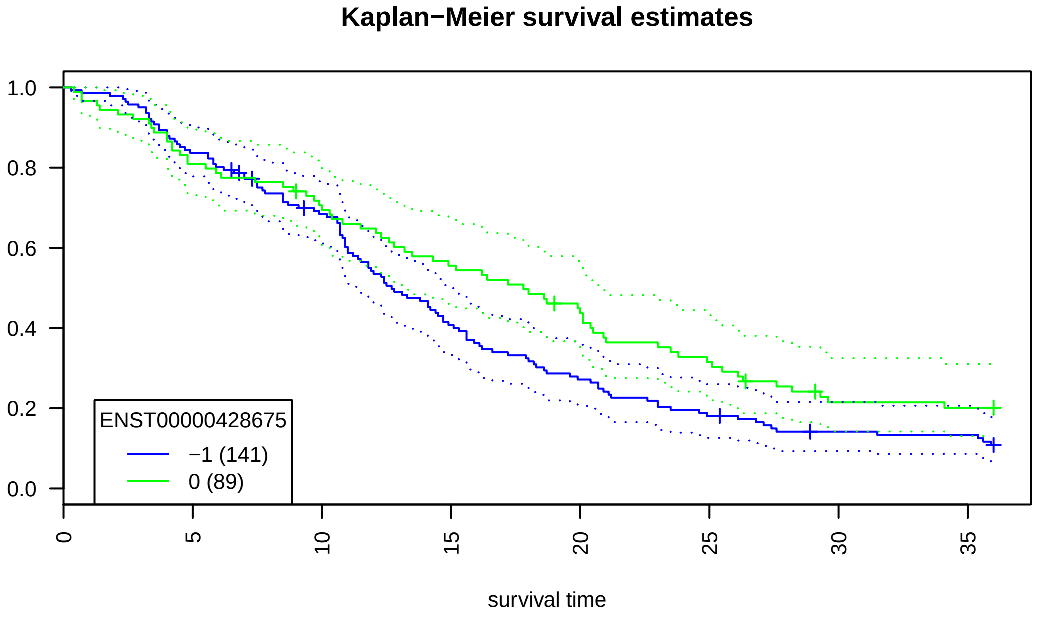

Figure 1283: Each curve represents the relative number of ENST00000428675 survived to the corresponding point of time. Dashed lines represent 0.95 confidence intervals for the curves with the corresponding color. Log-rank probability measure for the equality of these curves is 3.35975e-02. Additional annotations: *.GeneId*=ENSG00000115896, *.GeneName*=PLCL1, *.GeneDesc*=phospholipase C-like 1 [Source:HGNC Symbol;Acc:9063].

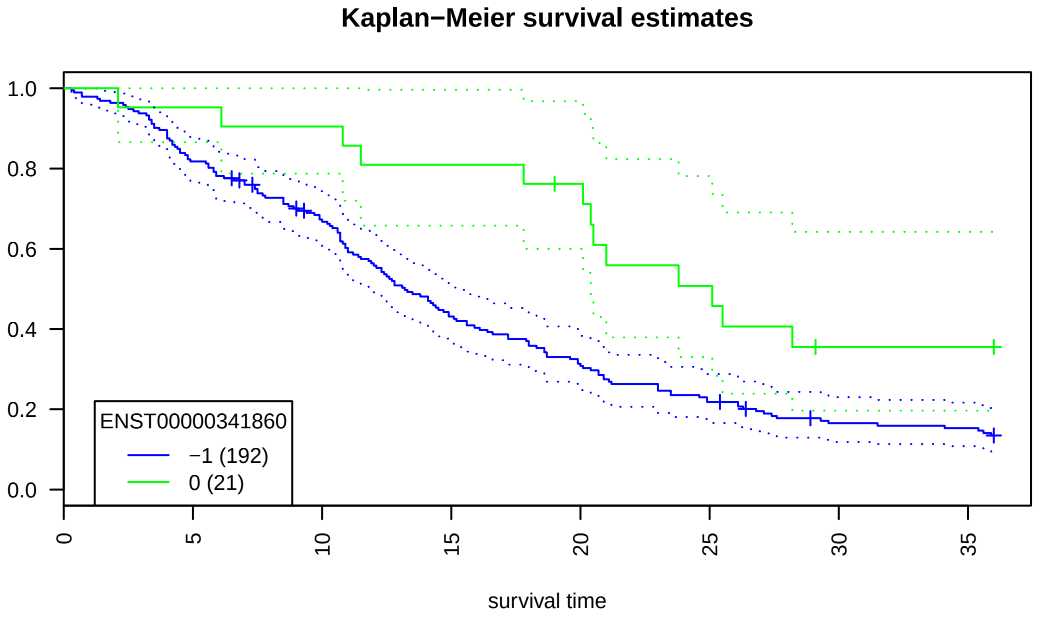

Figure 1284: Each curve represents the relative number of ENST00000341860 survived to the corresponding point of time. Dashed lines represent 0.95 confidence intervals for the curves with the corresponding color. Total of 17 observations deleted due to missingness. Log-rank probability measure for the equality of these curves is 5.60166e-03.

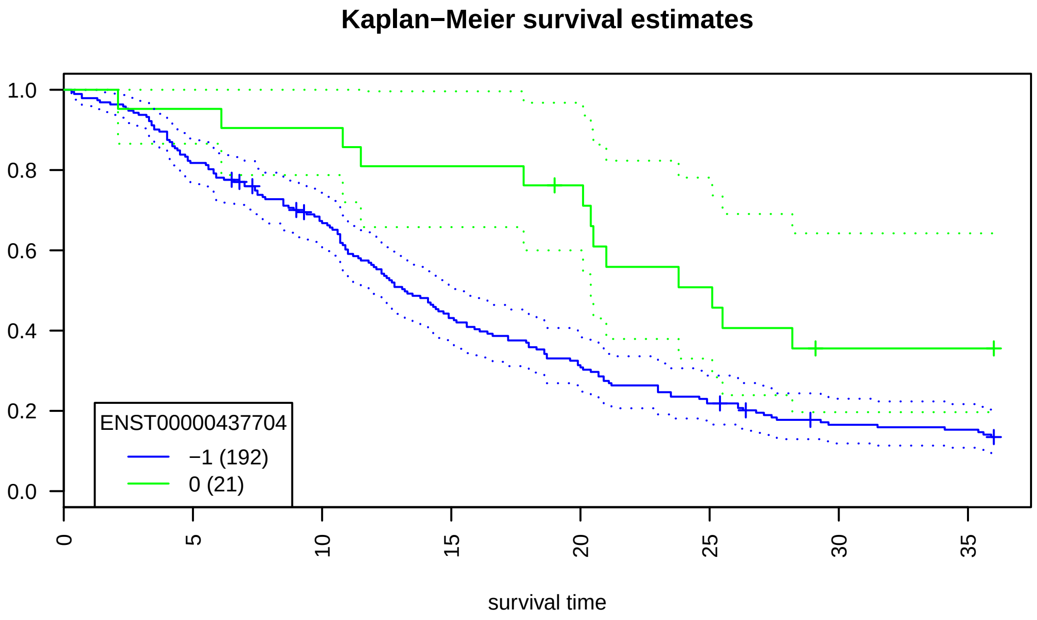

Figure 1285: Each curve represents the relative number of ENST00000437704 survived to the corresponding point of time. Dashed lines represent 0.95 confidence intervals for the curves with the corresponding color. Total of 17 observations deleted due to missingness. Log-rank probability measure for the equality of these curves is 5.60166e-03. Additional annotations: *.GeneId*=ENSG00000115896, *.GeneName*=PLCL1, *.GeneDesc*=phospholipase C-like 1 [Source:HGNC Symbol;Acc:9063].

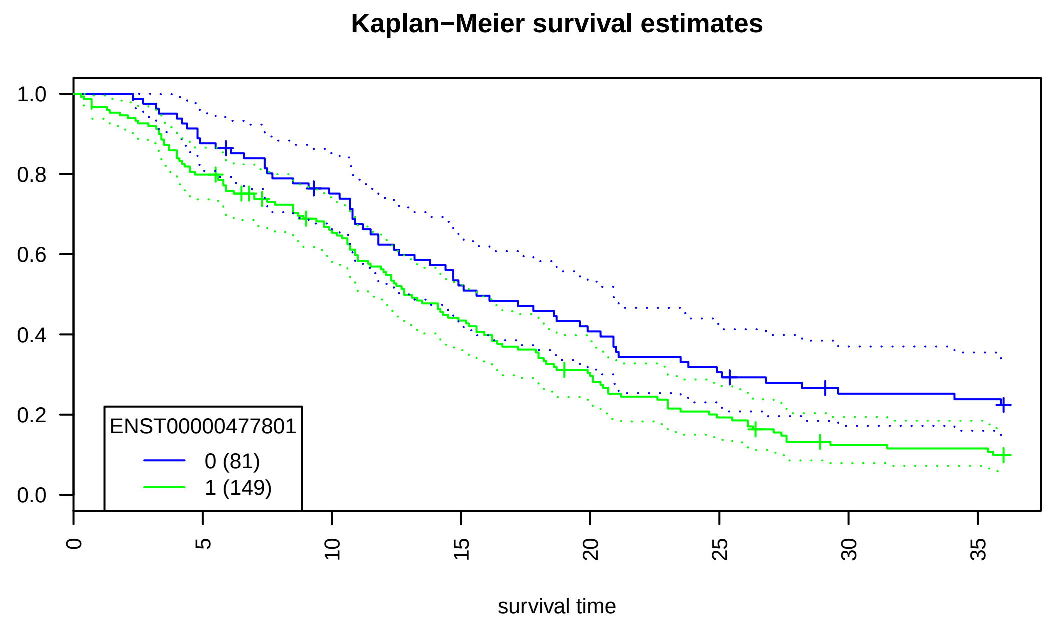

Figure 1286: Each curve represents the relative number of ENST00000477801 survived to the corresponding point of time. Dashed lines represent 0.95 confidence intervals for the curves with the corresponding color. Log-rank probability measure for the equality of these curves is 1.465941e-02. Additional annotations: *.GeneId*=ENSG00000143546, *.GeneName*=S100A8, *.GeneDesc*=S100 calcium binding protein A8 [Source:HGNC Symbol;Acc:10498].

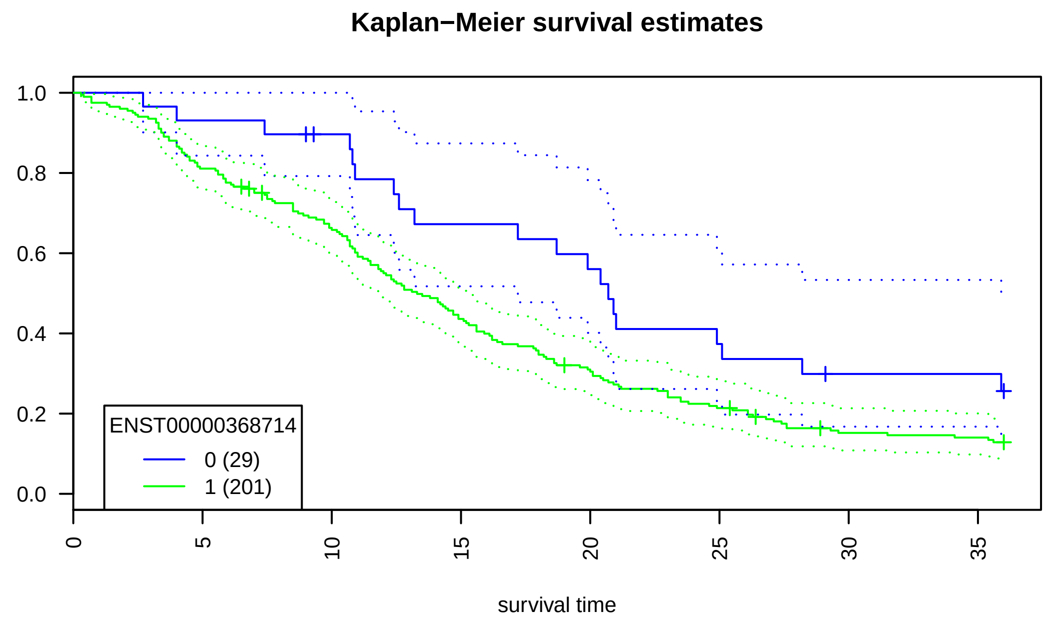

Figure 1287: Each curve represents the relative number of ENST00000368714 survived to the corresponding point of time. Dashed lines represent 0.95 confidence intervals for the curves with the corresponding color. Log-rank probability measure for the equality of these curves is 2.137738e-02. Additional annotations: *.GeneId*=ENSG00000196154, *.GeneName*=S100A4, *.GeneDesc*=S100 calcium binding protein A4 [Source:HGNC Symbol;Acc:10494].

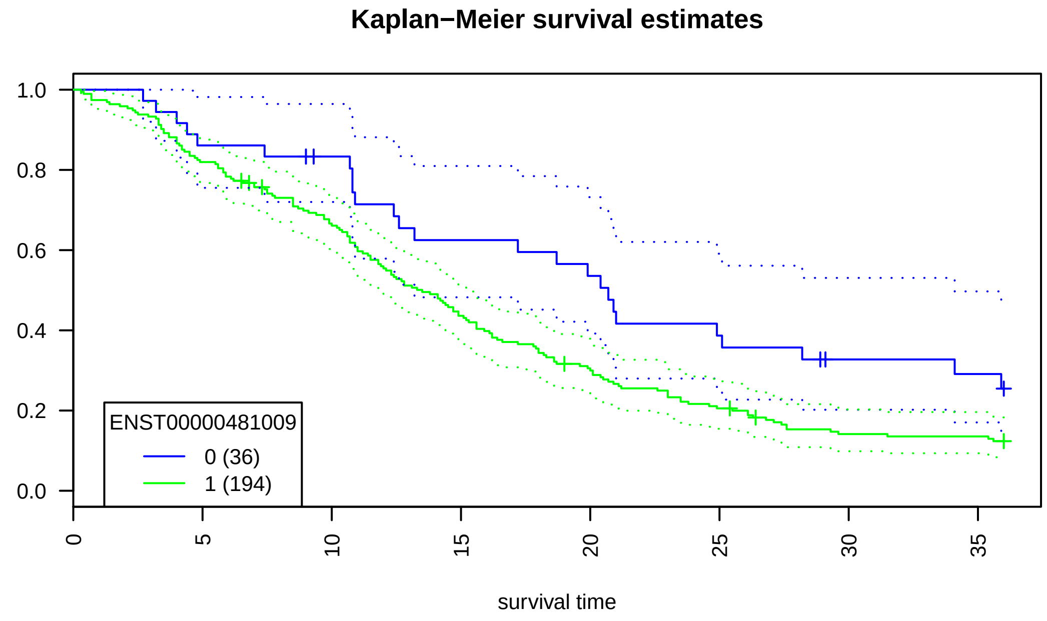

Figure 1288: Each curve represents the relative number of ENST00000481009 survived to the corresponding point of time. Dashed lines represent 0.95 confidence intervals for the curves with the corresponding color. Log-rank probability measure for the equality of these curves is 1.708128e-02. Additional annotations: *.GeneId*=ENSG00000196154, *.GeneName*=S100A4, *.GeneDesc*=S100 calcium binding protein A4 [Source:HGNC Symbol;Acc:10494].

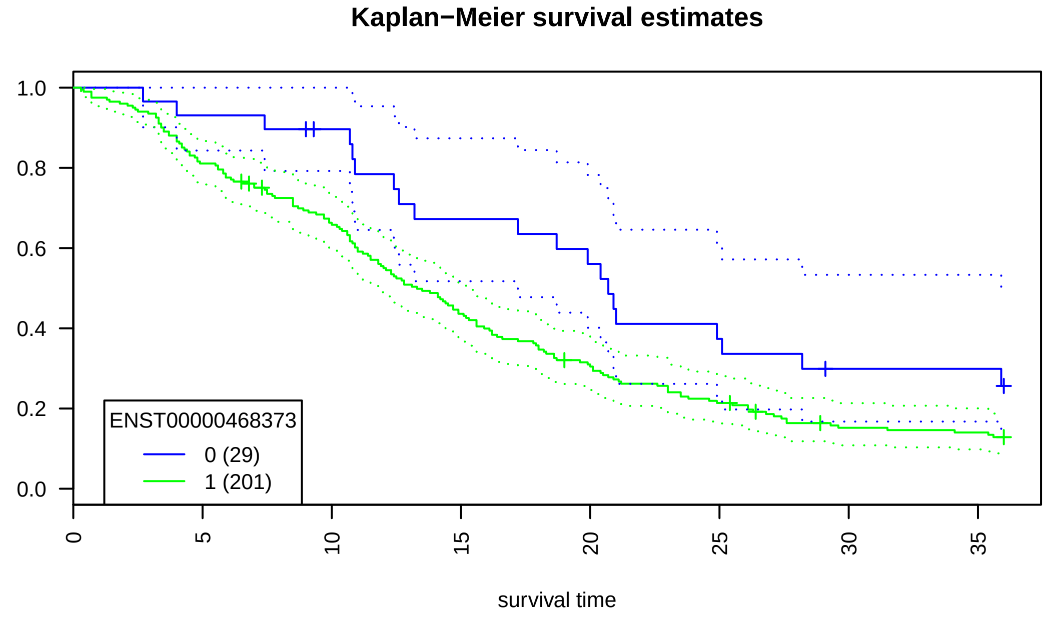

Figure 1289: Each curve represents the relative number of ENST00000468373 survived to the corresponding point of time. Dashed lines represent 0.95 confidence intervals for the curves with the corresponding color. Log-rank probability measure for the equality of these curves is 2.137738e-02. Additional annotations: *.GeneId*=ENSG00000196154, *.GeneName*=S100A4, *.GeneDesc*=S100 calcium binding protein A4 [Source:HGNC Symbol;Acc:10494].

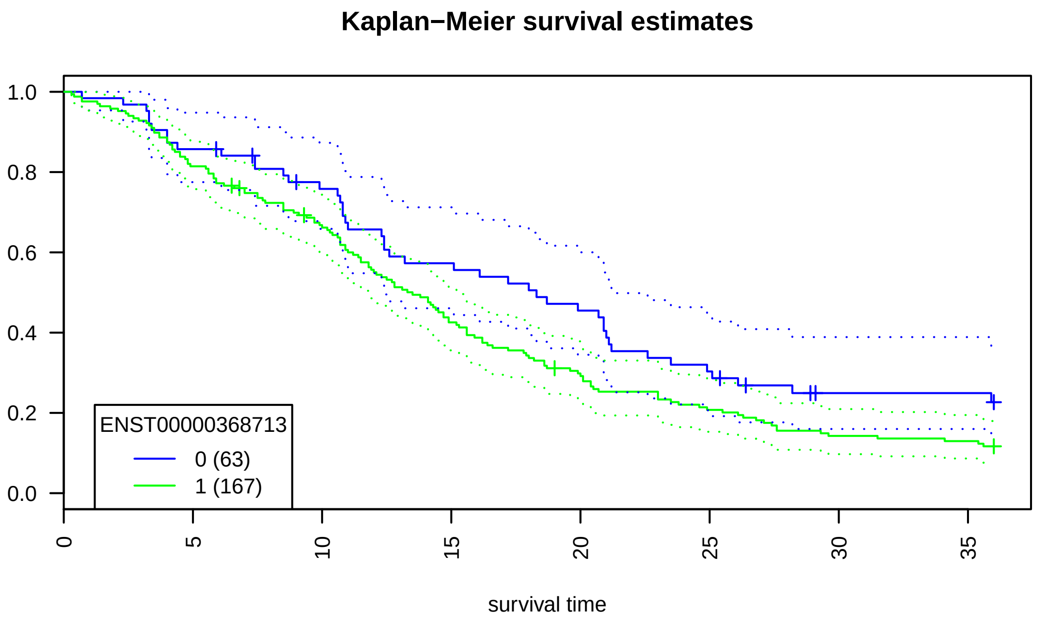

Figure 1290: Each curve represents the relative number of ENST00000368713 survived to the corresponding point of time. Dashed lines represent 0.95 confidence intervals for the curves with the corresponding color. Log-rank probability measure for the equality of these curves is 3.737583e-02. Additional annotations: *.GeneId*=ENSG00000188015, *.GeneName*=S100A3, *.GeneDesc*=S100 calcium binding protein A3 [Source:HGNC Symbol;Acc:10493].

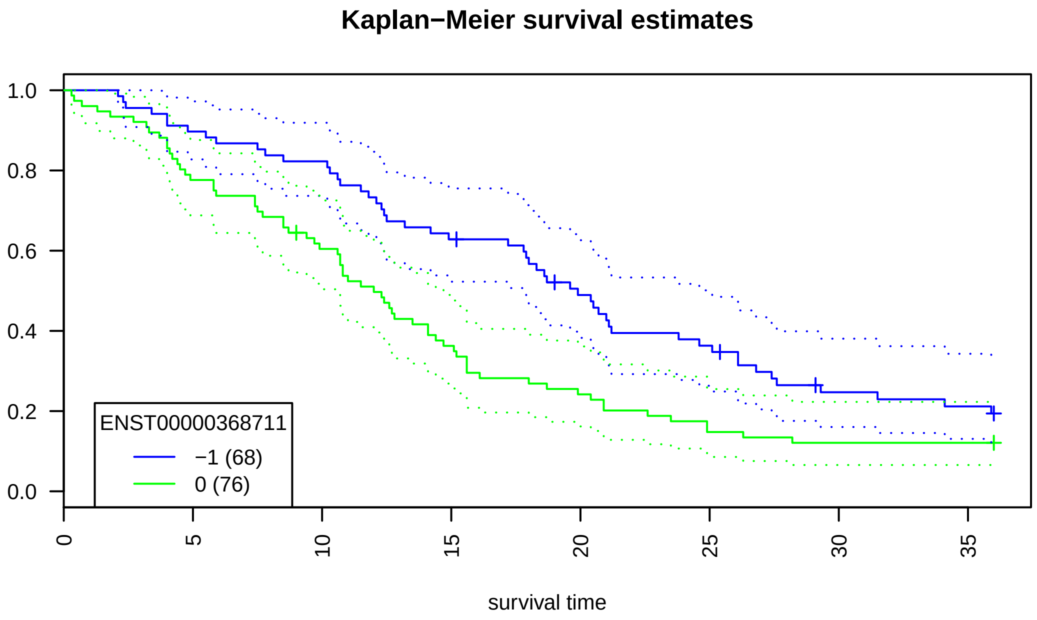

Figure 1291: Each curve represents the relative number of ENST00000368711 survived to the corresponding point of time. Dashed lines represent 0.95 confidence intervals for the curves with the corresponding color. Total of 86 observations deleted due to missingness. Log-rank probability measure for the equality of these curves is 4.25328e-03. Additional annotations: *.GeneId*=ENSG00000196754, *.GeneName*=S100A2, *.GeneDesc*=S100 calcium binding protein A2 [Source:HGNC Symbol;Acc:10492].

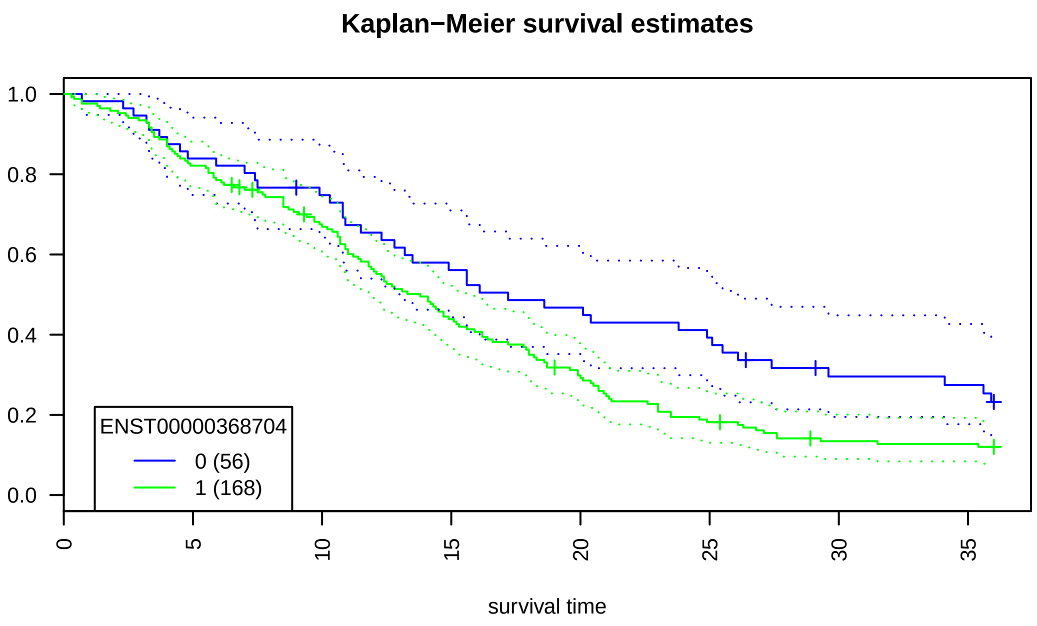

Figure 1292: Each curve represents the relative number of ENST00000368704 survived to the corresponding point of time. Dashed lines represent 0.95 confidence intervals for the curves with the corresponding color. Total of 6 observations deleted due to missingness. Log-rank probability measure for the equality of these curves is 2.270657e-02. Additional annotations: *.GeneId*=ENSG00000188643, *.GeneName*=S100A16, *.GeneDesc*=S100 calcium binding protein A16 [Source:HGNC Symbol;Acc:20441].

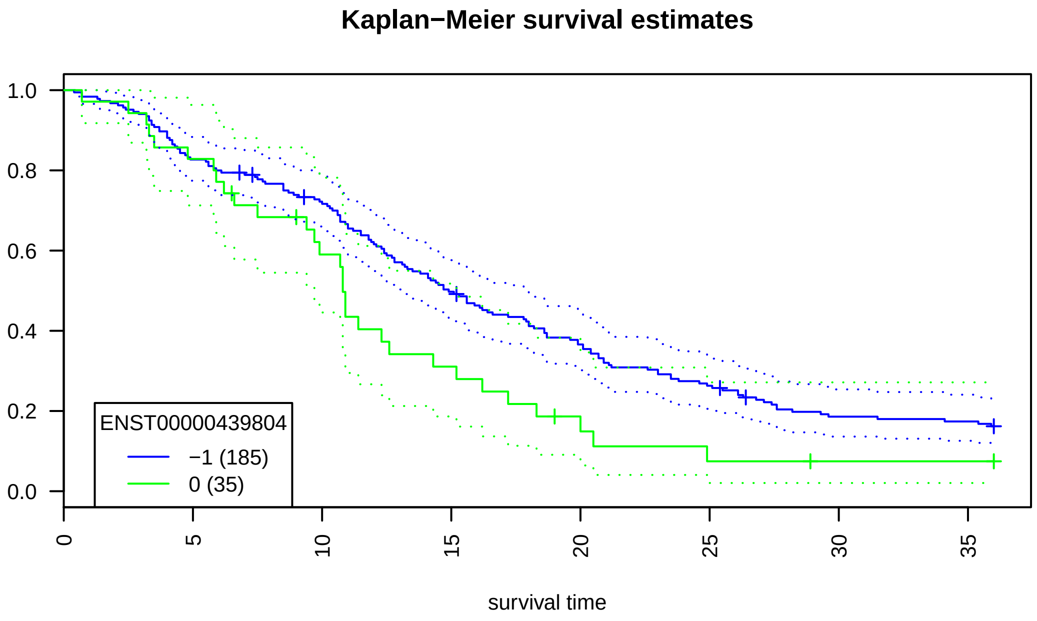

Figure 1293: Each curve represents the relative number of ENST00000439804 survived to the corresponding point of time. Dashed lines represent 0.95 confidence intervals for the curves with the corresponding color. Total of 10 observations deleted due to missingness. Log-rank probability measure for the equality of these curves is 1.270754e-02. Additional annotations: *.GeneId*=ENSG00000223812, *.GeneName*=RP11-197K6.1, *.GeneDesc*=[undefined].

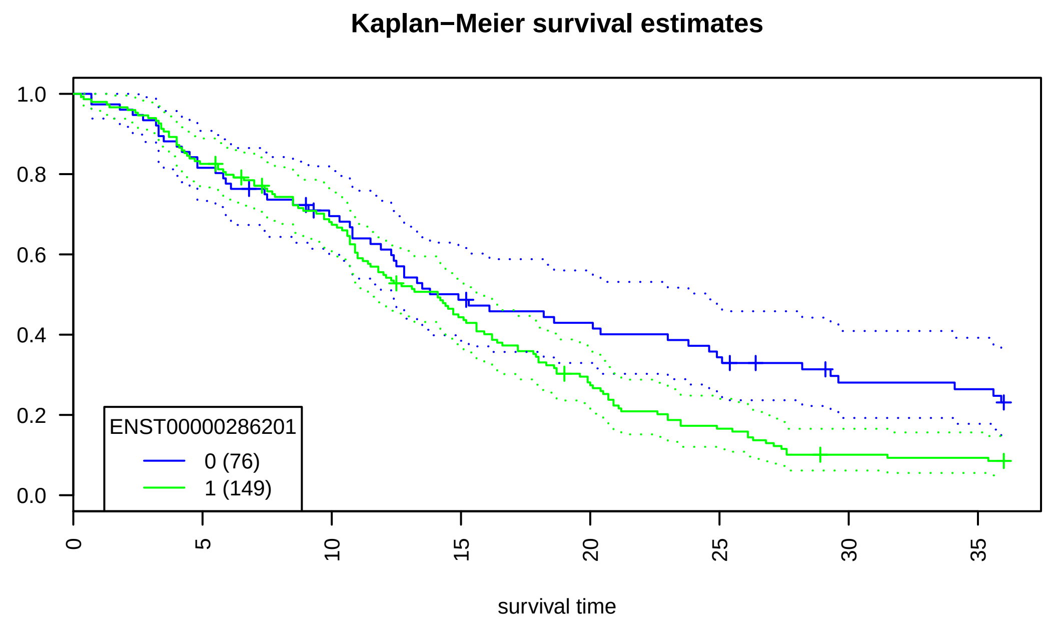

Figure 1294: Each curve represents the relative number of ENST00000286201 survived to the corresponding point of time. Dashed lines represent 0.95 confidence intervals for the curves with the corresponding color. Total of 5 observations deleted due to missingness. Log-rank probability measure for the equality of these curves is 1.218770e-02. Additional annotations: *.GeneId*=ENSG00000155760, *.GeneName*=FZD7, *.GeneDesc*=frizzled homolog 7 (Drosophila) [Source:HGNC Symbol;Acc:4045].

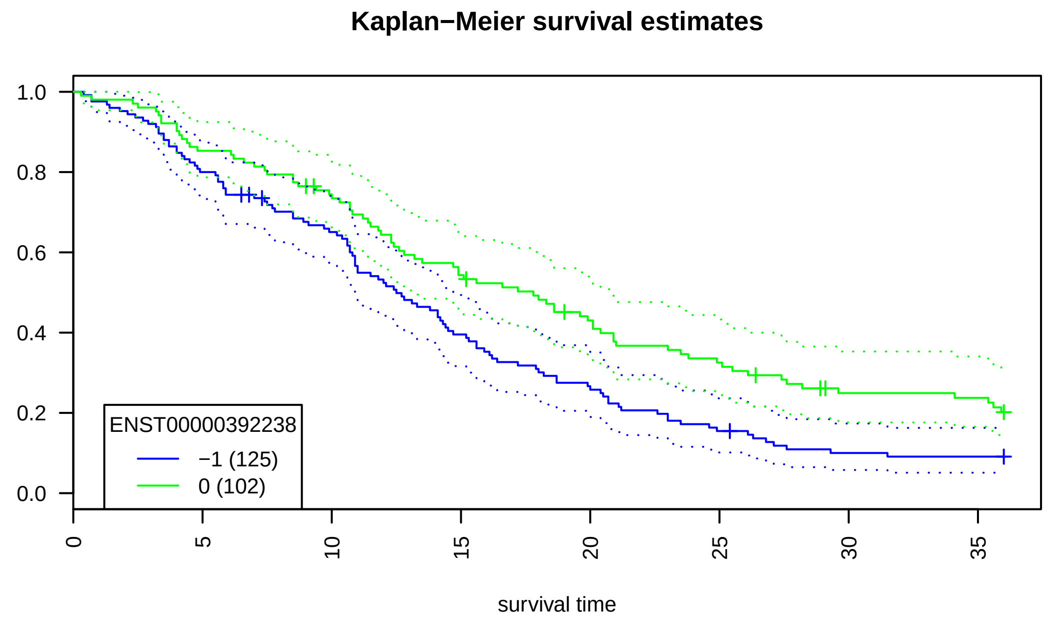

Figure 1295: Each curve represents the relative number of ENST00000392238 survived to the corresponding point of time. Dashed lines represent 0.95 confidence intervals for the curves with the corresponding color. Total of 3 observations deleted due to missingness. Log-rank probability measure for the equality of these curves is 3.0565e-03. Additional annotations: *.GeneId*=ENSG00000138439, *.GeneName*=FAM117B, *.GeneDesc*=family with sequence similarity 117, member B [Source:HGNC Symbol;Acc:14440].

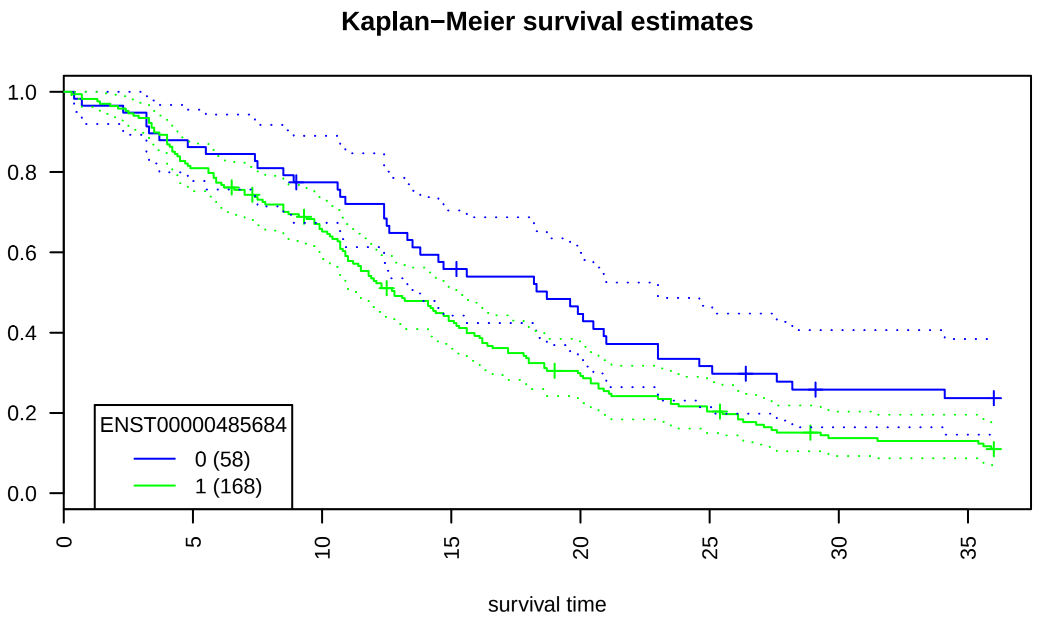

Figure 1296: Each curve represents the relative number of ENST00000485684 survived to the corresponding point of time. Dashed lines represent 0.95 confidence intervals for the curves with the corresponding color. Total of 4 observations deleted due to missingness. Log-rank probability measure for the equality of these curves is 1.956158e-02. Additional annotations: *.GeneId*=ENSG00000118257, *.GeneName*=NRP2, *.GeneDesc*=neuropilin 2 [Source:HGNC Symbol;Acc:8005].

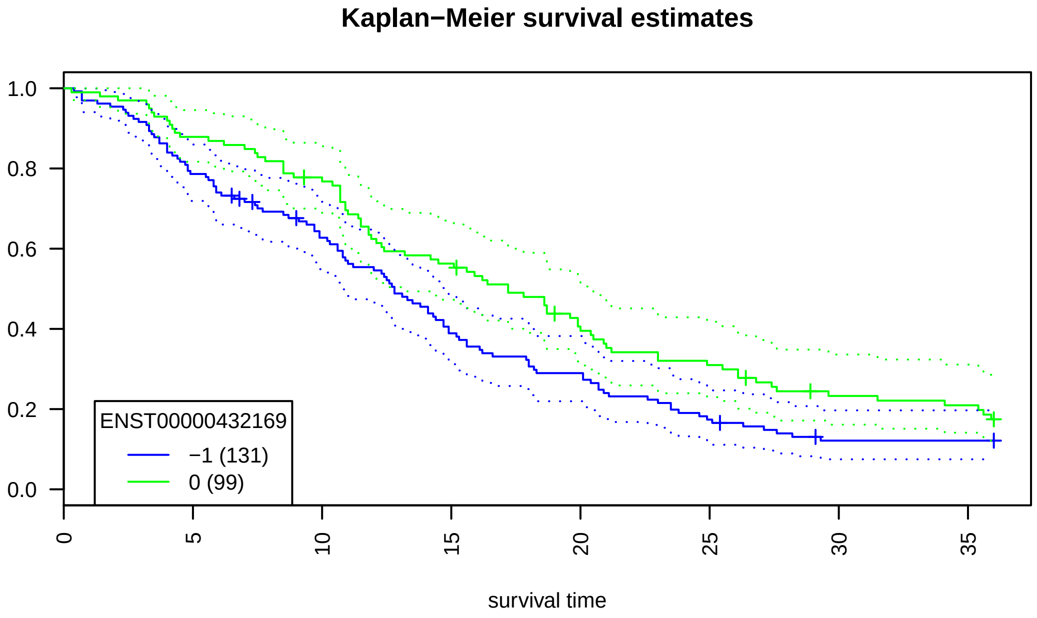

Figure 1297: Each curve represents the relative number of ENST00000432169 survived to the corresponding point of time. Dashed lines represent 0.95 confidence intervals for the curves with the corresponding color. Log-rank probability measure for the equality of these curves is 2.235225e-02. Additional annotations: *.GeneId*=ENSG00000023228, *.GeneName*=NDUFS1, *.GeneDesc*=NADH dehydrogenase (ubiquinone) Fe-S protein 1, 75kDa (NADH-coenzyme Q reductase) [Source:HGNC Symbol;Acc:7707].

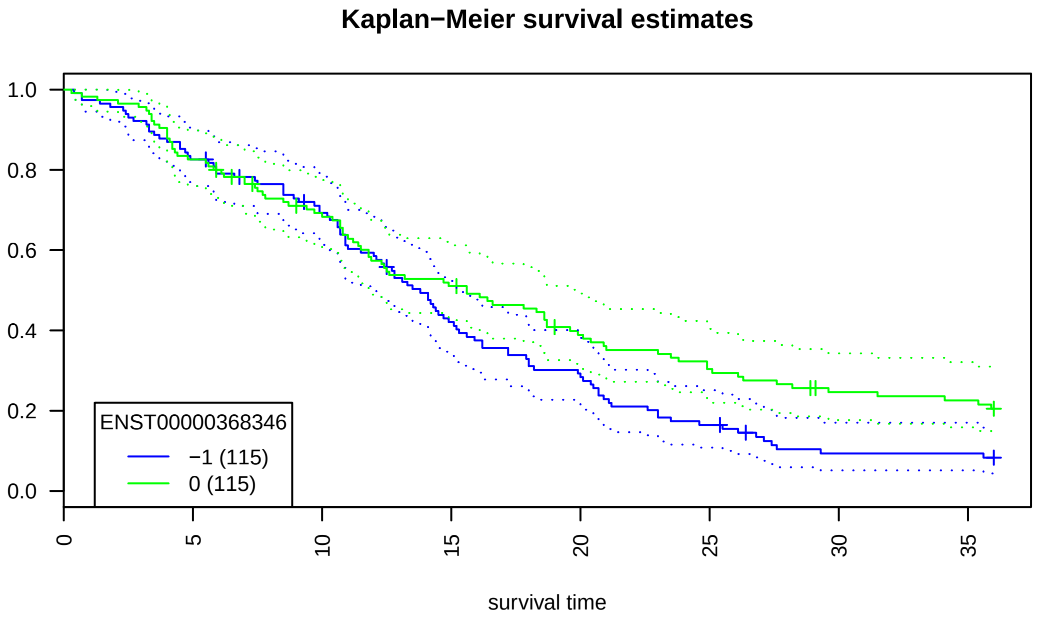

Figure 1298: Each curve represents the relative number of ENST00000368346 survived to the corresponding point of time. Dashed lines represent 0.95 confidence intervals for the curves with the corresponding color. Log-rank probability measure for the equality of these curves is 2.45289e-02. Additional annotations: *.GeneId*=ENSG00000116539, *.GeneName*=ASH1L, *.GeneDesc*=ash1 (absent, small, or homeotic)-like (Drosophila) [Source:HGNC Symbol;Acc:19088].

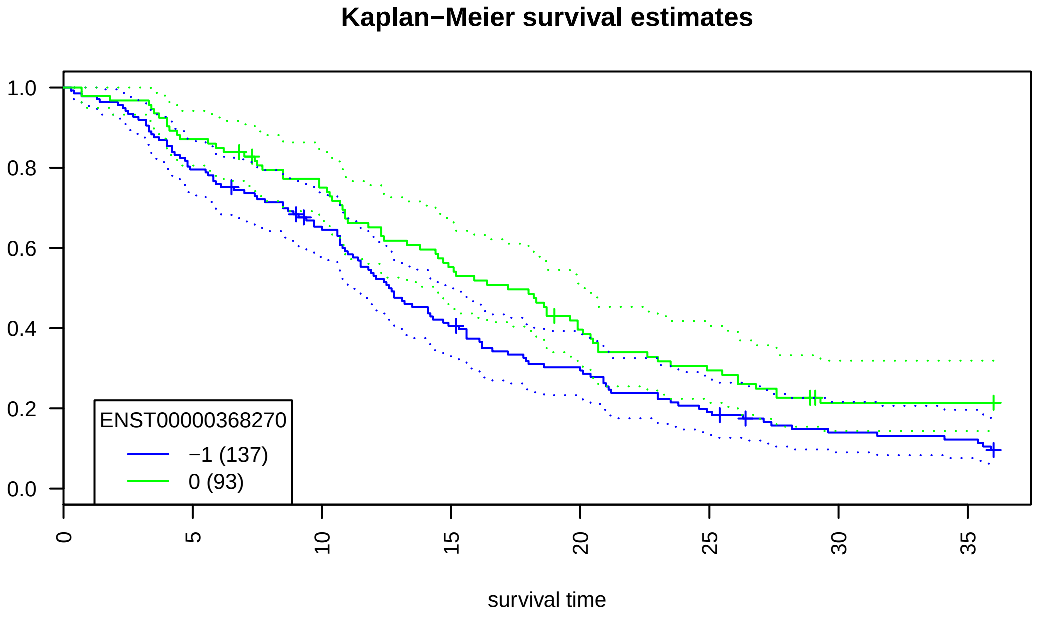

Figure 1299: Each curve represents the relative number of ENST00000368270 survived to the corresponding point of time. Dashed lines represent 0.95 confidence intervals for the curves with the corresponding color. Log-rank probability measure for the equality of these curves is 1.592736e-02. Additional annotations: *.GeneId*=ENSG00000160781, *.GeneName*=PAQR6, *.GeneDesc*=progestin and adipoQ receptor family member VI [Source:HGNC Symbol;Acc:30132].

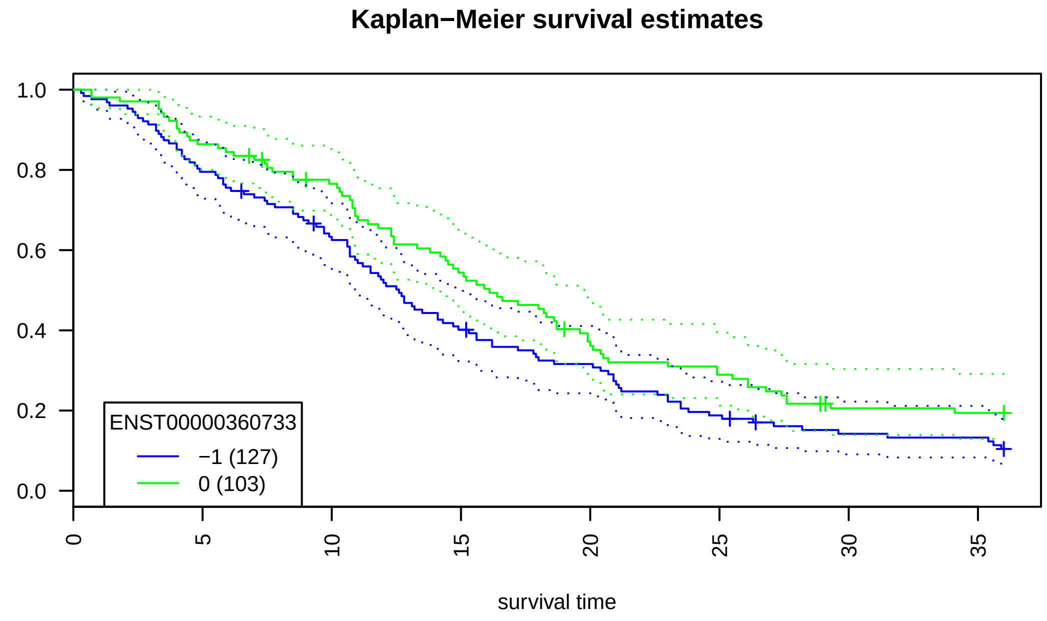

Figure 1300: Each curve represents the relative number of ENST00000360733 survived to the corresponding point of time. Dashed lines represent 0.95 confidence intervals for the curves with the corresponding color. Log-rank probability measure for the equality of these curves is 3.338611e-02. Additional annotations: .*GeneId*=ENSG00000160781, .*GeneName*=PAQR6, .*GeneDesc*=progestin and adipoQ receptor family member VI [Source:HGNC Symbol;Acc:30132].

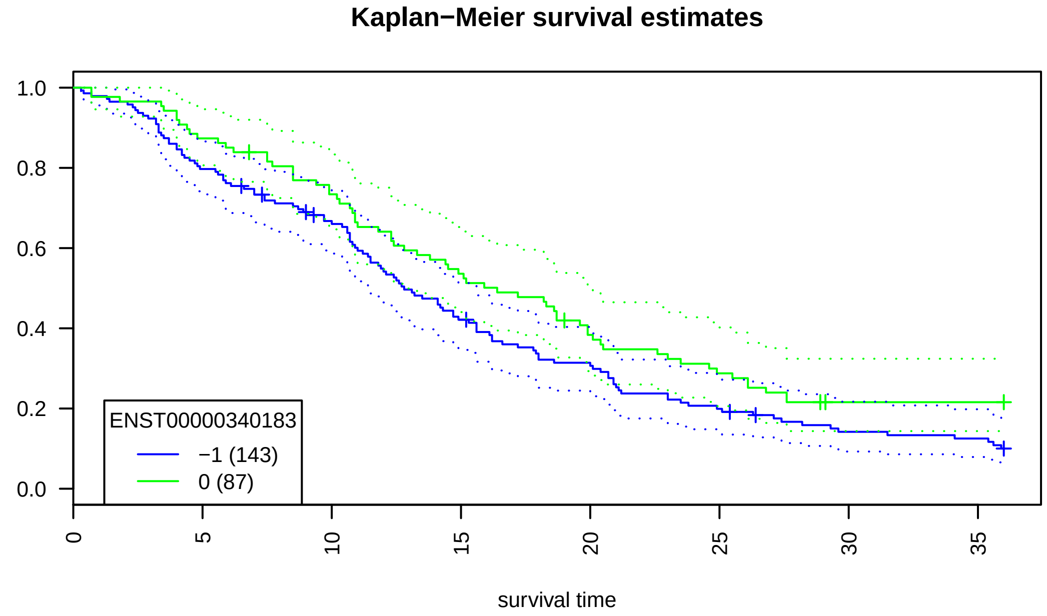

Figure 1301: Each curve represents the relative number of ENST00000340183 survived to the corresponding point of time. Dashed lines represent 0.95 confidence intervals for the curves with the corresponding color. Log-rank probability measure for the equality of these curves is 3.240058e-02. Additional annotations: .*GeneId*=ENSG00000160781, .*GeneName*=PAQR6, .*GeneDesc*=progestin and adipoQ receptor family member VI [Source:HGNC Symbol;Acc:30132].

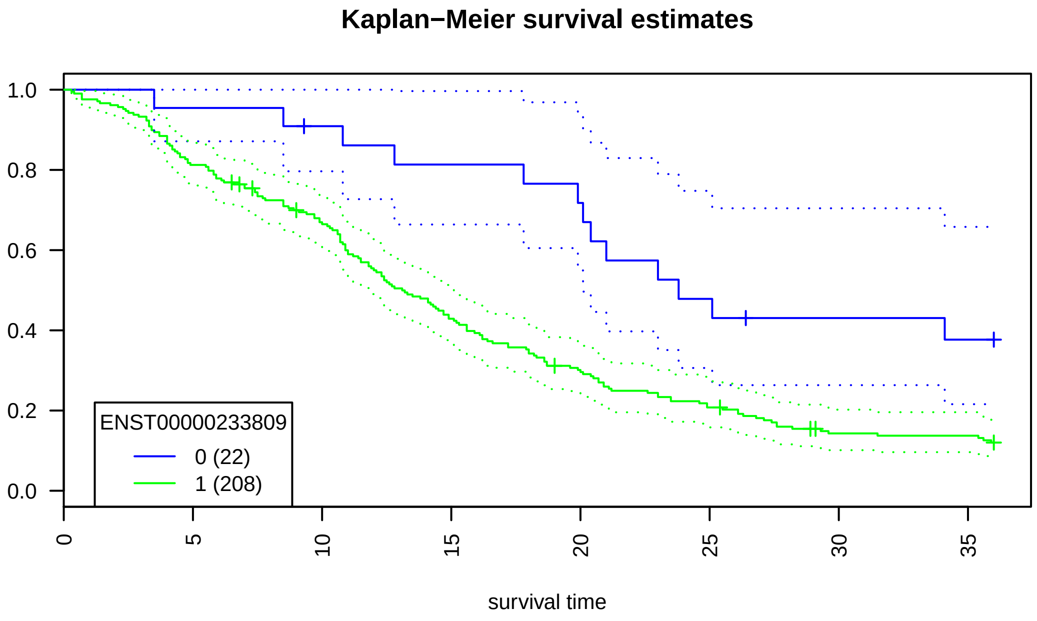

Figure 1302: Each curve represents the relative number of ENST00000233809 survived to the corresponding point of time. Dashed lines represent 0.95 confidence intervals for the curves with the corresponding color. Log-rank probability measure for the equality of these curves is 1.695202e-03. Additional annotations: *.GeneId*=ENSG00000115457, *.GeneName*=IGFBP2, *.GeneDesc*=insulin-like growth factor binding protein 2, 36kDa [Source:HGNC Symbol;Acc:5471].

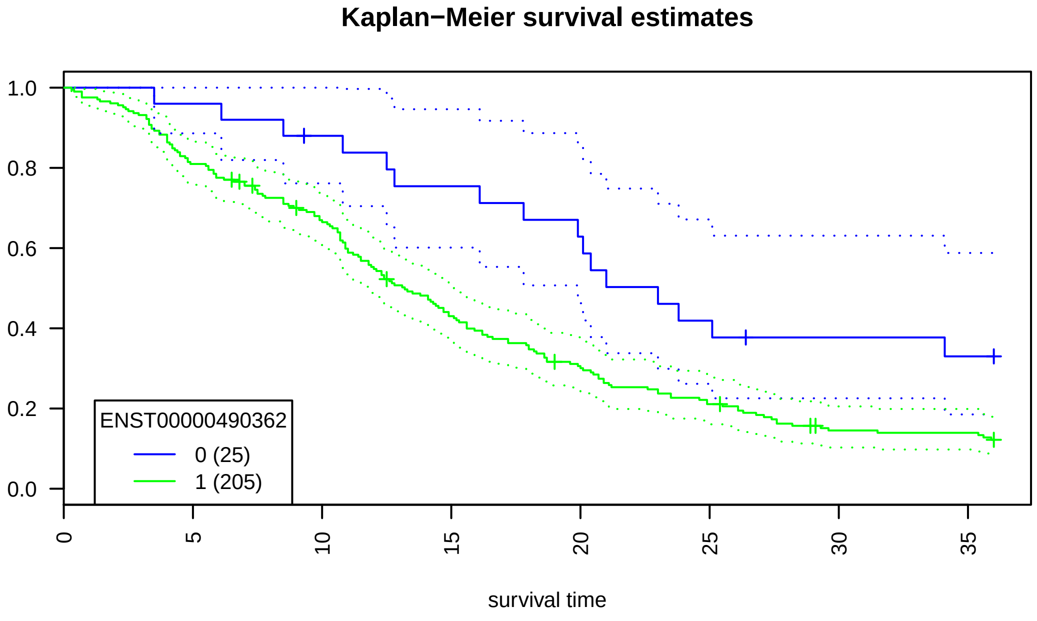

Figure 1303: Each curve represents the relative number of ENST00000490362 survived to the corresponding point of time. Dashed lines represent 0.95 confidence intervals for the curves with the corresponding color. Log-rank probability measure for the equality of these curves is 5.24001e-03. Additional annotations: *.GeneId*=ENSG00000115457, *.GeneName*=IGFBP2, *.GeneDesc*=insulin-like growth factor binding protein 2, 36kDa [Source:HGNC Symbol;Acc:5471].

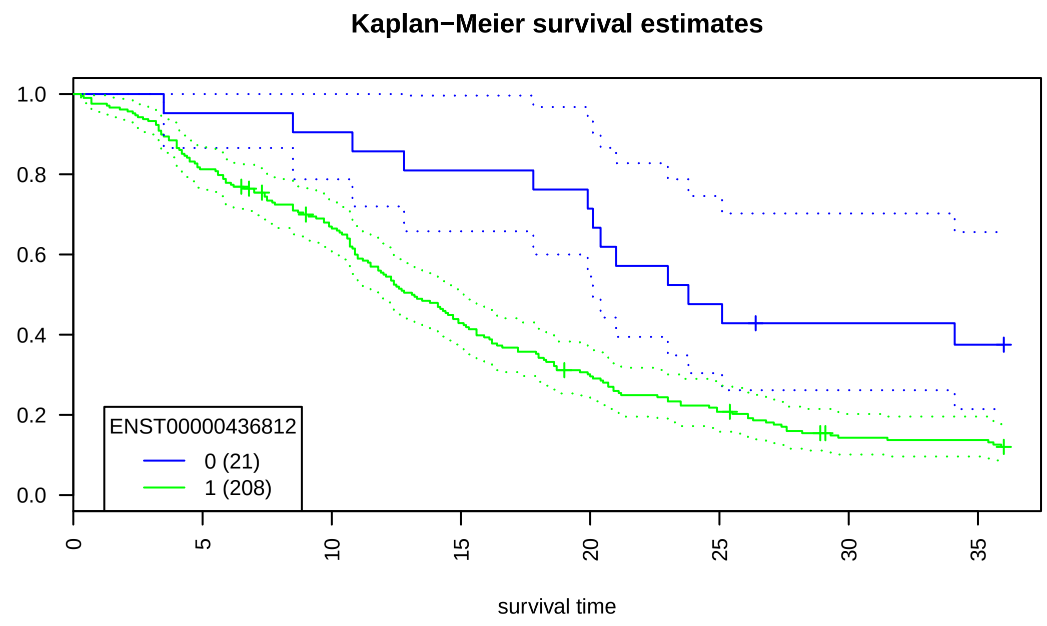

Figure 1304: Each curve represents the relative number of ENST00000436812 survived to the corresponding point of time. Dashed lines represent 0.95 confidence intervals for the curves with the corresponding color. Total of 1 observation deleted due to missingness. Log-rank probability measure for the equality of these curves is 1.982325e-03. Additional annotations: *.GeneId*=ENSG00000115457, *.GeneName*=IGFBP2, *.GeneDesc*=insulin-like growth factor binding protein 2, 36kDa [Source:HGNC Symbol;Acc:5471].

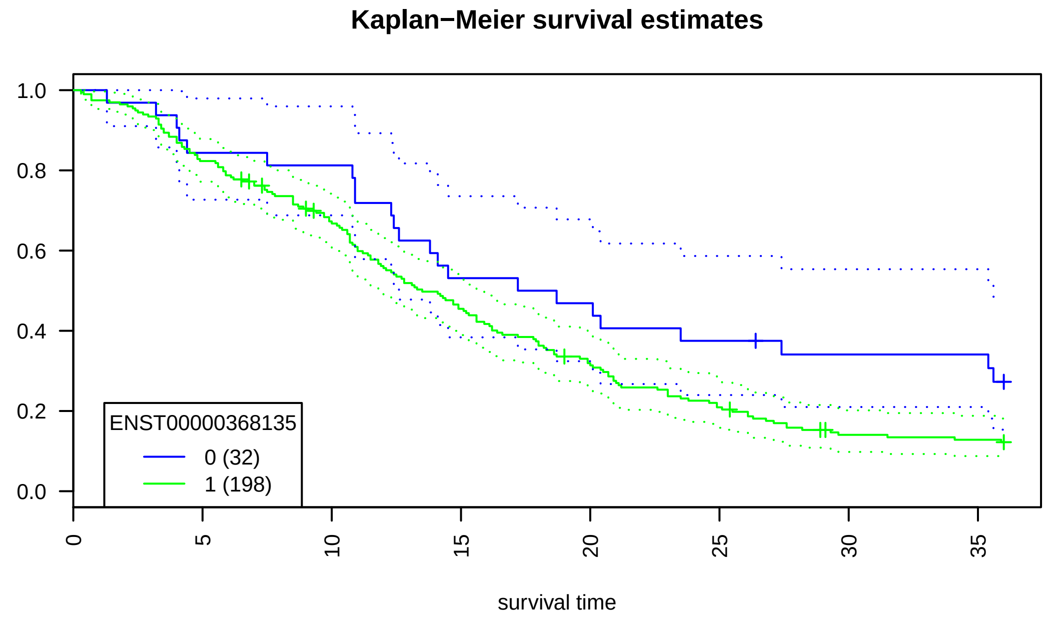

Figure 1305: Each curve represents the relative number of ENST00000368135 survived to the corresponding point of time. Dashed lines represent 0.95 confidence intervals for the curves with the corresponding color. Log-rank probability measure for the equality of these curves is 3.915032e-02. Additional annotations: *.GeneId*=ENSG00000163564, *.GeneName*=PYHIN1, *.GeneDesc*=pyrin and HIN domain family, member 1 [Source:HGNC Symbol;Acc:28894].

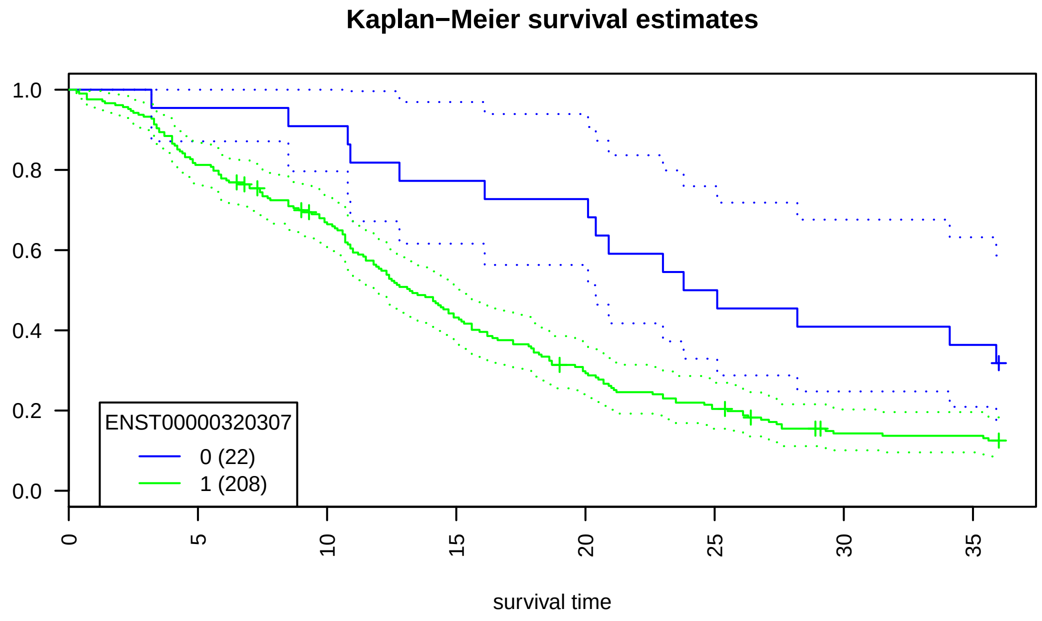

Figure 1306: Each curve represents the relative number of ENST00000320307 survived to the corresponding point of time. Dashed lines represent 0.95 confidence intervals for the curves with the corresponding color. Log-rank probability measure for the equality of these curves is 3.749044e-03. Additional annotations: *.GeneId*=ENSG00000158710, *.GeneName*=TAGLN2, *.GeneDesc*=transgelin 2 [Source:HGNC Symbol;Acc:11554].

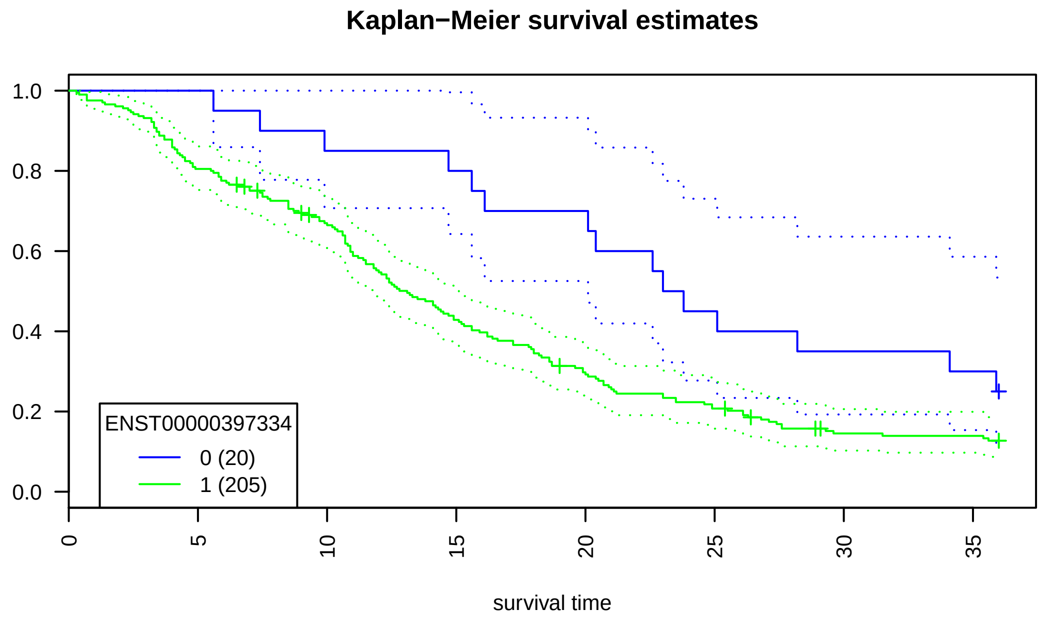

Figure 1307: Each curve represents the relative number of ENST00000397334 survived to the corresponding point of time. Dashed lines represent 0.95 confidence intervals for the curves with the corresponding color. Total of 5 observations deleted due to missingness. Log-rank probability measure for the equality of these curves is 1.556713e-02. Additional annotations: *.GeneId*=ENSG00000158710, *.GeneName*=TAGLN2, *.GeneDesc*=transgelin 2 [Source:HGNC Symbol;Acc:11554].

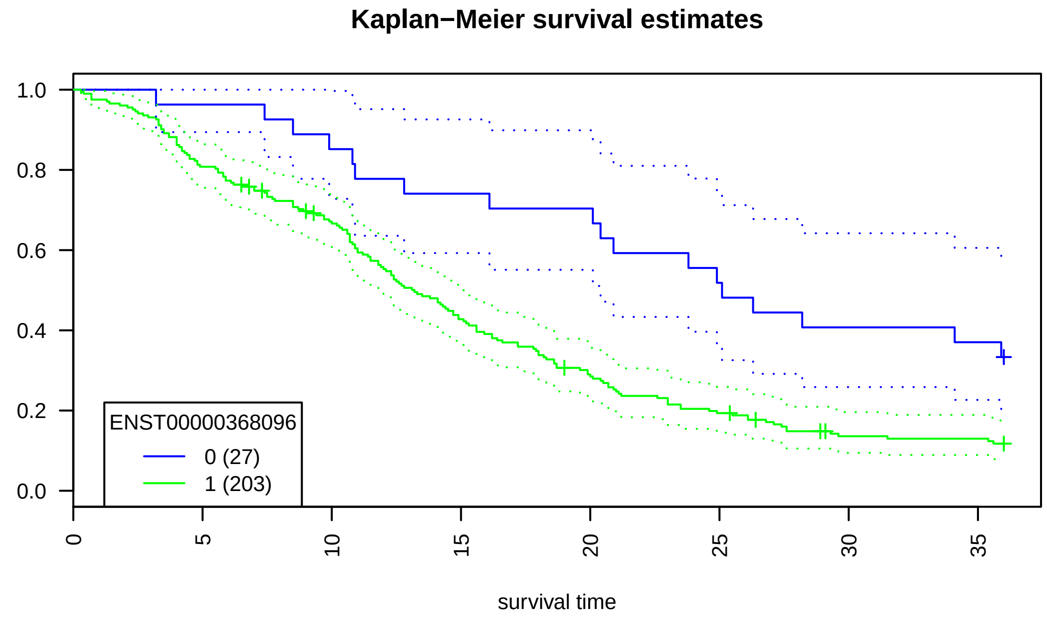

Figure 1308: Each curve represents the relative number of ENST00000368096 survived to the corresponding point of time. Dashed lines represent 0.95 confidence intervals for the curves with the corresponding color. Log-rank probability measure for the equality of these curves is 1.009707e-03. Additional annotations: *.GeneId*=ENSG00000158710, *.GeneName*=TAGLN2, *.GeneDesc*=transgelin 2 [Source:HGNC Symbol;Acc:11554].

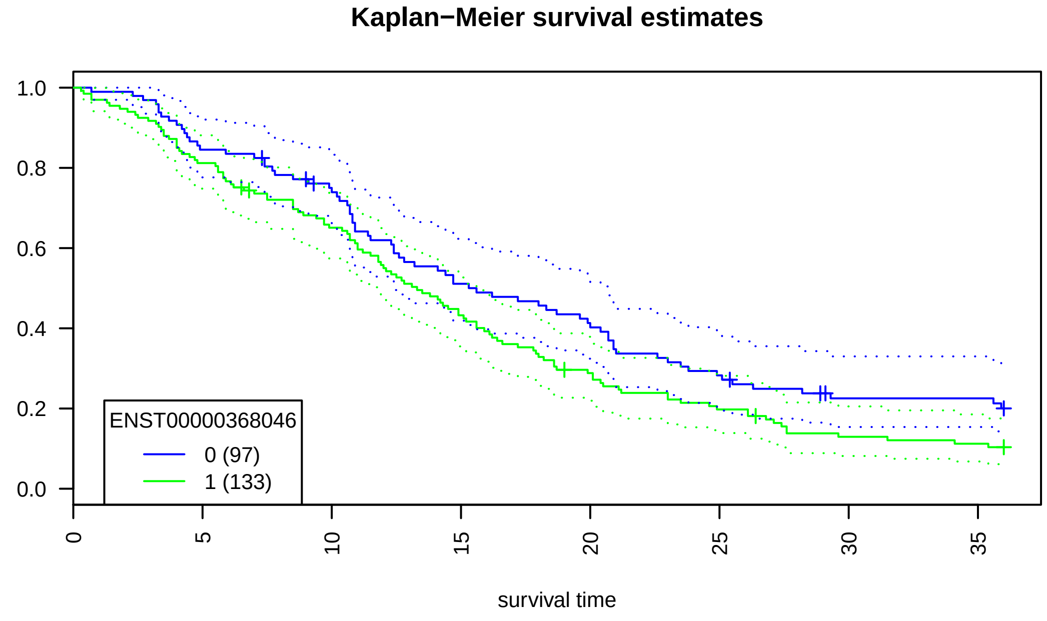

Figure 1309: Each curve represents the relative number of ENST00000368046 survived to the corresponding point of time. Dashed lines represent 0.95 confidence intervals for the curves with the corresponding color. Log-rank probability measure for the equality of these curves is 4.155966e-02. Additional annotations: *.GeneId*=ENSG00000117091, *.GeneName*=CD48, *.GeneDesc*=CD48 molecule [Source:HGNC Symbol;Acc:1683].

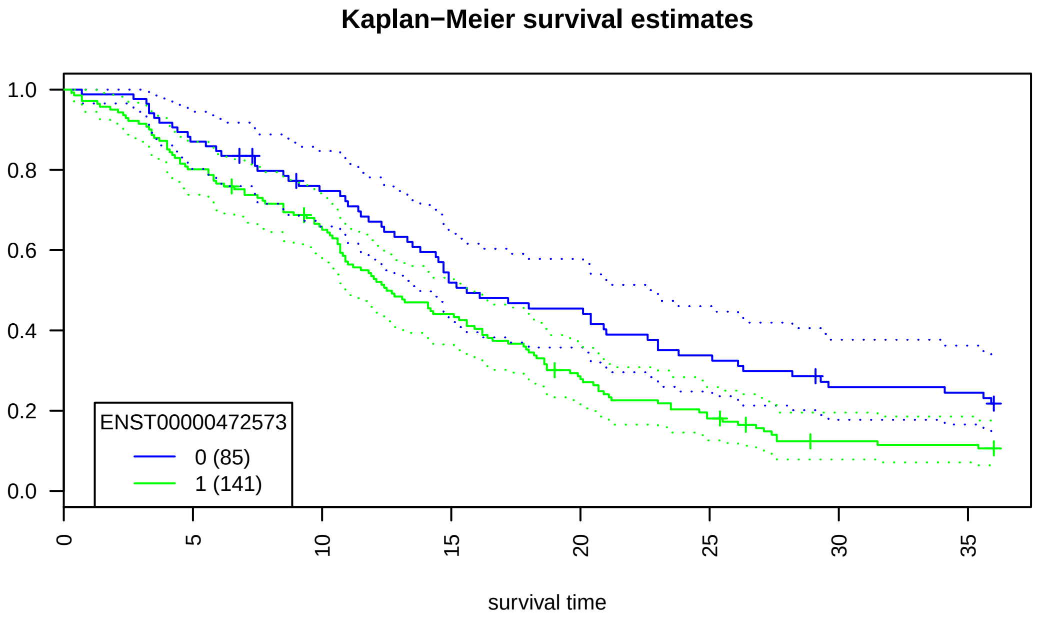

Figure 1310: Each curve represents the relative number of ENST00000472573 survived to the corresponding point of time. Dashed lines represent 0.95 confidence intervals for the curves with the corresponding color. Total of 4 observations deleted due to missingness. Log-rank probability measure for the equality of these curves is 8.766065e-03. Additional annotations: *.GeneId*=ENSG00000158769, *.GeneName*=F11R, *.GeneDesc*=F11 receptor [Source:HGNC Symbol;Acc:14685].

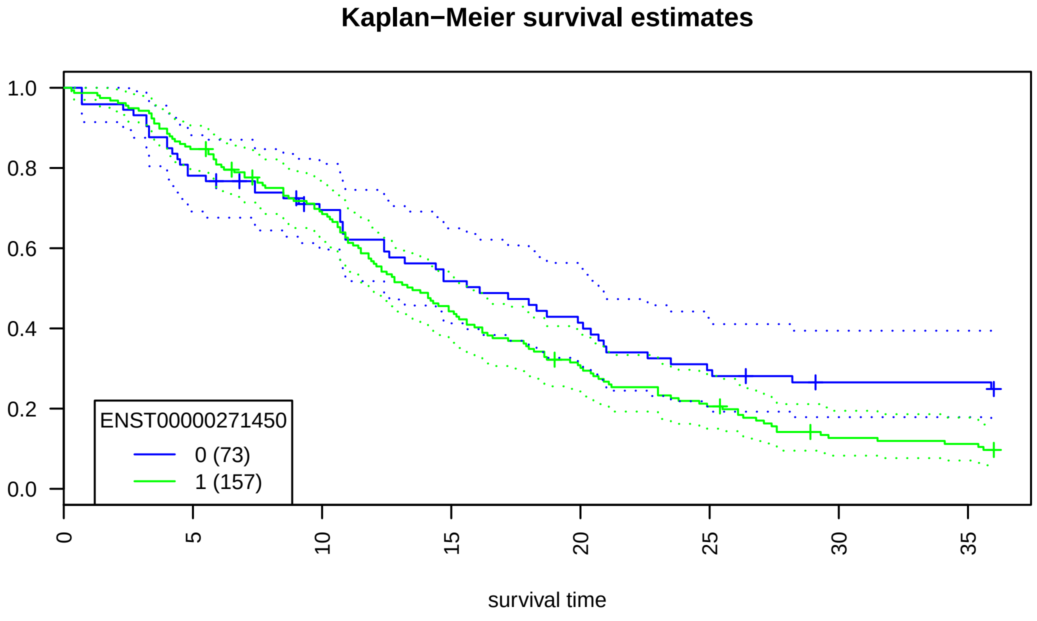

Figure 1311: Each curve represents the relative number of ENST00000271450 survived to the corresponding point of time. Dashed lines represent 0.95 confidence intervals for the curves with the corresponding color. Log-rank probability measure for the equality of these curves is 4.634527e-02. Additional annotations: *.GeneId*=ENSG00000143226, *.GeneName*=FCGR2A, *.GeneDesc*=Fc fragment of IgG, low affinity IIa, receptor (CD32) [Source:HGNC Symbol;Acc:3616].

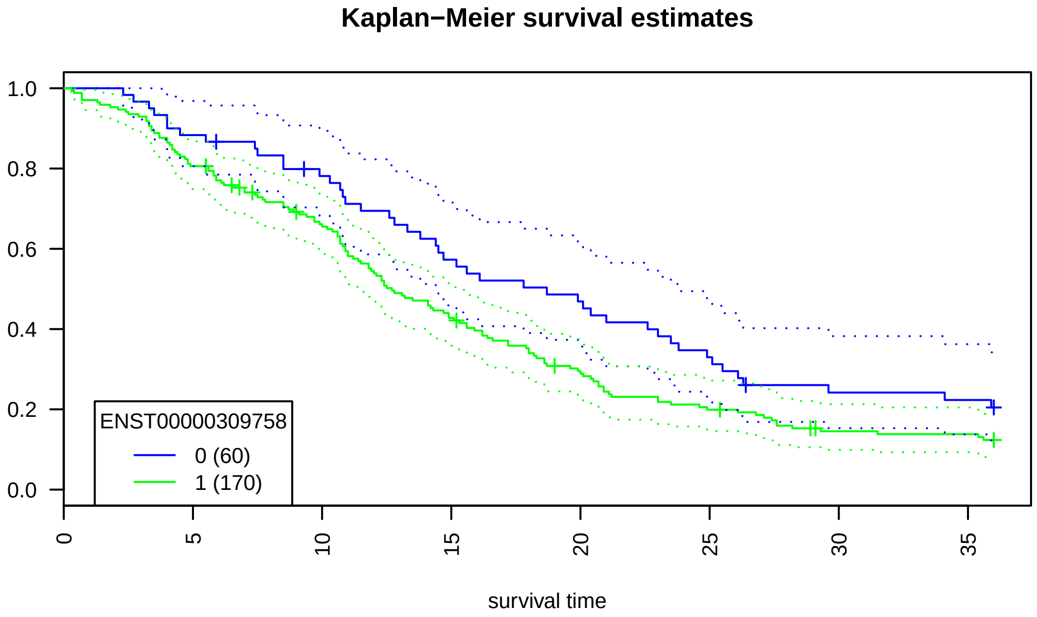

Figure 1312: Each curve represents the relative number of ENST00000309758 survived to the corresponding point of time. Dashed lines represent 0.95 confidence intervals for the curves with the corresponding color. Log-rank probability measure for the equality of these curves is 2.934139e-02. Additional annotations: *.GeneId*=ENSG00000173110, *.GeneName*=HSPA6, *.GeneDesc*=heat shock 70kDa protein 7 (HSP70B) [Source:HGNC Symbol;Acc:5240].

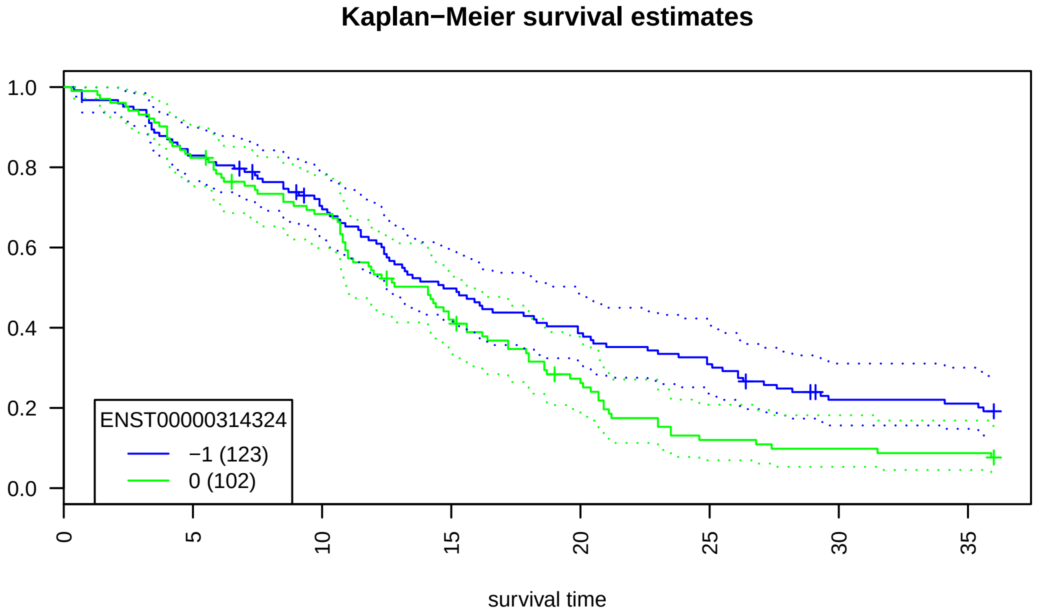

Figure 1313: Each curve represents the relative number of ENST00000314324 survived to the corresponding point of time. Dashed lines represent 0.95 confidence intervals for the curves with the corresponding color. Total of 5 observations deleted due to missingness. Log-rank probability measure for the equality of these curves is 1.827197e-02.

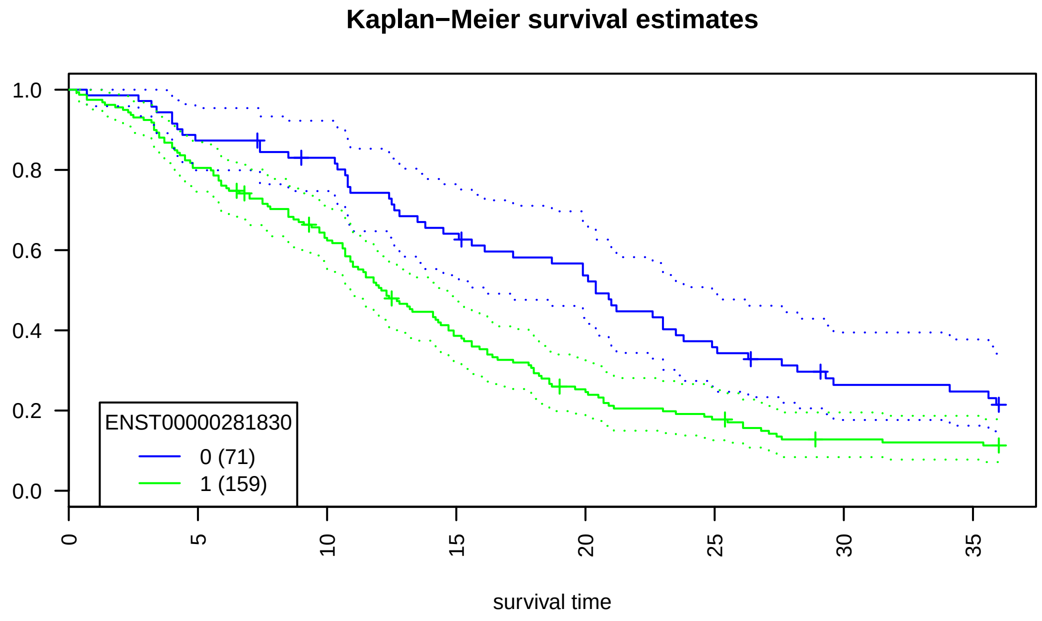

Figure 1314: Each curve represents the relative number of ENST00000281830 survived to the corresponding point of time. Dashed lines represent 0.95 confidence intervals for the curves with the corresponding color. Log-rank probability measure for the equality of these curves is 6.84277e-04. Additional annotations: *.GeneId*=ENSG00000152049, *.GeneName*=KCNE4, *.GeneDesc*=potassium voltage-gated channel, Isk-related family, member 4 [Source:HGNC Symbol;Acc:6244].

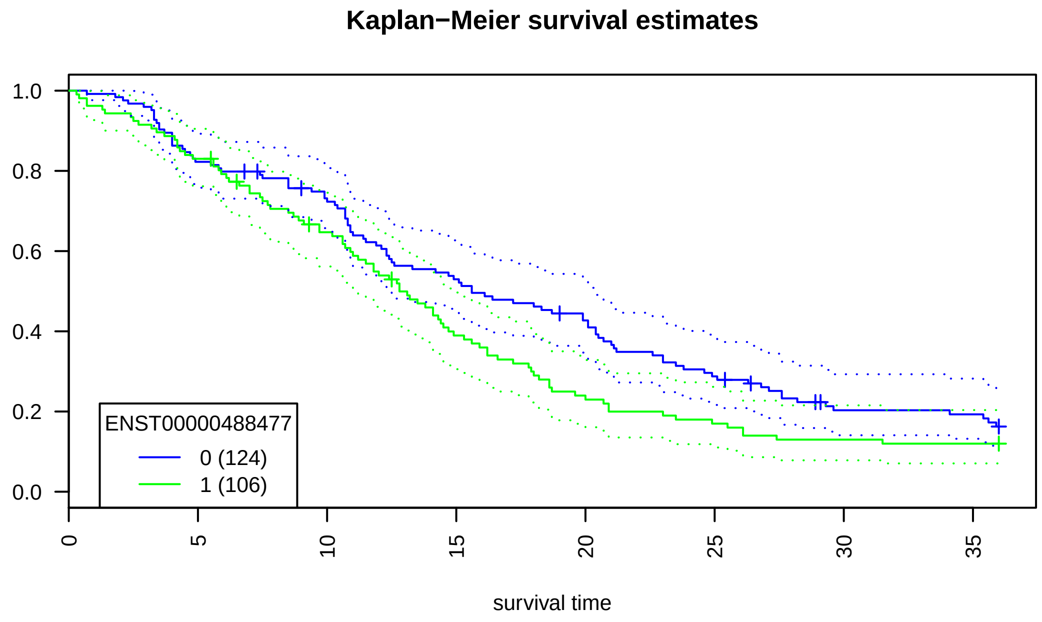

Figure 1315: Each curve represents the relative number of ENST00000488477 survived to the corresponding point of time. Dashed lines represent 0.95 confidence intervals for the curves with the corresponding color. Log-rank probability measure for the equality of these curves is 4.852626e-02. Additional annotations: *.GeneId*=ENSG00000152049, *.GeneName*=KCNE4, *.GeneDesc*=potassium voltage-gated channel, Isk-related family, member 4 [Source:HGNC Symbol;Acc:6244].

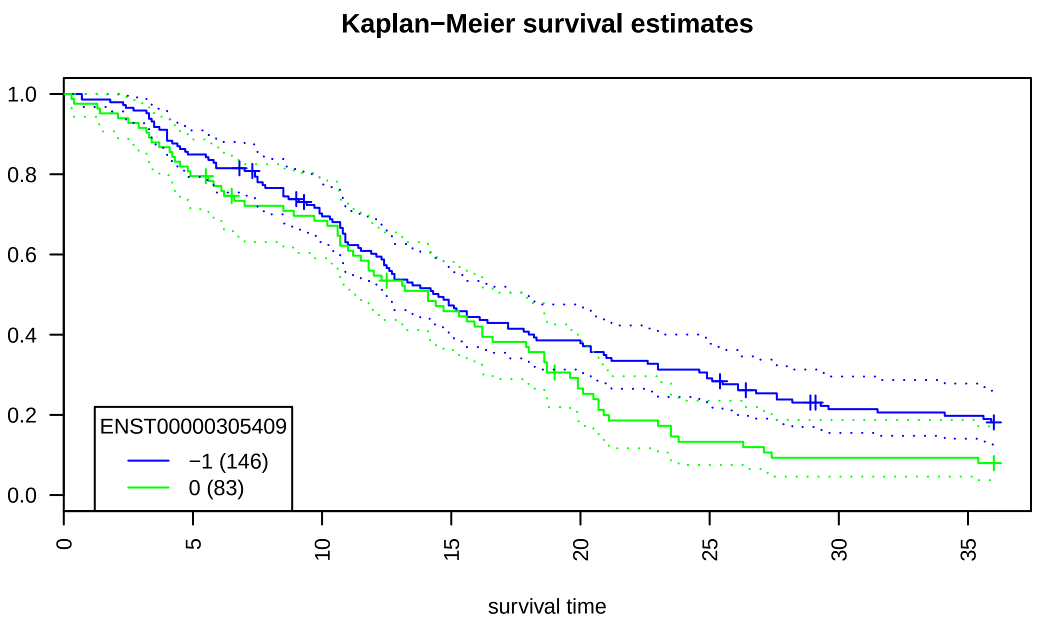

Figure 1316: Each curve represents the relative number of ENST00000305409 survived to the corresponding point of time. Dashed lines represent 0.95 confidence intervals for the curves with the corresponding color. Total of 1 observation deleted due to missingness. Log-rank probability measure for the equality of these curves is 4.418511e-02. Additional annotations: *.GeneId*=ENSG00000171951, *.GeneName*=SCG2, *.GeneDesc*=secretogranin II (chromogranin C) [Source:HGNC Symbol;Acc:10575].

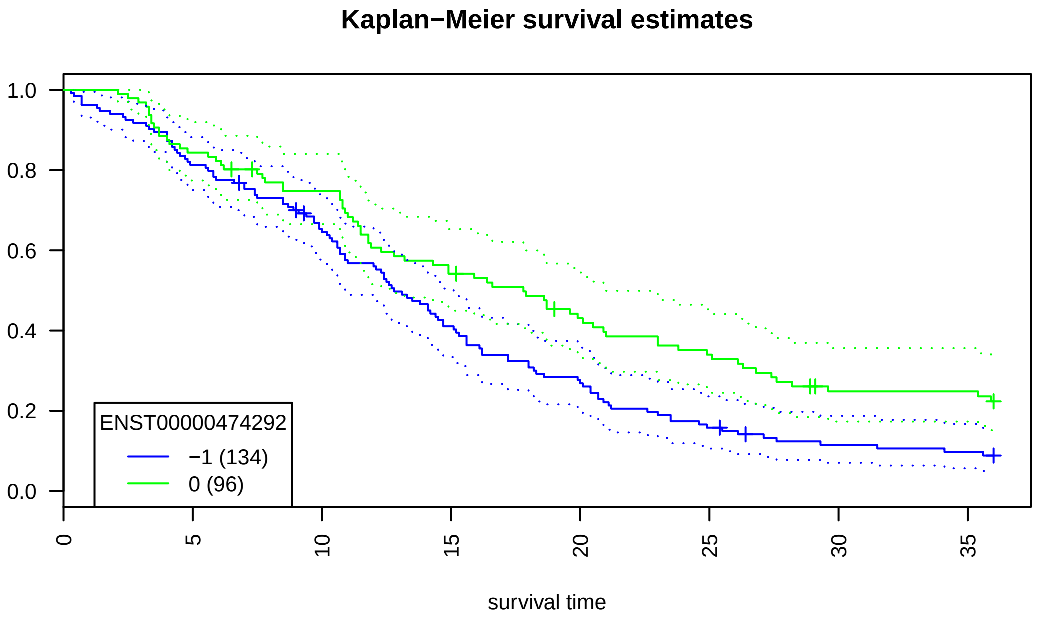

Figure 1317: Each curve represents the relative number of ENST00000474292 survived to the corresponding point of time. Dashed lines represent 0.95 confidence intervals for the curves with the corresponding color. Log-rank probability measure for the equality of these curves is 3.041997e-03. Additional annotations: *.GeneId*=ENSG00000075945, *.GeneName*=KIFAP3, *.GeneDesc*=kinesin-associated protein 3 [Source:HGNC Symbol;Acc:17060].

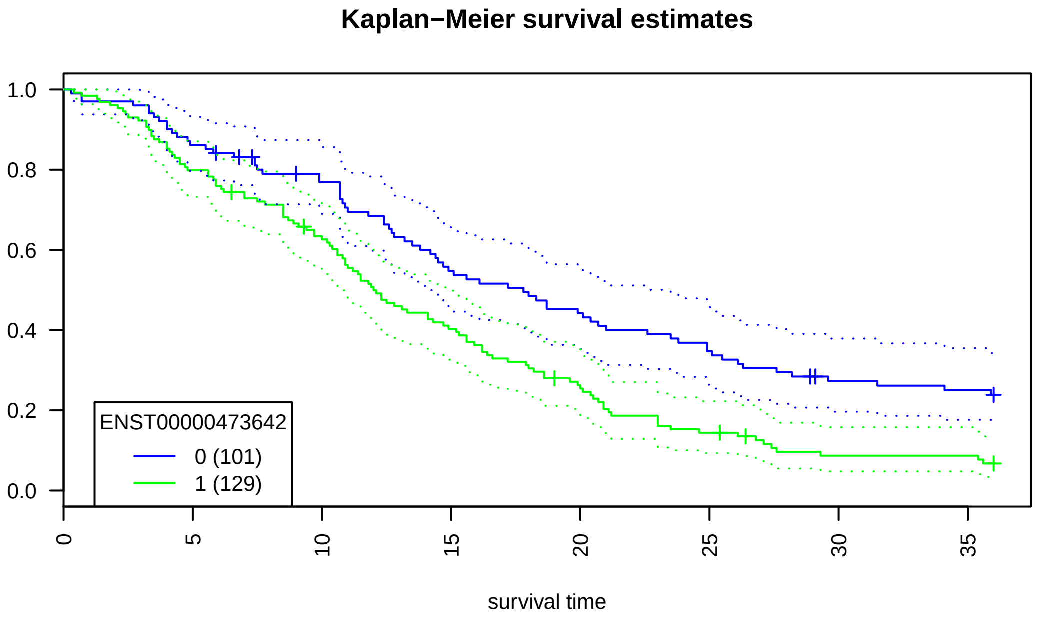

Figure 1318: Each curve represents the relative number of ENST00000473642 survived to the corresponding point of time. Dashed lines represent 0.95 confidence intervals for the curves with the corresponding color. Log-rank probability measure for the equality of these curves is 1.498395e-04. Additional annotations: *.GeneId*=ENSG00000115009, *.GeneName*=CCL20, *.GeneDesc*=chemokine (C-C motif) ligand 20 [Source:HGNC Symbol;Acc:10619].

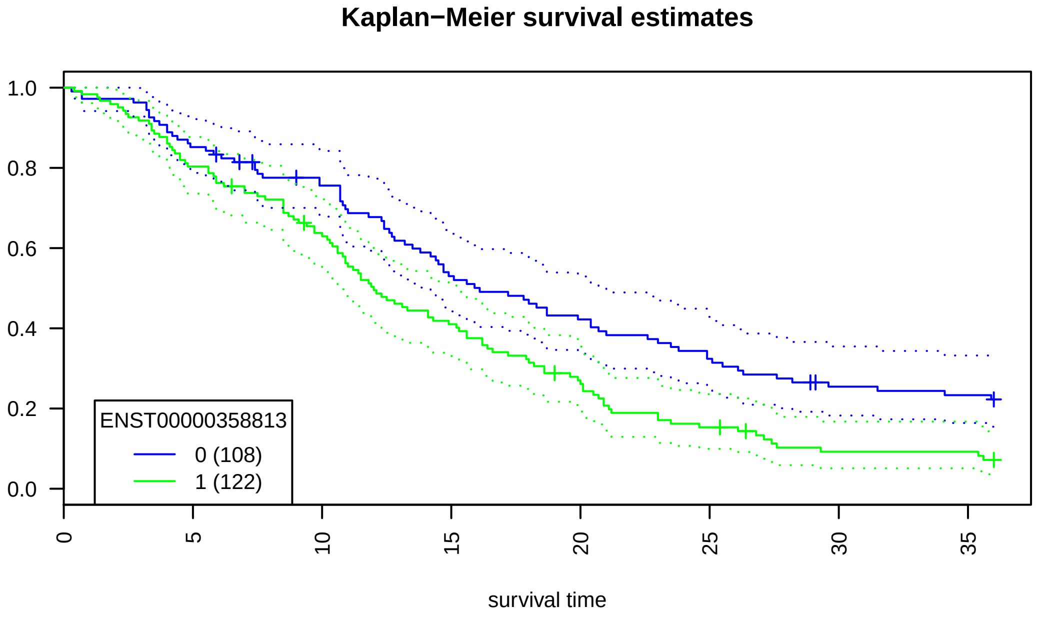

Figure 1319: Each curve represents the relative number of ENST00000358813 survived to the corresponding point of time. Dashed lines represent 0.95 confidence intervals for the curves with the corresponding color. Log-rank probability measure for the equality of these curves is 8.75448e-04. Additional annotations: *.GeneId*=ENSG00000115009, *.GeneName*=CCL20, *.GeneDesc*=chemokine (C-C motif) ligand 20 [Source:HGNC Symbol;Acc:10619].

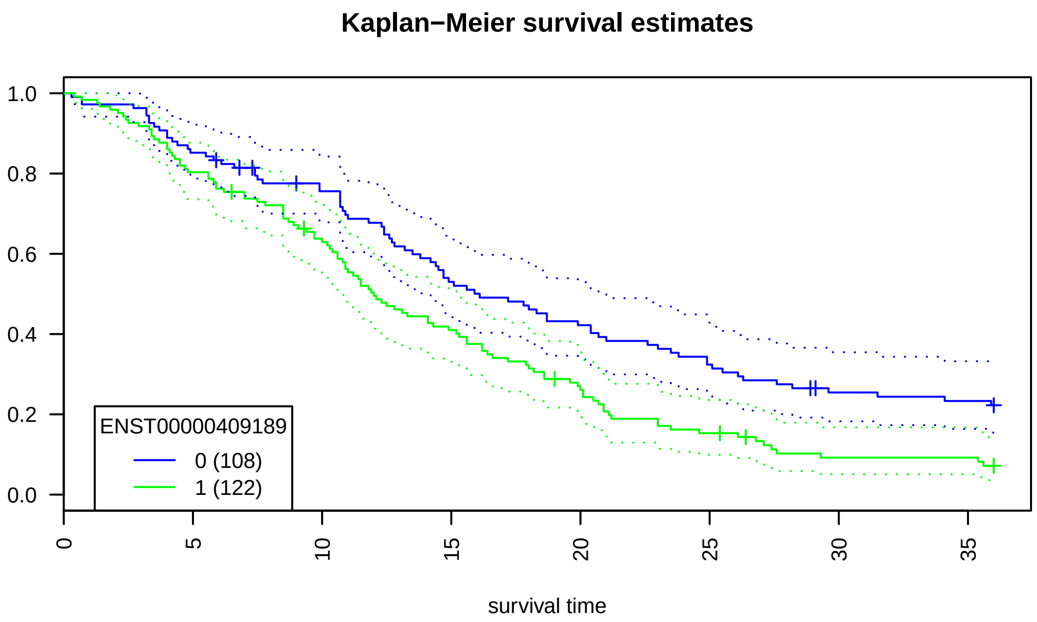

Figure 1320: Each curve represents the relative number of ENST00000409189 survived to the corresponding point of time. Dashed lines represent 0.95 confidence intervals for the curves with the corresponding color. Log-rank probability measure for the equality of these curves is 8.75448e-04. Additional annotations: *.GeneId*=ENSG00000115009, *.GeneName*=CCL20, *.GeneDesc*=chemokine (C-C motif) ligand 20 [Source:HGNC Symbol;Acc:10619].

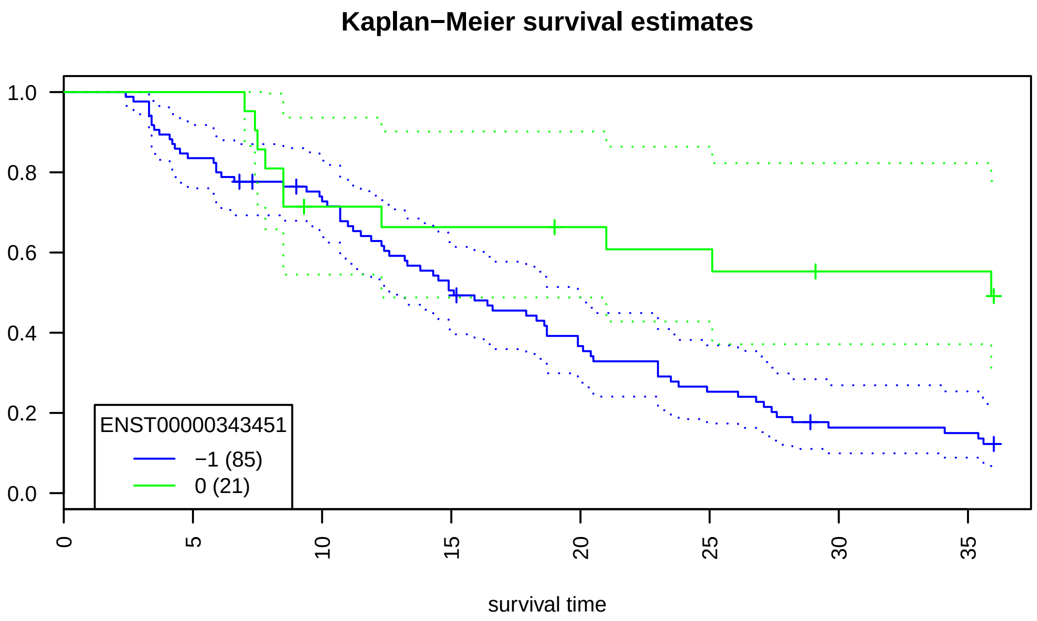

Figure 1321: Each curve represents the relative number of ENST00000343451 survived to the corresponding point of time. Dashed lines represent 0.95 confidence intervals for the curves with the corresponding color. Total of 124 observations deleted due to missingness. Log-rank probability measure for the equality of these curves is 4.228805e-03.

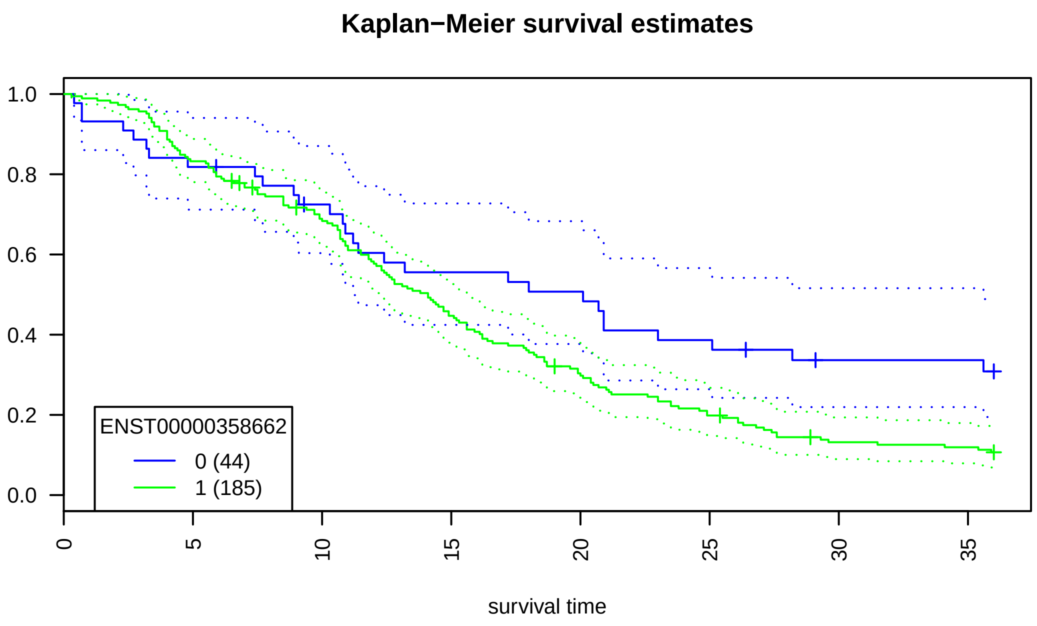

Figure 1322: Each curve represents the relative number of ENST00000358662 survived to the corresponding point of time. Dashed lines represent 0.95 confidence intervals for the curves with the corresponding color. Total of 1 observation deleted due to missingness. Log-rank probability measure for the equality of these curves is 1.481332e-02. Additional annotations: *.GeneId*=ENSG00000135899, *.GeneName*=SP110, *.GeneDesc*=SP110 nuclear body protein [Source:HGNC Symbol;Acc:5401].

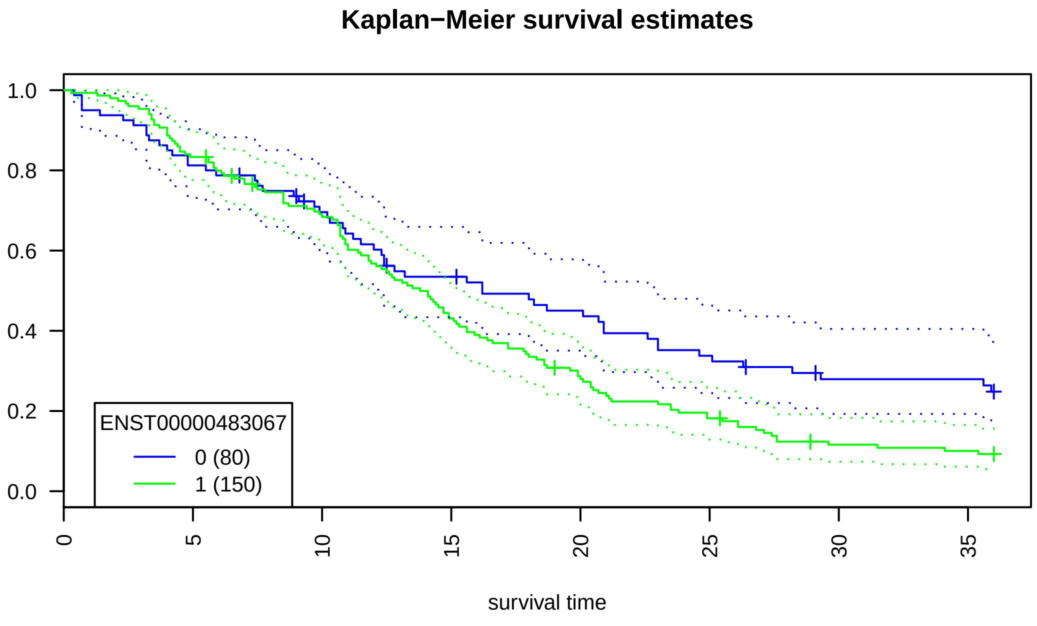

Figure 1323: Each curve represents the relative number of ENST00000483067 survived to the corresponding point of time. Dashed lines represent 0.95 confidence intervals for the curves with the corresponding color. Log-rank probability measure for the equality of these curves is 1.402725e-02. Additional annotations: *.GeneId*=ENSG00000135899, *.GeneName*=SP110, *.GeneDesc*=SP110 nuclear body protein [Source:HGNC Symbol;Acc:5401].

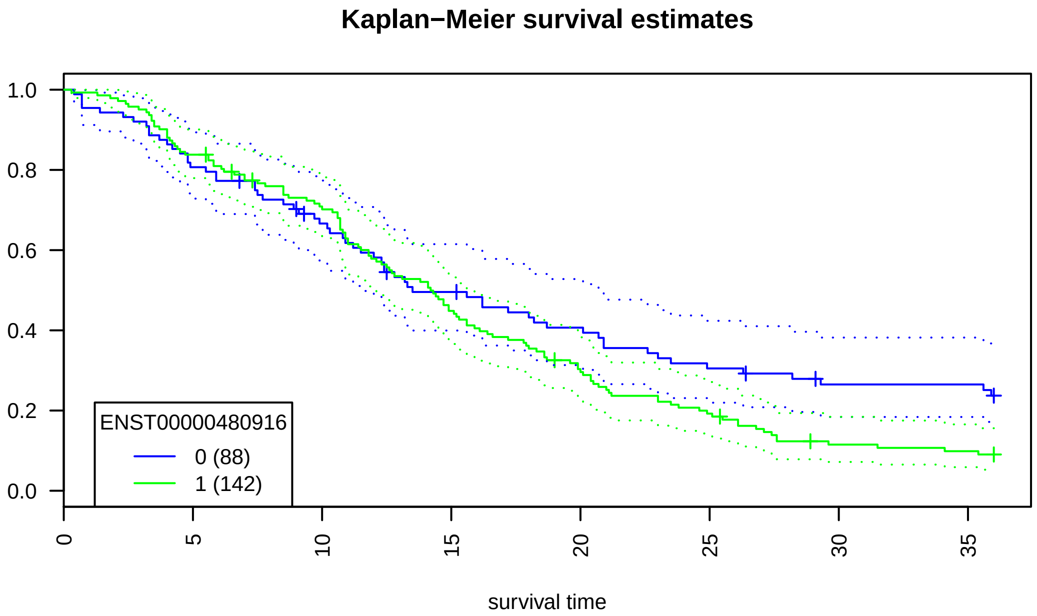

Figure 1324: Each curve represents the relative number of ENST00000480916 survived to the corresponding point of time. Dashed lines represent 0.95 confidence intervals for the curves with the corresponding color. Log-rank probability measure for the equality of these curves is 4.886543e-02. Additional annotations: *.GeneId*=ENSG00000135899, *.GeneName*=SP110, *.GeneDesc*=SP110 nuclear body protein [Source:HGNC Symbol;Acc:5401].

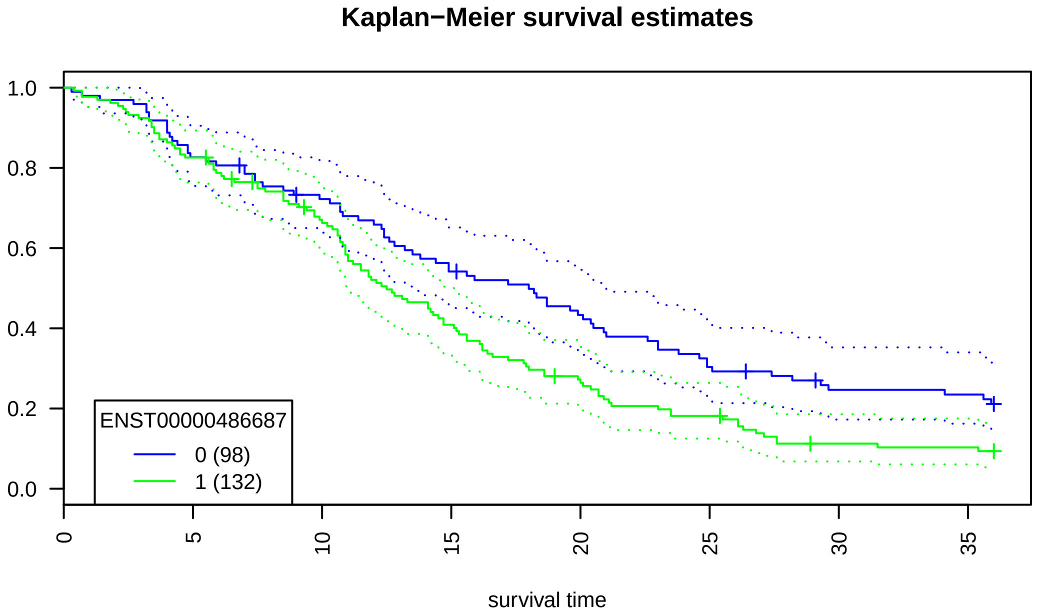

Figure 1325: Each curve represents the relative number of ENST00000486687 survived to the corresponding point of time. Dashed lines represent 0.95 confidence intervals for the curves with the corresponding color. Log-rank probability measure for the equality of these curves is 5.792757e-03. Additional annotations: *.GeneId*=ENSG00000079263, *.GeneName*=SP140, *.GeneDesc*=SP140 nuclear body protein [Source:HGNC Symbol;Acc:17133].

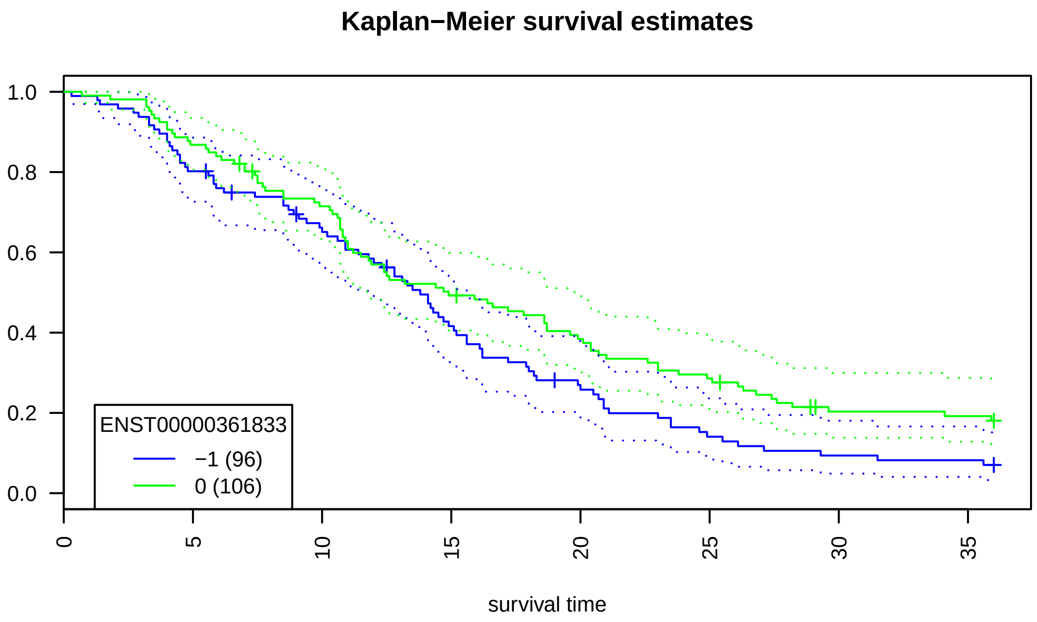

Figure 1326: Each curve represents the relative number of ENST00000361833 survived to the corresponding point of time. Dashed lines represent 0.95 confidence intervals for the curves with the corresponding color. Total of 28 observations deleted due to missingness. Log-rank probability measure for the equality of these curves is 2.818560e-02. Additional annotations: *.GeneId*=ENSG00000152092, *.GeneName*=ASTN1, *.GeneDesc*=astrotactin 1 [Source:HGNC Symbol;Acc:773].

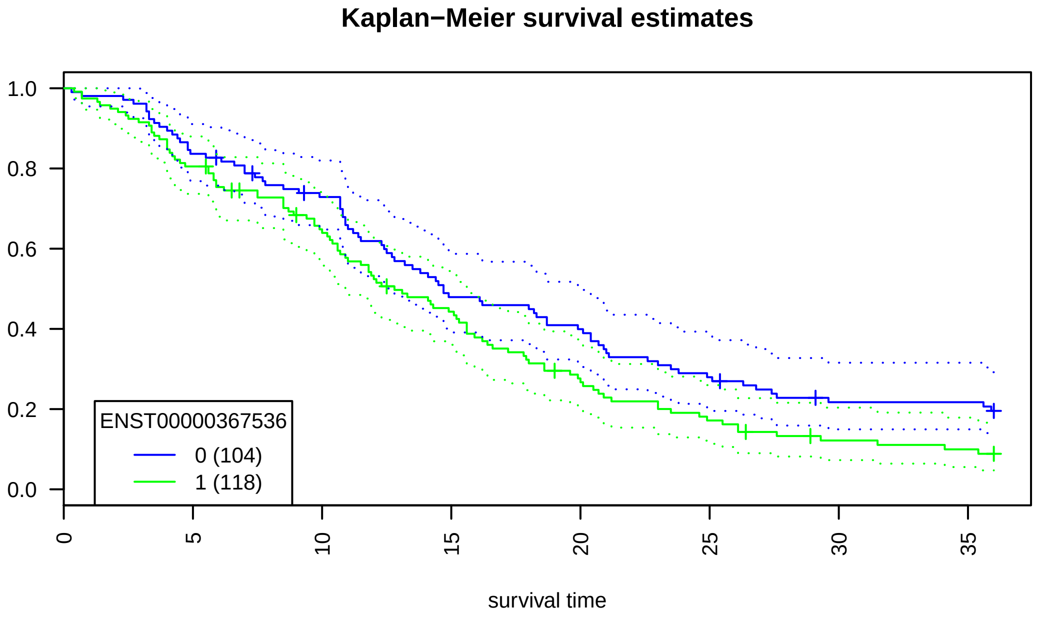

Figure 1327: Each curve represents the relative number of ENST00000367536 survived to the corresponding point of time. Dashed lines represent 0.95 confidence intervals for the curves with the corresponding color. Total of 8 observations deleted due to missingness. Log-rank probability measure for the equality of these curves is 2.803976e-02. Additional annotations: *.GeneId*=ENSG00000116701, *.GeneName*=NCF2, *.GeneDesc*=neutrophil cytosolic factor 2 [Source:HGNC Symbol;Acc:7661].

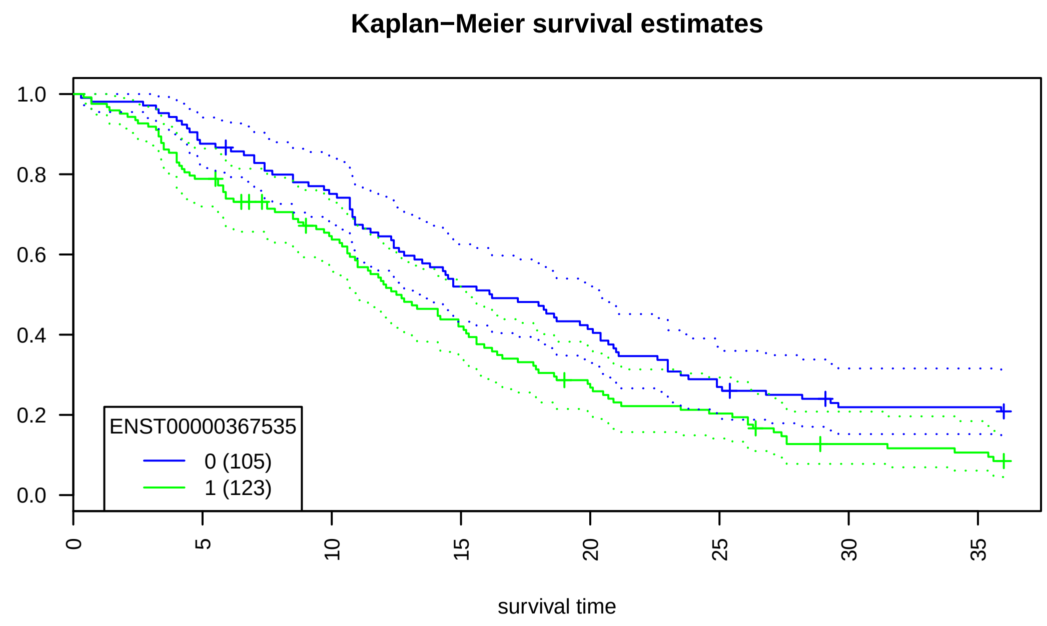

Figure 1328: Each curve represents the relative number of ENST00000367535 survived to the corresponding point of time. Dashed lines represent 0.95 confidence intervals for the curves with the corresponding color. Total of 2 observations deleted due to missingness. Log-rank probability measure for the equality of these curves is 8.126513e-03. Additional annotations: *.GeneId*=ENSG00000116701, *.GeneName*=NCF2, *.GeneDesc*=neutrophil cytosolic factor 2 [Source:HGNC Symbol;Acc:7661].

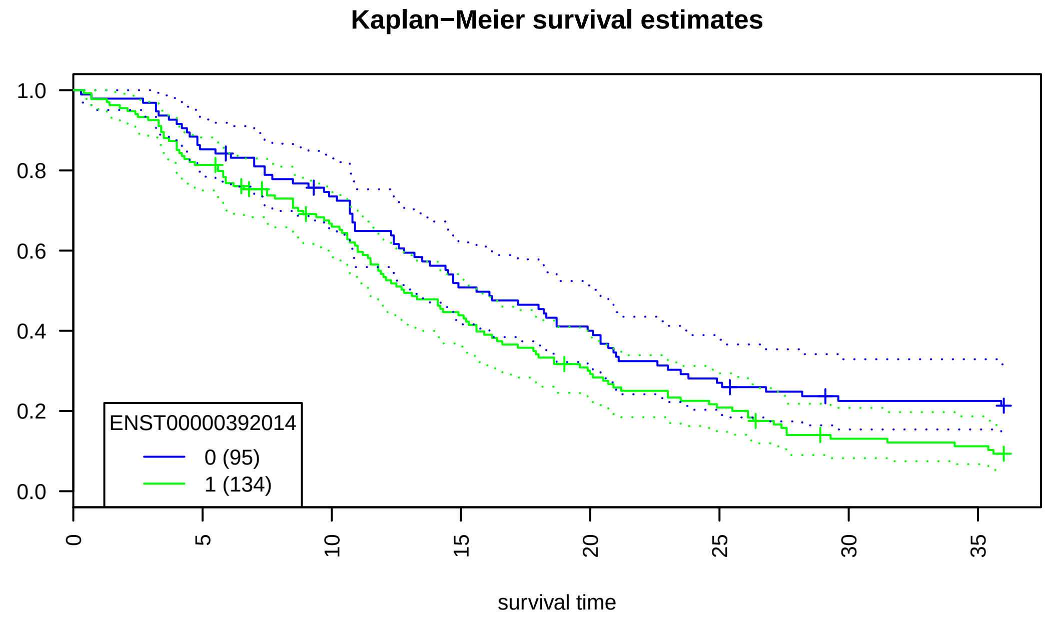

Figure 1329: Each curve represents the relative number of ENST00000392014 survived to the corresponding point of time. Dashed lines represent 0.95 confidence intervals for the curves with the corresponding color. Total of 1 observation deleted due to missingness. Log-rank probability measure for the equality of these curves is 3.245182e-02. Additional annotations: *.GeneId*=ENSG00000116701, *.GeneName*=NCF2, *.GeneDesc*=neutrophil cytosolic factor 2 [Source:HGNC Symbol;Acc:7661].

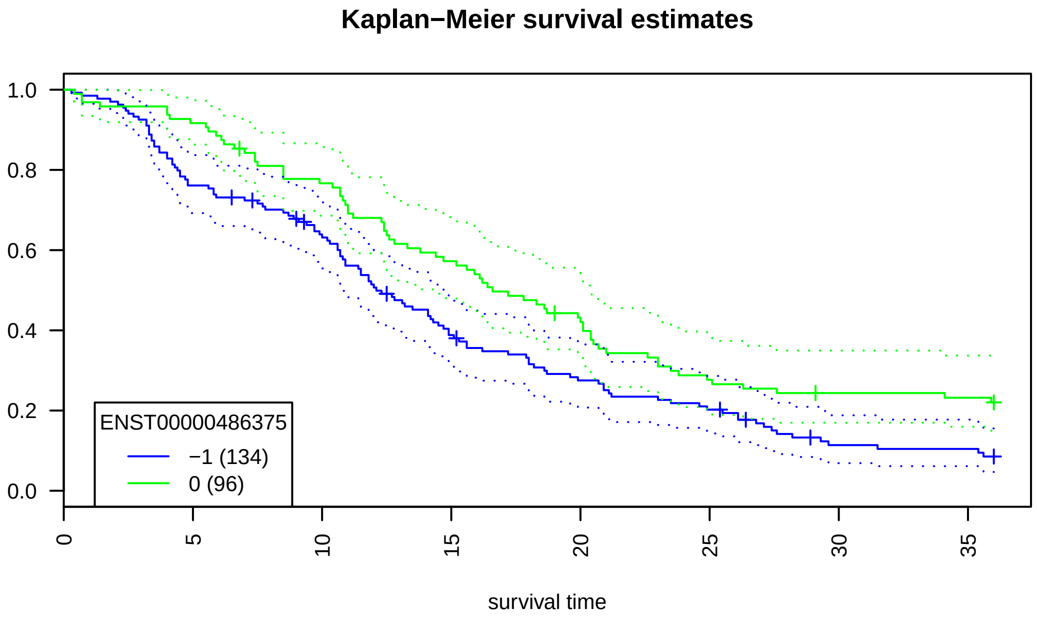

Figure 1330: Each curve represents the relative number of ENST00000486375 survived to the corresponding point of time. Dashed lines represent 0.95 confidence intervals for the curves with the corresponding color. Log-rank probability measure for the equality of these curves is 3.993083e-03. Additional annotations: .*GeneId*=ENSG00000198756, .*GeneName*=GLT25D2, .*GeneDesc*=glycosyltransferase 25 domain containing 2 [Source:HGNC Symbol;Acc:16790].

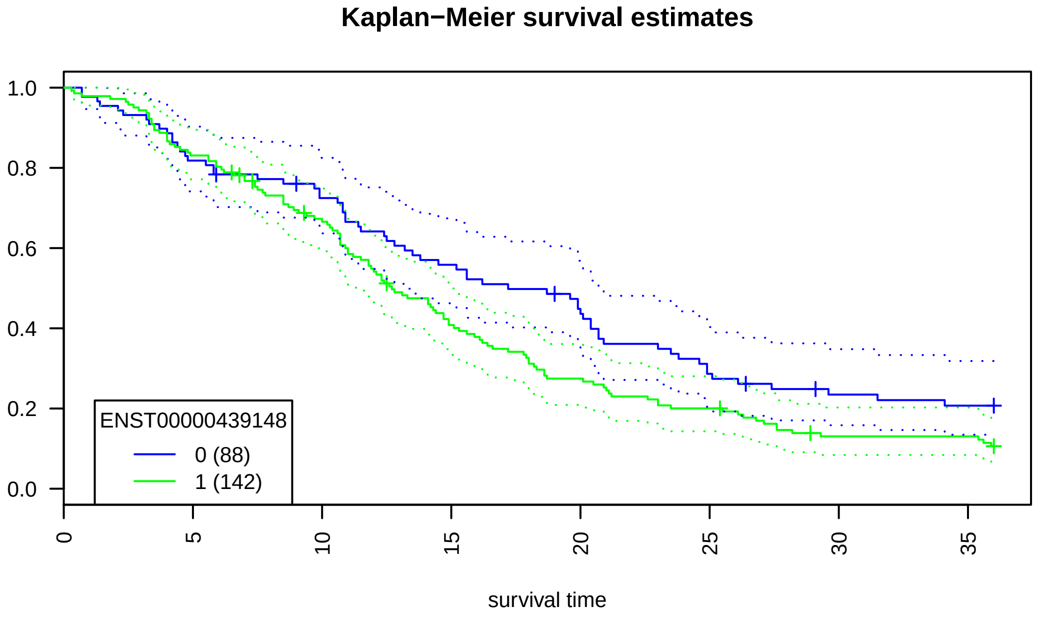

Figure 1331: Each curve represents the relative number of ENST00000439148 survived to the corresponding point of time. Dashed lines represent 0.95 confidence intervals for the curves with the corresponding color. Log-rank probability measure for the equality of these curves is 2.722888e-02. Additional annotations: .*GeneId*=ENSG00000144481, .*GeneName*=TRPM8, .*GeneDesc*=transient receptor potential cation channel, subfamily M, member 8 [Source:HGNC Symbol;Acc:17961].

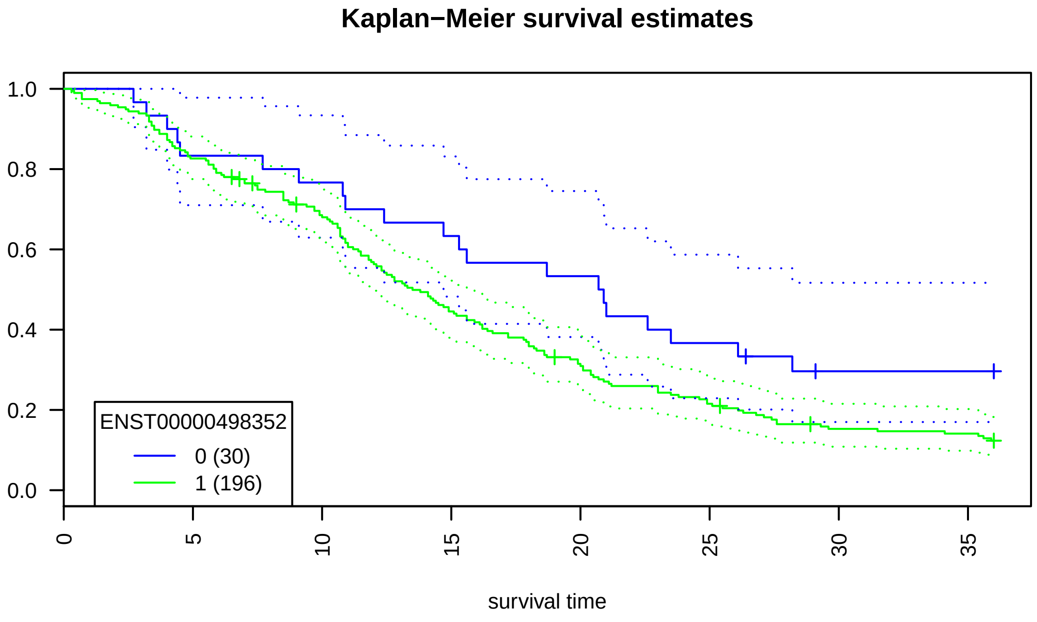

Figure 1332: Each curve represents the relative number of ENST00000498352 survived to the corresponding point of time. Dashed lines represent 0.95 confidence intervals for the curves with the corresponding color. Total of 4 observations deleted due to missingness. Log-rank probability measure for the equality of these curves is 3.205179e-02. Additional annotations: *.GeneId*=ENSG00000090104, *.GeneName*=RGS1, *.GeneDesc*=regulator of G-protein signaling 1 [Source:HGNC Symbol;Acc:9991].

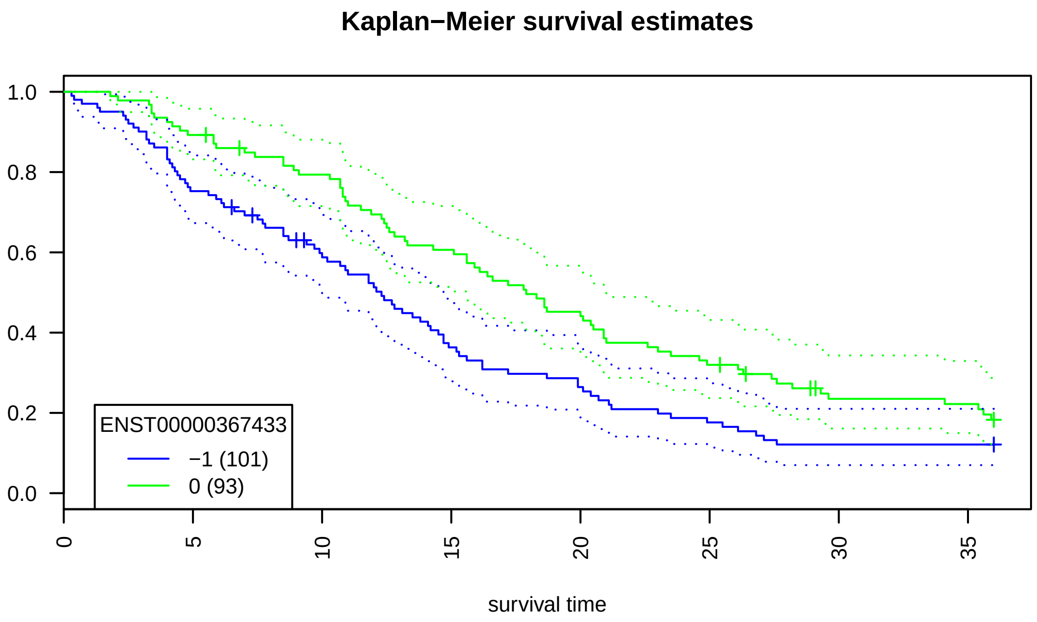

Figure 1333: Each curve represents the relative number of ENST00000367433 survived to the corresponding point of time. Dashed lines represent 0.95 confidence intervals for the curves with the corresponding color. Total of 36 observations deleted due to missingness. Log-rank probability measure for the equality of these curves is 5.56025e-03. Additional annotations: *.GeneId*=ENSG00000162687, *.GeneName*=KCNT2, *.GeneDesc*=potassium channel, subfamily T, member 2 [Source:HGNC Symbol;Acc:18866].

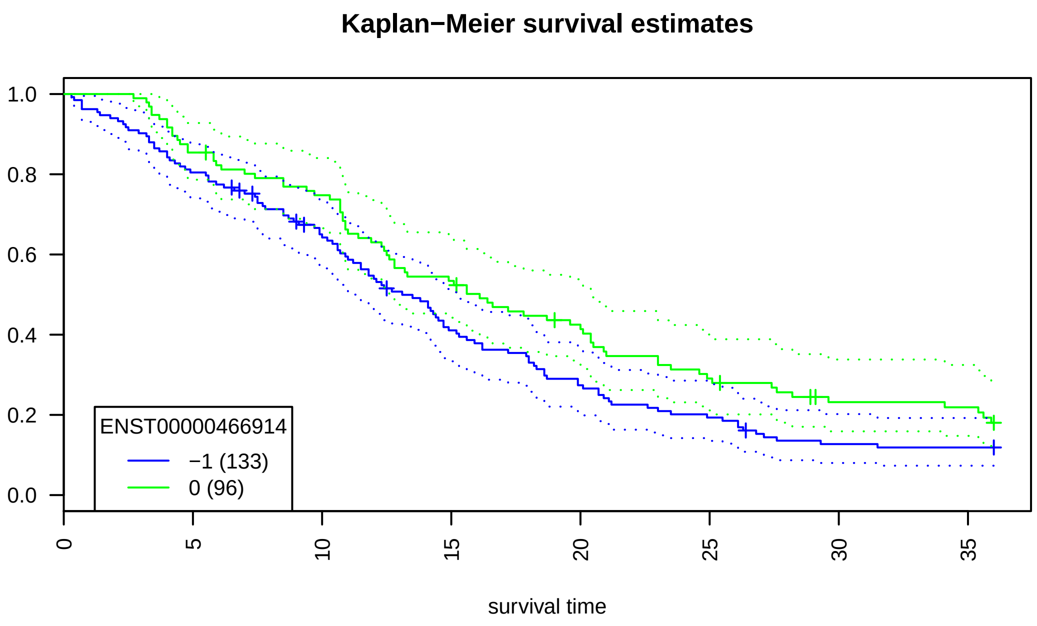

Figure 1334: Each curve represents the relative number of ENST00000466914 survived to the corresponding point of time. Dashed lines represent 0.95 confidence intervals for the curves with the corresponding color. Total of 1 observation deleted due to missingness. Log-rank probability measure for the equality of these curves is 4.374692e-02. Additional annotations: *.GeneId*=ENSG00000162687, *.GeneName*=KCNT2, *.GeneDesc*=potassium channel, subfamily T, member 2 [Source:HGNC Symbol;Acc:18866].

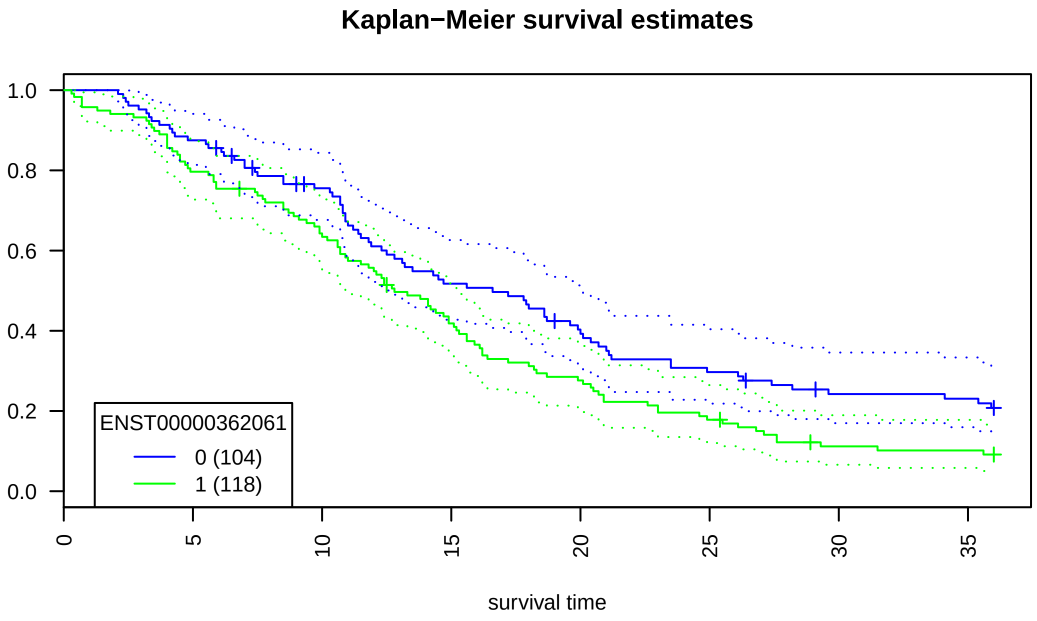

Figure 1335: Each curve represents the relative number of ENST00000362061 survived to the corresponding point of time. Dashed lines represent 0.95 confidence intervals for the curves with the corresponding color. Total of 8 observations deleted due to missingness. Log-rank probability measure for the equality of these curves is 1.194543e-02. Additional annotations: *.GeneId*=ENSG00000081248, *.GeneName*=CACNA1S, *.GeneDesc*=calcium channel, voltage-dependent, L type, alpha 1S subunit [Source:HGNC Symbol;Acc:1397].

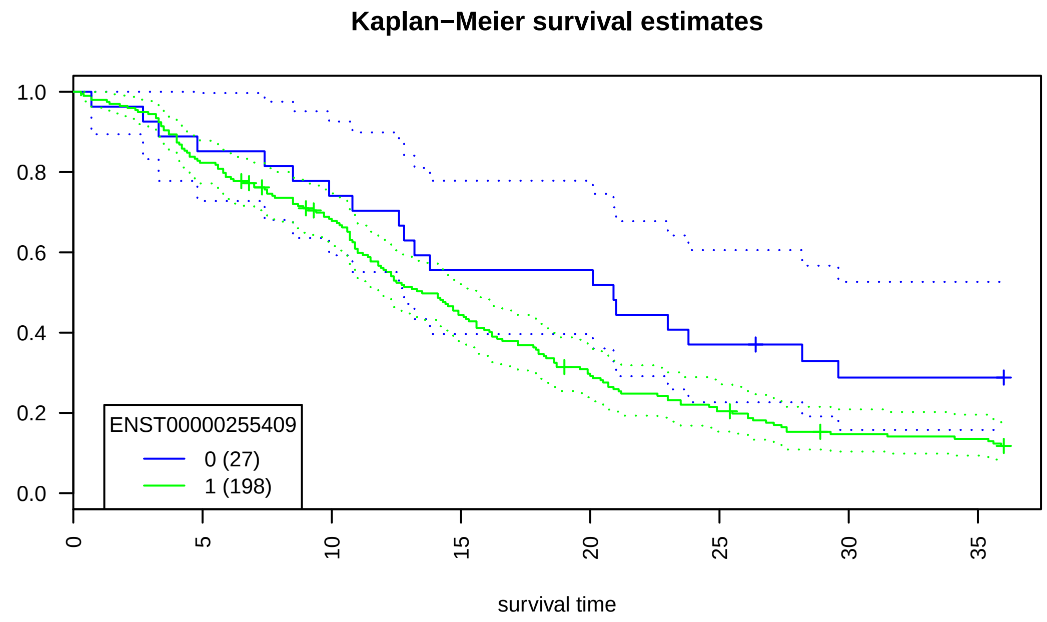

Figure 1336: Each curve represents the relative number of ENST00000255409 survived to the corresponding point of time. Dashed lines represent 0.95 confidence intervals for the curves with the corresponding color. Total of 5 observations deleted due to missingness. Log-rank probability measure for the equality of these curves is 3.398897e-02. Additional annotations: *.GeneId*=ENSG00000133048, *.GeneName*=CHI3L1, *.GeneDesc*=chitinase 3-like 1 (cartilage glycoprotein-39) [Source:HGNC Symbol;Acc:1932].

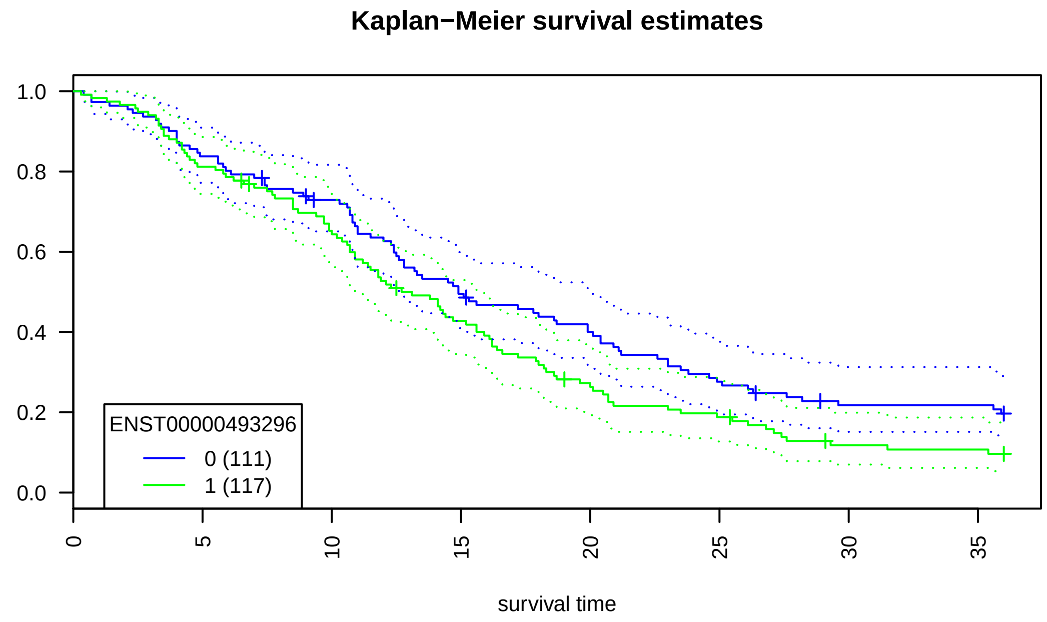

Figure 1337: Each curve represents the relative number of ENST00000493296 survived to the corresponding point of time. Dashed lines represent 0.95 confidence intervals for the curves with the corresponding color. Total of 2 observations deleted due to missingness. Log-rank probability measure for the equality of these curves is 3.740611e-02. Additional annotations: *.GeneId*=ENSG00000122176, *.GeneName*=FMOD, *.GeneDesc*=fibromodulin [Source:HGNC Symbol;Acc:3774].

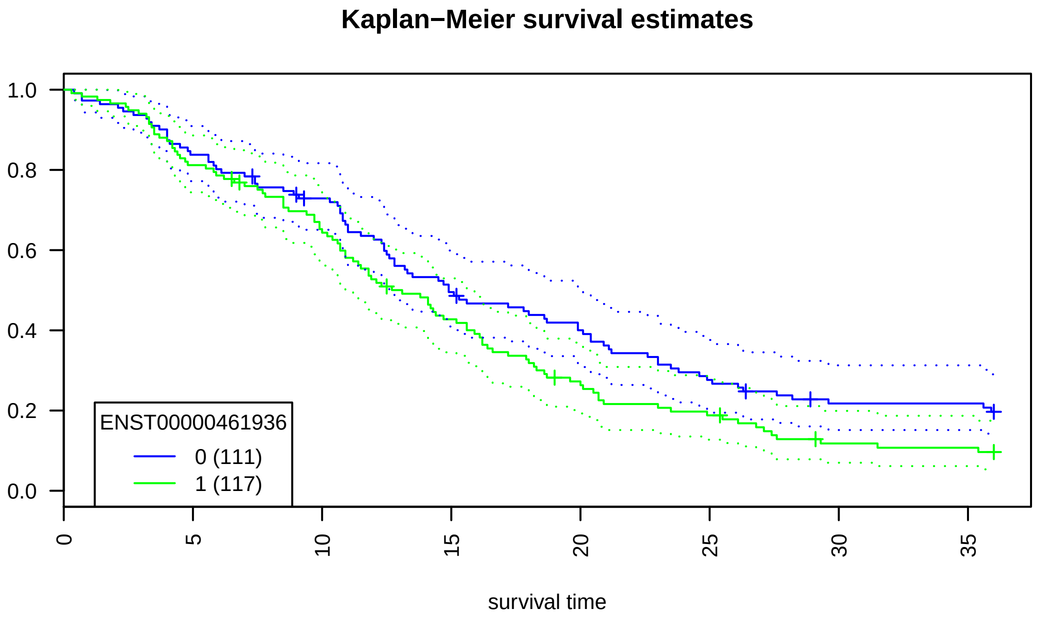

Figure 1338: Each curve represents the relative number of ENST00000461936 survived to the corresponding point of time. Dashed lines represent 0.95 confidence intervals for the curves with the corresponding color. Total of 2 observations deleted due to missingness. Log-rank probability measure for the equality of these curves is 3.740611e-02. Additional annotations: *.GeneId*=ENSG00000122176, *.GeneName*=FMOD, *.GeneDesc*=fibromodulin [Source:HGNC Symbol;Acc:3774].

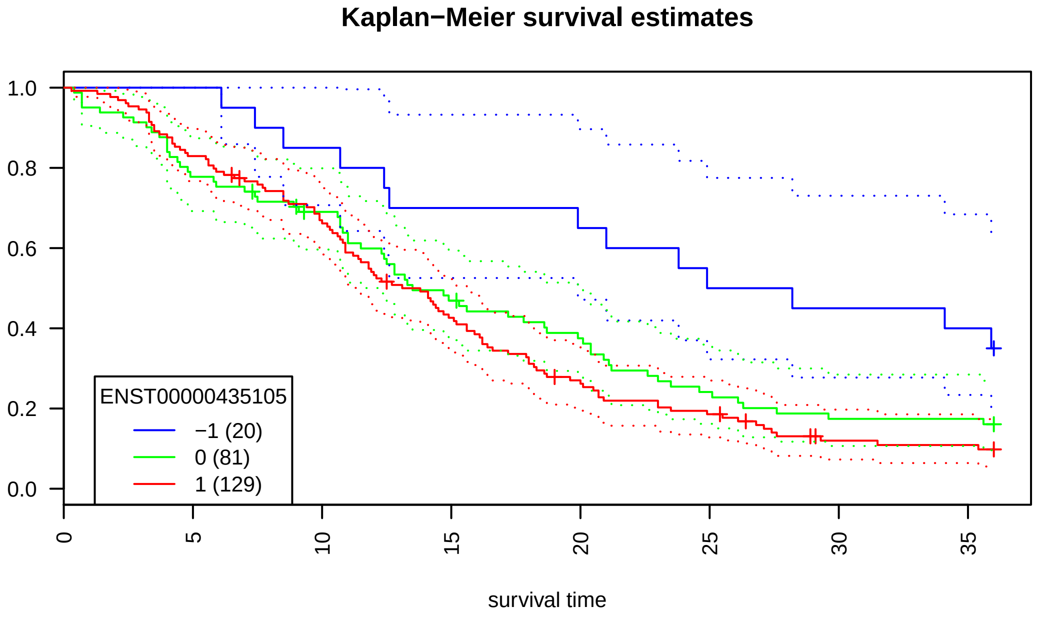

Figure 1339: Each curve represents the relative number of ENST00000435105 survived to the corresponding point of time. Dashed lines represent 0.95 confidence intervals for the curves with the corresponding color. Log-rank probability measure for the equality of these curves is 8.429941e-03. Additional annotations: *.GeneId*=ENSG00000122176, *.GeneName*=FMOD, *.GeneDesc*=fibromodulin [Source:HGNC Symbol;Acc:3774].

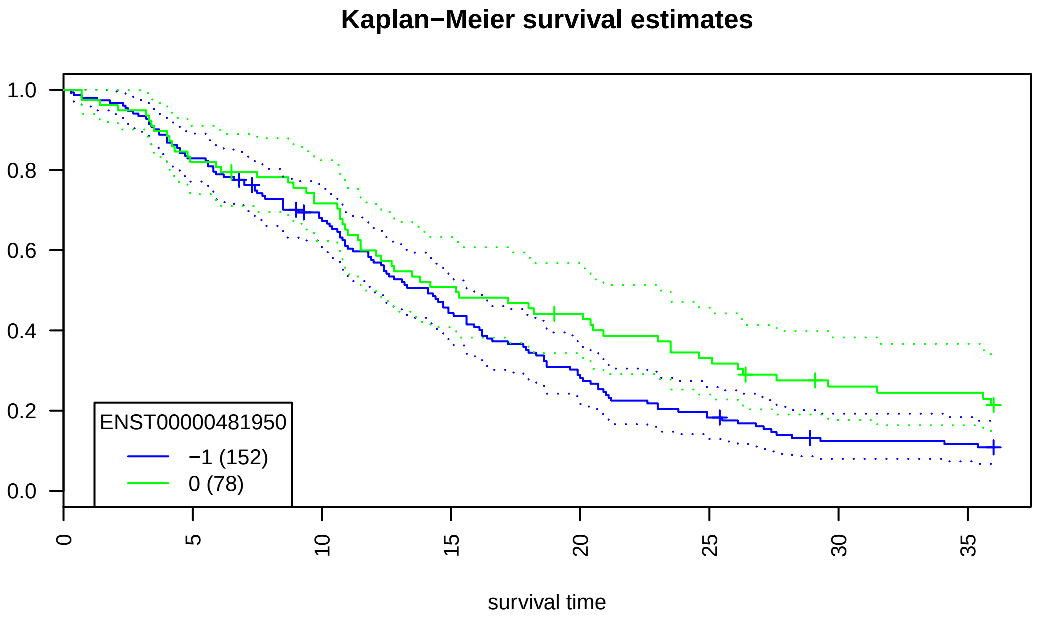

Figure 1340: Each curve represents the relative number of ENST00000481950 survived to the corresponding point of time. Dashed lines represent 0.95 confidence intervals for the curves with the corresponding color. Log-rank probability measure for the equality of these curves is 4.125127e-02. Additional annotations: *.GeneId*=ENSG00000133069, *.GeneName*=TMCC2, *.GeneDesc*=transmembrane and coiled-coil domain family 2 [Source:HGNC Symbol;Acc:24239].

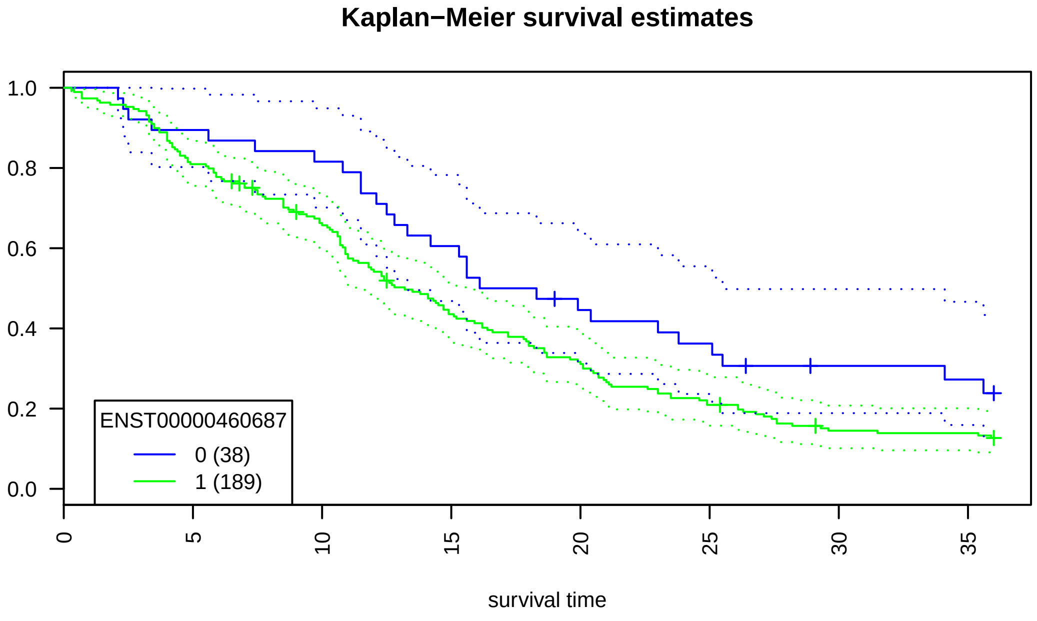

Figure 1341: Each curve represents the relative number of ENST00000460687 survived to the corresponding point of time. Dashed lines represent 0.95 confidence intervals for the curves with the corresponding color. Total of 3 observations deleted due to missingness. Log-rank probability measure for the equality of these curves is 4.45988e-02. Additional annotations: *.GeneId*=ENSG00000162873, *.GeneName*=KLHDC8A, *.GeneDesc*=kelch domain containing 8A [Source:HGNC Symbol;Acc:25573].

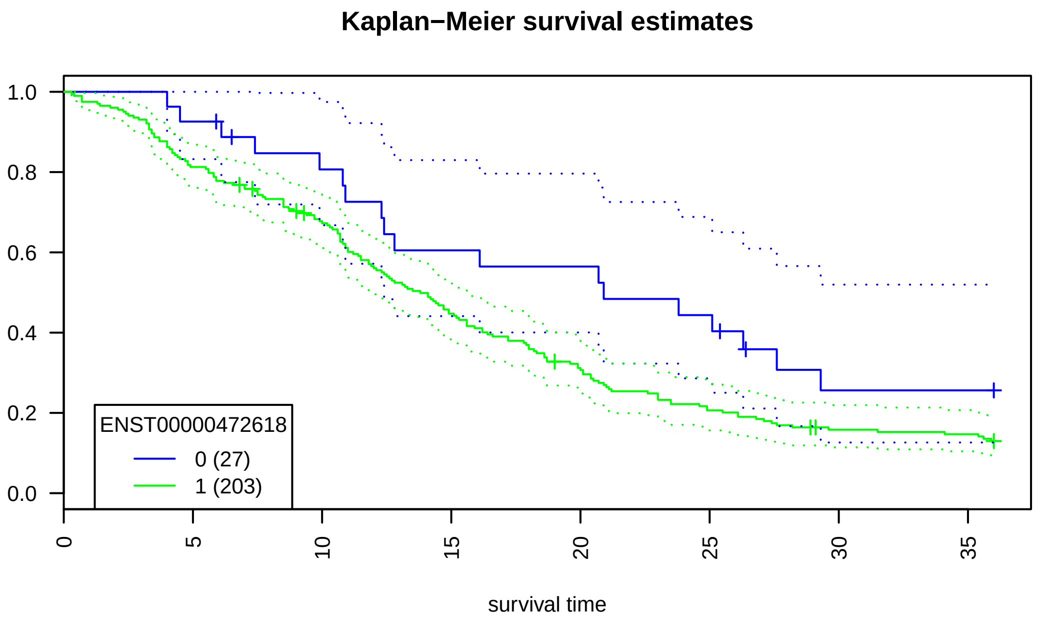

Figure 1342: Each curve represents the relative number of ENST00000472618 survived to the corresponding point of time. Dashed lines represent 0.95 confidence intervals for the curves with the corresponding color. Log-rank probability measure for the equality of these curves is 4.059585e-02. Additional annotations: *.GeneId*=ENSG00000168393, *.GeneName*=DTYMK, *.GeneDesc*=deoxythymidylate kinase (thymidylate kinase) [Source:HGNC Symbol;Acc:3061].

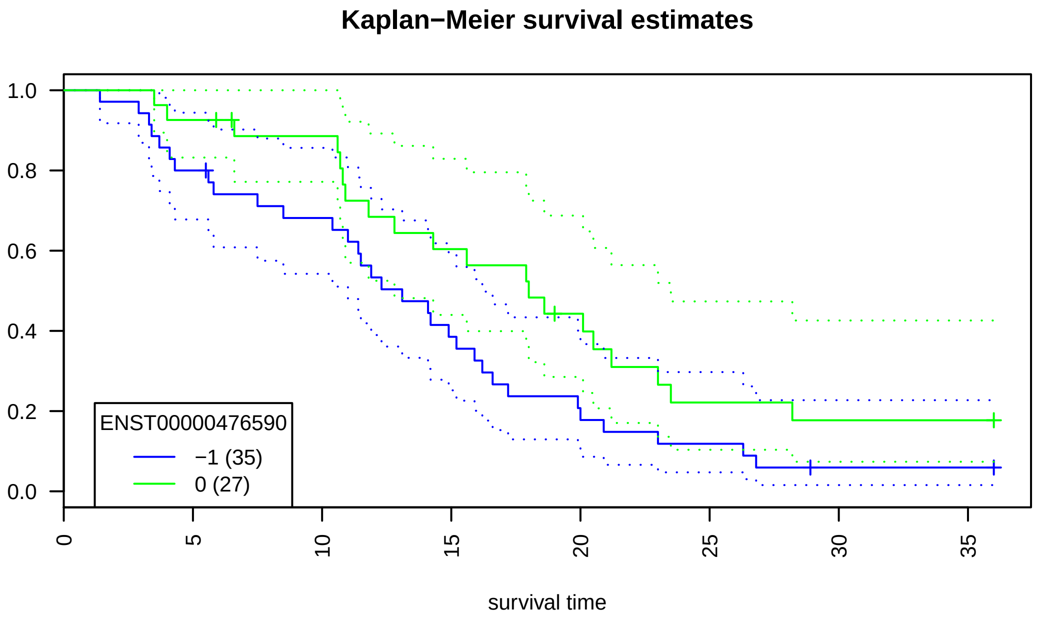

Figure 1343: Each curve represents the relative number of ENST00000476590 survived to the corresponding point of time. Dashed lines represent 0.95 confidence intervals for the curves with the corresponding color. Total of 168 observations deleted due to missingness. Log-rank probability measure for the equality of these curves is 4.232893e-02. Additional annotations: *.GeneId*=ENSG00000196352, *.GeneName*=CD55, *.GeneDesc*=CD55 molecule, decay accelerating factor for complement (Cromer blood group) [Source:HGNC Symbol;Acc:2665].

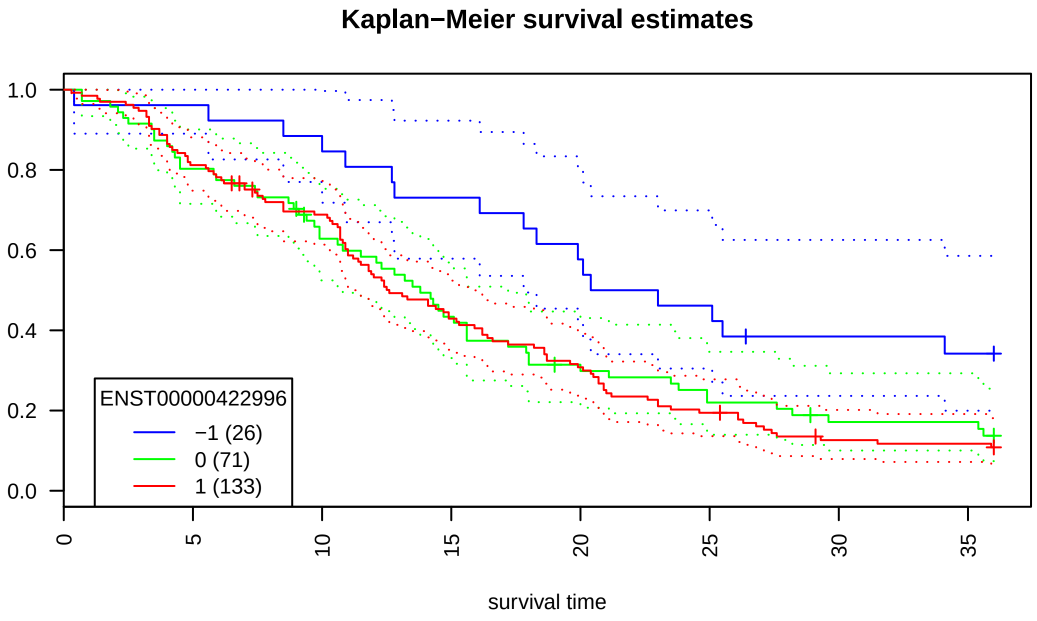

Figure 1344: Each curve represents the relative number of ENST00000422996 survived to the corresponding point of time. Dashed lines represent 0.95 confidence intervals for the curves with the corresponding color. Log-rank probability measure for the equality of these curves is 1.438412e-02. Additional annotations: *.GeneId*=ENSG00000225649, *.GeneName*=AC064875.2, *.GeneDesc*=[undefined].

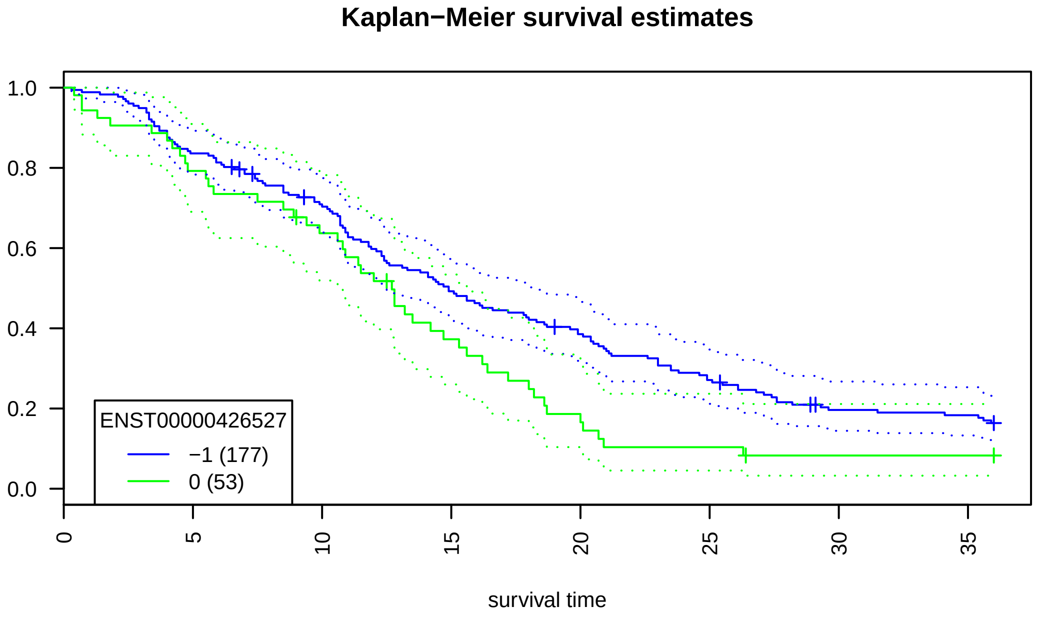

Figure 1345: Each curve represents the relative number of ENST00000426527 survived to the corresponding point of time. Dashed lines represent 0.95 confidence intervals for the curves with the corresponding color. Log-rank probability measure for the equality of these curves is 1.554613e-02. Additional annotations: *.GeneId*=ENSG00000223634, *.GeneName*=AC012506.3, *.GeneDesc*=[undefined].

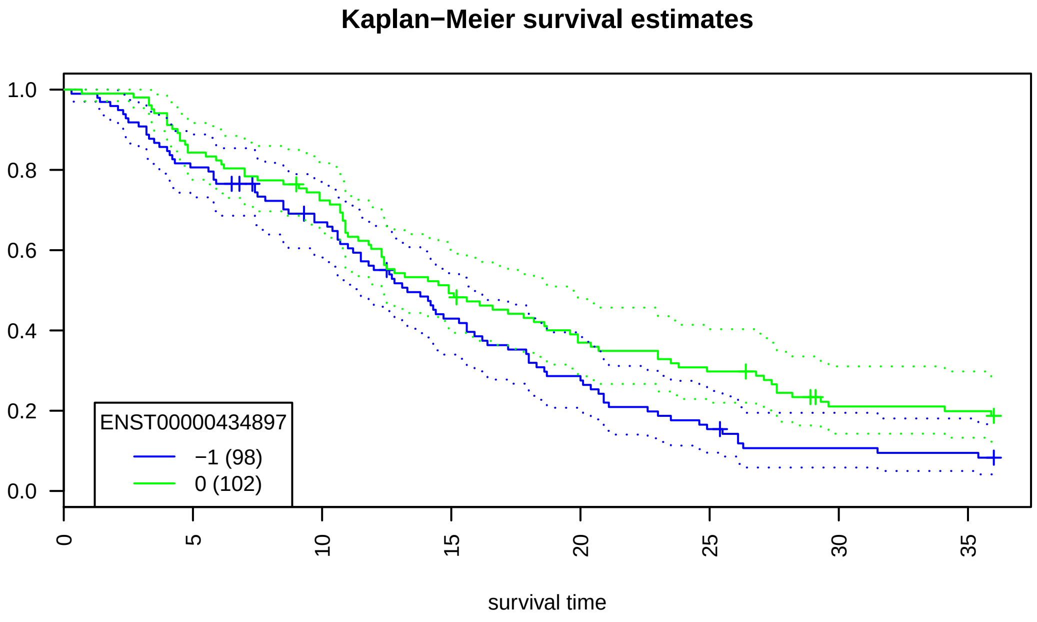

Figure 1346: Each curve represents the relative number of ENST00000434897 survived to the corresponding point of time. Dashed lines represent 0.95 confidence intervals for the curves with the corresponding color. Total of 30 observations deleted due to missingness. Log-rank probability measure for the equality of these curves is 2.823298e-02. Additional annotations: *.GeneId*=ENSG00000224165, *.GeneName*=AC013267.2, *.GeneDesc*=[undefined].

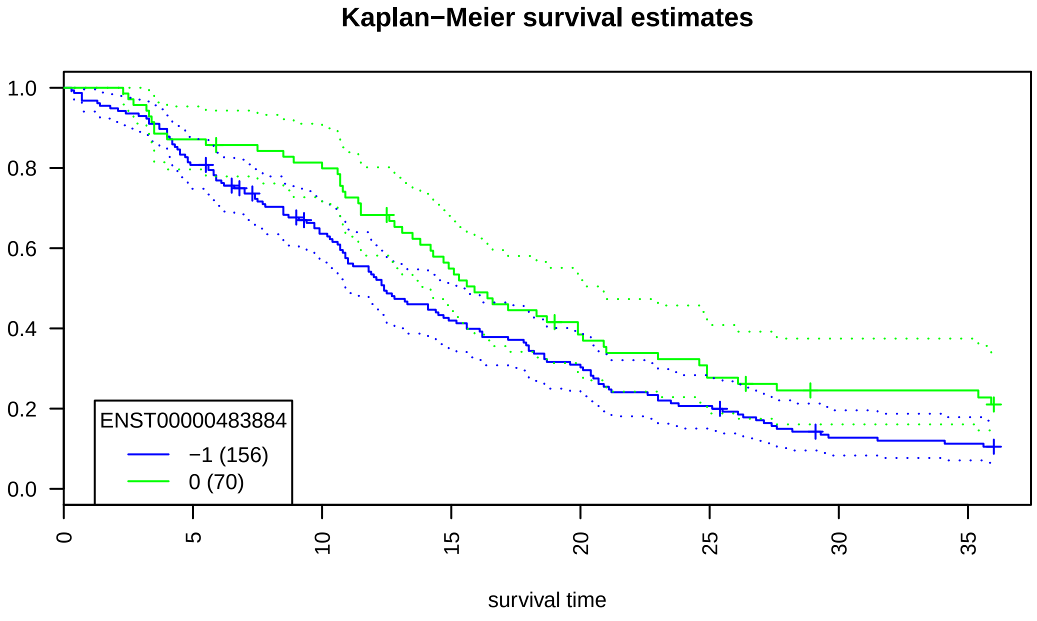

Figure 1347: Each curve represents the relative number of ENST00000483884 survived to the corresponding point of time. Dashed lines represent 0.95 confidence intervals for the curves with the corresponding color. Total of 4 observations deleted due to missingness. Log-rank probability measure for the equality of these curves is 2.619288e-02. Additional annotations: *.GeneId*=ENSG00000082497, *.GeneName*=SERTAD4, *.GeneDesc*=SERTA domain containing 4 [Source:HGNC Symbol;Acc:25236].

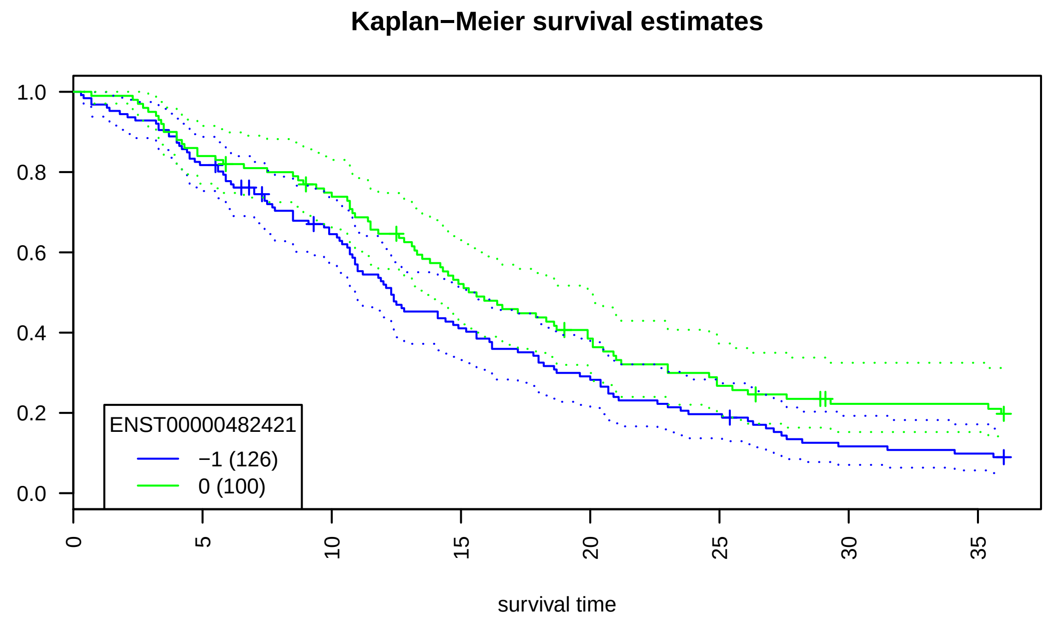

Figure 1348: Each curve represents the relative number of ENST00000482421 survived to the corresponding point of time. Dashed lines represent 0.95 confidence intervals for the curves with the corresponding color. Total of 4 observations deleted due to missingness. Log-rank probability measure for the equality of these curves is 2.191139e-02. Additional annotations: *.GeneId*=ENSG00000082497, *.GeneName*=SERTAD4, *.GeneDesc*=SERTA domain containing 4 [Source:HGNC Symbol;Acc:25236].

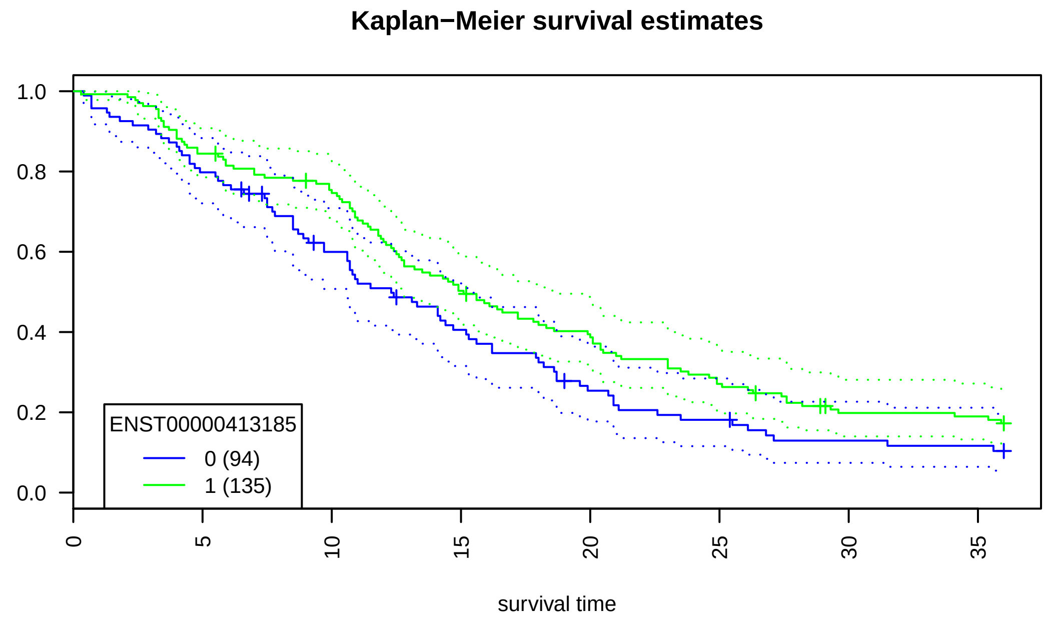

Figure 1349: Each curve represents the relative number of ENST00000413185 survived to the corresponding point of time. Dashed lines represent 0.95 confidence intervals for the curves with the corresponding color. Total of 1 observation deleted due to missingness. Log-rank probability measure for the equality of these curves is 3.528527e-02. Additional annotations: *.GeneId*=ENSG00000234690, *.GeneName*=AC073283.4, *.GeneDesc*=[undefined].

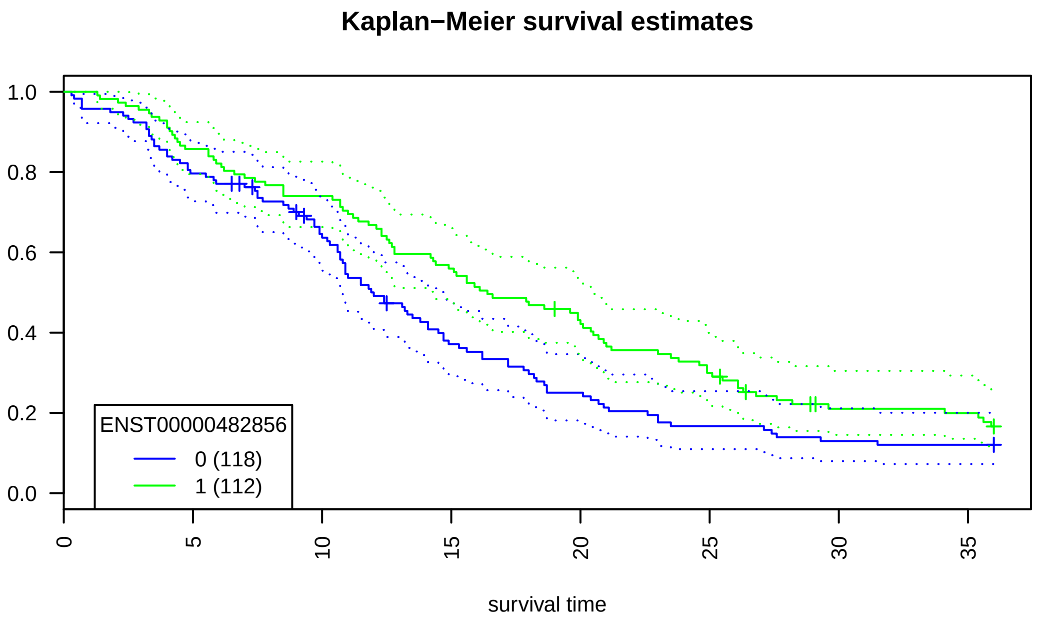

Figure 1350: Each curve represents the relative number of ENST00000482856 survived to the corresponding point of time. Dashed lines represent 0.95 confidence intervals for the curves with the corresponding color. Log-rank probability measure for the equality of these curves is 2.085306e-02. Additional annotations: *.GeneId*=ENSG00000154309, *.GeneName*=DISP1, *.GeneDesc*=dispatched homolog 1 (Drosophila) [Source:HGNC Symbol;Acc:19711].

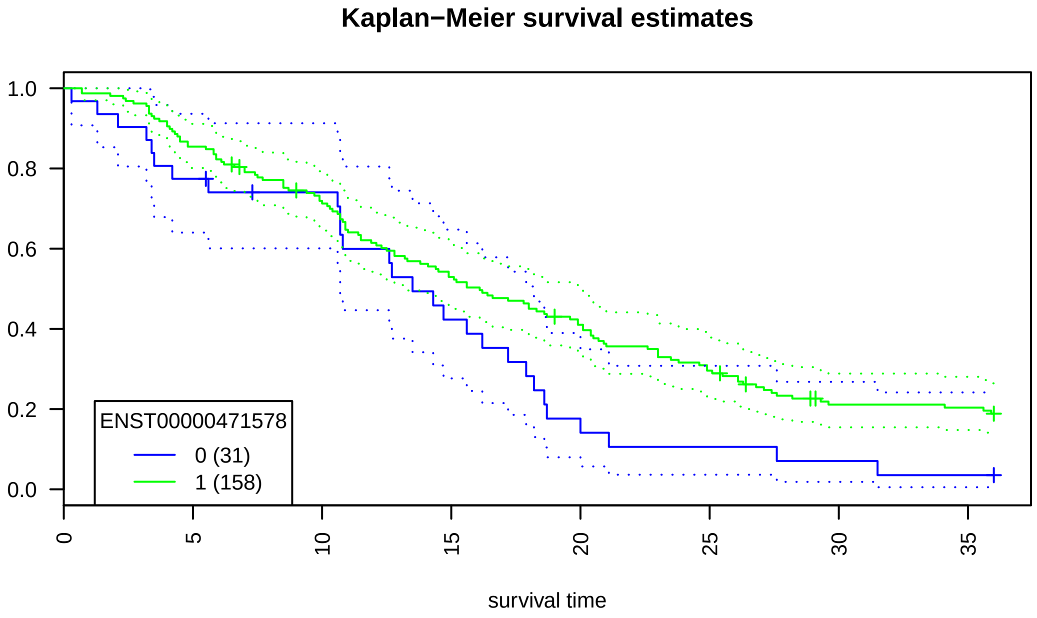

Figure 1351: Each curve represents the relative number of ENST00000471578 survived to the corresponding point of time. Dashed lines represent 0.95 confidence intervals for the curves with the corresponding color. Total of 41 observations deleted due to missingness. Log-rank probability measure for the equality of these curves is 1.735424e-02. Additional annotations: *.GeneId*=ENSG00000143786, *.GeneName*=CNIH3, *.GeneDesc*=cornichon homolog 3 (Drosophila) [Source:HGNC Symbol;Acc:26802].

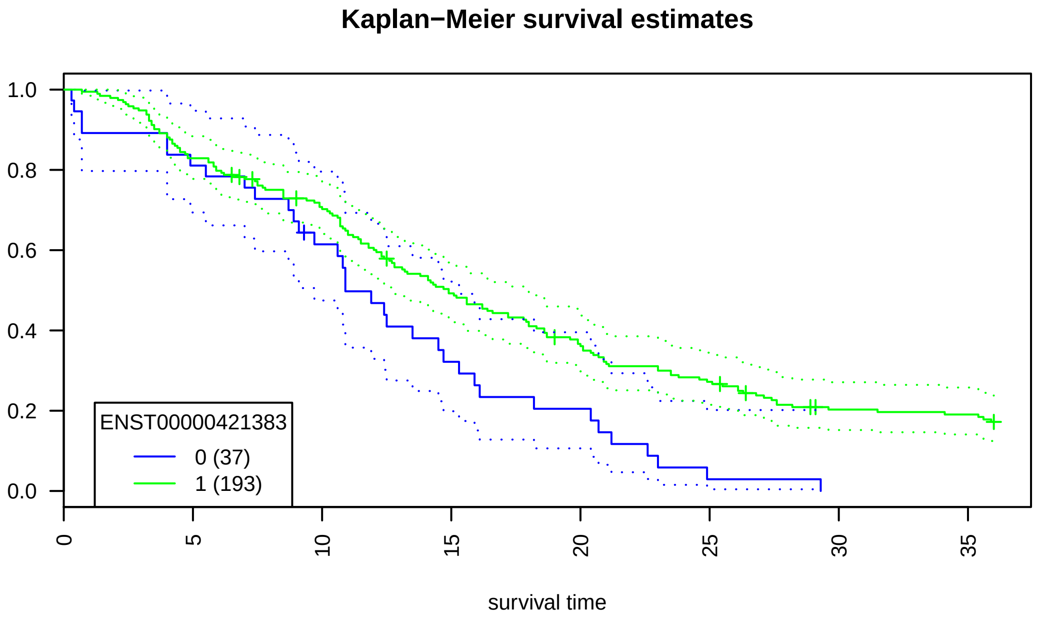

Figure 1352: Each curve represents the relative number of ENST00000421383 survived to the corresponding point of time. Dashed lines represent 0.95 confidence intervals for the curves with the corresponding color. Log-rank probability measure for the equality of these curves is 1.712460e-03. Additional annotations: *.GeneId*=ENSG00000143815, *.GeneName*=LBR, *.GeneDesc*=lamin B receptor [Source:HGNC Symbol;Acc:6518].

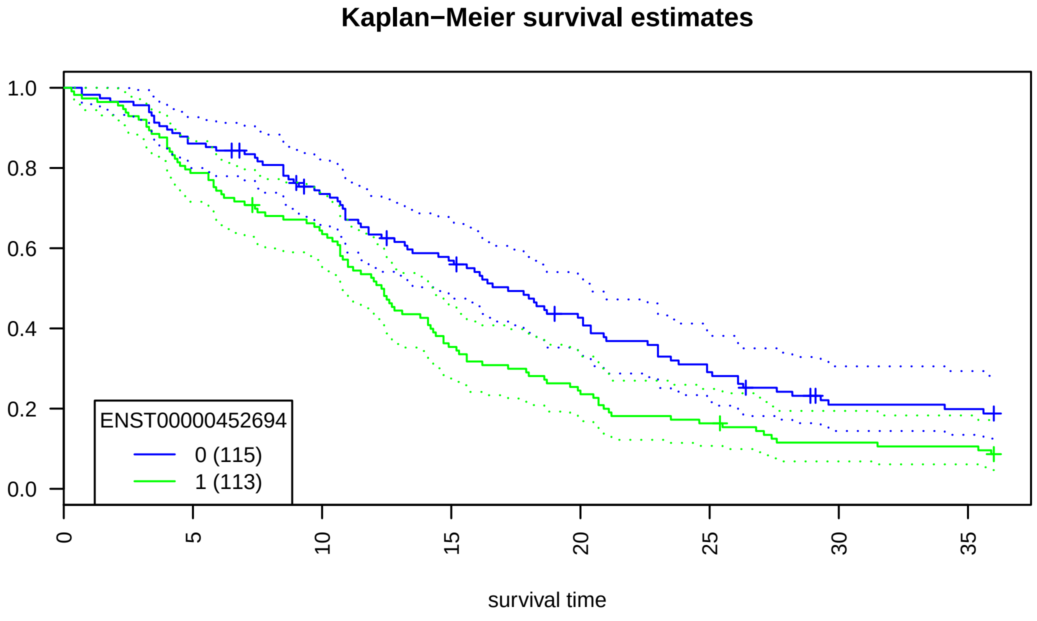

Figure 1353: Each curve represents the relative number of ENST00000452694 survived to the corresponding point of time. Dashed lines represent 0.95 confidence intervals for the curves with the corresponding color. Total of 2 observations deleted due to missingness. Log-rank probability measure for the equality of these curves is 2.826276e-03. Additional annotations: *.GeneId*=ENSG00000237288, *.GeneName*=AC133644.2, *.GeneDesc*=[undefined].

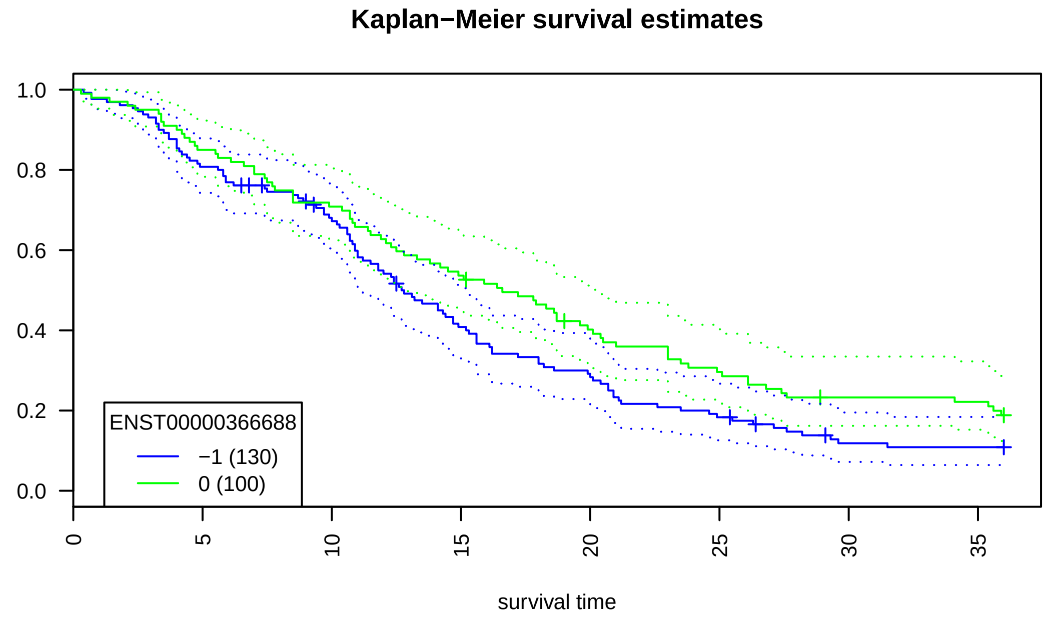

Figure 1354: Each curve represents the relative number of ENST00000366688 survived to the corresponding point of time. Dashed lines represent 0.95 confidence intervals for the curves with the corresponding color. Log-rank probability measure for the equality of these curves is 3.962687e-02. Additional annotations: *.GeneId*=ENSG00000213029, *.GeneName*=SPHAR, *.GeneDesc*=S-phase response (cyclin related) [Source:HGNC Symbol;Acc:16957].

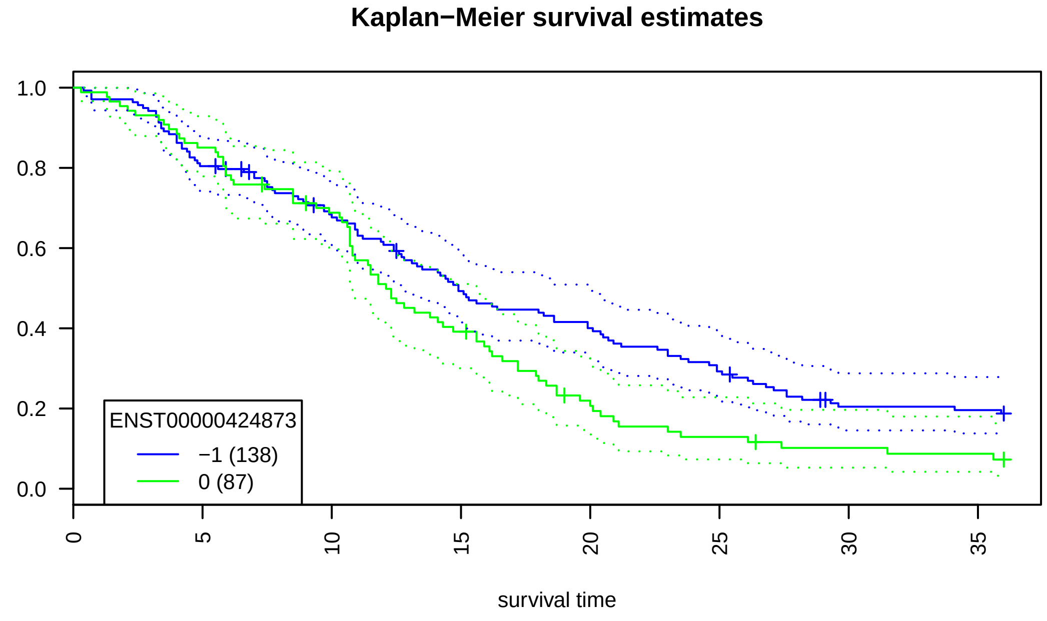

Figure 1355: Each curve represents the relative number of ENST00000424873 survived to the corresponding point of time. Dashed lines represent 0.95 confidence intervals for the curves with the corresponding color. Total of 5 observations deleted due to missingness. Log-rank probability measure for the equality of these curves is 1.042905e-02. Additional annotations: *.GeneId*=ENSG00000178162, *.GeneName*=AC140481.1, *.GeneDesc*=[undefined].

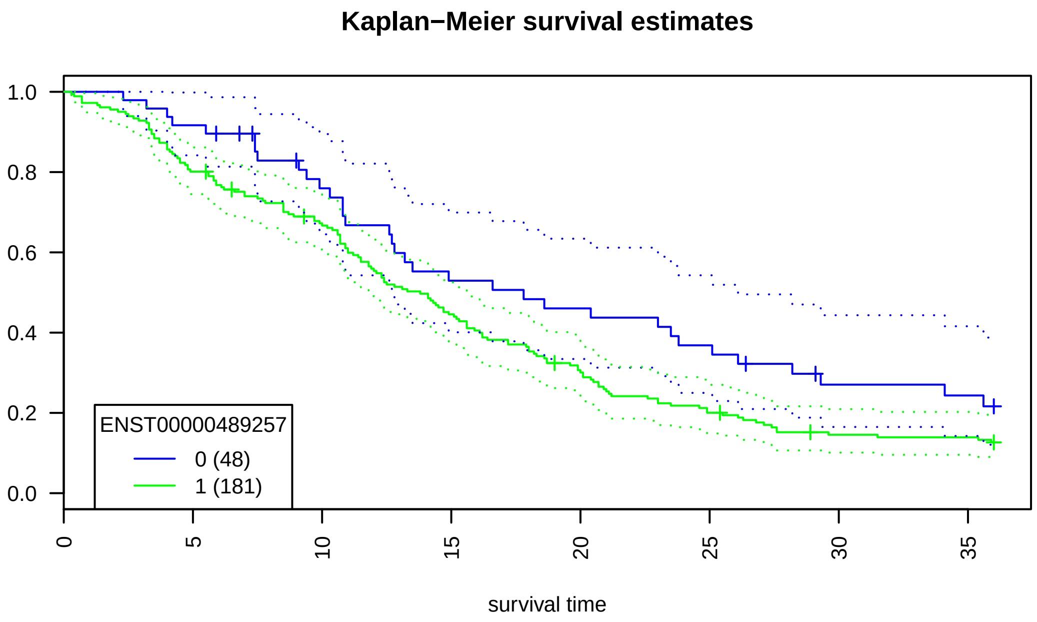

Figure 1356: Each curve represents the relative number of ENST00000489257 survived to the corresponding point of time. Dashed lines represent 0.95 confidence intervals for the curves with the corresponding color. Total of 1 observation deleted due to missingness. Log-rank probability measure for the equality of these curves is 4.337221e-02. Additional annotations: *.GeneId*=ENSG00000244020, *.GeneName*=MT1P2, *.GeneDesc*=metallothionein 1 pseudogene 2 [Source:HGNC Symbol;Acc:31864].

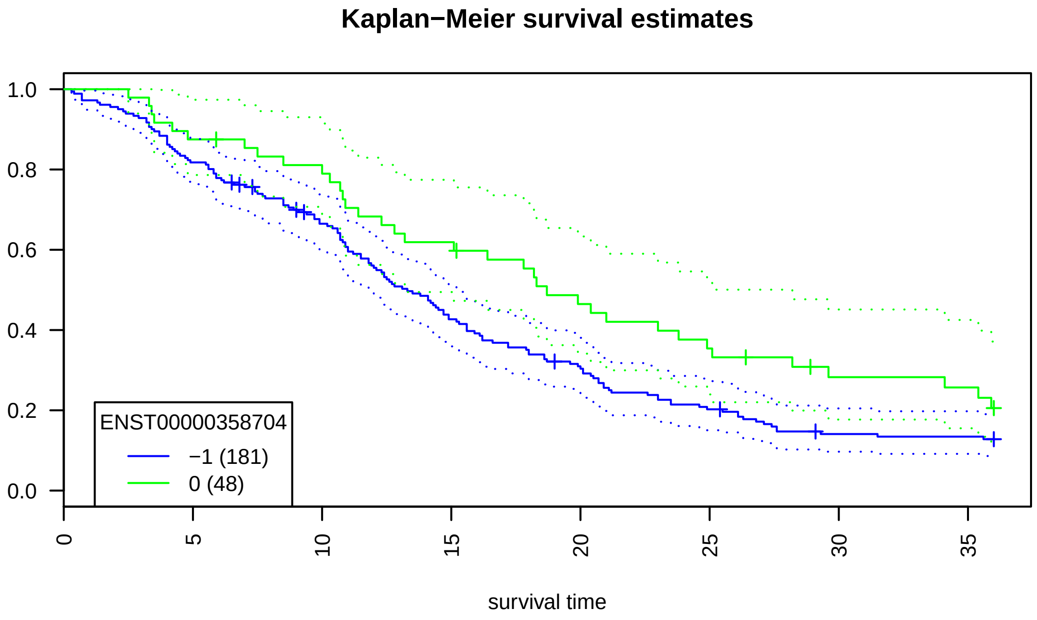

Figure 1357: Each curve represents the relative number of ENST00000358704 survived to the corresponding point of time. Dashed lines represent 0.95 confidence intervals for the curves with the corresponding color. Total of 1 observation deleted due to missingness. Log-rank probability measure for the equality of these curves is 3.011498e-02. Additional annotations: *.GeneId*=ENSG00000179456, *.GeneName*=ZNF238, *.GeneDesc*=zinc finger protein 238 [Source:HGNC Symbol;Acc:13030].

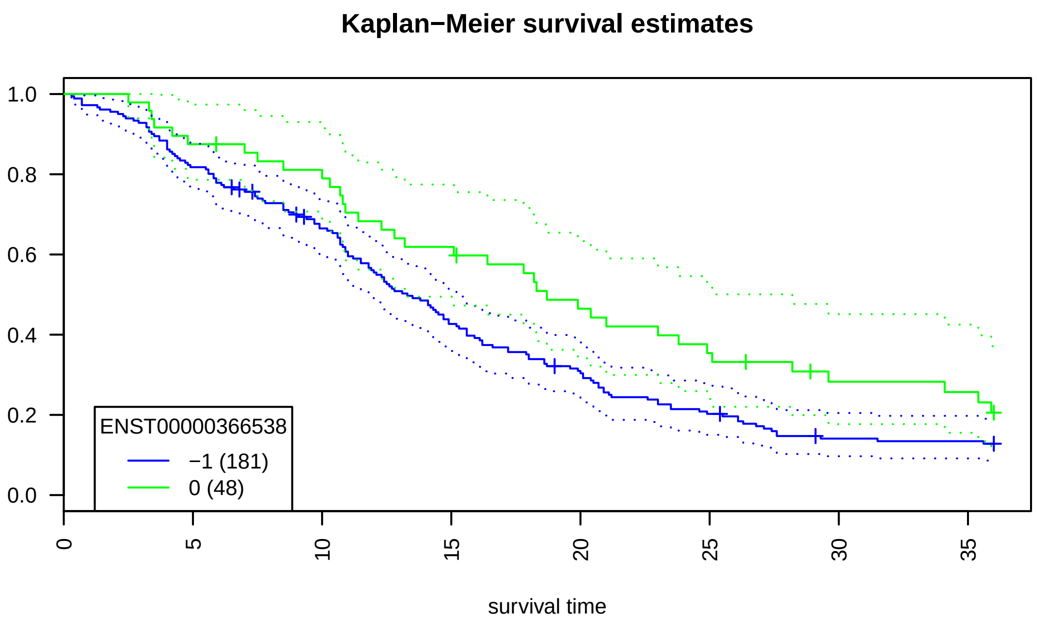

Figure 1358: Each curve represents the relative number of ENST00000366538 survived to the corresponding point of time. Dashed lines represent 0.95 confidence intervals for the curves with the corresponding color. Total of 1 observation deleted due to missingness. Log-rank probability measure for the equality of these curves is 3.011498e-02. Additional annotations: *.GeneId*=ENSG00000179456, *.GeneName*=ZNF238, *.GeneDesc*=zinc finger protein 238 [Source:HGNC Symbol;Acc:13030].

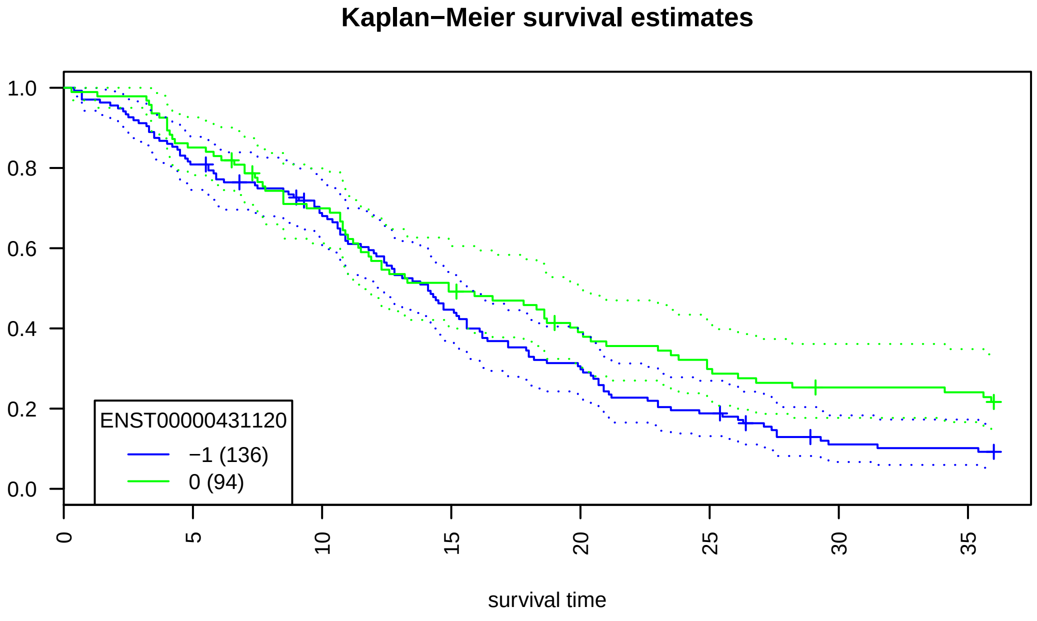

Figure 1359: Each curve represents the relative number of ENST00000431120 survived to the corresponding point of time. Dashed lines represent 0.95 confidence intervals for the curves with the corresponding color. Log-rank probability measure for the equality of these curves is 3.791588e-02. Additional annotations: *.GeneId*=ENSG00000237580, *.GeneName*=AC007383.5, *.GeneDesc*=[undefined].

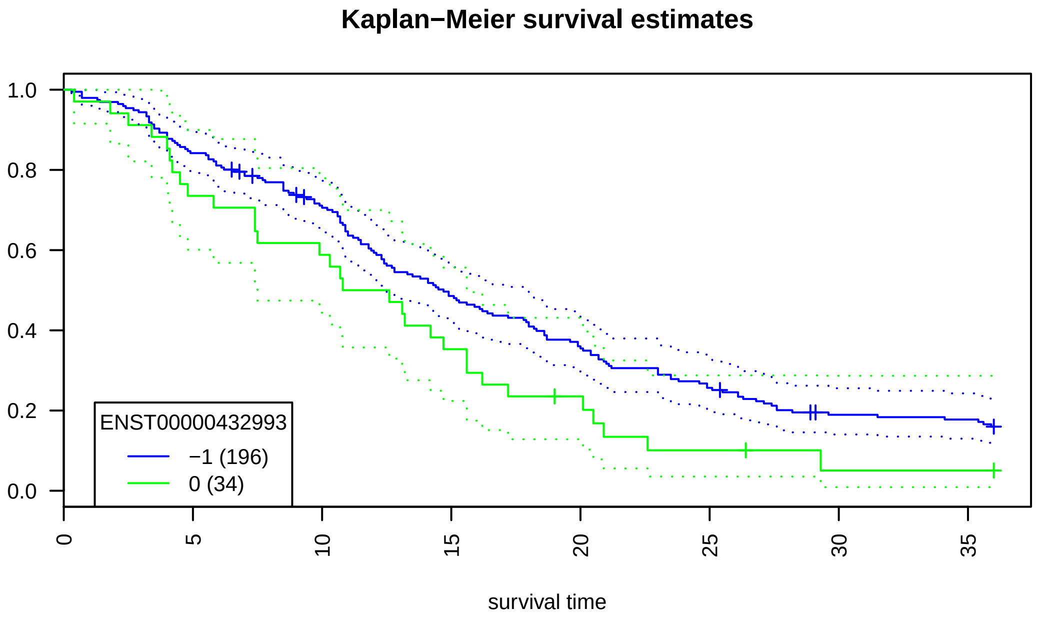

Figure 1360: Each curve represents the relative number of ENST00000432993 survived to the corresponding point of time. Dashed lines represent 0.95 confidence intervals for the curves with the corresponding color. Log-rank probability measure for the equality of these curves is 2.463584e-02. Additional annotations: *.GeneId*=ENSG00000239498, *.GeneName*=AC114765.1, *.GeneDesc*=[undefined].

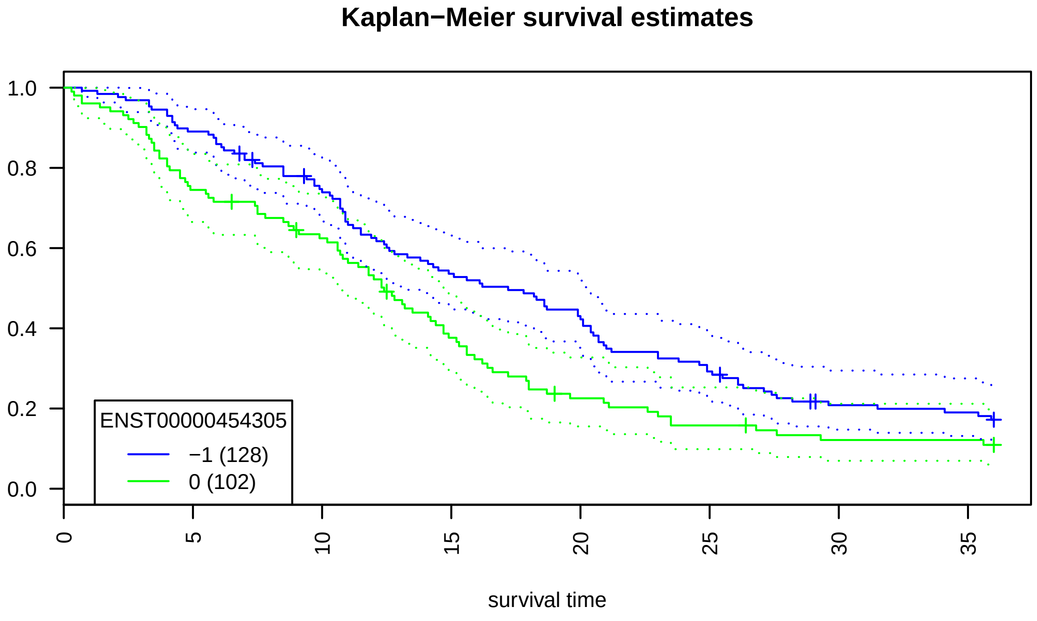

Figure 1361: Each curve represents the relative number of ENST00000454305 survived to the corresponding point of time. Dashed lines represent 0.95 confidence intervals for the curves with the corresponding color. Log-rank probability measure for the equality of these curves is 8.15649e-03. Additional annotations: *.GeneId*=ENSG00000230498, *.GeneName*=RP4-706A16.2, *.GeneDesc*=[undefined].

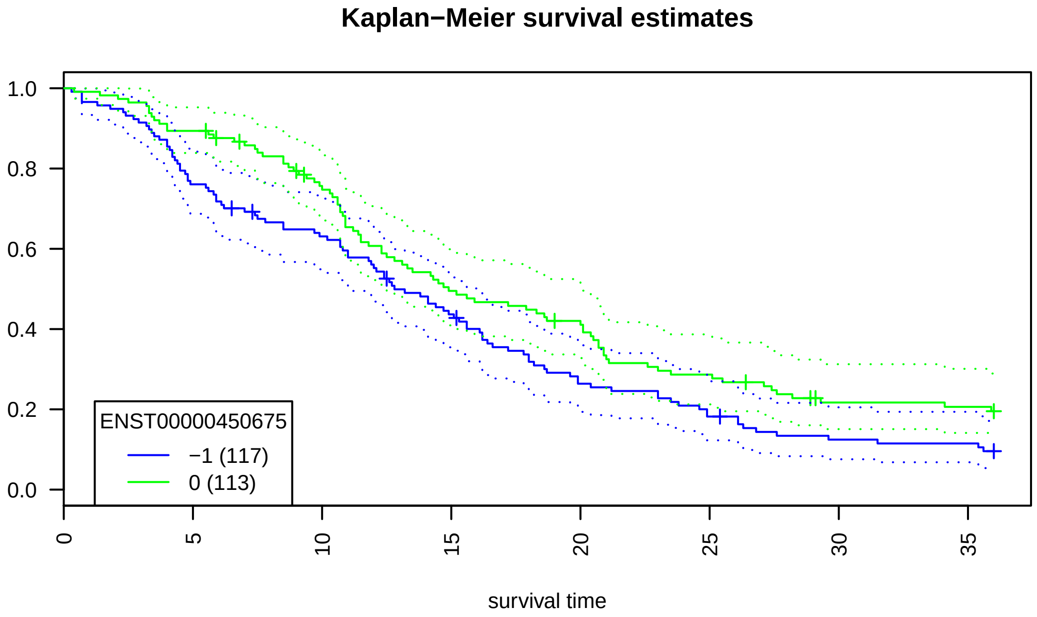

Figure 1362: Each curve represents the relative number of ENST00000450675 survived to the corresponding point of time. Dashed lines represent 0.95 confidence intervals for the curves with the corresponding color. Log-rank probability measure for the equality of these curves is 2.410483e-02. Additional annotations: *.GeneId*=ENSG00000242959, *.GeneName*=RP4-599G15.3, *.GeneDesc*=[undefined].

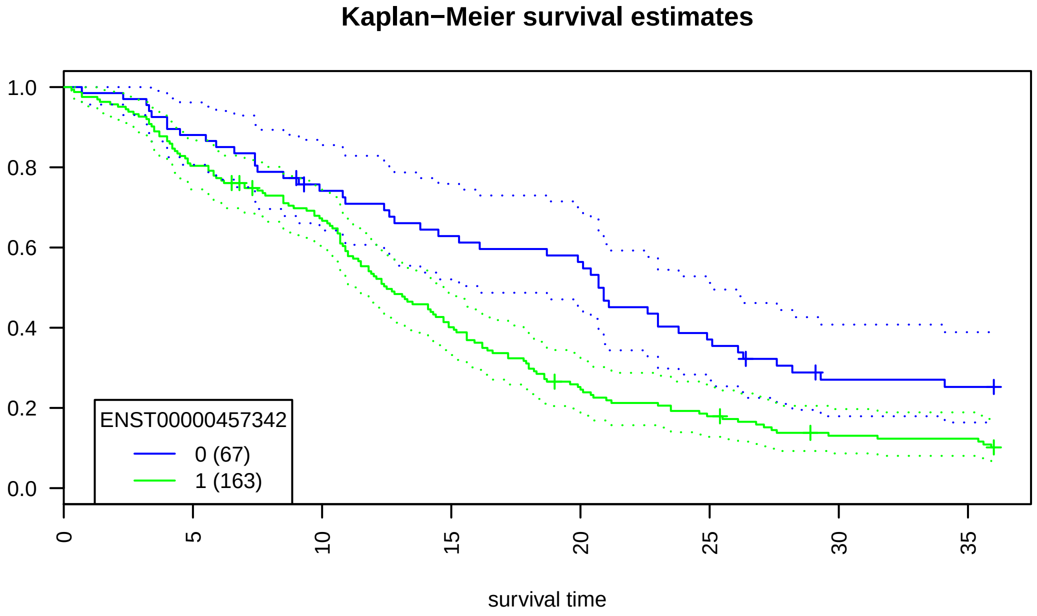

Figure 1363: Each curve represents the relative number of ENST00000457342 survived to the corresponding point of time. Dashed lines represent 0.95 confidence intervals for the curves with the corresponding color. Log-rank probability measure for the equality of these curves is 8.672919e-04.

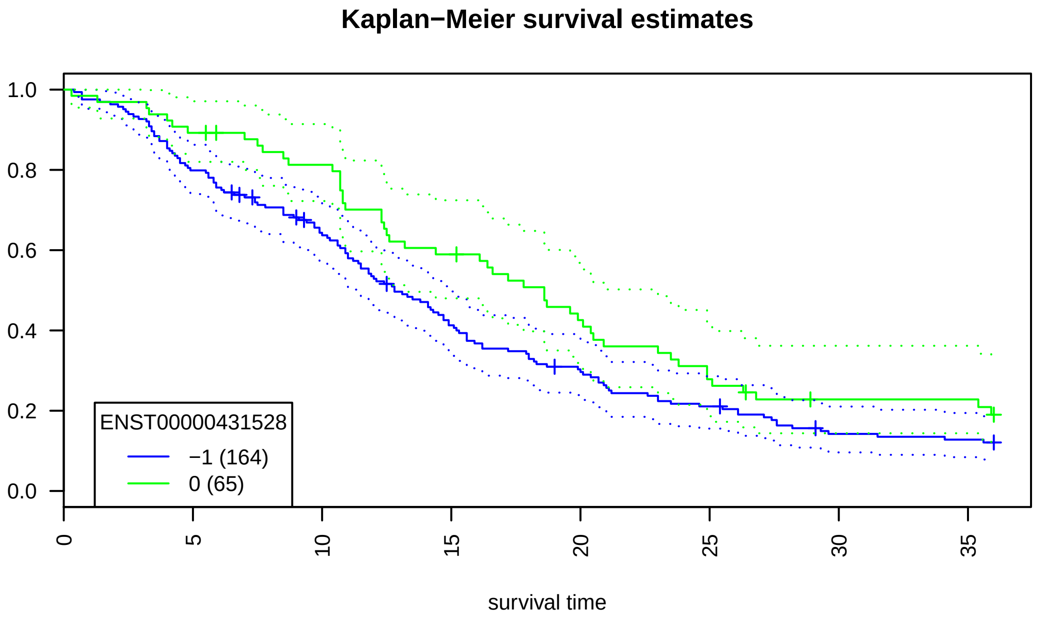

Figure 1364: Each curve represents the relative number of ENST00000431528 survived to the corresponding point of time. Dashed lines represent 0.95 confidence intervals for the curves with the corresponding color. Total of 1 observation deleted due to missingness. Log-rank probability measure for the equality of these curves is 4.113112e-02. Additional annotations: *.GeneId*=ENSG00000214837, *.GeneName*=RP11-261C10.3, *.GeneDesc*=[undefined].

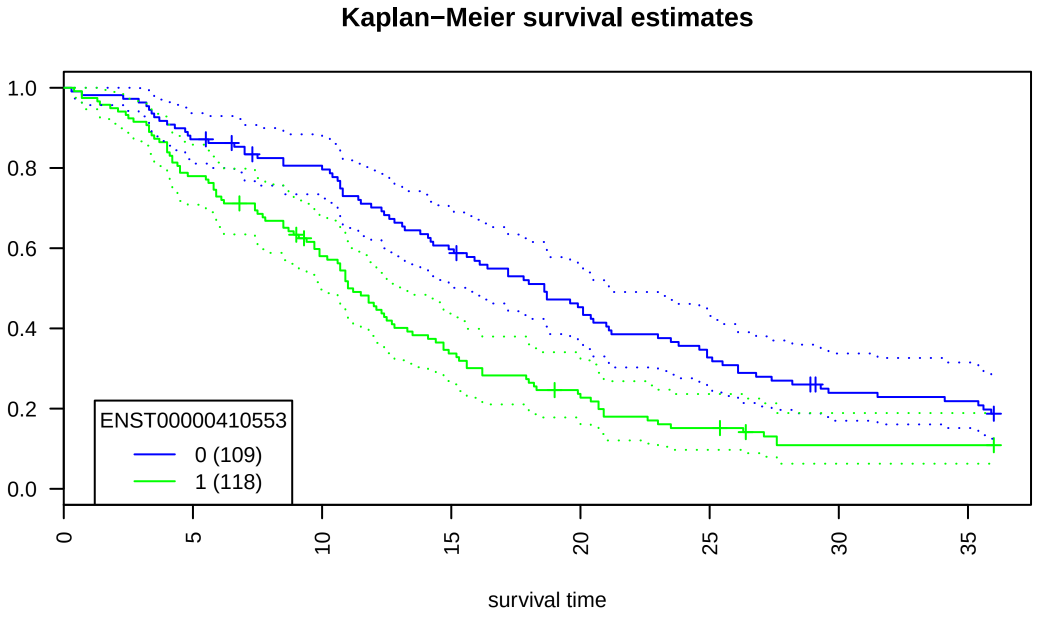

Figure 1365: Each curve represents the relative number of ENST00000410553 survived to the corresponding point of time. Dashed lines represent 0.95 confidence intervals for the curves with the corresponding color. Total of 3 observations deleted due to missingness. Log-rank probability measure for the equality of these curves is 3.597213e-04.

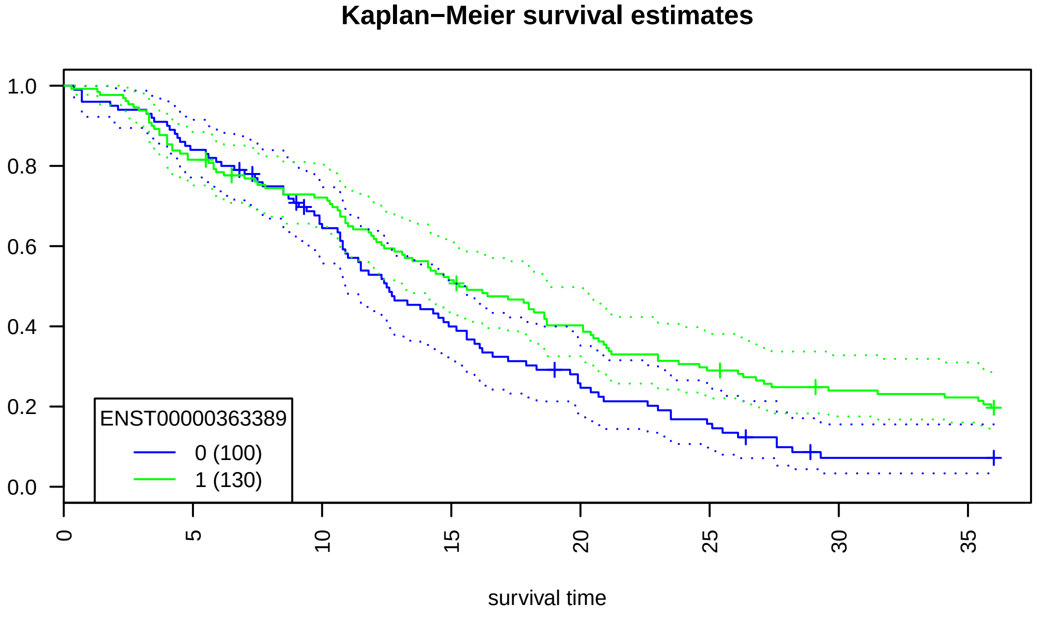

Figure 1366: Each curve represents the relative number of ENST00000363389 survived to the corresponding point of time. Dashed lines represent 0.95 confidence intervals for the curves with the corresponding color. Log-rank probability measure for the equality of these curves is 9.976898e-03. Additional annotations: *.GeneId*=ENSG00000200259, *.GeneName*=SNORD35A, *.GeneDesc*=small nucleolar RNA, C/D box 35A [Source:HGNC Symbol;Acc:10162].

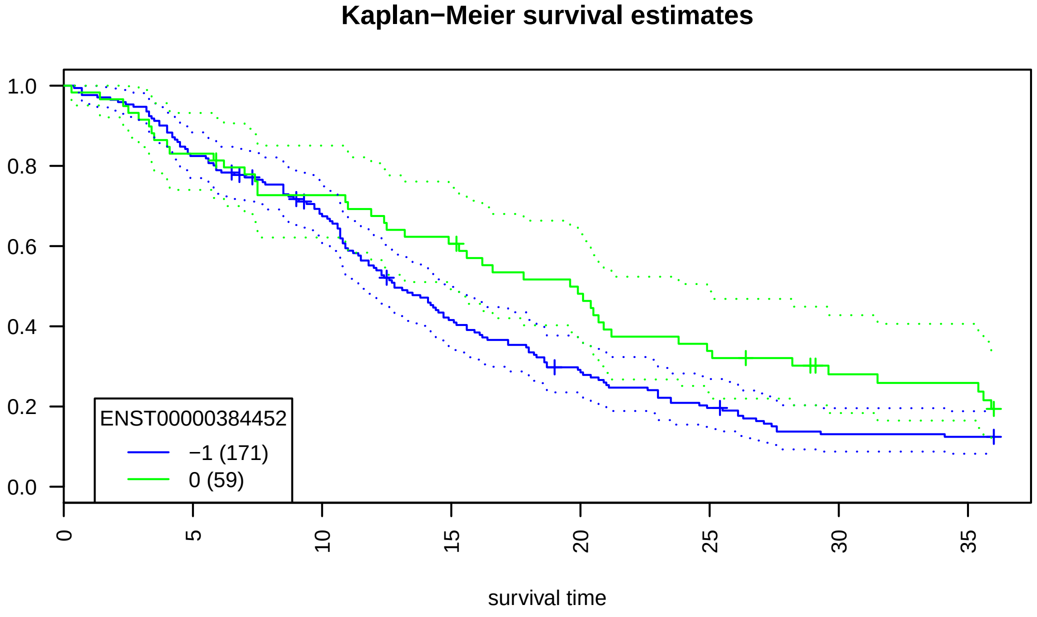

Figure 1367: Each curve represents the relative number of ENST00000384452 survived to the corresponding point of time. Dashed lines represent 0.95 confidence intervals for the curves with the corresponding color. Log-rank probability measure for the equality of these curves is 3.659371e-02. Additional annotations: *.GeneId*=ENSG00000207181, *.GeneName*=SNORA14B, *.GeneDesc*=small nucleolar RNA, H/ACA box 14B [Source:HGNC Symbol;Acc:32603].

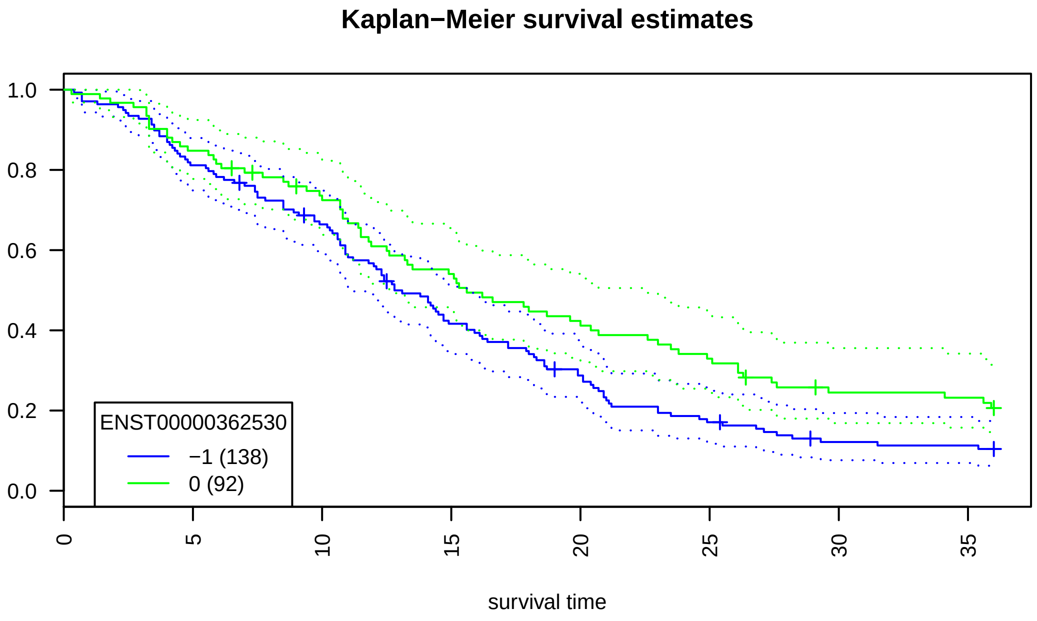

Figure 1368: Each curve represents the relative number of ENST00000362530 survived to the corresponding point of time. Dashed lines represent 0.95 confidence intervals for the curves with the corresponding color. Log-rank probability measure for the equality of these curves is 2.090396e-02. Additional annotations: *.GeneId*=ENSG00000199400, *.GeneName*=AC093171.1, *.GeneDesc*=[undefined].

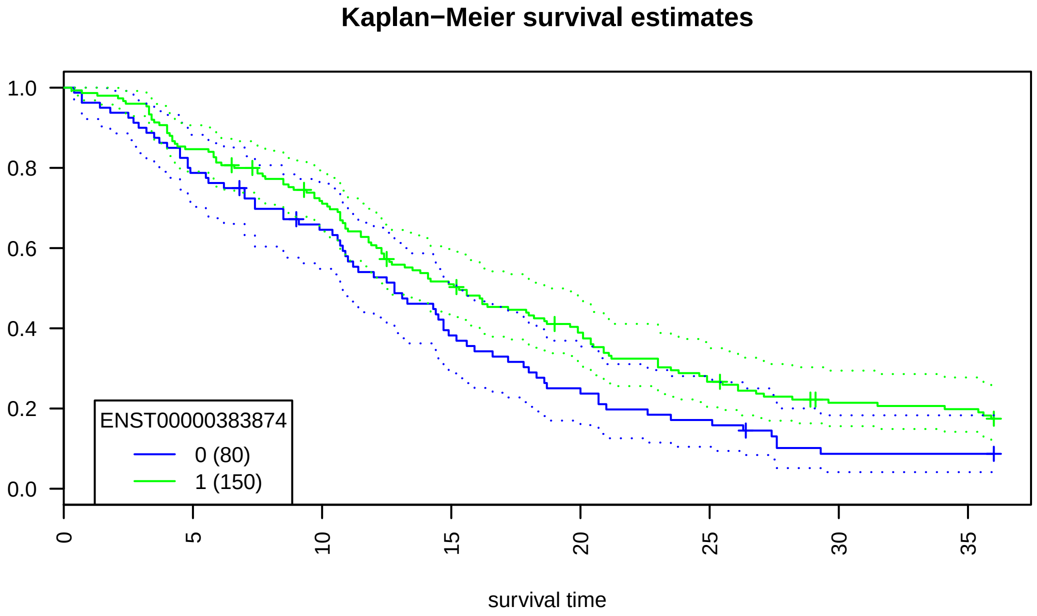

Figure 1369: Each curve represents the relative number of ENST00000383874 survived to the corresponding point of time. Dashed lines represent 0.95 confidence intervals for the curves with the corresponding color. Log-rank probability measure for the equality of these curves is 2.671710e-02. Additional annotations: *.GeneId*=ENSG00000206601, *.GeneName*=AC107071.1, *.GeneDesc*=[undefined].

Kaplan−Meier survival estimates

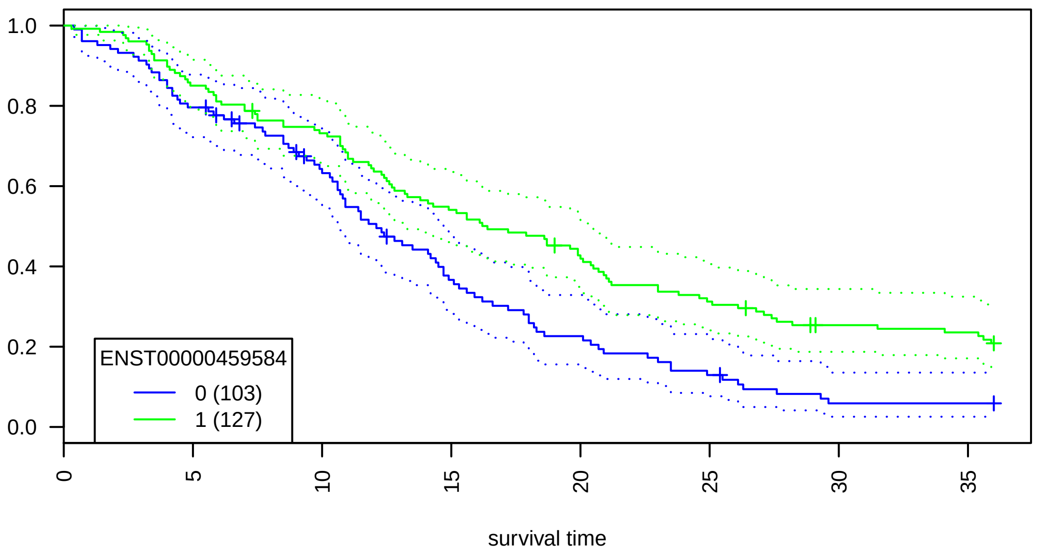

Figure 1370: Each curve represents the relative number of ENST00000459584 survived to the corresponding point of time. Dashed lines represent 0.95 confidence intervals for the curves with the corresponding color. Log-rank probability measure for the equality of these curves is 3.055553e-04. Additional annotations: *.GeneId*=ENSG00000238649, *.GeneName*=SNORD42A, *.GeneDesc*=small nucleolar RNA, C/D box 42A [Source:HGNC Symbol;Acc:10180].

Kaplan−Meier survival estimates

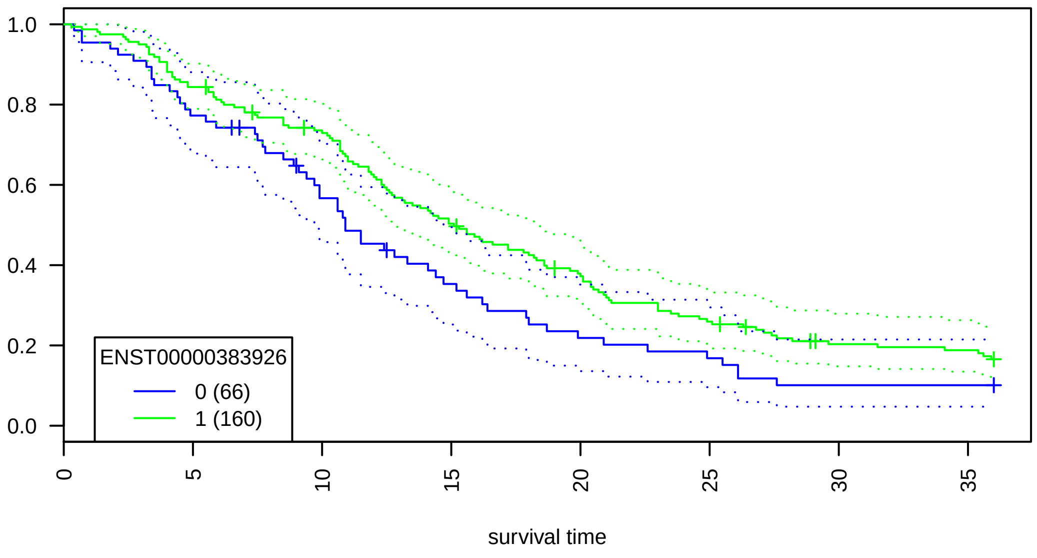

Figure 1371: Each curve represents the relative number of ENST00000383926 survived to the corresponding point of time. Dashed lines represent 0.95 confidence intervals for the curves with the corresponding color. Total of 4 observations deleted due to missingness. Log-rank probability measure for the equality of these curves is 2.524463e-02. Additional annotations: *.GeneId*=ENSG00000206653, *.GeneName*=SNORD29, *.GeneDesc*=small nucleolar RNA, C/D box 29 [Source:HGNC Symbol;Acc:10151].

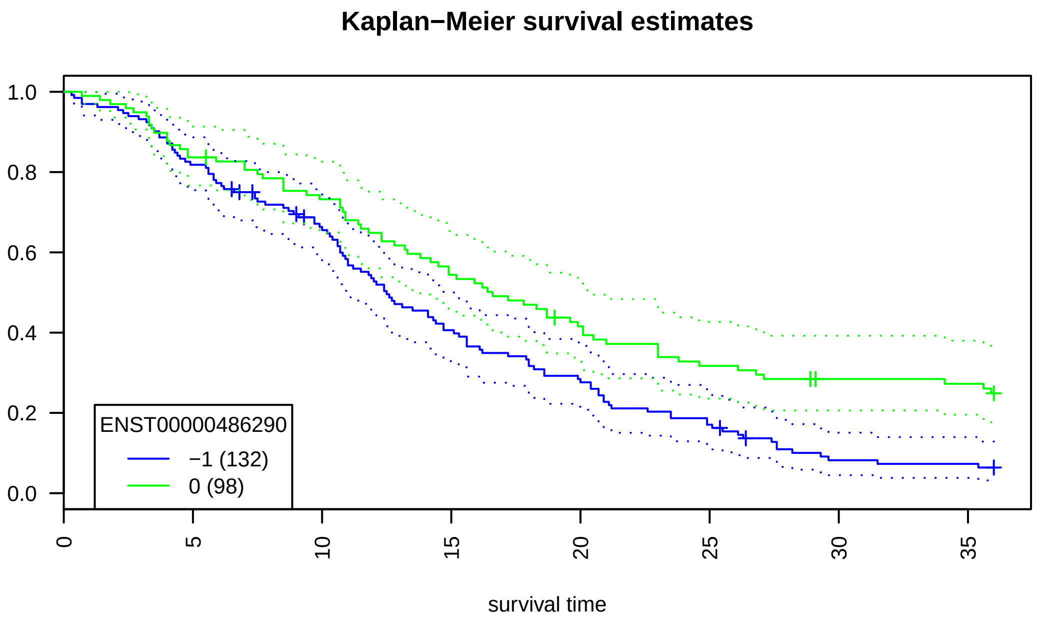

Figure 1372: Each curve represents the relative number of ENST00000486290 survived to the corresponding point of time. Dashed lines represent 0.95 confidence intervals for the curves with the corresponding color. Log-rank probability measure for the equality of these curves is 8.044088e-04. Additional annotations: *.GeneId*=ENSG00000151967, *.GeneName*=SCHIP1, *.GeneDesc*=schwannomin interacting protein 1 [Source:HGNC Symbol;Acc:15678].

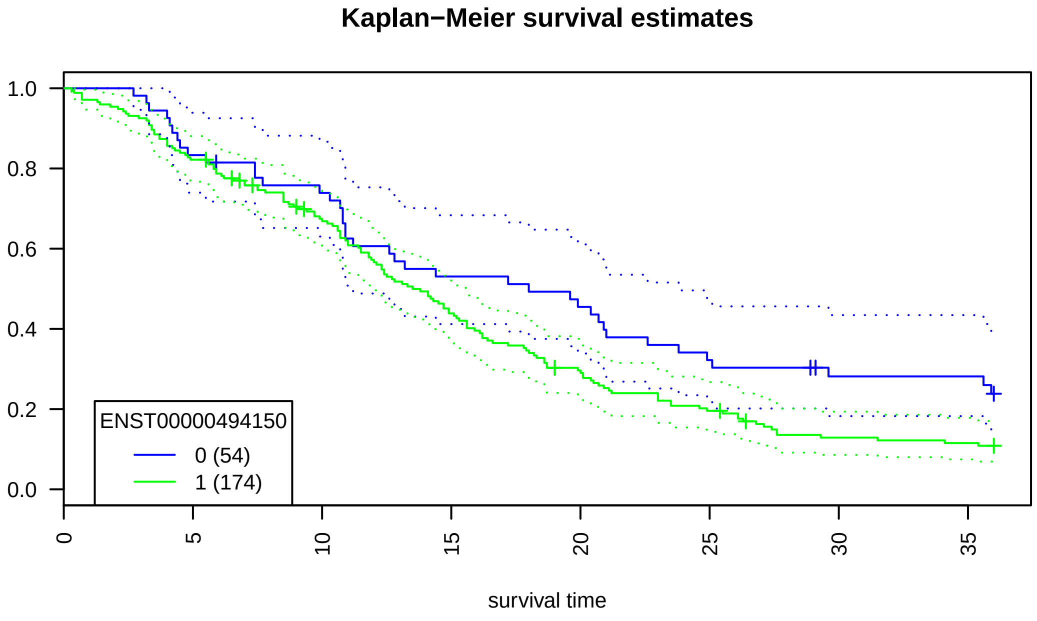

Figure 1373: Each curve represents the relative number of ENST00000494150 survived to the corresponding point of time. Dashed lines represent 0.95 confidence intervals for the curves with the corresponding color. Total of 2 observations deleted due to missingness. Log-rank probability measure for the equality of these curves is 2.788626e-02. Additional annotations: *.GeneId*=ENSG00000239713, *.GeneName*=APOBEC3G, *.GeneDesc*=apolipoprotein B mRNA editing enzyme, catalytic polypeptide-like 3G [Source:HGNC Symbol;Acc:17357].

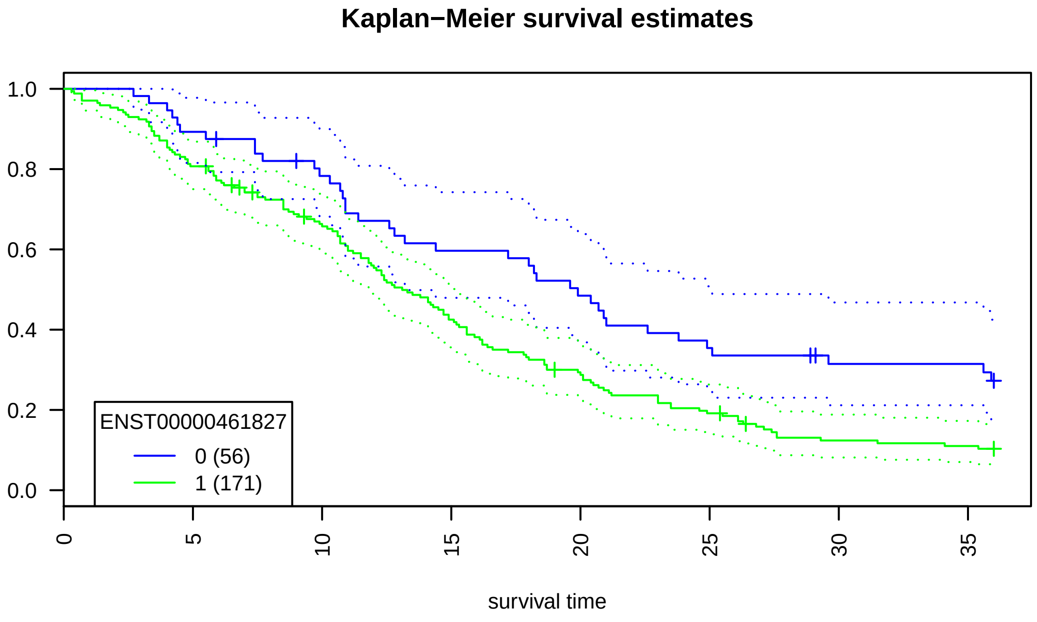

Figure 1374: Each curve represents the relative number of ENST00000461827 survived to the corresponding point of time. Dashed lines represent 0.95 confidence intervals for the curves with the corresponding color. Total of 3 observations deleted due to missingness. Log-rank probability measure for the equality of these curves is 2.257064e-03. Additional annotations: *.GeneId*=ENSG00000239713, *.GeneName*=APOBEC3G, *.GeneDesc*=apolipoprotein B mRNA editing enzyme, catalytic polypeptide-like 3G [Source:HGNC Symbol;Acc:17357].

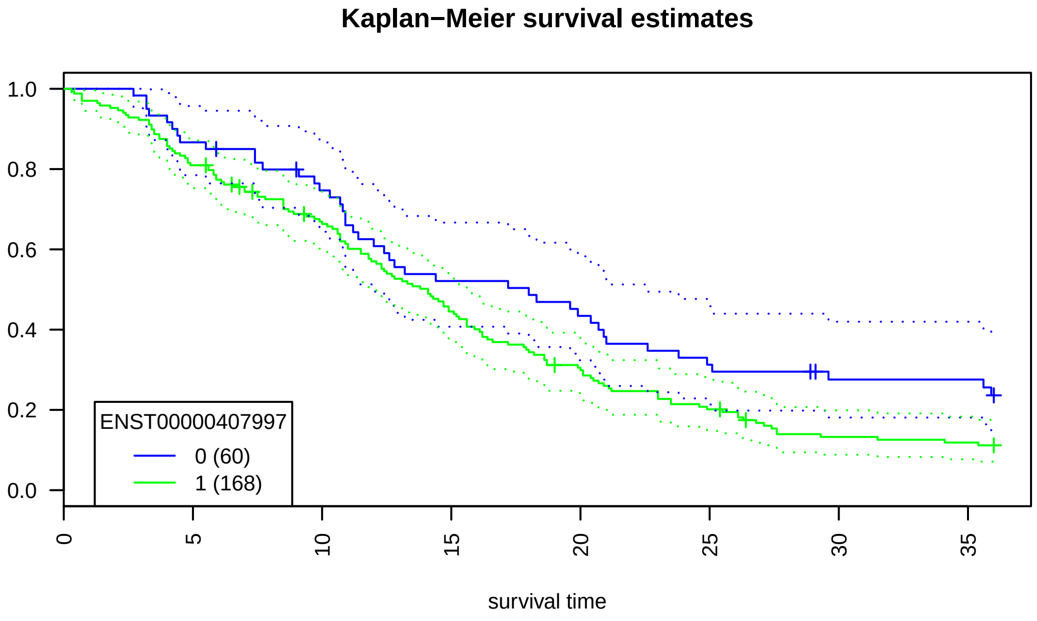

Figure 1375: Each curve represents the relative number of ENST00000407997 survived to the corresponding point of time. Dashed lines represent 0.95 confidence intervals for the curves with the corresponding color. Total of 2 observations deleted due to missingness. Log-rank probability measure for the equality of these curves is 3.058669e-02. Additional annotations: *.GeneId*=ENSG00000239713, *.GeneName*=APOBEC3G, *.GeneDesc*=apolipoprotein B mRNA editing enzyme, catalytic polypeptide-like 3G [Source:HGNC Symbol;Acc:17357].

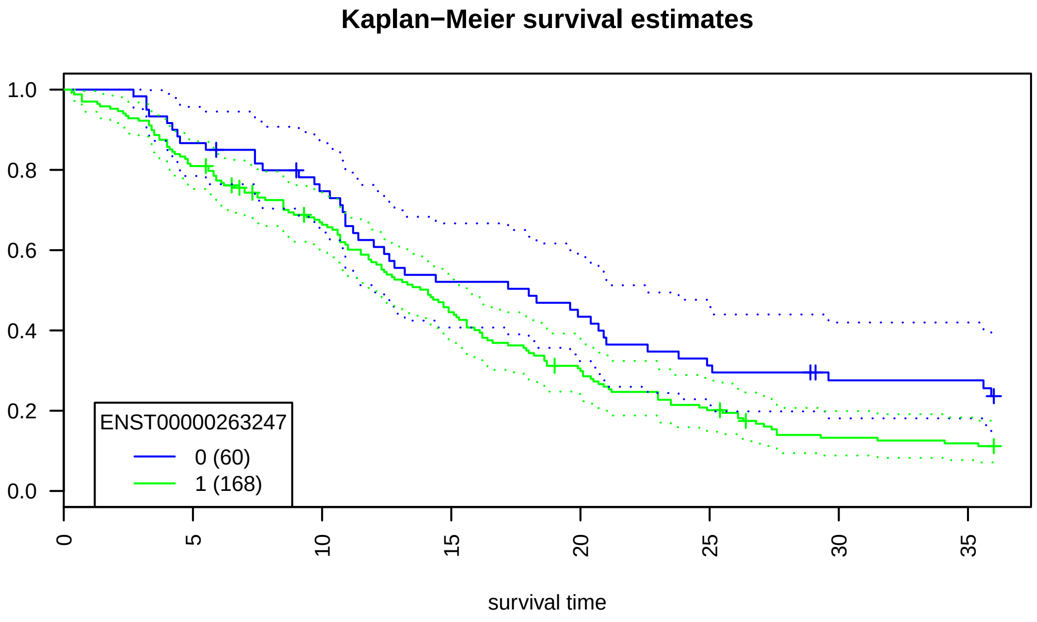

Figure 1376: Each curve represents the relative number of ENST00000263247 survived to the corresponding point of time. Dashed lines represent 0.95 confidence intervals for the curves with the corresponding color. Total of 2 observations deleted due to missingness. Log-rank probability measure for the equality of these curves is 3.058669e-02.

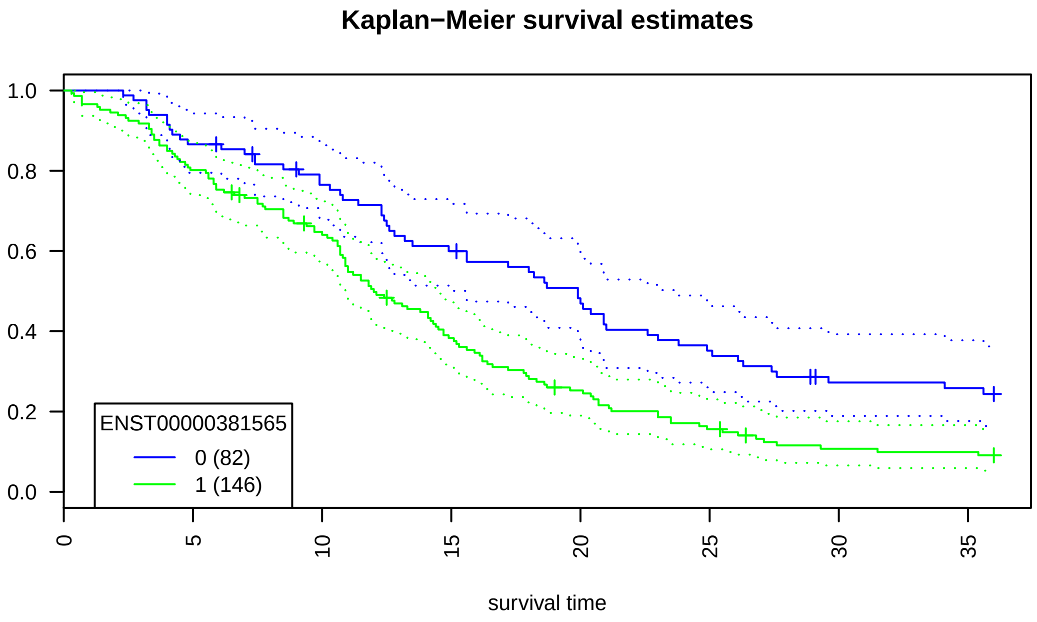

Figure 1377: Each curve represents the relative number of ENST00000381565 survived to the corresponding point of time. Dashed lines represent 0.95 confidence intervals for the curves with the corresponding color. Total of 2 observations deleted due to missingness. Log-rank probability measure for the equality of these curves is 2.68942e-04. Additional annotations: *.GeneId*=ENSG00000128394, *.GeneName*=APOBEC3F, *.GeneDesc*=apolipoprotein B mRNA editing enzyme, catalytic polypeptide-like 3F [Source:HGNC Symbol;Acc:17356].

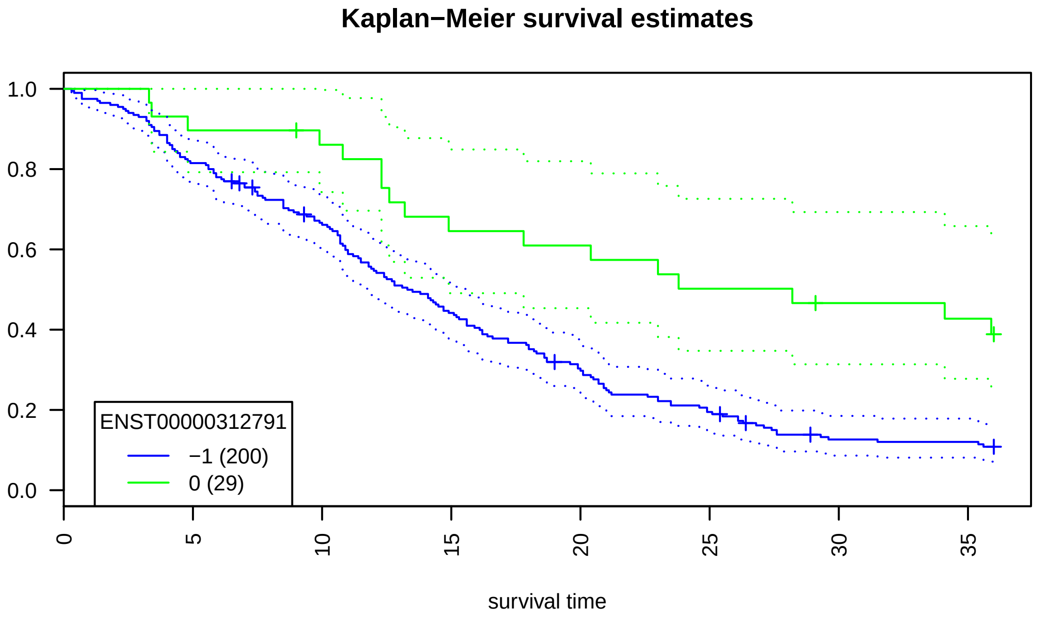

Figure 1378: Each curve represents the relative number of ENST00000312791 survived to the corresponding point of time. Dashed lines represent 0.95 confidence intervals for the curves with the corresponding color. Total of 1 observation deleted due to missingness. Log-rank probability measure for the equality of these curves is 4.385117e-04. Additional annotations: *.GeneId*=ENSG00000240891, *.GeneName*=PLCXD2, *.GeneDesc*=phosphatidylinositol-specific phospholipase C, X domain containing 2 [Source:HGNC Symbol;Acc:26462].

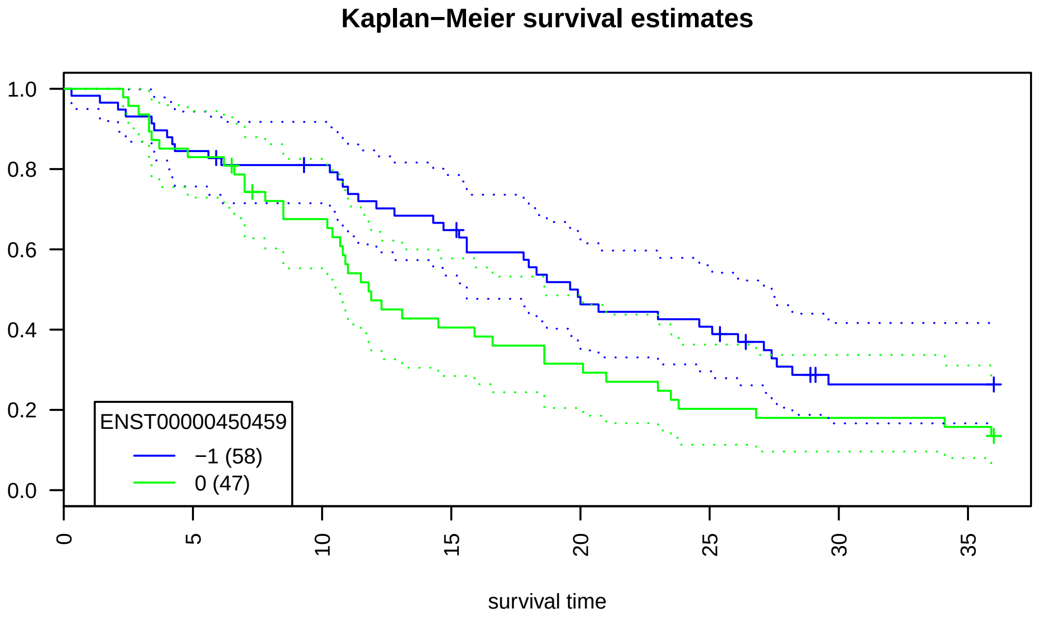

Figure 1379: Each curve represents the relative number of ENST00000450459 survived to the corresponding point of time. Dashed lines represent 0.95 confidence intervals for the curves with the corresponding color. Total of 125 observations deleted due to missingness. Log-rank probability measure for the equality of these curves is 3.871698e-02. Additional annotations: *.GeneId*=ENSG00000115561, *.GeneName*=VPS24, *.GeneDesc*=vacuolar protein sorting 24 homolog (S. cerevisiae) [Source:HGNC Symbol;Acc:29865].

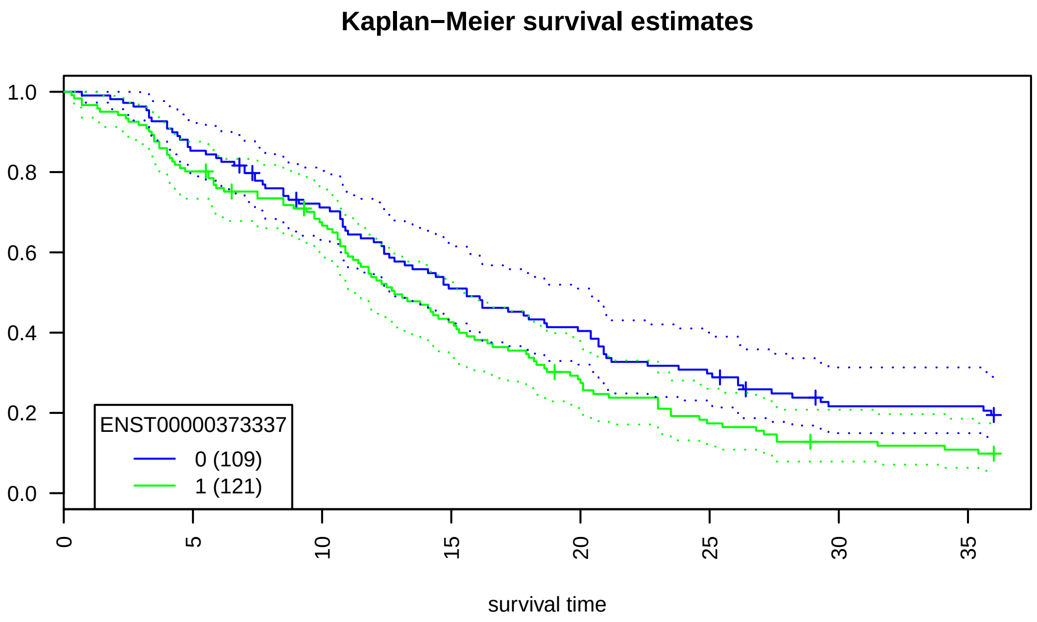

Figure 1380: Each curve represents the relative number of ENST00000373337 survived to the corresponding point of time. Dashed lines represent 0.95 confidence intervals for the curves with the corresponding color. Log-rank probability measure for the equality of these curves is 3.152006e-02. Additional annotations: *.GeneId*=ENSG00000243749, *.GeneName*=RP11-244H3.2, *.GeneDesc*=ZMYM6 proteinZinc finger, MYM-type 6, isoform CRA_a ; [Source:UniProtKB/TrEMBL;Acc:Q8NCS4].

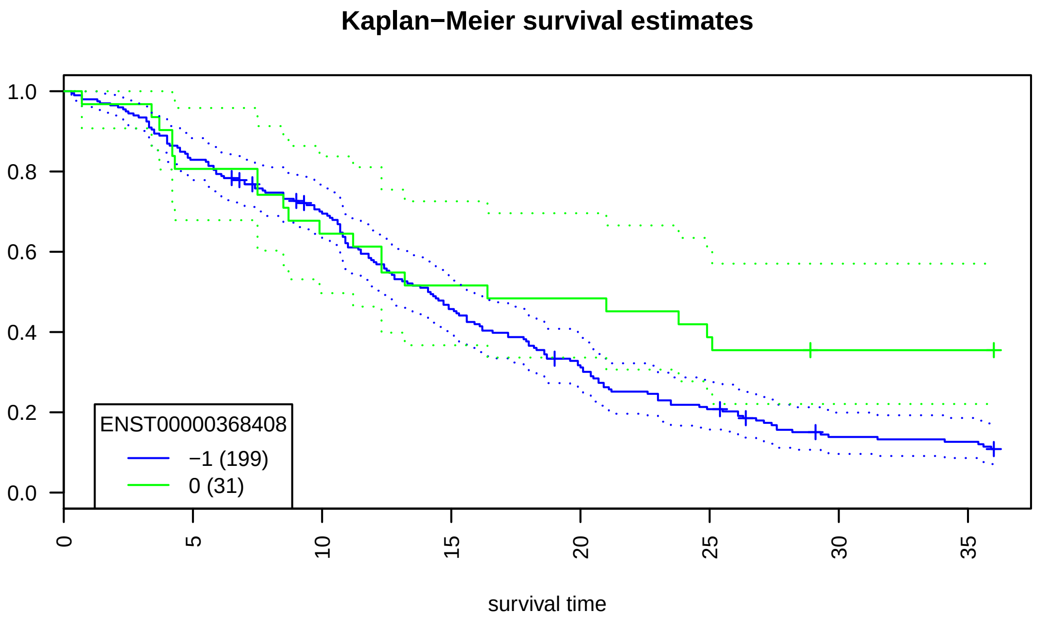

Figure 1381: Each curve represents the relative number of ENST00000368408 survived to the corresponding point of time. Dashed lines represent 0.95 confidence intervals for the curves with the corresponding color. Log-rank probability measure for the equality of these curves is 2.079650e-02. Additional annotations: *.GeneId*=ENSG00000143590, *.GeneName*=EFNA3, *.GeneDesc*=ephrin-A3 [Source:HGNC Symbol;Acc:3223].

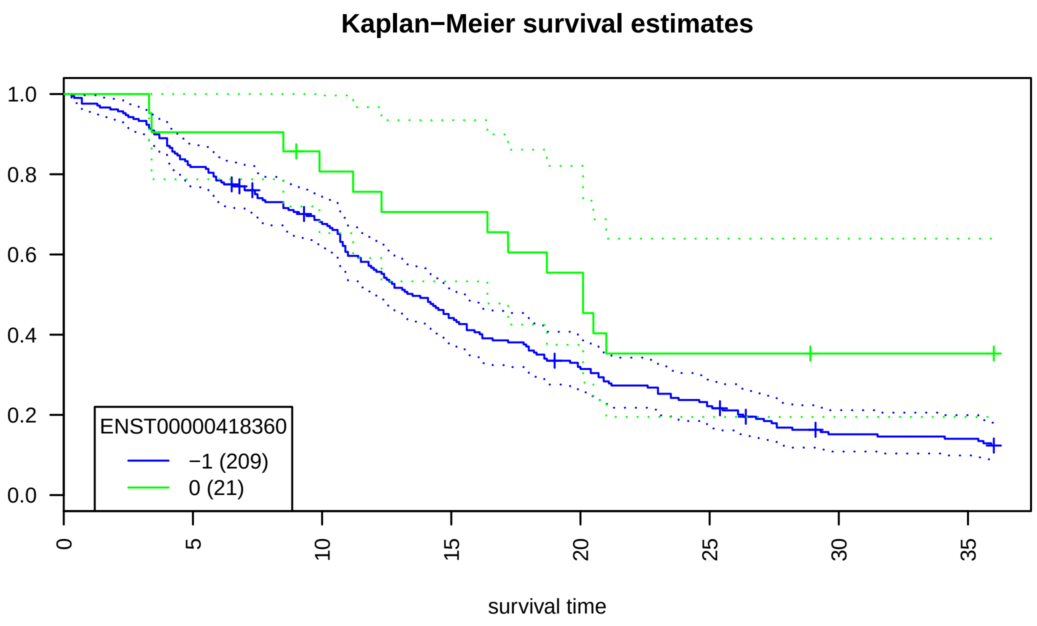

Figure 1382: Each curve represents the relative number of ENST00000418360 survived to the corresponding point of time. Dashed lines represent 0.95 confidence intervals for the curves with the corresponding color. Log-rank probability measure for the equality of these curves is 2.716051e-02. Additional annotations: *.GeneId*=ENSG00000143590, *.GeneName*=EFNA3, *.GeneDesc*=ephrin-A3 [Source:HGNC Symbol;Acc:3223].

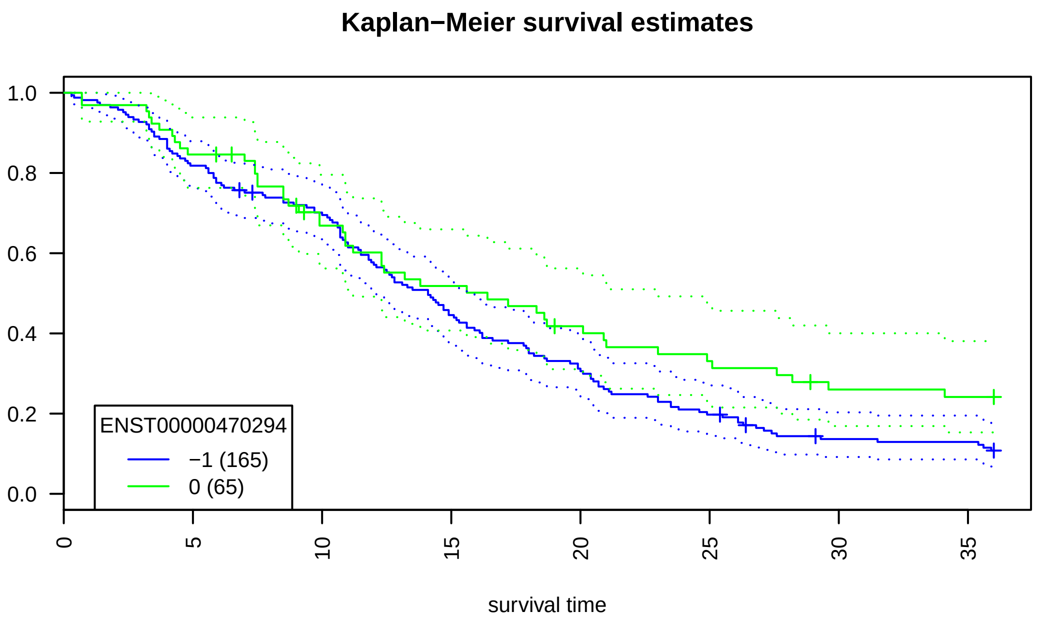

Figure 1383: Each curve represents the relative number of ENST00000470294 survived to the corresponding point of time. Dashed lines represent 0.95 confidence intervals for the curves with the corresponding color. Log-rank probability measure for the equality of these curves is 4.910685e-02. Additional annotations: *.GeneId*=ENSG00000143590, *.GeneName*=EFNA3, *.GeneDesc*=ephrin-A3 [Source:HGNC Symbol;Acc:3223].

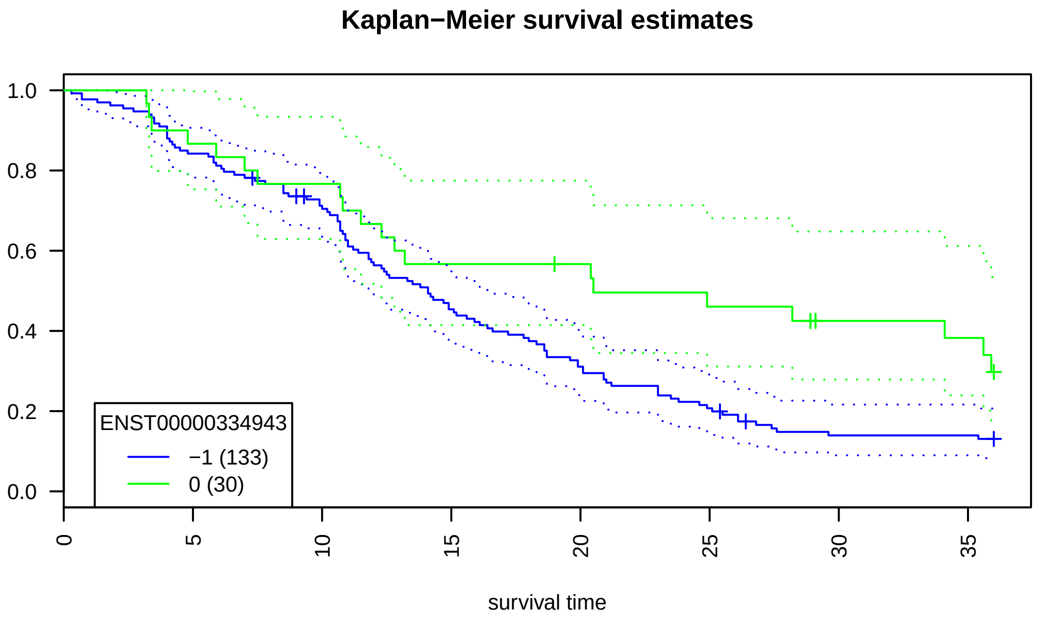

Figure 1384: Each curve represents the relative number of ENST00000334943 survived to the corresponding point of time. Dashed lines represent 0.95 confidence intervals for the curves with the corresponding color. Total of 67 observations deleted due to missingness. Log-rank probability measure for the equality of these curves is 2.090236e-02. Additional annotations: *.GeneId*=ENSG00000241697, *.GeneName*=TMEFF1, *.GeneDesc*=transmembrane protein with EGF-like and two follistatin-like domains 1 [Source:HGNC Symbol;Acc:11866].

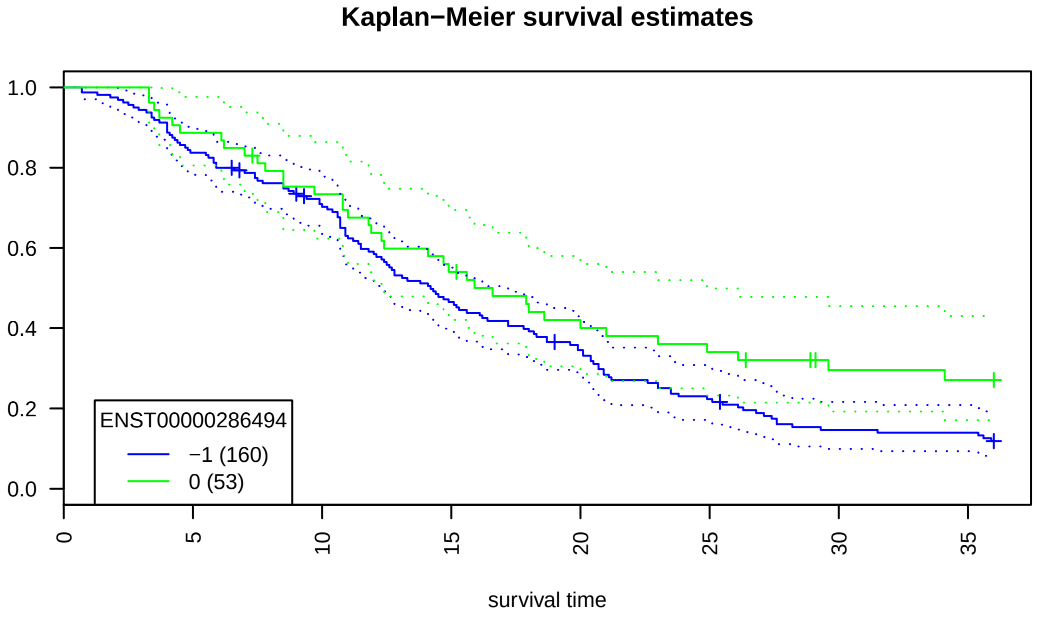

Figure 1385: Each curve represents the relative number of ENST00000286494 survived to the corresponding point of time. Dashed lines represent 0.95 confidence intervals for the curves with the corresponding color. Total of 17 observations deleted due to missingness. Log-rank probability measure for the equality of these curves is 4.736266e-02. Additional annotations: *.GeneId*=ENSG00000240771, *.GeneName*=AC025165.7, *.GeneDesc*=Guanine nucleotide exchange factor GEFT (RhoA/Rac/Cdc42 guanine nucleotide exchange factor GEFT)(Rac/Cdc42/Rho exchange factor GEFT)(p63RhoGEF) [Source:UniProtKB/Swiss-Prot;Acc:Q86VW2].

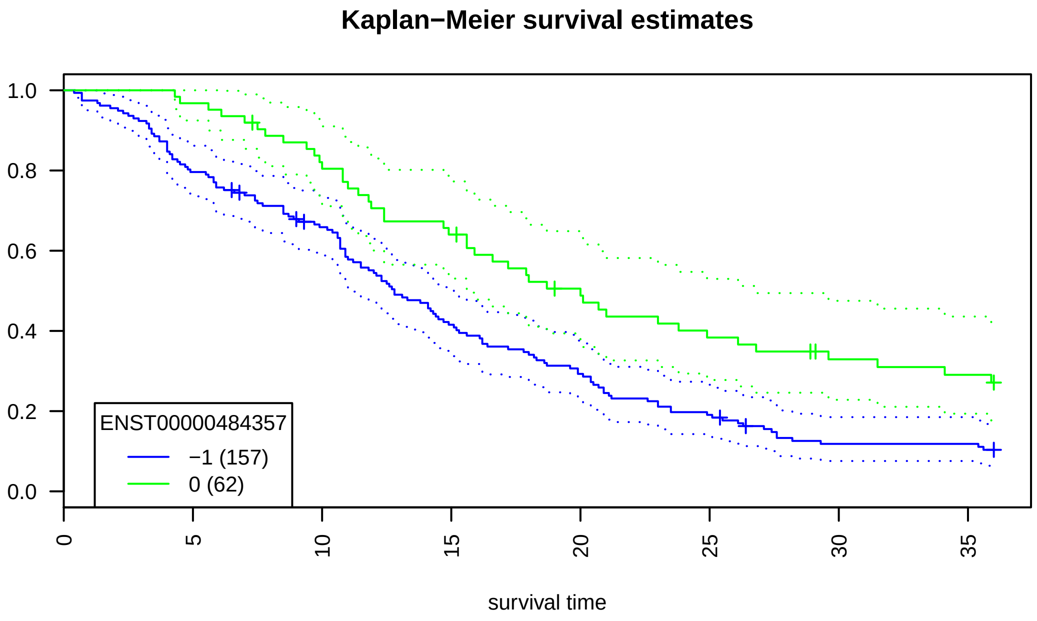

Figure 1386: Each curve represents the relative number of ENST00000484357 survived to the corresponding point of time. Dashed lines represent 0.95 confidence intervals for the curves with the corresponding color. Total of 11 observations deleted due to missingness. Log-rank probability measure for the equality of these curves is 4.404784e-04. Additional annotations: *.GeneId*=ENSG00000240771, *.GeneName*=AC025165.7, *.GeneDesc*=Guanine nucleotide exchange factor GEFT (RhoA/Rac/Cdc42 guanine nucleotide exchange factor GEFT)(Rac/Cdc42/Rho exchange factor GEFT)(p63RhoGEF) [Source:UniProtKB/Swiss-Prot;Acc:Q86VW2].

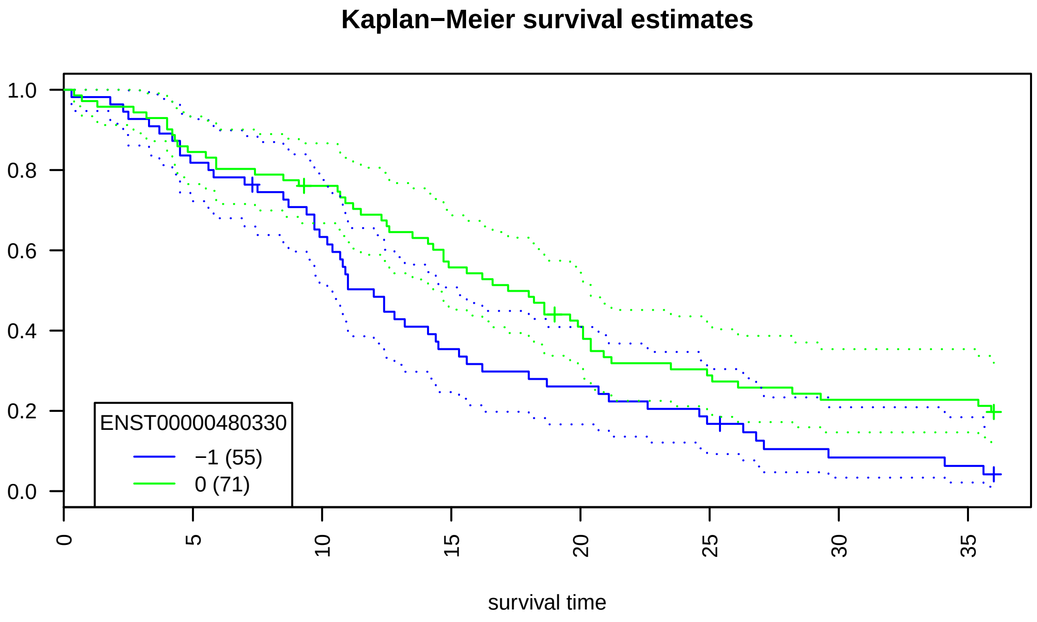

Figure 1387: Each curve represents the relative number of ENST00000480330 survived to the corresponding point of time. Dashed lines represent 0.95 confidence intervals for the curves with the corresponding color. Total of 104 observations deleted due to missingness. Log-rank probability measure for the equality of these curves is 9.759917e-03. Additional annotations: *.GeneId*=ENSG00000239474, *.GeneName*=KBTBD10, *.GeneDesc*=kelch repeat and BTB (POZ) domain containing 10 [Source:HGNC Symbol;Acc:16905].

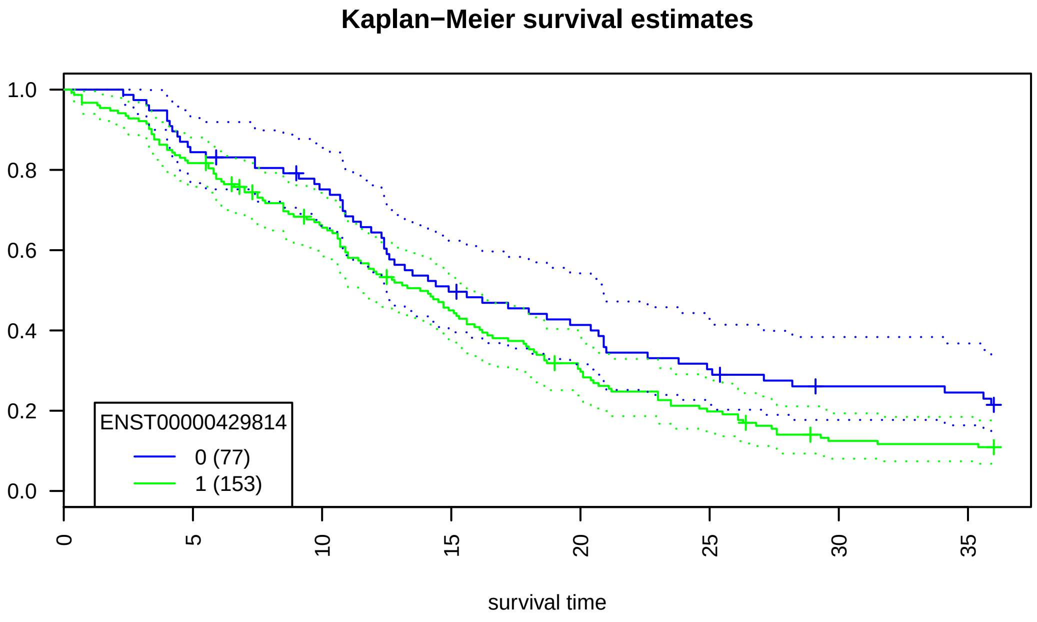

Figure 1388: Each curve represents the relative number of ENST00000429814 survived to the corresponding point of time. Dashed lines represent 0.95 confidence intervals for the curves with the corresponding color. Log-rank probability measure for the equality of these curves is 3.87553e-02. Additional annotations: *.GeneId*=ENSG00000242885, *.GeneName*=TRIM6-TRIM34, *.GeneDesc*=TRIM6-TRIM34 readthrough [Source:HGNC Symbol;Acc:33440].

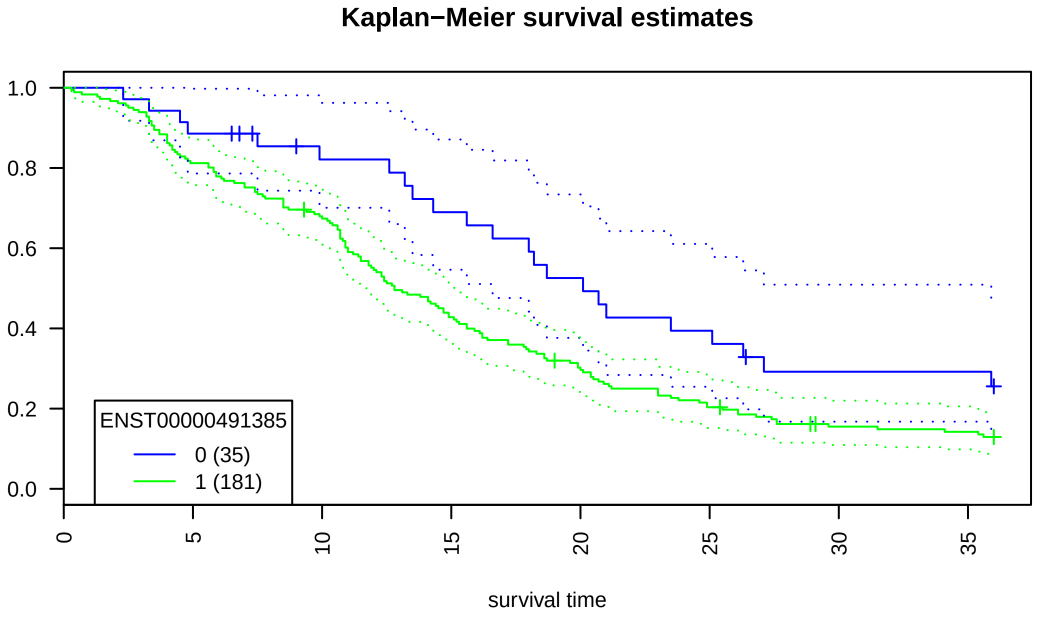

Figure 1389: Each curve represents the relative number of ENST00000491385 survived to the corresponding point of time. Dashed lines represent 0.95 confidence intervals for the curves with the corresponding color. Total of 14 observations deleted due to missingness. Log-rank probability measure for the equality of these curves is 1.693006e-02. Additional annotations: *.GeneId*=ENSG00000239456, *.GeneName*=TRIM34, *.GeneDesc*=tripartite motif-containing 34 [Source:HGNC Symbol;Acc:10063].

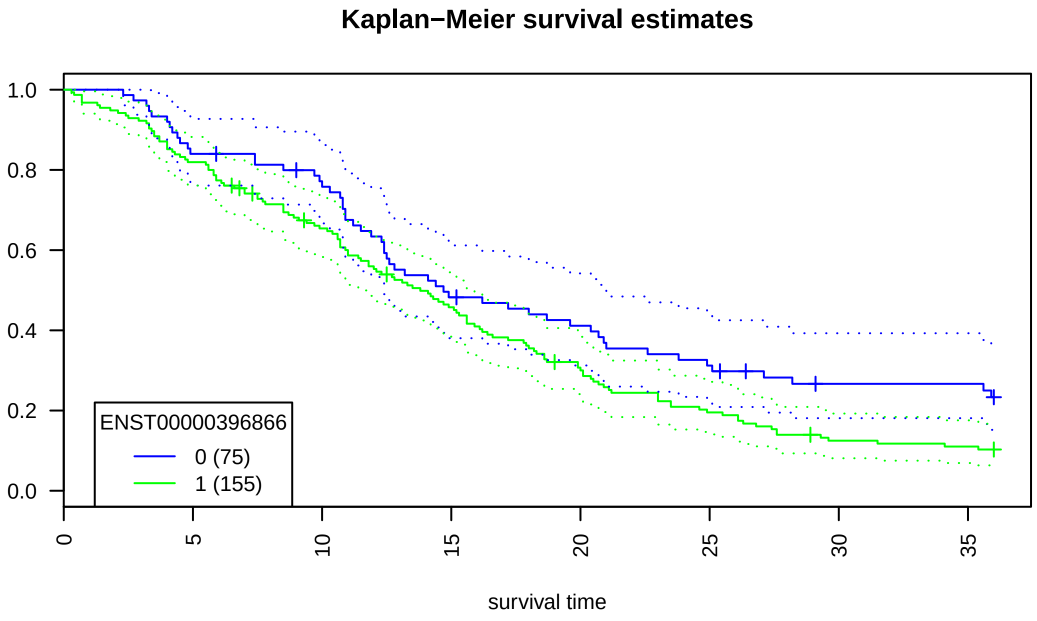

Figure 1390: Each curve represents the relative number of ENST00000396866 survived to the corresponding point of time. Dashed lines represent 0.95 confidence intervals for the curves with the corresponding color. Log-rank probability measure for the equality of these curves is 2.521565e-02. Additional annotations: .*GeneId*=ENSG00000242885, .*GeneName*=TRIM6-TRIM34, .*GeneDesc*=TRIM6-TRIM34 readthrough [Source:HGNC Symbol;Acc:33440].

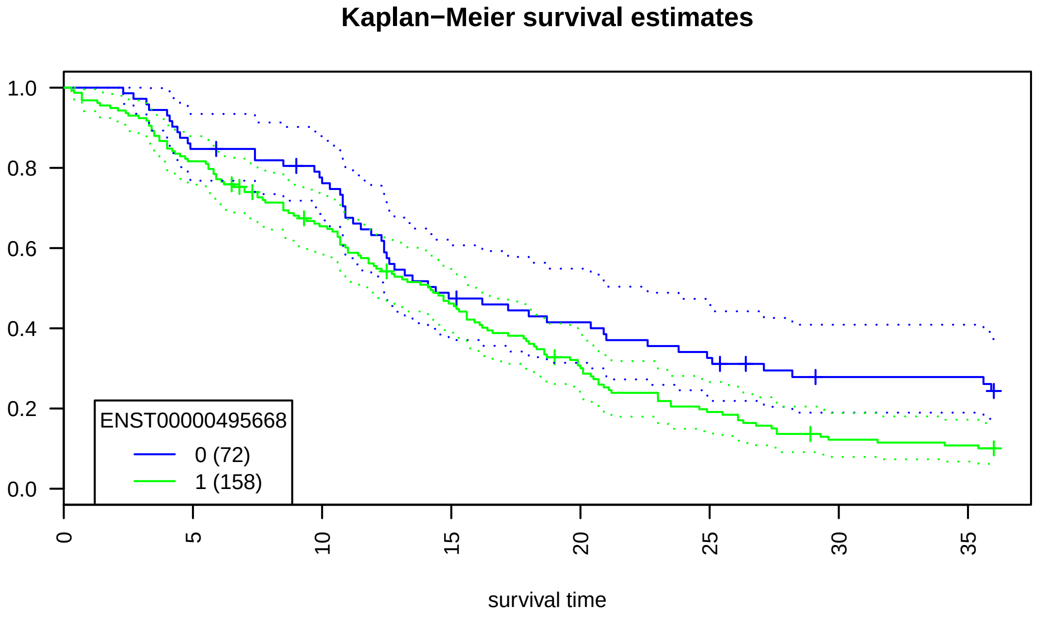

Figure 1391: Each curve represents the relative number of ENST00000495668 survived to the corresponding point of time. Dashed lines represent 0.95 confidence intervals for the curves with the corresponding color. Log-rank probability measure for the equality of these curves is 1.883848e-02. Additional annotations: .*GeneId*=ENSG00000239456, .*GeneName*=TRIM34, .*GeneDesc*=tripartite motif-containing 34 [Source:HGNC Symbol;Acc:10063].

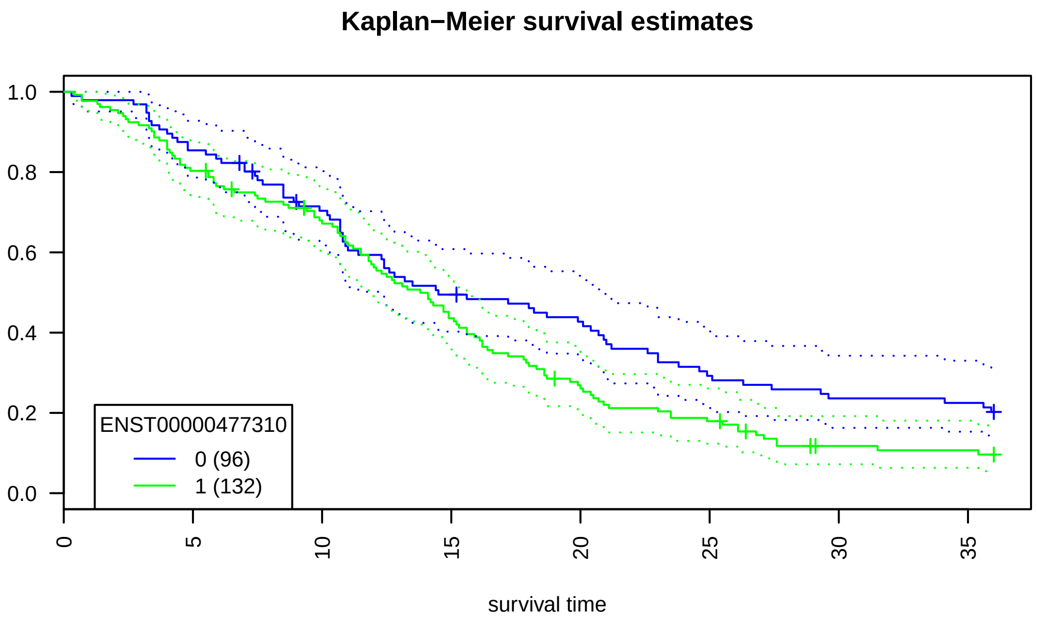

Figure 1392: Each curve represents the relative number of ENST00000477310 survived to the corresponding point of time. Dashed lines represent 0.95 confidence intervals for the curves with the corresponding color. Total of 2 observations deleted due to missingness. Log-rank probability measure for the equality of these curves is 2.937775e-02. Additional annotations: *.GeneId*=ENSG00000244255, *.GeneName*=XXbac-BPG116M5.17, *.GeneDesc*=cDNA FLJ55673, highly similar to Complement factor B (EC 3.4.21.47) [Source:UniProtKB/TrEMBL;Acc:B4E1Z4].

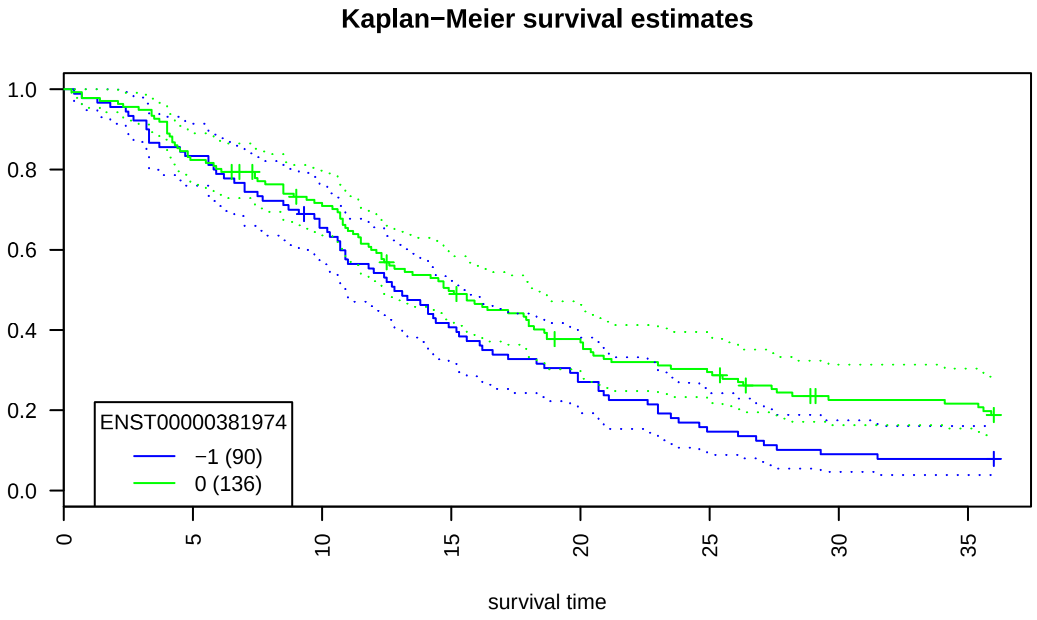

Figure 1393: Each curve represents the relative number of ENST00000381974 survived to the corresponding point of time. Dashed lines represent 0.95 confidence intervals for the curves with the corresponding color. Total of 4 observations deleted due to missingness. Log-rank probability measure for the equality of these curves is 2.557645e-02. Additional annotations: *.GeneId*=ENSG00000251172, *.GeneName*=RP11-529P9.1, *.GeneDesc*=[undefined].

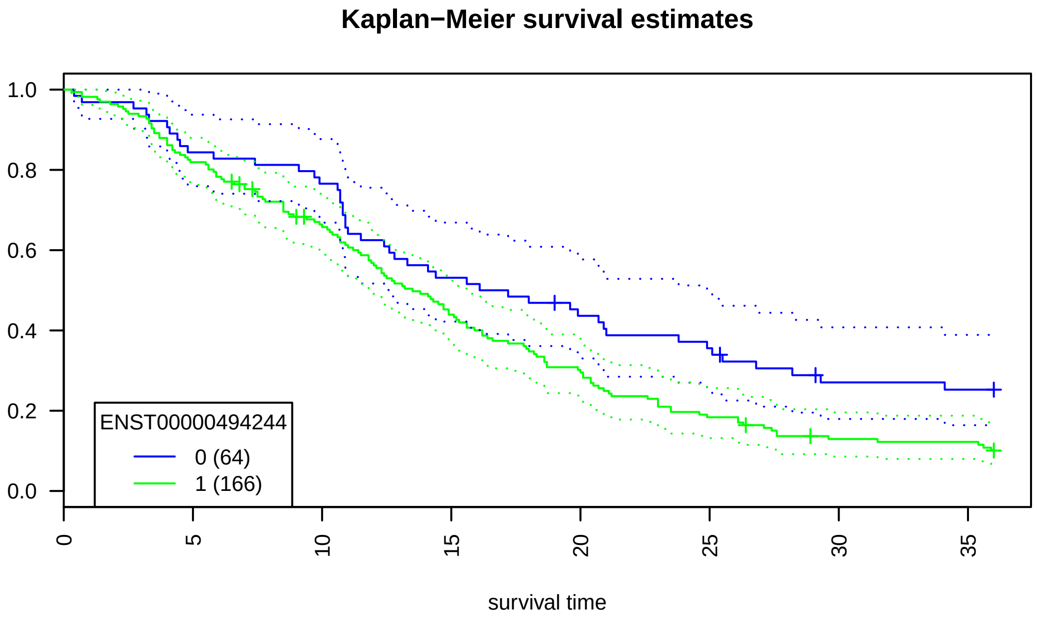

Figure 1394: Each curve represents the relative number of ENST00000494244 survived to the corresponding point of time. Dashed lines represent 0.95 confidence intervals for the curves with the corresponding color. Log-rank probability measure for the equality of these curves is 1.099402e-02. Additional annotations: *.GeneId*=ENSG00000120087, *.GeneName*=HOXB7, *.GeneDesc*=homeobox B7 [Source:HGNC Symbol;Acc:5118].

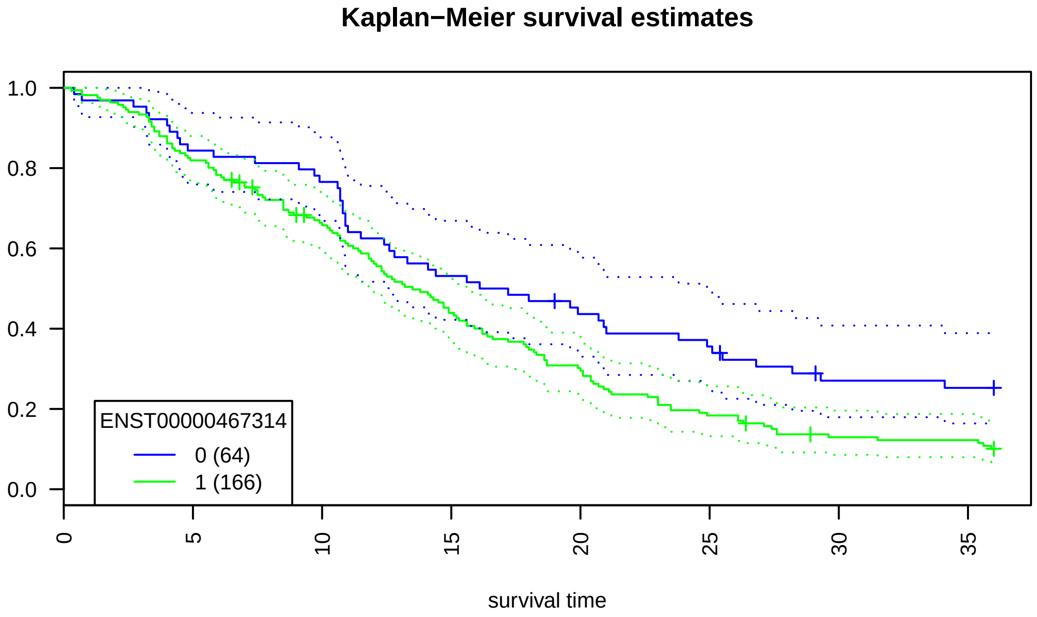

Figure 1395: Each curve represents the relative number of ENST00000467314 survived to the corresponding point of time. Dashed lines represent 0.95 confidence intervals for the curves with the corresponding color. Log-rank probability measure for the equality of these curves is 1.099402e-02. Additional annotations: *.GeneId*=ENSG00000120087, *.GeneName*=HOXB7, *.GeneDesc*=homeobox B7 [Source:HGNC Symbol;Acc:5118].

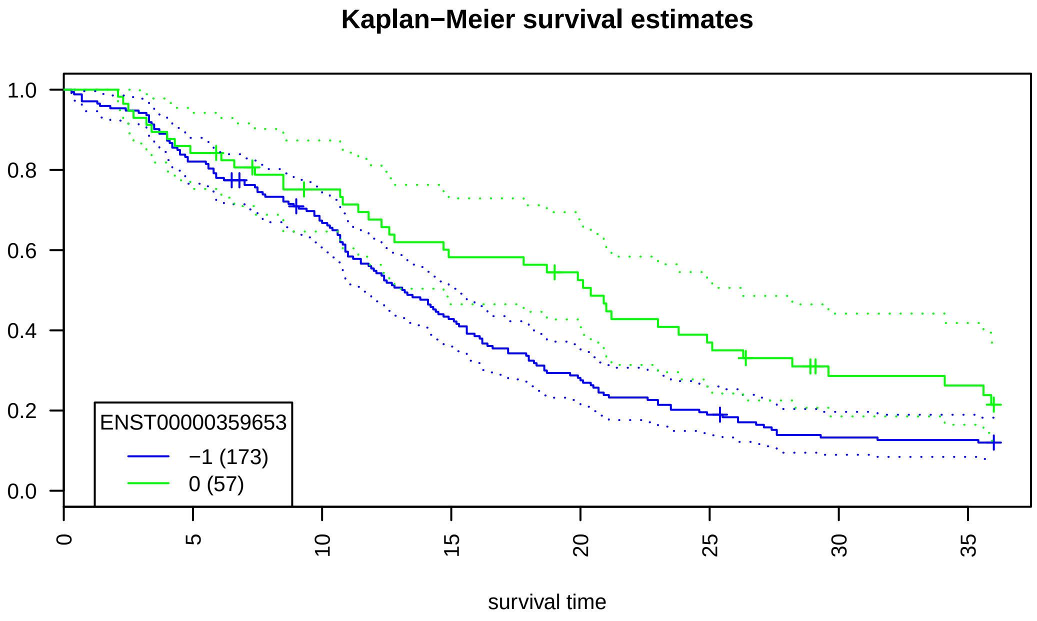

Figure 1396: Each curve represents the relative number of ENST00000359653 survived to the corresponding point of time. Dashed lines represent 0.95 confidence intervals for the curves with the corresponding color. Log-rank probability measure for the equality of these curves is 1.225054e-02. Additional annotations: .*GeneId*=ENSG00000019995, .*GeneName*=ZRANB1, .*GeneDesc*=zinc finger, RAN-binding domain containing 1 [Source:HGNC Symbol;Acc:18224].

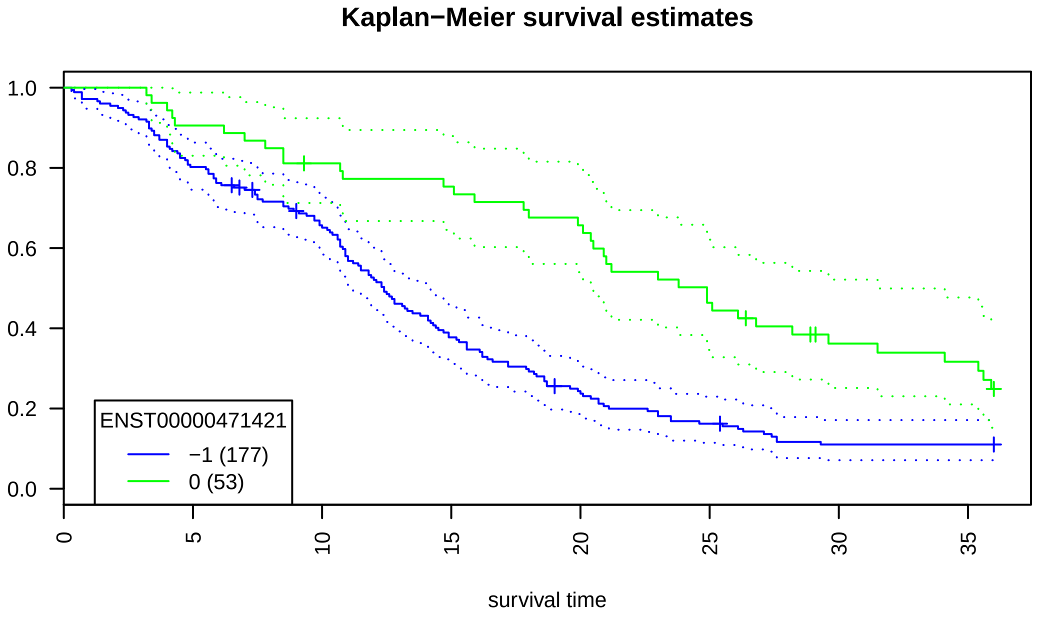

Figure 1397: Each curve represents the relative number of ENST00000471421 survived to the corresponding point of time. Dashed lines represent 0.95 confidence intervals for the curves with the corresponding color. Log-rank probability measure for the equality of these curves is 3.235572e-05. Additional annotations: .*GeneId*=ENSG00000019995, .*GeneName*=ZRANB1, .*GeneDesc*=zinc finger, RAN-binding domain containing 1 [Source:HGNC Symbol;Acc:18224].

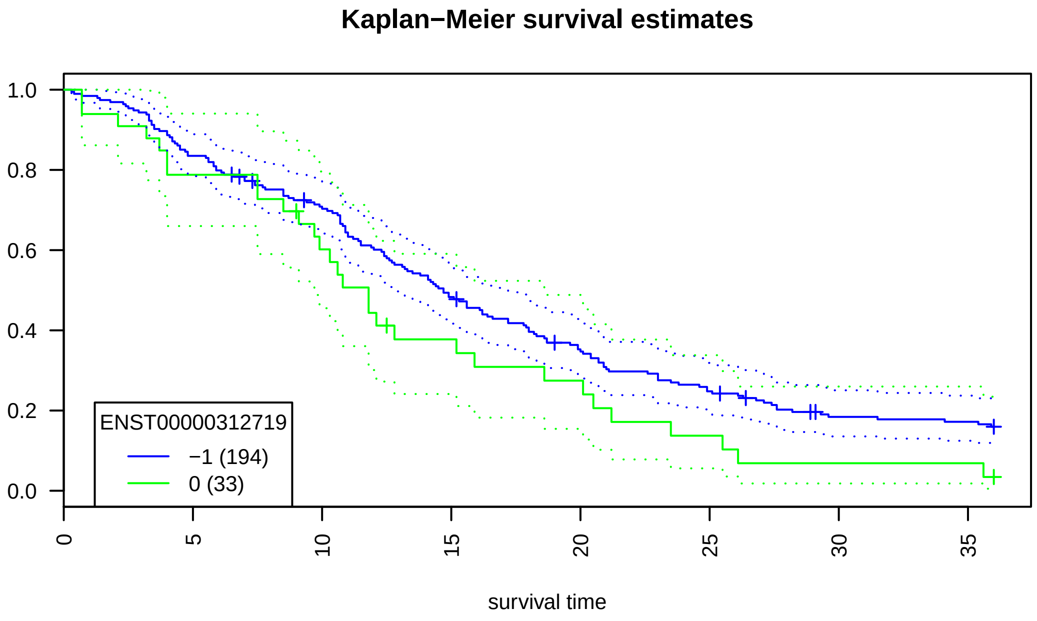

Figure 1398: Each curve represents the relative number of ENST00000312719 survived to the corresponding point of time. Dashed lines represent 0.95 confidence intervals for the curves with the corresponding color. Total of 3 observations deleted due to missingness. Log-rank probability measure for the equality of these curves is 4.081776e-02. Additional annotations: *.GeneId*=ENSG00000175818, *.GeneName*=RP11-112L15.1, *.GeneDesc*=[undefined].

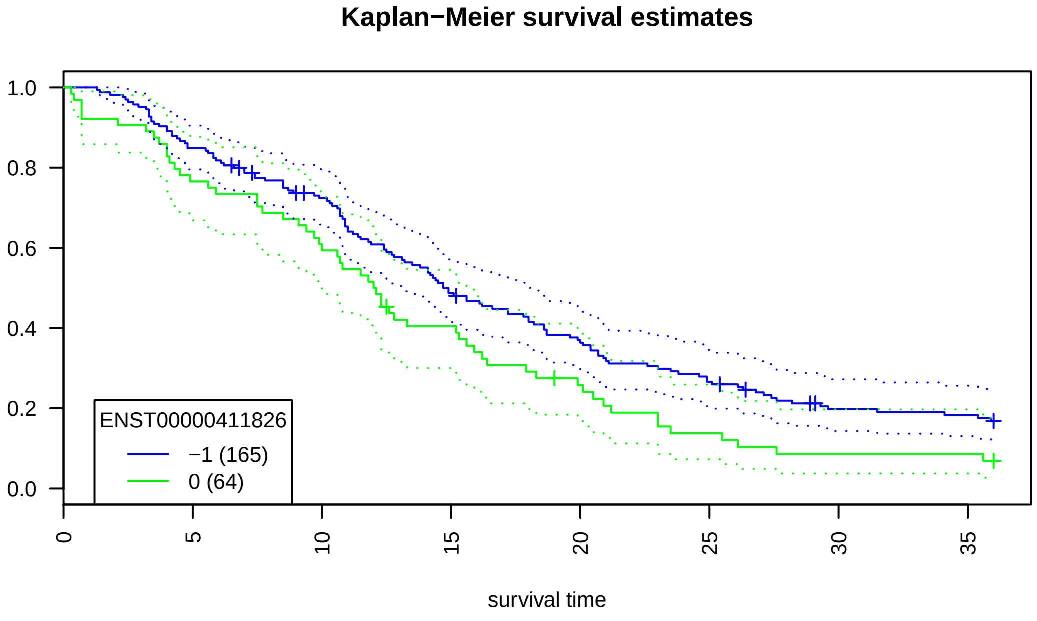

Figure 1399: Each curve represents the relative number of ENST00000411826 survived to the corresponding point of time. Dashed lines represent 0.95 confidence intervals for the curves with the corresponding color. Total of 1 observation deleted due to missingness. Log-rank probability measure for the equality of these curves is 1.539872e-02. Additional annotations: *.GeneId*=ENSG00000175818, *.GeneName*=RP11-112L15.1, *.GeneDesc*=[undefined].

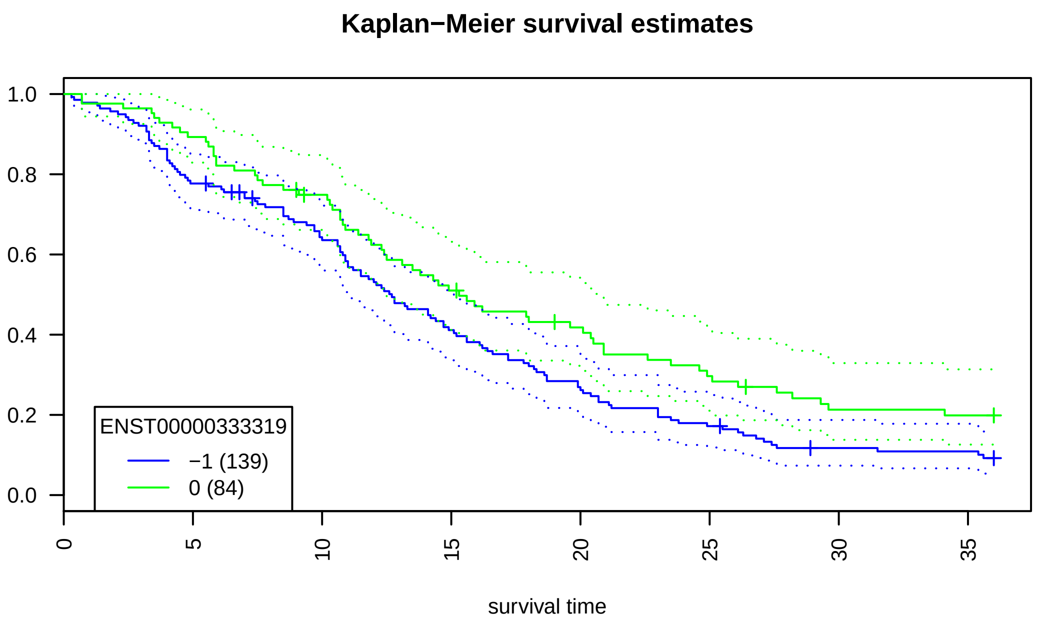

Figure 1400: Each curve represents the relative number of ENST00000333319 survived to the corresponding point of time. Dashed lines represent 0.95 confidence intervals for the curves with the corresponding color. Total of 7 observations deleted due to missingness. Log-rank probability measure for the equality of these curves is 1.812737e-02. Additional annotations: *.GeneId*=ENSG00000244291, *.GeneName*=C7orf13, *.GeneDesc*=Uncharacterized protein C7orf13 [Source:UniProtKB/Swiss-Prot;Acc:Q8NI28].

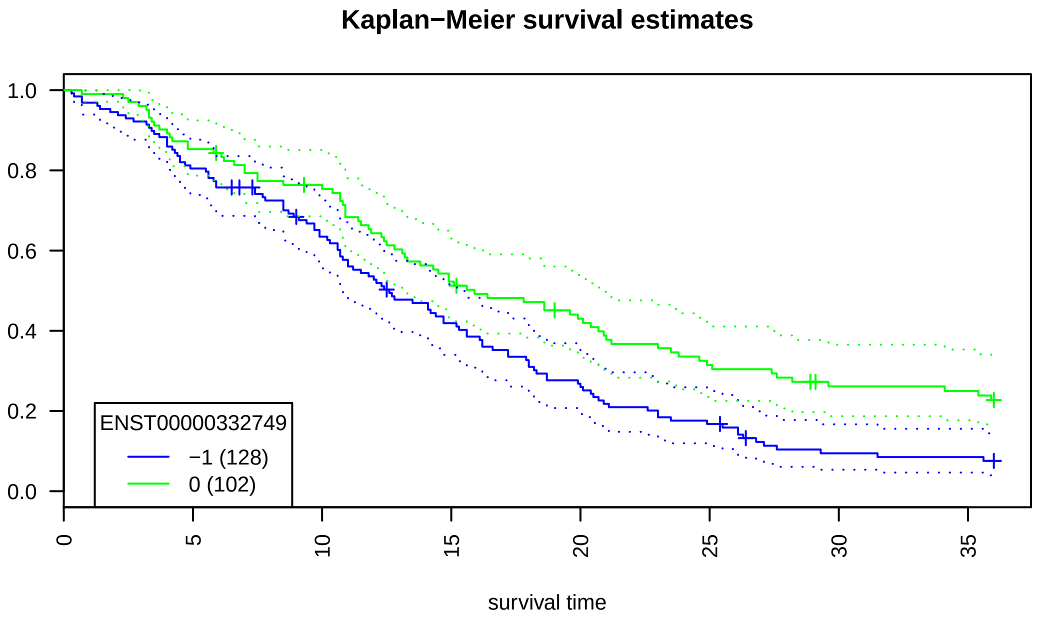

Figure 1401: Each curve represents the relative number of ENST00000332749 survived to the corresponding point of time. Dashed lines represent 0.95 confidence intervals for the curves with the corresponding color. Log-rank probability measure for the equality of these curves is 1.487513e-03.

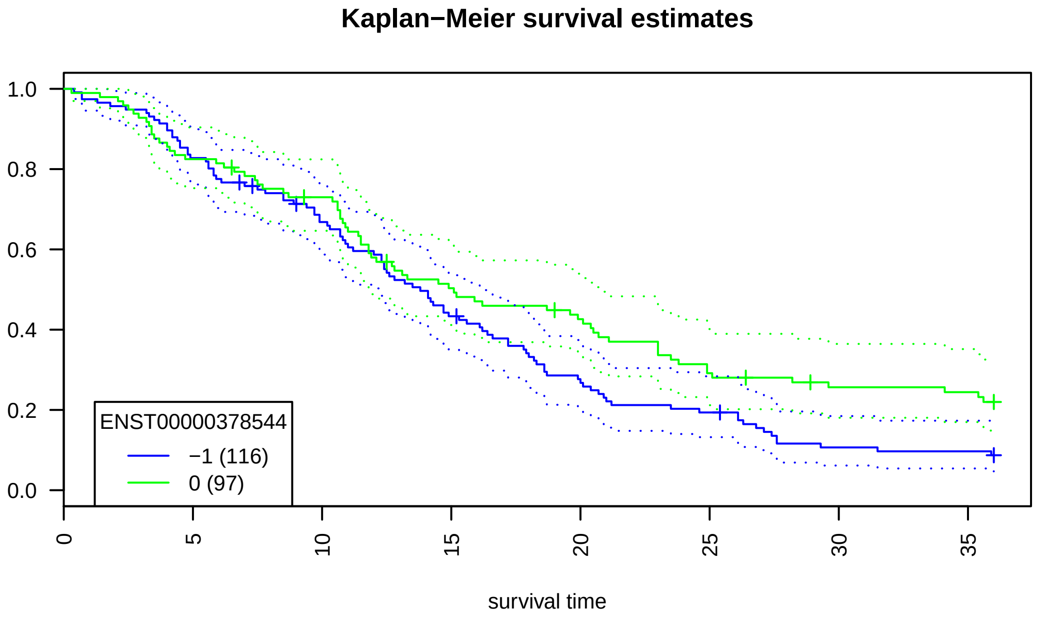

Figure 1402: Each curve represents the relative number of ENST00000378544 survived to the corresponding point of time. Dashed lines represent 0.95 confidence intervals for the curves with the corresponding color. Total of 17 observations deleted due to missingness. Log-rank probability measure for the equality of these curves is 2.764455e-02. Additional annotations: *.GeneId*=ENSG00000165630, *.GeneName*=PRPF18, *.GeneDesc*=PRP18 pre-mRNA processing factor 18 homolog (S. cerevisiae) [Source:HGNC Symbol;Acc:17351].

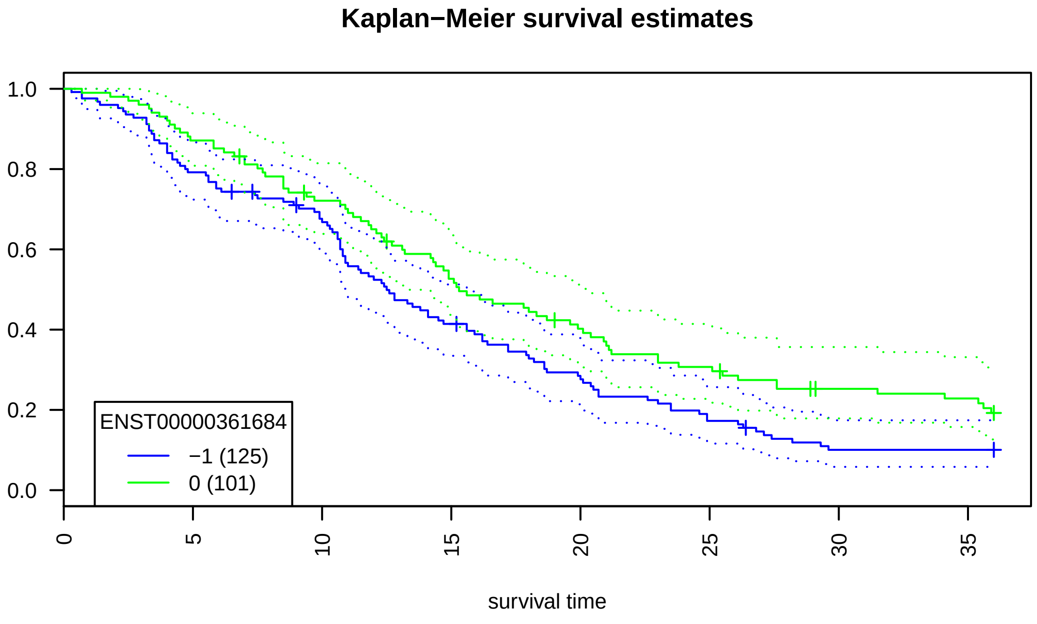

Figure 1403: Each curve represents the relative number of ENST00000361684 survived to the corresponding point of time. Dashed lines represent 0.95 confidence intervals for the curves with the corresponding color. Total of 4 observations deleted due to missingness. Log-rank probability measure for the equality of these curves is 1.653693e-02. Additional annotations: *.GeneId*=ENSG00000198792, *.GeneName*=TMEM184B, *.GeneDesc*=transmembrane protein 184B [Source:HGNC Symbol;Acc:1310].

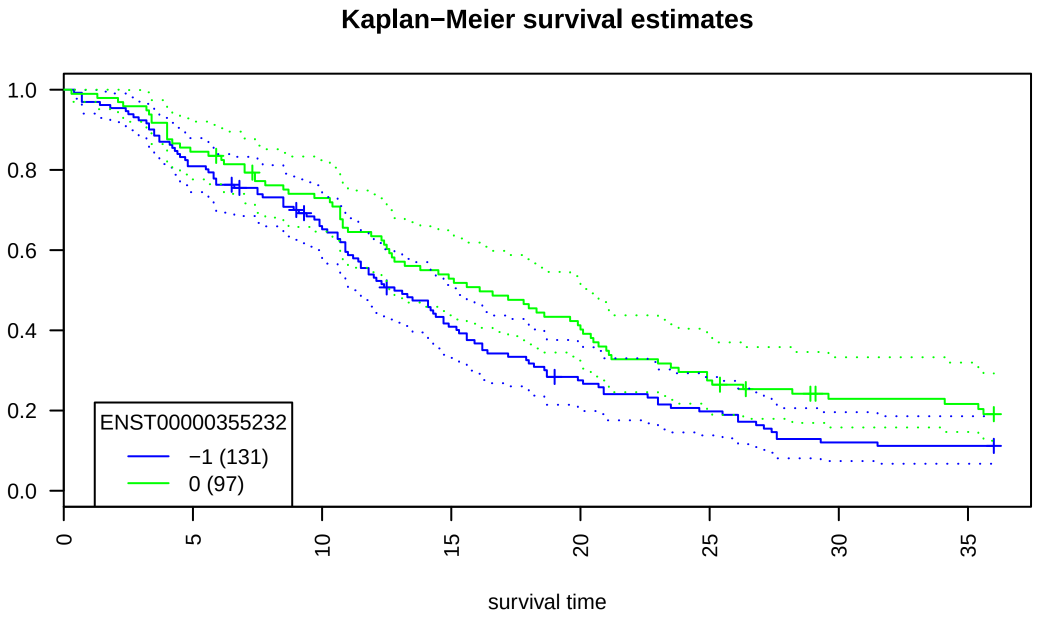

Figure 1404: Each curve represents the relative number of ENST00000355232 survived to the corresponding point of time. Dashed lines represent 0.95 confidence intervals for the curves with the corresponding color. Total of 2 observations deleted due to missingness. Log-rank probability measure for the equality of these curves is 4.71498e-02. Additional annotations: *.GeneId*=ENSG00000204172, *.GeneName*=AGAP10, *.GeneDesc*=ArfGAP with GTPase domain, ankyrin repeat and PH domain 10 [Source:HGNC Symbol;Acc:23462].

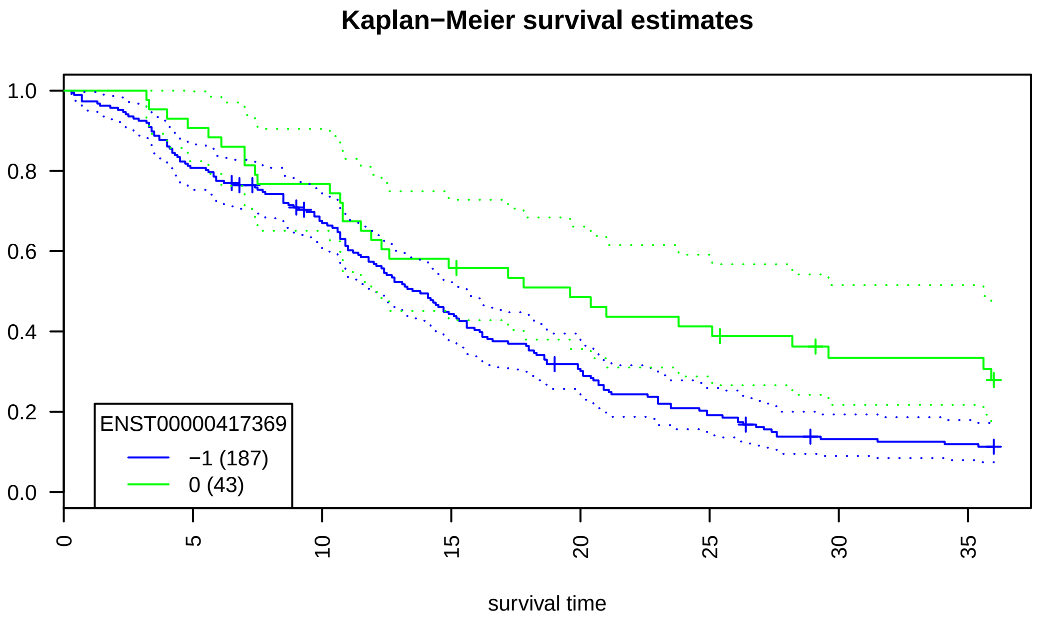

Figure 1405: Each curve represents the relative number of ENST00000417369 survived to the corresponding point of time. Dashed lines represent 0.95 confidence intervals for the curves with the corresponding color. Log-rank probability measure for the equality of these curves is 9.321319e-03.

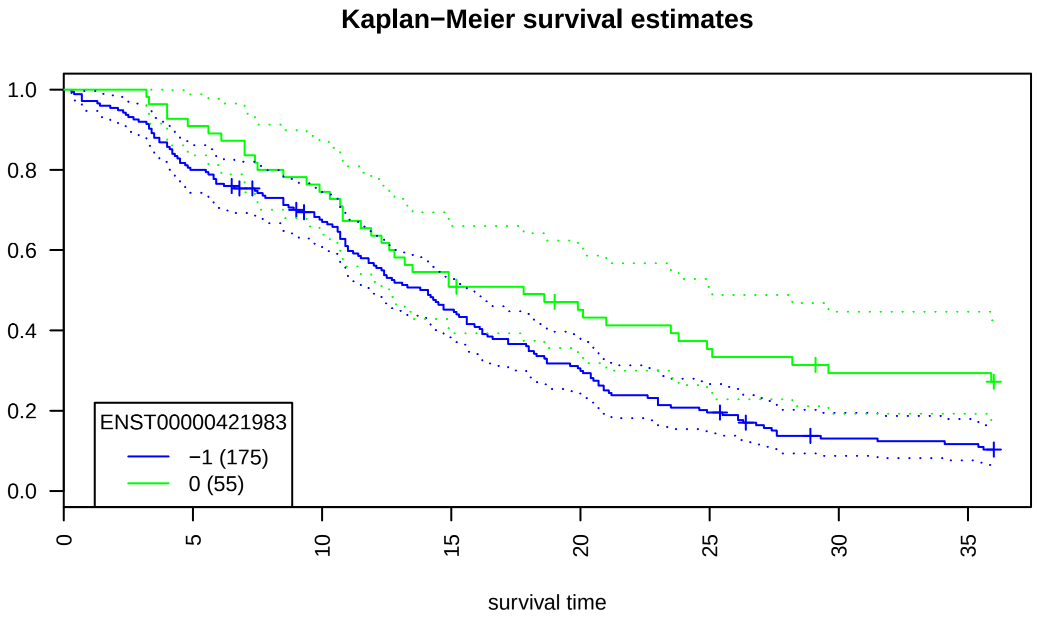

Figure 1406: Each curve represents the relative number of ENST00000421983 survived to the corresponding point of time. Dashed lines represent 0.95 confidence intervals for the curves with the corresponding color. Log-rank probability measure for the equality of these curves is 8.747126e-03. Additional annotations: *.GeneId*=ENSG00000120251, *.GeneName*=GRIA2, *.GeneDesc*=glutamate receptor, ionotropic, AMPA 2 [Source:HGNC Symbol;Acc:4572].

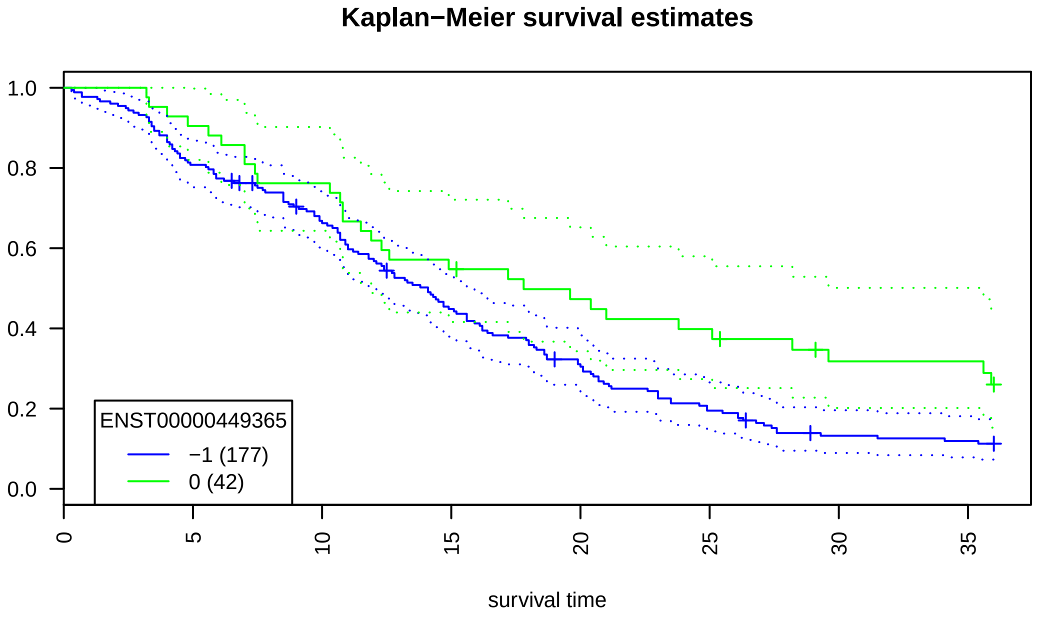

Figure 1407: Each curve represents the relative number of ENST00000449365 survived to the corresponding point of time. Dashed lines represent 0.95 confidence intervals for the curves with the corresponding color. Total of 11 observations deleted due to missingness. Log-rank probability measure for the equality of these curves is 1.842773e-02. Additional annotations: *.GeneId*=ENSG00000120251, *.GeneName*=GRIA2, *.GeneDesc*=glutamate receptor, ionotropic, AMPA 2 [Source:HGNC Symbol;Acc:4572].

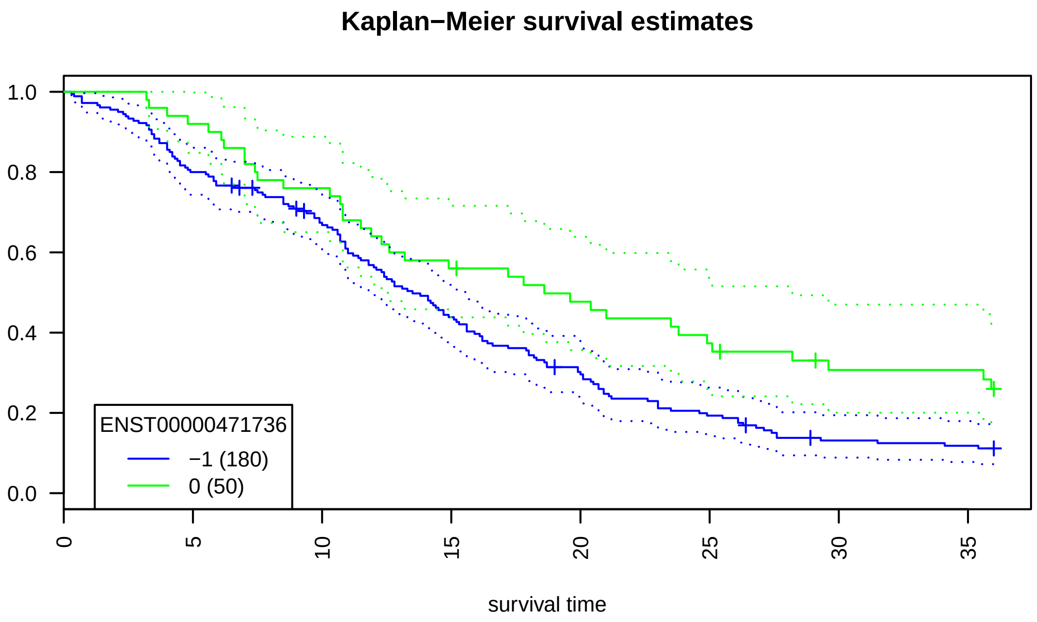

Figure 1408: Each curve represents the relative number of ENST00000471736 survived to the corresponding point of time. Dashed lines represent 0.95 confidence intervals for the curves with the corresponding color. Log-rank probability measure for the equality of these curves is 8.50402e-03. Additional annotations: *.GeneId*=ENSG00000120251, *.GeneName*=GRIA2, *.GeneDesc*=glutamate receptor, ionotropic, AMPA 2 [Source:HGNC Symbol;Acc:4572].

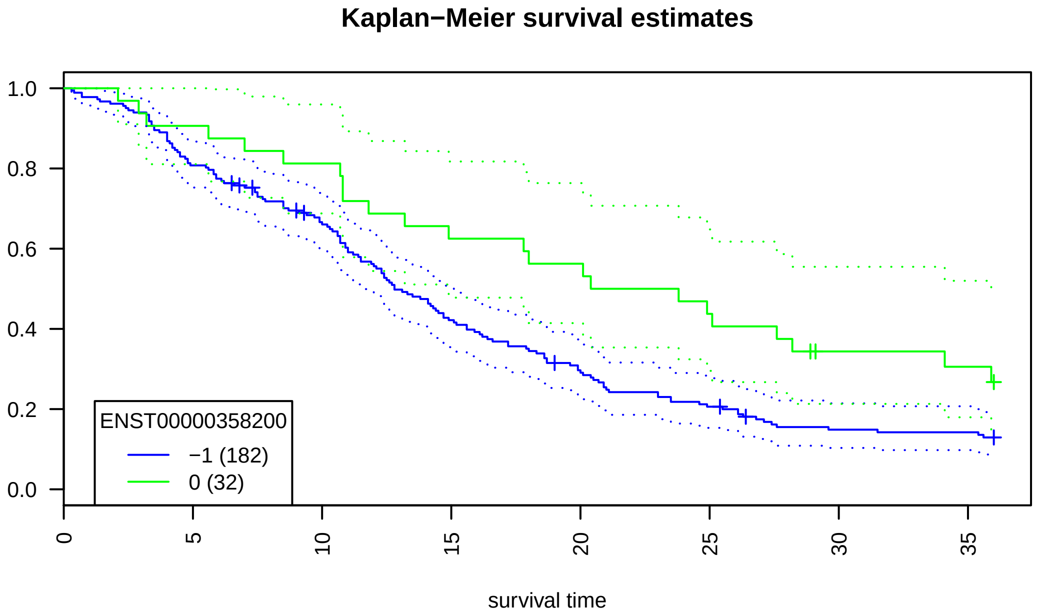

Figure 1409: Each curve represents the relative number of ENST00000358200 survived to the corresponding point of time. Dashed lines represent 0.95 confidence intervals for the curves with the corresponding color. Total of 16 observations deleted due to missingness. Log-rank probability measure for the equality of these curves is 1.379342e-02. Additional annotations: *.GeneId*=ENSG00000197147, *.GeneName*=LRRC8B, *.GeneDesc*=leucine rich repeat containing 8 family, member B [Source:HGNC Symbol;Acc:30692].

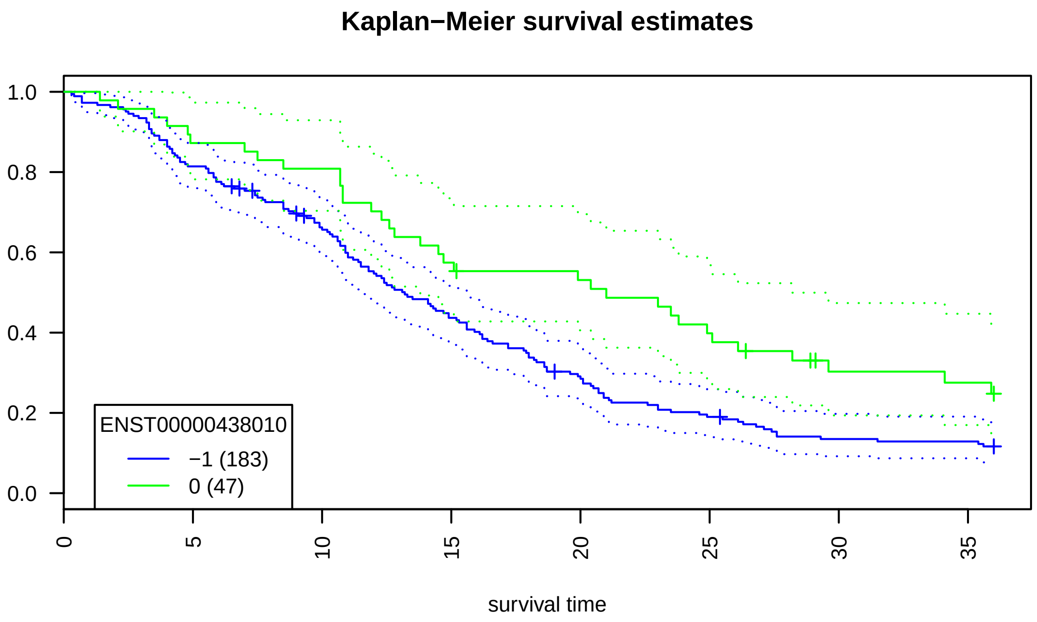

Figure 1410: Each curve represents the relative number of ENST00000438010 survived to the corresponding point of time. Dashed lines represent 0.95 confidence intervals for the curves with the corresponding color. Log-rank probability measure for the equality of these curves is 5.877241e-03. Additional annotations: *.GeneId*=ENSG00000227781, *.GeneName*=RP11-508M1.5, *.GeneDesc*=[undefined].

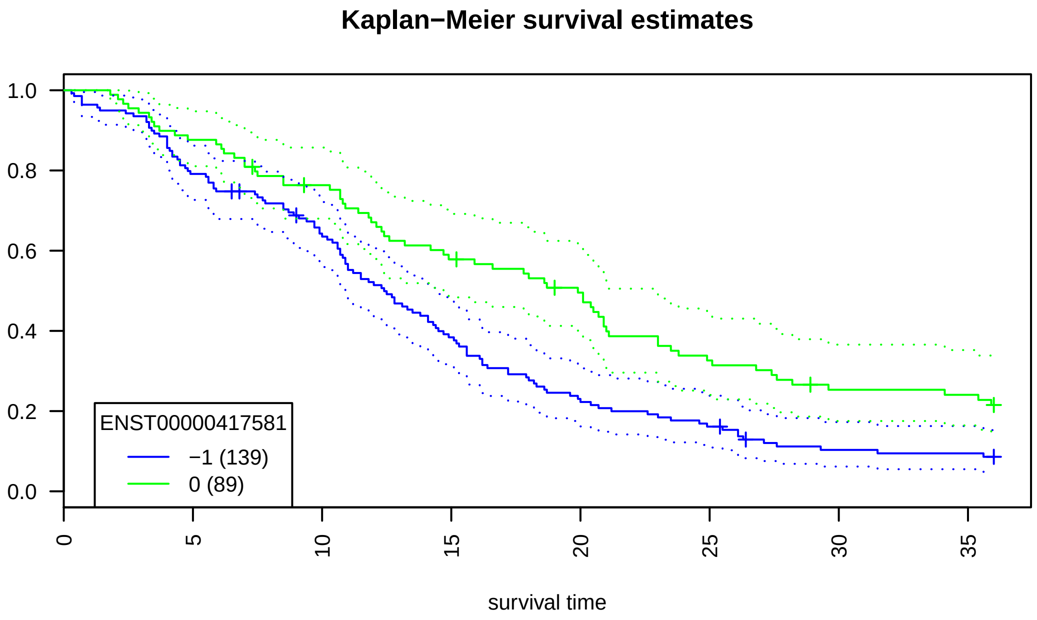

Figure 1411: Each curve represents the relative number of ENST00000417581 survived to the corresponding point of time. Dashed lines represent 0.95 confidence intervals for the curves with the corresponding color. Total of 2 observations deleted due to missingness. Log-rank probability measure for the equality of these curves is 5.189427e-04. Additional annotations: *.GeneId*=ENSG00000107951, *.GeneName*=MTPAP, *.GeneDesc*=mitochondrial poly(A) polymerase [Source:HGNC Symbol;Acc:25532].

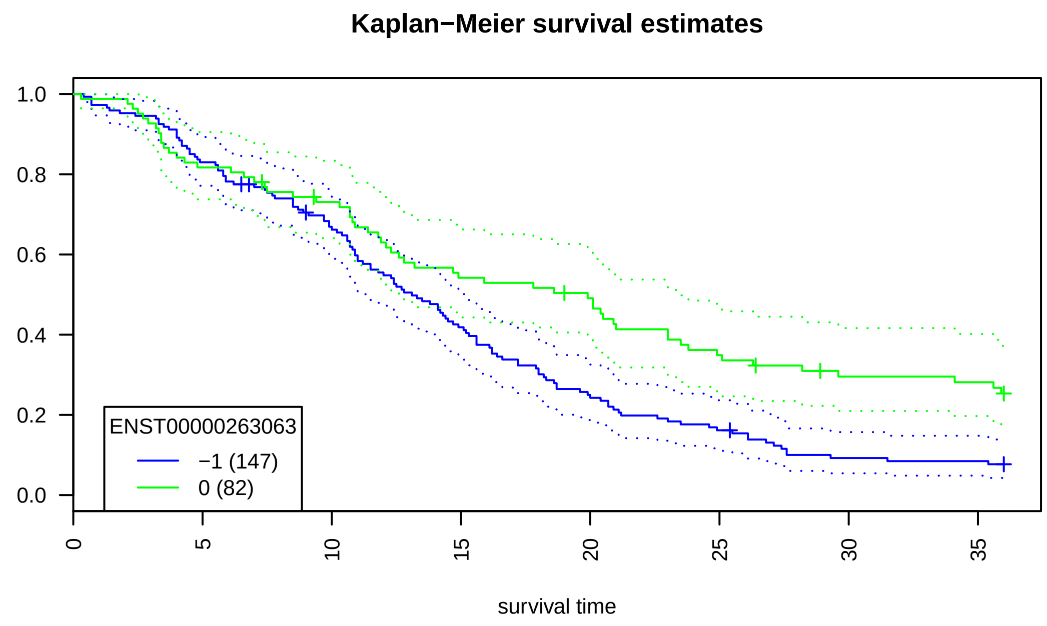

Figure 1412: Each curve represents the relative number of ENST00000263063 survived to the corresponding point of time. Dashed lines represent 0.95 confidence intervals for the curves with the corresponding color. Total of 1 observation deleted due to missingness. Log-rank probability measure for the equality of these curves is 8.57457e-04. Additional annotations: *.GeneId*=ENSG00000107951, *.GeneName*=MTPAP, *.GeneDesc*=mitochondrial poly(A) polymerase [Source:HGNC Symbol;Acc:25532].

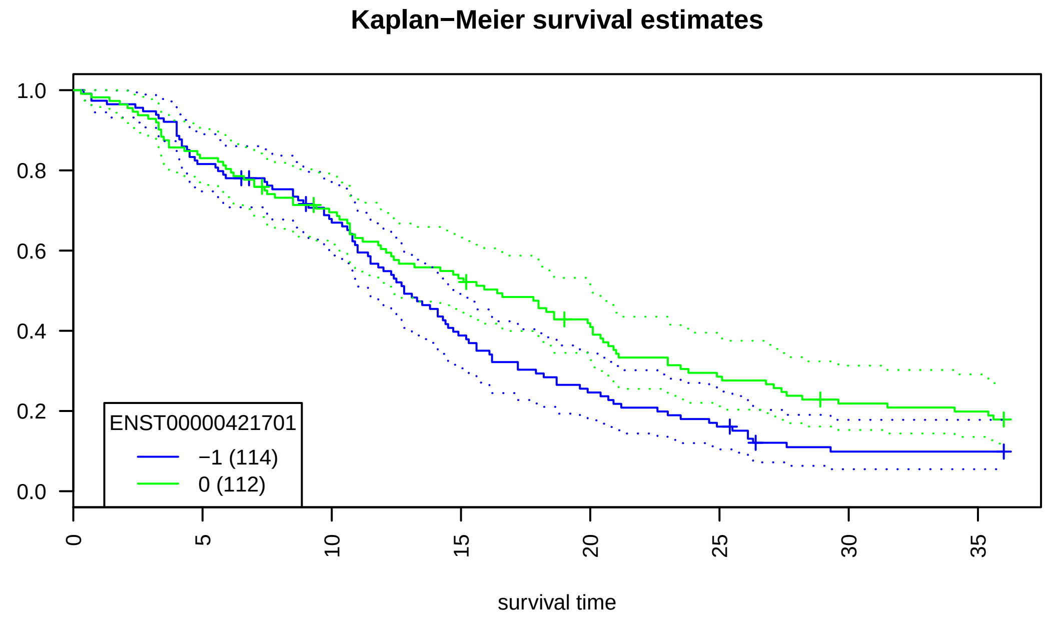

Figure 1413: Each curve represents the relative number of ENST00000421701 survived to the corresponding point of time. Dashed lines represent 0.95 confidence intervals for the curves with the corresponding color. Total of 4 observations deleted due to missingness. Log-rank probability measure for the equality of these curves is 3.431758e-02. Additional annotations: *.GeneId*=ENSG00000107951, *.GeneName*=MTPAP, *.GeneDesc*=mitochondrial poly(A) polymerase [Source:HGNC Symbol;Acc:25532].

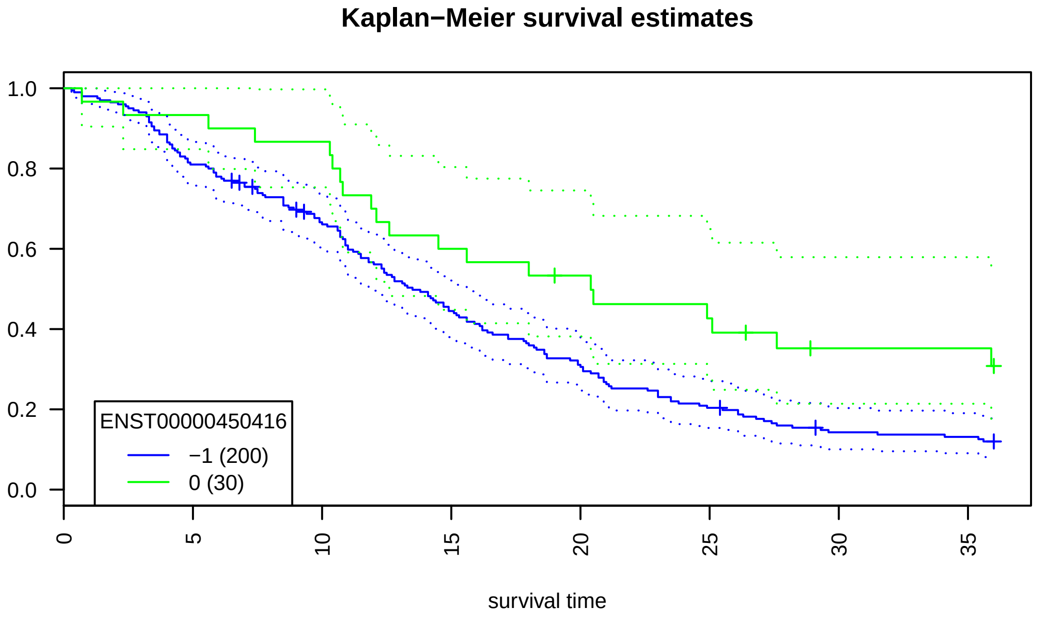

Figure 1414: Each curve represents the relative number of ENST00000450416 survived to the corresponding point of time. Dashed lines represent 0.95 confidence intervals for the curves with the corresponding color. Log-rank probability measure for the equality of these curves is 1.306926e-02.

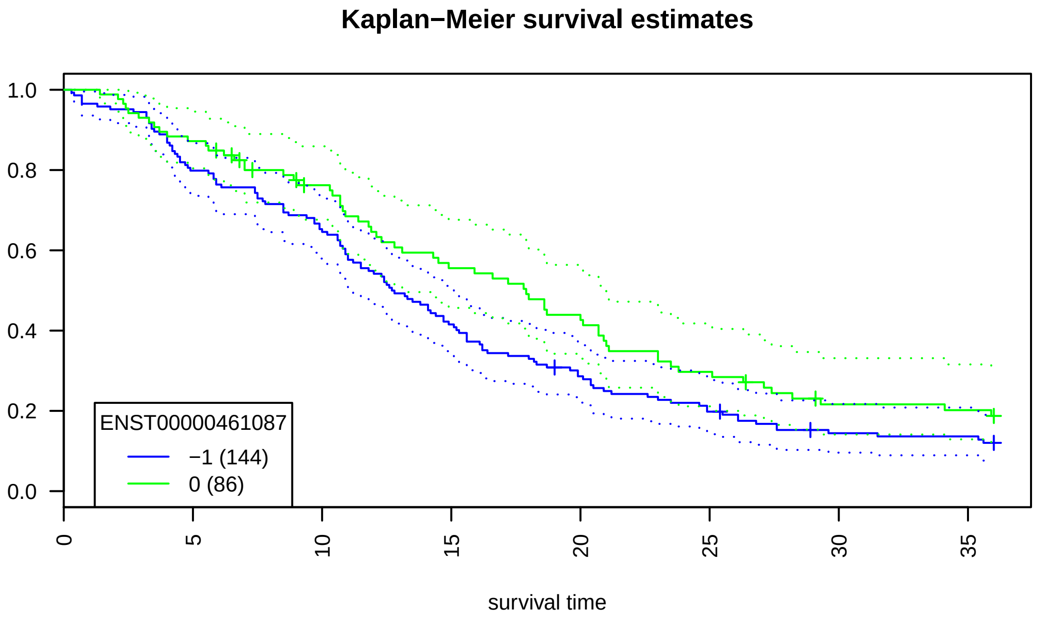

Figure 1415: Each curve represents the relative number of ENST00000461087 survived to the corresponding point of time. Dashed lines represent 0.95 confidence intervals for the curves with the corresponding color. Log-rank probability measure for the equality of these curves is 4.619339e-02. Additional annotations: $.GeneId$=ENSG00000240124, $.GeneName$=AL163636.2, $.GeneDesc$=[undefined].

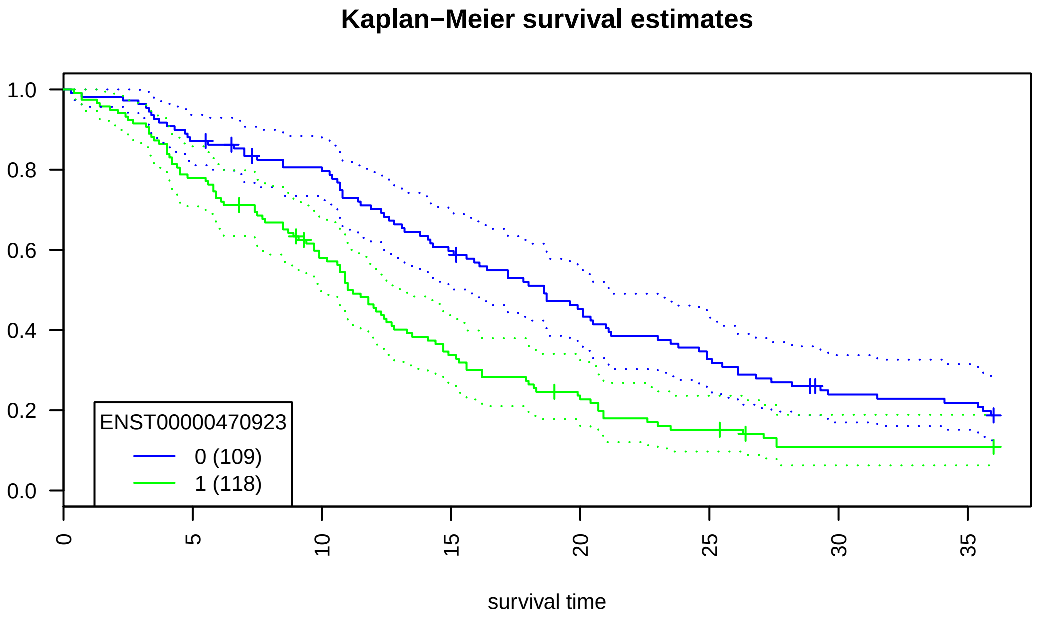

Figure 1416: Each curve represents the relative number of ENST00000470923 survived to the corresponding point of time. Dashed lines represent 0.95 confidence intervals for the curves with the corresponding color. Total of 3 observations deleted due to missingness. Log-rank probability measure for the equality of these curves is 3.597213e-04. Additional annotations: *.GeneId*=ENSG00000243125, *.GeneName*=AC068035.1, *.GeneDesc*=[undefined].

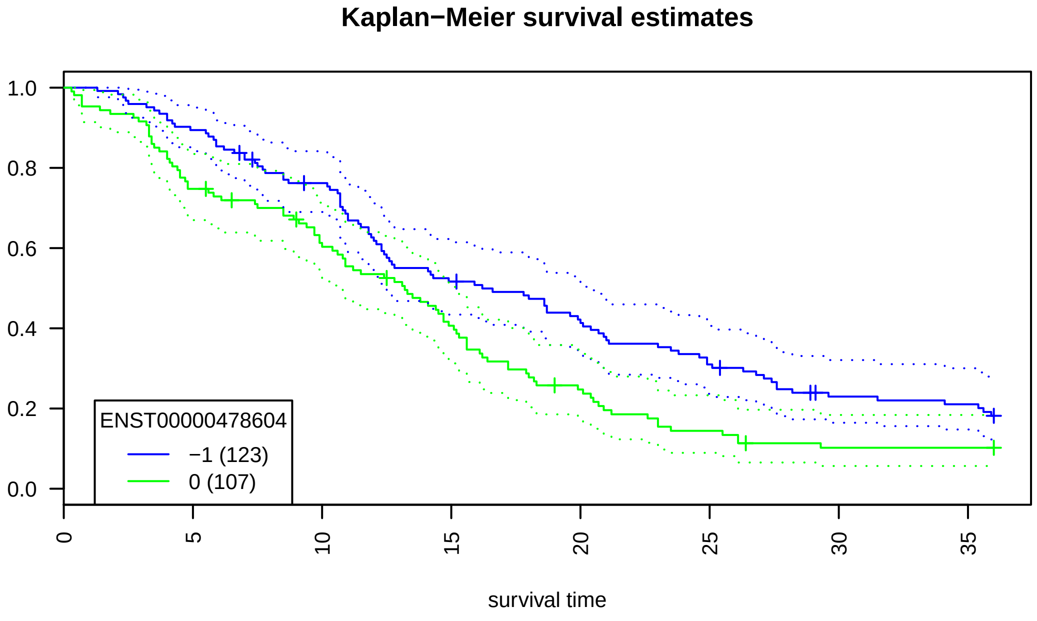

Figure 1417: Each curve represents the relative number of ENST00000478604 survived to the corresponding point of time. Dashed lines represent 0.95 confidence intervals for the curves with the corresponding color. Log-rank probability measure for the equality of these curves is 4.21561e-03. Additional annotations: *.GeneId*=ENSG00000240597, *.GeneName*=AL121932.1, *.GeneDesc*=[undefined].

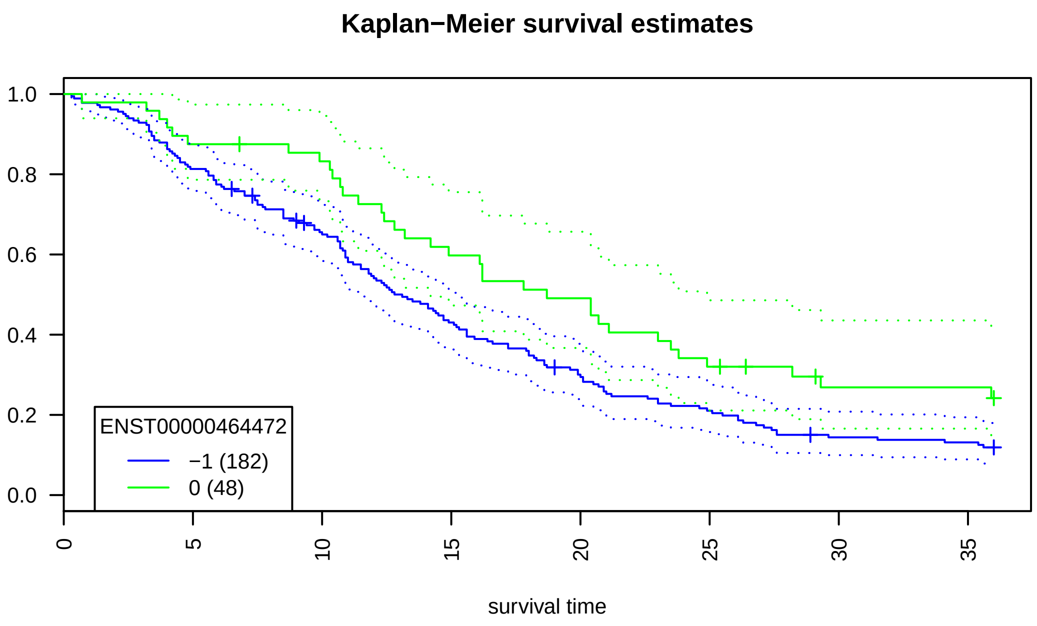

Figure 1418: Each curve represents the relative number of ENST00000464472 survived to the corresponding point of time. Dashed lines represent 0.95 confidence intervals for the curves with the corresponding color. Log-rank probability measure for the equality of these curves is 1.483216e-02. Additional annotations: *.GeneId*=ENSG00000244112, *.GeneName*=AC087885.1, *.GeneDesc*=[undefined].

6 Signalling pathway impact analysis for differentially expressed transcripts with survival p < 0.01

Table 5: SPIA analysis [24]. Name is the KEGG name for the pathway, and ID is the KEGG internal identication for the pathway. pSize is total number of genes in the pathway according to KEGG, where as NDE is the number of diffentially expressed genes on the pathway. pNDE is the p-value to observe NDE number of genes on the pathway by chance. tA is a perturbation factor defined by SPIA algorithm and pPERT is the probability of observing a more intense perturbation than tA by chance. pG is the combination of pPERT and pNDE. pGFdr is the false discovery rate and pGFWER the Bonferoni corrected p-value.

Name	ID	pSize	NDE	tA	pNDE	pPERT	pG	pGFdr	pGFWER	Status
Leukocyte transendothelial mig	hsa04670	87	7	5.4124	0.0000	0.464	0.0002	0.0064	0.0064	Activated
Regulation of actin cytoskelet	hsa04810	157	6	14.8743	0.0051	0.046	0.0022	0.0459	0.0918	Activated
Wnt signaling pathway	hsa04310	118	5	0.0000	0.0068	1	0.0405	0.5672	1.0000	Inhibited
Long-term depression	hsa04730	53	2	-4.8683	0.0993	0.114	0.0620	0.6512	1.0000	Inhibited
Melanoma	hsa05218	56	1	-9.5231	0.4326	0.048	0.1012	0.6862	1.0000	Inhibited
Focal adhesion	hsa04510	136	4	4.0383	0.0483	0.474	0.1094	0.6862	1.0000	Activated
Oocyte meiosis	hsa04114	87	3	-3.1264	0.0573	0.562	0.1428	0.6862	1.0000	Inhibited
MAPK signaling pathway	hsa04010	186	4	-2.3279	0.1179	0.32	0.1614	0.6862	1.0000	Inhibited
Chemokine signaling pathway	hsa04062	151	2	9.5111	0.4498	0.108	0.1955	0.6862	1.0000	Activated
Cytokine-cytokine receptor int	hsa04060	212	4	2.6052	0.1651	0.314	0.2053	0.6862	1.0000	Activated
Intestinal immune network for	hsa04672	38	2	0.0000	0.0557	1	0.2167	0.6862	1.0000	Inhibited
Long-term potentiation	hsa04720	53	2	3.1422	0.0993	0.6	0.2275	0.6862	1.0000	Activated
Amyotrophic lateral sclerosis	hsa05014	40	2	0.0000	0.0611	1	0.2318	0.6862	1.0000	Inhibited
VEGF signaling pathway	hsa04370	62	2	-2.0948	0.1287	0.508	0.2436	0.6862	1.0000	Inhibited
Arrhythmogenic right ventricul	hsa05412	45	2	0.0000	0.0750	1	0.2694	0.6862	1.0000	Inhibited
Natural killer cell mediated c	hsa04650	89	2	7.1016	0.2252	0.348	0.2779	0.6862	1.0000	Activated
Hedgehog signaling pathway	hsa04340	49	1	2.5454	0.3909	0.214	0.2912	0.6862	1.0000	Activated
Antigen processing and present	hsa04612	48	1	1.0450	0.3847	0.238	0.3105	0.6862	1.0000	Activated
Toll-like receptor signaling p	hsa04620	71	1	4.2258	0.5127	0.186	0.3195	0.6862	1.0000	Activated
Complement and coagulation cas	hsa04610	48	1	-4.9015	0.3847	0.256	0.3268	0.6862	1.0000	Inhibited
ECM-receptor interaction	hsa04512	59	2	0.0000	0.1186	1	0.3715	0.7040	1.0000	Inhibited
Alzheimer's disease	hsa05010	109	1	-1.0474	0.6689	0.18	0.3753	0.7040	1.0000	Inhibited
Dilated cardiomyopathy	hsa05414	61	2	0.0000	0.1253	1	0.3855	0.7040	1.0000	Inhibited
Systemic lupus erythematosus	hsa05322	70	2	0.0000	0.1562	1	0.4463	0.7259	1.0000	Inhibited
Fc gamma R-mediated phagocytos	hsa04666	68	1	-2.2502	0.4977	0.326	0.4573	0.7259	1.0000	Inhibited
Thyroid cancer	hsa05216	18	1	0.0000	0.1663	1	0.4647	0.7259	1.0000	Inhibited
Axon guidance	hsa04360	88	2	1.0846	0.2215	0.756	0.4667	0.7259	1.0000	Activated
Lysosome	hsa04142	86	2	0.0000	0.2141	1	0.5441	0.8161	1.0000	Inhibited
Prion diseases	hsa05020	32	1	0.0000	0.2765	1	0.6319	0.9001	1.0000	Inhibited
Apoptosis	hsa04210	64	1	-1.0474	0.4769	0.598	0.6430	0.9001	1.0000	Inhibited
Jak-STAT signaling pathway	hsa04630	122	2	0.0000	0.3476	1	0.7149	0.9217	1.0000	Inhibited
Neuroactive ligand-receptor in	hsa04080	213	3	0.0000	0.3618	1	0.7296	0.9217	1.0000	Inhibited
NOD-like receptor signaling pa	hsa04621	46	1	0.0000	0.3721	1	0.7400	0.9217	1.0000	Inhibited
p53 signaling pathway	hsa04115	47	1	0.0000	0.3784	1	0.7462	0.9217	1.0000	Inhibited
B cell receptor signaling path	hsa04662	52	1	0.0000	0.4092	1	0.7748	0.9298	1.0000	Inhibited
Fc epsilon RI signaling pathwa	hsa04664	60	1	0.0000	0.4552	1	0.8135	0.9469	1.0000	Inhibited
GnRH signaling pathway	hsa04912	76	1	0.0000	0.5369	1	0.8708	0.9469	1.0000	Inhibited
T cell receptor signaling path	hsa04660	79	1	0.0000	0.5508	1	0.8793	0.9469	1.0000	Inhibited
Vascular smooth muscle contrac	hsa04270	81	1	0.0000	0.5598	1	0.8846	0.9469	1.0000	Inhibited
Tight junction	hsa04530	90	1	0.0000	0.5983	1	0.9056	0.9469	1.0000	Inhibited
Insulin signaling pathway	hsa04910	100	1	0.0000	0.6371	1	0.9243	0.9469	1.0000	Inhibited
Calcium signaling pathway	hsa04020	134	1	0.0000	0.7433	1	0.9638	0.9638	1.0000	Inhibited

7 Summary of differentially expressed genes: fc > 2.0 and corrected p-value < 0.05

Gene set	Size
over	1311
under	2161

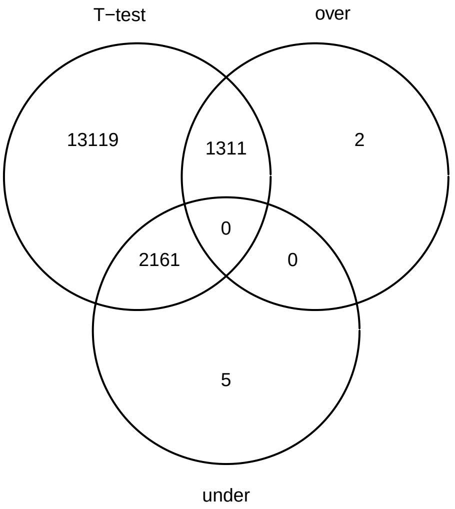

(a)

Figure 1419: Intersections of sets: (a) T-test, over and under.

7.1 Ordered transcript expression values

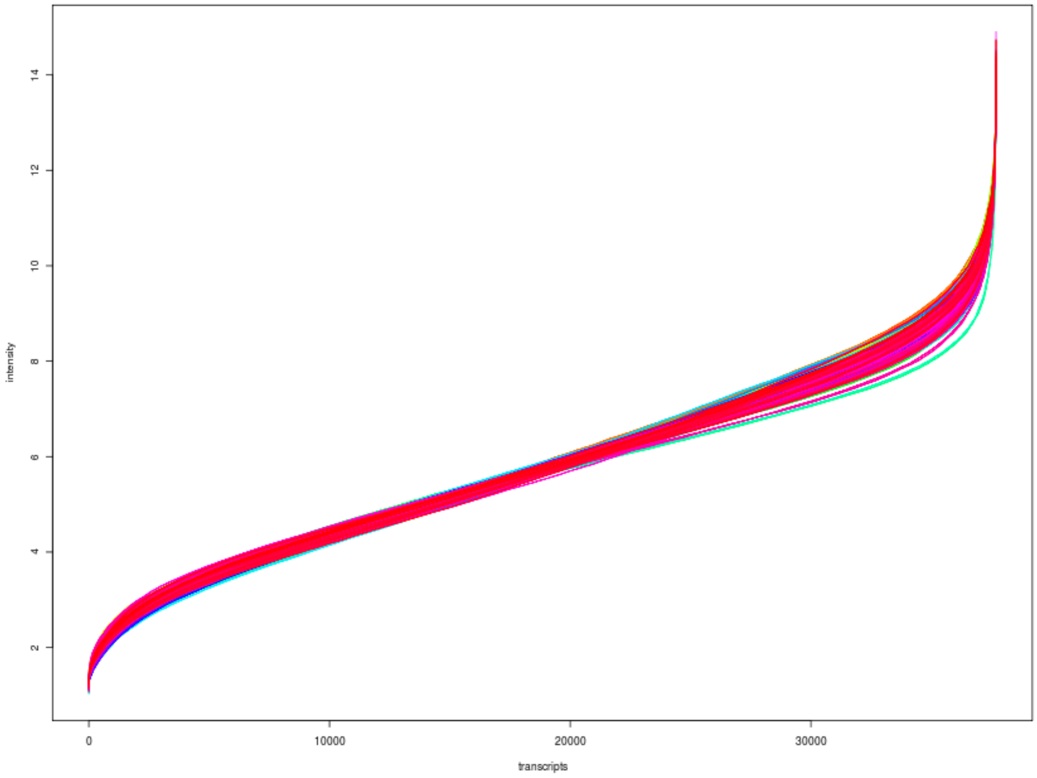

Figure 1420: Distribution of transcript expression values (log2) for the samples.

7.2 Multidimensional scaling of the samples

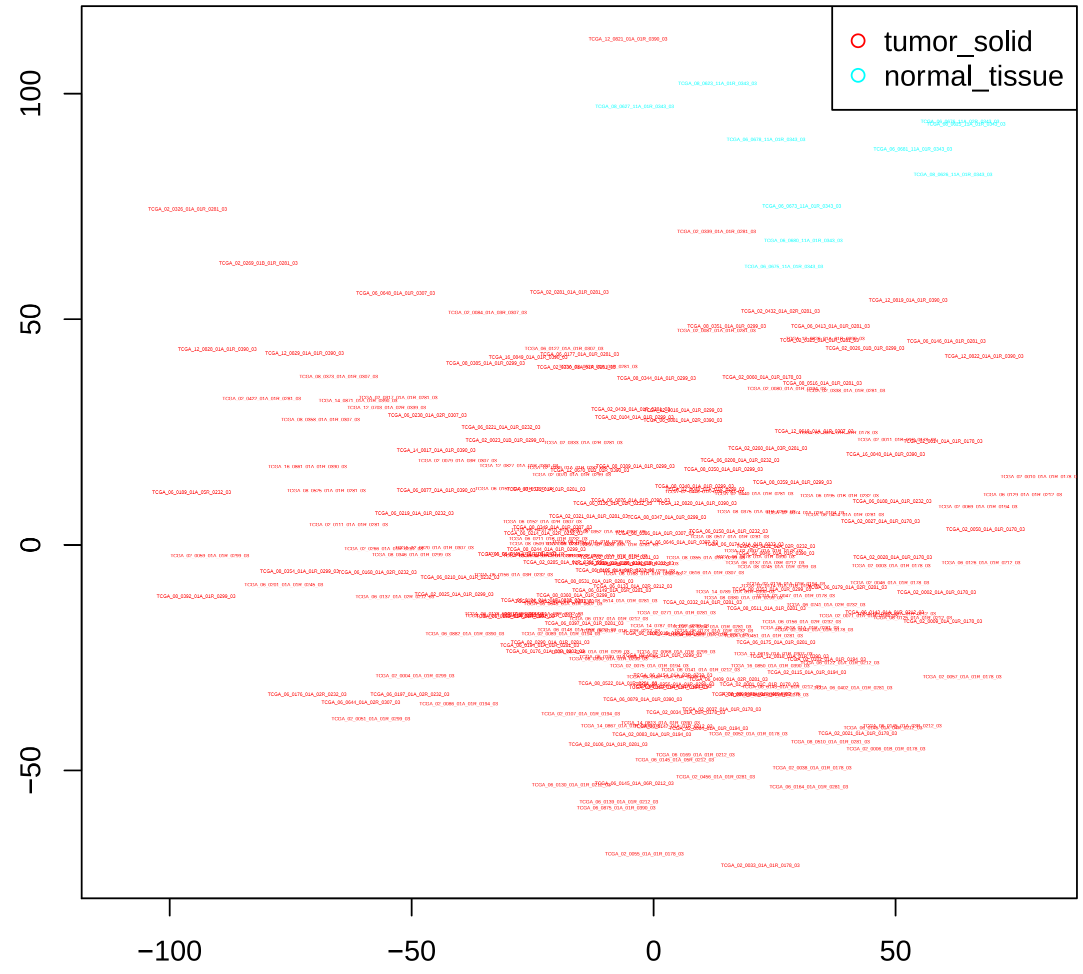

Figure 1421: Multidimensional Scaling

7.3 GO clustering

Gene products are clustered (grouped) based on their Gene Ontology (GO) annotations. Genes that have similar annotations belong to the same cluster. The distance between gene products is defined using information-theoretical semantic similarity measures.

In the table below, the most informative (most specific) common GO terms of each cluster are displayed. All gene products in the cluster are annotated with the GO term mentioned. A GO term is more informative if it occurs rarely in the GO annotations of the whole genome. The priori column contains the a priori probability p that a random gene product is annotated with the given GO term. Information is defined as $-\log_2 p$.

Generally, clusters with (1) a large number of gene products and (2) common GO terms with high information content are the most interesting.

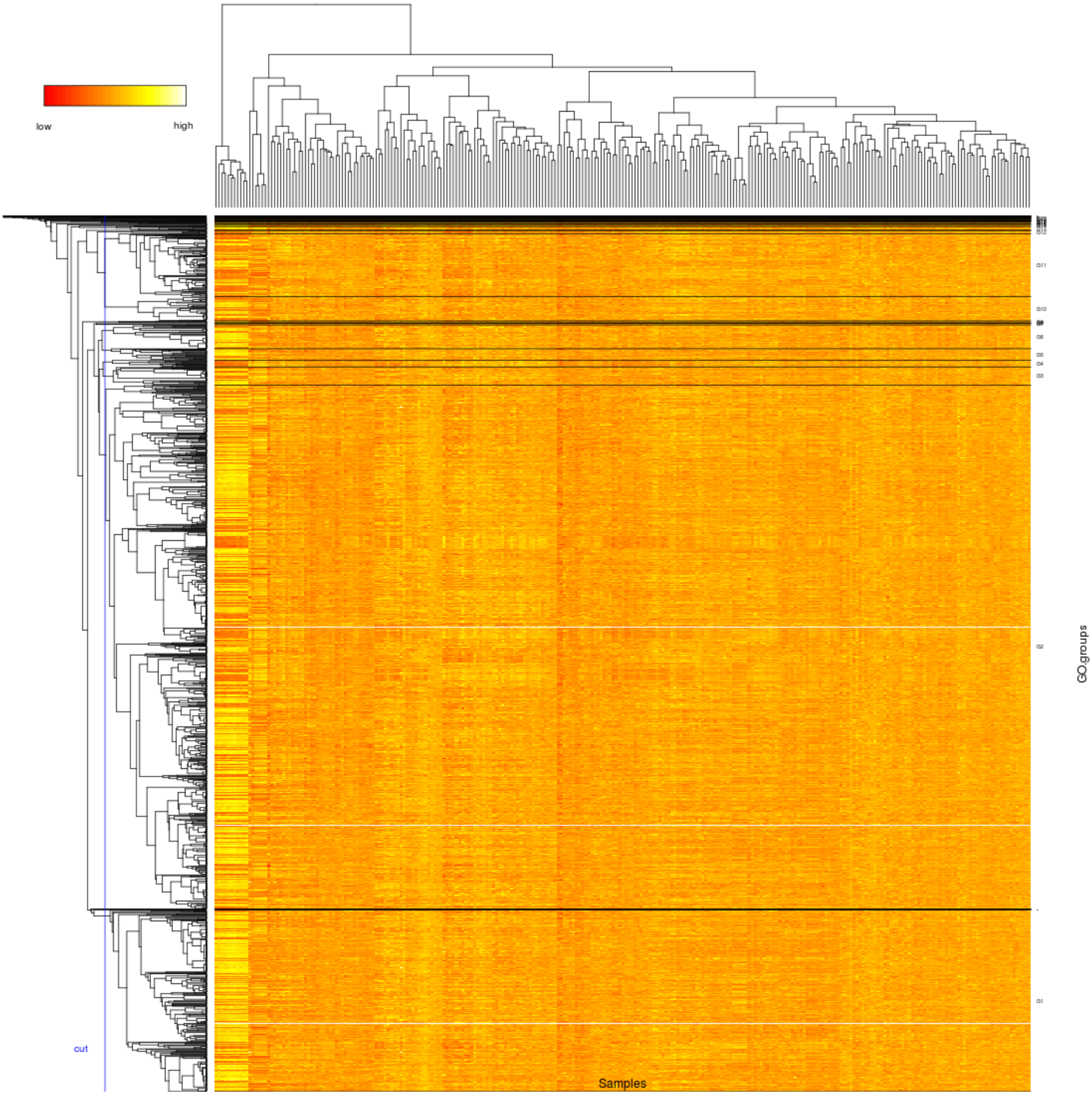

Figure 1422: GO clustering dendrogram and expression heatmap. The analysis is based on 2839 gene products. The median number of GO annotations per gene product is 10. In addition, 633 gene products had no GO annotation and were excluded from the analysis. The colors represent expression values of the gene products. The lower expression values are indicated with red and higher expressions with white. The left dendrogram corresponds to GO based clustering, while the top dendrogram uses expression values for clustering.

CLUSTER	SIZE	GOID	PRIORI	INFO	DESCRIPTION
G1	589				*[no interesting GO terms]*
G2	1697				*[no interesting GO terms]*
G3	59	GO:0044424	0.515	0.958	intracellular part
G4	22	GO:0009987	0.617	0.697	cellular process
G5	37	GO:0005737	0.295	1.763	cytoplasm
G6	77	GO:0005622	0.554	0.852	intracellular
G7	5	GO:0006986	0.001	9.707	response to unfolded protein
G8	2	GO:0005853	0	13.274	eukaryotic translation elongation factor 1 complex
		GO:0003746	0	11.254	translation elongation factor activity
		GO:0006414	0.002	9.142	translational elongation
G9	6	GO:0006810	0.096	3.38	transport
		GO:0009987	0.617	0.697	cellular process
G10	79				*[no interesting GO terms]*
G11	204				*[no interesting GO terms]*
G12	10	GO:0006629	0.026	5.288	lipid metabolic process
G13	12	GO:0005509	0.012	6.354	calcium ion binding
G14	4	GO:0043005	0.009	6.757	neuron projection
G15	4	GO:0031072	0.001	9.398	heat shock protein binding
G16	2	GO:0030156	0	14.646	benzodiazepine receptor binding
		GO:0005739	0.038	4.706	mitochondrion
G17	4	GO:0007417	0.011	6.464	central nervous system development
G18	3	GO:0016757	0.01	6.669	transferase activity, transferring glycosyl groups
		GO:0009058	0.119	3.071	biosynthetic process
G19	2	GO:0000079	0.001	9.966	regulation of cyclin-dependent protein kinase activity
		GO:0019887	0.002	9.076	protein kinase regulator activity
G20	2	GO:0016998	0	11.99	cell wall macromolecule catabolic process

8 Survival analysis for gene expression

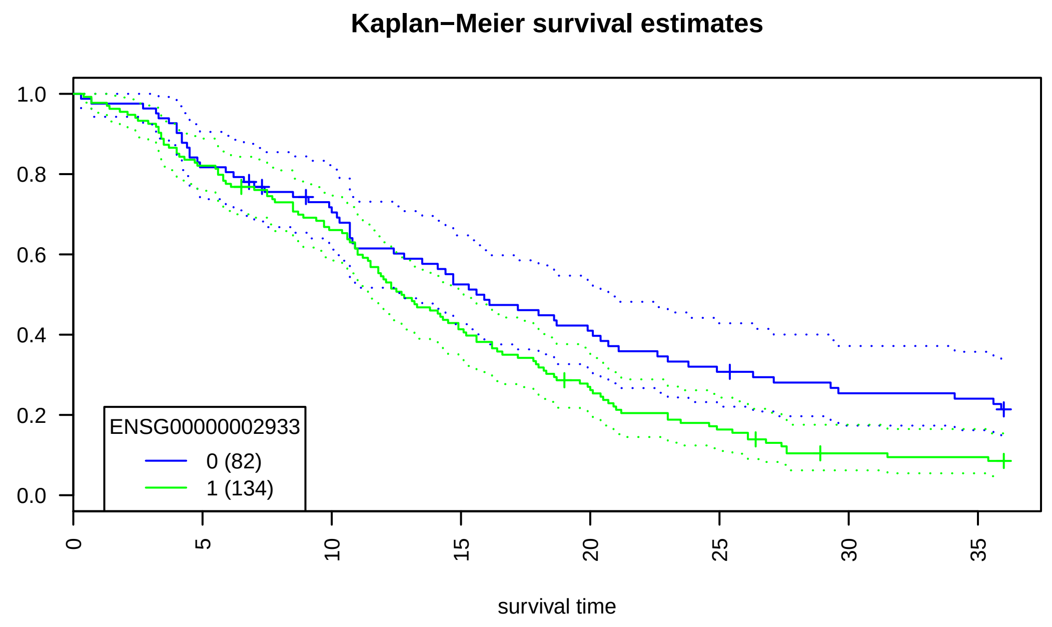

Figure 1423: Each curve represents the relative number of ENSG00000002933 survived to the corresponding point of time. Dashed lines represent 0.95 confidence intervals for the curves with the corresponding color. Total of 14 observations deleted due to missingness. Log-rank probability measure for the equality of these curves is 1.216144e-02. Additional annotations: *.GeneName*=TMEM176A, *.GeneDesc*=transmembrane protein 176A [Source:HGNC Symbol;Acc:24930].

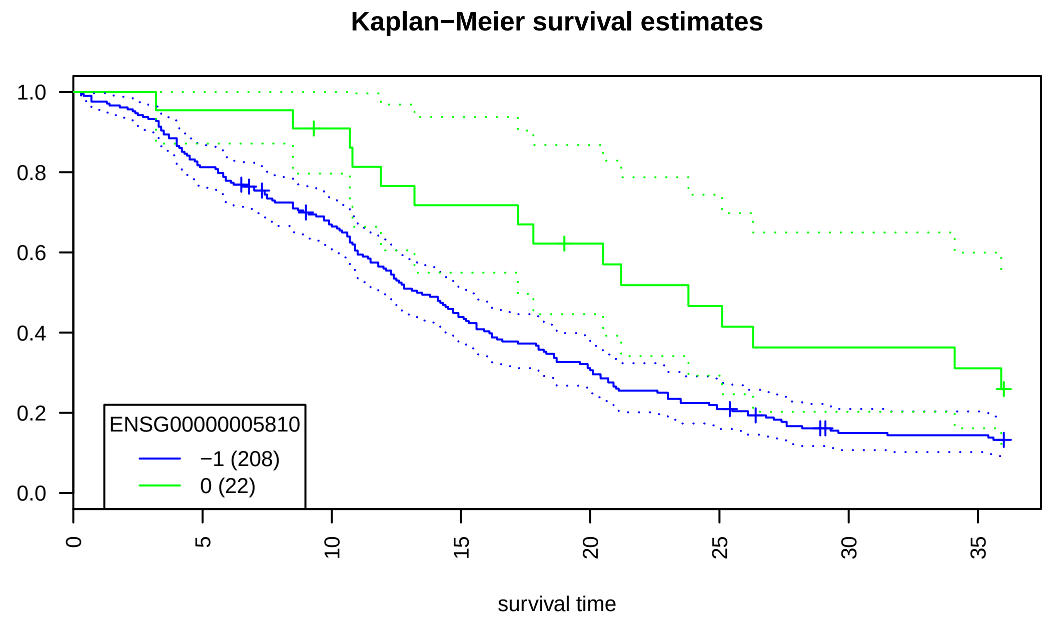

Figure 1424: Each curve represents the relative number of ENSG00000005810 survived to the corresponding point of time. Dashed lines represent 0.95 confidence intervals for the curves with the corresponding color. Log-rank probability measure for the equality of these curves is 2.182711e-02. Additional annotations: *.GeneName*=MYCBP2, *.GeneDesc*=MYC binding protein 2 [Source:HGNC Symbol;Acc:23386].

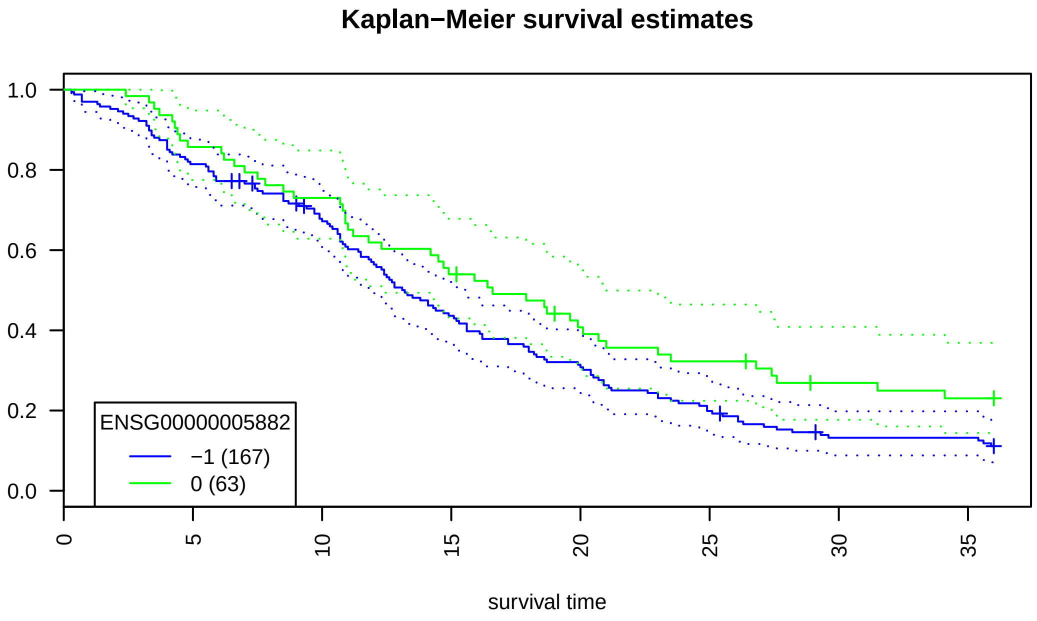

Figure 1425: Each curve represents the relative number of ENSG00000005882 survived to the corresponding point of time. Dashed lines represent 0.95 confidence intervals for the curves with the corresponding color. Log-rank probability measure for the equality of these curves is 3.392445e-02. Additional annotations: *.GeneName*=PDK2, *.GeneDesc*=pyruvate dehydrogenase kinase, isozyme 2 [Source:HGNC Symbol;Acc:8810].

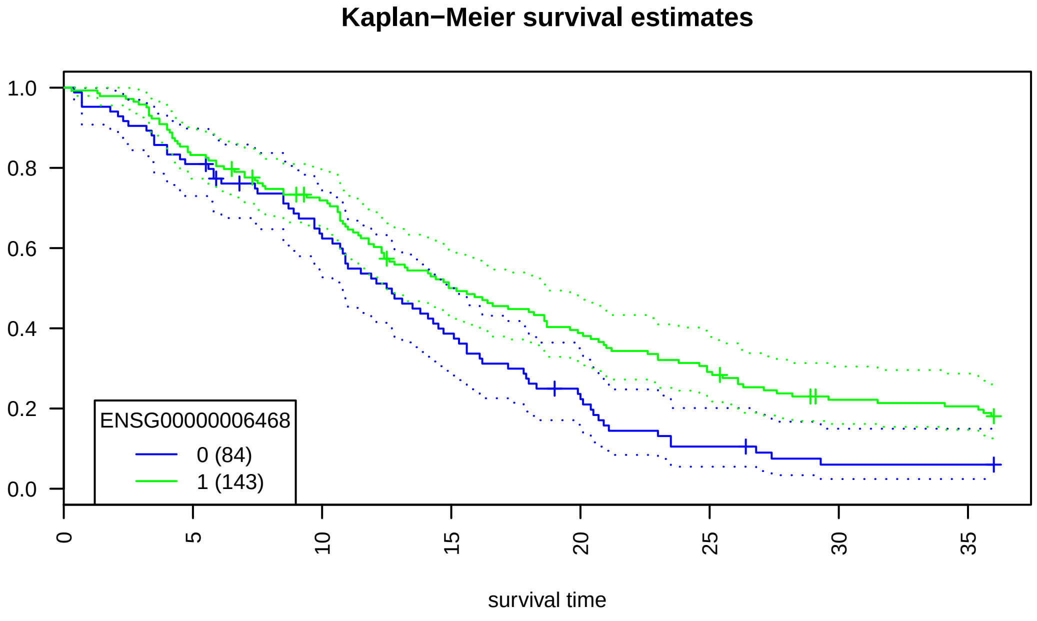

Figure 1426: Each curve represents the relative number of ENSG00000006468 survived to the corresponding point of time. Dashed lines represent 0.95 confidence intervals for the curves with the corresponding color. Total of 3 observations deleted due to missingness. Log-rank probability measure for the equality of these curves is 2.94152e-03. Additional annotations: *.GeneName*=ETV1, *.GeneDesc*=ets variant 1 [Source:HGNC Symbol;Acc:3490].

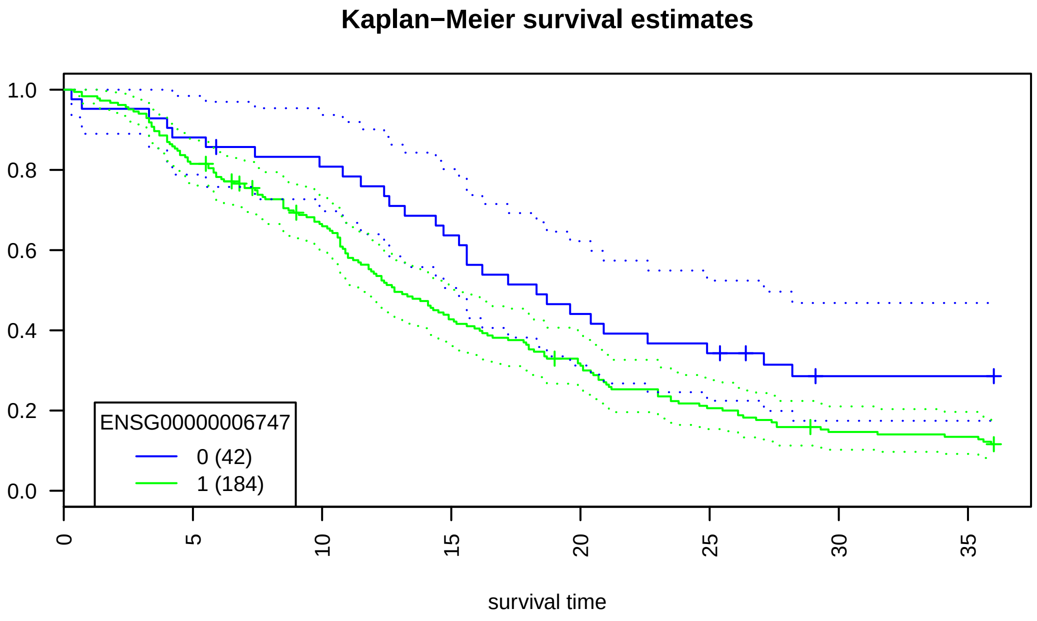

Figure 1427: Each curve represents the relative number of ENSG00000006747 survived to the corresponding point of time. Dashed lines represent 0.95 confidence intervals for the curves with the corresponding color. Total of 4 observations deleted due to missingness. Log-rank probability measure for the equality of these curves is 1.395398e-02. Additional annotations: *.GeneName*=SCIN, *.GeneDesc*=scinderin [Source:HGNC Symbol;Acc:21695].

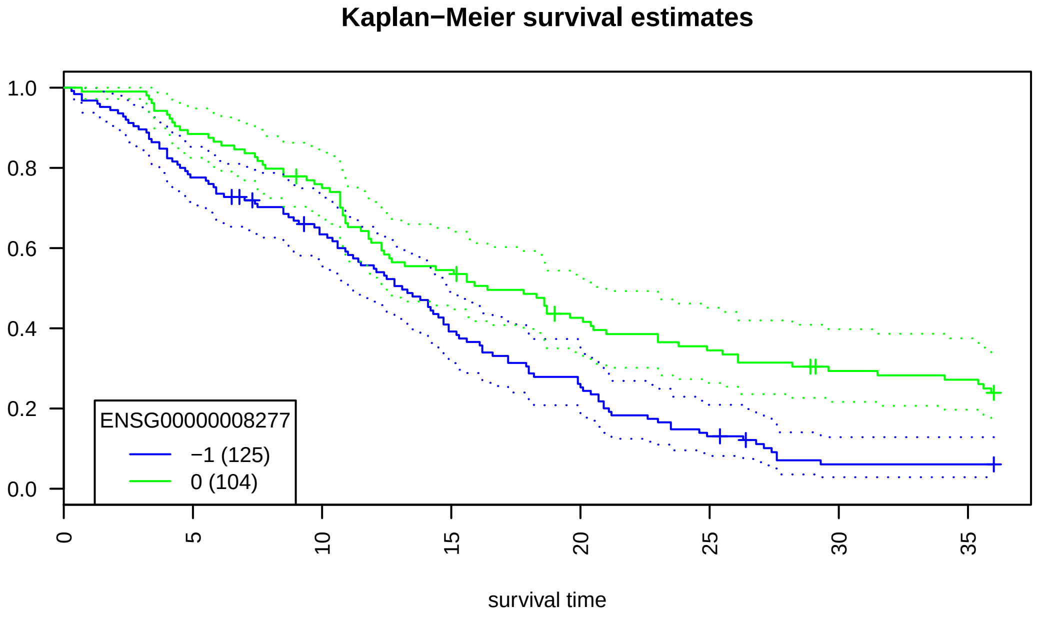

Figure 1428: Each curve represents the relative number of ENSG00000008277 survived to the corresponding point of time. Dashed lines represent 0.95 confidence intervals for the curves with the corresponding color. Total of 1 observation deleted due to missingness. Log-rank probability measure for the equality of these curves is 1.449672e-04. Additional annotations: *.GeneName*=ADAM22, *.GeneDesc*=ADAM metallopeptidase domain 22 [Source:HGNC Symbol;Acc:201].

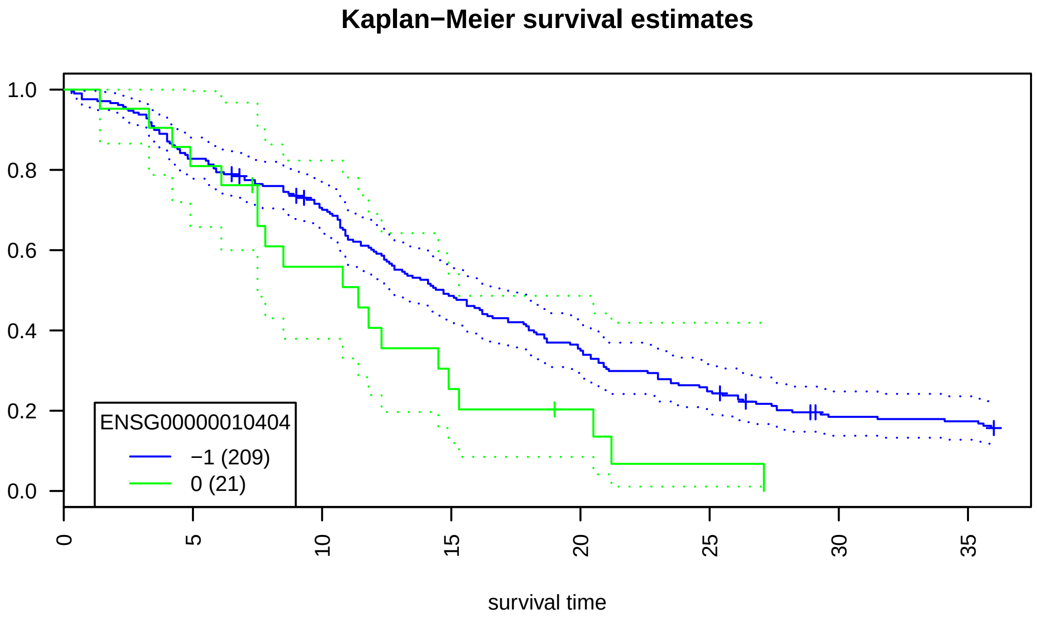

Figure 1429: Each curve represents the relative number of ENSG00000010404 survived to the corresponding point of time. Dashed lines represent 0.95 confidence intervals for the curves with the corresponding color. Log-rank probability measure for the equality of these curves is 2.014527e-02. Additional annotations: *.GeneName*=IDS, *.GeneDesc*=iduronate 2-sulfatase [Source:HGNC Symbol;Acc:5389].

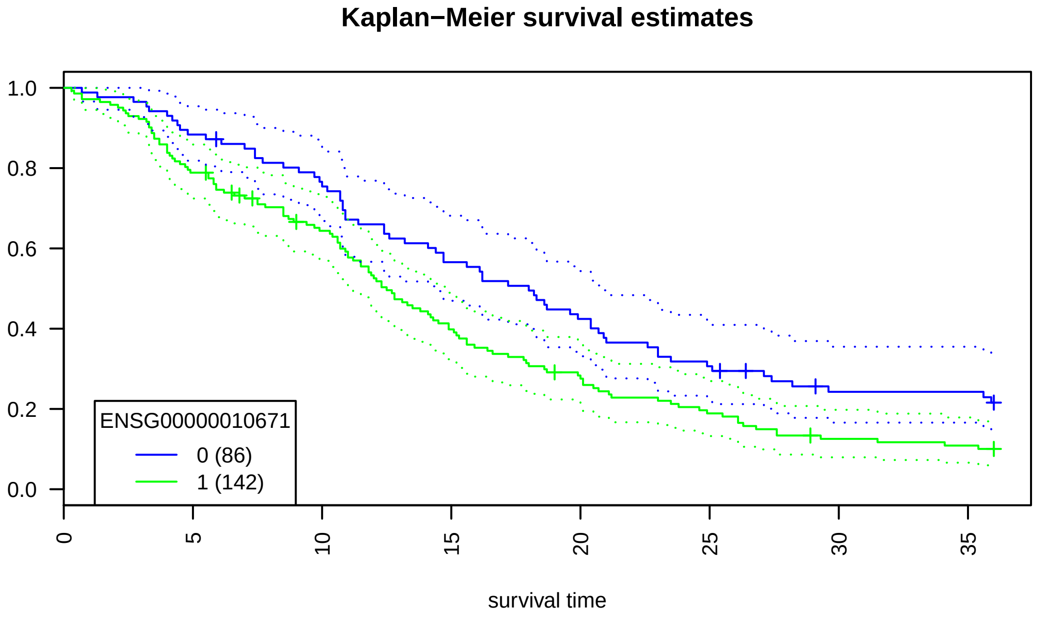

Figure 1430: Each curve represents the relative number of ENSG00000010671 survived to the corresponding point of time. Dashed lines represent 0.95 confidence intervals for the curves with the corresponding color. Total of 2 observations deleted due to missingness. Log-rank probability measure for the equality of these curves is 5.403651e-03. Additional annotations: *.GeneName*=BTK, *.GeneDesc*=Bruton agammaglobulinemia tyrosine kinase [Source:HGNC Symbol;Acc:1133].

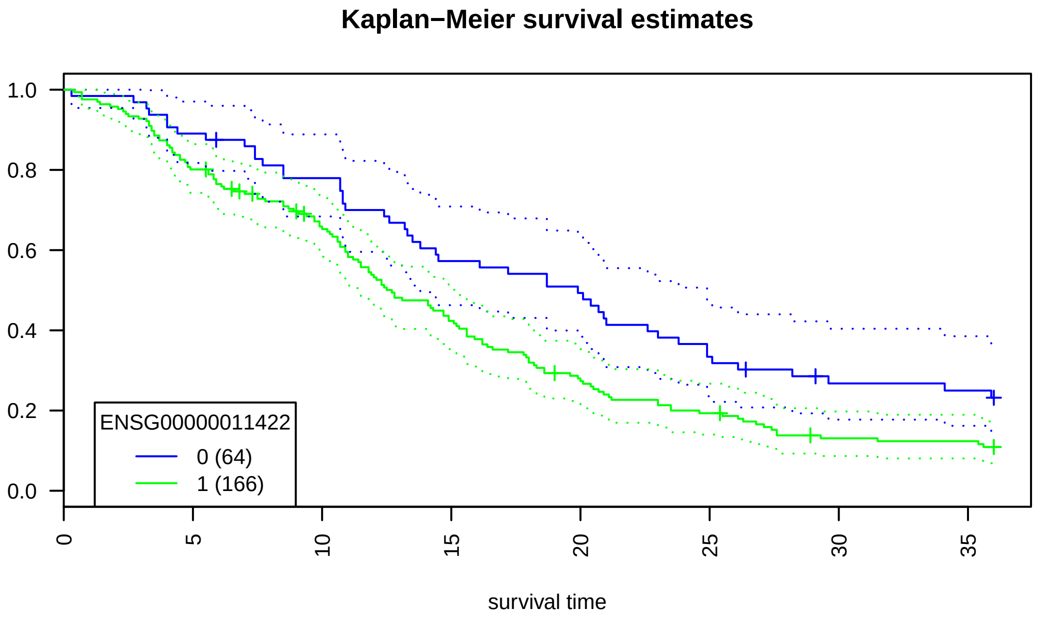

Figure 1431: Each curve represents the relative number of ENSG00000011422 survived to the corresponding point of time. Dashed lines represent 0.95 confidence intervals for the curves with the corresponding color. Log-rank probability measure for the equality of these curves is 5.115013e-03. Additional annotations: *.GeneName*=PLAUR, *.GeneDesc*=plasminogen activator, urokinase receptor [Source:HGNC Symbol;Acc:9053].

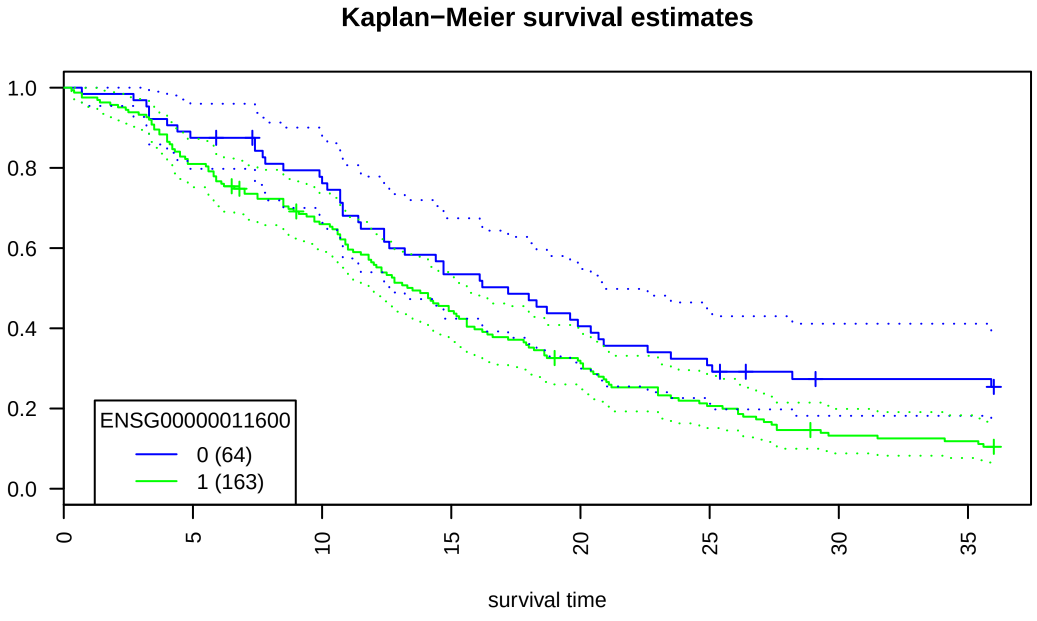

Figure 1432: Each curve represents the relative number of ENSG00000011600 survived to the corresponding point of time. Dashed lines represent 0.95 confidence intervals for the curves with the corresponding color. Total of 3 observations deleted due to missingness. Log-rank probability measure for the equality of these curves is 1.978366e-02. Additional annotations: *.GeneName*=TYROBP, *.GeneDesc*=TYRO protein tyrosine kinase binding protein [Source:HGNC Symbol;Acc:12449].

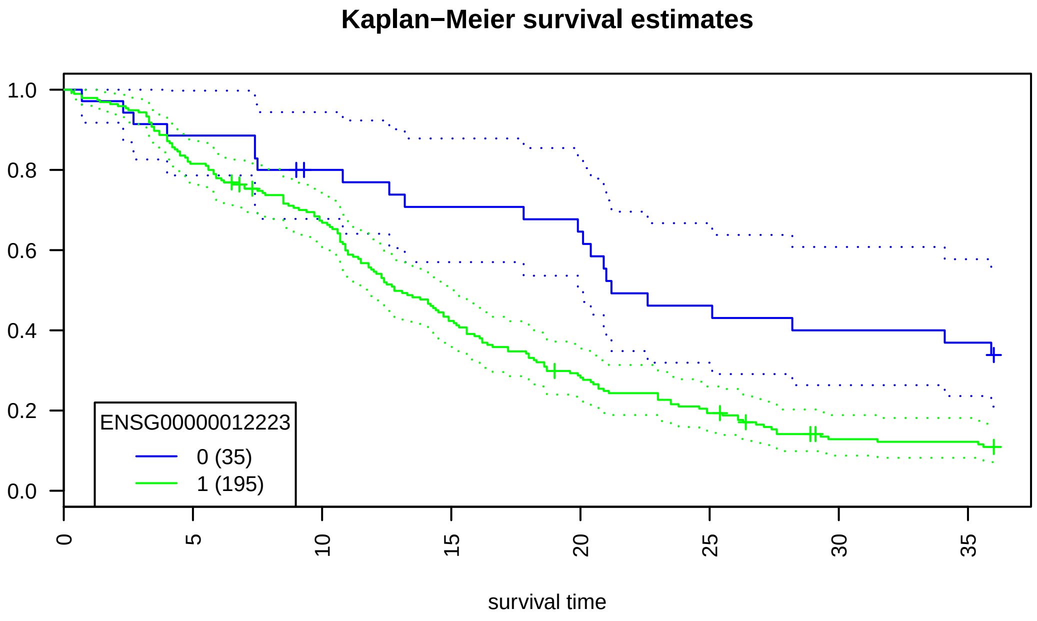

Figure 1433: Each curve represents the relative number of ENSG00000012223 survived to the corresponding point of time. Dashed lines represent 0.95 confidence intervals for the curves with the corresponding color. Log-rank probability measure for the equality of these curves is 8.23289e-04. Additional annotations: *.GeneName*=LTF, *.GeneDesc*=lactotransferrin [Source:HGNC Symbol;Acc:6720].

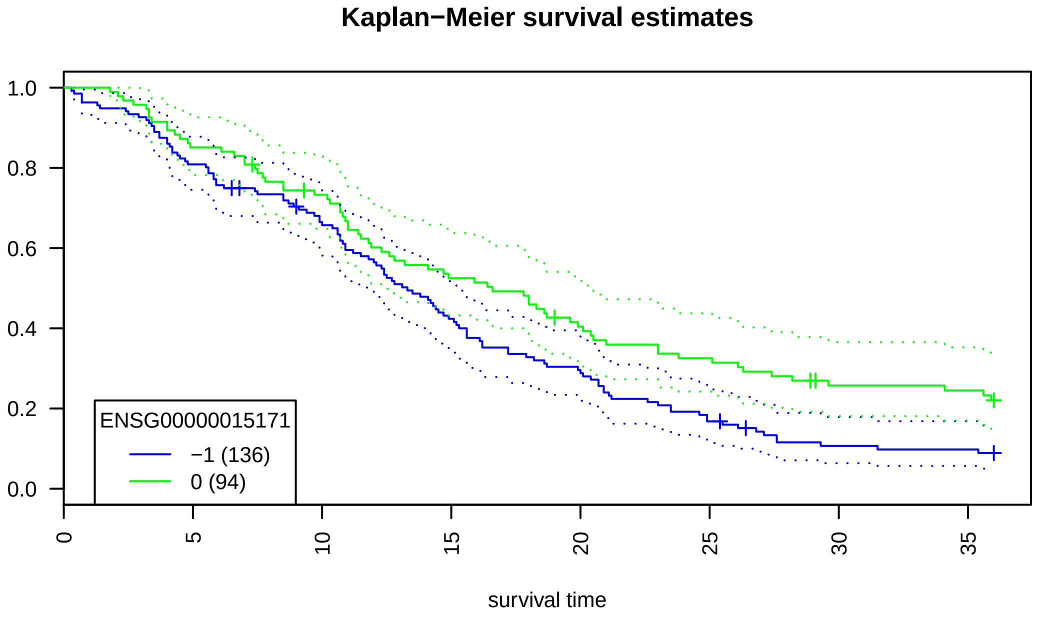

Figure 1434: Each curve represents the relative number of ENSG00000015171 survived to the corresponding point of time. Dashed lines represent 0.95 confidence intervals for the curves with the corresponding color. Log-rank probability measure for the equality of these curves is 9.132161e-03. Additional annotations: *.GeneName*=ZMYND11, *.GeneDesc*=zinc finger, MYND domain containing 11 [Source:HGNC Symbol;Acc:16966].

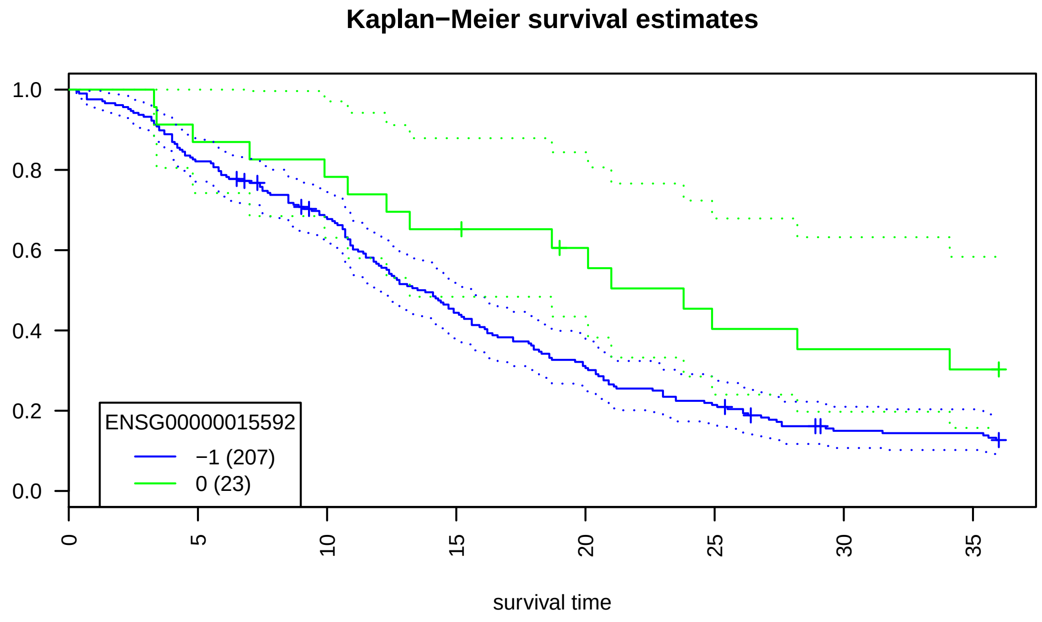

Figure 1435: Each curve represents the relative number of ENSG00000015592 survived to the corresponding point of time. Dashed lines represent 0.95 confidence intervals for the curves with the corresponding color. Log-rank probability measure for the equality of these curves is 2.283024e-02. Additional annotations: *.GeneName*=STMN4, *.GeneDesc*=stathmin-like 4 [Source:HGNC Symbol;Acc:16078].

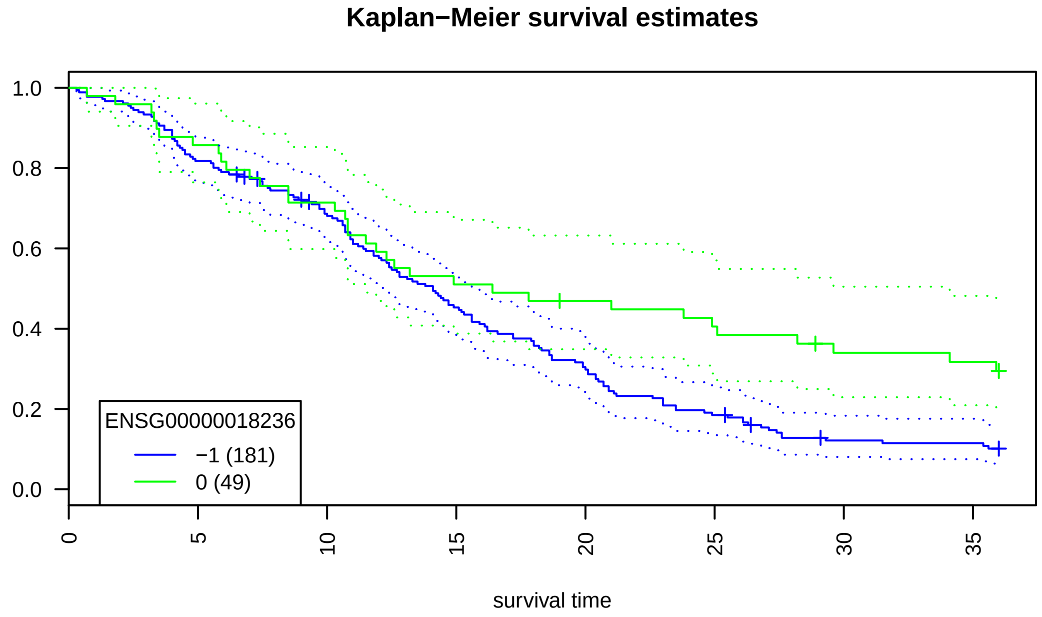

Figure 1436: Each curve represents the relative number of ENSG00000018236 survived to the corresponding point of time. Dashed lines represent 0.95 confidence intervals for the curves with the corresponding color. Log-rank probability measure for the equality of these curves is 7.799886e-03. Additional annotations: *.GeneName*=CNTN1, *.GeneDesc*=contactin 1 [Source:HGNC Symbol;Acc:2171].

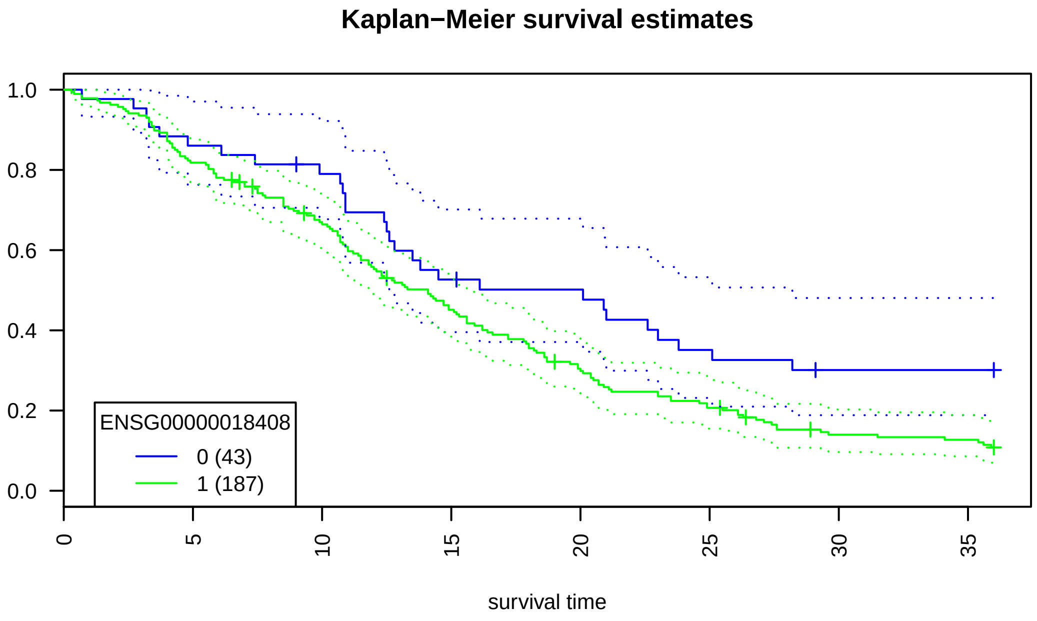

Figure 1437: Each curve represents the relative number of ENSG00000018408 survived to the corresponding point of time. Dashed lines represent 0.95 confidence intervals for the curves with the corresponding color. Log-rank probability measure for the equality of these curves is 1.391988e-02. Additional annotations: *.GeneName*=WWTR1, *.GeneDesc*=WW domain containing transcription regulator 1 [Source:HGNC Symbol;Acc:24042].

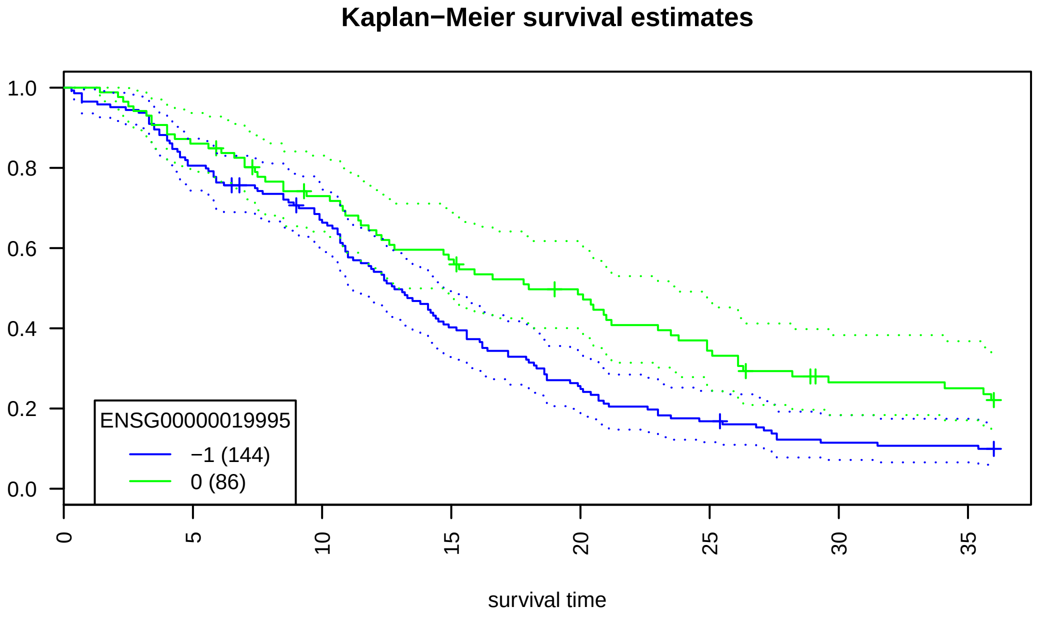

Figure 1438: Each curve represents the relative number of ENSG00000019995 survived to the corresponding point of time. Dashed lines represent 0.95 confidence intervals for the curves with the corresponding color. Log-rank probability measure for the equality of these curves is 3.141327e-03. Additional annotations: *.GeneName*=ZRANB1, *.GeneDesc*=zinc finger, RAN-binding domain containing 1 [Source:HGNC Symbol;Acc:18224].

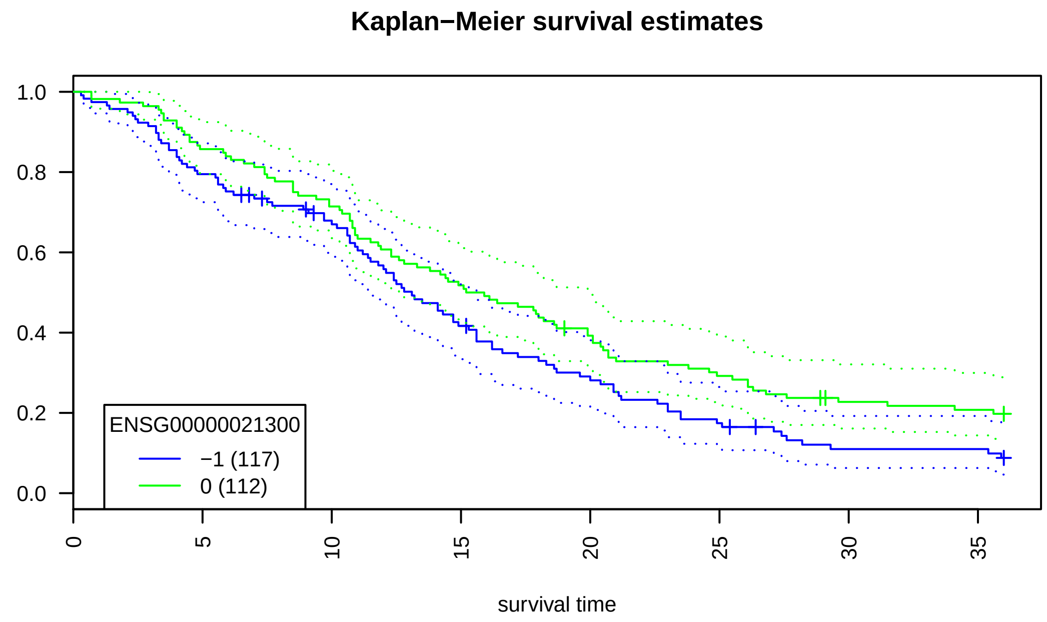

Figure 1439: Each curve represents the relative number of ENSG00000021300 survived to the corresponding point of time. Dashed lines represent 0.95 confidence intervals for the curves with the corresponding color. Total of 1 observation deleted due to missingness. Log-rank probability measure for the equality of these curves is 2.594816e-02. Additional annotations: *.GeneName*=PLEKHB1, *.GeneDesc*=pleckstrin homology domain containing, family B (evectins) member 1 [Source:HGNC Symbol;Acc:19079].

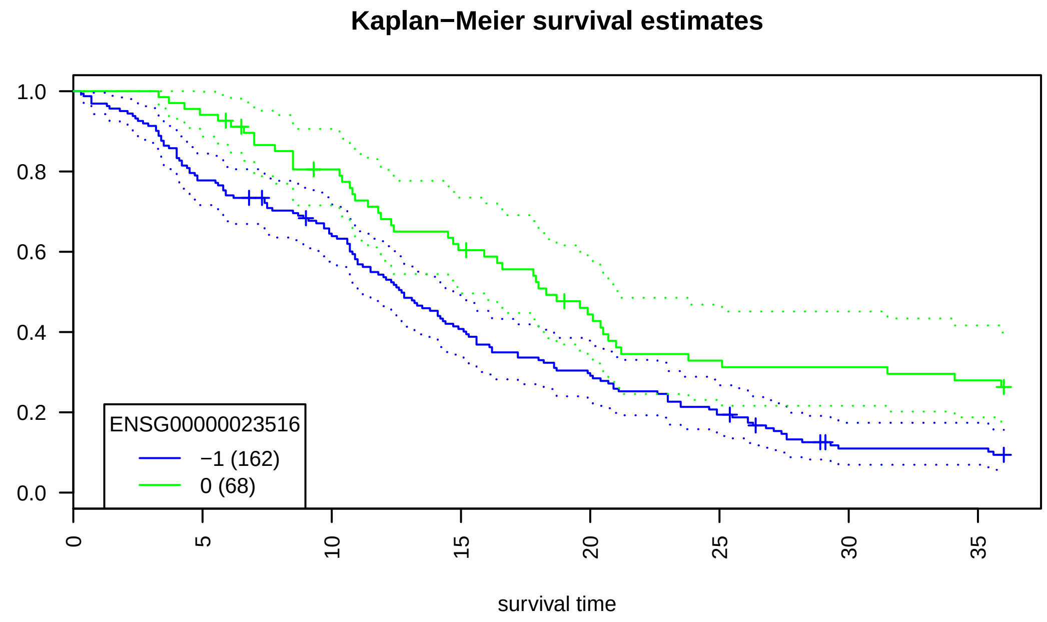

Figure 1440: Each curve represents the relative number of ENSG00000023516 survived to the corresponding point of time. Dashed lines represent 0.95 confidence intervals for the curves with the corresponding color. Log-rank probability measure for the equality of these curves is 1.170510e-03. Additional annotations: *.GeneName*=AKAP11, *.GeneDesc*=A kinase (PRKA) anchor protein 11 [Source:HGNC Symbol;Acc:369].

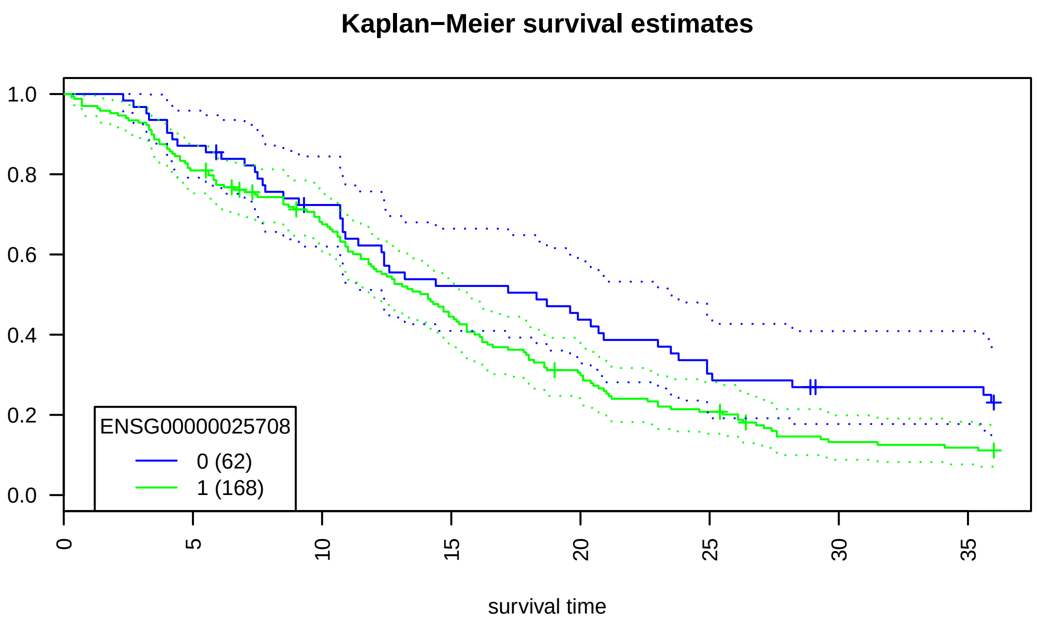

Figure 1441: Each curve represents the relative number of ENSG00000025708 survived to the corresponding point of time. Dashed lines represent 0.95 confidence intervals for the curves with the corresponding color. Log-rank probability measure for the equality of these curves is 3.812539e-02. Additional annotations: *.GeneName*=TYMP, *.GeneDesc*=thymidine phosphorylase [Source:HGNC Symbol;Acc:3148].

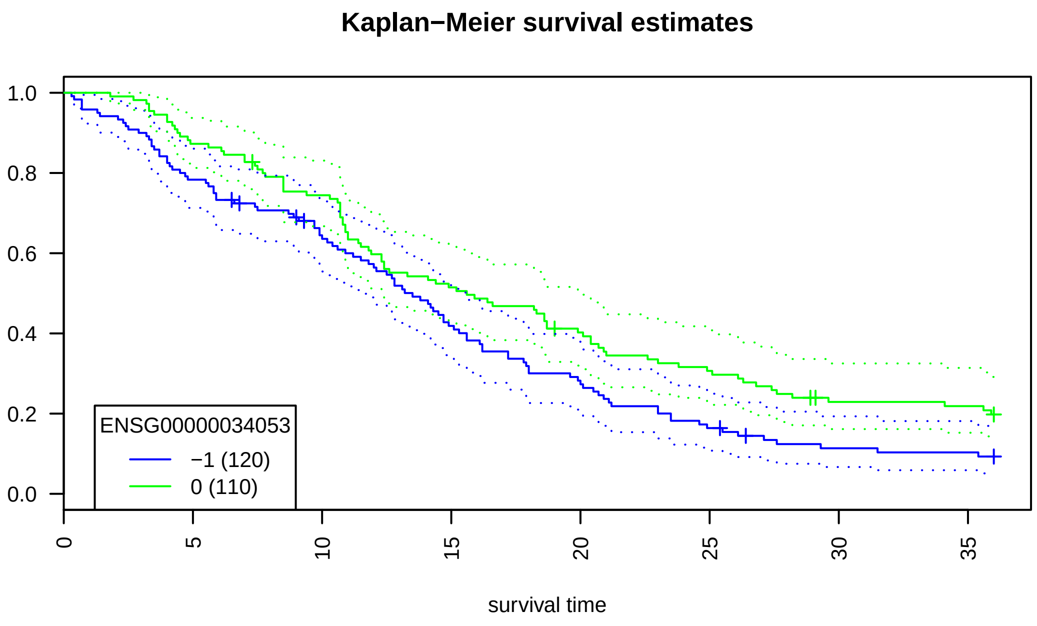

Figure 1442: Each curve represents the relative number of ENSG00000034053 survived to the corresponding point of time. Dashed lines represent 0.95 confidence intervals for the curves with the corresponding color. Log-rank probability measure for the equality of these curves is 1.365151e-02. Additional annotations: *.GeneName*=APBA2, *.GeneDesc*=amyloid beta (A4) precursor protein-binding, family A, member 2 [Source:HGNC Symbol;Acc:579].

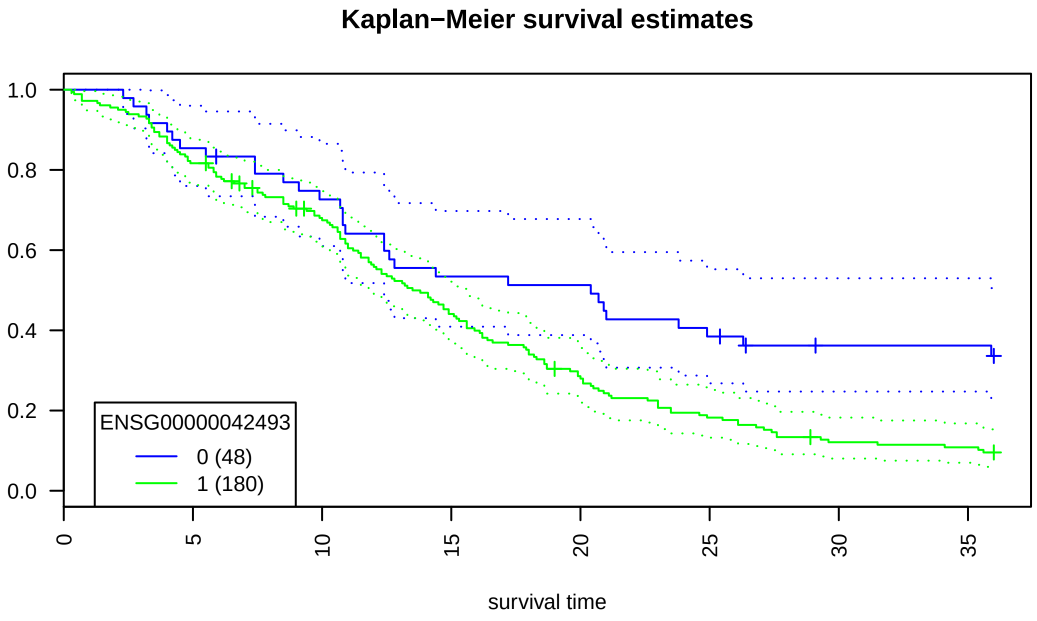

Figure 1443: Each curve represents the relative number of ENSG00000042493 survived to the corresponding point of time. Dashed lines represent 0.95 confidence intervals for the curves with the corresponding color. Total of 2 observations deleted due to missingness. Log-rank probability measure for the equality of these curves is 2.125349e-03. Additional annotations: *.GeneName*=CAPG, *.GeneDesc*=capping protein (actin filament), gelsolin-like [Source:HGNC Symbol;Acc:1474].

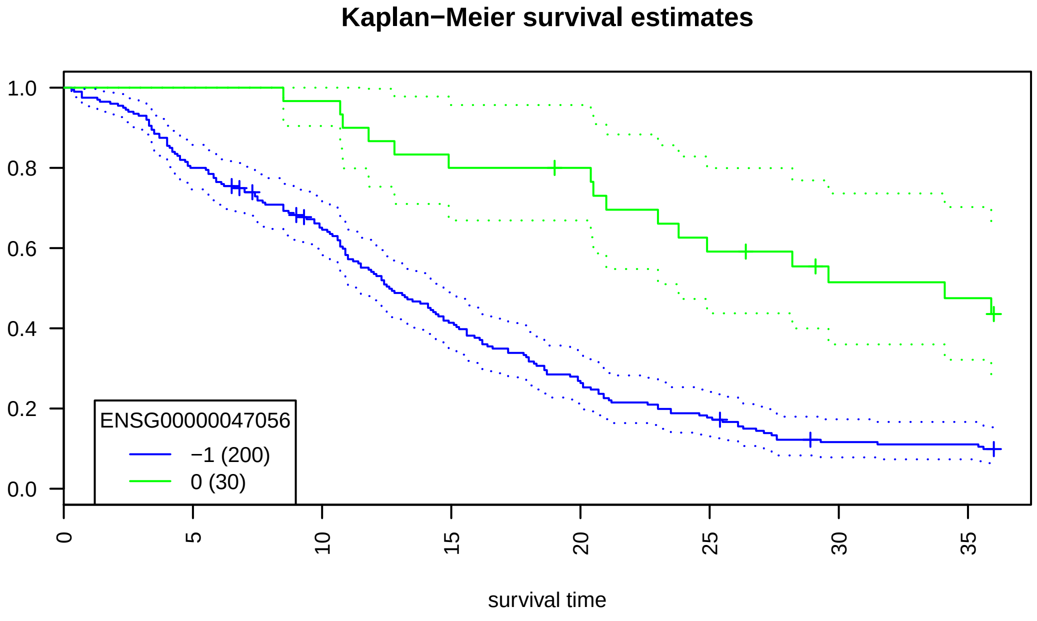

Figure 1444: Each curve represents the relative number of ENSG00000047056 survived to the corresponding point of time. Dashed lines represent 0.95 confidence intervals for the curves with the corresponding color. Log-rank probability measure for the equality of these curves is 1.366089e-06. Additional annotations: *.GeneName*=WDR37, *.GeneDesc*=WD repeat domain 37 [Source:HGNC Symbol;Acc:31406].

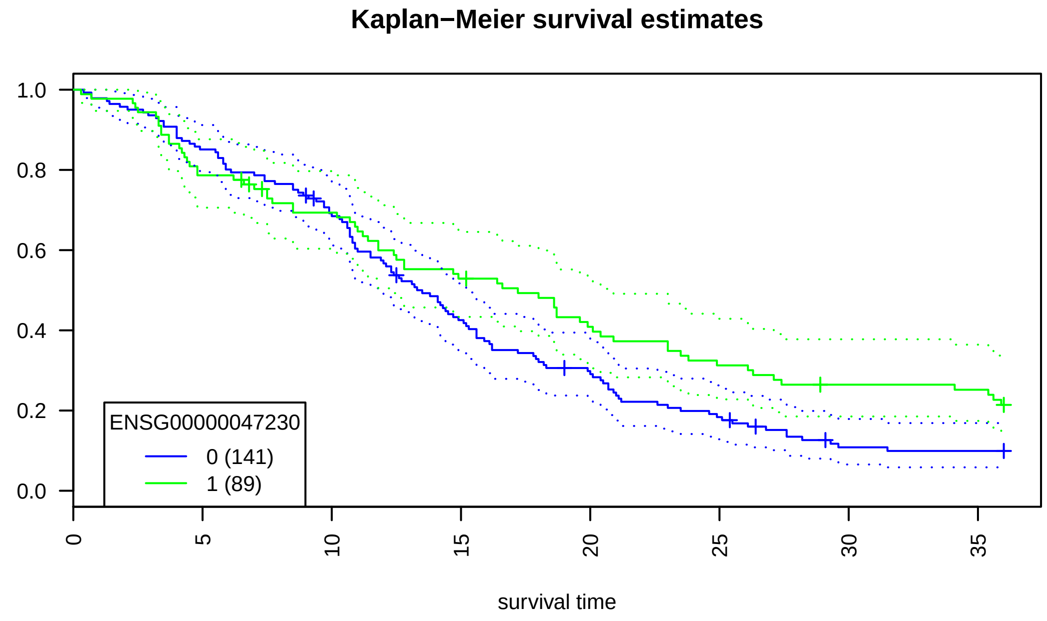

Figure 1445: Each curve represents the relative number of ENSG00000047230 survived to the corresponding point of time. Dashed lines represent 0.95 confidence intervals for the curves with the corresponding color. Log-rank probability measure for the equality of these curves is 3.066511e-02. Additional annotations: *.GeneName*=CTPS2, *.GeneDesc*=CTP synthase II [Source:HGNC Symbol;Acc:2520].

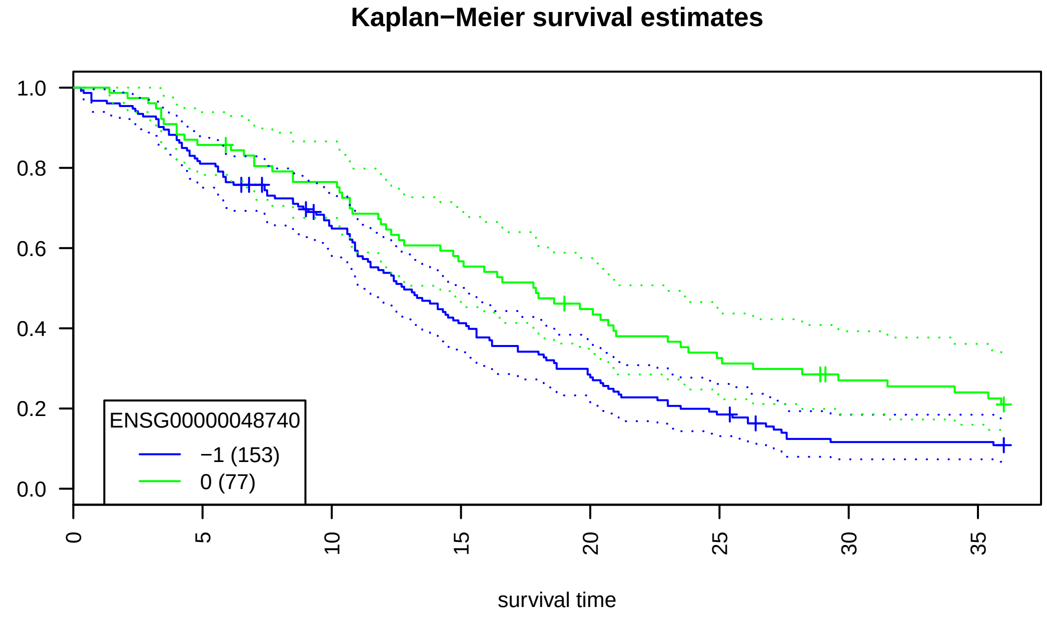

Figure 1446: Each curve represents the relative number of ENSG00000048740 survived to the corresponding point of time. Dashed lines represent 0.95 confidence intervals for the curves with the corresponding color. Log-rank probability measure for the equality of these curves is 1.101174e-02. Additional annotations: *.GeneName*=CUGBP2, *.GeneDesc*=CUGBP, Elav-like family member 2 [Source:HGNC Symbol;Acc:2550].

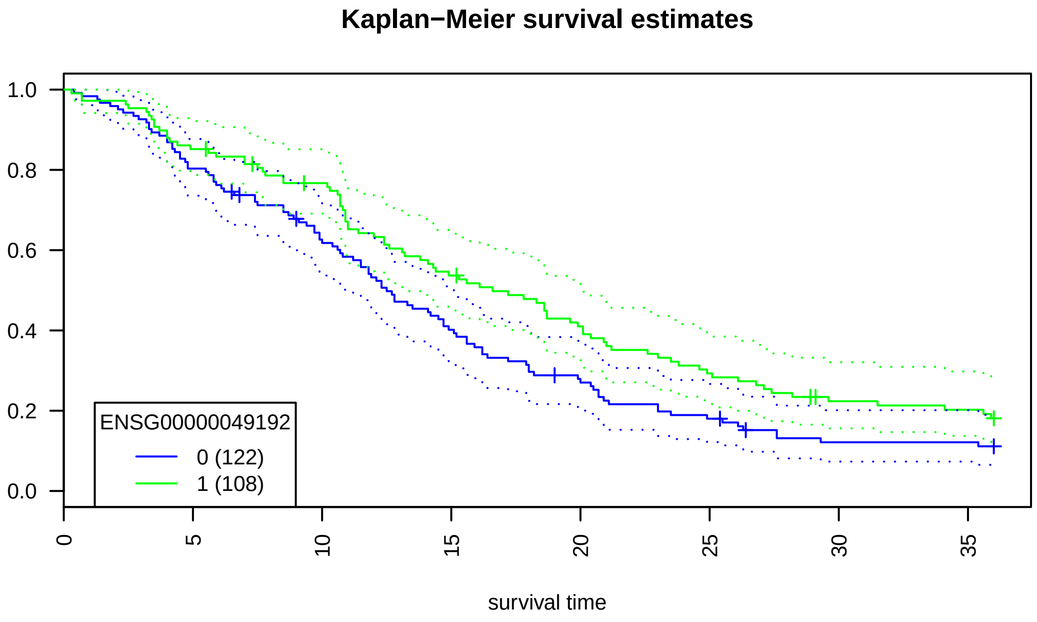

Figure 1447: Each curve represents the relative number of ENSG00000049192 survived to the corresponding point of time. Dashed lines represent 0.95 confidence intervals for the curves with the corresponding color. Log-rank probability measure for the equality of these curves is 2.254069e-02. Additional annotations: *.GeneName*=ADAMTS6, *.GeneDesc*=ADAM metallopeptidase with thrombospondin type 1 motif, 6 [Source:HGNC Symbol;Acc:222].

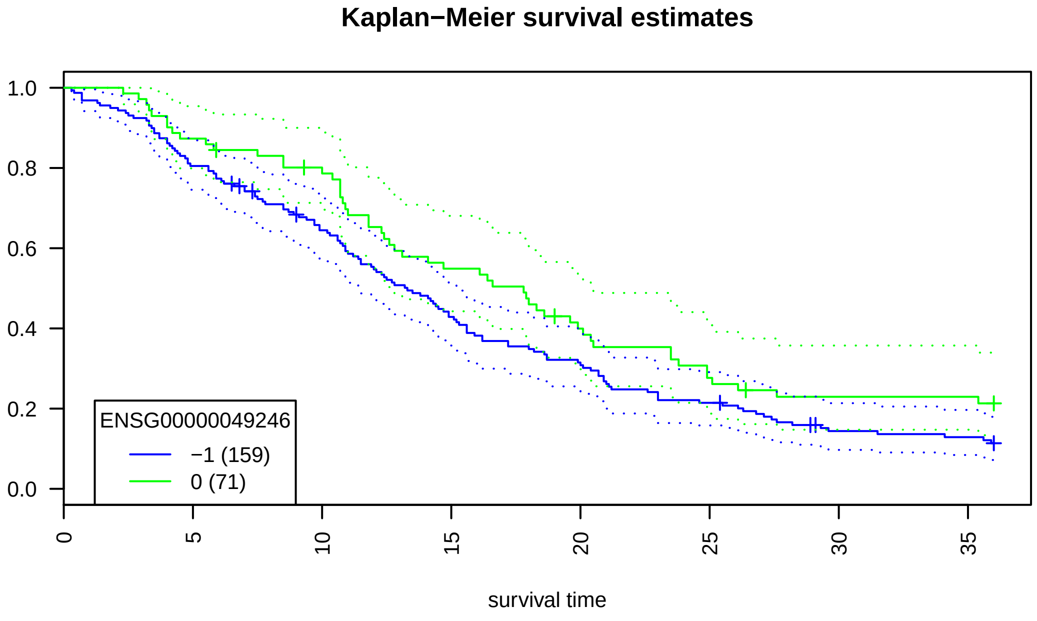

Figure 1448: Each curve represents the relative number of ENSG00000049246 survived to the corresponding point of time. Dashed lines represent 0.95 confidence intervals for the curves with the corresponding color. Log-rank probability measure for the equality of these curves is 4.89512e-02. Additional annotations: *.GeneName*=PER3, *.GeneDesc*=period homolog 3 (Drosophila) [Source:HGNC Symbol;Acc:8847].

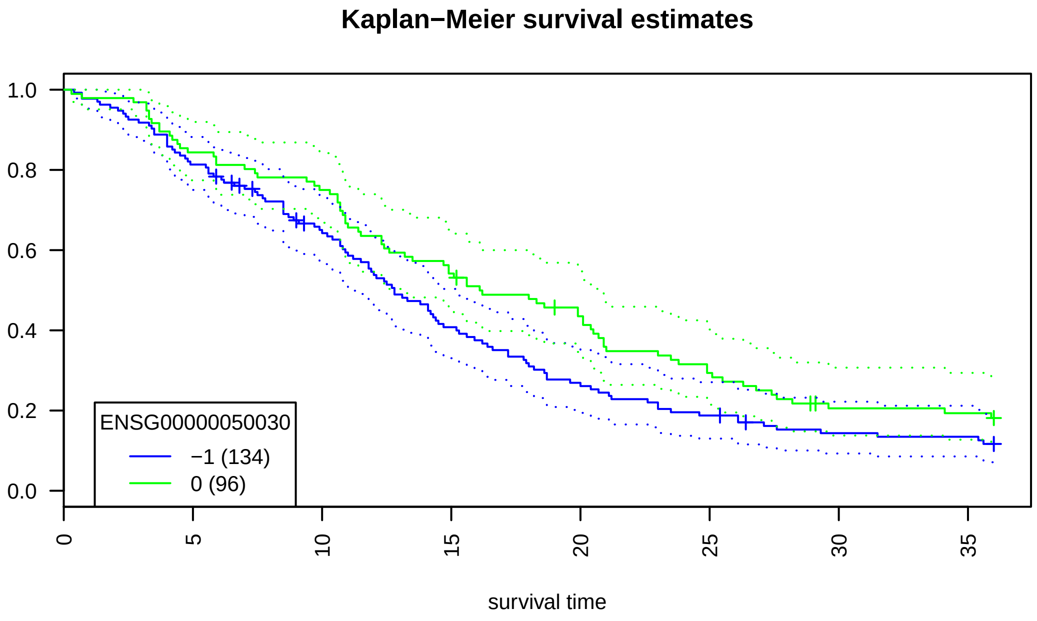

Figure 1449: Each curve represents the relative number of ENSG00000050030 survived to the corresponding point of time. Dashed lines represent 0.95 confidence intervals for the curves with the corresponding color. Log-rank probability measure for the equality of these curves is 4.257061e-02. Additional annotations: .*GeneName*=KIAA2022, .*GeneDesc*=KIAA2022 [Source:HGNC Symbol;Acc:29433].

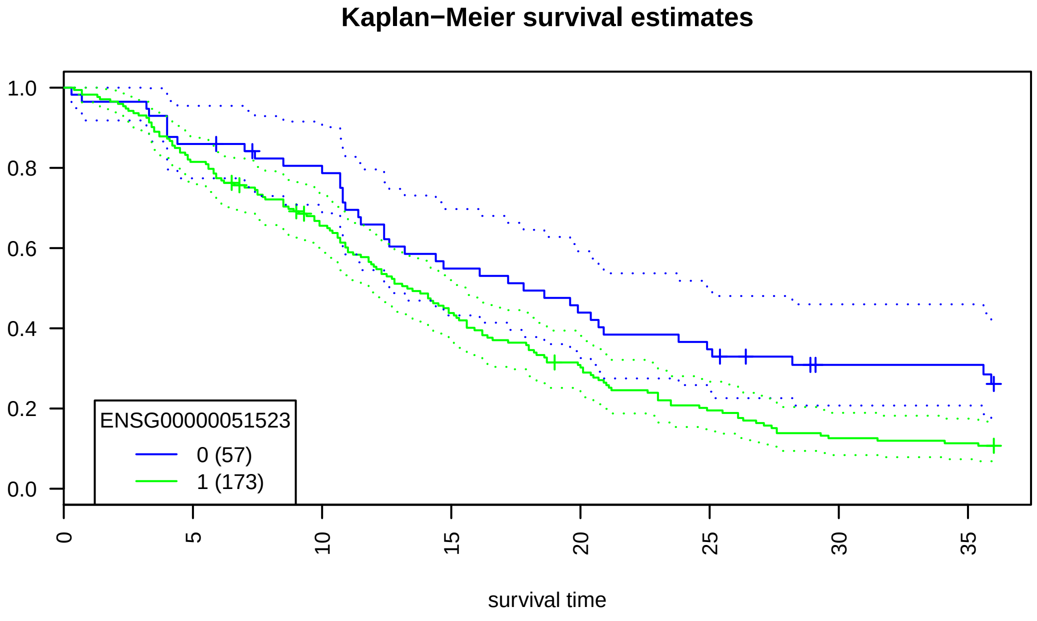

Figure 1450: Each curve represents the relative number of ENSG00000051523 survived to the corresponding point of time. Dashed lines represent 0.95 confidence intervals for the curves with the corresponding color. Log-rank probability measure for the equality of these curves is 9.795886e-03. Additional annotations: .*GeneName*=CYBA, .*GeneDesc*=cytochrome b-245, alpha polypeptide [Source:HGNC Symbol;Acc:2577].

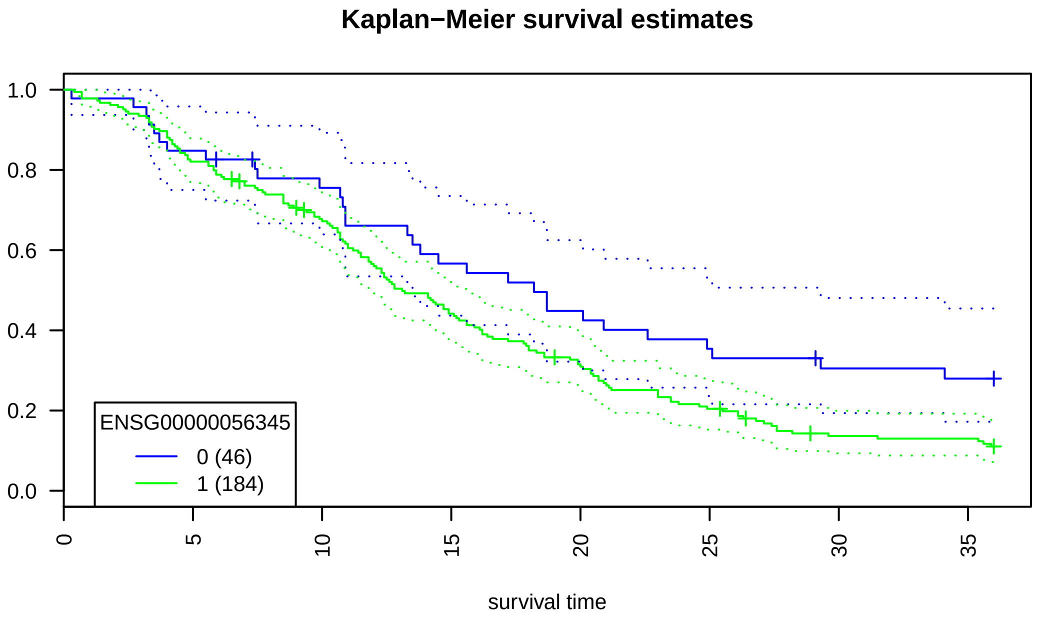

Figure 1451: Each curve represents the relative number of ENSG00000056345 survived to the corresponding point of time. Dashed lines represent 0.95 confidence intervals for the curves with the corresponding color. Log-rank probability measure for the equality of these curves is 2.017113e-02. Additional annotations: *.GeneName*=ITGB3, *.GeneDesc*=integrin, beta 3 (platelet glycoprotein IIIa, antigen CD61) [Source:HGNC Symbol;Acc:6156].

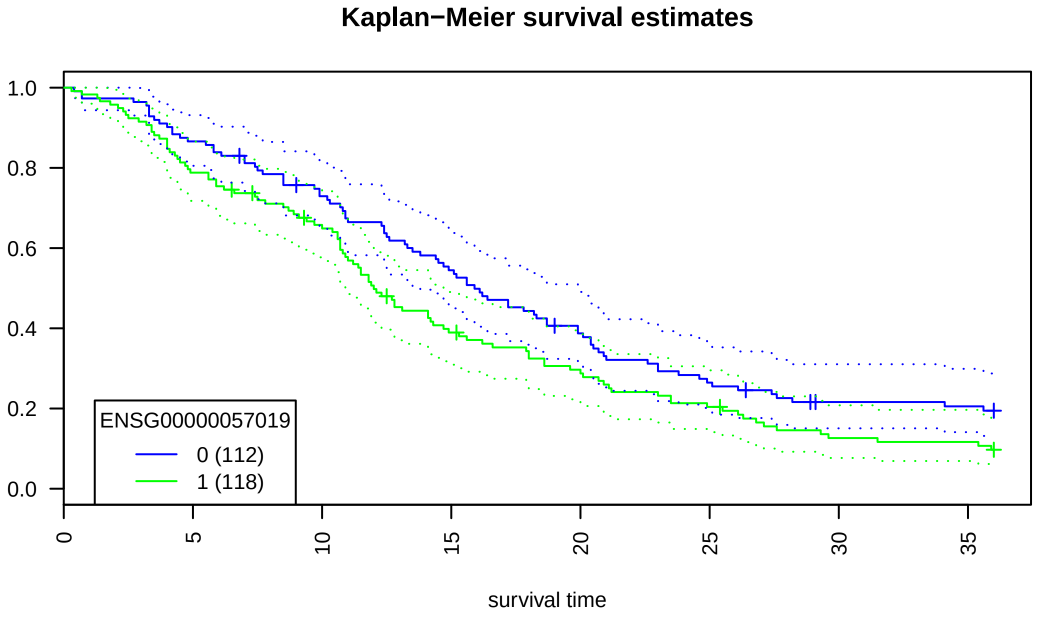

Figure 1452: Each curve represents the relative number of ENSG00000057019 survived to the corresponding point of time. Dashed lines represent 0.95 confidence intervals for the curves with the corresponding color. Log-rank probability measure for the equality of these curves is 2.878369e-02. Additional annotations: *.GeneName*=DCBLD2, *.GeneDesc*=discoidin, CUB and LCCL domain containing 2 [Source:HGNC Symbol;Acc:24627].

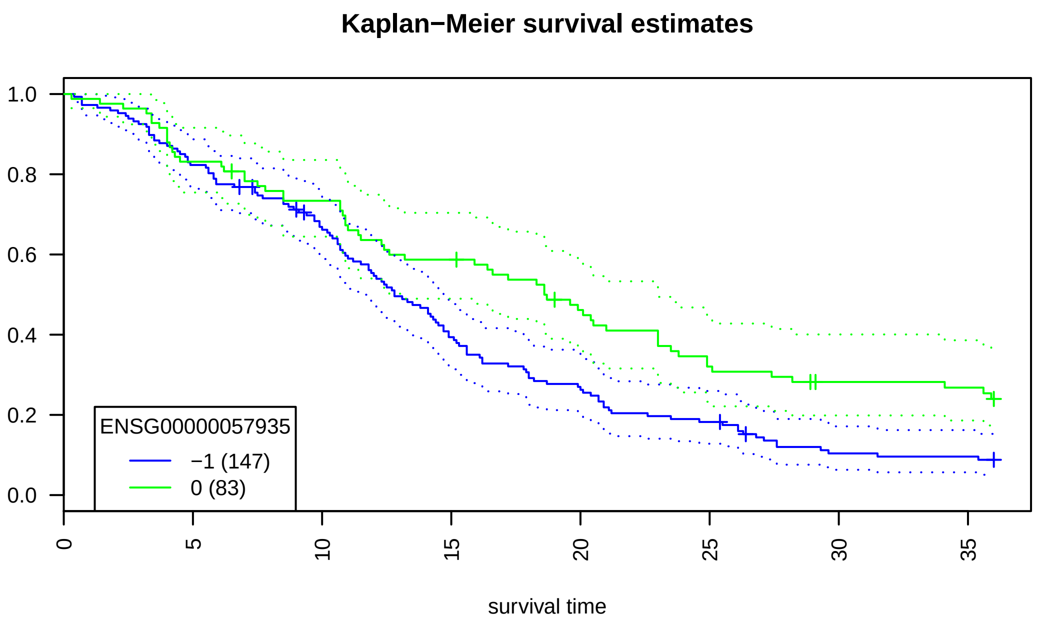

Figure 1453: Each curve represents the relative number of ENSG00000057935 survived to the corresponding point of time. Dashed lines represent 0.95 confidence intervals for the curves with the corresponding color. Log-rank probability measure for the equality of these curves is 1.732740e-03. Additional annotations: *.GeneName*=MTA3, *.GeneDesc*=metastasis associated 1 family, member 3 [Source:HGNC Symbol;Acc:23784].

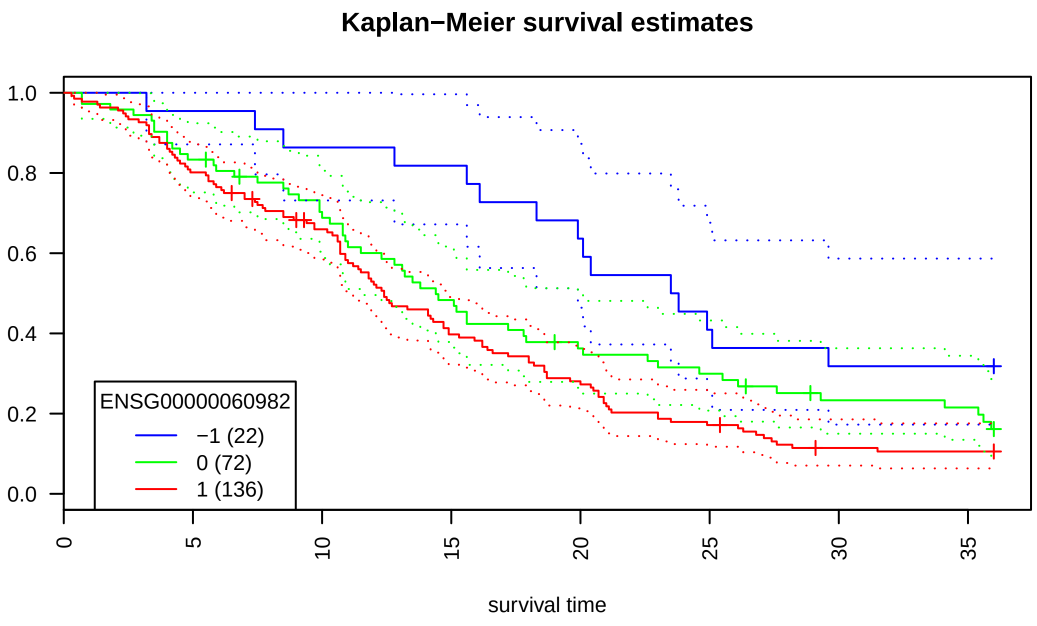

Figure 1454: Each curve represents the relative number of ENSG00000060982 survived to the corresponding point of time. Dashed lines represent 0.95 confidence intervals for the curves with the corresponding color. Log-rank probability measure for the equality of these curves is 6.516292e-03. Additional annotations: *.GeneName*=BCAT1, *.GeneDesc*=branched chain aminotransferase 1, cytosolic [Source:HGNC Symbol;Acc:976].

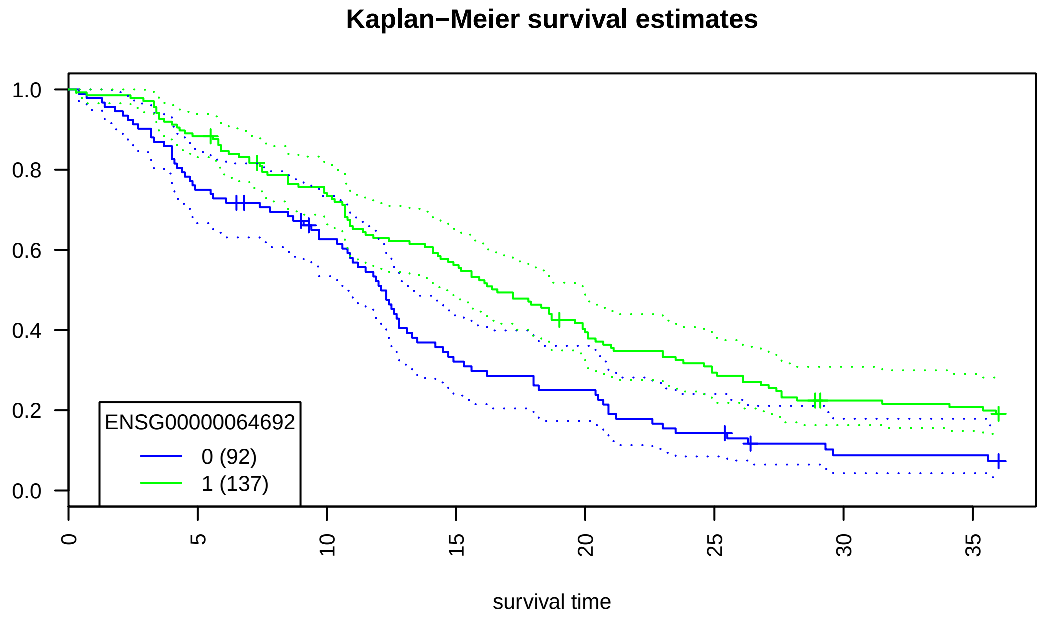

Figure 1455: Each curve represents the relative number of ENSG00000064692 survived to the corresponding point of time. Dashed lines represent 0.95 confidence intervals for the curves with the corresponding color. Total of 1 observation deleted due to missingness. Log-rank probability measure for the equality of these curves is 9.261328e-04. Additional annotations: *.GeneName*=SNCAIP, *.GeneDesc*=synuclein, alpha interacting protein [Source:HGNC Symbol;Acc:11139].

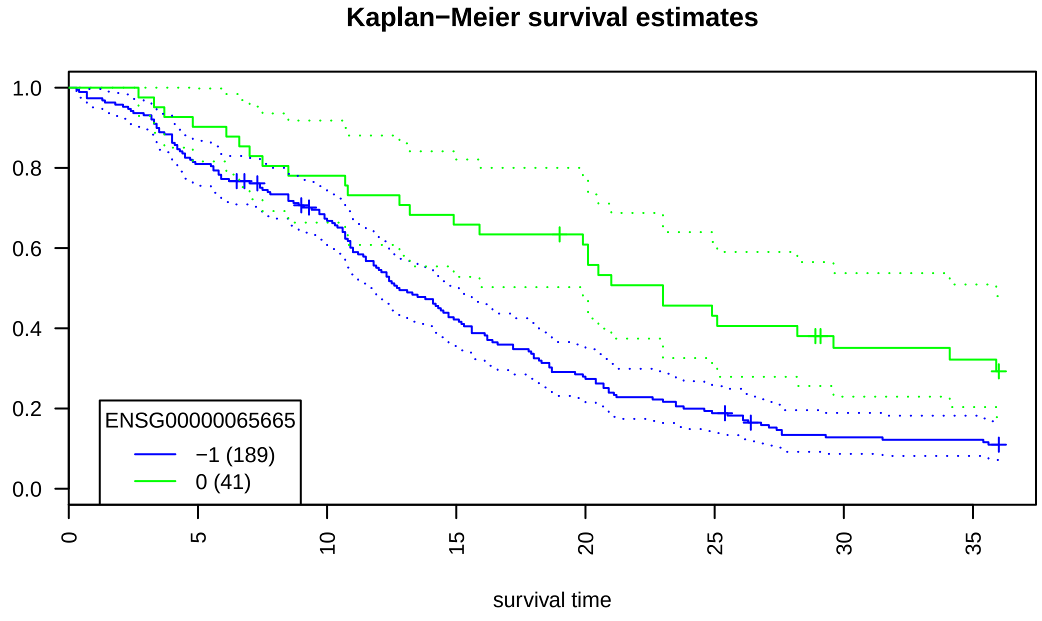

Figure 1456: Each curve represents the relative number of ENSG00000065665 survived to the corresponding point of time. Dashed lines represent 0.95 confidence intervals for the curves with the corresponding color. Log-rank probability measure for the equality of these curves is 9.163529e-04. Additional annotations: *.GeneName*=SEC61A2, *.GeneDesc*=Sec61 alpha 2 subunit (S. cerevisiae) [Source:HGNC Symbol;Acc:17702].

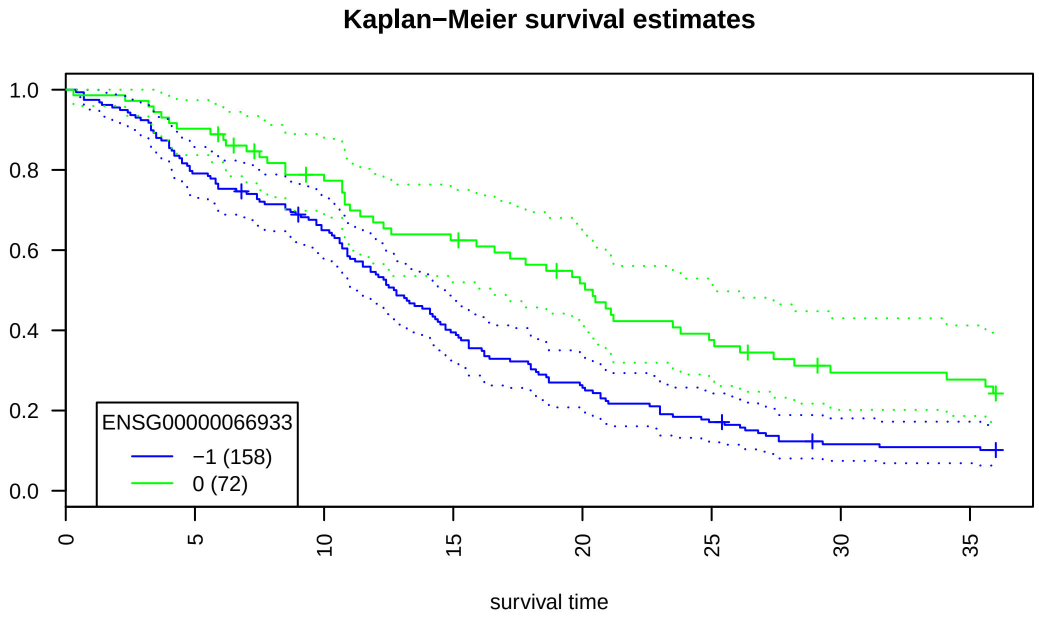

Figure 1457: Each curve represents the relative number of ENSG00000066933 survived to the corresponding point of time. Dashed lines represent 0.95 confidence intervals for the curves with the corresponding color. Log-rank probability measure for the equality of these curves is 5.383521e-04. Additional annotations: *.GeneName*=MYO9A, *.GeneDesc*=myosin IXA [Source:HGNC Symbol;Acc:7608].

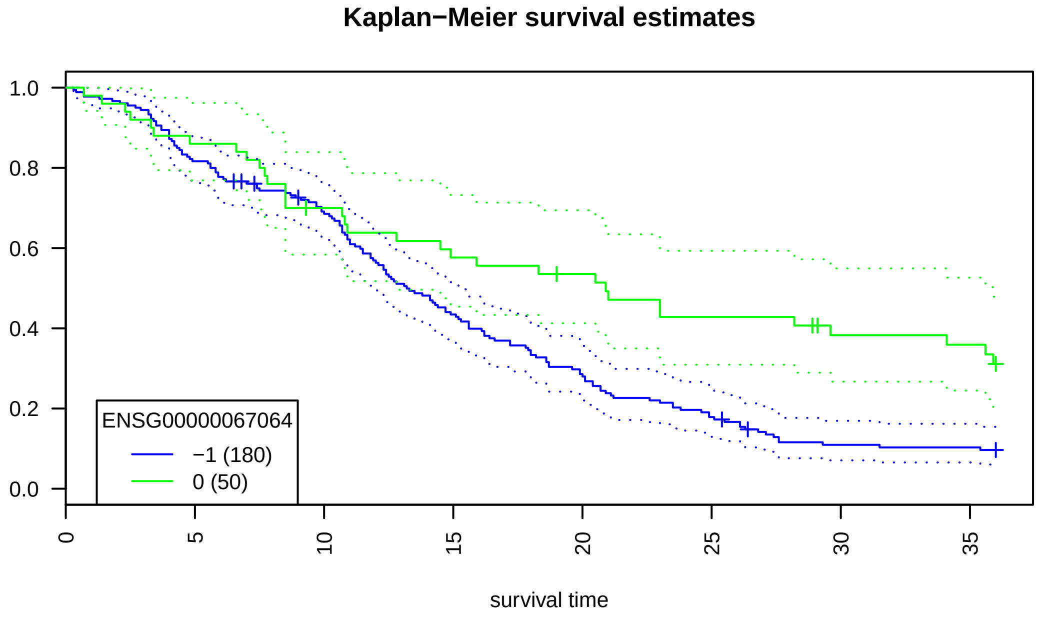

Figure 1458: Each curve represents the relative number of ENSG00000067064 survived to the corresponding point of time. Dashed lines represent 0.95 confidence intervals for the curves with the corresponding color. Log-rank probability measure for the equality of these curves is 9.392125e-04. Additional annotations: *.GeneName*=IDI1, *.GeneDesc*=isopentenyl-diphosphate delta isomerase 1 [Source:HGNC Symbol;Acc:5387].

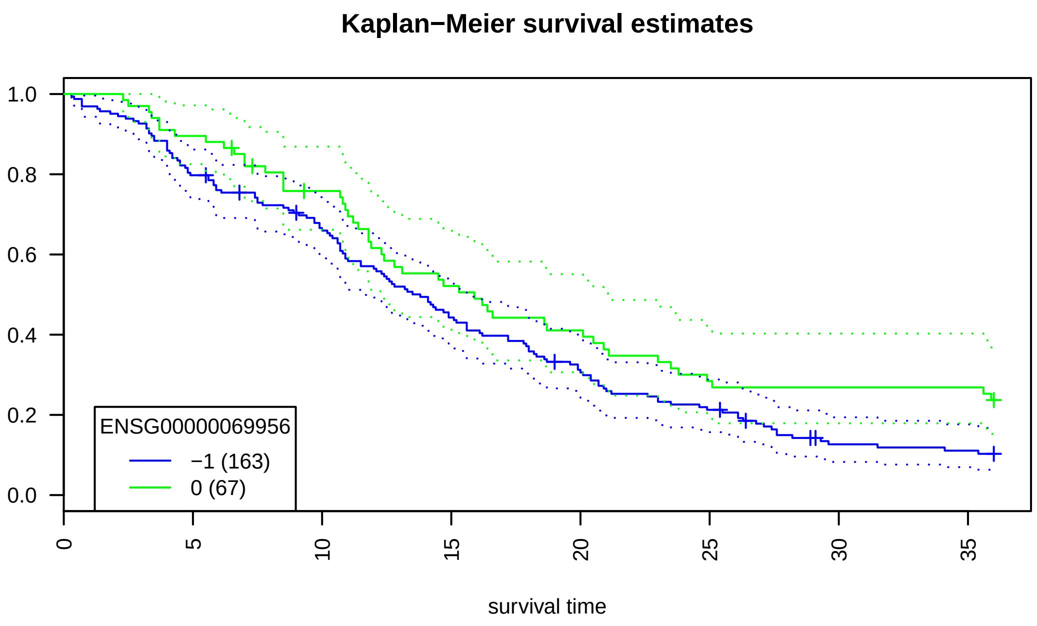

Figure 1459: Each curve represents the relative number of ENSG00000069956 survived to the corresponding point of time. Dashed lines represent 0.95 confidence intervals for the curves with the corresponding color. Log-rank probability measure for the equality of these curves is 3.298887e-02. Additional annotations: *.GeneName*=MAPK6, *.GeneDesc*=mitogen-activated protein kinase 6 [Source:HGNC Symbol;Acc:6879].

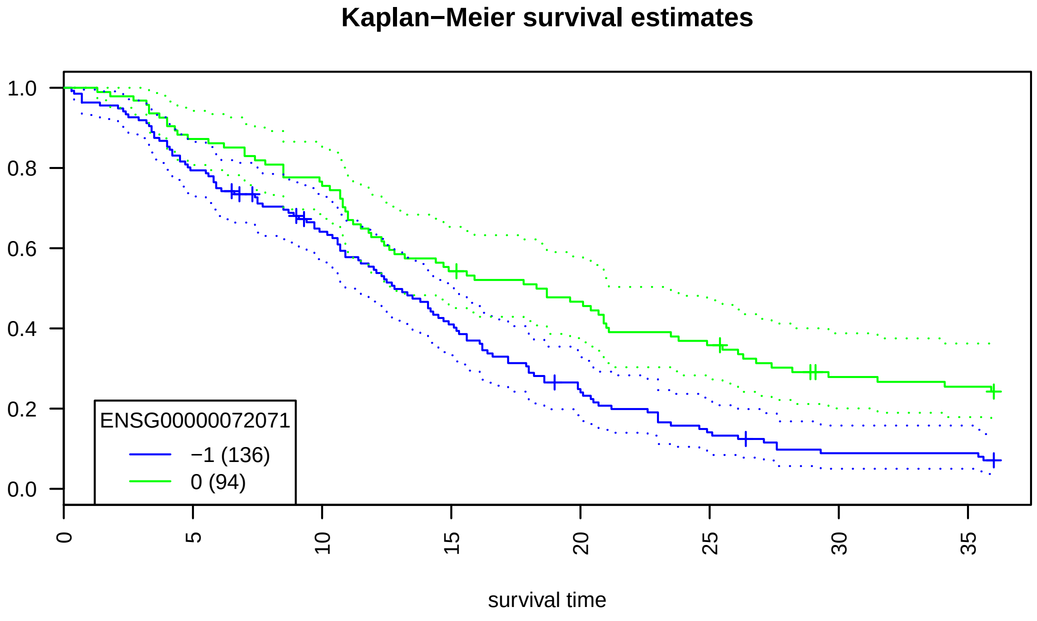

Figure 1460: Each curve represents the relative number of ENSG00000072071 survived to the corresponding point of time. Dashed lines represent 0.95 confidence intervals for the curves with the corresponding color. Log-rank probability measure for the equality of these curves is 1.957212e-04. Additional annotations: *.GeneName*=LPHN1, *.GeneDesc*=latrophilin 1 [Source:HGNC Symbol;Acc:20973].

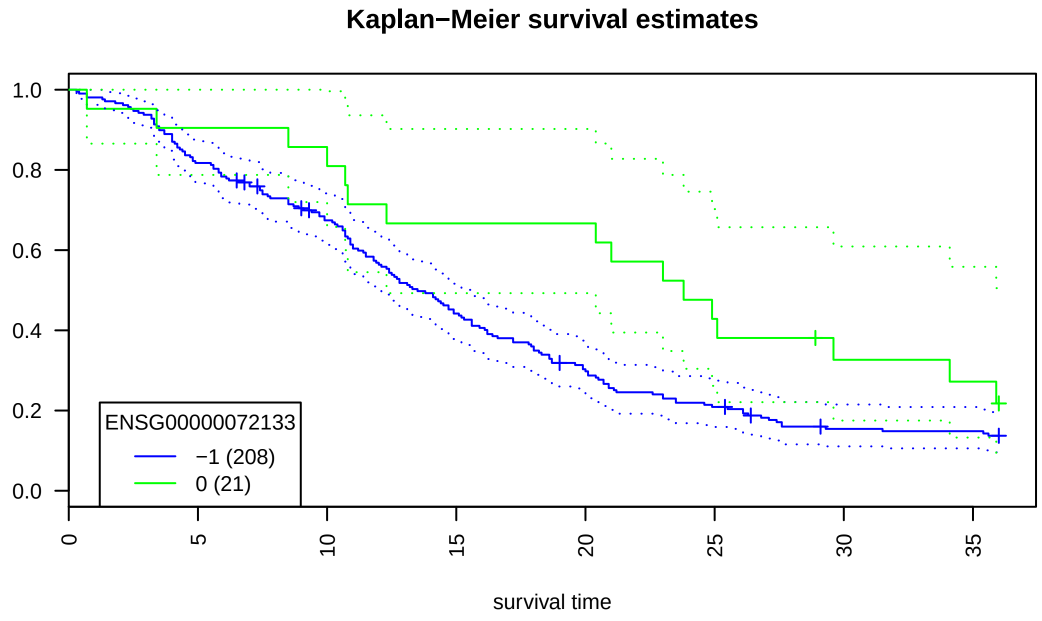

Figure 1461: Each curve represents the relative number of ENSG00000072133 survived to the corresponding point of time. Dashed lines represent 0.95 confidence intervals for the curves with the corresponding color. Total of 1 observation deleted due to missingness. Log-rank probability measure for the equality of these curves is 4.853603e-02. Additional annotations: .*GeneName*=RPS6KA6, .*GeneDesc*=ribosomal protein S6 kinase, 90kDa, polypeptide 6 [Source:HGNC Symbol;Acc:10435].

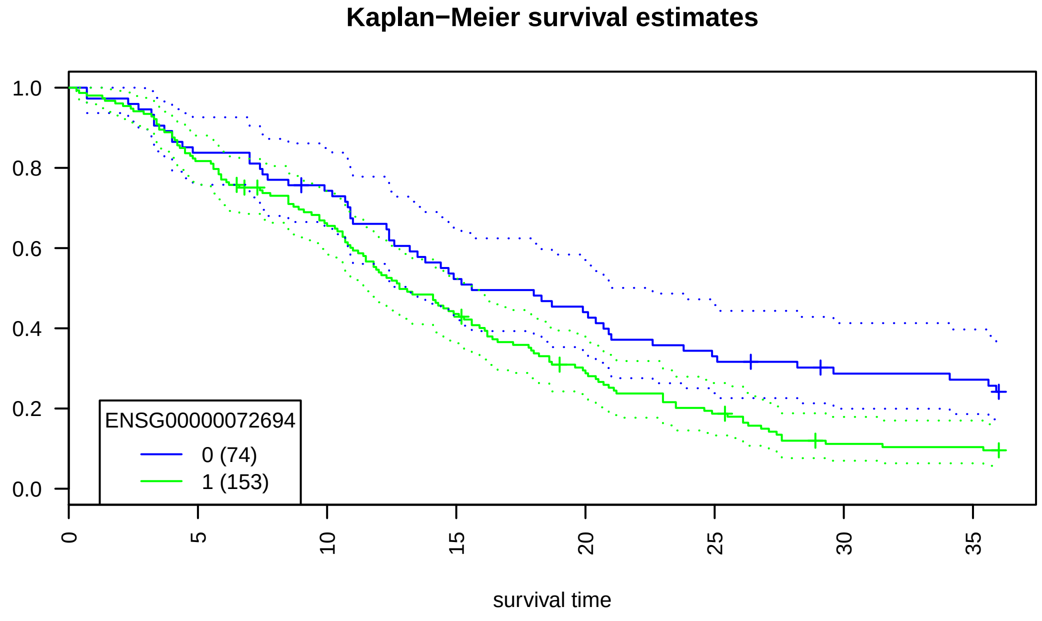

Figure 1462: Each curve represents the relative number of ENSG00000072694 survived to the corresponding point of time. Dashed lines represent 0.95 confidence intervals for the curves with the corresponding color. Total of 3 observations deleted due to missingness. Log-rank probability measure for the equality of these curves is 7.897738e-03. Additional annotations: .*GeneName*=FCGR2B, .*GeneDesc*=Fc fragment of IgG, low affinity IIb, receptor (CD32) [Source:HGNC Symbol;Acc:3618].

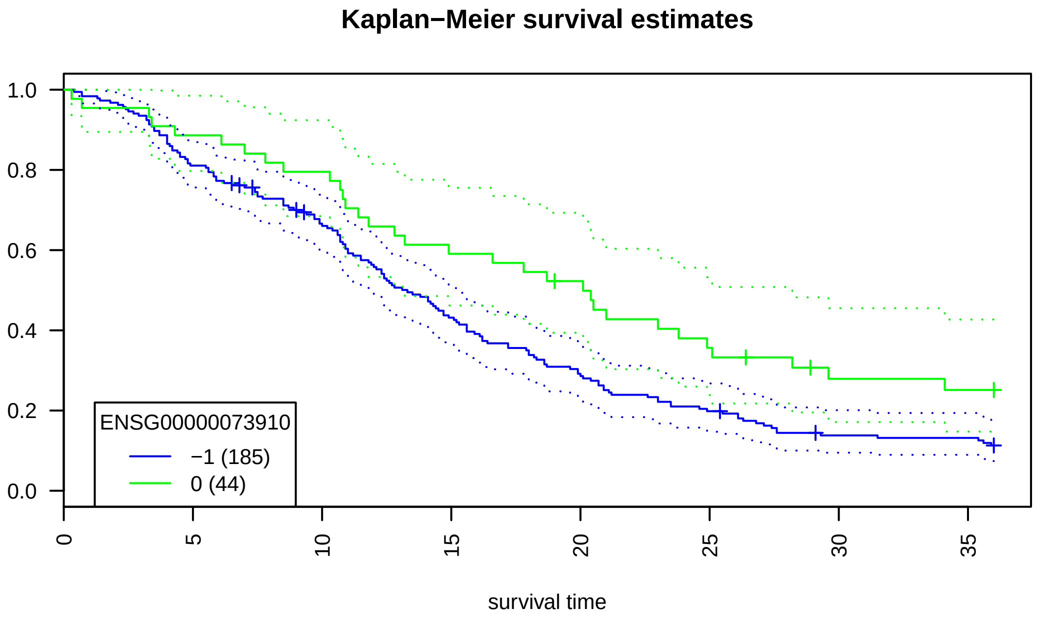

Figure 1463: Each curve represents the relative number of ENSG00000073910 survived to the corresponding point of time. Dashed lines represent 0.95 confidence intervals for the curves with the corresponding color. Total of 1 observation deleted due to missingness. Log-rank probability measure for the equality of these curves is 1.290161e-02. Additional annotations: *.GeneName*=FRY, *.GeneDesc*=furry homolog (Drosophila) [Source:HGNC Symbol;Acc:20367].

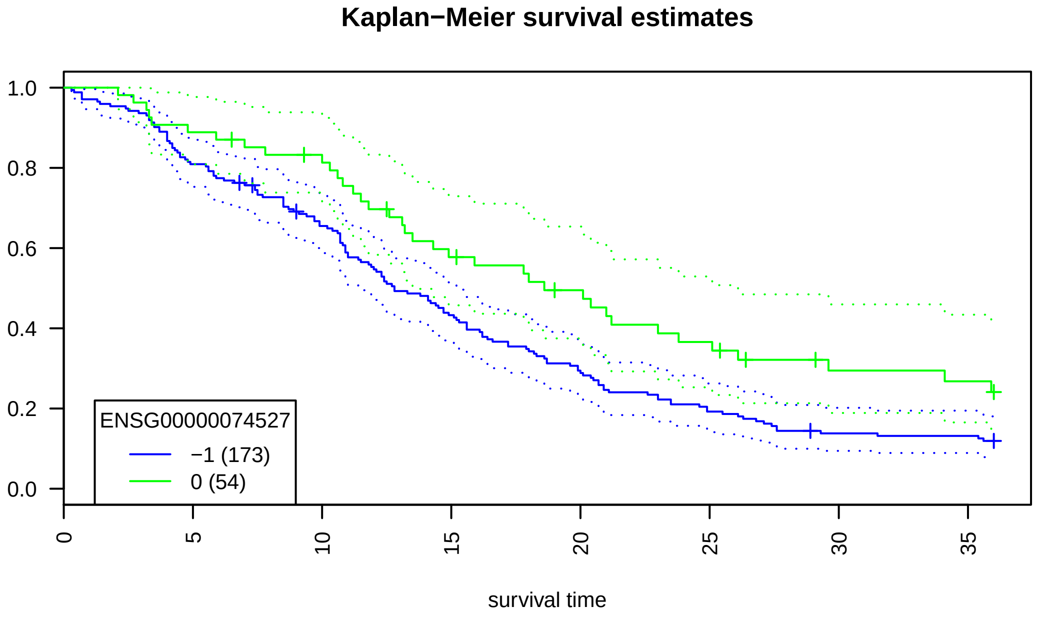

Figure 1464: Each curve represents the relative number of ENSG00000074527 survived to the corresponding point of time. Dashed lines represent 0.95 confidence intervals for the curves with the corresponding color. Total of 3 observations deleted due to missingness. Log-rank probability measure for the equality of these curves is 1.020493e-02. Additional annotations: *.GeneName*=NTN4, *.GeneDesc*=netrin 4 [Source:HGNC Symbol;Acc:13658].

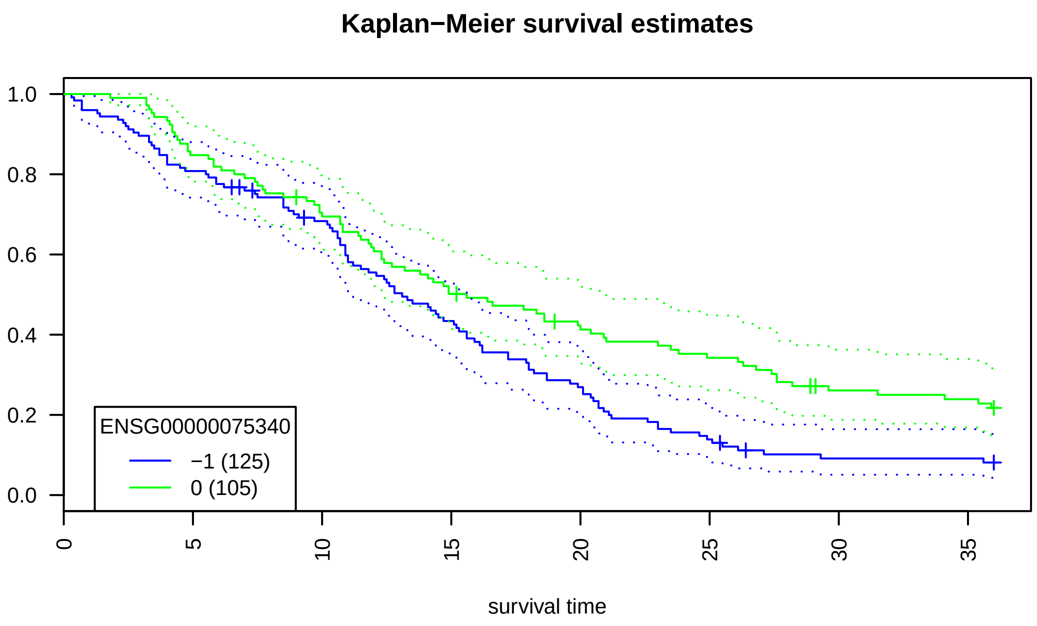

Figure 1465: Each curve represents the relative number of ENSG00000075340 survived to the corresponding point of time. Dashed lines represent 0.95 confidence intervals for the curves with the corresponding color. Log-rank probability measure for the equality of these curves is 2.594768e-03. Additional annotations: *.GeneName*=ADD2, *.GeneDesc*=adducin 2 (beta) [Source:HGNC Symbol;Acc:244].

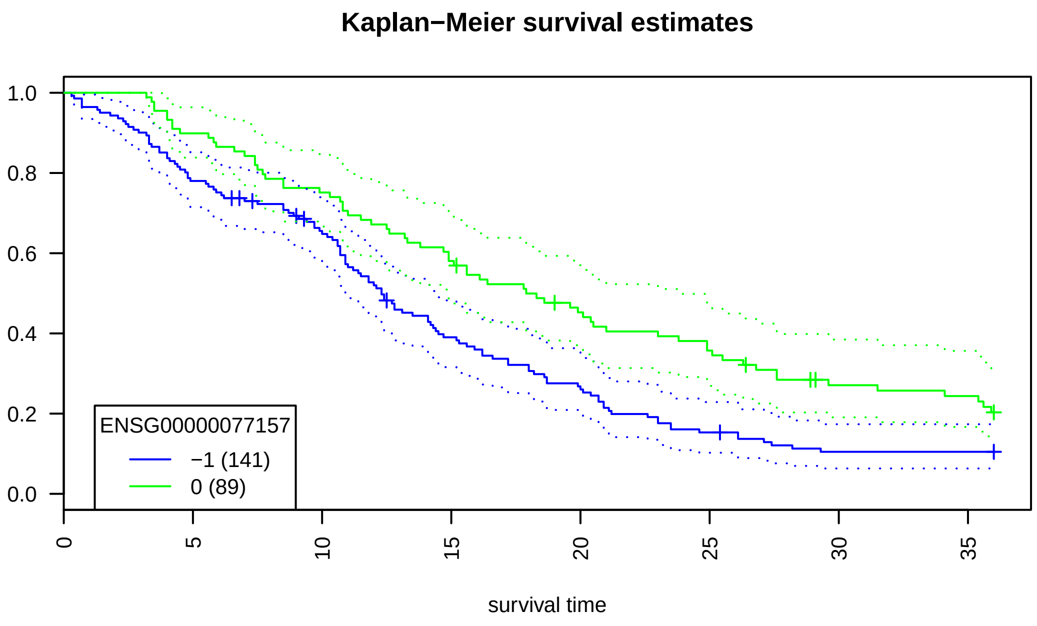

Figure 1466: Each curve represents the relative number of ENSG00000077157 survived to the corresponding point of time. Dashed lines represent 0.95 confidence intervals for the curves with the corresponding color. Log-rank probability measure for the equality of these curves is 1.375322e-03. Additional annotations: *.GeneName*=PPP1R12B, *.GeneDesc*=protein phosphatase 1, regulatory (inhibitor) subunit 12B [Source:HGNC Symbol;Acc:7619].

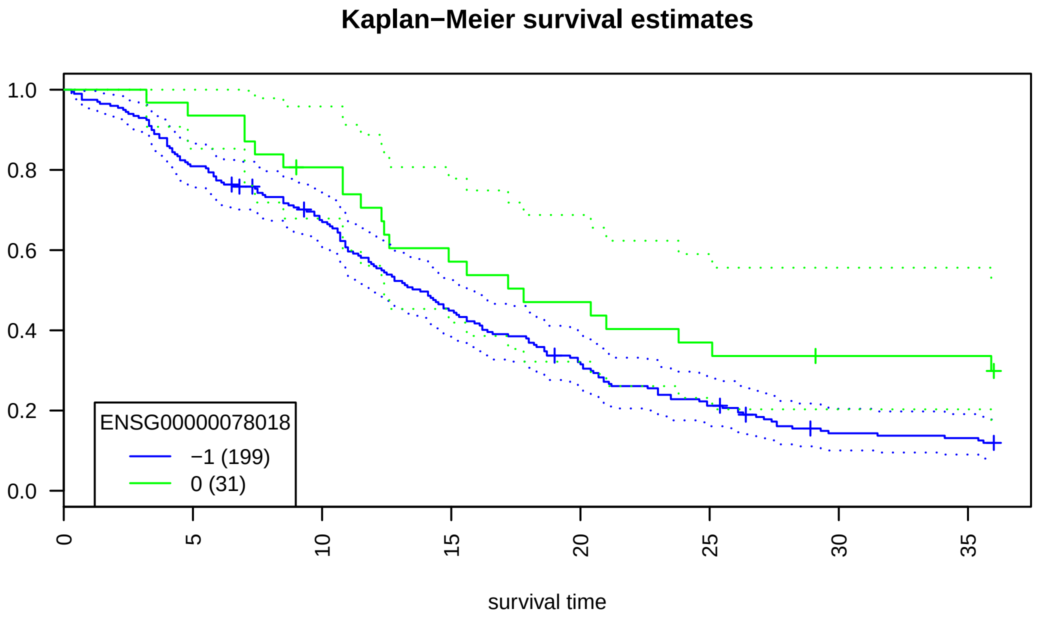

Figure 1467: Each curve represents the relative number of ENSG00000078018 survived to the corresponding point of time. Dashed lines represent 0.95 confidence intervals for the curves with the corresponding color. Log-rank probability measure for the equality of these curves is 2.696364e-02. Additional annotations: *.GeneName*=MAP2, *.GeneDesc*=microtubule-associated protein 2 [Source:HGNC Symbol;Acc:6839].

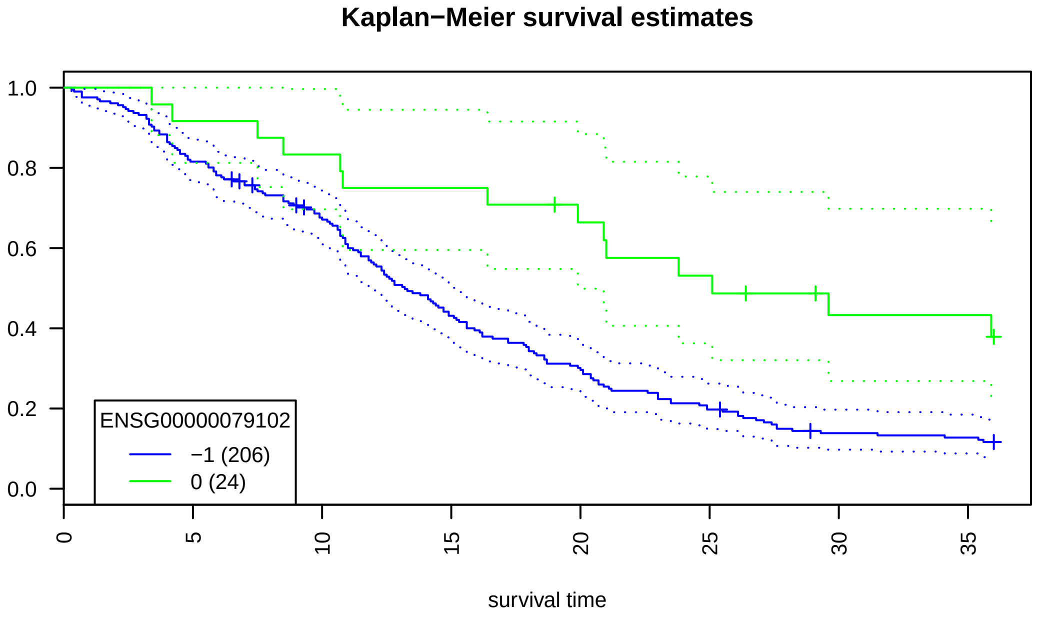

Figure 1468: Each curve represents the relative number of ENSG00000079102 survived to the corresponding point of time. Dashed lines represent 0.95 confidence intervals for the curves with the corresponding color. Log-rank probability measure for the equality of these curves is 1.077165e-03. Additional annotations: *.GeneName*=RUNX1T1, *.GeneDesc*=runt-related transcription factor 1; translocated to, 1 (cyclin D-related) [Source:HGNC Symbol;Acc:1535].

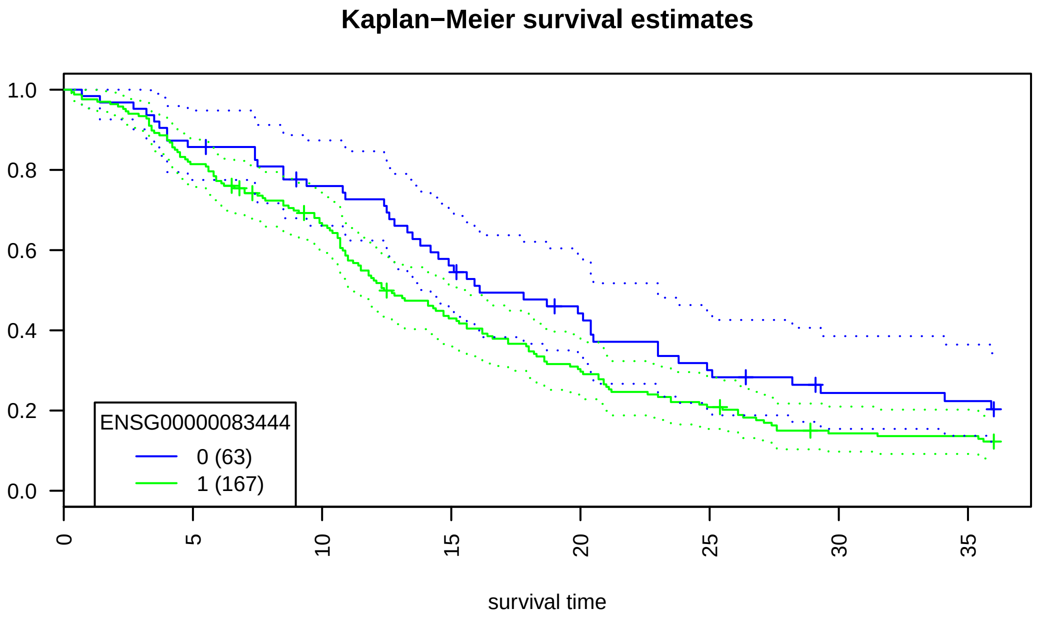

Figure 1469: Each curve represents the relative number of ENSG00000083444 survived to the corresponding point of time. Dashed lines represent 0.95 confidence intervals for the curves with the corresponding color. Log-rank probability measure for the equality of these curves is 4.521752e-02. Additional annotations: *.GeneName*=PLOD1, *.GeneDesc*=procollagen-lysine 1, 2-oxoglutarate 5-dioxygenase 1 [Source:HGNC Symbol;Acc:9081].

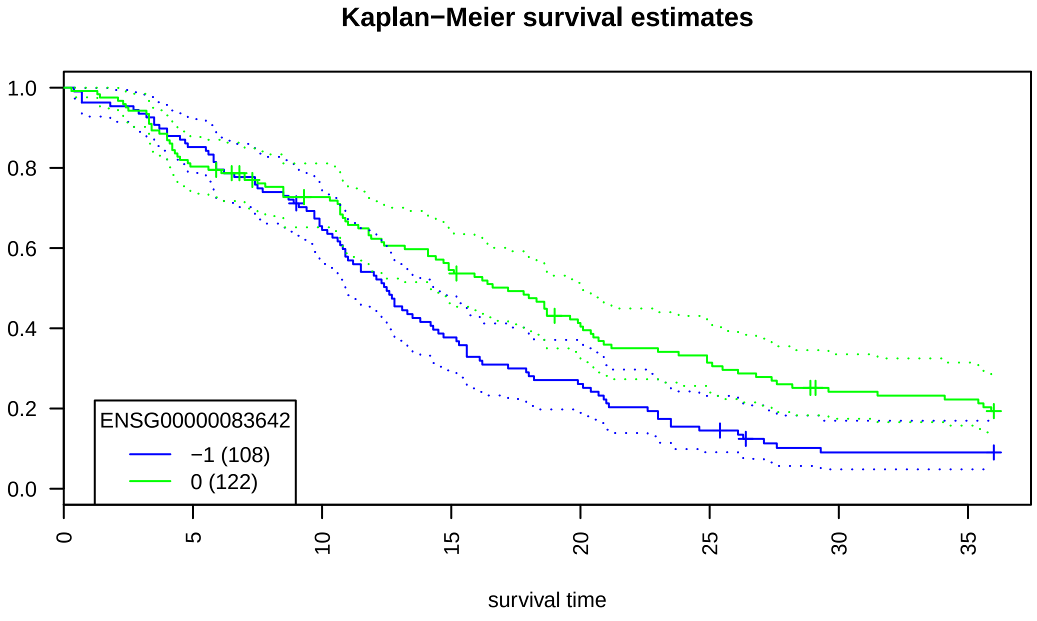

Figure 1470: Each curve represents the relative number of ENSG00000083642 survived to the corresponding point of time. Dashed lines represent 0.95 confidence intervals for the curves with the corresponding color. Log-rank probability measure for the equality of these curves is 5.964541e-03. Additional annotations: *.GeneName*=PDS5B, *.GeneDesc*=PDS5, regulator of cohesion maintenance, homolog B (S. cerevisiae) [Source:HGNC Symbol;Acc:20418].

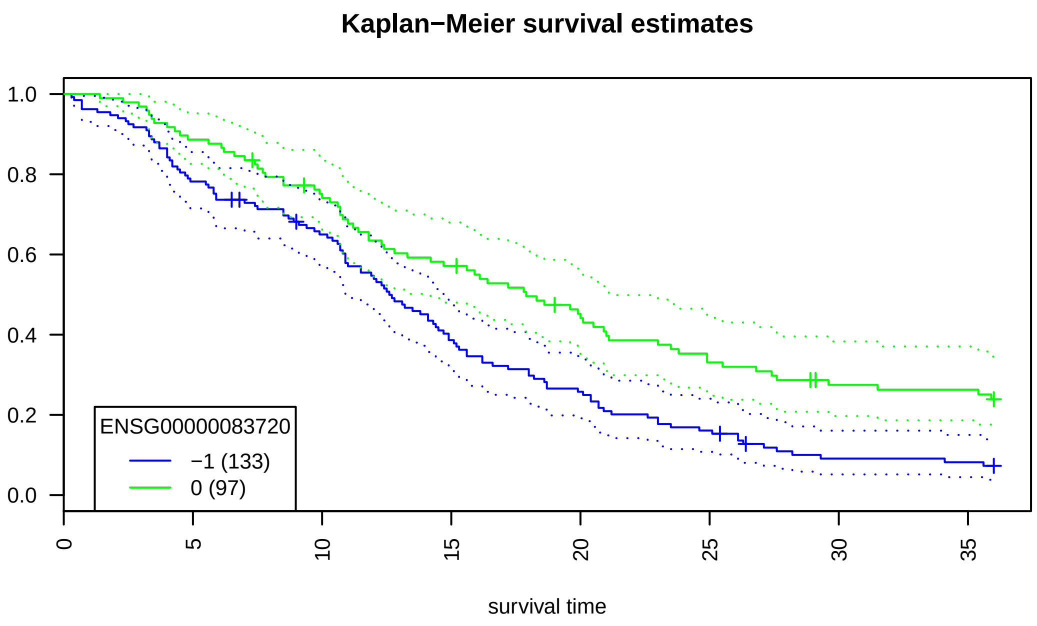

Figure 1471: Each curve represents the relative number of ENSG00000083720 survived to the corresponding point of time. Dashed lines represent 0.95 confidence intervals for the curves with the corresponding color. Log-rank probability measure for the equality of these curves is 2.808146e-04. Additional annotations: *.GeneName*=OXCT1, *.GeneDesc*=3-oxoacid CoA transferase 1 [Source:HGNC Symbol;Acc:8527].

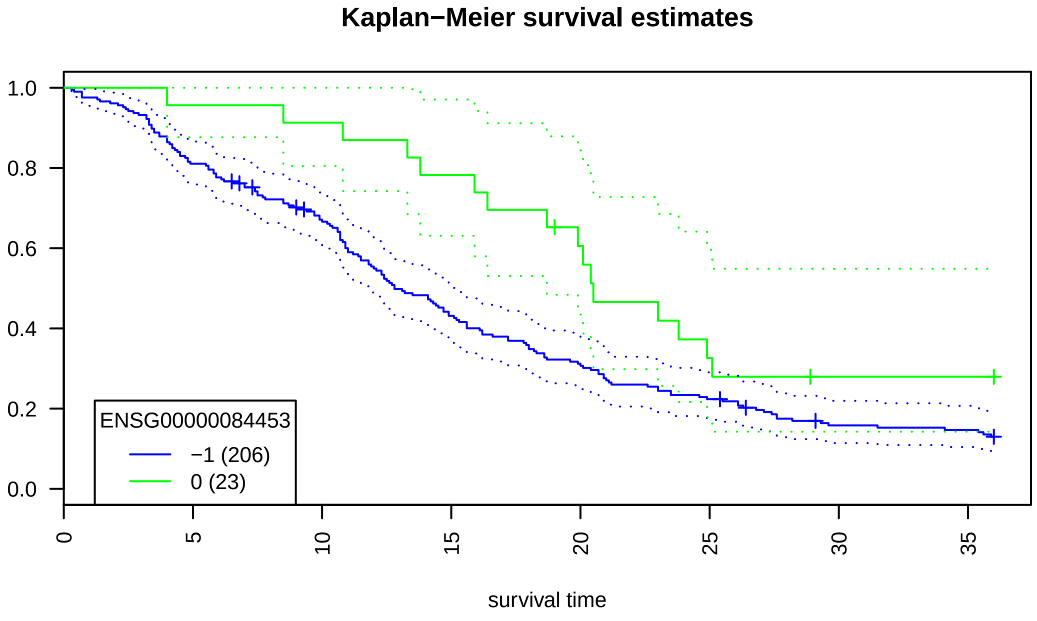

Figure 1472: Each curve represents the relative number of ENSG00000084453 survived to the corresponding point of time. Dashed lines represent 0.95 confidence intervals for the curves with the corresponding color. Total of 1 observation deleted due to missingness. Log-rank probability measure for the equality of these curves is 1.916107e-02. Additional annotations: *.GeneName*=SLCO1A2, *.GeneDesc*=solute carrier organic anion transporter family, member 1A2 [Source:HGNC Symbol;Acc:10956].

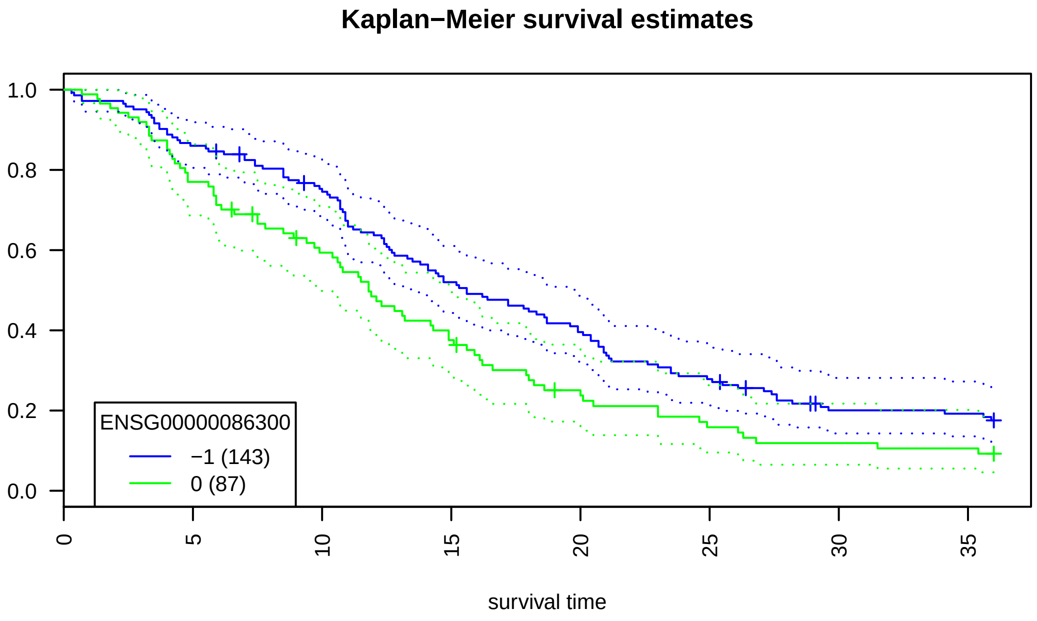

Figure 1473: Each curve represents the relative number of ENSG00000086300 survived to the corresponding point of time. Dashed lines represent 0.95 confidence intervals for the curves with the corresponding color. Log-rank probability measure for the equality of these curves is 8.220335e-03. Additional annotations: *.GeneName*=SNX10, *.GeneDesc*=sorting nexin 10 [Source:HGNC Symbol;Acc:14974].

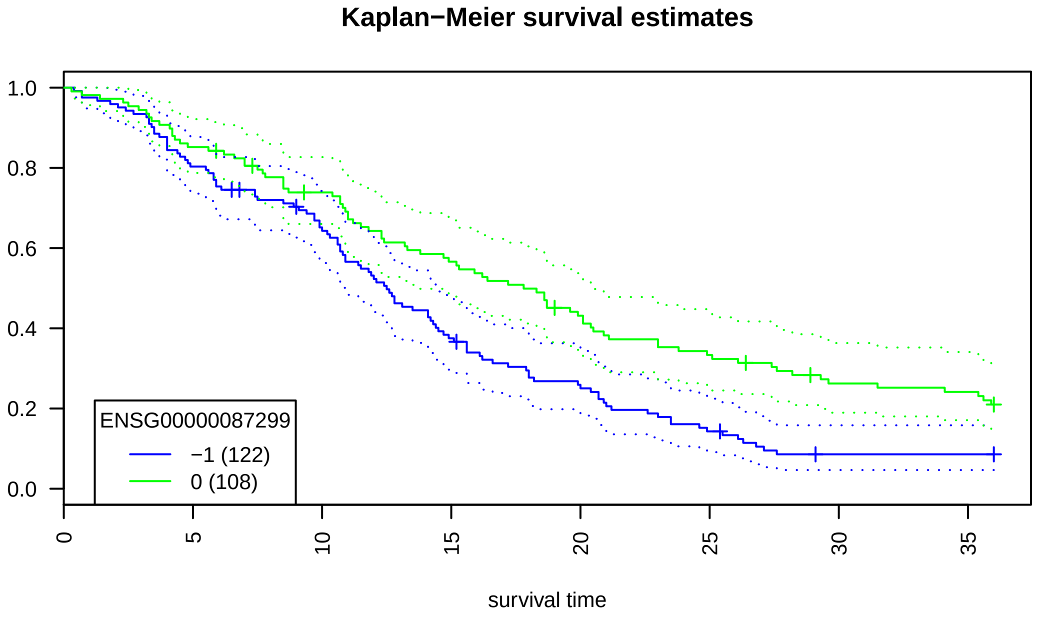

Figure 1474: Each curve represents the relative number of ENSG00000087299 survived to the corresponding point of time. Dashed lines represent 0.95 confidence intervals for the curves with the corresponding color. Log-rank probability measure for the equality of these curves is 8.768643e-04. Additional annotations: *.GeneName*=L2HGDH, *.GeneDesc*=L-2-hydroxyglutarate dehydrogenase [Source:HGNC Symbol;Acc:20499].

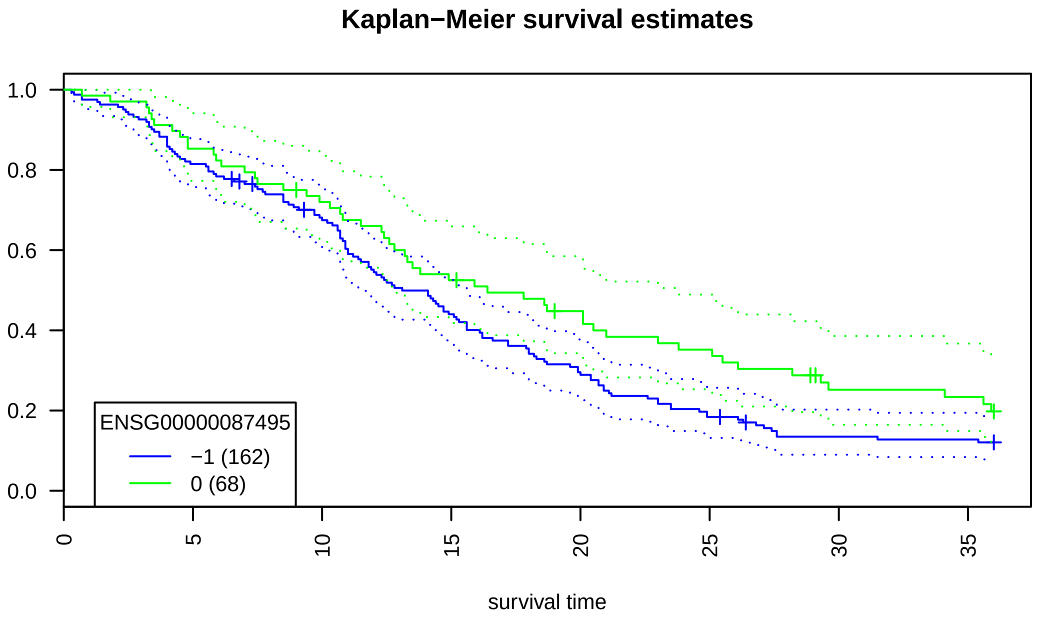

Figure 1475: Each curve represents the relative number of ENSG00000087495 survived to the corresponding point of time. Dashed lines represent 0.95 confidence intervals for the curves with the corresponding color. Log-rank probability measure for the equality of these curves is 4.580893e-02. Additional annotations: *.GeneName*=PHACTR3, *.GeneDesc*=phosphatase and actin regulator 3 [Source:HGNC Symbol;Acc:15833].

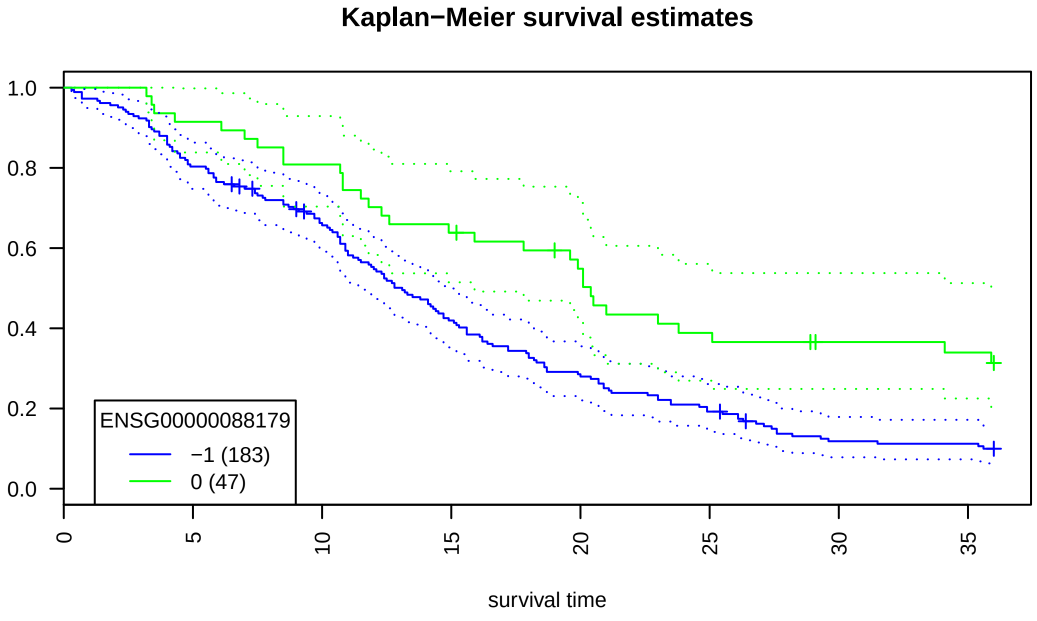

Figure 1476: Each curve represents the relative number of ENSG00000088179 survived to the corresponding point of time. Dashed lines represent 0.95 confidence intervals for the curves with the corresponding color. Log-rank probability measure for the equality of these curves is 6.033684e-04. Additional annotations: *.GeneName*=PTPN4, *.GeneDesc*=protein tyrosine phosphatase, non-receptor type 4 (megakaryocyte) [Source:HGNC Symbol;Acc:9656].

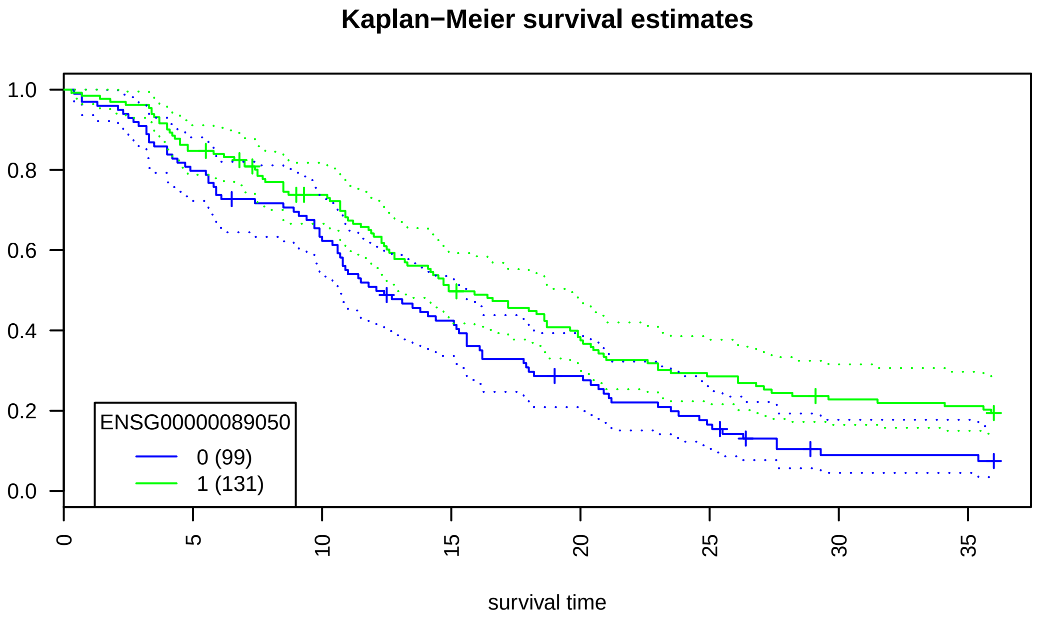

Figure 1477: Each curve represents the relative number of ENSG00000089050 survived to the corresponding point of time. Dashed lines represent 0.95 confidence intervals for the curves with the corresponding color. Log-rank probability measure for the equality of these curves is 8.204224e-03. Additional annotations: *.GeneName*=RBBP9, *.GeneDesc*=retinoblastoma binding protein 9 [Source:HGNC Symbol;Acc:9892].

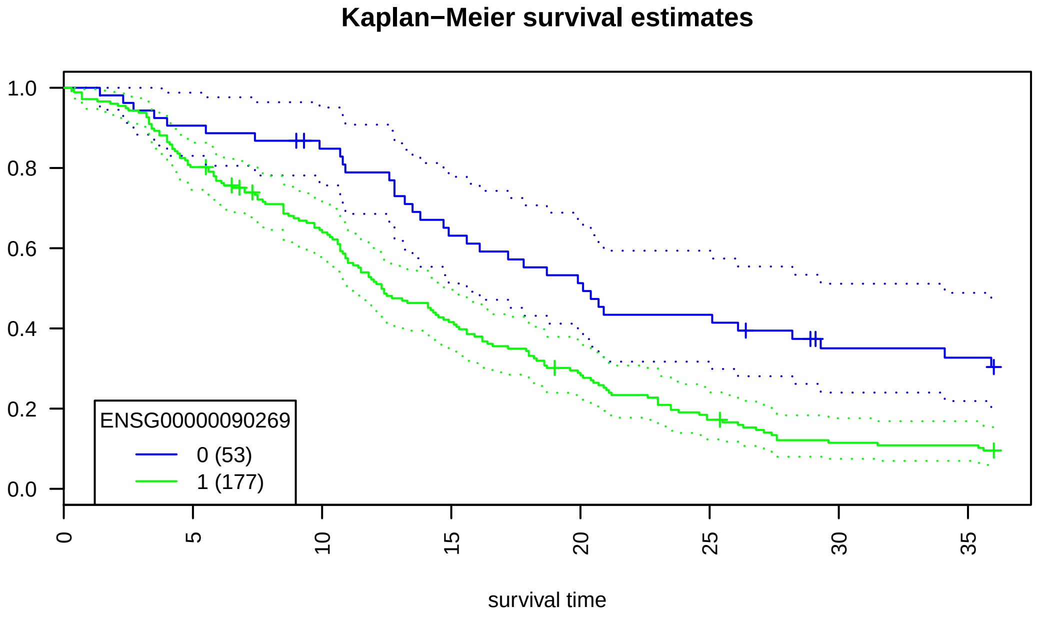

Figure 1478: Each curve represents the relative number of ENSG00000090269 survived to the corresponding point of time. Dashed lines represent 0.95 confidence intervals for the curves with the corresponding color. Log-rank probability measure for the equality of these curves is 1.568376e-04. Additional annotations: *.GeneName*=CLEC5A, *.GeneDesc*=C-type lectin domain family 5, member A [Source:HGNC Symbol;Acc:2054].

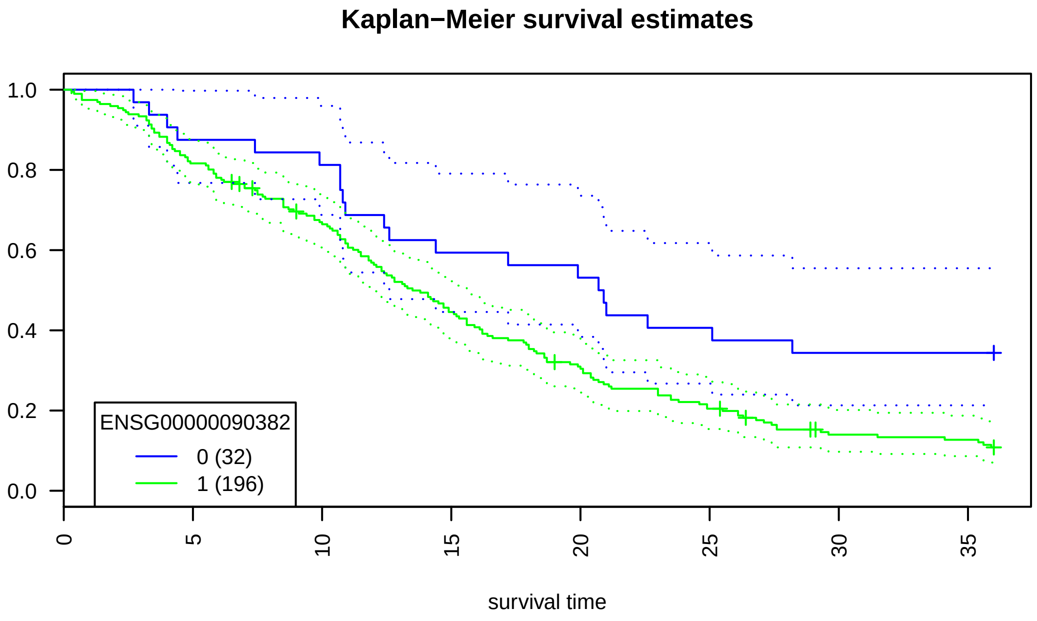

Figure 1479: Each curve represents the relative number of ENSG00000090382 survived to the corresponding point of time. Dashed lines represent 0.95 confidence intervals for the curves with the corresponding color. Total of 2 observations deleted due to missingness. Log-rank probability measure for the equality of these curves is 5.439553e-03. Additional annotations: *.GeneName*=LYZ, *.GeneDesc*=lysozyme (renal amyloidosis) [Source:HGNC Symbol;Acc:6740].

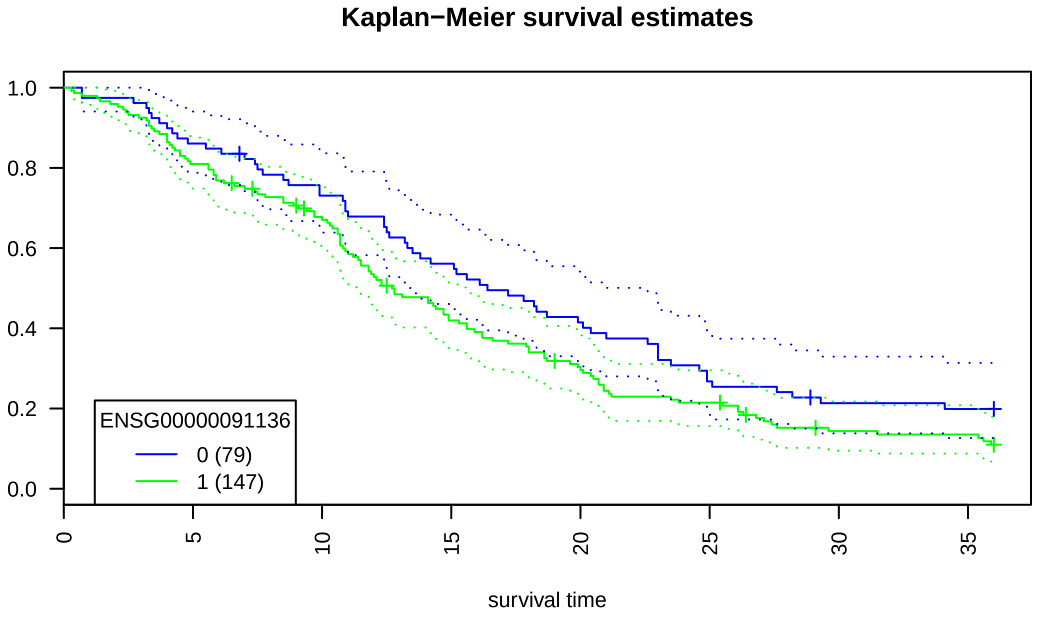

Figure 1480: Each curve represents the relative number of ENSG00000091136 survived to the corresponding point of time. Dashed lines represent 0.95 confidence intervals for the curves with the corresponding color. Total of 4 observations deleted due to missingness. Log-rank probability measure for the equality of these curves is 4.924821e-02. Additional annotations: *.GeneName*=LAMB1, *.GeneDesc*=laminin, beta 1 [Source:HGNC Symbol;Acc:6486].

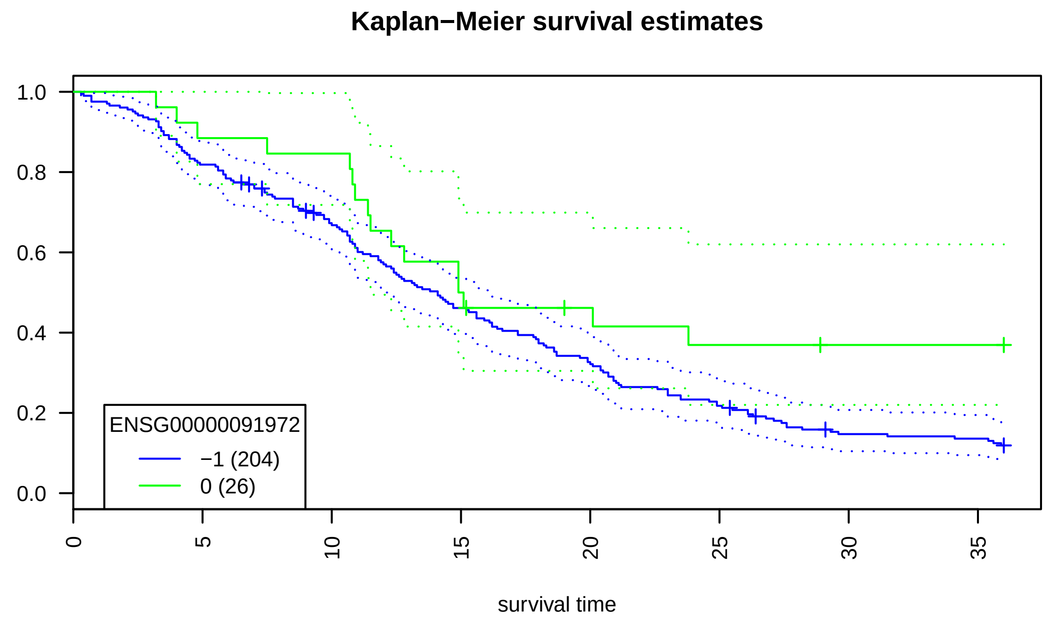

Figure 1481: Each curve represents the relative number of ENSG00000091972 survived to the corresponding point of time. Dashed lines represent 0.95 confidence intervals for the curves with the corresponding color. Log-rank probability measure for the equality of these curves is 3.515565e-02. Additional annotations: *.GeneName*=CD200, *.GeneDesc*=CD200 molecule [Source:HGNC Symbol;Acc:7203].

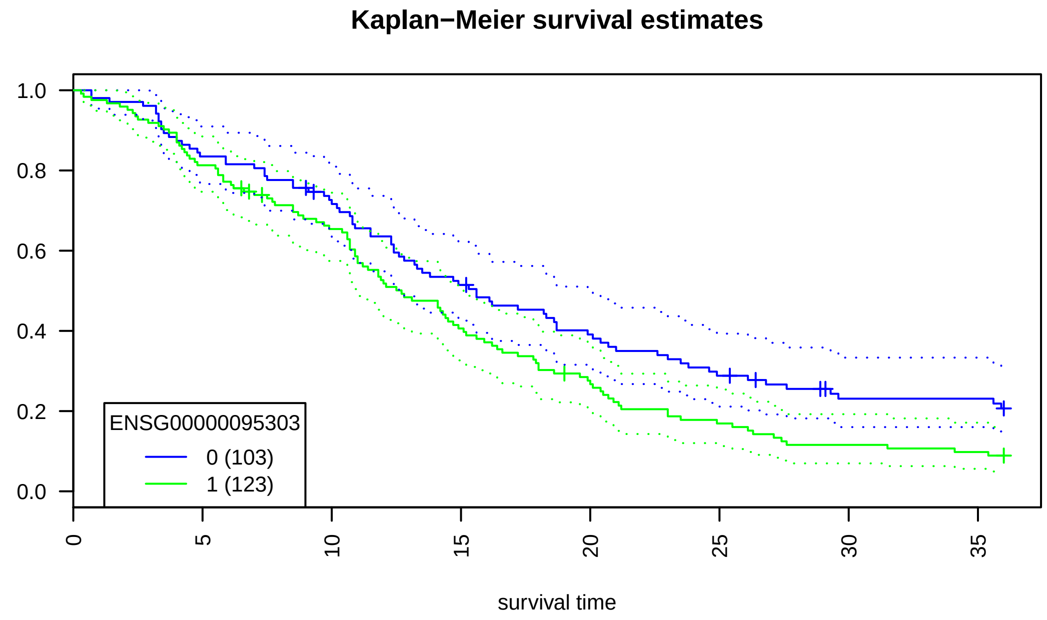

Figure 1482: Each curve represents the relative number of ENSG00000095303 survived to the corresponding point of time. Dashed lines represent 0.95 confidence intervals for the curves with the corresponding color. Total of 4 observations deleted due to missingness. Log-rank probability measure for the equality of these curves is 1.379500e-02. Additional annotations: *.GeneName*=PTGS1, *.GeneDesc*=prostaglandin-endoperoxide synthase 1 (prostaglandin G/H synthase and cyclooxygenase) [Source:HGNC Symbol;Acc:9604].

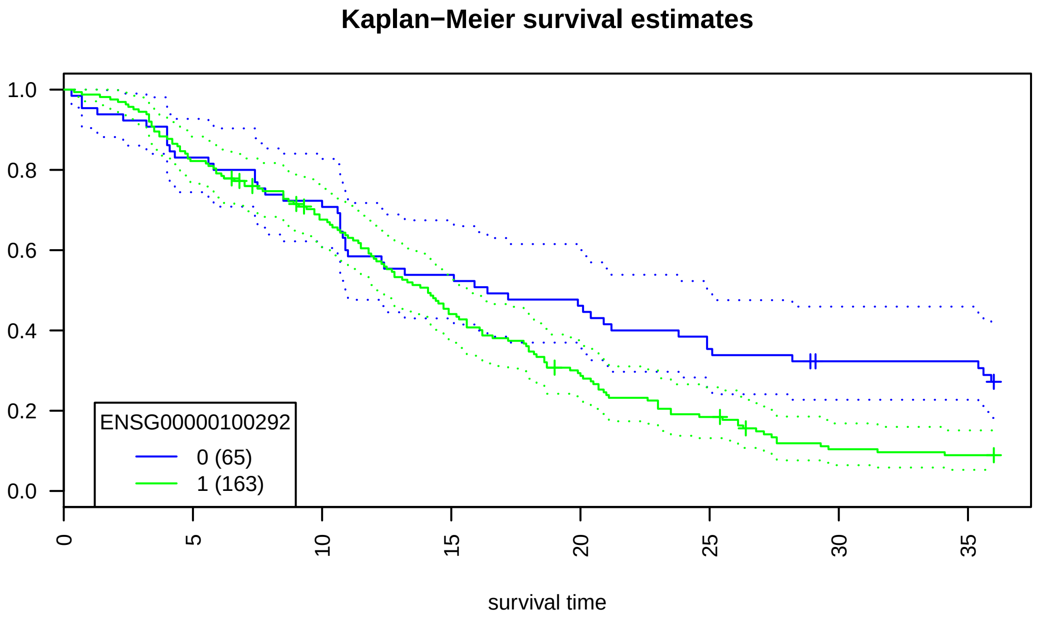

Figure 1483: Each curve represents the relative number of ENSG00000100292 survived to the corresponding point of time. Dashed lines represent 0.95 confidence intervals for the curves with the corresponding color. Total of 2 observations deleted due to missingness. Log-rank probability measure for the equality of these curves is 6.25857e-03. Additional annotations: *.GeneName*=HMOX1, *.GeneDesc*=heme oxygenase (decycling) 1 [Source:HGNC Symbol;Acc:5013].

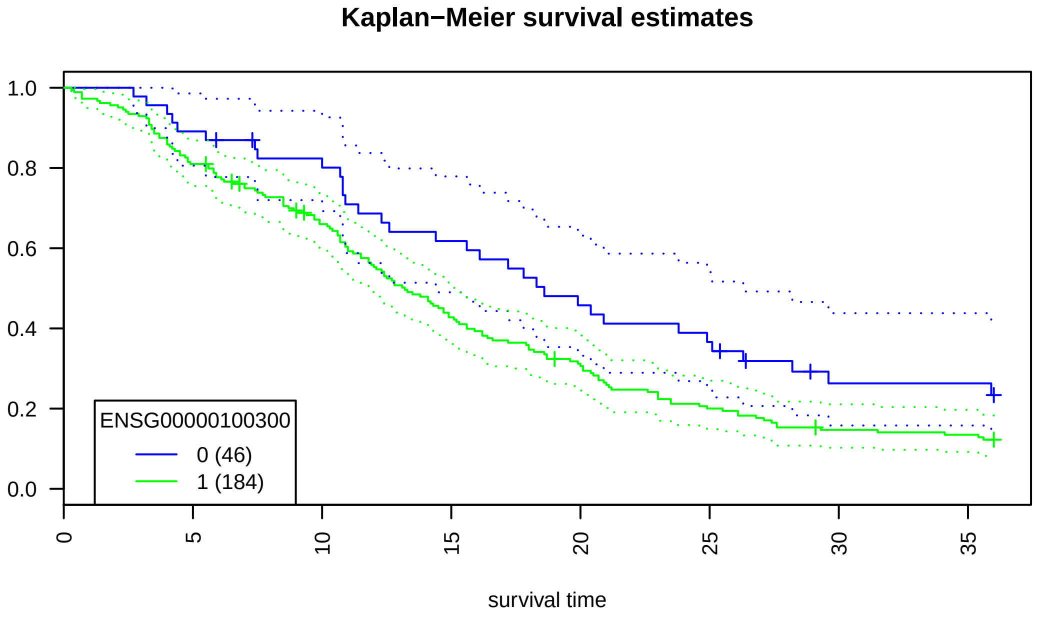

Figure 1484: Each curve represents the relative number of ENSG00000100300 survived to the corresponding point of time. Dashed lines represent 0.95 confidence intervals for the curves with the corresponding color. Log-rank probability measure for the equality of these curves is 2.219586e-02. Additional annotations: *.GeneName*=TSPO, *.GeneDesc*=translocator protein (18kDa) [Source:HGNC Symbol;Acc:1158].

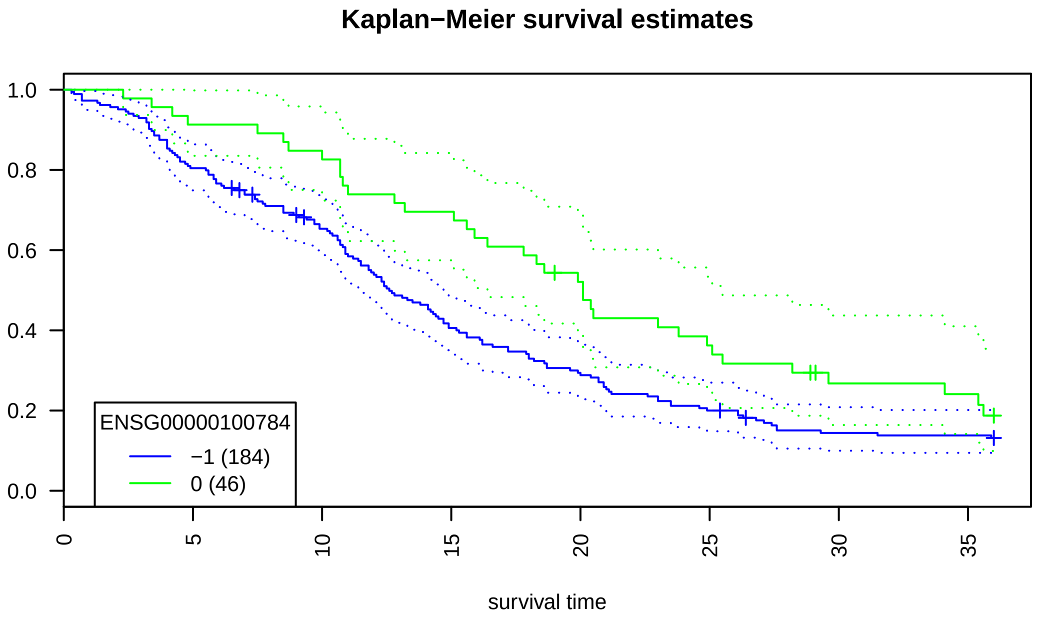

Figure 1485: Each curve represents the relative number of ENSG00000100784 survived to the corresponding point of time. Dashed lines represent 0.95 confidence intervals for the curves with the corresponding color. Log-rank probability measure for the equality of these curves is 1.622457e-02. Additional annotations: *.GeneName*=RPS6KA5, *.GeneDesc*=ribosomal protein S6 kinase, 90kDa, polypeptide 5 [Source:HGNC Symbol;Acc:10434].

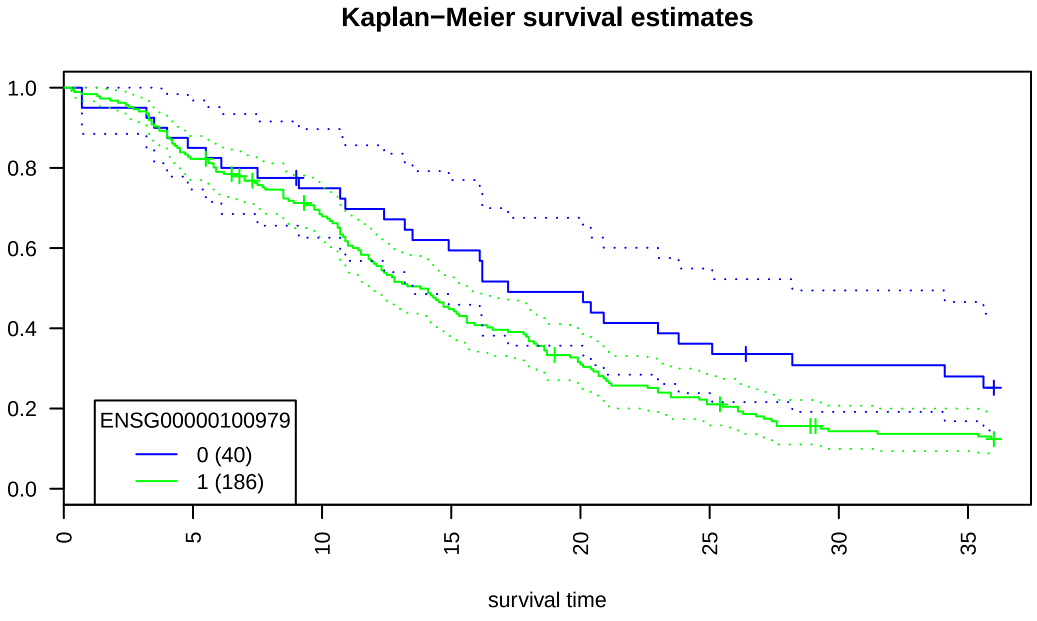

Figure 1486: Each curve represents the relative number of ENSG00000100979 survived to the corresponding point of time. Dashed lines represent 0.95 confidence intervals for the curves with the corresponding color. Total of 4 observations deleted due to missingness. Log-rank probability measure for the equality of these curves is 4.405087e-02. Additional annotations: *.GeneName*=PLTP, *.GeneDesc*=phospholipid transfer protein [Source:HGNC Symbol;Acc:9093].

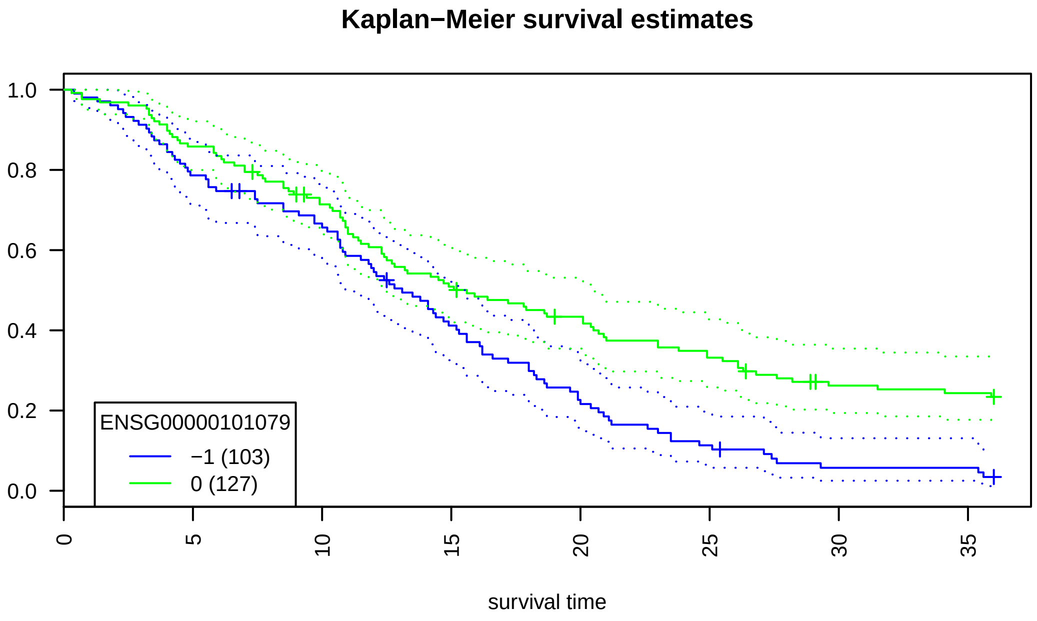

Figure 1487: Each curve represents the relative number of ENSG00000101079 survived to the corresponding point of time. Dashed lines represent 0.95 confidence intervals for the curves with the corresponding color. Log-rank probability measure for the equality of these curves is 1.418467e-04. Additional annotations: *.GeneName*=NDRG3, *.GeneDesc*=NDRG family member 3 [Source:HGNC Symbol;Acc:14462].

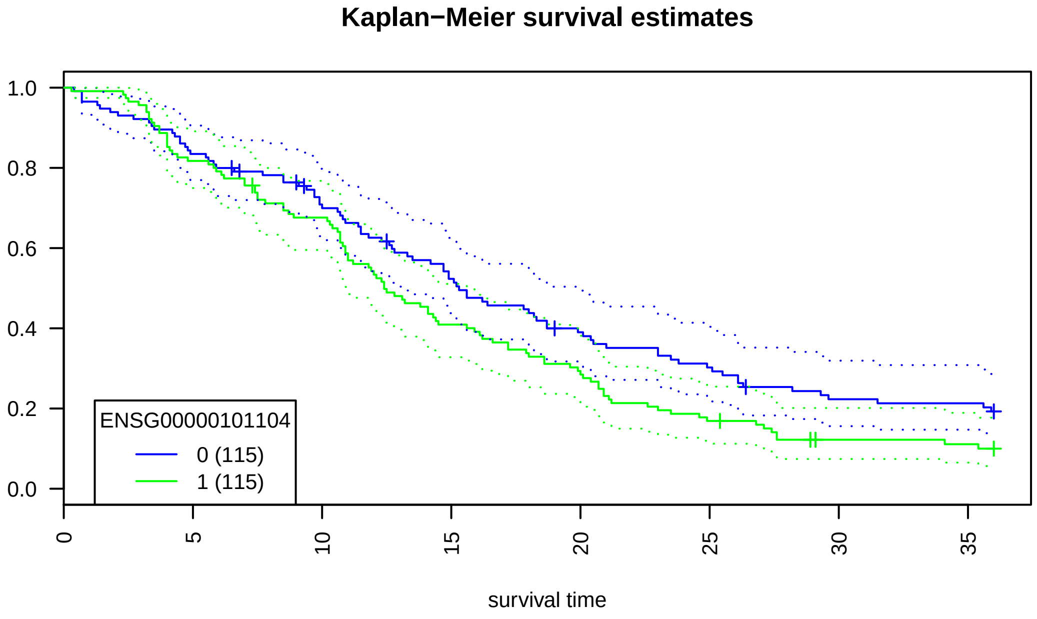

Figure 1488: Each curve represents the relative number of ENSG00000101104 survived to the corresponding point of time. Dashed lines represent 0.95 confidence intervals for the curves with the corresponding color. Log-rank probability measure for the equality of these curves is 3.257743e-02. Additional annotations: *.GeneName*=PABPC1L, *.GeneDesc*=poly(A) binding protein, cytoplasmic 1-like [Source:HGNC Symbol;Acc:15797].

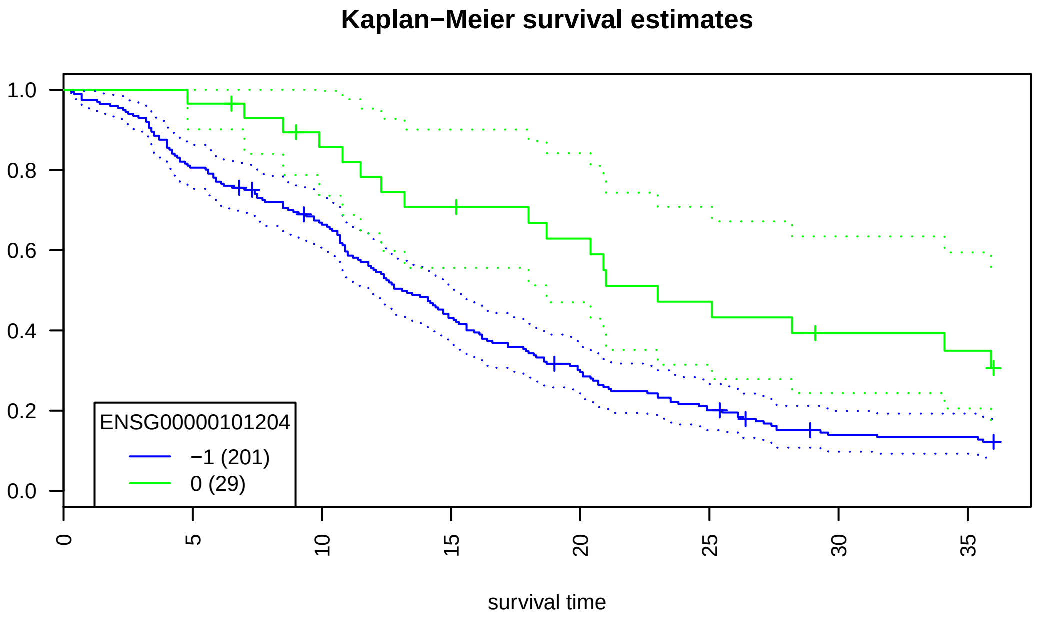

Figure 1489: Each curve represents the relative number of ENSG00000101204 survived to the corresponding point of time. Dashed lines represent 0.95 confidence intervals for the curves with the corresponding color. Log-rank probability measure for the equality of these curves is 2.8809e-03. Additional annotations: *.GeneName*=CHRNA4, *.GeneDesc*=cholinergic receptor, nicotinic, alpha 4 [Source:HGNC Symbol;Acc:1958].

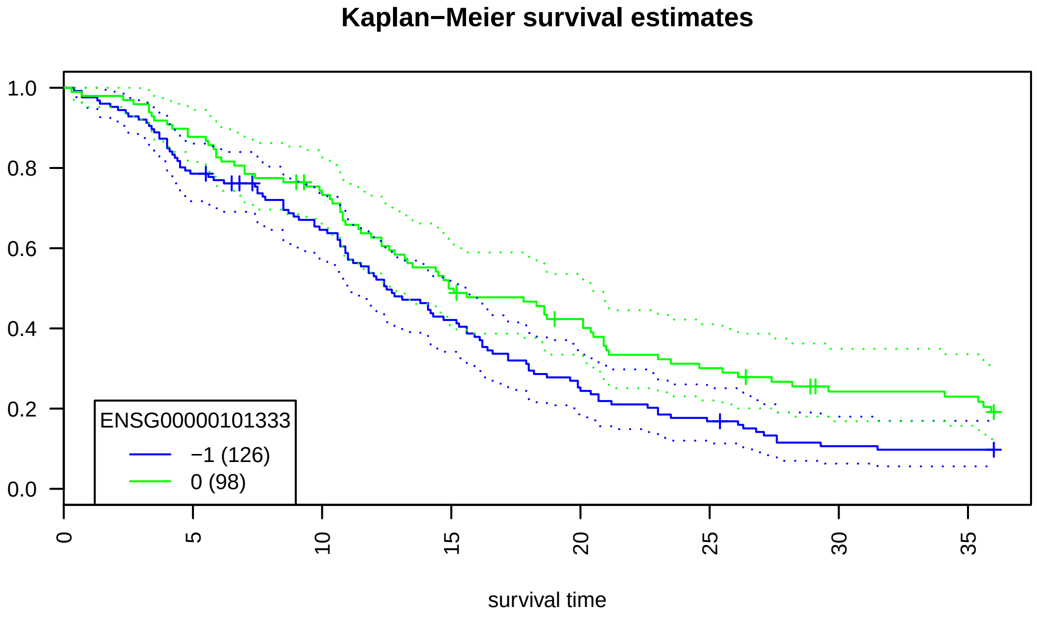

Figure 1490: Each curve represents the relative number of ENSG00000101333 survived to the corresponding point of time. Dashed lines represent 0.95 confidence intervals for the curves with the corresponding color. Total of 6 observations deleted due to missingness. Log-rank probability measure for the equality of these curves is 1.492107e-02. Additional annotations: *.GeneName*=PLCB4, *.GeneDesc*=phospholipase C, beta 4 [Source:HGNC Symbol;Acc:9059].

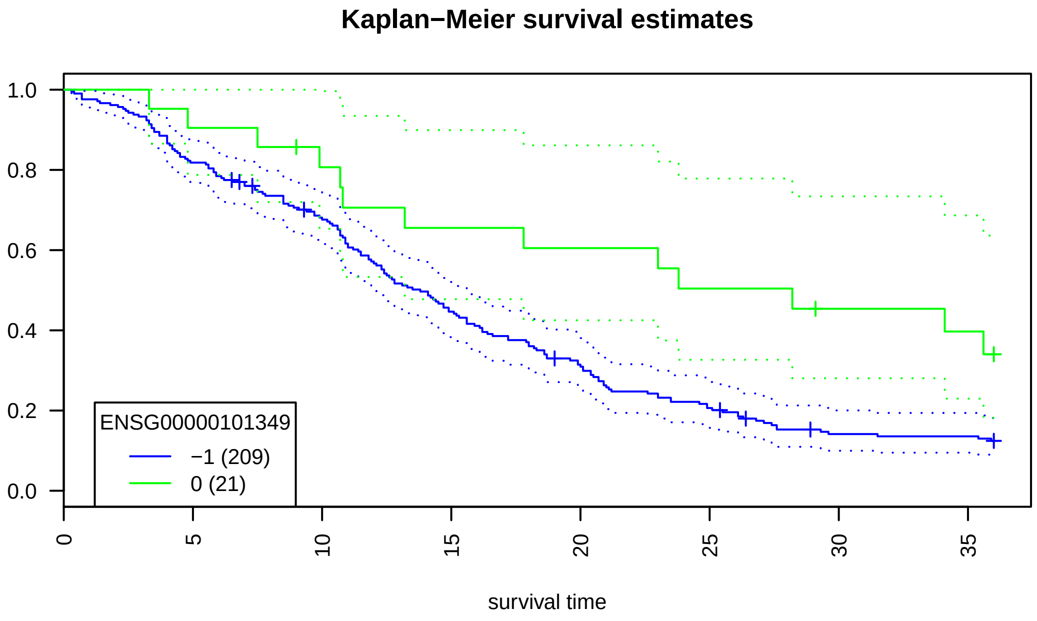

Figure 1491: Each curve represents the relative number of ENSG00000101349 survived to the corresponding point of time. Dashed lines represent 0.95 confidence intervals for the curves with the corresponding color. Log-rank probability measure for the equality of these curves is 9.2907e-03. Additional annotations: *.GeneName*=PAK7, *.GeneDesc*=p21 protein (Cdc42/Rac)-activated kinase 7 [Source:HGNC Symbol;Acc:15916].

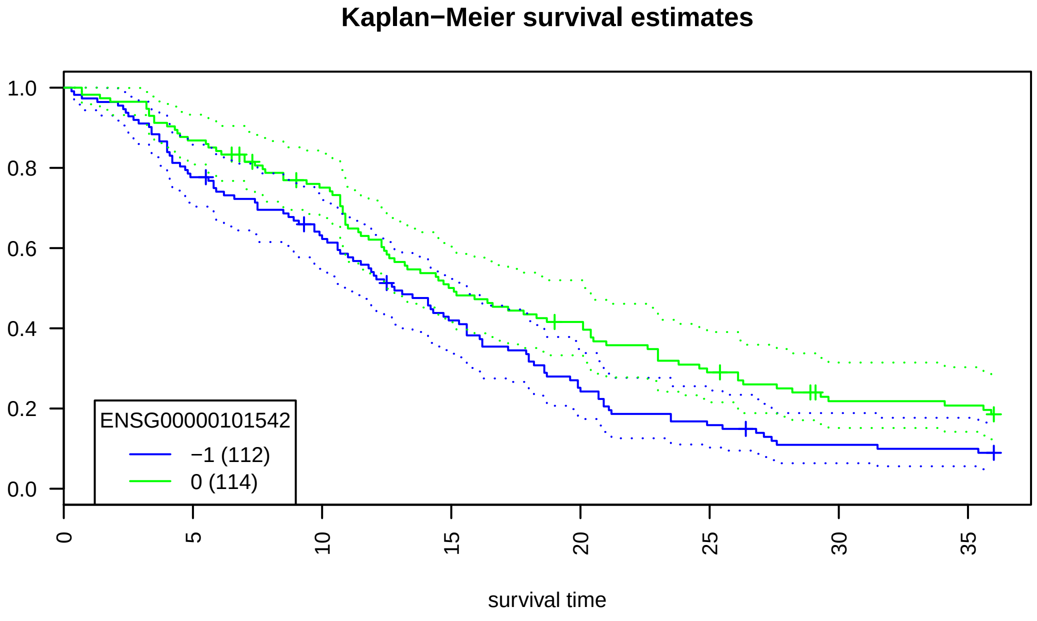

Figure 1492: Each curve represents the relative number of ENSG00000101542 survived to the corresponding point of time. Dashed lines represent 0.95 confidence intervals for the curves with the corresponding color. Total of 4 observations deleted due to missingness. Log-rank probability measure for the equality of these curves is 1.274535e-02. Additional annotations: *.GeneName*=CDH20, *.GeneDesc*=cadherin 20, type 2 [Source:HGNC Symbol;Acc:1760].

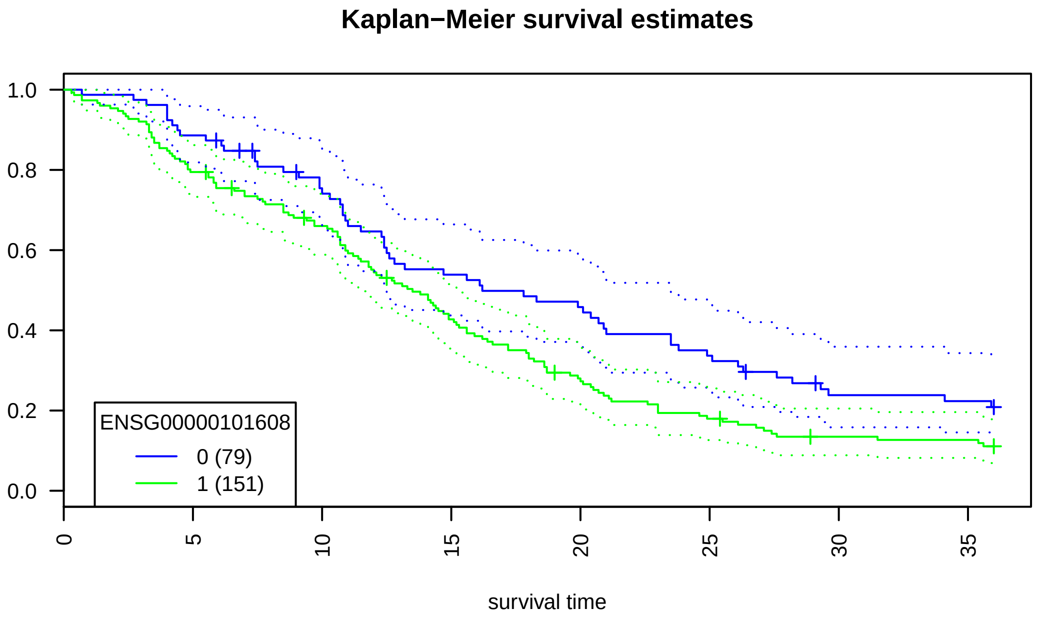

Figure 1493: Each curve represents the relative number of ENSG00000101608 survived to the corresponding point of time. Dashed lines represent 0.95 confidence intervals for the curves with the corresponding color. Log-rank probability measure for the equality of these curves is 1.336805e-02. Additional annotations: *.GeneName*=MYL12A, *.GeneDesc*=myosin, light chain 12A, regulatory, non-sarcomeric [Source:HGNC Symbol;Acc:16701].

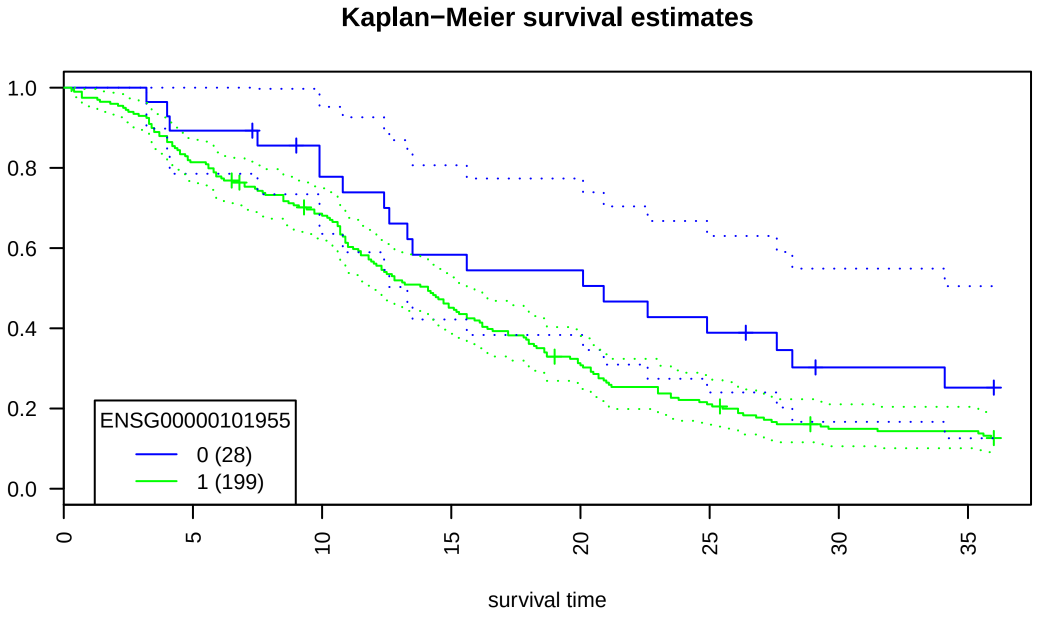

Figure 1494: Each curve represents the relative number of ENSG00000101955 survived to the corresponding point of time. Dashed lines represent 0.95 confidence intervals for the curves with the corresponding color. Total of 3 observations deleted due to missingness. Log-rank probability measure for the equality of these curves is 4.47232e-02. Additional annotations: *.GeneName*=SRPX, *.GeneDesc*=sushi-repeat-containing protein, X-linked [Source:HGNC Symbol;Acc:11309].

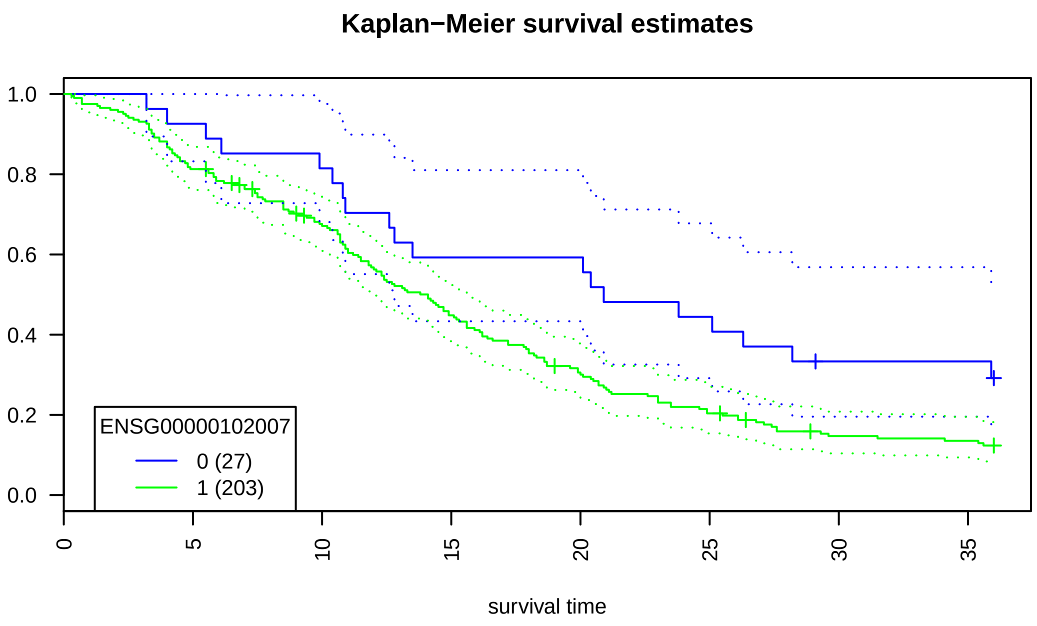

Figure 1495: Each curve represents the relative number of ENSG00000102007 survived to the corresponding point of time. Dashed lines represent 0.95 confidence intervals for the curves with the corresponding color. Log-rank probability measure for the equality of these curves is 1.939577e-02. Additional annotations: *.GeneName*=PLP2, *.GeneDesc*=proteolipid protein 2 (colonic epithelium-enriched) [Source:HGNC Symbol;Acc:9087].

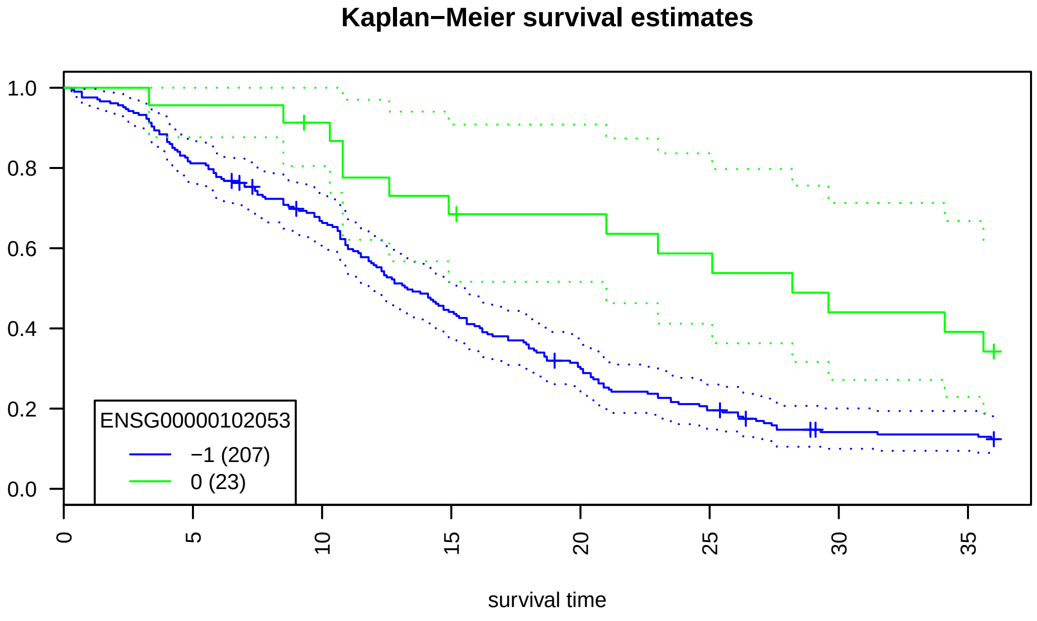

Figure 1496: Each curve represents the relative number of ENSG00000102053 survived to the corresponding point of time. Dashed lines represent 0.95 confidence intervals for the curves with the corresponding color. Log-rank probability measure for the equality of these curves is 2.525515e-03. Additional annotations: *.GeneName*=ZC3H12B, *.GeneDesc*=zinc finger CCCH-type containing 12B [Source:HGNC Symbol;Acc:17407].

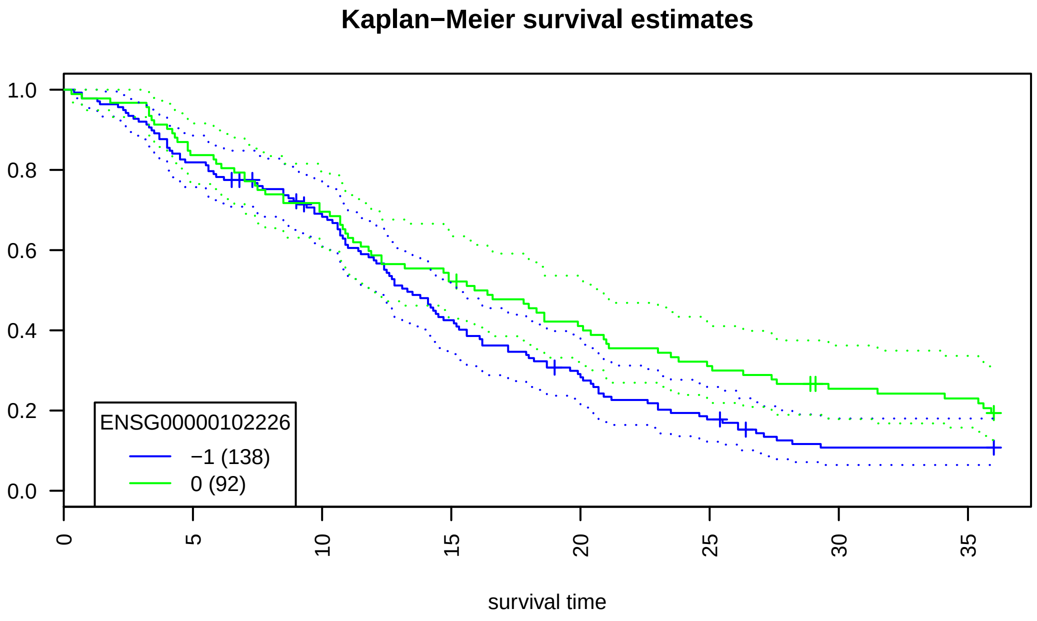

Figure 1497: Each curve represents the relative number of ENSG00000102226 survived to the corresponding point of time. Dashed lines represent 0.95 confidence intervals for the curves with the corresponding color. Log-rank probability measure for the equality of these curves is 3.958322e-02. Additional annotations: *.GeneName*=USP11, *.GeneDesc*=ubiquitin specific peptidase 11 [Source:HGNC Symbol;Acc:12609].

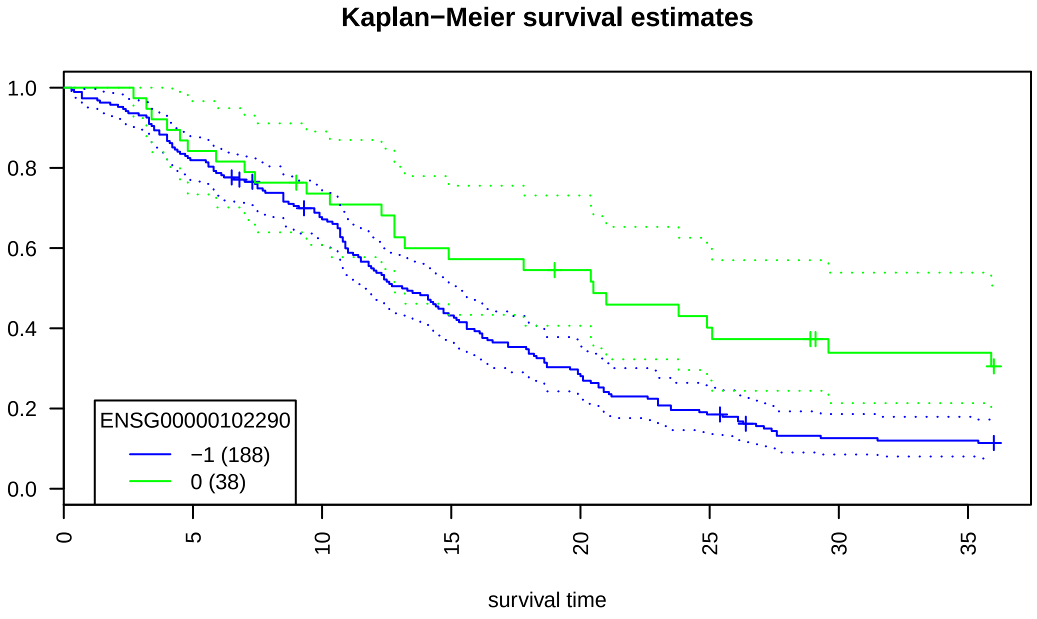

Figure 1498: Each curve represents the relative number of ENSG00000102290 survived to the corresponding point of time. Dashed lines represent 0.95 confidence intervals for the curves with the corresponding color. Total of 4 observations deleted due to missingness. Log-rank probability measure for the equality of these curves is 6.183014e-03. Additional annotations: *.GeneName*=PCDH11X, *.GeneDesc*=protocadherin 11 X-linked [Source:HGNC Symbol;Acc:8656].

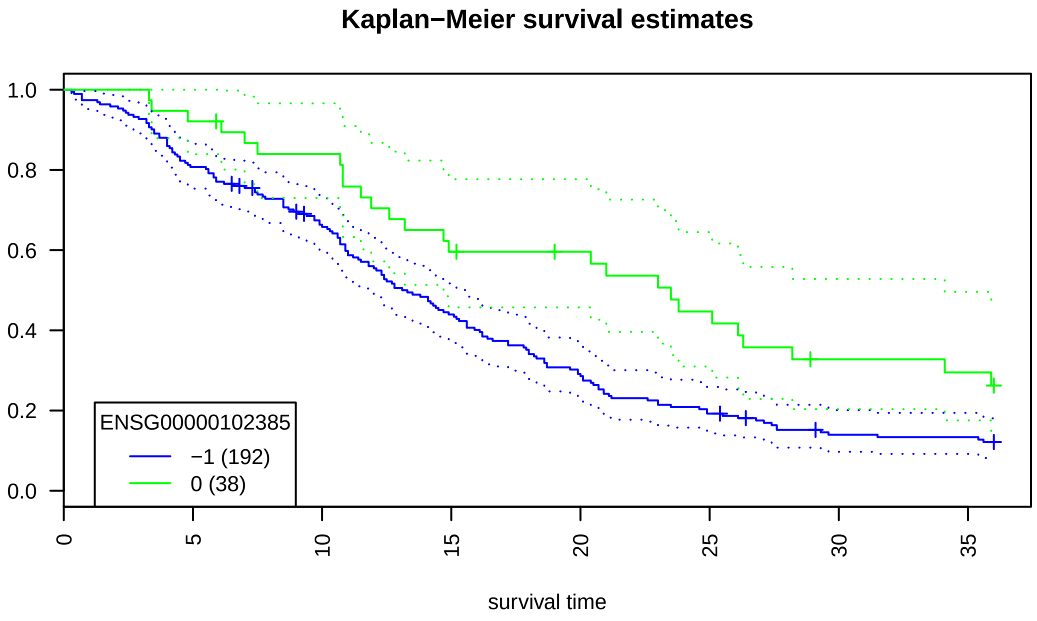

Figure 1499: Each curve represents the relative number of ENSG00000102385 survived to the corresponding point of time. Dashed lines represent 0.95 confidence intervals for the curves with the corresponding color. Log-rank probability measure for the equality of these curves is 5.444182e-03. Additional annotations: *.GeneName*=DRP2, *.GeneDesc*=dystrophin related protein 2 [Source:HGNC Symbol;Acc:3032].

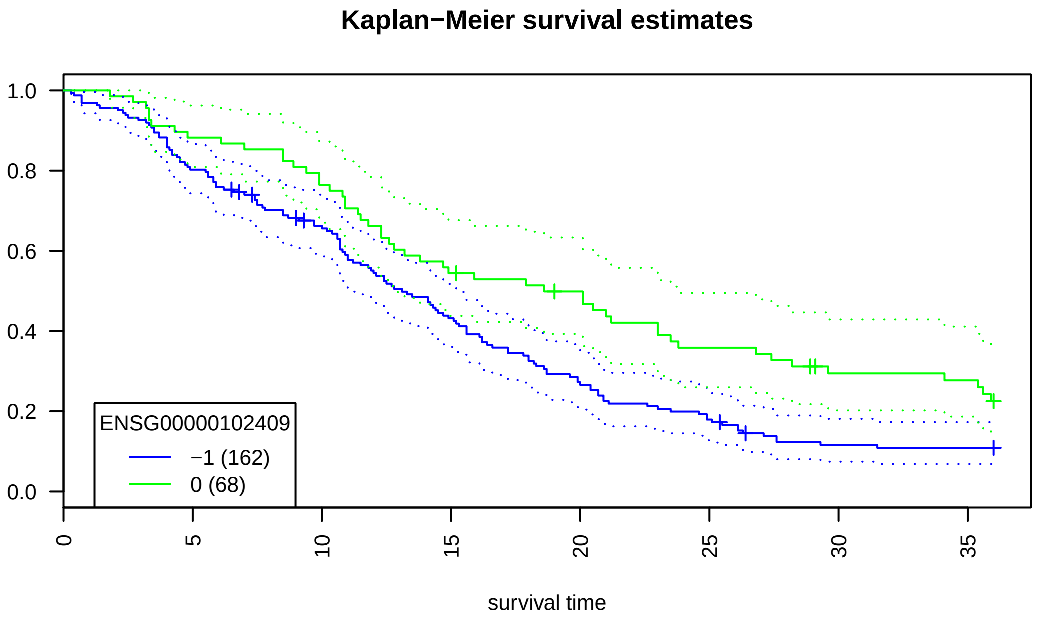

Figure 1500: Each curve represents the relative number of ENSG00000102409 survived to the corresponding point of time. Dashed lines represent 0.95 confidence intervals for the curves with the corresponding color. Log-rank probability measure for the equality of these curves is 3.790459e-03. Additional annotations: *.GeneName*=BEX4, *.GeneDesc*=brain expressed, X-linked 4 [Source:HGNC Symbol;Acc:25475].

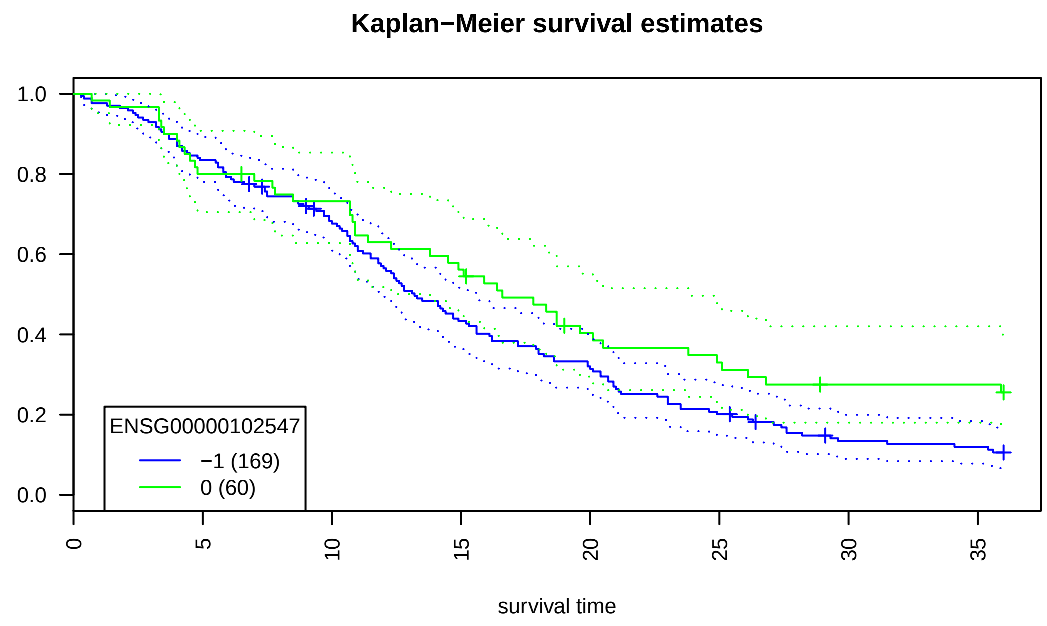

Figure 1501: Each curve represents the relative number of ENSG00000102547 survived to the corresponding point of time. Dashed lines represent 0.95 confidence intervals for the curves with the corresponding color. Total of 1 observation deleted due to missingness. Log-rank probability measure for the equality of these curves is 3.218196e-02. Additional annotations: *.GeneName*=CAB39L, *.GeneDesc*=calcium binding protein 39-like [Source:HGNC Symbol;Acc:20290].

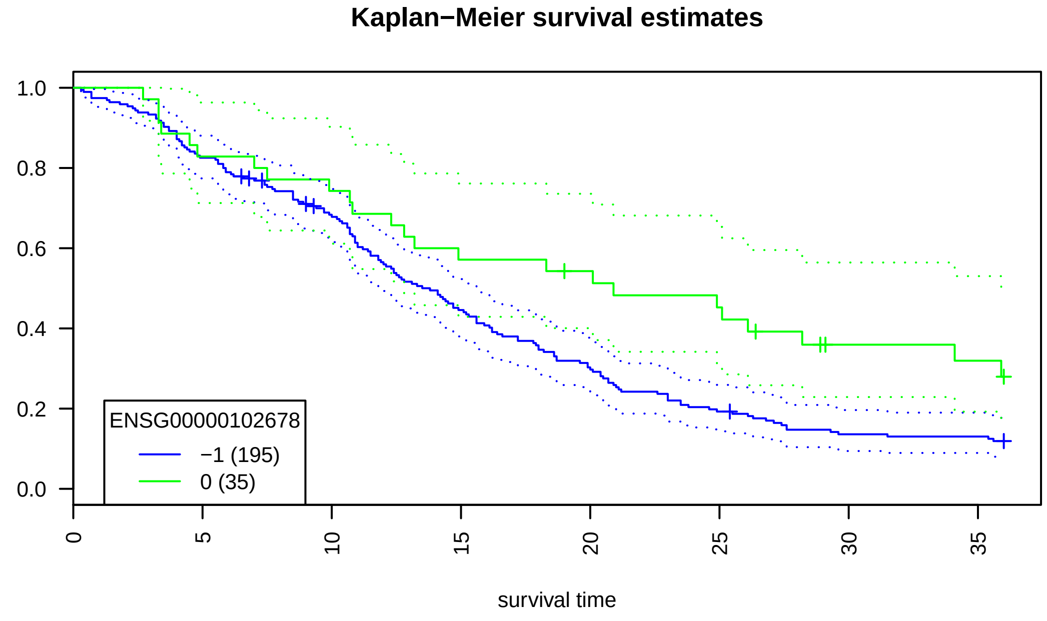

Figure 1502: Each curve represents the relative number of ENSG00000102678 survived to the corresponding point of time. Dashed lines represent 0.95 confidence intervals for the curves with the corresponding color. Log-rank probability measure for the equality of these curves is 1.243936e-02. Additional annotations: *.GeneName*=FGF9, *.GeneDesc*=fibroblast growth factor 9 (glia-activating factor) [Source:HGNC Symbol;Acc:3687].

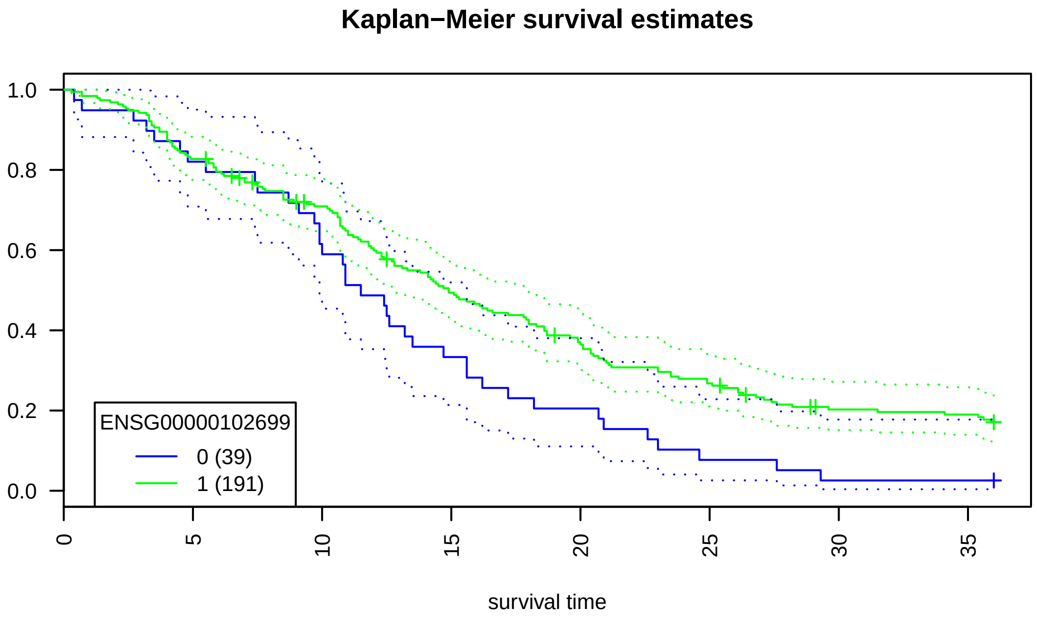

Figure 1503: Each curve represents the relative number of ENSG00000102699 survived to the corresponding point of time. Dashed lines represent 0.95 confidence intervals for the curves with the corresponding color. Log-rank probability measure for the equality of these curves is 5.96234e-03. Additional annotations: *.GeneName*=PARP4, *.GeneDesc*=poly (ADP-ribose) polymerase family, member 4 [Source:HGNC Symbol;Acc:271].

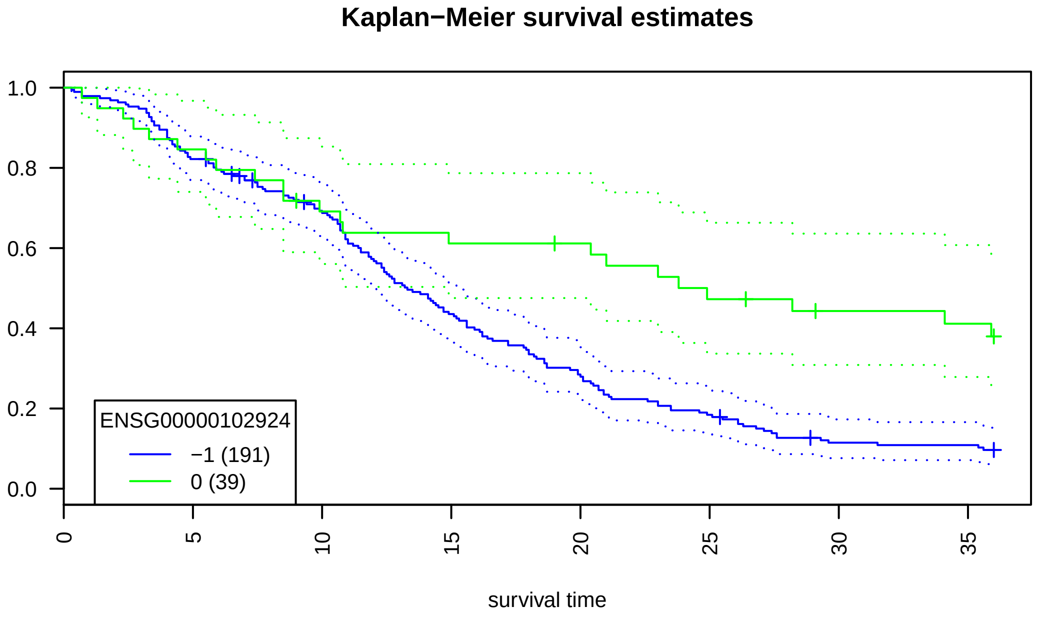

Figure 1504: Each curve represents the relative number of ENSG00000102924 survived to the corresponding point of time. Dashed lines represent 0.95 confidence intervals for the curves with the corresponding color. Log-rank probability measure for the equality of these curves is 3.448969e-04. Additional annotations: *.GeneName*=CBLN1, *.GeneDesc*=cerebellin 1 precursor [Source:HGNC Symbol;Acc:1543].

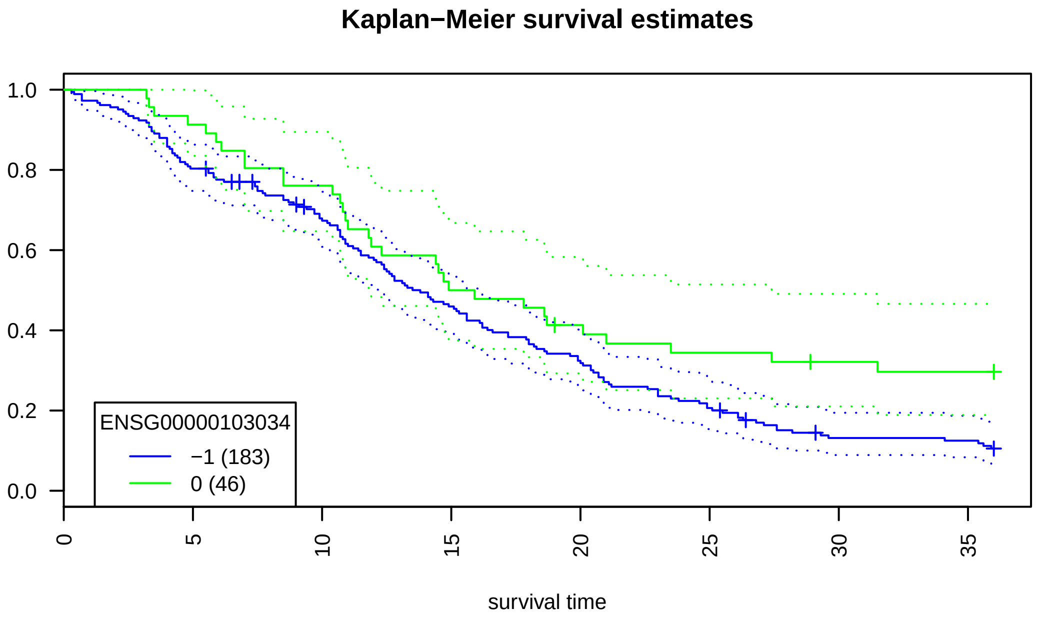

Figure 1505: Each curve represents the relative number of ENSG00000103034 survived to the corresponding point of time. Dashed lines represent 0.95 confidence intervals for the curves with the corresponding color. Total of 1 observation deleted due to missingness. Log-rank probability measure for the equality of these curves is 2.086024e-02. Additional annotations: *.GeneName*=NDRG4, *.GeneDesc*=NDRG family member 4 [Source:HGNC Symbol;Acc:14466].

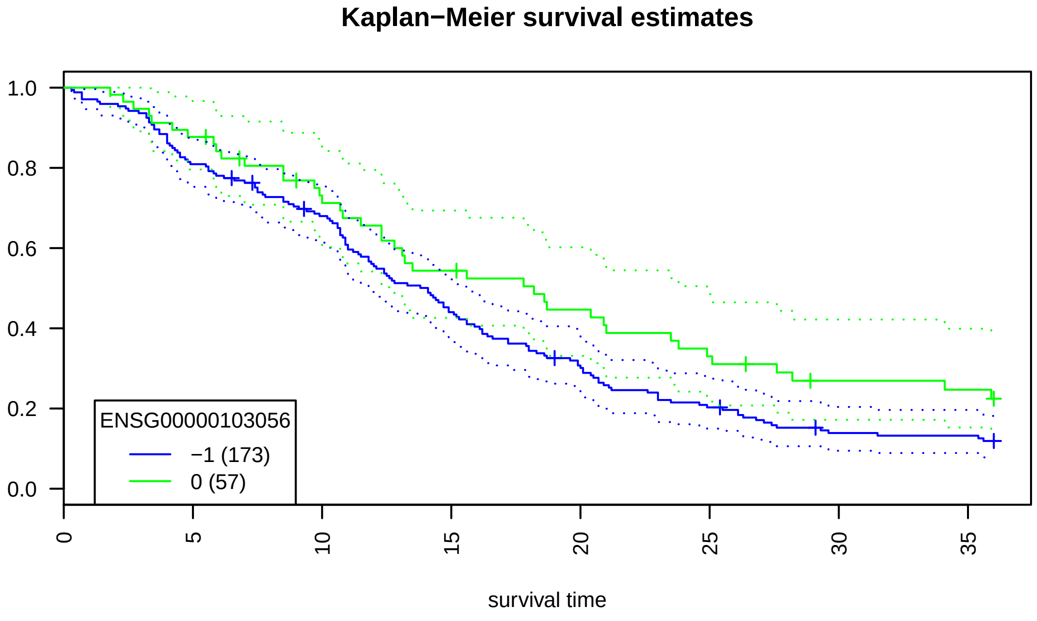

Figure 1506: Each curve represents the relative number of ENSG00000103056 survived to the corresponding point of time. Dashed lines represent 0.95 confidence intervals for the curves with the corresponding color. Log-rank probability measure for the equality of these curves is 4.397013e-02. Additional annotations: *.GeneName*=SMPD3, *.GeneDesc*=sphingomyelin phosphodiesterase 3, neutral membrane (neutral sphingomyelinase II) [Source:HGNC Symbol;Acc:14240].

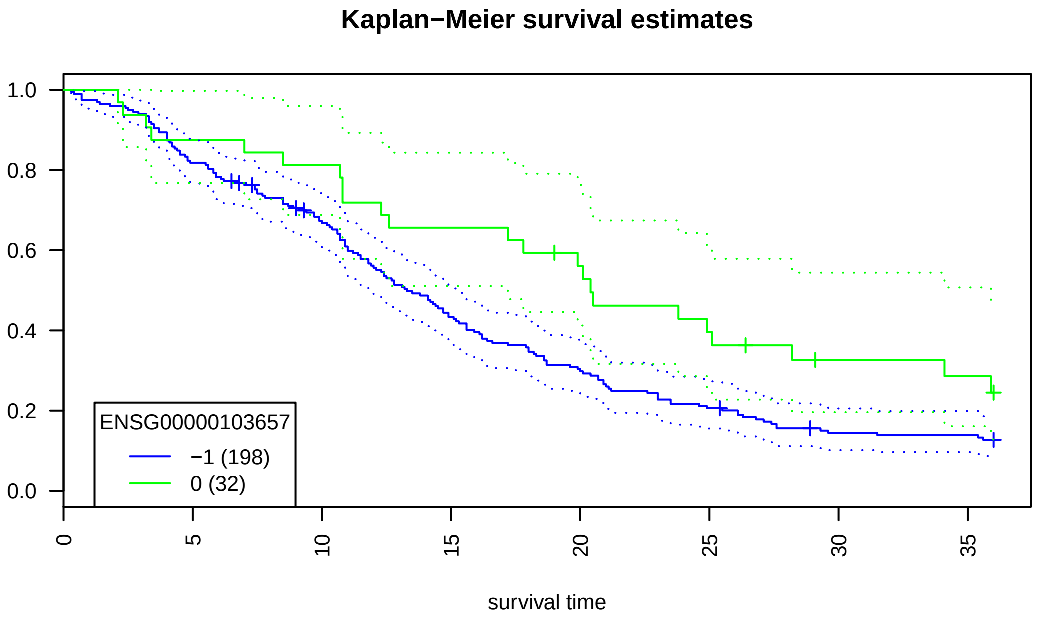

Figure 1507: Each curve represents the relative number of ENSG00000103657 survived to the corresponding point of time. Dashed lines represent 0.95 confidence intervals for the curves with the corresponding color. Log-rank probability measure for the equality of these curves is 2.269331e-02. Additional annotations: *.GeneName*=HERC1, *.GeneDesc*=hect (homologous to the E6-AP (UBE3A) carboxyl terminus) domain and RCC1 (CHC1)-like domain (RLD) 1 [Source:HGNC Symbol;Acc:4867].

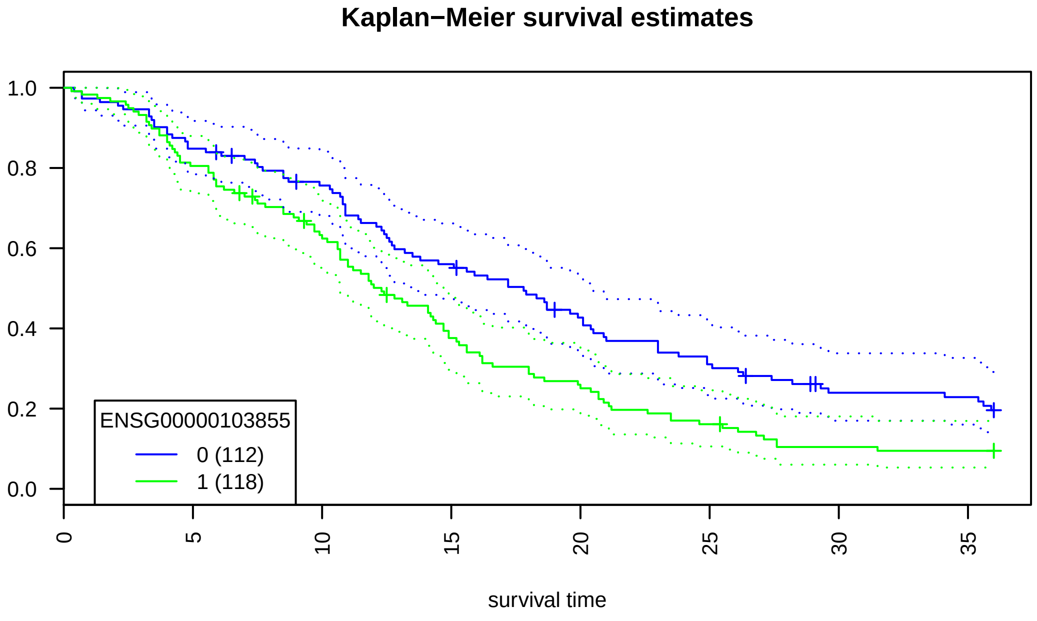

Figure 1508: Each curve represents the relative number of ENSG00000103855 survived to the corresponding point of time. Dashed lines represent 0.95 confidence intervals for the curves with the corresponding color. Log-rank probability measure for the equality of these curves is 2.950430e-03. Additional annotations: *.GeneName*=CD276, *.GeneDesc*=CD276 molecule [Source:HGNC Symbol;Acc:19137].

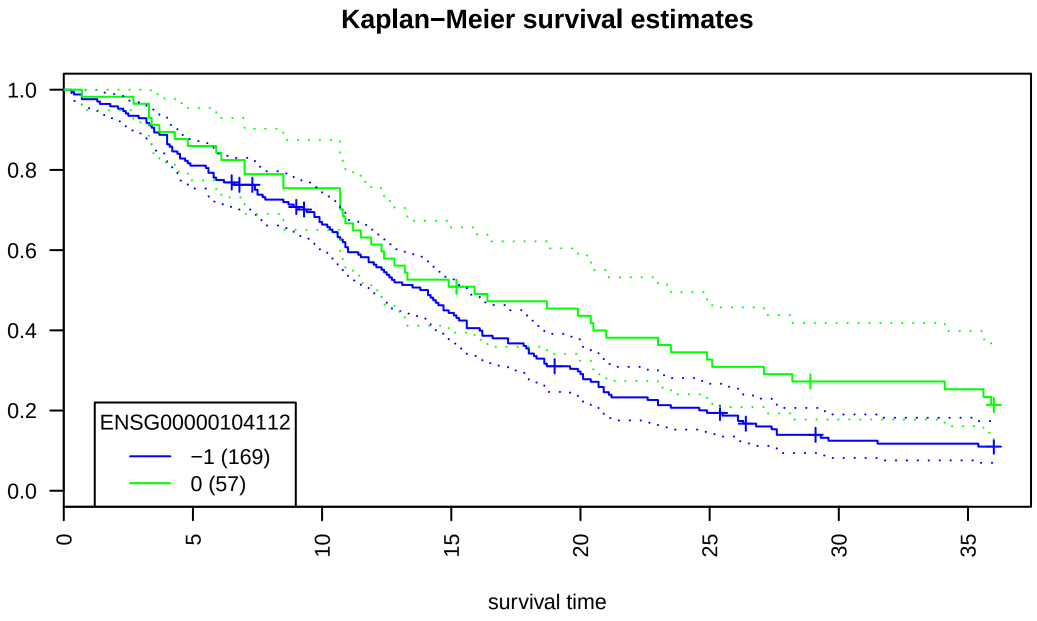

Figure 1509: Each curve represents the relative number of ENSG00000104112 survived to the corresponding point of time. Dashed lines represent 0.95 confidence intervals for the curves with the corresponding color. Total of 4 observations deleted due to missingness. Log-rank probability measure for the equality of these curves is 4.488712e-02. Additional annotations: *.GeneName*=SCG3, *.GeneDesc*=secretogranin III [Source:HGNC Symbol;Acc:13707].

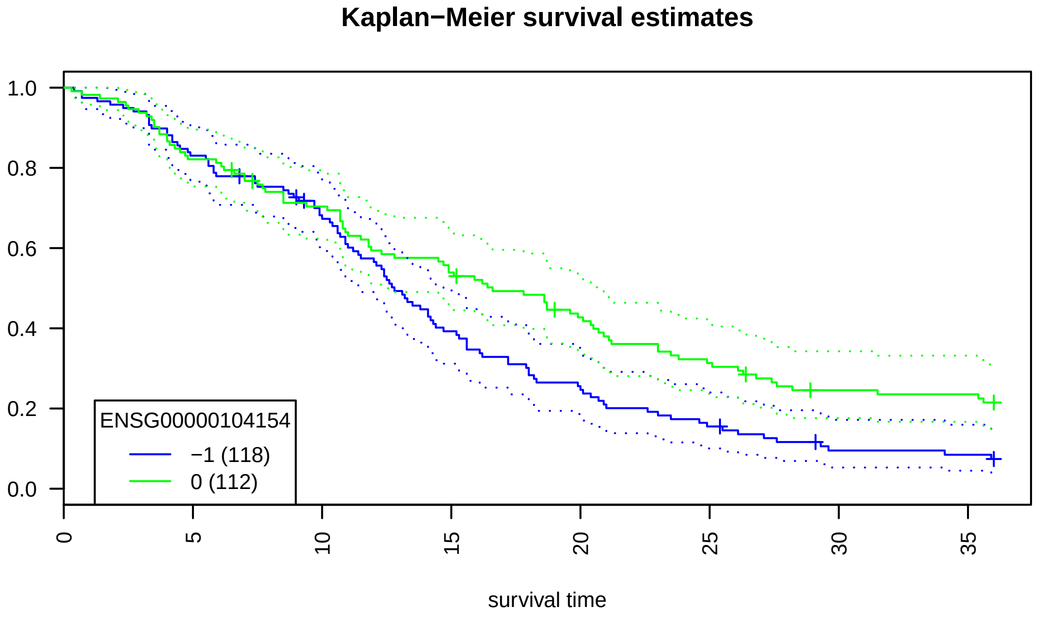

Figure 1510: Each curve represents the relative number of ENSG00000104154 survived to the corresponding point of time. Dashed lines represent 0.95 confidence intervals for the curves with the corresponding color. Log-rank probability measure for the equality of these curves is 4.402925e-03. Additional annotations: *.GeneName*=SLC30A4, *.GeneDesc*=solute carrier family 30 (zinc transporter), member 4 [Source:HGNC Symbol;Acc:11015].

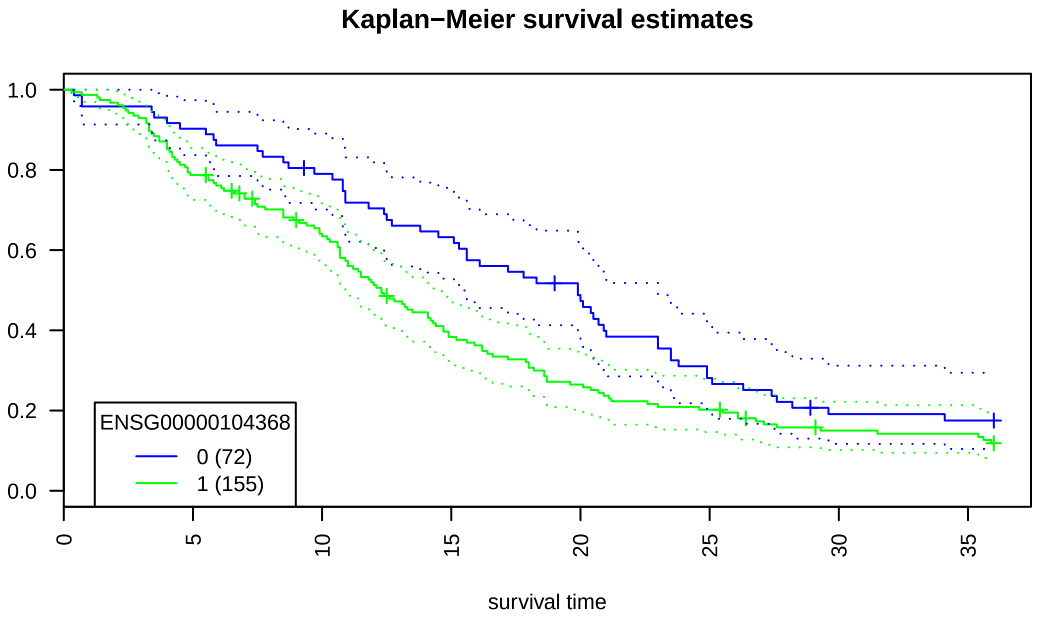

Figure 1511: Each curve represents the relative number of ENSG00000104368 survived to the corresponding point of time. Dashed lines represent 0.95 confidence intervals for the curves with the corresponding color. Total of 3 observations deleted due to missingness. Log-rank probability measure for the equality of these curves is 1.772383e-02. Additional annotations: *.GeneName*=PLAT, *.GeneDesc*=plasminogen activator, tissue [Source:HGNC Symbol;Acc:9051].

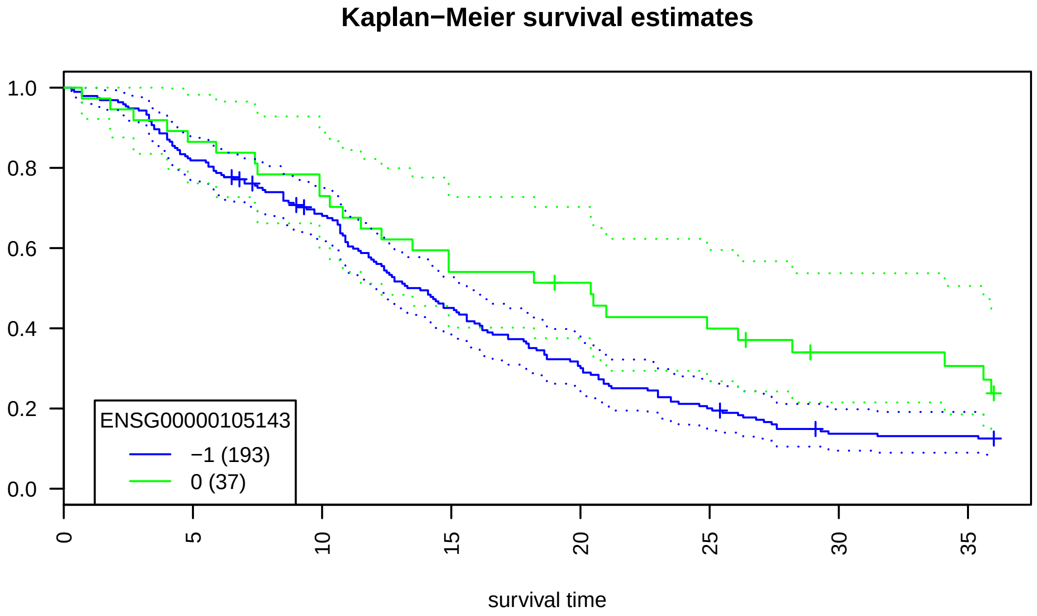

Figure 1512: Each curve represents the relative number of ENSG00000105143 survived to the corresponding point of time. Dashed lines represent 0.95 confidence intervals for the curves with the corresponding color. Log-rank probability measure for the equality of these curves is 3.671236e-02. Additional annotations: *.GeneName*=SLC1A6, *.GeneDesc*=solute carrier family 1 (high affinity aspartate/glutamate transporter), member 6 [Source:HGNC Symbol;Acc:10944].

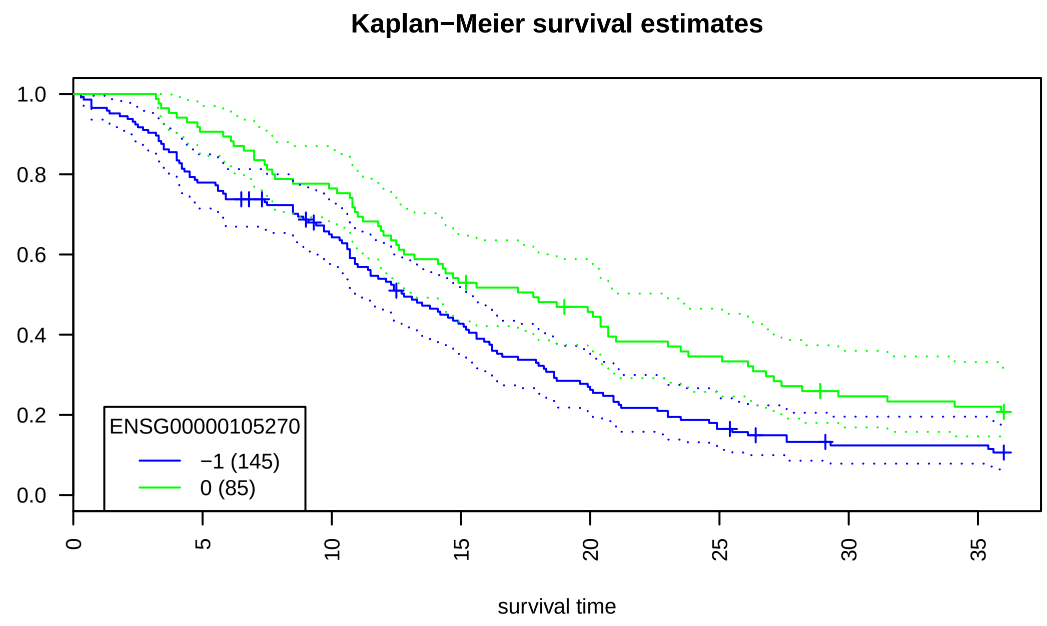

Figure 1513: Each curve represents the relative number of ENSG00000105270 survived to the corresponding point of time. Dashed lines represent 0.95 confidence intervals for the curves with the corresponding color. Log-rank probability measure for the equality of these curves is 5.381471e-03. Additional annotations: *.GeneName*=CLIP3, *.GeneDesc*=CAP-GLY domain containing linker protein 3 [Source:HGNC Symbol;Acc:24314].

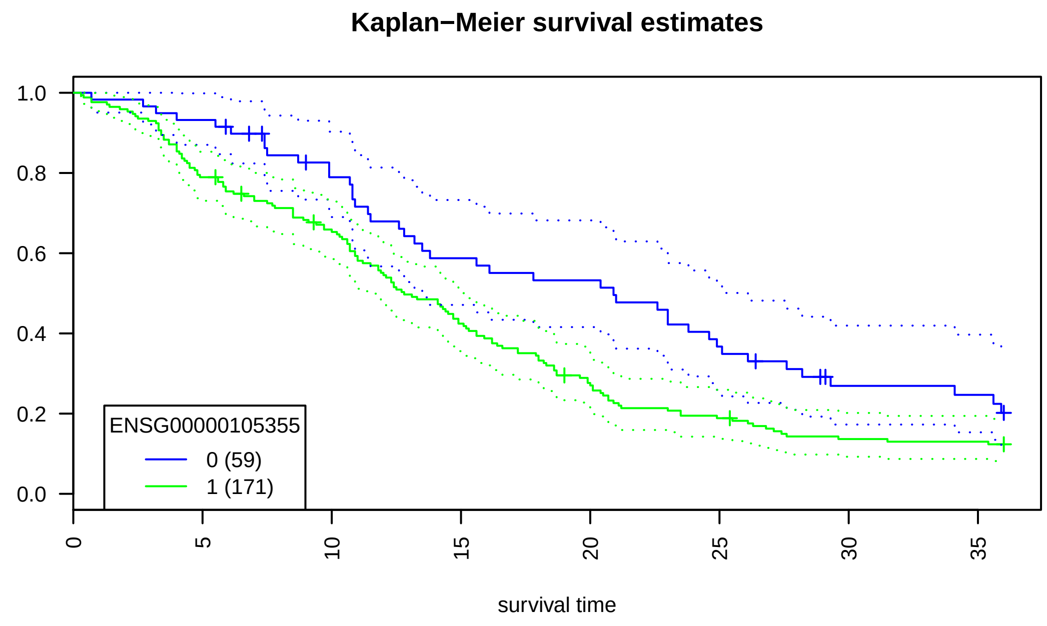

Figure 1514: Each curve represents the relative number of ENSG00000105355 survived to the corresponding point of time. Dashed lines represent 0.95 confidence intervals for the curves with the corresponding color. Log-rank probability measure for the equality of these curves is 7.909775e-03. Additional annotations: *.GeneName*=PLIN3, *.GeneDesc*=perilipin 3 [Source:HGNC Symbol;Acc:16893].

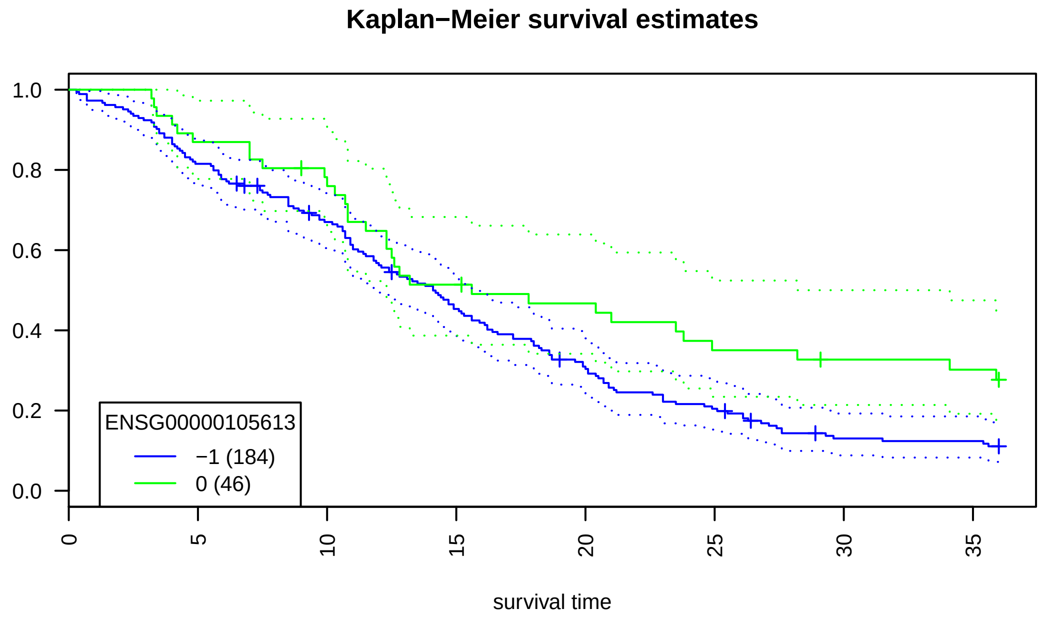

Figure 1515: Each curve represents the relative number of ENSG00000105613 survived to the corresponding point of time. Dashed lines represent 0.95 confidence intervals for the curves with the corresponding color. Log-rank probability measure for the equality of these curves is 1.916299e-02. Additional annotations: *.GeneName*=MAST1, *.GeneDesc*=microtubule associated serine/threonine kinase 1 [Source:HGNC Symbol;Acc:19034].

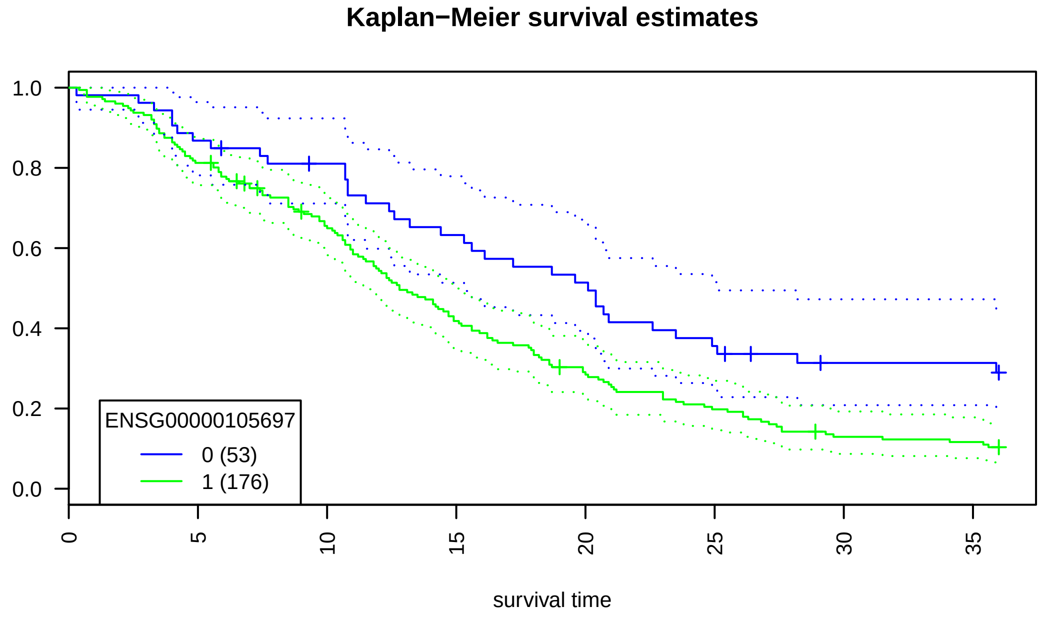

Figure 1516: Each curve represents the relative number of ENSG00000105697 survived to the corresponding point of time. Dashed lines represent 0.95 confidence intervals for the curves with the corresponding color. Total of 1 observation deleted due to missingness. Log-rank probability measure for the equality of these curves is 2.356614e-03. Additional annotations: *.GeneName*=HAMP, *.GeneDesc*=hepcidin antimicrobial peptide [Source:HGNC Symbol;Acc:15598].

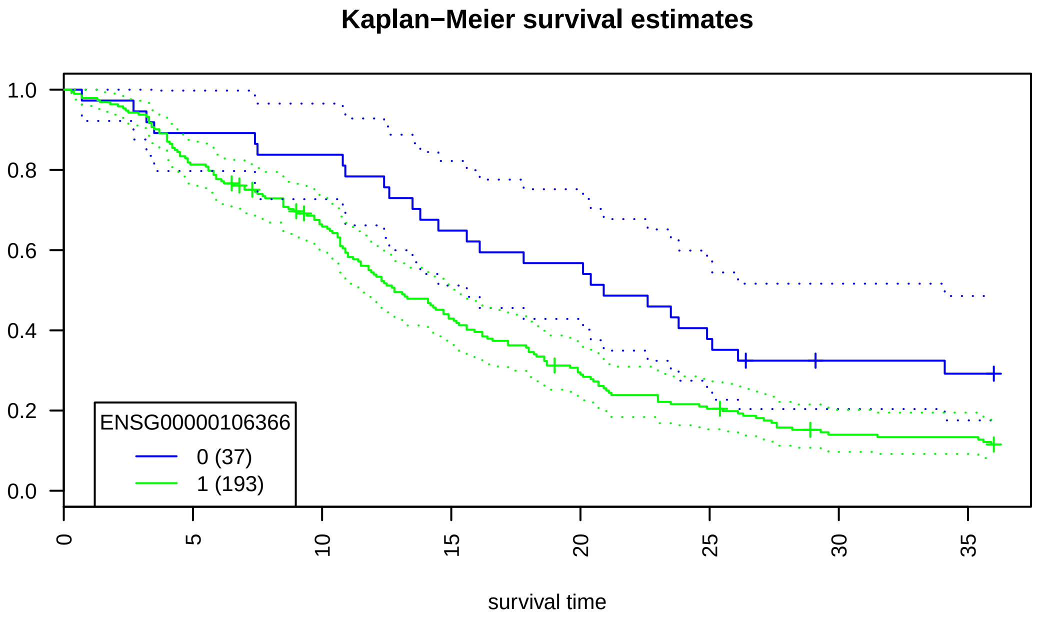

Figure 1517: Each curve represents the relative number of ENSG00000106366 survived to the corresponding point of time. Dashed lines represent 0.95 confidence intervals for the curves with the corresponding color. Log-rank probability measure for the equality of these curves is 5.341169e-03. Additional annotations: *.GeneName*=SERPINE1, *.GeneDesc*=serpin peptidase inhibitor, clade E (nexin, plasminogen activator inhibitor type 1), member 1 [Source:HGNC Symbol;Acc:8583].

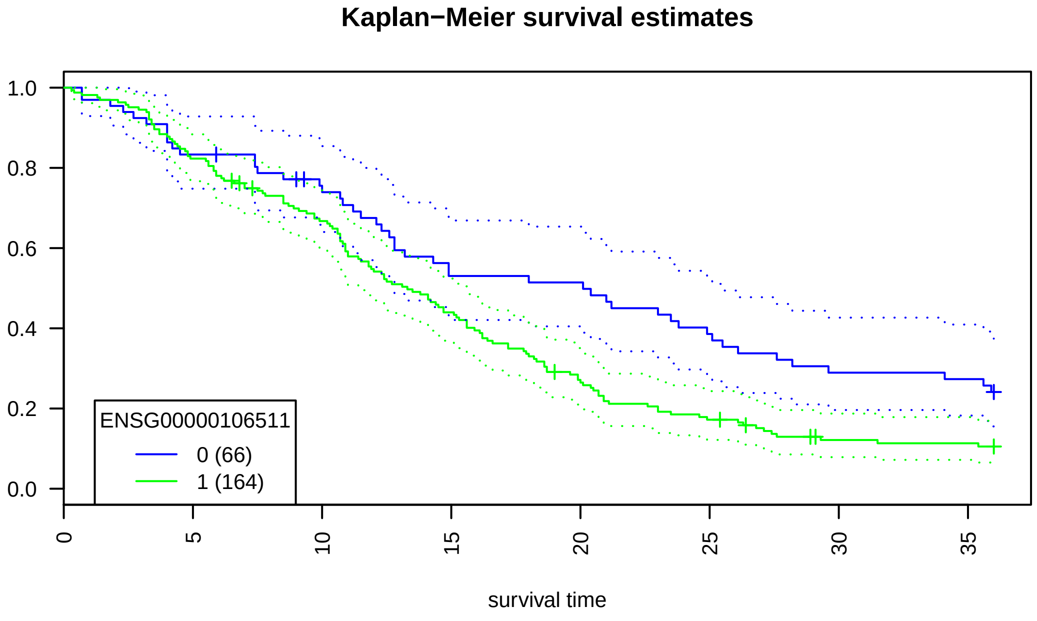

Figure 1518: Each curve represents the relative number of ENSG00000106511 survived to the corresponding point of time. Dashed lines represent 0.95 confidence intervals for the curves with the corresponding color. Log-rank probability measure for the equality of these curves is 4.015652e-03. Additional annotations: *.GeneName*=MEOX2, *.GeneDesc*=mesenchyme homeobox 2 [Source:HGNC Symbol;Acc:7014].

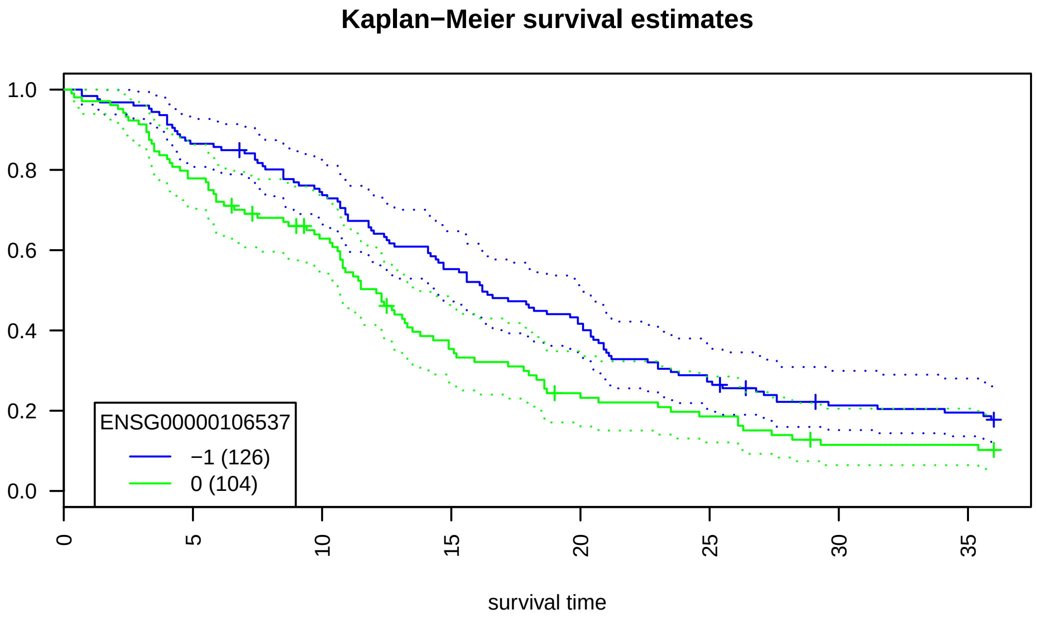

Figure 1519: Each curve represents the relative number of ENSG00000106537 survived to the corresponding point of time. Dashed lines represent 0.95 confidence intervals for the curves with the corresponding color. Log-rank probability measure for the equality of these curves is 7.016766e-03. Additional annotations: *.GeneName*=TSPAN13, *.GeneDesc*=tetraspanin 13 [Source:HGNC Symbol;Acc:21643].

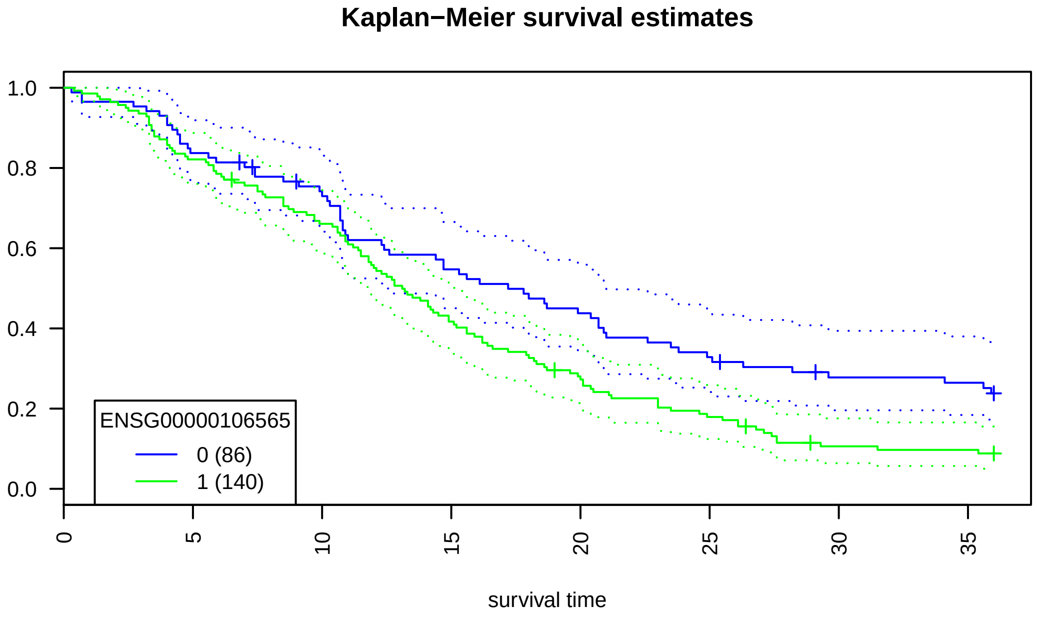

Figure 1520: Each curve represents the relative number of ENSG00000106565 survived to the corresponding point of time. Dashed lines represent 0.95 confidence intervals for the curves with the corresponding color. Total of 4 observations deleted due to missingness. Log-rank probability measure for the equality of these curves is 4.627145e-03. Additional annotations: *.GeneName*=TMEM176B, *.GeneDesc*=transmembrane protein 176B [Source:HGNC Symbol;Acc:29596].

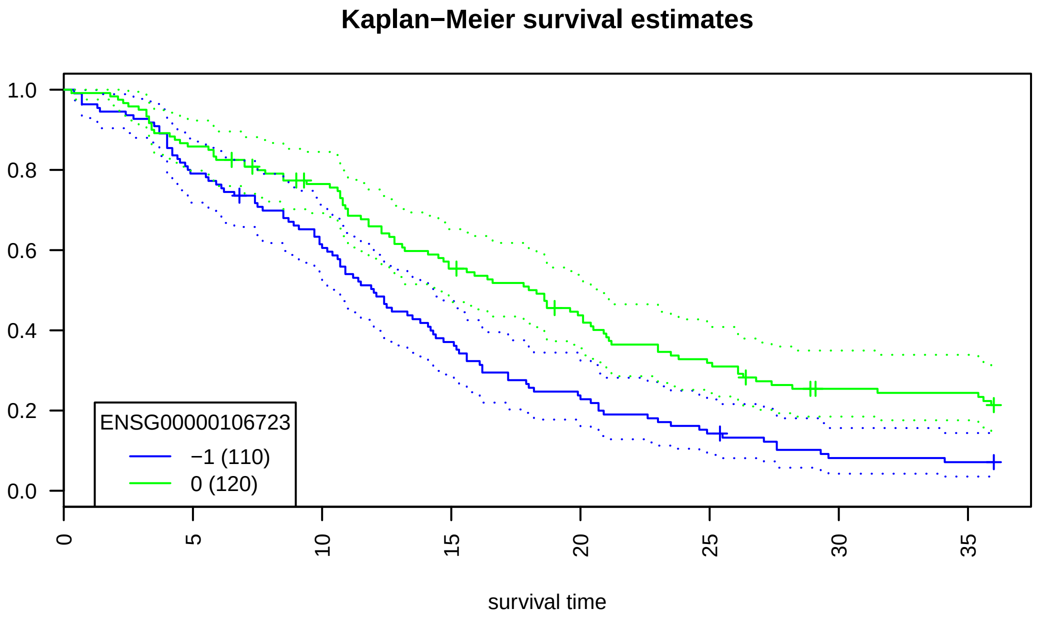

Figure 1521: Each curve represents the relative number of ENSG00000106723 survived to the corresponding point of time. Dashed lines represent 0.95 confidence intervals for the curves with the corresponding color. Log-rank probability measure for the equality of these curves is 2.444819e-04. Additional annotations: *.GeneName*=SPIN1, *.GeneDesc*=spindlin 1 [Source:HGNC Symbol;Acc:11243].

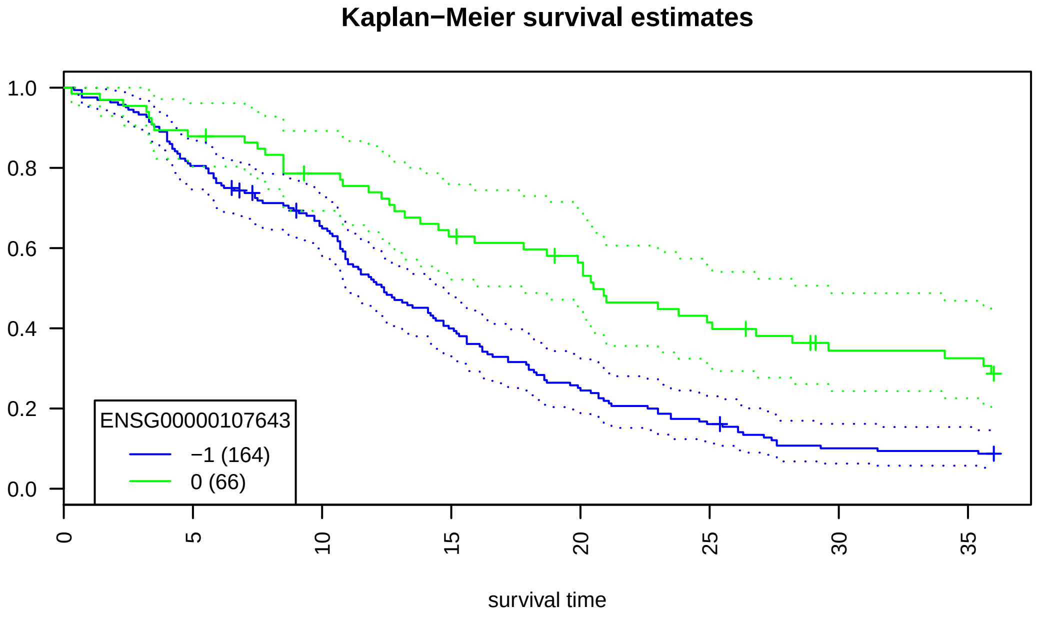

Figure 1522: Each curve represents the relative number of ENSG00000107643 survived to the corresponding point of time. Dashed lines represent 0.95 confidence intervals for the curves with the corresponding color. Log-rank probability measure for the equality of these curves is 2.803963e-05. Additional annotations: *.GeneName*=MAPK8, *.GeneDesc*=mitogen-activated protein kinase 8 [Source:HGNC Symbol;Acc:6881].

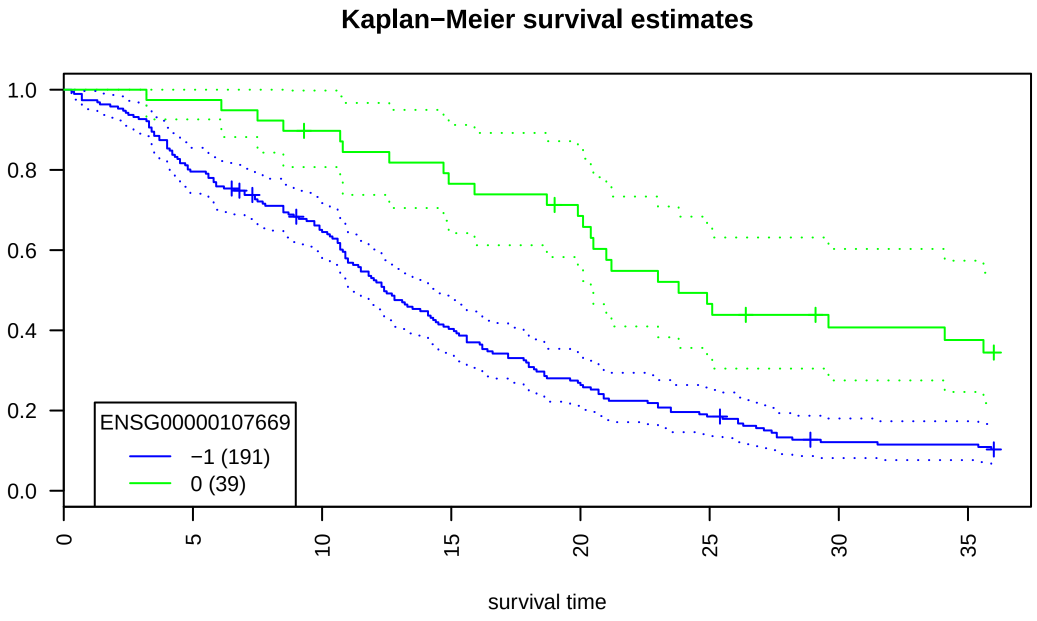

Figure 1523: Each curve represents the relative number of ENSG00000107669 survived to the corresponding point of time. Dashed lines represent 0.95 confidence intervals for the curves with the corresponding color. Log-rank probability measure for the equality of these curves is 1.911031e-05. Additional annotations: *.GeneName*=ATE1, *.GeneDesc*=arginyltransferase 1 [Source:HGNC Symbol;Acc:782].

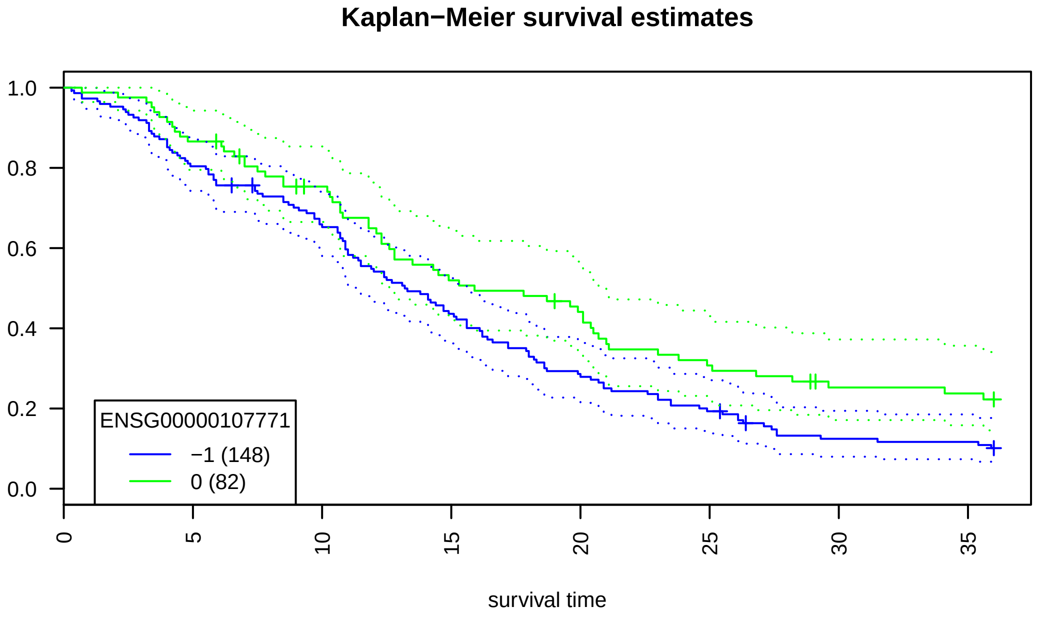

Figure 1524: Each curve represents the relative number of ENSG00000107771 survived to the corresponding point of time. Dashed lines represent 0.95 confidence intervals for the curves with the corresponding color. Log-rank probability measure for the equality of these curves is 1.66377e-02. Additional annotations: *.GeneName*=FAM190, *.GeneDesc*=family with sequence similarity 190, member B [Source:HGNC Symbol;Acc:29197].

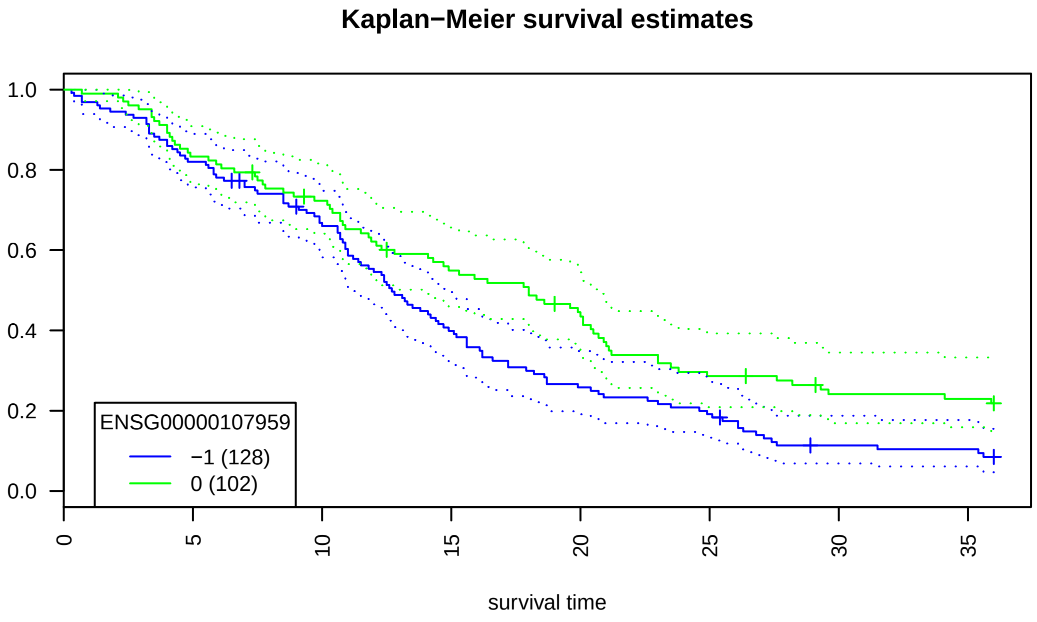

Figure 1525: Each curve represents the relative number of ENSG00000107959 survived to the corresponding point of time. Dashed lines represent 0.95 confidence intervals for the curves with the corresponding color. Log-rank probability measure for the equality of these curves is 5.982425e-03. Additional annotations: *.GeneName*=PITRM1, *.GeneDesc*=pitrilysin metallopeptidase 1 [Source:HGNC Symbol;Acc:17663].

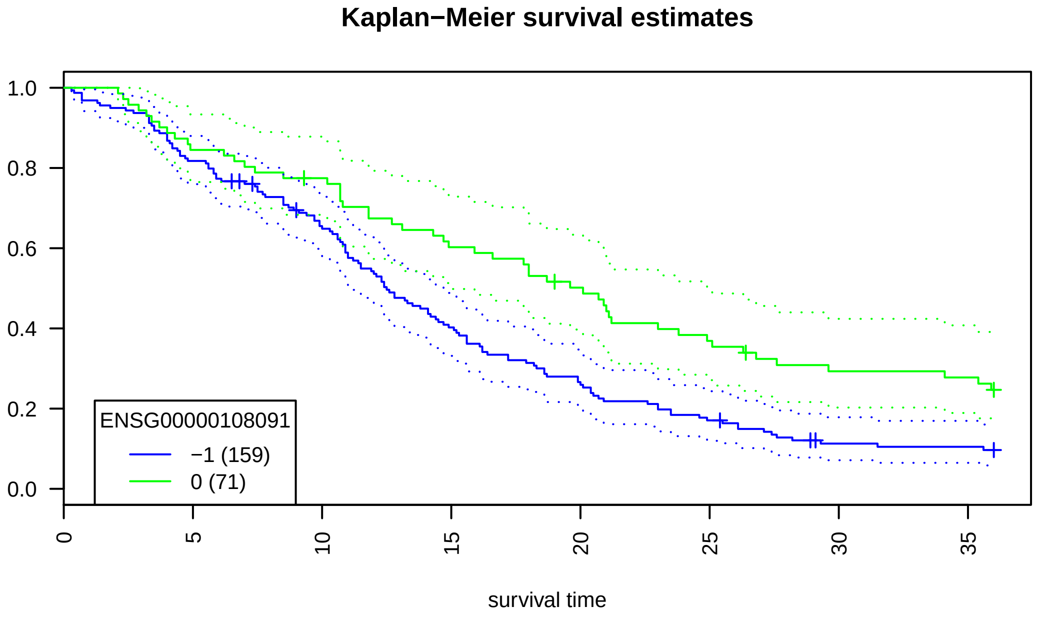

Figure 1526: Each curve represents the relative number of ENSG00000108091 survived to the corresponding point of time. Dashed lines represent 0.95 confidence intervals for the curves with the corresponding color. Log-rank probability measure for the equality of these curves is 7.665458e-04. Additional annotations: *.GeneName*=CCDC6, *.GeneDesc*=coiled-coil domain containing 6 [Source:HGNC Symbol;Acc:18782].

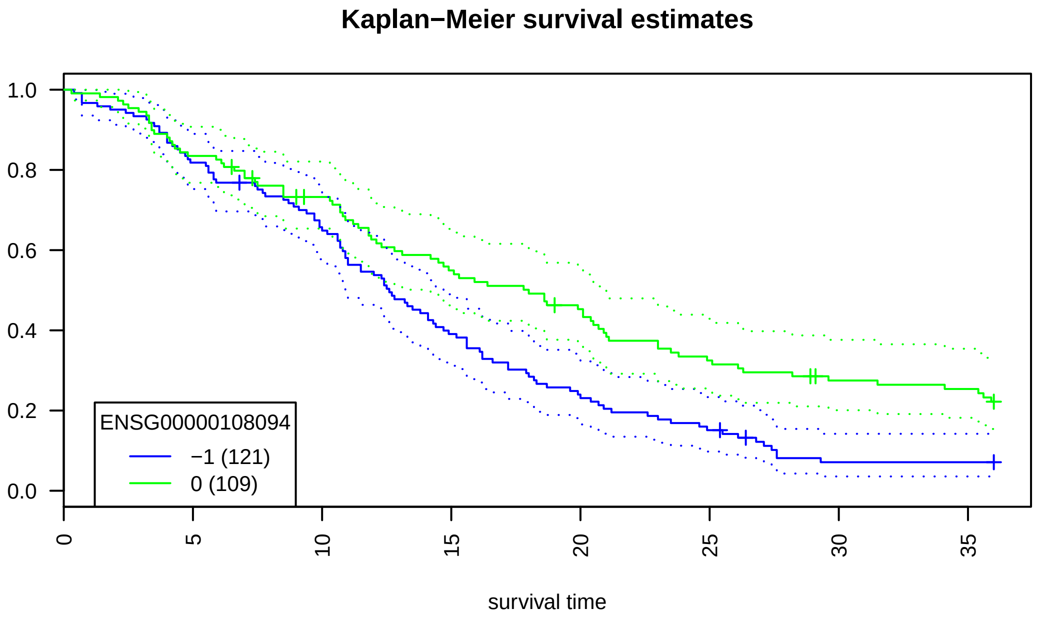

Figure 1527: Each curve represents the relative number of ENSG00000108094 survived to the corresponding point of time. Dashed lines represent 0.95 confidence intervals for the curves with the corresponding color. Log-rank probability measure for the equality of these curves is 7.745271e-04. Additional annotations: .*GeneName*=CUL2, .*GeneDesc*=cullin 2 [Source:HGNC Symbol;Acc:2552].

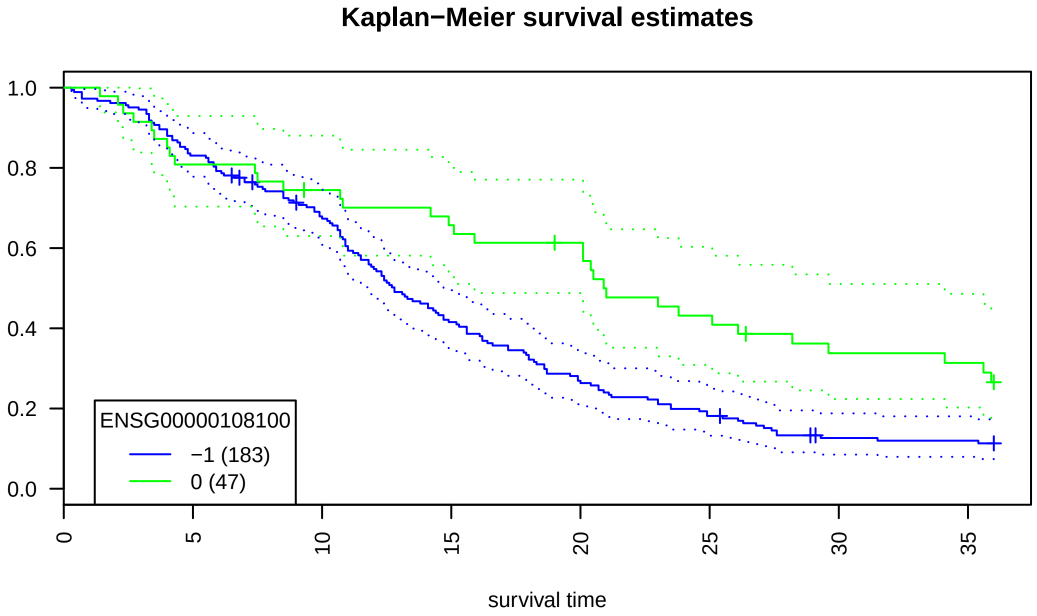

Figure 1528: Each curve represents the relative number of ENSG00000108100 survived to the corresponding point of time. Dashed lines represent 0.95 confidence intervals for the curves with the corresponding color. Log-rank probability measure for the equality of these curves is 2.370677e-03. Additional annotations: .*GeneName*=CCNY, .*GeneDesc*=cyclin Y [Source:HGNC Symbol;Acc:23354].

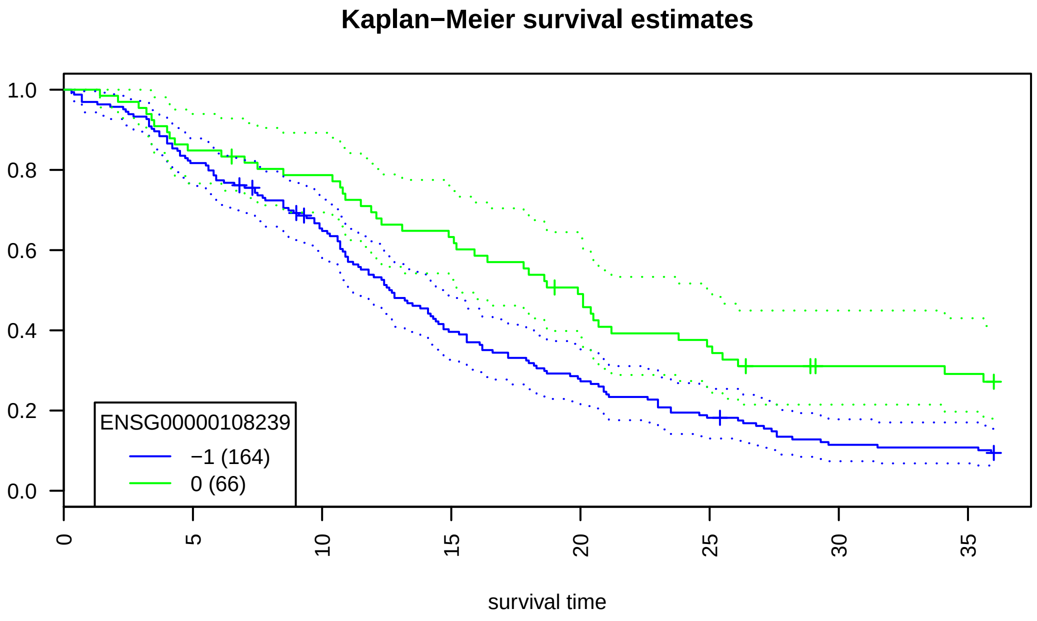

Figure 1529: Each curve represents the relative number of ENSG00000108239 survived to the corresponding point of time. Dashed lines represent 0.95 confidence intervals for the curves with the corresponding color. Log-rank probability measure for the equality of these curves is 1.041095e-03. Additional annotations: *.GeneName*=TBC1D12, *.GeneDesc*=TBC1 domain family, member 12 [Source:HGNC Symbol;Acc:29082].

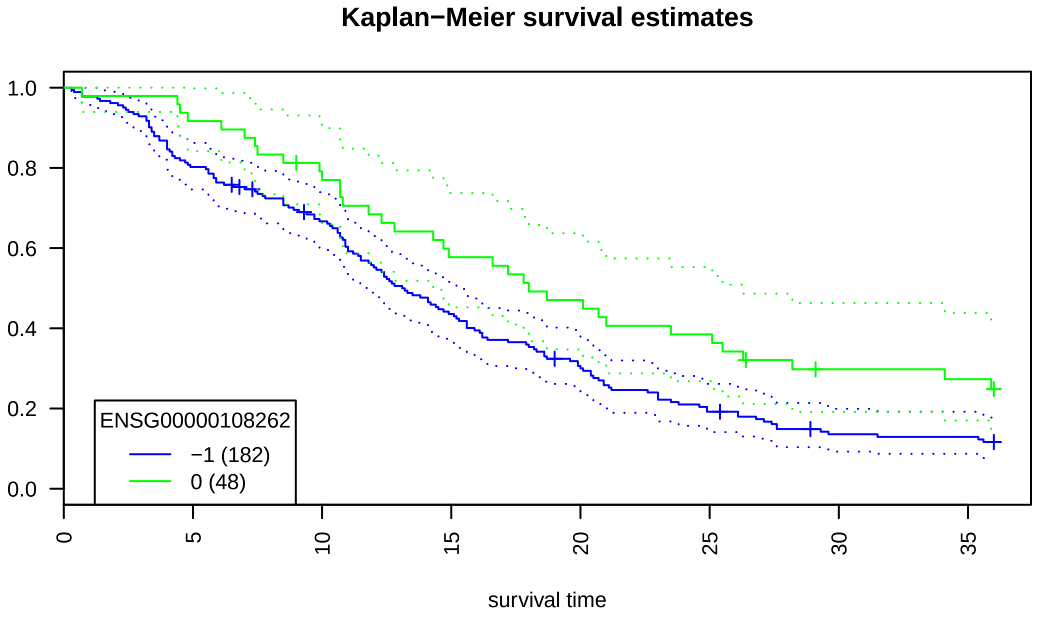

Figure 1530: Each curve represents the relative number of ENSG00000108262 survived to the corresponding point of time. Dashed lines represent 0.95 confidence intervals for the curves with the corresponding color. Log-rank probability measure for the equality of these curves is 1.226800e-02. Additional annotations: *.GeneName*=GIT1, *.GeneDesc*=G protein-coupled receptor kinase interacting ArfGAP 1 [Source:HGNC Symbol;Acc:4272].

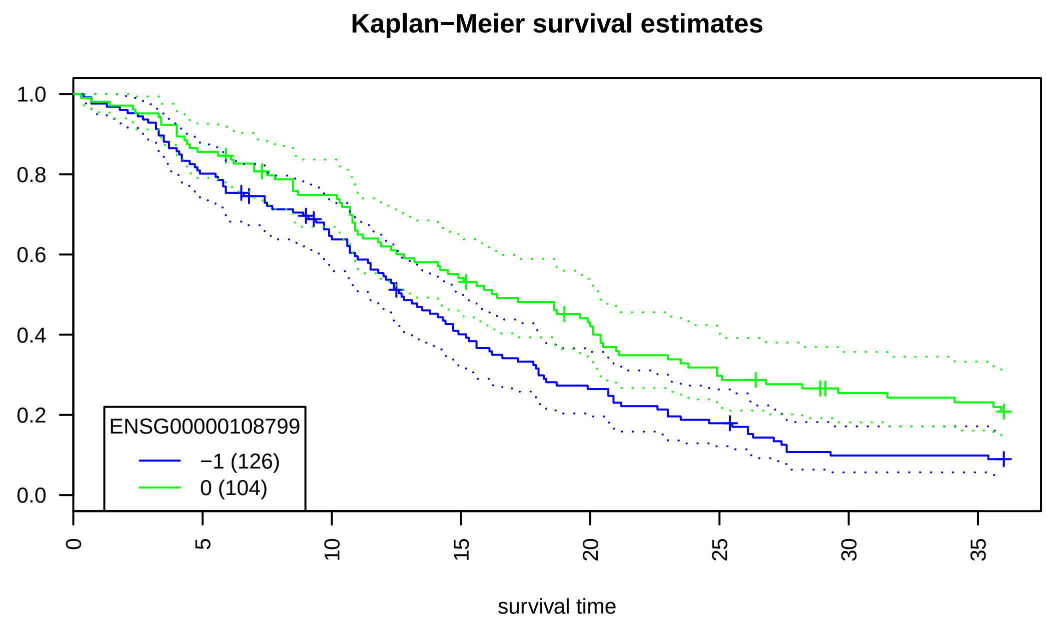

Figure 1531: Each curve represents the relative number of ENSG00000108799 survived to the corresponding point of time. Dashed lines represent 0.95 confidence intervals for the curves with the corresponding color. Log-rank probability measure for the equality of these curves is 6.091217e-03. Additional annotations: *.GeneName*=EZH1, *.GeneDesc*=enhancer of zeste homolog 1 (Drosophila) [Source:HGNC Symbol;Acc:3526].

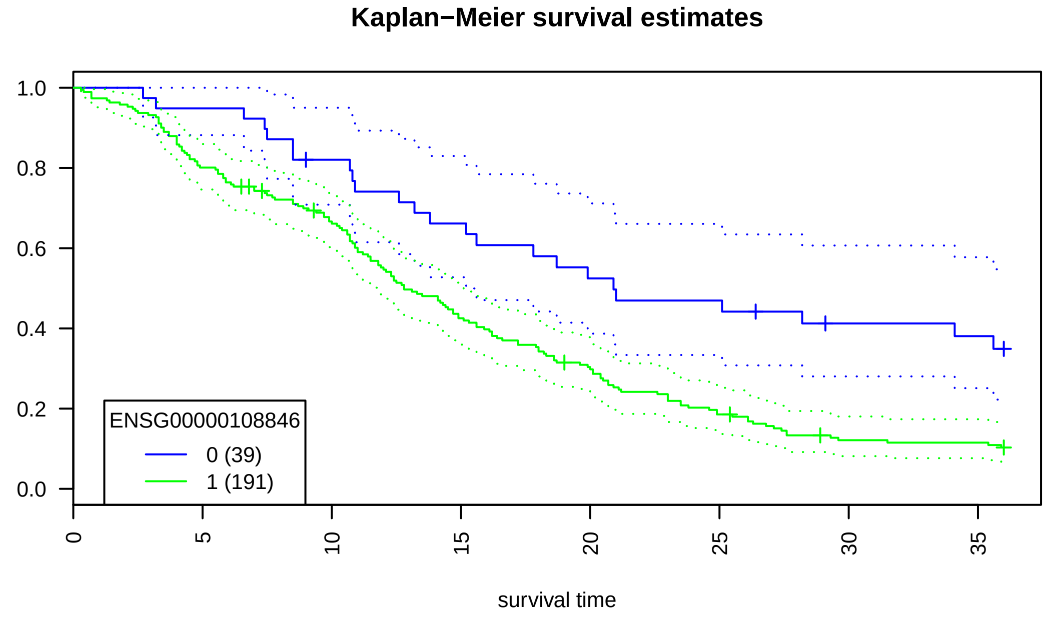

Figure 1532: Each curve represents the relative number of ENSG00000108846 survived to the corresponding point of time. Dashed lines represent 0.95 confidence intervals for the curves with the corresponding color. Log-rank probability measure for the equality of these curves is 4.575209e-04. Additional annotations: *.GeneName*=ABCC3, *.GeneDesc*=ATP-binding cassette, sub-family C (CFTR/MRP), member 3 [Source:HGNC Symbol;Acc:54].

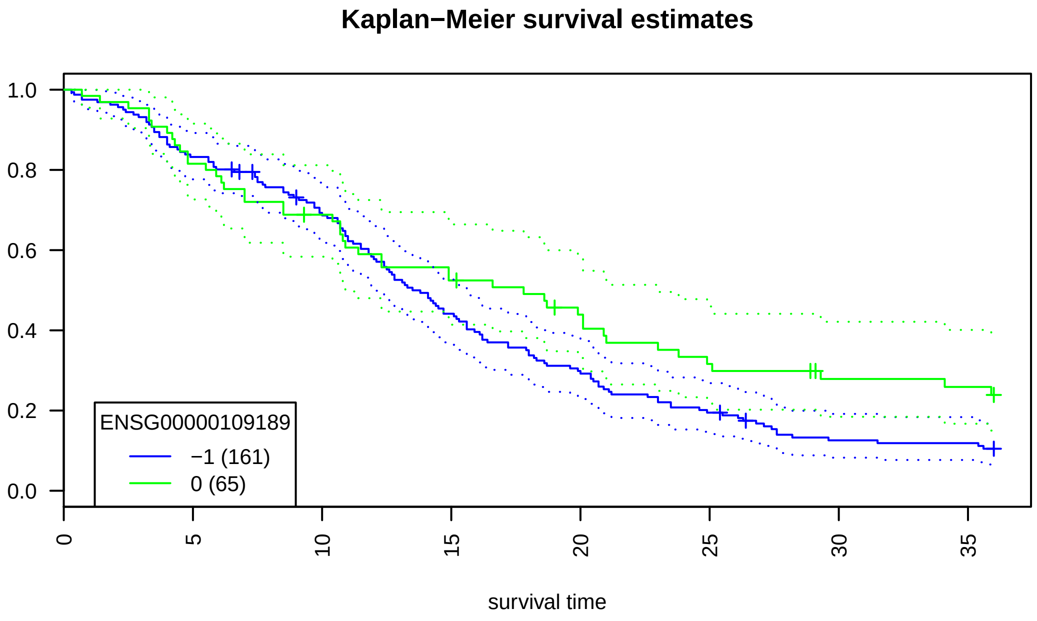

Figure 1533: Each curve represents the relative number of ENSG00000109189 survived to the corresponding point of time. Dashed lines represent 0.95 confidence intervals for the curves with the corresponding color. Total of 4 observations deleted due to missingness. Log-rank probability measure for the equality of these curves is 4.447509e-02. Additional annotations: $.GeneName$=USP46, $.GeneDesc$=ubiquitin specific peptidase 46 [Source:HGNC Symbol;Acc:20075].

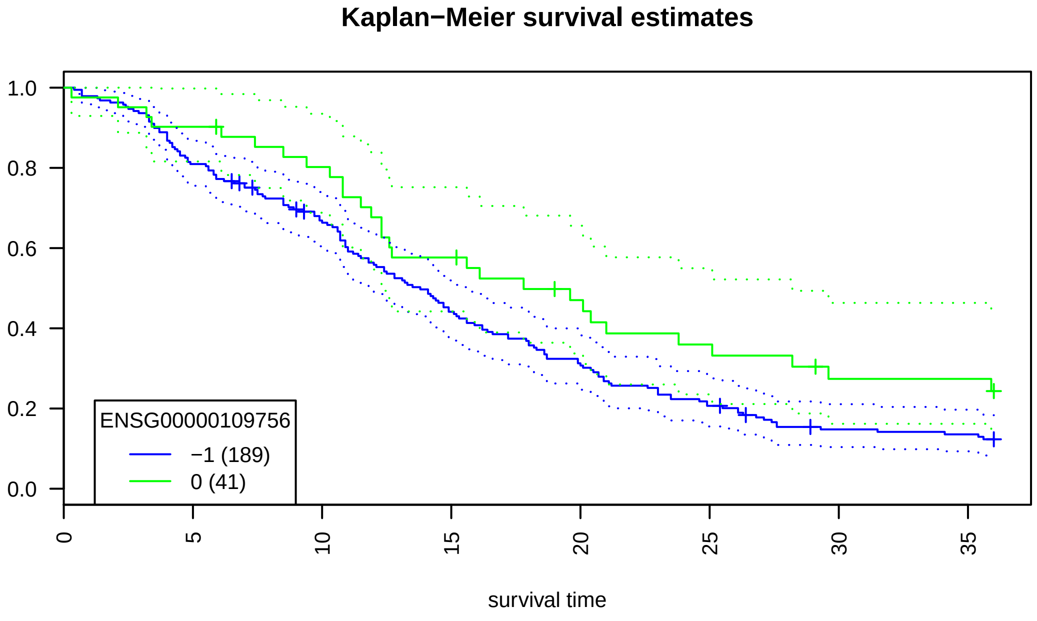

Figure 1534: Each curve represents the relative number of ENSG00000109756 survived to the corresponding point of time. Dashed lines represent 0.95 confidence intervals for the curves with the corresponding color. Log-rank probability measure for the equality of these curves is 4.210835e-02. Additional annotations: $.GeneName$=RAPGEF2, $.GeneDesc$=Rap guanine nucleotide exchange factor (GEF) 2 [Source:HGNC Symbol;Acc:16854].

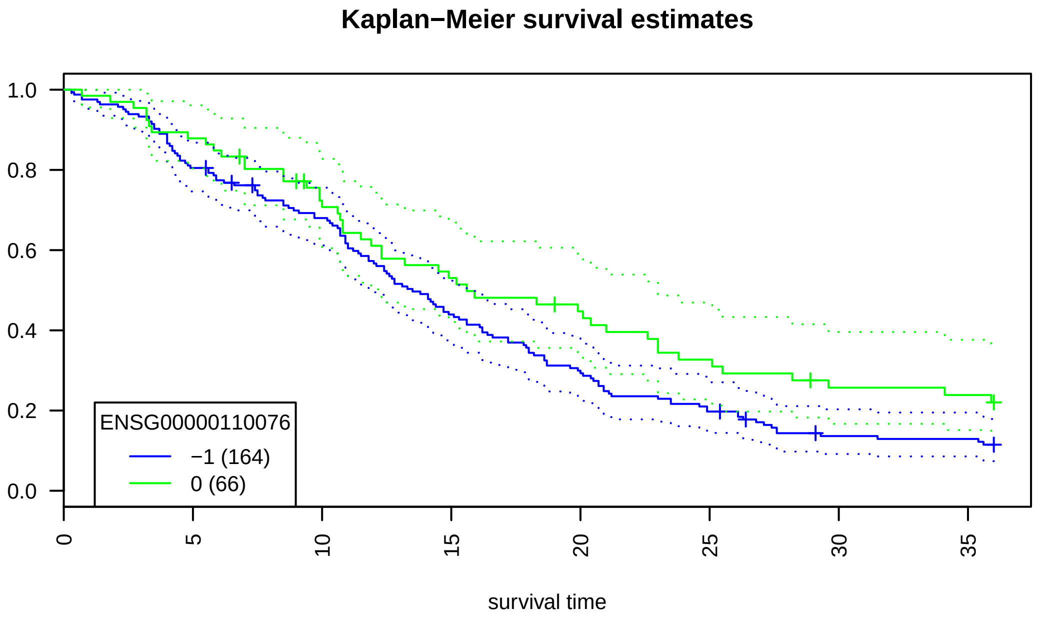

Figure 1535: Each curve represents the relative number of ENSG00000110076 survived to the corresponding point of time. Dashed lines represent 0.95 confidence intervals for the curves with the corresponding color. Log-rank probability measure for the equality of these curves is 4.681261e-02. Additional annotations: *.GeneName*=NRXN2, *.GeneDesc*=neurexin 2 [Source:HGNC Symbol;Acc:8009].

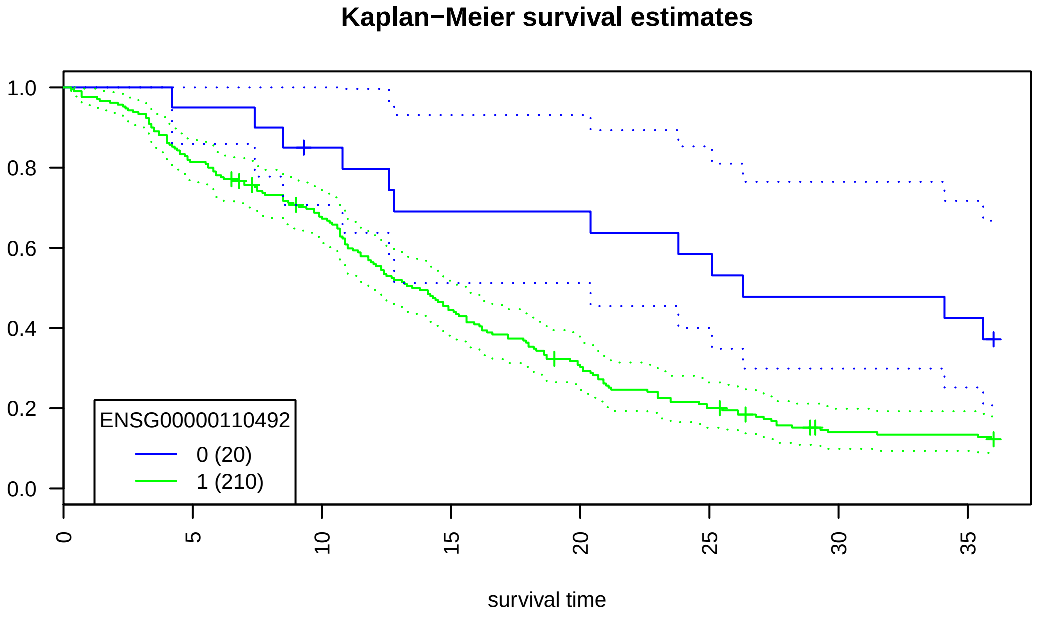

Figure 1536: Each curve represents the relative number of ENSG00000110492 survived to the corresponding point of time. Dashed lines represent 0.95 confidence intervals for the curves with the corresponding color. Log-rank probability measure for the equality of these curves is 3.86949e-03. Additional annotations: *.GeneName*=MDK, *.GeneDesc*=midkine (neurite growth-promoting factor 2) [Source:HGNC Symbol;Acc:6972].

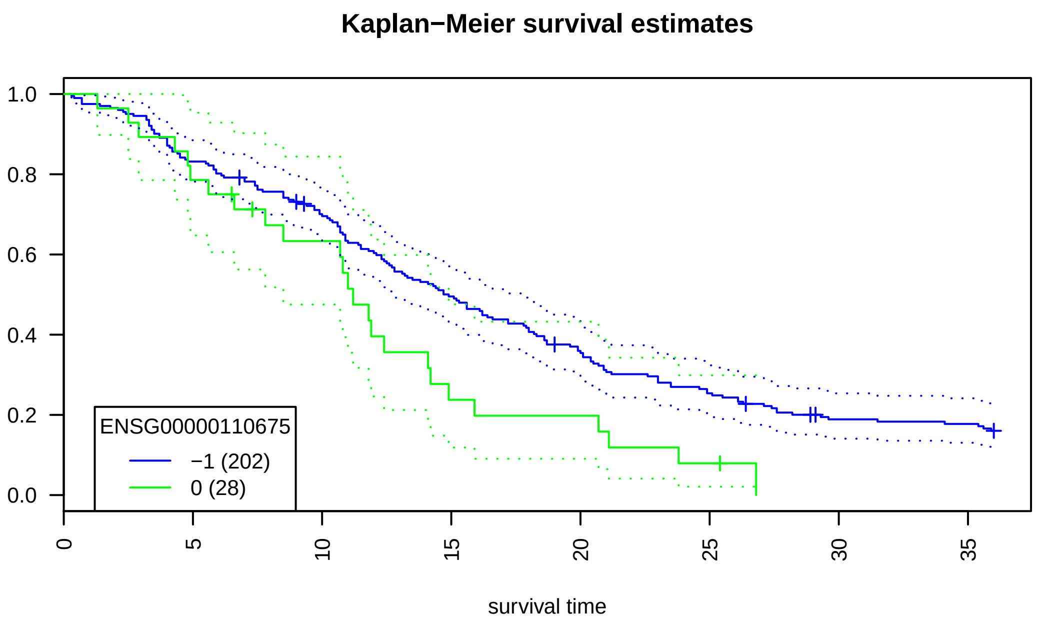

Figure 1537: Each curve represents the relative number of ENSG00000110675 survived to the corresponding point of time. Dashed lines represent 0.95 confidence intervals for the curves with the corresponding color. Log-rank probability measure for the equality of these curves is 1.116988e-02. Additional annotations: *.GeneName*=ELMOD1, *.GeneDesc*=ELMO/CED-12 domain containing 1 [Source:HGNC Symbol;Acc:25334].

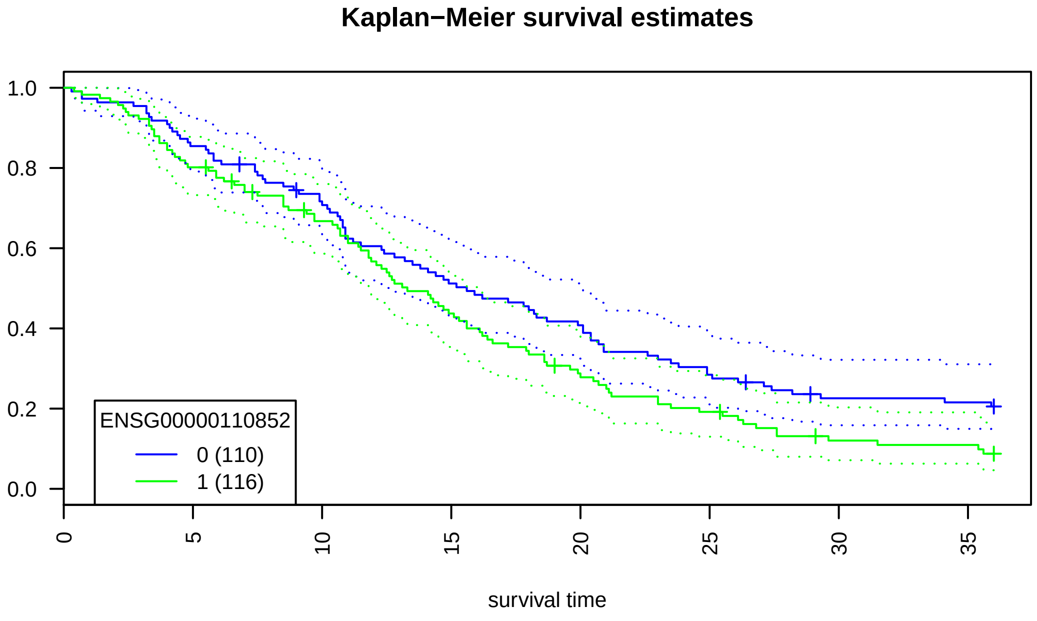

Figure 1538: Each curve represents the relative number of ENSG00000110852 survived to the corresponding point of time. Dashed lines represent 0.95 confidence intervals for the curves with the corresponding color. Total of 4 observations deleted due to missingness. Log-rank probability measure for the equality of these curves is 3.499775e-02. Additional annotations: *.GeneName*=CLEC2B, *.GeneDesc*=C-type lectin domain family 2, member B [Source:HGNC Symbol;Acc:2053].

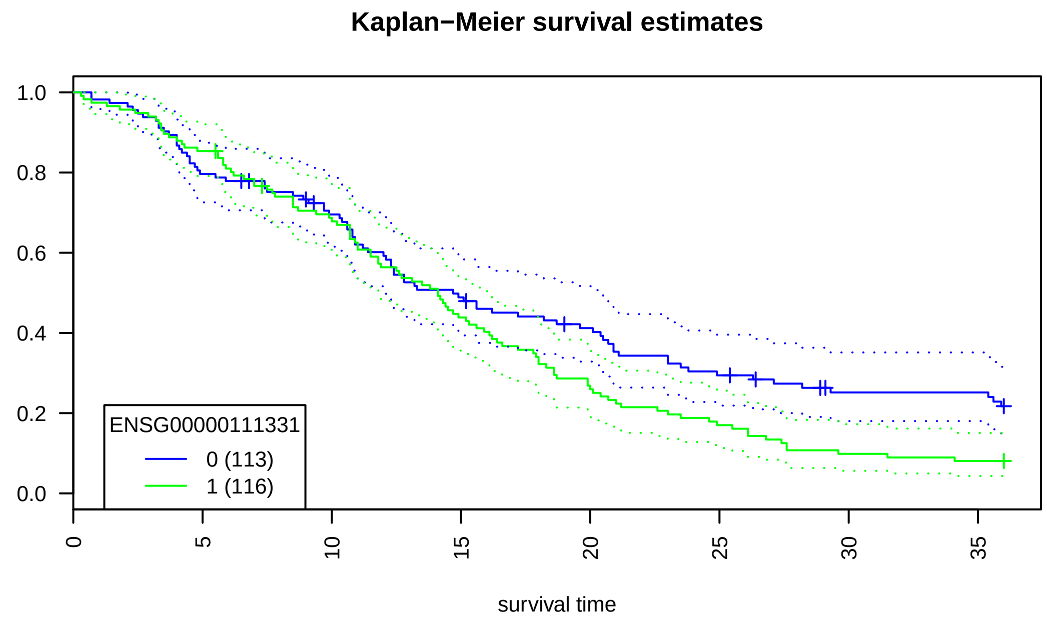

Figure 1539: Each curve represents the relative number of ENSG00000111331 survived to the corresponding point of time. Dashed lines represent 0.95 confidence intervals for the curves with the corresponding color. Total of 1 observation deleted due to missingness. Log-rank probability measure for the equality of these curves is 2.454137e-02. Additional annotations: *.GeneName*=OAS3, *.GeneDesc*=2'-5'-oligoadenylate synthetase 3, 100kDa [Source:HGNC Symbol;Acc:8088].

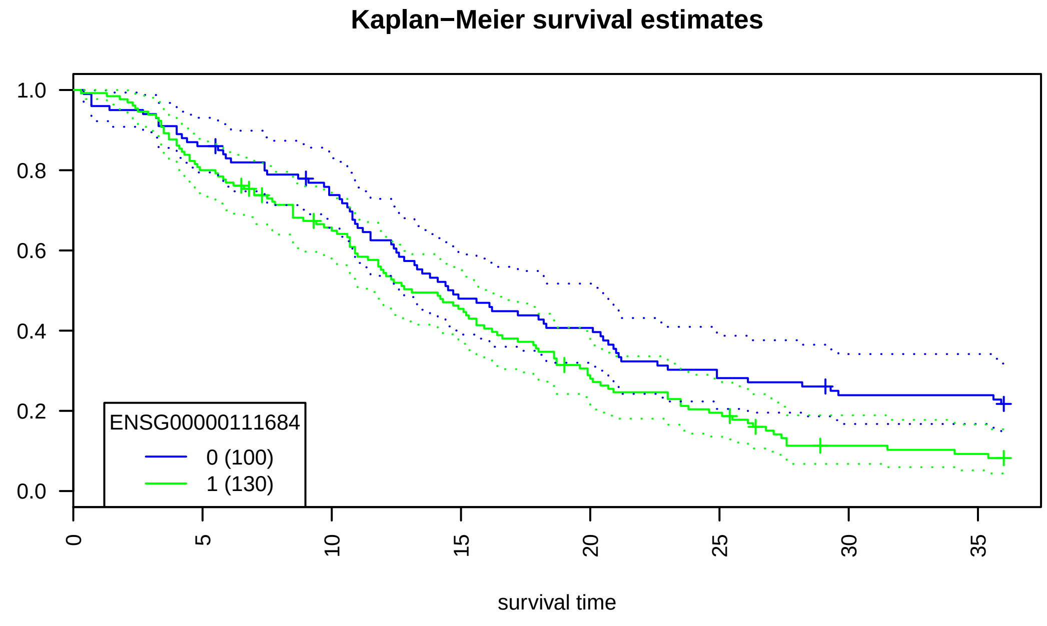

Figure 1540: Each curve represents the relative number of ENSG00000111684 survived to the corresponding point of time. Dashed lines represent 0.95 confidence intervals for the curves with the corresponding color. Log-rank probability measure for the equality of these curves is 2.058382e-02. Additional annotations: *.GeneName*=U47924.1, *.GeneDesc*=Lysophospholipid acyltransferase 5 (LPLAT 5)(EC 2.3.1.-)(1-acylglycerophosphocholine O-acyltransferase)(EC 2.3.1.23)(Lysophosphatidylcholine acyltransferase)(Lyso-PC acyltransferase)(LPCAT)(1-acylglycerophosphoserine O-acyltransferase)(EC 2.3.1.n6)(/.../osphatidylserine acyltransferase)(Lyso-PS acyltransferase)(LPSAT)(Membrane-bound O-acyltransferase domain-containing protein 5)(O-acyltransferase domain-containing protein 5)(Lysophosphatidylcholine acyltransferase 3)(Lyso-PC acyltransferase 3) [Source:UniProtKB/Swiss-Prot;Acc:Q6P1A2].

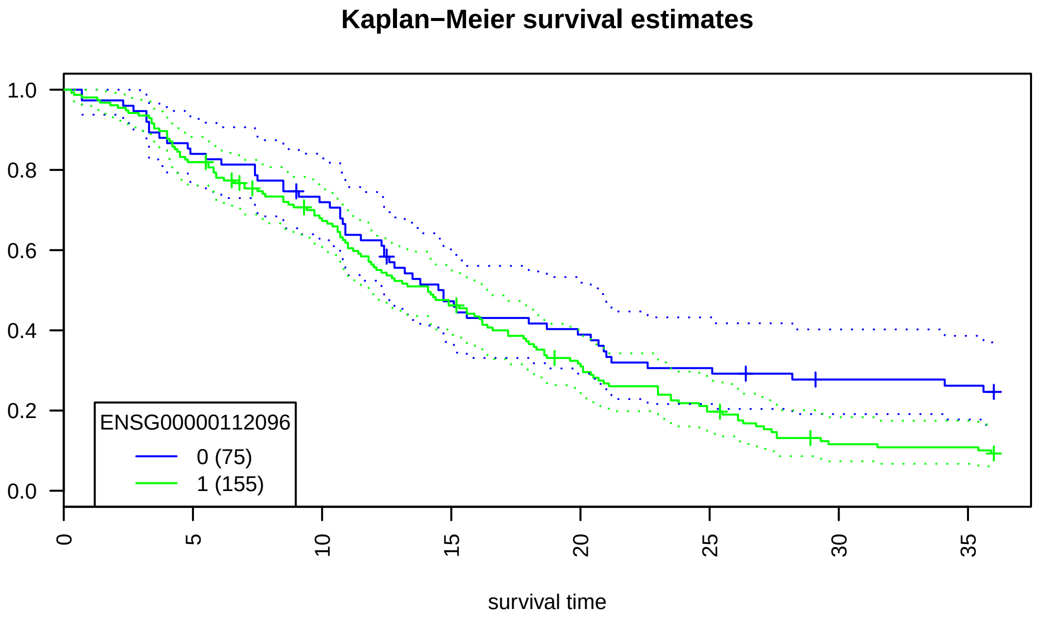

Figure 1541: Each curve represents the relative number of ENSG00000112096 survived to the corresponding point of time. Dashed lines represent 0.95 confidence intervals for the curves with the corresponding color. Log-rank probability measure for the equality of these curves is 4.214133e-02. Additional annotations: *.GeneName*=SOD2, *.GeneDesc*=superoxide dismutase 2, mitochondrial [Source:HGNC Symbol;Acc:11180].

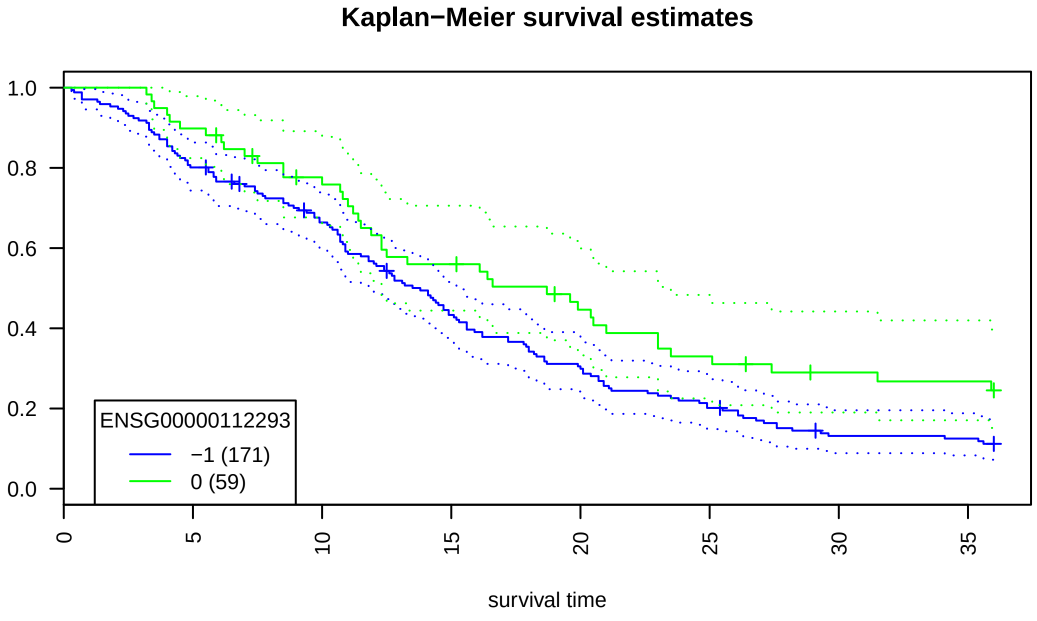

Figure 1542: Each curve represents the relative number of ENSG00000112293 survived to the corresponding point of time. Dashed lines represent 0.95 confidence intervals for the curves with the corresponding color. Log-rank probability measure for the equality of these curves is 1.850798e-02. Additional annotations: *.GeneName*=GPLD1, *.GeneDesc*=glycosylphosphatidylinositol specific phospholipase D1 [Source:HGNC Symbol;Acc:4459].

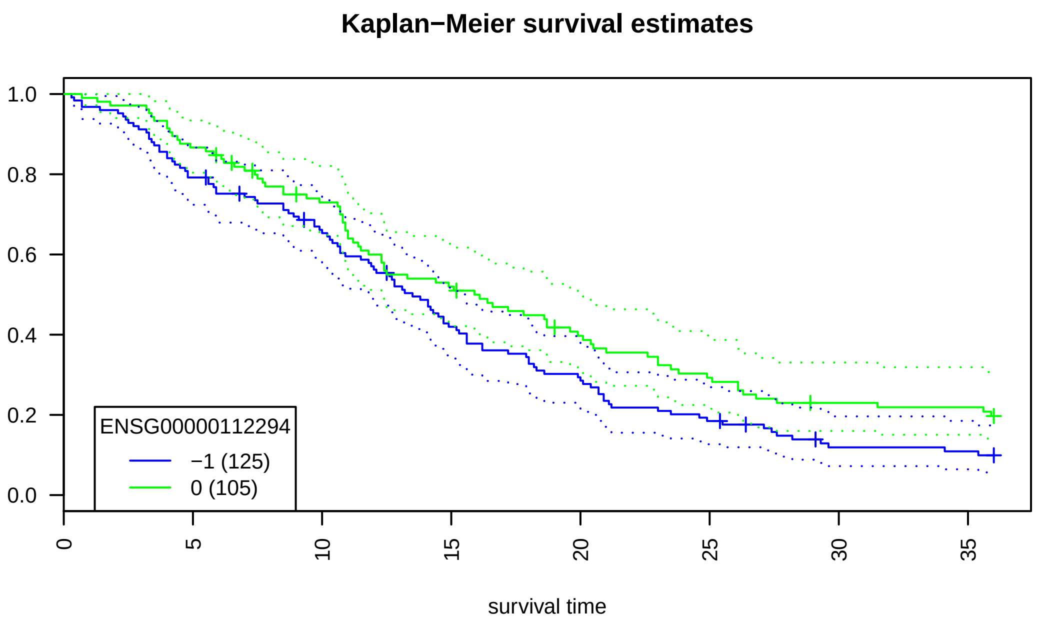

Figure 1543: Each curve represents the relative number of ENSG00000112294 survived to the corresponding point of time. Dashed lines represent 0.95 confidence intervals for the curves with the corresponding color. Log-rank probability measure for the equality of these curves is 3.59083e-02. Additional annotations: *.GeneName*=ALDH5A1, *.GeneDesc*=aldehyde dehydrogenase 5 family, member A1 [Source:HGNC Symbol;Acc:408].

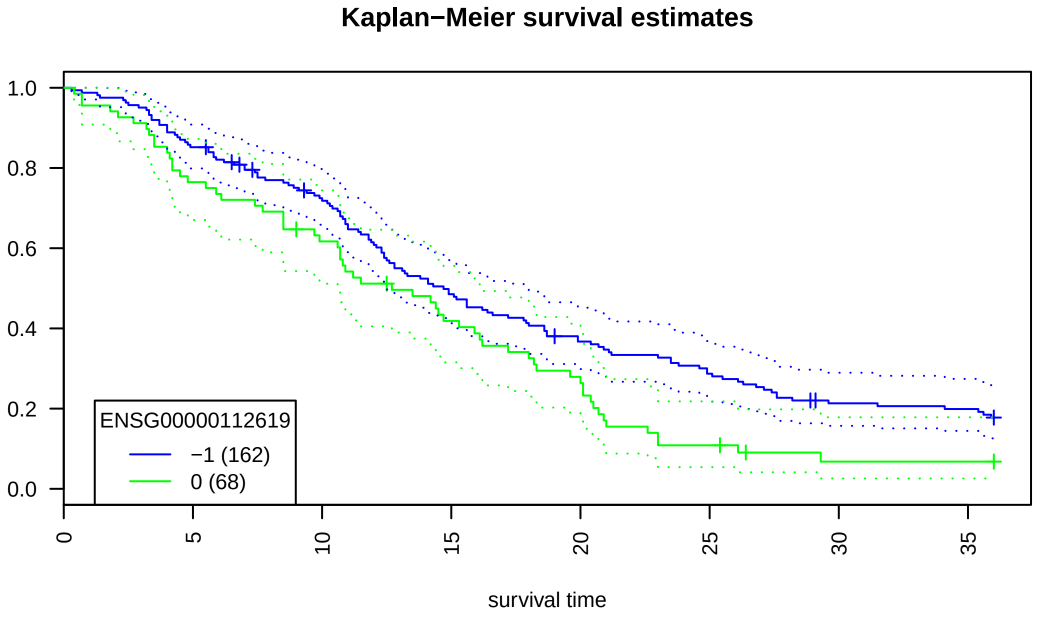

Figure 1544: Each curve represents the relative number of ENSG00000112619 survived to the corresponding point of time. Dashed lines represent 0.95 confidence intervals for the curves with the corresponding color. Log-rank probability measure for the equality of these curves is 9.508865e-03. Additional annotations: *.GeneName*=PRPH2, *.GeneDesc*=peripherin 2 (retinal degeneration, slow) [Source:HGNC Symbol;Acc:9942].

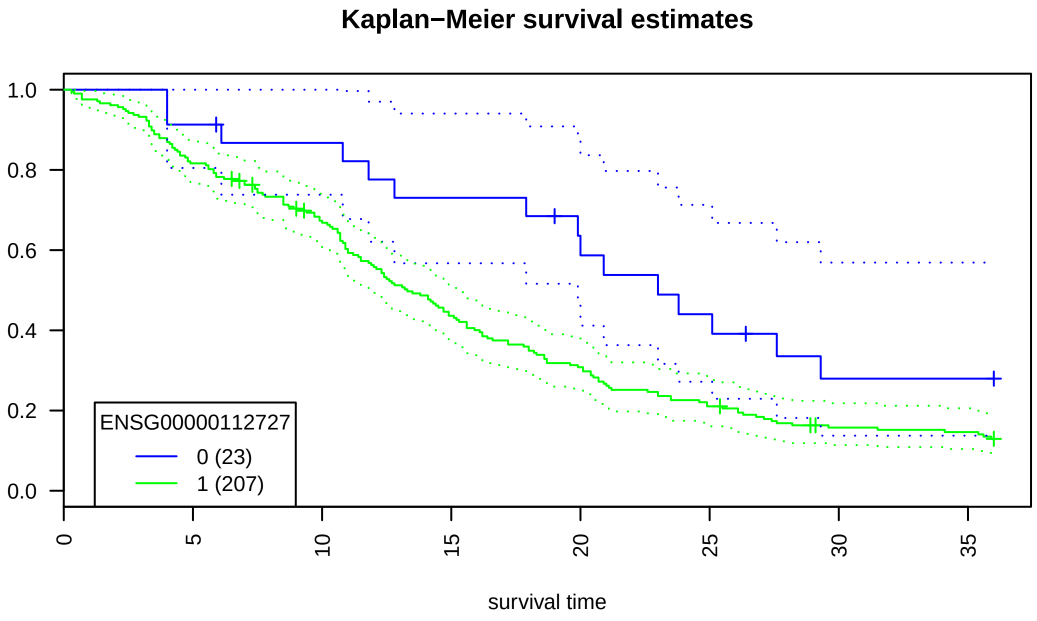

Figure 1545: Each curve represents the relative number of ENSG00000112727 survived to the corresponding point of time. Dashed lines represent 0.95 confidence intervals for the curves with the corresponding color. Log-rank probability measure for the equality of these curves is 1.648849e-02. Additional annotations: *.GeneName*=HIST1H3F, *.GeneDesc*=histone cluster 1, H3f [Source:HGNC Symbol;Acc:4773].

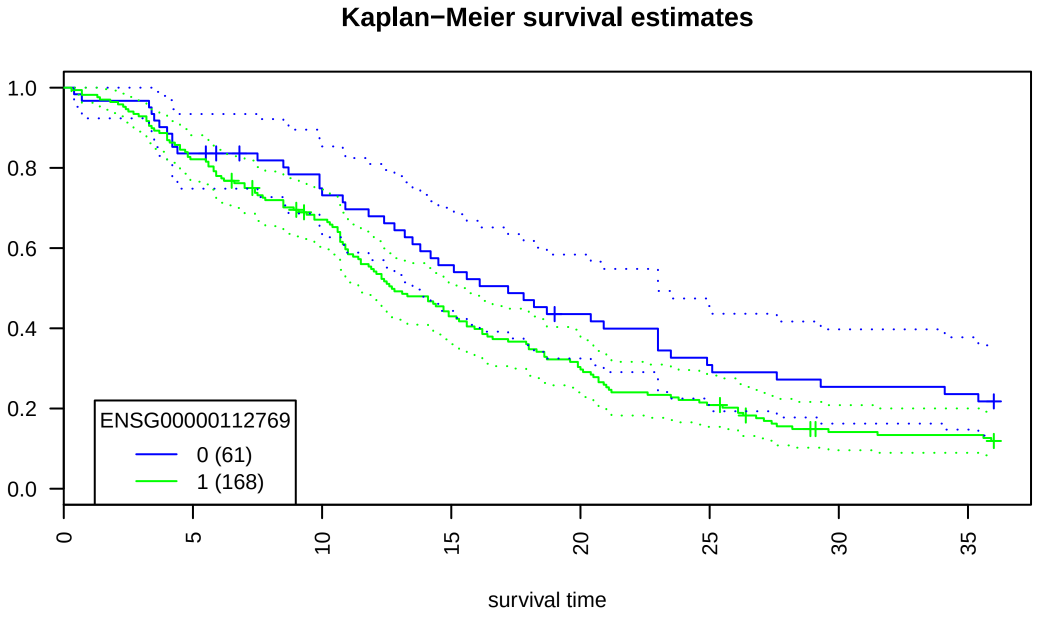

Figure 1546: Each curve represents the relative number of ENSG00000112769 survived to the corresponding point of time. Dashed lines represent 0.95 confidence intervals for the curves with the corresponding color. Total of 1 observation deleted due to missingness. Log-rank probability measure for the equality of these curves is 4.38616e-02. Additional annotations: *.GeneName*=LAMA4, *.GeneDesc*=laminin, alpha 4 [Source:HGNC Symbol;Acc:6484].

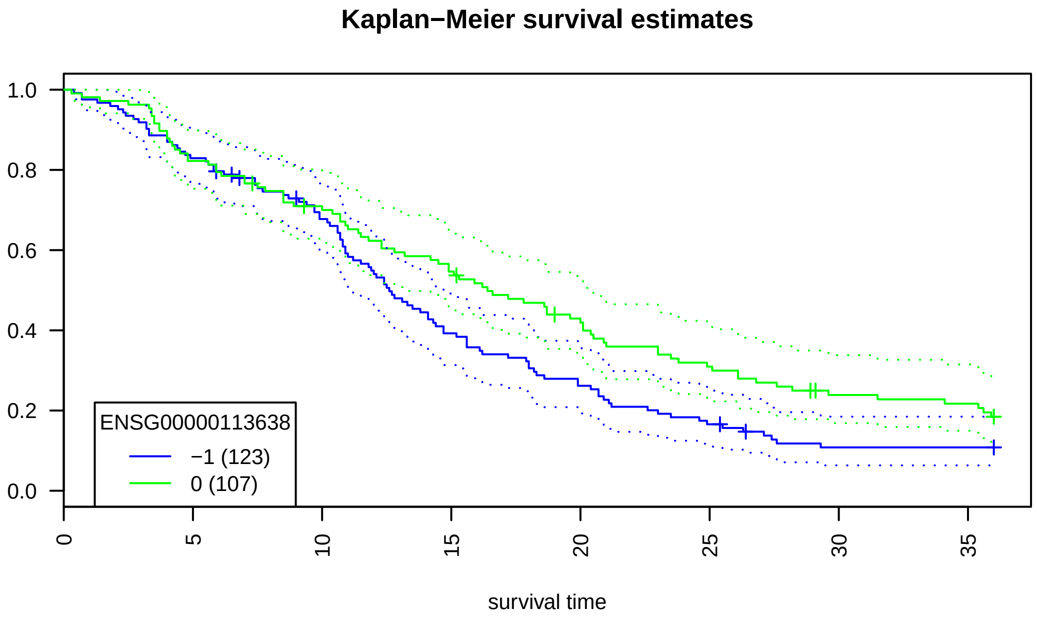

Figure 1547: Each curve represents the relative number of ENSG00000113638 survived to the corresponding point of time. Dashed lines represent 0.95 confidence intervals for the curves with the corresponding color. Log-rank probability measure for the equality of these curves is 2.338636e-02. Additional annotations: *.GeneName*=TTC33, *.GeneDesc*=tetratricopeptide repeat domain 33 [Source:HGNC Symbol;Acc:29959].

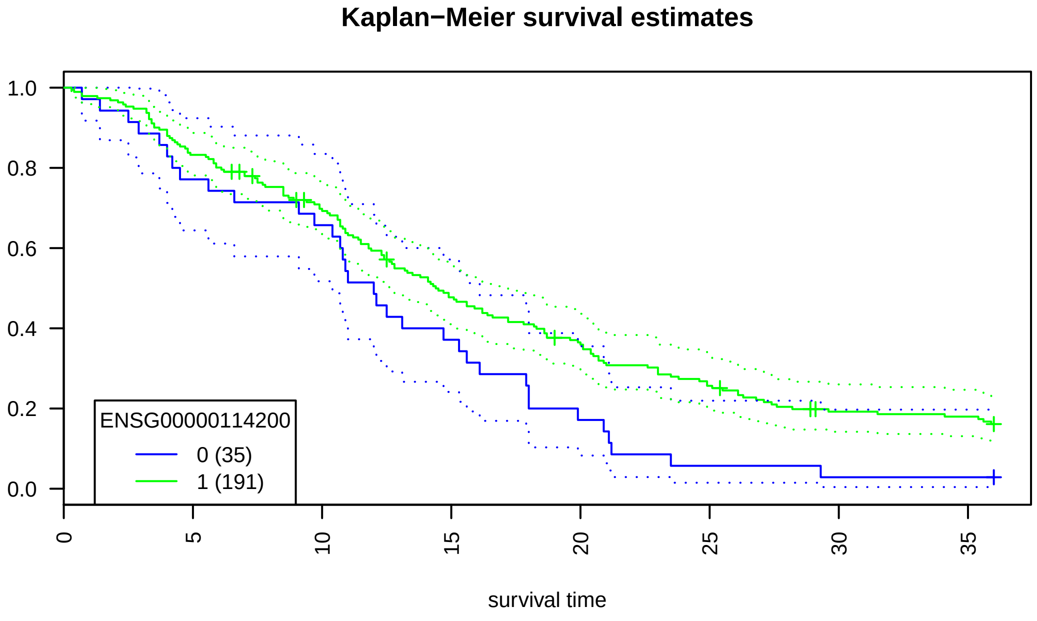

Figure 1548: Each curve represents the relative number of ENSG00000114200 survived to the corresponding point of time. Dashed lines represent 0.95 confidence intervals for the curves with the corresponding color. Total of 4 observations deleted due to missingness. Log-rank probability measure for the equality of these curves is 9.866055e-03. Additional annotations: *.GeneName*=BCHE, *.GeneDesc*=butyrylcholinesterase [Source:HGNC Symbol;Acc:983].

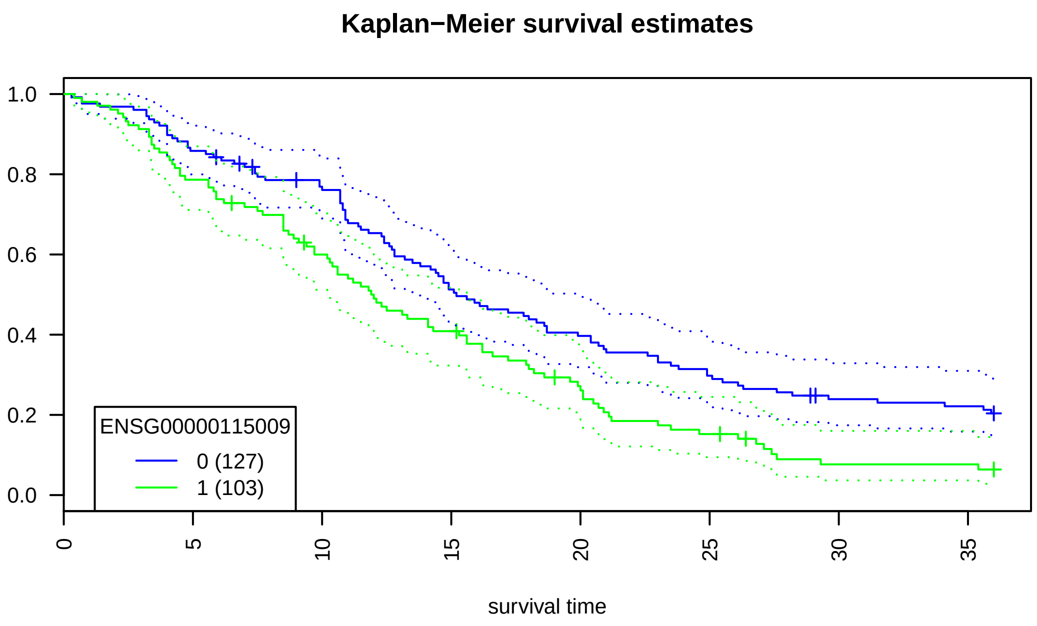

Figure 1549: Each curve represents the relative number of ENSG00000115009 survived to the corresponding point of time. Dashed lines represent 0.95 confidence intervals for the curves with the corresponding color. Log-rank probability measure for the equality of these curves is 1.344441e-03. Additional annotations: *.GeneName*=CCL20, *.GeneDesc*=chemokine (C-C motif) ligand 20 [Source:HGNC Symbol;Acc:10619].

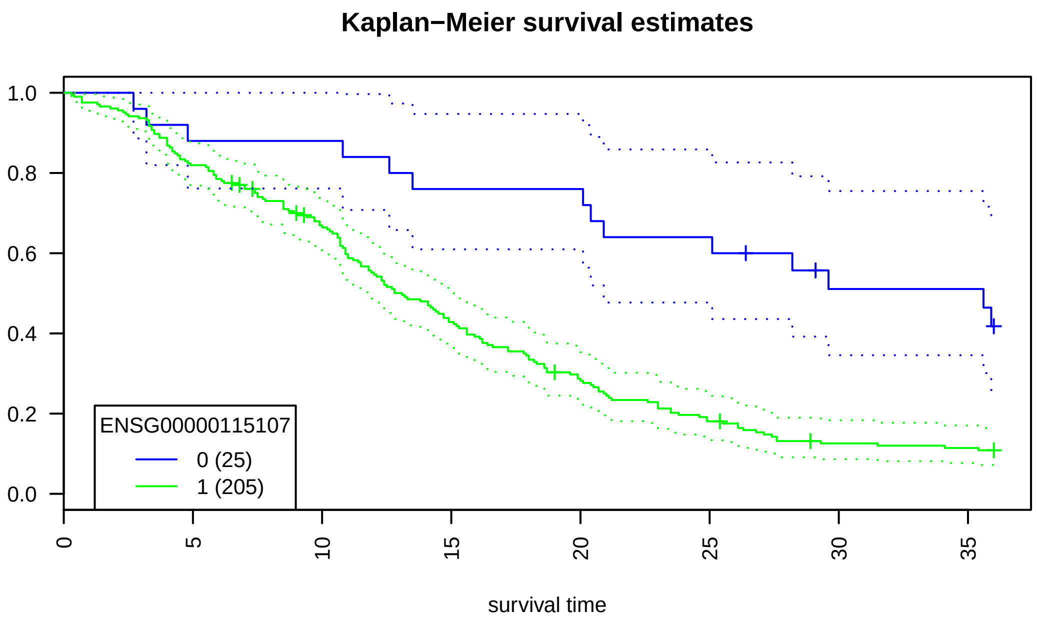

Figure 1550: Each curve represents the relative number of ENSG00000115107 survived to the corresponding point of time. Dashed lines represent 0.95 confidence intervals for the curves with the corresponding color. Log-rank probability measure for the equality of these curves is 7.250681e-05. Additional annotations: *.GeneName*=STEAP3, *.GeneDesc*=STEAP family member 3 [Source:HGNC Symbol;Acc:24592].

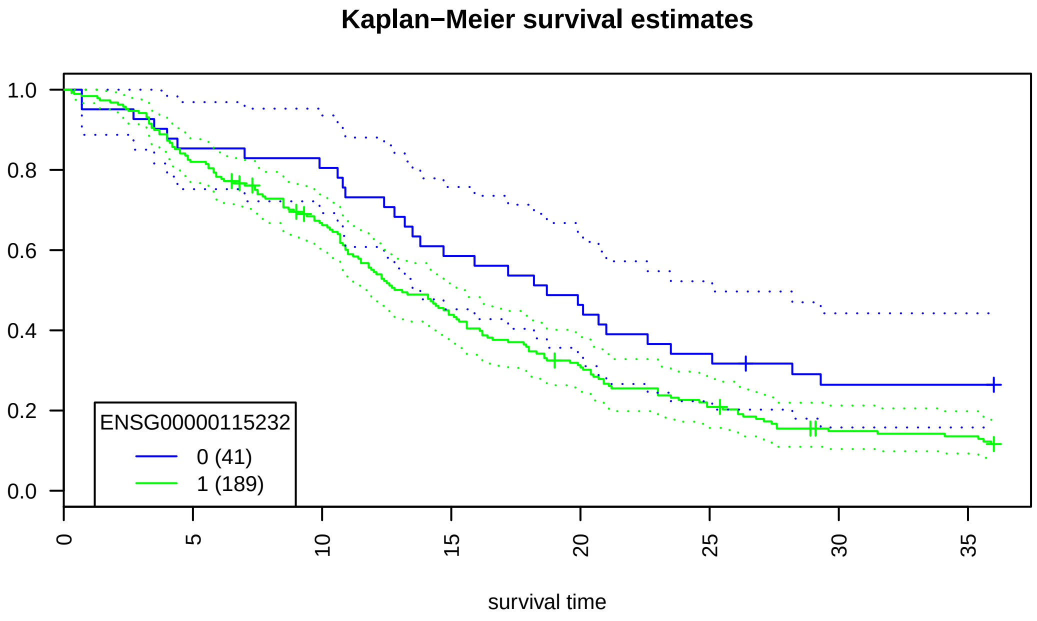

Figure 1551: Each curve represents the relative number of ENSG00000115232 survived to the corresponding point of time. Dashed lines represent 0.95 confidence intervals for the curves with the corresponding color. Log-rank probability measure for the equality of these curves is 2.403819e-02. Additional annotations: *.GeneName*=ITGA4, *.GeneDesc*=integrin, alpha 4 (antigen CD49D, alpha 4 subunit of VLA-4 receptor) [Source:HGNC Symbol;Acc:6140].

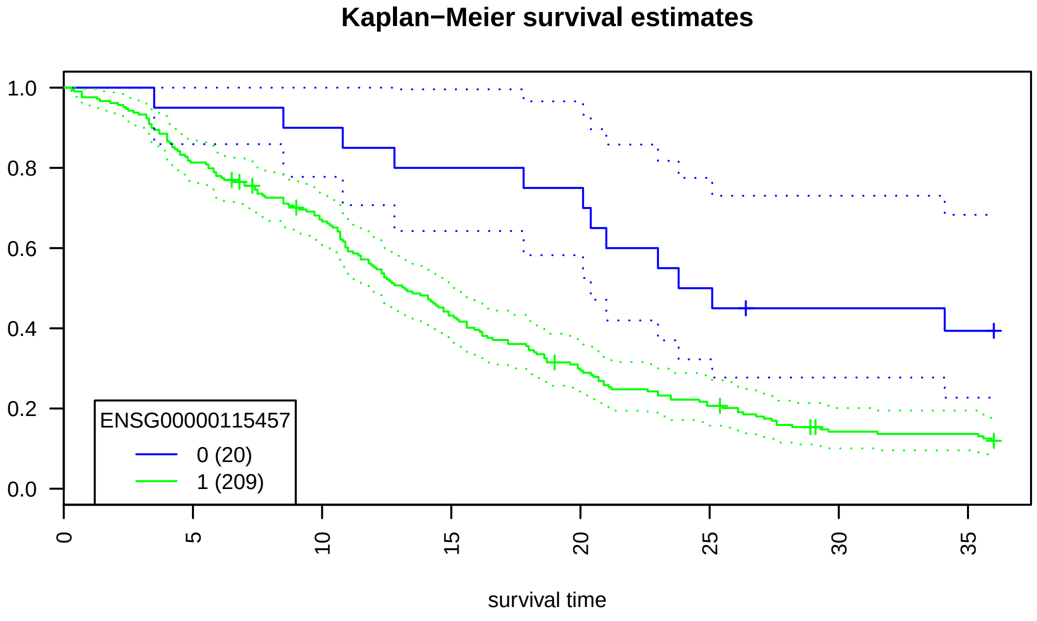

Figure 1552: Each curve represents the relative number of ENSG00000115457 survived to the corresponding point of time. Dashed lines represent 0.95 confidence intervals for the curves with the corresponding color. Total of 1 observation deleted due to missingness. Log-rank probability measure for the equality of these curves is 1.746184e-03. Additional annotations: *.GeneName*=IGFBP2, *.GeneDesc*=insulin-like growth factor binding protein 2, 36kDa [Source:HGNC Symbol;Acc:5471].

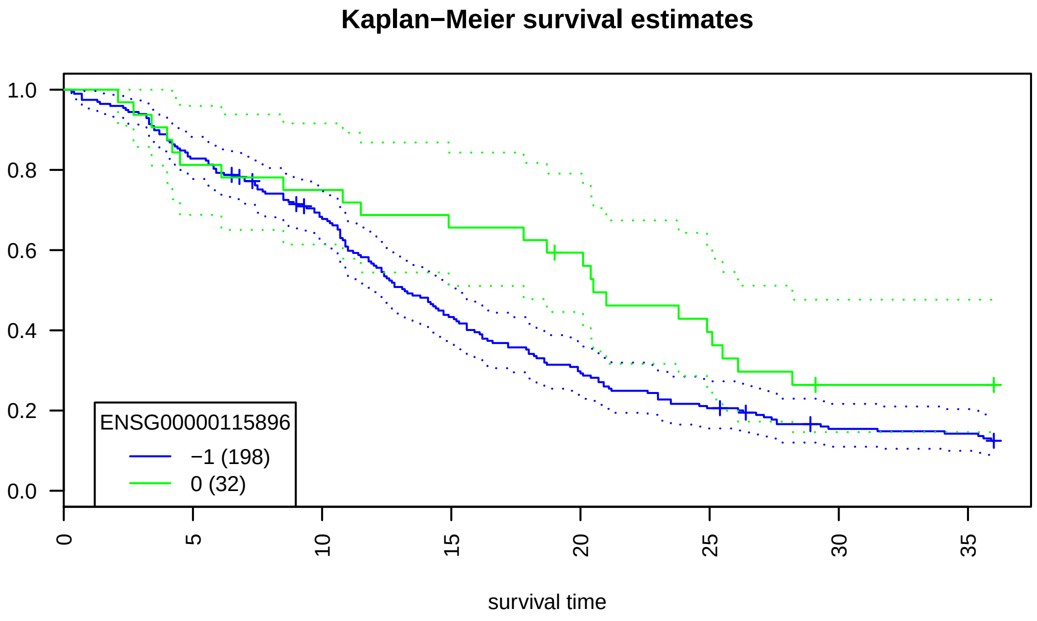

Figure 1553: Each curve represents the relative number of ENSG00000115896 survived to the corresponding point of time. Dashed lines represent 0.95 confidence intervals for the curves with the corresponding color. Log-rank probability measure for the equality of these curves is 2.764723e-02. Additional annotations: *.GeneName*=PLCL1, *.GeneDesc*=phospholipase C-like 1 [Source:HGNC Symbol;Acc:9063].

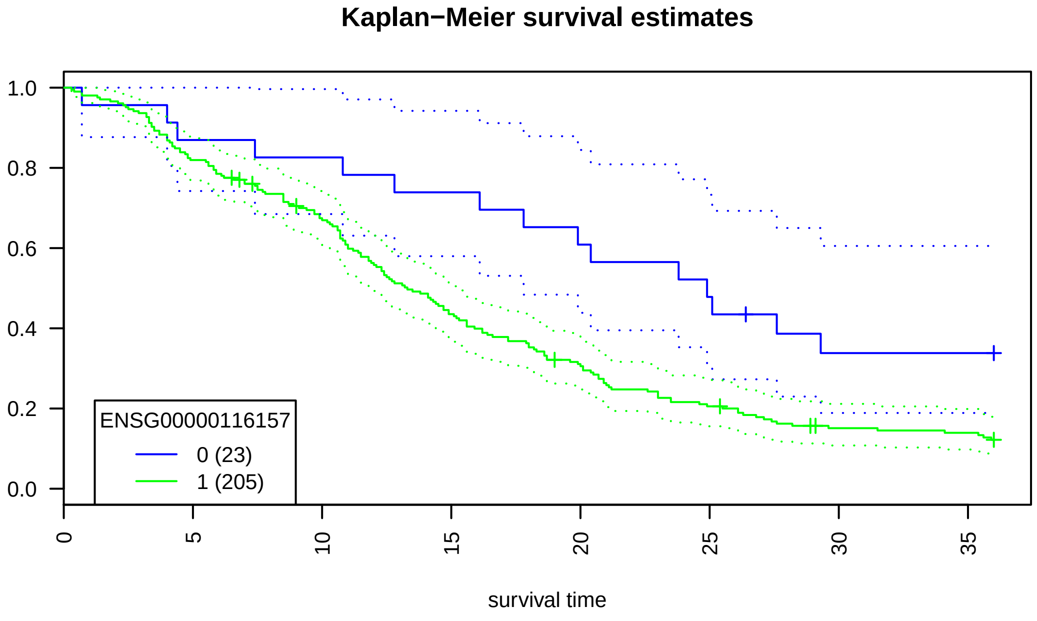

Figure 1554: Each curve represents the relative number of ENSG00000116157 survived to the corresponding point of time. Dashed lines represent 0.95 confidence intervals for the curves with the corresponding color. Total of 2 observations deleted due to missingness. Log-rank probability measure for the equality of these curves is 6.400122e-03. Additional annotations: *.GeneName*=GPX7, *.GeneDesc*=glutathione peroxidase 7 [Source:HGNC Symbol;Acc:4559].

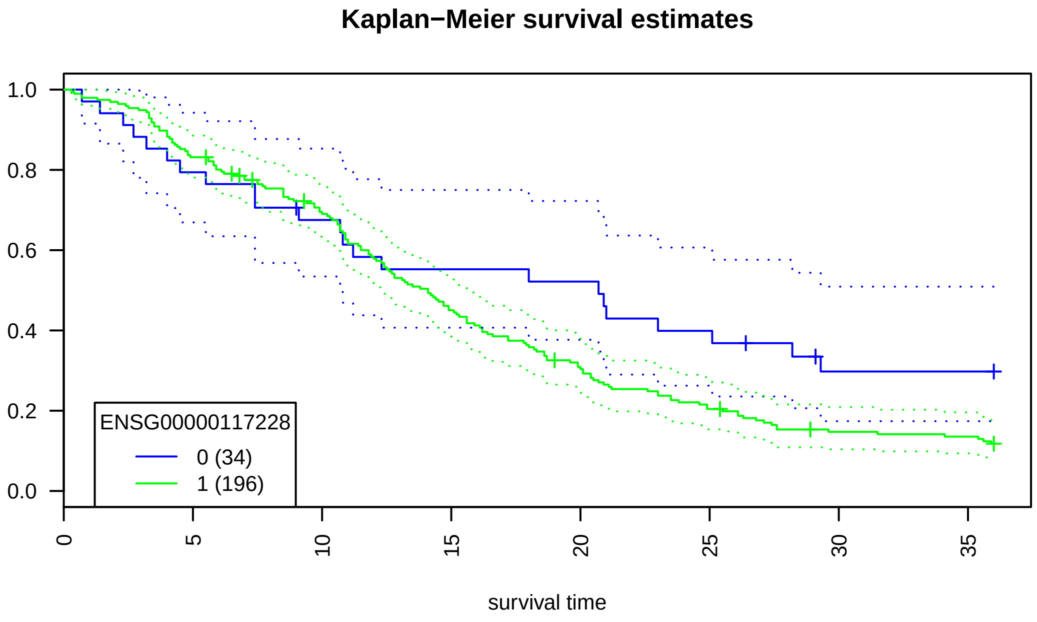

Figure 1555: Each curve represents the relative number of ENSG00000117228 survived to the corresponding point of time. Dashed lines represent 0.95 confidence intervals for the curves with the corresponding color. Log-rank probability measure for the equality of these curves is 4.879062e-02. Additional annotations: *.GeneName*=GBP1, *.GeneDesc*=guanylate binding protein 1, interferon-inducible, 67kDa [Source:HGNC Symbol;Acc:4182].

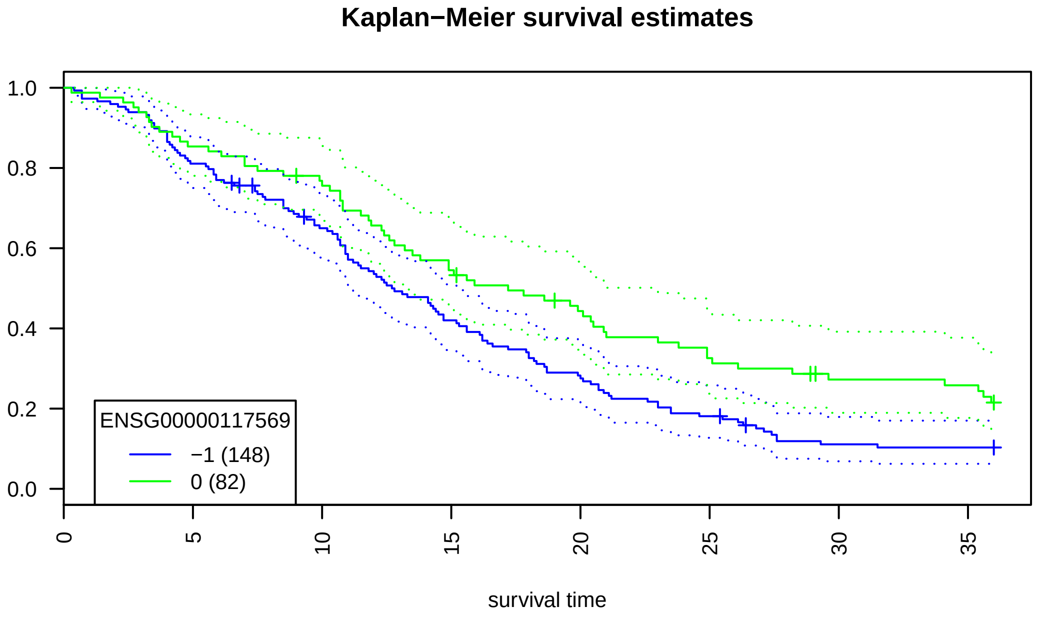

Figure 1556: Each curve represents the relative number of ENSG00000117569 survived to the corresponding point of time. Dashed lines represent 0.95 confidence intervals for the curves with the corresponding color. Log-rank probability measure for the equality of these curves is 7.791912e-03. Additional annotations: *.GeneName*=PTBP2, *.GeneDesc*=polypyrimidine tract binding protein 2 [Source:HGNC Symbol;Acc:17662].

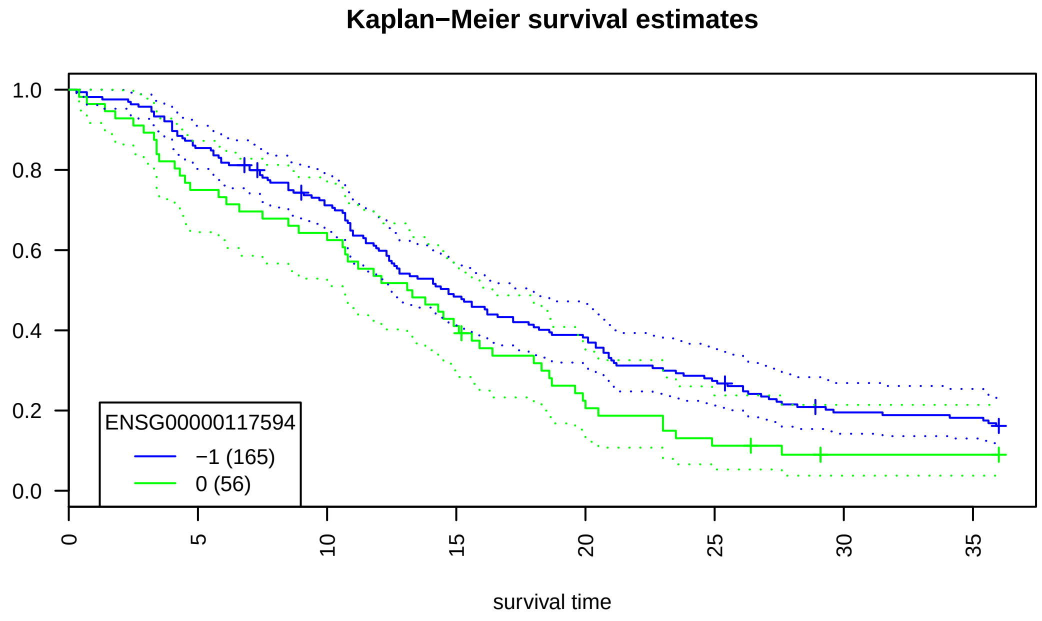

Figure 1557: Each curve represents the relative number of ENSG00000117594 survived to the corresponding point of time. Dashed lines represent 0.95 confidence intervals for the curves with the corresponding color. Total of 9 observations deleted due to missingness. Log-rank probability measure for the equality of these curves is 3.882734e-02. Additional annotations: *.GeneName*=HSD11B1, *.GeneDesc*=hydroxysteroid (11-beta) dehydrogenase 1 [Source:HGNC Symbol;Acc:5208].

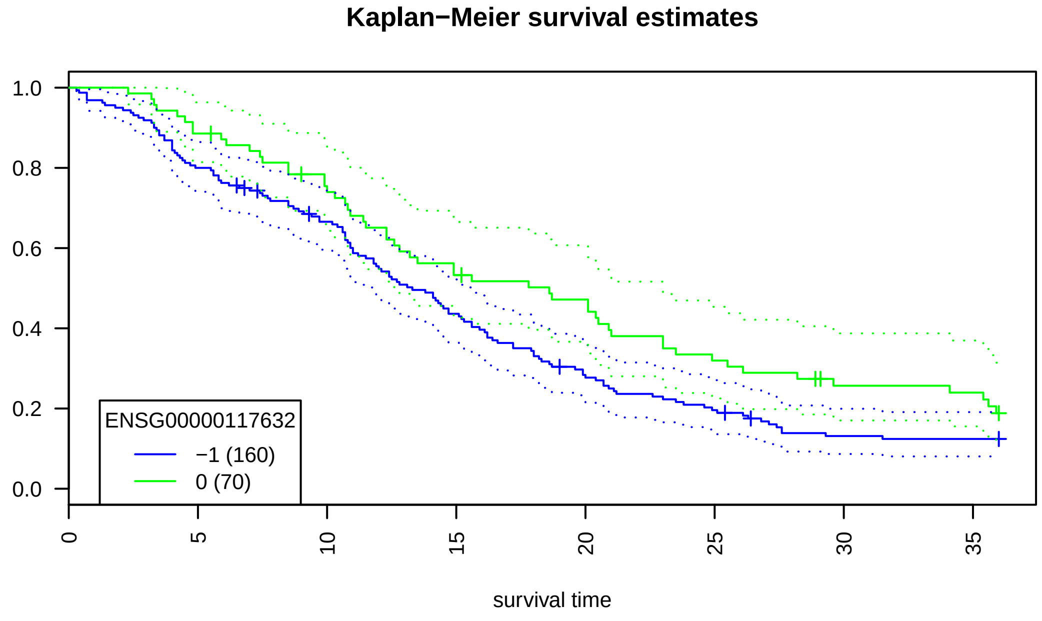

Figure 1558: Each curve represents the relative number of ENSG00000117632 survived to the corresponding point of time. Dashed lines represent 0.95 confidence intervals for the curves with the corresponding color. Log-rank probability measure for the equality of these curves is 3.549527e-02. Additional annotations: *.GeneName*=STMN1, *.GeneDesc*=stathmin 1 [Source:HGNC Symbol;Acc:6510].

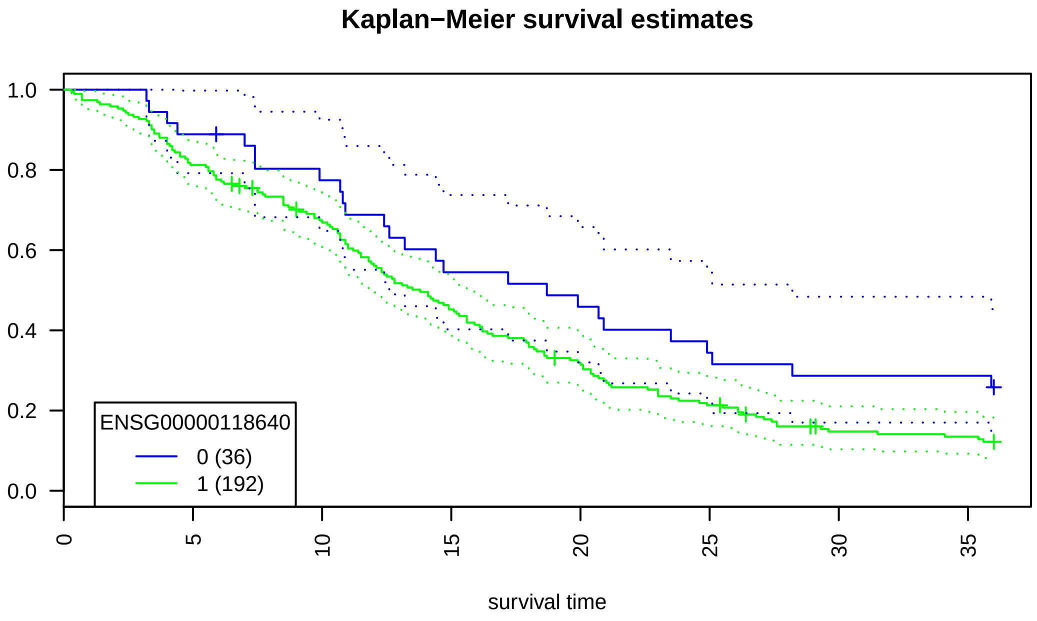

Figure 1559: Each curve represents the relative number of ENSG00000118640 survived to the corresponding point of time. Dashed lines represent 0.95 confidence intervals for the curves with the corresponding color. Total of 2 observations deleted due to missingness. Log-rank probability measure for the equality of these curves is 4.344173e-02. Additional annotations: *.GeneName*=VAMP8, *.GeneDesc*=vesicle-associated membrane protein 8 (endobrevin) [Source:HGNC Symbol;Acc:12647].

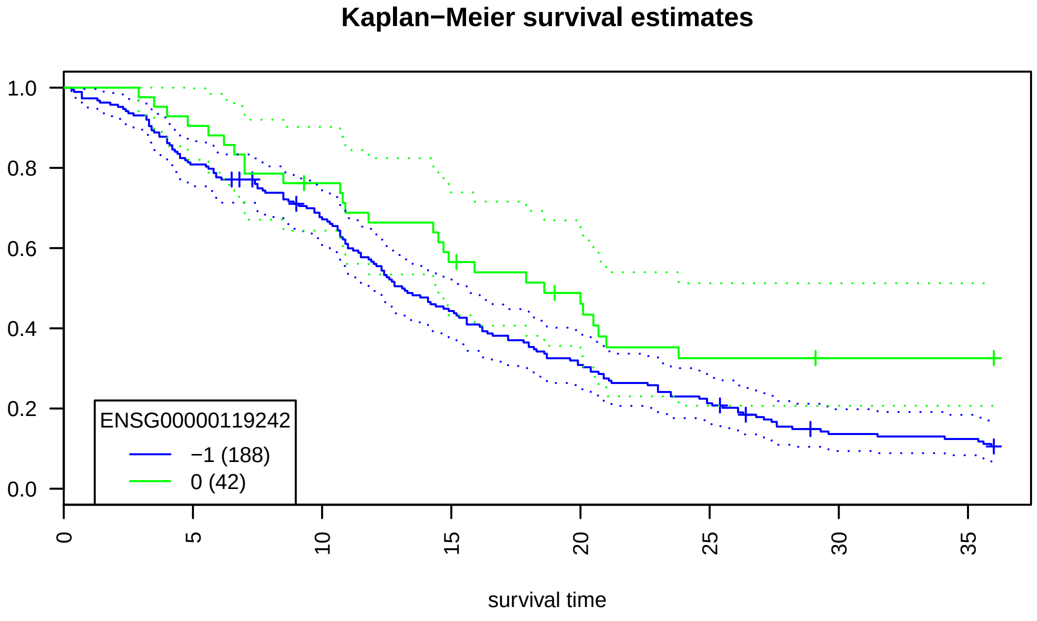

Figure 1560: Each curve represents the relative number of ENSG00000119242 survived to the corresponding point of time. Dashed lines represent 0.95 confidence intervals for the curves with the corresponding color. Log-rank probability measure for the equality of these curves is 9.82668e-03. Additional annotations: *.GeneName*=CCDC92, *.GeneDesc*=coiled-coil domain containing 92 [Source:HGNC Symbol;Acc:29563].

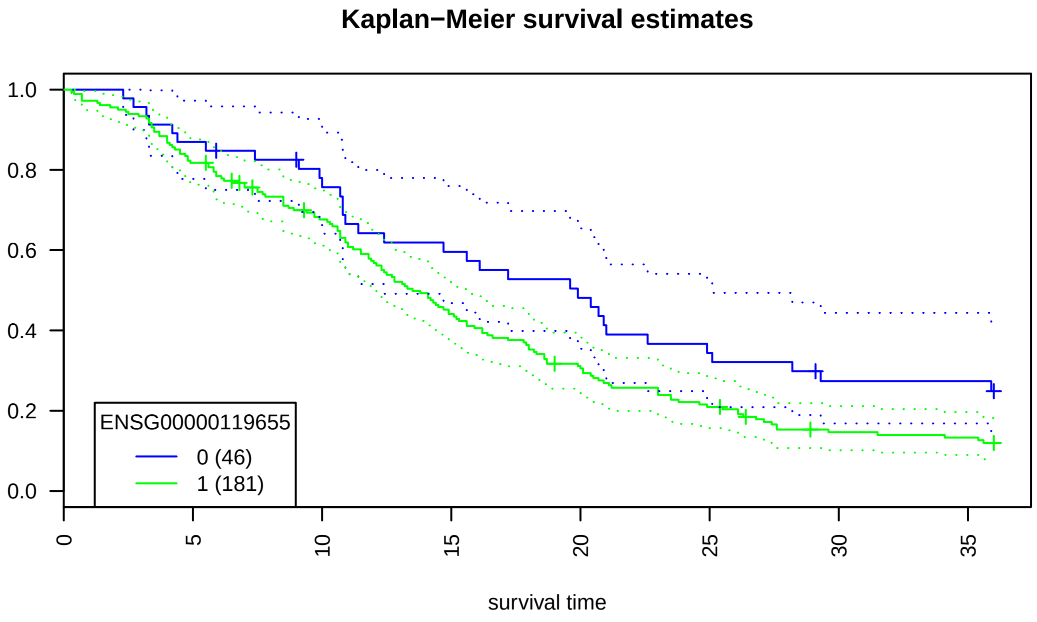

Figure 1561: Each curve represents the relative number of ENSG00000119655 survived to the corresponding point of time. Dashed lines represent 0.95 confidence intervals for the curves with the corresponding color. Total of 3 observations deleted due to missingness. Log-rank probability measure for the equality of these curves is 3.002792e-02. Additional annotations: *.GeneName*=NPC2, *.GeneDesc*=Niemann-Pick disease, type C2 [Source:HGNC Symbol;Acc:14537].

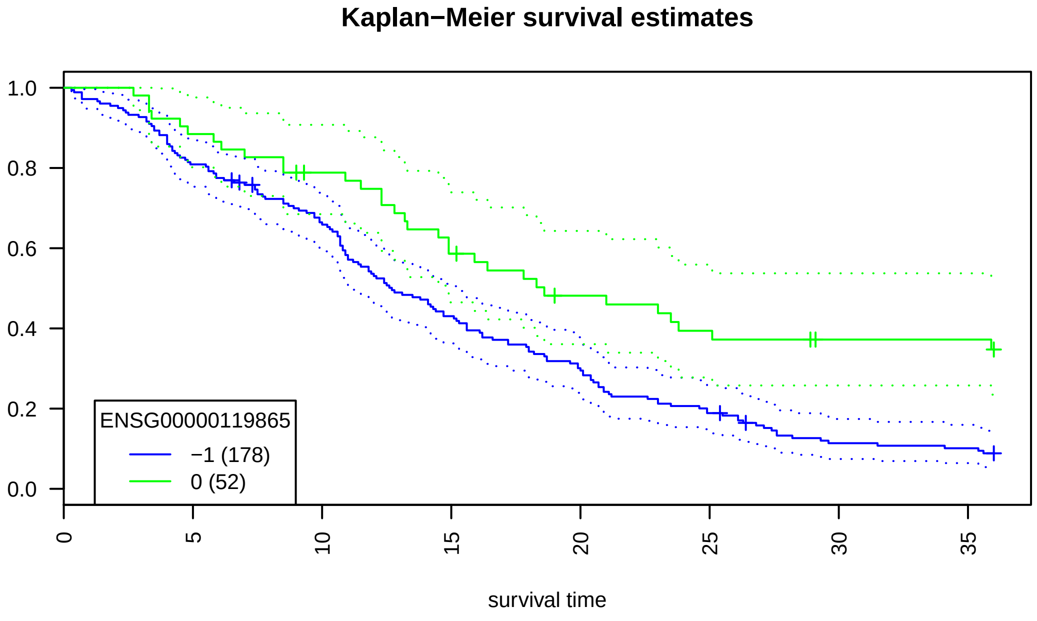

Figure 1562: Each curve represents the relative number of ENSG00000119865 survived to the corresponding point of time. Dashed lines represent 0.95 confidence intervals for the curves with the corresponding color. Log-rank probability measure for the equality of these curves is 2.967295e-04. Additional annotations: *.GeneName*=CNRIP1, *.GeneDesc*=cannabinoid receptor interacting protein 1 [Source:HGNC Symbol;Acc:24546].

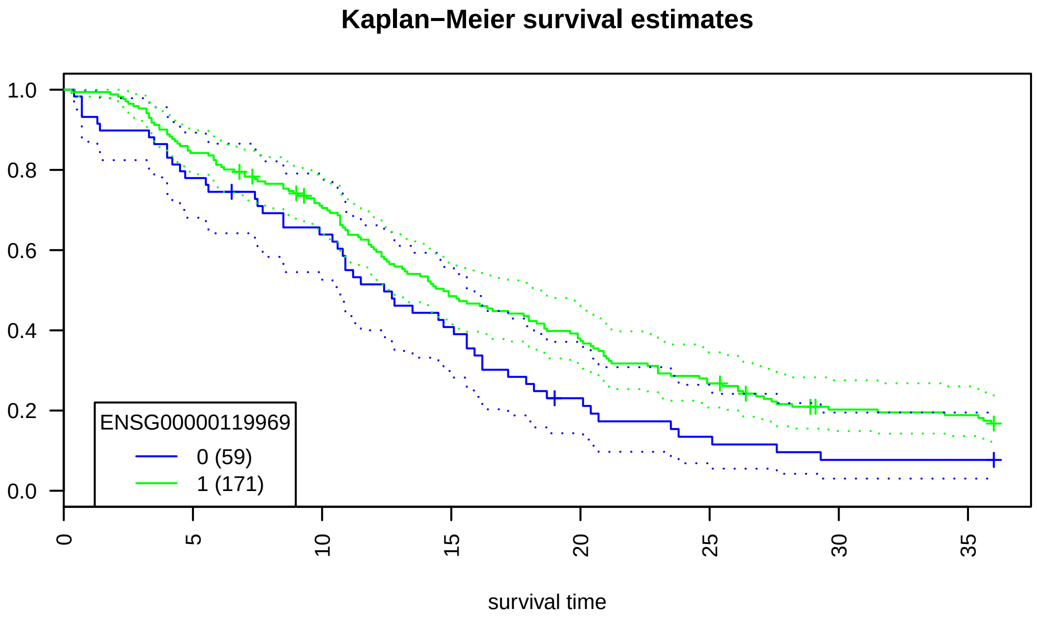

Figure 1563: Each curve represents the relative number of ENSG00000119969 survived to the corresponding point of time. Dashed lines represent 0.95 confidence intervals for the curves with the corresponding color. Log-rank probability measure for the equality of these curves is 2.252214e-02. Additional annotations: *.GeneName*=HELLS, *.GeneDesc*=helicase, lymphoid-specific [Source:HGNC Symbol;Acc:4861].

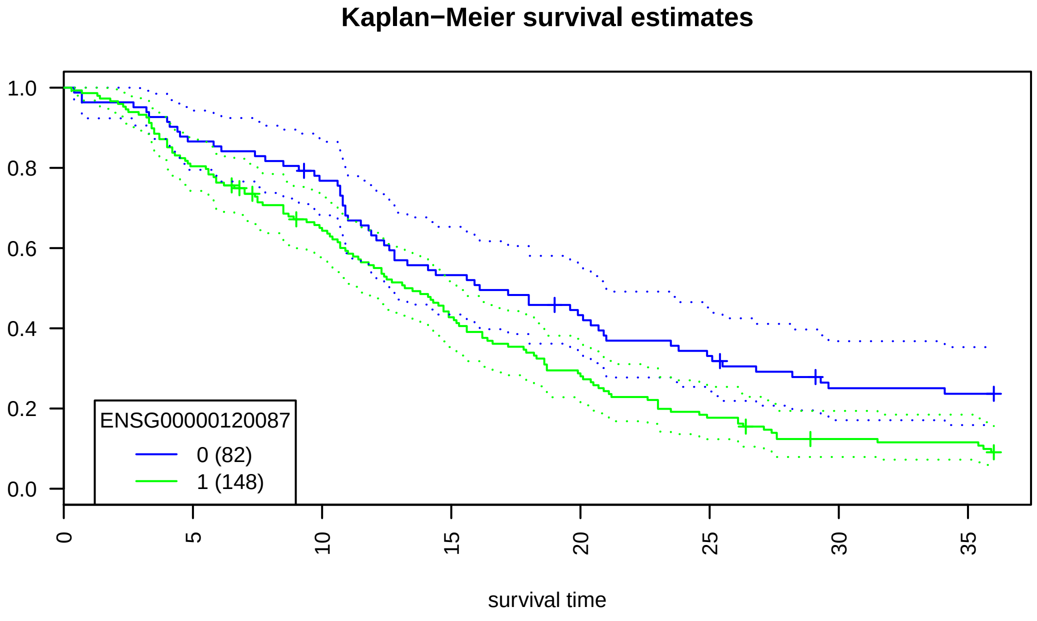

Figure 1564: Each curve represents the relative number of ENSG00000120087 survived to the corresponding point of time. Dashed lines represent 0.95 confidence intervals for the curves with the corresponding color. Log-rank probability measure for the equality of these curves is 5.464616e-03. Additional annotations: *.GeneName*=HOXB7, *.GeneDesc*=homeobox B7 [Source:HGNC Symbol;Acc:5118].

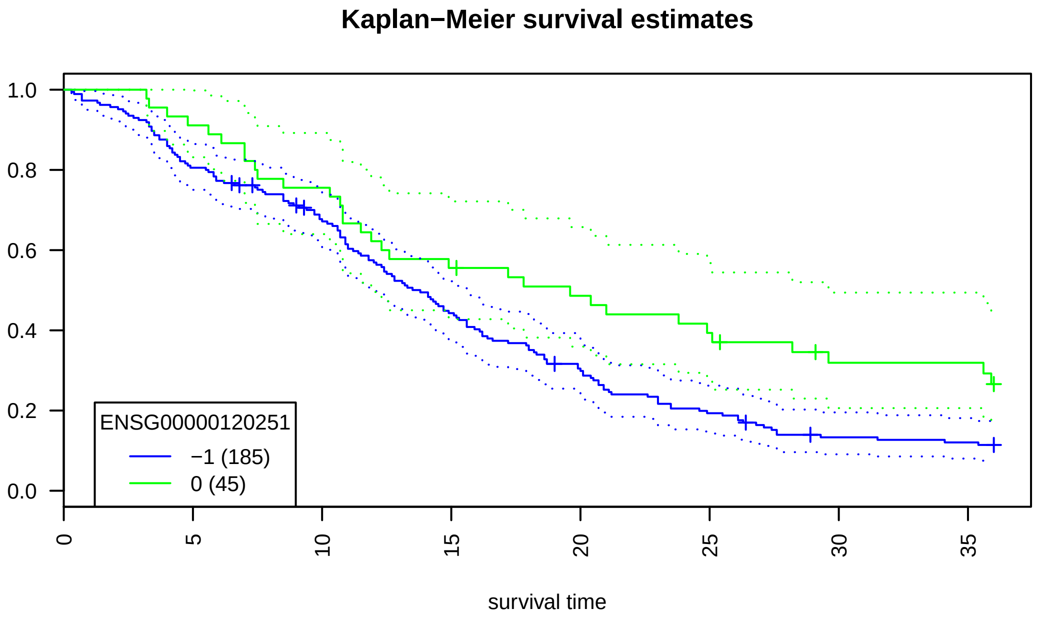

Figure 1565: Each curve represents the relative number of ENSG00000120251 survived to the corresponding point of time. Dashed lines represent 0.95 confidence intervals for the curves with the corresponding color. Log-rank probability measure for the equality of these curves is 1.160275e-02. Additional annotations: *.GeneName*=GRIA2, *.GeneDesc*=glutamate receptor, ionotropic, AMPA 2 [Source:HGNC Symbol;Acc:4572].

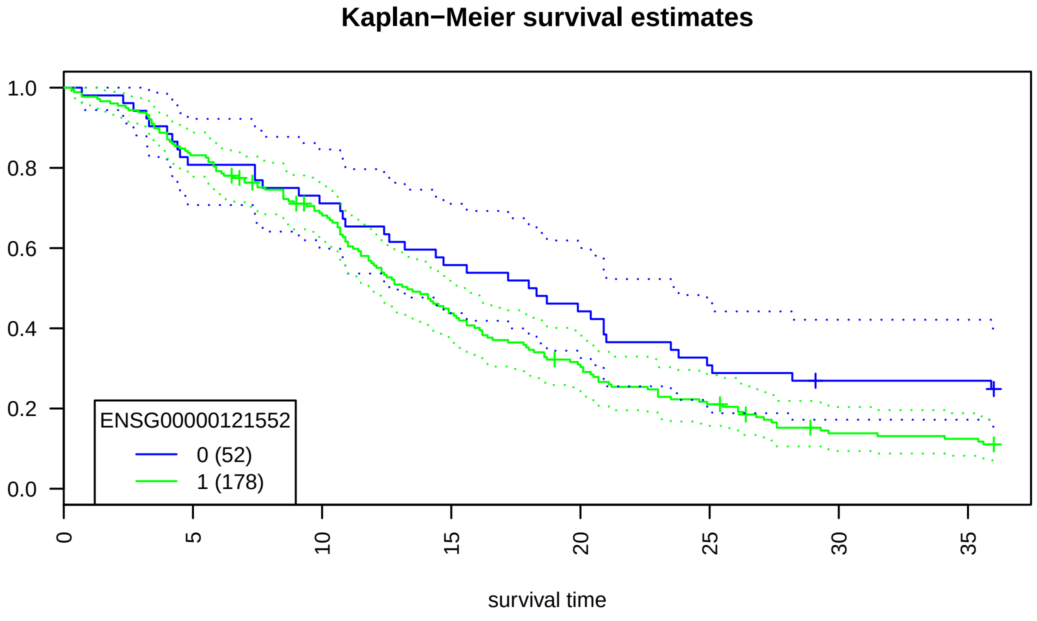

Figure 1566: Each curve represents the relative number of ENSG00000121552 survived to the corresponding point of time. Dashed lines represent 0.95 confidence intervals for the curves with the corresponding color. Log-rank probability measure for the equality of these curves is 3.638182e-02. Additional annotations: *.GeneName*=CSTA, *.GeneDesc*=cystatin A (stefin A) [Source:HGNC Symbol;Acc:2481].

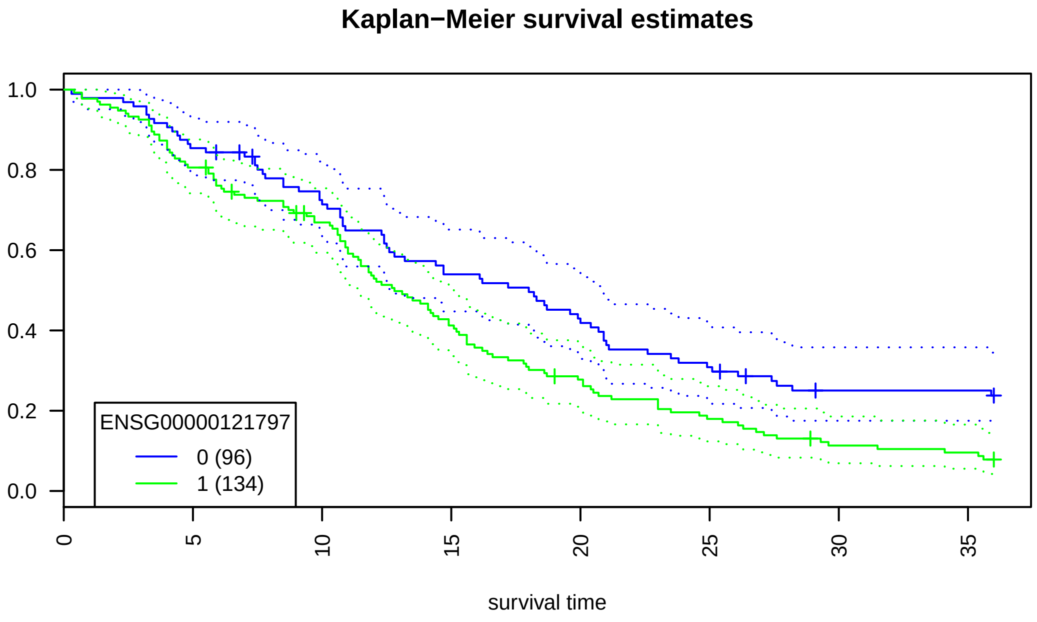

Figure 1567: Each curve represents the relative number of ENSG00000121797 survived to the corresponding point of time. Dashed lines represent 0.95 confidence intervals for the curves with the corresponding color. Log-rank probability measure for the equality of these curves is 3.145654e-03. Additional annotations: *.GeneName*=CCRL2, *.GeneDesc*=chemokine (C-C motif) receptor-like 2 [Source:HGNC Symbol;Acc:1612].

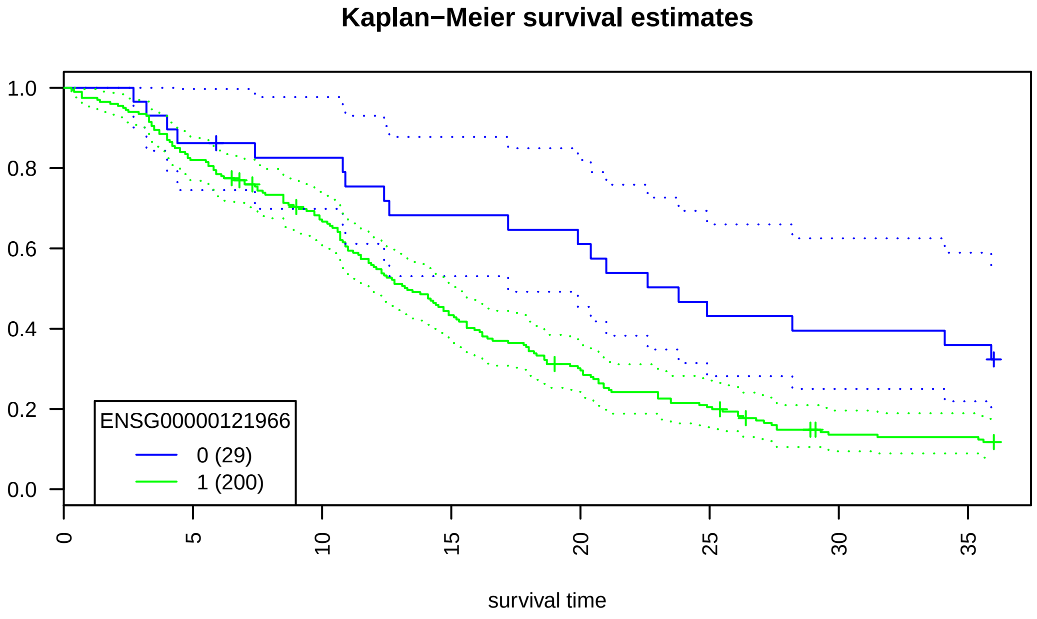

Figure 1568: Each curve represents the relative number of ENSG00000121966 survived to the corresponding point of time. Dashed lines represent 0.95 confidence intervals for the curves with the corresponding color. Total of 1 observation deleted due to missingness. Log-rank probability measure for the equality of these curves is 3.663795e-03. Additional annotations: *.GeneName*=CXCR4, *.GeneDesc*=chemokine (C-X-C motif) receptor 4 [Source:HGNC Symbol;Acc:2561].

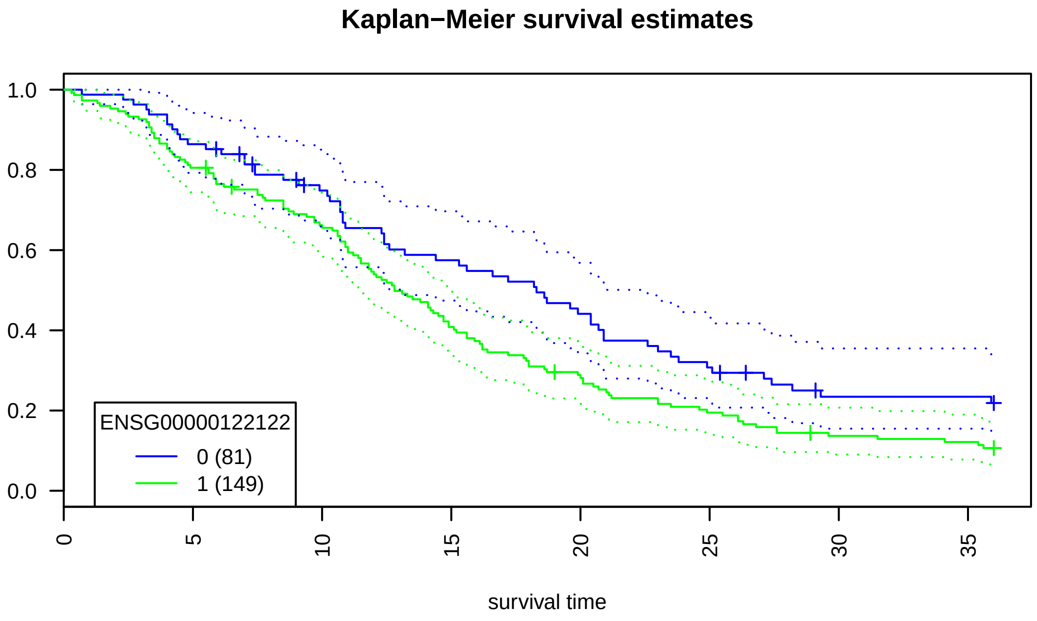

Figure 1569: Each curve represents the relative number of ENSG00000122122 survived to the corresponding point of time. Dashed lines represent 0.95 confidence intervals for the curves with the corresponding color. Log-rank probability measure for the equality of these curves is 1.375699e-02. Additional annotations: *.GeneName*=SASH3, *.GeneDesc*=SAM and SH3 domain containing 3 [Source:HGNC Symbol;Acc:15975].

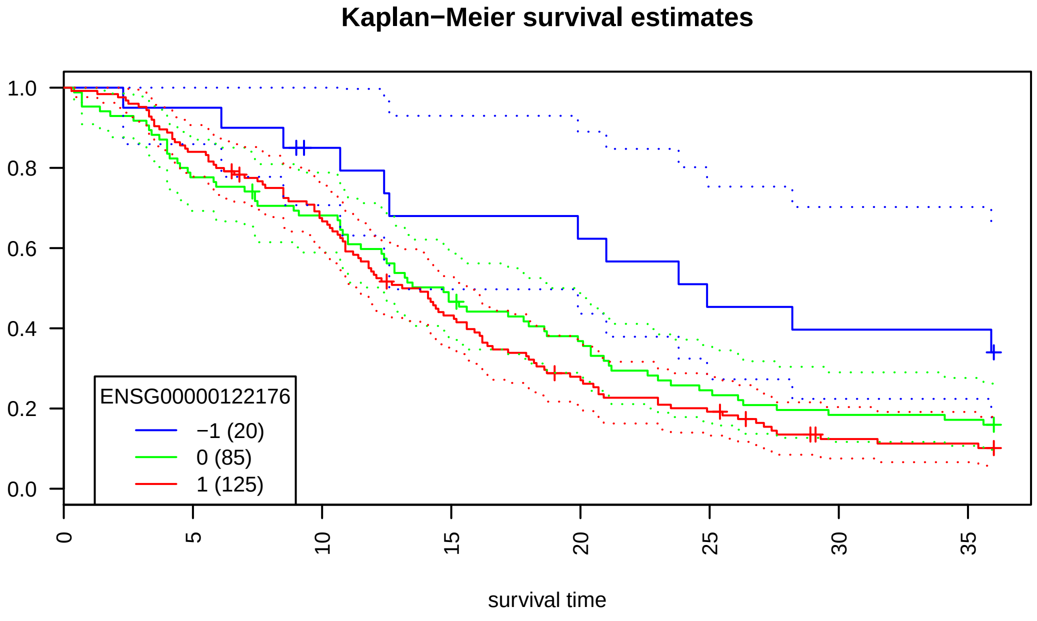

Figure 1570: Each curve represents the relative number of ENSG00000122176 survived to the corresponding point of time. Dashed lines represent 0.95 confidence intervals for the curves with the corresponding color. Log-rank probability measure for the equality of these curves is 2.520388e-02. Additional annotations: *.GeneName*=FMOD, *.GeneDesc*=fibromodulin [Source:HGNC Symbol;Acc:3774].

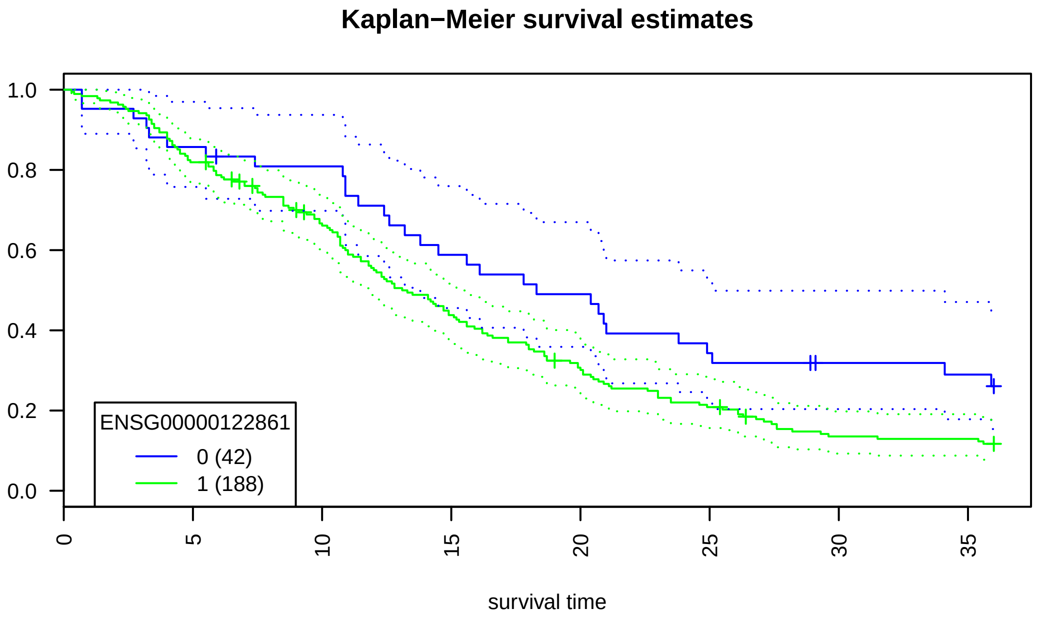

Figure 1571: Each curve represents the relative number of ENSG00000122861 survived to the corresponding point of time. Dashed lines represent 0.95 confidence intervals for the curves with the corresponding color. Log-rank probability measure for the equality of these curves is 2.167013e-02. Additional annotations: *.GeneName*=PLAU, *.GeneDesc*=plasminogen activator, urokinase [Source:HGNC Symbol;Acc:9052].

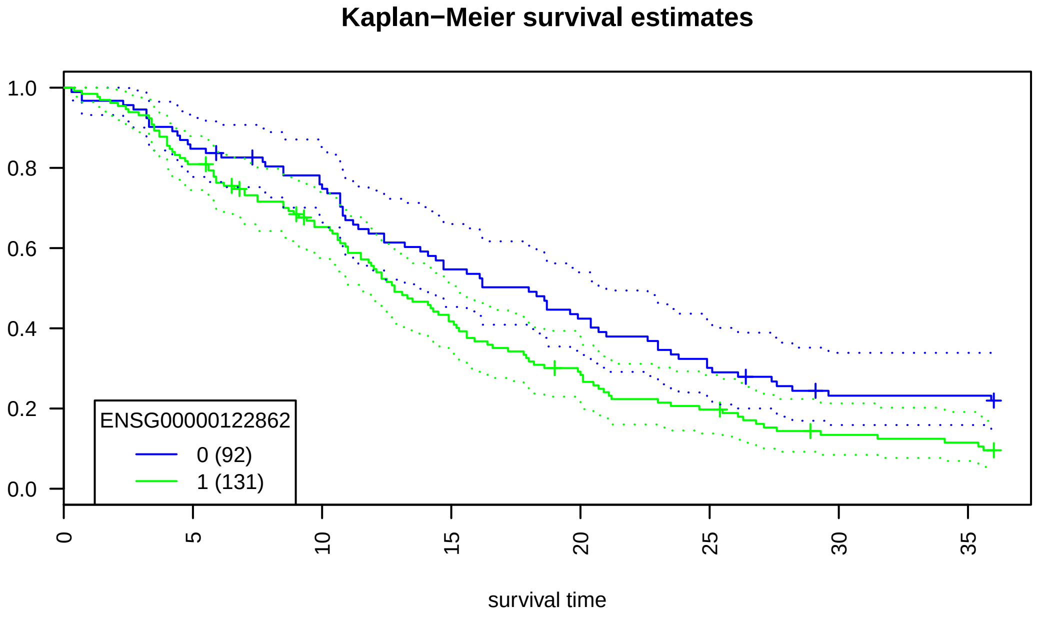

Figure 1572: Each curve represents the relative number of ENSG00000122862 survived to the corresponding point of time. Dashed lines represent 0.95 confidence intervals for the curves with the corresponding color. Total of 7 observations deleted due to missingness. Log-rank probability measure for the equality of these curves is 1.078041e-02. Additional annotations: *.GeneName*=SRGN, *.GeneDesc*=serglycin [Source:HGNC Symbol;Acc:9361].

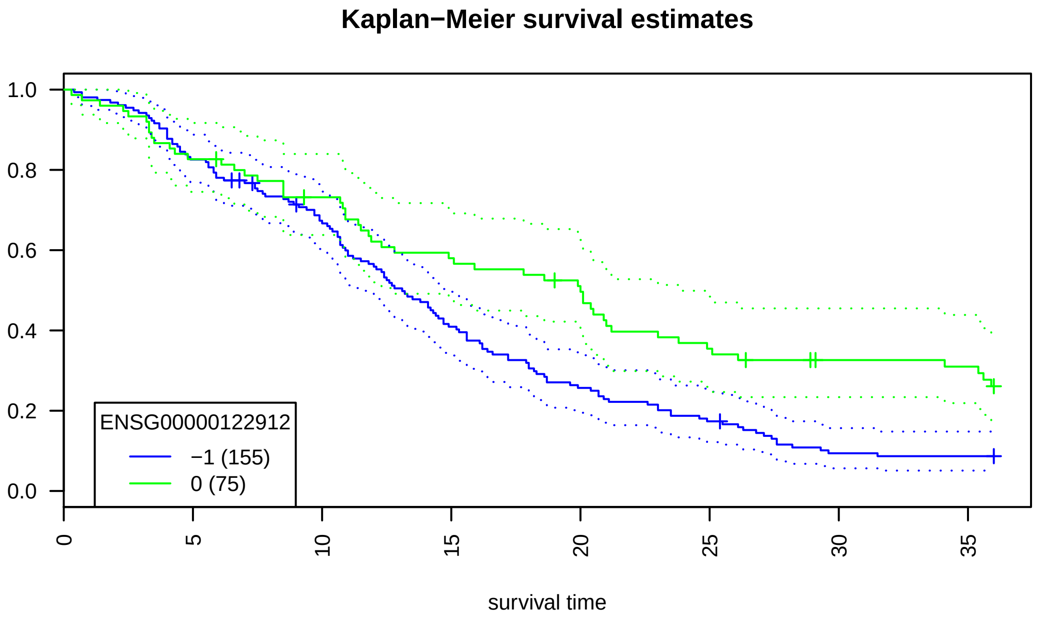

Figure 1573: Each curve represents the relative number of ENSG00000122912 survived to the corresponding point of time. Dashed lines represent 0.95 confidence intervals for the curves with the corresponding color. Log-rank probability measure for the equality of these curves is 1.123133e-03. Additional annotations: *.GeneName*=SLC25A16, *.GeneDesc*=solute carrier family 25 (mitochondrial carrier; Graves disease autoantigen), member 16 [Source:HGNC Symbol;Acc:10986].

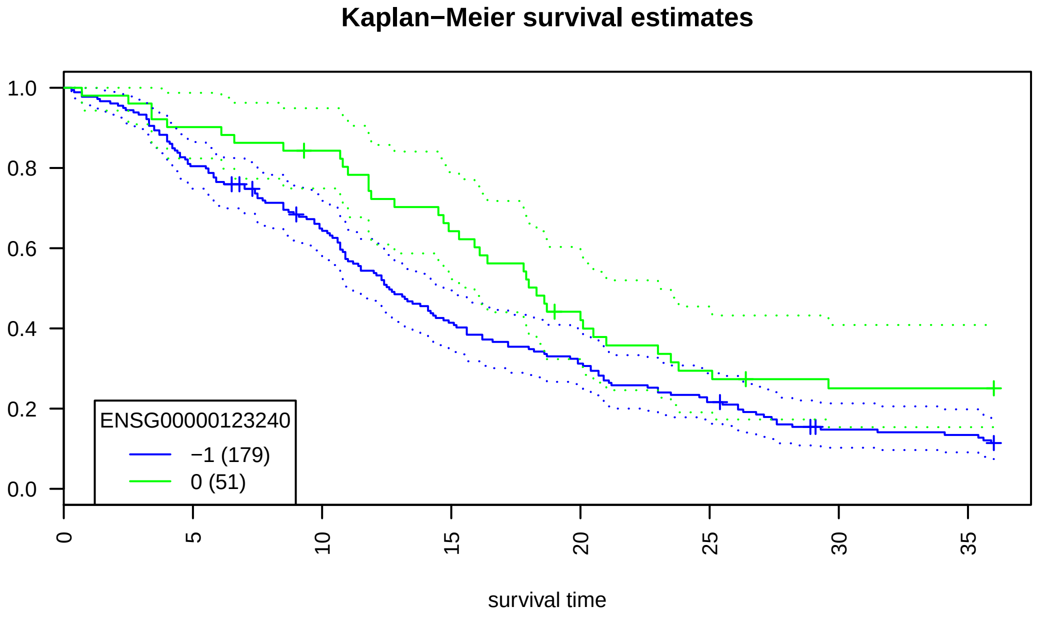

Figure 1574: Each curve represents the relative number of ENSG00000123240 survived to the corresponding point of time. Dashed lines represent 0.95 confidence intervals for the curves with the corresponding color. Log-rank probability measure for the equality of these curves is 1.405832e-02. Additional annotations: *.GeneName*=OPTN, *.GeneDesc*=optineurin [Source:HGNC Symbol;Acc:17142].

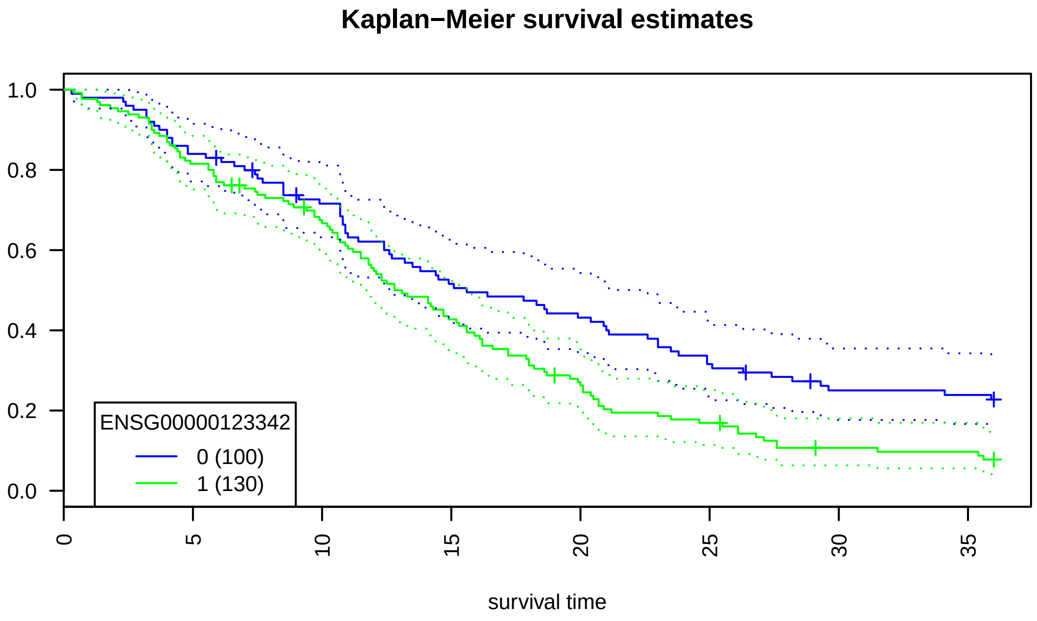

Figure 1575: Each curve represents the relative number of ENSG00000123342 survived to the corresponding point of time. Dashed lines represent 0.95 confidence intervals for the curves with the corresponding color. Log-rank probability measure for the equality of these curves is 3.877299e-03. Additional annotations: .*GeneName*=MMP19, .*GeneDesc*=matrix metallopeptidase 19 [Source:HGNC Symbol;Acc:7165].

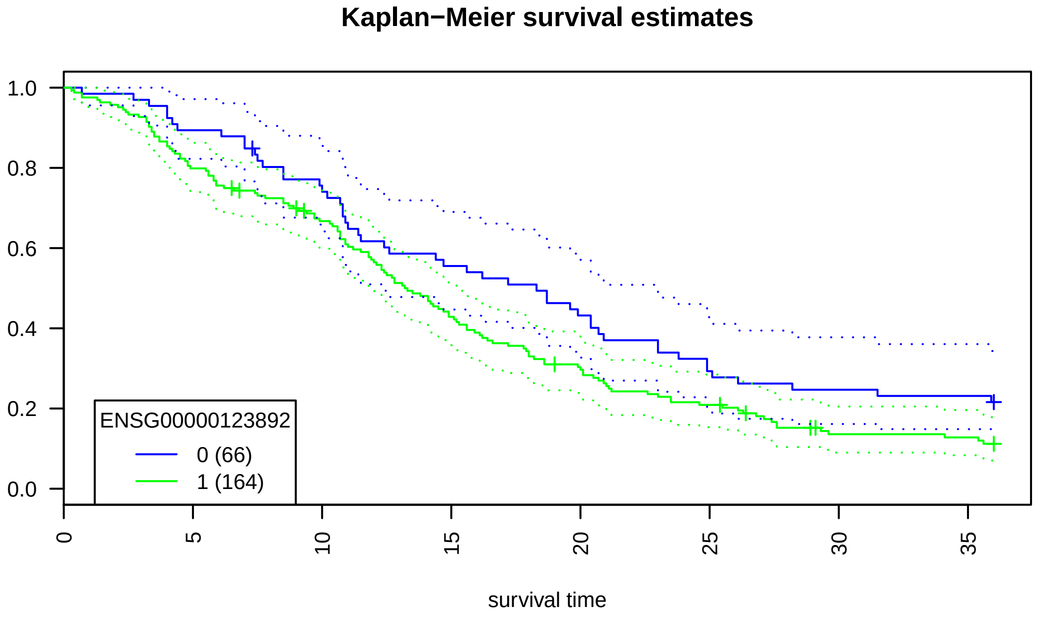

Figure 1576: Each curve represents the relative number of ENSG00000123892 survived to the corresponding point of time. Dashed lines represent 0.95 confidence intervals for the curves with the corresponding color. Log-rank probability measure for the equality of these curves is 3.340736e-02. Additional annotations: .*GeneName*=RAB38, .*GeneDesc*=RAB38, member RAS oncogene family [Source:HGNC Symbol;Acc:9776].

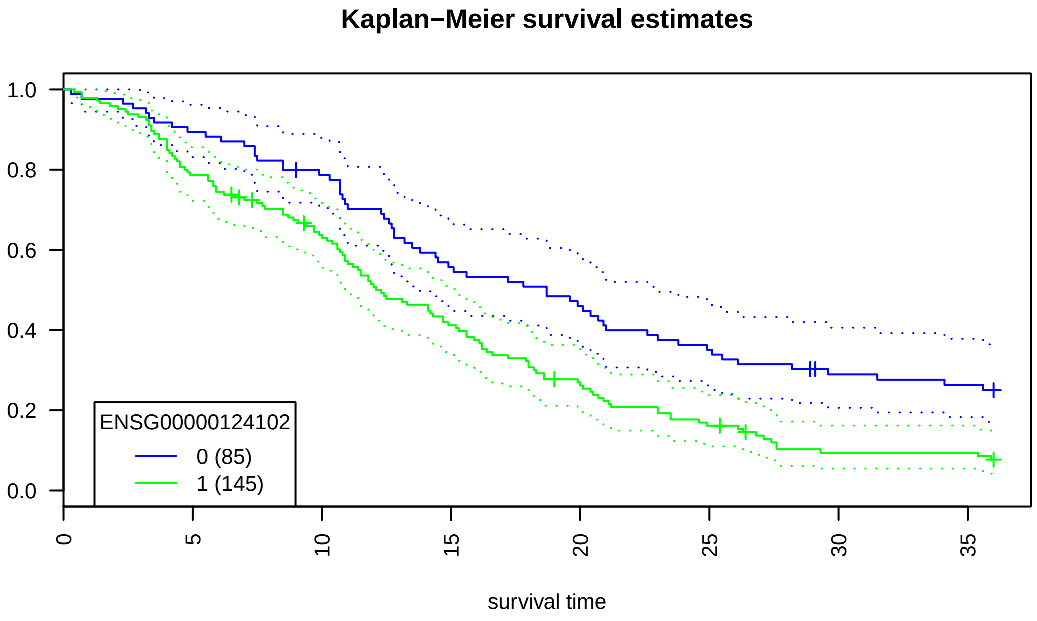

Figure 1577: Each curve represents the relative number of ENSG00000124102 survived to the corresponding point of time. Dashed lines represent 0.95 confidence intervals for the curves with the corresponding color. Log-rank probability measure for the equality of these curves is 2.541276e-04. Additional annotations: *.GeneName*=PI3, *.GeneDesc*=peptidase inhibitor 3, skin-derived [Source:HGNC Symbol;Acc:8947].

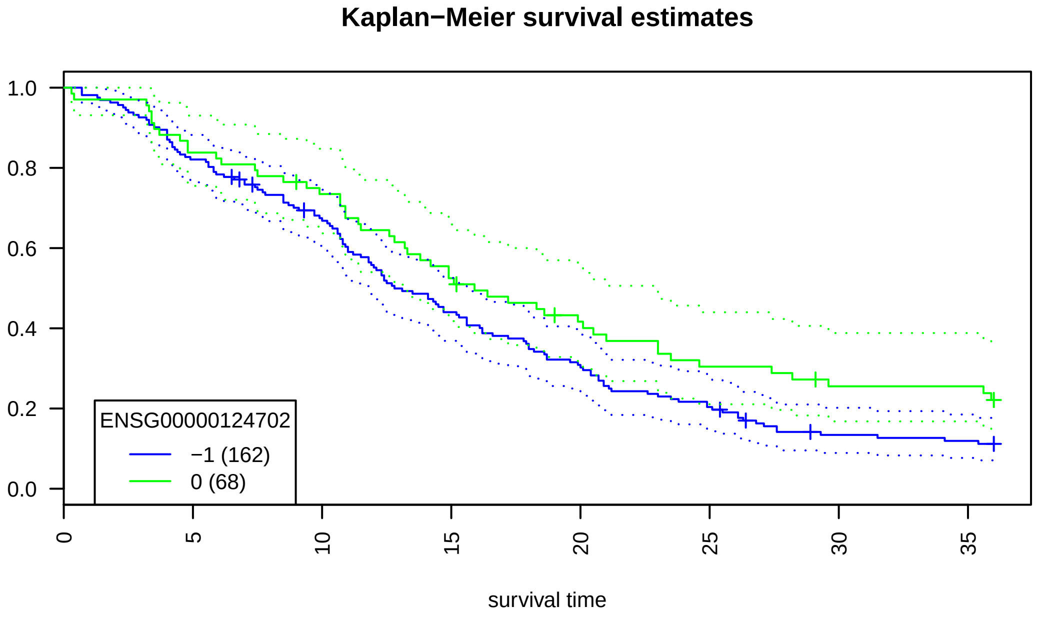

Figure 1578: Each curve represents the relative number of ENSG00000124702 survived to the corresponding point of time. Dashed lines represent 0.95 confidence intervals for the curves with the corresponding color. Log-rank probability measure for the equality of these curves is 3.980115e-02. Additional annotations: *.GeneName*=KLHDC3, *.GeneDesc*=kelch domain containing 3 [Source:HGNC Symbol;Acc:20704].

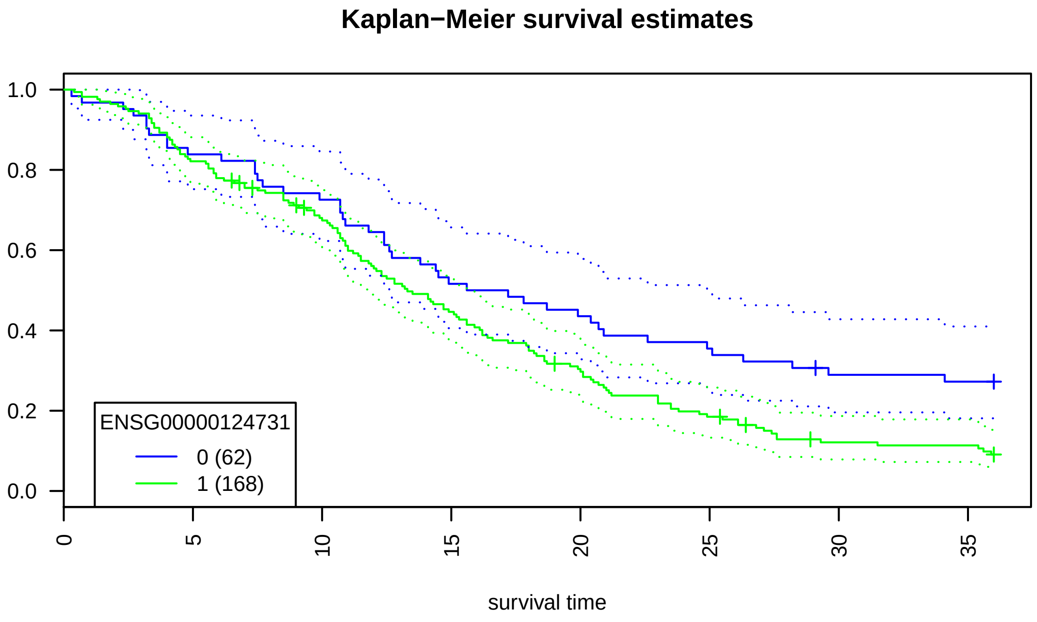

Figure 1579: Each curve represents the relative number of ENSG00000124731 survived to the corresponding point of time. Dashed lines represent 0.95 confidence intervals for the curves with the corresponding color. Log-rank probability measure for the equality of these curves is 7.668407e-03. Additional annotations: *.GeneName*=TREM1, *.GeneDesc*=triggering receptor expressed on myeloid cells 1 [Source:HGNC Symbol;Acc:17760].

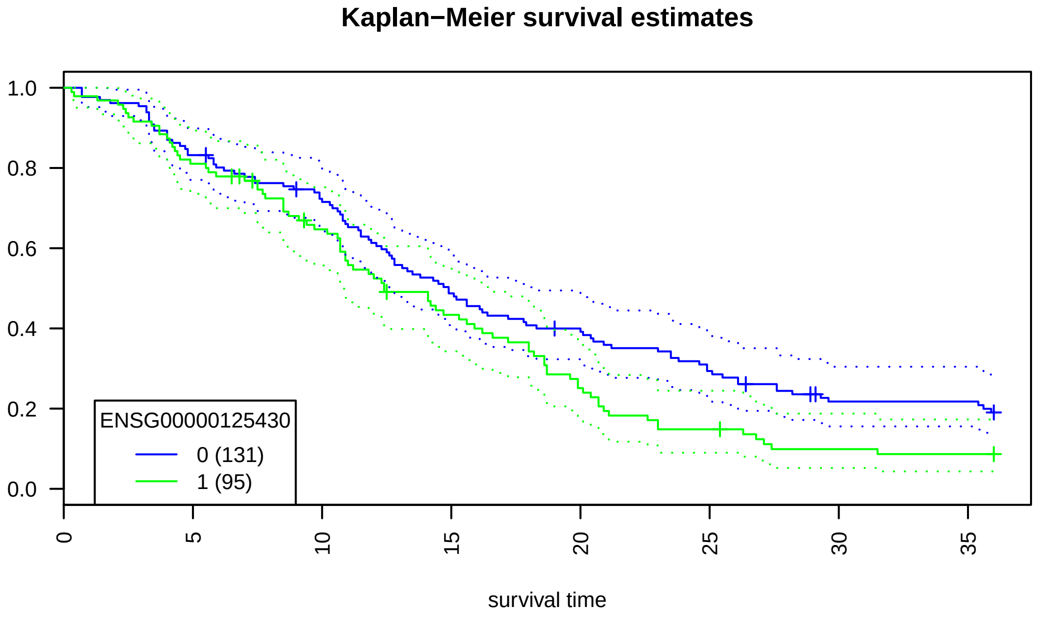

Figure 1580: Each curve represents the relative number of ENSG00000125430 survived to the corresponding point of time. Dashed lines represent 0.95 confidence intervals for the curves with the corresponding color. Total of 4 observations deleted due to missingness. Log-rank probability measure for the equality of these curves is 2.283634e-02. Additional annotations: *.GeneName*=HS3ST3B1, *.GeneDesc*=heparan sulfate (glucosamine) 3-O-sulfotransferase 3B1 [Source:HGNC Symbol;Acc:5198].

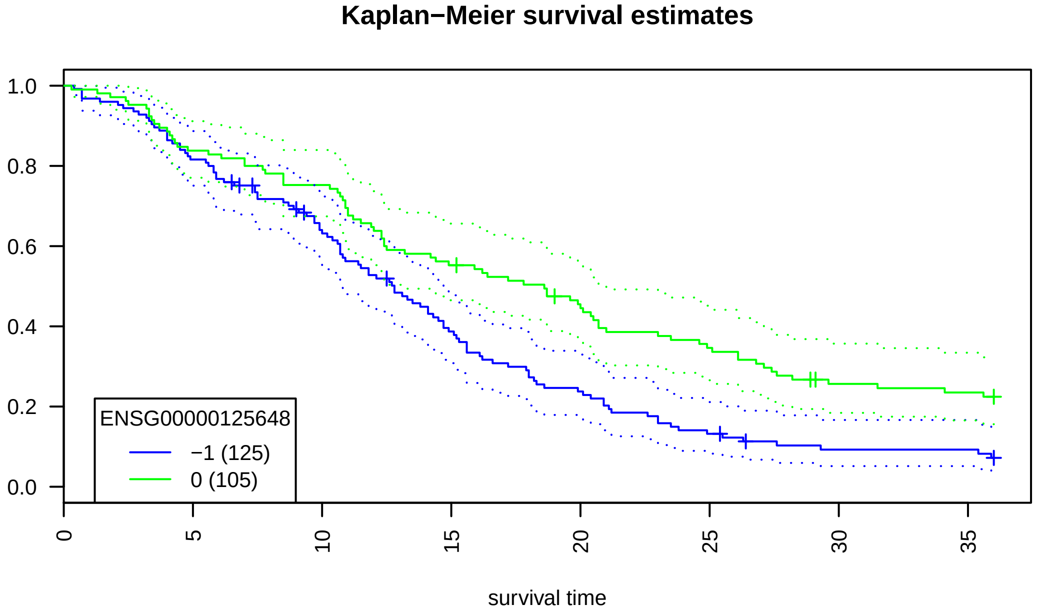

Figure 1581: Each curve represents the relative number of ENSG00000125648 survived to the corresponding point of time. Dashed lines represent 0.95 confidence intervals for the curves with the corresponding color. Log-rank probability measure for the equality of these curves is 3.536675e-04. Additional annotations: .*GeneName*=SLC25A23, .*GeneDesc*=solute carrier family 25 (mitochondrial carrier; phosphate carrier), member 23 [Source:HGNC Symbol;Acc:19375].

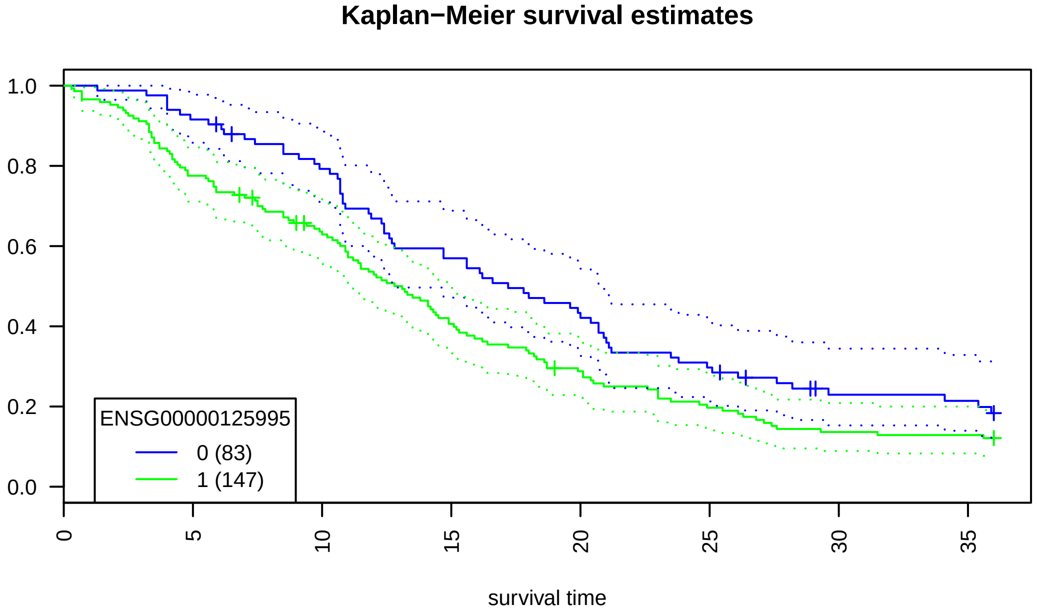

Figure 1582: Each curve represents the relative number of ENSG00000125995 survived to the corresponding point of time. Dashed lines represent 0.95 confidence intervals for the curves with the corresponding color. Log-rank probability measure for the equality of these curves is 2.036275e-02. Additional annotations: .*GeneName*=ROMO1, .*GeneDesc*=reactive oxygen species modulator 1 [Source:HGNC Symbol;Acc:16185].

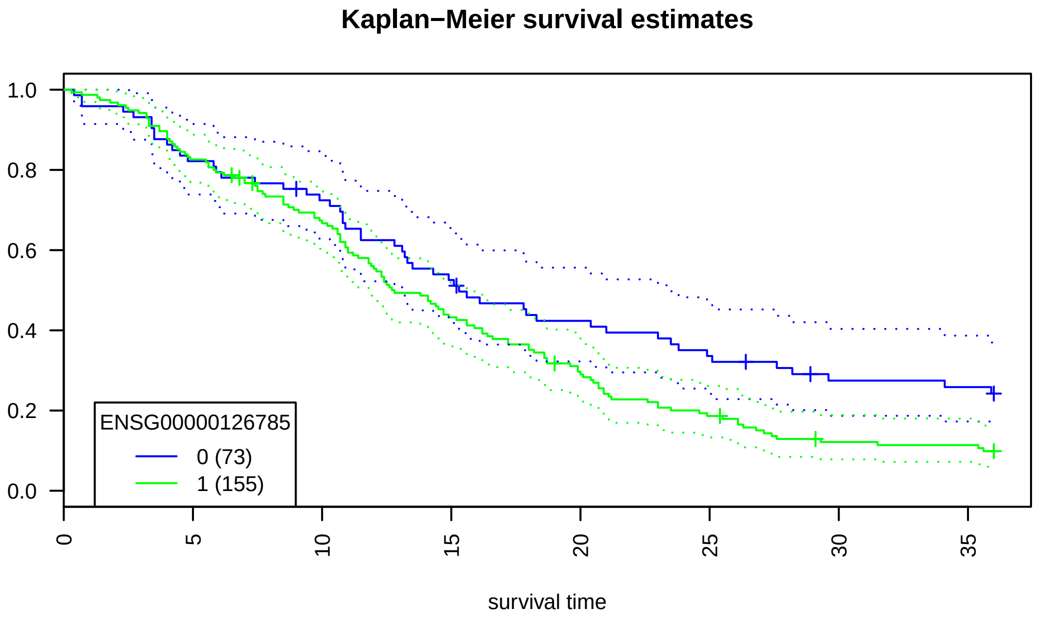

Figure 1583: Each curve represents the relative number of ENSG00000126785 survived to the corresponding point of time. Dashed lines represent 0.95 confidence intervals for the curves with the corresponding color. Total of 2 observations deleted due to missingness. Log-rank probability measure for the equality of these curves is 1.646242e-02. Additional annotations: *.GeneName*=RHOJ, *.GeneDesc*=ras homolog gene family, member J [Source:HGNC Symbol;Acc:688].

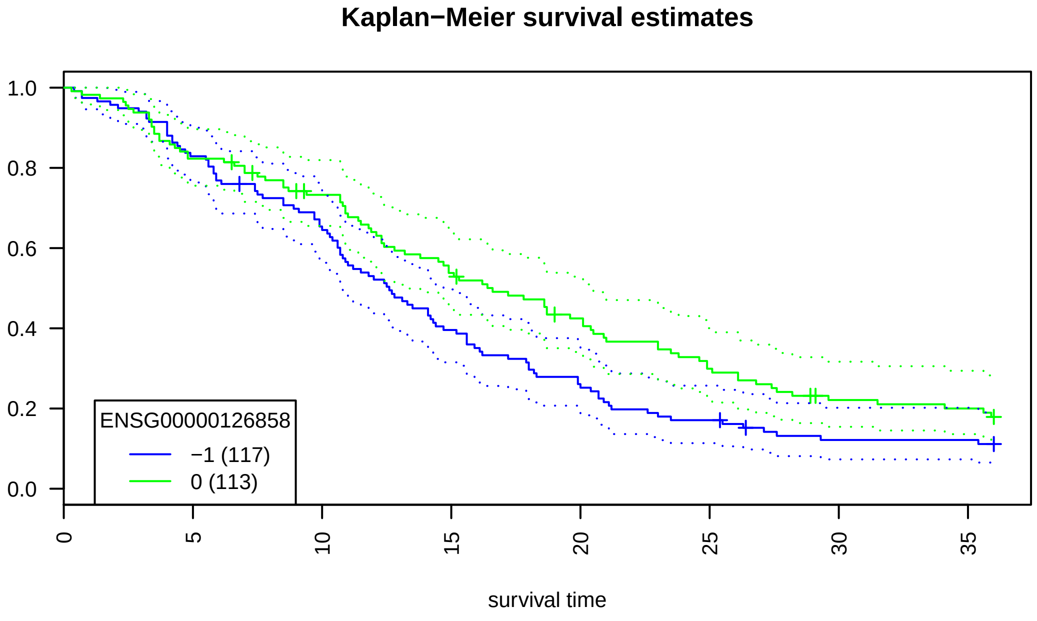

Figure 1584: Each curve represents the relative number of ENSG00000126858 survived to the corresponding point of time. Dashed lines represent 0.95 confidence intervals for the curves with the corresponding color. Log-rank probability measure for the equality of these curves is 2.115733e-02. Additional annotations: *.GeneName*=RHOT1, *.GeneDesc*=ras homolog gene family, member T1 [Source:HGNC Symbol;Acc:21168].

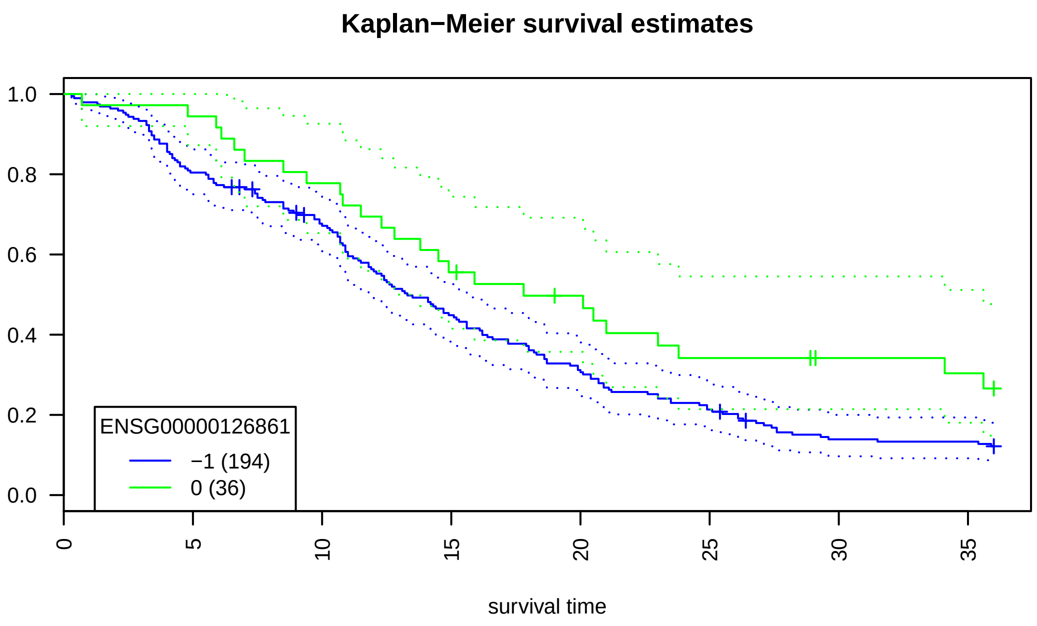

Figure 1585: Each curve represents the relative number of ENSG00000126861 survived to the corresponding point of time. Dashed lines represent 0.95 confidence intervals for the curves with the corresponding color. Log-rank probability measure for the equality of these curves is 2.922255e-02. Additional annotations: .*GeneName*=OMG, .*GeneDesc*=oligodendrocyte myelin glycoprotein [Source:HGNC Symbol;Acc:8135].

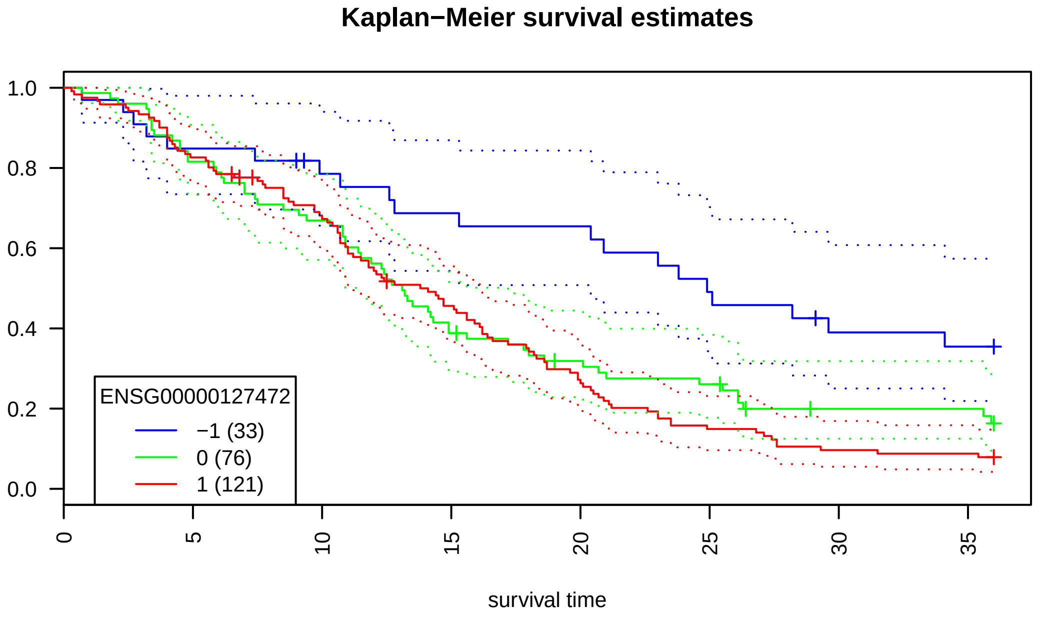

Figure 1586: Each curve represents the relative number of ENSG00000127472 survived to the corresponding point of time. Dashed lines represent 0.95 confidence intervals for the curves with the corresponding color. Log-rank probability measure for the equality of these curves is 2.054906e-03. Additional annotations: .*GeneName*=PLA2G5, .*GeneDesc*=phospholipase A2, group V [Source:HGNC Symbol;Acc:9038].

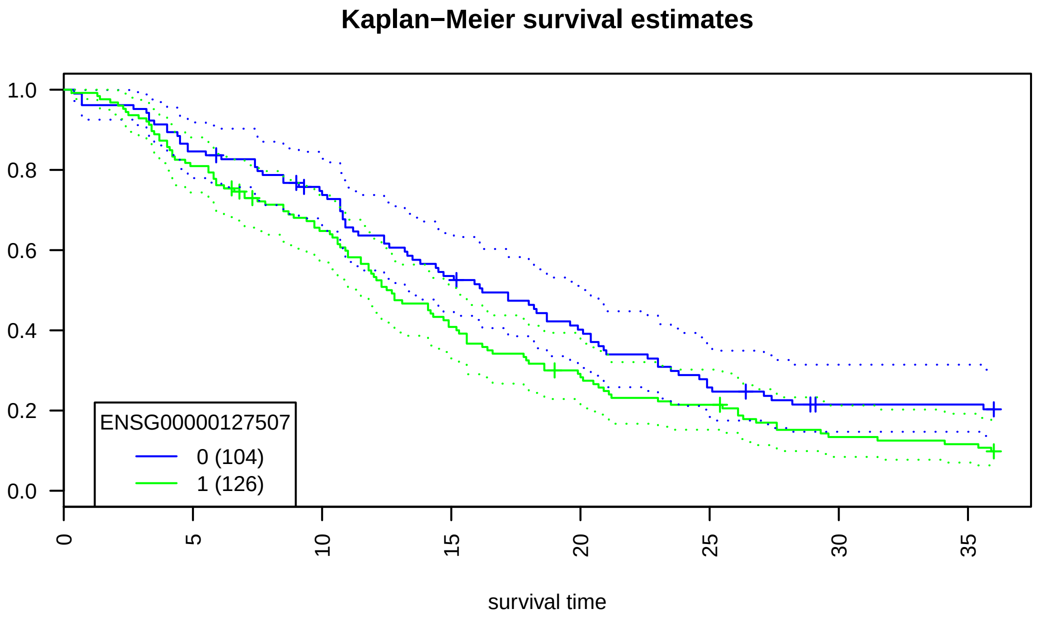

Figure 1587: Each curve represents the relative number of ENSG00000127507 survived to the corresponding point of time. Dashed lines represent 0.95 confidence intervals for the curves with the corresponding color. Log-rank probability measure for the equality of these curves is 3.176562e-02. Additional annotations: *.GeneName*=EMR2, *.GeneDesc*=egf-like module containing, mucin-like, hormone receptor-like 2 [Source:HGNC Symbol;Acc:3337].

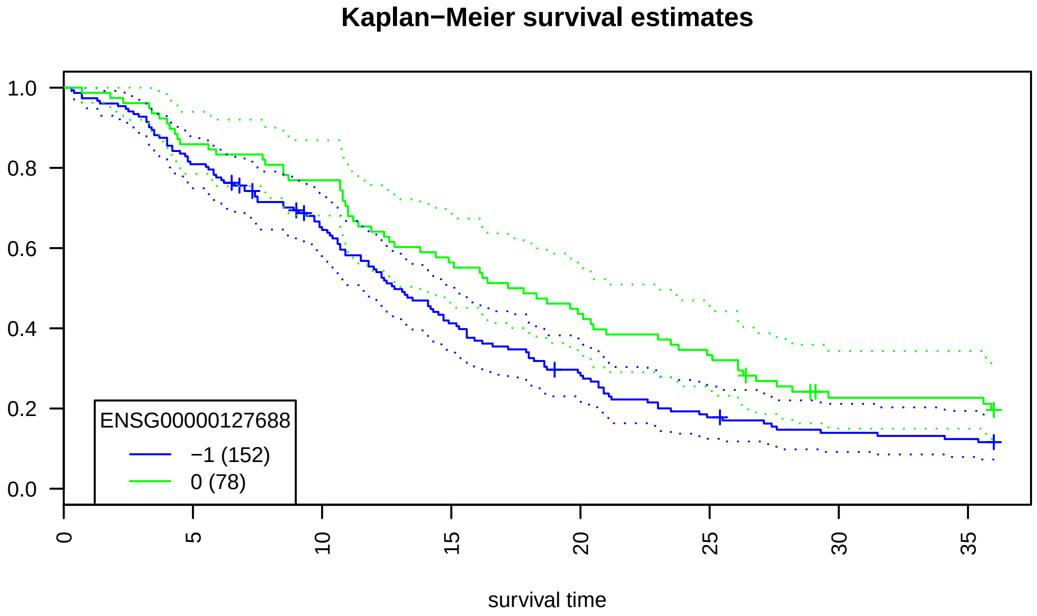

Figure 1588: Each curve represents the relative number of ENSG00000127688 survived to the corresponding point of time. Dashed lines represent 0.95 confidence intervals for the curves with the corresponding color. Log-rank probability measure for the equality of these curves is 1.993584e-02. Additional annotations: *.GeneName*=GAN, *.GeneDesc*=gigaxonin [Source:HGNC Symbol;Acc:4137].

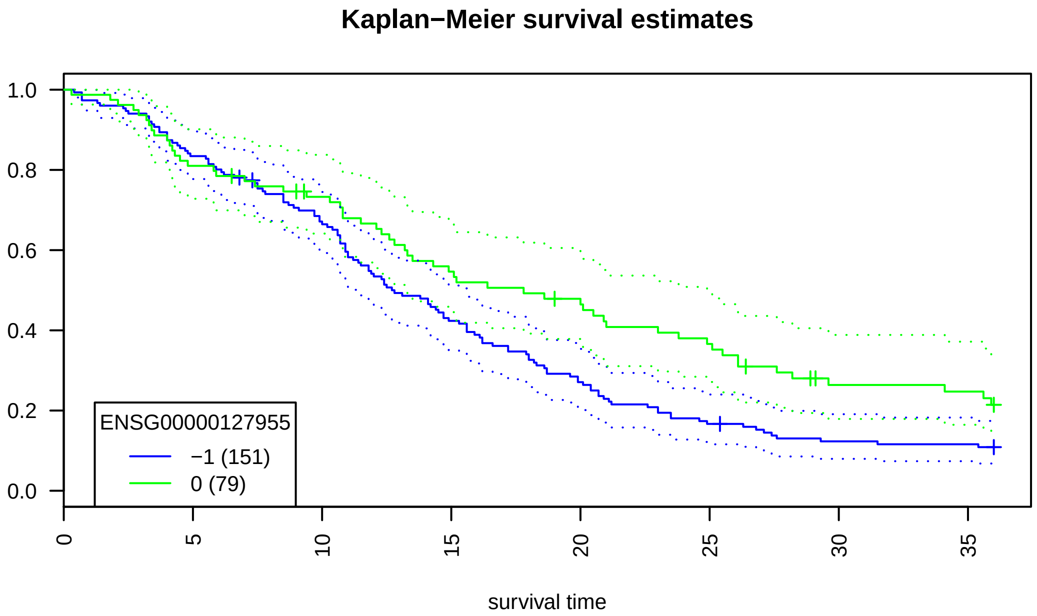

Figure 1589: Each curve represents the relative number of ENSG00000127955 survived to the corresponding point of time. Dashed lines represent 0.95 confidence intervals for the curves with the corresponding color. Log-rank probability measure for the equality of these curves is 1.235365e-02. Additional annotations: *.GeneName*=GNAI1, *.GeneDesc*=guanine nucleotide binding protein (G protein), alpha inhibiting activity polypeptide 1 [Source:HGNC Symbol;Acc:4384].

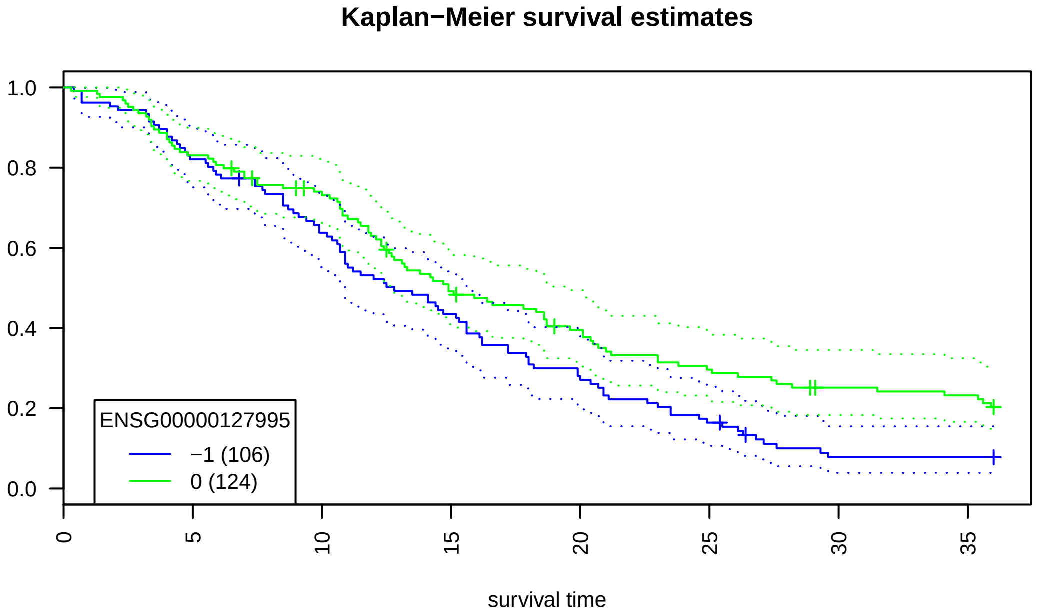

Figure 1590: Each curve represents the relative number of ENSG00000127995 survived to the corresponding point of time. Dashed lines represent 0.95 confidence intervals for the curves with the corresponding color. Log-rank probability measure for the equality of these curves is 1.219503e-02. Additional annotations: *.GeneName*=CASD1, *.GeneDesc*=CAS1 domain containing 1 [Source:HGNC Symbol;Acc:16014].

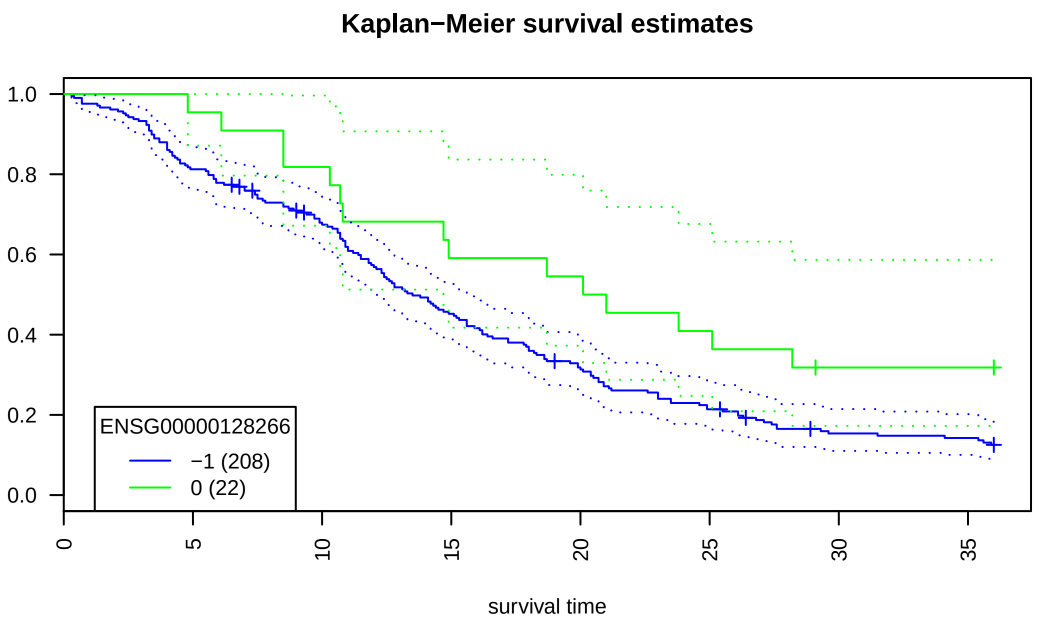

Figure 1591: Each curve represents the relative number of ENSG00000128266 survived to the corresponding point of time. Dashed lines represent 0.95 confidence intervals for the curves with the corresponding color. Log-rank probability measure for the equality of these curves is 3.199389e-02. Additional annotations: *.GeneName*=GNAZ, *.GeneDesc*=guanine nucleotide binding protein (G protein), alpha z polypeptide [Source:HGNC Symbol;Acc:4395].

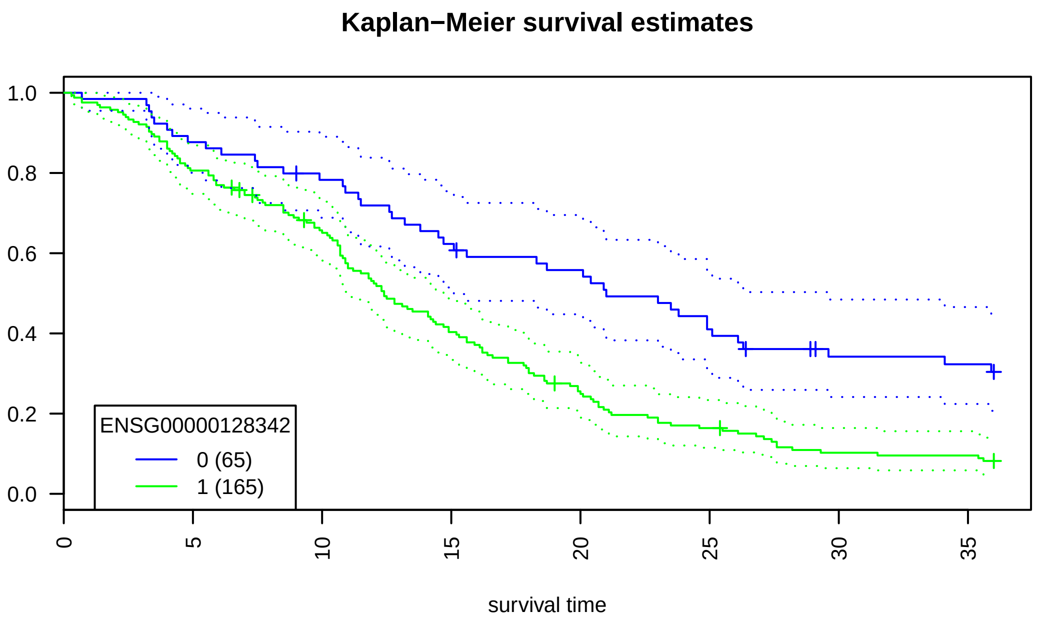

Figure 1592: Each curve represents the relative number of ENSG00000128342 survived to the corresponding point of time. Dashed lines represent 0.95 confidence intervals for the curves with the corresponding color. Log-rank probability measure for the equality of these curves is 2.41727e-05. Additional annotations: *.GeneName*=LIF, *.GeneDesc*=leukemia inhibitory factor (cholinergic differentiation factor) [Source:HGNC Symbol;Acc:6596].

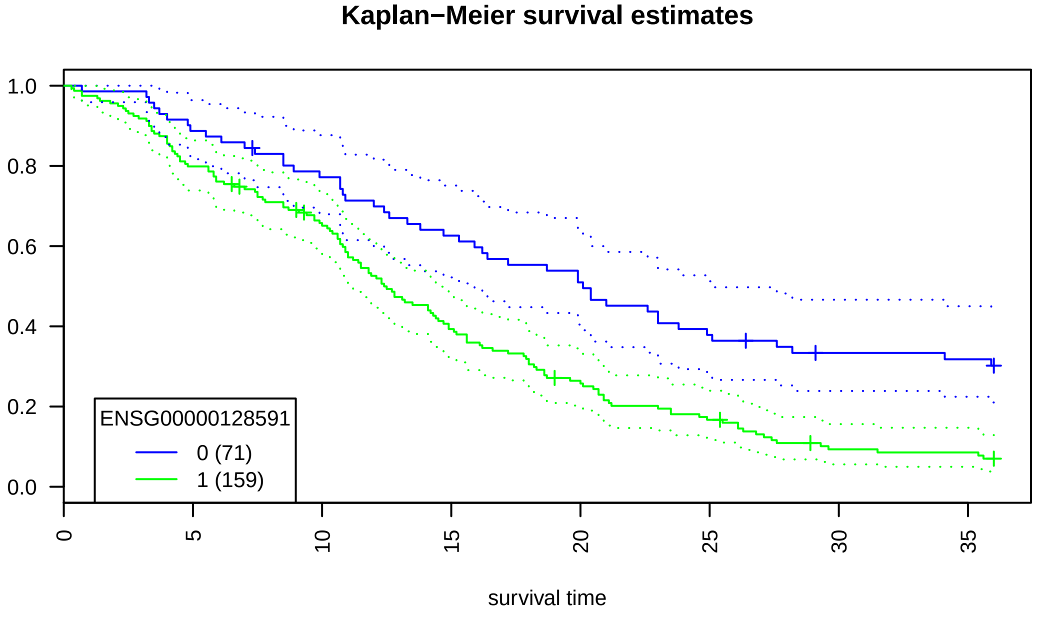

Figure 1593: Each curve represents the relative number of ENSG00000128591 survived to the corresponding point of time. Dashed lines represent 0.95 confidence intervals for the curves with the corresponding color. Log-rank probability measure for the equality of these curves is 1.991466e-05. Additional annotations: *.GeneName*=FLNC, *.GeneDesc*=filamin C, gamma [Source:HGNC Symbol;Acc:3756].

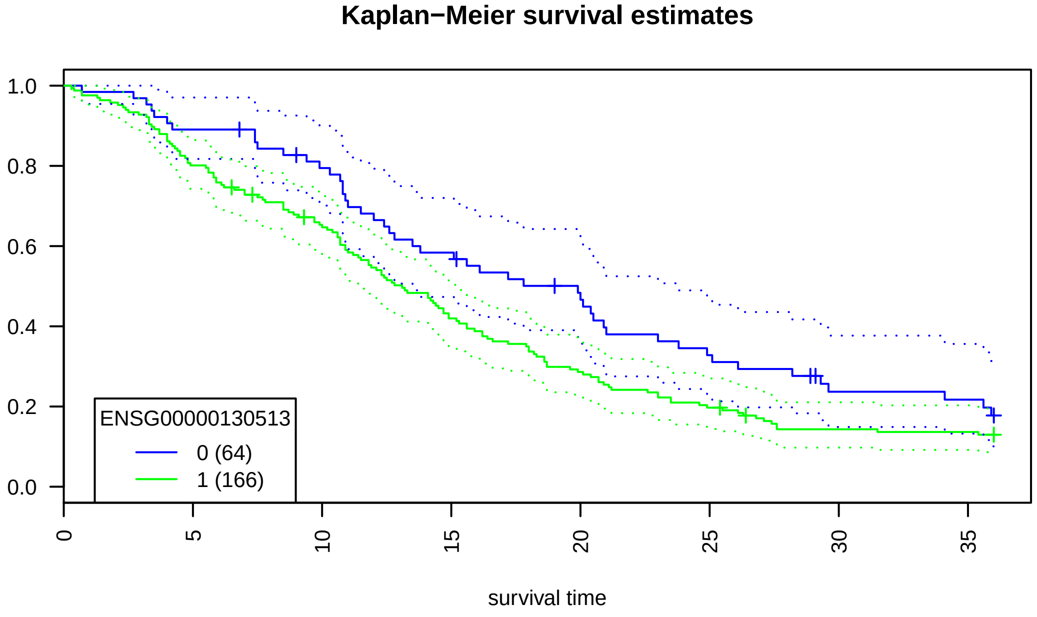

Figure 1594: Each curve represents the relative number of ENSG00000130513 survived to the corresponding point of time. Dashed lines represent 0.95 confidence intervals for the curves with the corresponding color. Log-rank probability measure for the equality of these curves is 3.578444e-02. Additional annotations: *.GeneName*=GDF15, *.GeneDesc*=growth differentiation factor 15 [Source:HGNC Symbol;Acc:30142].

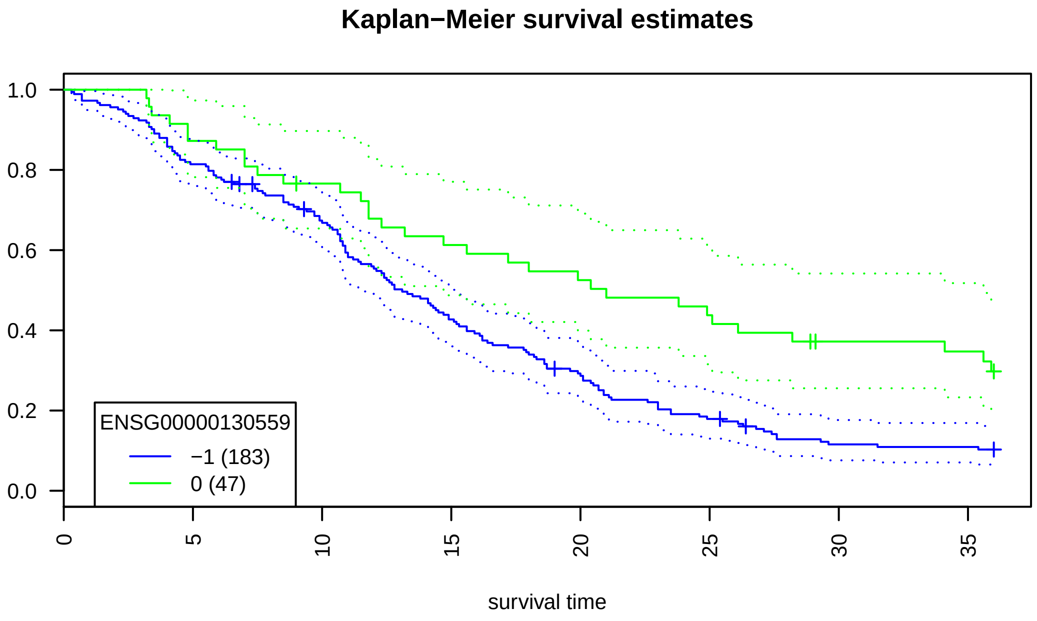

Figure 1595: Each curve represents the relative number of ENSG00000130559 survived to the corresponding point of time. Dashed lines represent 0.95 confidence intervals for the curves with the corresponding color. Log-rank probability measure for the equality of these curves is 9.376781e-04. Additional annotations: *.GeneName*=CAMSAP1, *.GeneDesc*=calmodulin regulated spectrin-associated protein 1 [Source:HGNC Symbol;Acc:19946].

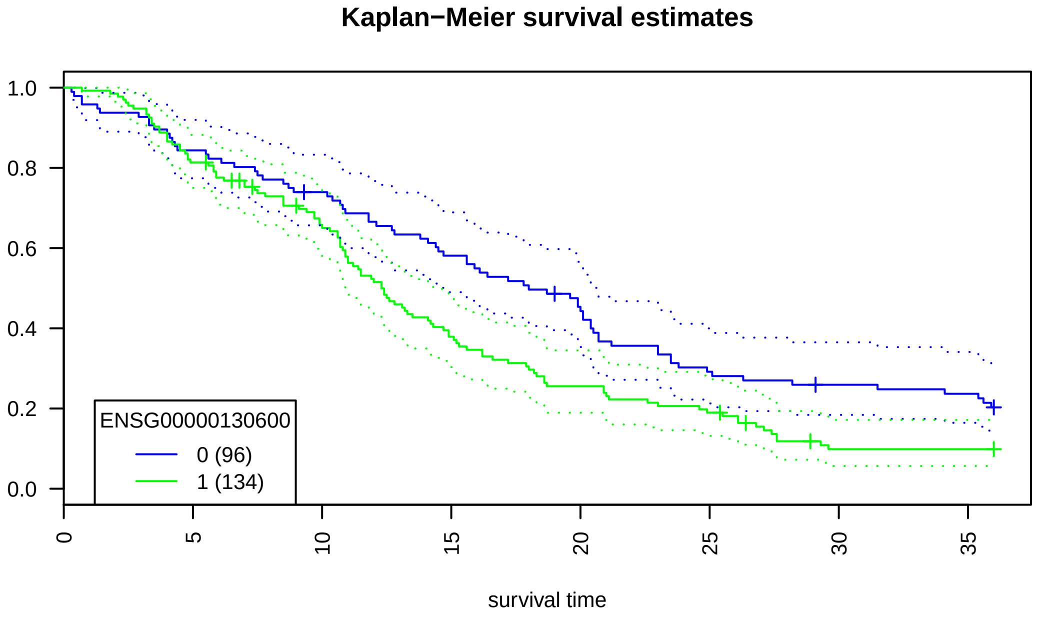

Figure 1596: Each curve represents the relative number of ENSG00000130600 survived to the corresponding point of time. Dashed lines represent 0.95 confidence intervals for the curves with the corresponding color. Log-rank probability measure for the equality of these curves is 6.144802e-03. Additional annotations: *.GeneName*=H19, *.GeneDesc*=microRNA 675 [Source:HGNC Symbol;Acc:33351].

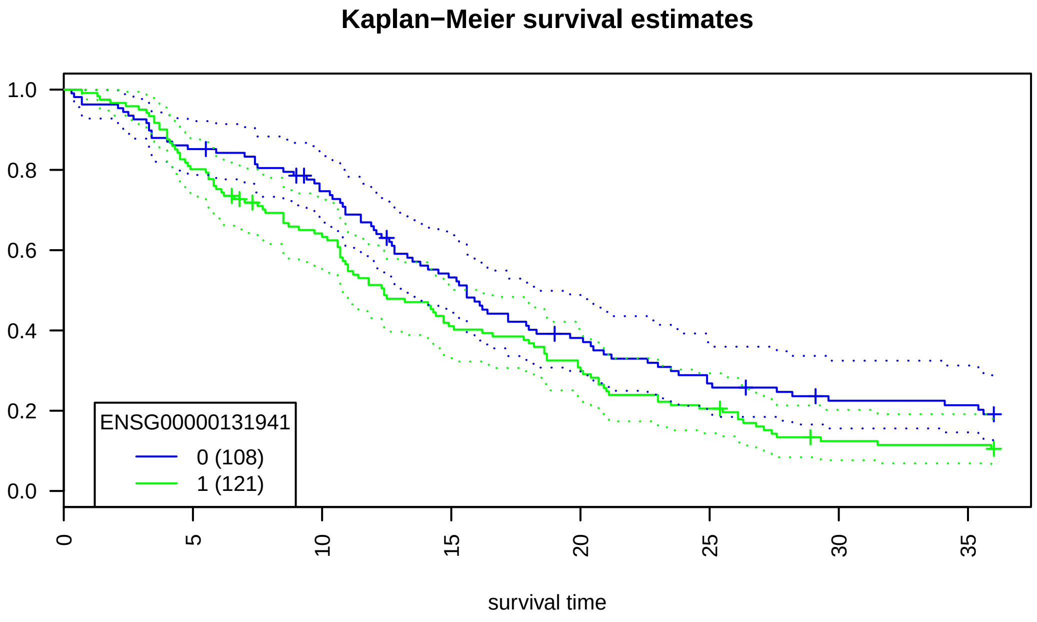

Figure 1597: Each curve represents the relative number of ENSG00000131941 survived to the corresponding point of time. Dashed lines represent 0.95 confidence intervals for the curves with the corresponding color. Total of 1 observation deleted due to missingness. Log-rank probability measure for the equality of these curves is 4.921426e-02. Additional annotations: *.GeneName*=RHPN2, *.GeneDesc*=rhophilin, Rho GTPase binding protein 2 [Source:HGNC Symbol;Acc:19974].

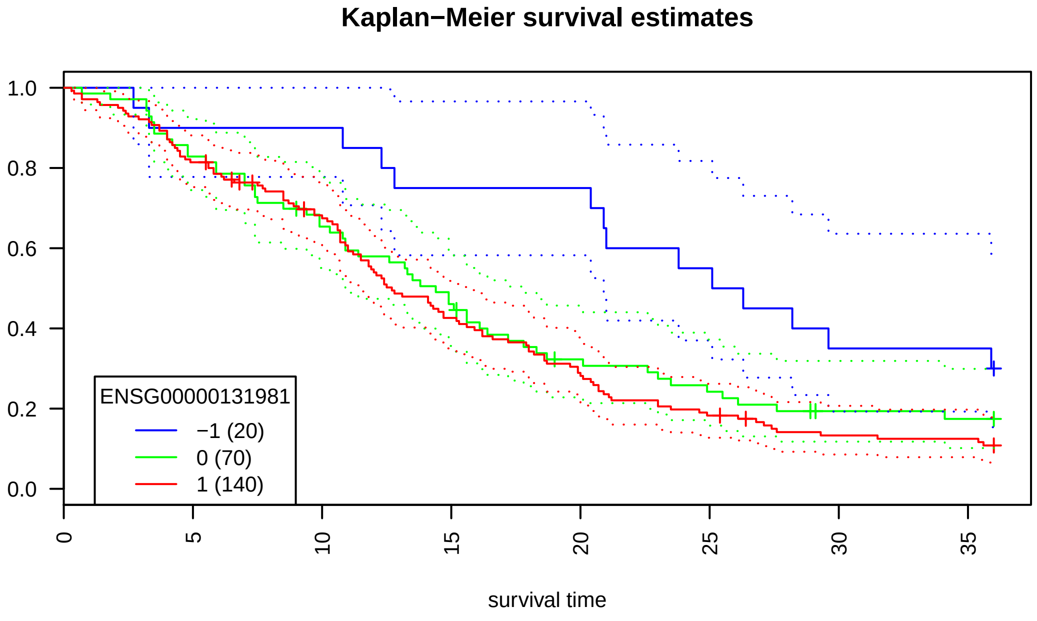

Figure 1598: Each curve represents the relative number of ENSG00000131981 survived to the corresponding point of time. Dashed lines represent 0.95 confidence intervals for the curves with the corresponding color. Log-rank probability measure for the equality of these curves is 1.805361e-02. Additional annotations: *.GeneName*=LGALS3, *.GeneDesc*=lectin, galactoside-binding, soluble, 3 [Source:HGNC Symbol;Acc:6563].

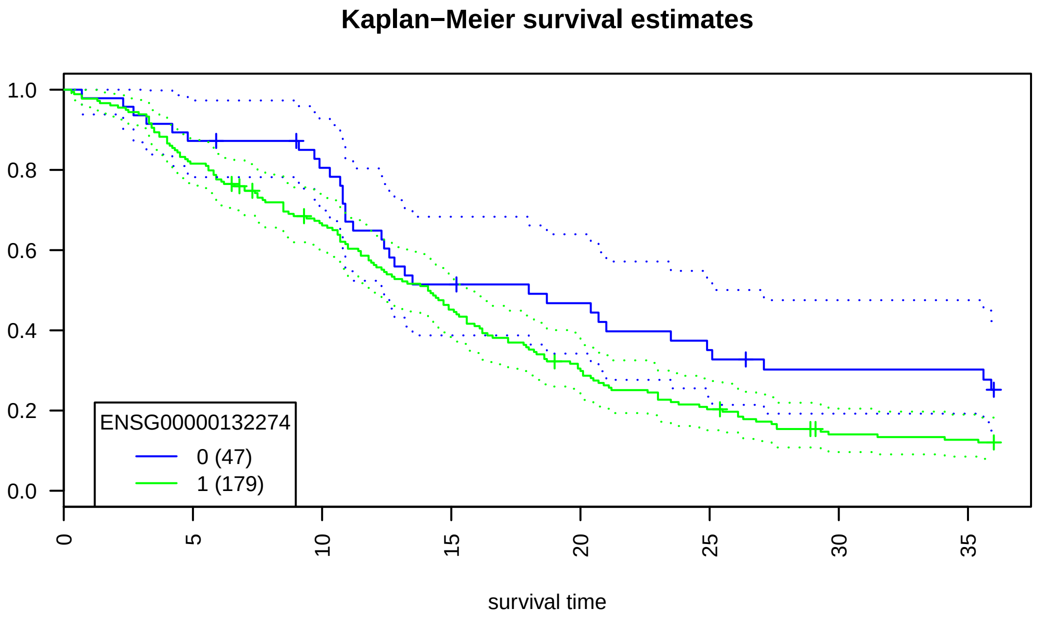

Figure 1599: Each curve represents the relative number of ENSG00000132274 survived to the corresponding point of time. Dashed lines represent 0.95 confidence intervals for the curves with the corresponding color. Total of 4 observations deleted due to missingness. Log-rank probability measure for the equality of these curves is 3.329186e-02. Additional annotations: *.GeneName*=TRIM22, *.GeneDesc*=tripartite motif-containing 22 [Source:HGNC Symbol;Acc:16379].

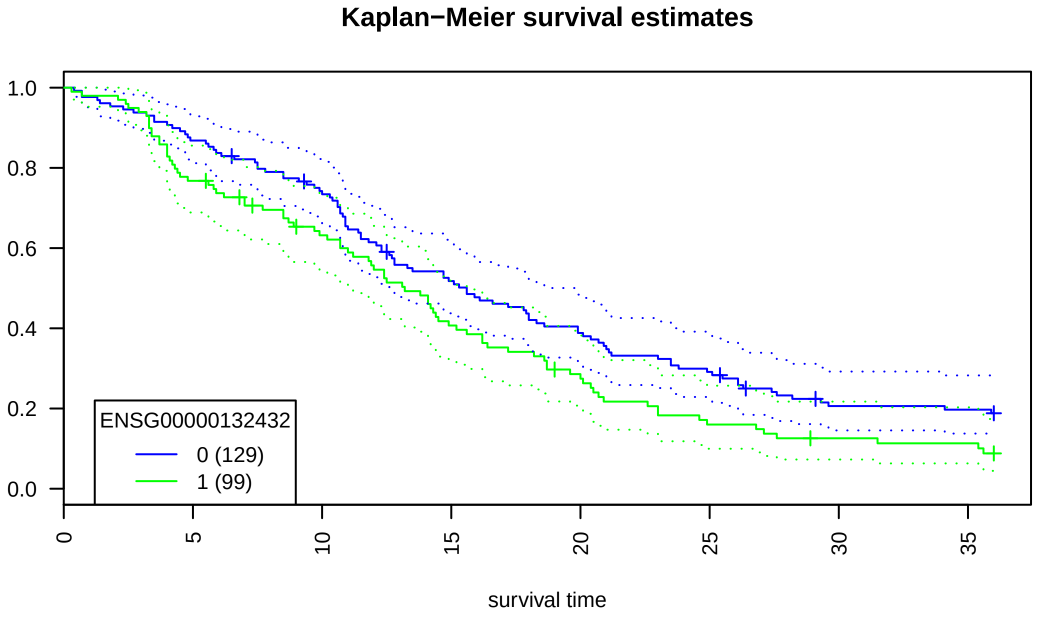

Figure 1600: Each curve represents the relative number of ENSG00000132432 survived to the corresponding point of time. Dashed lines represent 0.95 confidence intervals for the curves with the corresponding color. Total of 2 observations deleted due to missingness. Log-rank probability measure for the equality of these curves is 2.309038e-02. Additional annotations: *.GeneName*=SEC61G, *.GeneDesc*=Sec61 gamma subunit [Source:HGNC Symbol;Acc:18277].

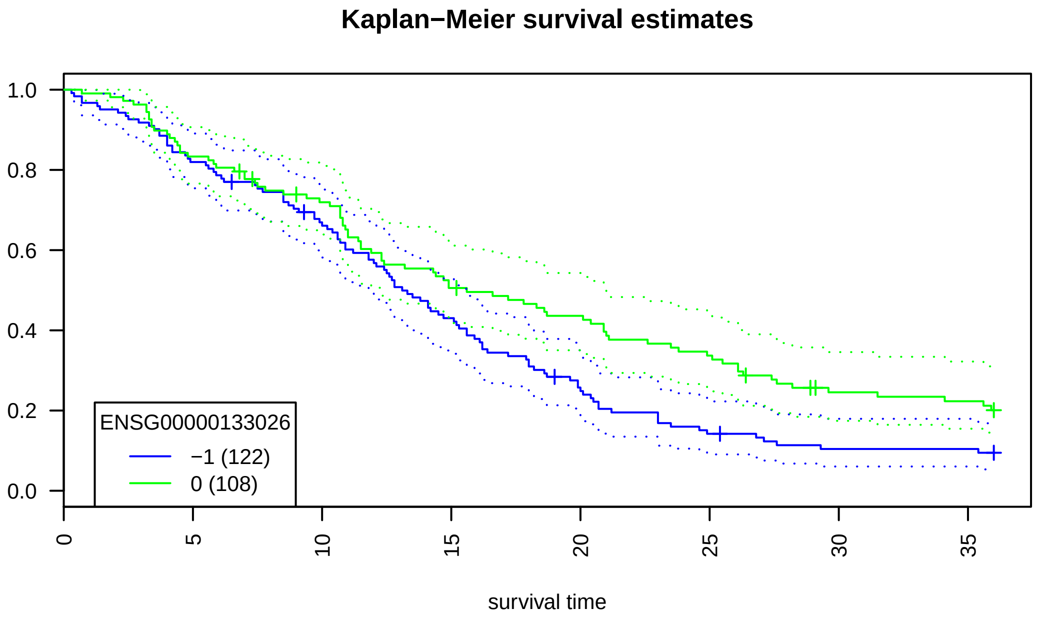

Figure 1601: Each curve represents the relative number of ENSG00000133026 survived to the corresponding point of time. Dashed lines represent 0.95 confidence intervals for the curves with the corresponding color. Log-rank probability measure for the equality of these curves is 1.049202e-02. Additional annotations: *.GeneName*=MYH10, *.GeneDesc*=myosin, heavy chain 10, non-muscle [Source:HGNC Symbol;Acc:7568].

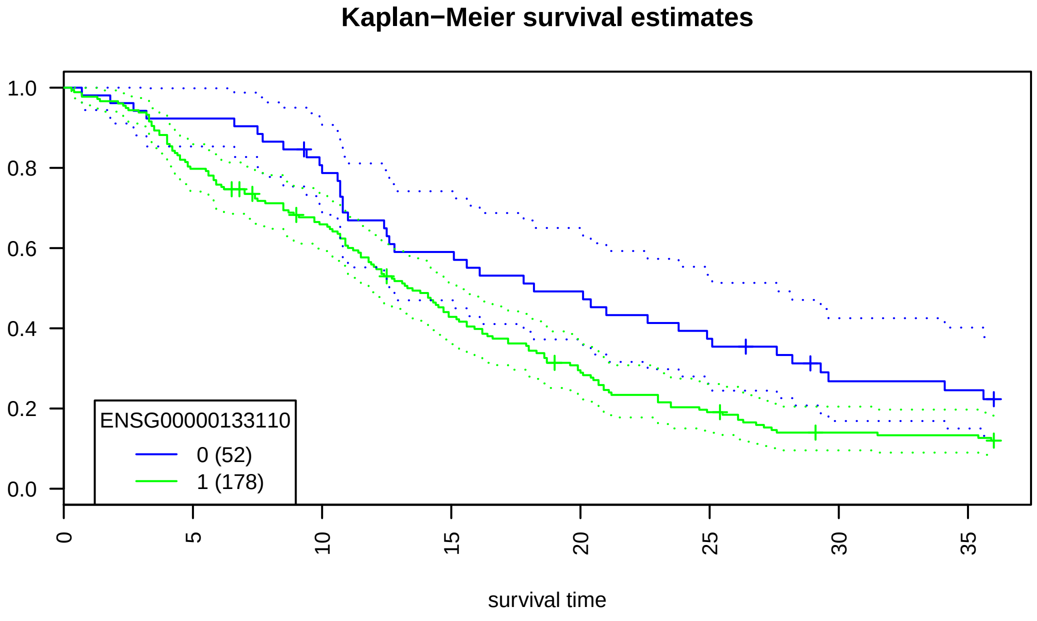

Figure 1602: Each curve represents the relative number of ENSG00000133110 survived to the corresponding point of time. Dashed lines represent 0.95 confidence intervals for the curves with the corresponding color. Log-rank probability measure for the equality of these curves is 1.44264e-02. Additional annotations: *.GeneName*=POSTN, *.GeneDesc*=periostin, osteoblast specific factor [Source:HGNC Symbol;Acc:16953].

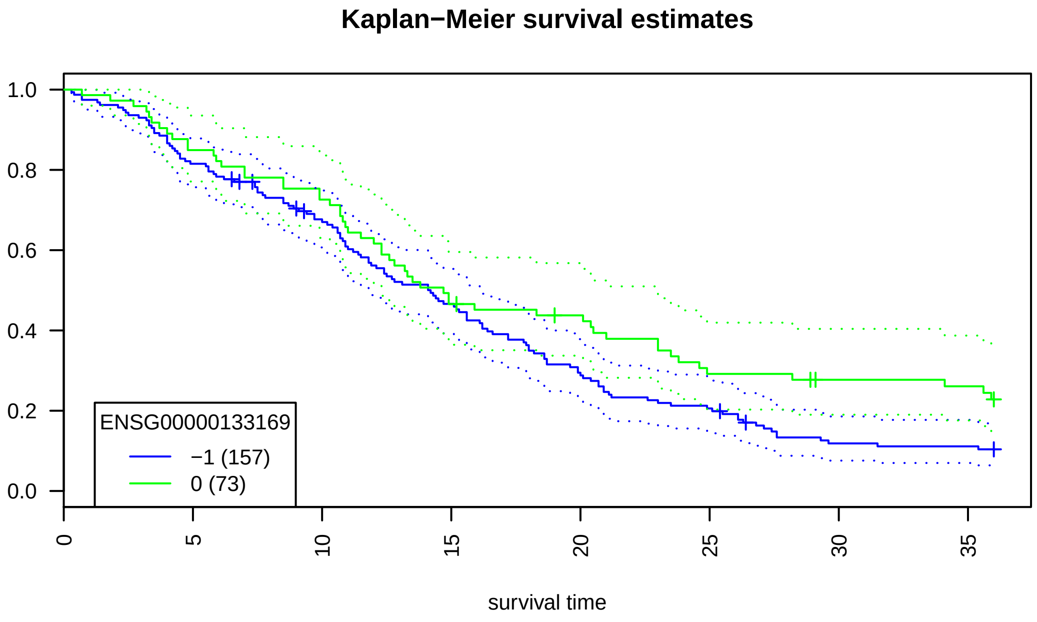

Figure 1603: Each curve represents the relative number of ENSG00000133169 survived to the corresponding point of time. Dashed lines represent 0.95 confidence intervals for the curves with the corresponding color. Log-rank probability measure for the equality of these curves is 3.761947e-02. Additional annotations: *.GeneName*=BEX1, *.GeneDesc*=brain expressed, X-linked 1 [Source:HGNC Symbol;Acc:1036].

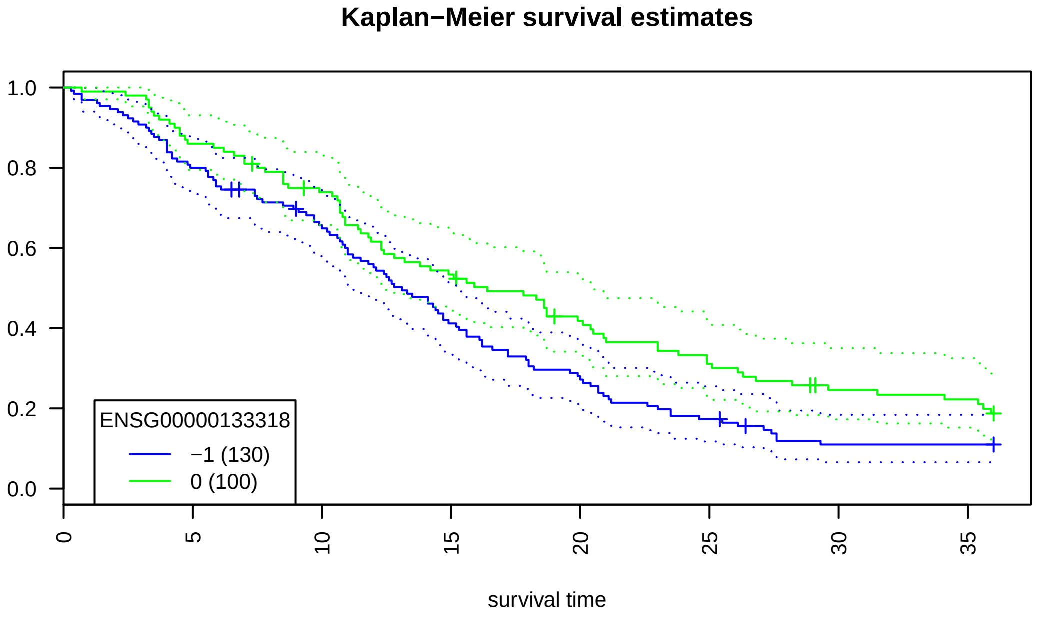

Figure 1604: Each curve represents the relative number of ENSG00000133318 survived to the corresponding point of time. Dashed lines represent 0.95 confidence intervals for the curves with the corresponding color. Log-rank probability measure for the equality of these curves is 1.87713e-02. Additional annotations: *.GeneName*=RTN3, *.GeneDesc*=reticulon 3 [Source:HGNC Symbol;Acc:10469].

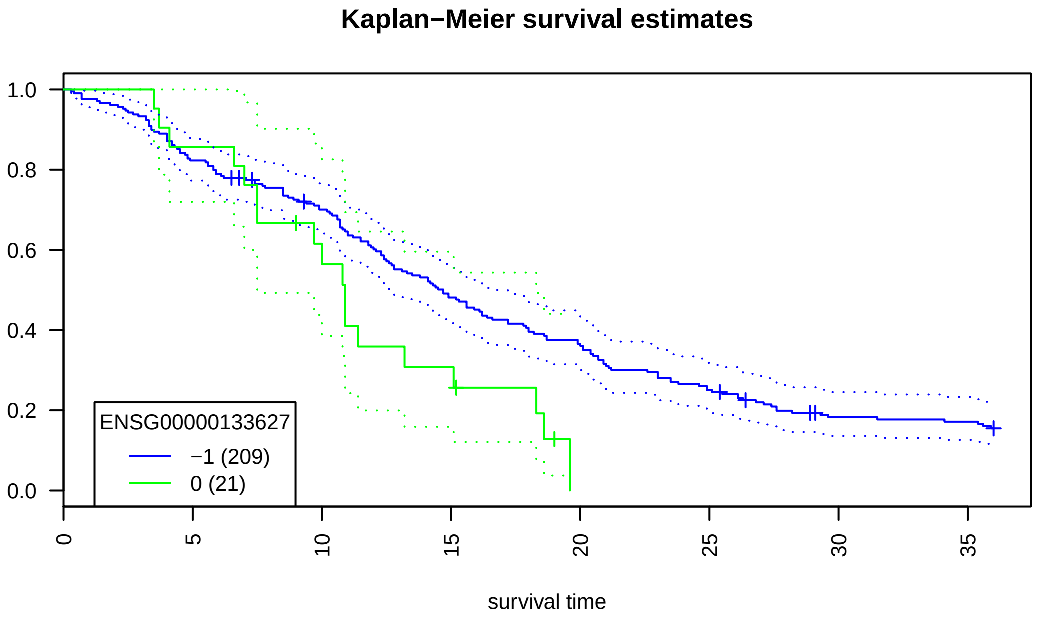

Figure 1605: Each curve represents the relative number of ENSG00000133627 survived to the corresponding point of time. Dashed lines represent 0.95 confidence intervals for the curves with the corresponding color. Log-rank probability measure for the equality of these curves is 1.729027e-02. Additional annotations: *.GeneName*=ACTR3B, *.GeneDesc*=ARP3 actin-related protein 3 homolog B (yeast) [Source:HGNC Symbol;Acc:17256].

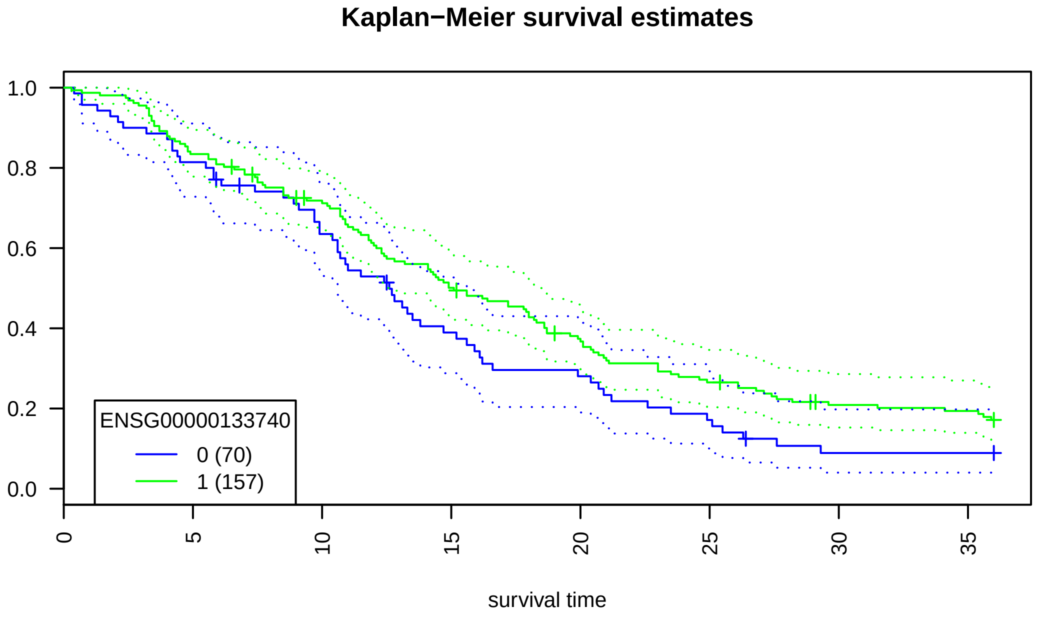

Figure 1606: Each curve represents the relative number of ENSG00000133740 survived to the corresponding point of time. Dashed lines represent 0.95 confidence intervals for the curves with the corresponding color. Total of 3 observations deleted due to missingness. Log-rank probability measure for the equality of these curves is 4.689818e-02. Additional annotations: *.GeneName*=E2F5, *.GeneDesc*=E2F transcription factor 5, p130-binding [Source:HGNC Symbol;Acc:3119].

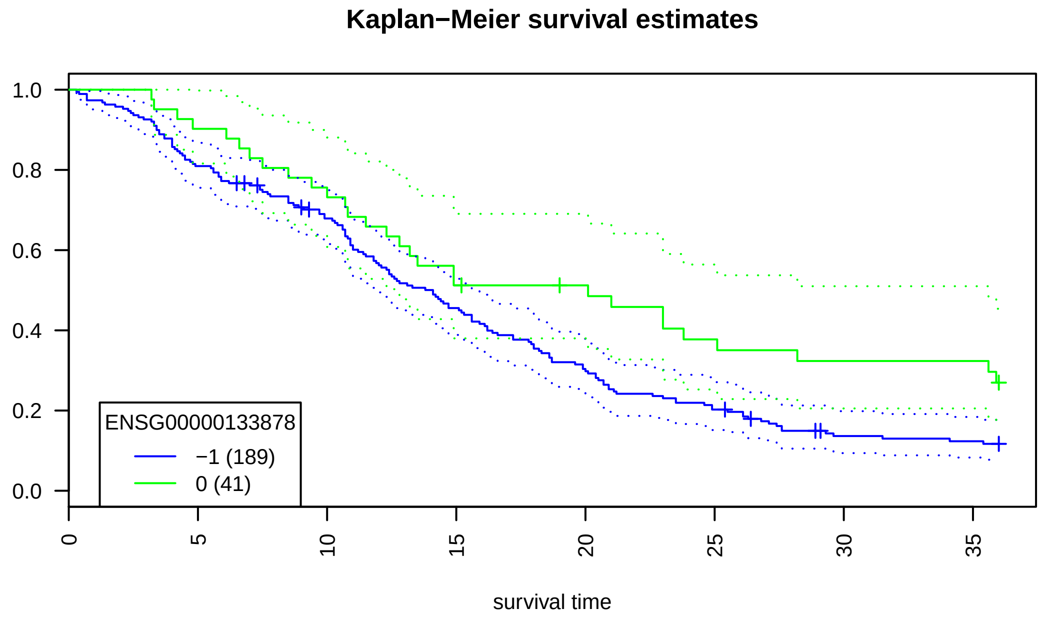

Figure 1607: Each curve represents the relative number of ENSG00000133878 survived to the corresponding point of time. Dashed lines represent 0.95 confidence intervals for the curves with the corresponding color. Log-rank probability measure for the equality of these curves is 1.980215e-02. Additional annotations: *.GeneName*=DUSP26, *.GeneDesc*=dual specificity phosphatase 26 (putative) [Source:HGNC Symbol;Acc:28161].

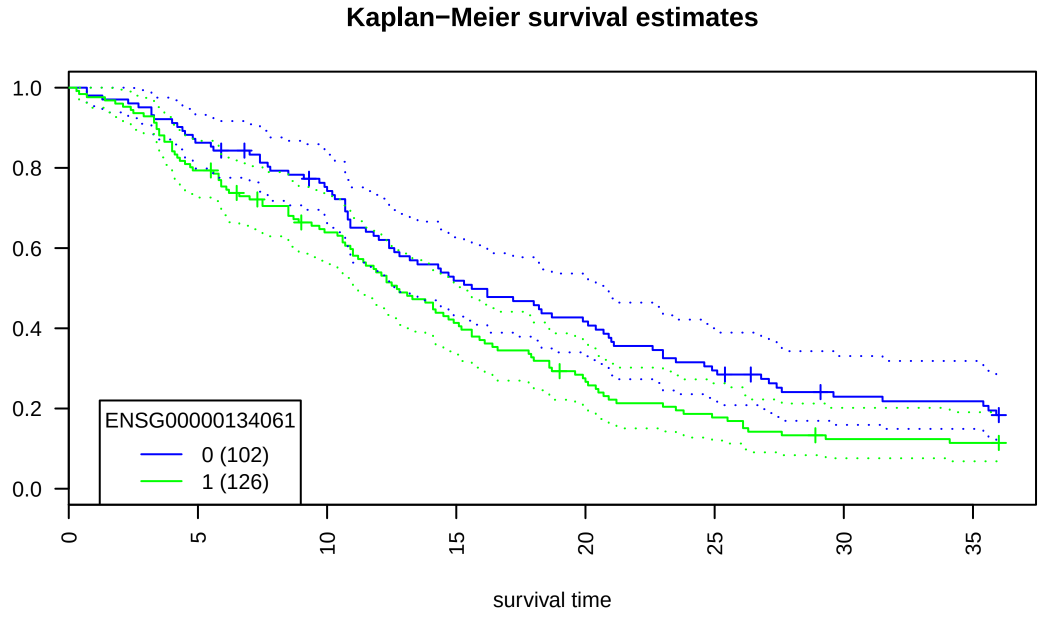

Figure 1608: Each curve represents the relative number of ENSG00000134061 survived to the corresponding point of time. Dashed lines represent 0.95 confidence intervals for the curves with the corresponding color. Total of 2 observations deleted due to missingness. Log-rank probability measure for the equality of these curves is 2.755332e-02. Additional annotations: *.GeneName*=CD180, *.GeneDesc*=CD180 molecule [Source:HGNC Symbol;Acc:6726].

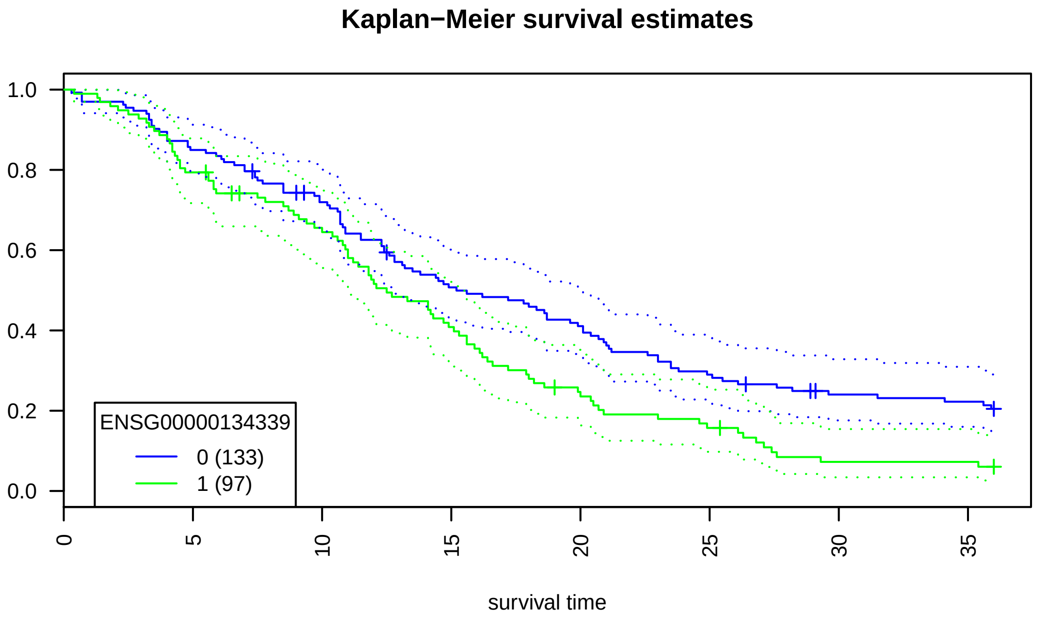

Figure 1609: Each curve represents the relative number of ENSG00000134339 survived to the corresponding point of time. Dashed lines represent 0.95 confidence intervals for the curves with the corresponding color. Log-rank probability measure for the equality of these curves is 2.819089e-03. Additional annotations: *.GeneName*=SAA2, *.GeneDesc*=serum amyloid A2 [Source:HGNC Symbol;Acc:10514].

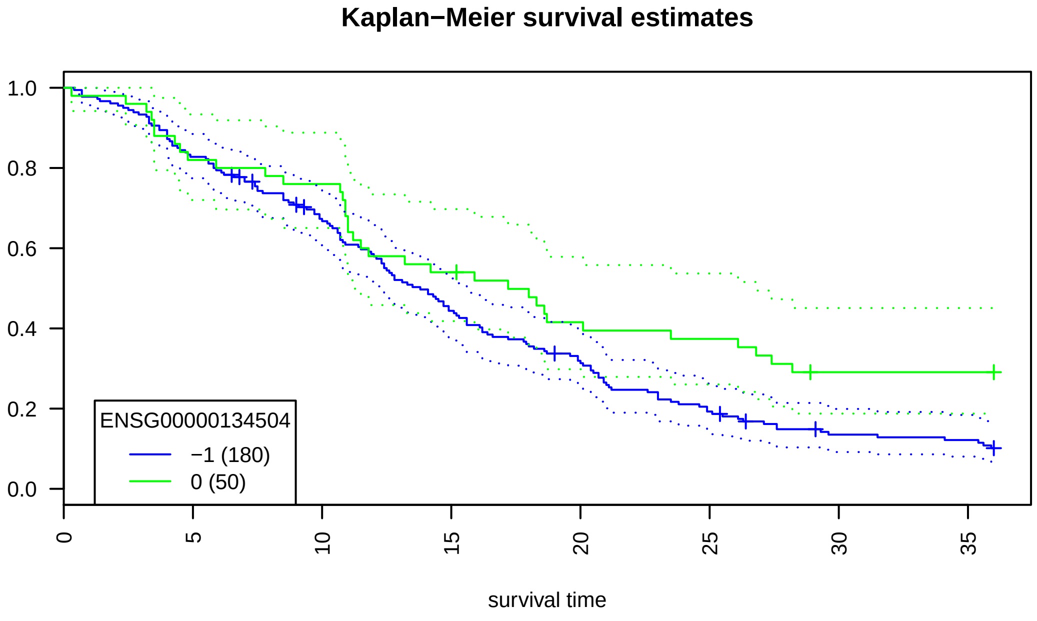

Figure 1610: Each curve represents the relative number of ENSG00000134504 survived to the corresponding point of time. Dashed lines represent 0.95 confidence intervals for the curves with the corresponding color. Log-rank probability measure for the equality of these curves is 1.621379e-02. Additional annotations: *.GeneName*=KCTD1, *.GeneDesc*=potassium channel tetramerisation domain containing 1 [Source:HGNC Symbol;Acc:18249].

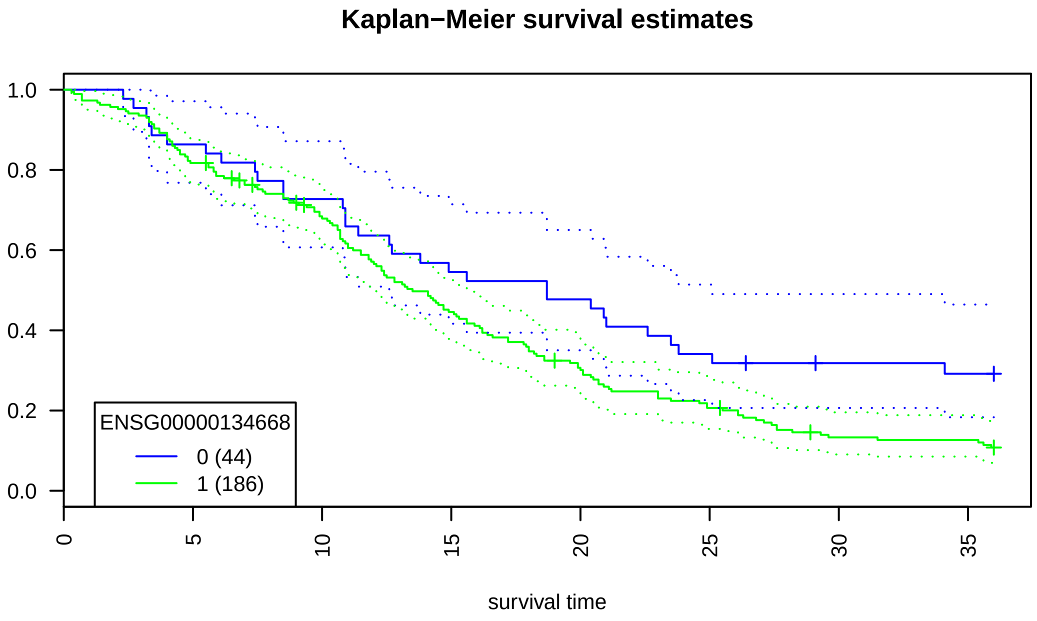

Figure 1611: Each curve represents the relative number of ENSG00000134668 survived to the corresponding point of time. Dashed lines represent 0.95 confidence intervals for the curves with the corresponding color. Log-rank probability measure for the equality of these curves is 1.810981e-02. Additional annotations: *.GeneName*=SPOCD1, *.GeneDesc*=SPOC domain containing 1 [Source:HGNC Symbol;Acc:26338].

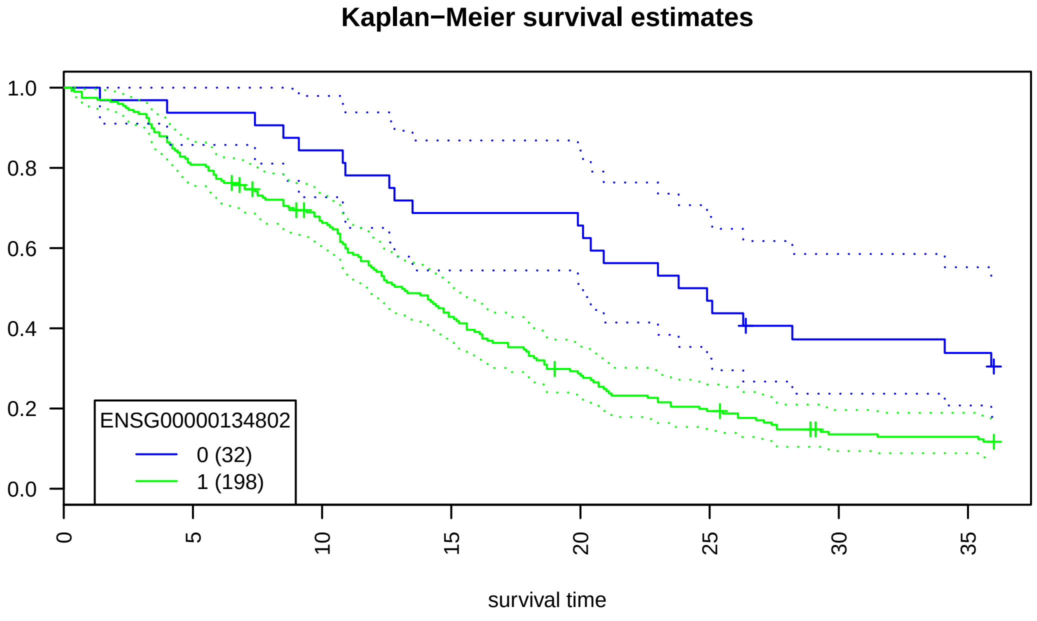

Figure 1612: Each curve represents the relative number of ENSG00000134802 survived to the corresponding point of time. Dashed lines represent 0.95 confidence intervals for the curves with the corresponding color. Log-rank probability measure for the equality of these curves is 1.296072e-03. Additional annotations: *.GeneName*=SLC43A3, *.GeneDesc*=solute carrier family 43, member 3 [Source:HGNC Symbol;Acc:17466].

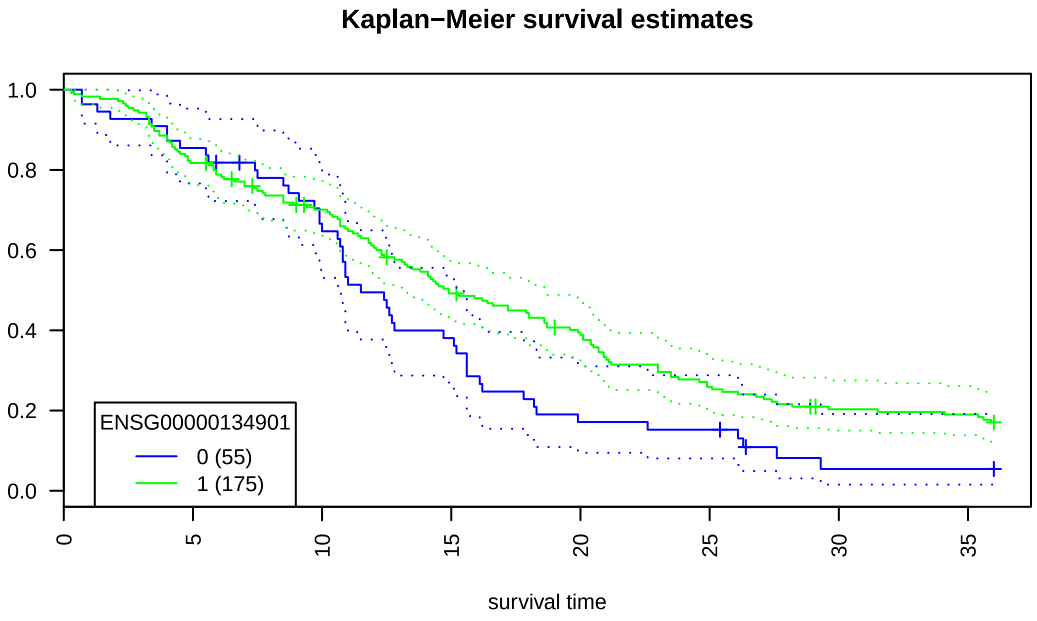

Figure 1613: Each curve represents the relative number of ENSG00000134901 survived to the corresponding point of time. Dashed lines represent 0.95 confidence intervals for the curves with the corresponding color. Log-rank probability measure for the equality of these curves is 1.571178e-02. Additional annotations: *.GeneName*=KDELC1, *.GeneDesc*=KDEL (Lys-Asp-Glu-Leu) containing 1 [Source:HGNC Symbol;Acc:19350].

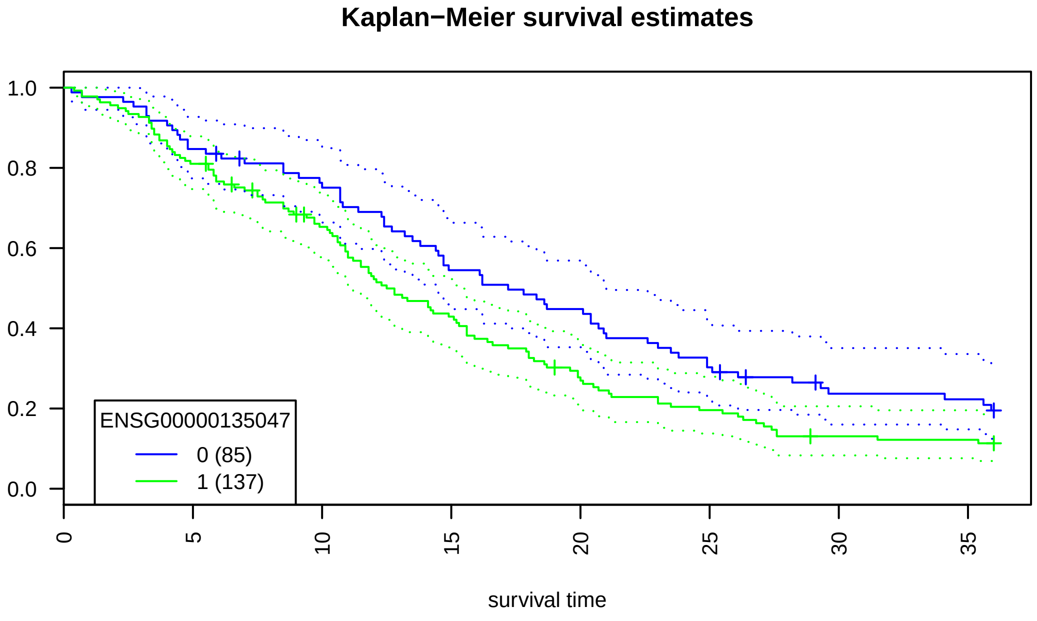

Figure 1614: Each curve represents the relative number of ENSG00000135047 survived to the corresponding point of time. Dashed lines represent 0.95 confidence intervals for the curves with the corresponding color. Total of 8 observations deleted due to missingness. Log-rank probability measure for the equality of these curves is 1.844830e-02. Additional annotations: *.GeneName*=CTSL1, *.GeneDesc*=cathepsin L1 [Source:HGNC Symbol;Acc:2537].

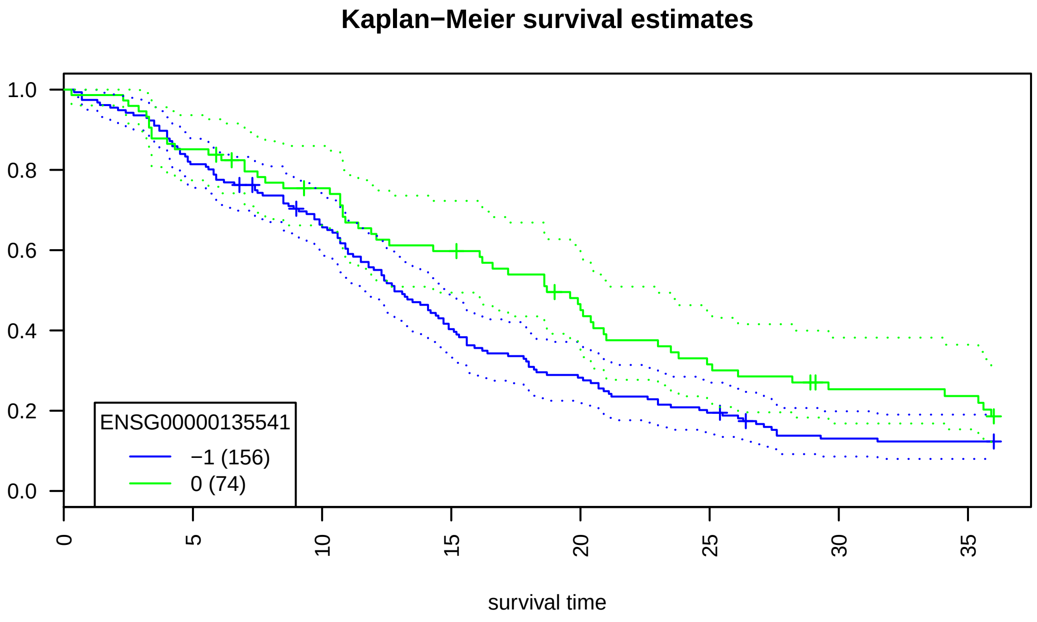

Figure 1615: Each curve represents the relative number of ENSG00000135541 survived to the corresponding point of time. Dashed lines represent 0.95 confidence intervals for the curves with the corresponding color. Log-rank probability measure for the equality of these curves is 3.040279e-02. Additional annotations: *.GeneName*=AHI1, *.GeneDesc*=Abelson helper integration site 1 [Source:HGNC Symbol;Acc:21575].

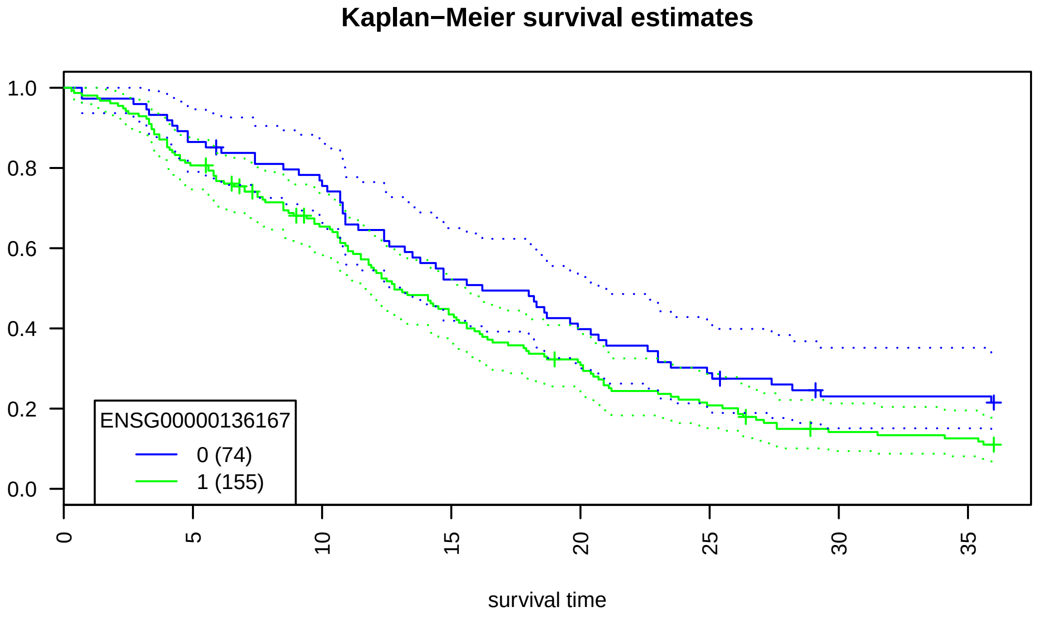

Figure 1616: Each curve represents the relative number of ENSG00000136167 survived to the corresponding point of time. Dashed lines represent 0.95 confidence intervals for the curves with the corresponding color. Total of 1 observation deleted due to missingness. Log-rank probability measure for the equality of these curves is 4.119938e-02. Additional annotations: *.GeneName*=LCP1, *.GeneDesc*=lymphocyte cytosolic protein 1 (L-plastin) [Source:HGNC Symbol;Acc:6528].

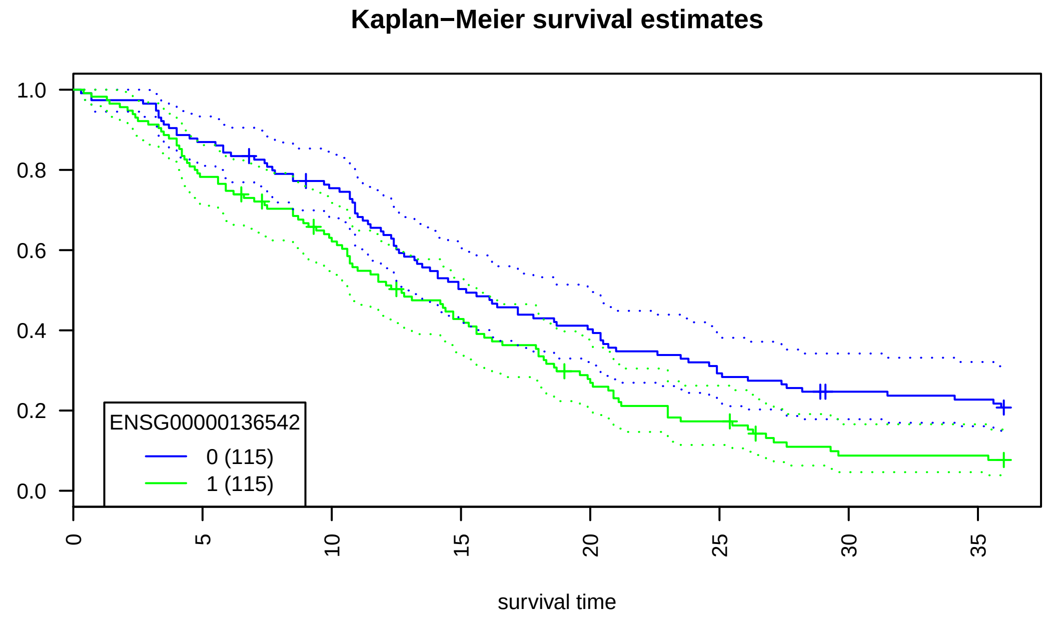

Figure 1617: Each curve represents the relative number of ENSG00000136542 survived to the corresponding point of time. Dashed lines represent 0.95 confidence intervals for the curves with the corresponding color. Log-rank probability measure for the equality of these curves is 5.558051e-03. Additional annotations: *.GeneName*=GALNT5, *.GeneDesc*=UDP-N-acetyl-alpha-D-galactosamine:polypeptide N-acetylgalactosaminyltransferase 5 (GalNAc-T5) [Source:HGNC Symbol;Acc:4127].

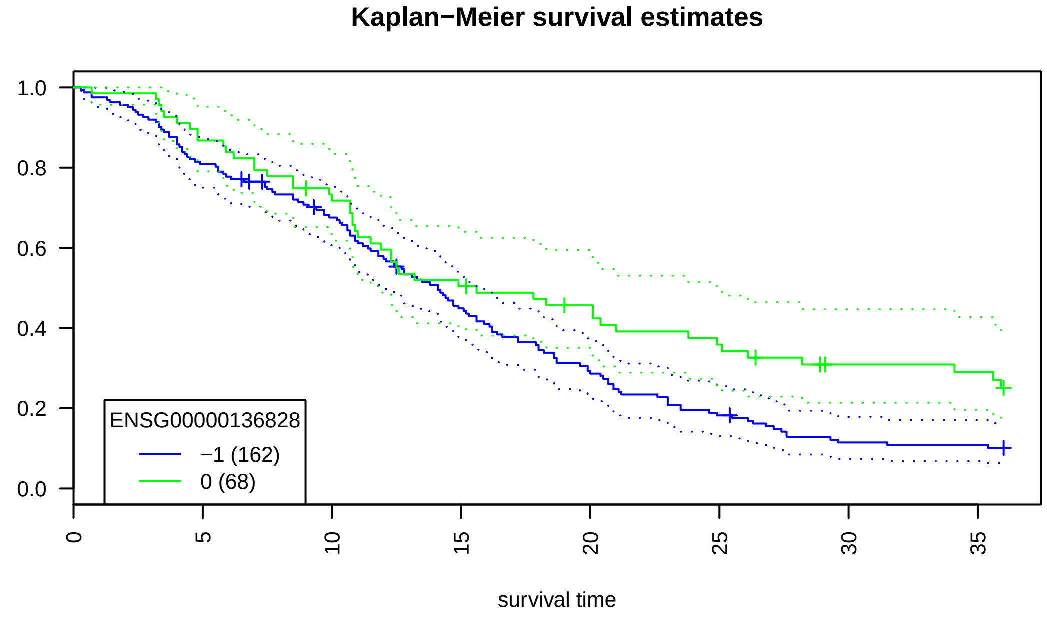

Figure 1618: Each curve represents the relative number of ENSG00000136828 survived to the corresponding point of time. Dashed lines represent 0.95 confidence intervals for the curves with the corresponding color. Log-rank probability measure for the equality of these curves is 1.307145e-02. Additional annotations: *.GeneName*=RALGPS1, *.GeneDesc*=Ral GEF with PH domain and SH3 binding motif 1 [Source:HGNC Symbol;Acc:16851].

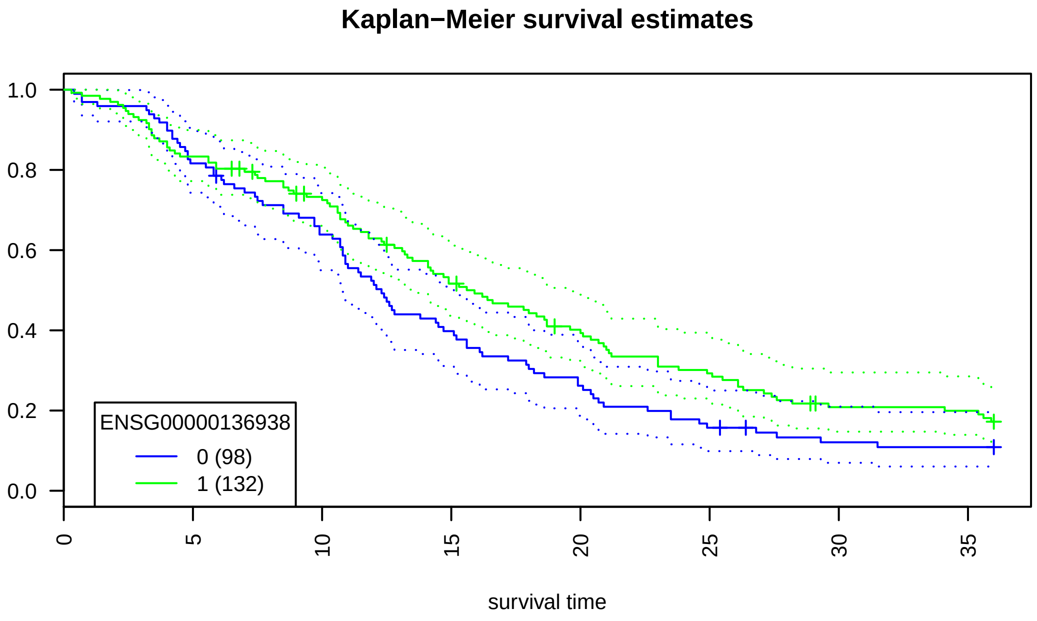

Figure 1619: Each curve represents the relative number of ENSG00000136938 survived to the corresponding point of time. Dashed lines represent 0.95 confidence intervals for the curves with the corresponding color. Log-rank probability measure for the equality of these curves is 3.908306e-02. Additional annotations: *.GeneName*=ANP32B, *.GeneDesc*=acidic (leucine-rich) nuclear phosphoprotein 32 family, member B [Source:HGNC Symbol;Acc:16677].

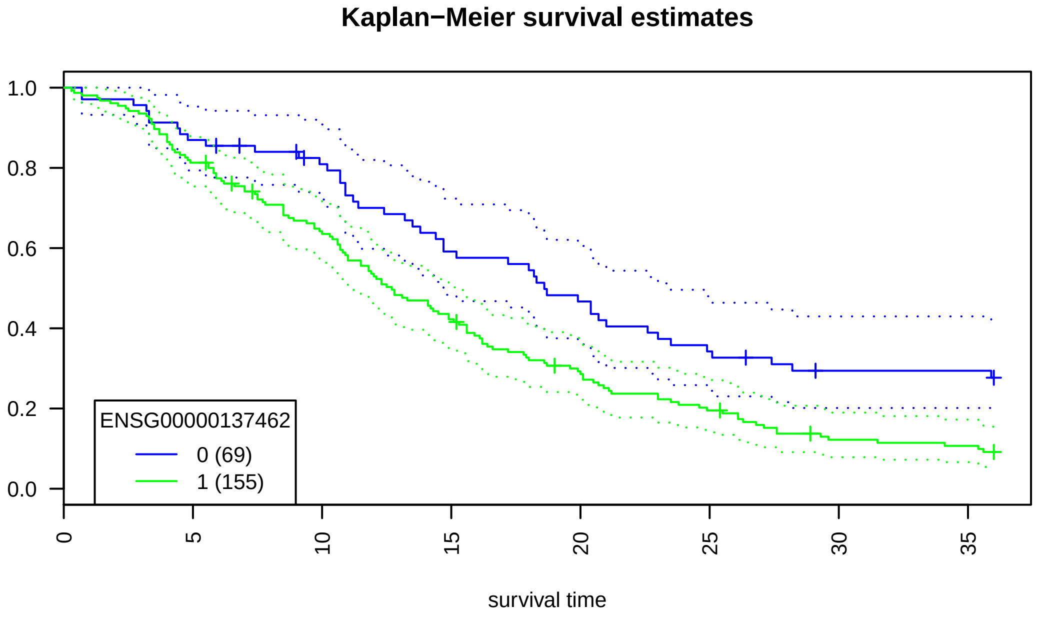

Figure 1620: Each curve represents the relative number of ENSG00000137462 survived to the corresponding point of time. Dashed lines represent 0.95 confidence intervals for the curves with the corresponding color. Total of 6 observations deleted due to missingness. Log-rank probability measure for the equality of these curves is 9.821117e-04. Additional annotations: *.GeneName*=TLR2, *.GeneDesc*=toll-like receptor 2 [Source:HGNC Symbol;Acc:11848].

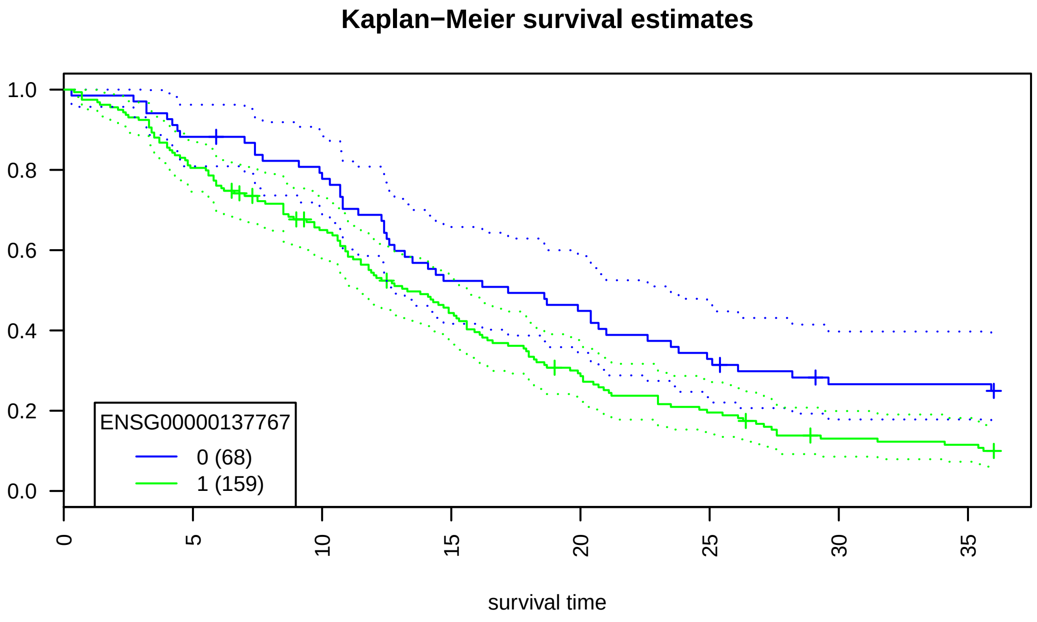

Figure 1621: Each curve represents the relative number of ENSG00000137767 survived to the corresponding point of time. Dashed lines represent 0.95 confidence intervals for the curves with the corresponding color. Total of 3 observations deleted due to missingness. Log-rank probability measure for the equality of these curves is 6.667793e-03. Additional annotations: *.GeneName*=SQRDL, *.GeneDesc*=sulfide quinone reductase-like (yeast) [Source:HGNC Symbol;Acc:20390].

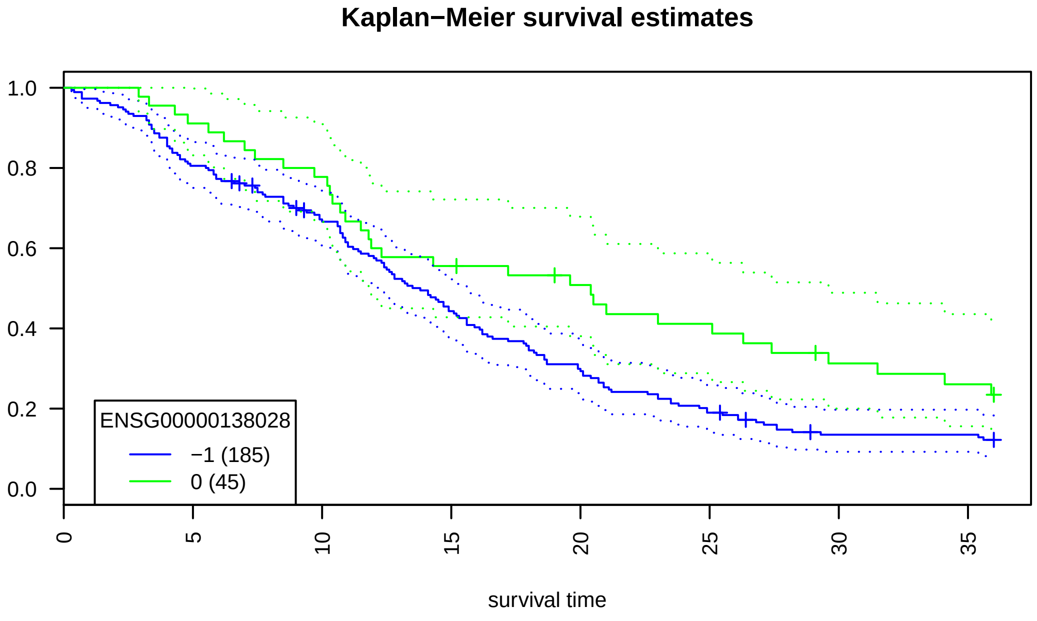

Figure 1622: Each curve represents the relative number of ENSG00000138028 survived to the corresponding point of time. Dashed lines represent 0.95 confidence intervals for the curves with the corresponding color. Log-rank probability measure for the equality of these curves is 2.181567e-02. Additional annotations: *.GeneName*=CGREF1, *.GeneDesc*=cell growth regulator with EF-hand domain 1 [Source:HGNC Symbol;Acc:16962].

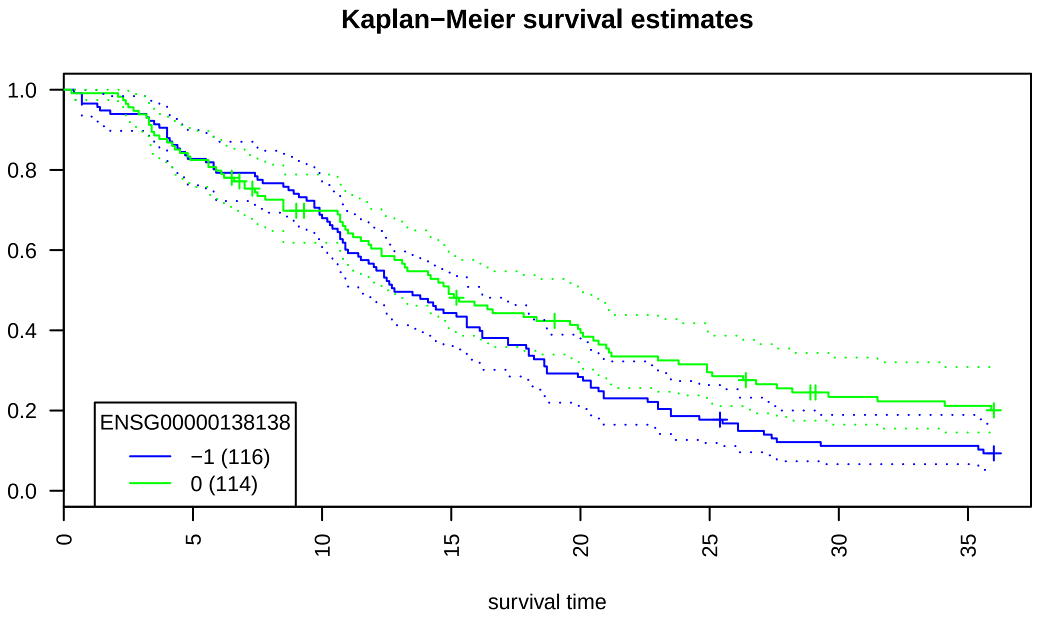

Figure 1623: Each curve represents the relative number of ENSG00000138138 survived to the corresponding point of time. Dashed lines represent 0.95 confidence intervals for the curves with the corresponding color. Log-rank probability measure for the equality of these curves is 4.84929e-02. Additional annotations: *.GeneName*=ATAD1, *.GeneDesc*=ATPase family, AAA domain containing 1 [Source:HGNC Symbol;Acc:25903].

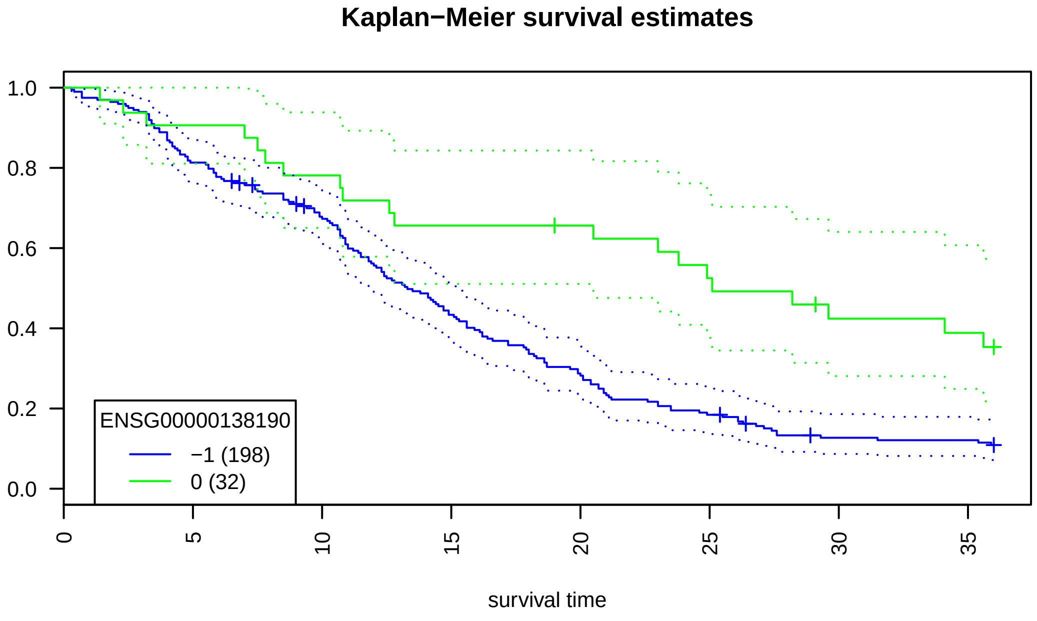

Figure 1624: Each curve represents the relative number of ENSG00000138190 survived to the corresponding point of time. Dashed lines represent 0.95 confidence intervals for the curves with the corresponding color. Log-rank probability measure for the equality of these curves is 4.521253e-04. Additional annotations: *.GeneName*=EXOC6, *.GeneDesc*=exocyst complex component 6 [Source:HGNC Symbol;Acc:23196].

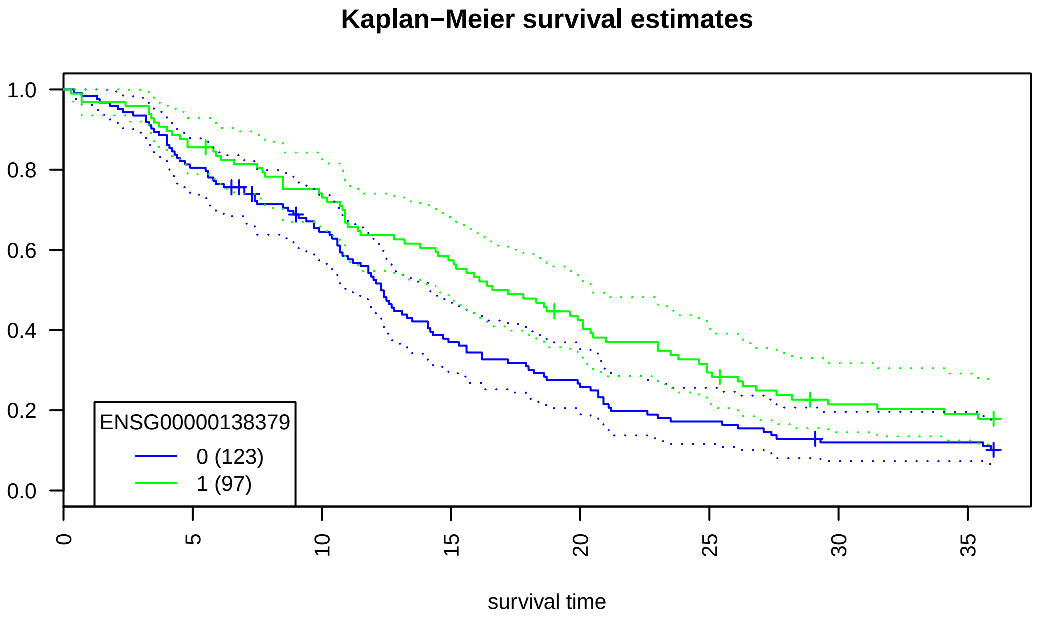

Figure 1625: Each curve represents the relative number of ENSG00000138379 survived to the corresponding point of time. Dashed lines represent 0.95 confidence intervals for the curves with the corresponding color. Total of 10 observations deleted due to missingness. Log-rank probability measure for the equality of these curves is 1.245509e-02. Additional annotations: *.GeneName*=MSTN, *.GeneDesc*=myostatin [Source:HGNC Symbol;Acc:4223].

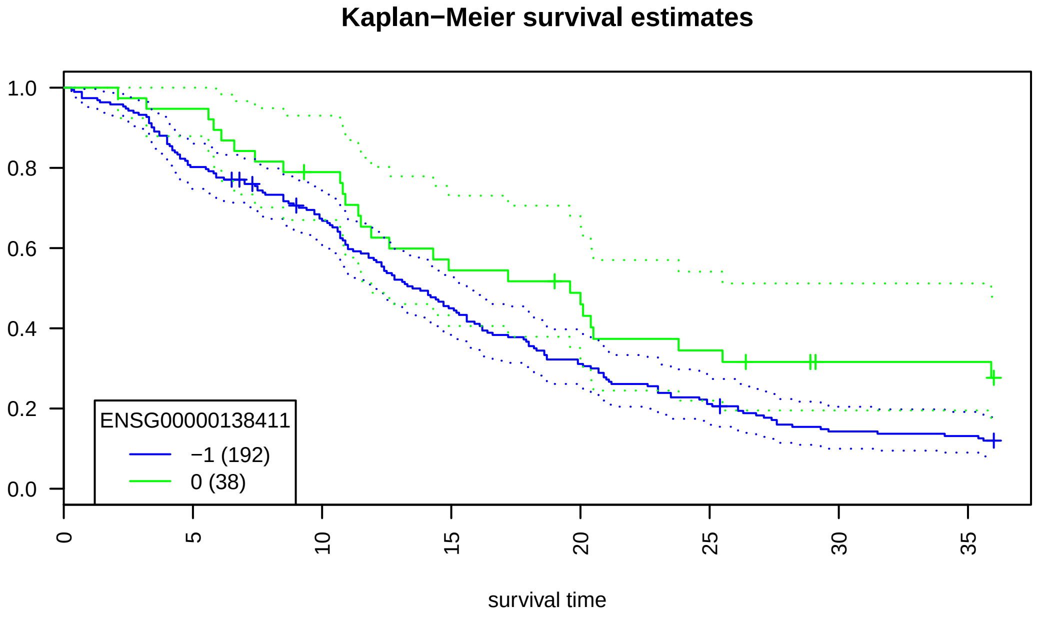

Figure 1626: Each curve represents the relative number of ENSG00000138411 survived to the corresponding point of time. Dashed lines represent 0.95 confidence intervals for the curves with the corresponding color. Log-rank probability measure for the equality of these curves is 3.553569e-02. Additional annotations: *.GeneName*=HECW2, *.GeneDesc*=HECT, C2 and WW domain containing E3 ubiquitin protein ligase 2 [Source:HGNC Symbol;Acc:29853].

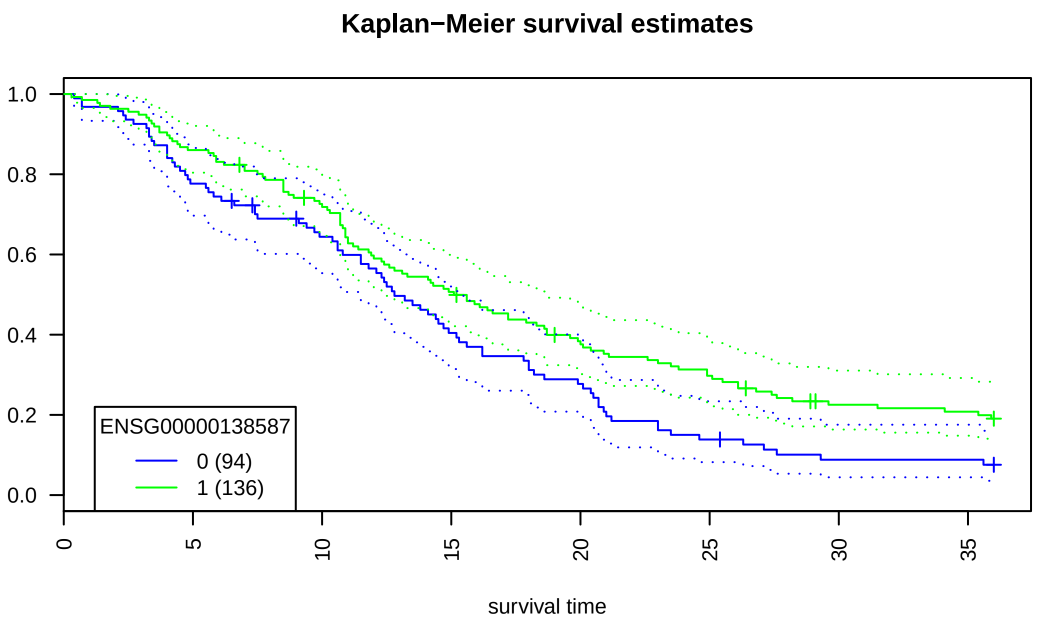

Figure 1627: Each curve represents the relative number of ENSG00000138587 survived to the corresponding point of time. Dashed lines represent 0.95 confidence intervals for the curves with the corresponding color. Log-rank probability measure for the equality of these curves is 1.103495e-02. Additional annotations: *.GeneName*=MNS1, *.GeneDesc*=meiosis-specific nuclear structural 1 [Source:HGNC Symbol;Acc:29636].

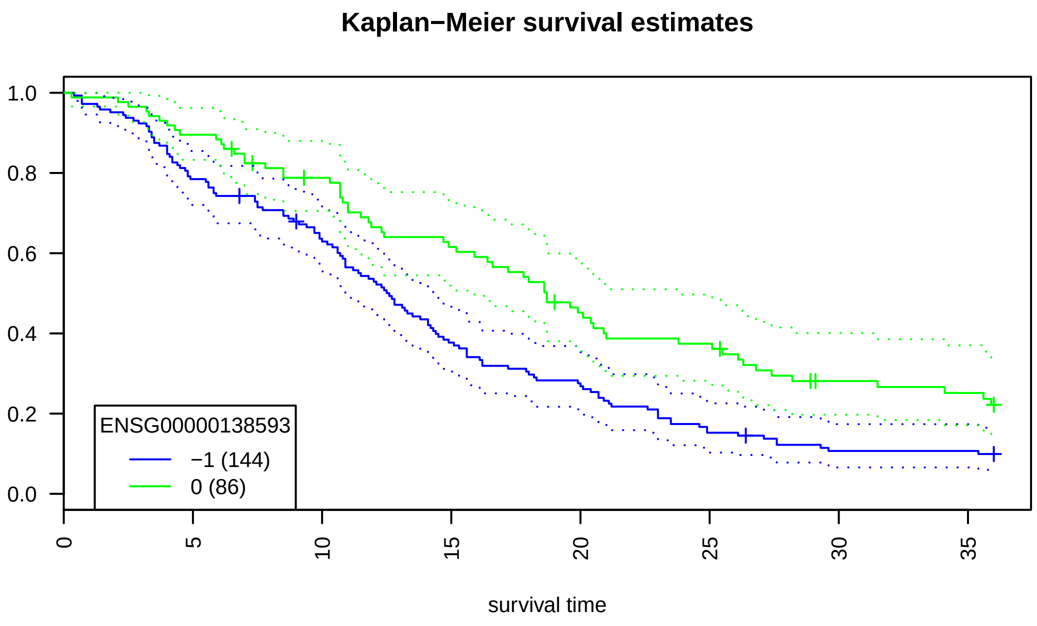

Figure 1628: Each curve represents the relative number of ENSG00000138593 survived to the corresponding point of time. Dashed lines represent 0.95 confidence intervals for the curves with the corresponding color. Log-rank probability measure for the equality of these curves is 7.430829e-04. Additional annotations: *.GeneName*=SECISBP2L, *.GeneDesc*=SECIS binding protein 2-like [Source:HGNC Symbol;Acc:28997].

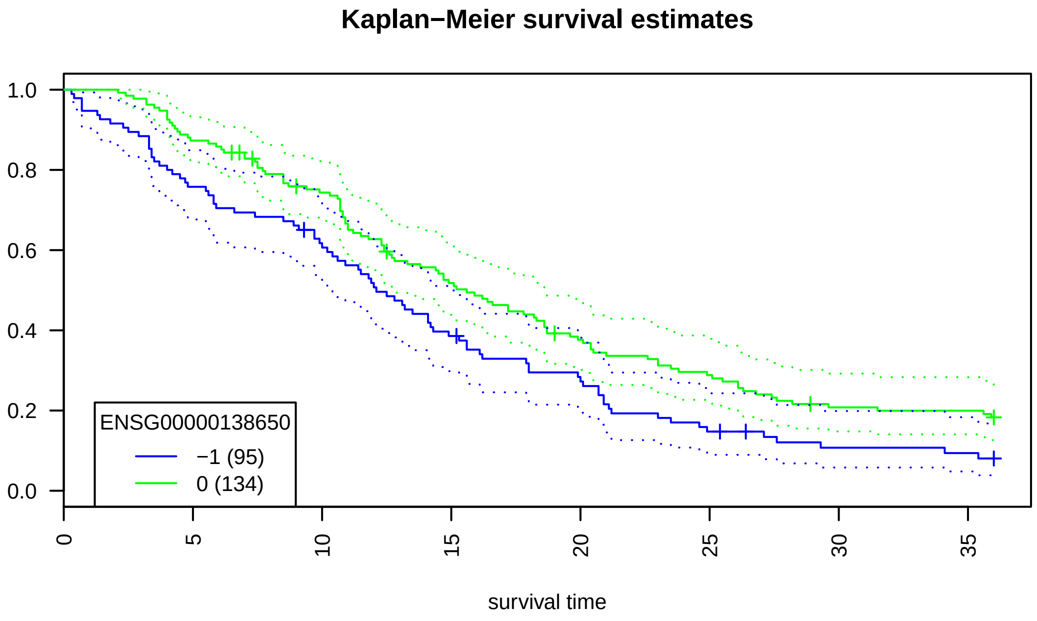

Figure 1629: Each curve represents the relative number of ENSG00000138650 survived to the corresponding point of time. Dashed lines represent 0.95 confidence intervals for the curves with the corresponding color. Total of 1 observation deleted due to missingness. Log-rank probability measure for the equality of these curves is 7.283282e-03. Additional annotations: *.GeneName*=PCDH10, *.GeneDesc*=protocadherin 10 [Source:HGNC Symbol;Acc:13404].

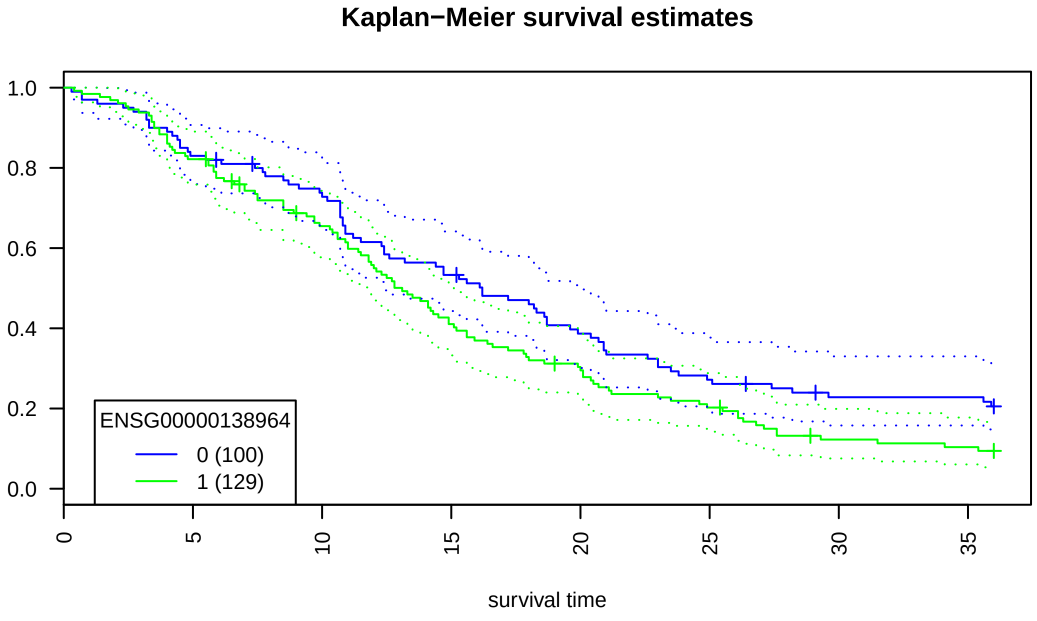

Figure 1630: Each curve represents the relative number of ENSG00000138964 survived to the corresponding point of time. Dashed lines represent 0.95 confidence intervals for the curves with the corresponding color. Total of 1 observation deleted due to missingness. Log-rank probability measure for the equality of these curves is 3.406036e-02. Additional annotations: *.GeneName*=PARVG, *.GeneDesc*=parvin, gamma [Source:HGNC Symbol;Acc:14654].

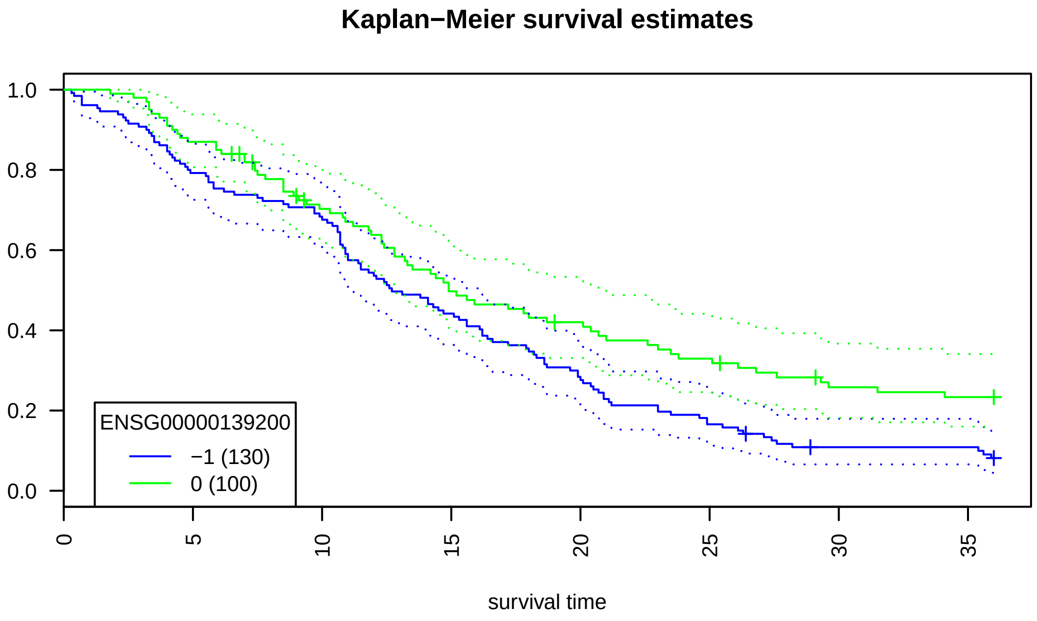

Figure 1631: Each curve represents the relative number of ENSG00000139200 survived to the corresponding point of time. Dashed lines represent 0.95 confidence intervals for the curves with the corresponding color. Log-rank probability measure for the equality of these curves is 5.056008e-03. Additional annotations: *.GeneName*=C12orf53, *.GeneDesc*=Uncharacterized protein C12orf53 Precursor [Source:UniProtKB/Swiss-Prot;Acc:Q8IYJ0].

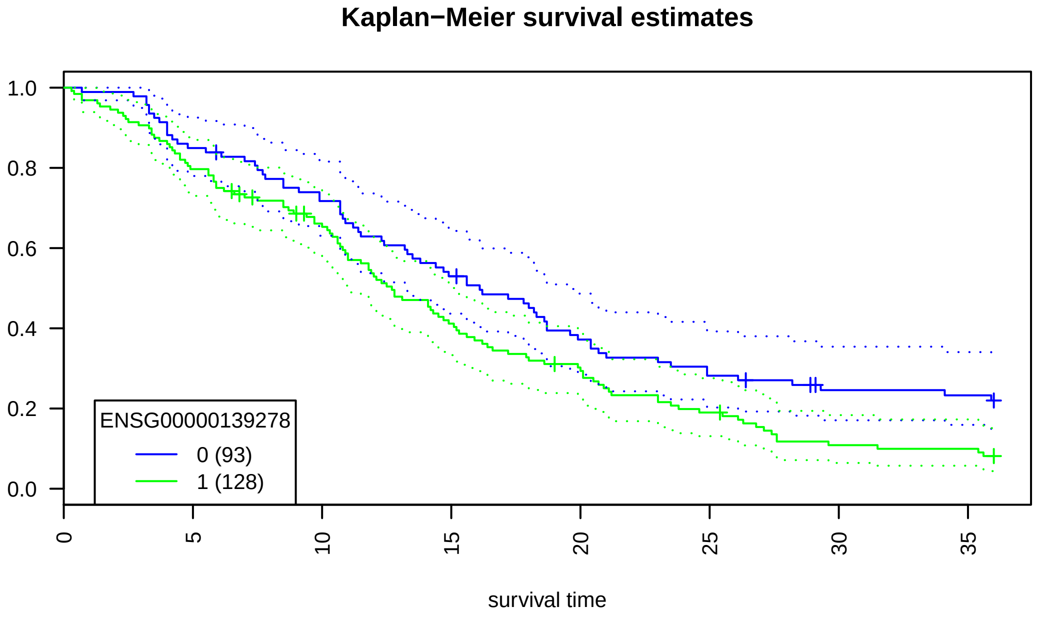

Figure 1632: Each curve represents the relative number of ENSG00000139278 survived to the corresponding point of time. Dashed lines represent 0.95 confidence intervals for the curves with the corresponding color. Total of 9 observations deleted due to missingness. Log-rank probability measure for the equality of these curves is 1.158008e-02. Additional annotations: *.GeneName*=GLIPR1, *.GeneDesc*=GLI pathogenesis-related 1 [Source:HGNC Symbol;Acc:17001].

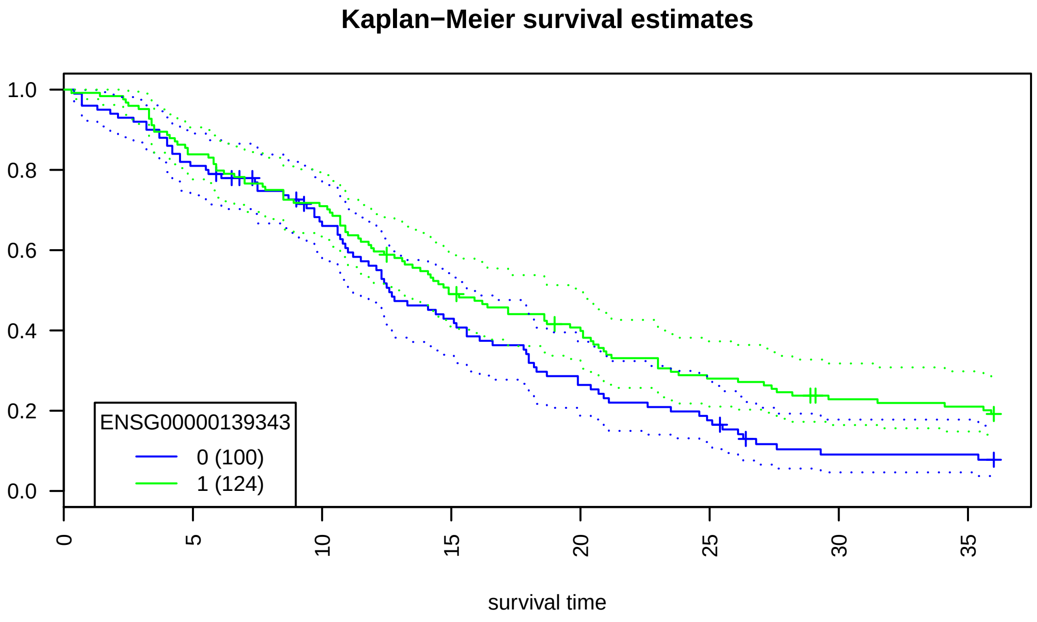

Figure 1633: Each curve represents the relative number of ENSG00000139343 survived to the corresponding point of time. Dashed lines represent 0.95 confidence intervals for the curves with the corresponding color. Total of 6 observations deleted due to missingness. Log-rank probability measure for the equality of these curves is 2.514322e-02. Additional annotations: *.GeneName*=SNRPF, *.GeneDesc*=small nuclear ribonucleoprotein polypeptide F [Source:HGNC Symbol;Acc:11162].

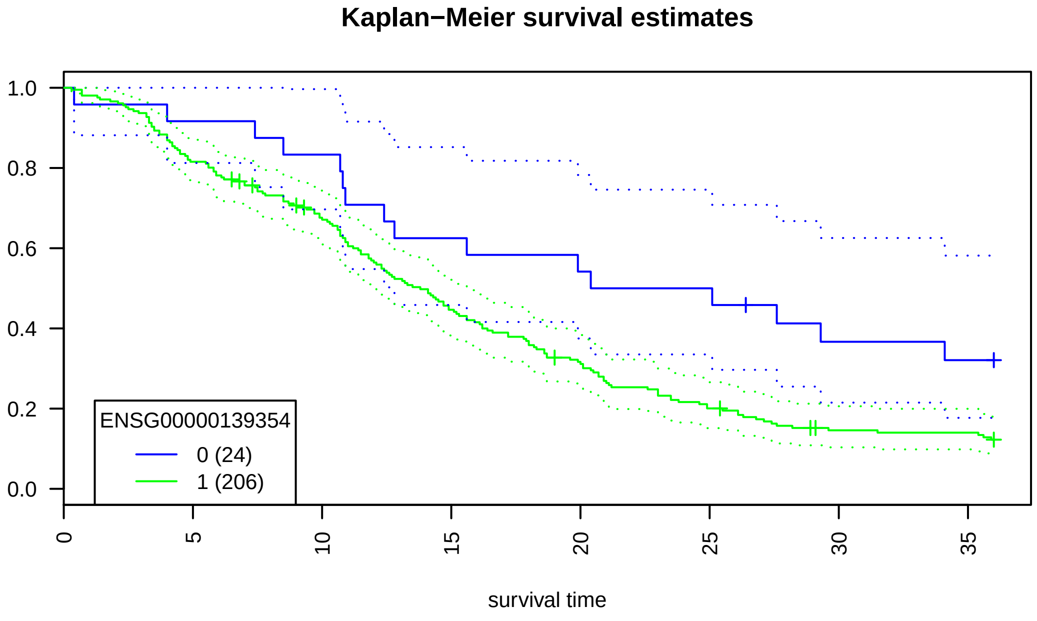

Figure 1634: Each curve represents the relative number of ENSG00000139354 survived to the corresponding point of time. Dashed lines represent 0.95 confidence intervals for the curves with the corresponding color. Log-rank probability measure for the equality of these curves is 1.354080e-02. Additional annotations: *.GeneName*=GAS2L3, *.GeneDesc*=growth arrest-specific 2 like 3 [Source:HGNC Symbol;Acc:27475].

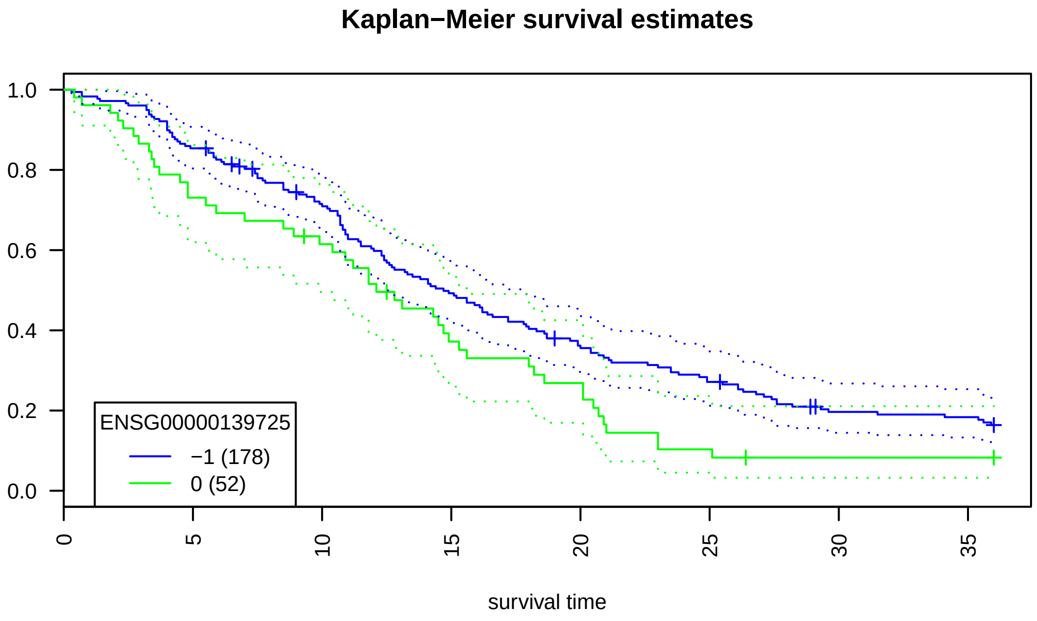

Figure 1635: Each curve represents the relative number of ENSG00000139725 survived to the corresponding point of time. Dashed lines represent 0.95 confidence intervals for the curves with the corresponding color. Log-rank probability measure for the equality of these curves is 2.047041e-02. Additional annotations: *.GeneName*=RHOF, *.GeneDesc*=ras homolog gene family, member F (in filopodia) [Source:HGNC Symbol;Acc:15703].

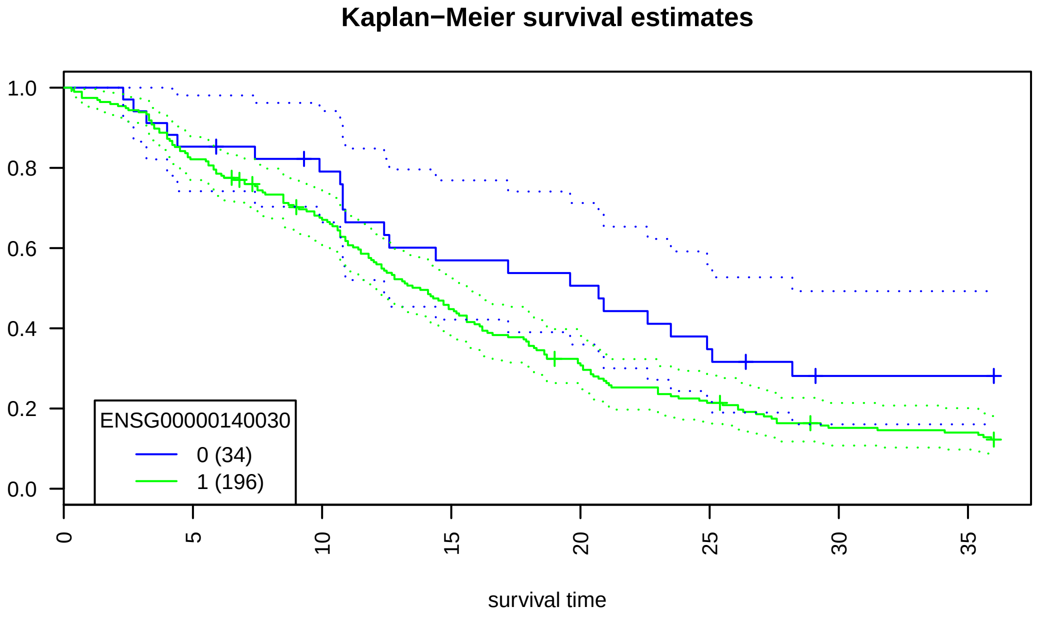

Figure 1636: Each curve represents the relative number of ENSG00000140030 survived to the corresponding point of time. Dashed lines represent 0.95 confidence intervals for the curves with the corresponding color. Log-rank probability measure for the equality of these curves is 4.276002e-02. Additional annotations: *.GeneName*=GPR65, *.GeneDesc*=G protein-coupled receptor 65 [Source:HGNC Symbol;Acc:4517].

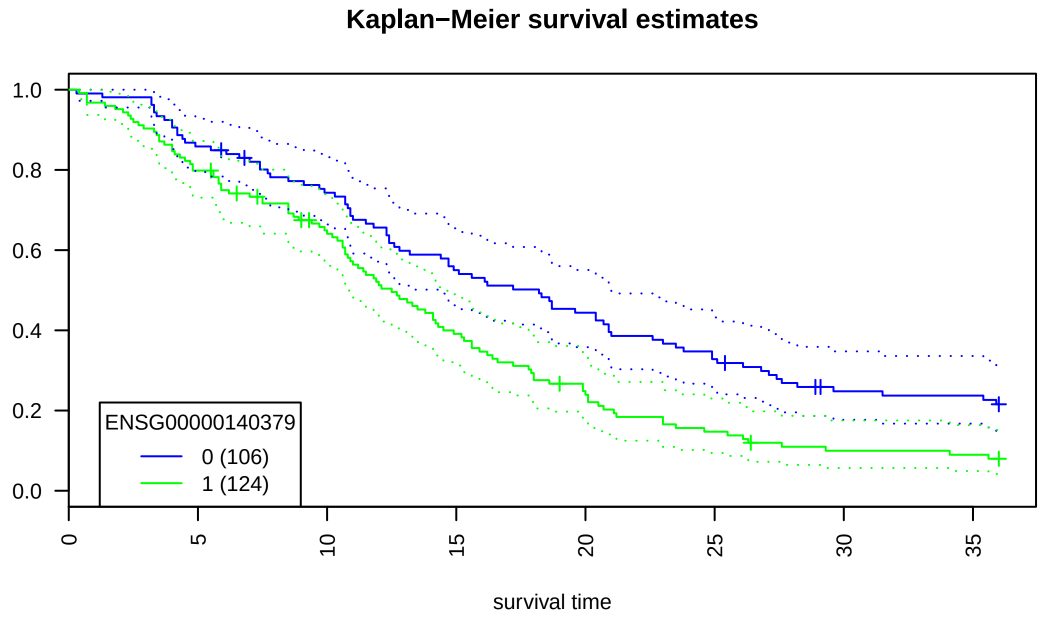

Figure 1637: Each curve represents the relative number of ENSG00000140379 survived to the corresponding point of time. Dashed lines represent 0.95 confidence intervals for the curves with the corresponding color. Log-rank probability measure for the equality of these curves is 6.295481e-04. Additional annotations: *.GeneName*=BCL2A1, *.GeneDesc*=BCL2-related protein A1 [Source:HGNC Symbol;Acc:991].

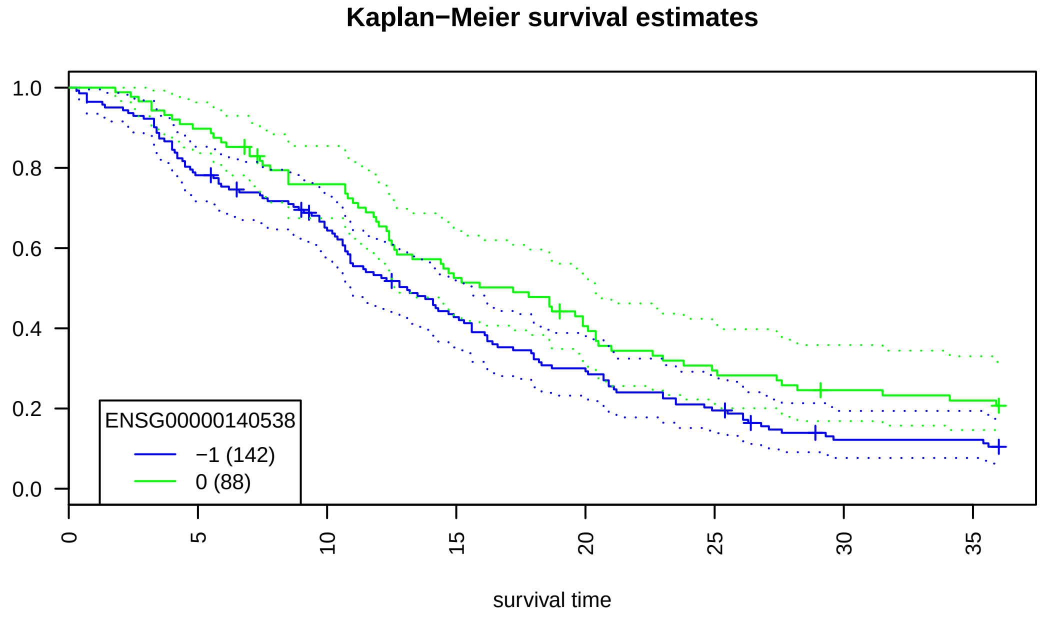

Figure 1638: Each curve represents the relative number of ENSG00000140538 survived to the corresponding point of time. Dashed lines represent 0.95 confidence intervals for the curves with the corresponding color. Log-rank probability measure for the equality of these curves is 1.819462e-02. Additional annotations: *.GeneName*=NTRK3, *.GeneDesc*=neurotrophic tyrosine kinase, receptor, type 3 [Source:HGNC Symbol;Acc:8033].

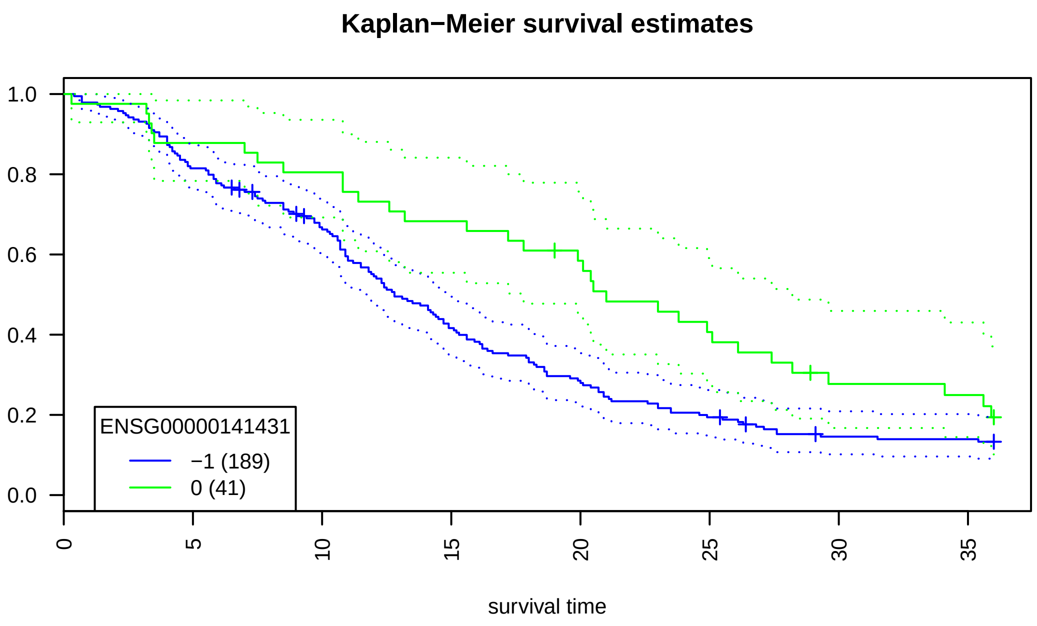

Figure 1639: Each curve represents the relative number of ENSG00000141431 survived to the corresponding point of time. Dashed lines represent 0.95 confidence intervals for the curves with the corresponding color. Log-rank probability measure for the equality of these curves is 1.551015e-02. Additional annotations: .*GeneName*=ASXL3, .*GeneDesc*=additional sex combs like 3 (Drosophila) [Source:HGNC Symbol;Acc:29357].

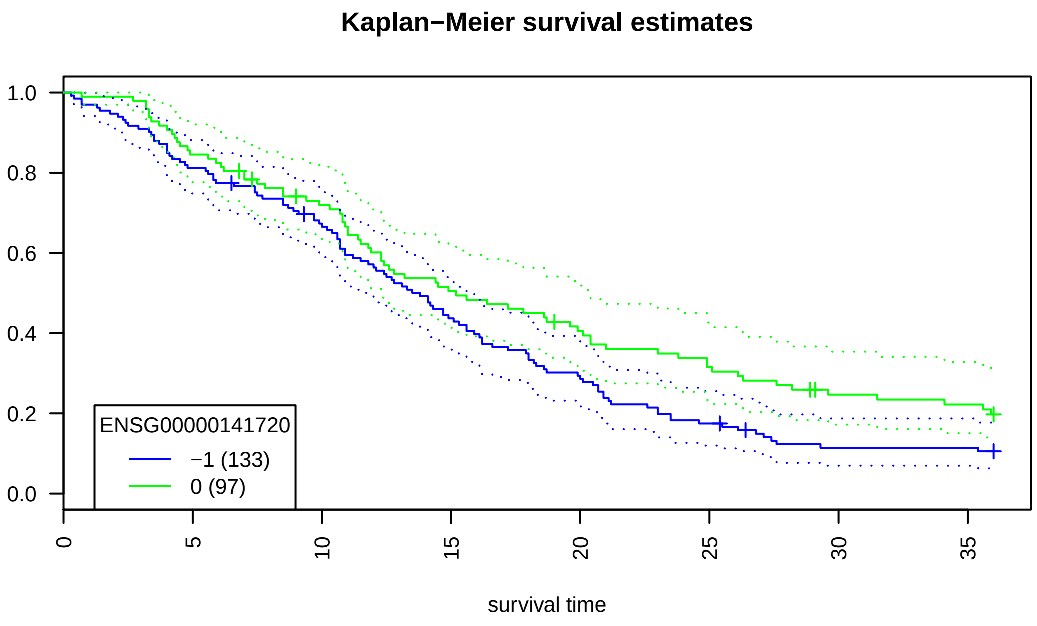

Figure 1640: Each curve represents the relative number of ENSG00000141720 survived to the corresponding point of time. Dashed lines represent 0.95 confidence intervals for the curves with the corresponding color. Log-rank probability measure for the equality of these curves is 3.386897e-02. Additional annotations: .*GeneName*=PIP5K2B, .*GeneDesc*=phosphatidylinositol-5-phosphate 4-kinase, type II, beta [Source:HGNC Symbol;Acc:8998].

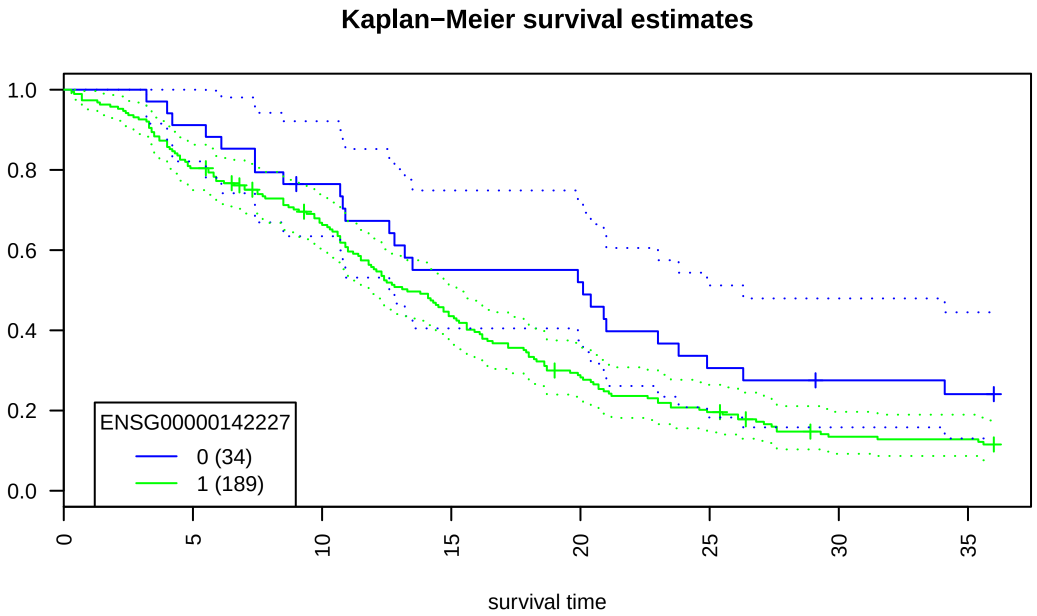

Figure 1641: Each curve represents the relative number of ENSG00000142227 survived to the corresponding point of time. Dashed lines represent 0.95 confidence intervals for the curves with the corresponding color. Total of 7 observations deleted due to missingness. Log-rank probability measure for the equality of these curves is 4.602256e-02. Additional annotations: *.GeneName*=EMP3, *.GeneDesc*=epithelial membrane protein 3 [Source:HGNC Symbol;Acc:3335].

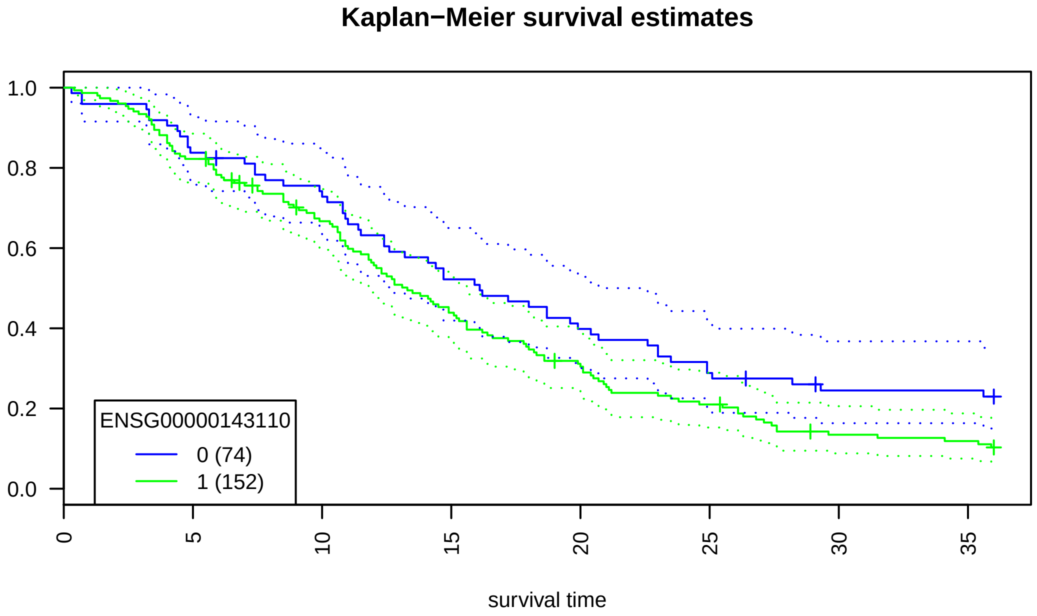

Figure 1642: Each curve represents the relative number of ENSG00000143110 survived to the corresponding point of time. Dashed lines represent 0.95 confidence intervals for the curves with the corresponding color. Total of 4 observations deleted due to missingness. Log-rank probability measure for the equality of these curves is 3.517997e-02. Additional annotations: *.GeneName*=C1orf162, *.GeneDesc*=Transmembrane protein C1orf162 [Source:UniProtKB/Swiss-Prot;Acc:Q8NEQ5].

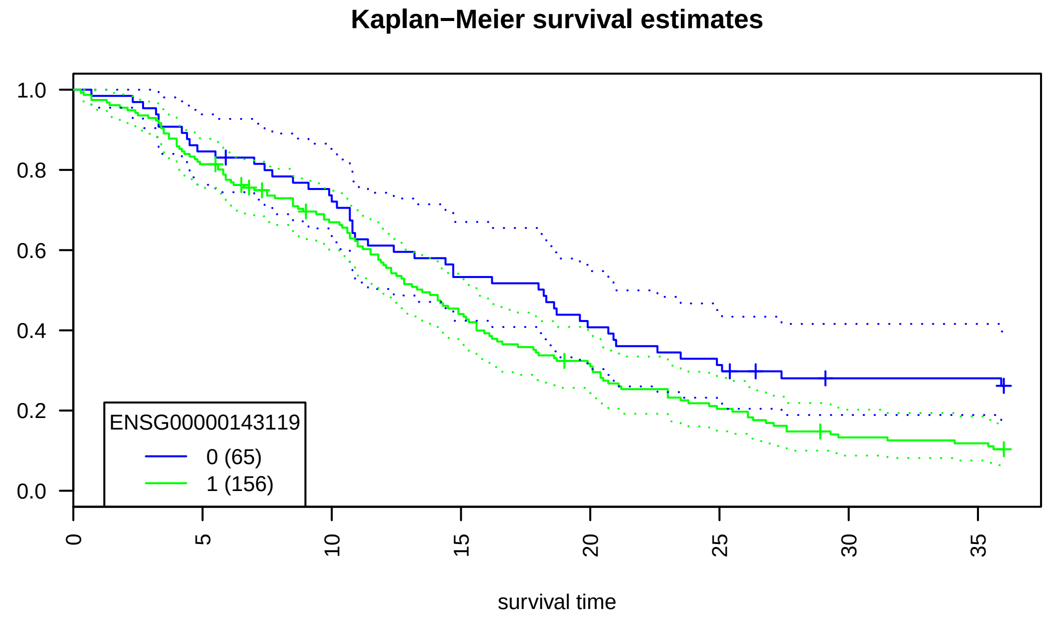

Figure 1643: Each curve represents the relative number of ENSG00000143119 survived to the corresponding point of time. Dashed lines represent 0.95 confidence intervals for the curves with the corresponding color. Total of 9 observations deleted due to missingness. Log-rank probability measure for the equality of these curves is 2.140309e-02. Additional annotations: *.GeneName*=CD53, *.GeneDesc*=CD53 molecule [Source:HGNC Symbol;Acc:1686].

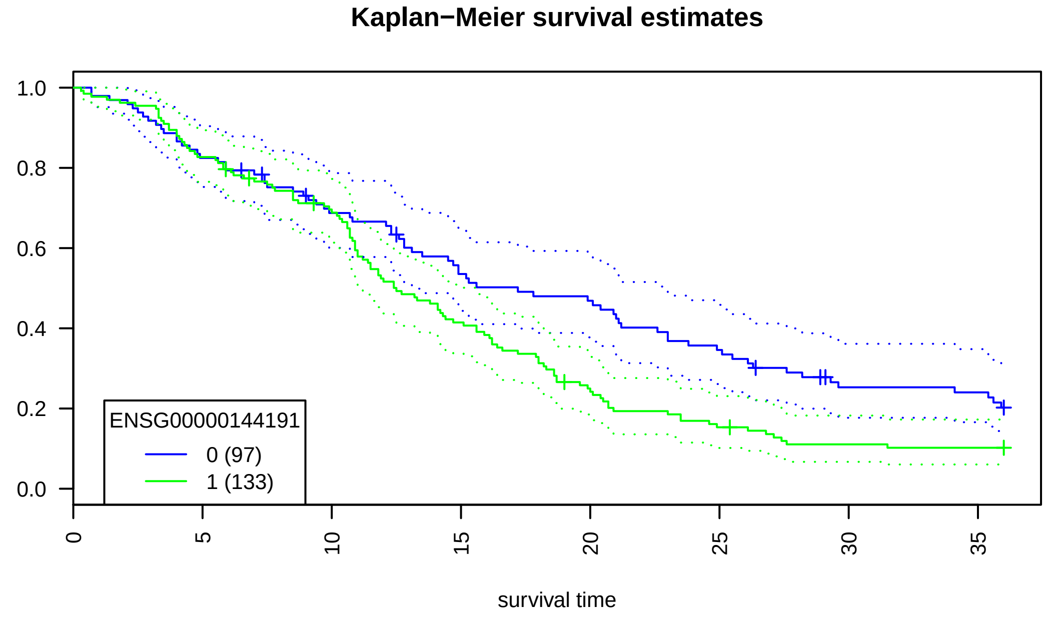

Figure 1644: Each curve represents the relative number of ENSG00000144191 survived to the corresponding point of time. Dashed lines represent 0.95 confidence intervals for the curves with the corresponding color. Log-rank probability measure for the equality of these curves is 6.718993e-03. Additional annotations: *.GeneName*=CNGA3, *.GeneDesc*=cyclic nucleotide gated channel alpha 3 [Source:HGNC Symbol;Acc:2150].

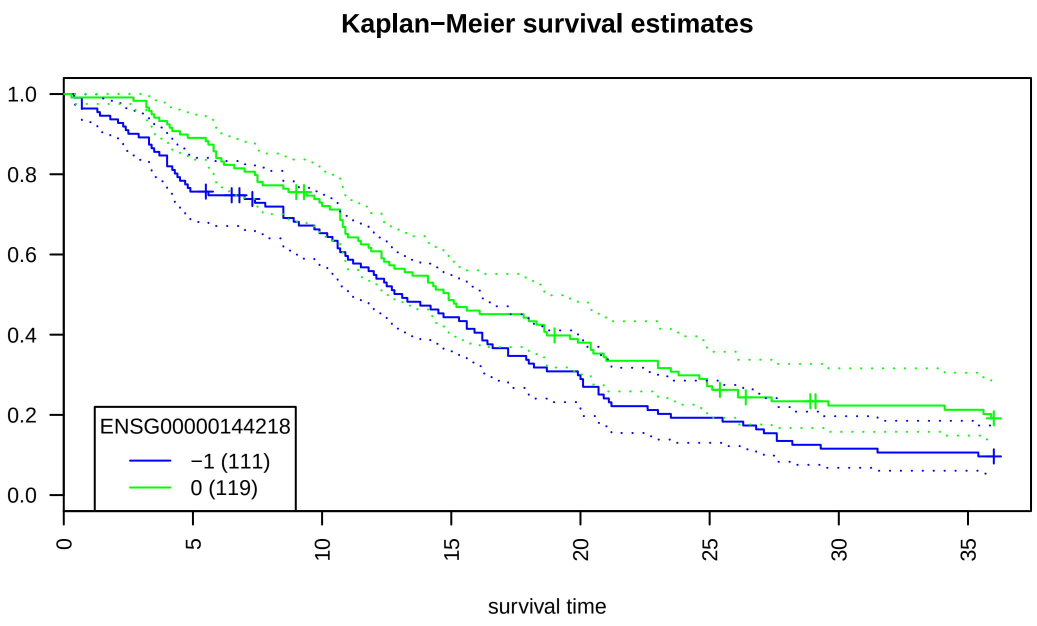

Figure 1645: Each curve represents the relative number of ENSG00000144218 survived to the corresponding point of time. Dashed lines represent 0.95 confidence intervals for the curves with the corresponding color. Log-rank probability measure for the equality of these curves is 3.890738e-02. Additional annotations: *.GeneName*=AFF3, *.GeneDesc*=AF4/FMR2 family, member 3 [Source:HGNC Symbol;Acc:6473].

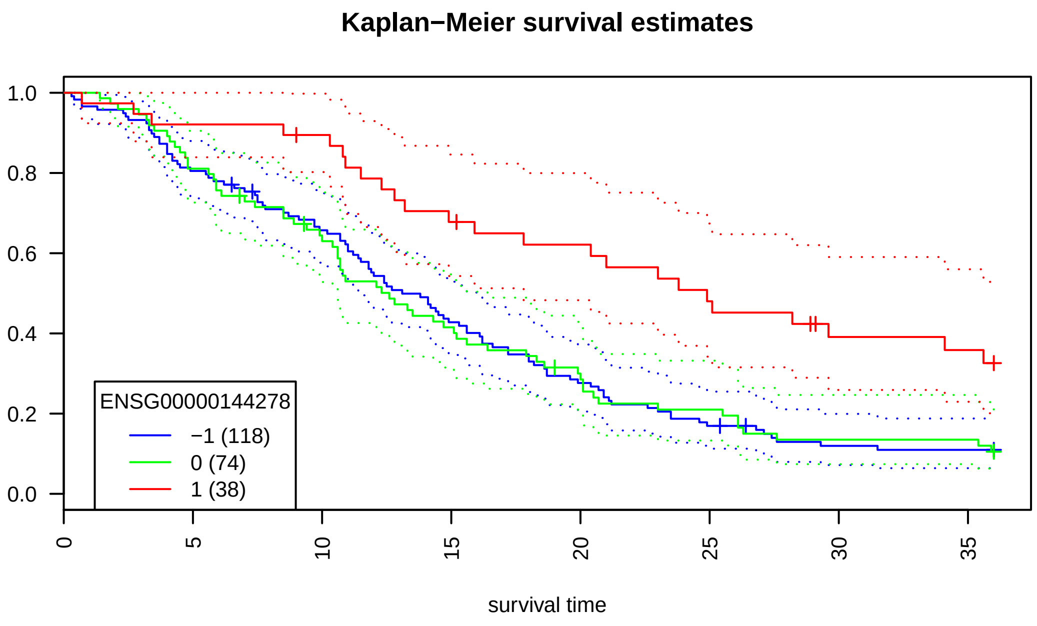

Figure 1646: Each curve represents the relative number of ENSG00000144278 survived to the corresponding point of time. Dashed lines represent 0.95 confidence intervals for the curves with the corresponding color. Log-rank probability measure for the equality of these curves is 1.057514e-03. Additional annotations: *.GeneName*=GALNT13, *.GeneDesc*=UDP-N-acetyl-alpha-D-galactosamine:polypeptide N-acetylgalactosaminyltransferase 13 (GalNAc-T13) [Source:HGNC Symbol;Acc:23242].

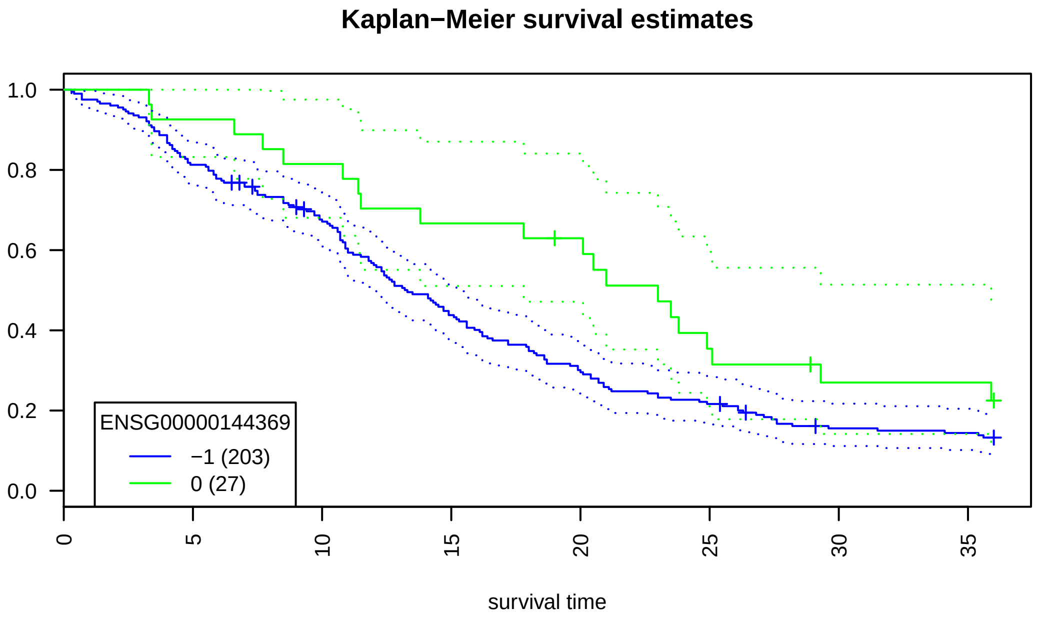

Figure 1647: Each curve represents the relative number of ENSG00000144369 survived to the corresponding point of time. Dashed lines represent 0.95 confidence intervals for the curves with the corresponding color. Log-rank probability measure for the equality of these curves is 3.684794e-02. Additional annotations: *.GeneName*=FAM171B, *.GeneDesc*=family with sequence similarity 171, member B [Source:HGNC Symbol;Acc:29412].

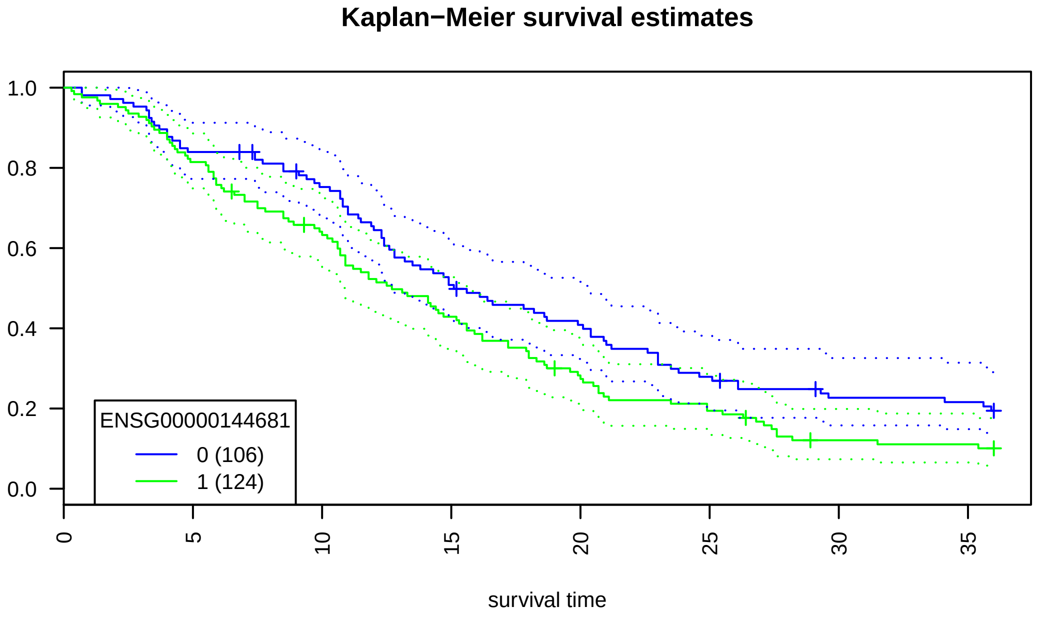

Figure 1648: Each curve represents the relative number of ENSG00000144681 survived to the corresponding point of time. Dashed lines represent 0.95 confidence intervals for the curves with the corresponding color. Log-rank probability measure for the equality of these curves is 2.468884e-02. Additional annotations: *.GeneName*=STAC, *.GeneDesc*=SH3 and cysteine rich domain [Source:HGNC Symbol;Acc:11353].

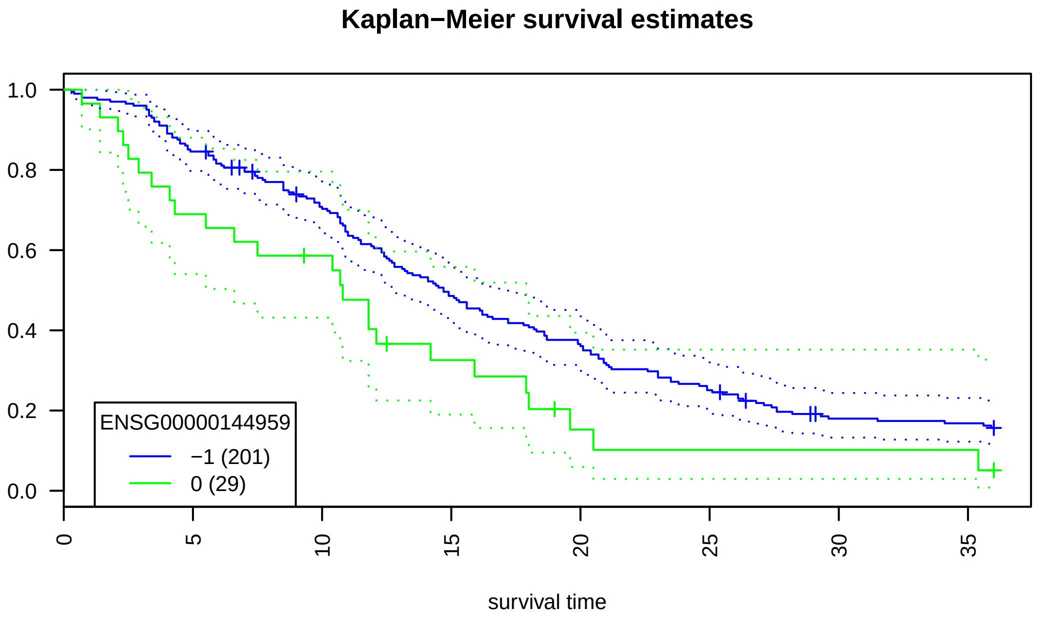

Figure 1649: Each curve represents the relative number of ENSG00000144959 survived to the corresponding point of time. Dashed lines represent 0.95 confidence intervals for the curves with the corresponding color. Log-rank probability measure for the equality of these curves is 1.168864e-02. Additional annotations: *.GeneName*=AADACL1, *.GeneDesc*=neutral cholesterol ester hydrolase 1 [Source:HGNC Symbol;Acc:29260].

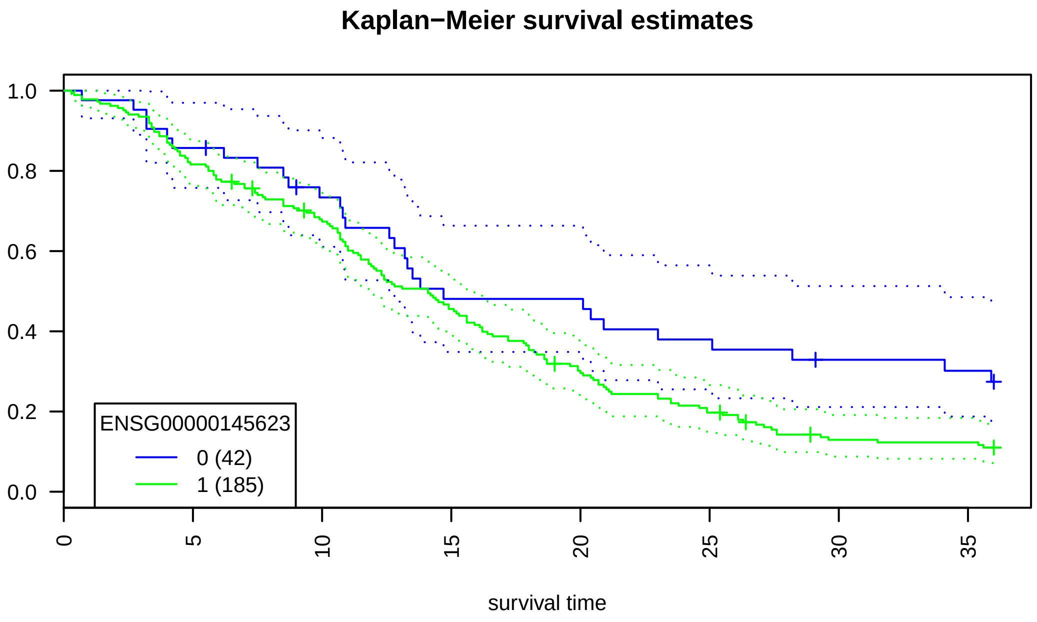

Figure 1650: Each curve represents the relative number of ENSG00000145623 survived to the corresponding point of time. Dashed lines represent 0.95 confidence intervals for the curves with the corresponding color. Total of 3 observations deleted due to missingness. Log-rank probability measure for the equality of these curves is 2.602943e-02. Additional annotations: *.GeneName*=OSMR, *.GeneDesc*=oncostatin M receptor [Source:HGNC Symbol;Acc:8507].

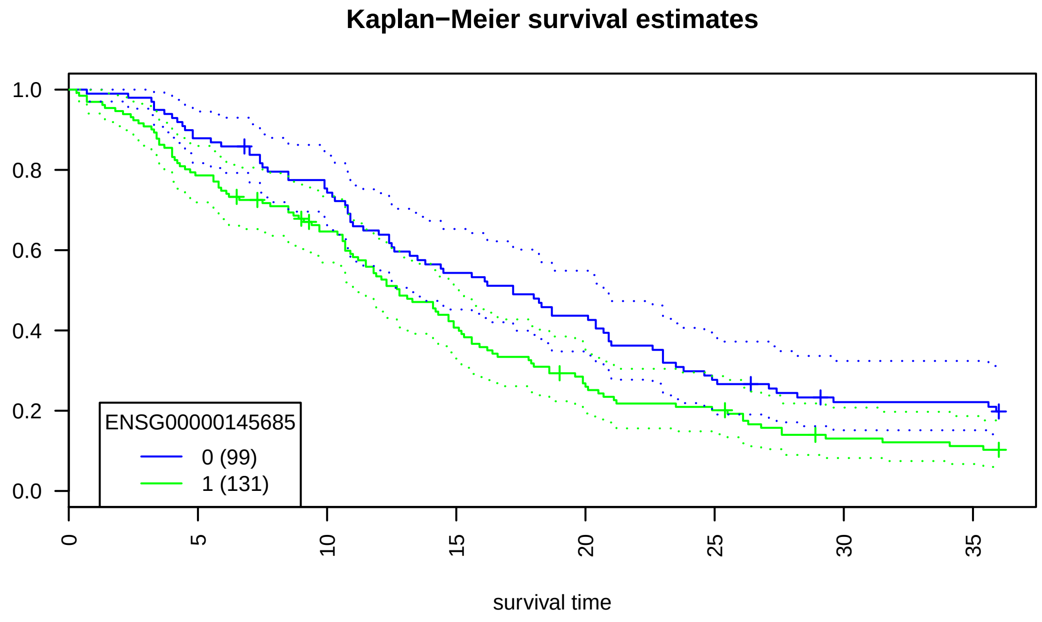

Figure 1651: Each curve represents the relative number of ENSG00000145685 survived to the corresponding point of time. Dashed lines represent 0.95 confidence intervals for the curves with the corresponding color. Log-rank probability measure for the equality of these curves is 1.353278e-02. Additional annotations: *.GeneName*=LHFPL2, *.GeneDesc*=lipoma HMGIC fusion partner-like 2 [Source:HGNC Symbol;Acc:6588].

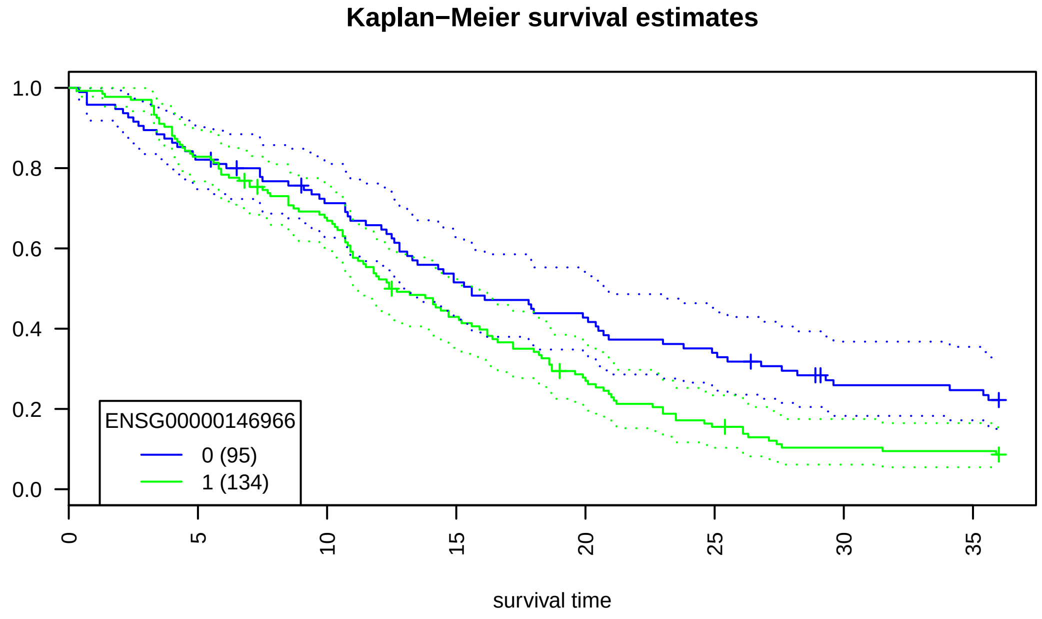

Figure 1652: Each curve represents the relative number of ENSG00000146966 survived to the corresponding point of time. Dashed lines represent 0.95 confidence intervals for the curves with the corresponding color. Total of 1 observation deleted due to missingness. Log-rank probability measure for the equality of these curves is 6.505693e-03. Additional annotations: *.GeneName*=DENND2A, *.GeneDesc*=DENN/MADD domain containing 2A [Source:HGNC Symbol;Acc:22212].

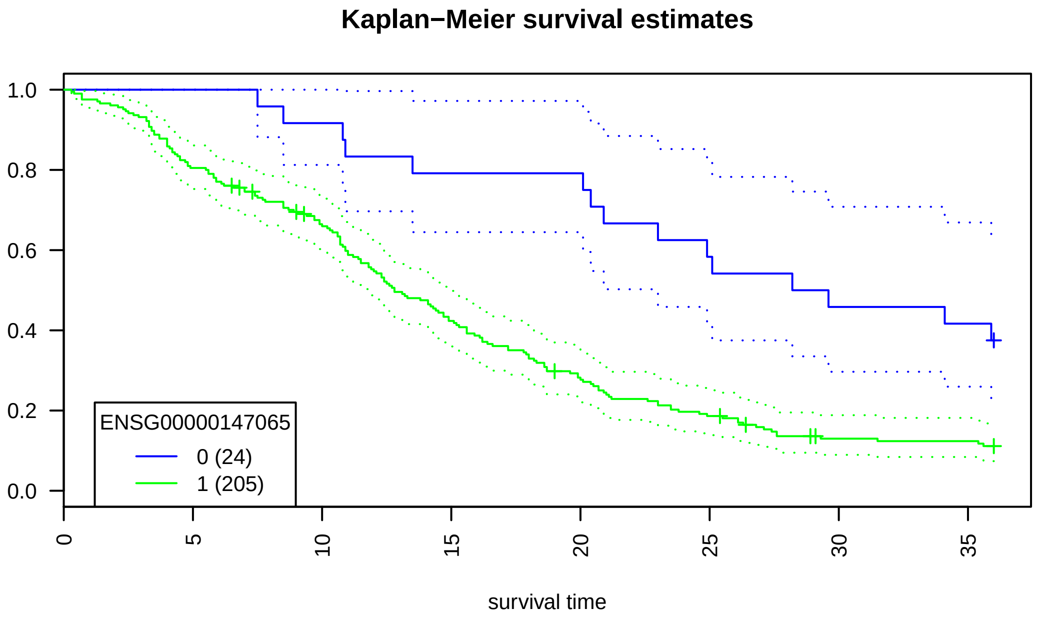

Figure 1653: Each curve represents the relative number of ENSG00000147065 survived to the corresponding point of time. Dashed lines represent 0.95 confidence intervals for the curves with the corresponding color. Total of 1 observation deleted due to missingness. Log-rank probability measure for the equality of these curves is 1.595617e-04. Additional annotations: *.GeneName*=MSN, *.GeneDesc*=moesin [Source:HGNC Symbol;Acc:7373].

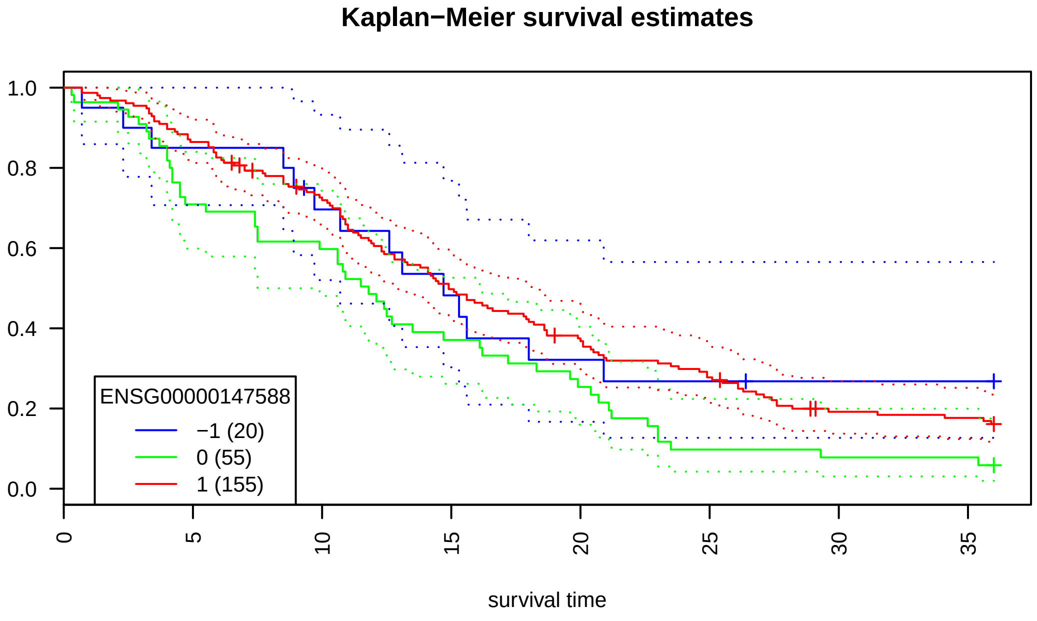

Figure 1654: Each curve represents the relative number of ENSG00000147588 survived to the corresponding point of time. Dashed lines represent 0.95 confidence intervals for the curves with the corresponding color. Log-rank probability measure for the equality of these curves is 2.664543e-02. Additional annotations: *.GeneName*=PMP2, *.GeneDesc*=peripheral myelin protein 2 [Source:HGNC Symbol;Acc:9117].

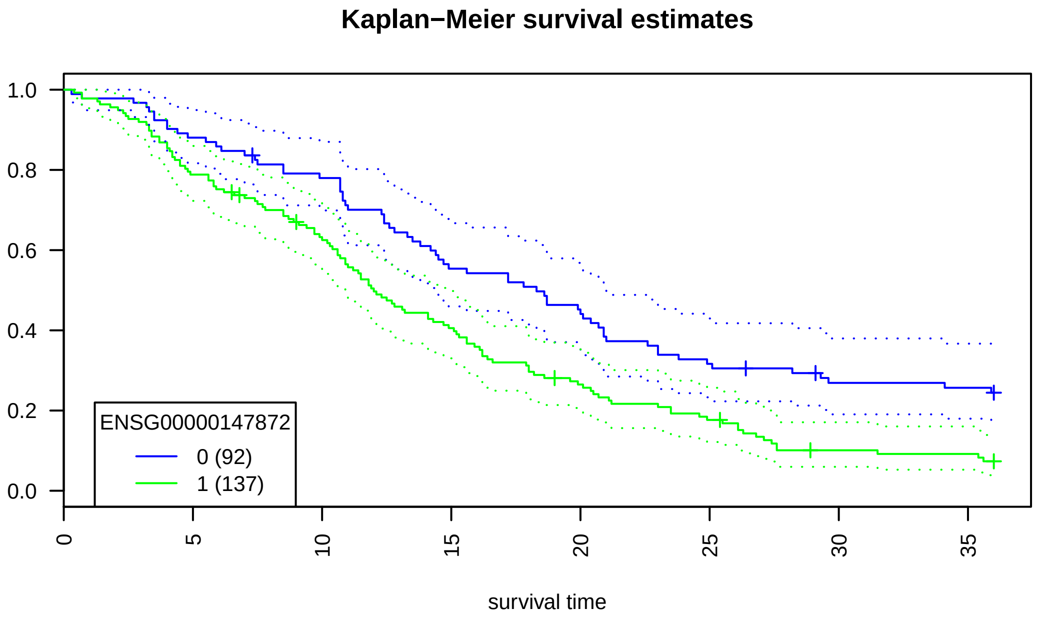

Figure 1655: Each curve represents the relative number of ENSG00000147872 survived to the corresponding point of time. Dashed lines represent 0.95 confidence intervals for the curves with the corresponding color. Total of 1 observation deleted due to missingness. Log-rank probability measure for the equality of these curves is 2.906491e-04. Additional annotations: *.GeneName*=PLIN2, *.GeneDesc*=perilipin 2 [Source:HGNC Symbol;Acc:248].

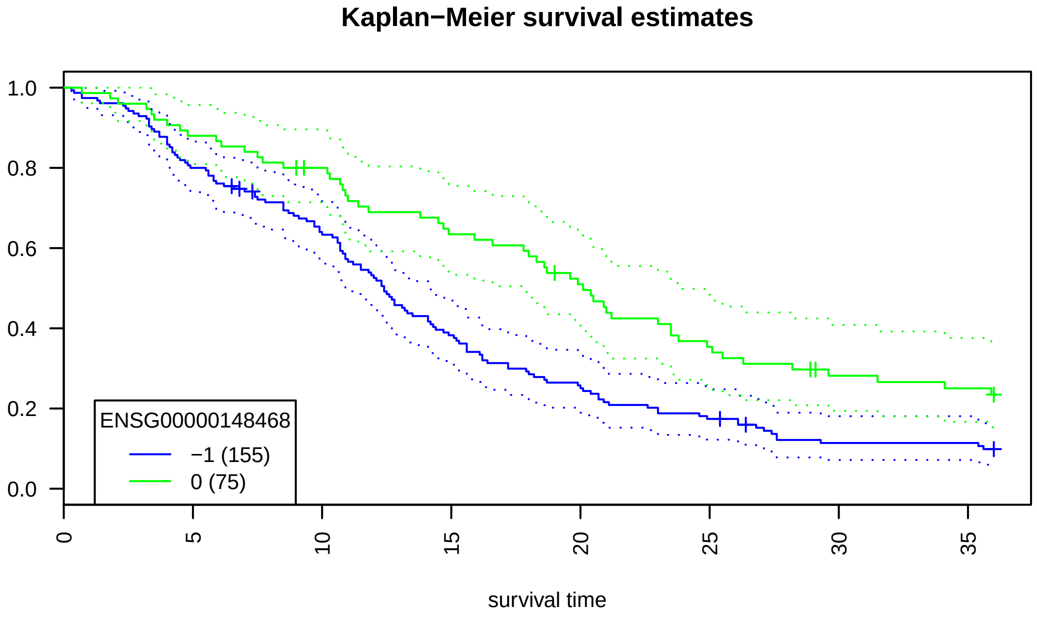

Figure 1656: Each curve represents the relative number of ENSG00000148468 survived to the corresponding point of time. Dashed lines represent 0.95 confidence intervals for the curves with the corresponding color. Log-rank probability measure for the equality of these curves is 3.424845e-04. Additional annotations: *.GeneName*=FAM171A1, *.GeneDesc*=family with sequence similarity 171, member A1 [Source:HGNC Symbol;Acc:23522].

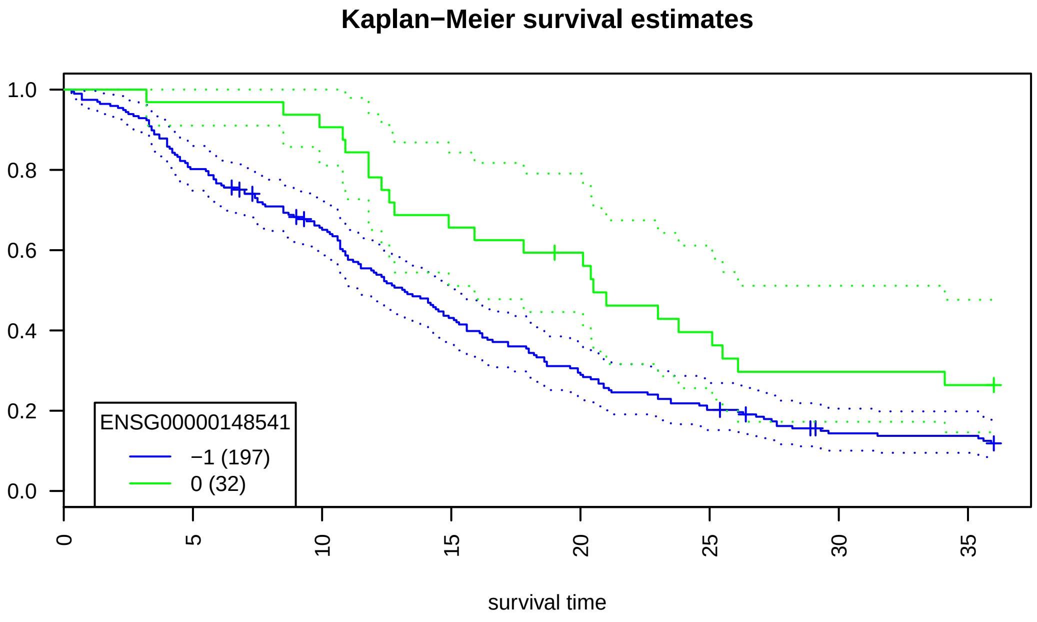

Figure 1657: Each curve represents the relative number of ENSG00000148541 survived to the corresponding point of time. Dashed lines represent 0.95 confidence intervals for the curves with the corresponding color. Total of 1 observation deleted due to missingness. Log-rank probability measure for the equality of these curves is 8.049463e-03. Additional annotations: *.GeneName*=FAM13C, *.GeneDesc*=family with sequence similarity 13, member C [Source:HGNC Symbol;Acc:19371].

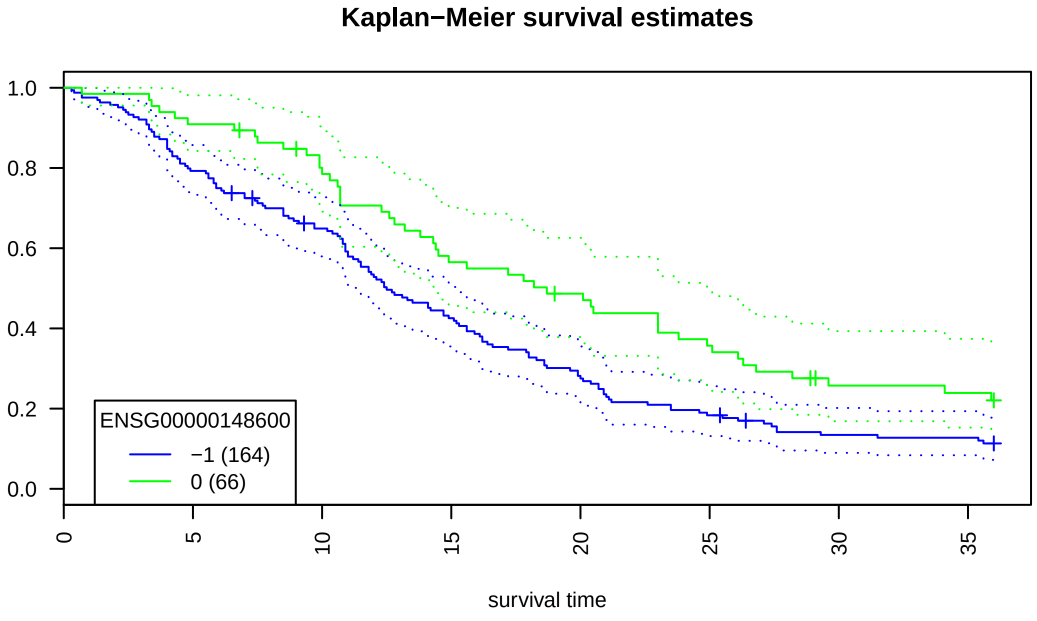

Figure 1658: Each curve represents the relative number of ENSG00000148600 survived to the corresponding point of time. Dashed lines represent 0.95 confidence intervals for the curves with the corresponding color. Log-rank probability measure for the equality of these curves is 4.821134e-03. Additional annotations: *.GeneName*=PCDH21, *.GeneDesc*=cadherin-related family member 1 [Source:HGNC Symbol;Acc:14550].

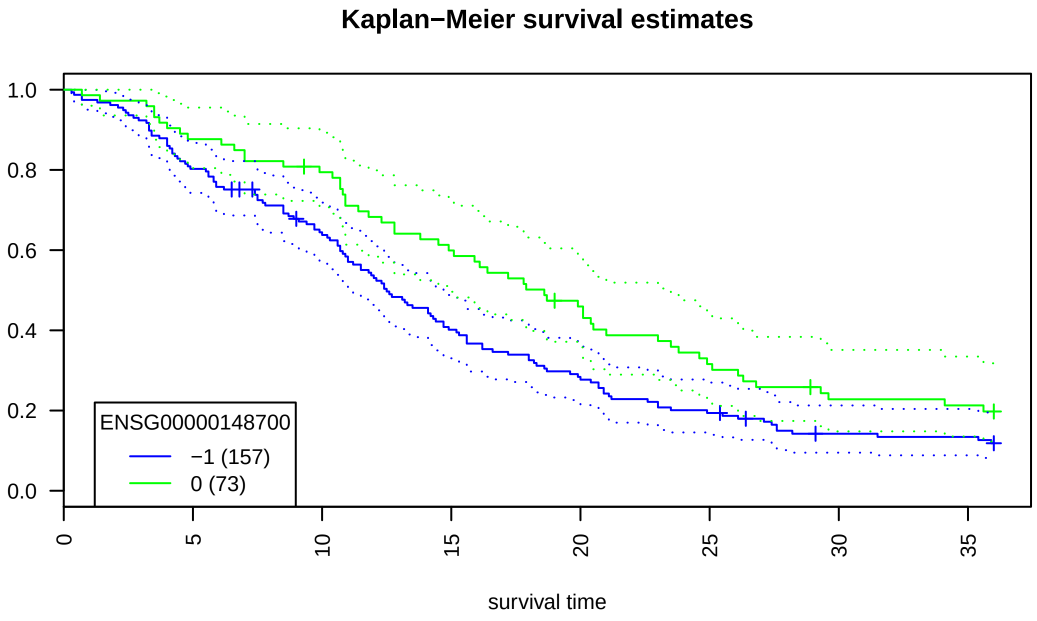

Figure 1659: Each curve represents the relative number of ENSG00000148700 survived to the corresponding point of time. Dashed lines represent 0.95 confidence intervals for the curves with the corresponding color. Log-rank probability measure for the equality of these curves is 1.415866e-02. Additional annotations: *.GeneName*=ADD3, *.GeneDesc*=adducin 3 (gamma) [Source:HGNC Symbol;Acc:245].

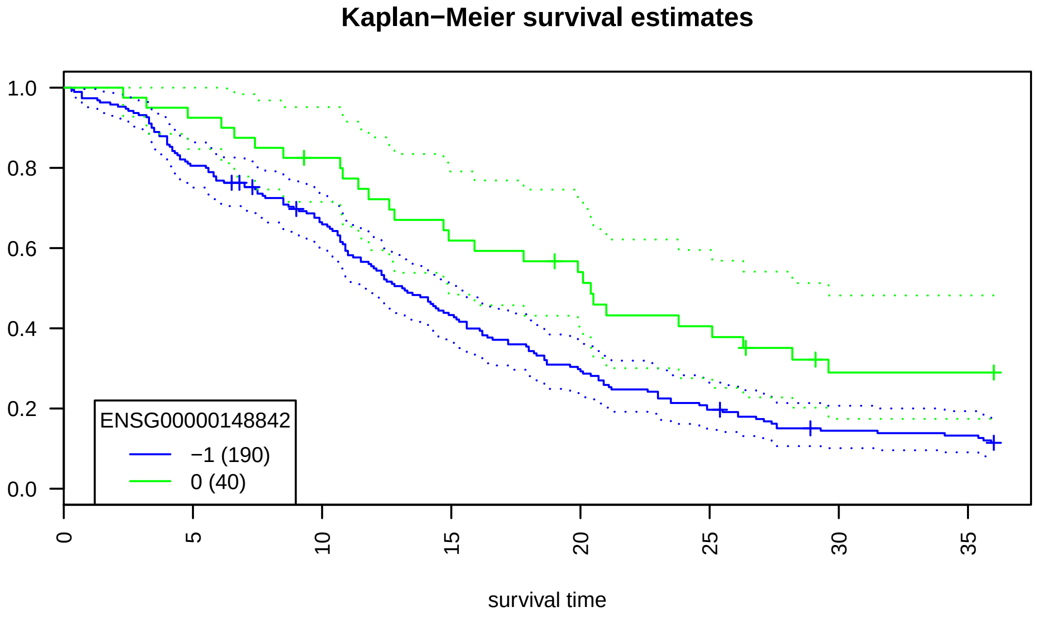

Figure 1660: Each curve represents the relative number of ENSG00000148842 survived to the corresponding point of time. Dashed lines represent 0.95 confidence intervals for the curves with the corresponding color. Log-rank probability measure for the equality of these curves is 4.882601e-03. Additional annotations: *.GeneName*=CNNM2, *.GeneDesc*=cyclin M2 [Source:HGNC Symbol;Acc:103].

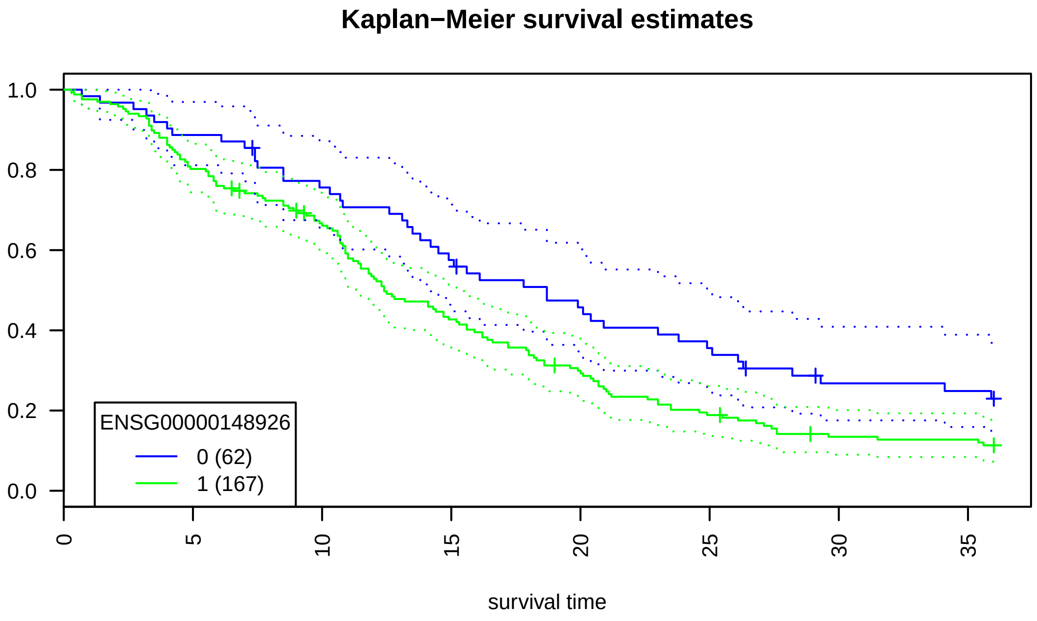

Figure 1661: Each curve represents the relative number of ENSG00000148926 survived to the corresponding point of time. Dashed lines represent 0.95 confidence intervals for the curves with the corresponding color. Total of 1 observation deleted due to missingness. Log-rank probability measure for the equality of these curves is 1.047888e-02. Additional annotations: *.GeneName*=ADM, *.GeneDesc*=adrenomedullin [Source:HGNC Symbol;Acc:259].

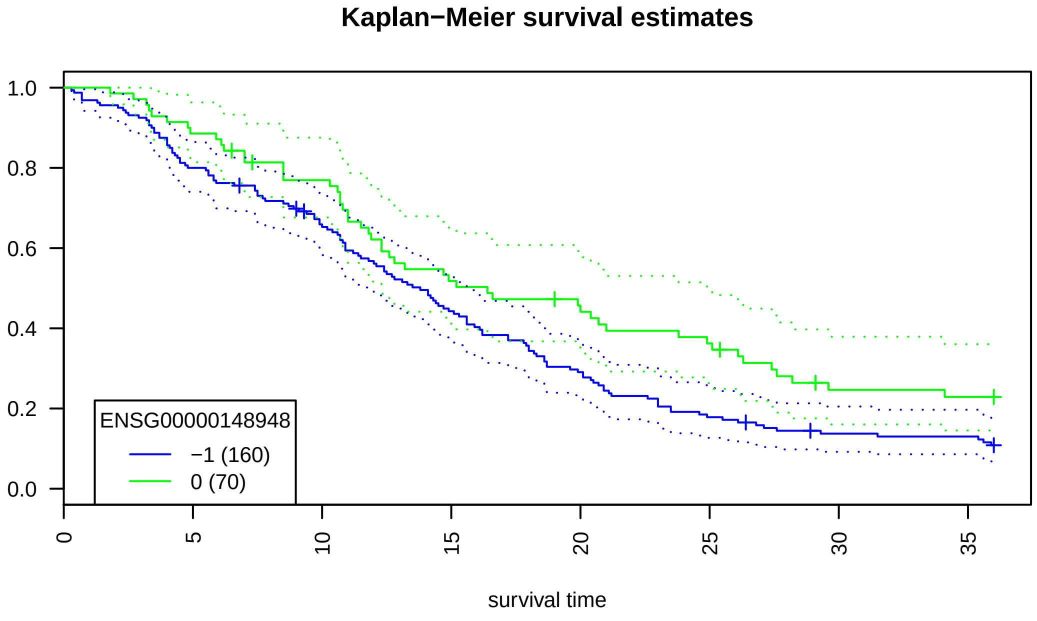

Figure 1662: Each curve represents the relative number of ENSG00000148948 survived to the corresponding point of time. Dashed lines represent 0.95 confidence intervals for the curves with the corresponding color. Log-rank probability measure for the equality of these curves is 1.633196e-02. Additional annotations: *.GeneName*=LRRC4C, *.GeneDesc*=leucine rich repeat containing 4C [Source:HGNC Symbol;Acc:29317].

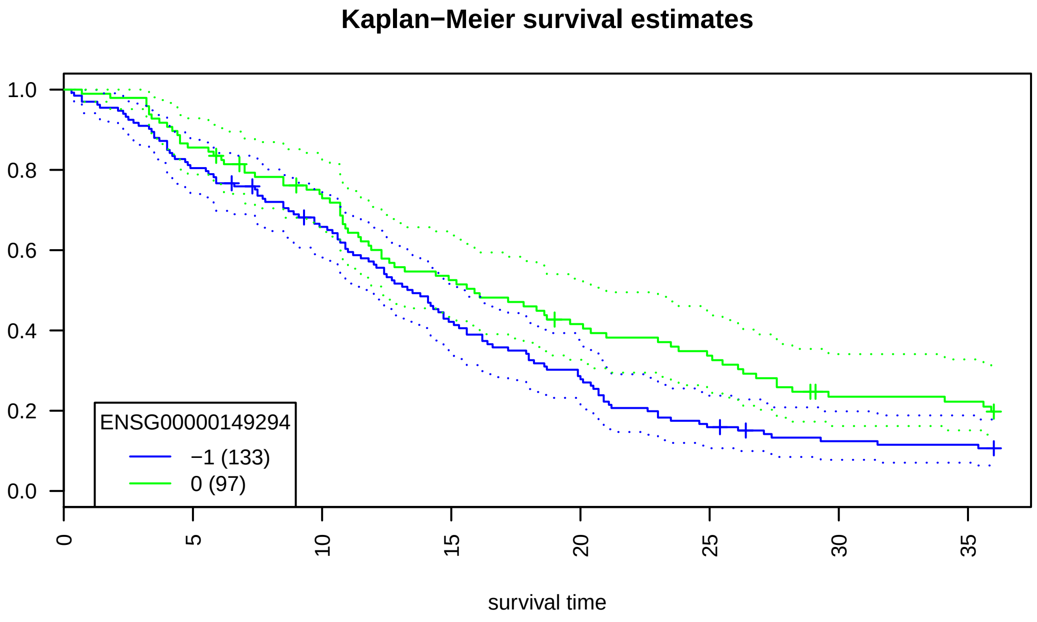

Figure 1663: Each curve represents the relative number of ENSG00000149294 survived to the corresponding point of time. Dashed lines represent 0.95 confidence intervals for the curves with the corresponding color. Log-rank probability measure for the equality of these curves is 2.035960e-02. Additional annotations: *.GeneName*=NCAM1, *.GeneDesc*=neural cell adhesion molecule 1 [Source:HGNC Symbol;Acc:7656].

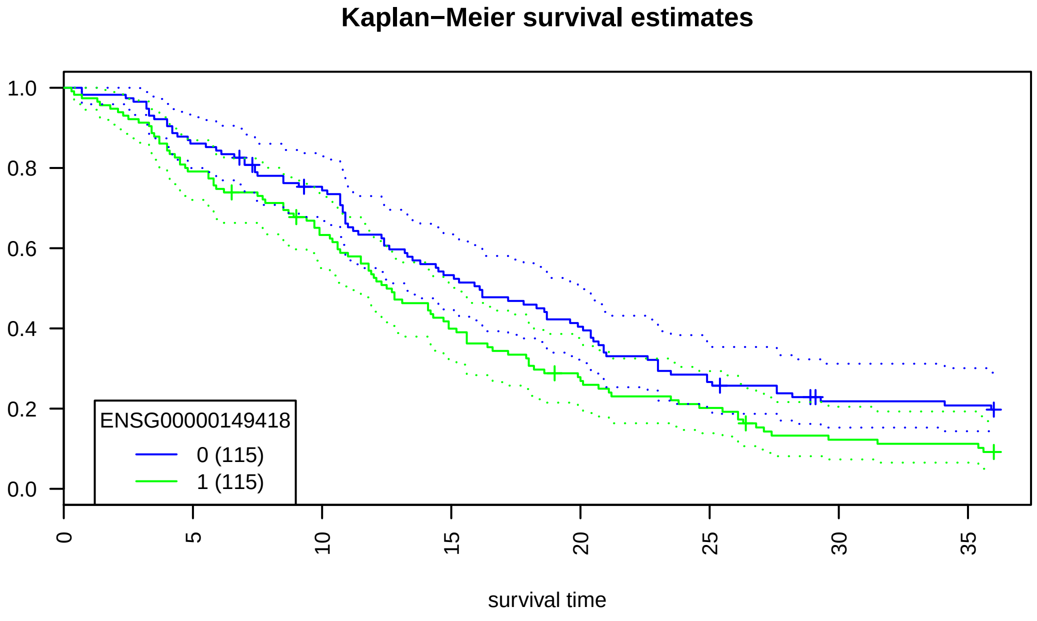

Figure 1664: Each curve represents the relative number of ENSG00000149418 survived to the corresponding point of time. Dashed lines represent 0.95 confidence intervals for the curves with the corresponding color. Log-rank probability measure for the equality of these curves is 1.786562e-02. Additional annotations: *.GeneName*=ST14, *.GeneDesc*=suppression of tumorigenicity 14 (colon carcinoma) [Source:HGNC Symbol;Acc:11344].

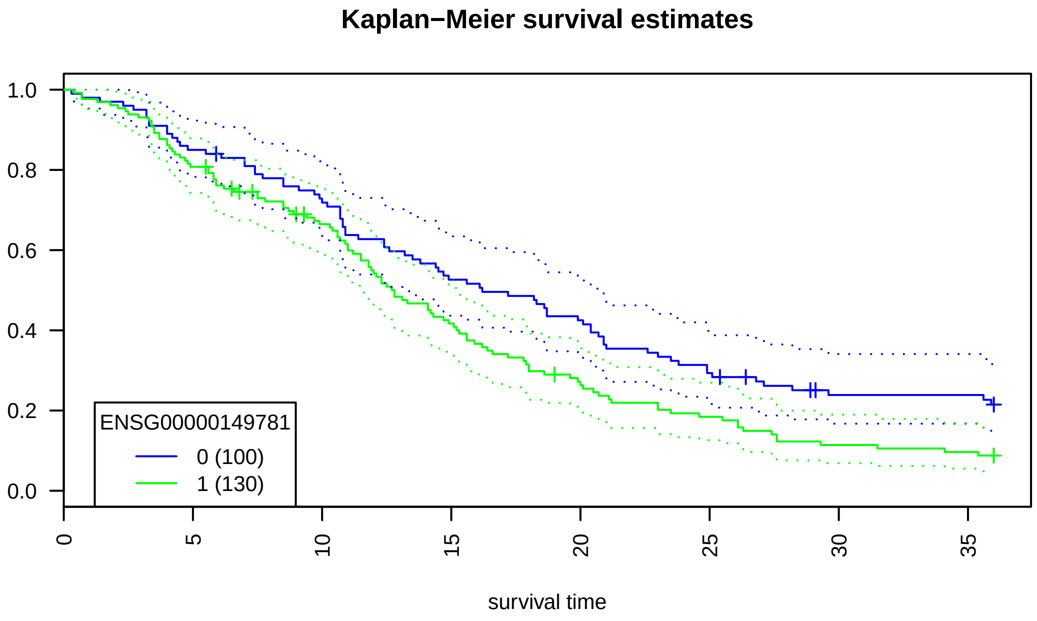

Figure 1665: Each curve represents the relative number of ENSG00000149781 survived to the corresponding point of time. Dashed lines represent 0.95 confidence intervals for the curves with the corresponding color. Log-rank probability measure for the equality of these curves is 8.709954e-03. Additional annotations: *.GeneName*=FERMT3, *.GeneDesc*=fermitin family homolog 3 (Drosophila) [Source:HGNC Symbol;Acc:23151].

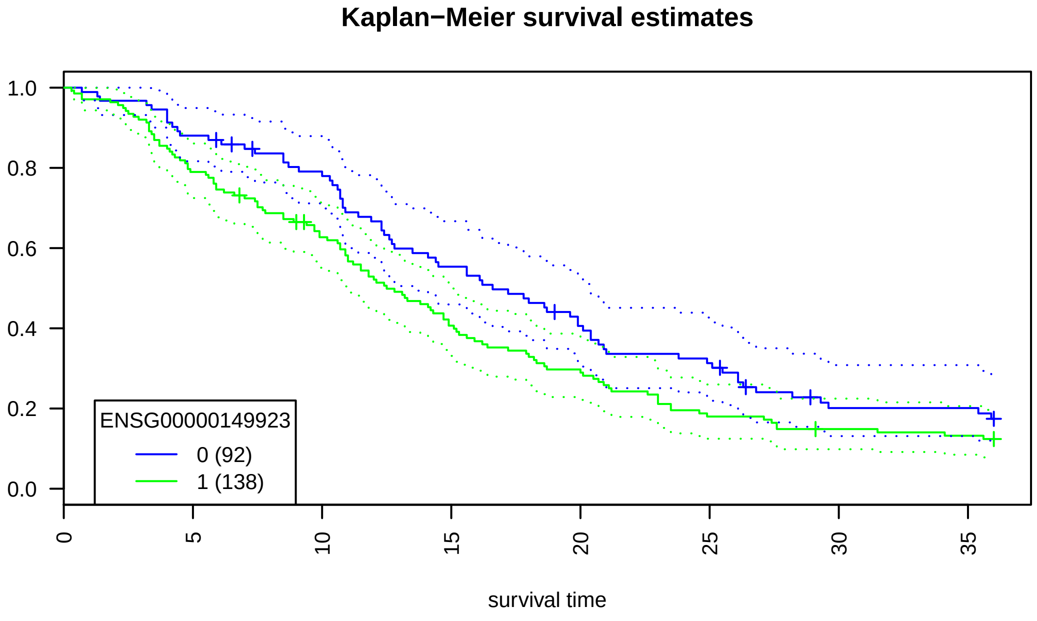

Figure 1666: Each curve represents the relative number of ENSG00000149923 survived to the corresponding point of time. Dashed lines represent 0.95 confidence intervals for the curves with the corresponding color. Log-rank probability measure for the equality of these curves is 4.023349e-02. Additional annotations: *.GeneName*=PPP4C, *.GeneDesc*=protein phosphatase 4, catalytic subunit [Source:HGNC Symbol;Acc:9319].

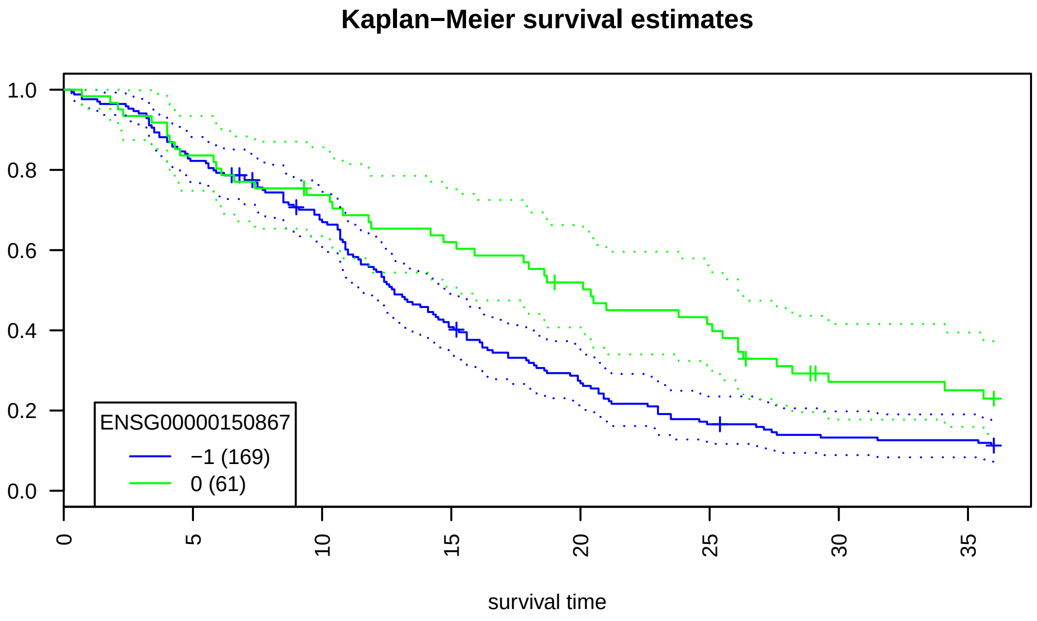

Figure 1667: Each curve represents the relative number of ENSG00000150867 survived to the corresponding point of time. Dashed lines represent 0.95 confidence intervals for the curves with the corresponding color. Log-rank probability measure for the equality of these curves is 5.063812e-03. Additional annotations: *.GeneName*=PIP5K2A, *.GeneDesc*=phosphatidylinositol-5-phosphate 4-kinase, type II, alpha [Source:HGNC Symbol;Acc:8997].

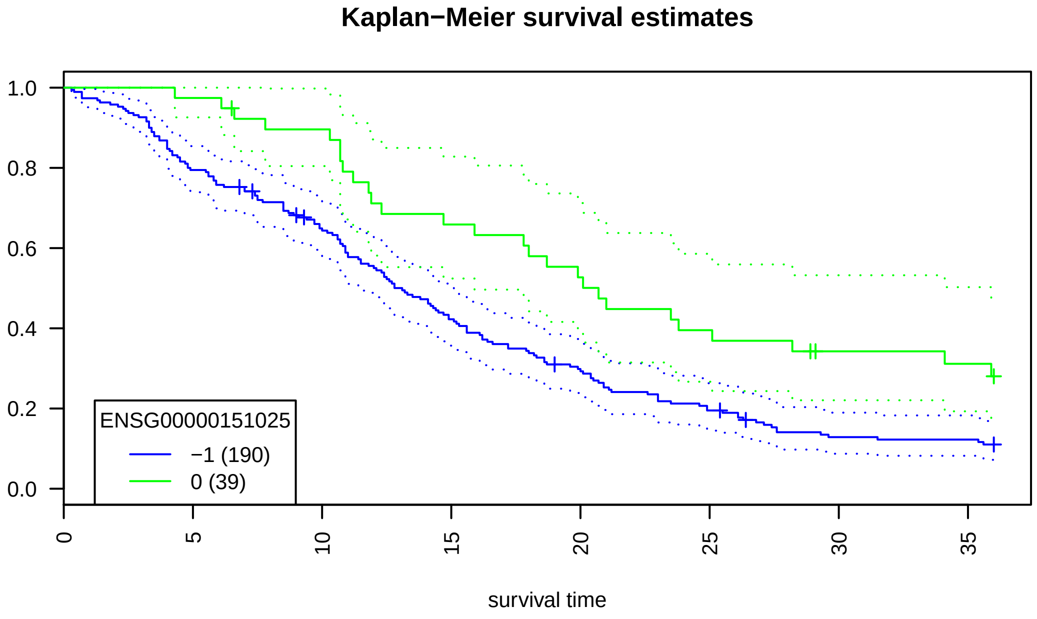

Figure 1668: Each curve represents the relative number of ENSG00000151025 survived to the corresponding point of time. Dashed lines represent 0.95 confidence intervals for the curves with the corresponding color. Total of 1 observation deleted due to missingness. Log-rank probability measure for the equality of these curves is 2.094242e-03. Additional annotations: *.GeneName*=GPR158, *.GeneDesc*=G protein-coupled receptor 158 [Source:HGNC Symbol;Acc:23689].

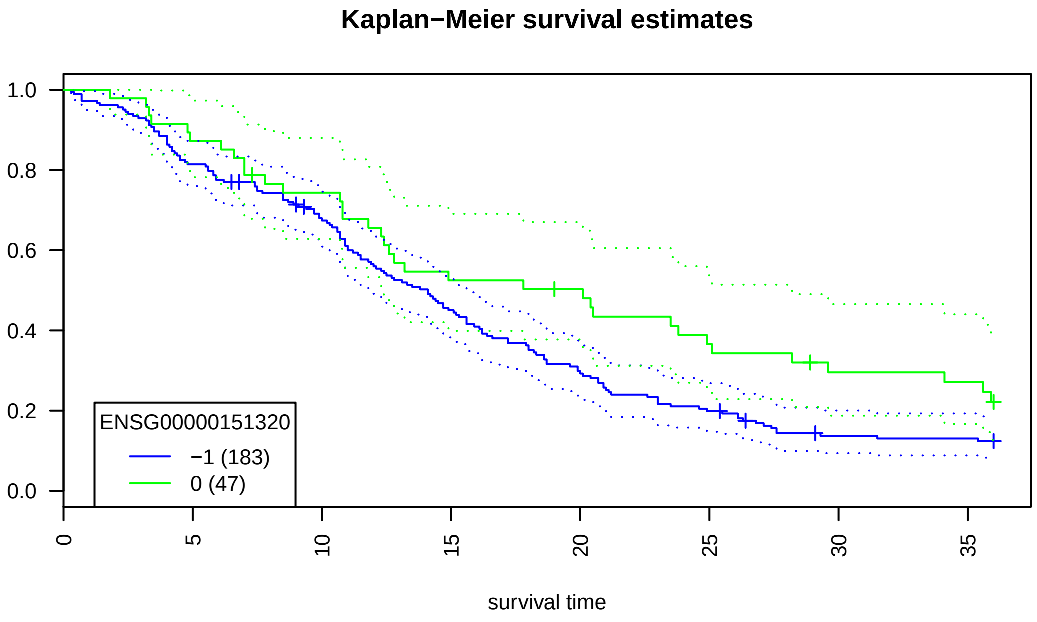

Figure 1669: Each curve represents the relative number of ENSG00000151320 survived to the corresponding point of time. Dashed lines represent 0.95 confidence intervals for the curves with the corresponding color. Log-rank probability measure for the equality of these curves is 3.958949e-02. Additional annotations: *.GeneName*=AKAP6, *.GeneDesc*=A kinase (PRKA) anchor protein 6 [Source:HGNC Symbol;Acc:376].

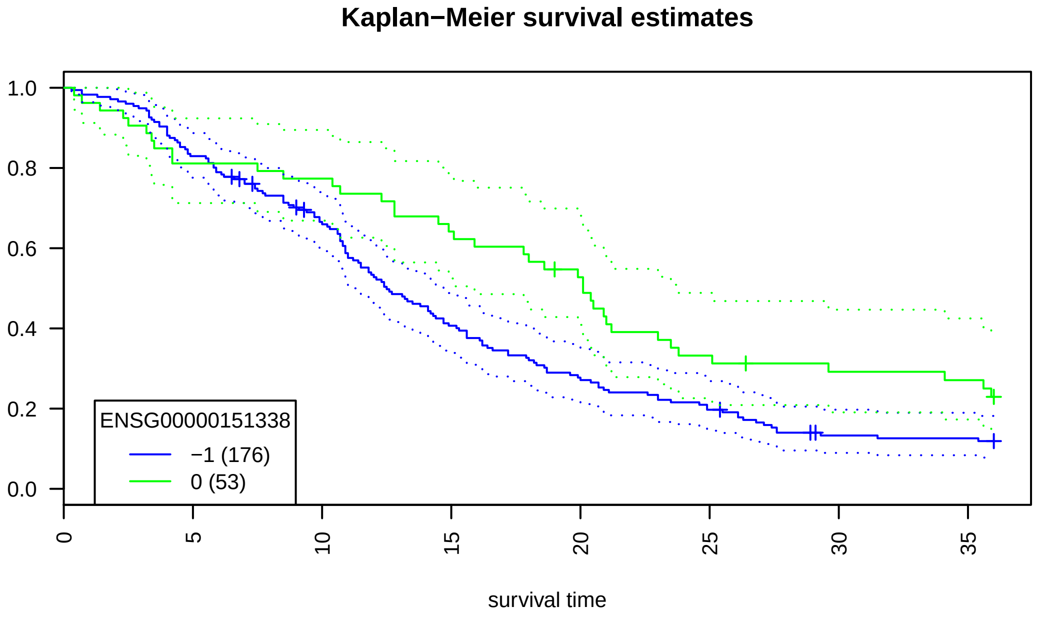

Figure 1670: Each curve represents the relative number of ENSG00000151338 survived to the corresponding point of time. Dashed lines represent 0.95 confidence intervals for the curves with the corresponding color. Total of 1 observation deleted due to missingness. Log-rank probability measure for the equality of these curves is 1.029698e-02. Additional annotations: *.GeneName*=MIPOL1, *.GeneDesc*=mirror-image polydactyly 1 [Source:HGNC Symbol;Acc:21460].

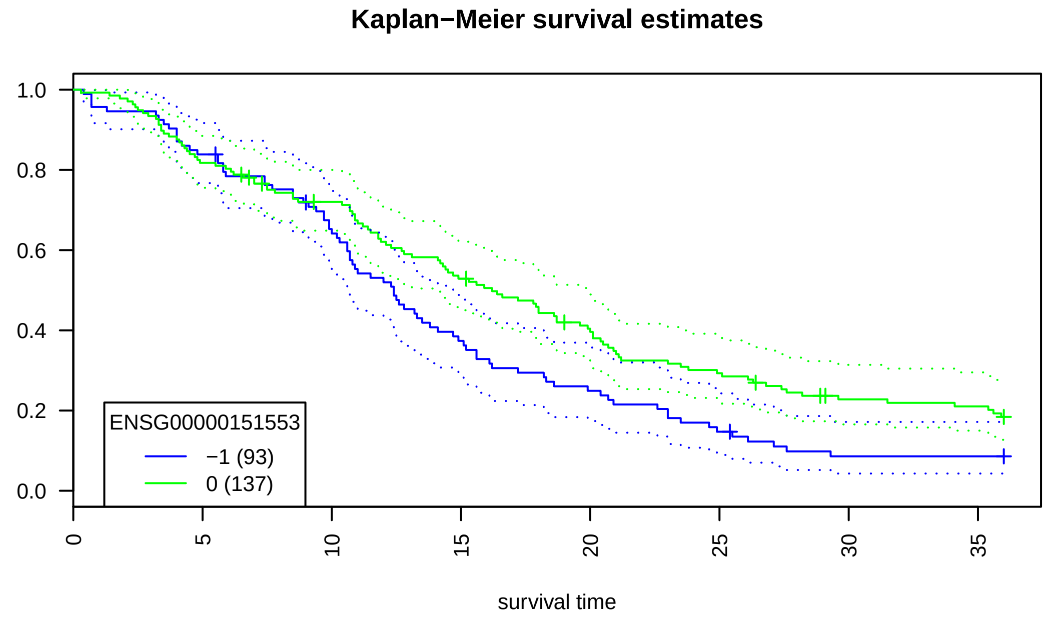

Figure 1671: Each curve represents the relative number of ENSG00000151553 survived to the corresponding point of time. Dashed lines represent 0.95 confidence intervals for the curves with the corresponding color. Log-rank probability measure for the equality of these curves is 1.023249e-02. Additional annotations: *.GeneName*=FAM160B1, *.GeneDesc*=family with sequence similarity 160, member B1 [Source:HGNC Symbol;Acc:29320].

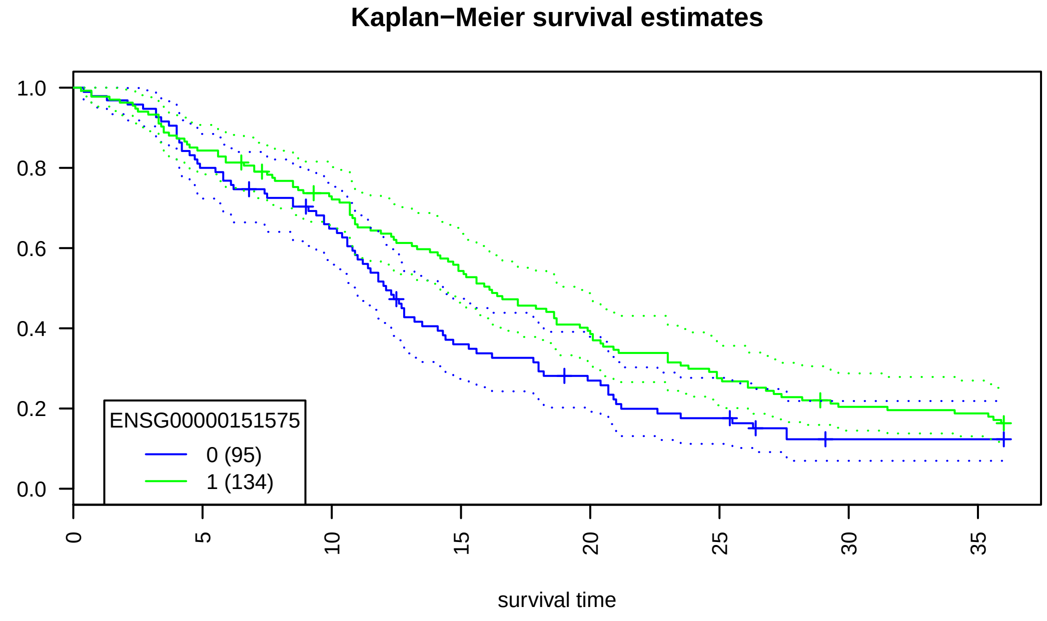

Figure 1672: Each curve represents the relative number of ENSG00000151575 survived to the corresponding point of time. Dashed lines represent 0.95 confidence intervals for the curves with the corresponding color. Total of 1 observation deleted due to missingness. Log-rank probability measure for the equality of these curves is 4.65876e-02. Additional annotations: *.GeneName*=TEX9, *.GeneDesc*=testis expressed 9 [Source:HGNC Symbol;Acc:29585].

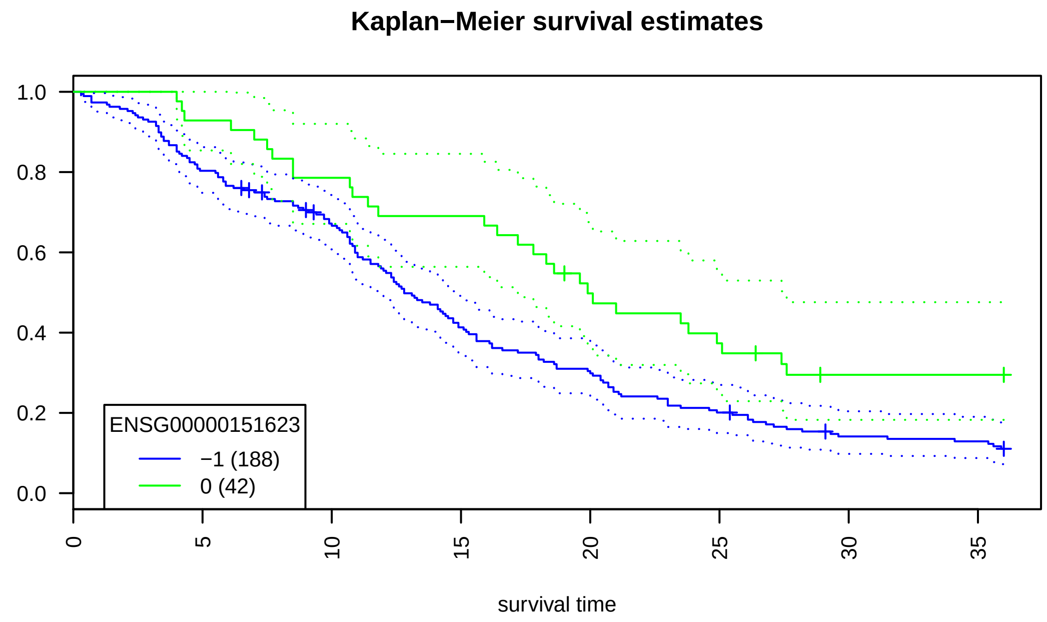

Figure 1673: Each curve represents the relative number of ENSG00000151623 survived to the corresponding point of time. Dashed lines represent 0.95 confidence intervals for the curves with the corresponding color. Log-rank probability measure for the equality of these curves is 3.092295e-03. Additional annotations: *.GeneName*=NR3C2, *.GeneDesc*=nuclear receptor subfamily 3, group C, member 2 [Source:HGNC Symbol;Acc:7979].

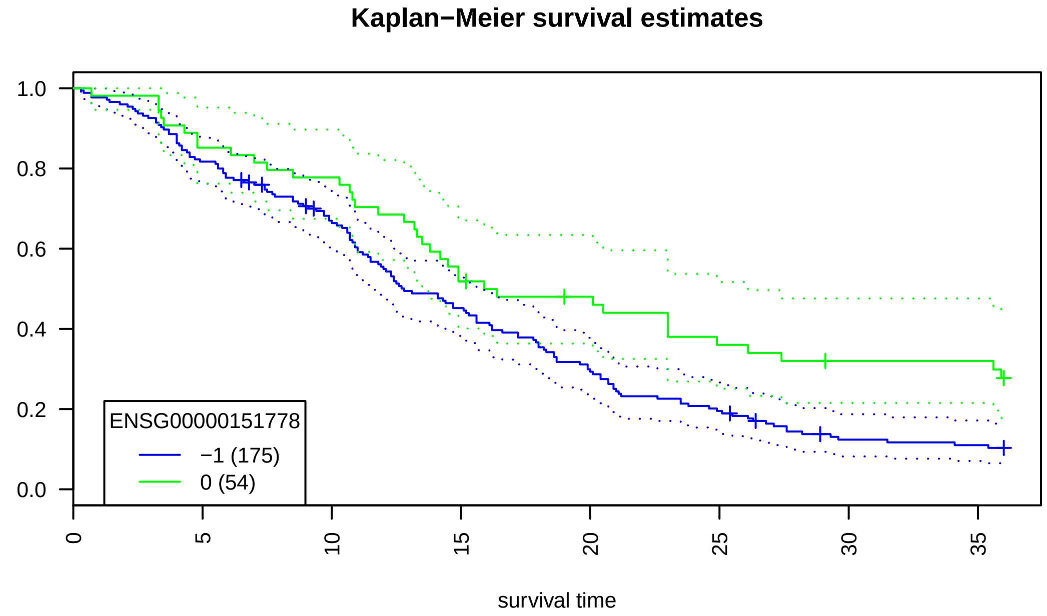

Figure 1674: Each curve represents the relative number of ENSG00000151778 survived to the corresponding point of time. Dashed lines represent 0.95 confidence intervals for the curves with the corresponding color. Total of 1 observation deleted due to missingness. Log-rank probability measure for the equality of these curves is 5.908576e-03. Additional annotations: *.GeneName*=SERP2, *.GeneDesc*=stress-associated endoplasmic reticulum protein family member 2 [Source:HGNC Symbol;Acc:20607].

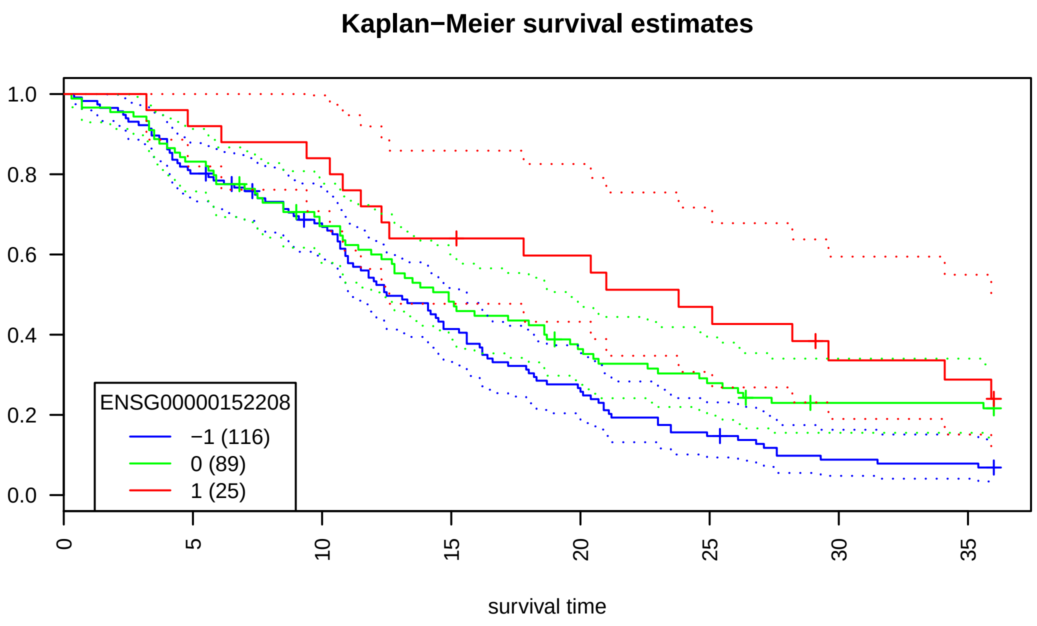

Figure 1675: Each curve represents the relative number of ENSG00000152208 survived to the corresponding point of time. Dashed lines represent 0.95 confidence intervals for the curves with the corresponding color. Log-rank probability measure for the equality of these curves is 5.240793e-03. Additional annotations: *.GeneName*=GRID2, *.GeneDesc*=glutamate receptor, ionotropic, delta 2 [Source:HGNC Symbol;Acc:4576].

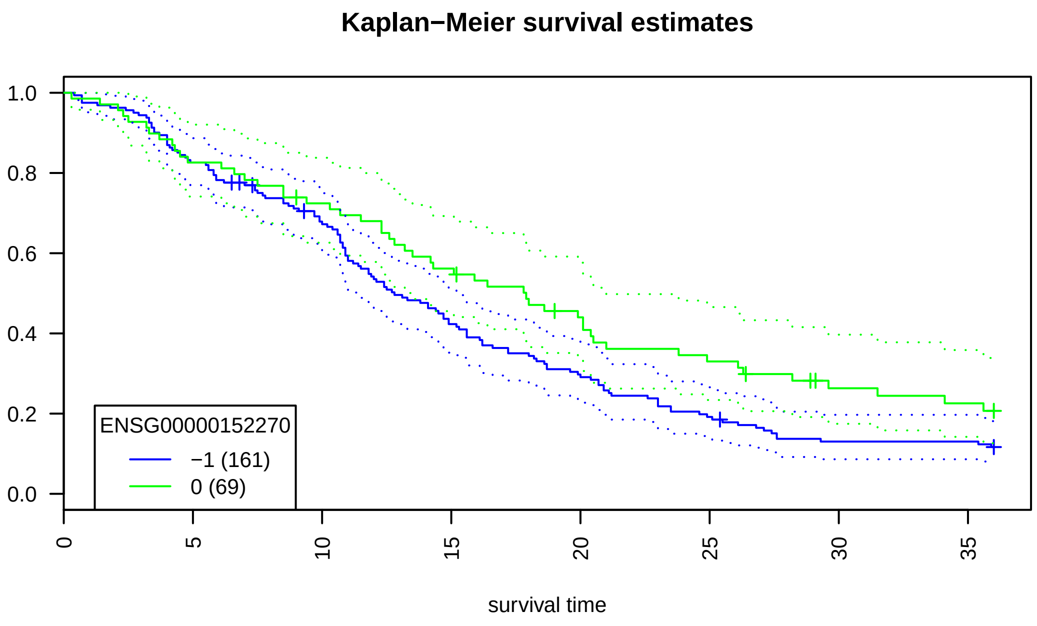

Figure 1676: Each curve represents the relative number of ENSG00000152270 survived to the corresponding point of time. Dashed lines represent 0.95 confidence intervals for the curves with the corresponding color. Log-rank probability measure for the equality of these curves is 3.738949e-02. Additional annotations: *.GeneName*=PDE3B, *.GeneDesc*=phosphodiesterase 3B, cGMP-inhibited [Source:HGNC Symbol;Acc:8779].

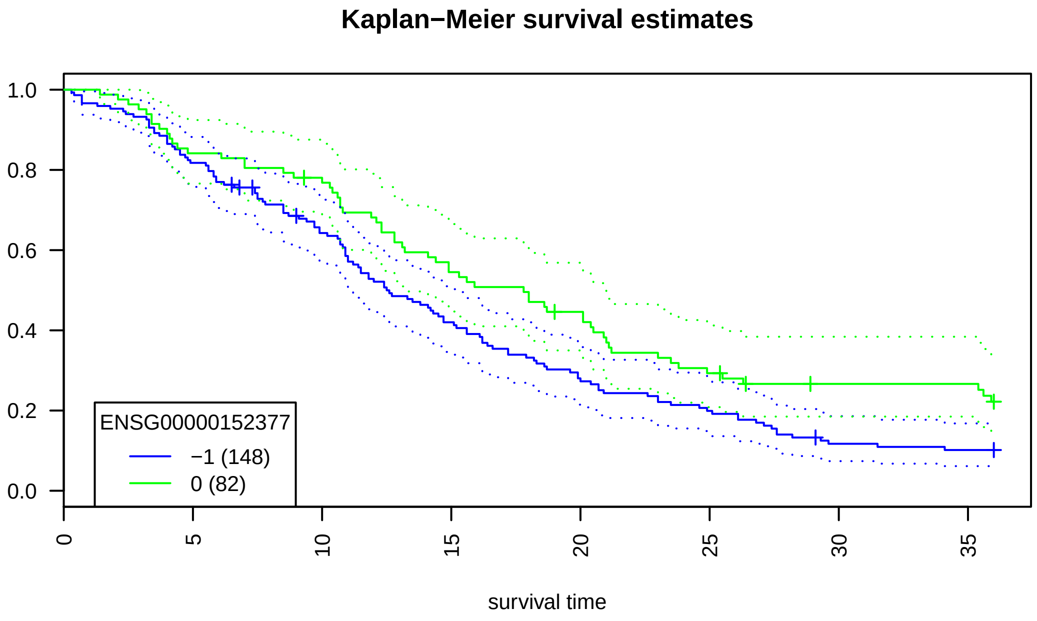

Figure 1677: Each curve represents the relative number of ENSG00000152377 survived to the corresponding point of time. Dashed lines represent 0.95 confidence intervals for the curves with the corresponding color. Log-rank probability measure for the equality of these curves is 1.153654e-02. Additional annotations: .*GeneName*=SPOCK1, .*GeneDesc*=sparc/osteonectin, cwcv and kazal-like domains proteoglycan (testican) 1 [Source:HGNC Symbol;Acc:11251].

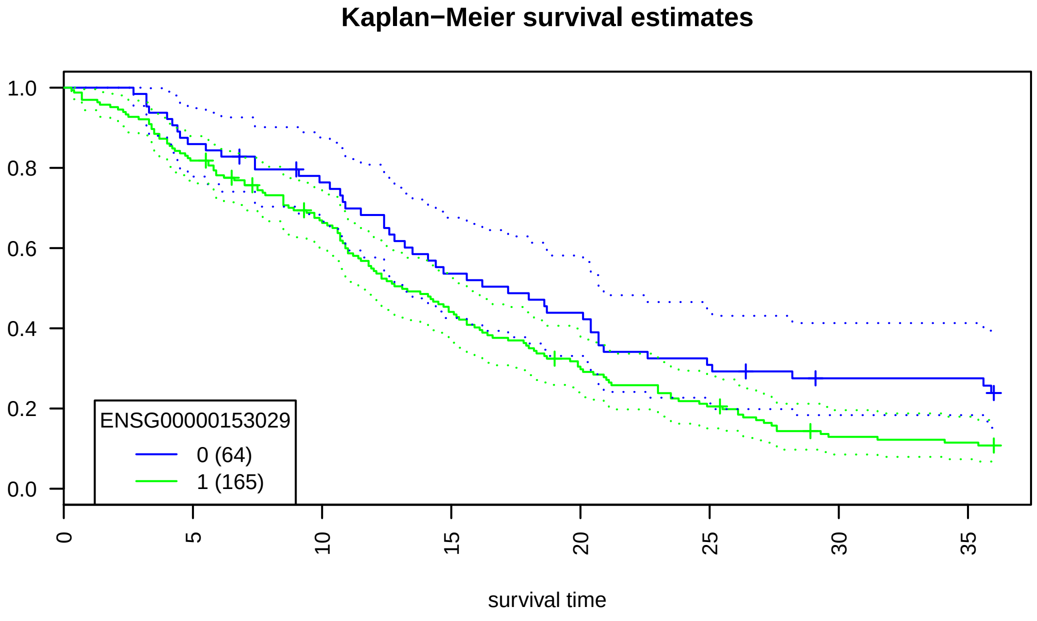

Figure 1678: Each curve represents the relative number of ENSG00000153029 survived to the corresponding point of time. Dashed lines represent 0.95 confidence intervals for the curves with the corresponding color. Total of 1 observation deleted due to missingness. Log-rank probability measure for the equality of these curves is 2.331544e-02. Additional annotations: .*GeneName*=MR1, .*GeneDesc*=major histocompatibility complex, class I-related [Source:HGNC Symbol;Acc:4975].

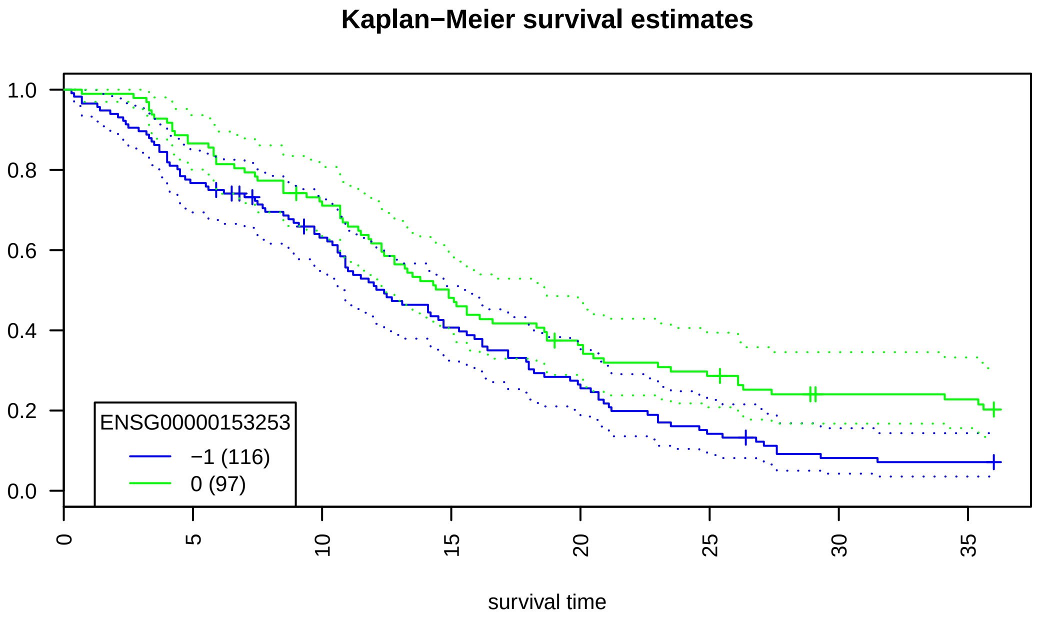

Figure 1679: Each curve represents the relative number of ENSG00000153253 survived to the corresponding point of time. Dashed lines represent 0.95 confidence intervals for the curves with the corresponding color. Total of 17 observations deleted due to missingness. Log-rank probability measure for the equality of these curves is 1.006917e-02. Additional annotations: *.GeneName*=SCN3A, *.GeneDesc*=sodium channel, voltage-gated, type III, alpha subunit [Source:HGNC Symbol;Acc:10590].

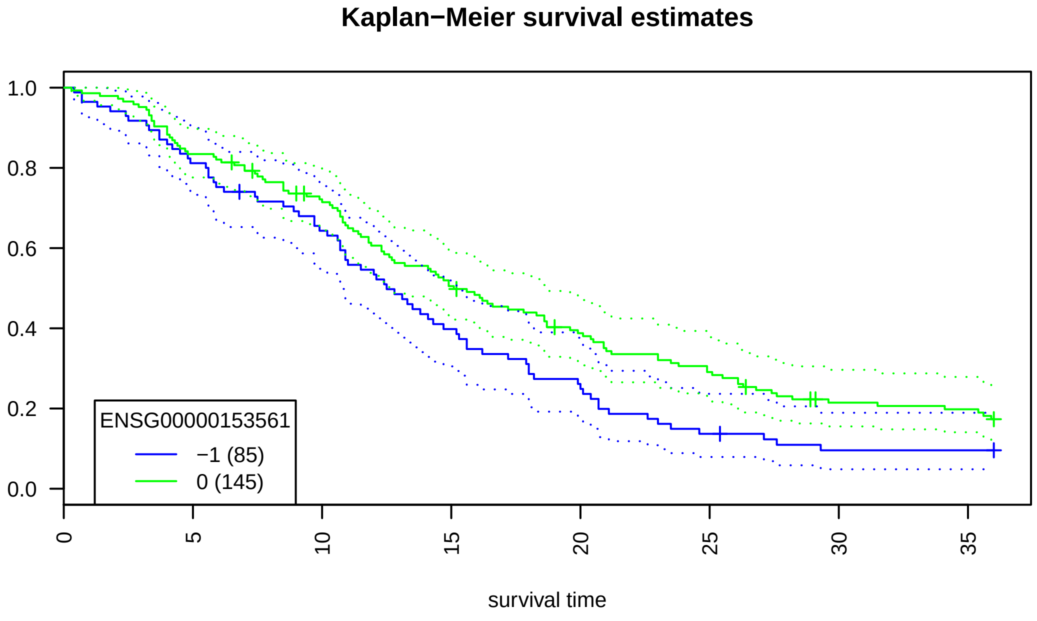

Figure 1680: Each curve represents the relative number of ENSG00000153561 survived to the corresponding point of time. Dashed lines represent 0.95 confidence intervals for the curves with the corresponding color. Log-rank probability measure for the equality of these curves is 2.238386e-02. Additional annotations: *.GeneName*=RMND5A, *.GeneDesc*=required for meiotic nuclear division 5 homolog A (S. cerevisiae) [Source:HGNC Symbol;Acc:25850].

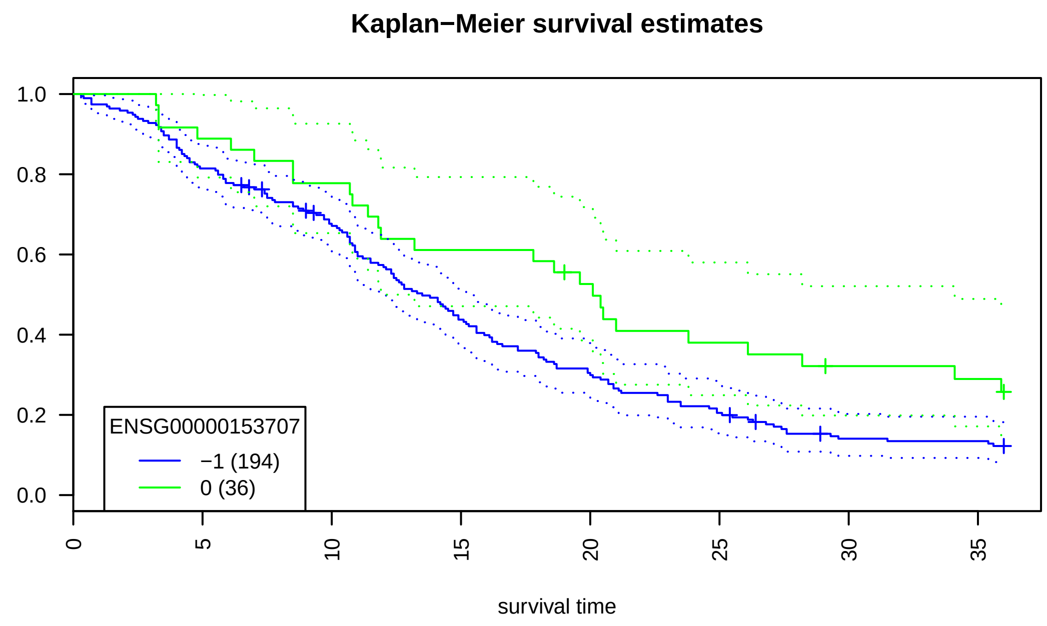

Figure 1681: Each curve represents the relative number of ENSG00000153707 survived to the corresponding point of time. Dashed lines represent 0.95 confidence intervals for the curves with the corresponding color. Log-rank probability measure for the equality of these curves is 2.189162e-02. Additional annotations: *.GeneName*=PTPRD, *.GeneDesc*=protein tyrosine phosphatase, receptor type, D [Source:HGNC Symbol;Acc:9668].

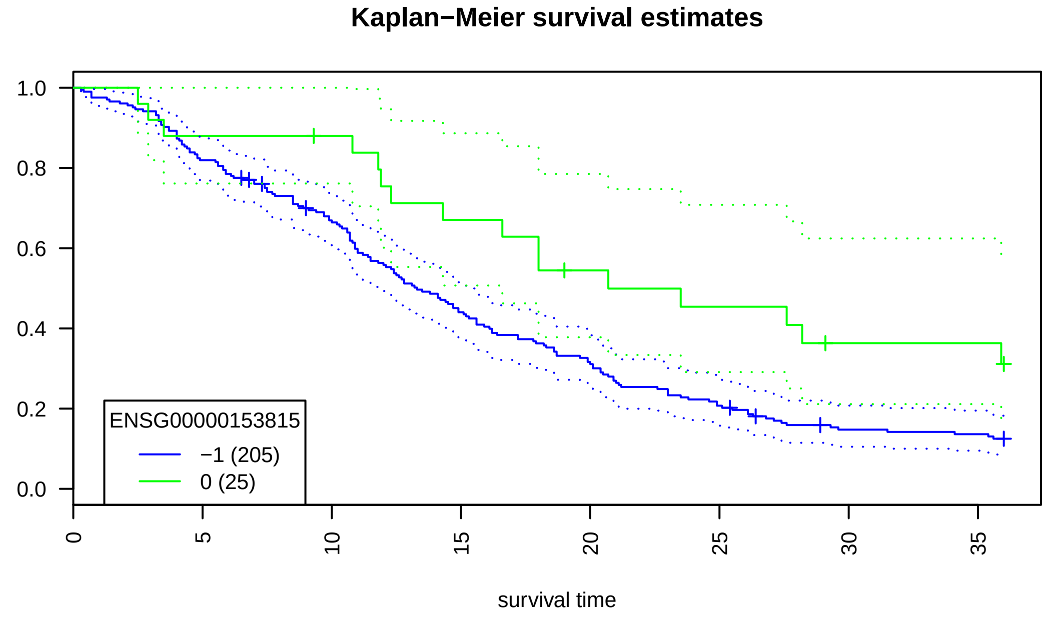

Figure 1682: Each curve represents the relative number of ENSG00000153815 survived to the corresponding point of time. Dashed lines represent 0.95 confidence intervals for the curves with the corresponding color. Log-rank probability measure for the equality of these curves is 1.225543e-02. Additional annotations: *.GeneName*=AC099524.1, *.GeneDesc*=C-Maf-inducing protein (c-Mip)(Truncated c-Maf-inducing protein)(Tc-Mip) [Source:UniProtKB/Swiss-Prot;Acc:Q8IY22].

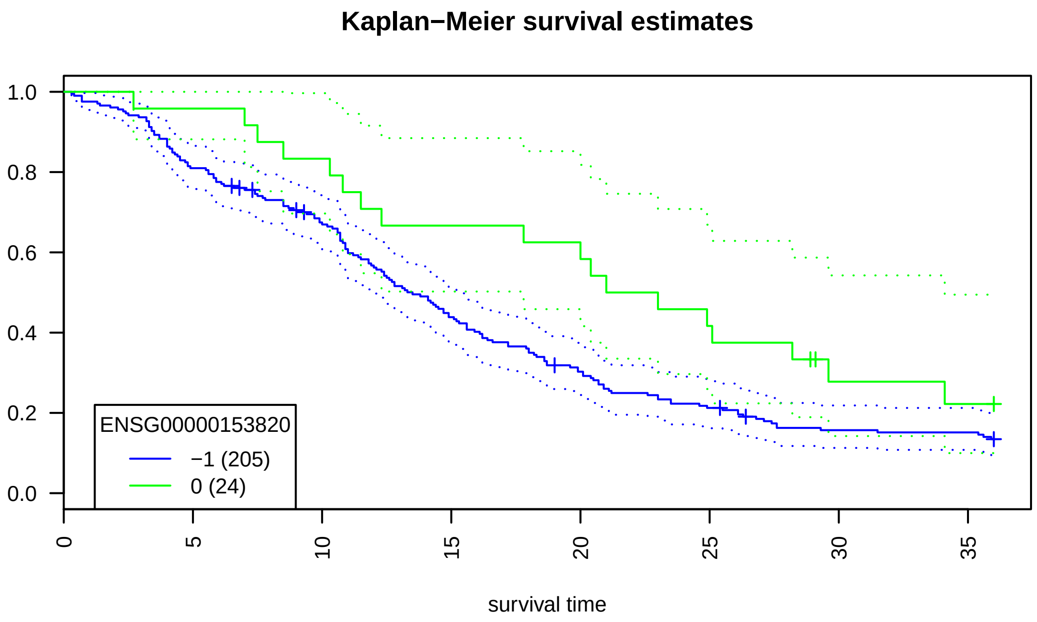

Figure 1683: Each curve represents the relative number of ENSG00000153820 survived to the corresponding point of time. Dashed lines represent 0.95 confidence intervals for the curves with the corresponding color. Total of 1 observation deleted due to missingness. Log-rank probability measure for the equality of these curves is 4.345836e-02. Additional annotations: *.GeneName*=SPHKAP, *.GeneDesc*=SPHK1 interactor, AKAP domain containing [Source:HGNC Symbol;Acc:30619].

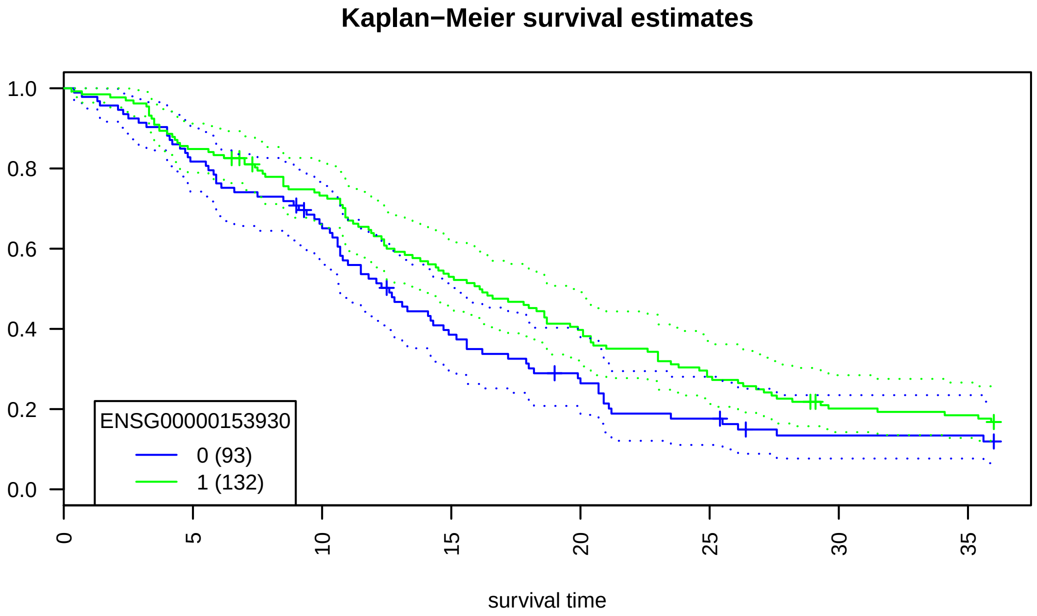

Figure 1684: Each curve represents the relative number of ENSG00000153930 survived to the corresponding point of time. Dashed lines represent 0.95 confidence intervals for the curves with the corresponding color. Total of 5 observations deleted due to missingness. Log-rank probability measure for the equality of these curves is 4.792641e-02. Additional annotations: *.GeneName*=ANKFN1, *.GeneDesc*=ankyrin-repeat and fibronectin type III domain containing 1 [Source:HGNC Symbol;Acc:26766].

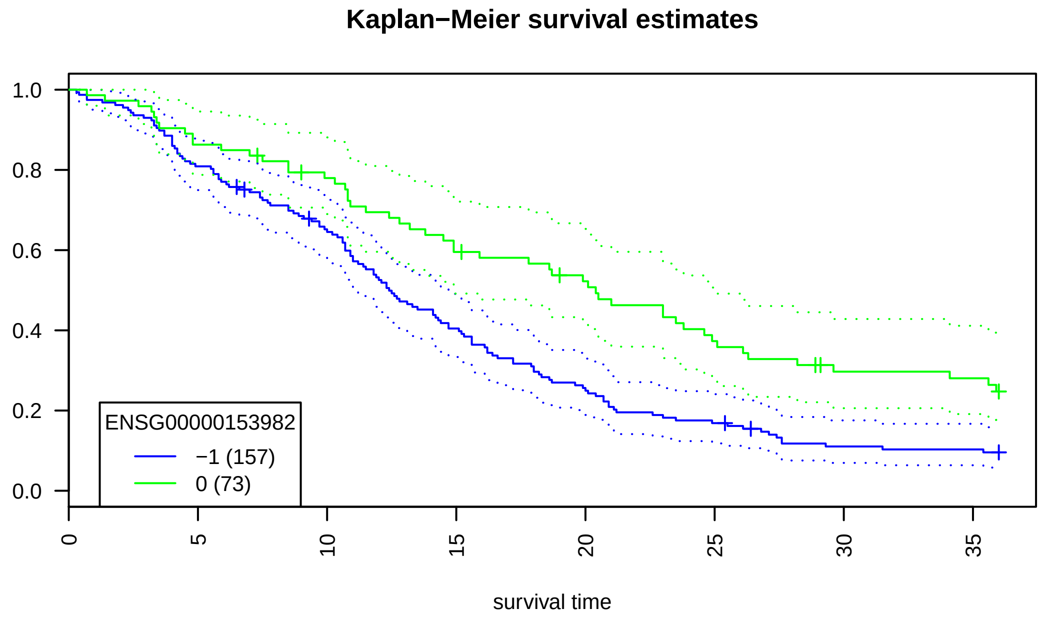

Figure 1685: Each curve represents the relative number of ENSG00000153982 survived to the corresponding point of time. Dashed lines represent 0.95 confidence intervals for the curves with the corresponding color. Log-rank probability measure for the equality of these curves is 2.924411e-04. Additional annotations: *.GeneName*=GDPD1, *.GeneDesc*=glycerophosphodiester phosphodiesterase domain containing 1 [Source:HGNC Symbol;Acc:20883].

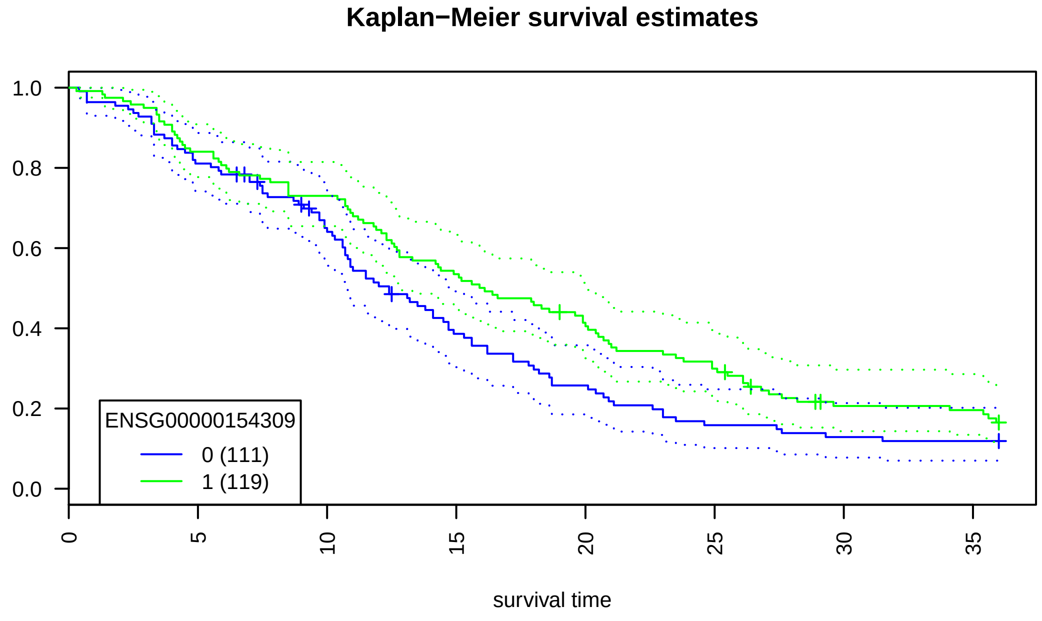

Figure 1686: Each curve represents the relative number of ENSG00000154309 survived to the corresponding point of time. Dashed lines represent 0.95 confidence intervals for the curves with the corresponding color. Log-rank probability measure for the equality of these curves is 3.975466e-02. Additional annotations: *.GeneName*=DISP1, *.GeneDesc*=dispatched homolog 1 (Drosophila) [Source:HGNC Symbol;Acc:19711].

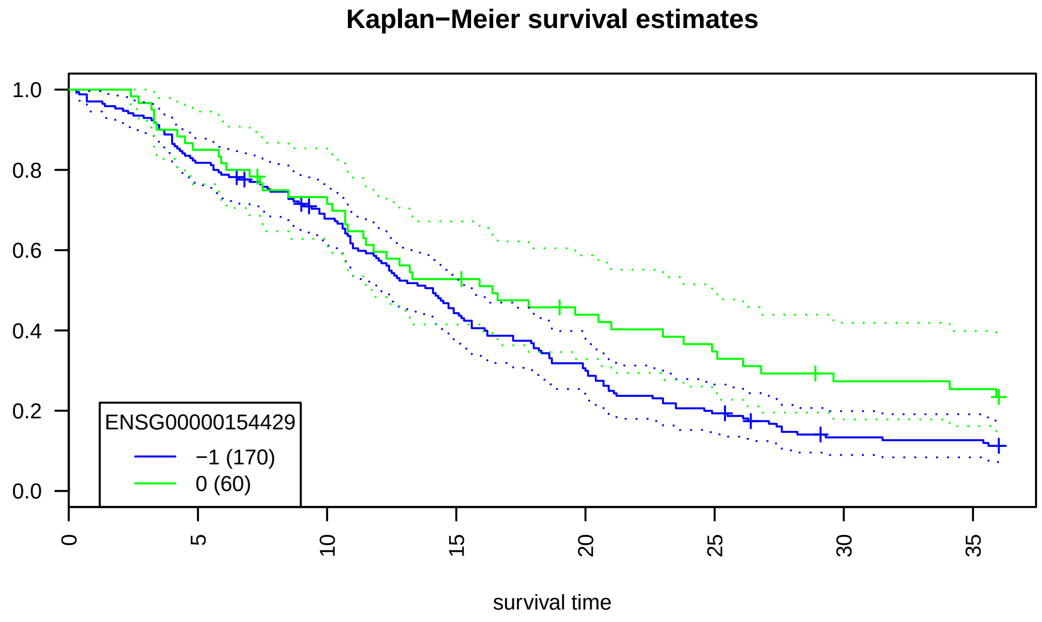

Figure 1687: Each curve represents the relative number of ENSG00000154429 survived to the corresponding point of time. Dashed lines represent 0.95 confidence intervals for the curves with the corresponding color. Log-rank probability measure for the equality of these curves is 4.223192e-02. Additional annotations: *.GeneName*=C1orf96, *.GeneDesc*=Uncharacterized protein C1orf96 [Source:UniProtKB/Swiss-Prot;Acc:Q6IQ19].

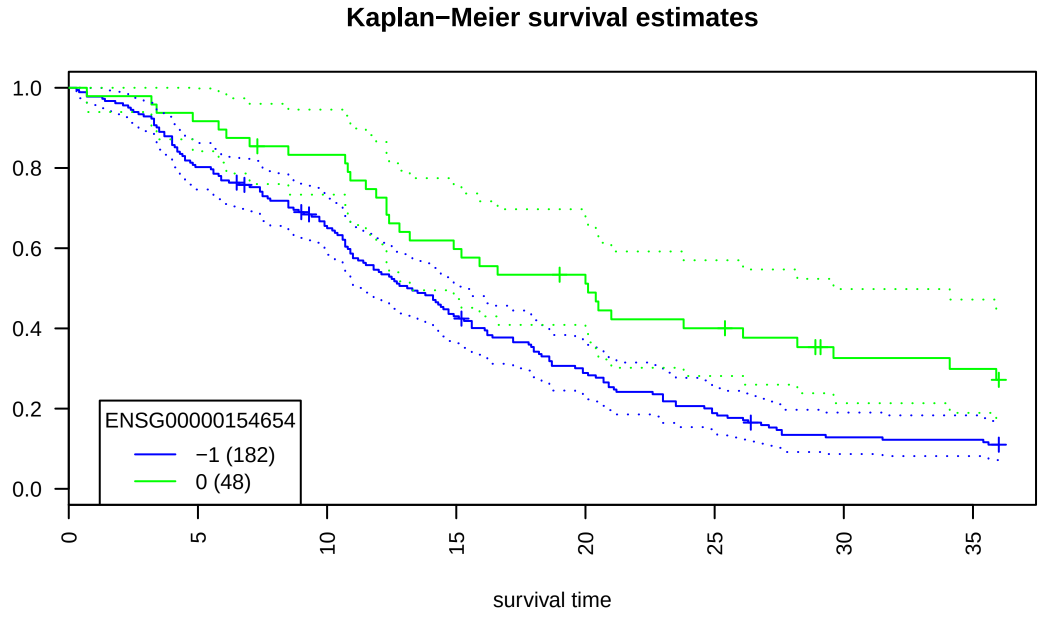

Figure 1688: Each curve represents the relative number of ENSG00000154654 survived to the corresponding point of time. Dashed lines represent 0.95 confidence intervals for the curves with the corresponding color. Log-rank probability measure for the equality of these curves is 2.757864e-03. Additional annotations: *.GeneName*=NCAM2, *.GeneDesc*=neural cell adhesion molecule 2 [Source:HGNC Symbol;Acc:7657].

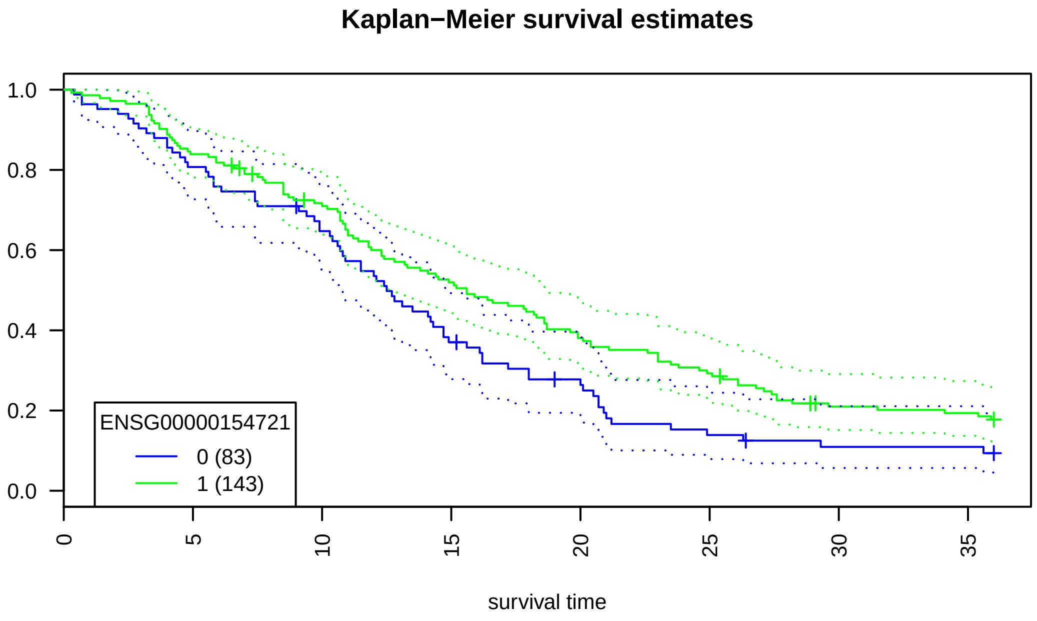

Figure 1689: Each curve represents the relative number of ENSG00000154721 survived to the corresponding point of time. Dashed lines represent 0.95 confidence intervals for the curves with the corresponding color. Total of 4 observations deleted due to missingness. Log-rank probability measure for the equality of these curves is 2.309269e-02. Additional annotations: *.GeneName*=JAM2, *.GeneDesc*=junctional adhesion molecule 2 [Source:HGNC Symbol;Acc:14686].

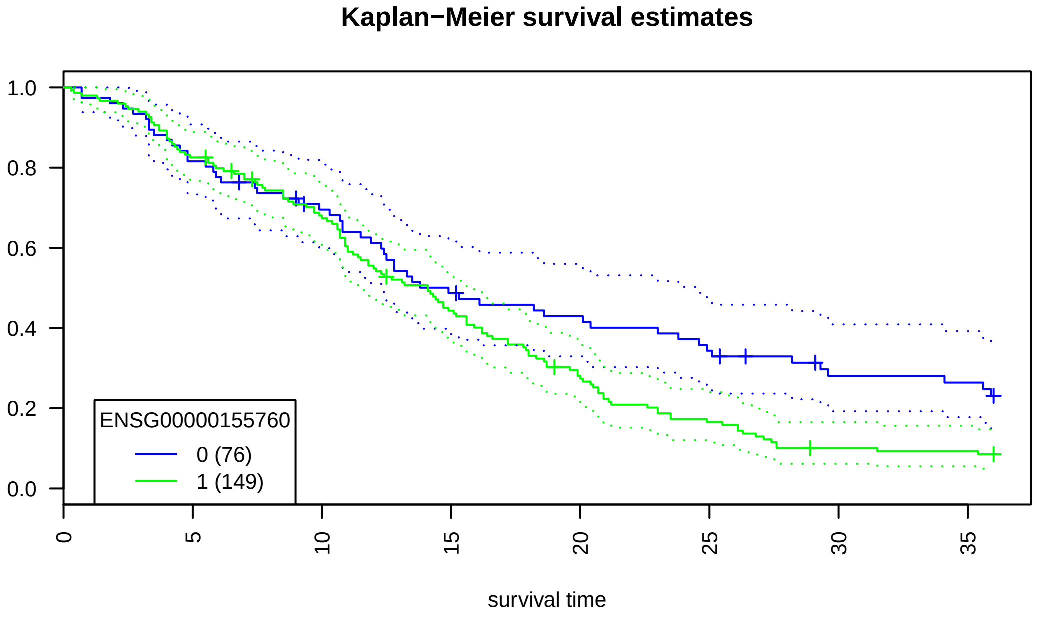

Figure 1690: Each curve represents the relative number of ENSG00000155760 survived to the corresponding point of time. Dashed lines represent 0.95 confidence intervals for the curves with the corresponding color. Total of 5 observations deleted due to missingness. Log-rank probability measure for the equality of these curves is 1.218770e-02. Additional annotations: *.GeneName*=FZD7, *.GeneDesc*=frizzled homolog 7 (Drosophila) [Source:HGNC Symbol;Acc:4045].

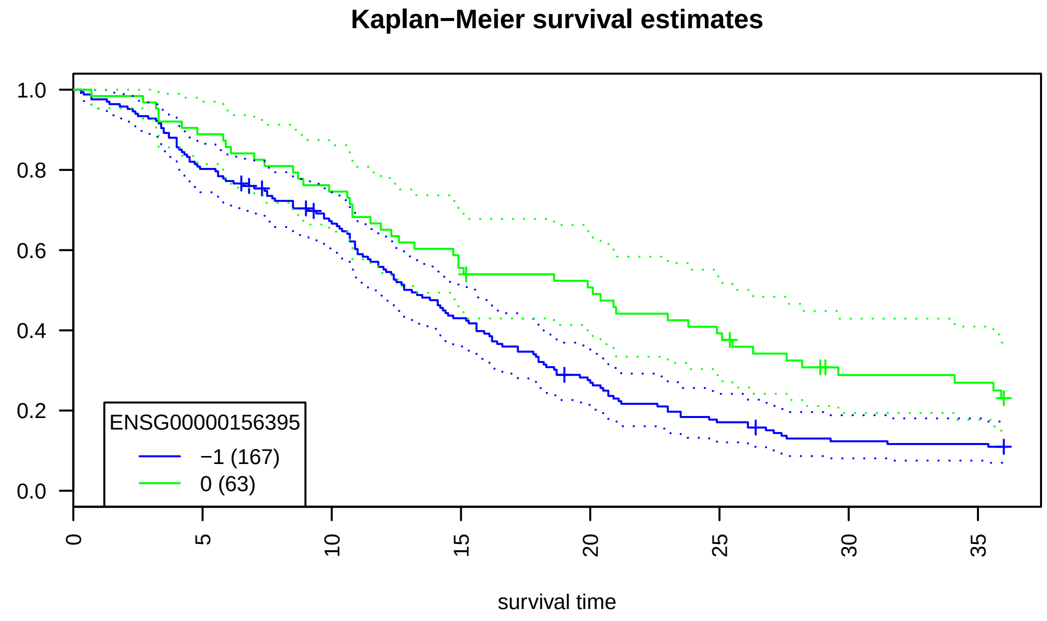

Figure 1691: Each curve represents the relative number of ENSG00000156395 survived to the corresponding point of time. Dashed lines represent 0.95 confidence intervals for the curves with the corresponding color. Log-rank probability measure for the equality of these curves is 4.064355e-03. Additional annotations: *.GeneName*=SORCS3, *.GeneDesc*=sortilin-related VPS10 domain containing receptor 3 [Source:HGNC Symbol;Acc:16699].

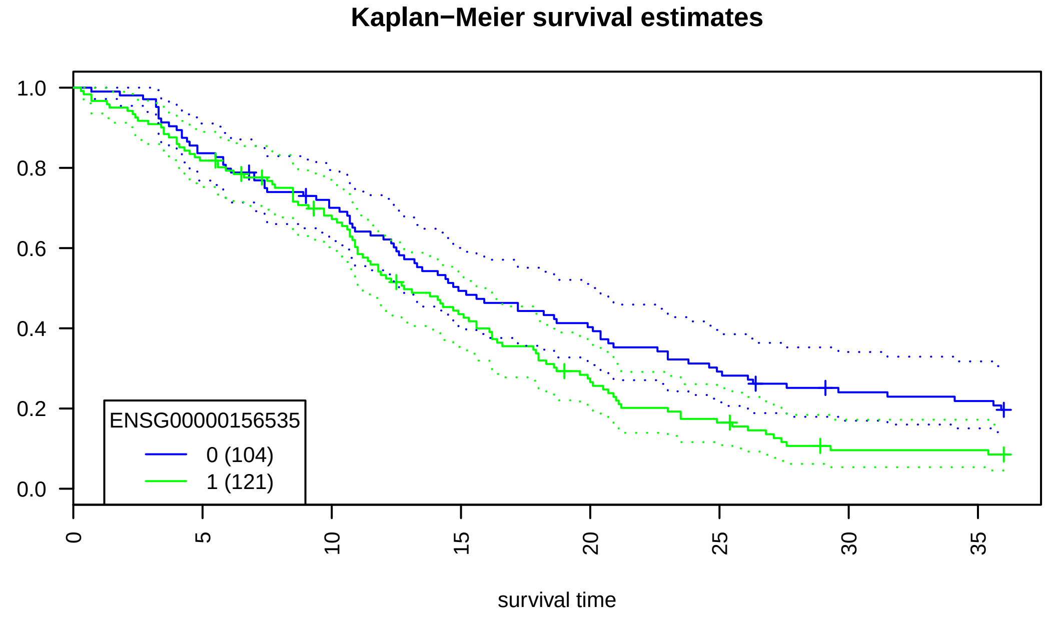

Figure 1692: Each curve represents the relative number of ENSG00000156535 survived to the corresponding point of time. Dashed lines represent 0.95 confidence intervals for the curves with the corresponding color. Total of 5 observations deleted due to missingness. Log-rank probability measure for the equality of these curves is 2.355082e-02. Additional annotations: *.GeneName*=CD109, *.GeneDesc*=CD109 molecule [Source:HGNC Symbol;Acc:21685].

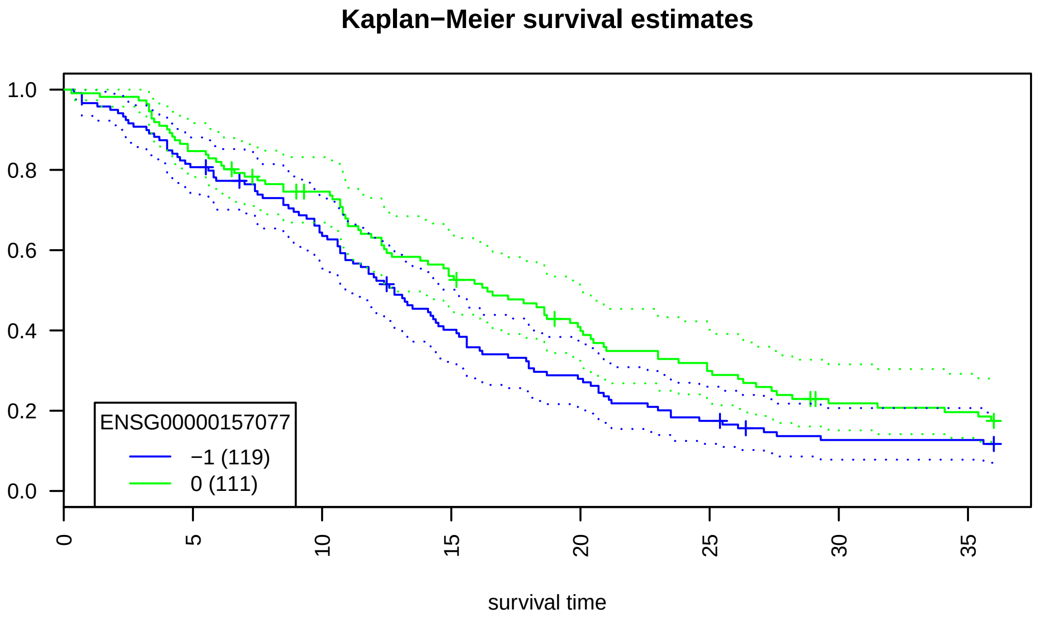

Figure 1693: Each curve represents the relative number of ENSG00000157077 survived to the corresponding point of time. Dashed lines represent 0.95 confidence intervals for the curves with the corresponding color. Log-rank probability measure for the equality of these curves is 3.770449e-02. Additional annotations: *.GeneName*=ZFYVE9, *.GeneDesc*=zinc finger, FYVE domain containing 9 [Source:HGNC Symbol;Acc:6775].

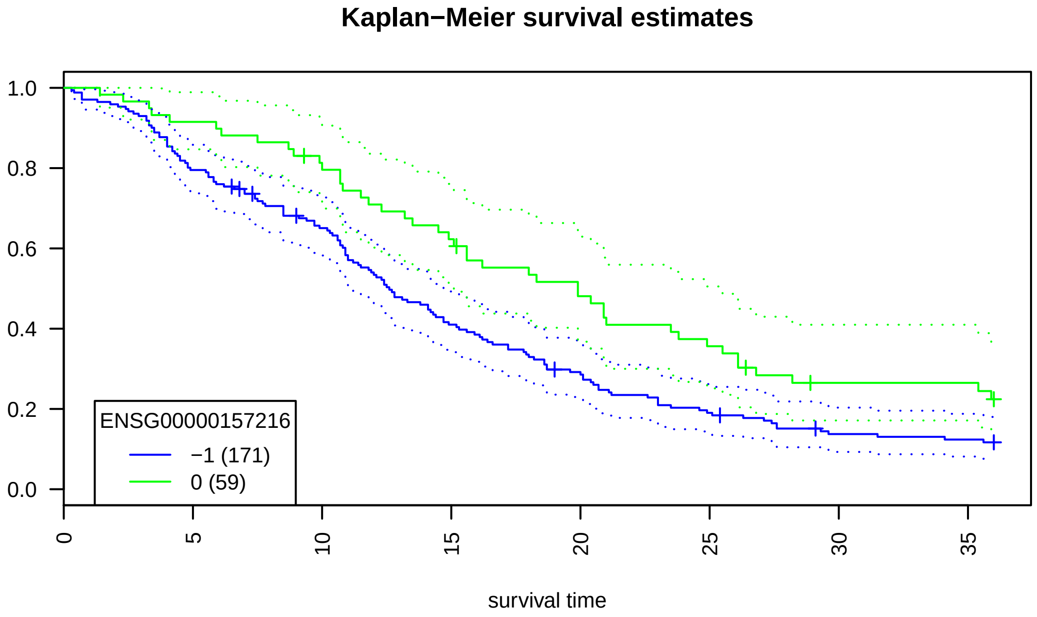

Figure 1694: Each curve represents the relative number of ENSG00000157216 survived to the corresponding point of time. Dashed lines represent 0.95 confidence intervals for the curves with the corresponding color. Log-rank probability measure for the equality of these curves is 6.075918e-03. Additional annotations: *.GeneName*=SSBP3, *.GeneDesc*=single stranded DNA binding protein 3 [Source:HGNC Symbol;Acc:15674].

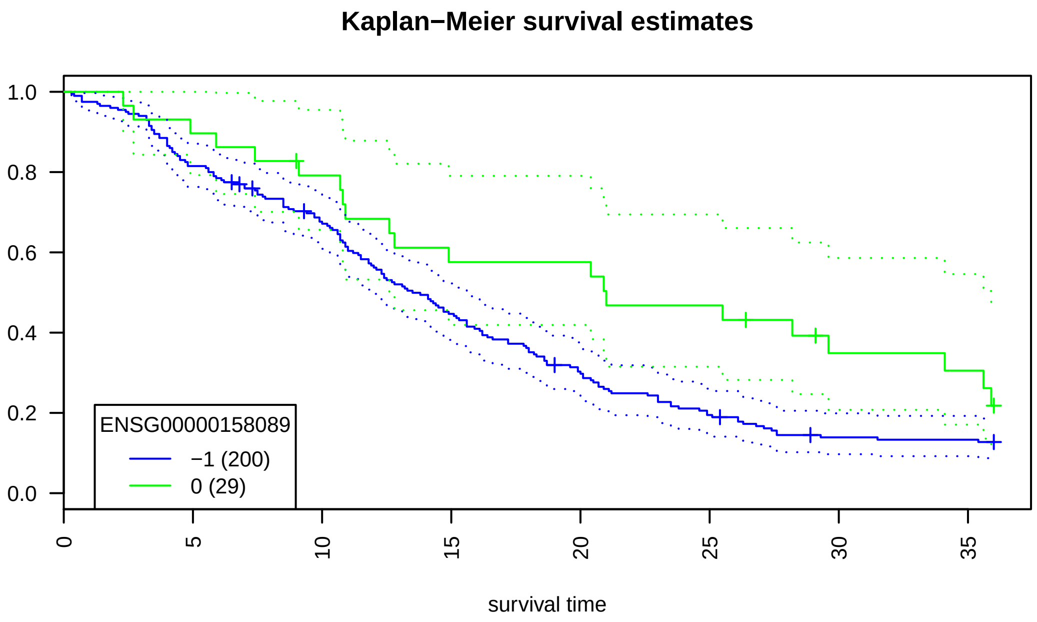

Figure 1695: Each curve represents the relative number of ENSG00000158089 survived to the corresponding point of time. Dashed lines represent 0.95 confidence intervals for the curves with the corresponding color. Total of 1 observation deleted due to missingness. Log-rank probability measure for the equality of these curves is 2.991704e-02. Additional annotations: *.GeneName*=GALNT14, *.GeneDesc*=UDP-N-acetyl-alpha-D-galactosamine:polypeptide N-acetylgalactosaminyltransferase 14 (GalNAc-T14) [Source:HGNC Symbol;Acc:22946].

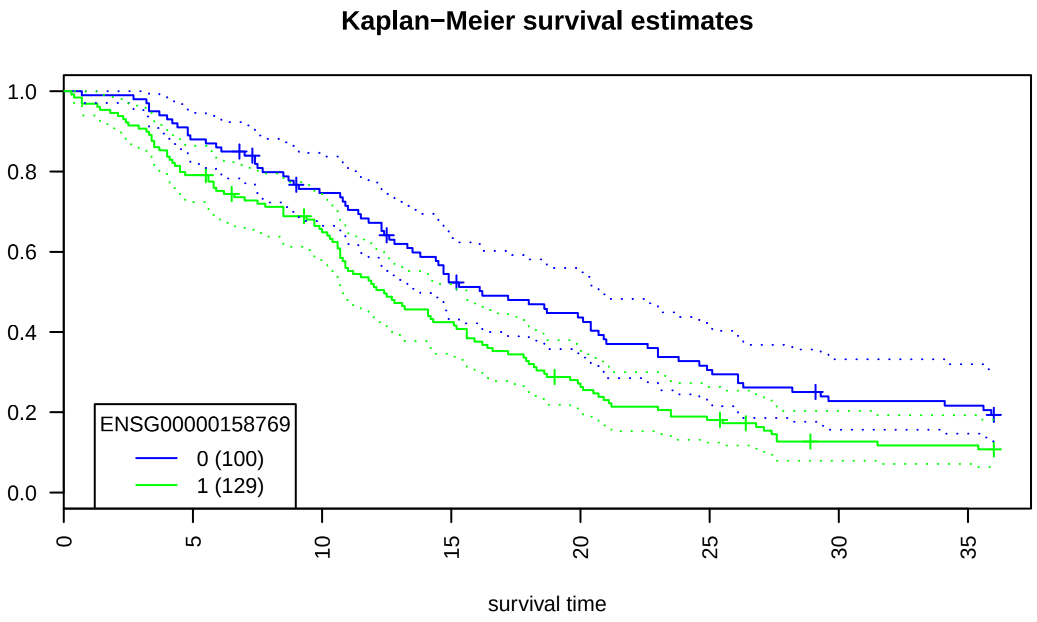

Figure 1696: Each curve represents the relative number of ENSG00000158769 survived to the corresponding point of time. Dashed lines represent 0.95 confidence intervals for the curves with the corresponding color. Total of 1 observation deleted due to missingness. Log-rank probability measure for the equality of these curves is 9.720599e-03. Additional annotations: *.GeneName*=F11R, *.GeneDesc*=F11 receptor [Source:HGNC Symbol;Acc:14685].

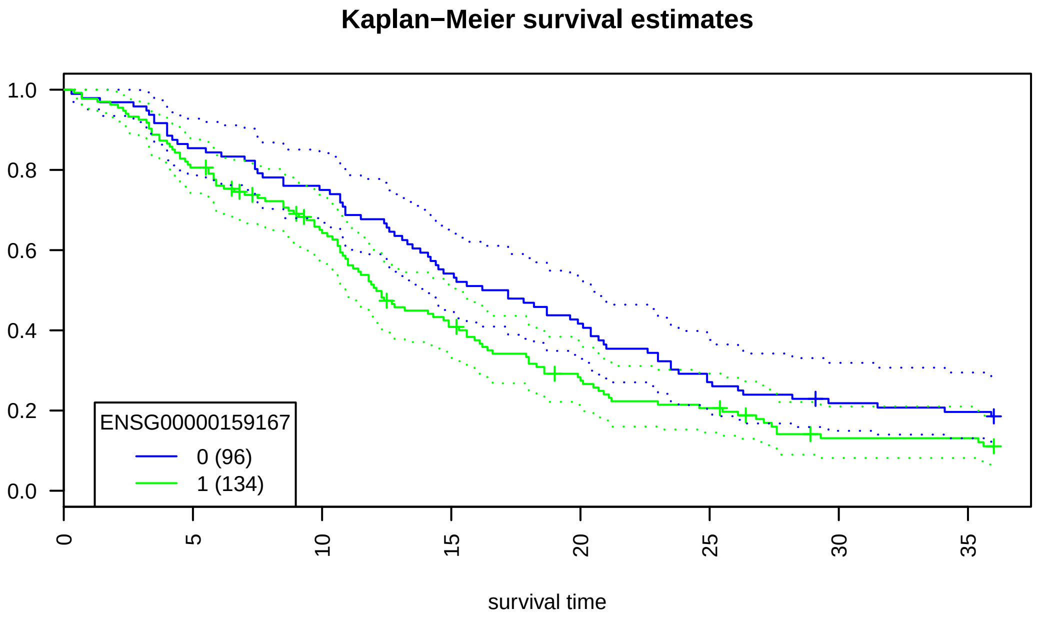

Figure 1697: Each curve represents the relative number of ENSG00000159167 survived to the corresponding point of time. Dashed lines represent 0.95 confidence intervals for the curves with the corresponding color. Log-rank probability measure for the equality of these curves is 3.139294e-02. Additional annotations: *.GeneName*=STC1, *.GeneDesc*=stanniocalcin 1 [Source:HGNC Symbol;Acc:11373].

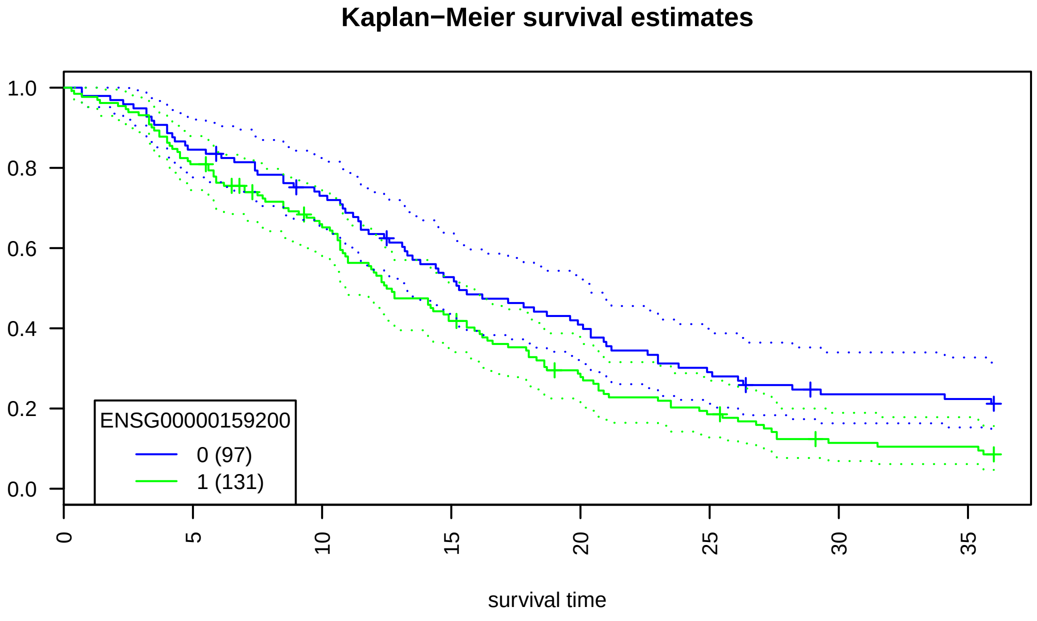

Figure 1698: Each curve represents the relative number of ENSG00000159200 survived to the corresponding point of time. Dashed lines represent 0.95 confidence intervals for the curves with the corresponding color. Total of 2 observations deleted due to missingness. Log-rank probability measure for the equality of these curves is 1.470387e-02. Additional annotations: *.GeneName*=RCAN1, *.GeneDesc*=regulator of calcineurin 1 [Source:HGNC Symbol;Acc:3040].

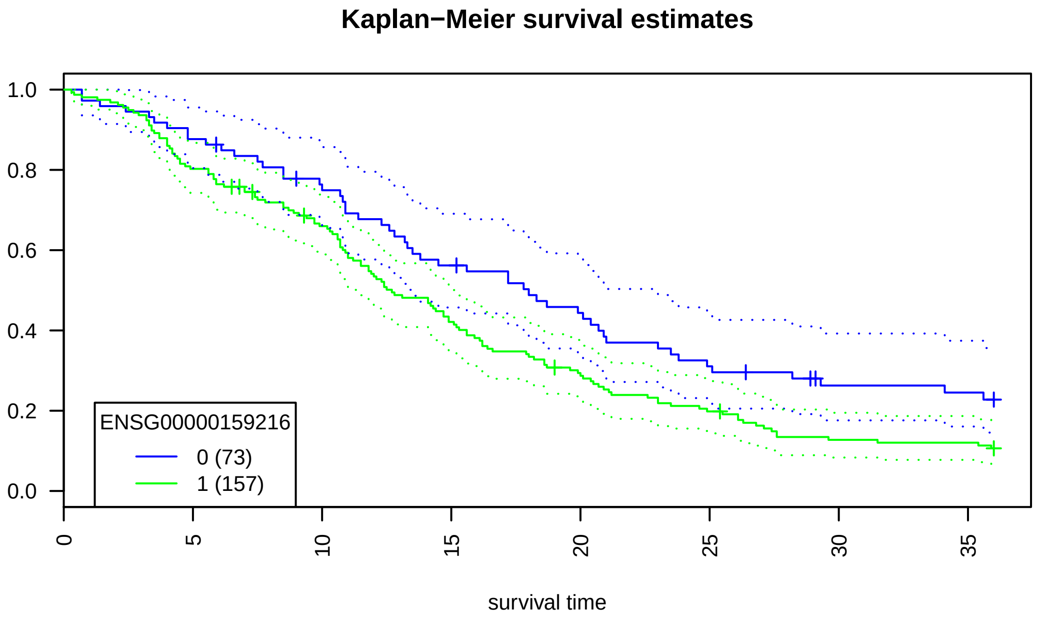

Figure 1699: Each curve represents the relative number of ENSG00000159216 survived to the corresponding point of time. Dashed lines represent 0.95 confidence intervals for the curves with the corresponding color. Log-rank probability measure for the equality of these curves is 1.084521e-02. Additional annotations: *.GeneName*=RUNX1, *.GeneDesc*=runt-related transcription factor 1 [Source:HGNC Symbol;Acc:10471].

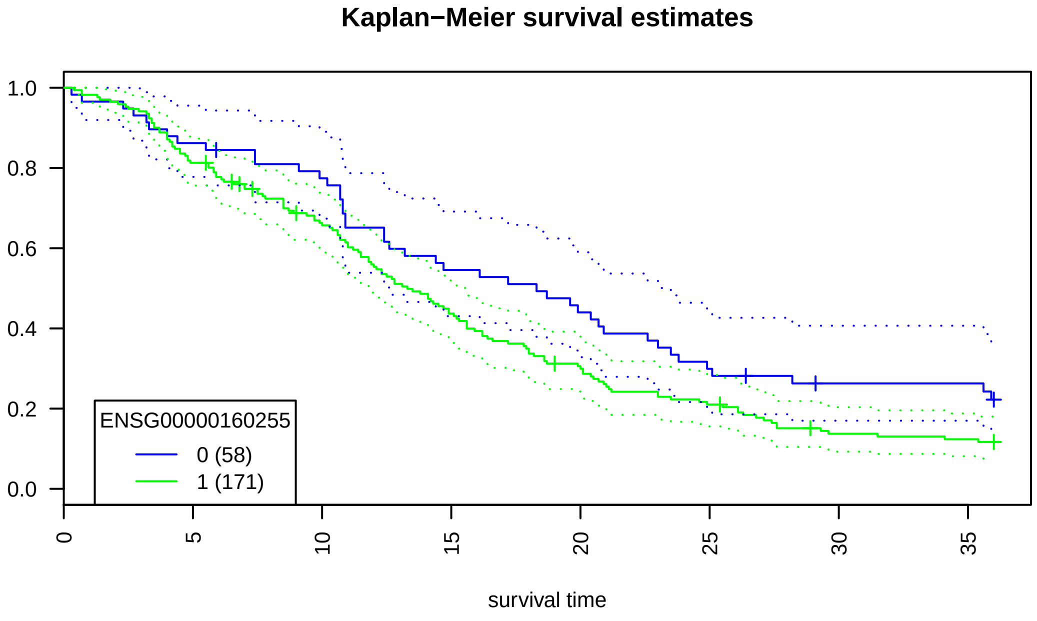

Figure 1700: Each curve represents the relative number of ENSG00000160255 survived to the corresponding point of time. Dashed lines represent 0.95 confidence intervals for the curves with the corresponding color. Total of 1 observation deleted due to missingness. Log-rank probability measure for the equality of these curves is 4.206882e-02. Additional annotations: *.GeneName*=ITGB2, *.GeneDesc*=integrin, beta 2 (complement component 3 receptor 3 and 4 subunit) [Source:HGNC Symbol;Acc:6155].

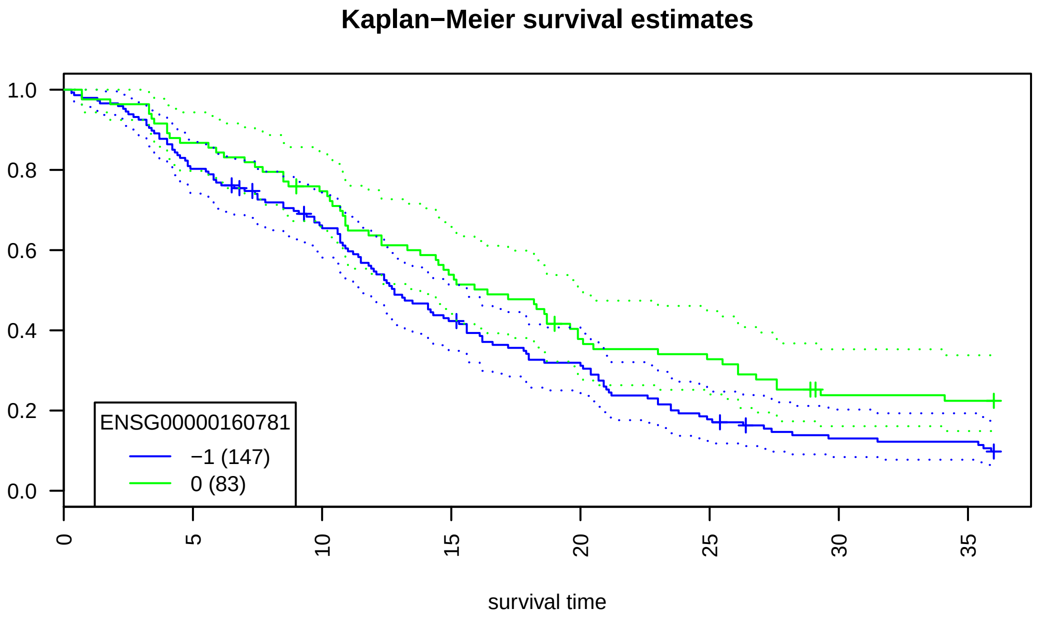

Figure 1701: Each curve represents the relative number of ENSG00000160781 survived to the corresponding point of time. Dashed lines represent 0.95 confidence intervals for the curves with the corresponding color. Log-rank probability measure for the equality of these curves is 1.777904e-02. Additional annotations: *.GeneName*=PAQR6, *.GeneDesc*=progestin and adipoQ receptor family member VI [Source:HGNC Symbol;Acc:30132].

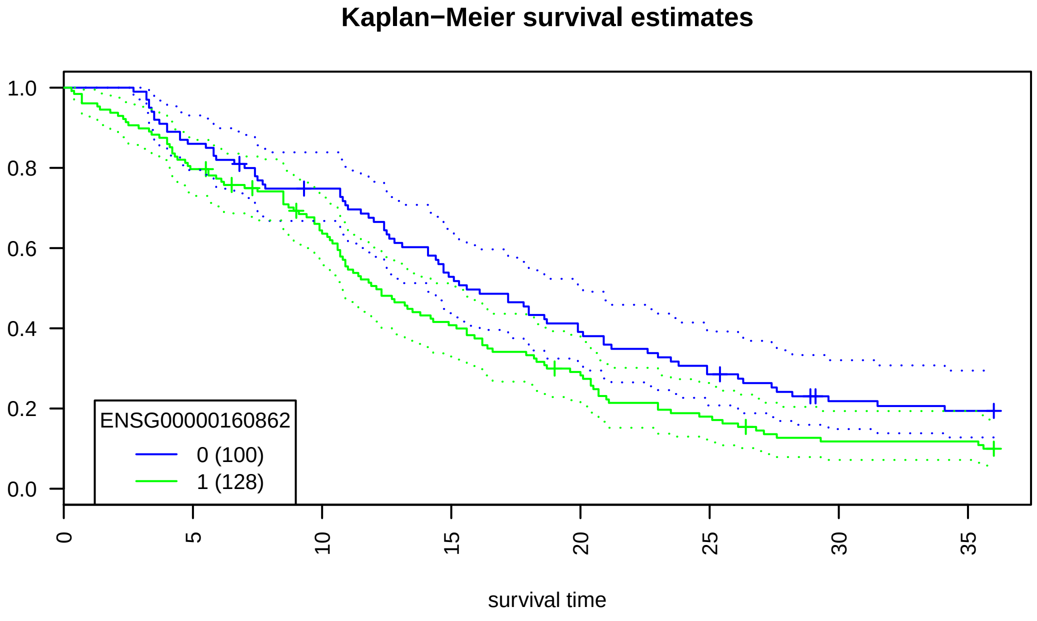

Figure 1702: Each curve represents the relative number of ENSG00000160862 survived to the corresponding point of time. Dashed lines represent 0.95 confidence intervals for the curves with the corresponding color. Total of 2 observations deleted due to missingness. Log-rank probability measure for the equality of these curves is 1.448947e-02. Additional annotations: *.GeneName*=AZGP1, *.GeneDesc*=alpha-2-glycoprotein 1, zinc-binding [Source:HGNC Symbol;Acc:910].

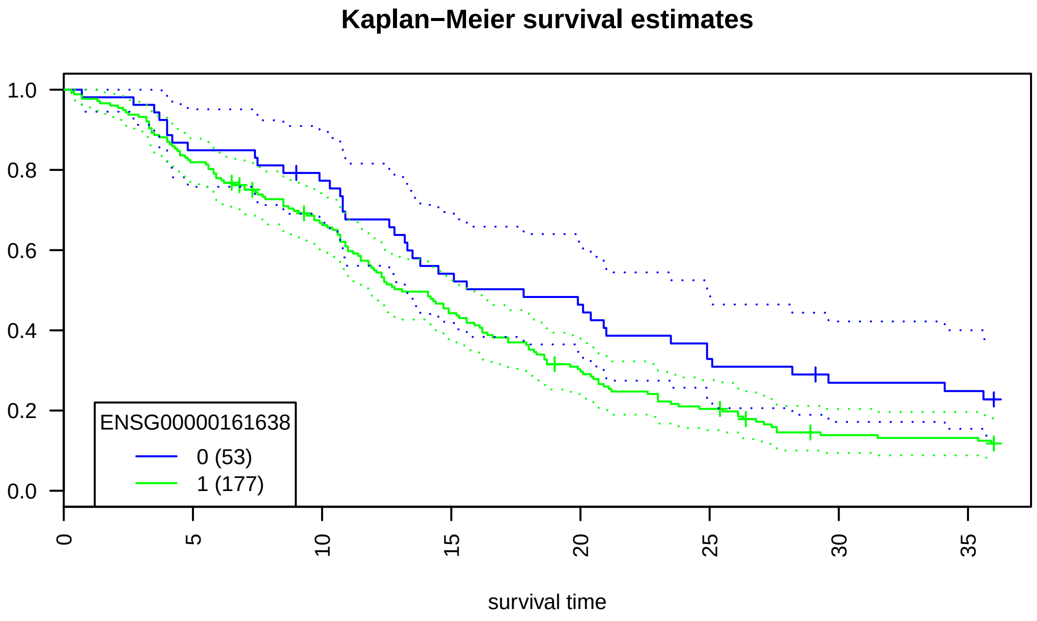

Figure 1703: Each curve represents the relative number of ENSG00000161638 survived to the corresponding point of time. Dashed lines represent 0.95 confidence intervals for the curves with the corresponding color. Log-rank probability measure for the equality of these curves is 3.279834e-02. Additional annotations: *.GeneName*=ITGA5, *.GeneDesc*=integrin, alpha 5 (fibronectin receptor, alpha polypeptide) [Source:HGNC Symbol;Acc:6141].

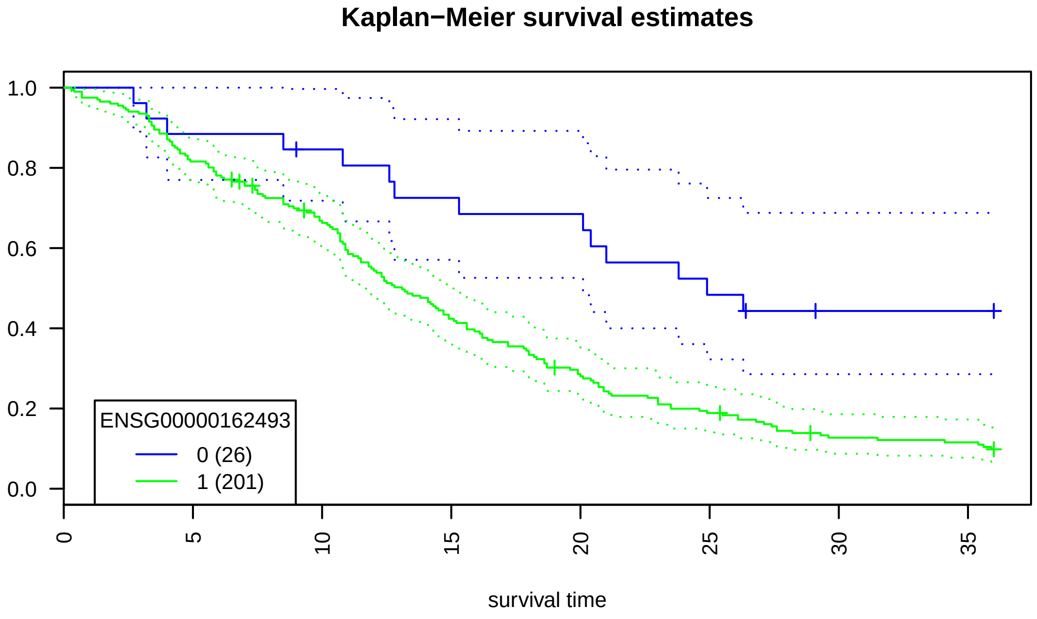

Figure 1704: Each curve represents the relative number of ENSG00000162493 survived to the corresponding point of time. Dashed lines represent 0.95 confidence intervals for the curves with the corresponding color. Total of 3 observations deleted due to missingness. Log-rank probability measure for the equality of these curves is 2.956349e-04. Additional annotations: *.GeneName*=PDPN, *.GeneDesc*=podoplanin [Source:HGNC Symbol;Acc:29602].

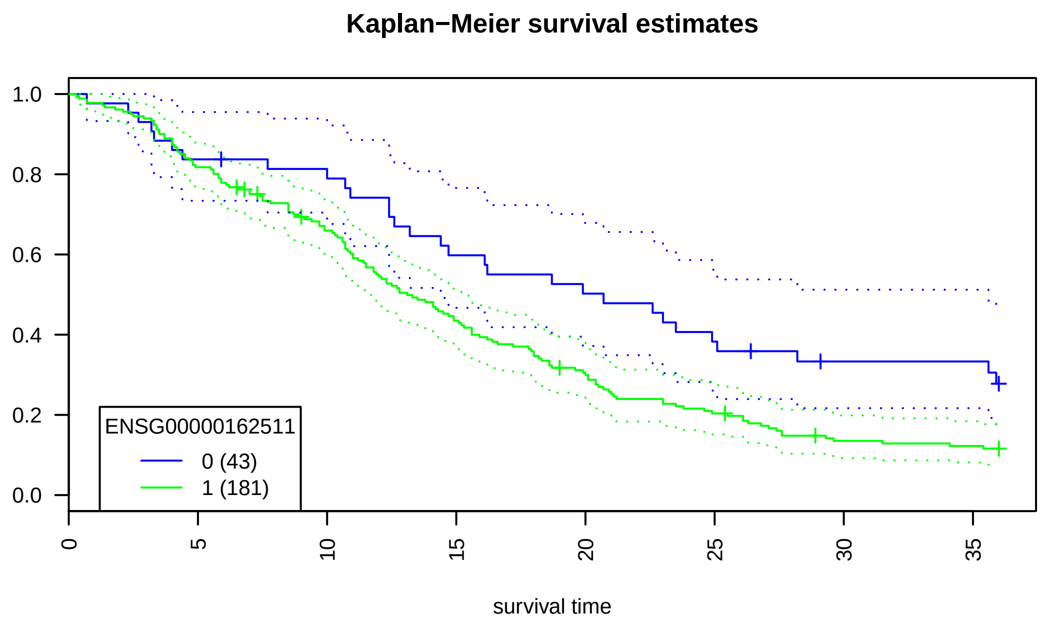

Figure 1705: Each curve represents the relative number of ENSG00000162511 survived to the corresponding point of time. Dashed lines represent 0.95 confidence intervals for the curves with the corresponding color. Total of 6 observations deleted due to missingness. Log-rank probability measure for the equality of these curves is 7.357856e-03. Additional annotations: *.GeneName*=LAPTM5, *.GeneDesc*=lysosomal protein transmembrane 5 [Source:HGNC Symbol;Acc:29612].

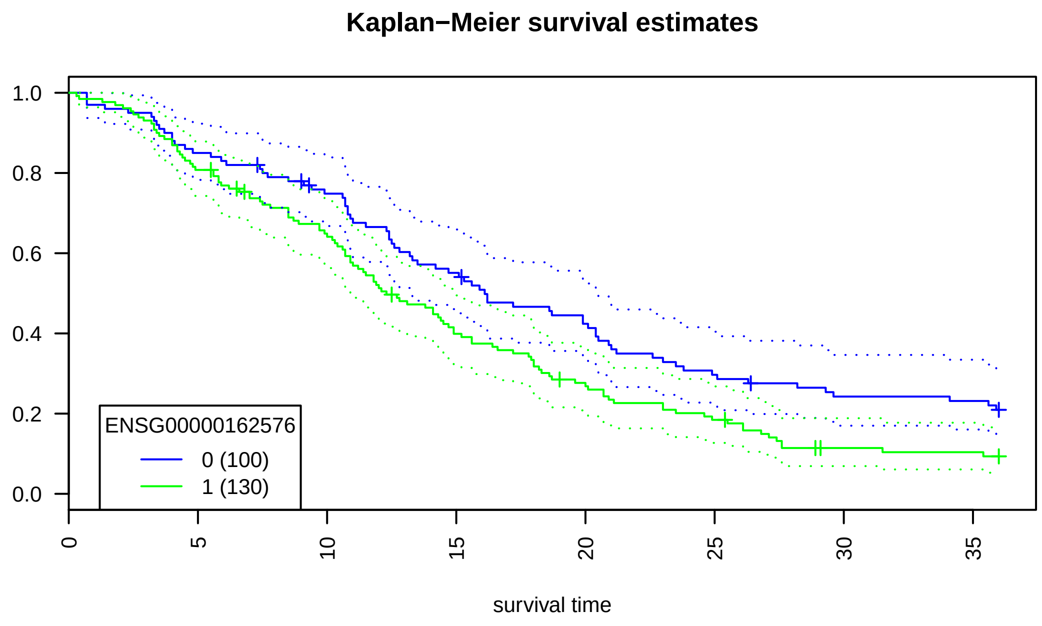

Figure 1706: Each curve represents the relative number of ENSG00000162576 survived to the corresponding point of time. Dashed lines represent 0.95 confidence intervals for the curves with the corresponding color. Log-rank probability measure for the equality of these curves is 7.802187e-03. Additional annotations: *.GeneName*=MXRA8, *.GeneDesc*=matrix-remodelling associated 8 [Source:HGNC Symbol;Acc:7542].

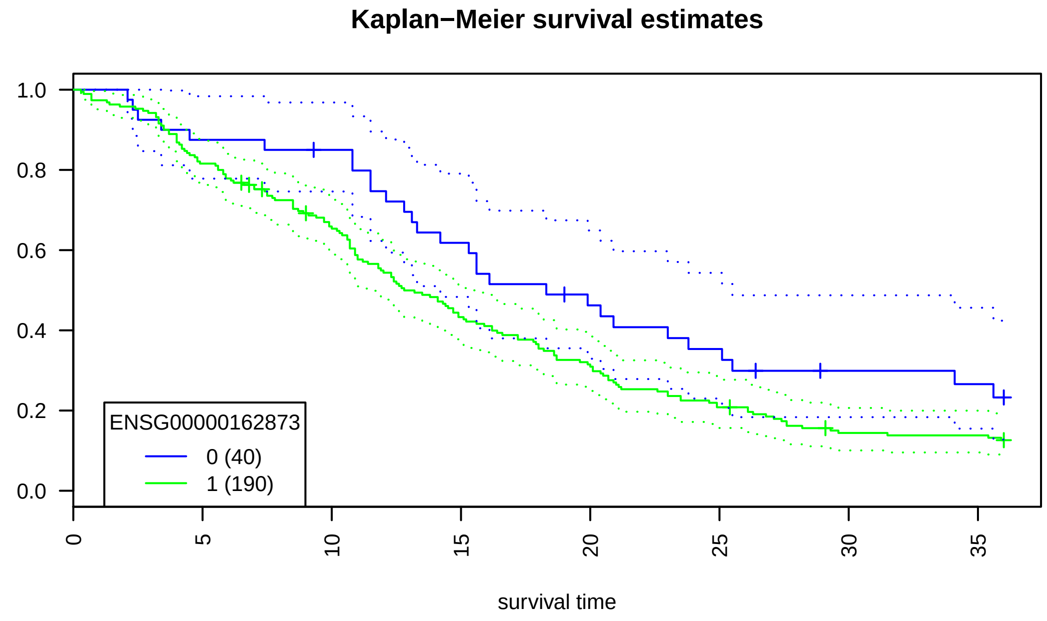

Figure 1707: Each curve represents the relative number of ENSG00000162873 survived to the corresponding point of time. Dashed lines represent 0.95 confidence intervals for the curves with the corresponding color. Log-rank probability measure for the equality of these curves is 3.640858e-02. Additional annotations: *.GeneName*=KLHDC8A, *.GeneDesc*=kelch domain containing 8A [Source:HGNC Symbol;Acc:25573].

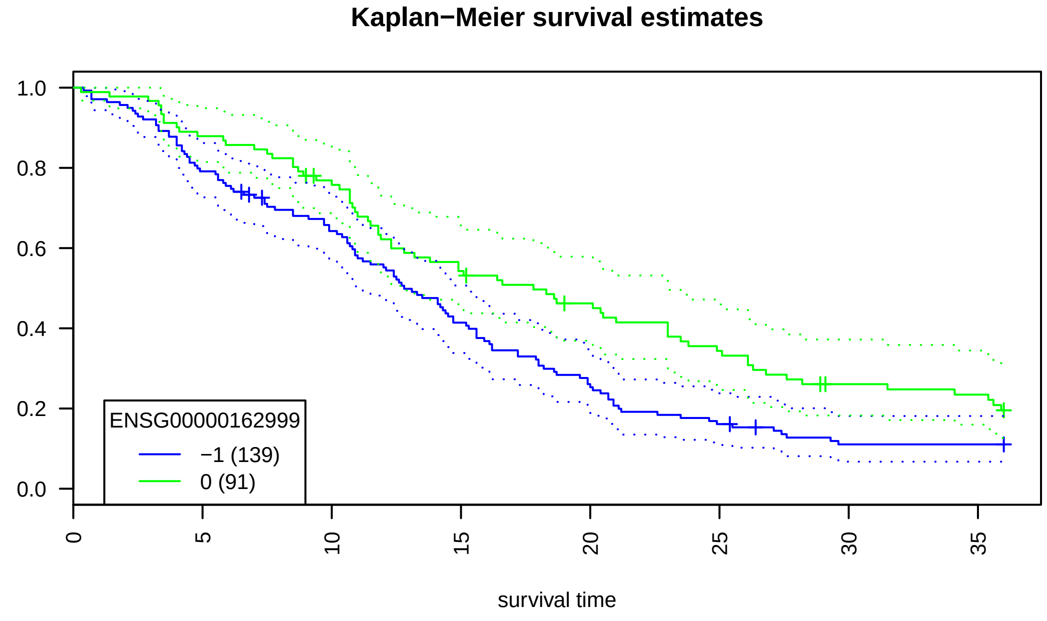

Figure 1708: Each curve represents the relative number of ENSG00000162999 survived to the corresponding point of time. Dashed lines represent 0.95 confidence intervals for the curves with the corresponding color. Log-rank probability measure for the equality of these curves is 5.962874e-03. Additional annotations: *.GeneName*=DUSP19, *.GeneDesc*=dual specificity phosphatase 19 [Source:HGNC Symbol;Acc:18894].

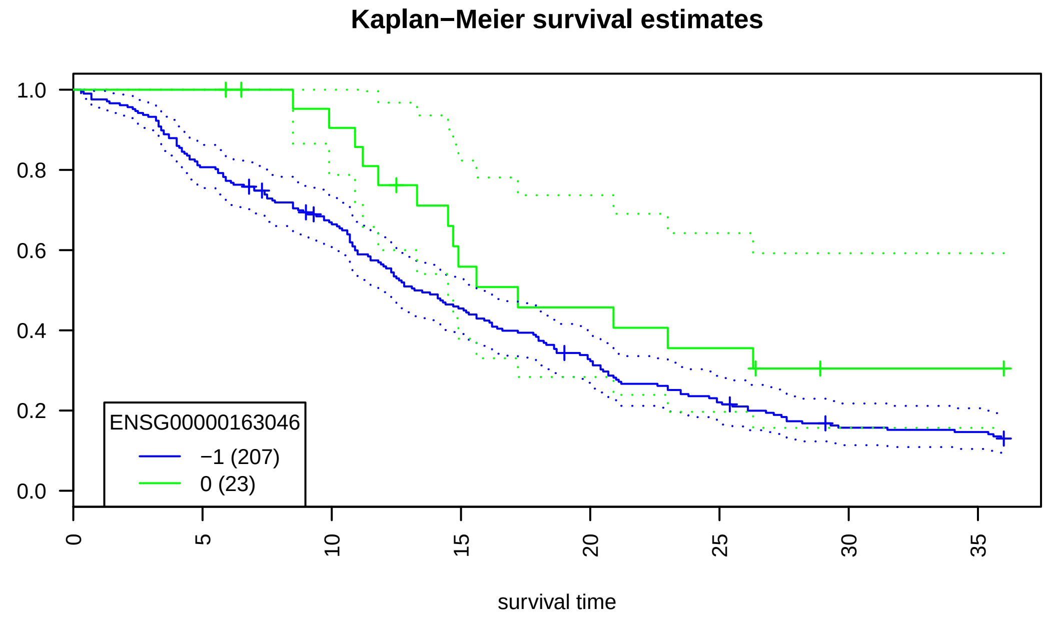

Figure 1709: Each curve represents the relative number of ENSG00000163046 survived to the corresponding point of time. Dashed lines represent 0.95 confidence intervals for the curves with the corresponding color. Log-rank probability measure for the equality of these curves is 4.825425e-02. Additional annotations: *.GeneName*=NCRNA00164, *.GeneDesc*=non-protein coding RNA 164 [Source:HGNC Symbol;Acc:35167].

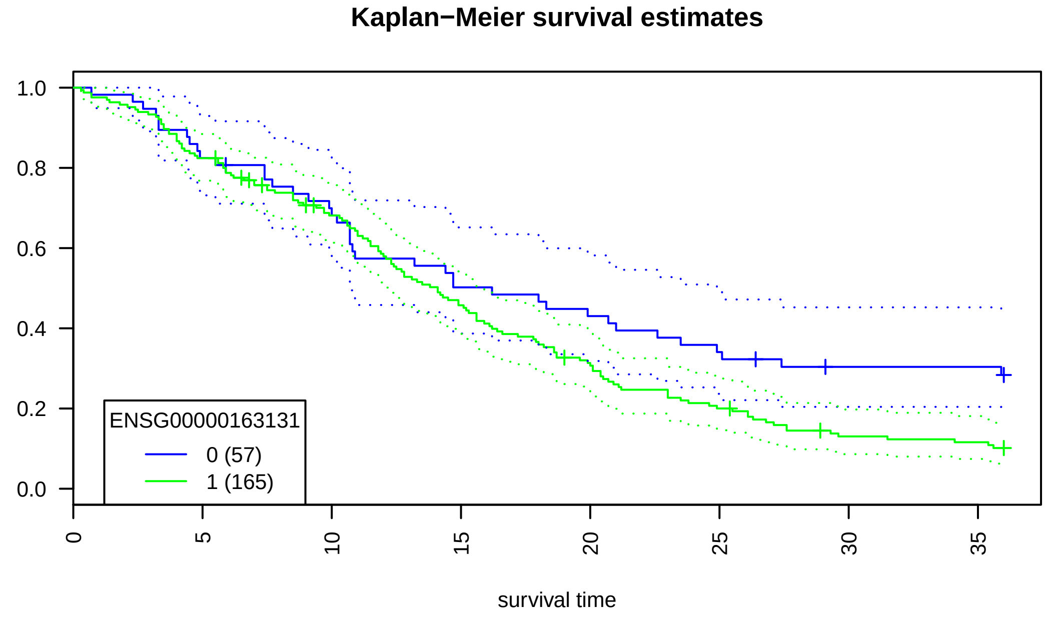

Figure 1710: Each curve represents the relative number of ENSG00000163131 survived to the corresponding point of time. Dashed lines represent 0.95 confidence intervals for the curves with the corresponding color. Total of 8 observations deleted due to missingness. Log-rank probability measure for the equality of these curves is 2.284763e-02. Additional annotations: *.GeneName*=CTSS, *.GeneDesc*=cathepsin S [Source:HGNC Symbol;Acc:2545].

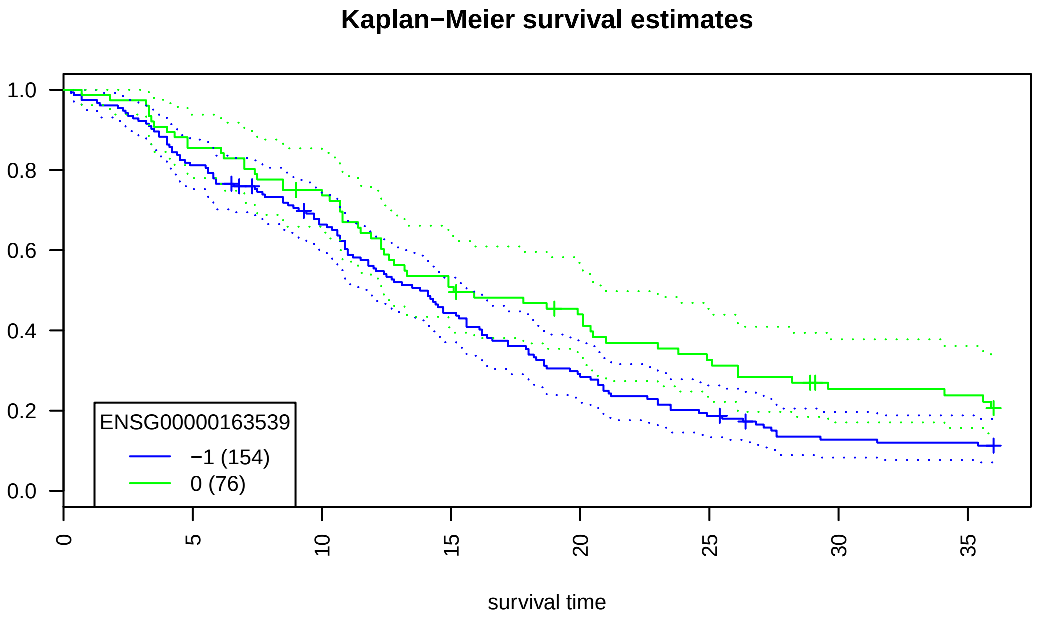

Figure 1711: Each curve represents the relative number of ENSG00000163539 survived to the corresponding point of time. Dashed lines represent 0.95 confidence intervals for the curves with the corresponding color. Log-rank probability measure for the equality of these curves is 3.947478e-02. Additional annotations: *.GeneName*=CLASP2, *.GeneDesc*=cytoplasmic linker associated protein 2 [Source:HGNC Symbol;Acc:17078].

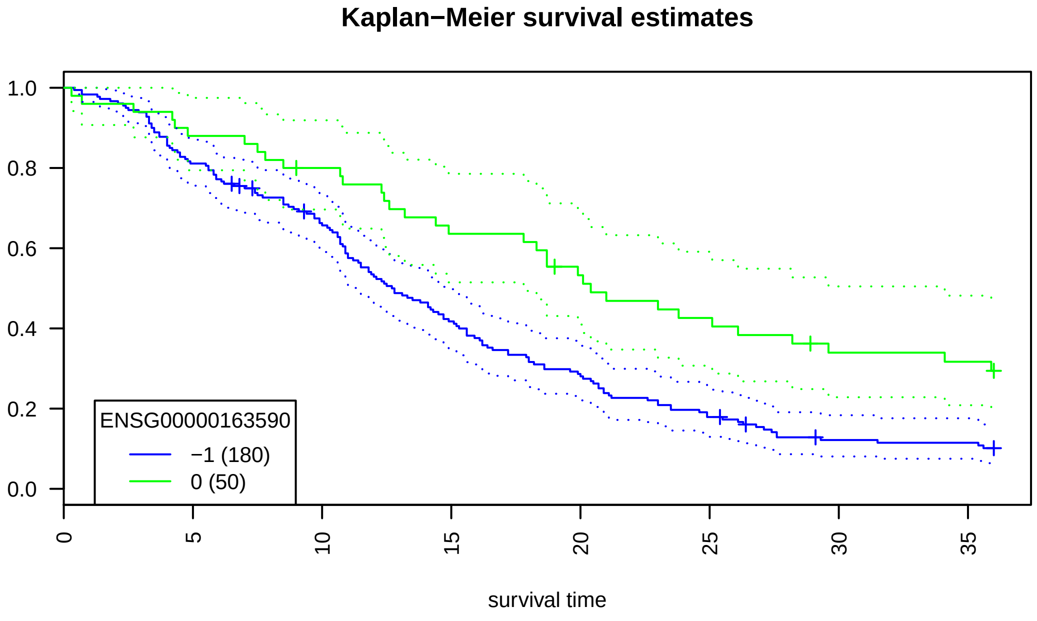

Figure 1712: Each curve represents the relative number of ENSG00000163590 survived to the corresponding point of time. Dashed lines represent 0.95 confidence intervals for the curves with the corresponding color. Log-rank probability measure for the equality of these curves is 4.231085e-04. Additional annotations: *.GeneName*=PPM1L, *.GeneDesc*=protein phosphatase, Mg2+/Mn2+ dependent, 1L [Source:HGNC Symbol;Acc:16381].

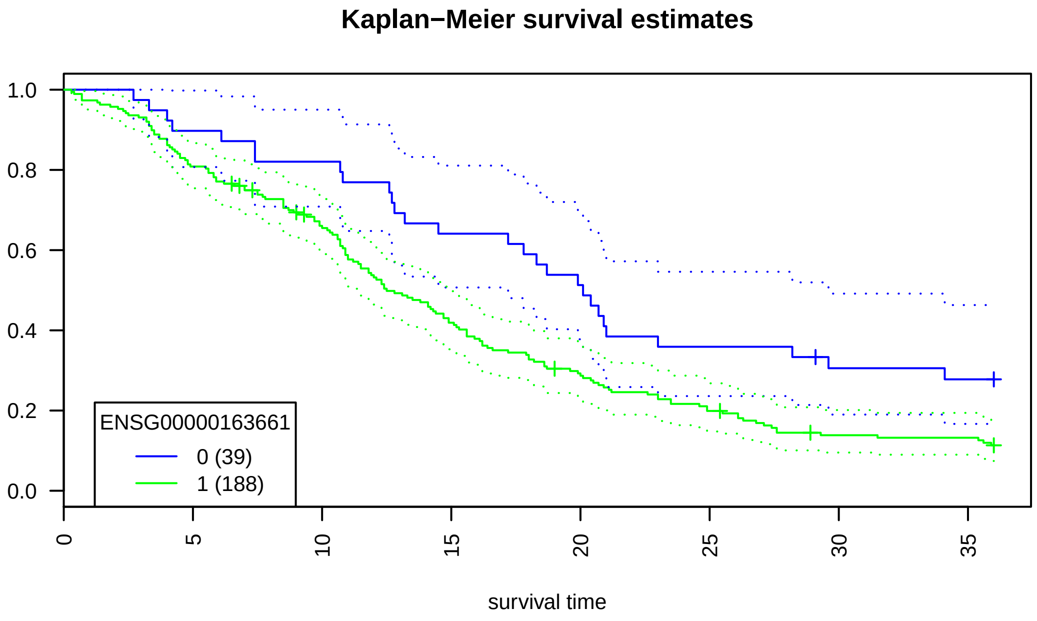

Figure 1713: Each curve represents the relative number of ENSG00000163661 survived to the corresponding point of time. Dashed lines represent 0.95 confidence intervals for the curves with the corresponding color. Total of 3 observations deleted due to missingness. Log-rank probability measure for the equality of these curves is 5.369629e-03. Additional annotations: *.GeneName*=PTX3, *.GeneDesc*=pentraxin-related gene, rapidly induced by IL-1 beta [Source:HGNC Symbol;Acc:9692].

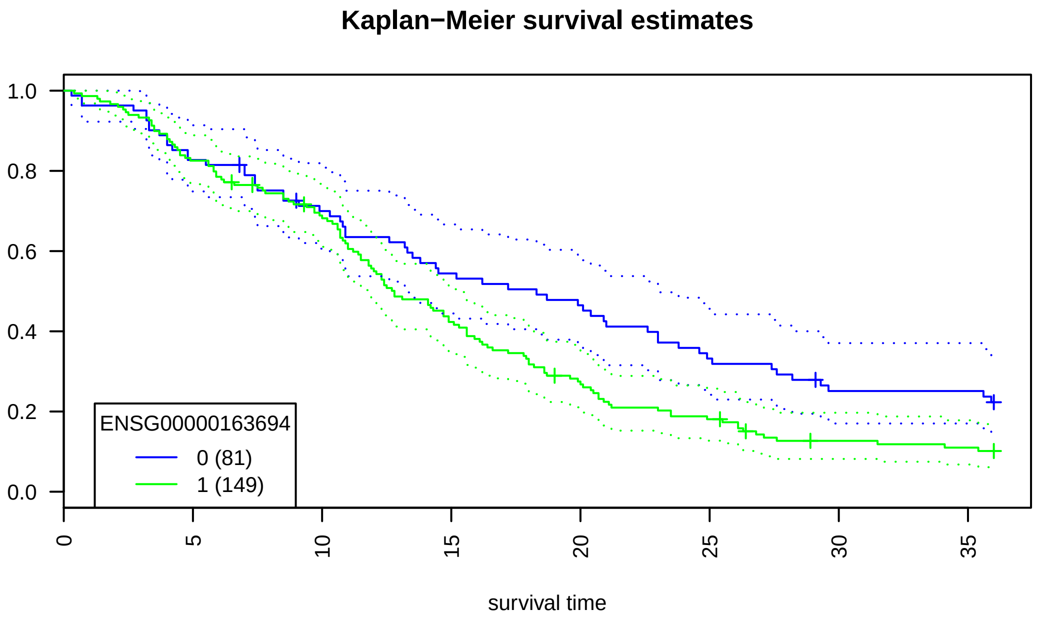

Figure 1714: Each curve represents the relative number of ENSG00000163694 survived to the corresponding point of time. Dashed lines represent 0.95 confidence intervals for the curves with the corresponding color. Log-rank probability measure for the equality of these curves is 1.025775e-02. Additional annotations: *.GeneName*=RBM47, *.GeneDesc*=RNA binding motif protein 47 [Source:HGNC Symbol;Acc:30358].

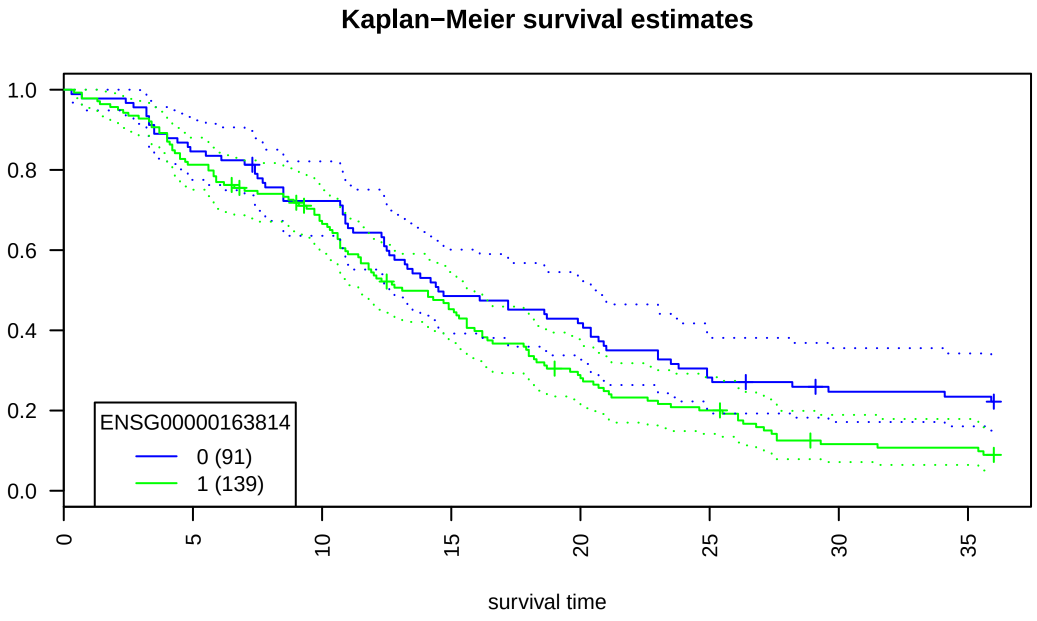

Figure 1715: Each curve represents the relative number of ENSG00000163814 survived to the corresponding point of time. Dashed lines represent 0.95 confidence intervals for the curves with the corresponding color. Log-rank probability measure for the equality of these curves is 2.219622e-02. Additional annotations: *.GeneName*=CDCP1, *.GeneDesc*=CUB domain containing protein 1 [Source:HGNC Symbol;Acc:24357].

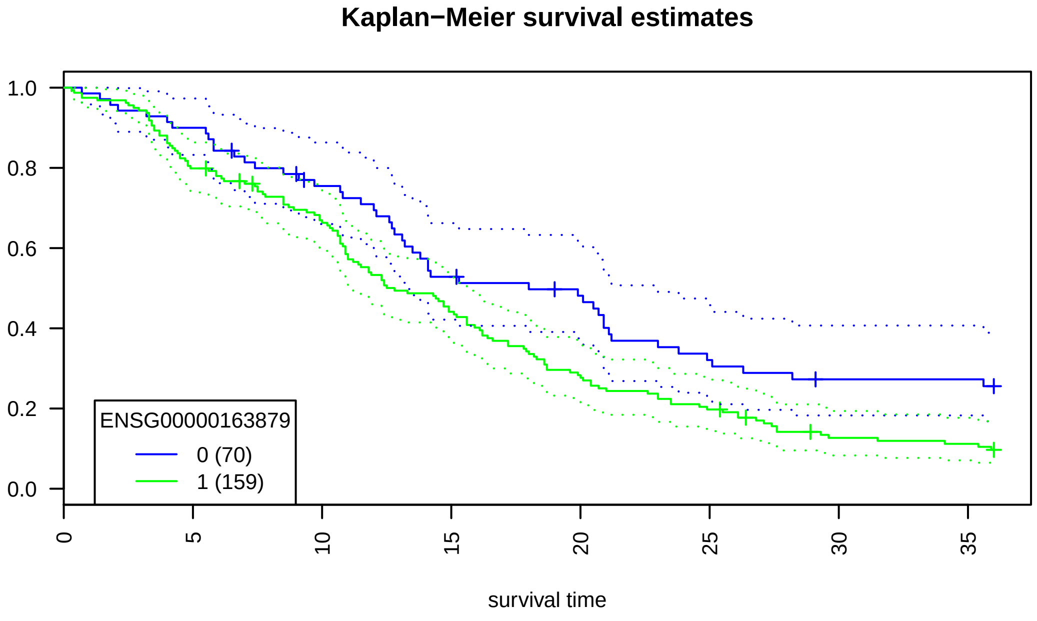

Figure 1716: Each curve represents the relative number of ENSG00000163879 survived to the corresponding point of time. Dashed lines represent 0.95 confidence intervals for the curves with the corresponding color. Total of 1 observation deleted due to missingness. Log-rank probability measure for the equality of these curves is 6.133266e-03. Additional annotations: *.GeneName*=DNALI1, *.GeneDesc*=dynein, axonemal, light intermediate chain 1 [Source:HGNC Symbol;Acc:14353].

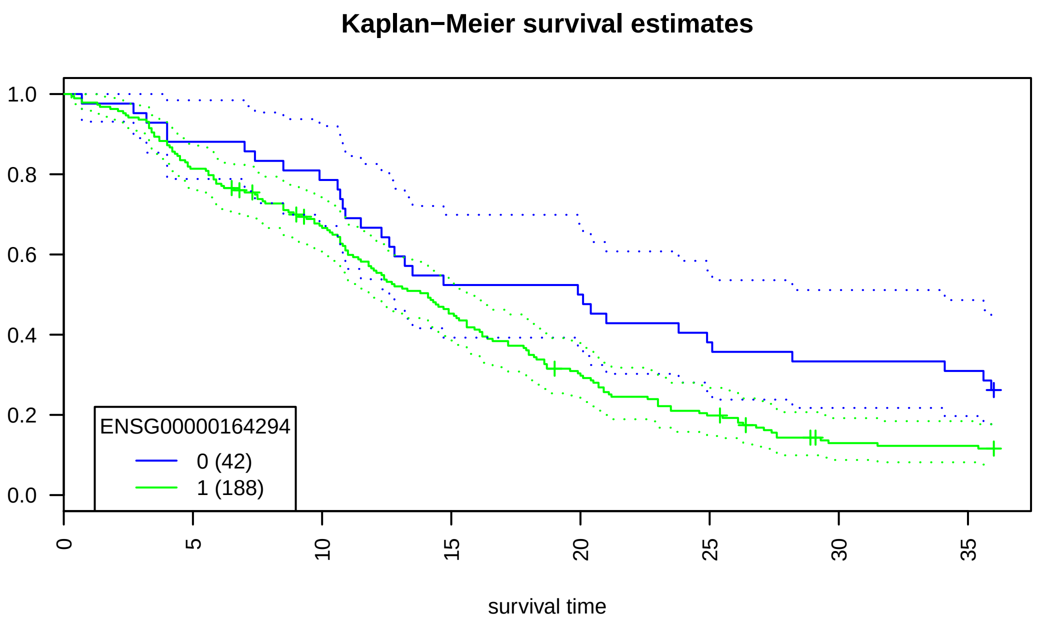

Figure 1717: Each curve represents the relative number of ENSG00000164294 survived to the corresponding point of time. Dashed lines represent 0.95 confidence intervals for the curves with the corresponding color. Log-rank probability measure for the equality of these curves is 1.598317e-02. Additional annotations: *.GeneName*=GPX8, *.GeneDesc*=glutathione peroxidase 8 (putative) [Source:HGNC Symbol;Acc:33100].

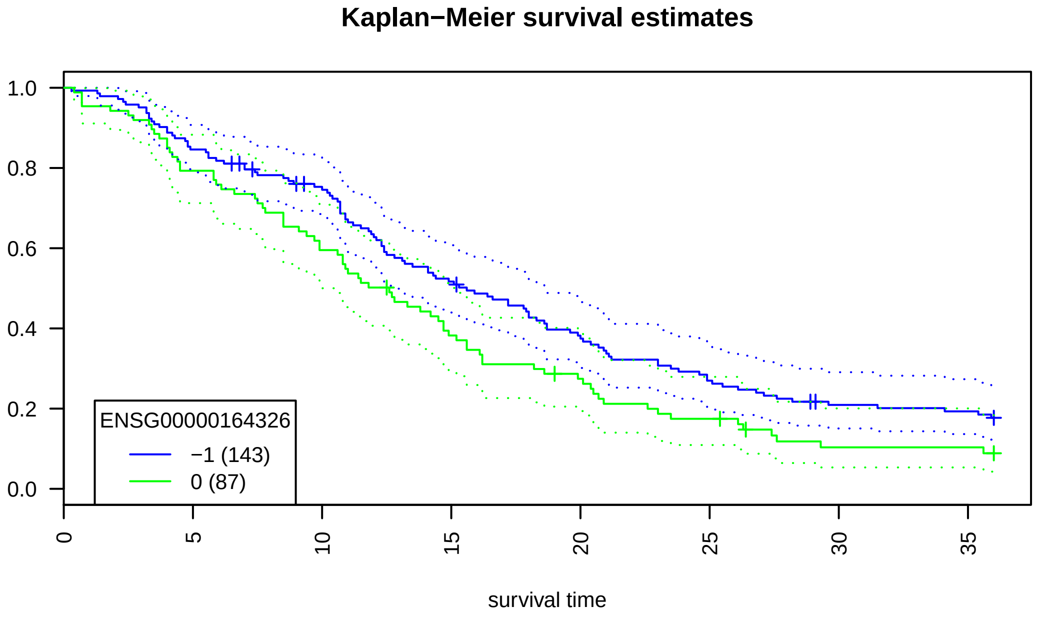

Figure 1718: Each curve represents the relative number of ENSG00000164326 survived to the corresponding point of time. Dashed lines represent 0.95 confidence intervals for the curves with the corresponding color. Log-rank probability measure for the equality of these curves is 2.138193e-02. Additional annotations: *.GeneName*=CARTPT, *.GeneDesc*=CART prepropeptide [Source:HGNC Symbol;Acc:24323].

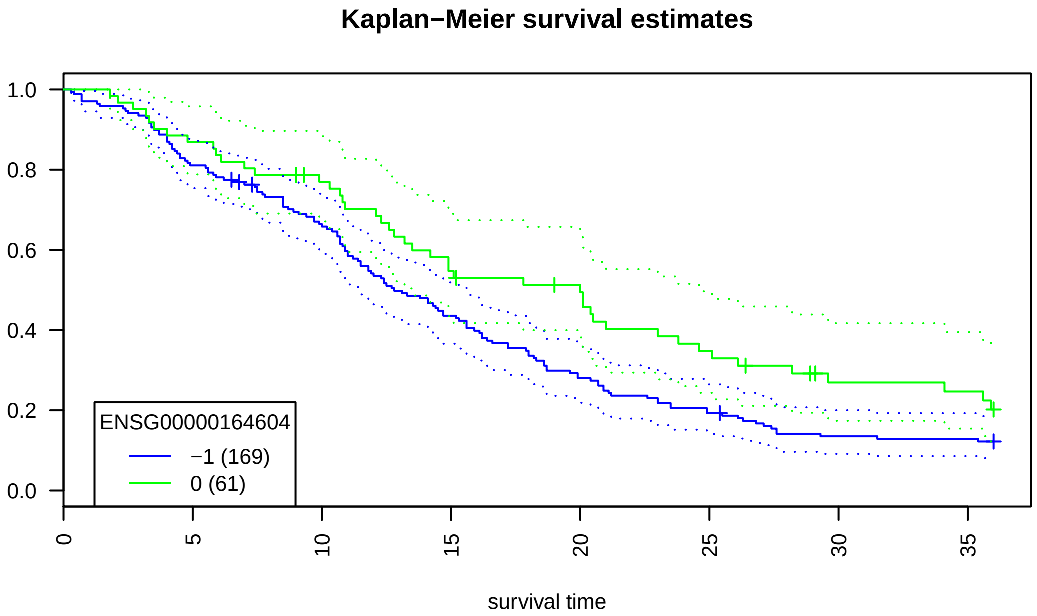

Figure 1719: Each curve represents the relative number of ENSG00000164604 survived to the corresponding point of time. Dashed lines represent 0.95 confidence intervals for the curves with the corresponding color. Log-rank probability measure for the equality of these curves is 2.514456e-02. Additional annotations: *.GeneName*=GPR85, *.GeneDesc*=G protein-coupled receptor 85 [Source:HGNC Symbol;Acc:4536].

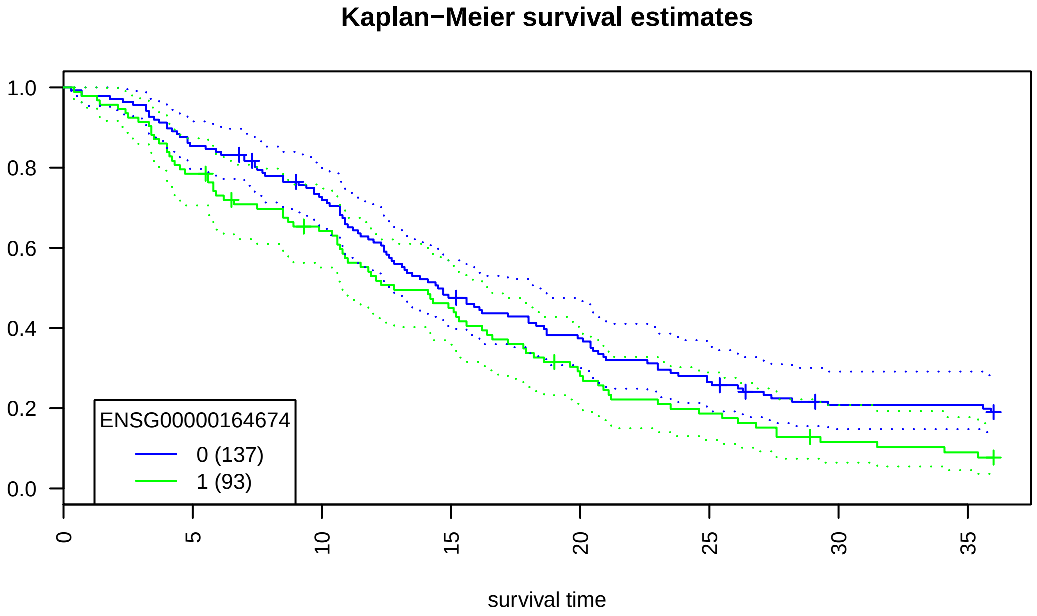

Figure 1720: Each curve represents the relative number of ENSG00000164674 survived to the corresponding point of time. Dashed lines represent 0.95 confidence intervals for the curves with the corresponding color. Log-rank probability measure for the equality of these curves is 3.766187e-02. Additional annotations: *.GeneName*=SYTL3, *.GeneDesc*=synaptotagmin-like 3 [Source:HGNC Symbol;Acc:15587].

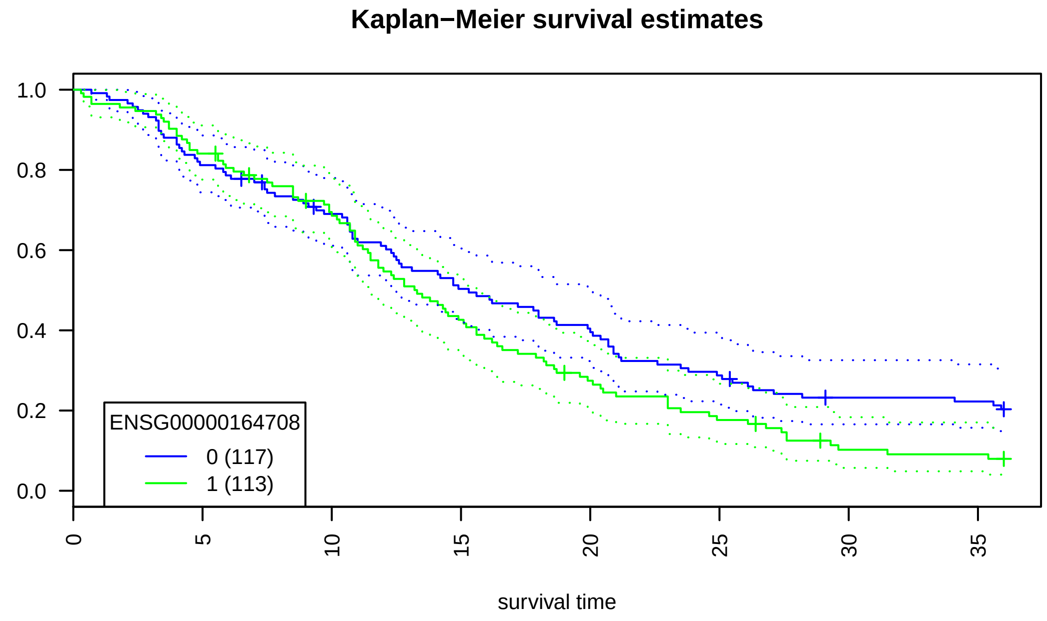

Figure 1721: Each curve represents the relative number of ENSG00000164708 survived to the corresponding point of time. Dashed lines represent 0.95 confidence intervals for the curves with the corresponding color. Log-rank probability measure for the equality of these curves is 4.274152e-02. Additional annotations: *.GeneName*=PGAM2, *.GeneDesc*=phosphoglycerate mutase 2 (muscle) [Source:HGNC Symbol;Acc:8889].

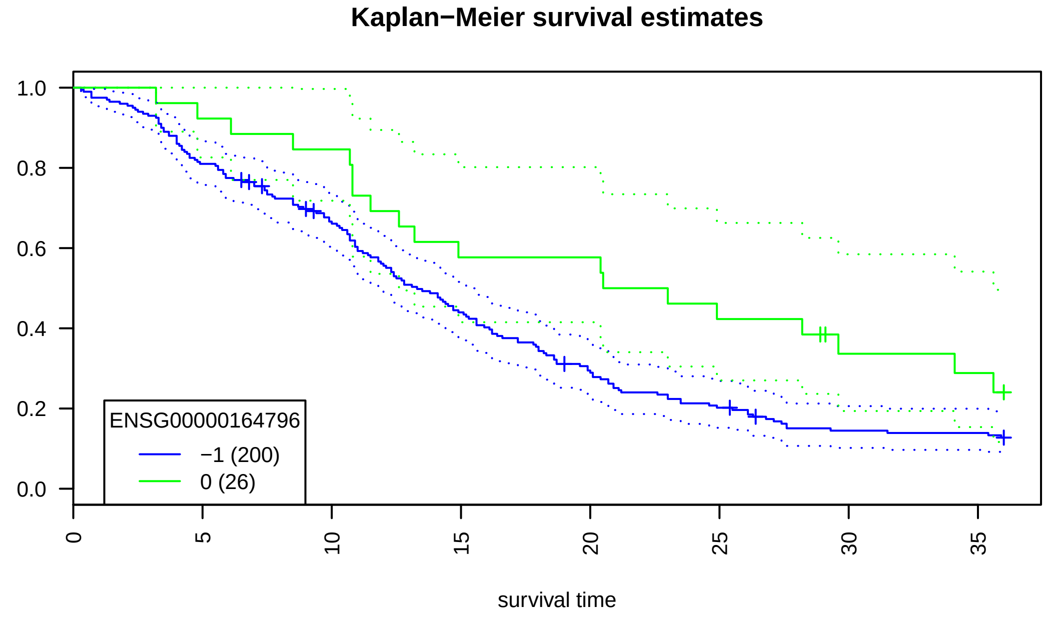

Figure 1722: Each curve represents the relative number of ENSG00000164796 survived to the corresponding point of time. Dashed lines represent 0.95 confidence intervals for the curves with the corresponding color. Total of 4 observations deleted due to missingness. Log-rank probability measure for the equality of these curves is 2.487633e-02. Additional annotations: *.GeneName*=CSMD3, *.GeneDesc*=CUB and Sushi multiple domains 3 [Source:HGNC Symbol;Acc:19291].

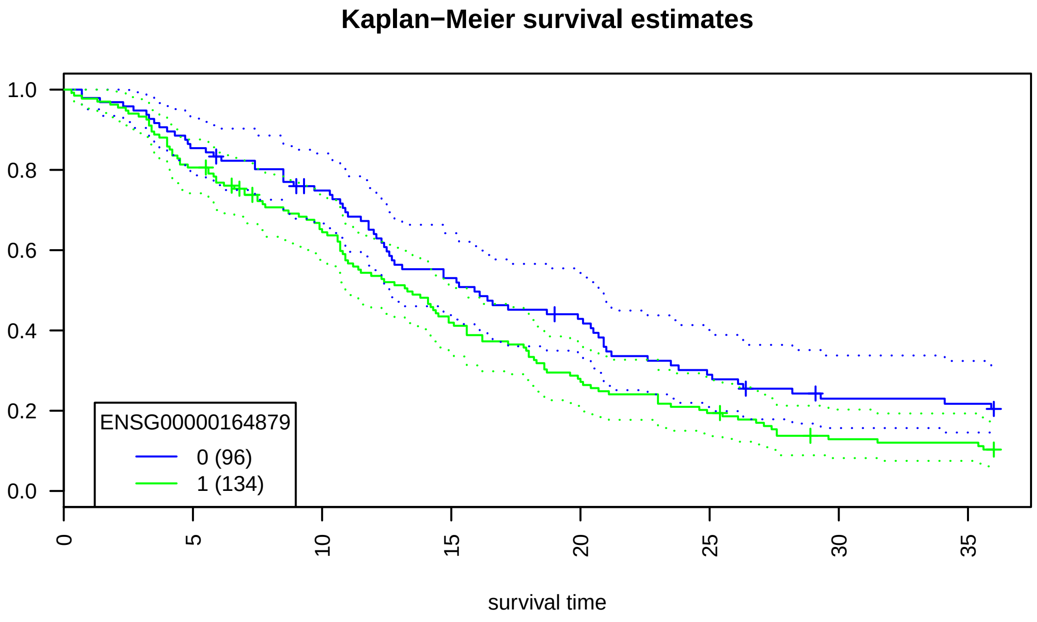

Figure 1723: Each curve represents the relative number of ENSG00000164879 survived to the corresponding point of time. Dashed lines represent 0.95 confidence intervals for the curves with the corresponding color. Log-rank probability measure for the equality of these curves is 2.790319e-02. Additional annotations: *.GeneName*=CA3, *.GeneDesc*=carbonic anhydrase III, muscle specific [Source:HGNC Symbol;Acc:1374].

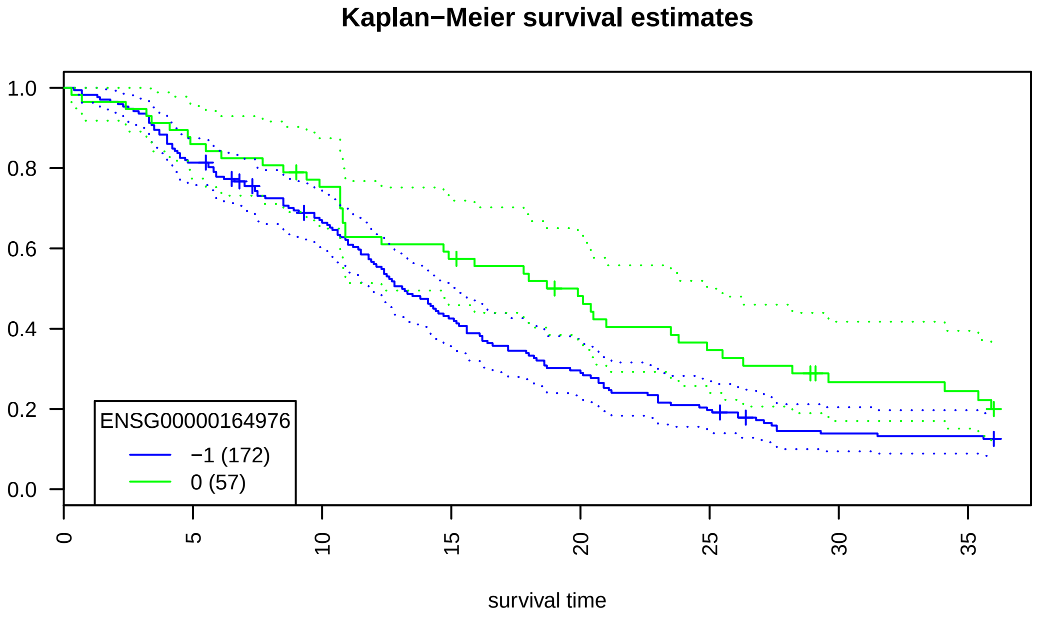

Figure 1724: Each curve represents the relative number of ENSG00000164976 survived to the corresponding point of time. Dashed lines represent 0.95 confidence intervals for the curves with the corresponding color. Total of 1 observation deleted due to missingness. Log-rank probability measure for the equality of these curves is 3.558239e-02. Additional annotations: *.GeneName*=KIAA1161, *.GeneDesc*=KIAA1161 [Source:HGNC Symbol;Acc:19918].

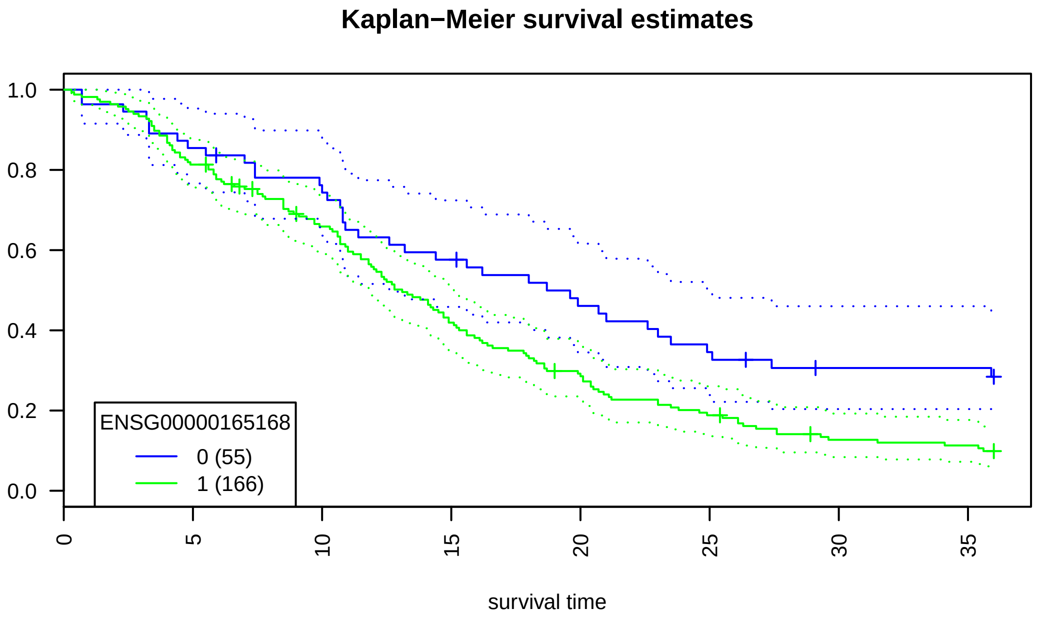

Figure 1725: Each curve represents the relative number of ENSG00000165168 survived to the corresponding point of time. Dashed lines represent 0.95 confidence intervals for the curves with the corresponding color. Total of 9 observations deleted due to missingness. Log-rank probability measure for the equality of these curves is 5.157321e-03. Additional annotations: *.GeneName*=CYBB, *.GeneDesc*=cytochrome b-245, beta polypeptide [Source:HGNC Symbol;Acc:2578].

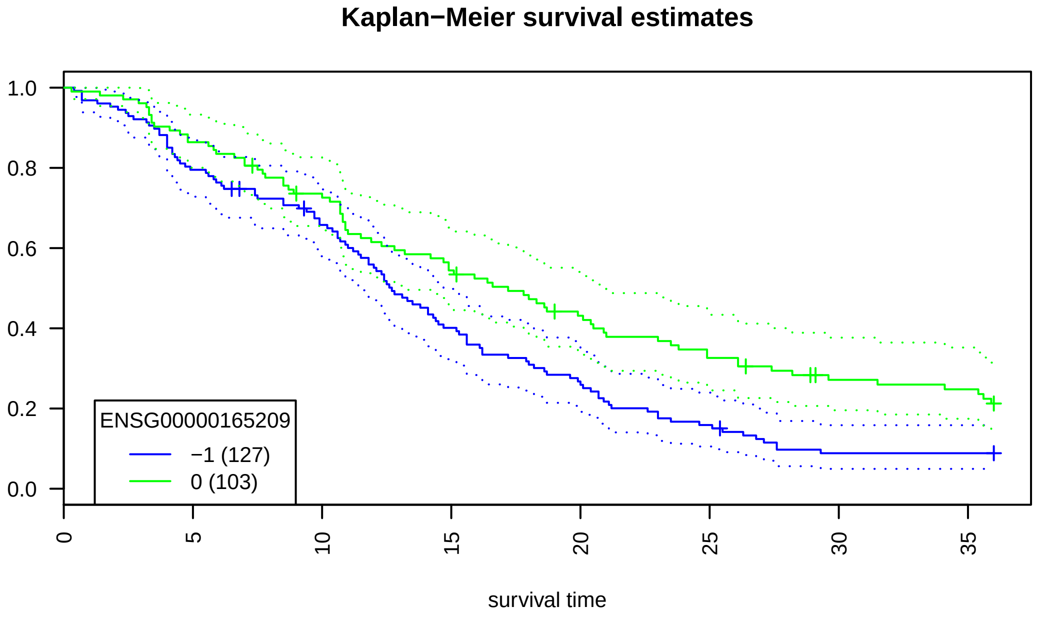

Figure 1726: Each curve represents the relative number of ENSG00000165209 survived to the corresponding point of time. Dashed lines represent 0.95 confidence intervals for the curves with the corresponding color. Log-rank probability measure for the equality of these curves is 2.467985e-03. Additional annotations: *.GeneName*=STRBP, *.GeneDesc*=spermatid perinuclear RNA binding protein [Source:HGNC Symbol;Acc:16462].

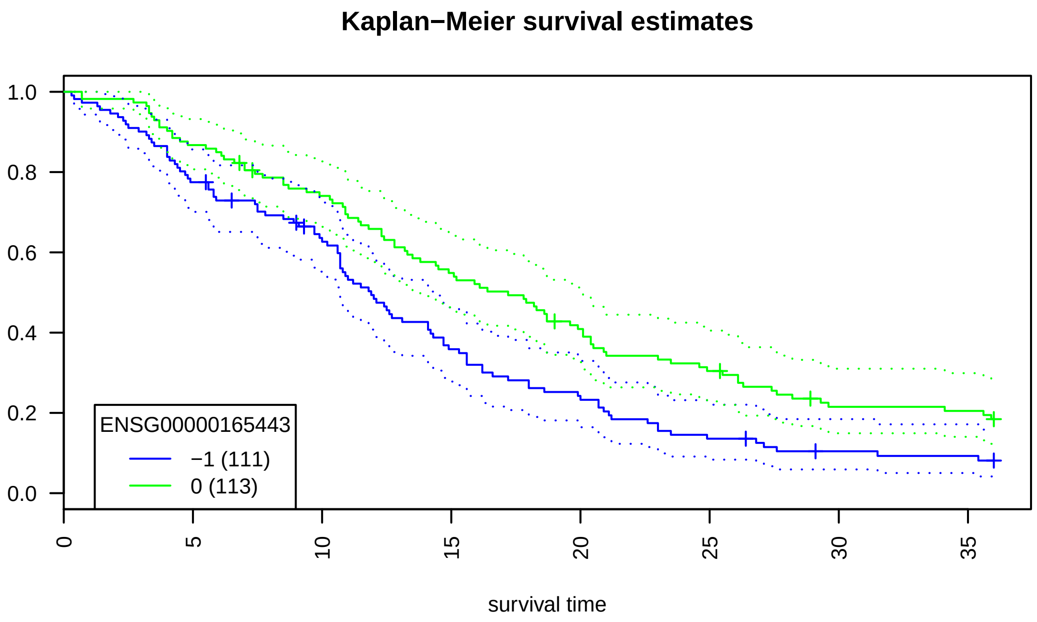

Figure 1727: Each curve represents the relative number of ENSG00000165443 survived to the corresponding point of time. Dashed lines represent 0.95 confidence intervals for the curves with the corresponding color. Total of 6 observations deleted due to missingness. Log-rank probability measure for the equality of these curves is 1.697675e-03. Additional annotations: *.GeneName*=PHYHIPL, *.GeneDesc*=phytanoyl-CoA 2-hydroxylase interacting protein-like [Source:HGNC Symbol;Acc:29378].

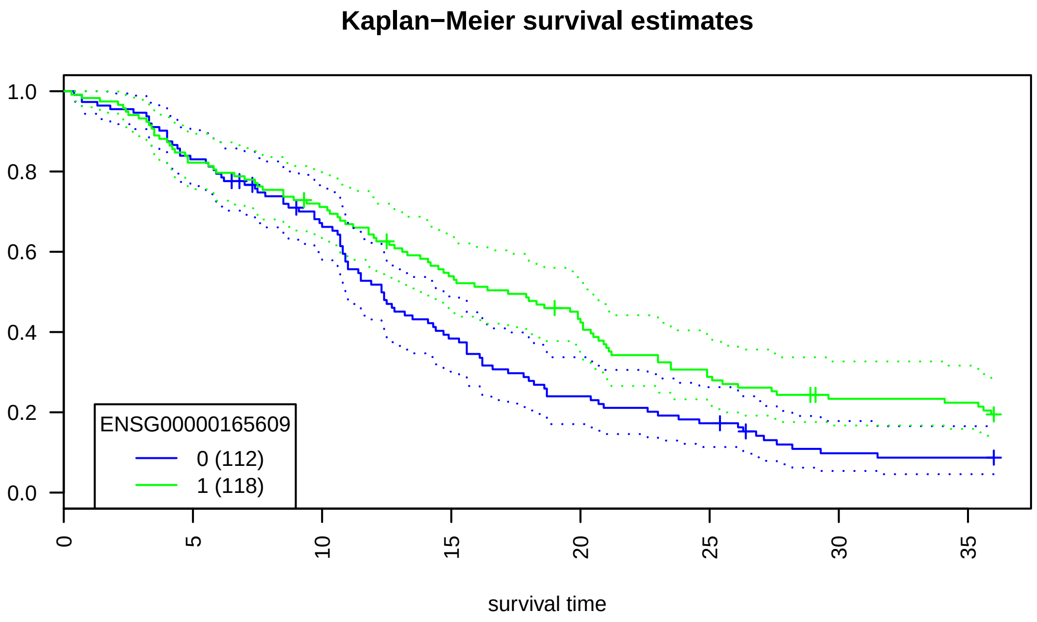

Figure 1728: Each curve represents the relative number of ENSG00000165609 survived to the corresponding point of time. Dashed lines represent 0.95 confidence intervals for the curves with the corresponding color. Log-rank probability measure for the equality of these curves is 8.105634e-03. Additional annotations: *.GeneName*=NUDT5, *.GeneDesc*=nudix (nucleoside diphosphate linked moiety X)-type motif 5 [Source:HGNC Symbol;Acc:8052].

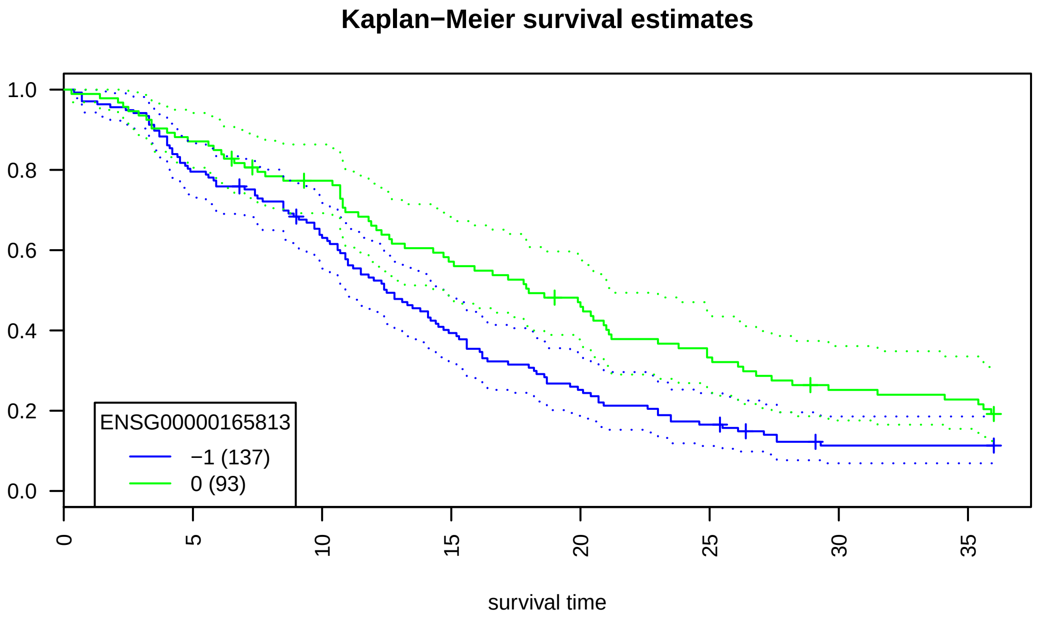

Figure 1729: Each curve represents the relative number of ENSG00000165813 survived to the corresponding point of time. Dashed lines represent 0.95 confidence intervals for the curves with the corresponding color. Log-rank probability measure for the equality of these curves is 5.222798e-03. Additional annotations: *.GeneName*=C10orf118, *.GeneDesc*=microRNA 2110 [Source:HGNC Symbol;Acc:37071].

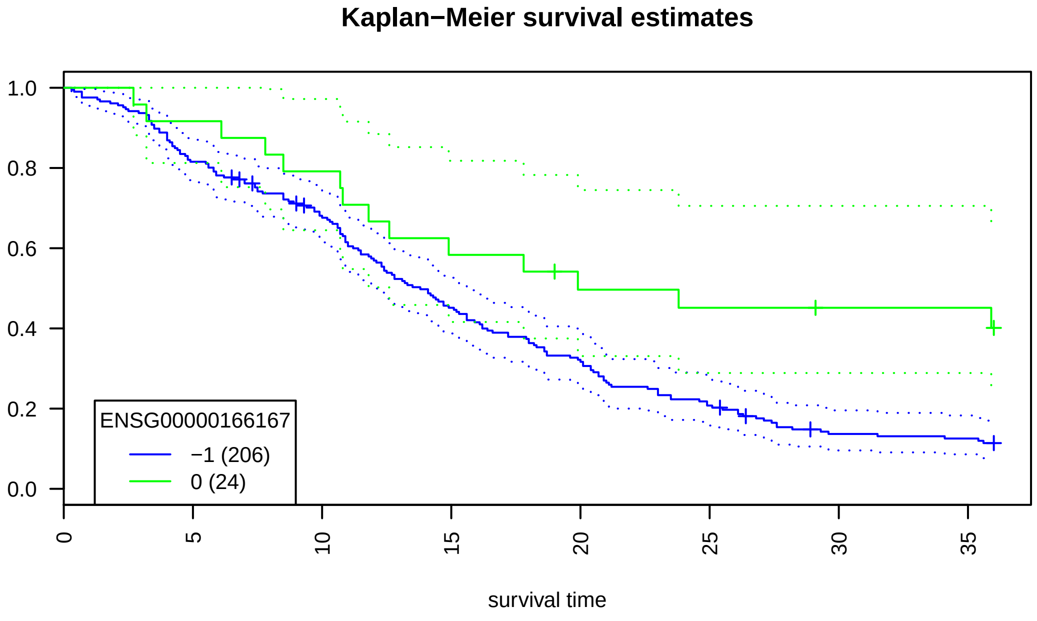

Figure 1730: Each curve represents the relative number of ENSG00000166167 survived to the corresponding point of time. Dashed lines represent 0.95 confidence intervals for the curves with the corresponding color. Log-rank probability measure for the equality of these curves is 6.11131e-03. Additional annotations: *.GeneName*=BTRC, *.GeneDesc*=beta-transducin repeat containing [Source:HGNC Symbol;Acc:1144].

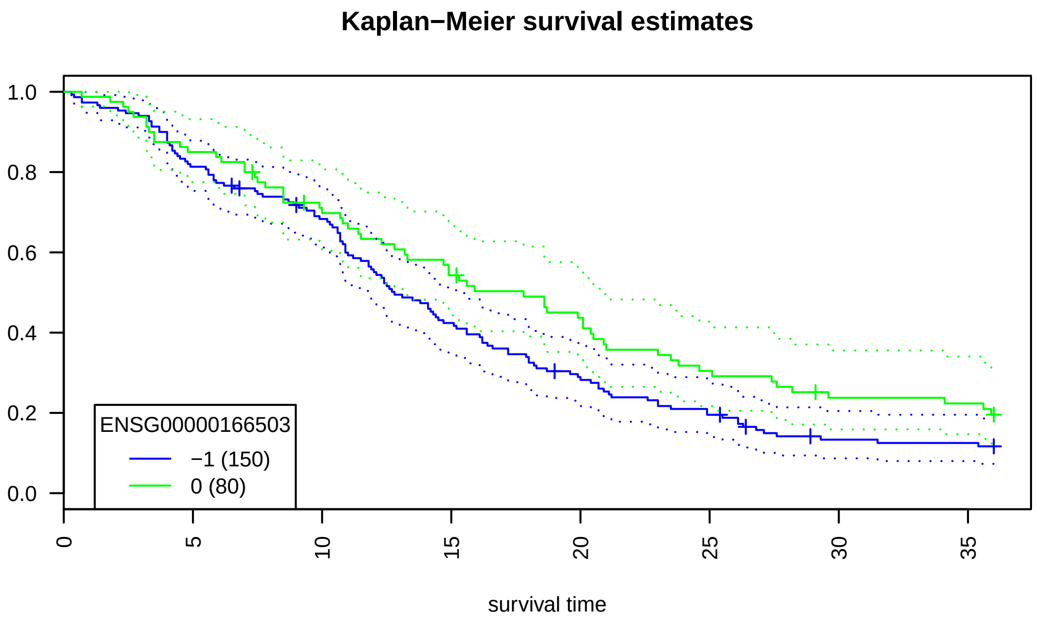

Figure 1731: Each curve represents the relative number of ENSG00000166503 survived to the corresponding point of time. Dashed lines represent 0.95 confidence intervals for the curves with the corresponding color. Log-rank probability measure for the equality of these curves is 4.796419e-02. Additional annotations: *.GeneName*=AC024270.1, *.GeneDesc*=Hepatoma-derived growth factor-related protein 3 (HRP-3)(Hepatoma-derived growth factor 2)(HDGF-2) [Source:UniProtKB/Swiss-Prot;Acc:Q9Y3E1].

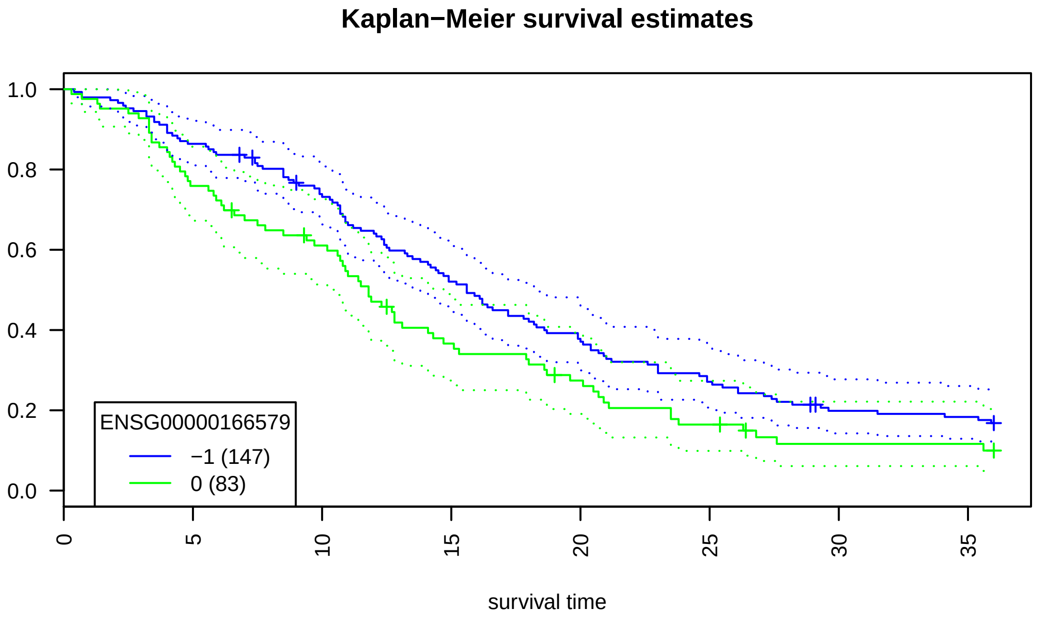

Figure 1732: Each curve represents the relative number of ENSG00000166579 survived to the corresponding point of time. Dashed lines represent 0.95 confidence intervals for the curves with the corresponding color. Log-rank probability measure for the equality of these curves is 2.195242e-02. Additional annotations: *.GeneName*=NDEL1, *.GeneDesc*=nudE nuclear distribution gene E homolog (A. nidulans)-like 1 [Source:HGNC Symbol;Acc:17620].

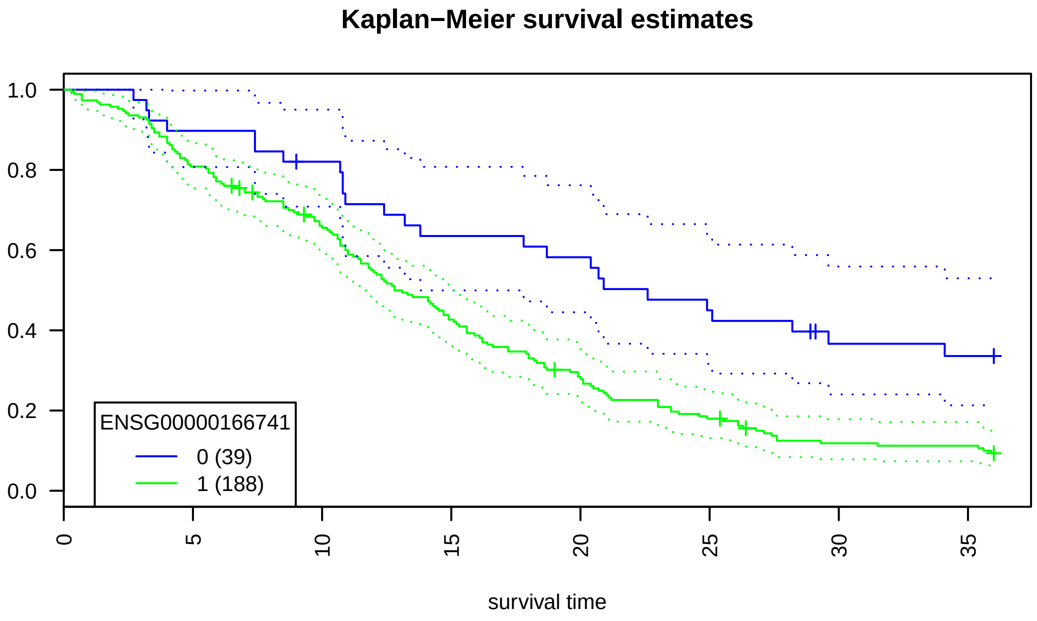

Figure 1733: Each curve represents the relative number of ENSG00000166741 survived to the corresponding point of time. Dashed lines represent 0.95 confidence intervals for the curves with the corresponding color. Total of 3 observations deleted due to missingness. Log-rank probability measure for the equality of these curves is 3.686735e-04. Additional annotations: *.GeneName*=NNMT, *.GeneDesc*=nicotinamide N-methyltransferase [Source:HGNC Symbol;Acc:7861].

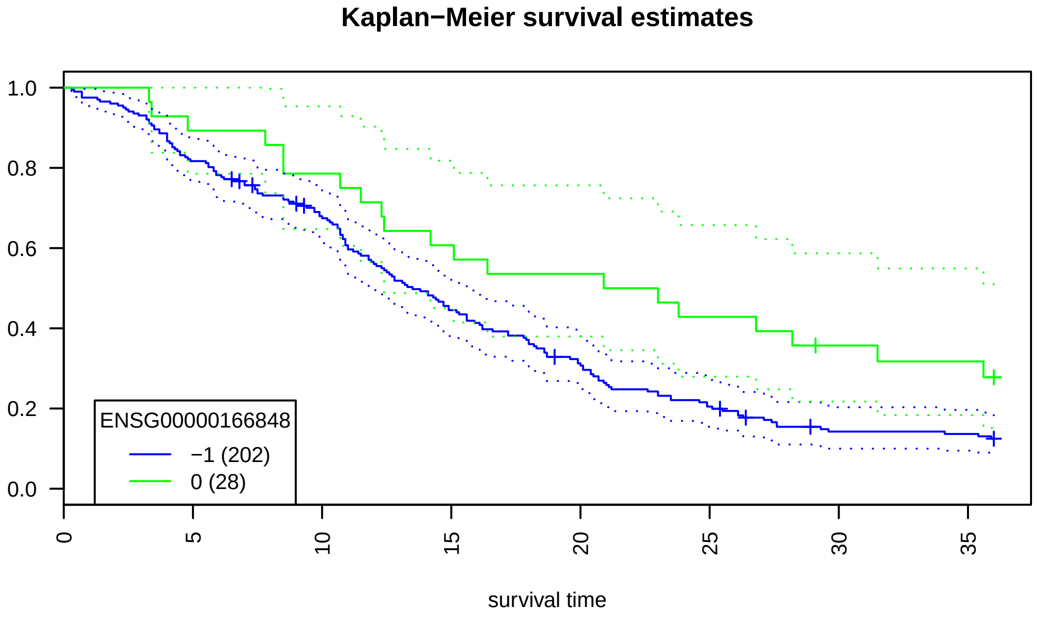

Figure 1734: Each curve represents the relative number of ENSG00000166848 survived to the corresponding point of time. Dashed lines represent 0.95 confidence intervals for the curves with the corresponding color. Log-rank probability measure for the equality of these curves is 2.167003e-02. Additional annotations: *.GeneName*=TERF2IP, *.GeneDesc*=telomeric repeat binding factor 2, interacting protein [Source:HGNC Symbol;Acc:19246].

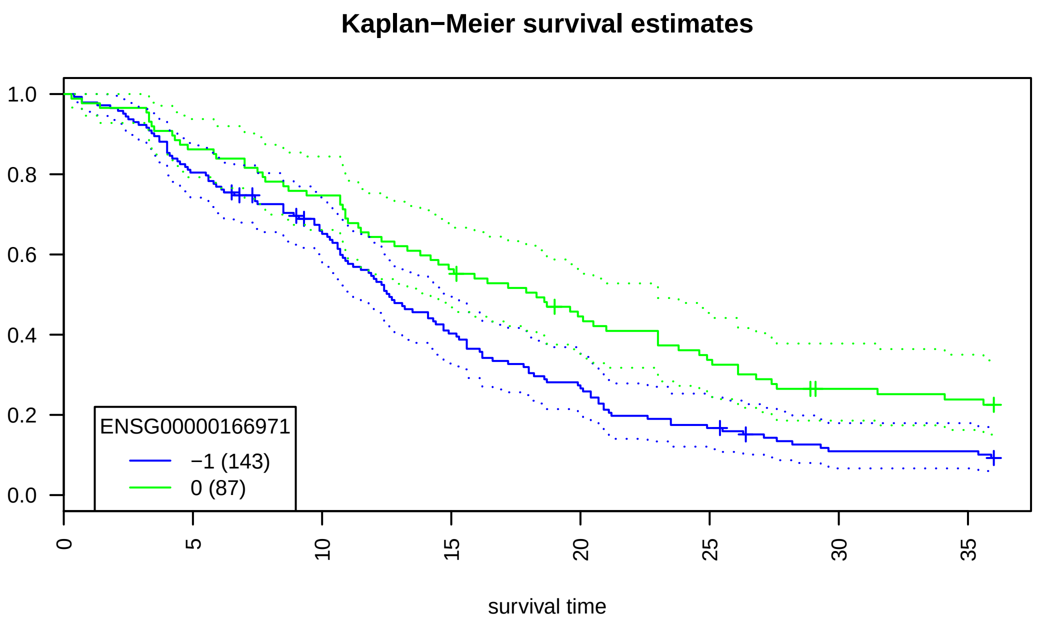

Figure 1735: Each curve represents the relative number of ENSG00000166971 survived to the corresponding point of time. Dashed lines represent 0.95 confidence intervals for the curves with the corresponding color. Log-rank probability measure for the equality of these curves is 2.272220e-03. Additional annotations: *.GeneName*=AKTIP, *.GeneDesc*=AKT interacting protein [Source:HGNC Symbol;Acc:16710].

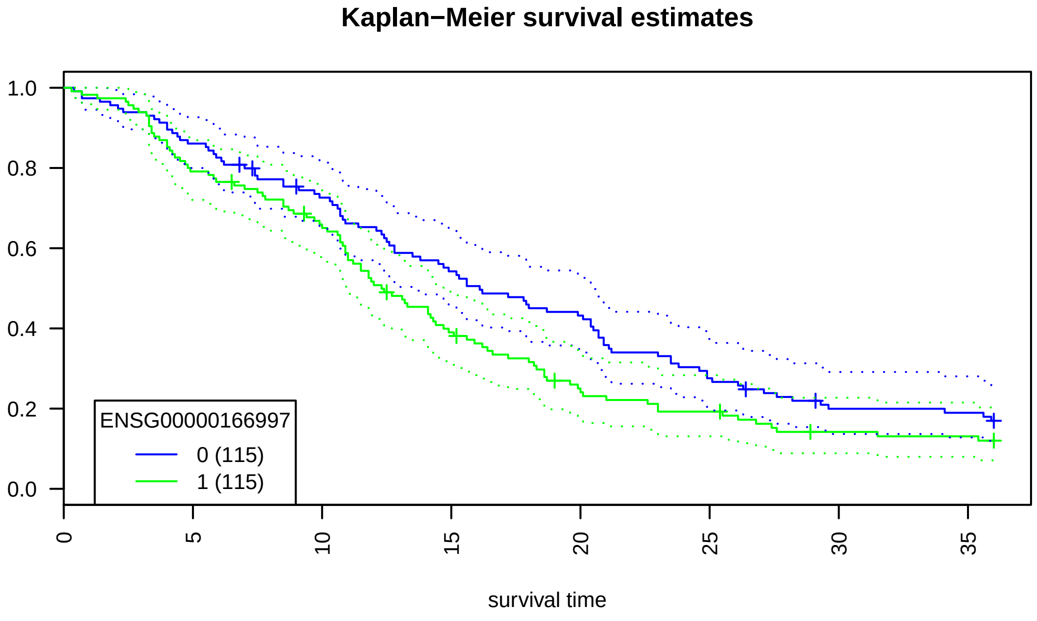

Figure 1736: Each curve represents the relative number of ENSG00000166997 survived to the corresponding point of time. Dashed lines represent 0.95 confidence intervals for the curves with the corresponding color. Log-rank probability measure for the equality of these curves is 4.53214e-02. Additional annotations: *.GeneName*=CNPY4, *.GeneDesc*=canopy 4 homolog (zebrafish) [Source:HGNC Symbol;Acc:28631].

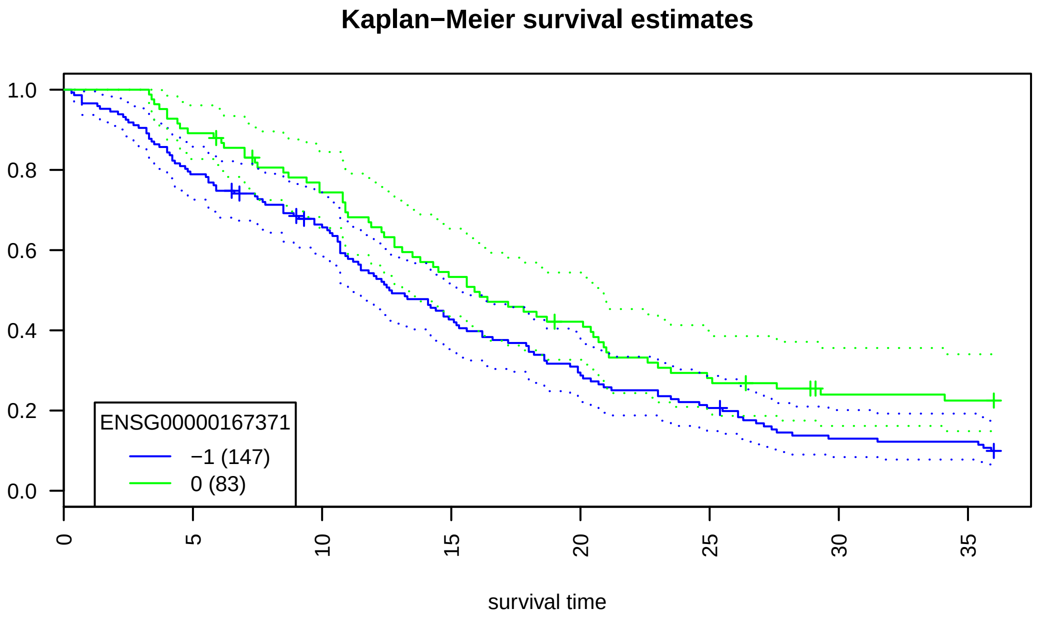

Figure 1737: Each curve represents the relative number of ENSG00000167371 survived to the corresponding point of time. Dashed lines represent 0.95 confidence intervals for the curves with the corresponding color. Log-rank probability measure for the equality of these curves is 1.676541e-02. Additional annotations: *.GeneName*=PRRT2, *.GeneDesc*=proline-rich transmembrane protein 2 [Source:HGNC Symbol;Acc:30500].

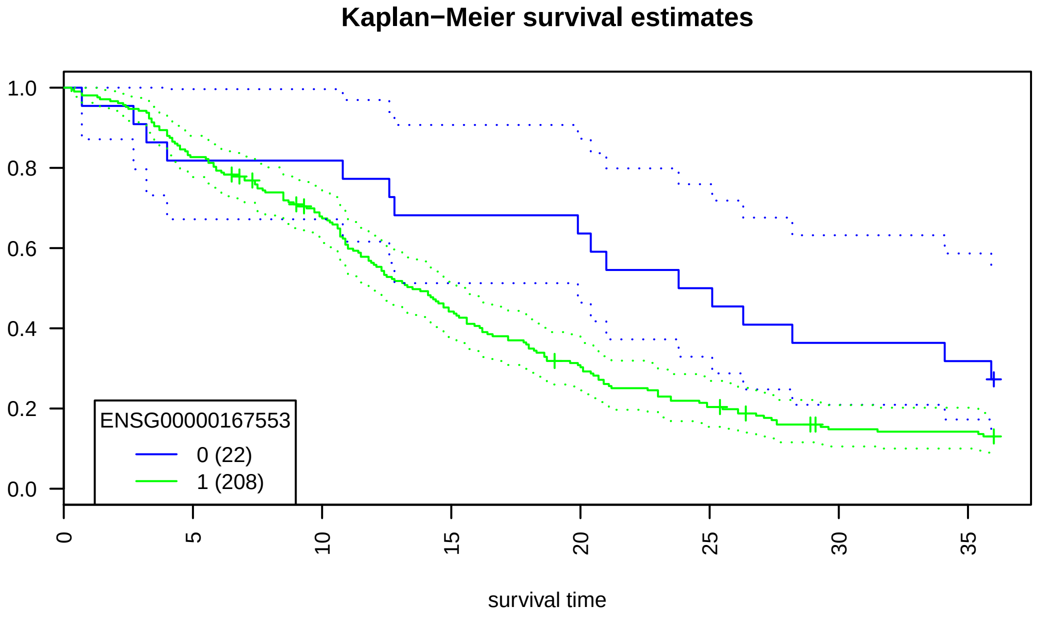

Figure 1738: Each curve represents the relative number of ENSG00000167553 survived to the corresponding point of time. Dashed lines represent 0.95 confidence intervals for the curves with the corresponding color. Log-rank probability measure for the equality of these curves is 2.376179e-02. Additional annotations: *.GeneName*=TUBA1C, *.GeneDesc*=tubulin, alpha 1c [Source:HGNC Symbol;Acc:20768].

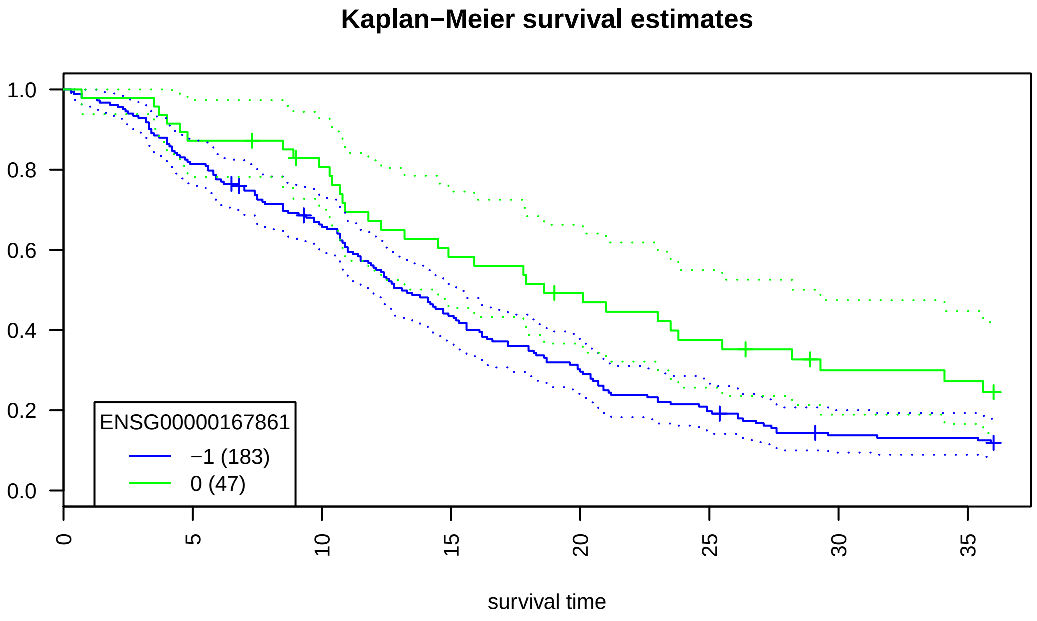

Figure 1739: Each curve represents the relative number of ENSG00000167861 survived to the corresponding point of time. Dashed lines represent 0.95 confidence intervals for the curves with the corresponding color. Log-rank probability measure for the equality of these curves is 1.224048e-02. Additional annotations: *.GeneName*=C17orf28, *.GeneDesc*=UPF0663 transmembrane protein C17orf28 (Down-regulated in multiple cancers 1) [Source:UniProtKB/Swiss-Prot;Acc:Q8IV36].

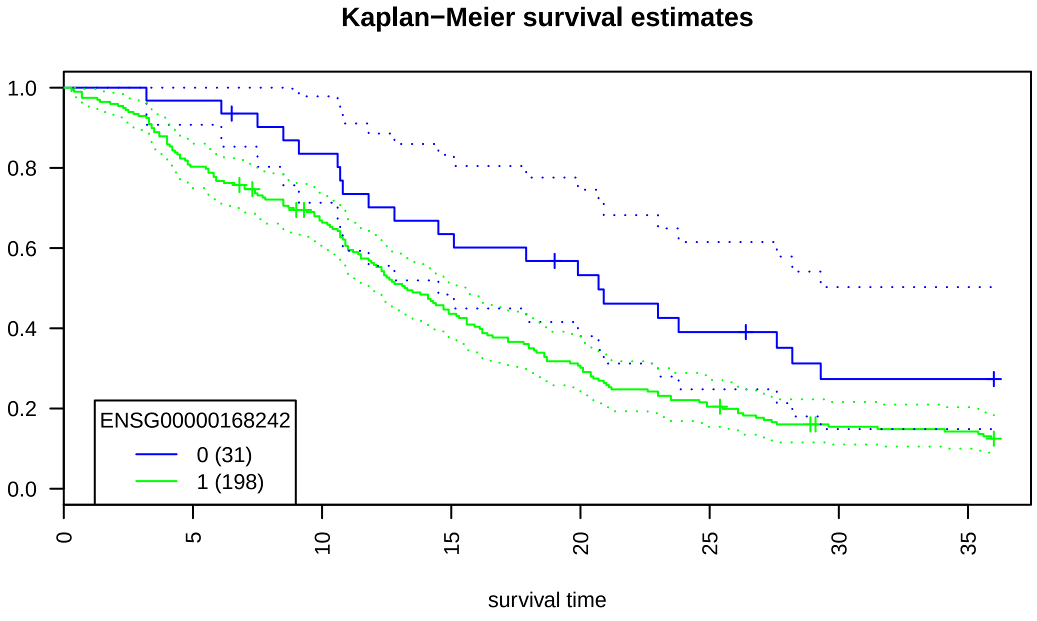

Figure 1740: Each curve represents the relative number of ENSG00000168242 survived to the corresponding point of time. Dashed lines represent 0.95 confidence intervals for the curves with the corresponding color. Total of 1 observation deleted due to missingness. Log-rank probability measure for the equality of these curves is 1.561564e-02. Additional annotations: *.GeneName*=HIST1H2BI, *.GeneDesc*=histone cluster 1, H2bi [Source:HGNC Symbol;Acc:4756].

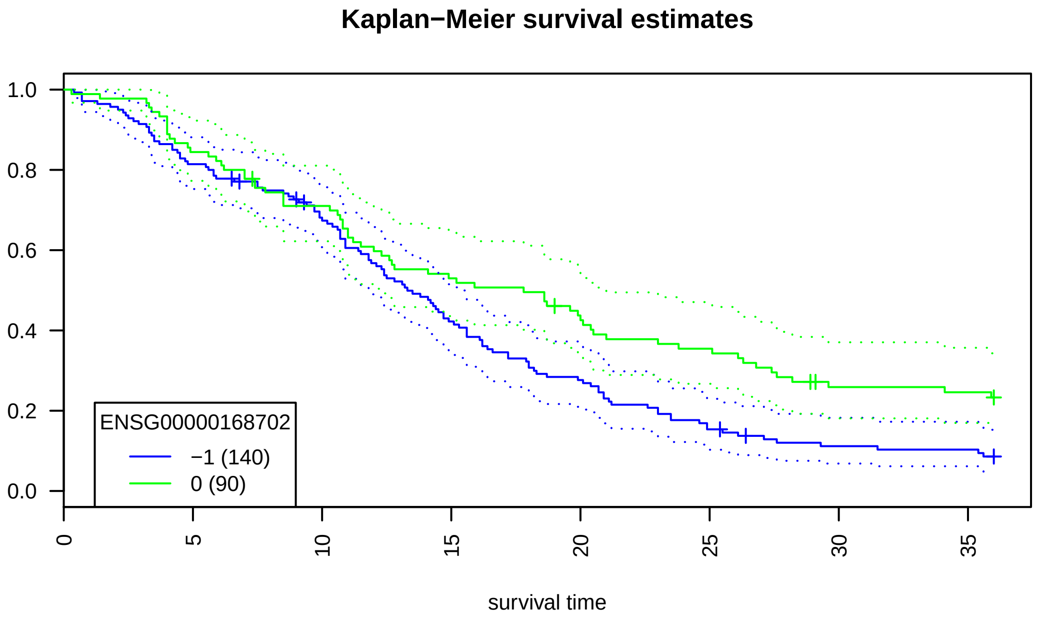

Figure 1741: Each curve represents the relative number of ENSG00000168702 survived to the corresponding point of time. Dashed lines represent 0.95 confidence intervals for the curves with the corresponding color. Log-rank probability measure for the equality of these curves is 5.197555e-03. Additional annotations: *.GeneName*=LRP1B, *.GeneDesc*=low density lipoprotein receptor-related protein 1B [Source:HGNC Symbol;Acc:6693].

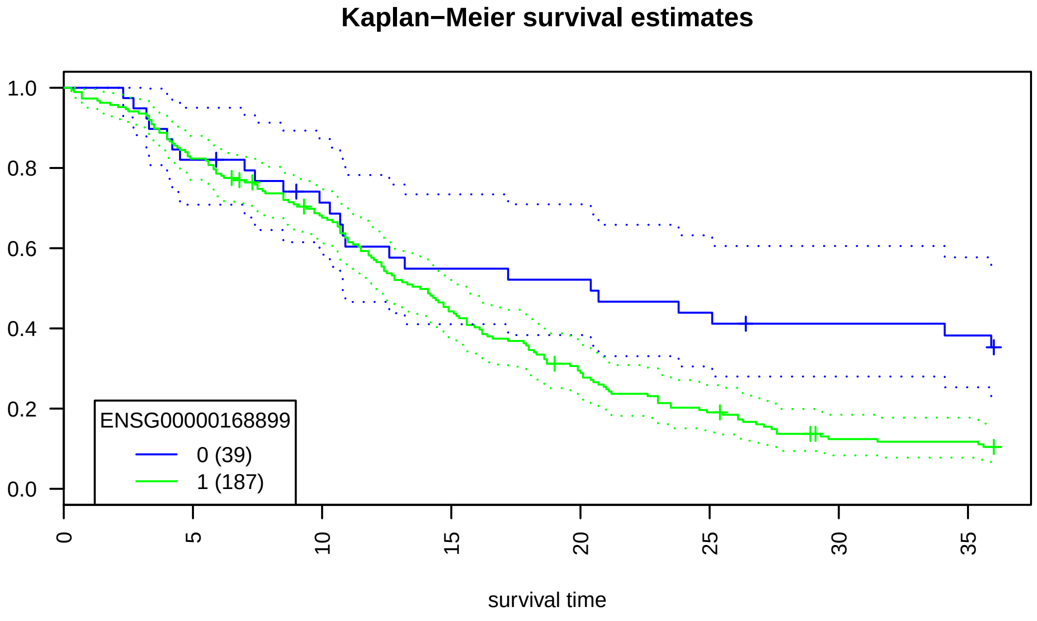

Figure 1742: Each curve represents the relative number of ENSG00000168899 survived to the corresponding point of time. Dashed lines represent 0.95 confidence intervals for the curves with the corresponding color. Total of 4 observations deleted due to missingness. Log-rank probability measure for the equality of these curves is 4.331675e-03. Additional annotations: *.GeneName*=VAMP5, *.GeneDesc*=vesicle-associated membrane protein 5 (myobrevin) [Source:HGNC Symbol;Acc:12646].

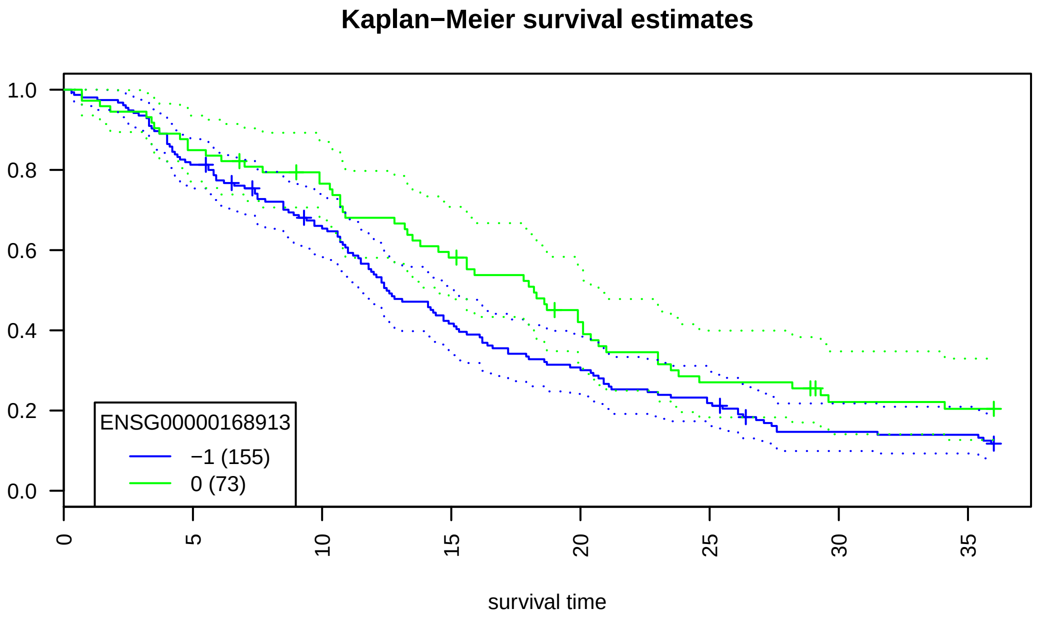

Figure 1743: Each curve represents the relative number of ENSG00000168913 survived to the corresponding point of time. Dashed lines represent 0.95 confidence intervals for the curves with the corresponding color. Total of 2 observations deleted due to missingness. Log-rank probability measure for the equality of these curves is 4.133012e-02. Additional annotations: *.GeneName*=ENHO, *.GeneDesc*=energy homeostasis associated [Source:HGNC Symbol;Acc:24838].

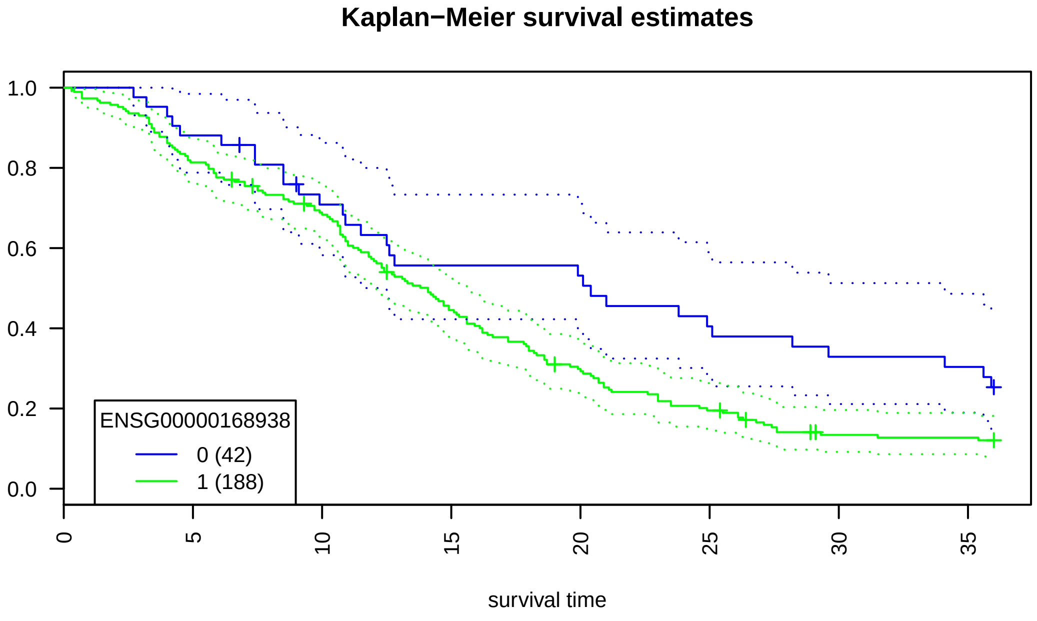

Figure 1744: Each curve represents the relative number of ENSG00000168938 survived to the corresponding point of time. Dashed lines represent 0.95 confidence intervals for the curves with the corresponding color. Log-rank probability measure for the equality of these curves is 1.784263e-02. Additional annotations: *.GeneName*=PPIC, *.GeneDesc*=peptidylprolyl isomerase C (cyclophilin C) [Source:HGNC Symbol;Acc:9256].

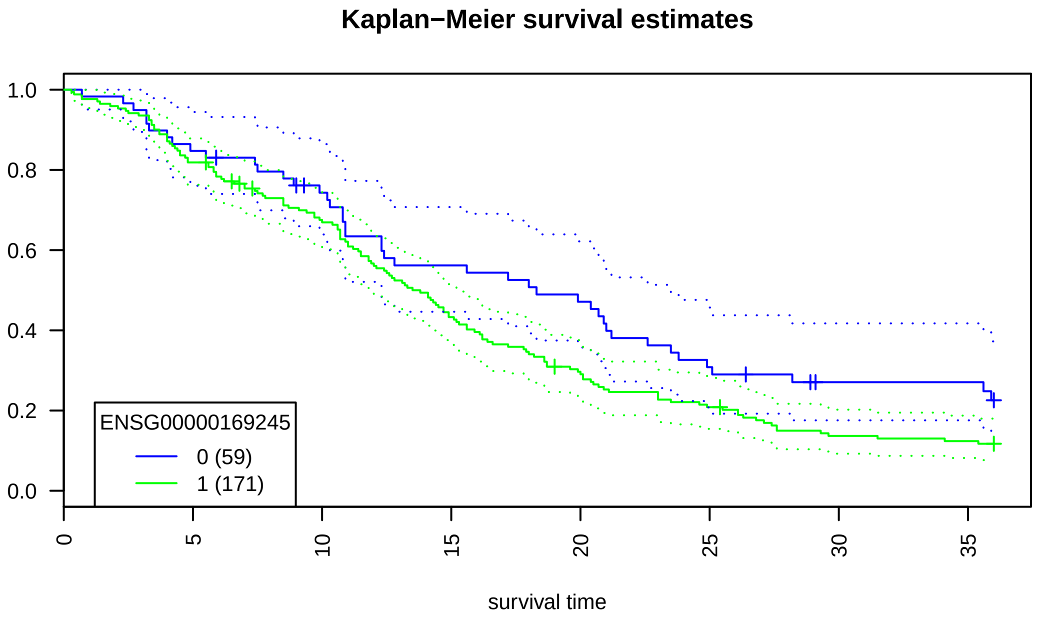

Figure 1745: Each curve represents the relative number of ENSG00000169245 survived to the corresponding point of time. Dashed lines represent 0.95 confidence intervals for the curves with the corresponding color. Log-rank probability measure for the equality of these curves is 4.181004e-02. Additional annotations: *.GeneName*=CXCL10, *.GeneDesc*=chemokine (C-X-C motif) ligand 10 [Source:HGNC Symbol;Acc:10637].

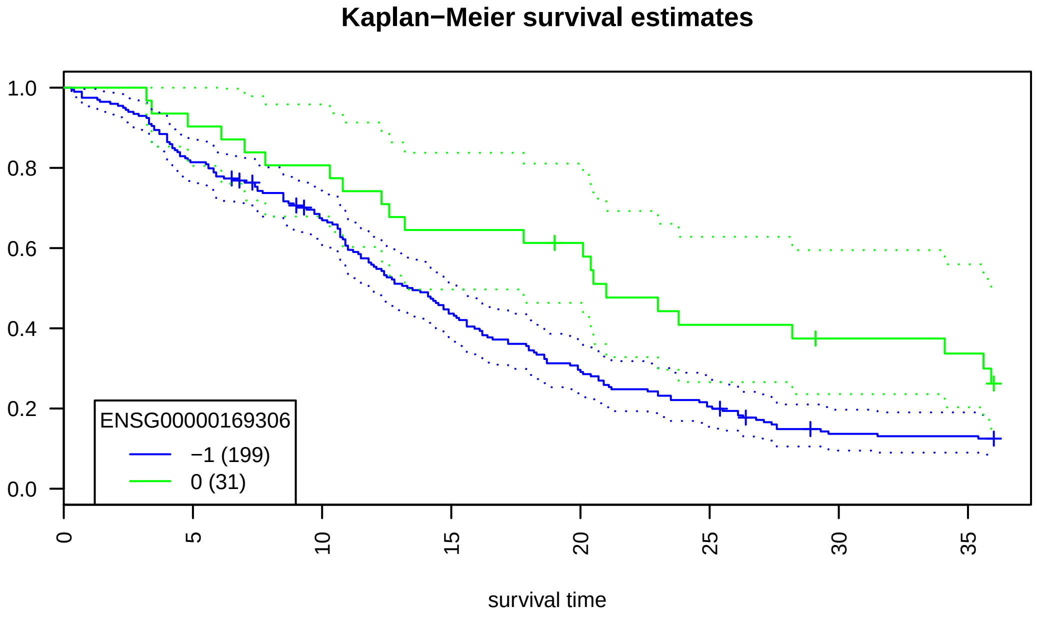

Figure 1746: Each curve represents the relative number of ENSG00000169306 survived to the corresponding point of time. Dashed lines represent 0.95 confidence intervals for the curves with the corresponding color. Log-rank probability measure for the equality of these curves is 1.125570e-02. Additional annotations: *.GeneName*=IL1RAPL1, *.GeneDesc*=interleukin 1 receptor accessory protein-like 1 [Source:HGNC Symbol;Acc:5996].

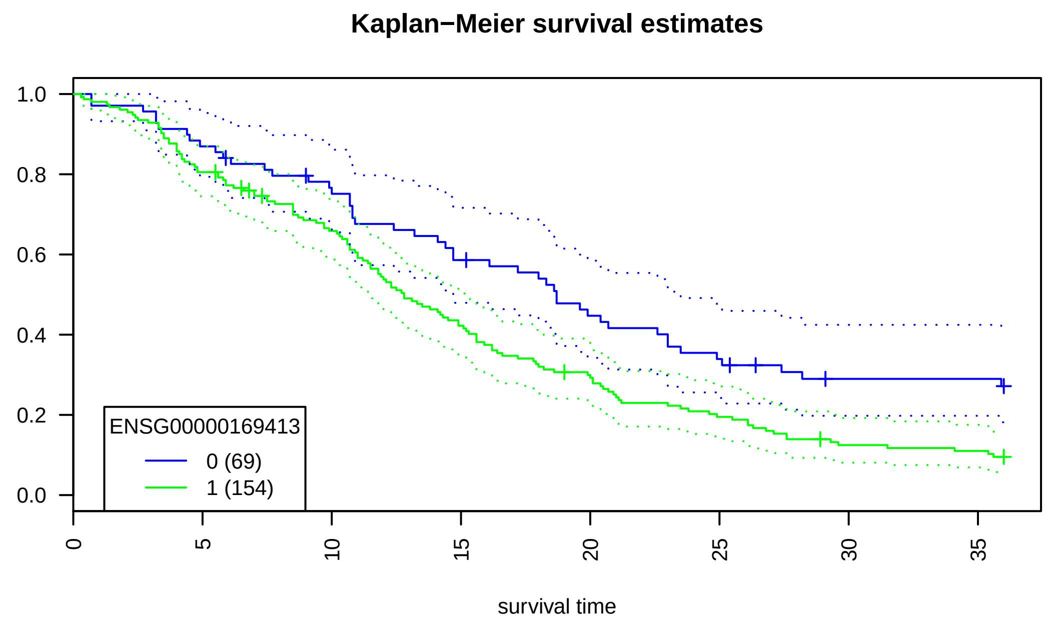

Figure 1747: Each curve represents the relative number of ENSG00000169413 survived to the corresponding point of time. Dashed lines represent 0.95 confidence intervals for the curves with the corresponding color. Total of 7 observations deleted due to missingness. Log-rank probability measure for the equality of these curves is 2.106621e-03. Additional annotations: *.GeneName*=RNASE6, *.GeneDesc*=ribonuclease, RNase A family, k6 [Source:HGNC Symbol;Acc:10048].

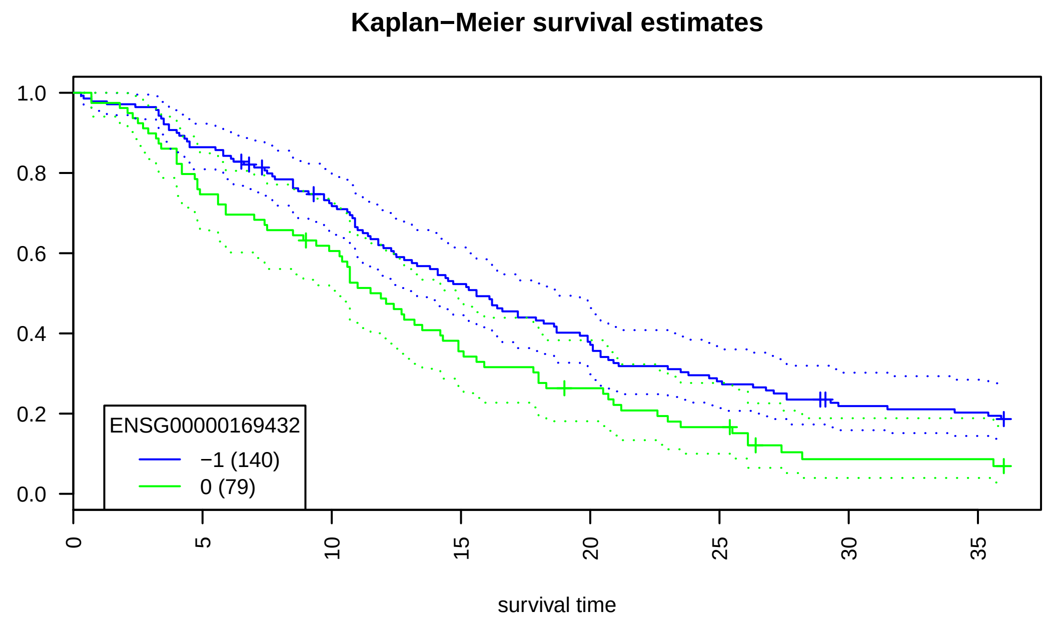

Figure 1748: Each curve represents the relative number of ENSG00000169432 survived to the corresponding point of time. Dashed lines represent 0.95 confidence intervals for the curves with the corresponding color. Total of 11 observations deleted due to missingness. Log-rank probability measure for the equality of these curves is 4.984125e-03. Additional annotations: *.GeneName*=SCN9A, *.GeneDesc*=sodium channel, voltage-gated, type IX, alpha subunit [Source:HGNC Symbol;Acc:10597].

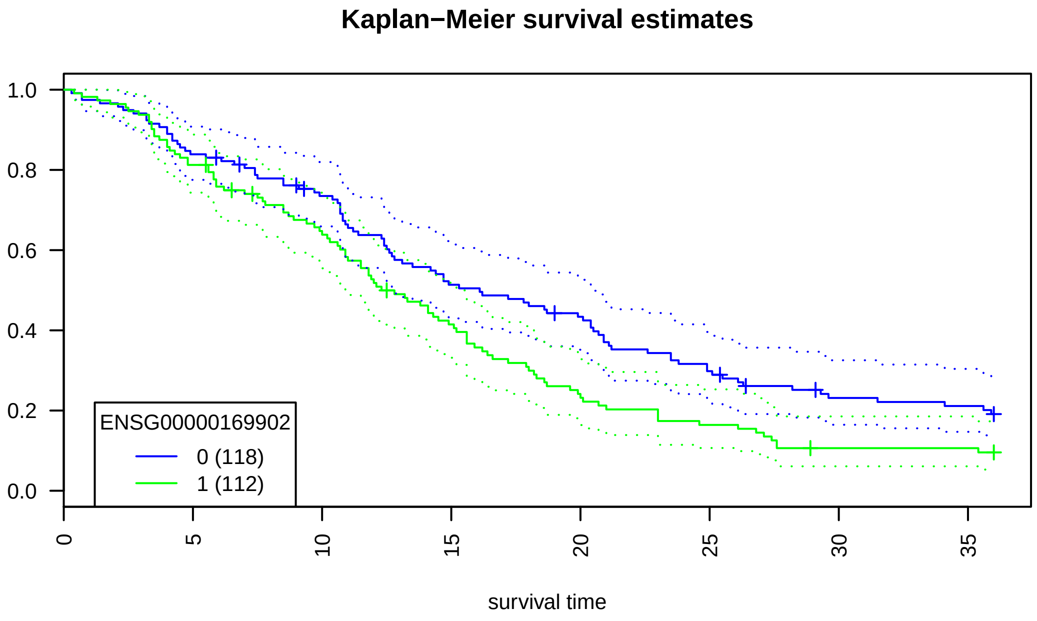

Figure 1749: Each curve represents the relative number of ENSG00000169902 survived to the corresponding point of time. Dashed lines represent 0.95 confidence intervals for the curves with the corresponding color. Log-rank probability measure for the equality of these curves is 9.602822e-03. Additional annotations: *.GeneName*=TPST1, *.GeneDesc*=tyrosylprotein sulfotransferase 1 [Source:HGNC Symbol;Acc:12020].

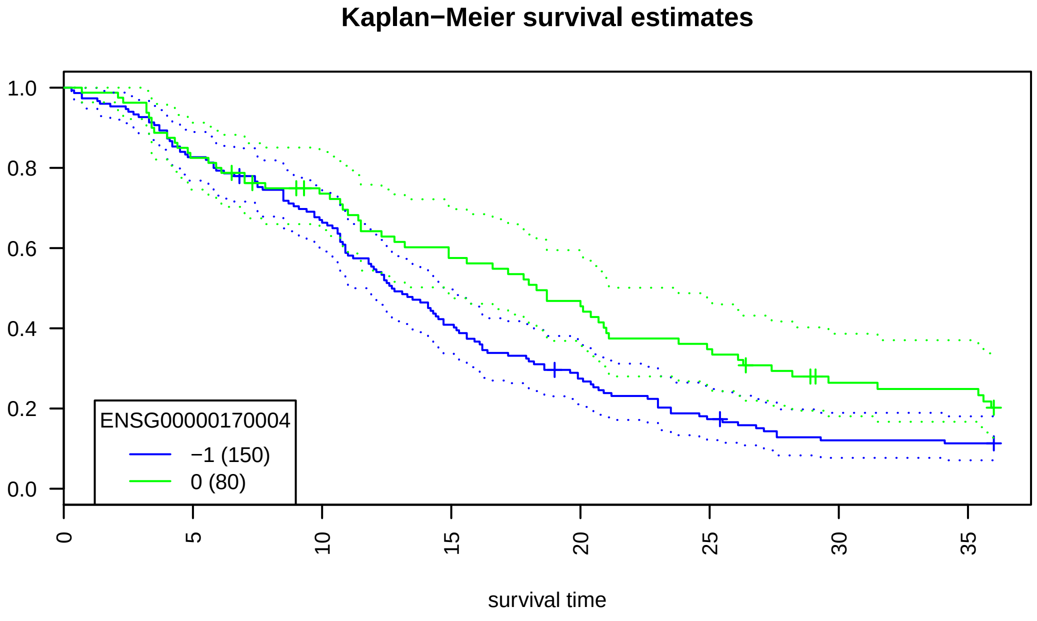

Figure 1750: Each curve represents the relative number of ENSG00000170004 survived to the corresponding point of time. Dashed lines represent 0.95 confidence intervals for the curves with the corresponding color. Log-rank probability measure for the equality of these curves is 1.353480e-02. Additional annotations: *.GeneName*=CHD3, *.GeneDesc*=chromodomain helicase DNA binding protein 3 [Source:HGNC Symbol;Acc:1918].

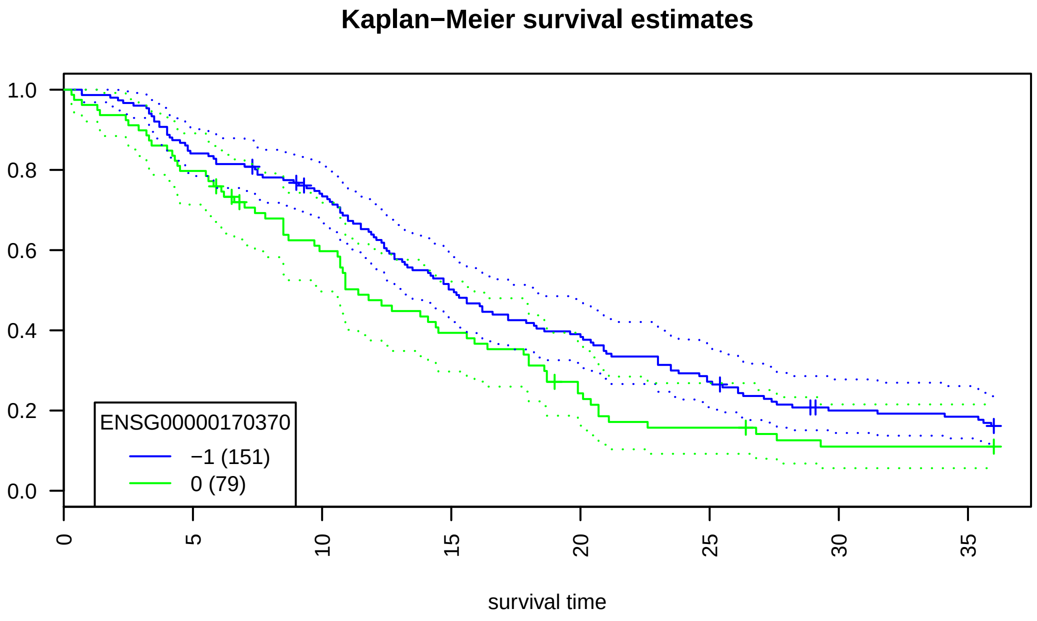

Figure 1751: Each curve represents the relative number of ENSG00000170370 survived to the corresponding point of time. Dashed lines represent 0.95 confidence intervals for the curves with the corresponding color. Log-rank probability measure for the equality of these curves is 3.431554e-02. Additional annotations: *.GeneName*=EMX2, *.GeneDesc*=empty spiracles homeobox 2 [Source:HGNC Symbol;Acc:3341].

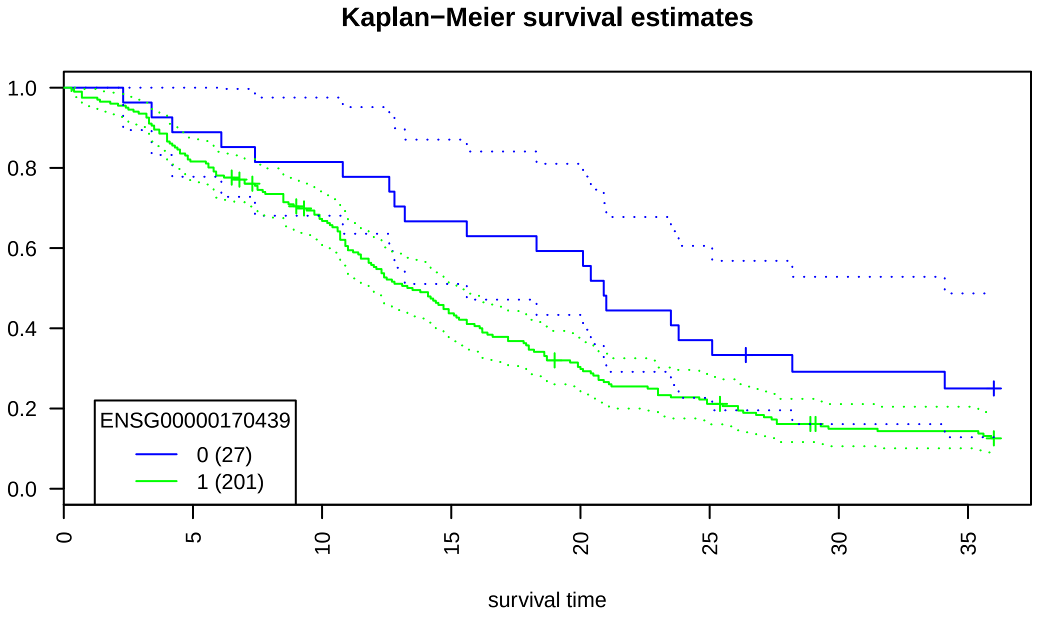

Figure 1752: Each curve represents the relative number of ENSG00000170439 survived to the corresponding point of time. Dashed lines represent 0.95 confidence intervals for the curves with the corresponding color. Total of 2 observations deleted due to missingness. Log-rank probability measure for the equality of these curves is 3.492120e-02. Additional annotations: *.GeneName*=METTL7B, *.GeneDesc*=methyltransferase like 7B [Source:HGNC Symbol;Acc:28276].

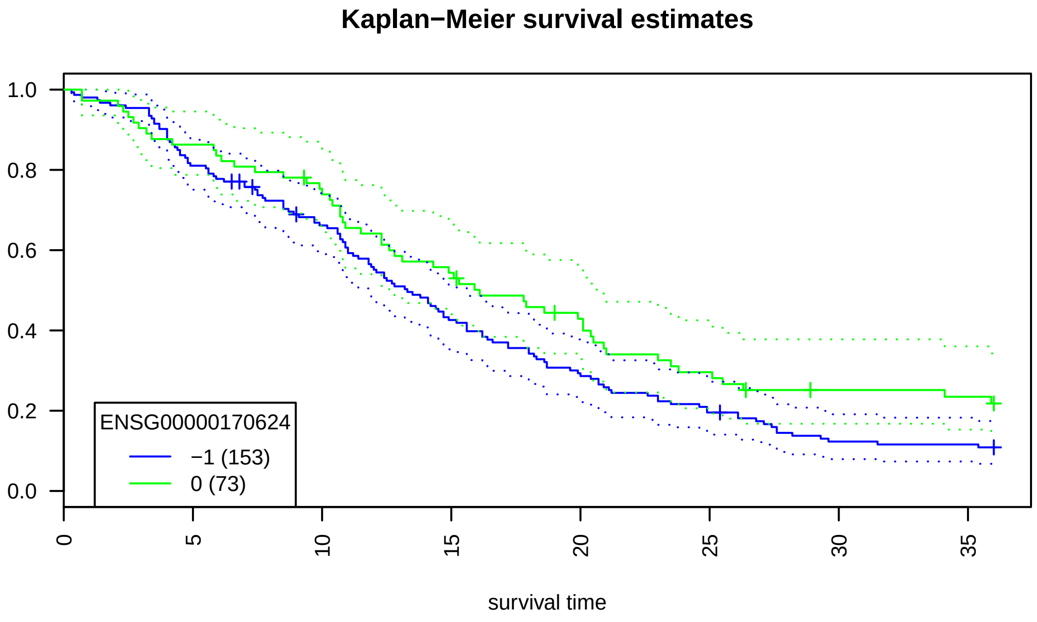

Figure 1753: Each curve represents the relative number of ENSG00000170624 survived to the corresponding point of time. Dashed lines represent 0.95 confidence intervals for the curves with the corresponding color. Total of 4 observations deleted due to missingness. Log-rank probability measure for the equality of these curves is 4.951182e-02. Additional annotations: *.GeneName*=SGCD, *.GeneDesc*=sarcoglycan, delta (35kDa dystrophin-associated glycoprotein) [Source:HGNC Symbol;Acc:10807].

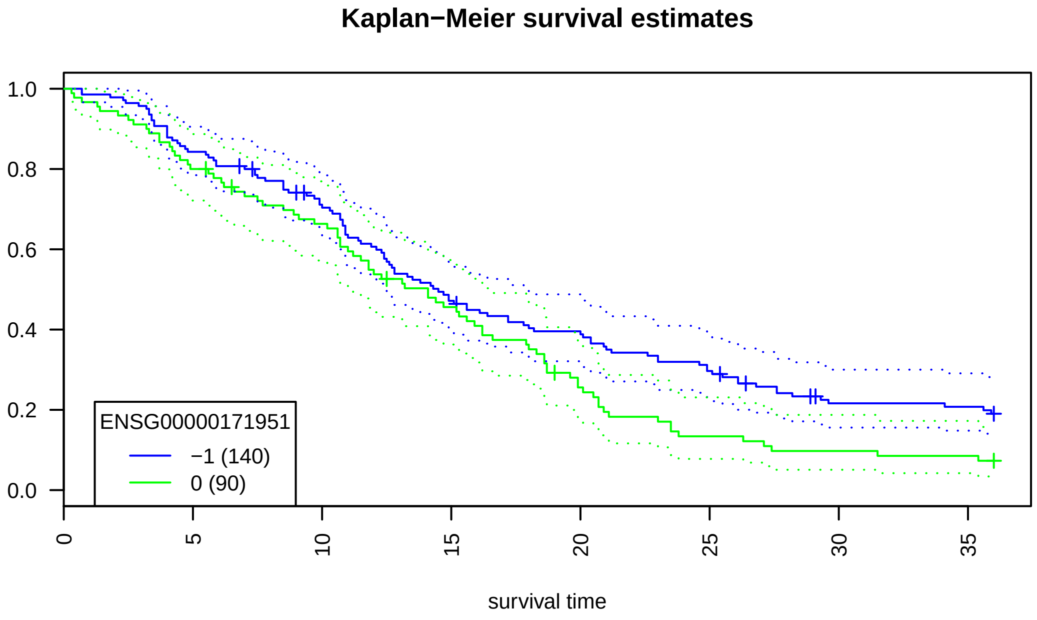

Figure 1754: Each curve represents the relative number of ENSG00000171951 survived to the corresponding point of time. Dashed lines represent 0.95 confidence intervals for the curves with the corresponding color. Log-rank probability measure for the equality of these curves is 2.089856e-02. Additional annotations: *.GeneName*=SCG2, *.GeneDesc*=secretogranin II (chromogranin C) [Source:HGNC Symbol;Acc:10575].

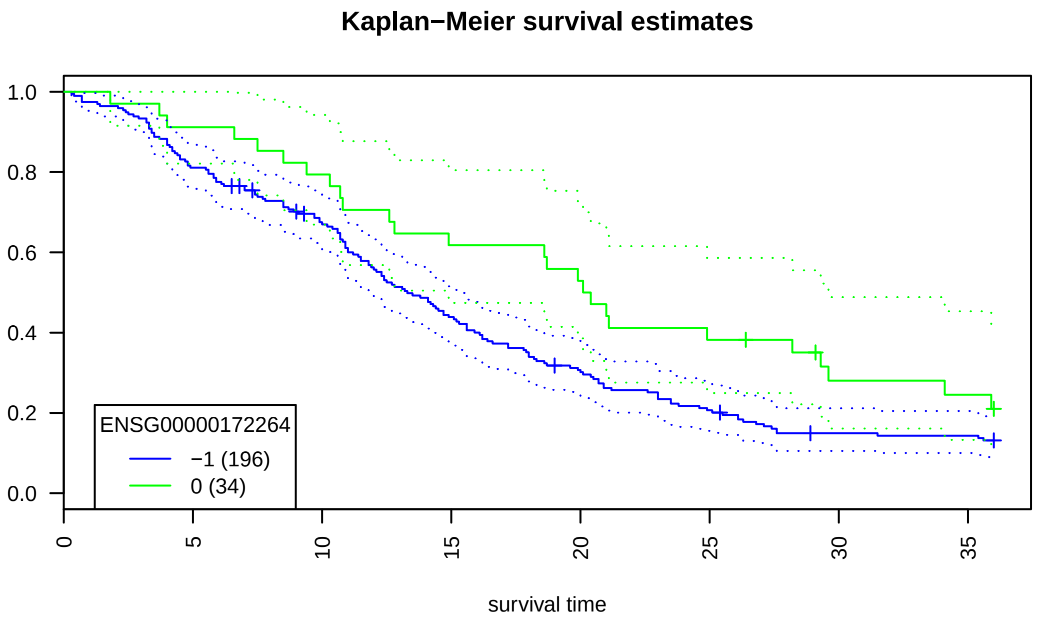

Figure 1755: Each curve represents the relative number of ENSG00000172264 survived to the corresponding point of time. Dashed lines represent 0.95 confidence intervals for the curves with the corresponding color. Log-rank probability measure for the equality of these curves is 3.292858e-02. Additional annotations: *.GeneName*=MACROD2, *.GeneDesc*=MACRO domain containing 2 [Source:HGNC Symbol;Acc:16126].

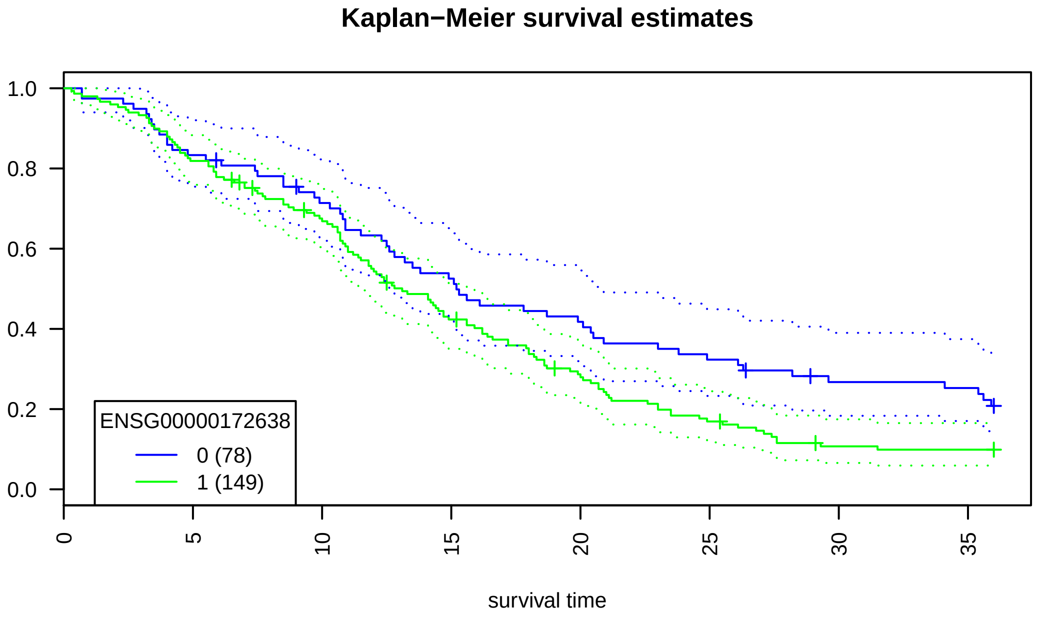

Figure 1756: Each curve represents the relative number of ENSG00000172638 survived to the corresponding point of time. Dashed lines represent 0.95 confidence intervals for the curves with the corresponding color. Total of 3 observations deleted due to missingness. Log-rank probability measure for the equality of these curves is 2.325566e-02. Additional annotations: *.GeneName*=EFEMP2, *.GeneDesc*=EGF-containing fibulin-like extracellular matrix protein 2 [Source:HGNC Symbol;Acc:3219].

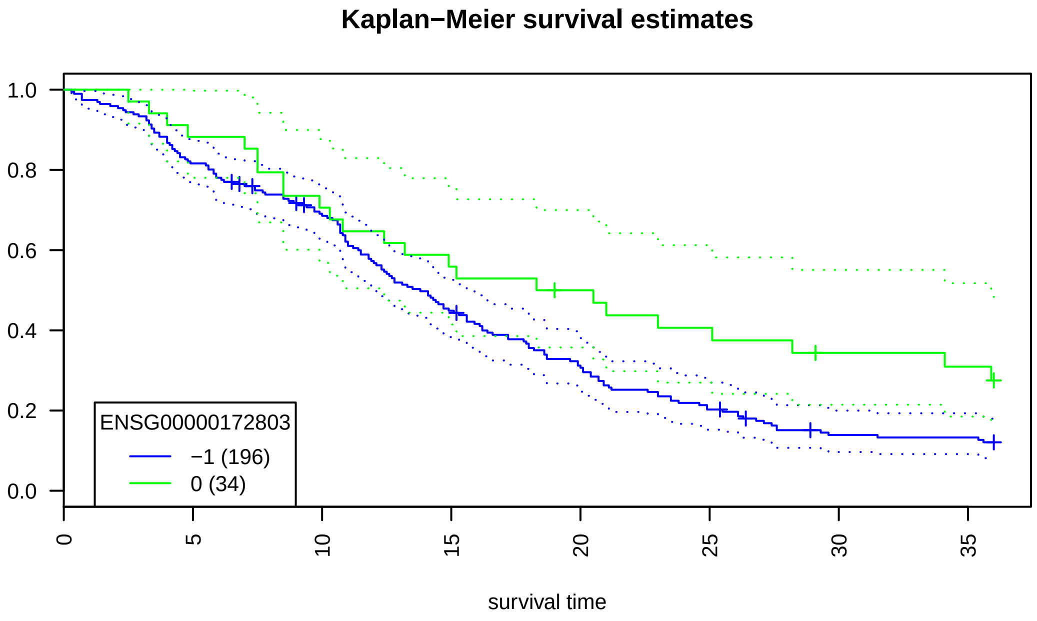

Figure 1757: Each curve represents the relative number of ENSG00000172803 survived to the corresponding point of time. Dashed lines represent 0.95 confidence intervals for the curves with the corresponding color. Log-rank probability measure for the equality of these curves is 3.094571e-02. Additional annotations: *.GeneName*=SNX32, *.GeneDesc*=sorting nexin 32 [Source:HGNC Symbol;Acc:26423].

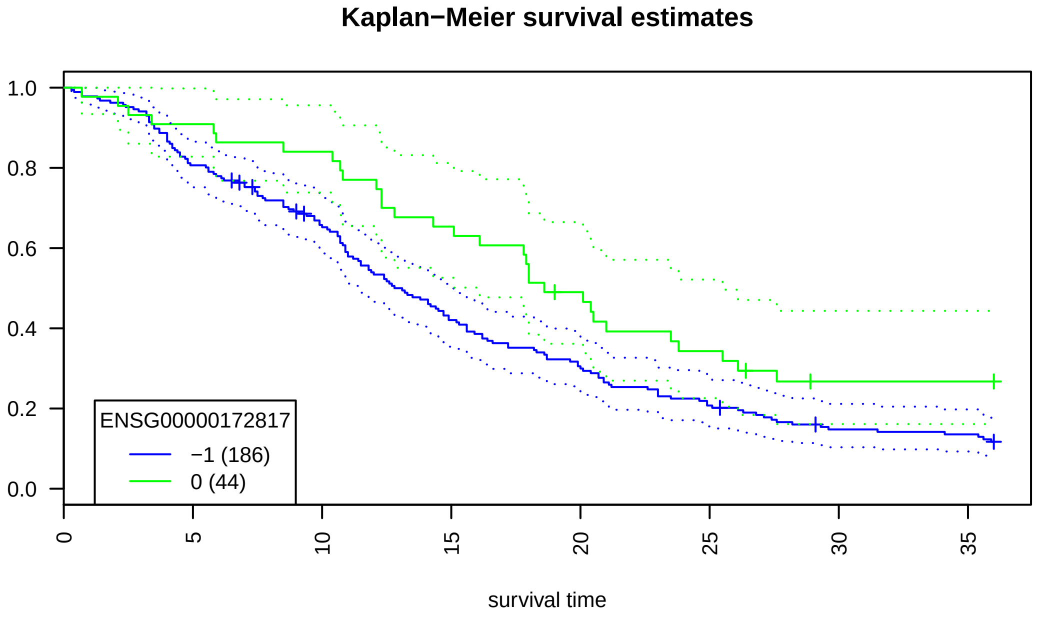

Figure 1758: Each curve represents the relative number of ENSG00000172817 survived to the corresponding point of time. Dashed lines represent 0.95 confidence intervals for the curves with the corresponding color. Log-rank probability measure for the equality of these curves is 1.354874e-02. Additional annotations: *.GeneName*=CYP7B1, *.GeneDesc*=cytochrome P450, family 7, subfamily B, polypeptide 1 [Source:HGNC Symbol;Acc:2652].

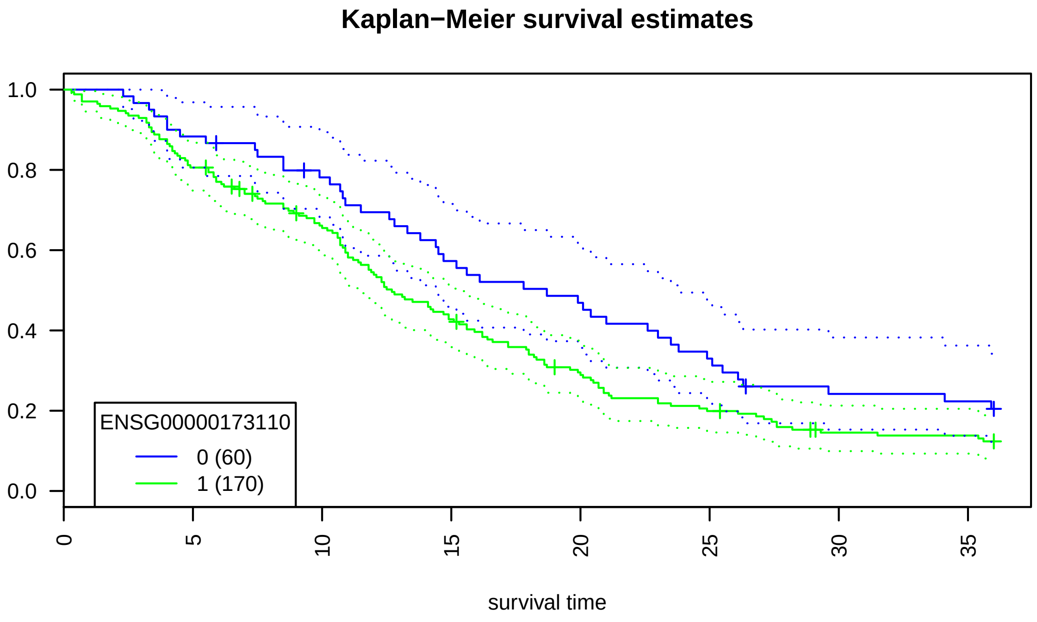

Figure 1759: Each curve represents the relative number of ENSG00000173110 survived to the corresponding point of time. Dashed lines represent 0.95 confidence intervals for the curves with the corresponding color. Log-rank probability measure for the equality of these curves is 2.934139e-02. Additional annotations: *.GeneName*=HSPA6, *.GeneDesc*=heat shock 70kDa protein 7 (HSP70B) [Source:HGNC Symbol;Acc:5240].

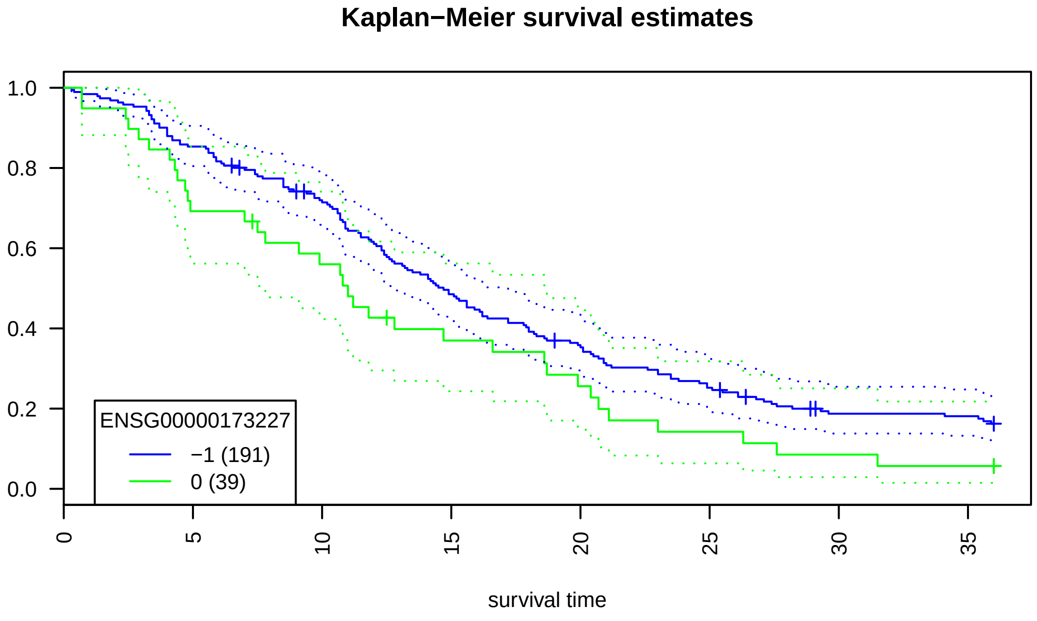

Figure 1760: Each curve represents the relative number of ENSG00000173227 survived to the corresponding point of time. Dashed lines represent 0.95 confidence intervals for the curves with the corresponding color. Log-rank probability measure for the equality of these curves is 3.310846e-02. Additional annotations: *.GeneName*=SYT12, *.GeneDesc*=synaptotagmin XII [Source:HGNC Symbol;Acc:18381].

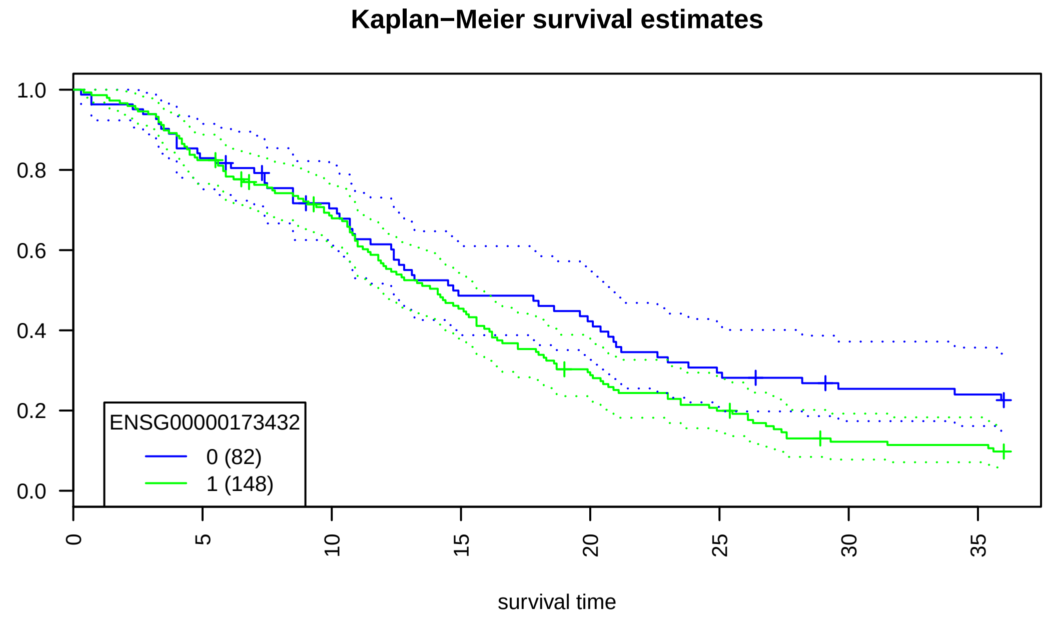

Figure 1761: Each curve represents the relative number of ENSG00000173432 survived to the corresponding point of time. Dashed lines represent 0.95 confidence intervals for the curves with the corresponding color. Log-rank probability measure for the equality of these curves is 4.249225e-02. Additional annotations: *.GeneName*=SAA1, *.GeneDesc*=serum amyloid A1 [Source:HGNC Symbol;Acc:10513].

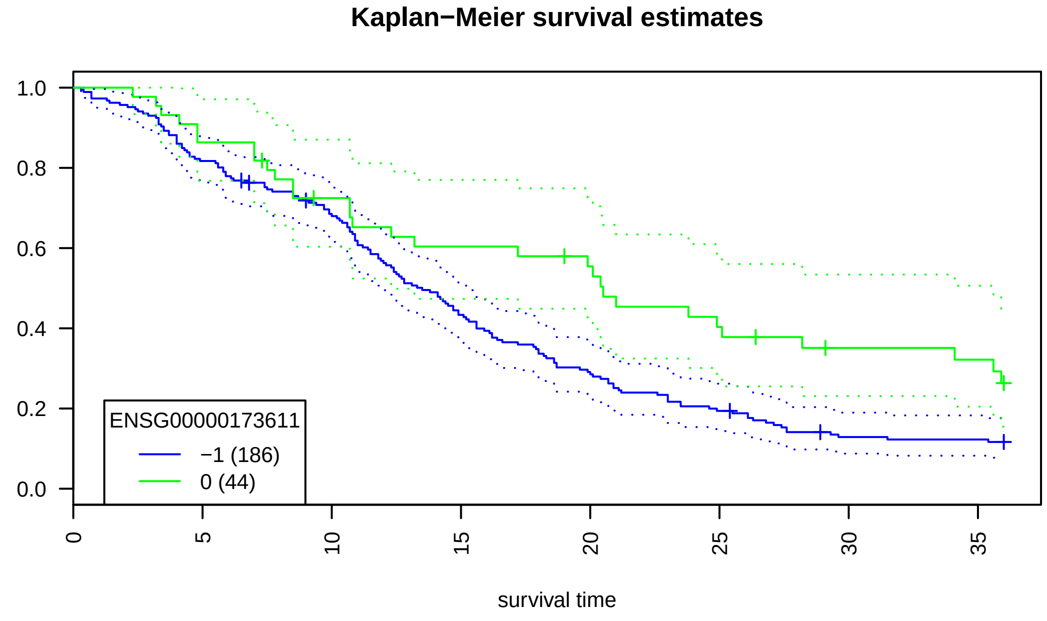

Figure 1762: Each curve represents the relative number of ENSG00000173611 survived to the corresponding point of time. Dashed lines represent 0.95 confidence intervals for the curves with the corresponding color. Log-rank probability measure for the equality of these curves is 8.61229e-03. Additional annotations: *.GeneName*=C9orf126, *.GeneDesc*=suppressor of cancer cell invasion [Source:HGNC Symbol;Acc:26709].

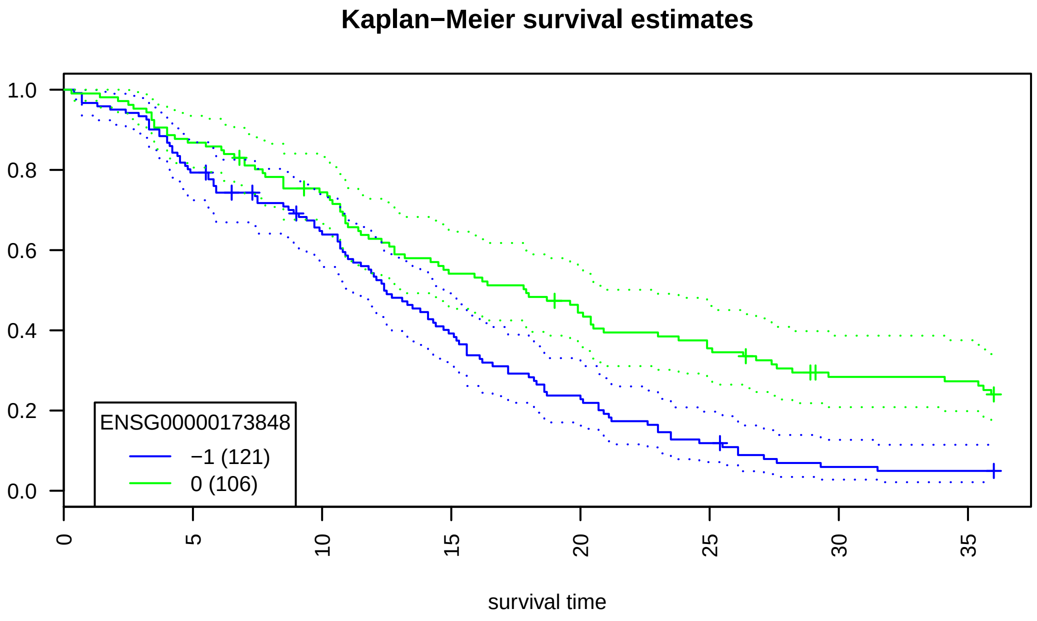

Figure 1763: Each curve represents the relative number of ENSG00000173848 survived to the corresponding point of time. Dashed lines represent 0.95 confidence intervals for the curves with the corresponding color. Total of 3 observations deleted due to missingness. Log-rank probability measure for the equality of these curves is 3.367379e-05. Additional annotations: *.GeneName*=NET1, *.GeneDesc*=neuroepithelial cell transforming 1 [Source:HGNC Symbol;Acc:14592].

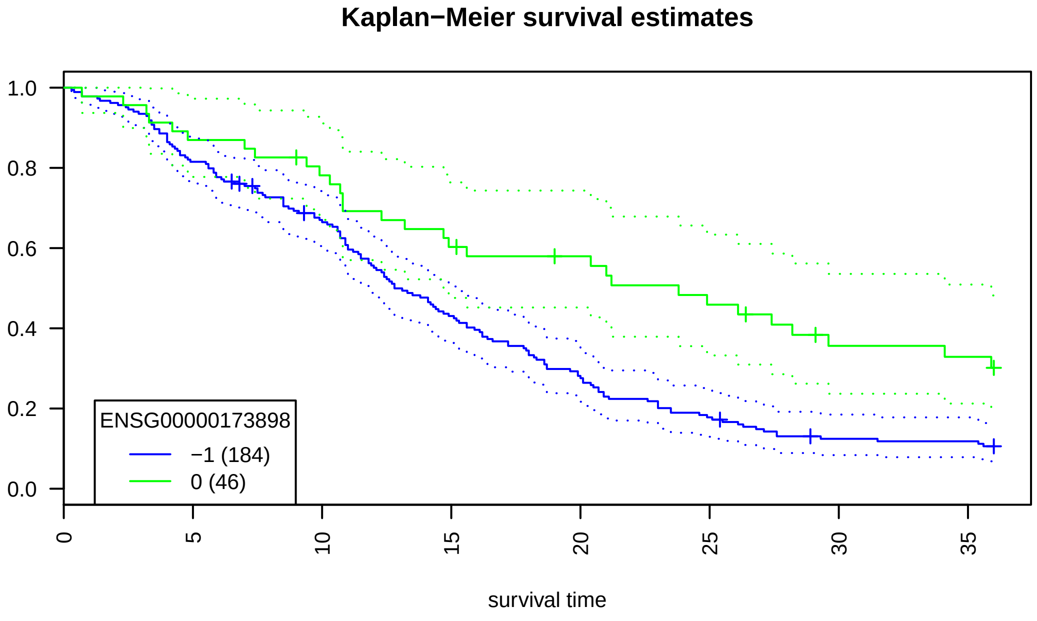

Figure 1764: Each curve represents the relative number of ENSG00000173898 survived to the corresponding point of time. Dashed lines represent 0.95 confidence intervals for the curves with the corresponding color. Log-rank probability measure for the equality of these curves is 7.190511e-04. Additional annotations: *.GeneName*=SPTBN2, *.GeneDesc*=spectrin, beta, non-erythrocytic 2 [Source:HGNC Symbol;Acc:11276].

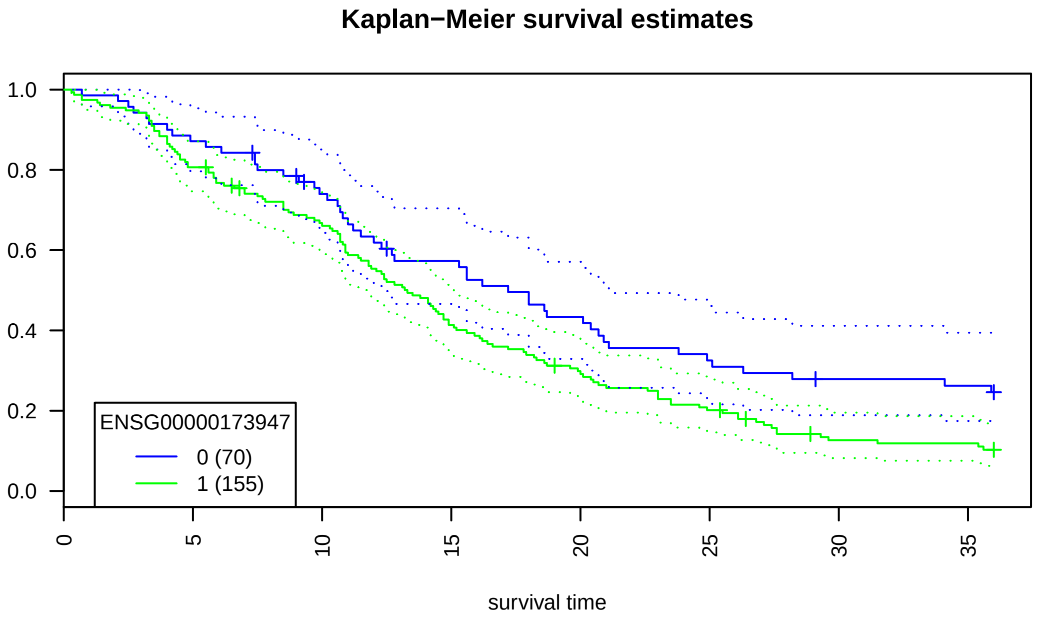

Figure 1765: Each curve represents the relative number of ENSG00000173947 survived to the corresponding point of time. Dashed lines represent 0.95 confidence intervals for the curves with the corresponding color. Total of 5 observations deleted due to missingness. Log-rank probability measure for the equality of these curves is 1.448885e-02. Additional annotations: *.GeneName*=C1orf88, *.GeneDesc*=Uncharacterized protein C1orf88 [Source:UniProtKB/Swiss-Prot;Acc:Q8TCI5].

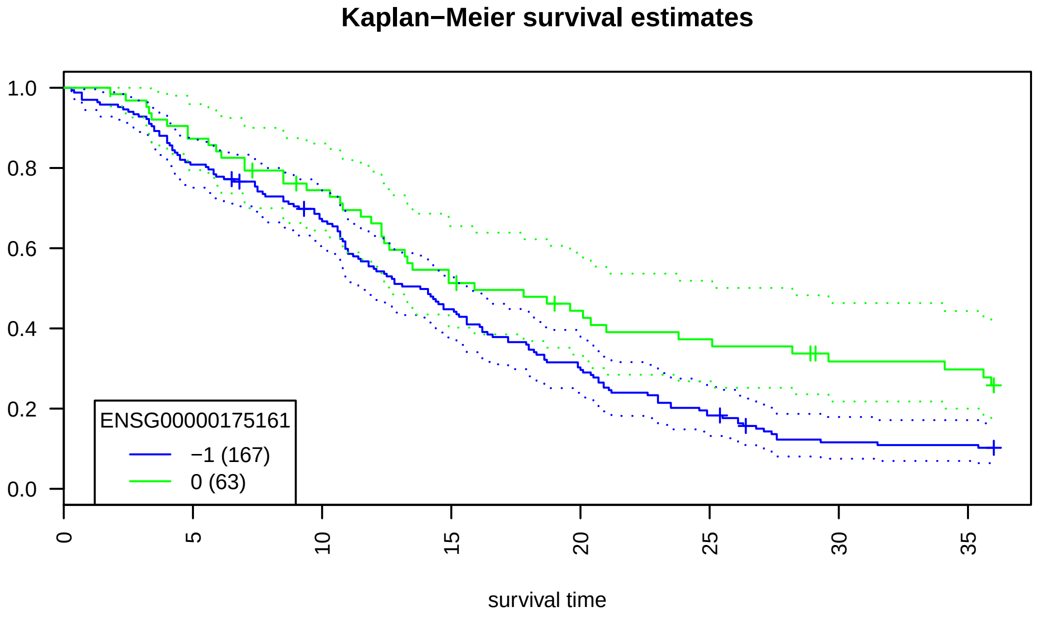

Figure 1766: Each curve represents the relative number of ENSG00000175161 survived to the corresponding point of time. Dashed lines represent 0.95 confidence intervals for the curves with the corresponding color. Log-rank probability measure for the equality of these curves is 7.629626e-03. Additional annotations: *.GeneName*=CADM2, *.GeneDesc*=cell adhesion molecule 2 [Source:HGNC Symbol;Acc:29849].

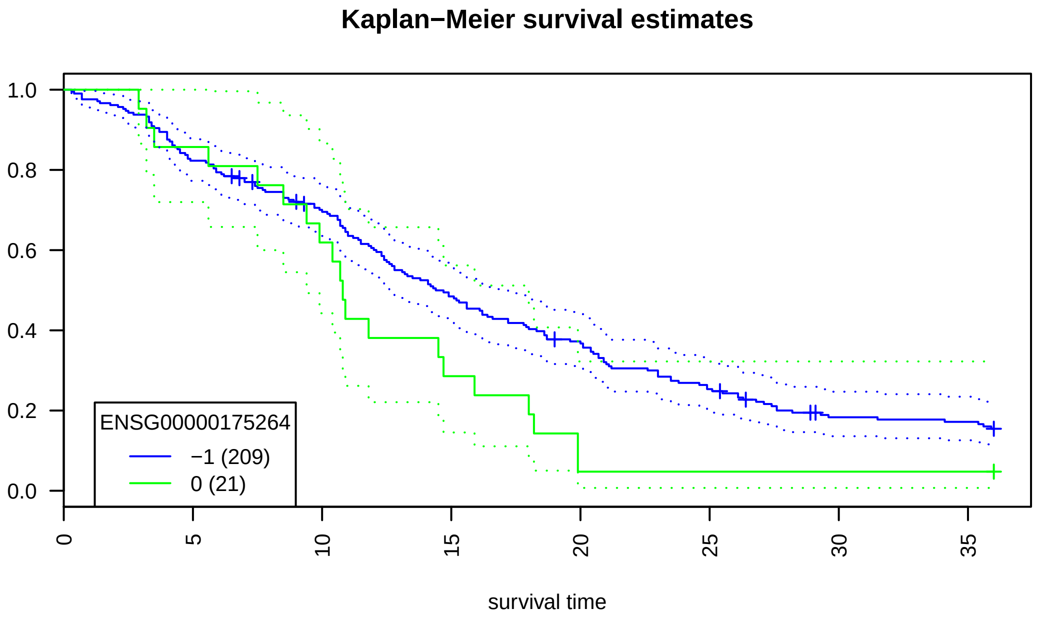

Figure 1767: Each curve represents the relative number of ENSG00000175264 survived to the corresponding point of time. Dashed lines represent 0.95 confidence intervals for the curves with the corresponding color. Log-rank probability measure for the equality of these curves is 2.128899e-02. Additional annotations: *.GeneName*=CHST1, *.GeneDesc*=carbohydrate (keratan sulfate Gal-6) sulfotransferase 1 [Source:HGNC Symbol;Acc:1969].

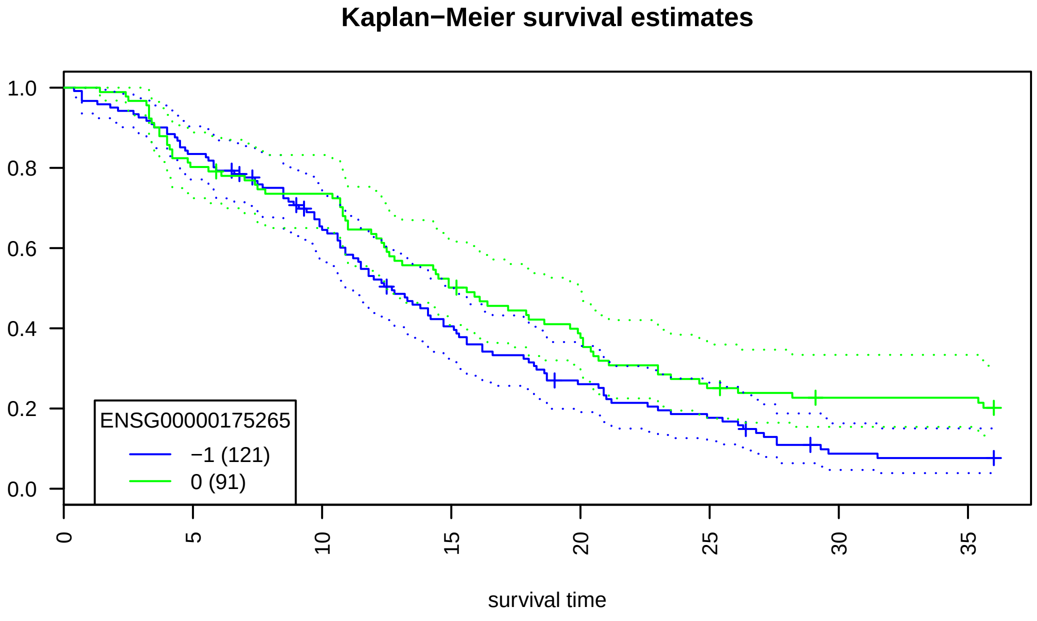

Figure 1768: Each curve represents the relative number of ENSG00000175265 survived to the corresponding point of time. Dashed lines represent 0.95 confidence intervals for the curves with the corresponding color. Total of 18 observations deleted due to missingness. Log-rank probability measure for the equality of these curves is 2.973444e-02. Additional annotations: *.GeneName*=GOLGA8A, *.GeneDesc*=golgin A8 family, member A [Source:HGNC Symbol;Acc:31972].

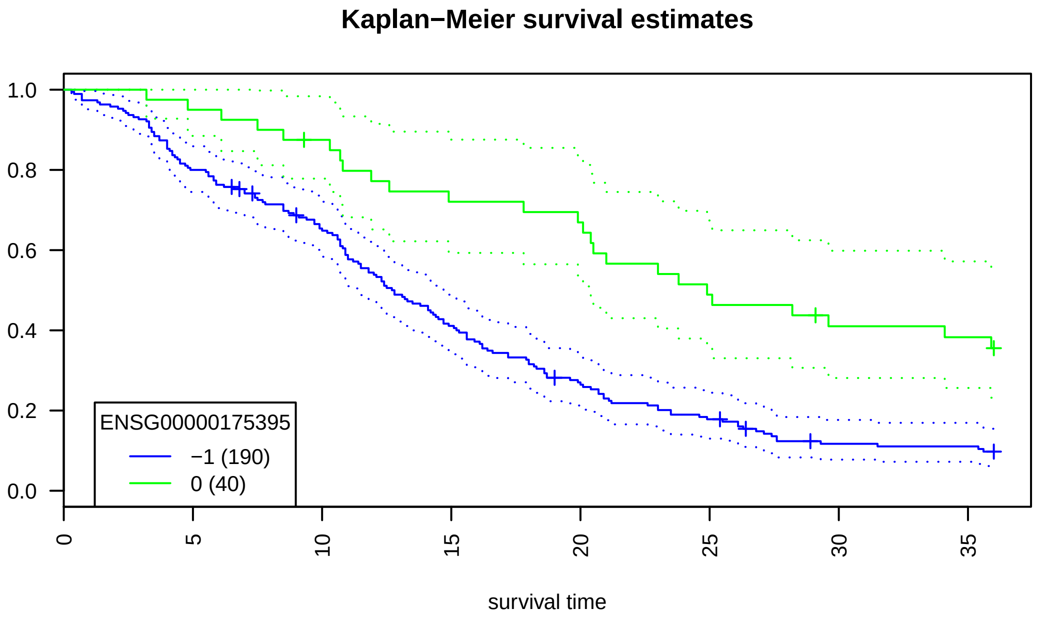

Figure 1769: Each curve represents the relative number of ENSG00000175395 survived to the corresponding point of time. Dashed lines represent 0.95 confidence intervals for the curves with the corresponding color. Log-rank probability measure for the equality of these curves is 1.780451e-05. Additional annotations: *.GeneName*=ZNF25, *.GeneDesc*=zinc finger protein 25 [Source:HGNC Symbol;Acc:13043].

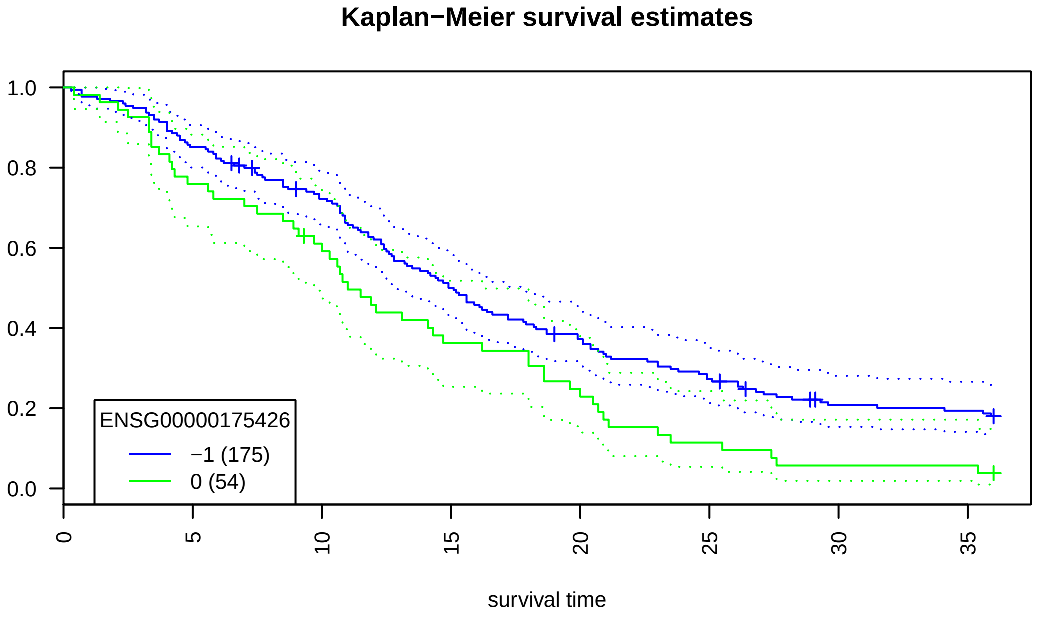

Figure 1770: Each curve represents the relative number of ENSG00000175426 survived to the corresponding point of time. Dashed lines represent 0.95 confidence intervals for the curves with the corresponding color. Total of 1 observation deleted due to missingness. Log-rank probability measure for the equality of these curves is 3.209387e-03. Additional annotations: *.GeneName*=PCSK1, *.GeneDesc*=proprotein convertase subtilisin/kexin type 1 [Source:HGNC Symbol;Acc:8743].

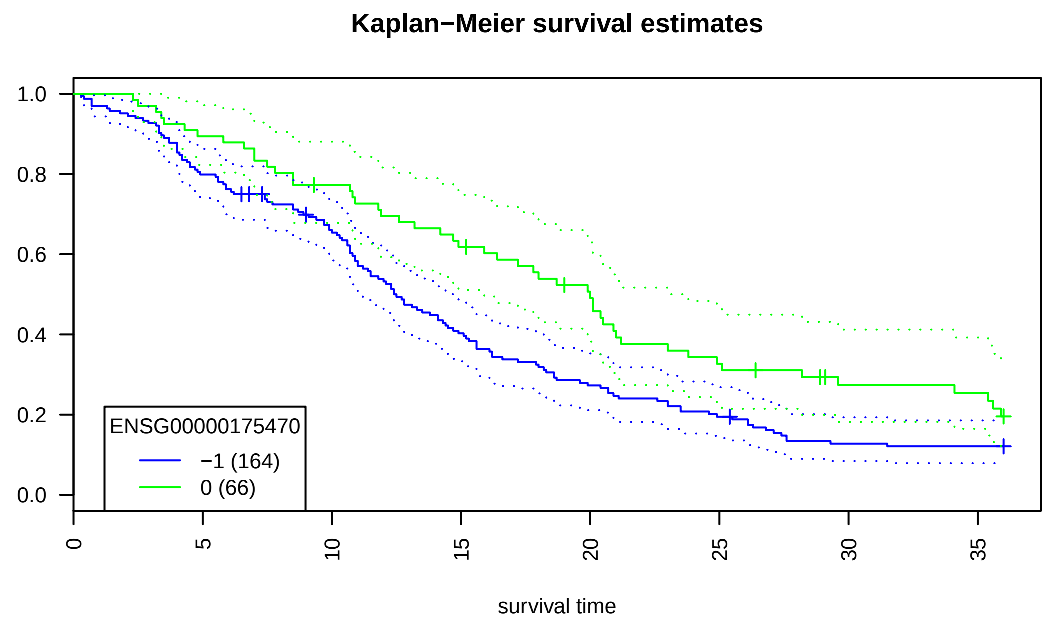

Figure 1771: Each curve represents the relative number of ENSG00000175470 survived to the corresponding point of time. Dashed lines represent 0.95 confidence intervals for the curves with the corresponding color. Log-rank probability measure for the equality of these curves is 7.841094e-03. Additional annotations: *.GeneName*=PPP2R2D, *.GeneDesc*=protein phosphatase 2, regulatory subunit B, delta isoform [Source:HGNC Symbol;Acc:23732].

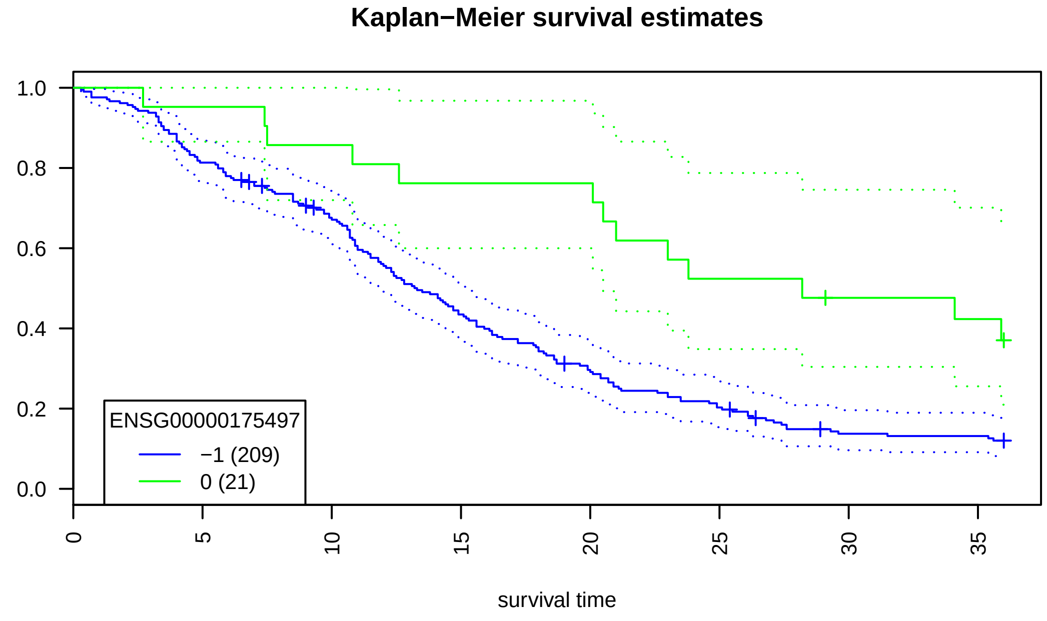

Figure 1772: Each curve represents the relative number of ENSG00000175497 survived to the corresponding point of time. Dashed lines represent 0.95 confidence intervals for the curves with the corresponding color. Log-rank probability measure for the equality of these curves is 1.480471e-03. Additional annotations: *.GeneName*=DPP10, *.GeneDesc*=dipeptidyl-peptidase 10 [Source:HGNC Symbol;Acc:20823].

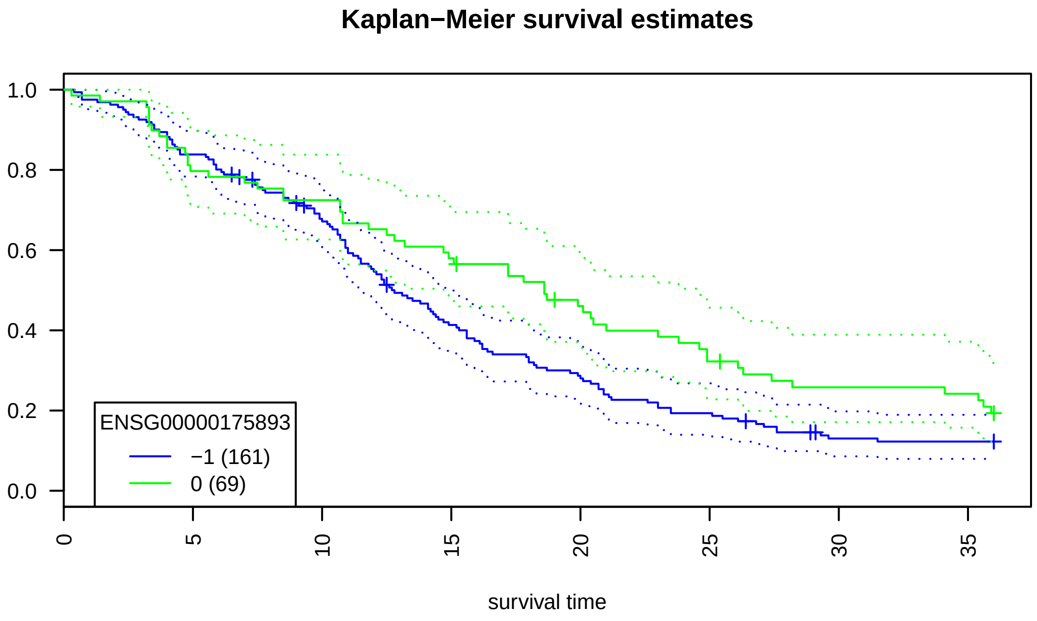

Figure 1773: Each curve represents the relative number of ENSG00000175893 survived to the corresponding point of time. Dashed lines represent 0.95 confidence intervals for the curves with the corresponding color. Log-rank probability measure for the equality of these curves is 3.304211e-02. Additional annotations: *.GeneName*=ZDHHC21, *.GeneDesc*=zinc finger, DHHC-type containing 21 [Source:HGNC Symbol;Acc:20750].

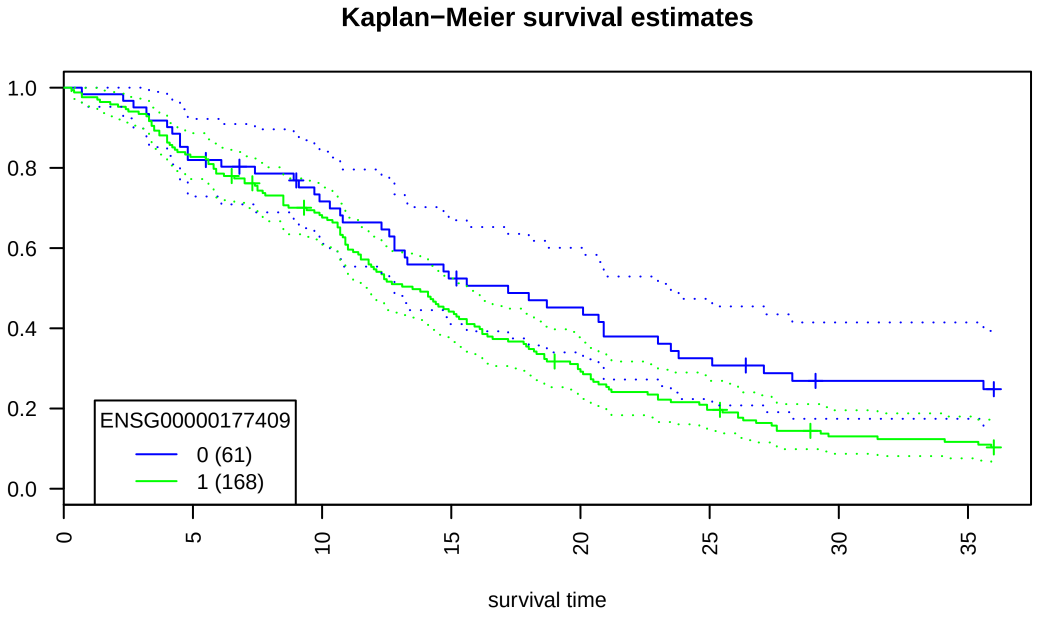

Figure 1774: Each curve represents the relative number of ENSG00000177409 survived to the corresponding point of time. Dashed lines represent 0.95 confidence intervals for the curves with the corresponding color. Total of 1 observation deleted due to missingness. Log-rank probability measure for the equality of these curves is 2.385983e-02. Additional annotations: *.GeneName*=SAMD9L, *.GeneDesc*=sterile alpha motif domain containing 9-like [Source:HGNC Symbol;Acc:1349].

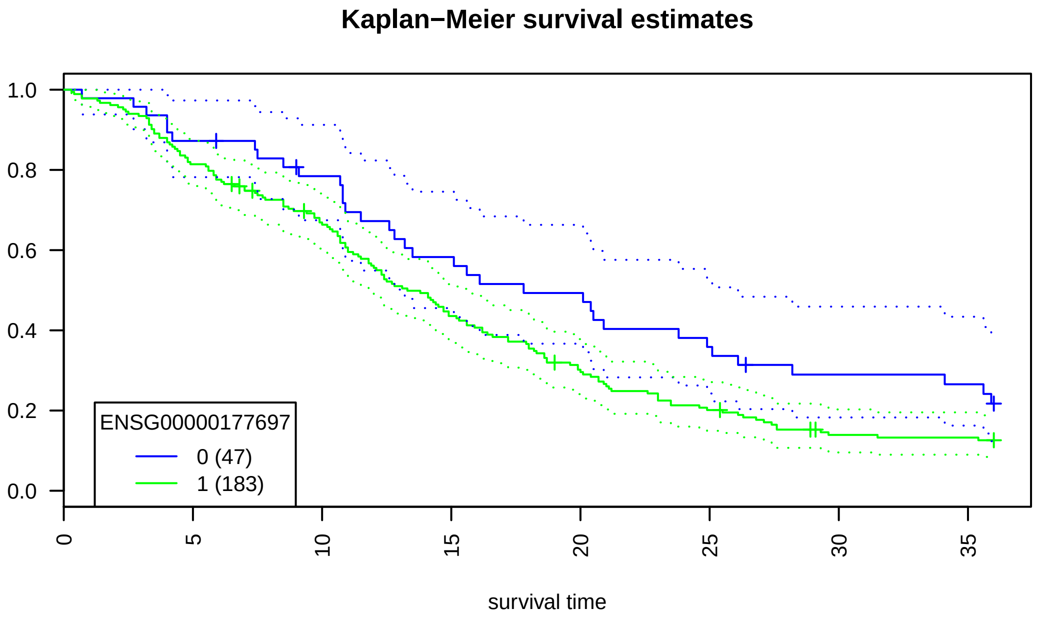

Figure 1775: Each curve represents the relative number of ENSG00000177697 survived to the corresponding point of time. Dashed lines represent 0.95 confidence intervals for the curves with the corresponding color. Log-rank probability measure for the equality of these curves is 4.172206e-02. Additional annotations: *.GeneName*=CD151, *.GeneDesc*=CD151 molecule (Raph blood group) [Source:HGNC Symbol;Acc:1630].

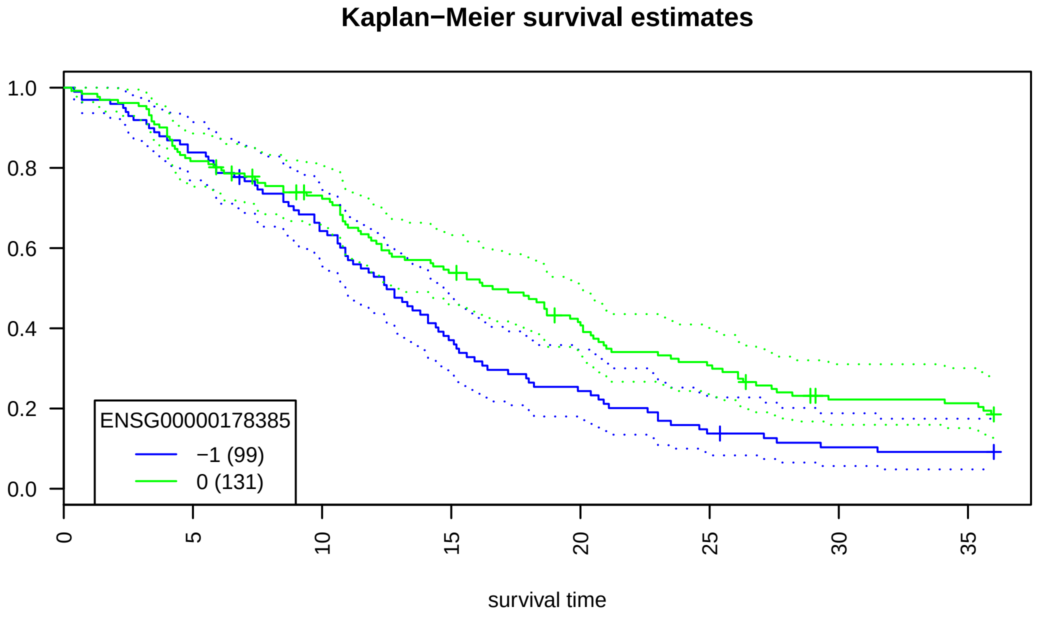

Figure 1776: Each curve represents the relative number of ENSG00000178385 survived to the corresponding point of time. Dashed lines represent 0.95 confidence intervals for the curves with the corresponding color. Log-rank probability measure for the equality of these curves is 8.963283e-03. Additional annotations: *.GeneName*=PLEKHM3, *.GeneDesc*=pleckstrin homology domain containing, family M, member 3 [Source:HGNC Symbol;Acc:34006].

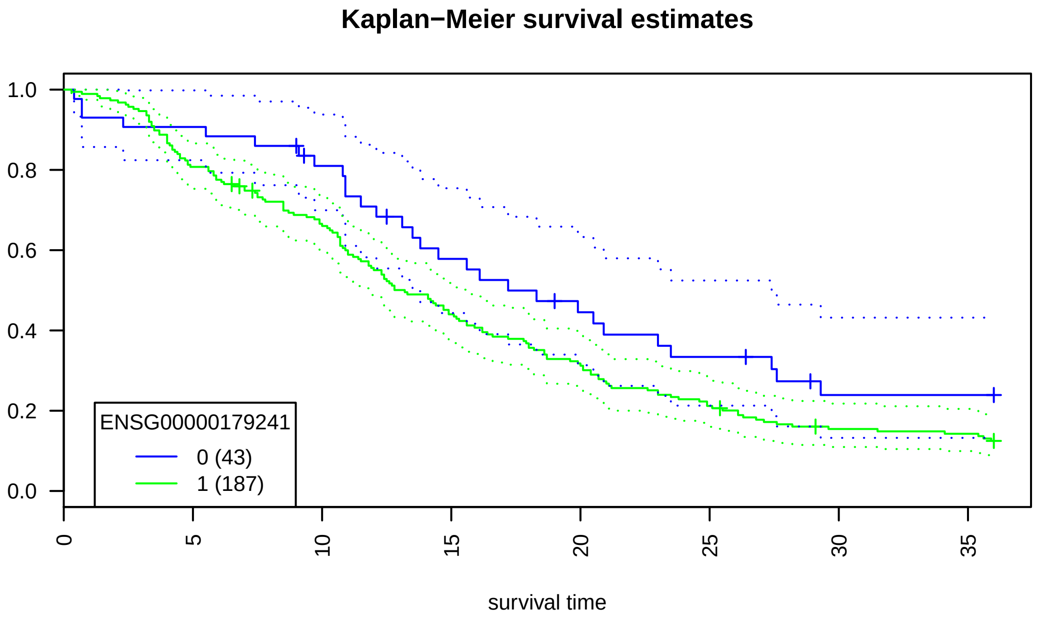

Figure 1777: Each curve represents the relative number of ENSG00000179241 survived to the corresponding point of time. Dashed lines represent 0.95 confidence intervals for the curves with the corresponding color. Log-rank probability measure for the equality of these curves is 4.594499e-02. Additional annotations: *.GeneName*=LDLRAD3, *.GeneDesc*=low density lipoprotein receptor class A domain containing 3 [Source:HGNC Symbol;Acc:27046].

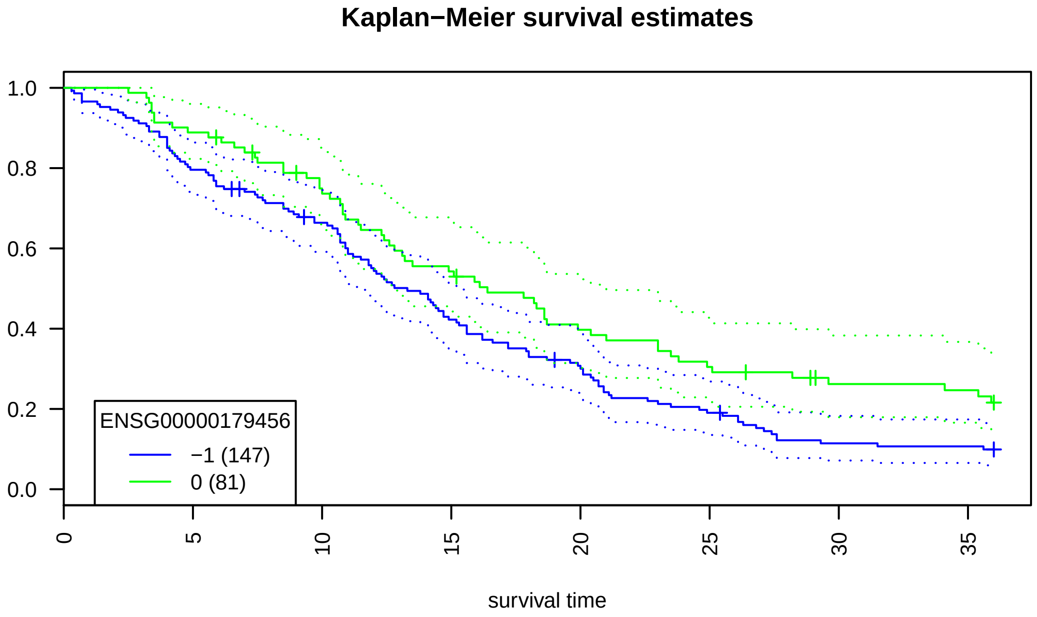

Figure 1778: Each curve represents the relative number of ENSG00000179456 survived to the corresponding point of time. Dashed lines represent 0.95 confidence intervals for the curves with the corresponding color. Total of 2 observations deleted due to missingness. Log-rank probability measure for the equality of these curves is 1.296593e-02. Additional annotations: *.GeneName*=ZNF238, *.GeneDesc*=zinc finger protein 238 [Source:HGNC Symbol;Acc:13030].

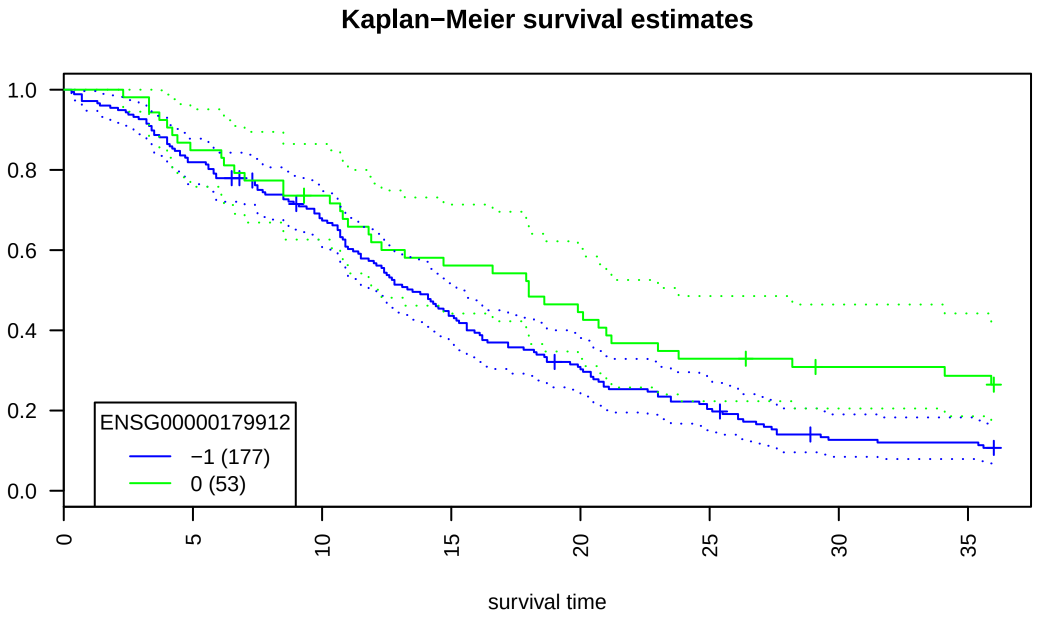

Figure 1779: Each curve represents the relative number of ENSG00000179912 survived to the corresponding point of time. Dashed lines represent 0.95 confidence intervals for the curves with the corresponding color. Log-rank probability measure for the equality of these curves is 1.676491e-02. Additional annotations: *.GeneName*=R3HDM2, *.GeneDesc*=R3H domain containing 2 [Source:HGNC Symbol;Acc:29167].

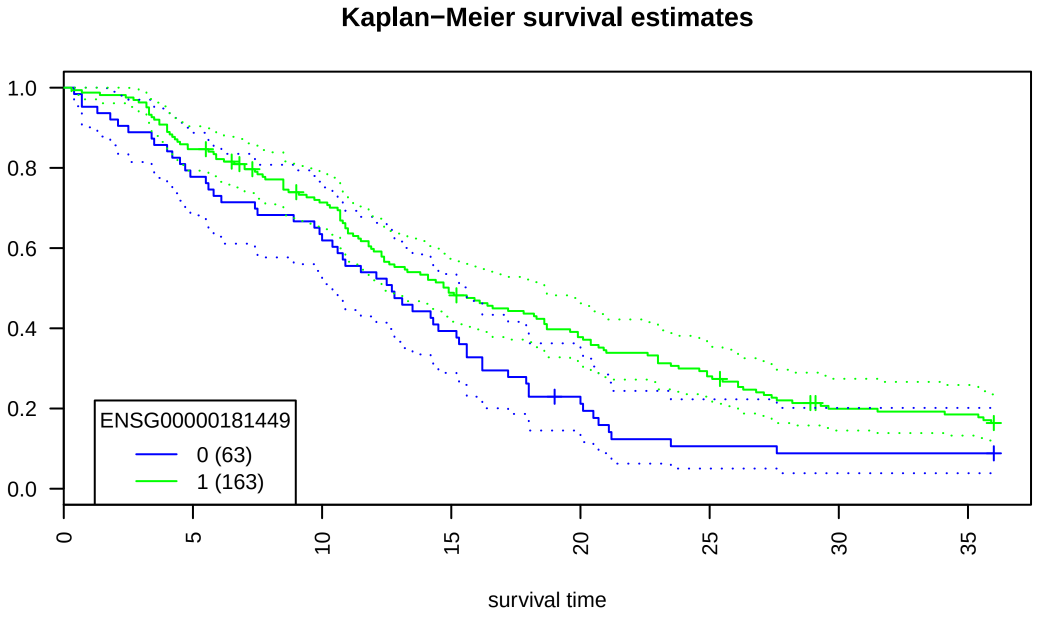

Figure 1780: Each curve represents the relative number of ENSG00000181449 survived to the corresponding point of time. Dashed lines represent 0.95 confidence intervals for the curves with the corresponding color. Total of 4 observations deleted due to missingness. Log-rank probability measure for the equality of these curves is 1.529828e-02. Additional annotations: *.GeneName*=SOX2, *.GeneDesc*=SRY (sex determining region Y)-box 2 [Source:HGNC Symbol;Acc:11195].

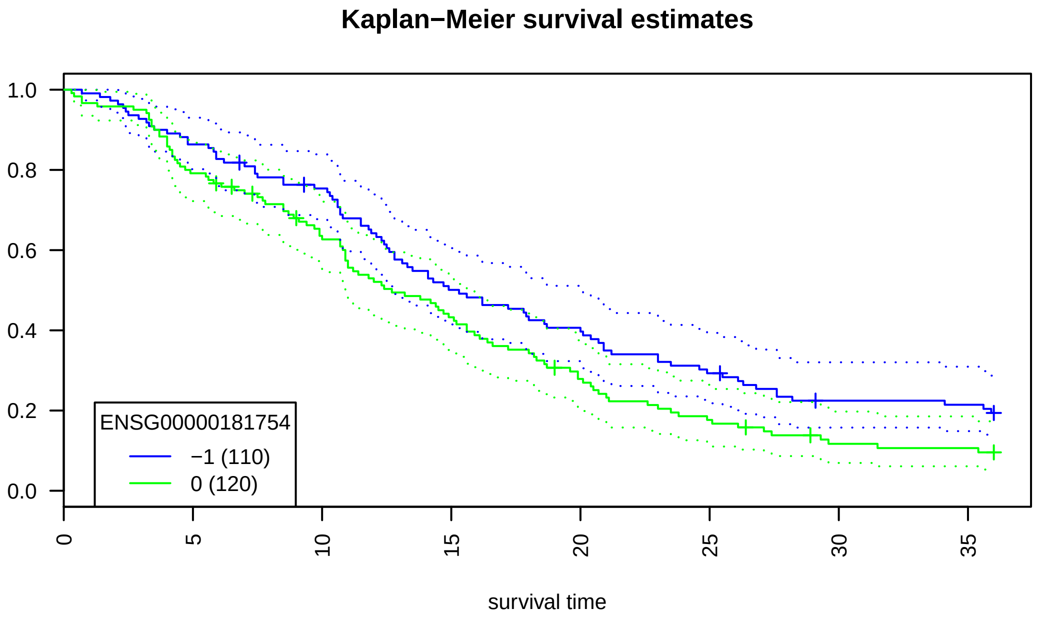

Figure 1781: Each curve represents the relative number of ENSG00000181754 survived to the corresponding point of time. Dashed lines represent 0.95 confidence intervals for the curves with the corresponding color. Log-rank probability measure for the equality of these curves is 2.640113e-02. Additional annotations: *.GeneName*=AMIGO1, *.GeneDesc*=adhesion molecule with Ig-like domain 1 [Source:HGNC Symbol;Acc:20824].

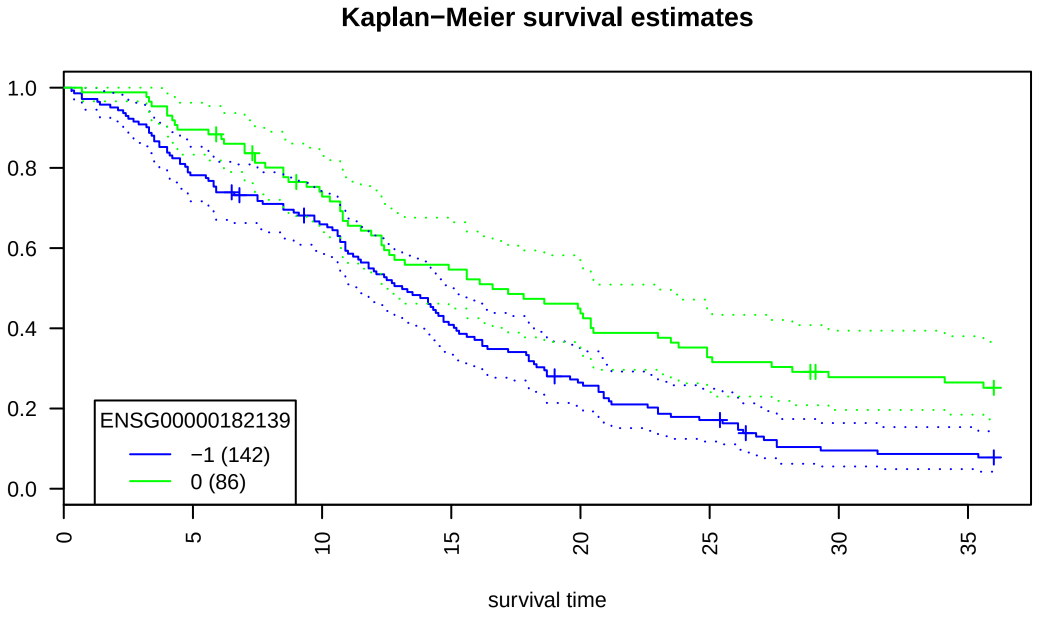

Figure 1782: Each curve represents the relative number of ENSG00000182139 survived to the corresponding point of time. Dashed lines represent 0.95 confidence intervals for the curves with the corresponding color. Total of 2 observations deleted due to missingness. Log-rank probability measure for the equality of these curves is 1.011498e-03. Additional annotations: *.GeneName*=AC123788.1, *.GeneDesc*=Putative uncharacterized protein [Source:UniProtKB/TrEMBL;Acc:Q96FU4].

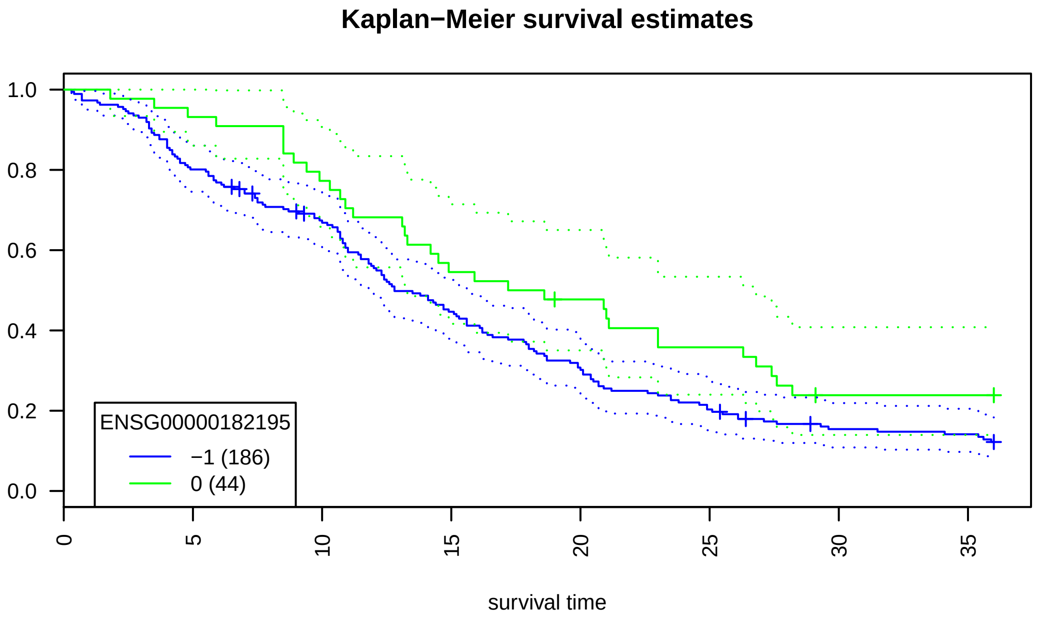

Figure 1783: Each curve represents the relative number of ENSG00000182195 survived to the corresponding point of time. Dashed lines represent 0.95 confidence intervals for the curves with the corresponding color. Log-rank probability measure for the equality of these curves is 3.171656e-02. Additional annotations: *.GeneName*=LDOC1, *.GeneDesc*=leucine zipper, down-regulated in cancer 1 [Source:HGNC Symbol;Acc:6548].

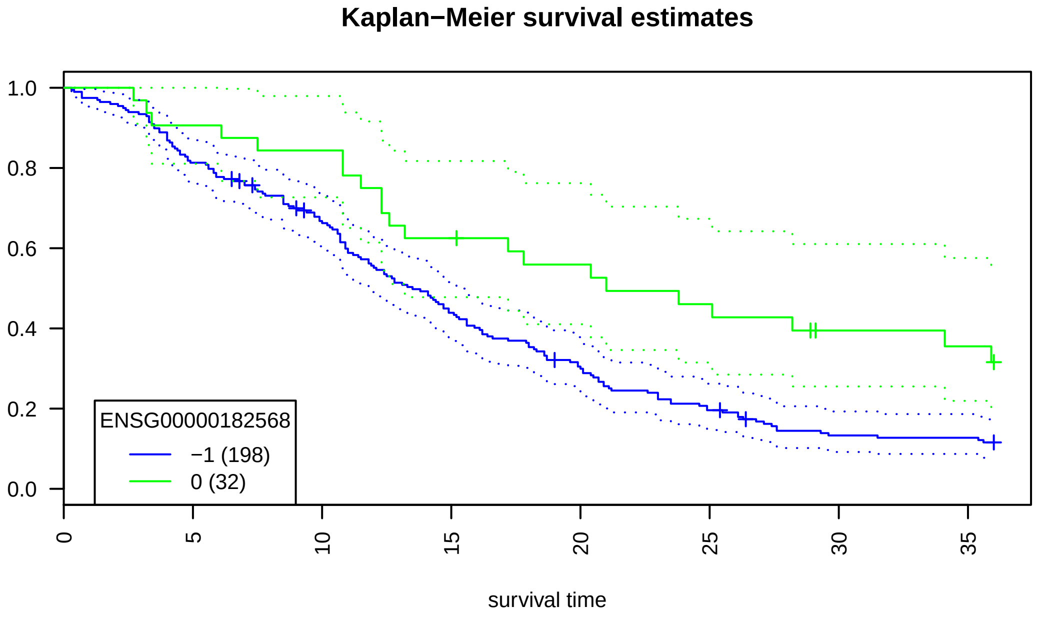

Figure 1784: Each curve represents the relative number of ENSG00000182568 survived to the corresponding point of time. Dashed lines represent 0.95 confidence intervals for the curves with the corresponding color. Log-rank probability measure for the equality of these curves is 3.944976e-03. Additional annotations: *.GeneName*=SATB1, *.GeneDesc*=SATB homeobox 1 [Source:HGNC Symbol;Acc:10541].

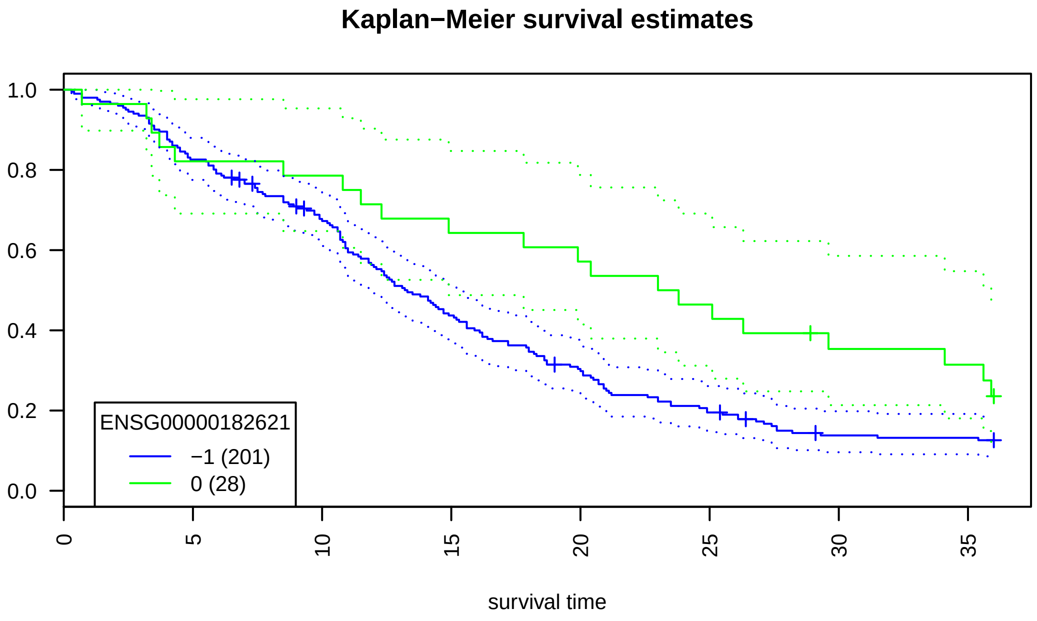

Figure 1785: Each curve represents the relative number of ENSG00000182621 survived to the corresponding point of time. Dashed lines represent 0.95 confidence intervals for the curves with the corresponding color. Total of 1 observation deleted due to missingness. Log-rank probability measure for the equality of these curves is 2.126563e-02. Additional annotations: *.GeneName*=PLCB1, *.GeneDesc*=phospholipase C, beta 1 (phosphoinositide-specific) [Source:HGNC Symbol;Acc:15917].

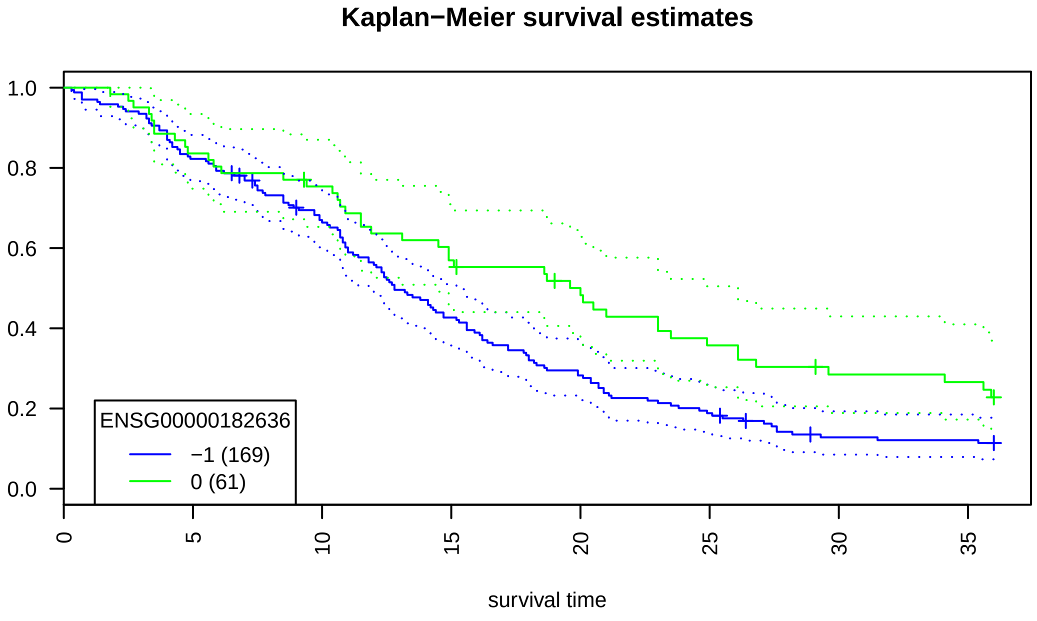

Figure 1786: Each curve represents the relative number of ENSG00000182636 survived to the corresponding point of time. Dashed lines represent 0.95 confidence intervals for the curves with the corresponding color. Log-rank probability measure for the equality of these curves is 1.10241e-02. Additional annotations: *.GeneName*=NDN, *.GeneDesc*=necdin homolog (mouse) [Source:HGNC Symbol;Acc:7675].

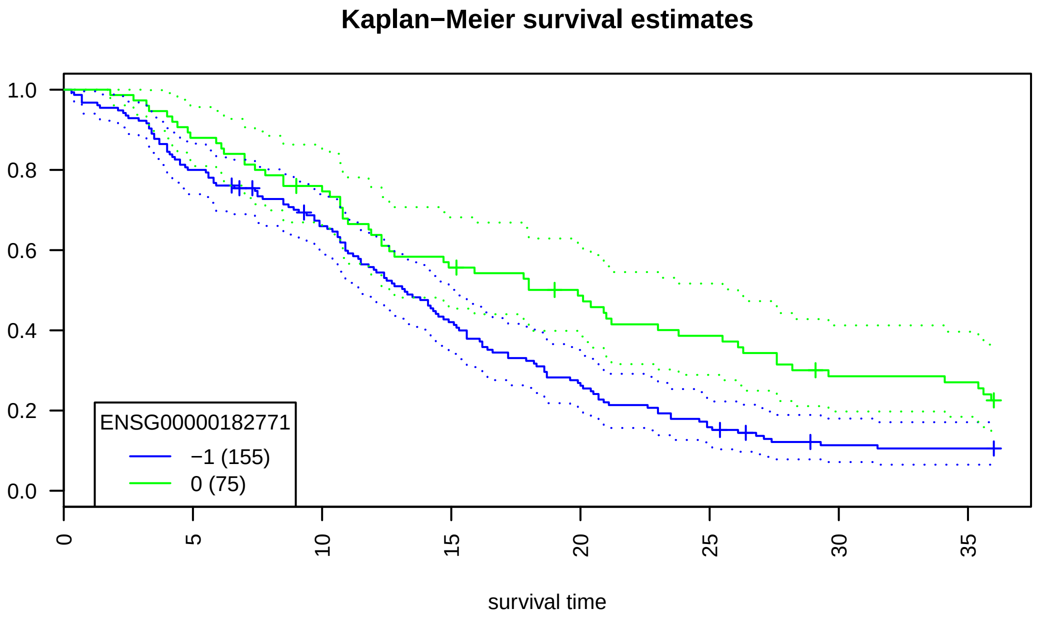

Figure 1787: Each curve represents the relative number of ENSG00000182771 survived to the corresponding point of time. Dashed lines represent 0.95 confidence intervals for the curves with the corresponding color. Log-rank probability measure for the equality of these curves is 2.435620e-03. Additional annotations: *.GeneName*=GRID1, *.GeneDesc*=glutamate receptor, ionotropic, delta 1 [Source:HGNC Symbol;Acc:4575].

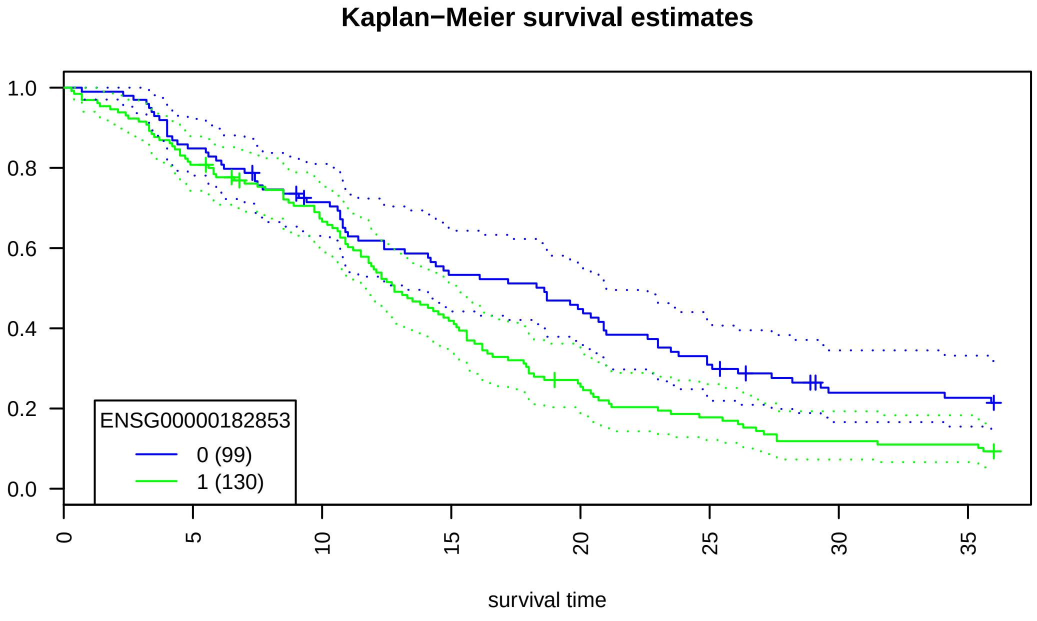

Figure 1788: Each curve represents the relative number of ENSG00000182853 survived to the corresponding point of time. Dashed lines represent 0.95 confidence intervals for the curves with the corresponding color. Total of 1 observation deleted due to missingness. Log-rank probability measure for the equality of these curves is 6.492584e-03. Additional annotations: *.GeneName*=VMO1, *.GeneDesc*=vitelline membrane outer layer 1 homolog (chicken) [Source:HGNC Symbol;Acc:30387].

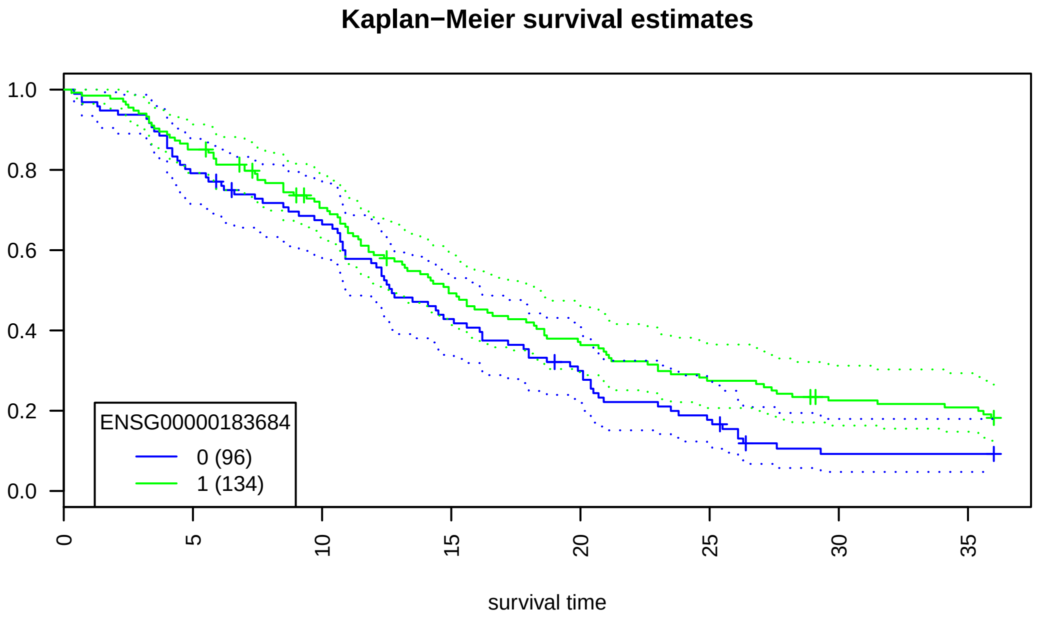

Figure 1789: Each curve represents the relative number of ENSG00000183684 survived to the corresponding point of time. Dashed lines represent 0.95 confidence intervals for the curves with the corresponding color. Log-rank probability measure for the equality of these curves is 4.896413e-02. Additional annotations: *.GeneName*=THOC4, *.GeneDesc*=THO complex 4 [Source:HGNC Symbol;Acc:19071].

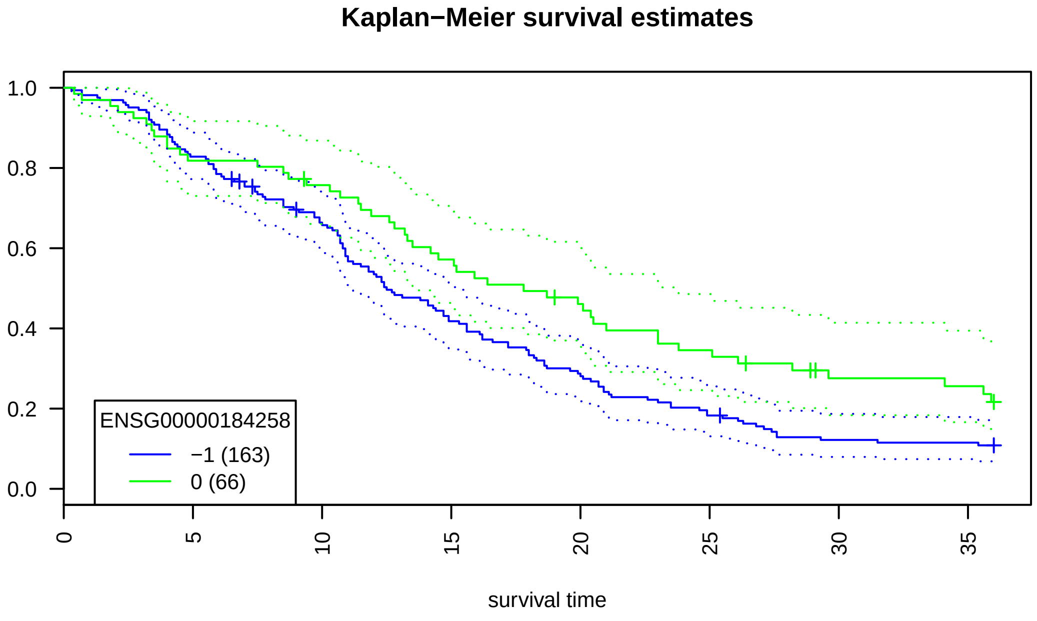

Figure 1790: Each curve represents the relative number of ENSG00000184258 survived to the corresponding point of time. Dashed lines represent 0.95 confidence intervals for the curves with the corresponding color. Total of 1 observation deleted due to missingness. Log-rank probability measure for the equality of these curves is 1.280911e-02. Additional annotations: *.GeneName*=CDR1, *.GeneDesc*=cerebellar degeneration-related protein 1, 34kDa [Source:HGNC Symbol;Acc:1798].

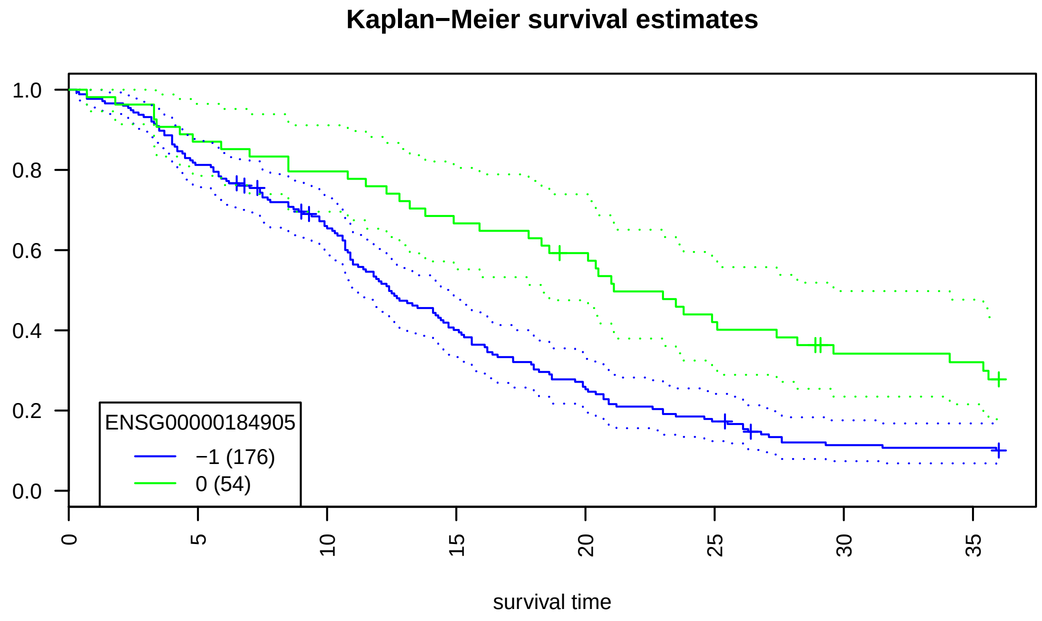

Figure 1791: Each curve represents the relative number of ENSG00000184905 survived to the corresponding point of time. Dashed lines represent 0.95 confidence intervals for the curves with the corresponding color. Log-rank probability measure for the equality of these curves is 1.121324e-04. Additional annotations: *.GeneName*=TCEAL2, *.GeneDesc*=transcription elongation factor A (SII)-like 2 [Source:HGNC Symbol;Acc:29818].

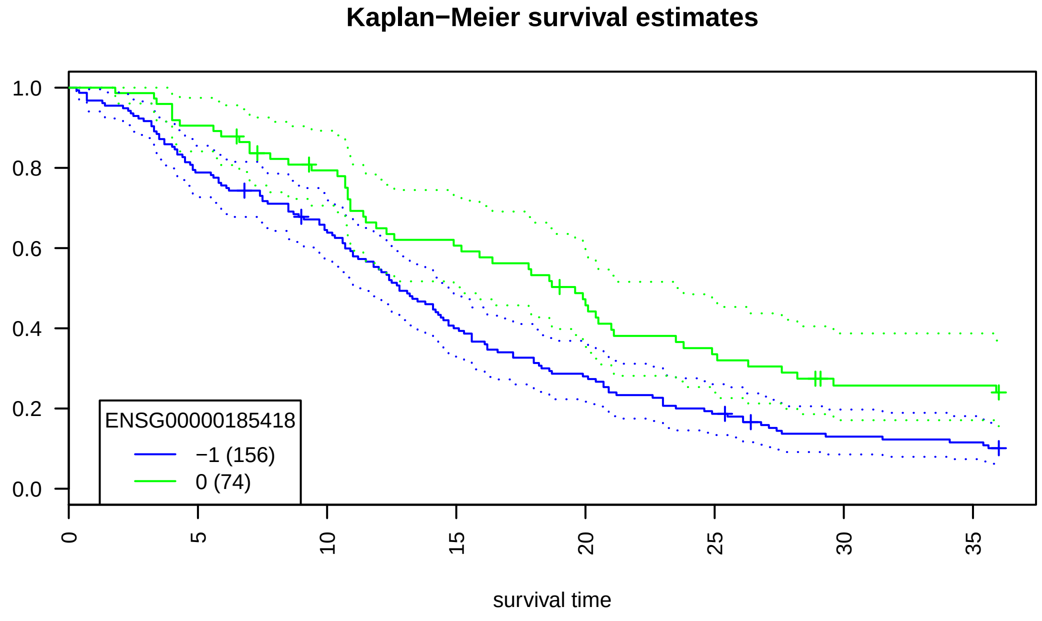

Figure 1792: Each curve represents the relative number of ENSG00000185418 survived to the corresponding point of time. Dashed lines represent 0.95 confidence intervals for the curves with the corresponding color. Log-rank probability measure for the equality of these curves is 2.300786e-03. Additional annotations: *.GeneName*=TARSL2, *.GeneDesc*=threonyl-tRNA synthetase-like 2 [Source:HGNC Symbol;Acc:24728].

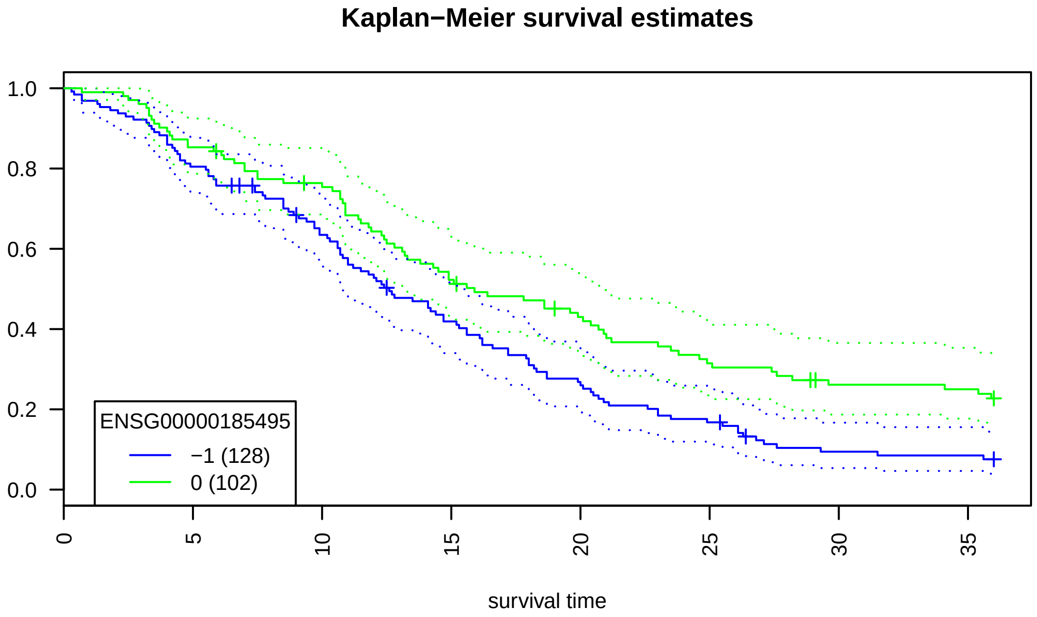

Figure 1793: Each curve represents the relative number of ENSG00000185495 survived to the corresponding point of time. Dashed lines represent 0.95 confidence intervals for the curves with the corresponding color. Log-rank probability measure for the equality of these curves is 1.487513e-03. Additional annotations: *.GeneName*=RP11-504P24.4, *.GeneDesc*=[undefined].

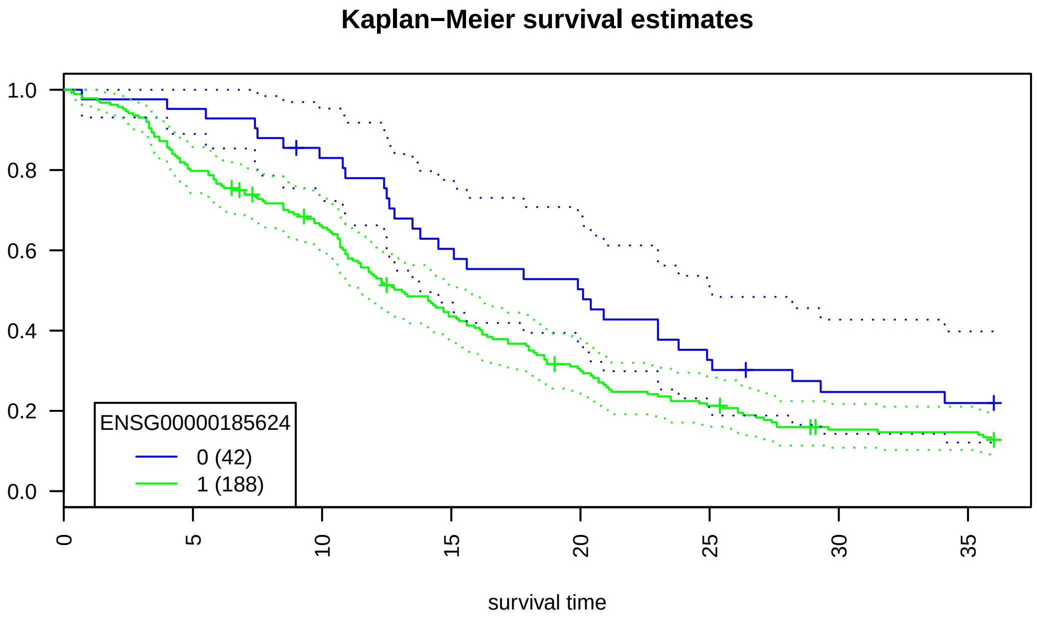

Figure 1794: Each curve represents the relative number of ENSG00000185624 survived to the corresponding point of time. Dashed lines represent 0.95 confidence intervals for the curves with the corresponding color. Log-rank probability measure for the equality of these curves is 2.934117e-02. Additional annotations: *.GeneName*=P4HB, *.GeneDesc*=prolyl 4-hydroxylase, beta polypeptide [Source:HGNC Symbol;Acc:8548].

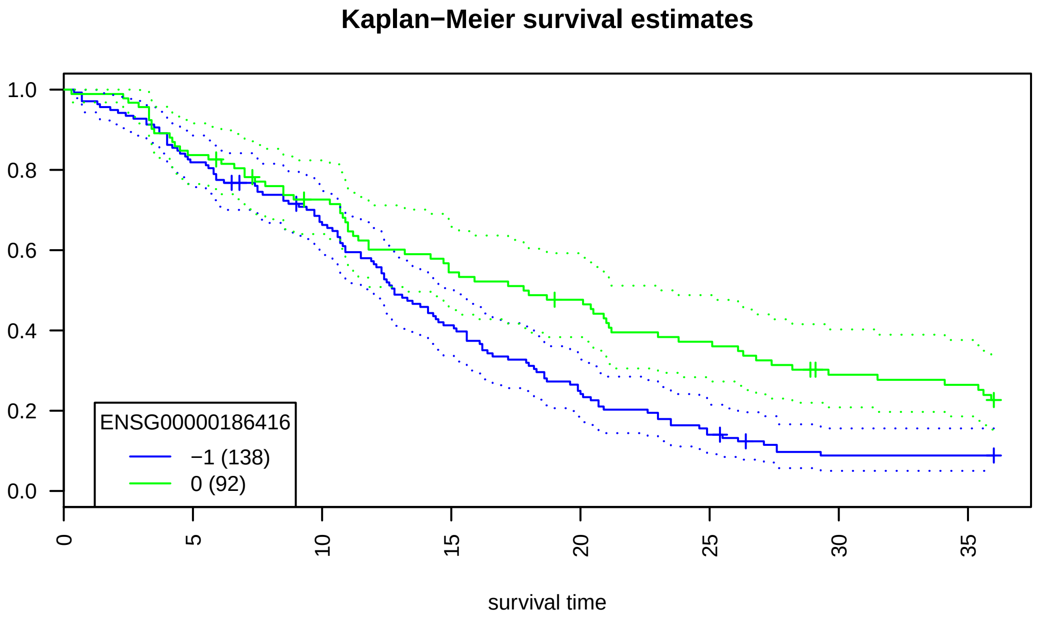

Figure 1795: Each curve represents the relative number of ENSG00000186416 survived to the corresponding point of time. Dashed lines represent 0.95 confidence intervals for the curves with the corresponding color. Log-rank probability measure for the equality of these curves is 1.320587e-03. Additional annotations: *.GeneName*=NKRF, *.GeneDesc*=NFKB repressing factor [Source:HGNC Symbol;Acc:19374].

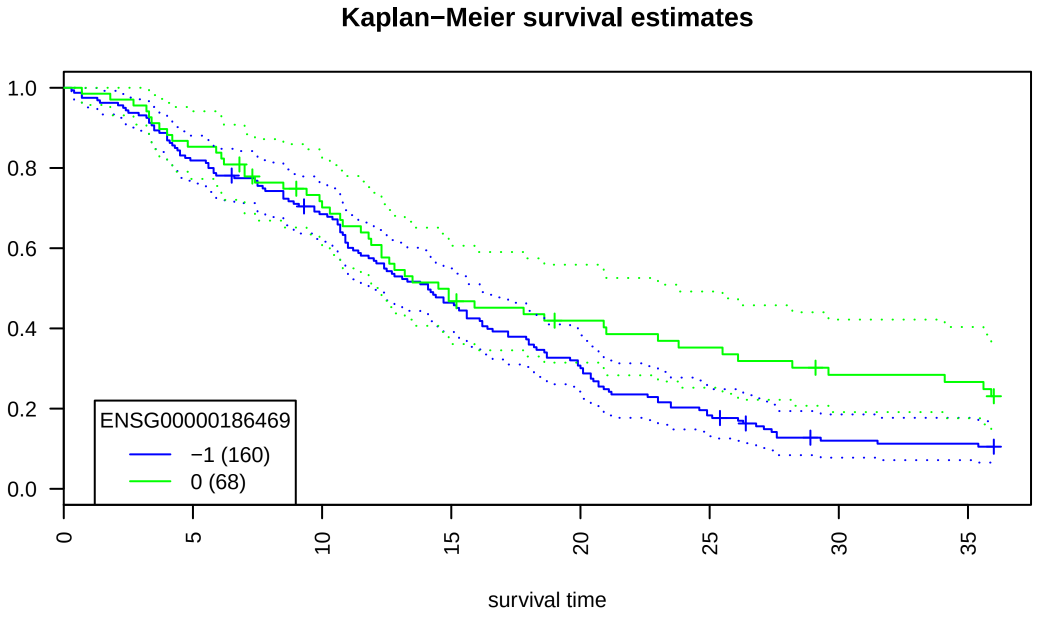

Figure 1796: Each curve represents the relative number of ENSG00000186469 survived to the corresponding point of time. Dashed lines represent 0.95 confidence intervals for the curves with the corresponding color. Total of 2 observations deleted due to missingness. Log-rank probability measure for the equality of these curves is 3.864008e-02. Additional annotations: *.GeneName*=GNG2, *.GeneDesc*=guanine nucleotide binding protein (G protein), gamma 2 [Source:HGNC Symbol;Acc:4404].

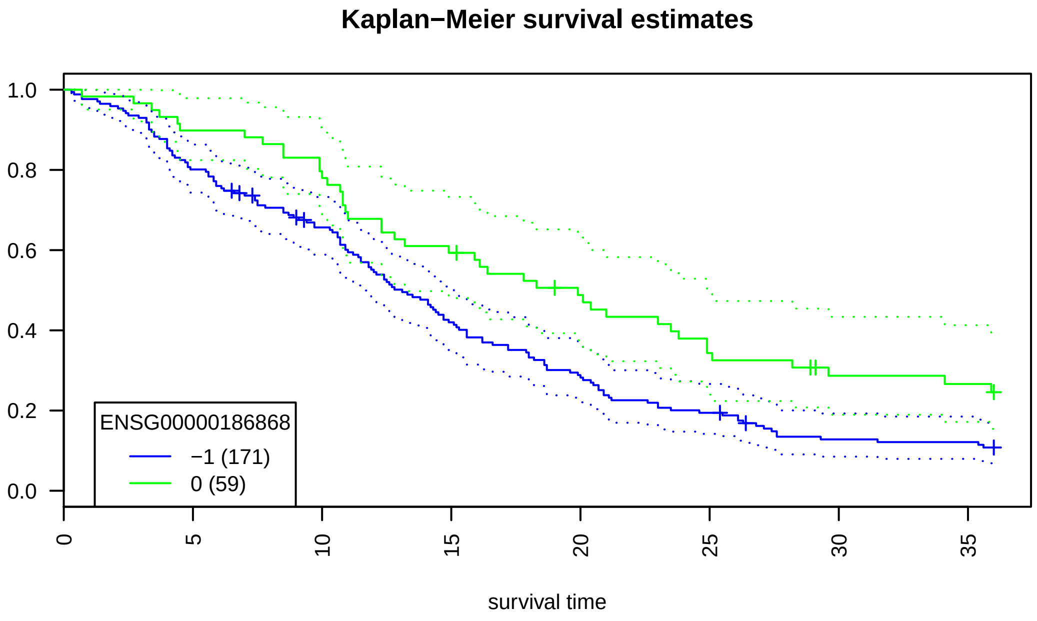

Figure 1797: Each curve represents the relative number of ENSG00000186868 survived to the corresponding point of time. Dashed lines represent 0.95 confidence intervals for the curves with the corresponding color. Log-rank probability measure for the equality of these curves is 3.780813e-03. Additional annotations: *.GeneName*=MAPT, *.GeneDesc*=microtubule-associated protein tau [Source:HGNC Symbol;Acc:6893].

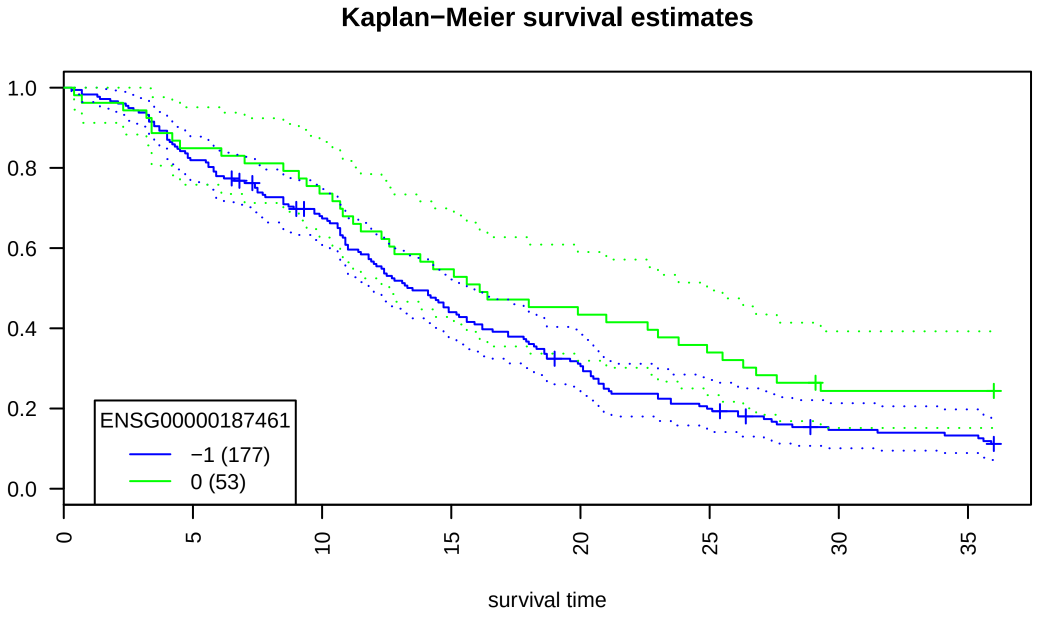

Figure 1798: Each curve represents the relative number of ENSG00000187461 survived to the corresponding point of time. Dashed lines represent 0.95 confidence intervals for the curves with the corresponding color. Log-rank probability measure for the equality of these curves is 3.927575e-02. Additional annotations: *.GeneName*=C6orf114, *.GeneDesc*=Uncharacterized protein C6orf114 (Protein ADG-90) [Source:UniProtKB/Swiss-Prot;Acc:Q96JD4].

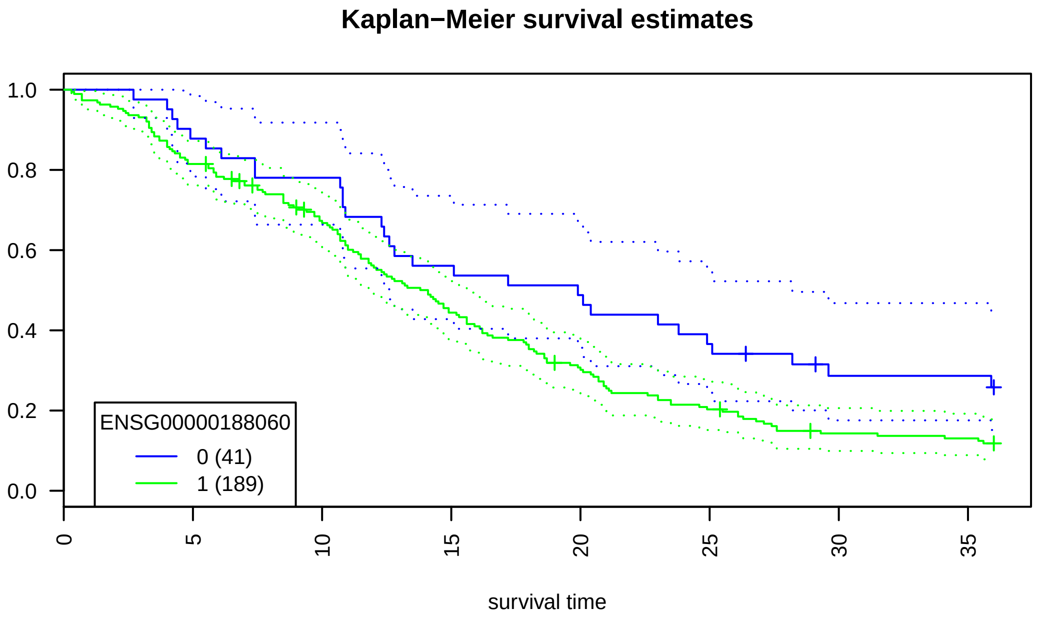

Figure 1799: Each curve represents the relative number of ENSG00000188060 survived to the corresponding point of time. Dashed lines represent 0.95 confidence intervals for the curves with the corresponding color. Log-rank probability measure for the equality of these curves is 2.357865e-02. Additional annotations: *.GeneName*=RAB42, *.GeneDesc*=RAB42, member RAS oncogene family [Source:HGNC Symbol;Acc:28702].

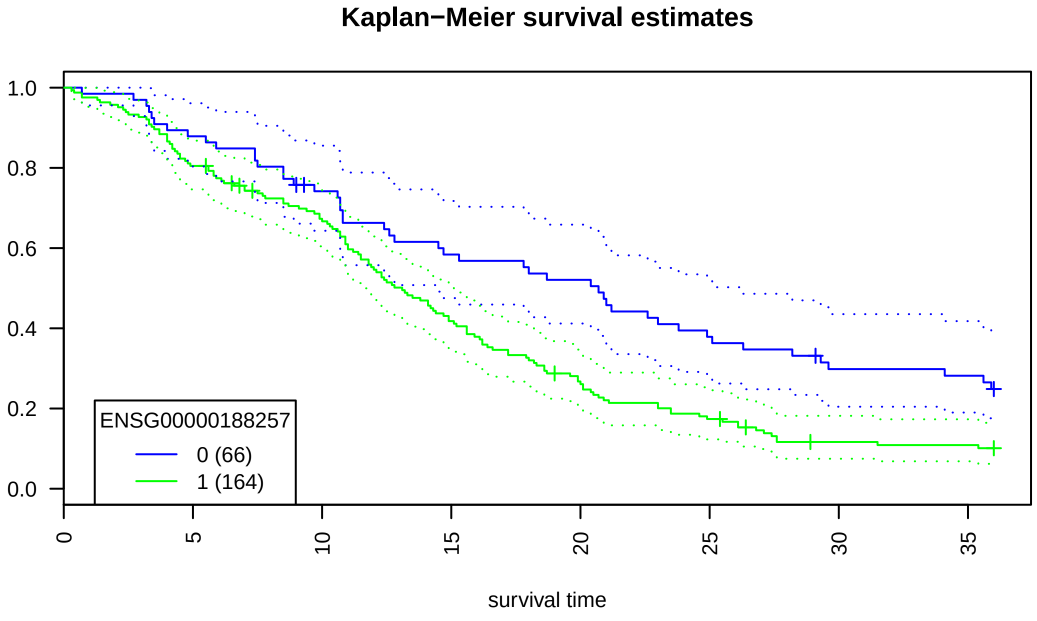

Figure 1800: Each curve represents the relative number of ENSG00000188257 survived to the corresponding point of time. Dashed lines represent 0.95 confidence intervals for the curves with the corresponding color. Log-rank probability measure for the equality of these curves is 1.398443e-03. Additional annotations: *.GeneName*=PLA2G2A, *.GeneDesc*=phospholipase A2, group IIA (platelets, synovial fluid) [Source:HGNC Symbol;Acc:9031].

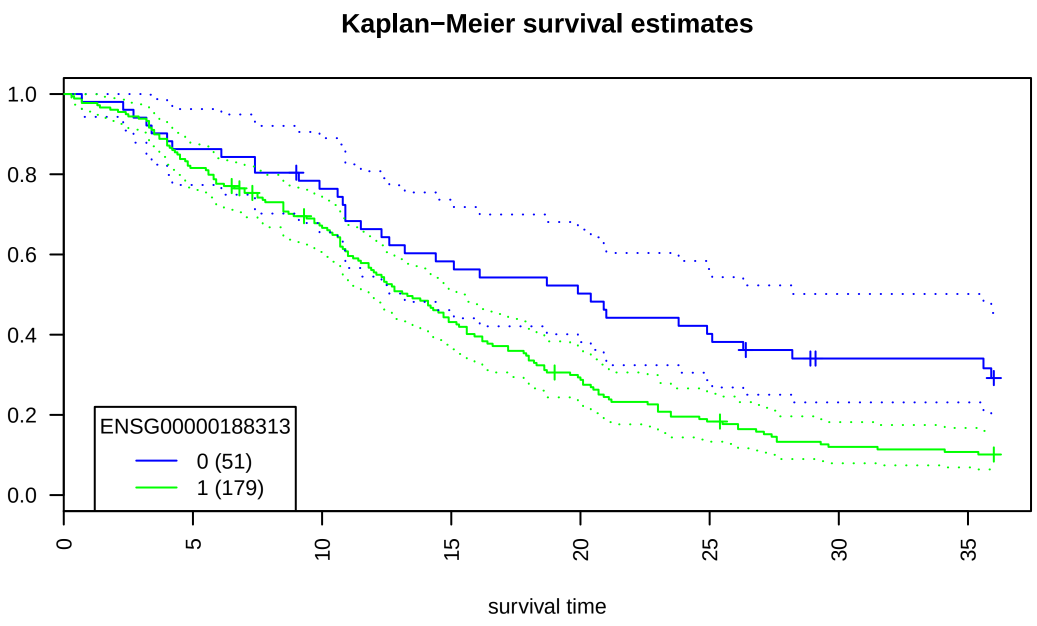

Figure 1801: Each curve represents the relative number of ENSG00000188313 survived to the corresponding point of time. Dashed lines represent 0.95 confidence intervals for the curves with the corresponding color. Log-rank probability measure for the equality of these curves is 2.206087e-03. Additional annotations: *.GeneName*=PLSCR1, *.GeneDesc*=phospholipid scramblase 1 [Source:HGNC Symbol;Acc:9092].

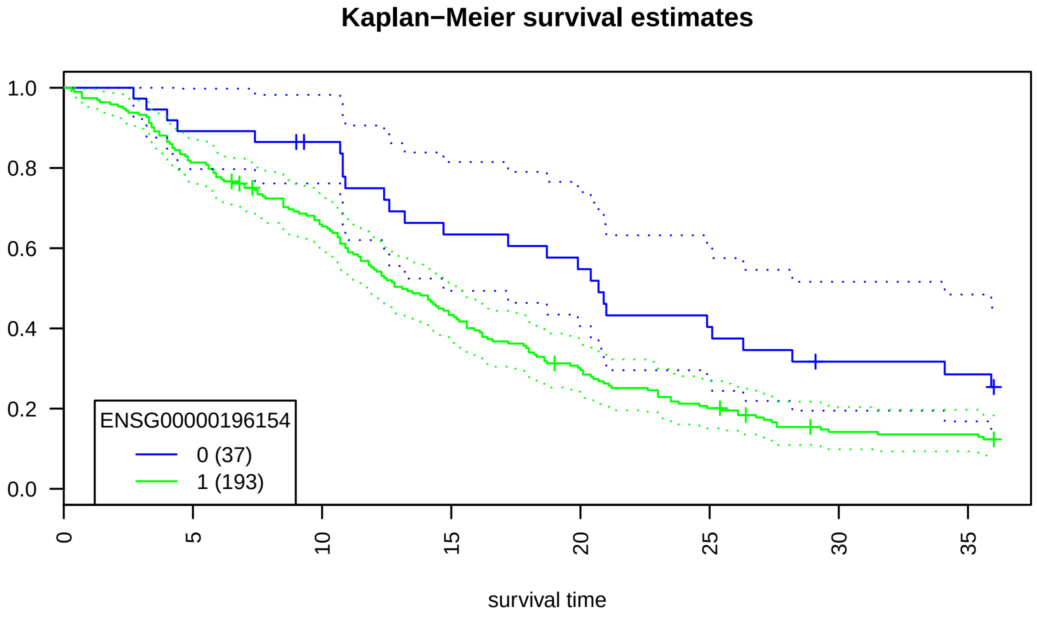

Figure 1802: Each curve represents the relative number of ENSG00000196154 survived to the corresponding point of time. Dashed lines represent 0.95 confidence intervals for the curves with the corresponding color. Log-rank probability measure for the equality of these curves is 1.012681e-02. Additional annotations: *.GeneName*=S100A4, *.GeneDesc*=S100 calcium binding protein A4 [Source:HGNC Symbol;Acc:10494].

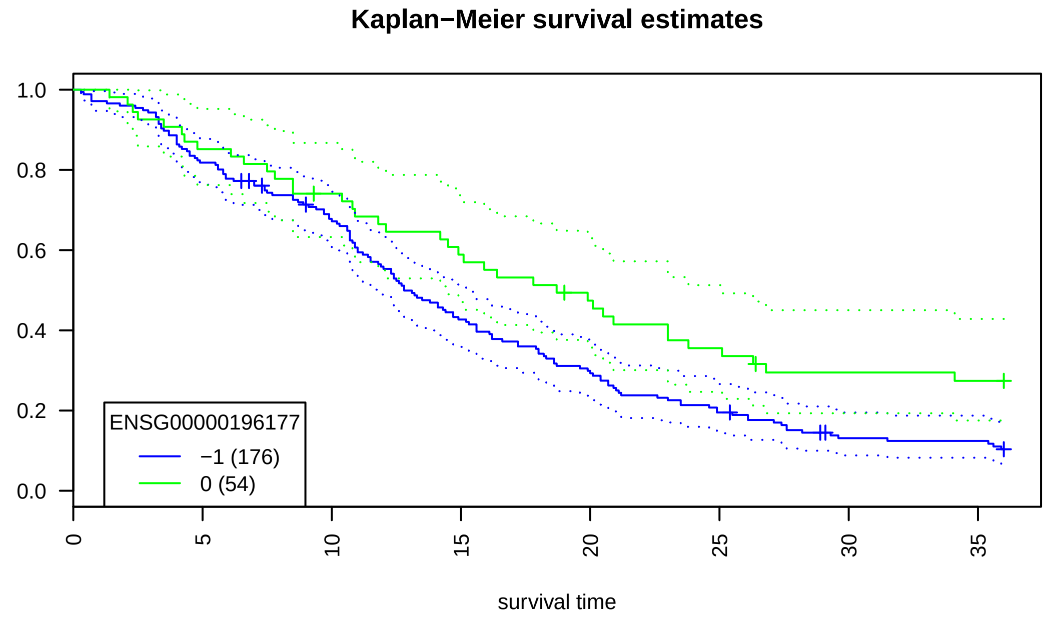

Figure 1803: Each curve represents the relative number of ENSG00000196177 survived to the corresponding point of time. Dashed lines represent 0.95 confidence intervals for the curves with the corresponding color. Log-rank probability measure for the equality of these curves is 8.519573e-03. Additional annotations: *.GeneName*=ACADSB, *.GeneDesc*=acyl-Coenzyme A dehydrogenase, short/branched chain [Source:HGNC Symbol;Acc:91].

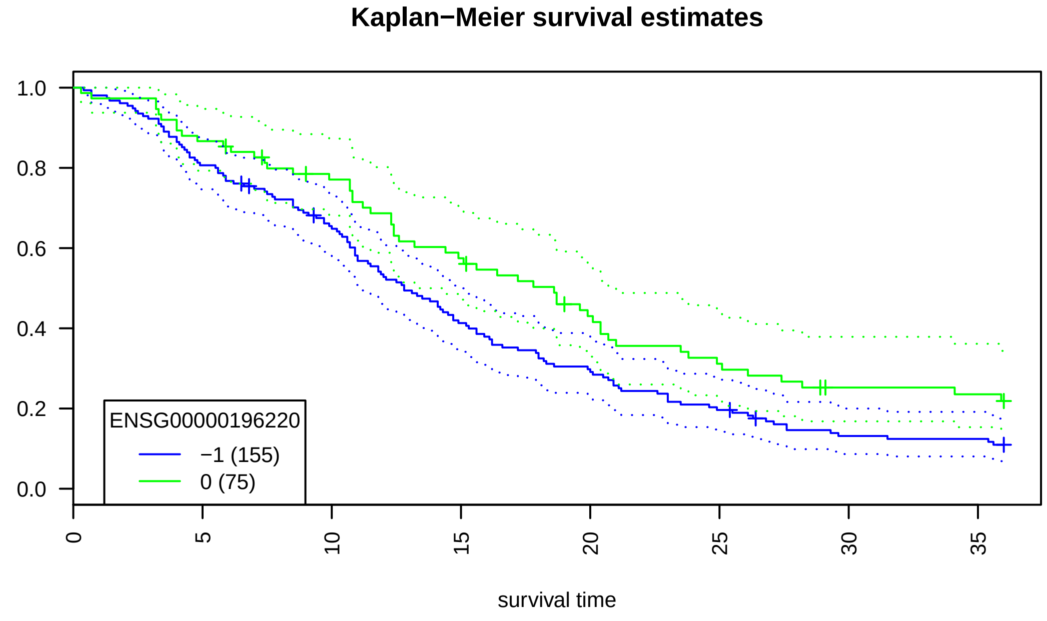

Figure 1804: Each curve represents the relative number of ENSG00000196220 survived to the corresponding point of time. Dashed lines represent 0.95 confidence intervals for the curves with the corresponding color. Log-rank probability measure for the equality of these curves is 1.532631e-02. Additional annotations: *.GeneName*=SRGAP3, *.GeneDesc*=SLIT-ROBO Rho GTPase activating protein 3 [Source:HGNC Symbol;Acc:19744].

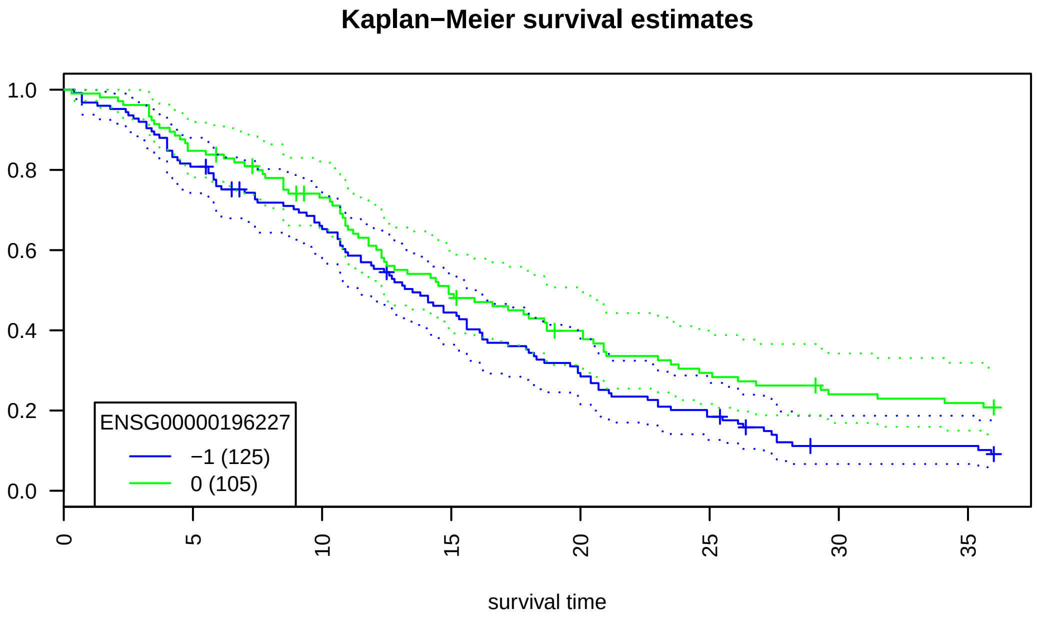

Figure 1805: Each curve represents the relative number of ENSG00000196227 survived to the corresponding point of time. Dashed lines represent 0.95 confidence intervals for the curves with the corresponding color. Log-rank probability measure for the equality of these curves is 2.959463e-02. Additional annotations: *.GeneName*=C20orf177, *.GeneDesc*=Uncharacterized protein C20orf177 [Source:UniProtKB/Swiss-Prot;Acc:Q9NTX9].

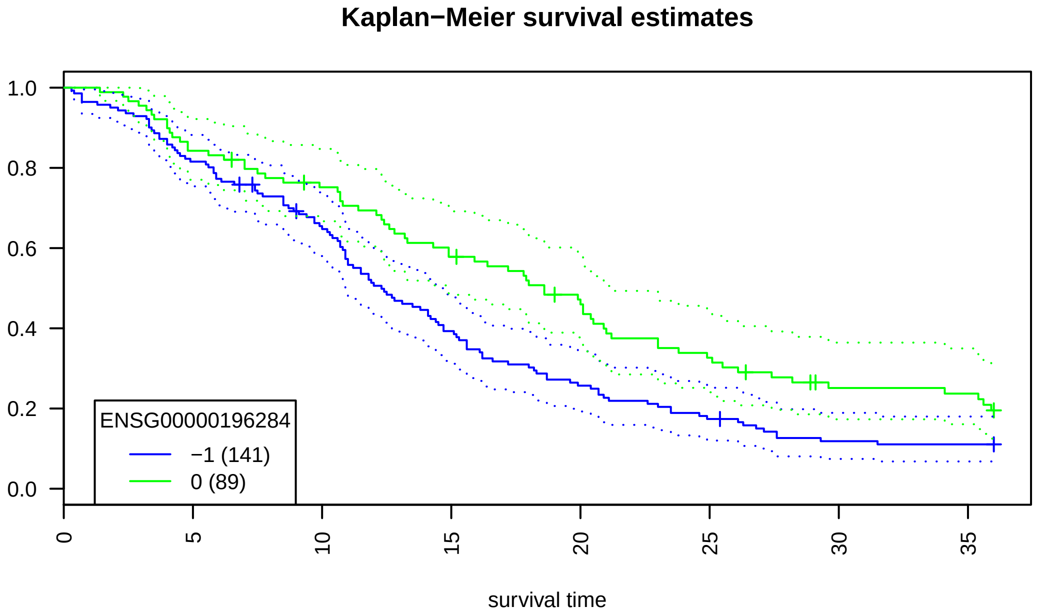

Figure 1806: Each curve represents the relative number of ENSG00000196284 survived to the corresponding point of time. Dashed lines represent 0.95 confidence intervals for the curves with the corresponding color. Log-rank probability measure for the equality of these curves is 5.529347e-03. Additional annotations: *.GeneName*=SUPT3H, *.GeneDesc*=suppressor of Ty 3 homolog (S. cerevisiae) [Source:HGNC Symbol;Acc:11466].

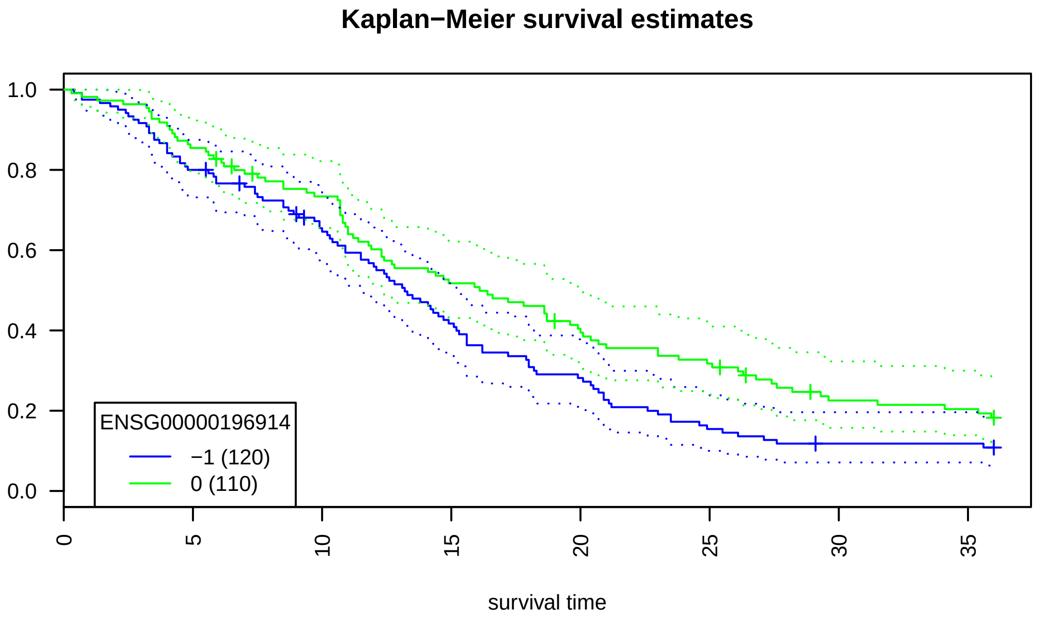

Figure 1807: Each curve represents the relative number of ENSG00000196914 survived to the corresponding point of time. Dashed lines represent 0.95 confidence intervals for the curves with the corresponding color. Log-rank probability measure for the equality of these curves is 2.609386e-02. Additional annotations: .*GeneName*=ARHGEF12, .*GeneDesc*=Rho guanine nucleotide exchange factor (GEF) 12 [Source:HGNC Symbol;Acc:14193].

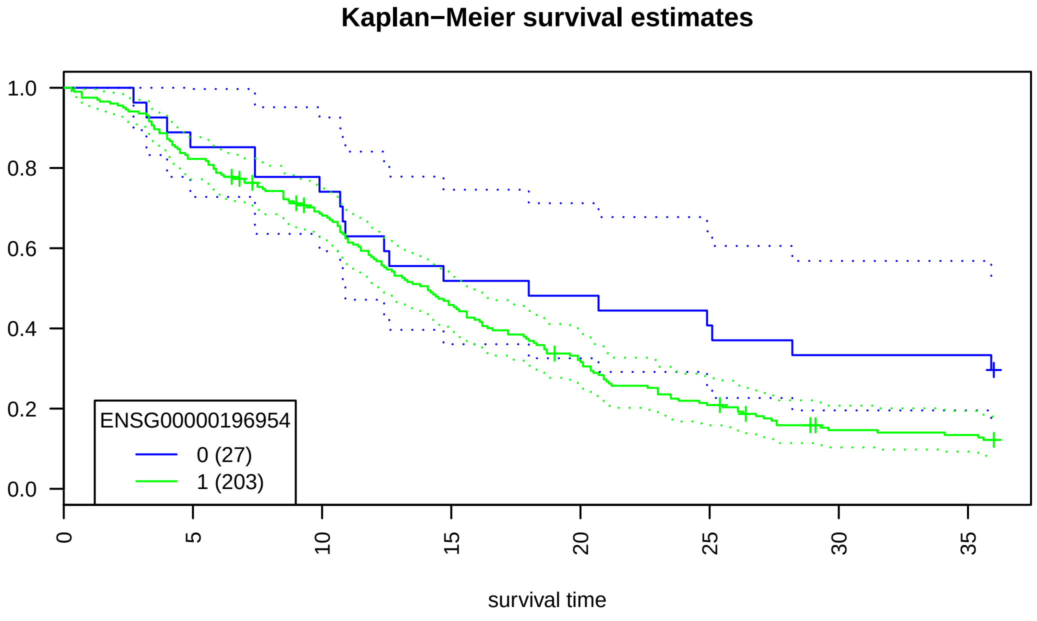

Figure 1808: Each curve represents the relative number of ENSG00000196954 survived to the corresponding point of time. Dashed lines represent 0.95 confidence intervals for the curves with the corresponding color. Log-rank probability measure for the equality of these curves is 4.92246e-02. Additional annotations: .*GeneName*=CASP4, .*GeneDesc*=caspase 4, apoptosis-related cysteine peptidase [Source:HGNC Symbol;Acc:1505].

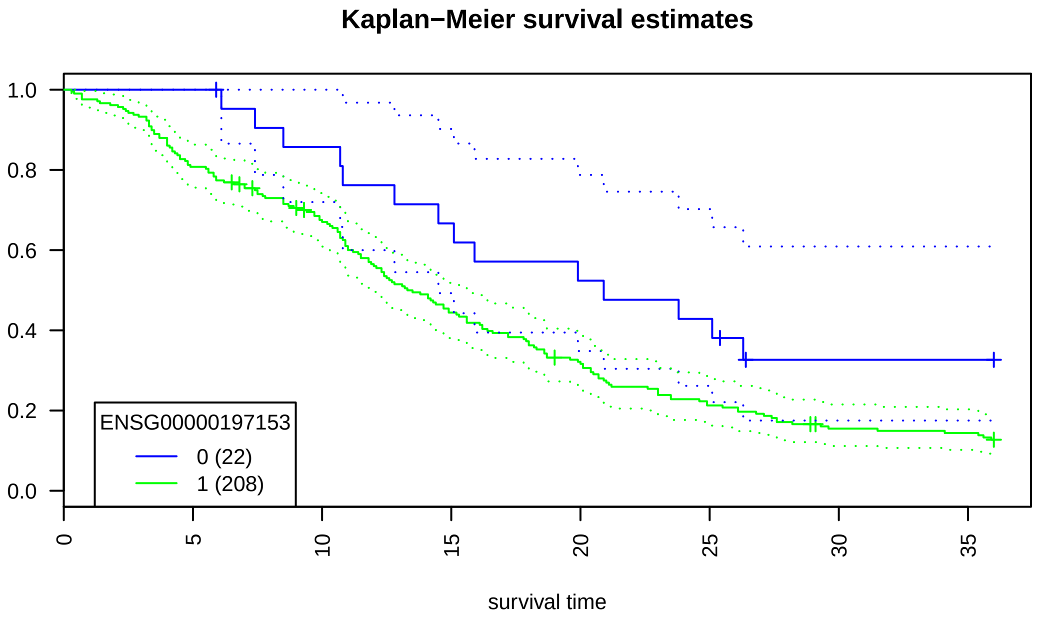

Figure 1809: Each curve represents the relative number of ENSG00000197153 survived to the corresponding point of time. Dashed lines represent 0.95 confidence intervals for the curves with the corresponding color. Log-rank probability measure for the equality of these curves is 2.649082e-02. Additional annotations: *.GeneName*=HIST1H3J, *.GeneDesc*=histone cluster 1, H3j [Source:HGNC Symbol;Acc:4774].

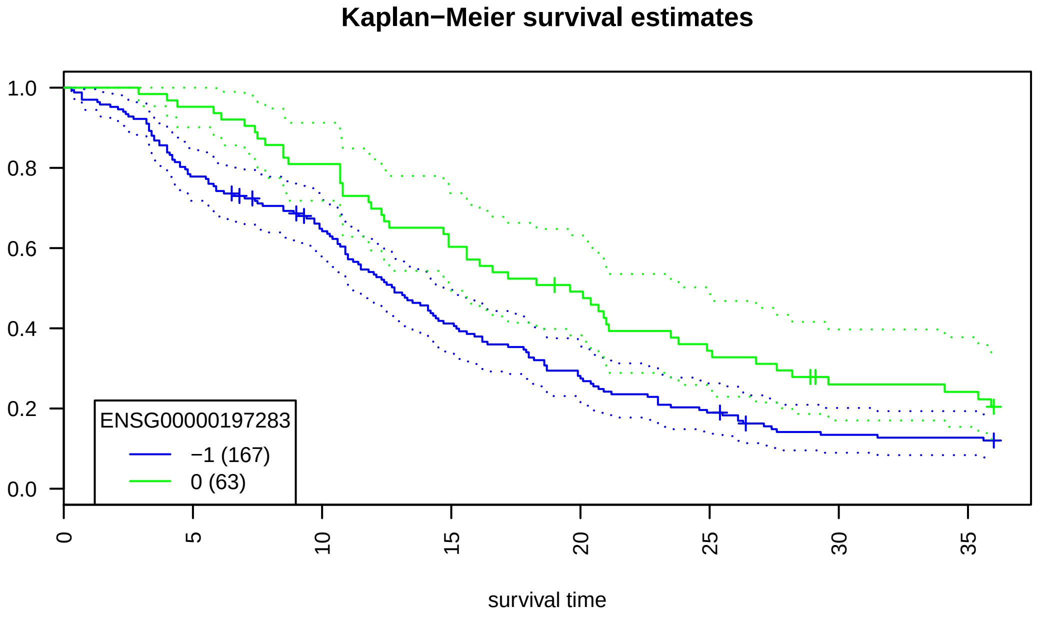

Figure 1810: Each curve represents the relative number of ENSG00000197283 survived to the corresponding point of time. Dashed lines represent 0.95 confidence intervals for the curves with the corresponding color. Log-rank probability measure for the equality of these curves is 5.942509e-03. Additional annotations: *.GeneName*=SYNGAP1, *.GeneDesc*=synaptic Ras GTPase activating protein 1 homolog (rat) [Source:HGNC Symbol;Acc:11497].

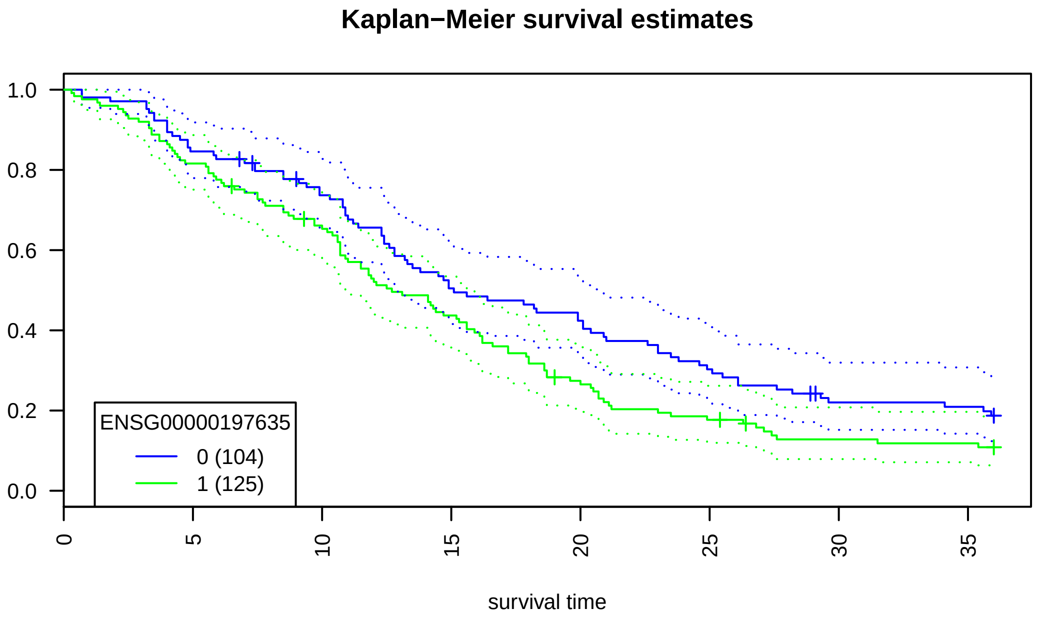

Figure 1811: Each curve represents the relative number of ENSG00000197635 survived to the corresponding point of time. Dashed lines represent 0.95 confidence intervals for the curves with the corresponding color. Total of 1 observation deleted due to missingness. Log-rank probability measure for the equality of these curves is 2.41707e-02. Additional annotations: *.GeneName*=DPP4, *.GeneDesc*=dipeptidyl-peptidase 4 [Source:HGNC Symbol;Acc:3009].

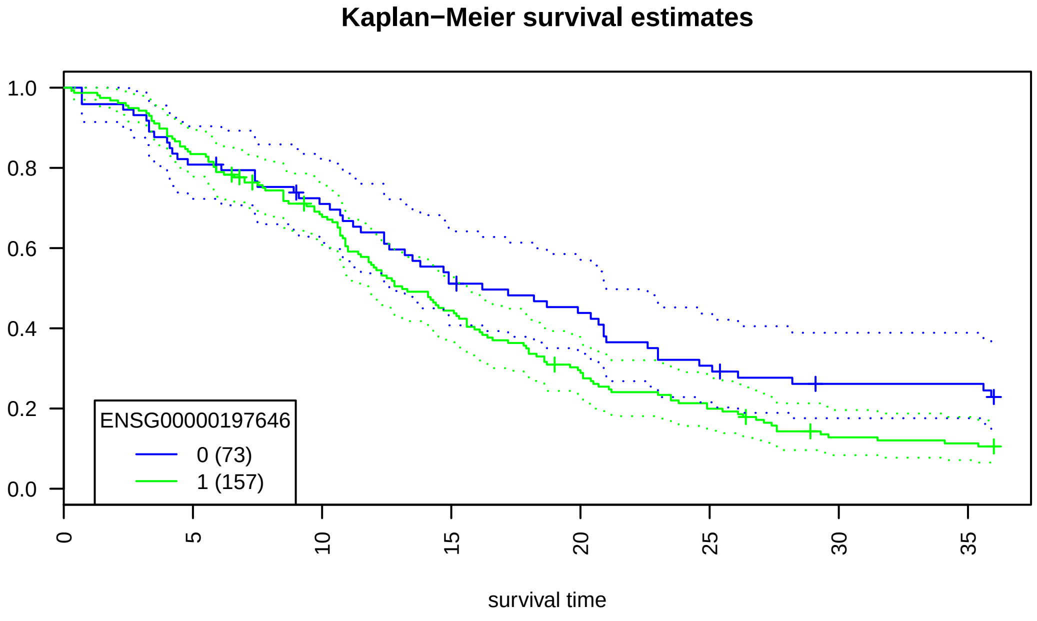

Figure 1812: Each curve represents the relative number of ENSG00000197646 survived to the corresponding point of time. Dashed lines represent 0.95 confidence intervals for the curves with the corresponding color. Log-rank probability measure for the equality of these curves is 3.819301e-02. Additional annotations: *.GeneName*=PDCD1LG2, *.GeneDesc*=programmed cell death 1 ligand 2 [Source:HGNC Symbol;Acc:18731].

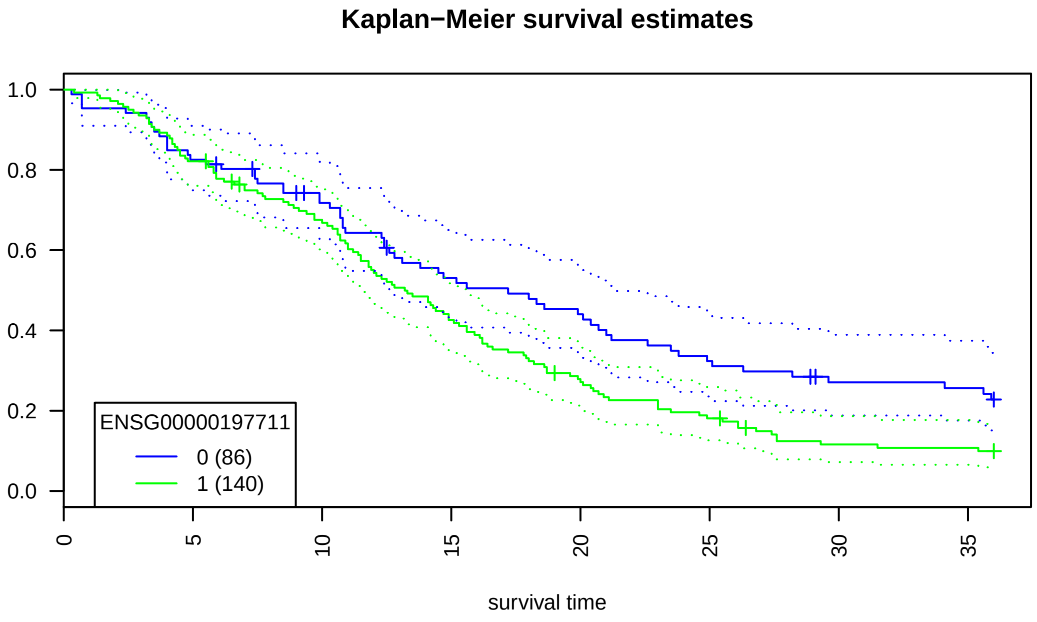

Figure 1813: Each curve represents the relative number of ENSG00000197711 survived to the corresponding point of time. Dashed lines represent 0.95 confidence intervals for the curves with the corresponding color. Total of 4 observations deleted due to missingness. Log-rank probability measure for the equality of these curves is 1.315246e-02. Additional annotations: *.GeneName*=HP, *.GeneDesc*=haptoglobin [Source:HGNC Symbol;Acc:5141].

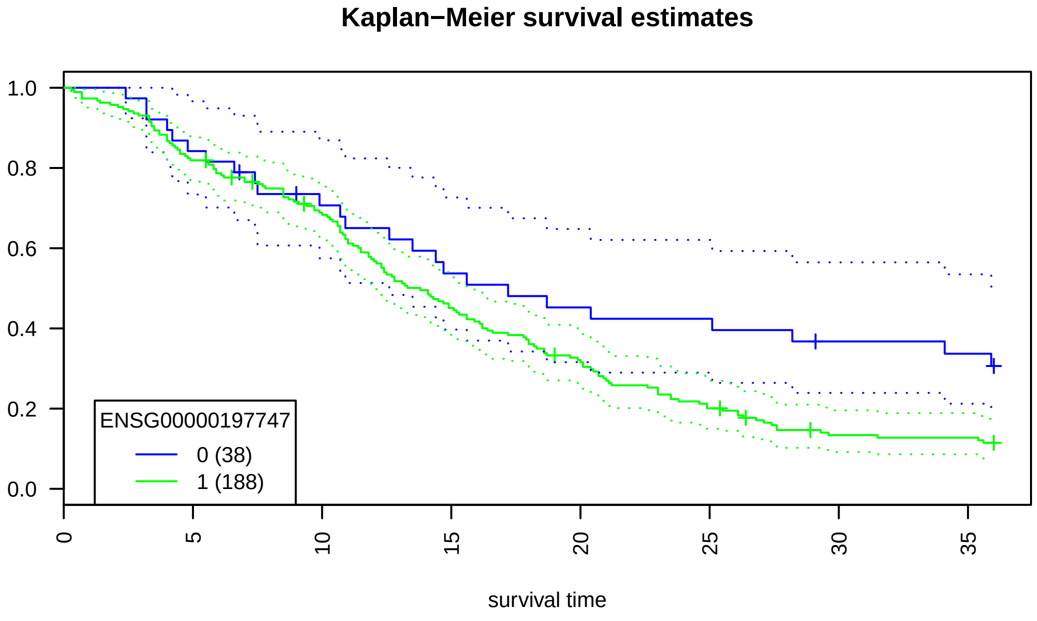

Figure 1814: Each curve represents the relative number of ENSG00000197747 survived to the corresponding point of time. Dashed lines represent 0.95 confidence intervals for the curves with the corresponding color. Total of 4 observations deleted due to missingness. Log-rank probability measure for the equality of these curves is 2.009475e-02. Additional annotations: *.GeneName*=S100A10, *.GeneDesc*=S100 calcium binding protein A10 [Source:HGNC Symbol;Acc:10487].

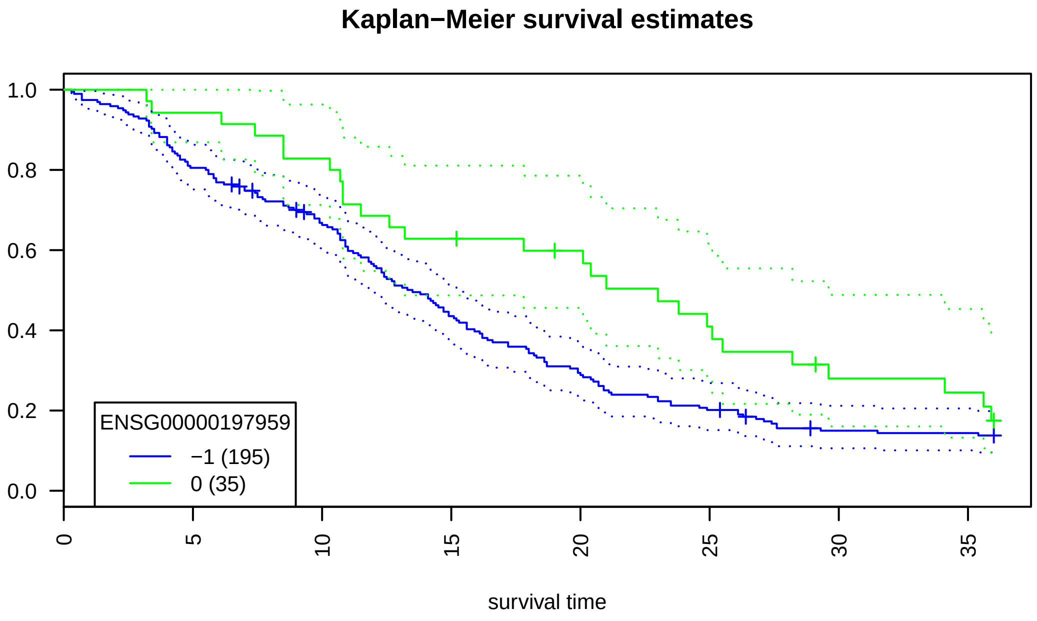

Figure 1815: Each curve represents the relative number of ENSG00000197959 survived to the corresponding point of time. Dashed lines represent 0.95 confidence intervals for the curves with the corresponding color. Log-rank probability measure for the equality of these curves is 3.798425e-02. Additional annotations: *.GeneName*=DNM3, *.GeneDesc*=dynamin 3 [Source:HGNC Symbol;Acc:29125].

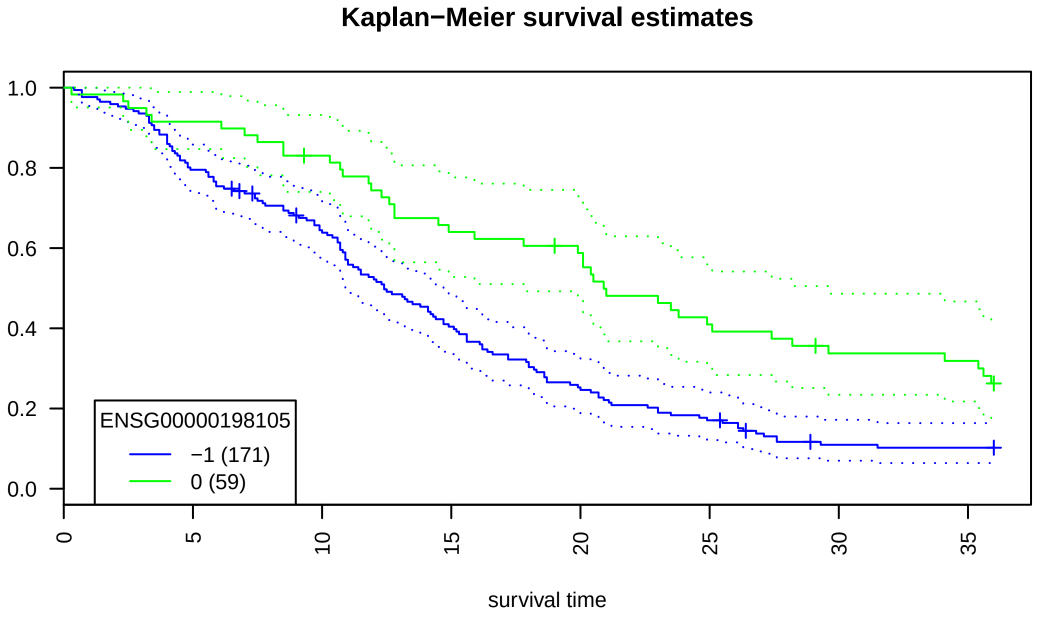

Figure 1816: Each curve represents the relative number of ENSG00000198105 survived to the corresponding point of time. Dashed lines represent 0.95 confidence intervals for the curves with the corresponding color. Log-rank probability measure for the equality of these curves is 1.050332e-04. Additional annotations: *.GeneName*=ZNF248, *.GeneDesc*=zinc finger protein 248 [Source:HGNC Symbol;Acc:13041].

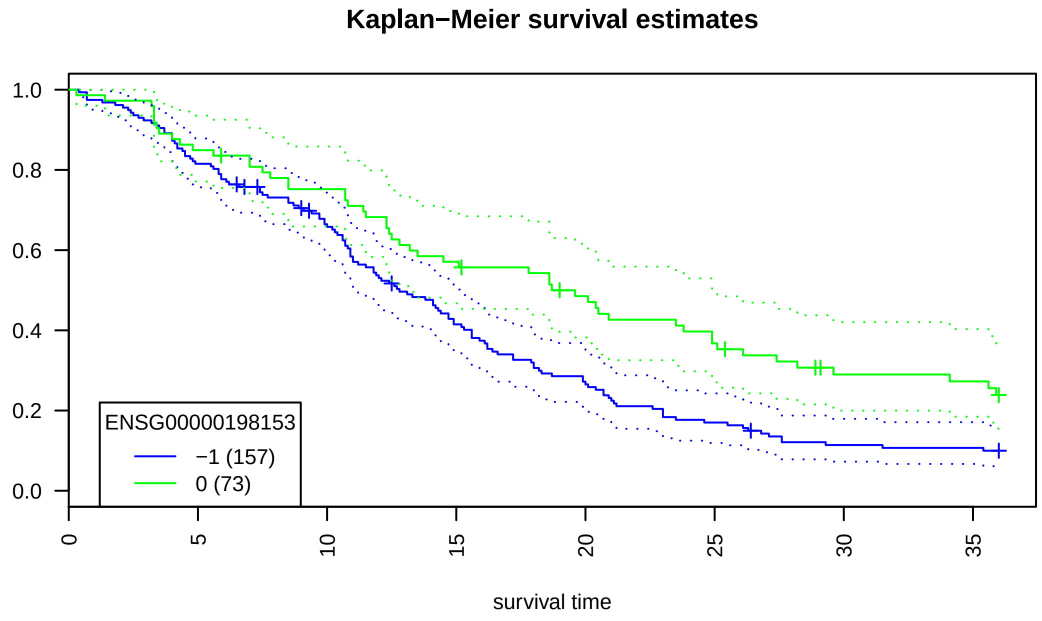

Figure 1817: Each curve represents the relative number of ENSG00000198153 survived to the corresponding point of time. Dashed lines represent 0.95 confidence intervals for the curves with the corresponding color. Log-rank probability measure for the equality of these curves is 1.841847e-03. Additional annotations: *.GeneName*=ZNF737, *.GeneDesc*=zinc finger protein 737 [Source:HGNC Symbol;Acc:32468].

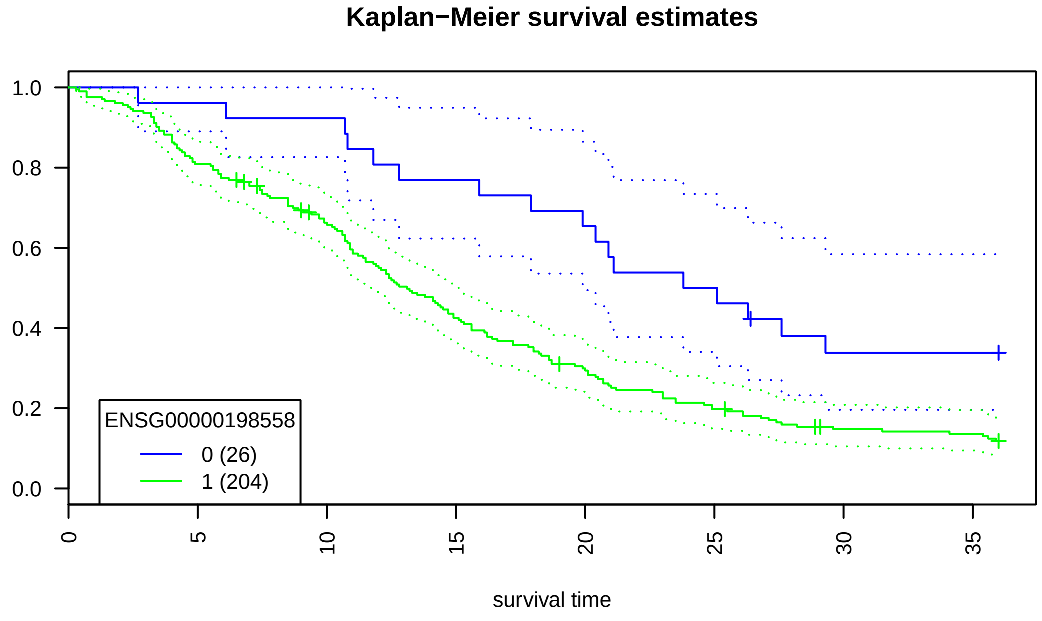

Figure 1818: Each curve represents the relative number of ENSG00000198558 survived to the corresponding point of time. Dashed lines represent 0.95 confidence intervals for the curves with the corresponding color. Log-rank probability measure for the equality of these curves is 1.562658e-03. Additional annotations: *.GeneName*=HIST1H4L, *.GeneDesc*=histone cluster 1, H4l [Source:HGNC Symbol;Acc:4791].

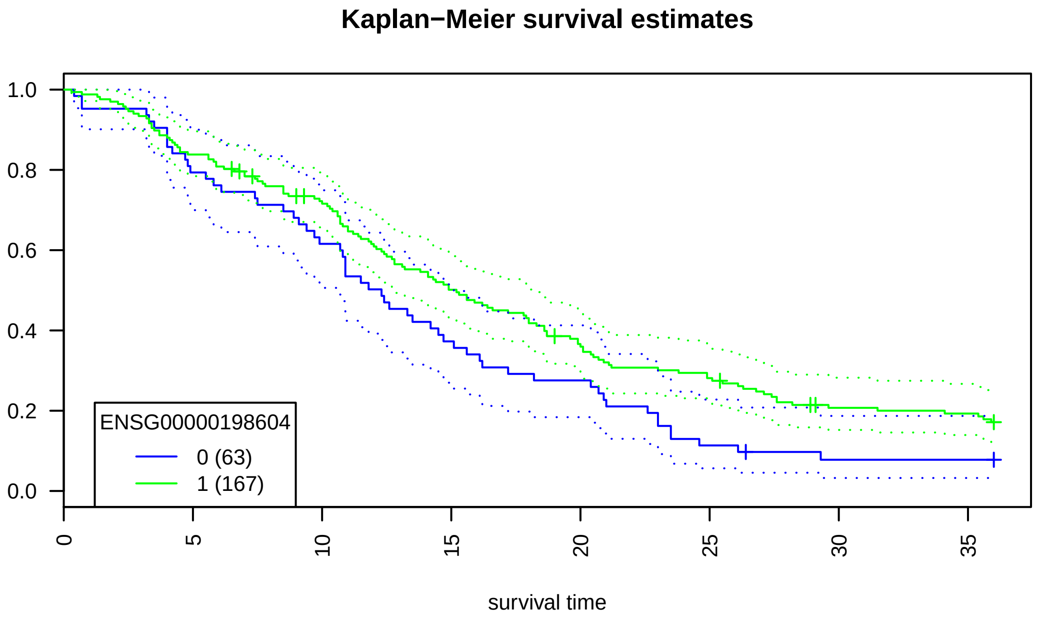

Figure 1819: Each curve represents the relative number of ENSG00000198604 survived to the corresponding point of time. Dashed lines represent 0.95 confidence intervals for the curves with the corresponding color. Log-rank probability measure for the equality of these curves is 2.187291e-02. Additional annotations: *.GeneName*=BAZ1A, *.GeneDesc*=bromodomain adjacent to zinc finger domain, 1A [Source:HGNC Symbol;Acc:960].

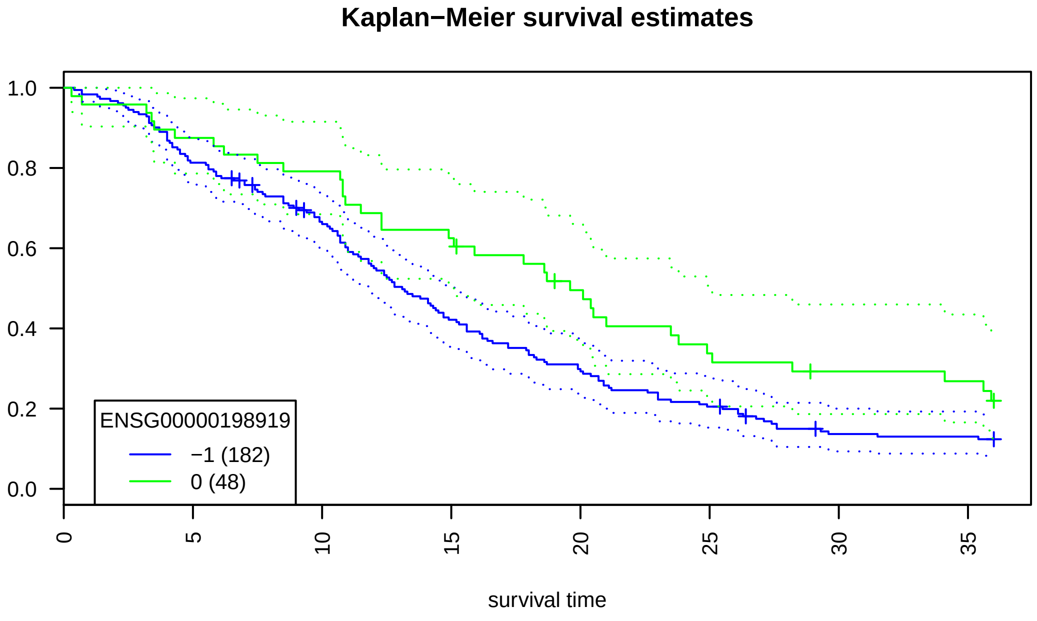

Figure 1820: Each curve represents the relative number of ENSG00000198919 survived to the corresponding point of time. Dashed lines represent 0.95 confidence intervals for the curves with the corresponding color. Log-rank probability measure for the equality of these curves is 2.324504e-02. Additional annotations: *.GeneName*=DZIP3, *.GeneDesc*=DAZ interacting protein 3, zinc finger [Source:HGNC Symbol;Acc:30938].

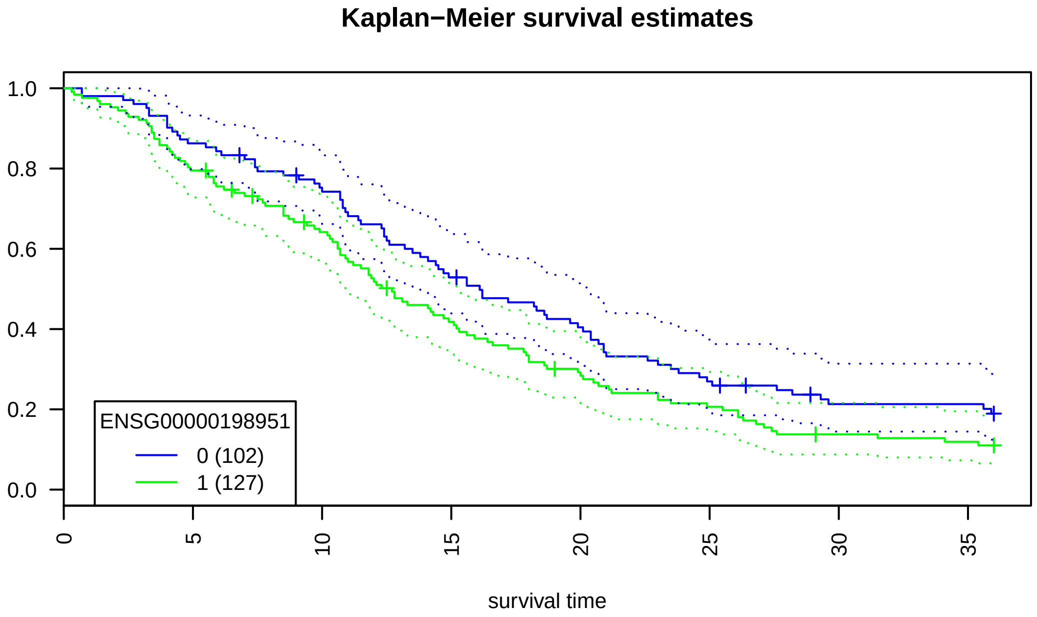

Figure 1821: Each curve represents the relative number of ENSG00000198951 survived to the corresponding point of time. Dashed lines represent 0.95 confidence intervals for the curves with the corresponding color. Total of 1 observation deleted due to missingness. Log-rank probability measure for the equality of these curves is 3.306933e-02. Additional annotations: *.GeneName*=NAGA, *.GeneDesc*=N-acetylgalactosaminidase, alpha- [Source:HGNC Symbol;Acc:7631].

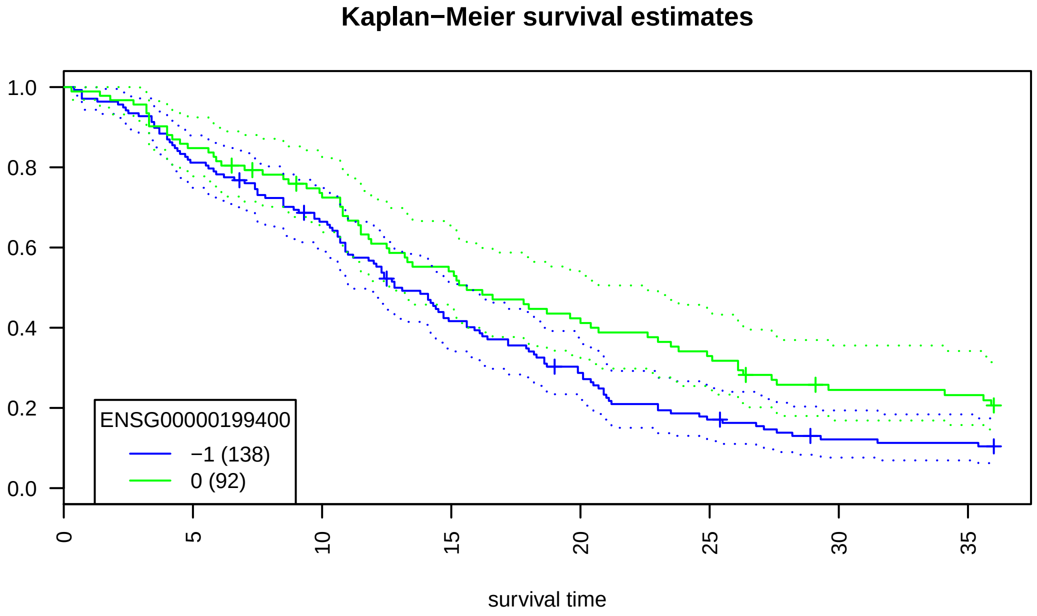

Figure 1822: Each curve represents the relative number of ENSG00000199400 survived to the corresponding point of time. Dashed lines represent 0.95 confidence intervals for the curves with the corresponding color. Log-rank probability measure for the equality of these curves is 2.090396e-02. Additional annotations: *.GeneName*=AC093171.1, *.GeneDesc*=[undefined].

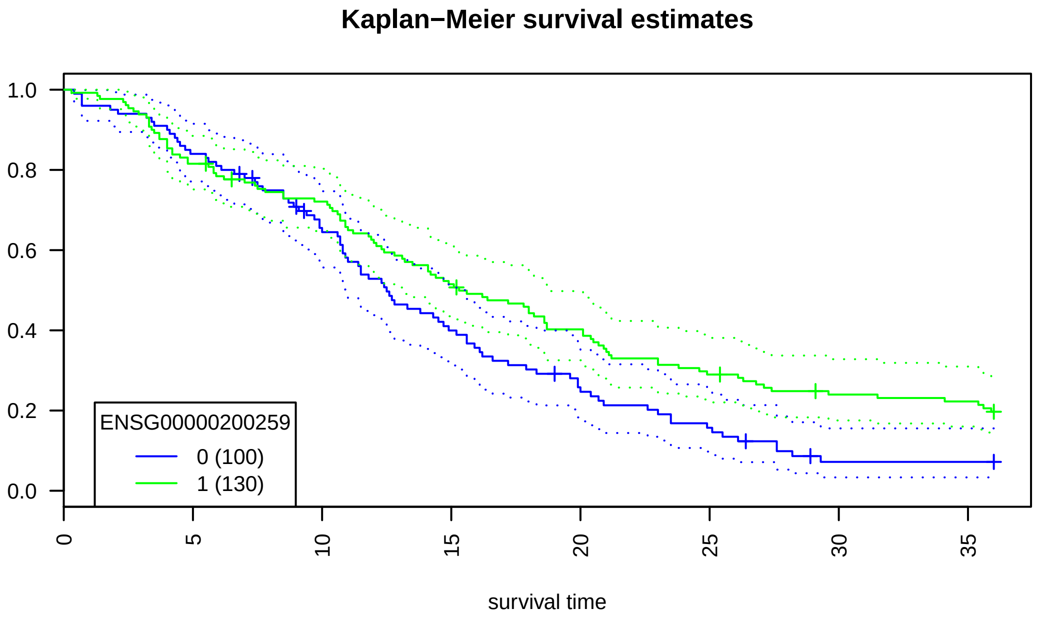

Figure 1823: Each curve represents the relative number of ENSG00000200259 survived to the corresponding point of time. Dashed lines represent 0.95 confidence intervals for the curves with the corresponding color. Log-rank probability measure for the equality of these curves is 9.976898e-03. Additional annotations: *.GeneName*=SNORD35A, *.GeneDesc*=small nucleolar RNA, C/D box 35A [Source:HGNC Symbol;Acc:10162].

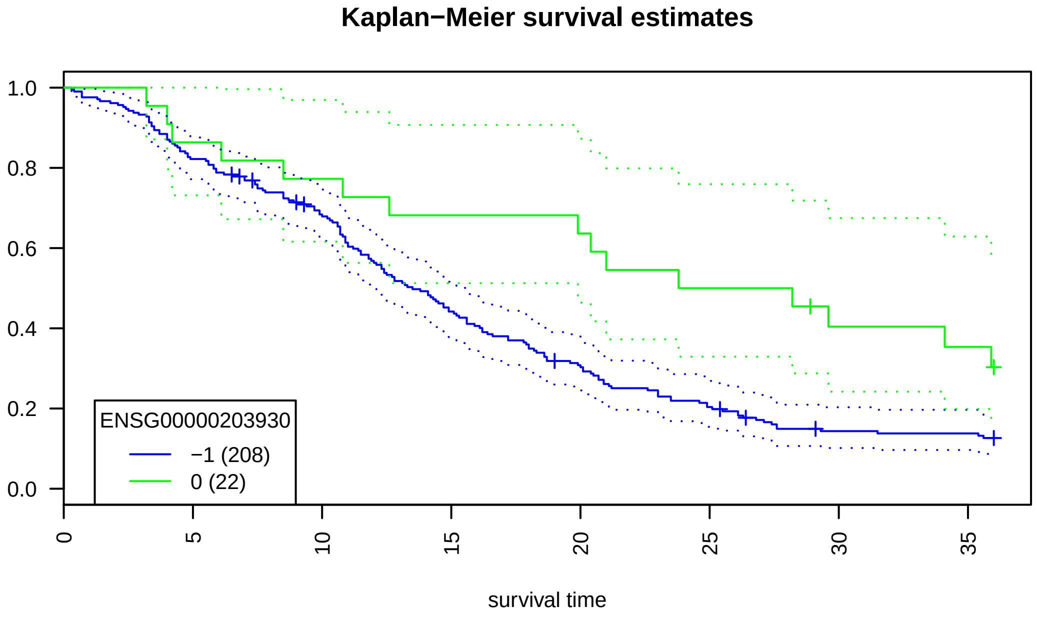

Figure 1824: Each curve represents the relative number of ENSG00000203930 survived to the corresponding point of time. Dashed lines represent 0.95 confidence intervals for the curves with the corresponding color. Log-rank probability measure for the equality of these curves is 1.075150e-02. Additional annotations: *.GeneName*=RP1-177G6.2, *.GeneDesc*=Novel protein [Source:UniProtKB/TrEMBL;Acc:Q5JPA2].

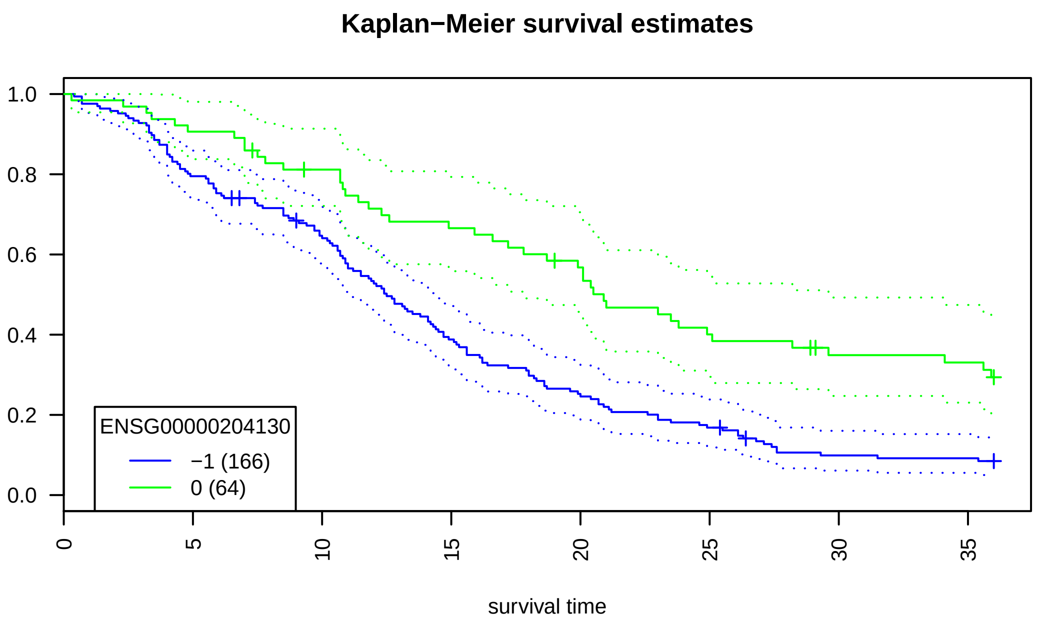

Figure 1825: Each curve represents the relative number of ENSG00000204130 survived to the corresponding point of time. Dashed lines represent 0.95 confidence intervals for the curves with the corresponding color. Log-rank probability measure for the equality of these curves is 1.568147e-05. Additional annotations: *.GeneName*=RUFY2, *.GeneDesc*=RUN and FYVE domain containing 2 [Source:HGNC Symbol;Acc:19761].

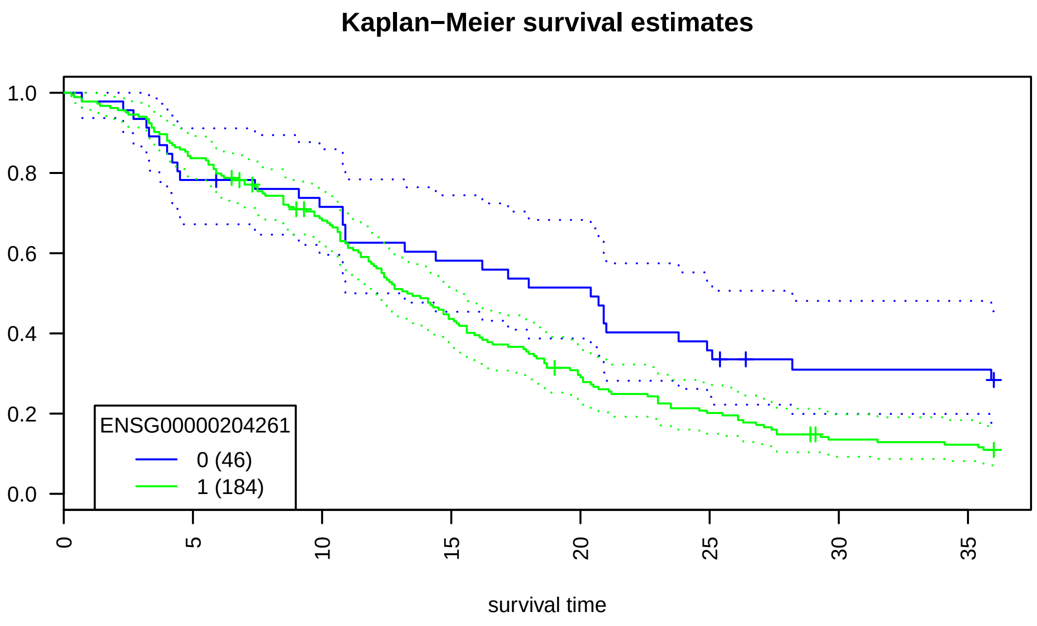

Figure 1826: Each curve represents the relative number of ENSG00000204261 survived to the corresponding point of time. Dashed lines represent 0.95 confidence intervals for the curves with the corresponding color. Log-rank probability measure for the equality of these curves is 1.604769e-02. Additional annotations: *.GeneName*=XXbac-BPG246D15.8, *.GeneDesc*=[undefined].

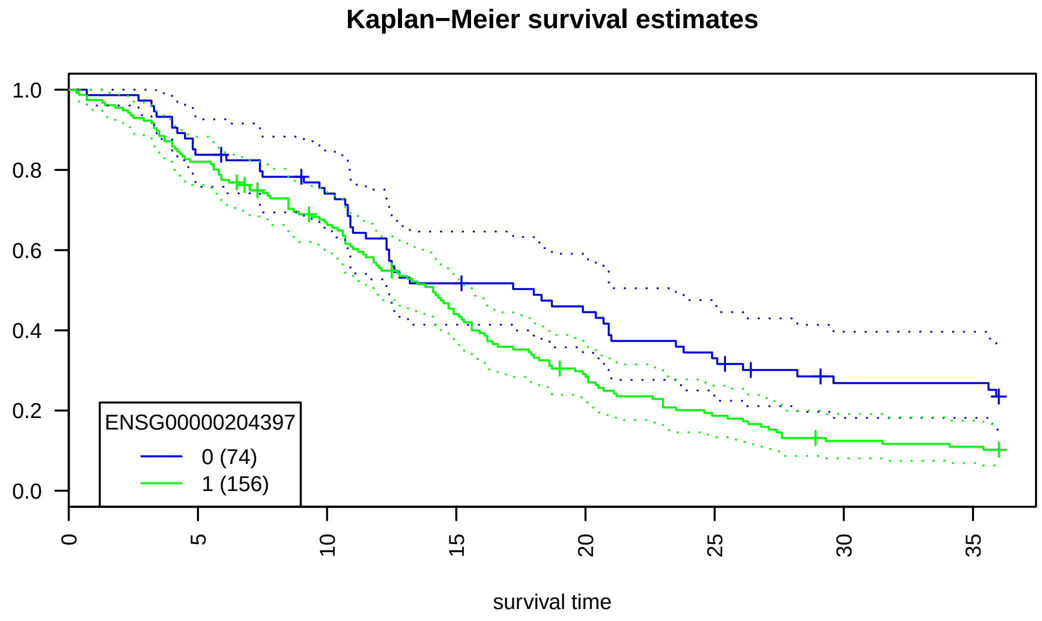

Figure 1827: Each curve represents the relative number of ENSG00000204397 survived to the corresponding point of time. Dashed lines represent 0.95 confidence intervals for the curves with the corresponding color. Log-rank probability measure for the equality of these curves is 1.409366e-02. Additional annotations: *.GeneName*=CARD16, *.GeneDesc*=caspase recruitment domain family, member 16 [Source:HGNC Symbol;Acc:33701].

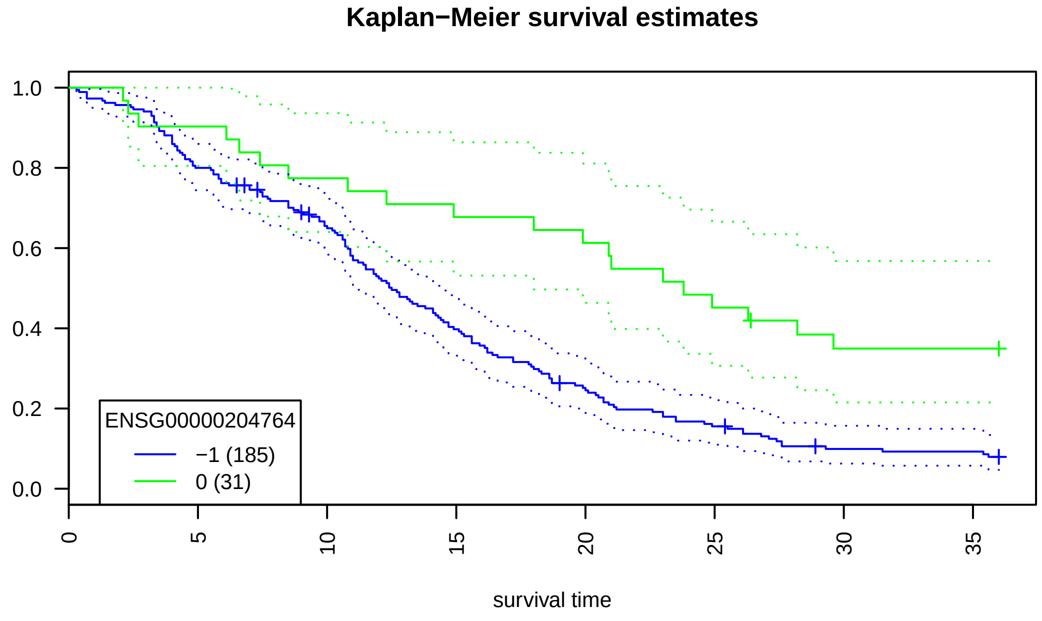

Figure 1828: Each curve represents the relative number of ENSG00000204764 survived to the corresponding point of time. Dashed lines represent 0.95 confidence intervals for the curves with the corresponding color. Total of 14 observations deleted due to missingness. Log-rank probability measure for the equality of these curves is 1.608405e-04. Additional annotations: *.GeneName*=RANBP17, *.GeneDesc*=RAN binding protein 17 [Source:HGNC Symbol;Acc:14428].

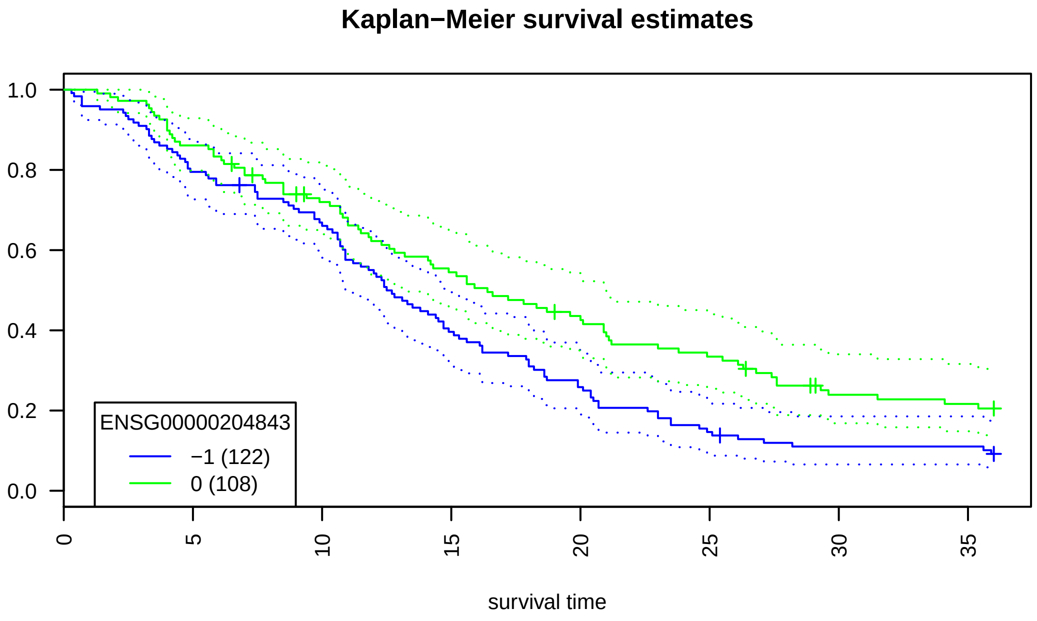

Figure 1829: Each curve represents the relative number of ENSG00000204843 survived to the corresponding point of time. Dashed lines represent 0.95 confidence intervals for the curves with the corresponding color. Log-rank probability measure for the equality of these curves is 4.097782e-03. Additional annotations: *.GeneName*=DCTN1, *.GeneDesc*=dynactin 1 (p150, glued homolog, Drosophila) [Source:HGNC Symbol;Acc:2711].

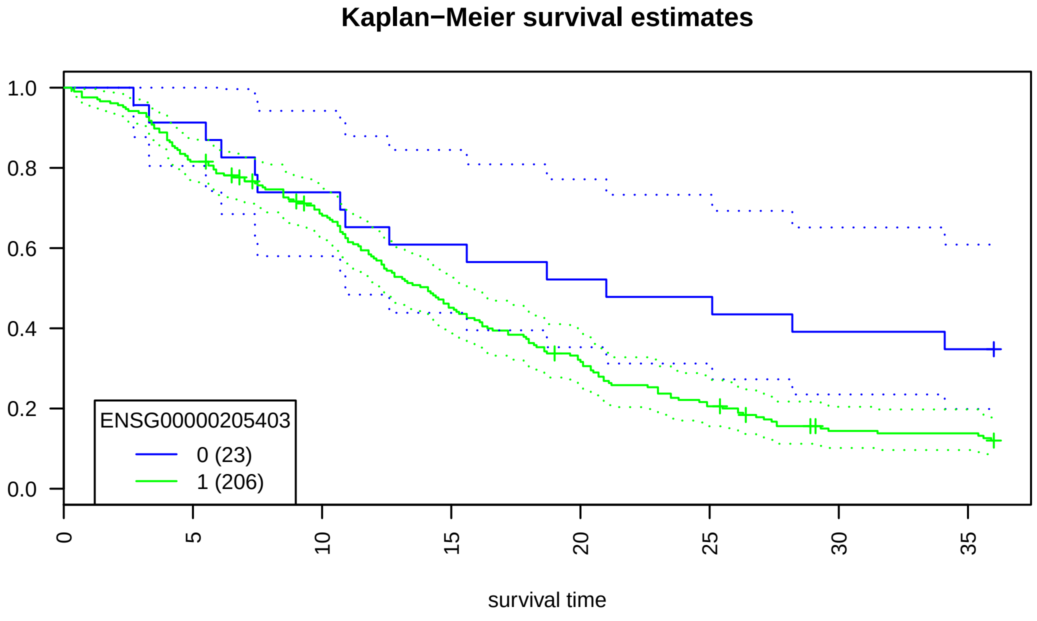

Figure 1830: Each curve represents the relative number of ENSG00000205403 survived to the corresponding point of time. Dashed lines represent 0.95 confidence intervals for the curves with the corresponding color. Total of 1 observation deleted due to missingness. Log-rank probability measure for the equality of these curves is 1.940991e-02. Additional annotations: *.GeneName*=CFI, *.GeneDesc*=complement factor I [Source:HGNC Symbol;Acc:5394].

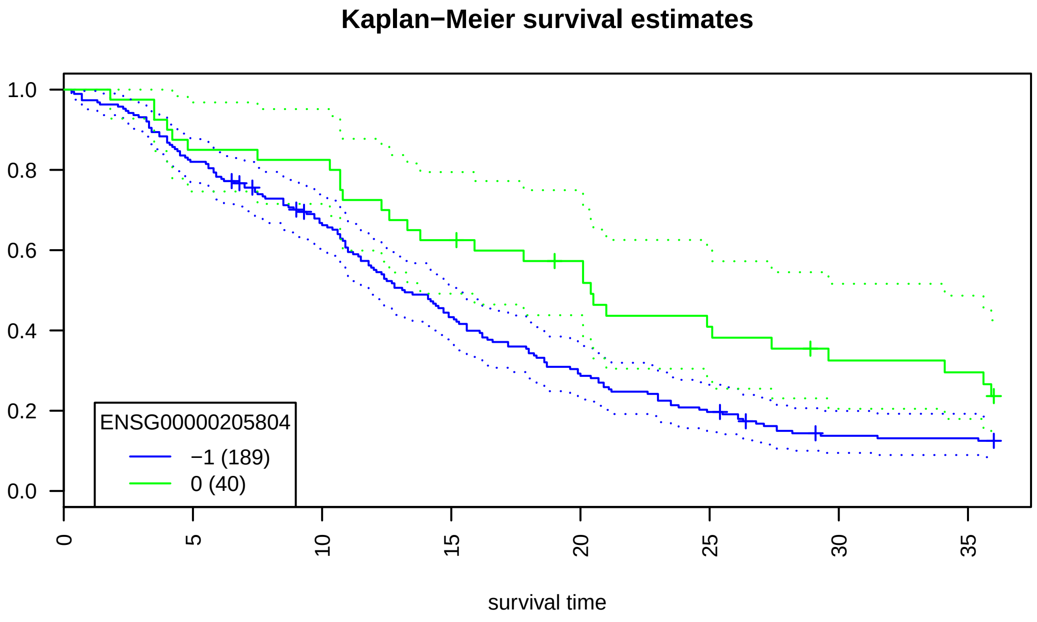

Figure 1831: Each curve represents the relative number of ENSG00000205804 survived to the corresponding point of time. Dashed lines represent 0.95 confidence intervals for the curves with the corresponding color. Total of 1 observation deleted due to missingness. Log-rank probability measure for the equality of these curves is 1.282063e-02. Additional annotations: *.GeneName*=TPTE2P1, *.GeneDesc*=transmembrane phosphoinositide 3-phosphatase and tensin homolog 2 pseudogene 1 [Source:HGNC Symbol;Acc:35196].

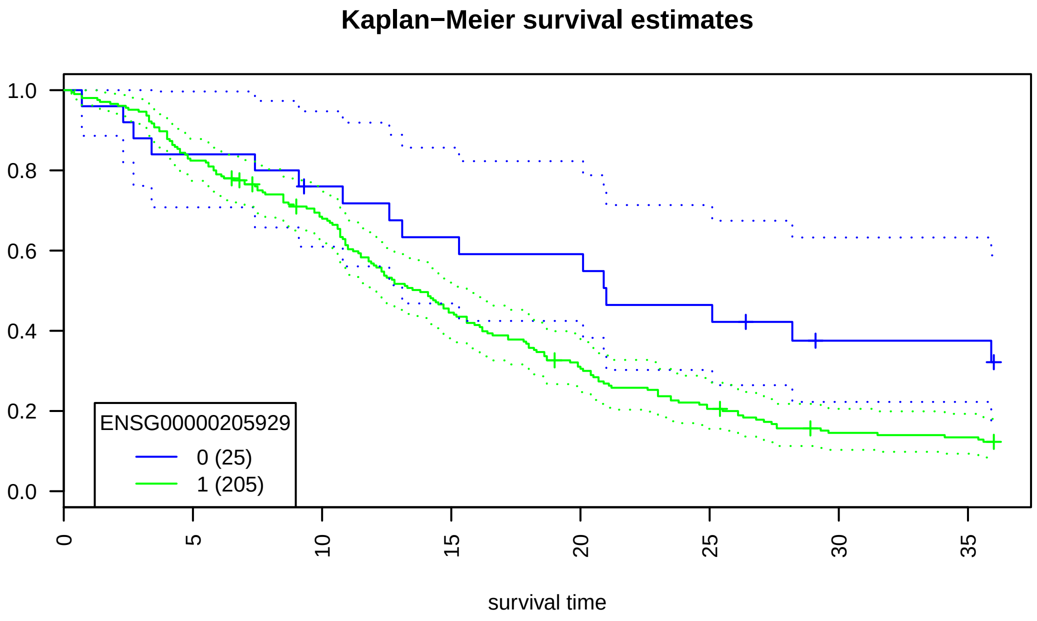

Figure 1832: Each curve represents the relative number of ENSG00000205929 survived to the corresponding point of time. Dashed lines represent 0.95 confidence intervals for the curves with the corresponding color. Log-rank probability measure for the equality of these curves is 2.125299e-02. Additional annotations: *.GeneName*=C21orf62, *.GeneDesc*=Uncharacterized protein C21orf62 (B37) [Source:UniProtKB/Swiss-Prot;Acc:Q9NYP8].

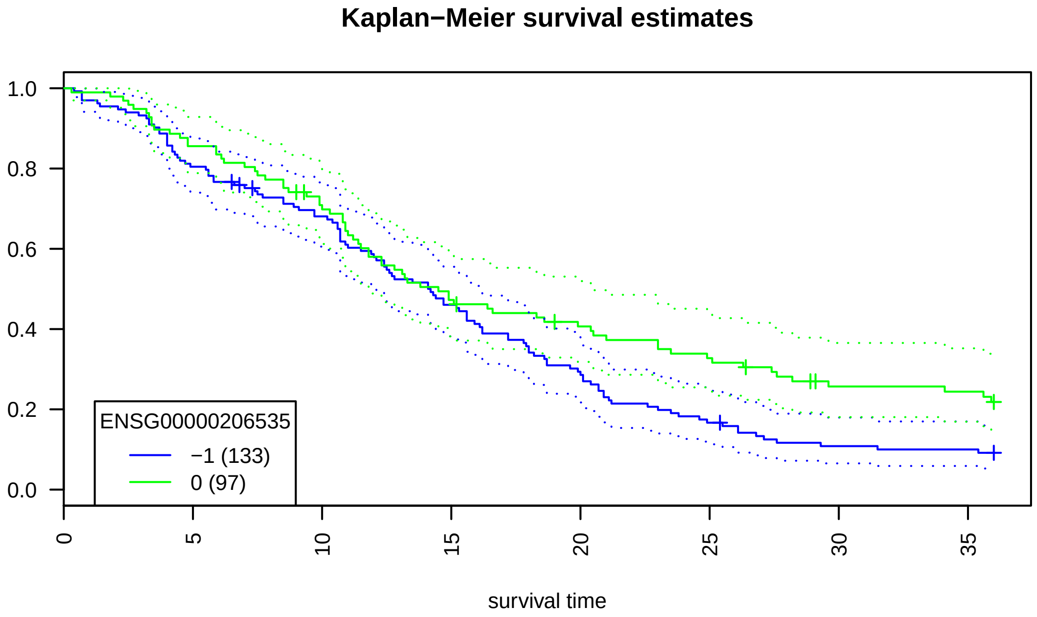

Figure 1833: Each curve represents the relative number of ENSG00000206535 survived to the corresponding point of time. Dashed lines represent 0.95 confidence intervals for the curves with the corresponding color. Log-rank probability measure for the equality of these curves is 2.069186e-02. Additional annotations: *.GeneName*=LNP1, *.GeneDesc*=leukemia NUP98 fusion partner 1 [Source:HGNC Symbol;Acc:28014].

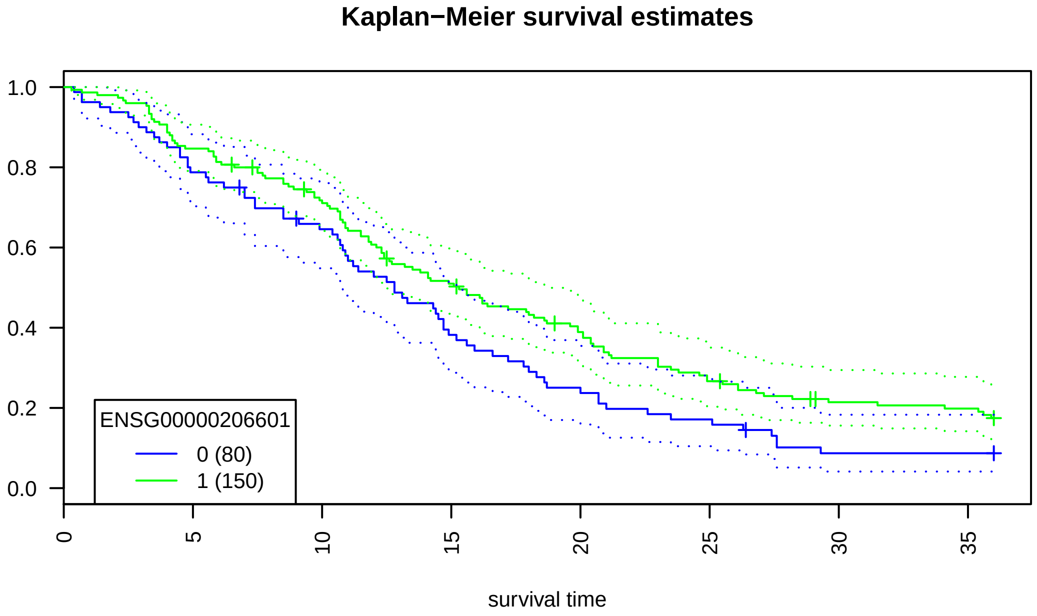

Figure 1834: Each curve represents the relative number of ENSG00000206601 survived to the corresponding point of time. Dashed lines represent 0.95 confidence intervals for the curves with the corresponding color. Log-rank probability measure for the equality of these curves is 2.671710e-02. Additional annotations: *.GeneName*=AC107071.1, *.GeneDesc*=[undefined].

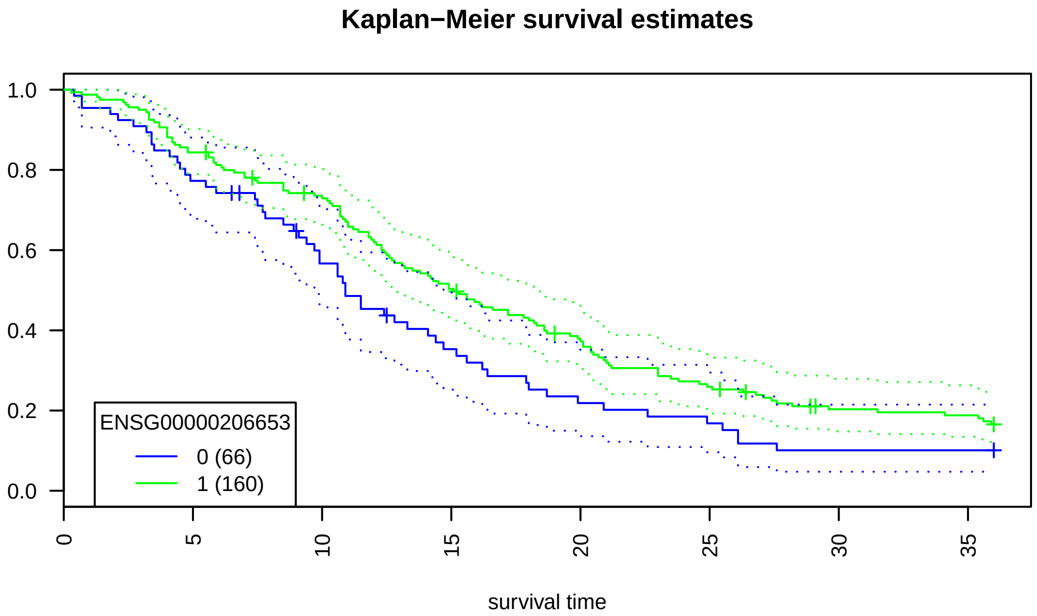

Figure 1835: Each curve represents the relative number of ENSG00000206653 survived to the corresponding point of time. Dashed lines represent 0.95 confidence intervals for the curves with the corresponding color. Total of 4 observations deleted due to missingness. Log-rank probability measure for the equality of these curves is 2.524463e-02. Additional annotations: *.GeneName*=SNORD29, *.GeneDesc*=small nucleolar RNA, C/D box 29 [Source:HGNC Symbol;Acc:10151].

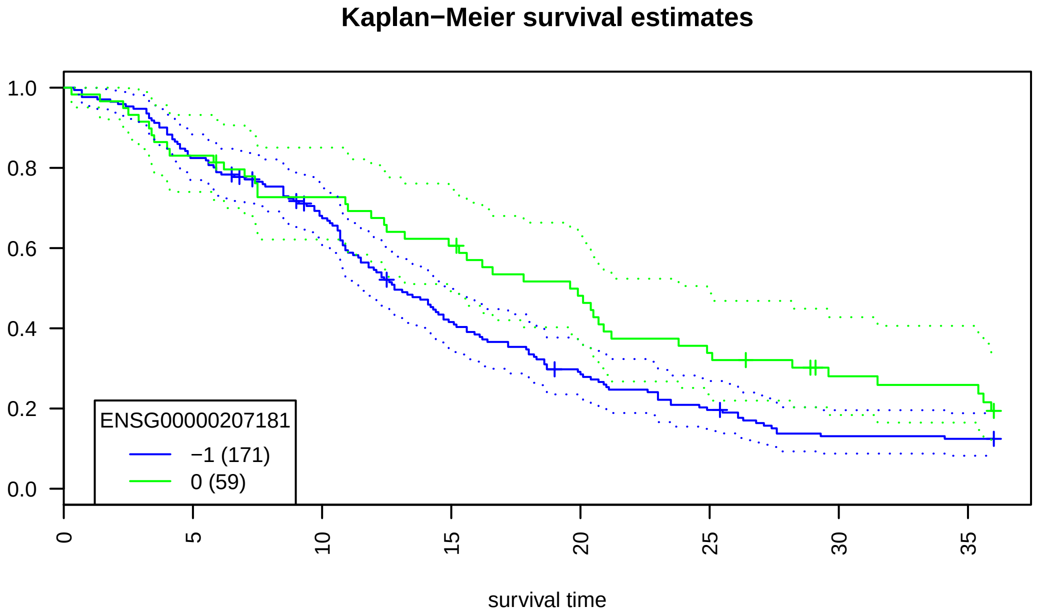

Figure 1836: Each curve represents the relative number of ENSG00000207181 survived to the corresponding point of time. Dashed lines represent 0.95 confidence intervals for the curves with the corresponding color. Log-rank probability measure for the equality of these curves is 3.659371e-02. Additional annotations: *.GeneName*=SNORA14B, *.GeneDesc*=small nucleolar RNA, H/ACA box 14B [Source:HGNC Symbol;Acc:32603].

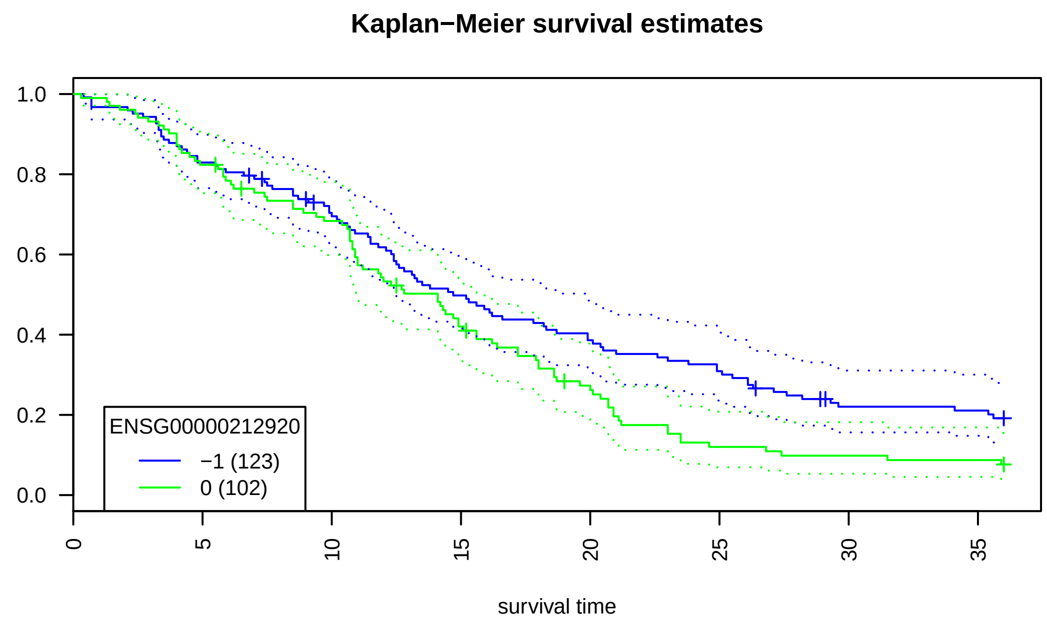

Figure 1837: Each curve represents the relative number of ENSG00000212920 survived to the corresponding point of time. Dashed lines represent 0.95 confidence intervals for the curves with the corresponding color. Total of 5 observations deleted due to missingness. Log-rank probability measure for the equality of these curves is 1.827197e-02.

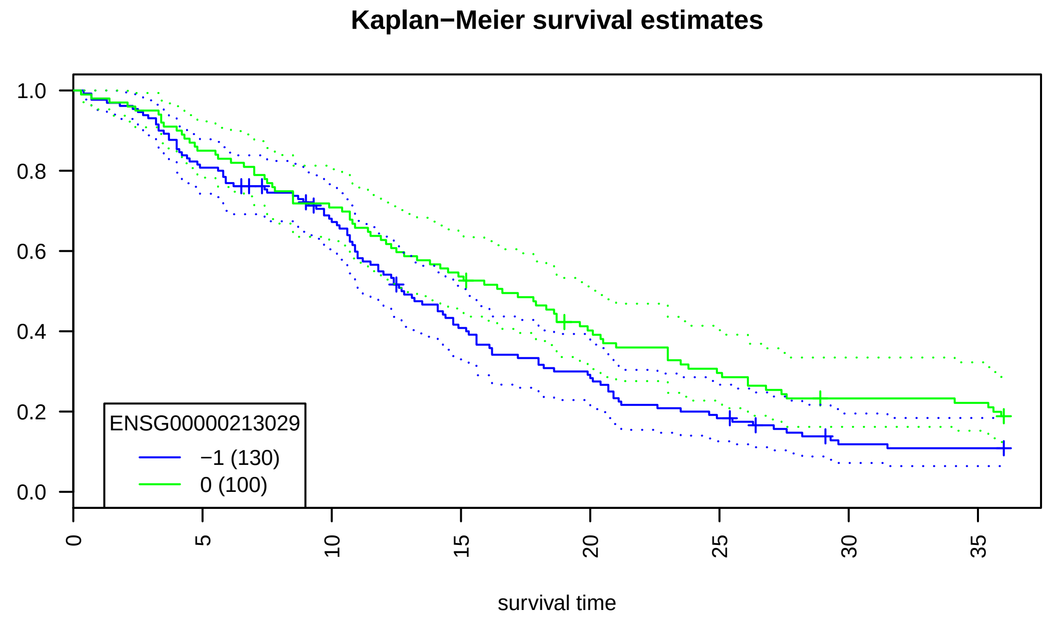

Figure 1838: Each curve represents the relative number of ENSG00000213029 survived to the corresponding point of time. Dashed lines represent 0.95 confidence intervals for the curves with the corresponding color. Log-rank probability measure for the equality of these curves is 3.962687e-02. Additional annotations: *.GeneName*=SPHAR, *.GeneDesc*=S-phase response (cyclin related) [Source:HGNC Symbol;Acc:16957].

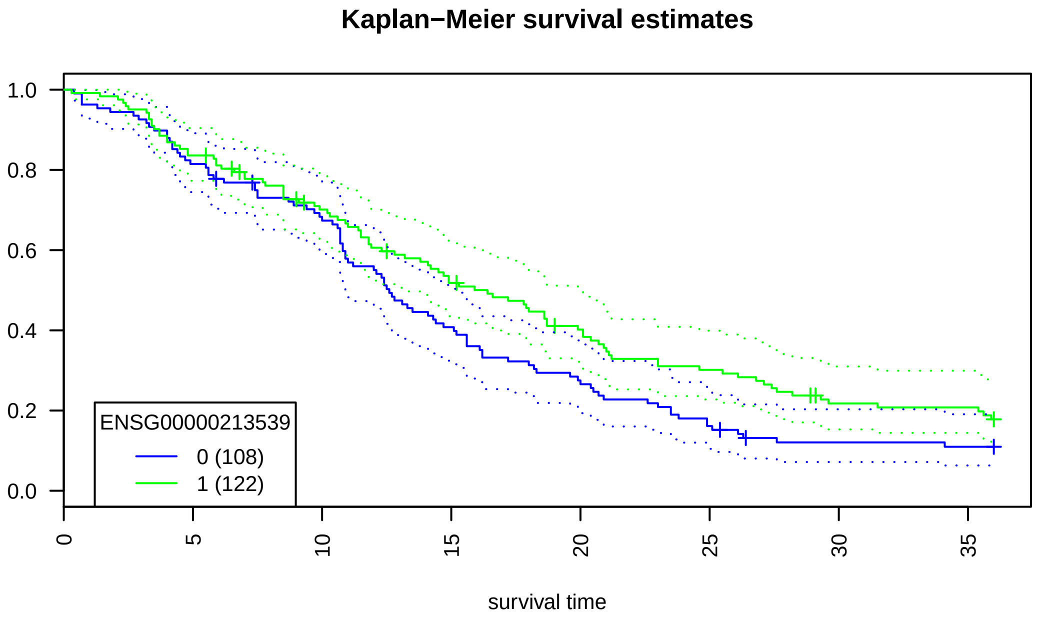

Figure 1839: Each curve represents the relative number of ENSG00000213539 survived to the corresponding point of time. Dashed lines represent 0.95 confidence intervals for the curves with the corresponding color. Log-rank probability measure for the equality of these curves is 3.753921e-02. Additional annotations: .*GeneName*=RP11-397F23.3, .*GeneDesc*=[undefined].

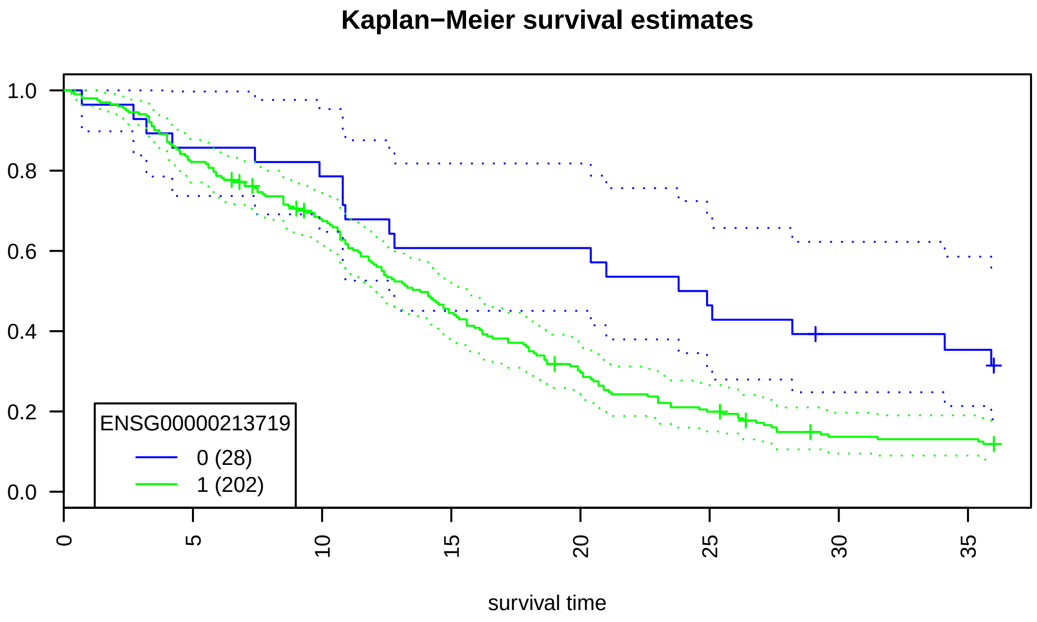

Figure 1840: Each curve represents the relative number of ENSG00000213719 survived to the corresponding point of time. Dashed lines represent 0.95 confidence intervals for the curves with the corresponding color. Log-rank probability measure for the equality of these curves is 7.868463e-03. Additional annotations: .*GeneName*=CLIC1, .*GeneDesc*=Chloride intracellular channel protein 1 (Nuclear chloride ion channel 27)(NCC27)(Chloride channel ABP)(Regulatory nuclear chloride ion channel protein)(hRNCC) [Source:UniProtKB/Swiss-Prot;Acc:O00299].

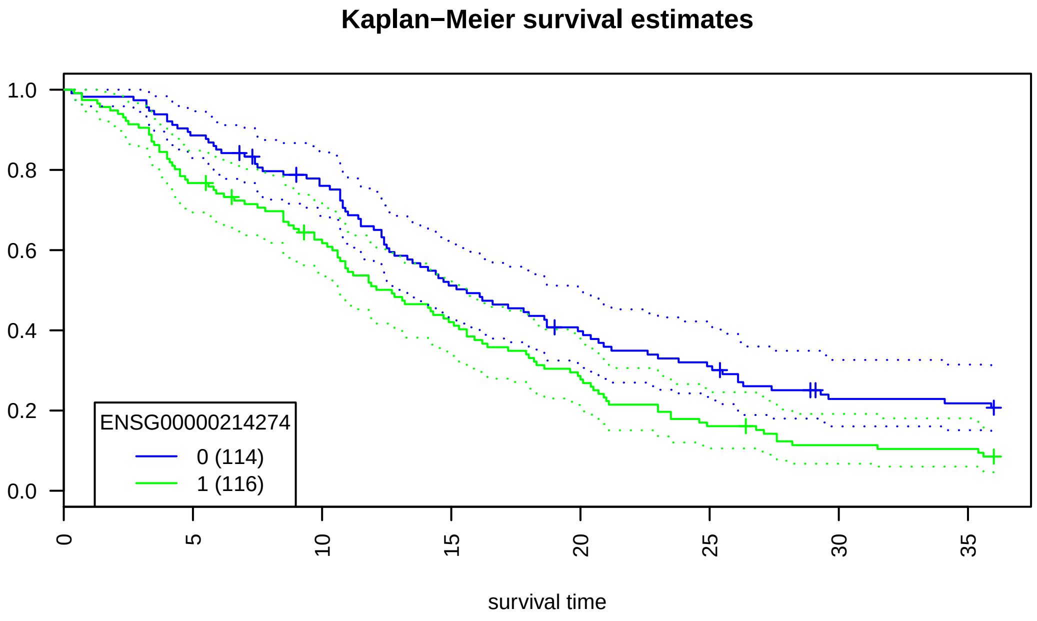

Figure 1841: Each curve represents the relative number of ENSG00000214274 survived to the corresponding point of time. Dashed lines represent 0.95 confidence intervals for the curves with the corresponding color. Log-rank probability measure for the equality of these curves is 5.164664e-03. Additional annotations: *.GeneName*=ANG, *.GeneDesc*=angiogenin, ribonuclease, RNase A family, 5 [Source:HGNC Symbol;Acc:483].

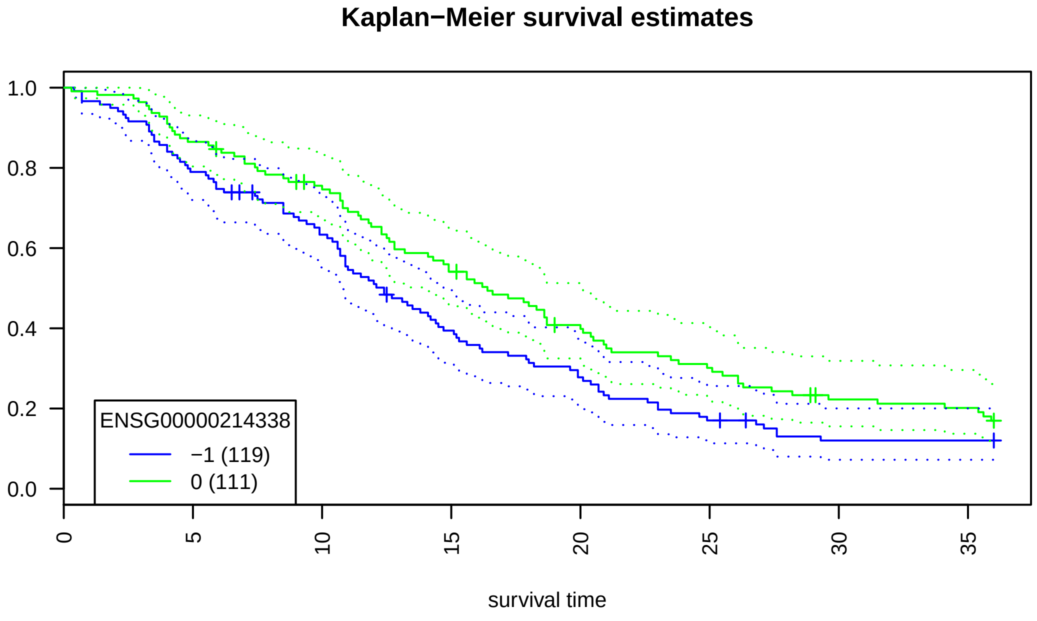

Figure 1842: Each curve represents the relative number of ENSG00000214338 survived to the corresponding point of time. Dashed lines represent 0.95 confidence intervals for the curves with the corresponding color. Log-rank probability measure for the equality of these curves is 2.776909e-02. Additional annotations: *.GeneName*=AL096711.1, *.GeneDesc*=Uncharacterized protein C6orf174 Precursor [Source:UniProtKB/Swiss-Prot;Acc:Q5TF21].

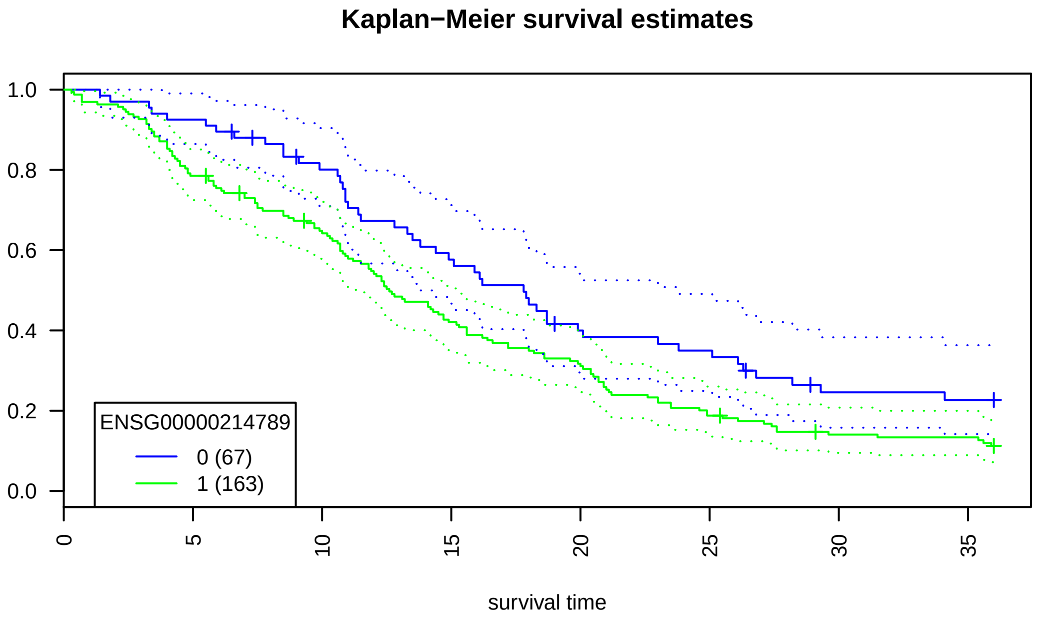

Figure 1843: Each curve represents the relative number of ENSG00000214789 survived to the corresponding point of time. Dashed lines represent 0.95 confidence intervals for the curves with the corresponding color. Log-rank probability measure for the equality of these curves is 1.271800e-02.

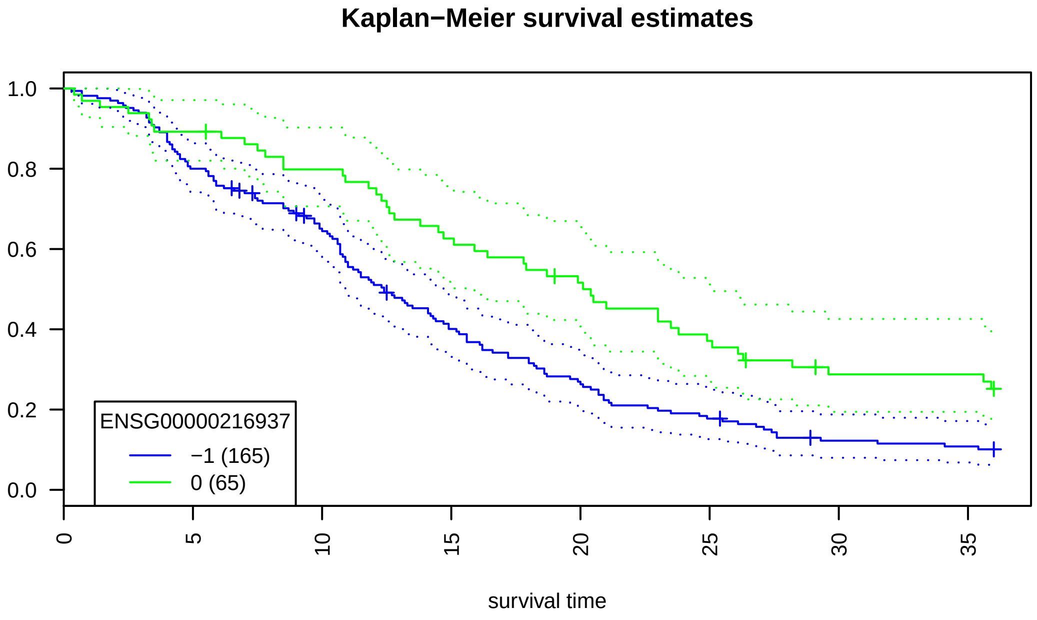

Figure 1844: Each curve represents the relative number of ENSG00000216937 survived to the corresponding point of time. Dashed lines represent 0.95 confidence intervals for the curves with the corresponding color. Log-rank probability measure for the equality of these curves is 5.650665e-04. Additional annotations: *.GeneName*=CCDC7, *.GeneDesc*=coiled-coil domain containing 7 [Source:HGNC Symbol;Acc:26533].

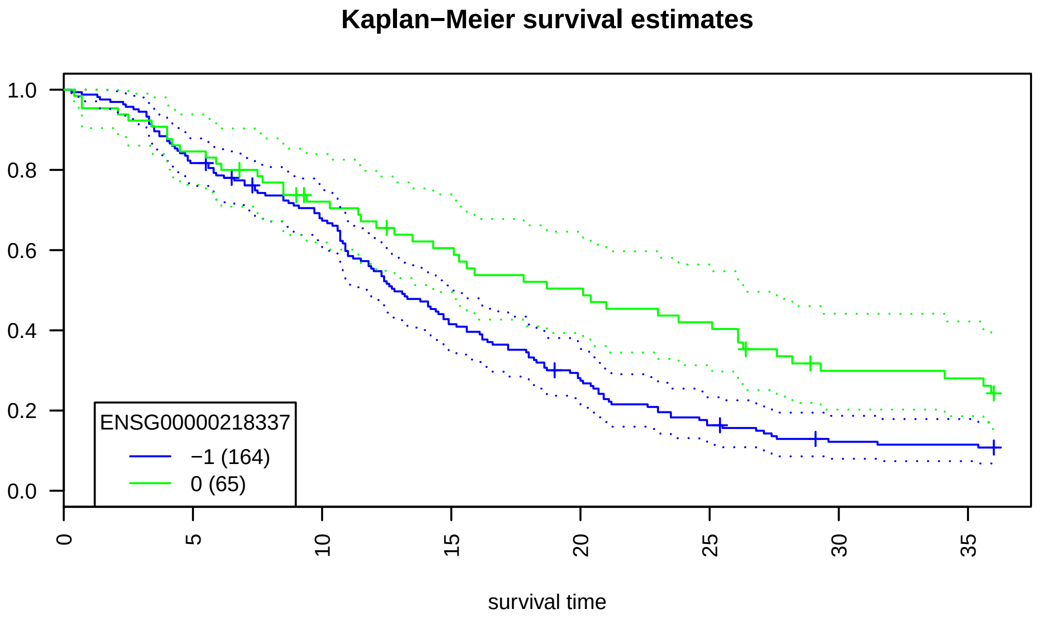

Figure 1845: Each curve represents the relative number of ENSG00000218337 survived to the corresponding point of time. Dashed lines represent 0.95 confidence intervals for the curves with the corresponding color. Total of 1 observation deleted due to missingness. Log-rank probability measure for the equality of these curves is 3.90062e-03. Additional annotations: *.GeneName*=RP4-724P12.1, *.GeneDesc*=[undefined].

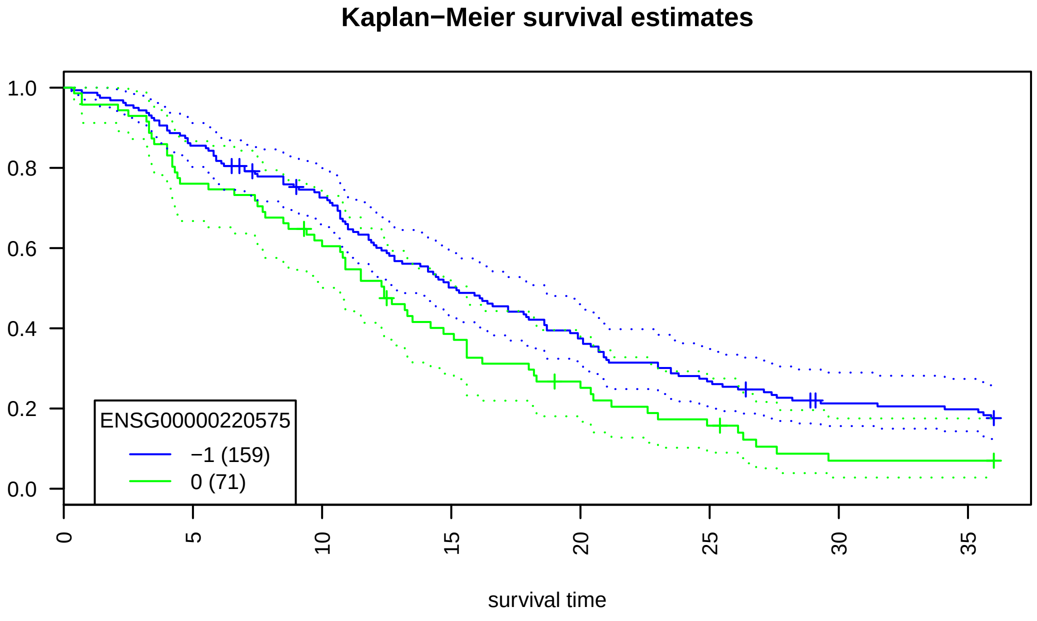

Figure 1846: Each curve represents the relative number of ENSG00000220575 survived to the corresponding point of time. Dashed lines represent 0.95 confidence intervals for the curves with the corresponding color. Log-rank probability measure for the equality of these curves is 1.315732e-02. Additional annotations: *.GeneName*=AC093726.4, *.GeneDesc*=cDNA FLJ50038 [Source:UniProtKB/TrEMBL;Acc:B7Z8E6].

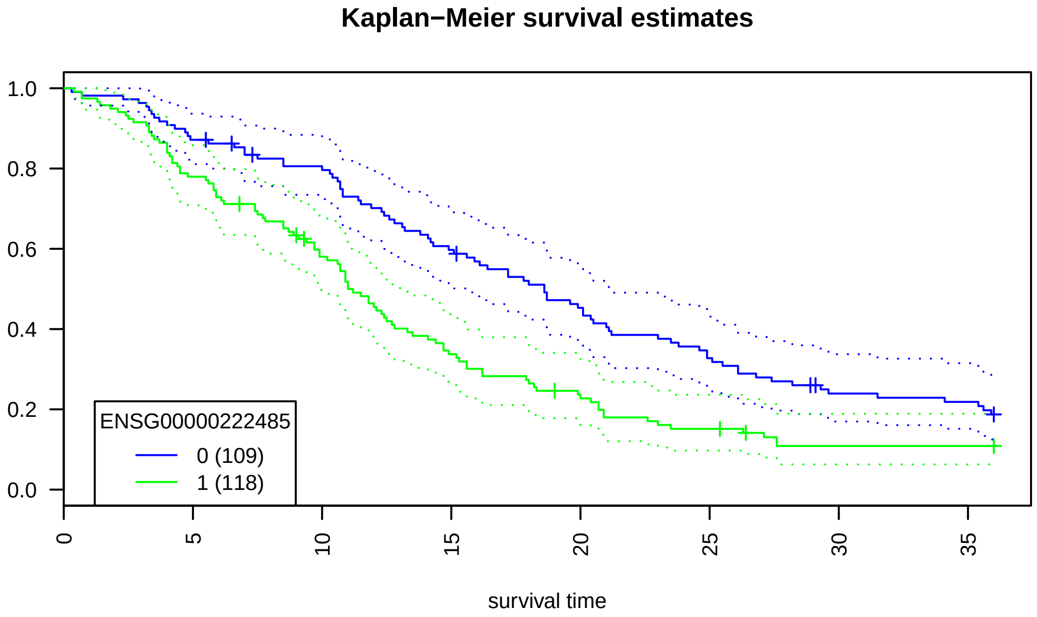

Figure 1847: Each curve represents the relative number of ENSG00000222485 survived to the corresponding point of time. Dashed lines represent 0.95 confidence intervals for the curves with the corresponding color. Total of 3 observations deleted due to missingness. Log-rank probability measure for the equality of these curves is 3.597213e-04.

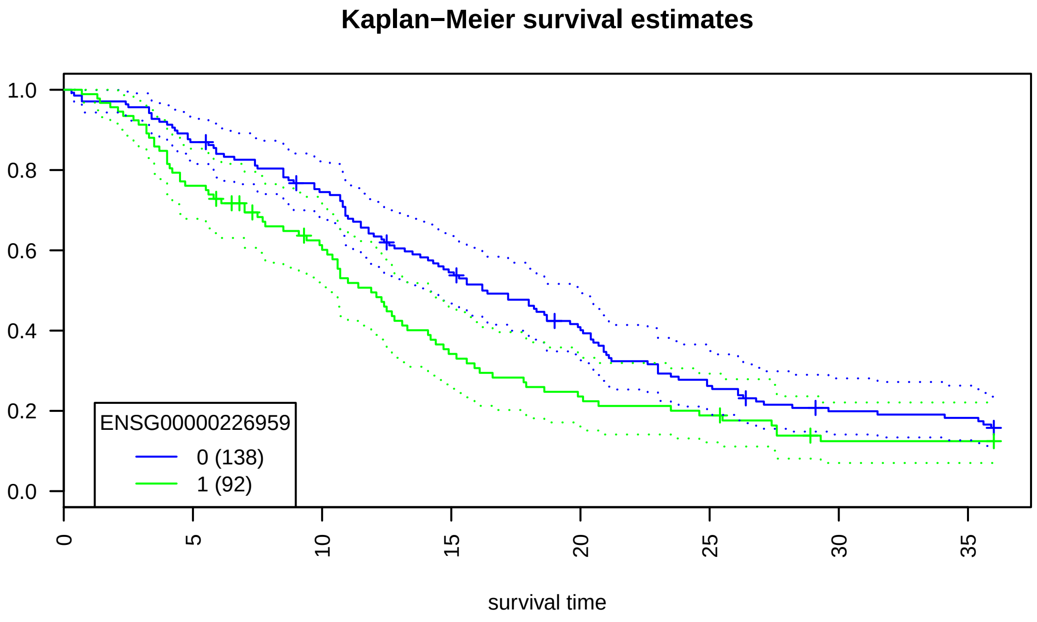

Figure 1848: Each curve represents the relative number of ENSG00000226959 survived to the corresponding point of time. Dashed lines represent 0.95 confidence intervals for the curves with the corresponding color. Log-rank probability measure for the equality of these curves is 2.174921e-02. Additional annotations: *.GeneName*=AC025423.2, *.GeneDesc*=cDNA, FLJ92387 [Source:UniProtKB/TrEMBL;Acc:B2R594].

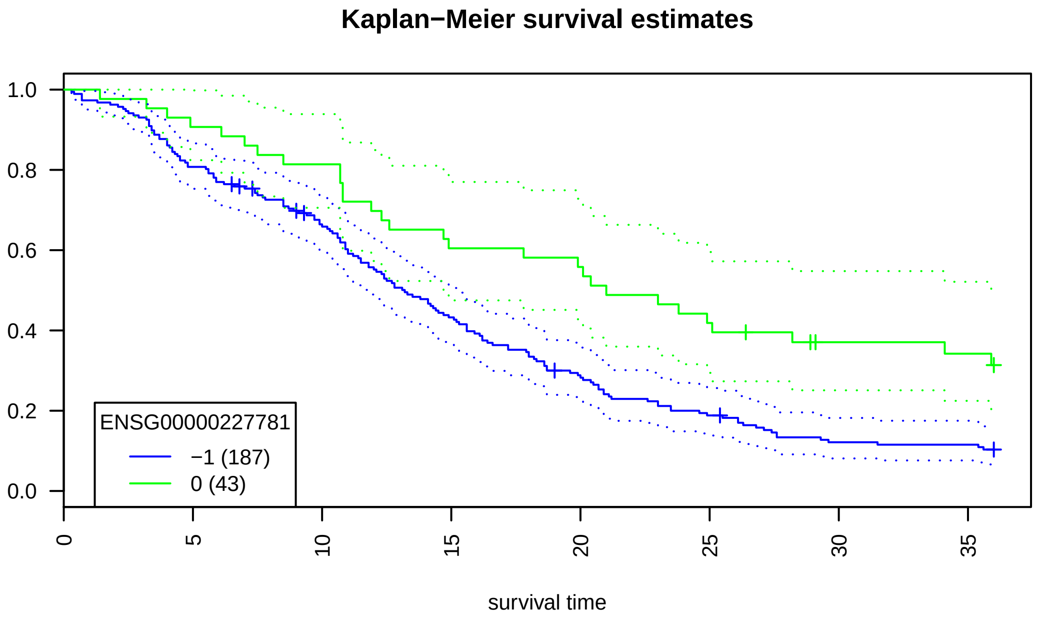

Figure 1849: Each curve represents the relative number of ENSG00000227781 survived to the corresponding point of time. Dashed lines represent 0.95 confidence intervals for the curves with the corresponding color. Log-rank probability measure for the equality of these curves is 7.690807e-04. Additional annotations: *.GeneName*=RP11-508M1.5, *.GeneDesc*=[undefined].

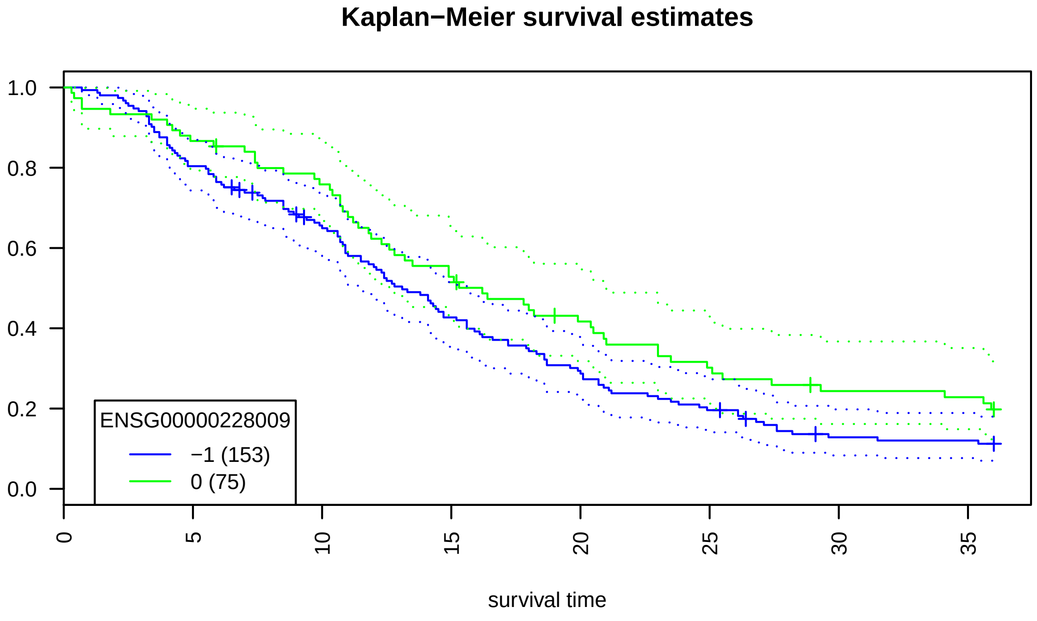

Figure 1850: Each curve represents the relative number of ENSG00000228009 survived to the corresponding point of time. Dashed lines represent 0.95 confidence intervals for the curves with the corresponding color. Total of 2 observations deleted due to missingness. Log-rank probability measure for the equality of these curves is 4.76141e-02. Additional annotations: *.GeneName*=RP11-756A22.7, *.GeneDesc*=[undefined].

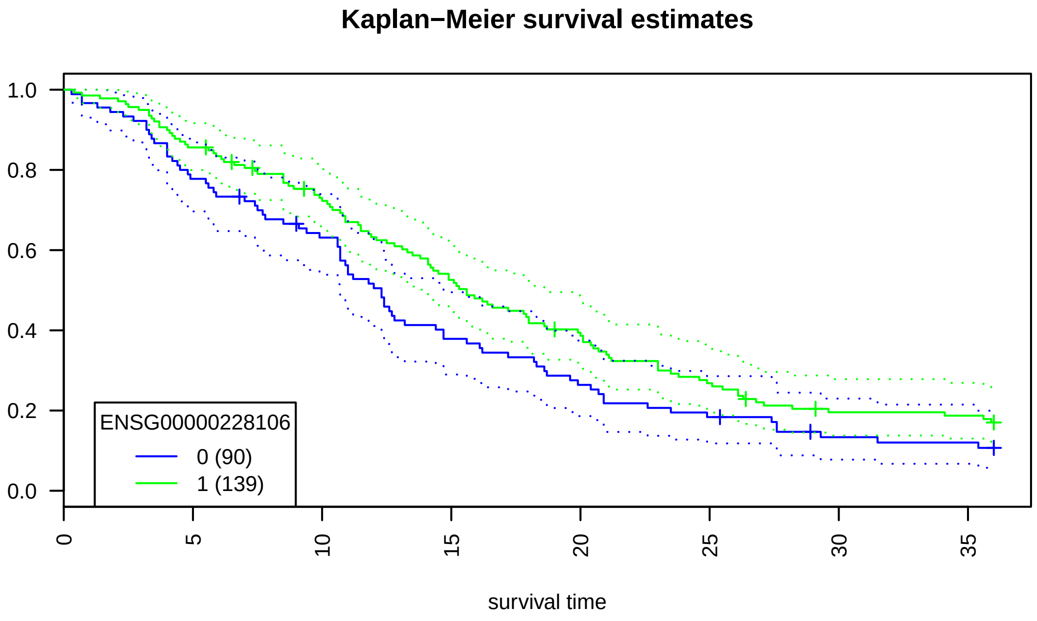

Figure 1851: Each curve represents the relative number of ENSG00000228106 survived to the corresponding point of time. Dashed lines represent 0.95 confidence intervals for the curves with the corresponding color. Total of 1 observation deleted due to missingness. Log-rank probability measure for the equality of these curves is 4.557913e-02. Additional annotations: *.GeneName*=RP11-452F19.3, *.GeneDesc*=[undefined].

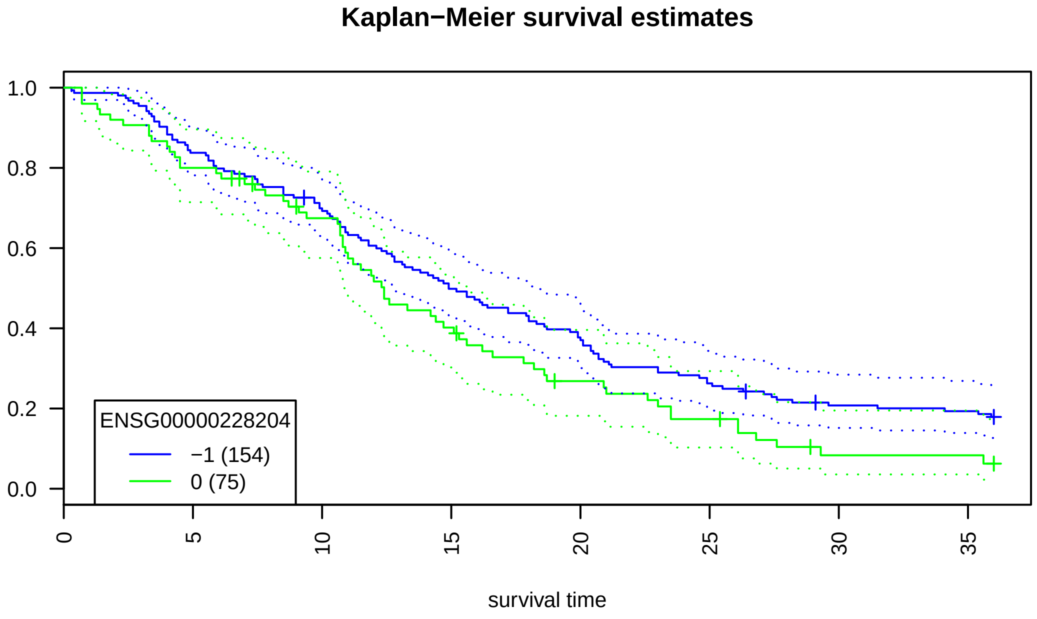

Figure 1852: Each curve represents the relative number of ENSG00000228204 survived to the corresponding point of time. Dashed lines represent 0.95 confidence intervals for the curves with the corresponding color. Total of 1 observation deleted due to missingness. Log-rank probability measure for the equality of these curves is 3.973413e-02. Additional annotations: *.GeneName*=RP4-724E13.2, *.GeneDesc*=[undefined].

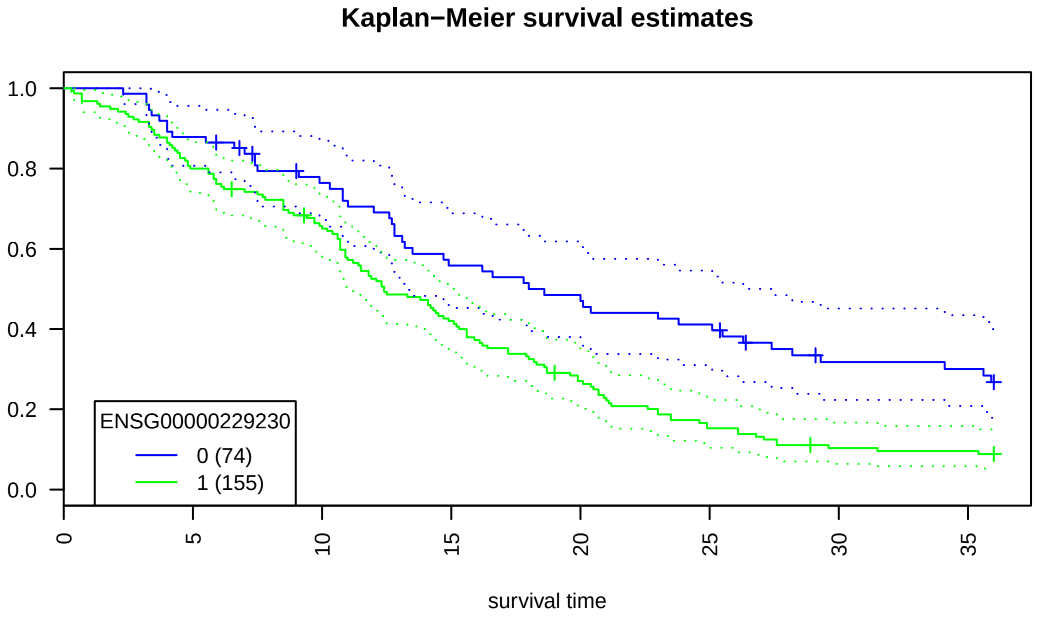

Figure 1853: Each curve represents the relative number of ENSG00000229230 survived to the corresponding point of time. Dashed lines represent 0.95 confidence intervals for the curves with the corresponding color. Total of 1 observation deleted due to missingness. Log-rank probability measure for the equality of these curves is 3.234496e-04. Additional annotations: *.GeneName*=C20orf127, *.GeneDesc*=[undefined].

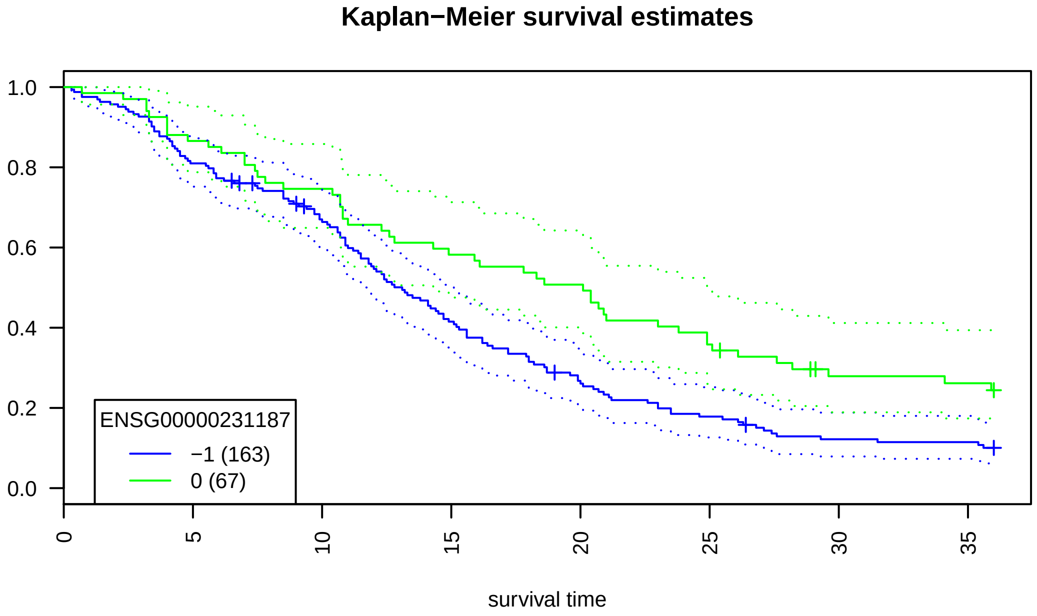

Figure 1854: Each curve represents the relative number of ENSG00000231187 survived to the corresponding point of time. Dashed lines represent 0.95 confidence intervals for the curves with the corresponding color. Log-rank probability measure for the equality of these curves is 2.765743e-03. Additional annotations: *.GeneName*=RP11-38L15.3, *.GeneDesc*=[undefined].

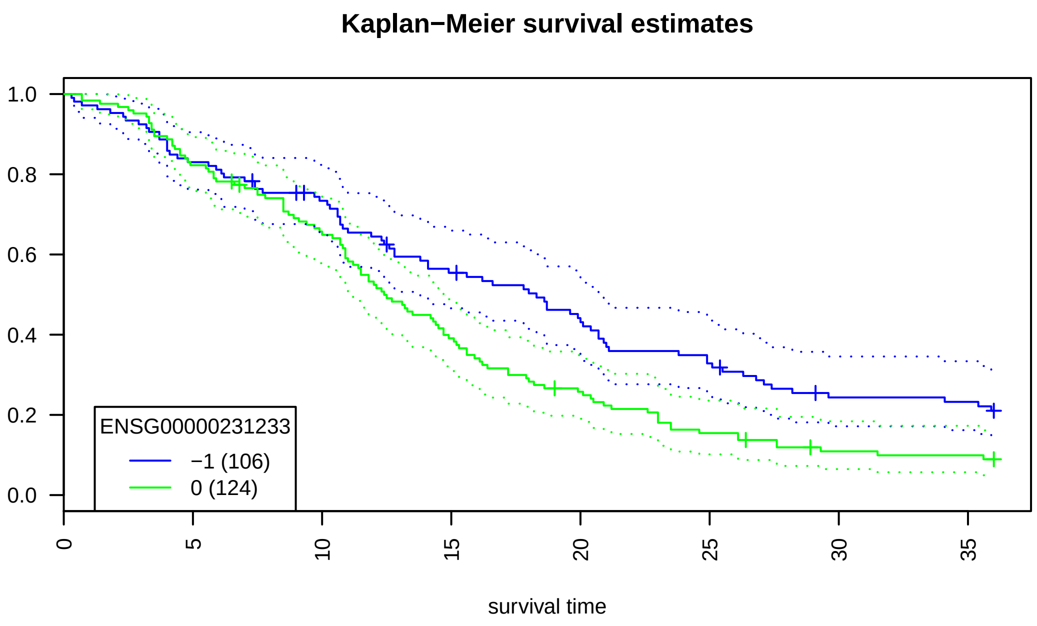

Figure 1855: Each curve represents the relative number of ENSG00000231233 survived to the corresponding point of time. Dashed lines represent 0.95 confidence intervals for the curves with the corresponding color. Log-rank probability measure for the equality of these curves is 3.970294e-03. Additional annotations: *.GeneName*=RP11-127L20.6, *.GeneDesc*=[undefined].

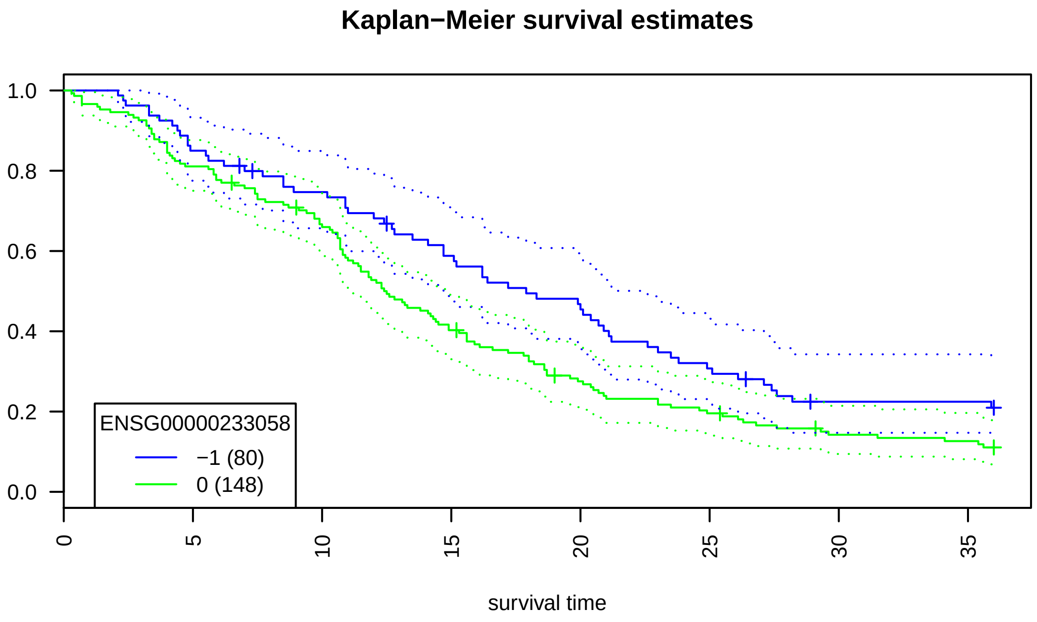

Figure 1856: Each curve represents the relative number of ENSG00000233058 survived to the corresponding point of time. Dashed lines represent 0.95 confidence intervals for the curves with the corresponding color. Total of 2 observations deleted due to missingness. Log-rank probability measure for the equality of these curves is 1.454171e-02. Additional annotations: *.GeneName*=AC046143.7, *.GeneDesc*=[undefined].

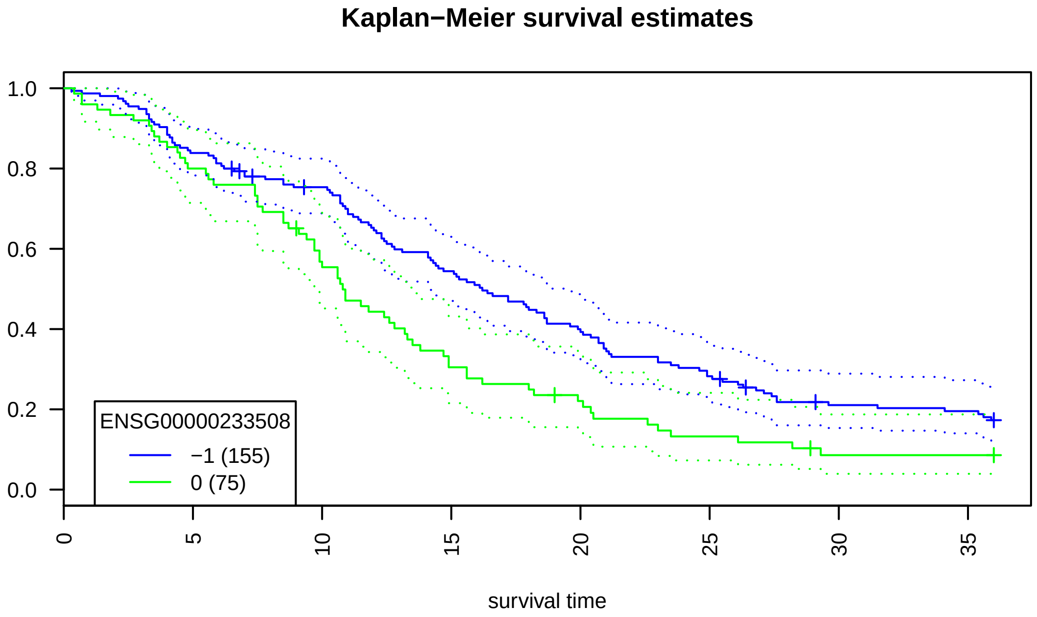

Figure 1857: Each curve represents the relative number of ENSG00000233508 survived to the corresponding point of time. Dashed lines represent 0.95 confidence intervals for the curves with the corresponding color. Log-rank probability measure for the equality of these curves is 2.051362e-03. Additional annotations: *.GeneName*=RP1-269M15.3, *.GeneDesc*=[undefined].

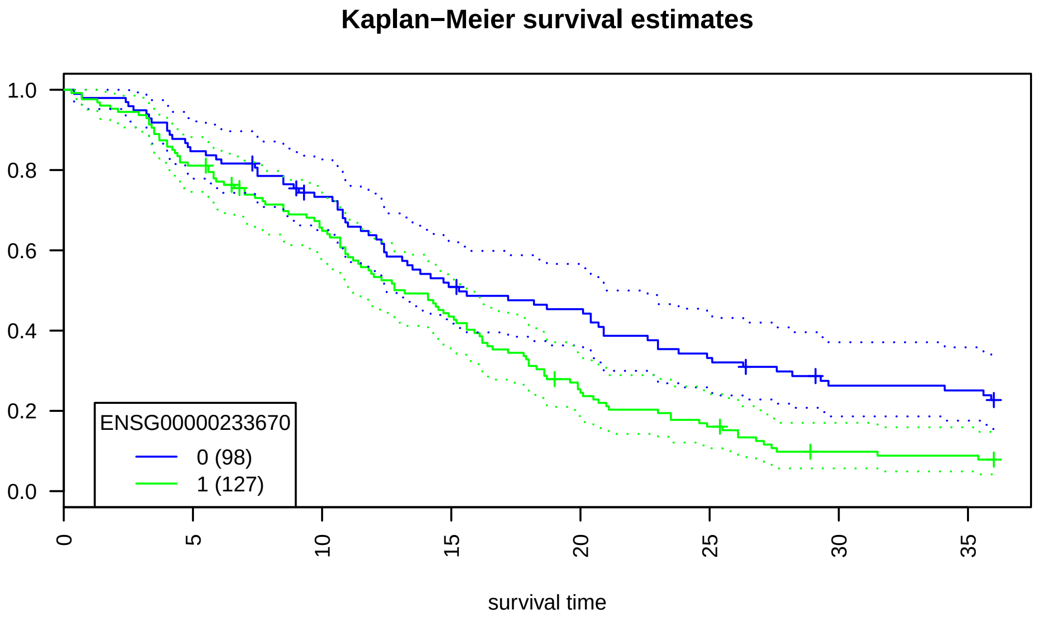

Figure 1858: Each curve represents the relative number of ENSG00000233670 survived to the corresponding point of time. Dashed lines represent 0.95 confidence intervals for the curves with the corresponding color. Total of 5 observations deleted due to missingness. Log-rank probability measure for the equality of these curves is 2.457165e-03. Additional annotations: *.GeneName*=AC015908.1, *.GeneDesc*=Phosphoinositide-interacting protein [Source:UniProtKB/Swiss-Prot;Acc:P0C851].

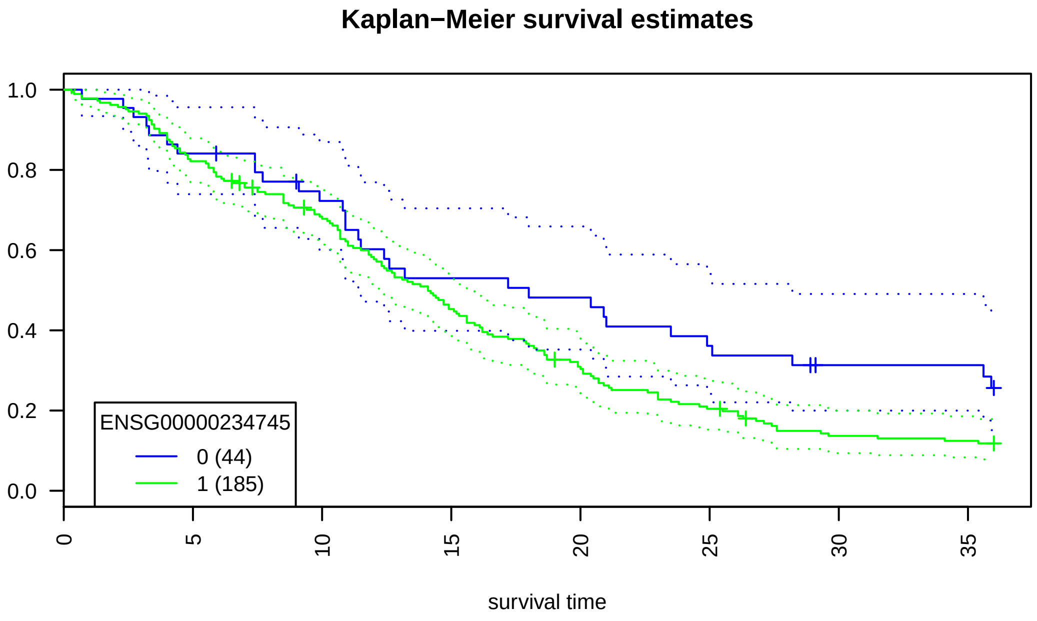

Figure 1859: Each curve represents the relative number of ENSG00000234745 survived to the corresponding point of time. Dashed lines represent 0.95 confidence intervals for the curves with the corresponding color. Total of 1 observation deleted due to missingness. Log-rank probability measure for the equality of these curves is 4.190537e-02. Additional annotations: *.GeneName*=HLA-B, *.GeneDesc*=HLA class I histocompatibility antigen, B-48 alpha chain Precursor (MHC class I antigen B*48)(Bw-48) [Source:UniProtKB/Swiss-Prot;Acc:P30486].

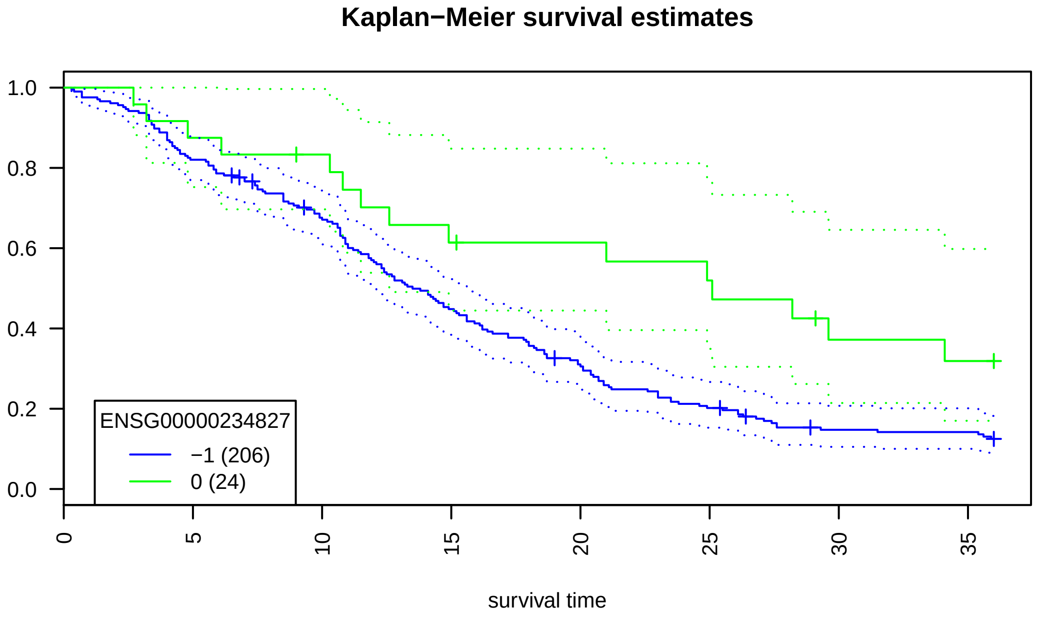

Figure 1860: Each curve represents the relative number of ENSG00000234827 survived to the corresponding point of time. Dashed lines represent 0.95 confidence intervals for the curves with the corresponding color. Log-rank probability measure for the equality of these curves is 1.212771e-02.

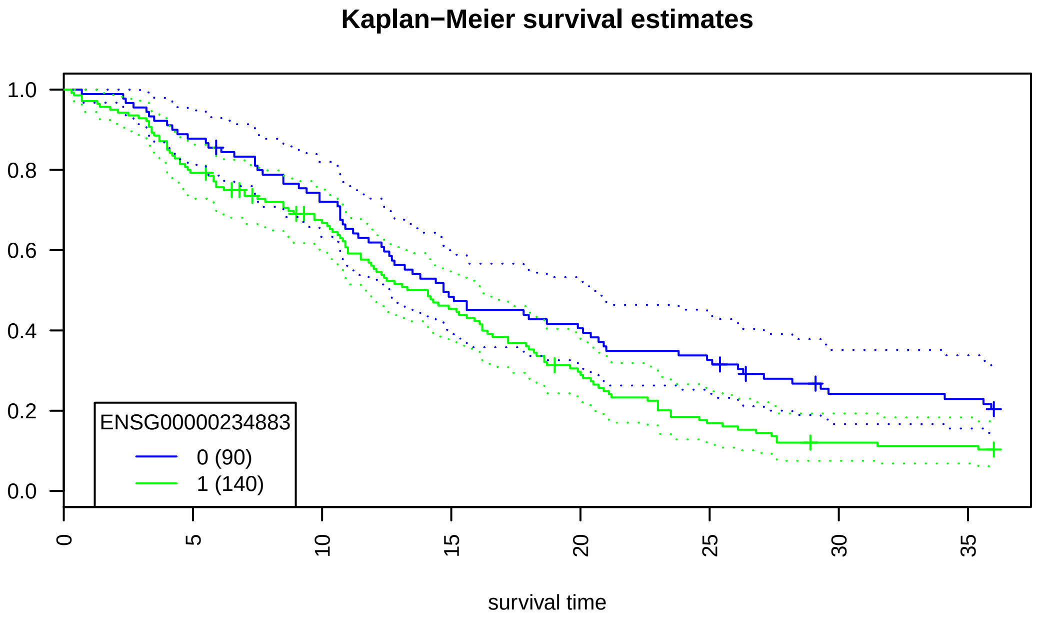

Figure 1861: Each curve represents the relative number of ENSG00000234883 survived to the corresponding point of time. Dashed lines represent 0.95 confidence intervals for the curves with the corresponding color. Log-rank probability measure for the equality of these curves is 3.022319e-02. Additional annotations: *.GeneName*=MIR155HG, *.GeneDesc*=microRNA 155 [Source:HGNC Symbol;Acc:31542].

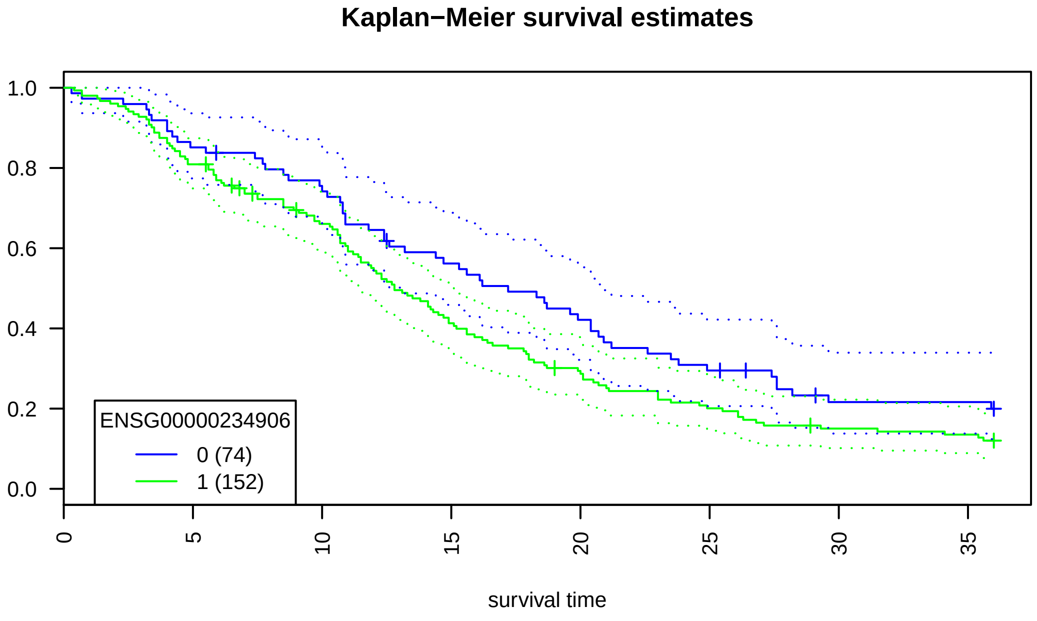

Figure 1862: Each curve represents the relative number of ENSG00000234906 survived to the corresponding point of time. Dashed lines represent 0.95 confidence intervals for the curves with the corresponding color. Total of 4 observations deleted due to missingness. Log-rank probability measure for the equality of these curves is 4.58449e-02. Additional annotations: *.GeneName*=APOC2, *.GeneDesc*=apolipoprotein C-II [Source:HGNC Symbol;Acc:609].

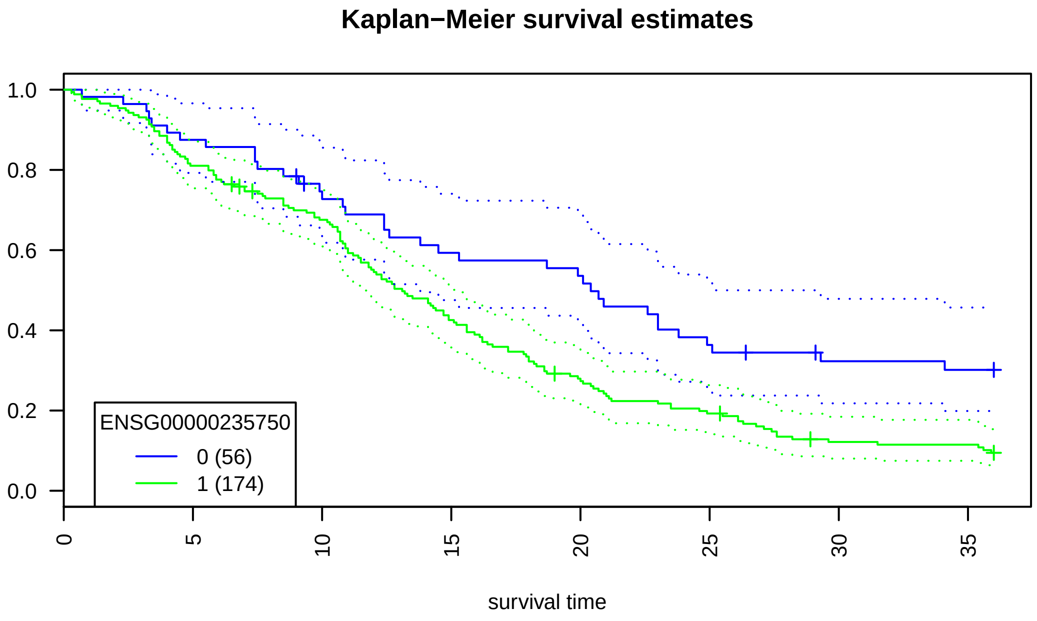

Figure 1863: Each curve represents the relative number of ENSG00000235750 survived to the corresponding point of time. Dashed lines represent 0.95 confidence intervals for the curves with the corresponding color. Log-rank probability measure for the equality of these curves is 1.142621e-03. Additional annotations: *.GeneName*=KIAA0040, *.GeneDesc*=KIAA0040 [Source:HGNC Symbol;Acc:28950].

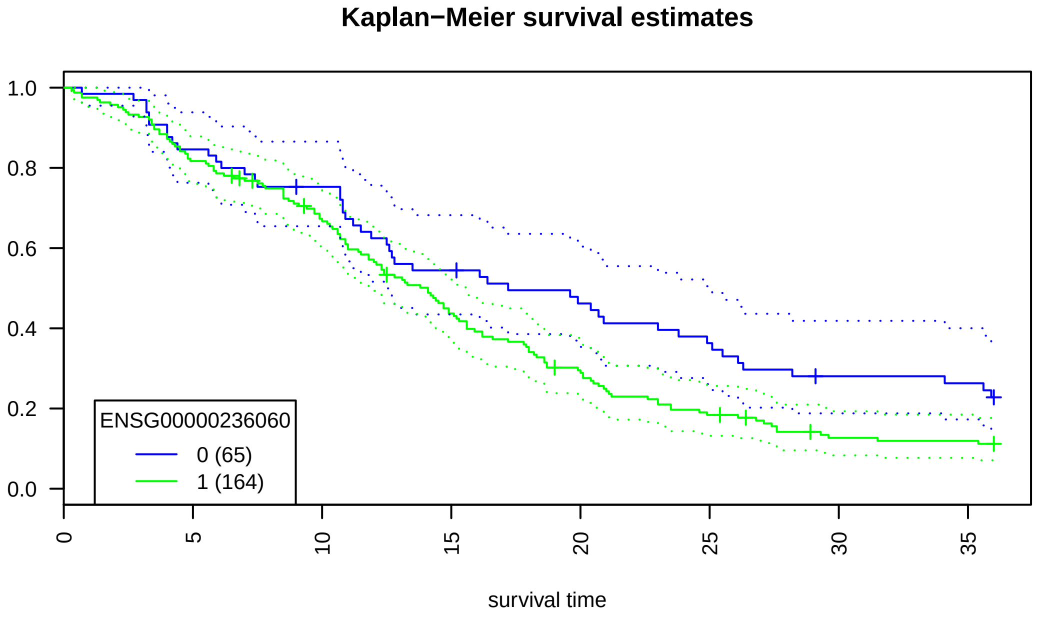

Figure 1864: Each curve represents the relative number of ENSG00000236060 survived to the corresponding point of time. Dashed lines represent 0.95 confidence intervals for the curves with the corresponding color. Total of 1 observation deleted due to missingness. Log-rank probability measure for the equality of these curves is 2.122252e-02. Additional annotations: *.GeneName*=RP11-346E17.4, *.GeneDesc*=[undefined].

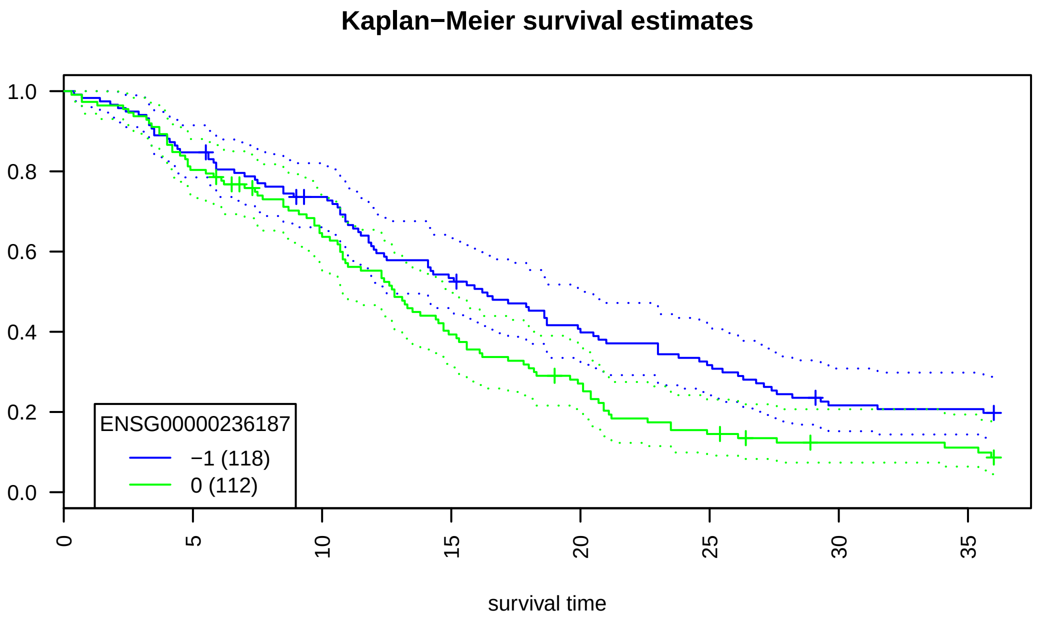

Figure 1865: Each curve represents the relative number of ENSG00000236187 survived to the corresponding point of time. Dashed lines represent 0.95 confidence intervals for the curves with the corresponding color. Log-rank probability measure for the equality of these curves is 8.914662e-03. Additional annotations: *.GeneName*=RP1-245G19.2, *.GeneDesc*=[undefined].

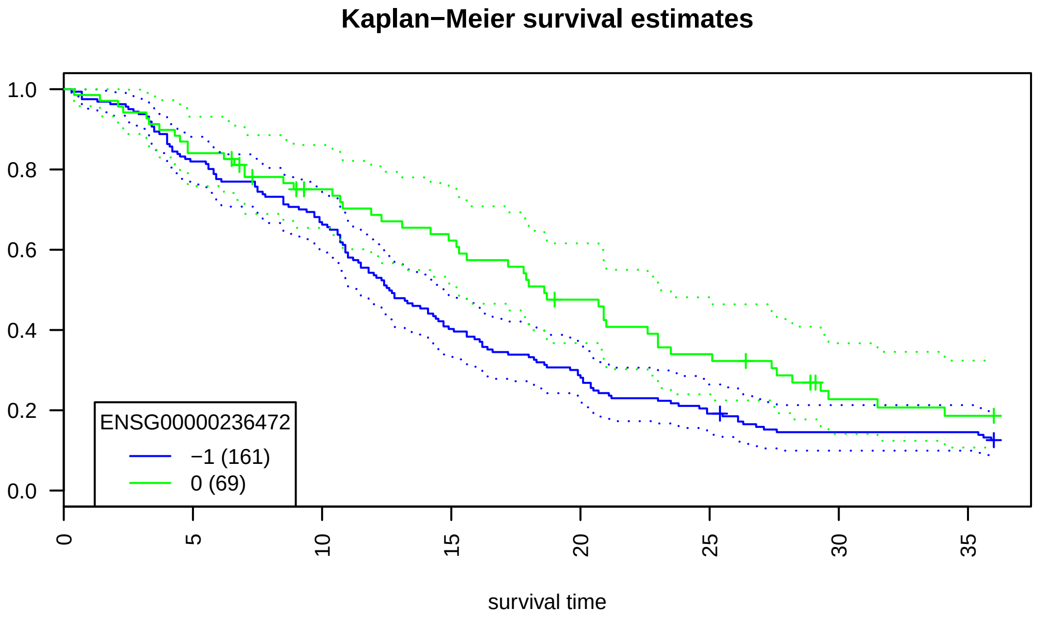

Figure 1866: Each curve represents the relative number of ENSG00000236472 survived to the corresponding point of time. Dashed lines represent 0.95 confidence intervals for the curves with the corresponding color. Log-rank probability measure for the equality of these curves is 2.642495e-02. Additional annotations: *.GeneName*=AC002401.1, *.GeneDesc*=[undefined].

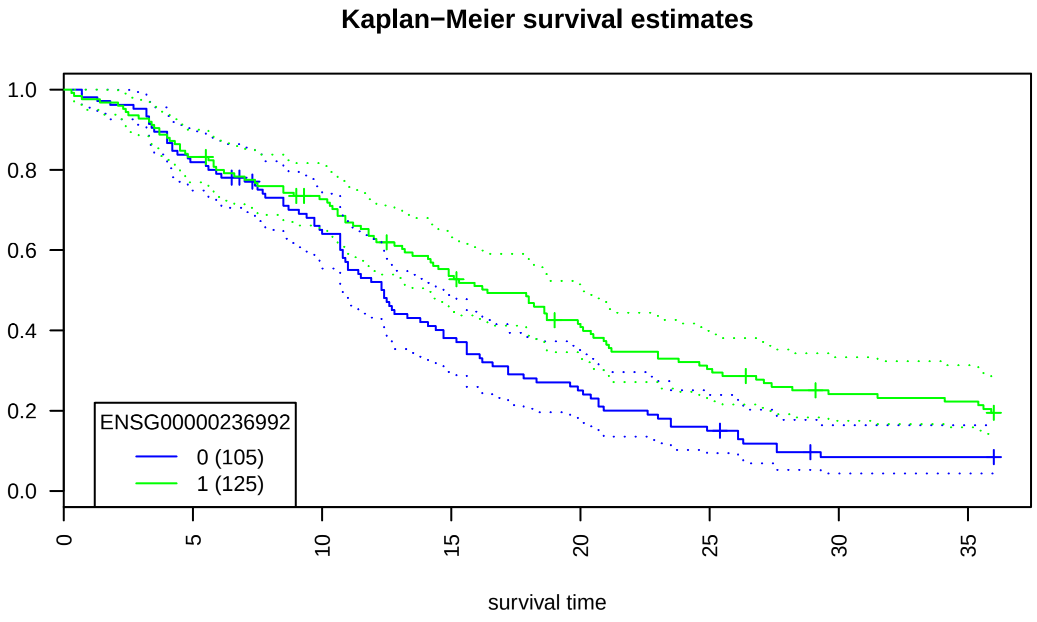

Figure 1867: Each curve represents the relative number of ENSG00000236992 survived to the corresponding point of time. Dashed lines represent 0.95 confidence intervals for the curves with the corresponding color. Log-rank probability measure for the equality of these curves is 4.338715e-03. Additional annotations: *.GeneName*=RPL12L3, *.GeneDesc*=[undefined].

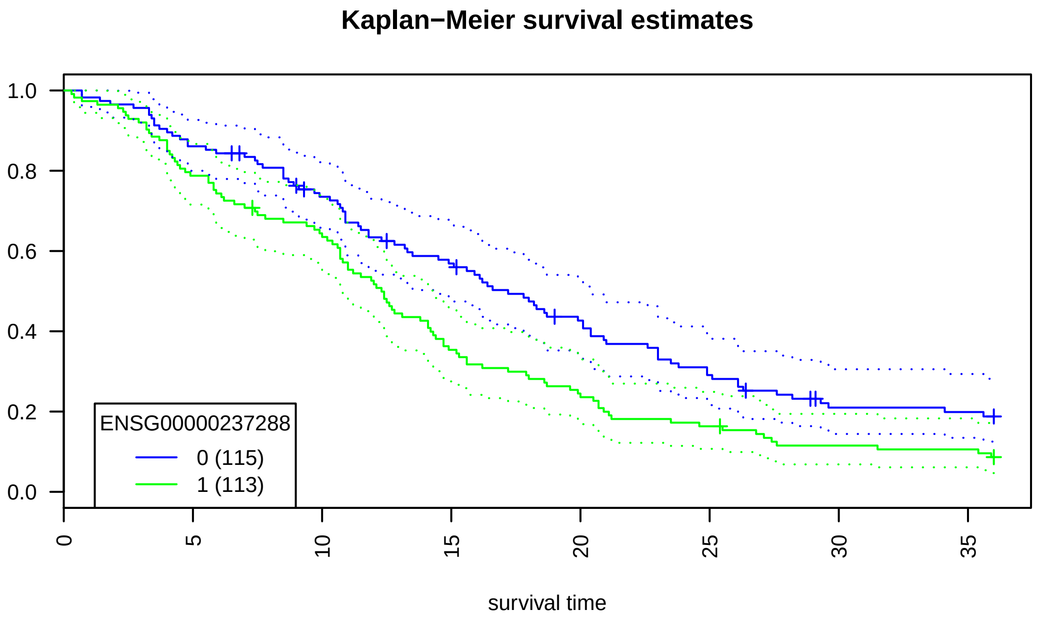

Figure 1868: Each curve represents the relative number of ENSG00000237288 survived to the corresponding point of time. Dashed lines represent 0.95 confidence intervals for the curves with the corresponding color. Total of 2 observations deleted due to missingness. Log-rank probability measure for the equality of these curves is 2.826276e-03. Additional annotations: *.GeneName*=AC133644.2, *.GeneDesc*=[undefined].

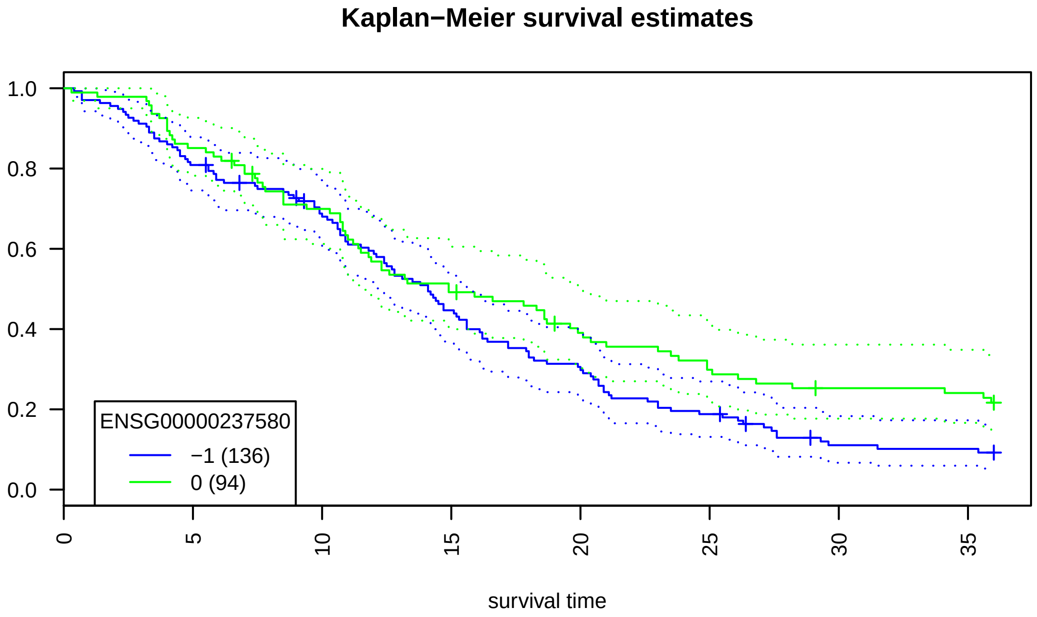

Figure 1869: Each curve represents the relative number of ENSG00000237580 survived to the corresponding point of time. Dashed lines represent 0.95 confidence intervals for the curves with the corresponding color. Log-rank probability measure for the equality of these curves is 3.791588e-02. Additional annotations: *.GeneName*=AC007383.5, *.GeneDesc*=[undefined].

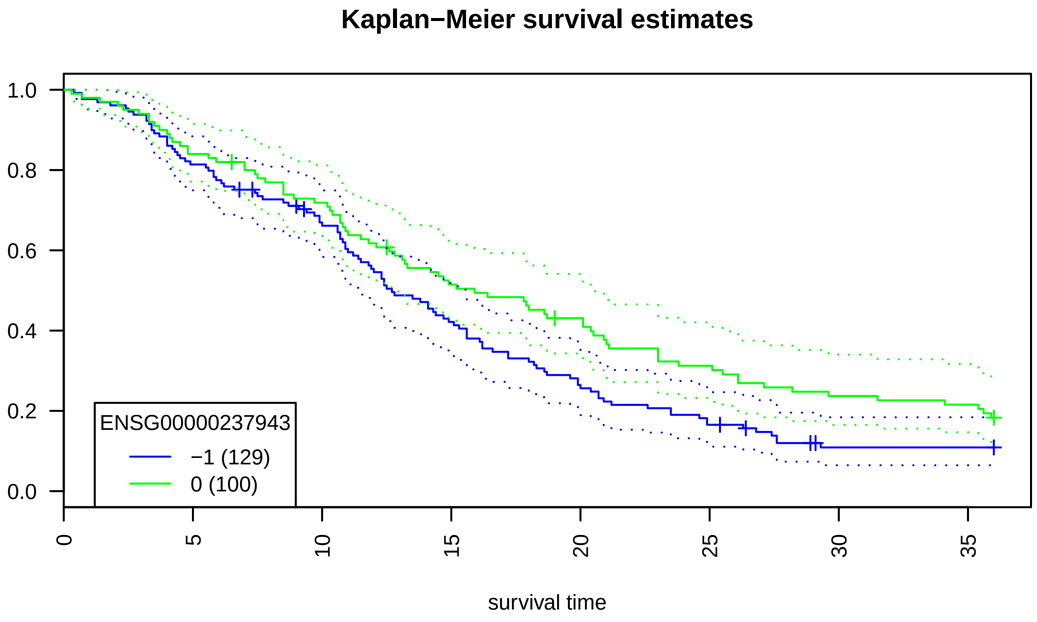

Figure 1870: Each curve represents the relative number of ENSG00000237943 survived to the corresponding point of time. Dashed lines represent 0.95 confidence intervals for the curves with the corresponding color. Total of 1 observation deleted due to missingness. Log-rank probability measure for the equality of these curves is 3.397386e-02. Additional annotations: *.GeneName*=RP11-5N23.2, *.GeneDesc*=[undefined].

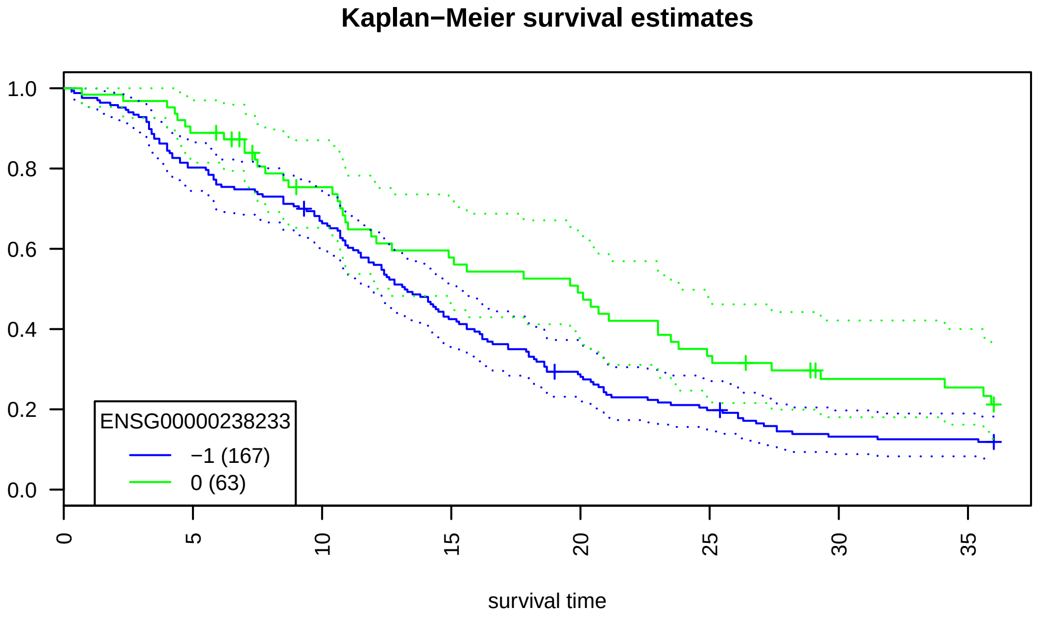

Figure 1871: Each curve represents the relative number of ENSG00000238233 survived to the corresponding point of time. Dashed lines represent 0.95 confidence intervals for the curves with the corresponding color. Log-rank probability measure for the equality of these curves is 1.723802e-02.

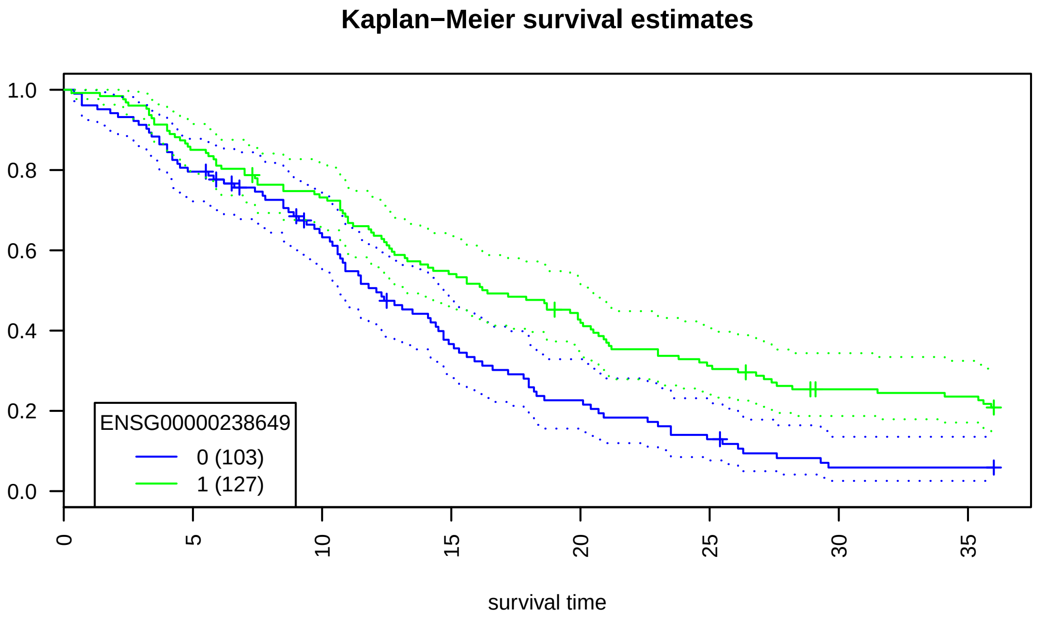

Figure 1872: Each curve represents the relative number of ENSG00000238649 survived to the corresponding point of time. Dashed lines represent 0.95 confidence intervals for the curves with the corresponding color. Log-rank probability measure for the equality of these curves is 3.055553e-04. Additional annotations: *.GeneName*=SNORD42A, *.GeneDesc*=small nucleolar RNA, C/D box 42A [Source:HGNC Symbol;Acc:10180].

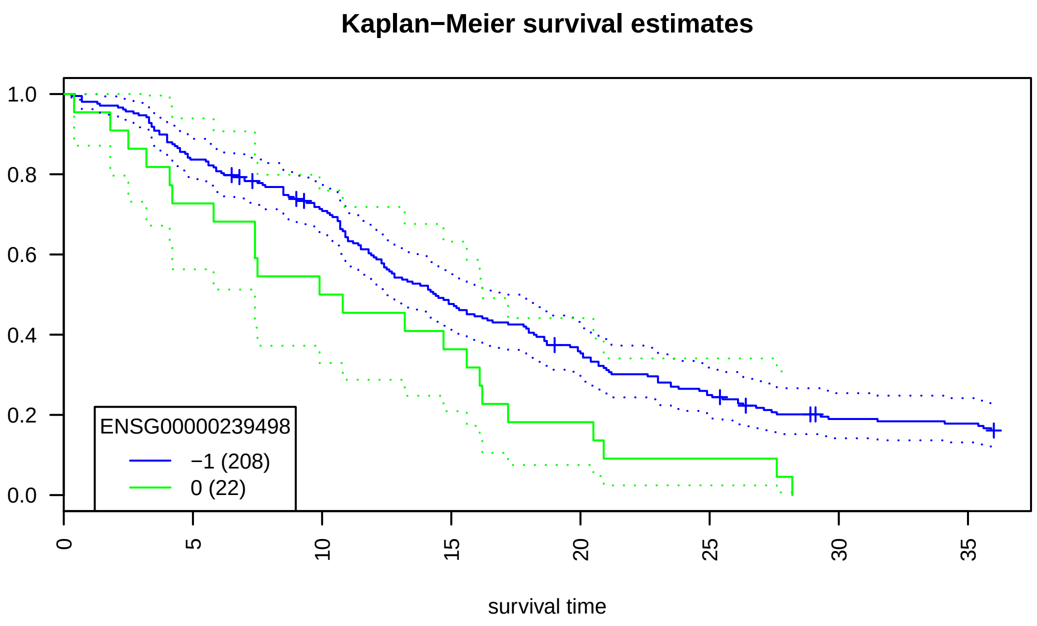

Figure 1873: Each curve represents the relative number of ENSG00000239498 survived to the corresponding point of time. Dashed lines represent 0.95 confidence intervals for the curves with the corresponding color. Log-rank probability measure for the equality of these curves is 7.678046e-03. Additional annotations: .*GeneName*=AC114765.1, .*GeneDesc*=[undefined].

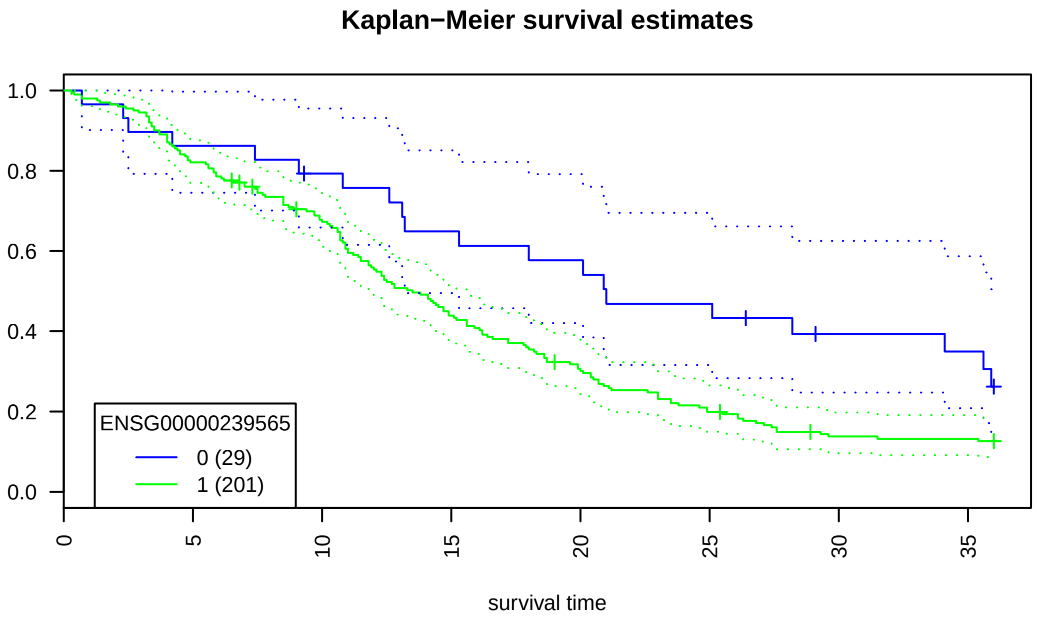

Figure 1874: Each curve represents the relative number of ENSG00000239565 survived to the corresponding point of time. Dashed lines represent 0.95 confidence intervals for the curves with the corresponding color. Log-rank probability measure for the equality of these curves is 1.622247e-02. Additional annotations: .*GeneName*=AP000280.66, .*GeneDesc*=[undefined].

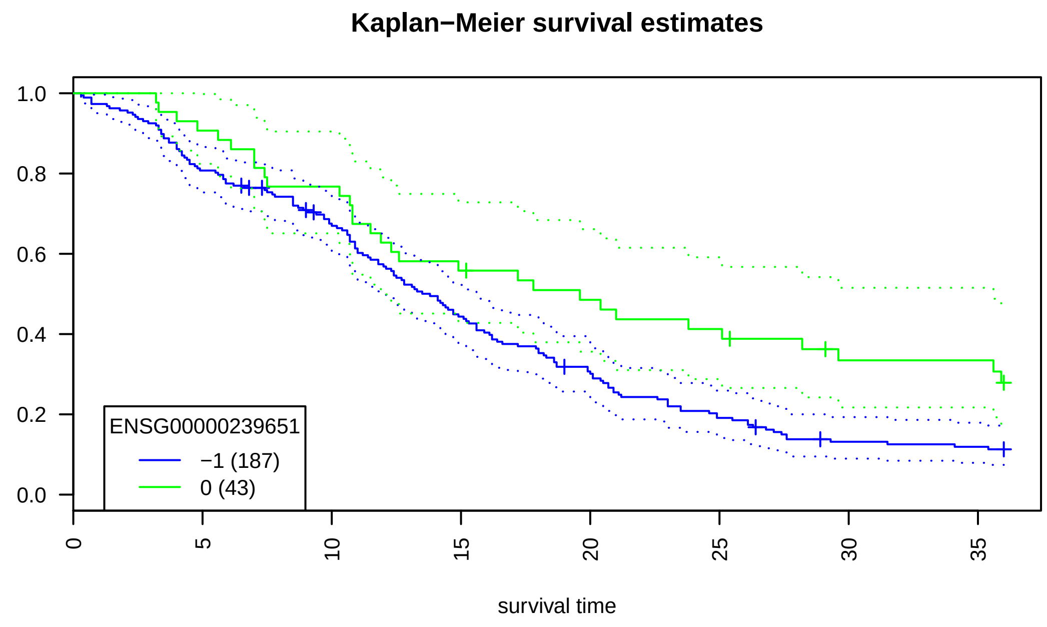

Figure 1875: Each curve represents the relative number of ENSG00000239651 survived to the corresponding point of time. Dashed lines represent 0.95 confidence intervals for the curves with the corresponding color. Log-rank probability measure for the equality of these curves is 9.321319e-03.

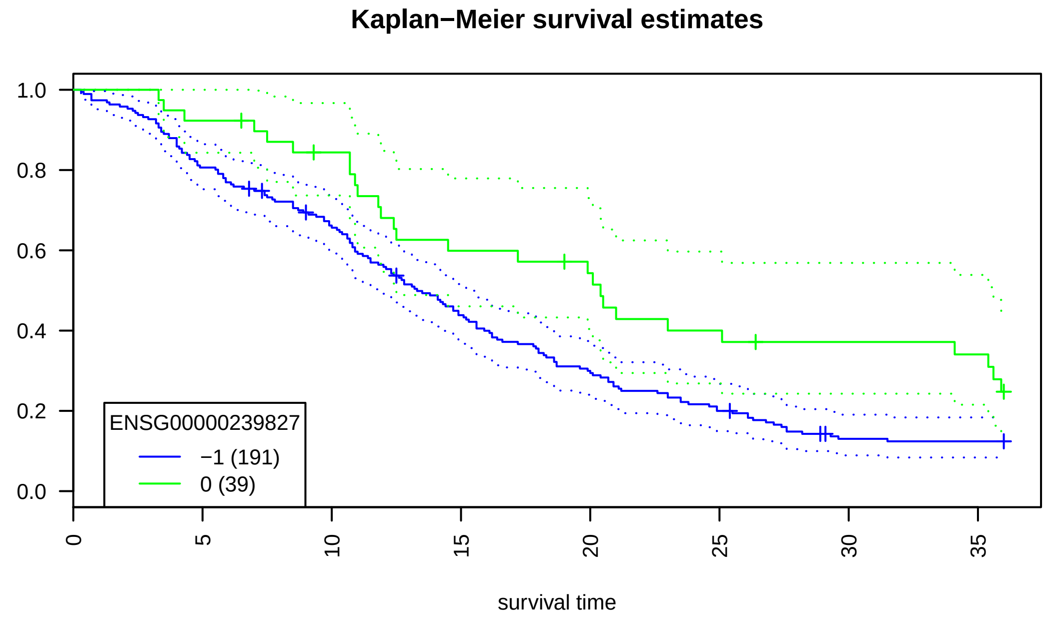

Figure 1876: Each curve represents the relative number of ENSG00000239827 survived to the corresponding point of time. Dashed lines represent 0.95 confidence intervals for the curves with the corresponding color. Log-rank probability measure for the equality of these curves is 1.120602e-02. Additional annotations: *.GeneName*=SUGT1L1, *.GeneDesc*=SGT1, suppressor of G2 allele of SKP1 like 1 (S. cerevisiae) [Source:HGNC Symbol;Acc:20513].

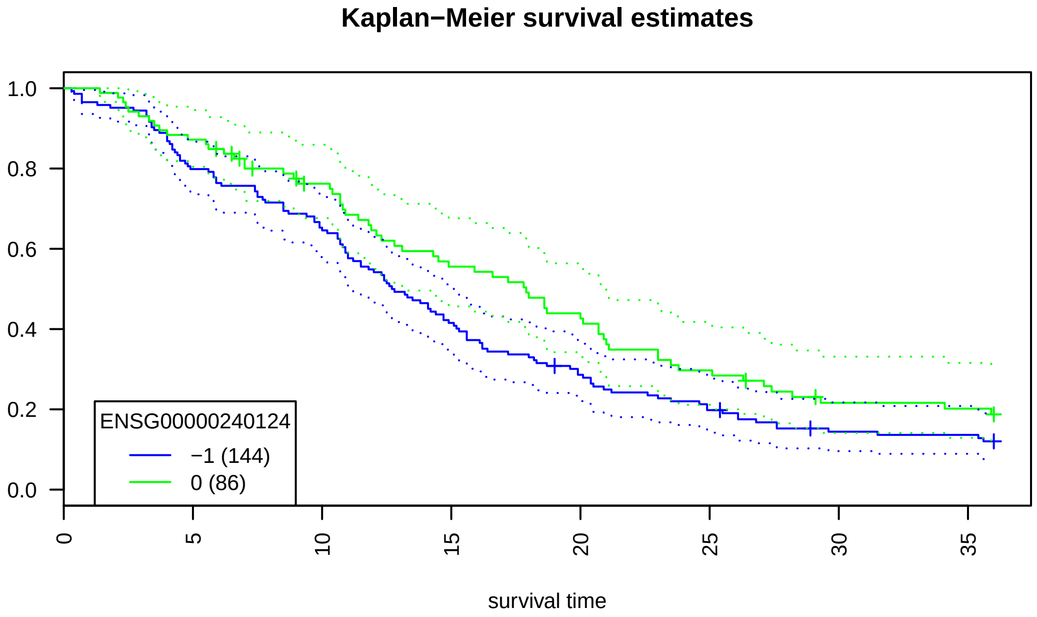

Figure 1877: Each curve represents the relative number of ENSG00000240124 survived to the corresponding point of time. Dashed lines represent 0.95 confidence intervals for the curves with the corresponding color. Log-rank probability measure for the equality of these curves is 4.619339e-02. Additional annotations: *.GeneName*=AL163636.2, *.GeneDesc*=[undefined].

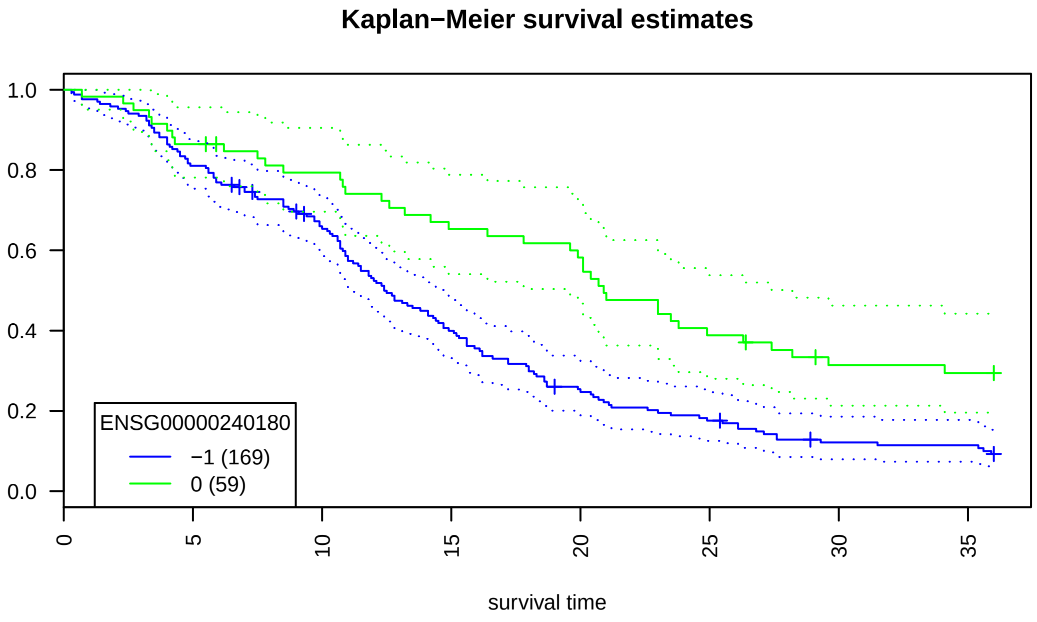

Figure 1878: Each curve represents the relative number of ENSG00000240180 survived to the corresponding point of time. Dashed lines represent 0.95 confidence intervals for the curves with the corresponding color. Total of 2 observations deleted due to missingness. Log-rank probability measure for the equality of these curves is 9.715046e-05. Additional annotations: *.GeneName*=RP11-318E3.4, *.GeneDesc*=[undefined].

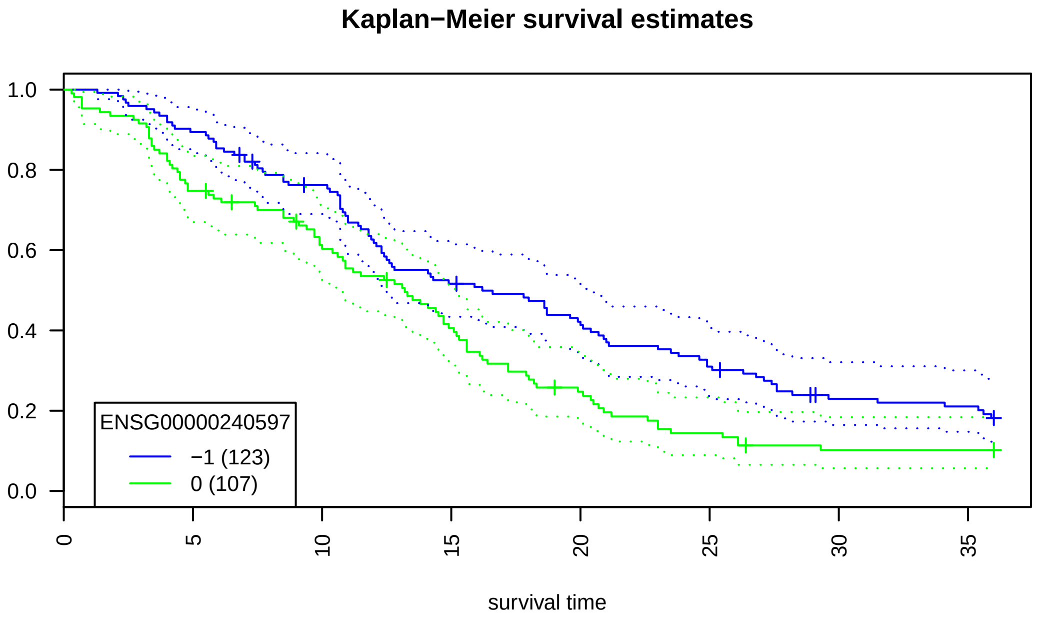

Figure 1879: Each curve represents the relative number of ENSG00000240597 survived to the corresponding point of time. Dashed lines represent 0.95 confidence intervals for the curves with the corresponding color. Log-rank probability measure for the equality of these curves is 4.21561e-03. Additional annotations: *.GeneName*=AL121932.1, *.GeneDesc*=[undefined].

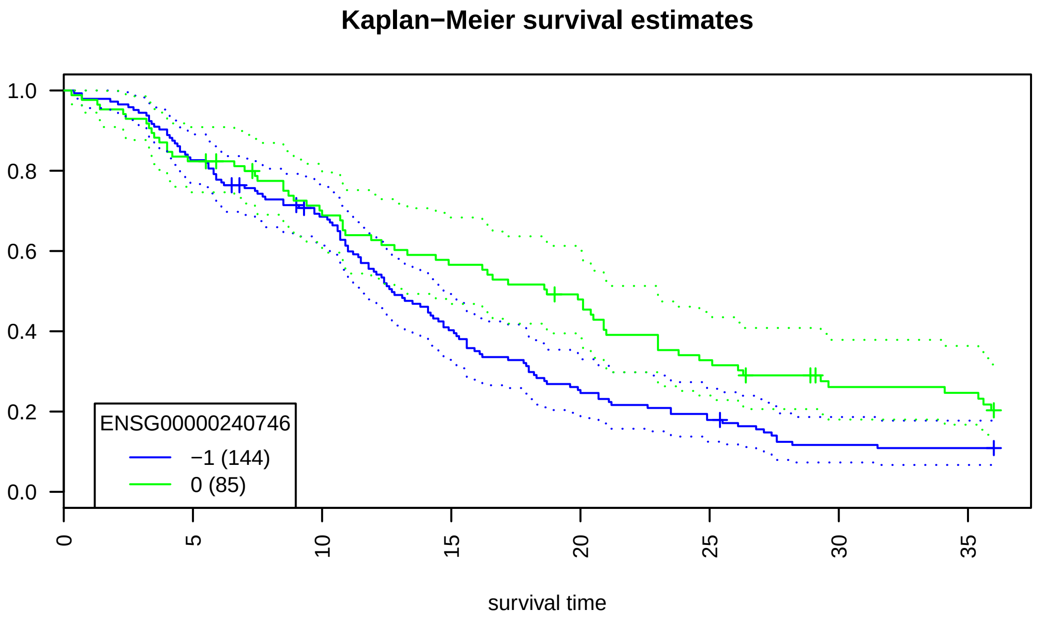

Figure 1880: Each curve represents the relative number of ENSG00000240746 survived to the corresponding point of time. Dashed lines represent 0.95 confidence intervals for the curves with the corresponding color. Total of 1 observation deleted due to missingness. Log-rank probability measure for the equality of these curves is 1.238118e-02. Additional annotations: *.GeneName*=AC026495.2, *.GeneDesc*=[undefined].

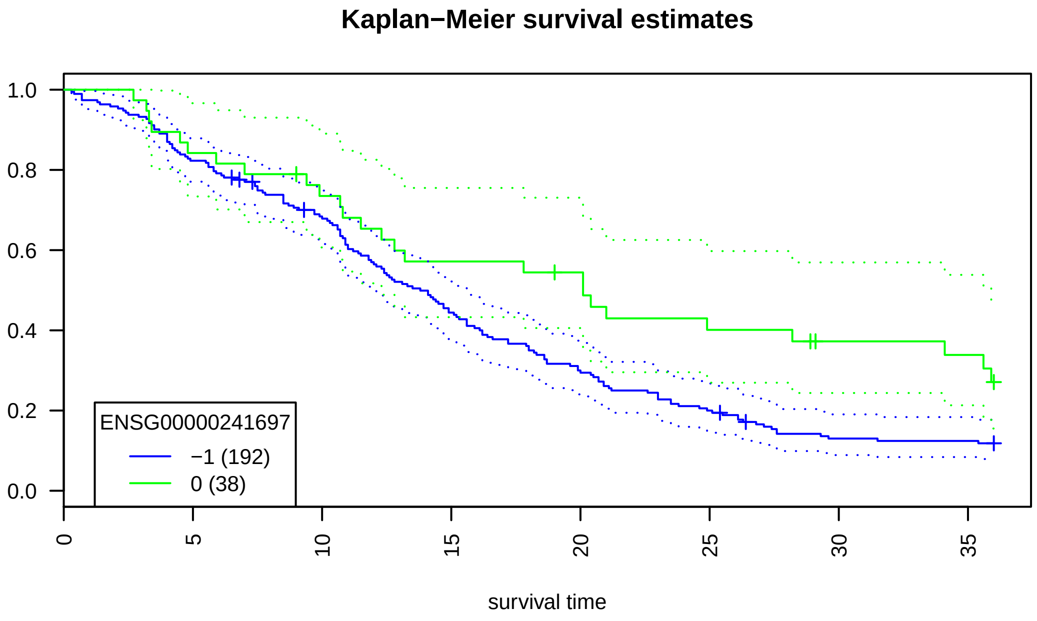

Figure 1881: Each curve represents the relative number of ENSG00000241697 survived to the corresponding point of time. Dashed lines represent 0.95 confidence intervals for the curves with the corresponding color. Log-rank probability measure for the equality of these curves is 1.534798e-02. Additional annotations: *.GeneName*=TMEFF1, *.GeneDesc*=transmembrane protein with EGF-like and two follistatin-like domains 1 [Source:HGNC Symbol;Acc:11866].

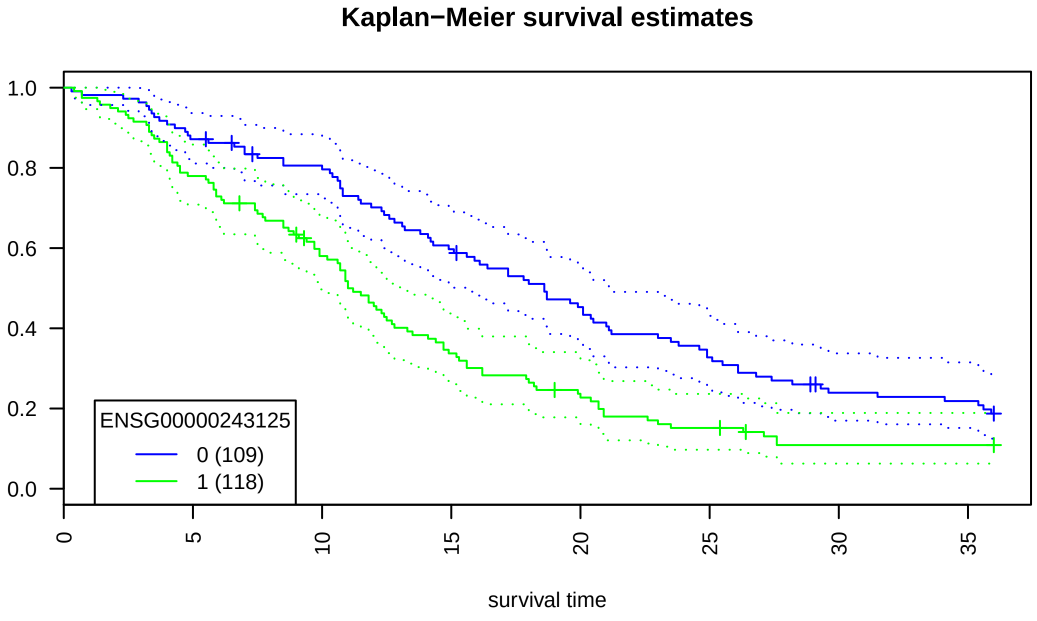

Figure 1882: Each curve represents the relative number of ENSG00000243125 survived to the corresponding point of time. Dashed lines represent 0.95 confidence intervals for the curves with the corresponding color. Total of 3 observations deleted due to missingness. Log-rank probability measure for the equality of these curves is 3.597213e-04. Additional annotations: *.GeneName*=AC068035.1, *.GeneDesc*=[undefined].

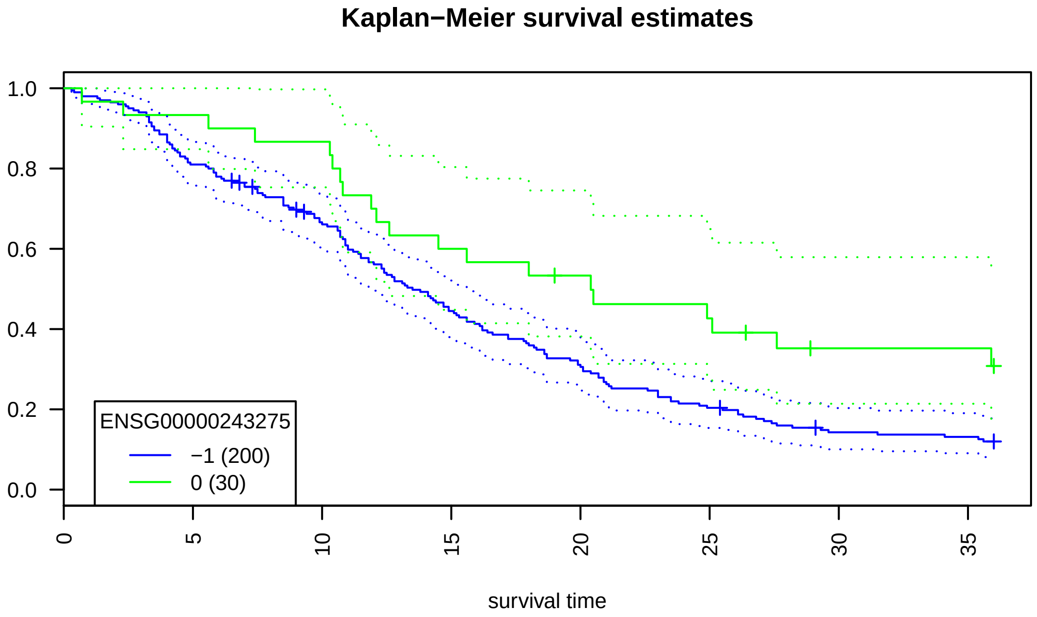

Figure 1883: Each curve represents the relative number of ENSG00000243275 survived to the corresponding point of time. Dashed lines represent 0.95 confidence intervals for the curves with the corresponding color. Log-rank probability measure for the equality of these curves is 1.306926e-02.

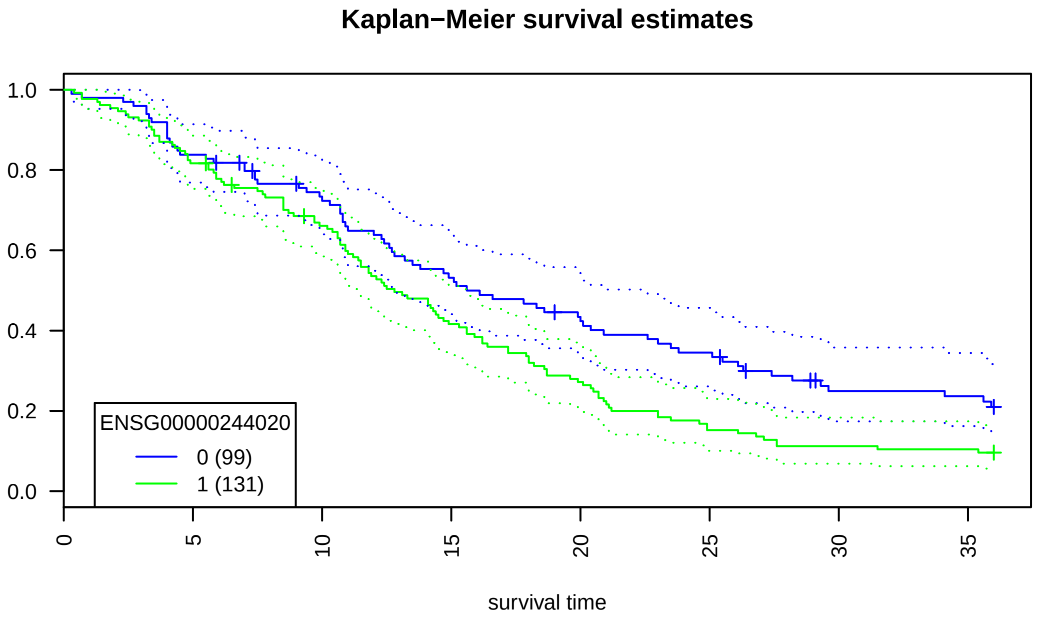

Figure 1884: Each curve represents the relative number of ENSG00000244020 survived to the corresponding point of time. Dashed lines represent 0.95 confidence intervals for the curves with the corresponding color. Log-rank probability measure for the equality of these curves is 7.684233e-03. Additional annotations: *.GeneName*=MT1P2, *.GeneDesc*=metallothionein 1 pseudogene 2 [Source:HGNC Symbol;Acc:31864].

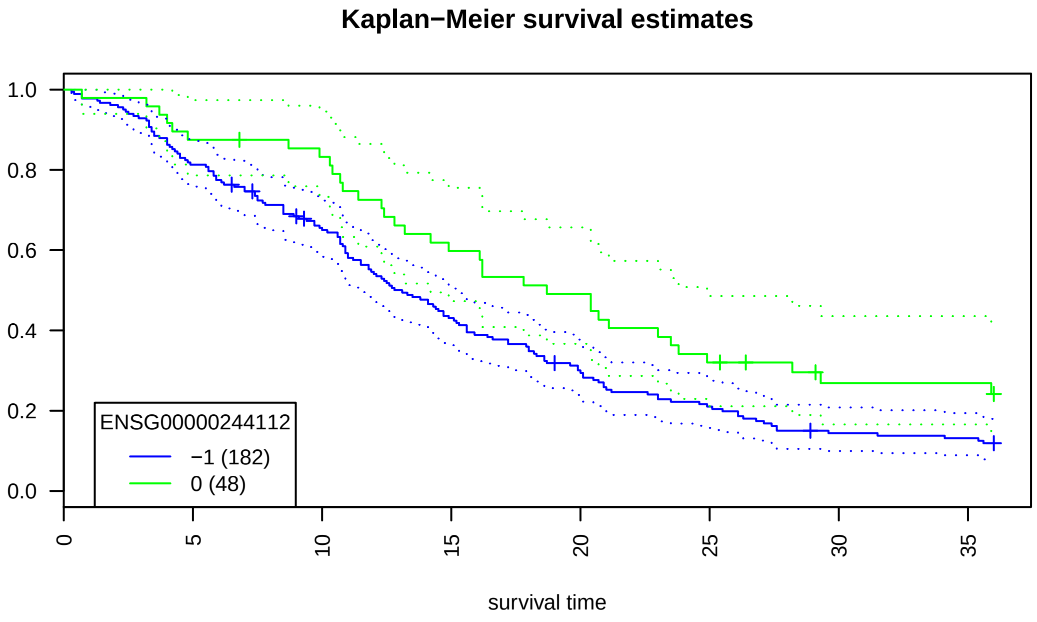

Figure 1885: Each curve represents the relative number of ENSG00000244112 survived to the corresponding point of time. Dashed lines represent 0.95 confidence intervals for the curves with the corresponding color. Log-rank probability measure for the equality of these curves is 1.483216e-02. Additional annotations: *.GeneName*=AC087885.1, *.GeneDesc*=[undefined].

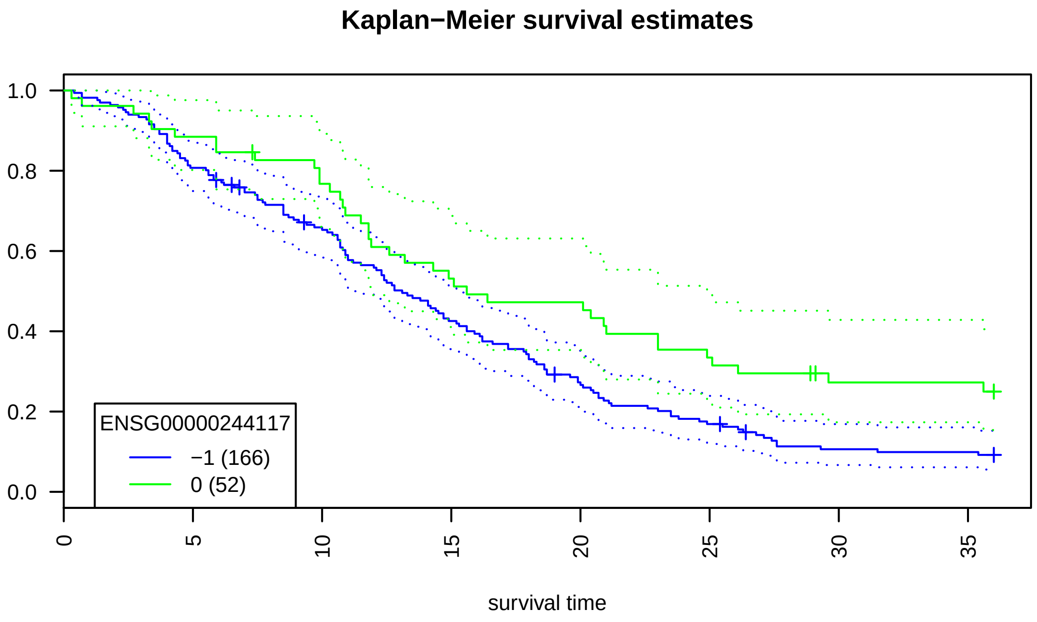

Figure 1886: Each curve represents the relative number of ENSG00000244117 survived to the corresponding point of time. Dashed lines represent 0.95 confidence intervals for the curves with the corresponding color. Total of 12 observations deleted due to missingness. Log-rank probability measure for the equality of these curves is 8.604916e-03. Additional annotations: *.GeneName*=RP11-3N2.1, *.GeneDesc*=[undefined].

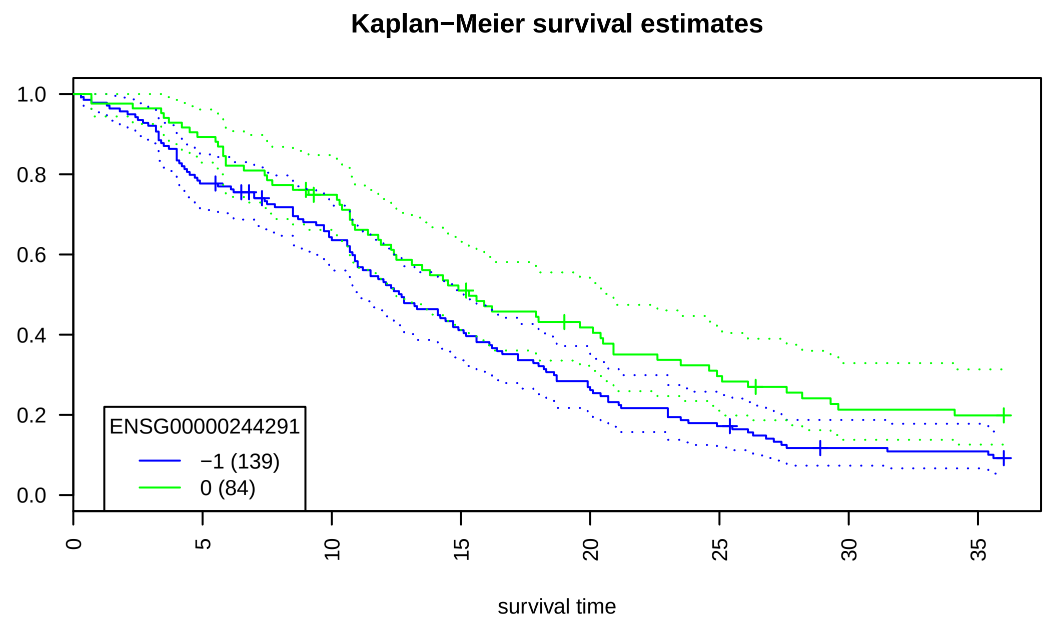

Figure 1887: Each curve represents the relative number of ENSG00000244291 survived to the corresponding point of time. Dashed lines represent 0.95 confidence intervals for the curves with the corresponding color. Total of 7 observations deleted due to missingness. Log-rank probability measure for the equality of these curves is 1.812737e-02. Additional annotations: *.GeneName*=C7orf13, *.GeneDesc*=Uncharacterized protein C7orf13 [Source:UniProtKB/Swiss-Prot;Acc:Q8NI28].

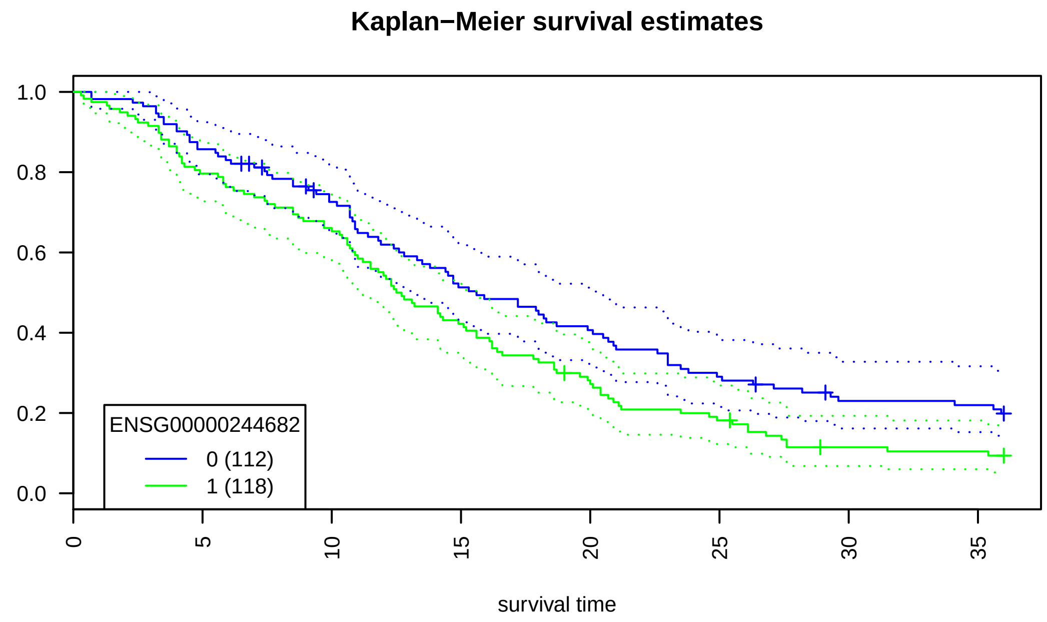

Figure 1888: Each curve represents the relative number of ENSG00000244682 survived to the corresponding point of time. Dashed lines represent 0.95 confidence intervals for the curves with the corresponding color. Log-rank probability measure for the equality of these curves is 1.437852e-02. Additional annotations: *.GeneName*=FCGR2C, *.GeneDesc*=Fc fragment of IgG, low affinity IIc, receptor for (CD32) [Source:HGNC Symbol;Acc:15626].

9 Signalling pathway impact analysis for differentially expressed genes evaluated from median exon expression with survival $p < 0.01$

Table 7: SPIA analysis [24]. Name is the KEGG name for the pathway, and ID is the KEGG internal identication for the pathway. pSize is total number of genes in the pathway according to KEGG, where as NDE is the number of diffentially expressed genes on the pathway. pNDE is the p-value to observe NDE number of genes on the pathway by chance. tA is a perturbation factor defined by SPIA algorithm and pPERT is the probability of observing a more intense perturbation than tA by chance. pG is the combination of pPERT and pNDE. pGFdr is the false discovery rate and pGFWER the Bonferoni corrected p-value.

Name	ID	pSize	NDE	tA	pNDE	pPERT	pG	pGFdr	pGFWER	Status
Leukocyte transendothelial mig	hsa04670	112	5	3.6525	0.0034	0.514	0.0130	0.5892	0.6616	Activated
Fc epsilon RI signaling pathwa	hsa04664	77	4	1.7535	0.0051	0.674	0.0231	0.5892	1.0000	Activated
Epithelial cell signaling in H	hsa05120	67	2	-3.3594	0.1221	0.148	0.0906	0.6778	1.0000	Inhibited
Long-term depression	hsa04730	68	3	0.6762	0.0234	0.942	0.1062	0.6778	1.0000	Activated
Tight junction	hsa04530	129	2	3.4799	0.3237	0.07	0.1085	0.6778	1.0000	Activated
Hedgehog signaling pathway	hsa04340	56	1	3.3091	0.3978	0.066	0.1218	0.6778	1.0000	Activated
Focal adhesion	hsa04510	195	3	8.9386	0.2568	0.126	0.1433	0.6778	1.0000	Activated
Aldosterone-regulated sodium r	hsa04960	41	1	-1.8974	0.3100	0.108	0.1472	0.6778	1.0000	Inhibited
Chemokine signaling pathway	hsa04062	185	2	9.8692	0.4979	0.08	0.1682	0.6778	1.0000	Activated
Toll-like receptor signaling p	hsa04620	96	2	4.2258	0.2140	0.198	0.1763	0.6778	1.0000	Activated
Cytokine-cytokine receptor int	hsa04060	254	3	2.8734	0.4012	0.11	0.1818	0.6778	1.0000	Activated
Vascular smooth muscle contrac	hsa04270	111	3	1.3051	0.0787	0.564	0.1827	0.6778	1.0000	Activated
Complement and coagulation cas	hsa04610	66	2	-4.8615	0.1191	0.39	0.1890	0.6778	1.0000	Inhibited
GnRH signaling pathway	hsa04912	98	3	-1.3438	0.0586	0.842	0.1979	0.6778	1.0000	Inhibited
MAPK signaling pathway	hsa04010	265	5	1.3645	0.0916	0.544	0.1994	0.6778	1.0000	Activated
Type II diabetes mellitus	hsa04930	47	1	2.6876	0.3466	0.214	0.2671	0.8230	1.0000	Activated
Acute myeloid leukemia	hsa05221	56	1	-1.8807	0.3978	0.196	0.2769	0.8230	1.0000	Inhibited
Regulation of actin cytoskelet	hsa04810	206	3	4.8621	0.2835	0.294	0.2905	0.8230	1.0000	Activated
Adipocytokine signaling pathwa	hsa04920	66	1	1.3438	0.4500	0.202	0.3089	0.8291	1.0000	Activated
Axon guidance	hsa04360	127	2	2.0849	0.3171	0.36	0.3619	0.8391	1.0000	Activated
p53 signaling pathway	hsa04115	68	2	0.0000	0.1251	1	0.3851	0.8391	1.0000	Inhibited
Renal cell carcinoma	hsa05211	69	2	0.0000	0.1281	1	0.3913	0.8391	1.0000	Inhibited
ErbB signaling pathway	hsa04012	87	2	-1.3438	0.1846	0.728	0.4041	0.8391	1.0000	Inhibited
B cell receptor signaling path	hsa04662	73	2	0.0000	0.1403	1	0.4158	0.8391	1.0000	Inhibited
Insulin signaling pathway	hsa04910	132	1	3.6829	0.6982	0.204	0.4200	0.8391	1.0000	Activated
VEGF signaling pathway	hsa04370	75	2	0.0000	0.1464	1	0.4278	0.8391	1.0000	Inhibited
Systemic lupus erythematosus	hsa05322	84	2	0.0000	0.1749	1	0.4799	0.9064	1.0000	Inhibited
Thyroid cancer	hsa05216	29	1	0.0000	0.2308	1	0.5692	0.9846	1.0000	Inhibited
Prion diseases	hsa05020	35	1	0.0000	0.2715	1	0.6255	0.9846	1.0000	Inhibited
SNARE interactions in vesicula	hsa04130	38	1	0.0000	0.2910	1	0.6503	0.9846	1.0000	Inhibited
Neurotrophin signaling pathway	hsa04722	124	1	-2.0157	0.6754	0.45	0.6659	0.9846	1.0000	Inhibited
Intestinal immune network for	hsa04672	44	1	0.0000	0.3285	1	0.6942	0.9846	1.0000	Inhibited
Vasopressin-regulated water re	hsa04962	44	1	0.0000	0.3285	1	0.6942	0.9846	1.0000	Inhibited
Amyotrophic lateral sclerosis	hsa05014	51	1	0.0000	0.3698	1	0.7377	0.9846	1.0000	Inhibited
Vibrio cholerae infection	hsa05110	53	1	0.0000	0.3812	1	0.7488	0.9846	1.0000	Inhibited
Wnt signaling pathway	hsa04310	150	2	0.0000	0.3919	1	0.7590	0.9846	1.0000	Inhibited
NOD-like receptor signaling pa	hsa04621	59	1	0.0000	0.4139	1	0.7790	0.9846	1.0000	Inhibited
Huntington's disease	hsa05016	165	2	0.0000	0.4387	1	0.8001	0.9846	1.0000	Inhibited
RIG-I-like receptor signaling	hsa04622	65	1	0.0000	0.4450	1	0.8053	0.9846	1.0000	Inhibited
Pancreatic cancer	hsa05212	72	1	0.0000	0.4791	1	0.8317	0.9846	1.0000	Inhibited
Progesterone-mediated oocyte m	hsa04914	83	1	0.0000	0.5287	1	0.8656	0.9846	1.0000	Inhibited
Colorectal cancer	hsa05210	84	1	0.0000	0.5329	1	0.8683	0.9846	1.0000	Inhibited
Fc gamma R-mediated phagocytos	hsa04666	92	1	0.0000	0.5657	1	0.8880	0.9846	1.0000	Inhibited
Oocyte meiosis	hsa04114	107	1	0.0000	0.6210	1	0.9169	0.9846	1.0000	Inhibited
T cell receptor signaling path	hsa04660	107	1	0.0000	0.6210	1	0.9169	0.9846	1.0000	Inhibited
Parkinson's disease	hsa05012	110	1	0.0000	0.6312	1	0.9217	0.9846	1.0000	Inhibited
Lysosome	hsa04142	117	1	0.0000	0.6540	1	0.9317	0.9846	1.0000	Inhibited
Neuroactive ligand-receptor in	hsa04080	251	2	0.0000	0.6630	1	0.9355	0.9846	1.0000	Inhibited
Jak-STAT signaling pathway	hsa04630	145	1	0.0000	0.7319	1	0.9603	0.9846	1.0000	Inhibited
Alzheimer's disease	hsa05010	152	1	0.0000	0.7485	1	0.9653	0.9846	1.0000	Inhibited
Olfactory transduction	hsa04740	355	1	2.0196	0.9609	0.93	0.9941	0.9941	1.0000	Activated

10 ArrayCGH: Segmentation Results

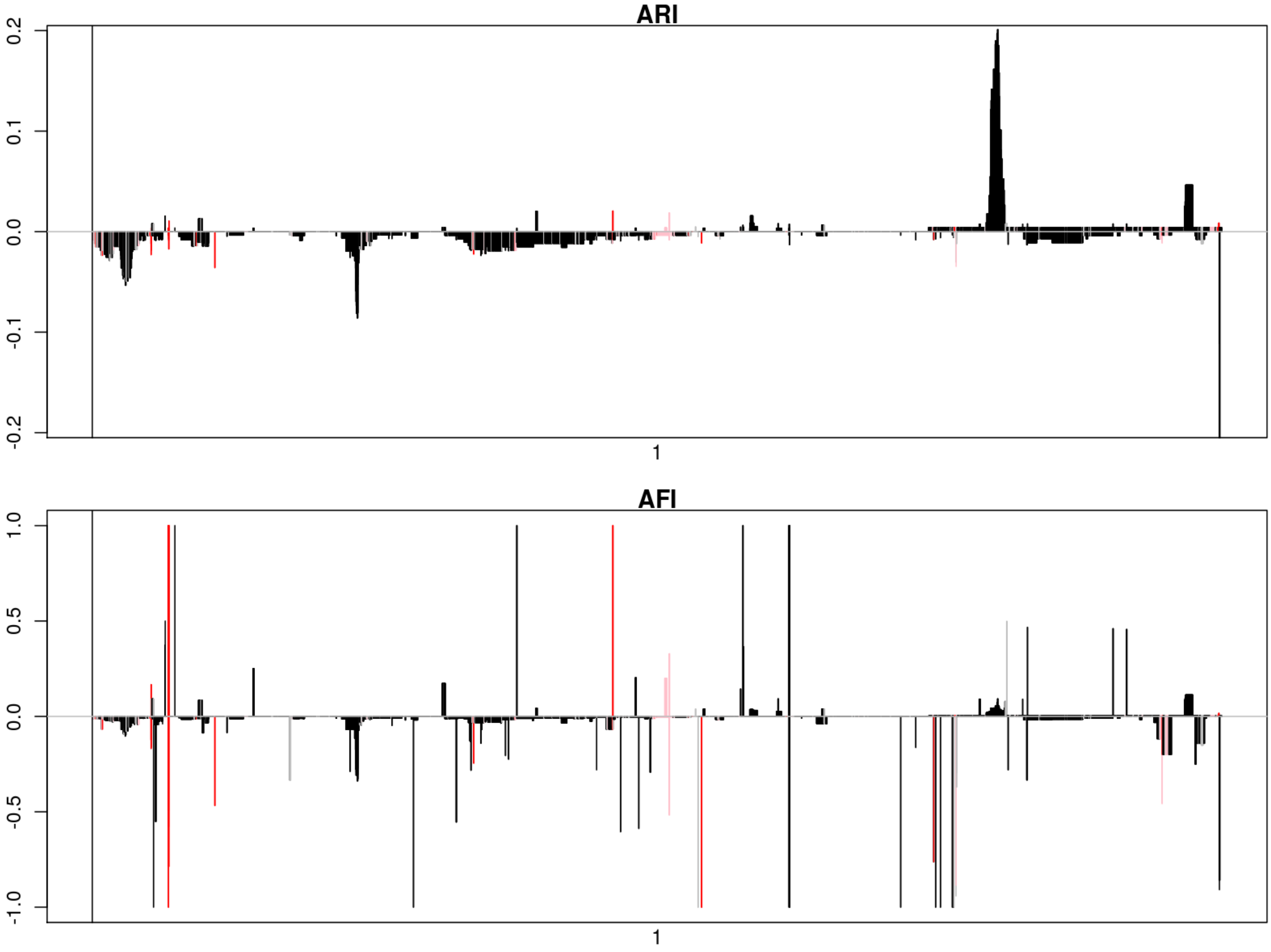

Figure 1889: Aberration Recurrence Index and Aberration Focality Index for gains and losses. Most interesting regions share a high AFI and ARI score. Y-axis represents chromosomal location of aberrations. The indeces are calculated over all samples with gains and losses calculated separetely. ARI is the sum of the segmented means over all the samples, AFI represents how the ARI score is distributed among the putative target genes in a region. Black areas have no reported CNV's according to Database of Genomic Variants, grey areas have 1, red areas have 3 or more. The Database of Genomic Variants contains copy number variation observed in healthy controls only.

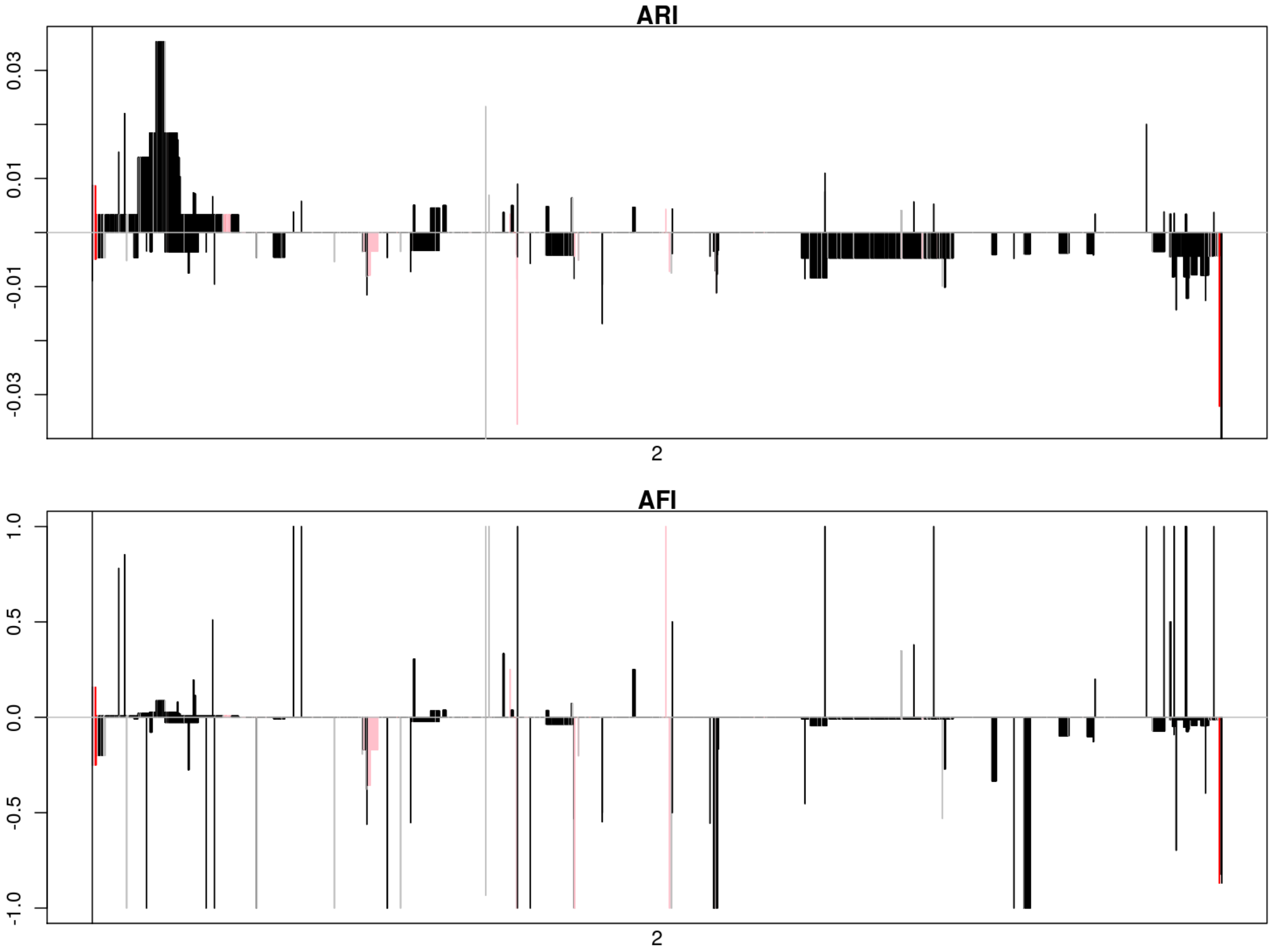

Figure 1890: Aberration Recurrence Index and Aberration Focality Index for gains and losses. Most interesting regions share a high AFI and ARI score. Y-axis represents chromosomal location of aberrations. The indeces are calculated over all samples with gains and losses calculated separetely. ARI is the sum of the segmented means over all the samples, AFI represents how the ARI score is distributed among the putative target genes in a region. Black areas have no reported CNV's according to Database of Genomic Variants, grey areas have 1, red areas have 3 or more. The Database of Genomic Variants contains copy number variation observed in healthy controls only.

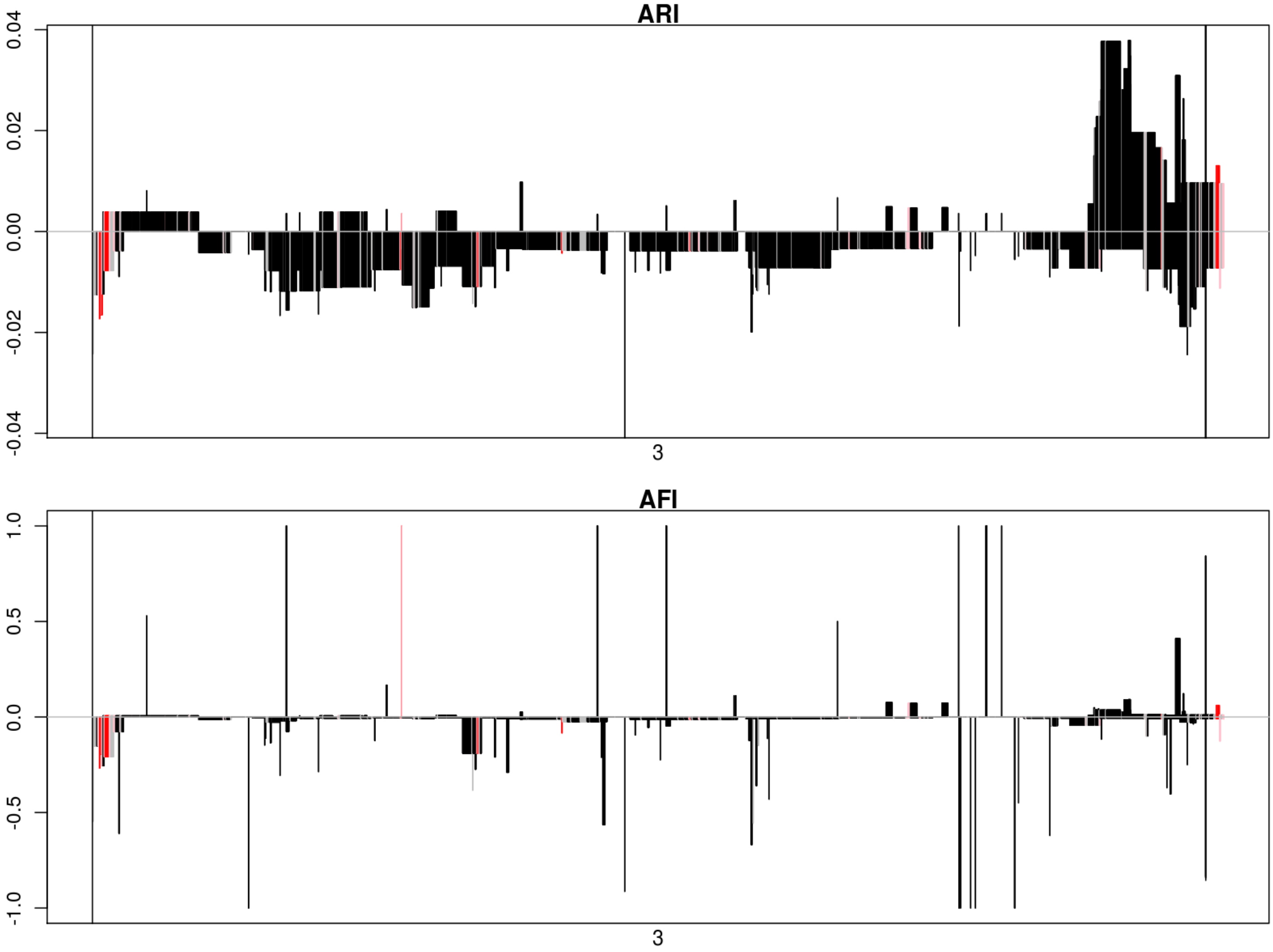

Figure 1891: Aberration Recurrence Index and Aberration Focality Index for gains and losses. Most interesting regions share a high AFI and ARI score. Y-axis represents chromosomal location of aberrations. The indeces are calculated over all samples with gains and losses calculated separetely. ARI is the sum of the segmented means over all the samples, AFI represents how the ARI score is distributed among the putative target genes in a region. Black areas have no reported CNV's according to Database of Genomic Variants, grey areas have 1, red areas have 3 or more. The Database of Genomic Variants contains copy number variation observed in healthy controls only.

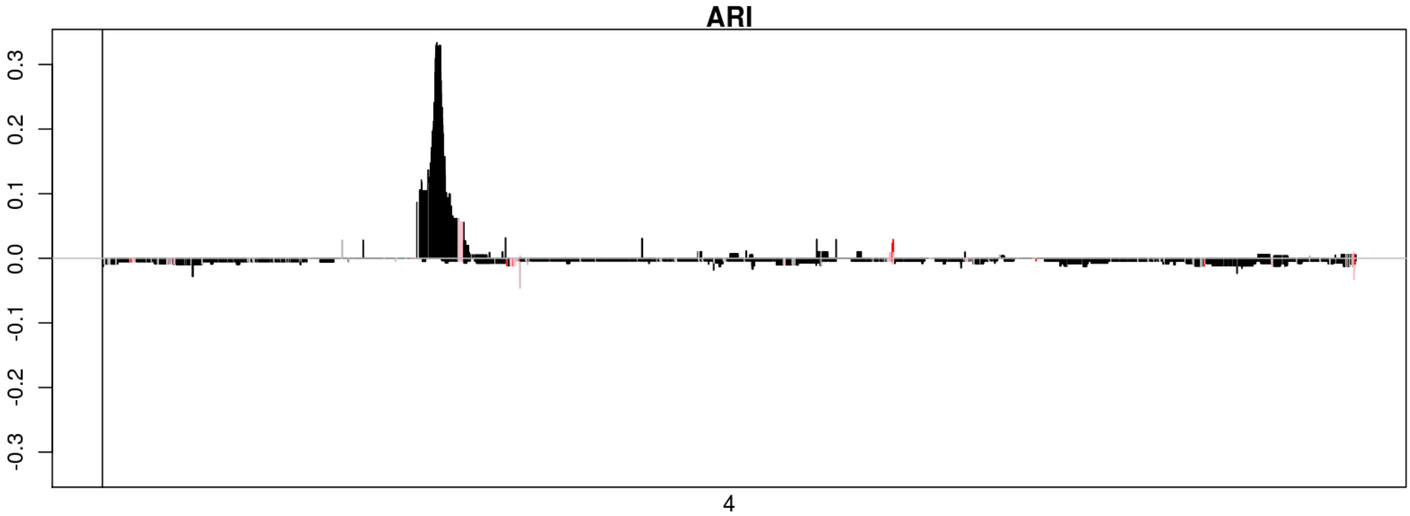

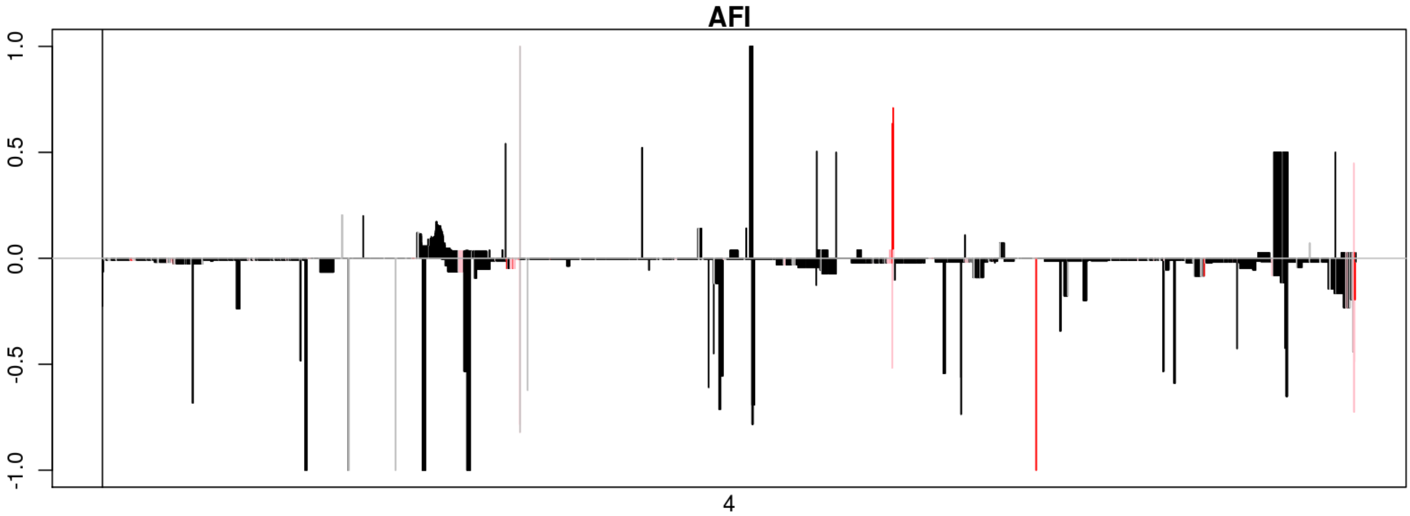

Figure 1892: Aberration Recurrence Index and Aberration Focality Index for gains and losses. Most interesting regions share a high AFI and ARI score. Y-axis represents chromosomal location of aberrations. The indeces are calculated over all samples with gains and losses calculated separetely. ARI is the sum of the segmented means over all the samples, AFI represents how the ARI score is distributed among the putative target genes in a region. Black areas have no reported CNV's according to Database of Genomic Variants, grey areas have 1, red areas have 3 or more. The Database of Genomic Variants contains copy number variation observed in healthy controls only.

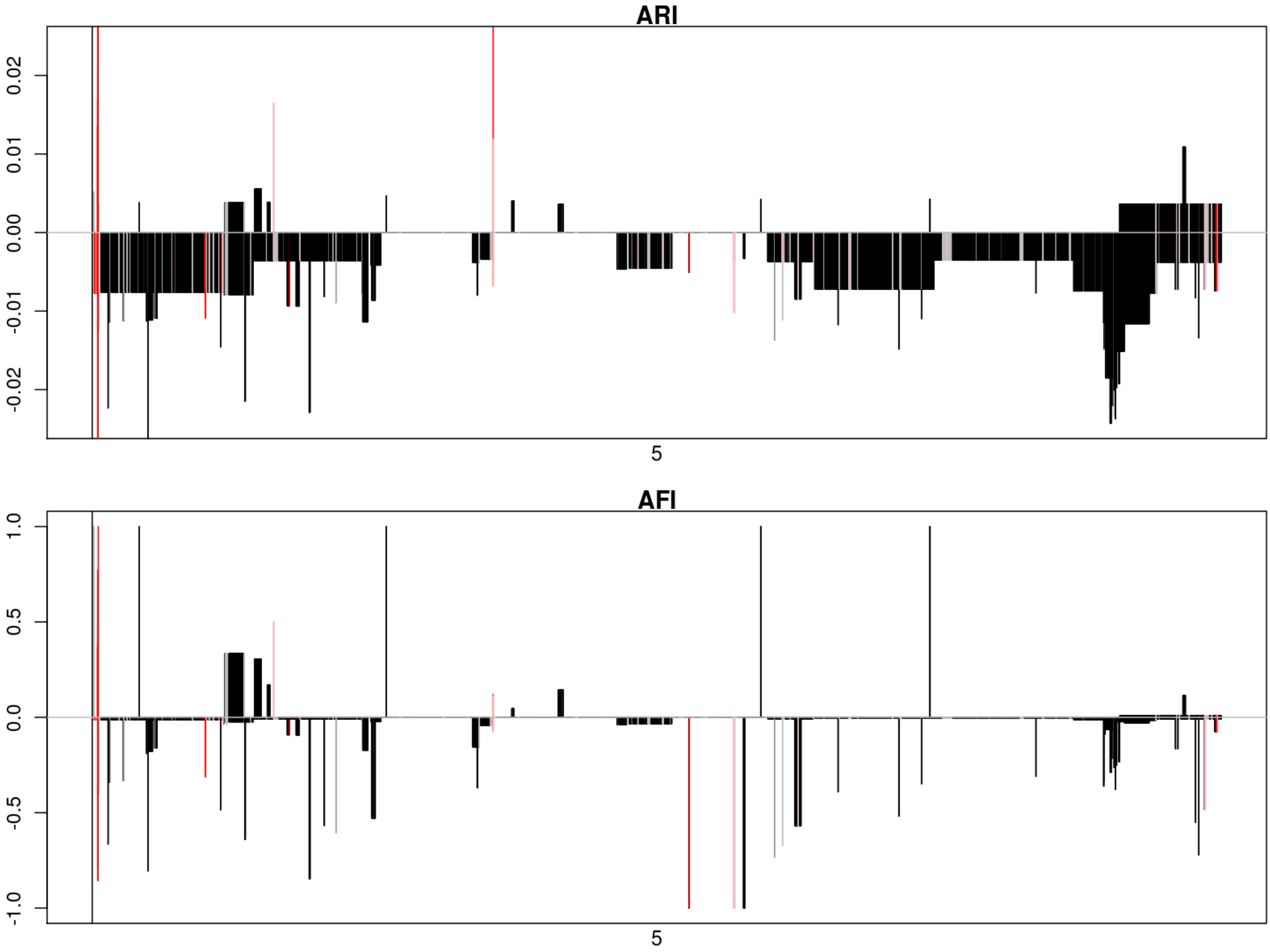

Figure 1893: Aberration Recurrence Index and Aberration Focality Index for gains and losses. Most interesting regions share a high AFI and ARI score. Y-axis represents chromosomal location of aberrations. The indeces are calculated over all samples with gains and losses calculated separetely. ARI is the sum of the segmented means over all the samples, AFI represents how the ARI score is distributed among the putative target genes in a region. Black areas have no reported CNV's according to Database of Genomic Variants, grey areas have 1, red areas have 3 or more. The Database of Genomic Variants contains copy number variation observed in healthy controls only.

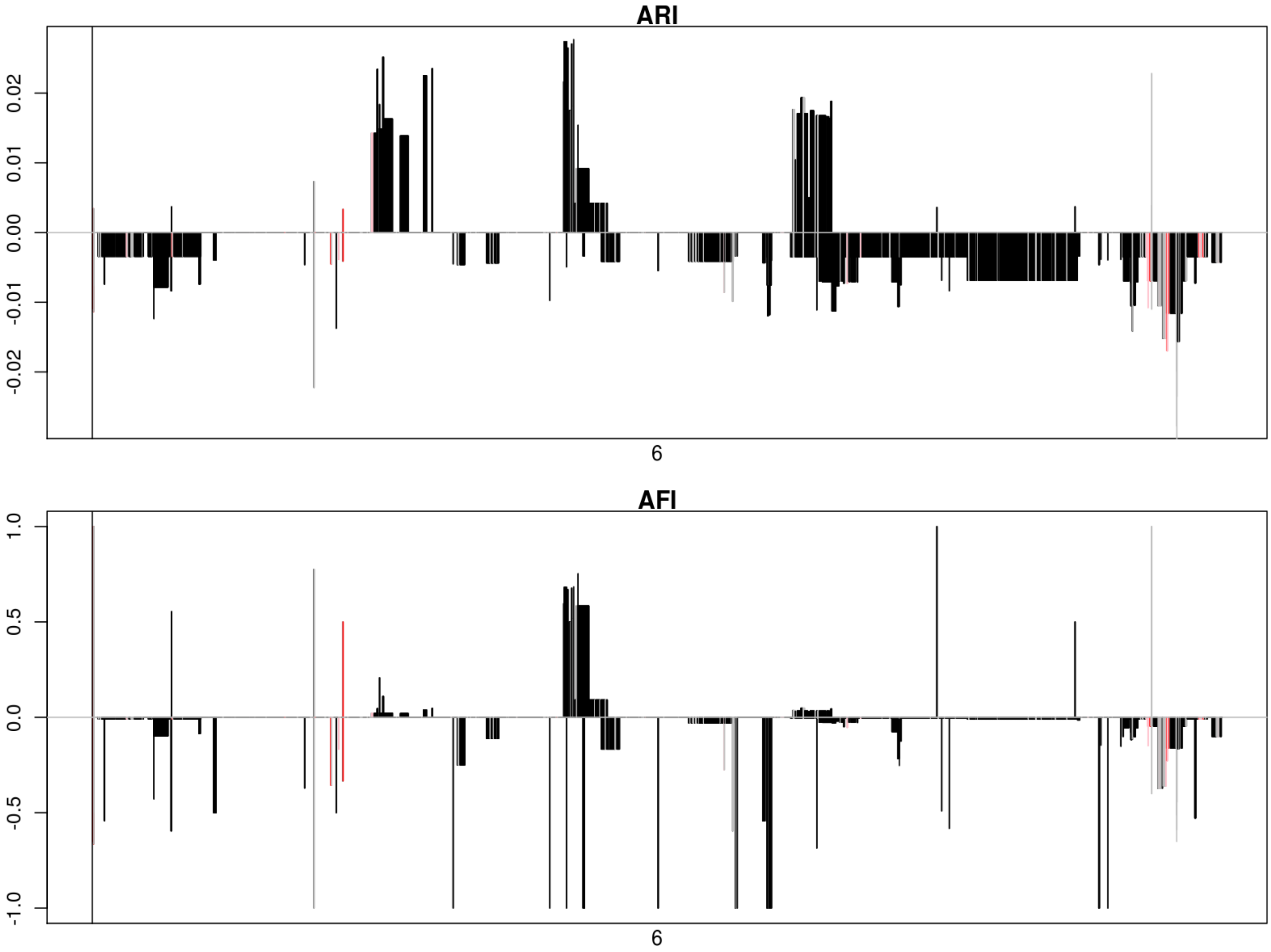

Figure 1894: Aberration Recurrence Index and Aberration Focality Index for gains and losses. Most interesting regions share a high AFI and ARI score. Y-axis represents chromosomal location of aberrations. The indeces are calculated over all samples with gains and losses calculated separetely. ARI is the sum of the segmented means over all the samples, AFI represents how the ARI score is distributed among the putative target genes in a region. Black areas have no reported CNV's according to Database of Genomic Variants, grey areas have 1, red areas have 3 or more. The Database of Genomic Variants contains copy number variation observed in healthy controls only.

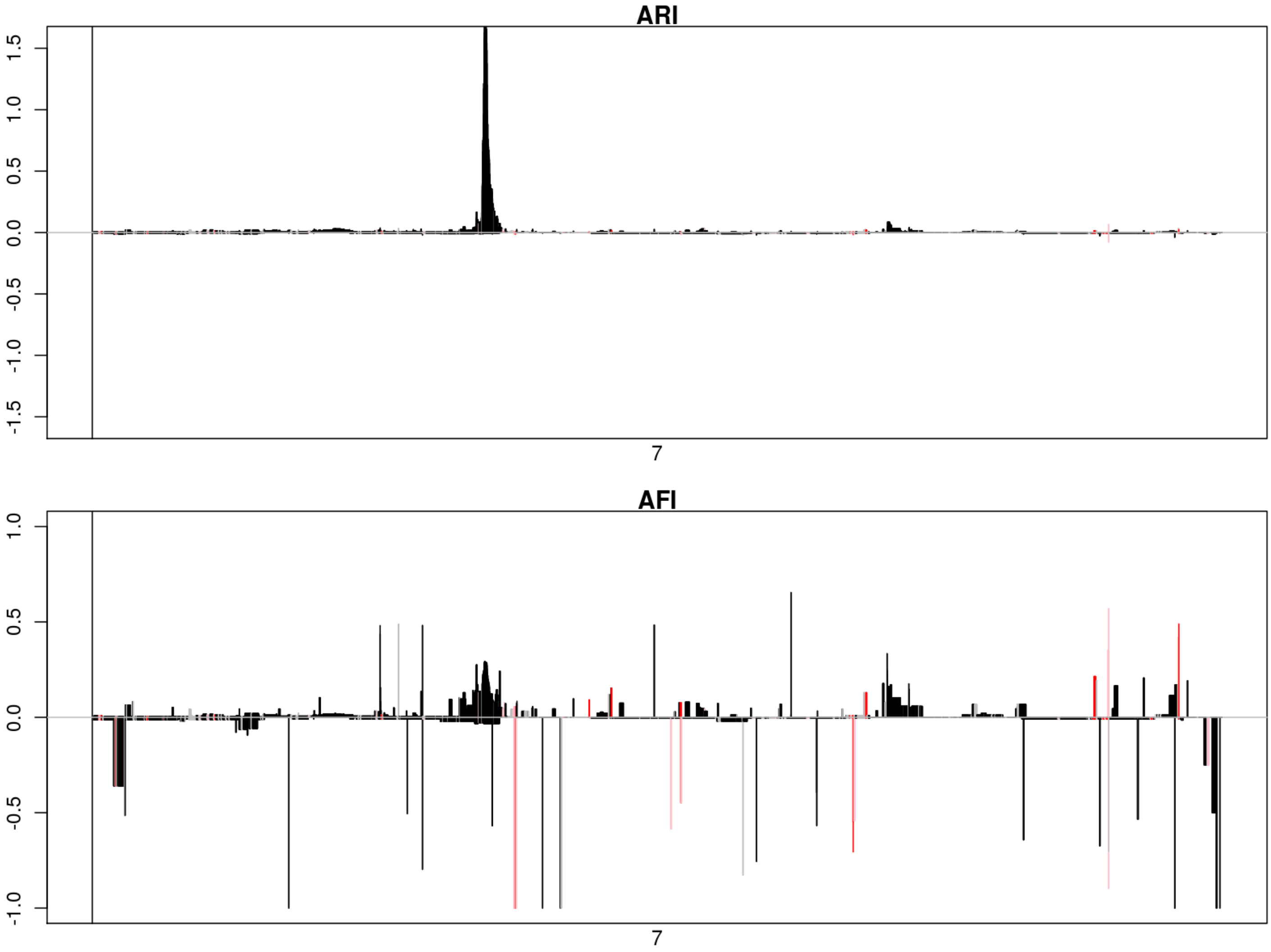

Figure 1895: Aberration Recurrence Index and Aberration Focality Index for gains and losses. Most interesting regions share a high AFI and ARI score. Y-axis represents chromosomal location of aberrations. The indeces are calculated over all samples with gains and losses calculated separetely. ARI is the sum of the segmented means over all the samples, AFI represents how the ARI score is distributed among the putative target genes in a region. Black areas have no reported CNV's according to Database of Genomic Variants, grey areas have 1, red areas have 3 or more. The Database of Genomic Variants contains copy number variation observed in healthy controls only.

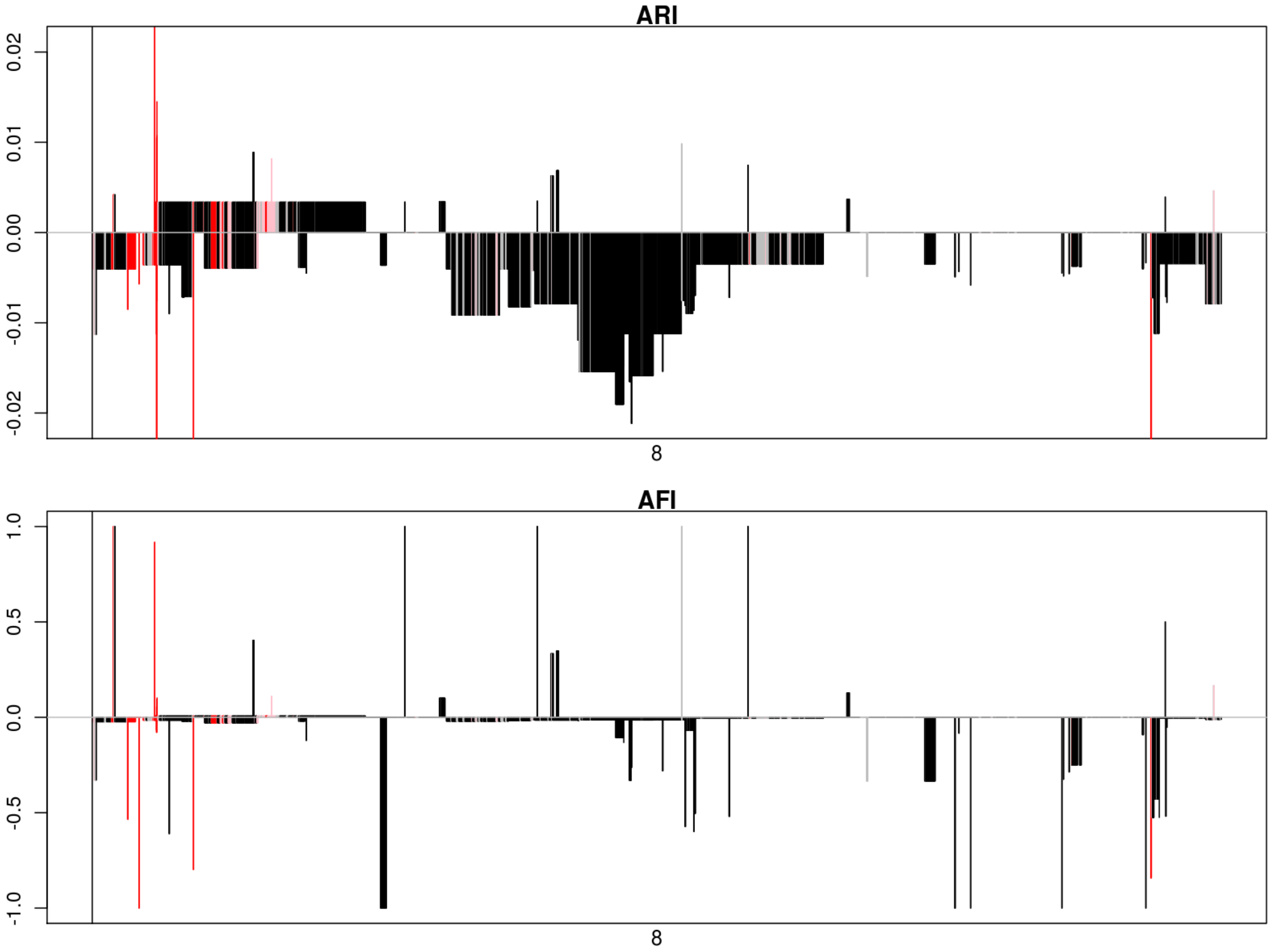

Figure 1896: Aberration Recurrence Index and Aberration Focality Index for gains and losses. Most interesting regions share a high AFI and ARI score. Y-axis represents chromosomal location of aberrations. The indeces are calculated over all samples with gains and losses calculated separetely. ARI is the sum of the segmented means over all the samples, AFI represents how the ARI score is distributed among the putative target genes in a region. Black areas have no reported CNV's according to Database of Genomic Variants, grey areas have 1, red areas have 3 or more. The Database of Genomic Variants contains copy number variation observed in healthy controls only.

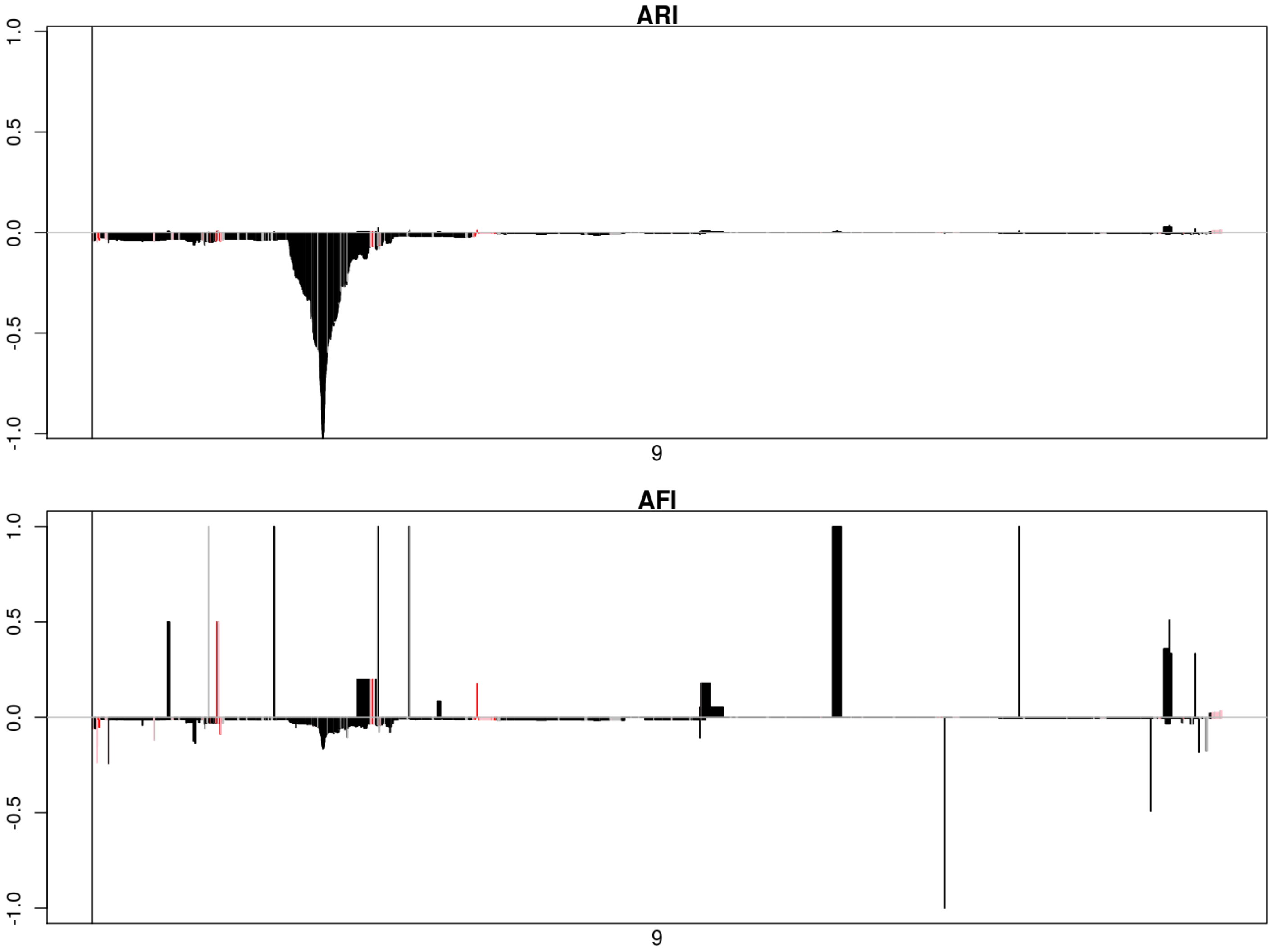

Figure 1897: Aberration Recurrence Index and Aberration Focality Index for gains and losses. Most interesting regions share a high AFI and ARI score. Y-axis represents chromosomal location of aberrations. The indeces are calculated over all samples with gains and losses calculated separetely. ARI is the sum of the segmented means over all the samples, AFI represents how the ARI score is distributed among the putative target genes in a region. Black areas have no reported CNV's according to Database of Genomic Variants, grey areas have 1, red areas have 3 or more. The Database of Genomic Variants contains copy number variation observed in healthy controls only.

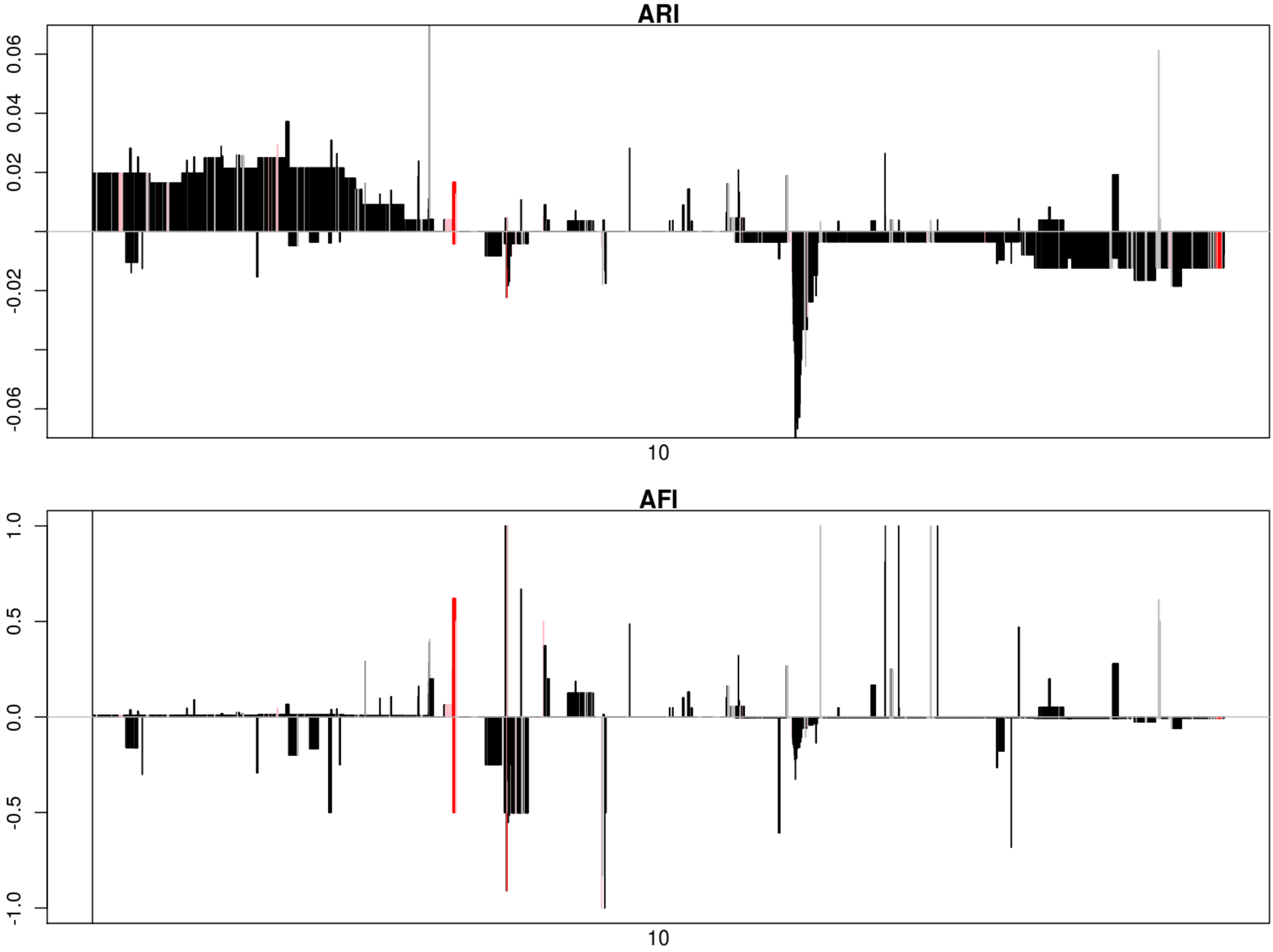

Figure 1898: Aberration Recurrence Index and Aberration Focality Index for gains and losses. Most interesting regions share a high AFI and ARI score. Y-axis represents chromosomal location of aberrations. The indeces are calculated over all samples with gains and losses calculated separetely. ARI is the sum of the segmented means over all the samples, AFI represents how the ARI score is distributed among the putative target genes in a region. Black areas have no reported CNV's according to Database of Genomic Variants, grey areas have 1, red areas have 3 or more. The Database of Genomic Variants contains copy number variation observed in healthy controls only.

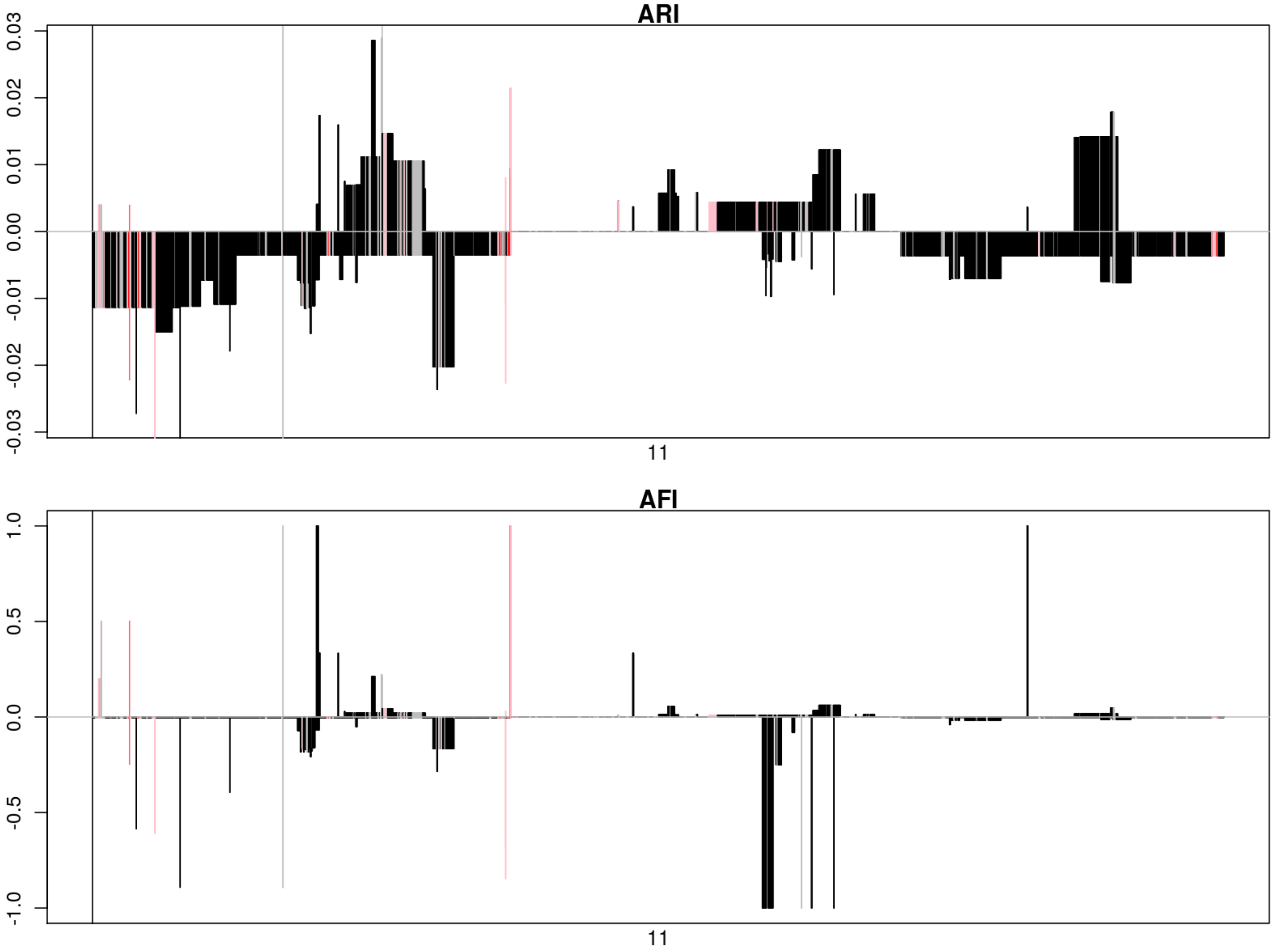

Figure 1899: Aberration Recurrence Index and Aberration Focality Index for gains and losses. Most interesting regions share a high AFI and ARI score. Y-axis represents chromosomal location of aberrations. The indeces are calculated over all samples with gains and losses calculated separetely. ARI is the sum of the segmented means over all the samples, AFI represents how the ARI score is distributed among the putative target genes in a region. Black areas have no reported CNV's according to Database of Genomic Variants, grey areas have 1, red areas have 3 or more. The Database of Genomic Variants contains copy number variation observed in healthy controls only.

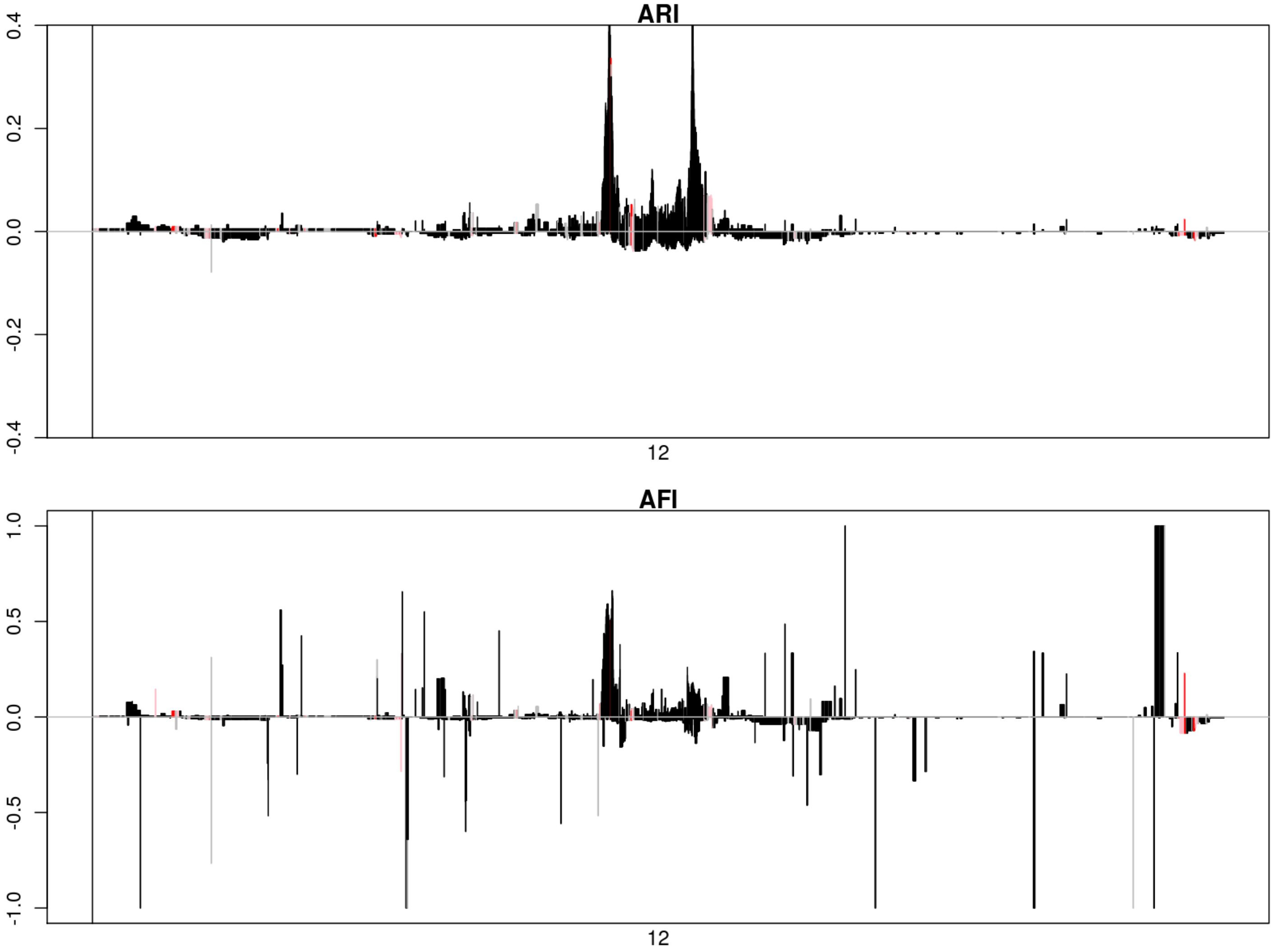

Figure 1900: Aberration Recurrence Index and Aberration Focality Index for gains and losses. Most interesting regions share a high AFI and ARI score. Y-axis represents chromosomal location of aberrations. The indeces are calculated over all samples with gains and losses calculated separetely. ARI is the sum of the segmented means over all the samples, AFI represents how the ARI score is distributed among the putative target genes in a region. Black areas have no reported CNV's according to Database of Genomic Variants, grey areas have 1, red areas have 3 or more. The Database of Genomic Variants contains copy number variation observed in healthy controls only.

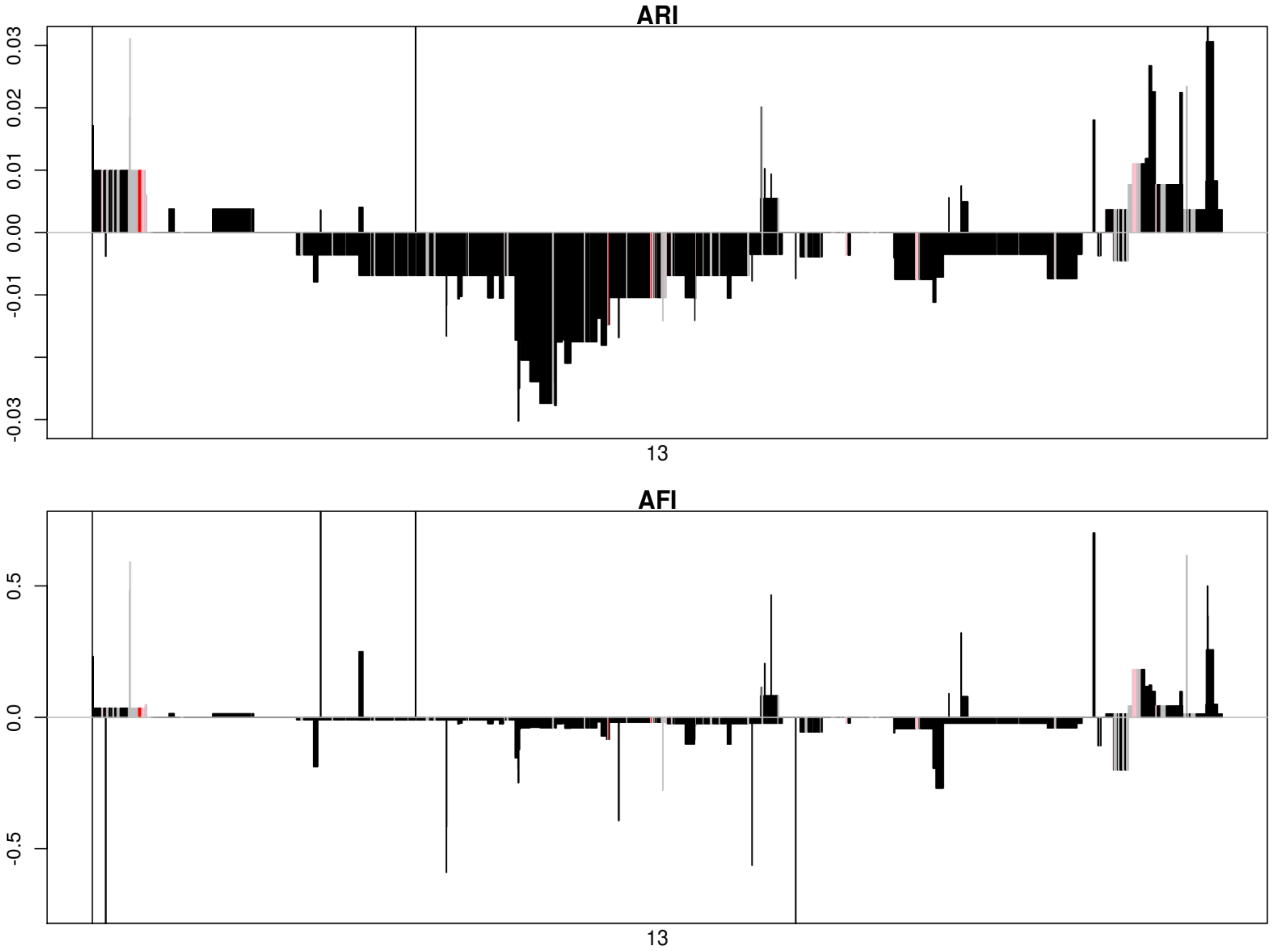

Figure 1901: Aberration Recurrence Index and Aberration Focality Index for gains and losses. Most interesting regions share a high AFI and ARI score. Y-axis represents chromosomal location of aberrations. The indeces are calculated over all samples with gains and losses calculated separetely. ARI is the sum of the segmented means over all the samples, AFI represents how the ARI score is distributed among the putative target genes in a region. Black areas have no reported CNV's according to Database of Genomic Variants, grey areas have 1, red areas have 3 or more. The Database of Genomic Variants contains copy number variation observed in healthy controls only.

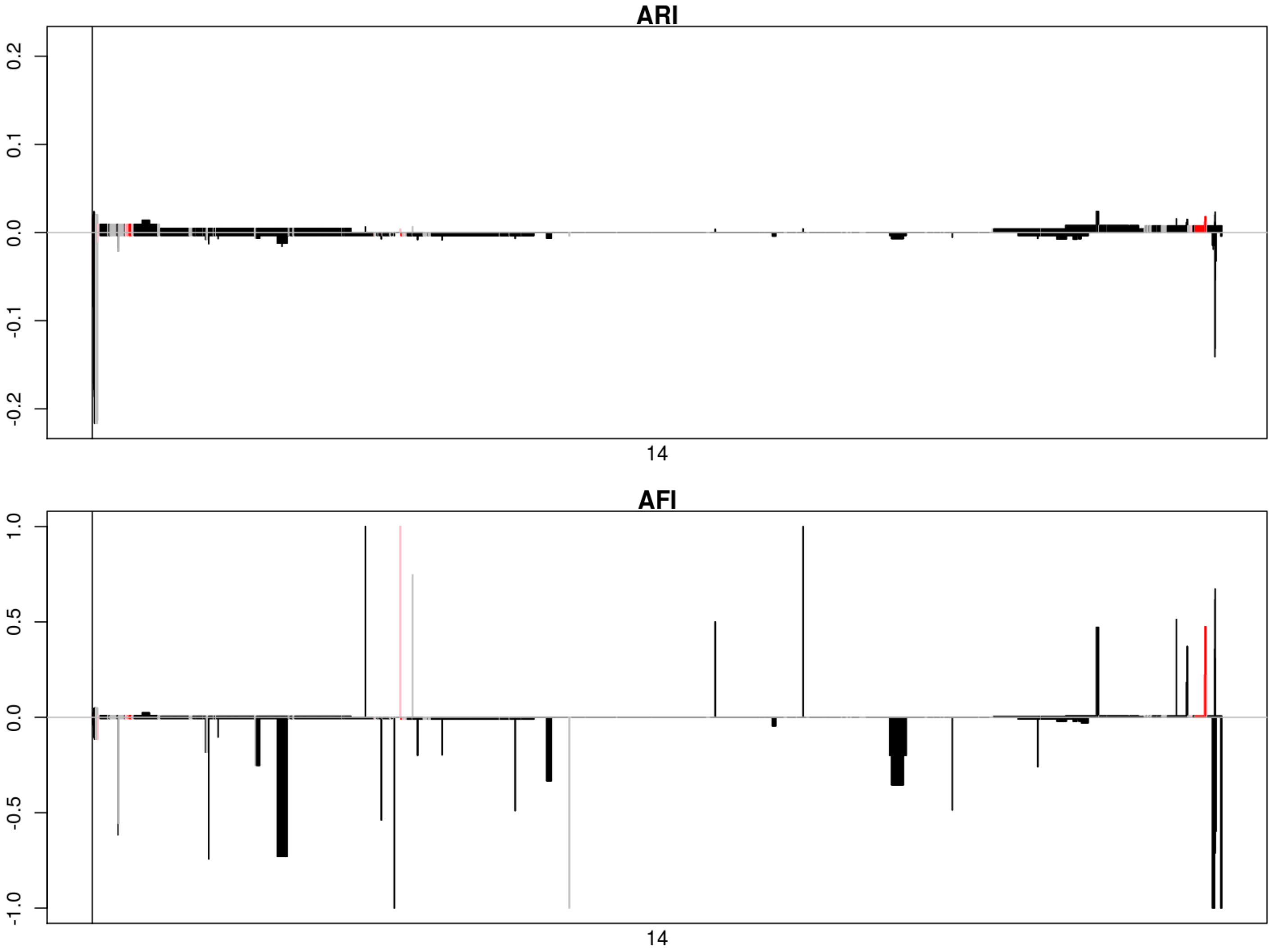

Figure 1902: Aberration Recurrence Index and Aberration Focality Index for gains and losses. Most interesting regions share a high AFI and ARI score. Y-axis represents chromosomal location of aberrations. The indeces are calculated over all samples with gains and losses calculated separetely. ARI is the sum of the segmented means over all the samples, AFI represents how the ARI score is distributed among the putative target genes in a region. Black areas have no reported CNV's according to Database of Genomic Variants, grey areas have 1, red areas have 3 or more. The Database of Genomic Variants contains copy number variation observed in healthy controls only.

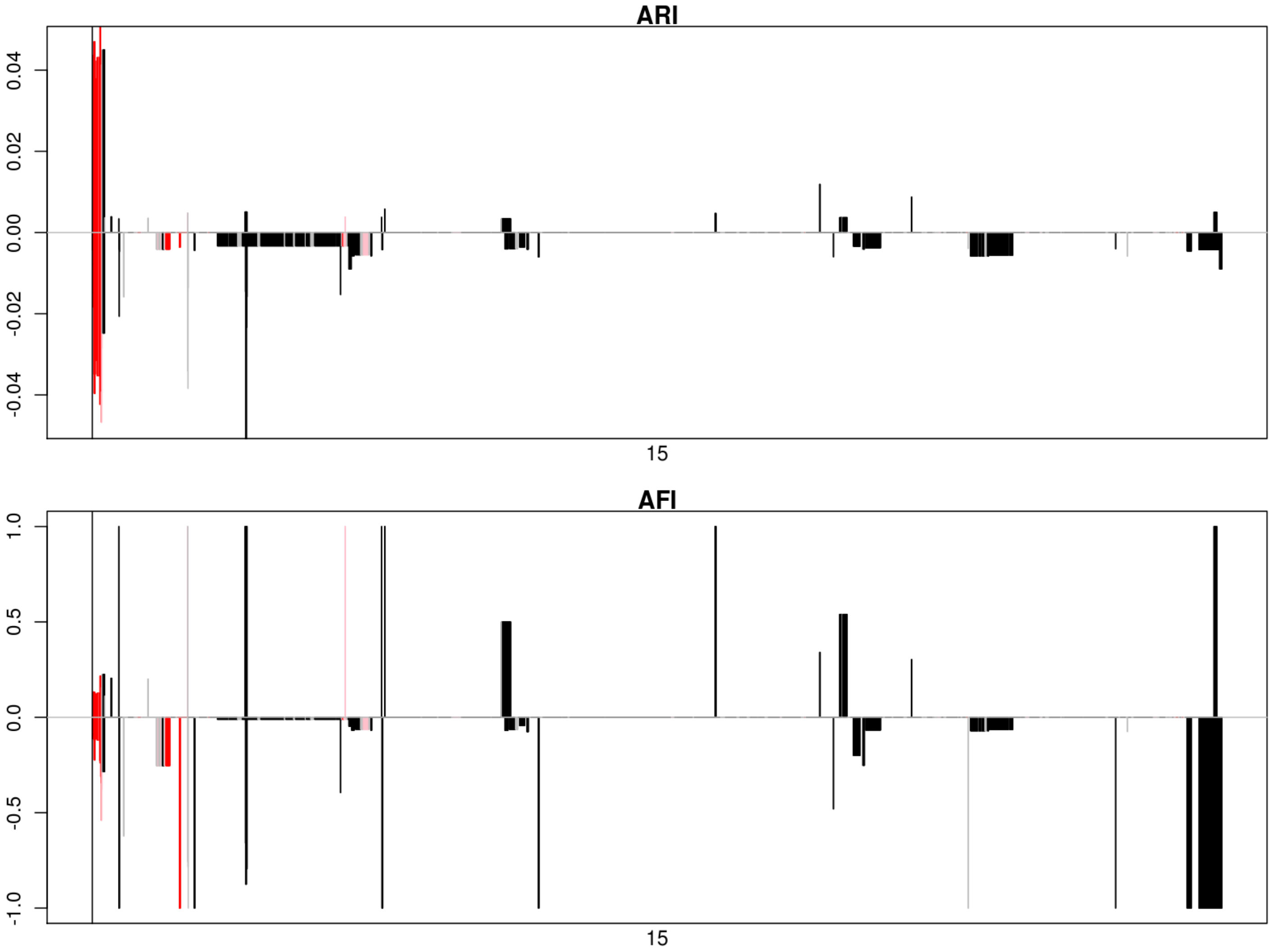

Figure 1903: Aberration Recurrence Index and Aberration Focality Index for gains and losses. Most interesting regions share a high AFI and ARI score. Y-axis represents chromosomal location of aberrations. The indeces are calculated over all samples with gains and losses calculated separetely. ARI is the sum of the segmented means over all the samples, AFI represents how the ARI score is distributed among the putative target genes in a region. Black areas have no reported CNV's according to Database of Genomic Variants, grey areas have 1, red areas have 3 or more. The Database of Genomic Variants contains copy number variation observed in healthy controls only.

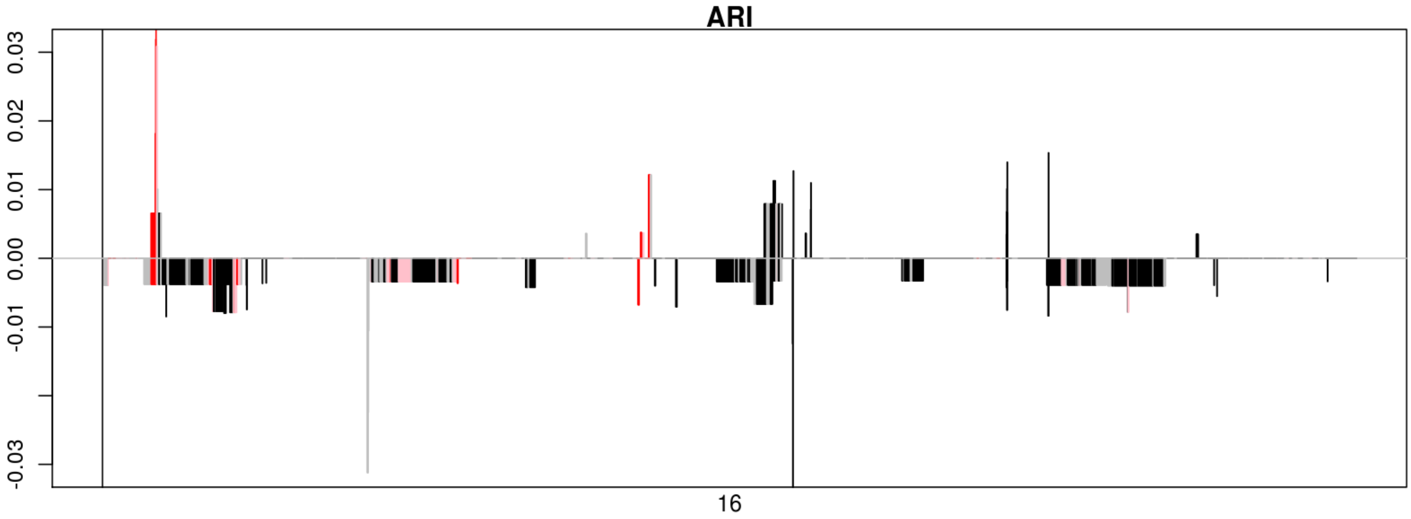

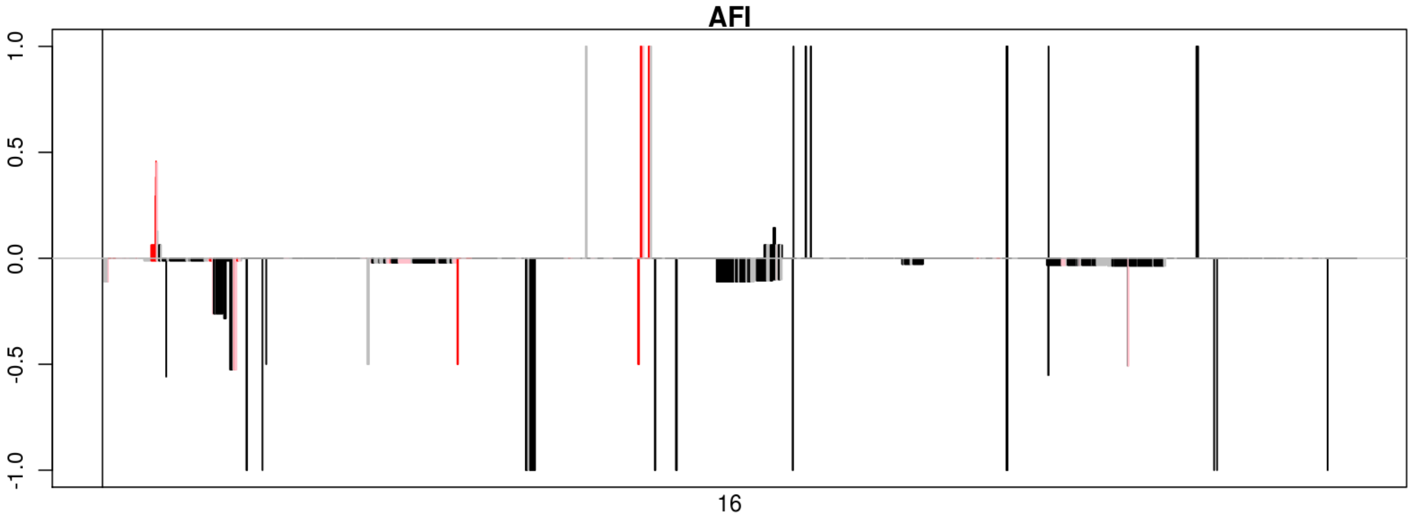

Figure 1904: Aberration Recurrence Index and Aberration Focality Index for gains and losses. Most interesting regions share a high AFI and ARI score. Y-axis represents chromosomal location of aberrations. The indeces are calculated over all samples with gains and losses calculated separetely. ARI is the sum of the segmented means over all the samples, AFI represents how the ARI score is distributed among the putative target genes in a region. Black areas have no reported CNV's according to Database of Genomic Variants, grey areas have 1, red areas have 3 or more. The Database of Genomic Variants contains copy number variation observed in healthy controls only.

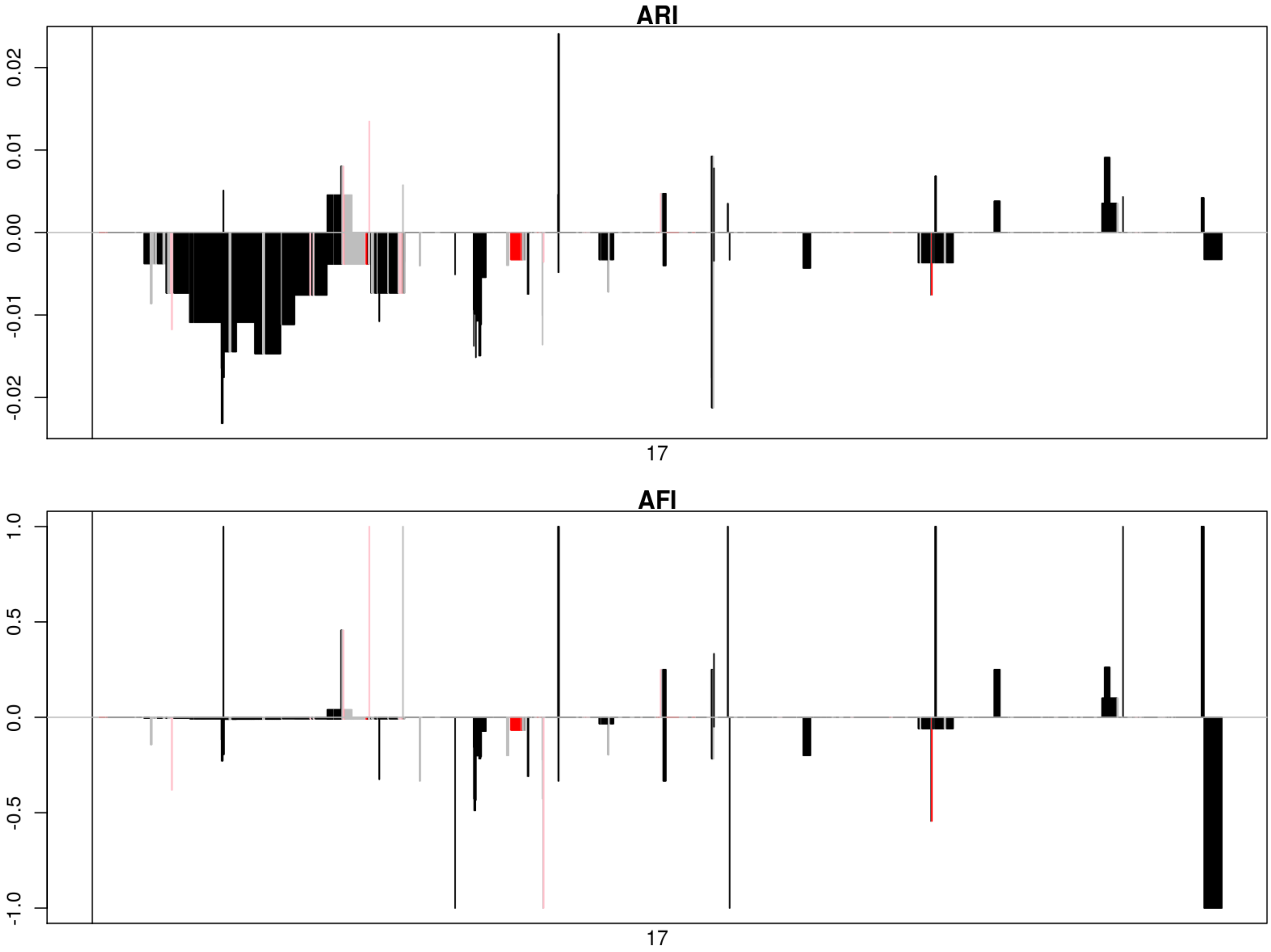

Figure 1905: Aberration Recurrence Index and Aberration Focality Index for gains and losses. Most interesting regions share a high AFI and ARI score. Y-axis represents chromosomal location of aberrations. The indeces are calculated over all samples with gains and losses calculated separetely. ARI is the sum of the segmented means over all the samples, AFI represents how the ARI score is distributed among the putative target genes in a region. Black areas have no reported CNV's according to Database of Genomic Variants, grey areas have 1, red areas have 3 or more. The Database of Genomic Variants contains copy number variation observed in healthy controls only.

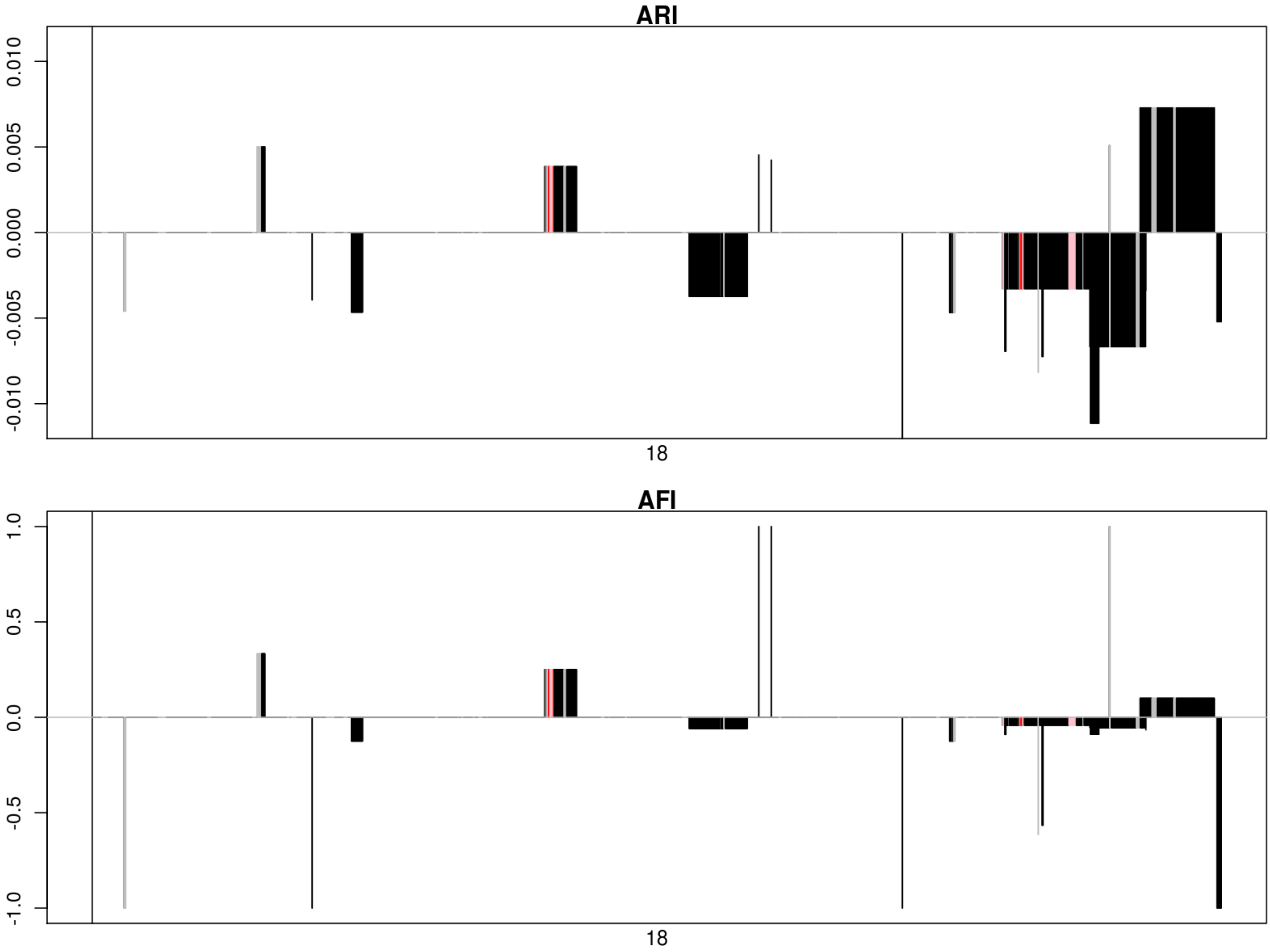

Figure 1906: Aberration Recurrence Index and Aberration Focality Index for gains and losses. Most interesting regions share a high AFI and ARI score. Y-axis represents chromosomal location of aberrations. The indeces are calculated over all samples with gains and losses calculated separetely. ARI is the sum of the segmented means over all the samples, AFI represents how the ARI score is distributed among the putative target genes in a region. Black areas have no reported CNV's according to Database of Genomic Variants, grey areas have 1, red areas have 3 or more. The Database of Genomic Variants contains copy number variation observed in healthy controls only.

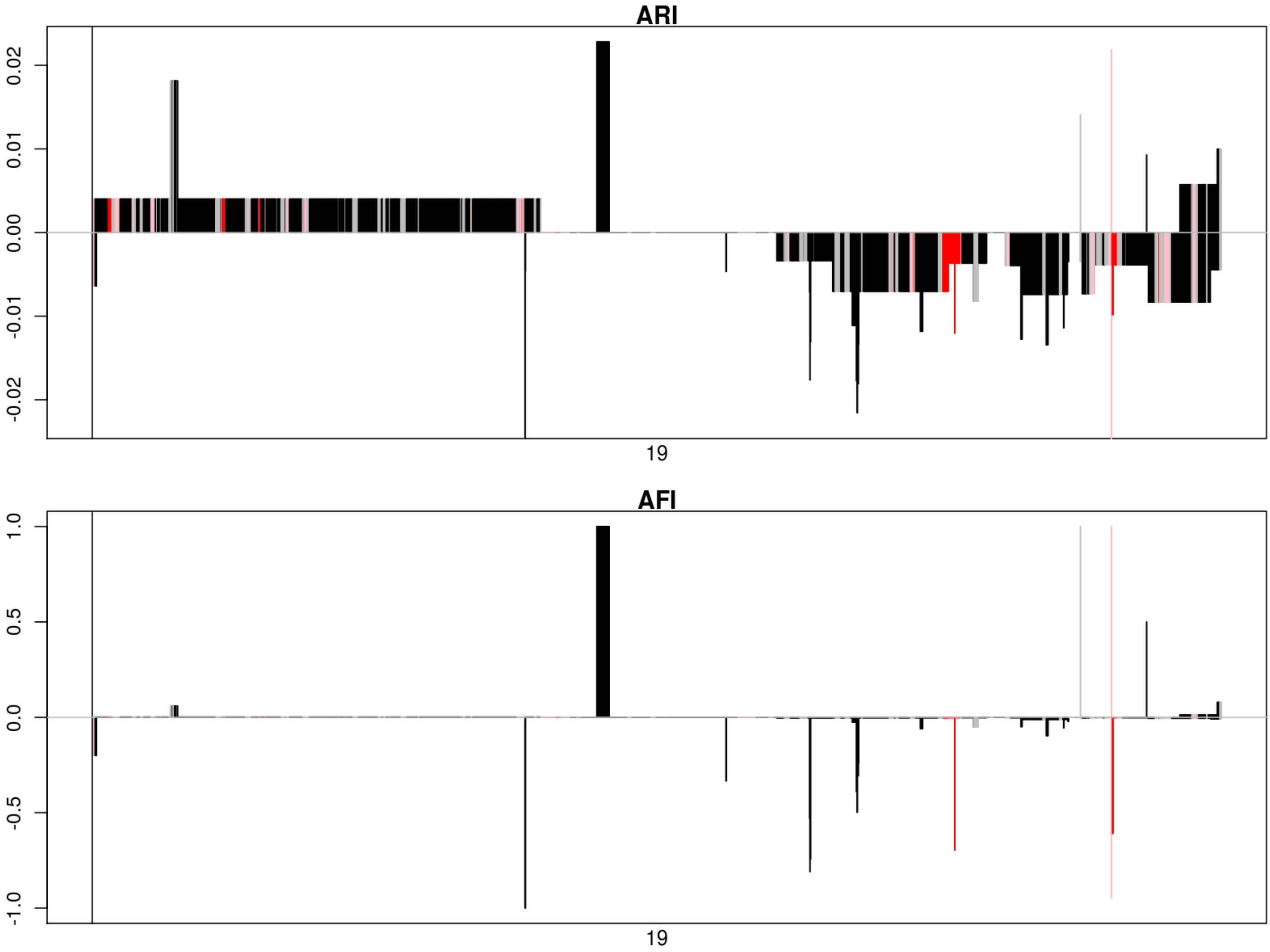

Figure 1907: Aberration Recurrence Index and Aberration Focality Index for gains and losses. Most interesting regions share a high AFI and ARI score. Y-axis represents chromosomal location of aberrations. The indeces are calculated over all samples with gains and losses calculated separetely. ARI is the sum of the segmented means over all the samples, AFI represents how the ARI score is distributed among the putative target genes in a region. Black areas have no reported CNV's according to Database of Genomic Variants, grey areas have 1, red areas have 3 or more. The Database of Genomic Variants contains copy number variation observed in healthy controls only.

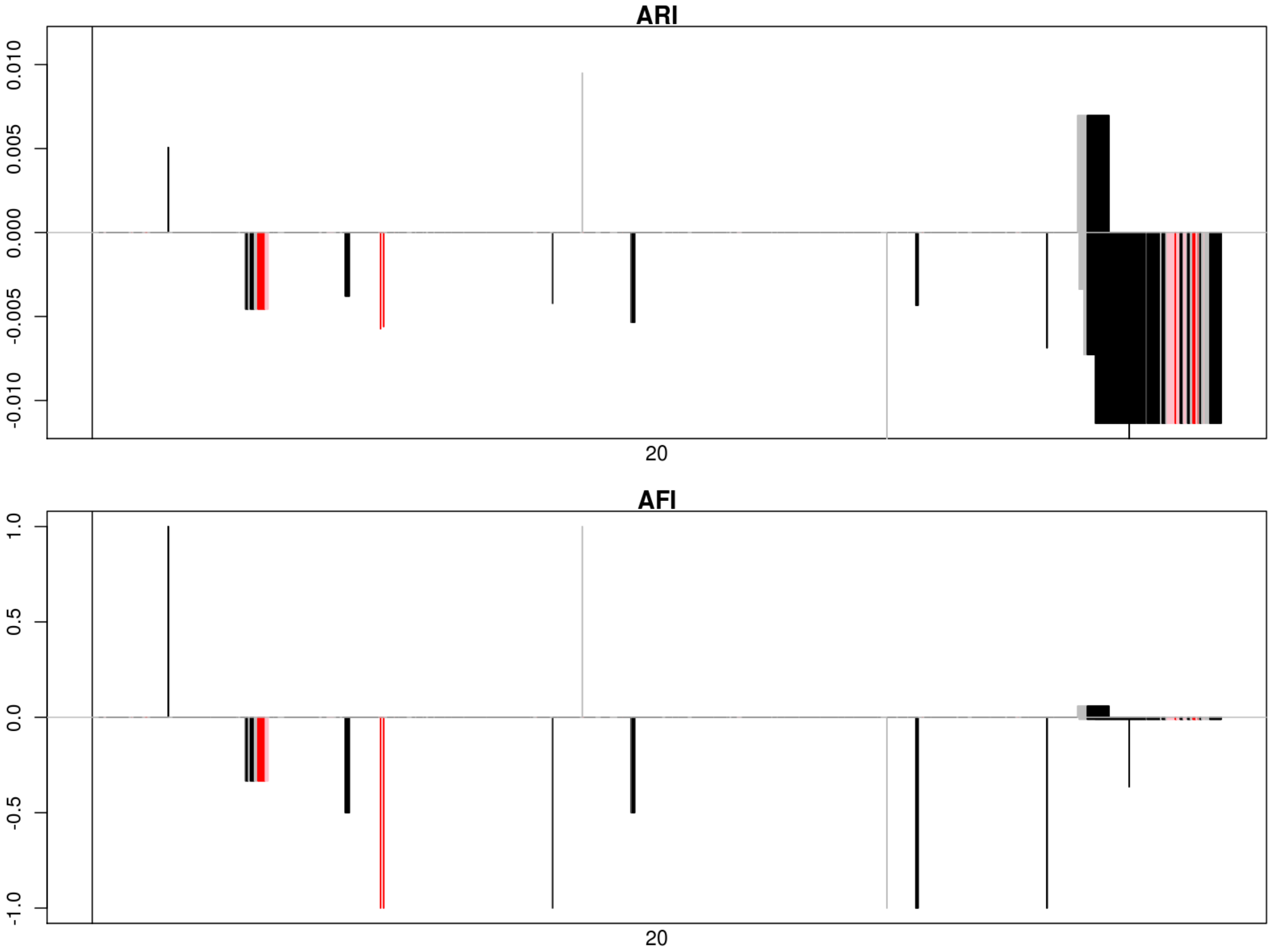

Figure 1908: Aberration Recurrence Index and Aberration Focality Index for gains and losses. Most interesting regions share a high AFI and ARI score. Y-axis represents chromosomal location of aberrations. The indeces are calculated over all samples with gains and losses calculated separetely. ARI is the sum of the segmented means over all the samples, AFI represents how the ARI score is distributed among the putative target genes in a region. Black areas have no reported CNV's according to Database of Genomic Variants, grey areas have 1, red areas have 3 or more. The Database of Genomic Variants contains copy number variation observed in healthy controls only.

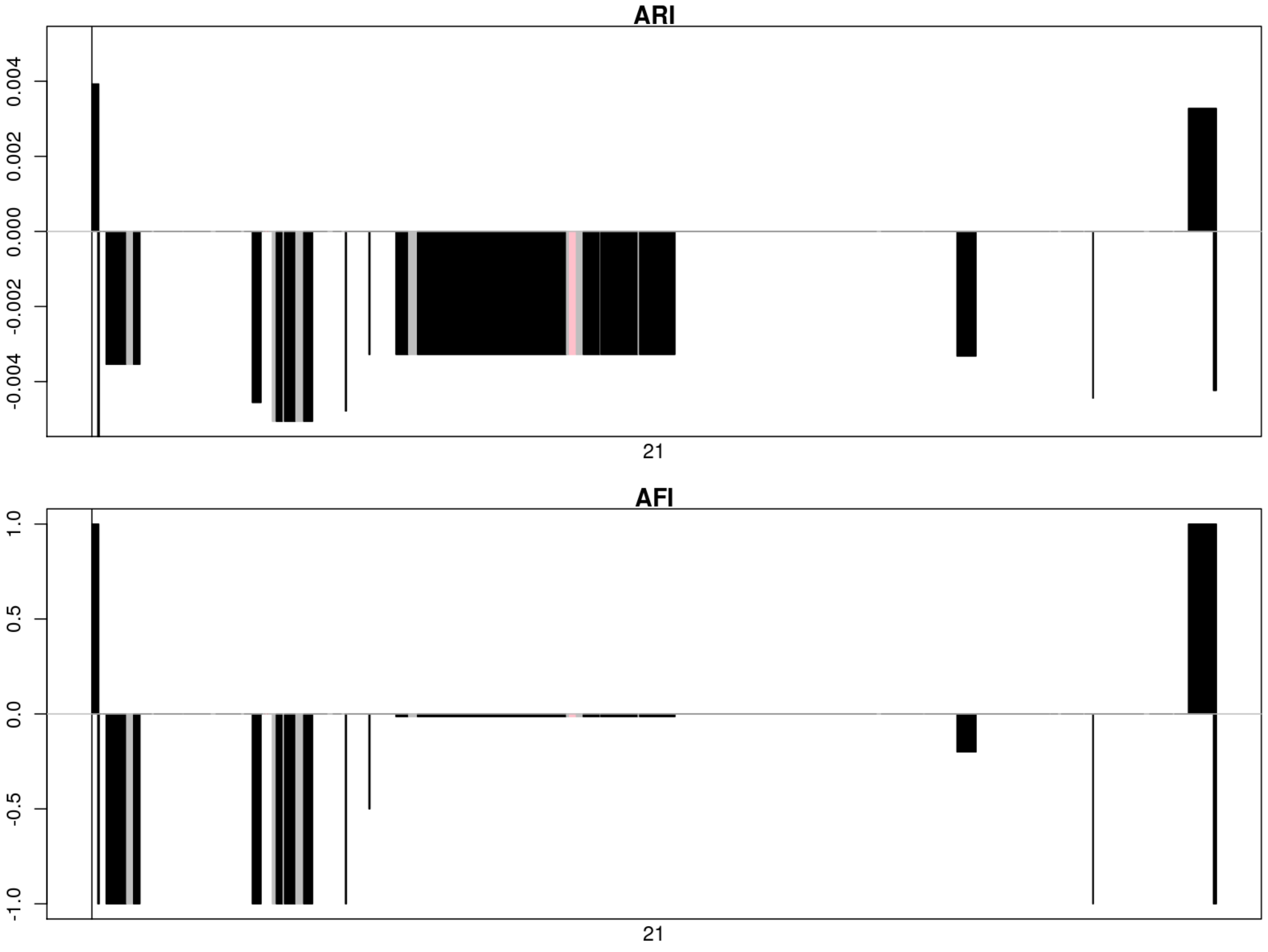

Figure 1909: Aberration Recurrence Index and Aberration Focality Index for gains and losses. Most interesting regions share a high AFI and ARI score. Y-axis represents chromosomal location of aberrations. The indeces are calculated over all samples with gains and losses calculated separetely. ARI is the sum of the segmented means over all the samples, AFI represents how the ARI score is distributed among the putative target genes in a region. Black areas have no reported CNV's according to Database of Genomic Variants, grey areas have 1, red areas have 3 or more. The Database of Genomic Variants contains copy number variation observed in healthy controls only.

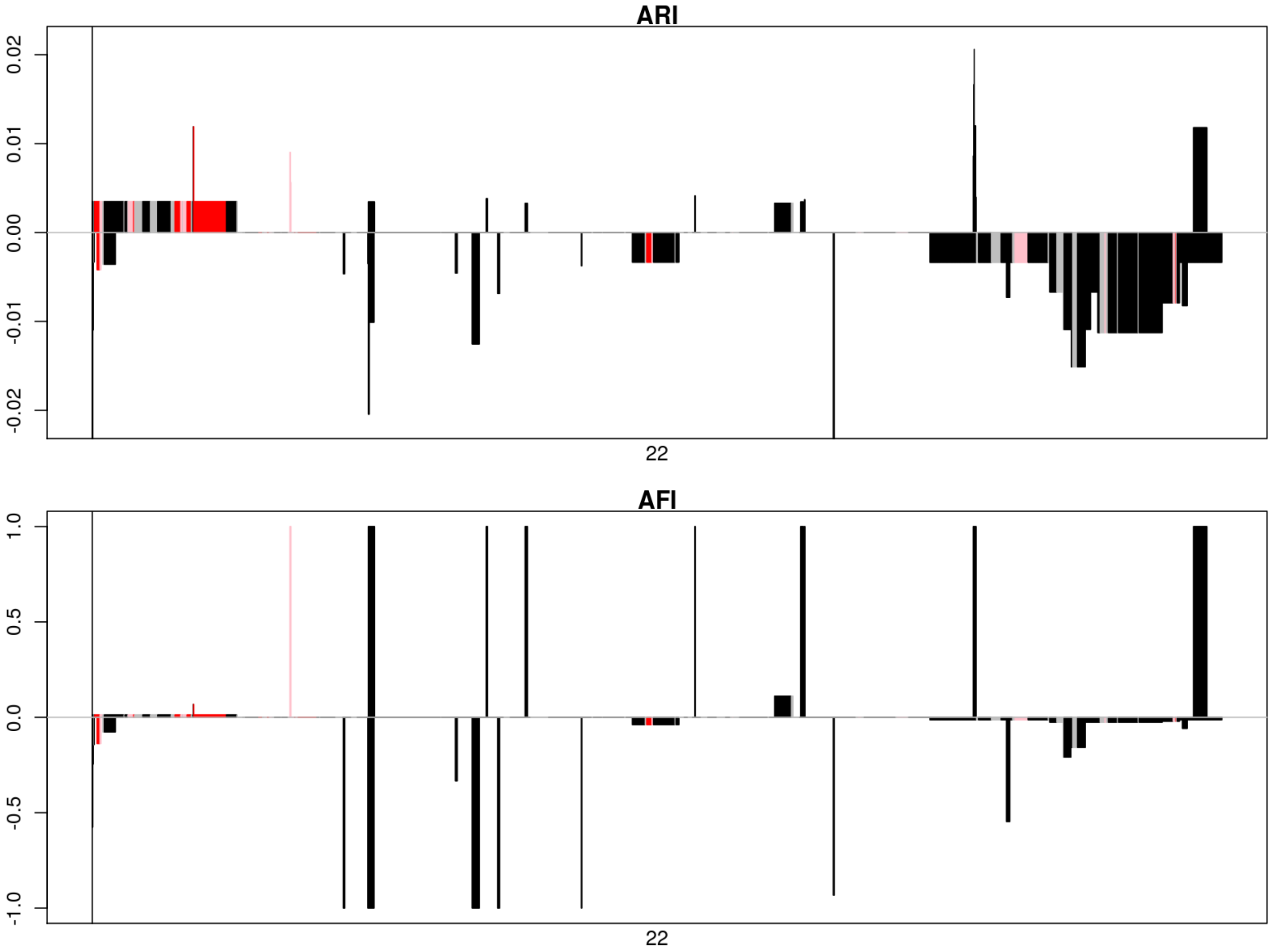

Figure 1910: Aberration Recurrence Index and Aberration Focality Index for gains and losses. Most interesting regions share a high AFI and ARI score. Y-axis represents chromosomal location of aberrations. The indeces are calculated over all samples with gains and losses calculated separetely. ARI is the sum of the segmented means over all the samples, AFI represents how the ARI score is distributed among the putative target genes in a region. Black areas have no reported CNV's according to Database of Genomic Variants, grey areas have 1, red areas have 3 or more. The Database of Genomic Variants contains copy number variation observed in healthy controls only.

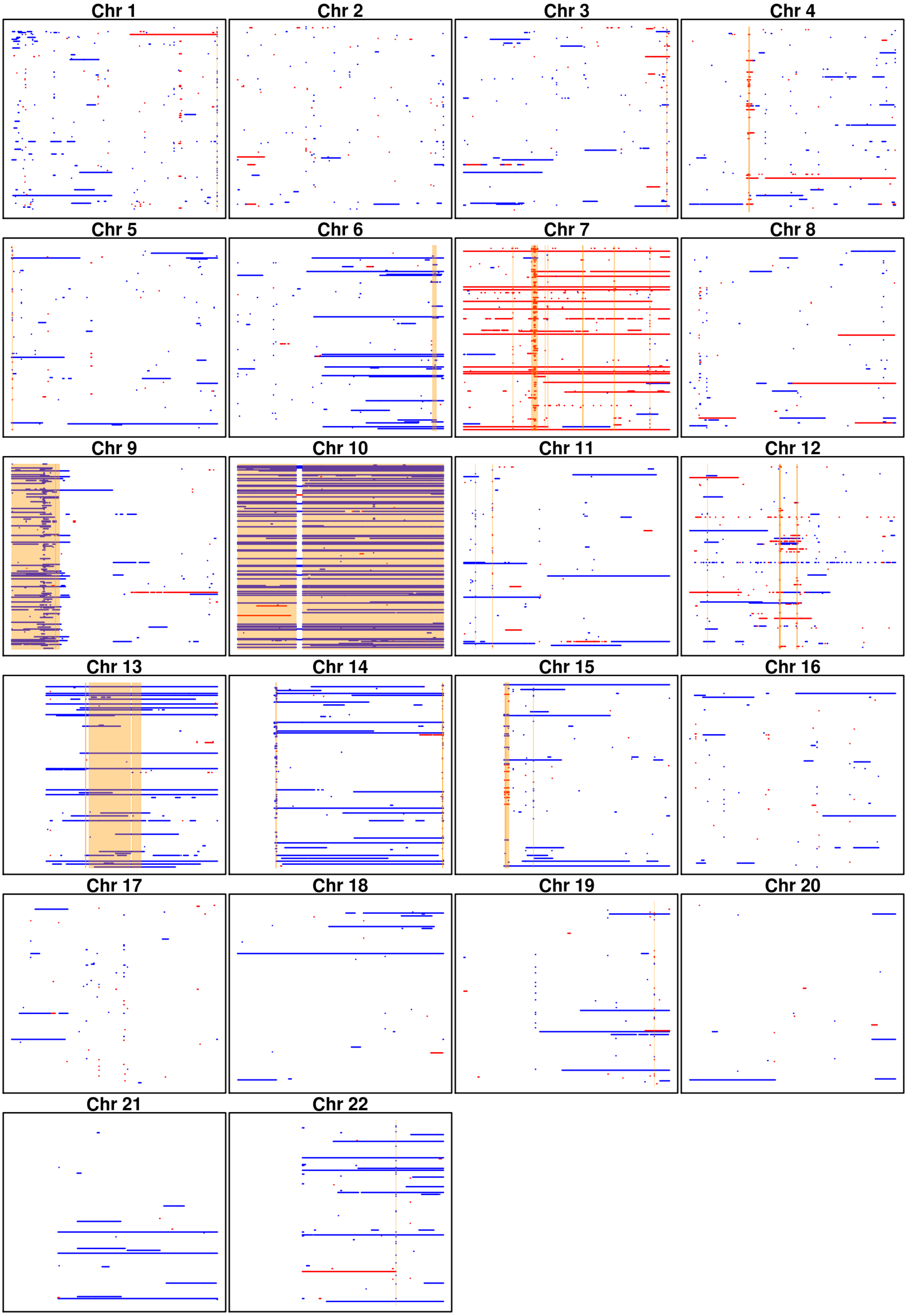

Figure 1911: Red lines represent CN gain segments, blue lines represent CN loss segments, areas highlighted with yellow have gains and losses in more than 0.1 of samples.

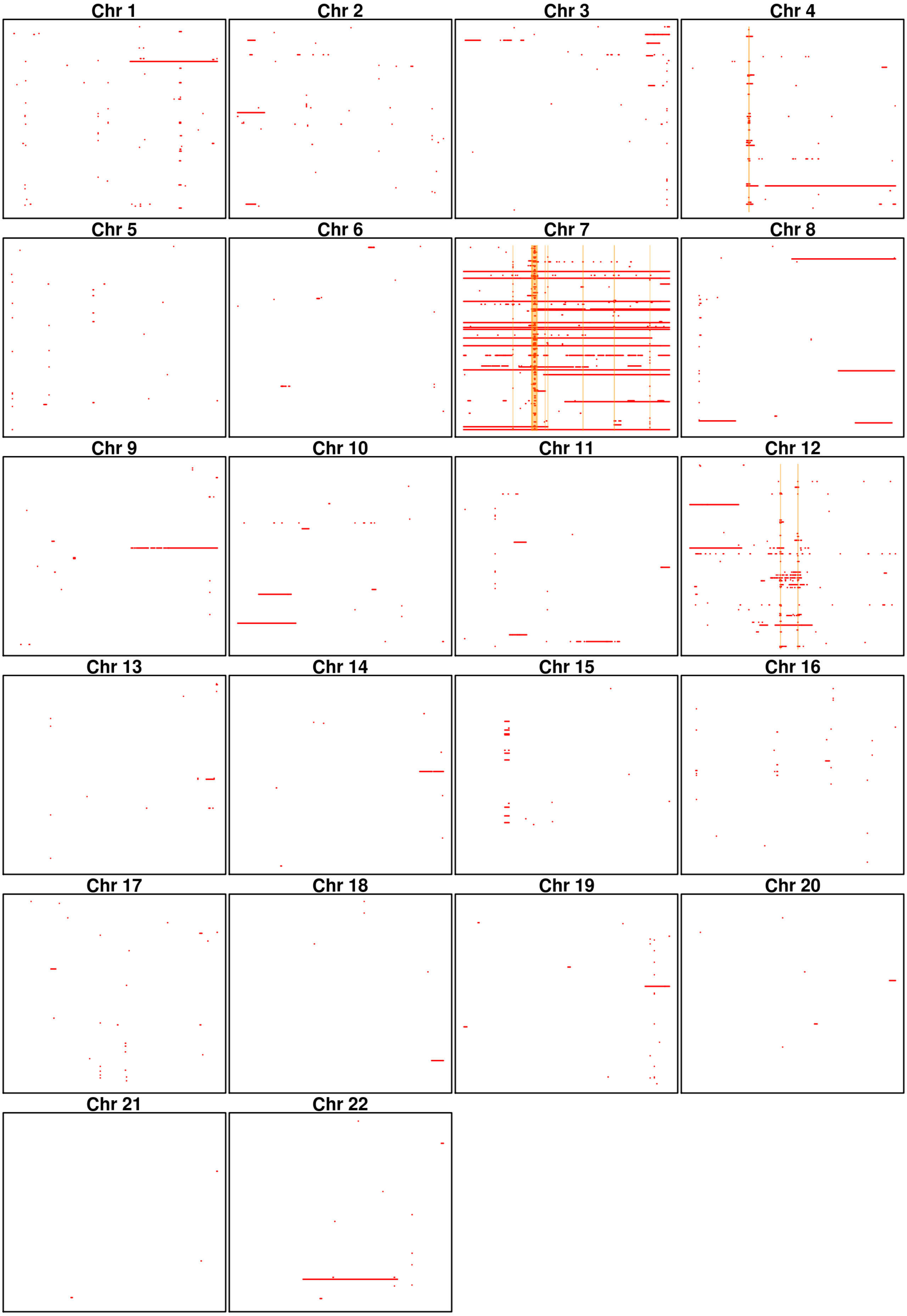

Figure 1912: Red lines represent CN gain segments, areas highlighted with yellow have gains in more than 0.1 of samples.

10.1 Interesting copy number 1 segments

Table 8: This table has 151 rows.

gene name	chr	.DNABand	.GeneDesc	rank
CPM	12:69191693-69257563	12q15	carboxypeptidase M [Source:HGNC Symbol;Acc:2311]	4.17132
MDM2	12:69191693-69257563	12q15	Mdm2 p53 binding protein homolog (mouse) [Source:HGNC Symbol;Acc:6973]	4.17132
AC025423.2	12:69191693-69257563	12q15	cDNA, FLJ92387 [Source:UniProtKB/TrEMBL;Acc:B2R594]	4.17132
CHCHD2	7:53125226-56191148	7p11.2	coiled-coil-helix-coiled-coil-helix domain containing 2 [Source:HGNC Symbol;Acc:21645]	3.43478
SUMF2	7:53125226-56191148	7p11.2	sulfatase modifying factor 2 [Source:HGNC Symbol;Acc:20415]	3.43478
SEC61G	7:53125226-56191148	7p11.2	Sec61 gamma subunit [Source:HGNC Symbol;Acc:18277]	3.43478
LANCL2	7:53125226-56191148	7p11.2	LanC lantibiotic synthetase component C-like 2 (bacterial) [Source:HGNC Symbol;Acc:6509]	3.43478
EGFR	7:53125226-56191148	7p11.2	epidermal growth factor receptor (erythroblastic leukemia viral (v-erb-b) oncogene homolog, avian) [Source:HGNC Symbol;Acc:3236]	3.43478
GBAS	7:53125226-56191148	7p11.2	glioblastoma amplified sequence [Source:HGNC Symbol;Acc:4179]	3.43478
CCT6A	7:53125226-56191148	7p11.2	chaperonin containing TCP1, subunit 6A (zeta 1) [Source:HGNC Symbol;Acc:1620]	3.43478
PSPH	7:53125226-56191148	7p11.2	phosphoserine phosphatase [Source:HGNC Symbol;Acc:9577]	3.43478
VOPP1	7:53125226-56191148	7p11.2	vesicular, overexpressed in cancer, prosurvival protein 1 [Source:HGNC Symbol;Acc:34518]	3.43478
SEPT14	7:53125226-56191148	7p11.2	septin 14 [Source:HGNC Symbol;Acc:33280]	3.43478
PHKG1	7:53125226-56191148	7p11.2	phosphorylase kinase, gamma 1 (muscle) [Source:HGNC Symbol;Acc:8930]	3.43478
VSTM2A	7:53125226-56191148	7p11.2	V-set and transmembrane domain containing 2A [Source:HGNC Symbol;Acc:28499]	3.43478
FKBP9L	7:53125226-56191148	7p11.2	FK506 binding protein 9-like [Source:HGNC Symbol;Acc:23568]	3.43478
ZNF713	7:53125226-56191148	7p11.2	zinc finger protein 713 [Source:HGNC Symbol;Acc:22043]	3.43478
RP4-725G10.1	7:53125226-56191148	7p11.2	[undefined]	3.43478
RNU1-14P	7:53125226-56191148	7p12.1	RNA, U1 small nuclear 14 (pseudogene) [Source:HGNC Symbol;Acc:10139]	3.43478
AC011228.1	7:53125226-56191148	7p11.2	[undefined]	3.43478
GS1-179L18.1	7:53125226-56191148	7p12.1	[undefined]	3.43478
AC092579.2	7:53125226-56191148	7p11.2	[undefined]	3.43478
AC092647.1	7:53125226-56191148	7p11.2	[undefined]	3.43478
SNORA15	7:53125226-56191148	7p11.2	small nucleolar RNA, H/ACA box 15 [Source:HGNC Symbol;Acc:32604]	3.43478
RP11-436F9.2	7:53125226-56191148	7p11.2	[undefined]	3.43478
RP4-791C19.1	7:53125226-56191148	7p11.2	[undefined]	3.43478
AC009468.1	7:53125226-56191148	7p12.1	[undefined]	3.43478
AC074348.1	7:53125226-56191148	7p12.1	[undefined]	3.43478
RP11-310H4.1	7:53125226-56191148	7p11.2	[undefined]	3.43478
RP5-1091E12.1	7:53125226-56191148	7p11.2	[undefined]	3.43478
GS1-18A18.1	7:53125226-56191148	7p11.2	[undefined]	3.43478
RP11-613E4.1	7:53125226-56191148	7p11.2	[undefined]	3.43478
RP11-419M24.5	7:53125226-56191148	7p11.2	[undefined]	3.43478
RP11-419M24.3	7:53125226-56191148	7p11.2	[undefined]	3.43478
RP11-613E4.3	7:53125226-56191148	7p11.2	[undefined]	3.43478
RP11-266F12.1	7:53125226-56191148	7p12.1	[undefined]	3.43478
RP11-419M24.4	7:53125226-56191148	7p11.2	[undefined]	3.43478
GS1-278J22.2	7:53125226-56191148	7p12.1	[undefined]	3.43478
GS1-18A18.2	7:53125226-56191148	7p11.2	[undefined]	3.43478
RP11-419M24.1	7:53125226-56191148	7p11.2	[undefined]	3.43478
RP11-339F13.1	7:53125226-56191148	7p11.2	[undefined]	3.43478
RP11-775L16.1	7:53125226-56191148	7p11.2	[undefined]	3.43478
RP11-310H4.6	7:53125226-56191148	7p11.2	[undefined]	3.43478
RP11-310H4.3	7:53125226-56191148	7p11.2	[undefined]	3.43478
RP11-436F9.1	7:53125226-56191148	7p11.2	[undefined]	3.43478
RP11-745J15.1	7:53125226-56191148	7p11.2	[undefined]	3.43478
RP11-678B3.1	7:53125226-56191148	7p12.1	[undefined]	3.43478
GS1-18A18.3	7:53125226-56191148	7p11.2	[undefined]	3.43478
RP11-745C15.1	7:53125226-56191148	7p11.2	[undefined]	3.43478
RP11-310H4.2	7:53125226-56191148	7p11.2	[undefined]	3.43478
GS1-278J22.1	7:53125226-56191148	7p12.1	[undefined]	3.43478
RP11-745C15.2	7:53125226-56191148	7p11.2	[undefined]	3.43478
RP11-310H4.5	7:53125226-56191148	7p11.2	[undefined]	3.43478
RP11-310H4.4	7:53125226-56191148	7p11.2	similar to tubulin, beta 5 (LOC442308), non-coding RNA [Source:RefSeq DNA;Acc:NR_003598]	3.43478
RP11-678B3.2	7:53125226-56191148	7p12.1	[undefined]	3.43478
RP11-419M24.2	7:53125226-56191148	7p11.2	[undefined]	3.43478
RP11-339F13.2	7:53125226-56191148	7p11.2	[undefined]	3.43478
RP11-613E4.2	7:53125226-56191148	7p11.2	[undefined]	3.43478
RP11-806J6.1	7:53125226-56191148	7p11.2 7p12.1	[undefined]	3.43478
AC073269.1	7:53125226-56191148	7p11.2	[undefined]	3.43478
AC006970.1	7:53125226-56191148	7p11.2	[undefined]	3.43478
MRPS17	7:53125226-56191148	7p11.2	mitochondrial ribosomal protein S17 [Source:HGNC Symbol;Acc:14047]	3.43478
AC091812.2	7:53125226-56191148	7p11.2	FK506 binding protein 9-like (FKBP9L), transcript variant 4, non-coding RNA [Source:RefSeq DNA;Acc:NR_003949]	3.43478
AC099681.2	7:53125226-56191148	7p11.2	[undefined]	3.43478
AC099681.1	7:53125226-56191148	7p11.2	[undefined]	3.43478
AC092579.1	7:53125226-56191148	7p11.2	[undefined]	3.43478
RP11-15K19.2	7:53125226-56191148	7p11.2	28S ribosomal protein S17, mitochondrial Precursor (S17mt)(MRP-S17) [Source:UniProtKB/Swiss-Prot;Acc:Q9Y2R5]	3.43478
AC092449.1	7:53125226-56191148	7p11.2	[undefined]	3.43478
AC091812.1	7:53125226-56191148	7p11.2	[undefined]	3.43478
METTL1	12:58034214-58257243	12q14.1	methyltransferase like 1 [Source:HGNC Symbol;Acc:7030]	2.93662
CYP27B1	12:58034214-58257243	12q14.1	cytochrome P450, family 27, subfamily B, polypeptide 1 [Source:HGNC Symbol;Acc:2606]	2.93662
TSFM	12:58034214-58257243	12q14.1	Ts translation elongation factor, mitochondrial [Source:HGNC Symbol;Acc:12367]	2.93662
FAM119B	12:58034214-58257243	12q14.1	family with sequence similarity 119, member B [Source:HGNC Symbol;Acc:24936]	2.93662
AVIL	12:58034214-58257243	12q14.1	advillin [Source:HGNC Symbol;Acc:14188]	2.93662
AGAP2	12:58034214-58257243	12q14.1	ArfGAP with GTPase domain, ankyrin repeat and PH domain 2 [Source:HGNC Symbol;Acc:16921]	2.93662
CDK4	12:58034214-58257243	12q14.1	cyclin-dependent kinase 4 [Source:HGNC Symbol;Acc:1773]	2.93662
TSPAN31	12:58034214-58257243	12q14.1	tetraspanin 31 [Source:HGNC Symbol;Acc:10539]	2.93662
OS9	12:58034214-58257243	12q13.3 12q14.1	osteosarcoma amplified 9, endoplasmic reticulum lectin [Source:HGNC Symbol;Acc:16994]	2.93662
MARCH9	12:58034214-58257243	12q14.1	membrane-associated ring finger (C3HC4) 9 [Source:HGNC Symbol;Acc:25139]	2.93662
CTDSP2	12:58034214-58257243	12q14.1	CTD (carboxy-terminal domain, RNA polymerase II, polypeptide A) small phosphatase 2 [Source:HGNC Symbol;Acc:17077]	2.93662
AC025165.1	12:58034214-58257243	12q14.1	[undefined]	2.93662
MIR26A2	12:58034214-58257243	12q14.1	microRNA 26a-2 [Source:HGNC Symbol;Acc:31611]	2.93662
AC025165.3	12:58034214-58257243	12q13.3	[undefined]	2.93662
AC083805.1	12:58034214-58257243	12q14.1	[undefined]	2.93662
RP11-571M6.1	12:58034214-58257243	12q13.3	[undefined]	2.93662
CHIC2	4:54821806-55526632	4q12	cysteine-rich hydrophobic domain 2 [Source:HGNC Symbol;Acc:1935]	2.57217
PDGFRA	4:54821806-55526632	4q12	platelet-derived growth factor receptor, alpha polypeptide [Source:HGNC Symbol;Acc:8803]	2.57217
FIP1L1	4:54821806-55526632	4q12	FIP1 like 1 (S. cerevisiae) [Source:HGNC Symbol;Acc:19124]	2.57217
KIT	4:54821806-55526632	4q12	v-kit Hardy-Zuckerman 4 feline sarcoma viral oncogene homolog [Source:HGNC Symbol;Acc:6342]	2.57217
GSX2	4:54821806-55526632	4q12	GS homeobox 2 [Source:HGNC Symbol;Acc:24959]	2.57217
AC110298.1	4:54821806-55526632	4q12	[undefined]	2.57217
RPL21P44	4:54821806-55526632	4q12	ribosomal protein L21 pseudogene 44 [Source:HGNC Symbol;Acc:33820]	2.57217
AC138607.1	4:54821806-55526632	4q12	[undefined]	2.57217
AC138779.1	4:54821806-55526632	4q12	[undefined]	2.57217
RP11-601I15.1	4:54821806-55526632	4q12	[undefined]	2.57217
RP11-231C18.1	4:54821806-55526632	4q12	[undefined]	2.57217
RP11-231C18.2	4:54821806-55526632	4q12	[undefined]	2.57217
RP11-571I18.3	4:54821806-55526632	4q12	[undefined]	2.57217
AC110792.1	4:54821806-55526632	4q12	[undefined]	2.57217
AC006552.1	4:54821806-55526632	4q12	[undefined]	2.57217
RP11-571I18.2	4:54821806-55526632	4q12	[undefined]	2.57217
RP11-545H22.1	4:54821806-55526632	4q12	[undefined]	2.57217
TNRC18C	7:56300336-57185777	7p11.2	trinucleotide repeat containing 18C (pseudogene) [Source:HGNC Symbol;Acc:34015]	1.81172
AC093392.2	7:56300336-57185777	7p11.2	[undefined]	1.81172
RP11-814E24.1	7:56300336-57185777	7p11.2	[undefined]	1.81172
RP11-760D2.10	7:56300336-57185777	7p11.2	[undefined]	1.81172
RP11-760D2.7	7:56300336-57185777	7p11.2	[undefined]	1.81172
RP11-220H4.1	7:56300336-57185777	7p11.2	[undefined]	1.81172
AC093392.1	7:56300336-57185777	7p11.2	[undefined]	1.81172
RP11-700P18.1	7:56300336-57185777	7p11.2	[undefined]	1.81172
RP11-760D2.4	7:56300336-57185777	7p11.2	[undefined]	1.81172

Continued on next page...

gene name	chr	.DNABand	.GeneDesc	rank
RP11-700P18.2	7:56300336-57185777	7p11.2	[undefined]	1.81172
RP11-814E24.3	7:56300336-57185777	7p11.2	[undefined]	1.81172
RP11-760D2.6	7:56300336-57185777	7p11.2	[undefined]	1.81172
RP11-220H4.2	7:56300336-57185777	7p11.2	[undefined]	1.81172
CICP8	7:56300336-57185777	7p11.2	capicua homolog (Drosophila) pseudogene 8 [Source:HGNC Symbol;Acc:37757]	1.81172
RP11-760D2.1	7:56300336-57185777	7p11.2	[undefined]	1.81172
RP11-760D2.2	7:56300336-57185777	7p11.2	[undefined]	1.81172
RP11-10F11.2	7:56300336-57185777	7p11.2	[undefined]	1.81172
RP11-220H4.4	7:56300336-57185777	7p11.2	[undefined]	1.81172
RP11-10F11.1	7:56300336-57185777	7p11.2	[undefined]	1.81172
RP11-419M24.6	7:56300336-57185777	7p11.2	[undefined]	1.81172
NMD3P2	7:56300336-57185777	7p11.2	nonsense-mediated mRNA 3 homolog (S. cerevisiae) pseudogene 2 [Source:HGNC Symbol;Acc:37820]	1.81172
RP13-492C18.1	7:56300336-57185777	7p11.2	[undefined]	1.81172
RP11-760D2.5	7:56300336-57185777	7p11.2	[undefined]	1.81172
RP13-580B18.1	7:56300336-57185777	7p11.2	[undefined]	1.81172
RP11-760D2.9	7:56300336-57185777	7p11.2	[undefined]	1.81172
RP13-492C18.2	7:56300336-57185777	7p11.2	[undefined]	1.81172
AC092423.1	7:56300336-57185777	7p11.2	[undefined]	1.81172
RP11-598O8.1	7:52638067-53094734	7p12.1	[undefined]	1.44739
RP11-398K14.1	7:52638067-53094734	7p12.1	[undefined]	1.44739
CAV2	7:116126525-116501861	7q31.2	caveolin 2 [Source:HGNC Symbol;Acc:1528]	1.33132
CAV1	7:116126525-116501861	7q31.2	caveolin 1, caveolae protein, 22kDa [Source:HGNC Symbol;Acc:1527]	1.33132
MET	7:116126525-116501861	7q31.2	met proto-oncogene (hepatocyte growth factor receptor) [Source:HGNC Symbol;Acc:7029]	1.33132
CAPZA2	7:116126525-116501861	7q31.2	capping protein (actin filament) muscle Z-line, alpha 2 [Source:HGNC Symbol;Acc:1490]	1.33132
AC006159.3	7:116126525-116501861	7q31.2	[undefined]	1.33132
AC006159.5	7:116126525-116501861	7q31.2	[undefined]	1.33132
AC002066.1	7:116126525-116501861	7q31.2	[undefined]	1.33132
AC006159.4	7:116126525-116501861	7q31.2	[undefined]	1.33132
ZNF734	7:62915687-62919814	7q11.21	zinc finger protein 734 [Source:HGNC Symbol;Acc:32465]	0.97618
ANKIB1	7:91988671-92327770	7q21.2	ankyrin repeat and IBR domain containing 1 [Source:HGNC Symbol;Acc:22215]	0.9478
CDK6	7:91988671-92327770	7q21.2	cyclin-dependent kinase 6 [Source:HGNC Symbol;Acc:1777]	0.9478
PEX1	7:91988671-92327770	7q21.2	peroxisomal biogenesis factor 1 [Source:HGNC Symbol;Acc:8850]	0.9478
C7orf64	7:91988671-92327770	7q21.2	UPF0712 protein C7orf64 [Source:UniProtKB/Swiss-Prot;Acc:Q5RL73]	0.9478
GATAD1	7:91988671-92327770	7q21.2	GATA zinc finger domain containing 1 [Source:HGNC Symbol;Acc:29941]	0.9478
AC007566.11	7:91988671-92327770	7q21.2	[undefined]	0.9478
FAM133B	7:91988671-92327770	7q21.2	family with sequence similarity 133, member B [Source:HGNC Symbol;Acc:28629]	0.9478
ERVWE1	7:91988671-92327770	7q21.2	endogenous retroviral family W, env(C7), member 1 [Source:HGNC Symbol;Acc:13525]	0.9478
AC007566.10	7:91988671-92327770	7q21.2	[undefined]	0.9478
AC004128.1	7:91988671-92327770	7q21.2	[undefined]	0.9478
AC104073.1	7:65030038-65030038	7q11.21	[undefined]	0.86769

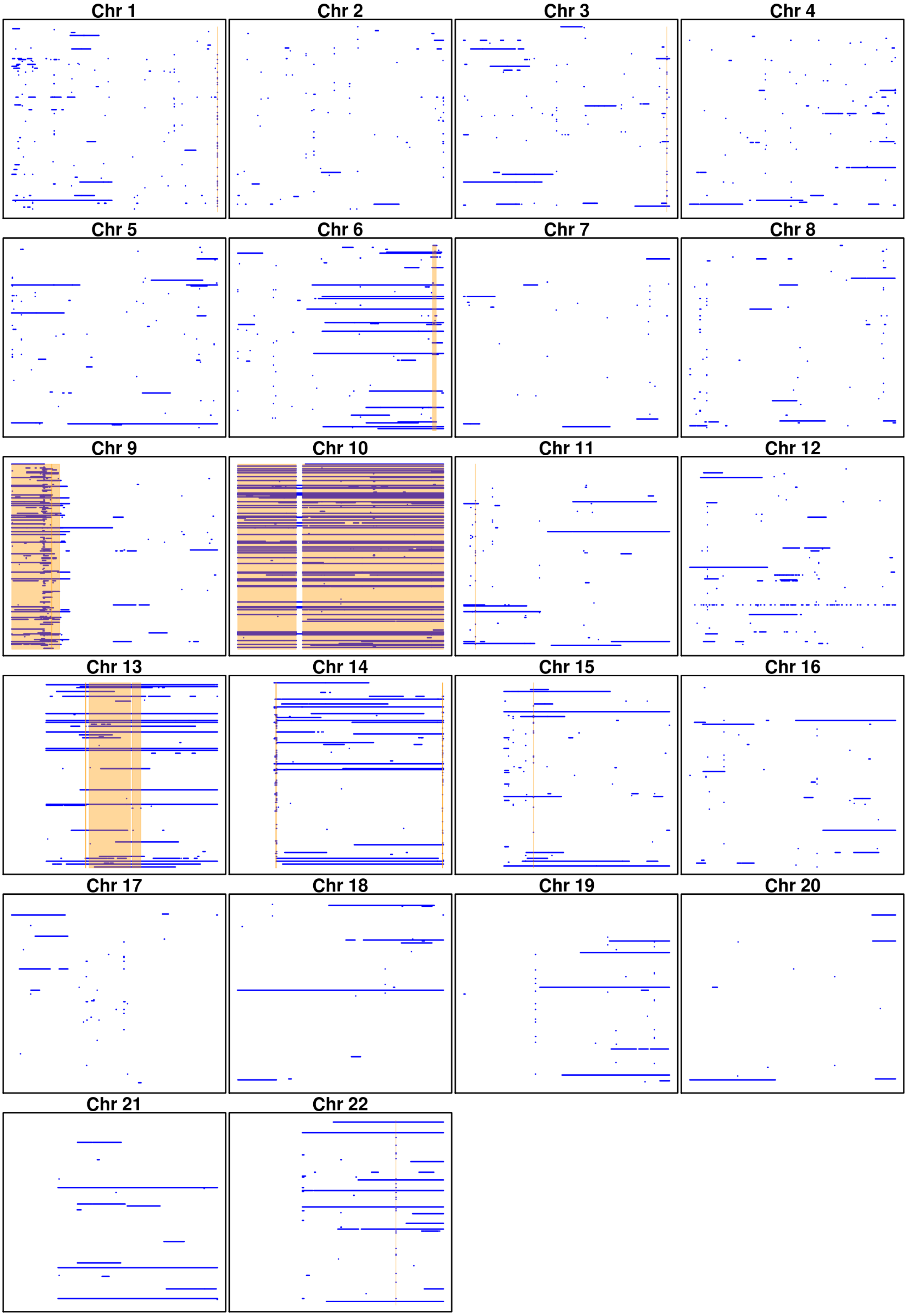

Figure 1913: Blue lines represent CN loss segments, areas highlighted with yellow have losses in more than 0.1 of samples.

10.2 Interesting copy number -1.0 segments

Table 9: This table has 2974 rows.

gene name	chr	.DNABand	.GeneDesc	rank
CNTLN	9:163131-27213502	9p22.2	centlein, centrosomal protein [Source:HGNC Symbol;Acc:23432]	1.40693
DMRT3	9:163131-27213502	9p24.3	doublesex and mab-3 related transcription factor 3 [Source:HGNC Symbol;Acc:13909]	1.40693
RFX3	9:163131-27213502	9p24.2	regulatory factor X, 3 (influences HLA class II expression) [Source:HGNC Symbol;Acc:9984]	1.40693
SMARCA2	9:163131-27213502	9p24.3	SWI/SNF related, matrix associated, actin dependent regulator of chromatin, subfamily a, member 2 [Source:HGNC Symbol;Acc:11098]	1.40693
KIAA0020	9:163131-27213502	9p24.2	KIAA0020 [Source:HGNC Symbol;Acc:29676]	1.40693
IFT74	9:163131-27213502	9p21.2	intraflagellar transport 74 homolog (Chlamydomonas) [Source:HGNC Symbol;Acc:21424]	1.40693
JAK2	9:163131-27213502	9p24.1	Janus kinase 2 [Source:HGNC Symbol;Acc:6192]	1.40693
ERMP1	9:163131-27213502	9p24.1	endoplasmic reticulum metallopeptidase 1 [Source:HGNC Symbol;Acc:23703]	1.40693
MTAP	9:163131-27213502	9p21.3	methylthioadenosine phosphorylase [Source:HGNC Symbol;Acc:7413]	1.40693
C9orf68	9:163131-27213502	9p24.1 9p24.2	Uncharacterized protein C9orf68 [Source:UniProtKB/Swiss-Prot;Acc:Q8N4H0]	1.40693
SLC1A1	9:163131-27213502	9p24.2	solute carrier family 1 (neuronal/epithelial high affinity glutamate transporter, system Xag), member 1 [Source:HGNC Symbol;Acc:10939]	1.40693
CDC37L1	9:163131-27213502	9p24.1	cell division cycle 37 homolog (S. cerevisiae)-like 1 [Source:HGNC Symbol;Acc:17179]	1.40693
RLN2	9:163131-27213502	9p24.1	relaxin 2 [Source:HGNC Symbol;Acc:10027]	1.40693
RLN1	9:163131-27213502	9p24.1	relaxin 1 [Source:HGNC Symbol;Acc:10026]	1.40693
C9orf46	9:163131-27213502	9p24.1	Transmembrane protein C9orf46 [Source:UniProtKB/Swiss-Prot;Acc:Q9HBL7]	1.40693
KIAA1432	9:163131-27213502	9p24.1	KIAA1432 [Source:HGNC Symbol;Acc:17686]	1.40693
KDM4C	9:163131-27213502	9p24.1	lysine (K)-specific demethylase 4C [Source:HGNC Symbol;Acc:17071]	1.40693
DOCK8	9:163131-27213502	9p24.3	dedicator of cytokinesis 8 [Source:HGNC Symbol;Acc:19191]	1.40693
KANK1	9:163131-27213502	9p24.3	KN motif and ankyrin repeat domains 1 [Source:HGNC Symbol;Acc:19309]	1.40693
ELAVL2	9:163131-27213502	9p21.3	ELAV (embryonic lethal, abnormal vision, Drosophila)-like 2 (Hu antigen B) [Source:HGNC Symbol;Acc:3313]	1.40693
TYRP1	9:163131-27213502	9p23	tyrosinase-related protein 1 [Source:HGNC Symbol;Acc:12450]	1.40693
MPDZ	9:163131-27213502	9p23	multiple PDZ domain protein [Source:HGNC Symbol;Acc:7208]	1.40693
GLIS3	9:163131-27213502	9p24.2	GLIS family zinc finger 3 [Source:HGNC Symbol;Acc:28510]	1.40693
SH3GL2	9:163131-27213502	9p22.2	SH3-domain GRB2-like 2 [Source:HGNC Symbol;Acc:10831]	1.40693
TEK	9:163131-27213502	9p21.2	TEK tyrosine kinase, endothelial [Source:HGNC Symbol;Acc:11724]	1.40693
RCL1	9:163131-27213502	9p24.1	RNA terminal phosphate cyclase-like 1 [Source:HGNC Symbol;Acc:17687]	1.40693
C9orf82	9:163131-27213502	9p21.2	Uncharacterized protein C9orf82 [Source:UniProtKB/Swiss-Prot;Acc:Q9H8G2]	1.40693
INSL6	9:163131-27213502	9p24.1	insulin-like 6 [Source:HGNC Symbol;Acc:6089]	1.40693
INSL4	9:163131-27213502	9p24.1	insulin-like 4 (placenta) [Source:HGNC Symbol;Acc:6087]	1.40693
MLANA	9:163131-27213502	9p24.1	melan-A [Source:HGNC Symbol;Acc:7124]	1.40693
CD274	9:163131-27213502	9p24.1	CD274 molecule [Source:HGNC Symbol;Acc:17635]	1.40693
IFNA6	9:163131-27213502	9p21.3	interferon, alpha 6 [Source:HGNC Symbol;Acc:5427]	1.40693
IFNA8	9:163131-27213502	9p21.3	interferon, alpha 8 [Source:HGNC Symbol;Acc:5429]	1.40693
IL33	9:163131-27213502	9p24.1	interleukin 33 [Source:HGNC Symbol;Acc:16028]	1.40693
C9orf123	9:163131-27213502	9p24.1	Transmembrane protein C9orf123 [Source:UniProtKB/Swiss-Prot;Acc:Q96GE9]	1.40693
RANBP6	9:163131-27213502	9p24.1	RAN binding protein 6 [Source:HGNC Symbol;Acc:9851]	1.40693
PLAA	9:163131-27213502	9p21.2	phospholipase A2-activating protein [Source:HGNC Symbol;Acc:9043]	1.40693
IFNA21	9:163131-27213502	9p21.3	interferon, alpha 21 [Source:HGNC Symbol;Acc:5424]	1.40693
DMRT1	9:163131-27213502	9p24.3	doublesex and mab-3 related transcription factor 1 [Source:HGNC Symbol;Acc:2934]	1.40693
DENND4C	9:163131-27213502	9p22.1	DENN/MADD domain containing 4C [Source:HGNC Symbol;Acc:26079]	1.40693
RPS6	9:163131-27213502	9p22.1	ribosomal protein S6 [Source:HGNC Symbol;Acc:10429]	1.40693
VLDLR	9:163131-27213502	9p24.2	very low density lipoprotein receptor [Source:HGNC Symbol;Acc:12698]	1.40693
AK3	9:163131-27213502	9p24.1	adenylate kinase 3 [Source:HGNC Symbol;Acc:17376]	1.40693
UHRF2	9:163131-27213502	9p24.1	ubiquitin-like with PHD and ring finger domains 2 [Source:HGNC Symbol;Acc:12557]	1.40693
NFIB	9:163131-27213502	9p22.3 9p23	nuclear factor I/B [Source:HGNC Symbol;Acc:7785]	1.40693
CER1	9:163131-27213502	9p22.3	cerberus 1, cysteine knot superfamily, homolog (Xenopus laevis) [Source:HGNC Symbol;Acc:1862]	1.40693
PLIN2	9:163131-27213502	9p22.1	perilipin 2 [Source:HGNC Symbol;Acc:248]	1.40693
IFNA5	9:163131-27213502	9p21.3	interferon, alpha 5 [Source:HGNC Symbol;Acc:5426]	1.40693
HAUS6	9:163131-27213502	9p22.1	HAUS augmin-like complex, subunit 6 [Source:HGNC Symbol;Acc:25948]	1.40693
CDKN2B	9:163131-27213502	9p21.3	cyclin-dependent kinase inhibitor 2B (p15, inhibits CDK4) [Source:HGNC Symbol;Acc:1788]	1.40693
IFNA16	9:163131-27213502	9p21.3	interferon, alpha 16 [Source:HGNC Symbol;Acc:5421]	1.40693
CDKN2A	9:163131-27213502	9p21.3	cyclin-dependent kinase inhibitor 2A (melanoma, p16, inhibits CDK4) [Source:HGNC Symbol;Acc:1787]	1.40693
PTPRD	9:163131-27213502	9p23 9p24.1	protein tyrosine phosphatase, receptor type, D [Source:HGNC Symbol;Acc:9668]	1.40693
C9orf150	9:163131-27213502	9p23	Uncharacterized protein C9orf150 [Source:UniProtKB/Swiss-Prot;Acc:Q8IV03]	1.40693
TTC39B	9:163131-27213502	9p22.3	tetratricopeptide repeat domain 39B [Source:HGNC Symbol;Acc:23704]	1.40693
FAM154A	9:163131-27213502	9p22.1	family with sequence similarity 154, member A [Source:HGNC Symbol;Acc:28566]	1.40693
RRAGA	9:163131-27213502	9p22.1	Ras-related GTP binding A [Source:HGNC Symbol;Acc:16963]	1.40693
SLC24A2	9:163131-27213502	9p22.1	solute carrier family 24 (sodium/potassium/calcium exchanger), member 2 [Source:HGNC Symbol;Acc:10976]	1.40693
FREM1	9:163131-27213502	9p22.3	FRAS1 related extracellular matrix 1 [Source:HGNC Symbol;Acc:23399]	1.40693
SNAPC3	9:163131-27213502	9p22.3	small nuclear RNA activating complex, polypeptide 3, 50kDa [Source:HGNC Symbol;Acc:11136]	1.40693
PSIP1	9:163131-27213502	9p22.3	PC4 and SFRS1 interacting protein 1 [Source:HGNC Symbol;Acc:9527]	1.40693
C9orf93	9:163131-27213502	9p22.3	Uncharacterized protein C9orf93 [Source:UniProtKB/Swiss-Prot;Acc:Q6TFL3]	1.40693
KCNV2	9:163131-27213502	9p24.2	potassium channel, subfamily V, member 2 [Source:HGNC Symbol;Acc:19698]	1.40693
TPD52L3	9:163131-27213502	9p24.1	tumor protein D52-like 3 [Source:HGNC Symbol;Acc:23382]	1.40693
MLLT3	9:163131-27213502	9p21.3	myeloid/lymphoid or mixed-lineage leukemia (trithorax homolog, Drosophila); translocated to, 3 [Source:HGNC Symbol;Acc:7136]	1.40693
IFNB1	9:163131-27213502	9p21.3	interferon, beta 1, fibroblast [Source:HGNC Symbol;Acc:5434]	1.40693
RP11-354P17.9	9:163131-27213502	9p21.3	[undefined]	1.40693
CBWD1	9:163131-27213502	9p24.3	COBW domain containing 1 [Source:HGNC Symbol;Acc:17134]	1.40693
BNC2	9:163131-27213502	9p22.2 9p22.3	basonuclin 2 [Source:HGNC Symbol;Acc:30988]	1.40693
DMRT2	9:163131-27213502	9p24.3	doublesex and mab-3 related transcription factor 2 [Source:HGNC Symbol;Acc:2935]	1.40693
ZDHHC21	9:163131-27213502	9p22.3	zinc finger, DHHC-type containing 21 [Source:HGNC Symbol;Acc:20750]	1.40693
DMRTA1	9:163131-27213502	9p21.3	DMRT-like family A1 [Source:HGNC Symbol;Acc:13826]	1.40693
IFNW1	9:163131-27213502	9p21.3	interferon, omega 1 [Source:HGNC Symbol;Acc:5448]	1.40693
ACER2	9:163131-27213502	9p22.1	alkaline ceramidase 2 [Source:HGNC Symbol;Acc:23675]	1.40693
ADAMTSL1	9:163131-27213502	9p22.1 9p22.2	ADAMTS-like 1 [Source:HGNC Symbol;Acc:14632]	1.40693
GLDC	9:163131-27213502	9p24.1	glycine dehydrogenase (decarboxylating) [Source:HGNC Symbol;Acc:4313]	1.40693
RP11-366I21.1	9:163131-27213502	9p24.1	[undefined]	1.40693
RP11-39K24.9	9:163131-27213502	9p24.1	[undefined]	1.40693
KIAA2026	9:163131-27213502	9p24.1	KIAA2026 [Source:HGNC Symbol;Acc:23378]	1.40693
C9orf66	9:163131-27213502	9p24.3	Uncharacterized protein C9orf66 [Source:UniProtKB/Swiss-Prot;Acc:Q5T8R8]	1.40693
LRRC19	9:163131-27213502	9p21.2	leucine rich repeat containing 19 [Source:HGNC Symbol;Acc:23379]	1.40693
IFNE	9:163131-27213502	9p21.3	interferon, epsilon [Source:HGNC Symbol;Acc:18163]	1.40693
IFNA10	9:163131-27213502	9p21.3	interferon, alpha 10 [Source:HGNC Symbol;Acc:5418]	1.40693
KIAA1797	9:163131-27213502	9p21.3	KIAA1797 [Source:HGNC Symbol;Acc:23377]	1.40693
IFNA2	9:163131-27213502	9p21.3	interferon, alpha 2 [Source:HGNC Symbol;Acc:5423]	1.40693
PTPLAD2	9:163131-27213502	9p21.3	protein tyrosine phosphatase-like A domain containing 2 [Source:HGNC Symbol;Acc:20920]	1.40693
AL356791.1	9:163131-27213502	9p21.2	Putative deoxyuridine 5'-triphosphate nucleotidohydrolase-like protein FLJ16323 (dUTPase-like protein) [Source:UniProtKB/Swiss-Prot;Acc:Q6ZN92]	1.40693
PDCD1LG2	9:163131-27213502	9p24.1	programmed cell death 1 ligand 2 [Source:HGNC Symbol;Acc:18731]	1.40693
IFNA1	9:163131-27213502	9p21.3	interferon, alpha 1 [Source:HGNC Symbol;Acc:5417]	1.40693
KLHL9	9:163131-27213502	9p21.3	kelch-like 9 (Drosophila) [Source:HGNC Symbol;Acc:18732]	1.40693
TUSC1	9:163131-27213502	9p21.2	tumor suppressor candidate 1 [Source:HGNC Symbol;Acc:31010]	1.40693
MIR101-2	9:163131-27213502	9p24.1	microRNA 101-2 [Source:HGNC Symbol;Acc:31489]	1.40693
MIR31	9:163131-27213502	9p21.3	microRNA 31 [Source:HGNC Symbol;Acc:31630]	1.40693
AL390882.1	9:163131-27213502	9p21.3	[undefined]	1.40693
AL512643.1	9:163131-27213502	9p22.3	[undefined]	1.40693
AL162419.1	9:163131-27213502	9p24.2	[undefined]	1.40693
AL591206.1	9:163131-27213502	9p22.1	[undefined]	1.40693
AL392089.1	9:163131-27213502	9p24.3	[undefined]	1.40693
AL445624.1	9:163131-27213502	9p21.3	[undefined]	1.40693
AL358934.1	9:163131-27213502	9p24.3	[undefined]	1.40693
AL365202.1	9:163131-27213502	9p24.2	Putative uncharacterized protein ENSP00000382846 Fragment [Source:UniProtKB/TrEMBL;Acc:A8MV70]	1.40693
C9orf134	9:163131-27213502	9p21.3	Uncharacterized protein C9orf134 Precursor [Source:UniProtKB/Swiss-Prot;Acc:Q5VZ72]	1.40693
C9orf92	9:163131-27213502	9p22.3	Putative uncharacterized protein C9orf92 [Source:UniProtKB/Swiss-Prot;Acc:A6NGG3]	1.40693
C9orf146	9:163131-27213502	9p23	[undefined]	1.40693
PPAPDC2	9:163131-27213502	9p24.1	phosphatidic acid phosphatase type 2 domain containing 2 [Source:HGNC Symbol;Acc:23682]	1.40693
RP11-106A1.2	9:163131-27213502	9p24.1	[undefined]	1.40693
AL359877.1	9:163131-27213502	9p22.3	[undefined]	1.40693

Continued on next page...

gene name	chr	.DNABand	.GeneDesc	rank
AL513423.1	9:163131-27213502	9p22.3	[undefined]	1.40693
MIR491	9:163131-27213502	9p21.3	microRNA 491 [Source:HGNC Symbol;Acc:32076]	1.40693
RP11-134K1.2	9:163131-27213502	9p24.1	[undefined]	1.40693
RPS26P3	9:163131-27213502	9p23	ribosomal protein S26 pseudogene 3 [Source:HGNC Symbol;Acc:23411]	1.40693
RP11-518K17.3	9:163131-27213502	9p22.3	[undefined]	1.40693
RP11-280O24.1	9:163131-27213502	9p23	[undefined]	1.40693
IFNA7	9:163131-27213502	9p21.3	interferon, alpha 7 [Source:HGNC Symbol;Acc:5428]	1.40693
RP11-54D18.1	9:163131-27213502	9p22.3	[undefined]	1.40693
RP11-382H24.3	9:163131-27213502	9p23	[undefined]	1.40693
RP11-12D24.7	9:163131-27213502	9p24.1	[undefined]	1.40693
RP11-12D24.3	9:163131-27213502	9p24.1	[undefined]	1.40693
UBA52P6	9:163131-27213502	9p21.3	ubiquitin A-52 residue ribosomal protein fusion product 1 pseudogene 6 [Source:HGNC Symbol;Acc:36763]	1.40693
RP11-490C5.1	9:163131-27213502	9p22.3	[undefined]	1.40693
RP11-54D18.2	9:163131-27213502	9p22.3	[undefined]	1.40693
IGHEP2	9:163131-27213502	9p24.1	immunoglobulin heavy constant epsilon P2 pseudogene [Source:HGNC Symbol;Acc:5524]	1.40693
RP11-62E14.2	9:163131-27213502	9p24.2	[undefined]	1.40693
AL157830.2	9:163131-27213502	9p21.3	[undefined]	1.40693
RP11-307I14.2	9:163131-27213502	9p24.1	[undefined]	1.40693
RP11-125K10.2	9:163131-27213502	9p24.1	[undefined]	1.40693
AL365202.2	9:163131-27213502	9p24.2	[undefined]	1.40693
AL353733.1	9:163131-27213502	9p23	[undefined]	1.40693
AL162419.2	9:163131-27213502	9p24.2	[undefined]	1.40693
AL512635.1	9:163131-27213502	9p21.3	[undefined]	1.40693
AL513498.1	9:163131-27213502	9p21.3	[undefined]	1.40693
AL162422.1	9:163131-27213502	9p23	[undefined]	1.40693
AL353711.1	9:163131-27213502	9p23	[undefined]	1.40693
AL627260.1	9:163131-27213502	9p21.3	[undefined]	1.40693
AL138755.1	9:163131-27213502	9p24.3	[undefined]	1.40693
AL157830.1	9:163131-27213502	9p21.3	[undefined]	1.40693
AL355433.1	9:163131-27213502	9p21.2	[undefined]	1.40693
AL158150.1	9:163131-27213502	9p22.1	[undefined]	1.40693
RP11-284P20.4	9:163131-27213502	9p23	[undefined]	1.40693
RP11-546N22.2	9:163131-27213502	9p24.1	[undefined]	1.40693
RP11-272P10.3	9:163131-27213502	9p23	[undefined]	1.40693
IFNWP18	9:163131-27213502	9p21.3	interferon, omega 18 (pseudogene) [Source:HGNC Symbol;Acc:5450]	1.40693
RP11-39K24.4	9:163131-27213502	9p24.1	[undefined]	1.40693
RP11-443B9.1	9:163131-27213502	9p24.3	[undefined]	1.40693
RP11-536D16.2	9:163131-27213502	9p22.3	[undefined]	1.40693
IFNA22P	9:163131-27213502	9p21.3	interferon, alpha 22, pseudogene [Source:HGNC Symbol;Acc:5431]	1.40693
RP11-492A12.2	9:163131-27213502	9p22.2	[undefined]	1.40693
RP11-370B11.3	9:163131-27213502	9p21.3	[undefined]	1.40693
RP11-341G2.3	9:163131-27213502	9p24.3	[undefined]	1.40693
C9orf53	9:163131-27213502	9p21.3	Susceptibility protein NSG-x [Source:UniProtKB/Swiss-Prot;Acc:Q9UH64]	1.40693
RP11-74C3.1	9:163131-27213502	9p23	[undefined]	1.40693
RP11-403H13.1	9:163131-27213502	9p24.1	[undefined]	1.40693
RP11-113D19.3	9:163131-27213502	9p21.3	[undefined]	1.40693
RP11-207C16.4	9:163131-27213502	9p24.1	[undefined]	1.40693
RP11-120J1.1	9:163131-27213502	9p22.3	[undefined]	1.40693
RP11-390F4.3	9:163131-27213502	9p24.1	[undefined]	1.40693
RP11-75C9.1	9:163131-27213502	9p24.1	[undefined]	1.40693
RP11-6J24.3	9:163131-27213502	9p24.1	[undefined]	1.40693
RP11-536O18.1	9:163131-27213502	9p23	[undefined]	1.40693
RP11-29B9.1	9:163131-27213502	9p24.1	[undefined]	1.40693
IFNP20	9:163131-27213502	9p21.3	interferon pseudogene 20 [Source:HGNC Symbol;Acc:5444]	1.40693
RP11-31F19.1	9:163131-27213502	9p24.3	[undefined]	1.40693
RP11-132E11.3	9:163131-27213502	9p22.2	[undefined]	1.40693
IFNWP9	9:163131-27213502	9p21.3	interferon, omega 9 (pseudogene) [Source:HGNC Symbol;Acc:5455]	1.40693
RP11-315I14.5	9:163131-27213502	9p21.3	[undefined]	1.40693
RP11-509J21.4	9:163131-27213502	9p24.2	[undefined]	1.40693
RP11-87N24.2	9:163131-27213502	9p23	[undefined]	1.40693
RP11-4E23.2	9:163131-27213502	9p21.3	[undefined]	1.40693
RP11-421B23.2	9:163131-27213502	9p23	[undefined]	1.40693
RP11-165F24.3	9:163131-27213502	9p24.3	[undefined]	1.40693
RP11-570H19.2	9:163131-27213502	9p22.2	[undefined]	1.40693
RP11-344A7.1	9:163131-27213502	9p21.3	[undefined]	1.40693
RP11-315I14.1	9:163131-27213502	9p21.3	[undefined]	1.40693
SNRPEL1	9:163131-27213502	9p24.1	small nuclear ribonucleoprotein polypeptide E-like 1 [Source:HGNC Symbol;Acc:20733]	1.40693
RP11-39K24.5	9:163131-27213502	9p24.1	[undefined]	1.40693
CARM1L	9:163131-27213502	9p24.2	coactivator associated arginine methyltransferase 1-like [Source:HGNC Symbol;Acc:23392]	1.40693
RP11-130C19.3	9:163131-27213502	9p24.3	[undefined]	1.40693
IFNA14	9:163131-27213502	9p21.3	interferon, alpha 14 [Source:HGNC Symbol;Acc:5420]	1.40693
RP11-39K24.12	9:163131-27213502	9p24.1	[undefined]	1.40693
RP11-165F24.5	9:163131-27213502	9p24.3	[undefined]	1.40693
RP11-279E1.1	9:163131-27213502	9p24.1	[undefined]	1.40693
RP11-125K10.5	9:163131-27213502	9p24.1	[undefined]	1.40693
RP11-39K24.11	9:163131-27213502	9p24.1	[undefined]	1.40693
RP11-358M14.2	9:163131-27213502	9p24.2	[undefined]	1.40693
RP11-218I7.2	9:163131-27213502	9p24.1	[undefined]	1.40693
RP11-147I11.1	9:163131-27213502	9p24.3	[undefined]	1.40693
RP11-163F8.3	9:163131-27213502	9p22.2	[undefined]	1.40693
RP11-408A13.1	9:163131-27213502	9p22.3	[undefined]	1.40693
RP11-106A1.3	9:163131-27213502	9p24.1	[undefined]	1.40693
RP11-255J3.3	9:163131-27213502	9p24.2	[undefined]	1.40693
RP11-284P20.2	9:163131-27213502	9p23	[undefined]	1.40693
RP11-70L8.1	9:163131-27213502	9p21.3	[undefined]	1.40693
RP11-390F4.10	9:163131-27213502	9p24.1	[undefined]	1.40693
RP11-473O3.1	9:163131-27213502	9p21.3	[undefined]	1.40693
RP11-130C19.1	9:163131-27213502	9p24.3	[undefined]	1.40693
RP11-70J12.1	9:163131-27213502	9p24.2	[undefined]	1.40693
RP11-87M1.1	9:163131-27213502	9p24.1	[undefined]	1.40693
RP11-113D19.6	9:163131-27213502	9p21.3	[undefined]	1.40693
RP11-39K24.15	9:163131-27213502	9p24.1	[undefined]	1.40693
RP11-23D5.1	9:163131-27213502	9p23	[undefined]	1.40693
RP11-390F4.8	9:163131-27213502	9p24.1	[undefined]	1.40693
RP11-421B23.1	9:163131-27213502	9p23	[undefined]	1.40693
RP11-62F24.1	9:163131-27213502	9p22.2	[undefined]	1.40693
RP11-315I14.3	9:163131-27213502	9p21.3	[undefined]	1.40693
RP11-527D15.1	9:163131-27213502	9p23	[undefined]	1.40693
RP11-91N2.3	9:163131-27213502	9p24.2	[undefined]	1.40693
RP11-12D24.8	9:163131-27213502	9p24.1	[undefined]	1.40693
RP11-390F4.9	9:163131-27213502	9p24.1	[undefined]	1.40693
RP11-147I11.2	9:163131-27213502	9p24.3	[undefined]	1.40693
IFNP11	9:163131-27213502	9p21.3	interferon pseudogene 11 [Source:HGNC Symbol;Acc:5442]	1.40693
HMGN2L	9:163131-27213502	9p22.3	high-mobility group nucleosomal binding domain 2-like [Source:HGNC Symbol;Acc:31330]	1.40693
RNF2P1	9:163131-27213502	9p24.1	ring finger protein 2 pseudogene 1 [Source:HGNC Symbol;Acc:33987]	1.40693
RP11-321L2.1	9:163131-27213502	9p21.3	[undefined]	1.40693
RP11-71E22.1	9:163131-27213502	9p23	[undefined]	1.40693
RP11-574F11.3	9:163131-27213502	9p24.1	[undefined]	1.40693
RP11-183I6.2	9:163131-27213502	9p22.3	[undefined]	1.40693
RP11-29B9.2	9:163131-27213502	9p24.1	[undefined]	1.40693
RP11-363E7.3	9:163131-27213502	9p22.1	[undefined]	1.40693
RP11-12D24.6	9:163131-27213502	9p24.1	[undefined]	1.40693
RP11-54D18.3	9:163131-27213502	9p22.3	[undefined]	1.40693
RP11-87N24.3	9:163131-27213502	9p23	[undefined]	1.40693
RP11-509J21.1	9:163131-27213502	9p24.2	[undefined]	1.40693
IFNWP5	9:163131-27213502	9p21.3	interferon, omega 5 (pseudogene) [Source:HGNC Symbol;Acc:5454]	1.40693
RP11-252O2.2	9:163131-27213502	9p22.1	[undefined]	1.40693
RP11-146N23.1	9:163131-27213502	9p22.1	[undefined]	1.40693
IFNWP15	9:163131-27213502	9p21.3	interferon, omega 15 (pseudogene) [Source:HGNC Symbol;Acc:5449]	1.40693
RP11-337A23.5	9:163131-27213502	9p21.2	[undefined]	1.40693
RP11-179D22.1	9:163131-27213502	9p21.2	[undefined]	1.40693
RP11-390F4.2	9:163131-27213502	9p24.1	[undefined]	1.40693
RP11-146N23.4	9:163131-27213502	9p22.1	[undefined]	1.40693

Continued on next page...

gene name	chr	.DNABand	.GeneDesc	rank
RP11-307I14.3	9:163131-27213502	9p24.1	[undefined]	1.40693
RP11-70L8.3	9:163131-27213502	9p21.3	[undefined]	1.40693
RP11-307L3.4	9:163131-27213502	9p24.1	[undefined]	1.40693
RP11-335H2.2	9:163131-27213502	9p22.2	[undefined]	1.40693
RP11-459D20.1	9:163131-27213502	9p24.3	[undefined]	1.40693
IFNA13	9:163131-27213502	9p21.3	interferon, alpha 13 [Source:HGNC Symbol;Acc:5419]	1.40693
RP11-307L3.2	9:163131-27213502	9p24.1	[undefined]	1.40693
RP11-315I14.2	9:163131-27213502	9p21.3	[undefined]	1.40693
RP11-75C9.2	9:163131-27213502	9p24.1	[undefined]	1.40693
RP11-109M17.1	9:163131-27213502	9p23	[undefined]	1.40693
RP11-54D18.4	9:163131-27213502	9p22.3	[undefined]	1.40693
RP11-87N24.1	9:163131-27213502	9p23	[undefined]	1.40693
RP11-39K24.2	9:163131-27213502	9p24.1	[undefined]	1.40693
RP11-382H24.2	9:163131-27213502	9p23	[undefined]	1.40693
AL589675.1	9:163131-27213502	9p24.2	[undefined]	1.40693
RP11-337A23.3	9:163131-27213502	9p21.2	[undefined]	1.40693
RP11-272P10.2	9:163131-27213502	9p23	[undefined]	1.40693
RP11-62F24.2	9:163131-27213502	9p22.2	[undefined]	1.40693
IFNA17	9:163131-27213502	9p21.3	interferon, alpha 17 [Source:HGNC Symbol;Acc:5422]	1.40693
RP11-399D6.2	9:163131-27213502	9p21.3	[undefined]	1.40693
RP11-513M16.5	9:163131-27213502	9p22.1	[undefined]	1.40693
RP11-280O24.3	9:163131-27213502	9p22.3	[undefined]	1.40693
IFNP12	9:163131-27213502	9p21.3	interferon pseudogene 12 [Source:HGNC Symbol;Acc:5443]	1.40693
RP11-509J21.3	9:163131-27213502	9p24.2	[undefined]	1.40693
RP11-165F24.2	9:163131-27213502	9p24.3	[undefined]	1.40693
RP11-134K1.3	9:163131-27213502	9p24.1	[undefined]	1.40693
RP11-3L8.3	9:163131-27213502	9p23	[undefined]	1.40693
RP11-284P20.3	9:163131-27213502	9p23	[undefined]	1.40693
RP11-59O6.3	9:163131-27213502	9p24.3	[undefined]	1.40693
RP11-39K24.14	9:163131-27213502	9p24.1	[undefined]	1.40693
RP11-264I13.2	9:163131-27213502	9p24.3	[undefined]	1.40693
RP11-39K24.8	9:163131-27213502	9p24.1	[undefined]	1.40693
RP11-33G16.1	9:163131-27213502	9p21.2	[undefined]	1.40693
RP11-125B21.2	9:163131-27213502	9p24.2	[undefined]	1.40693
RP11-255J3.2	9:163131-27213502	9p24.2	[undefined]	1.40693
RP11-32F11.2	9:163131-27213502	9p24.2	[undefined]	1.40693
RP11-390F4.7	9:163131-27213502	9p24.1	[undefined]	1.40693
RP11-39K24.7	9:163131-27213502	9p24.1	[undefined]	1.40693
RP11-341G2.1	9:163131-27213502	9p24.3	[undefined]	1.40693
IFNA4	9:163131-27213502	9p21.3	interferon, alpha 4 [Source:HGNC Symbol;Acc:5425]	1.40693
RP11-296P7.4	9:163131-27213502	9p22.1	[undefined]	1.40693
RP11-252M18.3	9:163131-27213502	9p24.2	[undefined]	1.40693
RP11-370B11.1	9:163131-27213502	9p21.3	[undefined]	1.40693
RP11-503K16.2	9:163131-27213502	9p22.1	[undefined]	1.40693
RP11-408N14.1	9:163131-27213502	9p21.3	[undefined]	1.40693
RP11-390F4.6	9:163131-27213502	9p24.1	[undefined]	1.40693
C9orf70	9:163131-27213502	9p24.2	[undefined]	1.40693
RP11-132E11.4	9:163131-27213502	9p22.2	[undefined]	1.40693
RP11-73E6.2	9:163131-27213502	9p21.3	[undefined]	1.40693
RP11-408A13.2	9:163131-27213502	9p22.3	[undefined]	1.40693
RP11-132E11.2	9:163131-27213502	9p22.2	[undefined]	1.40693
RP11-472F14.4	9:163131-27213502	9p24.1	[undefined]	1.40693
RP11-575C20.1	9:163131-27213502	9p24.1	[undefined]	1.40693
RP11-509J21.2	9:163131-27213502	9p24.2	[undefined]	1.40693
RP11-321L2.2	9:163131-27213502	9p21.3	[undefined]	1.40693
RP11-15P13.1	9:163131-27213502	9p21.3	[undefined]	1.40693
RP11-140C18.2	9:163131-27213502	9p24.1	[undefined]	1.40693
IFNWP2	9:163131-27213502	9p21.3	interferon, omega 2 (pseudogene) [Source:HGNC Symbol;Acc:5452]	1.40693
RP11-39K24.13	9:163131-27213502	9p24.1	[undefined]	1.40693
IFNWP19	9:163131-27213502	9p21.3	interferon, omega 19 (pseudogene) [Source:HGNC Symbol;Acc:5451]	1.40693
AL161909.1	9:163131-27213502	9p22.1	[undefined]	1.40693
AL158147.1	9:163131-27213502	9p24.1	[undefined]	1.40693
AL365360.1	9:163131-27213502	9p24.1	[undefined]	1.40693
AL451130.1	9:163131-27213502	9p24.1	[undefined]	1.40693
AL161450.7	9:163131-27213502	9p24.1	[undefined]	1.40693
AL161450.8	9:163131-27213502	9p24.1	[undefined]	1.40693
AL161450.11	9:163131-27213502	9p24.1	[undefined]	1.40693
AL356133.2	9:163131-27213502	9p21.2	[undefined]	1.40693
RP11-546N22.6	9:163131-27213502	9p24.1	[undefined]	1.40693
AL161450.10	9:163131-27213502	9p24.1	[undefined]	1.40693
AL356133.1	9:163131-27213502	9p21.2	[undefined]	1.40693
CDKN2BAS	9:163131-27213502	9p21.3	CDKN2B antisense RNA (non-protein coding) [Source:HGNC Symbol;Acc:34341]	1.40693
AL158206.1	9:163131-27213502	9p22.1	[undefined]	1.40693
AL513423.2	9:163131-27213502	9p22.3	[undefined]	1.40693
AL162725.1	9:163131-27213502	9p22.2	[undefined]	1.40693
AL161450.15	9:163131-27213502	9p24.1	[undefined]	1.40693
AL161450.3	9:163131-27213502	9p24.1	[undefined]	1.40693
RP11-513M16.2	9:163131-27213502	9p22.1	[undefined]	1.40693
AL161450.13	9:163131-27213502	9p24.1	[undefined]	1.40693
AL161450.14	9:163131-27213502	9p24.1	[undefined]	1.40693
AL161450.4	9:163131-27213502	9p24.1	[undefined]	1.40693
AL138753.2	9:163131-27213502	9p23	[undefined]	1.40693
AL161450.1	9:163131-27213502	9p24.1	[undefined]	1.40693
AL161450.6	9:163131-27213502	9p24.1	[undefined]	1.40693
AL161450.12	9:163131-27213502	9p24.1	[undefined]	1.40693
AL161450.16	9:163131-27213502	9p24.1	[undefined]	1.40693
RP11-125K10.4	9:163131-27213502	9p24.1	[undefined]	1.40693
AL161450.5	9:163131-27213502	9p24.1	[undefined]	1.40693
AL355679.1	9:163131-27213502	9p21.3	[undefined]	1.40693
AL161450.9	9:163131-27213502	9p24.1	[undefined]	1.40693
AL358117.1	9:163131-27213502	9p22.2	[undefined]	1.40693
AL138753.1	9:163131-27213502	9p23	[undefined]	1.40693
AL161450.2	9:163131-27213502	9p24.1	[undefined]	1.40693
AL358976.1	9:163131-27213502	9p24.3	[undefined]	1.40693
RP11-546N22.5	9:163131-27213502	9p24.1	[undefined]	1.40693
AL513423.3	9:163131-27213502	9p22.3	[undefined]	1.40693
RP11-39K24.10	9:163131-27213502	9p24.1	[undefined]	1.40693
AL596451.1	9:163131-27213502	9p24.1	[undefined]	1.40693
SCARNA8	9:163131-27213502	9p22.1	small Cajal body-specific RNA 8 [Source:HGNC Symbol;Acc:32564]	1.40693
AL441925.1	9:163131-27213502	9p22.3	[undefined]	1.40693
AL591393.1	9:163131-27213502	9p22.3	[undefined]	1.40693
AL353718.1	9:163131-27213502	9p24.1	[undefined]	1.40693
AL163193.1	9:163131-27213502	9p21.3	[undefined]	1.40693
AL451137.1	9:163131-27213502	9p21.2	[undefined]	1.40693
AL591206.2	9:163131-27213502	9p22.1	[undefined]	1.40693
AL449963.1	9:163131-27213502	9p22.1	[undefined]	1.40693
AL358976.2	9:163131-27213502	9p24.3	[undefined]	1.40693
MOBKL2B	9:27218901-32395746	9p21.2	MOB1, Mps One Binder kinase activator-like 2B (yeast) [Source:HGNC Symbol;Acc:23825]	1.18038
C9orf72	9:27218901-32395746	9p21.2	Uncharacterized protein C9orf72 [Source:UniProtKB/Swiss-Prot;Acc:Q96LT7]	1.18038
C9orf11	9:27218901-32395746	9p21.2	Acrosome formation-associated factor Precursor [Source:UniProtKB/Swiss-Prot;Acc:Q9NQ60]	1.18038
LINGO2	9:27218901-32395746	9p21.1 9p21.2	leucine rich repeat and Ig domain containing 2 [Source:HGNC Symbol;Acc:21207]	1.18038
ACO1	9:27218901-32395746	9p21.1	aconitase 1, soluble [Source:HGNC Symbol;Acc:117]	1.18038
RP11-560D2.1	9:27218901-32395746	9p21.1	[undefined]	1.18038
IFNK	9:27218901-32395746	9p21.2	interferon, kappa [Source:HGNC Symbol;Acc:21714]	1.18038
RP11-535I8.1	9:27218901-32395746	9p21.2	[undefined]	1.18038
AL590726.1	9:27218901-32395746	9p21.1	[undefined]	1.18038
MIR876	9:27218901-32395746	9p21.1	microRNA 876 [Source:HGNC Symbol;Acc:33653]	1.18038
RP11-255N24.3	9:27218901-32395746	9p21.1	[undefined]	1.18038
RP11-572H4.1	9:27218901-32395746	9p21.1	[undefined]	1.18038
MIR873	9:27218901-32395746	9p21.1	microRNA 873 [Source:HGNC Symbol;Acc:33663]	1.18038
RP11-255N24.2	9:27218901-32395746	9p21.1	[undefined]	1.18038
AL590726.2	9:27218901-32395746	9p21.1	[undefined]	1.18038
KRT18P36	9:27218901-32395746	9p21.1	keratin 18 pseudogene 36 [Source:HGNC Symbol;Acc:33405]	1.18038
RP11-32I2.1	9:27218901-32395746	9p21.1	[undefined]	1.18038

Continued on next page...

gene name	chr	.DNABand	.GeneDesc	rank
C9orf35	9:27218901-32395746	9p21.2	CDNA FLJ23651 fis, clone COL08363CDNA FLJ23916 fis, clone COL00117 ; [Source:UniProtKB/TrEMBL;Acc:Q8TEC6]	1.18038
RP11-550E18.1	9:27218901-32395746	9p21.1	[undefined]	1.18038
RP11-20P5.2	9:27218901-32395746	9p21.1	[undefined]	1.18038
RP11-291J9.1	9:27218901-32395746	9p21.1	[undefined]	1.18038
RP11-48L13.1	9:27218901-32395746	9p21.1	[undefined]	1.18038
RP11-560D2.2	9:27218901-32395746	9p21.1	[undefined]	1.18038
RP11-373A6.1	9:27218901-32395746	9p21.1	[undefined]	1.18038
RP11-291J9.2	9:27218901-32395746	9p21.1	[undefined]	1.18038
RP11-255N24.4	9:27218901-32395746	9p21.1	[undefined]	1.18038
RP11-460C6.1	9:27218901-32395746	9p21.1	[undefined]	1.18038
RPS26P2	9:27218901-32395746	9p21.1	ribosomal protein S26 pseudogene 2 [Source:HGNC Symbol;Acc:23662]	1.18038
NCRNA00032	9:27218901-32395746	9p21.2	non-protein coding RNA 32 [Source:HGNC Symbol;Acc:16506]	1.18038
RP11-441F11.1	9:27218901-32395746	9p21.1	[undefined]	1.18038
RP11-572H4.2	9:27218901-32395746	9p21.1	[undefined]	1.18038
AL390883.1	9:27218901-32395746	9p21.1	[undefined]	1.18038
RP11-402B2.1	9:27218901-32395746	9p21.1	[undefined]	1.18038
AL589825.1	9:27218901-32395746	9p21.1	[undefined]	1.18038
RP11-298E2.2	9:27218901-32395746	9p21.2	[undefined]	1.18038
AL353573.1	9:27218901-32395746	9p21.1	[undefined]	1.18038
RP11-255N24.1	9:27218901-32395746	9p21.1	[undefined]	1.18038
AL162396.1	9:27218901-32395746	9p21.2	[undefined]	1.18038
OR5P2	11:7817978-7826768	11p15.4	olfactory receptor, family 5, subfamily P, member 2 [Source:HGNC Symbol;Acc:14783]	1.10036
APOBEC3C	22:39361572-39379993	22q13.1	apolipoprotein B mRNA editing enzyme, catalytic polypeptide-like 3C [Source:HGNC Symbol;Acc:17353]	1.0937
APOBEC3B	22:39361572-39379993	22q13.1	apolipoprotein B mRNA editing enzyme, catalytic polypeptide-like 3B [Source:HGNC Symbol;Acc:17352]	1.0937
APOBEC3D	22:39361572-39379993	22q13.1	apolipoprotein B mRNA editing enzyme, catalytic polypeptide-like 3D [Source:HGNC Symbol;Acc:17354]	1.0937
ALOX5	10:42889244-135513631	10q11.21	arachidonate 5-lipoxygenase [Source:HGNC Symbol;Acc:435]	1.05119
COX15	10:42889244-135513631	10q24.2	COX15 homolog, cytochrome c oxidase assembly protein (yeast) [Source:HGNC Symbol;Acc:2263]	1.05119
ZRANB1	10:42889244-135513631	10q26.13	zinc finger, RAN-binding domain containing 1 [Source:HGNC Symbol;Acc:18224]	1.05119
ZDHHC6	10:42889244-135513631	10q25.2	zinc finger, DHHC-type containing 6 [Source:HGNC Symbol;Acc:19160]	1.05119
ABCC2	10:42889244-135513631	10q24.2	ATP-binding cassette, sub-family C (CFTR/MRP), member 2 [Source:HGNC Symbol;Acc:53]	1.05119
FAS	10:42889244-135513631	10q23.31	Fas (TNF receptor superfamily, member 6) [Source:HGNC Symbol;Acc:11920]	1.05119
VCL	10:42889244-135513631	10q22.2	vinculin [Source:HGNC Symbol;Acc:12665]	1.05119
AIFM2	10:42889244-135513631	10q22.1	apoptosis-inducing factor, mitochondrion-associated, 2 [Source:HGNC Symbol;Acc:21411]	1.05119
ADRB1	10:42889244-135513631	10q25.3	adrenergic, beta-1-, receptor [Source:HGNC Symbol;Acc:285]	1.05119
RRP12	10:42889244-135513631	10q24.1	ribosomal RNA processing 12 homolog (S. cerevisiae) [Source:HGNC Symbol;Acc:29100]	1.05119
MRPL43	10:42889244-135513631	10q24.31	mitochondrial ribosomal protein L43 [Source:HGNC Symbol;Acc:14517]	1.05119
ALDH18A1	10:42889244-135513631	10q24.1	aldehyde dehydrogenase 18 family, member A1 [Source:HGNC Symbol;Acc:9722]	1.05119
PSD	10:42889244-135513631	10q24.32	pleckstrin and Sec7 domain containing [Source:HGNC Symbol;Acc:9507]	1.05119
CCAR1	10:42889244-135513631	10q21.3	cell division cycle and apoptosis regulator 1 [Source:HGNC Symbol;Acc:24236]	1.05119
WAPAL	10:42889244-135513631	10q23.2	wings apart-like homolog (Drosophila) [Source:HGNC Symbol;Acc:23293]	1.05119
OAT	10:42889244-135513631	10q26.13	ornithine aminotransferase [Source:HGNC Symbol;Acc:8091]	1.05119
SLK	10:42889244-135513631	10q24.33	STE20-like kinase (yeast) [Source:HGNC Symbol;Acc:11088]	1.05119
COL17A1	10:42889244-135513631	10q24.33 10q25.1	collagen, type XVII, alpha 1 [Source:HGNC Symbol;Acc:2194]	1.05119
GSTO2	10:42889244-135513631	10q25.1	glutathione S-transferase omega 2 [Source:HGNC Symbol;Acc:23064]	1.05119
FGFR2	10:42889244-135513631	10q26.13	fibroblast growth factor receptor 2 [Source:HGNC Symbol;Acc:3689]	1.05119
INPP5A	10:42889244-135513631	10q26.3	inositol polyphosphate-5-phosphatase, 40kDa [Source:HGNC Symbol;Acc:6076]	1.05119
CHAT	10:42889244-135513631	10q11.23	choline acetyltransferase [Source:HGNC Symbol;Acc:1912]	1.05119
UBE2D1	10:42889244-135513631	10q21.1	ubiquitin-conjugating enzyme E2D 1 (UBC4/5 homolog, yeast) [Source:HGNC Symbol;Acc:12474]	1.05119
RHOBTB1	10:42889244-135513631	10q21.2	Rho-related BTB domain containing 1 [Source:HGNC Symbol;Acc:18738]	1.05119
ASAH2C	10:42889244-135513631	10q11.22	N-acylsphingosine amidohydrolase (non-lysosomal ceramidase) 2C [Source:HGNC Symbol;Acc:23457]	1.05119
TACR2	10:42889244-135513631	10q22.1	tachykinin receptor 2 [Source:HGNC Symbol;Acc:11527]	1.05119
WNT8B	10:42889244-135513631	10q24.31	wingless-type MMTV integration site family, member 8B [Source:HGNC Symbol;Acc:12789]	1.05119
SEC31B	10:42889244-135513631	10q24.31	SEC31 homolog B (S. cerevisiae) [Source:HGNC Symbol;Acc:23197]	1.05119
PAX2	10:42889244-135513631	10q24.31	paired box 2 [Source:HGNC Symbol;Acc:8616]	1.05119
NT5C2	10:42889244-135513631	10q24.32 10q24.33	5'-nucleotidase, cytosolic II [Source:HGNC Symbol;Acc:8022]	1.05119
TM9SF3	10:42889244-135513631	10q24.1	transmembrane 9 superfamily member 3 [Source:HGNC Symbol;Acc:21529]	1.05119
NFKB2	10:42889244-135513631	10q24.32	nuclear factor of kappa light polypeptide gene enhancer in B-cells 2 (p49/p100) [Source:HGNC Symbol;Acc:7795]	1.05119
SAR1A	10:42889244-135513631	10q22.1	SAR1 homolog A (S. cerevisiae) [Source:HGNC Symbol;Acc:10534]	1.05119
DUSP13	10:42889244-135513631	10q22.2	dual specificity phosphatase 13 [Source:HGNC Symbol;Acc:19681]	1.05119
DHX32	10:42889244-135513631	10q26.2	DEAH (Asp-Glu-Ala-His) box polypeptide 32 [Source:HGNC Symbol;Acc:16717]	1.05119
PDE6C	10:42889244-135513631	10q23.33	phosphodiesterase 6C, cGMP-specific, cone, alpha prime [Source:HGNC Symbol;Acc:8787]	1.05119
CWF19L1	10:42889244-135513631	10q24.31	CWF19-like 1, cell cycle control (S. pombe) [Source:HGNC Symbol;Acc:25613]	1.05119
SEMA4G	10:42889244-135513631	10q24.31	sema domain, immunoglobulin domain (Ig), transmembrane domain (TM) and short cytoplasmic domain, (semaphorin) 4G [Source:HGNC Symbol;Acc:10735]	1.05119
BTAF1	10:42889244-135513631	10q23.32	BTAF1 RNA polymerase II, B-TFIID transcription factor-associated, 170kDa (Mot1 homolog, S. cerevisiae) [Source:HGNC Symbol;Acc:17307]	1.05119
IKZF5	10:42889244-135513631	10q26.13	IKAROS family zinc finger 5 (Pegasus) [Source:HGNC Symbol;Acc:14283]	1.05119
BLNK	10:42889244-135513631	10q24.1	B-cell linker [Source:HGNC Symbol;Acc:14211]	1.05119
TLL2	10:42889244-135513631	10q24.1	tolloid-like 2 [Source:HGNC Symbol;Acc:11844]	1.05119
CYP26A1	10:42889244-135513631	10q23.33	cytochrome P450, family 26, subfamily A, polypeptide 1 [Source:HGNC Symbol;Acc:2603]	1.05119
TDRD1	10:42889244-135513631	10q25.3	tudor domain containing 1 [Source:HGNC Symbol;Acc:11712]	1.05119
SORBS1	10:42889244-135513631	10q24.1	sorbin and SH3 domain containing 1 [Source:HGNC Symbol;Acc:14565]	1.05119
CRTAC1	10:42889244-135513631	10q24.2	cartilage acidic protein 1 [Source:HGNC Symbol;Acc:14882]	1.05119
SIRT1	10:42889244-135513631	10q21.3	sirtuin (silent mating type information regulation 2 homolog) 1 (S. cerevisiae) [Source:HGNC Symbol;Acc:14929]	1.05119
HNRNPH3	10:42889244-135513631	10q21.3	heterogeneous nuclear ribonucleoprotein H3 (2H9) [Source:HGNC Symbol;Acc:5043]	1.05119
SCD	10:42889244-135513631	10q24.31	stearoyl-CoA desaturase (delta-9-desaturase) [Source:HGNC Symbol;Acc:10571]	1.05119
ABLIM1	10:42889244-135513631	10q25.3	actin binding LIM protein 1 [Source:HGNC Symbol;Acc:78]	1.05119
TSPAN15	10:42889244-135513631	10q22.1	tetraspanin 15 [Source:HGNC Symbol;Acc:23298]	1.05119
H2AFY2	10:42889244-135513631	10q22.1	H2A histone family, member Y2 [Source:HGNC Symbol;Acc:14453]	1.05119
FAM21A	10:42889244-135513631	10q11.23	family with sequence similarity 21, member A [Source:HGNC Symbol;Acc:23416]	1.05119
NOLA2P1	10:42889244-135513631	10q23.32	[undefined]	1.05119
PDLIM1	10:42889244-135513631	10q23.33 10q24.1	PDZ and LIM domain 1 [Source:HGNC Symbol;Acc:2067]	1.05119
CCNJ	10:42889244-135513631	10q24.1	cyclin J [Source:HGNC Symbol;Acc:23434]	1.05119
DNTT	10:42889244-135513631	10q24.1	deoxynucleotidyltransferase, terminal [Source:HGNC Symbol;Acc:2983]	1.05119
ATRNL1	10:42889244-135513631	10q25.3	attractin-like 1 [Source:HGNC Symbol;Acc:29063]	1.05119
HPS1	10:42889244-135513631	10q24.2	Hermansky-Pudlak syndrome 1 [Source:HGNC Symbol;Acc:5163]	1.05119
RASSF4	10:42889244-135513631	10q11.21	Ras association (RalGDS/AF-6) domain family member 4 [Source:HGNC Symbol;Acc:20793]	1.05119
DNMBP	10:42889244-135513631	10q24.2	dynamin binding protein [Source:HGNC Symbol;Acc:30373]	1.05119
RAB11FIP2	10:42889244-135513631	10q26.11	RAB11 family interacting protein 2 (class I) [Source:HGNC Symbol;Acc:29152]	1.05119
CXCL12	10:42889244-135513631	10q11.21	chemokine (C-X-C motif) ligand 12 (stromal cell-derived factor 1) [Source:HGNC Symbol;Acc:10672]	1.05119
ERLIN1	10:42889244-135513631	10q24.31	ER lipid raft associated 1 [Source:HGNC Symbol;Acc:16947]	1.05119
EIF3A	10:42889244-135513631	10q26.11	eukaryotic translation initiation factor 3, subunit A [Source:HGNC Symbol;Acc:3271]	1.05119
PKD2L1	10:42889244-135513631	10q24.31	polycystic kidney disease 2-like 1 [Source:HGNC Symbol;Acc:9011]	1.05119
RBP3	10:42889244-135513631	10q11.22	retinol binding protein 3, interstitial [Source:HGNC Symbol;Acc:9921]	1.05119
GDF10	10:42889244-135513631	10q11.22	growth differentiation factor 10 [Source:HGNC Symbol;Acc:4215]	1.05119
DDX50	10:42889244-135513631	10q22.1	DEAD (Asp-Glu-Ala-Asp) box polypeptide 50 [Source:HGNC Symbol;Acc:17906]	1.05119
MAPK8	10:42889244-135513631	10q11.22	mitogen-activated protein kinase 8 [Source:HGNC Symbol;Acc:6881]	1.05119
SEC23IP	10:42889244-135513631	10q26.11 10q26.12	SEC23 interacting protein [Source:HGNC Symbol;Acc:17018]	1.05119
ATE1	10:42889244-135513631	10q26.13	arginyltransferase 1 [Source:HGNC Symbol;Acc:782]	1.05119
NSMCE4A	10:42889244-135513631	10q26.13	non-SMC element 4 homolog A (S. cerevisiae) [Source:HGNC Symbol;Acc:25935]	1.05119
PLEKHA1	10:42889244-135513631	10q26.13	pleckstrin homology domain containing, family A (phosphoinositide binding specific) member 1 [Source:HGNC Symbol;Acc:14335]	1.05119
KIAA1274	10:42889244-135513631	10q22.1	KIAA1274 [Source:HGNC Symbol;Acc:23530]	1.05119
UNC5B	10:42889244-135513631	10q22.1	unc-5 homolog B (C. elegans) [Source:HGNC Symbol;Acc:12568]	1.05119
CDH23	10:42889244-135513631	10q22.1	cadherin-related 23 [Source:HGNC Symbol;Acc:13733]	1.05119
C10orf54	10:42889244-135513631	10q22.1	Platelet receptor Gi24 Precursor [Source:UniProtKB/Swiss-Prot;Acc:Q9H7M9]	1.05119
SPOCK2	10:42889244-135513631	10q22.1	sparc/osteonectin, cwcv and kazal-like domains proteoglycan (testican) 2 [Source:HGNC Symbol;Acc:13564]	1.05119
CBARA1	10:42889244-135513631	10q22.1	calcium binding atopy-related autoantigen 1 [Source:HGNC Symbol;Acc:1530]	1.05119
PPP3CB	10:42889244-135513631	10q22.2	protein phosphatase 3, catalytic subunit, beta isozyme [Source:HGNC Symbol;Acc:9315]	1.05119
FAM190	10:42889244-135513631	10q23.1	family with sequence similarity 190, member B [Source:HGNC Symbol;Acc:29197]	1.05119
BMPR1A	10:42889244-135513631	10q23.2	bone morphogenetic protein receptor, type IA [Source:HGNC Symbol;Acc:1076]	1.05119
MINPP1	10:42889244-135513631	10q23.2	multiple inositol polyphosphate histidine phosphatase, 1 [Source:HGNC Symbol;Acc:7102]	1.05119
ACTA2	10:42889244-135513631	10q23.31	actin, alpha 2, smooth muscle, aorta [Source:HGNC Symbol;Acc:130]	1.05119

Continued on next page...

gene name	chr	.DNABand	.GeneDesc	rank
LIPA	10:42889244-135513631	10q23.31	lipase A, lysosomal acid, cholesterol esterase [Source:HGNC Symbol;Acc:6617]	1.05119
TLX1	10:42889244-135513631	10q24.31	T-cell leukemia homeobox 1 [Source:HGNC Symbol;Acc:5056]	1.05119
C10orf2	10:42889244-135513631	10q24.31	Twinkle protein, mitochondrial Precursor (EC 3.6.1.-)(T7 gp4-like protein with intramitochondrial nucleoid localization)(T7-like mitochondrial DNA helicase)(Progressive external ophthalmoplegia 1 protein) [Source:UniProtKB/Swiss-Prot;Acc:Q96RR1]	1.05119
LZTS2	10:42889244-135513631	10q24.31	leucine zipper, putative tumor suppressor 2 [Source:HGNC Symbol;Acc:29381]	1.05119
SFXN3	10:42889244-135513631	10q24.31	sideroflexin 3 [Source:HGNC Symbol;Acc:16087]	1.05119
KAZALD1	10:42889244-135513631	10q24.31	Kazal-type serine peptidase inhibitor domain 1 [Source:HGNC Symbol;Acc:25460]	1.05119
FBXW4	10:42889244-135513631	10q24.32	F-box and WD repeat domain containing 4 [Source:HGNC Symbol;Acc:10847]	1.05119
FGF8	10:42889244-135513631	10q24.32	fibroblast growth factor 8 (androgen-induced) [Source:HGNC Symbol;Acc:3686]	1.05119
NPM3	10:42889244-135513631	10q24.32	nucleophosmin/nucleoplasmin 3 [Source:HGNC Symbol;Acc:7931]	1.05119
TNKS2	10:42889244-135513631	10q23.32	tankyrase, TRF1-interacting ankyrin-related ADP-ribose polymerase 2 [Source:HGNC Symbol;Acc:15677]	1.05119
PITX3	10:42889244-135513631	10q24.32	paired-like homeodomain 3 [Source:HGNC Symbol;Acc:9006]	1.05119
GBF1	10:42889244-135513631	10q24.32	golgi brefeldin A resistant guanine nucleotide exchange factor 1 [Source:HGNC Symbol;Acc:4181]	1.05119
CPEB3	10:42889244-135513631	10q23.32	cytoplasmic polyadenylation element binding protein 3 [Source:HGNC Symbol;Acc:21746]	1.05119
FBXL15	10:42889244-135513631	10q24.32	F-box and leucine-rich repeat protein 15 [Source:HGNC Symbol;Acc:28155]	1.05119
CUEDC2	10:42889244-135513631	10q24.32	CUE domain containing 2 [Source:HGNC Symbol;Acc:28352]	1.05119
SUFU	10:42889244-135513631	10q24.32	suppressor of fused homolog (Drosophila) [Source:HGNC Symbol;Acc:16466]	1.05119
LHPP	10:42889244-135513631	10q26.13	phospholysine phosphohistidine inorganic pyrophosphate phosphatase [Source:HGNC Symbol;Acc:30042]	1.05119
C10orf137	10:42889244-135513631	10q26.13	Erythroid differentiation-related factor 1 [Source:UniProtKB/Swiss-Prot;Acc:Q3B7T1]	1.05119
BCCIP	10:42889244-135513631	10q26.2	BRCA2 and CDKN1A interacting protein [Source:HGNC Symbol;Acc:978]	1.05119
NEURL	10:42889244-135513631	10q24.33	neuralized homolog (Drosophila) [Source:HGNC Symbol;Acc:7761]	1.05119
SH3PXD2A	10:42889244-135513631	10q24.33	SH3 and PX domains 2A [Source:HGNC Symbol;Acc:23664]	1.05119
OBFC1	10:42889244-135513631	10q24.33	oligonucleotide/oligosaccharide-binding fold containing 1 [Source:HGNC Symbol;Acc:26200]	1.05119
DKK1	10:42889244-135513631	10q21.1	dickkopf homolog 1 (Xenopus laevis) [Source:HGNC Symbol;Acc:2891]	1.05119
EBF3	10:42889244-135513631	10q26.3	early B-cell factor 3 [Source:HGNC Symbol;Acc:19087]	1.05119
GLRX3	10:42889244-135513631	10q26.3	glutaredoxin 3 [Source:HGNC Symbol;Acc:15987]	1.05119
SORCS1	10:42889244-135513631	10q25.1	sortilin-related VPS10 domain containing receptor 1 [Source:HGNC Symbol;Acc:16697]	1.05119
XPNPEP1	10:42889244-135513631	10q25.1	X-prolyl aminopeptidase (aminopeptidase P) 1, soluble [Source:HGNC Symbol;Acc:12822]	1.05119
SMC3	10:42889244-135513631	10q25.2	structural maintenance of chromosomes 3 [Source:HGNC Symbol;Acc:2468]	1.05119
SHOC2	10:42889244-135513631	10q25.2	soc-2 suppressor of clear homolog (C. elegans) [Source:HGNC Symbol;Acc:15454]	1.05119
TFAM	10:42889244-135513631	10q21.1	transcription factor A, mitochondrial [Source:HGNC Symbol;Acc:11741]	1.05119
CCDC6	10:42889244-135513631	10q21.2	coiled-coil domain containing 6 [Source:HGNC Symbol;Acc:18782]	1.05119
ZMIZ1	10:42889244-135513631	10q22.3	zinc finger, MIZ-type containing 1 [Source:HGNC Symbol;Acc:16493]	1.05119
DNAJC12	10:42889244-135513631	10q21.3	DnaJ (Hsp40) homolog, subfamily C, member 12 [Source:HGNC Symbol;Acc:28908]	1.05119
PPIF	10:42889244-135513631	10q22.3	peptidylprolyl isomerase F [Source:HGNC Symbol;Acc:9259]	1.05119
PBLD	10:42889244-135513631	10q21.3	phenazine biosynthesis-like protein domain containing [Source:HGNC Symbol;Acc:23301]	1.05119
TSPAN14	10:42889244-135513631	10q23.1	tetraspanin 14 [Source:HGNC Symbol;Acc:23303]	1.05119
LGI1	10:42889244-135513631	10q23.33	leucine-rich, glioma inactivated 1 [Source:HGNC Symbol;Acc:6572]	1.05119
TBC1D12	10:42889244-135513631	10q23.33	TBC1 domain family, member 12 [Source:HGNC Symbol;Acc:29082]	1.05119
CYP2C18	10:42889244-135513631	10q23.33	cytochrome P450, family 2, subfamily C, polypeptide 18 [Source:HGNC Symbol;Acc:2620]	1.05119
FAM178A	10:42889244-135513631	10q24.31	family with sequence similarity 178, member A [Source:HGNC Symbol;Acc:17814]	1.05119
IDE	10:42889244-135513631	10q23.33	insulin-degrading enzyme [Source:HGNC Symbol;Acc:5381]	1.05119
TECTB	10:42889244-135513631	10q25.2	tectorin beta [Source:HGNC Symbol;Acc:11721]	1.05119
ELOVL3	10:42889244-135513631	10q24.32	elongation of very long chain fatty acids (FEN1/Elo2, SUR4/Elo3, yeast)-like 3 [Source:HGNC Symbol;Acc:18047]	1.05119
IFIT3	10:42889244-135513631	10q23.31	interferon-induced protein with tetratricopeptide repeats 3 [Source:HGNC Symbol;Acc:5411]	1.05119
NKX2-3	10:42889244-135513631	10q24.2	NK2 transcription factor related, locus 3 (Drosophila) [Source:HGNC Symbol;Acc:7836]	1.05119
IFIT2	10:42889244-135513631	10q23.31	interferon-induced protein with tetratricopeptide repeats 2 [Source:HGNC Symbol;Acc:5409]	1.05119
GPAM	10:42889244-135513631	10q25.2	glycerol-3-phosphate acyltransferase, mitochondrial [Source:HGNC Symbol;Acc:24865]	1.05119
CUTC	10:42889244-135513631	10q24.2	cutC copper transporter homolog (E. coli) [Source:HGNC Symbol;Acc:24271]	1.05119
PPP1R3C	10:42889244-135513631	10q23.32	protein phosphatase 1, regulatory (inhibitor) subunit 3C [Source:HGNC Symbol;Acc:9293]	1.05119
PYROXD2	10:42889244-135513631	10q24.2	pyridine nucleotide-disulphide oxidoreductase domain 2 [Source:HGNC Symbol;Acc:23517]	1.05119
CNNM1	10:42889244-135513631	10q24.2	cyclin M1 [Source:HGNC Symbol;Acc:102]	1.05119
MXI1	10:42889244-135513631	10q25.2	MAX interactor 1 [Source:HGNC Symbol;Acc:7534]	1.05119
SMNDC1	10:42889244-135513631	10q25.2	survival motor neuron domain containing 1 [Source:HGNC Symbol;Acc:16900]	1.05119
C10orf88	10:42889244-135513631	10q26.13	Uncharacterized protein C10orf88 [Source:UniProtKB/Swiss-Prot;Acc:Q9H8K7]	1.05119
HELLS	10:42889244-135513631	10q23.33	helicase, lymphoid-specific [Source:HGNC Symbol;Acc:4861]	1.05119
PRLHR	10:42889244-135513631	10q26.11	prolactin releasing hormone receptor [Source:HGNC Symbol;Acc:4464]	1.05119
TCTN3	10:42889244-135513631	10q24.1	tectonic family member 3 [Source:HGNC Symbol;Acc:24519]	1.05119
FAM45A	10:42889244-135513631	10q26.11	family with sequence similarity 45, member A [Source:HGNC Symbol;Acc:31793]	1.05119
AVPI1	10:42889244-135513631	10q24.2	arginine vasopressin-induced 1 [Source:HGNC Symbol;Acc:30898]	1.05119
BRWD2	10:42889244-135513631	10q26.12	WD repeat domain 11 [Source:HGNC Symbol;Acc:13831]	1.05119
C10orf76	10:42889244-135513631	10q24.32	UPF0668 protein C10orf76 [Source:UniProtKB/Swiss-Prot;Acc:Q5T2E6]	1.05119
KCNIP2	10:42889244-135513631	10q24.32	Kv channel interacting protein 2 [Source:HGNC Symbol;Acc:15522]	1.05119
CCDC147	10:42889244-135513631	10q25.1	coiled-coil domain containing 147 [Source:HGNC Symbol;Acc:26676]	1.05119
GOT1	10:42889244-135513631	10q24.2	glutamic-oxaloacetic transaminase 1, soluble (aspartate aminotransferase 1) [Source:HGNC Symbol;Acc:4432]	1.05119
CPN1	10:42889244-135513631	10q24.2	carboxypeptidase N, polypeptide 1 [Source:HGNC Symbol;Acc:2312]	1.05119
C10orf95	10:42889244-135513631	10q24.32	Uncharacterized protein C10orf95 [Source:UniProtKB/Swiss-Prot;Acc:Q9H7T3]	1.05119
SFRP5	10:42889244-135513631	10q24.2	secreted frizzled-related protein 5 [Source:HGNC Symbol;Acc:10779]	1.05119
CPXM2	10:42889244-135513631	10q26.13	carboxypeptidase X (M14 family), member 2 [Source:HGNC Symbol;Acc:26977]	1.05119
ANXA11	10:42889244-135513631	10q22.3	annexin A11 [Source:HGNC Symbol;Acc:535]	1.05119
LDB3	10:42889244-135513631	10q23.2	LIM domain binding 3 [Source:HGNC Symbol;Acc:15710]	1.05119
OPN4	10:42889244-135513631	10q23.2	opsin 4 [Source:HGNC Symbol;Acc:14449]	1.05119
FAM35A	10:42889244-135513631	10q23.2	family with sequence similarity 35, member A [Source:HGNC Symbol;Acc:28773]	1.05119
C10orf57	10:42889244-135513631	10q23.1	Uncharacterized protein C10orf58 Precursor [Source:UniProtKB/Swiss-Prot;Acc:Q9BRX8]	1.05119
SFTPA1	10:42889244-135513631	10q22.3	surfactant protein A1 [Source:HGNC Symbol;Acc:10798]	1.05119
NEUROG3	10:42889244-135513631	10q22.1	neurogenin 3 [Source:HGNC Symbol;Acc:13806]	1.05119
PLAU	10:42889244-135513631	10q22.2	plasminogen activator, urokinase [Source:HGNC Symbol;Acc:9052]	1.05119
SRGN	10:42889244-135513631	10q22.1	serglycin [Source:HGNC Symbol;Acc:9361]	1.05119
CHST3	10:42889244-135513631	10q22.1	carbohydrate (chondroitin 6) sulfotransferase 3 [Source:HGNC Symbol;Acc:1971]	1.05119
BICC1	10:42889244-135513631	10q21.1	bicaudal C homolog 1 (Drosophila) [Source:HGNC Symbol;Acc:19351]	1.05119
ARL4P	10:42889244-135513631	10q21.2	ADP-ribosylation factor-like 4 pseudogene [Source:HGNC Symbol;Acc:17741]	1.05119
CISD1	10:42889244-135513631	10q21.1	CDGSH iron sulfur domain 1 [Source:HGNC Symbol;Acc:30880]	1.05119
EGR2	10:42889244-135513631	10q21.3	early growth response 2 [Source:HGNC Symbol;Acc:3239]	1.05119
ECD	10:42889244-135513631	10q22.1 10q22.2	ecdysoneless homolog (Drosophila) [Source:HGNC Symbol;Acc:17029]	1.05119
P4HA1	10:42889244-135513631	10q22.1	prolyl 4-hydroxylase, alpha polypeptide I [Source:HGNC Symbol;Acc:8546]	1.05119
SLC25A16	10:42889244-135513631	10q21.3	solute carrier family 25 (mitochondrial carrier; Graves disease autoantigen), member 16 [Source:HGNC Symbol;Acc:10986]	1.05119
ZWINT	10:42889244-135513631	10q21.1	ZW10 interactor [Source:HGNC Symbol;Acc:13195]	1.05119
VPS26A	10:42889244-135513631	10q22.1	vacuolar protein sorting 26 homolog A (S. pombe) [Source:HGNC Symbol;Acc:12711]	1.05119
PTPN20C	10:42889244-135513631	10q11.22	protein tyrosine phosphatase, non-receptor type 20C [Source:HGNC Symbol;Acc:23424]	1.05119
ECHS1	10:42889244-135513631	10q26.3	enoyl Coenzyme A hydratase, short chain, 1, mitochondrial [Source:HGNC Symbol;Acc:3151]	1.05119
GDF2	10:42889244-135513631	10q11.22	growth differentiation factor 2 [Source:HGNC Symbol;Acc:4217]	1.05119
ARHGAP22	10:42889244-135513631	10q11.22	Rho GTPase activating protein 22 [Source:HGNC Symbol;Acc:30320]	1.05119
WDFY4	10:42889244-135513631	10q11.22 10q11.23	WDFY family member 4 [Source:HGNC Symbol;Acc:29323]	1.05119
TUBGCP2	10:42889244-135513631	10q26.3	tubulin, gamma complex associated protein 2 [Source:HGNC Symbol;Acc:18599]	1.05119
CALY	10:42889244-135513631	10q26.3	calcyon neuron-specific vesicular protein [Source:HGNC Symbol;Acc:17938]	1.05119
CYP2E1	10:42889244-135513631	10q26.3	cytochrome P450, family 2, subfamily E, polypeptide 1 [Source:HGNC Symbol;Acc:2631]	1.05119
PTPRE	10:42889244-135513631	10q26.2	protein tyrosine phosphatase, receptor type, E [Source:HGNC Symbol;Acc:9669]	1.05119
SFTPD	10:42889244-135513631	10q22.3	surfactant protein D [Source:HGNC Symbol;Acc:10803]	1.05119
DYDC2	10:42889244-135513631	10q23.1	DPY30 domain containing 2 [Source:HGNC Symbol;Acc:23468]	1.05119
RP11-369J21.6	10:42889244-135513631	10q22.3	Transmembrane protein C10orf57 [Source:UniProtKB/Swiss-Prot;Acc:Q8TBM7]	1.05119
ACTR1A	10:42889244-135513631	10q24.32	ARP1 actin-related protein 1 homolog A, centractin alpha (yeast) [Source:HGNC Symbol;Acc:167]	1.05119
CYP2C9	10:42889244-135513631	10q23.33	cytochrome P450, family 2, subfamily C, polypeptide 9 [Source:HGNC Symbol;Acc:2623]	1.05119
TMEM180	10:42889244-135513631	10q24.32	transmembrane protein 180 [Source:HGNC Symbol;Acc:26196]	1.05119
CYP2C8	10:42889244-135513631	10q23.33	cytochrome P450, family 2, subfamily C, polypeptide 8 [Source:HGNC Symbol;Acc:2622]	1.05119
MYOF	10:42889244-135513631	10q23.33	myoferlin [Source:HGNC Symbol;Acc:3656]	1.05119
LOXL4	10:42889244-135513631	10q24.2	lysyl oxidase-like 4 [Source:HGNC Symbol;Acc:17171]	1.05119
STAMBPL1	10:42889244-135513631	10q23.31	STAM binding protein-like 1 [Source:HGNC Symbol;Acc:24105]	1.05119
CH25H	10:42889244-135513631	10q23.31	cholesterol 25-hydroxylase [Source:HGNC Symbol;Acc:1907]	1.05119
LBX1	10:42889244-135513631	10q24.31	ladybird homeobox 1 [Source:HGNC Symbol;Acc:16960]	1.05119
ATAD1	10:42889244-135513631	10q23.31	ATPase family, AAA domain containing 1 [Source:HGNC Symbol;Acc:25903]	1.05119
BTBD16	10:42889244-135513631	10q26.13	BTB (POZ) domain containing 16 [Source:HGNC Symbol;Acc:26340]	1.05119
KIF11	10:42889244-135513631	10q23.33	kinesin family member 11 [Source:HGNC Symbol;Acc:6388]	1.05119
CUZD1	10:42889244-135513631	10q26.13	CUB and zona pellucida-like domains 1 [Source:HGNC Symbol;Acc:17937]	1.05119
TACC2	10:42889244-135513631	10q26.13	transforming, acidic coiled-coil containing protein 2 [Source:HGNC Symbol;Acc:11523]	1.05119
DUSP5	10:42889244-135513631	10q25.2	dual specificity phosphatase 5 [Source:HGNC Symbol;Acc:3071]	1.05119
CALHM2	10:42889244-135513631	10q24.33	calcium homeostasis modulator 2 [Source:HGNC Symbol;Acc:23493]	1.05119

Continued on next page...

gene name	chr	.DNABand	.GeneDesc	rank
ARL3	10:42889244-135513631	10q24.32	ADP-ribosylation factor-like 3 [Source:HGNC Symbol;Acc:694]	1.05119
CEP55	10:42889244-135513631	10q23.33	centrosomal protein 55kDa [Source:HGNC Symbol;Acc:1161]	1.05119
KIF20B	10:42889244-135513631	10q23.31	kinesin family member 20B [Source:HGNC Symbol;Acc:7212]	1.05119
ENTPD1	10:42889244-135513631	10q24.1	ectonucleoside triphosphate diphosphohydrolase 1 [Source:HGNC Symbol;Acc:3363]	1.05119
EXOC6	10:42889244-135513631	10q23.33	exocyst complex component 6 [Source:HGNC Symbol;Acc:23196]	1.05119
PLCE1	10:42889244-135513631	10q23.33	phospholipase C, epsilon 1 [Source:HGNC Symbol;Acc:17175]	1.05119
RBP4	10:42889244-135513631	10q23.33	retinol binding protein 4, plasma [Source:HGNC Symbol;Acc:9922]	1.05119
ANXA7	10:42889244-135513631	10q22.2	annexin A7 [Source:HGNC Symbol;Acc:545]	1.05119
FAM149B1	10:42889244-135513631	10q22.2	family with sequence similarity 149, member B1 [Source:HGNC Symbol;Acc:29162]	1.05119
NCOA4	10:42889244-135513631	10q11.23	nuclear receptor coactivator 4 [Source:HGNC Symbol;Acc:7671]	1.05119
MSMB	10:42889244-135513631	10q11.23	microseminoprotein, beta- [Source:HGNC Symbol;Acc:7372]	1.05119
TIMM23	10:42889244-135513631	10q11.23	translocase of inner mitochondrial membrane 23 homolog (yeast) [Source:HGNC Symbol;Acc:17312]	1.05119
ASCC1	10:42889244-135513631	10q22.1	activating signal cointegrator 1 complex subunit 1 [Source:HGNC Symbol;Acc:24268]	1.05119
PLA2G12B	10:42889244-135513631	10q22.1	phospholipase A2, group XIIB [Source:HGNC Symbol;Acc:18555]	1.05119
ZNF365	10:42889244-135513631	10q21.2	zinc finger protein 365 [Source:HGNC Symbol;Acc:18194]	1.05119
OIT3	10:42889244-135513631	10q22.1	oncoprotein induced transcript 3 [Source:HGNC Symbol;Acc:29953]	1.05119
ADAMTS14	10:42889244-135513631	10q22.1	ADAM metallopeptidase with thrombospondin type 1 motif, 14 [Source:HGNC Symbol;Acc:14899]	1.05119
RPS24	10:42889244-135513631	10q22.3	ribosomal protein S24 [Source:HGNC Symbol;Acc:10411]	1.05119
TET1	10:42889244-135513631	10q21.3	tet oncogene 1 [Source:HGNC Symbol;Acc:29484]	1.05119
AL589863.1	10:42889244-135513631	10q22.2	[undefined]	1.05119
DNA2	10:42889244-135513631	10q21.3	DNA replication helicase 2 homolog (yeast) [Source:HGNC Symbol;Acc:2939]	1.05119
MYPN	10:42889244-135513631	10q21.3	myopalladin [Source:HGNC Symbol;Acc:23246]	1.05119
FAM13C	10:42889244-135513631	10q21.1	family with sequence similarity 13, member C [Source:HGNC Symbol;Acc:19371]	1.05119
NRBF2	10:42889244-135513631	10q21.3	nuclear receptor binding factor 2 [Source:HGNC Symbol;Acc:19692]	1.05119
A1CF	10:42889244-135513631	10q11.23	APOBEC1 complementation factor [Source:HGNC Symbol;Acc:24086]	1.05119
PCDH21	10:42889244-135513631	10q23.1	cadherin-related family member 1 [Source:HGNC Symbol;Acc:14550]	1.05119
LRIT1	10:42889244-135513631	10q23.1	leucine-rich repeat, immunoglobulin-like and transmembrane domains 1 [Source:HGNC Symbol;Acc:23404]	1.05119
RGR	10:42889244-135513631	10q23.1	retinal G protein coupled receptor [Source:HGNC Symbol;Acc:9990]	1.05119
POLR3A	10:42889244-135513631	10q22.3	polymerase (RNA) III (DNA directed) polypeptide A, 155kDa [Source:HGNC Symbol;Acc:30074]	1.05119
HERC4	10:42889244-135513631	10q21.3	hect domain and RLD 4 [Source:HGNC Symbol;Acc:24521]	1.05119
C10orf11	10:42889244-135513631	10q22.2 10q22.3	Leucine-rich repeat-containing protein C10orf11 [Source:UniProtKB/Swiss-Prot;Acc:Q9H2I8]	1.05119
CAMK2G	10:42889244-135513631	10q22.2	calcium/calmodulin-dependent protein kinase II gamma [Source:HGNC Symbol;Acc:1463]	1.05119
C10orf116	10:42889244-135513631	10q23.2	Adipose most abundant gene transcript 2 protein (apM-2) [Source:UniProtKB/Swiss-Prot;Acc:Q15847]	1.05119
GLUD1	10:42889244-135513631	10q23.2	glutamate dehydrogenase 1 [Source:HGNC Symbol;Acc:4335]	1.05119
ANKRD1	10:42889244-135513631	10q23.31	ankyrin repeat domain 1 (cardiac muscle) [Source:HGNC Symbol;Acc:15819]	1.05119
HTR7	10:42889244-135513631	10q23.31	5-hydroxytryptamine (serotonin) receptor 7 (adenylate cyclase-coupled) [Source:HGNC Symbol;Acc:5302]	1.05119
RPP30	10:42889244-135513631	10q23.31	ribonuclease P/MRP 30kDa subunit [Source:HGNC Symbol;Acc:17688]	1.05119
C10orf4	10:42889244-135513631	10q23.33	Protein FRA10AC1 [Source:UniProtKB/Swiss-Prot;Acc:Q70Z53]	1.05119
ADD3	10:42889244-135513631	10q25.1	adducin 3 (gamma) [Source:HGNC Symbol;Acc:245]	1.05119
HABP2	10:42889244-135513631	10q25.3	hyaluronan binding protein 2 [Source:HGNC Symbol;Acc:4798]	1.05119
VAX1	10:42889244-135513631	10q25.3	ventral anterior homeobox 1 [Source:HGNC Symbol;Acc:12660]	1.05119
DNAJB12	10:42889244-135513631	10q22.1	DnaJ (Hsp40) homolog, subfamily B, member 12 [Source:HGNC Symbol;Acc:14891]	1.05119
EIF4EBP2	10:42889244-135513631	10q22.1	eukaryotic translation initiation factor 4E binding protein 2 [Source:HGNC Symbol;Acc:3289]	1.05119
NPFFR1	10:42889244-135513631	10q22.1	neuropeptide FF receptor 1 [Source:HGNC Symbol;Acc:17425]	1.05119
C10orf81	10:42889244-135513631	10q25.3	PH domain-containing protein C10orf81 (Epididymis luminal protein 185)(hEL185) [Source:UniProtKB/Swiss-Prot;Acc:Q5SXH7]	1.05119
TCF7L2	10:42889244-135513631	10q25.2 10q25.3	transcription factor 7-like 2 (T-cell specific, HMG-box) [Source:HGNC Symbol;Acc:11641]	1.05119
MKI67	10:42889244-135513631	10q26.2	antigen identified by monoclonal antibody Ki-67 [Source:HGNC Symbol;Acc:7107]	1.05119
CYP17A1	10:42889244-135513631	10q24.32	cytochrome P450, family 17, subfamily A, polypeptide 1 [Source:HGNC Symbol;Acc:2593]	1.05119
INA	10:42889244-135513631	10q24.33	internexin neuronal intermediate filament protein, alpha [Source:HGNC Symbol;Acc:6057]	1.05119
C10orf125	10:42889244-135513631	10q26.3	Protein fucU homolog [Source:UniProtKB/Swiss-Prot;Acc:A2VDF0]	1.05119
LRRC27	10:42889244-135513631	10q26.3	leucine rich repeat containing 27 [Source:HGNC Symbol;Acc:29346]	1.05119
MTG1	10:42889244-135513631	10q26.3	mitochondrial GTPase 1 homolog (S. cerevisiae) [Source:HGNC Symbol;Acc:32159]	1.05119
NKX6-2	10:42889244-135513631	10q26.3	NK6 homeobox 2 [Source:HGNC Symbol;Acc:19321]	1.05119
PAOX	10:42889244-135513631	10q26.3	polyamine oxidase (exo-N4-amino) [Source:HGNC Symbol;Acc:20837]	1.05119
GSTO1	10:42889244-135513631	10q25.1	glutathione S-transferase omega 1 [Source:HGNC Symbol;Acc:13312]	1.05119
TAF5	10:42889244-135513631	10q24.33	TAF5 RNA polymerase II, TATA box binding protein (TBP)-associated factor, 100kDa [Source:HGNC Symbol;Acc:11539]	1.05119
PPRC1	10:42889244-135513631	10q24.32	peroxisome proliferator-activated receptor gamma, coactivator-related 1 [Source:HGNC Symbol;Acc:30025]	1.05119
ITPRIP	10:42889244-135513631	10q25.1	inositol 1,4,5-triphosphate receptor interacting protein [Source:HGNC Symbol;Acc:29370]	1.05119
CNNM2	10:42889244-135513631	10q24.32	cyclin M2 [Source:HGNC Symbol;Acc:103]	1.05119
PDCD11	10:42889244-135513631	10q24.33	programmed cell death 11 [Source:HGNC Symbol;Acc:13408]	1.05119
ADAM12	10:42889244-135513631	10q26.2	ADAM metallopeptidase domain 12 [Source:HGNC Symbol;Acc:190]	1.05119
RGS10	10:42889244-135513631	10q26.11	regulator of G-protein signaling 10 [Source:HGNC Symbol;Acc:9992]	1.05119
ANXA8L1	10:42889244-135513631	10q11.22	annexin A8-like 1 [Source:HGNC Symbol;Acc:23334]	1.05119
FRMPD2L2	10:42889244-135513631	10q11.22	FERM and PDZ domain containing 2 like 2 [Source:HGNC Symbol;Acc:23453]	1.05119
FXYD4	10:42889244-135513631	10q11.21	FXYD domain containing ion transport regulator 4 [Source:HGNC Symbol;Acc:4028]	1.05119
PCDH15	10:42889244-135513631	10q21.1	protocadherin-related 15 [Source:HGNC Symbol;Acc:14674]	1.05119
ARID5B	10:42889244-135513631	10q21.2	AT rich interactive domain 5B (MRF1-like) [Source:HGNC Symbol;Acc:17362]	1.05119
PDCD4	10:42889244-135513631	10q25.2	programmed cell death 4 (neoplastic transformation inhibitor) [Source:HGNC Symbol;Acc:8763]	1.05119
ADRA2A	10:42889244-135513631	10q25.2	adrenergic, alpha-2A-, receptor [Source:HGNC Symbol;Acc:281]	1.05119
DOCK1	10:42889244-135513631	10q26.2	dedicator of cytokinesis 1 [Source:HGNC Symbol;Acc:2987]	1.05119
ANK3	10:42889244-135513631	10q21.2	ankyrin 3, node of Ranvier (ankyrin G) [Source:HGNC Symbol;Acc:494]	1.05119
IPMK	10:42889244-135513631	10q21.1	inositol polyphosphate multikinase [Source:HGNC Symbol;Acc:20739]	1.05119
DLG5	10:42889244-135513631	10q22.3	discs, large homolog 5 (Drosophila) [Source:HGNC Symbol;Acc:2904]	1.05119
MAT1A	10:42889244-135513631	10q23.1	methionine adenosyltransferase I, alpha [Source:HGNC Symbol;Acc:6903]	1.05119
AGAP11	10:42889244-135513631	10q23.2	ankyrin repeat and GTPase domain Arf GTPase activating protein 11 [Source:HGNC Symbol;Acc:29421]	1.05119
VTI1A	10:42889244-135513631	10q25.2	vesicle transport through interaction with t-SNAREs homolog 1A (yeast) [Source:HGNC Symbol;Acc:17792]	1.05119
FAM160B1	10:42889244-135513631	10q25.3	family with sequence similarity 160, member B1 [Source:HGNC Symbol;Acc:29320]	1.05119
DPYSL4	10:42889244-135513631	10q26.3	dihydropyrimidinase-like 4 [Source:HGNC Symbol;Acc:3016]	1.05119
VENTX	10:42889244-135513631	10q26.3	VENT homeobox homolog (Xenopus laevis) [Source:HGNC Symbol;Acc:13639]	1.05119
ADAM8	10:42889244-135513631	10q26.3	ADAM metallopeptidase domain 8 [Source:HGNC Symbol;Acc:215]	1.05119
GFRA1	10:42889244-135513631	10q25.3	GDNF family receptor alpha 1 [Source:HGNC Symbol;Acc:4243]	1.05119
C10orf46	10:42889244-135513631	10q26.11	Uncharacterized protein C10orf46 [Source:UniProtKB/Swiss-Prot;Acc:Q86Y37]	1.05119
TIAL1	10:42889244-135513631	10q26.11	TIA1 cytotoxic granule-associated RNA binding protein-like 1 [Source:HGNC Symbol;Acc:11804]	1.05119
BAG3	10:42889244-135513631	10q26.11	BCL2-associated athanogene 3 [Source:HGNC Symbol;Acc:939]	1.05119
FAM21B	10:42889244-135513631	10q11.22	family with sequence similarity 21, member B [Source:HGNC Symbol;Acc:23417]	1.05119
ANKRD22	10:42889244-135513631	10q23.31	ankyrin repeat domain 22 [Source:HGNC Symbol;Acc:28321]	1.05119
IFIT5	10:42889244-135513631	10q23.31	interferon-induced protein with tetratricopeptide repeats 5 [Source:HGNC Symbol;Acc:13328]	1.05119
SLC16A12	10:42889244-135513631	10q23.31	solute carrier family 16, member 12 (monocarboxylic acid transporter 12) [Source:HGNC Symbol;Acc:23094]	1.05119
PANK1	10:42889244-135513631	10q23.31	pantothenate kinase 1 [Source:HGNC Symbol;Acc:8598]	1.05119
HHEX	10:42889244-135513631	10q23.33	hematopoietically expressed homeobox [Source:HGNC Symbol;Acc:4901]	1.05119
BUB3	10:42889244-135513631	10q26.13	budding uninhibited by benzimidazoles 3 homolog (yeast) [Source:HGNC Symbol;Acc:1151]	1.05119
GPR26	10:42889244-135513631	10q26.13	G protein-coupled receptor 26 [Source:HGNC Symbol;Acc:4481]	1.05119
MMP21	10:42889244-135513631	10q26.13	matrix metallopeptidase 21 [Source:HGNC Symbol;Acc:14357]	1.05119
C10orf90	10:42889244-135513631	10q26.2	Uncharacterized protein C10orf90 [Source:UniProtKB/Swiss-Prot;Acc:Q96M02]	1.05119
MMS19	10:42889244-135513631	10q24.1	MMS19 nucleotide excision repair homolog (S. cerevisiae) [Source:HGNC Symbol;Acc:13824]	1.05119
PI4K2A	10:42889244-135513631	10q24.2	phosphatidylinositol 4-kinase type 2 alpha [Source:HGNC Symbol;Acc:30031]	1.05119
MARVELD1	10:42889244-135513631	10q24.2	MARVEL domain containing 1 [Source:HGNC Symbol;Acc:28674]	1.05119
ZFYVE27	10:42889244-135513631	10q24.2	zinc finger, FYVE domain containing 27 [Source:HGNC Symbol;Acc:26559]	1.05119
GOLGA7B	10:42889244-135513631	10q24.2	golgin A7 family, member B [Source:HGNC Symbol;Acc:31668]	1.05119
SLC25A28	10:42889244-135513631	10q24.2	solute carrier family 25, member 28 [Source:HGNC Symbol;Acc:23472]	1.05119
PIK3AP1	10:42889244-135513631	10q24.1	phosphoinositide-3-kinase adaptor protein 1 [Source:HGNC Symbol;Acc:30034]	1.05119
C10orf12	10:42889244-135513631	10q24.1	Uncharacterized protein C10orf12 [Source:UniProtKB/Swiss-Prot;Acc:Q8N655]	1.05119
CCDC109A	10:42889244-135513631	10q22.1	coiled-coil domain containing 109A [Source:HGNC Symbol;Acc:23526]	1.05119
TTC18	10:42889244-135513631	10q22.2	tetratricopeptide repeat domain 18 [Source:HGNC Symbol;Acc:30726]	1.05119
ADK	10:42889244-135513631	10q22.2	adenosine kinase [Source:HGNC Symbol;Acc:257]	1.05119
KCNMA1	10:42889244-135513631	10q22.3	potassium large conductance calcium-activated channel, subfamily M, alpha member 1 [Source:HGNC Symbol;Acc:6284]	1.05119
PCGF6	10:42889244-135513631	10q24.33	polycomb group ring finger 6 [Source:HGNC Symbol;Acc:21156]	1.05119
C10orf78	10:42889244-135513631	10q25.1	Uncharacterized protein C10orf78 [Source:UniProtKB/Swiss-Prot;Acc:Q86XK3]	1.05119
SORCS3	10:42889244-135513631	10q25.1	sortilin-related VPS10 domain containing receptor 3 [Source:HGNC Symbol;Acc:16699]	1.05119

Continued on next page...

gene name	chr	.DNABand	.GeneDesc	rank
SFXN2	10:42889244-135513631	10q24.32	sideroflexin 2 [Source:HGNC Symbol;Acc:16086]	1.05119
SUPV3L1	10:42889244-135513631	10q22.1	suppressor of var1, 3-like 1 (S. cerevisiae) [Source:HGNC Symbol;Acc:11471]	1.05119
HKDC1	10:42889244-135513631	10q22.1	hexokinase domain containing 1 [Source:HGNC Symbol;Acc:23302]	1.05119
HK1	10:42889244-135513631	10q22.1	hexokinase 1 [Source:HGNC Symbol;Acc:4922]	1.05119
TYSND1	10:42889244-135513631	10q22.1	trypsin domain containing 1 [Source:HGNC Symbol;Acc:28531]	1.05119
NODAL	10:42889244-135513631	10q22.1	nodal homolog (mouse) [Source:HGNC Symbol;Acc:7865]	1.05119
MYST4	10:42889244-135513631	10q22.2	MYST histone acetyltransferase (monocytic leukemia) 4 [Source:HGNC Symbol;Acc:17582]	1.05119
SAMD8	10:42889244-135513631	10q22.2	sterile alpha motif domain containing 8 [Source:HGNC Symbol;Acc:26320]	1.05119
HECTD2	10:42889244-135513631	10q23.32	HECT domain containing 2 [Source:HGNC Symbol;Acc:26736]	1.05119
LRRC18	10:42889244-135513631	10q11.23	leucine rich repeat containing 18 [Source:HGNC Symbol;Acc:23199]	1.05119
ZNF488	10:42889244-135513631	10q11.22	zinc finger protein 488 [Source:HGNC Symbol;Acc:23535]	1.05119
ANXA8	10:42889244-135513631	10q11.22	annexin A8 [Source:HGNC Symbol;Acc:546]	1.05119
MARCH8	10:42889244-135513631	10q11.21	membrane-associated ring finger (C3HC4) 8 [Source:HGNC Symbol;Acc:23356]	1.05119
ZCCHC24	10:42889244-135513631	10q22.3	zinc finger, CCHC domain containing 24 [Source:HGNC Symbol;Acc:26911]	1.05119
PHYHIPL	10:42889244-135513631	10q21.1	phytanoyl-CoA 2-hydroxylase interacting protein-like [Source:HGNC Symbol;Acc:29378]	1.05119
SLC16A9	10:42889244-135513631	10q21.2	solute carrier family 16, member 9 (monocarboxylic acid transporter 9) [Source:HGNC Symbol;Acc:23520]	1.05119
MBL2	10:42889244-135513631	10q21.1	mannose-binding lectin (protein C) 2, soluble (opsonic defect) [Source:HGNC Symbol;Acc:6922]	1.05119
REEP3	10:42889244-135513631	10q21.3	receptor accessory protein 3 [Source:HGNC Symbol;Acc:23711]	1.05119
C10orf10	10:42889244-135513631	10q11.21	Protein DEPP (Decidual protein induced by progesterone)(Fasting-induced gene protein)(FIG) [Source:UniProtKB/Swiss-Prot;Acc:Q9NTK1]	1.05119
C10orf25	10:42889244-135513631	10q11.21	Uncharacterized protein C10orf25 Precursor [Source:UniProtKB/Swiss-Prot;Acc:Q5T742]	1.05119
ZNF22	10:42889244-135513631	10q11.21	zinc finger protein 22 (KOX 15) [Source:HGNC Symbol;Acc:13012]	1.05119
DRGX	10:42889244-135513631	10q11.23	dorsal root ganglia homeobox [Source:HGNC Symbol;Acc:21536]	1.05119
C10orf72	10:42889244-135513631	10q11.23	Uncharacterized Ig-like domain-containing protein C10orf72 Precursor [Source:UniProtKB/Swiss-Prot;Acc:Q8IW00]	1.05119
VDAC2	10:42889244-135513631	10q22.2	voltage-dependent anion channel 2 [Source:HGNC Symbol;Acc:12672]	1.05119
COMTD1	10:42889244-135513631	10q22.2	catechol-O-methyltransferase domain containing 1 [Source:HGNC Symbol;Acc:26309]	1.05119
SLC18A2	10:42889244-135513631	10q25.3	solute carrier family 18 (vesicular monoamine), member 2 [Source:HGNC Symbol;Acc:10935]	1.05119
PDZD8	10:42889244-135513631	10q25.3 10q26.11	PDZ domain containing 8 [Source:HGNC Symbol;Acc:26974]	1.05119
ZNF503	10:42889244-135513631	10q22.2	zinc finger protein 503 [Source:HGNC Symbol;Acc:23589]	1.05119
FAM175B	10:42889244-135513631	10q26.13	family with sequence similarity 175, member B [Source:HGNC Symbol;Acc:28975]	1.05119
C10orf84	10:42889244-135513631	10q26.11	Uncharacterized protein C10orf84 [Source:UniProtKB/Swiss-Prot;Acc:Q9H8W3]	1.05119
PRDX3	10:42889244-135513631	10q26.11	peroxiredoxin 3 [Source:HGNC Symbol;Acc:9354]	1.05119
GHITM	10:42889244-135513631	10q23.1	growth hormone inducible transmembrane protein [Source:HGNC Symbol;Acc:17281]	1.05119
STOX1	10:42889244-135513631	10q21.3 10q22.1	storkhead box 1 [Source:HGNC Symbol;Acc:23508]	1.05119
RET	10:42889244-135513631	10q11.21	ret proto-oncogene [Source:HGNC Symbol;Acc:9967]	1.05119
DDX21	10:42889244-135513631	10q22.1	DEAD (Asp-Glu-Ala-Asp) box polypeptide 21 [Source:HGNC Symbol;Acc:2744]	1.05119
BMS1	10:42889244-135513631	10q11.21	BMS1 homolog, ribosome assembly protein (yeast) [Source:HGNC Symbol;Acc:23505]	1.05119
STK32C	10:42889244-135513631	10q26.3	serine/threonine kinase 32C [Source:HGNC Symbol;Acc:21332]	1.05119
CASP7	10:42889244-135513631	10q25.3	caspase 7, apoptosis-related cysteine peptidase [Source:HGNC Symbol;Acc:1508]	1.05119
C10orf118	10:42889244-135513631	10q25.3	microRNA 2110 [Source:HGNC Symbol;Acc:37071]	1.05119
VWA2	10:42889244-135513631	10q25.3	von Willebrand factor A domain containing 2 [Source:HGNC Symbol;Acc:24709]	1.05119
PRAP1	10:42889244-135513631	10q26.3	proline-rich acidic protein 1 [Source:HGNC Symbol;Acc:23304]	1.05119
TRUB1	10:42889244-135513631	10q25.3	TruB pseudouridine (psi) synthase homolog 1 (E. coli) [Source:HGNC Symbol;Acc:16060]	1.05119
CYP2C19	10:42889244-135513631	10q23.33	cytochrome P450, family 2, subfamily C, polypeptide 19 [Source:HGNC Symbol;Acc:2621]	1.05119
PNLIPRP2	10:42889244-135513631	10q25.3	pancreatic lipase-related protein 2 [Source:HGNC Symbol;Acc:9157]	1.05119
C10orf82	10:42889244-135513631	10q25.3	Uncharacterized protein C10orf82 [Source:UniProtKB/Swiss-Prot;Acc:Q8WW14]	1.05119
HSPA12A	10:42889244-135513631	10q25.3	heat shock 70kDa protein 12A [Source:HGNC Symbol;Acc:19022]	1.05119
FAM35B	10:42889244-135513631	10q11.22	family with sequence similarity 35, member B [Source:HGNC Symbol;Acc:31425]	1.05119
FRAT1	10:42889244-135513631	10q24.1	frequently rearranged in advanced T-cell lymphomas [Source:HGNC Symbol;Acc:3944]	1.05119
UBTD1	10:42889244-135513631	10q24.1 10q24.2	ubiquitin domain containing 1 [Source:HGNC Symbol;Acc:25683]	1.05119
ANKRD2	10:42889244-135513631	10q24.2	ankyrin repeat domain 2 (stretch responsive muscle) [Source:HGNC Symbol;Acc:495]	1.05119
C10orf28	10:42889244-135513631	10q24.2	Growth inhibition and differentiation-related protein 88 (Putative mitochondrial space protein 32.1) [Source:UniProtKB/Swiss-Prot;Acc:Q7Z5L2]	1.05119
HTRA1	10:42889244-135513631	10q26.13	HtrA serine peptidase 1 [Source:HGNC Symbol;Acc:9476]	1.05119
HIF1AN	10:42889244-135513631	10q24.31	hypoxia inducible factor 1, alpha subunit inhibitor [Source:HGNC Symbol;Acc:17113]	1.05119
NDUFB8	10:42889244-135513631	10q24.31	NADH dehydrogenase (ubiquinone) 1 beta subcomplex, 8, 19kDa [Source:HGNC Symbol;Acc:7703]	1.05119
BTRC	10:42889244-135513631	10q24.32	beta-transducin repeat containing [Source:HGNC Symbol;Acc:1144]	1.05119
POLL	10:42889244-135513631	10q24.32	polymerase (DNA directed), lambda [Source:HGNC Symbol;Acc:9184]	1.05119
DPCD	10:42889244-135513631	10q24.32	deleted in primary ciliary dyskinesia homolog (mouse) [Source:HGNC Symbol;Acc:24542]	1.05119
HPS6	10:42889244-135513631	10q24.32	Hermansky-Pudlak syndrome 6 [Source:HGNC Symbol;Acc:18817]	1.05119
NOLC1	10:42889244-135513631	10q24.32	nucleolar and coiled-body phosphoprotein 1 [Source:HGNC Symbol;Acc:15608]	1.05119
C10orf27	10:42889244-135513631	10q22.1	Protein SPATIAL (Stromal protein associated with thymii and lymph node homolog) [Source:UniProtKB/Swiss-Prot;Acc:Q96M53]	1.05119
SGPL1	10:42889244-135513631	10q22.1	sphingosine-1-phosphate lyase 1 [Source:HGNC Symbol;Acc:10817]	1.05119
PCBD1	10:42889244-135513631	10q22.1	pterin-4 alpha-carbinolamine dehydratase/dimerization cofactor of hepatocyte nuclear factor 1 alpha [Source:HGNC Symbol;Acc:8646]	1.05119
C10orf26	10:42889244-135513631	10q24.32	Outcome predictor in acute leukemia 1 [Source:UniProtKB/Swiss-Prot;Acc:Q9NX94]	1.05119
C10orf32	10:42889244-135513631	10q24.32	UPF0693 protein C10orf32 [Source:UniProtKB/Swiss-Prot;Acc:Q96B45]	1.05119
C10orf104	10:42889244-135513631	10q22.1	UPF0448 protein C10orf104 [Source:UniProtKB/Swiss-Prot;Acc:Q96DE5]	1.05119
SYNPO2L	10:42889244-135513631	10q22.2	synaptopodin 2-like [Source:HGNC Symbol;Acc:23532]	1.05119
NUDT13	10:42889244-135513631	10q22.1	nudix (nucleoside diphosphate linked moiety X)-type motif 13 [Source:HGNC Symbol;Acc:18827]	1.05119
ZMYND17	10:42889244-135513631	10q22.2	zinc finger, MYND-type containing 17 [Source:HGNC Symbol;Acc:21000]	1.05119
USP54	10:42889244-135513631	10q22.2	ubiquitin specific peptidase 54 [Source:HGNC Symbol;Acc:23513]	1.05119
NDST2	10:42889244-135513631	10q22.2	N-deacetylase/N-sulfotransferase (heparan glucosaminyl) 2 [Source:HGNC Symbol;Acc:7681]	1.05119
RP13-49I15.3	10:42889244-135513631	10q26.3	[undefined]	1.05119
DDIT4	10:42889244-135513631	10q22.1	DNA-damage-inducible transcript 4 [Source:HGNC Symbol;Acc:24944]	1.05119
AFAP1L2	10:42889244-135513631	10q25.3	actin filament associated protein 1-like 2 [Source:HGNC Symbol;Acc:25901]	1.05119
ZNF32	10:42889244-135513631	10q11.21	zinc finger protein 32 [Source:HGNC Symbol;Acc:13095]	1.05119
HNRNPF	10:42889244-135513631	10q11.21	heterogeneous nuclear ribonucleoprotein F [Source:HGNC Symbol;Acc:5039]	1.05119
CSGALNACT2	10:42889244-135513631	10q11.21	chondroitin sulfate N-acetylgalactosaminyltransferase 2 [Source:HGNC Symbol;Acc:24292]	1.05119
CDK1	10:42889244-135513631	10q21.2	cyclin-dependent kinase 1 [Source:HGNC Symbol;Acc:1722]	1.05119
FRMPD2	10:42889244-135513631	10q11.22	FERM and PDZ domain containing 2 [Source:HGNC Symbol;Acc:28572]	1.05119
EMX2	10:42889244-135513631	10q26.11	empty spiracles homeobox 2 [Source:HGNC Symbol;Acc:3341]	1.05119
MGMT	10:42889244-135513631	10q26.3	O-6-methylguanine-DNA methyltransferase [Source:HGNC Symbol;Acc:7059]	1.05119
DYDC1	10:42889244-135513631	10q23.1	DPY30 domain containing 1 [Source:HGNC Symbol;Acc:23460]	1.05119
MORN4	10:42889244-135513631	10q24.2	MORN repeat containing 4 [Source:HGNC Symbol;Acc:24001]	1.05119
TRIM8	10:42889244-135513631	10q24.32	tripartite motif-containing 8 [Source:HGNC Symbol;Acc:15579]	1.05119
C10orf35	10:42889244-135513631	10q22.1	Uncharacterized protein C10orf35 [Source:UniProtKB/Swiss-Prot;Acc:Q96D05]	1.05119
ZDHHC16	10:42889244-135513631	10q24.1	zinc finger, DHHC-type containing 16 [Source:HGNC Symbol;Acc:20714]	1.05119
EXOSC1	10:42889244-135513631	10q24.1	exosome component 1 [Source:HGNC Symbol;Acc:17286]	1.05119
PGAM1	10:42889244-135513631	10q24.1	phosphoglycerate mutase 1 (brain) [Source:HGNC Symbol;Acc:8888]	1.05119
SYCE1	10:42889244-135513631	10q26.3	synaptonemal complex central element protein 1 [Source:HGNC Symbol;Acc:28852]	1.05119
UTF1	10:42889244-135513631	10q26.3	undifferentiated embryonic cell transcription factor 1 [Source:HGNC Symbol;Acc:12634]	1.05119
KNDC1	10:42889244-135513631	10q26.3	kinase non-catalytic C-lobe domain (KIND) containing 1 [Source:HGNC Symbol;Acc:29374]	1.05119
C10orf92	10:42889244-135513631	10q26.3	Uncharacterized protein C10orf92 [Source:UniProtKB/Swiss-Prot;Acc:Q8IYW2]	1.05119
PWWP2B	10:42889244-135513631	10q26.3	PWWP domain containing 2B [Source:HGNC Symbol;Acc:25150]	1.05119
PTEN	10:42889244-135513631	10q23.31	phosphatase and tensin homolog [Source:HGNC Symbol;Acc:9588]	1.05119
JMJD1C	10:42889244-135513631	10q21.3	jumonji domain containing 1C [Source:HGNC Symbol;Acc:12313]	1.05119
FAM170B	10:42889244-135513631	10q11.23	family with sequence similarity 170, member B [Source:HGNC Symbol;Acc:19736]	1.05119
CHCHD1	10:42889244-135513631	10q22.2	coiled-coil-helix-coiled-coil-helix domain containing 1 [Source:HGNC Symbol;Acc:23518]	1.05119
AGAP5	10:42889244-135513631	10q22.2	ArfGAP with GTPase domain, ankyrin repeat and PH domain 5 [Source:HGNC Symbol;Acc:23467]	1.05119
FAM21C	10:42889244-135513631	10q11.22	family with sequence similarity 21, member C [Source:HGNC Symbol;Acc:23414]	1.05119
ANUBL1	10:42889244-135513631	10q11.22	AN1, ubiquitin-like, homolog (Xenopus laevis) [Source:HGNC Symbol;Acc:23504]	1.05119
OR13A1	10:42889244-135513631	10q11.21	olfactory receptor, family 13, subfamily A, member 1 [Source:HGNC Symbol;Acc:14772]	1.05119
LRRC20	10:42889244-135513631	10q22.1	leucine rich repeat containing 20 [Source:HGNC Symbol;Acc:23421]	1.05119
HPSE2	10:42889244-135513631	10q24.2	heparanase 2 [Source:HGNC Symbol;Acc:18374]	1.05119
C10orf131	10:42889244-135513631	10q24.1	Uncharacterized protein C10orf131 [Source:UniProtKB/Swiss-Prot;Acc:A6NCD4]	1.05119
C10orf129	10:42889244-135513631	10q23.33	Acyl-coenzyme A synthetase ACSM6, mitochondrial Precursor (EC 6.2.1.2) [Source:UniProtKB/Swiss-Prot;Acc:Q6P461]	1.05119
NOC3L	10:42889244-135513631	10q23.33	nucleolar complex associated 3 homolog (S. cerevisiae) [Source:HGNC Symbol;Acc:24034]	1.05119
LIPM	10:42889244-135513631	10q23.31	lipase, family member M [Source:HGNC Symbol;Acc:23455]	1.05119
SNCG	10:42889244-135513631	10q23.2	synuclein, gamma (breast cancer-specific protein 1) [Source:HGNC Symbol;Acc:11141]	1.05119
MMRN2	10:42889244-135513631	10q23.2	multimerin 2 [Source:HGNC Symbol;Acc:19888]	1.05119
USMG5	10:42889244-135513631	10q24.33	up-regulated during skeletal muscle growth 5 homolog (mouse) [Source:HGNC Symbol;Acc:30889]	1.05119
AGAP8	10:42889244-135513631	10q11.23	ArfGAP with GTPase domain, ankyrin repeat and PH domain 8 [Source:HGNC Symbol;Acc:23464]	1.05119
FAM21D	10:42889244-135513631	10q11.23	family with sequence similarity 21, member D [Source:HGNC Symbol;Acc:23418]	1.05119

Continued on next page...

gene name	chr	.DNABand	.GeneDesc	rank
FGFBP3	10:42889244-135513631	10q23.32	fibroblast growth factor binding protein 3 [Source:HGNC Symbol;Acc:23428]	1.05119
C10orf122	10:42889244-135513631	10q26.13	Putative uncharacterized protein C10orf122 [Source:UniProtKB/Swiss-Prot;Acc:Q5VZQ5]	1.05119
CTBP2	10:42889244-135513631	10q26.13	C-terminal binding protein 2 [Source:HGNC Symbol;Acc:2495]	1.05119
PPP2R2D	10:42889244-135513631	10q26.3	protein phosphatase 2, regulatory subunit B, delta isoform [Source:HGNC Symbol;Acc:23732]	1.05119
PNLIP	10:42889244-135513631	10q25.3	pancreatic lipase [Source:HGNC Symbol;Acc:9155]	1.05119
BNIP3	10:42889244-135513631	10q26.3	BCL2/adenovirus E1B 19kDa interacting protein 3 [Source:HGNC Symbol;Acc:1084]	1.05119
TMEM20	10:42889244-135513631	10q23.33	transmembrane protein 20 [Source:HGNC Symbol;Acc:26607]	1.05119
RP11-107C16.2	10:42889244-135513631	10q26.13	[undefined]	1.05119
TCERG1L	10:42889244-135513631	10q26.3	transcription elongation regulator 1-like [Source:HGNC Symbol;Acc:23533]	1.05119
SEC24C	10:42889244-135513631	10q22.2	SEC24 family, member C (S. cerevisiae) [Source:HGNC Symbol;Acc:10705]	1.05119
C10orf85	10:42889244-135513631	10q26.12	Uncharacterized protein C10orf85 [Source:UniProtKB/Swiss-Prot;Acc:Q8N1V8]	1.05119
C10orf71	10:42889244-135513631	10q11.23	Uncharacterized protein C10orf71 [Source:UniProtKB/Swiss-Prot;Acc:Q711Q0]	1.05119
CSTF2T	10:42889244-135513631	10q21.1	cleavage stimulation factor, 3' pre-RNA, subunit 2, 64kDa, tau variant [Source:HGNC Symbol;Acc:17086]	1.05119
CASC2	10:42889244-135513631	10q26.11	cancer susceptibility candidate 2 [Source:HGNC Symbol;Acc:22933]	1.05119
MYOZ1	10:42889244-135513631	10q22.2	myozenin 1 [Source:HGNC Symbol;Acc:13752]	1.05119
ZNF518A	10:42889244-135513631	10q24.1	zinc finger protein 518A [Source:HGNC Symbol;Acc:29009]	1.05119
SH2D4B	10:42889244-135513631	10q23.1	SH2 domain containing 4B [Source:HGNC Symbol;Acc:31440]	1.05119
RPS3AP5	10:42889244-135513631	10q23.1	ribosomal protein S3A pseudogene 5 [Source:HGNC Symbol;Acc:23744]	1.05119
RP11-324H6.6	10:42889244-135513631	10q11.23	[undefined]	1.05119
C10orf53	10:42889244-135513631	10q11.23	UPF0728 protein C10orf53 [Source:UniProtKB/Swiss-Prot;Acc:Q8N6V4]	1.05119
AL391137.2	10:42889244-135513631	10q11.22	cDNA FLJ39396 fis, clone PLACE6008640 [Source:UniProtKB/TrEMBL;Acc:Q8N8J2]	1.05119
CTGLF12P	10:42889244-135513631	10q11.22	centaurin, gamma-like family, member 12 pseudogene [Source:HGNC Symbol;Acc:23661]	1.05119
AL445486.1	10:42889244-135513631	10q25.2	[undefined]	1.05119
ATOH7	10:42889244-135513631	10q21.3	atonal homolog 7 (Drosophila) [Source:HGNC Symbol;Acc:13907]	1.05119
PSTK	10:42889244-135513631	10q26.13	phosphoseryl-tRNA kinase [Source:HGNC Symbol;Acc:28578]	1.05119
C10orf91	10:42889244-135513631	10q26.3	Uncharacterized protein C10orf91 [Source:UniProtKB/Swiss-Prot;Acc:Q5T1B1]	1.05119
RP11-399O19.5	10:42889244-135513631	10q23.31	[undefined]	1.05119
RP11-179B15.2	10:42889244-135513631	10q21.1	[undefined]	1.05119
RP11-198M6.2	10:42889244-135513631	10q26.12	[undefined]	1.05119
SRP9L1	10:42889244-135513631	10q23.32	signal recognition particle 9-like 1 [Source:HGNC Symbol;Acc:23402]	1.05119
PCGF5	10:42889244-135513631	10q23.32	polycomb group ring finger 5 [Source:HGNC Symbol;Acc:28264]	1.05119
PRF1	10:42889244-135513631	10q22.1	perforin 1 (pore forming protein) [Source:HGNC Symbol;Acc:9360]	1.05119
CLRN3	10:42889244-135513631	10q26.2	clarin 3 [Source:HGNC Symbol;Acc:20795]	1.05119
PIPSL	10:42889244-135513631	10q23.33	PIP5K1A and PSMD4-like [Source:HGNC Symbol;Acc:23733]	1.05119
RP11-517P14.6	10:42889244-135513631	10q11.21	[undefined]	1.05119
PPA1	10:42889244-135513631	10q22.1	pyrophosphatase (inorganic) 1 [Source:HGNC Symbol;Acc:9226]	1.05119
FRAT2	10:42889244-135513631	10q24.1	frequently rearranged in advanced T-cell lymphomas 2 [Source:HGNC Symbol;Acc:16048]	1.05119
ADO	10:42889244-135513631	10q21.3	2-aminoethanethiol (cysteamine) dioxygenase [Source:HGNC Symbol;Acc:23506]	1.05119
RTKN2	10:42889244-135513631	10q21.2	rhotekin 2 [Source:HGNC Symbol;Acc:19364]	1.05119
CHST15	10:42889244-135513631	10q26.13	carbohydrate (N-acetylgalactosamine 4-sulfate 6-O) sulfotransferase 15 [Source:HGNC Symbol;Acc:18137]	1.05119
MRPS16	10:42889244-135513631	10q22.2	mitochondrial ribosomal protein S16 [Source:HGNC Symbol;Acc:14048]	1.05119
LIPF	10:42889244-135513631	10q23.31	lipase, gastric [Source:HGNC Symbol;Acc:6622]	1.05119
CCNYL2	10:42889244-135513631	10q11.21	cyclin Y-like 2 [Source:HGNC Symbol;Acc:23495]	1.05119
C10orf96	10:42889244-135513631	10q25.3	UPF0628 protein C10orf96 [Source:UniProtKB/Swiss-Prot;Acc:P0C7W6]	1.05119
GRID1	10:42889244-135513631	10q23.1 10q23.2	glutamate receptor, ionotropic, delta 1 [Source:HGNC Symbol;Acc:4575]	1.05119
RP11-79I23.2	10:42889244-135513631	10q21.1	[undefined]	1.05119
CALHM3	10:42889244-135513631	10q24.33	calcium homeostasis modulator 3 [Source:HGNC Symbol;Acc:23458]	1.05119
CTNNA3	10:42889244-135513631	10q21.3	catenin (cadherin-associated protein), alpha 3 [Source:HGNC Symbol;Acc:2511]	1.05119
C10orf107	10:42889244-135513631	10q21.2	Uncharacterized protein C10orf107 [Source:UniProtKB/Swiss-Prot;Acc:Q8IVU9]	1.05119
C10orf120	10:42889244-135513631	10q26.13	Uncharacterized protein C10orf120 [Source:UniProtKB/Swiss-Prot;Acc:Q5SQS8]	1.05119
SFXN4	10:42889244-135513631	10q26.11	sideroflexin 4 [Source:HGNC Symbol;Acc:16088]	1.05119
PTPN20B	10:42889244-135513631	10q11.22	protein tyrosine phosphatase, non-receptor type 20B [Source:HGNC Symbol;Acc:23423]	1.05119
RP11-366I13.3	10:42889244-135513631	10q23.33	[undefined]	1.05119
RNLS	10:42889244-135513631	10q23.31	renalase, FAD-dependent amine oxidase [Source:HGNC Symbol;Acc:25641]	1.05119
FAM22A	10:42889244-135513631	10q23.2	family with sequence similarity 22, member A [Source:HGNC Symbol;Acc:23438]	1.05119
AP3M1	10:42889244-135513631	10q22.2	adaptor-related protein complex 3, mu 1 subunit [Source:HGNC Symbol;Acc:569]	1.05119
SFTPA2	10:42889244-135513631	10q22.3	surfactant protein A2 [Source:HGNC Symbol;Acc:10799]	1.05119
PRKG1	10:42889244-135513631	10q11.23 10q21.1	protein kinase, cGMP-dependent, type I [Source:HGNC Symbol;Acc:9414]	1.05119
NRG3	10:42889244-135513631	10q23.1	neuregulin 3 [Source:HGNC Symbol;Acc:7999]	1.05119
IFIT1	10:42889244-135513631	10q23.31	interferon-induced protein with tetratricopeptide repeats 1 [Source:HGNC Symbol;Acc:5407]	1.05119
RP11-178A10.1	10:42889244-135513631	10q11.21	[undefined]	1.05119
CALHM1	10:42889244-135513631	10q24.33	calcium homeostasis modulator 1 [Source:HGNC Symbol;Acc:23494]	1.05119
GPR120	10:42889244-135513631	10q23.33	G protein-coupled receptor 120 [Source:HGNC Symbol;Acc:19061]	1.05119
FOXI2	10:42889244-135513631	10q26.2	forkhead box I2 [Source:HGNC Symbol;Acc:32448]	1.05119
KCNK18	10:42889244-135513631	10q25.3	potassium channel, subfamily K, member 18 [Source:HGNC Symbol;Acc:19439]	1.05119
ANXA8L2	10:42889244-135513631	10q11.22	annexin A8-like 2 [Source:HGNC Symbol;Acc:23335]	1.05119
PDZD7	10:42889244-135513631	10q24.31	PDZ domain containing 7 [Source:HGNC Symbol;Acc:26257]	1.05119
PNLIPRP1	10:42889244-135513631	10q25.3	pancreatic lipase-related protein 1 [Source:HGNC Symbol;Acc:9156]	1.05119
SLIT1	10:42889244-135513631	10q24.1	slit homolog 1 (Drosophila) [Source:HGNC Symbol;Acc:11085]	1.05119
KIAA1598	10:42889244-135513631	10q25.3	KIAA1598 [Source:HGNC Symbol;Acc:29319]	1.05119
CYP26C1	10:42889244-135513631	10q23.33	cytochrome P450, family 26, subfamily C, polypeptide 1 [Source:HGNC Symbol;Acc:20577]	1.05119
SLC18A3	10:42889244-135513631	10q11.23	solute carrier family 18 (vesicular acetylcholine), member 3 [Source:HGNC Symbol;Acc:10936]	1.05119
TMEM72	10:42889244-135513631	10q11.21	transmembrane protein 72 [Source:HGNC Symbol;Acc:31658]	1.05119
DMBT1	10:42889244-135513631	10q26.13	deleted in malignant brain tumors 1 [Source:HGNC Symbol;Acc:2926]	1.05119
FAM25A	10:42889244-135513631	10q23.2	family with sequence similarity 25, member A [Source:HGNC Symbol;Acc:23436]	1.05119
FAM22B	10:42889244-135513631	10q22.3	family with sequence similarity 22, member B [Source:HGNC Symbol;Acc:23445]	1.05119
AGAP4	10:42889244-135513631	10q11.22	ArfGAP with GTPase domain, ankyrin repeat and PH domain 4 [Source:HGNC Symbol;Acc:23459]	1.05119
FAM25C	10:42889244-135513631	10q11.22	family with sequence similarity 25, member C [Source:HGNC Symbol;Acc:23586]	1.05119
ENO4	10:42889244-135513631	10q25.3	enolase family member 4 [Source:HGNC Symbol;Acc:31670]	1.05119
C10orf99	10:42889244-135513631	10q23.1	Putative uncharacterized protein C10orf99 Precursor [Source:UniProtKB/Swiss-Prot;Acc:Q6UWK7]	1.05119
JAKMIP3	10:42889244-135513631	10q26.3	Janus kinase and microtubule interacting protein 3 [Source:HGNC Symbol;Acc:23523]	1.05119
ASAH2	10:42889244-135513631	10q11.23	N-acylsphingosine amidohydrolase (non-lysosomal ceramidase) 2 [Source:HGNC Symbol;Acc:18860]	1.05119
NANOS1	10:42889244-135513631	10q26.11	nanos homolog 1 (Drosophila) [Source:HGNC Symbol;Acc:23044]	1.05119
HMX3	10:42889244-135513631	10q26.13	H6 family homeobox 3 [Source:HGNC Symbol;Acc:5019]	1.05119
CC2D2B	10:42889244-135513631	10q24.1	coiled-coil and C2 domain containing 2B [Source:HGNC Symbol;Acc:31666]	1.05119
UROS	10:42889244-135513631	10q26.13 10q26.2	uroporphyrinogen III synthase [Source:HGNC Symbol;Acc:12592]	1.05119
DUPD1	10:42889244-135513631	10q22.2	dual specificity phosphatase and pro isomerase domain containing 1 [Source:HGNC Symbol;Acc:23481]	1.05119
AL391005.1	10:42889244-135513631	10q26.2	[undefined]	1.05119
HMX2	10:42889244-135513631	10q26.13	H6 family homeobox 2 [Source:HGNC Symbol;Acc:5018]	1.05119
FAM196A	10:42889244-135513631	10q26.2	family with sequence similarity 196, member A [Source:HGNC Symbol;Acc:33859]	1.05119
FAM35B2	10:42889244-135513631	10q11.22	family with sequence similarity 35, member B2 [Source:HGNC Symbol;Acc:34038]	1.05119
FAM25B	10:42889244-135513631	10q11.22	family with sequence similarity 25, member B [Source:HGNC Symbol;Acc:23584]	1.05119
PLAC9	10:42889244-135513631	10q22.3	placenta-specific 9 [Source:HGNC Symbol;Acc:19255]	1.05119
AL450307.1	10:42889244-135513631	10q26.3	cDNA FLJ46300 fis, clone TESTI4035989 [Source:UniProtKB/TrEMBL;Acc:Q6ZRK0]	1.05119
FAM53B	10:42889244-135513631	10q26.13	family with sequence similarity 53, member B [Source:HGNC Symbol;Acc:28968]	1.05119
AL513534.2	10:42889244-135513631	10q21.3	[undefined]	1.05119
BLOC1S2	10:42889244-135513631	10q24.31	biogenesis of lysosomal organelles complex-1, subunit 2 [Source:HGNC Symbol;Acc:20984]	1.05119
ACADSB	10:42889244-135513631	10q26.13	acyl-Coenzyme A dehydrogenase, short/branched chain [Source:HGNC Symbol;Acc:91]	1.05119
LCOR	10:42889244-135513631	10q24.1	ligand dependent nuclear receptor corepressor [Source:HGNC Symbol;Acc:29503]	1.05119
RP11-57C13.3	10:42889244-135513631	10q23.2	[undefined]	1.05119
ZNF33B	10:42889244-135513631	10q11.21	zinc finger protein 33B [Source:HGNC Symbol;Acc:13097]	1.05119
ZNF239	10:42889244-135513631	10q11.21	zinc finger protein 239 [Source:HGNC Symbol;Acc:13031]	1.05119
NHLRC2	10:42889244-135513631	10q25.3	NHL repeat containing 2 [Source:HGNC Symbol;Acc:24731]	1.05119
TMEM26	10:42889244-135513631	10q21.2	transmembrane protein 26 [Source:HGNC Symbol;Acc:28550]	1.05119
FUT11	10:42889244-135513631	10q22.2	fucosyltransferase 11 (alpha (1,3) fucosyltransferase) [Source:HGNC Symbol;Acc:19233]	1.05119
ACSL5	10:42889244-135513631	10q25.2	acyl-CoA synthetase long-chain family member 5 [Source:HGNC Symbol;Acc:16526]	1.05119
GPR123	10:42889244-135513631	10q26.3	G protein-coupled receptor 123 [Source:HGNC Symbol;Acc:13838]	1.05119
AC073585.1	10:42889244-135513631	10q26.13	Putative uncharacterized protein ENSP00000357890 Fragment [Source:UniProtKB/TrEMBL;Acc:A6NK05]	1.05119
OPALIN	10:42889244-135513631	10q24.1	oligodendrocytic myelin paranodal and inner loop protein [Source:HGNC Symbol;Acc:20707]	1.05119
OGDHL	10:42889244-135513631	10q11.23	oxoglutarate dehydrogenase-like [Source:HGNC Symbol;Acc:25590]	1.05119
COL13A1	10:42889244-135513631	10q22.1	collagen, type XIII, alpha 1 [Source:HGNC Symbol;Acc:2190]	1.05119
AL512662.1	10:42889244-135513631	10q22.3	Putative BMS1-like protein [Source:UniProtKB/Swiss-Prot;Acc:Q6IPT3]	1.05119
AL442003.2	10:42889244-135513631	10q11.23	[undefined]	1.05119
FRMPD2L1	10:42889244-135513631	10q11.22	FERM and PDZ domain containing 2 like 1 [Source:HGNC Symbol;Acc:16843]	1.05119
AL451069.1	10:42889244-135513631	10q26.3	Putative uncharacterized protein ENSP00000346801 Fragment [Source:UniProtKB/TrEMBL;Acc:A6NE69]	1.05119
PSAP	10:42889244-135513631	10q22.1	prosaposin [Source:HGNC Symbol;Acc:9498]	1.05119

Continued on next page...

gene name	chr	.DNABand	.GeneDesc	rank
C10orf79	10:42889244-135513631	10q25.1	WD repeat-containing protein C10orf79 [Source:UniProtKB/Swiss-Prot;Acc:Q8NDM7]	1.05119
C10orf119	10:42889244-135513631	10q26.11	UPF0557 protein C10orf119 [Source:UniProtKB/Swiss-Prot;Acc:Q9BTE3]	1.05119
NRAP	10:42889244-135513631	10q25.3	nebulin-related anchoring protein [Source:HGNC Symbol;Acc:7988]	1.05119
FAM25G	10:42889244-135513631	10q11.22	family with sequence similarity 25, member G [Source:HGNC Symbol;Acc:23590]	1.05119
ENTPD7	10:42889244-135513631	10q24.2	ectonucleoside triphosphate diphosphohydrolase 7 [Source:HGNC Symbol;Acc:19745]	1.05119
CTGLF6	10:42889244-135513631	10q11.22	ArfGAP with GTPase domain, ankyrin repeat and PH domain 9 [Source:HGNC Symbol;Acc:23463]	1.05119
MARCH5	10:42889244-135513631	10q23.32 10q23.33	membrane-associated ring finger (C3HC4) 5 [Source:HGNC Symbol;Acc:26025]	1.05119
SLC29A3	10:42889244-135513631	10q22.1	solute carrier family 29 (nucleoside transporters), member 3 [Source:HGNC Symbol;Acc:23096]	1.05119
ANTXRL	10:42889244-135513631	10q11.22	anthrax toxin receptor-like [Source:HGNC Symbol;Acc:27277]	1.05119
ZNF485	10:42889244-135513631	10q11.21	zinc finger protein 485 [Source:HGNC Symbol;Acc:23440]	1.05119
MGEA5	10:42889244-135513631	10q24.32	meningioma expressed antigen 5 (hyaluronidase) [Source:HGNC Symbol;Acc:7056]	1.05119
ZNF511	10:42889244-135513631	10q26.3	zinc finger protein 511 [Source:HGNC Symbol;Acc:28445]	1.05119
PAPSS2	10:42889244-135513631	10q23.2 10q23.31	3'-phosphoadenosine 5'-phosphosulfate synthase 2 [Source:HGNC Symbol;Acc:8604]	1.05119
LDB1	10:42889244-135513631	10q24.32	LIM domain binding 1 [Source:HGNC Symbol;Acc:6532]	1.05119
LRRTM3	10:42889244-135513631	10q21.3	leucine rich repeat transmembrane neuronal 3 [Source:HGNC Symbol;Acc:19410]	1.05119
INPP5F	10:42889244-135513631	10q26.11	inositol polyphosphate-5-phosphatase F [Source:HGNC Symbol;Acc:17054]	1.05119
GRK5	10:42889244-135513631	10q26.11	G protein-coupled receptor kinase 5 [Source:HGNC Symbol;Acc:4544]	1.05119
RASGEF1A	10:42889244-135513631	10q11.21	RasGEF domain family, member 1A [Source:HGNC Symbol;Acc:24246]	1.05119
DCLRE1A	10:42889244-135513631	10q25.3	DNA cross-link repair 1A (PSO2 homolog, S. cerevisiae) [Source:HGNC Symbol;Acc:17660]	1.05119
KIAA1279	10:42889244-135513631	10q22.1	KIAA1279 [Source:HGNC Symbol;Acc:23419]	1.05119
SGMS1	10:42889244-135513631	10q11.23	sphingomyelin synthase 1 [Source:HGNC Symbol;Acc:29799]	1.05119
MIR107	10:42889244-135513631	10q23.31	microRNA 107 [Source:HGNC Symbol;Acc:31496]	1.05119
MIR202	10:42889244-135513631	10q26.3	microRNA 202 [Source:HGNC Symbol;Acc:32080]	1.05119
MIR346	10:42889244-135513631	10q23.2	microRNA 346 [Source:HGNC Symbol;Acc:31780]	1.05119
AC016394.2	10:42889244-135513631	10q22.2	[undefined]	1.05119
AL512272.1	10:42889244-135513631	10q26.2	[undefined]	1.05119
AL136368.1	10:42889244-135513631	10q25.2	[undefined]	1.05119
AL590502.1	10:42889244-135513631	10q21.3	[undefined]	1.05119
AC068896.2	10:42889244-135513631	10q26.13	[undefined]	1.05119
AL731556.2	10:42889244-135513631	10q22.3	[undefined]	1.05119
AL136233.1	10:42889244-135513631	10q21.3	[undefined]	1.05119
AL450306.1	10:42889244-135513631	10q22.3	[undefined]	1.05119
AL158040.1	10:42889244-135513631	10q23.32	[undefined]	1.05119
AC022024.1	10:42889244-135513631	10q25.1	[undefined]	1.05119
AL607035.1	10:42889244-135513631	10q22.1	[undefined]	1.05119
AC025268.2	10:42889244-135513631	10q23.2	[undefined]	1.05119
AL138766.1	10:42889244-135513631	10q25.1	[undefined]	1.05119
AL391539.1	10:42889244-135513631	10q22.1	[undefined]	1.05119
AL136982.1	10:42889244-135513631	10q23.2	[undefined]	1.05119
AL607035.2	10:42889244-135513631	10q22.1	[undefined]	1.05119
AL512656.3	10:42889244-135513631	10q22.2	[undefined]	1.05119
AC079955.1	10:42889244-135513631	10q23.1	[undefined]	1.05119
AL160010.1	10:42889244-135513631	10q25.1	[undefined]	1.05119
AC021037.1	10:42889244-135513631	10q24.1	[undefined]	1.05119
AL355490.1	10:42889244-135513631	10q24.1	[undefined]	1.05119
AL589782.1	10:42889244-135513631	10q23.1	[undefined]	1.05119
AC022394.1	10:42889244-135513631	10q23.1	[undefined]	1.05119
AC063965.3	10:42889244-135513631	10q23.31	[undefined]	1.05119
AL731653.1	10:42889244-135513631	10q25.3	[undefined]	1.05119
AL731563.4	10:42889244-135513631	10q22.1	[undefined]	1.05119
AL358790.2	10:42889244-135513631	10q24.32	[undefined]	1.05119
AL365506.1	10:42889244-135513631	10q21.1	[undefined]	1.05119
AC012560.1	10:42889244-135513631	10q22.3	[undefined]	1.05119
AL590080.1	10:42889244-135513631	10q23.33	[undefined]	1.05119
AL139406.1	10:42889244-135513631	10q23.31	[undefined]	1.05119
AL365434.1	10:42889244-135513631	10q23.31	[undefined]	1.05119
AL513185.2	10:42889244-135513631	10q22.1	[undefined]	1.05119
AL731542.1	10:42889244-135513631	10q26.13	[undefined]	1.05119
AC012048.1	10:42889244-135513631	10q22.3	[undefined]	1.05119
AL138765.1	10:42889244-135513631	10q24.1	[undefined]	1.05119
AL592430.2	10:42889244-135513631	10q21.2	[undefined]	1.05119
BX842243.1	10:42889244-135513631	10q26.13	[undefined]	1.05119
AL161652.1	10:42889244-135513631	10q23.32	[undefined]	1.05119
AL772134.1	10:42889244-135513631	10q26.2	[undefined]	1.05119
AC091769.1	10:42889244-135513631	10q22.1	[undefined]	1.05119
MIR146B	10:42889244-135513631	10q24.32	microRNA 146b [Source:HGNC Symbol;Acc:32079]	1.05119
RP13-439H18.4	10:42889244-135513631	10q26.3	[undefined]	1.05119
RP11-500G10.5	10:42889244-135513631	10q26.3	[undefined]	1.05119
RP11-163F15.1	10:42889244-135513631	10q25.1	[undefined]	1.05119
AC068896.3	10:42889244-135513631	10q26.13	[undefined]	1.05119
RP11-313D6.4	10:42889244-135513631	10q25.2	[undefined]	1.05119
SPRN	10:42889244-135513631	10q26.3	shadow of prion protein homolog (zebrafish) [Source:HGNC Symbol;Acc:16871]	1.05119
AL589787.1	10:42889244-135513631	10q26.2	HCG2044975Putative uncharacterized protein ENSP00000357662 ; [Source:UniProtKB/TrEMBL;Acc:B7WP59]	1.05119
FANK1	10:42889244-135513631	10q26.2	fibronectin type III and ankyrin repeat domains 1 [Source:HGNC Symbol;Acc:23527]	1.05119
METTL10	10:42889244-135513631	10q26.13	methyltransferase like 10 [Source:HGNC Symbol;Acc:33787]	1.05119
FAM24A	10:42889244-135513631	10q26.13	family with sequence similarity 24, member A [Source:HGNC Symbol;Acc:23470]	1.05119
RP11-564D11.3	10:42889244-135513631	10q26.13	[undefined]	1.05119
PPAPDC1A	10:42889244-135513631	10q26.12	phosphatidic acid phosphatase type 2 domain containing 1A [Source:HGNC Symbol;Acc:23531]	1.05119
PNLIPRP3	10:42889244-135513631	10q25.3	pancreatic lipase-related protein 3 [Source:HGNC Symbol;Acc:23492]	1.05119
RBM20	10:42889244-135513631	10q25.2	RNA binding motif protein 20 [Source:HGNC Symbol;Acc:27424]	1.05119
RP11-451M19.3	10:42889244-135513631	10q25.1	Putative uncharacterized protein ENSP00000358670 [Source:UniProtKB/TrEMBL;Acc:A6NF56]	1.05119
CYP17A1OS	10:42889244-135513631	10q24.32	cytochrome P450, family 17, subfamily A, polypeptide 1 opposite strand [Source:HGNC Symbol;Acc:31671]	1.05119
C10orf62	10:42889244-135513631	10q24.2	Uncharacterized protein C10orf62 [Source:UniProtKB/Swiss-Prot;Acc:Q5T681]	1.05119
AL356155.1	10:42889244-135513631	10q24.1	Putative uncharacterized protein ENSP00000360241 Fragment [Source:UniProtKB/TrEMBL;Acc:B7WPP8]	1.05119
IFIT1L	10:42889244-135513631	10q23.31	interferon-induced protein with tetratricopeptide repeats 1-like [Source:HGNC Symbol;Acc:23442]	1.05119
LIPN	10:42889244-135513631	10q23.31	lipase, family member N [Source:HGNC Symbol;Acc:23452]	1.05119
LIPK	10:42889244-135513631	10q23.31	lipase, family member K [Source:HGNC Symbol;Acc:23444]	1.05119
LIPJ	10:42889244-135513631	10q23.31	lipase, family member J [Source:HGNC Symbol;Acc:21773]	1.05119
LRIT2	10:42889244-135513631	10q23.1	leucine-rich repeat, immunoglobulin-like and transmembrane domains 2 [Source:HGNC Symbol;Acc:23443]	1.05119
AL359195.1	10:42889244-135513631	10q23.1	cDNA FLJ46261 fis, clone TESTI4025062 [Source:UniProtKB/TrEMBL;Acc:Q6ZRL6]	1.05119
AL391421.4	10:42889244-135513631	10q22.3	cDNA FLJ43696 fis, clone TBAES2007964 [Source:UniProtKB/TrEMBL;Acc:Q6ZUH9]	1.05119
RUFY2	10:42889244-135513631	10q21.3	RUN and FYVE domain containing 2 [Source:HGNC Symbol;Acc:19761]	1.05119
ASAH2B	10:42889244-135513631	10q11.23	N-acylsphingosine amidohydrolase (non-lysosomal ceramidase) 2B [Source:HGNC Symbol;Acc:23456]	1.05119
AGAP6	10:42889244-135513631	10q11.23	ArfGAP with GTPase domain, ankyrin repeat and PH domain 6 [Source:HGNC Symbol;Acc:23466]	1.05119
CTGLF11P	10:42889244-135513631	10q11.22	centaurin, gamma-like family, member 11 pseudogene [Source:HGNC Symbol;Acc:23660]	1.05119
TIMM23B	10:42889244-135513631	10q11.23	translocase of inner mitochondrial membrane 23 homolog B (yeast) [Source:HGNC Symbol;Acc:23581]	1.05119
C10orf128	10:42889244-135513631	10q11.23	Putative uncharacterized protein C10orf128 Precursor [Source:UniProtKB/Swiss-Prot;Acc:Q5T292]	1.05119
RP11-508M1.3	10:42889244-135513631	10q11.22	BMS1 pseudogene 5 (BMS1P5), non-coding RNA [Source:RefSeq DNA;Acc:NR_003611]	1.05119
AGAP7	10:42889244-135513631	10q11.23	ArfGAP with GTPase domain, ankyrin repeat and PH domain 7 [Source:HGNC Symbol;Acc:23465]	1.05119
AGAP10	10:42889244-135513631	10q11.22	ArfGAP with GTPase domain, ankyrin repeat and PH domain 10 [Source:HGNC Symbol;Acc:23462]	1.05119
PPYR1	10:42889244-135513631	10q11.22	pancreatic polypeptide receptor 1 [Source:HGNC Symbol;Acc:9329]	1.05119
GPRIN2	10:42889244-135513631	10q11.22	G protein regulated inducer of neurite outgrowth 2 [Source:HGNC Symbol;Acc:23730]	1.05119
SYT15	10:42889244-135513631	10q11.22	synaptotagmin XV [Source:HGNC Symbol;Acc:17167]	1.05119
BMS1P1	10:42889244-135513631	10q11.22	BMS1 pseudogene 1 [Source:HGNC Symbol;Acc:23649]	1.05119
PTPN20A	10:42889244-135513631	10q11.22	protein tyrosine phosphatase, non-receptor type 20A [Source:HGNC Symbol;Acc:23422]	1.05119
C10orf136	10:42889244-135513631	10q11.21	Putative uncharacterized protein C10orf136 [Source:UniProtKB/Swiss-Prot;Acc:Q5W0B8]	1.05119
CTSLL6	10:42889244-135513631	10q22.3	cathepsin L-like 6 [Source:HGNC Symbol;Acc:23611]	1.05119
RP11-295O23.2	10:42889244-135513631	10q25.3	[undefined]	1.05119
AL389885.1	10:42889244-135513631	10q23.33	[undefined]	1.05119
AC012046.1	10:42889244-135513631	10q22.2	[undefined]	1.05119
AL359844.1	10:42889244-135513631	10q22.1	[undefined]	1.05119

Continued on next page...

gene name	chr	.DNABand	.GeneDesc	rank
AL157386.2	10:42889244-135513631	10q24.32	[undefined]	1.05119
AC026225.1	10:42889244-135513631	10q25.1	[undefined]	1.05119
AL512324.1	10:42889244-135513631	10q11.21	[undefined]	1.05119
AL391478.1	10:42889244-135513631	10q23.1	[undefined]	1.05119
AL358235.1	10:42889244-135513631	10q24.1	[undefined]	1.05119
AL359074.2	10:42889244-135513631	10q22.2	[undefined]	1.05119
AL138921.2	10:42889244-135513631	10q24.31	[undefined]	1.05119
AL713888.1	10:42889244-135513631	10q21.3	[undefined]	1.05119
SNORA19	10:42889244-135513631	10q26.11	small nucleolar RNA, H/ACA box 19 [Source:HGNC Symbol;Acc:32609]	1.05119
AL512656.1	10:42889244-135513631	10q22.2	[undefined]	1.05119
AC068896.1	10:42889244-135513631	10q26.13	[undefined]	1.05119
MIR608	10:42889244-135513631	10q24.31	microRNA 608 [Source:HGNC Symbol;Acc:32864]	1.05119
MIR606	10:42889244-135513631	10q22.2	microRNA 606 [Source:HGNC Symbol;Acc:32862]	1.05119
MIR605	10:42889244-135513631	10q21.1	microRNA 605 [Source:HGNC Symbol;Acc:32861]	1.05119
AL161652.2	10:42889244-135513631	10q23.33	[undefined]	1.05119
MIR607	10:42889244-135513631	10q24.1	microRNA 607 [Source:HGNC Symbol;Acc:32863]	1.05119
MIR609	10:42889244-135513631	10q25.1	microRNA 609 [Source:HGNC Symbol;Acc:32865]	1.05119
AC069540.1	10:42889244-135513631	10q23.1	[undefined]	1.05119
AL359759.1	10:42889244-135513631	10q24.31	[undefined]	1.05119
AL731569.1	10:42889244-135513631	10q23.2	[undefined]	1.05119
AL356214.1	10:42889244-135513631	10q23.33	[undefined]	1.05119
AL591408.1	10:42889244-135513631	10q24.33	[undefined]	1.05119
SNORA12	10:42889244-135513631	10q24.31	small nucleolar RNA, H/ACA box 12 [Source:HGNC Symbol;Acc:32600]	1.05119
AL133551.1	10:42889244-135513631	10q21.3	[undefined]	1.05119
AL592546.2	10:42889244-135513631	10q25.3	[undefined]	1.05119
HMGN2L8	10:42889244-135513631	10q23.1	high-mobility group nucleosomal binding domain 2-like 8 [Source:HGNC Symbol;Acc:31662]	1.05119
RP11-476E15.3	10:42889244-135513631	10q24.1	Novel protein (KIAA0894)Sorbin and SH3 domain containing 1, isoform CRA_e ; [Source:UniProtKB/TrEMBL;Acc:Q5T925]	1.05119
RP11-119F7.2	10:42889244-135513631	10q21.3	[undefined]	1.05119
RP11-753C18.4	10:42889244-135513631	10q24.32	[undefined]	1.05119
AL590105.1	10:42889244-135513631	10q26.3	Putative uncharacterized protein ENSP00000402881 Fragment [Source:UniProtKB/TrEMBL;Acc:C9JQA5]	1.05119
RP11-81B10.2	10:42889244-135513631	10q26.2	[undefined]	1.05119
FAM24B	10:42889244-135513631	10q26.13	family with sequence similarity 24, member B [Source:HGNC Symbol;Acc:23475]	1.05119
RP11-479A21.1	10:42889244-135513631	10q25.2	[undefined]	1.05119
RP11-45P22.1	10:42889244-135513631	10q25.1	[undefined]	1.05119
RP11-332O19.3	10:42889244-135513631	10q24.33	[undefined]	1.05119
CHUK	10:42889244-135513631	10q24.31	conserved helix-loop-helix ubiquitous kinase [Source:HGNC Symbol;Acc:1974]	1.05119
ARHGAP19	10:42889244-135513631	10q24.1	Rho GTPase activating protein 19 [Source:HGNC Symbol;Acc:23724]	1.05119
RP11-314P12.3	10:42889244-135513631	10q11.22	[undefined]	1.05119
RP11-624L12.1	10:42889244-135513631	10q23.33	[undefined]	1.05119
RP11-47G11.3	10:42889244-135513631	10q26.13	[undefined]	1.05119
GAPDHP28	10:42889244-135513631	10q23.32	glyceraldehyde 3 phosphate dehydrogenase pseudogene 28 [Source:HGNC Symbol;Acc:37780]	1.05119
RP11-360G10.3	10:42889244-135513631	10q23.32	[undefined]	1.05119
RP13-39P12.2	10:42889244-135513631	10q22.3	[undefined]	1.05119
RP11-428P16.2	10:42889244-135513631	10q22.3	[undefined]	1.05119
RP11-383B24.3	10:42889244-135513631	10q22.2	[undefined]	1.05119
XX-2136C111.2	10:42889244-135513631	10q26.3	[undefined]	1.05119
DNAJC9	10:42889244-135513631	10q22.2	DnaJ (Hsp40) homolog, subfamily C, member 9 [Source:HGNC Symbol;Acc:19123]	1.05119
RP11-498J9.1	10:42889244-135513631	10q26.11	[undefined]	1.05119
RP11-323N10.1	10:42889244-135513631	10q21.3	[undefined]	1.05119
RP11-170M17.2	10:42889244-135513631	10q21.3	[undefined]	1.05119
RP11-380G5.3	10:42889244-135513631	10q23.31	[undefined]	1.05119
RP11-346D6.4	10:42889244-135513631	10q21.1	[undefined]	1.05119
RP11-539E19.1	10:42889244-135513631	10q21.1	[undefined]	1.05119
RP11-564C4.8	10:42889244-135513631	10q11.23	[undefined]	1.05119
RP11-508M1.9	10:42889244-135513631	10q11.22	[undefined]	1.05119
RP11-344N10.3	10:42889244-135513631	10q22.1	[undefined]	1.05119
RP11-506B4.4	10:42889244-135513631	10q22.2	[undefined]	1.05119
RP11-549L6.2	10:42889244-135513631	10q25.2	[undefined]	1.05119
RP11-256K7.1	10:42889244-135513631	10q11.21	[undefined]	1.05119
RP11-108K14.4	10:42889244-135513631	10q26.3	Putative scavenger receptor cysteine-rich domain-containing protein LOC619207 Precursor [Source:UniProtKB/Swiss-Prot;Acc:Q4G0T1]	1.05119
NPS	10:42889244-135513631	10q26.2	neuropeptide S [Source:HGNC Symbol;Acc:33940]	1.05119
AL158835.1	10:42889244-135513631	10q26.13	Putative uncharacterized protein ENSP00000381127 Fragment [Source:UniProtKB/TrEMBL;Acc:A8MUE3]	1.05119
ALDOAP2	10:42889244-135513631	10q26.13	aldolase A, fructose-bisphosphate pseudogene 2 [Source:HGNC Symbol;Acc:416]	1.05119
RP11-151A10.2	10:42889244-135513631	10q26.13	[undefined]	1.05119
AL603764.1	10:42889244-135513631	10q26.13	Putative uncharacterized protein ENSP00000381147 Fragment [Source:UniProtKB/TrEMBL;Acc:A8MWX8]	1.05119
RP11-7D5.2	10:42889244-135513631	10q24.1	[undefined]	1.05119
NCRNA00081	10:42889244-135513631	10q25.2	non-protein coding RNA 81 [Source:HGNC Symbol;Acc:28093]	1.05119
AS3MT	10:42889244-135513631	10q24.32	arsenic (+3 oxidation state) methyltransferase [Source:HGNC Symbol;Acc:17452]	1.05119
RP11-360G10.2	10:42889244-135513631	10q23.32	[undefined]	1.05119
FAM22D	10:42889244-135513631	10q23.2	family with sequence similarity 22, member D [Source:HGNC Symbol;Acc:23447]	1.05119
RP11-479O17.2	10:42889244-135513631	10q22.3	[undefined]	1.05119
RP11-312P12.2	10:42889244-135513631	10q22.2	[undefined]	1.05119
RP11-506B4.3	10:42889244-135513631	10q22.2	[undefined]	1.05119
KIAA0913	10:42889244-135513631	10q22.2	KIAA0913 [Source:HGNC Symbol;Acc:23528]	1.05119
C10orf105	10:42889244-135513631	10q22.1	Uncharacterized protein C10orf105 [Source:UniProtKB/Swiss-Prot;Acc:Q8TEF2]	1.05119
RP11-182L21.4	10:42889244-135513631	10q22.3	[undefined]	1.05119
RP11-464O2.5	10:42889244-135513631	10q26.13	[undefined]	1.05119
RP11-314J18.5	10:42889244-135513631	10q22.1	[undefined]	1.05119
TMEM14D	10:42889244-135513631	10q21.3	transmembrane protein 14D [Source:HGNC Symbol;Acc:15660]	1.05119
RP11-481A12.5	10:42889244-135513631	10q11.23	[undefined]	1.05119
FAM25D	10:42889244-135513631	10q11.23	family with sequence similarity 25, member D [Source:HGNC Symbol;Acc:23588]	1.05119
RP11-446H13.2	10:42889244-135513631	10q25.1	[undefined]	1.05119
AL591684.2	10:42889244-135513631	10q11.22	Putative uncharacterized protein ENSP00000363376 [Source:UniProtKB/TrEMBL;Acc:A6NLY1]	1.05119
CTD-2173J9.3	10:42889244-135513631	10q11.22	[undefined]	1.05119
AL603965.1	10:42889244-135513631	10q11.22	Putative uncharacterized protein ENSP00000334090 [Source:UniProtKB/TrEMBL;Acc:A6NF06]	1.05119
RP11-292F22.3	10:42889244-135513631	10q11.22	[undefined]	1.05119
RP11-144G6.9	10:42889244-135513631	10q11.22	[undefined]	1.05119
RP11-522H2.3	10:42889244-135513631	10q22.1	[undefined]	1.05119
RP11-342C24.6	10:42889244-135513631	10q11.22	[undefined]	1.05119
RP11-106M7.2	10:42889244-135513631	10q25.3	[undefined]	1.05119
AL731568.1	10:42889244-135513631	10q22.2	[undefined]	1.05119
AL157788.1	10:42889244-135513631	10q26.11	[undefined]	1.05119
AL360176.1	10:42889244-135513631	10q26.2	[undefined]	1.05119
AL359832.1	10:42889244-135513631	10q22.1	[undefined]	1.05119
AC068062.1	10:42889244-135513631	10q21.1	[undefined]	1.05119
MIR936	10:42889244-135513631	10q25.1	microRNA 936 [Source:HGNC Symbol;Acc:33679]	1.05119
EIF5AP1	10:42889244-135513631	10q22.3	[undefined]	1.05119
ANXA2P3	10:42889244-135513631	10q21.3	annexin A2 pseudogene 3 [Source:HGNC Symbol;Acc:540]	1.05119
RP11-589B3.5	10:42889244-135513631	10q22.3	[undefined]	1.05119
RPL12P8	10:42889244-135513631	10q21.3	ribosomal protein L12 pseudogene 8 [Source:HGNC Symbol;Acc:23735]	1.05119
AL512662.2	10:42889244-135513631	10q22.3	Putative uncharacterized protein ENSP00000385074 [Source:UniProtKB/TrEMBL;Acc:B5MCL0]	1.05119
RP11-236B18.1	10:42889244-135513631	10q23.31	[undefined]	1.05119
RP11-572P18.1	10:42889244-135513631	10q26.12	[undefined]	1.05119
AL158040.2	10:42889244-135513631	10q23.32	[undefined]	1.05119
MIR1296	10:42889244-135513631	10q21.3	microRNA 1296 [Source:HGNC Symbol;Acc:35249]	1.05119
AL671972.1	10:42889244-135513631	10q21.2	[undefined]	1.05119
AL845259.1	10:42889244-135513631	10q26.3	[undefined]	1.05119
AL356741.1	10:42889244-135513631	10q21.3	[undefined]	1.05119
AC016394.1	10:42889244-135513631	10q22.1	[undefined]	1.05119
SNORD98	10:42889244-135513631	10q21.3	small nucleolar RNA, C/D box 98 [Source:HGNC Symbol;Acc:32761]	1.05119
MIR1254	10:42889244-135513631	10q21.3	microRNA 1254 [Source:HGNC Symbol;Acc:35319]	1.05119
MIR548E	10:42889244-135513631	10q25.2	microRNA 548e [Source:HGNC Symbol;Acc:35275]	1.05119
AC012047.1	10:42889244-135513631	10q22.3	[undefined]	1.05119
AC067742.1	10:42889244-135513631	10q21.2	[undefined]	1.05119
AC009987.1	10:42889244-135513631	10q26.13	[undefined]	1.05119
AL356214.2	10:42889244-135513631	10q23.33	[undefined]	1.05119

Continued on next page...

gene name	chr	.DNABand	.GeneDesc	rank
AL355512.1	10:42889244-135513631	10q25.2	[undefined]	1.05119
AL096864.1	10:42889244-135513631	10q23.1	[undefined]	1.05119
AL450326.2	10:42889244-135513631	10q11.21	[undefined]	1.05119
AL353664.1	10:42889244-135513631	10q26.12	[undefined]	1.05119
MIR1287	10:42889244-135513631	10q24.2	microRNA 1287 [Source:HGNC Symbol;Acc:35280]	1.05119
AC068058.1	10:42889244-135513631	10q26.13	[undefined]	1.05119
AL162502.1	10:42889244-135513631	10q24.1	[undefined]	1.05119
AC068707.1	10:42889244-135513631	10q11.21	[undefined]	1.05119
MIR548F1	10:42889244-135513631	10q21.1	microRNA 548f-1 [Source:HGNC Symbol;Acc:35305]	1.05119
AL583860.1	10:42889244-135513631	10q26.2	[undefined]	1.05119
MIR1307	10:42889244-135513631	10q24.33	microRNA 1307 [Source:HGNC Symbol;Acc:35372]	1.05119
AC016821.1	10:42889244-135513631	10q22.1	[undefined]	1.05119
RP11-137L10.6	10:42889244-135513631	10q22.2	[undefined]	1.05119
C10orf55	10:42889244-135513631	10q22.2	Uncharacterized protein C10orf55 [Source:UniProtKB/Swiss-Prot;Acc:Q5SWW7]	1.05119
AL627424.1	10:42889244-135513631	10q24.32	[undefined]	1.05119
AL138762.1	10:42889244-135513631	10q24.31	[undefined]	1.05119
AL672187.2	10:42889244-135513631	10q11.23	[undefined]	1.05119
AL442003.1	10:42889244-135513631	10q11.23	[undefined]	1.05119
AL138767.1	10:42889244-135513631	10q23.2	[undefined]	1.05119
AL359836.1	10:42889244-135513631	10q26.11	[undefined]	1.05119
AL133387.3	10:42889244-135513631	10q24.32	[undefined]	1.05119
AL590622.1	10:42889244-135513631	10q23.31	[undefined]	1.05119
AC024258.1	10:42889244-135513631	10q21.3	[undefined]	1.05119
AL731733.1	10:42889244-135513631	10q11.22	[undefined]	1.05119
AL133387.1	10:42889244-135513631	10q24.32	[undefined]	1.05119
AL391827.1	10:42889244-135513631	10q24.32	[undefined]	1.05119
AL713969.1	10:42889244-135513631	10q25.1	[undefined]	1.05119
AL139340.1	10:42889244-135513631	10q23.31	[undefined]	1.05119
AL121929.2	10:42889244-135513631	10q24.33	[undefined]	1.05119
AL355598.2	10:42889244-135513631	10q26.11	[undefined]	1.05119
AL360176.2	10:42889244-135513631	10q26.2	[undefined]	1.05119
AC073588.1	10:42889244-135513631	10q25.3	[undefined]	1.05119
AC022015.1	10:42889244-135513631	10q26.2	[undefined]	1.05119
AC009989.1	10:42889244-135513631	10q26.13	[undefined]	1.05119
AL355500.1	10:42889244-135513631	10q26.3	[undefined]	1.05119
AL731532.1	10:42889244-135513631	10q23.1	[undefined]	1.05119
AL138765.2	10:42889244-135513631	10q24.1	[undefined]	1.05119
AC027678.1	10:42889244-135513631	10q21.2	[undefined]	1.05119
AL450342.3	10:42889244-135513631	10q11.23	[undefined]	1.05119
AL672187.1	10:42889244-135513631	10q11.23	[undefined]	1.05119
AC074323.1	10:42889244-135513631	10q22.3	[undefined]	1.05119
RNU4-5P	10:42889244-135513631	10q25.1	RNA, U4 small nuclear 5 (pseudogene) [Source:HGNC Symbol;Acc:10198]	1.05119
AL359260.1	10:42889244-135513631	10q25.2	[undefined]	1.05119
RP11-655H13.2	10:42889244-135513631	10q25.1	[undefined]	1.05119
RP11-399K21.5	10:42889244-135513631	10q22.2	[undefined]	1.05119
RP11-62L18.3	10:42889244-135513631	10q26.13	[undefined]	1.05119
RP11-781P14.3	10:42889244-135513631	10q26.12	[undefined]	1.05119
RP11-285G1.9	10:42889244-135513631	10q11.21	[undefined]	1.05119
RP11-314P12.2	10:42889244-135513631	10q11.22	[undefined]	1.05119
RP11-322M19.1	10:42889244-135513631	10q23.2	[undefined]	1.05119
RP11-96B5.3	10:42889244-135513631	10q11.23	[undefined]	1.05119
RP11-223P11.3	10:42889244-135513631	10q26.2	[undefined]	1.05119
RPS15AP5	10:42889244-135513631	10q26.13	ribosomal protein S15a pseudogene 5 [Source:HGNC Symbol;Acc:23743]	1.05119
RP11-344L13.1	10:42889244-135513631	10q23.1	[undefined]	1.05119
RP11-272J7.1	10:42889244-135513631	10q11.21	[undefined]	1.05119
RP11-57C13.4	10:42889244-135513631	10q23.2	[undefined]	1.05119
RP11-598C10.2	10:42889244-135513631	10q21.1	[undefined]	1.05119
NCRNA00223	10:42889244-135513631	10q22.1	non-protein coding RNA 223 [Source:HGNC Symbol;Acc:31433]	1.05119
CFLP1	10:42889244-135513631	10q23.31	cofilin pseudogene 1 [Source:HGNC Symbol;Acc:28560]	1.05119
RP11-462G8.2	10:42889244-135513631	10q26.3	[undefined]	1.05119
RP11-448K10.1	10:42889244-135513631	10q21.1	[undefined]	1.05119
RPS27P1	10:42889244-135513631	10q23.32	ribosomal protein S27 pseudogene 1 [Source:HGNC Symbol;Acc:23740]	1.05119
AL450342.1	10:42889244-135513631	10q11.23	[undefined]	1.05119
C10orf44	10:42889244-135513631	10q11.21	[undefined]	1.05119
XX-2136C48.3	10:42889244-135513631	10q26.3	[undefined]	1.05119
RP11-168P8.4	10:42889244-135513631	10q11.21	[undefined]	1.05119
RP11-475D12.1	10:42889244-135513631	10q23.1	[undefined]	1.05119
AL672187.3	10:42889244-135513631	10q11.23	Uncharacterized protein PRO1102 [Source:UniProtKB/Swiss-Prot;Acc:Q9H374]	1.05119
RP11-383C5.4	10:42889244-135513631	10q26.13	[undefined]	1.05119
CTSLL2	10:42889244-135513631	10q11.22	cathepsin L-like 2 [Source:HGNC Symbol;Acc:2540]	1.05119
RP11-445N18.2	10:42889244-135513631	10q11.21	[undefined]	1.05119
RP11-272J7.3	10:42889244-135513631	10q11.21	[undefined]	1.05119
RP11-442O18.2	10:42889244-135513631	10q26.3	[undefined]	1.05119
RP11-574K11.5	10:42889244-135513631	10q22.2	[undefined]	1.05119
RP11-262I2.2	10:42889244-135513631	10q22.1	[undefined]	1.05119
RP11-78A18.2	10:42889244-135513631	10q26.13	[undefined]	1.05119
RP11-168L22.2	10:42889244-135513631	10q11.21	[undefined]	1.05119
RP11-149I23.5	10:42889244-135513631	10q23.31	[undefined]	1.05119
RP11-144G16.1	10:42889244-135513631	10q21.3	[undefined]	1.05119
RP11-302K17.3	10:42889244-135513631	10q24.32	[undefined]	1.05119
OR6D1P	10:42889244-135513631	10q11.21	olfactory receptor, family 6, subfamily D, member 1 pseudogene [Source:HGNC Symbol;Acc:14849]	1.05119
RP11-351O1.3	10:42889244-135513631	10q21.3	[undefined]	1.05119
RP11-182L21.2	10:42889244-135513631	10q22.3	[undefined]	1.05119
RP11-179H18.8	10:42889244-135513631	10q26.11	[undefined]	1.05119
RP11-655H13.1	10:42889244-135513631	10q25.1	[undefined]	1.05119
RP11-328K22.1	10:42889244-135513631	10q22.3	[undefined]	1.05119
RP11-475D12.2	10:42889244-135513631	10q23.1	[undefined]	1.05119
RP11-202P11.1	10:42889244-135513631	10q22.3	[undefined]	1.05119
RP11-100A13.1	10:42889244-135513631	10q21.1	[undefined]	1.05119
RP11-179K3.2	10:42889244-135513631	10q21.3	[undefined]	1.05119
C10orf37	10:42889244-135513631	10q22.1	[undefined]	1.05119
RP11-380G5.2	10:42889244-135513631	10q23.31	[undefined]	1.05119
RP11-94M14.2	10:42889244-135513631	10q23.31	[undefined]	1.05119
RP13-137A17.5	10:42889244-135513631	10q26.3	[undefined]	1.05119
RP11-369J21.2	10:42889244-135513631	10q22.3	[undefined]	1.05119
RP11-18M11.2	10:42889244-135513631	10q11.21	[undefined]	1.05119
RP11-190J1.3	10:42889244-135513631	10q24.32	[undefined]	1.05119
RP11-236B18.4	10:42889244-135513631	10q23.31	[undefined]	1.05119
RP11-119F19.4	10:42889244-135513631	10q22.3	[undefined]	1.05119
RP11-451M19.2	10:42889244-135513631	10q25.1	[undefined]	1.05119
RP11-322M19.2	10:42889244-135513631	10q23.2	[undefined]	1.05119
RP11-108L7.7	10:42889244-135513631	10q24.31	[undefined]	1.05119
RP11-144G6.4	10:42889244-135513631	10q11.22	[undefined]	1.05119
RP11-441O15.3	10:42889244-135513631	10q24.2	[undefined]	1.05119
RP11-443A13.4	10:42889244-135513631	10q22.3	[undefined]	1.05119
RP11-479O17.7	10:42889244-135513631	10q22.3	[undefined]	1.05119
RP11-477L16.2	10:42889244-135513631	10q23.33	[undefined]	1.05119
RP11-56A21.4	10:42889244-135513631	10q11.23	[undefined]	1.05119
RP11-338O1.2	10:42889244-135513631	10q26.13	[undefined]	1.05119
RP11-36N22.3	10:42889244-135513631	10q26.11	[undefined]	1.05119
RP11-107I14.2	10:42889244-135513631	10q24.32	[undefined]	1.05119
RP11-57H14.3	10:42889244-135513631	10q25.2	[undefined]	1.05119
RP11-344A5.1	10:42889244-135513631	10q21.3	[undefined]	1.05119
RP11-539I5.1	10:42889244-135513631	10q25.3	[undefined]	1.05119
RP11-512N4.2	10:42889244-135513631	10q11.23	[undefined]	1.05119
RP11-400G3.3	10:42889244-135513631	10q23.33	[undefined]	1.05119
RP11-122F14.3	10:42889244-135513631	10q23.1	[undefined]	1.05119
RP11-168C9.1	10:42889244-135513631	10q26.3	[undefined]	1.05119
RP11-525A16.4	10:42889244-135513631	10q25.2	[undefined]	1.05119
RP11-542E6.3	10:42889244-135513631	10q11.22	[undefined]	1.05119
RP11-773D16.1	10:42889244-135513631	10q22.3	[undefined]	1.05119
RP11-180I22.2	10:42889244-135513631	10q22.3	[undefined]	1.05119
RP11-236B18.2	10:42889244-135513631	10q23.31	[undefined]	1.05119

Continued on next page...

gene name	chr	.DNABand	.GeneDesc	rank
RP11-236B18.3	10:42889244-135513631	10q23.31	[undefined]	1.05119
RP11-310E22.2	10:42889244-135513631	10q23.33	[undefined]	1.05119
RP11-619F23.2	10:42889244-135513631	10q22.3	[undefined]	1.05119
C10orf100	10:42889244-135513631	10q23.1	[undefined]	1.05119
RP11-417O11.5	10:42889244-135513631	10q22.2	[undefined]	1.05119
RP11-127O4.3	10:42889244-135513631	10q25.1	[undefined]	1.05119
RP11-592B15.4	10:42889244-135513631	10q11.23	[undefined]	1.05119
RP11-589B3.3	10:42889244-135513631	10q22.3	[undefined]	1.05119
ERCC6	10:42889244-135513631	10q11.23	excision repair cross-complementing rodent repair deficiency, complementation group 6 [Source:HGNC Symbol;Acc:3438]	1.05119
RP11-80H5.6	10:42889244-135513631	10q23.31	[undefined]	1.05119
RP11-452K12.4	10:42889244-135513631	10q24.1	[undefined]	1.05119
FRG2B	10:42889244-135513631	10q26.3	FSHD region gene 2 family, member B [Source:HGNC Symbol;Acc:33518]	1.05119
RP11-57C13.6	10:42889244-135513631	10q23.2	[undefined]	1.05119
RP11-57G10.1	10:42889244-135513631	10q21.3	[undefined]	1.05119
RP11-122F14.4	10:42889244-135513631	10q23.1	[undefined]	1.05119
RP11-501J20.5	10:42889244-135513631	10q25.3	[undefined]	1.05119
RP11-190J1.10	10:42889244-135513631	10q24.32	[undefined]	1.05119
RP11-399K21.4	10:42889244-135513631	10q22.2	[undefined]	1.05119
RP11-463P17.1	10:42889244-135513631	10q11.22	[undefined]	1.05119
RP11-399K21.6	10:42889244-135513631	10q22.2	[undefined]	1.05119
RP11-444I9.4	10:42889244-135513631	10q21.3	[undefined]	1.05119
RP11-478K7.2	10:42889244-135513631	10q23.31	[undefined]	1.05119
RP11-167P22.3	10:42889244-135513631	10q22.1	[undefined]	1.05119
RP11-564C4.4	10:42889244-135513631	10q11.23	[undefined]	1.05119
RP11-50E11.3	10:42889244-135513631	10q11.23	[undefined]	1.05119
ZNF32OS1	10:42889244-135513631	10q11.21	zinc finger protein 32 opposite strand 1 [Source:HGNC Symbol;Acc:23577]	1.05119
MRPL35P3	10:42889244-135513631	10q22.2	mitochondrial ribosomal protein L35 pseudogene 3 [Source:HGNC Symbol;Acc:23731]	1.05119
RP11-71J2.1	10:42889244-135513631	10q21.1	[undefined]	1.05119
XX-2136C48.1	10:42889244-135513631	10q26.3	[undefined]	1.05119
RP11-474D14.2	10:42889244-135513631	10q21.3	[undefined]	1.05119
CTD-2173J9.4	10:42889244-135513631	10q11.22	[undefined]	1.05119
RP11-186O14.5	10:42889244-135513631	10q23.31	[undefined]	1.05119
RP11-119F19.2	10:42889244-135513631	10q22.3	[undefined]	1.05119
RP11-483B5.3	10:42889244-135513631	10q25.1	[undefined]	1.05119
RP11-507K13.4	10:42889244-135513631	10q11.23	[undefined]	1.05119
RP11-348J12.2	10:42889244-135513631	10q23.33	[undefined]	1.05119
RP11-174J11.1	10:42889244-135513631	10q21.3	[undefined]	1.05119
RP11-106M7.4	10:42889244-135513631	10q25.3	[undefined]	1.05119
RP11-416N2.3	10:42889244-135513631	10q24.33	[undefined]	1.05119
RP11-93L14.1	10:42889244-135513631	10q21.3	[undefined]	1.05119
RP11-417O11.6	10:42889244-135513631	10q22.2	[undefined]	1.05119
RP11-102H24.1	10:42889244-135513631	10q23.1	[undefined]	1.05119
RP11-524O24.2	10:42889244-135513631	10q21.3	[undefined]	1.05119
RP11-561G1.2	10:42889244-135513631	10q21.1	[undefined]	1.05119
RP11-512N4.3	10:42889244-135513631	10q11.23	[undefined]	1.05119
RP11-523O18.1	10:42889244-135513631	10q11.23	[undefined]	1.05119
RP11-411P18.2	10:42889244-135513631	10q25.3	[undefined]	1.05119
SLC9A3P3	10:42889244-135513631	10q11.23	solute carrier family 9 (sodium/hydrogen exchanger), member 3 pseudogene 3 [Source:HGNC Symbol;Acc:33492]	1.05119
RP11-137H2.4	10:42889244-135513631	10q23.1	[undefined]	1.05119
RP11-292F22.5	10:42889244-135513631	10q11.22	[undefined]	1.05119
RP11-589B3.6	10:42889244-135513631	10q22.3	[undefined]	1.05119
RP11-429G19.2	10:42889244-135513631	10q24.1	[undefined]	1.05119
RP11-122K13.7	10:42889244-135513631	10q26.3	[undefined]	1.05119
RP11-570G20.1	10:42889244-135513631	10q22.1	[undefined]	1.05119
RP11-252O7.2	10:42889244-135513631	10q25.1	[undefined]	1.05119
RP11-31L23.3	10:42889244-135513631	10q24.31	[undefined]	1.05119
RP11-272J7.5	10:42889244-135513631	10q11.21	[undefined]	1.05119
RP11-343J3.3	10:42889244-135513631	10q22.1	[undefined]	1.05119
RP11-168P8.2	10:42889244-135513631	10q11.21	[undefined]	1.05119
RP11-500G22.2	10:42889244-135513631	10q26.13	[undefined]	1.05119
XX-2136C48.7	10:42889244-135513631	10q26.3	[undefined]	1.05119
RP11-561G1.3	10:42889244-135513631	10q21.1	[undefined]	1.05119
RP11-298J20.3	10:42889244-135513631	10q26.13	[undefined]	1.05119
RP11-432J24.5	10:42889244-135513631	10q26.3	[undefined]	1.05119
RP11-443A13.5	10:42889244-135513631	10q22.3	[undefined]	1.05119
RP11-285G1.8	10:42889244-135513631	10q11.21	[undefined]	1.05119
RP11-575L16.2	10:42889244-135513631	10q11.22	[undefined]	1.05119
RP11-168P8.3	10:42889244-135513631	10q11.21	[undefined]	1.05119
RP11-34E5.4	10:42889244-135513631	10q24.1	[undefined]	1.05119
RP11-4C20.4	10:42889244-135513631	10q26.2	[undefined]	1.05119
RP11-295O23.4	10:42889244-135513631	10q25.3	[undefined]	1.05119
RP11-319F12.2	10:42889244-135513631	10q21.1	[undefined]	1.05119
RP11-107I14.1	10:42889244-135513631	10q24.31 10q24.32	[undefined]	1.05119
C10orf101	10:42889244-135513631	10q22.3	[undefined]	1.05119
RP11-159H3.2	10:42889244-135513631	10q26.12	[undefined]	1.05119
RP11-323P17.1	10:42889244-135513631	10q26.12	[undefined]	1.05119
MYL6P3	10:42889244-135513631	10q21.3	myosin, light chain 6 pseudogene 3 [Source:HGNC Symbol;Acc:37812]	1.05119
RP11-77G23.5	10:42889244-135513631	10q22.2	[undefined]	1.05119
GLUDP7	10:42889244-135513631	10q11.22	glutamate dehydrogenase 1 pseudogene 7 [Source:HGNC Symbol;Acc:23636]	1.05119
RP11-315E23.1	10:42889244-135513631	10q23.1	[undefined]	1.05119
RP11-322C8.2	10:42889244-135513631	10q21.2	[undefined]	1.05119
AC063965.1	10:42889244-135513631	10q23.31	Killin [Source:UniProtKB/Swiss-Prot;Acc:B2CW77]	1.05119
RP11-399K21.2	10:42889244-135513631	10q22.2	[undefined]	1.05119
RP11-95I16.2	10:42889244-135513631	10q26.12	[undefined]	1.05119
RP11-144G6.10	10:42889244-135513631	10q11.22	[undefined]	1.05119
RP11-443A13.3	10:42889244-135513631	10q22.3	[undefined]	1.05119
PARG	10:42889244-135513631	10q11.23	poly (ADP-ribose) glycohydrolase [Source:HGNC Symbol;Acc:8605]	1.05119
RP11-483F11.6	10:42889244-135513631	10q24.2	[undefined]	1.05119
RP11-459F3.3	10:42889244-135513631	10q24.2	[undefined]	1.05119
RP11-109A6.3	10:42889244-135513631	10q26.3	[undefined]	1.05119
RP11-152N13.8	10:42889244-135513631	10q22.2	[undefined]	1.05119
RP11-18M11.1	10:42889244-135513631	10q11.21	[undefined]	1.05119
RP11-179H18.5	10:42889244-135513631	10q26.11	[undefined]	1.05119
RP11-316M21.6	10:42889244-135513631	10q24.31	[undefined]	1.05119
RP11-369J21.4	10:42889244-135513631	10q22.3	[undefined]	1.05119
RP11-506B4.5	10:42889244-135513631	10q22.2	[undefined]	1.05119
RP11-152N13.5	10:42889244-135513631	10q22.2	[undefined]	1.05119
RP11-139K1.2	10:42889244-135513631	10q25.2	[undefined]	1.05119
RP11-119F7.3	10:42889244-135513631	10q21.3	[undefined]	1.05119
RP11-162A23.5	10:42889244-135513631	10q26.13	[undefined]	1.05119
RP11-445N18.5	10:42889244-135513631	10q11.21	[undefined]	1.05119
NCRNA00093	10:42889244-135513631	10q24.2	non-protein coding RNA 93 [Source:HGNC Symbol;Acc:20431]	1.05119
RP11-508M1.5	10:42889244-135513631	10q11.22	[undefined]	1.05119
RP11-248J23.5	10:42889244-135513631	10q24.1	[undefined]	1.05119
RP11-381K7.1	10:42889244-135513631	10q25.2	[undefined]	1.05119
RP11-59J5.1	10:42889244-135513631	10q21.2	[undefined]	1.05119
RP11-77P6.2	10:42889244-135513631	10q23.2	[undefined]	1.05119
RP11-380G5.4	10:42889244-135513631	10q23.31	[undefined]	1.05119
RP11-710A11.2	10:42889244-135513631	10q22.1	[undefined]	1.05119
RP11-346D6.5	10:42889244-135513631	10q21.1	[undefined]	1.05119
RP11-397J18.1	10:42889244-135513631	10q23.33	[undefined]	1.05119
RP11-383C5.3	10:42889244-135513631	10q26.13	[undefined]	1.05119
RP11-598C10.1	10:42889244-135513631	10q21.1	[undefined]	1.05119
RP11-399L7.2	10:42889244-135513631	10q23.2	[undefined]	1.05119
RP11-222A11.1	10:42889244-135513631	10q21.3	[undefined]	1.05119
RP11-119F7.4	10:42889244-135513631	10q21.3	[undefined]	1.05119
RP11-302K17.7	10:42889244-135513631	10q24.32	[undefined]	1.05119
RP11-417C21.2	10:42889244-135513631	10q22.3	[undefined]	1.05119
RP11-108K1.2	10:42889244-135513631	10q25.3	[undefined]	1.05119
RP11-498B4.7	10:42889244-135513631	10q25.3	[undefined]	1.05119
RP11-292F22.2	10:42889244-135513631	10q11.22	[undefined]	1.05119
ARMS2	10:42889244-135513631	10q26.13	age-related maculopathy susceptibility 2 [Source:HGNC Symbol;Acc:32685]	1.05119

Continued on next page...

gene name	chr	.DNABand	.GeneDesc	rank
RP11-127L20.3	10:42889244-135513631	10q25.1	[undefined]	1.05119
RP11-254K3.1	10:42889244-135513631	10q26.12	[undefined]	1.05119
RP11-367B6.2	10:42889244-135513631	10q22.2	[undefined]	1.05119
BX248123.1	10:42889244-135513631	10q22.3	Putative uncharacterized protein ENSP00000400080 Fragment [Source:UniProtKB/TrEMBL;Acc:C9J1A1]	1.05119
RP11-109G10.2	10:42889244-135513631	10q11.23	[undefined]	1.05119
RP11-563N6.6	10:42889244-135513631	10q11.23	[undefined]	1.05119
RP11-549L6.3	10:42889244-135513631	10q25.2	[undefined]	1.05119
CTSLL3	10:42889244-135513631	10q11.22	cathepsin L-like 3 [Source:HGNC Symbol;Acc:2541]	1.05119
RP11-402L1.11	10:42889244-135513631	10q11.21	[undefined]	1.05119
RP11-208C17.3	10:42889244-135513631	10q23.33	[undefined]	1.05119
RP11-445N18.3	10:42889244-135513631	10q11.21	[undefined]	1.05119
RP11-106M7.1	10:42889244-135513631	10q25.3	[undefined]	1.05119
RP11-567J24.4	10:42889244-135513631	10q26.11	[undefined]	1.05119
RP11-179B15.5	10:42889244-135513631	10q21.1	[undefined]	1.05119
RP11-391J2.3	10:42889244-135513631	10q23.33	[undefined]	1.05119
RP11-170M17.1	10:42889244-135513631	10q21.3	[undefined]	1.05119
FAM22E	10:42889244-135513631	10q22.3	family with sequence similarity 22, member E [Source:HGNC Symbol;Acc:23448]	1.05119
RP11-556E13.1	10:42889244-135513631	10q21.1	[undefined]	1.05119
RP11-170J3.2	10:42889244-135513631	10q24.32	[undefined]	1.05119
RP11-109A6.4	10:42889244-135513631	10q26.3	[undefined]	1.05119
RP11-354E23.2	10:42889244-135513631	10q22.1	[undefined]	1.05119
RP11-31E13.2	10:42889244-135513631	10q22.3	[undefined]	1.05119
RP11-251A15.4	10:42889244-135513631	10q23.32	[undefined]	1.05119
RP11-671E7.1	10:42889244-135513631	10q11.22	[undefined]	1.05119
RP11-178G16.2	10:42889244-135513631	10q22.2	[undefined]	1.05119
CTSLL5	10:42889244-135513631	10q11.22	cathepsin L-like 5 [Source:HGNC Symbol;Acc:23580]	1.05119
RP13-39P12.3	10:42889244-135513631	10q22.3	[undefined]	1.05119
RP11-534L6.3	10:42889244-135513631	10q11.23	[undefined]	1.05119
RP11-251A15.1	10:42889244-135513631	10q23.32	[undefined]	1.05119
RP11-129J12.1	10:42889244-135513631	10q24.2	[undefined]	1.05119
RP11-463P17.3	10:42889244-135513631	10q11.22	[undefined]	1.05119
RP11-478K7.1	10:42889244-135513631	10q23.31	[undefined]	1.05119
RP11-314J18.6	10:42889244-135513631	10q22.1	[undefined]	1.05119
RP11-432J24.6	10:42889244-135513631	10q26.3	[undefined]	1.05119
RP11-367H5.5	10:42889244-135513631	10q22.1	[undefined]	1.05119
RP11-20J15.3	10:42889244-135513631	10q11.21	[undefined]	1.05119
RP11-76P2.3	10:42889244-135513631	10q23.33	[undefined]	1.05119
RP11-227H15.7	10:42889244-135513631	10q22.1	[undefined]	1.05119
RP11-38L15.2	10:42889244-135513631	10q11.22	[undefined]	1.05119
RP11-332O19.4	10:42889244-135513631	10q24.33	[undefined]	1.05119
RP11-227H15.4	10:42889244-135513631	10q22.1	[undefined]	1.05119
RP11-498J9.2	10:42889244-135513631	10q26.11	[undefined]	1.05119
RP11-483F11.7	10:42889244-135513631	10q24.2	[undefined]	1.05119
RP11-198M6.5	10:42889244-135513631	10q26.11	[undefined]	1.05119
RP11-124L5.7	10:42889244-135513631	10q23.1	[undefined]	1.05119
RP11-44D15.3	10:42889244-135513631	10q24.1	[undefined]	1.05119
RP11-35J23.1	10:42889244-135513631	10q24.1	[undefined]	1.05119
RP13-238F13.4	10:42889244-135513631	10q26.13	[undefined]	1.05119
RP11-202D18.2	10:42889244-135513631	10q23.1	[undefined]	1.05119
RP11-481A12.6	10:42889244-135513631	10q11.23	[undefined]	1.05119
RP11-56I23.1	10:42889244-135513631	10q25.1	[undefined]	1.05119
RP11-90J7.2	10:42889244-135513631	10q22.3	[undefined]	1.05119
NKX1-2	10:42889244-135513631	10q26.13	NK1 homeobox 2 [Source:HGNC Symbol;Acc:31652]	1.05119
RP11-481G8.2	10:42889244-135513631	10q22.3	[undefined]	1.05119
RP11-292F22.4	10:42889244-135513631	10q11.22	[undefined]	1.05119
RP11-369J21.1	10:42889244-135513631	10q22.3	[undefined]	1.05119
AL590080.2	10:42889244-135513631	10q23.33	Putative uncharacterized protein ENSP00000396115 Fragment [Source:UniProtKB/TrEMBL;Acc:C9JET1]	1.05119
RP11-302K17.4	10:42889244-135513631	10q24.32	[undefined]	1.05119
RP11-124O11.2	10:42889244-135513631	10q11.21	[undefined]	1.05119
RP11-107I14.4	10:42889244-135513631	10q24.32	[undefined]	1.05119
RP11-345K20.2	10:42889244-135513631	10q22.2	[undefined]	1.05119
RP11-317F20.2	10:42889244-135513631	10q25.3	[undefined]	1.05119
RP11-449O16.2	10:42889244-135513631	10q11.23	[undefined]	1.05119
RP11-298H24.1	10:42889244-135513631	10q25.1	[undefined]	1.05119
RP11-190J1.7	10:42889244-135513631	10q24.32	[undefined]	1.05119
EMX2OS	10:42889244-135513631	10q26.11	EMX2 opposite strand (non-protein coding) [Source:HGNC Symbol;Acc:18511]	1.05119
RP11-507K13.6	10:42889244-135513631	10q11.23	[undefined]	1.05119
RP11-38L15.6	10:42889244-135513631	10q11.22	[undefined]	1.05119
RP11-96C23.10	10:42889244-135513631	10q23.2	[undefined]	1.05119
RP11-215N21.1	10:42889244-135513631	10q25.1	[undefined]	1.05119
RP11-479O17.5	10:42889244-135513631	10q22.3	[undefined]	1.05119
RP11-574K11.8	10:42889244-135513631	10q22.2	[undefined]	1.05119
CTSLL4	10:42889244-135513631	10q11.23	cathepsin L-like 4 [Source:HGNC Symbol;Acc:23645]	1.05119
RP11-481H12.1	10:42889244-135513631	10q25.3	[undefined]	1.05119
AL596137.1	10:42889244-135513631	10q11.23	[undefined]	1.05119
RP11-369J21.5	10:42889244-135513631	10q22.3	[undefined]	1.05119
RP11-462G8.3	10:42889244-135513631	10q26.3	[undefined]	1.05119
RP11-282I1.1	10:42889244-135513631	10q26.13	[undefined]	1.05119
RP11-498J9.4	10:42889244-135513631	10q26.11	[undefined]	1.05119
RP11-432B10.1	10:42889244-135513631	10q25.1	[undefined]	1.05119
CTB-1144G6.5	10:42889244-135513631	10q25.3	[undefined]	1.05119
RP11-526K21.2	10:42889244-135513631	10q11.23	[undefined]	1.05119
RP11-369J21.7	10:42889244-135513631	10q22.3	[undefined]	1.05119
RP11-508M1.8	10:42889244-135513631	10q11.22	[undefined]	1.05119
RP11-316M21.5	10:42889244-135513631	10q24.31	[undefined]	1.05119
RP11-90J7.4	10:42889244-135513631	10q22.3	[undefined]	1.05119
RP11-122K13.8	10:42889244-135513631	10q26.3	[undefined]	1.05119
RP11-466J14.3	10:42889244-135513631	10q23.33	[undefined]	1.05119
RP11-90J7.3	10:42889244-135513631	10q22.3	[undefined]	1.05119
AL022344.6	10:42889244-135513631	10q11.21	Putative uncharacterized protein ENSP00000403698 Fragment [Source:UniProtKB/TrEMBL;Acc:C9JBS0]	1.05119
XX-2136C77.1	10:42889244-135513631	10q26.3	[undefined]	1.05119
RP11-242G20.2	10:42889244-135513631	10q22.1	[undefined]	1.05119
RP11-432I13.1	10:42889244-135513631	10q11.21	[undefined]	1.05119
RPL7AP8	10:42889244-135513631	10q23.2	ribosomal protein L7a pseudogene 8 [Source:HGNC Symbol;Acc:23738]	1.05119
RP11-472G21.2	10:42889244-135513631	10q22.1	[undefined]	1.05119
RP11-481A12.2	10:42889244-135513631	10q11.23	[undefined]	1.05119
RP11-517P14.2	10:42889244-135513631	10q11.21	[undefined]	1.05119
ZNF32OS2	10:42889244-135513631	10q11.21	zinc finger protein 32 opposite strand 2 [Source:HGNC Symbol;Acc:23593]	1.05119
RP11-369F10.2	10:42889244-135513631	10q22.3	[undefined]	1.05119
RP11-167P22.4	10:42889244-135513631	10q22.1	[undefined]	1.05119
RP11-367H5.7	10:42889244-135513631	10q22.1	[undefined]	1.05119
AL022345.1	10:42889244-135513631	10q11.21	Putative uncharacterized protein ENSP00000400038 Fragment [Source:UniProtKB/TrEMBL;Acc:C9J1D3]	1.05119
RP11-104F15.7	10:42889244-135513631	10q22.1	[undefined]	1.05119
RP11-444I9.3	10:42889244-135513631	10q21.3	[undefined]	1.05119
RP11-343J3.7	10:42889244-135513631	10q22.1	[undefined]	1.05119
RP11-342C24.8	10:42889244-135513631	10q11.22	[undefined]	1.05119
AL161645.1	10:42889244-135513631	10q26.3	cDNA FLJ44653 fis, clone BRACE2046976 [Source:UniProtKB/TrEMBL;Acc:Q6ZTH4]	1.05119
RP11-309P22.1	10:42889244-135513631	10q25.2	[undefined]	1.05119
CTGLF10P	10:42889244-135513631	10q11.22	centaurin, gamma-like family, member 10 pseudogene [Source:HGNC Symbol;Acc:23659]	1.05119
AC022387.1	10:42889244-135513631	10q21.3	REEP3 protein [Source:UniProtKB/TrEMBL;Acc:Q8WV73]	1.05119
RP11-34A14.3	10:42889244-135513631	10q24.2	[undefined]	1.05119
RP11-113E21.1	10:42889244-135513631	10q23.1	[undefined]	1.05119
RP11-107I14.5	10:42889244-135513631	10q24.32	[undefined]	1.05119
AL022344.3	10:42889244-135513631	10q11.21	[undefined]	1.05119
RP11-175O19.4	10:42889244-135513631	10q24.1	[undefined]	1.05119
RP11-20E23.2	10:42889244-135513631	10q23.1	[undefined]	1.05119
RP11-514F8.2	10:42889244-135513631	10q23.1	[undefined]	1.05119
RP11-354M20.3	10:42889244-135513631	10q26.11	[undefined]	1.05119
FAM25E	10:42889244-135513631	10q11.22	family with sequence similarity 25, member E [Source:HGNC Symbol;Acc:23587]	1.05119
RP11-346D6.6	10:42889244-135513631	10q21.1	[undefined]	1.05119

Continued on next page...

gene name	chr	.DNABand	.GeneDesc	rank
RP11-40C11.2	10:42889244-135513631	10q21.1	[undefined]	1.05119
RP11-391M7.3	10:42889244-135513631	10q26.13	[undefined]	1.05119
RP11-343J3.4	10:42889244-135513631	10q22.1	[undefined]	1.05119
RP11-38L15.3	10:42889244-135513631	10q11.22	[undefined]	1.05119
RP11-34D15.2	10:42889244-135513631	10q24.31	[undefined]	1.05119
GLUDP6	10:42889244-135513631	10q11.22	glutamate dehydrogenase 1 pseudogene 6 [Source:HGNC Symbol;Acc:23610]	1.05119
AL133215.1	10:42889244-135513631	10q24.31	[undefined]	1.05119
RP11-127L20.6	10:42889244-135513631	10q25.1	[undefined]	1.05119
RP11-442O18.3	10:42889244-135513631	10q26.3	[undefined]	1.05119
RP11-564C4.6	10:42889244-135513631	10q11.23	[undefined]	1.05119
RP11-399N22.3	10:42889244-135513631	10q22.1	[undefined]	1.05119
RP11-375N9.2	10:42889244-135513631	10q24.1	[undefined]	1.05119
RP11-418N10.1	10:42889244-135513631	10q21.1	[undefined]	1.05119
RP11-167P22.2	10:42889244-135513631	10q22.1	[undefined]	1.05119
RP11-452K12.6	10:42889244-135513631	10q24.1	[undefined]	1.05119
RP11-399L7.3	10:42889244-135513631	10q23.2	[undefined]	1.05119
RP11-162K11.4	10:42889244-135513631	10q23.33	[undefined]	1.05119
RP11-50E11.2	10:42889244-135513631	10q11.23	[undefined]	1.05119
AL138921.1	10:42889244-135513631	10q24.31	[undefined]	1.05119
RP11-432J24.2	10:42889244-135513631	10q26.3	[undefined]	1.05119
RP11-227H15.5	10:42889244-135513631	10q22.1	[undefined]	1.05119
RP11-310E22.5	10:42889244-135513631	10q23.33	[undefined]	1.05119
RP11-541M12.3	10:42889244-135513631	10q11.22	[undefined]	1.05119
RP11-157J13.1	10:42889244-135513631	10q22.3	[undefined]	1.05119
RP11-67C2.2	10:42889244-135513631	10q11.21	[undefined]	1.05119
RP11-452K12.7	10:42889244-135513631	10q24.1	[undefined]	1.05119
RP11-402L1.1	10:42889244-135513631	10q11.21	[undefined]	1.05119
RP11-111N20.3	10:42889244-135513631	10q21.3	[undefined]	1.05119
RP11-33D13.2	10:42889244-135513631	10q25.3	[undefined]	1.05119
AL022344.2	10:42889244-135513631	10q11.21	[undefined]	1.05119
RP11-149I23.3	10:42889244-135513631	10q23.31	[undefined]	1.05119
RP11-123G9.1	10:42889244-135513631	10q21.1	[undefined]	1.05119
RP11-319I23.2	10:42889244-135513631	10q26.11	[undefined]	1.05119
RP11-367H5.8	10:42889244-135513631	10q22.1	[undefined]	1.05119
RP11-248C1.2	10:42889244-135513631	10q23.31	[undefined]	1.05119
RP11-316M21.8	10:42889244-135513631	10q24.2	[undefined]	1.05119
RP11-4C20.3	10:42889244-135513631	10q26.2	[undefined]	1.05119
RP11-47G11.2	10:42889244-135513631	10q26.13	[undefined]	1.05119
RP11-46O21.2	10:42889244-135513631	10q22.2	[undefined]	1.05119
RP11-508M1.7	10:42889244-135513631	10q11.22	[undefined]	1.05119
RP11-487I5.4	10:42889244-135513631	10q22.2	[undefined]	1.05119
CTSLL7	10:42889244-135513631	10q11.22	cathepsin L-like 7 [Source:HGNC Symbol;Acc:23635]	1.05119
RP11-13E1.5	10:42889244-135513631	10q11.22	[undefined]	1.05119
RP11-313D6.3	10:42889244-135513631	10q25.2	[undefined]	1.05119
RP11-445N18.4	10:42889244-135513631	10q11.21	[undefined]	1.05119
GLUDP8	10:42889244-135513631	10q11.22	glutamate dehydrogenase 1 pseudogene 8 [Source:HGNC Symbol;Acc:23637]	1.05119
RP11-344N10.4	10:42889244-135513631	10q22.1	[undefined]	1.05119
RP11-388P9.2	10:42889244-135513631	10q21.2	[undefined]	1.05119
RP11-369F10.3	10:42889244-135513631	10q22.3	[undefined]	1.05119
RP11-564C4.5	10:42889244-135513631	10q11.23	[undefined]	1.05119
RP11-543N17.2	10:42889244-135513631	10q23.33	[undefined]	1.05119
RP11-402N18.1	10:42889244-135513631	10q25.1	[undefined]	1.05119
RP11-404C6.3	10:42889244-135513631	10q22.1	[undefined]	1.05119
RP11-498B4.5	10:42889244-135513631	10q25.3	[undefined]	1.05119
RP11-280G19.1	10:42889244-135513631	10q23.33	[undefined]	1.05119
RP11-179H18.7	10:42889244-135513631	10q26.11	[undefined]	1.05119
RP11-153G4.2	10:42889244-135513631	10q24.1	[undefined]	1.05119
RP13-137A17.4	10:42889244-135513631	10q26.3	[undefined]	1.05119
RP11-429H9.4	10:42889244-135513631	10q23.33	[undefined]	1.05119
RP11-324O2.3	10:42889244-135513631	10q25.2	[undefined]	1.05119
RP11-223P11.2	10:42889244-135513631	10q26.2	[undefined]	1.05119
RP11-80H5.2	10:42889244-135513631	10q23.31	[undefined]	1.05119
RP11-36D19.4	10:42889244-135513631	10q23.1	[undefined]	1.05119
RP11-589B3.1	10:42889244-135513631	10q22.3	[undefined]	1.05119
RP11-442O18.1	10:42889244-135513631	10q26.3	[undefined]	1.05119
SLC9A3P1	10:42889244-135513631	10q11.23	solute carrier family 9 (sodium/hydrogen exchanger), member 3 pseudogene 1 [Source:HGNC Symbol;Acc:11074]	1.05119
RP11-432J9.4	10:42889244-135513631	10q22.1	[undefined]	1.05119
CTAGE7	10:42889244-135513631	10q26.3	CTAGE family, member 7 [Source:HGNC Symbol;Acc:25111]	1.05119
RP11-359B3.1	10:42889244-135513631	10q26.13	[undefined]	1.05119
RP11-537A6.9	10:42889244-135513631	10q22.2	[undefined]	1.05119
RP11-259F16.3	10:42889244-135513631	10q22.1	[undefined]	1.05119
RP11-96C23.8	10:42889244-135513631	10q23.2	[undefined]	1.05119
RP11-179H18.2	10:42889244-135513631	10q26.11	[undefined]	1.05119
RP11-122F14.2	10:42889244-135513631	10q23.1	[undefined]	1.05119
RP11-404C6.5	10:42889244-135513631	10q22.1	[undefined]	1.05119
RP11-219F10.1	10:42889244-135513631	10q23.1	[undefined]	1.05119
RP11-341B24.3	10:42889244-135513631	10q23.31	[undefined]	1.05119
HMGA1L5	10:42889244-135513631	10q22.2	high mobility group AT-hook 1-like 5 [Source:HGNC Symbol;Acc:19120]	1.05119
RP11-500G10.4	10:42889244-135513631	10q26.3	[undefined]	1.05119
RP11-464O2.2	10:42889244-135513631	10q26.13	[undefined]	1.05119
RP11-25C19.3	10:42889244-135513631	10q25.2	[undefined]	1.05119
RP11-324O2.4	10:42889244-135513631	10q25.2	[undefined]	1.05119
RP11-472N6.1	10:42889244-135513631	10q23.1	[undefined]	1.05119
RP11-208C17.2	10:42889244-135513631	10q23.33	[undefined]	1.05119
RP11-168P8.7	10:42889244-135513631	10q11.21	[undefined]	1.05119
RP11-295J3.2	10:42889244-135513631	10q26.2	[undefined]	1.05119
XX-2136C111.3	10:42889244-135513631	10q26.3	[undefined]	1.05119
RP11-45P22.2	10:42889244-135513631	10q25.1	[undefined]	1.05119
AL022344.4	10:42889244-135513631	10q11.21	[undefined]	1.05119
RP11-73H14.1	10:42889244-135513631	10q25.2	[undefined]	1.05119
RP11-57H14.2	10:42889244-135513631	10q25.2	[undefined]	1.05119
RP11-153K11.3	10:42889244-135513631	10q21.3	[undefined]	1.05119
RP11-491H19.1	10:42889244-135513631	10q21.2	[undefined]	1.05119
RP11-523O18.5	10:42889244-135513631	10q11.23	[undefined]	1.05119
RP11-108K14.7	10:42889244-135513631	10q26.3	[undefined]	1.05119
RP11-483F11.5	10:42889244-135513631	10q24.2	[undefined]	1.05119
RP11-20E23.1	10:42889244-135513631	10q23.1	[undefined]	1.05119
RP11-252C24.2	10:42889244-135513631	10q21.3	[undefined]	1.05119
AC010997.1	10:42889244-135513631	10q22.2	[undefined]	1.05119
RP11-44D15.2	10:42889244-135513631	10q24.1	[undefined]	1.05119
RP11-449J3.3	10:42889244-135513631	10q21.1	[undefined]	1.05119
EIF3LP2	10:42889244-135513631	10q11.21	eukaryotic translation initiation factor 3, subunit L pseudogene 2 [Source:HGNC Symbol;Acc:24657]	1.05119
RP11-126H7.3	10:42889244-135513631	10q22.3	[undefined]	1.05119
RP11-76F14.1	10:42889244-135513631	10q21.3	[undefined]	1.05119
RP11-344L13.2	10:42889244-135513631	10q23.1	[undefined]	1.05119
RP11-295J3.3	10:42889244-135513631	10q26.2	[undefined]	1.05119
RP11-214N15.5	10:42889244-135513631	10q25.2	[undefined]	1.05119
RP11-310E22.4	10:42889244-135513631	10q23.33	[undefined]	1.05119
RP11-56M3.1	10:42889244-135513631	10q23.32	[undefined]	1.05119
RP11-153K11.6	10:42889244-135513631	10q21.3	[undefined]	1.05119
RP11-379D21.3	10:42889244-135513631	10q24.2	[undefined]	1.05119
RPL13AP6	10:42889244-135513631	10q25.2	ribosomal protein L13a pseudogene 6 [Source:HGNC Symbol;Acc:23737]	1.05119
RP11-383C5.5	10:42889244-135513631	10q26.13	[undefined]	1.05119
RP11-77G23.2	10:42889244-135513631	10q22.2	[undefined]	1.05119
RP11-257I14.1	10:42889244-135513631	10q21.1	[undefined]	1.05119
RP11-57C13.5	10:42889244-135513631	10q23.2	[undefined]	1.05119
RP11-25C19.1	10:42889244-135513631	10q25.2	[undefined]	1.05119
RP11-399O19.6	10:42889244-135513631	10q23.31	[undefined]	1.05119
RP11-36N22.1	10:42889244-135513631	10q26.11	[undefined]	1.05119
RP11-432J24.3	10:42889244-135513631	10q26.3	[undefined]	1.05119
RP11-40F6.3	10:42889244-135513631	10q22.3	[undefined]	1.05119
RP11-444I9.2	10:42889244-135513631	10q21.3	[undefined]	1.05119

Continued on next page...

gene name	chr	.DNABand	.GeneDesc	rank
RP11-40F6.1	10:42889244-135513631	10q22.3 10q23.1	[undefined]	1.05119
RP11-211N11.5	10:42889244-135513631	10q25.3	[undefined]	1.05119
ZNF37B	10:42889244-135513631	10q11.21	zinc finger protein 37B (pseudogene) [Source:HGNC Symbol;Acc:13103]	1.05119
RP11-234G16.2	10:42889244-135513631	10q26.3	[undefined]	1.05119
RP11-168O10.6	10:42889244-135513631	10q23.31	[undefined]	1.05119
RP11-501J20.2	10:42889244-135513631	10q25.3	[undefined]	1.05119
ATQL4	10:42889244-135513631	10q21.3	antiquitin-like 4 [Source:HGNC Symbol;Acc:881]	1.05119
RP11-285G1.2	10:42889244-135513631	10q11.21	[undefined]	1.05119
RP11-572P18.2	10:42889244-135513631	10q26.12	[undefined]	1.05119
RP11-288G11.3	10:42889244-135513631	10q26.3	[undefined]	1.05119
RP11-282I1.2	10:42889244-135513631	10q26.13	[undefined]	1.05119
RP11-468L18.1	10:42889244-135513631	10q25.1	[undefined]	1.05119
RP11-179H18.4	10:42889244-135513631	10q26.11	[undefined]	1.05119
RP11-733D4.1	10:42889244-135513631	10q11.21	[undefined]	1.05119
RP11-175I17.2	10:42889244-135513631	10q11.22	[undefined]	1.05119
RP11-464F9.19	10:42889244-135513631	10q22.2	[undefined]	1.05119
RP11-126H7.2	10:42889244-135513631	10q22.3	[undefined]	1.05119
AL162407.1	10:42889244-135513631	10q25.3	CDNA FLJ20147 fis, clone COL07954HCG1781466 ; [Source:UniProtKB/TrEMBL;Acc:Q9NXN1]	1.05119
RP11-9E13.2	10:42889244-135513631	10q21.3	[undefined]	1.05119
RP11-264E18.1	10:42889244-135513631	10q26.2	[undefined]	1.05119
RP11-285A18.2	10:42889244-135513631	10q26.13	[undefined]	1.05119
RP11-225H22.4	10:42889244-135513631	10q24.33	[undefined]	1.05119
RP11-391J2.1	10:42889244-135513631	10q23.33	[undefined]	1.05119
RP11-507P23.3	10:42889244-135513631	10q11.23	[undefined]	1.05119
RP11-36D19.5	10:42889244-135513631	10q23.1	[undefined]	1.05119
RP11-120C12.3	10:42889244-135513631	10q21.2	[undefined]	1.05119
RP11-9E13.4	10:42889244-135513631	10q21.3	[undefined]	1.05119
RP11-453E2.2	10:42889244-135513631	10q24.1	[undefined]	1.05119
AL022344.5	10:42889244-135513631	10q11.21	[undefined]	1.05119
RP11-510K18.1	10:42889244-135513631	10q23.1	[undefined]	1.05119
RP11-93H12.2	10:42889244-135513631	10q23.1	[undefined]	1.05119
RP11-124O11.1	10:42889244-135513631	10q11.21	[undefined]	1.05119
RP11-328K15.1	10:42889244-135513631	10q26.11	[undefined]	1.05119
RP11-272J7.4	10:42889244-135513631	10q11.21	[undefined]	1.05119
RP11-432I13.3	10:42889244-135513631	10q11.21	[undefined]	1.05119
RP11-140A10.3	10:42889244-135513631	10q26.3	[undefined]	1.05119
RP11-80H5.4	10:42889244-135513631	10q23.31	[undefined]	1.05119
RP11-135D11.2	10:42889244-135513631	10q21.2	[undefined]	1.05119
RP11-186O14.4	10:42889244-135513631	10q23.31	[undefined]	1.05119
RP11-422P15.3	10:42889244-135513631	10q26.2	[undefined]	1.05119
CTA-109P11.1	10:42889244-135513631	10q26.11	[undefined]	1.05119
RP11-400G3.2	10:42889244-135513631	10q23.33	[undefined]	1.05119
RP11-122K13.12	10:42889244-135513631	10q26.3	[undefined]	1.05119
RP13-439H18.7	10:42889244-135513631	10q26.3	[undefined]	1.05119
RP11-753C18.8	10:42889244-135513631	10q24.32	[undefined]	1.05119
RP11-539E19.2	10:42889244-135513631	10q21.1	[undefined]	1.05119
RP11-574K11.20	10:42889244-135513631	10q22.2	[undefined]	1.05119
MIR107	10:42889244-135513631	10q23.31	microRNA 107 [Source:HGNC Symbol;Acc:31496]	1.05119
RP11-428G2.1	10:42889244-135513631	10q21.3	[undefined]	1.05119
RP11-332O19.5	10:42889244-135513631	10q24.33	[undefined]	1.05119
RP11-342M3.5	10:42889244-135513631	10q22.3	[undefined]	1.05119
RP11-135D11.3	10:42889244-135513631	10q21.2	[undefined]	1.05119
RP11-225H22.5	10:42889244-135513631	10q24.33	[undefined]	1.05119
RP11-386C23.1	10:42889244-135513631	10q21.3	[undefined]	1.05119
RP11-343J3.5	10:42889244-135513631	10q22.1	[undefined]	1.05119
RP11-324H6.5	10:42889244-135513631	10q11.23	[undefined]	1.05119
RP11-242G20.1	10:42889244-135513631	10q22.1	[undefined]	1.05119
RP11-34E5.2	10:42889244-135513631	10q24.1	[undefined]	1.05119
RP11-445N18.6	10:42889244-135513631	10q11.21	[undefined]	1.05119
RP11-342M3.2	10:42889244-135513631	10q22.3	[undefined]	1.05119
RP11-5G18.2	10:42889244-135513631	10q25.3	[undefined]	1.05119
RP11-119K6.4	10:42889244-135513631	10q23.33	[undefined]	1.05119
RP11-343J3.6	10:42889244-135513631	10q22.1	[undefined]	1.05119
RP11-550A9.1	10:42889244-135513631	10q21.1	[undefined]	1.05119
RP11-351O1.2	10:42889244-135513631	10q21.3	[undefined]	1.05119
C10orf75	10:42889244-135513631	10q24.31	[undefined]	1.05119
RP11-36D19.8	10:42889244-135513631	10q23.1	[undefined]	1.05119
RP11-525A16.1	10:42889244-135513631	10q25.2	[undefined]	1.05119
RP11-479O17.8	10:42889244-135513631	10q22.3	[undefined]	1.05119
C10orf40	10:42889244-135513631	10q21.2	Putative uncharacterized protein C10orf40 [Source:UniProtKB/Swiss-Prot;Acc:A4QN01]	1.05119
RP11-123B3.2	10:42889244-135513631	10q11.23	[undefined]	1.05119
XX-2136C48.6	10:42889244-135513631	10q26.3	[undefined]	1.05119
RP11-78C6.1	10:42889244-135513631	10q25.1	[undefined]	1.05119
RP11-215A21.2	10:42889244-135513631	10q26.11	[undefined]	1.05119
AL450342.2	10:42889244-135513631	10q11.23	Putative uncharacterized protein ENSP00000390764 Fragment [Source:UniProtKB/TrEMBL;Acc:C9JFN6]	1.05119
RP11-517P14.7	10:42889244-135513631	10q11.21	[undefined]	1.05119
RP11-343J3.2	10:42889244-135513631	10q22.1	[undefined]	1.05119
RP11-501J20.3	10:42889244-135513631	10q25.3	[undefined]	1.05119
RP11-255M1.1	10:42889244-135513631	10q11.23	[undefined]	1.05119
RP11-369J21.3	10:42889244-135513631	10q22.3	[undefined]	1.05119
RP11-540N6.1	10:42889244-135513631	10q26.3	[undefined]	1.05119
RP11-316M21.7	10:42889244-135513631	10q24.31	[undefined]	1.05119
TLX1NB	10:42889244-135513631	10q24.31	TLX1 neighbor [Source:HGNC Symbol;Acc:37183]	1.05119
RP11-574K11.16	10:42889244-135513631	10q22.2	[undefined]	1.05119
RP11-15K3.1	10:42889244-135513631	10q23.31	[undefined]	1.05119
RP11-474D14.3	10:42889244-135513631	10q21.3	[undefined]	1.05119
CTSLL1	10:42889244-135513631	10q23.2	cathepsin L-like 1 [Source:HGNC Symbol;Acc:2539]	1.05119
RP11-79M19.2	10:42889244-135513631	10q26.11	[undefined]	1.05119
RP11-445N18.8	10:42889244-135513631	10q11.21	[undefined]	1.05119
XX-2136C48.4	10:42889244-135513631	10q26.3	[undefined]	1.05119
RP11-443A13.2	10:42889244-135513631	10q22.3	[undefined]	1.05119
EIF2S2P3	10:42889244-135513631	10q23.33	eukaryotic translation initiation factor 2, subunit 2 beta pseudogene 3 [Source:HGNC Symbol;Acc:31664]	1.05119
RPL13AP5	10:42889244-135513631	10q24.1	ribosomal protein L13a pseudogene 5 [Source:HGNC Symbol;Acc:23736]	1.05119
RP11-443O13.3	10:42889244-135513631	10q21.1	[undefined]	1.05119
RP11-93H12.3	10:42889244-135513631	10q23.1	[undefined]	1.05119
RP11-108L7.4	10:42889244-135513631	10q24.31	[undefined]	1.05119
RP11-573I11.2	10:42889244-135513631	10q21.1	[undefined]	1.05119
RP11-168O22.1	10:42889244-135513631	10q21.1	[undefined]	1.05119
C10orf103	10:42889244-135513631	10q22.2	Putative uncharacterized protein C10orf103 [Source:UniProtKB/Swiss-Prot;Acc:A6NH13]	1.05119
RP11-159H3.1	10:42889244-135513631	10q26.12	[undefined]	1.05119
RP11-168P8.5	10:42889244-135513631	10q11.21	[undefined]	1.05119
RP11-592B15.6	10:42889244-135513631	10q11.23	[undefined]	1.05119
SPA17P1	10:42889244-135513631	10q22.2	sperm autoantigenic protein 17 pseudogene 1 [Source:HGNC Symbol;Acc:23224]	1.05119
RP11-383C6.2	10:42889244-135513631	10q25.3	[undefined]	1.05119
RP11-534L6.2	10:42889244-135513631	10q11.22	[undefined]	1.05119
RP11-379D21.2	10:42889244-135513631	10q24.2	[undefined]	1.05119
RP11-399K21.10	10:42889244-135513631	10q22.2	[undefined]	1.05119
RP11-506B4.2	10:42889244-135513631	10q22.2	[undefined]	1.05119
RP11-753C18.7	10:42889244-135513631	10q24.32	[undefined]	1.05119
RP11-96B5.2	10:42889244-135513631	10q11.23	[undefined]	1.05119
RP11-264A11.1	10:42889244-135513631	10q21.1	[undefined]	1.05119
RP11-383C5.7	10:42889244-135513631	10q26.13	[undefined]	1.05119
GAPDHP21	10:42889244-135513631	10q21.1	glyceraldehyde 3 phosphate dehydrogenase pseudogene 21 [Source:HGNC Symbol;Acc:37773]	1.05119
RP11-432J9.3	10:42889244-135513631	10q22.1	[undefined]	1.05119
AL157893.1	10:42889244-135513631	10q23.2	Putative uncharacterized protein ENSP00000416928 Fragment [Source:UniProtKB/TrEMBL;Acc:C9JJV9]	1.05119
RP11-137L10.5	10:42889244-135513631	10q22.2	[undefined]	1.05119
RP11-57G10.6	10:42889244-135513631	10q21.3	[undefined]	1.05119
C10orf41	10:42889244-135513631	10q22.2	Putative uncharacterized protein C10orf41 [Source:UniProtKB/Swiss-Prot;Acc:A6NEH8]	1.05119
RP11-452K12.3	10:42889244-135513631	10q24.1	[undefined]	1.05119
RP11-425M17.3	10:42889244-135513631	10q23.31	[undefined]	1.05119

Continued on next page...

gene name	chr	.DNABand	.GeneDesc	rank
RP11-109A6.2	10:42889244-135513631	10q26.3	[undefined]	1.05119
RP11-809M12.1	10:42889244-135513631	10q21.2	[undefined]	1.05119
RP11-563N6.4	10:42889244-135513631	10q11.23	[undefined]	1.05119
RP11-402N18.2	10:42889244-135513631	10q25.1	[undefined]	1.05119
RP11-181F12.1	10:42889244-135513631	10q23.1	[undefined]	1.05119
RP11-96C23.9	10:42889244-135513631	10q23.2	[undefined]	1.05119
RP11-500G10.6	10:42889244-135513631	10q26.3	[undefined]	1.05119
RP11-402L1.4	10:42889244-135513631	10q11.21	[undefined]	1.05119
SLC9A3P4	10:42889244-135513631	10q11.22	solute carrier family 9 (sodium/hydrogen exchanger), member 3 pseudogene 4 [Source:HGNC Symbol;Acc:33493]	1.05119
RP11-280G19.2	10:42889244-135513631	10q23.33	[undefined]	1.05119
RP11-500G10.1	10:42889244-135513631	10q26.3	[undefined]	1.05119
RP11-790G19.2	10:42889244-135513631	10q22.1	[undefined]	1.05119
RP11-40F6.2	10:42889244-135513631	10q22.3	[undefined]	1.05119
RP11-324L3.1	10:42889244-135513631	10q24.31	[undefined]	1.05119
RP11-20J15.2	10:42889244-135513631	10q11.21	[undefined]	1.05119
RP11-381K7.2	10:42889244-135513631	10q25.2	[undefined]	1.05119
XX-2136C48.8	10:42889244-135513631	10q26.3	[undefined]	1.05119
RP11-525A16.3	10:42889244-135513631	10q25.2	[undefined]	1.05119
RP11-118H17.1	10:42889244-135513631	10q26.13	[undefined]	1.05119
RP11-157H10.4	10:42889244-135513631	10q23.2	[undefined]	1.05119
RP11-127O4.2	10:42889244-135513631	10q25.1	[undefined]	1.05119
RP11-344N10.2	10:42889244-135513631	10q22.1	[undefined]	1.05119
RP11-332O19.2	10:42889244-135513631	10q24.32	[undefined]	1.05119
RP11-358L16.4	10:42889244-135513631	10q11.22	[undefined]	1.05119
RP11-298J20.1	10:42889244-135513631	10q26.13	[undefined]	1.05119
RP11-487I5.1	10:42889244-135513631	10q22.2	[undefined]	1.05119
RP11-208C17.4	10:42889244-135513631	10q23.33	[undefined]	1.05119
RP11-456A18.1	10:42889244-135513631	10q21.1	[undefined]	1.05119
RP11-258A12.3	10:42889244-135513631	10q25.2	[undefined]	1.05119
RP11-432J9.5	10:42889244-135513631	10q22.1	[undefined]	1.05119
RP11-167P22.5	10:42889244-135513631	10q22.1	[undefined]	1.05119
RP11-402L1.12	10:42889244-135513631	10q11.21	[undefined]	1.05119
RP11-245J24.1	10:42889244-135513631	10q26.11	[undefined]	1.05119
RP11-436D10.3	10:42889244-135513631	10q21.3	[undefined]	1.05119
RP11-94M14.3	10:42889244-135513631	10q23.31	[undefined]	1.05119
AC074322.1	10:42889244-135513631	10q21.1	[undefined]	1.05119
AL357042.1	10:42889244-135513631	10q25.3	[undefined]	1.05119
AL450334.1	10:42889244-135513631	10q11.22	[undefined]	1.05119
RNU7-38P	10:42889244-135513631	10q22.1	RNA, U7 small nuclear 38 pseudogene [Source:HGNC Symbol;Acc:34134]	1.05119
AC091487.1	10:42889244-135513631	10q23.1	[undefined]	1.05119
AL441886.1	10:42889244-135513631	10q24.2	[undefined]	1.05119
AL117341.1	10:42889244-135513631	10q11.23	[undefined]	1.05119
AL354873.1	10:42889244-135513631	10q25.3	[undefined]	1.05119
AL353719.1	10:42889244-135513631	10q24.2	[undefined]	1.05119
AL162742.1	10:42889244-135513631	10q25.1	[undefined]	1.05119
AC025039.1	10:42889244-135513631	10q21.1	[undefined]	1.05119
AL022344.1	10:42889244-135513631	10q11.21	[undefined]	1.05119
MIR2110	10:42889244-135513631	10q25.3	microRNA 2110 [Source:HGNC Symbol;Acc:37071]	1.05119
AL513185.1	10:42889244-135513631	10q22.1	[undefined]	1.05119
AL732479.1	10:42889244-135513631	10q23.2	[undefined]	1.05119
AL359384.1	10:42889244-135513631	10q22.1	[undefined]	1.05119
AL512640.1	10:42889244-135513631	10q11.21	[undefined]	1.05119
AC016396.1	10:42889244-135513631	10q21.1	[undefined]	1.05119
AL512656.2	10:42889244-135513631	10q22.2	[undefined]	1.05119
AL391318.1	10:42889244-135513631	10q23.31	[undefined]	1.05119
AC020642.1	10:42889244-135513631	10q21.3	[undefined]	1.05119
AL133387.5	10:42889244-135513631	10q24.32	[undefined]	1.05119
AL359260.2	10:42889244-135513631	10q25.2	[undefined]	1.05119
AC012044.2	10:42889244-135513631	10q11.22	[undefined]	1.05119
FAM25HP	10:42889244-135513631	10q11.22	family with sequence similarity 25, member H pseudogene [Source:HGNC Symbol;Acc:23591]	1.05119
AC073366.1	10:42889244-135513631	10q11.23	[undefined]	1.05119
RP11-459F3.6	10:42889244-135513631	10q24.2	[undefined]	1.05119
RP11-324H6.7	10:42889244-135513631	10q11.23	[undefined]	1.05119
RP11-175I17.4	10:42889244-135513631	10q11.22	[undefined]	1.05119
AL450311.9	10:42889244-135513631	10q22.1	[undefined]	1.05119
AL356608.1	10:42889244-135513631	10q24.32	[undefined]	1.05119
AL450311.4	10:42889244-135513631	10q22.1	[undefined]	1.05119
BMS1P3	10:42889244-135513631	10q23.2	BMS1 pseudogene 3 [Source:HGNC Symbol;Acc:23651]	1.05119
AL356056.1	10:42889244-135513631	10q11.22	[undefined]	1.05119
AL158165.1	10:42889244-135513631	10q24.1	[undefined]	1.05119
AL672187.4	10:42889244-135513631	10q11.23	[undefined]	1.05119
RP11-96C23.7	10:42889244-135513631	10q23.2	[undefined]	1.05119
RP11-429G19.3	10:42889244-135513631	10q24.1	[undefined]	1.05119
AL359385.2	10:42889244-135513631	10q24.1	[undefined]	1.05119
AC005879.1	10:42889244-135513631	10q26.11	[undefined]	1.05119
RP11-564C4.7	10:42889244-135513631	10q11.23	[undefined]	1.05119
RP13-137A17.6	10:42889244-135513631	10q26.3	[undefined]	1.05119
RP11-119K6.5	10:42889244-135513631	10q23.33	[undefined]	1.05119
AC024597.1	10:42889244-135513631	10q21.2	[undefined]	1.05119
RP11-80H5.7	10:42889244-135513631	10q23.31	tigger transposable element derived 2 pseudogene (FLJ37201), non-coding RNA [Source:RefSeq DNA;Acc:NR_026835]	1.05119
AL359074.1	10:42889244-135513631	10q22.2	[undefined]	1.05119
AL359385.1	10:42889244-135513631	10q24.1	[undefined]	1.05119
AL441886.2	10:42889244-135513631	10q24.2	[undefined]	1.05119
AL355598.1	10:42889244-135513631	10q26.11	[undefined]	1.05119
AL136982.2	10:42889244-135513631	10q23.2	[undefined]	1.05119
RP11-342M3.1	10:42889244-135513631	10q22.3	[undefined]	1.05119
RP11-342C24.1	10:42889244-135513631	10q11.22	[undefined]	1.05119
AL359747.1	10:42889244-135513631	10q26.13	[undefined]	1.05119
XX-2136C48.2	10:42889244-135513631	10q26.3	[undefined]	1.05119
RP11-523O18.7	10:42889244-135513631	10q11.23	[undefined]	1.05119
AC074322.3	10:42889244-135513631	10q21.1	[undefined]	1.05119
AC022016.2	10:42889244-135513631	10q23.31	[undefined]	1.05119
AL133387.2	10:42889244-135513631	10q24.32	[undefined]	1.05119
RP11-76P2.2	10:42889244-135513631	10q23.33	[undefined]	1.05119
DHDPSL	10:42889244-135513631	10q24.2	dihydrodipicolinate synthase-like, mitochondrial [Source:HGNC Symbol;Acc:25155]	1.05119
AL512654.1	10:42889244-135513631	10q11.21	[undefined]	1.05119
AC016542.1	10:42889244-135513631	10q22.1	[undefined]	1.05119
RP11-464F9.1	10:42889244-135513631	10q22.2	BMS1 pseudogene 4 (BMS1P4), non-coding RNA [Source:RefSeq DNA;Acc:NR_026592]	1.05119
AL157386.1	10:42889244-135513631	10q24.32	[undefined]	1.05119
BMS1P4	10:42889244-135513631	10q22.2	BMS1 pseudogene 4 [Source:HGNC Symbol;Acc:23652]	1.05119
AC074322.2	10:42889244-135513631	10q21.1	[undefined]	1.05119
AL450311.2	10:42889244-135513631	10q22.1	[undefined]	1.05119
AL592546.1	10:42889244-135513631	10q25.3	[undefined]	1.05119
AL139240.1	10:42889244-135513631	10q21.3	[undefined]	1.05119
MBL1P1	10:42889244-135513631	10q22.3	mannose-binding lectin (protein A) 1, pseudogene [Source:HGNC Symbol;Acc:6921]	1.05119
RP11-358L16.2	10:42889244-135513631	10q11.21	[undefined]	1.05119
AL391421.3	10:42889244-135513631	10q22.3	[undefined]	1.05119
AL158014.1	10:42889244-135513631	10q26.11	[undefined]	1.05119
RP11-445N18.9	10:42889244-135513631	10q11.21	[undefined]	1.05119
AL355861.1	10:42889244-135513631	10q26.11	[undefined]	1.05119
GLUDP2	10:42889244-135513631	10q11.22	glutamate dehydrogenase 1 pseudogene 2 [Source:HGNC Symbol;Acc:4337]	1.05119
AC063962.1	10:42889244-135513631	10q22.2	[undefined]	1.05119
AL133384.1	10:42889244-135513631	10q25.3	[undefined]	1.05119
AL513185.3	10:42889244-135513631	10q22.1	[undefined]	1.05119
AL135794.1	10:42889244-135513631	10q24.31	[undefined]	1.05119
AL450311.3	10:42889244-135513631	10q22.1	[undefined]	1.05119
AL731733.2	10:42889244-135513631	10q11.22	[undefined]	1.05119
AL365364.1	10:42889244-135513631	10q23.33	[undefined]	1.05119
PGBD3	10:42889244-135513631	10q11.23	piggyBac transposable element derived 3 [Source:HGNC Symbol;Acc:19400]	1.05119
CTGLF8P	10:42889244-135513631	10q11.22	centaurin, gamma-like family, member 8 pseudogene [Source:HGNC Symbol;Acc:23461]	1.05119
AL731543.1	10:42889244-135513631	10q26.13	deleted in malignant brain tumors 1 pseudogene (FLJ46361), non-coding RNA [Source:RefSeq DNA;Acc:NR_003570]	1.05119

Continued on next page...

gene name	chr	.DNABand	.GeneDesc	rank
GUCY2G	10:42889244-135513631	10q25.2	guanylate cyclase 2G homolog (mouse) pseudogene [Source:HGNC Symbol;Acc:31863]	1.05119
AL513534.4	10:42889244-135513631	10q21.3	[undefined]	1.05119
RP11-445N18.7	10:42889244-135513631	10q11.21	[undefined]	1.05119
AL450311.8	10:42889244-135513631	10q22.1	[undefined]	1.05119
AL731563.3	10:42889244-135513631	10q22.1	[undefined]	1.05119
AL391421.2	10:42889244-135513631	10q22.3	[undefined]	1.05119
AL391421.1	10:42889244-135513631	10q22.3	[undefined]	1.05119
RP11-292F22.6	10:42889244-135513631	10q11.22	[undefined]	1.05119
RP11-319I23.3	10:42889244-135513631	10q26.11	[undefined]	1.05119
AL450311.7	10:42889244-135513631	10q22.1	[undefined]	1.05119
AL359844.2	10:42889244-135513631	10q22.1	[undefined]	1.05119
AL356632.1	10:42889244-135513631	10q24.1	[undefined]	1.05119
RP11-18I14.7	10:42889244-135513631	10q24.32	[undefined]	1.05119
XX-2136C48.5	10:42889244-135513631	10q26.3	[undefined]	1.05119
ZNF487	10:42889244-135513631	10q11.21	zinc finger protein 487 [Source:HGNC Symbol;Acc:23488]	1.05119
RP11-574K11.18	10:42889244-135513631	10q22.2	[undefined]	1.05119
AC022532.1	10:42889244-135513631	10q22.1	[undefined]	1.05119
AC022016.1	10:42889244-135513631	10q23.31	[undefined]	1.05119
BMS1P7	10:42889244-135513631	10q11.22	BMS1 pseudogene 7 [Source:HGNC Symbol;Acc:23655]	1.05119
AL450311.6	10:42889244-135513631	10q22.1	[undefined]	1.05119
AL450311.1	10:42889244-135513631	10q22.1	[undefined]	1.05119
AL645992.1	10:42889244-135513631	10q23.2	[undefined]	1.05119
AL450311.5	10:42889244-135513631	10q22.1	[undefined]	1.05119
AL442635.1	10:42889244-135513631	10q22.1	[undefined]	1.05119
RP11-119K6.6	10:42889244-135513631	10q23.33	[undefined]	1.05119
AC022027.1	10:42889244-135513631	10q21.2	[undefined]	1.05119
RP11-152N13.12	10:42889244-135513631	10q22.2	[undefined]	1.05119
RP11-592B15.3	10:42889244-135513631	10q11.23	[undefined]	1.05119
C10orf43	10:42889244-135513631	10q11.22	[undefined]	1.05119
RP11-506M13.3	10:42889244-135513631	10q22.3	[undefined]	1.05119
AL139123.1	10:42889244-135513631	10q26.3	[undefined]	1.05119
AC022400.3	10:42889244-135513631	10q22.2	[undefined]	1.05119
AL360001.1	10:42889244-135513631	10q24.33	[undefined]	1.05119
AL121929.1	10:42889244-135513631	10q24.33	[undefined]	1.05119
AL121928.1	10:42889244-135513631	10q24.32	[undefined]	1.05119
AC063965.2	10:42889244-135513631	10q23.31	[undefined]	1.05119
AL355344.1	10:42889244-135513631	10q22.1	[undefined]	1.05119
AL359986.1	10:42889244-135513631	10q23.32	[undefined]	1.05119
AL162742.2	10:42889244-135513631	10q25.1	[undefined]	1.05119
AL731563.2	10:42889244-135513631	10q22.1	[undefined]	1.05119
AL513534.1	10:42889244-135513631	10q21.3	[undefined]	1.05119
AL671972.2	10:42889244-135513631	10q21.2	[undefined]	1.05119
RP11-13E1.3	10:42889244-135513631	10q11.22	[undefined]	1.05119
RP11-539I5.2	10:42889244-135513631	10q25.3	[undefined]	1.05119
AC027674.1	10:42889244-135513631	10q11.23	[undefined]	1.05119
AC022389.1	10:42889244-135513631	10q23.1	RPE-retinal G protein-coupled receptor isoform 3 [Source:RefSeq peptide;Acc:NP_001012740]	1.05119
RP11-541M12.5	10:42889244-135513631	10q11.22	[undefined]	1.05119
AL390920.1	10:42889244-135513631	10q26.2	[undefined]	1.05119
AC010789.1	10:42889244-135513631	10q24.32	[undefined]	1.05119
RP11-498B4.2	10:42889244-135513631	10q25.3	[undefined]	1.05119
AC012044.1	10:42889244-135513631	10q11.22	[undefined]	1.05119
AC025268.1	10:42889244-135513631	10q23.2	[undefined]	1.05119
RP11-152N13.11	10:42889244-135513631	10q22.2	[undefined]	1.05119
RP11-537A6.7	10:42889244-135513631	10q22.2	[undefined]	1.05119
RP11-298J20.4	10:42889244-135513631	10q26.13	[undefined]	1.05119
GLUDP3	10:42889244-135513631	10q22.2	glutamate dehydrogenase 1 pseudogene 3 [Source:HGNC Symbol;Acc:4338]	1.05119
BX005072.1	10:42889244-135513631	10q11.22	cDNA, FLJ94239 [Source:UniProtKB/TrEMBL;Acc:B2R966]	1.05119
RP11-164N7.1	10:42889244-135513631	10q11.22	[undefined]	1.05119
RP11-80H5.5	10:42889244-135513631	10q23.31	[undefined]	1.05119
RP11-548K23.11	10:42889244-135513631	10q24.2	cDNA FLJ56960, highly similar to Homo sapiens phosphatidylinositol 4-kinase type II (PI4KII), mRNA [Source:UniProtKB/TrEMBL;Acc:B4DEP8]	1.05119
AC016399.1	10:42889244-135513631	10q11.22	[undefined]	1.05119
AL355315.1	10:42889244-135513631	10q24.2	[undefined]	1.05119
AC010997.2	10:42889244-135513631	10q22.2	[undefined]	1.05119
XXyac-YR14BB7.1	10:42889244-135513631	10q11.22	[undefined]	1.05119
AL731556.1	10:42889244-135513631	10q22.3	[undefined]	1.05119
AL450334.3	10:42889244-135513631	10q11.22	Putative protein FRMPD2-like (PDZ domain-containing protein 5A) [Source:UniProtKB/Swiss-Prot;Acc:Q6IN97]	1.05119
AL592430.1	10:42889244-135513631	10q21.2	[undefined]	1.05119
AL731576.1	10:42889244-135513631	10q22.2	[undefined]	1.05119
AC022028.1	10:42889244-135513631	10q23.1	[undefined]	1.05119
RP11-574K11.12	10:42889244-135513631	10q22.2	Putative uncharacterized protein ENSP00000416940 Fragment [Source:UniProtKB/TrEMBL;Acc:C9JJU5]	1.05119
AL442003.3	10:42889244-135513631	10q11.23	[undefined]	1.05119
RP11-35J23.5	10:42889244-135513631	10q24.1	[undefined]	1.05119
BMS1P6	10:42889244-135513631	10q11.22	BMS1 pseudogene 6 [Source:HGNC Symbol;Acc:23654]	1.05119
BMS1P2	10:42889244-135513631	10q11.22	BMS1 pseudogene 2 [Source:HGNC Symbol;Acc:23650]	1.05119
AL731563.1	10:42889244-135513631	10q22.1	[undefined]	1.05119
AL450334.2	10:42889244-135513631	10q11.22	cDNA, FLJ94239 [Source:UniProtKB/TrEMBL;Acc:B2R966]	1.05119
AC044782.1	10:42889244-135513631	10q21.3	[undefined]	1.05119
RP11-534L6.5	10:42889244-135513631	10q11.22	[undefined]	1.05119
CTGLF9P	10:42889244-135513631	10q11.22	centaurin, gamma-like family, member 9 pseudogene [Source:HGNC Symbol;Acc:23499]	1.05119
BMS1P5	10:42889244-135513631	10q11.22	BMS1 pseudogene 5 [Source:HGNC Symbol;Acc:23653]	1.05119
RP11-464F9.9	10:42889244-135513631	10q22.2	[undefined]	1.05119
AL022345.2	10:42889244-135513631	10q11.21	[undefined]	1.05119
AL157388.1	10:42889244-135513631	10q26.11	[undefined]	1.05119
AL513534.3	10:42889244-135513631	10q21.3	[undefined]	1.05119
AL117341.2	10:42889244-135513631	10q11.23	[undefined]	1.05119
AL136368.2	10:42889244-135513631	10q25.2	[undefined]	1.05119
AC022400.2	10:42889244-135513631	10q22.2	[undefined]	1.05119
AL607042.1	10:42889244-135513631	10q21.1	[undefined]	1.05119
AL603966.1	10:42889244-135513631	10q11.22	[undefined]	1.05119
AC026223.1	10:42889244-135513631	10q21.1	[undefined]	1.05119
AC067750.1	10:42889244-135513631	10q23.2	[undefined]	1.05119
AC016819.1	10:42889244-135513631	10q21.3	[undefined]	1.05119
AL353784.1	10:42889244-135513631	10q21.1	[undefined]	1.05119
AC022028.2	10:42889244-135513631	10q23.1	[undefined]	1.05119
AL022344.2	10:42889244-135513631	10q11.21	[undefined]	1.05119
AL450326.1	10:42889244-135513631	10q11.21	[undefined]	1.05119
AL138766.2	10:42889244-135513631	10q25.1	[undefined]	1.05119
AL355340.1	10:42889244-135513631	10q25.3	[undefined]	1.05119
AL731532.2	10:42889244-135513631	10q23.1	[undefined]	1.05119
AL591684.1	10:42889244-135513631	10q11.22	[undefined]	1.05119
AL133387.4	10:42889244-135513631	10q24.32	[undefined]	1.05119
AL391137.1	10:42889244-135513631	10q11.22	[undefined]	1.05119
AC013738.1	10:42889244-135513631	10q22.3	[undefined]	1.05119
AL365510.1	10:42889244-135513631	10q23.33	[undefined]	1.05119
AL365398.1	10:42889244-135513631	10q23.32	[undefined]	1.05119
AL358790.1	10:42889244-135513631	10q24.32	[undefined]	1.05119
AC022538.1	10:42889244-135513631	10q21.3	[undefined]	1.05119
AC022400.1	10:42889244-135513631	10q22.2	[undefined]	1.05119
AC022023.1	10:42889244-135513631	10q25.3	[undefined]	1.05119
AL500527.1	10:42889244-135513631	10q24.32	[undefined]	1.05119
AHCTF1	1:247024176-247051615	1q44	AT hook containing transcription factor 1 [Source:HGNC Symbol;Acc:24618]	1.04269
AB019439.21	14:106786148-106810234	14q32.33	[undefined]	1.00528
IGHV3-30	14:106786148-106810234	14q32.33	Rheumatoid factor RF-IP20 Fragment [Source:UniProtKB/TrEMBL;Acc:A2J1N3]	1.00528
IGHVII-30-1	14:106786148-106810234	14q32.33	[undefined]	1.00528
IGHV3-30-2	14:106786148-106810234	14q32.33	[undefined]	1.00528
IGHV4-31	14:106786148-106810234	14q32.33	[undefined]	1.00528
IGHV3-29	14:106786148-106810234	14q32.33	[undefined]	1.00528
AC027139.2	15:34730221-34791191	15q14	Putative uncharacterized protein ENSP00000388079 Fragment [Source:UniProtKB/TrEMBL;Acc:C9JXI7]	0.93205
TDRD3	13:43317927-66491226	13q21.2	tudor domain containing 3 [Source:HGNC Symbol;Acc:20612]	0.92263
C13orf18	13:43317927-66491226	13q14.13	Uncharacterized protein C13orf18 [Source:UniProtKB/Swiss-Prot;Acc:Q9H714]	0.92263

Continued on next page...

gene name	chr	.DNABand	.GeneDesc	rank
CPB2	13:43317927-66491226	13q14.13	carboxypeptidase B2 (plasma) [Source:HGNC Symbol;Acc:2300]	0.92263
FNDC3A	13:43317927-66491226	13q14.2	fibronectin type III domain containing 3A [Source:HGNC Symbol;Acc:20296]	0.92263
NUFIP1	13:43317927-66491226	13q14.12	nuclear fragile X mental retardation protein interacting protein 1 [Source:HGNC Symbol;Acc:8057]	0.92263
CDADC1	13:43317927-66491226	13q14.2	cytidine and dCMP deaminase domain containing 1 [Source:HGNC Symbol;Acc:20299]	0.92263
HTR2A	13:43317927-66491226	13q14.2	5-hydroxytryptamine (serotonin) receptor 2A [Source:HGNC Symbol;Acc:5293]	0.92263
KPNA3	13:43317927-66491226	13q14.2	karyopherin alpha 3 (importin alpha 4) [Source:HGNC Symbol;Acc:6396]	0.92263
MLNR	13:43317927-66491226	13q14.2	motilin receptor [Source:HGNC Symbol;Acc:4495]	0.92263
DHRS12	13:43317927-66491226	13q14.3	dehydrogenase/reductase (SDR family) member 12 [Source:HGNC Symbol;Acc:25832]	0.92263
CAB39L	13:43317927-66491226	13q14.2	calcium binding protein 39-like [Source:HGNC Symbol;Acc:20290]	0.92263
OLFM4	13:43317927-66491226	13q14.3	olfactomedin 4 [Source:HGNC Symbol;Acc:17190]	0.92263
INTS6	13:43317927-66491226	13q14.3	integrator complex subunit 6 [Source:HGNC Symbol;Acc:14879]	0.92263
ENOX1	13:43317927-66491226	13q14.11	ecto-NOX disulfide-thiol exchanger 1 [Source:HGNC Symbol;Acc:25474]	0.92263
TSC22D1	13:43317927-66491226	13q14.11	TSC22 domain family, member 1 [Source:HGNC Symbol;Acc:16826]	0.92263
CCDC70	13:43317927-66491226	13q14.3	coiled-coil domain containing 70 [Source:HGNC Symbol;Acc:25303]	0.92263
PCDH17	13:43317927-66491226	13q21.1	protocadherin 17 [Source:HGNC Symbol;Acc:14267]	0.92263
EBPL	13:43317927-66491226	13q14.2	emopamil binding protein-like [Source:HGNC Symbol;Acc:18061]	0.92263
DNAJC15	13:43317927-66491226	13q14.11	DnaJ (Hsp40) homolog, subfamily C, member 15 [Source:HGNC Symbol;Acc:20325]	0.92263
ZC3H13	13:43317927-66491226	13q14.13	zinc finger CCCH-type containing 13 [Source:HGNC Symbol;Acc:20368]	0.92263
C13orf1	13:43317927-66491226	13q14.2	Chronic lymphocytic leukemia deletion region gene 6 protein (CLL deletion region gene 6 protein) [Source:UniProtKB/Swiss-Prot;Acc:Q5W111]	0.92263
EPSTI1	13:43317927-66491226	13q14.11	epithelial stromal interaction 1 (breast) [Source:HGNC Symbol;Acc:16465]	0.92263
ATP7B	13:43317927-66491226	13q14.3	ATPase, Cu++ transporting, beta polypeptide [Source:HGNC Symbol;Acc:870]	0.92263
KIAA1704	13:43317927-66491226	13q14.12	KIAA1704 [Source:HGNC Symbol;Acc:20298]	0.92263
GUCY1B2	13:43317927-66491226	13q14.3	guanylate cyclase 1, soluble, beta 2 [Source:HGNC Symbol;Acc:4686]	0.92263
PCDH8	13:43317927-66491226	13q14.3	protocadherin 8 [Source:HGNC Symbol;Acc:8660]	0.92263
TPT1	13:43317927-66491226	13q14.13	tumor protein, translationally-controlled 1 [Source:HGNC Symbol;Acc:12022]	0.92263
RNASEH2B	13:43317927-66491226	13q14.3	ribonuclease H2, subunit B [Source:HGNC Symbol;Acc:25671]	0.92263
NEK3	13:43317927-66491226	13q14.3	NIMA (never in mitosis gene a)-related kinase 3 [Source:HGNC Symbol;Acc:7746]	0.92263
LECT1	13:43317927-66491226	13q14.3	leukocyte cell derived chemotaxin 1 [Source:HGNC Symbol;Acc:17005]	0.92263
VPS36	13:43317927-66491226	13q14.3	vacuolar protein sorting 36 homolog (S. cerevisiae) [Source:HGNC Symbol;Acc:20312]	0.92263
LRCH1	13:43317927-66491226	13q14.13 13q14.2	leucine-rich repeats and calponin homology (CH) domain containing 1 [Source:HGNC Symbol;Acc:20309]	0.92263
CKAP2	13:43317927-66491226	13q14.3	cytoskeleton associated protein 2 [Source:HGNC Symbol;Acc:1990]	0.92263
RCBTB1	13:43317927-66491226	13q14.2	regulator of chromosome condensation (RCC1) and BTB (POZ) domain containing protein 1 [Source:HGNC Symbol;Acc:18243]	0.92263
THSD1	13:43317927-66491226	13q14.3	thrombospondin, type I, domain containing 1 [Source:HGNC Symbol;Acc:17754]	0.92263
PHF11	13:43317927-66491226	13q14.2	PHD finger protein 11 [Source:HGNC Symbol;Acc:17024]	0.92263
SUCLA2	13:43317927-66491226	13q14.2	succinate-CoA ligase, ADP-forming, beta subunit [Source:HGNC Symbol;Acc:11448]	0.92263
COG3	13:43317927-66491226	13q14.13	component of oligomeric golgi complex 3 [Source:HGNC Symbol;Acc:18619]	0.92263
MED4	13:43317927-66491226	13q14.2	mediator complex subunit 4 [Source:HGNC Symbol;Acc:17903]	0.92263
NUDT15	13:43317927-66491226	13q14.2	nudix (nucleoside diphosphate linked moiety X)-type motif 15 [Source:HGNC Symbol;Acc:23063]	0.92263
RP11-365K22.1	13:43317927-66491226	13q14.3	[undefined]	0.92263
LCP1	13:43317927-66491226	13q14.13	lymphocyte cytosolic protein 1 (L-plastin) [Source:HGNC Symbol;Acc:6528]	0.92263
ITM2B	13:43317927-66491226	13q14.2	integral membrane protein 2B [Source:HGNC Symbol;Acc:6174]	0.92263
RP11-478K15.4	13:43317927-66491226	13q14.11	Putative transmembrane protein MGC5590 [Source:UniProtKB/Swiss-Prot;Acc:Q9BVW6]	0.92263
RCBTB2	13:43317927-66491226	13q14.2	regulator of chromosome condensation (RCC1) and BTB (POZ) domain containing protein 2 [Source:HGNC Symbol;Acc:1914]	0.92263
HNRNPA1L2	13:43317927-66491226	13q14.3	heterogeneous nuclear ribonucleoprotein A1-like 2 [Source:HGNC Symbol;Acc:27067]	0.92263
SETDB2	13:43317927-66491226	13q14.2	SET domain, bifurcated 2 [Source:HGNC Symbol;Acc:20263]	0.92263
ESD	13:43317927-66491226	13q14.2	esterase D/formylglutathione hydrolase [Source:HGNC Symbol;Acc:3465]	0.92263
WDFY2	13:43317927-66491226	13q14.3	WD repeat and FYVE domain containing 2 [Source:HGNC Symbol;Acc:20482]	0.92263
DIAPH3	13:43317927-66491226	13q21.2	diaphanous homolog 3 (Drosophila) [Source:HGNC Symbol;Acc:15480]	0.92263
LPAR6	13:43317927-66491226	13q14.2	lysophosphatidic acid receptor 6 [Source:HGNC Symbol;Acc:15520]	0.92263
FAM124A	13:43317927-66491226	13q14.3	family with sequence similarity 124A [Source:HGNC Symbol;Acc:26413]	0.92263
RB1	13:43317927-66491226	13q14.2	retinoblastoma 1 [Source:HGNC Symbol;Acc:9884]	0.92263
SERP2	13:43317927-66491226	13q14.11	stress-associated endoplasmic reticulum protein family member 2 [Source:HGNC Symbol;Acc:20607]	0.92263
RP11-431O22.2	13:43317927-66491226	13q14.3	[undefined]	0.92263
ARL11	13:43317927-66491226	13q14.2	ADP-ribosylation factor-like 11 [Source:HGNC Symbol;Acc:24046]	0.92263
CCDC122	13:43317927-66491226	13q14.11	coiled-coil domain containing 122 [Source:HGNC Symbol;Acc:26478]	0.92263
FAM194B	13:43317927-66491226	13q14.13	family with sequence similarity 194, member B [Source:HGNC Symbol;Acc:26523]	0.92263
CYSLTR2	13:43317927-66491226	13q14.2	cysteinyl leukotriene receptor 2 [Source:HGNC Symbol;Acc:18274]	0.92263
LRRC63	13:43317927-66491226	13q14.13	leucine rich repeat containing 63 [Source:HGNC Symbol;Acc:34296]	0.92263
SUGT1	13:43317927-66491226	13q14.3	SGT1, suppressor of G2 allele of SKP1 (S. cerevisiae) [Source:HGNC Symbol;Acc:16987]	0.92263
SLC25A30	13:43317927-66491226	13q14.13	solute carrier family 25, member 30 [Source:HGNC Symbol;Acc:27371]	0.92263
XXyac-R12DG2.2	13:43317927-66491226	13q14.13	HCG1815546, isoform CRA_aPutative uncharacterized protein pp13759 ; [Source:UniProtKB/TrEMBL;Acc:Q8WYW1]	0.92263
RP11-432M24.4	13:43317927-66491226	13q14.2	[undefined]	0.92263
SPERT	13:43317927-66491226	13q14.13	spermatid associated [Source:HGNC Symbol;Acc:30720]	0.92263
RP11-5G9.4	13:43317927-66491226	13q14.11	[undefined]	0.92263
DLEU1	13:43317927-66491226	13q14.2 13q14.3	deleted in lymphocytic leukemia 1 (non-protein coding) [Source:HGNC Symbol;Acc:13747]	0.92263
C13orf30	13:43317927-66491226	13q14.11	Uncharacterized protein C13orf30 [Source:UniProtKB/Swiss-Prot;Acc:Q8N7L0]	0.92263
RP11-305D15.5	13:43317927-66491226	13q14.2	[undefined]	0.92263
RP11-236M15.1	13:43317927-66491226	13q14.2	CTAGE family, member 5 pseudogene (LOC220429), non-coding RNA [Source:RefSeq DNA;Acc:NR_003268]	0.92263
C13orf31	13:43317927-66491226	13q14.11	UPF0124 protein C13orf31 [Source:UniProtKB/Swiss-Prot;Acc:Q8IV20]	0.92263
GTF2F2	13:43317927-66491226	13q14.12 13q14.13	general transcription factor IIF, polypeptide 2, 30kDa [Source:HGNC Symbol;Acc:4653]	0.92263
KCTD4	13:43317927-66491226	13q14.12	potassium channel tetramerisation domain containing 4 [Source:HGNC Symbol;Acc:23227]	0.92263
PCDH20	13:43317927-66491226	13q21.2	protocadherin 20 [Source:HGNC Symbol;Acc:14257]	0.92263
DLEU7	13:43317927-66491226	13q14.3	deleted in lymphocytic leukemia, 7 [Source:HGNC Symbol;Acc:17567]	0.92263
KCNRG	13:43317927-66491226	13q14.2	potassium channel regulator [Source:HGNC Symbol;Acc:18893]	0.92263
NEK5	13:43317927-66491226	13q14.3	NIMA (never in mitosis gene a)-related kinase 5 [Source:HGNC Symbol;Acc:7748]	0.92263
AL359513.1	13:43317927-66491226	13q14.3	[undefined]	0.92263
RP11-78J21.5	13:43317927-66491226	13q14.3	[undefined]	0.92263
AL136123.2	13:43317927-66491226	13q14.2	[undefined]	0.92263
SNORA31	13:43317927-66491226	13q14.13	small nucleolar RNA, H/ACA box 31 [Source:HGNC Symbol;Acc:32621]	0.92263
AL137880.1	13:43317927-66491226	13q14.3	[undefined]	0.92263
AL137000.1	13:43317927-66491226	13q14.2	[undefined]	0.92263
AL161421.1	13:43317927-66491226	13q14.2	[undefined]	0.92263
AL139326.1	13:43317927-66491226	13q14.13	[undefined]	0.92263
AL356502.1	13:43317927-66491226	13q21.2	[undefined]	0.92263
AL138693.3	13:43317927-66491226	13q14.12	[undefined]	0.92263
AL445238.1	13:43317927-66491226	13q21.31	[undefined]	0.92263
AL355576.1	13:43317927-66491226	13q21.1	[undefined]	0.92263
AL135901.3	13:43317927-66491226	13q14.2	[undefined]	0.92263
AL157772.1	13:43317927-66491226	13q21.1	[undefined]	0.92263
AL135901.2	13:43317927-66491226	13q14.2	[undefined]	0.92263
AL135901.4	13:43317927-66491226	13q14.2	[undefined]	0.92263
PRR20B	13:43317927-66491226	13q21.1	proline rich 20B [Source:HGNC Symbol;Acc:37220]	0.92263
AL135901.5	13:43317927-66491226	13q14.2	[undefined]	0.92263
TRIM13	13:43317927-66491226	13q14.2	tripartite motif-containing 13 [Source:HGNC Symbol;Acc:9976]	0.92263
AL135901.1	13:43317927-66491226	13q14.2	[undefined]	0.92263
AL627344.1	13:43317927-66491226	13q21.2	[undefined]	0.92263
PRR20	13:43317927-66491226	13q21.1	proline rich 20A [Source:HGNC Symbol;Acc:24754]	0.92263
MIR15A	13:43317927-66491226	13q14.2	microRNA 15a [Source:HGNC Symbol;Acc:31543]	0.92263
RP11-189B4.3	13:43317927-66491226	13q14.13	[undefined]	0.92263
MIR759	13:43317927-66491226	13q14.3	microRNA 759 [Source:HGNC Symbol;Acc:37307]	0.92263
AL139183.2	13:43317927-66491226	13q14.3	[undefined]	0.92263
AL354764.1	13:43317927-66491226	13q21.2	[undefined]	0.92263
MIR16-1	13:43317927-66491226	13q14.2	microRNA 16-1 [Source:HGNC Symbol;Acc:31545]	0.92263
RP11-129M14.1	13:43317927-66491226	13q21.32	[undefined]	0.92263
AL596329.1	13:43317927-66491226	13q21.31	[undefined]	0.92263
RP11-334O13.1	13:43317927-66491226	13q21.1	[undefined]	0.92263
RP11-394A14.1	13:43317927-66491226	13q21.31	[undefined]	0.92263
RP11-339I24.1	13:43317927-66491226	13q21.1	[undefined]	0.92263
RP11-261A1.1	13:43317927-66491226	13q21.31	[undefined]	0.92263
XXyac-R12DG2.3	13:43317927-66491226	13q14.13	[undefined]	0.92263
UTP14C	13:43317927-66491226	13q14.3	UTP14, U3 small nucleolar ribonucleoprotein, homolog C (yeast) [Source:HGNC Symbol;Acc:20321]	0.92263
RP11-473M10.1	13:43317927-66491226	13q21.31	[undefined]	0.92263
RP11-34F20.4	13:43317927-66491226	13q14.2	[undefined]	0.92263
SERPINE3	13:43317927-66491226	13q14.3	serpin peptidase inhibitor, clade E (nexin, plasminogen activator inhibitor type 1), member 3 [Source:HGNC Symbol;Acc:24774]	0.92263

Continued on next page...

gene name	chr	.DNABand	.GeneDesc	rank
RP11-457D13.1	13:43317927-66491226	13q14.3	[undefined]	0.92263
SIAH3	13:43317927-66491226	13q14.13	seven in absentia homolog 3 (Drosophila) [Source:HGNC Symbol;Acc:30553]	0.92263
RP11-394A14.3	13:43317927-66491226	13q21.31	[undefined]	0.92263
RP11-394A14.2	13:43317927-66491226	13q21.31	[undefined]	0.92263
AL136218.1	13:43317927-66491226	13q14.2	Sarcoma antigen NY-SAR-79 Fragment [Source:UniProtKB/TrEMBL;Acc:Q86WE6]	0.92263
AL138963.1	13:43317927-66491226	13q14.13	[undefined]	0.92263
THSD1P	13:43317927-66491226	13q14.3	thrombospondin, type I, domain containing 1 pseudogene [Source:HGNC Symbol;Acc:31375]	0.92263
AL354775.1	13:43317927-66491226	13q21.32	[undefined]	0.92263
AL450423.1	13:43317927-66491226	13q14.3	[undefined]	0.92263
AL137118.1	13:43317927-66491226	13q14.2	[undefined]	0.92263
AL138696.1	13:43317927-66491226	13q14.3	[undefined]	0.92263
AL160157.1	13:43317927-66491226	13q14.3	[undefined]	0.92263
AL136123.1	13:43317927-66491226	13q14.2	[undefined]	0.92263
AL139801.1	13:43317927-66491226	13q14.13	[undefined]	0.92263
AL162716.1	13:43317927-66491226	13q21.1	[undefined]	0.92263
RP11-369L4.1	13:43317927-66491226	13q14.3	[undefined]	0.92263
AL161901.1	13:43317927-66491226	13q21.2	[undefined]	0.92263
RP11-156K23.2	13:43317927-66491226	13q14.2	[undefined]	0.92263
AL138693.1	13:43317927-66491226	13q14.12	[undefined]	0.92263
RP11-430I3.2	13:43317927-66491226	13q21.2	[undefined]	0.92263
RP11-522F22.1	13:43317927-66491226	13q21.1	[undefined]	0.92263
POLR2K	13:43317927-66491226	13q14.2	polymerase (RNA) II (DNA directed) polypeptide K, 7.0kDa [Source:HGNC Symbol;Acc:9198]	0.92263
RP11-321C24.1	13:43317927-66491226	13q14.12	[undefined]	0.92263
RP11-195L15.2	13:43317927-66491226	13q14.2	[undefined]	0.92263
RP11-39N8.2	13:43317927-66491226	13q14.3	[undefined]	0.92263
PSME2P2	13:43317927-66491226	13q14.2	proteasome activator subunit 2 pseudogene 2 [Source:HGNC Symbol;Acc:30160]	0.92263
RP11-83N23.2	13:43317927-66491226	13q14.2	[undefined]	0.92263
RP11-640E11.2	13:43317927-66491226	13q21.1	[undefined]	0.92263
RP11-550E22.4	13:43317927-66491226	13q14.3	[undefined]	0.92263
RP11-71C5.2	13:43317927-66491226	13q14.11	[undefined]	0.92263
RP11-521H3.1	13:43317927-66491226	13q21.2	[undefined]	0.92263
RP11-547C18.3	13:43317927-66491226	13q14.3	[undefined]	0.92263
RP11-24H2.1	13:43317927-66491226	13q14.3	[undefined]	0.92263
AL445989.1	13:43317927-66491226	13q21.31	[undefined]	0.92263
RP11-478K15.1	13:43317927-66491226	13q14.11	[undefined]	0.92263
PRR20D	13:43317927-66491226	13q21.1	proline rich 20D [Source:HGNC Symbol;Acc:37222]	0.92263
RP11-39N8.3	13:43317927-66491226	13q14.3	[undefined]	0.92263
RP11-359P14.1	13:43317927-66491226	13q21.2	[undefined]	0.92263
RP11-189B4.2	13:43317927-66491226	13q14.13	[undefined]	0.92263
RP11-442F12.2	13:43317927-66491226	13q21.2	[undefined]	0.92263
RP11-478K15.2	13:43317927-66491226	13q14.11	[undefined]	0.92263
RP11-168G22.3	13:43317927-66491226	13q21.31	[undefined]	0.92263
RP11-196P2.1	13:43317927-66491226	13q21.2	[undefined]	0.92263
RP11-528D24.3	13:43317927-66491226	13q14.2	[undefined]	0.92263
RP11-527N12.1	13:43317927-66491226	13q21.31	[undefined]	0.92263
RP11-279N8.1	13:43317927-66491226	13q14.2	[undefined]	0.92263
RP11-520F9.2	13:43317927-66491226	13q21.31	[undefined]	0.92263
RP11-234O23.1	13:43317927-66491226	13q21.31	[undefined]	0.92263
RP11-252B20.1	13:43317927-66491226	13q21.1	[undefined]	0.92263
RP11-290D2.3	13:43317927-66491226	13q14.13	[undefined]	0.92263
HNF4GP1	13:43317927-66491226	13q21.1	hepatocyte nuclear factor 4, gamma pseudogene 1 [Source:HGNC Symbol;Acc:35417]	0.92263
RP11-38J6.1	13:43317927-66491226	13q14.13	[undefined]	0.92263
RP11-154C3.1	13:43317927-66491226	13q14.13	[undefined]	0.92263
RP11-175B12.2	13:43317927-66491226	13q14.3	[undefined]	0.92263
RP11-90M2.4	13:43317927-66491226	13q14.2	[undefined]	0.92263
RP11-366O17.3	13:43317927-66491226	13q14.11	[undefined]	0.92263
RP11-168G22.2	13:43317927-66491226	13q21.31	[undefined]	0.92263
PRR20C	13:43317927-66491226	13q21.1	proline rich 20C [Source:HGNC Symbol;Acc:37221]	0.92263
RP11-214O11.2	13:43317927-66491226	13q14.2	[undefined]	0.92263
RP11-460G11.1	13:43317927-66491226	13q14.11	[undefined]	0.92263
RP11-71L7.1	13:43317927-66491226	13q21.31	[undefined]	0.92263
RP11-168G22.1	13:43317927-66491226	13q21.31	[undefined]	0.92263
RP11-550E22.5	13:43317927-66491226	13q14.3	[undefined]	0.92263
RP11-547C18.2	13:43317927-66491226	13q14.3	[undefined]	0.92263
RP11-100C24.1	13:43317927-66491226	13q21.1	[undefined]	0.92263
RP11-484D5.2	13:43317927-66491226	13q14.11	[undefined]	0.92263
FKBP1AP3	13:43317927-66491226	13q14.13	FK506 binding protein 1A, 12kDa pseudogene 3 [Source:HGNC Symbol;Acc:3716]	0.92263
RP11-473M10.2	13:43317927-66491226	13q21.31	[undefined]	0.92263
RP11-478K15.5	13:43317927-66491226	13q14.11	[undefined]	0.92263
DLEU2	13:43317927-66491226	13q14.2	deleted in lymphocytic leukemia 2 (non-protein coding) [Source:HGNC Symbol;Acc:13748]	0.92263
RP11-366O17.2	13:43317927-66491226	13q14.11	[undefined]	0.92263
RP11-189B4.6	13:43317927-66491226	13q14.13	[undefined]	0.92263
RP11-305D15.2	13:43317927-66491226	13q14.2	[undefined]	0.92263
RP11-34F20.5	13:43317927-66491226	13q14.2	[undefined]	0.92263
RP11-203I16.3	13:43317927-66491226	13q14.2	[undefined]	0.92263
RP11-480P3.1	13:43317927-66491226	13q14.2	[undefined]	0.92263
RP11-327P2.5	13:43317927-66491226	13q14.3	[undefined]	0.92263
RP11-105A24.1	13:43317927-66491226	13q21.2	[undefined]	0.92263
RP11-168G22.4	13:43317927-66491226	13q21.31	[undefined]	0.92263
RP11-327P2.3	13:43317927-66491226	13q14.3	[undefined]	0.92263
RP11-64J21.2	13:43317927-66491226	13q14.11	[undefined]	0.92263
RP11-165D7.3	13:43317927-66491226	13q14.2	[undefined]	0.92263
RP11-351K3.4	13:43317927-66491226	13q14.13	[undefined]	0.92263
RP11-165D7.4	13:43317927-66491226	13q14.2	[undefined]	0.92263
RP11-538C21.1	13:43317927-66491226	13q21.1	[undefined]	0.92263
RP11-65B7.1	13:43317927-66491226	13q14.11	[undefined]	0.92263
AL162377.2	13:43317927-66491226	13q14.3	Putative uncharacterized protein ENSP00000391598 Fragment [Source:UniProtKB/TrEMBL;Acc:C9JBL8]	0.92263
RP11-429G17.1	13:43317927-66491226	13q21.31	[undefined]	0.92263
RP11-40A8.3	13:43317927-66491226	13q14.3	[undefined]	0.92263
PRR20E	13:43317927-66491226	13q21.1	proline rich 20E [Source:HGNC Symbol;Acc:37223]	0.92263
RP11-191J12.1	13:43317927-66491226	13q14.11	[undefined]	0.92263
RP11-94N9.1	13:43317927-66491226	13q14.2	[undefined]	0.92263
RP11-214O11.1	13:43317927-66491226	13q14.2	[undefined]	0.92263
RP11-223F20.2	13:43317927-66491226	13q21.32	[undefined]	0.92263
RP11-640E11.1	13:43317927-66491226	13q21.1	[undefined]	0.92263
RP11-96M12.1	13:43317927-66491226	13q14.12	[undefined]	0.92263
RP11-279N8.2	13:43317927-66491226	13q14.2	[undefined]	0.92263
RP11-154C3.2	13:43317927-66491226	13q14.13	[undefined]	0.92263
RP11-168P6.1	13:43317927-66491226	13q14.3	[undefined]	0.92263
RP11-139H14.3	13:43317927-66491226	13q14.13	[undefined]	0.92263
RP11-478K15.3	13:43317927-66491226	13q14.11	[undefined]	0.92263
RP11-381L18.2	13:43317927-66491226	13q14.3	[undefined]	0.92263
RP11-78J21.3	13:43317927-66491226	13q14.3	[undefined]	0.92263
RP11-185C18.4	13:43317927-66491226	13q14.2	[undefined]	0.92263
COX7CP1	13:43317927-66491226	13q14.2	cytochrome c oxidase subunit VIIc pseudogene 1 [Source:HGNC Symbol;Acc:2293]	0.92263
RP11-550E22.3	13:43317927-66491226	13q14.3	[undefined]	0.92263
RP11-223F20.1	13:43317927-66491226	13q21.32	[undefined]	0.92263
RP11-233H19.2	13:43317927-66491226	13q14.3	[undefined]	0.92263
RP11-521H3.2	13:43317927-66491226	13q21.2	[undefined]	0.92263
RP11-473M10.3	13:43317927-66491226	13q21.31	[undefined]	0.92263
RP11-24H2.2	13:43317927-66491226	13q14.3	[undefined]	0.92263
NFYAP1	13:43317927-66491226	13q21.31	nuclear transcription factor Y, alpha pseudogene 1 [Source:HGNC Symbol;Acc:34078]	0.92263
RP11-269C23.3	13:43317927-66491226	13q14.11	[undefined]	0.92263
RP11-145J3.3	13:43317927-66491226	13q14.11	[undefined]	0.92263
RP11-442J21.1	13:43317927-66491226	13q14.12	[undefined]	0.92263
RP11-64J21.3	13:43317927-66491226	13q14.11	[undefined]	0.92263
RP11-154C3.3	13:43317927-66491226	13q14.13	[undefined]	0.92263
AL390375.1	13:43317927-66491226	13q21.1	[undefined]	0.92263
RP11-305D15.6	13:43317927-66491226	13q14.2	[undefined]	0.92263
AL356107.1	13:43317927-66491226	13q14.12	[undefined]	0.92263
RP11-359P14.2	13:43317927-66491226	13q21.2	[undefined]	0.92263
RP11-64P12.3	13:43317927-66491226	13q14.3	[undefined]	0.92263
AL137141.1	13:43317927-66491226	13q14.13	[undefined]	0.92263

Continued on next page...

gene name	chr	.DNABand	.GeneDesc	rank
AL136960.1	13:43317927-66491226	13q14.2	[undefined]	0.92263
AL356241.1	13:43317927-66491226	13q21.2	[undefined]	0.92263
AL137141.2	13:43317927-66491226	13q14.13	[undefined]	0.92263
AL160397.1	13:43317927-66491226	13q14.2	[undefined]	0.92263
AL136359.1	13:43317927-66491226	13q14.3	[undefined]	0.92263
AL355136.1	13:43317927-66491226	13q21.1	[undefined]	0.92263
AL137880.2	13:43317927-66491226	13q14.3	[undefined]	0.92263
RP11-189B4.1	13:43317927-66491226	13q14.13	[undefined]	0.92263
AL139183.1	13:43317927-66491226	13q14.3	[undefined]	0.92263
AL160397.2	13:43317927-66491226	13q14.2	[undefined]	0.92263
RP11-64P12.2	13:43317927-66491226	13q14.3	[undefined]	0.92263
AL590102.1	13:43317927-66491226	13q21.2	[undefined]	0.92263
AL354820.1	13:43317927-66491226	13q14.3	[undefined]	0.92263
RP11-189B4.5	13:43317927-66491226	13q14.13	[undefined]	0.92263
AL359180.1	13:43317927-66491226	13q14.2	[undefined]	0.92263
AL139320.1	13:43317927-66491226	13q14.13	[undefined]	0.92263
RP11-290D2.4	13:43317927-66491226	13q14.13	[undefined]	0.92263
RP11-78J21.4	13:43317927-66491226	13q14.3	[undefined]	0.92263
AL162377.1	13:43317927-66491226	13q14.3	[undefined]	0.92263
XXyac-R12DG2.1	13:43317927-66491226	13q14.13	[undefined]	0.92263
AL356107.2	13:43317927-66491226	13q14.11	[undefined]	0.92263
AL139082.1	13:43317927-66491226	13q14.3	[undefined]	0.92263
AL354820.3	13:43317927-66491226	13q14.3	integrator complex subunit 6 isoform c [Source:RefSeq peptide;Acc:NP_001035027]	0.92263
AL627107.1	13:43317927-66491226	13q14.13	[undefined]	0.92263
RP11-78J21.2	13:43317927-66491226	13q14.3	[undefined]	0.92263
AL139089.1	13:43317927-66491226	13q14.3	[undefined]	0.92263
XXyac-YMR12DG2.1	13:43317927-66491226	13q14.13	[undefined]	0.92263
RP11-248G5.3	13:43317927-66491226	13q14.3	[undefined]	0.92263
AL138693.2	13:43317927-66491226	13q14.12	[undefined]	0.92263
AL627107.2	13:43317927-66491226	13q14.13	[undefined]	0.92263
AL138686.1	13:43317927-66491226	13q14.13	[undefined]	0.92263
RP11-247M1.2	13:43317927-66491226	13q14.13	[undefined]	0.92263
AL627344.2	13:43317927-66491226	13q21.2	[undefined]	0.92263
AL354820.2	13:43317927-66491226	13q14.3	[undefined]	0.92263
AL137141.3	13:43317927-66491226	13q14.13	[undefined]	0.92263
AL138963.2	13:43317927-66491226	13q14.13	[undefined]	0.92263
ZMYND11	10:125544-38642259	10p15.3	zinc finger, MYND domain containing 11 [Source:HGNC Symbol;Acc:16966]	0.89874
VIM	10:125544-38642259	10p13	vimentin [Source:HGNC Symbol;Acc:12692]	0.89874
WDR37	10:125544-38642259	10p15.3	WD repeat domain 37 [Source:HGNC Symbol;Acc:31406]	0.89874
CUGBP2	10:125544-38642259	10p14	CUGBP, Elav-like family member 2 [Source:HGNC Symbol;Acc:2550]	0.89874
GDI2	10:125544-38642259	10p15.1	GDP dissociation inhibitor 2 [Source:HGNC Symbol;Acc:4227]	0.89874
MCM10	10:125544-38642259	10p13	minichromosome maintenance complex component 10 [Source:HGNC Symbol;Acc:18043]	0.89874
SEC61A2	10:125544-38642259	10p13 10p14	Sec61 alpha 2 subunit (S. cerevisiae) [Source:HGNC Symbol;Acc:17702]	0.89874
PRKCQ	10:125544-38642259	10p14 10p15.1	protein kinase C, theta [Source:HGNC Symbol;Acc:9410]	0.89874
FAM107B	10:125544-38642259	10p13	family with sequence similarity 107, member B [Source:HGNC Symbol;Acc:23726]	0.89874
PFKP	10:125544-38642259	10p15.2	phosphofructokinase, platelet [Source:HGNC Symbol;Acc:8878]	0.89874
IDI1	10:125544-38642259	10p15.3	isopentenyl-diphosphate delta isomerase 1 [Source:HGNC Symbol;Acc:5387]	0.89874
KLF6	10:125544-38642259	10p15.1	Kruppel-like factor 6 [Source:HGNC Symbol;Acc:2235]	0.89874
ZNF37A	10:125544-38642259	10p11.1	zinc finger protein 37A [Source:HGNC Symbol;Acc:13102]	0.89874
SPAG6	10:125544-38642259	10p12.2	sperm associated antigen 6 [Source:HGNC Symbol;Acc:11215]	0.89874
APBB1IP	10:125544-38642259	10p12.1	amyloid beta (A4) precursor protein-binding, family B, member 1 interacting protein [Source:HGNC Symbol;Acc:17379]	0.89874
ITGA8	10:125544-38642259	10p13	integrin, alpha 8 [Source:HGNC Symbol;Acc:6144]	0.89874
NEBL	10:125544-38642259	10p12.31	nebulette [Source:HGNC Symbol;Acc:16932]	0.89874
MLLT10	10:125544-38642259	10p12.31	myeloid/lymphoid or mixed-lineage leukemia (trithorax homolog, Drosophila); translocated to, 10 [Source:HGNC Symbol;Acc:16063]	0.89874
SEPHS1	10:125544-38642259	10p13	selenophosphate synthetase 1 [Source:HGNC Symbol;Acc:19685]	0.89874
BAMBI	10:125544-38642259	10p12.1	BMP and activin membrane-bound inhibitor homolog (Xenopus laevis) [Source:HGNC Symbol;Acc:30251]	0.89874
MYO3A	10:125544-38642259	10p12.1	myosin IIIA [Source:HGNC Symbol;Acc:7601]	0.89874
WAC	10:125544-38642259	10p12.1	WW domain containing adaptor with coiled-coil [Source:HGNC Symbol;Acc:17327]	0.89874
CREM	10:125544-38642259	10p11.21	cAMP responsive element modulator [Source:HGNC Symbol;Acc:2352]	0.89874
RAB18	10:125544-38642259	10p12.1	RAB18, member RAS oncogene family [Source:HGNC Symbol;Acc:14244]	0.89874
NRP1	10:125544-38642259	10p11.22	neuropilin 1 [Source:HGNC Symbol;Acc:8004]	0.89874
PRTFDC1	10:125544-38642259	10p12.1	phosphoribosyl transferase domain containing 1 [Source:HGNC Symbol;Acc:23333]	0.89874
GATA3	10:125544-38642259	10p14	GATA binding protein 3 [Source:HGNC Symbol;Acc:4172]	0.89874
PHYH	10:125544-38642259	10p13	phytanoyl-CoA 2-hydroxylase [Source:HGNC Symbol;Acc:8940]	0.89874
CUBN	10:125544-38642259	10p13	cubilin (intrinsic factor-cobalamin receptor) [Source:HGNC Symbol;Acc:2548]	0.89874
TRDMT1	10:125544-38642259	10p13	tRNA aspartic acid methyltransferase 1 [Source:HGNC Symbol;Acc:2977]	0.89874
ARHGAP21	10:125544-38642259	10p12.1	Rho GTPase activating protein 21 [Source:HGNC Symbol;Acc:23725]	0.89874
ANKRD26	10:125544-38642259	10p12.1	ankyrin repeat domain 26 [Source:HGNC Symbol;Acc:29186]	0.89874
ACBD5	10:125544-38642259	10p12.1	acyl-Coenzyme A binding domain containing 5 [Source:HGNC Symbol;Acc:23338]	0.89874
LARP4B	10:125544-38642259	10p15.3	La ribonucleoprotein domain family, member 4B [Source:HGNC Symbol;Acc:28987]	0.89874
GTPBP4	10:125544-38642259	10p15.3	GTP binding protein 4 [Source:HGNC Symbol;Acc:21535]	0.89874
MTPAP	10:125544-38642259	10p11.23	mitochondrial poly(A) polymerase [Source:HGNC Symbol;Acc:25532]	0.89874
PITRM1	10:125544-38642259	10p15.2	pitrilysin metallopeptidase 1 [Source:HGNC Symbol;Acc:17663]	0.89874
MAP3K8	10:125544-38642259	10p11.23	mitogen-activated protein kinase kinase kinase 8 [Source:HGNC Symbol;Acc:6860]	0.89874
C10orf18	10:125544-38642259	10p15.1	Uncharacterized protein C10orf18 [Source:UniProtKB/Swiss-Prot;Acc:Q5VWN6]	0.89874
CUL2	10:125544-38642259	10p11.21	cullin 2 [Source:HGNC Symbol;Acc:2552]	0.89874
CCNY	10:125544-38642259	10p11.21	cyclin Y [Source:HGNC Symbol;Acc:23354]	0.89874
MASTL	10:125544-38642259	10p12.1	microtubule associated serine/threonine kinase-like [Source:HGNC Symbol;Acc:19042]	0.89874
KIAA1217	10:125544-38642259	10p12.1 10p12.2	KIAA1217 [Source:HGNC Symbol;Acc:25428]	0.89874
LYZL1	10:125544-38642259	10p11.23 10p12.1	lysozyme-like 1 [Source:HGNC Symbol;Acc:30502]	0.89874
MRC1	10:125544-38642259	10p12.33	mannose receptor, C type 1 [Source:HGNC Symbol;Acc:7228]	0.89874
PLXDC2	10:125544-38642259	10p12.31	plexin domain containing 2 [Source:HGNC Symbol;Acc:21013]	0.89874
EPC1	10:125544-38642259	10p11.22	enhancer of polycomb homolog 1 (Drosophila) [Source:HGNC Symbol;Acc:19876]	0.89874
OPTN	10:125544-38642259	10p13	optineurin [Source:HGNC Symbol;Acc:17142]	0.89874
ITIH5	10:125544-38642259	10p14	inter-alpha (globulin) inhibitor H5 [Source:HGNC Symbol;Acc:21449]	0.89874
FBXO18	10:125544-38642259	10p15.1	F-box protein, helicase, 18 [Source:HGNC Symbol;Acc:13620]	0.89874
RBM17	10:125544-38642259	10p15.1	RNA binding motif protein 17 [Source:HGNC Symbol;Acc:16944]	0.89874
IL2RA	10:125544-38642259	10p15.1	interleukin 2 receptor, alpha [Source:HGNC Symbol;Acc:6008]	0.89874
ANKRD16	10:125544-38642259	10p15.1	ankyrin repeat domain 16 [Source:HGNC Symbol;Acc:23471]	0.89874
ECHDC3	10:125544-38642259	10p14	enoyl Coenzyme A hydratase domain containing 3 [Source:HGNC Symbol;Acc:23489]	0.89874
IL15RA	10:125544-38642259	10p15.1	interleukin 15 receptor, alpha [Source:HGNC Symbol;Acc:5978]	0.89874
STAM	10:125544-38642259	10p12.33	signal transducing adaptor molecule (SH3 domain and ITAM motif) 1 [Source:HGNC Symbol;Acc:11357]	0.89874
GAD2	10:125544-38642259	10p12.1	glutamate decarboxylase 2 (pancreatic islets and brain, 65kDa) [Source:HGNC Symbol;Acc:4093]	0.89874
ABI1	10:125544-38642259	10p12.1	abl-interactor 1 [Source:HGNC Symbol;Acc:11320]	0.89874
YME1L1	10:125544-38642259	10p12.1	YME1-like 1 (S. cerevisiae) [Source:HGNC Symbol;Acc:12843]	0.89874
DNAJC1	10:125544-38642259	10p12.31	DnaJ (Hsp40) homolog, subfamily C, member 1 [Source:HGNC Symbol;Acc:20090]	0.89874
IDI2	10:125544-38642259	10p15.3	isopentenyl-diphosphate delta isomerase 2 [Source:HGNC Symbol;Acc:23487]	0.89874
C10orf47	10:125544-38642259	10p14	Uncharacterized protein C10orf47 [Source:UniProtKB/Swiss-Prot;Acc:Q86WR7]	0.89874
USP6NL	10:125544-38642259	10p14	USP6 N-terminal like [Source:HGNC Symbol;Acc:16858]	0.89874
COMMD3	10:125544-38642259	10p12.2	COMM domain containing 3 [Source:HGNC Symbol;Acc:23332]	0.89874
MSRB2	10:125544-38642259	10p12.2	methionine sulfoxide reductase B2 [Source:HGNC Symbol;Acc:17061]	0.89874
PDSS1	10:125544-38642259	10p12.1	prenyl (decaprenyl) diphosphate synthase, subunit 1 [Source:HGNC Symbol;Acc:17759]	0.89874
FAM171A1	10:125544-38642259	10p13	family with sequence similarity 171, member A1 [Source:HGNC Symbol;Acc:23522]	0.89874
FAM188A	10:125544-38642259	10p13	family with sequence similarity 188, member A [Source:HGNC Symbol;Acc:23578]	0.89874
SLC39A12	10:125544-38642259	10p12.33	solute carrier family 39 (zinc transporter), member 12 [Source:HGNC Symbol;Acc:20860]	0.89874
FAM23A	10:125544-38642259	10p12.33	family with sequence similarity 23, member A [Source:HGNC Symbol;Acc:23473]	0.89874
RSU1	10:125544-38642259	10p13	Ras suppressor protein 1 [Source:HGNC Symbol;Acc:10464]	0.89874
ST8SIA6	10:125544-38642259	10p12.33	ST8 alpha-N-acetyl-neuraminide alpha-2,8-sialyltransferase 6 [Source:HGNC Symbol;Acc:23317]	0.89874
PARD3	10:125544-38642259	10p11.21 10p11.22	par-3 partitioning defective 3 homolog (C. elegans) [Source:HGNC Symbol;Acc:16051]	0.89874
ANKRD30A	10:125544-38642259	10p11.21	ankyrin repeat domain 30A [Source:HGNC Symbol;Acc:17234]	0.89874
ZEB1	10:125544-38642259	10p11.22	zinc finger E-box binding homeobox 1 [Source:HGNC Symbol;Acc:11642]	0.89874
MKX	10:125544-38642259	10p12.1	mohawk homeobox [Source:HGNC Symbol;Acc:23729]	0.89874
MPP7	10:125544-38642259	10p12.1	membrane protein, palmitoylated 7 (MAGUK p55 subfamily member 7) [Source:HGNC Symbol;Acc:26542]	0.89874
C10orf68	10:125544-38642259	10p11.22	Uncharacterized protein C10orf68 [Source:UniProtKB/Swiss-Prot;Acc:Q9H943]	0.89874
ITGB1	10:125544-38642259	10p11.22	integrin, beta 1 (fibronectin receptor, beta polypeptide, antigen CD29 includes MDF2, MSK12) [Source:HGNC Symbol;Acc:6153]	0.89874
PIP5K2A	10:125544-38642259	10p12.2	phosphatidylinositol-5-phosphate 4-kinase, type II, alpha [Source:HGNC Symbol;Acc:8997]	0.89874

Continued on next page...

gene name	chr	.DNABand	.GeneDesc	rank
ENKUR	10:125544-38642259	10p12.1	enkurin, TRPC channel interacting protein [Source:HGNC Symbol;Acc:28388]	0.89874
GPR158	10:125544-38642259	10p12.1	G protein-coupled receptor 158 [Source:HGNC Symbol;Acc:23689]	0.89874
LYZL2	10:125544-38642259	10p11.23	lysozyme-like 2 [Source:HGNC Symbol;Acc:29613]	0.89874
DIP2C	10:125544-38642259	10p15.3	DIP2 disco-interacting protein 2 homolog C (Drosophila) [Source:HGNC Symbol;Acc:29150]	0.89874
UPF2	10:125544-38642259	10p14	UPF2 regulator of nonsense transcripts homolog (yeast) [Source:HGNC Symbol;Acc:17854]	0.89874
CDC123	10:125544-38642259	10p13	cell division cycle 123 homolog (S. cerevisiae) [Source:HGNC Symbol;Acc:16827]	0.89874
CCDC3	10:125544-38642259	10p13	coiled-coil domain containing 3 [Source:HGNC Symbol;Acc:23813]	0.89874
FRMD4A	10:125544-38642259	10p13	FERM domain containing 4A [Source:HGNC Symbol;Acc:25491]	0.89874
RP11-397P14.3	10:125544-38642259	10p15.1	aldo-keto reductase, truncated (tAKR), non-coding RNA [Source:RefSeq DNA;Acc:NR_026743]	0.89874
AKR1C2	10:125544-38642259	10p15.1	aldo-keto reductase family 1, member C2 (dihydrodiol dehydrogenase 2; bile acid binding protein; 3-alpha hydroxysteroid dehydrogenase, type III) [Source:HGNC Symbol;Acc:385]	0.89874
ITIH2	10:125544-38642259	10p14	inter-alpha (globulin) inhibitor H2 [Source:HGNC Symbol;Acc:6167]	0.89874
KIN	10:125544-38642259	10p14	KIN, antigenic determinant of recA protein homolog (mouse) [Source:HGNC Symbol;Acc:6327]	0.89874
RP11-775A3.1	10:125544-38642259	10p11.1	[undefined]	0.89874
SUV39H2	10:125544-38642259	10p13	suppressor of variegation 3-9 homolog 2 (Drosophila) [Source:HGNC Symbol;Acc:17287]	0.89874
DCLRE1C	10:125544-38642259	10p13	DNA cross-link repair 1C (PSO2 homolog, S. cerevisiae) [Source:HGNC Symbol;Acc:17642]	0.89874
OLAH	10:125544-38642259	10p13	oleoyl-ACP hydrolase [Source:HGNC Symbol;Acc:25625]	0.89874
RPP38	10:125544-38642259	10p13	ribonuclease P/MRP 38kDa subunit [Source:HGNC Symbol;Acc:30329]	0.89874
NMT2	10:125544-38642259	10p13	N-myristoyltransferase 2 [Source:HGNC Symbol;Acc:7858]	0.89874
RP11-139J15.2	10:125544-38642259	10p12.31	[undefined]	0.89874
ARMC3	10:125544-38642259	10p12.2	armadillo repeat containing 3 [Source:HGNC Symbol;Acc:30964]	0.89874
OTUD1	10:125544-38642259	10p12.2	OTU domain containing 1 [Source:HGNC Symbol;Acc:27346]	0.89874
ARHGAP12	10:125544-38642259	10p11.22	Rho GTPase activating protein 12 [Source:HGNC Symbol;Acc:16348]	0.89874
AKR1E2	10:125544-38642259	10p15.1	aldo-keto reductase family 1, member E2 [Source:HGNC Symbol;Acc:23437]	0.89874
NUDT5	10:125544-38642259	10p13	nudix (nucleoside diphosphate linked moiety X)-type motif 5 [Source:HGNC Symbol;Acc:8052]	0.89874
UCMA	10:125544-38642259	10p13	upper zone of growth plate and cartilage matrix associated [Source:HGNC Symbol;Acc:25205]	0.89874
BEND7	10:125544-38642259	10p13	BEN domain containing 7 [Source:HGNC Symbol;Acc:23514]	0.89874
ATP5C1	10:125544-38642259	10p14	ATP synthase, H+ transporting, mitochondrial F1 complex, gamma polypeptide 1 [Source:HGNC Symbol;Acc:833]	0.89874
PRPF18	10:125544-38642259	10p13	PRP18 pre-mRNA processing factor 18 homolog (S. cerevisiae) [Source:HGNC Symbol;Acc:17351]	0.89874
TAF3	10:125544-38642259	10p14	TAF3 RNA polymerase II, TATA box binding protein (TBP)-associated factor, 140kDa [Source:HGNC Symbol;Acc:17303]	0.89874
KIAA1462	10:125544-38642259	10p11.23	KIAA1462 [Source:HGNC Symbol;Acc:29283]	0.89874
PTER	10:125544-38642259	10p13	phosphotriesterase related [Source:HGNC Symbol;Acc:9590]	0.89874
C1QL3	10:125544-38642259	10p13	complement component 1, q subcomponent-like 3 [Source:HGNC Symbol;Acc:19359]	0.89874
CACNB2	10:125544-38642259	10p12.31 10p12.32 10p12.33	calcium channel, voltage-dependent, beta 2 subunit [Source:HGNC Symbol;Acc:1402]	0.89874
PTPLA	10:125544-38642259	10p12.33	protein tyrosine phosphatase-like (proline instead of catalytic arginine), member A [Source:HGNC Symbol;Acc:9639]	0.89874
ARL5B	10:125544-38642259	10p12.31	ADP-ribosylation factor-like 5B [Source:HGNC Symbol;Acc:23052]	0.89874
PTF1A	10:125544-38642259	10p12.2	pancreas specific transcription factor, 1a [Source:HGNC Symbol;Acc:23734]	0.89874
BMI1	10:125544-38642259	10p12.2	BMI1 polycomb ring finger oncogene [Source:HGNC Symbol;Acc:1066]	0.89874
ARMC4	10:125544-38642259	10p12.1	armadillo repeat containing 4 [Source:HGNC Symbol;Acc:25583]	0.89874
PFKFB3	10:125544-38642259	10p15.1	6-phosphofructo-2-kinase/fructose-2,6-biphosphatase 3 [Source:HGNC Symbol;Acc:8874]	0.89874
KIF5B	10:125544-38642259	10p11.22	kinesin family member 5B [Source:HGNC Symbol;Acc:6324]	0.89874
NET1	10:125544-38642259	10p15.1	neuroepithelial cell transforming 1 [Source:HGNC Symbol;Acc:14592]	0.89874
ZNF25	10:125544-38642259	10p11.1	zinc finger protein 25 [Source:HGNC Symbol;Acc:13043]	0.89874
C10orf111	10:125544-38642259	10p13	Uncharacterized protein C10orf111 [Source:UniProtKB/Swiss-Prot;Acc:Q8N326]	0.89874
ACBD7	10:125544-38642259	10p13	acyl-Coenzyme A binding domain containing 7 [Source:HGNC Symbol;Acc:17715]	0.89874
FZD8	10:125544-38642259	10p11.21	frizzled homolog 8 (Drosophila) [Source:HGNC Symbol;Acc:4046]	0.89874
GJD4	10:125544-38642259	10p11.21	gap junction protein, delta 4, 40.1kDa [Source:HGNC Symbol;Acc:23296]	0.89874
CALML3	10:125544-38642259	10p15.1	calmodulin-like 3 [Source:HGNC Symbol;Acc:1452]	0.89874
CALML5	10:125544-38642259	10p15.1	calmodulin-like 5 [Source:HGNC Symbol;Acc:18180]	0.89874
TUBAL3	10:125544-38642259	10p15.1	tubulin, alpha-like 3 [Source:HGNC Symbol;Acc:23534]	0.89874
UCN3	10:125544-38642259	10p15.1	urocortin 3 (stresscopin) [Source:HGNC Symbol;Acc:17781]	0.89874
C10orf67	10:125544-38642259	10p12.2	Uncharacterized protein C10orf67 [Source:UniProtKB/Swiss-Prot;Acc:Q8IYJ2]	0.89874
C10orf108	10:125544-38642259	10p15.3	Putative uncharacterized protein C10orf108 [Source:UniProtKB/Swiss-Prot;Acc:Q8N8Z3]	0.89874
C10orf140	10:125544-38642259	10p12.31	Protein DLN-1 [Source:UniProtKB/Swiss-Prot;Acc:Q1XH10]	0.89874
DHTKD1	10:125544-38642259	10p14	dehydrogenase E1 and transketolase domain containing 1 [Source:HGNC Symbol;Acc:23537]	0.89874
AL512631.1	10:125544-38642259	10p14	Putative uncharacterized protein LOC439951 [Source:UniProtKB/Swiss-Prot;Acc:Q8NDZ9]	0.89874
C10orf31	10:125544-38642259	10p14	Putative uncharacterized protein C10orf31 Precursor [Source:UniProtKB/Swiss-Prot;Acc:Q8N7Q2]	0.89874
PTCHD3	10:125544-38642259	10p12.1	patched domain containing 3 [Source:HGNC Symbol;Acc:24776]	0.89874
RP11-271M1.1	10:125544-38642259	10p13	[undefined]	0.89874
CAMK1D	10:125544-38642259	10p13	calcium/calmodulin-dependent protein kinase ID [Source:HGNC Symbol;Acc:19341]	0.89874
ZNF438	10:125544-38642259	10p11.22 10p11.23	zinc finger protein 438 [Source:HGNC Symbol;Acc:21029]	0.89874
MRC1L1	10:125544-38642259	10p12.33	mannose receptor, C type 1-like 1 [Source:HGNC Symbol;Acc:23403]	0.89874
FAM23B	10:125544-38642259	10p12.33	family with sequence similarity 23, member B [Source:HGNC Symbol;Acc:23474]	0.89874
CDNF	10:125544-38642259	10p13	cerebral dopamine neurotrophic factor [Source:HGNC Symbol;Acc:24913]	0.89874
ADARB2	10:125544-38642259	10p15.3	adenosine deaminase, RNA-specific, B2 (RED2 homolog rat) [Source:HGNC Symbol;Acc:227]	0.89874
THNSL1	10:125544-38642259	10p12.1	threonine synthase-like 1 (S. cerevisiae) [Source:HGNC Symbol;Acc:26160]	0.89874
AKR1C1	10:125544-38642259	10p15.1	aldo-keto reductase family 1, member C1 (dihydrodiol dehydrogenase 1; 20-alpha (3-alpha)-hydroxysteroid dehydrogenase) [Source:HGNC Symbol;Acc:384]	0.89874
HSPA14	10:125544-38642259	10p13	heat shock 70kDa protein 14 [Source:HGNC Symbol;Acc:29526]	0.89874
ZNF33A	10:125544-38642259	10p11.1	zinc finger protein 33A [Source:HGNC Symbol;Acc:13096]	0.89874
AKR1C3	10:125544-38642259	10p15.1	aldo-keto reductase family 1, member C3 (3-alpha hydroxysteroid dehydrogenase, type II) [Source:HGNC Symbol;Acc:386]	0.89874
AKR1CL1	10:125544-38642259	10p15.1	aldo-keto reductase family 1, member C-like 1 [Source:HGNC Symbol;Acc:23469]	0.89874
ASB13	10:125544-38642259	10p15.1	ankyrin repeat and SOCS box-containing 13 [Source:HGNC Symbol;Acc:19765]	0.89874
AL117340.3	10:125544-38642259	10p11.22	Putative UPF0607 protein FLJ37424 [Source:UniProtKB/Swiss-Prot;Acc:Q8N9G6]	0.89874
RP11-379F12.7	10:125544-38642259	10p14	[undefined]	0.89874
SVIL	10:125544-38642259	10p11.23	supervillin [Source:HGNC Symbol;Acc:11480]	0.89874
MEIG1	10:125544-38642259	10p13	meiosis expressed gene 1 homolog (mouse) [Source:HGNC Symbol;Acc:23429]	0.89874
ZNF248	10:125544-38642259	10p11.1	zinc finger protein 248 [Source:HGNC Symbol;Acc:13041]	0.89874
AKR1C4	10:125544-38642259	10p15.1	aldo-keto reductase family 1, member C4 (chlordecone reductase; 3-alpha hydroxysteroid dehydrogenase, type I; dihydrodiol dehydrogenase 4) [Source:HGNC Symbol;Acc:387]	0.89874
SFMBT2	10:125544-38642259	10p14	Scm-like with four mbt domains 2 [Source:HGNC Symbol;Acc:20256]	0.89874
AL390766.2	10:125544-38642259	10p11.21	[undefined]	0.89874
AL358780.2	10:125544-38642259	10p12.31	[undefined]	0.89874
AL390248.1	10:125544-38642259	10p12.1	[undefined]	0.89874
AL157392.2	10:125544-38642259	10p13	[undefined]	0.89874
AL392104.1	10:125544-38642259	10p12.1	[undefined]	0.89874
AL160287.1	10:125544-38642259	10p12.1	[undefined]	0.89874
AL162272.1	10:125544-38642259	10p12.1	[undefined]	0.89874
AL117336.1	10:125544-38642259	10p11.21	[undefined]	0.89874
AC073160.2	10:125544-38642259	10p14	[undefined]	0.89874
AL355493.3	10:125544-38642259	10p12.1	[undefined]	0.89874
AL391839.2	10:125544-38642259	10p11.22	[undefined]	0.89874
AL157895.1	10:125544-38642259	10p12.31	[undefined]	0.89874
AL354916.1	10:125544-38642259	10p14	[undefined]	0.89874
AL353796.1	10:125544-38642259	10p11.23	[undefined]	0.89874
AL355376.1	10:125544-38642259	10p12.1	[undefined]	0.89874
AL355339.1	10:125544-38642259	10p13	[undefined]	0.89874
AL445431.1	10:125544-38642259	10p12.31	[undefined]	0.89874
AL161799.1	10:125544-38642259	10p12.31	[undefined]	0.89874
AL157395.1	10:125544-38642259	10p15.1	[undefined]	0.89874
AL158168.1	10:125544-38642259	10p13	[undefined]	0.89874
AL603831.2	10:125544-38642259	10p15.3	[undefined]	0.89874
AL590677.1	10:125544-38642259	10p13	[undefined]	0.89874
RP11-730A19.6	10:125544-38642259	10p13	[undefined]	0.89874
AL359697.1	10:125544-38642259	10p12.31	[undefined]	0.89874
RP13-259N13.2	10:125544-38642259	10p11.22	[undefined]	0.89874
C10orf126	10:125544-38642259	10p12.1	Putative uncharacterized protein C10orf126 [Source:UniProtKB/Swiss-Prot;Acc:Q8N4M7]	0.89874
C10orf115	10:125544-38642259	10p12.2	Putative uncharacterized protein C10orf115 [Source:UniProtKB/Swiss-Prot;Acc:Q5QP74]	0.89874
C10orf114	10:125544-38642259	10p12.31	Putative uncharacterized protein C10orf114 [Source:UniProtKB/Swiss-Prot;Acc:Q5T4H9]	0.89874
C10orf113	10:125544-38642259	10p12.31	Putative uncharacterized protein C10orf113 [Source:UniProtKB/Swiss-Prot;Acc:Q5VZT2]	0.89874
C10orf112	10:125544-38642259	10p12.31	MAM and LDL-receptor class A domain-containing protein C10orf112 [Source:UniProtKB/Swiss-Prot;Acc:Q5VYJ5]	0.89874
RP11-414K1.3	10:125544-38642259	10p12.33	[undefined]	0.89874
AL732437.1	10:125544-38642259	10p15.1	cDNA FLJ46692 fis, clone TRACH3012718 [Source:UniProtKB/TrEMBL;Acc:Q6ZR35]	0.89874
NCRNA00168	10:125544-38642259	10p15.3	non-protein coding RNA 168 [Source:HGNC Symbol;Acc:23299]	0.89874
AL359878.2	10:125544-38642259	10p15.3	cDNA FLJ27363 fis, clone UBA02185 [Source:UniProtKB/TrEMBL;Acc:Q6ZNQ2]	0.89874
RP11-455B2.8	10:125544-38642259	10p13	[undefined]	0.89874

Continued on next page...

gene name	chr	.DNABand	.GeneDesc	rank
AL139404.1	10:125544-38642259	10p12.1	[undefined]	0.89874
AL161932.1	10:125544-38642259	10p11.22	[undefined]	0.89874
AL357148.1	10:125544-38642259	10p11.21	[undefined]	0.89874
AL117337.1	10:125544-38642259	10p11.1	[undefined]	0.89874
AL359375.1	10:125544-38642259	10p12.2	[undefined]	0.89874
AC022535.1	10:125544-38642259	10p15.1	[undefined]	0.89874
AL355493.1	10:125544-38642259	10p12.1	[undefined]	0.89874
RNU6-15	10:125544-38642259	10p12.31	RNA, U6 small nuclear 15 [Source:HGNC Symbol;Acc:34259]	0.89874
AL354660.1	10:125544-38642259	10p11.21	[undefined]	0.89874
AL358780.4	10:125544-38642259	10p12.31	[undefined]	0.89874
RNU6-2	10:125544-38642259	10p13	RNA, U6 small nuclear 2 [Source:HGNC Symbol;Acc:10228]	0.89874
AL139125.1	10:125544-38642259	10p14	[undefined]	0.89874
AL161936.1	10:125544-38642259	10p12.1	[undefined]	0.89874
MIR604	10:125544-38642259	10p11.23	microRNA 604 [Source:HGNC Symbol;Acc:32860]	0.89874
MIR603	10:125544-38642259	10p12.2	microRNA 603 [Source:HGNC Symbol;Acc:32859]	0.89874
MIR511-2	10:125544-38642259	10p12.33	microRNA 511-2 [Source:HGNC Symbol;Acc:32078]	0.89874
MIR511-2	10:125544-38642259	10p12.33	microRNA 511-2 [Source:HGNC Symbol;Acc:32078]	0.89874
AL353147.6	10:125544-38642259	10p12.31	[undefined]	0.89874
AL590730.3	10:125544-38642259	10p11.21	[undefined]	0.89874
AL354747.1	10:125544-38642259	10p15.3	[undefined]	0.89874
AL713922.1	10:125544-38642259	10p15.3	[undefined]	0.89874
AL160060.1	10:125544-38642259	10p11.23	[undefined]	0.89874
AL353586.1	10:125544-38642259	10p13	[undefined]	0.89874
AL355333.1	10:125544-38642259	10p14	[undefined]	0.89874
AL137145.2	10:125544-38642259	10p15.1	Putative uncharacterized protein DKFZp667F0711 Fragment [Source:UniProtKB/TrEMBL;Acc:Q8TCQ9]	0.89874
RP11-51B10.4	10:125544-38642259	10p11.21	[undefined]	0.89874
RP11-748L13.3	10:125544-38642259	10p12.1	[undefined]	0.89874
RP11-543F8.1	10:125544-38642259	10p14	[undefined]	0.89874
RP11-449I17.7	10:125544-38642259	10p11.23	[undefined]	0.89874
RP11-414H17.5	10:125544-38642259	10p15.1	[undefined]	0.89874
RP11-414H17.2	10:125544-38642259	10p15.1	[undefined]	0.89874
RP11-449I17.4	10:125544-38642259	10p11.23	[undefined]	0.89874
AC026396.1	10:125544-38642259	10p15.3	Putative uncharacterized protein ENSP00000381534 Fragment [Source:UniProtKB/TrEMBL;Acc:A8MU16]	0.89874
RP11-8L18.2	10:125544-38642259	10p11.21	[undefined]	0.89874
RP11-308K19.2	10:125544-38642259	10p13	[undefined]	0.89874
AL137145.1	10:125544-38642259	10p15.1	cDNA FLJ46311 fis, clone TESTI4040197 [Source:UniProtKB/TrEMBL;Acc:Q6ZRJ4]	0.89874
RP11-445P17.4	10:125544-38642259	10p15.1	[undefined]	0.89874
RP11-748L13.2	10:125544-38642259	10p12.1	[undefined]	0.89874
MIR938	10:125544-38642259	10p11.23	microRNA 938 [Source:HGNC Symbol;Acc:33681]	0.89874
CCDC7	10:125544-38642259	10p11.22	coiled-coil domain containing 7 [Source:HGNC Symbol;Acc:26533]	0.89874
RP11-460H18.1	10:125544-38642259	10p11.22	[undefined]	0.89874
AL161651.3	10:125544-38642259	10p11.23	[undefined]	0.89874
AL512598.1	10:125544-38642259	10p12.1	[undefined]	0.89874
AL139815.1	10:125544-38642259	10p12.2	[undefined]	0.89874
MIR548Q	10:125544-38642259	10p13	microRNA 548q [Source:HGNC Symbol;Acc:37312]	0.89874
MIR1265	10:125544-38642259	10p13	microRNA 1265 [Source:HGNC Symbol;Acc:35332]	0.89874
MIR1915	10:125544-38642259	10p12.31	microRNA 1915 [Source:HGNC Symbol;Acc:35399]	0.89874
AL159170.1	10:125544-38642259	10p11.23	[undefined]	0.89874
AL157392.3	10:125544-38642259	10p13	[undefined]	0.89874
AL391839.3	10:125544-38642259	10p11.22	[undefined]	0.89874
AC022021.2	10:125544-38642259	10p12.1	[undefined]	0.89874
AL353769.1	10:125544-38642259	10p11.22	[undefined]	0.89874
AL161654.1	10:125544-38642259	10p12.1	[undefined]	0.89874
AL136452.1	10:125544-38642259	10p14	[undefined]	0.89874
AC022021.1	10:125544-38642259	10p12.1	[undefined]	0.89874
AC022021.3	10:125544-38642259	10p12.1	[undefined]	0.89874
AL450337.2	10:125544-38642259	10p11.21	[undefined]	0.89874
AL731546.2	10:125544-38642259	10p12.1	[undefined]	0.89874
AL139404.2	10:125544-38642259	10p12.1	[undefined]	0.89874
AL450337.1	10:125544-38642259	10p11.21	[undefined]	0.89874
AL139815.2	10:125544-38642259	10p12.2	[undefined]	0.89874
AL360230.2	10:125544-38642259	10p13	[undefined]	0.89874
RP11-490O24.2	10:125544-38642259	10p11.22	[undefined]	0.89874
RP11-393K12.2	10:125544-38642259	10p14	[undefined]	0.89874
RP11-108B14.3	10:125544-38642259	10p12.31	[undefined]	0.89874
RP11-554I8.2	10:125544-38642259	10p14	[undefined]	0.89874
RP11-428L9.2	10:125544-38642259	10p14	[undefined]	0.89874
RP11-472N13.2	10:125544-38642259	10p11.22	[undefined]	0.89874
AL355493.2	10:125544-38642259	10p12.1	Putative uncharacterized protein ENSP00000390206 Fragment [Source:UniProtKB/TrEMBL;Acc:C9JHR1]	0.89874
RP11-183E9.2	10:125544-38642259	10p12.2	[undefined]	0.89874
RP11-177H22.2	10:125544-38642259	10p12.31	[undefined]	0.89874
RP11-445P17.5	10:125544-38642259	10p15.1	[undefined]	0.89874
RP11-445P17.8	10:125544-38642259	10p15.1	[undefined]	0.89874
RP11-69D4.3	10:125544-38642259	10p15.1	[undefined]	0.89874
AL606469.2	10:125544-38642259	10p12.2	Putative uncharacterized protein ENSP00000408798 Fragment [Source:UniProtKB/TrEMBL;Acc:C9JLN5]	0.89874
RP11-371A19.2	10:125544-38642259	10p12.2	[undefined]	0.89874
RP11-499O7.7	10:125544-38642259	10p15.1	[undefined]	0.89874
AL157831.1	10:125544-38642259	10p12.2 10p12.31	Putative uncharacterized protein ENSP00000413132 Fragment [Source:UniProtKB/TrEMBL;Acc:C9K089]	0.89874
RP11-34C15.1	10:125544-38642259	10p15.1	[undefined]	0.89874
RP11-351M16.1	10:125544-38642259	10p12.1	[undefined]	0.89874
RP11-449I17.5	10:125544-38642259	10p11.23	[undefined]	0.89874
RP11-414H17.8	10:125544-38642259	10p15.1	[undefined]	0.89874
RP11-534G20.3	10:125544-38642259	10p11.23	patched domain containing 3 pseudogene (LOC387647), non-coding RNA [Source:RefSeq DNA;Acc:NR_003930]	0.89874
RP11-508N22.8	10:125544-38642259	10p11.1	[undefined]	0.89874
RP5-933E2.1	10:125544-38642259	10p14	[undefined]	0.89874
RP11-175J10.1	10:125544-38642259	10p11.21	[undefined]	0.89874
AL355591.1	10:125544-38642259	10p14	Putative uncharacterized protein ENSP00000396902 Fragment [Source:UniProtKB/TrEMBL;Acc:C9JHV4]	0.89874
GS1-756B1.1	10:125544-38642259	10p14	[undefined]	0.89874
RP11-295P9.6	10:125544-38642259	10p13	[undefined]	0.89874
RP11-809C18.3	10:125544-38642259	10p15.3	[undefined]	0.89874
RP11-162G10.4	10:125544-38642259	10p11.1	[undefined]	0.89874
RP11-197M22.2	10:125544-38642259	10p13	[undefined]	0.89874
RP11-730A19.2	10:125544-38642259	10p13	[undefined]	0.89874
RP11-117P22.2	10:125544-38642259	10p15.1	[undefined]	0.89874
RP11-175J10.2	10:125544-38642259	10p11.21	[undefined]	0.89874
RP13-331H15.1	10:125544-38642259	10p11.21	[undefined]	0.89874
SFTA1P	10:125544-38642259	10p14	surfactant associated 1 (pseudogene) [Source:HGNC Symbol;Acc:18383]	0.89874
RP11-499O7.5	10:125544-38642259	10p15.1	[undefined]	0.89874
RP11-178D16.1	10:125544-38642259	10p15.2	[undefined]	0.89874
RP11-139J15.4	10:125544-38642259	10p12.31	[undefined]	0.89874
RP11-383B4.4	10:125544-38642259	10p12.31	[undefined]	0.89874
RP11-401F24.4	10:125544-38642259	10p14	[undefined]	0.89874
RP11-554I8.1	10:125544-38642259	10p14	[undefined]	0.89874
RP11-508N22.6	10:125544-38642259	10p11.1	[undefined]	0.89874
RP11-192N10.2	10:125544-38642259	10p11.23	[undefined]	0.89874
RP11-464C19.3	10:125544-38642259	10p15.1	[undefined]	0.89874
RP11-218D6.2	10:125544-38642259	10p12.1	[undefined]	0.89874
RP11-110H10.2	10:125544-38642259	10p12.33	[undefined]	0.89874
RP11-508N22.4	10:125544-38642259	10p11.1	[undefined]	0.89874
RP11-672F9.1	10:125544-38642259	10p11.1	[undefined]	0.89874
RP11-461K13.1	10:125544-38642259	10p13	[undefined]	0.89874
RP11-69D4.5	10:125544-38642259	10p15.1	[undefined]	0.89874
AL138764.1	10:125544-38642259	10p13	[undefined]	0.89874
RP11-288D15.2	10:125544-38642259	10p12.31	[undefined]	0.89874
RP11-435M3.2	10:125544-38642259	10p12.1	[undefined]	0.89874
RP11-51B10.2	10:125544-38642259	10p11.21	[undefined]	0.89874
RP11-218D6.3	10:125544-38642259	10p12.1	[undefined]	0.89874

Continued on next page...

gene name	chr	.DNABand	.GeneDesc	rank
RP11-490O24.1	10:125544-38642259	10p11.22	[undefined]	0.89874
RP11-476F14.1	10:125544-38642259	10p11.22	[undefined]	0.89874
RP11-393J16.4	10:125544-38642259	10p11.1	[undefined]	0.89874
RP11-258F22.1	10:125544-38642259	10p11.1	[undefined]	0.89874
RP11-382K22.1	10:125544-38642259	10p11.21	[undefined]	0.89874
RP11-336A10.2	10:125544-38642259	10p15.1	[undefined]	0.89874
RP11-69C17.2	10:125544-38642259	10p15.3	[undefined]	0.89874
RP11-298K24.2	10:125544-38642259	10p15.1	[undefined]	0.89874
RP11-298E9.6	10:125544-38642259	10p15.2	[undefined]	0.89874
RP11-166N17.3	10:125544-38642259	10p11.22	[undefined]	0.89874
RP5-1119O21.2	10:125544-38642259	10p14	[undefined]	0.89874
RP11-543F8.2	10:125544-38642259	10p14	[undefined]	0.89874
RP11-433J20.2	10:125544-38642259	10p15.1	[undefined]	0.89874
RP11-214D15.2	10:125544-38642259	10p13	[undefined]	0.89874
RP11-166N17.1	10:125544-38642259	10p11.22	[undefined]	0.89874
RP11-309N24.1	10:125544-38642259	10p11.21	[undefined]	0.89874
RP11-195B3.1	10:125544-38642259	10p15.2	[undefined]	0.89874
RP11-108B14.4	10:125544-38642259	10p12.31	[undefined]	0.89874
RP11-307B23.2	10:125544-38642259	10p12.1	[undefined]	0.89874
RP11-295P9.2	10:125544-38642259	10p13	[undefined]	0.89874
RP11-810B23.1	10:125544-38642259	10p11.21	[undefined]	0.89874
RP11-215C7.3	10:125544-38642259	10p12.2	[undefined]	0.89874
RP11-264C14.3	10:125544-38642259	10p14	[undefined]	0.89874
RP11-298E9.4	10:125544-38642259	10p15.2	[undefined]	0.89874
RP11-49L2.1	10:125544-38642259	10p12.31	[undefined]	0.89874
AL392086.1	10:125544-38642259	10p14	[undefined]	0.89874
RP11-271F18.2	10:125544-38642259	10p14	[undefined]	0.89874
RP11-69C17.3	10:125544-38642259	10p15.3	[undefined]	0.89874
RP13-16H11.2	10:125544-38642259	10p12.1	[undefined]	0.89874
RP11-305E6.1	10:125544-38642259	10p11.23	[undefined]	0.89874
AL158209.1	10:125544-38642259	10p12.31	Putative uncharacterized protein ENSP00000408677 Fragment [Source:UniProtKB/TrEMBL;Acc:C9JM83]	0.89874
RP1-251M9.3	10:125544-38642259	10p14	[undefined]	0.89874
AC069542.2	10:125544-38642259	10p12.33	[undefined]	0.89874
RP11-455B2.9	10:125544-38642259	10p13	[undefined]	0.89874
RP11-165O3.2	10:125544-38642259	10p12.31	[undefined]	0.89874
RP11-445P17.6	10:125544-38642259	10p15.1	[undefined]	0.89874
RP11-186N15.3	10:125544-38642259	10p13	[undefined]	0.89874
RP11-455B2.3	10:125544-38642259	10p13	[undefined]	0.89874
RP11-730A19.5	10:125544-38642259	10p13	[undefined]	0.89874
RP11-337N19.1	10:125544-38642259	10p12.31	[undefined]	0.89874
RP11-184A2.2	10:125544-38642259	10p15.1	[undefined]	0.89874
RP11-183E9.3	10:125544-38642259	10p12.2	[undefined]	0.89874
RP11-271M1.3	10:125544-38642259	10p13	sterile alpha motif domain containing 14 (SAMD14), mRNA [Source:RefSeq DNA;Acc:NM_174920]	0.89874
AL357372.1	10:125544-38642259	10p12.31	Putative uncharacterized protein ENSP00000413808 Fragment [Source:UniProtKB/TrEMBL;Acc:C9JKQ4]	0.89874
RP5-1051H14.2	10:125544-38642259	10p14	[undefined]	0.89874
RP11-129O7.2	10:125544-38642259	10p12.1	[undefined]	0.89874
RP11-336A10.7	10:125544-38642259	10p15.1	[undefined]	0.89874
RP11-307B23.1	10:125544-38642259	10p12.1	[undefined]	0.89874
RP11-92J19.2	10:125544-38642259	10p11.21	[undefined]	0.89874
RP11-213K14.2	10:125544-38642259	10p12.31	[undefined]	0.89874
RP11-253D19.1	10:125544-38642259	10p11.23	[undefined]	0.89874
RP11-479G22.5	10:125544-38642259	10p11.22	[undefined]	0.89874
RP11-162G10.1	10:125544-38642259	10p11.1	[undefined]	0.89874
RP11-90B22.2	10:125544-38642259	10p11.22	[undefined]	0.89874
RP11-565H13.3	10:125544-38642259	10p12.31	[undefined]	0.89874
RP11-336A10.4	10:125544-38642259	10p15.1	[undefined]	0.89874
RP1-186E20.1	10:125544-38642259	10p14	[undefined]	0.89874
RP11-348G8.3	10:125544-38642259	10p14	[undefined]	0.89874
RP11-124N14.4	10:125544-38642259	10p13	[undefined]	0.89874
RP11-390B4.3	10:125544-38642259	10p12.33	[undefined]	0.89874
NCRNA00200	10:125544-38642259	10p15.3	non-protein coding RNA 200 [Source:HGNC Symbol;Acc:30974]	0.89874
RP11-397O4.1	10:125544-38642259	10p14	[undefined]	0.89874
RP11-8L18.3	10:125544-38642259	10p11.21	[undefined]	0.89874
RP1-251M9.1	10:125544-38642259	10p14	[undefined]	0.89874
RP11-414H17.7	10:125544-38642259	10p15.1	[undefined]	0.89874
RP11-135A24.2	10:125544-38642259	10p11.22	[undefined]	0.89874
RP11-492M23.2	10:125544-38642259	10p12.1	[undefined]	0.89874
NAMPTL	10:125544-38642259	10p11.21	nicotinamide phosphoribosyltransferase-like [Source:HGNC Symbol;Acc:17633]	0.89874
RP11-462L8.1	10:125544-38642259	10p11.22	[undefined]	0.89874
RP11-536K7.5	10:125544-38642259	10p15.1	[undefined]	0.89874
RP11-184A2.3	10:125544-38642259	10p15.1 10p15.2	[undefined]	0.89874
RP11-142M10.2	10:125544-38642259	10p13	[undefined]	0.89874
RP11-89C21.3	10:125544-38642259	10p14	[undefined]	0.89874
RP11-363N22.2	10:125544-38642259	10p15.3	[undefined]	0.89874
RP11-479G22.7	10:125544-38642259	10p11.22	[undefined]	0.89874
RP1-254P11.1	10:125544-38642259	10p14	[undefined]	0.89874
RP11-215C7.2	10:125544-38642259	10p12.2	[undefined]	0.89874
RP4-660B20.1	10:125544-38642259	10p14	[undefined]	0.89874
RP11-34C15.2	10:125544-38642259	10p15.1	[undefined]	0.89874
RP11-275N1.1	10:125544-38642259	10p12.31	[undefined]	0.89874
RP11-56H7.2	10:125544-38642259	10p12.31	[undefined]	0.89874
RP11-385N23.1	10:125544-38642259	10p14	[undefined]	0.89874
RP11-305E6.2	10:125544-38642259	10p11.23	[undefined]	0.89874
RP11-241I20.1	10:125544-38642259	10p11.22	[undefined]	0.89874
RP11-253D19.2	10:125544-38642259	10p11.23	[undefined]	0.89874
RP3-323N1.2	10:125544-38642259	10p14	[undefined]	0.89874
RP11-192P3.3	10:125544-38642259	10p11.22	[undefined]	0.89874
RP11-85G18.4	10:125544-38642259	10p12.1	[undefined]	0.89874
RP11-360I20.2	10:125544-38642259	10p12.1	[undefined]	0.89874
RP11-297A16.2	10:125544-38642259	10p11.21	[undefined]	0.89874
RP11-464C19.2	10:125544-38642259	10p15.1	[undefined]	0.89874
RP11-14C22.6	10:125544-38642259	10p11.23	[undefined]	0.89874
RP11-188P8.2	10:125544-38642259	10p12.31	[undefined]	0.89874
RP11-14C22.3	10:125544-38642259	10p11.23	[undefined]	0.89874
RP11-445P17.3	10:125544-38642259	10p15.1	[undefined]	0.89874
RP11-167O6.2	10:125544-38642259	10p11.22	[undefined]	0.89874
RP11-195O1.1	10:125544-38642259	10p11.22	[undefined]	0.89874
RP11-117P22.1	10:125544-38642259	10p15.1	[undefined]	0.89874
RP11-69C17.4	10:125544-38642259	10p15.3	[undefined]	0.89874
RP11-80K21.3	10:125544-38642259	10p12.1	[undefined]	0.89874
RP11-336A10.5	10:125544-38642259	10p15.1	[undefined]	0.89874
RP1-251M9.2	10:125544-38642259	10p14	[undefined]	0.89874
RP11-275N1.2	10:125544-38642259	10p12.31	[undefined]	0.89874
RP11-164C1.2	10:125544-38642259	10p15.3	[undefined]	0.89874
RP11-89K18.1	10:125544-38642259	10p15.3	[undefined]	0.89874
GLUDP5	10:125544-38642259	10p11.22	glutamate dehydrogenase 1 pseudogene 5 [Source:HGNC Symbol;Acc:4340]	0.89874
RP11-48B24.2	10:125544-38642259	10p12.1	[undefined]	0.89874
RP11-565H13.2	10:125544-38642259	10p12.31	[undefined]	0.89874
RP13-16H11.1	10:125544-38642259	10p12.1	[undefined]	0.89874
GS1-756B1.2	10:125544-38642259	10p14	[undefined]	0.89874
NCRNA00202	10:125544-38642259	10p12.1	non-protein coding RNA 202 [Source:HGNC Symbol;Acc:24672]	0.89874
IL9RP2	10:125544-38642259	10p15.3	interleukin 9 receptor pseudogene 2 [Source:HGNC Symbol;Acc:6032]	0.89874
RP11-108B14.2	10:125544-38642259	10p12.31	[undefined]	0.89874
RP11-51B10.3	10:125544-38642259	10p11.21	[undefined]	0.89874
RP11-414H17.6	10:125544-38642259	10p15.1	[undefined]	0.89874
RP5-1031D4.2	10:125544-38642259	10p14	[undefined]	0.89874
RP11-478H13.3	10:125544-38642259	10p12.1	[undefined]	0.89874
RP11-379F12.4	10:125544-38642259	10p14	[undefined]	0.89874
C10orf110	10:125544-38642259	10p15.3	[undefined]	0.89874
RP11-25G10.2	10:125544-38642259	10p13	[undefined]	0.89874
RP11-536K7.3	10:125544-38642259	10p15.1	[undefined]	0.89874

Continued on next page...

gene name	chr	.DNABand	.GeneDesc	rank
RP11-565H13.4	10:125544-38642259	10p12.31	[undefined]	0.89874
RP11-490E15.2	10:125544-38642259	10p15.3	[undefined]	0.89874
RP11-398B16.2	10:125544-38642259	10p15.3	[undefined]	0.89874
RP11-67B16.1	10:125544-38642259	10p15.1	[undefined]	0.89874
RP11-460H18.2	10:125544-38642259	10p11.22	[undefined]	0.89874
RP11-324I22.2	10:125544-38642259	10p11.21	[undefined]	0.89874
RP11-445K13.2	10:125544-38642259	10p13	[undefined]	0.89874
C10orf50	10:125544-38642259	10p12.1	[undefined]	0.89874
RP11-573G6.2	10:125544-38642259	10p12.31	[undefined]	0.89874
RP11-482E14.1	10:125544-38642259	10p15.2	[undefined]	0.89874
ATP6V1G1P4	10:125544-38642259	10p11.21	ATPase, H+ transporting, lysosomal 13kDa, V1 subunit G1 pseudogene 4 [Source:HGNC Symbol;Acc:37671]	0.89874
RP11-342D11.3	10:125544-38642259	10p11.22	[undefined]	0.89874
RP11-301N24.3	10:125544-38642259	10p12.2	[undefined]	0.89874
RP11-218D6.4	10:125544-38642259	10p12.1	[undefined]	0.89874
RP11-322I2.2	10:125544-38642259	10p11.21	[undefined]	0.89874
RP11-80K21.1	10:125544-38642259	10p12.1	[undefined]	0.89874
RP11-135A24.4	10:125544-38642259	10p11.22	[undefined]	0.89874
RP11-354E11.2	10:125544-38642259	10p12.31	[undefined]	0.89874
RP11-401E9.3	10:125544-38642259	10p14	[undefined]	0.89874
RP11-353M9.1	10:125544-38642259	10p13	[undefined]	0.89874
RP11-479G22.6	10:125544-38642259	10p11.22	[undefined]	0.89874
RP11-198D23.2	10:125544-38642259	10p11.21	[undefined]	0.89874
RP11-446F3.2	10:125544-38642259	10p15.3	[undefined]	0.89874
RP11-730A19.9	10:125544-38642259	10p13	[undefined]	0.89874
RP11-118K6.2	10:125544-38642259	10p15.2	[undefined]	0.89874
RP11-139J15.5	10:125544-38642259	10p12.31	[undefined]	0.89874
RP11-799O21.2	10:125544-38642259	10p14	[undefined]	0.89874
RP13-16H11.7	10:125544-38642259	10p12.1	[undefined]	0.89874
RP11-271F18.1	10:125544-38642259	10p14	[undefined]	0.89874
RP11-241I20.3	10:125544-38642259	10p11.22	[undefined]	0.89874
RP11-311B18.1	10:125544-38642259	10p15.3	[undefined]	0.89874
RP11-428L9.1	10:125544-38642259	10p14	[undefined]	0.89874
AL391992.1	10:125544-38642259	10p12.31	Putative uncharacterized protein ENSP00000399047 Fragment [Source:UniProtKB/TrEMBL;Acc:C9IZ90]	0.89874
RP13-16H11.6	10:125544-38642259	10p12.1	[undefined]	0.89874
RP11-241I20.5	10:125544-38642259	10p11.22	[undefined]	0.89874
RP11-288D15.1	10:125544-38642259	10p12.31	[undefined]	0.89874
RP11-14C22.4	10:125544-38642259	10p11.23	[undefined]	0.89874
RP11-20F24.2	10:125544-38642259	10p11.21	[undefined]	0.89874
RP11-508N22.11	10:125544-38642259	10p11.1	[undefined]	0.89874
RP11-124N14.3	10:125544-38642259	10p13	[undefined]	0.89874
RP11-69C17.1	10:125544-38642259	10p15.3	[undefined]	0.89874
RP11-109I13.2	10:125544-38642259	10p12.33	[undefined]	0.89874
RP11-241I20.4	10:125544-38642259	10p11.22	[undefined]	0.89874
RP5-1092K5.2	10:125544-38642259	10p14	[undefined]	0.89874
RP11-398C13.4	10:125544-38642259	10p13	[undefined]	0.89874
RP11-393J16.2	10:125544-38642259	10p11.1	[undefined]	0.89874
RP11-429A24.4	10:125544-38642259	10p12.1	[undefined]	0.89874
RP11-526P5.2	10:125544-38642259	10p15.3	[undefined]	0.89874
RP11-397C18.2	10:125544-38642259	10p13	[undefined]	0.89874
RP11-2K17.5	10:125544-38642259	10p13	[undefined]	0.89874
RP11-457D2.3	10:125544-38642259	10p12.33	[undefined]	0.89874
RP11-449I17.9	10:125544-38642259	10p11.23	[undefined]	0.89874
RP11-20F24.4	10:125544-38642259	10p11.21	[undefined]	0.89874
RP11-730A19.4	10:125544-38642259	10p13	[undefined]	0.89874
RP11-478H13.2	10:125544-38642259	10p12.1	[undefined]	0.89874
RP5-1051H14.1	10:125544-38642259	10p14	[undefined]	0.89874
RP13-16H11.5	10:125544-38642259	10p12.1	[undefined]	0.89874
RP11-56H7.3	10:125544-38642259	10p12.31	[undefined]	0.89874
RP11-462L8.2	10:125544-38642259	10p11.22	[undefined]	0.89874
RP11-508N22.5	10:125544-38642259	10p11.1	[undefined]	0.89874
RP11-14C22.5	10:125544-38642259	10p11.23	[undefined]	0.89874
RP11-508N22.10	10:125544-38642259	10p11.1	[undefined]	0.89874
RP11-20F24.6	10:125544-38642259	10p11.21	[undefined]	0.89874
RP11-33K5.1	10:125544-38642259	10p14	[undefined]	0.89874
RP11-7C6.1	10:125544-38642259	10p13	[undefined]	0.89874
RP11-162G10.5	10:125544-38642259	10p11.1	[undefined]	0.89874
RP11-390B4.4	10:125544-38642259	10p12.33	[undefined]	0.89874
RP11-92J19.1	10:125544-38642259	10p11.21	[undefined]	0.89874
RP11-478H13.1	10:125544-38642259	10p12.1	[undefined]	0.89874
RP11-108B14.1	10:125544-38642259	10p12.31	[undefined]	0.89874
RP11-482E14.2	10:125544-38642259	10p15.2	[undefined]	0.89874
RP11-128B16.3	10:125544-38642259	10p12.1	[undefined]	0.89874
RP11-265G8.3	10:125544-38642259	10p12.31	[undefined]	0.89874
RP11-298E9.5	10:125544-38642259	10p15.2	[undefined]	0.89874
C10orf52	10:125544-38642259	10p12.1	[undefined]	0.89874
RP11-433J20.1	10:125544-38642259	10p15.1	[undefined]	0.89874
RP11-92J19.3	10:125544-38642259	10p11.21	[undefined]	0.89874
RP11-398C13.2	10:125544-38642259	10p13	[undefined]	0.89874
RP11-192P3.1	10:125544-38642259	10p11.22	[undefined]	0.89874
RP11-351M16.3	10:125544-38642259	10p12.1	[undefined]	0.89874
RP11-330O11.2	10:125544-38642259	10p11.23	[undefined]	0.89874
RP11-298E9.3	10:125544-38642259	10p15.2	[undefined]	0.89874
RP11-271M1.2	10:125544-38642259	10p13	[undefined]	0.89874
RP11-526P5.1	10:125544-38642259	10p15.3	[undefined]	0.89874
RP11-730A19.7	10:125544-38642259	10p13	[undefined]	0.89874
RP11-351M16.2	10:125544-38642259	10p12.1	[undefined]	0.89874
RP11-472N13.3	10:125544-38642259	10p11.22	[undefined]	0.89874
RP11-499O7.4	10:125544-38642259	10p15.1	[undefined]	0.89874
RP11-37P5.1	10:125544-38642259	10p13	[undefined]	0.89874
RP11-224P11.1	10:125544-38642259	10p11.23	[undefined]	0.89874
RP11-5N23.2	10:125544-38642259	10p14	[undefined]	0.89874
RP11-748L13.4	10:125544-38642259	10p12.1	[undefined]	0.89874
RP11-20F24.5	10:125544-38642259	10p11.21	[undefined]	0.89874
RP1-33E13.1	10:125544-38642259	10p14	[undefined]	0.89874
RP11-748L13.1	10:125544-38642259	10p12.1	[undefined]	0.89874
PRINS	10:125544-38642259	10p12.2	psoriasis associated RNA induced by stress (non-protein coding) [Source:HGNC Symbol;Acc:34235]	0.89874
RP11-322I2.1	10:125544-38642259	10p11.21	[undefined]	0.89874
RP11-575A19.2	10:125544-38642259	10p12.31	[undefined]	0.89874
RP11-342D11.2	10:125544-38642259	10p11.22	[undefined]	0.89874
RP11-799O21.1	10:125544-38642259	10p14	[undefined]	0.89874
AL359960.1	10:125544-38642259	10p15.1	[undefined]	0.89874
AL139404.3	10:125544-38642259	10p12.1	[undefined]	0.89874
AL606469.1	10:125544-38642259	10p12.2	[undefined]	0.89874
AL360230.1	10:125544-38642259	10p13	[undefined]	0.89874
AC091817.1	10:125544-38642259	10p15.1	[undefined]	0.89874
AC073160.1	10:125544-38642259	10p14	[undefined]	0.89874
AC022536.1	10:125544-38642259	10p15.3	[undefined]	0.89874
AL590439.1	10:125544-38642259	10p13	[undefined]	0.89874
AL391427.2	10:125544-38642259	10p15.1	[undefined]	0.89874
AL391427.1	10:125544-38642259	10p15.1	[undefined]	0.89874
RP11-265G8.1	10:125544-38642259	10p12.31	[undefined]	0.89874
AL161651.2	10:125544-38642259	10p11.23	[undefined]	0.89874
AL353147.2	10:125544-38642259	10p12.31	[undefined]	0.89874
RP11-295P9.3	10:125544-38642259	10p13	[undefined]	0.89874
AL161932.2	10:125544-38642259	10p11.22	[undefined]	0.89874
AL590068.1	10:125544-38642259	10p11.23	[undefined]	0.89874
AL603831.1	10:125544-38642259	10p15.3	[undefined]	0.89874
RP11-318E3.4	10:125544-38642259	10p15.1	[undefined]	0.89874
RP11-499P20.2	10:125544-38642259	10p12.31	[undefined]	0.89874
AL590730.2	10:125544-38642259	10p11.21	[undefined]	0.89874
AL365356.1	10:125544-38642259	10p15.1	[undefined]	0.89874

Continued on next page...

gene name	chr	.DNABand	.GeneDesc	rank
RP11-20F24.3	10:125544-38642259	10p11.21	[undefined]	0.89874
NSUN6	10:125544-38642259	10p12.31	NOP2/Sun domain family, member 6 [Source:HGNC Symbol;Acc:23529]	0.89874
RP11-455B2.7	10:125544-38642259	10p13	[undefined]	0.89874
RP11-508N22.9	10:125544-38642259	10p11.1	[undefined]	0.89874
RP11-449I17.3	10:125544-38642259	10p11.23	[undefined]	0.89874
AL390766.1	10:125544-38642259	10p11.21	[undefined]	0.89874
AL355303.2	10:125544-38642259	10p15.1	[undefined]	0.89874
RP13-463N16.6	10:125544-38642259	10p15.1	[undefined]	0.89874
AL391499.1	10:125544-38642259	10p12.2	[undefined]	0.89874
RP11-809C18.1	10:125544-38642259	10p15.3	[undefined]	0.89874
RP11-89C21.2	10:125544-38642259	10p14	[undefined]	0.89874
AL590730.4	10:125544-38642259	10p11.21	[undefined]	0.89874
AL121927.1	10:125544-38642259	10p11.21	[undefined]	0.89874
AL353147.4	10:125544-38642259	10p12.31	[undefined]	0.89874
AL353147.5	10:125544-38642259	10p12.31	[undefined]	0.89874
RP11-379F12.3	10:125544-38642259	10p14	[undefined]	0.89874
AL590730.1	10:125544-38642259	10p11.21	[undefined]	0.89874
AL451107.1	10:125544-38642259	10p11.23	[undefined]	0.89874
AL359878.1	10:125544-38642259	10p15.3	[undefined]	0.89874
AL391839.1	10:125544-38642259	10p11.22	[undefined]	0.89874
RP11-479G22.3	10:125544-38642259	10p11.22	[undefined]	0.89874
AL353600.1	10:125544-38642259	10p11.22	[undefined]	0.89874
AL353147.1	10:125544-38642259	10p12.31	[undefined]	0.89874
AL157387.2	10:125544-38642259	10p11.21	[undefined]	0.89874
AL512641.1	10:125544-38642259	10p12.31	[undefined]	0.89874
AL160287.2	10:125544-38642259	10p12.1	[undefined]	0.89874
AL358780.3	10:125544-38642259	10p12.31	[undefined]	0.89874
AC026887.1	10:125544-38642259	10p14	[undefined]	0.89874
AL117340.2	10:125544-38642259	10p11.22	[undefined]	0.89874
AL157392.1	10:125544-38642259	10p13	[undefined]	0.89874
AL590365.1	10:125544-38642259	10p13	[undefined]	0.89874
AL158167.1	10:125544-38642259	10p11.23	[undefined]	0.89874
AL160060.2	10:125544-38642259	10p11.23	[undefined]	0.89874
DNM1P17	10:125544-38642259	10p11.23	DNM1 pseudogene 17 [Source:HGNC Symbol;Acc:21134]	0.89874
AL713867.1	10:125544-38642259	10p15.1	[undefined]	0.89874
AL731546.1	10:125544-38642259	10p12.1	[undefined]	0.89874
AL391427.3	10:125544-38642259	10p15.1	[undefined]	0.89874
AL358780.1	10:125544-38642259	10p12.31	[undefined]	0.89874
AL158164.1	10:125544-38642259	10p12.33	[undefined]	0.89874
AC024606.1	10:125544-38642259	10p12.1	[undefined]	0.89874
AL160291.1	10:125544-38642259	10p12.1	[undefined]	0.89874
AL355303.1	10:125544-38642259	10p15.1	[undefined]	0.89874
AL137186.1	10:125544-38642259	10p15.1	[undefined]	0.89874
AL138898.1	10:125544-38642259	10p14	[undefined]	0.89874
AC069542.1	10:125544-38642259	10p12.33	[undefined]	0.89874
AL157895.2	10:125544-38642259	10p12.31	[undefined]	0.89874
AL139281.1	10:125544-38642259	10p12.2	[undefined]	0.89874
AL512284.1	10:125544-38642259	10p13	[undefined]	0.89874
AL117337.2	10:125544-38642259	10p11.1	[undefined]	0.89874
AL161936.2	10:125544-38642259	10p12.1	[undefined]	0.89874
AL391314.1	10:125544-38642259	10p13	[undefined]	0.89874
AL117340.1	10:125544-38642259	10p11.22	[undefined]	0.89874
AL445071.1	10:125544-38642259	10p11.22	[undefined]	0.89874
AL358033.1	10:125544-38642259	10p13	[undefined]	0.89874
AL157387.1	10:125544-38642259	10p11.21	[undefined]	0.89874
AL359697.2	10:125544-38642259	10p12.31	[undefined]	0.89874
AL160291.2	10:125544-38642259	10p12.1	[undefined]	0.89874
AL353147.3	10:125544-38642259	10p12.31	[undefined]	0.89874
AL161651.1	10:125544-38642259	10p11.23	[undefined]	0.89874
AL451164.1	10:125544-38642259	10p15.2	[undefined]	0.89874
AL441943.1	10:125544-38642259	10p15.3	[undefined]	0.89874
AL157783.1	10:125544-38642259	10p11.21	[undefined]	0.89874
AL117336.2	10:125544-38642259	10p11.21	[undefined]	0.89874
PACRG	6:161937900-165004845	6q26	PARK2 co-regulated [Source:HGNC Symbol;Acc:19152]	0.86358
QKI	6:161937900-165004845	6q26	quaking homolog, KH domain RNA binding (mouse) [Source:HGNC Symbol;Acc:21100]	0.86358
PARK2	6:161937900-165004845	6q26	Parkinson disease (autosomal recessive, juvenile) 2, parkin [Source:HGNC Symbol;Acc:8607]	0.86358
RP3-495O10.1	6:161937900-165004845	6q26	[undefined]	0.86358
RP11-157L10.1	6:161937900-165004845	6q26	[undefined]	0.86358
AL035697.1	6:161937900-165004845	6q26	[undefined]	0.86358
RP1-257A15.1	6:161937900-165004845	6q26	[undefined]	0.86358
AL078585.1	6:161937900-165004845	6q26	cDNA FLJ58069 [Source:UniProtKB/TrEMBL;Acc:B4DJX3]	0.86358
RP11-347L18.1	6:161937900-165004845	6q27	[undefined]	0.86358
KB-1880F11.1	6:161937900-165004845	6q26	[undefined]	0.86358
RP5-826L7.1	6:161937900-165004845	6q26	[undefined]	0.86358
RP1-155D22.1	6:161937900-165004845	6q27	[undefined]	0.86358
RP1-230L10.1	6:161937900-165004845	6q26	[undefined]	0.86358
AL589952.1	6:161937900-165004845	6q27	Putative uncharacterized protein ENSP00000399158 [Source:UniProtKB/TrEMBL;Acc:C9IYB6]	0.86358
AL031121.1	6:161937900-165004845	6q26	[undefined]	0.86358
AL354942.1	6:161937900-165004845	6q26	[undefined]	0.86358
AP000886.1	6:161937900-165004845	6q26	[undefined]	0.86358
AL031121.2	6:161937900-165004845	6q26	[undefined]	0.86358
AL031781.1	6:161937900-165004845	6q26	[undefined]	0.86358
MUC20	3:195423082-195451559	3q29	mucin 20, cell surface associated [Source:HGNC Symbol;Acc:23282]	0.85831
MIR570	3:195423082-195451559	3q29	microRNA 570 [Source:HGNC Symbol;Acc:32826]	0.85831
AC069513.3	3:195423082-195451559	3q29	[undefined]	0.85831
OR4K2	14:19794561-20414173	14q11.2	olfactory receptor, family 4, subfamily K, member 2 [Source:HGNC Symbol;Acc:14728]	0.83323
OR4K3P	14:19794561-20414173	14q11.2	[undefined]	0.83323
OR4K1	14:19794561-20414173	14q11.2	olfactory receptor, family 4, subfamily K, member 1 [Source:HGNC Symbol;Acc:14726]	0.83323
OR4M1	14:19794561-20414173	14q11.2	olfactory receptor, family 4, subfamily M, member 1 [Source:HGNC Symbol;Acc:14735]	0.83323
OR4K5	14:19794561-20414173	14q11.2	olfactory receptor, family 4, subfamily K, member 5 [Source:HGNC Symbol;Acc:14745]	0.83323
OR4Q3	14:19794561-20414173	14q11.2	olfactory receptor, family 4, subfamily Q, member 3 [Source:HGNC Symbol;Acc:15426]	0.83323
OR4N2	14:19794561-20414173	14q11.2	olfactory receptor, family 4, subfamily N, member 2 [Source:HGNC Symbol;Acc:14742]	0.83323
AL589182.6	14:19794561-20414173	14q11.2	ANKRD26-like family C member ENSP00000349402 [Source:UniProtKB/Swiss-Prot;Acc:A6NI47]	0.83323
OR4H12P	14:19794561-20414173	14q11.2	olfactory receptor, family 4, subfamily H, member 12 pseudogene [Source:HGNC Symbol;Acc:19433]	0.83323
AL512310.2	14:19794561-20414173	14q11.2	[undefined]	0.83323
OR11H2	14:19794561-20414173	14q11.2	olfactory receptor, family 11, subfamily H, member 2 [Source:HGNC Symbol;Acc:14716]	0.83323
AL589182.2	14:19794561-20414173	14q11.2	[undefined]	0.83323
AL929601.3	14:19794561-20414173	14q11.2	[undefined]	0.83323
OR4K6P	14:19794561-20414173	14q11.2	olfactory receptor, family 4, subfamily K, member 6 pseudogene [Source:HGNC Symbol;Acc:14748]	0.83323
C14orf17	14:19794561-20414173	14q11.2	Full-length cDNA 5-PRIME end of clone CS0DK007YB08 of HeLa cells of Homo sapiens (human) [Source:UniProtKB/TrEMBL;Acc:Q86SX0]	0.83323
AL512310.3	14:19794561-20414173	14q11.2	Putative uncharacterized protein ENSP00000400691 Fragment [Source:UniProtKB/TrEMBL;Acc:C9J2A6]	0.83323
AL929601.1	14:19794561-20414173	14q11.2	Putative uncharacterized protein ENSP00000392148 [Source:UniProtKB/TrEMBL;Acc:C9IYV8]	0.83323
AL512310.4	14:19794561-20414173	14q11.2	[undefined]	0.83323
AL589182.1	14:19794561-20414173	14q11.2	Putative uncharacterized protein ENSP00000412483 Fragment [Source:UniProtKB/TrEMBL;Acc:C9IZS1]	0.83323
AL589182.5	14:19794561-20414173	14q11.2	[undefined]	0.83323
AL589743.1	14:19794561-20414173	14q11.2	[undefined]	0.83323
AL589182.3	14:19794561-20414173	14q11.2	[undefined]	0.83323
AL512310.1	14:19794561-20414173	14q11.2	[undefined]	0.83323
AL929601.2	14:19794561-20414173	14q11.2	[undefined]	0.83323
AL589182.4	14:19794561-20414173	14q11.2	[undefined]	0.83323
AL512310.5	14:19794561-20414173	14q11.2	[undefined]	0.83323
KLHL1	13:67406473-72359497	13q21.33	kelch-like 1 (Drosophila) [Source:HGNC Symbol;Acc:6352]	0.78858
PCDH9	13:67406473-72359497	13q21.32	protocadherin 9 [Source:HGNC Symbol;Acc:8661]	0.78858
AL356384.1	13:67406473-72359497	13q21.33	[undefined]	0.78858
DACH1	13:67406473-72359497	13q21.33	dachshund homolog 1 (Drosophila) [Source:HGNC Symbol;Acc:2663]	0.78858
RP11-459J23.2	13:67406473-72359497	13q21.33	[undefined]	0.78858

Continued on next page...

gene name	chr	.DNABand	.GeneDesc	rank
AL445264.1	13:67406473-72359497	13q21.33	[undefined]	0.78858
RP11-452P23.2	13:67406473-72359497	13q21.33	[undefined]	0.78858
RP11-346A3.2	13:67406473-72359497	13q21.32	[undefined]	0.78858
RP11-248N6.2	13:67406473-72359497	13q21.32	[undefined]	0.78858
RP11-138D23.2	13:67406473-72359497	13q21.32	[undefined]	0.78858
RP11-77P3.1	13:67406473-72359497	13q21.33	[undefined]	0.78858
RP11-209P2.1	13:67406473-72359497	13q21.33	[undefined]	0.78858
RP11-138D23.1	13:67406473-72359497	13q21.32	[undefined]	0.78858
RP11-184L18.1	13:67406473-72359497	13q21.33	[undefined]	0.78858
RP11-520F24.1	13:67406473-72359497	13q21.33	[undefined]	0.78858
RP11-248N6.3	13:67406473-72359497	13q21.32	[undefined]	0.78858
ATXN8OS	13:67406473-72359497	13q21.33	ATXN8 opposite strand (non-protein coding) [Source:HGNC Symbol;Acc:10561]	0.78858
RP11-430K22.2	13:67406473-72359497	13q21.33	[undefined]	0.78858
RP11-248N6.1	13:67406473-72359497	13q21.32	[undefined]	0.78858
RP11-631G24.1	13:67406473-72359497	13q21.32	[undefined]	0.78858
RP11-264M20.1	13:67406473-72359497	13q21.33	[undefined]	0.78858
RP11-430K22.1	13:67406473-72359497	13q21.33	[undefined]	0.78858
RP11-520F24.2	13:67406473-72359497	13q21.33	[undefined]	0.78858
RP11-206H15.2	13:67406473-72359497	13q21.33	[undefined]	0.78858
RP11-520F24.3	13:67406473-72359497	13q21.33	[undefined]	0.78858
RP11-138D23.3	13:67406473-72359497	13q21.32	[undefined]	0.78858
RP11-501G6.1	13:67406473-72359497	13q21.33	[undefined]	0.78858
RP11-459J23.1	13:67406473-72359497	13q21.33	[undefined]	0.78858
AL355611.2	13:67406473-72359497	13q21.33	[undefined]	0.78858
RP11-393H6.2	13:67406473-72359497	13q21.33	[undefined]	0.78858
AL355611.1	13:67406473-72359497	13q21.33	[undefined]	0.78858
RP11-393H6.3	13:67406473-72359497	13q21.33	[undefined]	0.78858
AL355611.3	13:67406473-72359497	13q21.33	[undefined]	0.78858
IGHV3-43	14:106906901-106931134	14q32.33	[undefined]	0.74955
IGHV3-42	14:106906901-106931134	14q32.33	[undefined]	0.74955
AB019438.63	14:106906901-106931134	14q32.33	[undefined]	0.74955
IGHVII-43-1	14:106906901-106931134	14q32.33	[undefined]	0.74955
ELF1	13:41431155-41661999	13q14.11	E74-like factor 1 (ets domain transcription factor) [Source:HGNC Symbol;Acc:3316]	0.74703
RP11-471M10.2	13:41431155-41661999	13q14.11	[undefined]	0.74703
WBP4	13:41431155-41661999	13q14.11	WW domain binding protein 4 (formin binding protein 21) [Source:HGNC Symbol;Acc:12739]	0.74703
SUGT1L1	13:41431155-41661999	13q14.11	SGT1, suppressor of G2 allele of SKP1 like 1 (S. cerevisiae) [Source:HGNC Symbol;Acc:20513]	0.74703
RP11-471M10.1	13:41431155-41661999	13q14.11	SGT1, suppressor of G2 allele of SKP1 like 1 (S. cerevisiae) (SUGT1L1), non-coding RNA [Source:RefSeq DNA;Acc:NR_003365]	0.74703
AL157877.1	13:41431155-41661999	13q14.11	[undefined]	0.74703
RP11-2P5.4	13:41431155-41661999	13q14.11	[undefined]	0.74703
RP11-346L13.3	13:41431155-41661999	13q14.11	[undefined]	0.74703
MRPS31	13:41229129-41345266	13q14.11	mitochondrial ribosomal protein S31 [Source:HGNC Symbol;Acc:16632]	0.74608
MIR320D1	13:41229129-41345266	13q14.11	microRNA 320d-1 [Source:HGNC Symbol;Acc:35386]	0.74608
FOXO1	13:41229129-41345266	13q14.11	forkhead box O1 [Source:HGNC Symbol;Acc:3819]	0.74608

10.3 Copy number abberations

Table 10: Results for the query

ID	chr	start	end	nroOfprobes	segmean	call
MSK_00006_251469322732_S01_CGH.v4_91.txt	1	147484815	147522198	3	-1.341	-1
MSK_00006_251469322732_S01_CGH.v4_91.txt	1	25489065	25536228	2	-0.6791	-1
MSK_00007_251469322733_S01_CGH.v4_91.txt	1	201535961	203933658	174	3.6226	1
MSK_00009_251469322626_S01_CGH.v4_91.txt	1	21686110	22237046	37	-0.6522	-1
MSK_00009_251469322626_S01_CGH.v4_91.txt	1	19496724	21598342	150	-0.6935	-1
MSK_00009_251469322626_S01_CGH.v4_91.txt	1	15779001	15921837	16	-0.7857	-1
MSK_00009_251469322626_S01_CGH.v4_91.txt	1	14503028	15556103	67	-0.7949	-1
MSK_00010_251469322627_S01_CGH.v4_91.txt	1	246987317	247051615	5	-0.777	-1
MSK_00010_251469322627_S01_CGH.v4_91.txt	1	154662565	155198046	49	0.6842	1
MSK_00010_251469322627_S01_CGH.v4_91.txt	1	148647113	148889867	19	0.699	1
MSK_00011_251469322628_S01_CGH.v4_91.txt	1	16253914	16260291	2	0.8122	1
MSK_00014_251469322629_S01_CGH.v4_91.txt	1	166087520	166454694	28	1.3827	1
MSK_00014_251469322629_S01_CGH.v4_91.txt	1	154734503	155020949	30	1.5307	1
MSK_00014_251469322629_S01_CGH.v4_91.txt	1	144518285	144577230	3	1.0289	1
MSK_00014_251469322629_S01_CGH.v4_91.txt	1	22586567	23236954	43	2.5991	1
MSK_WGA_0015.1_251469338754_S01_CGH.v4_91.txt	1	235142875	236443757	97	-0.7279	-1
MSK_WGA_0015.1_251469338754_S01_CGH.v4_91.txt	1	83904609	94988262	667	-0.6879	-1
MSK_WGA_0015.1_251469338754_S01_CGH.v4_91.txt	1	72286185	83536562	520	-0.7311	-1
MSK_WGA_0015.1_251469338754_S01_CGH.v4_91.txt	1	5091145	5210655	4	-0.7049	-1
MSK_WGA_0016.1_251469338755_S01_CGH.v4_91.txt	1	158644148	158717844	7	0.8417	1
MSK_WGA_0016.1_251469338755_S01_CGH.v4_91.txt	1	153532919	153591203	6	0.8623	1
MSK_WGA_0016.1_251469338755_S01_CGH.v4_91.txt	1	23270575	23406753	14	-0.6417	-1
MSK_00021_251469322630_S01_CGH.v4_91.txt	1	247024176	247051615	4	-0.781	-1
MSK_00021_251469322630_S01_CGH.v4_91.txt	1	232553850	232833503	26	-0.7935	-1
MSK_00021_251469322630_S01_CGH.v4_91.txt	1	208869126	210241206	101	-0.8212	-1
MSK_00021_251469322630_S01_CGH.v4_91.txt	1	150355014	151476900	95	-0.78	-1
MSK_00021_251469322630_S01_CGH.v4_91.txt	1	113153198	113338019	13	-0.7361	-1
MSK_00021_251469322630_S01_CGH.v4_91.txt	1	112772891	113115878	26	-0.7378	-1
MSK_00021_251469322630_S01_CGH.v4_91.txt	1	111831579	112015794	20	-0.8155	-1
MSK_00021_251469322630_S01_CGH.v4_91.txt	1	111612465	111763678	16	-0.8204	-1
MSK_00021_251469322630_S01_CGH.v4_91.txt	1	104354841	105092483	20	-0.8255	-1
MSK_00021_251469322630_S01_CGH.v4_91.txt	1	103621487	103944237	5	-0.7969	-1
MSK_00021_251469322630_S01_CGH.v4_91.txt	1	25489065	25536228	2	-0.6947	-1
MSK_00024_251469322721_S01_CGH.v4_91.txt	1	25489065	25536228	2	-0.9767	-1
MSK_WGA_0026.1_251469338758_S01_CGH.v4_91.txt	1	235633033	238787736	169	-0.6485	-1
MSK_00027_251469322705_S01_CGH.v4_91.txt	1	247024176	247051615	4	-0.7514	-1
MSK_00027_251469322705_S01_CGH.v4_91.txt	1	4741434	4748366	2	-0.6352	-1
MSK_00028_251469322706_S01_CGH.v4_91.txt	1	247024176	247051615	4	-0.8586	-1
MSK_00028_251469322706_S01_CGH.v4_91.txt	1	117132263	120430738	183	-0.9048	-1
MSK_00028_251469322706_S01_CGH.v4_91.txt	1	116122242	117029920	46	-0.8622	-1
MSK_00028_251469322706_S01_CGH.v4_91.txt	1	1751198	116111450	6686	-0.8568	-1
MSK_00033_251469322707_S01_CGH.v4_91.txt	1	18320651	18363139	4	0.7685	1
MSK_00037_251469322734_S01_CGH.v4_91.txt	1	247024176	247051615	4	-0.8518	-1
MSK_00037_251469322734_S01_CGH.v4_91.txt	1	147484815	147522198	3	-0.6366	-1
MSK_00037_251469322734_S01_CGH.v4_91.txt	1	14858867	14917755	6	-0.9138	-1
MSK_00043_251469322736_S01_CGH.v4_91.txt	1	228107521	228783692	30	-0.8658	-1
MSK_00043_251469322736_S01_CGH.v4_91.txt	1	151096005	151099276	2	-0.7674	-1
MSK_00043_251469322736_S01_CGH.v4_91.txt	1	83904609	99397749	903	-0.8809	-1
MSK_00043_251469322736_S01_CGH.v4_91.txt	1	75847000	83536562	369	-0.9502	-1
MSK_00043_251469322736_S01_CGH.v4_91.txt	1	21418776	23064565	97	-0.6646	-1
MSK_00043_251469322736_S01_CGH.v4_91.txt	1	5434399	6943232	81	-0.6888	-1
MSK_00057_251469323017_S01_CGH.v4_91.txt	1	246987317	247051615	5	-0.7483	-1
MSK_00058_251469323019_S01_CGH.v4_91.txt	1	16253914	16286752	3	0.7438	1
MSK_00060_251469322915_S01_CGH.v4_91.txt	1	160075224	160083915	2	-0.7963	-1
MSK_00069_251469322916_S01_CGH.v4_91.txt	1	247024176	247051615	4	-1.3007	-1
MSK_00069_251469322916_S01_CGH.v4_91.txt	1	208795858	208807566	2	0.6806	1
MSK_00069_251469322916_S01_CGH.v4_91.txt	1	208654239	208767324	10	-1.1941	-1
MSK_00069_251469322916_S01_CGH.v4_91.txt	1	187812439	188007496	7	-1.5346	-1
MSK_00069_251469322916_S01_CGH.v4_91.txt	1	72121896	72181341	5	-0.7378	-1
MSK_WGA_0070.1_251469338879_S01_CGH.v4_91.txt	1	222816561	222825864	2	-0.6445	-1
MSK_00071_251469322919_S01_CGH.v4_91.txt	1	201834539	203612400	121	1.7619	1
MSK_00071_251469322919_S01_CGH.v4_91.txt	1	201161532	201673478	42	1.7134	1
MSK_00071_251469322919_S01_CGH.v4_91.txt	1	16253914	16260291	2	0.8339	1
MSK_00074_251469322717_S01_CGH.v4_91.txt	1	247024176	247051615	4	-2.6384	-1
MSK_00074_251469322717_S01_CGH.v4_91.txt	1	51450036	52425736	65	-0.8117	-1
MSK_00074_251469322717_S01_CGH.v4_91.txt	1	51089216	51413716	27	-2.3824	-1
MSK_00074_251469322717_S01_CGH.v4_91.txt	1	50397795	51074161	38	-0.8654	-1

Continued on next page...

ID	chr	start	end	nroOfprobes	segmean	call
MSK_00083_251469322918_S01_CGH.v4_91.txt	1	247024176	247051615	4	-0.8322	-1
MSK_00083_251469322918_S01_CGH.v4_91.txt	1	116135518	120430738	234	-0.7884	-1
MSK_00083_251469322918_S01_CGH.v4_91.txt	1	110171335	115831758	372	-0.775	-1
MSK_00083_251469322918_S01_CGH.v4_91.txt	1	82010562	110123621	1459	-0.809	-1
MSK_00085_251469322917_S01_CGH.v4_91.txt	1	246987317	247051615	5	-0.8573	-1
MSK_00085_251469322917_S01_CGH.v4_91.txt	1	115088325	115409073	22	0.7381	1
MSK_0087.1_251469333362_S01_CGH.v4_91.txt	1	246987317	247051615	5	-0.8383	-1
MSK_0087.1_251469333362_S01_CGH.v4_91.txt	1	145457757	145467433	2	-0.9352	-1
MSK_0087.1_251469333362_S01_CGH.v4_91.txt	1	14231078	14274378	2	-0.6423	-1
MSK_00099_251469322903_S01_CGH.v4_91.txt	1	107666273	107677381	2	-1.017	-1
MSK_00102_251469322716_S01_CGH.v4_91.txt	1	104046963	104069935	3	1.1656	1
MSK_00102_251469322716_S01_CGH.v4_91.txt	1	76448586	78812135	166	-0.8062	-1
MSK_00107_251469322719_S01_CGH.v4_91.txt	1	246987317	247051615	5	-0.7524	-1
MSK_00107_251469322719_S01_CGH.v4_91.txt	1	180468826	180476622	2	-0.6595	-1
MSK_0111.1_251469337228_S01_CGH.v4_91.txt	1	247024176	247051615	4	-0.9329	-1
MSK_00114_251469322752_S01_CGH.v4_91.txt	1	25489065	25536228	2	-0.8376	-1
MSK_00114_251469322752_S01_CGH.v4_91.txt	1	15667329	15676772	3	-0.681	-1
MSK_00114_251469322752_S01_CGH.v4_91.txt	1	614405	5434399	208	-0.7179	-1
MSK_0258.1_251469336709_S01_CGH.v4_91.txt	1	247024176	247051615	4	-1.1543	-1
MSK_0269.1_251469333522_S01_CGH.v4_91.txt	1	61386861	62179701	47	-0.9539	-1
MSK_0269.1_251469333522_S01_CGH.v4_91.txt	1	60558901	61017528	16	-0.9259	-1
MSK_0269.1_251469333522_S01_CGH.v4_91.txt	1	48343089	53605650	352	-0.8996	-1
MSK_0269.1_251469333522_S01_CGH.v4_91.txt	1	23142686	24258674	84	-0.9363	-1
MSK_0269.1_251469333522_S01_CGH.v4_91.txt	1	21121240	23045114	115	-0.8708	-1
MSK_0271.1_251469333526_S01_CGH.v4_91.txt	1	116036079	116043769	5	1.0144	1
MSK_0281.1_251469331472_S01_CGH.v4_91.txt	1	160075224	160083915	2	-1.0931	-1
MSK_0285.1_251469337229_S01_CGH.v4_91.txt	1	246987317	247051615	5	-0.7685	-1
MSK_0285.1_251469337229_S01_CGH.v4_91.txt	1	195246619	195348373	6	-0.8594	-1
MSK_0289.1_251469337015_S01_CGH.v4_91.txt	1	12865571	13043941	5	-0.6741	-1
MSK_0290.1_251469336710_S01_CGH.v4_91.txt	1	9807485	10935520	78	-0.7116	-1
MSK_0290.1_251469336710_S01_CGH.v4_91.txt	1	8015260	9783199	119	-2.1693	-1
MSK_0290.1_251469336710_S01_CGH.v4_91.txt	1	4039041	8009450	204	-0.704	-1
MSK_0317.1_251469331584_S01_CGH.v4_91.txt	1	204602069	204607667	2	-0.7857	-1
MSK_0317.1_251469331584_S01_CGH.v4_91.txt	1	195290169	195356643	6	-0.7605	-1
MSK_0321.1_251469336723_S01_CGH.v4_91.txt	1	17086830	17088836	2	0.6813	1
MSK_0330.1_251469336713_S01_CGH.v4_91.txt	1	246987317	247051615	5	-0.6394	-1
MSK_0330.1_251469336713_S01_CGH.v4_91.txt	1	200984693	203000393	139	1.1034	1
MSK_0330.1_251469336713_S01_CGH.v4_91.txt	1	195290169	195356643	6	0.7757	1
MSK_0330.1_251469336713_S01_CGH.v4_91.txt	1	195244282	195246619	2	-0.9819	-1
MSK_0332.1_251469336711_S01_CGH.v4_91.txt	1	194798525	194862188	6	-0.9429	-1
MSK_0333.1_251469331585_S01_CGH.v4_91.txt	1	243535821	245200259	121	-0.7871	-1
MSK_0333.1_251469331585_S01_CGH.v4_91.txt	1	239835812	239928683	6	-0.7207	-1
MSK_0333.1_251469331585_S01_CGH.v4_91.txt	1	222638999	222811076	16	-0.7702	-1
MSK_0333.1_251469331585_S01_CGH.v4_91.txt	1	202875119	202972428	5	0.6538	1
MSK_0333.1_251469331585_S01_CGH.v4_91.txt	1	117079386	117104573	2	-0.7523	-1
MSK_0333.1_251469331585_S01_CGH.v4_91.txt	1	67808125	76059743	417	-0.8173	-1
MSK_0333.1_251469331585_S01_CGH.v4_91.txt	1	67041005	67743788	59	-0.8316	-1
MSK_0333.1_251469331585_S01_CGH.v4_91.txt	1	59442314	59503115	4	-0.8808	-1
MSK_0333.1_251469331585_S01_CGH.v4_91.txt	1	51681866	58748433	496	-0.7716	-1
MSK_0333.1_251469331585_S01_CGH.v4_91.txt	1	49170192	51663935	154	-2.6693	-1
MSK_0333.1_251469331585_S01_CGH.v4_91.txt	1	47172048	49144541	116	-0.7459	-1
MSK_0333.1_251469331585_S01_CGH.v4_91.txt	1	20726895	28540829	538	-0.6969	-1
MSK_0333.1_251469331585_S01_CGH.v4_91.txt	1	5310157	8015260	150	-0.6826	-1
MSK_0339.1_251469331462_S01_CGH.v4_91.txt	1	12865571	12929103	3	-0.8965	-1
MSK_0422.1_251469337230_S01_CGH.v4_91.txt	1	160038537	160105927	8	-0.6904	-1
MSK_0430.1_251469336714_S01_CGH.v4_91.txt	1	247024176	247051615	4	-0.7812	-1
MSK_0430.1_251469336714_S01_CGH.v4_91.txt	1	72561136	72567752	2	-0.9545	-1
MSK_0432.1_251469336712_S01_CGH.v4_91.txt	1	246987317	247051615	5	-0.877	-1
MSK_0446.1_251469333525_S01_CGH.v4_91.txt	1	51029935	51450036	32	-1.5508	-1
MSK_0446.1_251469333525_S01_CGH.v4_91.txt	1	50940542	50989866	6	-0.6744	-1
MSK_0446.1_251469333525_S01_CGH.v4_91.txt	1	8577720	8658721	9	-0.9456	-1
MSK_0451.1_251469333565_S01_CGH.v4_91.txt	1	160047409	160066072	4	1.04	1
MSK_00126.1_251469325171_S01_CGH.v4_91.txt	1	3578591	6489679	120	-0.7896	-1
MSK_00126.1_251469325171_S01_CGH.v4_91.txt	1	3097126	3285504	12	-1.0226	-1
MSK_00129.1_251469322907_S01_CGH.v4_91.txt	1	246987317	247051615	5	-0.6637	-1
MSK_0132.1_251469330459_S01_CGH.v4_91.txt	1	246987317	247051615	5	-0.8924	-1
MSK_00133_251469325752_S01_CGH.v4_91.txt	1	74949199	74966369	3	-0.8615	-1
MSK_00137.4_251469325162_S01_CGH.v4_91.txt	1	204602069	204607667	2	-0.9888	-1
MSK_00137.4_251469325162_S01_CGH.v4_91.txt	1	8377595	9303453	75	-1.8563	-1
MSK_00137.4_251469325162_S01_CGH.v4_91.txt	1	6544488	8355433	118	-0.6319	-1
MSK_00137.4_251469325162_S01_CGH.v4_91.txt	1	4274012	6516779	92	-0.7098	-1
MSK_00139.1_251469325164_S01_CGH.v4_91.txt	1	110589500	110609898	2	-0.9343	-1
MSK_00141.1_251469325753_S01_CGH.v4_91.txt	1	49861409	49927509	5	-1.0994	-1
MSK_00143.1_251469326154_S01_CGH.v4_91.txt	1	201770060	203162673	96	4.3314	1
MSK_0149.1_251469336715_S01_CGH.v4_91.txt	1	201935154	203880096	143	2.201	1
MSK_0152.1_251469337789_S01_CGH.v4_91.txt	1	208202085	221111908	691	-1.0928	-1
MSK_0157.1_251469329178_S01_CGH.v4_91.txt	1	203253129	203289326	3	4.5553	1
MSK_0157.1_251469329178_S01_CGH.v4_91.txt	1	202575472	203206938	42	5.4645	1
MSK_0157.1_251469329178_S01_CGH.v4_91.txt	1	202546006	202566927	3	4.8206	1
MSK_0158.1_251469329734_S01_CGH.v4_91.txt	1	247024176	247051615	4	-0.8187	-1
MSK_0158.1_251469329734_S01_CGH.v4_91.txt	1	8079620	8909658	56	-0.6711	-1
MSK_0162.1_251469336905_S01_CGH.v4_91.txt	1	153779296	153784237	3	1.3844	1
MSK_0164.1_251469331554_S01_CGH.v4_91.txt	1	247024176	247051615	4	-0.8868	-1
MSK_0164.1_251469331554_S01_CGH.v4_91.txt	1	204373940	204386812	3	0.7367	1
MSK_0166.1_251469329777_S01_CGH.v4_91.txt	1	247024176	247051615	4	-0.8224	-1
MSK_0166.1_251469329777_S01_CGH.v4_91.txt	1	17086830	17088836	2	0.7575	1
MSK_0173.1_251469330461_S01_CGH.v4_91.txt	1	246987317	247051615	5	-0.8743	-1
MSK_0173.1_251469330461_S01_CGH.v4_91.txt	1	241596645	242983714	90	5.0418	1
MSK_0173.1_251469330461_S01_CGH.v4_91.txt	1	241297646	241563409	7	4.1513	1
MSK_0173.1_251469330461_S01_CGH.v4_91.txt	1	13723390	14231078	23	1.5796	1
MSK_0174.1_251469330284_S01_CGH.v4_91.txt	1	246987317	247051615	5	-0.7173	-1
MSK_0175.1_251469336725_S01_CGH.v4_91.txt	1	183136927	183151136	2	-0.7766	-1
MSK_0175.1_251469336725_S01_CGH.v4_91.txt	1	90715508	100916016	577	-0.6326	-1
MSK_0175.1_251469336725_S01_CGH.v4_91.txt	1	27866293	27893341	3	-0.6669	-1
MSK_0175.1_251469336725_S01_CGH.v4_91.txt	1	7831480	8141137	32	-1.685	-1
MSK_0177.1_251469333358_S01_CGH.v4_91.txt	1	104046963	104069935	3	0.8979	1
MSK_0178.1_251469329154_S01_CGH.v4_91.txt	1	160029416	160105927	10	0.6427	1
MSK_0182.1_251469331464_S01_CGH.v4_91.txt	1	9908768	10744389	65	-0.695	-1
MSK_0184.1_251469329181_S01_CGH.v4_91.txt	1	247024176	247051615	4	-0.7962	-1
MSK_0185.1_251469329754_S01_CGH.v4_91.txt	1	203980984	204268757	25	0.6601	1
MSK_0185.1_251469329754_S01_CGH.v4_91.txt	1	202810999	202972428	12	0.6974	1
MSK_0187.1_251469330462_S01_CGH.v4_91.txt	1	51116509	51375541	21	-0.8016	-1
MSK_0188.1_251469330469_S01_CGH.v4_91.txt	1	104046963	104069935	3	0.9035	1
MSK_0188.1_251469330469_S01_CGH.v4_91.txt	1	51116509	51450036	25	-1.5365	-1
MSK_0189.1_251469330588_S01_CGH.v4_91.txt	1	188613373	188629218	2	-1.3856	-1
MSK_0189.1_251469330588_S01_CGH.v4_91.txt	1	104046963	104069935	3	0.6372	1
MSK_0194.1_251469336985_S01_CGH.v4_91.txt	1	195290169	195356643	6	-0.6865	-1
MSK_0194.1_251469336985_S01_CGH.v4_91.txt	1	51180925	51385084	17	-0.7588	-1
MSK_0195.1_251469329772_S01_CGH.v4_91.txt	1	224363007	224403248	6	0.833	1
MSK_0208.1_251469329182_S01_CGH.v4_91.txt	1	195244282	195246619	2	-0.7863	-1
MSK_0208.2_251469329755_S01_CGH.v4_91.txt	1	204602069	204607667	2	-0.7008	-1
MSK_0208.2_251469329755_S01_CGH.v4_91.txt	1	195244282	195246619	2	-0.8277	-1
MSK_0209.1_251469330463_S01_CGH.v4_91.txt	1	246987317	247051615	5	-0.7908	-1
MSK_0209.1_251469330463_S01_CGH.v4_91.txt	1	16964399	17088836	3	0.7434	1
MSK_0210.1_251469330448_S01_CGH.v4_91.txt	1	201770060	203310147	102	3.4215	1
MSK_0210.1_251469330448_S01_CGH.v4_91.txt	1	116036079	116043769	5	0.8611	1
MSK_0210.2_251469330589_S01_CGH.v4_91.txt	1	201770060	203310147	102	3.1013	1
MSK_0210.2_251469330589_S01_CGH.v4_91.txt	1	116036079	116043769	5	0.9887	1
MSK_0211.1_251469330227_S01_CGH.v4_91.txt	1	16900658	16921451	3	-0.7125	-1
MSK_0213.1_251469329174_S01_CGH.v4_91.txt	1	246987317	247051615	5	-0.8171	-1
MSK_0214.1_251469329168_S01_CGH.v4_91.txt	1	247024176	247051615	4	-0.8186	-1
MSK_0221.1_251469330192_S01_CGH.v4_91.txt	1	246987317	247051615	5	-0.7922	-1
MSK_0221.1_251469330192_S01_CGH.v4_91.txt	1	104046963	104069935	3	0.6371	1
MSK_0221.1_251469330192_S01_CGH.v4_91.txt	1	16253914	16260291	2	0.729	1

Continued on next page...

ID	chr	start	end	nroOfprobes	segmean	call
MSK_0237.1_251469330449_S01_CGH.v4_91.txt	1	247024176	247051615	4	-0.6444	-1
MSK_0237.1_251469330449_S01_CGH.v4_91.txt	1	112285914	112330968	5	-0.6395	-1
MSK_0237.1_251469330449_S01_CGH.v4_91.txt	1	16286752	16352344	4	0.7352	1
MSK_0241.1_251469329774_S01_CGH.v4_91.txt	1	229965059	230054033	6	0.6758	1
MSK_0241.1_251469329774_S01_CGH.v4_91.txt	1	104046963	104069935	3	0.7496	1
MSK_0241.1_251469329774_S01_CGH.v4_91.txt	1	51274960	51450036	11	-0.8374	-1
MSK_0397.1_251469330468_S01_CGH.v4_91.txt	1	101744563	104231689	84	-0.788	-1
MSK_0409.1_251469331555_S01_CGH.v4_91.txt	1	247024176	247051615	4	-0.7163	-1
MSK_0409.1_251469331555_S01_CGH.v4_91.txt	1	16961116	16964399	2	-1.6082	-1
MSK_0409.1_251469331555_S01_CGH.v4_91.txt	1	6517333	6525686	2	0.6392	1
MSK_0410.1_251469333359_S01_CGH.v4_91.txt	1	51074161	51385084	27	-3.0171	-1
MSK_0410.1_251469333359_S01_CGH.v4_91.txt	1	50907520	51060593	13	-0.8136	-1
MSK_0412.1_251469331490_S01_CGH.v4_91.txt	1	247024176	247051615	4	-0.8851	-1
MSK_0412.1_251469331490_S01_CGH.v4_91.txt	1	203012679	203699547	48	3.8737	1
MSK_0412.1_251469331490_S01_CGH.v4_91.txt	1	202810999	203000393	13	4.5408	1
MSK_0412.1_251469331490_S01_CGH.v4_91.txt	1	201770060	202795134	72	3.8772	1
MSK_0412.1_251469331490_S01_CGH.v4_91.txt	1	116036079	116043769	5	0.7778	1
MSK_0412.1_251469331490_S01_CGH.v4_91.txt	1	87111650	87361473	16	3.956	1
MSK_0413.1_251469331465_S01_CGH.v4_91.txt	1	200830689	200853125	4	-0.6511	-1
MSK_0413.1_251469331465_S01_CGH.v4_91.txt	1	116036079	116042877	4	-0.8546	-1
MSK_0414.1_251469336986_S01_CGH.v4_91.txt	1	17086830	17094607	3	0.8637	1
MSK_WGA_0348.1_251469338869_S01_CGH.v4_91.txt	1	104556978	104870294	9	-0.6745	-1
MSK_WGA_0348.1_251469338869_S01_CGH.v4_91.txt	1	87679692	104231689	859	-0.6718	-1
MSK_WGA_0348.1_251469338869_S01_CGH.v4_91.txt	1	87385844	87448176	7	-0.681	-1
MSK_WGA_0348.1_251469338869_S01_CGH.v4_91.txt	1	86391973	86597800	11	-0.7775	-1
MSK_WGA_0348.1_251469338869_S01_CGH.v4_91.txt	1	71124598	86314990	792	-0.7085	-1
MSK_WGA_0348.1_251469338869_S01_CGH.v4_91.txt	1	70399676	70771419	26	-0.6507	-1
MSK_0349.1_251469337892_S01_CGH.v4_91.txt	1	247024176	247051615	4	-0.6445	-1
MSK_0349.1_251469337892_S01_CGH.v4_91.txt	1	72200603	72242199	3	-0.6942	-1
MSK_WGA_0353.1_251469337506_S01_CGH.v4_91.txt	1	26789909	27531718	62	0.6919	1
MSK_WGA_0355.1_251469337508_S01_CGH.v4_91.txt	1	32982329	33108376	12	0.668	1
MSK_WGA_0355.1_251469337508_S01_CGH.v4_91.txt	1	2949375	3462999	27	0.6416	1
MSK_WGA_0356.1_251469337509_S01_CGH.v4_91.txt	1	71808632	76059743	204	-0.661	-1
MSK_WGA_0357.1_251469337522_S01_CGH.v4_91.txt	1	236423011	236443757	2	-0.8016	-1
MSK_WGA_0357.1_251469337522_S01_CGH.v4_91.txt	1	201632233	204059131	172	3.6449	1
MSK_WGA_0360.1_251469337524_S01_CGH.v4_91.txt	1	51180925	51385084	17	-0.7346	-1
MSK_WGA_0389.1_251469338874_S01_CGH.v4_91.txt	1	1579898	9610396	422	-0.6723	-1
MSK_WGA_0392.1_251469338876_S01_CGH.v4_91.txt	1	153798751	153835467	5	0.7753	1
MSK_0509.1_251469336717_S01_CGH.v4_91.txt	1	247024176	247051615	4	-4.3265	-1
MSK_0509.1_251469336717_S01_CGH.v4_91.txt	1	195244282	195246619	2	-0.9957	-1
MSK_0509.1_251469336717_S01_CGH.v4_91.txt	1	190291659	190316843	2	-1.1575	-1
MSK_0509.1_251469336717_S01_CGH.v4_91.txt	1	16926576	16964399	3	-0.9602	-1
MSK_0510.1_251469331581_S01_CGH.v4_91.txt	1	183136927	183151136	2	-0.983	-1
MSK_0510.1_251469331581_S01_CGH.v4_91.txt	1	166521793	166896562	29	-0.8551	-1
MSK_0510.1_251469331581_S01_CGH.v4_91.txt	1	165566035	166462354	62	-0.8506	-1
MSK_0510.1_251469331581_S01_CGH.v4_91.txt	1	165299088	165512622	23	-0.8521	-1
MSK_0510.1_251469331581_S01_CGH.v4_91.txt	1	25489065	25536228	2	-0.9232	-1
MSK_0510.1_251469331581_S01_CGH.v4_91.txt	1	7702225	8084866	40	-0.8799	-1
MSK_0511.1_251469336907_S01_CGH.v4_91.txt	1	99850234	99996754	4	-0.907	-1
MSK_0512.1_251469331556_S01_CGH.v4_91.txt	1	195244282	195246619	2	-0.6528	-1
MSK_0514.1_251469333360_S01_CGH.v4_91.txt	1	244485622	244672468	23	-0.8058	-1
MSK_0514.1_251469333360_S01_CGH.v4_91.txt	1	202228622	203554233	89	3.8387	1
MSK_0514.1_251469333360_S01_CGH.v4_91.txt	1	27678405	31159356	165	-0.6685	-1
MSK_0514.1_251469333360_S01_CGH.v4_91.txt	1	6544488	9638993	212	-0.6794	-1
MSK_0514.1_251469333360_S01_CGH.v4_91.txt	1	2625062	6516779	157	-0.6569	-1
MSK_0516.1_251469331469_S01_CGH.v4_91.txt	1	25489065	25536228	2	-0.8305	-1
MSK_0517.1_251469337226_S01_CGH.v4_91.txt	1	25489065	25536228	2	-0.8294	-1
MSK_0517.1_251469337226_S01_CGH.v4_91.txt	1	614405	6275651	233	-0.7575	-1
MSK_0518.1_251469337014_S01_CGH.v4_91.txt	1	66611959	67100599	35	0.7805	1
MSK_0518.1_251469337014_S01_CGH.v4_91.txt	1	5978302	8355433	152	-0.8551	-1
MSK_0520.1_251469336708_S01_CGH.v4_91.txt	1	81166299	81226128	3	-0.7882	-1
MSK_0520.1_251469336708_S01_CGH.v4_91.txt	1	68909829	68986098	4	-0.8642	-1
MSK_0520.1_251469336708_S01_CGH.v4_91.txt	1	19538295	28484369	621	-0.6352	-1
MSK_0520.1_251469336708_S01_CGH.v4_91.txt	1	9828376	10002036	13	-0.638	-1
MSK_0520.1_251469336708_S01_CGH.v4_91.txt	1	6544488	9783199	219	-0.6492	-1
MSK_0521.1_251469331582_S01_CGH.v4_91.txt	1	246987317	247051615	5	-0.8675	-1
MSK_0521.1_251469331582_S01_CGH.v4_91.txt	1	79748656	79792856	2	-0.6886	-1
MSK_0524.1_251469331557_S01_CGH.v4_91.txt	1	145056302	247367223	6120	0.8519	1
MSK_0524.1_251469331557_S01_CGH.v4_91.txt	1	142549947	144811606	44	0.8451	1
MSK_0524.1_251469331557_S01_CGH.v4_91.txt	1	25489065	25536228	2	-0.7054	-1
MSK_0524.1_251469331557_S01_CGH.v4_91.txt	1	16964399	17094607	4	0.6438	1
MSK_0524.1_251469331557_S01_CGH.v4_91.txt	1	12924248	13043941	4	-1.0056	-1
MSK_0525.1_251469333361_S01_CGH.v4_91.txt	1	104046963	104069935	3	0.7216	1
MSK_0525.1_251469333361_S01_CGH.v4_91.txt	1	23667522	24066043	39	-0.6451	-1
MSK_0529.1_251469331470_S01_CGH.v4_91.txt	1	246987317	247051615	5	-0.91	-1
MSK_0529.1_251469331470_S01_CGH.v4_91.txt	1	241715793	242983714	84	3.1325	1
MSK_0529.1_251469331470_S01_CGH.v4_91.txt	1	162181289	162412728	5	-0.6725	-1
MSK_0529.1_251469331470_S01_CGH.v4_91.txt	1	56945719	56958241	3	-0.6879	-1
MSK_0529.1_251469331470_S01_CGH.v4_91.txt	1	53625008	54361255	51	-0.7757	-1
MSK_0529.1_251469331470_S01_CGH.v4_91.txt	1	53243865	53442516	18	-0.8449	-1
MSK_0529.1_251469331470_S01_CGH.v4_91.txt	1	52102956	52886944	57	-0.8043	-1
MSK_0529.1_251469331470_S01_CGH.v4_91.txt	1	51663935	51697447	3	-0.8184	-1
MSK_0529.1_251469331470_S01_CGH.v4_91.txt	1	51128551	51385084	21	-0.7932	-1
MSK_0529.1_251469331470_S01_CGH.v4_91.txt	1	47196193	47229825	6	-0.7825	-1
MSK_0529.1_251469331470_S01_CGH.v4_91.txt	1	16921451	16964399	4	-0.7353	-1
MSK_0529.1_251469331470_S01_CGH.v4_91.txt	1	15409680	15912742	46	-0.7449	-1
MSK_0529.1_251469331470_S01_CGH.v4_91.txt	1	14712532	14787073	4	-0.7691	-1
MSK_0529.1_251469331470_S01_CGH.v4_91.txt	1	13931999	14063922	10	-0.8129	-1
MSK_0529.1_251469331470_S01_CGH.v4_91.txt	1	10935520	13787681	146	-0.7447	-1
MSK_0529.1_251469331470_S01_CGH.v4_91.txt	1	10083429	10452662	29	-0.7555	-1
MSK_0529.1_251469331470_S01_CGH.v4_91.txt	1	8623643	10022375	105	-0.7673	-1
MSK_0531.1_251469337227_S01_CGH.v4_91.txt	1	246832793	246875813	4	0.9213	1
MSK_0531.1_251469337227_S01_CGH.v4_91.txt	1	158432559	159141449	59	0.746	1
MSK_0531.1_251469337227_S01_CGH.v4_91.txt	1	154679235	155627268	89	1.0669	1
MSK_0531.1_251469337227_S01_CGH.v4_91.txt	1	115952912	116042877	9	-0.6728	-1
MSK_0531.1_251469337227_S01_CGH.v4_91.txt	1	41807463	42020411	15	-0.7968	-1
MSK_0531.1_251469337227_S01_CGH.v4_91.txt	1	41457783	41595773	13	-0.8402	-1
MSK_0531.1_251469337227_S01_CGH.v4_91.txt	1	19102654	24258674	354	-0.7739	-1
MSK_0531.1_251469337227_S01_CGH.v4_91.txt	1	11936819	11972951	5	-0.7948	-1
MSK_0531.1_251469337227_S01_CGH.v4_91.txt	1	11513843	11751143	11	-0.8858	-1
MSK_0531.1_251469337227_S01_CGH.v4_91.txt	1	9458789	11109589	107	-0.7954	-1
MSK_0531.1_251469337227_S01_CGH.v4_91.txt	1	8355433	9421527	87	-2.647	-1
MSK_0531.1_251469337227_S01_CGH.v4_91.txt	1	7798208	8294223	39	-0.8366	-1
MSK_0531.1_251469337227_S01_CGH.v4_91.txt	1	1220378	3838945	124	-0.6825	-1
MSK_0619.1_251469337893_S01_CGH.v4_91.txt	1	246987317	247051615	5	-3.9874	-1
MSK_0619.1_251469337893_S01_CGH.v4_91.txt	1	195244282	195246619	2	-1.0523	-1
MSK_00006_251469322732_S01_CGH.v4_91.txt	2	140836810	140891202	2	-0.8439	-1
MSK_00006_251469322732_S01_CGH.v4_91.txt	2	133129406	133140718	2	-0.7495	-1
MSK_00010_251469322627_S01_CGH.v4_91.txt	2	24859262	25079822	16	0.6921	1
MSK_00014_251469322629_S01_CGH.v4_91.txt	2	161104885	190627010	1765	-0.9131	-1
MSK_00014_251469322629_S01_CGH.v4_91.txt	2	39006420	41436663	132	-0.8845	-1
MSK_00014_251469322629_S01_CGH.v4_91.txt	2	20996660	21191446	10	1.3593	1
MSK_00014_251469322629_S01_CGH.v4_91.txt	2	10071015	20982160	480	2.0375	1
MSK_00014_251469322629_S01_CGH.v4_91.txt	2	9430029	10056030	47	-0.9035	-1
MSK_00014_251469322629_S01_CGH.v4_91.txt	2	30341	39193	2	-0.7818	-1
MSK_WGA_0015.1_251469338754_S01_CGH.v4_91.txt	2	240366800	240505975	11	-0.6941	-1
MSK_WGA_0015.1_251469338754_S01_CGH.v4_91.txt	2	238877296	240204516	72	-0.7073	-1
MSK_WGA_0015.1_251469338754_S01_CGH.v4_91.txt	2	236934328	238102427	66	-0.6878	-1
MSK_WGA_0015.1_251469338754_S01_CGH.v4_91.txt	2	235575928	236649612	33	-0.7996	-1
MSK_WGA_0015.1_251469338754_S01_CGH.v4_91.txt	2	211775548	213633978	117	-0.7569	-1
MSK_WGA_0016.1_251469338755_S01_CGH.v4_91.txt	2	142275732	142284899	2	-0.6371	-1
MSK_WGA_0016.1_251469338755_S01_CGH.v4_91.txt	2	114194296	114292619	3	-0.6536	-1
MSK_WGA_0016.1_251469338755_S01_CGH.v4_91.txt	2	56446902	60531352	170	-0.6645	-1
MSK_WGA_0016.1_251469338755_S01_CGH.v4_91.txt	2	56281343	56323250	4	-0.6839	-1

Continued on next page...

ID	chr	start	end	nroOfprobes	segmean	call
MSK_00033_251469322707_S01_CGH.v4_91.txt	2	50658390	50678386	3	-1.0817	-1
MSK_00038_251469322735_S01_CGH.v4_91.txt	2	66683288	71753293	330	-0.6699	-1
MSK_00038_251469322735_S01_CGH.v4_91.txt	2	66647448	66667094	2	-1.4283	-1
MSK_00043_251469322736_S01_CGH.v4_91.txt	2	683662	2119736	97	-0.9258	-1
MSK_WGA_0048.1_251469338742_S01_CGH.v4_91.txt	2	91677350	91693432	2	0.7406	1
MSK_00052_251469323033_S01_CGH.v4_91.txt	2	231537957	231547192	2	0.7098	1
MSK_00054_251469323034_S01_CGH.v4_91.txt	2	228365758	228373979	2	0.8274	1
MSK_00054_251469323034_S01_CGH.v4_91.txt	2	90090336	90292111	6	-0.9287	-1
MSK_00060_251469322915_S01_CGH.v4_91.txt	2	185881241	185887156	2	1.037	1
MSK_00074_251469322717_S01_CGH.v4_91.txt	2	17269001	25562499	386	-0.931	-1
MSK_0084.1_251469337921_S01_CGH.v4_91.txt	2	80897114	80907630	2	-1.5813	-1
MSK_00102_251469322716_S01_CGH.v4_91.txt	2	242997679	243095475	8	-0.7087	-1
MSK_WGA_0104.1_251469338880_S01_CGH.v4_91.txt	2	242997679	243095475	8	-0.7875	-1
MSK_WGA_0104.1_251469338880_S01_CGH.v4_91.txt	2	241481328	242613956	86	-0.7835	-1
MSK_WGA_0104.1_251469338880_S01_CGH.v4_91.txt	2	233387874	241414401	451	-0.8096	-1
MSK_WGA_0104.1_251469338880_S01_CGH.v4_91.txt	2	232479272	233302315	58	-0.8252	-1
MSK_WGA_0104.1_251469338880_S01_CGH.v4_91.txt	2	12114144	20600199	318	0.9175	1
MSK_0111.1_251469337228_S01_CGH.v4_91.txt	2	90163984	90316408	6	-0.8926	-1
MSK_00113_251469322906_S01_CGH.v4_91.txt	2	108280545	108364141	4	-0.727	-1
MSK_00114_251469322752_S01_CGH.v4_91.txt	2	30341	565575	45	-0.9497	-1
MSK_00116_251469322720_S01_CGH.v4_91.txt	2	107225036	121394547	678	-0.7123	-1
MSK_00116_251469322720_S01_CGH.v4_91.txt	2	99177196	107075624	447	-0.711	-1
MSK_00116_251469322720_S01_CGH.v4_91.txt	2	91677350	91693432	2	0.9326	1
MSK_0258.1_251469336709_S01_CGH.v4_91.txt	2	243084904	243095475	2	-0.9901	-1
MSK_0258.1_251469336709_S01_CGH.v4_91.txt	2	106889414	107075624	7	-0.7428	-1
MSK_0258.1_251469336709_S01_CGH.v4_91.txt	2	30341	31722427	1698	0.7389	1
MSK_0266.1_251469336909_S01_CGH.v4_91.txt	2	130939325	130942871	2	0.6749	1
MSK_0266.1_251469336909_S01_CGH.v4_91.txt	2	87434224	88063506	8	-0.7684	-1
MSK_0269.1_251469333522_S01_CGH.v4_91.txt	2	90090336	90292111	6	-0.8805	-1
MSK_0271.1_251469333526_S01_CGH.v4_91.txt	2	233703609	233718710	3	-1.9788	-1
MSK_0271.1_251469333526_S01_CGH.v4_91.txt	2	228365758	228373979	2	0.7865	1
MSK_0281.1_251469331472_S01_CGH.v4_91.txt	2	179100041	179222876	11	1.0482	1
MSK_0281.1_251469331472_S01_CGH.v4_91.txt	2	85934529	86098346	12	0.9818	1
MSK_0281.1_251469331472_S01_CGH.v4_91.txt	2	24622445	24756548	7	1.0483	1
MSK_0285.1_251469337229_S01_CGH.v4_91.txt	2	80897114	80907630	2	0.8875	1
MSK_0289.1_251469337015_S01_CGH.v4_91.txt	2	80897114	80907630	2	0.9205	1
MSK_0290.1_251469336710_S01_CGH.v4_91.txt	2	243084904	243095475	2	-0.8722	-1
MSK_0290.1_251469336710_S01_CGH.v4_91.txt	2	80897114	80907630	2	0.9444	1
MSK_0317.1_251469331584_S01_CGH.v4_91.txt	2	80897114	80907630	2	0.8209	1
MSK_0325.1_251469333563_S01_CGH.v4_91.txt	2	242997679	243095475	8	-0.7393	-1
MSK_0330.1_251469336713_S01_CGH.v4_91.txt	2	27822603	27832384	2	-0.7646	-1
MSK_0332.1_251469336711_S01_CGH.v4_91.txt	2	242997679	243095475	8	-0.8377	-1
MSK_0332.1_251469336711_S01_CGH.v4_91.txt	2	242560815	242567819	3	-0.8853	-1
MSK_0332.1_251469336711_S01_CGH.v4_91.txt	2	132212219	132235666	3	-0.6943	-1
MSK_0332.1_251469336711_S01_CGH.v4_91.txt	2	35924321	36119992	4	-0.9254	-1
MSK_0333.1_251469331585_S01_CGH.v4_91.txt	2	242997679	243095475	8	-0.6813	-1
MSK_0333.1_251469331585_S01_CGH.v4_91.txt	2	234929697	236294673	55	-0.746	-1
MSK_0333.1_251469331585_S01_CGH.v4_91.txt	2	216759179	217692109	68	-0.7691	-1
MSK_0338.1_251469333564_S01_CGH.v4_91.txt	2	80897114	80907630	2	0.8808	1
MSK_0339.1_251469331462_S01_CGH.v4_91.txt	2	90163984	91677350	7	-0.8624	-1
MSK_0430.1_251469336714_S01_CGH.v4_91.txt	2	165953862	165966136	3	0.8042	1
MSK_0446.1_251469333525_S01_CGH.v4_91.txt	2	64761046	64795279	3	-0.6601	-1
MSK_0446.1_251469333525_S01_CGH.v4_91.txt	2	57631133	57660291	2	-0.6411	-1
MSK_00122.1_251469325751_S01_CGH.v4_91.txt	2	233378916	233429462	5	0.6972	1
MSK_00122.1_251469325751_S01_CGH.v4_91.txt	2	141534028	141983037	36	-0.6506	-1
MSK_00122.1_251469325751_S01_CGH.v4_91.txt	2	7952066	8026172	3	-0.984	-1
MSK_0127.1_251469337891_S01_CGH.v4_91.txt	2	80897114	80907630	2	-1.5334	-1
MSK_00128.1_251469325161_S01_CGH.v4_91.txt	2	98105893	98116763	2	-0.9445	-1
MSK_00129.1_251469322907_S01_CGH.v4_91.txt	2	242997679	243095475	8	-0.7314	-1
MSK_0146.1_251469336983_S01_CGH.v4_91.txt	2	243084904	243095475	2	-0.8145	-1
MSK_0149.1_251469336715_S01_CGH.v4_91.txt	2	165953862	165966136	3	0.666	1
MSK_0149.1_251469336715_S01_CGH.v4_91.txt	2	90090336	90316408	7	-0.8352	-1
MSK_0162.1_251469336905_S01_CGH.v4_91.txt	2	203378715	203390961	2	-0.8619	-1
MSK_0171.1_251469329753_S01_CGH.v4_91.txt	2	87434224	88013008	6	0.6792	1
MSK_0173.1_251469330461_S01_CGH.v4_91.txt	2	242997679	243095475	8	-0.6696	-1
MSK_0173.1_251469330461_S01_CGH.v4_91.txt	2	242560815	242567819	3	-0.7871	-1
MSK_0174.1_251469330284_S01_CGH.v4_91.txt	2	235371747	235680908	15	0.6389	1
MSK_0174.1_251469330284_S01_CGH.v4_91.txt	2	199308691	200517526	51	-0.7985	-1
MSK_0175.1_251469336725_S01_CGH.v4_91.txt	2	242997679	243095475	8	-0.7622	-1
MSK_0175.1_251469336725_S01_CGH.v4_91.txt	2	242560815	242567819	3	-0.813	-1
MSK_0178.1_251469329154_S01_CGH.v4_91.txt	2	228365758	228373979	2	0.7023	1
MSK_0179.1_251469333566_S01_CGH.v4_91.txt	2	241626814	241640508	2	0.6937	1
MSK_0182.1_251469331464_S01_CGH.v4_91.txt	2	89540386	90316408	11	-0.9256	-1
MSK_0184.1_251469329181_S01_CGH.v4_91.txt	2	188691422	188886239	10	-0.982	-1
MSK_0185.1_251469329754_S01_CGH.v4_91.txt	2	228365758	228373979	2	0.6609	1
MSK_0187.1_251469330462_S01_CGH.v4_91.txt	2	161890889	161963624	3	-0.7496	-1
MSK_0188.1_251469330469_S01_CGH.v4_91.txt	2	242997679	243095475	8	-0.7907	-1
MSK_0188.1_251469330469_S01_CGH.v4_91.txt	2	242560815	242567819	3	-0.8437	-1
MSK_0190.1_251469330591_S01_CGH.v4_91.txt	2	90075480	90292111	7	-0.8823	-1
MSK_0197.1_251469329155_S01_CGH.v4_91.txt	2	27584011	27588426	2	0.6465	1
MSK_0208.1_251469329182_S01_CGH.v4_91.txt	2	82278185	82351769	3	0.6565	1
MSK_0208.1_251469329182_S01_CGH.v4_91.txt	2	26737686	26752597	2	-0.7032	-1
MSK_0208.2_251469329755_S01_CGH.v4_91.txt	2	82278185	82351769	3	0.6576	1
MSK_0209.1_251469330463_S01_CGH.v4_91.txt	2	181317467	181386573	3	1.0412	1
MSK_0209.1_251469330463_S01_CGH.v4_91.txt	2	121316705	121963272	25	0.8532	1
MSK_0209.1_251469330463_S01_CGH.v4_91.txt	2	67300524	67637342	16	0.9244	1
MSK_0210.1_251469330448_S01_CGH.v4_91.txt	2	7185322	7231001	6	2.0634	1
MSK_0210.1_251469330448_S01_CGH.v4_91.txt	2	5342165	5403688	2	2.2379	1
MSK_0210.2_251469330589_S01_CGH.v4_91.txt	2	7185322	7231001	6	1.5336	1
MSK_0211.1_251469330227_S01_CGH.v4_91.txt	2	242997679	243095475	8	-0.7952	-1
MSK_0211.1_251469330227_S01_CGH.v4_91.txt	2	242560815	242567819	3	-0.8083	-1
MSK_0211.2_251469329773_S01_CGH.v4_91.txt	2	242560815	242567819	3	-0.7455	-1
MSK_0211.2_251469329773_S01_CGH.v4_91.txt	2	237288039	237853588	30	-0.6937	-1
MSK_0219.1_251469329864_S01_CGH.v4_91.txt	2	239912309	239922475	2	-0.8648	-1
MSK_0221.1_251469330192_S01_CGH.v4_91.txt	2	30341	495052	39	1.0902	1
MSK_0237.1_251469330449_S01_CGH.v4_91.txt	2	23897416	24070299	11	-0.7934	-1
MSK_0241.1_251469329774_S01_CGH.v4_91.txt	2	132553683	132586206	3	-0.7557	-1
MSK_0397.1_251469330468_S01_CGH.v4_91.txt	2	132212219	132235666	3	-0.6513	-1
MSK_0410.1_251469333359_S01_CGH.v4_91.txt	2	132553683	132607158	4	-0.7207	-1
MSK_0410.1_251469333359_S01_CGH.v4_91.txt	2	132212219	132215312	2	-0.7546	-1
MSK_0412.1_251469331490_S01_CGH.v4_91.txt	2	114194296	114292619	3	-0.7412	-1
MSK_0646.1_251469340746_S01_CGH.v4_91.txt	2	62309884	62335237	5	-0.8513	-1
MSK_WGA_0244.1_251469338881_S01_CGH.v4_91.txt	2	141734138	142063211	25	-0.6801	-1
MSK_0345.1_251469337922_S01_CGH.v4_91.txt	2	80897114	80907630	2	-1.48	-1
MSK_WGA_0355.1_251469337508_S01_CGH.v4_91.txt	2	229595582	231761618	141	-0.7033	-1
MSK_WGA_0355.1_251469337508_S01_CGH.v4_91.txt	2	12232351	12658900	23	-0.708	-1
MSK_WGA_0356.1_251469337509_S01_CGH.v4_91.txt	2	43053689	43072694	3	0.713	1
MSK_0358.1_251469340789_S01_CGH.v4_91.txt	2	114194296	114436188	4	-0.8237	-1
MSK_WGA_0359.1_251469337523_S01_CGH.v4_91.txt	2	162773895	166248846	198	-0.7085	-1
MSK_WGA_0390.1_251469338875_S01_CGH.v4_91.txt	2	133129406	133154020	3	0.8311	1
MSK_WGA_0392.1_251469338876_S01_CGH.v4_91.txt	2	141868792	142085009	20	-0.755	-1
MSK_0509.1_251469336717_S01_CGH.v4_91.txt	2	27822603	27832384	2	-1.0118	-1
MSK_0517.1_251469337226_S01_CGH.v4_91.txt	2	204895896	205603781	28	0.65	1
MSK_0517.1_251469337226_S01_CGH.v4_91.txt	2	203496930	203731813	19	0.6478	1
MSK_0517.1_251469337226_S01_CGH.v4_91.txt	2	186255825	186334758	3	0.6376	1
MSK_0518.1_251469337014_S01_CGH.v4_91.txt	2	165960575	165966136	2	0.6333	1
MSK_0520.1_251469336708_S01_CGH.v4_91.txt	2	90163984	90292111	5	-0.8737	-1
MSK_0522.1_251469336908_S01_CGH.v4_91.txt	2	188167387	188206409	2	-0.7985	-1
MSK_0524.1_251469331557_S01_CGH.v4_91.txt	2	44580443	44596834	2	1.3093	1
MSK_0525.1_251469333361_S01_CGH.v4_91.txt	2	242560815	242567819	3	-0.7775	-1
MSK_0529.1_251469331470_S01_CGH.v4_91.txt	2	11428514	11440667	2	-0.6612	-1
MSK_0531.1_251469337227_S01_CGH.v4_91.txt	2	58119813	58500167	30	-0.9141	-1
MSK_0531.1_251469337227_S01_CGH.v4_91.txt	2	57522028	57856470	6	-0.9676	-1
MSK_0618.1_251469337983_S01_CGH.v4_91.txt	2	80897114	80907630	2	-1.372	-1

Continued on next page...

ID	chr	start	end	nroOfprobes	segmean	call
MSK_0619.1_251469337893_S01_CGH.v4_91.txt	2	106387698	106642129	15	1.337	1
MSK_0619.1_251469337893_S01_CGH.v4_91.txt	2	100562958	100961647	32	1.0339	1
MSK_0619.1_251469337893_S01_CGH.v4_91.txt	2	88448404	88874408	22	1.0742	1
MSK_0619.1_251469337893_S01_CGH.v4_91.txt	2	72831356	73226990	34	1.0749	1
MSK_0619.1_251469337893_S01_CGH.v4_91.txt	2	70377424	71811731	105	0.985	1
MSK_0619.1_251469337893_S01_CGH.v4_91.txt	2	14496255	17171439	106	3.3677	1
MSK_0620.1_251469337791_S01_CGH.v4_91.txt	2	114194296	114436188	4	-0.9934	-1
MSK_0620.1_251469337791_S01_CGH.v4_91.txt	2	80897114	80907630	2	-1.6119	-1
MSK_00003_251469322731_S01_CGH.v4_91.txt	3	48720857	48739359	4	0.642	1
MSK_00010_251469322627_S01_CGH.v4_91.txt	3	164766983	197840903	1976	-0.7679	-1
MSK_00011_251469322628_S01_CGH.v4_91.txt	3	195423082	195463451	5	0.7974	1
MSK_00011_251469322628_S01_CGH.v4_91.txt	3	89405719	89416743	2	-1.061	-1
MSK_00011_251469322628_S01_CGH.v4_91.txt	3	64066	1865637	116	-0.8618	-1
MSK_00014_251469322629_S01_CGH.v4_91.txt	3	55955540	55960993	2	-0.8772	-1
MSK_00014_251469322629_S01_CGH.v4_91.txt	3	50362319	54028295	268	-0.8347	-1
MSK_00014_251469322629_S01_CGH.v4_91.txt	3	31796776	50339735	1204	-0.8814	-1
MSK_WGA_0015.1_251469338754_S01_CGH.v4_91.txt	3	52575500	57886137	397	-0.6856	-1
MSK_WGA_0015.1_251469338754_S01_CGH.v4_91.txt	3	48687058	49673422	92	-0.6818	-1
MSK_WGA_0015.1_251469338754_S01_CGH.v4_91.txt	3	18500605	48603039	1694	-0.6871	-1
MSK_WGA_0015.1_251469338754_S01_CGH.v4_91.txt	3	6966433	18457493	789	-0.6701	-1
MSK_00021_251469322630_S01_CGH.v4_91.txt	3	190385460	195175217	265	-0.8175	-1
MSK_00021_251469322630_S01_CGH.v4_91.txt	3	125682711	128539901	171	-0.7782	-1
MSK_00021_251469322630_S01_CGH.v4_91.txt	3	114959491	125401676	666	-0.8084	-1
MSK_00021_251469322630_S01_CGH.v4_91.txt	3	67929319	67961054	24	-0.6735	-1
MSK_WGA_0023.1_251469338756_S01_CGH.v4_91.txt	3	190876790	192505217	81	-0.6342	-1
MSK_00033_251469322707_S01_CGH.v4_91.txt	3	195423082	195463451	5	0.7163	1
MSK_00038_251469322735_S01_CGH.v4_91.txt	3	195423082	195451559	4	-0.8247	-1
MSK_00043_251469322736_S01_CGH.v4_91.txt	3	195423082	195451559	4	-0.6556	-1
MSK_WGA_0048.1_251469338742_S01_CGH.v4_91.txt	3	187943262	188401988	41	1.8104	1
MSK_WGA_0048.1_251469338742_S01_CGH.v4_91.txt	3	177748816	187913140	603	2.286	1
MSK_WGA_0048.1_251469338742_S01_CGH.v4_91.txt	3	177530231	177710024	8	1.2628	1
MSK_WGA_0048.1_251469338742_S01_CGH.v4_91.txt	3	177288082	177479129	10	1.8331	1
MSK_WGA_0048.1_251469338742_S01_CGH.v4_91.txt	3	177090565	177246501	4	1.2385	1
MSK_WGA_0048.1_251469338742_S01_CGH.v4_91.txt	3	176180830	177010527	38	1.247	1
MSK_WGA_0048.1_251469338742_S01_CGH.v4_91.txt	3	25329398	25362375	3	-0.6874	-1
MSK_00055_251469323018_S01_CGH.v4_91.txt	3	195423082	195451559	4	-0.8133	-1
MSK_00057_251469323017_S01_CGH.v4_91.txt	3	195423082	195463451	5	0.7323	1
MSK_00083_251469322918_S01_CGH.v4_91.txt	3	195423082	195451559	4	-0.7136	-1
MSK_00083_251469322918_S01_CGH.v4_91.txt	3	68069930	75346301	383	-0.8151	-1
MSK_00083_251469322918_S01_CGH.v4_91.txt	3	50362319	67872509	1170	-0.7751	-1
MSK_00083_251469322918_S01_CGH.v4_91.txt	3	64066	50342171	3059	-0.7714	-1
MSK_00085_251469322917_S01_CGH.v4_91.txt	3	195423082	195463451	5	1.1038	1
MSK_00086_251469322749_S01_CGH.v4_91.txt	3	195423082	195463451	5	0.8082	1
MSK_00089_251469322904_S01_CGH.v4_91.txt	3	195423082	195451559	4	0.6573	1
MSK_WGA_0104.1_251469338880_S01_CGH.v4_91.txt	3	55125105	57779982	198	0.8124	1
MSK_WGA_0104.1_251469338880_S01_CGH.v4_91.txt	3	52564742	54825816	170	-0.8034	-1
MSK_WGA_0104.1_251469338880_S01_CGH.v4_91.txt	3	52041620	52542917	42	-0.8272	-1
MSK_WGA_0104.1_251469338880_S01_CGH.v4_91.txt	3	45608790	45887801	28	0.7886	1
MSK_WGA_0104.1_251469338880_S01_CGH.v4_91.txt	3	41390195	45313269	243	0.7838	1
MSK_WGA_0104.1_251469338880_S01_CGH.v4_91.txt	3	38490466	40348580	130	0.7714	1
MSK_WGA_0104.1_251469338880_S01_CGH.v4_91.txt	3	35483550	38455514	195	-0.7643	-1
MSK_WGA_0104.1_251469338880_S01_CGH.v4_91.txt	3	35399834	35444746	4	0.7459	1
MSK_WGA_0104.1_251469338880_S01_CGH.v4_91.txt	3	16190306	22229796	324	-0.7586	-1
MSK_WGA_0104.1_251469338880_S01_CGH.v4_91.txt	3	1635803	16178352	947	0.7764	1
MSK_WGA_0104.1_251469338880_S01_CGH.v4_91.txt	3	241076	1492662	90	-0.7575	-1
MSK_WGA_0104.1_251469338880_S01_CGH.v4_91.txt	3	64066	225925	7	-3.0178	-1
MSK_00114_251469322752_S01_CGH.v4_91.txt	3	195423082	195451559	4	-0.7173	-1
MSK_00114_251469322752_S01_CGH.v4_91.txt	3	75888543	85832518	442	-0.935	-1
MSK_00114_251469322752_S01_CGH.v4_91.txt	3	68069930	75346301	383	-0.9047	-1
MSK_00114_251469322752_S01_CGH.v4_91.txt	3	50362319	67872509	1170	-0.8555	-1
MSK_00114_251469322752_S01_CGH.v4_91.txt	3	37360517	50281837	900	-0.7864	-1
MSK_00116_251469322720_S01_CGH.v4_91.txt	3	28841451	34384842	312	-0.8518	-1
MSK_0266.1_251469336909_S01_CGH.v4_91.txt	3	195423082	195463451	5	0.6742	1
MSK_0266.1_251469336909_S01_CGH.v4_91.txt	3	89405719	89416743	2	-3.0134	-1
MSK_0271.1_251469333526_S01_CGH.v4_91.txt	3	195423082	195463451	5	0.6359	1
MSK_0271.1_251469333526_S01_CGH.v4_91.txt	3	151531707	151546281	3	-2.6737	-1
MSK_0281.1_251469331472_S01_CGH.v4_91.txt	3	195423082	195451559	4	-0.8018	-1
MSK_0281.1_251469331472_S01_CGH.v4_91.txt	3	89405719	89416743	2	-0.7106	-1
MSK_0281.1_251469331472_S01_CGH.v4_91.txt	3	65367725	65637656	22	-0.6875	-1
MSK_0289.1_251469337015_S01_CGH.v4_91.txt	3	135225987	146471448	583	-0.7501	-1
MSK_0289.1_251469337015_S01_CGH.v4_91.txt	3	129959535	135022569	322	-0.7576	-1
MSK_0289.1_251469337015_S01_CGH.v4_91.txt	3	125682711	129717274	266	-0.7956	-1
MSK_0289.1_251469337015_S01_CGH.v4_91.txt	3	116781727	125401676	565	-0.7597	-1
MSK_0290.1_251469336710_S01_CGH.v4_91.txt	3	151837066	151853508	2	-0.7249	-1
MSK_0290.1_251469336710_S01_CGH.v4_91.txt	3	151469103	151477188	2	0.7183	1
MSK_0317.1_251469331584_S01_CGH.v4_91.txt	3	177841657	178056903	4	-0.8499	-1
MSK_0317.1_251469331584_S01_CGH.v4_91.txt	3	172363489	177406569	298	-0.7294	-1
MSK_0317.1_251469331584_S01_CGH.v4_91.txt	3	169707250	170668371	72	-0.7372	-1
MSK_0317.1_251469331584_S01_CGH.v4_91.txt	3	84242618	84550338	8	0.6489	1
MSK_0326.1_251469331461_S01_CGH.v4_91.txt	3	3882385	3941397	7	-1.0018	-1
MSK_0330.1_251469336713_S01_CGH.v4_91.txt	3	195423082	195451559	4	-1.2743	-1
MSK_0333.1_251469331585_S01_CGH.v4_91.txt	3	197416450	197456505	5	-0.795	-1
MSK_0333.1_251469331585_S01_CGH.v4_91.txt	3	162895764	162919615	3	-0.9602	-1
MSK_0337.1_251469333524_S01_CGH.v4_91.txt	3	60544701	60642727	8	-0.7653	-1
MSK_0337.1_251469333524_S01_CGH.v4_91.txt	3	1117683	1232146	10	-0.9056	-1
MSK_0451.1_251469333565_S01_CGH.v4_91.txt	3	195423082	195451559	4	-0.7859	-1
MSK_0451.1_251469333565_S01_CGH.v4_91.txt	3	154395462	154465454	2	-1.5486	-1
MSK_00122.1_251469325751_S01_CGH.v4_91.txt	3	195429788	195451559	3	-0.7908	-1
MSK_00122.1_251469325751_S01_CGH.v4_91.txt	3	89405719	89416743	2	-0.9997	-1
MSK_0132.1_251469330459_S01_CGH.v4_91.txt	3	195423082	195436390	3	-0.6798	-1
MSK_0146.1_251469336983_S01_CGH.v4_91.txt	3	8825797	8835685	2	0.8082	1
MSK_0158.1_251469329734_S01_CGH.v4_91.txt	3	195423082	195451559	4	-0.767	-1
MSK_0164.1_251469331554_S01_CGH.v4_91.txt	3	169646348	169656220	2	-1.0606	-1
MSK_0173.1_251469330461_S01_CGH.v4_91.txt	3	195423082	195463451	5	0.8558	1
MSK_0174.1_251469330284_S01_CGH.v4_91.txt	3	189039769	189191248	7	-0.9595	-1
MSK_0175.1_251469336725_S01_CGH.v4_91.txt	3	190959475	196811852	352	-0.6363	-1
MSK_0175.1_251469336725_S01_CGH.v4_91.txt	3	159634854	159646735	2	0.6521	1
MSK_0177.1_251469333358_S01_CGH.v4_91.txt	3	60269909	60279030	2	-0.6325	-1
MSK_0182.1_251469331464_S01_CGH.v4_91.txt	3	31818566	31827265	2	-1.136	-1
MSK_0185.1_251469329754_S01_CGH.v4_91.txt	3	195423082	195451559	4	-0.6668	-1
MSK_0187.1_251469330462_S01_CGH.v4_91.txt	3	100308872	101039303	44	-0.7636	-1
MSK_0187.1_251469330462_S01_CGH.v4_91.txt	3	97605830	97714257	12	-0.7762	-1
MSK_0187.1_251469330462_S01_CGH.v4_91.txt	3	94738635	94777882	3	-0.8415	-1
MSK_0187.1_251469330462_S01_CGH.v4_91.txt	3	64066	4550622	308	-0.7516	-1
MSK_0190.1_251469330591_S01_CGH.v4_91.txt	3	155519925	155520514	2	-0.9007	-1
MSK_0190.1_251469330591_S01_CGH.v4_91.txt	3	151531707	151546281	3	-0.9738	-1
MSK_0194.1_251469336985_S01_CGH.v4_91.txt	3	89405719	89416743	2	-0.9535	-1
MSK_0197.1_251469329155_S01_CGH.v4_91.txt	3	195423082	195451559	4	-0.6841	-1
MSK_0209.1_251469330463_S01_CGH.v4_91.txt	3	189991961	190810987	44	4.858	1
MSK_0209.1_251469330463_S01_CGH.v4_91.txt	3	119610191	119615780	2	-1.0377	-1
MSK_0210.2_251469330589_S01_CGH.v4_91.txt	3	195423082	195451559	4	0.9527	1
MSK_0211.1_251469330227_S01_CGH.v4_91.txt	3	89405719	89416743	2	-0.883	-1
MSK_0213.1_251469329174_S01_CGH.v4_91.txt	3	175244331	175262511	3	0.6385	1
MSK_0213.1_251469329174_S01_CGH.v4_91.txt	3	89405719	89416743	2	-0.9118	-1
MSK_0219.1_251469329864_S01_CGH.v4_91.txt	3	192352414	192360809	2	-1.0757	-1
MSK_0221.1_251469330192_S01_CGH.v4_91.txt	3	17446385	17469931	2	-0.8448	-1
MSK_0237.1_251469330449_S01_CGH.v4_91.txt	3	117273475	117600150	14	-0.7642	-1
MSK_0237.1_251469330449_S01_CGH.v4_91.txt	3	85199016	85226892	3	-0.7555	-1
MSK_0237.1_251469330449_S01_CGH.v4_91.txt	3	47125594	47180832	6	-0.7135	-1
MSK_0237.1_251469330449_S01_CGH.v4_91.txt	3	29818707	29890378	8	-0.7038	-1
MSK_0238.1_251469340788_S01_CGH.v4_91.txt	3	85375339	85561010	23	-0.8712	-1
MSK_0241.1_251469329774_S01_CGH.v4_91.txt	3	190810987	193680517	159	-0.8489	-1
MSK_0241.1_251469329774_S01_CGH.v4_91.txt	3	75775649	75793214	3	-0.8307	-1
MSK_0397.1_251469330468_S01_CGH.v4_91.txt	3	188096924	188315419	22	-0.7744	-1

Continued on next page...

ID	chr	start	end	nroOfprobes	segmean	call
MSK_0397.1_251469330468_S01_CGH.v4_91.txt	3	116021997	116085280	6	-0.8163	-1
MSK_0397.1_251469330468_S01_CGH.v4_91.txt	3	115882996	116009976	12	-2.2634	-1
MSK_0397.1_251469330468_S01_CGH.v4_91.txt	3	115627388	115875144	21	-0.7099	-1
MSK_0402.1_251469336906_S01_CGH.v4_91.txt	3	191413767	192092045	28	1.727	1
MSK_0402.1_251469336906_S01_CGH.v4_91.txt	3	177804571	183564322	295	1.906	1
MSK_0413.1_251469331465_S01_CGH.v4_91.txt	3	195423082	195451559	4	1.0612	1
MSK_0413.1_251469331465_S01_CGH.v4_91.txt	3	89405719	89416743	2	-0.8925	-1
MSK_0645.1_251469340797_S01_CGH.v4_91.txt	3	100347505	100432966	7	0.9295	1
MSK_WGA_0246.1_251469338865_S01_CGH.v4_91.txt	3	116943394	117178355	7	-0.997	-1
MSK_0345.1_251469337922_S01_CGH.v4_91.txt	3	195423082	195451559	4	-0.7297	-1
MSK_0349.1_251469337892_S01_CGH.v4_91.txt	3	195423082	195451559	4	-0.8345	-1
MSK_WGA_0353.1_251469337506_S01_CGH.v4_91.txt	3	197402667	197899082	42	2.0513	1
MSK_WGA_0353.1_251469337506_S01_CGH.v4_91.txt	3	197377768	197389279	2	1.061	1
MSK_WGA_0353.1_251469337506_S01_CGH.v4_91.txt	3	191162117	197320957	373	2.0816	1
MSK_WGA_0353.1_251469337506_S01_CGH.v4_91.txt	3	186736591	191107366	265	1.3171	1
MSK_WGA_0353.1_251469337506_S01_CGH.v4_91.txt	3	183519296	186718384	242	1.9034	1
MSK_WGA_0353.1_251469337506_S01_CGH.v4_91.txt	3	182763285	183508394	64	2.4989	1
MSK_WGA_0353.1_251469337506_S01_CGH.v4_91.txt	3	176627840	182751819	274	1.7069	1
MSK_WGA_0353.1_251469337506_S01_CGH.v4_91.txt	3	174870033	176602262	85	1.2853	1
MSK_WGA_0353.1_251469337506_S01_CGH.v4_91.txt	3	67929319	67961054	24	2.1092	1
MSK_0373.1_251469340742_S01_CGH.v4_91.txt	3	195423082	195451559	4	-0.9149	-1
MSK_WGA_0390.1_251469338875_S01_CGH.v4_91.txt	3	115882996	116142196	21	-1.034	-1
MSK_WGA_0390.1_251469338875_S01_CGH.v4_91.txt	3	48603039	48687058	6	0.7	1
MSK_WGA_0392.1_251469338876_S01_CGH.v4_91.txt	3	195857780	197840903	156	0.66	1
MSK_WGA_0392.1_251469338876_S01_CGH.v4_91.txt	3	193281181	193503028	20	0.6613	1
MSK_WGA_0392.1_251469338876_S01_CGH.v4_91.txt	3	129267837	129333691	2	1.3815	1
MSK_WGA_0392.1_251469338876_S01_CGH.v4_91.txt	3	93538467	113867303	1063	-0.6377	-1
MSK_0509.1_251469336717_S01_CGH.v4_91.txt	3	195423082	195451559	4	-0.912	-1
MSK_0509.1_251469336717_S01_CGH.v4_91.txt	3	38309677	38326410	2	-0.8843	-1
MSK_0510.1_251469331581_S01_CGH.v4_91.txt	3	195423082	195463451	5	0.8585	1
MSK_0511.1_251469336907_S01_CGH.v4_91.txt	3	51613907	54185464	204	-0.6473	-1
MSK_0511.1_251469336907_S01_CGH.v4_91.txt	3	41944646	46601428	289	-0.7112	-1
MSK_0511.1_251469336907_S01_CGH.v4_91.txt	3	38842626	41915567	194	-0.7076	-1
MSK_0512.1_251469331556_S01_CGH.v4_91.txt	3	128338930	128442950	6	0.6601	1
MSK_0514.1_251469333360_S01_CGH.v4_91.txt	3	188490098	188509958	2	-0.8123	-1
MSK_0517.1_251469337226_S01_CGH.v4_91.txt	3	41944646	63378707	1518	-0.813	-1
MSK_0517.1_251469337226_S01_CGH.v4_91.txt	3	25683837	41915567	886	-0.8294	-1
MSK_0518.1_251469337014_S01_CGH.v4_91.txt	3	89405719	89416743	2	-1.0007	-1
MSK_0520.1_251469336708_S01_CGH.v4_91.txt	3	99610672	99616788	2	-0.8001	-1
MSK_0520.1_251469336708_S01_CGH.v4_91.txt	3	1293460	3090367	132	-0.7073	-1
MSK_0521.1_251469331582_S01_CGH.v4_91.txt	3	195423082	195463451	5	0.7482	1
MSK_0525.1_251469333361_S01_CGH.v4_91.txt	3	196896440	197320957	31	0.6982	1
MSK_0529.1_251469331470_S01_CGH.v4_91.txt	3	195423082	195451559	4	-0.9463	-1
MSK_0529.1_251469331470_S01_CGH.v4_91.txt	3	63284851	63378707	7	-0.8594	-1
MSK_0529.1_251469331470_S01_CGH.v4_91.txt	3	58665559	61330047	186	-0.8073	-1
MSK_0531.1_251469337227_S01_CGH.v4_91.txt	3	195463451	197899082	178	-0.8241	-1
MSK_0531.1_251469337227_S01_CGH.v4_91.txt	3	195423082	195451559	4	-1.501	-1
MSK_0531.1_251469337227_S01_CGH.v4_91.txt	3	193323445	195175217	118	-0.813	-1
MSK_0531.1_251469337227_S01_CGH.v4_91.txt	3	185307880	192870629	463	-0.8492	-1
MSK_0531.1_251469337227_S01_CGH.v4_91.txt	3	67929319	67961054	24	-0.7458	-1
MSK_0616.1_251469338022_S01_CGH.v4_91.txt	3	195423082	195451559	4	-0.798	-1
MSK_0618.1_251469337983_S01_CGH.v4_91.txt	3	175863131	181719687	264	1.892	1
MSK_0619.1_251469337893_S01_CGH.v4_91.txt	3	191742485	191861319	6	1.709	1
MSK_0619.1_251469337893_S01_CGH.v4_91.txt	3	183262086	183530311	22	1.23	1
MSK_0619.1_251469337893_S01_CGH.v4_91.txt	3	148708939	149514671	59	1.0492	1
MSK_0619.1_251469337893_S01_CGH.v4_91.txt	3	141625990	142811007	86	1.0376	1
MSK_0619.1_251469337893_S01_CGH.v4_91.txt	3	137953948	138855974	62	1.081	1
MSK_0619.1_251469337893_S01_CGH.v4_91.txt	3	113468703	113672520	21	1.322	1
MSK_0620.1_251469337791_S01_CGH.v4_91.txt	3	161435364	161537898	4	-1.0665	-1
MSK_00007_251469322733_S01_CGH.v4_91.txt	4	114432579	114519729	9	0.6767	1
MSK_00010_251469322627_S01_CGH.v4_91.txt	4	93528556	94482135	76	-0.6722	-1
MSK_00011_251469322628_S01_CGH.v4_91.txt	4	189128716	190907779	91	-0.8137	-1
MSK_00014_251469322629_S01_CGH.v4_91.txt	4	188817347	191021051	114	0.8212	1
MSK_00014_251469322629_S01_CGH.v4_91.txt	4	176193026	177879179	90	0.9237	1
MSK_00014_251469322629_S01_CGH.v4_91.txt	4	166100863	175482673	518	-0.9019	-1
MSK_00014_251469322629_S01_CGH.v4_91.txt	4	133969830	136800341	81	-1.0086	-1
MSK_00014_251469322629_S01_CGH.v4_91.txt	4	57332806	58721319	97	1.2088	1
MSK_00014_251469322629_S01_CGH.v4_91.txt	4	57092557	57320648	11	1.954	1
MSK_00014_251469322629_S01_CGH.v4_91.txt	4	52835272	57068520	266	2.634	1
MSK_00014_251469322629_S01_CGH.v4_91.txt	4	9874509	49229402	1871	-0.8272	-1
MSK_00014_251469322629_S01_CGH.v4_91.txt	4	4309294	8717501	284	-0.7496	-1
MSK_00014_251469322629_S01_CGH.v4_91.txt	4	51413	3886055	242	-0.6964	-1
MSK_WGA_0016.1_251469338755_S01_CGH.v4_91.txt	4	187893613	189946495	123	-0.6838	-1
MSK_00024_251469322721_S01_CGH.v4_91.txt	4	104005505	108707234	226	-1.4829	-1
MSK_00024_251469322721_S01_CGH.v4_91.txt	4	101936305	103944900	120	-1.4666	-1
MSK_00024_251469322721_S01_CGH.v4_91.txt	4	54985217	56943365	102	3.4635	1
MSK_00024_251469322721_S01_CGH.v4_91.txt	4	54595824	54976618	26	2.6977	1
MSK_00028_251469322706_S01_CGH.v4_91.txt	4	62122389	104944632	2471	-0.9368	-1
MSK_00028_251469322706_S01_CGH.v4_91.txt	4	55654694	56543534	47	-0.9015	-1
MSK_00033_251469322707_S01_CGH.v4_91.txt	4	70318717	70338618	2	0.646	1
MSK_00037_251469322734_S01_CGH.v4_91.txt	4	94217808	94601017	31	-0.9127	-1
MSK_00037_251469322734_S01_CGH.v4_91.txt	4	54898527	54989688	9	1.005	1
MSK_00043_251469322736_S01_CGH.v4_91.txt	4	112467115	123956953	564	-0.9256	-1
MSK_00043_251469322736_S01_CGH.v4_91.txt	4	16091639	16319455	15	-0.8734	-1
MSK_00046_251469322737_S01_CGH.v4_91.txt	4	119657747	119690950	2	1.1208	1
MSK_WGA_0048.1_251469338742_S01_CGH.v4_91.txt	4	148901153	149271478	29	-0.7382	-1
MSK_00055_251469323018_S01_CGH.v4_91.txt	4	185925364	186349819	31	0.6958	1
MSK_WGA_0068.1_251469338745_S01_CGH.v4_91.txt	4	158748953	159046648	7	-0.6471	-1
MSK_00069_251469322916_S01_CGH.v4_91.txt	4	70407754	191021051	6423	0.6522	1
MSK_00069_251469322916_S01_CGH.v4_91.txt	4	52835272	63403258	538	1.3581	1
MSK_00074_251469322717_S01_CGH.v4_91.txt	4	52835272	55905246	205	3.9466	1
MSK_0079.1_251469338020_S01_CGH.v4_91.txt	4	119204161	119756505	32	1.4454	1
MSK_0079.1_251469338020_S01_CGH.v4_91.txt	4	113544885	114046147	33	1.6515	1
MSK_0079.1_251469338020_S01_CGH.v4_91.txt	4	108571227	109525457	54	1.6487	1
MSK_0079.1_251469338020_S01_CGH.v4_91.txt	4	107941072	108303925	21	1.6436	1
MSK_0079.1_251469338020_S01_CGH.v4_91.txt	4	98170037	98247021	4	1.8817	1
MSK_0079.1_251469338020_S01_CGH.v4_91.txt	4	95695387	96442837	61	1.0675	1
MSK_0079.1_251469338020_S01_CGH.v4_91.txt	4	67201161	67289380	4	1.5949	1
MSK_0079.1_251469338020_S01_CGH.v4_91.txt	4	64421479	64513800	3	1.4727	1
MSK_0079.1_251469338020_S01_CGH.v4_91.txt	4	54225151	55707967	100	1.6556	1
MSK_00080_251469322748_S01_CGH.v4_91.txt	4	40221244	40290457	6	-1.0093	-1
MSK_00083_251469322918_S01_CGH.v4_91.txt	4	94217808	94386113	13	-0.8765	-1
MSK_00083_251469322918_S01_CGH.v4_91.txt	4	31341344	31643213	8	-0.8934	-1
MSK_00086_251469322749_S01_CGH.v4_91.txt	4	181833611	181863323	2	-0.8335	-1
MSK_WGA_0104.1_251469338880_S01_CGH.v4_91.txt	4	119739100	119918178	14	-0.6987	-1
MSK_WGA_0104.1_251469338880_S01_CGH.v4_91.txt	4	93693097	94153380	41	-0.7318	-1
MSK_WGA_0104.1_251469338880_S01_CGH.v4_91.txt	4	51413	129753	7	-0.7105	-1
MSK_0111.1_251469337228_S01_CGH.v4_91.txt	4	33084274	33120987	2	-0.7931	-1
MSK_00115_251469322751_S01_CGH.v4_91.txt	4	70313248	70338618	3	-1.2891	-1
MSK_00116_251469322720_S01_CGH.v4_91.txt	4	9874509	15168595	229	-0.9278	-1
MSK_00116_251469322720_S01_CGH.v4_91.txt	4	6748837	8717501	132	-0.7672	-1
MSK_0258.1_251469336709_S01_CGH.v4_91.txt	4	173872813	175848217	123	-0.802	-1
MSK_0266.1_251469336909_S01_CGH.v4_91.txt	4	13777234	13805118	4	-3.8392	-1
MSK_0281.1_251469331472_S01_CGH.v4_91.txt	4	119494963	119526655	2	-0.7333	-1
MSK_0289.1_251469337015_S01_CGH.v4_91.txt	4	190681400	190690401	2	0.7723	1
MSK_0289.1_251469337015_S01_CGH.v4_91.txt	4	183862569	184488656	38	-0.7401	-1
MSK_0289.1_251469337015_S01_CGH.v4_91.txt	4	163065586	163911399	33	-0.7926	-1
MSK_0289.1_251469337015_S01_CGH.v4_91.txt	4	109707067	110388162	58	-0.7669	-1
MSK_0289.1_251469337015_S01_CGH.v4_91.txt	4	108303925	109501568	58	-0.772	-1
MSK_0325.1_251469333563_S01_CGH.v4_91.txt	4	151457700	151779123	25	-0.8858	-1
MSK_0330.1_251469336713_S01_CGH.v4_91.txt	4	70313248	70338618	3	-1.0583	-1
MSK_0333.1_251469331585_S01_CGH.v4_91.txt	4	178453670	181994023	104	-0.86	-1
MSK_0333.1_251469331585_S01_CGH.v4_91.txt	4	167200014	169058044	79	-0.8419	-1
MSK_0339.1_251469331462_S01_CGH.v4_91.txt	4	54402894	55374569	73	3.0726	1

Continued on next page...

ID	chr	start	end	nroOfprobes	segmean	call
MSK_0422.1_251469337230_S01_CGH.v4_91.txt	4	70313248	70338618	3	-1.2129	-1
MSK_0440.1_251469336724_S01_CGH.v4_91.txt	4	54852513	55526632	45	4.2376	1
MSK_0440.1_251469336724_S01_CGH.v4_91.txt	4	54412335	54595824	17	1.9433	1
MSK_0440.1_251469336724_S01_CGH.v4_91.txt	4	54318330	54402894	8	4.4245	1
MSK_0440.1_251469336724_S01_CGH.v4_91.txt	4	54085752	54113797	3	4.1944	1
MSK_0446.1_251469333525_S01_CGH.v4_91.txt	4	107509404	107571174	2	-0.7244	-1
MSK_0446.1_251469333525_S01_CGH.v4_91.txt	4	57068520	59662650	132	2.1425	1
MSK_0446.1_251469333525_S01_CGH.v4_91.txt	4	54639275	57059343	134	4.3804	1
MSK_0446.1_251469333525_S01_CGH.v4_91.txt	4	54076814	54616654	45	2.2001	1
MSK_0451.1_251469333565_S01_CGH.v4_91.txt	4	70318717	70338618	2	-0.7852	-1
MSK_00129.1_251469322907_S01_CGH.v4_91.txt	4	150903520	190907779	2126	-0.7752	-1
MSK_00129.1_251469322907_S01_CGH.v4_91.txt	4	150177797	150233374	3	-0.6864	-1
MSK_00129.1_251469322907_S01_CGH.v4_91.txt	4	145513400	148620134	176	-0.8005	-1
MSK_00129.1_251469322907_S01_CGH.v4_91.txt	4	135333264	137469458	59	-0.7445	-1
MSK_00130.1_251469325022_S01_CGH.v4_91.txt	4	164700357	164804923	10	-0.9808	-1
MSK_00143.1_251469326154_S01_CGH.v4_91.txt	4	188017448	188032905	2	0.9422	1
MSK_00143.1_251469326154_S01_CGH.v4_91.txt	4	54878223	55905246	60	1.4471	1
MSK_0157.1_251469329178_S01_CGH.v4_91.txt	4	98928429	99003236	8	-0.9681	-1
MSK_0157.1_251469329178_S01_CGH.v4_91.txt	4	70318717	70338618	2	-0.818	-1
MSK_0166.1_251469329777_S01_CGH.v4_91.txt	4	71168447	71176354	2	-1.2162	-1
MSK_0166.1_251469329777_S01_CGH.v4_91.txt	4	52835272	59983624	404	0.6916	1
MSK_0173.1_251469330461_S01_CGH.v4_91.txt	4	190468262	190607433	7	-0.8398	-1
MSK_0173.1_251469330461_S01_CGH.v4_91.txt	4	119494963	119690950	9	2.6632	1
MSK_0173.1_251469330461_S01_CGH.v4_91.txt	4	54912187	55325323	35	0.7416	1
MSK_0174.1_251469330284_S01_CGH.v4_91.txt	4	93703322	93713738	2	-1.2819	-1
MSK_0174.1_251469330284_S01_CGH.v4_91.txt	4	61440892	64594261	120	-0.8627	-1
MSK_0174.1_251469330284_S01_CGH.v4_91.txt	4	56333782	59070476	166	-0.8091	-1
MSK_0174.1_251469330284_S01_CGH.v4_91.txt	4	56144036	56300329	5	4.9032	1
MSK_0174.1_251469330284_S01_CGH.v4_91.txt	4	55374569	56133406	31	0.9789	1
MSK_0174.1_251469330284_S01_CGH.v4_91.txt	4	54449724	55325323	69	4.8586	1
MSK_0174.1_251469330284_S01_CGH.v4_91.txt	4	53483750	54440331	75	0.8033	1
MSK_0174.1_251469330284_S01_CGH.v4_91.txt	4	53346231	53388454	3	4.9412	1
MSK_0174.1_251469330284_S01_CGH.v4_91.txt	4	52835272	53315640	36	5.7579	1
MSK_0175.1_251469336725_S01_CGH.v4_91.txt	4	189376099	189488652	8	-0.718	-1
MSK_0175.1_251469336725_S01_CGH.v4_91.txt	4	176517697	189234032	666	-0.6719	-1
MSK_0175.1_251469336725_S01_CGH.v4_91.txt	4	168037013	168059678	3	-0.6654	-1
MSK_0177.1_251469333358_S01_CGH.v4_91.txt	4	56813441	57811544	76	0.9902	1
MSK_0177.1_251469333358_S01_CGH.v4_91.txt	4	53054705	55490647	167	3.5823	1
MSK_0182.1_251469331464_S01_CGH.v4_91.txt	4	182591891	182711624	3	-0.7182	-1
MSK_0182.1_251469331464_S01_CGH.v4_91.txt	4	53533444	53843917	25	-1.0315	-1
MSK_0188.1_251469330469_S01_CGH.v4_91.txt	4	130840472	130884772	4	-0.9877	-1
MSK_0189.1_251469330588_S01_CGH.v4_91.txt	4	47150909	47165391	2	-0.8448	-1
MSK_0194.1_251469336985_S01_CGH.v4_91.txt	4	70313248	70338618	3	-0.6404	-1
MSK_0195.1_251469329772_S01_CGH.v4_91.txt	4	54821806	55687860	54	0.6364	1
MSK_0201.1_251469329757_S01_CGH.v4_91.txt	4	93703322	93713738	2	-0.9094	-1
MSK_0209.1_251469330463_S01_CGH.v4_91.txt	4	182476596	182542068	2	-0.6953	-1
MSK_0210.1_251469330448_S01_CGH.v4_91.txt	4	54513357	55759044	81	1.3006	1
MSK_0210.2_251469330589_S01_CGH.v4_91.txt	4	54513357	55759044	81	0.8383	1
MSK_0211.1_251469330227_S01_CGH.v4_91.txt	4	54821806	55687860	54	1.8577	1
MSK_0211.2_251469329773_S01_CGH.v4_91.txt	4	54821806	55687860	54	1.4136	1
MSK_0214.1_251469329168_S01_CGH.v4_91.txt	4	54527298	55429932	64	2.7582	1
MSK_0214.1_251469329168_S01_CGH.v4_91.txt	4	33489699	33666925	5	-0.9508	-1
MSK_0221.1_251469330192_S01_CGH.v4_91.txt	4	131822998	132020759	6	1.8069	1
MSK_0221.1_251469330192_S01_CGH.v4_91.txt	4	91800566	92078993	27	1.9629	1
MSK_0221.1_251469330192_S01_CGH.v4_91.txt	4	53483750	56429432	189	1.6058	1
MSK_0237.1_251469330449_S01_CGH.v4_91.txt	4	148477141	148584592	9	-0.7559	-1
MSK_0241.1_251469329774_S01_CGH.v4_91.txt	4	169894329	180417475	524	-0.8348	-1
MSK_0241.1_251469329774_S01_CGH.v4_91.txt	4	152246703	154361908	127	-0.7652	-1
MSK_0241.1_251469329774_S01_CGH.v4_91.txt	4	146384984	151975952	336	-0.8304	-1
MSK_0241.1_251469329774_S01_CGH.v4_91.txt	4	140983818	141932895	75	-0.8445	-1
MSK_0241.1_251469329774_S01_CGH.v4_91.txt	4	140438068	140461160	3	-0.6327	-1
MSK_0241.1_251469329774_S01_CGH.v4_91.txt	4	138666465	140219740	56	-0.7812	-1
MSK_0241.1_251469329774_S01_CGH.v4_91.txt	4	138267141	138507870	7	-0.8311	-1
MSK_0241.1_251469329774_S01_CGH.v4_91.txt	4	125738111	138083754	405	-0.875	-1
MSK_0241.1_251469329774_S01_CGH.v4_91.txt	4	123653155	123770404	9	-0.7797	-1
MSK_0241.1_251469329774_S01_CGH.v4_91.txt	4	86338338	86400049	3	-0.9437	-1
MSK_0241.1_251469329774_S01_CGH.v4_91.txt	4	55814204	56162814	15	5.6083	1
MSK_0241.1_251469329774_S01_CGH.v4_91.txt	4	55159469	55777977	29	6.2672	1
MSK_0241.1_251469329774_S01_CGH.v4_91.txt	4	54821806	55145285	31	5.4319	1
MSK_0402.1_251469336906_S01_CGH.v4_91.txt	4	98675681	98977377	23	0.9793	1
MSK_0409.1_251469331555_S01_CGH.v4_91.txt	4	145068372	145124568	3	-0.6413	-1
MSK_0410.1_251469333359_S01_CGH.v4_91.txt	4	98913450	99161054	20	-1.3727	-1
MSK_0410.1_251469333359_S01_CGH.v4_91.txt	4	53315640	58366261	328	3.1766	1
MSK_0414.1_251469336986_S01_CGH.v4_91.txt	4	127706156	128261977	14	-1.0526	-1
MSK_0414.1_251469336986_S01_CGH.v4_91.txt	4	124147758	124155366	2	-0.7881	-1
MSK_0648.1_251469338021_S01_CGH.v4_91.txt	4	173589463	173598293	2	-1.2902	-1
MSK_0648.1_251469338021_S01_CGH.v4_91.txt	4	20467426	20694039	25	-0.8599	-1
MSK_WGA_0246.1_251469338865_S01_CGH.v4_91.txt	4	182201663	182591891	10	-1.0545	-1
MSK_0345.1_251469337922_S01_CGH.v4_91.txt	4	190681400	190800684	6	-0.888	-1
MSK_WGA_0347.1_251469338868_S01_CGH.v4_91.txt	4	58418373	60353902	49	-0.6849	-1
MSK_WGA_0347.1_251469338868_S01_CGH.v4_91.txt	4	52835272	58366261	363	2.5166	1
MSK_0349.1_251469337892_S01_CGH.v4_91.txt	4	190681400	190800684	6	-0.8338	-1
MSK_0349.1_251469337892_S01_CGH.v4_91.txt	4	173589463	173598293	2	-1.0377	-1
MSK_0358.1_251469340789_S01_CGH.v4_91.txt	4	176804952	191021051	732	-0.8073	-1
MSK_0358.1_251469340789_S01_CGH.v4_91.txt	4	64667522	69369246	195	-0.8098	-1
MSK_WGA_0360.1_251469337524_S01_CGH.v4_91.txt	4	54888505	55026149	15	0.6723	1
MSK_0373.1_251469340742_S01_CGH.v4_91.txt	4	190681400	190809026	7	-0.9527	-1
MSK_0509.1_251469336717_S01_CGH.v4_91.txt	4	93047656	93073479	2	-1.0513	-1
MSK_0514.1_251469333360_S01_CGH.v4_91.txt	4	2877639	2967188	6	0.6445	1
MSK_0516.1_251469331469_S01_CGH.v4_91.txt	4	178592245	182759143	110	0.9081	1
MSK_0520.1_251469336708_S01_CGH.v4_91.txt	4	162889454	162951918	7	-0.8859	-1
MSK_0520.1_251469336708_S01_CGH.v4_91.txt	4	130851433	130884772	3	-1.1431	-1
MSK_0524.1_251469331557_S01_CGH.v4_91.txt	4	187396916	187790728	38	-0.9162	-1
MSK_0524.1_251469331557_S01_CGH.v4_91.txt	4	54687276	55844789	71	5.3665	1
MSK_0524.1_251469331557_S01_CGH.v4_91.txt	4	41840416	41846378	2	5.3715	1
MSK_0524.1_251469331557_S01_CGH.v4_91.txt	4	39600871	39624876	4	5.3992	1
MSK_0525.1_251469333361_S01_CGH.v4_91.txt	4	75717040	76226620	23	-1.0132	-1
MSK_0525.1_251469333361_S01_CGH.v4_91.txt	4	36653339	38896919	106	-1.3394	-1
MSK_0529.1_251469331470_S01_CGH.v4_91.txt	4	70313248	70338618	3	-1.4227	-1
MSK_0615.1_251469340783_S01_CGH.v4_91.txt	4	190681400	190800684	6	-1.1468	-1
MSK_0616.1_251469338022_S01_CGH.v4_91.txt	4	110388162	110414392	2	5.7016	1
MSK_0616.1_251469338022_S01_CGH.v4_91.txt	4	107721898	107755789	2	5.7523	1
MSK_0616.1_251469338022_S01_CGH.v4_91.txt	4	85308542	85344829	4	5.953	1
MSK_0616.1_251469338022_S01_CGH.v4_91.txt	4	68277073	68316265	2	6.1967	1
MSK_00001_251469322729_S01_CGH.v4_91.txt	5	686292	806176	7	0.7371	1
MSK_00006_251469322732_S01_CGH.v4_91.txt	5	164510588	164563294	3	-0.6756	-1
MSK_00010_251469322627_S01_CGH.v4_91.txt	5	90445843	96612017	358	-0.9934	-1
MSK_00010_251469322627_S01_CGH.v4_91.txt	5	88348147	90204368	80	-1.0136	-1
MSK_00011_251469322628_S01_CGH.v4_91.txt	5	49559920	180696747	7179	-0.7725	-1
MSK_00011_251469322628_S01_CGH.v4_91.txt	5	24666498	24856014	6	-3.1898	-1
MSK_00014_251469322629_S01_CGH.v4_91.txt	5	21574676	27187998	248	-0.9853	-1
MSK_00014_251469322629_S01_CGH.v4_91.txt	5	37692	21453181	1101	-0.91	-1
MSK_00038_251469322735_S01_CGH.v4_91.txt	5	174624578	175087401	23	-0.6898	-1
MSK_00046_251469322737_S01_CGH.v4_91.txt	5	134916418	134943207	3	-0.7991	-1
MSK_00060_251469322915_S01_CGH.v4_91.txt	5	41235047	41242781	2	-0.9923	-1
MSK_00060_251469322915_S01_CGH.v4_91.txt	5	759169	806176	4	1.4081	1
MSK_00069_251469322916_S01_CGH.v4_91.txt	5	27697683	29943023	55	0.6327	1
MSK_WGA_0070.1_251469338879_S01_CGH.v4_91.txt	5	178540792	178774657	12	-0.6844	-1
MSK_00074_251469322717_S01_CGH.v4_91.txt	5	69705562	70586959	3	0.9646	1
MSK_00083_251469322918_S01_CGH.v4_91.txt	5	177410262	177413892	2	-0.8903	-1
MSK_00083_251469322918_S01_CGH.v4_91.txt	5	175915860	176178142	22	1.3859	1
MSK_00083_251469322918_S01_CGH.v4_91.txt	5	166051337	166228856	6	-0.9046	-1
MSK_0084.1_251469337921_S01_CGH.v4_91.txt	5	109760507	109881401	9	-1.449	-1
MSK_00086_251469322749_S01_CGH.v4_91.txt	5	117375481	117392999	2	0.7401	1

Continued on next page...

ID	chr	start	end	nroOfprobes	segmean	call
MSK_00086_251469322749_S01_CGH.v4_91.txt	5	759169	806176	4	0.717	1
MSK_WGA_0104.1_251469338880_S01_CGH.v4_91.txt	5	162847124	180696747	977	-0.7997	-1
MSK_0106.1_251469331471_S01_CGH.v4_91.txt	5	133352105	133359854	2	0.6425	1
MSK_00107_251469322719_S01_CGH.v4_91.txt	5	32110734	32167579	6	0.6567	1
MSK_00113_251469322906_S01_CGH.v4_91.txt	5	759169	806176	4	2.2554	1
MSK_00114_251469322752_S01_CGH.v4_91.txt	5	114866969	139372091	1400	-0.8653	-1
MSK_00114_251469322752_S01_CGH.v4_91.txt	5	50123854	52434304	84	-0.9565	-1
MSK_00114_251469322752_S01_CGH.v4_91.txt	5	44924444	46115027	51	-0.9586	-1
MSK_00114_251469322752_S01_CGH.v4_91.txt	5	759169	806176	4	1.0088	1
MSK_00115_251469322751_S01_CGH.v4_91.txt	5	35166083	35788691	28	-1.1468	-1
MSK_00115_251469322751_S01_CGH.v4_91.txt	5	33913838	34341324	21	-1.1372	-1
MSK_0266.1_251469336909_S01_CGH.v4_91.txt	5	3239521	3850747	34	-0.6723	-1
MSK_0266.1_251469336909_S01_CGH.v4_91.txt	5	2048009	2374605	20	-0.8359	-1
MSK_0266.1_251469336909_S01_CGH.v4_91.txt	5	1450724	1566510	12	-0.6795	-1
MSK_0281.1_251469331472_S01_CGH.v4_91.txt	5	119449012	120800323	52	-0.8409	-1
MSK_0285.1_251469337229_S01_CGH.v4_91.txt	5	32110734	32167579	6	0.9941	1
MSK_0285.1_251469337229_S01_CGH.v4_91.txt	5	733455	766038	3	-1.4376	-1
MSK_0321.1_251469336723_S01_CGH.v4_91.txt	5	69705562	70586959	3	0.7177	1
MSK_0324.1_251469333523_S01_CGH.v4_91.txt	5	127077075	127082655	2	-0.7656	-1
MSK_0330.1_251469336713_S01_CGH.v4_91.txt	5	759169	806176	4	0.7212	1
MSK_0333.1_251469331585_S01_CGH.v4_91.txt	5	162871474	170564855	368	-0.8009	-1
MSK_0337.1_251469333524_S01_CGH.v4_91.txt	5	8725962	8747001	2	-1.1863	-1
MSK_0339.1_251469331462_S01_CGH.v4_91.txt	5	69705562	70308101	2	0.66	1
MSK_0339.1_251469331462_S01_CGH.v4_91.txt	5	21973548	46115027	1186	-0.9013	-1
MSK_0339.1_251469331462_S01_CGH.v4_91.txt	5	21584387	21921623	24	-0.9056	-1
MSK_0339.1_251469331462_S01_CGH.v4_91.txt	5	784253	21453181	1052	-0.9162	-1
MSK_0339.1_251469331462_S01_CGH.v4_91.txt	5	733455	766038	3	-1.647	-1
MSK_0339.1_251469331462_S01_CGH.v4_91.txt	5	37692	692859	46	-0.9477	-1
MSK_0432.1_251469336712_S01_CGH.v4_91.txt	5	20808528	20834116	2	-0.6533	-1
MSK_0439.1_251469331553_S01_CGH.v4_91.txt	5	69705562	70308101	2	1.2917	1
MSK_0439.1_251469331553_S01_CGH.v4_91.txt	5	4349436	4414148	6	-0.7566	-1
MSK_0439.1_251469331553_S01_CGH.v4_91.txt	5	2104553	2141433	4	-0.6558	-1
MSK_0439.1_251469331553_S01_CGH.v4_91.txt	5	784253	841957	3	-1.0169	-1
MSK_0439.1_251469331553_S01_CGH.v4_91.txt	5	759169	766038	2	-3.1464	-1
MSK_0446.1_251469333525_S01_CGH.v4_91.txt	5	137808567	137814208	2	-0.7167	-1
MSK_0451.1_251469333565_S01_CGH.v4_91.txt	5	20808528	20834116	2	-0.703	-1
MSK_00141.1_251469325753_S01_CGH.v4_91.txt	5	2048009	2059887	2	-1.0874	-1
MSK_0149.1_251469336715_S01_CGH.v4_91.txt	5	17479173	17506190	3	-0.6739	-1
MSK_0158.1_251469329734_S01_CGH.v4_91.txt	5	66492357	70680522	113	-0.6814	-1
MSK_0158.1_251469329734_S01_CGH.v4_91.txt	5	65758953	66418603	57	-0.7666	-1
MSK_0158.1_251469329734_S01_CGH.v4_91.txt	5	32110734	32167579	6	0.694	1
MSK_0173.1_251469330461_S01_CGH.v4_91.txt	5	165739832	165928260	6	-0.7401	-1
MSK_0175.1_251469336725_S01_CGH.v4_91.txt	5	162481732	165287964	85	-0.6778	-1
MSK_0177.1_251469333358_S01_CGH.v4_91.txt	5	23821646	30033060	179	-0.6771	-1
MSK_0179.1_251469333566_S01_CGH.v4_91.txt	5	69705562	70586959	3	-0.6901	-1
MSK_0179.1_251469333566_S01_CGH.v4_91.txt	5	733455	766038	3	-0.6898	-1
MSK_0182.1_251469331464_S01_CGH.v4_91.txt	5	733455	766038	3	-0.8045	-1
MSK_0185.1_251469329754_S01_CGH.v4_91.txt	5	164510588	164529560	2	-0.6953	-1
MSK_0185.1_251469329754_S01_CGH.v4_91.txt	5	8725962	8747001	2	-1.2981	-1
MSK_0185.1_251469329754_S01_CGH.v4_91.txt	5	733455	766038	3	-1.0783	-1
MSK_0189.1_251469330588_S01_CGH.v4_91.txt	5	686292	806176	7	0.9588	1
MSK_0190.1_251469330591_S01_CGH.v4_91.txt	5	32110734	32167579	6	0.8078	1
MSK_0194.1_251469336985_S01_CGH.v4_91.txt	5	733455	766038	3	-1.4008	-1
MSK_0208.1_251469329182_S01_CGH.v4_91.txt	5	177785791	177794120	2	-1.0026	-1
MSK_0208.2_251469329755_S01_CGH.v4_91.txt	5	177785791	177794120	2	-0.8817	-1
MSK_0211.1_251469330227_S01_CGH.v4_91.txt	5	8725962	8747001	2	-1.3953	-1
MSK_0211.2_251469329773_S01_CGH.v4_91.txt	5	162397933	162481732	4	-0.7918	-1
MSK_0211.2_251469329773_S01_CGH.v4_91.txt	5	8725962	8747001	2	-1.0658	-1
MSK_0219.1_251469329864_S01_CGH.v4_91.txt	5	39077634	39099986	3	-0.8586	-1
MSK_0237.1_251469330449_S01_CGH.v4_91.txt	5	151785263	151792610	2	-0.8435	-1
MSK_0237.1_251469330449_S01_CGH.v4_91.txt	5	113400680	113458757	3	0.7776	1
MSK_0237.1_251469330449_S01_CGH.v4_91.txt	5	66230098	66261992	4	-0.8396	-1
MSK_0237.1_251469330449_S01_CGH.v4_91.txt	5	45184041	46115027	48	-0.7374	-1
MSK_0409.1_251469331555_S01_CGH.v4_91.txt	5	115600160	115607519	2	-0.837	-1
MSK_0409.1_251469331555_S01_CGH.v4_91.txt	5	733455	841957	6	0.8669	1
MSK_0410.1_251469333359_S01_CGH.v4_91.txt	5	129471240	129652570	8	0.7061	1
MSK_0412.1_251469331490_S01_CGH.v4_91.txt	5	109708245	109907925	13	-0.6997	-1
MSK_0412.1_251469331490_S01_CGH.v4_91.txt	5	8725962	8747001	2	-1.0984	-1
MSK_0412.1_251469331490_S01_CGH.v4_91.txt	5	733455	766038	3	-0.7248	-1
MSK_0413.1_251469331465_S01_CGH.v4_91.txt	5	50399526	51451483	30	-0.9344	-1
MSK_0413.1_251469331465_S01_CGH.v4_91.txt	5	733455	766038	3	-0.6672	-1
MSK_0345.1_251469337922_S01_CGH.v4_91.txt	5	101034442	101218738	5	-1.026	-1
MSK_WGA_0355.1_251469337508_S01_CGH.v4_91.txt	5	166899718	167097555	6	-0.8186	-1
MSK_WGA_0356.1_251469337509_S01_CGH.v4_91.txt	5	110898076	111067210	12	-0.6628	-1
MSK_WGA_0357.1_251469337522_S01_CGH.v4_91.txt	5	7300005	7312537	2	0.7115	1
MSK_0358.1_251469340789_S01_CGH.v4_91.txt	5	2048009	2059887	2	-0.9546	-1
MSK_WGA_0359.1_251469337523_S01_CGH.v4_91.txt	5	9674190	10236900	21	-0.6476	-1
MSK_WGA_0359.1_251469337523_S01_CGH.v4_91.txt	5	9029950	9447841	33	-0.696	-1
MSK_WGA_0359.1_251469337523_S01_CGH.v4_91.txt	5	8111134	8142036	2	-0.7235	-1
MSK_0386.1_251469340798_S01_CGH.v4_91.txt	5	164020462	164329568	10	-1.2837	-1
MSK_WGA_0389.1_251469338874_S01_CGH.v4_91.txt	5	165287964	165847276	15	-0.9751	-1
MSK_WGA_0390.1_251469338875_S01_CGH.v4_91.txt	5	138719005	138725204	3	0.816	1
MSK_0510.1_251469331581_S01_CGH.v4_91.txt	5	69705562	70308101	2	1.3497	1
MSK_0511.1_251469336907_S01_CGH.v4_91.txt	5	165024838	165067453	2	-0.6964	-1
MSK_0516.1_251469331469_S01_CGH.v4_91.txt	5	157523565	171638493	701	-1.2131	-1
MSK_0516.1_251469331469_S01_CGH.v4_91.txt	5	733455	841957	6	-0.8089	-1
MSK_0517.1_251469337226_S01_CGH.v4_91.txt	5	174125810	180696747	411	-0.7466	-1
MSK_0517.1_251469337226_S01_CGH.v4_91.txt	5	153596376	173912734	1059	-0.7992	-1
MSK_0517.1_251469337226_S01_CGH.v4_91.txt	5	53368712	59982763	404	-0.8469	-1
MSK_0517.1_251469337226_S01_CGH.v4_91.txt	5	37272311	53332946	640	-0.8519	-1
MSK_0517.1_251469337226_S01_CGH.v4_91.txt	5	37187850	37253157	8	-4.1076	-1
MSK_0517.1_251469337226_S01_CGH.v4_91.txt	5	871638	37158302	1772	-0.8509	-1
MSK_0517.1_251469337226_S01_CGH.v4_91.txt	5	37692	672942	44	-0.7832	-1
MSK_0518.1_251469337014_S01_CGH.v4_91.txt	5	79191476	79751464	42	0.8433	1
MSK_0518.1_251469337014_S01_CGH.v4_91.txt	5	31421359	31790728	26	0.8971	1
MSK_0520.1_251469336708_S01_CGH.v4_91.txt	5	179911900	180261717	32	-0.6992	-1
MSK_0521.1_251469331582_S01_CGH.v4_91.txt	5	115600160	115607519	2	-0.9245	-1
MSK_0521.1_251469331582_S01_CGH.v4_91.txt	5	692859	806176	6	0.6753	1
MSK_0524.1_251469331557_S01_CGH.v4_91.txt	5	122672110	167777218	2613	-0.7633	-1
MSK_0529.1_251469331470_S01_CGH.v4_91.txt	5	117375481	117392999	2	-0.7753	-1
MSK_0529.1_251469331470_S01_CGH.v4_91.txt	5	733455	766038	3	-1.4954	-1
MSK_0531.1_251469337227_S01_CGH.v4_91.txt	5	759169	766038	2	-1.6896	-1
MSK_0618.1_251469337983_S01_CGH.v4_91.txt	5	151737	232775	7	0.9908	1
MSK_00003_251469322731_S01_CGH.v4_91.txt	6	116681021	171014731	3022	-0.7074	-1
MSK_00006_251469322732_S01_CGH.v4_91.txt	6	95525616	95554368	2	-1.1096	-1
MSK_00007_251469322733_S01_CGH.v4_91.txt	6	71099325	171014731	5159	-0.7993	-1
MSK_00014_251469322629_S01_CGH.v4_91.txt	6	106898112	150111395	2359	-0.9308	-1
MSK_WGA_0015.1_251469338754_S01_CGH.v4_91.txt	6	161520813	170628290	527	-0.7048	-1
MSK_WGA_0015.1_251469338754_S01_CGH.v4_91.txt	6	150432597	150621085	14	-0.6603	-1
MSK_WGA_0015.1_251469338754_S01_CGH.v4_91.txt	6	149699232	150304399	46	-0.6445	-1
MSK_WGA_0015.1_251469338754_S01_CGH.v4_91.txt	6	138894620	149564095	551	-0.7096	-1
MSK_00021_251469322630_S01_CGH.v4_91.txt	6	150432597	165004845	820	-0.794	-1
MSK_00021_251469322630_S01_CGH.v4_91.txt	6	133244259	150332931	925	-0.8311	-1
MSK_WGA_0023.1_251469338756_S01_CGH.v4_91.txt	6	32026687	32074296	5	-1.1414	-1
MSK_WGA_0026.1_251469338758_S01_CGH.v4_91.txt	6	1635349	1727928	6	-0.8536	-1
MSK_00033_251469322707_S01_CGH.v4_91.txt	6	160599768	160611295	2	-0.7089	-1
MSK_00033_251469322707_S01_CGH.v4_91.txt	6	31360179	31450100	7	-0.8291	-1
MSK_00033_251469322707_S01_CGH.v4_91.txt	6	29093912	29131790	4	-0.8489	-1
MSK_00034_251469322708_S01_CGH.v4_91.txt	6	102092268	102170720	6	-0.7601	-1
MSK_00038_251469322735_S01_CGH.v4_91.txt	6	94516233	126118391	1522	-0.7103	-1
MSK_WGA_0039.1_251469338741_S01_CGH.v4_91.txt	6	71879829	74041336	151	-1.2561	-1
MSK_00043_251469322736_S01_CGH.v4_91.txt	6	152641185	152724883	8	-0.9124	-1
MSK_00052_251469323033_S01_CGH.v4_91.txt	6	168419548	168633631	11	0.6445	1
MSK_00054_251469323034_S01_CGH.v4_91.txt	6	259528	293441	4	-0.689	-1

Continued on next page...

ID	chr	start	end	nroOfprobes	segmean	call
MSK_00055_251469323018_S01_CGH.v4_91.txt	6	62452970	62462125	2	-0.7153	-1
MSK_00057_251469323017_S01_CGH.v4_91.txt	6	153700671	153767422	2	-0.8259	-1
MSK_00058_251469323019_S01_CGH.v4_91.txt	6	167259259	171014731	203	-0.6987	-1
MSK_00058_251469323019_S01_CGH.v4_91.txt	6	166015142	166894682	68	-0.7125	-1
MSK_00058_251469323019_S01_CGH.v4_91.txt	6	160186932	165083904	297	-0.7031	-1
MSK_00058_251469323019_S01_CGH.v4_91.txt	6	158973080	160025455	61	-0.6646	-1
MSK_00058_251469323019_S01_CGH.v4_91.txt	6	158791500	158846772	6	-0.6862	-1
MSK_00058_251469323019_S01_CGH.v4_91.txt	6	131225654	158559055	1482	-0.7003	-1
MSK_00058_251469323019_S01_CGH.v4_91.txt	6	118718417	130805126	664	-0.7747	-1
MSK_00058_251469323019_S01_CGH.v4_91.txt	6	107449870	118551027	621	-0.7118	-1
MSK_00058_251469323019_S01_CGH.v4_91.txt	6	106793687	106898112	8	-0.7187	-1
MSK_00060_251469322915_S01_CGH.v4_91.txt	6	45220574	45227419	2	-0.8988	-1
MSK_WGA_0070.1_251469338879_S01_CGH.v4_91.txt	6	152544083	152557028	2	-0.634	-1
MSK_00071_251469322919_S01_CGH.v4_91.txt	6	29873951	29902255	2	-0.8752	-1
MSK_0087.1_251469333362_S01_CGH.v4_91.txt	6	62452970	62462125	2	-1.1044	-1
MSK_00102_251469322716_S01_CGH.v4_91.txt	6	93072079	93210103	6	-0.8947	-1
MSK_00102_251469322716_S01_CGH.v4_91.txt	6	31785578	31794839	3	-0.9403	-1
MSK_WGA_0104.1_251469338880_S01_CGH.v4_91.txt	6	69624855	69772817	14	-0.6617	-1
MSK_00114_251469322752_S01_CGH.v4_91.txt	6	169699210	170957352	79	-0.8377	-1
MSK_00116_251469322720_S01_CGH.v4_91.txt	6	109553063	169991091	3320	-0.8714	-1
MSK_00116_251469322720_S01_CGH.v4_91.txt	6	96436595	96681821	18	-0.9776	-1
MSK_00116_251469322720_S01_CGH.v4_91.txt	6	259528	293441	4	-0.7004	-1
MSK_0258.1_251469336709_S01_CGH.v4_91.txt	6	161132179	170957352	571	-0.9167	-1
MSK_0258.1_251469336709_S01_CGH.v4_91.txt	6	150432597	161079243	582	-0.8039	-1
MSK_0258.1_251469336709_S01_CGH.v4_91.txt	6	70351781	150353930	4041	-0.8215	-1
MSK_0260.1_251469331583_S01_CGH.v4_91.txt	6	7563912	7572032	2	-0.8821	-1
MSK_0266.1_251469336909_S01_CGH.v4_91.txt	6	259528	375890	11	-0.9795	-1
MSK_0289.1_251469337015_S01_CGH.v4_91.txt	6	157115229	168921339	689	-0.7823	-1
MSK_0289.1_251469337015_S01_CGH.v4_91.txt	6	10452629	14136143	249	-0.7754	-1
MSK_0289.1_251469337015_S01_CGH.v4_91.txt	6	6629072	10368034	176	-0.7769	-1
MSK_0289.1_251469337015_S01_CGH.v4_91.txt	6	815829	6035272	373	-0.7757	-1
MSK_0290.1_251469336710_S01_CGH.v4_91.txt	6	163115045	163451875	24	-0.6361	-1
MSK_0290.1_251469336710_S01_CGH.v4_91.txt	6	162798709	162849743	6	-1.0569	-1
MSK_0290.1_251469336710_S01_CGH.v4_91.txt	6	116977794	117062931	5	-0.7097	-1
MSK_0290.1_251469336710_S01_CGH.v4_91.txt	6	116432483	116784819	34	-0.6984	-1
MSK_0290.1_251469336710_S01_CGH.v4_91.txt	6	115374891	116289850	16	-0.6975	-1
MSK_0290.1_251469336710_S01_CGH.v4_91.txt	6	114713545	114925252	5	-0.7471	-1
MSK_0290.1_251469336710_S01_CGH.v4_91.txt	6	114203136	114277733	6	-0.7409	-1
MSK_0290.1_251469336710_S01_CGH.v4_91.txt	6	112990474	114163172	28	-0.6758	-1
MSK_0290.1_251469336710_S01_CGH.v4_91.txt	6	110959870	112420452	112	-0.7068	-1
MSK_0290.1_251469336710_S01_CGH.v4_91.txt	6	110550045	110814475	24	-0.6856	-1
MSK_0317.1_251469331584_S01_CGH.v4_91.txt	6	161132179	171014731	575	-0.7443	-1
MSK_0317.1_251469331584_S01_CGH.v4_91.txt	6	101764038	161003996	3161	-0.7198	-1
MSK_0317.1_251469331584_S01_CGH.v4_91.txt	6	97466938	101336193	196	-0.7197	-1
MSK_0317.1_251469331584_S01_CGH.v4_91.txt	6	80359021	97353188	732	-0.7131	-1
MSK_0317.1_251469331584_S01_CGH.v4_91.txt	6	72670292	80012785	348	-0.7126	-1
MSK_0324.1_251469333523_S01_CGH.v4_91.txt	6	163985691	164001232	3	-1.7846	-1
MSK_0333.1_251469331585_S01_CGH.v4_91.txt	6	165217583	165427858	9	-0.6512	-1
MSK_0333.1_251469331585_S01_CGH.v4_91.txt	6	163992468	164678941	22	-0.8033	-1
MSK_0333.1_251469331585_S01_CGH.v4_91.txt	6	158362782	158620251	21	-0.741	-1
MSK_0333.1_251469331585_S01_CGH.v4_91.txt	6	152871596	152877088	2	-0.7639	-1
MSK_0333.1_251469331585_S01_CGH.v4_91.txt	6	13788802	14087024	14	-0.7854	-1
MSK_0422.1_251469337230_S01_CGH.v4_91.txt	6	67164005	171014731	5330	-0.6962	-1
MSK_0422.1_251469337230_S01_CGH.v4_91.txt	6	66955441	67121954	3	4.9608	1
MSK_0422.1_251469337230_S01_CGH.v4_91.txt	6	66572087	66914376	11	3.1122	1
MSK_0422.1_251469337230_S01_CGH.v4_91.txt	6	66496293	66561198	6	4.838	1
MSK_0422.1_251469337230_S01_CGH.v4_91.txt	6	66112403	66488569	27	2.9936	1
MSK_0422.1_251469337230_S01_CGH.v4_91.txt	6	66072117	66104237	4	4.7269	1
MSK_0422.1_251469337230_S01_CGH.v4_91.txt	6	65947165	66044972	6	2.8892	1
MSK_0422.1_251469337230_S01_CGH.v4_91.txt	6	65228149	65926841	24	4.8999	1
MSK_0422.1_251469337230_S01_CGH.v4_91.txt	6	65017966	65192252	5	3.7867	1
MSK_0422.1_251469337230_S01_CGH.v4_91.txt	6	64263986	64976283	30	-0.6609	-1
MSK_0430.1_251469336714_S01_CGH.v4_91.txt	6	68712591	68739119	2	0.8293	1
MSK_0430.1_251469336714_S01_CGH.v4_91.txt	6	29873951	29902255	2	-0.9787	-1
MSK_0432.1_251469336712_S01_CGH.v4_91.txt	6	70351781	171014731	5205	-0.7668	-1
MSK_0451.1_251469333565_S01_CGH.v4_91.txt	6	161082375	161106317	2	-0.8065	-1
MSK_0456.1_251469331463_S01_CGH.v4_91.txt	6	130667106	130674659	2	-0.9742	-1
MSK_0456.1_251469331463_S01_CGH.v4_91.txt	6	259528	293441	4	-0.6393	-1
MSK_00126.1_251469325171_S01_CGH.v4_91.txt	6	65794022	65814264	2	-0.9295	-1
MSK_00128.1_251469325161_S01_CGH.v4_91.txt	6	161082375	161106317	2	1.162	1
MSK_00129.1_251469322907_S01_CGH.v4_91.txt	6	42734096	42761395	5	4.5755	1
MSK_00129.1_251469322907_S01_CGH.v4_91.txt	6	41846763	42169315	27	4.38	1
MSK_00129.1_251469322907_S01_CGH.v4_91.txt	6	38744528	39734163	70	2.7119	1
MSK_00129.1_251469322907_S01_CGH.v4_91.txt	6	36927874	37879884	73	3.1811	1
MSK_00129.1_251469322907_S01_CGH.v4_91.txt	6	36826534	36921731	9	4.892	1
MSK_00129.1_251469322907_S01_CGH.v4_91.txt	6	36401169	36822466	40	2.9045	1
MSK_00129.1_251469322907_S01_CGH.v4_91.txt	6	36300340	36392841	7	4.5551	1
MSK_00129.1_251469322907_S01_CGH.v4_91.txt	6	35681450	36294914	49	2.7807	1
MSK_00129.1_251469322907_S01_CGH.v4_91.txt	6	29873951	29902255	2	-1.5254	-1
MSK_00130.1_251469325022_S01_CGH.v4_91.txt	6	110243469	110293530	2	-1.6517	-1
MSK_0132.1_251469330459_S01_CGH.v4_91.txt	6	31785578	31794839	3	-0.9312	-1
MSK_00133_251469325752_S01_CGH.v4_91.txt	6	161082375	161106317	2	1.3314	1
MSK_0164.1_251469331554_S01_CGH.v4_91.txt	6	29873951	29902255	2	-1.862	-1
MSK_0174.1_251469330284_S01_CGH.v4_91.txt	6	7558320	10153921	119	-0.8742	-1
MSK_0175.1_251469336725_S01_CGH.v4_91.txt	6	135935242	147714893	640	-0.6743	-1
MSK_0175.1_251469336725_S01_CGH.v4_91.txt	6	133244259	134199163	56	-0.6779	-1
MSK_0175.1_251469336725_S01_CGH.v4_91.txt	6	132814183	132871673	5	-0.6468	-1
MSK_0178.1_251469329154_S01_CGH.v4_91.txt	6	259528	375890	11	-0.6664	-1
MSK_0182.1_251469331464_S01_CGH.v4_91.txt	6	129731188	129737392	2	-0.7098	-1
MSK_0182.1_251469331464_S01_CGH.v4_91.txt	6	29937554	29945375	2	-0.6876	-1
MSK_0185.1_251469329754_S01_CGH.v4_91.txt	6	161920060	166654859	272	-0.8274	-1
MSK_0187.1_251469330462_S01_CGH.v4_91.txt	6	63409218	171014731	5479	-0.7517	-1
MSK_0189.1_251469330588_S01_CGH.v4_91.txt	6	259528	375890	11	-0.6416	-1
MSK_0190.1_251469330591_S01_CGH.v4_91.txt	6	10472441	10529194	5	-0.9523	-1
MSK_0209.1_251469330463_S01_CGH.v4_91.txt	6	161722395	170148111	490	-0.6719	-1
MSK_0209.1_251469330463_S01_CGH.v4_91.txt	6	29867409	29902255	3	-0.8185	-1
MSK_0210.2_251469330589_S01_CGH.v4_91.txt	6	32411957	32565005	4	-0.8108	-1
MSK_0211.2_251469329773_S01_CGH.v4_91.txt	6	158381356	158929763	39	-0.7084	-1
MSK_0237.1_251469330449_S01_CGH.v4_91.txt	6	101025166	101258786	21	-0.7486	-1
MSK_0237.1_251469330449_S01_CGH.v4_91.txt	6	16489472	16740337	22	-0.6771	-1
MSK_0241.1_251469329774_S01_CGH.v4_91.txt	6	45938382	46619593	63	-0.8856	-1
MSK_0394.1_251469336716_S01_CGH.v4_91.txt	6	307939	375890	7	0.6593	1
MSK_0397.1_251469330468_S01_CGH.v4_91.txt	6	101858360	102347614	38	-0.8297	-1
MSK_0409.1_251469331555_S01_CGH.v4_91.txt	6	81589494	81619569	4	-1.1158	-1
MSK_0413.1_251469331465_S01_CGH.v4_91.txt	6	49927987	51970082	102	-0.9174	-1
MSK_0414.1_251469336986_S01_CGH.v4_91.txt	6	29867409	29923388	5	0.6558	1
MSK_WGA_0244.1_251469338881_S01_CGH.v4_91.txt	6	101829809	102307164	37	-0.6753	-1
MSK_WGA_0246.1_251469338865_S01_CGH.v4_91.txt	6	167842006	168494324	23	-0.8448	-1
MSK_WGA_0246.1_251469338865_S01_CGH.v4_91.txt	6	160548601	166156865	323	-1.0002	-1
MSK_0345.1_251469337922_S01_CGH.v4_91.txt	6	161082375	161106317	2	1.1303	1
MSK_WGA_0353.1_251469337506_S01_CGH.v4_91.txt	6	118231520	168829453	2806	-0.6337	-1
MSK_WGA_0353.1_251469337506_S01_CGH.v4_91.txt	6	152634	20932208	1235	-0.6614	-1
MSK_WGA_0355.1_251469337508_S01_CGH.v4_91.txt	6	124280383	169526769	2561	-0.722	-1
MSK_WGA_0355.1_251469337508_S01_CGH.v4_91.txt	6	123957926	124225801	15	-0.8811	-1
MSK_WGA_0356.1_251469337509_S01_CGH.v4_91.txt	6	163970717	163992468	3	-1.6611	-1
MSK_WGA_0357.1_251469337522_S01_CGH.v4_91.txt	6	167004389	167111518	10	-0.7566	-1
MSK_0358.1_251469340789_S01_CGH.v4_91.txt	6	57646751	171014731	5557	-0.8105	-1
MSK_WGA_0359.1_251469337523_S01_CGH.v4_91.txt	6	124341398	124424563	8	-0.7803	-1
MSK_WGA_0359.1_251469337523_S01_CGH.v4_91.txt	6	123003731	124203425	62	-0.7024	-1
MSK_WGA_0389.1_251469338874_S01_CGH.v4_91.txt	6	111930092	112684810	58	-0.8004	-1
MSK_WGA_0389.1_251469338874_S01_CGH.v4_91.txt	6	111759931	111922517	9	3.633	1
MSK_WGA_0389.1_251469338874_S01_CGH.v4_91.txt	6	111634619	111753124	9	3.1612	1
MSK_WGA_0389.1_251469338874_S01_CGH.v4_91.txt	6	111586765	111620297	6	0.9382	1
MSK_WGA_0389.1_251469338874_S01_CGH.v4_91.txt	6	111360508	111583447	18	3.2083	1

Continued on next page...

ID	chr	start	end	nroOfprobes	segmean	call
MSK_WGA_0389.1_251469338874_S01_CGH.v4_91.txt	6	110157265	111232953	77	3.2356	1
MSK_WGA_0389.1_251469338874_S01_CGH.v4_91.txt	6	109546220	109827552	29	3.3717	1
MSK_WGA_0389.1_251469338874_S01_CGH.v4_91.txt	6	109306827	109518892	16	0.9585	1
MSK_WGA_0389.1_251469338874_S01_CGH.v4_91.txt	6	108786005	109175413	24	3.2944	1
MSK_WGA_0389.1_251469338874_S01_CGH.v4_91.txt	6	108307273	108746493	32	3.7286	1
MSK_WGA_0389.1_251469338874_S01_CGH.v4_91.txt	6	107865086	108283798	30	3.2947	1
MSK_WGA_0389.1_251469338874_S01_CGH.v4_91.txt	6	107566704	107646625	8	2.0134	1
MSK_WGA_0389.1_251469338874_S01_CGH.v4_91.txt	6	107355252	107555834	16	3.3973	1
MSK_WGA_0389.1_251469338874_S01_CGH.v4_91.txt	6	87178844	95918819	369	-0.792	-1
MSK_WGA_0390.1_251469338875_S01_CGH.v4_91.txt	6	149757900	149777492	3	0.7211	1
MSK_WGA_0392.1_251469338876_S01_CGH.v4_91.txt	6	162363136	165494245	161	-1.087	-1
MSK_0510.1_251469331581_S01_CGH.v4_91.txt	6	31785578	31794839	3	-0.9262	-1
MSK_0510.1_251469331581_S01_CGH.v4_91.txt	6	29867409	29902255	3	-0.6683	-1
MSK_0510.1_251469331581_S01_CGH.v4_91.txt	6	259528	293441	4	-0.7413	-1
MSK_0514.1_251469333360_S01_CGH.v4_91.txt	6	158693998	159223848	32	-0.7072	-1
MSK_0514.1_251469333360_S01_CGH.v4_91.txt	6	158505624	158638495	11	-0.6981	-1
MSK_0514.1_251469333360_S01_CGH.v4_91.txt	6	156822605	157710262	50	-0.6836	-1
MSK_0514.1_251469333360_S01_CGH.v4_91.txt	6	155948467	155994826	3	-0.7489	-1
MSK_0514.1_251469333360_S01_CGH.v4_91.txt	6	101915498	101992355	7	-0.8554	-1
MSK_0517.1_251469337226_S01_CGH.v4_91.txt	6	62019880	171014731	5538	-0.8311	-1
MSK_0520.1_251469336708_S01_CGH.v4_91.txt	6	161937900	162686723	68	-0.6894	-1
MSK_0524.1_251469331557_S01_CGH.v4_91.txt	6	129202152	129222631	3	0.6824	1
MSK_00001_251469322729_S01_CGH.v4_91.txt	7	54185	158881735	9404	0.6828	1
MSK_00003_251469322731_S01_CGH.v4_91.txt	7	54591387	55188858	32	5.0135	1
MSK_00003_251469322731_S01_CGH.v4_91.txt	7	38111132	38159100	9	0.99	1
MSK_00006_251469322732_S01_CGH.v4_91.txt	7	143758646	143760132	2	1.0508	1
MSK_00007_251469322733_S01_CGH.v4_91.txt	7	102746406	112931994	654	-0.7556	-1
MSK_00007_251469322733_S01_CGH.v4_91.txt	7	97374991	102610350	396	-0.6909	-1
MSK_00007_251469322733_S01_CGH.v4_91.txt	7	76559230	97309455	1276	-0.7939	-1
MSK_00007_251469322733_S01_CGH.v4_91.txt	7	54185	65046028	3535	0.9529	1
MSK_00009_251469322626_S01_CGH.v4_91.txt	7	55030917	55134377	11	5.8383	1
MSK_00009_251469322626_S01_CGH.v4_91.txt	7	54924247	55016814	7	4.0455	1
MSK_00009_251469322626_S01_CGH.v4_91.txt	7	54411324	54904315	19	5.844	1
MSK_00010_251469322627_S01_CGH.v4_91.txt	7	116126525	121351812	292	1.0186	1
MSK_00011_251469322628_S01_CGH.v4_91.txt	7	143758646	143760132	2	1.0415	1
MSK_00011_251469322628_S01_CGH.v4_91.txt	7	11713815	12475448	40	-0.8169	-1
MSK_00011_251469322628_S01_CGH.v4_91.txt	7	5184469	11423049	395	-0.7811	-1
MSK_00011_251469322628_S01_CGH.v4_91.txt	7	54185	5060551	318	-0.7039	-1
MSK_WGA_0015.1_251469338754_S01_CGH.v4_91.txt	7	38116427	38145569	6	0.7635	1
MSK_WGA_0016.1_251469338755_S01_CGH.v4_91.txt	7	127928881	128383113	38	2.9258	1
MSK_WGA_0016.1_251469338755_S01_CGH.v4_91.txt	7	54678659	55111113	24	3.089	1
MSK_WGA_0016.1_251469338755_S01_CGH.v4_91.txt	7	38116427	38183216	9	1.2636	1
MSK_00021_251469322630_S01_CGH.v4_91.txt	7	143758646	143760132	2	1.4971	1
MSK_00021_251469322630_S01_CGH.v4_91.txt	7	62393280	62422002	4	1.2815	1
MSK_00021_251469322630_S01_CGH.v4_91.txt	7	55535145	55839876	25	4.6819	1
MSK_00021_251469322630_S01_CGH.v4_91.txt	7	54678659	55469426	47	5.9567	1
MSK_00021_251469322630_S01_CGH.v4_91.txt	7	54483191	54629892	6	4.926	1
MSK_WGA_0023.1_251469338756_S01_CGH.v4_91.txt	7	53798596	55382365	64	4.5246	1
MSK_WGA_0023.1_251469338756_S01_CGH.v4_91.txt	7	53324738	53773519	21	0.9776	1
MSK_WGA_0023.1_251469338756_S01_CGH.v4_91.txt	7	51305010	52193093	24	4.7039	1
MSK_WGA_0023.1_251469338756_S01_CGH.v4_91.txt	7	49837853	50044897	18	4.4896	1
MSK_00024_251469322721_S01_CGH.v4_91.txt	7	120967961	121351812	14	1.2177	1
MSK_00024_251469322721_S01_CGH.v4_91.txt	7	119795262	120932268	85	1.8527	1
MSK_00024_251469322721_S01_CGH.v4_91.txt	7	116126525	117469994	116	1.8571	1
MSK_00024_251469322721_S01_CGH.v4_91.txt	7	63859599	64232432	20	1.662	1
MSK_WGA_0025.1_251469338757_S01_CGH.v4_91.txt	7	38126727	38145569	4	0.7558	1
MSK_00028_251469322706_S01_CGH.v4_91.txt	7	133690858	149884175	1109	-0.8719	-1
MSK_00033_251469322707_S01_CGH.v4_91.txt	7	143758646	143760132	2	1.2409	1
MSK_00038_251469322735_S01_CGH.v4_91.txt	7	54678659	55241779	30	5.8185	1
MSK_00043_251469322736_S01_CGH.v4_91.txt	7	143758646	143760132	2	-1.7652	-1
MSK_00043_251469322736_S01_CGH.v4_91.txt	7	55589743	55661940	6	4.7185	1
MSK_00043_251469322736_S01_CGH.v4_91.txt	7	54678659	55361614	39	5.3602	1
MSK_00046_251469322737_S01_CGH.v4_91.txt	7	90088604	90491697	33	3.9575	1
MSK_00046_251469322737_S01_CGH.v4_91.txt	7	54031412	55722209	76	4.2912	1
MSK_00046_251469322737_S01_CGH.v4_91.txt	7	4425398	5060551	43	3.8035	1
MSK_WGA_0048.1_251469338742_S01_CGH.v4_91.txt	7	128900629	129423281	39	1.0431	1
MSK_WGA_0048.1_251469338742_S01_CGH.v4_91.txt	7	118238247	118534676	8	1.5149	1
MSK_WGA_0048.1_251469338742_S01_CGH.v4_91.txt	7	114031528	114185914	12	1.6103	1
MSK_WGA_0048.1_251469338742_S01_CGH.v4_91.txt	7	105821176	105859238	2	1.5157	1
MSK_WGA_0048.1_251469338742_S01_CGH.v4_91.txt	7	100212431	100280896	6	0.649	1
MSK_WGA_0048.1_251469338742_S01_CGH.v4_91.txt	7	91988671	92327770	27	1.4555	1
MSK_WGA_0048.1_251469338742_S01_CGH.v4_91.txt	7	83845413	84177017	15	0.6863	1
MSK_WGA_0048.1_251469338742_S01_CGH.v4_91.txt	7	77692578	77887977	23	1.4977	1
MSK_WGA_0048.1_251469338742_S01_CGH.v4_91.txt	7	73800949	73814245	2	0.7801	1
MSK_WGA_0048.1_251469338742_S01_CGH.v4_91.txt	7	71834419	72086290	11	0.7893	1
MSK_WGA_0048.1_251469338742_S01_CGH.v4_91.txt	7	66744402	67119520	10	0.6734	1
MSK_WGA_0048.1_251469338742_S01_CGH.v4_91.txt	7	65656405	66537662	57	1.41	1
MSK_WGA_0048.1_251469338742_S01_CGH.v4_91.txt	7	62792159	63094525	5	2.1362	1
MSK_WGA_0048.1_251469338742_S01_CGH.v4_91.txt	7	57432663	57690297	8	2.1362	1
MSK_WGA_0048.1_251469338742_S01_CGH.v4_91.txt	7	56636957	57384029	32	2.7971	1
MSK_WGA_0048.1_251469338742_S01_CGH.v4_91.txt	7	56300336	56601721	27	3.1819	1
MSK_WGA_0048.1_251469338742_S01_CGH.v4_91.txt	7	54355766	56191148	106	4.0031	1
MSK_WGA_0048.1_251469338742_S01_CGH.v4_91.txt	7	53773519	54261755	11	1.6754	1
MSK_WGA_0048.1_251469338742_S01_CGH.v4_91.txt	7	38111132	38183216	10	0.6888	1
MSK_00052_251469323033_S01_CGH.v4_91.txt	7	73586695	73634151	4	0.6719	1
MSK_00057_251469323017_S01_CGH.v4_91.txt	7	43823869	43861050	2	0.8809	1
MSK_00064_251469322718_S01_CGH.v4_91.txt	7	55030917	55607468	36	4.704	1
MSK_00064_251469322718_S01_CGH.v4_91.txt	7	54904315	55016814	8	0.7852	1
MSK_00064_251469322718_S01_CGH.v4_91.txt	7	54678659	54899138	7	4.5402	1
MSK_WGA_0068.1_251469338745_S01_CGH.v4_91.txt	7	55535145	55994300	44	0.7583	1
MSK_WGA_0068.1_251469338745_S01_CGH.v4_91.txt	7	54678659	55469426	47	5.0701	1
MSK_WGA_0068.1_251469338745_S01_CGH.v4_91.txt	7	50262996	54629892	147	0.8292	1
MSK_00071_251469322919_S01_CGH.v4_91.txt	7	55076166	55097996	4	3.8981	1
MSK_00071_251469322919_S01_CGH.v4_91.txt	7	54437140	55069561	27	4.9143	1
MSK_00071_251469322919_S01_CGH.v4_91.txt	7	54405478	54422628	4	3.8965	1
MSK_00071_251469322919_S01_CGH.v4_91.txt	7	52466580	52544984	6	3.8415	1
MSK_00071_251469322919_S01_CGH.v4_91.txt	7	47811704	48091367	23	3.8691	1
MSK_0079.1_251469338020_S01_CGH.v4_91.txt	7	110863584	111001312	14	-0.8234	-1
MSK_00080_251469322748_S01_CGH.v4_91.txt	7	78464558	158881735	5101	0.8508	1
MSK_00080_251469322748_S01_CGH.v4_91.txt	7	78194763	78246692	6	0.9962	1
MSK_00083_251469322918_S01_CGH.v4_91.txt	7	153167110	154429937	64	0.8049	1
MSK_00083_251469322918_S01_CGH.v4_91.txt	7	152927108	153094655	5	5.063	1
MSK_00083_251469322918_S01_CGH.v4_91.txt	7	151942754	152899064	28	0.8568	1
MSK_00083_251469322918_S01_CGH.v4_91.txt	7	149971095	151877220	160	0.9362	1
MSK_00083_251469322918_S01_CGH.v4_91.txt	7	126858741	131637622	338	0.9339	1
MSK_00083_251469322918_S01_CGH.v4_91.txt	7	86012852	86047180	3	5.0073	1
MSK_00083_251469322918_S01_CGH.v4_91.txt	7	55297487	55959849	52	0.8638	1
MSK_00083_251469322918_S01_CGH.v4_91.txt	7	54678659	55275549	33	4.4904	1
MSK_00083_251469322918_S01_CGH.v4_91.txt	7	50222459	50699318	27	0.8395	1
MSK_00083_251469322918_S01_CGH.v4_91.txt	7	30610870	32868322	158	0.6436	1
MSK_00083_251469322918_S01_CGH.v4_91.txt	7	3366980	5372492	139	0.8437	1
MSK_00089_251469322904_S01_CGH.v4_91.txt	7	54731478	55469426	45	4.9482	1
MSK_00102_251469322716_S01_CGH.v4_91.txt	7	143758646	143760132	2	1.2457	1
MSK_00102_251469322716_S01_CGH.v4_91.txt	7	55819226	55828507	3	-0.6441	-1
MSK_00102_251469322716_S01_CGH.v4_91.txt	7	54678659	55469426	47	6.0581	1
MSK_WGA_0104.1_251469338880_S01_CGH.v4_91.txt	7	158098754	158881735	54	-0.702	-1
MSK_WGA_0104.1_251469338880_S01_CGH.v4_91.txt	7	157714032	158076358	27	-2.5616	-1
MSK_WGA_0104.1_251469338880_S01_CGH.v4_91.txt	7	141226570	157696782	1049	-0.714	-1
MSK_0106.1_251469331471_S01_CGH.v4_91.txt	7	151942754	158881735	391	1.006	1
MSK_0106.1_251469331471_S01_CGH.v4_91.txt	7	97374991	151877220	3591	1.0259	1
MSK_0106.1_251469331471_S01_CGH.v4_91.txt	7	61837380	97309455	1995	1.0068	1
MSK_0106.1_251469331471_S01_CGH.v4_91.txt	7	55882462	55947269	9	1.0537	1
MSK_0106.1_251469331471_S01_CGH.v4_91.txt	7	55241779	55266145	3	1.0264	1
MSK_0106.1_251469331471_S01_CGH.v4_91.txt	7	54696117	55232820	28	4.4318	1

Continued on next page...

ID	chr	start	end	nroOfprobes	segmean	call
MSK_0106.1_251469331471_S01_CGH.v4_91.txt	7	876982	887896	2	1.472	1
MSK_00107_251469322719_S01_CGH.v4_91.txt	7	41722139	41746831	2	-0.6772	-1
MSK_00116_251469322720_S01_CGH.v4_91.txt	7	62792159	63094525	5	2.2194	1
MSK_00116_251469322720_S01_CGH.v4_91.txt	7	57690297	62635310	18	0.7088	1
MSK_00116_251469322720_S01_CGH.v4_91.txt	7	57514571	57662059	4	2.1816	1
MSK_00116_251469322720_S01_CGH.v4_91.txt	7	55444744	57471273	110	3.2129	1
MSK_00116_251469322720_S01_CGH.v4_91.txt	7	54613288	55430061	48	4.4516	1
MSK_0260.1_251469331583_S01_CGH.v4_91.txt	7	143758646	143760132	2	1.2267	1
MSK_0260.1_251469331583_S01_CGH.v4_91.txt	7	54355766	55097996	36	5.1327	1
MSK_0266.1_251469336909_S01_CGH.v4_91.txt	7	151942754	158881735	391	0.6558	1
MSK_0266.1_251469336909_S01_CGH.v4_91.txt	7	143881207	151877220	549	0.6547	1
MSK_0266.1_251469336909_S01_CGH.v4_91.txt	7	43890269	143677593	5709	0.6544	1
MSK_0266.1_251469336909_S01_CGH.v4_91.txt	7	54185	43806490	2748	0.655	1
MSK_0269.1_251469333522_S01_CGH.v4_91.txt	7	54678659	55134377	26	5.0346	1
MSK_0271.1_251469333526_S01_CGH.v4_91.txt	7	97374991	158881735	3985	0.7077	1
MSK_0271.1_251469333526_S01_CGH.v4_91.txt	7	54185	97309455	5417	0.6902	1
MSK_0281.1_251469331472_S01_CGH.v4_91.txt	7	154538135	154550194	3	2.9099	1
MSK_0281.1_251469331472_S01_CGH.v4_91.txt	7	148364334	148436956	6	3.0843	1
MSK_0281.1_251469331472_S01_CGH.v4_91.txt	7	142238186	142265061	4	2.9024	1
MSK_0281.1_251469331472_S01_CGH.v4_91.txt	7	141935711	142045293	14	3.1119	1
MSK_0281.1_251469331472_S01_CGH.v4_91.txt	7	116126525	116307096	18	2.9677	1
MSK_0281.1_251469331472_S01_CGH.v4_91.txt	7	5372492	5433334	6	0.9436	1
MSK_0285.1_251469337229_S01_CGH.v4_91.txt	7	143758646	143760132	2	1.3914	1
MSK_0285.1_251469337229_S01_CGH.v4_91.txt	7	88910675	89417291	18	1.221	1
MSK_0285.1_251469337229_S01_CGH.v4_91.txt	7	55188858	55535145	21	3.5571	1
MSK_0285.1_251469337229_S01_CGH.v4_91.txt	7	54731478	55154337	25	4.6544	1
MSK_0285.1_251469337229_S01_CGH.v4_91.txt	7	54591387	54696117	6	3.4451	1
MSK_0285.1_251469337229_S01_CGH.v4_91.txt	7	53773519	54513183	20	1.9577	1
MSK_0285.1_251469337229_S01_CGH.v4_91.txt	7	53468905	53717855	17	3.6714	1
MSK_0285.1_251469337229_S01_CGH.v4_91.txt	7	51305010	53412142	52	1.3495	1
MSK_0285.1_251469337229_S01_CGH.v4_91.txt	7	50417854	51194281	46	2.1448	1
MSK_0289.1_251469337015_S01_CGH.v4_91.txt	7	54591387	55275549	37	5.8153	1
MSK_0290.1_251469336710_S01_CGH.v4_91.txt	7	143758646	143760132	2	1.8726	1
MSK_0290.1_251469336710_S01_CGH.v4_91.txt	7	54513183	55275549	38	5.2209	1
MSK_0317.1_251469331584_S01_CGH.v4_91.txt	7	151942754	158881735	391	0.838	1
MSK_0317.1_251469331584_S01_CGH.v4_91.txt	7	143881207	151877220	549	0.8669	1
MSK_0317.1_251469331584_S01_CGH.v4_91.txt	7	143677593	143760132	3	1.5212	1
MSK_0317.1_251469331584_S01_CGH.v4_91.txt	7	97374991	143635040	3039	0.8811	1
MSK_0317.1_251469331584_S01_CGH.v4_91.txt	7	55232820	97309455	2128	0.8506	1
MSK_0317.1_251469331584_S01_CGH.v4_91.txt	7	54591387	55188858	32	4.4518	1
MSK_0317.1_251469331584_S01_CGH.v4_91.txt	7	43890269	54513183	507	0.86	1
MSK_0317.1_251469331584_S01_CGH.v4_91.txt	7	54185	43806490	2748	0.8789	1
MSK_0321.1_251469336723_S01_CGH.v4_91.txt	7	151942754	152964103	31	1.5738	1
MSK_0321.1_251469336723_S01_CGH.v4_91.txt	7	151274491	151877220	43	1.56	1
MSK_0321.1_251469336723_S01_CGH.v4_91.txt	7	142632558	142688051	6	-0.8882	-1
MSK_0326.1_251469331461_S01_CGH.v4_91.txt	7	38126727	38145569	4	0.7872	1
MSK_0330.1_251469336713_S01_CGH.v4_91.txt	7	110856135	110863584	2	-0.7719	-1
MSK_0333.1_251469331585_S01_CGH.v4_91.txt	7	156748014	157237415	41	-0.7653	-1
MSK_0333.1_251469331585_S01_CGH.v4_91.txt	7	119444004	119643449	8	5.3714	1
MSK_0333.1_251469331585_S01_CGH.v4_91.txt	7	115397322	115528585	10	5.1428	1
MSK_0333.1_251469331585_S01_CGH.v4_91.txt	7	55444744	55607468	6	4.5385	1
MSK_0333.1_251469331585_S01_CGH.v4_91.txt	7	54678659	55430061	45	5.2807	1
MSK_0337.1_251469333524_S01_CGH.v4_91.txt	7	75869696	76411062	15	0.7002	1
MSK_0422.1_251469337230_S01_CGH.v4_91.txt	7	54678659	55154337	27	4.5253	1
MSK_0430.1_251469336714_S01_CGH.v4_91.txt	7	54418648	55395451	52	5.2751	1
MSK_0432.1_251469336712_S01_CGH.v4_91.txt	7	38111132	38116427	2	1.3103	1
MSK_0432.1_251469336712_S01_CGH.v4_91.txt	7	5184469	24207665	1111	-0.6975	-1
MSK_0432.1_251469336712_S01_CGH.v4_91.txt	7	2832169	5060551	160	-0.7067	-1
MSK_0432.1_251469336712_S01_CGH.v4_91.txt	7	967775	1104275	11	-0.6899	-1
MSK_0432.1_251469336712_S01_CGH.v4_91.txt	7	54185	786726	27	-0.6452	-1
MSK_0439.1_251469331553_S01_CGH.v4_91.txt	7	49398527	55994300	293	1.1573	1
MSK_0451.1_251469333565_S01_CGH.v4_91.txt	7	62915687	63094525	4	1.7089	1
MSK_0451.1_251469333565_S01_CGH.v4_91.txt	7	56334822	57016361	29	2.8795	1
MSK_0451.1_251469333565_S01_CGH.v4_91.txt	7	55535145	55685694	9	2.3871	1
MSK_0451.1_251469333565_S01_CGH.v4_91.txt	7	55188858	55469426	20	3.0188	1
MSK_0451.1_251469333565_S01_CGH.v4_91.txt	7	54696117	55154337	26	5.2383	1
MSK_0451.1_251469333565_S01_CGH.v4_91.txt	7	53979897	54678659	20	4.5361	1
MSK_0456.1_251469331463_S01_CGH.v4_91.txt	7	143758646	143760132	2	-0.7604	-1
MSK_0456.1_251469331463_S01_CGH.v4_91.txt	7	38116427	38130640	4	0.6975	1
MSK_00122.1_251469325751_S01_CGH.v4_91.txt	7	55847664	57185777	64	1.0614	1
MSK_00122.1_251469325751_S01_CGH.v4_91.txt	7	54591387	55839876	76	4.5734	1
MSK_00125.1_251469324871_S01_CGH.v4_91.txt	7	54591387	55188858	32	5.1619	1
MSK_00126.1_251469325171_S01_CGH.v4_91.txt	7	55097996	55111113	2	4.9816	1
MSK_00126.1_251469325171_S01_CGH.v4_91.txt	7	54405478	55089018	34	6.0342	1
MSK_00126.1_251469325171_S01_CGH.v4_91.txt	7	53979897	54355766	7	4.5745	1
MSK_00126.1_251469325171_S01_CGH.v4_91.txt	7	38111132	38130640	5	1.2345	1
MSK_0127.1_251469337891_S01_CGH.v4_91.txt	7	54591387	55914655	82	4.4075	1
MSK_0127.1_251469337891_S01_CGH.v4_91.txt	7	54355766	54513183	9	1.1399	1
MSK_0127.1_251469337891_S01_CGH.v4_91.txt	7	43697511	43717705	5	4.5642	1
MSK_00129.1_251469322907_S01_CGH.v4_91.txt	7	147635243	147745034	8	-0.8015	-1
MSK_00129.1_251469322907_S01_CGH.v4_91.txt	7	143677593	143760132	3	0.7199	1
MSK_0132.1_251469330459_S01_CGH.v4_91.txt	7	54778609	55382365	38	3.7192	1
MSK_00133_251469325752_S01_CGH.v4_91.txt	7	55188858	55253581	4	3.9726	1
MSK_00133_251469325752_S01_CGH.v4_91.txt	7	54405478	55154337	39	5.106	1
MSK_00133_251469325752_S01_CGH.v4_91.txt	7	52491111	54355766	52	4.1329	1
MSK_00137.4_251469325162_S01_CGH.v4_91.txt	7	55030917	55607468	36	3.93	1
MSK_00137.4_251469325162_S01_CGH.v4_91.txt	7	53979897	54904315	27	4.0529	1
MSK_00139.1_251469325164_S01_CGH.v4_91.txt	7	143758646	143881207	3	-1.0436	-1
MSK_00143.1_251469326154_S01_CGH.v4_91.txt	7	54778609	55154337	24	5.0243	1
MSK_0149.1_251469336715_S01_CGH.v4_91.txt	7	54185	158881735	9404	0.7861	1
MSK_0152.1_251469337789_S01_CGH.v4_91.txt	7	54261755	55154337	41	6.0861	1
MSK_0157.1_251469329178_S01_CGH.v4_91.txt	7	54591387	55232820	33	4.5533	1
MSK_0158.1_251469329734_S01_CGH.v4_91.txt	7	91658945	96151807	328	0.7123	1
MSK_0158.1_251469329734_S01_CGH.v4_91.txt	7	86518643	90264363	241	0.6867	1
MSK_0158.1_251469329734_S01_CGH.v4_91.txt	7	56415210	84773027	1276	0.6688	1
MSK_0158.1_251469329734_S01_CGH.v4_91.txt	7	56334822	56399816	8	4.1887	1
MSK_0158.1_251469329734_S01_CGH.v4_91.txt	7	54483191	55188858	34	4.6552	1
MSK_0158.1_251469329734_S01_CGH.v4_91.txt	7	42877091	54459930	579	0.6475	1
MSK_0162.1_251469336905_S01_CGH.v4_91.txt	7	126961571	136836636	697	0.7514	1
MSK_0162.1_251469336905_S01_CGH.v4_91.txt	7	102641363	102735897	3	1.0331	1
MSK_0162.1_251469336905_S01_CGH.v4_91.txt	7	55589743	56191148	44	1.0008	1
MSK_0162.1_251469336905_S01_CGH.v4_91.txt	7	54620095	55154337	29	1.306	1
MSK_0162.1_251469336905_S01_CGH.v4_91.txt	7	54591387	54613288	2	0.7961	1
MSK_0162.1_251469336905_S01_CGH.v4_91.txt	7	52638067	53094734	12	2.6434	1
MSK_0162.1_251469336905_S01_CGH.v4_91.txt	7	52300922	52544984	8	3.4926	1
MSK_0162.1_251469336905_S01_CGH.v4_91.txt	7	52163169	52280711	4	2.4679	1
MSK_0162.1_251469336905_S01_CGH.v4_91.txt	7	51954146	52130054	7	0.8819	1
MSK_0162.1_251469336905_S01_CGH.v4_91.txt	7	44681774	44693555	2	0.7429	1
MSK_0162.1_251469336905_S01_CGH.v4_91.txt	7	42792936	42801204	2	2.1065	1
MSK_0162.1_251469336905_S01_CGH.v4_91.txt	7	33682780	34357387	47	2.387	1
MSK_0162.1_251469336905_S01_CGH.v4_91.txt	7	33572888	33641276	6	2.8796	1
MSK_0162.1_251469336905_S01_CGH.v4_91.txt	7	33472035	33564392	5	1.6914	1
MSK_0162.1_251469336905_S01_CGH.v4_91.txt	7	33332126	33457225	17	2.0852	1
MSK_0162.1_251469336905_S01_CGH.v4_91.txt	7	31538444	33324304	147	3.1561	1
MSK_0162.1_251469336905_S01_CGH.v4_91.txt	7	31515648	31522145	2	2.658	1
MSK_0162.1_251469336905_S01_CGH.v4_91.txt	7	31354886	31505092	15	1.902	1
MSK_0162.1_251469336905_S01_CGH.v4_91.txt	7	31230944	31346462	6	2.7467	1
MSK_0162.1_251469336905_S01_CGH.v4_91.txt	7	31039889	31198073	8	3.6417	1
MSK_0162.1_251469336905_S01_CGH.v4_91.txt	7	30801236	30979215	11	2.1064	1
MSK_0162.1_251469336905_S01_CGH.v4_91.txt	7	30595840	30770858	12	0.6597	1
MSK_0162.1_251469336905_S01_CGH.v4_91.txt	7	30408579	30586478	12	2.3474	1
MSK_0162.1_251469336905_S01_CGH.v4_91.txt	7	30304470	30390354	9	1.7062	1
MSK_0162.1_251469336905_S01_CGH.v4_91.txt	7	30160242	30217107	5	2.0989	1
MSK_0162.1_251469336905_S01_CGH.v4_91.txt	7	29497993	29885330	22	1.2071	1

Continued on next page...

ID	chr	start	end	nroOfprobes	segmean	call
MSK_0162.1_251469336905_S01_CGH.v4_91.txt	7	27457998	27811760	29	2.2029	1
MSK_0162.1_251469336905_S01_CGH.v4_91.txt	7	26446915	27172620	61	0.8037	1
MSK_0162.1_251469336905_S01_CGH.v4_91.txt	7	26119066	26233981	9	3.186	1
MSK_0162.1_251469336905_S01_CGH.v4_91.txt	7	25456173	26109455	30	2.4808	1
MSK_0162.1_251469336905_S01_CGH.v4_91.txt	7	25158835	25442785	8	1.8291	1
MSK_0162.1_251469336905_S01_CGH.v4_91.txt	7	24075299	25094133	70	2.2063	1
MSK_0162.1_251469336905_S01_CGH.v4_91.txt	7	24002664	24058993	4	1.8645	1
MSK_0162.1_251469336905_S01_CGH.v4_91.txt	7	23519247	23974394	29	0.8839	1
MSK_0162.1_251469336905_S01_CGH.v4_91.txt	7	23474468	23509553	4	2.2544	1
MSK_0162.1_251469336905_S01_CGH.v4_91.txt	7	21879790	23464784	115	3.304	1
MSK_0162.1_251469336905_S01_CGH.v4_91.txt	7	20517433	20918993	19	1.0685	1
MSK_0162.1_251469336905_S01_CGH.v4_91.txt	7	19142066	19879754	26	1.0139	1
MSK_0162.1_251469336905_S01_CGH.v4_91.txt	7	16154686	18460533	138	2.5293	1
MSK_0162.1_251469336905_S01_CGH.v4_91.txt	7	15421130	16142551	28	3.102	1
MSK_0162.1_251469336905_S01_CGH.v4_91.txt	7	14420660	15391233	64	1.091	1
MSK_0162.1_251469336905_S01_CGH.v4_91.txt	7	14010469	14072320	8	1.0316	1
MSK_00169.1_251469325174_S01_CGH.v4_91.txt	7	55030917	55122169	10	4.4526	1
MSK_00169.1_251469325174_S01_CGH.v4_91.txt	7	54989649	55016814	3	3.4815	1
MSK_00169.1_251469325174_S01_CGH.v4_91.txt	7	54613288	54981087	15	4.56	1
MSK_0171.1_251469329753_S01_CGH.v4_91.txt	7	143758646	143760132	2	0.8938	1
MSK_0173.1_251469330461_S01_CGH.v4_91.txt	7	54411324	55241779	41	4.4828	1
MSK_0174.1_251469330284_S01_CGH.v4_91.txt	7	54678659	55154337	27	1.3226	1
MSK_0175.1_251469336725_S01_CGH.v4_91.txt	7	143758646	143760132	2	1.1276	1
MSK_0177.1_251469333358_S01_CGH.v4_91.txt	7	102673453	102735897	2	1.7652	1
MSK_0179.1_251469333566_S01_CGH.v4_91.txt	7	143758646	143760132	2	0.9139	1
MSK_0179.1_251469333566_S01_CGH.v4_91.txt	7	54261755	55188858	42	3.5887	1
MSK_0182.1_251469331464_S01_CGH.v4_91.txt	7	64453516	64870313	20	-1.0101	-1
MSK_0182.1_251469331464_S01_CGH.v4_91.txt	7	54483191	55188858	34	5.3315	1
MSK_0184.1_251469329181_S01_CGH.v4_91.txt	7	150380381	158881735	512	0.9316	1
MSK_0184.1_251469329181_S01_CGH.v4_91.txt	7	136199036	138034974	127	0.7999	1
MSK_0184.1_251469329181_S01_CGH.v4_91.txt	7	134441949	135390996	77	0.7814	1
MSK_0184.1_251469329181_S01_CGH.v4_91.txt	7	128106930	132986129	340	1.0883	1
MSK_0184.1_251469329181_S01_CGH.v4_91.txt	7	127928881	128088836	7	0.7039	1
MSK_0184.1_251469329181_S01_CGH.v4_91.txt	7	127425095	127911988	36	1.2256	1
MSK_0184.1_251469329181_S01_CGH.v4_91.txt	7	126879621	127417343	47	0.802	1
MSK_0184.1_251469329181_S01_CGH.v4_91.txt	7	115988692	123294853	440	0.8501	1
MSK_0184.1_251469329181_S01_CGH.v4_91.txt	7	112893123	115501549	119	0.7533	1
MSK_0184.1_251469329181_S01_CGH.v4_91.txt	7	110248148	112850451	195	1.2115	1
MSK_0184.1_251469329181_S01_CGH.v4_91.txt	7	109845867	110237636	18	0.8046	1
MSK_0184.1_251469329181_S01_CGH.v4_91.txt	7	103663706	108226132	335	0.8252	1
MSK_0184.1_251469329181_S01_CGH.v4_91.txt	7	100505416	100945062	35	0.8026	1
MSK_0184.1_251469329181_S01_CGH.v4_91.txt	7	97374991	100284275	243	0.7783	1
MSK_0184.1_251469329181_S01_CGH.v4_91.txt	7	96383773	97309455	51	0.7414	1
MSK_0184.1_251469329181_S01_CGH.v4_91.txt	7	95461149	96304984	50	1.2743	1
MSK_0184.1_251469329181_S01_CGH.v4_91.txt	7	93830680	95451881	114	0.7562	1
MSK_0184.1_251469329181_S01_CGH.v4_91.txt	7	81595327	92407592	632	0.761	1
MSK_0184.1_251469329181_S01_CGH.v4_91.txt	7	79121388	80401376	71	0.8274	1
MSK_0184.1_251469329181_S01_CGH.v4_91.txt	7	64685843	65076523	10	0.6418	1
MSK_0184.1_251469329181_S01_CGH.v4_91.txt	7	63600299	64373673	37	0.8412	1
MSK_0184.1_251469329181_S01_CGH.v4_91.txt	7	61837380	63500612	49	0.7126	1
MSK_0184.1_251469329181_S01_CGH.v4_91.txt	7	56300336	57471273	62	0.7041	1
MSK_0184.1_251469329181_S01_CGH.v4_91.txt	7	55089018	56191148	72	1.1925	1
MSK_0184.1_251469329181_S01_CGH.v4_91.txt	7	52067966	55081527	95	0.7565	1
MSK_0184.1_251469329181_S01_CGH.v4_91.txt	7	51482429	52048958	14	1.2588	1
MSK_0184.1_251469329181_S01_CGH.v4_91.txt	7	50285879	51460356	57	0.8145	1
MSK_0184.1_251469329181_S01_CGH.v4_91.txt	7	32126265	37302948	373	1.2	1
MSK_0184.1_251469329181_S01_CGH.v4_91.txt	7	31039889	32094323	86	0.7664	1
MSK_0184.1_251469329181_S01_CGH.v4_91.txt	7	26109455	30979215	361	1.1681	1
MSK_0184.1_251469329181_S01_CGH.v4_91.txt	7	26030455	26076726	6	0.7452	1
MSK_0184.1_251469329181_S01_CGH.v4_91.txt	7	25396217	26004005	26	1.2141	1
MSK_0184.1_251469329181_S01_CGH.v4_91.txt	7	24496987	25358225	55	0.7763	1
MSK_0184.1_251469329181_S01_CGH.v4_91.txt	7	19910493	20918993	55	0.7682	1
MSK_0184.1_251469329181_S01_CGH.v4_91.txt	7	17060833	18887639	113	0.7903	1
MSK_0184.1_251469329181_S01_CGH.v4_91.txt	7	1642352	1933304	19	0.8016	1
MSK_0184.1_251469329181_S01_CGH.v4_91.txt	7	611576	1628964	67	1.2695	1
MSK_0184.1_251469329181_S01_CGH.v4_91.txt	7	54185	605496	14	0.7881	1
MSK_0185.1_251469329754_S01_CGH.v4_91.txt	7	55444744	55661940	10	3.7077	1
MSK_0185.1_251469329754_S01_CGH.v4_91.txt	7	54405478	55430061	57	4.4817	1
MSK_0185.1_251469329754_S01_CGH.v4_91.txt	7	38111132	38116427	2	1.4249	1
MSK_0187.1_251469330462_S01_CGH.v4_91.txt	7	54459930	55938247	88	5.6528	1
MSK_0189.1_251469330588_S01_CGH.v4_91.txt	7	143758646	143760132	2	1.0853	1
MSK_0194.1_251469336985_S01_CGH.v4_91.txt	7	54261755	55154337	41	1.2228	1
MSK_0194.1_251469336985_S01_CGH.v4_91.txt	7	38116427	38130640	4	0.8113	1
MSK_0195.1_251469329772_S01_CGH.v4_91.txt	7	56542075	56558461	3	1.2976	1
MSK_0195.1_251469329772_S01_CGH.v4_91.txt	7	56438233	56514706	10	3.0348	1
MSK_0195.1_251469329772_S01_CGH.v4_91.txt	7	55828507	56415210	33	0.6786	1
MSK_0195.1_251469329772_S01_CGH.v4_91.txt	7	55746262	55823102	12	3.0684	1
MSK_0195.1_251469329772_S01_CGH.v4_91.txt	7	55297487	55722209	25	0.9068	1
MSK_0195.1_251469329772_S01_CGH.v4_91.txt	7	55030917	55275549	18	3.2966	1
MSK_0195.1_251469329772_S01_CGH.v4_91.txt	7	54924247	55016814	7	1.277	1
MSK_0195.1_251469329772_S01_CGH.v4_91.txt	7	54411324	54904315	19	3.543	1
MSK_0195.1_251469329772_S01_CGH.v4_91.txt	7	53979897	54405478	8	2.3529	1
MSK_0206.1_251469329173_S01_CGH.v4_91.txt	7	151942754	158881735	391	0.6882	1
MSK_0206.1_251469329173_S01_CGH.v4_91.txt	7	54185	151877220	9010	0.6765	1
MSK_0208.1_251469329182_S01_CGH.v4_91.txt	7	76283523	76390622	4	0.9862	1
MSK_0208.1_251469329182_S01_CGH.v4_91.txt	7	65030038	65076523	3	1.4765	1
MSK_0208.1_251469329182_S01_CGH.v4_91.txt	7	53867158	55994300	112	3.9711	1
MSK_0208.2_251469329755_S01_CGH.v4_91.txt	7	143758646	143760132	2	0.9376	1
MSK_0208.2_251469329755_S01_CGH.v4_91.txt	7	76283523	76411062	5	1.015	1
MSK_0208.2_251469329755_S01_CGH.v4_91.txt	7	65030038	65076523	3	1.4151	1
MSK_0208.2_251469329755_S01_CGH.v4_91.txt	7	53867158	55994300	112	3.8319	1
MSK_0208.2_251469329755_S01_CGH.v4_91.txt	7	27594954	27604300	2	-0.7051	-1
MSK_0209.1_251469330463_S01_CGH.v4_91.txt	7	64453516	65003103	21	0.9388	1
MSK_0209.1_251469330463_S01_CGH.v4_91.txt	7	55552208	55813548	20	4.8389	1
MSK_0209.1_251469330463_S01_CGH.v4_91.txt	7	54964962	55535145	38	5.2928	1
MSK_0209.1_251469330463_S01_CGH.v4_91.txt	7	54899138	54953802	4	6.5017	1
MSK_0209.1_251469330463_S01_CGH.v4_91.txt	7	54261755	54883369	20	5.2272	1
MSK_0211.1_251469330227_S01_CGH.v4_91.txt	7	56438233	56558461	13	3.8675	1
MSK_0211.1_251469330227_S01_CGH.v4_91.txt	7	56370796	56415210	5	1.4388	1
MSK_0211.1_251469330227_S01_CGH.v4_91.txt	7	55722209	55823102	13	4.4546	1
MSK_0211.1_251469330227_S01_CGH.v4_91.txt	7	55297487	55701296	24	1.6367	1
MSK_0211.1_251469330227_S01_CGH.v4_91.txt	7	55030917	55275549	18	4.7069	1
MSK_0211.1_251469330227_S01_CGH.v4_91.txt	7	54924247	55016814	7	2.0556	1
MSK_0211.1_251469330227_S01_CGH.v4_91.txt	7	54678659	54904315	8	5.1868	1
MSK_0211.1_251469330227_S01_CGH.v4_91.txt	7	54411324	54629892	11	4.8134	1
MSK_0211.1_251469330227_S01_CGH.v4_91.txt	7	53979897	54405478	8	3.5292	1
MSK_0211.2_251469329773_S01_CGH.v4_91.txt	7	56542075	56558461	3	2.3319	1
MSK_0211.2_251469329773_S01_CGH.v4_91.txt	7	56438233	56514706	10	4.6268	1
MSK_0211.2_251469329773_S01_CGH.v4_91.txt	7	56370796	56415210	5	1.6164	1
MSK_0211.2_251469329773_S01_CGH.v4_91.txt	7	55828507	56359287	28	0.6407	1
MSK_0211.2_251469329773_S01_CGH.v4_91.txt	7	55722209	55823102	13	4.5895	1
MSK_0211.2_251469329773_S01_CGH.v4_91.txt	7	55297487	55701296	24	1.6663	1
MSK_0211.2_251469329773_S01_CGH.v4_91.txt	7	55030917	55275549	18	4.9801	1
MSK_0211.2_251469329773_S01_CGH.v4_91.txt	7	54924247	55016814	7	2.3135	1
MSK_0211.2_251469329773_S01_CGH.v4_91.txt	7	54678659	54904315	8	5.4449	1
MSK_0211.2_251469329773_S01_CGH.v4_91.txt	7	54411324	54629892	11	5.0701	1
MSK_0211.2_251469329773_S01_CGH.v4_91.txt	7	53979897	54405478	8	3.5929	1
MSK_0213.1_251469329174_S01_CGH.v4_91.txt	7	54185	145203752	8524	0.6705	1
MSK_0214.1_251469329168_S01_CGH.v4_91.txt	7	54591387	55111113	28	3.0154	1
MSK_0219.1_251469329864_S01_CGH.v4_91.txt	7	55030917	55325242	20	3.9127	1
MSK_0219.1_251469329864_S01_CGH.v4_91.txt	7	54989649	55016814	3	2.9433	1
MSK_0219.1_251469329864_S01_CGH.v4_91.txt	7	54355766	54981087	25	3.8323	1
MSK_0221.1_251469330192_S01_CGH.v4_91.txt	7	93936541	94003799	6	1.9221	1
MSK_0221.1_251469330192_S01_CGH.v4_91.txt	7	91563380	92327770	68	1.9435	1

Continued on next page...

ID	chr	start	end	nroOfprobes	segmean	call
MSK_0221.1_251469330192_S01_CGH.v4_91.txt	7	80076513	80598473	34	1.9524	1
MSK_0221.1_251469330192_S01_CGH.v4_91.txt	7	73473549	73506866	4	1.9989	1
MSK_0221.1_251469330192_S01_CGH.v4_91.txt	7	67871813	67978922	3	1.7145	1
MSK_0221.1_251469330192_S01_CGH.v4_91.txt	7	39473725	39604660	7	1.5884	1
MSK_0221.1_251469330192_S01_CGH.v4_91.txt	7	26403491	26609214	14	1.9034	1
MSK_0221.1_251469330192_S01_CGH.v4_91.txt	7	10747052	10851836	10	1.79	1
MSK_0237.1_251469330449_S01_CGH.v4_91.txt	7	142632558	142688051	6	-0.7501	-1
MSK_0237.1_251469330449_S01_CGH.v4_91.txt	7	62792159	62919814	3	1.2402	1
MSK_0237.1_251469330449_S01_CGH.v4_91.txt	7	56300336	57432663	60	1.2371	1
MSK_0237.1_251469330449_S01_CGH.v4_91.txt	7	53773519	56191148	117	2.5409	1
MSK_0394.1_251469336716_S01_CGH.v4_91.txt	7	143758646	143760132	2	-0.6724	-1
MSK_0394.1_251469336716_S01_CGH.v4_91.txt	7	128633944	128683978	4	0.7397	1
MSK_0397.1_251469330468_S01_CGH.v4_91.txt	7	133941756	133961610	4	-0.9411	-1
MSK_0397.1_251469330468_S01_CGH.v4_91.txt	7	38111132	38116427	2	1.4344	1
MSK_0397.1_251469330468_S01_CGH.v4_91.txt	7	4307266	4408693	8	-0.9966	-1
MSK_0402.1_251469336906_S01_CGH.v4_91.txt	7	91882320	92794516	63	2.9218	1
MSK_0402.1_251469336906_S01_CGH.v4_91.txt	7	91829833	91875068	6	1.3998	1
MSK_0402.1_251469336906_S01_CGH.v4_91.txt	7	88378244	88472861	9	2.7256	1
MSK_0402.1_251469336906_S01_CGH.v4_91.txt	7	78392772	78603109	23	1.4354	1
MSK_0402.1_251469336906_S01_CGH.v4_91.txt	7	77331770	78213843	82	1.8337	1
MSK_0402.1_251469336906_S01_CGH.v4_91.txt	7	67689642	68024809	9	3.6294	1
MSK_0402.1_251469336906_S01_CGH.v4_91.txt	7	56334822	56636957	27	3.7475	1
MSK_0402.1_251469336906_S01_CGH.v4_91.txt	7	55904056	56300336	18	1.207	1
MSK_0402.1_251469336906_S01_CGH.v4_91.txt	7	55097996	55895399	54	3.8717	1
MSK_0402.1_251469336906_S01_CGH.v4_91.txt	7	54678659	55089018	22	5.2402	1
MSK_0402.1_251469336906_S01_CGH.v4_91.txt	7	54031412	54629892	18	3.8478	1
MSK_0402.1_251469336906_S01_CGH.v4_91.txt	7	43100668	43297110	13	1.3102	1
MSK_0402.1_251469336906_S01_CGH.v4_91.txt	7	42780998	43074180	21	2.6061	1
MSK_0402.1_251469336906_S01_CGH.v4_91.txt	7	38568466	38918139	28	1.1883	1
MSK_0402.1_251469336906_S01_CGH.v4_91.txt	7	38537359	38559568	4	2.6965	1
MSK_0402.1_251469336906_S01_CGH.v4_91.txt	7	37642357	38494482	62	1.3574	1
MSK_0402.1_251469336906_S01_CGH.v4_91.txt	7	34357387	34658527	28	1.6629	1
MSK_0402.1_251469336906_S01_CGH.v4_91.txt	7	32524718	33699687	94	1.885	1
MSK_0402.1_251469336906_S01_CGH.v4_91.txt	7	28508027	28548622	5	2.8761	1
MSK_0402.1_251469336906_S01_CGH.v4_91.txt	7	24129247	24222824	9	2.3084	1
MSK_0402.1_251469336906_S01_CGH.v4_91.txt	7	16503407	16755084	25	1.7265	1
MSK_0402.1_251469336906_S01_CGH.v4_91.txt	7	13553778	13904056	17	3.6948	1
MSK_0409.1_251469331555_S01_CGH.v4_91.txt	7	87956073	88051593	4	-0.9298	-1
MSK_0409.1_251469331555_S01_CGH.v4_91.txt	7	54513183	55188858	33	4.9201	1
MSK_0410.1_251469333359_S01_CGH.v4_91.txt	7	144655835	145614295	27	3.0851	1
MSK_0410.1_251469333359_S01_CGH.v4_91.txt	7	133268635	134218685	75	1.5316	1
MSK_0410.1_251469333359_S01_CGH.v4_91.txt	7	116126525	116501861	35	1.4767	1
MSK_0410.1_251469333359_S01_CGH.v4_91.txt	7	112043292	112373132	25	3.0441	1
MSK_0410.1_251469333359_S01_CGH.v4_91.txt	7	78409248	78674884	26	3.0288	1
MSK_0410.1_251469333359_S01_CGH.v4_91.txt	7	3128655	4227688	87	-0.8768	-1
MSK_0412.1_251469331490_S01_CGH.v4_91.txt	7	151942754	158881735	391	0.7956	1
MSK_0412.1_251469331490_S01_CGH.v4_91.txt	7	57514571	151877220	5594	0.779	1
MSK_0412.1_251469331490_S01_CGH.v4_91.txt	7	55552208	57471273	107	1.411	1
MSK_0412.1_251469331490_S01_CGH.v4_91.txt	7	55188858	55535145	21	2.933	1
MSK_0412.1_251469331490_S01_CGH.v4_91.txt	7	55030917	55154337	12	4.165	1
MSK_0412.1_251469331490_S01_CGH.v4_91.txt	7	54981087	55016814	4	3.1037	1
MSK_0412.1_251469331490_S01_CGH.v4_91.txt	7	54678659	54964962	11	4.1744	1
MSK_0412.1_251469331490_S01_CGH.v4_91.txt	7	54185	54629892	3261	0.7866	1
MSK_0413.1_251469331465_S01_CGH.v4_91.txt	7	116088841	117315411	110	3.5222	1
MSK_0413.1_251469331465_S01_CGH.v4_91.txt	7	38116427	38130640	4	1.0208	1
MSK_0414.1_251469336986_S01_CGH.v4_91.txt	7	143758646	143760132	2	-0.7211	-1
MSK_0644.1_251469340741_S01_CGH.v4_91.txt	7	54185	158881735	9404	0.6468	1
MSK_0645.1_251469340797_S01_CGH.v4_91.txt	7	54355766	55325242	48	5.0005	1
MSK_0646.1_251469340746_S01_CGH.v4_91.txt	7	55030917	55134377	11	3.8016	1
MSK_0646.1_251469340746_S01_CGH.v4_91.txt	7	54678659	54924247	9	3.837	1
MSK_WGA_0244.1_251469338881_S01_CGH.v4_91.txt	7	55060233	55345073	20	4.0617	1
MSK_WGA_0244.1_251469338881_S01_CGH.v4_91.txt	7	54981087	55042974	6	3.3825	1
MSK_WGA_0244.1_251469338881_S01_CGH.v4_91.txt	7	54405478	54964962	23	4.1337	1
MSK_WGA_0246.1_251469338865_S01_CGH.v4_91.txt	7	65003103	65030038	3	0.9766	1
MSK_WGA_0246.1_251469338865_S01_CGH.v4_91.txt	7	54080556	55994300	108	3.7829	1
MSK_0345.1_251469337922_S01_CGH.v4_91.txt	7	116034821	116501861	39	3.8534	1
MSK_0349.1_251469337892_S01_CGH.v4_91.txt	7	52796403	55607468	107	3.9092	1
MSK_0349.1_251469337892_S01_CGH.v4_91.txt	7	49245546	50159777	54	3.7008	1
MSK_0352.1_251469337790_S01_CGH.v4_91.txt	7	71600913	158881735	5497	0.6578	1
MSK_0352.1_251469337790_S01_CGH.v4_91.txt	7	71476080	71585427	11	-0.8189	-1
MSK_0352.1_251469337790_S01_CGH.v4_91.txt	7	55469426	71450648	589	0.7091	1
MSK_0352.1_251469337790_S01_CGH.v4_91.txt	7	54778609	55444744	43	6.2797	1
MSK_WGA_0353.1_251469337506_S01_CGH.v4_91.txt	7	54437140	55154337	35	4.3898	1
MSK_WGA_0355.1_251469337508_S01_CGH.v4_91.txt	7	56300336	56378738	8	3.284	1
MSK_WGA_0355.1_251469337508_S01_CGH.v4_91.txt	7	55552208	56191148	45	3.5685	1
MSK_WGA_0355.1_251469337508_S01_CGH.v4_91.txt	7	54591387	55535145	52	4.2161	1
MSK_WGA_0355.1_251469337508_S01_CGH.v4_91.txt	7	54224937	54513183	11	3.383	1
MSK_WGA_0355.1_251469337508_S01_CGH.v4_91.txt	7	32783446	32818775	3	0.9332	1
MSK_WGA_0356.1_251469337509_S01_CGH.v4_91.txt	7	54459930	55069561	26	4.3142	1
MSK_WGA_0357.1_251469337522_S01_CGH.v4_91.txt	7	54678659	55122169	25	3.6638	1
MSK_0358.1_251469340789_S01_CGH.v4_91.txt	7	98026221	158881735	3951	0.8977	1
MSK_0358.1_251469340789_S01_CGH.v4_91.txt	7	55232820	93731811	1909	0.894	1
MSK_0358.1_251469340789_S01_CGH.v4_91.txt	7	55030917	55188858	13	5.5588	1
MSK_0358.1_251469340789_S01_CGH.v4_91.txt	7	54924247	55016814	7	1.8147	1
MSK_0358.1_251469340789_S01_CGH.v4_91.txt	7	54483191	54904315	14	5.5636	1
MSK_0358.1_251469340789_S01_CGH.v4_91.txt	7	54158308	54459930	10	6.4009	1
MSK_0358.1_251469340789_S01_CGH.v4_91.txt	7	53979897	54080556	3	5.5866	1
MSK_0358.1_251469340789_S01_CGH.v4_91.txt	7	53020322	53890341	30	1.0513	1
MSK_0358.1_251469340789_S01_CGH.v4_91.txt	7	52491111	52949178	15	5.4711	1
MSK_WGA_0360.1_251469337524_S01_CGH.v4_91.txt	7	55030917	55188858	13	1.4397	1
MSK_WGA_0360.1_251469337524_S01_CGH.v4_91.txt	7	54483191	54904315	14	1.4425	1
MSK_WGA_0360.1_251469337524_S01_CGH.v4_91.txt	7	54158308	54459930	10	1.9921	1
MSK_WGA_0360.1_251469337524_S01_CGH.v4_91.txt	7	53979897	54080556	3	1.4221	1
MSK_WGA_0360.1_251469337524_S01_CGH.v4_91.txt	7	52491111	52949178	15	1.3777	1
MSK_0386.1_251469340798_S01_CGH.v4_91.txt	7	22526599	23347514	71	-0.632	-1
MSK_0386.1_251469340798_S01_CGH.v4_91.txt	7	22178172	22288596	7	-0.7379	-1
MSK_0386.1_251469340798_S01_CGH.v4_91.txt	7	21072988	22100184	64	-0.6753	-1
MSK_WGA_0389.1_251469338874_S01_CGH.v4_91.txt	7	27065373	27090330	3	0.8764	1
MSK_WGA_0389.1_251469338874_S01_CGH.v4_91.txt	7	5372492	5433334	6	0.9993	1
MSK_WGA_0390.1_251469338875_S01_CGH.v4_91.txt	7	54355766	55241779	43	1.294	1
MSK_0509.1_251469336717_S01_CGH.v4_91.txt	7	12353144	12418696	5	0.8964	1
MSK_0511.1_251469336907_S01_CGH.v4_91.txt	7	55076166	55154337	8	2.2587	1
MSK_0511.1_251469336907_S01_CGH.v4_91.txt	7	54731478	55069561	17	4.168	1
MSK_0511.1_251469336907_S01_CGH.v4_91.txt	7	52440388	52544984	7	4.2301	1
MSK_0514.1_251469333360_S01_CGH.v4_91.txt	7	112240791	112278085	5	0.8069	1
MSK_0514.1_251469333360_S01_CGH.v4_91.txt	7	90604717	92363407	120	0.6319	1
MSK_0514.1_251469333360_S01_CGH.v4_91.txt	7	65030038	65076523	3	1.3376	1
MSK_0514.1_251469333360_S01_CGH.v4_91.txt	7	54158308	56074017	108	4.1549	1
MSK_0516.1_251469331469_S01_CGH.v4_91.txt	7	143758646	143760132	2	-1.2113	-1
MSK_0517.1_251469337226_S01_CGH.v4_91.txt	7	46412510	57130154	494	-0.8337	-1
MSK_0518.1_251469337014_S01_CGH.v4_91.txt	7	158229717	158881735	42	0.6966	1
MSK_0518.1_251469337014_S01_CGH.v4_91.txt	7	158191230	158218685	5	-0.6574	-1
MSK_0518.1_251469337014_S01_CGH.v4_91.txt	7	151942754	158172057	344	0.7071	1
MSK_0518.1_251469337014_S01_CGH.v4_91.txt	7	54696117	55089018	21	2.7881	1
MSK_0518.1_251469337014_S01_CGH.v4_91.txt	7	38111132	38126727	4	1.2516	1
MSK_0521.1_251469331582_S01_CGH.v4_91.txt	7	64498651	64870313	19	-0.9491	-1
MSK_0524.1_251469331557_S01_CGH.v4_91.txt	7	143758646	143760132	2	0.6324	1
MSK_0525.1_251469333361_S01_CGH.v4_91.txt	7	143758646	143760132	2	1.3274	1
MSK_0525.1_251469333361_S01_CGH.v4_91.txt	7	54513183	55134377	31	5.5216	1
MSK_0529.1_251469331470_S01_CGH.v4_91.txt	7	151942754	158881735	391	0.8785	1
MSK_0529.1_251469331470_S01_CGH.v4_91.txt	7	97374991	151877220	3591	0.8574	1
MSK_0529.1_251469331470_S01_CGH.v4_91.txt	7	55134377	97309455	2131	0.8377	1
MSK_0529.1_251469331470_S01_CGH.v4_91.txt	7	54678659	55122169	25	5.1961	1
MSK_0529.1_251469331470_S01_CGH.v4_91.txt	7	43890269	54629892	511	0.866	1

Continued on next page...

ID	chr	start	end	nroOfprobes	segmean	call
MSK_0529.1_251469331470_S01_CGH.v4_91.txt	7	54185	43806490	2748	0.8679	1
MSK_0531.1_251469337227_S01_CGH.v4_91.txt	7	54591387	55188858	32	5.7483	1
MSK_0615.1_251469340783_S01_CGH.v4_91.txt	7	55701296	57289571	90	2.3158	1
MSK_0615.1_251469340783_S01_CGH.v4_91.txt	7	53125226	55685694	103	4.3036	1
MSK_0615.1_251469340783_S01_CGH.v4_91.txt	7	52638067	53094734	12	2.3053	1
MSK_0615.1_251469340783_S01_CGH.v4_91.txt	7	52440388	52544984	7	3.804	1
MSK_0616.1_251469338022_S01_CGH.v4_91.txt	7	144202527	144424914	13	2.039	1
MSK_0616.1_251469338022_S01_CGH.v4_91.txt	7	133156865	133276505	13	1.9727	1
MSK_0616.1_251469338022_S01_CGH.v4_91.txt	7	119604087	119888514	23	1.9527	1
MSK_0616.1_251469338022_S01_CGH.v4_91.txt	7	116112896	116375854	25	2.9687	1
MSK_0616.1_251469338022_S01_CGH.v4_91.txt	7	115669696	115710769	4	2.8756	1
MSK_0616.1_251469338022_S01_CGH.v4_91.txt	7	109074970	109290325	3	1.7735	1
MSK_0616.1_251469338022_S01_CGH.v4_91.txt	7	101075728	101370763	15	2.7271	1
MSK_0616.1_251469338022_S01_CGH.v4_91.txt	7	100945062	101074105	9	2.0398	1
MSK_0616.1_251469338022_S01_CGH.v4_91.txt	7	98538861	98587094	5	2.1417	1
MSK_0616.1_251469338022_S01_CGH.v4_91.txt	7	70700729	70968480	24	1.9642	1
MSK_0616.1_251469338022_S01_CGH.v4_91.txt	7	68788431	68930864	10	1.4676	1
MSK_0616.1_251469338022_S01_CGH.v4_91.txt	7	57130154	57174141	6	5.7186	1
MSK_0616.1_251469338022_S01_CGH.v4_91.txt	7	53816770	55134377	48	2.5053	1
MSK_0616.1_251469338022_S01_CGH.v4_91.txt	7	52466580	52544984	6	6.0499	1
MSK_0616.1_251469338022_S01_CGH.v4_91.txt	7	41557554	41610138	5	1.958	1
MSK_0616.1_251469338022_S01_CGH.v4_91.txt	7	40144070	40157098	3	5.779	1
MSK_0616.1_251469338022_S01_CGH.v4_91.txt	7	37600733	38041017	27	2.0459	1
MSK_0616.1_251469338022_S01_CGH.v4_91.txt	7	37364273	37395669	3	1.9716	1
MSK_0616.1_251469338022_S01_CGH.v4_91.txt	7	30304470	30332085	4	2.6135	1
MSK_0616.1_251469338022_S01_CGH.v4_91.txt	7	21198314	21242002	2	2.4852	1
MSK_0619.1_251469337893_S01_CGH.v4_91.txt	7	54355766	55154337	40	4.8942	1
MSK_00006_251469322732_S01_CGH.v4_91.txt	8	12231807	12241093	2	-0.8117	-1
MSK_00009_251469322626_S01_CGH.v4_91.txt	8	112413776	112475488	3	-0.9242	-1
MSK_00009_251469322626_S01_CGH.v4_91.txt	8	26462520	26475578	3	-0.9603	-1
MSK_00009_251469322626_S01_CGH.v4_91.txt	8	25536511	26276943	43	-0.8376	-1
MSK_00009_251469322626_S01_CGH.v4_91.txt	8	5176317	12241093	347	-0.7785	-1
MSK_00009_251469322626_S01_CGH.v4_91.txt	8	186477	4388650	292	-0.8731	-1
MSK_00014_251469322629_S01_CGH.v4_91.txt	8	144567236	146294042	108	-0.751	-1
MSK_00014_251469322629_S01_CGH.v4_91.txt	8	117738338	143802541	1376	0.7229	1
MSK_00014_251469322629_S01_CGH.v4_91.txt	8	77161734	78130209	46	-0.9536	-1
MSK_WGA_0015.1_251469338754_S01_CGH.v4_91.txt	8	137748193	139272809	59	-0.7482	-1
MSK_00021_251469322630_S01_CGH.v4_91.txt	8	13745681	20489386	367	-0.8423	-1
MSK_00021_251469322630_S01_CGH.v4_91.txt	8	11114632	11860510	57	-0.7652	-1
MSK_00021_251469322630_S01_CGH.v4_91.txt	8	10902509	11055986	16	-0.7724	-1
MSK_00024_251469322721_S01_CGH.v4_91.txt	8	137748193	146294042	481	-0.6627	-1
MSK_00024_251469322721_S01_CGH.v4_91.txt	8	64217587	96272640	1662	-0.6692	-1
MSK_00024_251469322721_S01_CGH.v4_91.txt	8	6879755	32632456	1433	0.648	1
MSK_00024_251469322721_S01_CGH.v4_91.txt	8	6828426	6837287	2	1.8	1
MSK_00027_251469322705_S01_CGH.v4_91.txt	8	6828426	6837287	2	0.8426	1
MSK_00037_251469322734_S01_CGH.v4_91.txt	8	61695761	61862737	14	1.6247	1
MSK_00037_251469322734_S01_CGH.v4_91.txt	8	60996825	61329030	16	1.5128	1
MSK_00037_251469322734_S01_CGH.v4_91.txt	8	60616906	60971490	9	0.8967	1
MSK_00038_251469322735_S01_CGH.v4_91.txt	8	68531363	69318174	56	-0.7144	-1
MSK_00043_251469322736_S01_CGH.v4_91.txt	8	4685617	4693679	2	-1.1282	-1
MSK_WGA_0048.1_251469338742_S01_CGH.v4_91.txt	8	76224650	76293856	3	1.8915	1
MSK_00052_251469323033_S01_CGH.v4_91.txt	8	7268819	7691901	3	-0.6926	-1
MSK_00054_251469323034_S01_CGH.v4_91.txt	8	7268819	7691901	3	-0.8199	-1
MSK_WGA_0068.1_251469338745_S01_CGH.v4_91.txt	8	137748193	137850126	5	-1.572	-1
MSK_WGA_0068.1_251469338745_S01_CGH.v4_91.txt	8	12586413	14498658	115	-0.645	-1
MSK_00069_251469322916_S01_CGH.v4_91.txt	8	78961227	79008182	3	-0.734	-1
MSK_00074_251469322717_S01_CGH.v4_91.txt	8	6828426	6837287	2	0.8357	1
MSK_0079.1_251469338020_S01_CGH.v4_91.txt	8	76794376	76967024	4	-0.8412	-1
MSK_00083_251469322918_S01_CGH.v4_91.txt	8	58878306	76137983	996	-0.8185	-1
MSK_00083_251469322918_S01_CGH.v4_91.txt	8	12231807	12241093	2	-1.2765	-1
MSK_00086_251469322749_S01_CGH.v4_91.txt	8	134369939	134408685	2	-0.9074	-1
MSK_00086_251469322749_S01_CGH.v4_91.txt	8	3235094	3889536	64	-0.8831	-1
MSK_0087.1_251469333362_S01_CGH.v4_91.txt	8	12231807	12241093	2	-0.6655	-1
MSK_WGA_0104.1_251469338880_S01_CGH.v4_91.txt	8	72670565	146294042	3865	0.6921	1
MSK_WGA_0104.1_251469338880_S01_CGH.v4_91.txt	8	70078197	72639724	150	-0.6612	-1
MSK_WGA_0104.1_251469338880_S01_CGH.v4_91.txt	8	47673716	54344864	324	-0.7558	-1
MSK_0106.1_251469331471_S01_CGH.v4_91.txt	8	12231807	12241093	2	-2.4496	-1
MSK_0106.1_251469331471_S01_CGH.v4_91.txt	8	176452	753353	39	-0.633	-1
MSK_0111.1_251469337228_S01_CGH.v4_91.txt	8	6828426	6837287	2	0.6642	1
MSK_00114_251469322752_S01_CGH.v4_91.txt	8	12231807	12241093	2	-1.2583	-1
MSK_00115_251469322751_S01_CGH.v4_91.txt	8	137748193	137850126	5	-1.1498	-1
MSK_00115_251469322751_S01_CGH.v4_91.txt	8	7053186	7691901	4	-1.1186	-1
MSK_0266.1_251469336909_S01_CGH.v4_91.txt	8	7053186	7691901	4	-0.8506	-1
MSK_0289.1_251469337015_S01_CGH.v4_91.txt	8	138516741	139260098	36	-0.7754	-1
MSK_0289.1_251469337015_S01_CGH.v4_91.txt	8	127620374	127978921	13	-0.7447	-1
MSK_0289.1_251469337015_S01_CGH.v4_91.txt	8	126550861	127365404	40	-0.7305	-1
MSK_0289.1_251469337015_S01_CGH.v4_91.txt	8	126261517	126272510	3	-0.892	-1
MSK_0289.1_251469337015_S01_CGH.v4_91.txt	8	125665105	125676673	2	-0.9422	-1
MSK_0324.1_251469333023_S01_CGH.v4_91.txt	8	125513023	125518106	2	-0.9646	-1
MSK_0325.1_251469333563_S01_CGH.v4_91.txt	8	7268819	7691901	3	-0.9847	-1
MSK_0330.1_251469336713_S01_CGH.v4_91.txt	8	7268819	8079861	4	0.6912	1
MSK_0333.1_251469331585_S01_CGH.v4_91.txt	8	140186854	140220687	2	-0.8538	-1
MSK_0333.1_251469331585_S01_CGH.v4_91.txt	8	136170963	136328739	8	-0.7956	-1
MSK_0333.1_251469331585_S01_CGH.v4_91.txt	8	7268819	7691901	3	-1.1198	-1
MSK_0422.1_251469337230_S01_CGH.v4_91.txt	8	12231807	12241093	2	-0.7462	-1
MSK_0422.1_251469337230_S01_CGH.v4_91.txt	8	7268819	7691901	3	-0.9025	-1
MSK_0430.1_251469336714_S01_CGH.v4_91.txt	8	19796691	19873053	6	0.9025	1
MSK_0439.1_251469331553_S01_CGH.v4_91.txt	8	6828426	6837287	2	0.7344	1
MSK_00122.1_251469325751_S01_CGH.v4_91.txt	8	73838929	73856003	3	-0.7357	-1
MSK_00128.1_251469325161_S01_CGH.v4_91.txt	8	113921426	113929740	2	-0.9967	-1
MSK_00137.4_251469325162_S01_CGH.v4_91.txt	8	137748193	137850126	5	-0.8809	-1
MSK_00139.3_251469325756_S01_CGH.v4_91.txt	8	6828426	6837287	2	1.0703	1
MSK_0146.1_251469336983_S01_CGH.v4_91.txt	8	105704867	145516226	2037	0.635	1
MSK_0146.1_251469336983_S01_CGH.v4_91.txt	8	6828426	6837287	2	0.6789	1
MSK_0152.1_251469337789_S01_CGH.v4_91.txt	8	323560	516813	15	-1.4014	-1
MSK_0174.1_251469330284_S01_CGH.v4_91.txt	8	83674849	83826808	5	-0.7221	-1
MSK_0175.1_251469336725_S01_CGH.v4_91.txt	8	35175343	35756013	39	-0.7206	-1
MSK_0177.1_251469333358_S01_CGH.v4_91.txt	8	9499483	9510736	3	-0.845	-1
MSK_0178.1_251469329154_S01_CGH.v4_91.txt	8	137748193	137850126	5	-1.0977	-1
MSK_0179.1_251469333566_S01_CGH.v4_91.txt	8	12231807	12241093	2	-0.7894	-1
MSK_0194.1_251469336985_S01_CGH.v4_91.txt	8	12231807	12241093	2	-0.6542	-1
MSK_0197.1_251469329155_S01_CGH.v4_91.txt	8	78440358	78493886	3	-0.9706	-1
MSK_0201.1_251469329757_S01_CGH.v4_91.txt	8	6828426	6837287	2	1.0723	1
MSK_0208.2_251469329755_S01_CGH.v4_91.txt	8	136619177	136649617	2	-0.661	-1
MSK_0211.1_251469330227_S01_CGH.v4_91.txt	8	86366702	86381457	3	0.7874	1
MSK_0211.2_251469329773_S01_CGH.v4_91.txt	8	86366702	86381457	3	0.7099	1
MSK_0221.1_251469330192_S01_CGH.v4_91.txt	8	7274008	8079861	3	0.7189	1
MSK_0238.1_251469340788_S01_CGH.v4_91.txt	8	137748193	137850126	5	-0.7919	-1
MSK_0241.1_251469329774_S01_CGH.v4_91.txt	8	7268819	7691901	3	-1.1701	-1
MSK_0397.1_251469330468_S01_CGH.v4_91.txt	8	12231807	12241093	2	-0.8477	-1
MSK_0397.1_251469330468_S01_CGH.v4_91.txt	8	6828426	6837287	2	0.7225	1
MSK_0409.1_251469331555_S01_CGH.v4_91.txt	8	7274008	8094729	4	0.7091	1
MSK_0412.1_251469331490_S01_CGH.v4_91.txt	8	12231807	12241093	2	-1.6002	-1
MSK_0414.1_251469336986_S01_CGH.v4_91.txt	8	12231807	12241093	2	0.7139	1
MSK_0414.1_251469336986_S01_CGH.v4_91.txt	8	7274008	7691901	2	0.7995	1
MSK_0644.1_251469340741_S01_CGH.v4_91.txt	8	100561391	100690551	8	-0.9132	-1
MSK_WGA_0244.1_251469338881_S01_CGH.v4_91.txt	8	145516091	145564111	3	0.9041	1
MSK_WGA_0244.1_251469338881_S01_CGH.v4_91.txt	8	22448738	22477853	3	0.9438	1
MSK_WGA_0246.1_251469338865_S01_CGH.v4_91.txt	8	30648800	30921916	21	-0.9428	-1
MSK_WGA_0356.1_251469337509_S01_CGH.v4_91.txt	8	136984563	137882907	29	-0.6355	-1
MSK_0358.1_251469340789_S01_CGH.v4_91.txt	8	42898744	58143705	578	-0.8333	-1
MSK_0358.1_251469340789_S01_CGH.v4_91.txt	8	7268819	8079861	4	-0.8232	-1
MSK_WGA_0359.1_251469337523_S01_CGH.v4_91.txt	8	139904014	139920550	3	-0.7172	-1
MSK_0386.1_251469340798_S01_CGH.v4_91.txt	8	69811413	70215963	18	-1.0581	-1

Continued on next page...

ID	chr	start	end	nroOfprobes	segmean	call
MSK_WGA_0390.1_251469338875_S01_CGH.v4_91.txt	8	59048870	59076145	3	0.6794	1
MSK_WGA_0392.1_251469338876_S01_CGH.v4_91.txt	8	107025536	108497287	72	-0.7814	-1
MSK_0510.1_251469331581_S01_CGH.v4_91.txt	8	137748193	137850126	5	-1.4194	-1
MSK_0512.1_251469331556_S01_CGH.v4_91.txt	8	137748193	137850126	5	-0.9367	-1
MSK_0514.1_251469333360_S01_CGH.v4_91.txt	8	3639669	3689391	6	-0.8217	-1
MSK_0517.1_251469337226_S01_CGH.v4_91.txt	8	64028340	76630648	727	-0.838	-1
MSK_0517.1_251469337226_S01_CGH.v4_91.txt	8	12231807	12241093	2	-1.985	-1
MSK_0518.1_251469337014_S01_CGH.v4_91.txt	8	12231807	12241093	2	-0.7705	-1
MSK_0524.1_251469331557_S01_CGH.v4_91.txt	8	111341001	111447022	4	-0.968	-1
MSK_0529.1_251469331470_S01_CGH.v4_91.txt	8	126073160	145905403	1031	-0.7815	-1
MSK_0529.1_251469331470_S01_CGH.v4_91.txt	8	125325797	125390194	6	-0.7659	-1
MSK_0529.1_251469331470_S01_CGH.v4_91.txt	8	114945534	125102423	531	-0.8044	-1
MSK_00001_251469322729_S01_CGH.v4_91.txt	9	22281686	28143347	223	-0.8334	-1
MSK_00001_251469322729_S01_CGH.v4_91.txt	9	21909332	22246765	22	-1.402	-1
MSK_00007_251469322733_S01_CGH.v4_91.txt	9	20176373	24386546	233	-0.8332	-1
MSK_00009_251469322626_S01_CGH.v4_91.txt	9	32882737	33513496	46	-0.6857	-1
MSK_00009_251469322626_S01_CGH.v4_91.txt	9	30968682	32506630	54	-0.778	-1
MSK_00009_251469322626_S01_CGH.v4_91.txt	9	30357226	30634087	11	-0.8752	-1
MSK_00009_251469322626_S01_CGH.v4_91.txt	9	28733296	30052434	31	-0.841	-1
MSK_00009_251469322626_S01_CGH.v4_91.txt	9	27284759	28098731	47	-0.7582	-1
MSK_00009_251469322626_S01_CGH.v4_91.txt	9	26600444	26889915	14	-0.7574	-1
MSK_00009_251469322626_S01_CGH.v4_91.txt	9	25449089	26070321	22	-0.9221	-1
MSK_00009_251469322626_S01_CGH.v4_91.txt	9	23132133	24289661	42	-3.0302	-1
MSK_00009_251469322626_S01_CGH.v4_91.txt	9	22945656	23102507	7	-0.8365	-1
MSK_00009_251469322626_S01_CGH.v4_91.txt	9	19748331	22915506	200	-2.8981	-1
MSK_00009_251469322626_S01_CGH.v4_91.txt	9	17186326	19726199	137	-0.8044	-1
MSK_00009_251469322626_S01_CGH.v4_91.txt	9	15267912	17057595	115	-0.8104	-1
MSK_00009_251469322626_S01_CGH.v4_91.txt	9	14253220	14762351	36	-0.8333	-1
MSK_00009_251469322626_S01_CGH.v4_91.txt	9	13192268	14128789	33	-0.7981	-1
MSK_00009_251469322626_S01_CGH.v4_91.txt	9	11904249	12342481	14	0.9914	1
MSK_00009_251469322626_S01_CGH.v4_91.txt	9	10306637	11829736	47	-0.8601	-1
MSK_00009_251469322626_S01_CGH.v4_91.txt	9	9672075	9925681	17	-0.9629	-1
MSK_00009_251469322626_S01_CGH.v4_91.txt	9	8472415	9335203	67	-0.8444	-1
MSK_00009_251469322626_S01_CGH.v4_91.txt	9	6475338	6592113	11	-0.7895	-1
MSK_00009_251469322626_S01_CGH.v4_91.txt	9	6264454	6460788	14	0.993	1
MSK_00009_251469322626_S01_CGH.v4_91.txt	9	1157109	6072942	338	-0.839	-1
MSK_00009_251469322626_S01_CGH.v4_91.txt	9	556625	827900	23	-0.8192	-1
MSK_00009_251469322626_S01_CGH.v4_91.txt	9	163131	459017	18	-0.801	-1
MSK_00010_251469322627_S01_CGH.v4_91.txt	9	163131	22382290	1301	-0.8114	-1
MSK_00014_251469322629_S01_CGH.v4_91.txt	9	123131952	126181460	238	-0.8653	-1
MSK_00014_251469322629_S01_CGH.v4_91.txt	9	120138074	120540756	19	-0.885	-1
MSK_00014_251469322629_S01_CGH.v4_91.txt	9	68824215	80958918	681	-0.945	-1
MSK_00014_251469322629_S01_CGH.v4_91.txt	9	25046145	31104145	254	-0.9646	-1
MSK_00014_251469322629_S01_CGH.v4_91.txt	9	21213769	24996233	154	-4.3406	-1
MSK_00014_251469322629_S01_CGH.v4_91.txt	9	21178022	21203565	3	-2.2403	-1
MSK_00014_251469322629_S01_CGH.v4_91.txt	9	163131	21174435	1222	-0.9027	-1
MSK_WGA_0015.1_251469338754_S01_CGH.v4_91.txt	9	22451145	37573876	759	-0.6806	-1
MSK_WGA_0015.1_251469338754_S01_CGH.v4_91.txt	9	19770116	22431085	184	-2.1958	-1
MSK_WGA_0015.1_251469338754_S01_CGH.v4_91.txt	9	163131	19756132	1119	-0.6941	-1
MSK_WGA_0016.1_251469338755_S01_CGH.v4_91.txt	9	21832728	22052080	19	-1.8534	-1
MSK_WGA_0016.1_251469338755_S01_CGH.v4_91.txt	9	163131	21817082	1270	-0.6359	-1
MSK_00021_251469322630_S01_CGH.v4_91.txt	9	34289555	34481318	18	-0.6978	-1
MSK_00021_251469322630_S01_CGH.v4_91.txt	9	29788581	29849166	3	-3.0201	-1
MSK_00021_251469322630_S01_CGH.v4_91.txt	9	27941962	29667438	94	-0.9068	-1
MSK_00021_251469322630_S01_CGH.v4_91.txt	9	24548868	27464336	113	-1.3463	-1
MSK_00021_251469322630_S01_CGH.v4_91.txt	9	23981151	24505765	12	-2.9761	-1
MSK_00021_251469322630_S01_CGH.v4_91.txt	9	23771266	23933298	11	-1.1306	-1
MSK_00021_251469322630_S01_CGH.v4_91.txt	9	23326739	23761947	19	-2.8849	-1
MSK_00021_251469322630_S01_CGH.v4_91.txt	9	22416549	23288698	27	-1.1966	-1
MSK_00021_251469322630_S01_CGH.v4_91.txt	9	22246765	22382290	6	-2.8844	-1
MSK_00021_251469322630_S01_CGH.v4_91.txt	9	21978346	22212553	14	-1.296	-1
MSK_00021_251469322630_S01_CGH.v4_91.txt	9	21817082	21968041	12	-2.794	-1
MSK_00021_251469322630_S01_CGH.v4_91.txt	9	21603915	21790832	6	-0.9752	-1
MSK_00021_251469322630_S01_CGH.v4_91.txt	9	21476463	21558354	11	-2.8538	-1
MSK_00021_251469322630_S01_CGH.v4_91.txt	9	21352256	21470816	15	-1.0421	-1
MSK_00021_251469322630_S01_CGH.v4_91.txt	9	20764842	21002866	21	-0.8823	-1
MSK_00021_251469322630_S01_CGH.v4_91.txt	9	19700843	20636167	56	-0.6761	-1
MSK_00021_251469322630_S01_CGH.v4_91.txt	9	19470403	19576468	9	-0.7431	-1
MSK_WGA_0023.1_251469338756_S01_CGH.v4_91.txt	9	85027629	85823681	44	-0.6623	-1
MSK_WGA_0023.1_251469338756_S01_CGH.v4_91.txt	9	27841723	28435170	49	-0.6884	-1
MSK_WGA_0023.1_251469338756_S01_CGH.v4_91.txt	9	21381392	24083476	121	-1.8902	-1
MSK_00024_251469322721_S01_CGH.v4_91.txt	9	23486450	38648149	793	-0.6911	-1
MSK_00024_251469322721_S01_CGH.v4_91.txt	9	21504568	23348535	74	-2.8879	-1
MSK_00024_251469322721_S01_CGH.v4_91.txt	9	15461943	21497001	385	-0.6412	-1
MSK_00027_251469322705_S01_CGH.v4_91.txt	9	21241471	22302223	71	-1.6847	-1
MSK_00027_251469322705_S01_CGH.v4_91.txt	9	20346094	20932979	58	-1.6855	-1
MSK_00033_251469322707_S01_CGH.v4_91.txt	9	8903382	9028125	14	-1.0104	-1
MSK_00034_251469322708_S01_CGH.v4_91.txt	9	21978346	22125405	11	-0.8839	-1
MSK_00037_251469322734_S01_CGH.v4_91.txt	9	33819009	33998731	16	-0.7001	-1
MSK_00037_251469322734_S01_CGH.v4_91.txt	9	29849166	32073962	65	-0.8706	-1
MSK_00037_251469322734_S01_CGH.v4_91.txt	9	24083476	29485125	229	-0.8281	-1
MSK_00037_251469322734_S01_CGH.v4_91.txt	9	21944037	24063497	84	-2.5556	-1
MSK_00037_251469322734_S01_CGH.v4_91.txt	9	19521081	21936007	177	-0.8125	-1
MSK_00037_251469322734_S01_CGH.v4_91.txt	9	274517	12229427	697	-0.8034	-1
MSK_00038_251469322735_S01_CGH.v4_91.txt	9	22017835	32802206	417	-0.7238	-1
MSK_00038_251469322735_S01_CGH.v4_91.txt	9	19470403	22008167	189	-2.0926	-1
MSK_00038_251469322735_S01_CGH.v4_91.txt	9	8368946	19458798	578	-0.7202	-1
MSK_00038_251469322735_S01_CGH.v4_91.txt	9	314969	370184	5	-0.83	-1
MSK_00043_251469322736_S01_CGH.v4_91.txt	9	92954345	105102467	698	-0.8482	-1
MSK_00043_251469322736_S01_CGH.v4_91.txt	9	25779222	26321885	19	-1.0132	-1
MSK_00043_251469322736_S01_CGH.v4_91.txt	9	21002866	25713810	192	-3.7896	-1
MSK_00043_251469322736_S01_CGH.v4_91.txt	9	20971336	20993329	4	-2.0581	-1
MSK_00043_251469322736_S01_CGH.v4_91.txt	9	19892574	20946612	76	-3.7673	-1
MSK_00043_251469322736_S01_CGH.v4_91.txt	9	17930993	19856967	99	-0.8819	-1
MSK_00047_251469322738_S01_CGH.v4_91.txt	9	21292858	22146626	63	-1.6714	-1
MSK_WGA_0048.1_251469338742_S01_CGH.v4_91.txt	9	32955146	33478480	41	-0.6555	-1
MSK_WGA_0048.1_251469338742_S01_CGH.v4_91.txt	9	32205770	32572552	26	-0.6638	-1
MSK_WGA_0048.1_251469338742_S01_CGH.v4_91.txt	9	31218658	32172607	31	-2.6695	-1
MSK_WGA_0048.1_251469338742_S01_CGH.v4_91.txt	9	30618952	31191538	19	-0.7442	-1
MSK_WGA_0048.1_251469338742_S01_CGH.v4_91.txt	9	29667438	30576082	21	-2.1531	-1
MSK_WGA_0048.1_251469338742_S01_CGH.v4_91.txt	9	29165476	29646351	10	-0.8186	-1
MSK_WGA_0048.1_251469338742_S01_CGH.v4_91.txt	9	28777359	29085216	11	-2.2121	-1
MSK_WGA_0048.1_251469338742_S01_CGH.v4_91.txt	9	28233219	28744987	45	-0.7319	-1
MSK_WGA_0048.1_251469338742_S01_CGH.v4_91.txt	9	27975909	28226376	23	-2.8027	-1
MSK_WGA_0048.1_251469338742_S01_CGH.v4_91.txt	9	27941962	27970580	4	-0.6352	-1
MSK_WGA_0048.1_251469338742_S01_CGH.v4_91.txt	9	27661511	27899652	8	-2.8262	-1
MSK_WGA_0048.1_251469338742_S01_CGH.v4_91.txt	9	27392491	27626876	17	-0.6986	-1
MSK_WGA_0048.1_251469338742_S01_CGH.v4_91.txt	9	27218901	27384534	14	-2.7169	-1
MSK_WGA_0048.1_251469338742_S01_CGH.v4_91.txt	9	26746903	27213502	35	-0.771	-1
MSK_WGA_0048.1_251469338742_S01_CGH.v4_91.txt	9	25856578	26690984	27	-2.5504	-1
MSK_WGA_0048.1_251469338742_S01_CGH.v4_91.txt	9	25387669	25806667	14	-0.7412	-1
MSK_WGA_0048.1_251469338742_S01_CGH.v4_91.txt	9	21763782	25346912	119	-2.7206	-1
MSK_WGA_0048.1_251469338742_S01_CGH.v4_91.txt	9	17466848	21743410	265	-0.7243	-1
MSK_00054_251469323034_S01_CGH.v4_91.txt	9	163131	22086798	1291	-0.6573	-1
MSK_00064_251469322718_S01_CGH.v4_91.txt	9	27686969	27841723	6	-1.456	-1
MSK_00064_251469322718_S01_CGH.v4_91.txt	9	25527395	27129951	68	-1.394	-1
MSK_00064_251469322718_S01_CGH.v4_91.txt	9	24289661	25325601	20	-1.2803	-1
MSK_00064_251469322718_S01_CGH.v4_91.txt	9	19902639	23981151	232	-1.4143	-1
MSK_00064_251469322718_S01_CGH.v4_91.txt	9	19555983	19756132	17	-1.248	-1
MSK_WGA_0068.1_251469338745_S01_CGH.v4_91.txt	9	21967548	22472725	26	-1.5407	-1
MSK_WGA_0068.1_251469338745_S01_CGH.v4_91.txt	9	12515544	12600504	4	-1.599	-1
MSK_WGA_0070.1_251469338879_S01_CGH.v4_91.txt	9	133470676	133760988	21	-0.6351	-1
MSK_WGA_0070.1_251469338879_S01_CGH.v4_91.txt	9	31654235	31712883	3	-0.6978	-1
MSK_WGA_0070.1_251469338879_S01_CGH.v4_91.txt	9	24146307	24386546	7	-1.3452	-1

Continued on next page...

ID	chr	start	end	nroOfprobes	segmean	call
MSK_WGA_0070.1_251469338879_S01_CGH.v4_91.txt	9	23675998	23750648	7	-1.3352	-1
MSK_WGA_0070.1_251469338879_S01_CGH.v4_91.txt	9	21944037	21968041	4	-1.2798	-1
MSK_00071_251469322919_S01_CGH.v4_91.txt	9	28233219	32368928	144	-0.7193	-1
MSK_00071_251469322919_S01_CGH.v4_91.txt	9	21817082	28226376	259	-1.7	-1
MSK_00083_251469322918_S01_CGH.v4_91.txt	9	28165086	32418146	156	-0.8859	-1
MSK_00083_251469322918_S01_CGH.v4_91.txt	9	27830251	27949969	5	-0.8498	-1
MSK_00083_251469322918_S01_CGH.v4_91.txt	9	15812255	24883989	480	-0.8325	-1
MSK_00083_251469322918_S01_CGH.v4_91.txt	9	340083	11140881	664	-0.8417	-1
MSK_00085_251469322917_S01_CGH.v4_91.txt	9	21763782	28670178	300	-0.8888	-1
MSK_00085_251469322917_S01_CGH.v4_91.txt	9	20021931	20066456	3	-1.8312	-1
MSK_00086_251469322749_S01_CGH.v4_91.txt	9	22069983	33370763	446	-0.7816	-1
MSK_00086_251469322749_S01_CGH.v4_91.txt	9	21957735	22052080	11	-1.3898	-1
MSK_00086_251469322749_S01_CGH.v4_91.txt	9	163131	21944037	1278	-0.7574	-1
MSK_00089_251469322904_S01_CGH.v4_91.txt	9	26225945	26423210	7	-1.5399	-1
MSK_00089_251469322904_S01_CGH.v4_91.txt	9	23569089	23731004	11	-1.4613	-1
MSK_00089_251469322904_S01_CGH.v4_91.txt	9	23132133	23348535	6	-1.5919	-1
MSK_00089_251469322904_S01_CGH.v4_91.txt	9	20862647	21993401	89	-1.5147	-1
MSK_00089_251469322904_S01_CGH.v4_91.txt	9	19441347	20704720	85	-1.4747	-1
MSK_00102_251469322716_S01_CGH.v4_91.txt	9	134018345	134021518	2	0.7336	1
MSK_WGA_0104.1_251469338880_S01_CGH.v4_91.txt	9	138974808	139161878	12	1.3099	1
MSK_WGA_0104.1_251469338880_S01_CGH.v4_91.txt	9	138406197	138932589	44	0.6955	1
MSK_WGA_0104.1_251469338880_S01_CGH.v4_91.txt	9	24996233	26649384	48	-0.8258	-1
MSK_WGA_0104.1_251469338880_S01_CGH.v4_91.txt	9	21967548	24883989	99	-3.0651	-1
MSK_WGA_0104.1_251469338880_S01_CGH.v4_91.txt	9	434742	21957735	1265	-0.8217	-1
MSK_WGA_0104.1_251469338880_S01_CGH.v4_91.txt	9	163131	421985	14	-2.9053	-1
MSK_0106.1_251469331471_S01_CGH.v4_91.txt	9	21133314	22512565	91	-2.8452	-1
MSK_00107_251469322719_S01_CGH.v4_91.txt	9	24613645	24677137	2	-1.2074	-1
MSK_0111.1_251469337228_S01_CGH.v4_91.txt	9	134018345	134021518	2	0.7573	1
MSK_0111.1_251469337228_S01_CGH.v4_91.txt	9	21817082	27284759	201	-1.034	-1
MSK_00114_251469322752_S01_CGH.v4_91.txt	9	163131	39365550	2133	-0.8645	-1
MSK_00115_251469322751_S01_CGH.v4_91.txt	9	19521081	22281686	197	-1.1212	-1
MSK_00116_251469322720_S01_CGH.v4_91.txt	9	33473213	38741378	383	-0.7272	-1
MSK_00116_251469322720_S01_CGH.v4_91.txt	9	26042329	26501363	18	-0.9226	-1
MSK_00116_251469322720_S01_CGH.v4_91.txt	9	23326739	23911804	29	-0.9138	-1
MSK_00116_251469322720_S01_CGH.v4_91.txt	9	21241471	23288698	101	-3.247	-1
MSK_00116_251469322720_S01_CGH.v4_91.txt	9	21196354	21218548	4	-2.3275	-1
MSK_00116_251469322720_S01_CGH.v4_91.txt	9	20988259	21178022	19	-3.1158	-1
MSK_00116_251469322720_S01_CGH.v4_91.txt	9	2661697	20977887	1046	-0.8772	-1
MSK_00116_251469322720_S01_CGH.v4_91.txt	9	2303563	2390613	4	-0.8901	-1
MSK_0258.1_251469336709_S01_CGH.v4_91.txt	9	120866226	139161878	1241	-0.8805	-1
MSK_0258.1_251469336709_S01_CGH.v4_91.txt	9	76103791	76479768	10	-0.7556	-1
MSK_0258.1_251469336709_S01_CGH.v4_91.txt	9	21920518	25449089	113	-1.1757	-1
MSK_0260.1_251469331583_S01_CGH.v4_91.txt	9	21241471	26831029	204	-3.1099	-1
MSK_0260.1_251469331583_S01_CGH.v4_91.txt	9	21196354	21218548	4	-1.9806	-1
MSK_0260.1_251469331583_S01_CGH.v4_91.txt	9	20447302	21178022	70	-3.0574	-1
MSK_0266.1_251469336909_S01_CGH.v4_91.txt	9	106236751	139161878	2106	0.9534	1
MSK_0266.1_251469336909_S01_CGH.v4_91.txt	9	106152096	106192091	5	0.8515	1
MSK_0266.1_251469336909_S01_CGH.v4_91.txt	9	105935015	106061629	5	0.9432	1
MSK_0266.1_251469336909_S01_CGH.v4_91.txt	9	105196155	105723575	47	0.9133	1
MSK_0266.1_251469336909_S01_CGH.v4_91.txt	9	104232903	104503121	13	0.8897	1
MSK_0266.1_251469336909_S01_CGH.v4_91.txt	9	102927194	103774514	32	0.8789	1
MSK_0266.1_251469336909_S01_CGH.v4_91.txt	9	101298732	101501974	16	0.9095	1
MSK_0266.1_251469336909_S01_CGH.v4_91.txt	9	99940064	101142328	77	0.9364	1
MSK_0266.1_251469336909_S01_CGH.v4_91.txt	9	98075196	99529291	107	0.9515	1
MSK_0266.1_251469336909_S01_CGH.v4_91.txt	9	94199975	96882216	162	1.0351	1
MSK_0266.1_251469336909_S01_CGH.v4_91.txt	9	92226369	92495851	10	0.8698	1
MSK_0266.1_251469336909_S01_CGH.v4_91.txt	9	91461537	91943403	30	1.0013	1
MSK_0266.1_251469336909_S01_CGH.v4_91.txt	9	90492411	91294413	36	0.905	1
MSK_0266.1_251469336909_S01_CGH.v4_91.txt	9	84451517	90131737	298	0.8314	1
MSK_0266.1_251469336909_S01_CGH.v4_91.txt	9	83093417	83572663	15	0.9668	1
MSK_0266.1_251469336909_S01_CGH.v4_91.txt	9	80897811	82116035	29	0.8647	1
MSK_0269.1_251469333522_S01_CGH.v4_91.txt	9	21909332	22382290	27	-1.1158	-1
MSK_0269.1_251469333522_S01_CGH.v4_91.txt	9	21334395	21743410	33	-1.2141	-1
MSK_0271.1_251469333526_S01_CGH.v4_91.txt	9	556625	569898	3	-0.8482	-1
MSK_0281.1_251469331472_S01_CGH.v4_91.txt	9	21920518	22086798	16	-3.7497	-1
MSK_0285.1_251469337229_S01_CGH.v4_91.txt	9	21872531	22017835	13	-1.2594	-1
MSK_0289.1_251469337015_S01_CGH.v4_91.txt	9	133776802	134045710	27	-0.7779	-1
MSK_0289.1_251469337015_S01_CGH.v4_91.txt	9	21658291	22028273	23	-2.457	-1
MSK_0289.1_251469337015_S01_CGH.v4_91.txt	9	21241471	21504568	30	-2.0882	-1
MSK_0289.1_251469337015_S01_CGH.v4_91.txt	9	9861130	9880763	3	-2.2533	-1
MSK_0289.1_251469337015_S01_CGH.v4_91.txt	9	9308181	9411421	9	-2.292	-1
MSK_0289.1_251469337015_S01_CGH.v4_91.txt	9	8903382	8972137	8	-2.2944	-1
MSK_0290.1_251469336710_S01_CGH.v4_91.txt	9	103381656	103389814	2	-0.8157	-1
MSK_0290.1_251469336710_S01_CGH.v4_91.txt	9	33819009	36442195	219	-0.7149	-1
MSK_0290.1_251469336710_S01_CGH.v4_91.txt	9	29085216	33513496	165	-0.687	-1
MSK_0290.1_251469336710_S01_CGH.v4_91.txt	9	20558744	29066874	422	-2.1524	-1
MSK_0290.1_251469336710_S01_CGH.v4_91.txt	9	163131	20548249	1161	-0.6703	-1
MSK_0317.1_251469331584_S01_CGH.v4_91.txt	9	33819009	35851460	174	-0.7204	-1
MSK_0317.1_251469331584_S01_CGH.v4_91.txt	9	27356909	28866361	105	1.009	1
MSK_0317.1_251469331584_S01_CGH.v4_91.txt	9	22700103	23177135	15	-0.7605	-1
MSK_0317.1_251469331584_S01_CGH.v4_91.txt	9	21909332	22685780	37	-2.4566	-1
MSK_0317.1_251469331584_S01_CGH.v4_91.txt	9	21448637	21872531	25	-1.1386	-1
MSK_0317.1_251469331584_S01_CGH.v4_91.txt	9	21241471	21441614	22	-2.4751	-1
MSK_0317.1_251469331584_S01_CGH.v4_91.txt	9	21196354	21218548	4	-1.5053	-1
MSK_0317.1_251469331584_S01_CGH.v4_91.txt	9	21093730	21178022	8	-2.4559	-1
MSK_0317.1_251469331584_S01_CGH.v4_91.txt	9	19401873	21082470	124	-0.7495	-1
MSK_0324.1_251469333523_S01_CGH.v4_91.txt	9	134990841	135435066	10	-0.6998	-1
MSK_0324.1_251469333523_S01_CGH.v4_91.txt	9	92464625	92473105	2	-0.9821	-1
MSK_0324.1_251469333523_S01_CGH.v4_91.txt	9	22008167	22017835	2	-0.7261	-1
MSK_0324.1_251469333523_S01_CGH.v4_91.txt	9	21909332	22003651	10	-2.0979	-1
MSK_0325.1_251469333563_S01_CGH.v4_91.txt	9	26133251	26850969	21	-0.7788	-1
MSK_0325.1_251469333563_S01_CGH.v4_91.txt	9	23634959	25583028	54	-0.7992	-1
MSK_0325.1_251469333563_S01_CGH.v4_91.txt	9	22382290	22512565	7	-0.8822	-1
MSK_0325.1_251469333563_S01_CGH.v4_91.txt	9	21603915	22176172	31	-0.83	-1
MSK_0326.1_251469331461_S01_CGH.v4_91.txt	9	20422504	22125405	142	-1.0872	-1
MSK_0326.1_251469331461_S01_CGH.v4_91.txt	9	19748331	20048412	16	-1.0464	-1
MSK_0333.1_251469331585_S01_CGH.v4_91.txt	9	116102649	116706446	25	-0.8531	-1
MSK_0333.1_251469331585_S01_CGH.v4_91.txt	9	33819009	36442195	219	-0.7266	-1
MSK_0333.1_251469331585_S01_CGH.v4_91.txt	9	23177135	33370763	410	-0.8284	-1
MSK_0333.1_251469331585_S01_CGH.v4_91.txt	9	21241471	23132133	98	-2.8942	-1
MSK_0333.1_251469331585_S01_CGH.v4_91.txt	9	21196354	21218548	4	-1.5845	-1
MSK_0333.1_251469331585_S01_CGH.v4_91.txt	9	19668116	21178022	112	-2.8483	-1
MSK_0333.1_251469331585_S01_CGH.v4_91.txt	9	163131	19661342	1111	-0.8012	-1
MSK_0338.1_251469333564_S01_CGH.v4_91.txt	9	132462387	132482110	3	-0.6442	-1
MSK_0339.1_251469331462_S01_CGH.v4_91.txt	9	115122507	115126017	2	-0.771	-1
MSK_0339.1_251469331462_S01_CGH.v4_91.txt	9	163131	39177063	2132	-0.8953	-1
MSK_0422.1_251469337230_S01_CGH.v4_91.txt	9	68824215	75447275	399	-0.6962	-1
MSK_0422.1_251469337230_S01_CGH.v4_91.txt	9	34234206	34248723	2	-0.7983	-1
MSK_0422.1_251469337230_S01_CGH.v4_91.txt	9	25504416	26690984	38	-0.7658	-1
MSK_0422.1_251469337230_S01_CGH.v4_91.txt	9	22722626	25227246	72	-0.7233	-1
MSK_0422.1_251469337230_S01_CGH.v4_91.txt	9	21046758	22700103	101	-2.2017	-1
MSK_0422.1_251469337230_S01_CGH.v4_91.txt	9	20993329	21036673	6	-0.7263	-1
MSK_0422.1_251469337230_S01_CGH.v4_91.txt	9	20679665	20988259	31	-2.2377	-1
MSK_0422.1_251469337230_S01_CGH.v4_91.txt	9	20456703	20671715	20	-0.7287	-1
MSK_0422.1_251469337230_S01_CGH.v4_91.txt	9	19726199	20447302	38	-2.1978	-1
MSK_0422.1_251469337230_S01_CGH.v4_91.txt	9	17656958	19713883	101	-0.7011	-1
MSK_0422.1_251469337230_S01_CGH.v4_91.txt	9	8871565	17533269	450	-0.7093	-1
MSK_0422.1_251469337230_S01_CGH.v4_91.txt	9	5366453	8756447	205	-0.7061	-1
MSK_0422.1_251469337230_S01_CGH.v4_91.txt	9	4768744	5216903	39	-0.6649	-1
MSK_0422.1_251469337230_S01_CGH.v4_91.txt	9	4329063	4649634	24	-0.7507	-1
MSK_0422.1_251469337230_S01_CGH.v4_91.txt	9	2724158	4065626	92	-0.7401	-1
MSK_0422.1_251469337230_S01_CGH.v4_91.txt	9	2230300	2354226	7	-0.7968	-1
MSK_0422.1_251469337230_S01_CGH.v4_91.txt	9	1491277	2081979	31	-0.6662	-1
MSK_0422.1_251469337230_S01_CGH.v4_91.txt	9	1040254	1288054	11	-0.6871	-1

Continued on next page...

ID	chr	start	end	nroOfprobes	segmean	call
MSK_0422.1_251469337230_S01_CGH.v4_91.txt	9	163131	973803	64	-0.7818	-1
MSK_0430.1_251469336714_S01_CGH.v4_91.txt	9	136217889	136533609	16	-0.773	-1
MSK_0430.1_251469336714_S01_CGH.v4_91.txt	9	22852920	23213099	12	-1.1391	-1
MSK_0430.1_251469336714_S01_CGH.v4_91.txt	9	22625986	22778706	7	-2.6124	-1
MSK_0430.1_251469336714_S01_CGH.v4_91.txt	9	22017835	22592720	23	-1.7394	-1
MSK_0430.1_251469336714_S01_CGH.v4_91.txt	9	21837873	22008167	14	-2.7053	-1
MSK_0430.1_251469336714_S01_CGH.v4_91.txt	9	21241471	21832728	44	-1.2216	-1
MSK_0430.1_251469336714_S01_CGH.v4_91.txt	9	10101388	21218548	592	-0.6821	-1
MSK_0432.1_251469336712_S01_CGH.v4_91.txt	9	163131	35814206	1936	-0.7026	-1
MSK_0439.1_251469331553_S01_CGH.v4_91.txt	9	29849166	30694198	22	-0.6596	-1
MSK_0440.1_251469336724_S01_CGH.v4_91.txt	9	26464989	33427475	319	-0.8141	-1
MSK_0440.1_251469336724_S01_CGH.v4_91.txt	9	25909346	26423210	20	-2.7193	-1
MSK_0440.1_251469336724_S01_CGH.v4_91.txt	9	24505765	25856578	32	-0.9608	-1
MSK_0440.1_251469336724_S01_CGH.v4_91.txt	9	21957735	24386546	91	-2.7501	-1
MSK_0440.1_251469336724_S01_CGH.v4_91.txt	9	21855114	21944037	6	-0.8478	-1
MSK_0440.1_251469336724_S01_CGH.v4_91.txt	9	3955653	17802294	783	-0.7809	-1
MSK_0446.1_251469333525_S01_CGH.v4_91.txt	9	21292858	22973881	89	-1.5353	-1
MSK_0451.1_251469333565_S01_CGH.v4_91.txt	9	20325891	23810056	211	-1.5033	-1
MSK_0456.1_251469331463_S01_CGH.v4_91.txt	9	10320424	10328756	2	0.9896	1
MSK_00122.1_251469325751_S01_CGH.v4_91.txt	9	21832728	22778706	45	-1.7451	-1
MSK_00125.1_251469324871_S01_CGH.v4_91.txt	9	21133314	28345772	322	-2.9053	-1
MSK_00126.1_251469325171_S01_CGH.v4_91.txt	9	22685780	25387669	77	-0.6973	-1
MSK_00126.1_251469325171_S01_CGH.v4_91.txt	9	21708371	22674760	45	-2.1706	-1
MSK_00126.1_251469325171_S01_CGH.v4_91.txt	9	163131	21658291	1265	-0.6918	-1
MSK_0127.1_251469337891_S01_CGH.v4_91.txt	9	30908283	32395746	47	-1.2917	-1
MSK_0127.1_251469337891_S01_CGH.v4_91.txt	9	30144402	30478862	9	-1.1871	-1
MSK_0127.1_251469337891_S01_CGH.v4_91.txt	9	21352256	29918743	363	-1.2364	-1
MSK_00129.1_251469322907_S01_CGH.v4_91.txt	9	86338698	93031575	339	-0.7509	-1
MSK_00129.1_251469322907_S01_CGH.v4_91.txt	9	68824215	84027540	788	-0.767	-1
MSK_00129.1_251469322907_S01_CGH.v4_91.txt	9	4498207	4516438	3	-1.0327	-1
MSK_0132.1_251469330459_S01_CGH.v4_91.txt	9	132462387	132482110	3	-0.7458	-1
MSK_00133_251469325752_S01_CGH.v4_91.txt	9	21763782	22326165	32	-1.1399	-1
MSK_00137.4_251469325162_S01_CGH.v4_91.txt	9	19497546	25953049	305	-1.9014	-1
MSK_00143.1_251469326154_S01_CGH.v4_91.txt	9	21658291	22302223	34	-1.5506	-1
MSK_0146.1_251469336983_S01_CGH.v4_91.txt	9	115122507	115126017	2	-0.733	-1
MSK_0146.1_251469336983_S01_CGH.v4_91.txt	9	33732828	33788378	7	0.8295	1
MSK_0158.1_251469329734_S01_CGH.v4_91.txt	9	22382290	22685780	11	-2.1588	-1
MSK_0158.1_251469329734_S01_CGH.v4_91.txt	9	21133314	22086798	75	-2.073	-1
MSK_0164.1_251469331554_S01_CGH.v4_91.txt	9	21957735	22722626	35	-1.1464	-1
MSK_0166.1_251469329777_S01_CGH.v4_91.txt	9	28038897	28219132	17	-0.8891	-1
MSK_0166.1_251469329777_S01_CGH.v4_91.txt	9	21967548	22069983	11	-0.889	-1
MSK_00169.1_251469325174_S01_CGH.v4_91.txt	9	21396491	27343824	230	-0.9689	-1
MSK_0171.1_251469329753_S01_CGH.v4_91.txt	9	21470816	22416549	51	-1.4742	-1
MSK_0173.1_251469330461_S01_CGH.v4_91.txt	9	134018345	134021518	2	0.8931	1
MSK_0173.1_251469330461_S01_CGH.v4_91.txt	9	28932391	29813526	19	-1.032	-1
MSK_0173.1_251469330461_S01_CGH.v4_91.txt	9	27941962	28435170	47	-0.9552	-1
MSK_0173.1_251469330461_S01_CGH.v4_91.txt	9	21872531	24754409	102	-0.9409	-1
MSK_0173.1_251469330461_S01_CGH.v4_91.txt	9	1157109	17930993	956	-0.9154	-1
MSK_0175.1_251469336725_S01_CGH.v4_91.txt	9	22052080	39365550	845	-0.6351	-1
MSK_0175.1_251469336725_S01_CGH.v4_91.txt	9	20250034	22036446	150	-1.8349	-1
MSK_0175.1_251469336725_S01_CGH.v4_91.txt	9	163131	20195733	1138	-0.6603	-1
MSK_0182.1_251469331464_S01_CGH.v4_91.txt	9	137864514	138030897	11	-0.764	-1
MSK_0182.1_251469331464_S01_CGH.v4_91.txt	9	137135234	137193210	4	-0.778	-1
MSK_0185.1_251469329754_S01_CGH.v4_91.txt	9	19630553	22176172	187	-2.7419	-1
MSK_0187.1_251469330462_S01_CGH.v4_91.txt	9	31104145	39365550	501	-0.7109	-1
MSK_0187.1_251469330462_S01_CGH.v4_91.txt	9	29165476	29575728	8	4.547	1
MSK_0187.1_251469330462_S01_CGH.v4_91.txt	9	26070321	26357039	10	-0.7263	-1
MSK_0187.1_251469330462_S01_CGH.v4_91.txt	9	22176172	25856578	108	-0.7983	-1
MSK_0187.1_251469330462_S01_CGH.v4_91.txt	9	21763782	22146626	26	-2.3826	-1
MSK_0187.1_251469330462_S01_CGH.v4_91.txt	9	163131	21743410	1267	-0.7429	-1
MSK_0188.1_251469330469_S01_CGH.v4_91.txt	9	26600444	31943737	238	-0.7013	-1
MSK_0188.1_251469330469_S01_CGH.v4_91.txt	9	26042329	26501363	18	-2.2457	-1
MSK_0188.1_251469330469_S01_CGH.v4_91.txt	9	24207831	26015964	42	-0.7119	-1
MSK_0188.1_251469330469_S01_CGH.v4_91.txt	9	21920518	24146307	88	-2.214	-1
MSK_0190.1_251469330591_S01_CGH.v4_91.txt	9	21241471	21292858	4	-1.131	-1
MSK_0194.1_251469336985_S01_CGH.v4_91.txt	9	21872531	22146626	20	-1.0473	-1
MSK_0197.1_251469329155_S01_CGH.v4_91.txt	9	17595185	17630872	4	0.7274	1
MSK_0201.1_251469329757_S01_CGH.v4_91.txt	9	21558354	24814793	115	-0.8167	-1
MSK_0206.1_251469329173_S01_CGH.v4_91.txt	9	5308149	5335065	4	-0.944	-1
MSK_0208.1_251469329182_S01_CGH.v4_91.txt	9	19511524	26321885	318	-1.8235	-1
MSK_0208.2_251469329755_S01_CGH.v4_91.txt	9	19511524	26321885	318	-1.8021	-1
MSK_0208.2_251469329755_S01_CGH.v4_91.txt	9	163131	19497546	1099	-0.6385	-1
MSK_0210.1_251469330448_S01_CGH.v4_91.txt	9	21241471	23213099	100	-1.5419	-1
MSK_0210.1_251469330448_S01_CGH.v4_91.txt	9	21196354	21218548	4	-1.0456	-1
MSK_0210.1_251469330448_S01_CGH.v4_91.txt	9	20902540	21178022	26	-1.5148	-1
MSK_0211.1_251469330227_S01_CGH.v4_91.txt	9	41907475	43007249	2	0.7965	1
MSK_0211.1_251469330227_S01_CGH.v4_91.txt	9	28339106	30144402	68	-0.6848	-1
MSK_0211.1_251469330227_S01_CGH.v4_91.txt	9	27984198	28328562	30	-1.4602	-1
MSK_0211.1_251469330227_S01_CGH.v4_91.txt	9	20627643	26162115	246	-1.5003	-1
MSK_0211.1_251469330227_S01_CGH.v4_91.txt	9	9925681	9963296	2	-1.5124	-1
MSK_0211.2_251469329773_S01_CGH.v4_91.txt	9	41907475	43007249	2	0.9705	1
MSK_0211.2_251469329773_S01_CGH.v4_91.txt	9	28339106	30144402	68	-0.6585	-1
MSK_0211.2_251469329773_S01_CGH.v4_91.txt	9	27984198	28328562	30	-1.6506	-1
MSK_0211.2_251469329773_S01_CGH.v4_91.txt	9	20617752	26162115	247	-1.7513	-1
MSK_0211.2_251469329773_S01_CGH.v4_91.txt	9	9925681	9963296	2	-2.1224	-1
MSK_0214.1_251469329168_S01_CGH.v4_91.txt	9	23486450	24883989	48	-1.4423	-1
MSK_0214.1_251469329168_S01_CGH.v4_91.txt	9	23288698	23348535	3	-0.6418	-1
MSK_0214.1_251469329168_S01_CGH.v4_91.txt	9	21708371	23213099	62	-1.4482	-1
MSK_0214.1_251469329168_S01_CGH.v4_91.txt	9	9817232	12859897	112	-1.4647	-1
MSK_0214.1_251469329168_S01_CGH.v4_91.txt	9	8404560	9036005	57	-1.4439	-1
MSK_0219.1_251469329864_S01_CGH.v4_91.txt	9	21936007	22008167	9	-1.3506	-1
MSK_0221.1_251469330192_S01_CGH.v4_91.txt	9	163131	29003990	1582	-0.6563	-1
MSK_0241.1_251469329774_S01_CGH.v4_91.txt	9	78318312	84316294	243	-0.8495	-1
MSK_0241.1_251469329774_S01_CGH.v4_91.txt	9	73513606	75160867	57	-0.8621	-1
MSK_0241.1_251469329774_S01_CGH.v4_91.txt	9	71060280	71285322	15	-0.8239	-1
MSK_0241.1_251469329774_S01_CGH.v4_91.txt	9	70420982	70732636	17	-0.8739	-1
MSK_0241.1_251469329774_S01_CGH.v4_91.txt	9	38648149	39059899	6	-0.7986	-1
MSK_0241.1_251469329774_S01_CGH.v4_91.txt	9	36917114	38255139	92	-0.8129	-1
MSK_0241.1_251469329774_S01_CGH.v4_91.txt	9	25779222	27003783	49	-0.8212	-1
MSK_0241.1_251469329774_S01_CGH.v4_91.txt	9	22069983	25504416	101	-0.8781	-1
MSK_0241.1_251469329774_S01_CGH.v4_91.txt	9	21944037	22052080	12	-3.1796	-1
MSK_0241.1_251469329774_S01_CGH.v4_91.txt	9	19511524	21936007	178	-0.833	-1
MSK_0394.1_251469336716_S01_CGH.v4_91.txt	9	26301109	26423210	6	-1.0654	-1
MSK_0394.1_251469336716_S01_CGH.v4_91.txt	9	21978346	22052080	8	-0.9789	-1
MSK_0397.1_251469330468_S01_CGH.v4_91.txt	9	22176172	22722626	20	-0.7044	-1
MSK_0397.1_251469330468_S01_CGH.v4_91.txt	9	21978346	22146626	12	-2.0975	-1
MSK_0397.1_251469330468_S01_CGH.v4_91.txt	9	163131	21968041	1281	-0.754	-1
MSK_0402.1_251469336906_S01_CGH.v4_91.txt	9	544450	563286	3	-1.0388	-1
MSK_0410.1_251469333359_S01_CGH.v4_91.txt	9	21909332	22086798	17	-2.8968	-1
MSK_0412.1_251469331490_S01_CGH.v4_91.txt	9	19902639	22302223	171	-1.8425	-1
MSK_0412.1_251469331490_S01_CGH.v4_91.txt	9	163131	19892574	1127	-0.6392	-1
MSK_0413.1_251469331465_S01_CGH.v4_91.txt	9	21312633	23486450	100	-2.9057	-1
MSK_0644.1_251469340741_S01_CGH.v4_91.txt	9	26133251	26746903	16	-1.232	-1
MSK_0644.1_251469340741_S01_CGH.v4_91.txt	9	20874659	25806667	205	-1.2448	-1
MSK_0646.1_251469340746_S01_CGH.v4_91.txt	9	21978346	22382290	20	-2.2923	-1
MSK_0648.1_251469338021_S01_CGH.v4_91.txt	9	25481638	25979531	16	-0.9611	-1
MSK_0648.1_251469338021_S01_CGH.v4_91.txt	9	23675998	24310474	28	-0.8576	-1
MSK_0648.1_251469338021_S01_CGH.v4_91.txt	9	22625986	23288698	20	-0.8516	-1
MSK_0648.1_251469338021_S01_CGH.v4_91.txt	9	21658291	22052080	25	-2.8796	-1
MSK_0648.1_251469338021_S01_CGH.v4_91.txt	9	21396491	21603915	21	-0.7955	-1
MSK_WGA_0244.1_251469338881_S01_CGH.v4_91.txt	9	22086798	22592720	18	-0.7413	-1
MSK_WGA_0244.1_251469338881_S01_CGH.v4_91.txt	9	21920518	22069983	15	-2.163	-1
MSK_WGA_0244.1_251469338881_S01_CGH.v4_91.txt	9	17512109	21909332	268	-0.7219	-1
MSK_WGA_0244.1_251469338881_S01_CGH.v4_91.txt	9	13115036	17332539	252	-0.7225	-1

Continued on next page...

ID	chr	start	end	nroOfprobes	segmean	call
MSK_WGA_0246.1_251469338865_S01_CGH.v4_91.txt	9	22176172	22625986	16	-0.9157	-1
MSK_WGA_0246.1_251469338865_S01_CGH.v4_91.txt	9	21936007	22146626	17	-1.8535	-1
MSK_0345.1_251469337922_S01_CGH.v4_91.txt	9	21174435	22069983	69	-1.2951	-1
MSK_WGA_0346.1_251469338867_S01_CGH.v4_91.txt	9	21241471	22302223	71	-1.2503	-1
MSK_WGA_0347.1_251469338868_S01_CGH.v4_91.txt	9	23081027	31615752	331	-0.6484	-1
MSK_WGA_0347.1_251469338868_S01_CGH.v4_91.txt	9	21763782	23059932	55	-1.6514	-1
MSK_WGA_0347.1_251469338868_S01_CGH.v4_91.txt	9	19576468	19814638	20	-0.6777	-1
MSK_WGA_0348.1_251469338869_S01_CGH.v4_91.txt	9	26070321	31712883	249	-0.7124	-1
MSK_WGA_0348.1_251469338869_S01_CGH.v4_91.txt	9	25713810	26059517	11	-2.0058	-1
MSK_WGA_0348.1_251469338869_S01_CGH.v4_91.txt	9	25387669	25702847	11	-0.841	-1
MSK_WGA_0348.1_251469338869_S01_CGH.v4_91.txt	9	20946612	25346912	185	-1.9859	-1
MSK_WGA_0348.1_251469338869_S01_CGH.v4_91.txt	9	10440655	20932979	549	-0.6941	-1
MSK_WGA_0348.1_251469338869_S01_CGH.v4_91.txt	9	10268438	10328756	5	-0.7346	-1
MSK_WGA_0353.1_251469337506_S01_CGH.v4_91.txt	9	22945656	23569089	18	-2.857	-1
MSK_WGA_0353.1_251469337506_S01_CGH.v4_91.txt	9	22281686	22915506	21	-0.7447	-1
MSK_WGA_0353.1_251469337506_S01_CGH.v4_91.txt	9	20470847	22246765	141	-2.6672	-1
MSK_WGA_0353.1_251469337506_S01_CGH.v4_91.txt	9	10328756	20456703	509	-0.6656	-1
MSK_WGA_0355.1_251469337508_S01_CGH.v4_91.txt	9	21743410	22512565	41	-2.2665	-1
MSK_WGA_0355.1_251469337508_S01_CGH.v4_91.txt	9	659280	720090	5	-2.0939	-1
MSK_WGA_0355.1_251469337508_S01_CGH.v4_91.txt	9	434742	459017	4	-2.2943	-1
MSK_WGA_0356.1_251469337509_S01_CGH.v4_91.txt	9	122308580	122371290	3	0.663	1
MSK_WGA_0356.1_251469337509_S01_CGH.v4_91.txt	9	27777632	30357226	111	-0.6359	-1
MSK_WGA_0356.1_251469337509_S01_CGH.v4_91.txt	9	23613549	23839468	17	-0.6658	-1
MSK_WGA_0356.1_251469337509_S01_CGH.v4_91.txt	9	21241471	23515762	105	-0.6416	-1
MSK_WGA_0356.1_251469337509_S01_CGH.v4_91.txt	9	20853789	21093730	23	-0.6894	-1
MSK_WGA_0357.1_251469337522_S01_CGH.v4_91.txt	9	22472725	27661511	190	-0.7455	-1
MSK_WGA_0357.1_251469337522_S01_CGH.v4_91.txt	9	21743410	22451145	38	-2.1157	-1
MSK_WGA_0357.1_251469337522_S01_CGH.v4_91.txt	9	21441614	21708371	18	-0.7268	-1
MSK_0358.1_251469340789_S01_CGH.v4_91.txt	9	34704344	68241218	308	-0.7651	-1
MSK_0358.1_251469340789_S01_CGH.v4_91.txt	9	33912029	34664682	65	-0.7605	-1
MSK_0358.1_251469340789_S01_CGH.v4_91.txt	9	33100287	33676369	39	-0.7165	-1
MSK_0358.1_251469340789_S01_CGH.v4_91.txt	9	24289661	33037699	347	-0.8431	-1
MSK_0358.1_251469340789_S01_CGH.v4_91.txt	9	21150819	24242825	147	-3.2906	-1
MSK_0358.1_251469340789_S01_CGH.v4_91.txt	9	20916836	21140864	20	-0.8497	-1
MSK_0358.1_251469340789_S01_CGH.v4_91.txt	9	20728837	20902540	17	-3.301	-1
MSK_0358.1_251469340789_S01_CGH.v4_91.txt	9	15192527	20720169	328	-0.7848	-1
MSK_0358.1_251469340789_S01_CGH.v4_91.txt	9	10558927	14750215	166	-0.7976	-1
MSK_0358.1_251469340789_S01_CGH.v4_91.txt	9	163131	10381123	651	-0.8125	-1
MSK_WGA_0359.1_251469337523_S01_CGH.v4_91.txt	9	122308580	122371290	3	0.6698	1
MSK_WGA_0359.1_251469337523_S01_CGH.v4_91.txt	9	22246765	22700103	17	-0.755	-1
MSK_WGA_0359.1_251469337523_S01_CGH.v4_91.txt	9	19425150	22212553	202	-1.7989	-1
MSK_WGA_0359.1_251469337523_S01_CGH.v4_91.txt	9	16030267	16722823	40	-0.6926	-1
MSK_WGA_0359.1_251469337523_S01_CGH.v4_91.txt	9	12213622	12455191	11	-0.6466	-1
MSK_WGA_0359.1_251469337523_S01_CGH.v4_91.txt	9	9803920	11829736	73	-0.7647	-1
MSK_WGA_0360.1_251469337524_S01_CGH.v4_91.txt	9	21909332	22146626	19	-0.7661	-1
MSK_WGA_0392.1_251469338876_S01_CGH.v4_91.txt	9	21978346	22176172	13	-2.1988	-1
MSK_WGA_0392.1_251469338876_S01_CGH.v4_91.txt	9	163131	21968041	1281	-0.688	-1
MSK_0509.1_251469336717_S01_CGH.v4_91.txt	9	20916836	22778706	117	-0.8975	-1
MSK_0510.1_251469331581_S01_CGH.v4_91.txt	9	21140864	26914780	222	-1.5646	-1
MSK_0511.1_251469336907_S01_CGH.v4_91.txt	9	35059634	35756179	65	-0.6762	-1
MSK_0511.1_251469336907_S01_CGH.v4_91.txt	9	23515762	24548868	40	-0.7053	-1
MSK_0511.1_251469336907_S01_CGH.v4_91.txt	9	20663398	23486450	159	-2.1413	-1
MSK_0511.1_251469336907_S01_CGH.v4_91.txt	9	19213822	20650349	91	-0.7007	-1
MSK_0511.1_251469336907_S01_CGH.v4_91.txt	9	9985359	16565150	310	-0.7196	-1
MSK_0512.1_251469331556_S01_CGH.v4_91.txt	9	21855114	22176172	22	-0.7306	-1
MSK_0514.1_251469333360_S01_CGH.v4_91.txt	9	33819009	34556560	62	-0.6503	-1
MSK_0514.1_251469333360_S01_CGH.v4_91.txt	9	22176172	33370763	442	-0.6484	-1
MSK_0514.1_251469333360_S01_CGH.v4_91.txt	9	21909332	22146626	19	-2.3372	-1
MSK_0514.1_251469333360_S01_CGH.v4_91.txt	9	1592306	21872531	1181	-0.6319	-1
MSK_0514.1_251469333360_S01_CGH.v4_91.txt	9	1491277	1565527	4	-3.5394	-1
MSK_0514.1_251469333360_S01_CGH.v4_91.txt	9	163131	1444560	89	-0.6764	-1
MSK_0517.1_251469337226_S01_CGH.v4_91.txt	9	33819009	39177063	366	-0.7167	-1
MSK_0517.1_251469337226_S01_CGH.v4_91.txt	9	26052554	33783609	358	-0.7582	-1
MSK_0517.1_251469337226_S01_CGH.v4_91.txt	9	21708371	26042329	141	-3.5237	-1
MSK_0517.1_251469337226_S01_CGH.v4_91.txt	9	163131	21658291	1265	-0.7644	-1
MSK_0518.1_251469337014_S01_CGH.v4_91.txt	9	22246765	25387669	92	-0.9012	-1
MSK_0518.1_251469337014_S01_CGH.v4_91.txt	9	21150819	22212553	77	-3.7819	-1
MSK_0518.1_251469337014_S01_CGH.v4_91.txt	9	20487253	21140864	61	-0.9303	-1
MSK_0518.1_251469337014_S01_CGH.v4_91.txt	9	19700843	20290211	27	-0.8116	-1
MSK_0520.1_251469336708_S01_CGH.v4_91.txt	9	22146626	22356407	8	-0.6878	-1
MSK_0520.1_251469336708_S01_CGH.v4_91.txt	9	21547336	22125405	31	-2.2023	-1
MSK_0520.1_251469336708_S01_CGH.v4_91.txt	9	8751167	21531275	710	-0.6997	-1
MSK_0520.1_251469336708_S01_CGH.v4_91.txt	9	163131	8595863	538	-0.6831	-1
MSK_0521.1_251469331582_S01_CGH.v4_91.txt	9	21936007	22281686	21	-2.0355	-1
MSK_0522.1_251469336908_S01_CGH.v4_91.txt	9	26301109	26423210	6	-2.3007	-1
MSK_0522.1_251469336908_S01_CGH.v4_91.txt	9	21909332	22146626	19	-1.3419	-1
MSK_0525.1_251469333361_S01_CGH.v4_91.txt	9	30694198	38815416	509	-0.6921	-1
MSK_0525.1_251469333361_S01_CGH.v4_91.txt	9	21708371	30660176	353	-2.6393	-1
MSK_0525.1_251469333361_S01_CGH.v4_91.txt	9	163131	21658291	1265	-0.7374	-1
MSK_0529.1_251469331470_S01_CGH.v4_91.txt	9	136659723	136672384	3	3.3908	1
MSK_0529.1_251469331470_S01_CGH.v4_91.txt	9	134038123	134159611	12	5.1802	1
MSK_0529.1_251469331470_S01_CGH.v4_91.txt	9	134018345	134026204	3	4.0154	1
MSK_0529.1_251469331470_S01_CGH.v4_91.txt	9	133622545	134012880	31	5.3846	1
MSK_0529.1_251469331470_S01_CGH.v4_91.txt	9	22125405	22176172	3	-0.7694	-1
MSK_0529.1_251469331470_S01_CGH.v4_91.txt	9	21832728	22086798	21	-2.6397	-1
MSK_0529.1_251469331470_S01_CGH.v4_91.txt	9	19470403	21817082	174	-0.7899	-1
MSK_0529.1_251469331470_S01_CGH.v4_91.txt	9	2854940	5699927	199	-0.7965	-1
MSK_0531.1_251469337227_S01_CGH.v4_91.txt	9	115122507	115126017	2	-0.7841	-1
MSK_0531.1_251469337227_S01_CGH.v4_91.txt	9	26746903	26980599	17	-0.8669	-1
MSK_0531.1_251469337227_S01_CGH.v4_91.txt	9	21133314	26690984	211	-2.7386	-1
MSK_0531.1_251469337227_S01_CGH.v4_91.txt	9	19497546	21093730	118	-0.8198	-1
MSK_0615.1_251469340783_S01_CGH.v4_91.txt	9	21790832	27547786	223	-2.3889	-1
MSK_0615.1_251469340783_S01_CGH.v4_91.txt	9	163131	21763782	1268	-0.6779	-1
MSK_0620.1_251469337791_S01_CGH.v4_91.txt	9	21252517	23853392	127	-1.5633	-1
MSK_0620.1_251469337791_S01_CGH.v4_91.txt	9	544450	563286	3	-1.2549	-1
MSK_00003_251469322731_S01_CGH.v4_91.txt	10	127622813	135513631	409	-0.7934	-1
MSK_00003_251469322731_S01_CGH.v4_91.txt	10	42889244	127573288	4568	-0.81	-1
MSK_00003_251469322731_S01_CGH.v4_91.txt	10	125544	38629530	2112	-0.818	-1
MSK_00006_251469322732_S01_CGH.v4_91.txt	10	89348185	90784282	91	-1.3029	-1
MSK_00007_251469322733_S01_CGH.v4_91.txt	10	127622813	135513631	409	-0.7601	-1
MSK_00007_251469322733_S01_CGH.v4_91.txt	10	51798898	127573288	4271	-0.7814	-1
MSK_00007_251469322733_S01_CGH.v4_91.txt	10	48334407	51094982	122	-0.7607	-1
MSK_00009_251469322626_S01_CGH.v4_91.txt	10	68339177	135513631	3884	-0.7875	-1
MSK_00009_251469322626_S01_CGH.v4_91.txt	10	68304169	68318967	2	-3.315	-1
MSK_00009_251469322626_S01_CGH.v4_91.txt	10	42889244	68292412	1095	-0.8445	-1
MSK_00009_251469322626_S01_CGH.v4_91.txt	10	125544	38629530	2112	-0.7991	-1
MSK_00010_251469322627_S01_CGH.v4_91.txt	10	66896411	88868493	1237	-0.7225	-1
MSK_00014_251469322629_S01_CGH.v4_91.txt	10	127622813	135513631	409	-0.851	-1
MSK_00014_251469322629_S01_CGH.v4_91.txt	10	123381269	127573288	251	-0.8532	-1
MSK_00014_251469322629_S01_CGH.v4_91.txt	10	122878197	123370157	38	3.4983	1
MSK_00014_251469322629_S01_CGH.v4_91.txt	10	118485280	122842027	254	-0.8585	-1
MSK_00014_251469322629_S01_CGH.v4_91.txt	10	112839536	118131256	290	-0.8606	-1
MSK_00014_251469322629_S01_CGH.v4_91.txt	10	89266047	112647582	1426	-0.8707	-1
MSK_00014_251469322629_S01_CGH.v4_91.txt	10	88598797	88745329	13	-0.8713	-1
MSK_00014_251469322629_S01_CGH.v4_91.txt	10	88481842	88573523	9	3.4468	1
MSK_00014_251469322629_S01_CGH.v4_91.txt	10	81660274	88408032	326	-0.9065	-1
MSK_WGA_0015.1_251469338754_S01_CGH.v4_91.txt	10	102467993	102524217	4	0.6346	1
MSK_WGA_0015.1_251469338754_S01_CGH.v4_91.txt	10	97135762	98741284	117	-0.6777	-1
MSK_WGA_0015.1_251469338754_S01_CGH.v4_91.txt	10	94570903	97035821	167	-0.6849	-1
MSK_WGA_0015.1_251469338754_S01_CGH.v4_91.txt	10	89266047	91010550	107	-0.7166	-1
MSK_WGA_0015.1_251469338754_S01_CGH.v4_91.txt	10	88526998	88745329	18	-0.6827	-1
MSK_WGA_0015.1_251469338754_S01_CGH.v4_91.txt	10	73396915	79258860	383	-0.6705	-1
MSK_WGA_0015.1_251469338754_S01_CGH.v4_91.txt	10	31726807	31799621	7	-0.6574	-1
MSK_WGA_0015.1_251469338754_S01_CGH.v4_91.txt	10	24883967	28596527	225	-0.7025	-1

Continued on next page...

ID	chr	start	end	nroOfprobes	segmean	call
MSK_WGA_0015.1_251469338754_S01_CGH.v4_91.txt	10	22898400	23369938	26	-0.6892	-1
MSK_WGA_0016.1_251469338755_S01_CGH.v4_91.txt	10	82027464	135513631	3046	-0.6477	-1
MSK_WGA_0016.1_251469338755_S01_CGH.v4_91.txt	10	125544	81925897	4047	-0.6635	-1
MSK_00021_251469322630_S01_CGH.v4_91.txt	10	42889244	135513631	4981	-0.8012	-1
MSK_00021_251469322630_S01_CGH.v4_91.txt	10	30708381	38629530	399	-0.7814	-1
MSK_00021_251469322630_S01_CGH.v4_91.txt	10	125544	30611362	1707	-0.8078	-1
MSK_WGA_0023.1_251469338756_S01_CGH.v4_91.txt	10	129013164	130279345	65	-1.8011	-1
MSK_00028_251469322706_S01_CGH.v4_91.txt	10	124891202	127375323	133	-0.8579	-1
MSK_00028_251469322706_S01_CGH.v4_91.txt	10	4022815	4049156	2	-0.706	-1
MSK_00037_251469322734_S01_CGH.v4_91.txt	10	127622813	135513631	409	-0.8172	-1
MSK_00037_251469322734_S01_CGH.v4_91.txt	10	42889244	127588249	4569	-0.7684	-1
MSK_00037_251469322734_S01_CGH.v4_91.txt	10	125544	38629530	2112	-0.7822	-1
MSK_00038_251469322735_S01_CGH.v4_91.txt	10	125544	135513631	7098	-0.6796	-1
MSK_WGA_0039.1_251469338741_S01_CGH.v4_91.txt	10	90301277	90323021	2	-1.2283	-1
MSK_WGA_0039.1_251469338741_S01_CGH.v4_91.txt	10	89564473	90010043	18	-1.1823	-1
MSK_WGA_0039.1_251469338741_S01_CGH.v4_91.txt	10	68087319	68109984	3	-1.3658	-1
MSK_WGA_0039.1_251469338741_S01_CGH.v4_91.txt	10	27613431	27695851	6	-1.1038	-1
MSK_00043_251469322736_S01_CGH.v4_91.txt	10	98433832	135513631	2126	-0.8564	-1
MSK_00043_251469322736_S01_CGH.v4_91.txt	10	89664975	98376433	560	-0.8626	-1
MSK_00043_251469322736_S01_CGH.v4_91.txt	10	89610886	89625664	2	-3.8164	-1
MSK_00043_251469322736_S01_CGH.v4_91.txt	10	42889244	89583182	2291	-0.886	-1
MSK_00043_251469322736_S01_CGH.v4_91.txt	10	125544	38629530	2112	-0.8655	-1
MSK_00046_251469322737_S01_CGH.v4_91.txt	10	127622813	135513631	409	-0.7795	-1
MSK_00046_251469322737_S01_CGH.v4_91.txt	10	42889244	127573288	4568	-0.7846	-1
MSK_00046_251469322737_S01_CGH.v4_91.txt	10	125544	38629530	2112	-0.7853	-1
MSK_WGA_0048.1_251469338742_S01_CGH.v4_91.txt	10	42889244	135513631	4981	-0.725	-1
MSK_WGA_0048.1_251469338742_S01_CGH.v4_91.txt	10	125544	38545869	2108	-0.7281	-1
MSK_00052_251469323033_S01_CGH.v4_91.txt	10	128858972	135406557	342	-0.6359	-1
MSK_00069_251469322916_S01_CGH.v4_91.txt	10	125544	34944133	1953	1.3026	1
MSK_00074_251469322717_S01_CGH.v4_91.txt	10	127622813	135513631	409	-0.6931	-1
MSK_00074_251469322717_S01_CGH.v4_91.txt	10	104757591	127573288	1271	-0.7454	-1
MSK_00074_251469322717_S01_CGH.v4_91.txt	10	42889244	103686381	3225	-0.743	-1
MSK_00083_251469322918_S01_CGH.v4_91.txt	10	46091262	135513631	4845	-0.7892	-1
MSK_0087.1_251469333362_S01_CGH.v4_91.txt	10	30258402	32185412	109	0.6898	1
MSK_0087.1_251469333362_S01_CGH.v4_91.txt	10	12671321	30247574	989	1.1856	1
MSK_00089_251469322904_S01_CGH.v4_91.txt	10	98399254	98428161	2	0.634	1
MSK_00089_251469322904_S01_CGH.v4_91.txt	10	26397492	26581496	16	-1.28	-1
MSK_00102_251469322716_S01_CGH.v4_91.txt	10	127630986	135513631	408	-0.8348	-1
MSK_00102_251469322716_S01_CGH.v4_91.txt	10	126084061	127573288	90	-0.7467	-1
MSK_00102_251469322716_S01_CGH.v4_91.txt	10	47702528	125987000	4311	-0.7957	-1
MSK_00102_251469322716_S01_CGH.v4_91.txt	10	46984913	47633840	21	-1.582	-1
MSK_00102_251469322716_S01_CGH.v4_91.txt	10	42889244	46951237	144	-0.7553	-1
MSK_00102_251469322716_S01_CGH.v4_91.txt	10	125544	38629530	2112	-0.7866	-1
MSK_WGA_0104.1_251469338880_S01_CGH.v4_91.txt	10	127622813	135513631	409	-0.8449	-1
MSK_WGA_0104.1_251469338880_S01_CGH.v4_91.txt	10	89266047	127573288	2298	-0.7801	-1
MSK_WGA_0104.1_251469338880_S01_CGH.v4_91.txt	10	87450570	88745329	85	-0.8233	-1
MSK_00114_251469322752_S01_CGH.v4_91.txt	10	55405420	55421070	2	-1.0275	-1
MSK_00116_251469322720_S01_CGH.v4_91.txt	10	127622813	135513631	409	-0.8763	-1
MSK_00116_251469322720_S01_CGH.v4_91.txt	10	70406089	127573288	3315	-0.8129	-1
MSK_00116_251469322720_S01_CGH.v4_91.txt	10	70377991	70389653	2	1.3356	1
MSK_00116_251469322720_S01_CGH.v4_91.txt	10	46951237	70362914	1108	-0.8984	-1
MSK_00116_251469322720_S01_CGH.v4_91.txt	10	42889244	46148409	140	-0.83	-1
MSK_00116_251469322720_S01_CGH.v4_91.txt	10	20825532	38629530	949	-0.8113	-1
MSK_00116_251469322720_S01_CGH.v4_91.txt	10	125544	20784333	1159	-0.8372	-1
MSK_0258.1_251469336709_S01_CGH.v4_91.txt	10	112956647	135416364	1269	-0.9209	-1
MSK_0260.1_251469331583_S01_CGH.v4_91.txt	10	127622813	135513631	409	-0.8052	-1
MSK_0260.1_251469331583_S01_CGH.v4_91.txt	10	117807303	127573288	594	-0.815	-1
MSK_0260.1_251469331583_S01_CGH.v4_91.txt	10	99599558	115365445	875	-0.8205	-1
MSK_0260.1_251469331583_S01_CGH.v4_91.txt	10	94835565	99514801	334	-0.8009	-1
MSK_0260.1_251469331583_S01_CGH.v4_91.txt	10	94110864	94633099	29	-0.8286	-1
MSK_0260.1_251469331583_S01_CGH.v4_91.txt	10	76409730	93977161	912	-0.8201	-1
MSK_0260.1_251469331583_S01_CGH.v4_91.txt	10	74702311	76285088	113	-0.8961	-1
MSK_0260.1_251469331583_S01_CGH.v4_91.txt	10	74415642	74605631	16	-0.8069	-1
MSK_0260.1_251469331583_S01_CGH.v4_91.txt	10	42889244	74322745	1502	-0.8254	-1
MSK_0260.1_251469331583_S01_CGH.v4_91.txt	10	125544	38629530	2112	-0.8369	-1
MSK_0269.1_251469333522_S01_CGH.v4_91.txt	10	127622813	135513631	409	-0.8488	-1
MSK_0269.1_251469333522_S01_CGH.v4_91.txt	10	42889244	127573288	4568	-0.8397	-1
MSK_0269.1_251469333522_S01_CGH.v4_91.txt	10	125544	38545869	2108	-0.846	-1
MSK_0289.1_251469337015_S01_CGH.v4_91.txt	10	42889244	135513631	4981	-0.7756	-1
MSK_0289.1_251469337015_S01_CGH.v4_91.txt	10	125544	38580770	2110	-0.7794	-1
MSK_0290.1_251469336710_S01_CGH.v4_91.txt	10	127622813	135513631	409	-0.7304	-1
MSK_0290.1_251469336710_S01_CGH.v4_91.txt	10	89266047	127573288	2298	-0.7011	-1
MSK_0290.1_251469336710_S01_CGH.v4_91.txt	10	47540379	88868493	2101	-0.7048	-1
MSK_0290.1_251469336710_S01_CGH.v4_91.txt	10	42889244	46148409	140	-0.687	-1
MSK_0290.1_251469336710_S01_CGH.v4_91.txt	10	125544	38580770	2110	-0.7122	-1
MSK_0317.1_251469331584_S01_CGH.v4_91.txt	10	127622813	135513631	409	-0.7582	-1
MSK_0317.1_251469331584_S01_CGH.v4_91.txt	10	42889244	127573288	4568	-0.7394	-1
MSK_0317.1_251469331584_S01_CGH.v4_91.txt	10	28167059	38580770	532	-0.746	-1
MSK_0317.1_251469331584_S01_CGH.v4_91.txt	10	125544	18338506	1038	-0.7538	-1
MSK_0333.1_251469331585_S01_CGH.v4_91.txt	10	116512729	135513631	1091	-0.7635	-1
MSK_0333.1_251469331585_S01_CGH.v4_91.txt	10	42889244	116361961	3880	-0.7882	-1
MSK_0333.1_251469331585_S01_CGH.v4_91.txt	10	125544	38629530	2112	-0.7913	-1
MSK_0337.1_251469333524_S01_CGH.v4_91.txt	10	46984913	47148490	17	-0.8968	-1
MSK_0422.1_251469337230_S01_CGH.v4_91.txt	10	48334407	135513631	4815	-0.7035	-1
MSK_0422.1_251469337230_S01_CGH.v4_91.txt	10	125544	46234711	2260	-0.6959	-1
MSK_0430.1_251469336714_S01_CGH.v4_91.txt	10	60166778	135513631	4291	-0.7821	-1
MSK_0430.1_251469336714_S01_CGH.v4_91.txt	10	42889244	60129836	686	-0.8009	-1
MSK_0430.1_251469336714_S01_CGH.v4_91.txt	10	37435255	38629530	48	-0.7169	-1
MSK_0430.1_251469336714_S01_CGH.v4_91.txt	10	30708381	37385253	348	-0.8009	-1
MSK_0430.1_251469336714_S01_CGH.v4_91.txt	10	125544	30642892	1710	-0.7897	-1
MSK_0440.1_251469336724_S01_CGH.v4_91.txt	10	42889244	135513631	4981	-0.7708	-1
MSK_0440.1_251469336724_S01_CGH.v4_91.txt	10	32310146	38629530	307	-0.7859	-1
MSK_0440.1_251469336724_S01_CGH.v4_91.txt	10	125544	32215628	1802	-0.7798	-1
MSK_0446.1_251469333525_S01_CGH.v4_91.txt	10	87604497	87687361	9	-1.5969	-1
MSK_0451.1_251469333565_S01_CGH.v4_91.txt	10	55725985	57345079	107	-1.351	-1
MSK_0451.1_251469333565_S01_CGH.v4_91.txt	10	55606975	55714219	10	-0.6356	-1
MSK_0451.1_251469333565_S01_CGH.v4_91.txt	10	54433547	55587065	35	-1.3481	-1
MSK_00125.1_251469324871_S01_CGH.v4_91.txt	10	125544	135513631	7098	-0.6486	-1
MSK_00126.1_251469325171_S01_CGH.v4_91.txt	10	30708381	135513631	5385	-0.6866	-1
MSK_00126.1_251469325171_S01_CGH.v4_91.txt	10	125544	30642892	1710	-0.6744	-1
MSK_00128.1_251469325161_S01_CGH.v4_91.txt	10	98399254	98428161	2	0.7707	1
MSK_00130.1_251469325022_S01_CGH.v4_91.txt	10	67996049	68019573	2	-1.1383	-1
MSK_00137.4_251469325162_S01_CGH.v4_91.txt	10	125544	135513631	7098	-0.6521	-1
MSK_00141.1_251469325753_S01_CGH.v4_91.txt	10	68087319	68109984	3	-0.9358	-1
MSK_0146.1_251469336983_S01_CGH.v4_91.txt	10	80632628	82860481	88	0.7153	1
MSK_0149.1_251469336715_S01_CGH.v4_91.txt	10	89564473	89625664	4	-1.7224	-1
MSK_0157.1_251469329178_S01_CGH.v4_91.txt	10	127622813	135513631	409	-0.7652	-1
MSK_0157.1_251469329178_S01_CGH.v4_91.txt	10	82027464	127573288	2633	-0.7635	-1
MSK_0157.1_251469329178_S01_CGH.v4_91.txt	10	61257610	81962119	1184	-0.7553	-1
MSK_0157.1_251469329178_S01_CGH.v4_91.txt	10	42889244	60565796	714	-0.7808	-1
MSK_0157.1_251469329178_S01_CGH.v4_91.txt	10	125544	38629530	2112	-0.7746	-1
MSK_0158.1_251469329734_S01_CGH.v4_91.txt	10	127622813	135513631	409	-0.683	-1
MSK_0158.1_251469329734_S01_CGH.v4_91.txt	10	47540379	127573288	4407	-0.6939	-1
MSK_0158.1_251469329734_S01_CGH.v4_91.txt	10	46984913	47148490	17	-1.3026	-1
MSK_0158.1_251469329734_S01_CGH.v4_91.txt	10	42889244	46951237	144	-0.6675	-1
MSK_0158.1_251469329734_S01_CGH.v4_91.txt	10	125544	38629530	2112	-0.6935	-1
MSK_0164.1_251469331554_S01_CGH.v4_91.txt	10	57033399	57046937	2	-1.041	-1
MSK_0173.1_251469330461_S01_CGH.v4_91.txt	10	127622813	135513631	409	-0.8133	-1
MSK_0173.1_251469330461_S01_CGH.v4_91.txt	10	42889244	127573288	4568	-0.7524	-1
MSK_0173.1_251469330461_S01_CGH.v4_91.txt	10	125544	38629530	2112	-0.7515	-1
MSK_0174.1_251469330284_S01_CGH.v4_91.txt	10	127622813	135513631	409	-0.7092	-1
MSK_0174.1_251469330284_S01_CGH.v4_91.txt	10	42889244	127573288	4568	-0.7189	-1
MSK_0174.1_251469330284_S01_CGH.v4_91.txt	10	125544	38629530	2112	-0.7297	-1
MSK_0175.1_251469336725_S01_CGH.v4_91.txt	10	42889244	135513631	4981	-0.6494	-1

Continued on next page...

ID	chr	start	end	nroOfprobes	segmean	call
MSK_0175.1_251469336725_S01_CGH.v4_91.txt	10	125544	38629530	2112	-0.6536	-1
MSK_0182.1_251469331464_S01_CGH.v4_91.txt	10	92181531	135513631	2531	-0.8576	-1
MSK_0182.1_251469331464_S01_CGH.v4_91.txt	10	87268974	92004149	282	-0.8731	-1
MSK_0182.1_251469331464_S01_CGH.v4_91.txt	10	57236467	76942755	1074	-0.8428	-1
MSK_0182.1_251469331464_S01_CGH.v4_91.txt	10	55126356	57109153	121	-0.9342	-1
MSK_0182.1_251469331464_S01_CGH.v4_91.txt	10	43028988	54999982	467	-0.8779	-1
MSK_0182.1_251469331464_S01_CGH.v4_91.txt	10	38355949	38545869	11	-0.902	-1
MSK_0182.1_251469331464_S01_CGH.v4_91.txt	10	30799936	37636362	359	-0.8505	-1
MSK_0182.1_251469331464_S01_CGH.v4_91.txt	10	29684956	30301171	38	-0.8452	-1
MSK_0182.1_251469331464_S01_CGH.v4_91.txt	10	19775141	29467479	544	-0.86	-1
MSK_0182.1_251469331464_S01_CGH.v4_91.txt	10	19043104	19353135	9	-0.8396	-1
MSK_0185.1_251469329754_S01_CGH.v4_91.txt	10	127622813	135513631	409	-0.789	-1
MSK_0185.1_251469329754_S01_CGH.v4_91.txt	10	42889244	127573288	4568	-0.8047	-1
MSK_0185.1_251469329754_S01_CGH.v4_91.txt	10	125544	38629530	2112	-0.8075	-1
MSK_0187.1_251469330462_S01_CGH.v4_91.txt	10	82027464	135513631	3046	-0.7431	-1
MSK_0187.1_251469330462_S01_CGH.v4_91.txt	10	42889244	81962119	1933	-0.7616	-1
MSK_0187.1_251469330462_S01_CGH.v4_91.txt	10	125544	38677773	2114	-0.7625	-1
MSK_0208.1_251469329182_S01_CGH.v4_91.txt	10	127622813	135513631	409	-0.6663	-1
MSK_0208.1_251469329182_S01_CGH.v4_91.txt	10	82027464	127588249	2634	-0.6354	-1
MSK_0208.1_251469329182_S01_CGH.v4_91.txt	10	56472408	81962119	1360	-0.6341	-1
MSK_0208.1_251469329182_S01_CGH.v4_91.txt	10	42889244	56442061	570	-0.6544	-1
MSK_0208.1_251469329182_S01_CGH.v4_91.txt	10	125544	38629530	2112	-0.6424	-1
MSK_0208.2_251469329755_S01_CGH.v4_91.txt	10	42889244	56442061	570	-0.6361	-1
MSK_0210.1_251469330448_S01_CGH.v4_91.txt	10	22088552	23041802	61	-1.4561	-1
MSK_0211.2_251469329773_S01_CGH.v4_91.txt	10	82027464	135513631	3046	-0.7889	-1
MSK_0211.2_251469329773_S01_CGH.v4_91.txt	10	64257328	81962119	1018	-0.786	-1
MSK_0211.2_251469329773_S01_CGH.v4_91.txt	10	42889244	64161461	911	-0.8081	-1
MSK_0211.2_251469329773_S01_CGH.v4_91.txt	10	30708381	38629530	399	-0.7951	-1
MSK_0211.2_251469329773_S01_CGH.v4_91.txt	10	125544	30642892	1710	-0.796	-1
MSK_0213.1_251469329174_S01_CGH.v4_91.txt	10	109819022	109988413	6	-0.6325	-1
MSK_0214.1_251469329168_S01_CGH.v4_91.txt	10	89321293	90266472	54	-1.4167	-1
MSK_0219.1_251469329864_S01_CGH.v4_91.txt	10	89664975	90148976	26	-1.3714	-1
MSK_0237.1_251469330449_S01_CGH.v4_91.txt	10	125544	38629530	2112	-0.6568	-1
MSK_0241.1_251469329774_S01_CGH.v4_91.txt	10	42889244	135513631	4981	-0.819	-1
MSK_0241.1_251469329774_S01_CGH.v4_91.txt	10	125544	38629530	2112	-0.8248	-1
MSK_0397.1_251469330468_S01_CGH.v4_91.txt	10	24561107	25347459	55	-0.7446	-1
MSK_0402.1_251469336906_S01_CGH.v4_91.txt	10	82080348	135513631	3041	-0.7666	-1
MSK_0402.1_251469336906_S01_CGH.v4_91.txt	10	82049115	82058378	3	0.9728	1
MSK_0402.1_251469336906_S01_CGH.v4_91.txt	10	79891073	80212289	13	2.3763	1
MSK_0402.1_251469336906_S01_CGH.v4_91.txt	10	76179520	79784391	229	-0.6943	-1
MSK_0402.1_251469336906_S01_CGH.v4_91.txt	10	75984560	76142631	12	2.0457	1
MSK_0402.1_251469336906_S01_CGH.v4_91.txt	10	75533870	75603764	9	0.9985	1
MSK_0402.1_251469336906_S01_CGH.v4_91.txt	10	70377991	70389653	2	1.5705	1
MSK_0402.1_251469336906_S01_CGH.v4_91.txt	10	36826794	70362914	1321	-0.7847	-1
MSK_0402.1_251469336906_S01_CGH.v4_91.txt	10	36753426	36819854	5	2.1086	1
MSK_0402.1_251469336906_S01_CGH.v4_91.txt	10	26737690	36621432	540	-0.689	-1
MSK_0402.1_251469336906_S01_CGH.v4_91.txt	10	26552439	26719205	7	1.0813	1
MSK_0402.1_251469336906_S01_CGH.v4_91.txt	10	22107910	26547138	262	-0.6958	-1
MSK_0402.1_251469336906_S01_CGH.v4_91.txt	10	21777017	22094887	20	2.2974	1
MSK_0402.1_251469336906_S01_CGH.v4_91.txt	10	4867087	21743500	924	-0.8052	-1
MSK_0402.1_251469336906_S01_CGH.v4_91.txt	10	4049156	4737992	40	-0.7991	-1
MSK_0402.1_251469336906_S01_CGH.v4_91.txt	10	3909933	4022815	9	0.9135	1
MSK_0402.1_251469336906_S01_CGH.v4_91.txt	10	125544	3882398	234	-0.7272	-1
MSK_0412.1_251469331490_S01_CGH.v4_91.txt	10	42889244	135513631	4981	-0.6322	-1
MSK_0412.1_251469331490_S01_CGH.v4_91.txt	10	4680909	38629530	1833	-0.6341	-1
MSK_0413.1_251469331465_S01_CGH.v4_91.txt	10	103161204	103166659	2	0.7103	1
MSK_0414.1_251469336986_S01_CGH.v4_91.txt	10	89922387	90184362	19	-2.5293	-1
MSK_0414.1_251469336986_S01_CGH.v4_91.txt	10	89564473	89698253	7	-2.5554	-1
MSK_0414.1_251469336986_S01_CGH.v4_91.txt	10	89480321	89550153	4	-0.8503	-1
MSK_0414.1_251469336986_S01_CGH.v4_91.txt	10	89266047	89474512	15	-2.5169	-1
MSK_0414.1_251469336986_S01_CGH.v4_91.txt	10	5191182	5196840	2	-3.0472	-1
MSK_0414.1_251469336986_S01_CGH.v4_91.txt	10	4760398	5174667	28	-0.6808	-1
MSK_0414.1_251469336986_S01_CGH.v4_91.txt	10	4238998	4752025	30	-2.6469	-1
MSK_0414.1_251469336986_S01_CGH.v4_91.txt	10	3469802	4114978	39	-2.6405	-1
MSK_0414.1_251469336986_S01_CGH.v4_91.txt	10	125544	3448109	210	-0.6375	-1
MSK_0645.1_251469340797_S01_CGH.v4_91.txt	10	89610886	90236197	35	-1.4229	-1
MSK_0645.1_251469340797_S01_CGH.v4_91.txt	10	89521012	89583182	4	-0.7153	-1
MSK_0645.1_251469340797_S01_CGH.v4_91.txt	10	89437172	89488862	6	-1.6134	-1
MSK_0646.1_251469340746_S01_CGH.v4_91.txt	10	89729442	135513631	2684	-0.6702	-1
MSK_0646.1_251469340746_S01_CGH.v4_91.txt	10	89488862	89706666	11	-2.3675	-1
MSK_0646.1_251469340746_S01_CGH.v4_91.txt	10	125544	89480321	4403	-0.6976	-1
MSK_0648.1_251469338021_S01_CGH.v4_91.txt	10	90700722	135513631	2624	-0.7591	-1
MSK_0648.1_251469338021_S01_CGH.v4_91.txt	10	90668512	90688198	3	-3.1502	-1
MSK_0648.1_251469338021_S01_CGH.v4_91.txt	10	90323021	90639624	23	-0.9184	-1
MSK_0648.1_251469338021_S01_CGH.v4_91.txt	10	89851273	90301277	30	-2.8424	-1
MSK_0648.1_251469338021_S01_CGH.v4_91.txt	10	89664975	89801087	8	-0.9042	-1
MSK_0648.1_251469338021_S01_CGH.v4_91.txt	10	89564473	89625664	4	-2.8778	-1
MSK_0648.1_251469338021_S01_CGH.v4_91.txt	10	42889244	89550153	2289	-0.7793	-1
MSK_0648.1_251469338021_S01_CGH.v4_91.txt	10	125544	38642259	2113	-0.7494	-1
MSK_WGA_0244.1_251469338881_S01_CGH.v4_91.txt	10	42889244	135513631	4981	-0.6927	-1
MSK_WGA_0244.1_251469338881_S01_CGH.v4_91.txt	10	125544	38677773	2114	-0.7035	-1
MSK_WGA_0347.1_251469338868_S01_CGH.v4_91.txt	10	89480321	89664975	9	-1.5726	-1
MSK_WGA_0348.1_251469338869_S01_CGH.v4_91.txt	10	42889244	135513631	4981	-0.6456	-1
MSK_WGA_0348.1_251469338869_S01_CGH.v4_91.txt	10	125544	38580770	2110	-0.6484	-1
MSK_0352.1_251469337790_S01_CGH.v4_91.txt	10	38741782	42737548	3	0.9392	1
MSK_WGA_0353.1_251469337506_S01_CGH.v4_91.txt	10	127630986	135513631	408	-0.6503	-1
MSK_WGA_0355.1_251469337508_S01_CGH.v4_91.txt	10	127622813	135513631	409	-0.721	-1
MSK_WGA_0355.1_251469337508_S01_CGH.v4_91.txt	10	42889244	127573288	4568	-0.6842	-1
MSK_WGA_0355.1_251469337508_S01_CGH.v4_91.txt	10	125544	38545869	2108	-0.6624	-1
MSK_WGA_0357.1_251469337522_S01_CGH.v4_91.txt	10	127622813	135513631	409	-0.6982	-1
MSK_WGA_0357.1_251469337522_S01_CGH.v4_91.txt	10	42889244	127573288	4568	-0.6872	-1
MSK_WGA_0357.1_251469337522_S01_CGH.v4_91.txt	10	125544	38619030	2111	-0.6895	-1
MSK_0358.1_251469340789_S01_CGH.v4_91.txt	10	43224719	135513631	4960	-0.7968	-1
MSK_0358.1_251469340789_S01_CGH.v4_91.txt	10	36826794	38629530	65	-0.7525	-1
MSK_0358.1_251469340789_S01_CGH.v4_91.txt	10	33508859	36786266	168	-0.9039	-1
MSK_0358.1_251469340789_S01_CGH.v4_91.txt	10	11817848	33407746	1229	-0.8165	-1
MSK_0358.1_251469340789_S01_CGH.v4_91.txt	10	11065086	11767759	41	-0.7987	-1
MSK_0358.1_251469340789_S01_CGH.v4_91.txt	10	125544	11017489	591	-0.8154	-1
MSK_WGA_0359.1_251469337523_S01_CGH.v4_91.txt	10	55586719	55587065	2	-1.4715	-1
MSK_0386.1_251469340798_S01_CGH.v4_91.txt	10	91604949	135513631	2551	-0.6561	-1
MSK_0386.1_251469340798_S01_CGH.v4_91.txt	10	89583182	91586568	140	-2.8809	-1
MSK_0386.1_251469340798_S01_CGH.v4_91.txt	10	47555991	89564473	2128	-0.6722	-1
MSK_0386.1_251469340798_S01_CGH.v4_91.txt	10	18370499	46234711	1221	-0.6673	-1
MSK_0386.1_251469340798_S01_CGH.v4_91.txt	10	18254209	18348220	10	-3.638	-1
MSK_0386.1_251469340798_S01_CGH.v4_91.txt	10	125544	17891703	1029	-0.664	-1
MSK_WGA_0389.1_251469338874_S01_CGH.v4_91.txt	10	42889244	135513631	4981	-0.7785	-1
MSK_WGA_0389.1_251469338874_S01_CGH.v4_91.txt	10	125544	38580770	2110	-0.7751	-1
MSK_WGA_0392.1_251469338876_S01_CGH.v4_91.txt	10	44629347	135513631	4908	-0.6832	-1
MSK_0511.1_251469336907_S01_CGH.v4_91.txt	10	127622813	135513631	409	-0.6974	-1
MSK_0511.1_251469336907_S01_CGH.v4_91.txt	10	47540379	127573288	4407	-0.7152	-1
MSK_0511.1_251469336907_S01_CGH.v4_91.txt	10	42889244	46148409	140	-0.6728	-1
MSK_0511.1_251469336907_S01_CGH.v4_91.txt	10	125544	38629530	2112	-0.7116	-1
MSK_0512.1_251469331556_S01_CGH.v4_91.txt	10	89564473	90236197	37	-0.7689	-1
MSK_0512.1_251469331556_S01_CGH.v4_91.txt	10	68087319	68109984	3	-1.9158	-1
MSK_0514.1_251469333360_S01_CGH.v4_91.txt	10	42889244	135513631	4981	-0.7261	-1
MSK_0514.1_251469333360_S01_CGH.v4_91.txt	10	125544	38629530	2112	-0.7369	-1
MSK_0516.1_251469331469_S01_CGH.v4_91.txt	10	111789106	111796578	2	-1.4122	-1
MSK_0517.1_251469337226_S01_CGH.v4_91.txt	10	114791004	135513631	1191	-0.8366	-1
MSK_0517.1_251469337226_S01_CGH.v4_91.txt	10	54676148	55236904	15	0.895	1
MSK_0518.1_251469337014_S01_CGH.v4_91.txt	10	126345336	135513631	490	-0.9108	-1
MSK_0518.1_251469337014_S01_CGH.v4_91.txt	10	96012242	125987000	1790	-0.8983	-1
MSK_0518.1_251469337014_S01_CGH.v4_91.txt	10	88481842	95533324	418	-0.902	-1
MSK_0518.1_251469337014_S01_CGH.v4_91.txt	10	68456288	77483984	606	-0.8926	-1
MSK_0518.1_251469337014_S01_CGH.v4_91.txt	10	68381502	68449522	6	-3.9362	-1

Continued on next page...

ID	chr	start	end	nroOfprobes	segmean	call
MSK_0518.1_251469337014_S01_CGH.v4_91.txt	10	64995980	68348397	148	-0.9207	-1
MSK_0518.1_251469337014_S01_CGH.v4_91.txt	10	42889244	52760816	348	-0.896	-1
MSK_0518.1_251469337014_S01_CGH.v4_91.txt	10	27201098	38580770	583	-0.9002	-1
MSK_0520.1_251469336708_S01_CGH.v4_91.txt	10	55725985	135513631	4471	-0.6762	-1
MSK_0520.1_251469336708_S01_CGH.v4_91.txt	10	55307945	55714219	23	-2.281	-1
MSK_0520.1_251469336708_S01_CGH.v4_91.txt	10	54048725	55236904	40	-0.7695	-1
MSK_0520.1_251469336708_S01_CGH.v4_91.txt	10	52760816	54025077	100	-2.2851	-1
MSK_0520.1_251469336708_S01_CGH.v4_91.txt	10	42889244	52709143	347	-0.6833	-1
MSK_0520.1_251469336708_S01_CGH.v4_91.txt	10	125544	38580770	2110	-0.7066	-1
MSK_0525.1_251469333361_S01_CGH.v4_91.txt	10	42889244	135513631	4981	-0.7217	-1
MSK_0525.1_251469333361_S01_CGH.v4_91.txt	10	30708381	38456859	392	-0.7342	-1
MSK_0525.1_251469333361_S01_CGH.v4_91.txt	10	14250207	30642892	902	-0.7267	-1
MSK_0529.1_251469331470_S01_CGH.v4_91.txt	10	112647582	135513631	1286	-0.7862	-1
MSK_0529.1_251469331470_S01_CGH.v4_91.txt	10	42889244	112564493	3690	-0.8037	-1
MSK_0529.1_251469331470_S01_CGH.v4_91.txt	10	125544	38629530	2112	-0.8029	-1
MSK_0531.1_251469337227_S01_CGH.v4_91.txt	10	42889244	135513631	4981	-0.8317	-1
MSK_0531.1_251469337227_S01_CGH.v4_91.txt	10	21962681	38580770	887	-0.8478	-1
MSK_0531.1_251469337227_S01_CGH.v4_91.txt	10	21687711	21846097	8	-0.7726	-1
MSK_0531.1_251469337227_S01_CGH.v4_91.txt	10	5915729	9971860	201	-0.8318	-1
MSK_0615.1_251469340783_S01_CGH.v4_91.txt	10	91253623	135513631	2579	-0.6574	-1
MSK_0615.1_251469340783_S01_CGH.v4_91.txt	10	89365134	91234850	125	-2.425	-1
MSK_0615.1_251469340783_S01_CGH.v4_91.txt	10	125544	89348185	4394	-0.679	-1
MSK_0616.1_251469338022_S01_CGH.v4_91.txt	10	125544	135513631	7098	-0.7169	-1
MSK_0618.1_251469337983_S01_CGH.v4_91.txt	10	98448665	135513631	2125	-0.7775	-1
MSK_0618.1_251469337983_S01_CGH.v4_91.txt	10	55126356	98376433	2369	-0.7912	-1
MSK_0618.1_251469337983_S01_CGH.v4_91.txt	10	54978530	55105503	5	-4.3205	-1
MSK_0618.1_251469337983_S01_CGH.v4_91.txt	10	125544	54889675	2596	-0.7883	-1
MSK_0619.1_251469337893_S01_CGH.v4_91.txt	10	109411801	110328294	33	-1.7138	-1
MSK_0619.1_251469337893_S01_CGH.v4_91.txt	10	109218072	109381493	9	-0.6365	-1
MSK_0619.1_251469337893_S01_CGH.v4_91.txt	10	109098962	109188508	6	-1.9343	-1
MSK_0619.1_251469337893_S01_CGH.v4_91.txt	10	91393301	91429083	4	-1.85	-1
MSK_0619.1_251469337893_S01_CGH.v4_91.txt	10	90354456	90855045	35	-1.4934	-1
MSK_0619.1_251469337893_S01_CGH.v4_91.txt	10	89737383	89761762	2	-2.2844	-1
MSK_00006_251469322732_S01_CGH.v4_91.txt	11	18949929	18959295	2	0.7145	1
MSK_00007_251469322733_S01_CGH.v4_91.txt	11	77702127	103013413	1323	-0.759	-1
MSK_00007_251469322733_S01_CGH.v4_91.txt	11	57505048	77620741	1423	-0.6926	-1
MSK_00009_251469322626_S01_CGH.v4_91.txt	11	7817978	7826768	2	-0.9676	-1
MSK_00010_251469322627_S01_CGH.v4_91.txt	11	43686719	46582062	171	-0.8735	-1
MSK_00010_251469322627_S01_CGH.v4_91.txt	11	40195189	43661611	143	-3.6021	-1
MSK_00010_251469322627_S01_CGH.v4_91.txt	11	192372	40182121	2301	-0.7867	-1
MSK_00014_251469322629_S01_CGH.v4_91.txt	11	96904201	134904004	2186	-0.851	-1
MSK_00014_251469322629_S01_CGH.v4_91.txt	11	92145588	93480430	77	0.9274	1
MSK_00014_251469322629_S01_CGH.v4_91.txt	11	90717726	90754950	5	0.9213	1
MSK_00014_251469322629_S01_CGH.v4_91.txt	11	86260460	89066727	146	2.199	1
MSK_00014_251469322629_S01_CGH.v4_91.txt	11	85606130	86235279	40	1.4851	1
MSK_00014_251469322629_S01_CGH.v4_91.txt	11	73584463	85566581	702	0.6854	1
MSK_00014_251469322629_S01_CGH.v4_91.txt	11	72355343	72523601	15	0.9698	1
MSK_00014_251469322629_S01_CGH.v4_91.txt	11	70153580	70224372	8	0.8595	1
MSK_00014_251469322629_S01_CGH.v4_91.txt	11	67773863	70019477	119	0.9529	1
MSK_00014_251469322629_S01_CGH.v4_91.txt	11	64040895	64094324	6	0.7277	1
MSK_00014_251469322629_S01_CGH.v4_91.txt	11	49714985	49732266	2	1.3949	1
MSK_00014_251469322629_S01_CGH.v4_91.txt	11	11181300	13784787	150	-0.8479	-1
MSK_00014_251469322629_S01_CGH.v4_91.txt	11	192372	7070967	540	-0.8583	-1
MSK_WGA_0016.1_251469338755_S01_CGH.v4_91.txt	11	80296996	80489256	6	-1.0363	-1
MSK_00024_251469322721_S01_CGH.v4_91.txt	11	88372284	88377766	2	-2.085	-1
MSK_00027_251469322705_S01_CGH.v4_91.txt	11	644391	766488	10	0.7716	1
MSK_00028_251469322706_S01_CGH.v4_91.txt	11	81768536	82541219	38	-0.8857	-1
MSK_00033_251469322707_S01_CGH.v4_91.txt	11	7817978	7826768	2	-0.9456	-1
MSK_00037_251469322734_S01_CGH.v4_91.txt	11	33641632	37873727	221	1.2967	1
MSK_00037_251469322734_S01_CGH.v4_91.txt	11	32508093	33627789	72	2.0842	1
MSK_00037_251469322734_S01_CGH.v4_91.txt	11	29357768	32496452	183	1.4114	1
MSK_00037_251469322734_S01_CGH.v4_91.txt	11	28746189	29210783	14	1.3999	1
MSK_00037_251469322734_S01_CGH.v4_91.txt	11	27756601	28388185	37	1.3931	1
MSK_00037_251469322734_S01_CGH.v4_91.txt	11	27554852	27601415	4	1.5128	1
MSK_00052_251469323033_S01_CGH.v4_91.txt	11	4267917	4367260	2	-0.7501	-1
MSK_00060_251469322915_S01_CGH.v4_91.txt	11	78827491	78887529	5	-1.0135	-1
MSK_00064_251469322718_S01_CGH.v4_91.txt	11	7817978	7826768	2	-1.1069	-1
MSK_00069_251469322916_S01_CGH.v4_91.txt	11	22277048	22726957	31	-1.2338	-1
MSK_00086_251469322749_S01_CGH.v4_91.txt	11	40107045	40895121	48	-0.7429	-1
MSK_00099_251469322903_S01_CGH.v4_91.txt	11	40559091	40742235	8	-0.643	-1
MSK_00113_251469322906_S01_CGH.v4_91.txt	11	49714985	49732266	2	-1.3957	-1
MSK_00114_251469322752_S01_CGH.v4_91.txt	11	7846994	50381158	2237	-0.8432	-1
MSK_00114_251469322752_S01_CGH.v4_91.txt	11	7817978	7826768	2	-2.3945	-1
MSK_00114_251469322752_S01_CGH.v4_91.txt	11	5893184	7793639	169	-0.8787	-1
MSK_00114_251469322752_S01_CGH.v4_91.txt	11	5876105	5882369	2	-2.3255	-1
MSK_00114_251469322752_S01_CGH.v4_91.txt	11	192372	5871372	421	-0.7782	-1
MSK_00115_251469322751_S01_CGH.v4_91.txt	11	7817978	7826768	2	-3.9116	-1
MSK_00116_251469322720_S01_CGH.v4_91.txt	11	50261071	50279940	3	1.1474	1
MSK_0271.1_251469333526_S01_CGH.v4_91.txt	11	4262817	4367260	3	-1.0545	-1
MSK_0285.1_251469337229_S01_CGH.v4_91.txt	11	18949929	18959295	2	-0.7236	-1
MSK_0290.1_251469336710_S01_CGH.v4_91.txt	11	30372157	37509447	427	0.8043	1
MSK_0317.1_251469331584_S01_CGH.v4_91.txt	11	18949929	18959295	2	-0.7228	-1
MSK_0325.1_251469333563_S01_CGH.v4_91.txt	11	4813909	4820227	2	-0.6887	-1
MSK_0326.1_251469331461_S01_CGH.v4_91.txt	11	5876105	5882369	2	-1.0714	-1
MSK_0330.1_251469336713_S01_CGH.v4_91.txt	11	7817978	7826768	2	-0.7767	-1
MSK_0330.1_251469336713_S01_CGH.v4_91.txt	11	5876105	5882369	2	-0.9146	-1
MSK_0332.1_251469336711_S01_CGH.v4_91.txt	11	4813909	4820227	2	-0.9146	-1
MSK_0333.1_251469331585_S01_CGH.v4_91.txt	11	65212489	65303260	7	0.7115	1
MSK_0339.1_251469331462_S01_CGH.v4_91.txt	11	71627541	134904004	3626	-0.9009	-1
MSK_0339.1_251469331462_S01_CGH.v4_91.txt	11	55079853	71286776	1176	-0.8937	-1
MSK_0430.1_251469336714_S01_CGH.v4_91.txt	11	18949929	18959295	2	1.0722	1
MSK_0432.1_251469336712_S01_CGH.v4_91.txt	11	18949929	18959295	2	0.6367	1
MSK_0439.1_251469331553_S01_CGH.v4_91.txt	11	7817978	7826768	2	-0.8638	-1
MSK_0446.1_251469333525_S01_CGH.v4_91.txt	11	84412975	84419443	2	-0.6926	-1
MSK_0451.1_251469333565_S01_CGH.v4_91.txt	11	74573985	74622526	4	-0.6887	-1
MSK_0127.1_251469337891_S01_CGH.v4_91.txt	11	50269877	50279940	2	2.301	1
MSK_00128.1_251469325161_S01_CGH.v4_91.txt	11	192372	41340434	2371	-0.6361	-1
MSK_00129.1_251469322907_S01_CGH.v4_91.txt	11	29457987	29772573	10	-0.8328	-1
MSK_00129.1_251469322907_S01_CGH.v4_91.txt	11	27088028	27407012	21	-0.7501	-1
MSK_00129.1_251469322907_S01_CGH.v4_91.txt	11	192372	13810524	971	-0.7635	-1
MSK_00133_251469325752_S01_CGH.v4_91.txt	11	85431879	85453367	4	-1.1208	-1
MSK_0149.1_251469336715_S01_CGH.v4_91.txt	11	18949929	18959295	2	0.8522	1
MSK_0157.1_251469329178_S01_CGH.v4_91.txt	11	18949929	18959295	2	-0.8775	-1
MSK_0158.1_251469329734_S01_CGH.v4_91.txt	11	4262817	4367260	3	-1.0853	-1
MSK_0164.1_251469331554_S01_CGH.v4_91.txt	11	18949929	18959295	2	0.7244	1
MSK_0166.1_251469329777_S01_CGH.v4_91.txt	11	81514761	82194574	30	-0.7153	-1
MSK_0166.1_251469329777_S01_CGH.v4_91.txt	11	79913276	81010170	25	-0.7453	-1
MSK_0173.1_251469330461_S01_CGH.v4_91.txt	11	7817978	7826768	2	-1.0129	-1
MSK_0177.1_251469333358_S01_CGH.v4_91.txt	11	49699091	49732266	3	-0.9696	-1
MSK_0179.1_251469333566_S01_CGH.v4_91.txt	11	4813909	4820227	2	-0.9778	-1
MSK_0187.1_251469330462_S01_CGH.v4_91.txt	11	18949929	18959295	2	1.044	1
MSK_0188.1_251469330469_S01_CGH.v4_91.txt	11	18949929	18959295	2	0.8303	1
MSK_0195.1_251469329772_S01_CGH.v4_91.txt	11	118852173	123434662	255	1.9127	1
MSK_0195.1_251469329772_S01_CGH.v4_91.txt	11	118828782	118837096	2	1.1242	1
MSK_0195.1_251469329772_S01_CGH.v4_91.txt	11	118277831	118822061	36	1.8897	1
MSK_0201.1_251469329757_S01_CGH.v4_91.txt	11	49699091	49732266	3	-0.6844	-1
MSK_0208.1_251469329182_S01_CGH.v4_91.txt	11	7817978	7826768	2	-0.7157	-1
MSK_0208.2_251469329755_S01_CGH.v4_91.txt	11	7817978	7826768	2	-1.0777	-1
MSK_0214.1_251469329168_S01_CGH.v4_91.txt	11	7817978	7826768	2	-0.7492	-1
MSK_0221.1_251469330192_S01_CGH.v4_91.txt	11	105360035	109866905	246	-0.6482	-1
MSK_0221.1_251469330192_S01_CGH.v4_91.txt	11	103346716	104846694	54	-0.6457	-1
MSK_0221.1_251469330192_S01_CGH.v4_91.txt	11	103169844	103256987	8	-0.6806	-1
MSK_0221.1_251469330192_S01_CGH.v4_91.txt	11	18949929	18959295	2	0.8368	1
MSK_0237.1_251469330449_S01_CGH.v4_91.txt	11	80753138	80965983	7	-0.8229	-1

Continued on next page...

ID	chr	start	end	nroOfprobes	segmean	call
MSK_0237.1_251469330449_S01_CGH.v4_91.txt	11	79534785	79747028	5	-0.6732	-1
MSK_0237.1_251469330449_S01_CGH.v4_91.txt	11	7817978	7826768	2	-0.7746	-1
MSK_0241.1_251469329774_S01_CGH.v4_91.txt	11	78580435	78861431	26	-0.818	-1
MSK_0241.1_251469329774_S01_CGH.v4_91.txt	11	12923522	12937460	2	-1.3686	-1
MSK_0241.1_251469329774_S01_CGH.v4_91.txt	11	7817978	7826768	2	-1.0204	-1
MSK_0394.1_251469336716_S01_CGH.v4_91.txt	11	5876105	5882369	2	-0.9894	-1
MSK_0409.1_251469331555_S01_CGH.v4_91.txt	11	18939590	18959295	3	-0.7933	-1
MSK_0409.1_251469331555_S01_CGH.v4_91.txt	11	7817978	7826768	2	-1.0349	-1
MSK_0410.1_251469333359_S01_CGH.v4_91.txt	11	49714985	49732266	2	-1.5998	-1
MSK_0412.1_251469331490_S01_CGH.v4_91.txt	11	18949929	18959295	2	-0.9574	-1
MSK_0413.1_251469331465_S01_CGH.v4_91.txt	11	18949929	18959295	2	0.7302	1
MSK_0646.1_251469340746_S01_CGH.v4_91.txt	11	18949929	18959295	2	-1.4405	-1
MSK_0648.1_251469338021_S01_CGH.v4_91.txt	11	79792666	80489256	17	-0.8596	-1
MSK_0358.1_251469340789_S01_CGH.v4_91.txt	11	21004906	22320827	72	-0.7732	-1
MSK_0509.1_251469336717_S01_CGH.v4_91.txt	11	7817978	7826768	2	-0.8307	-1
MSK_0509.1_251469336717_S01_CGH.v4_91.txt	11	894906	938872	4	0.7782	1
MSK_0510.1_251469331581_S01_CGH.v4_91.txt	11	18949929	18959295	2	0.9276	1
MSK_0511.1_251469336907_S01_CGH.v4_91.txt	11	4813909	4820227	2	-0.691	-1
MSK_0514.1_251469333360_S01_CGH.v4_91.txt	11	22399439	23527639	48	-0.723	-1
MSK_0514.1_251469333360_S01_CGH.v4_91.txt	11	22063590	22351592	17	-0.7178	-1
MSK_0514.1_251469333360_S01_CGH.v4_91.txt	11	21286707	21537277	18	-0.741	-1
MSK_0514.1_251469333360_S01_CGH.v4_91.txt	11	21045963	21190457	15	-0.6611	-1
MSK_0514.1_251469333360_S01_CGH.v4_91.txt	11	20662255	20985806	20	-0.7369	-1
MSK_0517.1_251469337226_S01_CGH.v4_91.txt	11	83645252	83867590	18	-0.8294	-1
MSK_0517.1_251469337226_S01_CGH.v4_91.txt	11	192372	9715791	731	-0.7665	-1
MSK_0520.1_251469336708_S01_CGH.v4_91.txt	11	71647973	126223710	3139	-0.6394	-1
MSK_0521.1_251469331582_S01_CGH.v4_91.txt	11	7817978	7826768	2	-0.8716	-1
MSK_0529.1_251469331470_S01_CGH.v4_91.txt	11	18949929	18959295	2	-0.8208	-1
MSK_0531.1_251469337227_S01_CGH.v4_91.txt	11	122751331	124440919	122	-0.8418	-1
MSK_0531.1_251469337227_S01_CGH.v4_91.txt	11	121012856	122364739	68	-0.7998	-1
MSK_0616.1_251469338022_S01_CGH.v4_91.txt	11	32439100	32508093	7	3.1311	1
MSK_0616.1_251469338022_S01_CGH.v4_91.txt	11	31401096	31642266	23	3.0684	1
MSK_0616.1_251469338022_S01_CGH.v4_91.txt	11	26780501	26849941	4	2.7788	1
MSK_0616.1_251469338022_S01_CGH.v4_91.txt	11	23445008	23769409	8	3.0485	1
MSK_00003_251469322731_S01_CGH.v4_91.txt	12	111434437	111519057	9	-0.8074	-1
MSK_00003_251469322731_S01_CGH.v4_91.txt	12	57827519	58565440	65	3.7794	1
MSK_00006_251469322732_S01_CGH.v4_91.txt	12	72746155	72771807	3	3.1672	1
MSK_00006_251469322732_S01_CGH.v4_91.txt	12	69500737	70464916	66	2.7995	1
MSK_00006_251469322732_S01_CGH.v4_91.txt	12	69005602	69450888	35	3.2715	1
MSK_00006_251469322732_S01_CGH.v4_91.txt	12	60674338	61752761	34	2.9744	1
MSK_00006_251469322732_S01_CGH.v4_91.txt	12	57783438	59906948	119	3.0567	1
MSK_00009_251469322626_S01_CGH.v4_91.txt	12	34257362	34313088	2	-0.6427	-1
MSK_00010_251469322627_S01_CGH.v4_91.txt	12	88622244	88878411	8	-0.6872	-1
MSK_00010_251469322627_S01_CGH.v4_91.txt	12	87253546	87363263	3	0.6872	1
MSK_00010_251469322627_S01_CGH.v4_91.txt	12	85015986	85691886	40	-0.8676	-1
MSK_00010_251469322627_S01_CGH.v4_91.txt	12	83244589	84726562	49	-0.8615	-1
MSK_00011_251469322628_S01_CGH.v4_91.txt	12	112169344	112287209	11	0.7211	1
MSK_00014_251469322629_S01_CGH.v4_91.txt	12	74566455	75807423	57	-0.8639	-1
MSK_00014_251469322629_S01_CGH.v4_91.txt	12	29649498	34756150	235	-0.9287	-1
MSK_00014_251469322629_S01_CGH.v4_91.txt	12	9020831	16144372	516	-0.903	-1
MSK_00038_251469322735_S01_CGH.v4_91.txt	12	10311621	17888245	488	-0.6948	-1
MSK_WGA_0039.1_251469338741_S01_CGH.v4_91.txt	12	128407230	128498521	3	2.7518	1
MSK_WGA_0039.1_251469338741_S01_CGH.v4_91.txt	12	111434437	111479994	6	2.78	1
MSK_WGA_0039.1_251469338741_S01_CGH.v4_91.txt	12	81462714	81600764	15	2.6596	1
MSK_WGA_0039.1_251469338741_S01_CGH.v4_91.txt	12	78546224	78562297	2	2.7114	1
MSK_WGA_0039.1_251469338741_S01_CGH.v4_91.txt	12	69153009	69284898	9	2.8478	1
MSK_WGA_0039.1_251469338741_S01_CGH.v4_91.txt	12	56861431	56877674	3	2.8933	1
MSK_00046_251469322737_S01_CGH.v4_91.txt	12	80739749	80872059	7	-1.0338	-1
MSK_00046_251469322737_S01_CGH.v4_91.txt	12	80714388	80734851	2	4.121	1
MSK_00046_251469322737_S01_CGH.v4_91.txt	12	80611434	80700850	10	-1.0366	-1
MSK_00046_251469322737_S01_CGH.v4_91.txt	12	68650912	68845648	16	-0.8089	-1
MSK_00046_251469322737_S01_CGH.v4_91.txt	12	62364216	63094237	65	-0.7958	-1
MSK_00046_251469322737_S01_CGH.v4_91.txt	12	61005970	61359518	11	-1.024	-1
MSK_00046_251469322737_S01_CGH.v4_91.txt	12	58069216	58629215	35	3.8262	1
MSK_00046_251469322737_S01_CGH.v4_91.txt	12	47372931	49165096	113	-0.7626	-1
MSK_00046_251469322737_S01_CGH.v4_91.txt	12	45069044	47116562	129	-0.7919	-1
MSK_00046_251469322737_S01_CGH.v4_91.txt	12	43637837	43960070	21	2.6523	1
MSK_00046_251469322737_S01_CGH.v4_91.txt	12	42826087	43005992	17	2.5323	1
MSK_00046_251469322737_S01_CGH.v4_91.txt	12	41634082	42812858	64	-0.8068	-1
MSK_00052_251469323033_S01_CGH.v4_91.txt	12	69077506	69293033	17	5.6017	1
MSK_00052_251469323033_S01_CGH.v4_91.txt	12	68748013	68926252	11	5.5143	1
MSK_00055_251469323018_S01_CGH.v4_91.txt	12	12674271	12868234	12	-1.0476	-1
MSK_00060_251469322915_S01_CGH.v4_91.txt	12	71687560	78328520	330	1.2879	1
MSK_00060_251469322915_S01_CGH.v4_91.txt	12	54705067	71670617	1032	1.9247	1
MSK_00060_251469322915_S01_CGH.v4_91.txt	12	45832517	49886981	262	1.4728	1
MSK_00060_251469322915_S01_CGH.v4_91.txt	12	44926469	45586293	48	1.0241	1
MSK_00060_251469322915_S01_CGH.v4_91.txt	12	11513386	11539620	2	1.0288	1
MSK_00069_251469322916_S01_CGH.v4_91.txt	12	39105049	39287752	16	-1.1449	-1
MSK_00069_251469322916_S01_CGH.v4_91.txt	12	4865832	4872405	2	-1.3052	-1
MSK_00071_251469322919_S01_CGH.v4_91.txt	12	69191693	69354696	15	3.9212	1
MSK_00071_251469322919_S01_CGH.v4_91.txt	12	46469478	46623328	8	3.768	1
MSK_00071_251469322919_S01_CGH.v4_91.txt	12	44199445	44220870	4	3.6779	1
MSK_00071_251469322919_S01_CGH.v4_91.txt	12	40951185	41050720	3	3.9873	1
MSK_00071_251469322919_S01_CGH.v4_91.txt	12	40250838	40278119	4	3.8178	1
MSK_00074_251469322717_S01_CGH.v4_91.txt	12	57969687	58479176	41	3.4237	1
MSK_0087.1_251469333362_S01_CGH.v4_91.txt	12	3930993	4819170	55	0.9928	1
MSK_00099_251469322903_S01_CGH.v4_91.txt	12	69981761	70015475	5	4.0256	1
MSK_00099_251469322903_S01_CGH.v4_91.txt	12	69035784	69347063	26	4.0311	1
MSK_00099_251469322903_S01_CGH.v4_91.txt	12	68915949	69003943	8	3.8117	1
MSK_00099_251469322903_S01_CGH.v4_91.txt	12	68343398	68548894	10	3.9724	1
MSK_00099_251469322903_S01_CGH.v4_91.txt	12	67027909	67104358	6	4.0701	1
MSK_00099_251469322903_S01_CGH.v4_91.txt	12	64930207	65312366	25	3.6189	1
MSK_00099_251469322903_S01_CGH.v4_91.txt	12	64444991	64680140	17	4.0784	1
MSK_00099_251469322903_S01_CGH.v4_91.txt	12	63610470	64432436	31	1.3199	1
MSK_00099_251469322903_S01_CGH.v4_91.txt	12	63413457	63588668	9	3.9924	1
MSK_00099_251469322903_S01_CGH.v4_91.txt	12	63078335	63389750	21	4.9372	1
MSK_00099_251469322903_S01_CGH.v4_91.txt	12	62144076	62868552	57	4.0302	1
MSK_00099_251469322903_S01_CGH.v4_91.txt	12	58313157	58339411	4	3.8578	1
MSK_00099_251469322903_S01_CGH.v4_91.txt	12	58158768	58257243	12	1.5061	1
MSK_00099_251469322903_S01_CGH.v4_91.txt	12	58069216	58154016	10	3.9422	1
MSK_00099_251469322903_S01_CGH.v4_91.txt	12	57861005	57958213	18	3.2294	1
MSK_00099_251469322903_S01_CGH.v4_91.txt	12	57676614	57811618	7	2.9092	1
MSK_00102_251469322716_S01_CGH.v4_91.txt	12	71391910	71950366	38	-0.8652	-1
MSK_00102_251469322716_S01_CGH.v4_91.txt	12	69989640	71347992	84	4.1258	1
MSK_00102_251469322716_S01_CGH.v4_91.txt	12	69827339	69981761	12	5.1375	1
MSK_00102_251469322716_S01_CGH.v4_91.txt	12	69642075	69802523	15	4.1881	1
MSK_00102_251469322716_S01_CGH.v4_91.txt	12	69109435	69614654	31	5.7411	1
MSK_00102_251469322716_S01_CGH.v4_91.txt	12	66240148	69103785	159	-0.7409	-1
MSK_00102_251469322716_S01_CGH.v4_91.txt	12	66213612	66232315	3	4.0888	1
MSK_00102_251469322716_S01_CGH.v4_91.txt	12	58313157	66202776	384	-0.8392	-1
MSK_00102_251469322716_S01_CGH.v4_91.txt	12	58115294	58257243	18	4.0749	1
MSK_00102_251469322716_S01_CGH.v4_91.txt	12	38527748	58101958	1411	-0.7334	-1
MSK_WGA_0104.1_251469338880_S01_CGH.v4_91.txt	12	9735469	46668941	1918	-0.7607	-1
MSK_WGA_0104.1_251469338880_S01_CGH.v4_91.txt	12	8608171	9497763	65	-0.7843	-1
MSK_WGA_0104.1_251469338880_S01_CGH.v4_91.txt	12	7078074	8310174	96	-0.8155	-1
MSK_0106.1_251469331471_S01_CGH.v4_91.txt	12	11513386	11539620	2	-1.1812	-1
MSK_00107_251469322719_S01_CGH.v4_91.txt	12	57717539	58257243	63	0.8732	1
MSK_00116_251469322720_S01_CGH.v4_91.txt	12	37873948	37983586	2	1.0941	1
MSK_00116_251469322720_S01_CGH.v4_91.txt	12	31356945	31392964	4	0.7535	1
MSK_00116_251469322720_S01_CGH.v4_91.txt	12	11513386	11539620	2	-1.1511	-1
MSK_0258.1_251469336709_S01_CGH.v4_91.txt	12	71148030	71439095	18	0.6384	1
MSK_0260.1_251469331583_S01_CGH.v4_91.txt	12	85528043	85691886	12	-0.8346	-1
MSK_0266.1_251469336909_S01_CGH.v4_91.txt	12	86963330	90279329	141	-0.8834	-1
MSK_0266.1_251469336909_S01_CGH.v4_91.txt	12	76148019	86665400	574	-0.8964	-1

Continued on next page...

ID	chr	start	end	nroOfprobes	segmean	call
MSK_0266.1_251469336909_S01_CGH.v4_91.txt	12	75683436	76107468	25	1.6544	1
MSK_0266.1_251469336909_S01_CGH.v4_91.txt	12	74077811	75671400	62	-0.8858	-1
MSK_0266.1_251469336909_S01_CGH.v4_91.txt	12	73879477	74055454	7	1.4403	1
MSK_0266.1_251469336909_S01_CGH.v4_91.txt	12	70938332	71249769	24	1.4449	1
MSK_0266.1_251469336909_S01_CGH.v4_91.txt	12	60181368	70832735	585	-0.8581	-1
MSK_0266.1_251469336909_S01_CGH.v4_91.txt	12	58565440	60168852	63	1.2232	1
MSK_0266.1_251469336909_S01_CGH.v4_91.txt	12	57783438	58479176	71	2.5436	1
MSK_0266.1_251469336909_S01_CGH.v4_91.txt	12	57640585	57744187	6	1.2146	1
MSK_0266.1_251469336909_S01_CGH.v4_91.txt	12	57485677	57627832	13	1.9534	1
MSK_0266.1_251469336909_S01_CGH.v4_91.txt	12	56022748	57468471	142	1.172	1
MSK_0266.1_251469336909_S01_CGH.v4_91.txt	12	53593789	54130825	52	0.9017	1
MSK_0266.1_251469336909_S01_CGH.v4_91.txt	12	51442701	51715483	28	0.7113	1
MSK_0266.1_251469336909_S01_CGH.v4_91.txt	12	163593	33310529	2068	1.1611	1
MSK_0269.1_251469333522_S01_CGH.v4_91.txt	12	11513386	11539620	2	-1.3162	-1
MSK_0281.1_251469331472_S01_CGH.v4_91.txt	12	41220522	41223674	2	0.8692	1
MSK_0281.1_251469331472_S01_CGH.v4_91.txt	12	31346226	31392964	5	0.7879	1
MSK_0317.1_251469331584_S01_CGH.v4_91.txt	12	49682098	49685582	2	0.9352	1
MSK_0321.1_251469336723_S01_CGH.v4_91.txt	12	71050556	71551409	32	3.5503	1
MSK_0321.1_251469336723_S01_CGH.v4_91.txt	12	69029607	69802523	53	3.5905	1
MSK_0321.1_251469336723_S01_CGH.v4_91.txt	12	68538088	68629833	11	3.6279	1
MSK_0321.1_251469336723_S01_CGH.v4_91.txt	12	68090833	68343398	8	3.6141	1
MSK_0321.1_251469336723_S01_CGH.v4_91.txt	12	67311058	67983325	29	3.5836	1
MSK_0330.1_251469336713_S01_CGH.v4_91.txt	12	69318803	69657484	18	0.7722	1
MSK_0330.1_251469336713_S01_CGH.v4_91.txt	12	69050993	69309063	20	1.5742	1
MSK_0332.1_251469336711_S01_CGH.v4_91.txt	12	57921837	58230626	36	2.4396	1
MSK_0337.1_251469333524_S01_CGH.v4_91.txt	12	69191693	69269947	6	4.6543	1
MSK_0337.1_251469333524_S01_CGH.v4_91.txt	12	68877446	69050993	16	4.1978	1
MSK_0338.1_251469333564_S01_CGH.v4_91.txt	12	21898257	21909036	2	0.771	1
MSK_0422.1_251469337230_S01_CGH.v4_91.txt	12	86652563	87894085	55	-0.6619	-1
MSK_0422.1_251469337230_S01_CGH.v4_91.txt	12	76816469	86463830	523	-0.7021	-1
MSK_0422.1_251469337230_S01_CGH.v4_91.txt	12	76199996	76294211	6	-0.6885	-1
MSK_0439.1_251469331553_S01_CGH.v4_91.txt	12	46469478	46668941	12	-0.7023	-1
MSK_0439.1_251469331553_S01_CGH.v4_91.txt	12	42903007	42970317	7	-0.6826	-1
MSK_0446.1_251469333525_S01_CGH.v4_91.txt	12	11513386	11539620	2	-1.1919	-1
MSK_0451.1_251469333565_S01_CGH.v4_91.txt	12	89186534	89218109	2	0.9308	1
MSK_00126.1_251469325171_S01_CGH.v4_91.txt	12	11513386	11539620	2	-0.9336	-1
MSK_00128.1_251469325161_S01_CGH.v4_91.txt	12	58079280	58591960	33	3.4319	1
MSK_00128.1_251469325161_S01_CGH.v4_91.txt	12	43947646	43960070	2	-0.8909	-1
MSK_00129.1_251469322907_S01_CGH.v4_91.txt	12	132450967	132598627	15	-0.7354	-1
MSK_00129.1_251469322907_S01_CGH.v4_91.txt	12	131692281	132244904	18	-0.9153	-1
MSK_00129.1_251469322907_S01_CGH.v4_91.txt	12	130912997	131076779	14	-0.8917	-1
MSK_00129.1_251469322907_S01_CGH.v4_91.txt	12	130454810	130554967	8	-0.9253	-1
MSK_00129.1_251469322907_S01_CGH.v4_91.txt	12	129355893	129479215	7	4.4913	1
MSK_00129.1_251469322907_S01_CGH.v4_91.txt	12	127468043	128836586	40	-0.8219	-1
MSK_00129.1_251469322907_S01_CGH.v4_91.txt	12	127174648	127328438	7	-0.9846	-1
MSK_00129.1_251469322907_S01_CGH.v4_91.txt	12	126707903	126940790	10	-0.8712	-1
MSK_00129.1_251469322907_S01_CGH.v4_91.txt	12	124971796	125299921	10	-0.7027	-1
MSK_00129.1_251469322907_S01_CGH.v4_91.txt	12	124612951	124737038	5	1.1412	1
MSK_00129.1_251469322907_S01_CGH.v4_91.txt	12	124151767	124269653	11	1.6418	1
MSK_00129.1_251469322907_S01_CGH.v4_91.txt	12	123568371	123729128	17	0.9249	1
MSK_00129.1_251469322907_S01_CGH.v4_91.txt	12	119277428	119803599	31	-0.8414	-1
MSK_00129.1_251469322907_S01_CGH.v4_91.txt	12	115123750	115181671	3	4.4501	1
MSK_00129.1_251469322907_S01_CGH.v4_91.txt	12	114741236	115105812	12	-0.787	-1
MSK_00129.1_251469322907_S01_CGH.v4_91.txt	12	109799265	110230539	28	-0.727	-1
MSK_00129.1_251469322907_S01_CGH.v4_91.txt	12	108105854	108268416	8	-0.7327	-1
MSK_00129.1_251469322907_S01_CGH.v4_91.txt	12	103806082	104018415	15	-0.8431	-1
MSK_00129.1_251469322907_S01_CGH.v4_91.txt	12	103527280	103606738	7	-0.8878	-1
MSK_00129.1_251469322907_S01_CGH.v4_91.txt	12	100928062	101298379	25	-0.7728	-1
MSK_00129.1_251469322907_S01_CGH.v4_91.txt	12	97894254	98324164	20	-0.8036	-1
MSK_00129.1_251469322907_S01_CGH.v4_91.txt	12	96143871	96253592	11	-0.6694	-1
MSK_00129.1_251469322907_S01_CGH.v4_91.txt	12	94660321	95306840	33	-0.6336	-1
MSK_00129.1_251469322907_S01_CGH.v4_91.txt	12	93237034	93469229	12	-0.7916	-1
MSK_00129.1_251469322907_S01_CGH.v4_91.txt	12	91578735	92468502	23	-0.788	-1
MSK_00129.1_251469322907_S01_CGH.v4_91.txt	12	91320614	91336168	3	4.6092	1
MSK_00129.1_251469322907_S01_CGH.v4_91.txt	12	90542726	91090723	12	-0.7843	-1
MSK_00129.1_251469322907_S01_CGH.v4_91.txt	12	88466065	88917000	23	-0.8365	-1
MSK_00129.1_251469322907_S01_CGH.v4_91.txt	12	82951468	83118374	7	-0.8544	-1
MSK_00129.1_251469322907_S01_CGH.v4_91.txt	12	80714388	81493912	48	-0.8068	-1
MSK_00129.1_251469322907_S01_CGH.v4_91.txt	12	75617499	76920379	75	-0.7452	-1
MSK_00129.1_251469322907_S01_CGH.v4_91.txt	12	75158010	75424084	7	-0.6506	-1
MSK_00129.1_251469322907_S01_CGH.v4_91.txt	12	72971524	73026570	5	-0.845	-1
MSK_00129.1_251469322907_S01_CGH.v4_91.txt	12	71950366	72874417	60	-0.7018	-1
MSK_00129.1_251469322907_S01_CGH.v4_91.txt	12	70107961	70178509	6	1.3941	1
MSK_00129.1_251469322907_S01_CGH.v4_91.txt	12	66562822	68372468	90	-0.799	-1
MSK_00129.1_251469322907_S01_CGH.v4_91.txt	12	66107973	66264851	18	-0.8443	-1
MSK_00129.1_251469322907_S01_CGH.v4_91.txt	12	65151924	65919364	49	-0.7508	-1
MSK_00129.1_251469322907_S01_CGH.v4_91.txt	12	62327908	63850935	100	-0.7757	-1
MSK_00129.1_251469322907_S01_CGH.v4_91.txt	12	59435802	61300950	56	-0.8256	-1
MSK_00129.1_251469322907_S01_CGH.v4_91.txt	12	58479176	59106645	22	-0.6989	-1
MSK_00129.1_251469322907_S01_CGH.v4_91.txt	12	58200223	58459020	17	1.3165	1
MSK_00129.1_251469322907_S01_CGH.v4_91.txt	12	58101958	58186836	11	4.2623	1
MSK_00129.1_251469322907_S01_CGH.v4_91.txt	12	57921837	58092061	20	0.6516	1
MSK_00129.1_251469322907_S01_CGH.v4_91.txt	12	57783438	57912112	22	1.3538	1
MSK_00129.1_251469322907_S01_CGH.v4_91.txt	12	57640585	57744187	6	4.2513	1
MSK_00129.1_251469322907_S01_CGH.v4_91.txt	12	54969140	55050461	10	-0.683	-1
MSK_00129.1_251469322907_S01_CGH.v4_91.txt	12	51913343	52550063	45	-0.6884	-1
MSK_00129.1_251469322907_S01_CGH.v4_91.txt	12	50801245	51127870	29	1.738	1
MSK_00129.1_251469322907_S01_CGH.v4_91.txt	12	48057274	48366961	27	-0.8207	-1
MSK_00129.1_251469322907_S01_CGH.v4_91.txt	12	46161236	46180029	3	1.6131	1
MSK_00129.1_251469322907_S01_CGH.v4_91.txt	12	45619934	45945968	26	-0.7826	-1
MSK_00129.1_251469322907_S01_CGH.v4_91.txt	12	42554178	44238388	81	-0.7019	-1
MSK_00129.1_251469322907_S01_CGH.v4_91.txt	12	39213726	39363979	9	-0.641	-1
MSK_00129.1_251469322907_S01_CGH.v4_91.txt	12	34164347	34387199	8	-0.7791	-1
MSK_00129.1_251469322907_S01_CGH.v4_91.txt	12	32318906	32598017	19	0.7134	1
MSK_00129.1_251469322907_S01_CGH.v4_91.txt	12	30493844	31183253	28	-0.8019	-1
MSK_00129.1_251469322907_S01_CGH.v4_91.txt	12	21511315	21644501	14	-0.6375	-1
MSK_00129.1_251469322907_S01_CGH.v4_91.txt	12	20643583	21356582	54	-0.7167	-1
MSK_00129.1_251469322907_S01_CGH.v4_91.txt	12	17146091	17495103	9	-0.7755	-1
MSK_00129.1_251469322907_S01_CGH.v4_91.txt	12	15040826	16012974	73	-0.7418	-1
MSK_00129.1_251469322907_S01_CGH.v4_91.txt	12	14501752	14834290	28	-0.6824	-1
MSK_00129.1_251469322907_S01_CGH.v4_91.txt	12	12599418	13040479	38	-0.6452	-1
MSK_00129.1_251469322907_S01_CGH.v4_91.txt	12	10703177	10862802	17	-0.7732	-1
MSK_00129.1_251469322907_S01_CGH.v4_91.txt	12	8884892	9241766	27	-0.6605	-1
MSK_00129.1_251469322907_S01_CGH.v4_91.txt	12	8740418	8844275	7	-0.7053	-1
MSK_00129.1_251469322907_S01_CGH.v4_91.txt	12	7480237	7514300	3	-0.7481	-1
MSK_00129.1_251469322907_S01_CGH.v4_91.txt	12	3818353	3867619	6	1.2171	1
MSK_00129.1_251469322907_S01_CGH.v4_91.txt	12	3374336	3483345	7	-0.9444	-1
MSK_00133_251469325752_S01_CGH.v4_91.txt	12	11513386	11539620	2	0.7158	1
MSK_00137.4_251469325162_S01_CGH.v4_91.txt	12	81577021	81600764	4	-0.9978	-1
MSK_00139.1_251469325164_S01_CGH.v4_91.txt	12	94147321	94171947	3	-0.8037	-1
MSK_0149.1_251469336715_S01_CGH.v4_91.txt	12	17373342	17571573	6	-0.818	-1
MSK_0152.1_251469337789_S01_CGH.v4_91.txt	12	99962821	100071709	10	-0.8078	-1
MSK_0152.1_251469337789_S01_CGH.v4_91.txt	12	83505976	83559728	3	0.9701	1
MSK_0152.1_251469337789_S01_CGH.v4_91.txt	12	70610813	70672013	6	5.6901	1
MSK_0152.1_251469337789_S01_CGH.v4_91.txt	12	69756561	70015475	21	5.4986	1
MSK_0152.1_251469337789_S01_CGH.v4_91.txt	12	69694860	69751850	6	1.4009	1
MSK_0152.1_251469337789_S01_CGH.v4_91.txt	12	69642075	69664161	4	5.6878	1
MSK_0152.1_251469337789_S01_CGH.v4_91.txt	12	69269947	69450888	14	1.0024	1
MSK_0152.1_251469337789_S01_CGH.v4_91.txt	12	69121126	69257563	10	5.8526	1
MSK_0152.1_251469337789_S01_CGH.v4_91.txt	12	67233917	67377488	6	0.881	1
MSK_0152.1_251469337789_S01_CGH.v4_91.txt	12	65604663	65632426	3	5.6765	1
MSK_0152.1_251469337789_S01_CGH.v4_91.txt	12	64515975	64699585	12	6.0671	1
MSK_0152.1_251469337789_S01_CGH.v4_91.txt	12	62459847	62567274	10	0.962	1
MSK_0152.1_251469337789_S01_CGH.v4_91.txt	12	58139960	58168949	6	5.0954	1

Continued on next page...

ID	chr	start	end	nroOfprobes	segmean	call
MSK_0157.1_251469329178_S01_CGH.v4_91.txt	12	124327765	124350304	3	-0.9825	-1
MSK_0157.1_251469329178_S01_CGH.v4_91.txt	12	58339411	58709249	13	2.6456	1
MSK_0157.1_251469329178_S01_CGH.v4_91.txt	12	58003407	58334813	31	3.4793	1
MSK_0157.1_251469329178_S01_CGH.v4_91.txt	12	57912112	57997061	12	2.465	1
MSK_0157.1_251469329178_S01_CGH.v4_91.txt	12	3259072	4423802	70	2.1601	1
MSK_0162.1_251469336905_S01_CGH.v4_91.txt	12	74949059	75237648	5	0.8237	1
MSK_0162.1_251469336905_S01_CGH.v4_91.txt	12	73171928	73267362	4	0.7653	1
MSK_0162.1_251469336905_S01_CGH.v4_91.txt	12	73075286	73109595	3	3.326	1
MSK_0162.1_251469336905_S01_CGH.v4_91.txt	12	72539532	73068013	36	4.091	1
MSK_0162.1_251469336905_S01_CGH.v4_91.txt	12	72401652	72492985	7	3.569	1
MSK_0162.1_251469336905_S01_CGH.v4_91.txt	12	72188972	72388322	15	1.8843	1
MSK_0162.1_251469336905_S01_CGH.v4_91.txt	12	71569617	71720829	9	1.9091	1
MSK_0162.1_251469336905_S01_CGH.v4_91.txt	12	70704645	71551409	51	0.6556	1
MSK_0162.1_251469336905_S01_CGH.v4_91.txt	12	68775835	69293033	40	2.9932	1
MSK_0162.1_251469336905_S01_CGH.v4_91.txt	12	67076274	68564885	63	2.2794	1
MSK_0162.1_251469336905_S01_CGH.v4_91.txt	12	66689811	67062346	30	3.3199	1
MSK_0162.1_251469336905_S01_CGH.v4_91.txt	12	66478344	66648976	13	2.7149	1
MSK_0162.1_251469336905_S01_CGH.v4_91.txt	12	66178358	66196255	4	1.1014	1
MSK_0162.1_251469336905_S01_CGH.v4_91.txt	12	65901241	66069850	11	1.0986	1
MSK_0162.1_251469336905_S01_CGH.v4_91.txt	12	65151924	65857603	47	2.3553	1
MSK_0162.1_251469336905_S01_CGH.v4_91.txt	12	65096005	65140569	5	1.0233	1
MSK_0162.1_251469336905_S01_CGH.v4_91.txt	12	65013102	65078601	4	2.6602	1
MSK_0162.1_251469336905_S01_CGH.v4_91.txt	12	64680140	64991622	19	1.8408	1
MSK_0162.1_251469336905_S01_CGH.v4_91.txt	12	64515975	64669163	10	2.5163	1
MSK_0162.1_251469336905_S01_CGH.v4_91.txt	12	64240554	64497916	21	2.9027	1
MSK_0162.1_251469336905_S01_CGH.v4_91.txt	12	64055954	64199093	7	1.9241	1
MSK_0162.1_251469336905_S01_CGH.v4_91.txt	12	63691824	63947732	8	2.7002	1
MSK_0162.1_251469336905_S01_CGH.v4_91.txt	12	59612211	59851910	6	2.0158	1
MSK_0162.1_251469336905_S01_CGH.v4_91.txt	12	57958213	59543464	83	2.973	1
MSK_0173.1_251469330461_S01_CGH.v4_91.txt	12	69279573	69392739	10	-0.6374	-1
MSK_0173.1_251469330461_S01_CGH.v4_91.txt	12	69136361	69269947	9	5.9373	1
MSK_0173.1_251469330461_S01_CGH.v4_91.txt	12	69003943	69128502	14	-0.6979	-1
MSK_0173.1_251469330461_S01_CGH.v4_91.txt	12	68405197	68818839	32	-0.7447	-1
MSK_0173.1_251469330461_S01_CGH.v4_91.txt	12	67689672	68185230	21	-0.8046	-1
MSK_0173.1_251469330461_S01_CGH.v4_91.txt	12	66901439	67027909	12	-0.8202	-1
MSK_0173.1_251469330461_S01_CGH.v4_91.txt	12	66648976	66788272	10	-0.7277	-1
MSK_0173.1_251469330461_S01_CGH.v4_91.txt	12	66092929	66264851	19	-0.8343	-1
MSK_0173.1_251469330461_S01_CGH.v4_91.txt	12	66011023	66069850	5	5.9532	1
MSK_0173.1_251469330461_S01_CGH.v4_91.txt	12	61428699	64521330	172	-0.7774	-1
MSK_0173.1_251469330461_S01_CGH.v4_91.txt	12	61300950	61403869	3	5.7302	1
MSK_0173.1_251469330461_S01_CGH.v4_91.txt	12	59075529	61242130	75	-0.6422	-1
MSK_0173.1_251469330461_S01_CGH.v4_91.txt	12	58752602	59055024	9	2.6562	1
MSK_0173.1_251469330461_S01_CGH.v4_91.txt	12	58125890	58235206	15	3.4197	1
MSK_0173.1_251469330461_S01_CGH.v4_91.txt	12	57948906	58115294	16	0.9287	1
MSK_0173.1_251469330461_S01_CGH.v4_91.txt	12	57871624	57921837	9	4.3788	1
MSK_0173.1_251469330461_S01_CGH.v4_91.txt	12	57570668	57866181	26	0.8744	1
MSK_0173.1_251469330461_S01_CGH.v4_91.txt	12	55519787	55789222	24	-0.7989	-1
MSK_0173.1_251469330461_S01_CGH.v4_91.txt	12	47707209	47862888	2	4.2748	1
MSK_0173.1_251469330461_S01_CGH.v4_91.txt	12	47051643	47068155	2	5.8766	1
MSK_0173.1_251469330461_S01_CGH.v4_91.txt	12	46737959	46986552	15	-0.8138	-1
MSK_0173.1_251469330461_S01_CGH.v4_91.txt	12	46623328	46721743	7	5.7385	1
MSK_0173.1_251469330461_S01_CGH.v4_91.txt	12	46161236	46601229	26	-0.7672	-1
MSK_0173.1_251469330461_S01_CGH.v4_91.txt	12	46068039	46146012	6	0.9205	1
MSK_0173.1_251469330461_S01_CGH.v4_91.txt	12	45775524	46047112	16	5.8306	1
MSK_0173.1_251469330461_S01_CGH.v4_91.txt	12	43715174	45761461	145	-0.8171	-1
MSK_0174.1_251469330284_S01_CGH.v4_91.txt	12	69642075	69694860	5	0.7039	1
MSK_0174.1_251469330284_S01_CGH.v4_91.txt	12	69500737	69614654	6	3.0001	1
MSK_0174.1_251469330284_S01_CGH.v4_91.txt	12	69347063	69450888	6	5.3009	1
MSK_0174.1_251469330284_S01_CGH.v4_91.txt	12	69191693	69336208	13	6.0922	1
MSK_0174.1_251469330284_S01_CGH.v4_91.txt	12	69103785	69153009	7	4.8845	1
MSK_0174.1_251469330284_S01_CGH.v4_91.txt	12	64610281	69077506	265	-0.9038	-1
MSK_0174.1_251469330284_S01_CGH.v4_91.txt	12	64545790	64588486	3	3.2857	1
MSK_0174.1_251469330284_S01_CGH.v4_91.txt	12	64183868	64521330	26	-0.9188	-1
MSK_0174.1_251469330284_S01_CGH.v4_91.txt	12	61459381	63947732	141	-0.9217	-1
MSK_0174.1_251469330284_S01_CGH.v4_91.txt	12	61152793	61428699	7	2.0135	1
MSK_0174.1_251469330284_S01_CGH.v4_91.txt	12	58357953	61129383	95	-0.9798	-1
MSK_0174.1_251469330284_S01_CGH.v4_91.txt	12	58242482	58339411	6	3.4101	1
MSK_0174.1_251469330284_S01_CGH.v4_91.txt	12	57997061	58235206	27	4.5525	1
MSK_0174.1_251469330284_S01_CGH.v4_91.txt	12	57717539	57988882	34	3.4475	1
MSK_0175.1_251469336725_S01_CGH.v4_91.txt	12	82874631	83520188	37	-0.6707	-1
MSK_0175.1_251469336725_S01_CGH.v4_91.txt	12	9735469	18471000	562	-0.6539	-1
MSK_0177.1_251469333358_S01_CGH.v4_91.txt	12	71347992	71509916	6	3.3761	1
MSK_0177.1_251469333358_S01_CGH.v4_91.txt	12	71221977	71249769	3	3.2573	1
MSK_0177.1_251469333358_S01_CGH.v4_91.txt	12	71012436	71216003	14	2.7421	1
MSK_0177.1_251469333358_S01_CGH.v4_91.txt	12	70464916	70689691	11	2.9405	1
MSK_0177.1_251469333358_S01_CGH.v4_91.txt	12	69995267	70223360	20	3.1736	1
MSK_0177.1_251469333358_S01_CGH.v4_91.txt	12	69546489	69895635	24	3.2337	1
MSK_0177.1_251469333358_S01_CGH.v4_91.txt	12	69191693	69241143	4	4.9326	1
MSK_0177.1_251469333358_S01_CGH.v4_91.txt	12	68807554	68971415	11	4.9322	1
MSK_0177.1_251469333358_S01_CGH.v4_91.txt	12	67269436	67519392	11	3.4691	1
MSK_0177.1_251469333358_S01_CGH.v4_91.txt	12	67163538	67233917	4	5.1361	1
MSK_0177.1_251469333358_S01_CGH.v4_91.txt	12	66841204	66857245	3	2.9477	1
MSK_0177.1_251469333358_S01_CGH.v4_91.txt	12	66213612	66478344	14	2.8586	1
MSK_0177.1_251469333358_S01_CGH.v4_91.txt	12	65702345	65756764	6	3.1098	1
MSK_0177.1_251469333358_S01_CGH.v4_91.txt	12	65471182	65557000	7	4.9368	1
MSK_0177.1_251469333358_S01_CGH.v4_91.txt	12	64712841	64868025	12	3.9205	1
MSK_0177.1_251469333358_S01_CGH.v4_91.txt	12	64344494	64361243	3	5.1695	1
MSK_0177.1_251469333358_S01_CGH.v4_91.txt	12	62979117	63094237	15	3.363	1
MSK_0177.1_251469333358_S01_CGH.v4_91.txt	12	62483861	62500913	3	3.1977	1
MSK_0177.1_251469333358_S01_CGH.v4_91.txt	12	62193749	62267513	7	4.8453	1
MSK_0177.1_251469333358_S01_CGH.v4_91.txt	12	61091958	62173328	36	0.8432	1
MSK_0177.1_251469333358_S01_CGH.v4_91.txt	12	60975888	61056959	6	4.8368	1
MSK_0177.1_251469333358_S01_CGH.v4_91.txt	12	59290447	59307732	4	5.0276	1
MSK_0177.1_251469333358_S01_CGH.v4_91.txt	12	58591960	58734484	7	3.9685	1
MSK_0177.1_251469333358_S01_CGH.v4_91.txt	12	58139960	58257243	16	4.7894	1
MSK_0177.1_251469333358_S01_CGH.v4_91.txt	12	57861005	57912112	10	3.2295	1
MSK_0177.1_251469333358_S01_CGH.v4_91.txt	12	57233907	57403830	14	4.9575	1
MSK_0177.1_251469333358_S01_CGH.v4_91.txt	12	56277519	56303379	4	4.927	1
MSK_0177.1_251469333358_S01_CGH.v4_91.txt	12	56135092	56143565	3	3.4629	1
MSK_0177.1_251469333358_S01_CGH.v4_91.txt	12	55794260	55919934	16	2.7573	1
MSK_0177.1_251469333358_S01_CGH.v4_91.txt	12	55734542	55789222	5	4.8188	1
MSK_0177.1_251469333358_S01_CGH.v4_91.txt	12	55589411	55627771	5	2.6535	1
MSK_0177.1_251469333358_S01_CGH.v4_91.txt	12	55192723	55302884	8	4.5545	1
MSK_0177.1_251469333358_S01_CGH.v4_91.txt	12	54476879	54598301	7	3.0131	1
MSK_0177.1_251469333358_S01_CGH.v4_91.txt	12	54354005	54462999	15	5.1411	1
MSK_0177.1_251469333358_S01_CGH.v4_91.txt	12	54101839	54347286	14	3.357	1
MSK_0177.1_251469333358_S01_CGH.v4_91.txt	12	53151159	53416224	29	3.7542	1
MSK_0177.1_251469333358_S01_CGH.v4_91.txt	12	52905085	53146793	28	1.2123	1
MSK_0177.1_251469333358_S01_CGH.v4_91.txt	12	52669810	52892550	26	4.858	1
MSK_0177.1_251469333358_S01_CGH.v4_91.txt	12	52517178	52628042	10	2.9741	1
MSK_0177.1_251469333358_S01_CGH.v4_91.txt	12	52380943	52426511	8	5.0902	1
MSK_0177.1_251469333358_S01_CGH.v4_91.txt	12	52173991	52370191	16	3.3074	1
MSK_0177.1_251469333358_S01_CGH.v4_91.txt	12	51880647	52067220	11	3.1165	1
MSK_0178.1_251469329154_S01_CGH.v4_91.txt	12	11513386	11539620	2	-1.0297	-1
MSK_0179.1_251469333566_S01_CGH.v4_91.txt	12	21565934	21580444	2	-0.9493	-1
MSK_0179.1_251469333566_S01_CGH.v4_91.txt	12	11513386	11539620	2	-1.5864	-1
MSK_0182.1_251469331464_S01_CGH.v4_91.txt	12	81999798	82005348	2	-1.0585	-1
MSK_0182.1_251469331464_S01_CGH.v4_91.txt	12	72719768	72836609	9	-0.9211	-1
MSK_0182.1_251469331464_S01_CGH.v4_91.txt	12	70804662	71012436	10	3.2	1
MSK_0182.1_251469331464_S01_CGH.v4_91.txt	12	70768530	70794059	3	-0.9269	-1
MSK_0182.1_251469331464_S01_CGH.v4_91.txt	12	70539442	70754323	15	3.5963	1
MSK_0182.1_251469331464_S01_CGH.v4_91.txt	12	69746040	70515973	53	-0.7921	-1
MSK_0182.1_251469331464_S01_CGH.v4_91.txt	12	69409718	69664161	13	3.6251	1
MSK_0182.1_251469331464_S01_CGH.v4_91.txt	12	69050993	69392739	26	5.526	1

Continued on next page...

ID	chr	start	end	nroOfprobes	segmean	call
MSK_0182.1_251469331464_S01_CGH.v4_91.txt	12	68998145	69045795	8	3.5721	1
MSK_0182.1_251469331464_S01_CGH.v4_91.txt	12	67960348	68971415	58	-0.8646	-1
MSK_0182.1_251469331464_S01_CGH.v4_91.txt	12	67771223	67923107	5	1.5489	1
MSK_0182.1_251469331464_S01_CGH.v4_91.txt	12	67604008	67725945	10	4.8427	1
MSK_0182.1_251469331464_S01_CGH.v4_91.txt	12	67104358	67581627	19	3.2182	1
MSK_0182.1_251469331464_S01_CGH.v4_91.txt	12	66689811	67082368	32	-0.8336	-1
MSK_0182.1_251469331464_S01_CGH.v4_91.txt	12	66605320	66648976	5	3.4986	1
MSK_0182.1_251469331464_S01_CGH.v4_91.txt	12	66126955	66572501	31	-0.8547	-1
MSK_0182.1_251469331464_S01_CGH.v4_91.txt	12	66011023	66112680	8	1.5332	1
MSK_0182.1_251469331464_S01_CGH.v4_91.txt	12	65901241	65988835	6	4.8782	1
MSK_0182.1_251469331464_S01_CGH.v4_91.txt	12	65013102	65857603	56	-0.833	-1
MSK_0182.1_251469331464_S01_CGH.v4_91.txt	12	64727134	64991622	16	2.2738	1
MSK_0182.1_251469331464_S01_CGH.v4_91.txt	12	64398631	64712841	24	5.4406	1
MSK_0182.1_251469331464_S01_CGH.v4_91.txt	12	62538599	64361243	103	-0.7469	-1
MSK_0182.1_251469331464_S01_CGH.v4_91.txt	12	62400136	62524473	11	2.7743	1
MSK_0182.1_251469331464_S01_CGH.v4_91.txt	12	62038772	62387168	26	-0.8547	-1
MSK_0182.1_251469331464_S01_CGH.v4_91.txt	12	61653231	61974158	12	5.3193	1
MSK_0182.1_251469331464_S01_CGH.v4_91.txt	12	60338750	61638823	35	-0.8715	-1
MSK_0182.1_251469331464_S01_CGH.v4_91.txt	12	60199266	60304894	3	4.346	1
MSK_0182.1_251469331464_S01_CGH.v4_91.txt	12	59543464	60181368	24	-0.8381	-1
MSK_0182.1_251469331464_S01_CGH.v4_91.txt	12	59244767	59516660	15	4.8404	1
MSK_0182.1_251469331464_S01_CGH.v4_91.txt	12	58174446	59208299	45	-0.7291	-1
MSK_0182.1_251469331464_S01_CGH.v4_91.txt	12	58034214	58168949	13	4.5368	1
MSK_0182.1_251469331464_S01_CGH.v4_91.txt	12	57939182	58025318	12	-0.7383	-1
MSK_0182.1_251469331464_S01_CGH.v4_91.txt	12	57861005	57935573	14	3.2396	1
MSK_0182.1_251469331464_S01_CGH.v4_91.txt	12	56510821	57844861	114	-0.7899	-1
MSK_0182.1_251469331464_S01_CGH.v4_91.txt	12	56467393	56498888	3	1.5062	1
MSK_0182.1_251469331464_S01_CGH.v4_91.txt	12	55525168	56456048	103	-0.7889	-1
MSK_0182.1_251469331464_S01_CGH.v4_91.txt	12	55468272	55519787	4	3.2516	1
MSK_0182.1_251469331464_S01_CGH.v4_91.txt	12	55274474	55335008	5	-0.9526	-1
MSK_0182.1_251469331464_S01_CGH.v4_91.txt	12	55011735	55029636	3	-0.8703	-1
MSK_0182.1_251469331464_S01_CGH.v4_91.txt	12	54739001	54751886	4	-0.8183	-1
MSK_0185.1_251469329754_S01_CGH.v4_91.txt	12	124454986	125877320	67	0.803	1
MSK_0187.1_251469330462_S01_CGH.v4_91.txt	12	129783584	131975367	148	-0.7708	-1
MSK_0187.1_251469330462_S01_CGH.v4_91.txt	12	80611434	80700850	10	-1.1663	-1
MSK_0187.1_251469330462_S01_CGH.v4_91.txt	12	75237648	75278126	2	2.2165	1
MSK_0187.1_251469330462_S01_CGH.v4_91.txt	12	69191693	70832735	106	4.9002	1
MSK_0187.1_251469330462_S01_CGH.v4_91.txt	12	69109435	69153009	6	2.0396	1
MSK_0187.1_251469330462_S01_CGH.v4_91.txt	12	68538088	68564885	4	2.1675	1
MSK_0187.1_251469330462_S01_CGH.v4_91.txt	12	66689811	67923107	66	2.4801	1
MSK_0187.1_251469330462_S01_CGH.v4_91.txt	12	66112680	66317012	20	2.0827	1
MSK_0187.1_251469330462_S01_CGH.v4_91.txt	12	65224807	65257890	4	2.5236	1
MSK_0187.1_251469330462_S01_CGH.v4_91.txt	12	60883724	60909141	2	2.0527	1
MSK_0190.1_251469330591_S01_CGH.v4_91.txt	12	11513386	11539620	2	-1.2439	-1
MSK_0195.1_251469329772_S01_CGH.v4_91.txt	12	9735469	50152114	2160	-0.7882	-1
MSK_0195.1_251469329772_S01_CGH.v4_91.txt	12	163593	9497763	664	-0.7475	-1
MSK_0208.1_251469329182_S01_CGH.v4_91.txt	12	83156313	83186520	5	-0.9743	-1
MSK_0208.2_251469329755_S01_CGH.v4_91.txt	12	83156313	83186520	5	-1.0169	-1
MSK_0209.1_251469330463_S01_CGH.v4_91.txt	12	69071794	69962862	58	5.241	1
MSK_0209.1_251469330463_S01_CGH.v4_91.txt	12	58115294	58186836	10	5.6618	1
MSK_0209.1_251469330463_S01_CGH.v4_91.txt	12	19592050	19683074	8	5.7685	1
MSK_0211.2_251469329773_S01_CGH.v4_91.txt	12	129064186	130520009	105	-0.7482	-1
MSK_0211.2_251469329773_S01_CGH.v4_91.txt	12	77240026	77245285	2	-0.9416	-1
MSK_0214.1_251469329168_S01_CGH.v4_91.txt	12	69121126	69354696	20	1.496	1
MSK_0221.1_251469330192_S01_CGH.v4_91.txt	12	131561835	131674156	8	1.506	1
MSK_0221.1_251469330192_S01_CGH.v4_91.txt	12	127968232	128135593	7	1.8612	1
MSK_0221.1_251469330192_S01_CGH.v4_91.txt	12	123041378	123214030	15	0.8935	1
MSK_0221.1_251469330192_S01_CGH.v4_91.txt	12	114126586	114587218	27	1.8404	1
MSK_0221.1_251469330192_S01_CGH.v4_91.txt	12	96317310	96385364	5	1.5658	1
MSK_0221.1_251469330192_S01_CGH.v4_91.txt	12	76640073	76908977	26	1.1773	1
MSK_0221.1_251469330192_S01_CGH.v4_91.txt	12	71986856	72355558	27	1.8094	1
MSK_0221.1_251469330192_S01_CGH.v4_91.txt	12	68793573	68933776	9	1.3296	1
MSK_0221.1_251469330192_S01_CGH.v4_91.txt	12	64091277	64561751	31	0.7502	1
MSK_0221.1_251469330192_S01_CGH.v4_91.txt	12	63889089	64055954	5	1.2645	1
MSK_0221.1_251469330192_S01_CGH.v4_91.txt	12	58698888	59635040	39	1.3925	1
MSK_0221.1_251469330192_S01_CGH.v4_91.txt	12	58101958	58339411	23	2.0859	1
MSK_0221.1_251469330192_S01_CGH.v4_91.txt	12	57877356	58092061	25	1.587	1
MSK_0221.1_251469330192_S01_CGH.v4_91.txt	12	55574629	55652936	9	1.0213	1
MSK_0221.1_251469330192_S01_CGH.v4_91.txt	12	54825566	54929841	8	1.5431	1
MSK_0221.1_251469330192_S01_CGH.v4_91.txt	12	51675447	52517178	64	1.6433	1
MSK_0221.1_251469330192_S01_CGH.v4_91.txt	12	50370384	51097891	62	1.5687	1
MSK_0221.1_251469330192_S01_CGH.v4_91.txt	12	38614239	38935907	10	0.9197	1
MSK_0221.1_251469330192_S01_CGH.v4_91.txt	12	31392964	31660960	18	1.2992	1
MSK_0221.1_251469330192_S01_CGH.v4_91.txt	12	21471727	21880916	38	1.2556	1
MSK_0221.1_251469330192_S01_CGH.v4_91.txt	12	16739078	16785646	4	1.0251	1
MSK_0221.1_251469330192_S01_CGH.v4_91.txt	12	13070906	13186898	12	1.5506	1
MSK_0221.1_251469330192_S01_CGH.v4_91.txt	12	10114069	10357913	27	1.2458	1
MSK_0221.1_251469330192_S01_CGH.v4_91.txt	12	6536343	7382028	85	0.7056	1
MSK_0221.1_251469330192_S01_CGH.v4_91.txt	12	3953990	5811257	113	1.3453	1
MSK_0238.1_251469340788_S01_CGH.v4_91.txt	12	57861005	58369517	56	3.1935	1
MSK_0241.1_251469329774_S01_CGH.v4_91.txt	12	78157790	83347575	336	-0.8629	-1
MSK_0241.1_251469329774_S01_CGH.v4_91.txt	12	11513386	11539620	2	-1.0793	-1
MSK_0241.1_251469329774_S01_CGH.v4_91.txt	12	8013738	8117977	10	-0.6585	-1
MSK_0394.1_251469336716_S01_CGH.v4_91.txt	12	11513386	11539620	2	-1.0836	-1
MSK_0402.1_251469336906_S01_CGH.v4_91.txt	12	57877356	58406759	52	3.2621	1
MSK_0409.1_251469331555_S01_CGH.v4_91.txt	12	57717539	59950173	122	1.7446	1
MSK_0410.1_251469333359_S01_CGH.v4_91.txt	12	46104165	46230365	9	-1.2035	-1
MSK_0412.1_251469331490_S01_CGH.v4_91.txt	12	57811618	58655482	72	0.7385	1
MSK_0648.1_251469338021_S01_CGH.v4_91.txt	12	69614654	69731033	8	1.4714	1
MSK_0648.1_251469338021_S01_CGH.v4_91.txt	12	69500737	69603754	5	5.1376	1
MSK_0648.1_251469338021_S01_CGH.v4_91.txt	12	69191693	69336208	13	5.0852	1
MSK_0648.1_251469338021_S01_CGH.v4_91.txt	12	69103785	69153009	7	-0.8248	-1
MSK_0648.1_251469338021_S01_CGH.v4_91.txt	12	69035784	69077506	5	3.8783	1
MSK_0648.1_251469338021_S01_CGH.v4_91.txt	12	68807554	69029607	17	-0.663	-1
MSK_0648.1_251469338021_S01_CGH.v4_91.txt	12	68755553	68793573	3	5.1435	1
MSK_0648.1_251469338021_S01_CGH.v4_91.txt	12	68482726	68748013	22	-0.688	-1
MSK_0648.1_251469338021_S01_CGH.v4_91.txt	12	68405197	68474277	5	4.8701	1
MSK_0648.1_251469338021_S01_CGH.v4_91.txt	12	68185230	68372468	6	-0.6414	-1
MSK_0648.1_251469338021_S01_CGH.v4_91.txt	12	68070195	68141857	4	5.0507	1
MSK_0648.1_251469338021_S01_CGH.v4_91.txt	12	67555171	67794618	14	5.1045	1
MSK_WGA_0244.1_251469338881_S01_CGH.v4_91.txt	12	54439060	54462999	4	-0.7695	-1
MSK_WGA_0347.1_251469338868_S01_CGH.v4_91.txt	12	60883724	61034785	8	-0.633	-1
MSK_WGA_0348.1_251469338869_S01_CGH.v4_91.txt	12	9735469	21171894	704	-0.6736	-1
MSK_WGA_0348.1_251469338869_S01_CGH.v4_91.txt	12	9075867	9497763	30	-0.703	-1
MSK_WGA_0357.1_251469337522_S01_CGH.v4_91.txt	12	100723721	100904678	13	-0.6376	-1
MSK_WGA_0389.1_251469338874_S01_CGH.v4_91.txt	12	6789435	7048968	28	0.6535	1
MSK_WGA_0390.1_251469338875_S01_CGH.v4_91.txt	12	34427533	34756150	4	0.7167	1
MSK_WGA_0390.1_251469338875_S01_CGH.v4_91.txt	12	6425743	6444786	4	0.8069	1
MSK_0512.1_251469331556_S01_CGH.v4_91.txt	12	11513386	11539620	2	-1.195	-1
MSK_0516.1_251469331469_S01_CGH.v4_91.txt	12	11553454	31374385	1134	0.6962	1
MSK_0516.1_251469331469_S01_CGH.v4_91.txt	12	8608171	11502206	213	0.6564	1
MSK_0516.1_251469331469_S01_CGH.v4_91.txt	12	163593	8310174	596	0.686	1
MSK_0518.1_251469337014_S01_CGH.v4_91.txt	12	10941170	17541875	432	-0.8877	-1
MSK_0520.1_251469336708_S01_CGH.v4_91.txt	12	98937709	99154171	19	-0.7016	-1
MSK_0521.1_251469331582_S01_CGH.v4_91.txt	12	122598997	122603314	2	-0.9178	-1
MSK_0522.1_251469336908_S01_CGH.v4_91.txt	12	62261096	62267513	2	-0.6668	-1
MSK_0531.1_251469337227_S01_CGH.v4_91.txt	12	57336270	57374407	4	-0.9152	-1
MSK_0616.1_251469338022_S01_CGH.v4_91.txt	12	88418868	88546778	14	5.9476	1
MSK_0616.1_251469338022_S01_CGH.v4_91.txt	12	69191693	69279573	7	5.6966	1
MSK_0616.1_251469338022_S01_CGH.v4_91.txt	12	58969423	59021245	2	6.1424	1
MSK_0616.1_251469338022_S01_CGH.v4_91.txt	12	58139960	58145773	3	6.236	1
MSK_0616.1_251469338022_S01_CGH.v4_91.txt	12	57907421	58125890	25	1.3556	1
MSK_0616.1_251469338022_S01_CGH.v4_91.txt	12	52669810	52814707	16	5.6319	1
MSK_0618.1_251469337983_S01_CGH.v4_91.txt	12	57907421	58479176	51	4.1533	1

Continued on next page...

ID	chr	start	end	nroOfprobes	segmean	call
MSK_00001_251469322729_S01_CGH.v4_91.txt	13	87478072	91586295	135	-0.6872	-1
MSK_00001_251469322729_S01_CGH.v4_91.txt	13	46358466	87193811	1812	-0.6605	-1
MSK_00007_251469322733_S01_CGH.v4_91.txt	13	53271486	105522667	2479	-0.8196	-1
MSK_00007_251469322733_S01_CGH.v4_91.txt	13	42037972	53215883	801	-0.781	-1
MSK_00007_251469322733_S01_CGH.v4_91.txt	13	30691008	41992239	769	-0.7834	-1
MSK_00007_251469322733_S01_CGH.v4_91.txt	13	25356020	27862329	182	-0.8238	-1
MSK_00007_251469322733_S01_CGH.v4_91.txt	13	23030179	24944560	113	-0.8197	-1
MSK_00009_251469322626_S01_CGH.v4_91.txt	13	55256678	56828636	40	-0.9007	-1
MSK_00009_251469322626_S01_CGH.v4_91.txt	13	51890576	54471605	154	-0.7903	-1
MSK_00011_251469322628_S01_CGH.v4_91.txt	13	42037972	115105760	3838	-0.8018	-1
MSK_00011_251469322628_S01_CGH.v4_91.txt	13	19463637	41992239	1531	-0.7638	-1
MSK_00024_251469322721_S01_CGH.v4_91.txt	13	102202049	103740713	126	-0.7913	-1
MSK_00024_251469322721_S01_CGH.v4_91.txt	13	96149846	98814392	196	-0.7645	-1
MSK_00024_251469322721_S01_CGH.v4_91.txt	13	94784224	94832156	6	-0.7774	-1
MSK_00024_251469322721_S01_CGH.v4_91.txt	13	79247354	93905737	561	-0.8207	-1
MSK_00024_251469322721_S01_CGH.v4_91.txt	13	59191713	64159869	177	-0.83	-1
MSK_00024_251469322721_S01_CGH.v4_91.txt	13	53915149	57128062	89	-0.8622	-1
MSK_00024_251469322721_S01_CGH.v4_91.txt	13	42037972	53359853	815	-0.7068	-1
MSK_00024_251469322721_S01_CGH.v4_91.txt	13	41229129	41992239	63	-0.6421	-1
MSK_00024_251469322721_S01_CGH.v4_91.txt	13	27598439	28021442	41	-0.7044	-1
MSK_00024_251469322721_S01_CGH.v4_91.txt	13	21742457	21746471	2	0.6518	1
MSK_WGA_0026.1_251469338758_S01_CGH.v4_91.txt	13	58286456	66491226	264	-0.6655	-1
MSK_WGA_0026.1_251469338758_S01_CGH.v4_91.txt	13	58228647	58246434	3	-1.4648	-1
MSK_00028_251469322706_S01_CGH.v4_91.txt	13	104114620	104743122	15	-1.0035	-1
MSK_00028_251469322706_S01_CGH.v4_91.txt	13	49138704	74250467	1089	-0.9421	-1
MSK_WGA_0039.1_251469338741_S01_CGH.v4_91.txt	13	74397413	74402798	2	-1.2987	-1
MSK_00043_251469322736_S01_CGH.v4_91.txt	13	106991469	108269687	72	-0.9366	-1
MSK_00060_251469322915_S01_CGH.v4_91.txt	13	43996597	44032254	3	-0.9177	-1
MSK_00069_251469322916_S01_CGH.v4_91.txt	13	63058261	93270733	1263	-1.2701	-1
MSK_00069_251469322916_S01_CGH.v4_91.txt	13	62839986	62962420	5	-0.668	-1
MSK_00069_251469322916_S01_CGH.v4_91.txt	13	58778987	62818552	159	-1.2458	-1
MSK_00069_251469322916_S01_CGH.v4_91.txt	13	58170527	58476267	16	-1.3849	-1
MSK_0079.1_251469338020_S01_CGH.v4_91.txt	13	21735975	21746471	4	0.6349	1
MSK_00080_251469322748_S01_CGH.v4_91.txt	13	20228647	20248867	3	-0.7779	-1
MSK_WGA_0104.1_251469338880_S01_CGH.v4_91.txt	13	114918896	115105760	22	-0.8177	-1
MSK_WGA_0104.1_251469338880_S01_CGH.v4_91.txt	13	99863695	102820897	243	-0.7669	-1
MSK_WGA_0104.1_251469338880_S01_CGH.v4_91.txt	13	95651213	95675368	3	0.9802	1
MSK_WGA_0104.1_251469338880_S01_CGH.v4_91.txt	13	76655782	88575780	453	-0.7514	-1
MSK_WGA_0104.1_251469338880_S01_CGH.v4_91.txt	13	66688023	74997958	406	-0.7434	-1
MSK_WGA_0104.1_251469338880_S01_CGH.v4_91.txt	13	49015448	66510423	742	-0.7543	-1
MSK_WGA_0104.1_251469338880_S01_CGH.v4_91.txt	13	48809347	49004123	18	-2.4611	-1
MSK_WGA_0104.1_251469338880_S01_CGH.v4_91.txt	13	42029800	48799122	473	-0.7672	-1
MSK_WGA_0104.1_251469338880_S01_CGH.v4_91.txt	13	41431155	41992239	50	-0.7523	-1
MSK_WGA_0104.1_251469338880_S01_CGH.v4_91.txt	13	35703014	41345266	394	-0.7458	-1
MSK_WGA_0104.1_251469338880_S01_CGH.v4_91.txt	13	28666238	35674045	449	-0.7562	-1
MSK_WGA_0104.1_251469338880_S01_CGH.v4_91.txt	13	21667710	22784035	74	-0.7845	-1
MSK_00114_251469322752_S01_CGH.v4_91.txt	13	84559543	85158231	21	-0.8896	-1
MSK_00114_251469322752_S01_CGH.v4_91.txt	13	67872540	68080143	7	-0.9032	-1
MSK_00114_251469322752_S01_CGH.v4_91.txt	13	42037972	64913466	1191	-0.8791	-1
MSK_00114_251469322752_S01_CGH.v4_91.txt	13	33439717	41992239	566	-0.8808	-1
MSK_0258.1_251469336709_S01_CGH.v4_91.txt	13	49872637	77291002	1202	-0.9137	-1
MSK_0258.1_251469336709_S01_CGH.v4_91.txt	13	28666238	28734431	6	-0.8154	-1
MSK_0260.1_251469331583_S01_CGH.v4_91.txt	13	43996597	44016774	2	-0.96	-1
MSK_0333.1_251469331585_S01_CGH.v4_91.txt	13	100955119	102660564	142	-0.8271	-1
MSK_0333.1_251469331585_S01_CGH.v4_91.txt	13	91668526	94253664	201	-0.8549	-1
MSK_0333.1_251469331585_S01_CGH.v4_91.txt	13	78402537	80458078	105	-0.8243	-1
MSK_0339.1_251469331462_S01_CGH.v4_91.txt	13	42037972	115105760	3838	-0.898	-1
MSK_0339.1_251469331462_S01_CGH.v4_91.txt	13	19296527	41992239	1538	-0.8905	-1
MSK_0430.1_251469336714_S01_CGH.v4_91.txt	13	42037972	56302443	907	-0.7913	-1
MSK_0430.1_251469336714_S01_CGH.v4_91.txt	13	37938026	41992239	269	-0.7849	-1
MSK_0440.1_251469336724_S01_CGH.v4_91.txt	13	42037972	115105760	3838	-0.796	-1
MSK_0440.1_251469336724_S01_CGH.v4_91.txt	13	19463637	41992239	1531	-0.7795	-1
MSK_0146.1_251469336983_S01_CGH.v4_91.txt	13	111967284	112091743	12	2.8275	1
MSK_0146.1_251469336983_S01_CGH.v4_91.txt	13	109777396	110433062	31	2.8489	1
MSK_0146.1_251469336983_S01_CGH.v4_91.txt	13	75104102	75223758	5	2.7998	1
MSK_0146.1_251469336983_S01_CGH.v4_91.txt	13	72156599	72359497	18	-0.7286	-1
MSK_0146.1_251469336983_S01_CGH.v4_91.txt	13	67406473	67872540	47	-0.7131	-1
MSK_0152.1_251469337789_S01_CGH.v4_91.txt	13	78103167	78115380	2	-1.642	-1
MSK_0157.1_251469329178_S01_CGH.v4_91.txt	13	29652400	41609345	799	-0.7764	-1
MSK_0157.1_251469329178_S01_CGH.v4_91.txt	13	21751522	28708506	461	-0.7832	-1
MSK_0157.1_251469329178_S01_CGH.v4_91.txt	13	19463637	21729782	170	-0.7602	-1
MSK_0158.1_251469329734_S01_CGH.v4_91.txt	13	64394991	115105760	2662	-0.7212	-1
MSK_0158.1_251469329734_S01_CGH.v4_91.txt	13	64344325	64382388	2	-1.3461	-1
MSK_0158.1_251469329734_S01_CGH.v4_91.txt	13	42037972	64302868	1174	-0.7207	-1
MSK_0158.1_251469329734_S01_CGH.v4_91.txt	13	21751522	41992239	1357	-0.705	-1
MSK_0158.1_251469329734_S01_CGH.v4_91.txt	13	19456969	21729782	171	-0.6552	-1
MSK_0174.1_251469330284_S01_CGH.v4_91.txt	13	64344325	64382388	2	-0.7326	-1
MSK_0177.1_251469333358_S01_CGH.v4_91.txt	13	42016686	42029800	2	0.697	1
MSK_0188.1_251469330469_S01_CGH.v4_91.txt	13	42037972	115105760	3838	-0.6647	-1
MSK_0188.1_251469330469_S01_CGH.v4_91.txt	13	38378222	41992239	240	-0.6342	-1
MSK_0210.1_251469330448_S01_CGH.v4_91.txt	13	112695781	112728632	4	2.0614	1
MSK_0210.1_251469330448_S01_CGH.v4_91.txt	13	109577180	112168912	181	0.7444	1
MSK_0210.1_251469330448_S01_CGH.v4_91.txt	13	108700386	109562442	58	1.3843	1
MSK_0210.1_251469330448_S01_CGH.v4_91.txt	13	108269687	108607097	20	0.7433	1
MSK_0210.1_251469330448_S01_CGH.v4_91.txt	13	103622601	103732231	9	2.0158	1
MSK_0210.2_251469330589_S01_CGH.v4_91.txt	13	112695781	112728632	4	1.6643	1
MSK_0210.2_251469330589_S01_CGH.v4_91.txt	13	103622601	103732231	9	1.3765	1
MSK_0397.1_251469330468_S01_CGH.v4_91.txt	13	94108394	94641682	50	-0.7899	-1
MSK_0413.1_251469331465_S01_CGH.v4_91.txt	13	59115828	60867570	79	-0.8663	-1
MSK_0413.1_251469331465_S01_CGH.v4_91.txt	13	42037972	58844015	985	-0.849	-1
MSK_0413.1_251469331465_S01_CGH.v4_91.txt	13	39810046	41992239	149	-0.8564	-1
MSK_0414.1_251469336986_S01_CGH.v4_91.txt	13	49015448	51313822	178	-2.6255	-1
MSK_WGA_0348.1_251469338869_S01_CGH.v4_91.txt	13	114056632	114076944	3	0.6553	1
MSK_WGA_0348.1_251469338869_S01_CGH.v4_91.txt	13	42126212	57454474	938	-0.6695	-1
MSK_WGA_0348.1_251469338869_S01_CGH.v4_91.txt	13	25225538	41992239	1156	-0.6728	-1
MSK_0349.1_251469337892_S01_CGH.v4_91.txt	13	19296527	115105760	5379	-0.6489	-1
MSK_WGA_0357.1_251469337522_S01_CGH.v4_91.txt	13	84348567	85347837	41	-0.7887	-1
MSK_WGA_0357.1_251469337522_S01_CGH.v4_91.txt	13	82280104	82344565	2	-0.9363	-1
MSK_WGA_0357.1_251469337522_S01_CGH.v4_91.txt	13	42037972	78611971	1848	-0.7149	-1
MSK_WGA_0357.1_251469337522_S01_CGH.v4_91.txt	13	36521477	41992239	371	-0.6985	-1
MSK_WGA_0359.1_251469337523_S01_CGH.v4_91.txt	13	38458696	115105760	4078	-0.655	-1
MSK_0373.1_251469340742_S01_CGH.v4_91.txt	13	48986319	49004123	4	-1.3699	-1
MSK_WGA_0389.1_251469338874_S01_CGH.v4_91.txt	13	114550958	115105760	41	-0.6976	-1
MSK_WGA_0389.1_251469338874_S01_CGH.v4_91.txt	13	113948016	114537324	35	3.5145	1
MSK_WGA_0389.1_251469338874_S01_CGH.v4_91.txt	13	42037972	113928770	3762	-0.8373	-1
MSK_WGA_0389.1_251469338874_S01_CGH.v4_91.txt	13	19456969	41992239	1532	-0.7942	-1
MSK_WGA_0390.1_251469338875_S01_CGH.v4_91.txt	13	114056632	114087664	4	1.1361	1
MSK_0511.1_251469336907_S01_CGH.v4_91.txt	13	43317927	102749224	3062	-0.7144	-1
MSK_0511.1_251469336907_S01_CGH.v4_91.txt	13	41515037	41661999	16	-0.7595	-1
MSK_0511.1_251469336907_S01_CGH.v4_91.txt	13	33184693	38143352	323	-0.749	-1
MSK_0511.1_251469336907_S01_CGH.v4_91.txt	13	21742457	21746471	2	0.9869	1
MSK_0514.1_251469333360_S01_CGH.v4_91.txt	13	53424239	55256678	57	-0.6585	-1
MSK_0514.1_251469333360_S01_CGH.v4_91.txt	13	51815712	52272652	38	-0.7315	-1
MSK_0514.1_251469333360_S01_CGH.v4_91.txt	13	51261527	51711349	37	-0.804	-1
MSK_0514.1_251469333360_S01_CGH.v4_91.txt	13	50343314	51088020	62	-0.7401	-1
MSK_0514.1_251469333360_S01_CGH.v4_91.txt	13	47292707	47435953	20	-0.7745	-1
MSK_0514.1_251469333360_S01_CGH.v4_91.txt	13	46584549	46842656	27	-0.7581	-1
MSK_0514.1_251469333360_S01_CGH.v4_91.txt	13	46144603	46543702	27	-0.6915	-1
MSK_0514.1_251469333360_S01_CGH.v4_91.txt	13	45163141	46057467	66	-0.6791	-1
MSK_0514.1_251469333360_S01_CGH.v4_91.txt	13	44942113	45001201	8	-0.7163	-1
MSK_0514.1_251469333360_S01_CGH.v4_91.txt	13	44651172	44772423	8	-0.7913	-1
MSK_0517.1_251469337226_S01_CGH.v4_91.txt	13	42037972	115105760	3838	-0.8193	-1
MSK_0517.1_251469337226_S01_CGH.v4_91.txt	13	19296527	41992239	1538	-0.8454	-1
MSK_0520.1_251469336708_S01_CGH.v4_91.txt	13	102979340	115105760	667	-0.7047	-1

Continued on next page...

ID	chr	start	end	nroOfprobes	segmean	call
MSK_0520.1_251469336708_S01_CGH.v4_91.txt	13	99247656	102971613	299	-1.4773	-1
MSK_0520.1_251469336708_S01_CGH.v4_91.txt	13	99208237	99229893	3	-0.6972	-1
MSK_0520.1_251469336708_S01_CGH.v4_91.txt	13	91837820	99203711	590	-1.4758	-1
MSK_0520.1_251469336708_S01_CGH.v4_91.txt	13	49065281	91792318	1785	-0.731	-1
MSK_0520.1_251469336708_S01_CGH.v4_91.txt	13	48964423	49039119	9	-2.34	-1
MSK_0520.1_251469336708_S01_CGH.v4_91.txt	13	42037972	48947531	485	-0.7116	-1
MSK_0520.1_251469336708_S01_CGH.v4_91.txt	13	19456969	41992239	1532	-0.712	-1
MSK_0522.1_251469336908_S01_CGH.v4_91.txt	13	21735975	21746471	4	0.6332	1
MSK_0531.1_251469337227_S01_CGH.v4_91.txt	13	34939999	35518021	21	-0.9069	-1
MSK_0615.1_251469340783_S01_CGH.v4_91.txt	13	19296527	115105760	5379	-0.652	-1
MSK_00003_251469322731_S01_CGH.v4_91.txt	14	22520967	22942423	37	0.9082	1
MSK_00007_251469322733_S01_CGH.v4_91.txt	14	23534265	107278711	5122	-0.781	-1
MSK_00011_251469322628_S01_CGH.v4_91.txt	14	104027507	107278711	219	-0.6811	-1
MSK_00011_251469322628_S01_CGH.v4_91.txt	14	20427183	103980914	5199	-0.7533	-1
MSK_00011_251469322628_S01_CGH.v4_91.txt	14	20198936	20414173	21	-1.4064	-1
MSK_00014_251469322629_S01_CGH.v4_91.txt	14	107209370	107278711	7	-0.7945	-1
MSK_WGA_0016.1_251469338755_S01_CGH.v4_91.txt	14	46007366	78334820	2194	-0.6621	-1
MSK_WGA_0016.1_251469338755_S01_CGH.v4_91.txt	14	37110257	45821442	408	-0.6769	-1
MSK_WGA_0016.1_251469338755_S01_CGH.v4_91.txt	14	22996302	36600695	833	-0.6532	-1
MSK_00024_251469322721_S01_CGH.v4_91.txt	14	46007366	104564554	3732	-0.6871	-1
MSK_00024_251469322721_S01_CGH.v4_91.txt	14	20231319	45821442	1533	-0.7065	-1
MSK_00028_251469322706_S01_CGH.v4_91.txt	14	20198936	20414173	21	-0.7041	-1
MSK_00034_251469322708_S01_CGH.v4_91.txt	14	79759523	80651806	59	-0.9949	-1
MSK_00037_251469322734_S01_CGH.v4_91.txt	14	106786148	106801964	2	-0.706	-1
MSK_00037_251469322734_S01_CGH.v4_91.txt	14	19794561	20414173	23	-0.6546	-1
MSK_00038_251469322735_S01_CGH.v4_91.txt	14	87927177	88492470	30	-0.7561	-1
MSK_00038_251469322735_S01_CGH.v4_91.txt	14	24674119	46390736	1151	-0.71	-1
MSK_00043_251469322736_S01_CGH.v4_91.txt	14	20227886	20414173	17	-0.6692	-1
MSK_WGA_0048.1_251469338742_S01_CGH.v4_91.txt	14	106272105	107278711	72	-0.6746	-1
MSK_WGA_0048.1_251469338742_S01_CGH.v4_91.txt	14	104586953	106011715	80	-0.7065	-1
MSK_WGA_0048.1_251469338742_S01_CGH.v4_91.txt	14	104027507	104564554	50	-0.7441	-1
MSK_WGA_0048.1_251469338742_S01_CGH.v4_91.txt	14	22976257	103980914	4949	-0.7313	-1
MSK_WGA_0048.1_251469338742_S01_CGH.v4_91.txt	14	19554383	22489904	237	-0.7064	-1
MSK_00058_251469323019_S01_CGH.v4_91.txt	14	60821344	107278711	2974	-0.6455	-1
MSK_00060_251469322915_S01_CGH.v4_91.txt	14	106786148	106827629	4	-1.9091	-1
MSK_00060_251469322915_S01_CGH.v4_91.txt	14	20198936	20414173	21	-1.2301	-1
MSK_00064_251469322718_S01_CGH.v4_91.txt	14	106906901	106931134	2	-0.7474	-1
MSK_00064_251469322718_S01_CGH.v4_91.txt	14	20198936	20414173	21	-0.6911	-1
MSK_00071_251469322919_S01_CGH.v4_91.txt	14	19794561	20414173	23	-0.6926	-1
MSK_00083_251469322918_S01_CGH.v4_91.txt	14	106786148	106810234	3	0.7423	1
MSK_00102_251469322716_S01_CGH.v4_91.txt	14	106906901	106931134	2	-0.7903	-1
MSK_WGA_0104.1_251469338880_S01_CGH.v4_91.txt	14	106671750	107278711	46	-0.7462	-1
MSK_WGA_0104.1_251469338880_S01_CGH.v4_91.txt	14	92951930	97173911	307	-0.7914	-1
MSK_WGA_0104.1_251469338880_S01_CGH.v4_91.txt	14	68713288	86329199	1108	-0.7659	-1
MSK_00107_251469322719_S01_CGH.v4_91.txt	14	106786148	106827629	4	-1.0993	-1
MSK_0111.1_251469337228_S01_CGH.v4_91.txt	14	106723116	106728216	2	-0.862	-1
MSK_0111.1_251469337228_S01_CGH.v4_91.txt	14	19554383	20414173	25	-0.8986	-1
MSK_00114_251469322752_S01_CGH.v4_91.txt	14	106786148	106827629	4	-0.6835	-1
MSK_00115_251469322751_S01_CGH.v4_91.txt	14	46537594	46669931	4	-0.8317	-1
MSK_00116_251469322720_S01_CGH.v4_91.txt	14	86554957	86571497	2	-1.0734	-1
MSK_00116_251469322720_S01_CGH.v4_91.txt	14	20198936	20414173	21	-0.6627	-1
MSK_0258.1_251469336709_S01_CGH.v4_91.txt	14	106272105	107278711	72	-1.0655	-1
MSK_0258.1_251469336709_S01_CGH.v4_91.txt	14	56919643	106011715	3147	-1.0726	-1
MSK_0258.1_251469336709_S01_CGH.v4_91.txt	14	56318997	56845801	25	-1.9026	-1
MSK_0258.1_251469336709_S01_CGH.v4_91.txt	14	54956593	56291535	112	-0.9055	-1
MSK_0258.1_251469336709_S01_CGH.v4_91.txt	14	20198936	20414173	21	-0.8413	-1
MSK_0260.1_251469331583_S01_CGH.v4_91.txt	14	106786148	106810234	3	-1.5993	-1
MSK_0271.1_251469333526_S01_CGH.v4_91.txt	14	20198936	20414173	21	-0.8055	-1
MSK_0281.1_251469331472_S01_CGH.v4_91.txt	14	19079473	107278711	5447	-0.6792	-1
MSK_0289.1_251469337015_S01_CGH.v4_91.txt	14	106786148	106827629	4	-0.9149	-1
MSK_0289.1_251469337015_S01_CGH.v4_91.txt	14	28917556	28939998	2	-0.7327	-1
MSK_0289.1_251469337015_S01_CGH.v4_91.txt	14	20203449	20414173	20	-0.7111	-1
MSK_0290.1_251469336710_S01_CGH.v4_91.txt	14	79686499	80820275	82	-0.6622	-1
MSK_0321.1_251469336723_S01_CGH.v4_91.txt	14	20227886	20414173	17	-0.6877	-1
MSK_0325.1_251469333563_S01_CGH.v4_91.txt	14	106786148	106827629	4	-1.0214	-1
MSK_0325.1_251469333563_S01_CGH.v4_91.txt	14	19554383	20414173	25	-0.8699	-1
MSK_0326.1_251469331461_S01_CGH.v4_91.txt	14	19554383	20414173	25	-0.8219	-1
MSK_0330.1_251469336713_S01_CGH.v4_91.txt	14	106786148	106810234	3	-1.4787	-1
MSK_0330.1_251469336713_S01_CGH.v4_91.txt	14	20227886	20414173	17	-1.0911	-1
MSK_0332.1_251469336711_S01_CGH.v4_91.txt	14	106786148	106810234	3	-1.1683	-1
MSK_0332.1_251469336711_S01_CGH.v4_91.txt	14	20231319	20414173	16	-0.9367	-1
MSK_0333.1_251469331585_S01_CGH.v4_91.txt	14	106906901	106931134	2	-0.8112	-1
MSK_0337.1_251469333524_S01_CGH.v4_91.txt	14	106786148	106827629	4	-0.7708	-1
MSK_0337.1_251469333524_S01_CGH.v4_91.txt	14	106072662	106175161	7	1.127	1
MSK_0337.1_251469333524_S01_CGH.v4_91.txt	14	48817641	48891586	3	-0.9614	-1
MSK_0337.1_251469333524_S01_CGH.v4_91.txt	14	20198936	20414173	21	-0.7747	-1
MSK_0339.1_251469331462_S01_CGH.v4_91.txt	14	106786148	106810234	3	-0.6738	-1
MSK_0339.1_251469331462_S01_CGH.v4_91.txt	14	20224136	20414173	18	-0.8154	-1
MSK_0432.1_251469336712_S01_CGH.v4_91.txt	14	60539315	107278711	3001	-0.7237	-1
MSK_0440.1_251469336724_S01_CGH.v4_91.txt	14	44540364	44623010	4	-0.8198	-1
MSK_0440.1_251469336724_S01_CGH.v4_91.txt	14	43881311	43920723	3	-0.8909	-1
MSK_0440.1_251469336724_S01_CGH.v4_91.txt	14	42120879	42320960	15	-0.7949	-1
MSK_0440.1_251469336724_S01_CGH.v4_91.txt	14	41710066	41847694	4	-0.9308	-1
MSK_0440.1_251469336724_S01_CGH.v4_91.txt	14	20198936	40143568	1336	-0.7569	-1
MSK_00128.1_251469325161_S01_CGH.v4_91.txt	14	20198936	20414173	21	-0.9376	-1
MSK_0132.1_251469330459_S01_CGH.v4_91.txt	14	20198936	20414173	21	-0.9297	-1
MSK_00133_251469325752_S01_CGH.v4_91.txt	14	106786148	106801964	2	-1.182	-1
MSK_0146.1_251469336983_S01_CGH.v4_91.txt	14	106637164	106827629	16	-0.9105	-1
MSK_0149.1_251469336715_S01_CGH.v4_91.txt	14	21375760	21407536	4	-0.803	-1
MSK_0149.1_251469336715_S01_CGH.v4_91.txt	14	20227886	20414173	17	-0.6431	-1
MSK_0162.1_251469336905_S01_CGH.v4_91.txt	14	27563966	27579531	2	-2.2782	-1
MSK_0164.1_251469331554_S01_CGH.v4_91.txt	14	19794561	20414173	23	-0.7394	-1
MSK_0171.1_251469329753_S01_CGH.v4_91.txt	14	20198936	20414173	21	-1.0013	-1
MSK_0173.1_251469330461_S01_CGH.v4_91.txt	14	20198936	20414173	21	-0.6761	-1
MSK_0174.1_251469330284_S01_CGH.v4_91.txt	14	20224136	20414173	18	-0.7397	-1
MSK_0178.1_251469329154_S01_CGH.v4_91.txt	14	106801964	106827629	3	0.6364	1
MSK_0178.1_251469329154_S01_CGH.v4_91.txt	14	20198936	20414173	21	-0.7296	-1
MSK_0179.1_251469333566_S01_CGH.v4_91.txt	14	106786148	106810234	3	-1.2876	-1
MSK_0179.1_251469333566_S01_CGH.v4_91.txt	14	19572252	20414173	24	-0.9564	-1
MSK_0182.1_251469331464_S01_CGH.v4_91.txt	14	19572252	20414173	24	-0.8493	-1
MSK_0184.1_251469329181_S01_CGH.v4_91.txt	14	20198936	20414173	21	-0.7103	-1
MSK_0189.1_251469330588_S01_CGH.v4_91.txt	14	54156082	54241359	3	-0.6321	-1
MSK_0190.1_251469330591_S01_CGH.v4_91.txt	14	20198936	20414173	21	0.7768	1
MSK_0197.1_251469329155_S01_CGH.v4_91.txt	14	106906901	106931134	2	-0.8213	-1
MSK_0197.1_251469329155_S01_CGH.v4_91.txt	14	19572252	20414173	24	-0.6929	-1
MSK_0206.1_251469329173_S01_CGH.v4_91.txt	14	19554383	20414173	25	-0.6832	-1
MSK_0208.1_251469329182_S01_CGH.v4_91.txt	14	106906901	106931134	2	-0.8569	-1
MSK_0208.1_251469329182_S01_CGH.v4_91.txt	14	19794561	20414173	23	-0.7086	-1
MSK_0208.2_251469329755_S01_CGH.v4_91.txt	14	106906901	106931134	2	-0.7328	-1
MSK_0209.1_251469330463_S01_CGH.v4_91.txt	14	106906901	106931134	2	-0.6603	-1
MSK_0209.1_251469330463_S01_CGH.v4_91.txt	14	20198936	20414173	21	-0.7495	-1
MSK_0210.1_251469330448_S01_CGH.v4_91.txt	14	106906901	106931134	2	-0.6643	-1
MSK_0213.1_251469329174_S01_CGH.v4_91.txt	14	106786148	106810234	3	-0.9487	-1
MSK_0219.1_251469329864_S01_CGH.v4_91.txt	14	19794561	20414173	23	-1.2597	-1
MSK_0221.1_251469330192_S01_CGH.v4_91.txt	14	106786148	106931134	8	-0.687	-1
MSK_0221.1_251469330192_S01_CGH.v4_91.txt	14	20198936	20427183	22	-0.8486	-1
MSK_0237.1_251469330449_S01_CGH.v4_91.txt	14	102113978	107278711	372	0.8311	1
MSK_0237.1_251469330449_S01_CGH.v4_91.txt	14	100766234	101367153	45	0.8762	1
MSK_0237.1_251469330449_S01_CGH.v4_91.txt	14	94908699	100707490	305	0.883	1
MSK_0238.1_251469340788_S01_CGH.v4_91.txt	14	19079473	20414173	27	-0.7689	-1
MSK_0241.1_251469329774_S01_CGH.v4_91.txt	14	19079473	107278711	5447	-0.8165	-1
MSK_0394.1_251469336716_S01_CGH.v4_91.txt	14	20198936	20414173	21	-0.7082	-1
MSK_0397.1_251469330468_S01_CGH.v4_91.txt	14	20470454	70845024	3153	-0.7355	-1
MSK_0409.1_251469331555_S01_CGH.v4_91.txt	14	106786148	106810234	3	-1.445	-1

Continued on next page...

ID	chr	start	end	nroOfprobes	segmean	call
MSK_0409.1_251469331555_S01_CGH.v4_91.txt	14	20227886	20414173	17	-1.1132	-1
MSK_0410.1_251469333359_S01_CGH.v4_91.txt	14	20198936	20414173	21	-1.2723	-1
MSK_0412.1_251469331490_S01_CGH.v4_91.txt	14	106827629	107278711	32	-0.7127	-1
MSK_0412.1_251469331490_S01_CGH.v4_91.txt	14	106786148	106810234	3	-2.5918	-1
MSK_0412.1_251469331490_S01_CGH.v4_91.txt	14	19079473	20414173	27	-0.8026	-1
MSK_0413.1_251469331465_S01_CGH.v4_91.txt	14	95874684	96587231	48	-0.7793	-1
MSK_0413.1_251469331465_S01_CGH.v4_91.txt	14	95513392	95740974	20	-0.9015	-1
MSK_0413.1_251469331465_S01_CGH.v4_91.txt	14	94414937	94947190	48	-0.8387	-1
MSK_0413.1_251469331465_S01_CGH.v4_91.txt	14	19554383	20414173	25	-0.9341	-1
MSK_0414.1_251469336986_S01_CGH.v4_91.txt	14	106786148	106810234	3	-1.5969	-1
MSK_0645.1_251469340797_S01_CGH.v4_91.txt	14	19079473	20414173	27	-0.7977	-1
MSK_0646.1_251469340746_S01_CGH.v4_91.txt	14	34000168	107278711	4506	-0.6585	-1
MSK_0646.1_251469340746_S01_CGH.v4_91.txt	14	33483234	33958025	49	-2.3762	-1
MSK_0646.1_251469340746_S01_CGH.v4_91.txt	14	19079473	33461101	892	-0.6342	-1
MSK_WGA_0347.1_251469338868_S01_CGH.v4_91.txt	14	48308433	48397273	4	-0.7343	-1
MSK_WGA_0347.1_251469338868_S01_CGH.v4_91.txt	14	48054867	48128839	8	-0.7215	-1
MSK_WGA_0347.1_251469338868_S01_CGH.v4_91.txt	14	45765800	45821442	4	-0.6338	-1
MSK_WGA_0347.1_251469338868_S01_CGH.v4_91.txt	14	43361622	45400572	67	-0.6757	-1
MSK_0349.1_251469337892_S01_CGH.v4_91.txt	14	58235815	58251907	3	-0.7092	-1
MSK_0358.1_251469340789_S01_CGH.v4_91.txt	14	106786148	106827629	4	-0.9882	-1
MSK_0373.1_251469340742_S01_CGH.v4_91.txt	14	106786148	106827629	4	-1.1094	-1
MSK_0386.1_251469340798_S01_CGH.v4_91.txt	14	91984721	96023053	306	-0.6487	-1
MSK_0386.1_251469340798_S01_CGH.v4_91.txt	14	44934977	55585497	643	-0.6707	-1
MSK_WGA_0392.1_251469338876_S01_CGH.v4_91.txt	14	46007366	68377393	1456	-0.7018	-1
MSK_WGA_0392.1_251469338876_S01_CGH.v4_91.txt	14	19079473	45821442	1544	-0.6893	-1
MSK_0509.1_251469336717_S01_CGH.v4_91.txt	14	106786148	106810234	3	-0.9126	-1
MSK_0509.1_251469336717_S01_CGH.v4_91.txt	14	20227886	20414173	17	-0.7561	-1
MSK_0511.1_251469336907_S01_CGH.v4_91.txt	14	20198936	20414173	21	-0.7559	-1
MSK_0512.1_251469331556_S01_CGH.v4_91.txt	14	20198936	20414173	21	-1.0488	-1
MSK_0516.1_251469331469_S01_CGH.v4_91.txt	14	44836428	44915828	5	0.7426	1
MSK_0516.1_251469331469_S01_CGH.v4_91.txt	14	19794561	20414173	23	-0.8311	-1
MSK_0517.1_251469337226_S01_CGH.v4_91.txt	14	104027507	107278711	219	-0.7252	-1
MSK_0517.1_251469337226_S01_CGH.v4_91.txt	14	59681167	103980914	2844	-0.8015	-1
MSK_0517.1_251469337226_S01_CGH.v4_91.txt	14	39687768	39709629	3	0.8064	1
MSK_0517.1_251469337226_S01_CGH.v4_91.txt	14	19079473	20414173	27	-0.7501	-1
MSK_0518.1_251469337014_S01_CGH.v4_91.txt	14	106786148	106810234	3	-1.1041	-1
MSK_0518.1_251469337014_S01_CGH.v4_91.txt	14	20198936	20414173	21	-0.7605	-1
MSK_0520.1_251469336708_S01_CGH.v4_91.txt	14	46007366	107278711	3901	-0.6872	-1
MSK_0520.1_251469336708_S01_CGH.v4_91.txt	14	28339878	45821442	956	-0.7172	-1
MSK_0520.1_251469336708_S01_CGH.v4_91.txt	14	21411840	28192943	457	-0.6464	-1
MSK_0520.1_251469336708_S01_CGH.v4_91.txt	14	21354078	21407536	5	-3.2953	-1
MSK_0520.1_251469336708_S01_CGH.v4_91.txt	14	20427183	21298763	97	-0.6439	-1
MSK_0520.1_251469336708_S01_CGH.v4_91.txt	14	20227886	20414173	17	-1.417	-1
MSK_0521.1_251469331582_S01_CGH.v4_91.txt	14	20227886	20414173	17	-0.7805	-1
MSK_0524.1_251469331557_S01_CGH.v4_91.txt	14	20203449	43436387	1440	-0.8708	-1
MSK_0525.1_251469333361_S01_CGH.v4_91.txt	14	106786148	106810234	3	-1.2823	-1
MSK_0525.1_251469333361_S01_CGH.v4_91.txt	14	27059848	27438608	16	-0.8245	-1
MSK_0525.1_251469333361_S01_CGH.v4_91.txt	14	20198936	20427183	22	-0.6718	-1
MSK_0529.1_251469331470_S01_CGH.v4_91.txt	14	72351950	72581173	19	-0.8092	-1
MSK_0529.1_251469331470_S01_CGH.v4_91.txt	14	19794561	20414173	23	-0.9195	-1
MSK_0531.1_251469337227_S01_CGH.v4_91.txt	14	19554383	20414173	25	-0.9491	-1
MSK_0615.1_251469340783_S01_CGH.v4_91.txt	14	97086979	97311415	13	2.7036	1
MSK_0615.1_251469340783_S01_CGH.v4_91.txt	14	38419348	97038430	3592	-0.6794	-1
MSK_0616.1_251469338022_S01_CGH.v4_91.txt	14	106774613	107278711	36	-0.7898	-1
MSK_0616.1_251469338022_S01_CGH.v4_91.txt	14	106637164	106768394	11	-2.5001	-1
MSK_0618.1_251469337983_S01_CGH.v4_91.txt	14	106786148	106827629	4	-1.2616	-1
MSK_00003_251469322731_S01_CGH.v4_91.txt	15	66153441	102383414	2431	-0.8039	-1
MSK_00003_251469322731_S01_CGH.v4_91.txt	15	20102541	66048309	2993	-0.8031	-1
MSK_00010_251469322627_S01_CGH.v4_91.txt	15	22285291	22578571	24	-0.6741	-1
MSK_00011_251469322628_S01_CGH.v4_91.txt	15	91159545	96588108	297	-0.7821	-1
MSK_00011_251469322628_S01_CGH.v4_91.txt	15	79084484	82438837	230	-0.7358	-1
MSK_00011_251469322628_S01_CGH.v4_91.txt	15	34803351	78920553	3188	-0.7436	-1
MSK_00011_251469322628_S01_CGH.v4_91.txt	15	34730221	34791191	5	-1.9814	-1
MSK_00011_251469322628_S01_CGH.v4_91.txt	15	30954726	34724183	276	-0.7687	-1
MSK_00011_251469322628_S01_CGH.v4_91.txt	15	30931844	30943903	2	-3.2077	-1
MSK_00011_251469322628_S01_CGH.v4_91.txt	15	29222089	30819465	80	-0.749	-1
MSK_WGA_0016.1_251469338755_S01_CGH.v4_91.txt	15	40410733	44310732	343	-0.6336	-1
MSK_WGA_0016.1_251469338755_S01_CGH.v4_91.txt	15	35240530	40379081	309	-0.6537	-1
MSK_00024_251469322721_S01_CGH.v4_91.txt	15	32931428	41782642	647	-0.7151	-1
MSK_00024_251469322721_S01_CGH.v4_91.txt	15	31695322	32510804	47	-0.736	-1
MSK_WGA_0026.1_251469338758_S01_CGH.v4_91.txt	15	91341437	91352363	3	-0.734	-1
MSK_WGA_0026.1_251469338758_S01_CGH.v4_91.txt	15	80694072	80878740	16	-0.6355	-1
MSK_WGA_0026.1_251469338758_S01_CGH.v4_91.txt	15	20588199	20861743	5	-1.2143	-1
MSK_00033_251469322707_S01_CGH.v4_91.txt	15	43923363	43988761	5	-0.7498	-1
MSK_00033_251469322707_S01_CGH.v4_91.txt	15	30931844	30954726	3	-0.8174	-1
MSK_00038_251469322735_S01_CGH.v4_91.txt	15	49070495	49734407	64	-0.6896	-1
MSK_00038_251469322735_S01_CGH.v4_91.txt	15	42295658	48935353	460	-0.6701	-1
MSK_00038_251469322735_S01_CGH.v4_91.txt	15	31115047	42062528	773	-0.6784	-1
MSK_00055_251469323018_S01_CGH.v4_91.txt	15	21082791	22285291	13	-1.3867	-1
MSK_00055_251469323018_S01_CGH.v4_91.txt	15	20955412	21040742	8	-0.6553	-1
MSK_00055_251469323018_S01_CGH.v4_91.txt	15	20549990	20935292	8	-1.3066	-1
MSK_00069_251469322916_S01_CGH.v4_91.txt	15	93949528	93968530	2	-0.7467	-1
MSK_00107_251469322719_S01_CGH.v4_91.txt	15	34730221	34791191	5	-0.7904	-1
MSK_00114_251469322752_S01_CGH.v4_91.txt	15	101055828	102383414	111	-0.8374	-1
MSK_0260.1_251469331583_S01_CGH.v4_91.txt	15	75852782	75954558	11	-0.8354	-1
MSK_0260.1_251469331583_S01_CGH.v4_91.txt	15	75276096	75738257	33	-0.6988	-1
MSK_0260.1_251469331583_S01_CGH.v4_91.txt	15	27556183	28535007	66	-0.8392	-1
MSK_0266.1_251469336909_S01_CGH.v4_91.txt	15	41507997	41516559	2	-2.4791	-1
MSK_0266.1_251469336909_S01_CGH.v4_91.txt	15	25092274	25108806	3	-3.2215	-1
MSK_0285.1_251469337229_S01_CGH.v4_91.txt	15	34730221	34791191	5	-0.7229	-1
MSK_0290.1_251469336710_S01_CGH.v4_91.txt	15	30943903	30954726	2	-0.843	-1
MSK_0317.1_251469331584_S01_CGH.v4_91.txt	15	20462581	22578571	55	0.7847	1
MSK_0325.1_251469333563_S01_CGH.v4_91.txt	15	34730221	34791191	5	-0.6456	-1
MSK_0326.1_251469331461_S01_CGH.v4_91.txt	15	34724183	34841387	9	-0.7331	-1
MSK_0326.1_251469331461_S01_CGH.v4_91.txt	15	22364080	22385476	6	-0.7274	-1
MSK_0333.1_251469331585_S01_CGH.v4_91.txt	15	100300119	100605357	22	-0.8626	-1
MSK_0333.1_251469331585_S01_CGH.v4_91.txt	15	20462581	22669052	56	0.7178	1
MSK_0339.1_251469331462_S01_CGH.v4_91.txt	15	22318597	22578571	23	1.1095	1
MSK_0339.1_251469331462_S01_CGH.v4_91.txt	15	20397374	20624422	9	0.9059	1
MSK_0430.1_251469336714_S01_CGH.v4_91.txt	15	21040742	22578571	37	1.0225	1
MSK_0430.1_251469336714_S01_CGH.v4_91.txt	15	20462581	20935292	11	0.863	1
MSK_0432.1_251469336712_S01_CGH.v4_91.txt	15	30931844	30954726	3	-0.8691	-1
MSK_0432.1_251469336712_S01_CGH.v4_91.txt	15	22385476	22578571	14	0.6689	1
MSK_0432.1_251469336712_S01_CGH.v4_91.txt	15	20397374	22368341	39	0.7042	1
MSK_0439.1_251469331553_S01_CGH.v4_91.txt	15	57362597	57555423	19	-1.1581	-1
MSK_0439.1_251469331553_S01_CGH.v4_91.txt	15	34730221	34841387	8	-1.5866	-1
MSK_0446.1_251469333525_S01_CGH.v4_91.txt	15	34730221	34791191	5	-2.0897	-1
MSK_0451.1_251469333565_S01_CGH.v4_91.txt	15	24699878	24721688	2	-0.8245	-1
MSK_0451.1_251469333565_S01_CGH.v4_91.txt	15	20462581	22578571	55	0.6393	1
MSK_00128.1_251469325161_S01_CGH.v4_91.txt	15	34695166	34841387	11	0.6615	1
MSK_00130.1_251469325022_S01_CGH.v4_91.txt	15	20397374	22578571	56	0.9023	1
MSK_00133_251469325752_S01_CGH.v4_91.txt	15	44093455	44101897	3	1.0777	1
MSK_00139.3_251469325756_S01_CGH.v4_91.txt	15	30819465	30931844	2	0.949	1
MSK_0149.1_251469336715_S01_CGH.v4_91.txt	15	34730221	34791191	5	-0.6805	-1
MSK_0149.1_251469336715_S01_CGH.v4_91.txt	15	20462581	22578571	55	0.8284	1
MSK_0157.1_251469329178_S01_CGH.v4_91.txt	15	24699878	24721688	2	-0.762	-1
MSK_0158.1_251469329734_S01_CGH.v4_91.txt	15	24699878	24721688	2	-0.7211	-1
MSK_0162.1_251469336905_S01_CGH.v4_91.txt	15	22318597	22578571	23	0.6369	1
MSK_0164.1_251469331554_S01_CGH.v4_91.txt	15	43895645	43895965	2	0.729	1
MSK_0166.1_251469329777_S01_CGH.v4_91.txt	15	101946251	102108739	15	1.0777	1
MSK_00169.1_251469325174_S01_CGH.v4_91.txt	15	20397374	22578571	56	0.8331	1
MSK_0171.1_251469329753_S01_CGH.v4_91.txt	15	30931844	30943903	2	-0.9653	-1
MSK_0171.1_251469329753_S01_CGH.v4_91.txt	15	22318597	22578571	23	-0.7345	-1
MSK_0171.1_251469329753_S01_CGH.v4_91.txt	15	20549990	22285291	29	-1.2554	-1

Continued on next page...

ID	chr	start	end	nroOfprobes	segmean	call
MSK_0171.1_251469329753_S01_CGH.v4_91.txt	15	20397374	20530575	4	-0.6824	-1
MSK_0175.1_251469336725_S01_CGH.v4_91.txt	15	34730221	34791191	5	-0.6767	-1
MSK_0177.1_251469333358_S01_CGH.v4_91.txt	15	94576237	99010526	207	-0.687	-1
MSK_0177.1_251469333358_S01_CGH.v4_91.txt	15	20102541	48613238	1695	-0.6599	-1
MSK_0178.1_251469329154_S01_CGH.v4_91.txt	15	83614855	83619707	2	-0.7557	-1
MSK_0179.1_251469333566_S01_CGH.v4_91.txt	15	31356897	31381559	4	-0.8698	-1
MSK_0182.1_251469331464_S01_CGH.v4_91.txt	15	95021892	95023559	2	-0.9558	-1
MSK_0182.1_251469331464_S01_CGH.v4_91.txt	15	85143113	86726256	112	-0.9098	-1
MSK_0182.1_251469331464_S01_CGH.v4_91.txt	15	83710631	84789775	88	-0.951	-1
MSK_0182.1_251469331464_S01_CGH.v4_91.txt	15	43262676	43324728	5	-0.9415	-1
MSK_0182.1_251469331464_S01_CGH.v4_91.txt	15	42397759	43190404	68	-0.8885	-1
MSK_0182.1_251469331464_S01_CGH.v4_91.txt	15	41972352	42342097	27	-0.937	-1
MSK_0195.1_251469329772_S01_CGH.v4_91.txt	15	22378297	22381347	2	-0.752	-1
MSK_0201.1_251469329757_S01_CGH.v4_91.txt	15	74019106	74025790	2	-1.2201	-1
MSK_0201.1_251469329757_S01_CGH.v4_91.txt	15	20462581	22578571	55	-0.7392	-1
MSK_0213.1_251469329174_S01_CGH.v4_91.txt	15	55140392	55229664	4	-1.5605	-1
MSK_0214.1_251469329168_S01_CGH.v4_91.txt	15	81996959	82089696	3	0.6404	1
MSK_0221.1_251469330192_S01_CGH.v4_91.txt	15	20397374	22578571	56	-0.6412	-1
MSK_0237.1_251469330449_S01_CGH.v4_91.txt	15	29896747	29953893	5	-0.8216	-1
MSK_0238.1_251469340788_S01_CGH.v4_91.txt	15	20549990	22368341	35	-1.5386	-1
MSK_0397.1_251469330468_S01_CGH.v4_91.txt	15	24699878	24738180	3	-0.9338	-1
MSK_0410.1_251469333359_S01_CGH.v4_91.txt	15	22364080	22409503	7	-0.7353	-1
MSK_0413.1_251469331465_S01_CGH.v4_91.txt	15	54050047	54280754	8	-0.8777	-1
MSK_0413.1_251469331465_S01_CGH.v4_91.txt	15	53103000	53908354	25	-0.7769	-1
MSK_0413.1_251469331465_S01_CGH.v4_91.txt	15	52564821	52975164	42	-0.8494	-1
MSK_0413.1_251469331465_S01_CGH.v4_91.txt	15	52327909	52498069	16	-0.8664	-1
MSK_0413.1_251469331465_S01_CGH.v4_91.txt	15	20194036	22578571	57	-0.7757	-1
MSK_0648.1_251469338021_S01_CGH.v4_91.txt	15	34730221	34791191	5	-1.9211	-1
MSK_WGA_0348.1_251469338869_S01_CGH.v4_91.txt	15	27141405	72884545	3226	-0.6399	-1
MSK_WGA_0348.1_251469338869_S01_CGH.v4_91.txt	15	23699701	27108247	173	-0.6684	-1
MSK_WGA_0348.1_251469338869_S01_CGH.v4_91.txt	15	20102541	23300238	90	-0.751	-1
MSK_0349.1_251469337892_S01_CGH.v4_91.txt	15	22378297	22381347	2	-2.9243	-1
MSK_0349.1_251469337892_S01_CGH.v4_91.txt	15	20102541	22373326	44	-0.9105	-1
MSK_WGA_0355.1_251469337508_S01_CGH.v4_91.txt	15	33294222	42117159	645	-0.6724	-1
MSK_WGA_0356.1_251469337509_S01_CGH.v4_91.txt	15	72921369	72968355	3	0.6683	1
MSK_0373.1_251469340742_S01_CGH.v4_91.txt	15	30931844	30943903	2	-1.4599	-1
MSK_0509.1_251469336717_S01_CGH.v4_91.txt	15	22409503	22578571	13	0.7093	1
MSK_0509.1_251469336717_S01_CGH.v4_91.txt	15	22318597	22358752	4	0.78	1
MSK_0512.1_251469331556_S01_CGH.v4_91.txt	15	102310595	102383414	12	-0.8732	-1
MSK_0516.1_251469331469_S01_CGH.v4_91.txt	15	34730221	34791191	5	-1.1872	-1
MSK_0517.1_251469337226_S01_CGH.v4_91.txt	15	34724183	34817794	8	-0.6788	-1
MSK_0518.1_251469337014_S01_CGH.v4_91.txt	15	20462581	22578571	55	0.6507	1
MSK_0520.1_251469336708_S01_CGH.v4_91.txt	15	76083998	76989204	73	-0.7173	-1
MSK_0522.1_251469336908_S01_CGH.v4_91.txt	15	24612800	24721688	3	-0.7296	-1
MSK_0524.1_251469331557_S01_CGH.v4_91.txt	15	34730221	34791191	5	-0.7036	-1
MSK_0525.1_251469333361_S01_CGH.v4_91.txt	15	45516681	50271941	295	-0.7274	-1
MSK_0525.1_251469333361_S01_CGH.v4_91.txt	15	41803148	45319178	290	-0.7055	-1
MSK_0525.1_251469333361_S01_CGH.v4_91.txt	15	28485606	41782642	845	-0.7156	-1
MSK_0525.1_251469333361_S01_CGH.v4_91.txt	15	24612800	24721688	3	-0.7428	-1
MSK_0529.1_251469331470_S01_CGH.v4_91.txt	15	34724183	34817794	8	-0.7063	-1
MSK_0529.1_251469331470_S01_CGH.v4_91.txt	15	22285291	22578571	24	-0.6835	-1
MSK_0618.1_251469337983_S01_CGH.v4_91.txt	15	20102541	102383414	5430	-0.7874	-1
MSK_00001_251469322729_S01_CGH.v4_91.txt	16	55785964	55822333	3	-0.7739	-1
MSK_00006_251469322732_S01_CGH.v4_91.txt	16	46580883	46609457	4	-0.7127	-1
MSK_00009_251469322626_S01_CGH.v4_91.txt	16	22831266	28225941	333	-0.7594	-1
MSK_00009_251469322626_S01_CGH.v4_91.txt	16	20885656	22430533	94	-0.7086	-1
MSK_00009_251469322626_S01_CGH.v4_91.txt	16	3052343	6950959	274	-0.6827	-1
MSK_00010_251469322627_S01_CGH.v4_91.txt	16	70152776	70193889	5	0.8073	1
MSK_00014_251469322629_S01_CGH.v4_91.txt	16	6254607	6328916	8	-0.8075	-1
MSK_00014_251469322629_S01_CGH.v4_91.txt	16	5255595	6191513	37	-0.759	-1
MSK_00037_251469322734_S01_CGH.v4_91.txt	16	55802482	55822333	2	-1.332	-1
MSK_00038_251469322735_S01_CGH.v4_91.txt	16	71881249	82821702	623	-0.659	-1
MSK_00038_251469322735_S01_CGH.v4_91.txt	16	63736586	65919594	75	-0.6933	-1
MSK_WGA_0039.1_251469338741_S01_CGH.v4_91.txt	16	56700986	56704216	2	0.7252	1
MSK_00052_251469323033_S01_CGH.v4_91.txt	16	82197554	82201853	2	-0.8043	-1
MSK_00052_251469323033_S01_CGH.v4_91.txt	16	47531322	47588954	5	-1.4121	-1
MSK_00057_251469323017_S01_CGH.v4_91.txt	16	29664529	29707373	5	0.7368	1
MSK_00060_251469322915_S01_CGH.v4_91.txt	16	15048676	15081354	5	-0.7115	-1
MSK_WGA_0068.1_251469338745_S01_CGH.v4_91.txt	16	56700986	56704216	2	0.7991	1
MSK_00086_251469322749_S01_CGH.v4_91.txt	16	10592140	10626916	5	0.6443	1
MSK_00102_251469322716_S01_CGH.v4_91.txt	16	70188386	70193889	2	0.8051	1
MSK_00114_251469322752_S01_CGH.v4_91.txt	16	58400467	90163070	1770	-0.8132	-1
MSK_0260.1_251469331583_S01_CGH.v4_91.txt	16	55850718	55865111	2	0.7176	1
MSK_0266.1_251469336909_S01_CGH.v4_91.txt	16	15048676	15081354	5	-0.9018	-1
MSK_0271.1_251469333526_S01_CGH.v4_91.txt	16	34482042	34747529	10	0.7039	1
MSK_0290.1_251469336710_S01_CGH.v4_91.txt	16	53690422	55223047	64	1.5332	1
MSK_0317.1_251469331584_S01_CGH.v4_91.txt	16	15048676	15081354	5	-0.7666	-1
MSK_0321.1_251469336723_S01_CGH.v4_91.txt	16	78499163	79123527	47	-0.6802	-1
MSK_0325.1_251469333563_S01_CGH.v4_91.txt	16	7934683	7964279	2	-0.6959	-1
MSK_0330.1_251469336713_S01_CGH.v4_91.txt	16	72095998	72107306	2	1.4773	1
MSK_0330.1_251469336713_S01_CGH.v4_91.txt	16	15048676	15089859	6	-0.6628	-1
MSK_0339.1_251469331462_S01_CGH.v4_91.txt	16	70174866	70196733	4	-0.6623	-1
MSK_0339.1_251469331462_S01_CGH.v4_91.txt	16	33373258	33409140	4	0.6974	1
MSK_0422.1_251469337230_S01_CGH.v4_91.txt	16	55802482	55822333	2	-0.7789	-1
MSK_0430.1_251469336714_S01_CGH.v4_91.txt	16	21540592	21585411	4	-0.719	-1
MSK_0440.1_251469336724_S01_CGH.v4_91.txt	16	34482042	34747529	10	0.8107	1
MSK_0440.1_251469336724_S01_CGH.v4_91.txt	16	15048676	15073743	4	-0.8178	-1
MSK_0451.1_251469333565_S01_CGH.v4_91.txt	16	2652858	2732073	8	0.8814	1
MSK_0456.1_251469331463_S01_CGH.v4_91.txt	16	55850718	55865111	2	0.6655	1
MSK_0456.1_251469331463_S01_CGH.v4_91.txt	16	55802482	55822333	2	-1.0719	-1
MSK_0132.1_251469330459_S01_CGH.v4_91.txt	16	8629329	8688156	2	-0.6391	-1
MSK_00139.1_251469325164_S01_CGH.v4_91.txt	16	82675959	82685994	2	-1.0258	-1
MSK_0158.1_251469329734_S01_CGH.v4_91.txt	16	76787577	76911272	4	-0.7191	-1
MSK_0162.1_251469336905_S01_CGH.v4_91.txt	16	72085550	72107306	3	-0.7588	-1
MSK_0164.1_251469331554_S01_CGH.v4_91.txt	16	15048676	15073743	4	-0.9386	-1
MSK_0171.1_251469329753_S01_CGH.v4_91.txt	16	70152776	70193889	5	-1.0633	-1
MSK_0173.1_251469330461_S01_CGH.v4_91.txt	16	2698642	2732073	6	0.8364	1
MSK_0174.1_251469330284_S01_CGH.v4_91.txt	16	75584465	79037032	203	-0.8146	-1
MSK_0174.1_251469330284_S01_CGH.v4_91.txt	16	72043868	75531393	209	-0.7936	-1
MSK_0175.1_251469336725_S01_CGH.v4_91.txt	16	50189782	54022780	196	-0.6543	-1
MSK_0185.1_251469329754_S01_CGH.v4_91.txt	16	8629329	8688156	2	-0.6867	-1
MSK_0201.1_251469329757_S01_CGH.v4_91.txt	16	55850718	55865111	2	0.9308	1
MSK_0201.1_251469329757_S01_CGH.v4_91.txt	16	55785964	55822333	3	-0.8748	-1
MSK_0208.1_251469329182_S01_CGH.v4_91.txt	16	53256573	54993254	98	-0.6378	-1
MSK_0209.1_251469330463_S01_CGH.v4_91.txt	16	70174866	70196733	4	0.6422	1
MSK_0210.1_251469330448_S01_CGH.v4_91.txt	16	55802482	55822333	2	-1.0907	-1
MSK_0210.2_251469330589_S01_CGH.v4_91.txt	16	55802482	55822333	2	-1.4208	-1
MSK_0211.1_251469330227_S01_CGH.v4_91.txt	16	7186763	7203207	3	-0.6756	-1
MSK_0211.2_251469329773_S01_CGH.v4_91.txt	16	7180401	7203207	4	-0.8018	-1
MSK_0213.1_251469329174_S01_CGH.v4_91.txt	16	34482042	34747529	10	0.6433	1
MSK_0213.1_251469329174_S01_CGH.v4_91.txt	16	2705484	2732073	5	0.8833	1
MSK_0213.1_251469329174_S01_CGH.v4_91.txt	16	134248	270646	15	-0.7397	-1
MSK_0219.1_251469329864_S01_CGH.v4_91.txt	16	2698642	2732073	6	0.7711	1
MSK_0237.1_251469330449_S01_CGH.v4_91.txt	16	34482042	34747529	10	0.7695	1
MSK_0237.1_251469330449_S01_CGH.v4_91.txt	16	33599996	33625939	3	0.6788	1
MSK_0237.1_251469330449_S01_CGH.v4_91.txt	16	5937863	15404452	533	-0.6523	-1
MSK_0238.1_251469340788_S01_CGH.v4_91.txt	16	2456644	2911218	37	1.325	1
MSK_0241.1_251469329774_S01_CGH.v4_91.txt	16	32470625	32637849	5	-1.2884	-1
MSK_0409.1_251469331555_S01_CGH.v4_91.txt	16	72095998	72107306	2	1.6698	1
MSK_0410.1_251469333359_S01_CGH.v4_91.txt	16	54082799	54162243	8	0.8265	1
MSK_0644.1_251469340741_S01_CGH.v4_91.txt	16	2698642	2705484	2	1.1293	1
MSK_WGA_0356.1_251469337509_S01_CGH.v4_91.txt	16	56692814	56704216	3	0.7757	1
MSK_WGA_0357.1_251469337522_S01_CGH.v4_91.txt	16	6476966	6685640	24	-0.7716	-1
MSK_0386.1_251469340798_S01_CGH.v4_91.txt	16	25281764	25972139	34	-0.7108	-1

Continued on next page...

ID	chr	start	end	nroOfprobes	segmean	call
MSK_0510.1_251469331581_S01_CGH.v4_91.txt	16	81197195	81293284	8	0.7493	1
MSK_0514.1_251469333360_S01_CGH.v4_91.txt	16	78814326	79264809	36	-0.7113	-1
MSK_0514.1_251469333360_S01_CGH.v4_91.txt	16	5774602	28117693	1215	-0.6998	-1
MSK_0517.1_251469337226_S01_CGH.v4_91.txt	16	2145917	6876930	341	-0.7401	-1
MSK_0520.1_251469336708_S01_CGH.v4_91.txt	16	46500741	90163070	2452	-0.6625	-1
MSK_0520.1_251469336708_S01_CGH.v4_91.txt	16	33631522	34625122	22	-0.6594	-1
MSK_0520.1_251469336708_S01_CGH.v4_91.txt	16	15048676	15089859	6	-0.7517	-1
MSK_00007_251469322733_S01_CGH.v4_91.txt	17	50028931	50983095	32	-0.7668	-1
MSK_00010_251469322627_S01_CGH.v4_91.txt	17	44628091	44694224	2	0.8875	1
MSK_WGA_0016.1_251469338755_S01_CGH.v4_91.txt	17	60549587	60629995	6	1.3193	1
MSK_00021_251469322630_S01_CGH.v4_91.txt	17	34432532	34480451	6	0.9263	1
MSK_00024_251469322721_S01_CGH.v4_91.txt	17	44628091	44694224	2	0.8686	1
MSK_00028_251469322706_S01_CGH.v4_91.txt	17	34437475	34480451	5	0.752	1
MSK_00033_251469322707_S01_CGH.v4_91.txt	17	45711352	45724120	2	-0.7972	-1
MSK_00038_251469322735_S01_CGH.v4_91.txt	17	34437475	34480451	5	0.9884	1
MSK_00043_251469322736_S01_CGH.v4_91.txt	17	44171888	44279759	11	0.6424	1
MSK_WGA_0048.1_251469338742_S01_CGH.v4_91.txt	17	21835219	21872022	2	1.1437	1
MSK_00054_251469323034_S01_CGH.v4_91.txt	17	34437475	34480451	5	0.7675	1
MSK_00055_251469323018_S01_CGH.v4_91.txt	17	29521987	29639181	12	-1.1726	-1
MSK_00074_251469322717_S01_CGH.v4_91.txt	17	30340135	30361777	2	0.6699	1
MSK_00086_251469322749_S01_CGH.v4_91.txt	17	74211086	74251888	4	0.8107	1
MSK_00089_251469322904_S01_CGH.v4_91.txt	17	3507046	3558334	7	-0.9025	-1
MSK_00102_251469322716_S01_CGH.v4_91.txt	17	44171888	44279759	11	0.6857	1
MSK_WGA_0104.1_251469338880_S01_CGH.v4_91.txt	17	80783514	81058462	17	-0.6471	-1
MSK_WGA_0104.1_251469338880_S01_CGH.v4_91.txt	17	59739	21123689	1308	-0.6698	-1
MSK_0111.1_251469337228_S01_CGH.v4_91.txt	17	44188441	44351093	11	-0.6467	-1
MSK_0258.1_251469336709_S01_CGH.v4_91.txt	17	26622742	26658596	5	-0.9368	-1
MSK_0266.1_251469336909_S01_CGH.v4_91.txt	17	44628091	44694224	2	0.6648	1
MSK_0324.1_251469333523_S01_CGH.v4_91.txt	17	29414451	29533283	10	-0.8351	-1
MSK_0339.1_251469331462_S01_CGH.v4_91.txt	17	44188441	44351093	11	-0.6545	-1
MSK_0339.1_251469331462_S01_CGH.v4_91.txt	17	18752653	22205762	148	-0.8687	-1
MSK_0339.1_251469331462_S01_CGH.v4_91.txt	17	15281888	17215065	107	0.6844	1
MSK_0339.1_251469331462_S01_CGH.v4_91.txt	17	3094716	15234397	788	-0.92	-1
MSK_0422.1_251469337230_S01_CGH.v4_91.txt	17	38232610	38269365	3	-0.7525	-1
MSK_0440.1_251469336724_S01_CGH.v4_91.txt	17	45640221	45664546	3	0.6931	1
MSK_0446.1_251469333525_S01_CGH.v4_91.txt	17	4673088	4681356	3	-0.7821	-1
MSK_00122.1_251469325751_S01_CGH.v4_91.txt	17	44171888	44351093	13	1.1733	1
MSK_00128.1_251469325161_S01_CGH.v4_91.txt	17	44171888	44344985	12	0.7986	1
MSK_00133_251469325752_S01_CGH.v4_91.txt	17	33718541	33763283	3	-1.0146	-1
MSK_0152.1_251469337789_S01_CGH.v4_91.txt	17	73156693	73490406	24	0.9515	1
MSK_0152.1_251469337789_S01_CGH.v4_91.txt	17	41114693	41334568	24	0.7888	1
MSK_0158.1_251469329734_S01_CGH.v4_91.txt	17	34437475	34480451	5	0.7441	1
MSK_0162.1_251469336905_S01_CGH.v4_91.txt	17	28347727	28353223	2	-0.8661	-1
MSK_0166.1_251469329777_S01_CGH.v4_91.txt	17	34437475	34462427	4	-0.8133	-1
MSK_0166.1_251469329777_S01_CGH.v4_91.txt	17	29657353	29805908	11	-0.982	-1
MSK_0173.1_251469330461_S01_CGH.v4_91.txt	17	16416040	16532736	12	0.957	1
MSK_0174.1_251469330284_S01_CGH.v4_91.txt	17	31311751	31427641	9	-0.7291	-1
MSK_0179.1_251469333566_S01_CGH.v4_91.txt	17	44188441	44351093	11	-0.6845	-1
MSK_0187.1_251469330462_S01_CGH.v4_91.txt	17	44221743	44351093	8	-0.7387	-1
MSK_0187.1_251469330462_S01_CGH.v4_91.txt	17	41181107	41334568	13	-0.9057	-1
MSK_0197.1_251469329155_S01_CGH.v4_91.txt	17	45640221	45664546	3	-0.6543	-1
MSK_0206.1_251469329173_S01_CGH.v4_91.txt	17	44188441	44694224	13	-0.9494	-1
MSK_0206.1_251469329173_S01_CGH.v4_91.txt	17	29400334	29959640	54	-1.3332	-1
MSK_0208.1_251469329182_S01_CGH.v4_91.txt	17	32380137	32521894	8	-0.7347	-1
MSK_0208.1_251469329182_S01_CGH.v4_91.txt	17	31502602	32313321	67	-0.6601	-1
MSK_0208.2_251469329755_S01_CGH.v4_91.txt	17	32380137	32521894	8	-0.7267	-1
MSK_0213.1_251469329174_S01_CGH.v4_91.txt	17	7541707	7589646	8	-1.0683	-1
MSK_0237.1_251469330449_S01_CGH.v4_91.txt	17	33718541	33763283	3	-0.8608	-1
MSK_0237.1_251469330449_S01_CGH.v4_91.txt	17	7626890	11056416	242	-0.6455	-1
MSK_0397.1_251469330468_S01_CGH.v4_91.txt	17	44221743	44351093	8	-0.6355	-1
MSK_0397.1_251469330468_S01_CGH.v4_91.txt	17	29652842	29768369	9	-0.744	-1
MSK_0397.1_251469330468_S01_CGH.v4_91.txt	17	29400334	29414451	2	-0.7667	-1
MSK_0412.1_251469331490_S01_CGH.v4_91.txt	17	44203115	44351093	10	-0.686	-1
MSK_0413.1_251469331465_S01_CGH.v4_91.txt	17	44188441	44351093	11	-0.6345	-1
MSK_0646.1_251469340746_S01_CGH.v4_91.txt	17	7548064	7614115	12	-1.1906	-1
MSK_WGA_0244.1_251469338881_S01_CGH.v4_91.txt	17	59390454	61763646	151	-0.697	-1
MSK_WGA_0389.1_251469338874_S01_CGH.v4_91.txt	17	18571906	18574169	2	2.6991	1
MSK_WGA_0392.1_251469338876_S01_CGH.v4_91.txt	17	7604068	7607336	2	1.0806	1
MSK_0516.1_251469331469_S01_CGH.v4_91.txt	17	44188441	44351093	11	-0.743	-1
MSK_0517.1_251469337226_S01_CGH.v4_91.txt	17	76152064	76289300	14	0.6509	1
MSK_0520.1_251469336708_S01_CGH.v4_91.txt	17	60123520	60251509	6	-0.7112	-1
MSK_0524.1_251469331557_S01_CGH.v4_91.txt	17	9323149	22154515	646	-0.8505	-1
MSK_0525.1_251469333361_S01_CGH.v4_91.txt	17	34450405	34480451	3	0.6456	1
MSK_0531.1_251469337227_S01_CGH.v4_91.txt	17	73049166	73915332	70	0.6466	1
MSK_0615.1_251469340783_S01_CGH.v4_91.txt	17	79763081	79921395	12	0.9342	1
MSK_00011_251469322628_S01_CGH.v4_91.txt	18	131700	14733811	870	-0.7787	-1
MSK_00014_251469322629_S01_CGH.v4_91.txt	18	19371445	20373531	42	-0.9166	-1
MSK_00069_251469322916_S01_CGH.v4_91.txt	18	73242160	77718809	274	1.373	1
MSK_WGA_0070.1_251469338879_S01_CGH.v4_91.txt	18	13222407	13232028	2	-0.6404	-1
MSK_00083_251469322918_S01_CGH.v4_91.txt	18	43139702	46583389	213	-0.7858	-1
MSK_WGA_0104.1_251469338880_S01_CGH.v4_91.txt	18	77834981	78009973	17	-0.7996	-1
MSK_0258.1_251469336709_S01_CGH.v4_91.txt	18	59081050	59519007	21	-0.9085	-1
MSK_0269.1_251469333522_S01_CGH.v4_91.txt	18	55931229	55936488	2	-1.1149	-1
MSK_0333.1_251469331585_S01_CGH.v4_91.txt	18	71832632	71889295	4	0.9605	1
MSK_0339.1_251469331462_S01_CGH.v4_91.txt	18	55931229	55936488	2	-0.8321	-1
MSK_0439.1_251469331553_S01_CGH.v4_91.txt	18	65866260	65891840	2	-0.9765	-1
MSK_0211.1_251469330227_S01_CGH.v4_91.txt	18	44795853	44807831	2	-0.6478	-1
MSK_0214.1_251469329168_S01_CGH.v4_91.txt	18	55931229	55936488	2	-0.9753	-1
MSK_0237.1_251469330449_S01_CGH.v4_91.txt	18	14316	78009973	4106	-0.6609	-1
MSK_0645.1_251469340797_S01_CGH.v4_91.txt	18	1736753	1838882	7	-0.9381	-1
MSK_WGA_0246.1_251469338865_S01_CGH.v4_91.txt	18	47807143	47810513	2	0.6786	1
MSK_WGA_0357.1_251469337522_S01_CGH.v4_91.txt	18	69912892	73658861	205	-0.7267	-1
MSK_WGA_0357.1_251469337522_S01_CGH.v4_91.txt	18	62337958	62551519	6	-0.7779	-1
MSK_WGA_0359.1_251469337523_S01_CGH.v4_91.txt	18	34614266	74510864	2064	-0.6325	-1
MSK_WGA_0360.1_251469337524_S01_CGH.v4_91.txt	18	13222407	13232028	2	-1.0623	-1
MSK_WGA_0392.1_251469338876_S01_CGH.v4_91.txt	18	47807143	47810513	2	0.8392	1
MSK_0512.1_251469331556_S01_CGH.v4_91.txt	18	28972140	29049537	7	0.6551	1
MSK_0514.1_251469333360_S01_CGH.v4_91.txt	18	62067046	73631047	522	-0.7502	-1
MSK_0517.1_251469337226_S01_CGH.v4_91.txt	18	69959399	70759394	32	-0.8582	-1
MSK_0518.1_251469337014_S01_CGH.v4_91.txt	18	47694240	78009973	1648	-0.9182	-1
MSK_0518.1_251469337014_S01_CGH.v4_91.txt	18	41068861	44540594	203	-0.9121	-1
MSK_0520.1_251469336708_S01_CGH.v4_91.txt	18	66350129	66413688	5	-0.7578	-1
MSK_00006_251469322732_S01_CGH.v4_91.txt	19	55445098	55504718	4	0.707	1
MSK_00009_251469322626_S01_CGH.v4_91.txt	19	52031498	52103681	9	-0.7219	-1
MSK_00010_251469322627_S01_CGH.v4_91.txt	19	56347564	59083522	235	-0.6856	-1
MSK_WGA_0015.1_251469338754_S01_CGH.v4_91.txt	19	45730679	45743290	3	0.8282	1
MSK_WGA_0016.1_251469338755_S01_CGH.v4_91.txt	19	4141570	4470639	25	2.9355	1
MSK_00021_251469322630_S01_CGH.v4_91.txt	19	54735206	54742347	2	1.0555	1
MSK_00021_251469322630_S01_CGH.v4_91.txt	19	53527584	53545649	2	1.6596	1
MSK_00024_251469322721_S01_CGH.v4_91.txt	19	54735206	54742347	2	0.8937	1
MSK_00024_251469322721_S01_CGH.v4_91.txt	19	41448821	41525312	8	0.6618	1
MSK_00028_251469322706_S01_CGH.v4_91.txt	19	28272497	59083522	2052	-0.7625	-1
MSK_00033_251469322707_S01_CGH.v4_91.txt	19	20630736	20682577	3	-0.7992	-1
MSK_00054_251469323034_S01_CGH.v4_91.txt	19	54735206	54742347	2	0.7299	1
MSK_00058_251469323019_S01_CGH.v4_91.txt	19	54735206	54742347	2	-2.0945	-1
MSK_00060_251469322915_S01_CGH.v4_91.txt	19	43708938	43757708	3	-0.7086	-1
MSK_00085_251469322917_S01_CGH.v4_91.txt	19	53527584	53545649	2	0.8522	1
MSK_0106.1_251469331471_S01_CGH.v4_91.txt	19	43689971	43757708	5	-1.2251	-1
MSK_0111.1_251469337228_S01_CGH.v4_91.txt	19	54735206	54742347	2	-1.4429	-1
MSK_00114_251469322752_S01_CGH.v4_91.txt	19	53603269	58800403	413	-0.7483	-1
MSK_00114_251469322752_S01_CGH.v4_91.txt	19	50313000	52949327	201	-0.7633	-1
MSK_00114_251469322752_S01_CGH.v4_91.txt	19	42368983	49570621	495	-0.7115	-1
MSK_0258.1_251469336709_S01_CGH.v4_91.txt	19	21884259	59060208	2178	-0.8007	-1
MSK_0260.1_251469331583_S01_CGH.v4_91.txt	19	57743893	59083522	134	1.5666	1

Continued on next page...

ID	chr	start	end	nroOfprobes	segmean	call
MSK_0260.1_251469331583_S01_CGH.v4_91.txt	19	52116806	57724877	421	0.7531	1
MSK_0271.1_251469333526_S01_CGH.v4_91.txt	19	20630736	20682577	3	-2.4816	-1
MSK_0317.1_251469331584_S01_CGH.v4_91.txt	19	20630736	20682577	3	-1.012	-1
MSK_0324.1_251469333523_S01_CGH.v4_91.txt	19	46530393	46630666	9	-0.8904	-1
MSK_0326.1_251469331461_S01_CGH.v4_91.txt	19	54735206	54742347	2	0.6479	1
MSK_0330.1_251469336713_S01_CGH.v4_91.txt	19	54735206	54742347	2	-1.1657	-1
MSK_0332.1_251469336711_S01_CGH.v4_91.txt	19	54735206	54742347	2	-1.3603	-1
MSK_0338.1_251469333564_S01_CGH.v4_91.txt	19	20630736	20682577	3	-0.7643	-1
MSK_0422.1_251469337230_S01_CGH.v4_91.txt	19	20630736	20682577	3	-0.9824	-1
MSK_0432.1_251469336712_S01_CGH.v4_91.txt	19	33544241	59060208	1853	-0.7001	-1
MSK_0440.1_251469336724_S01_CGH.v4_91.txt	19	54735206	54742347	2	0.6483	1
MSK_0446.1_251469333525_S01_CGH.v4_91.txt	19	43808401	43859610	3	-0.827	-1
MSK_00126.1_251469325171_S01_CGH.v4_91.txt	19	43808401	43864443	4	-0.7194	-1
MSK_00128.1_251469325161_S01_CGH.v4_91.txt	19	37845388	37903211	3	-0.6956	-1
MSK_00129.1_251469322907_S01_CGH.v4_91.txt	19	56220371	56228225	2	1.9353	1
MSK_00129.1_251469322907_S01_CGH.v4_91.txt	19	20630736	20682577	3	-0.716	-1
MSK_00139.1_251469325164_S01_CGH.v4_91.txt	19	54735206	54742347	2	-1.0959	-1
MSK_00139.3_251469325756_S01_CGH.v4_91.txt	19	54735206	54742347	2	-1.0877	-1
MSK_00141.1_251469325753_S01_CGH.v4_91.txt	19	20630736	20738612	4	-0.6632	-1
MSK_0146.1_251469336983_S01_CGH.v4_91.txt	19	113418	972298	57	0.6587	1
MSK_0157.1_251469329178_S01_CGH.v4_91.txt	19	54735206	54742347	2	1.2014	1
MSK_0162.1_251469336905_S01_CGH.v4_91.txt	19	20630736	20682577	3	-0.6503	-1
MSK_0171.1_251469329753_S01_CGH.v4_91.txt	19	20630736	20682577	3	-1.0268	-1
MSK_0175.1_251469336725_S01_CGH.v4_91.txt	19	52762472	52783656	3	-0.681	-1
MSK_0187.1_251469330462_S01_CGH.v4_91.txt	19	20630736	20682577	3	-1.0269	-1
MSK_0197.1_251469329155_S01_CGH.v4_91.txt	19	20630736	20682577	3	-0.9287	-1
MSK_0208.1_251469329182_S01_CGH.v4_91.txt	19	54735206	54742347	2	-1.3718	-1
MSK_0208.2_251469329755_S01_CGH.v4_91.txt	19	54735206	54742347	2	-1.3793	-1
MSK_0213.1_251469329174_S01_CGH.v4_91.txt	19	54735206	54742347	2	0.6775	1
MSK_0213.1_251469329174_S01_CGH.v4_91.txt	19	113418	444207	15	-1.2523	-1
MSK_0214.1_251469329168_S01_CGH.v4_91.txt	19	54735206	54742347	2	0.7602	1
MSK_0219.1_251469329864_S01_CGH.v4_91.txt	19	41356006	41387063	5	-0.6338	-1
MSK_0221.1_251469330192_S01_CGH.v4_91.txt	19	20630736	20682577	3	-0.8314	-1
MSK_0237.1_251469330449_S01_CGH.v4_91.txt	19	51026143	53031718	155	-0.7109	-1
MSK_0394.1_251469336716_S01_CGH.v4_91.txt	19	54735206	54742347	2	0.7423	1
MSK_0349.1_251469337892_S01_CGH.v4_91.txt	19	29946716	30630215	41	4.0415	1
MSK_0509.1_251469336717_S01_CGH.v4_91.txt	19	54735206	54742347	2	-1.1163	-1
MSK_0511.1_251469336907_S01_CGH.v4_91.txt	19	41364029	41387063	4	-0.7382	-1
MSK_0512.1_251469331556_S01_CGH.v4_91.txt	19	53527584	53545649	2	0.938	1
MSK_0516.1_251469331469_S01_CGH.v4_91.txt	19	54759847	54775285	3	-1.2009	-1
MSK_0517.1_251469337226_S01_CGH.v4_91.txt	19	41884555	59083522	1280	-0.7557	-1
MSK_0518.1_251469337014_S01_CGH.v4_91.txt	19	54735206	54742347	2	0.6819	1
MSK_0520.1_251469336708_S01_CGH.v4_91.txt	19	53527584	53545649	2	0.6384	1
MSK_0522.1_251469336908_S01_CGH.v4_91.txt	19	41364029	41371517	2	-0.7776	-1
MSK_0524.1_251469331557_S01_CGH.v4_91.txt	19	58977339	59083522	14	1.0875	1
MSK_00011_251469322628_S01_CGH.v4_91.txt	20	70580	26208026	1578	-0.7682	-1
MSK_00014_251469322629_S01_CGH.v4_91.txt	20	56747422	62908615	405	-0.8269	-1
MSK_00027_251469322705_S01_CGH.v4_91.txt	20	14700040	14713027	2	-1.0872	-1
MSK_WGA_0048.1_251469338742_S01_CGH.v4_91.txt	20	25891919	25918045	2	1.0596	1
MSK_00052_251469323033_S01_CGH.v4_91.txt	20	23851932	23976911	4	-0.8202	-1
MSK_00055_251469323018_S01_CGH.v4_91.txt	20	44350693	44361886	2	-0.6477	-1
MSK_WGA_0104.1_251469338880_S01_CGH.v4_91.txt	20	55728636	62908615	457	-0.7411	-1
MSK_00116_251469322720_S01_CGH.v4_91.txt	20	25891919	25918045	2	1.3461	1
MSK_0258.1_251469336709_S01_CGH.v4_91.txt	20	6974518	8497393	74	-0.8355	-1
MSK_0289.1_251469337015_S01_CGH.v4_91.txt	20	55638915	57315378	102	1.8484	1
MSK_0324.1_251469333523_S01_CGH.v4_91.txt	20	44350693	44361886	2	-1.0971	-1
MSK_0333.1_251469331585_S01_CGH.v4_91.txt	20	32009788	32096800	9	1.0115	1
MSK_0339.1_251469331462_S01_CGH.v4_91.txt	20	31764009	31830278	13	-1.0514	-1
MSK_0157.1_251469329178_S01_CGH.v4_91.txt	20	34758178	35522559	53	0.7088	1
MSK_0188.1_251469330469_S01_CGH.v4_91.txt	20	58244975	58252845	2	-1.3279	-1
MSK_0516.1_251469331469_S01_CGH.v4_91.txt	20	14808927	14849501	3	-0.7617	-1
MSK_0517.1_251469337226_S01_CGH.v4_91.txt	20	55929059	62908615	442	-0.81	-1
MSK_0615.1_251469340783_S01_CGH.v4_91.txt	20	3005064	3027701	3	1.0517	1
MSK_00010_251469322627_S01_CGH.v4_91.txt	21	48021759	48090258	7	-0.7854	-1
MSK_00011_251469322628_S01_CGH.v4_91.txt	21	10874759	48090258	2294	-0.7634	-1
MSK_00014_251469322629_S01_CGH.v4_91.txt	21	10874759	11123010	15	0.7677	1
MSK_WGA_0015.1_251469338754_S01_CGH.v4_91.txt	21	19019415	25612873	290	-0.7423	-1
MSK_WGA_0015.1_251469338754_S01_CGH.v4_91.txt	21	15412671	18825591	216	-0.7001	-1
MSK_00043_251469322736_S01_CGH.v4_91.txt	21	45180840	47809486	203	-0.7957	-1
MSK_00043_251469322736_S01_CGH.v4_91.txt	21	36149648	45119709	734	-0.8562	-1
MSK_00080_251469322748_S01_CGH.v4_91.txt	21	21170003	22782651	83	-0.8841	-1
MSK_00083_251469322918_S01_CGH.v4_91.txt	21	11112564	11145542	3	-0.7217	-1
MSK_00114_251469322752_S01_CGH.v4_91.txt	21	10874759	48090258	2294	-0.7665	-1
MSK_0258.1_251469336709_S01_CGH.v4_91.txt	21	27015874	34687736	570	-0.8448	-1
MSK_0266.1_251469336909_S01_CGH.v4_91.txt	21	18912147	26538484	337	-0.9021	-1
MSK_0266.1_251469336909_S01_CGH.v4_91.txt	21	15412671	18745533	208	-0.8099	-1
MSK_0330.1_251469336713_S01_CGH.v4_91.txt	21	24398456	24437333	3	-0.8688	-1
MSK_0338.1_251469333564_S01_CGH.v4_91.txt	21	44987273	44992766	2	-0.8594	-1
MSK_0339.1_251469331462_S01_CGH.v4_91.txt	21	10874759	48090258	2294	-0.9065	-1
MSK_0432.1_251469336712_S01_CGH.v4_91.txt	21	37484659	37612933	16	0.635	1
MSK_00126.1_251469325171_S01_CGH.v4_91.txt	21	15385803	25509366	518	-0.7127	-1
MSK_00129.1_251469322907_S01_CGH.v4_91.txt	21	34614154	34687736	8	0.6492	1
MSK_0158.1_251469329734_S01_CGH.v4_91.txt	21	36023224	40330597	343	-0.6848	-1
MSK_0158.1_251469329734_S01_CGH.v4_91.txt	21	35583820	35888934	26	-0.643	-1
MSK_0221.1_251469330192_S01_CGH.v4_91.txt	21	15336388	16243615	70	-0.7827	-1
MSK_0402.1_251469336906_S01_CGH.v4_91.txt	21	11096027	11123010	3	-1.3372	-1
MSK_0517.1_251469337226_S01_CGH.v4_91.txt	21	20039810	20494465	19	-0.8776	-1
MSK_00007_251469322733_S01_CGH.v4_91.txt	22	23817481	51162228	1933	-0.7284	-1
MSK_00007_251469322733_S01_CGH.v4_91.txt	22	22104736	23758400	109	-0.7588	-1
MSK_00011_251469322628_S01_CGH.v4_91.txt	22	39361572	39379993	3	-0.7615	-1
MSK_00011_251469322628_S01_CGH.v4_91.txt	22	20306495	20703444	2	0.7574	1
MSK_00011_251469322628_S01_CGH.v4_91.txt	22	16124218	16498852	3	-0.7017	-1
MSK_00028_251469322706_S01_CGH.v4_91.txt	22	31942002	31959483	2	-0.6858	-1
MSK_WGA_0039.1_251469338741_S01_CGH.v4_91.txt	22	39361572	39379993	3	-0.9855	-1
MSK_00043_251469322736_S01_CGH.v4_91.txt	22	46398590	51162228	276	-0.8185	-1
MSK_00052_251469323033_S01_CGH.v4_91.txt	22	38600415	38604006	2	0.7813	1
MSK_00054_251469323034_S01_CGH.v4_91.txt	22	42902341	42912783	3	0.883	1
MSK_00058_251469323019_S01_CGH.v4_91.txt	22	39361572	39379993	3	-0.8209	-1
MSK_00069_251469322916_S01_CGH.v4_91.txt	22	20727981	39348124	1329	0.6976	1
MSK_00069_251469322916_S01_CGH.v4_91.txt	22	20306495	20703444	2	1.5568	1
MSK_00069_251469322916_S01_CGH.v4_91.txt	22	16124218	20301326	210	0.6611	1
MSK_00074_251469322717_S01_CGH.v4_91.txt	22	38513368	38655606	14	0.8182	1
MSK_00074_251469322717_S01_CGH.v4_91.txt	22	23468300	23608984	10	0.6772	1
MSK_00083_251469322918_S01_CGH.v4_91.txt	22	17423213	17861727	27	-0.6959	-1
MSK_00083_251469322918_S01_CGH.v4_91.txt	22	17143118	17304073	9	-0.8118	-1
MSK_00086_251469322749_S01_CGH.v4_91.txt	22	39361572	39379993	3	-1.7135	-1
MSK_0111.1_251469337228_S01_CGH.v4_91.txt	22	39361572	39379993	3	-0.9594	-1
MSK_00116_251469322720_S01_CGH.v4_91.txt	22	43782432	43844034	8	-0.6861	-1
MSK_0289.1_251469337015_S01_CGH.v4_91.txt	22	39361572	39379993	3	-0.8683	-1
MSK_0317.1_251469331584_S01_CGH.v4_91.txt	22	39361572	39379993	3	-2.4877	-1
MSK_0324.1_251469333523_S01_CGH.v4_91.txt	22	28792916	29007499	5	-0.772	-1
MSK_0330.1_251469336713_S01_CGH.v4_91.txt	22	39361572	39379993	3	-0.8703	-1
MSK_0332.1_251469336711_S01_CGH.v4_91.txt	22	35762222	35771995	2	0.8022	1
MSK_0332.1_251469336711_S01_CGH.v4_91.txt	22	16124218	16411562	2	-0.8038	-1
MSK_0333.1_251469331585_S01_CGH.v4_91.txt	22	18971453	51162228	2236	-0.6953	-1
MSK_0333.1_251469331585_S01_CGH.v4_91.txt	22	16124218	18656278	105	-0.692	-1
MSK_0339.1_251469331462_S01_CGH.v4_91.txt	22	39361572	39379993	3	-1.0866	-1
MSK_0339.1_251469331462_S01_CGH.v4_91.txt	22	16124218	16411562	2	-0.6981	-1
MSK_0430.1_251469336714_S01_CGH.v4_91.txt	22	45084698	48773196	235	-0.8004	-1
MSK_0430.1_251469336714_S01_CGH.v4_91.txt	22	33293000	34983426	98	-0.7982	-1
MSK_0430.1_251469336714_S01_CGH.v4_91.txt	22	19114638	19365769	20	-0.6828	-1
MSK_00122.1_251469325751_S01_CGH.v4_91.txt	22	42902341	42912783	3	0.8212	1
MSK_00133_251469325752_S01_CGH.v4_91.txt	22	25076189	25101818	4	-0.882	-1
MSK_00141.1_251469325753_S01_CGH.v4_91.txt	22	39361572	39379993	3	-0.994	-1

Continued on next page...

ID	chr	start	end	nroOfprobes	segmean	call
MSK_00143.1_251469326154_S01_CGH.v4_91.txt	22	39361572	39379993	3	-1.0489	-1
MSK_00169.1_251469325174_S01_CGH.v4_91.txt	22	42906396	42968226	6	0.793	1
MSK_0178.1_251469329154_S01_CGH.v4_91.txt	22	39361572	39379993	3	-0.7425	-1
MSK_0182.1_251469331464_S01_CGH.v4_91.txt	22	50216506	50253878	4	-0.7952	-1
MSK_0182.1_251469331464_S01_CGH.v4_91.txt	22	49768404	49893072	11	-1.1195	-1
MSK_0182.1_251469331464_S01_CGH.v4_91.txt	22	46613083	49659299	171	-1.0584	-1
MSK_0182.1_251469331464_S01_CGH.v4_91.txt	22	45736966	46429030	41	-0.9892	-1
MSK_0182.1_251469331464_S01_CGH.v4_91.txt	22	39361572	39379993	3	-0.9946	-1
MSK_0185.1_251469329754_S01_CGH.v4_91.txt	22	30769362	51162228	1442	-0.746	-1
MSK_0185.1_251469329754_S01_CGH.v4_91.txt	22	24936804	30060423	337	-0.6683	-1
MSK_0188.1_251469330469_S01_CGH.v4_91.txt	22	25741802	25841566	10	-1.7726	-1
MSK_0188.1_251469330469_S01_CGH.v4_91.txt	22	25659172	25720904	3	-2.9675	-1
MSK_0195.1_251469329772_S01_CGH.v4_91.txt	22	41912121	51162228	608	-0.9132	-1
MSK_0201.1_251469329757_S01_CGH.v4_91.txt	22	23914983	23973857	11	0.643	1
MSK_0206.1_251469329173_S01_CGH.v4_91.txt	22	16124218	16411562	2	-0.9136	-1
MSK_0211.1_251469330227_S01_CGH.v4_91.txt	22	42906396	42963635	5	0.6917	1
MSK_0211.2_251469329773_S01_CGH.v4_91.txt	22	43522704	51162228	467	-0.8422	-1
MSK_0213.1_251469329174_S01_CGH.v4_91.txt	22	25659172	25720904	3	-0.8006	-1
MSK_0219.1_251469329864_S01_CGH.v4_91.txt	22	39361572	39379993	3	-1.3891	-1
MSK_0219.1_251469329864_S01_CGH.v4_91.txt	22	16124218	16498852	3	-0.9952	-1
MSK_0237.1_251469330449_S01_CGH.v4_91.txt	22	39361572	39379993	3	-1.9497	-1
MSK_0241.1_251469329774_S01_CGH.v4_91.txt	22	16124218	51162228	2350	-0.7601	-1
MSK_0397.1_251469330468_S01_CGH.v4_91.txt	22	30008726	51162228	1502	-0.6607	-1
MSK_0397.1_251469330468_S01_CGH.v4_91.txt	22	29965724	30000461	5	-1.9728	-1
MSK_0402.1_251469336906_S01_CGH.v4_91.txt	22	39361572	39379993	3	-2.7671	-1
MSK_0410.1_251469333359_S01_CGH.v4_91.txt	22	29429381	29545584	16	-2.7673	-1
MSK_0410.1_251469333359_S01_CGH.v4_91.txt	22	16124218	16498852	3	-0.8566	-1
MSK_0412.1_251469331490_S01_CGH.v4_91.txt	22	16124218	16890151	5	-0.6383	-1
MSK_0645.1_251469340797_S01_CGH.v4_91.txt	22	39361572	39379993	3	-0.7218	-1
MSK_0648.1_251469338021_S01_CGH.v4_91.txt	22	50122206	50652539	29	2.2223	1
MSK_WGA_0244.1_251469338881_S01_CGH.v4_91.txt	22	39390969	51162228	806	-0.6403	-1
MSK_WGA_0244.1_251469338881_S01_CGH.v4_91.txt	22	39361572	39379993	3	-1.6494	-1
MSK_WGA_0244.1_251469338881_S01_CGH.v4_91.txt	22	16124218	39348124	1541	-0.6476	-1
MSK_0358.1_251469340789_S01_CGH.v4_91.txt	22	39361572	39379993	3	-0.7793	-1
MSK_WGA_0389.1_251469338874_S01_CGH.v4_91.txt	22	23800823	51162228	1936	-0.6508	-1
MSK_WGA_0390.1_251469338875_S01_CGH.v4_91.txt	22	29692992	29718420	4	0.965	1
MSK_0514.1_251469333360_S01_CGH.v4_91.txt	22	43066898	51162228	504	-0.6781	-1
MSK_0517.1_251469337226_S01_CGH.v4_91.txt	22	25648623	25841566	14	-0.6485	-1
MSK_0518.1_251469337014_S01_CGH.v4_91.txt	22	39361572	39379993	3	-1.0568	-1
MSK_0524.1_251469331557_S01_CGH.v4_91.txt	22	16124218	16498852	3	-0.6811	-1
MSK_0525.1_251469333361_S01_CGH.v4_91.txt	22	39361572	39379993	3	-0.7043	-1
MSK_0529.1_251469331470_S01_CGH.v4_91.txt	22	39361572	39379993	3	-1.0312	-1

11 Quality Control

11.1 Box plots

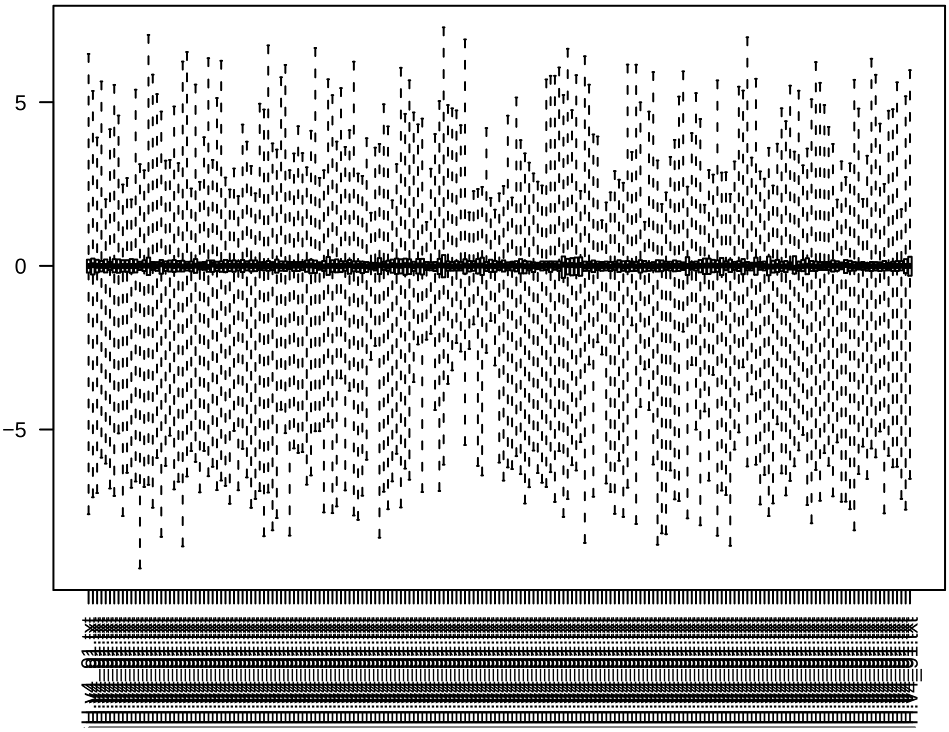

Figure 1914: . The bold line shows the location of median. The filled rectangle contains values between 25'th and 75'th percentile. The extremes show locations of minimum and maximum.

12 Array CGH: integration results for CN gain regions and expression

Table 11: Top 100 integration results of gene level transcript expression values with copy number aberration regions. The weight indicates the correlation in gene level transcript expression between aberrated and non-aberrated samples.

GeneName	Gene	band	weight	alpha
ING3	ENSG00000071243	7q31.31	3.50073	0.00000
RP11-472N13.2	ENSG00000223834	10p11.22	3.13570	0.00000
KCNS3	ENSG00000170745	2p24.2	2.75636	0.00000
RP11-462L8.1	ENSG00000229656	10p11.22	2.06007	0.00000
KCND2	ENSG00000184408	7q31.31	1.97660	0.00000
WAC	ENSG00000095787	10p12.1	1.01137	0.00000
LRRC66	ENSG00000188993	4q12	0.72222	0.00000
EGFR	ENSG00000146648	7p11.2	0.30530	0.00000
CTTNBP2	ENSG00000077063	7q31.2 7q31.31	2.30603	0.00100
KIAA1462	ENSG00000165757	10p11.23	2.14413	0.00100
RP11-192P3.1	ENSG00000237036	10p11.22	1.99606	0.00100
ZNF438	ENSG00000183621	10p11.22 10p11.23	1.94071	0.00100
ANKRD16	ENSG00000134461	10p15.1	0.90675	0.00100
RP11-745C15.2	ENSG00000234707	7p11.2	0.25376	0.00100
ARHGAP12	ENSG00000165322	10p11.22	2.01537	0.00200
TSPAN12	ENSG00000106025	7q31.31	1.87358	0.00200
CAPZA2	ENSG00000198898	7q31.2	0.76363	0.00200
LNX1	ENSG00000072201	4q12	0.41563	0.00200
AVIL	ENSG00000135407	12q14.1	0.35526	0.00200
LANCL2	ENSG00000132434	7p11.2	0.34696	0.00200
CYP27B1	ENSG00000111012	12q14.1	0.33718	0.00200
VOPP1	ENSG00000154978	7p11.2	0.32498	0.00200
RNF44	ENSG00000146083	5q35.2	1.58432	0.00300
BAMBI	ENSG00000095739	10p12.1	0.93382	0.00300
TAF3	ENSG00000165632	10p14	0.78086	0.00300
ABI1	ENSG00000136754	10p12.1	0.76701	0.00300
EPC1	ENSG00000120616	10p11.22	1.98979	0.00400
C12orf28	ENSG00000166268	12q15	0.87520	0.00400
AC064846.1	ENSG00000230448	2p24.3	0.93847	0.00600
RP11-455B2.7	ENSG00000241107	10p13	0.78044	0.00600
ACBD5	ENSG00000107897	10p12.1	0.72550	0.00600
HSPA14	ENSG00000187522	10p13	0.69165	0.00600
NMU	ENSG00000109255	4q12	0.59360	0.00600
PDCL2	ENSG00000163440	4q12	0.53649	0.00600
TPCN2	ENSG00000162341	11q13.3	1.63430	0.00700
SGCB	ENSG00000163069	4q12	0.62433	0.00700
FAM119B	ENSG00000123427	12q14.1	0.30929	0.00700
ZEB1	ENSG00000148516	10p11.22	1.52307	0.00900
RP11-1217F2.4	ENSG00000225295	7p11.2	1.42699	0.00900
KIT	ENSG00000157404	4q12	0.36671	0.00900
DCDC5	ENSG00000170959	11p13 11p14.1	1.20817	0.01000
RP11-534G20.3	ENSG00000224597	10p11.23	0.97445	0.01000
ST7	ENSG00000004866	7q31.2	0.96042	0.01000
RAB18	ENSG00000099246	10p12.1	0.65884	0.01000
SLC35E3	ENSG00000175782	12q15	0.33318	0.01000
DIRAS2	ENSG00000165023	9q22.2	1.38270	0.01100
MTPAP	ENSG00000107951	10p11.23	0.79702	0.01100
TSPAN31	ENSG00000135452	12q14.1	0.28802	0.01100
FAM171A1	ENSG00000148468	10p13	0.64112	0.01200
PRR5L	ENSG00000135362	11p12 11p13	1.28808	0.01400
RASSF3	ENSG00000153179	12q14.2	0.86013	0.01400
ASB13	ENSG00000196372	10p15.1	0.59400	0.01400
FAM188A	ENSG00000148481	10p13	0.58470	0.01400
TSFM	ENSG00000123297	12q14.1	0.29601	0.01400
AC073333.8	ENSG00000235837	7p21.1	1.54493	0.01600
KIF5B	ENSG00000170759	10p11.22	1.29611	0.01600
MYO1A	ENSG00000166866	12q13.3	1.24956	0.01600
ITGB1	ENSG00000150093	10p11.22	1.23027	0.01600
C13orf16	ENSG00000153495	13q34	1.18345	0.01600
BMI1	ENSG00000168283	10p12.2	0.64453	0.01600
USP15	ENSG00000135655	12q14.1	1.08627	0.01700
MASTL	ENSG00000120539	10p12.1	0.62996	0.01700
RP11-499P20.2	ENSG00000240291	10p12.31	0.52086	0.01800
CACNB2	ENSG00000165995	10p12.31 10p12.32 10p12.33	0.55888	0.01900
BEND7	ENSG00000165626	10p13	0.54340	0.02000
EFCAB3	ENSG00000240758	7q32.1	1.33305	0.02100
ST7OT4	ENSG00000214188	7q31.2	0.84512	0.02100
CTDSP2	ENSG00000175215	12q14.1	0.29094	0.02100
FBXO18	ENSG00000134452	10p15.1	0.70899	0.02200
PRTFDC1	ENSG00000099256	10p12.1	0.64124	0.02200
ANKRD52	ENSG00000139645	12q13.3	1.24318	0.02400
RBM17	ENSG00000134453	10p15.1	0.71289	0.02500
C12orf56	ENSG00000185306	12q14.2	0.46706	0.02500
SEC61A2	ENSG00000065665	10p13 10p14	0.58883	0.02600
PDGFRA	ENSG00000134853	4q12	0.27327	0.02700
OS9	ENSG00000135506	12q13.3 12q14.1	0.25786	0.02700
AC233309.1	ENSG00000205885	12p13.31	1.25047	0.02800
GHRHR	ENSG00000106128	7p14.3	1.20629	0.02800
DIP2B	ENSG00000066084	12q13.12	1.10199	0.02800
MLLT10	ENSG00000078403	10p12.31	0.48177	0.02800
AGAP2	ENSG00000135439	12q14.1	0.26680	0.02800
C10orf68	ENSG00000150076	10p11.22	1.12779	0.02900
STAM	ENSG00000136738	10p12.33	0.53635	0.02900
KRT83	ENSG00000170523	12q13.13	1.17004	0.03000
FRS2	ENSG00000166225	12q15	0.46553	0.03000
USP46	ENSG00000109189	4q12	0.36623	0.03000
CLOCK	ENSG00000134852	4q12	0.38795	0.03100
ARL5B	ENSG00000165997	10p12.31	0.51392	0.03300
SCFD2	ENSG00000184178	4q12	0.33060	0.03300
RCN1	ENSG00000049449	11p13	1.10113	0.03400
ACBD7	ENSG00000176244	10p13	0.57291	0.03600
SEPHS1	ENSG00000086475	10p13	0.49543	0.03600
DHTKD1	ENSG00000181192	10p14	0.56326	0.03700
IFNG	ENSG00000111537	12q15	0.53952	0.03700
NSUN6	ENSG00000241058	10p12.31	0.47009	0.03700
RPP38	ENSG00000152464	10p13	0.51763	0.03800
HS1BP3	ENSG00000118960	2p24.1	1.17906	0.04000
RP11-330O11.2	ENSG00000237135	10p11.23	0.90333	0.04000
YME1L1	ENSG00000136758	10p12.1	0.54149	0.04000
XRCC6BP1	ENSG00000166896	12q14.1	0.29091	0.04000

13 Array CGH: integration results for CN loss regions and expression

Table 12: Top 100 integration results of gene level transcript expression values with copy number aberration regions. The weight indicates the correlation in gene level transcript expression between aberrated and non-aberrated samples.

GeneName	Gene	band	weight	alpha
DKK3	ENSG00000050165	11p15.3	2.01165	0.00000
PTPRE	ENSG00000132334	10q26.2	1.44464	0.00000
SMU1	ENSG00000122692	9p21.1	0.98914	0.00000
C9orf82	ENSG00000120159	9p21.2	0.41159	0.00000

Continued on next page...

GeneName	Gene	band	weight	alpha
KIAA1797	ENSG00000188352	9p21.3	0.35286	0.00000
KLHL9	ENSG00000198642	9p21.3	0.28912	0.00000
CTRC	ENSG00000162438	1p36.21	2.32831	0.00100
KDM4C	ENSG00000107077	9p24.1	0.63767	0.00100
PLAA	ENSG00000137055	9p21.2	0.33669	0.00100
MLLT3	ENSG00000171843	9p21.3	0.31114	0.00100
PARP10	ENSG00000178685	8q24.3	1.96014	0.00200
NPM1	ENSG00000181163	5q35.1	1.76931	0.00200
HDAC7	ENSG00000061273	12q13.11	1.56636	0.00200
ARFGEF1	ENSG00000066777	8q13.2	0.93306	0.00200
SMARCA2	ENSG00000080503	9p24.3	0.59865	0.00200
RP1-21O18.1	ENSG00000189337	1p36.21	1.83056	0.00300
CYB5D1	ENSG00000182224	17p13.1	1.72425	0.00300
RANGRF	ENSG00000108961	17p13.1	1.66344	0.00300
RP1-287H17.1	ENSG00000236389	6q23.2	1.50276	0.00300
WNT2B	ENSG00000134245	1p13.2	1.23442	0.00300
API5	ENSG00000166181	11p12	1.18542	0.00300
STYK1	ENSG00000060140	12p13.2	0.92661	0.00300
TPM2	ENSG00000198467	9p13.3	0.75489	0.00300
IFT74	ENSG00000096872	9p21.2	0.38519	0.00300
DERL2	ENSG00000072849	17p13.2	1.54863	0.00400
RTEL1	ENSG00000026036	20q13.33	1.28270	0.00400
APTX	ENSG00000137074	9p21.1	0.94389	0.00400
TMEM110	ENSG00000213533	3p21.1	0.91253	0.00400
NPR2	ENSG00000159899	9p13.3	0.83627	0.00400
MSH4	ENSG00000057468	1p31.1	0.83143	0.00400
TLN1	ENSG00000137076	9p13.3	0.73953	0.00400
USP33	ENSG00000077254	1p31.1	0.72191	0.00400
SLC39A8	ENSG00000138821	4q24	1.65824	0.00500
ZFHX4	ENSG00000091656	8q21.11	1.58161	0.00500
FYTTD1	ENSG00000122068	3q29	1.43196	0.00500
BAP1	ENSG00000163930	3p21.1	0.86756	0.00500
RRAGA	ENSG00000155876	9p22.1	0.58640	0.00500
SH3TC1	ENSG00000125089	4p16.1	1.67166	0.00600
SCRIB	ENSG00000180900	8q24.3	1.48335	0.00600
TMEM56	ENSG00000152078	1p21.3	1.18861	0.00600
LRRFIP2	ENSG00000093167	3p22.2	1.17241	0.00600
YTHDF3	ENSG00000185728	8q12.3	1.12145	0.00600
MICAL2	ENSG00000133816	11p15.3	1.03210	0.00600
PIGO	ENSG00000165282	9p13.3	0.74853	0.00600
MTAP	ENSG00000099810	9p21.3	0.20952	0.00600
PPP1R16A	ENSG00000160972	8q24.3	1.46259	0.00700
FAM26F	ENSG00000188820	6q22.1	1.41847	0.00700
PARVA	ENSG00000197702	11p15.3	0.92578	0.00700
RNLS	ENSG00000184719	10q23.31	0.57715	0.00700
INCA1	ENSG00000196388	17p13.2	1.41267	0.00800
C8orf14	ENSG00000170983	8p23.1	1.39556	0.00800
COMMD10	ENSG00000145781	5q23.1	1.03224	0.00800
OSBPL2	ENSG00000130703	20q13.33	0.95604	0.00800
CMTM7	ENSG00000153551	3p22.3	0.90391	0.00800
MSMP	ENSG00000215183	9p13.3	0.58569	0.00800
JAK2	ENSG00000096968	9p24.1	0.49789	0.00800
SLC12A7	ENSG00000113504	5p15.33	1.57323	0.00900
ZBTB4	ENSG00000174282	17p13.1	1.33684	0.00900
STT3B	ENSG00000163527	3p23	1.17005	0.00900
PEX14	ENSG00000142655	1p36.22	1.13472	0.00900
RAB22A	ENSG00000124209	20q13.32	1.05703	0.00900
TPD52L2	ENSG00000101150	20q13.33	0.94710	0.00900
DNAH1	ENSG00000114841	3p21.1	0.82597	0.00900
FAM69A	ENSG00000154511	1p22.1	0.79250	0.00900
RRS1	ENSG00000179041	8q13.1	0.76761	0.00900
PRKACB	ENSG00000142875	1p31.1	0.72502	0.00900
NOL6	ENSG00000165271	9p13.3	0.70958	0.00900
ATP5E	ENSG00000124172	20q13.32	0.90979	0.01000
DLEU7	ENSG00000186047	13q14.3	0.67081	0.01000
CD72	ENSG00000137101	9p13.3	0.65012	0.01000
FAM154A	ENSG00000155875	9p22.1	0.50136	0.01000
KIAA1432	ENSG00000107036	9p24.1	0.46114	0.01000
CDKN2A	ENSG00000147889	9p21.3	0.19950	0.01000
NRBP2	ENSG00000185189	8q24.3	1.45826	0.01100
SLMO2	ENSG00000101166	20q13.32	0.95535	0.01100
TRIM13	ENSG00000204977	13q14.2	0.67450	0.01100
RP11-513M16.2	ENSG00000242215	9p22.1	0.53131	0.01100
THAP4	ENSG00000176946	2q37.3	0.50722	0.01100
GNAI2	ENSG00000114353	3p21.31	1.56797	0.01200
PDCD6IP	ENSG00000170248	3p22.3	1.02064	0.01200
EXOSC7	ENSG00000075914	3p21.31	0.92838	0.01200
TRIM6	ENSG00000121236	11p15.4	0.90762	0.01200
C8orf44	ENSG00000213865	8q13.1	0.74231	0.01200
HINT2	ENSG00000137133	9p13.3	0.64306	0.01200
DFFB	ENSG00000169598	1p36.32	0.56579	0.01200
TSPAN19	ENSG00000231738	12q21.31	0.85282	0.01300
DDX58	ENSG00000107201	9p21.1	0.63101	0.01300
TMEM19	ENSG00000139291	12q21.1	1.25287	0.01400
STOML3	ENSG00000133115	13q13.3	1.04879	0.01400
PLIN2	ENSG00000147872	9p22.1	0.47642	0.01400
UHRF2	ENSG00000147854	9p24.1	0.46541	0.01400
CLDN7	ENSG00000181885	17p13.1	1.27102	0.01500
MRVI1	ENSG00000072952	11p15.3 11p15.4	1.17200	0.01500
AP002004.1	ENSG00000235505	11q22.3	1.55481	0.01600
C22orf9	ENSG00000100364	22q13.31	1.27746	0.01600
STX8	ENSG00000170310	17p13.1	1.21625	0.01600
PLD2	ENSG00000129219	17p13.2	1.31594	0.01700
KIAA1107	ENSG00000069712	1p22.1	0.91848	0.01700
DENND4C	ENSG00000137145	9p22.1	0.51264	0.01700
PDCD1LG2	ENSG00000197646	9p24.1	0.43754	0.01700

14 Summary of differentially expressed miRNAs

Gene set	Size
over	31
under	32

14.1 Venn diagram of differentially expressed and statistically significant miRNAs

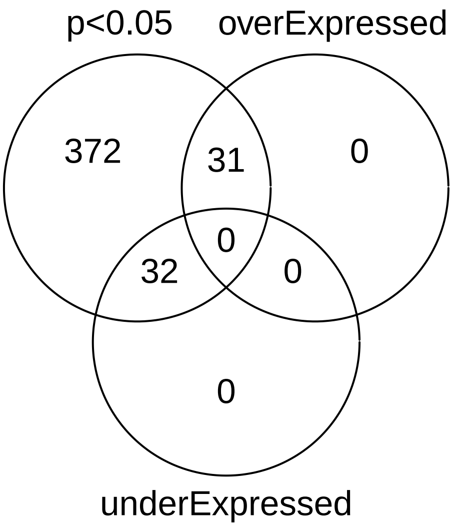

(a)

Figure 1915: Intersections of sets: (a) p<0.05, overExpressed and underExpressed.

14.2 Expression quality control

14.2.1 Ordered miRNA expression values

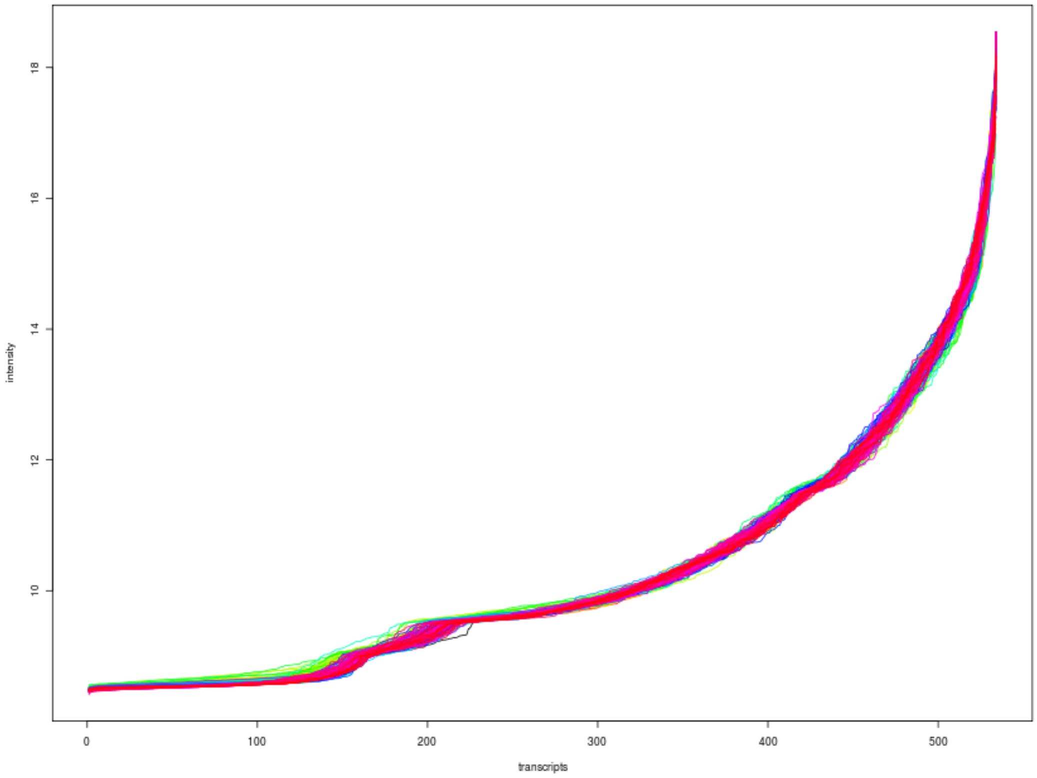

Figure 1916: Cumulative distributions of miRNA expression values for the samples.

14.2.2 Multidimensional scaling of the samples

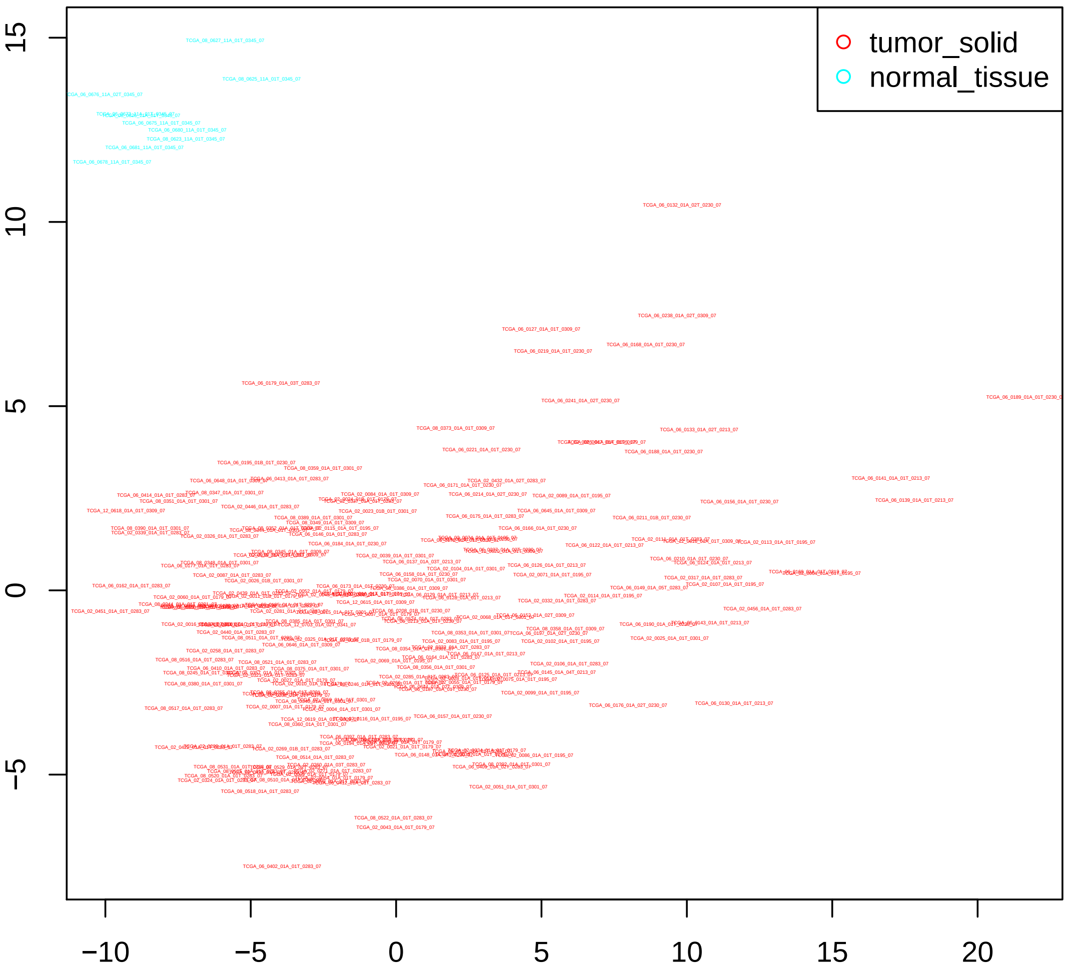

Figure 1917: Multidimensional Scaling

15 Survival analysis for miRNA expression

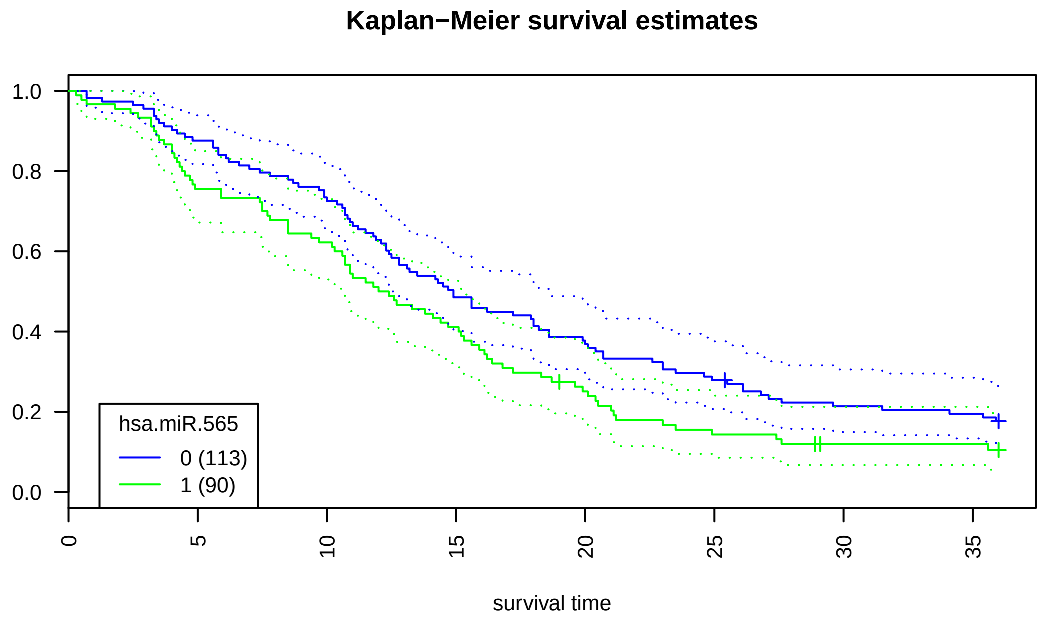

Figure 1918: Each curve represents the relative number of hsa.miR.565 survived to the corresponding point of time. Dashed lines represent 0.95 confidence intervals for the curves with the corresponding color. Total of 2 observations deleted due to missingness. Log-rank probability measure for the equality of these curves is 3.033808e-02.

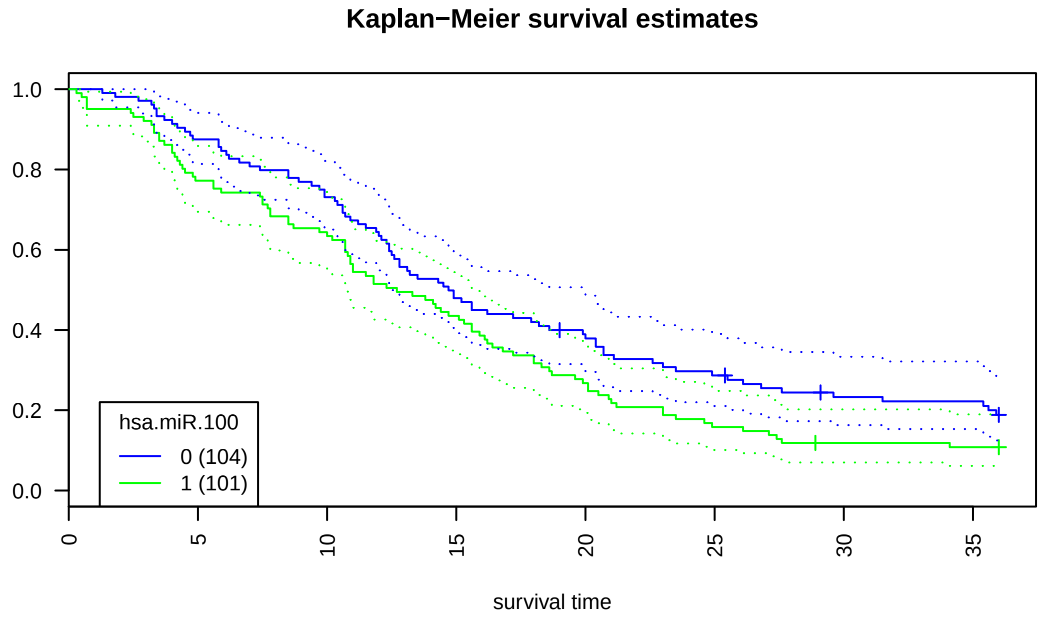

Figure 1919: Each curve represents the relative number of hsa.miR.100 survived to the corresponding point of time. Dashed lines represent 0.95 confidence intervals for the curves with the corresponding color. Log-rank probability measure for the equality of these curves is 3.954936e-02.

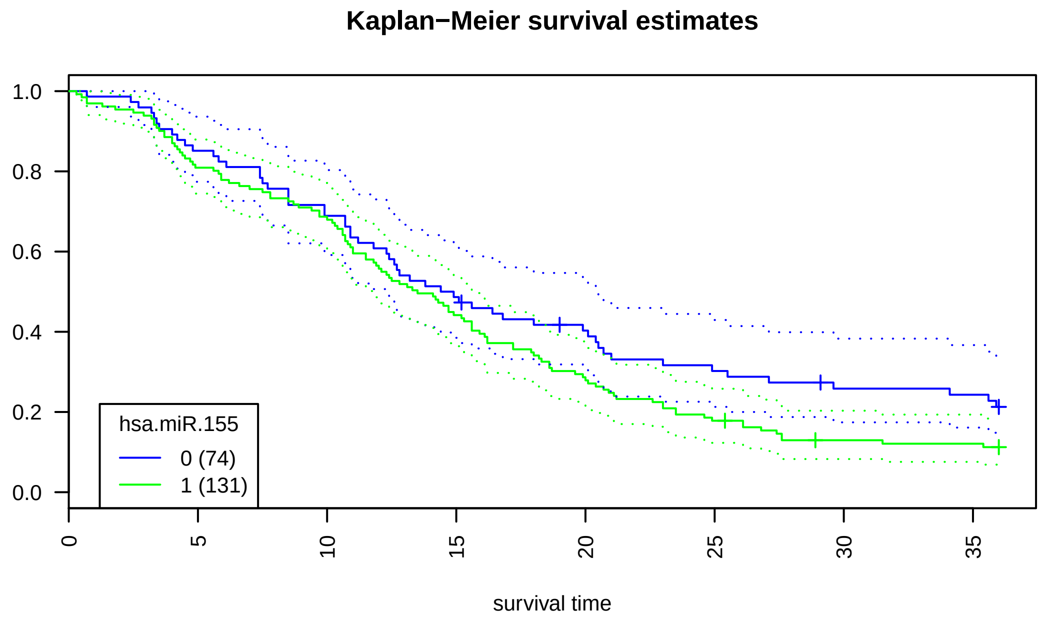

Figure 1920: Each curve represents the relative number of hsa.miR.155 survived to the corresponding point of time. Dashed lines represent 0.95 confidence intervals for the curves with the corresponding color. Log-rank probability measure for the equality of these curves is 8.024321e-02.

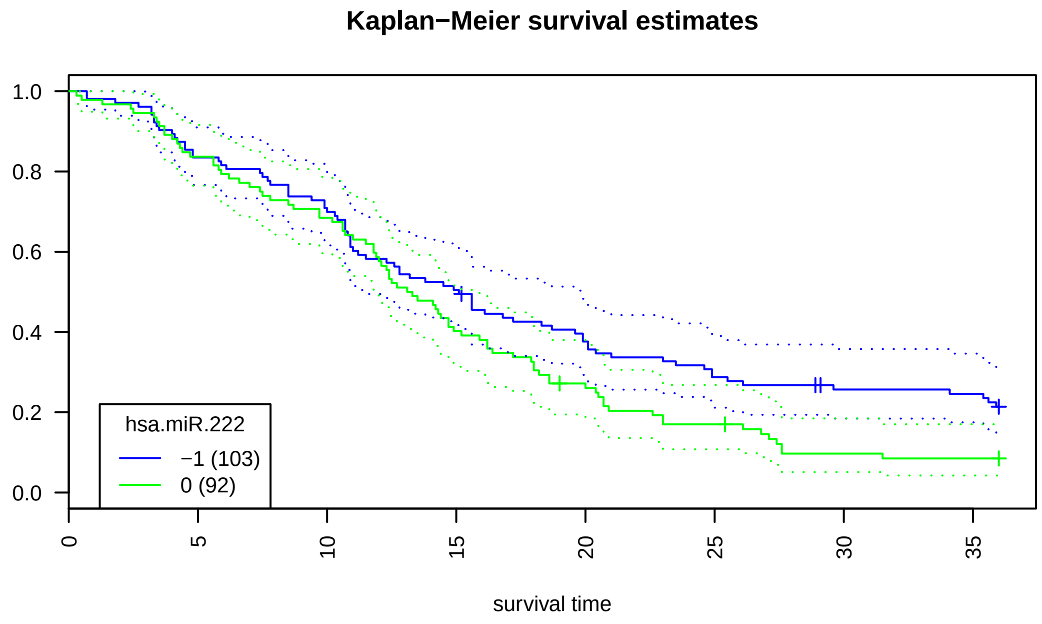

Figure 1921: Each curve represents the relative number of hsa.miR.222 survived to the corresponding point of time. Dashed lines represent 0.95 confidence intervals for the curves with the corresponding color. Total of 10 observations deleted due to missingness. Log-rank probability measure for the equality of these curves is 3.200286e-02.

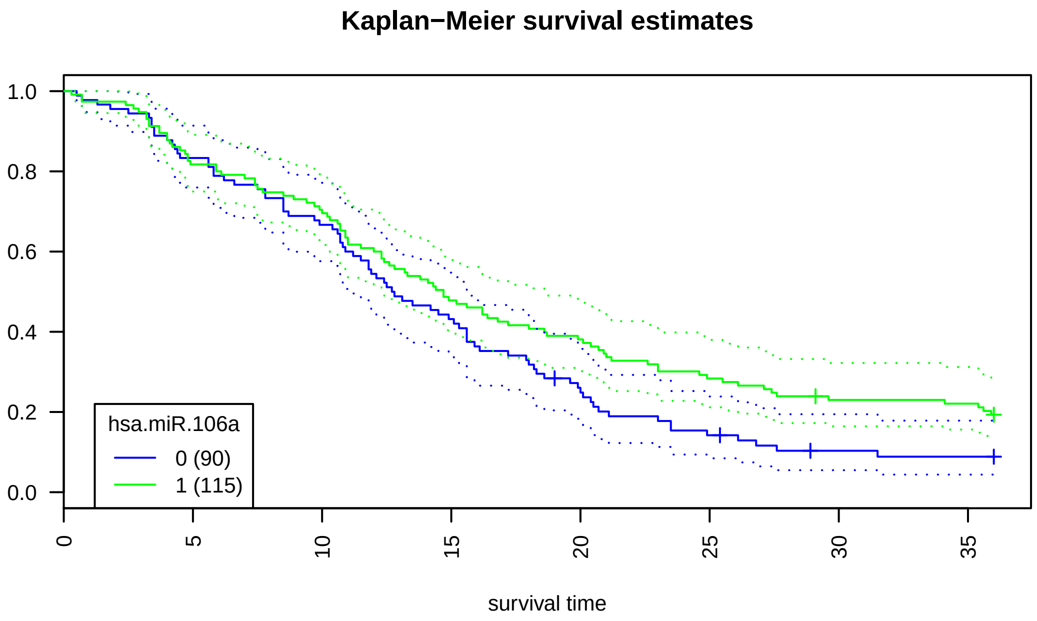

Figure 1922: Each curve represents the relative number of hsa.miR.106a survived to the corresponding point of time. Dashed lines represent 0.95 confidence intervals for the curves with the corresponding color. Log-rank probability measure for the equality of these curves is 4.125438e-02.

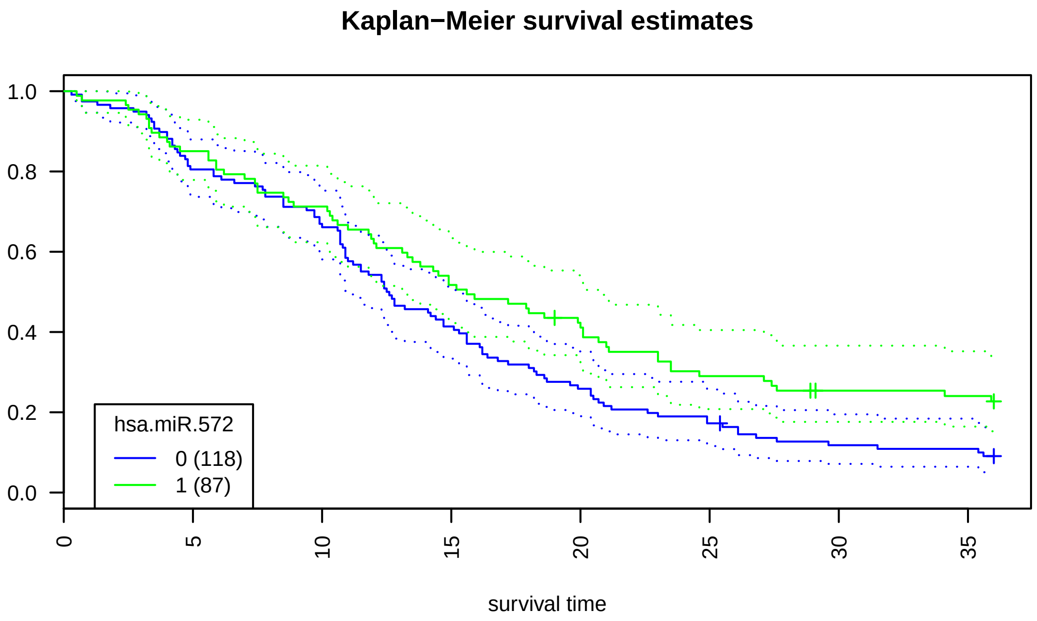

Figure 1923: Each curve represents the relative number of hsa.miR.572 survived to the corresponding point of time. Dashed lines represent 0.95 confidence intervals for the curves with the corresponding color. Log-rank probability measure for the equality of these curves is 1.394632e-02.

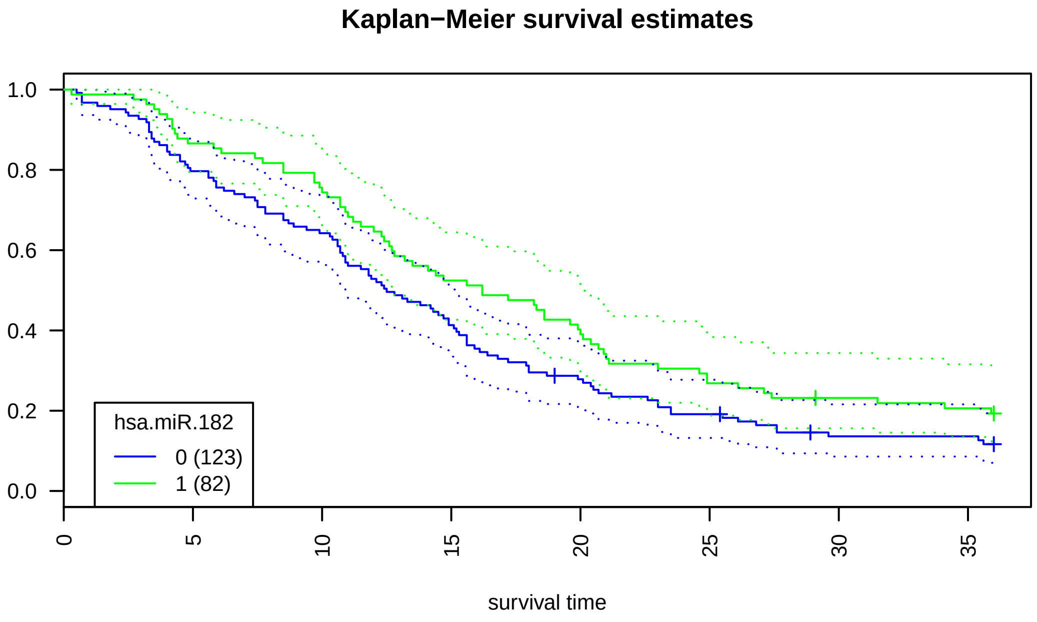

Figure 1924: Each curve represents the relative number of hsa.miR.182 survived to the corresponding point of time. Dashed lines represent 0.95 confidence intervals for the curves with the corresponding color. Log-rank probability measure for the equality of these curves is 4.254161e-02.

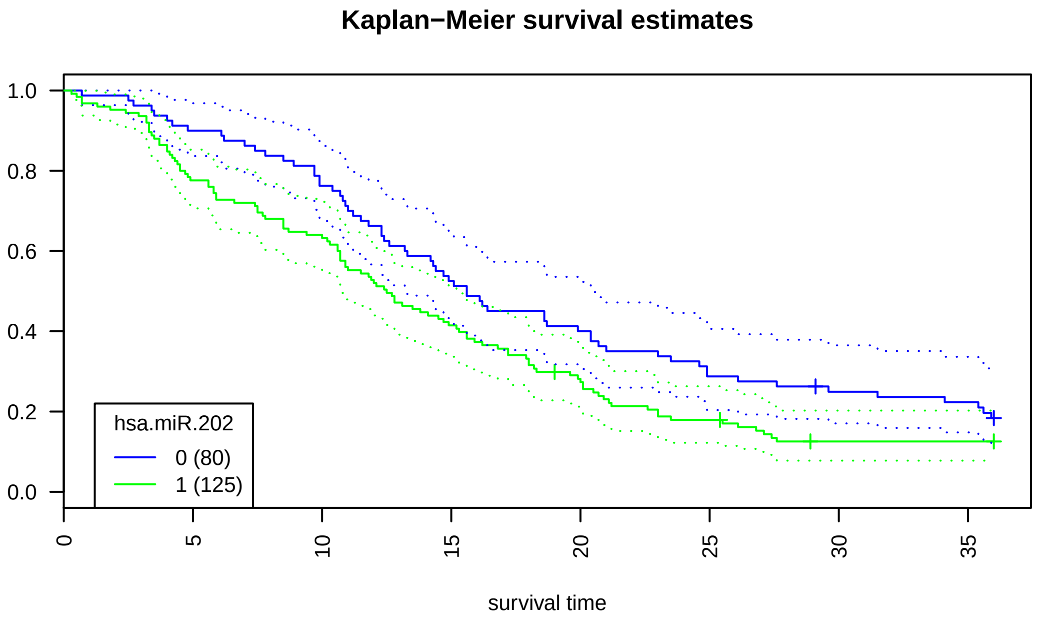

Figure 1925: Each curve represents the relative number of hsa.miR.202 survived to the corresponding point of time. Dashed lines represent 0.95 confidence intervals for the curves with the corresponding color. Log-rank probability measure for the equality of these curves is 2.979511e-02.

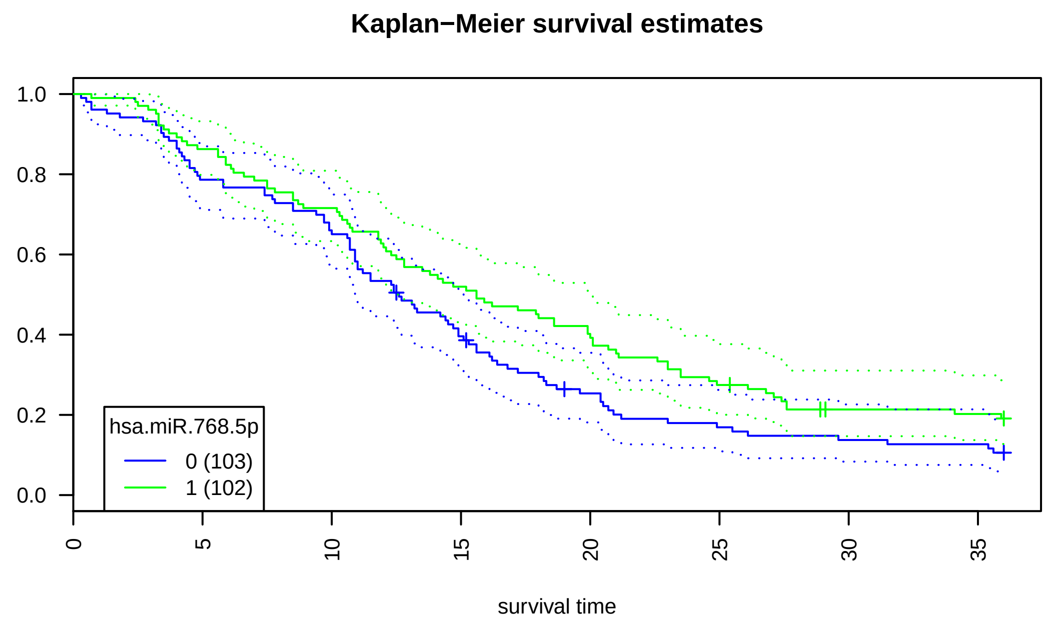

Figure 1926: Each curve represents the relative number of hsa.miR.768.5p survived to the corresponding point of time. Dashed lines represent 0.95 confidence intervals for the curves with the corresponding color. Log-rank probability measure for the equality of these curves is 3.690504e-02.

16 List of differentially expressed genes

Overexpressed genes are sorted with the most overexpressed first and underexpressed genes with the most underexpressed first. Genes go by the column first and then by row.

16.1 Gene set: over

Number of genes: 31

hsa-miR-21	hsa-miR-25	hsa-miR-630	hsa-miR-339	hsa-miR-320	hsa-miR-93	hsa-miR-106a	hsa-miR-768-5p
hsa-miR-10b	hsa-miR-15b	hsa-miR-92	hsa-miR-92b	hsa-miR-130b	hsa-miR-20a	hsa-miR-370	hsa-miR-202
hsa-miR-210	hsa-miR-23a	hsa-miR-106b	hsa-miR-494	hsa-miR-16	hsa-miR-182	hsa-miR-565	hsa-miR-19b
hsa-miR-27a	hsa-miR-638	hsa-miR-155	hsa-miR-34a	hsa-miR-572	hsa-miR-519e*	hsa-miR-100	

16.2 Gene set: under

Number of genes: 32

hsa-miR-124a	hsa-miR-218	hsa-miR-128b	hsa-miR-138	hsa-miR-379	hsa-miR-33	hsa-miR-222	hsa-miR-432
hsa-miR-139	hsa-miR-338	hsa-miR-136	hsa-miR-29b	hsa-miR-132	hsa-miR-127	hsa-miR-769-5p	hsa-miR-383
hsa-miR-137	hsa-miR-129	hsa-miR-410	hsa-miR-29c	hsa-miR-491	hsa-miR-330	hsa-miR-101	hsa-miR-107
hsa-miR-7	hsa-miR-219	hsa-miR-128a	hsa-miR-377	hsa-miR-149	hsa-miR-323	hsa-miR-342	hsa-miR-381

17 Genotype Specific Survival Estimates

17.1 Survival analysis of rs7536561

This marker has a genotype specific effect with a p-value of 4.254600e-05.

Odds ratios have been estimated using logistic regression at $t \approx 36$:
$\frac{P(event|AT)/P(\neg event|AT)}{P(event|AA)/P(\neg event|AA)} \approx 0.349$ and $\frac{P(event|TT)/P(\neg event|TT)}{P(event|AA)/P(\neg event|AA)} \approx 0.313$.

Table 15: Summary of genotype frequencies with their proportions in parenthesis.

genotype	total N	events	censored
AA	54	48 (0.889)	6 (0.111)
AT	91	67 (0.736)	24 (0.264)
TT	49	35 (0.714)	14 (0.286)

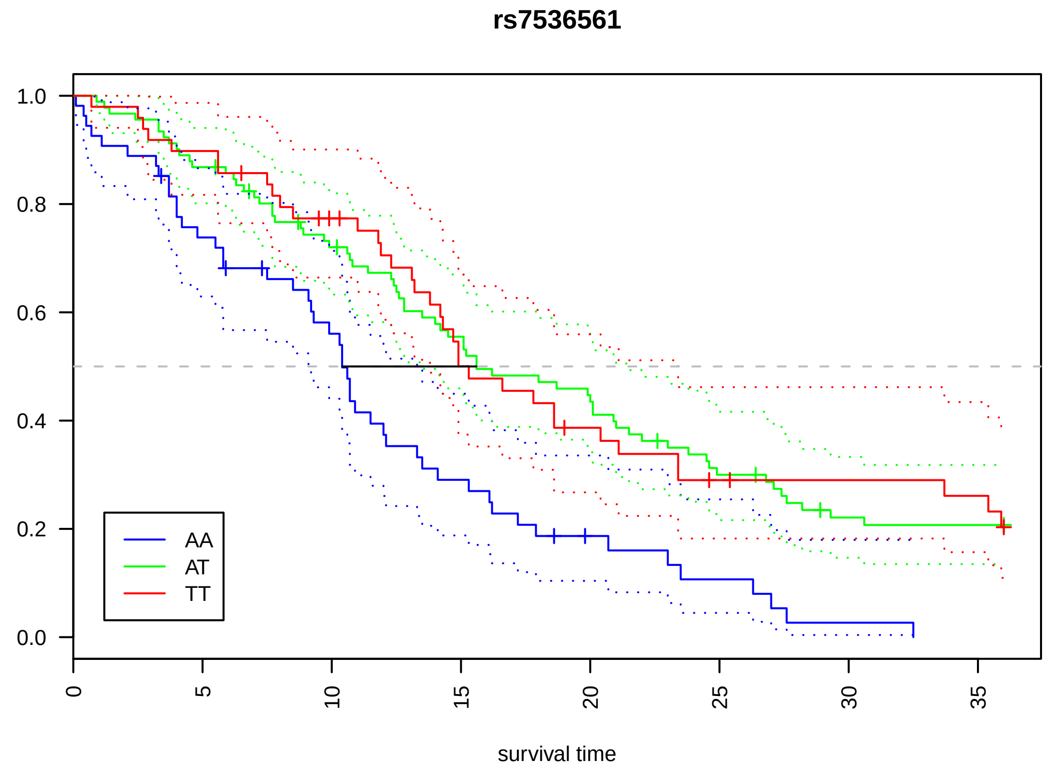

Figure 1927: Kaplan-Meier curves for the selected genotypes. Dashed lines represent 0.95 confidence intervals for the curves with the corresponding color. Black horizontal line $(15.60 - 10.40 = 5.20)$ indicates the maximal survival difference at $y = 0.50$.

Additional annotations:

Gene name	*Description*	*DNABand*	*protei*
LHX4,ACBD6	LIM homeobox 4 [Source:HGNC Symbol;Acc:21734],acyl-Coenzyme A binding domain containing 6 [Source:HGNC Symbol;Acc:23339]	1q25.2	LHX4_HUMAN,AC

17.2 Survival analysis of rs10506216

This marker has a genotype specific effect with a p-value of 9.835901e-05.

Odds ratios have been estimated using logistic regression at $t \approx 36$:
$\frac{P(event|AT,TT)/P(\neg event|AT,TT)}{P(event|AA)/P(\neg event|AA)} \approx 5.923$.

Table 16: Summary of genotype frequencies with their proportions in parenthesis.

genotype	total N	events	censored
AA	159	117 (0.736)	42 (0.264)
AT,TT	35	33 (0.943)	2 (0.0571)

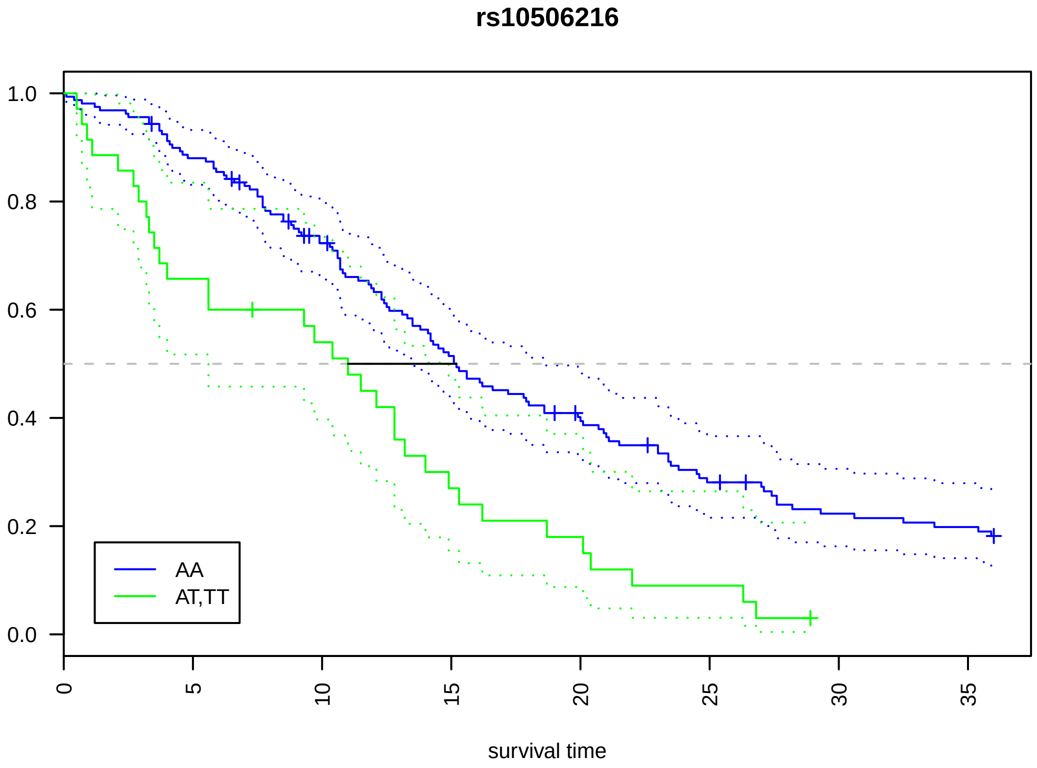

Figure 1928: Kaplan-Meier curves for the selected genotypes. Dashed lines represent 0.95 confidence intervals for the curves with the corresponding color. Black horizontal line $(15.20 - 11.00 = 4.20)$ indicates the maximal survival difference at $y = 0.50$.

17.3 Survival analysis of rs1919793

This marker has a genotype specific effect with a p-value of 2.388316e-06. Total of 9 observations deleted due to missingness. Odds ratios have been estimated using logistic regression at $t \approx 36$:
$\frac{P(event|AT,TT)/P(\neg event|AT,TT)}{P(event|AA)/P(\neg event|AA)} \approx 3.563$.

Table 17: Summary of genotype frequencies with their proportions in parenthesis.

genotype	total N	events	censored
AA	127	90 (0.709)	37 (0.291)
AT,TT	58	52 (0.897)	6 (0.103)

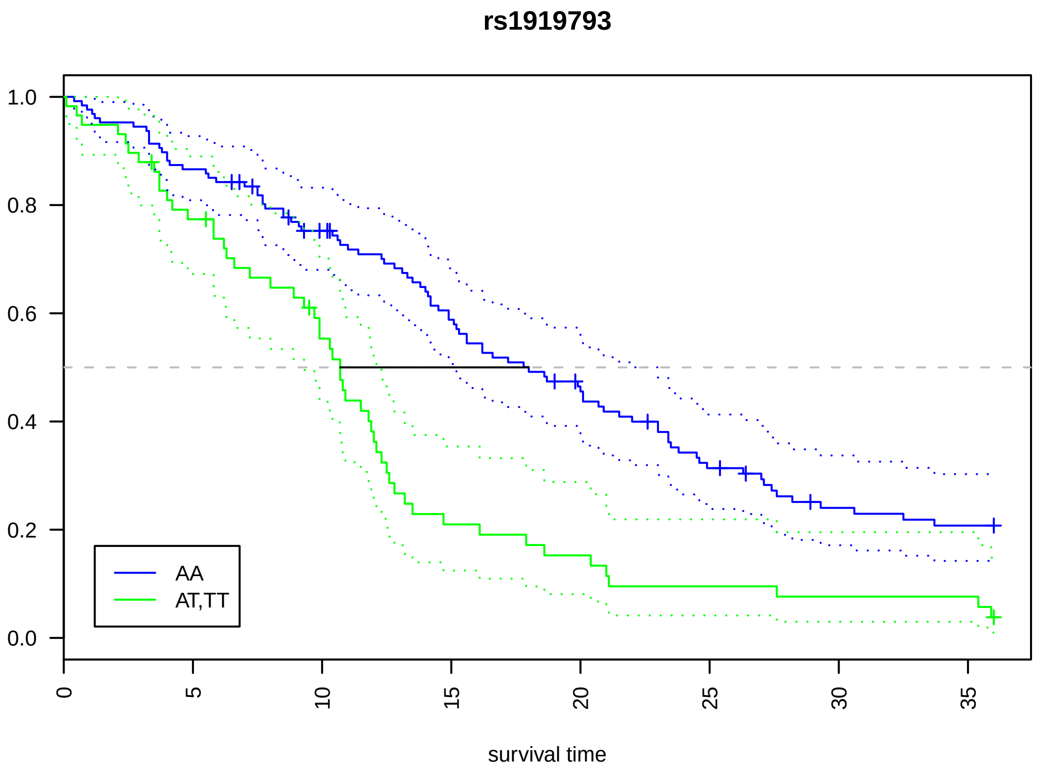

Figure 1929: Kaplan-Meier curves for the selected genotypes. Dashed lines represent 0.95 confidence intervals for the curves with the corresponding color. Black horizontal line $(18.00 - 10.70 = 7.30)$ indicates the maximal survival difference at $y = 0.50$.

17.4 Survival analysis of rs2881513

This marker has a genotype specific effect with a p-value of 2.295630e-05. Total of 1 observation deleted due to missingness. Odds ratios have been estimated using logistic regression at $t \approx 36$:
$\frac{P(event|AT,AA)/P(\neg event|AT,AA)}{P(event|TT)/P(\neg event|TT)} \approx 5.34.$

Table 18: Summary of genotype frequencies with their proportions in parenthesis.

genotype	total N	events	censored
TT	160	119 (0.744)	41 (0.256)
AT,AA	33	31 (0.939)	2 (0.0606)

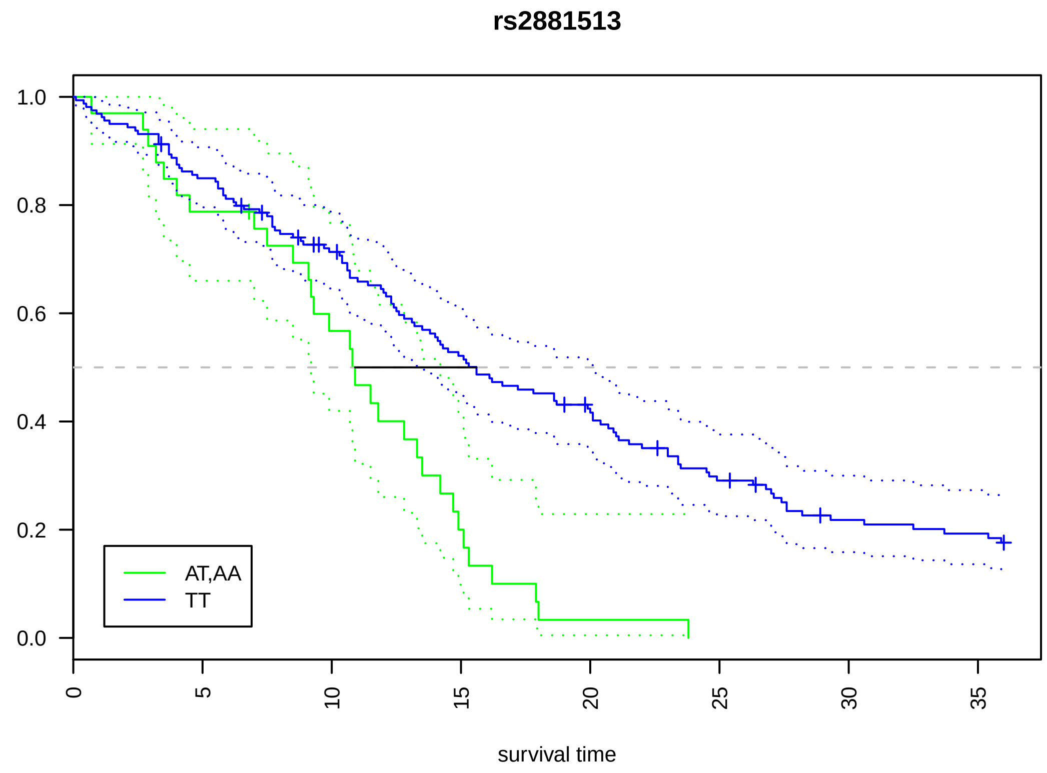

Figure 1930: Kaplan-Meier curves for the selected genotypes. Dashed lines represent 0.95 confidence intervals for the curves with the corresponding color. Black horizontal line ($15.60 - 10.90 = 4.70$) indicates the maximal survival difference at $y = 0.50$.

Additional annotations:

Gene name	*Description*	*DNABand*	*protein*
RFTN1	raftlin, lipid raft linker 1 [Source:HGNC Symbol;Acc:30278]	3p24.3,3p25.1	RFTN1_HUMAN

17.5 Survival analysis of rs555206

This marker has a genotype specific effect with a p-value of 8.148400e-05. Total of 1 observation deleted due to missingness. Odds ratios have been estimated using logistic regression at $t \approx 36$:
$\frac{P(event|AT)/P(\neg event|AT)}{P(event|TT)/P(\neg event|TT)} \approx 1.62$ and $\frac{P(event|AA)/P(\neg event|AA)}{P(event|TT)/P(\neg event|TT)} \approx 0.325$.

Table 19: Summary of genotype frequencies with their proportions in parenthesis.

genotype	total N	events	censored
TT	60	47 (0.783)	13 (0.217)
AT	96	82 (0.854)	14 (0.146)
AA	37	20 (0.541)	17 (0.459)

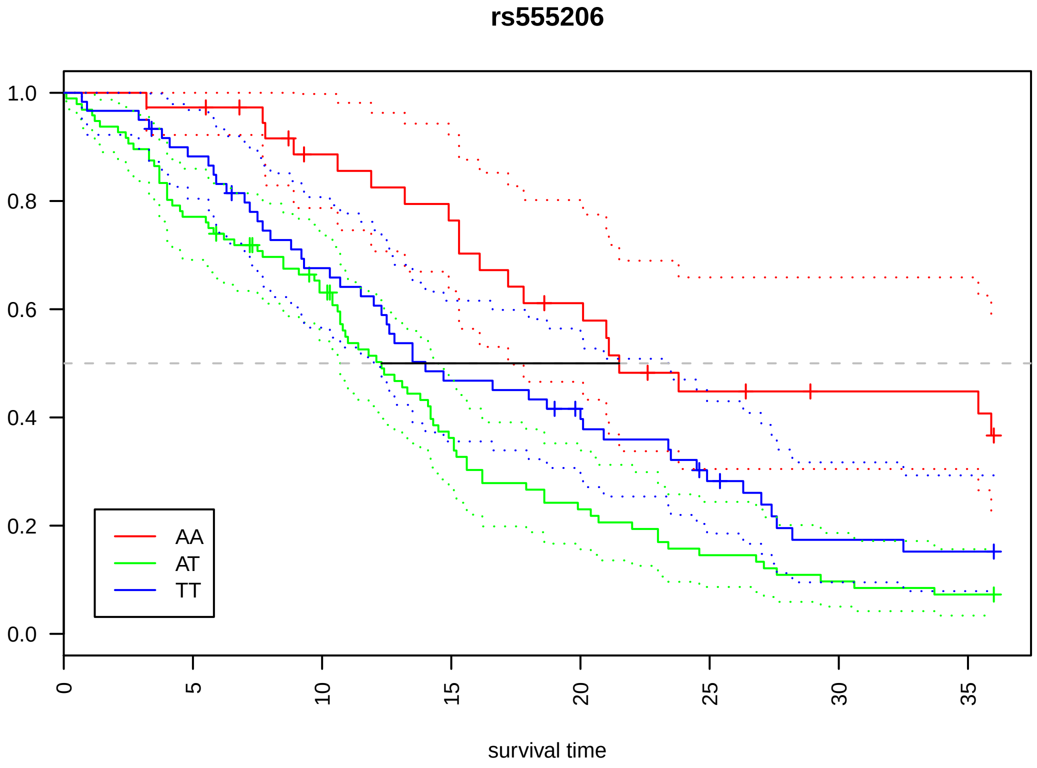

Figure 1931: Kaplan-Meier curves for the selected genotypes. Dashed lines represent 0.95 confidence intervals for the curves with the corresponding color. Black horizontal line ($21.50 - 12.30 = 9.20$) indicates the maximal survival difference at $y = 0.50$.

Additional annotations:

Gene name	*Description*	*DNABand*	*protein*
FAM129A	family with sequence similarity 129, member A [Source:HGNC Symbol;Acc:16784]	1q25.3	NIBAN_HUMAN

17.6 Survival analysis of rs1479852

This marker has a genotype specific effect with a p-value of 6.639866e-05.

Odds ratios have been estimated using logistic regression at $t \approx 36$:
$\frac{P(event|AT)/P(\neg event|AT)}{P(event|TT)/P(\neg event|TT)} \approx 0.59$ and $\frac{P(event|AA)/P(\neg event|AA)}{P(event|TT)/P(\neg event|TT)} \approx 5574.015$.

Table 20: Summary of genotype frequencies with their proportions in parenthesis.

genotype	total N	events	censored
TT	77	61 (0.792)	16 (0.208)
AT	91	63 (0.692)	28 (0.308)
AA	26	26 (1)	0 (0)

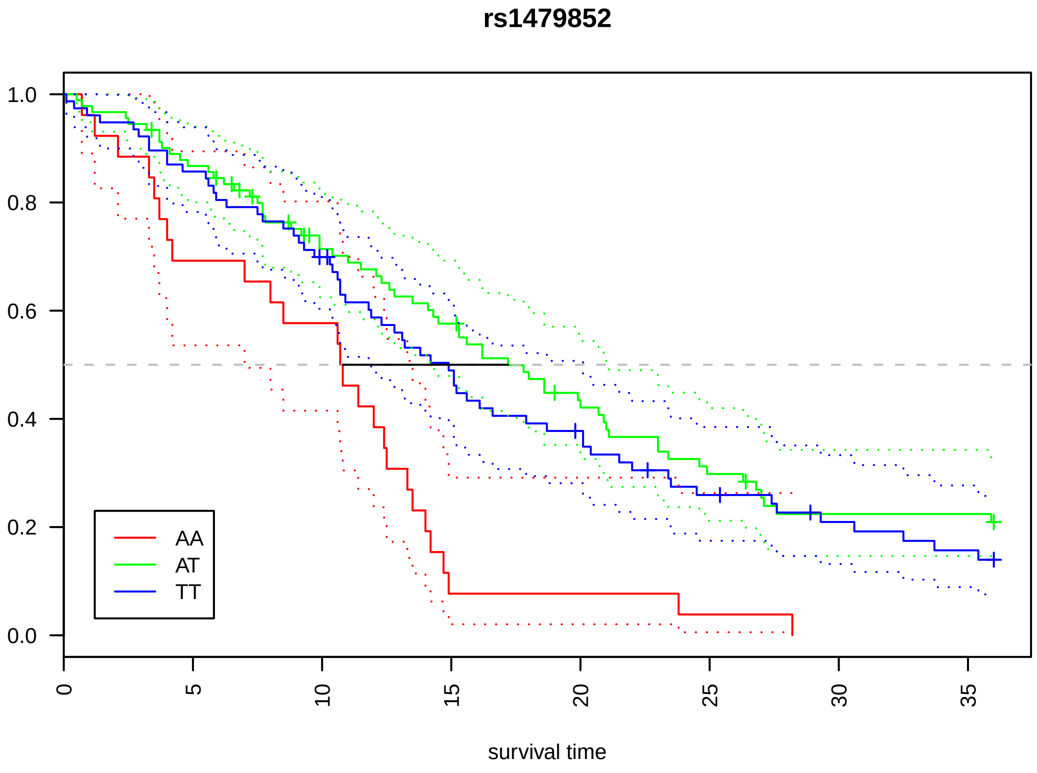

Figure 1932: Kaplan-Meier curves for the selected genotypes. Dashed lines represent 0.95 confidence intervals for the curves with the corresponding color. Black horizontal line ($17.20 - 10.80 = 6.40$) indicates the maximal survival difference at $y = 0.50$.

Additional annotations:

Gene name	*Description*	*DNABand*	*protein*
CLSTN2	calsyntenin 2 [Source:HGNC Symbol;Acc:17448]	3q23	CSTN2_HUMAN

17.7 Survival analysis of rs4382455

This marker has a genotype specific effect with a p-value of 3.867850e-05.

Odds ratios have been estimated using logistic regression at $t \approx 36$:
$\frac{P(event|AT,TT)/P(\neg event|AT,TT)}{P(event|AA)/P(\neg event|AA)} \approx 0.37$.

Table 21: Summary of genotype frequencies with their proportions in parenthesis.

genotype	total N	events	censored
AA	107	91 (0.85)	16 (0.15)
AT,TT	87	59 (0.678)	28 (0.322)

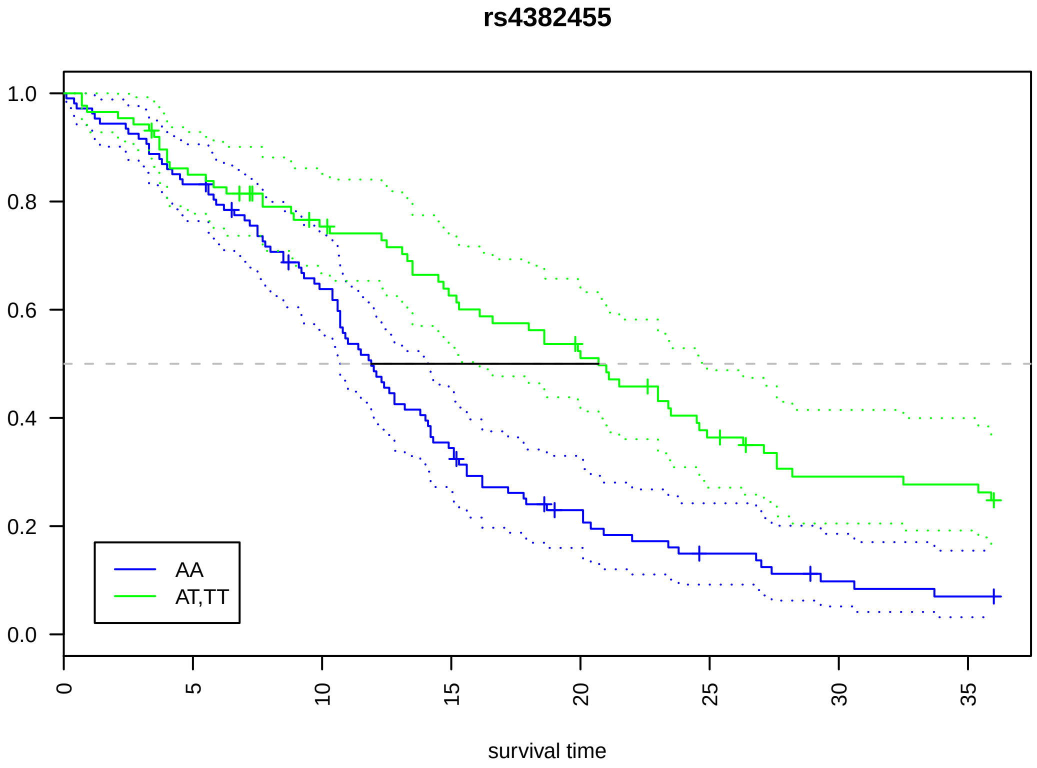

Figure 1933: Kaplan-Meier curves for the selected genotypes. Dashed lines represent 0.95 confidence intervals for the curves with the corresponding color. Black horizontal line $(20.70 - 11.90 = 8.80)$ indicates the maximal survival difference at $y = 0.50$.

Additional annotations:

Gene name	*Description*	*DNABand*	*protein*
WISP1	WNT1 inducible signaling pathway protein 1 [Source:HGNC Symbol;Acc:12769]	8q24.22	WISP1_HUMAN

17.8 Survival analysis of rs9395780

This marker has a genotype specific effect with a p-value of 1.660070e-05. Total of 1 observation deleted due to missingness. Odds ratios have been estimated using logistic regression at $t \approx 36$:
$\frac{P(event|AT,AA)/P(\neg event|AT,AA)}{P(event|TT)/P(\neg event|TT)} \approx 3.574.$

Table 22: Summary of genotype frequencies with their proportions in parenthesis.

genotype	total N	events	censored
TT	101	68 (0.673)	33 (0.327)
AT,AA	92	81 (0.88)	11 (0.12)

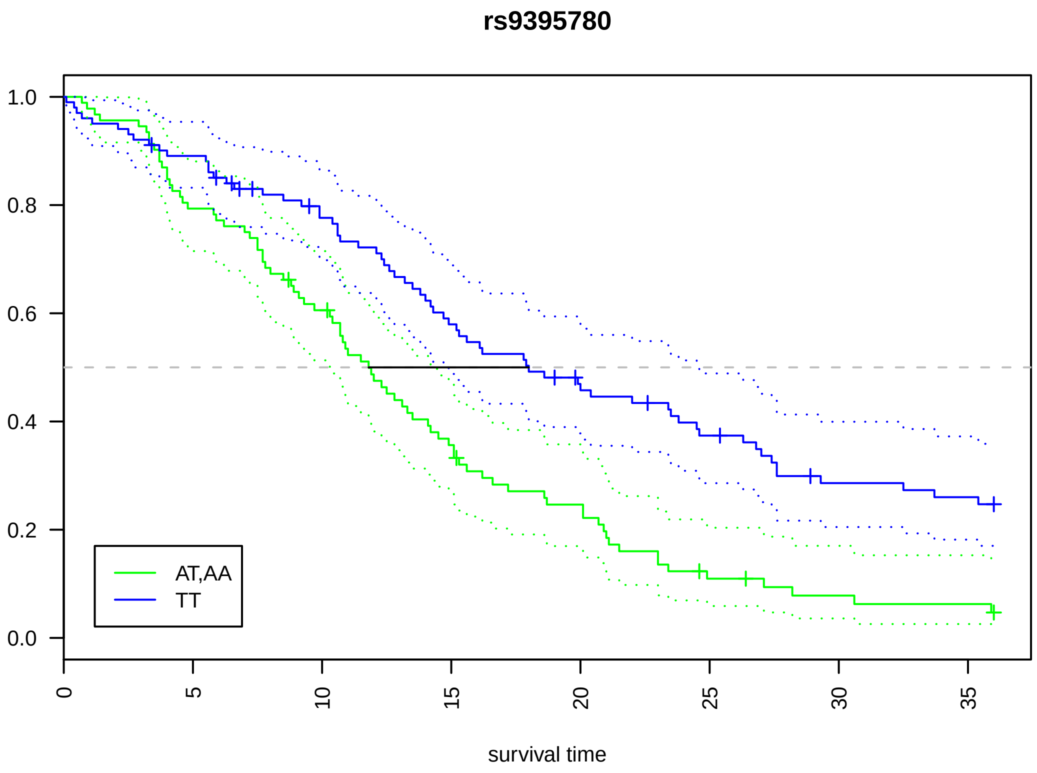

Figure 1934: Kaplan-Meier curves for the selected genotypes. Dashed lines represent 0.95 confidence intervals for the curves with the corresponding color. Black horizontal line $(18.00 - 11.80 = 6.20)$ indicates the maximal survival difference at $y = 0.50$.

17.9 Survival analysis of rs1349345

This marker has a genotype specific effect with a p-value of 8.070943e-05.

Odds ratios have been estimated using logistic regression at $t \approx 36$:
$\frac{P(event|AT)/P(\neg event|AT)}{P(event|AA)/P(\neg event|AA)} \approx 0.984$ and $\frac{P(event|TT)/P(\neg event|TT)}{P(event|AA)/P(\neg event|AA)} \approx 5829.903$.

Table 23: Summary of genotype frequencies with their proportions in parenthesis.

genotype	total N	events	censored
AA	86	64 (0.744)	22 (0.256)
AT	85	63 (0.741)	22 (0.259)
TT	23	23 (1)	0 (0)

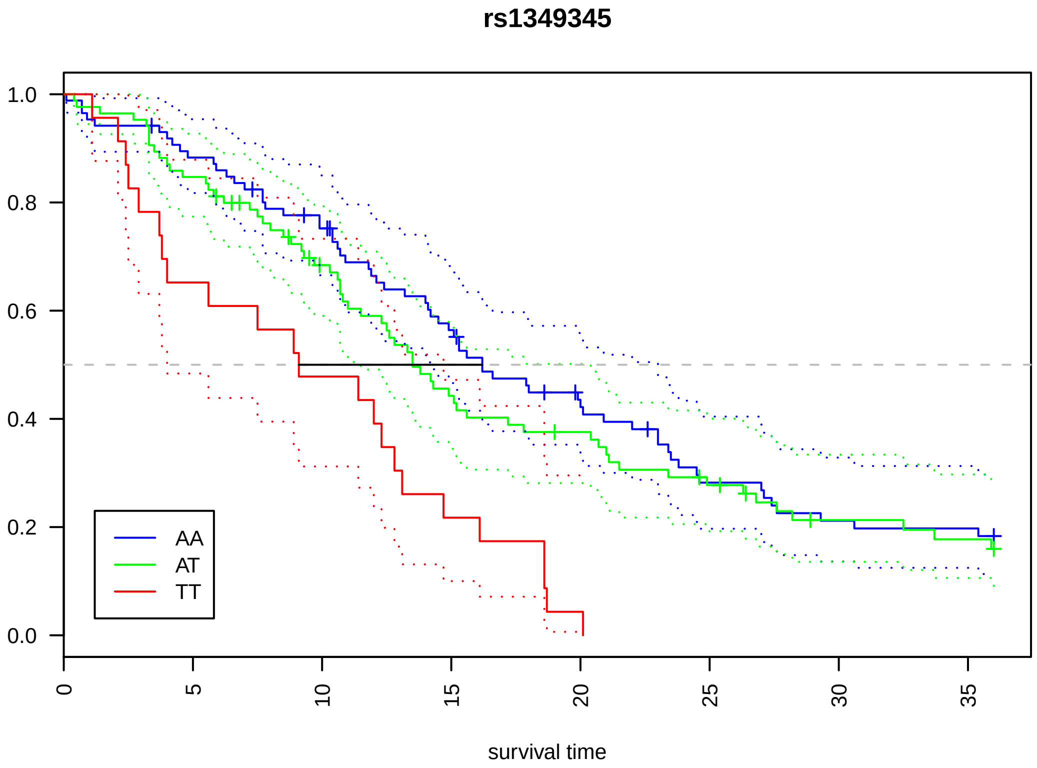

Figure 1935: Kaplan-Meier curves for the selected genotypes. Dashed lines represent 0.95 confidence intervals for the curves with the corresponding color. Black horizontal line ($16.20 - 9.10 = 7.10$) indicates the maximal survival difference at $y = 0.50$.

17.10 Survival analysis of rs7892835

This marker has a genotype specific effect with a p-value of 1.481628e-05. Total of 1 observation deleted due to missingness. Odds ratios have been estimated using logistic regression at $t \approx 36$:
$\frac{P(event|AT)/P(\neg event|AT)}{P(event|TT)/P(\neg event|TT)} \approx 6.071$ and $\frac{P(event|AA)/P(\neg event|AA)}{P(event|TT)/P(\neg event|TT)} \approx 3.75$.

Table 24: Summary of genotype frequencies with their proportions in parenthesis.

genotype	total N	events	censored
TT	152	112 (0.737)	40 (0.263)
AT	18	17 (0.944)	1 (0.0556)
AA	23	21 (0.913)	2 (0.087)

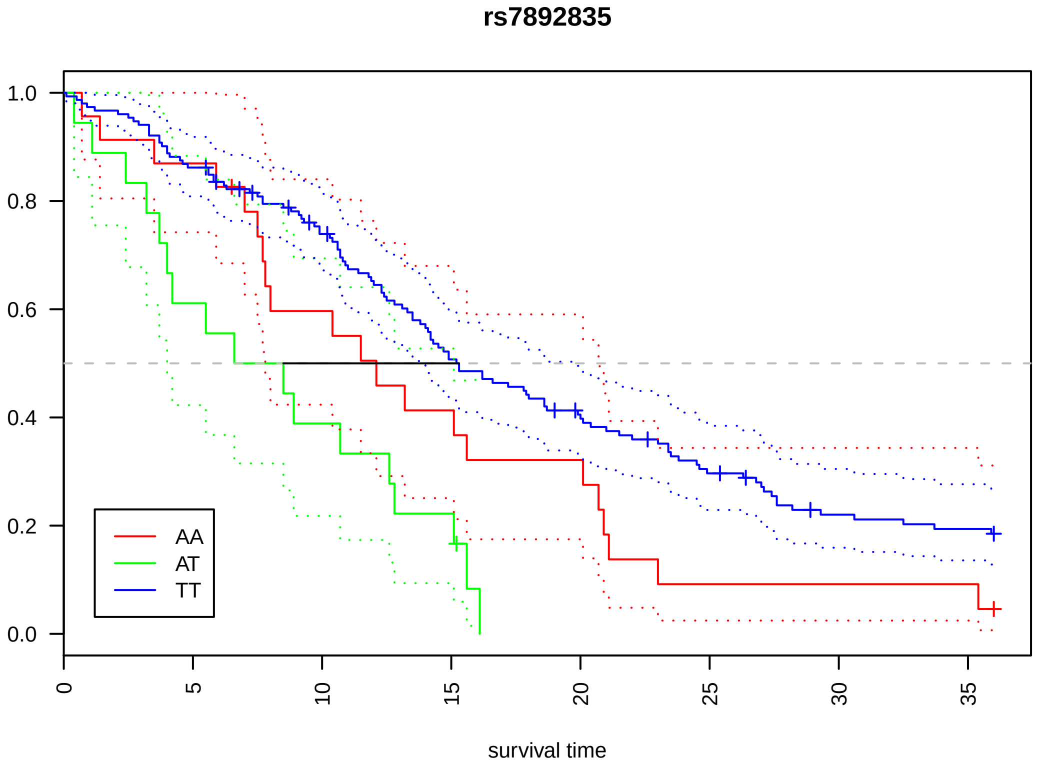

Figure 1936: Kaplan-Meier curves for the selected genotypes. Dashed lines represent 0.95 confidence intervals for the curves with the corresponding color. Black horizontal line ($15.30 - 8.50 = 6.80$) indicates the maximal survival difference at $y = 0.50$.

17.11 Survival analysis of rs7572149

This marker has a genotype specific effect with a p-value of 8.703489e-05. Total of 1 observation deleted due to missingness. Odds ratios have been estimated using logistic regression at $t \approx 36$:
$\frac{P(event|AT,AA)/P(\neg event|AT,AA)}{P(event|TT)/P(\neg event|TT)} \approx 2.967$.

Table 25: Summary of genotype frequencies with their proportions in parenthesis.

genotype	total N	events	censored
TT	154	115 (0.747)	39 (0.253)
AT,AA	39	35 (0.897)	4 (0.103)

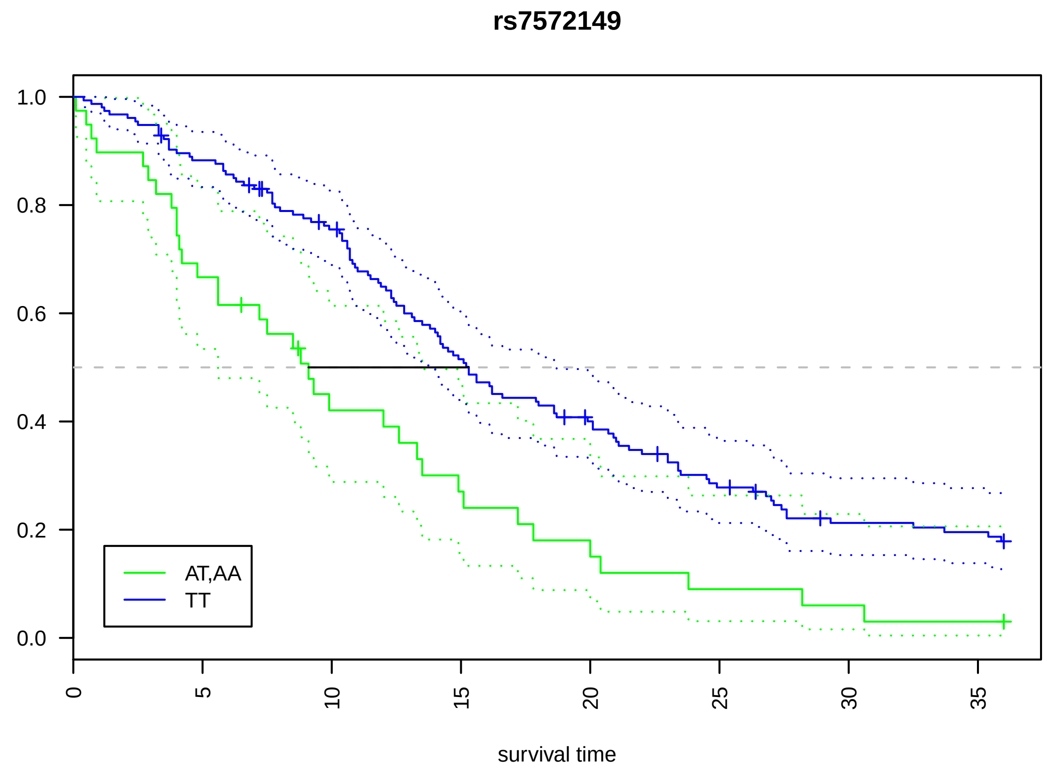

Figure 1937: Kaplan-Meier curves for the selected genotypes. Dashed lines represent 0.95 confidence intervals for the curves with the corresponding color. Black horizontal line ($15.30 - 9.10 = 6.20$) indicates the maximal survival difference at $y = 0.50$.

17.12 Survival analysis of rs2326613

This marker has a genotype specific effect with a p-value of 1.814547e-06.

Odds ratios have been estimated using logistic regression at $t \approx 36$:
$\frac{P(event|AT)/P(\neg event|AT)}{P(event|TT)/P(\neg event|TT)} \approx 0.927$ and $\frac{P(event|AA)/P(\neg event|AA)}{P(event|TT)/P(\neg event|TT)} \approx 5652.314$.

Table 26: Summary of genotype frequencies with their proportions in parenthesis.

genotype	total N	events	censored
TT	85	64 (0.753)	21 (0.247)
AT	88	65 (0.739)	23 (0.261)
AA	21	21 (1)	0 (0)

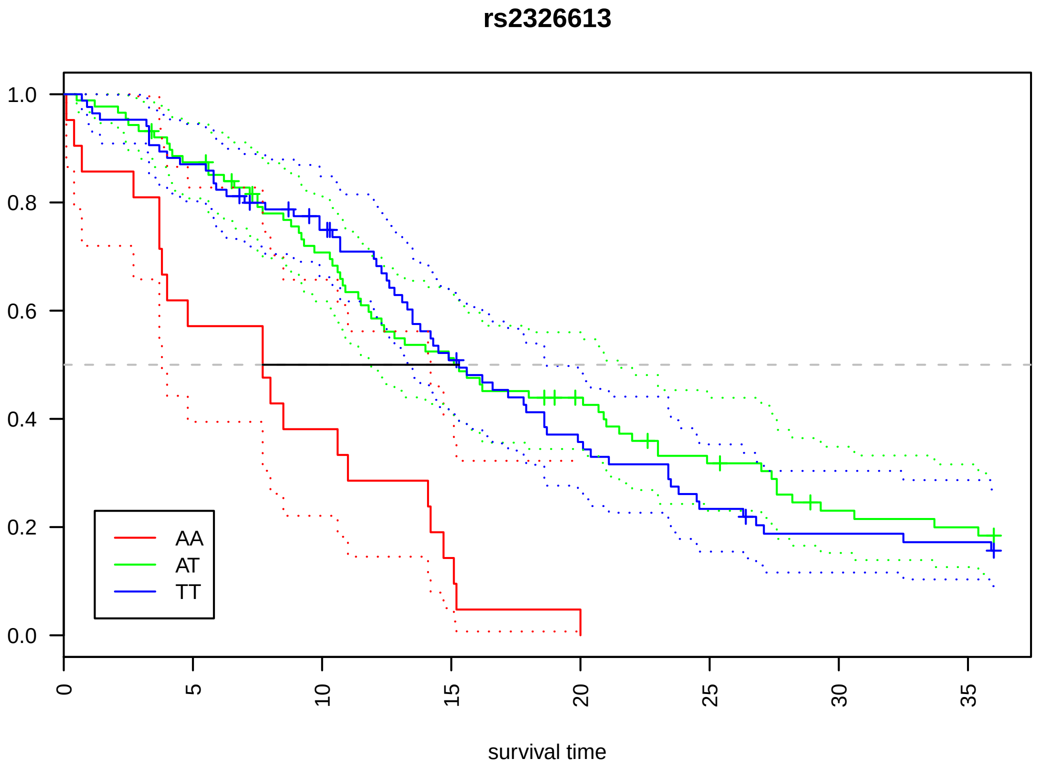

Figure 1938: Kaplan-Meier curves for the selected genotypes. Dashed lines represent 0.95 confidence intervals for the curves with the corresponding color. Black horizontal line (15.30 − 7.70 = 7.60) indicates the maximal survival difference at $y = 0.50$.

Additional annotations:

Gene name	*Description*	*DNABand*	*protein*
FARS2	phenylalanyl-tRNA synthetase 2, mitochondrial [Source:HGNC Symbol;Acc:21062]	6p25.1	SYFM_HUMAN

17.13 Survival analysis of rs826911

This marker has a genotype specific effect with a p-value of 1.321085e-05. Total of 3 observations deleted due to missingness. Odds ratios have been estimated using logistic regression at $t \approx 36$:
$\frac{P(event|AT,TT)/P(\neg event|AT,TT)}{P(event|AA)/P(\neg event|AA)} \approx 3.068$.

Table 27: Summary of genotype frequencies with their proportions in parenthesis.

genotype	total N	events	censored
AA	122	87 (0.713)	35 (0.287)
AT,TT	69	61 (0.884)	8 (0.116)

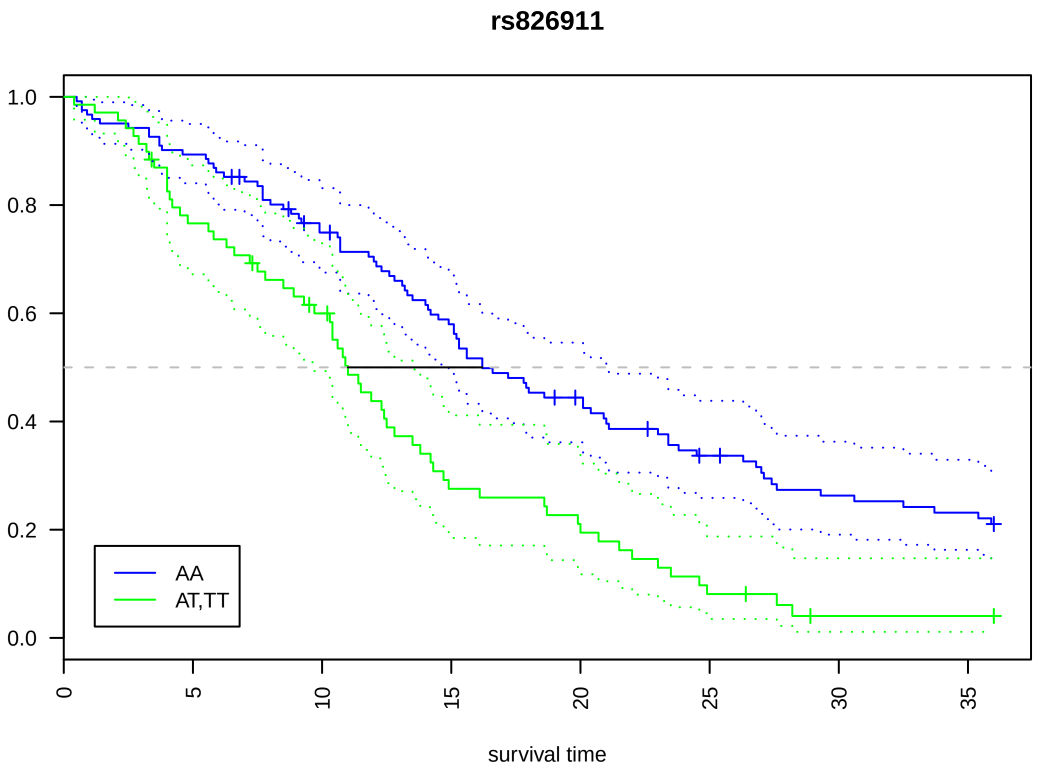

Figure 1939: Kaplan-Meier curves for the selected genotypes. Dashed lines represent 0.95 confidence intervals for the curves with the corresponding color. Black horizontal line $(16.20 - 11.00 = 5.20)$ indicates the maximal survival difference at $y = 0.50$.

17.14 Survival analysis of rs17258085

This marker has a genotype specific effect with a p-value of 2.622032e-05.

Odds ratios have been estimated using logistic regression at $t \approx 36$:
$\frac{P(event|AT,TT)/P(\neg event|AT,TT)}{P(event|AA)/P(\neg event|AA)} \approx 2.725$.

Table 28: Summary of genotype frequencies with their proportions in parenthesis.

genotype	total N	events	censored
AA	101	70 (0.693)	31 (0.307)
AT,TT	93	80 (0.86)	13 (0.14)

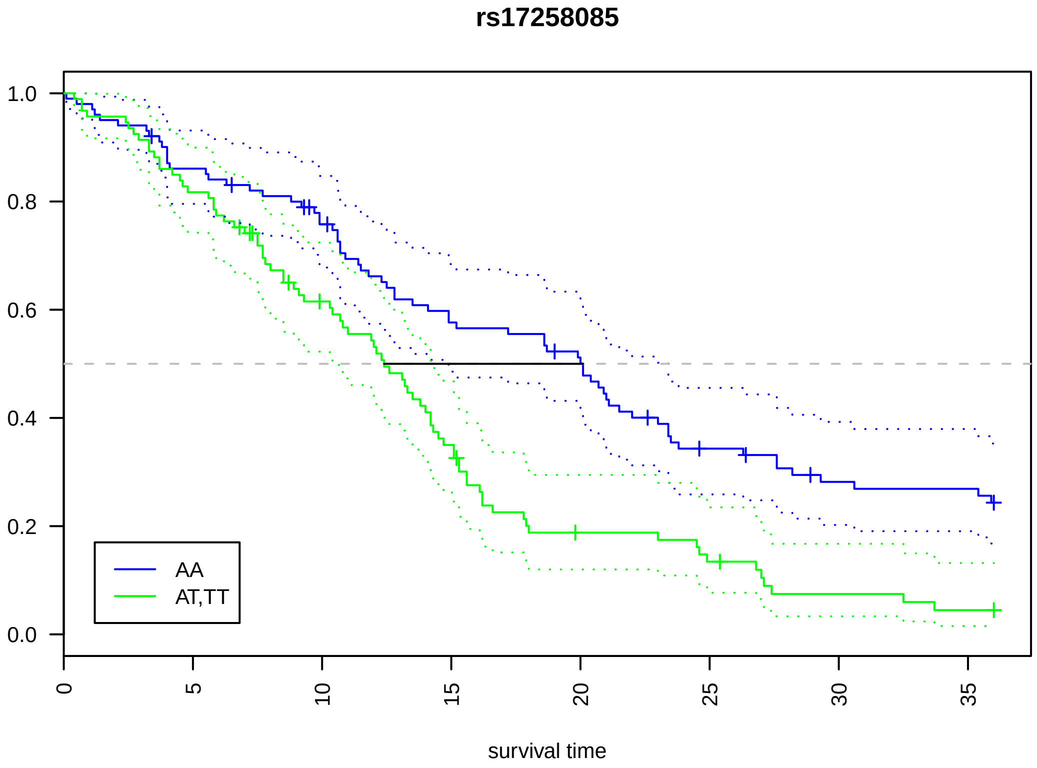

Figure 1940: Kaplan-Meier curves for the selected genotypes. Dashed lines represent 0.95 confidence intervals for the curves with the corresponding color. Black horizontal line ($20.10 - 12.40 = 7.70$) indicates the maximal survival difference at $y = 0.50$.

Additional annotations:

Gene name	*Description*	*DNABand*	*protein*
ODZ3	odz, odd Oz/ten-m homolog 3 (Drosophila) [Source:HGNC Symbol;Acc:29944]	4q35.1	TEN3_HUMAN

17.15 Survival analysis of rs12642867

This marker has a genotype specific effect with a p-value of 9.524763e-05.

Odds ratios have been estimated using logistic regression at $t \approx 36$:
$\frac{P(event|AT,AA)/P(\neg event|AT,AA)}{P(event|TT)/P(\neg event|TT)} \approx 3.559$.

Table 29: Summary of genotype frequencies with their proportions in parenthesis.

genotype	total N	events	censored
TT	142	103 (0.725)	39 (0.275)
AT,AA	52	47 (0.904)	5 (0.0962)

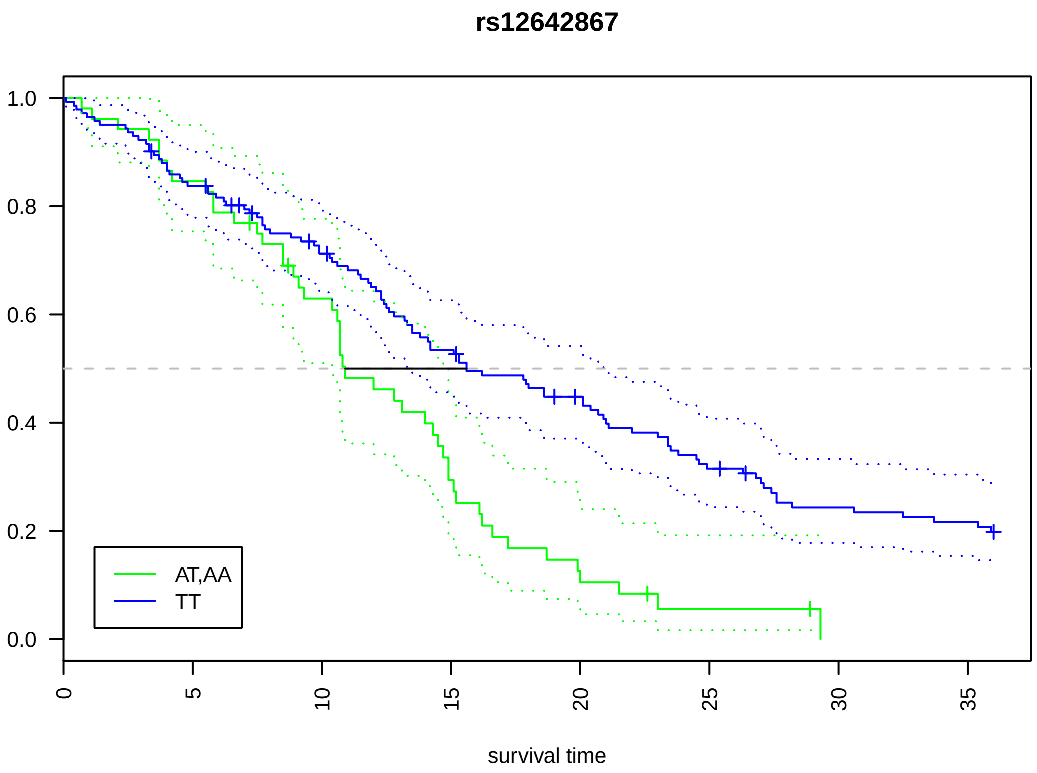

Figure 1941: Kaplan-Meier curves for the selected genotypes. Dashed lines represent 0.95 confidence intervals for the curves with the corresponding color. Black horizontal line $(15.60 - 10.90 = 4.70)$ indicates the maximal survival difference at $y = 0.50$.

17.16 Survival analysis of rs1159766

This marker has a genotype specific effect with a p-value of 2.631947e-05.

Odds ratios have been estimated using logistic regression at $t \approx 36$:
$\frac{P(event|AT)/P(\neg event|AT)}{P(event|AA)/P(\neg event|AA)} \approx 1.07$ and $\frac{P(event|TT)/P(\neg event|TT)}{P(event|AA)/P(\neg event|AA)} \approx 1.312$.

Table 30: Summary of genotype frequencies with their proportions in parenthesis.

genotype	total N	events	censored
AA	106	81 (0.764)	25 (0.236)
AT	67	52 (0.776)	15 (0.224)
TT	21	17 (0.81)	4 (0.19)

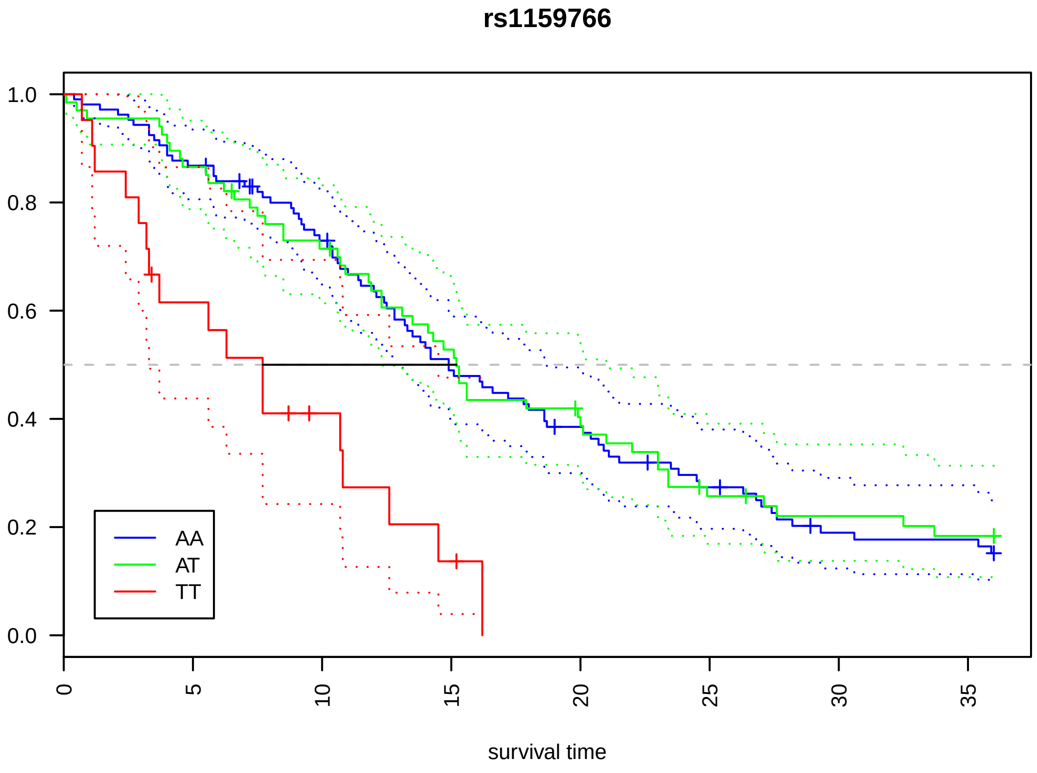

Figure 1942: Kaplan-Meier curves for the selected genotypes. Dashed lines represent 0.95 confidence intervals for the curves with the corresponding color. Black horizontal line $(15.20 - 7.70 = 7.50)$ indicates the maximal survival difference at $y = 0.50$.

17.17 Survival analysis of rs1159764

This marker has a genotype specific effect with a p-value of 2.631947e-05.

Odds ratios have been estimated using logistic regression at $t \approx 36$:
$\frac{P(event|AT)/P(\neg event|AT)}{P(event|TT)/P(\neg event|TT)} \approx 1.07$ and $\frac{P(event|AA)/P(\neg event|AA)}{P(event|TT)/P(\neg event|TT)} \approx 1.312$.

Table 31: Summary of genotype frequencies with their proportions in parenthesis.

genotype	total N	events	censored
TT	106	81 (0.764)	25 (0.236)
AT	67	52 (0.776)	15 (0.224)
AA	21	17 (0.81)	4 (0.19)

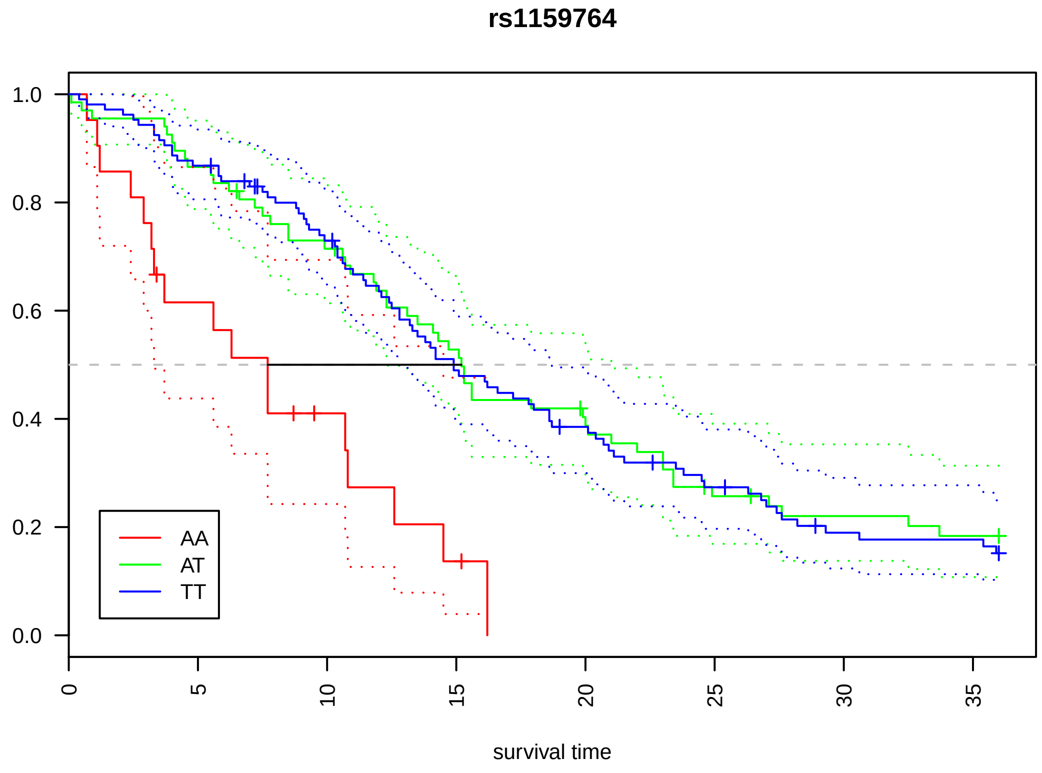

Figure 1943: Kaplan-Meier curves for the selected genotypes. Dashed lines represent 0.95 confidence intervals for the curves with the corresponding color. Black horizontal line $(15.20-7.70 = 7.50)$ indicates the maximal survival difference at $y = 0.50$.

17.18 Survival analysis of rs17022010

This marker has a genotype specific effect with a p-value of 4.159426e-05.

Odds ratios have been estimated using logistic regression at $t \approx 36$:
$\frac{P(event|AT,TT)/P(\neg event|AT,TT)}{P(event|AA)/P(\neg event|AA)} \approx 2.712$.

Table 32: Summary of genotype frequencies with their proportions in parenthesis.

genotype	total N	events	censored
AA	158	118 (0.747)	40 (0.253)
AT,TT	36	32 (0.889)	4 (0.111)

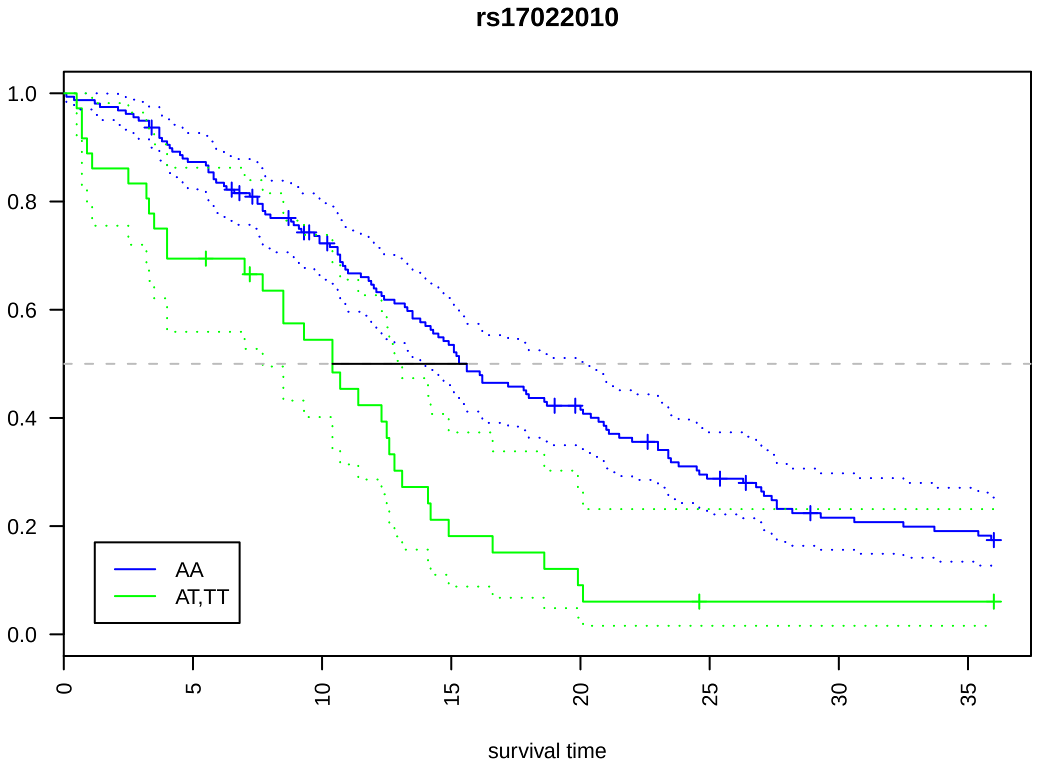

Figure 1944: Kaplan-Meier curves for the selected genotypes. Dashed lines represent 0.95 confidence intervals for the curves with the corresponding color. Black horizontal line $(15.60 - 10.40 = 5.20)$ indicates the maximal survival difference at $y = 0.50$.

Additional annotations:

Gene name	*Description*	*DNABand*	*protein*
CYP1B1	cytochrome P450, family 1, subfamily B, polypeptide 1 [Source:HGNC Symbol;Acc:2597]	2p22.2	CP1B1_HUMAN

17.19 Survival analysis of rs4378238

This marker has a genotype specific effect with a p-value of 6.727180e-05.

Odds ratios have been estimated using logistic regression at $t \approx 36$:
$\frac{P(event|AT,AA)/P(\neg event|AT,AA)}{P(event|TT)/P(\neg event|TT)} \approx 4.475$.

Table 33: Summary of genotype frequencies with their proportions in parenthesis.

genotype	total N	events	censored
TT	154	113 (0.734)	41 (0.266)
AT,AA	40	37 (0.925)	3 (0.075)

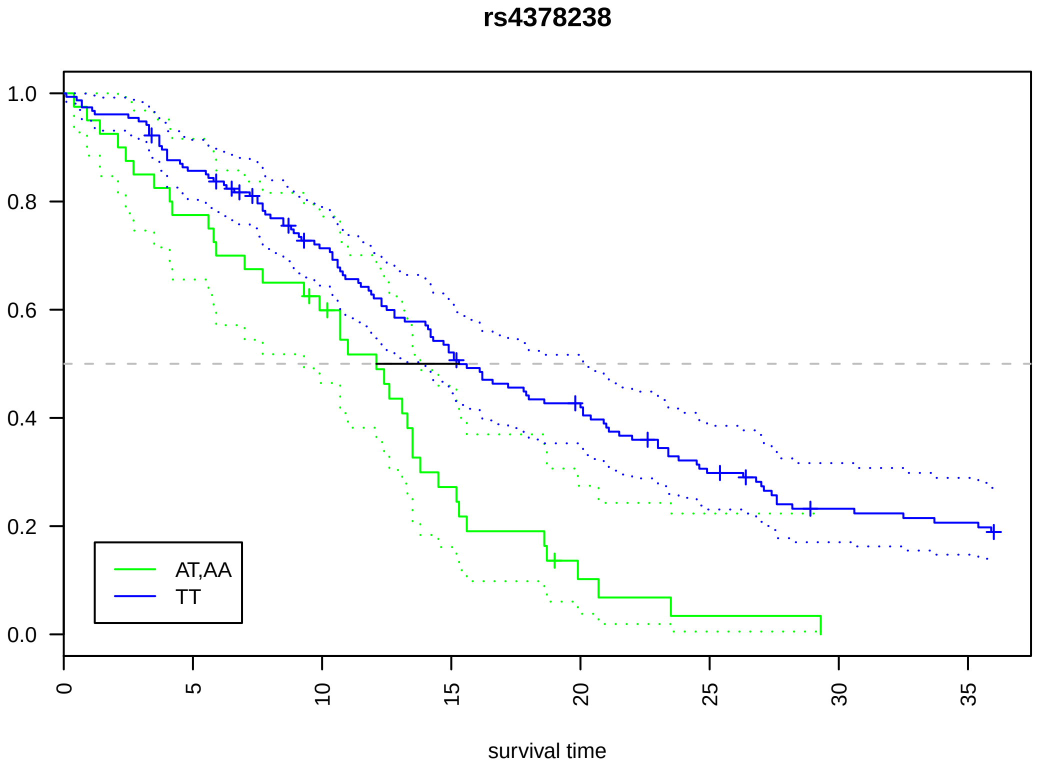

Figure 1945: Kaplan-Meier curves for the selected genotypes. Dashed lines represent 0.95 confidence intervals for the curves with the corresponding color. Black horizontal line ($15.30 - 12.10 = 3.20$) indicates the maximal survival difference at $y = 0.50$.

Additional annotations:

Gene name	*Description*	*DNABand*
RP11-92G12.1	[undefined]	1q32.1

17.20 Survival analysis of rs1376946

This marker has a genotype specific effect with a p-value of 5.339445e-06. Total of 3 observations deleted due to missingness. Odds ratios have been estimated using logistic regression at $t \approx 36$: $\frac{P(event|AT)/P(\neg event|AT)}{P(event|AA)/P(\neg event|AA)} \approx 5.545$ and $\frac{P(event|TT)/P(\neg event|TT)}{P(event|AA)/P(\neg event|AA)} \approx 1.141$.

Table 34: Summary of genotype frequencies with their proportions in parenthesis.

genotype	total N	events	censored
AA	65	42 (0.646)	23 (0.354)
AT	89	81 (0.91)	8 (0.0899)
TT	37	25 (0.676)	12 (0.324)

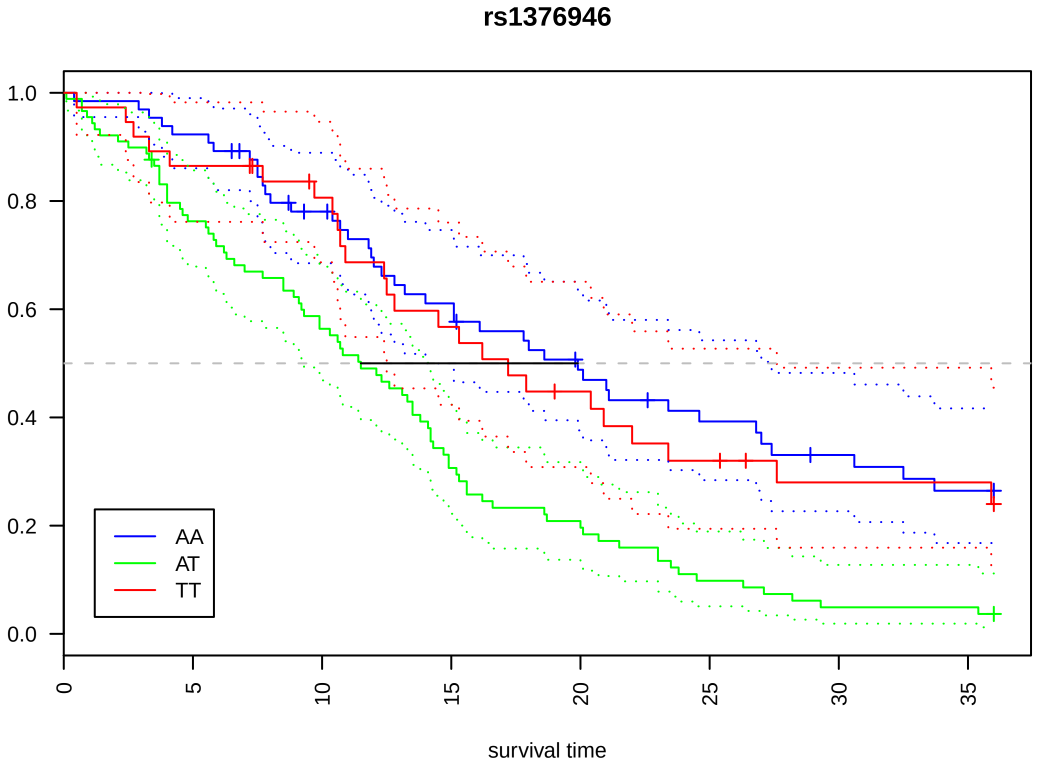

Figure 1946: Kaplan-Meier curves for the selected genotypes. Dashed lines represent 0.95 confidence intervals for the curves with the corresponding color. Black horizontal line $(19.90 - 11.50 = 8.40)$ indicates the maximal survival difference at $y = 0.50$.

Additional annotations:

Gene name	*Description*	*DNABand*	*protein*
CCDC81	coiled-coil domain containing 81 [Source:HGNC Symbol;Acc:26281]	11q14.2	CCD81_HUMAN

17.21 Survival analysis of rs10810961

This marker has a genotype specific effect with a p-value of 1.302697e-05.

Odds ratios have been estimated using logistic regression at $t \approx 36$:
$\frac{P(event|AT,TT)/P(\neg event|AT,TT)}{P(event|AA)/P(\neg event|AA)} \approx 7.125$.

Table 35: Summary of genotype frequencies with their proportions in parenthesis.

genotype	total N	events	censored
AA	154	112 (0.727)	42 (0.273)
AT,TT	40	38 (0.95)	2 (0.05)

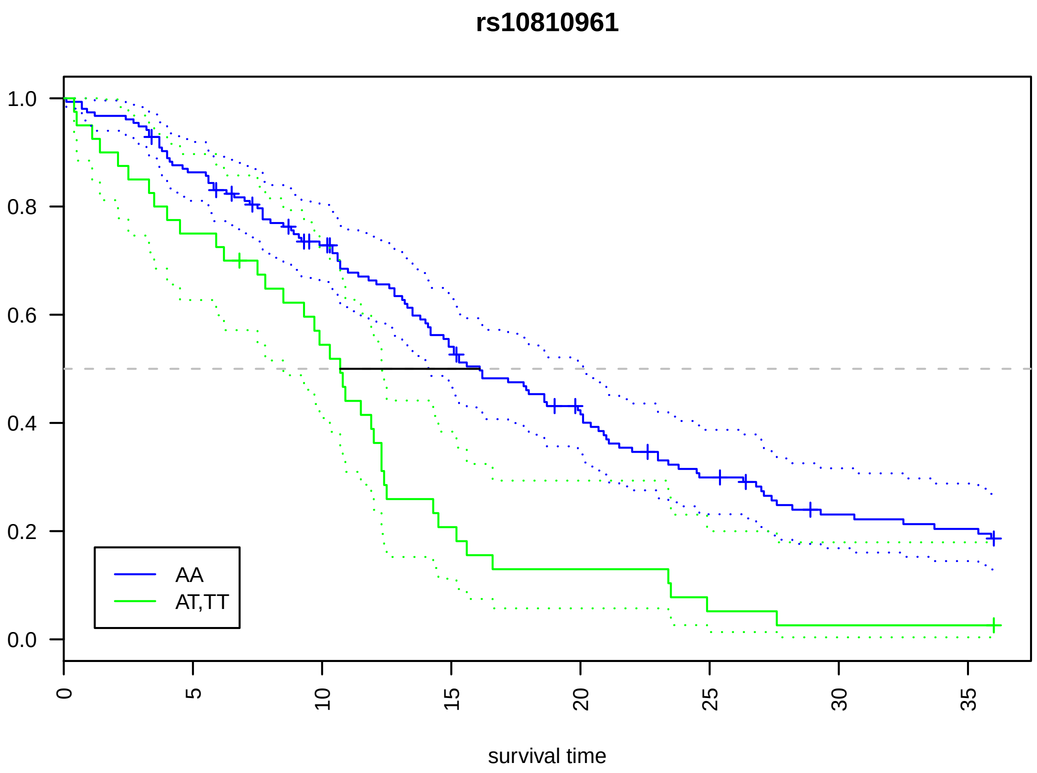

Figure 1947: Kaplan-Meier curves for the selected genotypes. Dashed lines represent 0.95 confidence intervals for the curves with the corresponding color. Black horizontal line $(16.10 - 10.70 = 5.40)$ indicates the maximal survival difference at $y = 0.50$.

17.22 Survival analysis of rs11664633

This marker has a genotype specific effect with a p-value of 3.562126e-05. Total of 1 observation deleted due to missingness. Odds ratios have been estimated using logistic regression at $t \approx 36$:
$\frac{P(event|AT)/P(\neg event|AT)}{P(event|AA)/P(\neg event|AA)} \approx 0.271$ and $\frac{P(event|TT)/P(\neg event|TT)}{P(event|AA)/P(\neg event|AA)} \approx 0.345$.

Table 36: Summary of genotype frequencies with their proportions in parenthesis.

genotype	total N	events	censored
AA	43	39 (0.907)	4 (0.093)
AT	102	74 (0.725)	28 (0.275)
TT	48	37 (0.771)	11 (0.229)

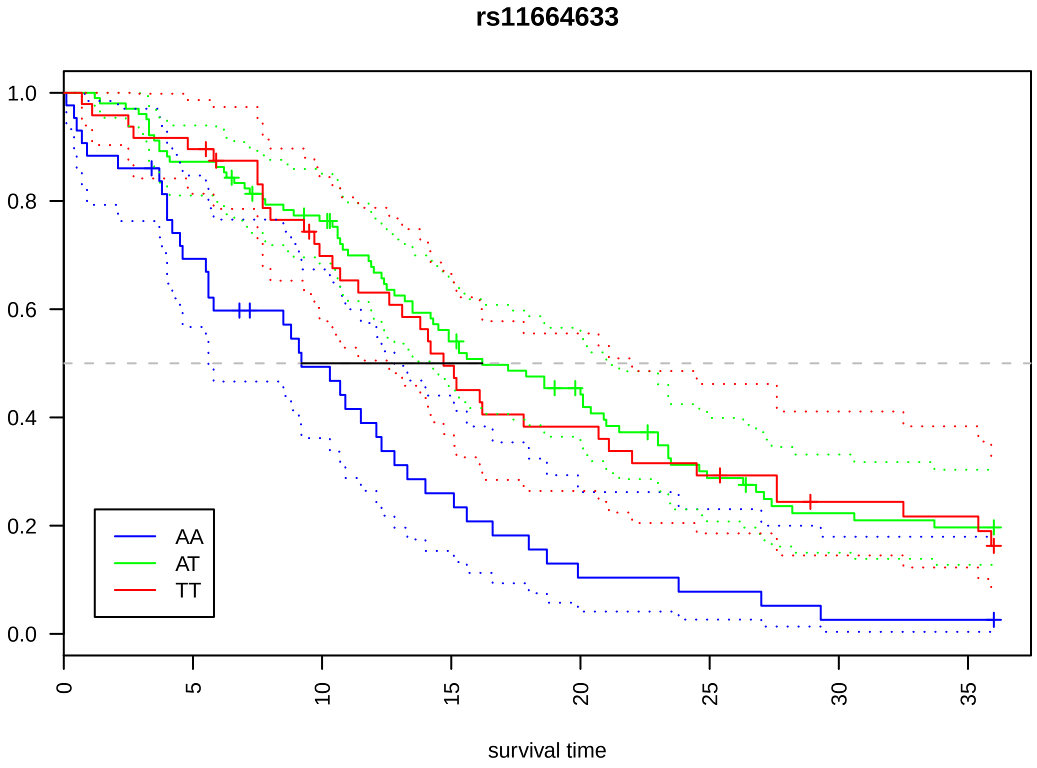

Figure 1948: Kaplan-Meier curves for the selected genotypes. Dashed lines represent 0.95 confidence intervals for the curves with the corresponding color. Black horizontal line ($16.20 - 9.20 = 7.00$) indicates the maximal survival difference at $y = 0.50$.

Additional annotations:

Gene name	*Description*	*DNABand*	*protein*
C18orf22	Putative ribosome-binding factor A, mitochondrial Precursor [Source:UniProtKB/Swiss-Prot;Acc:Q8N0V3]	18q23	RBFAL_HUMAN

17.23 Survival analysis of rs9920861

This marker has a genotype specific effect with a p-value of 3.094617e-05. Total of 6 observations deleted due to missingness. Odds ratios have been estimated using logistic regression at $t \approx 36$:
$\frac{P(event|AT,AA)/P(\neg event|AT,AA)}{P(event|TT)/P(\neg event|TT)} \approx 0.415$.

Table 37: Summary of genotype frequencies with their proportions in parenthesis.

genotype	total N	events	censored
TT	120	99 (0.825)	21 (0.175)
AT,AA	68	45 (0.662)	23 (0.338)

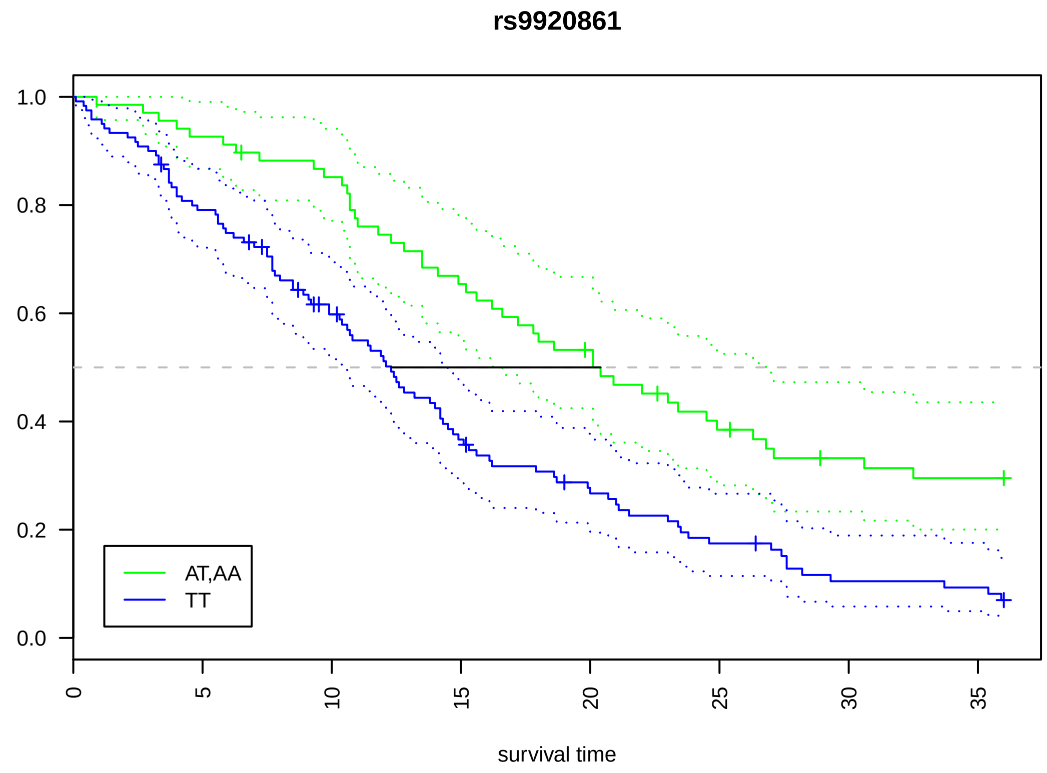

Figure 1949: Kaplan-Meier curves for the selected genotypes. Dashed lines represent 0.95 confidence intervals for the curves with the corresponding color. Black horizontal line $(20.40 - 12.30 = 8.10)$ indicates the maximal survival difference at $y = 0.50$.

Additional annotations:

Gene name	*Description*	*DNABand*	*protein*
ANXA2	annexin A2 pseudogene 1 [Source:HGNC Symbol;Acc:538]	15q22.2	ANXA2_HUMAN,AXA2L_HUMAN

17.24 Survival analysis of rs5928288

This marker has a genotype specific effect with a p-value of 3.805540e-06. Total of 5 observations deleted due to missingness. Odds ratios have been estimated using logistic regression at $t \approx 36$:
$\frac{P(event|AT)/P(\neg event|AT)}{P(event|AA)/P(\neg event|AA)} \approx 669.9$ and $\frac{P(event|TT)/P(\neg event|TT)}{P(event|AA)/P(\neg event|AA)} \approx 6.942$.

Table 38: Summary of genotype frequencies with their proportions in parenthesis.

genotype	total N	events	censored
AA	163	121 (0.742)	42 (0.258)
AT	5	5 (1)	0 (0)
TT	21	20 (0.952)	1 (0.0476)

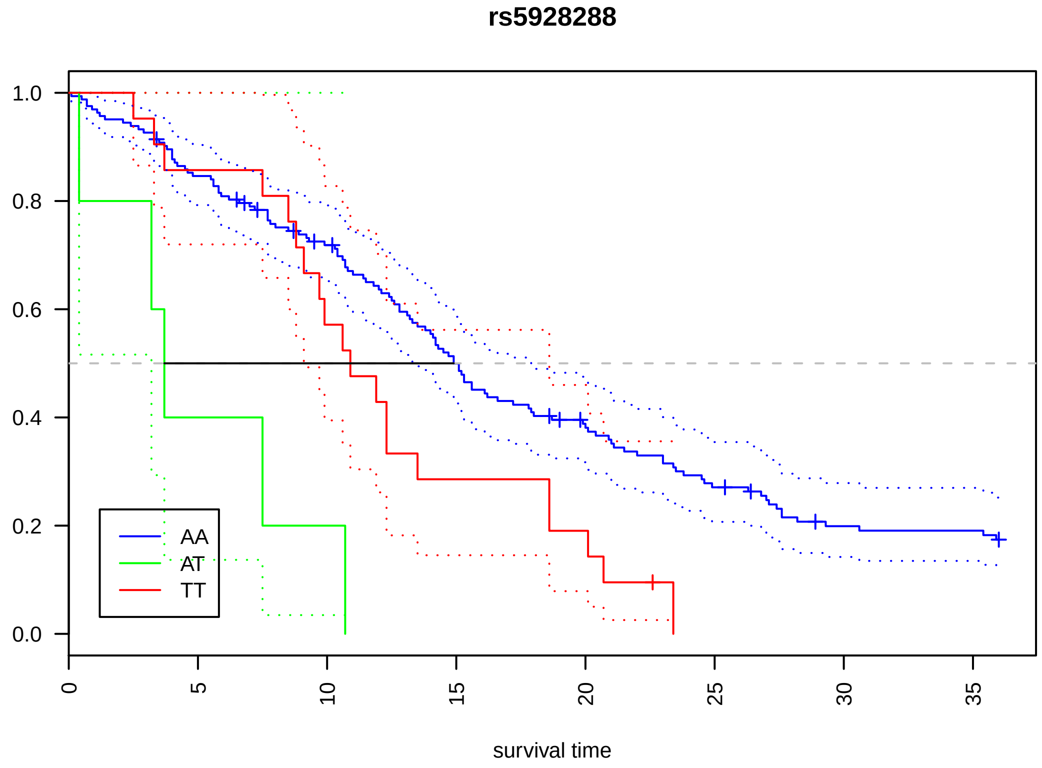

Figure 1950: Kaplan-Meier curves for the selected genotypes. Dashed lines represent 0.95 confidence intervals for the curves with the corresponding color. Black horizontal line $(14.90 - 3.70 = 11.20)$ indicates the maximal survival difference at $y = 0.50$.

17.25 Survival analysis of rs11664762

This marker has a genotype specific effect with a p-value of 4.904374e-05.

Odds ratios have been estimated using logistic regression at $t \approx 36$:
$\frac{P(event|AT)/P(\neg event|AT)}{P(event|AA)/P(\neg event|AA)} \approx 0.248$ and $\frac{P(event|TT)/P(\neg event|TT)}{P(event|AA)/P(\neg event|AA)} \approx 0.345$.

Table 39: Summary of genotype frequencies with their proportions in parenthesis.

genotype	total N	events	censored
AA	44	40 (0.909)	4 (0.0909)
AT	101	72 (0.713)	29 (0.287)
TT	49	38 (0.776)	11 (0.224)

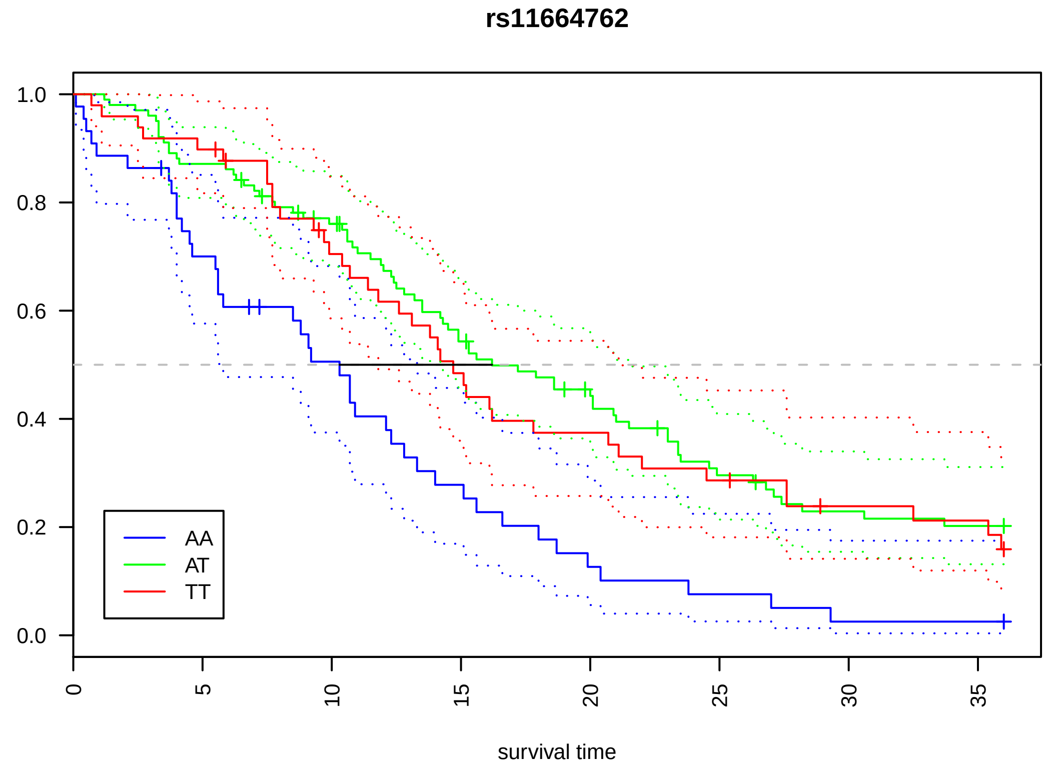

Figure 1951: Kaplan-Meier curves for the selected genotypes. Dashed lines represent 0.95 confidence intervals for the curves with the corresponding color. Black horizontal line ($16.20 - 10.30 = 5.90$) indicates the maximal survival difference at $y = 0.50$.

17.26 Survival analysis of rs11102233

This marker has a genotype specific effect with a p-value of 3.463888e-05.

Odds ratios have been estimated using logistic regression at $t \approx 36$:
$\frac{P(event|AT,AA)/P(\neg event|AT,AA)}{P(event|TT)/P(\neg event|TT)} \approx 0.287$.

Table 40: Summary of genotype frequencies with their proportions in parenthesis.

genotype	total N	events	censored
TT	102	89 (0.873)	13 (0.127)
AT,AA	92	61 (0.663)	31 (0.337)

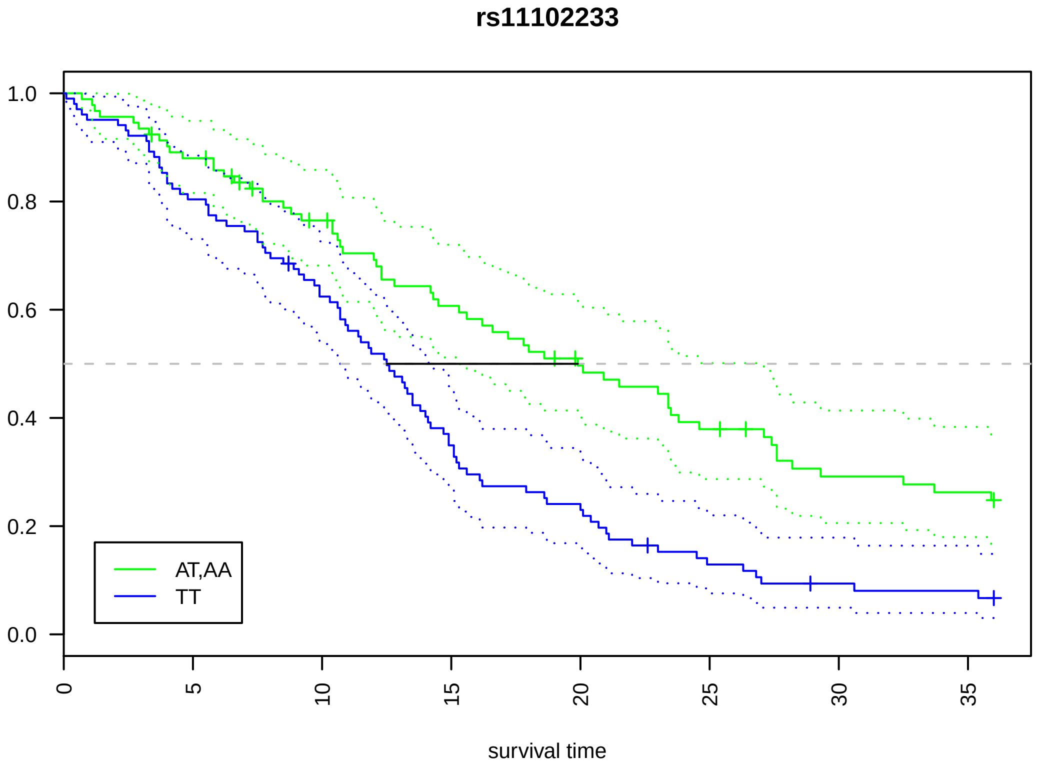

Figure 1952: Kaplan-Meier curves for the selected genotypes. Dashed lines represent 0.95 confidence intervals for the curves with the corresponding color. Black horizontal line $(19.90 - 12.50 = 7.40)$ indicates the maximal survival difference at $y = 0.50$.

Additional annotations:

Gene name	*Description*	*DNABand*
RP11-165H20.3	CHIA-like pseudogene (LOC149620), non-coding RNA [Source:RefSeq DNA;Acc:NR_003928]	1p13.2

17.27 Survival analysis of rs4644918

This marker has a genotype specific effect with a p-value of 3.787668e-05.

Odds ratios have been estimated using logistic regression at $t \approx 36$:
$\frac{P(event|AT)/P(\neg event|AT)}{P(event|TT)/P(\neg event|TT)} \approx 0.251$ and $\frac{P(event|AA)/P(\neg event|AA)}{P(event|TT)/P(\neg event|TT)} \approx 0.301$.

Table 41: Summary of genotype frequencies with their proportions in parenthesis.

genotype	total N	events	censored
TT	45	41 (0.911)	4 (0.0889)
AT	100	72 (0.72)	28 (0.28)
AA	49	37 (0.755)	12 (0.245)

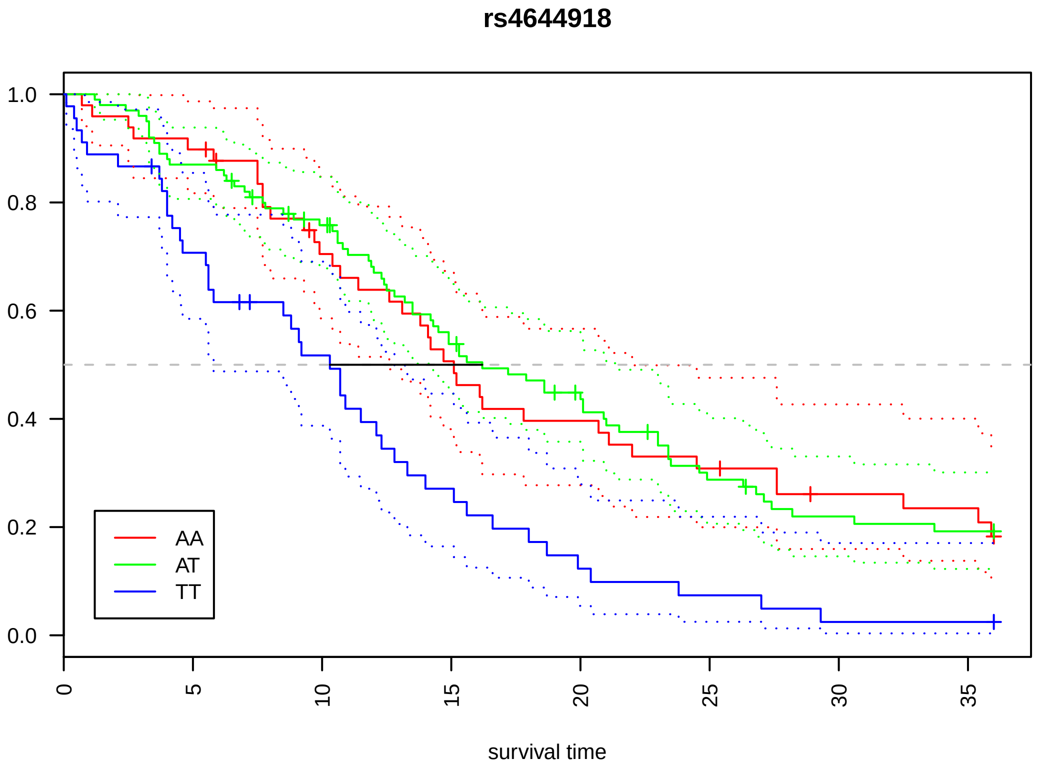

Figure 1953: Kaplan-Meier curves for the selected genotypes. Dashed lines represent 0.95 confidence intervals for the curves with the corresponding color. Black horizontal line $(16.20 - 10.30 = 5.90)$ indicates the maximal survival difference at $y = 0.50$.

17.28 Survival analysis of rs12964779

This marker has a genotype specific effect with a p-value of 4.104984e-05.

Odds ratios have been estimated using logistic regression at $t \approx 36$:
$\frac{P(event|AT)/P(\neg event|AT)}{P(event|AA)/P(\neg event|AA)} \approx 0.236$ and $\frac{P(event|TT)/P(\neg event|TT)}{P(event|AA)/P(\neg event|AA)} \approx 0.312$.

Table 42: Summary of genotype frequencies with their proportions in parenthesis.

genotype	total N	events	censored
AA	46	42 (0.913)	4 (0.087)
AT	101	72 (0.713)	29 (0.287)
TT	47	36 (0.766)	11 (0.234)

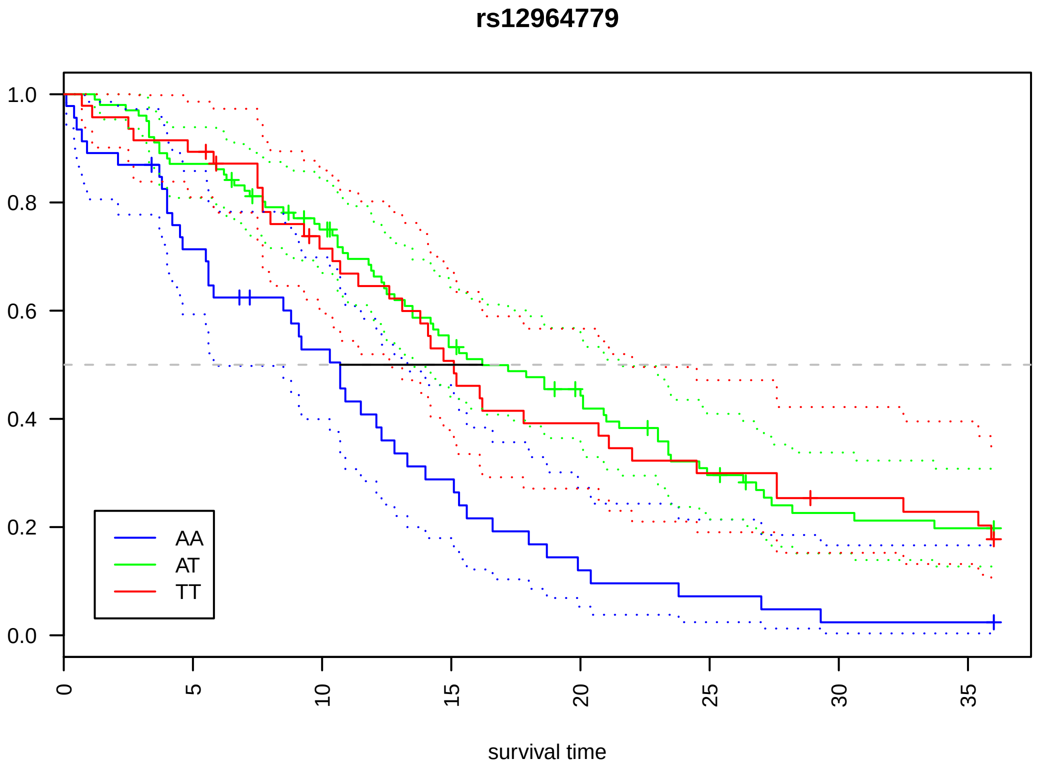

Figure 1954: Kaplan-Meier curves for the selected genotypes. Dashed lines represent 0.95 confidence intervals for the curves with the corresponding color. Black horizontal line ($16.20 - 10.70 = 5.50$) indicates the maximal survival difference at $y = 0.50$.

17.29 Survival analysis of rs8034783

This marker has a genotype specific effect with a p-value of 3.473147e-06.

Odds ratios have been estimated using logistic regression at $t \approx 36$:
$\frac{P(event|AT,AA)/P(\neg event|AT,AA)}{P(event|TT)/P(\neg event|TT)} \approx 2.286$.

Table 43: Summary of genotype frequencies with their proportions in parenthesis.

genotype	total N	events	censored
TT	155	116 (0.748)	39 (0.252)
AT,AA	39	34 (0.872)	5 (0.128)

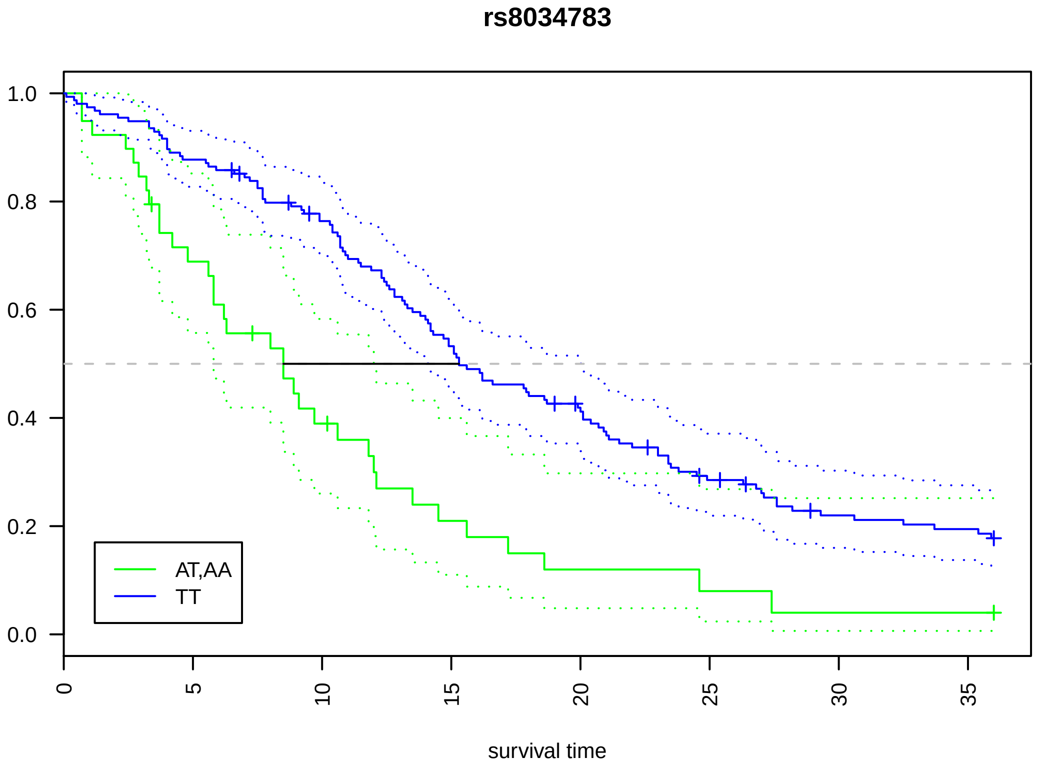

Figure 1955: Kaplan-Meier curves for the selected genotypes. Dashed lines represent 0.95 confidence intervals for the curves with the corresponding color. Black horizontal line ($15.30 - 8.50 = 6.80$) indicates the maximal survival difference at $y = 0.50$.

17.30 Survival analysis of rs12101080

This marker has a genotype specific effect with a p-value of 9.956857e-05.

Odds ratios have been estimated using logistic regression at $t \approx 36$:
$\frac{P(event|AT,AA)/P(\neg event|AT,AA)}{P(event|TT)/P(\neg event|TT)} \approx 2.918$.

Table 44: Summary of genotype frequencies with their proportions in parenthesis.

genotype	total N	events	censored
TT	127	91 (0.717)	36 (0.283)
AT,AA	67	59 (0.881)	8 (0.119)

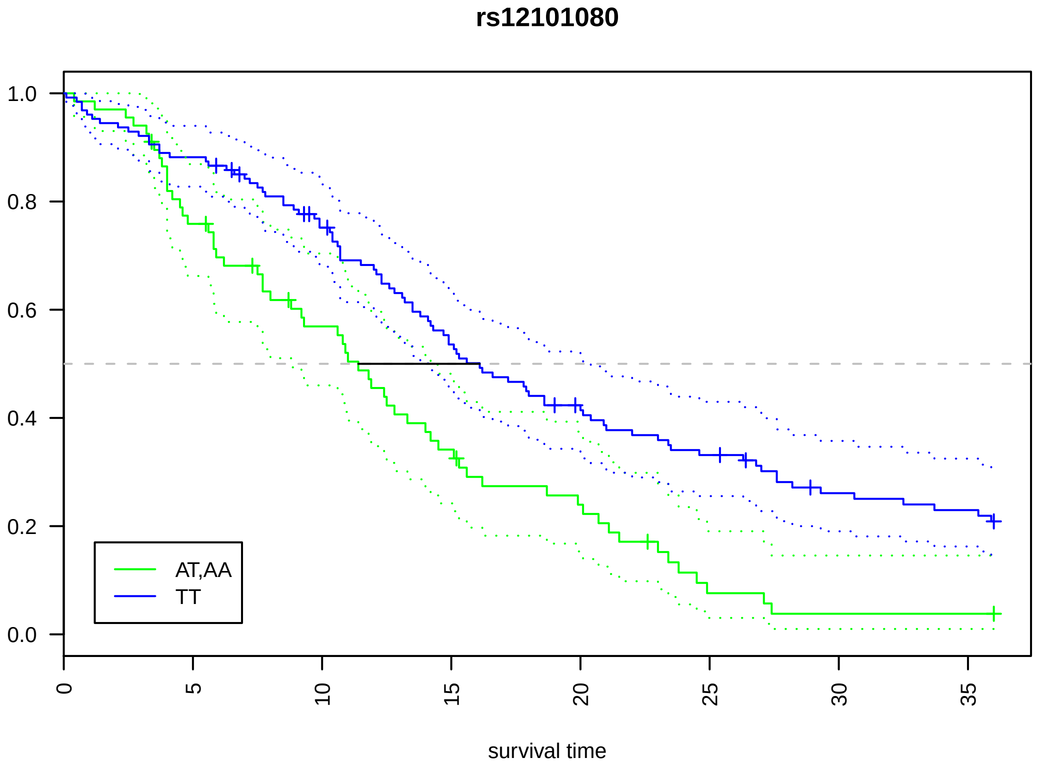

Figure 1956: Kaplan-Meier curves for the selected genotypes. Dashed lines represent 0.95 confidence intervals for the curves with the corresponding color. Black horizontal line $(16.10 - 11.40 = 4.70)$ indicates the maximal survival difference at $y = 0.50$.

Additional annotations:

Gene name	*Description*	*DNABand*	*protein*
ITPK1	inositol 1,3,4-triphosphate 5/6 kinase [Source:HGNC Symbol;Acc:6177]	14q32.12	ITPK1_HUMAN

17.31 Survival analysis of rs11708378

This marker has a genotype specific effect with a p-value of 8.074122e-05.

Odds ratios have been estimated using logistic regression at $t \approx 36$:
$\frac{P(event|AT,AA)/P(\neg event|AT,AA)}{P(event|TT)/P(\neg event|TT)} \approx 2.937$.

Table 45: Summary of genotype frequencies with their proportions in parenthesis.

genotype	total N	events	censored
TT	84	56 (0.667)	28 (0.333)
AT,AA	110	94 (0.855)	16 (0.145)

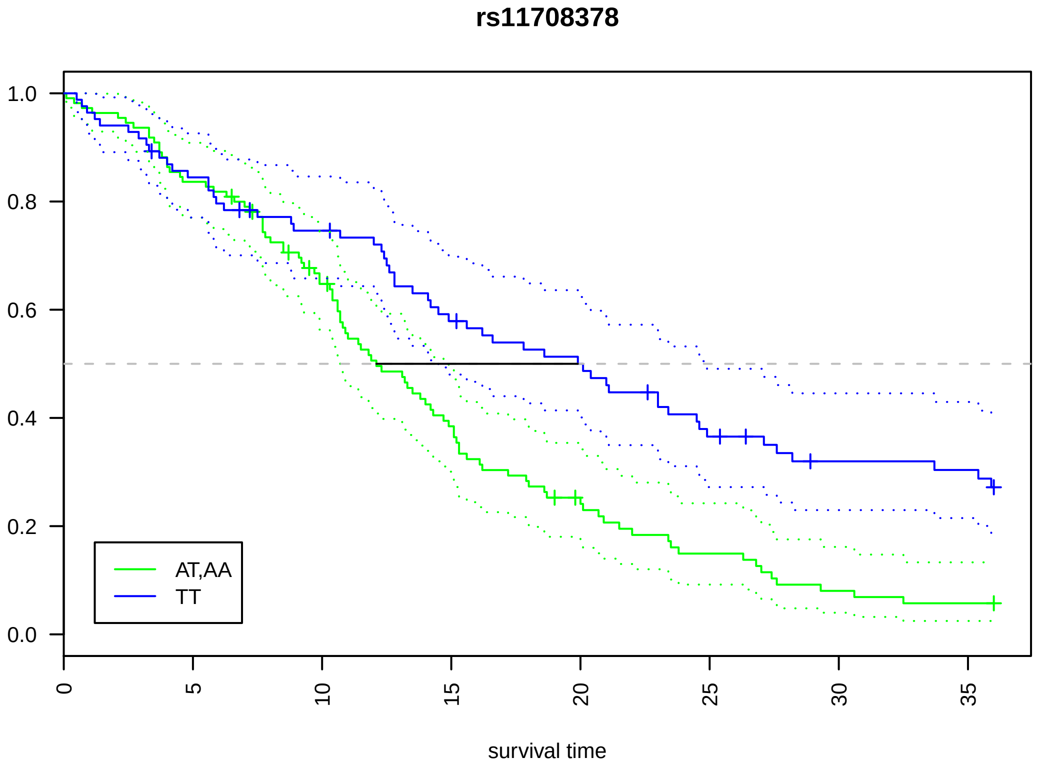

Figure 1957: Kaplan-Meier curves for the selected genotypes. Dashed lines represent 0.95 confidence intervals for the curves with the corresponding color. Black horizontal line $(19.90 - 12.10 = 7.80)$ indicates the maximal survival difference at $y = 0.50$.

Additional annotations:

Gene name	Description	DNABand	protein
SLC6A11	solute carrier family 6 (neurotransmitter transporter, GABA), member 11 [Source:HGNC Symbol;Acc:11044]	3p25.3	S6A11_HUMAN

17.32 Survival analysis of rs6004869

This marker has a genotype specific effect with a p-value of 2.113252e-05.

Odds ratios have been estimated using logistic regression at $t \approx 36$:
$\frac{P(event|AT)/P(\neg event|AT)}{P(event|AA)/P(\neg event|AA)} \approx 2.542$ and $\frac{P(event|TT)/P(\neg event|TT)}{P(event|AA)/P(\neg event|AA)} \approx 2.444$.

Table 46: Summary of genotype frequencies with their proportions in parenthesis.

genotype	total N	events	censored
AA	85	58 (0.682)	27 (0.318)
AT	84	71 (0.845)	13 (0.155)
TT	25	21 (0.84)	4 (0.16)

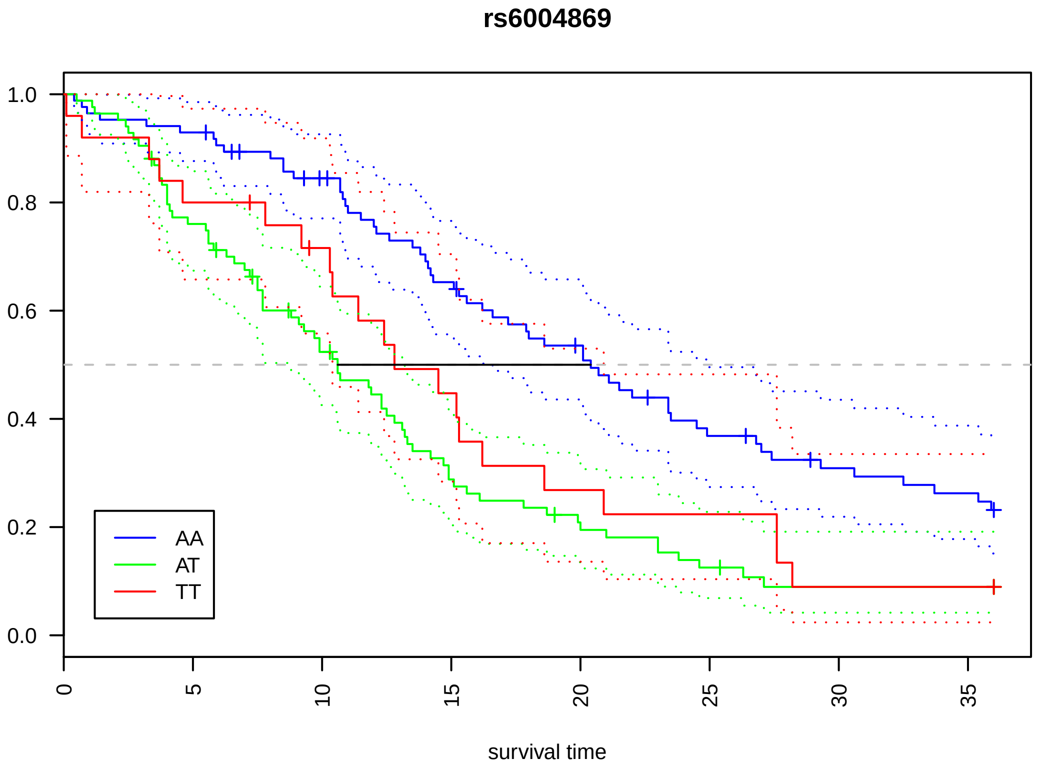

Figure 1958: Kaplan-Meier curves for the selected genotypes. Dashed lines represent 0.95 confidence intervals for the curves with the corresponding color. Black horizontal line ($20.40 - 10.60 = 9.80$) indicates the maximal survival difference at $y = 0.50$.

Additional annotations:

Gene name	*Description*	*DNABand*	*protein*
MYO18B	myosin XVIIIB [Source:HGNC Symbol;Acc:18150]	22q12.1	MY18B_HUMAN

17.33 Survival analysis of rs4906048

This marker has a genotype specific effect with a p-value of 9.445397e-05. Total of 8 observations deleted due to missingness. Odds ratios have been estimated using logistic regression at $t \approx 36$:
$\frac{P(event|AT,AA)/P(\neg event|AT,AA)}{P(event|TT)/P(\neg event|TT)} \approx 0.387$.

Table 47: Summary of genotype frequencies with their proportions in parenthesis.

genotype	total N	events	censored
TT	139	114 (0.82)	25 (0.18)
AT,AA	47	30 (0.638)	17 (0.362)

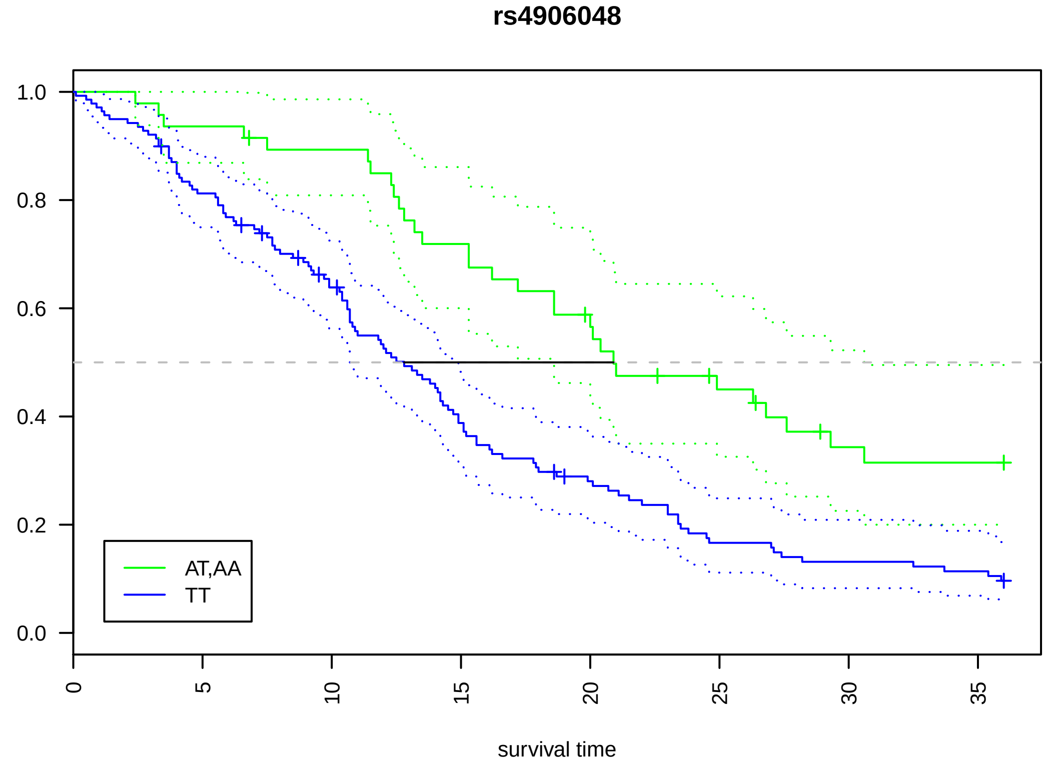

Figure 1959: Kaplan-Meier curves for the selected genotypes. Dashed lines represent 0.95 confidence intervals for the curves with the corresponding color. Black horizontal line $(20.90 - 12.80 = 8.10)$ indicates the maximal survival difference at $y = 0.50$.

17.34 Survival analysis of rs10803885

This marker has a genotype specific effect with a p-value of 9.625245e-05.

Odds ratios have been estimated using logistic regression at $t \approx 36$:
$\frac{P(event|AT)/P(\neg event|AT)}{P(event|AA)/P(\neg event|AA)} \approx 0.559$ and $\frac{P(event|TT)/P(\neg event|TT)}{P(event|AA)/P(\neg event|AA)} \approx 2.288$.

Table 48: Summary of genotype frequencies with their proportions in parenthesis.

genotype	total N	events	censored
AA	74	59 (0.797)	15 (0.203)
AT	80	55 (0.688)	25 (0.312)
TT	40	36 (0.9)	4 (0.1)

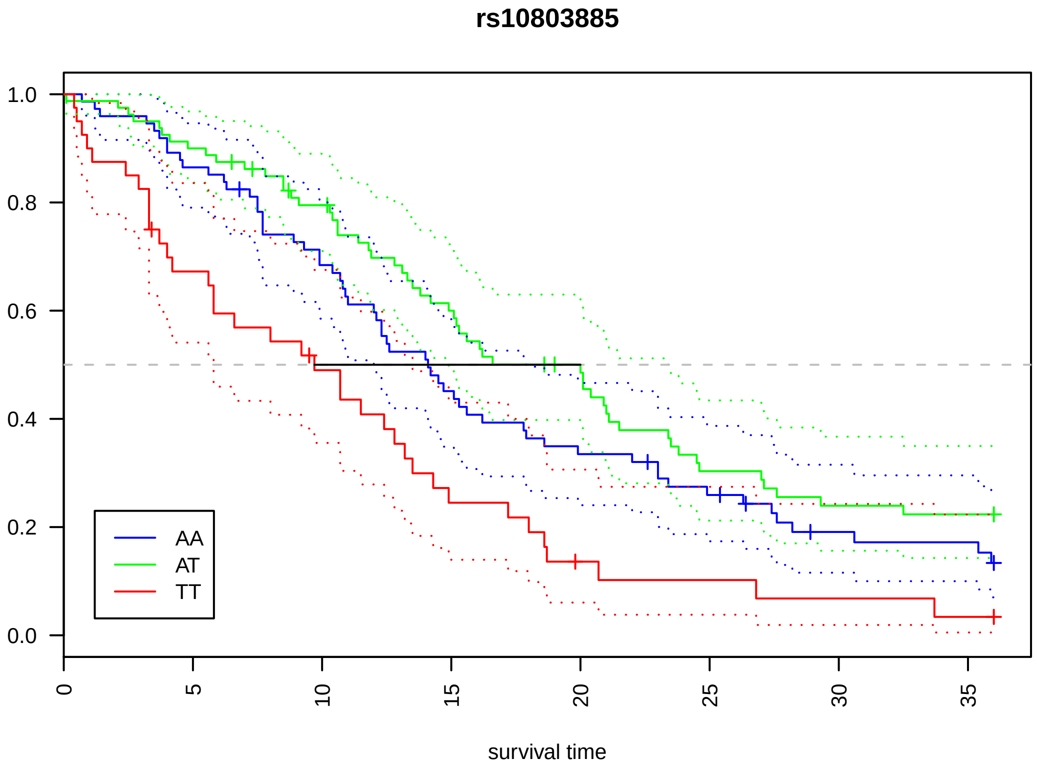

Figure 1960: Kaplan-Meier curves for the selected genotypes. Dashed lines represent 0.95 confidence intervals for the curves with the corresponding color. Black horizontal line $(20.00 - 9.70 = 10.30)$ indicates the maximal survival difference at $y = 0.50$.

Additional annotations:

Gene name	Description	DNABand
AC018890.2	[undefined]	2q31.1

17.35 Survival analysis of rs7902084

This marker has a genotype specific effect with a p-value of 2.439848e-05.

Odds ratios have been estimated using logistic regression at $t \approx 36$:
$\frac{P(event|AT)/P(\neg event|AT)}{P(event|TT)/P(\neg event|TT)} \approx 1.884$ and $\frac{P(event|AA)/P(\neg event|AA)}{P(event|TT)/P(\neg event|TT)} \approx 12.723$.

Table 49: Summary of genotype frequencies with their proportions in parenthesis.

genotype	total N	events	censored
TT	70	47 (0.671)	23 (0.329)
AT	97	77 (0.794)	20 (0.206)
AA	27	26 (0.963)	1 (0.037)

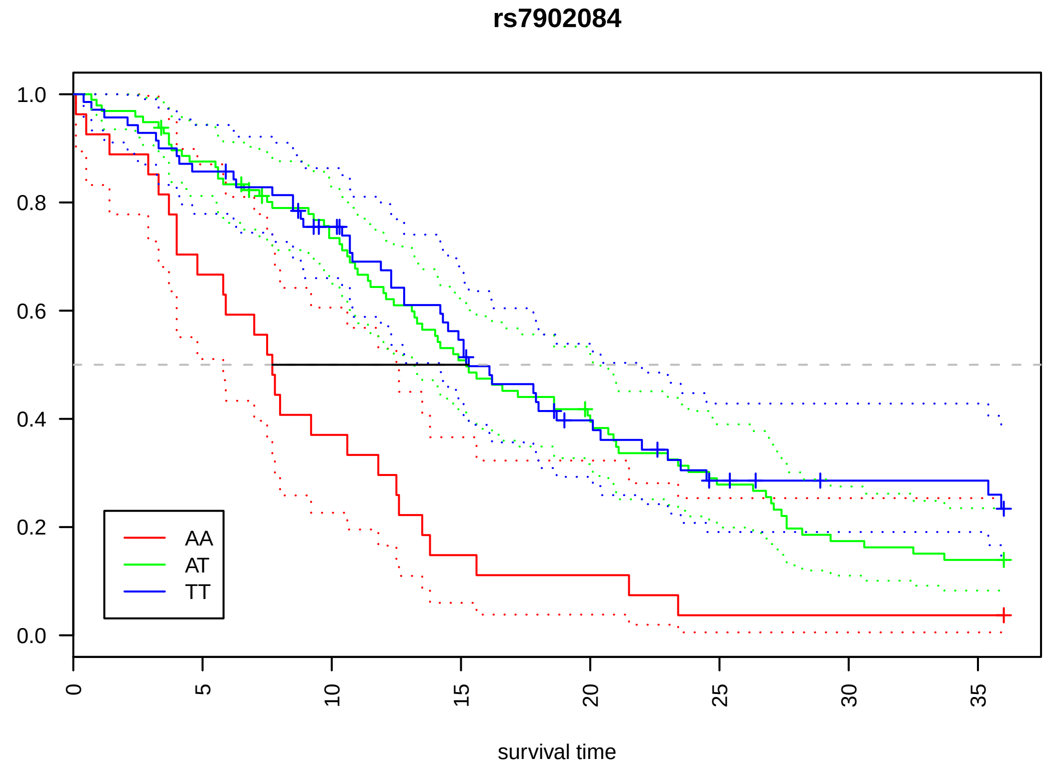

Figure 1961: Kaplan-Meier curves for the selected genotypes. Dashed lines represent 0.95 confidence intervals for the curves with the corresponding color. Black horizontal line $(15.30 - 7.70 = 7.60)$ indicates the maximal survival difference at $y = 0.50$.

Additional annotations:

Gene name	*Description*	*DNABand*	*protein*
C10orf81	PH domain-containing protein C10orf81 (Epididymis luminal protein 185)(hEL185) [Source:UniProtKB/Swiss-Prot;Acc:Q5SXH7]	10q25.3	CJ081_HUMAN

17.36 Survival analysis of rs306220

This marker has a genotype specific effect with a p-value of 4.598385e-06. Total of 18 observations deleted due to missingness. Odds ratios have been estimated using logistic regression at $t \approx 36$:
$\frac{P(event|AT,AA)/P(\neg event|AT,AA)}{P(event|TT)/P(\neg event|TT)} \approx 0.235$.

Table 50: Summary of genotype frequencies with their proportions in parenthesis.

genotype	total N	events	censored
TT	127	108 (0.85)	19 (0.15)
AT,AA	49	28 (0.571)	21 (0.429)

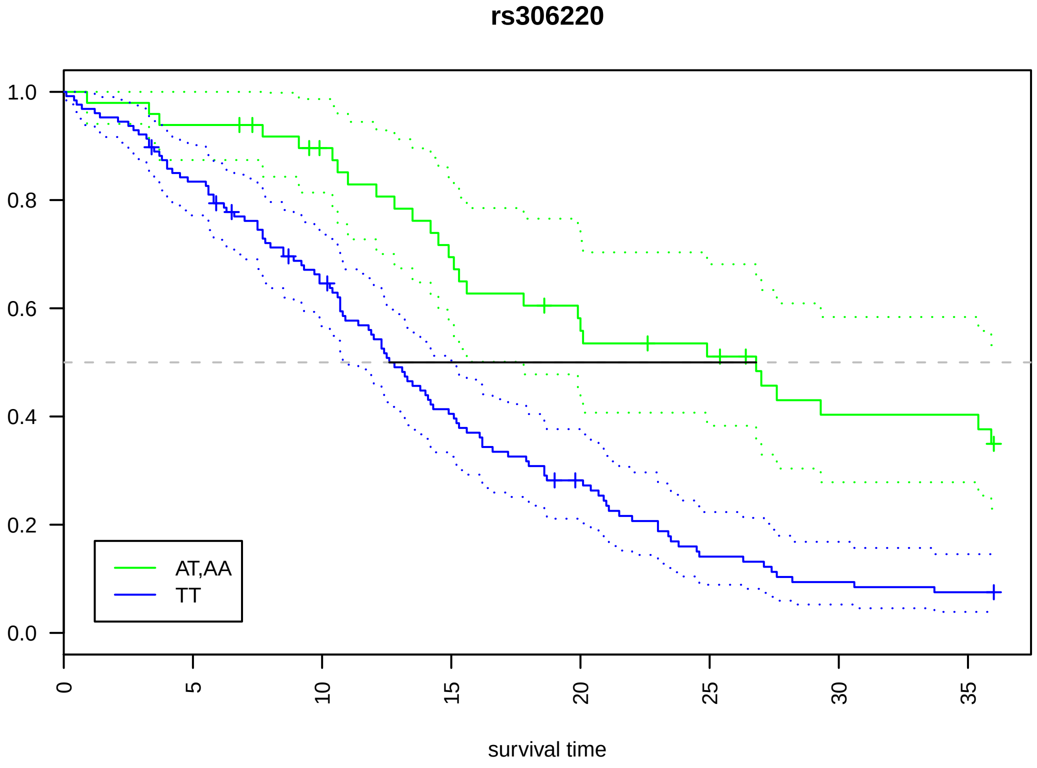

Figure 1962: Kaplan-Meier curves for the selected genotypes. Dashed lines represent 0.95 confidence intervals for the curves with the corresponding color. Black horizontal line ($26.80 - 12.60 = 14.20$) indicates the maximal survival difference at $y = 0.50$.

17.37 Survival analysis of rs4489589

This marker has a genotype specific effect with a p-value of 8.072571e-05. Total of 1 observation deleted due to missingness. Odds ratios have been estimated using logistic regression at $t \approx 36$:
$\frac{P(event|AT,AA)/P(\neg event|AT,AA)}{P(event|TT)/P(\neg event|TT)} \approx 12.233$.

Table 51: Summary of genotype frequencies with their proportions in parenthesis.

genotype	total N	events	censored
TT	159	116 (0.73)	43 (0.27)
AT,AA	34	33 (0.971)	1 (0.0294)

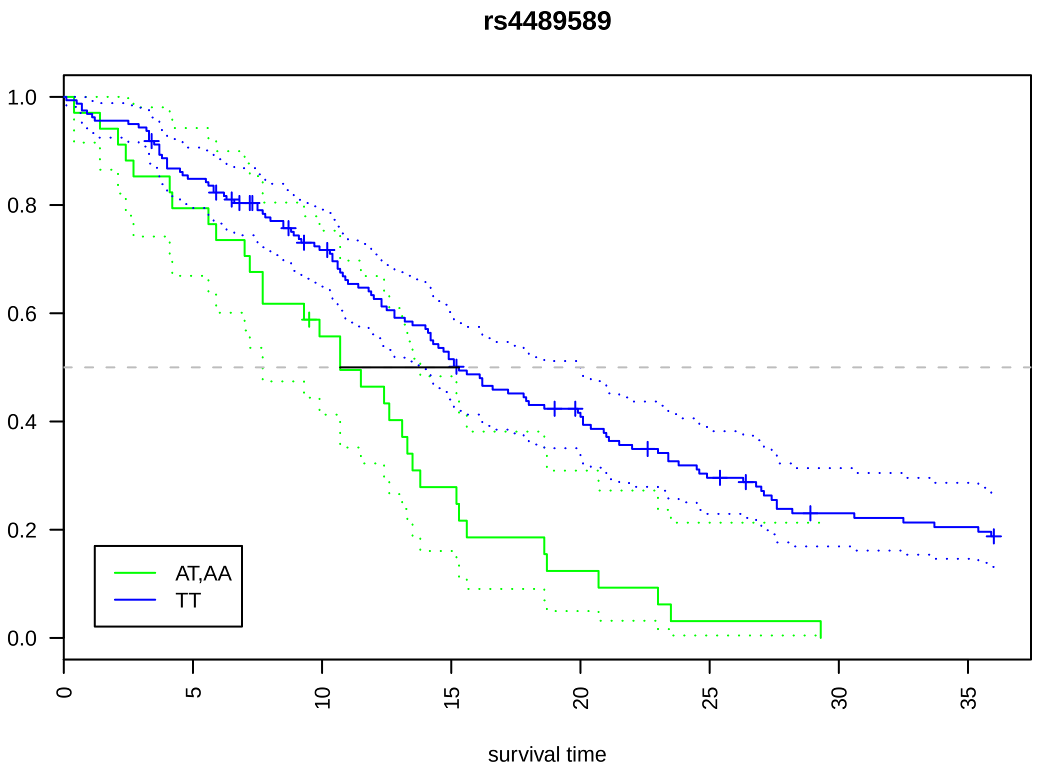

Figure 1963: Kaplan-Meier curves for the selected genotypes. Dashed lines represent 0.95 confidence intervals for the curves with the corresponding color. Black horizontal line $(15.30 - 10.70 = 4.60)$ indicates the maximal survival difference at $y = 0.50$.

17.38 Survival analysis of rs10221769

This marker has a genotype specific effect with a p-value of 2.350979e-05.

Odds ratios have been estimated using logistic regression at $t \approx 36$:
$\frac{P(event|AT)/P(\neg event|AT)}{P(event|AA)/P(\neg event|AA)} \approx 0.984$ and $\frac{P(event|TT)/P(\neg event|TT)}{P(event|AA)/P(\neg event|AA)} \approx 1.269$.

Table 52: Summary of genotype frequencies with their proportions in parenthesis.

genotype	total N	events	censored
AA	100	77 (0.77)	23 (0.23)
AT	73	56 (0.767)	17 (0.233)
TT	21	17 (0.81)	4 (0.19)

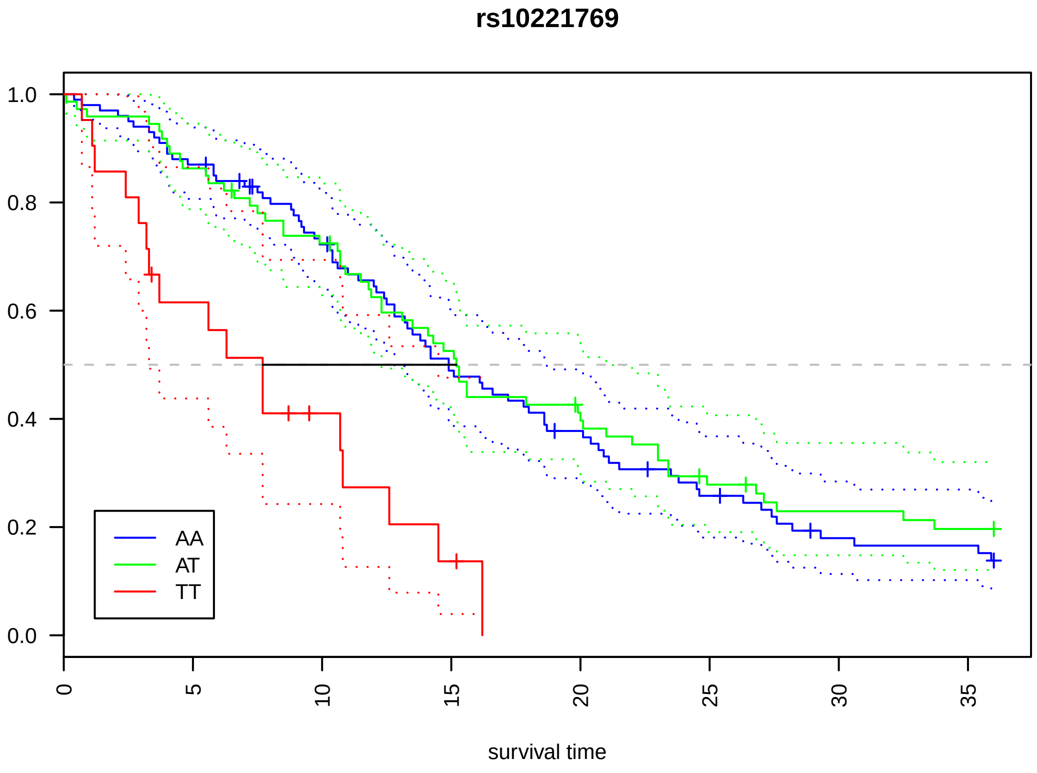

Figure 1964: Kaplan-Meier curves for the selected genotypes. Dashed lines represent 0.95 confidence intervals for the curves with the corresponding color. Black horizontal line $(15.20 - 7.70 = 7.50)$ indicates the maximal survival difference at $y = 0.50$.

Additional annotations:

Gene name	*Description*	*DNABand*	*protein*
EXOC6B	exocyst complex component 6B [Source:HGNC Symbol;Acc:17085]	2p13.2	EXC6B_HUMAN

17.39 Survival analysis of rs12891139

This marker has a genotype specific effect with a p-value of 4.708383e-05.

Odds ratios have been estimated using logistic regression at $t \approx 36$:
$\frac{P(event|AT,AA)/P(\neg event|AT,AA)}{P(event|TT)/P(\neg event|TT)} \approx 0.367$.

Table 53: Summary of genotype frequencies with their proportions in parenthesis.

genotype	total N	events	censored
TT	98	84 (0.857)	14 (0.143)
AT,AA	96	66 (0.688)	30 (0.312)

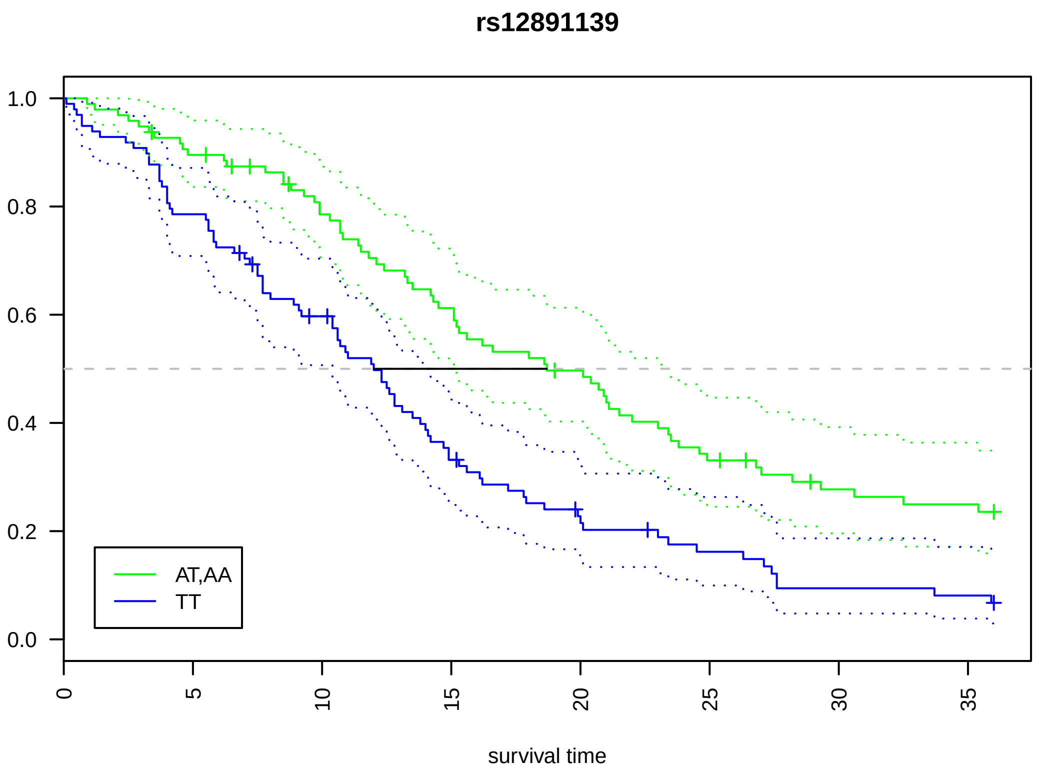

Figure 1965: Kaplan-Meier curves for the selected genotypes. Dashed lines represent 0.95 confidence intervals for the curves with the corresponding color. Black horizontal line ($18.70 - 12.00 = 6.70$) indicates the maximal survival difference at $y = 0.50$.

17.40 Survival analysis of rs10827154

This marker has a genotype specific effect with a p-value of 1.200712e-05.

Odds ratios have been estimated using logistic regression at $t \approx 36$:
$\frac{P(event|AT,AA)/P(\neg event|AT,AA)}{P(event|TT)/P(\neg event|TT)} \approx 2.723$.

Table 54: Summary of genotype frequencies with their proportions in parenthesis.

genotype	total N	events	censored
TT	136	99 (0.728)	37 (0.272)
AT,AA	58	51 (0.879)	7 (0.121)

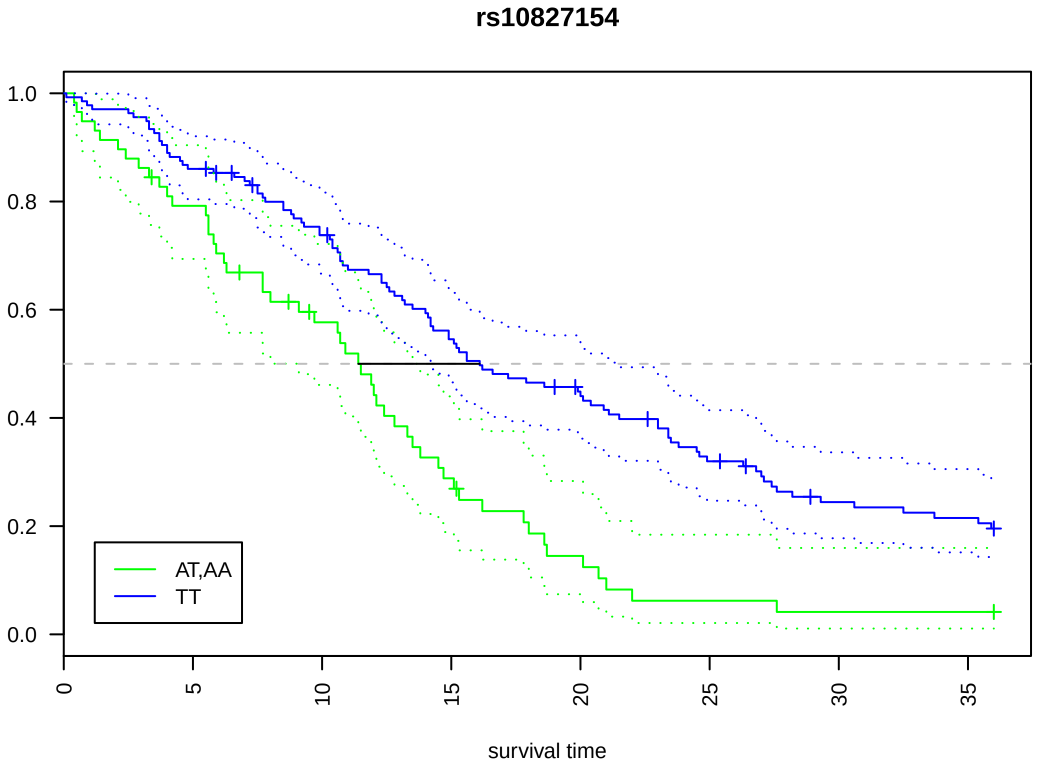

Figure 1966: Kaplan-Meier curves for the selected genotypes. Dashed lines represent 0.95 confidence intervals for the curves with the corresponding color. Black horizontal line $(16.10 - 11.40 = 4.70)$ indicates the maximal survival difference at $y = 0.50$.

Additional annotations:

Gene name	Description	DNABand	protein
C10orf112	MAM and LDL-receptor class A domain-containing protein C10orf112 [Source:UniProtKB/Swiss-Prot;Acc:Q5VYJ5]	10p12.31	CJ112_HUMAN

17.41 Survival analysis of rs13168015

This marker has a genotype specific effect with a p-value of 7.710583e-05.

Odds ratios have been estimated using logistic regression at $t \approx 36$:
$\frac{P(event|AT,AA)/P(\neg event|AT,AA)}{P(event|TT)/P(\neg event|TT)} \approx 2.712$.

Table 55: Summary of genotype frequencies with their proportions in parenthesis.

genotype	total N	events	censored
TT	158	118 (0.747)	40 (0.253)
AT,AA	36	32 (0.889)	4 (0.111)

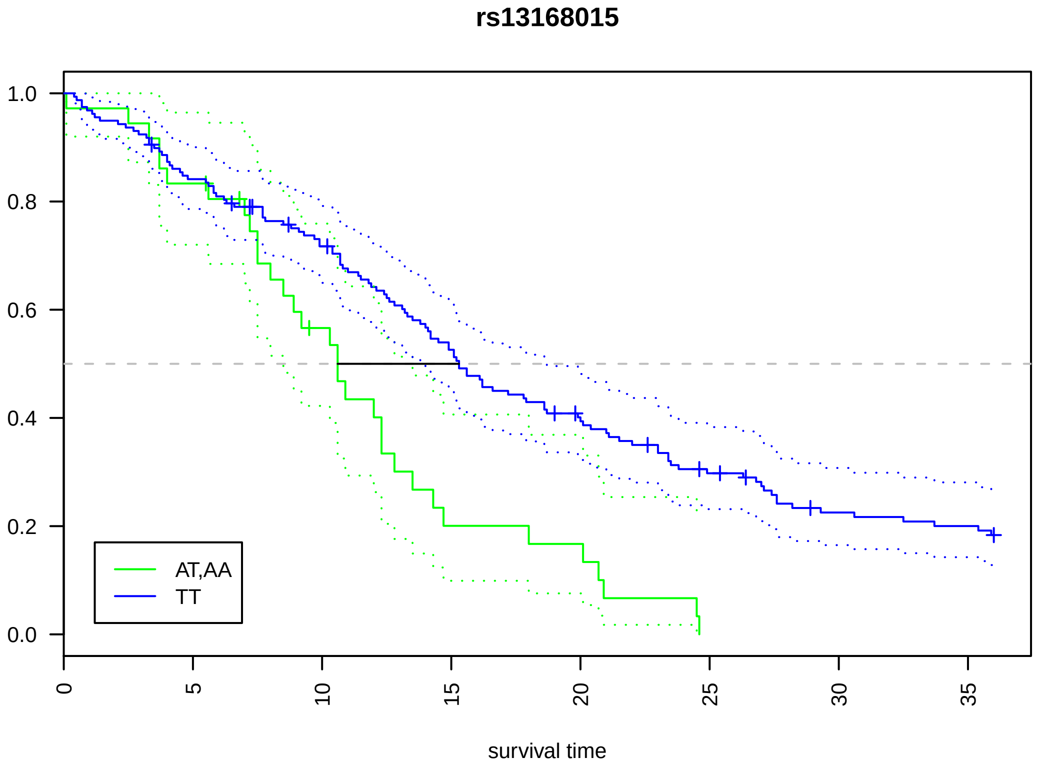

Figure 1967: Kaplan-Meier curves for the selected genotypes. Dashed lines represent 0.95 confidence intervals for the curves with the corresponding color. Black horizontal line ($15.30 - 10.60 = 4.70$) indicates the maximal survival difference at $y = 0.50$.

Additional annotations:

Gene name	*Description*	*DNABand*
U6	U6 spliceosomal RNA [Source:RFAM;Acc:RF00026]	5q31.1

17.42 Survival analysis of rs11692586

This marker has a genotype specific effect with a p-value of 2.799598e-05.

Odds ratios have been estimated using logistic regression at $t \approx 36$:
$\frac{P(event|AT)/P(\neg event|AT)}{P(event|AA)/P(\neg event|AA)} \approx 2.522$ and $\frac{P(event|TT)/P(\neg event|TT)}{P(event|AA)/P(\neg event|AA)} \approx 2.371$.

Table 56: Summary of genotype frequencies with their proportions in parenthesis.

genotype	total N	events	censored
AA	90	62 (0.689)	28 (0.311)
AT	79	67 (0.848)	12 (0.152)
TT	25	21 (0.84)	4 (0.16)

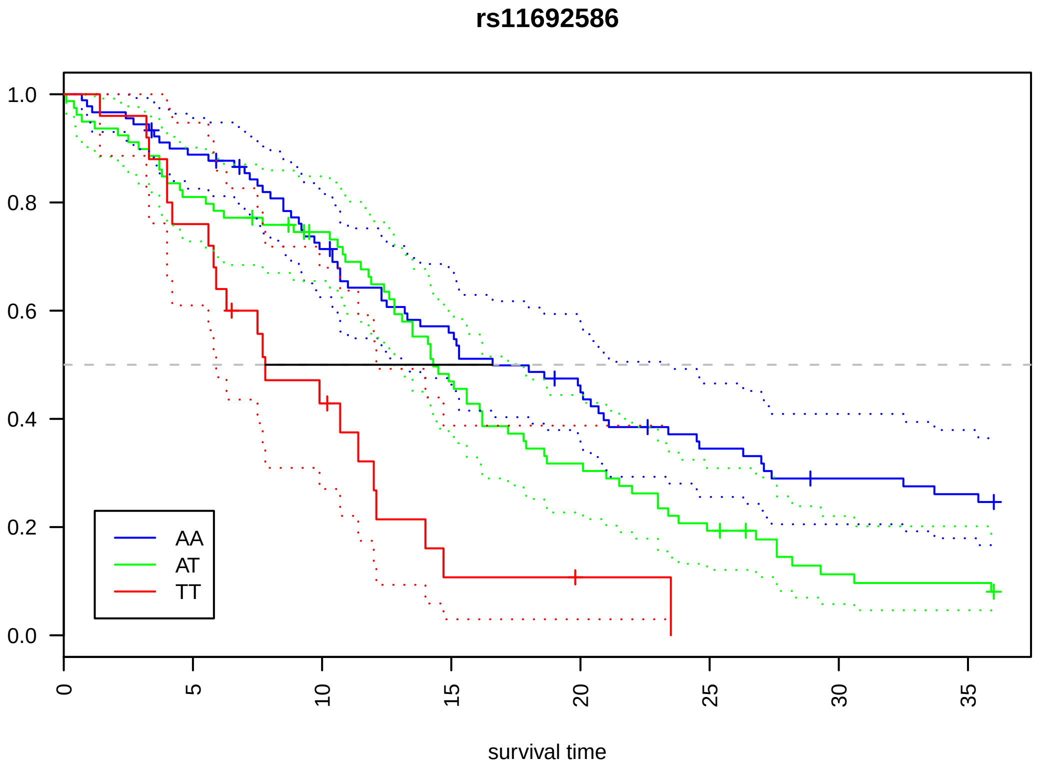

Figure 1968: Kaplan-Meier curves for the selected genotypes. Dashed lines represent 0.95 confidence intervals for the curves with the corresponding color. Black horizontal line ($16.60 - 7.80 = 8.80$) indicates the maximal survival difference at $y = 0.50$.

Additional annotations:

Gene name	*Description*	*DNABand*	*protein*
MGAT5	mannosyl (alpha-1,6-)-glycoprotein beta-1,6-N-acetyl-glucosaminyltransferase [Source:HGNC Symbol;Acc:7049]	2q21.2,2q21.3	MGT5A_HUMAN

17.43 Survival analysis of rs10793491

This marker has a genotype specific effect with a p-value of 6.340249e-05.

Odds ratios have been estimated using logistic regression at $t \approx 36$:
$\frac{P(event|AT,AA)/P(\neg event|AT,AA)}{P(event|TT)/P(\neg event|TT)} \approx 0.304$.

Table 57: Summary of genotype frequencies with their proportions in parenthesis.

genotype	total N	events	censored
TT	100	87 (0.87)	13 (0.13)
AT,AA	94	63 (0.67)	31 (0.33)

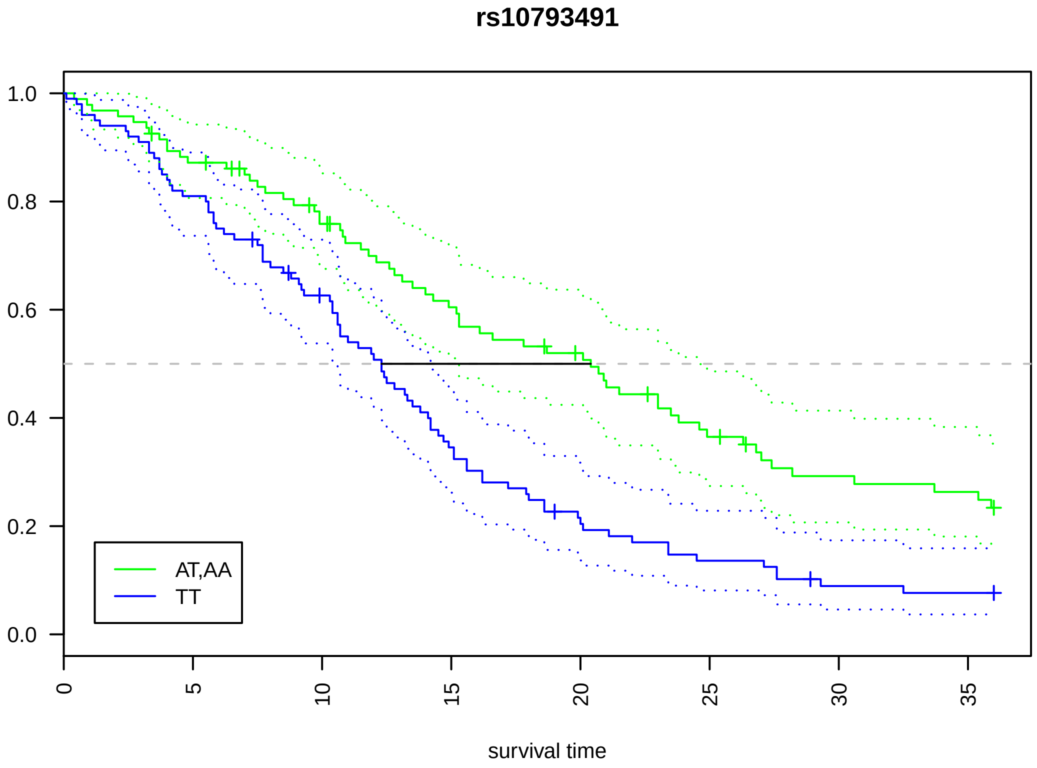

Figure 1969: Kaplan-Meier curves for the selected genotypes. Dashed lines represent 0.95 confidence intervals for the curves with the corresponding color. Black horizontal line ($20.40 - 12.30 = 8.10$) indicates the maximal survival difference at $y = 0.50$.

Additional annotations:

Gene name	Description	DNABand
RP11-272J7.1	[undefined]	10q11.21

17.44 Survival analysis of rs11007253

This marker has a genotype specific effect with a p-value of 2.315311e-05.

Odds ratios have been estimated using logistic regression at $t \approx 36$:
$\frac{P(event|AT,TT)/P(\neg event|AT,TT)}{P(event|AA)/P(\neg event|AA)} \approx 2.225$.

Table 58: Summary of genotype frequencies with their proportions in parenthesis.

genotype	total N	events	censored
AA	149	111 (0.745)	38 (0.255)
AT,TT	45	39 (0.867)	6 (0.133)

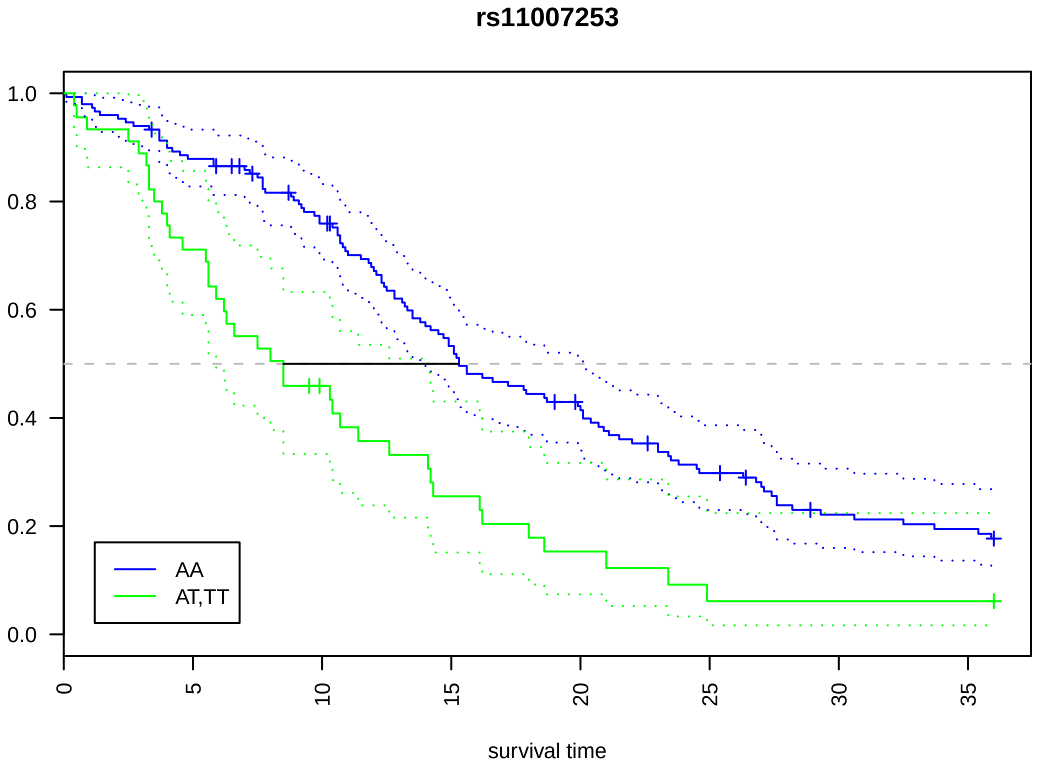

Figure 1970: Kaplan-Meier curves for the selected genotypes. Dashed lines represent 0.95 confidence intervals for the curves with the corresponding color. Black horizontal line ($15.30 - 8.50 = 6.80$) indicates the maximal survival difference at $y = 0.50$.

Additional annotations:

Gene name	*Description*	*DNABand*
RP11-478H13.3	[undefined]	10p12.1

17.45 Survival analysis of rs4498833

This marker has a genotype specific effect with a p-value of 3.193456e-05.

Odds ratios have been estimated using logistic regression at $t \approx 36$:
$\frac{P(event|AT,TT)/P(\neg event|AT,TT)}{P(event|AA)/P(\neg event|AA)} \approx 4.637$.

Table 59: Summary of genotype frequencies with their proportions in parenthesis.

genotype	total N	events	censored
AA	153	112 (0.732)	41 (0.268)
AT,TT	41	38 (0.927)	3 (0.0732)

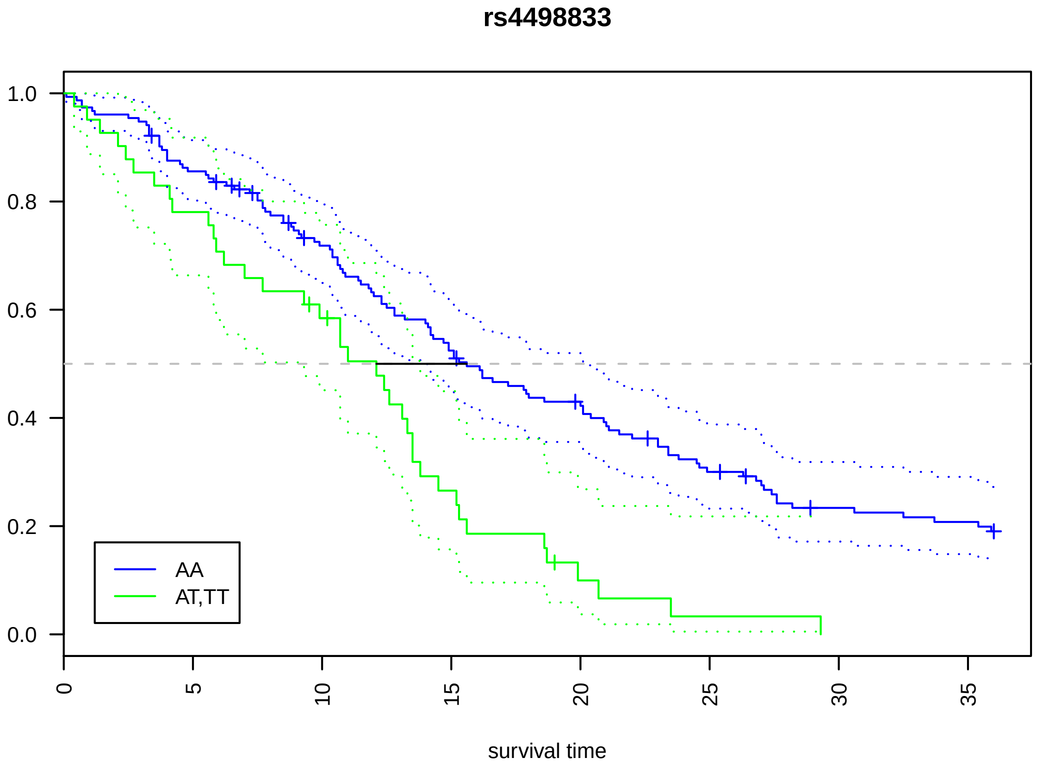

Figure 1971: Kaplan-Meier curves for the selected genotypes. Dashed lines represent 0.95 confidence intervals for the curves with the corresponding color. Black horizontal line $(15.60 - 12.10 = 3.50)$ indicates the maximal survival difference at $y = 0.50$.

17.46 Survival analysis of rs1436094

This marker has a genotype specific effect with a p-value of 5.800780e-06.

Odds ratios have been estimated using logistic regression at $t \approx 36$:
$\frac{P(event|AT)/P(\neg event|AT)}{P(event|AA)/P(\neg event|AA)} \approx 0.523$ and $\frac{P(event|TT)/P(\neg event|TT)}{P(event|AA)/P(\neg event|AA)} \approx 5.918$.

Table 60: Summary of genotype frequencies with their proportions in parenthesis.

genotype	total N	events	censored
AA	91	73 (0.802)	18 (0.198)
AT	78	53 (0.679)	25 (0.321)
TT	25	24 (0.96)	1 (0.04)

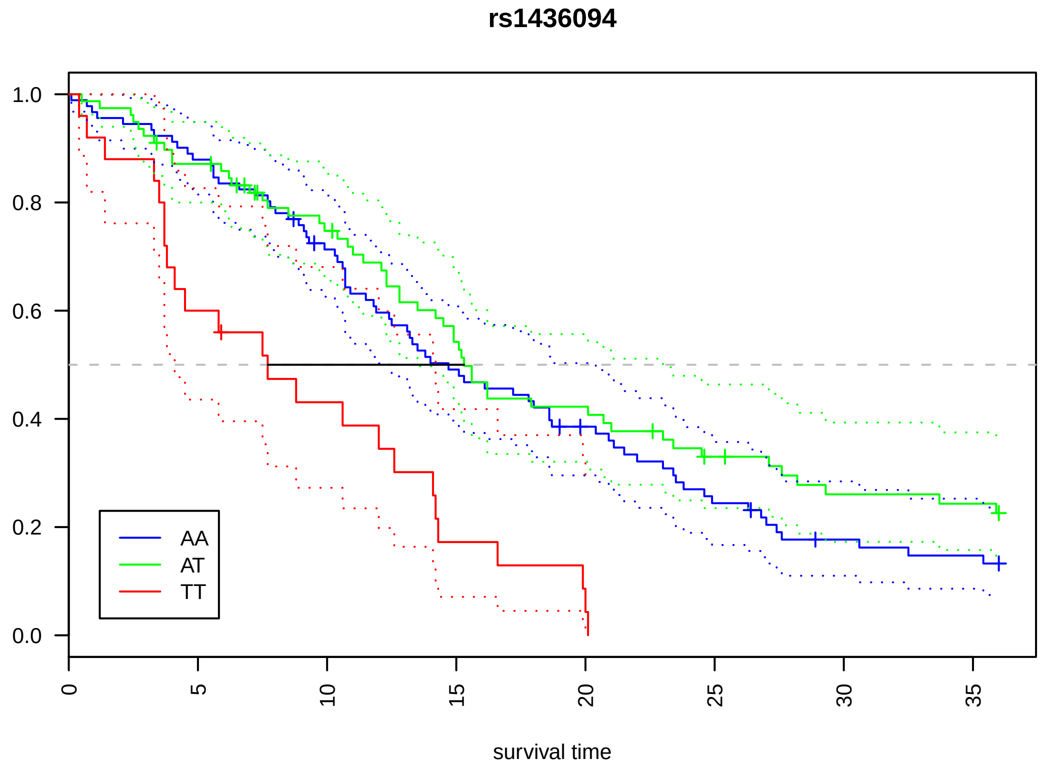

Figure 1972: Kaplan-Meier curves for the selected genotypes. Dashed lines represent 0.95 confidence intervals for the curves with the corresponding color. Black horizontal line ($15.30 - 7.70 = 7.60$) indicates the maximal survival difference at $y = 0.50$.

17.47 Survival analysis of rs9403788

This marker has a genotype specific effect with a p-value of 2.283060e-06.

Odds ratios have been estimated using logistic regression at $t \approx 36$:
$\frac{P(event|AT,TT)/P(\neg event|AT,TT)}{P(event|AA)/P(\neg event|AA)} \approx 8.45$.

Table 61: Summary of genotype frequencies with their proportions in parenthesis.

genotype	total N	events	censored
AA	111	72 (0.649)	39 (0.351)
AT,TT	83	78 (0.94)	5 (0.0602)

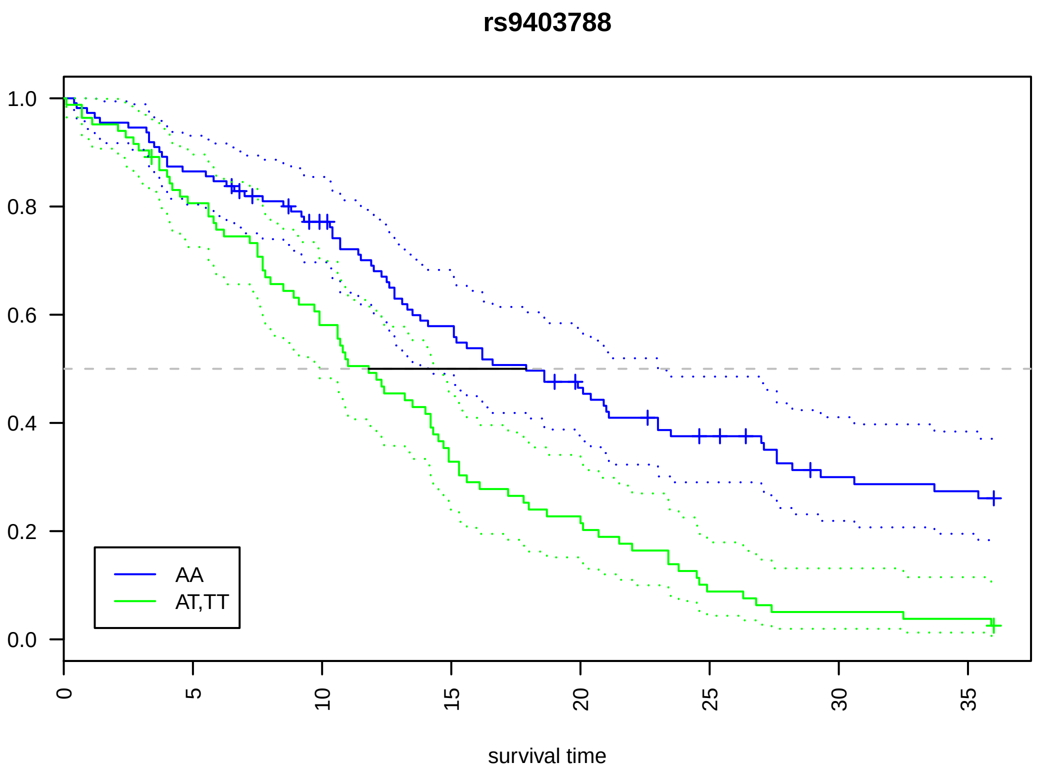

Figure 1973: Kaplan-Meier curves for the selected genotypes. Dashed lines represent 0.95 confidence intervals for the curves with the corresponding color. Black horizontal line ($17.90 - 11.80 = 6.10$) indicates the maximal survival difference at $y = 0.50$.

17.48 Survival analysis of rs6077364

This marker has a genotype specific effect with a p-value of 2.917788e-05.

Odds ratios have been estimated using logistic regression at $t \approx 36$:
$\frac{P(event|AT)/P(\neg event|AT)}{P(event|AA)/P(\neg event|AA)} \approx 0.312$ and $\frac{P(event|TT)/P(\neg event|TT)}{P(event|AA)/P(\neg event|AA)} \approx 0.375$.

Table 62: Summary of genotype frequencies with their proportions in parenthesis.

genotype	total N	events	censored
AA	69	61 (0.884)	8 (0.116)
AT	98	69 (0.704)	29 (0.296)
TT	27	20 (0.741)	7 (0.259)

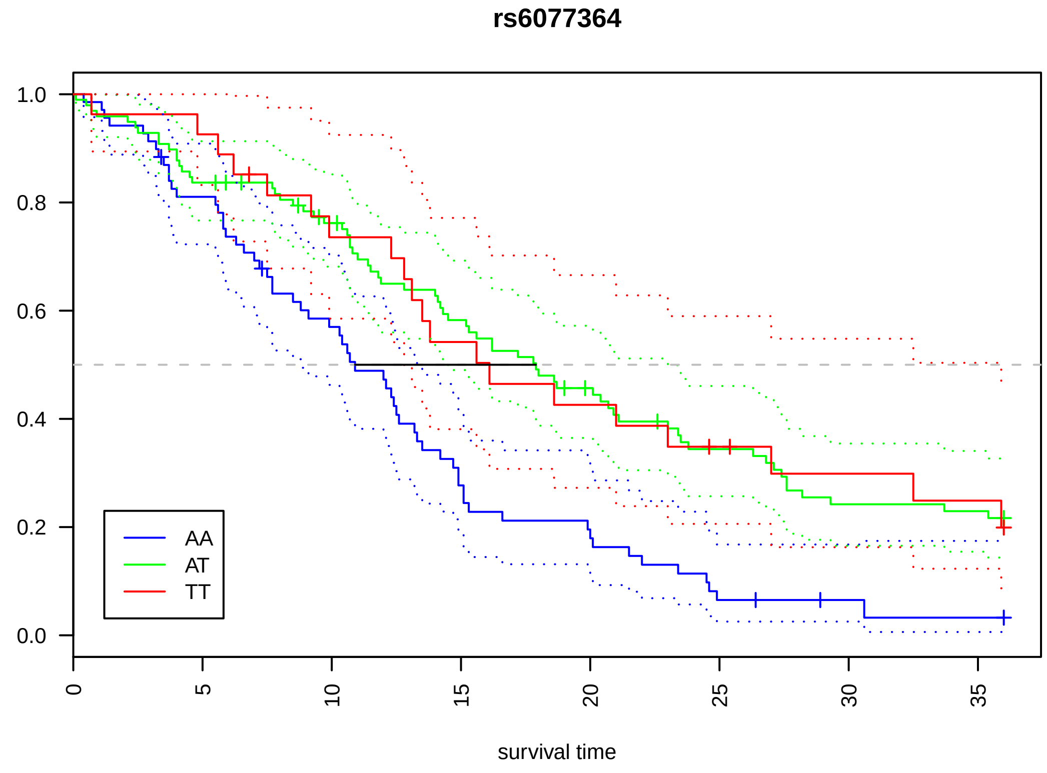

Figure 1974: Kaplan-Meier curves for the selected genotypes. Dashed lines represent 0.95 confidence intervals for the curves with the corresponding color. Black horizontal line ($17.90 - 10.90 = 7.00$) indicates the maximal survival difference at $y = 0.50$.

Additional annotations:

Gene name	*Description*	*DNABand*	*protein*
PLCB1	phospholipase C, beta 1 (phosphoinositide-specific) [Source:HGNC Symbol;Acc:15917]	20p12.3	PLCB1_HUMAN

17.49 Survival analysis of rs182174

This marker has a genotype specific effect with a p-value of 5.044725e-06.

Odds ratios have been estimated using logistic regression at $t \approx 36$:
$\frac{P(event|AT)/P(\neg event|AT)}{P(event|AA)/P(\neg event|AA)} \approx 1.19$ and $\frac{P(event|TT)/P(\neg event|TT)}{P(event|AA)/P(\neg event|AA)} \approx 2.868$.

Table 63: Summary of genotype frequencies with their proportions in parenthesis.

genotype	total N	events	censored
AA	72	53 (0.736)	19 (0.264)
AT	95	73 (0.768)	22 (0.232)
TT	27	24 (0.889)	3 (0.111)

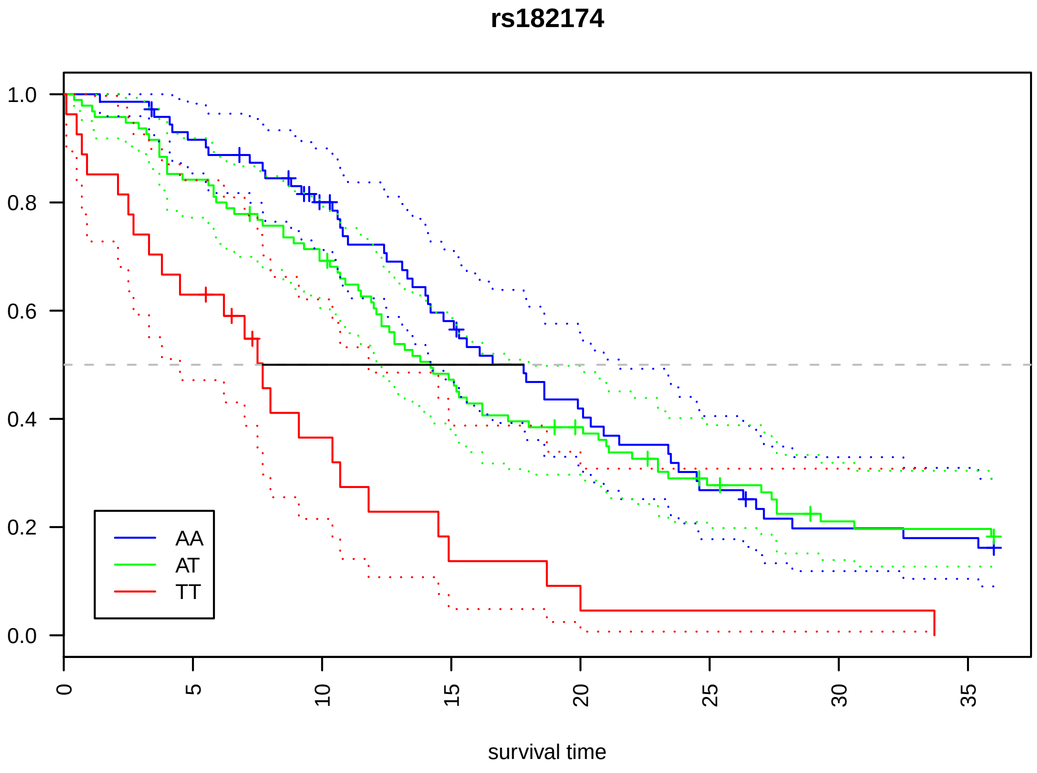

Figure 1975: Kaplan-Meier curves for the selected genotypes. Dashed lines represent 0.95 confidence intervals for the curves with the corresponding color. Black horizontal line ($17.80 - 7.70 = 10.10$) indicates the maximal survival difference at $y = 0.50$.

Additional annotations:

Gene name	*Description*	*DNABand*
AP000457.1	[undefined]	21q21.1

17.50 Survival analysis of rs10516167

This marker has a genotype specific effect with a p-value of 4.443498e-05. Total of 2 observations deleted due to missingness. Odds ratios have been estimated using logistic regression at $t \approx 36$:
$\frac{P(event|AT,AA)/P(\neg event|AT,AA)}{P(event|TT)/P(\neg event|TT)} \approx 0.312$.

Table 64: Summary of genotype frequencies with their proportions in parenthesis.

genotype	total N	events	censored
TT	122	104 (0.852)	18 (0.148)
AT,AA	70	45 (0.643)	25 (0.357)

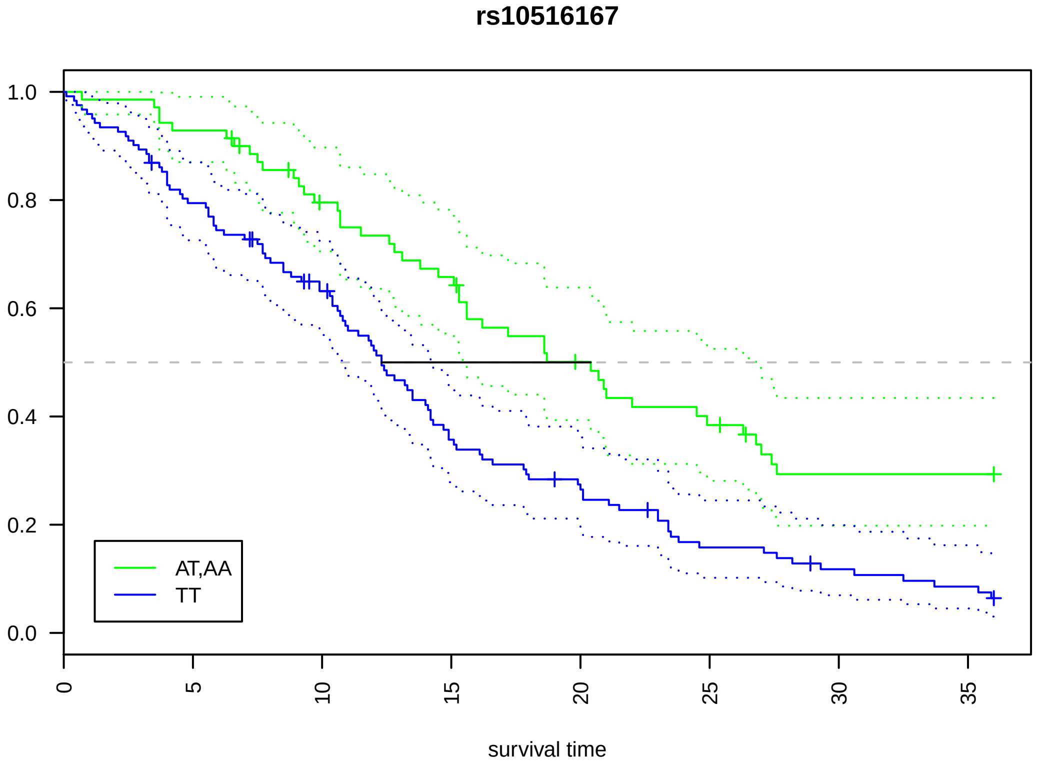

Figure 1976: Kaplan-Meier curves for the selected genotypes. Dashed lines represent 0.95 confidence intervals for the curves with the corresponding color. Black horizontal line $(20.40 - 12.30 = 8.10)$ indicates the maximal survival difference at $y = 0.50$.

Additional annotations:

Gene name	*Description*	*DNABand*	*protein*
STX18	syntaxin 18 [Source:HGNC Symbol;Acc:15942]	4p16.2,4p16.3	STX18_HUMAN

17.51 Survival analysis of rs701405

This marker has a genotype specific effect with a p-value of 9.650369e-05.

Odds ratios have been estimated using logistic regression at $t \approx 36$:
$\frac{P(event|AT,TT)/P(\neg event|AT,TT)}{P(event|AA)/P(\neg event|AA)} \approx 2.6$.

Table 65: Summary of genotype frequencies with their proportions in parenthesis.

genotype	total N	events	censored
AA	119	85 (0.714)	34 (0.286)
AT,TT	75	65 (0.867)	10 (0.133)

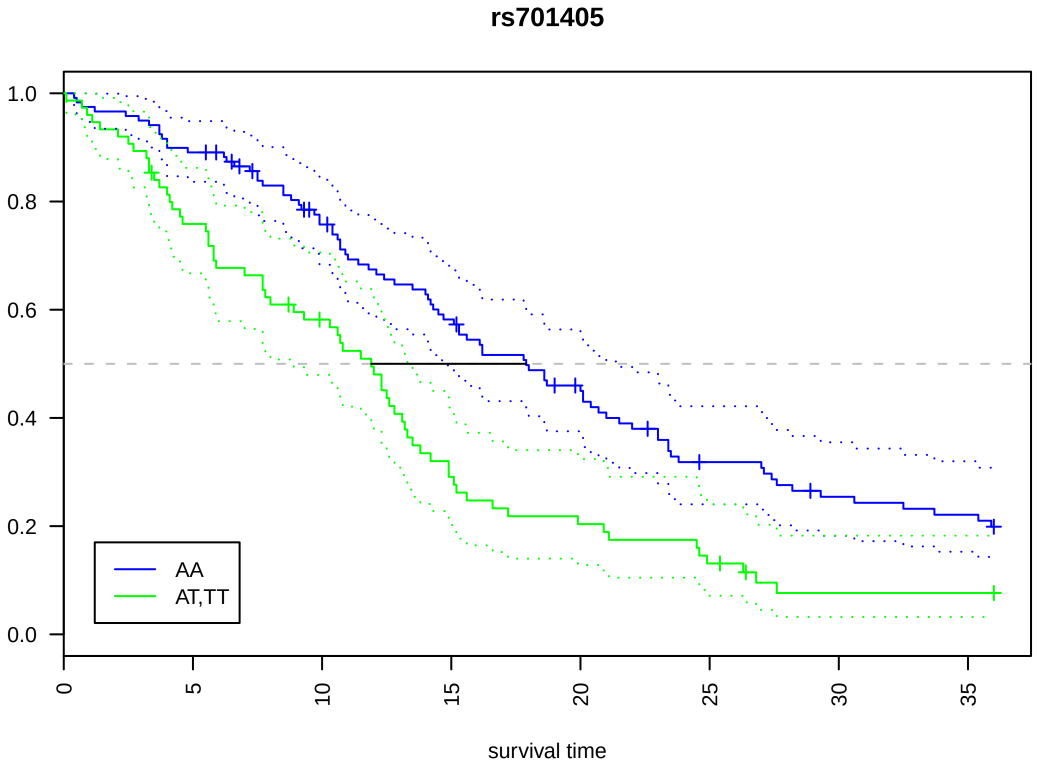

Figure 1977: Kaplan-Meier curves for the selected genotypes. Dashed lines represent 0.95 confidence intervals for the curves with the corresponding color. Black horizontal line $(17.90 - 11.90 = 6.00)$ indicates the maximal survival difference at $y = 0.50$.

Additional annotations:

Gene name	*Description*	*DNABand*	*protein*
ATP6AP1L	ATPase, H+ transporting, lysosomal accessory protein 1-like [Source:HGNC Symbol;Acc:28091]	5q14.2	VAS1L_HUMAN

17.52 Survival analysis of rs10107890

This marker has a genotype specific effect with a p-value of 4.593918e-05.

Odds ratios have been estimated using logistic regression at $t \approx 36$:
$\frac{P(event|AT,TT)/P(\neg event|AT,TT)}{P(event|AA)/P(\neg event|AA)} \approx 13.087$.

Table 66: Summary of genotype frequencies with their proportions in parenthesis.

genotype	total N	events	censored
AA	158	115 (0.728)	43 (0.272)
AT,TT	36	35 (0.972)	1 (0.0278)

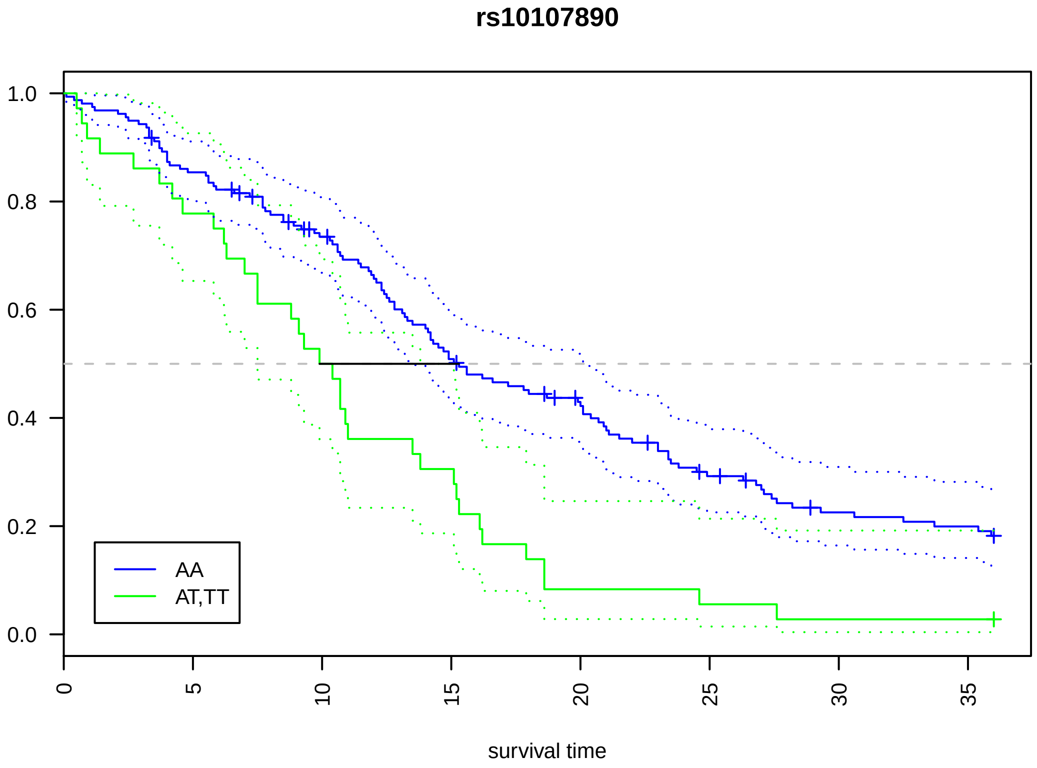

Figure 1978: Kaplan-Meier curves for the selected genotypes. Dashed lines represent 0.95 confidence intervals for the curves with the corresponding color. Black horizontal line ($15.30-9.90 = 5.40$) indicates the maximal survival difference at $y = 0.50$.

17.53 Survival analysis of rs6728529

This marker has a genotype specific effect with a p-value of 5.951195e-05. Total of 6 observations deleted due to missingness. Odds ratios have been estimated using logistic regression at $t \approx 36$:
$\frac{P(event|AT)/P(\neg event|AT)}{P(event|TT)/P(\neg event|TT)} \approx 0.121$ and $\frac{P(event|AA)/P(\neg event|AA)}{P(event|TT)/P(\neg event|TT)} \approx 0.116$.

Table 67: Summary of genotype frequencies with their proportions in parenthesis.

genotype	total N	events	censored
TT	45	43 (0.956)	2 (0.0444)
AT	101	73 (0.723)	28 (0.277)
AA	42	30 (0.714)	12 (0.286)

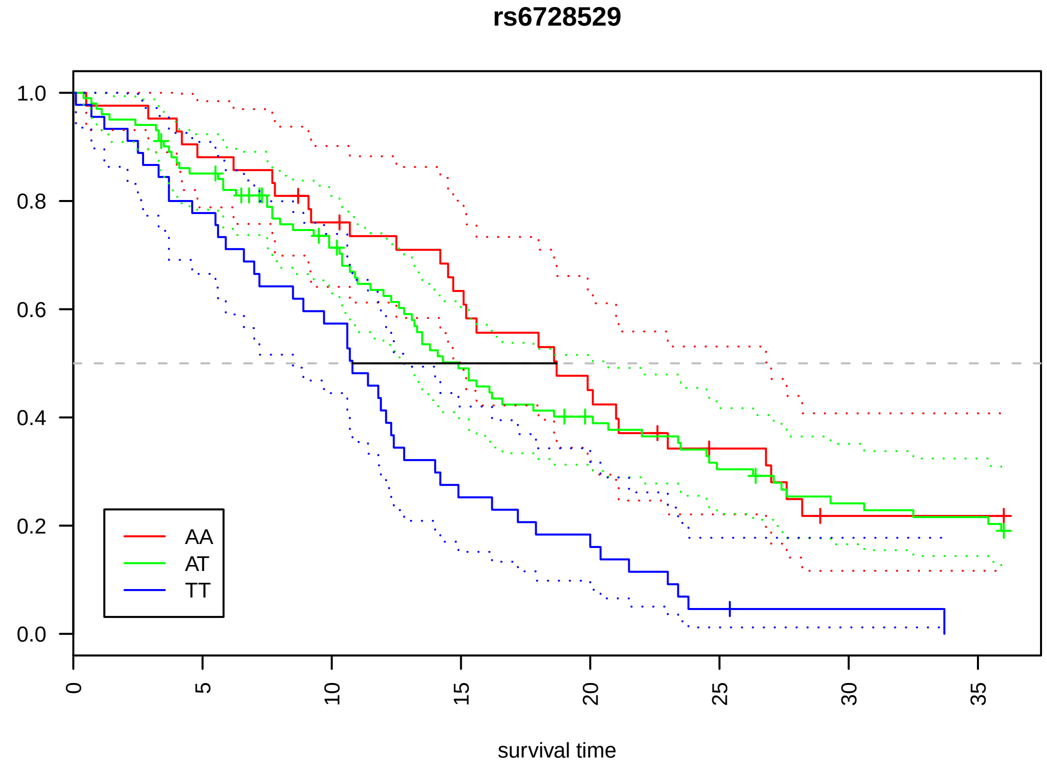

Figure 1979: Kaplan-Meier curves for the selected genotypes. Dashed lines represent 0.95 confidence intervals for the curves with the corresponding color. Black horizontal line $(18.70 - 10.80 = 7.90)$ indicates the maximal survival difference at $y = 0.50$.

Additional annotations:

Gene name	*Description*	*DNABand*
AC012508.2	[undefined]	2q14.3

17.54 Survival analysis of rs2521498

This marker has a genotype specific effect with a p-value of 1.041813e-05.

Odds ratios have been estimated using logistic regression at $t \approx 36$:
$\frac{P(event|AT)/P(\neg event|AT)}{P(event|TT)/P(\neg event|TT)} \approx 0.821$ and $\frac{P(event|AA)/P(\neg event|AA)}{P(event|TT)/P(\neg event|TT)} \approx 5.292$.

Table 68: Summary of genotype frequencies with their proportions in parenthesis.

genotype	total N	events	censored
TT	70	53 (0.757)	17 (0.243)
AT	89	64 (0.719)	25 (0.281)
AA	35	33 (0.943)	2 (0.0571)

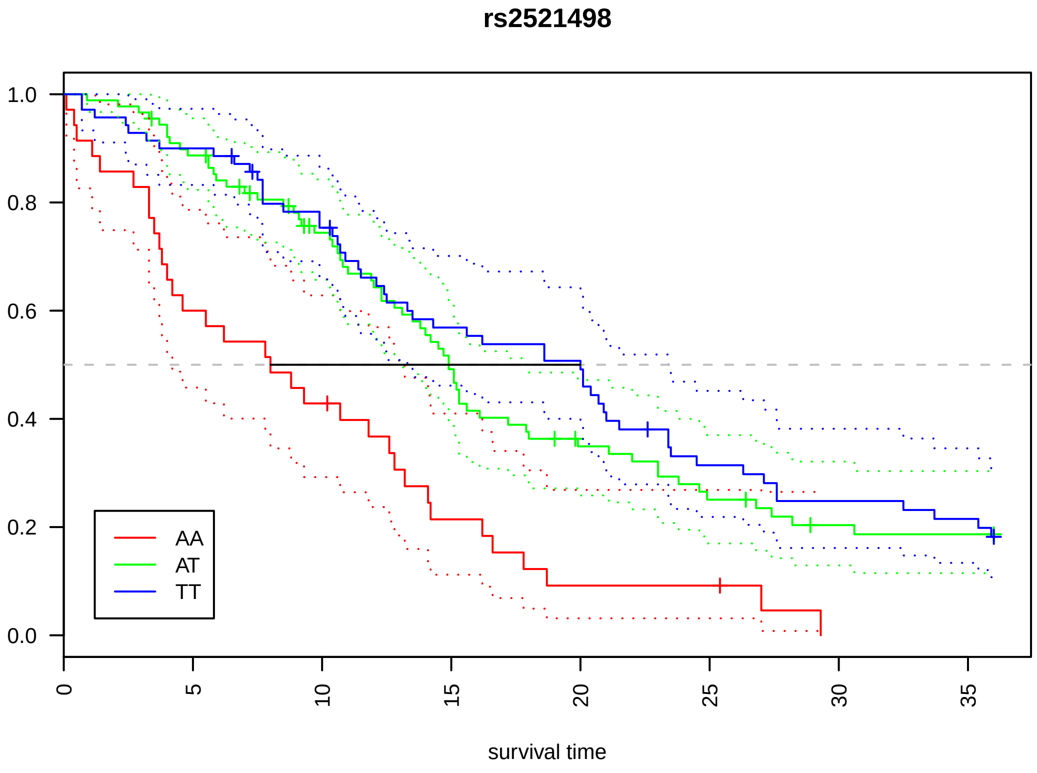

Figure 1980: Kaplan-Meier curves for the selected genotypes. Dashed lines represent 0.95 confidence intervals for the curves with the corresponding color. Black horizontal line $(20.00 - 8.00 = 12.00)$ indicates the maximal survival difference at $y = 0.50$.

Additional annotations:

Gene name	*Description*	*DNABand*	*protein*
FES	feline sarcoma oncogene [Source:HGNC Symbol;Acc:3657]	15q26.1	FES_HUMAN

17.55 Survival analysis of rs17403255

This marker has a genotype specific effect with a p-value of 4.034304e-06. Total of 1 observation deleted due to missingness. Odds ratios have been estimated using logistic regression at $t \approx 36$:
$\frac{P(event|AT,AA)/P(\neg event|AT,AA)}{P(event|TT)/P(\neg event|TT)} \approx 11.846$.

Table 69: Summary of genotype frequencies with their proportions in parenthesis.

genotype	total N	events	censored
TT	159	117 (0.736)	42 (0.264)
AT,AA	34	33 (0.971)	1 (0.0294)

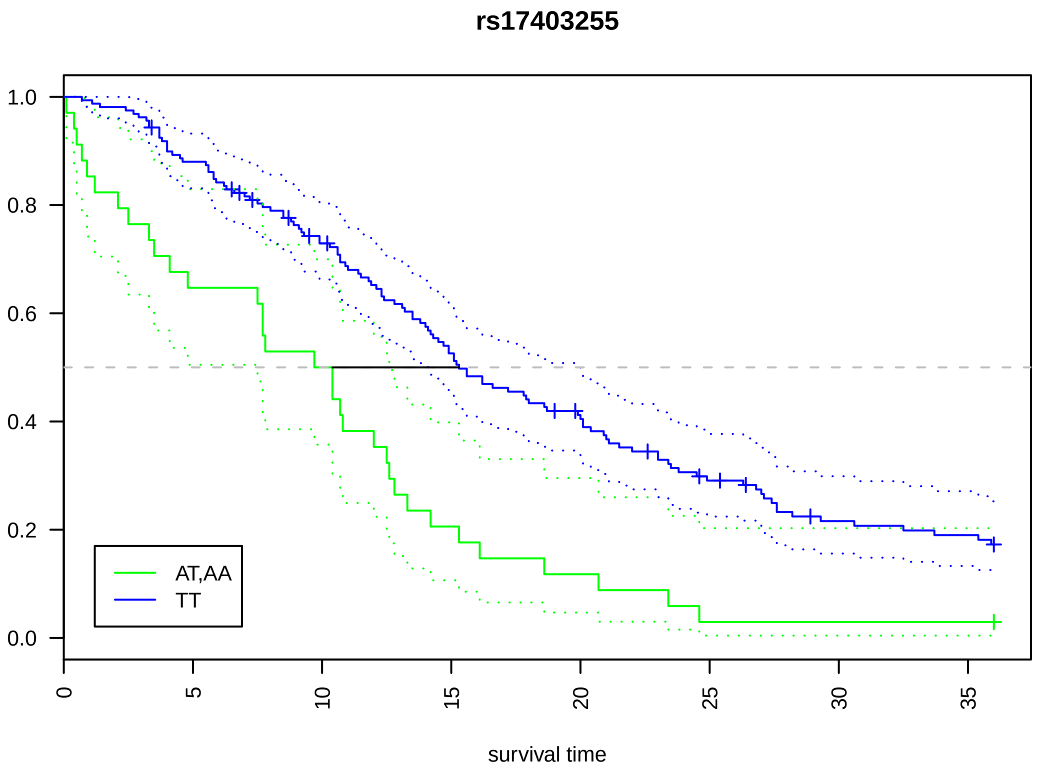

Figure 1981: Kaplan-Meier curves for the selected genotypes. Dashed lines represent 0.95 confidence intervals for the curves with the corresponding color. Black horizontal line ($15.30 - 10.40 = 4.90$) indicates the maximal survival difference at $y = 0.50$.

Additional annotations:

Gene name	*Description*	*DNABand*	*protein*
DTWD1	DTW domain containing 1 [Source:HGNC Symbol;Acc:30926]	15q21.2	DTWD1_HUMAN

17.56 Survival analysis of rs6671217

This marker has a genotype specific effect with a p-value of 4.065477e-05.

Odds ratios have been estimated using logistic regression at $t \approx 36$:
$\frac{P(event|AT,AA)/P(\neg event|AT,AA)}{P(event|TT)/P(\neg event|TT)} \approx 2.151$.

Table 70: Summary of genotype frequencies with their proportions in parenthesis.

genotype	total N	events	censored
TT	100	71 (0.71)	29 (0.29)
AT,AA	94	79 (0.84)	15 (0.16)

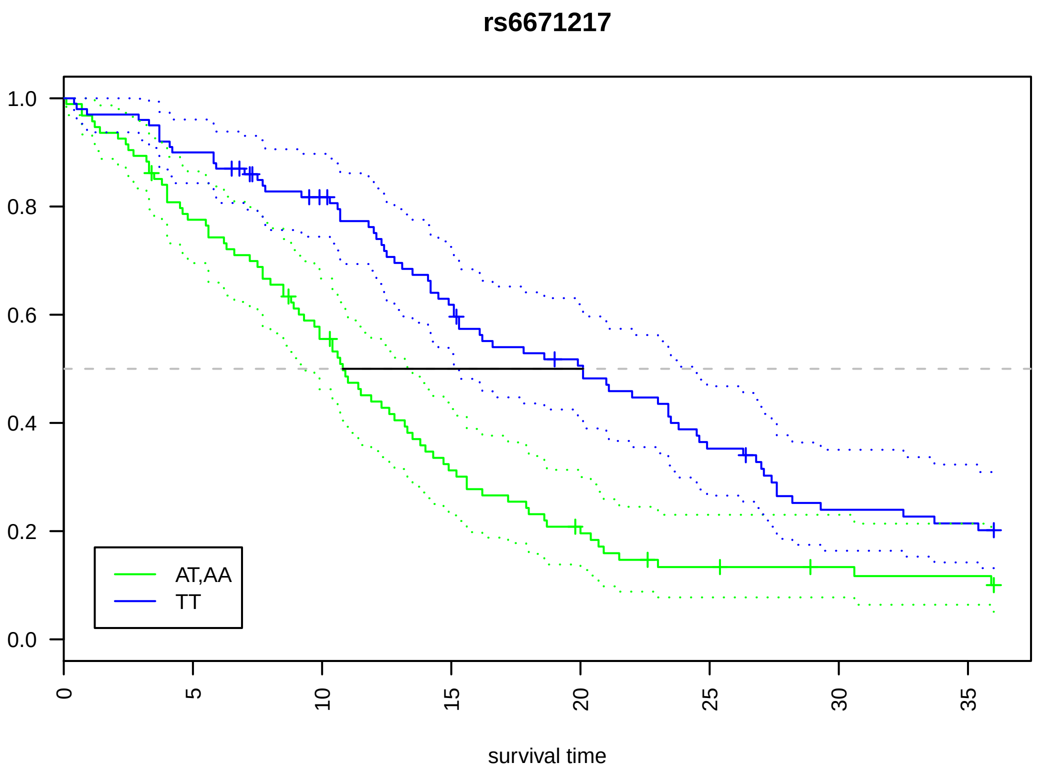

Figure 1982: Kaplan-Meier curves for the selected genotypes. Dashed lines represent 0.95 confidence intervals for the curves with the corresponding color. Black horizontal line $(20.10 - 10.80 = 9.30)$ indicates the maximal survival difference at $y = 0.50$.

17.57 Survival analysis of rs1506390

This marker has a genotype specific effect with a p-value of 4.829523e-05. Total of 7 observations deleted due to missingness. Odds ratios have been estimated using logistic regression at $t \approx 36$:
$\frac{P(event|AT)/P(\neg event|AT)}{P(event|AA)/P(\neg event|AA)} \approx 0.367$ and $\frac{P(event|TT)/P(\neg event|TT)}{P(event|AA)/P(\neg event|AA)} \approx 1.172$.

Table 71: Summary of genotype frequencies with their proportions in parenthesis.

genotype	total N	events	censored
AA	72	61 (0.847)	11 (0.153)
AT	85	57 (0.671)	28 (0.329)
TT	30	26 (0.867)	4 (0.133)

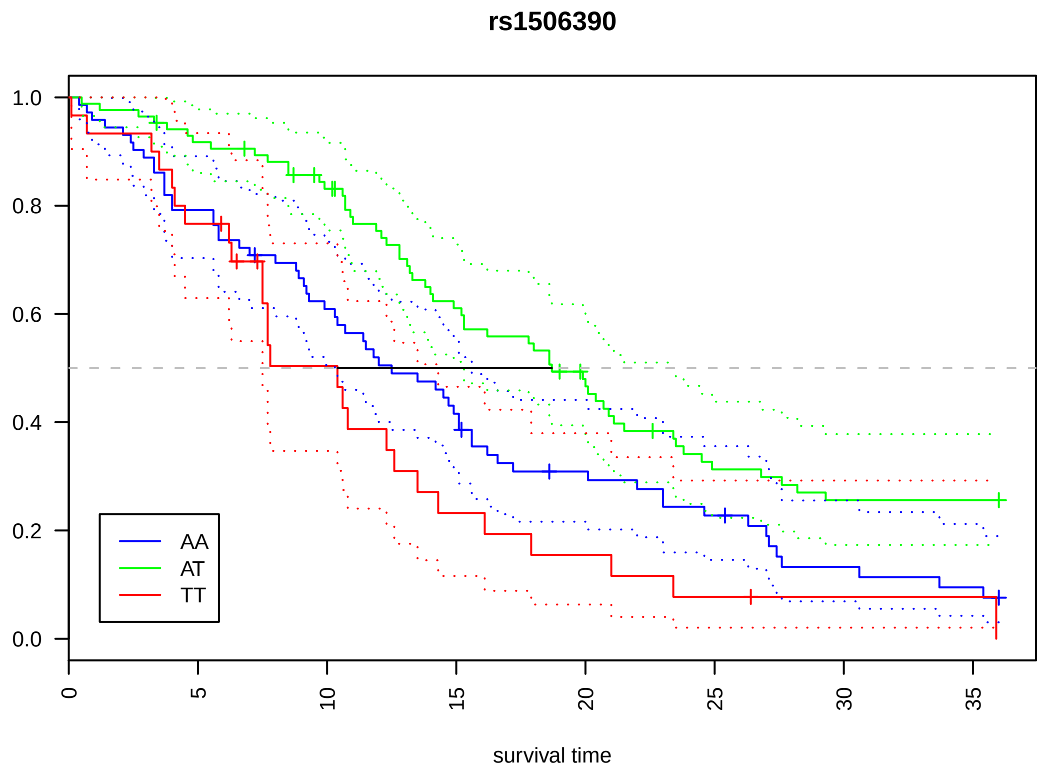

Figure 1983: Kaplan-Meier curves for the selected genotypes. Dashed lines represent 0.95 confidence intervals for the curves with the corresponding color. Black horizontal line $(18.70 - 10.40 = 8.30)$ indicates the maximal survival difference at $y = 0.50$.

17.58 Survival analysis of rs563460

This marker has a genotype specific effect with a p-value of 8.323634e-05. Total of 1 observation deleted due to missingness. Odds ratios have been estimated using logistic regression at $t \approx 36$:
$\frac{P(event|AT,AA)/P(\neg event|AT,AA)}{P(event|TT)/P(\neg event|TT)} \approx 0.335$.

Table 72: Summary of genotype frequencies with their proportions in parenthesis.

genotype	total N	events	censored
TT	130	109 (0.838)	21 (0.162)
AT,AA	63	40 (0.635)	23 (0.365)

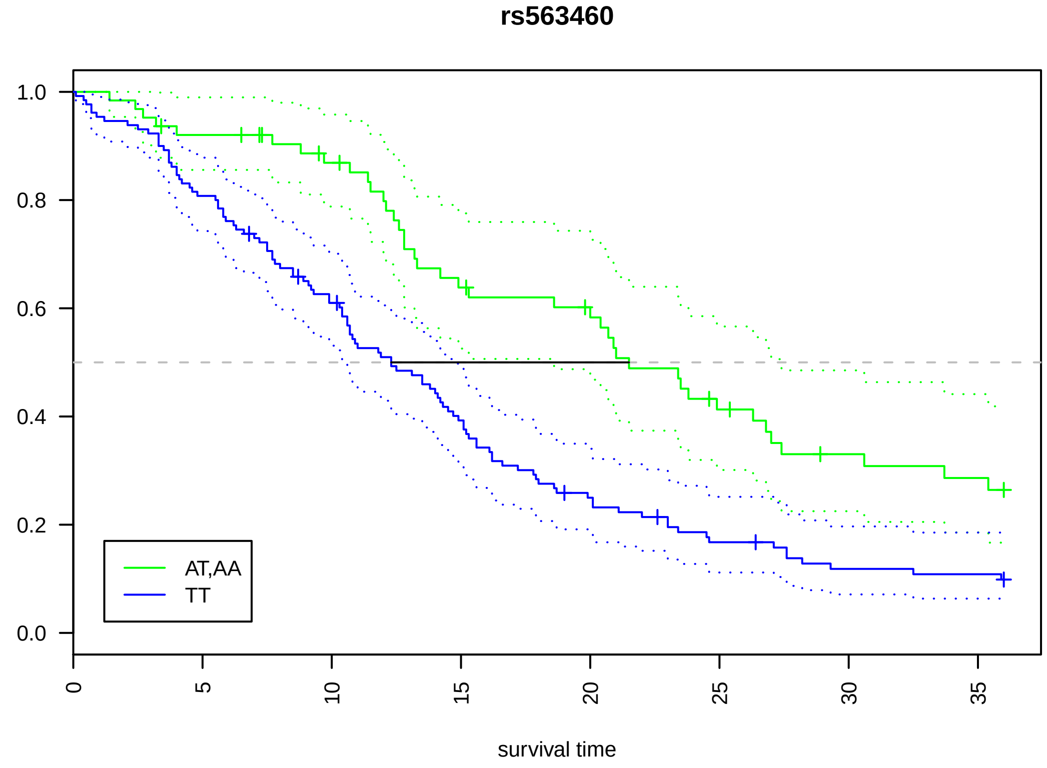

Figure 1984: Kaplan-Meier curves for the selected genotypes. Dashed lines represent 0.95 confidence intervals for the curves with the corresponding color. Black horizontal line (21.50 − 12.30 = 9.20) indicates the maximal survival difference at $y = 0.50$.

Additional annotations:

Gene name	*Description*	*DNABand*	*protein*
AP000872.1	Rho GTPase-activating protein 10-like [Source:UniProtKB/Swiss-Prot;Acc:A6NI28]	11q22.1	RH10L_HUMAN,YK021_HUMAN

17.59 Survival analysis of rs11599561

This marker has a genotype specific effect with a p-value of 6.717819e-06.

Odds ratios have been estimated using logistic regression at $t \approx 36$:
$\frac{P(event|AT,TT)/P(\neg event|AT,TT)}{P(event|AA)/P(\neg event|AA)} \approx 12.603$.

Table 73: Summary of genotype frequencies with their proportions in parenthesis.

genotype	total N	events	censored
AA	159	116 (0.73)	43 (0.27)
AT,TT	35	34 (0.971)	1 (0.0286)

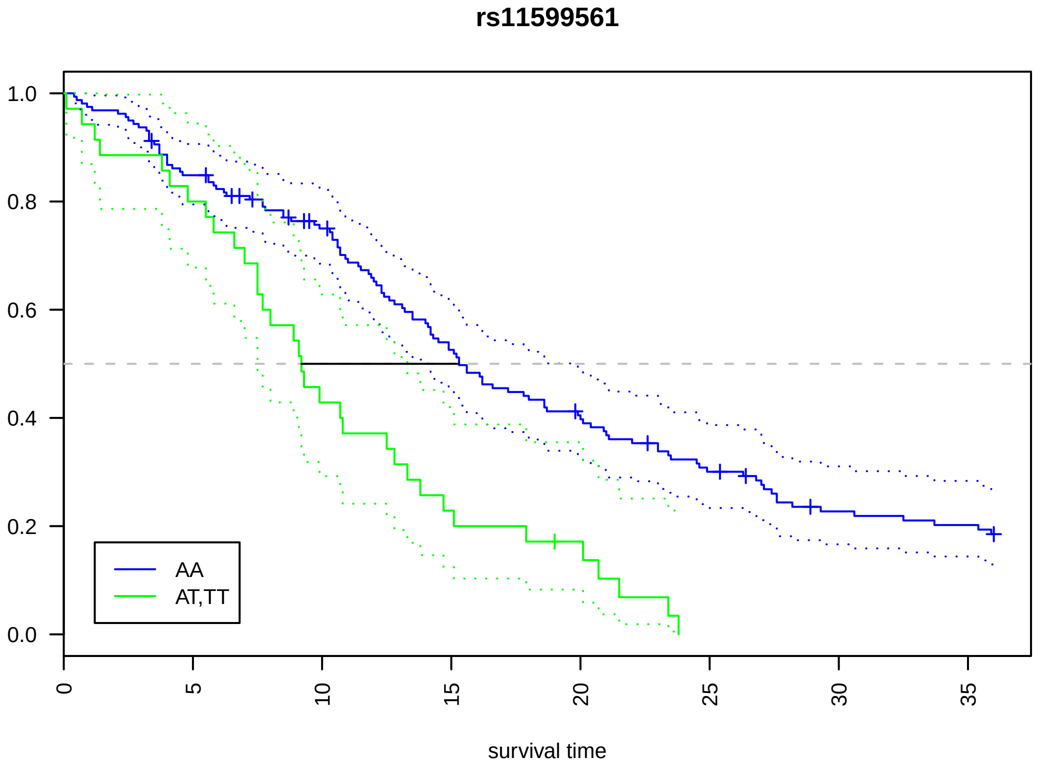

Figure 1985: Kaplan-Meier curves for the selected genotypes. Dashed lines represent 0.95 confidence intervals for the curves with the corresponding color. Black horizontal line ($15.30 - 9.20 = 6.10$) indicates the maximal survival difference at $y = 0.50$.

17.60 Survival analysis of rs7863546

This marker has a genotype specific effect with a p-value of 6.495046e-05.

Odds ratios have been estimated using logistic regression at $t \approx 36$:
$\frac{P(event|AT)/P(\neg event|AT)}{P(event|AA)/P(\neg event|AA)} \approx 4.454$ and $\frac{P(event|TT)/P(\neg event|TT)}{P(event|AA)/P(\neg event|AA)} \approx 1.685$.

Table 74: Summary of genotype frequencies with their proportions in parenthesis.

genotype	total N	events	censored
AA	74	48 (0.649)	26 (0.351)
AT	83	74 (0.892)	9 (0.108)
TT	37	28 (0.757)	9 (0.243)

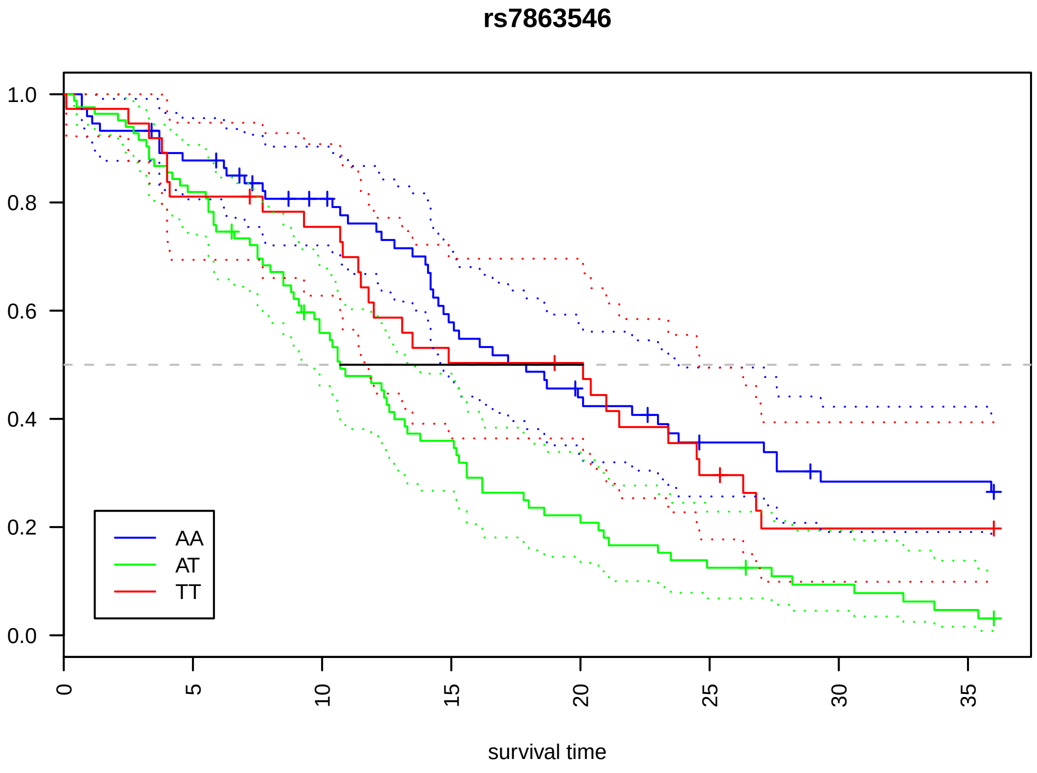

Figure 1986: Kaplan-Meier curves for the selected genotypes. Dashed lines represent 0.95 confidence intervals for the curves with the corresponding color. Black horizontal line ($20.10 - 10.70 = 9.40$) indicates the maximal survival difference at $y = 0.50$.

Additional annotations:

Gene name	*Description*	*DNABand*	*protein*
FUBP3	far upstream element (FUSE) binding protein 3 [Source:HGNC Symbol;Acc:4005]	9q34.11,9q34.12	FUBP3_HUMAN

17.61 Survival analysis of rs2784761

This marker has a genotype specific effect with a p-value of 9.420432e-06.

Odds ratios have been estimated using logistic regression at $t \approx 36$:
$\frac{P(event|AT,TT)/P(\neg event|AT,TT)}{P(event|AA)/P(\neg event|AA)} \approx 5.464$.

Table 75: Summary of genotype frequencies with their proportions in parenthesis.

genotype	total N	events	censored
AA	137	97 (0.708)	40 (0.292)
AT,TT	57	53 (0.93)	4 (0.0702)

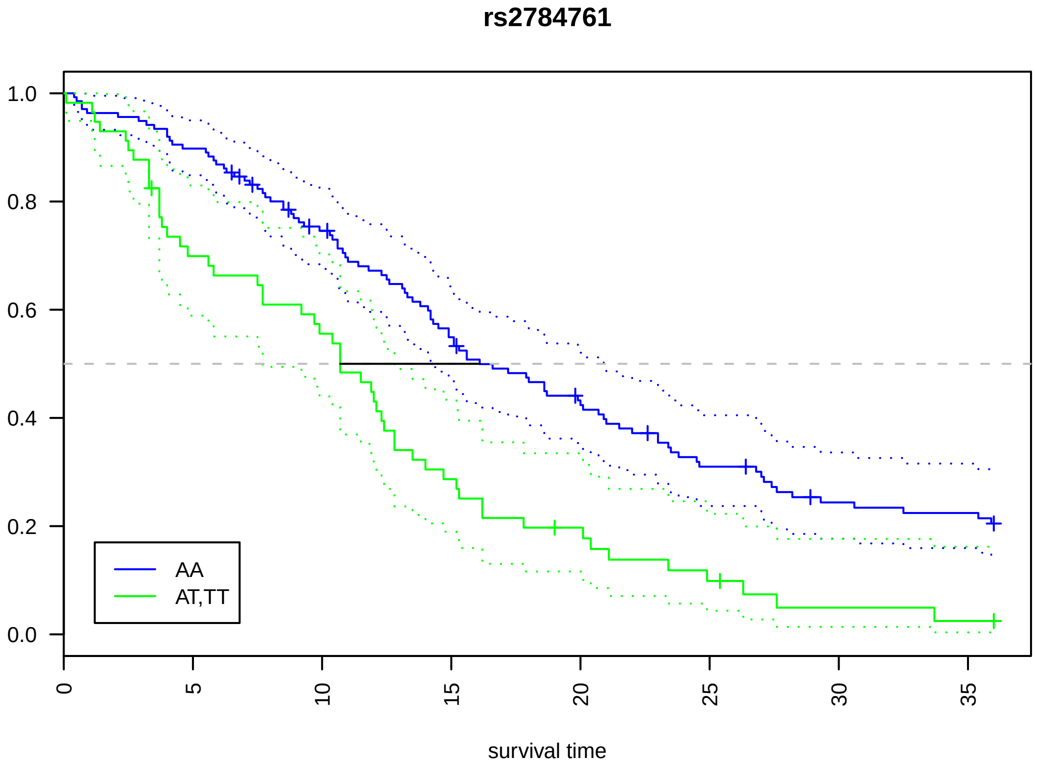

Figure 1987: Kaplan-Meier curves for the selected genotypes. Dashed lines represent 0.95 confidence intervals for the curves with the corresponding color. Black horizontal line $(16.10 - 10.70 = 5.40)$ indicates the maximal survival difference at $y = 0.50$.

Additional annotations:

Gene name	Description	DNABand
RP11-369J21.4	[undefined]	10q22.3

17.62 Survival analysis of rs12468464

This marker has a genotype specific effect with a p-value of 9.604163e-05.

Odds ratios have been estimated using logistic regression at $t \approx 36$:
$\frac{P(event|AT,AA)/P(\neg event|AT,AA)}{P(event|TT)/P(\neg event|TT)} \approx 0.329$.

Table 76: Summary of genotype frequencies with their proportions in parenthesis.

genotype	total N	events	censored
TT	97	84 (0.866)	13 (0.134)
AT,AA	97	66 (0.68)	31 (0.32)

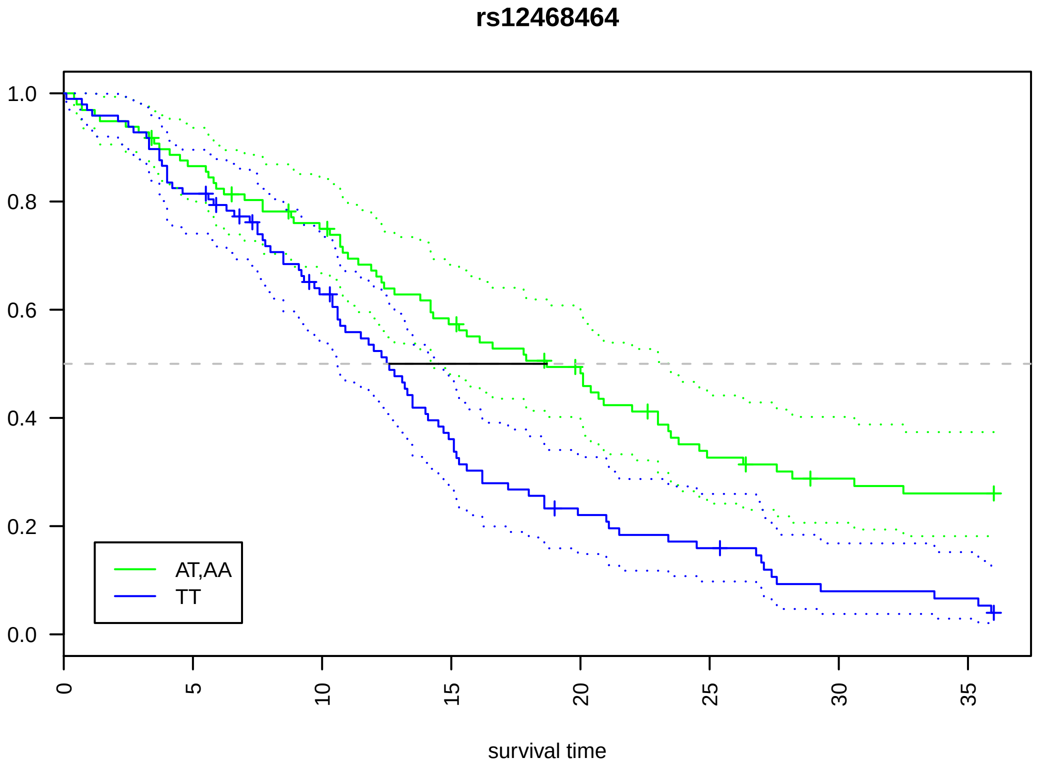

Figure 1988: Kaplan-Meier curves for the selected genotypes. Dashed lines represent 0.95 confidence intervals for the curves with the corresponding color. Black horizontal line $(18.70 - 12.60 = 6.10)$ indicates the maximal survival difference at $y = 0.50$.

17.63 Survival analysis of rs13091723

This marker has a genotype specific effect with a p-value of 2.779694e-05.

Odds ratios have been estimated using logistic regression at $t \approx 36$:
$\frac{P(event|AT)/P(\neg event|AT)}{P(event|AA)/P(\neg event|AA)} \approx 3.979$ and $\frac{P(event|TT)/P(\neg event|TT)}{P(event|AA)/P(\neg event|AA)} \approx 0.829$.

Table 77: Summary of genotype frequencies with their proportions in parenthesis.

genotype	total N	events	censored
AA	56	38 (0.679)	18 (0.321)
AT	94	84 (0.894)	10 (0.106)
TT	44	28 (0.636)	16 (0.364)

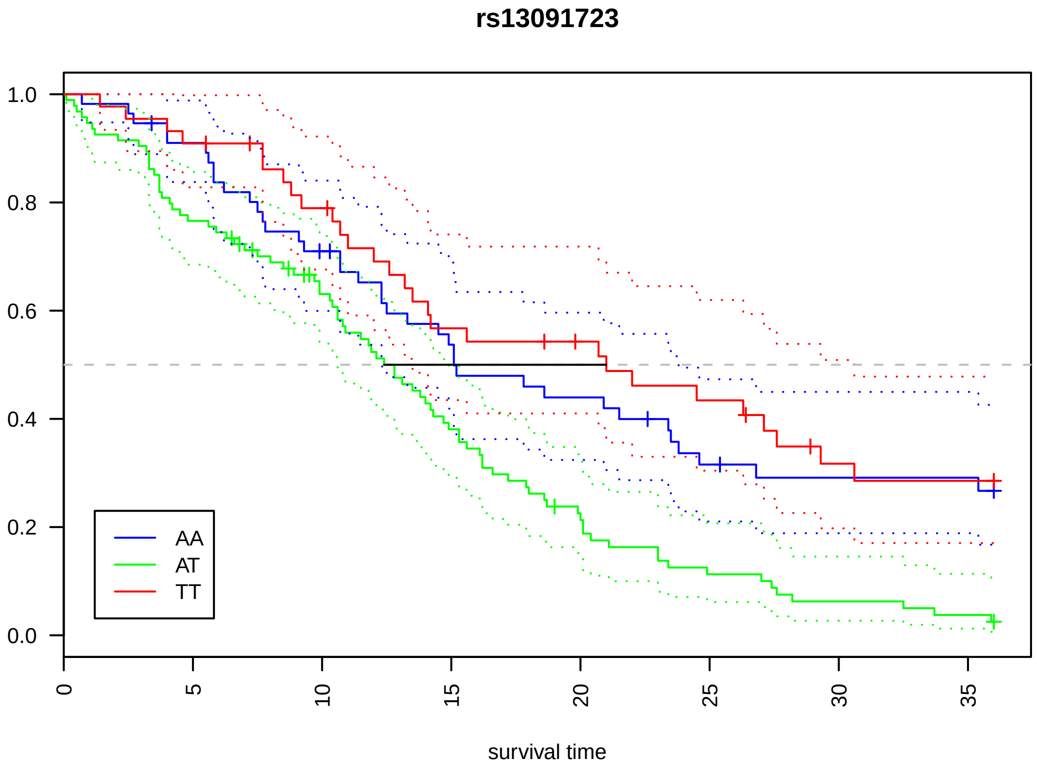

Figure 1989: Kaplan-Meier curves for the selected genotypes. Dashed lines represent 0.95 confidence intervals for the curves with the corresponding color. Black horizontal line $(21.00 - 12.40 = 8.60)$ indicates the maximal survival difference at $y = 0.50$.

17.64 Survival analysis of rs6846040

This marker has a genotype specific effect with a p-value of 1.144608e-06.

Odds ratios have been estimated using logistic regression at $t \approx 36$:
$\frac{P(event|AT)/P(\neg event|AT)}{P(event|TT)/P(\neg event|TT)} \approx 1.713$ and $\frac{P(event|AA)/P(\neg event|AA)}{P(event|TT)/P(\neg event|TT)} \approx 7.892$.

Table 78: Summary of genotype frequencies with their proportions in parenthesis.

genotype	total N	events	censored
TT	92	65 (0.707)	27 (0.293)
AT	82	66 (0.805)	16 (0.195)
AA	20	19 (0.95)	1 (0.05)

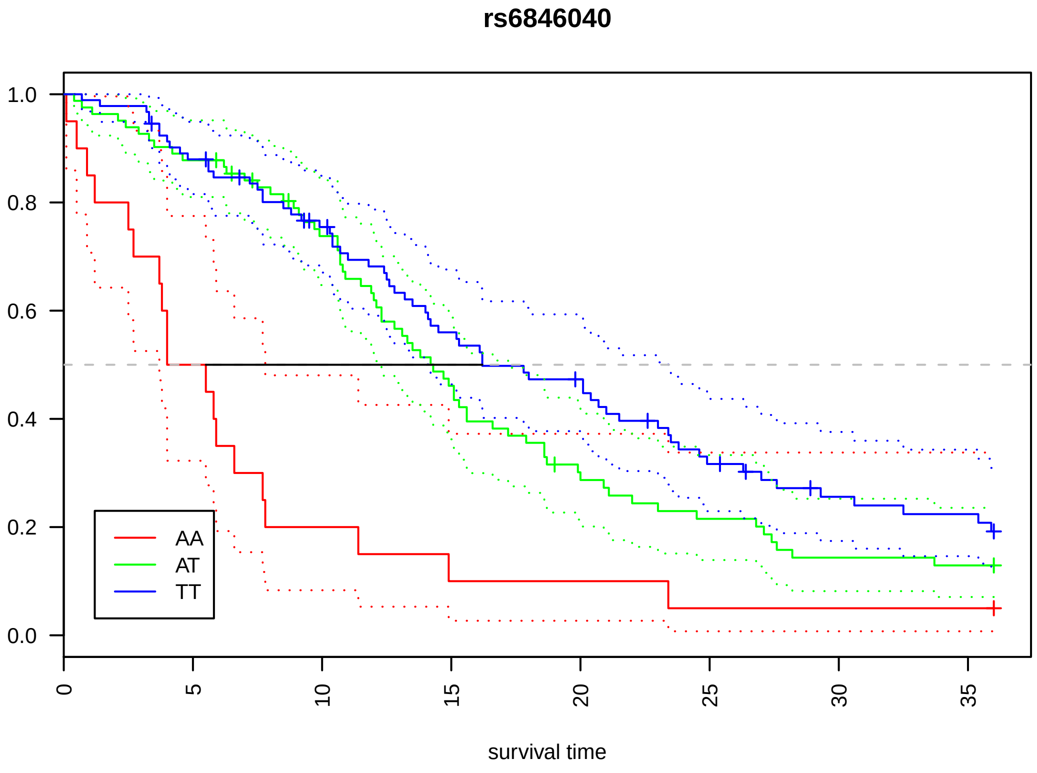

Figure 1990: Kaplan-Meier curves for the selected genotypes. Dashed lines represent 0.95 confidence intervals for the curves with the corresponding color. Black horizontal line $(16.20 - 5.50 = 10.70)$ indicates the maximal survival difference at $y = 0.50$.

17.65 Survival analysis of rs2600077

This marker has a genotype specific effect with a p-value of 4.112009e-05.

Odds ratios have been estimated using logistic regression at $t \approx 36$:
$\frac{P(event|AT,AA)/P(\neg event|AT,AA)}{P(event|TT)/P(\neg event|TT)} \approx 3.204$.

Table 79: Summary of genotype frequencies with their proportions in parenthesis.

genotype	total N	events	censored
TT	95	64 (0.674)	31 (0.326)
AT,AA	99	86 (0.869)	13 (0.131)

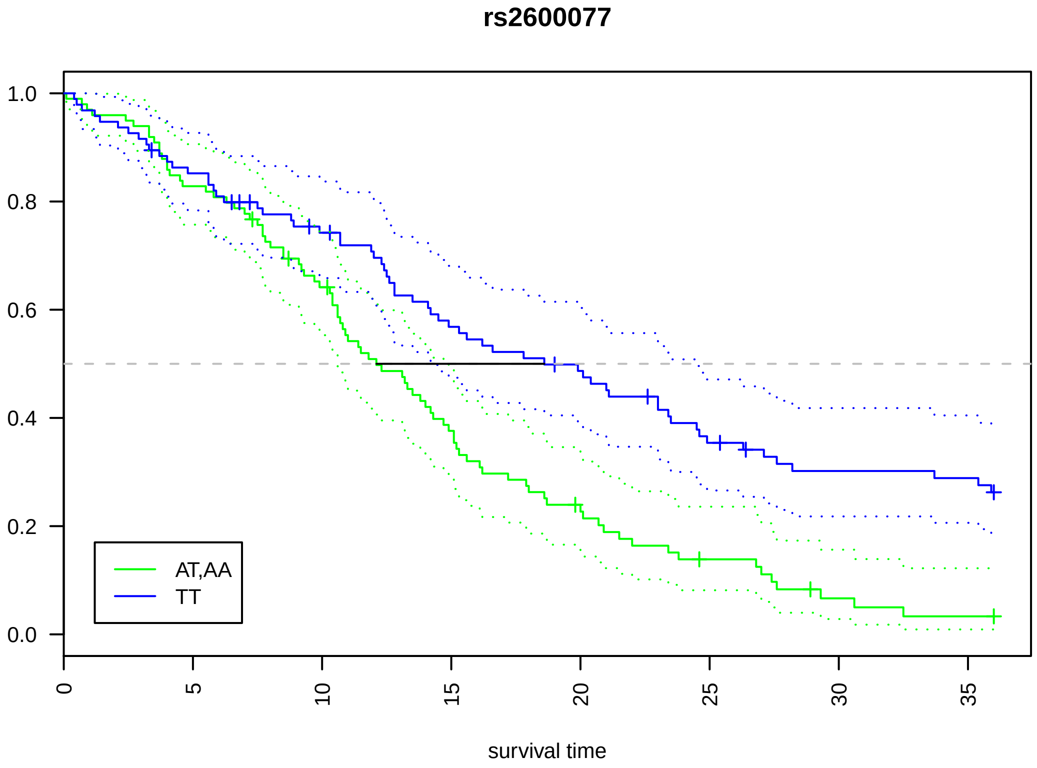

Figure 1991: Kaplan-Meier curves for the selected genotypes. Dashed lines represent 0.95 confidence intervals for the curves with the corresponding color. Black horizontal line $(18.60 - 12.10 = 6.50)$ indicates the maximal survival difference at $y = 0.50$.

17.66 Survival analysis of rs10932321

This marker has a genotype specific effect with a p-value of 4.551966e-06.

Odds ratios have been estimated using logistic regression at $t \approx 36$:
$\frac{P(event|AT)/P(\neg event|AT)}{P(event|AA)/P(\neg event|AA)} \approx 1.775$ and $\frac{P(event|TT)/P(\neg event|TT)}{P(event|AA)/P(\neg event|AA)} \approx 6.19$.

Table 80: Summary of genotype frequencies with their proportions in parenthesis.

genotype	total N	events	censored
AA	62	42 (0.677)	20 (0.323)
AT	104	82 (0.788)	22 (0.212)
TT	28	26 (0.929)	2 (0.0714)

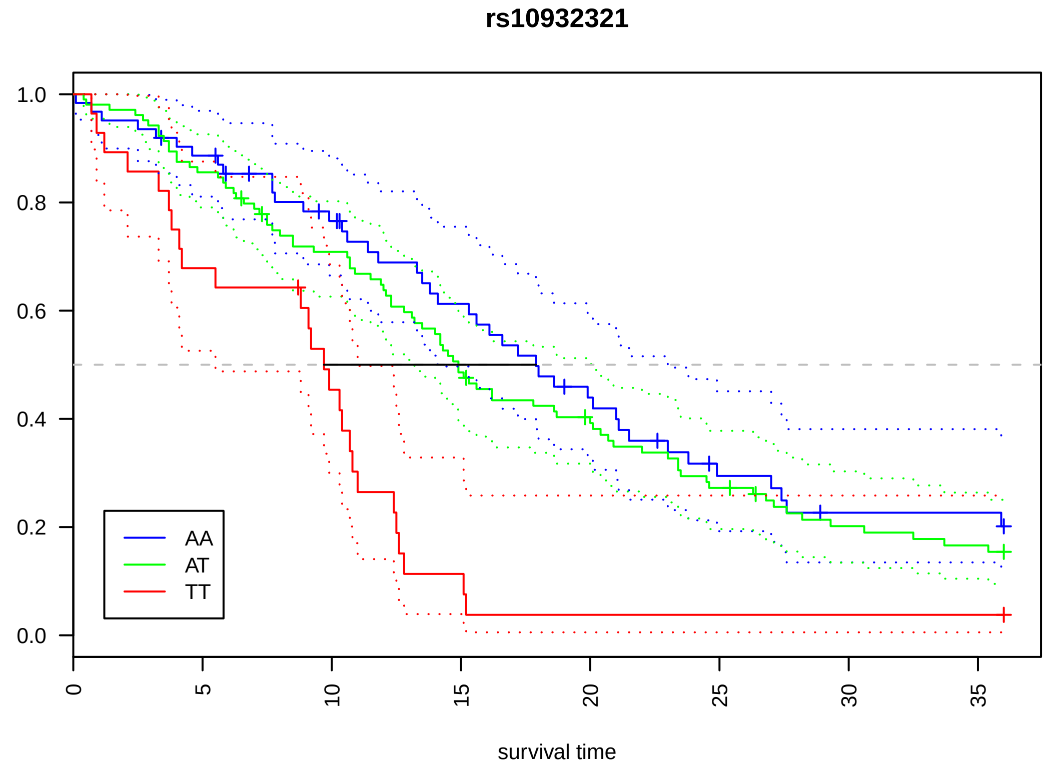

Figure 1992: Kaplan-Meier curves for the selected genotypes. Dashed lines represent 0.95 confidence intervals for the curves with the corresponding color. Black horizontal line ($17.90 - 9.70 = 8.20$) indicates the maximal survival difference at $y = 0.50$.

17.67 Survival analysis of rs1011127

This marker has a genotype specific effect with a p-value of 2.176563e-05.

Odds ratios have been estimated using logistic regression at $t \approx 36$:
$\frac{P(event|AT)/P(\neg event|AT)}{P(event|TT)/P(\neg event|TT)} \approx 0.505$ and $\frac{P(event|AA)/P(\neg event|AA)}{P(event|TT)/P(\neg event|TT)} \approx 6.51$.

Table 81: Summary of genotype frequencies with their proportions in parenthesis.

genotype	total N	events	censored
TT	60	49 (0.817)	11 (0.183)
AT	104	72 (0.692)	32 (0.308)
AA	30	29 (0.967)	1 (0.0333)

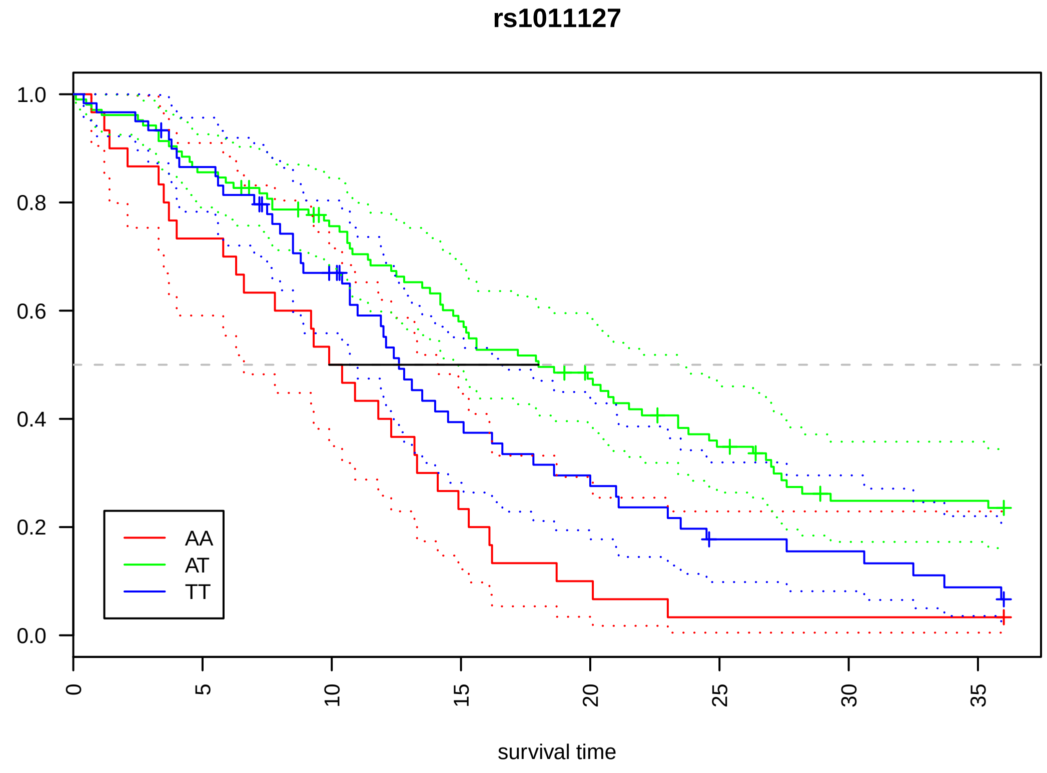

Figure 1993: Kaplan-Meier curves for the selected genotypes. Dashed lines represent 0.95 confidence intervals for the curves with the corresponding color. Black horizontal line (18.00 − 9.90 = 8.10) indicates the maximal survival difference at $y = 0.50$.

Additional annotations:

Gene name	*Description*	*DNABand*	*protein*
CAMTA1	calmodulin binding transcription activator 1 [Source:HGNC Symbol;Acc:18806]	1p36.23,1p36.31	CMTA1_HUMAN

17.68 Survival analysis of rs464420

This marker has a genotype specific effect with a p-value of 2.757139e-06. Total of 1 observation deleted due to missingness. Odds ratios have been estimated using logistic regression at $t \approx 36$:
$\frac{P(event|AT)/P(\neg event|AT)}{P(event|AA)/P(\neg event|AA)} \approx 0.365$ and $\frac{P(event|TT)/P(\neg event|TT)}{P(event|AA)/P(\neg event|AA)} \approx 4.162$.

Table 82: Summary of genotype frequencies with their proportions in parenthesis.

genotype	total N	events	censored
AA	88	74 (0.841)	14 (0.159)
AT	82	54 (0.659)	28 (0.341)
TT	23	22 (0.957)	1 (0.0435)

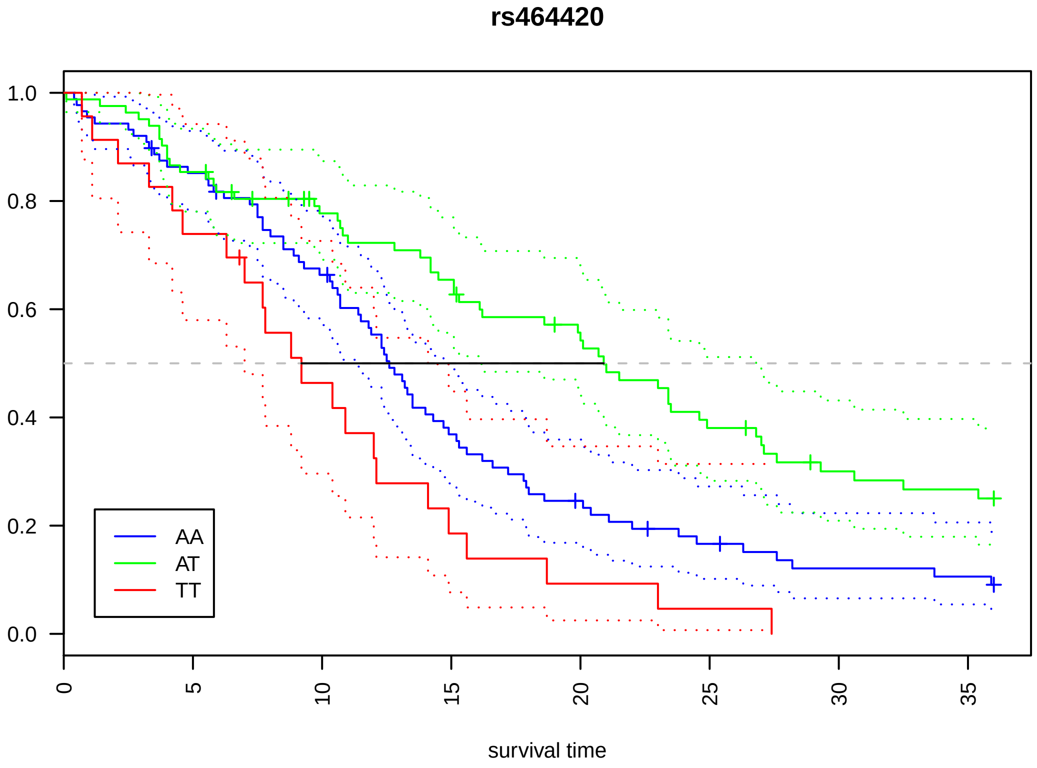

Figure 1994: Kaplan-Meier curves for the selected genotypes. Dashed lines represent 0.95 confidence intervals for the curves with the corresponding color. Black horizontal line $(20.90 - 9.20 = 11.70)$ indicates the maximal survival difference at $y = 0.50$.

Additional annotations:

Gene name	Description	DNABand	protein
RASGRF2	Ras protein-specific guanine nucleotide-releasing factor 2 [Source:HGNC Symbol;Acc:9876]	5q14.1	RGRF2_HUMAN

17.69 Survival analysis of rs8093433

This marker has a genotype specific effect with a p-value of 6.026063e-06.

Odds ratios have been estimated using logistic regression at $t \approx 36$:
$\frac{P(event|AT)/P(\neg event|AT)}{P(event|TT)/P(\neg event|TT)} \approx 0.234$ and $\frac{P(event|AA)/P(\neg event|AA)}{P(event|TT)/P(\neg event|TT)} \approx 0.437$.

Table 83: Summary of genotype frequencies with their proportions in parenthesis.

genotype	total N	events	censored
TT	52	47 (0.904)	5 (0.0962)
AT	96	66 (0.688)	30 (0.312)
AA	46	37 (0.804)	9 (0.196)

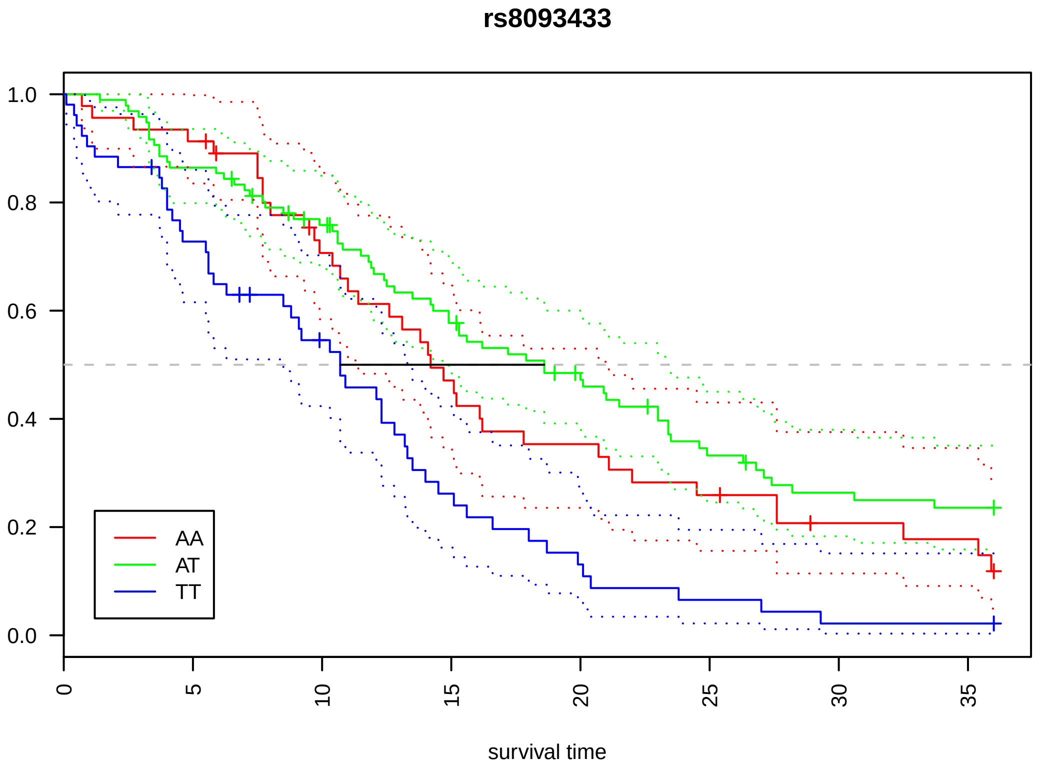

Figure 1995: Kaplan-Meier curves for the selected genotypes. Dashed lines represent 0.95 confidence intervals for the curves with the corresponding color. Black horizontal line $(18.60 - 10.70 = 7.90)$ indicates the maximal survival difference at $y = 0.50$.

17.70 Survival analysis of rs4921056

This marker has a genotype specific effect with a p-value of 9.074254e-05. Total of 1 observation deleted due to missingness. Odds ratios have been estimated using logistic regression at $t \approx 36$:
$\frac{P(event|AT)/P(\neg event|AT)}{P(event|TT)/P(\neg event|TT)} \approx 0.635$ and $\frac{P(event|AA)/P(\neg event|AA)}{P(event|TT)/P(\neg event|TT)} \approx 5.675$.

Table 84: Summary of genotype frequencies with their proportions in parenthesis.

genotype	total N	events	censored
TT	94	74 (0.787)	20 (0.213)
AT	77	54 (0.701)	23 (0.299)
AA	22	21 (0.955)	1 (0.0455)

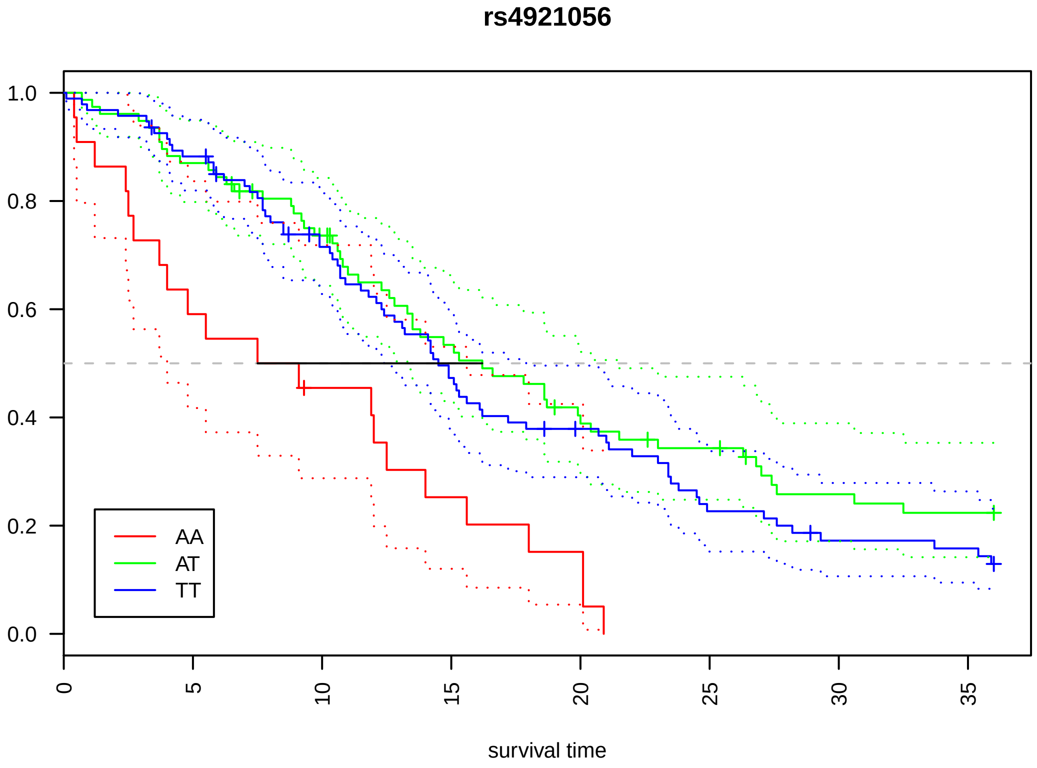

Figure 1996: Kaplan-Meier curves for the selected genotypes. Dashed lines represent 0.95 confidence intervals for the curves with the corresponding color. Black horizontal line ($16.20-7.50 = 8.70$) indicates the maximal survival difference at $y = 0.50$.

Additional annotations:

Gene name	*Description*	*DNABand*	*protein*
COMMD10	COMM domain containing 10 [Source:HGNC Symbol;Acc:30201]	5q23.1	COMDA_HUMAN

17.71 Survival analysis of rs6427839

This marker has a genotype specific effect with a p-value of 3.193456e-05.

Odds ratios have been estimated using logistic regression at $t \approx 36$:
$\frac{P(event|AT,AA)/P(\neg event|AT,AA)}{P(event|TT)/P(\neg event|TT)} \approx 4.637$.

Table 85: Summary of genotype frequencies with their proportions in parenthesis.

genotype	total N	events	censored
TT	153	112 (0.732)	41 (0.268)
AT,AA	41	38 (0.927)	3 (0.0732)

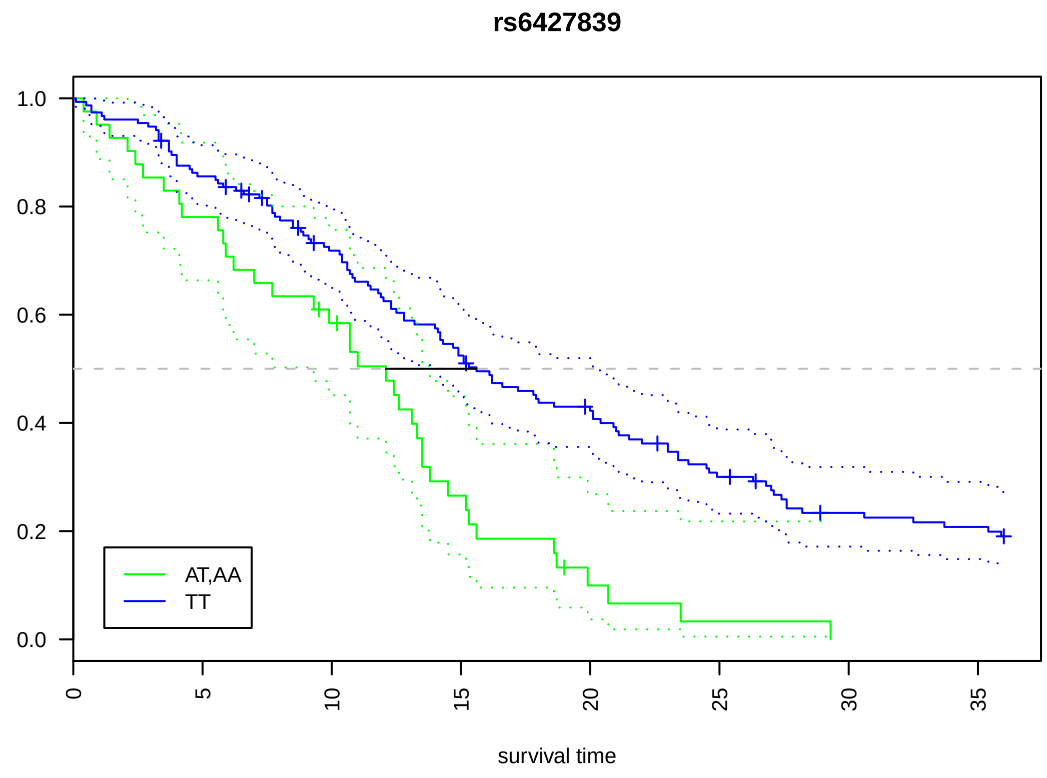

Figure 1997: Kaplan-Meier curves for the selected genotypes. Dashed lines represent 0.95 confidence intervals for the curves with the corresponding color. Black horizontal line ($15.60 - 12.10 = 3.50$) indicates the maximal survival difference at $y = 0.50$.

17.72 Survival analysis of rs7069891

This marker has a genotype specific effect with a p-value of 6.717819e-06.

Odds ratios have been estimated using logistic regression at $t \approx 36$:
$\frac{P(event|AT,TT)/P(\neg event|AT,TT)}{P(event|AA)/P(\neg event|AA)} \approx 12.603$.

Table 86: Summary of genotype frequencies with their proportions in parenthesis.

genotype	total N	events	censored
AA	159	116 (0.73)	43 (0.27)
AT,TT	35	34 (0.971)	1 (0.0286)

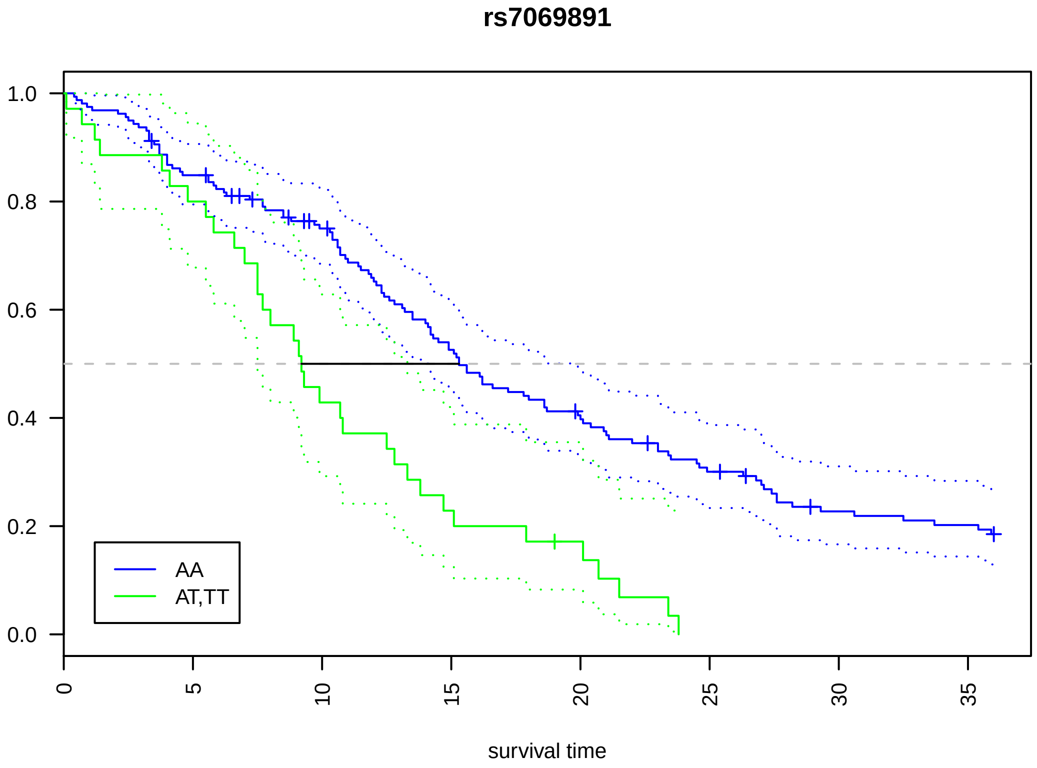

Figure 1998: Kaplan-Meier curves for the selected genotypes. Dashed lines represent 0.95 confidence intervals for the curves with the corresponding color. Black horizontal line ($15.30 - 9.20 = 6.10$) indicates the maximal survival difference at $y = 0.50$.

17.73 Survival analysis of rs9635999

This marker has a genotype specific effect with a p-value of 5.694096e-05. Total of 1 observation deleted due to missingness. Odds ratios have been estimated using logistic regression at $t \approx 36$:
$\frac{P(event|AT)/P(\neg event|AT)}{P(event|AA)/P(\neg event|AA)} \approx 0.5$ and $\frac{P(event|TT)/P(\neg event|TT)}{P(event|AA)/P(\neg event|AA)} \approx 2.823$.

Table 87: Summary of genotype frequencies with their proportions in parenthesis.

genotype	total N	events	censored
AA	76	62 (0.816)	14 (0.184)
AT	90	62 (0.689)	28 (0.311)
TT	27	25 (0.926)	2 (0.0741)

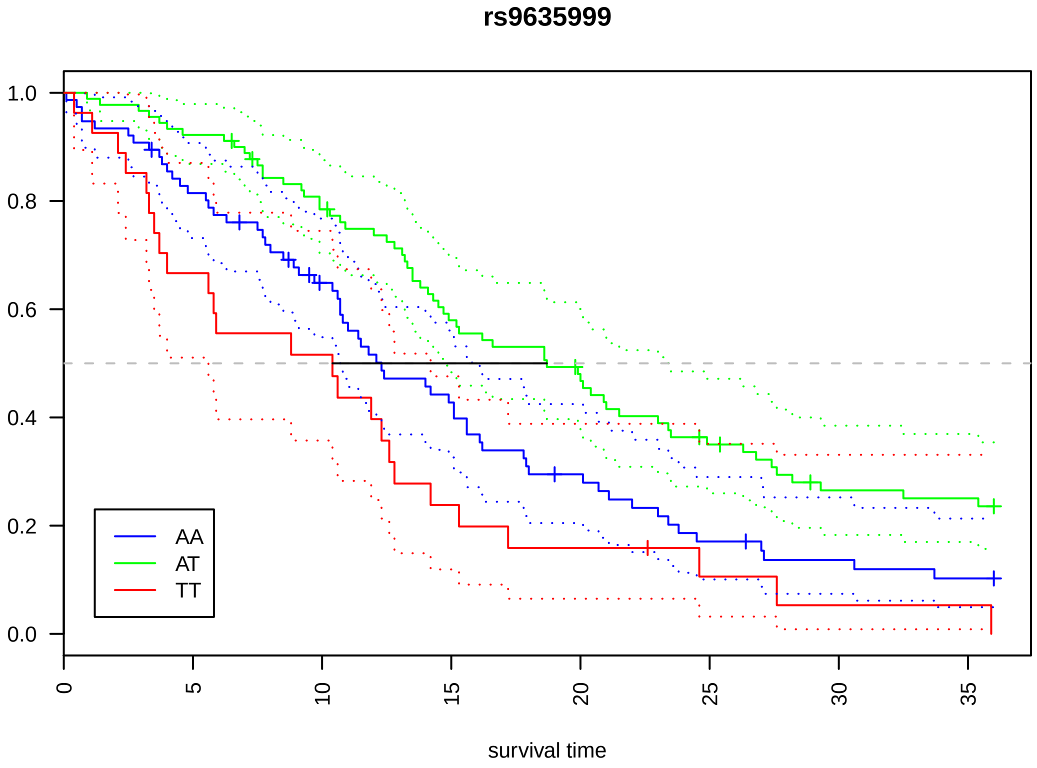

Figure 1999: Kaplan-Meier curves for the selected genotypes. Dashed lines represent 0.95 confidence intervals for the curves with the corresponding color. Black horizontal line ($18.70 - 10.40 = 8.30$) indicates the maximal survival difference at $y = 0.50$.

17.74 Survival analysis of rs7571480

This marker has a genotype specific effect with a p-value of 3.570650e-05.

Odds ratios have been estimated using logistic regression at $t \approx 36$:
$\frac{P(event|AT)/P(\neg event|AT)}{P(event|TT)/P(\neg event|TT)} \approx 0.066$ and $\frac{P(event|AA)/P(\neg event|AA)}{P(event|TT)/P(\neg event|TT)} \approx 0.053$.

Table 88: Summary of genotype frequencies with their proportions in parenthesis.

genotype	total N	events	censored
TT	42	41 (0.976)	1 (0.0238)
AT	111	81 (0.73)	30 (0.27)
AA	41	28 (0.683)	13 (0.317)

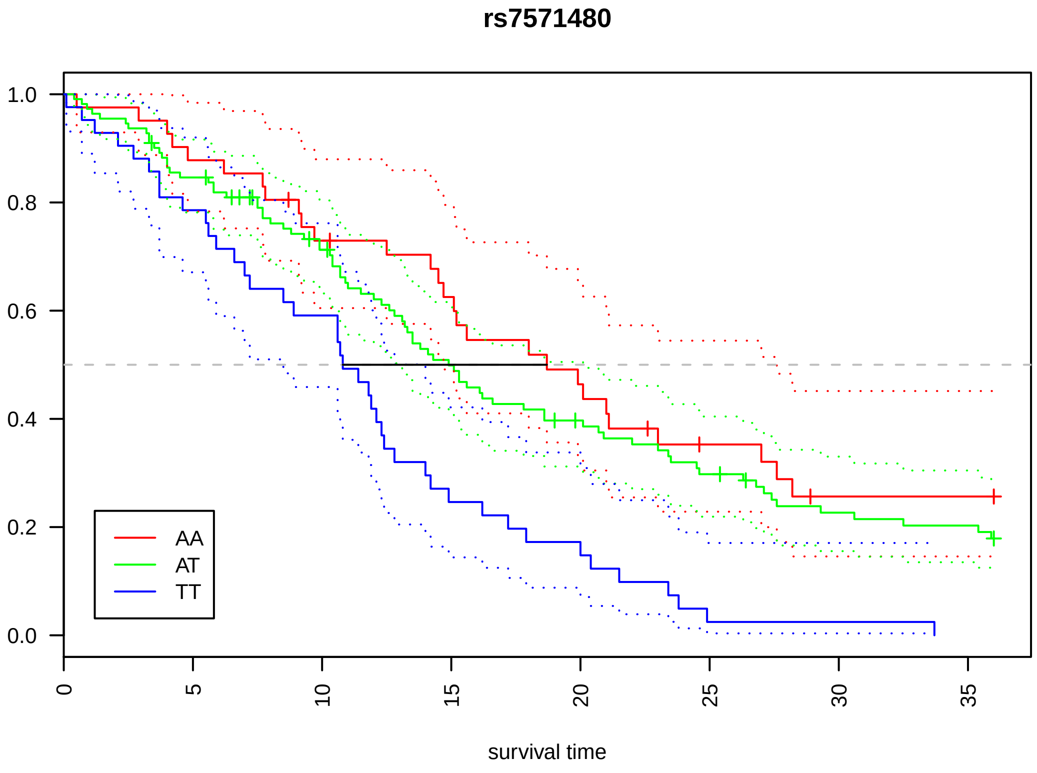

Figure 2000: Kaplan-Meier curves for the selected genotypes. Dashed lines represent 0.95 confidence intervals for the curves with the corresponding color. Black horizontal line $(18.70 - 10.80 = 7.90)$ indicates the maximal survival difference at $y = 0.50$.

17.75 Survival analysis of rs13423908

This marker has a genotype specific effect with a p-value of 6.120453e-05. Total of 13 observations deleted due to missingness. Odds ratios have been estimated using logistic regression at $t \approx 36$:
$\frac{P(event|AT)/P(\neg event|AT)}{P(event|AA)/P(\neg event|AA)} \approx 1.424$ and $\frac{P(event|TT)/P(\neg event|TT)}{P(event|AA)/P(\neg event|AA)} \approx 6.676$.

Table 89: Summary of genotype frequencies with their proportions in parenthesis.

genotype	total N	events	censored
AA	56	37 (0.661)	19 (0.339)
AT	83	61 (0.735)	22 (0.265)
TT	42	39 (0.929)	3 (0.0714)

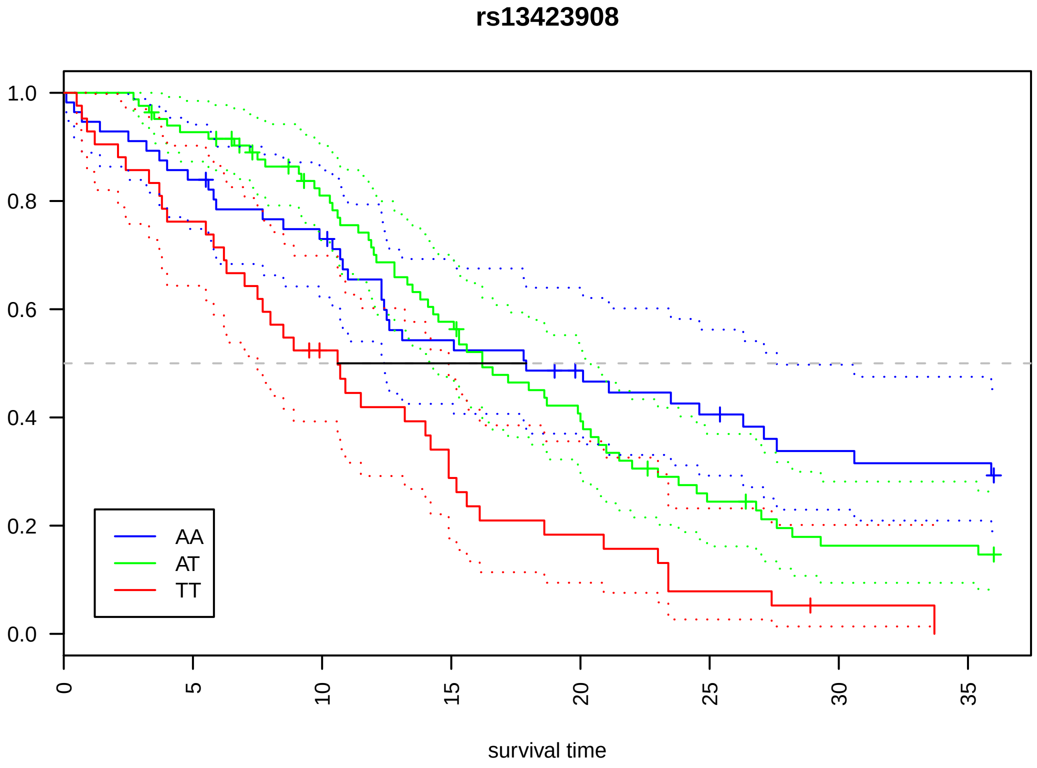

Figure 2001: Kaplan-Meier curves for the selected genotypes. Dashed lines represent 0.95 confidence intervals for the curves with the corresponding color. Black horizontal line $(17.90 - 10.60 = 7.30)$ indicates the maximal survival difference at $y = 0.50$.

Additional annotations:

Gene name	*Description*	*DNABand*	*protein*
MYO3B	myosin IIIB [Source:HGNC Symbol;Acc:15576]	2q31.1	MYO3B_HUMAN

17.76 Survival analysis of rs10749145

This marker has a genotype specific effect with a p-value of 2.430398e-05.

Odds ratios have been estimated using logistic regression at $t \approx 36$:
$\frac{P(event|AT)/P(\neg event|AT)}{P(event|AA)/P(\neg event|AA)} \approx 1.95$ and $\frac{P(event|TT)/P(\neg event|TT)}{P(event|AA)/P(\neg event|AA)} \approx 13$.

Table 90: Summary of genotype frequencies with their proportions in parenthesis.

genotype	total N	events	censored
AA	69	46 (0.667)	23 (0.333)
AT	98	78 (0.796)	20 (0.204)
TT	27	26 (0.963)	1 (0.037)

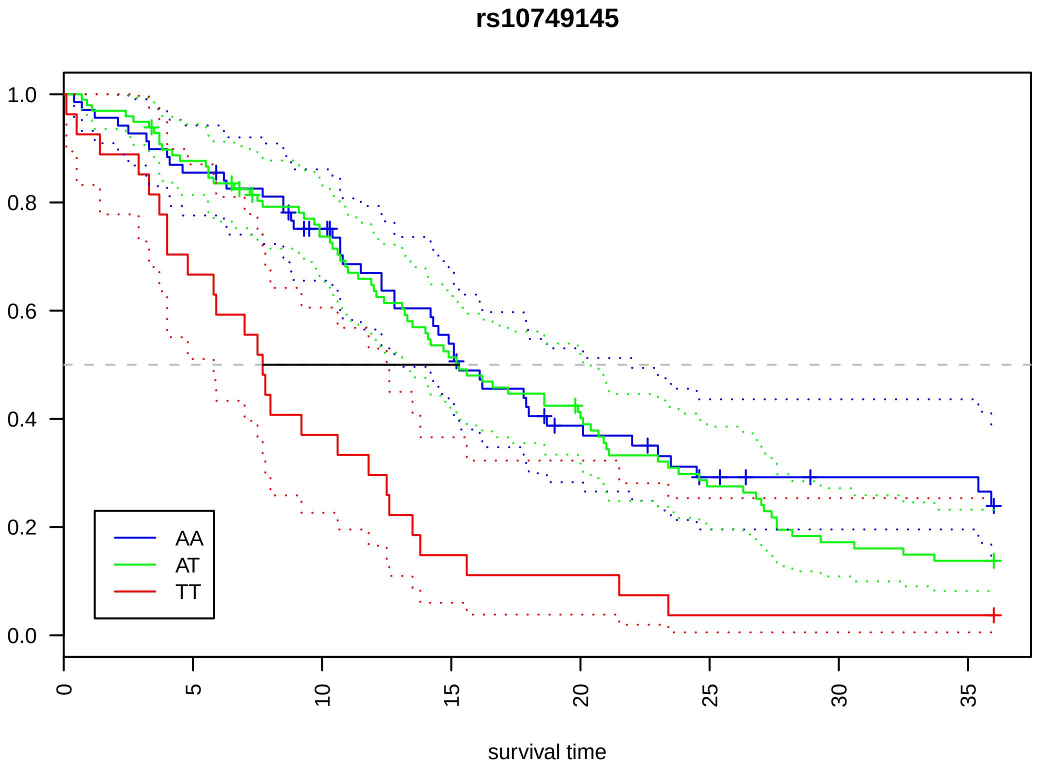

Figure 2002: Kaplan-Meier curves for the selected genotypes. Dashed lines represent 0.95 confidence intervals for the curves with the corresponding color. Black horizontal line ($15.30 - 7.70 = 7.60$) indicates the maximal survival difference at $y = 0.50$.

17.77 Survival analysis of rs2581221

This marker has a genotype specific effect with a p-value of 3.309370e-05. Total of 1 observation deleted due to missingness. Odds ratios have been estimated using logistic regression at $t \approx 36$:
$\frac{P(event|AT,AA)/P(\neg event|AT,AA)}{P(event|TT)/P(\neg event|TT)} \approx 3.082$.

Table 91: Summary of genotype frequencies with their proportions in parenthesis.

genotype	total N	events	censored
TT	96	65 (0.677)	31 (0.323)
AT,AA	97	84 (0.866)	13 (0.134)

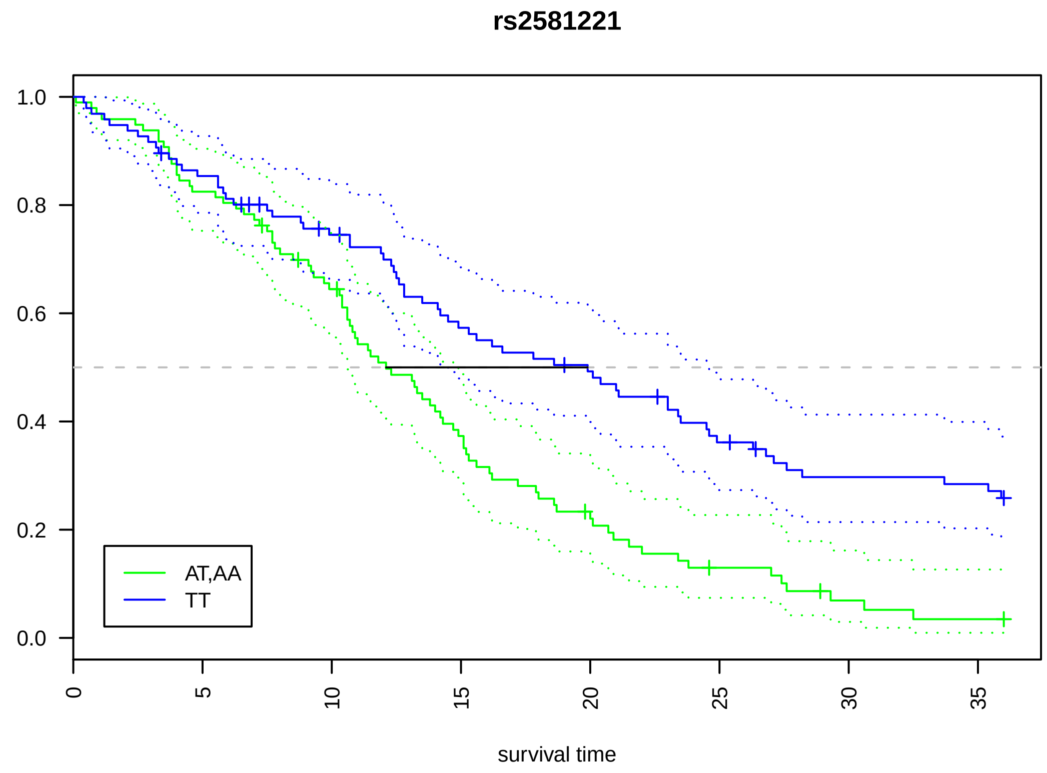

Figure 2003: Kaplan-Meier curves for the selected genotypes. Dashed lines represent 0.95 confidence intervals for the curves with the corresponding color. Black horizontal line $(19.90 - 12.10 = 7.80)$ indicates the maximal survival difference at $y = 0.50$.

17.78 Survival analysis of rs11897287

This marker has a genotype specific effect with a p-value of 2.301869e-05. Total of 5 observations deleted due to missingness. Odds ratios have been estimated using logistic regression at $t \approx 36$:
$\frac{P(event|AT,AA)/P(\neg event|AT,AA)}{P(event|TT)/P(\neg event|TT)} \approx 0.257$.

Table 92: Summary of genotype frequencies with their proportions in parenthesis.

genotype	total N	events	censored
TT	98	87 (0.888)	11 (0.112)
AT,AA	91	61 (0.67)	30 (0.33)

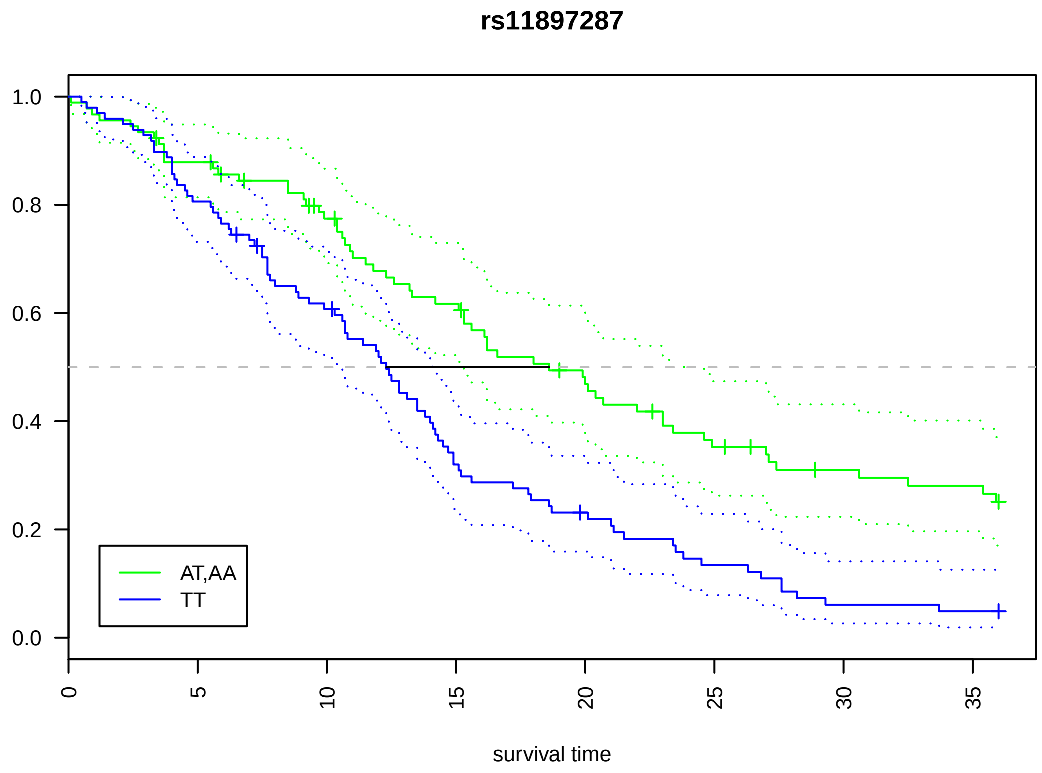

Figure 2004: Kaplan-Meier curves for the selected genotypes. Dashed lines represent 0.95 confidence intervals for the curves with the corresponding color. Black horizontal line $(18.60 - 12.30 = 6.30)$ indicates the maximal survival difference at $y = 0.50$.

17.79 Survival analysis of rs6756452

This marker has a genotype specific effect with a p-value of 8.744074e-05.

Odds ratios have been estimated using logistic regression at $t \approx 36$:
$\frac{P(event|AT,TT)/P(\neg event|AT,TT)}{P(event|AA)/P(\neg event|AA)} \approx 2.397$.

Table 93: Summary of genotype frequencies with their proportions in parenthesis.

genotype	total N	events	censored
AA	161	121 (0.752)	40 (0.248)
AT,TT	33	29 (0.879)	4 (0.121)

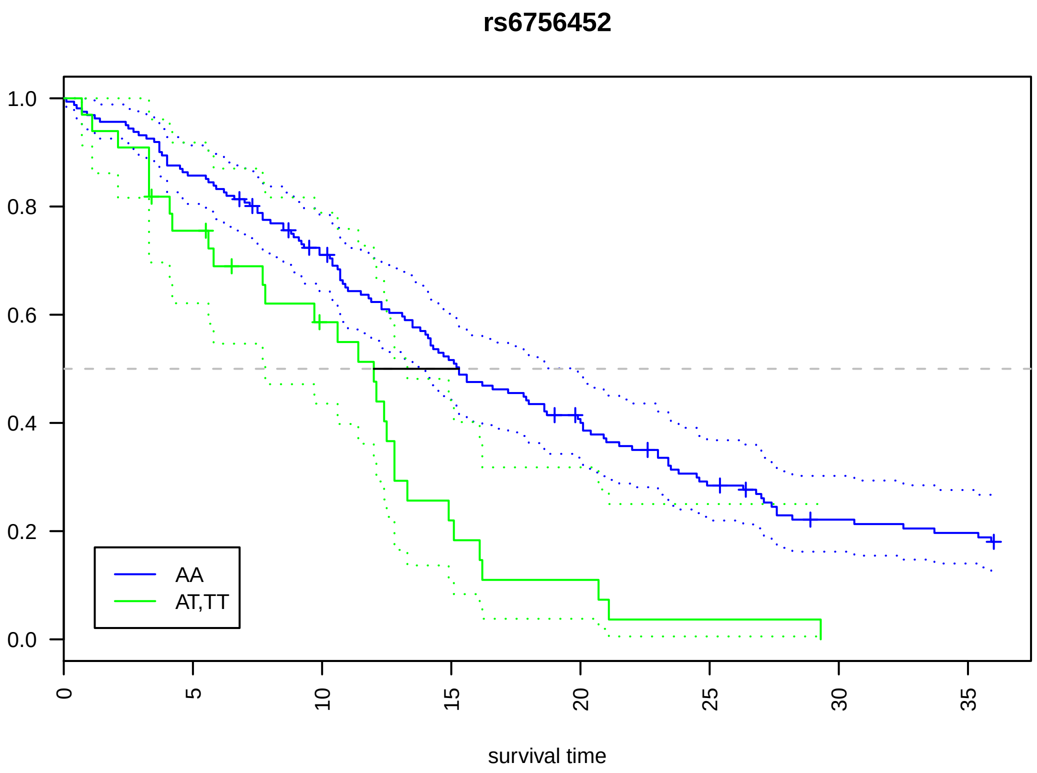

Figure 2005: Kaplan-Meier curves for the selected genotypes. Dashed lines represent 0.95 confidence intervals for the curves with the corresponding color. Black horizontal line ($15.30 - 12.00 = 3.30$) indicates the maximal survival difference at $y = 0.50$.

Additional annotations:

Gene name	Description	DNABand	protein
PRKCE	protein kinase C, epsilon [Source:HGNC Symbol;Acc:9401]	2p21	KPCE_HUMAN

17.80 Survival analysis of rs10169218

This marker has a genotype specific effect with a p-value of 2.360952e-05. Total of 1 observation deleted due to missingness. Odds ratios have been estimated using logistic regression at $t \approx 36$:
$\frac{P(event|AT,AA)/P(\neg event|AT,AA)}{P(event|TT)/P(\neg event|TT)} \approx 2.765$.

Table 94: Summary of genotype frequencies with their proportions in parenthesis.

genotype	total N	events	censored
TT	149	110 (0.738)	39 (0.262)
AT,AA	44	39 (0.886)	5 (0.114)

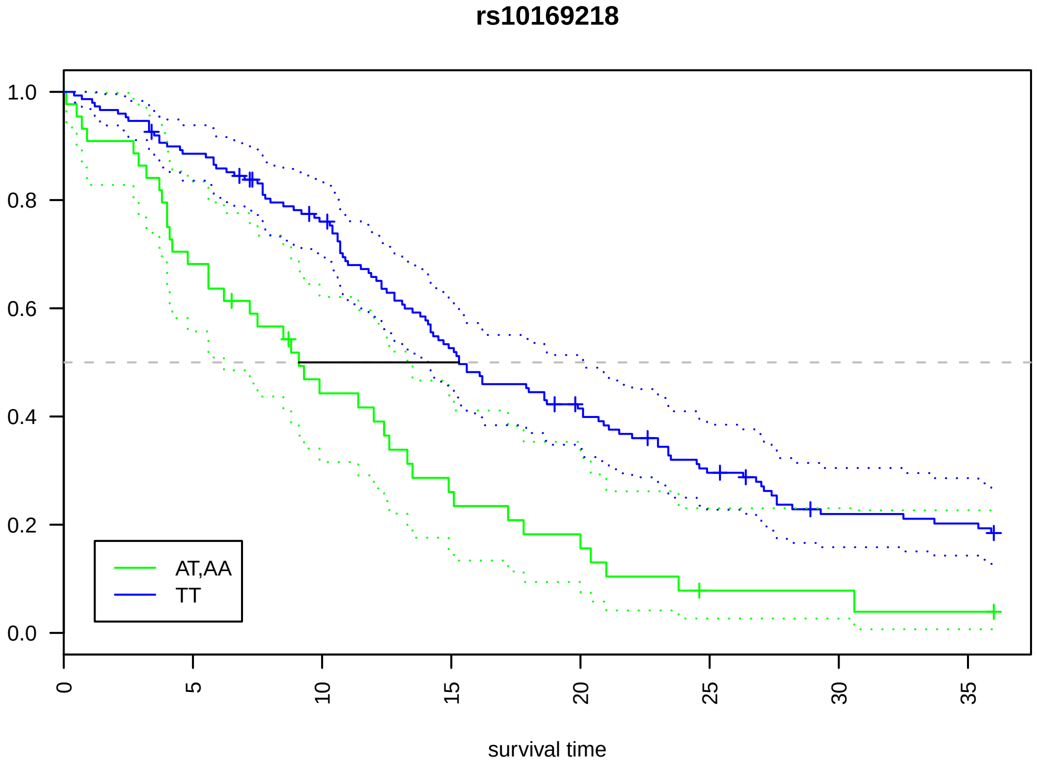

Figure 2006: Kaplan-Meier curves for the selected genotypes. Dashed lines represent 0.95 confidence intervals for the curves with the corresponding color. Black horizontal line $(15.30-9.10 = 6.20)$ indicates the maximal survival difference at $y = 0.50$.

17.81 Survival analysis of rs4962722

This marker has a genotype specific effect with a p-value of 9.285929e-05. Total of 5 observations deleted due to missingness. Odds ratios have been estimated using logistic regression at $t \approx 36$:
$\frac{P(event|AT)/P(\neg event|AT)}{P(event|AA)/P(\neg event|AA)} \approx 0.538$ and $\frac{P(event|TT)/P(\neg event|TT)}{P(event|AA)/P(\neg event|AA)} \approx 2.078$.

Table 95: Summary of genotype frequencies with their proportions in parenthesis.

genotype	total N	events	censored
AA	78	64 (0.821)	14 (0.179)
AT	90	64 (0.711)	26 (0.289)
TT	21	19 (0.905)	2 (0.0952)

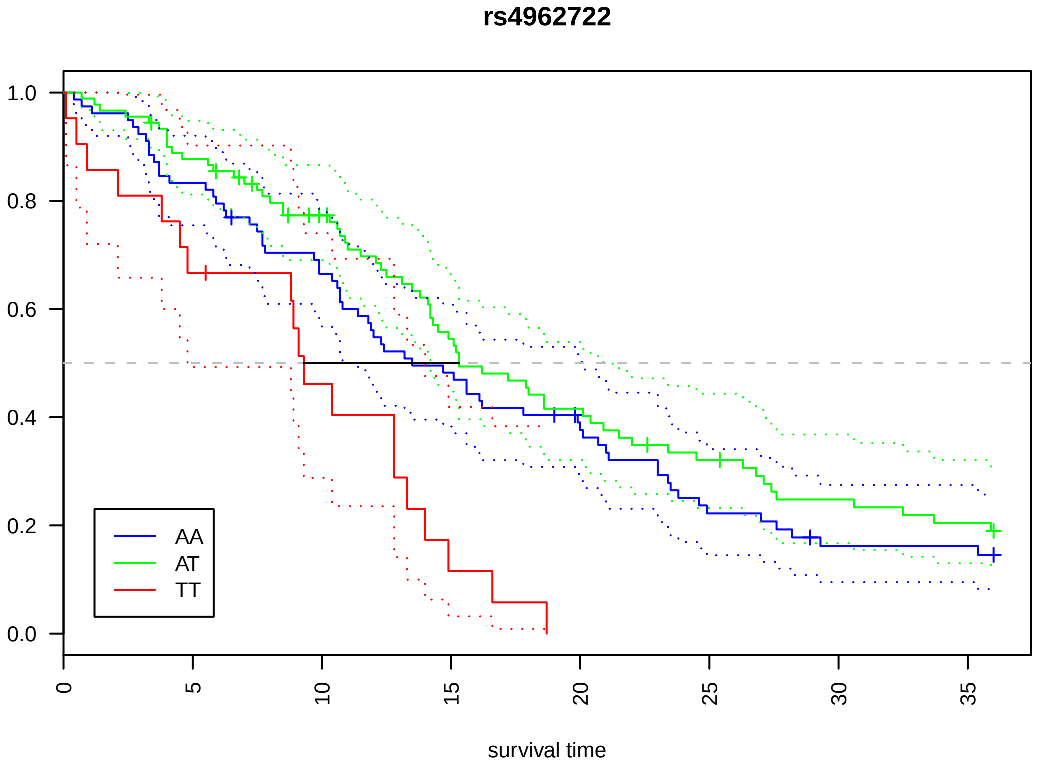

Figure 2007: Kaplan-Meier curves for the selected genotypes. Dashed lines represent 0.95 confidence intervals for the curves with the corresponding color. Black horizontal line $(15.30 - 9.30 = 6.00)$ indicates the maximal survival difference at $y = 0.50$.

Additional annotations:

Gene name	*Description*	*DNABand*	*protein*
CTBP2	C-terminal binding protein 2 [Source:HGNC Symbol;Acc:2495]	10q26.13	CTBP2_HUMAN

17.82 Survival analysis of rs588115

This marker has a genotype specific effect with a p-value of 4.025021e-05.

Odds ratios have been estimated using logistic regression at $t \approx 36$:
$\frac{P(event|AT,AA)/P(\neg event|AT,AA)}{P(event|TT)/P(\neg event|TT)} \approx 0.262$.

Table 96: Summary of genotype frequencies with their proportions in parenthesis.

genotype	total N	events	censored
TT	95	84 (0.884)	11 (0.116)
AT,AA	99	66 (0.667)	33 (0.333)

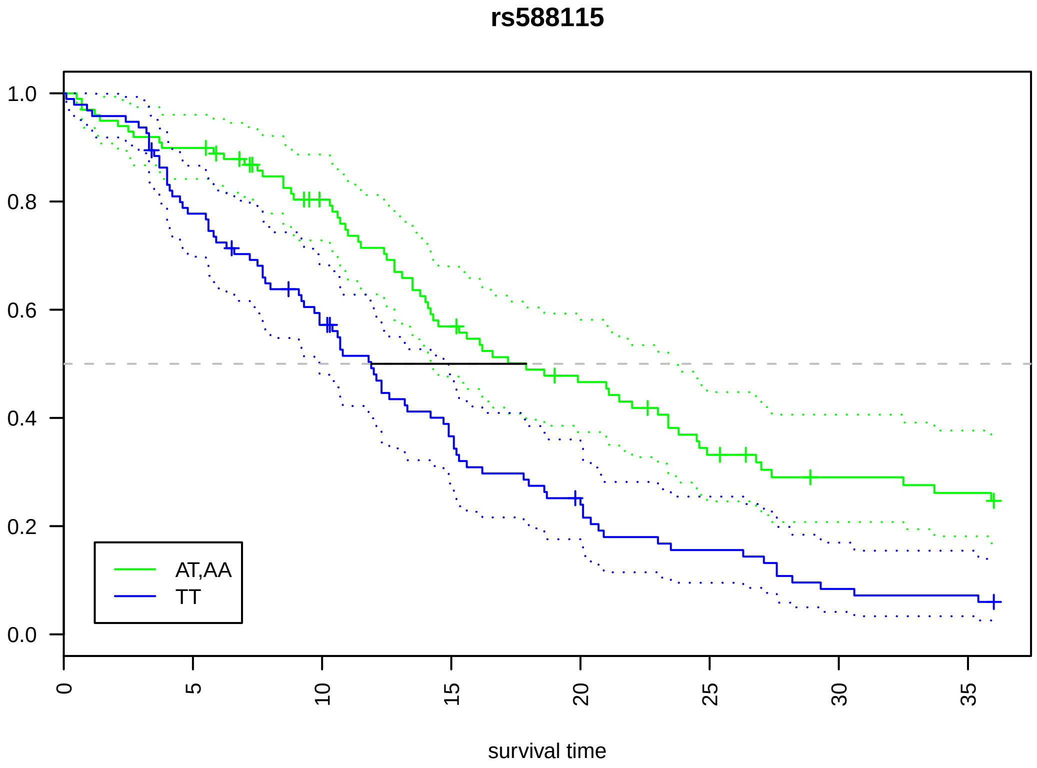

Figure 2008: Kaplan-Meier curves for the selected genotypes. Dashed lines represent 0.95 confidence intervals for the curves with the corresponding color. Black horizontal line $(17.90 - 11.90 = 6.00)$ indicates the maximal survival difference at $y = 0.50$.

Additional annotations:

Gene name	*Description*	*DNABand*	*protein*
FIZ1	FLT3-interacting zinc finger 1 [Source:HGNC Symbol;Acc:25917]	19q13.42	FIZ1_HUMAN

17.83 Survival analysis of rs2285218

This marker has a genotype specific effect with a p-value of 2.100634e-05.

Odds ratios have been estimated using logistic regression at $t \approx 36$:
$\frac{P(event|AT,AA)/P(\neg event|AT,AA)}{P(event|TT)/P(\neg event|TT)} \approx 8.439$.

Table 97: Summary of genotype frequencies with their proportions in parenthesis.

genotype	total N	events	censored
TT	149	107 (0.718)	42 (0.282)
AT,AA	45	43 (0.956)	2 (0.0444)

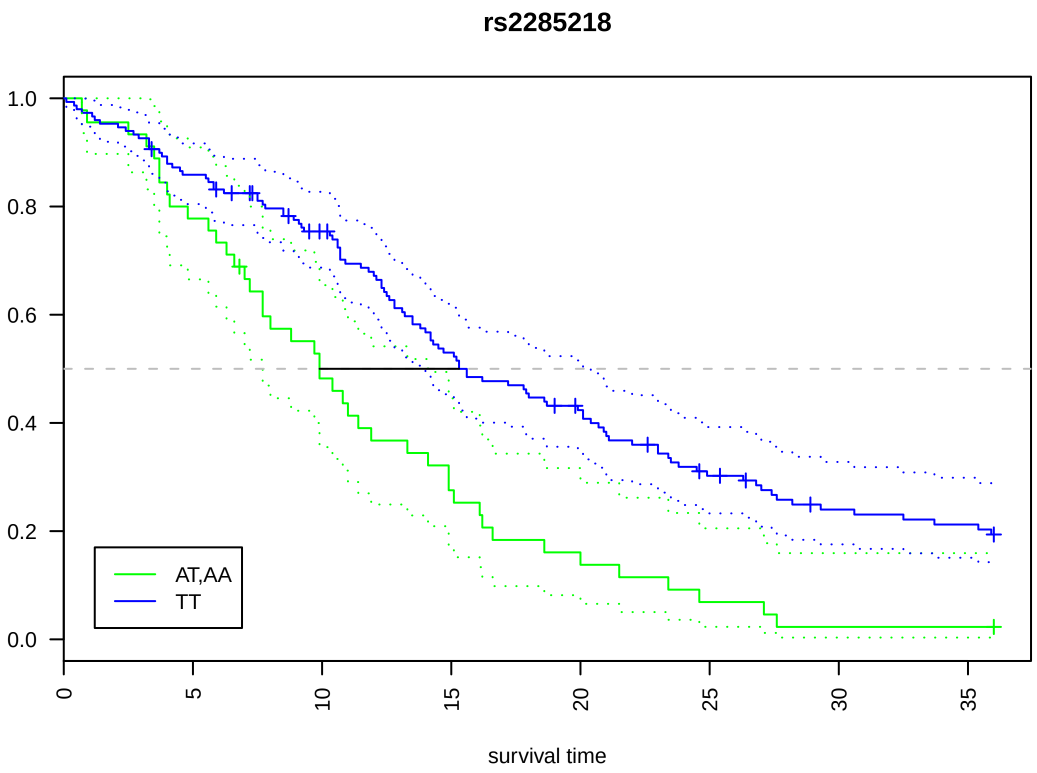

Figure 2009: Kaplan-Meier curves for the selected genotypes. Dashed lines represent 0.95 confidence intervals for the curves with the corresponding color. Black horizontal line ($15.30 - 9.90 = 5.40$) indicates the maximal survival difference at $y = 0.50$.

Additional annotations:

Gene name	*Description*	*DNABand*
KIAA0040	KIAA0040 [Source:HGNC Symbol;Acc:28950]	1q25.1

17.84 Survival analysis of rs9925903

This marker has a genotype specific effect with a p-value of 8.002366e-05. Total of 1 observation deleted due to missingness. Odds ratios have been estimated using logistic regression at $t \approx 36$:
$\frac{P(event|AT)/P(\neg event|AT)}{P(event|TT)/P(\neg event|TT)} \approx 3.487$ and $\frac{P(event|AA)/P(\neg event|AA)}{P(event|TT)/P(\neg event|TT)} \approx 2.287$.

Table 98: Summary of genotype frequencies with their proportions in parenthesis.

genotype	total N	events	censored
TT	72	47 (0.653)	25 (0.347)
AT	68	59 (0.868)	9 (0.132)
AA	53	43 (0.811)	10 (0.189)

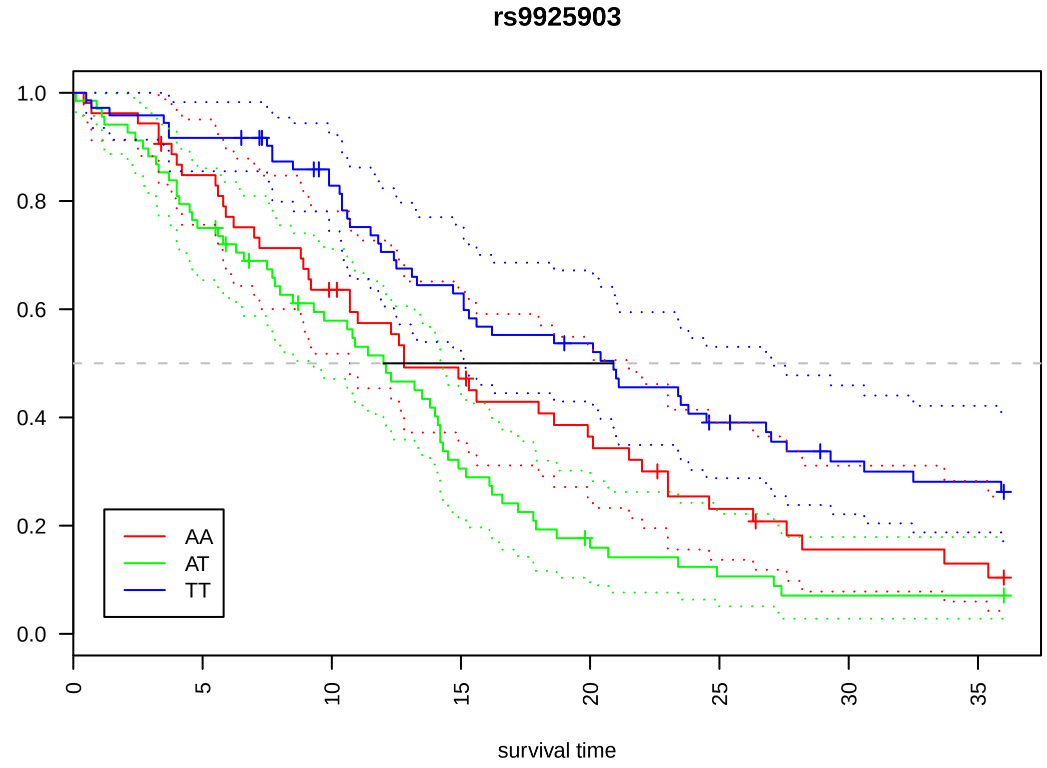

Figure 2010: Kaplan-Meier curves for the selected genotypes. Dashed lines represent 0.95 confidence intervals for the curves with the corresponding color. Black horizontal line $(20.90 - 12.00 = 8.90)$ indicates the maximal survival difference at $y = 0.50$.

Additional annotations:

Gene name	*Description*	*DNABand*	*protein*
CDH13	cadherin 13, H-cadherin (heart) [Source:HGNC Symbol;Acc:1753]	16q23.3	CAD13_HUMAN

17.85 Survival analysis of rs3779936

This marker has a genotype specific effect with a p-value of 2.791142e-05.

Odds ratios have been estimated using logistic regression at $t \approx 36$:
$\frac{P(event|AT,AA)/P(\neg event|AT,AA)}{P(event|TT)/P(\neg event|TT)} \approx 0.302$.

Table 99: Summary of genotype frequencies with their proportions in parenthesis.

genotype	total N	events	censored
TT	147	122 (0.83)	25 (0.17)
AT,AA	47	28 (0.596)	19 (0.404)

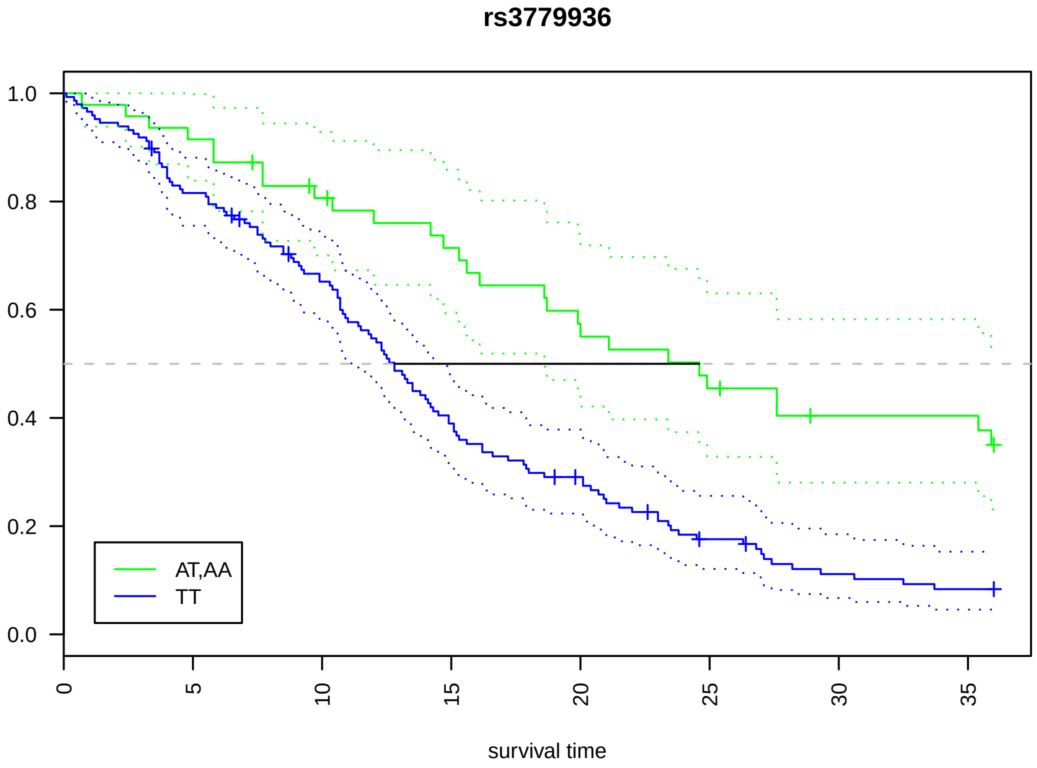

Figure 2011: Kaplan-Meier curves for the selected genotypes. Dashed lines represent 0.95 confidence intervals for the curves with the corresponding color. Black horizontal line $(24.60 - 12.80 = 11.80)$ indicates the maximal survival difference at $y = 0.50$.

Additional annotations:

Gene name	*Description*	*DNABand*	*protein*
NDRG1	N-myc downstream regulated 1 [Source:HGNC Symbol;Acc:7679]	8q24.22	NDRG1_HUMAN

17.86 Survival analysis of rs7833024

This marker has a genotype specific effect with a p-value of 1.328508e-06. Total of 1 observation deleted due to missingness. Odds ratios have been estimated using logistic regression at $t \approx 36$:
$\frac{P(event|AT)/P(\neg event|AT)}{P(event|TT)/P(\neg event|TT)} \approx 0.731$ and $\frac{P(event|AA)/P(\neg event|AA)}{P(event|TT)/P(\neg event|TT)} \approx 2.877$.

Table 100: Summary of genotype frequencies with their proportions in parenthesis.

genotype	total N	events	censored
TT	93	73 (0.785)	20 (0.215)
AT	77	56 (0.727)	21 (0.273)
AA	23	21 (0.913)	2 (0.087)

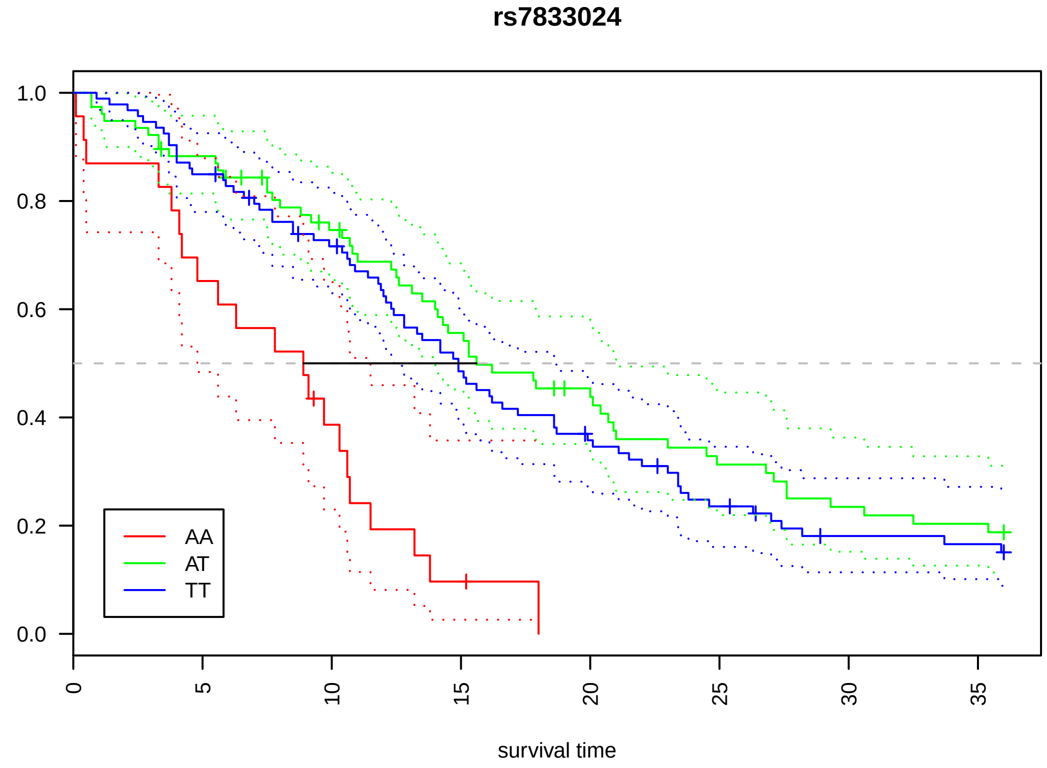

Figure 2012: Kaplan-Meier curves for the selected genotypes. Dashed lines represent 0.95 confidence intervals for the curves with the corresponding color. Black horizontal line ($15.60 - 8.90 = 6.70$) indicates the maximal survival difference at $y = 0.50$.

17.87 Survival analysis of rs1379149

This marker has a genotype specific effect with a p-value of 4.864788e-07. Total of 2 observations deleted due to missingness. Odds ratios have been estimated using logistic regression at $t \approx 36$:
$\frac{P(event|AT,AA)/P(\neg event|AT,AA)}{P(event|TT)/P(\neg event|TT)} \approx 17.037$.

Table 101: Summary of genotype frequencies with their proportions in parenthesis.

genotype	total N	events	censored
TT	149	106 (0.711)	43 (0.289)
AT,AA	43	42 (0.977)	1 (0.0233)

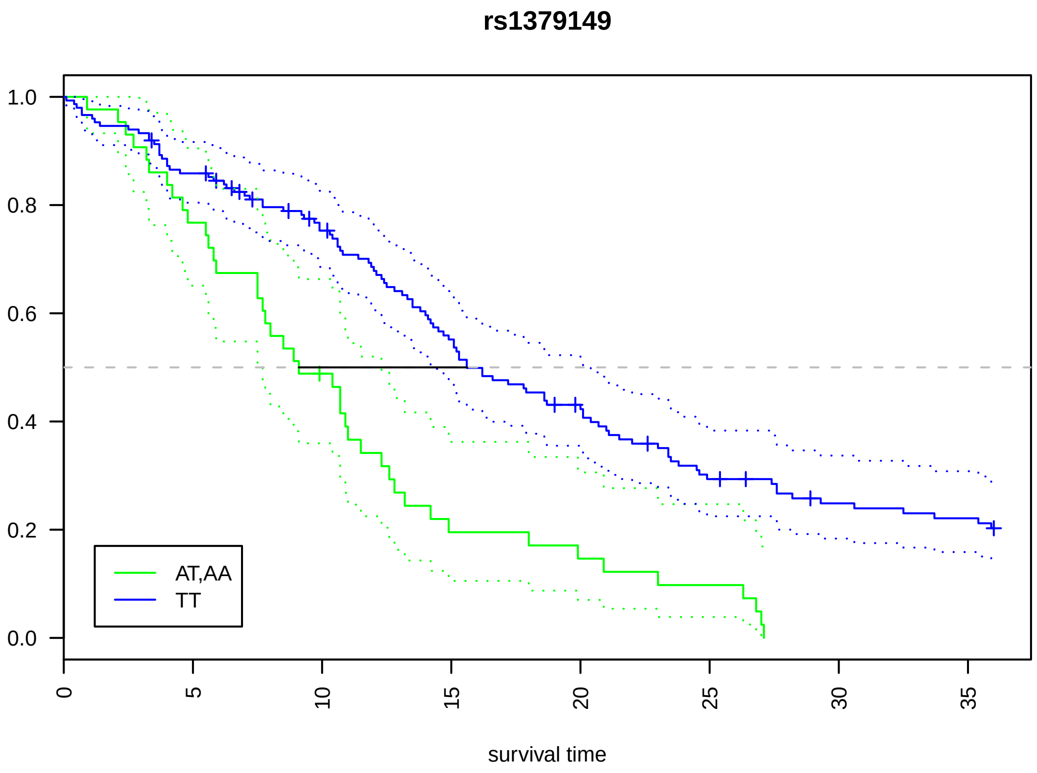

Figure 2013: Kaplan-Meier curves for the selected genotypes. Dashed lines represent 0.95 confidence intervals for the curves with the corresponding color. Black horizontal line $(15.60 - 9.10 = 6.50)$ indicates the maximal survival difference at $y = 0.50$.

17.88 Survival analysis of rs7903448

This marker has a genotype specific effect with a p-value of 2.141943e-05.

Odds ratios have been estimated using logistic regression at $t \approx 36$:
$\frac{P(event|AT,TT)/P(\neg event|AT,TT)}{P(event|AA)/P(\neg event|AA)} \approx 3.891$.

Table 102: Summary of genotype frequencies with their proportions in parenthesis.

genotype	total N	events	censored
AA	93	61 (0.656)	32 (0.344)
AT,TT	101	89 (0.881)	12 (0.119)

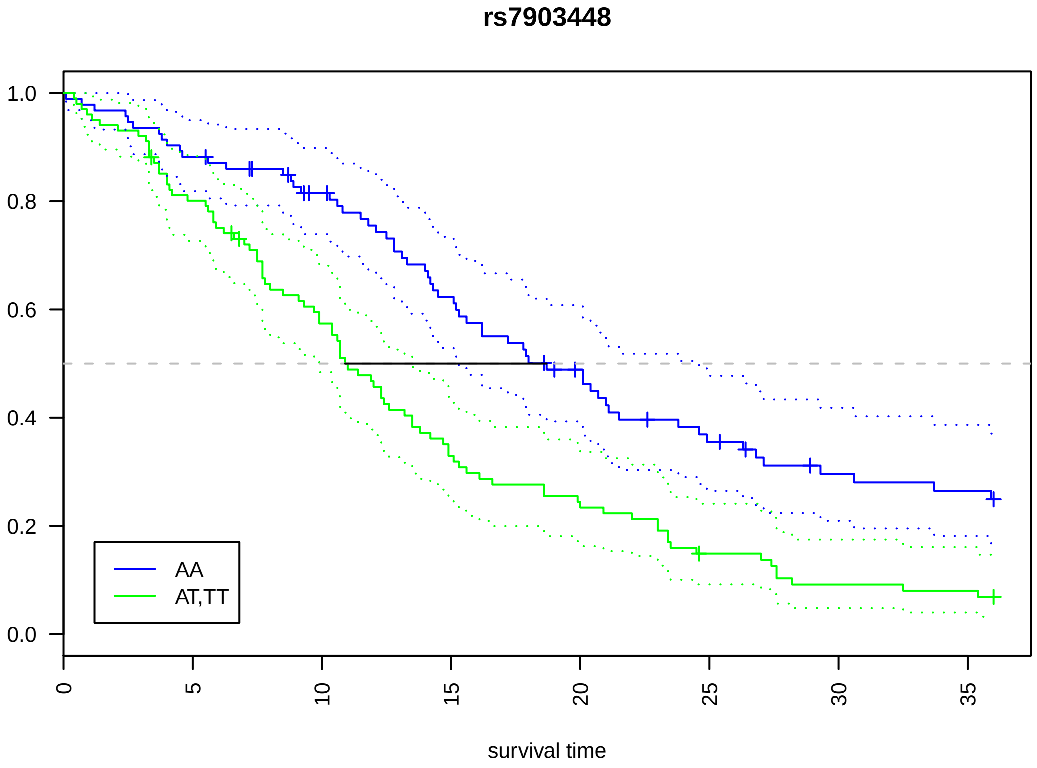

Figure 2014: Kaplan-Meier curves for the selected genotypes. Dashed lines represent 0.95 confidence intervals for the curves with the corresponding color. Black horizontal line $(18.70 - 10.90 = 7.80)$ indicates the maximal survival difference at $y = 0.50$.

17.89 Survival analysis of rs1554336

This marker has a genotype specific effect with a p-value of 6.033549e-05.

Odds ratios have been estimated using logistic regression at $t \approx 36$:
$\frac{P(event|AT,TT)/P(\neg event|AT,TT)}{P(event|AA)/P(\neg event|AA)} \approx 3.833$.

Table 103: Summary of genotype frequencies with their proportions in parenthesis.

genotype	total N	events	censored
AA	117	81 (0.692)	36 (0.308)
AT,TT	77	69 (0.896)	8 (0.104)

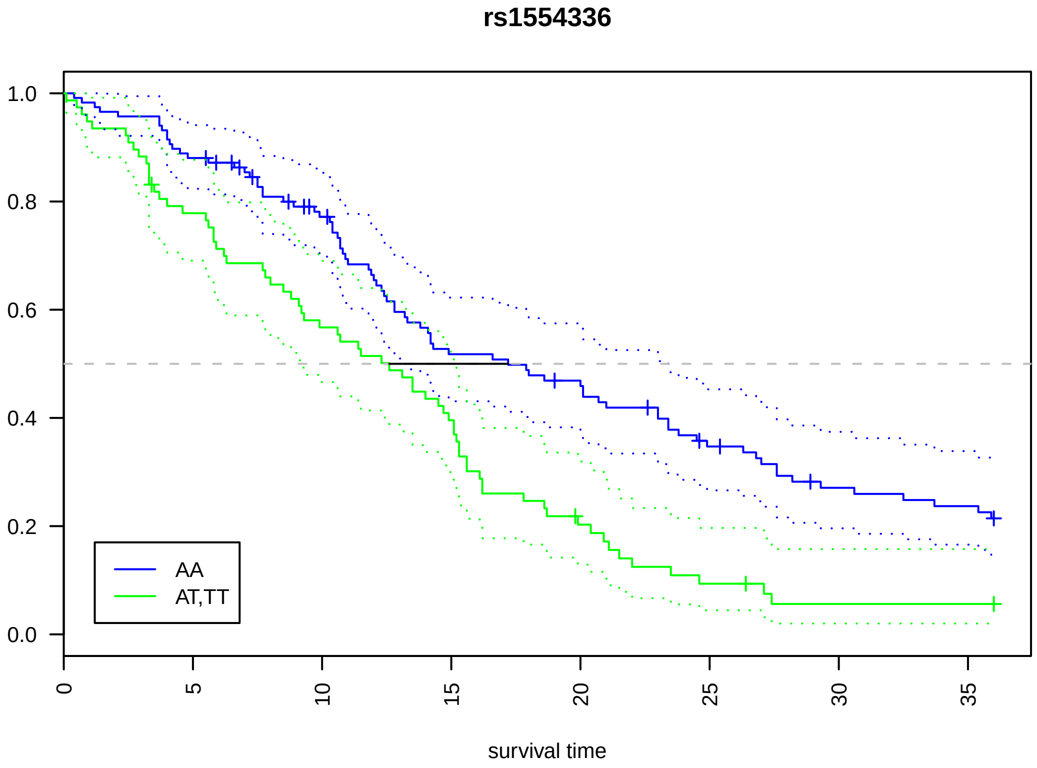

Figure 2015: Kaplan-Meier curves for the selected genotypes. Dashed lines represent 0.95 confidence intervals for the curves with the corresponding color. Black horizontal line $(17.20 - 12.60 = 4.60)$ indicates the maximal survival difference at $y = 0.50$.

Additional annotations:

Gene name	*Description*	*DNABand*	*protein*
ACSL1	acyl-CoA synthetase long-chain family member 1 [Source:HGNC Symbol;Acc:3569]	4q35.1	ACSL1_HUMAN

17.90 Survival analysis of rs13041611

This marker has a genotype specific effect with a p-value of 9.678715e-05.

Odds ratios have been estimated using logistic regression at $t \approx 36$:
$\frac{P(event|AT)/P(\neg event|AT)}{P(event|AA)/P(\neg event|AA)} \approx 0.3$ and $\frac{P(event|TT)/P(\neg event|TT)}{P(event|AA)/P(\neg event|AA)} \approx 0.354$.

Table 104: Summary of genotype frequencies with their proportions in parenthesis.

genotype	total N	events	censored
AA	63	56 (0.889)	7 (0.111)
AT	85	60 (0.706)	25 (0.294)
TT	46	34 (0.739)	12 (0.261)

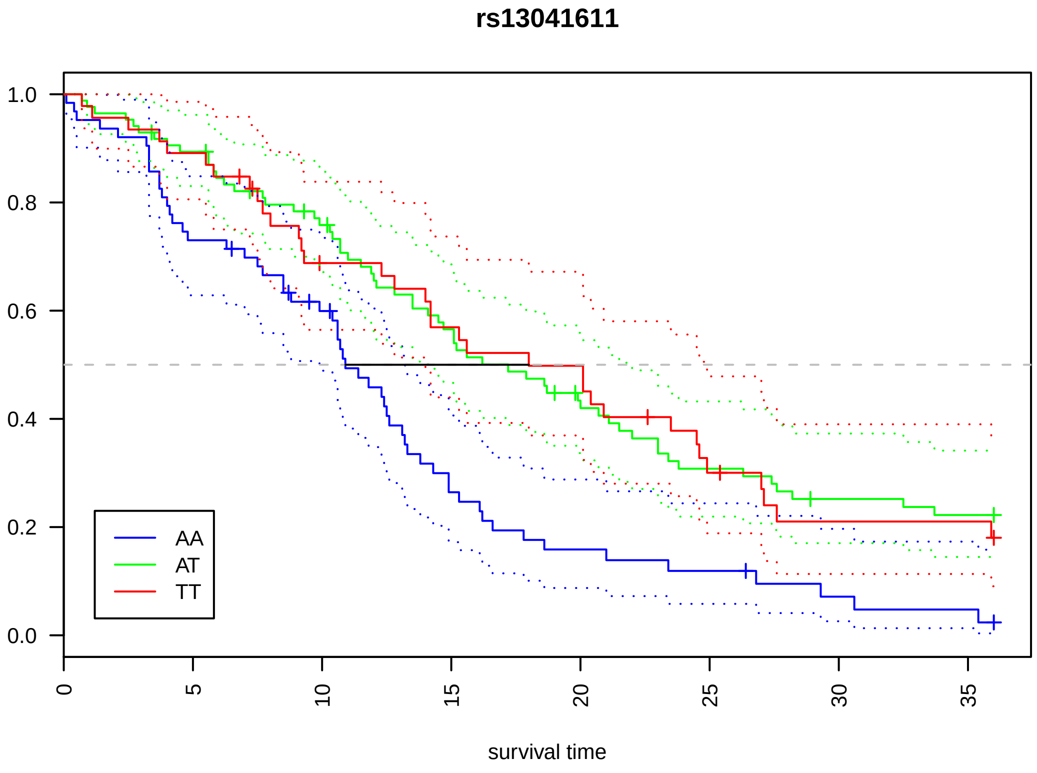

Figure 2016: Kaplan-Meier curves for the selected genotypes. Dashed lines represent 0.95 confidence intervals for the curves with the corresponding color. Black horizontal line ($18.00 - 10.90 = 7.10$) indicates the maximal survival difference at $y = 0.50$.

Additional annotations:

Gene name	*Description*	*DNABand*	*protein*
EYA2	eyes absent homolog 2 (Drosophila) [Source:HGNC Symbol;Acc:3520]	20q13.12	EYA2_HUMAN

17.91 Survival analysis of rs12010722

This marker has a genotype specific effect with a p-value of 9.397800e-05. Total of 1 observation deleted due to missingness. Odds ratios have been estimated using logistic regression at $t \approx 36$:
$\frac{P(event|AT)/P(\neg event|AT)}{P(event|AA)/P(\neg event|AA)} \approx 5.99$ and $\frac{P(event|TT)/P(\neg event|TT)}{P(event|AA)/P(\neg event|AA)} \approx 1.586$.

Table 105: Summary of genotype frequencies with their proportions in parenthesis.

genotype	total N	events	censored
AA	142	105 (0.739)	37 (0.261)
AT	18	17 (0.944)	1 (0.0556)
TT	33	27 (0.818)	6 (0.182)

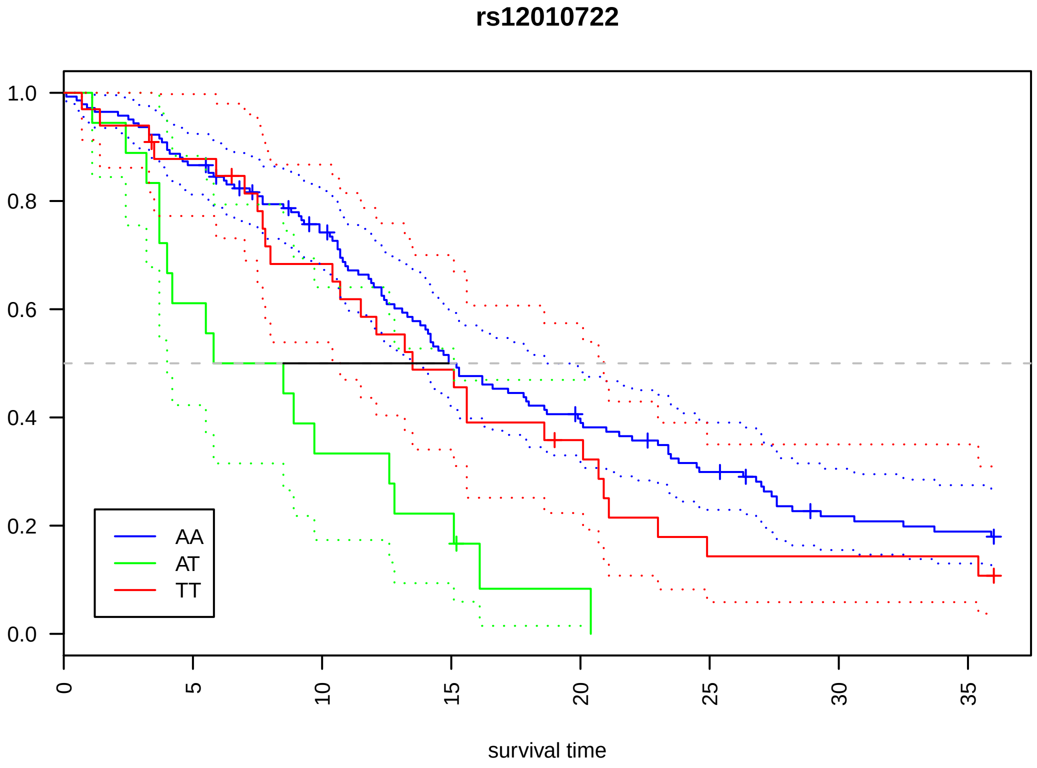

Figure 2017: Kaplan-Meier curves for the selected genotypes. Dashed lines represent 0.95 confidence intervals for the curves with the corresponding color. Black horizontal line $(14.90-8.50 = 6.40)$ indicates the maximal survival difference at $y = 0.50$.

Additional annotations:

Gene name	*Description*	*DNABand*	*protein*
RPS6KA3	ribosomal protein S6 kinase, 90kDa, polypeptide 3 [Source:HGNC Symbol;Acc:10432]	Xp22.12	KS6A3_HUMAN

17.92 Survival analysis of rs17676096

This marker has a genotype specific effect with a p-value of 1.410959e-05.

Odds ratios have been estimated using logistic regression at $t \approx 36$:
$\frac{P(event|AT,TT)/P(\neg event|AT,TT)}{P(event|AA)/P(\neg event|AA)} \approx 0.208$.

Table 106: Summary of genotype frequencies with their proportions in parenthesis.

genotype	total N	events	censored
AA	92	83 (0.902)	9 (0.0978)
AT,TT	102	67 (0.657)	35 (0.343)

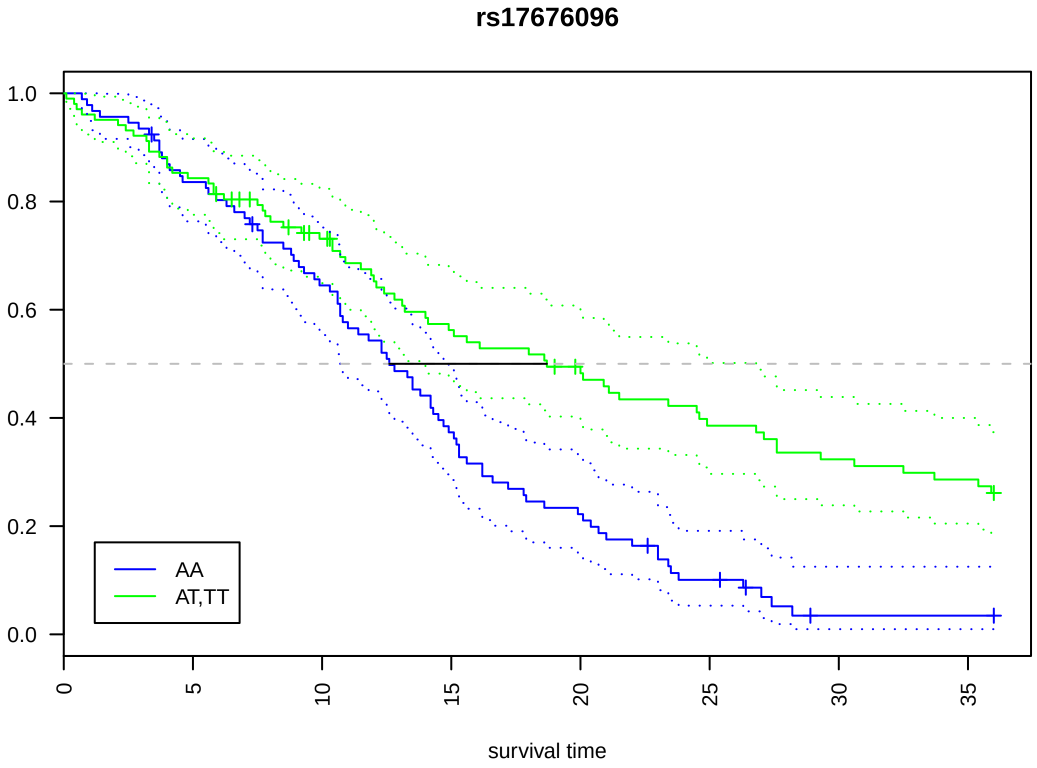

Figure 2018: Kaplan-Meier curves for the selected genotypes. Dashed lines represent 0.95 confidence intervals for the curves with the corresponding color. Black horizontal line $(18.70 - 12.60 = 6.10)$ indicates the maximal survival difference at $y = 0.50$.

Additional annotations:

Gene name	*Description*	*DNABand*
CTD-2230D16.1	[undefined]	3p14.1

17.93 Survival analysis of rs7064020

This marker has a genotype specific effect with a p-value of 4.049752e-05.

Odds ratios have been estimated using logistic regression at $t \approx 36$:
$\frac{P(event|AT)/P(\neg event|AT)}{P(event|TT)/P(\neg event|TT)} \approx 5.089$ and $\frac{P(event|AA)/P(\neg event|AA)}{P(event|TT)/P(\neg event|TT)} \approx 1.561$.

Table 107: Summary of genotype frequencies with their proportions in parenthesis.

genotype	total N	events	censored
TT	150	112 (0.747)	38 (0.253)
AT	16	15 (0.938)	1 (0.0625)
AA	28	23 (0.821)	5 (0.179)

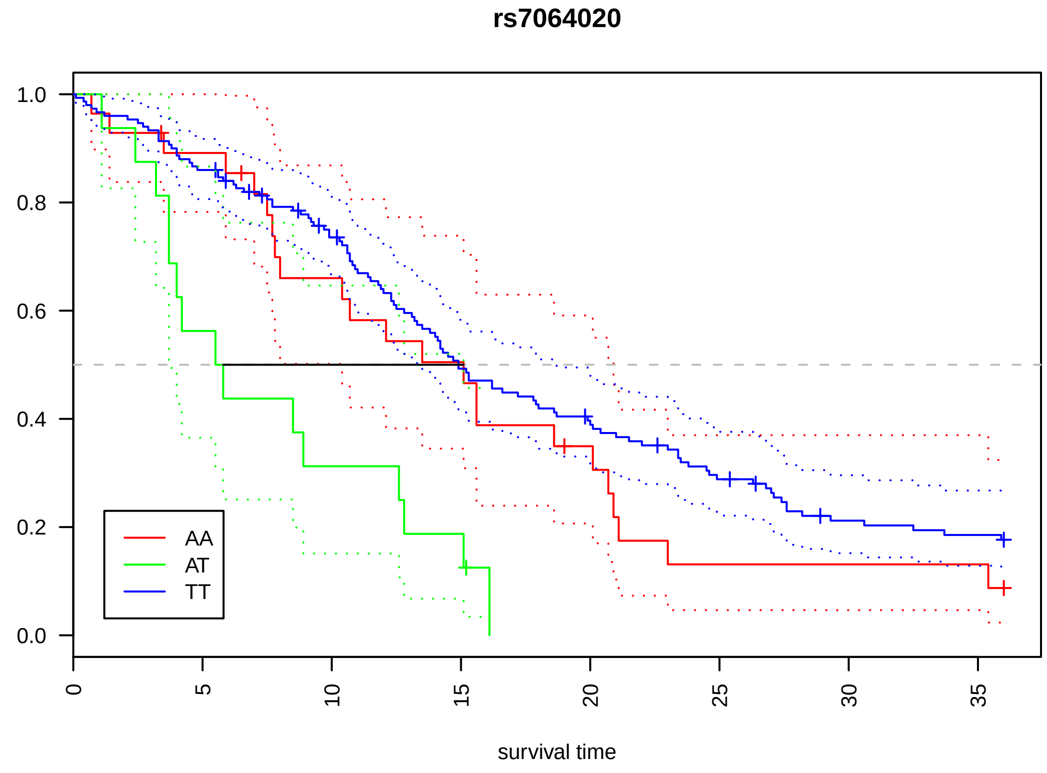

Figure 2019: Kaplan-Meier curves for the selected genotypes. Dashed lines represent 0.95 confidence intervals for the curves with the corresponding color. Black horizontal line ($15.10 - 5.80 = 9.30$) indicates the maximal survival difference at $y = 0.50$.

Listing 1: This SQL query has been used to select clinical parameters for the survival calculations.

```sql
-- Select samples and columns for the survival analysis
SELECT "PatientID"                                  AS "sample",
       "SurvivalEndTime"                            AS "endTime",
       0                                            AS "startTime",
       CAST ("VitalStatus"='DECEASED' AS INT) AS "hasEvent"
FROM   table1
WHERE  ("SurvivalEndTime" IS NOT NULL)
       -- No additional restrictions
```

Table 108: Gene annotations and the loci of the survival affecting markers

hitmarker	chr:locus	band	gene	gene description
fig rs1011127	1:7536187	1p36.23 1p36.31	AL596210.1	Putative uncharacterized protein ENSP00000403793 Fragment [Source:UniProtKB/TrEMBL;Acc:C9JJ20]
fig rs1011127	1:7536187	1p36.23 1p36.31	CAMTA1	calmodulin binding transcription activator 1 [Source:HGNC Symbol;Acc:18806]
fig rs6671217	1:79770059			
fig rs11102233	1:111827749	1p13.2	RP11-165H20.1	CHIA-like pseudogene (LOC149620), non-coding RNA [Source:RefSeq DNA;Acc:NR_003928]
fig rs2285218	1:175132079	1q25.1	RP5-1141O19.2	[undefined]
fig rs2285218	1:175132079	1q25.1	KIAA0040	KIAA0040 [Source:HGNC Symbol;Acc:28950]
fig rs7536561	1:180243524	1q25.2 1q25.3	ACBD6	acyl-Coenzyme A binding domain containing 6 [Source:HGNC Symbol;Acc:23339]
fig rs7536561	1:180243524	1q25.2	RP5-1180C10.2	[undefined]
fig rs7536561	1:180243524	1q25.2	LHX4	LIM homeobox 4 [Source:HGNC Symbol;Acc:21734]
fig rs555206	1:184842453	1q25.3	FAM129A	family with sequence similarity 129, member A [Source:HGNC Symbol;Acc:16784]
fig rs4489589	1:200651209	1q32.1	RP11-92G12.3	[undefined]
fig rs4378238	1:200652600	1q32.1	RP11-92G12.3	[undefined]
fig rs4498833	1:200652693	1q32.1	RP11-92G12.3	[undefined]
fig rs6427839	1:200653450	1q32.1	RP11-92G12.3	[undefined]
fig rs7903448	10:9808662			
fig rs10827154	10:19540391	10p12.31	C10orf112	MAM and LDL-receptor class A domain-containing protein C10orf112 [Source:UniProtKB/Swiss-Prot;Acc:Q5VYJ5]
fig rs11007253	10:29100054	10p12.1	RP11-478H13.3	[undefined]
fig rs10793491	10:44278726	10q11.21	RP11-272J7.4	[undefined]
fig rs11599561	10:44833031			
fig rs7069891	10:44838464			
fig rs2784761	10:81885914	10q22.3	RP11-369J21.7	[undefined]
fig rs7902084	10:115547965	10q25.3	C10orf81	PH domain-containing protein C10orf81 (Epididymis luminal protein 185)(hEL185) [Source:UniProtKB/Swiss-Prot;Acc:Q5SXH7]
fig rs10749145	10:115551895			
fig rs4962722	10:126702698	10q26.13	CTBP2	C-terminal binding protein 2 [Source:HGNC Symbol;Acc:2495]
fig rs1436094	11:30092081			
fig rs1376946	11:86094005	11q14.2	CCDC81	coiled-coil domain containing 81 [Source:HGNC Symbol;Acc:26281]
fig rs563460	11:100830693	11q22.1	AP000872.1	Rho GTPase-activating protein 10-like [Source:UniProtKB/Swiss-Prot;Acc:A6NI28]
fig rs1506390	12:30631079			
fig rs10506216	12:43130885			
fig rs826911	12:130559833			
fig rs12891139	14:89942068			
fig rs12101080	14:93562855	14q32.12	ITPK1	inositol 1,3,4-triphosphate 5/6 kinase [Source:HGNC Symbol;Acc:6177]
fig rs4906048	14:101585002			
fig rs8034783	15:47763726			
fig rs17403255	15:49938470	15q21.2	DTWD1	DTW domain containing 1 [Source:HGNC Symbol;Acc:30926]
fig rs9920861	15:60673690	15q22.2	ANXA2	annexin A2 [Source:HGNC Symbol;Acc:537]
fig rs2521498	15:91442152	15q26.1	FES	feline sarcoma oncogene [Source:HGNC Symbol;Acc:3657]
fig rs2521498	15:91442152	15q26.1	AC068831.2	[undefined]
fig rs9925903	16:83508437	16q23.3	CDH13	cadherin 13, H-cadherin (heart) [Source:HGNC Symbol;Acc:1753]
fig rs9635999	18:28350472			
fig rs306220	18:60298738			
fig rs11664762	18:77780683			
fig rs12964779	18:77794810	18q23	C18orf22	Putative ribosome-binding factor A, mitochondrial Precursor [Source:UniProtKB/Swiss-Prot;Acc:Q8N0V3]
fig rs4644918	18:77795232	18q23	C18orf22	Putative ribosome-binding factor A, mitochondrial Precursor [Source:UniProtKB/Swiss-Prot;Acc:Q8N0V3]
fig rs11664633	18:77803750	18q23	C18orf22	Putative ribosome-binding factor A, mitochondrial Precursor [Source:UniProtKB/Swiss-Prot;Acc:Q8N0V3]
fig rs8093433	18:77828600			
fig rs588115	19:56098935	19q13.42	FIZ1	FLT3-interacting zinc finger 1 [Source:HGNC Symbol;Acc:25917]
fig rs17022010	2:38331931	2p22.2	C2orf58	[undefined]
fig rs17022010	2:38331931	2p22.2	CYP1B1	cytochrome P450, family 1, subfamily B, polypeptide 1 [Source:HGNC Symbol;Acc:2597]
fig rs6756452	2:46077503	2p21	PRKCE	protein kinase C, epsilon [Source:HGNC Symbol;Acc:9401]
fig rs1159766	2:72464241	2p13.2	EXOC6B	exocyst complex component 6B [Source:HGNC Symbol;Acc:17085]
fig rs1159764	2:72464366	2p13.2	EXOC6B	exocyst complex component 6B [Source:HGNC Symbol;Acc:17085]
fig rs10221769	2:72479054	2p13.2	EXOC6B	exocyst complex component 6B [Source:HGNC Symbol;Acc:17085]
fig rs7572149	2:104848853			
fig rs10169218	2:104885689			
fig rs12468464	2:110447542			
fig rs6728529	2:127785959			
fig rs7571480	2:127786758			
fig rs11897287	2:134913954	2q21.2 2q21.3	MGAT5	mannosyl (alpha-1,6-)-glycoprotein beta-1,6-N-acetyl-glucosaminyltransferase [Source:HGNC Symbol;Acc:7049]
fig rs11692586	2:134917005	2q21.2 2q21.3	MGAT5	mannosyl (alpha-1,6-)-glycoprotein beta-1,6-N-acetyl-glucosaminyltransferase [Source:HGNC Symbol;Acc:7049]
fig rs13423908	2:171136568	2q31.1	MYO3B	myosin IIIB [Source:HGNC Symbol;Acc:15576]
fig rs10803885	2:175590615	2q31.1	AC018890.6	[undefined]
fig rs10932321	2:211138809			
fig rs6077364	20:8463481	20p12.3	PLCB1	phospholipase C, beta 1 (phosphoinositide-specific) [Source:HGNC Symbol;Acc:15917]
fig rs13041611	20:45600309	20q13.12	EYA2	eyes absent homolog 2 (Drosophila) [Source:HGNC Symbol;Acc:3520]
fig rs182174	21:18580354	21q21.1	AP000457.1	[undefined]
fig rs6004869	22:26380870	22q12.1	CTA-125H2.1	[undefined]
fig rs6004869	22:26380870	22q12.1	MYO18B	myosin XVIIIB [Source:HGNC Symbol;Acc:18150]
fig rs2600077	3:10841528			
fig rs2581221	3:10845417			
fig rs11708378	3:10886818	3p25.3	SLC6A11	solute carrier family 6 (neurotransmitter transporter, GABA), member 11 [Source:HGNC Symbol;Acc:11044]
fig rs2881513	3:16551213	3p24.3 3p25.1	RFTN1	raftlin, lipid raft linker 1 [Source:HGNC Symbol;Acc:30278]
fig rs13091723	3:35310758			
fig rs17676096	3:64715467	3p14.1	CTD-2230D16.1	[undefined]
fig rs1479852	3:140093954	3q23	CLSTN2	calsyntenin 2 [Source:HGNC Symbol;Acc:17448]
fig rs10516167	4:4548477	4p16.2	RP11-323F5.2	[undefined]
fig rs10516167	4:4548477	4p16.2 4p16.3	STX18	syntaxin 18 [Source:HGNC Symbol;Acc:15942]

Continued on next page...

hitmarker	chr:locus	band	gene	gene description
fig rs1379149	4:64649227			
fig rs6846040	4:121067686	4q27	RP11-679C8.2	[undefined]
fig rs1349345	4:139490191			
fig rs17258085	4:183479255	4q34.3 4q35.1	ODZ3	odz, odd Oz/ten-m homolog 3 (Drosophila) [Source:HGNC Symbol;Acc:29944]
fig rs1554336	4:185744820	4q35.1	RP11-701P16.2	[undefined]
fig rs1554336	4:185744820	4q35.1	ACSL1	acyl-CoA synthetase long-chain family member 1 [Source:HGNC Symbol;Acc:3569]
fig rs12642867	4:190092277			
fig rs464420	5:80402998	5q14.1	RASGRF2	Ras protein-specific guanine nucleotide-releasing factor 2 [Source:HGNC Symbol;Acc:9876]
fig rs701405	5:81593600	5q14.2	ATP6AP1L	ATPase, H+ transporting, lysosomal accessory protein 1-like [Source:HGNC Symbol;Acc:28091]
fig rs4921056	5:115422934	5q23.1	COMMD10	COMM domain containing 10 [Source:HGNC Symbol;Acc:30201]
fig rs13168015	5:133840309	5q31.1	AC005355.2	[undefined]
fig rs13168015	5:133840309	5q31.1	AC005355.1	[undefined]
fig rs2326613	6:5545046	6p25.1	FARS2	phenylalanyl-tRNA synthetase 2, mitochondrial [Source:HGNC Symbol;Acc:21062]
fig rs9395780	6:52178604			
fig rs9403788	6:146785984			
fig rs1919793	7:9353973			
fig rs10107890	8:83957451			
fig rs4382455	8:134214191	8q24.22	WISP1	WNT1 inducible signaling pathway protein 1 [Source:HGNC Symbol;Acc:12769]
fig rs3779936	8:134265748	8q24.22	NDRG1	N-myc downstream regulated 1 [Source:HGNC Symbol;Acc:7679]
fig rs7833024	8:137278411			
fig rs10810961	9:18371966			
fig rs7863546	9:133474146	9q34.11 9q34.12	FUBP3	far upstream element (FUSE) binding protein 3 [Source:HGNC Symbol;Acc:4005]
fig rs12010722	X:20170527	Xp22.12	RPS6KA3	ribosomal protein S6 kinase, 90kDa, polypeptide 3 [Source:HGNC Symbol;Acc:10432]
fig rs7064020	X:20171056	Xp22.12	RPS6KA3	ribosomal protein S6 kinase, 90kDa, polypeptide 3 [Source:HGNC Symbol;Acc:10432]
fig rs7892835	X:20184037	Xp22.12	RPS6KA3	ribosomal protein S6 kinase, 90kDa, polypeptide 3 [Source:HGNC Symbol;Acc:10432]
fig rs5928288	X:33503541			

17.94 Gene rank

Table 109: A list of survival affecting genes and the marker counts. P-value is the lowest value for the corresponding markers.

gene	markers	p-value	description
RP11-92G12.3	4	3.19346E-5	[undefined]
RPS6KA3	3	1.48163E-5	ribosomal protein S6 kinase, 90kDa, polypeptide 3 [Source:HGNC Symbol;Acc:10432]
EXOC6B	3	2.35098E-5	exocyst complex component 6B [Source:HGNC Symbol;Acc:17085]
C18orf22	3	3.56213E-5	Putative ribosome-binding factor A, mitochondrial Precursor [Source:UniProtKB/Swiss-Prot;Acc:Q8N0V3]
MGAT5	2	2.30187E-5	mannosyl (alpha-1,6-)-glycoprotein beta-1,6-N-acetyl-glucosaminyltransferase [Source:HGNC Symbol;Acc:7049]
RP11-679C8.2	1	1.14461E-6	[undefined]
FARS2	1	1.81455E-6	phenylalanyl-tRNA synthetase 2, mitochondrial [Source:HGNC Symbol;Acc:21062]
RASGRF2	1	2.75714E-6	Ras protein-specific guanine nucleotide-releasing factor 2 [Source:HGNC Symbol;Acc:9876]
DTWD1	1	4.0343E-6	DTW domain containing 1 [Source:HGNC Symbol;Acc:30926]
AP000457.1	1	5.04472E-6	[undefined]
CCDC81	1	5.33945E-6	coiled-coil domain containing 81 [Source:HGNC Symbol;Acc:26281]
RP11-369J21.7	1	9.42043E-6	[undefined]
AC068831.2	1	1.04181E-5	[undefined]
FES	1	1.04181E-5	feline sarcoma oncogene [Source:HGNC Symbol;Acc:3657]
C10orf112	1	1.20071E-5	MAM and LDL-receptor class A domain-containing protein C10orf112 [Source:UniProtKB/Swiss-Prot;Acc:Q5VYJ5]
CTD-2230D16.1	1	1.41096E-5	[undefined]
KIAA0040	1	2.10063E-5	KIAA0040 [Source:HGNC Symbol;Acc:28950]
RP5-1141O19.2	1	2.10063E-5	[undefined]
CTA-125H2.1	1	2.11325E-5	[undefined]
MYO18B	1	2.11325E-5	myosin XVIIIB [Source:HGNC Symbol;Acc:18150]
AL596210.1	1	2.17656E-5	Putative uncharacterized protein ENSP00000403793 Fragment [Source:UniProtKB/TrEMBL;Acc:C9JJ20]
CAMTA1	1	2.17656E-5	calmodulin binding transcription activator 1 [Source:HGNC Symbol;Acc:18806]
RFTN1	1	2.29563E-5	raftlin, lipid raft linker 1 [Source:HGNC Symbol;Acc:30278]
RP11-478H13.3	1	2.31531E-5	[undefined]
C10orf81	1	2.43985E-5	PH domain-containing protein C10orf81 (Epididymis luminal protein 185)(hEL185) [Source:UniProtKB/Swiss-Prot;Acc:Q5SXH7]
ODZ3	1	2.62203E-5	odz, odd Oz/ten-m homolog 3 (Drosophila) [Source:HGNC Symbol;Acc:29944]
NDRG1	1	2.79114E-5	N-myc downstream regulated 1 [Source:HGNC Symbol;Acc:7679]
PLCB1	1	2.91779E-5	phospholipase C, beta 1 (phosphoinositide-specific) [Source:HGNC Symbol;Acc:15917]
ANXA2	1	3.09462E-5	annexin A2 [Source:HGNC Symbol;Acc:537]
RP11-165H20.1	1	3.46389E-5	CHIA-like pseudogene (LOC149620), non-coding RNA [Source:RefSeq DNA;Acc:NR_003928]
WISP1	1	3.86785E-5	WNT1 inducible signaling pathway protein 1 [Source:HGNC Symbol;Acc:12769]
FIZ1	1	4.02502E-5	FLT3-interacting zinc finger 1 [Source:HGNC Symbol;Acc:25917]
C2orf58	1	4.15943E-5	[undefined]
CYP1B1	1	4.15943E-5	cytochrome P450, family 1, subfamily B, polypeptide 1 [Source:HGNC Symbol;Acc:2597]
ACBD6	1	4.2546E-5	acyl-Coenzyme A binding domain containing 6 [Source:HGNC Symbol;Acc:23339]
LHX4	1	4.2546E-5	LIM homeobox 4 [Source:HGNC Symbol;Acc:21734]
RP5-1180C10.2	1	4.2546E-5	[undefined]
RP11-323F5.2	1	4.4435E-5	[undefined]
STX18	1	4.4435E-5	syntaxin 18 [Source:HGNC Symbol;Acc:15942]
ACSL1	1	6.03355E-5	acyl-CoA synthetase long-chain family member 1 [Source:HGNC Symbol;Acc:3569]
RP11-701P16.2	1	6.03355E-5	[undefined]
MYO3B	1	6.12045E-5	myosin IIIB [Source:HGNC Symbol;Acc:15576]
RP11-272J7.4	1	6.34025E-5	[undefined]
FUBP3	1	6.49505E-5	far upstream element (FUSE) binding protein 3 [Source:HGNC Symbol;Acc:4005]
CLSTN2	1	6.63987E-5	calsyntenin 2 [Source:HGNC Symbol;Acc:17448]
AC005355.1	1	7.71058E-5	[undefined]
AC005355.2	1	7.71058E-5	[undefined]
CDH13	1	8.00237E-5	cadherin 13, H-cadherin (heart) [Source:HGNC Symbol;Acc:1753]
SLC6A11	1	8.07412E-5	solute carrier family 6 (neurotransmitter transporter, GABA), member 11 [Source:HGNC Symbol;Acc:11044]
FAM129A	1	8.1484E-5	family with sequence similarity 129, member A [Source:HGNC Symbol;Acc:16784]
AP000872.1	1	8.32363E-5	Rho GTPase-activating protein 10-like [Source:UniProtKB/Swiss-Prot;Acc:A6NI28]
PRKCE	1	8.74407E-5	protein kinase C, epsilon [Source:HGNC Symbol;Acc:9401]
COMMD10	1	9.07425E-5	COMM domain containing 10 [Source:HGNC Symbol;Acc:30201]
CTBP2	1	9.28593E-5	C-terminal binding protein 2 [Source:HGNC Symbol;Acc:2495]
AC018890.6	1	9.62524E-5	[undefined]
ATP6AP1L	1	9.65037E-5	ATPase, H+ transporting, lysosomal accessory protein 1-like [Source:HGNC Symbol;Acc:28091]
EYA2	1	9.67872E-5	eyes absent homolog 2 (Drosophila) [Source:HGNC Symbol;Acc:3520]
ITPK1	1	9.95686E-5	inositol 1,3,4-triphosphate 5/6 kinase [Source:HGNC Symbol;Acc:6177]

17.95 SNP database configuration

The following table shows the properties of **genotypeDB** component.

property	value
`dir.genotypes`	/opt/sandbox/snpDb/repository
`dir.suffix.length`	5
`file.input`	
`file.markernames`	/opt/sandbox/snpDb/catenated.lst
`file.output`	output.snpr
`file.samplenames`	/opt/sandbox/snpDb/samples.lst
`version`	2.22

Table 110: Distribution of the missing values in case samples

numberOfNAs	freq
0	40
1	50
2	29
3	14
4	12
5	12
6	9
7	9
8	5
9	4
10	3
11	5
13	1
15	1
19	1
54	1

18 Gene set information

18.1 GO clustering

Gene products are clustered (grouped) based on their Gene Ontology (GO) annotations. Genes that have similar annotations belong to the same cluster. The distance between gene products is defined using information-theoretical semantic similarity measures.

In the table below, the most informative (most specific) common GO terms of each cluster are displayed. All gene products in the cluster are annotated with the GO term mentioned. A GO term is more informative if it occurs rarely in the GO annotations of the whole genome. The priori column contains the a priori probability p that a random gene product is annotated with the given GO term. Information is defined as $-\log_2 p$.

Generally, clusters with (1) a large number of gene products and (2) common GO terms with high information content are the most interesting.

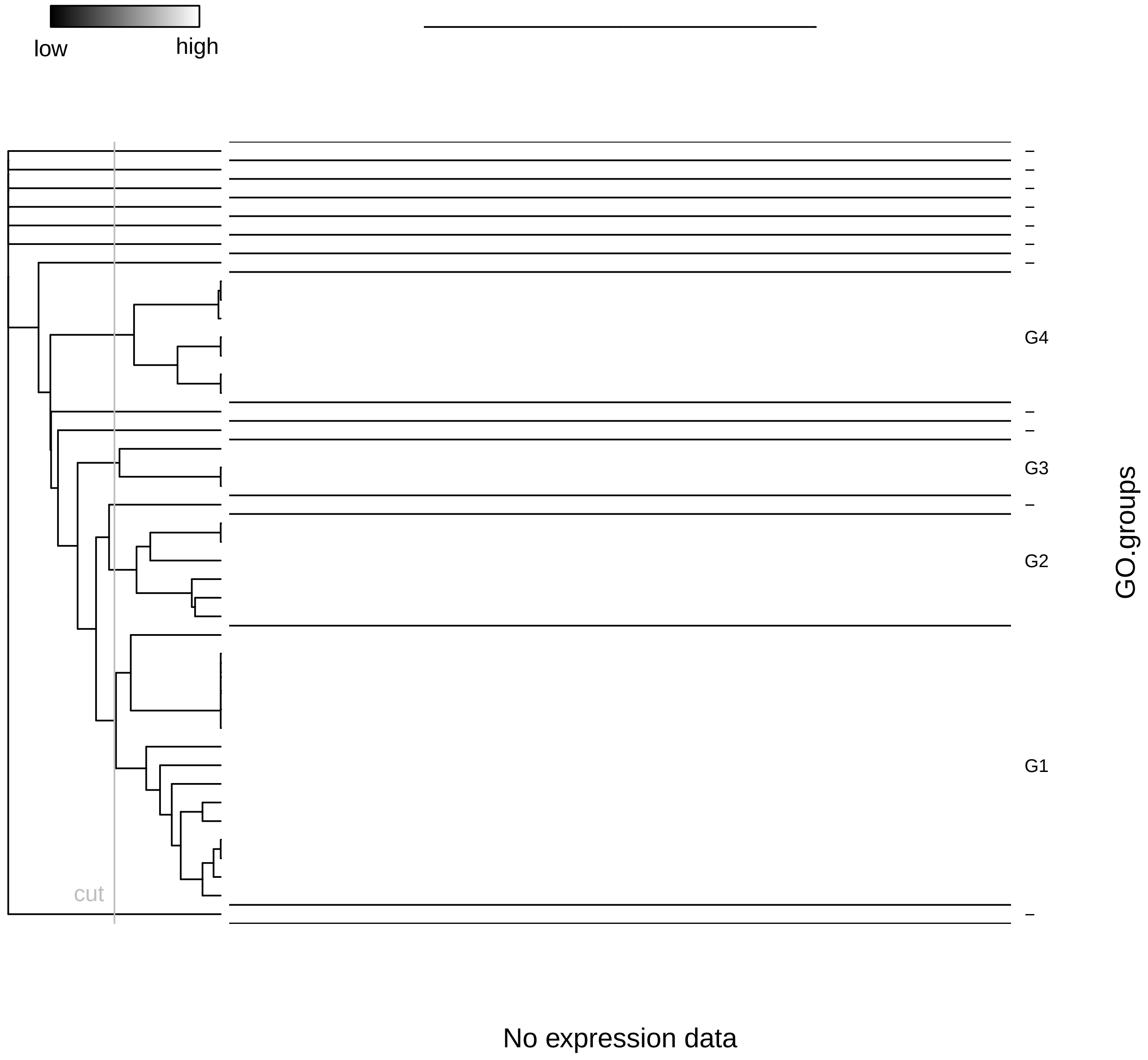

Figure 2020: GO clustering dendrogram and expression heatmap. The analysis is based on 42 gene products. The median number of GO annotations per gene product is 6. The colors represent expression values of the gene products. The lower expression values are indicated with black and the higher expressions with white. The left dendrogram corresponds to GO based clustering, while the top dendrogram uses expression values for clustering.

CLUSTER	SIZE	GOID	PRIORI	INFO	DESCRIPTION
G1	15				*[no interesting GO terms]*
G2	6	GO:0003824	0.228	2.13	catalytic activity
		GO:0008152	0.286	1.805	metabolic process
G3	3	GO:0050794	0.277	1.85	regulation of cellular process
		GO:0005737	0.295	1.763	cytoplasm
G4	7	GO:0016021	0.119	3.076	integral to membrane

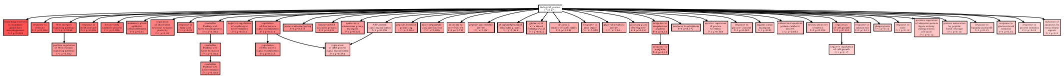

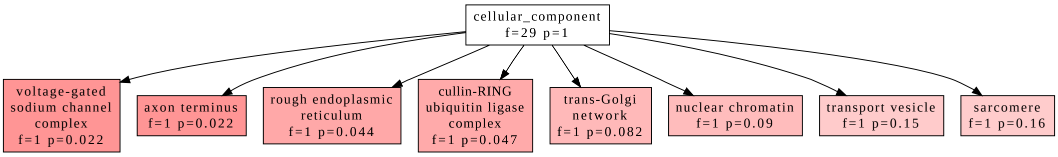

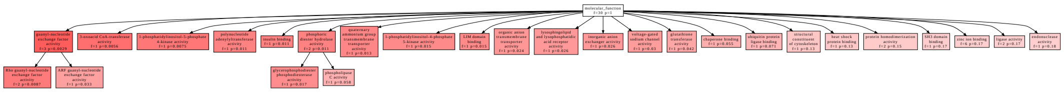

Table 112: KEGG pathways

Pathway	Name	QueryProteins
hsa00072	Synthesis and degradation of ketone bodies	P55809
hsa00280	Valine, leucine and isoleucine degradation	P55809
hsa00480	Glutathione metabolism	O15217
hsa00562	Inositol phosphate metabolism	P48426
hsa00650	Butanoate metabolism	P55809
hsa00980	Metabolism of xenobiotics by cytochrome P450	O15217
hsa00982	Drug metabolism	O15217
hsa04070	Phosphatidylinositol signaling system	P48426
hsa04114	Oocyte meiosis	Q9Y297
hsa04120	Ubiquitin mediated proteolysis	Q9Y297,Q9BYZ6
hsa04310	Wnt signaling pathway	Q9Y297
hsa04340	Hedgehog signaling pathway	Q9Y297
hsa04810	Regulation of actin cytoskeleton	P48426
hsa05131	Shigellosis	Q9Y297
hsa05200	Pathways in cancer	Q16204
hsa05216	Thyroid cancer	Q16204

19 Pipeline configuration

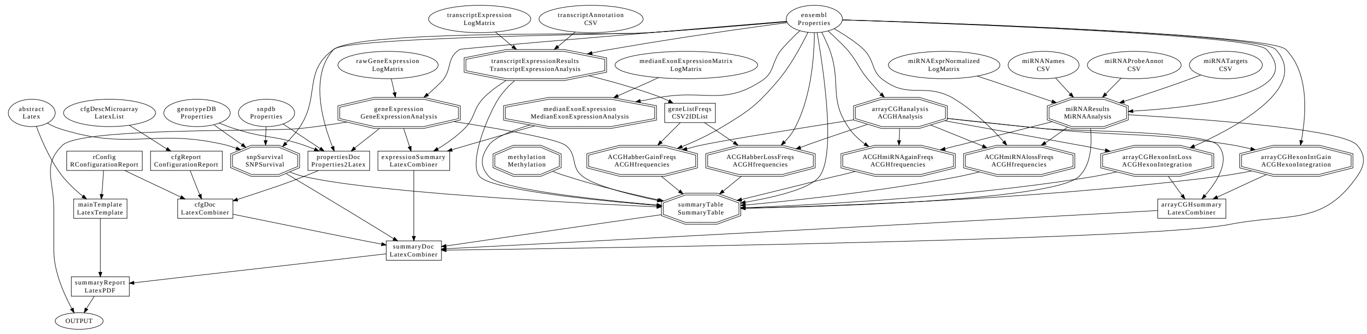

Figure 2021: Network topology.

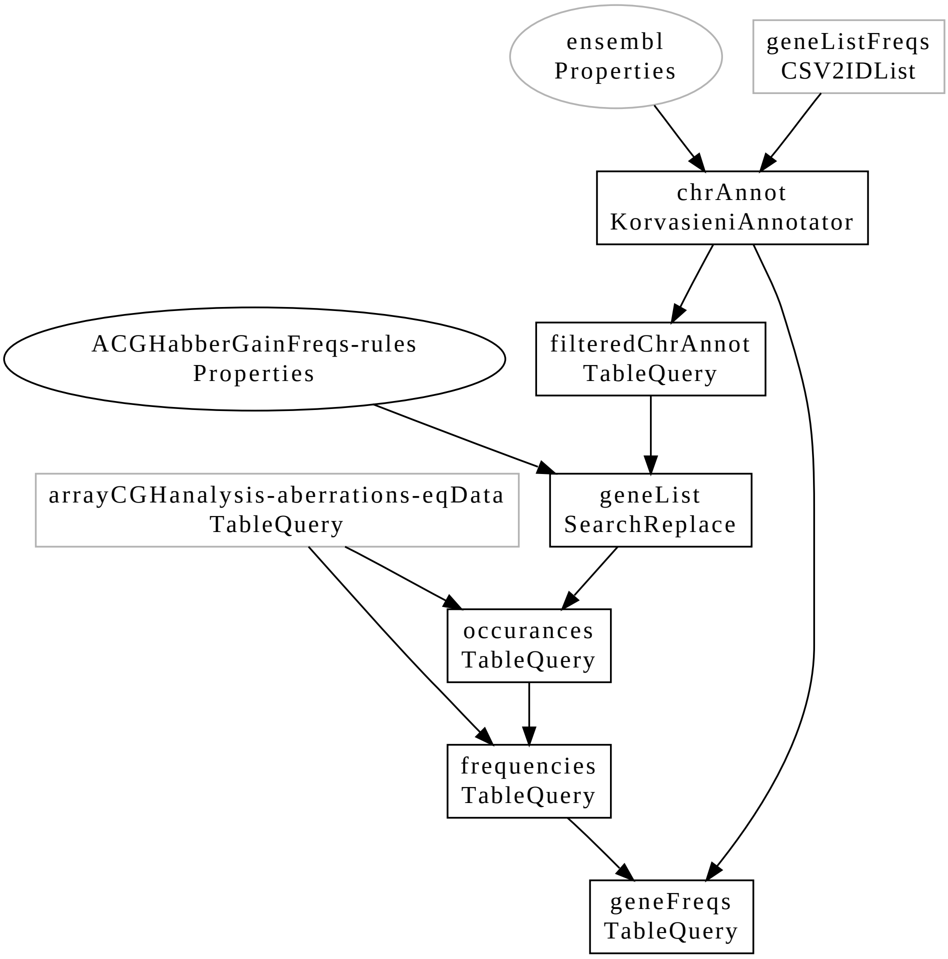

Figure 2022: Network topology for the subnetwork *ACGHabberGainFreqs*.

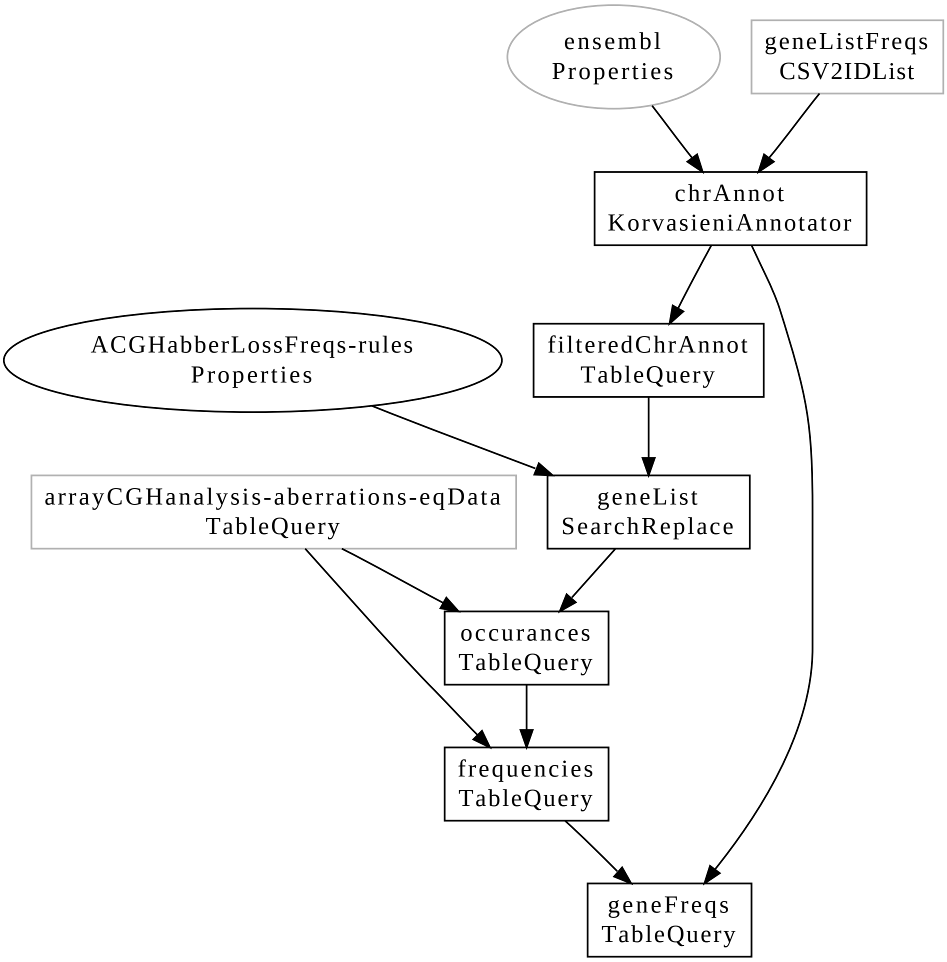

Figure 2023: Network topology for the subnetwork *ACGHabberLossFreqs*.

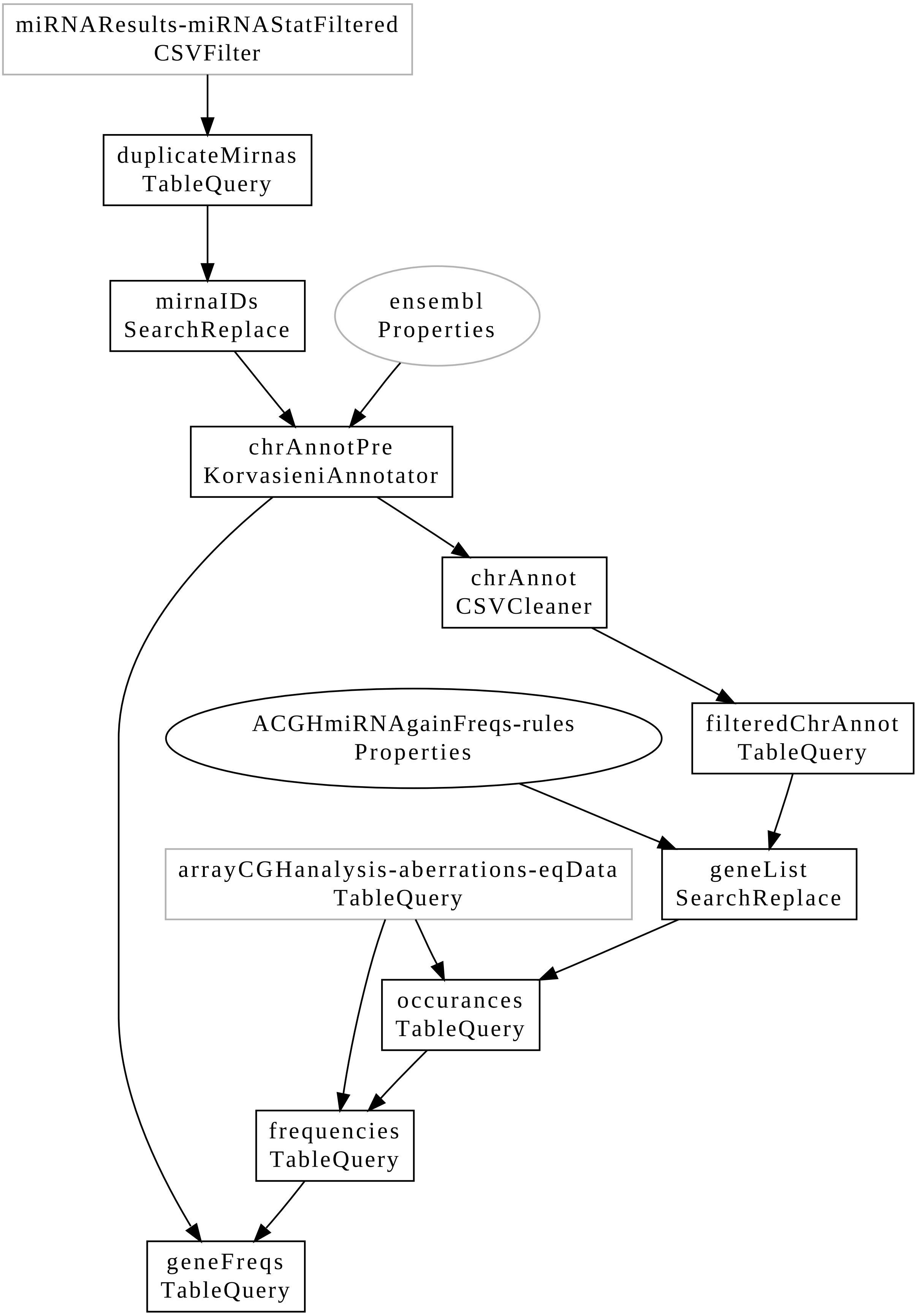

Figure 2024: Network topology for the subnetwork *ACGHmiRNAgainFreqs*.

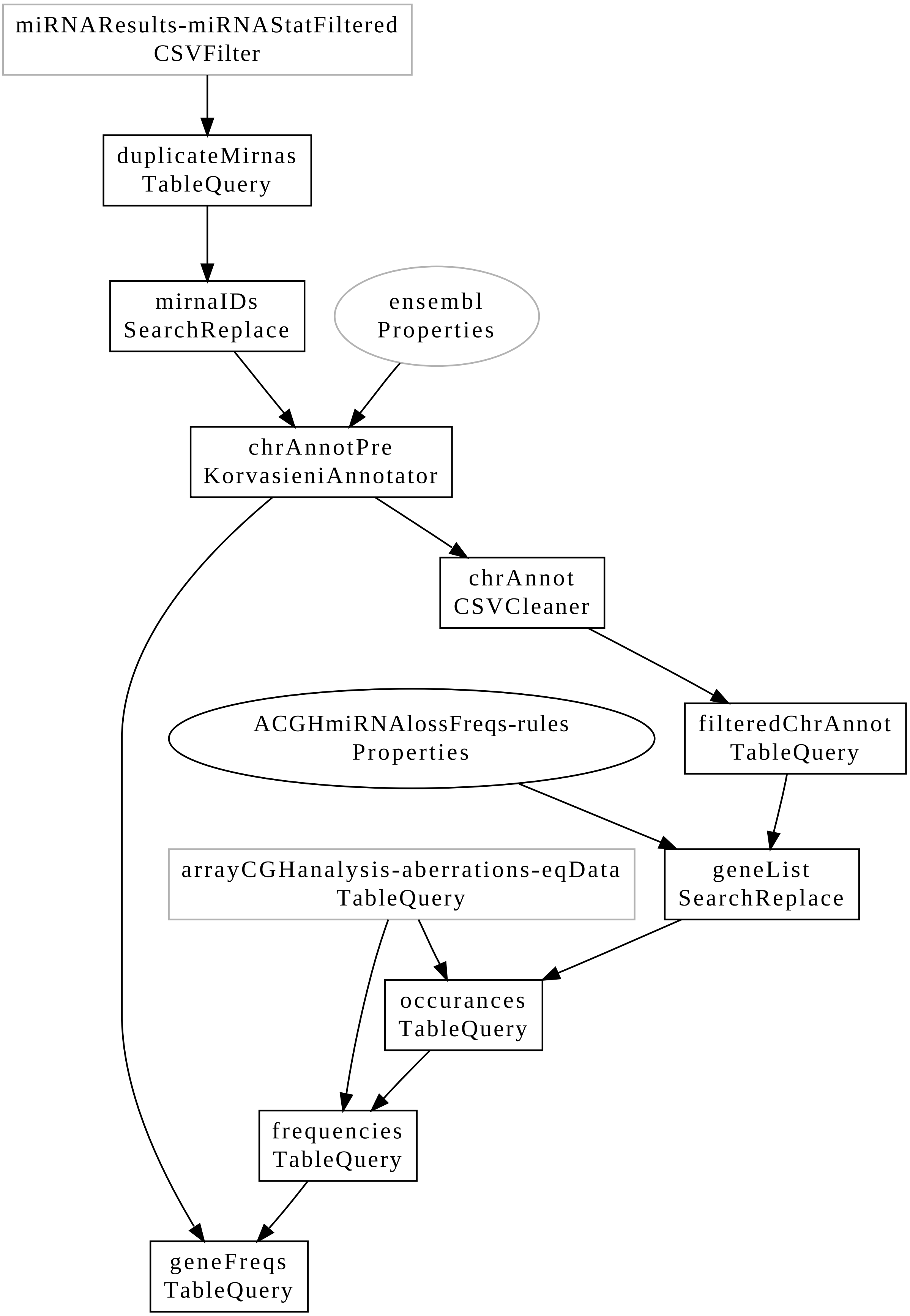

Figure 2025: Network topology for the subnetwork *ACGHmiRNAlossFreqs*.

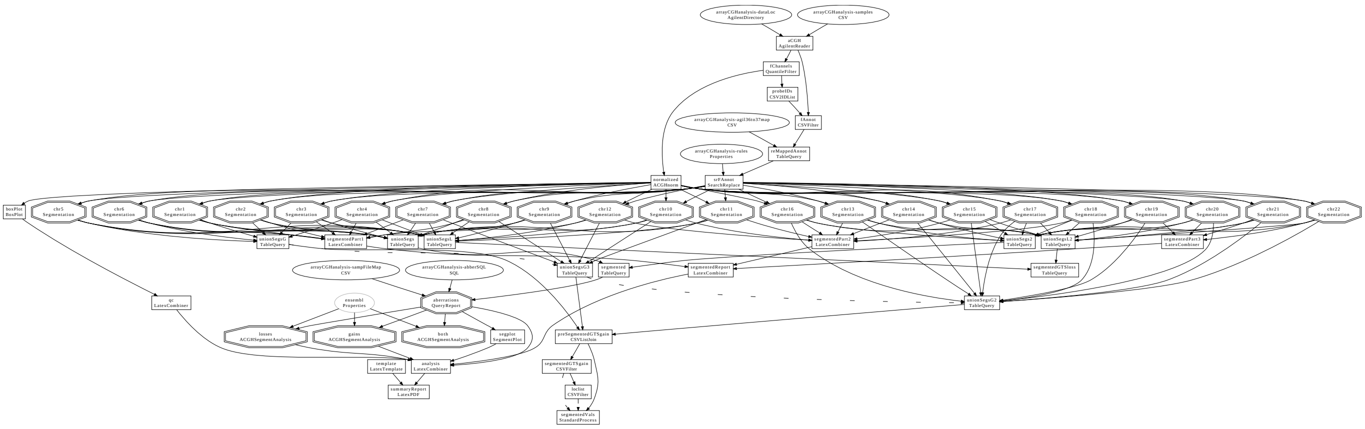

Figure 2026: Network topology for the subnetwork *arrayCGHanalysis*.

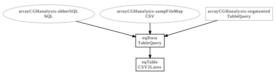

Figure 2027: Network topology for the subnetwork *arrayCGHanalysis-aberrations*.

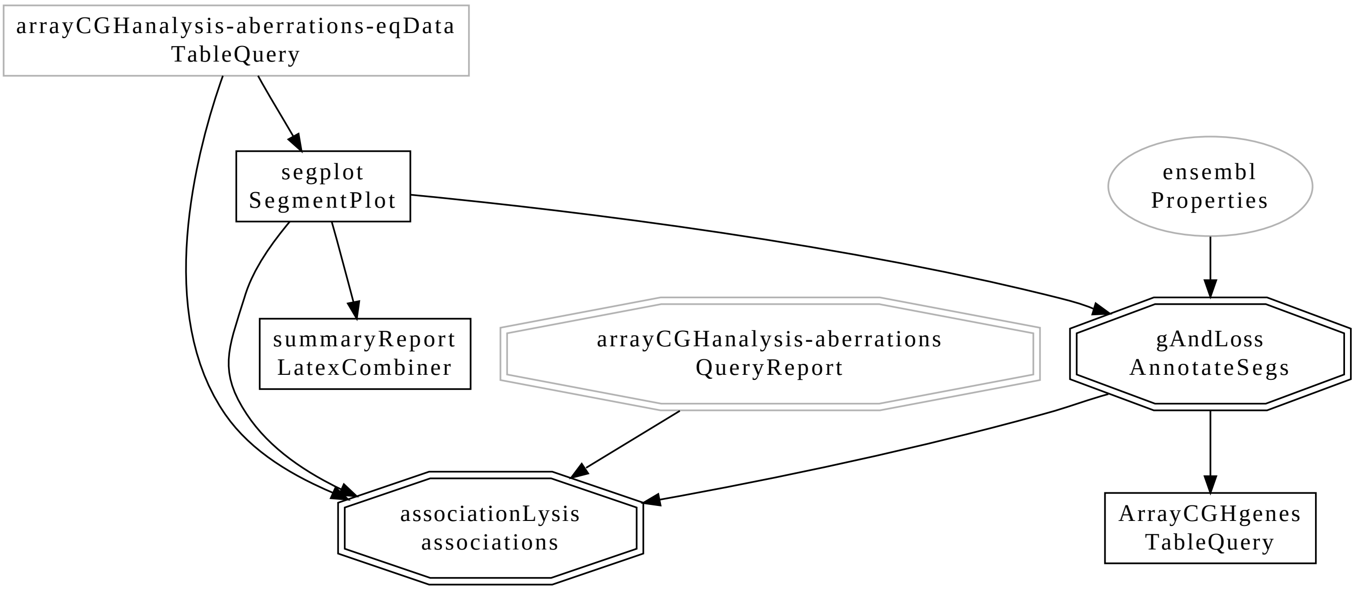

Figure 2028: Network topology for the subnetwork *arrayCGHanalysis-both*.

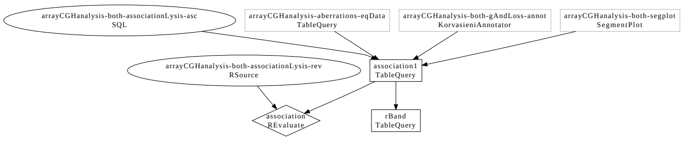

Figure 2029: Network topology for the subnetwork *arrayCGHanalysis-both-associationLysis*.

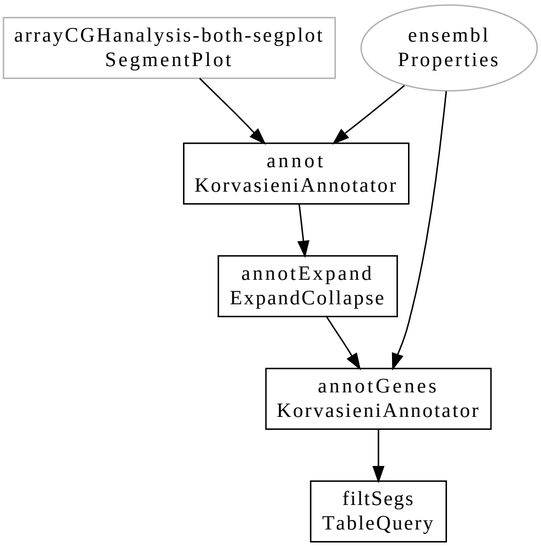

Figure 2030: Network topology for the subnetwork *arrayCGHanalysis-both-gAndLoss*.

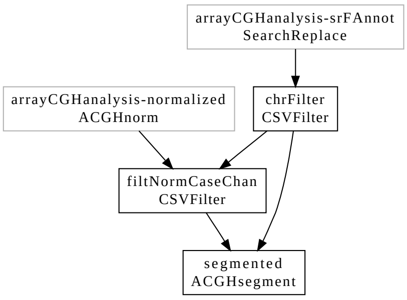

Figure 2031: Network topology for the subnetwork *arrayCGHanalysis-chr1*.

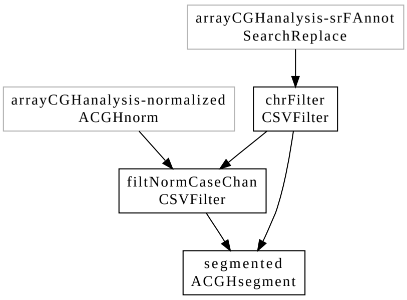

Figure 2032: Network topology for the subnetwork *arrayCGHanalysis-chr10*.

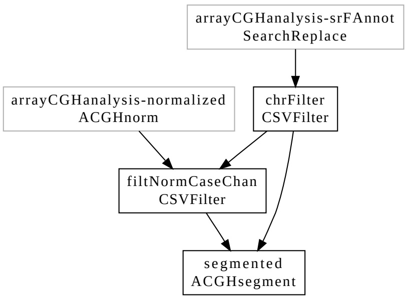

Figure 2033: Network topology for the subnetwork *arrayCGHanalysis-chr11*.

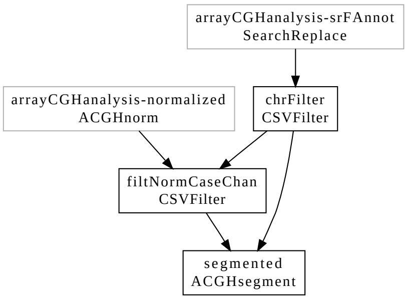

Figure 2034: Network topology for the subnetwork *arrayCGHanalysis-chr12*.

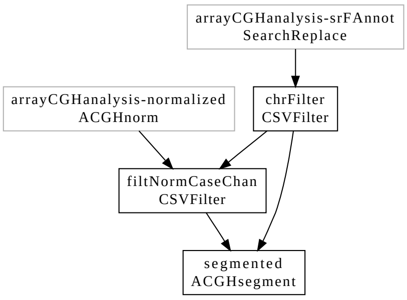

Figure 2035: Network topology for the subnetwork *arrayCGHanalysis-chr13*.

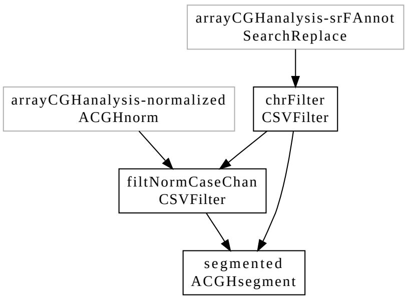

Figure 2036: Network topology for the subnetwork *arrayCGHanalysis-chr14*.

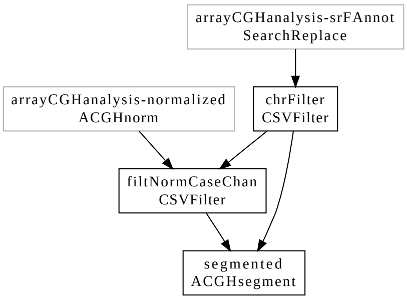

Figure 2037: Network topology for the subnetwork *arrayCGHanalysis-chr15*.

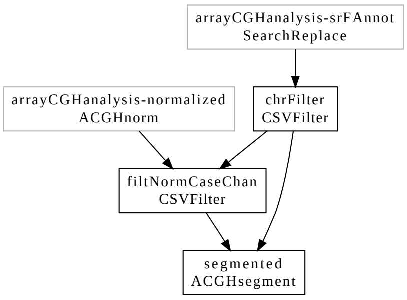

Figure 2038: Network topology for the subnetwork *arrayCGHanalysis-chr16*.

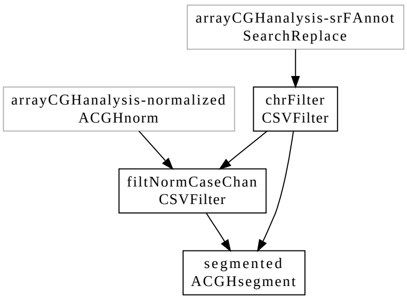

Figure 2039: Network topology for the subnetwork *arrayCGHanalysis-chr17*.

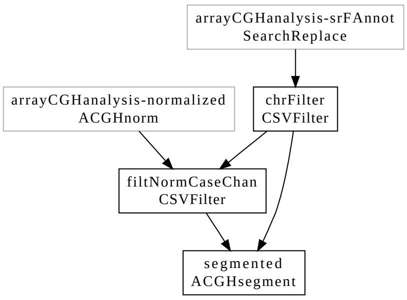

Figure 2040: Network topology for the subnetwork *arrayCGHanalysis-chr18*.

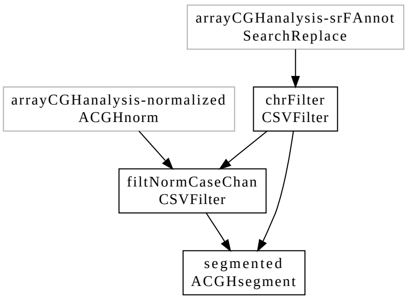

Figure 2041: Network topology for the subnetwork *arrayCGHanalysis-chr19*.

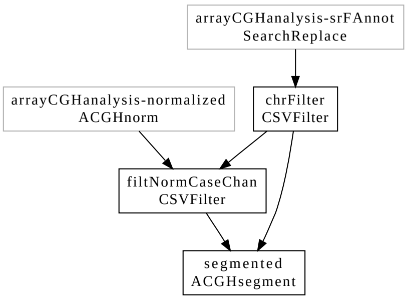

Figure 2042: Network topology for the subnetwork *arrayCGHanalysis-chr2*.

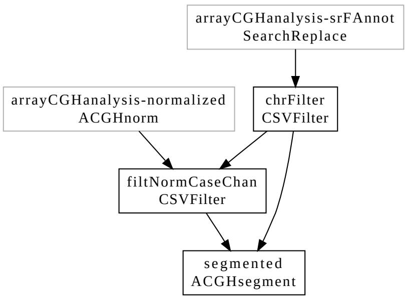

Figure 2043: Network topology for the subnetwork *arrayCGHanalysis-chr20*.

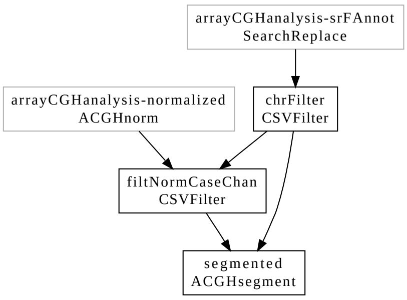

Figure 2044: Network topology for the subnetwork *arrayCGHanalysis-chr21*.

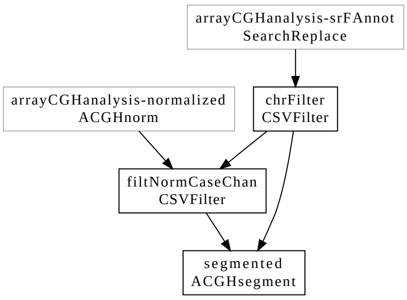

Figure 2045: Network topology for the subnetwork *arrayCGHanalysis-chr22*.

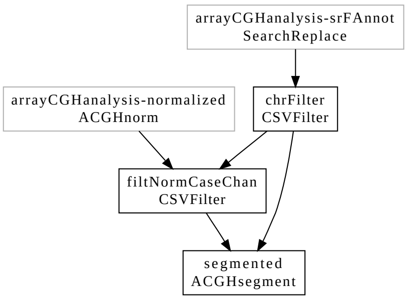

Figure 2046: Network topology for the subnetwork *arrayCGHanalysis-chr3*.

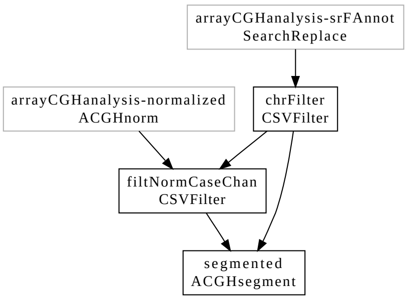

Figure 2047: Network topology for the subnetwork *arrayCGHanalysis-chr4*.

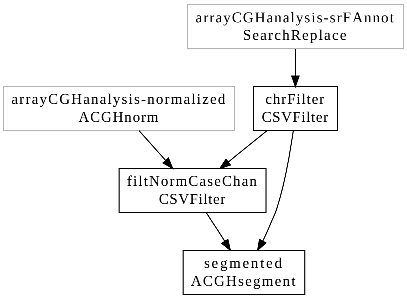

Figure 2048: Network topology for the subnetwork *arrayCGHanalysis-chr5*.

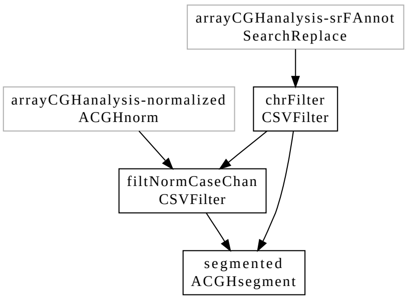

Figure 2049: Network topology for the subnetwork *arrayCGHanalysis-chr6*.

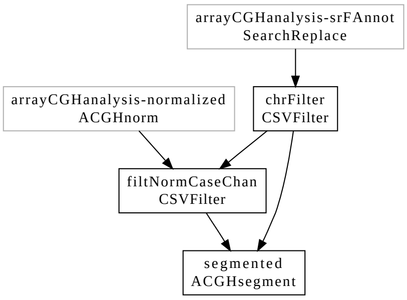

Figure 2050: Network topology for the subnetwork *arrayCGHanalysis-chr7*.

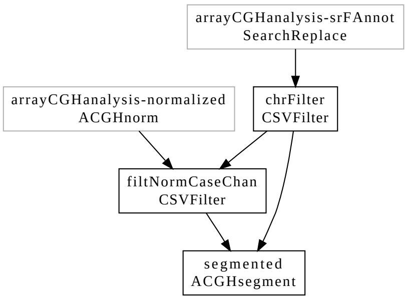

Figure 2051: Network topology for the subnetwork *arrayCGHanalysis-chr8*.

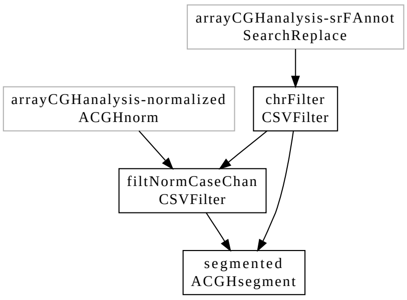

Figure 2052: Network topology for the subnetwork *arrayCGHanalysis-chr9*.

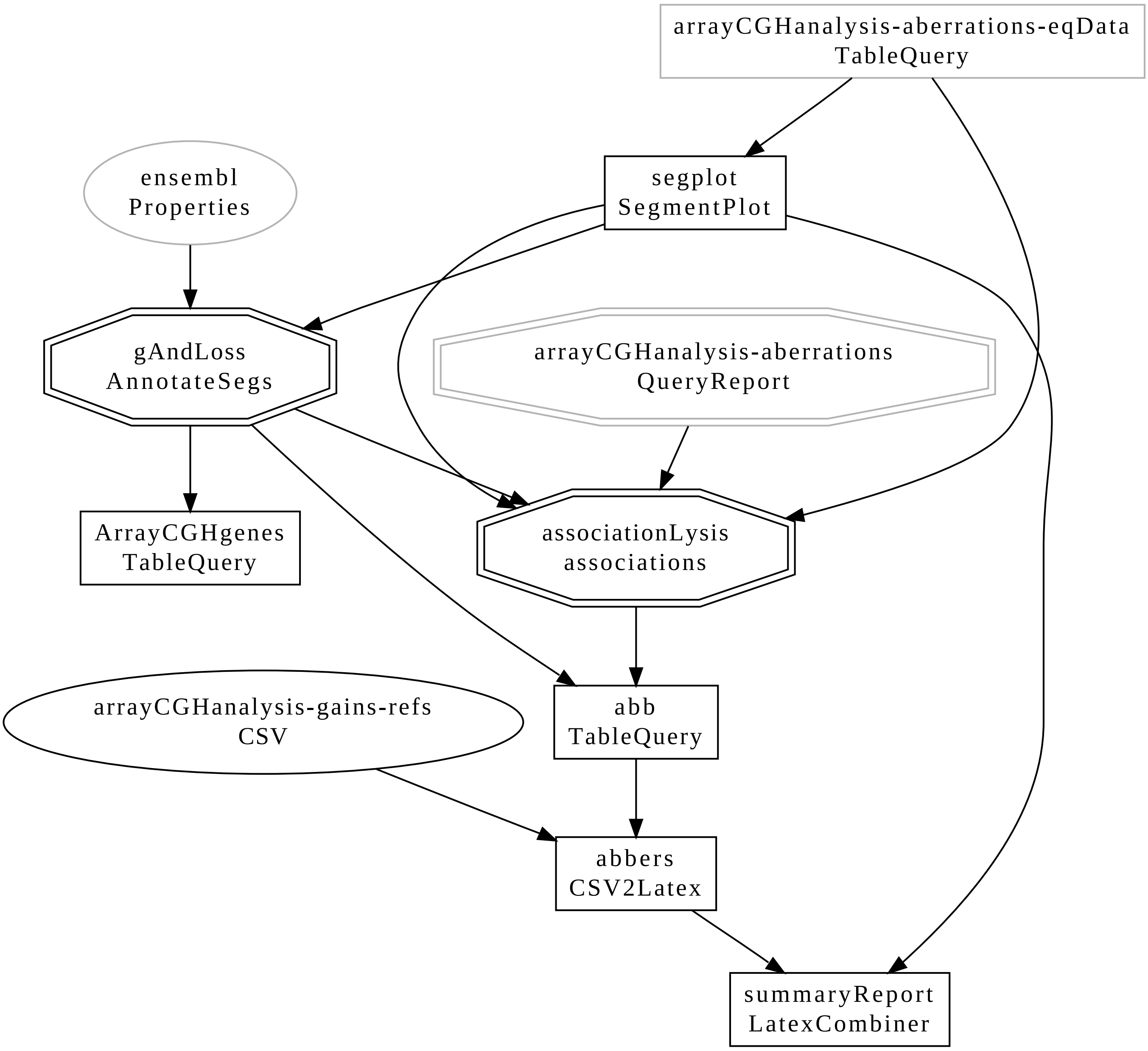

Figure 2053: Network topology for the subnetwork *arrayCGHanalysis-gains*.

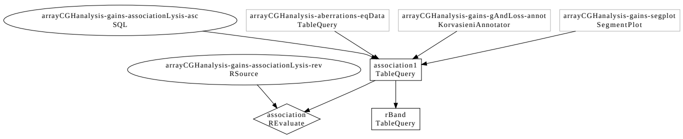

Figure 2054: Network topology for the subnetwork *arrayCGHanalysis-gains-associationLysis*.

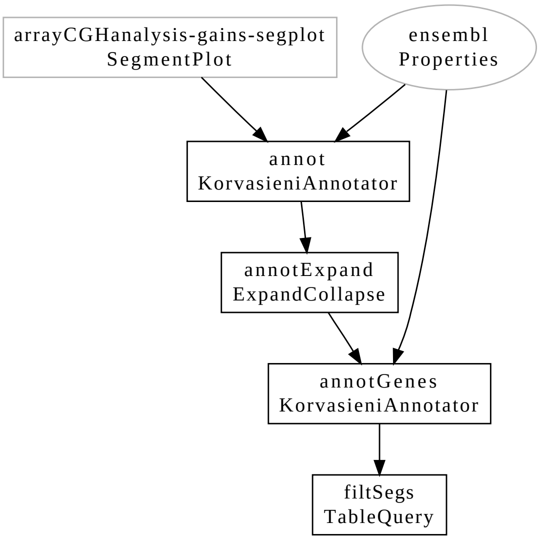

Figure 2055: Network topology for the subnetwork *arrayCGHanalysis-gains-gAndLoss*.

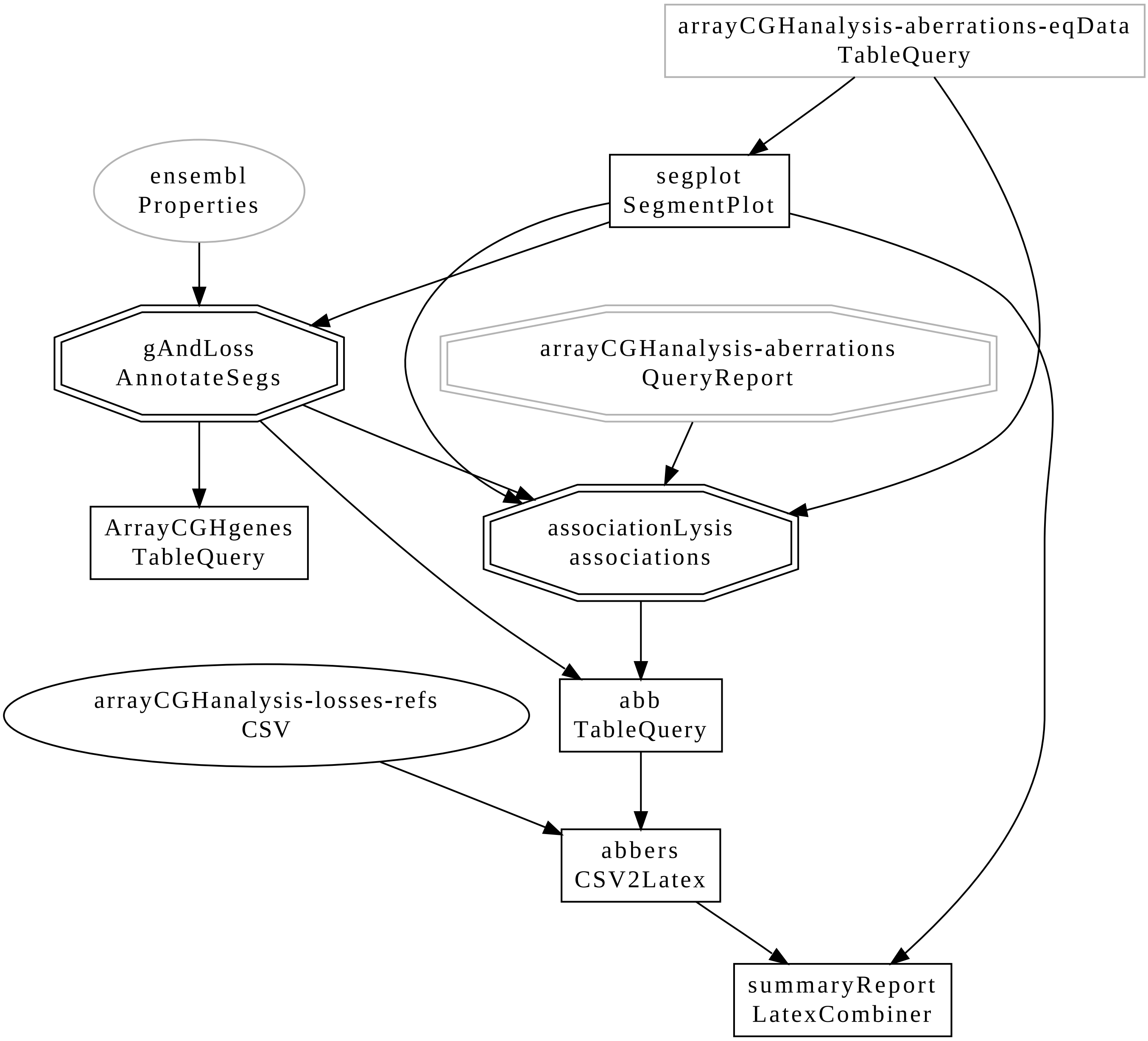

Figure 2056: Network topology for the subnetwork *arrayCGHanalysis-losses*.

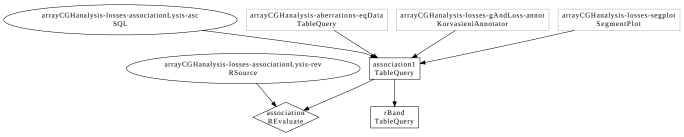

Figure 2057: Network topology for the subnetwork *arrayCGHanalysis-losses-associationLysis*.

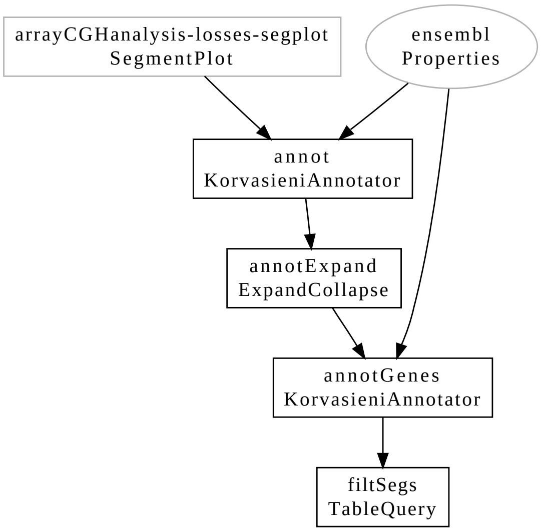

Figure 2058: Network topology for the subnetwork *arrayCGHanalysis-losses-gAndLoss*.

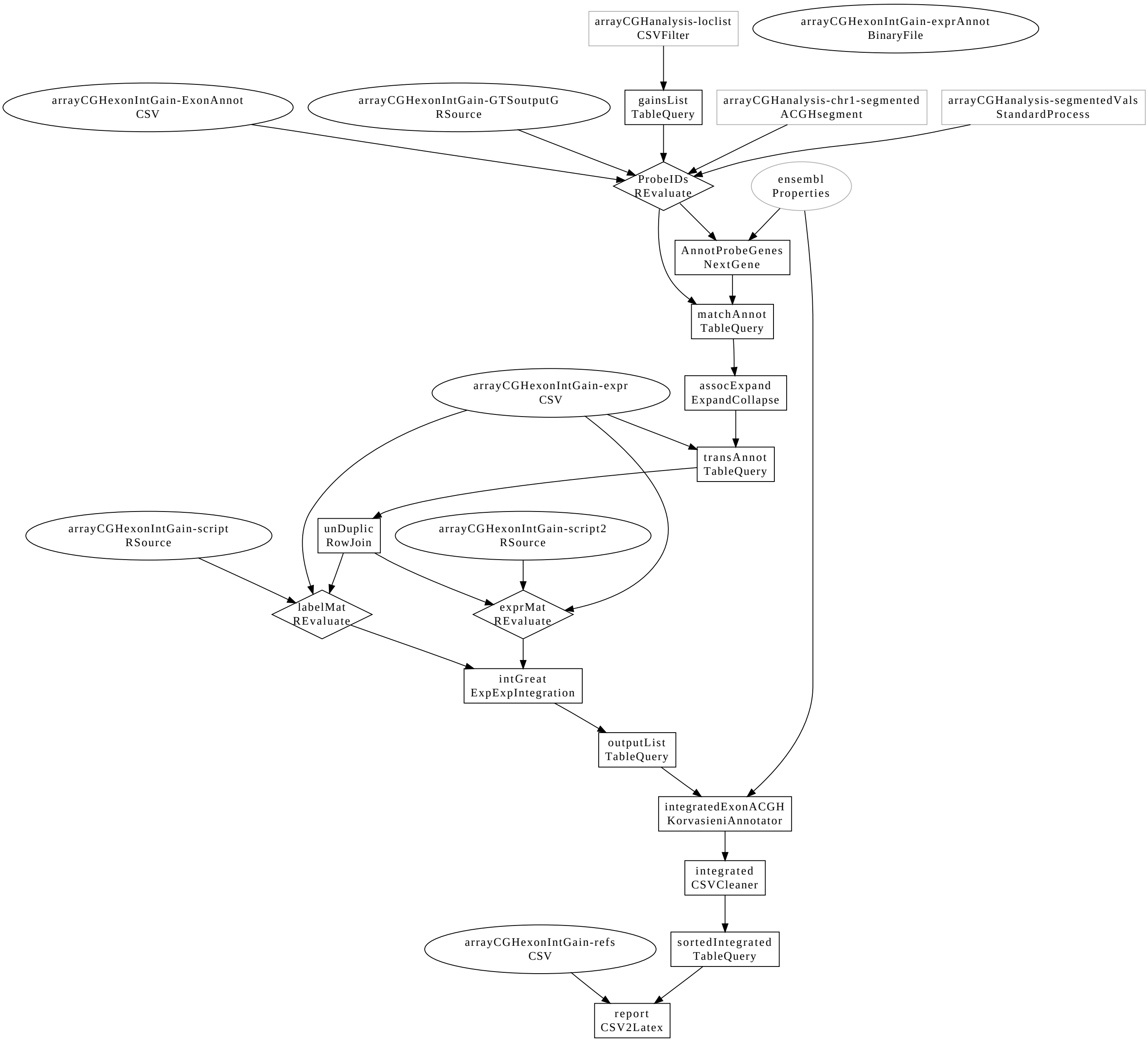

Figure 2059: Network topology for the subnetwork *arrayCGHexonIntGain*.

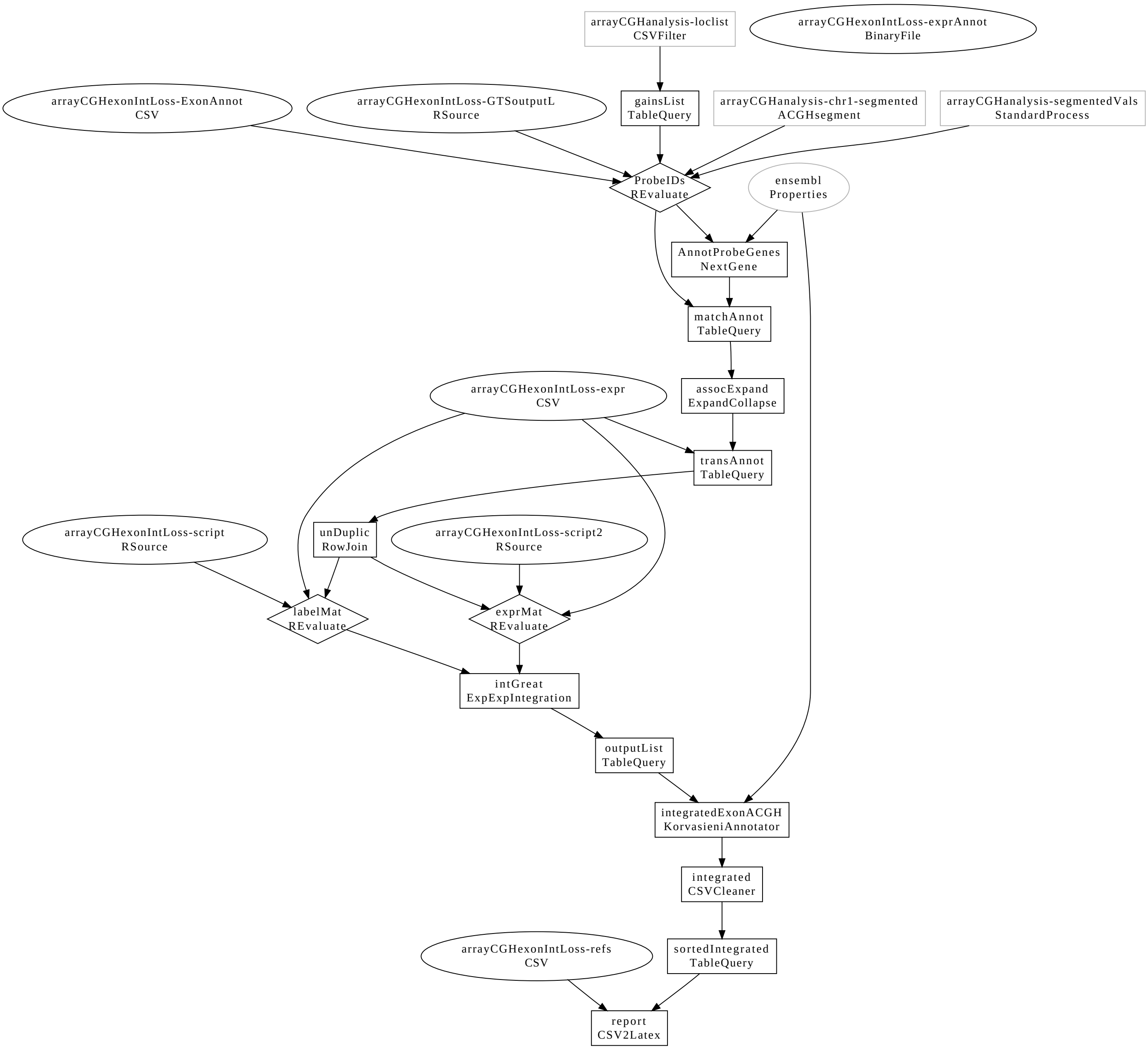

Figure 2060: Network topology for the subnetwork *arrayCGHexonIntLoss*.

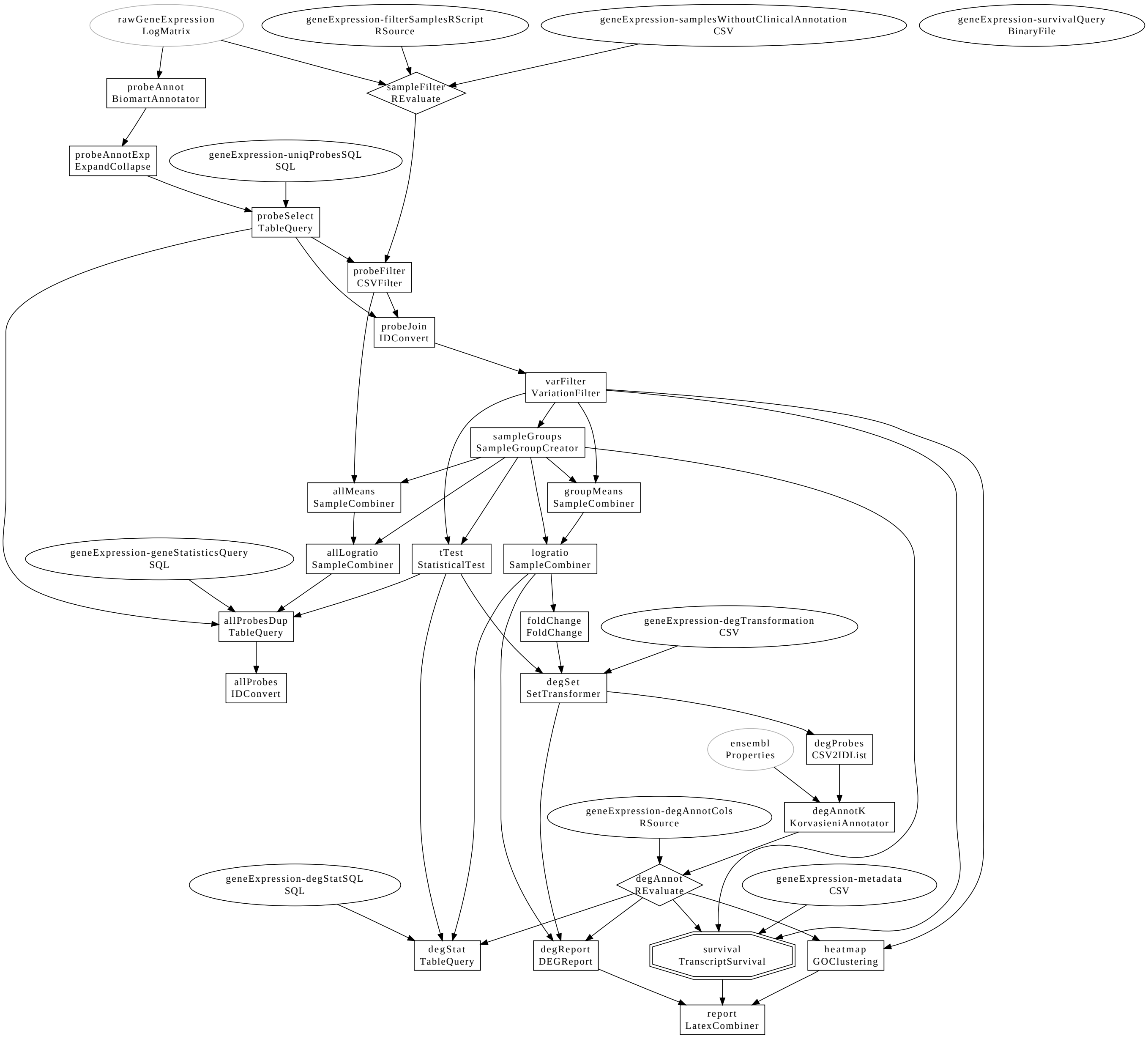

Figure 2061: Network topology for the subnetwork *geneExpression*.

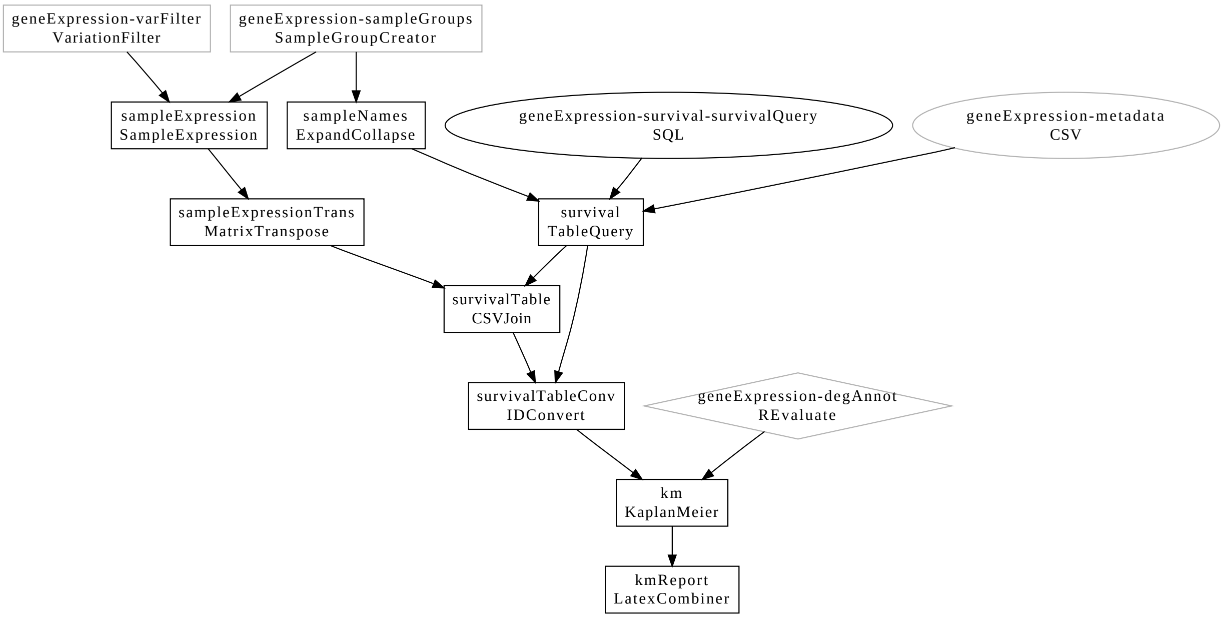

Figure 2062: Network topology for the subnetwork *geneExpression-survival*.

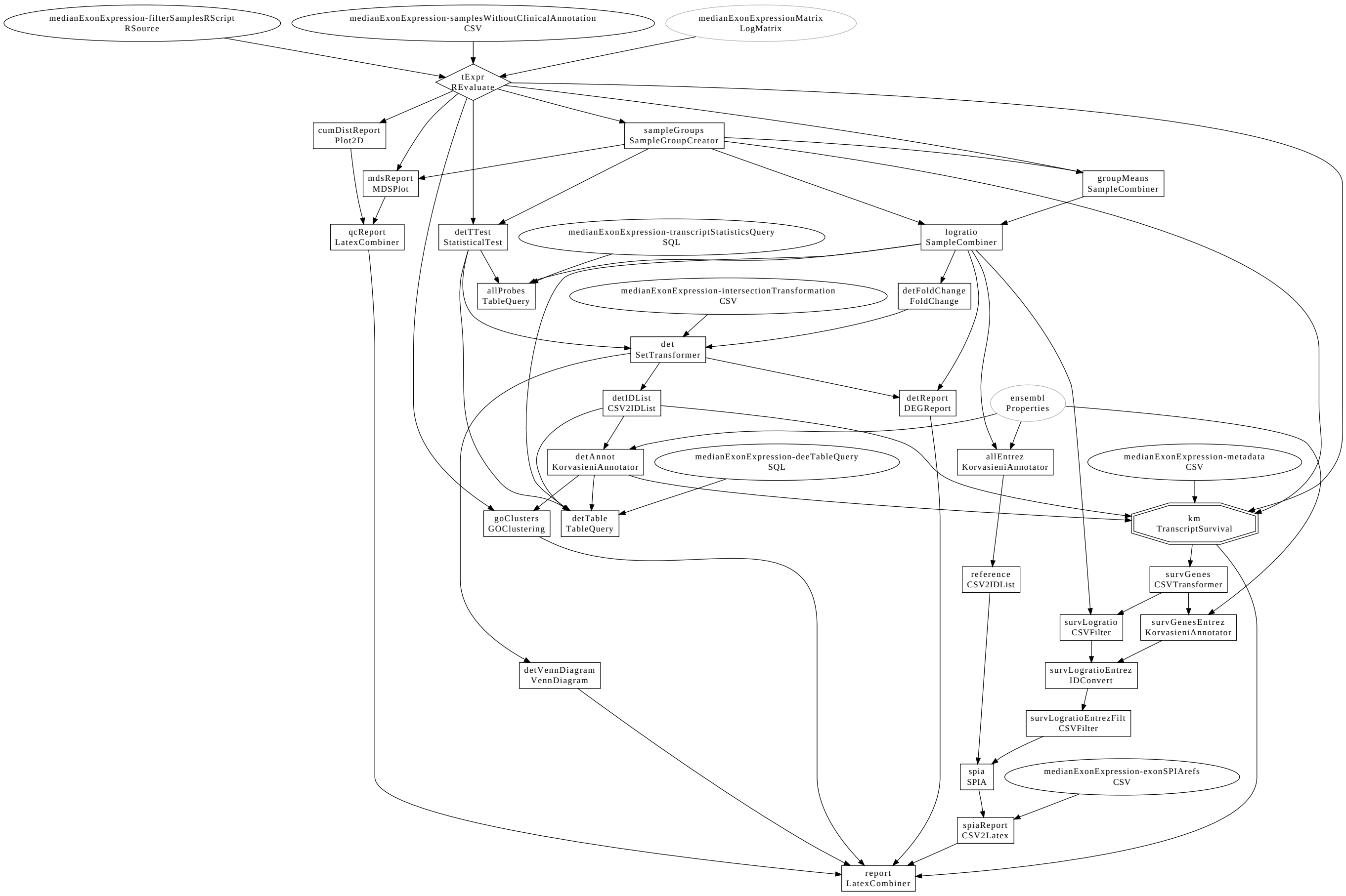

Figure 2063: Network topology for the subnetwork *medianExonExpression*.

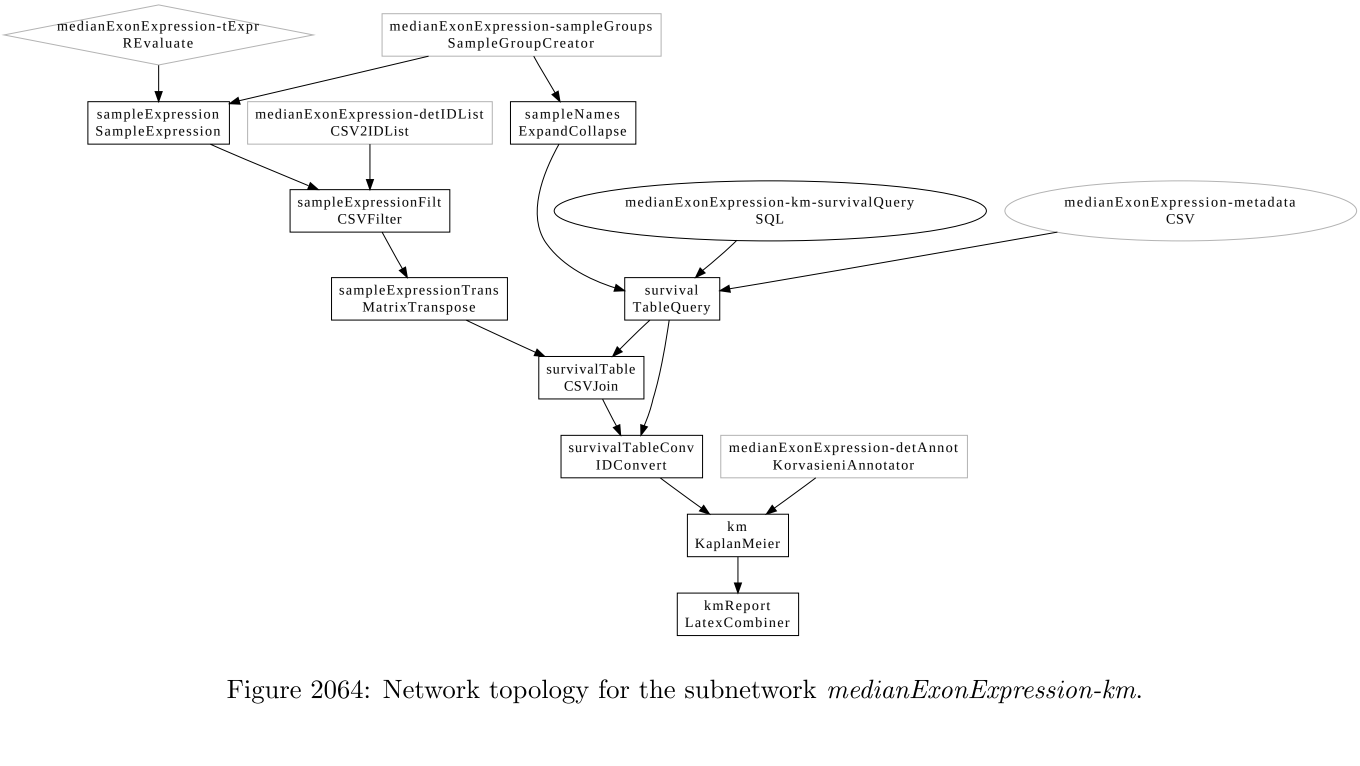

Figure 2064: Network topology for the subnetwork *medianExonExpression-km*.

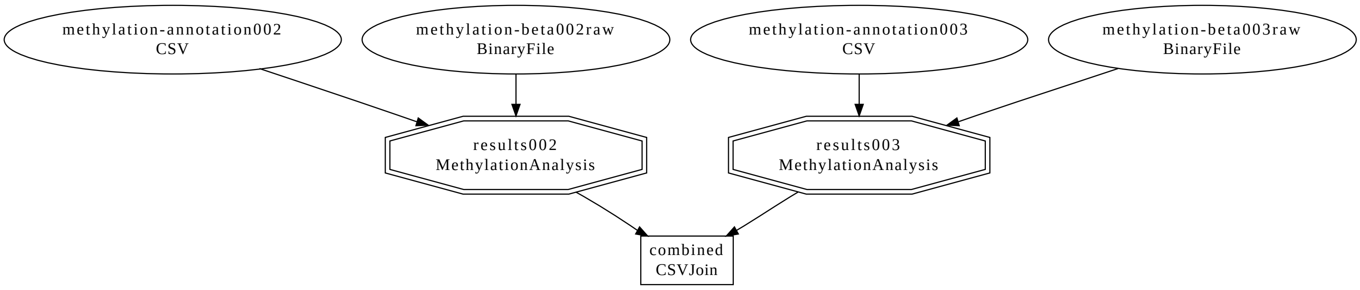

Figure 2065: Network topology for the subnetwork *methylation*.

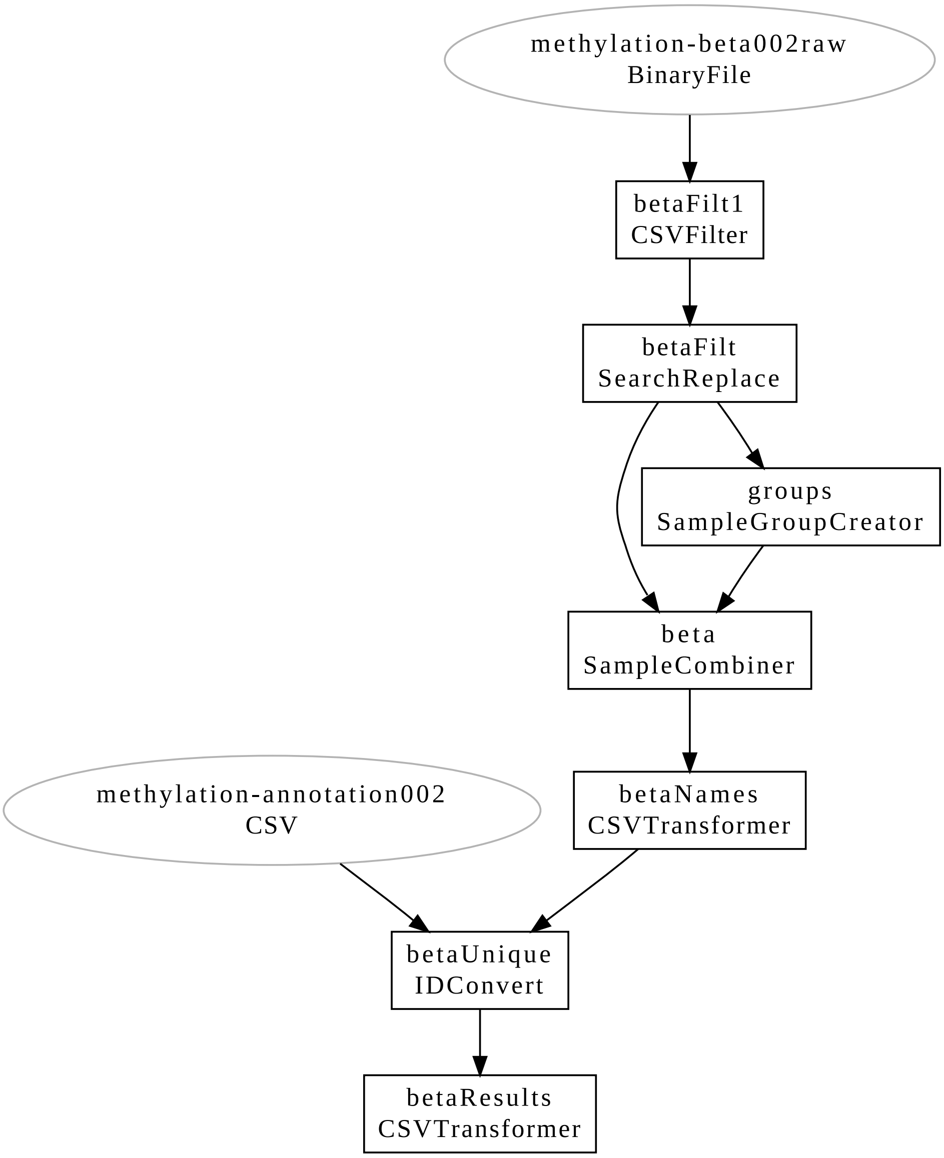

Figure 2066: Network topology for the subnetwork *methylation-results002*.

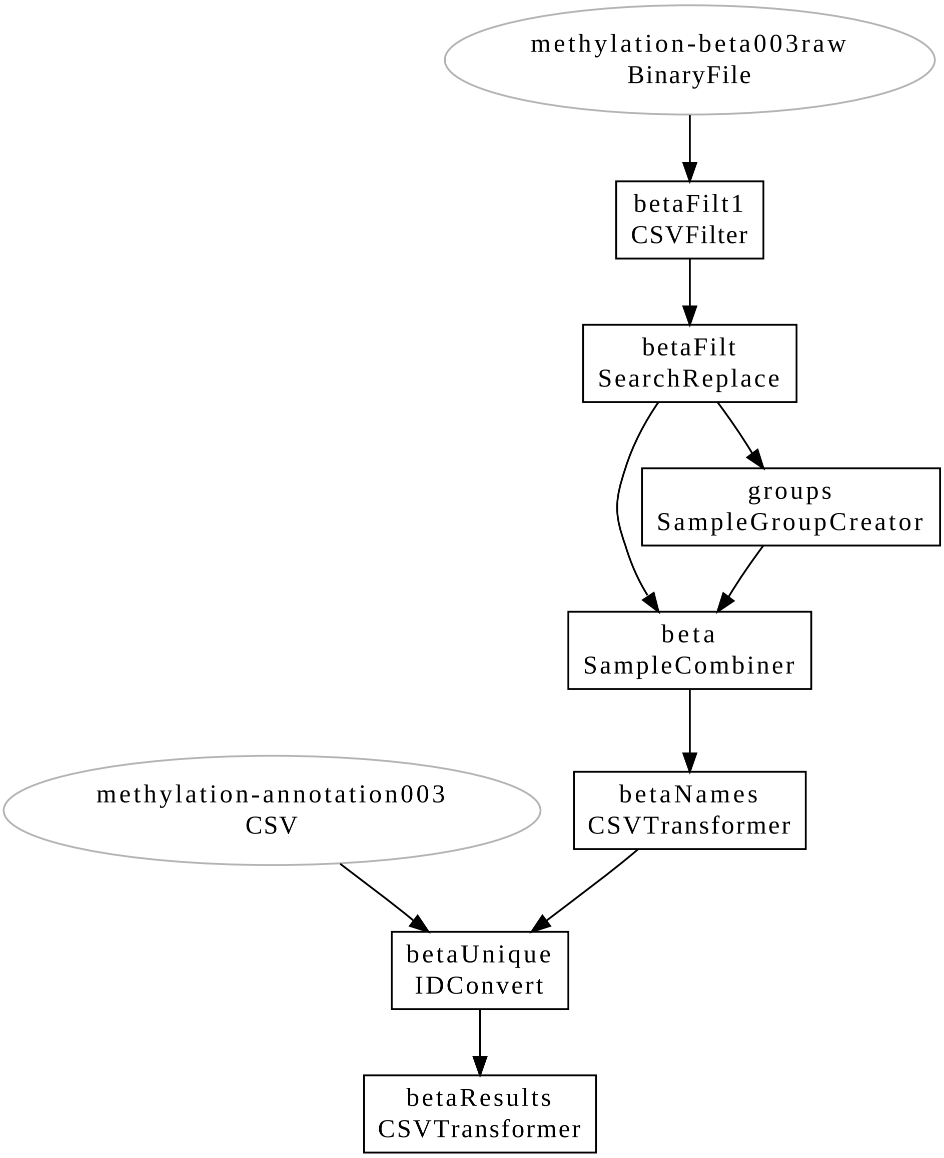

Figure 2067: Network topology for the subnetwork *methylation-results003*.

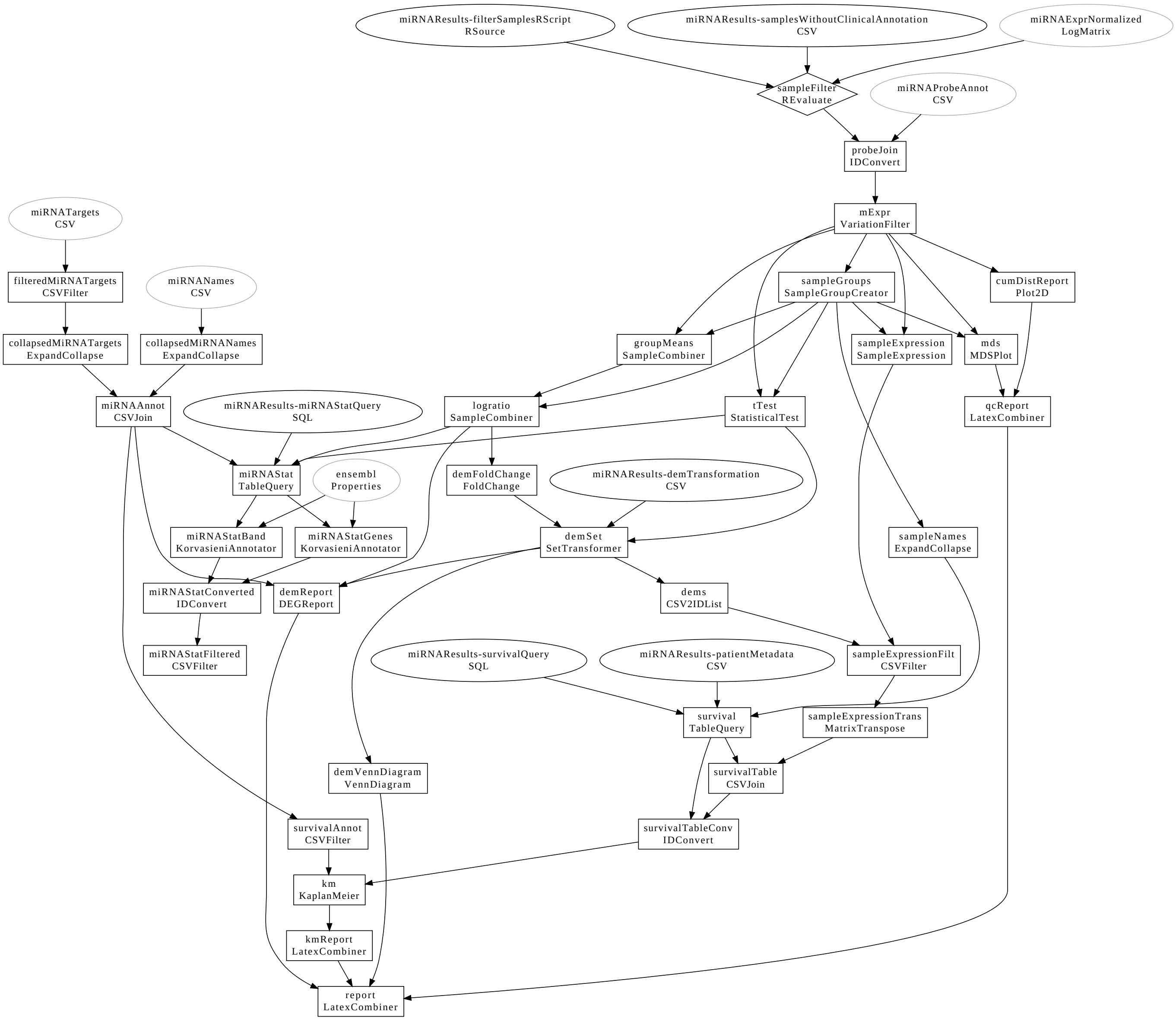

Figure 2068: Network topology for the subnetwork *miRNAResults*.

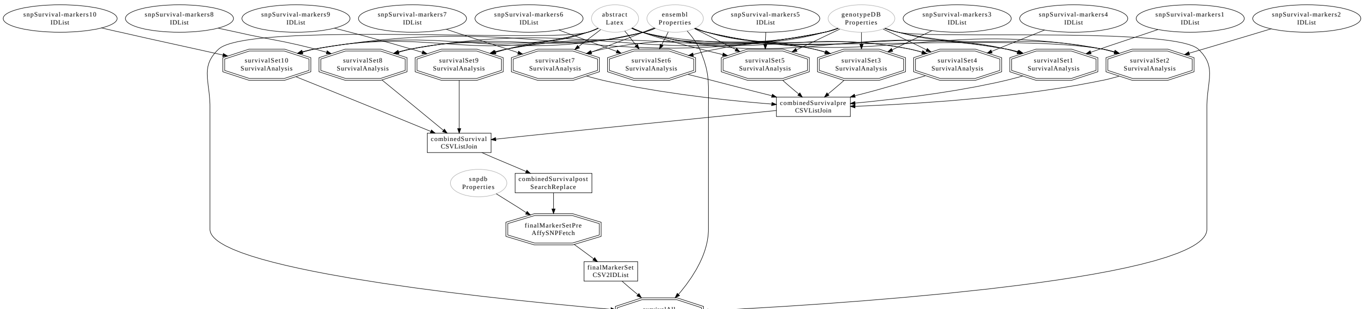

Figure 2069: Network topology for the subnetwork *snpSurvival*.

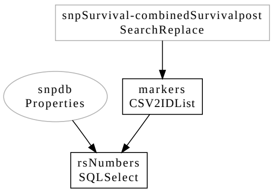

Figure 2070: Network topology for the subnetwork *snpSurvival-finalMarkerSetPre*.

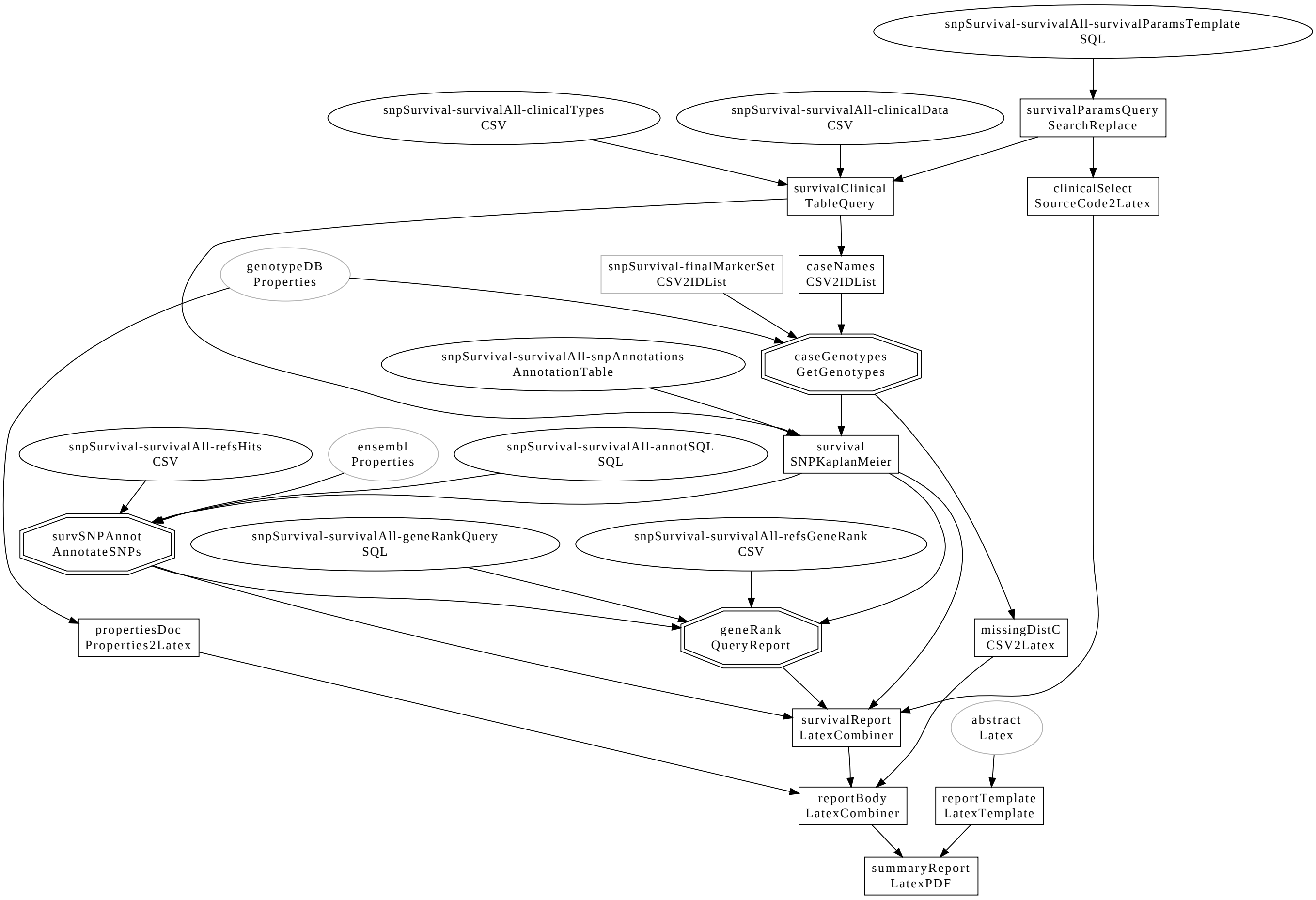

Figure 2071: Network topology for the subnetwork *snpSurvival-survivalAll*.

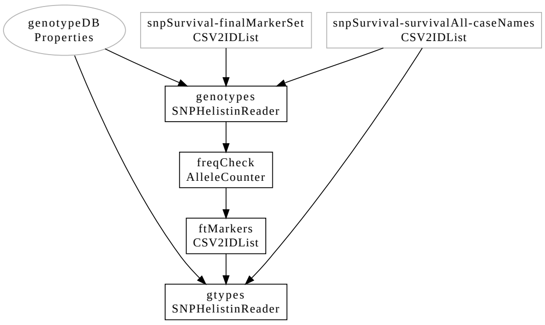

Figure 2072: Network topology for the subnetwork *snpSurvival-survivalAll-caseGenotypes*.

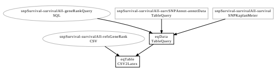

Figure 2073: Network topology for the subnetwork *snpSurvival-survivalAll-geneRank*.

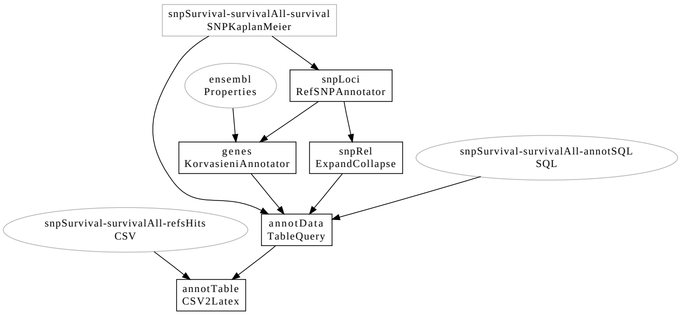

Figure 2074: Network topology for the subnetwork *snpSurvival-survivalAll-survSNPAnnot*.

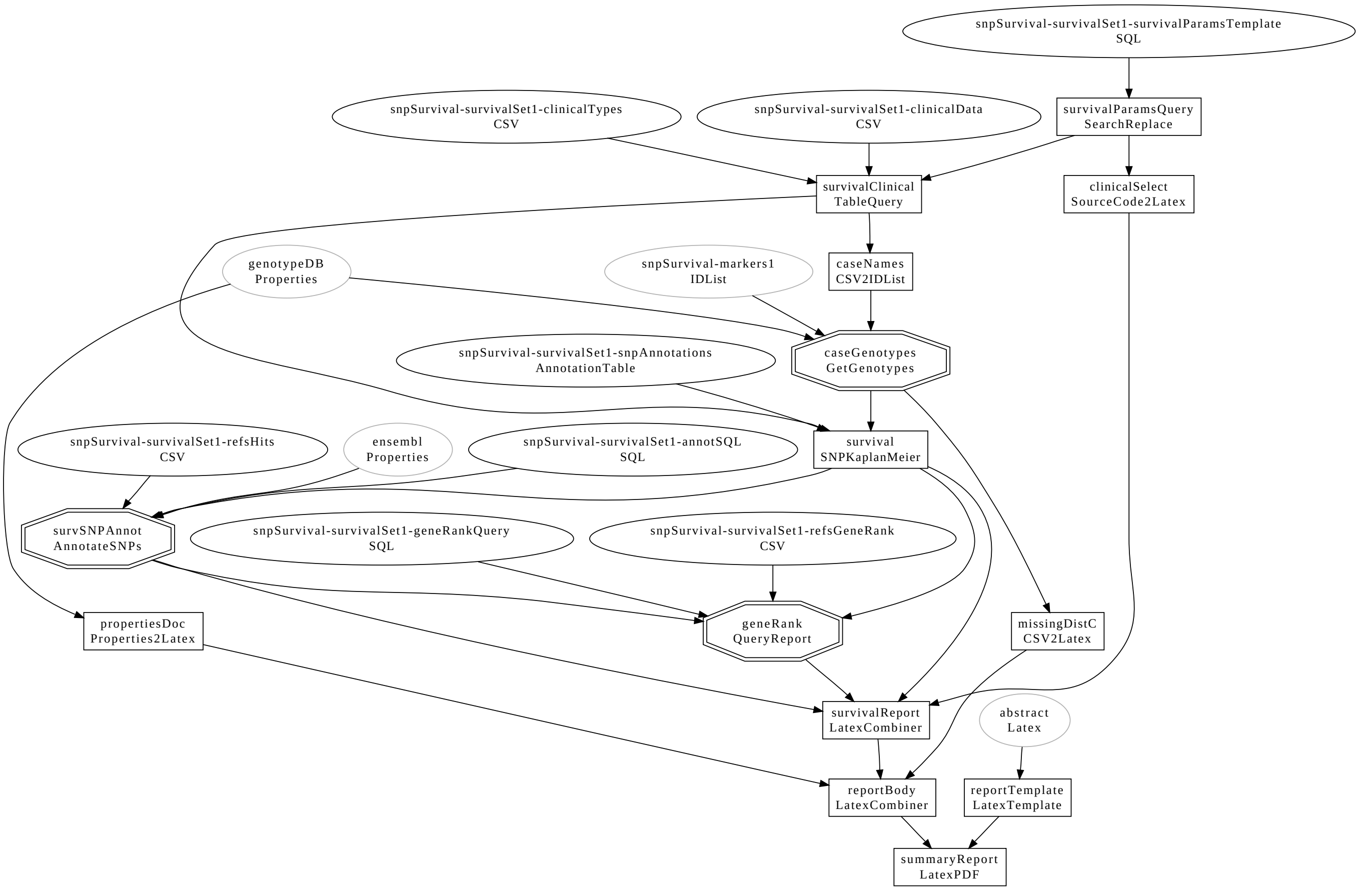

Figure 2075: Network topology for the subnetwork *snpSurvival-survivalSet1*.

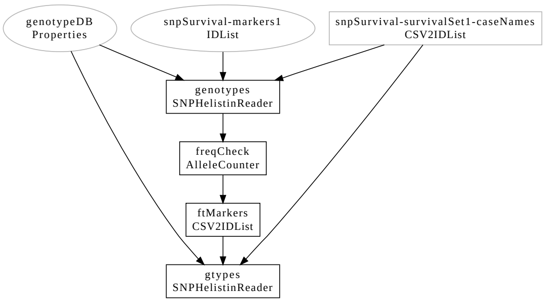

Figure 2076: Network topology for the subnetwork *snpSurvival-survivalSet1-caseGenotypes*.

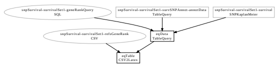

Figure 2077: Network topology for the subnetwork *snpSurvival-survivalSet1-geneRank*.

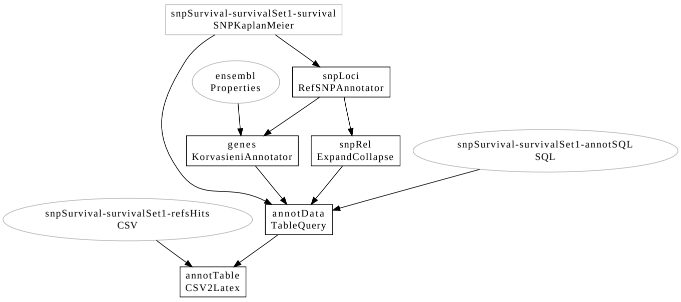

Figure 2078: Network topology for the subnetwork *snpSurvival-survivalSet1-survSNPAnnot*.

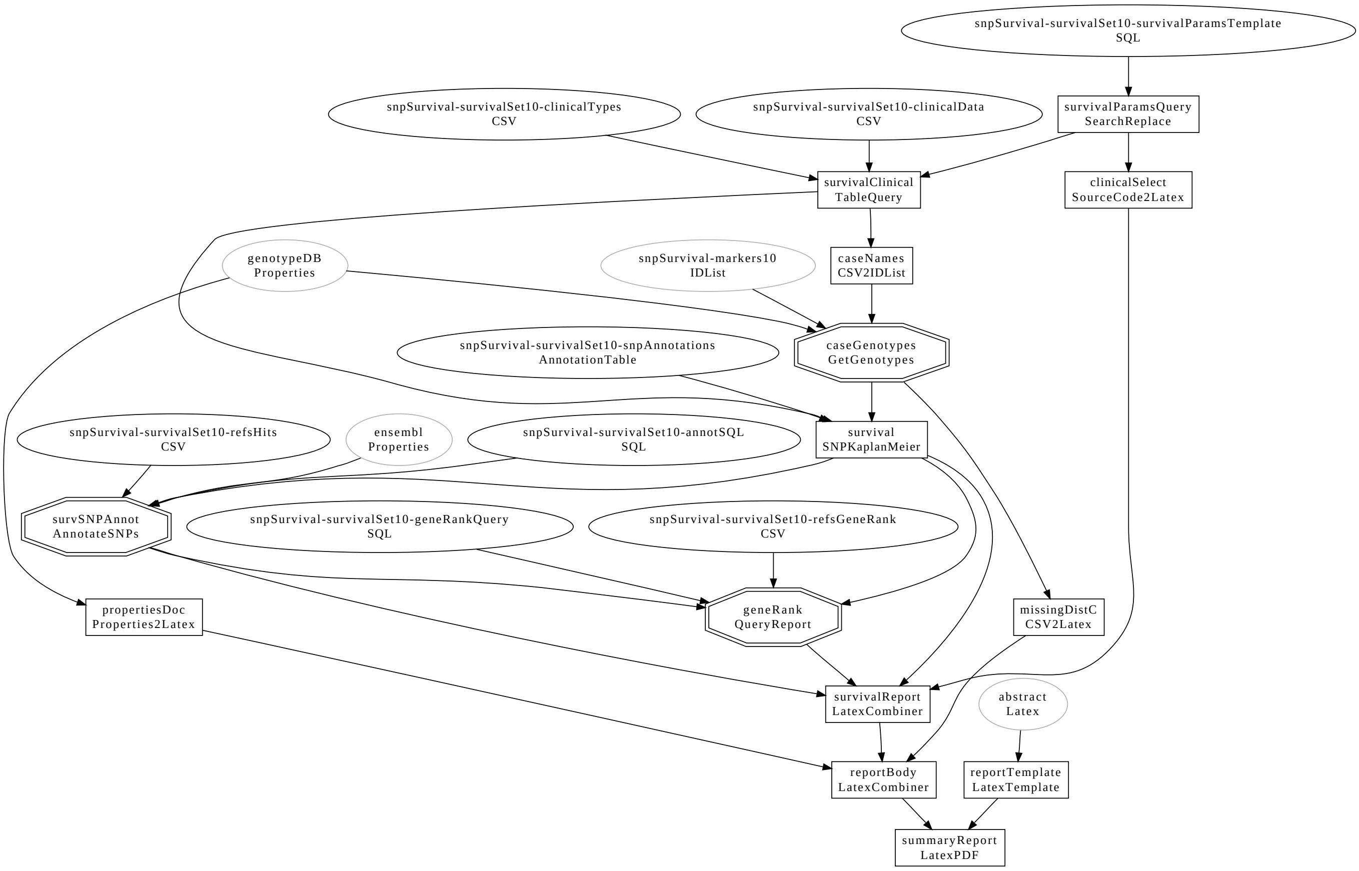

Figure 2079: Network topology for the subnetwork *snpSurvival-survivalSet10*.

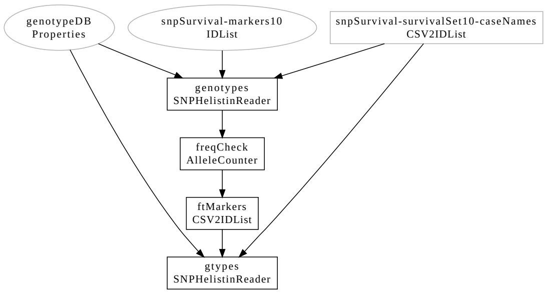

Figure 2080: Network topology for the subnetwork *snpSurvival-survivalSet10-caseGenotypes*.

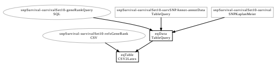

Figure 2081: Network topology for the subnetwork *snpSurvival-survivalSet10-geneRank*.

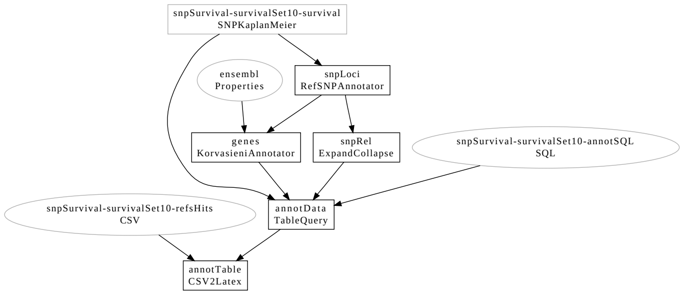

Figure 2082: Network topology for the subnetwork *snpSurvival-survivalSet10-survSNPAnnot*.

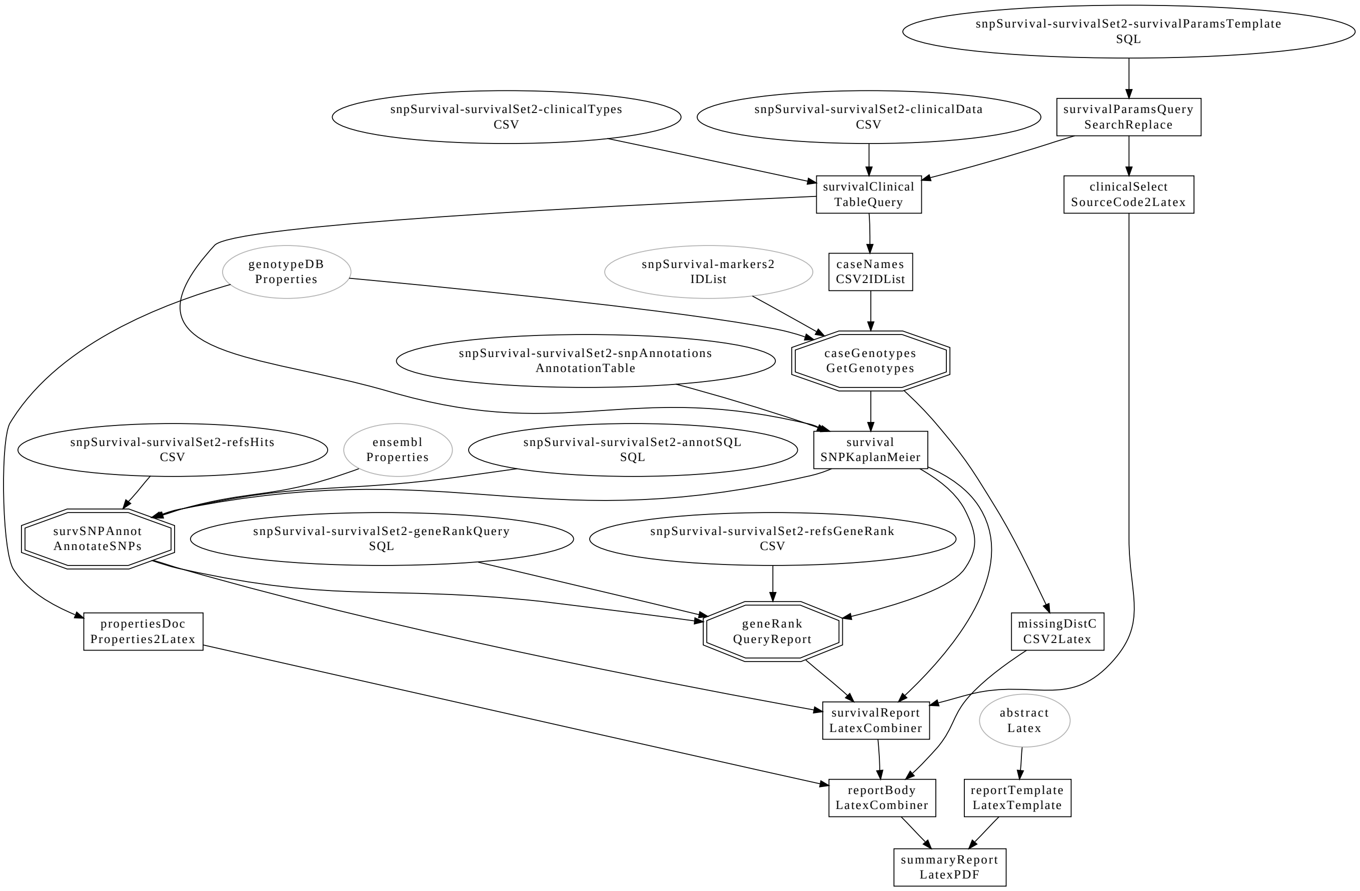

Figure 2083: Network topology for the subnetwork *snpSurvival-survivalSet2*.

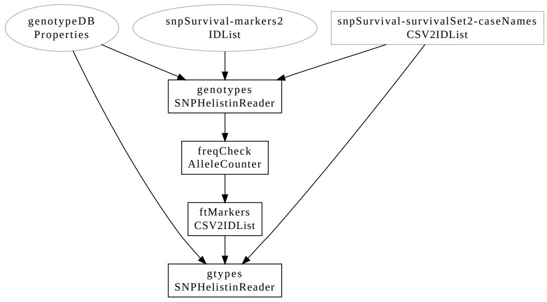

Figure 2084: Network topology for the subnetwork *snpSurvival-survivalSet2-caseGenotypes*.

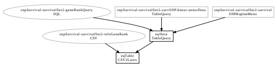

Figure 2085: Network topology for the subnetwork *snpSurvival-survivalSet2-geneRank*.

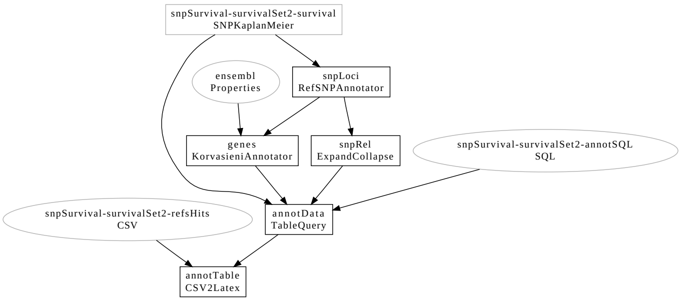

Figure 2086: Network topology for the subnetwork *snpSurvival-survivalSet2-survSNPAnnot*.

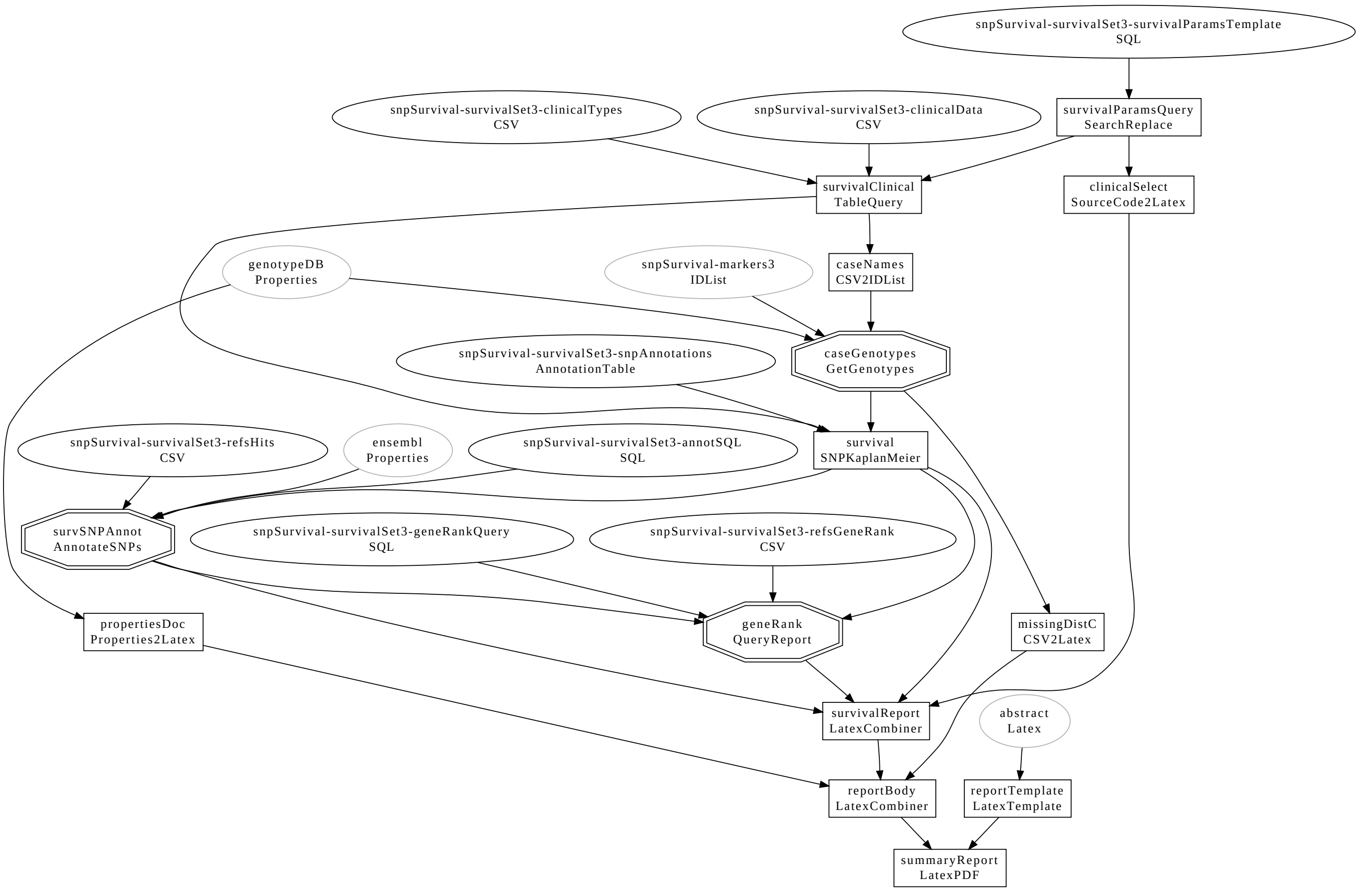

Figure 2087: Network topology for the subnetwork *snpSurvival-survivalSet3*.

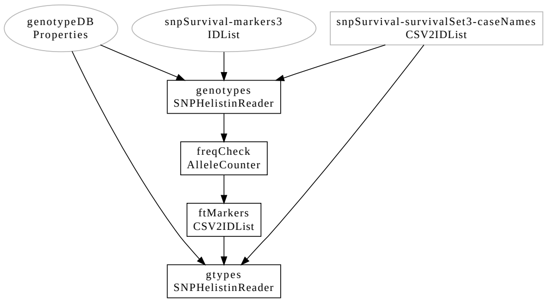

Figure 2088: Network topology for the subnetwork *snpSurvival-survivalSet3-caseGenotypes*.

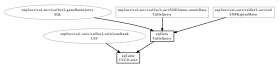

Figure 2089: Network topology for the subnetwork *snpSurvival-survivalSet3-geneRank*.

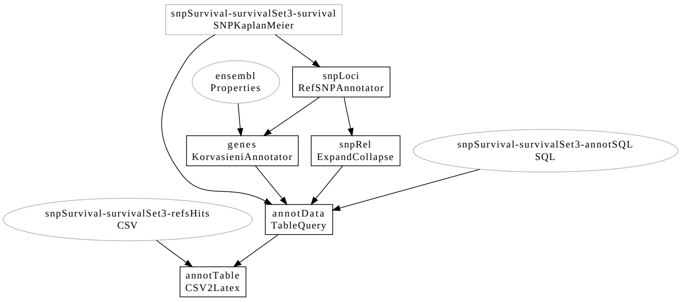

Figure 2090: Network topology for the subnetwork *snpSurvival-survivalSet3-survSNPAnnot*.

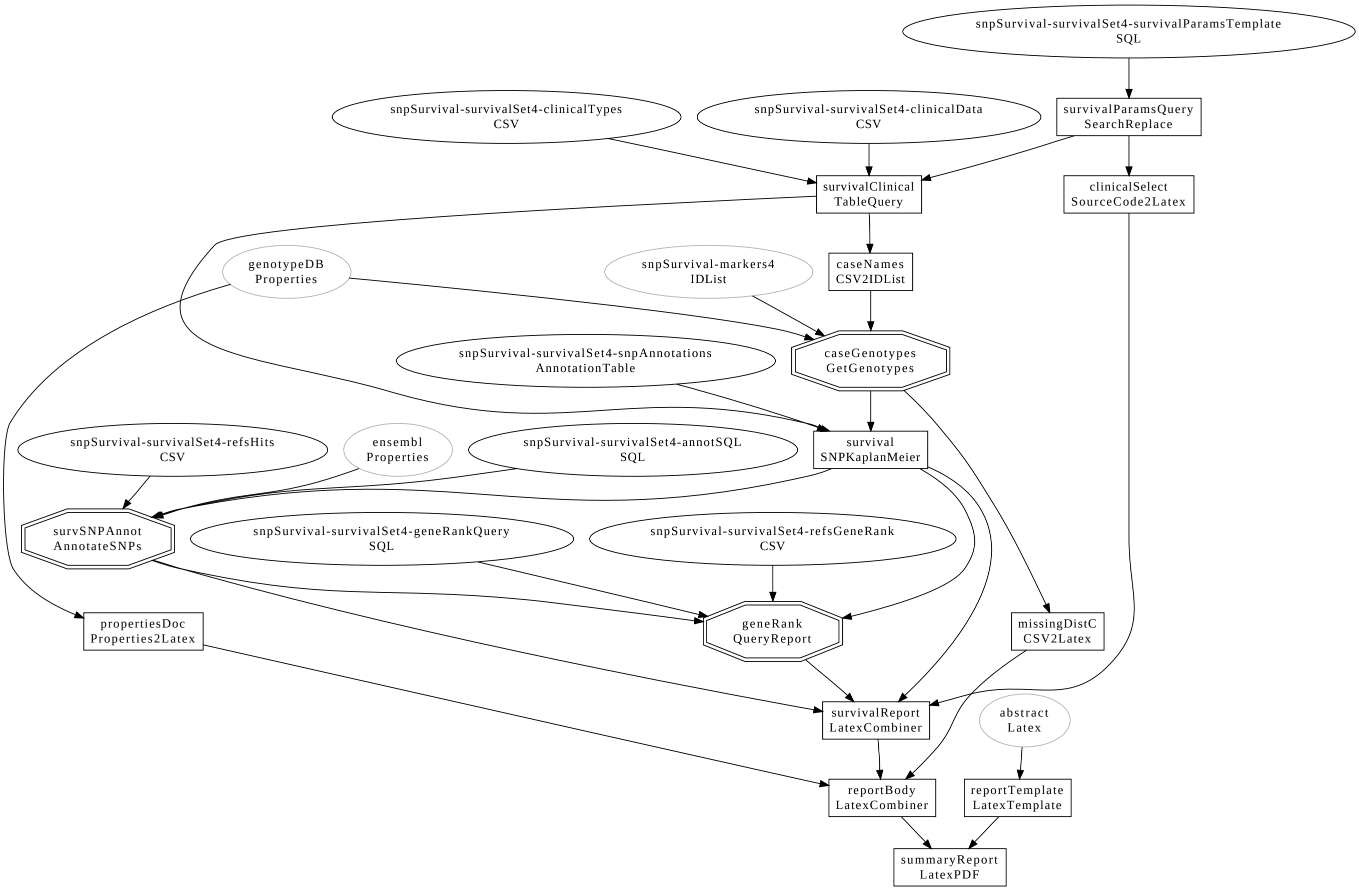

Figure 2091: Network topology for the subnetwork *snpSurvival-survivalSet4*.

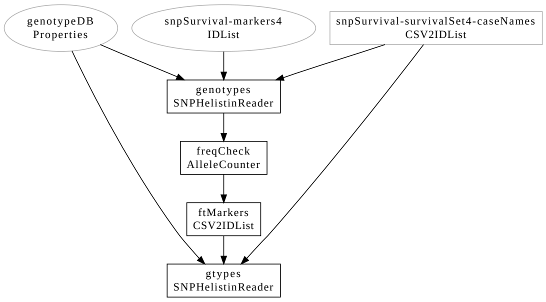

Figure 2092: Network topology for the subnetwork *snpSurvival-survivalSet4-caseGenotypes*.

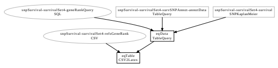

Figure 2093: Network topology for the subnetwork *snpSurvival-survivalSet4-geneRank*.

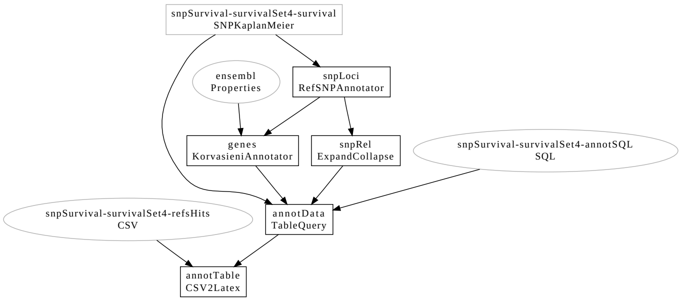

Figure 2094: Network topology for the subnetwork *snpSurvival-survivalSet4-survSNPAnnot*.

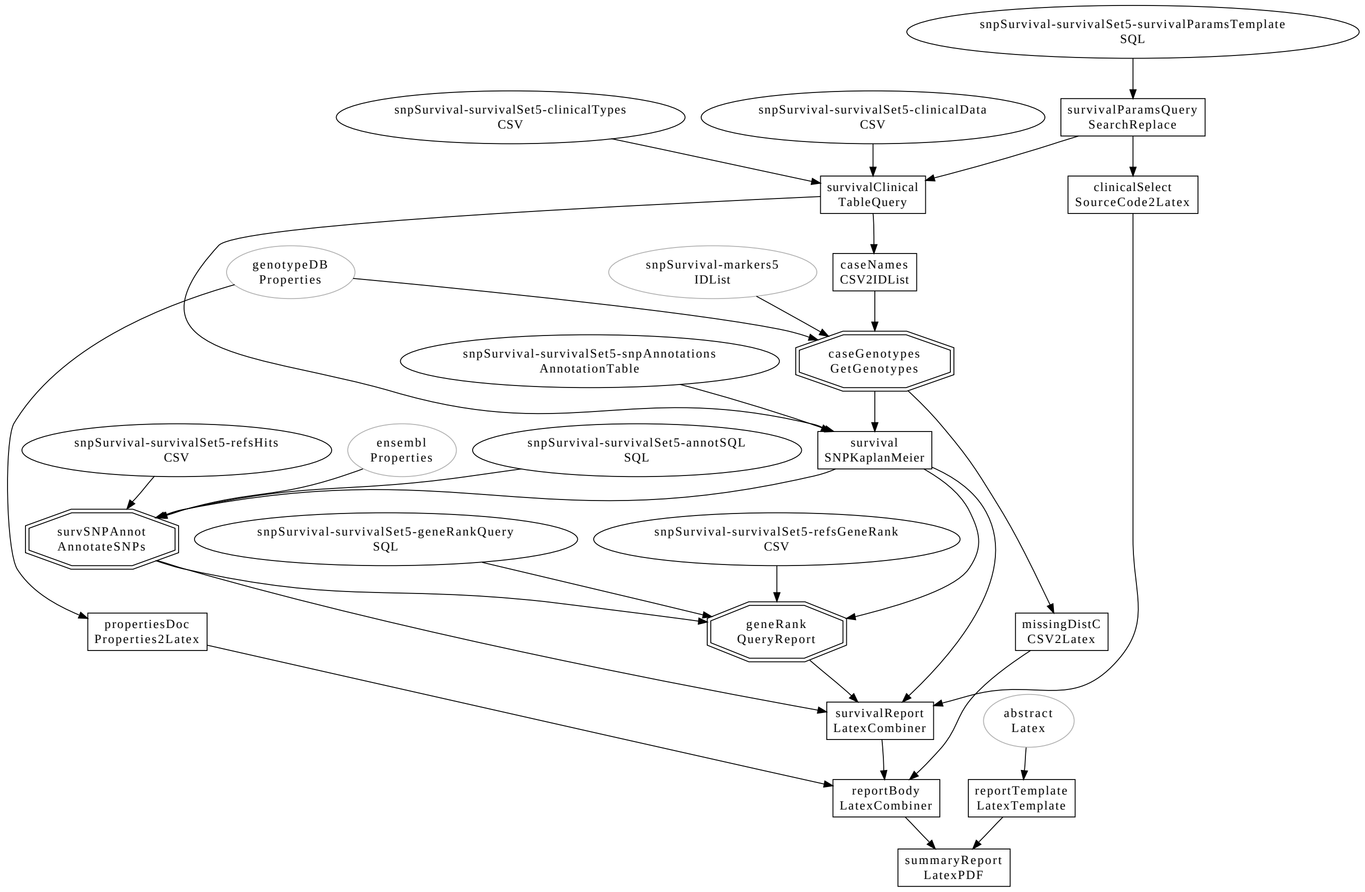

Figure 2095: Network topology for the subnetwork *snpSurvival-survivalSet5*.

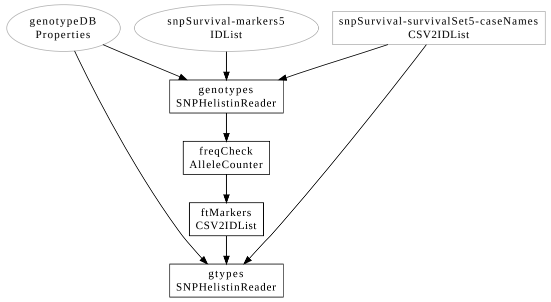

Figure 2096: Network topology for the subnetwork *snpSurvival-survivalSet5-caseGenotypes*.

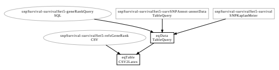

Figure 2097: Network topology for the subnetwork *snpSurvival-survivalSet5-geneRank*.

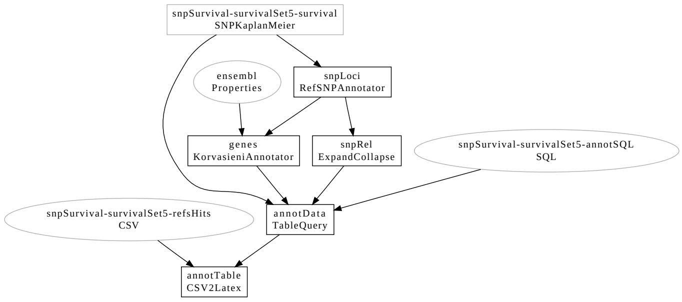

Figure 2098: Network topology for the subnetwork *snpSurvival-survivalSet5-survSNPAnnot*.

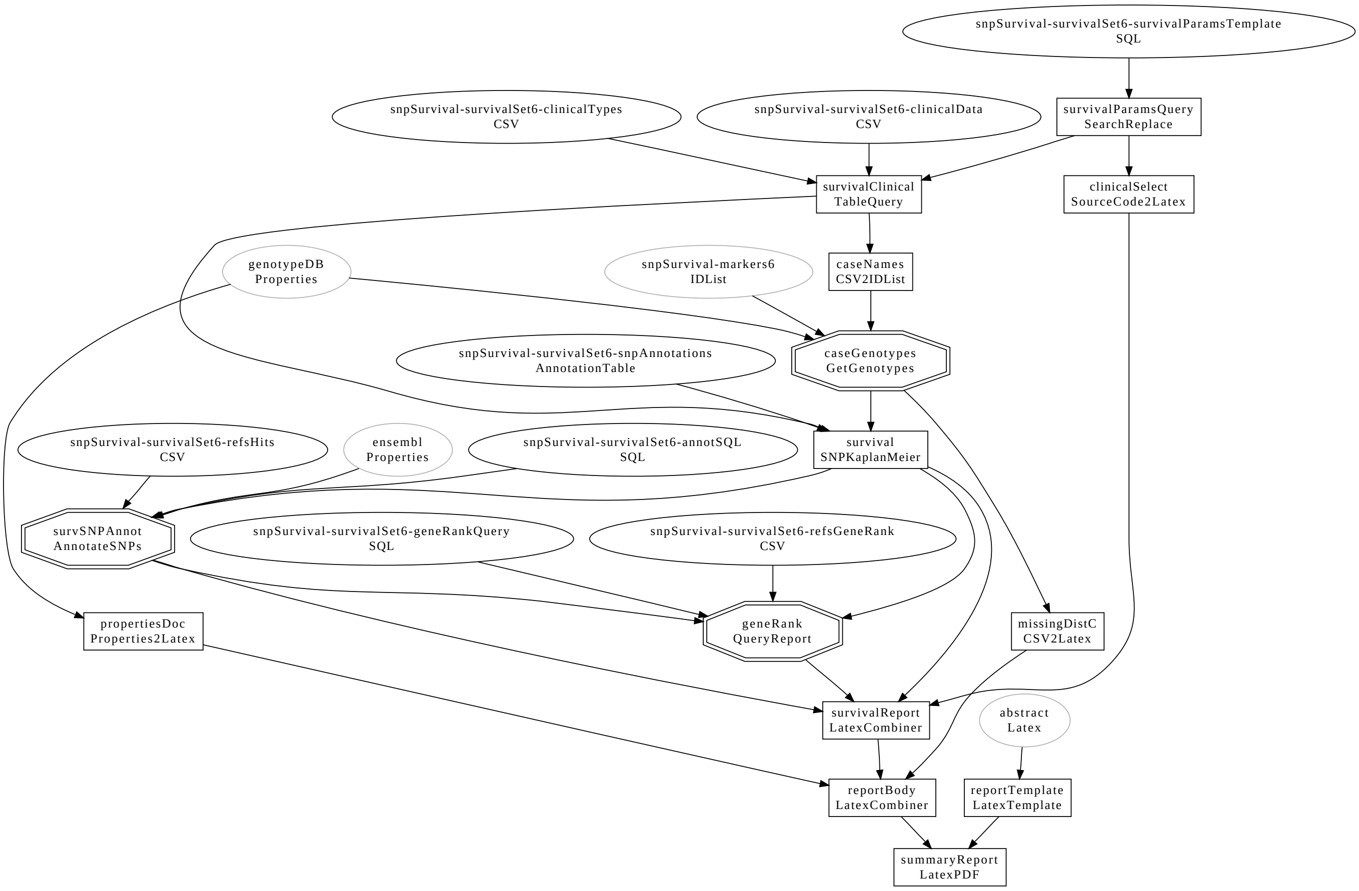

Figure 2099: Network topology for the subnetwork *snpSurvival-survivalSet6*.

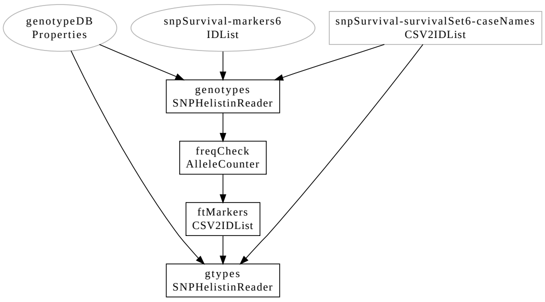

Figure 2100: Network topology for the subnetwork *snpSurvival-survivalSet6-caseGenotypes*.

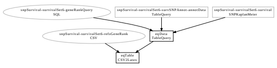

Figure 2101: Network topology for the subnetwork *snpSurvival-survivalSet6-geneRank*.

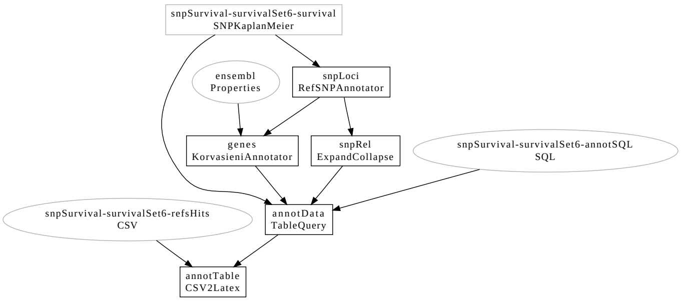

Figure 2102: Network topology for the subnetwork *snpSurvival-survivalSet6-survSNPAnnot*.

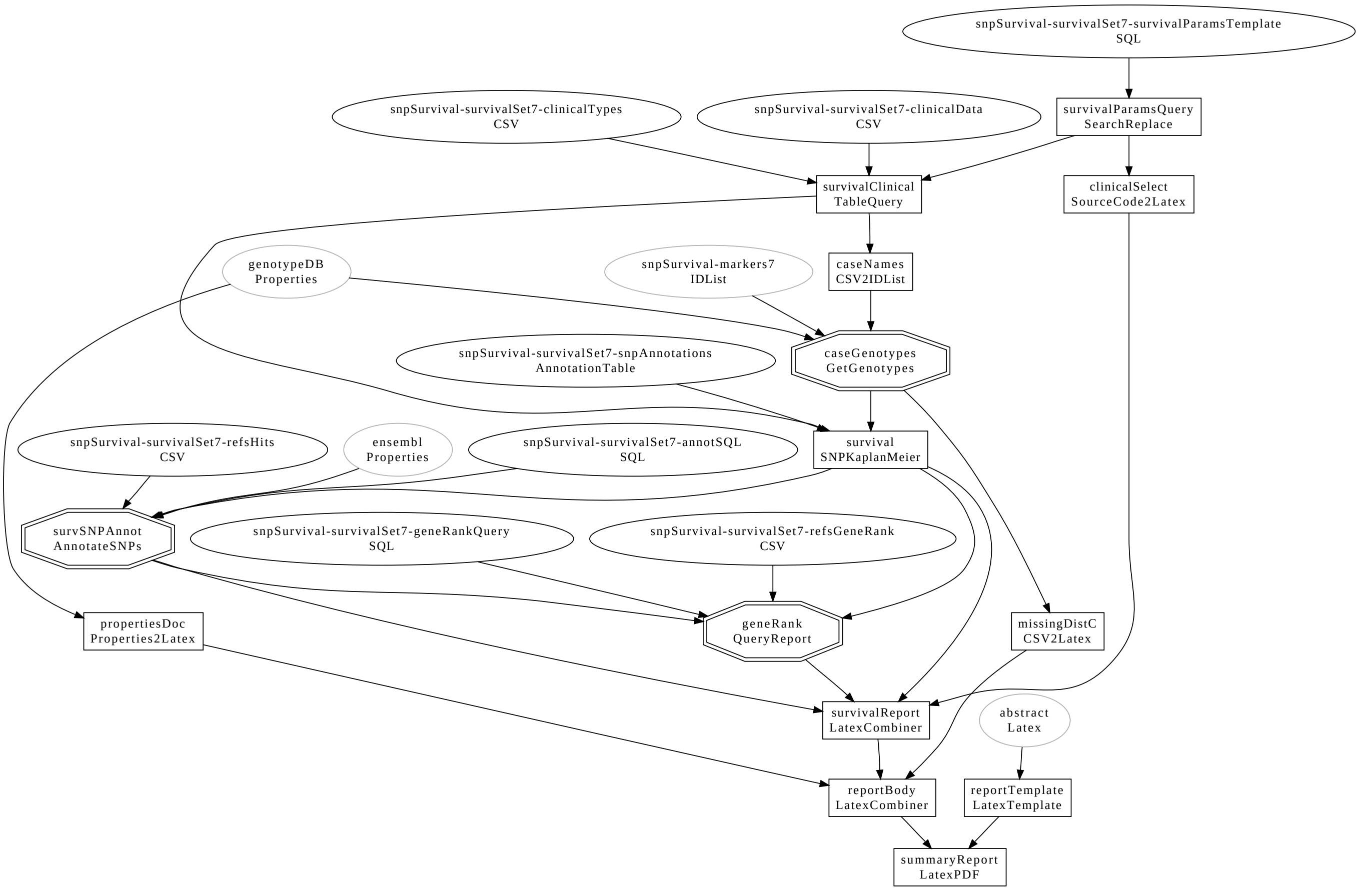

Figure 2103: Network topology for the subnetwork *snpSurvival-survivalSet7*.

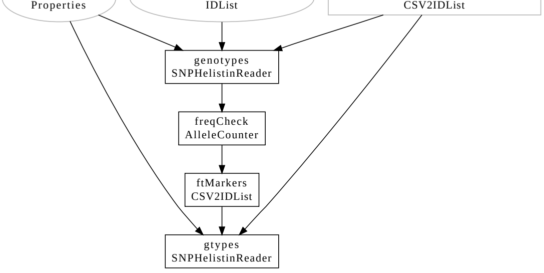

Figure 2104: Network topology for the subnetwork *snpSurvival-survivalSet7-caseGenotypes*.

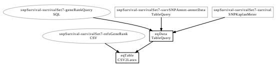

Figure 2105: Network topology for the subnetwork *snpSurvival-survivalSet7-geneRank*.

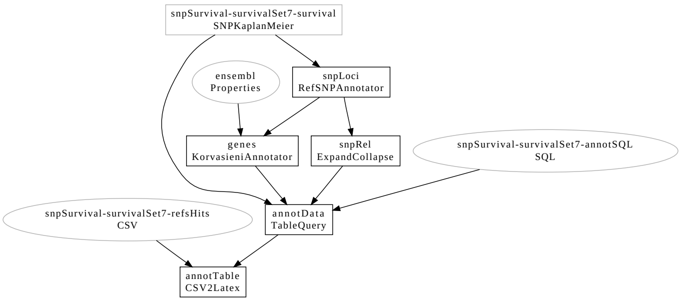

Figure 2106: Network topology for the subnetwork *snpSurvival-survivalSet7-survSNPAnnot*.

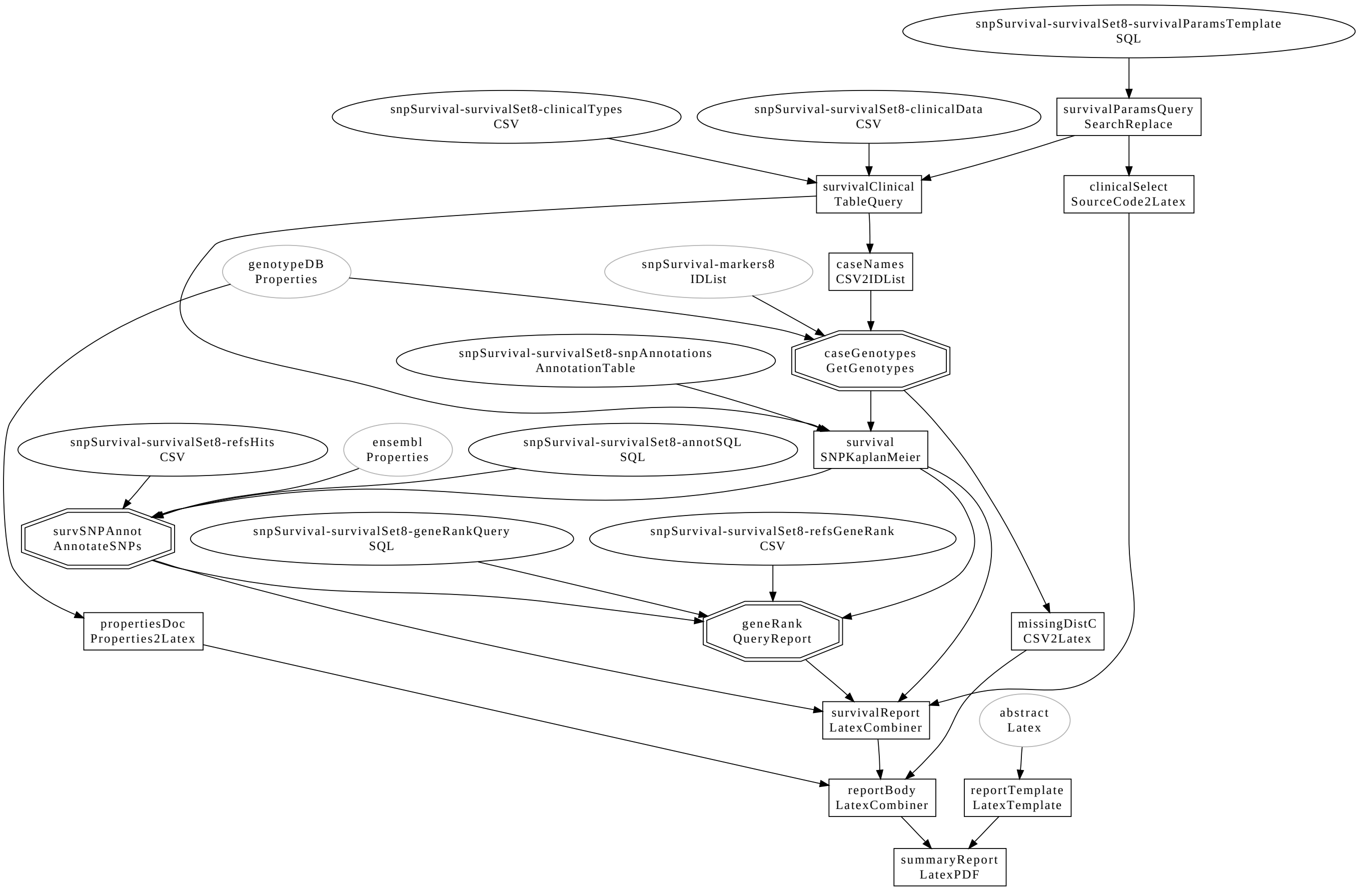

Figure 2107: Network topology for the subnetwork *snpSurvival-survivalSet8*.

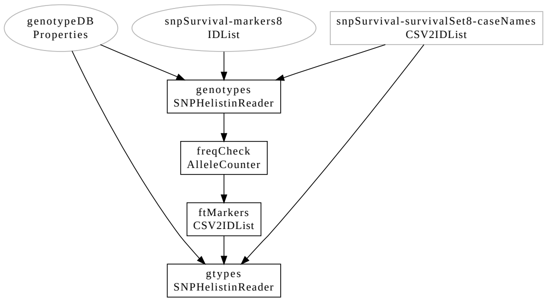

Figure 2108: Network topology for the subnetwork *snpSurvival-survivalSet8-caseGenotypes*.

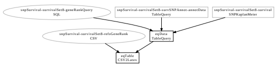

Figure 2109: Network topology for the subnetwork *snpSurvival-survivalSet8-geneRank*.

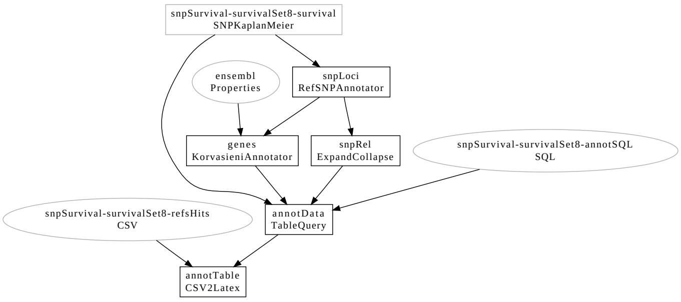

Figure 2110: Network topology for the subnetwork *snpSurvival-survivalSet8-survSNPAnnot*.

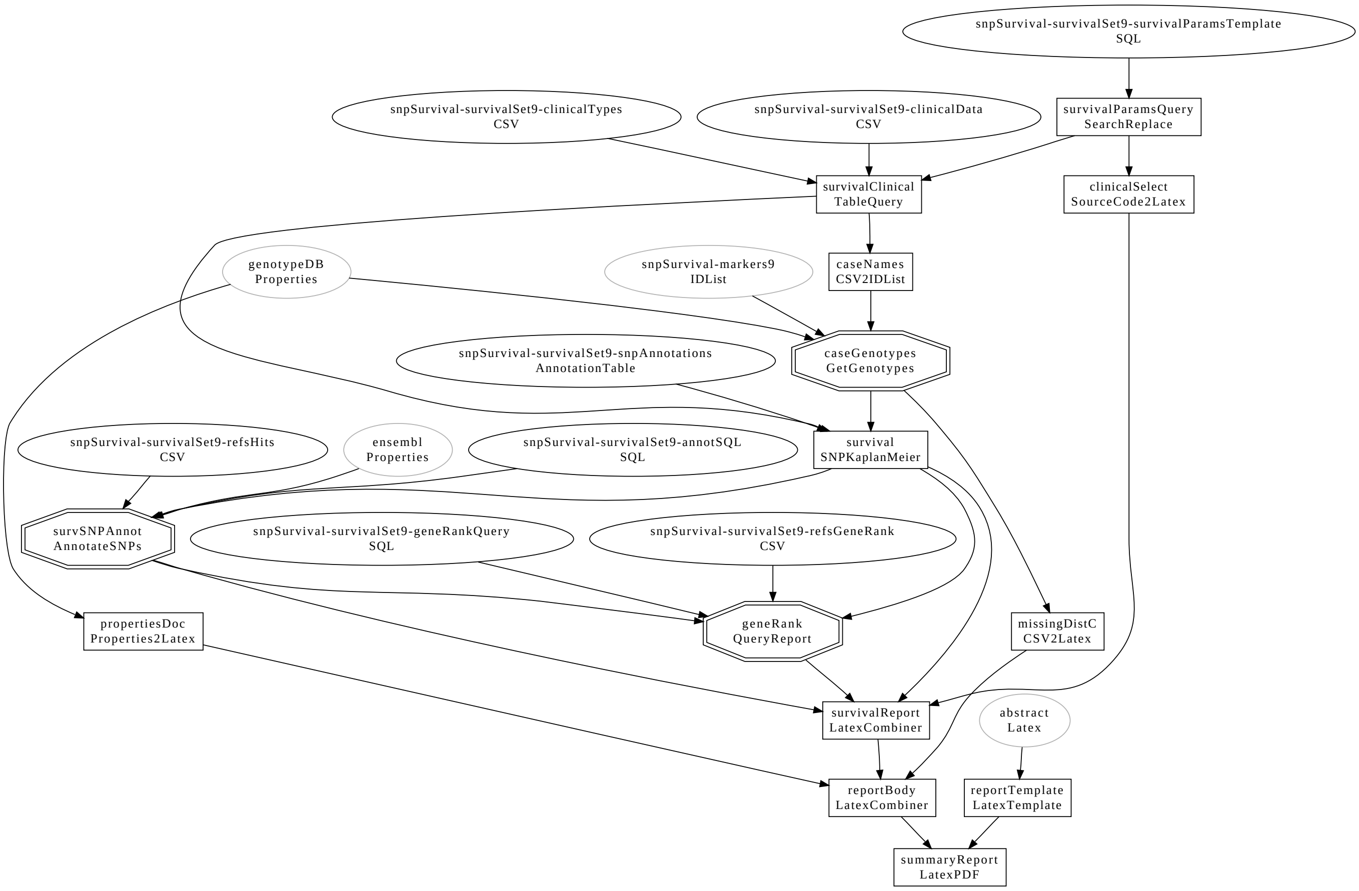

Figure 2111: Network topology for the subnetwork *snpSurvival-survivalSet9*.

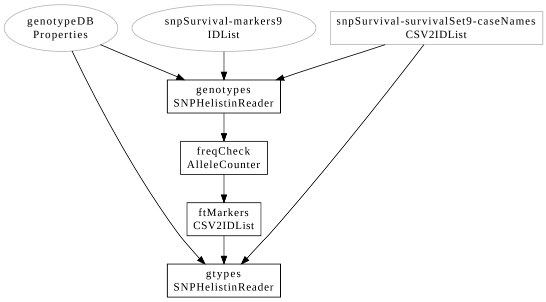

Figure 2112: Network topology for the subnetwork *snpSurvival-survivalSet9-caseGenotypes*.

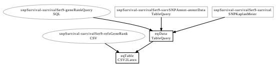

Figure 2113: Network topology for the subnetwork *snpSurvival-survivalSet9-geneRank*.

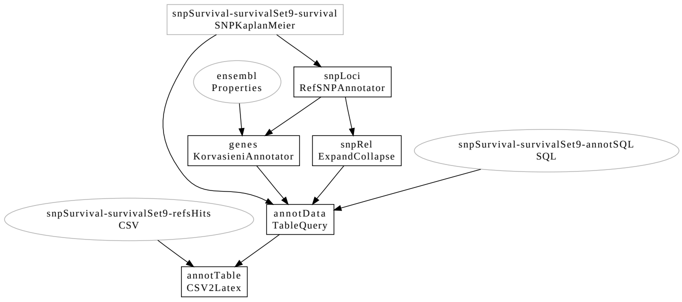

Figure 2114: Network topology for the subnetwork *snpSurvival-survivalSet9-survSNPAnnot*.

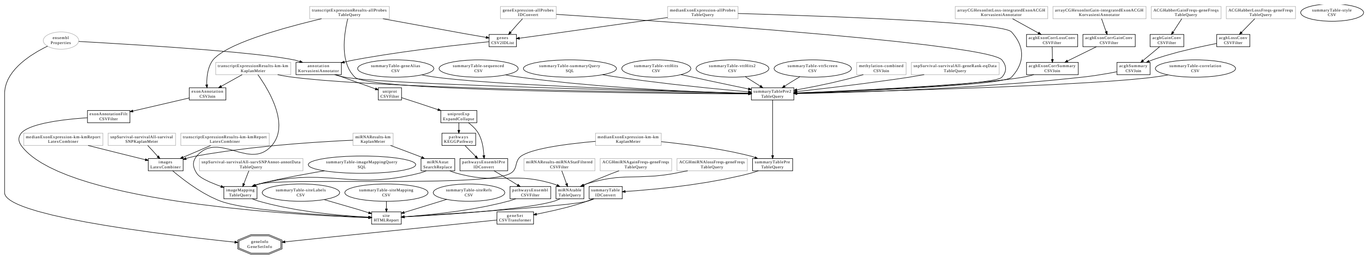

Figure 2115: Network topology for the subnetwork *summaryTable*.

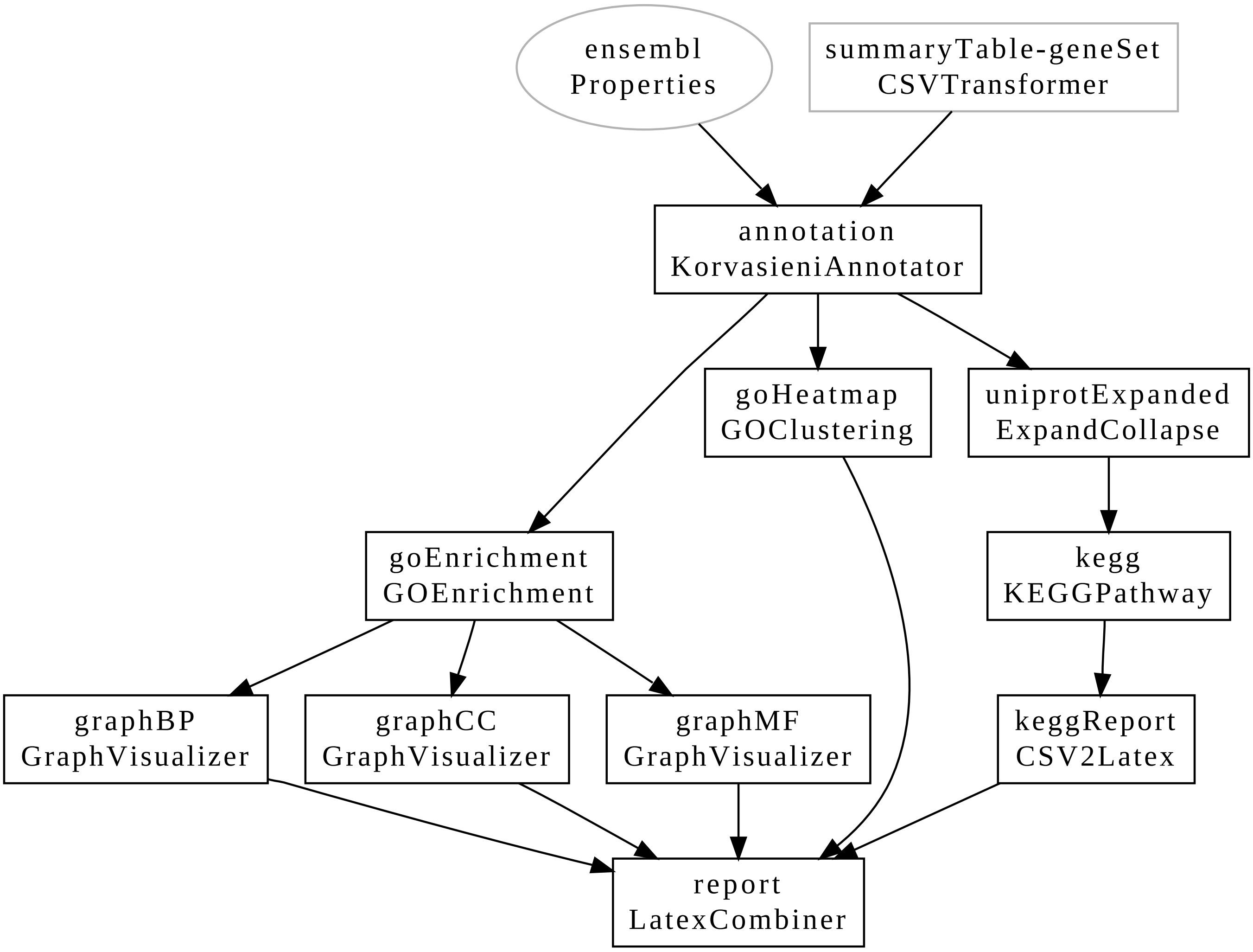

Figure 2116: Network topology for the subnetwork *summaryTable-geneInfo*.

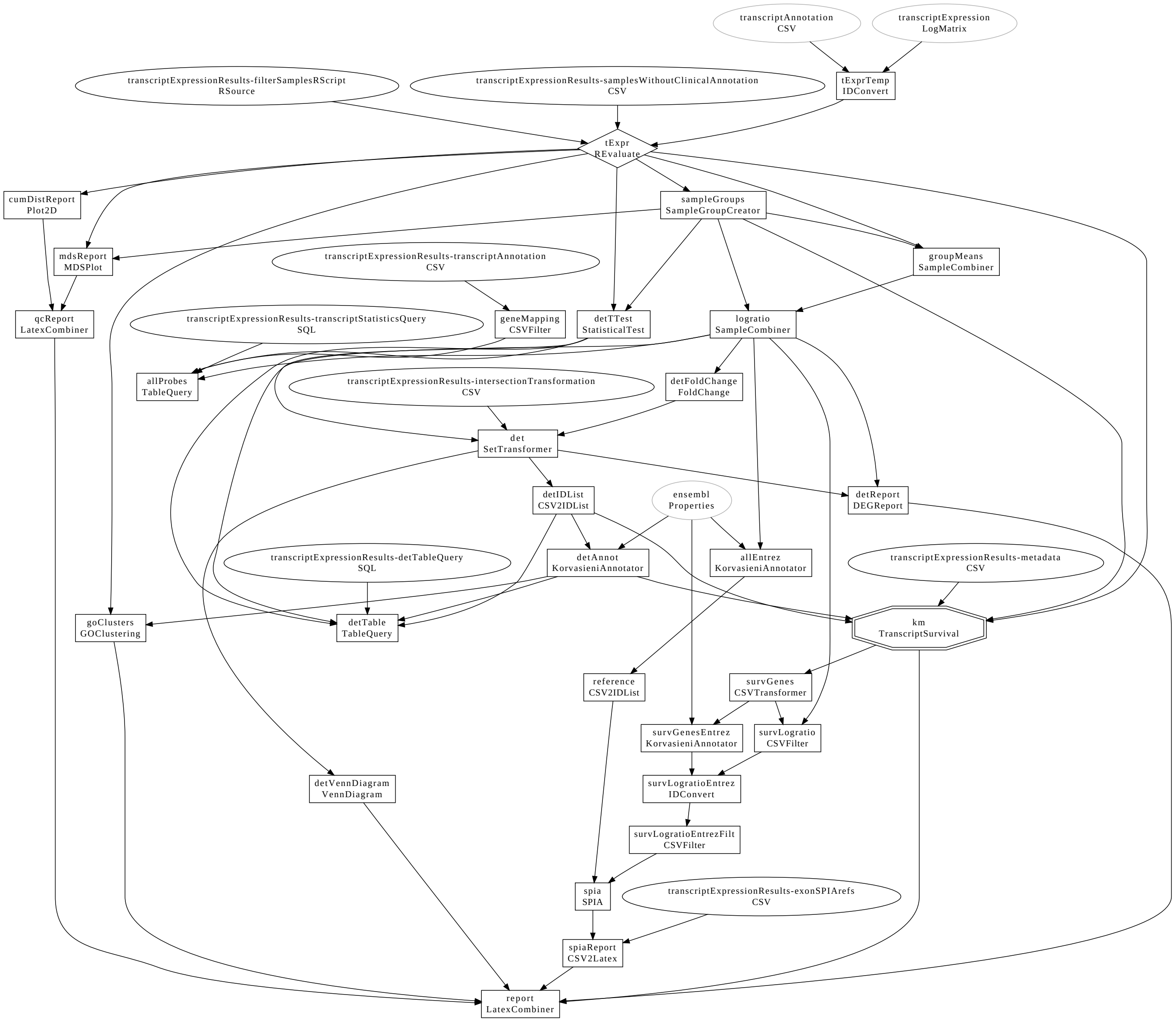

Figure 2117: Network topology for the subnetwork *transcriptExpressionResults*.

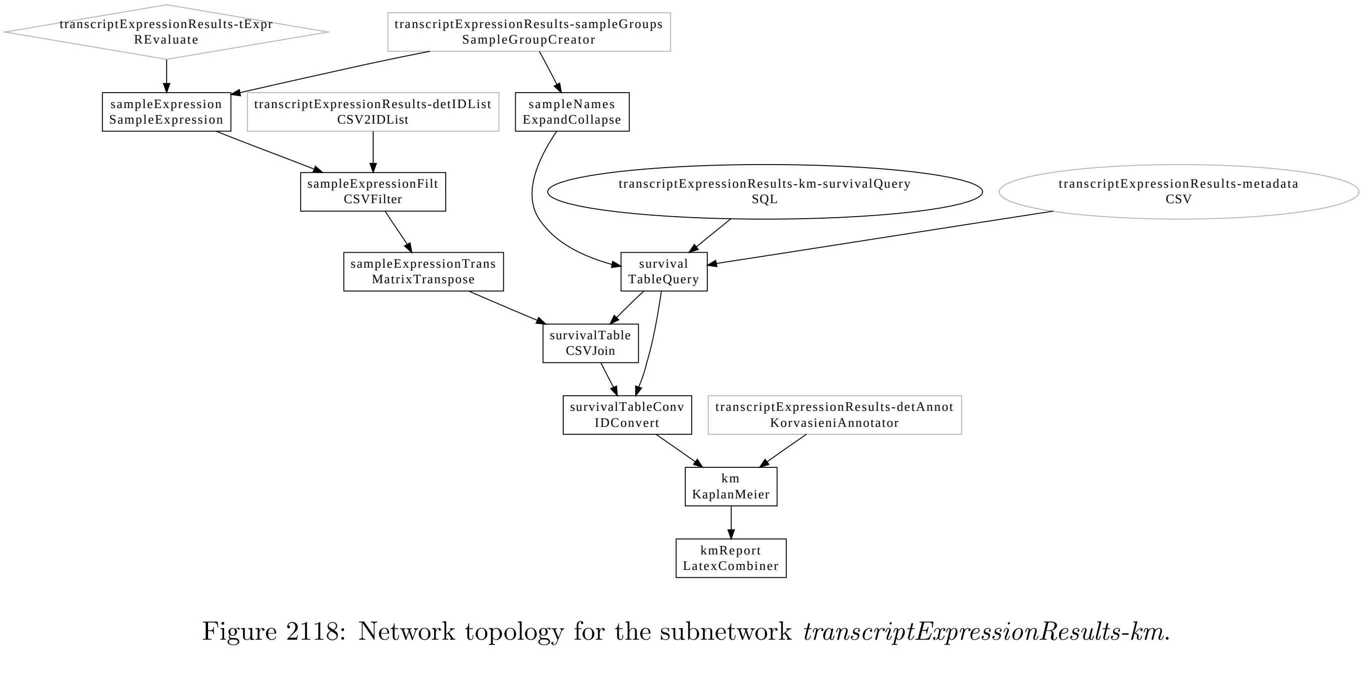

Figure 2118: Network topology for the subnetwork *transcriptExpressionResults-km*.

19.1 ACGHabberGainFreqs (ACGHfrequencies)

Calculate copy number gain aberration frequencies for each gene individually over the sample set.

Input name	Source
segments	arrayCGHanalysis.aberrations 19.43

Parameter name	Value
gainOrLoss	1
miRNAs	false

19.2 ACGHabberGainFreqs-chrAnnot (KorvasieniAnnotator)

Korvasieni is an Ensembl [12] based converter for database identifiers and it can be used to convert genome location, gene, transcript, and translation identifiers across multiple biological databases. The selected `keyColumn` may contain entries with comma separated lists of values in which case `isListKey` parameter may be used to obtain each value separately and to process them as they were given in consecutive lines.

Author:	Marko Laakso (Marko.Laakso@Helsinki.FI)
Version:	1.7
Categories:	Annotation, GO

Input name	Source	Description
sourceKeys	geneListFreqs.ids 19.319	A list of source database keys.
connection	ensembl.in 19.273	Database connection can be defined using this file. The definition of parameters: database.url, database.user, database.password, database.timeout, database.recycle, and database.driver can be found from the documentation of Korvasieni.

Parameter name	Value	Description
echoColumns		A comma separated list of column names for the columns that will be copied to the output. An asterisk (*) can be used to denote all columns execpt the keyColumn.
inputDB	.GeneId	Type of input keys. This must be a database supported by Korvasieni. If the parameter is omitted, the component tries to derive the database from the type of geneID. If this is not possible, an error is returned. You may define three columns in form of chromosome:start-end in case the inputDB is .DNARegion. This format provides a comfortable compatibility with DNARegion datatype.
inputType	Gene	Ensembl object type for the input keys (Any, Gene, Transcript, Translation)
isListKey	false	Enables the automatic value splits for the comma separated key column
keyColumn	GeneID	Name of the key column withing sourceKeys file or an empty string for the first column
maxHits	100000	Maximum number of target identifiers for a single source identifier
rename	GeneID=.GeneId	Comma separated list of column renaming rules (oldname=newname)
skipLevel	never	Skip result rows if the source identifier is unknown or target identifiers are not available. Possible values are: never (no filtering), source (skip if the source ID is unknown), target (skip if no target IDs are found), any (skip if any of the target IDs is missing).
targetDB	.DNARegion	Comma-separated list of annotation types. Possible values are all databases supported by Korvasieni.
unique	true	This flag can be turned on in order to eliminate duplicate annotations.

19.3 ACGHabberGainFreqs-filteredChrAnnot (TableQuery)

Execute an SQL query on input relations and create a result table. TableQuery uses HSQLDB (Hyperthreaded Structured Query Language Database [25]) for executing the query. Consequently, the syntax of the query is defined by HSQLDB.

Author: Kristian Ovaska (Kristian.Ovaska@Helsinki.FI)
Version: 1.2
Categories: convert, Filter

Input name	Source	Description
table1	ACGHabberGainFreqs-chrAnnot .bioAnnotation 19.2	CSV table 1. The table is referred to as 'table1' in the SQL query.

Parameter name	Value	Description
numIndices		Comma-separated list of index counts for input tables. All indices are single-column indices, running from column 1 to N, where N is retrieved from this parameter. If empty, the default number (1) is used. For example, ",2,,2" sets two indices for table2 and table4 and one index for the rest.
query	SELECT * FROM table1 WHERE ".DNARegion" IS NOT NULL	SQL query. Either this parameter or the query input must be provided, but not both.

19.4 ACGHabberGainFreqs-frequencies (TableQuery)

Execute an SQL query on input relations and create a result table. TableQuery uses HSQLDB (Hyper-threaded Structured Query Language Database [25]) for executing the query. Consequently, the syntax of the query is defined by HSQLDB.

Author: Kristian Ovaska (Kristian.Ovaska@Helsinki.FI)
Version: 1.2
Categories: convert, Filter

Input name	Source	Description
table1	arrayCGHanalysis-aberrations-eq Data.table 19.47	CSV table 1. The table is referred to as 'table1' in the SQL query.
table2	ACGHabberGainFreqs-occurances .table 19.7	CSV table 2. The table is referred to as 'table2' in the SQL query.

Parameter name	Value	Description
numIndices		Comma-separated list of index counts for input tables. All indices are single-column indices, running from column 1 to N, where N is retrieved from this parameter. If empty, the default number (1) is used. For example, ",2,,2" sets two indices for table2 and table4 and one index for the rest.
query	SELECT T2.".GeneId", (CAST(T2."count" AS DOUBLE) / (SELECT COUNT(DISTINCT T1."ID") FROM table1 T1)) AS "freq" FROM table2 T2	SQL query. Either this parameter or the query input must be provided, but not both.

19.5 ACGHabberGainFreqs-geneFreqs (TableQuery)

Execute an SQL query on input relations and create a result table. TableQuery uses HSQLDB (Hyper-threaded Structured Query Language Database [25]) for executing the query. Consequently, the syntax of the query is defined by HSQLDB.

Author: Kristian Ovaska (Kristian.Ovaska@Helsinki.FI)
Version: 1.2
Categories: convert, Filter

Continued on next page...

Input name	Source	Description

Input name	Source	Description
table1	ACGHabberGainFreqs -frequencies.table 19.4	CSV table 1. The table is referred to as 'table1' in the SQL query.
table2	ACGHabberGainFreqs-chrAnnot .bioAnnotation 19.2	CSV table 2. The table is referred to as 'table2' in the SQL query.

Parameter name	Value	Description
numIndices		Comma-separated list of index counts for input tables. All indices are single-column indices, running from column 1 to N, where N is retrieved from this parameter. If empty, the default number (1) is used. For example, ",2,,2" sets two indices for table2 and table4 and one index for the rest.
query	SELECT table2.".GeneId", table1."freq" FROM table2 LEFT OUTER JOIN table1 ON (table1. ".GeneId" = table2.".GeneId")	SQL query. Either this parameter or the query input must be provided, but not both.

19.6 ACGHabberGainFreqs-geneList (SearchReplace)

Replaces the occurences of the given regular expressions with the corresponding regular expressions. This component is used for transforming given patterns of the input file to a changed form specified by the regular expressions .

Author: Marko Laakso (Marko.Laakso@Helsinki.FI)
Version: 1.0
Category: convert

Input name	Source	Description
file	ACGHabberGainFreqs-filteredChr Annot.table 19.3	Source file to be modified
rules	ACGHabberGainFreqs-rules .in 19.8	A set of search keys

Parameter name	Value	Description
key00		Search key 0
key01		Search key 1
key02		Search key 2
key03		Search key 3
key04		Search key 4
key05		Search key 5
key06		Search key 6
key07		Search key 7
key08		Search key 8
key09		Search key 9
value00		Replacement 0
value01		Replacement 1
value02		Replacement 2
value03		Replacement 3
value04		Replacement 4
value05		Replacement 5
value06		Replacement 6
value07		Replacement 7
value08		Replacement 8
value09		Replacement 9

19.7 ACGHabberGainFreqs-occurances (TableQuery)

Execute an SQL query on input relations and create a result table. TableQuery uses HSQLDB (Hyper-threaded Structured Query Language Database [25]) for executing the query. Consequently, the syntax of the query is defined by HSQLDB.

Author:	Kristian Ovaska (Kristian.Ovaska@Helsinki.FI)
Version:	1.2
Categories:	convert, Filter

Input name	Source	Description
table1	arrayCGHanalysis-aberrations-eqData.table 19.47	CSV table 1. The table is referred to as 'table1' in the SQL query.
table2	ACGHabberGainFreqs-geneList.file 19.6	CSV table 2. The table is referred to as 'table2' in the SQL query.

Parameter name	Value	Description
numIndices		Comma-separated list of index counts for input tables. All indices are single-column indices, running from column 1 to N, where N is retrieved from this parameter. If empty, the default number (1) is used. For example, ",2,,2" sets two indices for table2 and table4 and one index for the rest.
query	SELECT T2.".GeneId", COUNT(T1."Sample") AS "count" FROM table1 T1, table2 T2 WHERE (T1."chr" = T2."chr") AND (T1."start" <= T2."end") AND (T1."end" >= T2."start") AND (T1."call" = 1) GROUP BY T2.".GeneId"	SQL query. Either this parameter or the query input must be provided, but not both.

19.8 ACGHabberGainFreqs-rules (INPUT)

Rules for string replacement in chromosome annotations.

Category: Internal

Parameter name	Value	Description
path	/gpfs/homes/mlaakso/asserSVN/andurilCaseStudy/csv/ACGHfreqRules.csv	Path (filename) of the input resource.

19.9 ACGHabberLossFreqs (ACGHfrequencies)

Calculate copy number loss aberration frequencies for each gene individually over the sample set.

Input name	Source
segments	arrayCGHanalysis.aberrations 19.43

Parameter name	Value
gainOrLoss	-1
miRNAs	false

19.10 ACGHabberLossFreqs-chrAnnot (KorvasieniAnnotator)

Korvasieni is an Ensembl [12] based converter for database identifiers and it can be used to convert genome location, gene, transcript, and translation identifiers across multiple biological databases. The selected `keyColumn` may contain entries with comma separated lists of values in which case `isListKey` parameter may be used to obtain each value separately and to process them as they were given in consecutive lines.

Author: Marko Laakso (Marko.Laakso@Helsinki.FI)
Version: 1.7
Categories: Annotation, GO

Input name	Source	Description
sourceKeys	geneListFreqs.ids 19.319	A list of source database keys.
connection	ensembl.in 19.273	Database connection can be defined using this file. The definition of parameters: database.url, database.user, database.password, database.timeout, database.recycle, and database.driver can be found from the documentation of Korvasieni.

Parameter name	Value	Description
echoColumns		A comma separated list of column names for the columns that will be copied to the output. An asterisk (*) can be used to denote all columns execpt the keyColumn.
inputDB	.GeneId	Type of input keys. This must be a database supported by Korvasieni. If the parameter is omitted, the component tries to derive the database from the type of geneID. If this is not possible, an error is returned. You may define three columns in form of chromosome:start-end in case the inputDB is .DNARegion. This format provides a comfortable compatibility with DNARegion datatype.
inputType	Gene	Ensembl object type for the input keys (Any, Gene, Transcript, Translation)
isListKey	false	Enables the automatic value splits for the comma separated key column
keyColumn	GeneID	Name of the key column withing sourceKeys file or an empty string for the first column
maxHits	100000	Maximum number of target identifiers for a single source identifier
rename	GeneID=.GeneId	Comma separated list of column renaming rules (oldname=newname)
skipLevel	never	Skip result rows if the source identifier is unknown or target identifiers are not available. Possible values are: never (no filtering), source (skip if the source ID is unknown), target (skip if no target IDs are found), any (skip if any of the target IDs is missing).
targetDB	.DNARegion	Comma-separated list of annotation types. Possible values are all databases supported by Korvasieni.
unique	true	This flag can be turned on in order to eliminate duplicate annotations.

19.11 ACGHabberLossFreqs-filteredChrAnnot (TableQuery)

Execute an SQL query on input relations and create a result table. TableQuery uses HSQLDB (Hyperthreaded Structured Query Language Database [25]) for executing the query. Consequently, the syntax of the query is defined by HSQLDB.

Author: Kristian Ovaska (Kristian.Ovaska@Helsinki.FI)
Version: 1.2
Categories: convert, Filter

Input name	Source	Description
table1	ACGHabberLossFreqs-chrAnnot .bioAnnotation 19.10	CSV table 1. The table is referred to as 'table1' in the SQL query.

Parameter name	Value	Description
numIndices		Comma-separated list of index counts for input tables. All indices are single-column indices, running from column 1 to N, where N is retrieved from this parameter. If empty, the default number (1) is used. For example, ",2,,2" sets two indices for table2 and table4 and one index for the rest.
query	SELECT * FROM table1 WHERE ".DNARegion" IS NOT NULL	SQL query. Either this parameter or the query input must be provided, but not both.

19.12 ACGHabberLossFreqs-frequencies (TableQuery)

Execute an SQL query on input relations and create a result table. TableQuery uses HSQLDB (Hyperthreaded Structured Query Language Database [25]) for executing the query. Consequently, the syntax of the query is defined by HSQLDB.

Author: Kristian Ovaska (Kristian.Ovaska@Helsinki.FI)
Version: 1.2
Categories: convert, Filter

Input name	Source	Description
table1	arrayCGHanalysis-aberrations-eqData.table 19.47	CSV table 1. The table is referred to as 'table1' in the SQL query.
table2	ACGHabberLossFreqs-occurances.table 19.15	CSV table 2. The table is referred to as 'table2' in the SQL query.

Parameter name	Value	Description
numIndices		Comma-separated list of index counts for input tables. All indices are single-column indices, running from column 1 to N, where N is retrieved from this parameter. If empty, the default number (1) is used. For example, ",2,,2" sets two indices for table2 and table4 and one index for the rest.
query	SELECT T2.".GeneId", (CAST(T2."count" AS DOUBLE) / (SELECT COUNT(DISTINCT T1."ID") FROM table1 T1)) AS "freq" FROM table2 T2	SQL query. Either this parameter or the query input must be provided, but not both.

19.13 ACGHabberLossFreqs-geneFreqs (TableQuery)

Execute an SQL query on input relations and create a result table. TableQuery uses HSQLDB (Hyperthreaded Structured Query Language Database [25]) for executing the query. Consequently, the syntax of the query is defined by HSQLDB.

Author: Kristian Ovaska (Kristian.Ovaska@Helsinki.FI)
Version: 1.2
Categories: convert, Filter

Input name	Source	Description
table1	ACGHabberLossFreqs-frequencies.table 19.12	CSV table 1. The table is referred to as 'table1' in the SQL query.
table2	ACGHabberLossFreqs-chrAnnot.bioAnnotation 19.10	CSV table 2. The table is referred to as 'table2' in the SQL query.

Parameter name	Value	Description
numIndices		Comma-separated list of index counts for input tables. All indices are single-column indices, running from column 1 to N, where N is retrieved from this parameter. If empty, the default number (1) is used. For example, ",2,,2" sets two indices for table2 and table4 and one index for the rest.
query	SELECT table2.".GeneId", table1."freq" FROM table2 LEFT OUTER JOIN table1 ON (table1.".GeneId" = table2.".GeneId")	SQL query. Either this parameter or the query input must be provided, but not both.

19.14 ACGHabberLossFreqs-geneList (SearchReplace)

Replaces the occurences of the given regular expressions with the corresponding regular expressions. This component is used for transforming given patterns of the input file to a changed form specified by the regular expressions .

Author: Marko Laakso (Marko.Laakso@Helsinki.FI)
Version: 1.0
Category: convert

Input name	Source	Description
file	ACGHabberLossFreqs-filteredChrAnnot.table 19.11	Source file to be modified
rules	ACGHabberLossFreqs-rules.in 19.16	A set of search keys

Parameter name	Value	Description
key00		Search key 0
key01		Search key 1
key02		Search key 2
key03		Search key 3
key04		Search key 4
key05		Search key 5
key06		Search key 6
key07		Search key 7
key08		Search key 8
key09		Search key 9
value00		Replacement 0
value01		Replacement 1
value02		Replacement 2
value03		Replacement 3
value04		Replacement 4
value05		Replacement 5
value06		Replacement 6
value07		Replacement 7
value08		Replacement 8
value09		Replacement 9

19.15 ACGHabberLossFreqs-occurances (TableQuery)

Execute an SQL query on input relations and create a result table. TableQuery uses HSQLDB (Hyper-threaded Structured Query Language Database [25]) for executing the query. Consequently, the syntax of the query is defined by HSQLDB.

Author: Kristian Ovaska (Kristian.Ovaska@Helsinki.FI)
Version: 1.2
Categories: convert, Filter

Input name	Source	Description
table1	arrayCGHanalysis-aberrations-eqData.table 19.47	CSV table 1. The table is referred to as 'table1' in the SQL query.
table2	ACGHabberLossFreqs-geneList.file 19.14	CSV table 2. The table is referred to as 'table2' in the SQL query.

Parameter name	Value	Description
numIndices		Comma-separated list of index counts for input tables. All indices are single-column indices, running from column 1 to N, where N is retrieved from this parameter. If empty, the default number (1) is used. For example, ",2,,2" sets two indices for table2 and table4 and one index for the rest.
query	SELECT T2.".GeneId", COUNT(T1."Sample") AS "count" FROM table1 T1, table2 T2 WHERE (T1."chr" = T2."chr") AND (T1. "start" <= T2."end") AND (T1."end" >= T2."start") AND (T1."call" = -1.0) GROUP BY T2.".GeneId"	SQL query. Either this parameter or the query input must be provided, but not both.

19.16 ACGHabberLossFreqs-rules (INPUT)

Rules for string replacement in chromosome annotations.

Category: Internal

Parameter name	Value	Description
path	/gpfs/homes/mlaakso/asserSVN/ andurilCaseStudy/csv/ ACGHfreqRules.csv	Path (filename) of the input resource.

19.17 ACGHmiRNAgainFreqs (ACGHfrequencies)

Calculate copy number gain aberration frequencies for each microRNA individually over the sample set.

Input name	Source
genes	miRNAResults.dems 19.394
segments	arrayCGHanalysis.aberrations 19.43

Parameter name	Value
gainOrLoss	1
miRNAs	true

19.18 ACGHmiRNAgainFreqs-chrAnnot (CSVCleaner)

CSVCleaner can be used to clean up Anduril outputs and to make them more suitable for other applications such as spreadsheets or text editors. The component is capable of removing file headers, quotations, and unused columns. Can be used to reorder and rename columns and their delimiters. Missing value symbols may be defined independently for the input and output.

Author: Marko Laakso (Marko.Laakso@Helsinki.FI)
Version: 1.6
Category: convert

Input name	Source	Description
original	ACGHmiRNAgainFreqs-chrAnnot Pre.bioAnnotation 19.19	Input file to be cleaned

Parameter name	Value	Description
columns	.GeneId,.DNARegion	Comma separated list of column selections for the output. An asterisk (*) may be used for all columns.
delimIn	\t	Column delimiter for the input
delimSymbol	\t	Column delimiter for the output
naIn	NA	Missing value symbol for the input
naSymbol	NA	Missing value symbol for the output
rename		Comma separated list of column renaming rules (oldname=newname)
rowSkip	0	Skip this many lines from the input before reading the tabular.
skipQuota		Comma separated list of output columns names that should not have quotation marks. An asterisk (*) may be used for all columns.

19.19 ACGHmiRNAgainFreqs-chrAnnotPre (KorvasieniAnnotator)

Korvasieni is an Ensembl [12] based converter for database identifiers and it can be used to convert genome location, gene, transcript, and translation identifiers across multiple biological databases. The selected `keyColumn` may contain entries with comma separated lists of values in which case `isListKey` parameter

may be used to obtain each value separately and to process them as they were given in consecutive lines.

Author: Marko Laakso (Marko.Laakso@Helsinki.FI)
Version: 1.7
Categories: Annotation, GO

Input name	Source	Description
sourceKeys	ACGHmiRNAgainFreqs-mirnaIDs.file 19.25	A list of source database keys.
connection	ensembl.in 19.273	Database connection can be defined using this file. The definition of parameters: database.url, database.user, database.password, database.timeout, database.recycle, and database.driver can be found from the documentation of Korvasieni.

Parameter name	Value	Description
echoColumns	miRNAName	A comma separated list of column names for the columns that will be copied to the output. An asterisk (*) can be used to denote all columns execpt the keyColumn.
inputDB	.GeneName	Type of input keys. This must be a database supported by Korvasieni. If the parameter is omitted, the component tries to derive the database from the type of geneID. If this is not possible, an error is returned. You may define three columns in form of chromosome:start-end in case the inputDB is .DNARegion. This format provides a comfortable compatibility with DNARegion datatype.
inputType	Gene	Ensembl object type for the input keys (Any, Gene, Transcript, Translation)
isListKey	false	Enables the automatic value splits for the comma separated key column
keyColumn	miRNANameTrans	Name of the key column withing sourceKeys file or an empty string for the first column
maxHits	100000	Maximum number of target identifiers for a single source identifier
rename	.DNABand=DNABand	Comma separated list of column renaming rules (oldname=newname)
skipLevel	never	Skip result rows if the source identifier is unknown or target identifiers are not available. Possible values are: never (no filtering), source (skip if the source ID is unknown), target (skip if no target IDs are found), any (skip if any of the target IDs is missing).
targetDB	.GeneId,.DNARegion,.DNABand	Comma-separated list of annotation types. Possible values are all databases supported by Korvasieni.
unique	true	This flag can be turned on in order to eliminate duplicate annotations.

19.20 ACGHmiRNAgainFreqs-duplicateMirnas (TableQuery)

Execute an SQL query on input relations and create a result table. TableQuery uses HSQLDB (Hyperthreaded Structured Query Language Database [25]) for executing the query. Consequently, the syntax of the query is defined by HSQLDB.

Author: Kristian Ovaska (Kristian.Ovaska@Helsinki.FI)
Version: 1.2
Categories: convert, Filter

Input name	Source	Description
table1	miRNAResults-miRNAStatFiltered.csv 19.416	CSV table 1. The table is referred to as 'table1' in the SQL query.

Parameter name	Value	Description
numIndices		Comma-separated list of index counts for input tables. All indices are single-column indices, running from column 1 to N, where N is retrieved from this parameter. If empty, the default number (1) is used. For example, ",2,,2" sets two indices for table2 and table4 and one index for the rest.
query	SELECT "miRNAName", UCASE("miRNAName") AS "miRNANameTrans" FROM table1	SQL query. Either this parameter or the query input must be provided, but not both.

19.21 ACGHmiRNAgainFreqs-filteredChrAnnot (TableQuery)

Execute an SQL query on input relations and create a result table. TableQuery uses HSQLDB (Hyperthreaded Structured Query Language Database [25]) for executing the query. Consequently, the syntax of the query is defined by HSQLDB.

Author: Kristian Ovaska (Kristian.Ovaska@Helsinki.FI)
Version: 1.2
Categories: convert, Filter

Input name	Source	Description
table1	ACGHmiRNAgainFreqs-chrAnnot .table 19.18	CSV table 1. The table is referred to as 'table1' in the SQL query.

Parameter name	Value	Description
numIndices		Comma-separated list of index counts for input tables. All indices are single-column indices, running from column 1 to N, where N is retrieved from this parameter. If empty, the default number (1) is used. For example, ",2,,2" sets two indices for table2 and table4 and one index for the rest.
query	SELECT * FROM table1 WHERE ".DNARegion" IS NOT NULL	SQL query. Either this parameter or the query input must be provided, but not both.

19.22 ACGHmiRNAgainFreqs-frequencies (TableQuery)

Execute an SQL query on input relations and create a result table. TableQuery uses HSQLDB (Hyperthreaded Structured Query Language Database [25]) for executing the query. Consequently, the syntax of the query is defined by HSQLDB.

Author: Kristian Ovaska (Kristian.Ovaska@Helsinki.FI)
Version: 1.2
Categories: convert, Filter

Input name	Source	Description
table1	arrayCGHanalysis-aberrations-eq Data.table 19.47	CSV table 1. The table is referred to as 'table1' in the SQL query.
table2	ACGHmiRNAgainFreqs -occurances.table 19.26	CSV table 2. The table is referred to as 'table2' in the SQL query.

Parameter name	Value	Description
numIndices		Comma-separated list of index counts for input tables. All indices are single-column indices, running from column 1 to N, where N is retrieved from this parameter. If empty, the default number (1) is used. For example, ",2,,2" sets two indices for table2 and table4 and one index for the rest.
query	SELECT T2.".GeneId", (CAST(T2."count" AS DOUBLE) / (SELECT COUNT(DISTINCT T1."ID") FROM table1 T1)) AS "freq" FROM table2 T2	SQL query. Either this parameter or the query input must be provided, but not both.

19.23 ACGHmiRNAgainFreqs-geneFreqs (TableQuery)

Execute an SQL query on input relations and create a result table. TableQuery uses HSQLDB (Hyperthreaded Structured Query Language Database [25]) for executing the query. Consequently, the syntax of the query is defined by HSQLDB.

Author: Kristian Ovaska (Kristian.Ovaska@Helsinki.FI)
Version: 1.2
Categories: convert, Filter

Input name	Source	Description
table1	ACGHmiRNAgainFreqs -frequencies.table 19.22	CSV table 1. The table is referred to as 'table1' in the SQL query.
table2	ACGHmiRNAgainFreqs-chrAnnot Pre.bioAnnotation 19.19	CSV table 2. The table is referred to as 'table2' in the SQL query.

Parameter name	Value	Description
numIndices		Comma-separated list of index counts for input tables. All indices are single-column indices, running from column 1 to N, where N is retrieved from this parameter. If empty, the default number (1) is used. For example, ",2,,2" sets two indices for table2 and table4 and one index for the rest.
query	SELECT table2."miRNAName", table2.".GeneId", table2. "DNABand", table1."freq" FROM table2 LEFT OUTER JOIN table1 ON (table1.". GeneId" = table2.".GeneId")	SQL query. Either this parameter or the query input must be provided, but not both.

19.24 ACGHmiRNAgainFreqs-geneList (SearchReplace)

Replaces the occurences of the given regular expressions with the corresponding regular expressions. This component is used for transforming given patterns of the input file to a changed form specified by the regular expressions .

Author: Marko Laakso (Marko.Laakso@Helsinki.FI)
Version: 1.0
Category: convert

Input name	Source	Description
file	ACGHmiRNAgainFreqs-filtered ChrAnnot.table 19.21	Source file to be modified
rules	ACGHmiRNAgainFreqs-rules .in 19.27	A set of search keys

Parameter name	Value	Description
key00		Search key 0
key01		Search key 1
key02		Search key 2
key03		Search key 3
key04		Search key 4
key05		Search key 5
key06		Search key 6
key07		Search key 7
key08		Search key 8
key09		Search key 9
value00		Replacement 0
value01		Replacement 1
value02		Replacement 2
value03		Replacement 3
value04		Replacement 4
value05		Replacement 5
value06		Replacement 6
value07		Replacement 7
value08		Replacement 8
value09		Replacement 9

19.25 ACGHmiRNAgainFreqs-mirnaIDs (SearchReplace)

Replaces the occurences of the given regular expressions with the corresponding regular expressions. This component is used for transforming given patterns of the input file to a changed form specified by the regular expressions .

Author: Marko Laakso (Marko.Laakso@Helsinki.FI)
Version: 1.0
Category: convert

Input name	Source	Description
file	ACGHmiRNAgainFreqs-duplicate Mirnas.table 19.20	Source file to be modified

Parameter name	Value	Description
key00	HSA-MIR-	Search key 0
key01		Search key 1
key02		Search key 2
key03		Search key 3
key04		Search key 4
key05		Search key 5
key06		Search key 6
key07		Search key 7
key08		Search key 8
key09		Search key 9
value00	MIR	Replacement 0
value01		Replacement 1
value02		Replacement 2
value03		Replacement 3
value04		Replacement 4
value05		Replacement 5
value06		Replacement 6
value07		Replacement 7
value08		Replacement 8
value09		Replacement 9

19.26 ACGHmiRNAgainFreqs-occurances (TableQuery)

Execute an SQL query on input relations and create a result table. TableQuery uses HSQLDB (Hyperthreaded Structured Query Language Database [25]) for executing the query. Consequently, the syntax of the query is defined by HSQLDB.

Author: Kristian Ovaska (Kristian.Ovaska@Helsinki.FI)
Version: 1.2
Categories: convert, Filter

Input name	Source	Description
table1	arrayCGHanalysis-aberrations-eq Data.table 19.47	CSV table 1. The table is referred to as 'table1' in the SQL query.
table2	ACGHmiRNAgainFreqs-geneList .file 19.24	CSV table 2. The table is referred to as 'table2' in the SQL query.

Parameter name	Value	Description
numIndices		Comma-separated list of index counts for input tables. All indices are single-column indices, running from column 1 to N, where N is retrieved from this parameter. If empty, the default number (1) is used. For example, ",2,,2" sets two indices for table2 and table4 and one index for the rest.

Continued on next page...

Parameter name	Value	Description
query	SELECT T2.".GeneId", COUNT(T1."Sample") AS "count" FROM table1 T1, table2 T2 WHERE (T1."chr" = T2."chr") AND (T1. "start" <= T2."end") AND (T1."end" >= T2."start") AND (T1."call" = 1) GROUP BY T2.". GeneId"	SQL query. Either this parameter or the query input must be provided, but not both.

19.27 ACGHmiRNAgainFreqs-rules (INPUT)

Rules for string replacement in chromosome annotations.

Category: Internal

Parameter name	Value	Description
path	/gpfs/homes/mlaakso/asserSVN/ andurilCaseStudy/csv/ ACGHfreqRules.csv	Path (filename) of the input resource.

19.28 ACGHmiRNAlossFreqs (ACGHfrequencies)

Calculate copy number loss aberration frequencies for each microRNA individually over the sample set.

Input name	Source
genes	miRNAResults.dems 19.394
segments	arrayCGHanalysis.aberrations 19.43

Parameter name	Value
gainOrLoss	-1
miRNAs	true

19.29 ACGHmiRNAlossFreqs-chrAnnot (CSVCleaner)

CSVCleaner can be used to clean up Anduril outputs and to make them more suitable for other applications such as spreadsheets or text editors. The component is capable of removing file headers, quotations, and unused columns. Can be used to reorder and rename columns and their delimiters. Missing value symbols may be defined independently for the input and output.

Author: Marko Laakso (Marko.Laakso@Helsinki.FI)
Version: 1.6
Category: convert

Input name	Source	Description
original	ACGHmiRNAlossFreqs-chrAnnot Pre.bioAnnotation 19.30	Input file to be cleaned

Parameter name	Value	Description
columns	.GeneId,.DNARegion	Comma separated list of column selections for the output. An asterisk (*) may be used for all columns.
delimIn	\t	Column delimiter for the input
delimSymbol	\t	Column delimiter for the output
naIn	NA	Missing value symbol for the input
naSymbol	NA	Missing value symbol for the output
rename		Comma separated list of column renaming rules (oldname=newname)

Continued on next page...

Parameter name	Value	Description
rowSkip	0	Skip this many lines from the input before reading the tabular.
skipQuota		Comma separated list of output columns names that should not have quotation marks. An asterisk (*) may be used for all columns.

19.30 ACGHmiRNAlossFreqs-chrAnnotPre (KorvasieniAnnotator)

Korvasieni is an Ensembl [12] based converter for database identifiers and it can be used to convert genome location, gene, transcript, and translation identifiers across multiple biological databases. The selected `keyColumn` may contain entries with comma separated lists of values in which case `isListKey` parameter may be used to obtain each value separately and to process them as they were given in consecutive lines.

Author: Marko Laakso (Marko.Laakso@Helsinki.FI)
Version: 1.7
Categories: Annotation, GO

Input name	Source	Description
sourceKeys	ACGHmiRNAlossFreqs-mirnaIDs.file 19.36	A list of source database keys.
connection	ensembl.in 19.273	Database connection can be defined using this file. The definition of parameters: database.url, database.user, database.password, database.timeout, database.recycle, and database.driver can be found from the documentation of Korvasieni.

Parameter name	Value	Description
echoColumns	miRNAName	A comma separated list of column names for the columns that will be copied to the output. An asterisk (*) can be used to denote all columns execpt the keyColumn.
inputDB	.GeneName	Type of input keys. This must be a database supported by Korvasieni. If the parameter is omitted, the component tries to derive the database from the type of geneID. If this is not possible, an error is returned. You may define three columns in form of chromosome:start-end in case the inputDB is .DNARegion. This format provides a comfortable compatibility with DNARegion datatype.
inputType	Gene	Ensembl object type for the input keys (Any, Gene, Transcript, Translation)
isListKey	false	Enables the automatic value splits for the comma separated key column
keyColumn	miRNANameTrans	Name of the key column withing sourceKeys file or an empty string for the first column
maxHits	100000	Maximum number of target identifiers for a single source identifier
rename	.DNABand=DNABand	Comma separated list of column renaming rules (oldname=newname)
skipLevel	never	Skip result rows if the source identifier is unknown or target identifiers are not available. Possible values are: never (no filtering), source (skip if the source ID is unknown), target (skip if no target IDs are found), any (skip if any of the target IDs is missing).
targetDB	.GeneId,.DNARegion,.DNABand	Comma-separated list of annotation types. Possible values are all databases supported by Korvasieni.
unique	true	This flag can be turned on in order to eliminate duplicate annotations.

19.31 ACGHmiRNAlossFreqs-duplicateMirnas (TableQuery)

Execute an SQL query on input relations and create a result table. TableQuery uses HSQLDB (Hyper-threaded Structured Query Language Database [25]) for executing the query. Consequently, the syntax of the query is defined by HSQLDB.

Author: Kristian Ovaska (Kristian.Ovaska@Helsinki.FI)
Version: 1.2
Categories: convert, Filter

Input name	Source	Description
table1	miRNAResults-miRNAStat Filtered.csv 19.416	CSV table 1. The table is referred to as 'table1' in the SQL query.

Parameter name	Value	Description
numIndices		Comma-separated list of index counts for input tables. All indices are single-column indices, running from column 1 to N, where N is retrieved from this parameter. If empty, the default number (1) is used. For example, ",2,,2" sets two indices for table2 and table4 and one index for the rest.
query	SELECT "miRNAName", UCASE("miRNAName") AS "miRNANameTrans" FROM table1	SQL query. Either this parameter or the query input must be provided, but not both.

19.32 ACGHmiRNAlossFreqs-filteredChrAnnot (TableQuery)

Execute an SQL query on input relations and create a result table. TableQuery uses HSQLDB (Hyperthreaded Structured Query Language Database [25]) for executing the query. Consequently, the syntax of the query is defined by HSQLDB.

Author: Kristian Ovaska (Kristian.Ovaska@Helsinki.FI)
Version: 1.2
Categories: convert, Filter

Input name	Source	Description
table1	ACGHmiRNAlossFreqs-chrAnnot .table 19.29	CSV table 1. The table is referred to as 'table1' in the SQL query.

Parameter name	Value	Description
numIndices		Comma-separated list of index counts for input tables. All indices are single-column indices, running from column 1 to N, where N is retrieved from this parameter. If empty, the default number (1) is used. For example, ",2,,2" sets two indices for table2 and table4 and one index for the rest.
query	SELECT * FROM table1 WHERE ".DNARegion" IS NOT NULL	SQL query. Either this parameter or the query input must be provided, but not both.

19.33 ACGHmiRNAlossFreqs-frequencies (TableQuery)

Execute an SQL query on input relations and create a result table. TableQuery uses HSQLDB (Hyperthreaded Structured Query Language Database [25]) for executing the query. Consequently, the syntax of the query is defined by HSQLDB.

Author: Kristian Ovaska (Kristian.Ovaska@Helsinki.FI)
Version: 1.2
Categories: convert, Filter

Input name	Source	Description
table1	arrayCGHanalysis-aberrations-eq Data.table 19.47	CSV table 1. The table is referred to as 'table1' in the SQL query.
table2	ACGHmiRNAlossFreqs -occurances.table 19.37	CSV table 2. The table is referred to as 'table2' in the SQL query.

Parameter name	Value	Description
numIndices		Comma-separated list of index counts for input tables. All indices are single-column indices, running from column 1 to N, where N is retrieved from this parameter. If empty, the default number (1) is used. For example, ",2,,2" sets two indices for table2 and table4 and one index for the rest.
query	SELECT T2.".GeneId", (CAST(T2."count" AS DOUBLE) / (SELECT COUNT(DISTINCT T1."ID") FROM table1 T1)) AS "freq" FROM table2 T2	SQL query. Either this parameter or the query input must be provided, but not both.

Continued on next page...

Parameter name	Value	Description

19.34 ACGHmiRNAlossFreqs-geneFreqs (TableQuery)

Execute an SQL query on input relations and create a result table. TableQuery uses HSQLDB (Hyper-threaded Structured Query Language Database [25]) for executing the query. Consequently, the syntax of the query is defined by HSQLDB.

Author: Kristian Ovaska (Kristian.Ovaska@Helsinki.FI)
Version: 1.2
Categories: convert, Filter

Input name	Source	Description
table1	ACGHmiRNAlossFreqs -frequencies.table 19.33	CSV table 1. The table is referred to as 'table1' in the SQL query.
table2	ACGHmiRNAlossFreqs-chrAnnot Pre.bioAnnotation 19.30	CSV table 2. The table is referred to as 'table2' in the SQL query.

Parameter name	Value	Description
numIndices		Comma-separated list of index counts for input tables. All indices are single-column indices, running from column 1 to N, where N is retrieved from this parameter. If empty, the default number (1) is used. For example, ",2,,2" sets two indices for table2 and table4 and one index for the rest.
query	SELECT table2."miRNAName", table2.".GeneId", table2. "DNABand", table1."freq" FROM table2 LEFT OUTER JOIN table1 ON (table1.". GeneId" = table2.".GeneId")	SQL query. Either this parameter or the query input must be provided, but not both.

19.35 ACGHmiRNAlossFreqs-geneList (SearchReplace)

Replaces the occurences of the given regular expressions with the corresponding regular expressions. This component is used for transforming given patterns of the input file to a changed form specified by the regular expressions .

Author: Marko Laakso (Marko.Laakso@Helsinki.FI)
Version: 1.0
Category: convert

Input name	Source	Description
file	ACGHmiRNAlossFreqs-filtered ChrAnnot.table 19.32	Source file to be modified
rules	ACGHmiRNAlossFreqs-rules .in 19.38	A set of search keys

Parameter name	Value	Description
key00		Search key 0
key01		Search key 1
key02		Search key 2
key03		Search key 3
key04		Search key 4
key05		Search key 5
key06		Search key 6
key07		Search key 7
key08		Search key 8
key09		Search key 9
value00		Replacement 0
value01		Replacement 1

Continued on next page...

Parameter name	Value	Description
value02		Replacement 2
value03		Replacement 3
value04		Replacement 4
value05		Replacement 5
value06		Replacement 6
value07		Replacement 7
value08		Replacement 8
value09		Replacement 9

19.36 ACGHmiRNAlossFreqs-mirnaIDs (SearchReplace)

Replaces the occurences of the given regular expressions with the corresponding regular expressions. This component is used for transforming given patterns of the input file to a changed form specified by the regular expressions .

Author: Marko Laakso (Marko.Laakso@Helsinki.FI)
Version: 1.0
Category: convert

Input name	Source	Description
file	ACGHmiRNAlossFreqs-duplicateMirnas.table 19.31	Source file to be modified

Parameter name	Value	Description
key00	HSA-MIR-	Search key 0
key01		Search key 1
key02		Search key 2
key03		Search key 3
key04		Search key 4
key05		Search key 5
key06		Search key 6
key07		Search key 7
key08		Search key 8
key09		Search key 9
value00	MIR	Replacement 0
value01		Replacement 1
value02		Replacement 2
value03		Replacement 3
value04		Replacement 4
value05		Replacement 5
value06		Replacement 6
value07		Replacement 7
value08		Replacement 8
value09		Replacement 9

19.37 ACGHmiRNAlossFreqs-occurances (TableQuery)

Execute an SQL query on input relations and create a result table. TableQuery uses HSQLDB (Hyperthreaded Structured Query Language Database [25]) for executing the query. Consequently, the syntax of the query is defined by HSQLDB.

Author: Kristian Ovaska (Kristian.Ovaska@Helsinki.FI)
Version: 1.2
Categories: convert, Filter

Input name	Source	Description
table1	arrayCGHanalysis-aberrations-eqData.table 19.47	CSV table 1. The table is referred to as 'table1' in the SQL query.
table2	ACGHmiRNAlossFreqs-geneList.file 19.35	CSV table 2. The table is referred to as 'table2' in the SQL query.

Parameter name	Value	Description
numIndices		Comma-separated list of index counts for input tables. All indices are single-column indices, running from column 1 to N, where N is retrieved from this parameter. If empty, the default number (1) is used. For example, ",2,,2" sets two indices for table2 and table4 and one index for the rest.
query	SELECT T2.".GeneId", COUNT(T1."Sample") AS "count" FROM table1 T1, table2 T2 WHERE (T1."chr" = T2."chr") AND (T1. "start" <= T2."end") AND (T1."end" >= T2."start") AND (T1."call" = -1.0) GROUP BY T2.".GeneId"	SQL query. Either this parameter or the query input must be provided, but not both.

19.38 ACGHmiRNAlossFreqs-rules (INPUT)

Rules for string replacement in chromosome annotations.

Category: Internal

Parameter name	Value	Description
path	/gpfs/homes/mlaakso/asserSVN/andurilCaseStudy/csv/ACGHfreqRules.csv	Path (filename) of the input resource.

19.39 OUTPUT1 (OUTPUT)

Technical description of the complete analysis.

Category: Internal

Input name	Source	Description
out	summaryReport.document 19.834	The file or directory to be exported.

19.40 OUTPUT2 (OUTPUT)

Differentially expressed genes with their annotations, fold change information and p-values.

Category: Internal

Input name	Source	Description
out	geneExpression-degStat.table 19.286	The file or directory to be exported.

19.41 OUTPUT3 (OUTPUT)

Summary table of the entire integrated glioblastoma analysis in Excel spreadsheet format.

Category: Internal

Input name	Source	Description
out	summaryTable-excel.excelFile 19.844	The file or directory to be exported.

19.42 abstract (INPUT)

A very short description of this study

Category: Internal

Parameter name	Value	Description
path	/gpfs/homes/mlaakso/asserSVN/ andurilCaseStudy/abstract	Path (filename) of the input resource.

19.43 arrayCGHanalysis (ACGHAnalysis)

Performs copy number aberration analysis from array CGH data.

Parameter name	Value
datadir	data/acgh/acgh-data/cangem/DataFiles/

19.44 arrayCGHanalysis-aCGH (AgilentReader)

AgilentReader is a Anduril component that extracts gene expression measurements from the given set of Agilent gene expression measurements. The processing of input files is implemented using [23].

Author: Kristian Ovaska (kristian.ovaska@helsinki.fi)
Version: 1.0.1
Categories: Data Import, Agilent

Input name	Source	Description
agilent	arrayCGHanalysis-dataLoc .in 19.155	Agilent source file directory.
sampleNames	arrayCGHanalysis-samples .in 19.203	Sample definitions. The table contains the columns GreenSampleID (sample ID for the sample on green channel), GreenDescription (human-readable description for the sample), RedSampleID, RedDescription, Filename (key; relative to the Agilent source directory).

Parameter name	Value	Description
channelColumns	gProcessedSignal,gMedianSignal, rProcessedSignal,rMedianSignal	Column names for matrix extraction, in the order green, green2, red, red2. Empty values may be omitted, so "col1" is the same as "col1,,,". The default values, for Agilent two-channel arrays, extract preprocessed values into "green" and "red" and raw values into "green2" and "red2".
combineProbes	true	If true, duplicate probes (having the same sequence) are combined into one using median. If false, duplicate probes are present in the output, with unique internal names.
filter	ControlType!=0 || (gIsSaturated= =1 && rIsSaturated==1) || gIsFeatNonUnifOL!=0 || rIsFeatNonUnifOL!=0 || gIsBGNonUnifOL!=0 || rIsBGNonUnifOL!=0 || gIsFeatPopnOL!=0 || rIsFeatPopnOL!=0 || IsManualFlag!=0 || gIsPosAndSignif!=1 || rIsPosAndSignif!=1 || SystematicName=="unmapped"	Rows in source files matching this Boolean expression are excluded from the result. The expression can refer to any cell value of the current row using column names. Boolean and arithmetic operators and parenthesis as defined in Java are available. For example, "ControlType!=0 || (gIsSaturated==1 && rIsSaturated==1)" removes probes that are either control probes or are saturated on both green and red channels. ControlType, gIsSaturated and rIsSaturated must be valid columns in input files.
idColumn	ProbeName	Column name in input CSV files that gives the probe ID. Features having the same probe ID are assumed to be copies of the same probe.
probeAnnotation	SystematicName,Row,Col	Comma-separated list of column names in input CSV files that contain probe annotation. These are extracted to the probeAnnotation output.
sampleAnnotation	SampleID,Saturated,Channel	Comma-separated list of target sample annotation columns. These column names appear in the sampleAnnotation output. These columns need not be present in input CSV files.

Continued on next page...

Parameter name	Value	Description
sampleAnnotation Channel1		Comma-separated list of sample annotation columns for channel 1 in the input files. The value for channel 1 is extracted from these columns. The list must have equal length to the sampleAnnotation list.
sampleAnnotation Channel2		Comma-separated list of sample annotation columns for channel 2 in the input files. The value for channel 2 is extracted from these columns. The list must have equal length to the sampleAnnotation list. If empty, it is assumed that the array has one channel and annotations for channel 2 are not processed.
startPattern	\"?FEATURES\"?\t	Regular expression that identifies the start of content in input CSV files. This allows to skip some content from the beginning of files. The pattern is matched to the start of each line. The matching line must be a header that contains column names.

19.45 arrayCGHanalysis-abberSQL (INPUT)

SQL query for aberration pattern extraction.

Category: Internal

Parameter name	Value	Description
path	/gpfs/homes/mlaakso/asserSVN/ andurilCaseStudy/sql/ ACGHabber.sql	Path (filename) of the input resource.

19.46 arrayCGHanalysis-aberrations (QueryReport)

Print out aberration patterns.

Author: Marko Laakso (Marko.Laakso@Helsinki.FI)
Version: 1.1
Category: Latex

Parameter name	Value	Description
colFormat	lp{1cm}llp{2cm}p{1cm}p{1cm}	LaTeX tabular formatting for the result columns
listCols		Comma separated list of column names. Columns of this list may contain several values separated with commas and the delimiters will be replaced with spaces.
resTableRef	the previous table	Textual reference from the SQL representation to the result table
section	Copy number abberations	Subsection title for the output document
showCols	ID,chr,start,end,nroOfprobes, segmean,call	Comma separated list of column selections for the output. The empty default will use all columns.
showQuery	false	This flag tells if the SQL shall be shown on the report
tableCaption	Results for the query	Description for the result table

19.47 arrayCGHanalysis-aberrations-eqData (TableQuery)

Execute an SQL query on input relations and create a result table. TableQuery uses HSQLDB (Hyperthreaded Structured Query Language Database [25]) for executing the query. Consequently, the syntax of the query is defined by HSQLDB.

Author: Kristian Ovaska (Kristian.Ovaska@Helsinki.FI)
Version: 1.2
Categories: convert, Filter

Input name	Source	Description
table1	arrayCGHanalysis-segmented .table 19.204	CSV table 1. The table is referred to as 'table1' in the SQL query.
table2	arrayCGHanalysis-sampFileMap .in 19.202	CSV table 2. The table is referred to as 'table2' in the SQL query.
query	arrayCGHanalysis-abberSQL. in 19.45	SQL query. Either this file or the query parameter must be provided, but not both.

Continued on next page...

Input name	Source	Description

Parameter name	Value	Description
numIndices		Comma-separated list of index counts for input tables. All indices are single-column indices, running from column 1 to N, where N is retrieved from this parameter. If empty, the default number (1) is used. For example, ",2,,2" sets two indices for table2 and table4 and one index for the rest.
query		SQL query. Either this parameter or the query input must be provided, but not both.

19.48 arrayCGHanalysis-aberrations-eqTable (CSV2Latex)

This component converts a tabular CSV file into a printable LaTeX table.

Author: Marko Laakso (Marko.Laakso@Helsinki.FI)
Version: 2.1
Categories: convert, Latex

Input name	Source	Description
tabledata	arrayCGHanalysis-aberrations-eq Data.table 19.47	Table content

Parameter name	Value	Description
attach	false	Include the original data as an attachment
caption	Results for the query	Caption text for the table.
colFormat	lp{1cm}llp{2cm}p{1cm}p{1cm}	LaTeX tabular format for the columns. Default produces a centered layout for all columns.
columns	ID,chr,start,end,nroOfprobes, segmean,call	Comma separated list of column selections for the output. The empty default will use all columns.
countRows	false	Include a row count to the table caption.
dropMissing	true	This flag can be turned off in order to generate links with missing texts. Link text are substituted with target identifiers.
listCols		Comma separated list of column names. Columns of this list may contain several values separated with commas and the delimiters will be replaced with spaces.
numberFormat		A comma separated list of decimal formats for the columns. Each entry consists of the column name and the Java DecimalFormal pattern separated with equal sign. For example, rounding to three decimals can be done like: myColumn=#0.000
pageBreak	false	Use clear page after the table.
rename		Comma separated list of column renaming rules (oldname=newname). New names are used in table header but they do not affect the other behaviour of this component.
ruler	{}	Latex command for the row separating rulers
section	Copy number abberations	Section title for the table container or an empty string if no section should be generated.
sectionType	subsection	Type of LaTeX section: usually one of: section, subsection, or subsubsection. No section statement is written if section title is empty.
skipEmpty	false	This flag can be used to replace empty tables with a simple LaTeX comment.

19.49 arrayCGHanalysis-agil36to37map (INPUT)

Agilent probe mappings from human genome 36 to 37.

Category: Internal

Parameter name	Value	Description
path	/gpfs/homes/mlaakso/asserSVN/ andurilCaseStudy/data/acgh/ agilentProbes37.csv	Path (filename) of the input resource.

19.50 arrayCGHanalysis-analysis (LatexCombiner)

LatexCombiner joins the given subdocuments together into one LaTeX file.

Authors:	Kristian Ovaska (Kristian.Ovaska@Helsinki.FI), Marko Laakso (Marko.Laakso@Helsinki.FI)
Version:	1.3
Category:	Latex

Input name	Source	Description
latex1	arrayCGHanalysis-segmented Report.document 19.210	LaTeX fragment 1.
latex2	arrayCGHanalysis-segplot .plot 19.212	LaTeX fragment 2.
latex3	arrayCGHanalysis-gains-summary Report.document 19.175	LaTeX fragment 3.
latex4	arrayCGHanalysis-losses -summaryReport.document 19.194	LaTeX fragment 4.
latex5	arrayCGHanalysis-aberrations-eq Table.report 19.48	LaTeX fragment 5.
latex6	arrayCGHanalysis-qc .document 19.199	LaTeX fragment 6.

Parameter name	Value	Description
pagebreak	false	Determines if the result document should start with a page break.
sectionTitle	ArrayCGH: Segmentation Results	If non-empty, a declaration of a new section with the given name is inserted to the beginning of the combined document. This is a convenience feature to make it easy to compile subsections into a section.
sectionType	section	Type of LaTeX section: usually one of section, subsection or subsubsection. No section statement is written if sectionTitle is empty.
strictBorders	true	Enables the flushing of all document elements before each fragment

19.51 arrayCGHanalysis-both (ACGHSegmentAnalysis)

Analyze interesting regions from segmented Array CGH regions exhibiting copy number gain and loss.

Input name	Source
segments	arrayCGHanalysis-aberrations.data 19.46

Parameter name	Value
annotateRegs	false
gainOrLoss	0
threshold	0.2

19.52 arrayCGHanalysis-both-ArrayCGHgenes (TableQuery)

Execute an SQL query on input relations and create a result table. TableQuery uses HSQLDB (Hyper-threaded Structured Query Language Database [25]) for executing the query. Consequently, the syntax of the query is defined by HSQLDB.

Author:	Kristian Ovaska (Kristian.Ovaska@Helsinki.FI)
Version:	1.2
Categories:	convert, Filter

Input name	Source	Description
table1	arrayCGHanalysis-both-gAndLoss -annotGenes.bioAnnotation 19.62	CSV table 1. The table is referred to as 'table1' in the SQL query.

Parameter name	Value	Description
numIndices		Comma-separated list of index counts for input tables. All indices are single-column indices, running from column 1 to N, where N is retrieved from this parameter. If empty, the default number (1) is used. For example, ",2,,2" sets two indices for table2 and table4 and one index for the rest.
query	SELECT ".GeneId", "freq" FROM table1	SQL query. Either this parameter or the query input must be provided, but not both.

19.53 arrayCGHanalysis-both-associationLysis (associations)

Perform data transformation to enable regionwise association analysis.

Input name	Source
regions	arrayCGHanalysis-both-gAndLoss.regions 19.59
segments	arrayCGHanalysis-aberrations.data 19.46

19.54 arrayCGHanalysis-both-associationLysis-asc (INPUT)

SQL query for ACGH region association manipulation.

Category: Internal

Parameter name	Value	Description
path	/gpfs/homes/mlaakso/asserSVN/ andurilCaseStudy/sql/ ACGHassoc.sql	Path (filename) of the input resource.

19.55 arrayCGHanalysis-both-associationLysis-association (REvaluate)

This component produces some outputs by executing an R [20] script with the given input data. The exact functionality of this component depends on the given script.

The component is also capable of making branch decisions on Anduril workflow. Two alternative options (*altCase* and *defCase*) are provided.

Authors: Marko Laakso (Marko.Laakso@Helsinki.FI), Erkka Valo (erkka.valo@tkk.fi)
Version: 2.2
Categories: External, Latex, Network Control

Input name	Source	Description
script	arrayCGHanalysis-both -associationLysis-rev.in 19.58	Data transformation
table1	arrayCGHanalysis-both -associationLysis-association1 .table 19.56	Data matrix 1 (data.frame/file table1)

Parameter name	Value	Description
asConnection	false	If true, assign file connections (class file) to the inputs to variables of the same name. This is useful e.g. if you want to read large data files row by row.
param1		Value of this parameter will be assigned to param1 variable
param10		Value of this parameter will be assigned to param10 variable
param2		Value of this parameter will be assigned to param2 variable
param3		Value of this parameter will be assigned to param3 variable
param4		Value of this parameter will be assigned to param4 variable
param5		Value of this parameter will be assigned to param5 variable
param6		Value of this parameter will be assigned to param6 variable
param7		Value of this parameter will be assigned to param7 variable
param8		Value of this parameter will be assigned to param8 variable
param9		Value of this parameter will be assigned to param9 variable

19.56 arrayCGHanalysis-both-associationLysis-association1 (TableQuery)

Execute an SQL query on input relations and create a result table. TableQuery uses HSQLDB (Hyper-threaded Structured Query Language Database [25]) for executing the query. Consequently, the syntax of the query is defined by HSQLDB.

Author: Kristian Ovaska (Kristian.Ovaska@Helsinki.FI)
Version: 1.2
Categories: convert, Filter

Input name	Source	Description
table1	arrayCGHanalysis-aberrations-eqData.table 19.47	CSV table 1. The table is referred to as 'table1' in the SQL query.
table2	arrayCGHanalysis-both-segplot.SignRegion 19.64	CSV table 2. The table is referred to as 'table2' in the SQL query.
table3	arrayCGHanalysis-both-gAndLoss-annot.bioAnnotation 19.60	CSV table 3. The table is referred to as 'table3' in the SQL query.
query	arrayCGHanalysis-both-associationLysis-asc.in 19.54	SQL query. Either this file or the query parameter must be provided, but not both.

Parameter name	Value	Description
numIndices		Comma-separated list of index counts for input tables. All indices are single-column indices, running from column 1 to N, where N is retrieved from this parameter. If empty, the default number (1) is used. For example, ",2,,2" sets two indices for table2 and table4 and one index for the rest.
query		SQL query. Either this parameter or the query input must be provided, but not both.

19.57 arrayCGHanalysis-both-associationLysis-rBand (TableQuery)

Execute an SQL query on input relations and create a result table. TableQuery uses HSQLDB (Hyper-threaded Structured Query Language Database [25]) for executing the query. Consequently, the syntax of the query is defined by HSQLDB.

Author: Kristian Ovaska (Kristian.Ovaska@Helsinki.FI)
Version: 1.2
Categories: convert, Filter

Input name	Source	Description
table1	arrayCGHanalysis-both-associationLysis-association1.table 19.56	CSV table 1. The table is referred to as 'table1' in the SQL query.

Parameter name	Value	Description
numIndices		Comma-separated list of index counts for input tables. All indices are single-column indices, running from column 1 to N, where N is retrieved from this parameter. If empty, the default number (1) is used. For example, ",2,,2" sets two indices for table2 and table4 and one index for the rest.
query	SELECT DISTINCT("Region"), "band", "chr", "end", "start", SUM("rank")/COUNT("Region") AS "rank" FROM table1 GROUP BY "Region","band","chr","end", "start"	SQL query. Either this parameter or the query input must be provided, but not both.

19.58 arrayCGHanalysis-both-associationLysis-rev (INPUT)

R script for ACGH region association manipulation.

Category: Internal

Parameter name	Value	Description
path	/gpfs/homes/mlaakso/asserSVN/ andurilCaseStudy/r/ACGHreval.r	Path (filename) of the input resource.

19.59 arrayCGHanalysis-both-gAndLoss (AnnotateSegs)

This function annotates interesting genomic regions with ensembl genes.

19.60 arrayCGHanalysis-both-gAndLoss-annot (KorvasieniAnnotator)

Korvasieni is an Ensembl [12] based converter for database identifiers and it can be used to convert genome location, gene, transcript, and translation identifiers across multiple biological databases. The selected `keyColumn` may contain entries with comma separated lists of values in which case `isListKey` parameter may be used to obtain each value separately and to process them as they were given in consecutive lines.

Author: Marko Laakso (Marko.Laakso@Helsinki.FI)
Version: 1.7
Categories: Annotation, GO

Input name	Source	Description
sourceKeys	arrayCGHanalysis-both-segplot.S ignRegion 19.64	A list of source database keys.
connection	ensembl.in 19.273	Database connection can be defined using this file. The definition of parameters: database.url, database.user, database.password, database.timeout, database.recycle, and database.driver can be found from the documentation of Korvasieni.

Parameter name	Value	Description
echoColumns	ID,freq,chromosome	A comma separated list of column names for the columns that will be copied to the output. An asterisk (*) can be used to denote all columns execpt the keyColumn.
inputDB	.DNARegion	Type of input keys. This must be a database supported by Korvasieni. If the parameter is omitted, the component tries to derive the database from the type of geneID. If this is not possible, an error is returned. You may define three columns in form of chromosome:start-end in case the inputDB is .DNARegion. This format provides a comfortable compatibility with DNARegion datatype.
inputType	Gene	Ensembl object type for the input keys (Any, Gene, Transcript, Translation)
isListKey	false	Enables the automatic value splits for the comma separated key column
keyColumn	chromosome:start-end	Name of the key column withing sourceKeys file or an empty string for the first column
maxHits	100000	Maximum number of target identifiers for a single source identifier
rename	chromosome:start-end=chr	Comma separated list of column renaming rules (oldname=newname)
skipLevel	never	Skip result rows if the source identifier is unknown or target identifiers are not available. Possible values are: never (no filtering), source (skip if the source ID is unknown), target (skip if no target IDs are found), any (skip if any of the target IDs is missing).
targetDB	.GeneId,.DNABand	Comma-separated list of annotation types. Possible values are all databases supported by Korvasieni.
unique	true	This flag can be turned on in order to eliminate duplicate annotations.

19.61 arrayCGHanalysis-both-gAndLoss-annotExpand (ExpandCollapse)

Converts between the two possible representations of a relations with multivalued columns. By default this component splits rows with multivalued (comma separated lists) columns into multiple rows each representing a single value. The input may contain several columns with comma separated values and all permutations of column values are shown as individual single-valued rows. The alternative mode recovers the original comma separated values from the expanded relations. This is performed by joining values of

the their list columns into comma separated lists.

Author: Marko Laakso (Marko.Laakso@Helsinki.FI)
Version: 1.3
Category: convert

Input name	Source	Description
relation	arrayCGHanalysis-both-gAndLoss-annot.bioAnnotation 19.60	The mandatory input relation

Parameter name	Value	Description
delim	,	Value delimiter between the list column values. Special characters can be encoded as specified in fi.helsinki.ltdk.csbl.asser.ArgumentEncoding.
expand	true	Action mode that is expand (true) or collapse (false)
listCols	.GeneId	A comma separated list of column names of input (expand) or output (collapse) columns that may contain delim separated values. The asterisk refers to every column of the input relation.

19.62 arrayCGHanalysis-both-gAndLoss-annotGenes (KorvasieniAnnotator)

Korvasieni is an Ensembl [12] based converter for database identifiers and it can be used to convert genome location, gene, transcript, and translation identifiers across multiple biological databases. The selected `keyColumn` may contain entries with comma separated lists of values in which case `isListKey` parameter may be used to obtain each value separately and to process them as they were given in consecutive lines.

Author: Marko Laakso (Marko.Laakso@Helsinki.FI)
Version: 1.7
Categories: Annotation, GO

Input name	Source	Description
sourceKeys	arrayCGHanalysis-both-gAndLoss-annotExpand.relation 19.61	A list of source database keys.
connection	ensembl.in 19.273	Database connection can be defined using this file. The definition of parameters: database.url, database.user, database.password, database.timeout, database.recycle, and database.driver can be found from the documentation of Korvasieni.

Parameter name	Value	Description
echoColumns	freq,chr,chromosome,ID	A comma separated list of column names for the columns that will be copied to the output. An asterisk (*) can be used to denote all columns execpt the keyColumn.
inputDB	.GeneId	Type of input keys. This must be a database supported by Korvasieni. If the parameter is omitted, the component tries to derive the database from the type of geneID. If this is not possible, an error is returned. You may define three columns in form of chromosome:start-end in case the inputDB is .DNARegion. This format provides a comfortable compatibility with DNARegion datatype.
inputType	Gene	Ensembl object type for the input keys (Any, Gene, Transcript, Translation)
isListKey	false	Enables the automatic value splits for the comma separated key column
keyColumn	.GeneId	Name of the key column withing sourceKeys file or an empty string for the first column
maxHits	100000	Maximum number of target identifiers for a single source identifier
rename	.GeneName=gene name,ID=Region	Comma separated list of column renaming rules (oldname=newname)
skipLevel	never	Skip result rows if the source identifier is unknown or target identifiers are not available. Possible values are: never (no filtering), source (skip if the source ID is unknown), target (skip if no target IDs are found), any (skip if any of the target IDs is missing).
targetDB	.GeneName,.GeneDesc,.DNABand,.DNARegion,GO	Comma-separated list of annotation types. Possible values are all databases supported by Korvasieni.
unique	true	This flag can be turned on in order to eliminate duplicate annotations.

19.63 arrayCGHanalysis-both-gAndLoss-filtSegs (TableQuery)

Execute an SQL query on input relations and create a result table. TableQuery uses HSQLDB (Hyper-threaded Structured Query Language Database [25]) for executing the query. Consequently, the syntax of the query is defined by HSQLDB.

Author:	Kristian Ovaska (Kristian.Ovaska@Helsinki.FI)
Version:	1.2
Categories:	convert, Filter

Input name	Source	Description
table1	arrayCGHanalysis-both-gAndLoss -annotGenes.bioAnnotation 19.62	CSV table 1. The table is referred to as 'table1' in the SQL query.

Parameter name	Value	Description
numIndices		Comma-separated list of index counts for input tables. All indices are single-column indices, running from column 1 to N, where N is retrieved from this parameter. If empty, the default number (1) is used. For example, ",2,,2" sets two indices for table2 and table4 and one index for the rest.
query	SELECT * FROM table1 ORDER BY "freq" DESC	SQL query. Either this parameter or the query input must be provided, but not both.

19.64 arrayCGHanalysis-both-segplot (SegmentPlot)

This component has two purposes: first, to plot segmented chromosomal data; second, call frequently occuring regions therein. Any chromosomal regions defined with chromosome, start and end locations can be plotted, but the main purpose of this component is to visualize Array CGH segmentation results. It can also be utilized for ChIPonChip analysis pipeline in a subset of use cases.

Deleted and gained regions can be analyzed separately or jointly. The input table must contain the following columns: Sample, chromosome, starting basepair, ending basepair. Segment call and score are optional columns. These are used to distinguish between duplications and deletions and to assign confidences or ranks to the calls, respectively.

Authors:	Riku Louhimo (Riku.Louhimo@Helsinki.FI), Marko Laakso (Marko.Laakso@Helsinki.FI)
Version:	1.5
Categories:	Latex, Copy Number Analysis

Input name	Source	Description
chrSegments	arrayCGHanalysis-aberrations-eq Data.table 19.47	Chromosomal segments, usually obtained from an Array CGH experiment after segmentation.

Parameter name	Value	Description
bpEndColumn	end	The name of the chromosomal end location column in the input file.
bpStartColumn	start	The name of the chromosomal start location column in the input file.
callColumn	call	The name of the call column in the input file. All calls are considered to be 1 if this parameter value is empty.
chrColumn	chr	The name of the chromosomal number column in the input file.
excludeChr	23,24,X,Y,M	Chromosomes that are not used in the analysis. Defaults to excluding mitochondrial and sex chromosomes.
gainsOrLosses	0	Determine whether to plot gains alone (1) or losses alone (-1) or both (default).
lineWidth	1	Line width in output plot
sThold	0.2	The significance threshold. Please note that this is just the fraction of samples we want the segment to be present in.
sampleColumn	ID	The name of the sample name column in the input file.
scoreColumn		The name of the score column in the input file. You may leave this parameter empty if the data do not contain such information.
scoreMerge	max	Defines an R function that is used to summarize the score values of all input calls that are contributing to an output regions.

19.65 arrayCGHanalysis-both-summaryReport (LatexCombiner)

LatexCombiner joins the given subdocuments together into one LaTeX file.

Authors:	Kristian Ovaska (Kristian.Ovaska@Helsinki.FI), Marko Laakso (Marko.Laakso@Helsinki.FI)
Version:	1.3
Category:	Latex

Input name	Source	Description
latex1	arrayCGHanalysis-both-segplot .plot 19.64	LaTeX fragment 1.

Parameter name	Value	Description
pagebreak	false	Determines if the result document should start with a page break.
sectionTitle		If non-empty, a declaration of a new section with the given name is inserted to the beginning of the combined document. This is a convenience feature to make it easy to compile subsections into a section.
sectionType	section	Type of LaTeX section: usually one of section, subsection or subsubsection. No section statement is written if sectionTitle is empty.
strictBorders	true	Enables the flushing of all document elements before each fragment

19.66 arrayCGHanalysis-boxPlot (BoxPlot)

BoxPlot generates box-and-whisker diagrams for the given sets of expression data. Each diagram shows the smallest expression, lower quartile, median, upper quartile, and largest expression value for each gene across all samples.

Authors:	Kristian Ovaska (Kristian.Ovaska@Helsinki.FI), Marko Laakso (Marko.Laakso@Helsinki.FI)
Version:	1.0.1
Categories:	Plot, Quality Control, Latex

Input name	Source	Description
matr1	arrayCGHanalysis-normalized .casechannel 19.196	Matrix data 1.

Parameter name	Value	Description
height	11	Height of each plot in cm.
pagebreak	false	Tells if the result document should start with a page break.
plotType	boxplot	Choices are: boxplot, violin.
title1		Main title for plot 1, e.g. "Log Ratio".
title2		Main title for plot 2, e.g. "Log Ratio".
title3		Main title for plot 3, e.g. "Log Ratio".
violinWidth	1.2	Multiplier for the violin plot widths.
width	14	Width of each plot in cm.
xvertical	true	X axis labels are printed in vertical direction.
ylabel1		Label for y axis in plot 1.
ylabel2		Label for y axis in plot 2.
ylabel3		Label for y axis in plot 3.

19.67 arrayCGHanalysis-chr1 (Segmentation)

No description

Parameter name	Value
chr	1

19.68 arrayCGHanalysis-chr1-chrFilter (CSVFilter)

Filters columns and/or rows from CSV files using flexible criteria. Default settings do not filter out anything. The *negate* parameter reverses all inclusion criteria.

Columns are filtered based on column names (see *includeColumns*); it is also possible to rename columns. Columns can be reordered using *includeColumns*.

A row is included in output if all conditions are satisfied. Rows having too many missing values are filtered using the *nonMissing* parameter. The parameters *regexp*, *lowBound* and *highBound* filter rows based on cell contents. If the *auxiliary* input is given, the result contains those rows from the input whose values match those from the auxiliary file.

Authors:	Kristian Ovaska (kristian.ovaska@helsinki.fi), Marko Laakso (Marko.Laakso@Helsinki.FI)
Version:	1.1
Category:	Filter

Input name	Source	Description
csv	arrayCGHanalysis-srFAnnot. file 19.213	CSV file to be filtered.

Parameter name	Value	Description
highBound		A row is included if the value in given numeric columns are at most as large as given numeric bounds. The parameter has a format COLNAME1=HIGH1,COLNAME2=HIGH2.
idColumn		Column name in "csv" that contains ID values. If the parameter is empty, the first column is used.
includeColumns	*	Comma-separated list of column names that should be included in the result file. The special value * includes all columns. The order of column names is significant: the output columns are in the order given. See also includeColumns input.
lowBound		A row is included if the value in given numeric columns are at least as large as given numeric bounds. The parameter has a format COLNAME1=LOW1,COLNAME2=LOW2. For example, "col=5" includes rows where the column col has a value of at least 5.
matchColumn		Column name in "auxiliary" containing values that must match the ID column in "csv" (specified using "idColumn"). If empty, the first column of "auxiliary" is used.
negate	false	If true, all inclusion criteria are negated so that rows or columns are excluded instead of included if they match the criteria. For example, includeColumns=* then excludes all columns (which would be an error since at least one column must be included).
nonMissing	0	Include those rows that have non-missing (non-NA) values in at least this many columns. Only those columns that are part of the output are counted.
regexp	Chr=1	Row filtering based on regular expression. A row is included in the result if values in the given columns match given regular expressions. The parameter has a format COLNAME1=EXPRESSION,COLNAME2=EXPRESSION2 where COLNAMEs are column names in "csv" and EXPRESSIONs are regular expressions using Java syntax. For example, "col=a|b" includes rows where the column col has a value of "a" or "b". Commas have to be escaped with "\\" if they are used in column names or regular expressions.
rename		Comma-separated list of column renaming rules (OLDNAME=NEWNAME). Parameters that refer to column names refer to old names; renaming is done last.

19.69 arrayCGHanalysis-chr1-filtNormCaseChan (CSVFilter)

Filters columns and/or rows from CSV files using flexible criteria. Default settings do not filter out anything. The *negate* parameter reverses all inclusion criteria.

Columns are filtered based on column names (see *includeColumns*); it is also possible to rename columns. Columns can be reordered using *includeColumns*.

A row is included in output if all conditions are satisfied. Rows having too many missing values are filtered using the *nonMissing* parameter. The parameters *regexp*, *lowBound* and *highBound* filter rows based on

cell contents. If the *auxiliary* input is given, the result contains those rows from the input whose values match those from the auxiliary file.

Authors:	Kristian Ovaska (kristian.ovaska@helsinki.fi), Marko Laakso (Marko.Laakso@Helsinki.FI)
Version:	1.1
Category:	Filter

Input name	Source	Description
csv	arrayCGHanalysis-normalized .casechannel 19.196	CSV file to be filtered.
auxiliary	arrayCGHanalysis-chr1-chrFilter .csv 19.68	If given, contains one column (see "matchColumn") whose values are matched to a column in the "csv" input (see "idColumn").

Parameter name	Value	Description
highBound		A row is included if the value in given numeric columns are at most as large as given numeric bounds. The parameter has a format COLNAME1=HIGH1,COLNAME2=HIGH2.
idColumn		Column name in "csv" that contains ID values. If the parameter is empty, the first column is used.
includeColumns	*	Comma-separated list of column names that should be included in the result file. The special value * includes all columns. The order of column names is significant: the output columns are in the order given. See also includeColumns input.
lowBound		A row is included if the value in given numeric columns are at least as large as given numeric bounds. The parameter has a format COLNAME1=LOW1,COLNAME2=LOW2. For example, "col=5" includes rows where the column col has a value of at least 5.
matchColumn	ProbeName	Column name in "auxiliary" containing values that must match the ID column in "csv" (specified using "idColumn"). If empty, the first column of "auxiliary" is used.
negate	false	If true, all inclusion criteria are negated so that rows or columns are excluded instead of included if they match the criteria. For example, includeColumns=* then excludes all columns (which would be an error since at least one column must be included).
nonMissing	0	Include those rows that have non-missing (non-NA) values in at least this many columns. Only those columns that are part of the output are counted.
regexp		Row filtering based on regular expression. A row is included in the result if values in the given columns match given regular expressions. The parameter has a format COLNAME1=EXPRESSION,COLNAME2=EXPRESSION2 where COLNAMEs are column names in "csv" and EXPRESSIONs are regular expressions using Java syntax. For example, "col=a|b" includes rows where the column col has a value of "a" or "b". Commas have to be escaped with "\\" if they are used in column names or regular expressions.
rename		Comma-separated list of column renaming rules (OLDNAME=NEWNAME). Parameters that refer to column names refer to old names; renaming is done last.

19.70 arrayCGHanalysis-chr1-segmented (ACGHsegment)

ACGHsegment segments normalized array CGH data, and calls aberrations in one of two ways: by classification of probe data discretely to gain, normal or loss, or computing probabilities to of a gain, normal or loss state for each probe. Segmentation utilizes the Circular Binary Segmentation algorithm [15].

Two ways of analysing Array CGH data post-segmentation are included. The first one, the Genome Topography Scan [27] computes scores over all the samples to calculate the most recurrent aberrations over the sample set. The second one outputs all the regions in all the samples whose segmented means are more than two standard deviations apart from the mean of all the intensities. Plots from the GTS as well as from the segmented intensities are output depending on the parameter setting.

Probabilities for copy number aberrations can be computed with two algorithms. The first, FastCall [3], is faster while the second, CGHcall [26], is more accurate.

Author: Riku Louhimo (Riku.Louhimo@Helsinki.FI)
Version: 1.3
Categories: Agilent, Copy Number Analysis

Input name	Source	Description
caseChan	arrayCGHanalysis-chr1-filtNorm CaseChan.csv 19.69	CSV file containing the normalized probe intensities for the case channel. First row should have the probenames and samples should be columnwise.
geneAnnotation	arrayCGHanalysis-chr1-chrFilter .csv 19.68	Probewise annotations as produced by the AgilenReader component. Only the probes present in the casechannel csv can be included. Use CSVFilter if the probes do not otherwise match.

Parameter name	Value	Description
CGHCallPrior	auto	Only used if callProbMethod=CGHCall. Set the method to determine prior probabilities to CGHCall algorithm. Must be one of "auto", "all", "not all".
analysisAlgorithm	0	1 means that only the GTS algorithm is run, 2 that only the standard deviation test is run. 0 indicates that both are run.
callProbMethod	CGHCall	Method with which to call CNA segment probabilities. Must be either CGHCall or FastCall. FastCall is significantly (30000 times) faster while CGHCall is more accurate.
callProbabilities	false	Enabling this will make the component estimate probabilties for CNA segments via the CGHcall package. Only guaranteed to work when multiple chromosomes are analyzed simultaneously.
lowerLimit	-0.631759089316322	Use this as the intensity limit for calling a segment lossed. Default value computes the mean of the sample set and estimates the lower threshold for CNA call to be two standard deviations from it.
nSegFit	3000	Maximum number of segments used for fitting the mixture model in probability calculations. Disabled if callProbabilities=false. Decreasing this lowers accuracy but can speed computation significantly.
plotChromosomes	0	Defines the plots that the user wants to output as a comma separated list. The whole genome is plotted by default for each sample. Inputting a value different than 0 will generate additional plots for these specific chromosomes from each sample.
plotEverySample	false	Enabling this will make the component print each sample separately.
undoSplits	sdundo	A character string specifying how change-points are to be undone, if at all. Undoing change-points decreases the sensitivity of segmentation. Choices are "none","prune", which uses a sum of squares criterion, and "sdundo" (default), which undoes splits that are not at least this many SDs apart. SD by default is 3.
upperLimit	0.631759089316322	Use this as the intensity limit for calling a segment gained. Default value computes the mean of the sample set and estimates the upper threshold for CNA call to be two standard deviations from it.

19.71 arrayCGHanalysis-chr10 (Segmentation)

No description

Parameter name	Value
chr	10

19.72 arrayCGHanalysis-chr10-chrFilter (CSVFilter)

Filters columns and/or rows from CSV files using flexible criteria. Default settings do not filter out anything. The *negate* parameter reverses all inclusion criteria.

Columns are filtered based on column names (see *includeColumns*); it is also possible to rename columns. Columns can be reordered using *includeColumns*.

A row is included in output if all conditions are satisfied. Rows having too many missing values are filtered using the *nonMissing* parameter. The parameters *regexp*, *lowBound* and *highBound* filter rows based on cell contents. If the *auxiliary* input is given, the result contains those rows from the input whose values match those from the auxiliary file.

Authors: Kristian Ovaska (kristian.ovaska@helsinki.fi), Marko Laakso (Marko.Laakso@Helsinki.FI)
Version: 1.1
Category: Filter

Input name	Source	Description
csv	arrayCGHanalysis-srFAnnot.file 19.213	CSV file to be filtered.

Parameter name	Value	Description
highBound		A row is included if the value in given numeric columns are at most as large as given numeric bounds. The parameter has a format COLNAME1=HIGH1,COLNAME2=HIGH2.
idColumn		Column name in "csv" that contains ID values. If the parameter is empty, the first column is used.
includeColumns	*	Comma-separated list of column names that should be included in the result file. The special value * includes all columns. The order of column names is significant: the output columns are in the order given. See also includeColumns input.
lowBound		A row is included if the value in given numeric columns are at least as large as given numeric bounds. The parameter has a format COLNAME1=LOW1,COLNAME2=LOW2. For example, "col=5" includes rows where the column col has a value of at least 5.
matchColumn		Column name in "auxiliary" containing values that must match the ID column in "csv" (specified using "idColumn"). If empty, the first column of "auxiliary" is used.
negate	false	If true, all inclusion criteria are negated so that rows or columns are excluded instead of included if they match the criteria. For example, includeColumns=* then excludes all columns (which would be an error since at least one column must be included).
nonMissing	0	Include those rows that have non-missing (non-NA) values in at least this many columns. Only those columns that are part of the output are counted.
regexp	Chr=10	Row filtering based on regular expression. A row is included in the result if values in the given columns match given regular expressions. The parameter has a format COLNAME1=EXPRESSION,COLNAME2=EXPRESSION2 where COLNAMEs are column names in "csv" and EXPRESSIONs are regular expressions using Java syntax. For example, "col=a|b" includes rows where the column col has a value of "a" or "b". Commas have to be escaped with "\\" if they are used in column names or regular expressions.
rename		Comma-separated list of column renaming rules (OLDNAME=NEWNAME). Parameters that refer to column names refer to old names; renaming is done last.

19.73 arrayCGHanalysis-chr10-filtNormCaseChan (CSVFilter)

Filters columns and/or rows from CSV files using flexible criteria. Default settings do not filter out anything. The *negate* parameter reverses all inclusion criteria.

Columns are filtered based on column names (see *includeColumns*); it is also possible to rename columns. Columns can be reordered using *includeColumns*.

A row is included in output if all conditions are satisfied. Rows having too many missing values are filtered using the *nonMissing* parameter. The parameters *regexp*, *lowBound* and *highBound* filter rows based on cell contents. If the *auxiliary* input is given, the result contains those rows from the input whose values match those from the auxiliary file.

Authors: Kristian Ovaska (kristian.ovaska@helsinki.fi), Marko Laakso (Marko.Laakso@Helsinki.FI)
Version: 1.1
Category: Filter

Input name	Source	Description
csv	arrayCGHanalysis-normalized.casechannel 19.196	CSV file to be filtered.
auxiliary	arrayCGHanalysis-chr10-chrFilter.csv 19.72	If given, contains one column (see "matchColumn") whose values are matched to a column in the "csv" input (see "idColumn").

Parameter name	Value	Description
highBound		A row is included if the value in given numeric columns are at most as large as given numeric bounds. The parameter has a format COLNAME1=HIGH1,COLNAME2=HIGH2.
idColumn		Column name in "csv" that contains ID values. If the parameter is empty, the first column is used.
includeColumns	*	Comma-separated list of column names that should be included in the result file. The special value * includes all columns. The order of column names is significant: the output columns are in the order given. See also includeColumns input.
lowBound		A row is included if the value in given numeric columns are at least as large as given numeric bounds. The parameter has a format COLNAME1=LOW1,COLNAME2=LOW2. For example, "col=5" includes rows where the column col has a value of at least 5.
matchColumn	ProbeName	Column name in "auxiliary" containing values that must match the ID column in "csv" (specified using "idColumn"). If empty, the first column of "auxiliary" is used.
negate	false	If true, all inclusion criteria are negated so that rows or columns are excluded instead of included if they match the criteria. For example, includeColumns=* then excludes all columns (which would be an error since at least one column must be included).
nonMissing	0	Include those rows that have non-missing (non-NA) values in at least this many columns. Only those columns that are part of the output are counted.
regexp		Row filtering based on regular expression. A row is included in the result if values in the given columns match given regular expressions. The parameter has a format COLNAME1=EXPRESSION,COLNAME2=EXPRESSION2 where COLNAMEs are column names in "csv" and EXPRESSIONs are regular expressions using Java syntax. For example, "col=a|b" includes rows where the column col has a value of "a" or "b". Commas have to be escaped with "\\" if they are used in column names or regular expressions.
rename		Comma-separated list of column renaming rules (OLDNAME=NEWNAME). Parameters that refer to column names refer to old names; renaming is done last.

19.74 arrayCGHanalysis-chr10-segmented (ACGHsegment)

ACGHsegment segments normalized array CGH data, and calls aberrations in one of two ways: by classification of probe data discretely to gain, normal or loss, or computing probabilities to of a gain, normal or loss state for each probe. Segmentation utilizes the Circular Binary Segmentation algorithm [15].

Two ways of analysing Array CGH data post-segmentation are included. The first one, the Genome Topography Scan [27] computes scores over all the samples to calculate the most recurrent aberrations over the sample set. The second one outputs all the regions in all the samples whose segmented means are more than two standard deviations apart from the mean of all the intensities. Plots from the GTS as well as from the segmented intensities are output depending on the parameter setting.

Probabilities for copy number aberrations can be computed with two algorithms. The first, FastCall [3], is faster while the second, CGHcall [26], is more accurate.

Author:	Riku Louhimo (Riku.Louhimo@Helsinki.FI)
Version:	1.3
Categories:	Agilent, Copy Number Analysis

Input name	Source	Description
caseChan	arrayCGHanalysis-chr10-filtNorm CaseChan.csv 19.73	CSV file containing the normalized probe intensities for the case channel. First row should have the probenames and samples should be columnwise.
geneAnnotation	arrayCGHanalysis-chr10-chrFilter .csv 19.72	Probewise annotations as produced by the AgilenReader component. Only the probes present in the casechannel csv can be included. Use CSVFilter if the probes do not otherwise match.

Parameter name	Value	Description
CGHCallPrior	auto	Only used if callProbMethod=CGHCall. Set the method to determine prior probabilities to CGHCall algorithm. Must be one of "auto", "all", "not all".
analysisAlgorithm	0	1 means that only the GTS algorithm is run, 2 that only the standard deviation test is run. 0 indicates that both are run.

Continued on next page...

Parameter name	Value	Description
callProbMethod	CGHCall	Method with which to call CNA segment probabilities. Must be either CGHCall or FastCall. FastCall is significantly (30000 times) faster while CGHCall is more accurate.
callProbabilities	false	Enabling this will make the component estimate probabilties for CNA segments via the CGHcall package. Only guaranteed to work when multiple chromosomes are analyzed simultaneously.
lowerLimit	-0.631759089316322	Use this as the intensity limit for calling a segment lossed. Default value computes the mean of the sample set and estimates the lower threshold for CNA call to be two standard deviations from it.
nSegFit	3000	Maximum number of segments used for fitting the mixture model in probability calculations. Disabled if callProbabilities=false. Decreasing this lowers accuracy but can speed computation significantly.
plotChromosomes	0	Defines the plots that the user wants to output as a comma separated list. The whole genome is plotted by default for each sample. Inputting a value different than 0 will generate additional plots for these specific chromosomes from each sample.
plotEverySample	false	Enabling this will make the component print each sample separately.
undoSplits	sdundo	A character string specifying how change-points are to be undone, if at all. Undoing change-points decreases the sensitivity of segmentation. Choices are "none","prune", which uses a sum of squares criterion, and "sdundo" (default), which undoes splits that are not at least this many SDs apart. SD by default is 3.
upperLimit	0.631759089316322	Use this as the intensity limit for calling a segment gained. Default value computes the mean of the sample set and estimates the upper threshold for CNA call to be two standard deviations from it.

19.75 arrayCGHanalysis-chr11 (Segmentation)

No description

Parameter name	Value
chr	11

19.76 arrayCGHanalysis-chr11-chrFilter (CSVFilter)

Filters columns and/or rows from CSV files using flexible criteria. Default settings do not filter out anything. The *negate* parameter reverses all inclusion criteria.

Columns are filtered based on column names (see *includeColumns*); it is also possible to rename columns. Columns can be reordered using *includeColumns*.

A row is included in output if all conditions are satisfied. Rows having too many missing values are filtered using the *nonMissing* parameter. The parameters *regexp*, *lowBound* and *highBound* filter rows based on cell contents. If the *auxiliary* input is given, the result contains those rows from the input whose values match those from the auxiliary file.

Authors:	Kristian Ovaska (kristian.ovaska@helsinki.fi), Marko Laakso (Marko.Laakso@Helsinki.FI)
Version:	1.1
Category:	Filter

Input name	Source	Description
csv	arrayCGHanalysis-srFAnnot.file 19.213	CSV file to be filtered.

Parameter name	Value	Description
highBound		A row is included if the value in given numeric columns are at most as large as given numeric bounds. The parameter has a format COLNAME1=HIGH1,COLNAME2=HIGH2.
idColumn		Column name in "csv" that contains ID values. If the parameter is empty, the first column is used.

Continued on next page...

Parameter name	Value	Description
includeColumns	*	Comma-separated list of column names that should be included in the result file. The special value * includes all columns. The order of column names is significant: the output columns are in the order given. See also includeColumns input.
lowBound		A row is included if the value in given numeric columns are at least as large as given numeric bounds. The parameter has a format COLNAME1=LOW1,COLNAME2=LOW2. For example, "col=5" includes rows where the column col has a value of at least 5.
matchColumn		Column name in "auxiliary" containing values that must match the ID column in "csv" (specified using "idColumn"). If empty, the first column of "auxiliary" is used.
negate	false	If true, all inclusion criteria are negated so that rows or columns are excluded instead of included if they match the criteria. For example, includeColumns=* then excludes all columns (which would be an error since at least one column must be included).
nonMissing	0	Include those rows that have non-missing (non-NA) values in at least this many columns. Only those columns that are part of the output are counted.
regexp	Chr=11	Row filtering based on regular expression. A row is included in the result if values in the given columns match given regular expressions. The parameter has a format COLNAME1=EXPRESSION,COLNAME2=EXPRESSION2 where COLNAMEs are column names in "csv" and EXPRESSIONs are regular expressions using Java syntax. For example, "col=a|b" includes rows where the column col has a value of "a" or "b". Commas have to be escaped with "\\" if they are used in column names or regular expressions.
rename		Comma-separated list of column renaming rules (OLDNAME=NEWNAME). Parameters that refer to column names refer to old names; renaming is done last.

19.77 arrayCGHanalysis-chr11-filtNormCaseChan (CSVFilter)

Filters columns and/or rows from CSV files using flexible criteria. Default settings do not filter out anything. The *negate* parameter reverses all inclusion criteria.

Columns are filtered based on column names (see *includeColumns*); it is also possible to rename columns. Columns can be reordered using *includeColumns*.

A row is included in output if all conditions are satisfied. Rows having too many missing values are filtered using the *nonMissing* parameter. The parameters *regexp*, *lowBound* and *highBound* filter rows based on cell contents. If the *auxiliary* input is given, the result contains those rows from the input whose values match those from the auxiliary file.

Authors: Kristian Ovaska (kristian.ovaska@helsinki.fi), Marko Laakso (Marko.Laakso@Helsinki.FI)
Version: 1.1
Category: Filter

Input name	Source	Description
csv	arrayCGHanalysis-normalized .casechannel 19.196	CSV file to be filtered.
auxiliary	arrayCGHanalysis-chr11-chrFilter .csv 19.76	If given, contains one column (see "matchColumn") whose values are matched to a column in the "csv" input (see "idColumn").

Parameter name	Value	Description
highBound		A row is included if the value in given numeric columns are at most as large as given numeric bounds. The parameter has a format COLNAME1=HIGH1,COLNAME2=HIGH2.
idColumn		Column name in "csv" that contains ID values. If the parameter is empty, the first column is used.
includeColumns	*	Comma-separated list of column names that should be included in the result file. The special value * includes all columns. The order of column names is significant: the output columns are in the order given. See also includeColumns input.
lowBound		A row is included if the value in given numeric columns are at least as large as given numeric bounds. The parameter has a format COLNAME1=LOW1,COLNAME2=LOW2. For example, "col=5" includes rows where the column col has a value of at least 5.

Continued on next page...

Parameter name	Value	Description
matchColumn	ProbeName	Column name in "auxiliary" containing values that must match the ID column in "csv" (specified using "idColumn"). If empty, the first column of "auxiliary" is used.
negate	false	If true, all inclusion criteria are negated so that rows or columns are excluded instead of included if they match the criteria. For example, includeColumns=* then excludes all columns (which would be an error since at least one column must be included).
nonMissing	0	Include those rows that have non-missing (non-NA) values in at least this many columns. Only those columns that are part of the output are counted.
regexp		Row filtering based on regular expression. A row is included in the result if values in the given columns match given regular expressions. The parameter has a format COLNAME1=EXPRESSION,COLNAME2=EXPRESSION2 where COLNAMEs are column names in "csv" and EXPRESSIONs are regular expressions using Java syntax. For example, "col=a|b" includes rows where the column col has a value of "a" or "b". Commas have to be escaped with "\\" if they are used in column names or regular expressions.
rename		Comma-separated list of column renaming rules (OLDNAME=NEWNAME). Parameters that refer to column names refer to old names; renaming is done last.

19.78 arrayCGHanalysis-chr11-segmented (ACGHsegment)

ACGHsegment segments normalized array CGH data, and calls aberrations in one of two ways: by classification of probe data discretely to gain, normal or loss, or computing probabilities to of a gain, normal or loss state for each probe. Segmentation utilizes the Circular Binary Segmentation algorithm [15].

Two ways of analysing Array CGH data post-segmentation are included. The first one, the Genome Topography Scan [27] computes scores over all the samples to calculate the most recurrent aberrations over the sample set. The second one outputs all the regions in all the samples whose segmented means are more than two standard deviations apart from the mean of all the intensities. Plots from the GTS as well as from the segmented intensities are output depending on the parameter setting.

Probabilities for copy number aberrations can be computed with two algorithms. The first, FastCall [3], is faster while the second, CGHcall [26], is more accurate.

Author:	Riku Louhimo (Riku.Louhimo@Helsinki.FI)
Version:	1.3
Categories:	Agilent, Copy Number Analysis

Input name	Source	Description
caseChan	arrayCGHanalysis-chr11-filtNorm CaseChan.csv 19.77	CSV file containing the normalized probe intensities for the case channel. First row should have the probenames and samples should be columnwise.
geneAnnotation	arrayCGHanalysis-chr11-chrFilter .csv 19.76	Probewise annotations as produced by the AgilenReader component. Only the probes present in the casechannel csv can be included. Use CSVFilter if the probes do not otherwise match.

Parameter name	Value	Description
CGHCallPrior	auto	Only used if callProbMethod=CGHCall. Set the method to determine prior probabilities to CGHCall algorithm. Must be one of "auto", "all", "not all".
analysisAlgorithm	0	1 means that only the GTS algorithm is run, 2 that only the standard deviation test is run. 0 indicates that both are run.
callProbMethod	CGHCall	Method with which to call CNA segment probabilities. Must be either CGHCall or FastCall. FastCall is significantly (30000 times) faster while CGHCall is more accurate.
callProbabilities	false	Enabling this will make the component estimate probabilties for CNA segments via the CGHcall package. Only guaranteed to work when multiple chromosomes are analyzed simultaneously.
lowerLimit	-0.631759089316322	Use this as the intensity limit for calling a segment lossed. Default value computes the mean of the sample set and estimates the lower threshold for CNA call to be two standard deviations from it.
nSegFit	3000	Maximum number of segments used for fitting the mixture model in probability calculations. Disabled if callProbabilities=false. Decreasing this lowers accuracy but can speed computation significantly.

Continued on next page...

Parameter name	Value	Description
plotChromosomes	0	Defines the plots that the user wants to output as a comma separated list. The whole genome is plotted by default for each sample. Inputting a value different than 0 will generate additional plots for these specific chromosomes from each sample.
plotEverySample	false	Enabling this will make the component print each sample separately.
undoSplits	sdundo	A character string specifying how change-points are to be undone, if at all. Undoing change-points decreases the sensitivity of segmentation. Choices are "none","prune", which uses a sum of squares criterion, and "sdundo" (default), which undoes splits that are not at least this many SDs apart. SD by default is 3.
upperLimit	0.631759089316322	Use this as the intensity limit for calling a segment gained. Default value computes the mean of the sample set and estimates the upper threshold for CNA call to be two standard deviations from it.

19.79 arrayCGHanalysis-chr12 (Segmentation)

No description

Parameter name	Value
chr	12

19.80 arrayCGHanalysis-chr12-chrFilter (CSVFilter)

Filters columns and/or rows from CSV files using flexible criteria. Default settings do not filter out anything. The *negate* parameter reverses all inclusion criteria.

Columns are filtered based on column names (see *includeColumns*); it is also possible to rename columns. Columns can be reordered using *includeColumns*.

A row is included in output if all conditions are satisfied. Rows having too many missing values are filtered using the *nonMissing* parameter. The parameters *regexp*, *lowBound* and *highBound* filter rows based on cell contents. If the *auxiliary* input is given, the result contains those rows from the input whose values match those from the auxiliary file.

Authors:	Kristian Ovaska (kristian.ovaska@helsinki.fi), Marko Laakso (Marko.Laakso@Helsinki.FI)
Version:	1.1
Category:	Filter

Input name	Source	Description
csv	arrayCGHanalysis-srFAnnot. file 19.213	CSV file to be filtered.

Parameter name	Value	Description
highBound		A row is included if the value in given numeric columns are at most as large as given numeric bounds. The parameter has a format COLNAME1=HIGH1,COLNAME2=HIGH2.
idColumn		Column name in "csv" that contains ID values. If the parameter is empty, the first column is used.
includeColumns	*	Comma-separated list of column names that should be included in the result file. The special value * includes all columns. The order of column names is significant: the output columns are in the order given. See also includeColumns input.
lowBound		A row is included if the value in given numeric columns are at least as large as given numeric bounds. The parameter has a format COLNAME1=LOW1,COLNAME2=LOW2. For example, "col=5" includes rows where the column col has a value of at least 5.
matchColumn		Column name in "auxiliary" containing values that must match the ID column in "csv" (specified using "idColumn"). If empty, the first column of "auxiliary" is used.
negate	false	If true, all inclusion criteria are negated so that rows or columns are excluded instead of included if they match the criteria. For example, includeColumns=* then excludes all columns (which would be an error since at least one column must be included).

Continued on next page...

Parameter name	Value	Description
nonMissing	0	Include those rows that have non-missing (non-NA) values in at least this many columns. Only those columns that are part of the output are counted.
regexp	Chr=12	Row filtering based on regular expression. A row is included in the result if values in the given columns match given regular expressions. The parameter has a format COLNAME1=EXPRESSION,COLNAME2=EXPRESSION2 where COLNAMEs are column names in "csv" and EXPRESSIONs are regular expressions using Java syntax. For example, "col=a|b" includes rows where the column col has a value of "a" or "b". Commas have to be escaped with "\\" if they are used in column names or regular expressions.
rename		Comma-separated list of column renaming rules (OLDNAME=NEWNAME). Parameters that refer to column names refer to old names; renaming is done last.

19.81 arrayCGHanalysis-chr12-filtNormCaseChan (CSVFilter)

Filters columns and/or rows from CSV files using flexible criteria. Default settings do not filter out anything. The *negate* parameter reverses all inclusion criteria.

Columns are filtered based on column names (see *includeColumns*); it is also possible to rename columns. Columns can be reordered using *includeColumns*.

A row is included in output if all conditions are satisfied. Rows having too many missing values are filtered using the *nonMissing* parameter. The parameters *regexp*, *lowBound* and *highBound* filter rows based on cell contents. If the *auxiliary* input is given, the result contains those rows from the input whose values match those from the auxiliary file.

Authors: Kristian Ovaska (kristian.ovaska@helsinki.fi), Marko Laakso (Marko.Laakso@Helsinki.FI)
Version: 1.1
Category: Filter

Input name	Source	Description
csv	arrayCGHanalysis-normalized .casechannel 19.196	CSV file to be filtered.
auxiliary	arrayCGHanalysis-chr12-chrFilter .csv 19.80	If given, contains one column (see "matchColumn") whose values are matched to a column in the "csv" input (see "idColumn").

Parameter name	Value	Description
highBound		A row is included if the value in given numeric columns are at most as large as given numeric bounds. The parameter has a format COLNAME1=HIGH1,COLNAME2=HIGH2.
idColumn		Column name in "csv" that contains ID values. If the parameter is empty, the first column is used.
includeColumns	*	Comma-separated list of column names that should be included in the result file. The special value * includes all columns. The order of column names is significant: the output columns are in the order given. See also includeColumns input.
lowBound		A row is included if the value in given numeric columns are at least as large as given numeric bounds. The parameter has a format COLNAME1=LOW1,COLNAME2=LOW2. For example, "col=5" includes rows where the column col has a value of at least 5.
matchColumn	ProbeName	Column name in "auxiliary" containing values that must match the ID column in "csv" (specified using "idColumn"). If empty, the first column of "auxiliary" is used.
negate	false	If true, all inclusion criteria are negated so that rows or columns are excluded instead of included if they match the criteria. For example, includeColumns=* then excludes all columns (which would be an error since at least one column must be included).
nonMissing	0	Include those rows that have non-missing (non-NA) values in at least this many columns. Only those columns that are part of the output are counted.
regexp		Row filtering based on regular expression. A row is included in the result if values in the given columns match given regular expressions. The parameter has a format COLNAME1=EXPRESSION,COLNAME2=EXPRESSION2 where COLNAMEs are column names in "csv" and EXPRESSIONs are regular expressions using Java syntax. For example, "col=a|b" includes rows where the column col has a value of "a" or "b". Commas have to be escaped with "\\" if they are used in column names or regular expressions.

Continued on next page...

Parameter name	Value	Description
rename		Comma-separated list of column renaming rules (OLDNAME=NEWNAME). Parameters that refer to column names refer to old names; renaming is done last.

19.82 arrayCGHanalysis-chr12-segmented (ACGHsegment)

ACGHsegment segments normalized array CGH data, and calls aberrations in one of two ways: by classification of probe data discretely to gain, normal or loss, or computing probabilities to of a gain, normal or loss state for each probe. Segmentation utilizes the Circular Binary Segmentation algorithm [15].

Two ways of analysing Array CGH data post-segmentation are included. The first one, the Genome Topography Scan [27] computes scores over all the samples to calculate the most recurrent aberrations over the sample set. The second one outputs all the regions in all the samples whose segmented means are more than two standard deviations apart from the mean of all the intensities. Plots from the GTS as well as from the segmented intensities are output depending on the parameter setting.

Probabilities for copy number aberrations can be computed with two algorithms. The first, FastCall [3], is faster while the second, CGHcall [26], is more accurate.

Author:	Riku Louhimo (Riku.Louhimo@Helsinki.FI)
Version:	1.3
Categories:	Agilent, Copy Number Analysis

Input name	Source	Description
caseChan	arrayCGHanalysis-chr12-filtNorm CaseChan.csv 19.81	CSV file containing the normalized probe intensities for the case channel. First row should have the probenames and samples should be columnwise.
geneAnnotation	arrayCGHanalysis-chr12-chrFilter .csv 19.80	Probewise annotations as produced by the AgilenReader component. Only the probes present in the casechannel csv can be included. Use CSVFilter if the probes do not otherwise match.

Parameter name	Value	Description
CGHCallPrior	auto	Only used if callProbMethod=CGHCall. Set the method to determine prior probabilities to CGHCall algorithm. Must be one of "auto", "all", "not all".
analysisAlgorithm	0	1 means that only the GTS algorithm is run, 2 that only the standard deviation test is run. 0 indicates that both are run.
callProbMethod	CGHCall	Method with which to call CNA segment probabilities. Must be either CGHCall or FastCall. FastCall is significantly (30000 times) faster while CGHCall is more accurate.
callProbabilities	false	Enabling this will make the component estimate probabilties for CNA segments via the CGHcall package. Only guaranteed to work when multiple chromosomes are analyzed simultaneously.
lowerLimit	-0.631759089316322	Use this as the intensity limit for calling a segment lossed. Default value computes the mean of the sample set and estimates the lower threshold for CNA call to be two standard deviations from it.
nSegFit	3000	Maximum number of segments used for fitting the mixture model in probability calculations. Disabled if callProbabilities=false. Decreasing this lowers accuracy but can speed computation significantly.
plotChromosomes	0	Defines the plots that the user wants to output as a comma separated list. The whole genome is plotted by default for each sample. Inputting a value different than 0 will generate additional plots for these specific chromosomes from each sample.
plotEverySample	false	Enabling this will make the component print each sample separately.
undoSplits	sdundo	A character string specifying how change-points are to be undone, if at all. Undoing change-points decreases the sensitivity of segmentation. Choices are "none","prune", which uses a sum of squares criterion, and "sdundo" (default), which undoes splits that are not at least this many SDs apart. SD by default is 3.
upperLimit	0.631759089316322	Use this as the intensity limit for calling a segment gained. Default value computes the mean of the sample set and estimates the upper threshold for CNA call to be two standard deviations from it.

19.83 arrayCGHanalysis-chr13 (Segmentation)

No description

Parameter name	Value
chr	13

19.84 arrayCGHanalysis-chr13-chrFilter (CSVFilter)

Filters columns and/or rows from CSV files using flexible criteria. Default settings do not filter out anything. The *negate* parameter reverses all inclusion criteria.

Columns are filtered based on column names (see *includeColumns*); it is also possible to rename columns. Columns can be reordered using *includeColumns*.

A row is included in output if all conditions are satisfied. Rows having too many missing values are filtered using the *nonMissing* parameter. The parameters *regexp*, *lowBound* and *highBound* filter rows based on cell contents. If the *auxiliary* input is given, the result contains those rows from the input whose values match those from the auxiliary file.

Authors:	Kristian Ovaska (kristian.ovaska@helsinki.fi), Marko Laakso (Marko.Laakso@Helsinki.FI)
Version:	1.1
Category:	Filter

Input name	Source	Description
csv	arrayCGHanalysis-srFAnnot.file 19.213	CSV file to be filtered.

Parameter name	Value	Description
highBound		A row is included if the value in given numeric columns are at most as large as given numeric bounds. The parameter has a format COLNAME1=HIGH1,COLNAME2=HIGH2.
idColumn		Column name in "csv" that contains ID values. If the parameter is empty, the first column is used.
includeColumns	*	Comma-separated list of column names that should be included in the result file. The special value * includes all columns. The order of column names is significant: the output columns are in the order given. See also includeColumns input.
lowBound		A row is included if the value in given numeric columns are at least as large as given numeric bounds. The parameter has a format COLNAME1=LOW1,COLNAME2=LOW2. For example, "col=5" includes rows where the column col has a value of at least 5.
matchColumn		Column name in "auxiliary" containing values that must match the ID column in "csv" (specified using "idColumn"). If empty, the first column of "auxiliary" is used.
negate	false	If true, all inclusion criteria are negated so that rows or columns are excluded instead of included if they match the criteria. For example, includeColumns=* then excludes all columns (which would be an error since at least one column must be included).
nonMissing	0	Include those rows that have non-missing (non-NA) values in at least this many columns. Only those columns that are part of the output are counted.
regexp	Chr=13	Row filtering based on regular expression. A row is included in the result if values in the given columns match given regular expressions. The parameter has a format COLNAME1=EXPRESSION,COLNAME2=EXPRESSION2 where COLNAMEs are column names in "csv" and EXPRESSIONs are regular expressions using Java syntax. For example, "col=a|b" includes rows where the column col has a value of "a" or "b". Commas have to be escaped with "\\" if they are used in column names or regular expressions.
rename		Comma-separated list of column renaming rules (OLDNAME=NEWNAME). Parameters that refer to column names refer to old names; renaming is done last.

19.85 arrayCGHanalysis-chr13-filtNormCaseChan (CSVFilter)

Filters columns and/or rows from CSV files using flexible criteria. Default settings do not filter out anything. The *negate* parameter reverses all inclusion criteria.

Columns are filtered based on column names (see *includeColumns*); it is also possible to rename columns. Columns can be reordered using *includeColumns*.

A row is included in output if all conditions are satisfied. Rows having too many missing values are filtered using the *nonMissing* parameter. The parameters *regexp*, *lowBound* and *highBound* filter rows based on cell contents. If the *auxiliary* input is given, the result contains those rows from the input whose values match those from the auxiliary file.

Authors:	Kristian Ovaska (kristian.ovaska@helsinki.fi), Marko Laakso (Marko.Laakso@Helsinki.FI)
Version:	1.1
Category:	Filter

Input name	Source	Description
csv	arrayCGHanalysis-normalized .casechannel 19.196	CSV file to be filtered.
auxiliary	arrayCGHanalysis-chr13-chrFilter .csv 19.84	If given, contains one column (see "matchColumn") whose values are matched to a column in the "csv" input (see "idColumn").

Parameter name	Value	Description
highBound		A row is included if the value in given numeric columns are at most as large as given numeric bounds. The parameter has a format COLNAME1=HIGH1,COLNAME2=HIGH2.
idColumn		Column name in "csv" that contains ID values. If the parameter is empty, the first column is used.
includeColumns	*	Comma-separated list of column names that should be included in the result file. The special value * includes all columns. The order of column names is significant: the output columns are in the order given. See also includeColumns input.
lowBound		A row is included if the value in given numeric columns are at least as large as given numeric bounds. The parameter has a format COLNAME1=LOW1,COLNAME2=LOW2. For example, "col=5" includes rows where the column col has a value of at least 5.
matchColumn	ProbeName	Column name in "auxiliary" containing values that must match the ID column in "csv" (specified using "idColumn"). If empty, the first column of "auxiliary" is used.
negate	false	If true, all inclusion criteria are negated so that rows or columns are excluded instead of included if they match the criteria. For example, includeColumns=* then excludes all columns (which would be an error since at least one column must be included).
nonMissing	0	Include those rows that have non-missing (non-NA) values in at least this many columns. Only those columns that are part of the output are counted.
regexp		Row filtering based on regular expression. A row is included in the result if values in the given columns match given regular expressions. The parameter has a format COLNAME1=EXPRESSION,COLNAME2=EXPRESSION2 where COLNAMEs are column names in "csv" and EXPRESSIONs are regular expressions using Java syntax. For example, "col=a|b" includes rows where the column col has a value of "a" or "b". Commas have to be escaped with "\\" if they are used in column names or regular expressions.
rename		Comma-separated list of column renaming rules (OLDNAME=NEWNAME). Parameters that refer to column names refer to old names; renaming is done last.

19.86 arrayCGHanalysis-chr13-segmented (ACGHsegment)

ACGHsegment segments normalized array CGH data, and calls aberrations in one of two ways: by classification of probe data discretely to gain, normal or loss, or computing probabilities to of a gain, normal or loss state for each probe. Segmentation utilizes the Circular Binary Segmentation algorithm [15].

Two ways of analysing Array CGH data post-segmentation are included. The first one, the Genome Topography Scan [27] computes scores over all the samples to calculate the most recurrent aberrations over the sample set. The second one outputs all the regions in all the samples whose segmented means

are more than two standard deviations apart from the mean of all the intensities. Plots from the GTS as well as from the segmented intensities are output depending on the parameter setting.

Probabilities for copy number aberrations can be computed with two algorithms. The first, FastCall [3], is faster while the second, CGHcall [26], is more accurate.

Author:	Riku Louhimo (Riku.Louhimo@Helsinki.FI)
Version:	1.3
Categories:	Agilent, Copy Number Analysis

Input name	Source	Description
caseChan	arrayCGHanalysis-chr13-filtNorm CaseChan.csv 19.85	CSV file containing the normalized probe intensities for the case channel. First row should have the probenames and samples should be columnwise.
geneAnnotation	arrayCGHanalysis-chr13-chrFilter .csv 19.84	Probewise annotations as produced by the AgilenReader component. Only the probes present in the casechannel csv can be included. Use CSVFilter if the probes do not otherwise match.

Parameter name	Value	Description
CGHCallPrior	auto	Only used if callProbMethod=CGHCall. Set the method to determine prior probabilities to CGHCall algorithm. Must be one of "auto", "all", "not all".
analysisAlgorithm	0	1 means that only the GTS algorithm is run, 2 that only the standard deviation test is run. 0 indicates that both are run.
callProbMethod	CGHCall	Method with which to call CNA segment probabilities. Must be either CGHCall or FastCall. FastCall is significantly (30000 times) faster while CGHCall is more accurate.
callProbabilities	false	Enabling this will make the component estimate probabilties for CNA segments via the CGHcall package. Only guaranteed to work when multiple chromosomes are analyzed simultaneously.
lowerLimit	-0.631759089316322	Use this as the intensity limit for calling a segment lossed. Default value computes the mean of the sample set and estimates the lower threshold for CNA call to be two standard deviations from it.
nSegFit	3000	Maximum number of segments used for fitting the mixture model in probability calculations. Disabled if callProbabilities=false. Decreasing this lowers accuracy but can speed computation significantly.
plotChromosomes	0	Defines the plots that the user wants to output as a comma separated list. The whole genome is plotted by default for each sample. Inputting a value different than 0 will generate additional plots for these specific chromosomes from each sample.
plotEverySample	false	Enabling this will make the component print each sample separately.
undoSplits	sdundo	A character string specifying how change-points are to be undone, if at all. Undoing change-points decreases the sensitivity of segmentation. Choices are "none","prune", which uses a sum of squares criterion, and "sdundo" (default), which undoes splits that are not at least this many SDs apart. SD by default is 3.
upperLimit	0.631759089316322	Use this as the intensity limit for calling a segment gained. Default value computes the mean of the sample set and estimates the upper threshold for CNA call to be two standard deviations from it.

19.87 arrayCGHanalysis-chr14 (Segmentation)

No description

Parameter name	Value
chr	14

19.88 arrayCGHanalysis-chr14-chrFilter (CSVFilter)

Filters columns and/or rows from CSV files using flexible criteria. Default settings do not filter out anything. The *negate* parameter reverses all inclusion criteria.

Columns are filtered based on column names (see *includeColumns*); it is also possible to rename columns. Columns can be reordered using *includeColumns*.

A row is included in output if all conditions are satisfied. Rows having too many missing values are filtered

using the *nonMissing* parameter. The parameters *regexp*, *lowBound* and *highBound* filter rows based on cell contents. If the *auxiliary* input is given, the result contains those rows from the input whose values match those from the auxiliary file.

Authors:	Kristian Ovaska (kristian.ovaska@helsinki.fi), Marko Laakso (Marko.Laakso@Helsinki.FI)
Version:	1.1
Category:	Filter

Input name	Source	Description
csv	arrayCGHanalysis-srFAnnot.file 19.213	CSV file to be filtered.

Parameter name	Value	Description
highBound		A row is included if the value in given numeric columns are at most as large as given numeric bounds. The parameter has a format COLNAME1=HIGH1,COLNAME2=HIGH2.
idColumn		Column name in "csv" that contains ID values. If the parameter is empty, the first column is used.
includeColumns	*	Comma-separated list of column names that should be included in the result file. The special value * includes all columns. The order of column names is significant: the output columns are in the order given. See also includeColumns input.
lowBound		A row is included if the value in given numeric columns are at least as large as given numeric bounds. The parameter has a format COLNAME1=LOW1,COLNAME2=LOW2. For example, "col=5" includes rows where the column col has a value of at least 5.
matchColumn		Column name in "auxiliary" containing values that must match the ID column in "csv" (specified using "idColumn"). If empty, the first column of "auxiliary" is used.
negate	false	If true, all inclusion criteria are negated so that rows or columns are excluded instead of included if they match the criteria. For example, includeColumns=* then excludes all columns (which would be an error since at least one column must be included).
nonMissing	0	Include those rows that have non-missing (non-NA) values in at least this many columns. Only those columns that are part of the output are counted.
regexp	Chr=14	Row filtering based on regular expression. A row is included in the result if values in the given columns match given regular expressions. The parameter has a format COLNAME1=EXPRESSION,COLNAME2=EXPRESSION2 where COLNAMEs are column names in "csv" and EXPRESSIONs are regular expressions using Java syntax. For example, "col=a|b" includes rows where the column col has a value of "a" or "b". Commas have to be escaped with "\\" if they are used in column names or regular expressions.
rename		Comma-separated list of column renaming rules (OLDNAME=NEWNAME). Parameters that refer to column names refer to old names; renaming is done last.

19.89 arrayCGHanalysis-chr14-filtNormCaseChan (CSVFilter)

Filters columns and/or rows from CSV files using flexible criteria. Default settings do not filter out anything. The *negate* parameter reverses all inclusion criteria.

Columns are filtered based on column names (see *includeColumns*); it is also possible to rename columns. Columns can be reordered using *includeColumns*.

A row is included in output if all conditions are satisfied. Rows having too many missing values are filtered using the *nonMissing* parameter. The parameters *regexp*, *lowBound* and *highBound* filter rows based on cell contents. If the *auxiliary* input is given, the result contains those rows from the input whose values match those from the auxiliary file.

Authors:	Kristian Ovaska (kristian.ovaska@helsinki.fi), Marko Laakso (Marko.Laakso@Helsinki.FI)
Version:	1.1
Category:	Filter

Input name	Source	Description
csv	arrayCGHanalysis-normalized .casechannel 19.196	CSV file to be filtered.
auxiliary	arrayCGHanalysis-chr14-chrFilter .csv 19.88	If given, contains one column (see "matchColumn") whose values are matched to a column in the "csv" input (see "idColumn").

Parameter name	Value	Description
highBound		A row is included if the value in given numeric columns are at most as large as given numeric bounds. The parameter has a format COLNAME1=HIGH1,COLNAME2=HIGH2.
idColumn		Column name in "csv" that contains ID values. If the parameter is empty, the first column is used.
includeColumns	*	Comma-separated list of column names that should be included in the result file. The special value * includes all columns. The order of column names is significant: the output columns are in the order given. See also includeColumns input.
lowBound		A row is included if the value in given numeric columns are at least as large as given numeric bounds. The parameter has a format COLNAME1=LOW1,COLNAME2=LOW2. For example, "col=5" includes rows where the column col has a value of at least 5.
matchColumn	ProbeName	Column name in "auxiliary" containing values that must match the ID column in "csv" (specified using "idColumn"). If empty, the first column of "auxiliary" is used.
negate	false	If true, all inclusion criteria are negated so that rows or columns are excluded instead of included if they match the criteria. For example, includeColumns=* then excludes all columns (which would be an error since at least one column must be included).
nonMissing	0	Include those rows that have non-missing (non-NA) values in at least this many columns. Only those columns that are part of the output are counted.
regexp		Row filtering based on regular expression. A row is included in the result if values in the given columns match given regular expressions. The parameter has a format COLNAME1=EXPRESSION,COLNAME2=EXPRESSION2 where COLNAMEs are column names in "csv" and EXPRESSIONs are regular expressions using Java syntax. For example, "col=a|b" includes rows where the column col has a value of "a" or "b". Commas have to be escaped with "\\" if they are used in column names or regular expressions.
rename		Comma-separated list of column renaming rules (OLDNAME=NEWNAME). Parameters that refer to column names refer to old names; renaming is done last.

19.90 arrayCGHanalysis-chr14-segmented (ACGHsegment)

ACGHsegment segments normalized array CGH data, and calls aberrations in one of two ways: by classification of probe data discretely to gain, normal or loss, or computing probabilities to of a gain, normal or loss state for each probe. Segmentation utilizes the Circular Binary Segmentation algorithm [15].

Two ways of analysing Array CGH data post-segmentation are included. The first one, the Genome Topography Scan [27] computes scores over all the samples to calculate the most recurrent aberrations over the sample set. The second one outputs all the regions in all the samples whose segmented means are more than two standard deviations apart from the mean of all the intensities. Plots from the GTS as well as from the segmented intensities are output depending on the parameter setting.

Probabilities for copy number aberrations can be computed with two algorithms. The first, FastCall [3], is faster while the second, CGHcall [26], is more accurate.

Author:	Riku Louhimo (Riku.Louhimo@Helsinki.FI)
Version:	1.3
Categories:	Agilent, Copy Number Analysis

Input name	Source	Description
caseChan	arrayCGHanalysis-chr14-filtNorm CaseChan.csv 19.89	CSV file containing the normalized probe intensities for the case channel. First row should have the probenames and samples should be columnwise.
geneAnnotation	arrayCGHanalysis-chr14-chrFilter .csv 19.88	Probewise annotations as produced by the AgilenReader component. Only the probes present in the casechannel csv can be included. Use CSVFilter if the probes do not otherwise match.

Parameter name	Value	Description
CGHCallPrior	auto	Only used if callProbMethod=CGHCall. Set the method to determine prior probabilities to CGHCall algorithm. Must be one of "auto", "all", "not all".
analysisAlgorithm	0	1 means that only the GTS algorithm is run, 2 that only the standard deviation test is run. 0 indicates that both are run.
callProbMethod	CGHCall	Method with which to call CNA segment probabilities. Must be either CGHCall or FastCall. FastCall is significantly (30000 times) faster while CGHCall is more accurate.
callProbabilities	false	Enabling this will make the component estimate probabilties for CNA segments via the CGHcall package. Only guaranteed to work when multiple chromosomes are analyzed simultaneously.
lowerLimit	-0.631759089316322	Use this as the intensity limit for calling a segment lossed. Default value computes the mean of the sample set and estimates the lower threshold for CNA call to be two standard deviations from it.
nSegFit	3000	Maximum number of segments used for fitting the mixture model in probability calculations. Disabled if callProbabilities=false. Decreasing this lowers accuracy but can speed computation significantly.
plotChromosomes	0	Defines the plots that the user wants to output as a comma separated list. The whole genome is plotted by default for each sample. Inputting a value different than 0 will generate additional plots for these specific chromosomes from each sample.
plotEverySample	false	Enabling this will make the component print each sample separately.
undoSplits	sdundo	A character string specifying how change-points are to be undone, if at all. Undoing change-points decreases the sensitivity of segmentation. Choices are "none","prune", which uses a sum of squares criterion, and "sdundo" (default), which undoes splits that are not at least this many SDs apart. SD by default is 3.
upperLimit	0.631759089316322	Use this as the intensity limit for calling a segment gained. Default value computes the mean of the sample set and estimates the upper threshold for CNA call to be two standard deviations from it.

19.91 arrayCGHanalysis-chr15 (Segmentation)

No description

Parameter name	Value
chr	15

19.92 arrayCGHanalysis-chr15-chrFilter (CSVFilter)

Filters columns and/or rows from CSV files using flexible criteria. Default settings do not filter out anything. The *negate* parameter reverses all inclusion criteria.

Columns are filtered based on column names (see *includeColumns*); it is also possible to rename columns. Columns can be reordered using *includeColumns*.

A row is included in output if all conditions are satisfied. Rows having too many missing values are filtered using the *nonMissing* parameter. The parameters *regexp*, *lowBound* and *highBound* filter rows based on cell contents. If the *auxiliary* input is given, the result contains those rows from the input whose values match those from the auxiliary file.

Authors: Kristian Ovaska (kristian.ovaska@helsinki.fi), Marko Laakso (Marko.Laakso@Helsinki.FI)
Version: 1.1
Category: Filter

Input name	Source	Description
csv	arrayCGHanalysis-srFAnnot.file 19.213	CSV file to be filtered.

Parameter name	Value	Description
highBound		A row is included if the value in given numeric columns are at most as large as given numeric bounds. The parameter has a format COLNAME1=HIGH1,COLNAME2=HIGH2.

Continued on next page...

Parameter name	Value	Description
idColumn		Column name in "csv" that contains ID values. If the parameter is empty, the first column is used.
includeColumns	*	Comma-separated list of column names that should be included in the result file. The special value * includes all columns. The order of column names is significant: the output columns are in the order given. See also includeColumns input.
lowBound		A row is included if the value in given numeric columns are at least as large as given numeric bounds. The parameter has a format COLNAME1=LOW1,COLNAME2=LOW2. For example, "col=5" includes rows where the column col has a value of at least 5.
matchColumn		Column name in "auxiliary" containing values that must match the ID column in "csv" (specified using "idColumn"). If empty, the first column of "auxiliary" is used.
negate	false	If true, all inclusion criteria are negated so that rows or columns are excluded instead of included if they match the criteria. For example, includeColumns=* then excludes all columns (which would be an error since at least one column must be included).
nonMissing	0	Include those rows that have non-missing (non-NA) values in at least this many columns. Only those columns that are part of the output are counted.
regexp	Chr=15	Row filtering based on regular expression. A row is included in the result if values in the given columns match given regular expressions. The parameter has a format COLNAME1=EXPRESSION,COLNAME2=EXPRESSION2 where COLNAMEs are column names in "csv" and EXPRESSIONs are regular expressions using Java syntax. For example, "col=a|b" includes rows where the column col has a value of "a" or "b". Commas have to be escaped with "\\" if they are used in column names or regular expressions.
rename		Comma-separated list of column renaming rules (OLDNAME=NEWNAME). Parameters that refer to column names refer to old names; renaming is done last.

19.93 arrayCGHanalysis-chr15-filtNormCaseChan (CSVFilter)

Filters columns and/or rows from CSV files using flexible criteria. Default settings do not filter out anything. The *negate* parameter reverses all inclusion criteria.

Columns are filtered based on column names (see *includeColumns*); it is also possible to rename columns. Columns can be reordered using *includeColumns*.

A row is included in output if all conditions are satisfied. Rows having too many missing values are filtered using the *nonMissing* parameter. The parameters *regexp*, *lowBound* and *highBound* filter rows based on cell contents. If the *auxiliary* input is given, the result contains those rows from the input whose values match those from the auxiliary file.

Authors: Kristian Ovaska (kristian.ovaska@helsinki.fi), Marko Laakso (Marko.Laakso@Helsinki.FI)
Version: 1.1
Category: Filter

Input name	Source	Description
csv	arrayCGHanalysis-normalized .casechannel 19.196	CSV file to be filtered.
auxiliary	arrayCGHanalysis-chr15-chrFilter .csv 19.92	If given, contains one column (see "matchColumn") whose values are matched to a column in the "csv" input (see "idColumn").

Parameter name	Value	Description
highBound		A row is included if the value in given numeric columns are at most as large as given numeric bounds. The parameter has a format COLNAME1=HIGH1,COLNAME2=HIGH2.
idColumn		Column name in "csv" that contains ID values. If the parameter is empty, the first column is used.
includeColumns	*	Comma-separated list of column names that should be included in the result file. The special value * includes all columns. The order of column names is significant: the output columns are in the order given. See also includeColumns input.
lowBound		A row is included if the value in given numeric columns are at least as large as given numeric bounds. The parameter has a format COLNAME1=LOW1,COLNAME2=LOW2. For example, "col=5" includes rows where the column col has a value of at least 5.

Continued on next page...

Parameter name	Value	Description
matchColumn	ProbeName	Column name in "auxiliary" containing values that must match the ID column in "csv" (specified using "idColumn"). If empty, the first column of "auxiliary" is used.
negate	false	If true, all inclusion criteria are negated so that rows or columns are excluded instead of included if they match the criteria. For example, includeColumns=* then excludes all columns (which would be an error since at least one column must be included).
nonMissing	0	Include those rows that have non-missing (non-NA) values in at least this many columns. Only those columns that are part of the output are counted.
regexp		Row filtering based on regular expression. A row is included in the result if values in the given columns match given regular expressions. The parameter has a format COLNAME1=EXPRESSION,COLNAME2=EXPRESSION2 where COLNAMEs are column names in "csv" and EXPRESSIONs are regular expressions using Java syntax. For example, "col=a|b" includes rows where the column col has a value of "a" or "b". Commas have to be escaped with "\\" if they are used in column names or regular expressions.
rename		Comma-separated list of column renaming rules (OLDNAME=NEWNAME). Parameters that refer to column names refer to old names; renaming is done last.

19.94 arrayCGHanalysis-chr15-segmented (ACGHsegment)

ACGHsegment segments normalized array CGH data, and calls aberrations in one of two ways: by classification of probe data discretely to gain, normal or loss, or computing probabilities to of a gain, normal or loss state for each probe. Segmentation utilizes the Circular Binary Segmentation algorithm [15].

Two ways of analysing Array CGH data post-segmentation are included. The first one, the Genome Topography Scan [27] computes scores over all the samples to calculate the most recurrent aberrations over the sample set. The second one outputs all the regions in all the samples whose segmented means are more than two standard deviations apart from the mean of all the intensities. Plots from the GTS as well as from the segmented intensities are output depending on the parameter setting.

Probabilities for copy number aberrations can be computed with two algorithms. The first, FastCall [3], is faster while the second, CGHcall [26], is more accurate.

Author:	Riku Louhimo (Riku.Louhimo@Helsinki.FI)
Version:	1.3
Categories:	Agilent, Copy Number Analysis

Input name	Source	Description
caseChan	arrayCGHanalysis-chr15-filtNorm CaseChan.csv 19.93	CSV file containing the normalized probe intensities for the case channel. First row should have the probenames and samples should be columnwise.
geneAnnotation	arrayCGHanalysis-chr15-chrFilter .csv 19.92	Probewise annotations as produced by the AgilenReader component. Only the probes present in the casechannel csv can be included. Use CSVFilter if the probes do not otherwise match.

Parameter name	Value	Description
CGHCallPrior	auto	Only used if callProbMethod=CGHCall. Set the method to determine prior probabilities to CGHCall algorithm. Must be one of "auto", "all", "not all".
analysisAlgorithm	0	1 means that only the GTS algorithm is run, 2 that only the standard deviation test is run. 0 indicates that both are run.
callProbMethod	CGHCall	Method with which to call CNA segment probabilities. Must be either CGHCall or FastCall. FastCall is significantly (30000 times) faster while CGHCall is more accurate.
callProbabilities	false	Enabling this will make the component estimate probabilties for CNA segments via the CGHcall package. Only guaranteed to work when multiple chromosomes are analyzed simultaneously.
lowerLimit	-0.631759089316322	Use this as the intensity limit for calling a segment lossed. Default value computes the mean of the sample set and estimates the lower threshold for CNA call to be two standard deviations from it.
nSegFit	3000	Maximum number of segments used for fitting the mixture model in probability calculations. Disabled if callProbabilities=false. Decreasing this lowers accuracy but can speed computation significantly.

Continued on next page...

Parameter name	Value	Description
plotChromosomes	0	Defines the plots that the user wants to output as a comma separated list. The whole genome is plotted by default for each sample. Inputting a value different than 0 will generate additional plots for these specific chromosomes from each sample.
plotEverySample	false	Enabling this will make the component print each sample separately.
undoSplits	sdundo	A character string specifying how change-points are to be undone, if at all. Undoing change-points decreases the sensitivity of segmentation. Choices are "none","prune", which uses a sum of squares criterion, and "sdundo" (default), which undoes splits that are not at least this many SDs apart. SD by default is 3.
upperLimit	0.631759089316322	Use this as the intensity limit for calling a segment gained. Default value computes the mean of the sample set and estimates the upper threshold for CNA call to be two standard deviations from it.

19.95 arrayCGHanalysis-chr16 (Segmentation)

No description

Parameter name	Value
chr	16

19.96 arrayCGHanalysis-chr16-chrFilter (CSVFilter)

Filters columns and/or rows from CSV files using flexible criteria. Default settings do not filter out anything. The *negate* parameter reverses all inclusion criteria.

Columns are filtered based on column names (see *includeColumns*); it is also possible to rename columns. Columns can be reordered using *includeColumns*.

A row is included in output if all conditions are satisfied. Rows having too many missing values are filtered using the *nonMissing* parameter. The parameters *regexp*, *lowBound* and *highBound* filter rows based on cell contents. If the *auxiliary* input is given, the result contains those rows from the input whose values match those from the auxiliary file.

Authors:	Kristian Ovaska (kristian.ovaska@helsinki.fi), Marko Laakso (Marko.Laakso@Helsinki.FI)
Version:	1.1
Category:	Filter

Input name	Source	Description
csv	arrayCGHanalysis-srFAnnot. file 19.213	CSV file to be filtered.

Parameter name	Value	Description
highBound		A row is included if the value in given numeric columns are at most as large as given numeric bounds. The parameter has a format COLNAME1=HIGH1,COLNAME2=HIGH2.
idColumn		Column name in "csv" that contains ID values. If the parameter is empty, the first column is used.
includeColumns	*	Comma-separated list of column names that should be included in the result file. The special value * includes all columns. The order of column names is significant: the output columns are in the order given. See also includeColumns input.
lowBound		A row is included if the value in given numeric columns are at least as large as given numeric bounds. The parameter has a format COLNAME1=LOW1,COLNAME2=LOW2. For example, "col=5" includes rows where the column col has a value of at least 5.
matchColumn		Column name in "auxiliary" containing values that must match the ID column in "csv" (specified using "idColumn"). If empty, the first column of "auxiliary" is used.
negate	false	If true, all inclusion criteria are negated so that rows or columns are excluded instead of included if they match the criteria. For example, includeColumns=* then excludes all columns (which would be an error since at least one column must be included).

Continued on next page...

Parameter name	Value	Description
nonMissing	0	Include those rows that have non-missing (non-NA) values in at least this many columns. Only those columns that are part of the output are counted.
regexp	Chr=16	Row filtering based on regular expression. A row is included in the result if values in the given columns match given regular expressions. The parameter has a format COLNAME1=EXPRESSION,COLNAME2=EXPRESSION2 where COLNAMEs are column names in "csv" and EXPRESSIONs are regular expressions using Java syntax. For example, "col=a|b" includes rows where the column col has a value of "a" or "b". Commas have to be escaped with "\\" if they are used in column names or regular expressions.
rename		Comma-separated list of column renaming rules (OLDNAME=NEWNAME). Parameters that refer to column names refer to old names; renaming is done last.

19.97 arrayCGHanalysis-chr16-filtNormCaseChan (CSVFilter)

Filters columns and/or rows from CSV files using flexible criteria. Default settings do not filter out anything. The *negate* parameter reverses all inclusion criteria.

Columns are filtered based on column names (see *includeColumns*); it is also possible to rename columns. Columns can be reordered using *includeColumns*.

A row is included in output if all conditions are satisfied. Rows having too many missing values are filtered using the *nonMissing* parameter. The parameters *regexp*, *lowBound* and *highBound* filter rows based on cell contents. If the *auxiliary* input is given, the result contains those rows from the input whose values match those from the auxiliary file.

Authors:	Kristian Ovaska (kristian.ovaska@helsinki.fi), Marko Laakso (Marko.Laakso@Helsinki.FI)
Version:	1.1
Category:	Filter

Input name	Source	Description
csv	arrayCGHanalysis-normalized .casechannel 19.196	CSV file to be filtered.
auxiliary	arrayCGHanalysis-chr16-chrFilter .csv 19.96	If given, contains one column (see "matchColumn") whose values are matched to a column in the "csv" input (see "idColumn").

Parameter name	Value	Description
highBound		A row is included if the value in given numeric columns are at most as large as given numeric bounds. The parameter has a format COLNAME1=HIGH1,COLNAME2=HIGH2.
idColumn		Column name in "csv" that contains ID values. If the parameter is empty, the first column is used.
includeColumns	*	Comma-separated list of column names that should be included in the result file. The special value * includes all columns. The order of column names is significant: the output columns are in the order given. See also includeColumns input.
lowBound		A row is included if the value in given numeric columns are at least as large as given numeric bounds. The parameter has a format COLNAME1=LOW1,COLNAME2=LOW2. For example, "col=5" includes rows where the column col has a value of at least 5.
matchColumn	ProbeName	Column name in "auxiliary" containing values that must match the ID column in "csv" (specified using "idColumn"). If empty, the first column of "auxiliary" is used.
negate	false	If true, all inclusion criteria are negated so that rows or columns are excluded instead of included if they match the criteria. For example, includeColumns=* then excludes all columns (which would be an error since at least one column must be included).
nonMissing	0	Include those rows that have non-missing (non-NA) values in at least this many columns. Only those columns that are part of the output are counted.
regexp		Row filtering based on regular expression. A row is included in the result if values in the given columns match given regular expressions. The parameter has a format COLNAME1=EXPRESSION,COLNAME2=EXPRESSION2 where COLNAMEs are column names in "csv" and EXPRESSIONs are regular expressions using Java syntax. For example, "col=a|b" includes rows where the column col has a value of "a" or "b". Commas have to be escaped with "\\" if they are used in column names or regular expressions.

Continued on next page...

Parameter name	Value	Description
rename		Comma-separated list of column renaming rules (OLDNAME=NEWNAME). Parameters that refer to column names refer to old names; renaming is done last.

19.98 arrayCGHanalysis-chr16-segmented (ACGHsegment)

ACGHsegment segments normalized array CGH data, and calls aberrations in one of two ways: by classification of probe data discretely to gain, normal or loss, or computing probabilities to of a gain, normal or loss state for each probe. Segmentation utilizes the Circular Binary Segmentation algorithm [15].

Two ways of analysing Array CGH data post-segmentation are included. The first one, the Genome Topography Scan [27] computes scores over all the samples to calculate the most recurrent aberrations over the sample set. The second one outputs all the regions in all the samples whose segmented means are more than two standard deviations apart from the mean of all the intensities. Plots from the GTS as well as from the segmented intensities are output depending on the parameter setting.

Probabilities for copy number aberrations can be computed with two algorithms. The first, FastCall [3], is faster while the second, CGHcall [26], is more accurate.

Author:	Riku Louhimo (Riku.Louhimo@Helsinki.FI)
Version:	1.3
Categories:	Agilent, Copy Number Analysis

Input name	Source	Description
caseChan	arrayCGHanalysis-chr16-filtNorm CaseChan.csv 19.97	CSV file containing the normalized probe intensities for the case channel. First row should have the probenames and samples should be columnwise.
geneAnnotation	arrayCGHanalysis-chr16-chrFilter .csv 19.96	Probewise annotations as produced by the AgilenReader component. Only the probes present in the casechannel csv can be included. Use CSVFilter if the probes do not otherwise match.

Parameter name	Value	Description
CGHCallPrior	auto	Only used if callProbMethod=CGHCall. Set the method to determine prior probabilities to CGHCall algorithm. Must be one of "auto", "all", "not all".
analysisAlgorithm	0	1 means that only the GTS algorithm is run, 2 that only the standard deviation test is run. 0 indicates that both are run.
callProbMethod	CGHCall	Method with which to call CNA segment probabilities. Must be either CGHCall or FastCall. FastCall is significantly (30000 times) faster while CGHCall is more accurate.
callProbabilities	false	Enabling this will make the component estimate probabilties for CNA segments via the CGHcall package. Only guaranteed to work when multiple chromosomes are analyzed simultaneously.
lowerLimit	-0.631759089316322	Use this as the intensity limit for calling a segment lossed. Default value computes the mean of the sample set and estimates the lower threshold for CNA call to be two standard deviations from it.
nSegFit	3000	Maximum number of segments used for fitting the mixture model in probability calculations. Disabled if callProbabilities=false. Decreasing this lowers accuracy but can speed computation significantly.
plotChromosomes	0	Defines the plots that the user wants to output as a comma separated list. The whole genome is plotted by default for each sample. Inputting a value different than 0 will generate additional plots for these specific chromosomes from each sample.
plotEverySample	false	Enabling this will make the component print each sample separately.
undoSplits	sdundo	A character string specifying how change-points are to be undone, if at all. Undoing change-points decreases the sensitivity of segmentation. Choices are "none","prune", which uses a sum of squares criterion, and "sdundo" (default), which undoes splits that are not at least this many SDs apart. SD by default is 3.
upperLimit	0.631759089316322	Use this as the intensity limit for calling a segment gained. Default value computes the mean of the sample set and estimates the upper threshold for CNA call to be two standard deviations from it.

19.99 arrayCGHanalysis-chr17 (Segmentation)

No description

Parameter name	Value
chr	17

19.100 arrayCGHanalysis-chr17-chrFilter (CSVFilter)

Filters columns and/or rows from CSV files using flexible criteria. Default settings do not filter out anything. The *negate* parameter reverses all inclusion criteria.

Columns are filtered based on column names (see *includeColumns*); it is also possible to rename columns. Columns can be reordered using *includeColumns*.

A row is included in output if all conditions are satisfied. Rows having too many missing values are filtered using the *nonMissing* parameter. The parameters *regexp*, *lowBound* and *highBound* filter rows based on cell contents. If the *auxiliary* input is given, the result contains those rows from the input whose values match those from the auxiliary file.

Authors: Kristian Ovaska (kristian.ovaska@helsinki.fi), Marko Laakso (Marko.Laakso@Helsinki.FI)
Version: 1.1
Category: Filter

Input name	Source	Description
csv	arrayCGHanalysis-srFAnnot.<br>file 19.213	CSV file to be filtered.

Parameter name	Value	Description
highBound		A row is included if the value in given numeric columns are at most as large as given numeric bounds. The parameter has a format COLNAME1=HIGH1,COLNAME2=HIGH2.
idColumn		Column name in "csv" that contains ID values. If the parameter is empty, the first column is used.
includeColumns	*	Comma-separated list of column names that should be included in the result file. The special value * includes all columns. The order of column names is significant: the output columns are in the order given. See also includeColumns input.
lowBound		A row is included if the value in given numeric columns are at least as large as given numeric bounds. The parameter has a format COLNAME1=LOW1,COLNAME2=LOW2. For example, "col=5" includes rows where the column col has a value of at least 5.
matchColumn		Column name in "auxiliary" containing values that must match the ID column in "csv" (specified using "idColumn"). If empty, the first column of "auxiliary" is used.
negate	false	If true, all inclusion criteria are negated so that rows or columns are excluded instead of included if they match the criteria. For example, includeColumns=* then excludes all columns (which would be an error since at least one column must be included).
nonMissing	0	Include those rows that have non-missing (non-NA) values in at least this many columns. Only those columns that are part of the output are counted.
regexp	Chr=17	Row filtering based on regular expression. A row is included in the result if values in the given columns match given regular expressions. The parameter has a format COLNAME1=EXPRESSION,COLNAME2=EXPRESSION2 where COLNAMEs are column names in "csv" and EXPRESSIONs are regular expressions using Java syntax. For example, "col=a|b" includes rows where the column col has a value of "a" or "b". Commas have to be escaped with "\\" if they are used in column names or regular expressions.
rename		Comma-separated list of column renaming rules (OLDNAME=NEWNAME). Parameters that refer to column names refer to old names; renaming is done last.

19.101 arrayCGHanalysis-chr17-filtNormCaseChan (CSVFilter)

Filters columns and/or rows from CSV files using flexible criteria. Default settings do not filter out anything. The *negate* parameter reverses all inclusion criteria.

Columns are filtered based on column names (see *includeColumns*); it is also possible to rename columns. Columns can be reordered using *includeColumns*.

A row is included in output if all conditions are satisfied. Rows having too many missing values are filtered using the *nonMissing* parameter. The parameters *regexp*, *lowBound* and *highBound* filter rows based on cell contents. If the *auxiliary* input is given, the result contains those rows from the input whose values match those from the auxiliary file.

Authors:	Kristian Ovaska (kristian.ovaska@helsinki.fi), Marko Laakso (Marko.Laakso@Helsinki.FI)
Version:	1.1
Category:	Filter

Input name	Source	Description
csv	arrayCGHanalysis-normalized .casechannel 19.196	CSV file to be filtered.
auxiliary	arrayCGHanalysis-chr17-chrFilter .csv 19.100	If given, contains one column (see "matchColumn") whose values are matched to a column in the "csv" input (see "idColumn").

Parameter name	Value	Description
highBound		A row is included if the value in given numeric columns are at most as large as given numeric bounds. The parameter has a format COLNAME1=HIGH1,COLNAME2=HIGH2.
idColumn		Column name in "csv" that contains ID values. If the parameter is empty, the first column is used.
includeColumns	*	Comma-separated list of column names that should be included in the result file. The special value * includes all columns. The order of column names is significant: the output columns are in the order given. See also includeColumns input.
lowBound		A row is included if the value in given numeric columns are at least as large as given numeric bounds. The parameter has a format COLNAME1=LOW1,COLNAME2=LOW2. For example, "col=5" includes rows where the column col has a value of at least 5.
matchColumn	ProbeName	Column name in "auxiliary" containing values that must match the ID column in "csv" (specified using "idColumn"). If empty, the first column of "auxiliary" is used.
negate	false	If true, all inclusion criteria are negated so that rows or columns are excluded instead of included if they match the criteria. For example, includeColumns=* then excludes all columns (which would be an error since at least one column must be included).
nonMissing	0	Include those rows that have non-missing (non-NA) values in at least this many columns. Only those columns that are part of the output are counted.
regexp		Row filtering based on regular expression. A row is included in the result if values in the given columns match given regular expressions. The parameter has a format COLNAME1=EXPRESSION,COLNAME2=EXPRESSION2 where COLNAMEs are column names in "csv" and EXPRESSIONs are regular expressions using Java syntax. For example, "col=a|b" includes rows where the column col has a value of "a" or "b". Commas have to be escaped with "\\" if they are used in column names or regular expressions.
rename		Comma-separated list of column renaming rules (OLDNAME=NEWNAME). Parameters that refer to column names refer to old names; renaming is done last.

19.102 arrayCGHanalysis-chr17-segmented (ACGHsegment)

ACGHsegment segments normalized array CGH data, and calls aberrations in one of two ways: by classification of probe data discretely to gain, normal or loss, or computing probabilities to of a gain, normal or loss state for each probe. Segmentation utilizes the Circular Binary Segmentation algorithm [15].

Two ways of analysing Array CGH data post-segmentation are included. The first one, the Genome Topography Scan [27] computes scores over all the samples to calculate the most recurrent aberrations over the sample set. The second one outputs all the regions in all the samples whose segmented means

are more than two standard deviations apart from the mean of all the intensities. Plots from the GTS as well as from the segmented intensities are output depending on the parameter setting.

Probabilities for copy number aberrations can be computed with two algorithms. The first, FastCall [3], is faster while the second, CGHcall [26], is more accurate.

Author:	Riku Louhimo (Riku.Louhimo@Helsinki.FI)
Version:	1.3
Categories:	Agilent, Copy Number Analysis

Input name	Source	Description
caseChan	arrayCGHanalysis-chr17-filtNorm CaseChan.csv 19.101	CSV file containing the normalized probe intensities for the case channel. First row should have the probenames and samples should be columnwise.
geneAnnotation	arrayCGHanalysis-chr17-chrFilter .csv 19.100	Probewise annotations as produced by the AgilenReader component. Only the probes present in the casechannel csv can be included. Use CSVFilter if the probes do not otherwise match.

Parameter name	Value	Description
CGHCallPrior	auto	Only used if callProbMethod=CGHCall. Set the method to determine prior probabilities to CGHCall algorithm. Must be one of "auto", "all", "not all".
analysisAlgorithm	0	1 means that only the GTS algorithm is run, 2 that only the standard deviation test is run. 0 indicates that both are run.
callProbMethod	CGHCall	Method with which to call CNA segment probabilities. Must be either CGHCall or FastCall. FastCall is significantly (30000 times) faster while CGHCall is more accurate.
callProbabilities	false	Enabling this will make the component estimate probabilties for CNA segments via the CGHcall package. Only guaranteed to work when multiple chromosomes are analyzed simultaneously.
lowerLimit	-0.631759089316322	Use this as the intensity limit for calling a segment lossed. Default value computes the mean of the sample set and estimates the lower threshold for CNA call to be two standard deviations from it.
nSegFit	3000	Maximum number of segments used for fitting the mixture model in probability calculations. Disabled if callProbabilities=false. Decreasing this lowers accuracy but can speed computation significantly.
plotChromosomes	0	Defines the plots that the user wants to output as a comma separated list. The whole genome is plotted by default for each sample. Inputting a value different than 0 will generate additional plots for these specific chromosomes from each sample.
plotEverySample	false	Enabling this will make the component print each sample separately.
undoSplits	sdundo	A character string specifying how change-points are to be undone, if at all. Undoing change-points decreases the sensitivity of segmentation. Choices are "none","prune", which uses a sum of squares criterion, and "sdundo" (default), which undoes splits that are not at least this many SDs apart. SD by default is 3.
upperLimit	0.631759089316322	Use this as the intensity limit for calling a segment gained. Default value computes the mean of the sample set and estimates the upper threshold for CNA call to be two standard deviations from it.

19.103 arrayCGHanalysis-chr18 (Segmentation)

No description

Parameter name	Value
chr	18

19.104 arrayCGHanalysis-chr18-chrFilter (CSVFilter)

Filters columns and/or rows from CSV files using flexible criteria. Default settings do not filter out anything. The *negate* parameter reverses all inclusion criteria.

Columns are filtered based on column names (see *includeColumns*); it is also possible to rename columns. Columns can be reordered using *includeColumns*.

A row is included in output if all conditions are satisfied. Rows having too many missing values are filtered

using the *nonMissing* parameter. The parameters *regexp*, *lowBound* and *highBound* filter rows based on cell contents. If the *auxiliary* input is given, the result contains those rows from the input whose values match those from the auxiliary file.

Authors:	Kristian Ovaska (kristian.ovaska@helsinki.fi), Marko Laakso (Marko.Laakso@Helsinki.FI)
Version:	1.1
Category:	Filter

Input name	Source	Description
csv	arrayCGHanalysis-srFAnnot.file 19.213	CSV file to be filtered.

Parameter name	Value	Description
highBound		A row is included if the value in given numeric columns are at most as large as given numeric bounds. The parameter has a format COLNAME1=HIGH1,COLNAME2=HIGH2.
idColumn		Column name in "csv" that contains ID values. If the parameter is empty, the first column is used.
includeColumns	*	Comma-separated list of column names that should be included in the result file. The special value * includes all columns. The order of column names is significant: the output columns are in the order given. See also includeColumns input.
lowBound		A row is included if the value in given numeric columns are at least as large as given numeric bounds. The parameter has a format COLNAME1=LOW1,COLNAME2=LOW2. For example, "col=5" includes rows where the column col has a value of at least 5.
matchColumn		Column name in "auxiliary" containing values that must match the ID column in "csv" (specified using "idColumn"). If empty, the first column of "auxiliary" is used.
negate	false	If true, all inclusion criteria are negated so that rows or columns are excluded instead of included if they match the criteria. For example, includeColumns=* then excludes all columns (which would be an error since at least one column must be included).
nonMissing	0	Include those rows that have non-missing (non-NA) values in at least this many columns. Only those columns that are part of the output are counted.
regexp	Chr=18	Row filtering based on regular expression. A row is included in the result if values in the given columns match given regular expressions. The parameter has a format COLNAME1=EXPRESSION,COLNAME2=EXPRESSION2 where COLNAMEs are column names in "csv" and EXPRESSIONs are regular expressions using Java syntax. For example, "col=a|b" includes rows where the column col has a value of "a" or "b". Commas have to be escaped with "\\" if they are used in column names or regular expressions.
rename		Comma-separated list of column renaming rules (OLDNAME=NEWNAME). Parameters that refer to column names refer to old names; renaming is done last.

19.105 arrayCGHanalysis-chr18-filtNormCaseChan (CSVFilter)

Filters columns and/or rows from CSV files using flexible criteria. Default settings do not filter out anything. The *negate* parameter reverses all inclusion criteria.

Columns are filtered based on column names (see *includeColumns*); it is also possible to rename columns. Columns can be reordered using *includeColumns*.

A row is included in output if all conditions are satisfied. Rows having too many missing values are filtered using the *nonMissing* parameter. The parameters *regexp*, *lowBound* and *highBound* filter rows based on cell contents. If the *auxiliary* input is given, the result contains those rows from the input whose values match those from the auxiliary file.

Authors:	Kristian Ovaska (kristian.ovaska@helsinki.fi), Marko Laakso (Marko.Laakso@Helsinki.FI)
Version:	1.1
Category:	Filter

Input name	Source	Description
csv	arrayCGHanalysis-normalized .casechannel 19.196	CSV file to be filtered.
auxiliary	arrayCGHanalysis-chr18-chrFilter .csv 19.104	If given, contains one column (see "matchColumn") whose values are matched to a column in the "csv" input (see "idColumn").

Parameter name	Value	Description
highBound		A row is included if the value in given numeric columns are at most as large as given numeric bounds. The parameter has a format COLNAME1=HIGH1,COLNAME2=HIGH2.
idColumn		Column name in "csv" that contains ID values. If the parameter is empty, the first column is used.
includeColumns	*	Comma-separated list of column names that should be included in the result file. The special value * includes all columns. The order of column names is significant: the output columns are in the order given. See also includeColumns input.
lowBound		A row is included if the value in given numeric columns are at least as large as given numeric bounds. The parameter has a format COLNAME1=LOW1,COLNAME2=LOW2. For example, "col=5" includes rows where the column col has a value of at least 5.
matchColumn	ProbeName	Column name in "auxiliary" containing values that must match the ID column in "csv" (specified using "idColumn"). If empty, the first column of "auxiliary" is used.
negate	false	If true, all inclusion criteria are negated so that rows or columns are excluded instead of included if they match the criteria. For example, includeColumns=* then excludes all columns (which would be an error since at least one column must be included).
nonMissing	0	Include those rows that have non-missing (non-NA) values in at least this many columns. Only those columns that are part of the output are counted.
regexp		Row filtering based on regular expression. A row is included in the result if values in the given columns match given regular expressions. The parameter has a format COLNAME1=EXPRESSION,COLNAME2=EXPRESSION2 where COLNAMEs are column names in "csv" and EXPRESSIONs are regular expressions using Java syntax. For example, "col=a|b" includes rows where the column col has a value of "a" or "b". Commas have to be escaped with "\\" if they are used in column names or regular expressions.
rename		Comma-separated list of column renaming rules (OLDNAME=NEWNAME). Parameters that refer to column names refer to old names; renaming is done last.

19.106 arrayCGHanalysis-chr18-segmented (ACGHsegment)

ACGHsegment segments normalized array CGH data, and calls aberrations in one of two ways: by classification of probe data discretely to gain, normal or loss, or computing probabilities to of a gain, normal or loss state for each probe. Segmentation utilizes the Circular Binary Segmentation algorithm [15].

Two ways of analysing Array CGH data post-segmentation are included. The first one, the Genome Topography Scan [27] computes scores over all the samples to calculate the most recurrent aberrations over the sample set. The second one outputs all the regions in all the samples whose segmented means are more than two standard deviations apart from the mean of all the intensities. Plots from the GTS as well as from the segmented intensities are output depending on the parameter setting.

Probabilities for copy number aberrations can be computed with two algorithms. The first, FastCall [3], is faster while the second, CGHcall [26], is more accurate.

Author:	Riku Louhimo (Riku.Louhimo@Helsinki.FI)
Version:	1.3
Categories:	Agilent, Copy Number Analysis

Input name	Source	Description
caseChan	arrayCGHanalysis-chr18-filtNorm CaseChan.csv 19.105	CSV file containing the normalized probe intensities for the case channel. First row should have the probenames and samples should be columnwise.
geneAnnotation	arrayCGHanalysis-chr18-chrFilter .csv 19.104	Probewise annotations as produced by the AgilenReader component. Only the probes present in the casechannel csv can be included. Use CSVFilter if the probes do not otherwise match.

Parameter name	Value	Description
CGHCallPrior	auto	Only used if callProbMethod=CGHCall. Set the method to determine prior probabilities to CGHCall algorithm. Must be one of "auto", "all", "not all".
analysisAlgorithm	0	1 means that only the GTS algorithm is run, 2 that only the standard deviation test is run. 0 indicates that both are run.
callProbMethod	CGHCall	Method with which to call CNA segment probabilities. Must be either CGHCall or FastCall. FastCall is significantly (30000 times) faster while CGHCall is more accurate.
callProbabilities	false	Enabling this will make the component estimate probabilties for CNA segments via the CGHcall package. Only guaranteed to work when multiple chromosomes are analyzed simultaneously.
lowerLimit	-0.631759089316322	Use this as the intensity limit for calling a segment lossed. Default value computes the mean of the sample set and estimates the lower threshold for CNA call to be two standard deviations from it.
nSegFit	3000	Maximum number of segments used for fitting the mixture model in probability calculations. Disabled if callProbabilities=false. Decreasing this lowers accuracy but can speed computation significantly.
plotChromosomes	0	Defines the plots that the user wants to output as a comma separated list. The whole genome is plotted by default for each sample. Inputting a value different than 0 will generate additional plots for these specific chromosomes from each sample.
plotEverySample	false	Enabling this will make the component print each sample separately.
undoSplits	sdundo	A character string specifying how change-points are to be undone, if at all. Undoing change-points decreases the sensitivity of segmentation. Choices are "none","prune", which uses a sum of squares criterion, and "sdundo" (default), which undoes splits that are not at least this many SDs apart. SD by default is 3.
upperLimit	0.631759089316322	Use this as the intensity limit for calling a segment gained. Default value computes the mean of the sample set and estimates the upper threshold for CNA call to be two standard deviations from it.

19.107 arrayCGHanalysis-chr19 (Segmentation)

No description

Parameter name	Value
chr	19

19.108 arrayCGHanalysis-chr19-chrFilter (CSVFilter)

Filters columns and/or rows from CSV files using flexible criteria. Default settings do not filter out anything. The *negate* parameter reverses all inclusion criteria.

Columns are filtered based on column names (see *includeColumns*); it is also possible to rename columns. Columns can be reordered using *includeColumns*.

A row is included in output if all conditions are satisfied. Rows having too many missing values are filtered using the *nonMissing* parameter. The parameters *regexp*, *lowBound* and *highBound* filter rows based on cell contents. If the *auxiliary* input is given, the result contains those rows from the input whose values match those from the auxiliary file.

Authors: Kristian Ovaska (kristian.ovaska@helsinki.fi), Marko Laakso (Marko.Laakso@Helsinki.FI)
Version: 1.1
Category: Filter

Input name	Source	Description
csv	arrayCGHanalysis-srFAnnot. file 19.213	CSV file to be filtered.

Parameter name	Value	Description
highBound		A row is included if the value in given numeric columns are at most as large as given numeric bounds. The parameter has a format COLNAME1=HIGH1,COLNAME2=HIGH2.

Continued on next page...

Parameter name	Value	Description
idColumn		Column name in "csv" that contains ID values. If the parameter is empty, the first column is used.
includeColumns	*	Comma-separated list of column names that should be included in the result file. The special value * includes all columns. The order of column names is significant: the output columns are in the order given. See also includeColumns input.
lowBound		A row is included if the value in given numeric columns are at least as large as given numeric bounds. The parameter has a format COLNAME1=LOW1,COLNAME2=LOW2. For example, "col=5" includes rows where the column col has a value of at least 5.
matchColumn		Column name in "auxiliary" containing values that must match the ID column in "csv" (specified using "idColumn"). If empty, the first column of "auxiliary" is used.
negate	false	If true, all inclusion criteria are negated so that rows or columns are excluded instead of included if they match the criteria. For example, includeColumns=* then excludes all columns (which would be an error since at least one column must be included).
nonMissing	0	Include those rows that have non-missing (non-NA) values in at least this many columns. Only those columns that are part of the output are counted.
regexp	Chr=19	Row filtering based on regular expression. A row is included in the result if values in the given columns match given regular expressions. The parameter has a format COLNAME1=EXPRESSION,COLNAME2=EXPRESSION2 where COLNAMEs are column names in "csv" and EXPRESSIONs are regular expressions using Java syntax. For example, "col=a|b" includes rows where the column col has a value of "a" or "b". Commas have to be escaped with "\\" if they are used in column names or regular expressions.
rename		Comma-separated list of column renaming rules (OLDNAME=NEWNAME). Parameters that refer to column names refer to old names; renaming is done last.

19.109 arrayCGHanalysis-chr19-filtNormCaseChan (CSVFilter)

Filters columns and/or rows from CSV files using flexible criteria. Default settings do not filter out anything. The *negate* parameter reverses all inclusion criteria.

Columns are filtered based on column names (see *includeColumns*); it is also possible to rename columns. Columns can be reordered using *includeColumns*.

A row is included in output if all conditions are satisfied. Rows having too many missing values are filtered using the *nonMissing* parameter. The parameters *regexp*, *lowBound* and *highBound* filter rows based on cell contents. If the *auxiliary* input is given, the result contains those rows from the input whose values match those from the auxiliary file.

Authors: Kristian Ovaska (kristian.ovaska@helsinki.fi), Marko Laakso (Marko.Laakso@Helsinki.FI)
Version: 1.1
Category: Filter

Input name	Source	Description
csv	arrayCGHanalysis-normalized .casechannel 19.196	CSV file to be filtered.
auxiliary	arrayCGHanalysis-chr19-chrFilter .csv 19.108	If given, contains one column (see "matchColumn") whose values are matched to a column in the "csv" input (see "idColumn").

Parameter name	Value	Description
highBound		A row is included if the value in given numeric columns are at most as large as given numeric bounds. The parameter has a format COLNAME1=HIGH1,COLNAME2=HIGH2.
idColumn		Column name in "csv" that contains ID values. If the parameter is empty, the first column is used.
includeColumns	*	Comma-separated list of column names that should be included in the result file. The special value * includes all columns. The order of column names is significant: the output columns are in the order given. See also includeColumns input.
lowBound		A row is included if the value in given numeric columns are at least as large as given numeric bounds. The parameter has a format COLNAME1=LOW1,COLNAME2=LOW2. For example, "col=5" includes rows where the column col has a value of at least 5.

Continued on next page...

Parameter name	Value	Description
matchColumn	ProbeName	Column name in "auxiliary" containing values that must match the ID column in "csv" (specified using "idColumn"). If empty, the first column of "auxiliary" is used.
negate	false	If true, all inclusion criteria are negated so that rows or columns are excluded instead of included if they match the criteria. For example, includeColumns=* then excludes all columns (which would be an error since at least one column must be included).
nonMissing	0	Include those rows that have non-missing (non-NA) values in at least this many columns. Only those columns that are part of the output are counted.
regexp		Row filtering based on regular expression. A row is included in the result if values in the given columns match given regular expressions. The parameter has a format COLNAME1=EXPRESSION,COLNAME2=EXPRESSION2 where COLNAMEs are column names in "csv" and EXPRESSIONs are regular expressions using Java syntax. For example, "col=a|b" includes rows where the column col has a value of "a" or "b". Commas have to be escaped with "\\" if they are used in column names or regular expressions.
rename		Comma-separated list of column renaming rules (OLDNAME=NEWNAME). Parameters that refer to column names refer to old names; renaming is done last.

19.110 arrayCGHanalysis-chr19-segmented (ACGHsegment)

ACGHsegment segments normalized array CGH data, and calls aberrations in one of two ways: by classification of probe data discretely to gain, normal or loss, or computing probabilities to of a gain, normal or loss state for each probe. Segmentation utilizes the Circular Binary Segmentation algorithm [15].

Two ways of analysing Array CGH data post-segmentation are included. The first one, the Genome Topography Scan [27] computes scores over all the samples to calculate the most recurrent aberrations over the sample set. The second one outputs all the regions in all the samples whose segmented means are more than two standard deviations apart from the mean of all the intensities. Plots from the GTS as well as from the segmented intensities are output depending on the parameter setting.

Probabilities for copy number aberrations can be computed with two algorithms. The first, FastCall [3], is faster while the second, CGHcall [26], is more accurate.

Author:	Riku Louhimo (Riku.Louhimo@Helsinki.FI)
Version:	1.3
Categories:	Agilent, Copy Number Analysis

Input name	Source	Description
caseChan	arrayCGHanalysis-chr19-filtNorm CaseChan.csv 19.109	CSV file containing the normalized probe intensities for the case channel. First row should have the probenames and samples should be columnwise.
geneAnnotation	arrayCGHanalysis-chr19-chrFilter .csv 19.108	Probewise annotations as produced by the AgilenReader component. Only the probes present in the casechannel csv can be included. Use CSVFilter if the probes do not otherwise match.

Parameter name	Value	Description
CGHCallPrior	auto	Only used if callProbMethod=CGHCall. Set the method to determine prior probabilities to CGHCall algorithm. Must be one of "auto", "all", "not all".
analysisAlgorithm	0	1 means that only the GTS algorithm is run, 2 that only the standard deviation test is run. 0 indicates that both are run.
callProbMethod	CGHCall	Method with which to call CNA segment probabilities. Must be either CGHCall or FastCall. FastCall is significantly (30000 times) faster while CGHCall is more accurate.
callProbabilities	false	Enabling this will make the component estimate probabilties for CNA segments via the CGHcall package. Only guaranteed to work when multiple chromosomes are analyzed simultaneously.
lowerLimit	-0.631759089316322	Use this as the intensity limit for calling a segment lossed. Default value computes the mean of the sample set and estimates the lower threshold for CNA call to be two standard deviations from it.
nSegFit	3000	Maximum number of segments used for fitting the mixture model in probability calculations. Disabled if callProbabilities=false. Decreasing this lowers accuracy but can speed computation significantly.

Continued on next page...

Parameter name	Value	Description
plotChromosomes	0	Defines the plots that the user wants to output as a comma separated list. The whole genome is plotted by default for each sample. Inputting a value different than 0 will generate additional plots for these specific chromosomes from each sample.
plotEverySample	false	Enabling this will make the component print each sample separately.
undoSplits	sdundo	A character string specifying how change-points are to be undone, if at all. Undoing change-points decreases the sensitivity of segmentation. Choices are "none","prune", which uses a sum of squares criterion, and "sdundo" (default), which undoes splits that are not at least this many SDs apart. SD by default is 3.
upperLimit	0.631759089316322	Use this as the intensity limit for calling a segment gained. Default value computes the mean of the sample set and estimates the upper threshold for CNA call to be two standard deviations from it.

19.111 arrayCGHanalysis-chr2 (Segmentation)

No description

Parameter name	Value
chr	2

19.112 arrayCGHanalysis-chr2-chrFilter (CSVFilter)

Filters columns and/or rows from CSV files using flexible criteria. Default settings do not filter out anything. The *negate* parameter reverses all inclusion criteria.

Columns are filtered based on column names (see *includeColumns*); it is also possible to rename columns. Columns can be reordered using *includeColumns*.

A row is included in output if all conditions are satisfied. Rows having too many missing values are filtered using the *nonMissing* parameter. The parameters *regexp*, *lowBound* and *highBound* filter rows based on cell contents. If the *auxiliary* input is given, the result contains those rows from the input whose values match those from the auxiliary file.

Authors:	Kristian Ovaska (kristian.ovaska@helsinki.fi), Marko Laakso (Marko.Laakso@Helsinki.FI)
Version:	1.1
Category:	Filter

Input name	Source	Description
csv	arrayCGHanalysis-srFAnnot. file 19.213	CSV file to be filtered.

Parameter name	Value	Description
highBound		A row is included if the value in given numeric columns are at most as large as given numeric bounds. The parameter has a format COLNAME1=HIGH1,COLNAME2=HIGH2.
idColumn		Column name in "csv" that contains ID values. If the parameter is empty, the first column is used.
includeColumns	*	Comma-separated list of column names that should be included in the result file. The special value * includes all columns. The order of column names is significant: the output columns are in the order given. See also includeColumns input.
lowBound		A row is included if the value in given numeric columns are at least as large as given numeric bounds. The parameter has a format COLNAME1=LOW1,COLNAME2=LOW2. For example, "col=5" includes rows where the column col has a value of at least 5.
matchColumn		Column name in "auxiliary" containing values that must match the ID column in "csv" (specified using "idColumn"). If empty, the first column of "auxiliary" is used.
negate	false	If true, all inclusion criteria are negated so that rows or columns are excluded instead of included if they match the criteria. For example, includeColumns=* then excludes all columns (which would be an error since at least one column must be included).

Continued on next page...

Parameter name	Value	Description
nonMissing	0	Include those rows that have non-missing (non-NA) values in at least this many columns. Only those columns that are part of the output are counted.
regexp	Chr=2	Row filtering based on regular expression. A row is included in the result if values in the given columns match given regular expressions. The parameter has a format COLNAME1=EXPRESSION,COLNAME2=EXPRESSION2 where COLNAMEs are column names in "csv" and EXPRESSIONs are regular expressions using Java syntax. For example, "col=a|b" includes rows where the column col has a value of "a" or "b". Commas have to be escaped with "\\" if they are used in column names or regular expressions.
rename		Comma-separated list of column renaming rules (OLDNAME=NEWNAME). Parameters that refer to column names refer to old names; renaming is done last.

19.113 arrayCGHanalysis-chr2-filtNormCaseChan (CSVFilter)

Filters columns and/or rows from CSV files using flexible criteria. Default settings do not filter out anything. The *negate* parameter reverses all inclusion criteria.

Columns are filtered based on column names (see *includeColumns*); it is also possible to rename columns. Columns can be reordered using *includeColumns*.

A row is included in output if all conditions are satisfied. Rows having too many missing values are filtered using the *nonMissing* parameter. The parameters *regexp*, *lowBound* and *highBound* filter rows based on cell contents. If the *auxiliary* input is given, the result contains those rows from the input whose values match those from the auxiliary file.

Authors: Kristian Ovaska (kristian.ovaska@helsinki.fi), Marko Laakso (Marko.Laakso@Helsinki.FI)
Version: 1.1
Category: Filter

Input name	Source	Description
csv	arrayCGHanalysis-normalized .casechannel 19.196	CSV file to be filtered.
auxiliary	arrayCGHanalysis-chr2-chrFilter .csv 19.112	If given, contains one column (see "matchColumn") whose values are matched to a column in the "csv" input (see "idColumn").

Parameter name	Value	Description
highBound		A row is included if the value in given numeric columns are at most as large as given numeric bounds. The parameter has a format COLNAME1=HIGH1,COLNAME2=HIGH2.
idColumn		Column name in "csv" that contains ID values. If the parameter is empty, the first column is used.
includeColumns	*	Comma-separated list of column names that should be included in the result file. The special value * includes all columns. The order of column names is significant: the output columns are in the order given. See also includeColumns input.
lowBound		A row is included if the value in given numeric columns are at least as large as given numeric bounds. The parameter has a format COLNAME1=LOW1,COLNAME2=LOW2. For example, "col=5" includes rows where the column col has a value of at least 5.
matchColumn	ProbeName	Column name in "auxiliary" containing values that must match the ID column in "csv" (specified using "idColumn"). If empty, the first column of "auxiliary" is used.
negate	false	If true, all inclusion criteria are negated so that rows or columns are excluded instead of included if they match the criteria. For example, includeColumns=* then excludes all columns (which would be an error since at least one column must be included).
nonMissing	0	Include those rows that have non-missing (non-NA) values in at least this many columns. Only those columns that are part of the output are counted.
regexp		Row filtering based on regular expression. A row is included in the result if values in the given columns match given regular expressions. The parameter has a format COLNAME1=EXPRESSION,COLNAME2=EXPRESSION2 where COLNAMEs are column names in "csv" and EXPRESSIONs are regular expressions using Java syntax. For example, "col=a|b" includes rows where the column col has a value of "a" or "b". Commas have to be escaped with "\\" if they are used in column names or regular expressions.

Continued on next page...

Parameter name	Value	Description
rename		Comma-separated list of column renaming rules (OLDNAME=NEWNAME). Parameters that refer to column names refer to old names; renaming is done last.

19.114 arrayCGHanalysis-chr2-segmented (ACGHsegment)

ACGHsegment segments normalized array CGH data, and calls aberrations in one of two ways: by classification of probe data discretely to gain, normal or loss, or computing probabilities to of a gain, normal or loss state for each probe. Segmentation utilizes the Circular Binary Segmentation algorithm [15].

Two ways of analysing Array CGH data post-segmentation are included. The first one, the Genome Topography Scan [27] computes scores over all the samples to calculate the most recurrent aberrations over the sample set. The second one outputs all the regions in all the samples whose segmented means are more than two standard deviations apart from the mean of all the intensities. Plots from the GTS as well as from the segmented intensities are output depending on the parameter setting.

Probabilities for copy number aberrations can be computed with two algorithms. The first, FastCall [3], is faster while the second, CGHcall [26], is more accurate.

Author: Riku Louhimo (Riku.Louhimo@Helsinki.FI)
Version: 1.3
Categories: Agilent, Copy Number Analysis

Input name	Source	Description
caseChan	arrayCGHanalysis-chr2-filtNorm CaseChan.csv 19.113	CSV file containing the normalized probe intensities for the case channel. First row should have the probenames and samples should be columnwise.
geneAnnotation	arrayCGHanalysis-chr2-chrFilter .csv 19.112	Probewise annotations as produced by the AgilenReader component. Only the probes present in the casechannel csv can be included. Use CSVFilter if the probes do not otherwise match.

Parameter name	Value	Description
CGHCallPrior	auto	Only used if callProbMethod=CGHCall. Set the method to determine prior probabilities to CGHCall algorithm. Must be one of "auto", "all", "not all".
analysisAlgorithm	0	1 means that only the GTS algorithm is run, 2 that only the standard deviation test is run. 0 indicates that both are run.
callProbMethod	CGHCall	Method with which to call CNA segment probabilities. Must be either CGHCall or FastCall. FastCall is significantly (30000 times) faster while CGHCall is more accurate.
callProbabilities	false	Enabling this will make the component estimate probabilties for CNA segments via the CGHcall package. Only guaranteed to work when multiple chromosomes are analyzed simultaneously.
lowerLimit	-0.631759089316322	Use this as the intensity limit for calling a segment lossed. Default value computes the mean of the sample set and estimates the lower threshold for CNA call to be two standard deviations from it.
nSegFit	3000	Maximum number of segments used for fitting the mixture model in probability calculations. Disabled if callProbabilities=false. Decreasing this lowers accuracy but can speed computation significantly.
plotChromosomes	0	Defines the plots that the user wants to output as a comma separated list. The whole genome is plotted by default for each sample. Inputting a value different than 0 will generate additional plots for these specific chromosomes from each sample.
plotEverySample	false	Enabling this will make the component print each sample separately.
undoSplits	sdundo	A character string specifying how change-points are to be undone, if at all. Undoing change-points decreases the sensitivity of segmentation. Choices are "none","prune", which uses a sum of squares criterion, and "sdundo" (default), which undoes splits that are not at least this many SDs apart. SD by default is 3.
upperLimit	0.631759089316322	Use this as the intensity limit for calling a segment gained. Default value computes the mean of the sample set and estimates the upper threshold for CNA call to be two standard deviations from it.

19.115 arrayCGHanalysis-chr20 (Segmentation)

No description

Parameter name	Value
chr	20

19.116 arrayCGHanalysis-chr20-chrFilter (CSVFilter)

Filters columns and/or rows from CSV files using flexible criteria. Default settings do not filter out anything. The *negate* parameter reverses all inclusion criteria.

Columns are filtered based on column names (see *includeColumns*); it is also possible to rename columns. Columns can be reordered using *includeColumns*.

A row is included in output if all conditions are satisfied. Rows having too many missing values are filtered using the *nonMissing* parameter. The parameters *regexp*, *lowBound* and *highBound* filter rows based on cell contents. If the *auxiliary* input is given, the result contains those rows from the input whose values match those from the auxiliary file.

Authors: Kristian Ovaska (kristian.ovaska@helsinki.fi), Marko Laakso (Marko.Laakso@Helsinki.FI)
Version: 1.1
Category: Filter

Input name	Source	Description
csv	arrayCGHanalysis-srFAnnot. file 19.213	CSV file to be filtered.

Parameter name	Value	Description
highBound		A row is included if the value in given numeric columns are at most as large as given numeric bounds. The parameter has a format COLNAME1=HIGH1,COLNAME2=HIGH2.
idColumn		Column name in "csv" that contains ID values. If the parameter is empty, the first column is used.
includeColumns	*	Comma-separated list of column names that should be included in the result file. The special value * includes all columns. The order of column names is significant: the output columns are in the order given. See also includeColumns input.
lowBound		A row is included if the value in given numeric columns are at least as large as given numeric bounds. The parameter has a format COLNAME1=LOW1,COLNAME2=LOW2. For example, "col=5" includes rows where the column col has a value of at least 5.
matchColumn		Column name in "auxiliary" containing values that must match the ID column in "csv" (specified using "idColumn"). If empty, the first column of "auxiliary" is used.
negate	false	If true, all inclusion criteria are negated so that rows or columns are excluded instead of included if they match the criteria. For example, includeColumns=* then excludes all columns (which would be an error since at least one column must be included).
nonMissing	0	Include those rows that have non-missing (non-NA) values in at least this many columns. Only those columns that are part of the output are counted.
regexp	Chr=20	Row filtering based on regular expression. A row is included in the result if values in the given columns match given regular expressions. The parameter has a format COLNAME1=EXPRESSION,COLNAME2=EXPRESSION2 where COLNAMEs are column names in "csv" and EXPRESSIONs are regular expressions using Java syntax. For example, "col=a|b" includes rows where the column col has a value of "a" or "b". Commas have to be escaped with "\\" if they are used in column names or regular expressions.
rename		Comma-separated list of column renaming rules (OLDNAME=NEWNAME). Parameters that refer to column names refer to old names; renaming is done last.

19.117 arrayCGHanalysis-chr20-filtNormCaseChan (CSVFilter)

Filters columns and/or rows from CSV files using flexible criteria. Default settings do not filter out anything. The *negate* parameter reverses all inclusion criteria.

Columns are filtered based on column names (see *includeColumns*); it is also possible to rename columns. Columns can be reordered using *includeColumns*.

A row is included in output if all conditions are satisfied. Rows having too many missing values are filtered using the *nonMissing* parameter. The parameters *regexp*, *lowBound* and *highBound* filter rows based on cell contents. If the *auxiliary* input is given, the result contains those rows from the input whose values match those from the auxiliary file.

Authors:	Kristian Ovaska (kristian.ovaska@helsinki.fi), Marko Laakso (Marko.Laakso@Helsinki.FI)
Version:	1.1
Category:	Filter

Input name	Source	Description
csv	arrayCGHanalysis-normalized .casechannel 19.196	CSV file to be filtered.
auxiliary	arrayCGHanalysis-chr20-chrFilter .csv 19.116	If given, contains one column (see "matchColumn") whose values are matched to a column in the "csv" input (see "idColumn").

Parameter name	Value	Description
highBound		A row is included if the value in given numeric columns are at most as large as given numeric bounds. The parameter has a format COLNAME1=HIGH1,COLNAME2=HIGH2.
idColumn		Column name in "csv" that contains ID values. If the parameter is empty, the first column is used.
includeColumns	*	Comma-separated list of column names that should be included in the result file. The special value * includes all columns. The order of column names is significant: the output columns are in the order given. See also includeColumns input.
lowBound		A row is included if the value in given numeric columns are at least as large as given numeric bounds. The parameter has a format COLNAME1=LOW1,COLNAME2=LOW2. For example, "col=5" includes rows where the column col has a value of at least 5.
matchColumn	ProbeName	Column name in "auxiliary" that must match the ID column in "csv" (specified using "idColumn"). If empty, the first column of "auxiliary" is used.
negate	false	If true, all inclusion criteria are negated so that rows or columns are excluded instead of included if they match the criteria. For example, includeColumns=* then excludes all columns (which would be an error since at least one column must be included).
nonMissing	0	Include those rows that have non-missing (non-NA) values in at least this many columns. Only those columns that are part of the output are counted.
regexp		Row filtering based on regular expression. A row is included in the result if values in the given columns match given regular expressions. The parameter has a format COLNAME1=EXPRESSION,COLNAME2=EXPRESSION2 where COLNAMEs are column names in "csv" and EXPRESSIONs are regular expressions using Java syntax. For example, "col=a|b" includes rows where the column col has a value of "a" or "b". Commas have to be escaped with "\\" if they are used in column names or regular expressions.
rename		Comma-separated list of column renaming rules (OLDNAME=NEWNAME). Parameters that refer to column names refer to old names; renaming is done last.

19.118 arrayCGHanalysis-chr20-segmented (ACGHsegment)

ACGHsegment segments normalized array CGH data, and calls aberrations in one of two ways: by classification of probe data discretely to gain, normal or loss, or computing probabilities to of a gain, normal or loss state for each probe. Segmentation utilizes the Circular Binary Segmentation algorithm [15].

Two ways of analysing Array CGH data post-segmentation are included. The first one, the Genome Topography Scan [27] computes scores over all the samples to calculate the most recurrent aberrations over the sample set. The second one outputs all the regions in all the samples whose segmented means

are more than two standard deviations apart from the mean of all the intensities. Plots from the GTS as well as from the segmented intensities are output depending on the parameter setting.

Probabilities for copy number aberrations can be computed with two algorithms. The first, FastCall [3], is faster while the second, CGHcall [26], is more accurate.

Author:	Riku Louhimo (Riku.Louhimo@Helsinki.FI)
Version:	1.3
Categories:	Agilent, Copy Number Analysis

Input name	Source	Description
caseChan	arrayCGHanalysis-chr20-filtNorm CaseChan.csv 19.117	CSV file containing the normalized probe intensities for the case channel. First row should have the probenames and samples should be columnwise.
geneAnnotation	arrayCGHanalysis-chr20-chrFilter .csv 19.116	Probewise annotations as produced by the AgilenReader component. Only the probes present in the casechannel csv can be included. Use CSVFilter if the probes do not otherwise match.

Parameter name	Value	Description
CGHCallPrior	auto	Only used if callProbMethod=CGHCall. Set the method to determine prior probabilities to CGHCall algorithm. Must be one of "auto", "all", "not all".
analysisAlgorithm	0	1 means that only the GTS algorithm is run, 2 that only the standard deviation test is run. 0 indicates that both are run.
callProbMethod	CGHCall	Method with which to call CNA segment probabilities. Must be either CGHCall or FastCall. FastCall is significantly (30000 times) faster while CGHCall is more accurate.
callProbabilities	false	Enabling this will make the component estimate probabilties for CNA segments via the CGHcall package. Only guaranteed to work when multiple chromosomes are analyzed simultaneously.
lowerLimit	-0.631759089316322	Use this as the intensity limit for calling a segment lossed. Default value computes the mean of the sample set and estimates the lower threshold for CNA call to be two standard deviations from it.
nSegFit	3000	Maximum number of segments used for fitting the mixture model in probability calculations. Disabled if callProbabilities=false. Decreasing this lowers accuracy but can speed computation significantly.
plotChromosomes	0	Defines the plots that the user wants to output as a comma separated list. The whole genome is plotted by default for each sample. Inputting a value different than 0 will generate additional plots for these specific chromosomes from each sample.
plotEverySample	false	Enabling this will make the component print each sample separately.
undoSplits	sdundo	A character string specifying how change-points are to be undone, if at all. Undoing change-points decreases the sensitivity of segmentation. Choices are "none","prune", which uses a sum of squares criterion, and "sdundo" (default), which undoes splits that are not at least this many SDs apart. SD by default is 3.
upperLimit	0.631759089316322	Use this as the intensity limit for calling a segment gained. Default value computes the mean of the sample set and estimates the upper threshold for CNA call to be two standard deviations from it.

19.119 arrayCGHanalysis-chr21 (Segmentation)

No description

Parameter name	Value
chr	21

19.120 arrayCGHanalysis-chr21-chrFilter (CSVFilter)

Filters columns and/or rows from CSV files using flexible criteria. Default settings do not filter out anything. The *negate* parameter reverses all inclusion criteria.

Columns are filtered based on column names (see *includeColumns*); it is also possible to rename columns. Columns can be reordered using *includeColumns*.

A row is included in output if all conditions are satisfied. Rows having too many missing values are filtered

using the *nonMissing* parameter. The parameters *regexp*, *lowBound* and *highBound* filter rows based on cell contents. If the *auxiliary* input is given, the result contains those rows from the input whose values match those from the auxiliary file.

Authors:	Kristian Ovaska (kristian.ovaska@helsinki.fi), Marko Laakso (Marko.Laakso@Helsinki.FI)
Version:	1.1
Category:	Filter

Input name	Source	Description
csv	arrayCGHanalysis-srFAnnot.file 19.213	CSV file to be filtered.

Parameter name	Value	Description
highBound		A row is included if the value in given numeric columns are at most as large as given numeric bounds. The parameter has a format COLNAME1=HIGH1,COLNAME2=HIGH2.
idColumn		Column name in "csv" that contains ID values. If the parameter is empty, the first column is used.
includeColumns	*	Comma-separated list of column names that should be included in the result file. The special value * includes all columns. The order of column names is significant: the output columns are in the order given. See also includeColumns input.
lowBound		A row is included if the value in given numeric columns are at least as large as given numeric bounds. The parameter has a format COLNAME1=LOW1,COLNAME2=LOW2. For example, "col=5" includes rows where the column col has a value of at least 5.
matchColumn		Column name in "auxiliary" containing values that must match the ID column in "csv" (specified using "idColumn"). If empty, the first column of "auxiliary" is used.
negate	false	If true, all inclusion criteria are negated so that rows or columns are excluded instead of included if they match the criteria. For example, includeColumns=* then excludes all columns (which would be an error since at least one column must be included).
nonMissing	0	Include those rows that have non-missing (non-NA) values in at least this many columns. Only those columns that are part of the output are counted.
regexp	Chr=21	Row filtering based on regular expression. A row is included in the result if values in the given columns match given regular expressions. The parameter has a format COLNAME1=EXPRESSION,COLNAME2=EXPRESSION2 where COLNAMEs are column names in "csv" and EXPRESSIONs are regular expressions using Java syntax. For example, "col=a|b" includes rows where the column col has a value of "a" or "b". Commas have to be escaped with "\\" if they are used in column names or regular expressions.
rename		Comma-separated list of column renaming rules (OLDNAME=NEWNAME). Parameters that refer to column names refer to old names; renaming is done last.

19.121 arrayCGHanalysis-chr21-filtNormCaseChan (CSVFilter)

Filters columns and/or rows from CSV files using flexible criteria. Default settings do not filter out anything. The *negate* parameter reverses all inclusion criteria.

Columns are filtered based on column names (see *includeColumns*); it is also possible to rename columns. Columns can be reordered using *includeColumns*.

A row is included in output if all conditions are satisfied. Rows having too many missing values are filtered using the *nonMissing* parameter. The parameters *regexp*, *lowBound* and *highBound* filter rows based on cell contents. If the *auxiliary* input is given, the result contains those rows from the input whose values match those from the auxiliary file.

Authors:	Kristian Ovaska (kristian.ovaska@helsinki.fi), Marko Laakso (Marko.Laakso@Helsinki.FI)
Version:	1.1
Category:	Filter

Input name	Source	Description
csv	arrayCGHanalysis-normalized .casechannel 19.196	CSV file to be filtered.
auxiliary	arrayCGHanalysis-chr21-chrFilter .csv 19.120	If given, contains one column (see "matchColumn") whose values are matched to a column in the "csv" input (see "idColumn").

Parameter name	Value	Description
highBound		A row is included if the value in given numeric columns are at most as large as given numeric bounds. The parameter has a format COLNAME1=HIGH1,COLNAME2=HIGH2.
idColumn		Column name in "csv" that contains ID values. If the parameter is empty, the first column is used.
includeColumns	*	Comma-separated list of column names that should be included in the result file. The special value * includes all columns. The order of column names is significant: the output columns are in the order given. See also includeColumns input.
lowBound		A row is included if the value in given numeric columns are at least as large as given numeric bounds. The parameter has a format COLNAME1=LOW1,COLNAME2=LOW2. For example, "col=5" includes rows where the column col has a value of at least 5.
matchColumn	ProbeName	Column name in "auxiliary" containing values that must match the ID column in "csv" (specified using "idColumn"). If empty, the first column of "auxiliary" is used.
negate	false	If true, all inclusion criteria are negated so that rows or columns are excluded instead of included if they match the criteria. For example, includeColumns=* then excludes all columns (which would be an error since at least one column must be included).
nonMissing	0	Include those rows that have non-missing (non-NA) values in at least this many columns. Only those columns that are part of the output are counted.
regexp		Row filtering based on regular expression. A row is included in the result if values in the given columns match given regular expressions. The parameter has a format COLNAME1=EXPRESSION,COLNAME2=EXPRESSION2 where COLNAMEs are column names in "csv" and EXPRESSIONs are regular expressions using Java syntax. For example, "col=a|b" includes rows where the column col has a value of "a" or "b". Commas have to be escaped with "\\" if they are used in column names or regular expressions.
rename		Comma-separated list of column renaming rules (OLDNAME=NEWNAME). Parameters that refer to column names refer to old names; renaming is done last.

19.122 arrayCGHanalysis-chr21-segmented (ACGHsegment)

ACGHsegment segments normalized array CGH data, and calls aberrations in one of two ways: by classification of probe data discretely to gain, normal or loss, or computing probabilities to of a gain, normal or loss state for each probe. Segmentation utilizes the Circular Binary Segmentation algorithm [15].

Two ways of analysing Array CGH data post-segmentation are included. The first one, the Genome Topography Scan [27] computes scores over all the samples to calculate the most recurrent aberrations over the sample set. The second one outputs all the regions in all the samples whose segmented means are more than two standard deviations apart from the mean of all the intensities. Plots from the GTS as well as from the segmented intensities are output depending on the parameter setting.

Probabilities for copy number aberrations can be computed with two algorithms. The first, FastCall [3], is faster while the second, CGHcall [26], is more accurate.

Author:	Riku Louhimo (Riku.Louhimo@Helsinki.FI)
Version:	1.3
Categories:	Agilent, Copy Number Analysis

Input name	Source	Description
caseChan	arrayCGHanalysis-chr21-filtNorm CaseChan.csv 19.121	CSV file containing the normalized probe intensities for the case channel. First row should have the probenames and samples should be columnwise.
geneAnnotation	arrayCGHanalysis-chr21-chrFilter .csv 19.120	Probewise annotations as produced by the AgilenReader component. Only the probes present in the casechannel csv can be included. Use CSVFilter if the probes do not otherwise match.

Parameter name	Value	Description
CGHCallPrior	auto	Only used if callProbMethod=CGHCall. Set the method to determine prior probabilities to CGHCall algorithm. Must be one of "auto", "all", "not all".
analysisAlgorithm	0	1 means that only the GTS algorithm is run, 2 that only the standard deviation test is run. 0 indicates that both are run.
callProbMethod	CGHCall	Method with which to call CNA segment probabilities. Must be either CGHCall or FastCall. FastCall is significantly (30000 times) faster while CGHCall is more accurate.
callProbabilities	false	Enabling this will make the component estimate probabilties for CNA segments via the CGHcall package. Only guaranteed to work when multiple chromosomes are analyzed simultaneously.
lowerLimit	-0.631759089316322	Use this as the intensity limit for calling a segment lossed. Default value computes the mean of the sample set and estimates the lower threshold for CNA call to be two standard deviations from it.
nSegFit	3000	Maximum number of segments used for fitting the mixture model in probability calculations. Disabled if callProbabilities=false. Decreasing this lowers accuracy but can speed computation significantly.
plotChromosomes	0	Defines the plots that the user wants to output as a comma separated list. The whole genome is plotted by default for each sample. Inputting a value different than 0 will generate additional plots for these specific chromosomes from each sample.
plotEverySample	false	Enabling this will make the component print each sample separately.
undoSplits	sdundo	A character string specifying how change-points are to be undone, if at all. Undoing change-points decreases the sensitivity of segmentation. Choices are "none","prune", which uses a sum of squares criterion, and "sdundo" (default), which undoes splits that are not at least this many SDs apart. SD by default is 3.
upperLimit	0.631759089316322	Use this as the intensity limit for calling a segment gained. Default value computes the mean of the sample set and estimates the upper threshold for CNA call to be two standard deviations from it.

19.123 arrayCGHanalysis-chr22 (Segmentation)

No description

Parameter name	Value
chr	22

19.124 arrayCGHanalysis-chr22-chrFilter (CSVFilter)

Filters columns and/or rows from CSV files using flexible criteria. Default settings do not filter out anything. The *negate* parameter reverses all inclusion criteria.

Columns are filtered based on column names (see *includeColumns*); it is also possible to rename columns. Columns can be reordered using *includeColumns*.

A row is included in output if all conditions are satisfied. Rows having too many missing values are filtered using the *nonMissing* parameter. The parameters *regexp*, *lowBound* and *highBound* filter rows based on cell contents. If the *auxiliary* input is given, the result contains those rows from the input whose values match those from the auxiliary file.

Authors: Kristian Ovaska (kristian.ovaska@helsinki.fi), Marko Laakso (Marko.Laakso@Helsinki.FI)
Version: 1.1
Category: Filter

Input name	Source	Description
csv	arrayCGHanalysis-srFAnnot. file 19.213	CSV file to be filtered.

Parameter name	Value	Description
highBound		A row is included if the value in given numeric columns are at most as large as given numeric bounds. The parameter has a format COLNAME1=HIGH1,COLNAME2=HIGH2.

Continued on next page...

Parameter name	Value	Description
idColumn		Column name in "csv" that contains ID values. If the parameter is empty, the first column is used.
includeColumns	*	Comma-separated list of column names that should be included in the result file. The special value * includes all columns. The order of column names is significant: the output columns are in the order given. See also includeColumns input.
lowBound		A row is included if the value in given numeric columns are at least as large as given numeric bounds. The parameter has a format COLNAME1=LOW1,COLNAME2=LOW2. For example, "col=5" includes rows where the column col has a value of at least 5.
matchColumn		Column name in "auxiliary" containing values that must match the ID column in "csv" (specified using "idColumn"). If empty, the first column of "auxiliary" is used.
negate	false	If true, all inclusion criteria are negated so that rows or columns are excluded instead of included if they match the criteria. For example, includeColumns=* then excludes all columns (which would be an error since at least one column must be included).
nonMissing	0	Include those rows that have non-missing (non-NA) values in at least this many columns. Only those columns that are part of the output are counted.
regexp	Chr=22	Row filtering based on regular expression. A row is included in the result if values in the given columns match given regular expressions. The parameter has a format COLNAME1=EXPRESSION,COLNAME2=EXPRESSION2 where COLNAMEs are column names in "csv" and EXPRESSIONs are regular expressions using Java syntax. For example, "col=a|b" includes rows where the column col has a value of "a" or "b". Commas have to be escaped with "\\" if they are used in column names or regular expressions.
rename		Comma-separated list of column renaming rules (OLDNAME=NEWNAME). Parameters that refer to column names refer to old names; renaming is done last.

19.125 arrayCGHanalysis-chr22-filtNormCaseChan (CSVFilter)

Filters columns and/or rows from CSV files using flexible criteria. Default settings do not filter out anything. The *negate* parameter reverses all inclusion criteria.

Columns are filtered based on column names (see *includeColumns*); it is also possible to rename columns. Columns can be reordered using *includeColumns*.

A row is included in output if all conditions are satisfied. Rows having too many missing values are filtered using the *nonMissing* parameter. The parameters *regexp*, *lowBound* and *highBound* filter rows based on cell contents. If the *auxiliary* input is given, the result contains those rows from the input whose values match those from the auxiliary file.

Authors: Kristian Ovaska (kristian.ovaska@helsinki.fi), Marko Laakso (Marko.Laakso@Helsinki.FI)
Version: 1.1
Category: Filter

Input name	Source	Description
csv	arrayCGHanalysis-normalized .casechannel 19.196	CSV file to be filtered.
auxiliary	arrayCGHanalysis-chr22-chrFilter .csv 19.124	If given, contains one column (see "matchColumn") whose values are matched to a column in the "csv" input (see "idColumn").

Parameter name	Value	Description
highBound		A row is included if the value in given numeric columns are at most as large as given numeric bounds. The parameter has a format COLNAME1=HIGH1,COLNAME2=HIGH2.
idColumn		Column name in "csv" that contains ID values. If the parameter is empty, the first column is used.
includeColumns	*	Comma-separated list of column names that should be included in the result file. The special value * includes all columns. The order of column names is significant: the output columns are in the order given. See also includeColumns input.
lowBound		A row is included if the value in given numeric columns are at least as large as given numeric bounds. The parameter has a format COLNAME1=LOW1,COLNAME2=LOW2. For example, "col=5" includes rows where the column col has a value of at least 5.

Continued on next page...

Parameter name	Value	Description
matchColumn	ProbeName	Column name in "auxiliary" containing values that must match the ID column in "csv" (specified using "idColumn"). If empty, the first column of "auxiliary" is used.
negate	false	If true, all inclusion criteria are negated so that rows or columns are excluded instead of included if they match the criteria. For example, includeColumns=* then excludes all columns (which would be an error since at least one column must be included).
nonMissing	0	Include those rows that have non-missing (non-NA) values in at least this many columns. Only those columns that are part of the output are counted.
regexp		Row filtering based on regular expression. A row is included in the result if values in the given columns match given regular expressions. The parameter has a format COLNAME1=EXPRESSION,COLNAME2=EXPRESSION2 where COLNAMEs are column names in "csv" and EXPRESSIONs are regular expressions using Java syntax. For example, "col=a|b" includes rows where the column col has a value of "a" or "b". Commas have to be escaped with "\\" if they are used in column names or regular expressions.
rename		Comma-separated list of column renaming rules (OLDNAME=NEWNAME). Parameters that refer to column names refer to old names; renaming is done last.

19.126 arrayCGHanalysis-chr22-segmented (ACGHsegment)

ACGHsegment segments normalized array CGH data, and calls aberrations in one of two ways: by classification of probe data discretely to gain, normal or loss, or computing probabilities to of a gain, normal or loss state for each probe. Segmentation utilizes the Circular Binary Segmentation algorithm [15].

Two ways of analysing Array CGH data post-segmentation are included. The first one, the Genome Topography Scan [27] computes scores over all the samples to calculate the most recurrent aberrations over the sample set. The second one outputs all the regions in all the samples whose segmented means are more than two standard deviations apart from the mean of all the intensities. Plots from the GTS as well as from the segmented intensities are output depending on the parameter setting.

Probabilities for copy number aberrations can be computed with two algorithms. The first, FastCall [3], is faster while the second, CGHcall [26], is more accurate.

Author:	Riku Louhimo (Riku.Louhimo@Helsinki.FI)
Version:	1.3
Categories:	Agilent, Copy Number Analysis

Input name	Source	Description
caseChan	arrayCGHanalysis-chr22-filtNorm CaseChan.csv 19.125	CSV file containing the normalized probe intensities for the case channel. First row should have the probenames and samples should be columnwise.
geneAnnotation	arrayCGHanalysis-chr22-chrFilter .csv 19.124	Probewise annotations as produced by the AgilenReader component. Only the probes present in the casechannel csv can be included. Use CSVFilter if the probes do not otherwise match.

Parameter name	Value	Description
CGHCallPrior	auto	Only used if callProbMethod=CGHCall. Set the method to determine prior probabilities to CGHCall algorithm. Must be one of "auto", "all", "not all".
analysisAlgorithm	0	1 means that only the GTS algorithm is run, 2 that only the standard deviation test is run. 0 indicates that both are run.
callProbMethod	CGHCall	Method with which to call CNA segment probabilities. Must be either CGHCall or FastCall. FastCall is significantly (30000 times) faster while CGHCall is more accurate.
callProbabilities	false	Enabling this will make the component estimate probabilties for CNA segments via the CGHcall package. Only guaranteed to work when multiple chromosomes are analyzed simultaneously.
lowerLimit	-0.631759089316322	Use this as the intensity limit for calling a segment lossed. Default value computes the mean of the sample set and estimates the lower threshold for CNA call to be two standard deviations from it.
nSegFit	3000	Maximum number of segments used for fitting the mixture model in probability calculations. Disabled if callProbabilities=false. Decreasing this lowers accuracy but can speed computation significantly.

Continued on next page...

Parameter name	Value	Description
plotChromosomes	0	Defines the plots that the user wants to output as a comma separated list. The whole genome is plotted by default for each sample. Inputting a value different than 0 will generate additional plots for these specific chromosomes from each sample.
plotEverySample	false	Enabling this will make the component print each sample separately.
undoSplits	sdundo	A character string specifying how change-points are to be undone, if at all. Undoing change-points decreases the sensitivity of segmentation. Choices are "none","prune", which uses a sum of squares criterion, and "sdundo" (default), which undoes splits that are not at least this many SDs apart. SD by default is 3.
upperLimit	0.631759089316322	Use this as the intensity limit for calling a segment gained. Default value computes the mean of the sample set and estimates the upper threshold for CNA call to be two standard deviations from it.

19.127 arrayCGHanalysis-chr3 (Segmentation)

No description

Parameter name	Value
chr	3

19.128 arrayCGHanalysis-chr3-chrFilter (CSVFilter)

Filters columns and/or rows from CSV files using flexible criteria. Default settings do not filter out anything. The *negate* parameter reverses all inclusion criteria.

Columns are filtered based on column names (see *includeColumns*); it is also possible to rename columns. Columns can be reordered using *includeColumns*.

A row is included in output if all conditions are satisfied. Rows having too many missing values are filtered using the *nonMissing* parameter. The parameters *regexp*, *lowBound* and *highBound* filter rows based on cell contents. If the *auxiliary* input is given, the result contains those rows from the input whose values match those from the auxiliary file.

Authors:	Kristian Ovaska (kristian.ovaska@helsinki.fi), Marko Laakso (Marko.Laakso@Helsinki.FI)
Version:	1.1
Category:	Filter

Input name	Source	Description
csv	arrayCGHanalysis-srFAnnot.file 19.213	CSV file to be filtered.

Parameter name	Value	Description
highBound		A row is included if the value in given numeric columns are at most as large as given numeric bounds. The parameter has a format COLNAME1=HIGH1,COLNAME2=HIGH2.
idColumn		Column name in "csv" that contains ID values. If the parameter is empty, the first column is used.
includeColumns	*	Comma-separated list of column names that should be included in the result file. The special value * includes all columns. The order of column names is significant: the output columns are in the order given. See also includeColumns input.
lowBound		A row is included if the value in given numeric columns are at least as large as given numeric bounds. The parameter has a format COLNAME1=LOW1,COLNAME2=LOW2. For example, "col=5" includes rows where the column col has a value of at least 5.
matchColumn		Column name in "auxiliary" containing values that must match the ID column in "csv" (specified using "idColumn"). If empty, the first column of "auxiliary" is used.
negate	false	If true, all inclusion criteria are negated so that rows or columns are excluded instead of included if they match the criteria. For example, includeColumns=* then excludes all columns (which would be an error since at least one column must be included).

Continued on next page...

Parameter name	Value	Description
nonMissing	0	Include those rows that have non-missing (non-NA) values in at least this many columns. Only those columns that are part of the output are counted.
regexp	Chr=3	Row filtering based on regular expression. A row is included in the result if values in the given columns match given regular expressions. The parameter has a format COLNAME1=EXPRESSION,COLNAME2=EXPRESSION2 where COLNAMEs are column names in "csv" and EXPRESSIONs are regular expressions using Java syntax. For example, "col=a|b" includes rows where the column col has a value of "a" or "b". Commas have to be escaped with "\\" if they are used in column names or regular expressions.
rename		Comma-separated list of column renaming rules (OLDNAME=NEWNAME). Parameters that refer to column names refer to old names; renaming is done last.

19.129 arrayCGHanalysis-chr3-filtNormCaseChan (CSVFilter)

Filters columns and/or rows from CSV files using flexible criteria. Default settings do not filter out anything. The *negate* parameter reverses all inclusion criteria.

Columns are filtered based on column names (see *includeColumns*); it is also possible to rename columns. Columns can be reordered using *includeColumns*.

A row is included in output if all conditions are satisfied. Rows having too many missing values are filtered using the *nonMissing* parameter. The parameters *regexp*, *lowBound* and *highBound* filter rows based on cell contents. If the *auxiliary* input is given, the result contains those rows from the input whose values match those from the auxiliary file.

Authors: Kristian Ovaska (kristian.ovaska@helsinki.fi), Marko Laakso (Marko.Laakso@Helsinki.FI)
Version: 1.1
Category: Filter

Input name	Source	Description
csv	arrayCGHanalysis-normalized .casechannel 19.196	CSV file to be filtered.
auxiliary	arrayCGHanalysis-chr3-chrFilter .csv 19.128	If given, contains one column (see "matchColumn") whose values are matched to a column in the "csv" input (see "idColumn").

Parameter name	Value	Description
highBound		A row is included if the value in given numeric columns are at most as large as given numeric bounds. The parameter has a format COLNAME1=HIGH1,COLNAME2=HIGH2.
idColumn		Column name in "csv" that contains ID values. If the parameter is empty, the first column is used.
includeColumns	*	Comma-separated list of column names that should be included in the result file. The special value * includes all columns. The order of column names is significant: the output columns are in the order given. See also includeColumns input.
lowBound		A row is included if the value in given numeric columns are at least as large as given numeric bounds. The parameter has a format COLNAME1=LOW1,COLNAME2=LOW2. For example, "col=5" includes rows where the column col has a value of at least 5.
matchColumn	ProbeName	Column name in "auxiliary" containing values that must match the ID column in "csv" (specified using "idColumn"). If empty, the first column of "auxiliary" is used.
negate	false	If true, all inclusion criteria are negated so that rows or columns are excluded instead of included if they match the criteria. For example, includeColumns=* then excludes all columns (which would be an error since at least one column must be included).
nonMissing	0	Include those rows that have non-missing (non-NA) values in at least this many columns. Only those columns that are part of the output are counted.
regexp		Row filtering based on regular expression. A row is included in the result if values in the given columns match given regular expressions. The parameter has a format COLNAME1=EXPRESSION,COLNAME2=EXPRESSION2 where COLNAMEs are column names in "csv" and EXPRESSIONs are regular expressions using Java syntax. For example, "col=a|b" includes rows where the column col has a value of "a" or "b". Commas have to be escaped with "\\" if they are used in column names or regular expressions.

Continued on next page...

Parameter name	Value	Description
rename		Comma-separated list of column renaming rules (OLDNAME=NEWNAME). Parameters that refer to column names refer to old names; renaming is done last.

19.130 arrayCGHanalysis-chr3-segmented (ACGHsegment)

ACGHsegment segments normalized array CGH data, and calls aberrations in one of two ways: by classification of probe data discretely to gain, normal or loss, or computing probabilities to of a gain, normal or loss state for each probe. Segmentation utilizes the Circular Binary Segmentation algorithm [15].

Two ways of analysing Array CGH data post-segmentation are included. The first one, the Genome Topography Scan [27] computes scores over all the samples to calculate the most recurrent aberrations over the sample set. The second one outputs all the regions in all the samples whose segmented means are more than two standard deviations apart from the mean of all the intensities. Plots from the GTS as well as from the segmented intensities are output depending on the parameter setting.

Probabilities for copy number aberrations can be computed with two algorithms. The first, FastCall [3], is faster while the second, CGHcall [26], is more accurate.

Author: Riku Louhimo (Riku.Louhimo@Helsinki.FI)
Version: 1.3
Categories: Agilent, Copy Number Analysis

Input name	Source	Description
caseChan	arrayCGHanalysis-chr3-filtNorm CaseChan.csv 19.129	CSV file containing the normalized probe intensities for the case channel. First row should have the probenames and samples should be columnwise.
geneAnnotation	arrayCGHanalysis-chr3-chrFilter .csv 19.128	Probewise annotations as produced by the AgilenReader component. Only the probes present in the casechannel csv can be included. Use CSVFilter if the probes do not otherwise match.

Parameter name	Value	Description
CGHCallPrior	auto	Only used if callProbMethod=CGHCall. Set the method to determine prior probabilities to CGHCall algorithm. Must be one of "auto", "all", "not all".
analysisAlgorithm	0	1 means that only the GTS algorithm is run, 2 that only the standard deviation test is run. 0 indicates that both are run.
callProbMethod	CGHCall	Method with which to call CNA segment probabilities. Must be either CGHCall or FastCall. FastCall is significantly (30000 times) faster while CGHCall is more accurate.
callProbabilities	false	Enabling this will make the component estimate probabilties for CNA segments via the CGHcall package. Only guaranteed to work when multiple chromosomes are analyzed simultaneously.
lowerLimit	-0.631759089316322	Use this as the intensity limit for calling a segment lossed. Default value computes the mean of the sample set and estimates the lower threshold for CNA call to be two standard deviations from it.
nSegFit	3000	Maximum number of segments used for fitting the mixture model in probability calculations. Disabled if callProbabilities=false. Decreasing this lowers accuracy but can speed computation significantly.
plotChromosomes	0	Defines the plots that the user wants to output as a comma separated list. The whole genome is plotted by default for each sample. Inputting a value different than 0 will generate additional plots for these specific chromosomes from each sample.
plotEverySample	false	Enabling this will make the component print each sample separately.
undoSplits	sdundo	A character string specifying how change-points are to be undone, if at all. Undoing change-points decreases the sensitivity of segmentation. Choices are "none","prune", which uses a sum of squares criterion, and "sdundo" (default), which undoes splits that are not at least this many SDs apart. SD by default is 3.
upperLimit	0.631759089316322	Use this as the intensity limit for calling a segment gained. Default value computes the mean of the sample set and estimates the upper threshold for CNA call to be two standard deviations from it.

19.131 arrayCGHanalysis-chr4 (Segmentation)

No description

Parameter name	Value
chr	4

19.132 arrayCGHanalysis-chr4-chrFilter (CSVFilter)

Filters columns and/or rows from CSV files using flexible criteria. Default settings do not filter out anything. The *negate* parameter reverses all inclusion criteria.

Columns are filtered based on column names (see *includeColumns*); it is also possible to rename columns. Columns can be reordered using *includeColumns*.

A row is included in output if all conditions are satisfied. Rows having too many missing values are filtered using the *nonMissing* parameter. The parameters *regexp*, *lowBound* and *highBound* filter rows based on cell contents. If the *auxiliary* input is given, the result contains those rows from the input whose values match those from the auxiliary file.

Authors: Kristian Ovaska (kristian.ovaska@helsinki.fi), Marko Laakso (Marko.Laakso@Helsinki.FI)
Version: 1.1
Category: Filter

Input name	Source	Description
csv	arrayCGHanalysis-srFAnnot.file 19.213	CSV file to be filtered.

Parameter name	Value	Description
highBound		A row is included if the value in given numeric columns are at most as large as given numeric bounds. The parameter has a format COLNAME1=HIGH1,COLNAME2=HIGH2.
idColumn		Column name in "csv" that contains ID values. If the parameter is empty, the first column is used.
includeColumns	*	Comma-separated list of column names that should be included in the result file. The special value * includes all columns. The order of column names is significant: the output columns are in the order given. See also includeColumns input.
lowBound		A row is included if the value in given numeric columns are at least as large as given numeric bounds. The parameter has a format COLNAME1=LOW1,COLNAME2=LOW2. For example, "col=5" includes rows where the column col has a value of at least 5.
matchColumn		Column name in "auxiliary" containing values that must match the ID column in "csv" (specified using "idColumn"). If empty, the first column of "auxiliary" is used.
negate	false	If true, all inclusion criteria are negated so that rows or columns are excluded instead of included if they match the criteria. For example, includeColumns=* then excludes all columns (which would be an error since at least one column must be included).
nonMissing	0	Include those rows that have non-missing (non-NA) values in at least this many columns. Only those columns that are part of the output are counted.
regexp	Chr=4	Row filtering based on regular expression. A row is included in the result if values in the given columns match given regular expressions. The parameter has a format COLNAME1=EXPRESSION,COLNAME2=EXPRESSION2 where COLNAMEs are column names in "csv" and EXPRESSIONs are regular expressions using Java syntax. For example, "col=a|b" includes rows where the column col has a value of "a" or "b". Commas have to be escaped with "\\" if they are used in column names or regular expressions.
rename		Comma-separated list of column renaming rules (OLDNAME=NEWNAME). Parameters that refer to column names refer to old names; renaming is done last.

19.133 arrayCGHanalysis-chr4-filtNormCaseChan (CSVFilter)

Filters columns and/or rows from CSV files using flexible criteria. Default settings do not filter out anything. The *negate* parameter reverses all inclusion criteria.

Columns are filtered based on column names (see *includeColumns*); it is also possible to rename columns. Columns can be reordered using *includeColumns*.

A row is included in output if all conditions are satisfied. Rows having too many missing values are filtered using the *nonMissing* parameter. The parameters *regexp*, *lowBound* and *highBound* filter rows based on cell contents. If the *auxiliary* input is given, the result contains those rows from the input whose values match those from the auxiliary file.

Authors: Kristian Ovaska (kristian.ovaska@helsinki.fi), Marko Laakso (Marko.Laakso@Helsinki.FI)
Version: 1.1
Category: Filter

Input name	Source	Description
csv	arrayCGHanalysis-normalized .casechannel 19.196	CSV file to be filtered.
auxiliary	arrayCGHanalysis-chr4-chrFilter .csv 19.132	If given, contains one column (see "matchColumn") whose values are matched to a column in the "csv" input (see "idColumn").

Parameter name	Value	Description
highBound		A row is included if the value in given numeric columns are at most as large as given numeric bounds. The parameter has a format COLNAME1=HIGH1,COLNAME2=HIGH2.
idColumn		Column name in "csv" that contains ID values. If the parameter is empty, the first column is used.
includeColumns	*	Comma-separated list of column names that should be included in the result file. The special value * includes all columns. The order of column names is significant: the output columns are in the order given. See also includeColumns input.
lowBound		A row is included if the value in given numeric columns are at least as large as given numeric bounds. The parameter has a format COLNAME1=LOW1,COLNAME2=LOW2. For example, "col=5" includes rows where the column col has a value of at least 5.
matchColumn	ProbeName	Column name in "auxiliary" containing values that must match the ID column in "csv" (specified using "idColumn"). If empty, the first column of "auxiliary" is used.
negate	false	If true, all inclusion criteria are negated so that rows or columns are excluded instead of included if they match the criteria. For example, includeColumns=* then excludes all columns (which would be an error since at least one column must be included).
nonMissing	0	Include those rows that have non-missing (non-NA) values in at least this many columns. Only those columns that are part of the output are counted.
regexp		Row filtering based on regular expression. A row is included in the result if values in the given columns match given regular expressions. The parameter has a format COLNAME1=EXPRESSION,COLNAME2=EXPRESSION2 where COLNAMEs are column names in "csv" and EXPRESSIONs are regular expressions using Java syntax. For example, "col=a|b" includes rows where the column col has a value of "a" or "b". Commas have to be escaped with "\\" if they are used in column names or regular expressions.
rename		Comma-separated list of column renaming rules (OLDNAME=NEWNAME). Parameters that refer to column names refer to old names; renaming is done last.

19.134 arrayCGHanalysis-chr4-segmented (ACGHsegment)

ACGHsegment segments normalized array CGH data, and calls aberrations in one of two ways: by classification of probe data discretely to gain, normal or loss, or computing probabilities to of a gain, normal or loss state for each probe. Segmentation utilizes the Circular Binary Segmentation algorithm [15].

Two ways of analysing Array CGH data post-segmentation are included. The first one, the Genome Topography Scan [27] computes scores over all the samples to calculate the most recurrent aberrations over the sample set. The second one outputs all the regions in all the samples whose segmented means

are more than two standard deviations apart from the mean of all the intensities. Plots from the GTS as well as from the segmented intensities are output depending on the parameter setting.

Probabilities for copy number aberrations can be computed with two algorithms. The first, FastCall [3], is faster while the second, CGHcall [26], is more accurate.

Author:	Riku Louhimo (Riku.Louhimo@Helsinki.FI)
Version:	1.3
Categories:	Agilent, Copy Number Analysis

Input name	Source	Description
caseChan	arrayCGHanalysis-chr4-filtNorm CaseChan.csv 19.133	CSV file containing the normalized probe intensities for the case channel. First row should have the probenames and samples should be columnwise.
geneAnnotation	arrayCGHanalysis-chr4-chrFilter .csv 19.132	Probewise annotations as produced by the AgilenReader component. Only the probes present in the casechannel csv can be included. Use CSVFilter if the probes do not otherwise match.

Parameter name	Value	Description
CGHCallPrior	auto	Only used if callProbMethod=CGHCall. Set the method to determine prior probabilities to CGHCall algorithm. Must be one of "auto", "all", "not all".
analysisAlgorithm	0	1 means that only the GTS algorithm is run, 2 that only the standard deviation test is run. 0 indicates that both are run.
callProbMethod	CGHCall	Method with which to call CNA segment probabilities. Must be either CGHCall or FastCall. FastCall is significantly (30000 times) faster while CGHCall is more accurate.
callProbabilities	false	Enabling this will make the component estimate probabilties for CNA segments via the CGHcall package. Only guaranteed to work when multiple chromosomes are analyzed simultaneously.
lowerLimit	-0.631759089316322	Use this as the intensity limit for calling a segment lossed. Default value computes the mean of the sample set and estimates the lower threshold for CNA call to be two standard deviations from it.
nSegFit	3000	Maximum number of segments used for fitting the mixture model in probability calculations. Disabled if callProbabilities=false. Decreasing this lowers accuracy but can speed computation significantly.
plotChromosomes	0	Defines the plots that the user wants to output as a comma separated list. The whole genome is plotted by default for each sample. Inputting a value different than 0 will generate additional plots for these specific chromosomes from each sample.
plotEverySample	false	Enabling this will make the component print each sample separately.
undoSplits	sdundo	A character string specifying how change-points are to be undone, if at all. Undoing change-points decreases the sensitivity of segmentation. Choices are "none","prune", which uses a sum of squares criterion, and "sdundo" (default), which undoes splits that are not at least this many SDs apart. SD by default is 3.
upperLimit	0.631759089316322	Use this as the intensity limit for calling a segment gained. Default value computes the mean of the sample set and estimates the upper threshold for CNA call to be two standard deviations from it.

19.135 arrayCGHanalysis-chr5 (Segmentation)

No description

Parameter name	Value
chr	5

19.136 arrayCGHanalysis-chr5-chrFilter (CSVFilter)

Filters columns and/or rows from CSV files using flexible criteria. Default settings do not filter out anything. The *negate* parameter reverses all inclusion criteria.

Columns are filtered based on column names (see *includeColumns*); it is also possible to rename columns. Columns can be reordered using *includeColumns*.

A row is included in output if all conditions are satisfied. Rows having too many missing values are filtered

using the *nonMissing* parameter. The parameters *regexp*, *lowBound* and *highBound* filter rows based on cell contents. If the *auxiliary* input is given, the result contains those rows from the input whose values match those from the auxiliary file.

Authors:	Kristian Ovaska (kristian.ovaska@helsinki.fi), Marko Laakso (Marko.Laakso@Helsinki.FI)
Version:	1.1
Category:	Filter

Input name	Source	Description
csv	arrayCGHanalysis-srFAnnot. file 19.213	CSV file to be filtered.

Parameter name	Value	Description
highBound		A row is included if the value in given numeric columns are at most as large as given numeric bounds. The parameter has a format COLNAME1=HIGH1,COLNAME2=HIGH2.
idColumn		Column name in "csv" that contains ID values. If the parameter is empty, the first column is used.
includeColumns	*	Comma-separated list of column names that should be included in the result file. The special value * includes all columns. The order of column names is significant: the output columns are in the order given. See also includeColumns input.
lowBound		A row is included if the value in given numeric columns are at least as large as given numeric bounds. The parameter has a format COLNAME1=LOW1,COLNAME2=LOW2. For example, "col=5" includes rows where the column col has a value of at least 5.
matchColumn		Column name in "auxiliary" containing values that must match the ID column in "csv" (specified using "idColumn"). If empty, the first column of "auxiliary" is used.
negate	false	If true, all inclusion criteria are negated so that rows or columns are excluded instead of included if they match the criteria. For example, includeColumns=* then excludes all columns (which would be an error since at least one column must be included).
nonMissing	0	Include those rows that have non-missing (non-NA) values in at least this many columns. Only those columns that are part of the output are counted.
regexp	Chr=5	Row filtering based on regular expression. A row is included in the result if values in the given columns match given regular expressions. The parameter has a format COLNAME1=EXPRESSION,COLNAME2=EXPRESSION2 where COLNAMEs are column names in "csv" and EXPRESSIONs are regular expressions using Java syntax. For example, "col=a|b" includes rows where the column col has a value of "a" or "b". Commas have to be escaped with "\\" if they are used in column names or regular expressions.
rename		Comma-separated list of column renaming rules (OLDNAME=NEWNAME). Parameters that refer to column names refer to old names; renaming is done last.

19.137 arrayCGHanalysis-chr5-filtNormCaseChan (CSVFilter)

Filters columns and/or rows from CSV files using flexible criteria. Default settings do not filter out anything. The *negate* parameter reverses all inclusion criteria.

Columns are filtered based on column names (see *includeColumns*); it is also possible to rename columns. Columns can be reordered using *includeColumns*.

A row is included in output if all conditions are satisfied. Rows having too many missing values are filtered using the *nonMissing* parameter. The parameters *regexp*, *lowBound* and *highBound* filter rows based on cell contents. If the *auxiliary* input is given, the result contains those rows from the input whose values match those from the auxiliary file.

Authors:	Kristian Ovaska (kristian.ovaska@helsinki.fi), Marko Laakso (Marko.Laakso@Helsinki.FI)
Version:	1.1
Category:	Filter

Input name	Source	Description
csv	arrayCGHanalysis-normalized .casechannel 19.196	CSV file to be filtered.
auxiliary	arrayCGHanalysis-chr5-chrFilter .csv 19.136	If given, contains one column (see "matchColumn") whose values are matched to a column in the "csv" input (see "idColumn").

Parameter name	Value	Description
highBound		A row is included if the value in given numeric columns are at most as large as given numeric bounds. The parameter has a format COLNAME1=HIGH1,COLNAME2=HIGH2.
idColumn		Column name in "csv" that contains ID values. If the parameter is empty, the first column is used.
includeColumns	*	Comma-separated list of column names that should be included in the result file. The special value * includes all columns. The order of column names is significant: the output columns are in the order given. See also includeColumns input.
lowBound		A row is included if the value in given numeric columns are at least as large as given numeric bounds. The parameter has a format COLNAME1=LOW1,COLNAME2=LOW2. For example, "col=5" includes rows where the column col has a value of at least 5.
matchColumn	ProbeName	Column name in "auxiliary" containing values that must match the ID column in "csv" (specified using "idColumn"). If empty, the first column of "auxiliary" is used.
negate	false	If true, all inclusion criteria are negated so that rows or columns are excluded instead of included if they match the criteria. For example, includeColumns=* then excludes all columns (which would be an error since at least one column must be included).
nonMissing	0	Include those rows that have non-missing (non-NA) values in at least this many columns. Only those columns that are part of the output are counted.
regexp		Row filtering based on regular expression. A row is included in the result if values in the given columns match given regular expressions. The parameter has a format COLNAME1=EXPRESSION,COLNAME2=EXPRESSION2 where COLNAMEs are column names in "csv" and EXPRESSIONs are regular expressions using Java syntax. For example, "col=a|b" includes rows where the column col has a value of "a" or "b". Commas have to be escaped with "\\" if they are used in column names or regular expressions.
rename		Comma-separated list of column renaming rules (OLDNAME=NEWNAME). Parameters that refer to column names refer to old names; renaming is done last.

19.138 arrayCGHanalysis-chr5-segmented (ACGHsegment)

ACGHsegment segments normalized array CGH data, and calls aberrations in one of two ways: by classification of probe data discretely to gain, normal or loss, or computing probabilities to of a gain, normal or loss state for each probe. Segmentation utilizes the Circular Binary Segmentation algorithm [15].

Two ways of analysing Array CGH data post-segmentation are included. The first one, the Genome Topography Scan [27] computes scores over all the samples to calculate the most recurrent aberrations over the sample set. The second one outputs all the regions in all the samples whose segmented means are more than two standard deviations apart from the mean of all the intensities. Plots from the GTS as well as from the segmented intensities are output depending on the parameter setting.

Probabilities for copy number aberrations can be computed with two algorithms. The first, FastCall [3], is faster while the second, CGHcall [26], is more accurate.

Author: Riku Louhimo (Riku.Louhimo@Helsinki.FI)
Version: 1.3
Categories: Agilent, Copy Number Analysis

Input name	Source	Description
caseChan	arrayCGHanalysis-chr5-filtNorm CaseChan.csv 19.137	CSV file containing the normalized probe intensities for the case channel. First row should have the probenames and samples should be columnwise.
geneAnnotation	arrayCGHanalysis-chr5-chrFilter .csv 19.136	Probewise annotations as produced by the AgilenReader component. Only the probes present in the casechannel csv can be included. Use CSVFilter if the probes do not otherwise match.

Parameter name	Value	Description
CGHCallPrior	auto	Only used if callProbMethod=CGHCall. Set the method to determine prior probabilities to CGHCall algorithm. Must be one of "auto", "all", "not all".
analysisAlgorithm	0	1 means that only the GTS algorithm is run, 2 that only the standard deviation test is run. 0 indicates that both are run.
callProbMethod	CGHCall	Method with which to call CNA segment probabilities. Must be either CGHCall or FastCall. FastCall is significantly (30000 times) faster while CGHCall is more accurate.
callProbabilities	false	Enabling this will make the component estimate probabilties for CNA segments via the CGHcall package. Only guaranteed to work when multiple chromosomes are analyzed simultaneously.
lowerLimit	-0.631759089316322	Use this as the intensity limit for calling a segment lossed. Default value computes the mean of the sample set and estimates the lower threshold for CNA call to be two standard deviations from it.
nSegFit	3000	Maximum number of segments used for fitting the mixture model in probability calculations. Disabled if callProbabilities=false. Decreasing this lowers accuracy but can speed computation significantly.
plotChromosomes	0	Defines the plots that the user wants to output as a comma separated list. The whole genome is plotted by default for each sample. Inputting a value different than 0 will generate additional plots for these specific chromosomes from each sample.
plotEverySample	false	Enabling this will make the component print each sample separately.
undoSplits	sdundo	A character string specifying how change-points are to be undone, if at all. Undoing change-points decreases the sensitivity of segmentation. Choices are "none","prune", which uses a sum of squares criterion, and "sdundo" (default), which undoes splits that are not at least this many SDs apart. SD by default is 3.
upperLimit	0.631759089316322	Use this as the intensity limit for calling a segment gained. Default value computes the mean of the sample set and estimates the upper threshold for CNA call to be two standard deviations from it.

19.139 arrayCGHanalysis-chr6 (Segmentation)

No description

Parameter name	Value
chr	6

19.140 arrayCGHanalysis-chr6-chrFilter (CSVFilter)

Filters columns and/or rows from CSV files using flexible criteria. Default settings do not filter out anything. The *negate* parameter reverses all inclusion criteria.

Columns are filtered based on column names (see *includeColumns*); it is also possible to rename columns. Columns can be reordered using *includeColumns*.

A row is included in output if all conditions are satisfied. Rows having too many missing values are filtered using the *nonMissing* parameter. The parameters *regexp*, *lowBound* and *highBound* filter rows based on cell contents. If the *auxiliary* input is given, the result contains those rows from the input whose values match those from the auxiliary file.

Authors: Kristian Ovaska (kristian.ovaska@helsinki.fi), Marko Laakso (Marko.Laakso@Helsinki.FI)
Version: 1.1
Category: Filter

Input name	Source	Description
csv	arrayCGHanalysis-srFAnnot.file 19.213	CSV file to be filtered.

Parameter name	Value	Description
highBound		A row is included if the value in given numeric columns are at most as large as given numeric bounds. The parameter has a format COLNAME1=HIGH1,COLNAME2=HIGH2.

Continued on next page...

Parameter name	Value	Description
idColumn		Column name in "csv" that contains ID values. If the parameter is empty, the first column is used.
includeColumns	*	Comma-separated list of column names that should be included in the result file. The special value * includes all columns. The order of column names is significant: the output columns are in the order given. See also includeColumns input.
lowBound		A row is included if the value in given numeric columns are at least as large as given numeric bounds. The parameter has a format COLNAME1=LOW1,COLNAME2=LOW2. For example, "col=5" includes rows where the column col has a value of at least 5.
matchColumn		Column name in "auxiliary" containing values that must match the ID column in "csv" (specified using "idColumn"). If empty, the first column of "auxiliary" is used.
negate	false	If true, all inclusion criteria are negated so that rows or columns are excluded instead of included if they match the criteria. For example, includeColumns=* then excludes all columns (which would be an error since at least one column must be included).
nonMissing	0	Include those rows that have non-missing (non-NA) values in at least this many columns. Only those columns that are part of the output are counted.
regexp	Chr=6	Row filtering based on regular expression. A row is included in the result if values in the given columns match given regular expressions. The parameter has a format COLNAME1=EXPRESSION,COLNAME2=EXPRESSION2 where COLNAMEs are column names in "csv" and EXPRESSIONs are regular expressions using Java syntax. For example, "col=a|b" includes rows where the column col has a value of "a" or "b". Commas have to be escaped with "\\" if they are used in column names or regular expressions.
rename		Comma-separated list of column renaming rules (OLDNAME=NEWNAME). Parameters that refer to column names refer to old names; renaming is done last.

19.141 arrayCGHanalysis-chr6-filtNormCaseChan (CSVFilter)

Filters columns and/or rows from CSV files using flexible criteria. Default settings do not filter out anything. The *negate* parameter reverses all inclusion criteria.

Columns are filtered based on column names (see *includeColumns*); it is also possible to rename columns. Columns can be reordered using *includeColumns*.

A row is included in output if all conditions are satisfied. Rows having too many missing values are filtered using the *nonMissing* parameter. The parameters *regexp*, *lowBound* and *highBound* filter rows based on cell contents. If the *auxiliary* input is given, the result contains those rows from the input whose values match those from the auxiliary file.

Authors: Kristian Ovaska (kristian.ovaska@helsinki.fi), Marko Laakso (Marko.Laakso@Helsinki.FI)
Version: 1.1
Category: Filter

Input name	Source	Description
csv	arrayCGHanalysis-normalized .casechannel 19.196	CSV file to be filtered.
auxiliary	arrayCGHanalysis-chr6-chrFilter .csv 19.140	If given, contains one column (see "matchColumn") whose values are matched to a column in the "csv" input (see "idColumn").

Parameter name	Value	Description
highBound		A row is included if the value in given numeric columns are at most as large as given numeric bounds. The parameter has a format COLNAME1=HIGH1,COLNAME2=HIGH2.
idColumn		Column name in "csv" that contains ID values. If the parameter is empty, the first column is used.
includeColumns	*	Comma-separated list of column names that should be included in the result file. The special value * includes all columns. The order of column names is significant: the output columns are in the order given. See also includeColumns input.
lowBound		A row is included if the value in given numeric columns are at least as large as given numeric bounds. The parameter has a format COLNAME1=LOW1,COLNAME2=LOW2. For example, "col=5" includes rows where the column col has a value of at least 5.

Continued on next page...

Parameter name	Value	Description
matchColumn	ProbeName	Column name in "auxiliary" containing values that must match the ID column in "csv" (specified using "idColumn"). If empty, the first column of "auxiliary" is used.
negate	false	If true, all inclusion criteria are negated so that rows or columns are excluded instead of included if they match the criteria. For example, includeColumns=* then excludes all columns (which would be an error since at least one column must be included).
nonMissing	0	Include those rows that have non-missing (non-NA) values in at least this many columns. Only those columns that are part of the output are counted.
regexp		Row filtering based on regular expression. A row is included in the result if values in the given columns match given regular expressions. The parameter has a format COLNAME1=EXPRESSION,COLNAME2=EXPRESSION2 where COLNAMEs are column names in "csv" and EXPRESSIONs are regular expressions using Java syntax. For example, "col=a|b" includes rows where the column col has a value of "a" or "b". Commas have to be escaped with "\\" if they are used in column names or regular expressions.
rename		Comma-separated list of column renaming rules (OLDNAME=NEWNAME). Parameters that refer to column names refer to old names; renaming is done last.

19.142 arrayCGHanalysis-chr6-segmented (ACGHsegment)

ACGHsegment segments normalized array CGH data, and calls aberrations in one of two ways: by classification of probe data discretely to gain, normal or loss, or computing probabilities to of a gain, normal or loss state for each probe. Segmentation utilizes the Circular Binary Segmentation algorithm [15].

Two ways of analysing Array CGH data post-segmentation are included. The first one, the Genome Topography Scan [27] computes scores over all the samples to calculate the most recurrent aberrations over the sample set. The second one outputs all the regions in all the samples whose segmented means are more than two standard deviations apart from the mean of all the intensities. Plots from the GTS as well as from the segmented intensities are output depending on the parameter setting.

Probabilities for copy number aberrations can be computed with two algorithms. The first, FastCall [3], is faster while the second, CGHcall [26], is more accurate.

Author:	Riku Louhimo (Riku.Louhimo@Helsinki.FI)
Version:	1.3
Categories:	Agilent, Copy Number Analysis

Input name	Source	Description
caseChan	arrayCGHanalysis-chr6-filtNorm CaseChan.csv 19.141	CSV file containing the normalized probe intensities for the case channel. First row should have the probenames and samples should be columnwise.
geneAnnotation	arrayCGHanalysis-chr6-chrFilter .csv 19.140	Probewise annotations as produced by the AgilenReader component. Only the probes present in the casechannel csv can be included. Use CSVFilter if the probes do not otherwise match.

Parameter name	Value	Description
CGHCallPrior	auto	Only used if callProbMethod=CGHCall. Set the method to determine prior probabilities to CGHCall algorithm. Must be one of "auto", "all", "not all".
analysisAlgorithm	0	1 means that only the GTS algorithm is run, 2 that only the standard deviation test is run. 0 indicates that both are run.
callProbMethod	CGHCall	Method with which to call CNA segment probabilities. Must be either CGHCall or FastCall. FastCall is significantly (30000 times) faster while CGHCall is more accurate.
callProbabilities	false	Enabling this will make the component estimate probabilties for CNA segments via the CGHcall package. Only guaranteed to work when multiple chromosomes are analyzed simultaneously.
lowerLimit	-0.631759089316322	Use this as the intensity limit for calling a segment lossed. Default value computes the mean of the sample set and estimates the lower threshold for CNA call to be two standard deviations from it.
nSegFit	3000	Maximum number of segments used for fitting the mixture model in probability calculations. Disabled if callProbabilities=false. Decreasing this lowers accuracy but can speed computation significantly.

Continued on next page. . .

Parameter name	Value	Description
plotChromosomes	0	Defines the plots that the user wants to output as a comma separated list. The whole genome is plotted by default for each sample. Inputting a value different than 0 will generate additional plots for these specific chromosomes from each sample.
plotEverySample	false	Enabling this will make the component print each sample separately.
undoSplits	sdundo	A character string specifying how change-points are to be undone, if at all. Undoing change-points decreases the sensitivity of segmentation. Choices are "none","prune", which uses a sum of squares criterion, and "sdundo" (default), which undoes splits that are not at least this many SDs apart. SD by default is 3.
upperLimit	0.631759089316322	Use this as the intensity limit for calling a segment gained. Default value computes the mean of the sample set and estimates the upper threshold for CNA call to be two standard deviations from it.

19.143 arrayCGHanalysis-chr7 (Segmentation)

No description

Parameter name	Value
chr	7

19.144 arrayCGHanalysis-chr7-chrFilter (CSVFilter)

Filters columns and/or rows from CSV files using flexible criteria. Default settings do not filter out anything. The *negate* parameter reverses all inclusion criteria.

Columns are filtered based on column names (see *includeColumns*); it is also possible to rename columns. Columns can be reordered using *includeColumns*.

A row is included in output if all conditions are satisfied. Rows having too many missing values are filtered using the *nonMissing* parameter. The parameters *regexp*, *lowBound* and *highBound* filter rows based on cell contents. If the *auxiliary* input is given, the result contains those rows from the input whose values match those from the auxiliary file.

Authors:	Kristian Ovaska (kristian.ovaska@helsinki.fi), Marko Laakso (Marko.Laakso@Helsinki.FI)
Version:	1.1
Category:	Filter

Input name	Source	Description
csv	arrayCGHanalysis-srFAnnot. file 19.213	CSV file to be filtered.

Parameter name	Value	Description
highBound		A row is included if the value in given numeric columns are at most as large as given numeric bounds. The parameter has a format COLNAME1=HIGH1,COLNAME2=HIGH2.
idColumn		Column name in "csv" that contains ID values. If the parameter is empty, the first column is used.
includeColumns	*	Comma-separated list of column names that should be included in the result file. The special value * includes all columns. The order of column names is significant: the output columns are in the order given. See also includeColumns input.
lowBound		A row is included if the value in given numeric columns are at least as large as given numeric bounds. The parameter has a format COLNAME1=LOW1,COLNAME2=LOW2. For example, "col=5" includes rows where the column col has a value of at least 5.
matchColumn		Column name in "auxiliary" containing values that must match the ID column in "csv" (specified using "idColumn"). If empty, the first column of "auxiliary" is used.
negate	false	If true, all inclusion criteria are negated so that rows or columns are excluded instead of included if they match the criteria. For example, includeColumns=* then excludes all columns (which would be an error since at least one column must be included).

Continued on next page...

Parameter name	Value	Description
nonMissing	0	Include those rows that have non-missing (non-NA) values in at least this many columns. Only those columns that are part of the output are counted.
regexp	Chr=7	Row filtering based on regular expression. A row is included in the result if values in the given columns match given regular expressions. The parameter has a format COLNAME1=EXPRESSION,COLNAME2=EXPRESSION2 where COLNAMEs are column names in "csv" and EXPRESSIONs are regular expressions using Java syntax. For example, "col=a|b" includes rows where the column col has a value of "a" or "b". Commas have to be escaped with "\\" if they are used in column names or regular expressions.
rename		Comma-separated list of column renaming rules (OLDNAME=NEWNAME). Parameters that refer to column names refer to old names; renaming is done last.

19.145 arrayCGHanalysis-chr7-filtNormCaseChan (CSVFilter)

Filters columns and/or rows from CSV files using flexible criteria. Default settings do not filter out anything. The *negate* parameter reverses all inclusion criteria.

Columns are filtered based on column names (see *includeColumns*); it is also possible to rename columns. Columns can be reordered using *includeColumns*.

A row is included in output if all conditions are satisfied. Rows having too many missing values are filtered using the *nonMissing* parameter. The parameters *regexp*, *lowBound* and *highBound* filter rows based on cell contents. If the *auxiliary* input is given, the result contains those rows from the input whose values match those from the auxiliary file.

Authors: Kristian Ovaska (kristian.ovaska@helsinki.fi), Marko Laakso (Marko.Laakso@Helsinki.FI)
Version: 1.1
Category: Filter

Input name	Source	Description
csv	arrayCGHanalysis-normalized .casechannel 19.196	CSV file to be filtered.
auxiliary	arrayCGHanalysis-chr7-chrFilter .csv 19.144	If given, contains one column (see "matchColumn") whose values are matched to a column in the "csv" input (see "idColumn").

Parameter name	Value	Description
highBound		A row is included if the value in given numeric columns are at most as large as given numeric bounds. The parameter has a format COLNAME1=HIGH1,COLNAME2=HIGH2.
idColumn		Column name in "csv" that contains ID values. If the parameter is empty, the first column is used.
includeColumns	*	Comma-separated list of column names that should be included in the result file. The special value * includes all columns. The order of column names is significant: the output columns are in the order given. See also includeColumns input.
lowBound		A row is included if the value in given numeric columns are at least as large as given numeric bounds. The parameter has a format COLNAME1=LOW1,COLNAME2=LOW2. For example, "col=5" includes rows where the column col has a value of at least 5.
matchColumn	ProbeName	Column name in "auxiliary" containing values that must match the ID column in "csv" (specified using "idColumn"). If empty, the first column of "auxiliary" is used.
negate	false	If true, all inclusion criteria are negated so that rows or columns are excluded instead of included if they match the criteria. For example, includeColumns=* then excludes all columns (which would be an error since at least one column must be included).
nonMissing	0	Include those rows that have non-missing (non-NA) values in at least this many columns. Only those columns that are part of the output are counted.
regexp		Row filtering based on regular expression. A row is included in the result if values in the given columns match given regular expressions. The parameter has a format COLNAME1=EXPRESSION,COLNAME2=EXPRESSION2 where COLNAMEs are column names in "csv" and EXPRESSIONs are regular expressions using Java syntax. For example, "col=a|b" includes rows where the column col has a value of "a" or "b". Commas have to be escaped with "\\" if they are used in column names or regular expressions.

Continued on next page...

Parameter name	Value	Description
rename		Comma-separated list of column renaming rules (OLDNAME=NEWNAME). Parameters that refer to column names refer to old names; renaming is done last.

19.146 arrayCGHanalysis-chr7-segmented (ACGHsegment)

ACGHsegment segments normalized array CGH data, and calls aberrations in one of two ways: by classification of probe data discretely to gain, normal or loss, or computing probabilities to of a gain, normal or loss state for each probe. Segmentation utilizes the Circular Binary Segmentation algorithm [15].

Two ways of analysing Array CGH data post-segmentation are included. The first one, the Genome Topography Scan [27] computes scores over all the samples to calculate the most recurrent aberrations over the sample set. The second one outputs all the regions in all the samples whose segmented means are more than two standard deviations apart from the mean of all the intensities. Plots from the GTS as well as from the segmented intensities are output depending on the parameter setting.

Probabilities for copy number aberrations can be computed with two algorithms. The first, FastCall [3], is faster while the second, CGHcall [26], is more accurate.

Author: Riku Louhimo (Riku.Louhimo@Helsinki.FI)
Version: 1.3
Categories: Agilent, Copy Number Analysis

Input name	Source	Description
caseChan	arrayCGHanalysis-chr7-filtNorm CaseChan.csv 19.145	CSV file containing the normalized probe intensities for the case channel. First row should have the probenames and samples should be columnwise.
geneAnnotation	arrayCGHanalysis-chr7-chrFilter .csv 19.144	Probewise annotations as produced by the AgilenReader component. Only the probes present in the casechannel csv can be included. Use CSVFilter if the probes do not otherwise match.

Parameter name	Value	Description
CGHCallPrior	auto	Only used if callProbMethod=CGHCall. Set the method to determine prior probabilities to CGHCall algorithm. Must be one of ”auto”, ”all”, ”not all”.
analysisAlgorithm	0	1 means that only the GTS algorithm is run, 2 that only the standard deviation test is run. 0 indicates that both are run.
callProbMethod	CGHCall	Method with which to call CNA segment probabilities. Must be either CGHCall or FastCall. FastCall is significantly (30000 times) faster while CGHCall is more accurate.
callProbabilities	false	Enabling this will make the component estimate probabilties for CNA segments via the CGHcall package. Only guaranteed to work when multiple chromosomes are analyzed simultaneously.
lowerLimit	-0.631759089316322	Use this as the intensity limit for calling a segment lossed. Default value computes the mean of the sample set and estimates the lower threshold for CNA call to be two standard deviations from it.
nSegFit	3000	Maximum number of segments used for fitting the mixture model in probability calculations. Disabled if callProbabilities=false. Decreasing this lowers accuracy but can speed computation significantly.
plotChromosomes	0	Defines the plots that the user wants to output as a comma separated list. The whole genome is plotted by default for each sample. Inputting a value different than 0 will generate additional plots for these specific chromosomes from each sample.
plotEverySample	false	Enabling this will make the component print each sample separately.
undoSplits	sdundo	A character string specifying how change-points are to be undone, if at all. Undoing change-points decreases the sensitivity of segmentation. Choices are ”none”,”prune”, which uses a sum of squares criterion, and ”sdundo” (default), which undoes splits that are not at least this many SDs apart. SD by default is 3.
upperLimit	0.631759089316322	Use this as the intensity limit for calling a segment gained. Default value computes the mean of the sample set and estimates the upper threshold for CNA call to be two standard deviations from it.

19.147 arrayCGHanalysis-chr8 (Segmentation)

No description

Parameter name	Value
chr	8

19.148 arrayCGHanalysis-chr8-chrFilter (CSVFilter)

Filters columns and/or rows from CSV files using flexible criteria. Default settings do not filter out anything. The *negate* parameter reverses all inclusion criteria.

Columns are filtered based on column names (see *includeColumns*); it is also possible to rename columns. Columns can be reordered using *includeColumns*.

A row is included in output if all conditions are satisfied. Rows having too many missing values are filtered using the *nonMissing* parameter. The parameters *regexp*, *lowBound* and *highBound* filter rows based on cell contents. If the *auxiliary* input is given, the result contains those rows from the input whose values match those from the auxiliary file.

Authors:	Kristian Ovaska (kristian.ovaska@helsinki.fi), Marko Laakso (Marko.Laakso@Helsinki.FI)
Version:	1.1
Category:	Filter

Input name	Source	Description
csv	arrayCGHanalysis-srFAnnot. file 19.213	CSV file to be filtered.

Parameter name	Value	Description
highBound		A row is included if the value in given numeric columns are at most as large as given numeric bounds. The parameter has a format COLNAME1=HIGH1,COLNAME2=HIGH2.
idColumn		Column name in "csv" that contains ID values. If the parameter is empty, the first column is used.
includeColumns	*	Comma-separated list of column names that should be included in the result file. The special value * includes all columns. The order of column names is significant: the output columns are in the order given. See also includeColumns input.
lowBound		A row is included if the value in given numeric columns are at least as large as given numeric bounds. The parameter has a format COLNAME1=LOW1,COLNAME2=LOW2. For example, "col=5" includes rows where the column col has a value of at least 5.
matchColumn		Column name in "auxiliary" containing values that must match the ID column in "csv" (specified using "idColumn"). If empty, the first column of "auxiliary" is used.
negate	false	If true, all inclusion criteria are negated so that rows or columns are excluded instead of included if they match the criteria. For example, includeColumns=* then excludes all columns (which would be an error since at least one column must be included).
nonMissing	0	Include those rows that have non-missing (non-NA) values in at least this many columns. Only those columns that are part of the output are counted.
regexp	Chr=8	Row filtering based on regular expression. A row is included in the result if values in the given columns match given regular expressions. The parameter has a format COLNAME1=EXPRESSION,COLNAME2=EXPRESSION2 where COLNAMEs are column names in "csv" and EXPRESSIONs are regular expressions using Java syntax. For example, "col=a|b" includes rows where the column col has a value of "a" or "b". Commas have to be escaped with "\\" if they are used in column names or regular expressions.
rename		Comma-separated list of column renaming rules (OLDNAME=NEWNAME). Parameters that refer to column names refer to old names; renaming is done last.

19.149 arrayCGHanalysis-chr8-filtNormCaseChan (CSVFilter)

Filters columns and/or rows from CSV files using flexible criteria. Default settings do not filter out anything. The *negate* parameter reverses all inclusion criteria.

Columns are filtered based on column names (see *includeColumns*); it is also possible to rename columns. Columns can be reordered using *includeColumns*.

A row is included in output if all conditions are satisfied. Rows having too many missing values are filtered using the *nonMissing* parameter. The parameters *regexp*, *lowBound* and *highBound* filter rows based on cell contents. If the *auxiliary* input is given, the result contains those rows from the input whose values match those from the auxiliary file.

Authors: Kristian Ovaska (kristian.ovaska@helsinki.fi), Marko Laakso (Marko.Laakso@Helsinki.FI)
Version: 1.1
Category: Filter

Input name	Source	Description
csv	arrayCGHanalysis-normalized .casechannel 19.196	CSV file to be filtered.
auxiliary	arrayCGHanalysis-chr8-chrFilter .csv 19.148	If given, contains one column (see "matchColumn") whose values are matched to a column in the "csv" input (see "idColumn").

Parameter name	Value	Description
highBound		A row is included if the value in given numeric columns are at most as large as given numeric bounds. The parameter has a format COLNAME1=HIGH1,COLNAME2=HIGH2.
idColumn		Column name in "csv" that contains ID values. If the parameter is empty, the first column is used.
includeColumns	*	Comma-separated list of column names that should be included in the result file. The special value * includes all columns. The order of column names is significant: the output columns are in the order given. See also includeColumns input.
lowBound		A row is included if the value in given numeric columns are at least as large as given numeric bounds. The parameter has a format COLNAME1=LOW1,COLNAME2=LOW2. For example, "col=5" includes rows where the column col has a value of at least 5.
matchColumn	ProbeName	Column name in "auxiliary" containing values that must match the ID column in "csv" (specified using "idColumn"). If empty, the first column of "auxiliary" is used.
negate	false	If true, all inclusion criteria are negated so that rows or columns are excluded instead of included if they match the criteria. For example, includeColumns=* then excludes all columns (which would be an error since at least one column must be included).
nonMissing	0	Include those rows that have non-missing (non-NA) values in at least this many columns. Only those columns that are part of the output are counted.
regexp		Row filtering based on regular expression. A row is included in the result if values in the given columns match given regular expressions. The parameter has a format COLNAME1=EXPRESSION,COLNAME2=EXPRESSION2 where COLNAMEs are column names in "csv" and EXPRESSIONs are regular expressions using Java syntax. For example, "col=a|b" includes rows where the column col has a value of "a" or "b". Commas have to be escaped with "\\" if they are used in column names or regular expressions.
rename		Comma-separated list of column renaming rules (OLDNAME=NEWNAME). Parameters that refer to column names refer to old names; renaming is done last.

19.150 arrayCGHanalysis-chr8-segmented (ACGHsegment)

ACGHsegment segments normalized array CGH data, and calls aberrations in one of two ways: by classification of probe data discretely to gain, normal or loss, or computing probabilities to of a gain, normal or loss state for each probe. Segmentation utilizes the Circular Binary Segmentation algorithm [15].

Two ways of analysing Array CGH data post-segmentation are included. The first one, the Genome Topography Scan [27] computes scores over all the samples to calculate the most recurrent aberrations over the sample set. The second one outputs all the regions in all the samples whose segmented means

are more than two standard deviations apart from the mean of all the intensities. Plots from the GTS as well as from the segmented intensities are output depending on the parameter setting.

Probabilities for copy number aberrations can be computed with two algorithms. The first, FastCall [3], is faster while the second, CGHcall [26], is more accurate.

Author:	Riku Louhimo (Riku.Louhimo@Helsinki.FI)
Version:	1.3
Categories:	Agilent, Copy Number Analysis

Input name	Source	Description
caseChan	arrayCGHanalysis-chr8-filtNorm CaseChan.csv 19.149	CSV file containing the normalized probe intensities for the case channel. First row should have the probenames and samples should be columnwise.
geneAnnotation	arrayCGHanalysis-chr8-chrFilter .csv 19.148	Probewise annotations as produced by the AgilenReader component. Only the probes present in the casechannel csv can be included. Use CSVFilter if the probes do not otherwise match.

Parameter name	Value	Description
CGHCallPrior	auto	Only used if callProbMethod=CGHCall. Set the method to determine prior probabilities to CGHCall algorithm. Must be one of "auto", "all", "not all".
analysisAlgorithm	0	1 means that only the GTS algorithm is run, 2 that only the standard deviation test is run. 0 indicates that both are run.
callProbMethod	CGHCall	Method with which to call CNA segment probabilities. Must be either CGHCall or FastCall. FastCall is significantly (30000 times) faster while CGHCall is more accurate.
callProbabilities	false	Enabling this will make the component estimate probabilties for CNA segments via the CGHcall package. Only guaranteed to work when multiple chromosomes are analyzed simultaneously.
lowerLimit	-0.631759089316322	Use this as the intensity limit for calling a segment lossed. Default value computes the mean of the sample set and estimates the lower threshold for CNA call to be two standard deviations from it.
nSegFit	3000	Maximum number of segments used for fitting the mixture model in probability calculations. Disabled if callProbabilities=false. Decreasing this lowers accuracy but can speed computation significantly.
plotChromosomes	0	Defines the plots that the user wants to output as a comma separated list. The whole genome is plotted by default for each sample. Inputting a value different than 0 will generate additional plots for these specific chromosomes from each sample.
plotEverySample	false	Enabling this will make the component print each sample separately.
undoSplits	sdundo	A character string specifying how change-points are to be undone, if at all. Undoing change-points decreases the sensitivity of segmentation. Choices are "none","prune", which uses a sum of squares criterion, and "sdundo" (default), which undoes splits that are not at least this many SDs apart. SD by default is 3.
upperLimit	0.631759089316322	Use this as the intensity limit for calling a segment gained. Default value computes the mean of the sample set and estimates the upper threshold for CNA call to be two standard deviations from it.

19.151 arrayCGHanalysis-chr9 (Segmentation)

No description

Parameter name	Value
chr	9

19.152 arrayCGHanalysis-chr9-chrFilter (CSVFilter)

Filters columns and/or rows from CSV files using flexible criteria. Default settings do not filter out anything. The *negate* parameter reverses all inclusion criteria.

Columns are filtered based on column names (see *includeColumns*); it is also possible to rename columns. Columns can be reordered using *includeColumns*.

A row is included in output if all conditions are satisfied. Rows having too many missing values are filtered

using the *nonMissing* parameter. The parameters *regexp*, *lowBound* and *highBound* filter rows based on cell contents. If the *auxiliary* input is given, the result contains those rows from the input whose values match those from the auxiliary file.

Authors:	Kristian Ovaska (kristian.ovaska@helsinki.fi), Marko Laakso (Marko.Laakso@Helsinki.FI)
Version:	1.1
Category:	Filter

Input name	Source	Description
csv	arrayCGHanalysis-srFAnnot.file 19.213	CSV file to be filtered.

Parameter name	Value	Description
highBound		A row is included if the value in given numeric columns are at most as large as given numeric bounds. The parameter has a format COLNAME1=HIGH1,COLNAME2=HIGH2.
idColumn		Column name in "csv" that contains ID values. If the parameter is empty, the first column is used.
includeColumns	*	Comma-separated list of column names that should be included in the result file. The special value * includes all columns. The order of column names is significant: the output columns are in the order given. See also includeColumns input.
lowBound		A row is included if the value in given numeric columns are at least as large as given numeric bounds. The parameter has a format COLNAME1=LOW1,COLNAME2=LOW2. For example, "col=5" includes rows where the column col has a value of at least 5.
matchColumn		Column name in "auxiliary" containing values that must match the ID column in "csv" (specified using "idColumn"). If empty, the first column of "auxiliary" is used.
negate	false	If true, all inclusion criteria are negated so that rows or columns are excluded instead of included if they match the criteria. For example, includeColumns=* then excludes all columns (which would be an error since at least one column must be included).
nonMissing	0	Include those rows that have non-missing (non-NA) values in at least this many columns. Only those columns that are part of the output are counted.
regexp	Chr=9	Row filtering based on regular expression. A row is included in the result if values in the given columns match given regular expressions. The parameter has a format COLNAME1=EXPRESSION,COLNAME2=EXPRESSION2 where COLNAMEs are column names in "csv" and EXPRESSIONs are regular expressions using Java syntax. For example, "col=a|b" includes rows where the column col has a value of "a" or "b". Commas have to be escaped with "\\" if they are used in column names or regular expressions.
rename		Comma-separated list of column renaming rules (OLDNAME=NEWNAME). Parameters that refer to column names refer to old names; renaming is done last.

19.153 arrayCGHanalysis-chr9-filtNormCaseChan (CSVFilter)

Filters columns and/or rows from CSV files using flexible criteria. Default settings do not filter out anything. The *negate* parameter reverses all inclusion criteria.

Columns are filtered based on column names (see *includeColumns*); it is also possible to rename columns. Columns can be reordered using *includeColumns*.

A row is included in output if all conditions are satisfied. Rows having too many missing values are filtered using the *nonMissing* parameter. The parameters *regexp*, *lowBound* and *highBound* filter rows based on cell contents. If the *auxiliary* input is given, the result contains those rows from the input whose values match those from the auxiliary file.

Authors:	Kristian Ovaska (kristian.ovaska@helsinki.fi), Marko Laakso (Marko.Laakso@Helsinki.FI)
Version:	1.1
Category:	Filter

Input name	Source	Description
csv	arrayCGHanalysis-normalized .casechannel 19.196	CSV file to be filtered.
auxiliary	arrayCGHanalysis-chr9-chrFilter .csv 19.152	If given, contains one column (see "matchColumn") whose values are matched to a column in the "csv" input (see "idColumn").

Parameter name	Value	Description
highBound		A row is included if the value in given numeric columns are at most as large as given numeric bounds. The parameter has a format COLNAME1=HIGH1,COLNAME2=HIGH2.
idColumn		Column name in "csv" that contains ID values. If the parameter is empty, the first column is used.
includeColumns	*	Comma-separated list of column names that should be included in the result file. The special value * includes all columns. The order of column names is significant: the output columns are in the order given. See also includeColumns input.
lowBound		A row is included if the value in given numeric columns are at least as large as given numeric bounds. The parameter has a format COLNAME1=LOW1,COLNAME2=LOW2. For example, "col=5" includes rows where the column col has a value of at least 5.
matchColumn	ProbeName	Column name in "auxiliary" containing values that must match the ID column in "csv" (specified using "idColumn"). If empty, the first column of "auxiliary" is used.
negate	false	If true, all inclusion criteria are negated so that rows or columns are excluded instead of included if they match the criteria. For example, includeColumns=* then excludes all columns (which would be an error since at least one column must be included).
nonMissing	0	Include those rows that have non-missing (non-NA) values in at least this many columns. Only those columns that are part of the output are counted.
regexp		Row filtering based on regular expression. A row is included in the result if values in the given columns match given regular expressions. The parameter has a format COLNAME1=EXPRESSION,COLNAME2=EXPRESSION2 where COLNAMEs are column names in "csv" and EXPRESSIONs are regular expressions using Java syntax. For example, "col=a|b" includes rows where the column col has a value of "a" or "b". Commas have to be escaped with "\\" if they are used in column names or regular expressions.
rename		Comma-separated list of column renaming rules (OLDNAME=NEWNAME). Parameters that refer to column names refer to old names; renaming is done last.

19.154 arrayCGHanalysis-chr9-segmented (ACGHsegment)

ACGHsegment segments normalized array CGH data, and calls aberrations in one of two ways: by classification of probe data discretely to gain, normal or loss, or computing probabilities to of a gain, normal or loss state for each probe. Segmentation utilizes the Circular Binary Segmentation algorithm [15].

Two ways of analysing Array CGH data post-segmentation are included. The first one, the Genome Topography Scan [27] computes scores over all the samples to calculate the most recurrent aberrations over the sample set. The second one outputs all the regions in all the samples whose segmented means are more than two standard deviations apart from the mean of all the intensities. Plots from the GTS as well as from the segmented intensities are output depending on the parameter setting.

Probabilities for copy number aberrations can be computed with two algorithms. The first, FastCall [3], is faster while the second, CGHcall [26], is more accurate.

Author:	Riku Louhimo (Riku.Louhimo@Helsinki.FI)
Version:	1.3
Categories:	Agilent, Copy Number Analysis

Input name	Source	Description
caseChan	arrayCGHanalysis-chr9-filtNorm CaseChan.csv 19.153	CSV file containing the normalized probe intensities for the case channel. First row should have the probenames and samples should be columnwise.
geneAnnotation	arrayCGHanalysis-chr9-chrFilter .csv 19.152	Probewise annotations as produced by the AgilenReader component. Only the probes present in the casechannel csv can be included. Use CSVFilter if the probes do not otherwise match.

Parameter name	Value	Description
CGHCallPrior	auto	Only used if callProbMethod=CGHCall. Set the method to determine prior probabilities to CGHCall algorithm. Must be one of "auto", "all", "not all".
analysisAlgorithm	0	1 means that only the GTS algorithm is run, 2 that only the standard deviation test is run. 0 indicates that both are run.
callProbMethod	CGHCall	Method with which to call CNA segment probabilities. Must be either CGHCall or FastCall. FastCall is significantly (30000 times) faster while CGHCall is more accurate.
callProbabilities	false	Enabling this will make the component estimate probabilties for CNA segments via the CGHcall package. Only guaranteed to work when multiple chromosomes are analyzed simultaneously.
lowerLimit	-0.631759089316322	Use this as the intensity limit for calling a segment lossed. Default value computes the mean of the sample set and estimates the lower threshold for CNA call to be two standard deviations from it.
nSegFit	3000	Maximum number of segments used for fitting the mixture model in probability calculations. Disabled if callProbabilities=false. Decreasing this lowers accuracy but can speed computation significantly.
plotChromosomes	0	Defines the plots that the user wants to output as a comma separated list. The whole genome is plotted by default for each sample. Inputting a value different than 0 will generate additional plots for these specific chromosomes from each sample.
plotEverySample	false	Enabling this will make the component print each sample separately.
undoSplits	sdundo	A character string specifying how change-points are to be undone, if at all. Undoing change-points decreases the sensitivity of segmentation. Choices are "none","prune", which uses a sum of squares criterion, and "sdundo" (default), which undoes splits that are not at least this many SDs apart. SD by default is 3.
upperLimit	0.631759089316322	Use this as the intensity limit for calling a segment gained. Default value computes the mean of the sample set and estimates the upper threshold for CNA call to be two standard deviations from it.

19.155 arrayCGHanalysis-dataLoc (INPUT)

Physical location of array CGH data in the file system.

Category: Internal

Parameter name	Value	Description
path	/gpfs/homes/mlaakso/asserSVN/ andurilCaseStudy/data/acgh/ acgh-data/cangem/DataFiles	Path (filename) of the input resource.

19.156 arrayCGHanalysis-fAnnot (CSVFilter)

Filters columns and/or rows from CSV files using flexible criteria. Default settings do not filter out anything. The *negate* parameter reverses all inclusion criteria.

Columns are filtered based on column names (see *includeColumns*); it is also possible to rename columns. Columns can be reordered using *includeColumns*.

A row is included in output if all conditions are satisfied. Rows having too many missing values are filtered using the *nonMissing* parameter. The parameters *regexp*, *lowBound* and *highBound* filter rows based on cell contents. If the *auxiliary* input is given, the result contains those rows from the input whose values match those from the auxiliary file.

Authors: Kristian Ovaska (kristian.ovaska@helsinki.fi), Marko Laakso (Marko.Laakso@Helsinki.FI)
Version: 1.1
Category: Filter

Input name	Source	Description
csv	arrayCGHanalysis-aCGH.probeA nnotation 19.44	CSV file to be filtered.
auxiliary	arrayCGHanalysis-probeIDs. ids 19.198	If given, contains one column (see "matchColumn") whose values are matched to a column in the "csv" input (see "idColumn").

Parameter name	Value	Description
highBound		A row is included if the value in given numeric columns are at most as large as given numeric bounds. The parameter has a format COLNAME1=HIGH1,COLNAME2=HIGH2.
idColumn		Column name in "csv" that contains ID values. If the parameter is empty, the first column is used.
includeColumns	*	Comma-separated list of column names that should be included in the result file. The special value * includes all columns. The order of column names is significant: the output columns are in the order given. See also includeColumns input.
lowBound		A row is included if the value in given numeric columns are at least as large as given numeric bounds. The parameter has a format COLNAME1=LOW1,COLNAME2=LOW2. For example, "col=5" includes rows where the column col has a value of at least 5.
matchColumn		Column name in "auxiliary" containing values that must match the ID column in "csv" (specified using "idColumn"). If empty, the first column of "auxiliary" is used.
negate	false	If true, all inclusion criteria are negated so that rows or columns are excluded instead of included if they match the criteria. For example, includeColumns=* then excludes all columns (which would be an error since at least one column must be included).
nonMissing	0	Include those rows that have non-missing (non-NA) values in at least this many columns. Only those columns that are part of the output are counted.
regexp		Row filtering based on regular expression. A row is included in the result if values in the given columns match given regular expressions. The parameter has a format COLNAME1=EXPRESSION,COLNAME2=EXPRESSION2 where COLNAMEs are column names in "csv" and EXPRESSIONs are regular expressions using Java syntax. For example, "col=a|b" includes rows where the column col has a value of "a" or "b". Commas have to be escaped with "\\" if they are used in column names or regular expressions.
rename		Comma-separated list of column renaming rules (OLDNAME=NEWNAME). Parameters that refer to column names refer to old names; renaming is done last.

19.157 arrayCGHanalysis-fChannels (QuantileFilter)

Filter out bad quality probes from raw data channels.

Author: Kristian Ovaska (kristian.ovaska@helsinki.fi)
Version: 1.0
Category: Filter

Input name	Source	Description
matrix	arrayCGHanalysis-aCGH. green 19.44	Input matrix 1.
matrix2	arrayCGHanalysis-aCGH. red 19.44	Input matrix 2.

Parameter name	Value	Description
highQuantile	1	High threshold, between 0 and 1 inclusive. Set to 1 to disable high threshold filtering.
lowQuantile	0.05	Low threshold, between 0 and 1 inclusive. Set to 0 to disable low threshold filtering.
removeMissing	true	If true, do not include rows that have only missing values in the output. If false, include the rows but print NA's for all columns.

19.158 arrayCGHanalysis-gains (ACGHSegmentAnalysis)

Analyze interesting regions from segmented Array CGH regions exhibiting copy number gain.

Input name	Source
segments	arrayCGHanalysis-aberrations.data 19.46

Parameter name	Value
annotateRegs	true
gainOrLoss	1
threshold	0.1

19.159 arrayCGHanalysis-gains-ArrayCGHgenes (TableQuery)

Execute an SQL query on input relations and create a result table. TableQuery uses HSQLDB (Hyper-threaded Structured Query Language Database [25]) for executing the query. Consequently, the syntax of the query is defined by HSQLDB.

Author: Kristian Ovaska (Kristian.Ovaska@Helsinki.FI)
Version: 1.2
Categories: convert, Filter

Input name	Source	Description
table1	arrayCGHanalysis-gains-gAndLoss-annotGenes.bioAnnotation 19.171	CSV table 1. The table is referred to as 'table1' in the SQL query.

Parameter name	Value	Description
numIndices		Comma-separated list of index counts for input tables. All indices are single-column indices, running from column 1 to N, where N is retrieved from this parameter. If empty, the default number (1) is used. For example, ",2,,2" sets two indices for table2 and table4 and one index for the rest.
query	SELECT ".GeneId", "freq" FROM table1	SQL query. Either this parameter or the query input must be provided, but not both.

19.160 arrayCGHanalysis-gains-abb (TableQuery)

Execute an SQL query on input relations and create a result table. TableQuery uses HSQLDB (Hyper-threaded Structured Query Language Database [25]) for executing the query. Consequently, the syntax of the query is defined by HSQLDB.

Author: Kristian Ovaska (Kristian.Ovaska@Helsinki.FI)
Version: 1.2
Categories: convert, Filter

Input name	Source	Description
table1	arrayCGHanalysis-gains-gAndLoss-filtSegs.table 19.172	CSV table 1. The table is referred to as 'table1' in the SQL query.
table2	arrayCGHanalysis-gains-associationLysis-rBand.table 19.166	CSV table 2. The table is referred to as 'table2' in the SQL query.

Parameter name	Value	Description
numIndices		Comma-separated list of index counts for input tables. All indices are single-column indices, running from column 1 to N, where N is retrieved from this parameter. If empty, the default number (1) is used. For example, ",2,,2" sets two indices for table2 and table4 and one index for the rest.
query	SELECT T1.*, ROUND(T2."rank", 5) AS "rank" FROM table1 T1, table2 T2 WHERE (T1."Region" = T2."Region") ORDER BY "rank" DESC	SQL query. Either this parameter or the query input must be provided, but not both.

19.161 arrayCGHanalysis-gains-abbers (CSV2Latex)

This component converts a tabular CSV file into a printable LaTeX table.

Author: Marko Laakso (Marko.Laakso@Helsinki.FI)
Version: 2.1
Categories: convert, Latex

Input name	Source	Description
tabledata	arrayCGHanalysis-gains-abb .table 19.160	Table content
refs	arrayCGHanalysis-gains-refs .in 19.173	Reference rules for the hyperlinks

Parameter name	Value	Description
attach	false	Include the original data as an attachment
caption		Caption text for the table.
colFormat	llp{2cm}p{10cm}r	LaTeX tabular format for the columns. Default produces a centered layout for all columns.
columns	gene name,chr,.DNABand,. GeneDesc,rank	Comma separated list of column selections for the output. The empty default will use all columns.
countRows	true	Include a row count to the table caption.
dropMissing	true	This flag can be turned off in order to generate links with missing texts. Link text are substituted with target identifiers.
listCols	.DNABand	Comma separated list of column names. Columns of this list may contain several values separated with commas and the delimiters will be replaced with spaces.
numberFormat		A comma separated list of decimal formats for the columns. Each entry consists of the column name and the Java DecimalFormal pattern separated with equal sign. For example, rounding to three decimals can be done like: myColumn=#0.000
pageBreak	false	Use clear page after the table.
rename		Comma separated list of column renaming rules (oldname=newname). New names are used in table header but they do not affect the other behaviour of this component.
ruler	{}	Latex command for the row separating rulers
section	Interesting copy number 1 segments	Section title for the table container or an empty string if no section should be generated.
sectionType	subsection	Type of LaTeX section: usually one of: section, subsection, or subsubsection. No section statement is written if section title is empty.
skipEmpty	false	This flag can be used to replace empty tables with a simple LaTeX comment.

19.162 arrayCGHanalysis-gains-associationLysis (associations)

Perform data transformation to enable regionwise association analysis.

Input name	Source
regions	arrayCGHanalysis-gains-gAndLoss.regions 19.168
segments	arrayCGHanalysis-aberrations.data 19.46

19.163 arrayCGHanalysis-gains-associationLysis-asc (INPUT)

SQL query for ACGH region association manipulation.

Category: Internal

Parameter name	Value	Description
path	/gpfs/homes/mlaakso/asserSVN/ andurilCaseStudy/sql/ ACGHassoc.sql	Path (filename) of the input resource.

19.164 arrayCGHanalysis-gains-associationLysis-association (REvaluate)

This component produces some outputs by executing an R [20] script with the given input data. The exact functionality of this component depends on the given script.

The component is also capable of making branch decisions on Anduril workflow. Two alternative options (*altCase* and *defCase*) are provided.

Authors:	Marko Laakso (Marko.Laakso@Helsinki.FI), Erkka Valo (erkka.valo@tkk.fi)
Version:	2.2
Categories:	External, Latex, Network Control

Input name	Source	Description
script	arrayCGHanalysis-gains -associationLysis-rev.in 19.167	Data transformation
table1	arrayCGHanalysis-gains -associationLysis-association1 .table 19.165	Data matrix 1 (data.frame/file table1)

Parameter name	Value	Description
asConnection	false	If true, assign file connections (class file) to the inputs to variables of the same name. This is useful e.g. if you want to read large data files row by row.
param1		Value of this parameter will be assigned to param1 variable
param10		Value of this parameter will be assigned to param10 variable
param2		Value of this parameter will be assigned to param2 variable
param3		Value of this parameter will be assigned to param3 variable
param4		Value of this parameter will be assigned to param4 variable
param5		Value of this parameter will be assigned to param5 variable
param6		Value of this parameter will be assigned to param6 variable
param7		Value of this parameter will be assigned to param7 variable
param8		Value of this parameter will be assigned to param8 variable
param9		Value of this parameter will be assigned to param9 variable

19.165 arrayCGHanalysis-gains-associationLysis-association1 (TableQuery)

Execute an SQL query on input relations and create a result table. TableQuery uses HSQLDB (Hyper-threaded Structured Query Language Database [25]) for executing the query. Consequently, the syntax of the query is defined by HSQLDB.

Author:	Kristian Ovaska (Kristian.Ovaska@Helsinki.FI)
Version:	1.2
Categories:	convert, Filter

Input name	Source	Description
table1	arrayCGHanalysis-aberrations-eq Data.table 19.47	CSV table 1. The table is referred to as 'table1' in the SQL query.
table2	arrayCGHanalysis-gains-segplot.S ignRegion 19.174	CSV table 2. The table is referred to as 'table2' in the SQL query.
table3	arrayCGHanalysis-gains-gAnd Loss-annot.bioAnnotation 19.169	CSV table 3. The table is referred to as 'table3' in the SQL query.
query	arrayCGHanalysis-gains -associationLysis-asc.in 19.163	SQL query. Either this file or the query parameter must be provided, but not both.

Parameter name	Value	Description
numIndices		Comma-separated list of index counts for input tables. All indices are single-column indices, running from column 1 to N, where N is retrieved from this parameter. If empty, the default number (1) is used. For example, ",2,,2" sets two indices for table2 and table4 and one index for the rest.
query		SQL query. Either this parameter or the query input must be provided, but not both.

19.166 arrayCGHanalysis-gains-associationLysis-rBand (TableQuery)

Execute an SQL query on input relations and create a result table. TableQuery uses HSQLDB (Hyper-threaded Structured Query Language Database [25]) for executing the query. Consequently, the syntax of the query is defined by HSQLDB.

Author: Kristian Ovaska (Kristian.Ovaska@Helsinki.FI)
Version: 1.2
Categories: convert, Filter

Input name	Source	Description
table1	arrayCGHanalysis-gains -associationLysis-association1 .table 19.165	CSV table 1. The table is referred to as 'table1' in the SQL query.

Parameter name	Value	Description
numIndices		Comma-separated list of index counts for input tables. All indices are single-column indices, running from column 1 to N, where N is retrieved from this parameter. If empty, the default number (1) is used. For example, ",2,,2" sets two indices for table2 and table4 and one index for the rest.
query	SELECT DISTINCT("Region"), "band", "chr", "end", "start", SUM("rank")/COUNT("Region") AS "rank" FROM table1 GROUP BY "Region","band","chr","end", "start"	SQL query. Either this parameter or the query input must be provided, but not both.

19.167 arrayCGHanalysis-gains-associationLysis-rev (INPUT)

R script for ACGH region association manipulation.

Category: Internal

Parameter name	Value	Description
path	/gpfs/homes/mlaakso/asserSVN/ andurilCaseStudy/r/ACGHreval.r	Path (filename) of the input resource.

19.168 arrayCGHanalysis-gains-gAndLoss (AnnotateSegs)

This function annotates interesting genomic regions with ensembl genes.

19.169 arrayCGHanalysis-gains-gAndLoss-annot (KorvasieniAnnotator)

Korvasieni is an Ensembl [12] based converter for database identifiers and it can be used to convert genome location, gene, transcript, and translation identifiers across multiple biological databases. The selected `keyColumn` may contain entries with comma separated lists of values in which case `isListKey` parameter may be used to obtain each value separately and to process them as they were given in consecutive lines.

Author: Marko Laakso (Marko.Laakso@Helsinki.FI)
Version: 1.7
Categories: Annotation, GO

Input name	Source	Description
sourceKeys	arrayCGHanalysis-gains-segplot.S ignRegion 19.174	A list of source database keys.

Continued on next page...

Input name	Source	Description
connection	ensembl.in 19.273	Database connection can be defined using this file. The definition of parameters: database.url, database.user, database.password, database.timeout, database.recycle, and database.driver can be found from the documentation of Korvasieni.

Parameter name	Value	Description
echoColumns	ID,freq,chromosome	A comma separated list of column names for the columns that will be copied to the output. An asterisk (*) can be used to denote all columns execpt the keyColumn.
inputDB	.DNARegion	Type of input keys. This must be a database supported by Korvasieni. If the parameter is omitted, the component tries to derive the database from the type of geneID. If this is not possible, an error is returned. You may define three columns in form of chromosome:start-end in case the inputDB is .DNARegion. This format provides a comfortable compatibility with DNARegion datatype.
inputType	Gene	Ensembl object type for the input keys (Any, Gene, Transcript, Translation)
isListKey	false	Enables the automatic value splits for the comma separated key column
keyColumn	chromosome:start-end	Name of the key column withing sourceKeys file or an empty string for the first column
maxHits	100000	Maximum number of target identifiers for a single source identifier
rename	chromosome:start-end=chr	Comma separated list of column renaming rules (oldname=newname)
skipLevel	never	Skip result rows if the source identifier is unknown or target identifiers are not available. Possible values are: never (no filtering), source (skip if the source ID is unknown), target (skip if no target IDs are found), any (skip if any of the target IDs is missing).
targetDB	.GeneId,.DNABand	Comma-separated list of annotation types. Possible values are all databases supported by Korvasieni.
unique	true	This flag can be turned on in order to eliminate duplicate annotations.

19.170 arrayCGHanalysis-gains-gAndLoss-annotExpand (ExpandCollapse)

Converts between the two possible representations of a relations with multivalued columns. By default this component splits rows with multivalued (comma separated lists) columns into multiple rows each representing a single value. The input may contain several columns with comma separated values and all permutations of column values are shown as individual single-valued rows. The alternative mode recovers the original comma separated values from the expanded relations. This is performed by joining values of the their list columns into comma separated lists.

Author: Marko Laakso (Marko.Laakso@Helsinki.FI)
Version: 1.3
Category: convert

Input name	Source	Description
relation	arrayCGHanalysis-gains-gAndLoss-annot.bioAnnotation 19.169	The mandatory input relation

Parameter name	Value	Description
delim	,	Value delimiter between the list column values. Special characters can be encoded as specified in fi.helsinki.ltdk.csbl.asser.ArgumentEncoding.
expand	true	Action mode that is expand (true) or collapse (false)
listCols	.GeneId	A comma separated list of column names of input (expand) or output (collapse) columns that may contain delim separated values. The asterisk refers to every column of the input relation.

19.171 arrayCGHanalysis-gains-gAndLoss-annotGenes (KorvasieniAnnotator)

Korvasieni is an Ensembl [12] based converter for database identifiers and it can be used to convert genome location, gene, transcript, and translation identifiers across multiple biological databases. The selected `keyColumn` may contain entries with comma separated lists of values in which case `isListKey` parameter may be used to obtain each value separately and to process them as they were given in consecutive lines.

Author: Marko Laakso (Marko.Laakso@Helsinki.FI)
Version: 1.7
Categories: Annotation, GO

Input name	Source	Description
sourceKeys	arrayCGHanalysis-gains-gAnd Loss-annotExpand.relation 19.170	A list of source database keys.
connection	ensembl.in 19.273	Database connection can be defined using this file. The definition of parameters: database.url, database.user, database.password, database.timeout, database.recycle, and database.driver can be found from the documentation of Korvasieni.

Parameter name	Value	Description
echoColumns	freq,chr,chromosome,ID	A comma separated list of column names for the columns that will be copied to the output. An asterisk (*) can be used to denote all columns execpt the keyColumn.
inputDB	.GeneId	Type of input keys. This must be a database supported by Korvasieni. If the parameter is omitted, the component tries to derive the database from the type of geneID. If this is not possible, an error is returned. You may define three columns in form of chromosome:start-end in case the inputDB is .DNARegion. This format provides a comfortable compatibility with DNARegion datatype.
inputType	Gene	Ensembl object type for the input keys (Any, Gene, Transcript, Translation)
isListKey	false	Enables the automatic value splits for the comma separated key column
keyColumn	.GeneId	Name of the key column withing sourceKeys file or an empty string for the first column
maxHits	100000	Maximum number of target identifiers for a single source identifier
rename	.GeneName=gene name,ID= Region	Comma separated list of column renaming rules (oldname=newname)
skipLevel	never	Skip result rows if the source identifier is unknown or target identifiers are not available. Possible values are: never (no filtering), source (skip if the source ID is unknown), target (skip if no target IDs are found), any (skip if any of the target IDs is missing).
targetDB	.GeneName,.GeneDesc,. DNABand,.DNARegion,GO	Comma-separated list of annotation types. Possible values are all databases supported by Korvasieni.
unique	true	This flag can be turned on in order to eliminate duplicate annotations.

19.172 arrayCGHanalysis-gains-gAndLoss-filtSegs (TableQuery)

Execute an SQL query on input relations and create a result table. TableQuery uses HSQLDB (Hyperthreaded Structured Query Language Database [25]) for executing the query. Consequently, the syntax of the query is defined by HSQLDB.

Author: Kristian Ovaska (Kristian.Ovaska@Helsinki.FI)
Version: 1.2
Categories: convert, Filter

Input name	Source	Description
table1	arrayCGHanalysis-gains-gAnd Loss-annotGenes.bio Annotation 19.171	CSV table 1. The table is referred to as 'table1' in the SQL query.

Parameter name	Value	Description
numIndices		Comma-separated list of index counts for input tables. All indices are single-column indices, running from column 1 to N, where N is retrieved from this parameter. If empty, the default number (1) is used. For example, ",2,,2" sets two indices for table2 and table4 and one index for the rest.
query	SELECT * FROM table1 ORDER BY "freq" DESC	SQL query. Either this parameter or the query input must be provided, but not both.

19.173 arrayCGHanalysis-gains-refs (INPUT)

(Technical documentation) Imports an external input file or directory to the pipeline. This is the only way external files are imported. File path is given with the parameter "path".

Category: Internal

Parameter name	Value	Description
path	/gpfs/homes/mlaakso/asserSVN/ andurilCaseStudy/csv/ refsSegRank.csv	Path (filename) of the input resource.

19.174 arrayCGHanalysis-gains-segplot (SegmentPlot)

This component has two purposes: first, to plot segmented chromosomal data; second, call frequently occuring regions therein. Any chromosomal regions defined with chromosome, start and end locations can be plotted, but the main purpose of this component is to visualize Array CGH segmentation results. It can also be utilized for ChIPonChip analysis pipeline in a subset of use cases.

Deleted and gained regions can be analyzed separately or jointly. The input table must contain the following columns: Sample, chromosome, starting basepair, ending basepair. Segment call and score are optional columns. These are used to distinguish between duplications and deletions and to assign confidences or ranks to the calls, respectively.

Authors: Riku Louhimo (Riku.Louhimo@Helsinki.FI), Marko Laakso (Marko.Laakso@Helsinki.FI)
Version: 1.5
Categories: Latex, Copy Number Analysis

Input name	Source	Description
chrSegments	arrayCGHanalysis-aberrations-eq Data.table 19.47	Chromosomal segments, usually obtained from an Array CGH experiment after segmentation.

Parameter name	Value	Description
bpEndColumn	end	The name of the chromosomal end location column in the input file.
bpStartColumn	start	The name of the chromosomal start location column in the input file.
callColumn	call	The name of the call column in the input file. All calls are considered to be 1 if this parameter value is empty.
chrColumn	chr	The name of the chromosomal number column in the input file.
excludeChr	23,24,X,Y,M	Chromosomes that are not used in the analysis. Defaults to excluding mitochondrial and sex chromosomes.
gainsOrLosses	1	Determine whether to plot gains alone (1) or losses alone (-1) or both (default).
lineWidth	1	Line width in output plot
sThold	0.1	The significance threshold. Please note that this is just the fraction of samples we want the segment to be present in.
sampleColumn	ID	The name of the sample name column in the input file.
scoreColumn		The name of the score column in the input file. You may leave this parameter empty if the data do not contain such information.
scoreMerge	max	Defines an R function that is used to summarize the score values of all input calls that are contributing to an output regions.

19.175 arrayCGHanalysis-gains-summaryReport (LatexCombiner)

LatexCombiner joins the given subdocuments together into one LaTeX file.

Authors: Kristian Ovaska (Kristian.Ovaska@Helsinki.FI), Marko Laakso (Marko.Laakso@Helsinki.FI)
Version: 1.3
Category: Latex

Input name	Source	Description
latex1	arrayCGHanalysis-gains-segplot .plot 19.174	LaTeX fragment 1.
latex2	arrayCGHanalysis-gains-abbers .report 19.161	LaTeX fragment 2.

Parameter name	Value	Description
pagebreak	false	Determines if the result document should start with a page break.
sectionTitle		If non-empty, a declaration of a new section with the given name is inserted to the beginning of the combined document. This is a convenience feature to make it easy to compile subsections into a section.
sectionType	section	Type of LaTeX section: usually one of section, subsection or subsubsection. No section statement is written if sectionTitle is empty.
strictBorders	true	Enables the flushing of all document elements before each fragment

19.176 arrayCGHanalysis-loclist (CSVFilter)

Filters columns and/or rows from CSV files using flexible criteria. Default settings do not filter out anything. The *negate* parameter reverses all inclusion criteria.

Columns are filtered based on column names (see *includeColumns*); it is also possible to rename columns. Columns can be reordered using *includeColumns*.

A row is included in output if all conditions are satisfied. Rows having too many missing values are filtered using the *nonMissing* parameter. The parameters *regexp*, *lowBound* and *highBound* filter rows based on cell contents. If the *auxiliary* input is given, the result contains those rows from the input whose values match those from the auxiliary file.

Authors: Kristian Ovaska (kristian.ovaska@helsinki.fi), Marko Laakso (Marko.Laakso@Helsinki.FI)
Version: 1.1
Category: Filter

Input name	Source	Description
csv	arrayCGHanalysis-segmentedGT Sgain.csv 19.205	CSV file to be filtered.

Parameter name	Value	Description
highBound		A row is included if the value in given numeric columns are at most as large as given numeric bounds. The parameter has a format COLNAME1=HIGH1,COLNAME2=HIGH2.
idColumn		Column name in "csv" that contains ID values. If the parameter is empty, the first column is used.
includeColumns	gts.chr,gts.pos	Comma-separated list of column names that should be included in the result file. The special value * includes all columns. The order of column names is significant: the output columns are in the order given. See also includeColumns input.
lowBound		A row is included if the value in given numeric columns are at least as large as given numeric bounds. The parameter has a format COLNAME1=LOW1,COLNAME2=LOW2. For example, "col=5" includes rows where the column col has a value of at least 5.
matchColumn		Column name in "auxiliary" containing values that must match the ID column in "csv" (specified using "idColumn"). If empty, the first column of "auxiliary" is used.
negate	false	If true, all inclusion criteria are negated so that rows or columns are excluded instead of included if they match the criteria. For example, includeColumns=* then excludes all columns (which would be an error since at least one column must be included).
nonMissing	0	Include those rows that have non-missing (non-NA) values in at least this many columns. Only those columns that are part of the output are counted.
regexp		Row filtering based on regular expression. A row is included in the result if values in the given columns match given regular expressions. The parameter has a format COLNAME1=EXPRESSION,COLNAME2=EXPRESSION2 where COLNAMEs are column names in "csv" and EXPRESSIONs are regular expressions using Java syntax. For example, "col=a|b" includes rows where the column col has a value of "a" or "b". Commas have to be escaped with "\\" if they are used in column names or regular expressions.

Continued on next page...

Parameter name	Value	Description
rename		Comma-separated list of column renaming rules (OLDNAME=NEWNAME). Parameters that refer to column names refer to old names; renaming is done last.

19.177 arrayCGHanalysis-losses (ACGHSegmentAnalysis)

Analyze interesting regions from segmented Array CGH regions exhibiting copy number loss.

Input name	Source
segments	arrayCGHanalysis-aberrations.data 19.46

Parameter name	Value
annotateRegs	true
gainOrLoss	-1
threshold	0.1

19.178 arrayCGHanalysis-losses-ArrayCGHgenes (TableQuery)

Execute an SQL query on input relations and create a result table. TableQuery uses HSQLDB (Hyperthreaded Structured Query Language Database [25]) for executing the query. Consequently, the syntax of the query is defined by HSQLDB.

Author:	Kristian Ovaska (Kristian.Ovaska@Helsinki.FI)
Version:	1.2
Categories:	convert, Filter

Input name	Source	Description
table1	arrayCGHanalysis-losses-gAndLoss-annotGenes.bioAnnotation 19.190	CSV table 1. The table is referred to as 'table1' in the SQL query.

Parameter name	Value	Description
numIndices		Comma-separated list of index counts for input tables. All indices are single-column indices, running from column 1 to N, where N is retrieved from this parameter. If empty, the default number (1) is used. For example, ",2,,2" sets two indices for table2 and table4 and one index for the rest.
query	SELECT ".GeneId", "freq" FROM table1	SQL query. Either this parameter or the query input must be provided, but not both.

19.179 arrayCGHanalysis-losses-abb (TableQuery)

Execute an SQL query on input relations and create a result table. TableQuery uses HSQLDB (Hyperthreaded Structured Query Language Database [25]) for executing the query. Consequently, the syntax of the query is defined by HSQLDB.

Author:	Kristian Ovaska (Kristian.Ovaska@Helsinki.FI)
Version:	1.2
Categories:	convert, Filter

Input name	Source	Description
table1	arrayCGHanalysis-losses-gAndLoss-filtSegs.table 19.191	CSV table 1. The table is referred to as 'table1' in the SQL query.

Continued on next page...

Input name	Source	Description
table2	arrayCGHanalysis-losses-associationLysis-rBand.table 19.185	CSV table 2. The table is referred to as 'table2' in the SQL query.

Parameter name	Value	Description
numIndices		Comma-separated list of index counts for input tables. All indices are single-column indices, running from column 1 to N, where N is retrieved from this parameter. If empty, the default number (1) is used. For example, ",2,,2" sets two indices for table2 and table4 and one index for the rest.
query	SELECT T1.*, ROUND(T2."rank", 5) AS "rank" FROM table1 T1, table2 T2 WHERE (T1."Region" = T2."Region") ORDER BY "rank" DESC	SQL query. Either this parameter or the query input must be provided, but not both.

19.180 arrayCGHanalysis-losses-abbers (CSV2Latex)

This component converts a tabular CSV file into a printable LaTeX table.

Author: Marko Laakso (Marko.Laakso@Helsinki.FI)
Version: 2.1
Categories: convert, Latex

Input name	Source	Description
tabledata	arrayCGHanalysis-losses-abb.table 19.179	Table content
refs	arrayCGHanalysis-losses-refs.in 19.192	Reference rules for the hyperlinks

Parameter name	Value	Description
attach	false	Include the original data as an attachment
caption		Caption text for the table.
colFormat	llp{2cm}p{10cm}r	LaTeX tabular format for the columns. Default produces a centered layout for all columns.
columns	gene name,chr,.DNABand,.GeneDesc,rank	Comma separated list of column selections for the output. The empty default will use all columns.
countRows	true	Include a row count to the table caption.
dropMissing	true	This flag can be turned off in order to generate links with missing texts. Link text are substituted with target identifiers.
listCols	.DNABand	Comma separated list of column names. Columns of this list may contain several values separated with commas and the delimiters will be replaced with spaces.
numberFormat		A comma separated list of decimal formats for the columns. Each entry consists of the column name and the Java DecimalFormal pattern separated with equal sign. For example, rounding to three decimals can be done like: myColumn=#0.000
pageBreak	false	Use clear page after the table.
rename		Comma separated list of column renaming rules (oldname=newname). New names are used in table header but they do not affect the other behaviour of this component.
ruler	{}	Latex command for the row separating rulers
section	Interesting copy number -1.0 segments	Section title for the table container or an empty string if no section should be generated.
sectionType	subsection	Type of LaTeX section: usually one of: section, subsection, or subsubsection. No section statement is written if section title is empty.
skipEmpty	false	This flag can be used to replace empty tables with a simple LaTeX comment.

19.181 arrayCGHanalysis-losses-associationLysis (associations)

Perform data transformation to enable regionwise association analysis.

Input name	Source
regions	arrayCGHanalysis-losses-gAndLoss.regions 19.187

Continued on next page...

Input name	Source
segments	arrayCGHanalysis-aberrations.data 19.46

19.182 arrayCGHanalysis-losses-associationLysis-asc (INPUT)

SQL query for ACGH region association manipulation.

Category: Internal

Parameter name	Value	Description
path	/gpfs/homes/mlaakso/asserSVN/anduril CaseStudy/sql/ACGHassoc.sql	Path (filename) of the input resource.

19.183 arrayCGHanalysis-losses-associationLysis-association (REvaluate)

This component produces some outputs by executing an R [20] script with the given input data. The exact functionality of this component depends on the given script.

The component is also capable of making branch decisions on Anduril workflow. Two alternative options (*altCase* and *defCase*) are provided.

Authors: Marko Laakso (Marko.Laakso@Helsinki.FI), Erkka Valo (erkka.valo@tkk.fi)
Version: 2.2
Categories: External, Latex, Network Control

Input name	Source	Description
script	arrayCGHanalysis-losses -associationLysis-rev.in 19.186	Data transformation
table1	arrayCGHanalysis-losses -associationLysis-association1 .table 19.184	Data matrix 1 (data.frame/file table1)

Parameter name	Value	Description
asConnection	false	If true, assign file connections (class file) to the inputs to variables of the same name. This is useful e.g. if you want to read large data files row by row.
param1		Value of this parameter will be assigned to param1 variable
param10		Value of this parameter will be assigned to param10 variable
param2		Value of this parameter will be assigned to param2 variable
param3		Value of this parameter will be assigned to param3 variable
param4		Value of this parameter will be assigned to param4 variable
param5		Value of this parameter will be assigned to param5 variable
param6		Value of this parameter will be assigned to param6 variable
param7		Value of this parameter will be assigned to param7 variable
param8		Value of this parameter will be assigned to param8 variable
param9		Value of this parameter will be assigned to param9 variable

19.184 arrayCGHanalysis-losses-associationLysis-association1 (TableQuery)

Execute an SQL query on input relations and create a result table. TableQuery uses HSQLDB (Hyper-threaded Structured Query Language Database [25]) for executing the query. Consequently, the syntax of the query is defined by HSQLDB.

Author: Kristian Ovaska (Kristian.Ovaska@Helsinki.FI)
Version: 1.2
Categories: convert, Filter

Input name	Source	Description
table1	arrayCGHanalysis-aberrations-eqData.table 19.47	CSV table 1. The table is referred to as 'table1' in the SQL query.
table2	arrayCGHanalysis-losses-segplot.SignRegion 19.193	CSV table 2. The table is referred to as 'table2' in the SQL query.
table3	arrayCGHanalysis-losses-gAndLoss-annot.bioAnnotation 19.188	CSV table 3. The table is referred to as 'table3' in the SQL query.
query	arrayCGHanalysis-losses-associationLysis-asc.in 19.182	SQL query. Either this file or the query parameter must be provided, but not both.

Parameter name	Value	Description
numIndices		Comma-separated list of index counts for input tables. All indices are single-column indices, running from column 1 to N, where N is retrieved from this parameter. If empty, the default number (1) is used. For example, ",2,,2" sets two indices for table2 and table4 and one index for the rest.
query		SQL query. Either this parameter or the query input must be provided, but not both.

19.185 arrayCGHanalysis-losses-associationLysis-rBand (TableQuery)

Execute an SQL query on input relations and create a result table. TableQuery uses HSQLDB (Hyperthreaded Structured Query Language Database [25]) for executing the query. Consequently, the syntax of the query is defined by HSQLDB.

Author:	Kristian Ovaska (Kristian.Ovaska@Helsinki.FI)
Version:	1.2
Categories:	convert, Filter

Input name	Source	Description
table1	arrayCGHanalysis-losses-associationLysis-association1.table 19.184	CSV table 1. The table is referred to as 'table1' in the SQL query.

Parameter name	Value	Description
numIndices		Comma-separated list of index counts for input tables. All indices are single-column indices, running from column 1 to N, where N is retrieved from this parameter. If empty, the default number (1) is used. For example, ",2,,2" sets two indices for table2 and table4 and one index for the rest.
query	SELECT DISTINCT("Region"), "band", "chr", "end", "start", SUM("rank")/COUNT("Region") AS "rank" FROM table1 GROUP BY "Region","band","chr","end", "start"	SQL query. Either this parameter or the query input must be provided, but not both.

19.186 arrayCGHanalysis-losses-associationLysis-rev (INPUT)

R script for ACGH region association manipulation.

Category:	Internal

Parameter name	Value	Description
path	/gpfs/homes/mlaakso/asserSVN/andurilCaseStudy/r/ACGHreval.r	Path (filename) of the input resource.

19.187 arrayCGHanalysis-losses-gAndLoss (AnnotateSegs)

This function annotates interesting genomic regions with ensembl genes.

19.188 arrayCGHanalysis-losses-gAndLoss-annot (KorvasieniAnnotator)

Korvasieni is an Ensembl [12] based converter for database identifiers and it can be used to convert genome location, gene, transcript, and translation identifiers across multiple biological databases. The selected `keyColumn` may contain entries with comma separated lists of values in which case `isListKey` parameter may be used to obtain each value separately and to process them as they were given in consecutive lines.

Author:	Marko Laakso (Marko.Laakso@Helsinki.FI)
Version:	1.7
Categories:	Annotation, GO

Input name	Source	Description
sourceKeys	arrayCGHanalysis-losses-segplot.SignRegion 19.193	A list of source database keys.
connection	ensembl.in 19.273	Database connection can be defined using this file. The definition of parameters: database.url, database.user, database.password, database.timeout, database.recycle, and database.driver can be found from the documentation of Korvasieni.

Parameter name	Value	Description
echoColumns	ID,freq,chromosome	A comma separated list of column names for the columns that will be copied to the output. An asterisk (*) can be used to denote all columns execpt the keyColumn.
inputDB	.DNARegion	Type of input keys. This must be a database supported by Korvasieni. If the parameter is omitted, the component tries to derive the database from the type of geneID. If this is not possible, an error is returned. You may define three columns in form of chromosome:start-end in case the inputDB is .DNARegion. This format provides a comfortable compatibility with DNARegion datatype.
inputType	Gene	Ensembl object type for the input keys (Any, Gene, Transcript, Translation)
isListKey	false	Enables the automatic value splits for the comma separated key column
keyColumn	chromosome:start-end	Name of the key column withing sourceKeys file or an empty string for the first column
maxHits	100000	Maximum number of target identifiers for a single source identifier
rename	chromosome:start-end=chr	Comma separated list of column renaming rules (oldname=newname)
skipLevel	never	Skip result rows if the source identifier is unknown or target identifiers are not available. Possible values are: never (no filtering), source (skip if the source ID is unknown), target (skip if no target IDs are found), any (skip if any of the target IDs is missing).
targetDB	.GeneId,.DNABand	Comma-separated list of annotation types. Possible values are all databases supported by Korvasieni.
unique	true	This flag can be turned on in order to eliminate duplicate annotations.

19.189 arrayCGHanalysis-losses-gAndLoss-annotExpand (ExpandCollapse)

Converts between the two possible representations of a relations with multivalued columns. By default this component splits rows with multivalued (comma separated lists) columns into multiple rows each representing a single value. The input may contain several columns with comma separated values and all permutations of column values are shown as individual single-valued rows. The alternative mode recovers the original comma separated values from the expanded relations. This is performed by joining values of the their list columns into comma separated lists.

Author:	Marko Laakso (Marko.Laakso@Helsinki.FI)
Version:	1.3
Category:	convert

Input name	Source	Description
relation	arrayCGHanalysis-losses-gAndLoss-annot.bioAnnotation 19.188	The mandatory input relation

Parameter name	Value	Description
delim	,	Value delimiter between the list column values. Special characters can be encoded as specified in fi.helsinki.ltdk.csbl.asser.ArgumentEncoding.
expand	true	Action mode that is expand (true) or collapse (false)
listCols	.GeneId	A comma separated list of column names of input (expand) or output (collapse) columns that may contain delim separated values. The asterisk refers to every column of the input relation.

19.190 arrayCGHanalysis-losses-gAndLoss-annotGenes (KorvasieniAnnotator)

Korvasieni is an Ensembl [12] based converter for database identifiers and it can be used to convert genome location, gene, transcript, and translation identifiers across multiple biological databases. The selected `keyColumn` may contain entries with comma separated lists of values in which case `isListKey` parameter may be used to obtain each value separately and to process them as they were given in consecutive lines.

Author: Marko Laakso (Marko.Laakso@Helsinki.FI)
Version: 1.7
Categories: Annotation, GO

Input name	Source	Description
sourceKeys	arrayCGHanalysis-losses-gAndLoss-annotExpand.relation 19.189	A list of source database keys.
connection	ensembl.in 19.273	Database connection can be defined using this file. The definition of parameters: database.url, database.user, database.password, database.timeout, database.recycle, and database.driver can be found from the documentation of Korvasieni.

Parameter name	Value	Description
echoColumns	freq,chr,chromosome,ID	A comma separated list of column names for the columns that will be copied to the output. An asterisk (*) can be used to denote all columns execpt the keyColumn.
inputDB	.GeneId	Type of input keys. This must be a database supported by Korvasieni. If the parameter is omitted, the component tries to derive the database from the type of geneID. If this is not possible, an error is returned. You may define three columns in form of chromosome:start-end in case the inputDB is .DNARegion. This format provides a comfortable compatibility with DNARegion datatype.
inputType	Gene	Ensembl object type for the input keys (Any, Gene, Transcript, Translation)
isListKey	false	Enables the automatic value splits for the comma separated key column
keyColumn	.GeneId	Name of the key column withing sourceKeys file or an empty string for the first column
maxHits	100000	Maximum number of target identifiers for a single source identifier
rename	.GeneName=gene name,ID=Region	Comma separated list of column renaming rules (oldname=newname)
skipLevel	never	Skip result rows if the source identifier is unknown or target identifiers are not available. Possible values are: never (no filtering), source (skip if the source ID is unknown), target (skip if no target IDs are found), any (skip if any of the target IDs is missing).
targetDB	.GeneName,.GeneDesc,.DNABand,.DNARegion,GO	Comma-separated list of annotation types. Possible values are all databases supported by Korvasieni.
unique	true	This flag can be turned on in order to eliminate duplicate annotations.

19.191 arrayCGHanalysis-losses-gAndLoss-filtSegs (TableQuery)

Execute an SQL query on input relations and create a result table. TableQuery uses HSQLDB (Hyperthreaded Structured Query Language Database [25]) for executing the query. Consequently, the syntax of the query is defined by HSQLDB.

Author: Kristian Ovaska (Kristian.Ovaska@Helsinki.FI)
Version: 1.2
Categories: convert, Filter

Input name	Source	Description
table1	arrayCGHanalysis-losses-gAnd Loss-annotGenes.bio Annotation 19.190	CSV table 1. The table is referred to as 'table1' in the SQL query.

Parameter name	Value	Description
numIndices		Comma-separated list of index counts for input tables. All indices are single-column indices, running from column 1 to N, where N is retrieved from this parameter. If empty, the default number (1) is used. For example, ",2,,2" sets two indices for table2 and table4 and one index for the rest.
query	SELECT * FROM table1 ORDER BY "freq" DESC	SQL query. Either this parameter or the query input must be provided, but not both.

19.192 arrayCGHanalysis-losses-refs (INPUT)

(Technical documentation) Imports an external input file or directory to the pipeline. This is the only way external files are imported. File path is given with the parameter "path".

Category: Internal

Parameter name	Value	Description
path	/gpfs/homes/mlaakso/asserSVN/ andurilCaseStudy/csv/ refsSegRank.csv	Path (filename) of the input resource.

19.193 arrayCGHanalysis-losses-segplot (SegmentPlot)

This component has two purposes: first, to plot segmented chromosomal data; second, call frequently occuring regions therein. Any chromosomal regions defined with chromosome, start and end locations can be plotted, but the main purpose of this component is to visualize Array CGH segmentation results. It can also be utilized for ChIPonChip analysis pipeline in a subset of use cases.

Deleted and gained regions can be analyzed separately or jointly. The input table must contain the following columns: Sample, chromosome, starting basepair, ending basepair. Segment call and score are optional columns. These are used to distinguish between duplications and deletions and to assign confidences or ranks to the calls, respectively.

Authors: Riku Louhimo (Riku.Louhimo@Helsinki.FI), Marko Laakso (Marko.Laakso@Helsinki.FI)
Version: 1.5
Categories: Latex, Copy Number Analysis

Input name	Source	Description
chrSegments	arrayCGHanalysis-aberrations-eq Data.table 19.47	Chromosomal segments, usually obtained from an Array CGH experiment after segmentation.

Parameter name	Value	Description
bpEndColumn	end	The name of the chromosomal end location column in the input file.
bpStartColumn	start	The name of the chromosomal start location column in the input file.
callColumn	call	The name of the call column in the input file. All calls are considered to be 1 if this parameter value is empty.
chrColumn	chr	The name of the chromosomal number column in the input file.
excludeChr	23,24,X,Y,M	Chromosomes that are not used in the analysis. Defaults to excluding mitochondrial and sex chromosomes.
gainsOrLosses	-1	Determine whether to plot gains alone (1) or losses alone (-1) or both (default).
lineWidth	1	Line width in output plot
sThold	0.1	The significance threshold. Please note that this is just the fraction of samples we want the segment to be present in.
sampleColumn	ID	The name of the sample name column in the input file.

Continued on next page...

Parameter name	Value	Description
scoreColumn		The name of the score column in the input file. You may leave this parameter empty if the data do not contain such information.
scoreMerge	max	Defines an R function that is used to summarize the score values of all input calls that are contributing to an output regions.

19.194 arrayCGHanalysis-losses-summaryReport (LatexCombiner)

LatexCombiner joins the given subdocuments together into one LaTeX file.

Authors: Kristian Ovaska (Kristian.Ovaska@Helsinki.FI), Marko Laakso (Marko.Laakso@Helsinki.FI)
Version: 1.3
Category: Latex

Input name	Source	Description
latex1	arrayCGHanalysis-losses-segplot .plot 19.193	LaTeX fragment 1.
latex2	arrayCGHanalysis-losses-abbers .report 19.180	LaTeX fragment 2.

Parameter name	Value	Description
pagebreak	false	Determines if the result document should start with a page break.
sectionTitle		If non-empty, a declaration of a new section with the given name is inserted to the beginning of the combined document. This is a convenience feature to make it easy to compile subsections into a section.
sectionType	section	Type of LaTeX section: usually one of section, subsection or subsubsection. No section statement is written if sectionTitle is empty.
strictBorders	true	Enables the flushing of all document elements before each fragment

19.195 arrayCGHanalysis-maPlot (Plot2D)

Create scatter or line plots having a dot for each supplied (Y, X) coordinate pair. Coordinates for the Y axis must always be provided; values for the X axis are optional. If X coordinates are missing, integers 1 to N are used.

Authors: Kristian Ovaska (kristian.ovaska@helsinki.fi), Marko Laakso (Marko.Laakso@Helsinki.FI), Sirkku Karinen (sirkku.karinen@Helsinki.FI)
Version: 1.0
Categories: Latex, Plot

Input name	Source	Description
y	arrayCGHanalysis-normalized .casechannel 19.196	Coordinates on the Y axis. Columns from this file are selected for plotting using "yColumns".
x	arrayCGHanalysis-normalized .controlchannel 19.196	Coordinates on the X axis. Columns from this file are selected for plotting using "xColumns". There must as many rows in "x" as there are in "y".

Parameter name	Value	Description
caption	MA plot for normalized values (%s vs %s)	LaTeX caption of each figure. The caption may contain one or two %s placeholders, which are replaced with the current column names in "y" and "x" (if defined). Column names are not used when multiple plots are created to the same image (imageType = 'single').
colorFunction		Custom function for colors. Example c('red', 'blue', 'green') or rainbow(3) or rainbow(%d), in which %d is replaced with the number of different labels or columns in the plot. Default colors start from black and continue by rainbow()-function until each label or column is assigned with own color.
commonScale	true	If true, all plots have the same limits for X and Y axes. This makes it easier to compare plots. Only relevant when multiple columns have been selected.
dpCm	60	Dots per centimeter. Used for png-images.
drawX	true	If false, do not print X axis ticks and numbers.
height	11	Height of each plot in cm.

Continued on next page...

Parameter name	Value	Description
imageType	multiple	Plot multiple images = 'multiple', single image = 'single' or a pairs plot = 'pairs'
labelColumn		Columns in input "label" that is used. If not defined, uses the first column.
legendPosition	off	Legend position 'bottomleft' or 'topright' etc (Can not be used with the pairs plot)
nInf	-2e100	Substitution value for infinitely small values
pInf	2e100	Substitution value for infinitely great values
plotType	p	Plot type p=points, l=lines, h=bars
pngImage	true	Switch image format between png and pdf
sectionTitle	Two dimensional plot	Title of the LaTeX section or an empty string to omit the section
sectionType	subsection	Type of LaTeX section: usually one of section, subsection or subsubsection. No section statement is written if sectionTitle is empty.
sort	false	If true, sort values before plotting. This creates a cumulative-like distribution plot. (Can not be used with pairs plot)
title	%s vs %s	Image title. The caption may contain one or two %s placeholders, which are replaced with the current column names in "y" and "x" (if defined). Column names are not used when multiple plots are created to the same image (imageType = 'single').
width	14	Width of each plot in cm.
xColumns	*	Columns in input "x" that are plotted. The special value * includes all columns except non-numeric ones. There must be as many selected columns in X as there are in Y. Note that the files "y" and "x" may still contain a different number of columns.
xHigh	-0.000001	High bound of the X axis. The special value -0.000001 means that the bound is computed from the data.
xLabel	Fit curve	X axis label. The label may contain one %s placeholder, which is replaced with the current column name in "x", or if not defined, in "y".
xLow	-0.000001	Low bound of the X axis. The special value -0.000001 means that the bound is computed from the data.
xTransformation		Numeric R expression that is used to transform X values. The X values are available as a numeric matrix (or vector) under the name "x". This is analogous to "yTransformation".
yColumns	*	Columns in input "y" that are plotted. The special value * includes all columns except non-numeric ones.
yHigh	-0.000001	High bound of the Y axis. The special value -0.000001 means that the bound is computed from the data.
yLabel	Residuals	X axis label. The label may contain one %s placeholder, which is replaced with the current column name in "y".
yLow	-0.000001	Low bound of the Y axis. The special value -0.000001 means that the bound is computed from the data.
yTransformation		Numeric R expression that is used to transform Y values. The Y values are available as a numeric matrix (or vector) under the name "y". Only those columns are present that are selected using "yColumns". For example, "-log10(y)" transforms Y values using a negative base 10 logarithm (defined only for positive values). The expression may be any legal R expression that takes a numeric matrix (or vector) and produces a matrix (or vector) of the same size.

19.196 arrayCGHanalysis-normalized (ACGHnorm)

(Technical documentation) Normalizes two channel array CGH data with LOESS. Tested with Agilent 244A Array CGH data.

Author: Riku Louhimo (Riku.Louhimo@Helsinki.FI)
Version: 1.0
Categories: Normalization, Preprocessing, Copy Number Analysis

Input name	Source	Description
casechannel	arrayCGHanalysis-fChannels .matrix2 19.157	Probe intensities of the case channel of an Array CGH experiment, preferrably as output by AgilentReader.
control	arrayCGHanalysis-fChannels .matrix 19.157	Probe intensities of the control channel of an Array CGH experiment, preferrably as output by AgilentReader.

Parameter name	Value	Description
iterations	4	Number of iterations of loess fit.
span	0.3	Numeric parameter between 0 and 1 specifying proportion of data to be used in the local regression moving window. Larger numbers give smoother fits.

19.197 arrayCGHanalysis-preSegmentedGTSgain (CSVListJoin)

Combines the content of the input files into a single CSV file. The columns of the output file will form a union of all column names of the input files plus an additional column for the names of the source files.

Author: Marko Laakso (Marko.Laakso@Helsinki.FI)
Version: 2.1
Category: convert

Input name	Source	Description
file1	arrayCGHanalysis-unionSegsG.table 19.218	An optional input file
file2	arrayCGHanalysis-unionSegsG3.table 19.220	An optional input file
file3	arrayCGHanalysis-unionSegsG2.table 19.219	An optional input file

Parameter name	Value	Description
acceptExt		File extension that selects the files of interest. Add a dot (like .csv or .motif) into this parameter if the extension has it. Empty value refers to all files.
fileCol		Column name for the source file names. The whole column will be omitted if this string is empty.
stripExt	true	This flag can be used to remove file extensions from the source file names.

19.198 arrayCGHanalysis-probeIDs (CSV2IDList)

Filter out probeIDs from the geneAnnotation table.

Author: Marko Laakso (Marko.Laakso@Helsinki.FI)
Version: 1.3
Category: convert

Input name	Source	Description
table1	arrayCGHanalysis-fChannels.matrix2 19.157	The mandatory input relation

Parameter name	Value	Description
acceptMissing	false	Files with missing columnIn are accepted as empty if this is true.
columnIn	ProbeName	A comma separated list of column names for the IDs of interest in each file. Empty values refer to the first column of the file.
columnOut		Name of the only column of the output list. Empty input refers to the name of the input column.
constants		A comma separated list of values that are always included into the output
isList	false	True if the seleted column contains a comma separated list of values to be splitted
quotation	false	Indicator that can be used to disable quotation of the output values
regexp1		Regular expression for the row filtering in table1. A row is included in the result if this parameter is empty or if values in the given columns match given regular expressions. The parameter has a format COLNAME1=EXPRESSION,COLNAME2=EXPRESSION2 where COLNAMEs are column names in ”csv” and EXPRESSIONs are regular expressions using Java syntax. For example, ”col=a|b” includes rows where the column col has a value of ”a” or ”b”.
regexp2		Regular expression for the row filtering in table2.
regexp3		Regular expression for the row filtering in table3.
regexp4		Regular expression for the row filtering in table4.
regexp5		Regular expression for the row filtering in table5.
regexp6		Regular expression for the row filtering in table6.
regexp7		Regular expression for the row filtering in table7.
regexp8		Regular expression for the row filtering in table8.
regexp9		Regular expression for the row filtering in table9.

19.199 arrayCGHanalysis-qc (LatexCombiner)

LatexCombiner joins the given subdocuments together into one LaTeX file.

Authors:	Kristian Ovaska (Kristian.Ovaska@Helsinki.FI), Marko Laakso (Marko.Laakso@Helsinki.FI)
Version:	1.3
Category:	Latex

Input name	Source	Description
latex1	arrayCGHanalysis-boxPlot .report 19.66	LaTeX fragment 1.

Parameter name	Value	Description
pagebreak	false	Determines if the result document should start with a page break.
sectionTitle	Quality Control	If non-empty, a declaration of a new section with the given name is inserted to the beginning of the combined document. This is a convenience feature to make it easy to compile subsections into a section.
sectionType	section	Type of LaTeX section: usually one of section, subsection or subsubsection. No section statement is written if sectionTitle is empty.
strictBorders	true	Enables the flushing of all document elements before each fragment

19.200 arrayCGHanalysis-reMappedAnnot (TableQuery)

Execute an SQL query on input relations and create a result table. TableQuery uses HSQLDB (Hyperthreaded Structured Query Language Database [25]) for executing the query. Consequently, the syntax of the query is defined by HSQLDB.

Author:	Kristian Ovaska (Kristian.Ovaska@Helsinki.FI)
Version:	1.2
Categories:	convert, Filter

Input name	Source	Description
table1	arrayCGHanalysis-agil36to37map .in 19.49	CSV table 1. The table is referred to as 'table1' in the SQL query.
table2	arrayCGHanalysis-fAnnot .csv 19.156	CSV table 2. The table is referred to as 'table2' in the SQL query.

Parameter name	Value	Description
numIndices		Comma-separated list of index counts for input tables. All indices are single-column indices, running from column 1 to N, where N is retrieved from this parameter. If empty, the default number (1) is used. For example, ",2,,2" sets two indices for table2 and table4 and one index for the rest.
query	SELECT table2."ProbeName", table1."Chr", table1."start", table1."end" FROM table1, table2 WHERE table1."ProbeName" = table2."ProbeName"	SQL query. Either this parameter or the query input must be provided, but not both.

19.201 arrayCGHanalysis-rules (INPUT)

Rules for search and replace of DNA region annotations.

Category:	Internal

Continued on next page...

Parameter name	Value	Description

Parameter name	Value	Description
path	/gpfs/homes/mlaakso/asserSVN/ andurilCaseStudy/csv/ ACGHchr2.rules	Path (filename) of the input resource.

19.202 arrayCGHanalysis-sampFileMap (INPUT)

Filename to patient id mappings.

Category: Internal

Parameter name	Value	Description
path	/gpfs/homes/mlaakso/asserSVN/ andurilCaseStudy/csv/ ACGHsample2file-map.lst	Path (filename) of the input resource.

19.203 arrayCGHanalysis-samples (INPUT)

Sample Annotations for array CGH data.

Category: Internal

Parameter name	Value	Description
path	/gpfs/homes/mlaakso/asserSVN/ andurilCaseStudy/csv/ ACGHsamples.lst	Path (filename) of the input resource.

19.204 arrayCGHanalysis-segmented (TableQuery)

Execute an SQL query on input relations and create a result table. TableQuery uses HSQLDB (Hyper-threaded Structured Query Language Database [25]) for executing the query. Consequently, the syntax of the query is defined by HSQLDB.

Author: Kristian Ovaska (Kristian.Ovaska@Helsinki.FI)
Version: 1.2
Categories: convert, Filter

Input name	Source	Description
table1	arrayCGHanalysis-unionSegs .table 19.216	CSV table 1. The table is referred to as 'table1' in the SQL query.
table2	arrayCGHanalysis-unionSegs2 .table 19.217	CSV table 2. The table is referred to as 'table2' in the SQL query.

Parameter name	Value	Description
numIndices		Comma-separated list of index counts for input tables. All indices are single-column indices, running from column 1 to N, where N is retrieved from this parameter. If empty, the default number (1) is used. For example, ",2,,2" sets two indices for table2 and table4 and one index for the rest.
query	SELECT * FROM table1 UNION SELECT * FROM table2	SQL query. Either this parameter or the query input must be provided, but not both.

19.205 arrayCGHanalysis-segmentedGTSgain (CSVFilter)

Filters columns and/or rows from CSV files using flexible criteria. Default settings do not filter out anything. The *negate* parameter reverses all inclusion criteria.

Columns are filtered based on column names (see *includeColumns*); it is also possible to rename columns. Columns can be reordered using *includeColumns*.

A row is included in output if all conditions are satisfied. Rows having too many missing values are filtered using the *nonMissing* parameter. The parameters *regexp*, *lowBound* and *highBound* filter rows based on cell contents. If the *auxiliary* input is given, the result contains those rows from the input whose values match those from the auxiliary file.

Authors:	Kristian Ovaska (kristian.ovaska@helsinki.fi), Marko Laakso (Marko.Laakso@Helsinki.FI)
Version:	1.1
Category:	Filter

Input name	Source	Description
csv	arrayCGHanalysis-preSegmented GTSgain.join 19.197	CSV file to be filtered.

Parameter name	Value	Description
highBound		A row is included if the value in given numeric columns are at most as large as given numeric bounds. The parameter has a format COLNAME1=HIGH1,COLNAME2=HIGH2.
idColumn		Column name in "csv" that contains ID values. If the parameter is empty, the first column is used.
includeColumns	gts.chr,gts.pos,gts.ARI.gain,gts.AFI.gain	Comma-separated list of column names that should be included in the result file. The special value * includes all columns. The order of column names is significant: the output columns are in the order given. See also includeColumns input.
lowBound		A row is included if the value in given numeric columns are at least as large as given numeric bounds. The parameter has a format COLNAME1=LOW1,COLNAME2=LOW2. For example, "col=5" includes rows where the column col has a value of at least 5.
matchColumn		Column name in "auxiliary" containing values that must match the ID column in "csv" (specified using "idColumn"). If empty, the first column of "auxiliary" is used.
negate	false	If true, all inclusion criteria are negated so that rows or columns are excluded instead of included if they match the criteria. For example, includeColumns=* then excludes all columns (which would be an error since at least one column must be included).
nonMissing	0	Include those rows that have non-missing (non-NA) values in at least this many columns. Only those columns that are part of the output are counted.
regexp		Row filtering based on regular expression. A row is included in the result if values in the given columns match given regular expressions. The parameter has a format COLNAME1=EXPRESSION,COLNAME2=EXPRESSION2 where COLNAMEs are column names in "csv" and EXPRESSIONs are regular expressions using Java syntax. For example, "col=a|b" includes rows where the column col has a value of "a" or "b". Commas have to be escaped with "\\" if they are used in column names or regular expressions.
rename		Comma-separated list of column renaming rules (OLDNAME=NEWNAME). Parameters that refer to column names refer to old names; renaming is done last.

19.206 arrayCGHanalysis-segmentedGTSloss (TableQuery)

Execute an SQL query on input relations and create a result table. TableQuery uses HSQLDB (Hyperthreaded Structured Query Language Database [25]) for executing the query. Consequently, the syntax of the query is defined by HSQLDB.

Author:	Kristian Ovaska (Kristian.Ovaska@Helsinki.FI)
Version:	1.2
Categories:	convert, Filter

Input name	Source	Description
table1	arrayCGHanalysis-unionSegsL.table 19.221	CSV table 1. The table is referred to as 'table1' in the SQL query.
table2	arrayCGHanalysis-unionSegsL2.table 19.222	CSV table 2. The table is referred to as 'table2' in the SQL query.

Parameter name	Value	Description
numIndices		Comma-separated list of index counts for input tables. All indices are single-column indices, running from column 1 to N, where N is retrieved from this parameter. If empty, the default number (1) is used. For example, ",2,,2" sets two indices for table2 and table4 and one index for the rest.
query	SELECT "gts.chr","gts.pos","gts.ARI.loss","gts.AFI.loss","gts.cnv" FROM table1 UNION SELECT "gts.chr","gts.pos","gts.ARI.loss","gts.AFI.loss","gts.cnv" FROM table2	SQL query. Either this parameter or the query input must be provided, but not both.

19.207 arrayCGHanalysis-segmentedPart1 (LatexCombiner)

LatexCombiner joins the given subdocuments together into one LaTeX file.

Authors: Kristian Ovaska (Kristian.Ovaska@Helsinki.FI), Marko Laakso (Marko.Laakso@Helsinki.FI)
Version: 1.3
Category: Latex

Input name	Source	Description
latex1	arrayCGHanalysis-chr1-segmented.report 19.70	LaTeX fragment 1.
latex2	arrayCGHanalysis-chr2-segmented.report 19.114	LaTeX fragment 2.
latex3	arrayCGHanalysis-chr3-segmented.report 19.130	LaTeX fragment 3.
latex4	arrayCGHanalysis-chr4-segmented.report 19.134	LaTeX fragment 4.
latex5	arrayCGHanalysis-chr5-segmented.report 19.138	LaTeX fragment 5.
latex6	arrayCGHanalysis-chr6-segmented.report 19.142	LaTeX fragment 6.
latex7	arrayCGHanalysis-chr7-segmented.report 19.146	LaTeX fragment 7.
latex8	arrayCGHanalysis-chr8-segmented.report 19.150	LaTeX fragment 8.
latex9	arrayCGHanalysis-chr9-segmented.report 19.154	LaTeX fragment 9.

Parameter name	Value	Description
pagebreak	false	Determines if the result document should start with a page break.
sectionTitle		If non-empty, a declaration of a new section with the given name is inserted to the beginning of the combined document. This is a convenience feature to make it easy to compile subsections into a section.
sectionType	section	Type of LaTeX section: usually one of section, subsection or subsubsection. No section statement is written if sectionTitle is empty.
strictBorders	true	Enables the flushing of all document elements before each fragment

19.208 arrayCGHanalysis-segmentedPart2 (LatexCombiner)

LatexCombiner joins the given subdocuments together into one LaTeX file.

Authors: Kristian Ovaska (Kristian.Ovaska@Helsinki.FI), Marko Laakso (Marko.Laakso@Helsinki.FI)
Version: 1.3
Category: Latex

Input name	Source	Description
latex1	arrayCGHanalysis-chr10 -segmented.report 19.74	LaTeX fragment 1.
latex2	arrayCGHanalysis-chr11 -segmented.report 19.78	LaTeX fragment 2.
latex3	arrayCGHanalysis-chr12 -segmented.report 19.82	LaTeX fragment 3.
latex4	arrayCGHanalysis-chr13 -segmented.report 19.86	LaTeX fragment 4.
latex5	arrayCGHanalysis-chr14 -segmented.report 19.90	LaTeX fragment 5.
latex6	arrayCGHanalysis-chr15 -segmented.report 19.94	LaTeX fragment 6.
latex7	arrayCGHanalysis-chr16 -segmented.report 19.98	LaTeX fragment 7.
latex8	arrayCGHanalysis-chr17 -segmented.report 19.102	LaTeX fragment 8.
latex9	arrayCGHanalysis-chr18 -segmented.report 19.106	LaTeX fragment 9.

Parameter name	Value	Description
pagebreak	false	Determines if the result document should start with a page break.
sectionTitle		If non-empty, a declaration of a new section with the given name is inserted to the beginning of the combined document. This is a convenience feature to make it easy to compile subsections into a section.
sectionType	section	Type of LaTeX section: usually one of section, subsection or subsubsection. No section statement is written if sectionTitle is empty.
strictBorders	true	Enables the flushing of all document elements before each fragment

19.209 arrayCGHanalysis-segmentedPart3 (LatexCombiner)

LatexCombiner joins the given subdocuments together into one LaTeX file.

Authors: Kristian Ovaska (Kristian.Ovaska@Helsinki.FI), Marko Laakso (Marko.Laakso@Helsinki.FI)
Version: 1.3
Category: Latex

Input name	Source	Description
latex1	arrayCGHanalysis-chr19 -segmented.report 19.110	LaTeX fragment 1.
latex2	arrayCGHanalysis-chr20 -segmented.report 19.118	LaTeX fragment 2.
latex3	arrayCGHanalysis-chr21 -segmented.report 19.122	LaTeX fragment 3.
latex4	arrayCGHanalysis-chr22 -segmented.report 19.126	LaTeX fragment 4.

Parameter name	Value	Description
pagebreak	false	Determines if the result document should start with a page break.
sectionTitle		If non-empty, a declaration of a new section with the given name is inserted to the beginning of the combined document. This is a convenience feature to make it easy to compile subsections into a section.
sectionType	section	Type of LaTeX section: usually one of section, subsection or subsubsection. No section statement is written if sectionTitle is empty.
strictBorders	true	Enables the flushing of all document elements before each fragment

19.210 arrayCGHanalysis-segmentedReport (LatexCombiner)

LatexCombiner joins the given subdocuments together into one LaTeX file.

Authors: Kristian Ovaska (Kristian.Ovaska@Helsinki.FI), Marko Laakso (Marko.Laakso@Helsinki.FI)
Version: 1.3
Category: Latex

Input name	Source	Description
latex1	arrayCGHanalysis-segmented Part1.document 19.207	LaTeX fragment 1.
latex2	arrayCGHanalysis-segmented Part2.document 19.208	LaTeX fragment 2.
latex3	arrayCGHanalysis-segmented Part3.document 19.209	LaTeX fragment 3.

Parameter name	Value	Description
pagebreak	false	Determines if the result document should start with a page break.
sectionTitle		If non-empty, a declaration of a new section with the given name is inserted to the beginning of the combined document. This is a convenience feature to make it easy to compile subsections into a section.
sectionType	section	Type of LaTeX section: usually one of section, subsection or subsubsection. No section statement is written if sectionTitle is empty.
strictBorders	true	Enables the flushing of all document elements before each fragment

19.211 arrayCGHanalysis-segmentedVals (StandardProcess)

Execute the given command. The given input is delivered to the standard input stream and the output streams are bind to the output ports.

Author: Marko Laakso (Marko.Laakso@Helsinki.FI)
Version: 1.2
Category: External

Input name	Source	Description
input	arrayCGHanalysis-preSegmented GTSgain.join 19.197	Standard input stream for the executable

Parameter name	Value	Description
command	cut -f 7-	Command to be lauched
wd		Working directory for the executable if not the component folder

19.212 arrayCGHanalysis-segplot (SegmentPlot)

This component has two purposes: first, to plot segmented chromosomal data; second, call frequently occuring regions therein. Any chromosomal regions defined with chromosome, start and end locations can be plotted, but the main purpose of this component is to visualize Array CGH segmentation results. It can also be utilized for ChIPonChip analysis pipeline in a subset of use cases.

Deleted and gained regions can be analyzed separately or jointly. The input table must contain the following columns: Sample, chromosome, starting basepair, ending basepair. Segment call and score are optional columns. These are used to distinguish between duplications and deletions and to assign confidences or ranks to the calls, respectively.

Authors: Riku Louhimo (Riku.Louhimo@Helsinki.FI), Marko Laakso (Marko.Laakso@Helsinki.FI)
Version: 1.5
Categories: Latex, Copy Number Analysis

Input name	Source	Description
chrSegments	arrayCGHanalysis-aberrations-eq Data.table 19.47	Chromosomal segments, usually obtained from an Array CGH experiment after segmentation.

Parameter name	Value	Description
bpEndColumn	end	The name of the chromosomal end location column in the input file.
bpStartColumn	start	The name of the chromosomal start location column in the input file.
callColumn	call	The name of the call column in the input file. All calls are considered to be 1 if this parameter value is empty.
chrColumn	chr	The name of the chromosomal number column in the input file.
excludeChr	23,24,X,Y,M	Chromosomes that are not used in the analysis. Defaults to excluding mitochondrial and sex chromosomes.
gainsOrLosses	0	Determine whether to plot gains alone (1) or losses alone (-1) or both (default).
lineWidth	1	Line width in output plot
sThold	0.1	The significance threshold. Please note that this is just the fraction of samples we want the segment to be present in.
sampleColumn	Sample	The name of the sample name column in the input file.
scoreColumn		The name of the score column in the input file. You may leave this parameter empty if the data do not contain such information.
scoreMerge	max	Defines an R function that is used to summarize the score values of all input calls that are contributing to an output regions.

19.213 arrayCGHanalysis-srFAnnot (SearchReplace)

Replaces the occurences of the given regular expressions with the corresponding regular expressions. This component is used for transforming given patterns of the input file to a changed form specified by the regular expressions .

Author:	Marko Laakso (Marko.Laakso@Helsinki.FI)
Version:	1.0
Category:	convert

Input name	Source	Description
file	arrayCGHanalysis-reMapped Annot.table 19.200	Source file to be modified
rules	arrayCGHanalysis-rules.in 19.201	A set of search keys

Parameter name	Value	Description
key00		Search key 0
key01		Search key 1
key02		Search key 2
key03		Search key 3
key04		Search key 4
key05		Search key 5
key06		Search key 6
key07		Search key 7
key08		Search key 8
key09		Search key 9
value00		Replacement 0
value01		Replacement 1
value02		Replacement 2
value03		Replacement 3
value04		Replacement 4
value05		Replacement 5
value06		Replacement 6
value07		Replacement 7
value08		Replacement 8
value09		Replacement 9

19.214 arrayCGHanalysis-summaryReport (LatexPDF)

This component is a LaTeX compiler that generates a portable document format (PDF) file from the given Anduril document.

Author: Kristian Ovaska (Kristian.Ovaska@Helsinki.FI)
Version: 1.0
Category: Latex

Input name	Source	Description
document	arrayCGHanalysis-analysis .document 19.50	Body of the LaTeX document.
header	arrayCGHanalysis-template .header 19.215	LaTeX header that is written to the start of the document. If missing, a default header is used.
footer	arrayCGHanalysis-template .footer 19.215	LaTeX footer that is written to the end of the document. If missing, a default header is used.

Parameter name	Value	Description
bibtexExec	bibtex	Executable command for BibTeX.
latexExec	pdflatex	Executable command for pdflatex.
useRefs	false	This flag can be used to activate BibTeX compiler

19.215 arrayCGHanalysis-template (LatexTemplate)

This component generates the standard layout configuration for Anduril output documents. The component produces a header and a footer fragment that can be used to build LaTeX documents. The header fragment takes care of the page geometry and other technical details such as library imports and function declarations. Reference list is generated by the footer.

Author: Marko Laakso (Marko.Laakso@Helsinki.FI)
Version: 1.4
Category: Latex

Parameter name	Value	Description
authors	Riku Louhimo, Sampsa Hautaniemi	A comma-separated list of document authors on the title page. If authors is empty but title is non-empty, a title page with no author name is printed.
baselineskip	1.2	Baselineskip factor that indicates the line spacing
bibstyle	abbrv	BibTeX reference formatting style
printTOC	false	If true, print Table Of Contents.
title	Glioblastoma Array CGH analysis	Document title on the title page. If empty, no title page is printed. Also see author.
usepackage		Comma-separated list of LaTeX packages that are used in the document. Each package generates a \usepackage{X} line in the header.

19.216 arrayCGHanalysis-unionSegs (TableQuery)

Execute an SQL query on input relations and create a result table. TableQuery uses HSQLDB (Hyperthreaded Structured Query Language Database [25]) for executing the query. Consequently, the syntax of the query is defined by HSQLDB.

Author: Kristian Ovaska (Kristian.Ovaska@Helsinki.FI)
Version: 1.2
Categories: convert, Filter

Input name	Source	Description
table1	arrayCGHanalysis-chr1-segmented .segments 19.70	CSV table 1. The table is referred to as 'table1' in the SQL query.
table2	arrayCGHanalysis-chr2-segmented .segments 19.114	CSV table 2. The table is referred to as 'table2' in the SQL query.
table3	arrayCGHanalysis-chr3-segmented .segments 19.130	CSV table 3. The table is referred to as 'table3' in the SQL query.
table4	arrayCGHanalysis-chr4-segmented .segments 19.134	CSV table 4. The table is referred to as 'table4' in the SQL query.

Continued on next page...

Input name	Source	Description
table5	arrayCGHanalysis-chr5-segmented .segments 19.138	CSV table 5. The table is referred to as 'table5' in the SQL query.
table6	arrayCGHanalysis-chr6-segmented .segments 19.142	CSV table 6. The table is referred to as 'table6' in the SQL query.
table7	arrayCGHanalysis-chr7-segmented .segments 19.146	CSV table 7. The table is referred to as 'table7' in the SQL query.
table8	arrayCGHanalysis-chr8-segmented .segments 19.150	CSV table 8. The table is referred to as 'table8' in the SQL query.
table9	arrayCGHanalysis-chr9-segmented .segments 19.154	CSV table 9. The table is referred to as 'table9' in the SQL query.
table10	arrayCGHanalysis-chr10 -segmented.segments 19.74	CSV table 10. The table is referred to as 'table10' in the SQL query.
table11	arrayCGHanalysis-chr11 -segmented.segments 19.78	CSV table 11. The table is referred to as 'table11' in the SQL query.
table12	arrayCGHanalysis-chr12 -segmented.segments 19.82	CSV table 12. The table is referred to as 'table12' in the SQL query.

Parameter name	Value	Description
numIndices		Comma-separated list of index counts for input tables. All indices are single-column indices, running from column 1 to N, where N is retrieved from this parameter. If empty, the default number (1) is used. For example, ",2,,2" sets two indices for table2 and table4 and one index for the rest.
query	SELECT * FROM table1 UNION SELECT * FROM table2 UNION SELECT * FROM table3 UNION SELECT * FROM table4 UNION SELECT * FROM table5 UNION SELECT * FROM table6 UNION SELECT * FROM table7 UNION SELECT * FROM table8 UNION SELECT * FROM table9 UNION SELECT * FROM table10 UNION SELECT * FROM table11 UNION SELECT * FROM table12	SQL query. Either this parameter or the query input must be provided, but not both.

19.217 arrayCGHanalysis-unionSegs2 (TableQuery)

Execute an SQL query on input relations and create a result table. TableQuery uses HSQLDB (Hyperthreaded Structured Query Language Database [25]) for executing the query. Consequently, the syntax of the query is defined by HSQLDB.

Author: Kristian Ovaska (Kristian.Ovaska@Helsinki.FI)
Version: 1.2
Categories: convert, Filter

Input name	Source	Description
table1	arrayCGHanalysis-chr13 -segmented.segments 19.86	CSV table 1. The table is referred to as 'table1' in the SQL query.
table2	arrayCGHanalysis-chr14 -segmented.segments 19.90	CSV table 2. The table is referred to as 'table2' in the SQL query.
table3	arrayCGHanalysis-chr15 -segmented.segments 19.94	CSV table 3. The table is referred to as 'table3' in the SQL query.
table4	arrayCGHanalysis-chr16 -segmented.segments 19.98	CSV table 4. The table is referred to as 'table4' in the SQL query.
table5	arrayCGHanalysis-chr17 -segmented.segments 19.102	CSV table 5. The table is referred to as 'table5' in the SQL query.
table6	arrayCGHanalysis-chr18 -segmented.segments 19.106	CSV table 6. The table is referred to as 'table6' in the SQL query.
table7	arrayCGHanalysis-chr19 -segmented.segments 19.110	CSV table 7. The table is referred to as 'table7' in the SQL query.
table8	arrayCGHanalysis-chr20 -segmented.segments 19.118	CSV table 8. The table is referred to as 'table8' in the SQL query.
table9	arrayCGHanalysis-chr21 -segmented.segments 19.122	CSV table 9. The table is referred to as 'table9' in the SQL query.
table10	arrayCGHanalysis-chr22 -segmented.segments 19.126	CSV table 10. The table is referred to as 'table10' in the SQL query.

Parameter name	Value	Description
numIndices		Comma-separated list of index counts for input tables. All indices are single-column indices, running from column 1 to N, where N is retrieved from this parameter. If empty, the default number (1) is used. For example, ",2,,2" sets two indices for table2 and table4 and one index for the rest.
query	SELECT * FROM table1 UNION SELECT * FROM table2 UNION SELECT * FROM table3 UNION SELECT * FROM table4 UNION SELECT * FROM table5 UNION SELECT * FROM table6 UNION SELECT * FROM table7 UNION SELECT * FROM table8 UNION SELECT * FROM table9 UNION SELECT * FROM table10	SQL query. Either this parameter or the query input must be provided, but not both.

19.218 arrayCGHanalysis-unionSegsG (TableQuery)

Execute an SQL query on input relations and create a result table. TableQuery uses HSQLDB (Hyperthreaded Structured Query Language Database [25]) for executing the query. Consequently, the syntax of the query is defined by HSQLDB.

Author: Kristian Ovaska (Kristian.Ovaska@Helsinki.FI)
Version: 1.2
Categories: convert, Filter

Input name	Source	Description
table1	arrayCGHanalysis-chr1-segmented .GTSgain 19.70	CSV table 1. The table is referred to as 'table1' in the SQL query.
table2	arrayCGHanalysis-chr2-segmented .GTSgain 19.114	CSV table 2. The table is referred to as 'table2' in the SQL query.
table3	arrayCGHanalysis-chr3-segmented .GTSgain 19.130	CSV table 3. The table is referred to as 'table3' in the SQL query.
table4	arrayCGHanalysis-chr4-segmented .GTSgain 19.134	CSV table 4. The table is referred to as 'table4' in the SQL query.
table5	arrayCGHanalysis-chr5-segmented .GTSgain 19.138	CSV table 5. The table is referred to as 'table5' in the SQL query.
table6	arrayCGHanalysis-chr6-segmented .GTSgain 19.142	CSV table 6. The table is referred to as 'table6' in the SQL query.

Parameter name	Value	Description
numIndices		Comma-separated list of index counts for input tables. All indices are single-column indices, running from column 1 to N, where N is retrieved from this parameter. If empty, the default number (1) is used. For example, ",2,,2" sets two indices for table2 and table4 and one index for the rest.
query	SELECT * FROM table1 UNION SELECT * FROM table2 UNION SELECT * FROM table3 UNION SELECT * FROM table4 UNION SELECT * FROM table5 UNION SELECT * FROM table6	SQL query. Either this parameter or the query input must be provided, but not both.

19.219 arrayCGHanalysis-unionSegsG2 (TableQuery)

Execute an SQL query on input relations and create a result table. TableQuery uses HSQLDB (Hyperthreaded Structured Query Language Database [25]) for executing the query. Consequently, the syntax of the query is defined by HSQLDB.

Author: Kristian Ovaska (Kristian.Ovaska@Helsinki.FI)
Version: 1.2
Categories: convert, Filter

Input name	Source	Description
table1	arrayCGHanalysis-chr13 -segmented.GTSgain 19.86	CSV table 1. The table is referred to as 'table1' in the SQL query.
table2	arrayCGHanalysis-chr14 -segmented.GTSgain 19.90	CSV table 2. The table is referred to as 'table2' in the SQL query.
table3	arrayCGHanalysis-chr15 -segmented.GTSgain 19.94	CSV table 3. The table is referred to as 'table3' in the SQL query.
table4	arrayCGHanalysis-chr16 -segmented.GTSgain 19.98	CSV table 4. The table is referred to as 'table4' in the SQL query.
table5	arrayCGHanalysis-chr17 -segmented.GTSgain 19.102	CSV table 5. The table is referred to as 'table5' in the SQL query.
table6	arrayCGHanalysis-chr18 -segmented.GTSgain 19.106	CSV table 6. The table is referred to as 'table6' in the SQL query.
table7	arrayCGHanalysis-chr19 -segmented.GTSgain 19.110	CSV table 7. The table is referred to as 'table7' in the SQL query.
table8	arrayCGHanalysis-chr20 -segmented.GTSgain 19.118	CSV table 8. The table is referred to as 'table8' in the SQL query.
table9	arrayCGHanalysis-chr21 -segmented.GTSgain 19.122	CSV table 9. The table is referred to as 'table9' in the SQL query.
table10	arrayCGHanalysis-chr22 -segmented.GTSgain 19.126	CSV table 10. The table is referred to as 'table10' in the SQL query.

Parameter name	Value	Description
numIndices		Comma-separated list of index counts for input tables. All indices are single-column indices, running from column 1 to N, where N is retrieved from this parameter. If empty, the default number (1) is used. For example, ",2,,2" sets two indices for table2 and table4 and one index for the rest.
query	SELECT * FROM table1 UNION SELECT * FROM table2 UNION SELECT * FROM table3 UNION SELECT * FROM table4 UNION SELECT * FROM table5 UNION SELECT * FROM table6 UNION SELECT * FROM table7 UNION SELECT * FROM table8 UNION SELECT * FROM table9 UNION SELECT * FROM table10	SQL query. Either this parameter or the query input must be provided, but not both.

19.220 arrayCGHanalysis-unionSegsG3 (TableQuery)

Execute an SQL query on input relations and create a result table. TableQuery uses HSQLDB (Hyperthreaded Structured Query Language Database [25]) for executing the query. Consequently, the syntax of the query is defined by HSQLDB.

Author:	Kristian Ovaska (Kristian.Ovaska@Helsinki.FI)
Version:	1.2
Categories:	convert, Filter

Input name	Source	Description
table1	arrayCGHanalysis-chr7-segmented .GTSgain 19.146	CSV table 1. The table is referred to as 'table1' in the SQL query.
table2	arrayCGHanalysis-chr8-segmented .GTSgain 19.150	CSV table 2. The table is referred to as 'table2' in the SQL query.
table3	arrayCGHanalysis-chr9-segmented .GTSgain 19.154	CSV table 3. The table is referred to as 'table3' in the SQL query.
table4	arrayCGHanalysis-chr10 -segmented.GTSgain 19.74	CSV table 4. The table is referred to as 'table4' in the SQL query.
table5	arrayCGHanalysis-chr11 -segmented.GTSgain 19.78	CSV table 5. The table is referred to as 'table5' in the SQL query.
table6	arrayCGHanalysis-chr12 -segmented.GTSgain 19.82	CSV table 6. The table is referred to as 'table6' in the SQL query.

Parameter name	Value	Description
numIndices		Comma-separated list of index counts for input tables. All indices are single-column indices, running from column 1 to N, where N is retrieved from this parameter. If empty, the default number (1) is used. For example, ",2,,2" sets two indices for table2 and table4 and one index for the rest.

Continued on next page...

Parameter name	Value	Description
query	SELECT * FROM table1 UNION SELECT * FROM table2 UNION SELECT * FROM table3 UNION SELECT * FROM table4 UNION SELECT * FROM table5 UNION SELECT * FROM table6	SQL query. Either this parameter or the query input must be provided, but not both.

19.221 arrayCGHanalysis-unionSegsL (TableQuery)

Execute an SQL query on input relations and create a result table. TableQuery uses HSQLDB (Hyperthreaded Structured Query Language Database [25]) for executing the query. Consequently, the syntax of the query is defined by HSQLDB.

Author: Kristian Ovaska (Kristian.Ovaska@Helsinki.FI)
Version: 1.2
Categories: convert, Filter

Input name	Source	Description
table1	arrayCGHanalysis-chr1-segmented .GTSloss 19.70	CSV table 1. The table is referred to as 'table1' in the SQL query.
table2	arrayCGHanalysis-chr2-segmented .GTSloss 19.114	CSV table 2. The table is referred to as 'table2' in the SQL query.
table3	arrayCGHanalysis-chr3-segmented .GTSloss 19.130	CSV table 3. The table is referred to as 'table3' in the SQL query.
table4	arrayCGHanalysis-chr4-segmented .GTSloss 19.134	CSV table 4. The table is referred to as 'table4' in the SQL query.
table5	arrayCGHanalysis-chr5-segmented .GTSloss 19.138	CSV table 5. The table is referred to as 'table5' in the SQL query.
table6	arrayCGHanalysis-chr6-segmented .GTSloss 19.142	CSV table 6. The table is referred to as 'table6' in the SQL query.
table7	arrayCGHanalysis-chr7-segmented .GTSloss 19.146	CSV table 7. The table is referred to as 'table7' in the SQL query.
table8	arrayCGHanalysis-chr8-segmented .GTSloss 19.150	CSV table 8. The table is referred to as 'table8' in the SQL query.
table9	arrayCGHanalysis-chr9-segmented .GTSloss 19.154	CSV table 9. The table is referred to as 'table9' in the SQL query.
table10	arrayCGHanalysis-chr10 -segmented.GTSloss 19.74	CSV table 10. The table is referred to as 'table10' in the SQL query.
table11	arrayCGHanalysis-chr11 -segmented.GTSloss 19.78	CSV table 11. The table is referred to as 'table11' in the SQL query.
table12	arrayCGHanalysis-chr12 -segmented.GTSloss 19.82	CSV table 12. The table is referred to as 'table12' in the SQL query.

Parameter name	Value	Description
numIndices		Comma-separated list of index counts for input tables. All indices are single-column indices, running from column 1 to N, where N is retrieved from this parameter. If empty, the default number (1) is used. For example, ",2,,2" sets two indices for table2 and table4 and one index for the rest.
query	SELECT * FROM table1 UNION SELECT * FROM table2 UNION SELECT * FROM table3 UNION SELECT * FROM table4 UNION SELECT * FROM table5 UNION SELECT * FROM table6 UNION SELECT * FROM table7 UNION SELECT * FROM table8 UNION SELECT * FROM table9 UNION SELECT * FROM table10 UNION SELECT * FROM table11 UNION SELECT * FROM table12	SQL query. Either this parameter or the query input must be provided, but not both.

19.222 arrayCGHanalysis-unionSegsL2 (TableQuery)

Execute an SQL query on input relations and create a result table. TableQuery uses HSQLDB (Hyperthreaded Structured Query Language Database [25]) for executing the query. Consequently, the syntax of the query is defined by HSQLDB.

Author: Kristian Ovaska (Kristian.Ovaska@Helsinki.FI)
Version: 1.2
Categories: convert, Filter

Input name	Source	Description
table1	arrayCGHanalysis-chr13 -segmented.GTSloss 19.86	CSV table 1. The table is referred to as 'table1' in the SQL query.
table2	arrayCGHanalysis-chr14 -segmented.GTSloss 19.90	CSV table 2. The table is referred to as 'table2' in the SQL query.
table3	arrayCGHanalysis-chr15 -segmented.GTSloss 19.94	CSV table 3. The table is referred to as 'table3' in the SQL query.
table4	arrayCGHanalysis-chr16 -segmented.GTSloss 19.98	CSV table 4. The table is referred to as 'table4' in the SQL query.
table5	arrayCGHanalysis-chr17 -segmented.GTSloss 19.102	CSV table 5. The table is referred to as 'table5' in the SQL query.
table6	arrayCGHanalysis-chr18 -segmented.GTSloss 19.106	CSV table 6. The table is referred to as 'table6' in the SQL query.
table7	arrayCGHanalysis-chr19 -segmented.GTSloss 19.110	CSV table 7. The table is referred to as 'table7' in the SQL query.
table8	arrayCGHanalysis-chr20 -segmented.GTSloss 19.118	CSV table 8. The table is referred to as 'table8' in the SQL query.
table9	arrayCGHanalysis-chr21 -segmented.GTSloss 19.122	CSV table 9. The table is referred to as 'table9' in the SQL query.
table10	arrayCGHanalysis-chr22 -segmented.GTSloss 19.126	CSV table 10. The table is referred to as 'table10' in the SQL query.

Parameter name	Value	Description
numIndices		Comma-separated list of index counts for input tables. All indices are single-column indices, running from column 1 to N, where N is retrieved from this parameter. If empty, the default number (1) is used. For example, ",2,,2" sets two indices for table2 and table4 and one index for the rest.
query	SELECT * FROM table1 UNION SELECT * FROM table2 UNION SELECT * FROM table3 UNION SELECT * FROM table4 UNION SELECT * FROM table5 UNION SELECT * FROM table6 UNION SELECT * FROM table7 UNION SELECT * FROM table8 UNION SELECT * FROM table9 UNION SELECT * FROM table10	SQL query. Either this parameter or the query input must be provided, but not both.

19.223 arrayCGHexonIntGain (ACGHexonIntegration)

Integrates array CGH results with transcript expression intensities for amplified regions.

Input name	Source
GTSsegm	arrayCGHanalysis.segmentedVals 19.43
gtsOutput	arrayCGHanalysis.loclist 19.43
tholds	arrayCGHanalysis.tholds 19.43

Parameter name	Value
gain	true

19.224 arrayCGHexonIntGain-AnnotProbeGenes (NextGene)

Finds the near by genes, exons, or transcripts for the given loci. The search for the closest element of interest is restricted into a window that expands the locus independently into both directions along the DNA strand. An element is assigned to the given locus if (assuming it is the nearest or all elements within the window are accepted) and only if it is overlapping with $[p-bpBefore, p+bpAfter]$ at forward strand or the similarly constructed site at the backward strand. The overlap refers to the whole element if signDist is false and to the beginning of the element if it is true. The information about the element loci is fetched from Ensembl [12] database.

Author: Marko Laakso (Marko.Laakso@Helsinki.FI)
Version: 1.5
Categories: Annotation, Short-read Sequencing, SNP

Input name	Source	Description
sourceKeys	arrayCGHexonIntGain-ProbeIDs .table 19.227	Locations of interest
connection	ensembl.in 19.273	Database connection can be defined using this file. The definition of parameters: database.url, database.timeout, database.recycle, and database.driver can be found from the documentation of Korvasieni.

Parameter name	Value	Description
baseColumn	loci	Name of the base pair loci column in input.
bothStrands	false	Take the nearest separately for both DNA strands. The same nextDist limit applies to both strands that is the strand with the closest gene determines the distance limit.
bpAfter	1	Number of base pairs that are looked after the given loci
bpBefore	0	Number of base pairs that are looked before the given loci
chrColumn	chr	Name of the chromosome column in input if the base column does not include this information.
echoColumns		A comma separated list of column names for the columns that will be copied to the output.
elementType	gene	Type of elements of interest (gene, exon, or transcript)
idColumn	ID	Name of the identifier column in input.
locationDef	average	Definition of the reference point if the input is in region format (chr:start-end). Options are: start=start base pair, average=(start+end)/2, and end=end base pair.
nearestOnly	false	Take only that gene that is the closest for the given locus
nextDist	-1000	If this value is greater than zero then the nearest gene is reported separately for both directions along the strand. The value determines the maximum number of base pairs that are allowed after the nearest gene to accept the second hit from the other side of the site or those from the complement strand (see bothStrands).
signDist	false	True means that the distance is gene.start-loci and false means that it is max(0, gene.start-loci, loci-gene.end).

19.225 arrayCGHexonIntGain-ExonAnnot (INPUT)

Sample ID mappings between array CGH and exon data.

Category: Internal

Parameter name	Value	Description
path	/gpfs/homes/mlaakso/asserSVN/ andurilCaseStudy/csv/ ACGHexonAnnot.csv	Path (filename) of the input resource.

19.226 arrayCGHexonIntGain-GTSoutputG (INPUT)

Filter script for segmented array CGH data (gain regions) from GTS algorithm.

Category: Internal

Parameter name	Value	Description
path	/gpfs/homes/mlaakso/asserSVN/ andurilCaseStudy/r/ ExonIntegrationG.r	Path (filename) of the input resource.

19.227 arrayCGHexonIntGain-ProbeIDs (REvaluate)

This component produces some outputs by executing an R [20] script with the given input data. The exact functionality of this component depends on the given script.

The component is also capable of making branch decisions on Anduril workflow. Two alternative options (*altCase* and *defCase*) are provided.

Authors: Marko Laakso (Marko.Laakso@Helsinki.FI), Erkka Valo (erkka.valo@tkk.fi)
Version: 2.2
Categories: External, Latex, Network Control

Input name	Source	Description
script	arrayCGHexonIntGain-GTS outputG.in 19.226	Data transformation
table1	arrayCGHanalysis-segmentedVals .stdout 19.211	Data matrix 1 (data.frame/file table1)
table2	arrayCGHexonIntGain-ExonA nnot.in 19.225	Data matrix 2 (data.frame/file table2)
table3	arrayCGHexonIntGain-gainsList .table 19.232	Data matrix 3 (data.frame/file table3)
table4	arrayCGHanalysis-chr1-segmented .tholds 19.70	Data matrix 4 (data.frame/file table4)

Parameter name	Value	Description
asConnection	false	If true, assign file connections (class file) to the inputs to variables of the same name. This is useful e.g. if you want to read large data files row by row.
param1		Value of this parameter will be assigned to param1 variable
param10		Value of this parameter will be assigned to param10 variable
param2		Value of this parameter will be assigned to param2 variable
param3		Value of this parameter will be assigned to param3 variable
param4		Value of this parameter will be assigned to param4 variable
param5		Value of this parameter will be assigned to param5 variable
param6		Value of this parameter will be assigned to param6 variable
param7		Value of this parameter will be assigned to param7 variable
param8		Value of this parameter will be assigned to param8 variable
param9		Value of this parameter will be assigned to param9 variable

19.228 arrayCGHexonIntGain-assocExpand (ExpandCollapse)

Converts between the two possible representations of a relations with multivalued columns. By default this component splits rows with multivalued (comma separated lists) columns into multiple rows each representing a single value. The input may contain several columns with comma separated values and all permutations of column values are shown as individual single-valued rows. The alternative mode recovers the original comma separated values from the expanded relations. This is performed by joining values of the their list columns into comma separated lists.

Author: Marko Laakso (Marko.Laakso@Helsinki.FI)
Version: 1.3
Category: convert

Input name	Source	Description
relation	arrayCGHexonIntGain-match Annot.table 19.237	The mandatory input relation

Parameter name	Value	Description
delim	,	Value delimiter between the list column values. Special characters can be encoded as specified in fi.helsinki.ltdk.csbl.asser.ArgumentEncoding.
expand	false	Action mode that is expand (true) or collapse (false)
listCols	ID,chr,loci	A comma separated list of column names of input (expand) or output (collapse) columns that may contain delim separated values. The asterisk refers to every column of the input relation.

19.229 arrayCGHexonIntGain-expr (INPUT)

Transcript expression matrix.

Category: Internal

Parameter name	Value	Description
path	/gpfs/homes/mlaakso/asserSVN/ andurilCaseStudy/data/exon-normalized/Expression-Exon/ LBL_HuEx-1_0-st-v2/Level_2/ GeneExpression_v56	Path (filename) of the input resource.

19.230 arrayCGHexonIntGain-exprAnnot (INPUT)

Transcript expression annotations.

Category: Internal

Parameter name	Value	Description
path	/gpfs/homes/mlaakso/asserSVN/ andurilCaseStudy/data/exon-normalized/Expression-Exon/ LBL_HuEx-1_0-st-v2/Level_2/ TranscriptExprAnnotation_v56	Path (filename) of the input resource.

19.231 arrayCGHexonIntGain-exprMat (REvaluate)

This component produces some outputs by executing an R [20] script with the given input data. The exact functionality of this component depends on the given script.

The component is also capable of making branch decisions on Anduril workflow. Two alternative options (*altCase* and *defCase*) are provided.

Authors: Marko Laakso (Marko.Laakso@Helsinki.FI), Erkka Valo (erkka.valo@tkk.fi)
Version: 2.2
Categories: External, Latex, Network Control

Input name	Source	Description
script	arrayCGHexonIntGain-script2 .in 19.242	Data transformation
table1	arrayCGHexonIntGain-unDuplic .table 19.245	Data matrix 1 (data.frame/file table1)
table2	arrayCGHexonIntGain-expr .in 19.229	Data matrix 2 (data.frame/file table2)

Parameter name	Value	Description
asConnection	false	If true, assign file connections (class file) to the inputs to variables of the same name. This is useful e.g. if you want to read large data files row by row.
param1		Value of this parameter will be assigned to param1 variable
param10		Value of this parameter will be assigned to param10 variable
param2		Value of this parameter will be assigned to param2 variable
param3		Value of this parameter will be assigned to param3 variable
param4		Value of this parameter will be assigned to param4 variable
param5		Value of this parameter will be assigned to param5 variable
param6		Value of this parameter will be assigned to param6 variable
param7		Value of this parameter will be assigned to param7 variable
param8		Value of this parameter will be assigned to param8 variable
param9		Value of this parameter will be assigned to param9 variable

19.232 arrayCGHexonIntGain-gainsList (TableQuery)

Execute an SQL query on input relations and create a result table. TableQuery uses HSQLDB (Hyperthreaded Structured Query Language Database [25]) for executing the query. Consequently, the syntax of the query is defined by HSQLDB.

Author: Kristian Ovaska (Kristian.Ovaska@Helsinki.FI)
Version: 1.2
Categories: convert, Filter

Input name	Source	Description
table1	arrayCGHanalysis-loclist .csv 19.176	CSV table 1. The table is referred to as 'table1' in the SQL query.

Parameter name	Value	Description
numIndices		Comma-separated list of index counts for input tables. All indices are single-column indices, running from column 1 to N, where N is retrieved from this parameter. If empty, the default number (1) is used. For example, ",2,,2" sets two indices for table2 and table4 and one index for the rest.
query	SELECT SUBSTR("gts.chr", 4) AS "gts.chr", "gts.pos" FROM table1	SQL query. Either this parameter or the query input must be provided, but not both.

19.233 arrayCGHexonIntGain-intGreat (ExpExpIntegration)

ExpExpIntegration component enables the integration of gene/transcript/other expression data with binary explanatory data derived in some way (eg. ACGH). The input matrices are two N x M matrices that must have their columns (samples) and rows in the same order. Each row shall in the exprMatrix has the gene, transcript or other such expression values samplewise. The labelMatrix has must have a vector 0 and 1 on each row. This represents a partition of the samples rowwise.

The output of the component contains a score ("weight") for each gene shared by the explanatory and expression data. Genes in expression data have output weights as NA. The alpha value is the result of permutation test denoting the probability that H0: "Large weight is due to random event" is erroneously rejected. The weight is calculated for each row such that that the vector of the labelMatrix is used to partition the values of exprMatrix into two groups. Then, the mean of the expression values of the group labeled with 1 is subtracted from the mean of the other group. This is divided by the sum of the standard deviations of these two groups to obtain final weight.

This component implements the data integration method from Hautaniemi et al. [11]. Please refer to this article for a detailed description of the method, and an extended description of the inputs.

Author: Riku Louhimo (Riku.Louhimo@Helsinki.FI)
Version: 1.0
Category: Integration

Input name	Source	Description
labelMatrix	arrayCGHexonIntGain-labelMat .table 19.236	The binary explanatory values.
exprMatrix	arrayCGHexonIntGain-exprMat .table 19.231	The samplewise expression values.

Parameter name	Value	Description
exprNALimit	0.1	Defines the percentage of missing measurements in the expression matrix we are willing to allow.
gainData	true	Defines the which type of CNV data is being used.
idColumn	1	Defines the idColumn. The output idColumn is the column name of idColumn in the label matrix.
nroOfperms	1000	Defines the number of permutations applied during permutation testing.

19.234 arrayCGHexonIntGain-integrated (CSVCleaner)

CSVCleaner can be used to clean up Anduril outputs and to make them more suitable for other applications such as spreadsheets or text editors. The component is capable of removing file headers, quotations, and unused columns. Can be used to reorder and rename columns and their delimiters. Missing value symbols may be defined independently for the input and output.

Author: Marko Laakso (Marko.Laakso@Helsinki.FI)
Version: 1.6
Category: convert

Input name	Source	Description
original	arrayCGHexonIntGain-integrated ExonACGH.bioAnnotation 19.235	Input file to be cleaned

Parameter name	Value	Description
columns	.GeneName,Gene,.DNABand ,weight,alpha	Comma separated list of column selections for the output. An asterisk (*) may be used for all columns.
delimIn	\t	Column delimiter for the input
delimSymbol	\t	Column delimiter for the output
naIn	NA	Missing value symbol for the input
naSymbol	NA	Missing value symbol for the output
rename	.GeneName=GeneName, .DNABand=band	Comma separated list of column renaming rules (oldname=newname)
rowSkip	0	Skip this many lines from the input before reading the tabular.
skipQuota		Comma separated list of output columns names that should not have quotation marks. An asterisk (*) may be used for all columns.

19.235 arrayCGHexonIntGain-integratedExonACGH (KorvasieniAnnotator)

Korvasieni is an Ensembl [12] based converter for database identifiers and it can be used to convert genome location, gene, transcript, and translation identifiers across multiple biological databases. The selected `keyColumn` may contain entries with comma separated lists of values in which case `isListKey` parameter may be used to obtain each value separately and to process them as they were given in consecutive lines.

Author: Marko Laakso (Marko.Laakso@Helsinki.FI)
Version: 1.7
Categories: Annotation, GO

Input name	Source	Description
sourceKeys	arrayCGHexonIntGain-outputList .table 19.238	A list of source database keys.

Continued on next page...

Input name	Source	Description
connection	ensembl.in 19.273	Database connection can be defined using this file. The definition of parameters: database.url, database.user, database.password, database.timeout, database.recycle, and database.driver can be found from the documentation of Korvasieni.

Parameter name	Value	Description
echoColumns	Gene,weight,alpha	A comma separated list of column names for the columns that will be copied to the output. An asterisk (*) can be used to denote all columns execpt the keyColumn.
inputDB	.GeneId	Type of input keys. This must be a database supported by Korvasieni. If the parameter is omitted, the component tries to derive the database from the type of geneID. If this is not possible, an error is returned. You may define three columns in form of chromosome:start-end in case the inputDB is .DNARegion. This format provides a comfortable compatibility with DNARegion datatype.
inputType	Gene	Ensembl object type for the input keys (Any, Gene, Transcript, Translation)
isListKey	false	Enables the automatic value splits for the comma separated key column
keyColumn	Gene	Name of the key column withing sourceKeys file or an empty string for the first column
maxHits	100000	Maximum number of target identifiers for a single source identifier
rename		Comma separated list of column renaming rules (oldname=newname)
skipLevel	never	Skip result rows if the source identifier is unknown or target identifiers are not available. Possible values are: never (no filtering), source (skip if the source ID is unknown), target (skip if no target IDs are found), any (skip if any of the target IDs is missing).
targetDB	.GeneName,.DNABand,GO	Comma-separated list of annotation types. Possible values are all databases supported by Korvasieni.
unique	false	This flag can be turned on in order to eliminate duplicate annotations.

19.236 arrayCGHexonIntGain-labelMat (REvaluate)

This component produces some outputs by executing an R [20] script with the given input data. The exact functionality of this component depends on the given script.

The component is also capable of making branch decisions on Anduril workflow. Two alternative options (*altCase* and *defCase*) are provided.

Authors:	Marko Laakso (Marko.Laakso@Helsinki.FI), Erkka Valo (erkka.valo@tkk.fi)
Version:	2.2
Categories:	External, Latex, Network Control

Input name	Source	Description
script	arrayCGHexonIntGain-script .in 19.241	Data transformation
table1	arrayCGHexonIntGain-unDuplic .table 19.245	Data matrix 1 (data.frame/file table1)
table2	arrayCGHexonIntGain-expr .in 19.229	Data matrix 2 (data.frame/file table2)

Parameter name	Value	Description
asConnection	false	If true, assign file connections (class file) to the inputs to variables of the same name. This is useful e.g. if you want to read large data files row by row.
param1		Value of this parameter will be assigned to param1 variable
param10		Value of this parameter will be assigned to param10 variable
param2		Value of this parameter will be assigned to param2 variable
param3		Value of this parameter will be assigned to param3 variable
param4		Value of this parameter will be assigned to param4 variable
param5		Value of this parameter will be assigned to param5 variable
param6		Value of this parameter will be assigned to param6 variable
param7		Value of this parameter will be assigned to param7 variable
param8		Value of this parameter will be assigned to param8 variable
param9		Value of this parameter will be assigned to param9 variable

19.237 arrayCGHexonIntGain-matchAnnot (TableQuery)

Execute an SQL query on input relations and create a result table. TableQuery uses HSQLDB (Hyper-threaded Structured Query Language Database [25]) for executing the query. Consequently, the syntax of the query is defined by HSQLDB.

Author: Kristian Ovaska (Kristian.Ovaska@Helsinki.FI)
Version: 1.2
Categories: convert, Filter

Input name	Source	Description
table1	arrayCGHexonIntGain-ProbeIDs .table 19.227	CSV table 1. The table is referred to as 'table1' in the SQL query.
table2	arrayCGHexonIntGain-AnnotP robeGenes.bioAnnotation 19.224	CSV table 2. The table is referred to as 'table2' in the SQL query.

Parameter name	Value	Description
numIndices		Comma-separated list of index counts for input tables. All indices are single-column indices, running from column 1 to N, where N is retrieved from this parameter. If empty, the default number (1) is used. For example, ",2,,2" sets two indices for table2 and table4 and one index for the rest.
query	SELECT ".GeneId", table1.* FROM table1 LEFT OUTER JOIN table2 ON (table2."ID" = table1."ID")	SQL query. Either this parameter or the query input must be provided, but not both.

19.238 arrayCGHexonIntGain-outputList (TableQuery)

Process output from copy number analysis and exon integration together.

Author: Kristian Ovaska (Kristian.Ovaska@Helsinki.FI)
Version: 1.2
Categories: convert, Filter

Input name	Source	Description
table1	arrayCGHexonIntGain-intGreat.V alues 19.233	CSV table 1. The table is referred to as 'table1' in the SQL query.

Parameter name	Value	Description
numIndices		Comma-separated list of index counts for input tables. All indices are single-column indices, running from column 1 to N, where N is retrieved from this parameter. If empty, the default number (1) is used. For example, ",2,,2" sets two indices for table2 and table4 and one index for the rest.
query	SELECT "GeneID" AS "Gene", "weight", "alpha" FROM table1	SQL query. Either this parameter or the query input must be provided, but not both.

19.239 arrayCGHexonIntGain-refs (INPUT)

(Technical documentation) Imports an external input file or directory to the pipeline. This is the only way external files are imported. File path is given with the parameter "path".

Category: Internal

Parameter name	Value	Description
path	/gpfs/homes/mlaakso/asserSVN/ andurilCaseStudy/csv/ ACGHexonRefs.csv	Path (filename) of the input resource.

Continued on next page...

Parameter name	Value	Description

19.240 arrayCGHexonIntGain-report (CSV2Latex)

This component converts a tabular CSV file into a printable LaTeX table.

Author: Marko Laakso (Marko.Laakso@Helsinki.FI)
Version: 2.1
Categories: convert, Latex

Input name	Source	Description
tabledata	arrayCGHexonIntGain-sorted Integrated.table 19.243	Table content
refs	arrayCGHexonIntGain-refs .in 19.239	Reference rules for the hyperlinks

Parameter name	Value	Description
attach	false	Include the original data as an attachment
caption	Top 100 integration results of gene level transcript expression values with copy number aberration regions. The weight indicates the correlation in gene level transcript expression between aberrated and non-aberrated samples.	Caption text for the table.
colFormat	lp{2.5cm}p{2cm}p{2cm}p{2cm}	LaTeX tabular format for the columns. Default produces a centered layout for all columns.
columns	GeneName,Gene,band,weight, alpha	Comma separated list of column selections for the output. The empty default will use all columns.
countRows	false	Include a row count to the table caption.
dropMissing	true	This flag can be turned off in order to generate links with missing texts. Link text are substituted with target identifiers.
listCols	band	Comma separated list of column names. Columns of this list may contain several values separated with commas and the delimiters will be replaced with spaces.
numberFormat	weight=#0.00000,alpha=#0. 00000	A comma separated list of decimal formats for the columns. Each entry consists of the column name and the Java DecimalFormal pattern separated with equal sign. For example, rounding to three decimals can be done like: myColumn=#0.000
pageBreak	false	Use clear page after the table.
rename		Comma separated list of column renaming rules (oldname=newname). New names are used in table header but they do not affect the other behaviour of this component.
ruler	{}	Latex command for the row separating rulers
section	Array CGH: integration results for CN gain regions and expression	Section title for the table container or an empty string if no section should be generated.
sectionType	section	Type of LaTeX section: usually one of: section, subsection, or subsubsection. No section statement is written if section title is empty.
skipEmpty	false	This flag can be used to replace empty tables with a simple LaTeX comment.

19.241 arrayCGHexonIntGain-script (INPUT)

R script that compares two matrices and orders them column- and rowwise in the same order.

Category: Internal

Parameter name	Value	Description
path	/gpfs/homes/mlaakso/asserSVN/ andurilCaseStudy/r/ ACGHcheckInput.r	Path (filename) of the input resource.

19.242 arrayCGHexonIntGain-script2 (INPUT)

R script that compares two matrices and orders them column- and rowwise in the same order.

Category: Internal

Parameter name	Value	Description
path	/gpfs/homes/mlaakso/asserSVN/andurilCaseStudy/r/ACGHcheckInput2.r	Path (filename) of the input resource.

19.243 arrayCGHexonIntGain-sortedIntegrated (TableQuery)

Execute an SQL query on input relations and create a result table. TableQuery uses HSQLDB (Hyperthreaded Structured Query Language Database [25]) for executing the query. Consequently, the syntax of the query is defined by HSQLDB.

Author: Kristian Ovaska (Kristian.Ovaska@Helsinki.FI)
Version: 1.2
Categories: convert, Filter

Input name	Source	Description
table1	arrayCGHexonIntGain-integrated.table 19.234	CSV table 1. The table is referred to as 'table1' in the SQL query.

Parameter name	Value	Description
numIndices		Comma-separated list of index counts for input tables. All indices are single-column indices, running from column 1 to N, where N is retrieved from this parameter. If empty, the default number (1) is used. For example, ",2,,2" sets two indices for table2 and table4 and one index for the rest.
query	SELECT * FROM table1 WHERE "alpha" IS NOT NULL AND "weight" > 0 ORDER BY "alpha","weight" DESC LIMIT 100	SQL query. Either this parameter or the query input must be provided, but not both.

19.244 arrayCGHexonIntGain-transAnnot (TableQuery)

Execute an SQL query on input relations and create a result table. TableQuery uses HSQLDB (Hyperthreaded Structured Query Language Database [25]) for executing the query. Consequently, the syntax of the query is defined by HSQLDB.

Author: Kristian Ovaska (Kristian.Ovaska@Helsinki.FI)
Version: 1.2
Categories: convert, Filter

Input name	Source	Description
table1	arrayCGHexonIntGain-assocExpand.relation 19.228	CSV table 1. The table is referred to as 'table1' in the SQL query.
table2	arrayCGHexonIntGain-expr.in 19.229	CSV table 2. The table is referred to as 'table2' in the SQL query.

Parameter name	Value	Description
numIndices		Comma-separated list of index counts for input tables. All indices are single-column indices, running from column 1 to N, where N is retrieved from this parameter. If empty, the default number (1) is used. For example, ",2,,2" sets two indices for table2 and table4 and one index for the rest.

Continued on next page...

Parameter name	Value	Description
query	SELECT "GeneID", table1.* FROM table2 LEFT OUTER JOIN table1 ON (table2. "GeneID" = table1.".GeneId")	SQL query. Either this parameter or the query input must be provided, but not both.

19.245 arrayCGHexonIntGain-unDuplic (RowJoin)

(Technical documentation) Join duplicate id rows from a numeric matrix based on the frequency of some given value or impute row wise based on column wise mean value of matrix rows with identical IDs.

Author: Riku Louhimo (Riku.Louhimo@Helsinki.FI)
Version: 0.1
Category: Convert

Input name	Source	Description
matrix	arrayCGHexonIntGain-trans Annot.table 19.244	Matrix that needs rows joined.

Parameter name	Value	Description
idColumn	1	Name of the column that contains IDs. Empty defaults to the first column of the matrix.
impute	false	Collapse row values for rows with identical IDs. The collapsed values are column wise means. NA values are treated as zeros when calculating means.
matchMe	1	Defines which feature is used to calculate the frequency. Ignored if impute=true.
startColumn	6	Defines start of data in the matrix if the input contains more columns than the data and ID.

19.246 arrayCGHexonIntLoss (ACGHexonIntegration)

Integrates array CGH results with transcript expression intensities for lossed regions.

Input name	Source
GTSsegm	arrayCGHanalysis.segmentedVals 19.43
gtsOutput	arrayCGHanalysis.loclist 19.43
tholds	arrayCGHanalysis.tholds 19.43

Parameter name	Value
gain	false

19.247 arrayCGHexonIntLoss-AnnotProbeGenes (NextGene)

Finds the near by genes, exons, or transcripts for the given loci. The search for the closest element of interest is restricted into a window that expands the locus independently into both directions along the DNA strand. An element is assigned to the given locus if (assuming it is the nearest or all elements within the window are accepted) and only if it is overlapping with $[p-bpBefore, p+bpAfter]$ at forward strand or the similarly constructed site at the backward strand. The overlap refers to the whole element if signDist is false and to the beginning of the element if it is true. The information about the element loci is fetched from Ensembl [12] database.

Author: Marko Laakso (Marko.Laakso@Helsinki.FI)
Version: 1.5
Categories: Annotation, Short-read Sequencing, SNP

Input name	Source	Description
sourceKeys	arrayCGHexonIntLoss-ProbeIDs .table 19.250	Locations of interest
connection	ensembl.in 19.273	Database connection can be defined using this file. The definition of parameters: database.url, database.timeout, database.recycle, and database.driver can be found from the documentation of Korvasieni.

Parameter name	Value	Description
baseColumn	loci	Name of the base pair loci column in input.
bothStrands	false	Take the nearest separately for both DNA strands. The same nextDist limit applies to both strands that is the strand with the closest gene determines the distance limit.
bpAfter	1	Number of base pairs that are looked after the given loci
bpBefore	0	Number of base pairs that are looked before the given loci
chrColumn	chr	Name of the chromosome column in input if the base column does not include this information.
echoColumns		A comma separated list of column names for the columns that will be copied to the output.
elementType	gene	Type of elements of interest (gene, exon, or transcript)
idColumn	ID	Name of the identifier column in input.
locationDef	average	Definition of the reference point if the input is in region format (chr:start-end). Options are: start=start base pair, average=(start+end)/2, and end=end base pair.
nearestOnly	false	Take only that gene that is the closest for the given locus
nextDist	-1000	If this value is greater than zero then the nearest gene is reported separately for both directions along the strand. The value determines the maximum number of base pairs that are allowed after the nearest gene to accept the second hit from the other side of the site or those from the complement strand (see bothStrands).
signDist	false	True means that the distance is gene.start-loci and false means that it is max(0, gene.start-loci, loci-gene.end).

19.248 arrayCGHexonIntLoss-ExonAnnot (INPUT)

Sample ID mappings between array CGH and exon data.

Category: Internal

Parameter name	Value	Description
path	/gpfs/homes/mlaakso/asserSVN/ anduril CaseStudy/csv/ ACGHexonAnnot.csv	Path (filename) of the input resource.

19.249 arrayCGHexonIntLoss-GTSoutputL (INPUT)

Filter script for segmented array CGH data (loss regions) from GTS algorithm.

Category: Internal

Parameter name	Value	Description
path	/gpfs/homes/mlaakso/asserSVN/ anduril CaseStudy/r/ ExonIntegrationL.r	Path (filename) of the input resource.

19.250 arrayCGHexonIntLoss-ProbeIDs (REvaluate)

This component produces some outputs by executing an R [20] script with the given input data. The exact functionality of this component depends on the given script.

The component is also capable of making branch decisions on Anduril workflow. Two alternative options (*altCase* and *defCase*) are provided.

Authors: Marko Laakso (Marko.Laakso@Helsinki.FI), Erkka Valo (erkka.valo@tkk.fi)
Version: 2.2
Categories: External, Latex, Network Control

Input name	Source	Description
script	arrayCGHexonIntLoss-GTS outputL.in 19.249	Data transformation
table1	arrayCGHanalysis-segmentedVals .stdout 19.211	Data matrix 1 (data.frame/file table1)
table2	arrayCGHexonIntLoss-ExonA nnot.in 19.248	Data matrix 2 (data.frame/file table2)
table3	arrayCGHexonIntLoss-gainsList .table 19.255	Data matrix 3 (data.frame/file table3)
table4	arrayCGHanalysis-chr1-segmented .tholds 19.70	Data matrix 4 (data.frame/file table4)

Parameter name	Value	Description
asConnection	false	If true, assign file connections (class file) to the inputs to variables of the same name. This is useful e.g. if you want to read large data files row by row.
param1		Value of this parameter will be assigned to param1 variable
param10		Value of this parameter will be assigned to param10 variable
param2		Value of this parameter will be assigned to param2 variable
param3		Value of this parameter will be assigned to param3 variable
param4		Value of this parameter will be assigned to param4 variable
param5		Value of this parameter will be assigned to param5 variable
param6		Value of this parameter will be assigned to param6 variable
param7		Value of this parameter will be assigned to param7 variable
param8		Value of this parameter will be assigned to param8 variable
param9		Value of this parameter will be assigned to param9 variable

19.251 arrayCGHexonIntLoss-assocExpand (ExpandCollapse)

Converts between the two possible representations of a relations with multivalued columns. By default this component splits rows with multivalued (comma separated lists) columns into multiple rows each representing a single value. The input may contain several columns with comma separated values and all permutations of column values are shown as individual single-valued rows. The alternative mode recovers the original comma separated values from the expanded relations. This is performed by joining values of the their list columns into comma separated lists.

Author: Marko Laakso (Marko.Laakso@Helsinki.FI)
Version: 1.3
Category: convert

Input name	Source	Description
relation	arrayCGHexonIntLoss-match Annot.table 19.260	The mandatory input relation

Parameter name	Value	Description
delim	,	Value delimiter between the list column values. Special characters can be encoded as specified in fi.helsinki.ltdk.csbl.asser.ArgumentEncoding.
expand	false	Action mode that is expand (true) or collapse (false)
listCols	ID,chr,loci	A comma separated list of column names of input (expand) or output (collapse) columns that may contain delim separated values. The asterisk refers to every column of the input relation.

19.252 arrayCGHexonIntLoss-expr (INPUT)

Transcript expression matrix.

Category: Internal

Parameter name	Value	Description
path	/gpfs/homes/mlaakso/asserSVN/andurilCaseStudy/data/exon-normalized/Expression-Exon/LBL_HuEx-1_0-st-v2/Level_2/GeneExpression_v56	Path (filename) of the input resource.

19.253 arrayCGHexonIntLoss-exprAnnot (INPUT)

Transcript expression annotations.

Category: Internal

Parameter name	Value	Description
path	/gpfs/homes/mlaakso/asserSVN/andurilCaseStudy/data/exon-normalized/Expression-Exon/LBL_HuEx-1_0-st-v2/Level_2/TranscriptExprAnnotation_v56	Path (filename) of the input resource.

19.254 arrayCGHexonIntLoss-exprMat (REvaluate)

This component produces some outputs by executing an R [20] script with the given input data. The exact functionality of this component depends on the given script.

The component is also capable of making branch decisions on Anduril workflow. Two alternative options (*altCase* and *defCase*) are provided.

Authors: Marko Laakso (Marko.Laakso@Helsinki.FI), Erkka Valo (erkka.valo@tkk.fi)
Version: 2.2
Categories: External, Latex, Network Control

Input name	Source	Description
script	arrayCGHexonIntLoss-script2 .in 19.265	Data transformation
table1	arrayCGHexonIntLoss-unDuplic .table 19.268	Data matrix 1 (data.frame/file table1)
table2	arrayCGHexonIntLoss-expr .in 19.252	Data matrix 2 (data.frame/file table2)

Parameter name	Value	Description
asConnection	false	If true, assign file connections (class file) to the inputs to variables of the same name. This is useful e.g. if you want to read large data files row by row.
param1		Value of this parameter will be assigned to param1 variable
param10		Value of this parameter will be assigned to param10 variable
param2		Value of this parameter will be assigned to param2 variable
param3		Value of this parameter will be assigned to param3 variable
param4		Value of this parameter will be assigned to param4 variable
param5		Value of this parameter will be assigned to param5 variable
param6		Value of this parameter will be assigned to param6 variable
param7		Value of this parameter will be assigned to param7 variable
param8		Value of this parameter will be assigned to param8 variable
param9		Value of this parameter will be assigned to param9 variable

19.255 arrayCGHexonIntLoss-gainsList (TableQuery)

Execute an SQL query on input relations and create a result table. TableQuery uses HSQLDB (Hyperthreaded Structured Query Language Database [25]) for executing the query. Consequently, the syntax of the query is defined by HSQLDB.

Author: Kristian Ovaska (Kristian.Ovaska@Helsinki.FI)
Version: 1.2
Categories: convert, Filter

Input name	Source	Description
table1	arrayCGHanalysis-loclist .csv 19.176	CSV table 1. The table is referred to as 'table1' in the SQL query.

Parameter name	Value	Description
numIndices		Comma-separated list of index counts for input tables. All indices are single-column indices, running from column 1 to N, where N is retrieved from this parameter. If empty, the default number (1) is used. For example, ",2,,2" sets two indices for table2 and table4 and one index for the rest.
query	SELECT SUBSTR("gts.chr", 4) AS "gts.chr", "gts.pos" FROM table1	SQL query. Either this parameter or the query input must be provided, but not both.

19.256 arrayCGHexonIntLoss-intGreat (ExpExpIntegration)

ExpExpIntegration component enables the integration of gene/transcript/other expression data with binary explanatory data derived in some way (eg. ACGH). The input matrices are two N x M matrices that must have their columns (samples) and rows in the same order. Each row shall in the exprMatrix has the gene, transcript or other such expression values samplewise. The labelMatrix has must have a vector 0 and 1 on each row. This represents a partition of the samples rowwise.

The output of the component contains a score ("weight") for each gene shared by the explanatory and expression data. Genes in expression data have output weights as NA. The alpha value is the result of permutation test denoting the probability that H0: "Large weight is due to random event" is erroneously rejected. The weight is calculated for each row such that that the vector of the labelMatrix is used to partition the values of exprMatrix into two groups. Then, the mean of the expression values of the group labeled with 1 is subtracted from the mean of the other group. This is divided by the sum of the standard deviations of these two groups to obtain final weight.

This component implements the data integration method from Hautaniemi et al. [11]. Please refer to this article for a detailed description of the method, and an extended description of the inputs.

Author: Riku Louhimo (Riku.Louhimo@Helsinki.FI)
Version: 1.0
Category: Integration

Input name	Source	Description
labelMatrix	arrayCGHexonIntLoss-labelMat .table 19.259	The binary explanatory values.
exprMatrix	arrayCGHexonIntLoss-exprMat .table 19.254	The samplewise expression values.

Parameter name	Value	Description
exprNALimit	0.1	Defines the percentage of missing measurements in the expression matrix we are willing to allow.
gainData	false	Defines the which type of CNV data is being used.
idColumn	1	Defines the idColumn. The output idColumn is the column name of idColumn in the label matrix.

Continued on next page...

Parameter name	Value	Description
nroOfperms	1000	Defines the number of permutations applied during permutation testing.

19.257 arrayCGHexonIntLoss-integrated (CSVCleaner)

CSVCleaner can be used to clean up Anduril outputs and to make them more suitable for other applications such as spreadsheets or text editors. The component is capable of removing file headers, quotations, and unused columns. Can be used to reorder and rename columns and their delimiters. Missing value symbols may be defined independently for the input and output.

Author: Marko Laakso (Marko.Laakso@Helsinki.FI)
Version: 1.6
Category: convert

Input name	Source	Description
original	arrayCGHexonIntLoss-integrated ExonACGH.bioAnnotation 19.258	Input file to be cleaned

Parameter name	Value	Description
columns	.GeneName,Gene,.DNABand ,weight,alpha	Comma separated list of column selections for the output. An asterisk (*) may be used for all columns.
delimIn	\t	Column delimiter for the input
delimSymbol	\t	Column delimiter for the output
naIn	NA	Missing value symbol for the input
naSymbol	NA	Missing value symbol for the output
rename	.GeneName=GeneName, .DNABand=band	Comma separated list of column renaming rules (oldname=newname)
rowSkip	0	Skip this many lines from the input before reading the tabular.
skipQuota		Comma separated list of output columns names that should not have quotation marks. An asterisk (*) may be used for all columns.

19.258 arrayCGHexonIntLoss-integratedExonACGH (KorvasieniAnnotator)

Korvasieni is an Ensembl [12] based converter for database identifiers and it can be used to convert genome location, gene, transcript, and translation identifiers across multiple biological databases. The selected `keyColumn` may contain entries with comma separated lists of values in which case `isListKey` parameter may be used to obtain each value separately and to process them as they were given in consecutive lines.

Author: Marko Laakso (Marko.Laakso@Helsinki.FI)
Version: 1.7
Categories: Annotation, GO

Input name	Source	Description
sourceKeys	arrayCGHexonIntLoss-outputList .table 19.261	A list of source database keys.
connection	ensembl.in 19.273	Database connection can be defined using this file. The definition of parameters: database.url, database.user, database.password, database.timeout, database.recycle, and database.driver can be found from the documentation of Korvasieni.

Parameter name	Value	Description
echoColumns	Gene,weight,alpha	A comma separated list of column names for the columns that will be copied to the output. An asterisk (*) can be used to denote all columns execpt the keyColumn.

Continued on next page...

Parameter name	Value	Description
inputDB	.GeneId	Type of input keys. This must be a database supported by Korvasieni. If the parameter is omitted, the component tries to derive the database from the type of geneID. If this is not possible, an error is returned. You may define three columns in form of chromosome:start-end in case the inputDB is .DNARegion. This format provides a comfortable compatibility with DNARegion datatype.
inputType	Gene	Ensembl object type for the input keys (Any, Gene, Transcript, Translation)
isListKey	false	Enables the automatic value splits for the comma separated key column
keyColumn	Gene	Name of the key column withing sourceKeys file or an empty string for the first column
maxHits	100000	Maximum number of target identifiers for a single source identifier
rename		Comma separated list of column renaming rules (oldname=newname)
skipLevel	never	Skip result rows if the source identifier is unknown or target identifiers are not available. Possible values are: never (no filtering), source (skip if the source ID is unknown), target (skip if no target IDs are found), any (skip if any of the target IDs is missing).
targetDB	.GeneName,.DNABand,GO	Comma-separated list of annotation types. Possible values are all databases supported by Korvasieni.
unique	false	This flag can be turned on in order to eliminate duplicate annotations.

19.259 arrayCGHexonIntLoss-labelMat (REvaluate)

This component produces some outputs by executing an R [20] script with the given input data. The exact functionality of this component depends on the given script.

The component is also capable of making branch decisions on Anduril workflow. Two alternative options (*altCase* and *defCase*) are provided.

Authors: Marko Laakso (Marko.Laakso@Helsinki.FI), Erkka Valo (erkka.valo@tkk.fi)
Version: 2.2
Categories: External, Latex, Network Control

Input name	Source	Description
script	arrayCGHexonIntLoss-script .in 19.264	Data transformation
table1	arrayCGHexonIntLoss-unDuplic .table 19.268	Data matrix 1 (data.frame/file table1)
table2	arrayCGHexonIntLoss-expr .in 19.252	Data matrix 2 (data.frame/file table2)

Parameter name	Value	Description
asConnection	false	If true, assign file connections (class file) to the inputs to variables of the same name. This is useful e.g. if you want to read large data files row by row.
param1		Value of this parameter will be assigned to param1 variable
param10		Value of this parameter will be assigned to param10 variable
param2		Value of this parameter will be assigned to param2 variable
param3		Value of this parameter will be assigned to param3 variable
param4		Value of this parameter will be assigned to param4 variable
param5		Value of this parameter will be assigned to param5 variable
param6		Value of this parameter will be assigned to param6 variable
param7		Value of this parameter will be assigned to param7 variable
param8		Value of this parameter will be assigned to param8 variable
param9		Value of this parameter will be assigned to param9 variable

19.260 arrayCGHexonIntLoss-matchAnnot (TableQuery)

Execute an SQL query on input relations and create a result table. TableQuery uses HSQLDB (Hyperthreaded Structured Query Language Database [25]) for executing the query. Consequently, the syntax of the query is defined by HSQLDB.

Author: Kristian Ovaska (Kristian.Ovaska@Helsinki.FI)
Version: 1.2
Categories: convert, Filter

Input name	Source	Description
table1	arrayCGHexonIntLoss-ProbeIDs .table 19.250	CSV table 1. The table is referred to as 'table1' in the SQL query.
table2	arrayCGHexonIntLoss-AnnotP robeGenes.bioAnnotation 19.247	CSV table 2. The table is referred to as 'table2' in the SQL query.

Parameter name	Value	Description
numIndices		Comma-separated list of index counts for input tables. All indices are single-column indices, running from column 1 to N, where N is retrieved from this parameter. If empty, the default number (1) is used. For example, ",2,,2" sets two indices for table2 and table4 and one index for the rest.
query	SELECT ".GeneId", table1.* FROM table1 LEFT OUTER JOIN table2 ON (table2."ID" = table1."ID")	SQL query. Either this parameter or the query input must be provided, but not both.

19.261 arrayCGHexonIntLoss-outputList (TableQuery)

Process output from copy number analysis and exon integration together.

Author:	Kristian Ovaska (Kristian.Ovaska@Helsinki.FI)
Version:	1.2
Categories:	convert, Filter

Input name	Source	Description
table1	arrayCGHexonIntLoss-intGreat.V alues 19.256	CSV table 1. The table is referred to as 'table1' in the SQL query.

Parameter name	Value	Description
numIndices		Comma-separated list of index counts for input tables. All indices are single-column indices, running from column 1 to N, where N is retrieved from this parameter. If empty, the default number (1) is used. For example, ",2,,2" sets two indices for table2 and table4 and one index for the rest.
query	SELECT "GeneID" AS "Gene", "weight", "alpha" FROM table1	SQL query. Either this parameter or the query input must be provided, but not both.

19.262 arrayCGHexonIntLoss-refs (INPUT)

(Technical documentation) Imports an external input file or directory to the pipeline. This is the only way external files are imported. File path is given with the parameter "path".

Category:	Internal

Parameter name	Value	Description
path	/gpfs/homes/mlaakso/asserSVN/ andurilCaseStudy/csv/ ACGHexonRefs.csv	Path (filename) of the input resource.

19.263 arrayCGHexonIntLoss-report (CSV2Latex)

This component converts a tabular CSV file into a printable LaTeX table.

Author:	Marko Laakso (Marko.Laakso@Helsinki.FI)
Version:	2.1
Categories:	convert, Latex

Input name	Source	Description
tabledata	arrayCGHexonIntLoss-sorted Integrated.table 19.266	Table content
refs	arrayCGHexonIntLoss-refs .in 19.262	Reference rules for the hyperlinks

Parameter name	Value	Description
attach	false	Include the original data as an attachment
caption	Top 100 integration results of gene level transcript expression values with copy number aberration regions. The weight indicates the correlation in gene level transcript expression between aberrated and non-aberrated samples.	Caption text for the table.
colFormat	lp{2.5cm}p{2cm}p{2cm}p{2cm}	LaTeX tabular format for the columns. Default produces a centered layout for all columns.
columns	GeneName,Gene,band,weight, alpha	Comma separated list of column selections for the output. The empty default will use all columns.
countRows	false	Include a row count to the table caption.
dropMissing	true	This flag can be turned off in order to generate links with missing texts. Link text are substituted with target identifiers.
listCols	band	Comma separated list of column names. Columns of this list may contain several values separated with commas and the delimiters will be replaced with spaces.
numberFormat	weight=#0.00000,alpha=#0. 00000	A comma separated list of decimal formats for the columns. Each entry consists of the column name and the Java DecimalFormal pattern separated with equal sign. For example, rounding to three decimals can be done like: myColumn=#0.000
pageBreak	false	Use clear page after the table.
rename		Comma separated list of column renaming rules (oldname=newname). New names are used in table header but they do not affect the other behaviour of this component.
ruler	{}	Latex command for the row separating rulers
section	Array CGH: integration results for CN loss regions and expression	Section title for the table container or an empty string if no section should be generated.
sectionType	section	Type of LaTeX section: usually one of: section, subsection, or subsubsection. No section statement is written if section title is empty.
skipEmpty	false	This flag can be used to replace empty tables with a simple LaTeX comment.

19.264 arrayCGHexonIntLoss-script (INPUT)

R script that compares two matrices and orders them column- and rowwise in the same order.

Category: Internal

Parameter name	Value	Description
path	/gpfs/homes/mlaakso/asserSVN/ andurilCaseStudy/r/ ACGHcheckInput.r	Path (filename) of the input resource.

19.265 arrayCGHexonIntLoss-script2 (INPUT)

R script that compares two matrices and orders them column- and rowwise in the same order.

Category: Internal

Parameter name	Value	Description
path	/gpfs/homes/mlaakso/asserSVN/ andurilCaseStudy/r/ ACGHcheckInput2.r	Path (filename) of the input resource.

19.266 arrayCGHexonIntLoss-sortedIntegrated (TableQuery)

Execute an SQL query on input relations and create a result table. TableQuery uses HSQLDB (Hyperthreaded Structured Query Language Database [25]) for executing the query. Consequently, the syntax of the query is defined by HSQLDB.

Author: Kristian Ovaska (Kristian.Ovaska@Helsinki.FI)
Version: 1.2
Categories: convert, Filter

Input name	Source	Description
table1	arrayCGHexonIntLoss-integrated .table 19.257	CSV table 1. The table is referred to as 'table1' in the SQL query.

Parameter name	Value	Description
numIndices		Comma-separated list of index counts for input tables. All indices are single-column indices, running from column 1 to N, where N is retrieved from this parameter. If empty, the default number (1) is used. For example, ",2,,2" sets two indices for table2 and table4 and one index for the rest.
query	SELECT * FROM table1 WHERE "alpha" IS NOT NULL AND "weight" > 0 ORDER BY "alpha","weight" DESC LIMIT 100	SQL query. Either this parameter or the query input must be provided, but not both.

19.267 arrayCGHexonIntLoss-transAnnot (TableQuery)

Execute an SQL query on input relations and create a result table. TableQuery uses HSQLDB (Hyperthreaded Structured Query Language Database [25]) for executing the query. Consequently, the syntax of the query is defined by HSQLDB.

Author: Kristian Ovaska (Kristian.Ovaska@Helsinki.FI)
Version: 1.2
Categories: convert, Filter

Input name	Source	Description
table1	arrayCGHexonIntLoss-assoc Expand.relation 19.251	CSV table 1. The table is referred to as 'table1' in the SQL query.
table2	arrayCGHexonIntLoss-expr .in 19.252	CSV table 2. The table is referred to as 'table2' in the SQL query.

Parameter name	Value	Description
numIndices		Comma-separated list of index counts for input tables. All indices are single-column indices, running from column 1 to N, where N is retrieved from this parameter. If empty, the default number (1) is used. For example, ",2,,2" sets two indices for table2 and table4 and one index for the rest.
query	SELECT "GeneID", table1.* FROM table2 LEFT OUTER JOIN table1 ON (table2. "GeneID" = table1.".GeneId")	SQL query. Either this parameter or the query input must be provided, but not both.

19.268 arrayCGHexonIntLoss-unDuplic (RowJoin)

(Technical documentation) Join duplicate id rows from a numeric matrix based on the frequency of some given value or impute row wise based on column wise mean value of matrix rows with identical IDs.

Author: Riku Louhimo (Riku.Louhimo@Helsinki.FI)
Version: 0.1
Category: Convert

Input name	Source	Description
matrix	arrayCGHexonIntLoss-trans Annot.table 19.267	Matrix that needs rows joined.

Parameter name	Value	Description
idColumn	1	Name of the column that contains IDs. Empty defaults to the first column of the matrix.
impute	false	Collapse row values for rows with identical IDs. The collapsed values are column wise means. NA values are treated as zeros when calculating means.
matchMe	1	Defines which feature is used to calculate the frequency. Ignored if impute=true.
startColumn	6	Defines start of data in the matrix if the input contains more columns than the data and ID.

19.269 arrayCGHsummary (LatexCombiner)

LatexCombiner joins the given subdocuments together into one LaTeX file.

Authors: Kristian Ovaska (Kristian.Ovaska@Helsinki.FI), Marko Laakso (Marko.Laakso@Helsinki.FI)
Version: 1.3
Category: Latex

Input name	Source	Description
latex1	arrayCGHanalysis-analysis .document 19.50	LaTeX fragment 1.
latex2	arrayCGHexonIntGain-report .report 19.240	LaTeX fragment 2.
latex3	arrayCGHexonIntLoss-report .report 19.263	LaTeX fragment 3.

Parameter name	Value	Description
pagebreak	false	Determines if the result document should start with a page break.
sectionTitle		If non-empty, a declaration of a new section with the given name is inserted to the beginning of the combined document. This is a convenience feature to make it easy to compile subsections into a section.
sectionType	section	Type of LaTeX section: usually one of section, subsection or subsubsection. No section statement is written if sectionTitle is empty.
strictBorders	true	Enables the flushing of all document elements before each fragment

19.270 cfgDescMicroarray (INPUT)

Standard descriptions for Anduril components

Category: Internal

Parameter name	Value	Description
path	/usr/share/anduril/trunk/ microarray/components/report-descriptions	Path (filename) of the input resource.

19.271 cfgDoc (LatexCombiner)

LatexCombiner joins the given subdocuments together into one LaTeX file.

Authors: Kristian Ovaska (Kristian.Ovaska@Helsinki.FI), Marko Laakso (Marko.Laakso@Helsinki.FI)
Version: 1.3
Category: Latex

Input name	Source	Description
latex1	cfgReport.report 19.272	LaTeX fragment 1.
latex2	rConfig.report 19.438	LaTeX fragment 2.
latex3	propertiesDoc.report 19.437	LaTeX fragment 3.

Parameter name	Value	Description
pagebreak	true	Determines if the result document should start with a page break.
sectionTitle		If non-empty, a declaration of a new section with the given name is inserted to the beginning of the combined document. This is a convenience feature to make it easy to compile subsections into a section.
sectionType	section	Type of LaTeX section: usually one of section, subsection or subsubsection. No section statement is written if sectionTitle is empty.
strictBorders	true	Enables the flushing of all document elements before each fragment

19.272 cfgReport (ConfigurationReport)

This component generates these descriptions about the Anduril components and the steps of the analysis.

Authors: Kristian Ovaska (Kristian.Ovaska@Helsinki.FI), Marko Laakso (Marko.Laakso@Helsinki.FI)
Version: 1.0
Category: Latex

Input name	Source	Description
descriptions1	cfgDescMicroarray.in 19.270	Component descriptions for end users. The directory should contain a LaTeX source file XXX.tex for each component named XXX. The contents of XXX.tex are inserted into the section for component XXX. LaTeX files are not complete documents but fragments that are inserted inside a section. Hence, they should not use section commands. Supplementary files should be named like XXX-file1 to avoid filename conflicts.

Parameter name	Value	Description
dot	dot	Graphviz/dot execution command
includeSelf	true	Include the ConfigurationReport instance inself in the report
paramDescWidth	10.5	Length of the parameter description column in centimeters
ps2pdf	ps2pdf	PS2PDF execution command
showCategories	true	Include category lists of each component into their descriptions
showVersions	true	Include version numbers of each component into their descriptions

19.273 ensembl (INPUT)

Database configurations for Ensemble database [8]. The exact content can be seen in Section 19.937.

Category: Internal

Parameter name	Value	Description
path	/gpfs/homes/mlaakso/asserSVN/andurilCaseStudy/ensemblHS.properties	Path (filename) of the input resource.

19.274 expressionSummary (LatexCombiner)

LatexCombiner joins the given subdocuments together into one LaTeX file.

Authors: Kristian Ovaska (Kristian.Ovaska@Helsinki.FI), Marko Laakso (Marko.Laakso@Helsinki.FI)
Version: 1.3
Category: Latex

Input name	Source	Description
latex1	geneExpression-report .document 19.301	LaTeX fragment 1.
latex2	transcriptExpressionResults -report.document 19.922	LaTeX fragment 2.
latex3	medianExonExpression-report .document 19.356	LaTeX fragment 3.

Parameter name	Value	Description
pagebreak	false	Determines if the result document should start with a page break.
sectionTitle		If non-empty, a declaration of a new section with the given name is inserted to the beginning of the combined document. This is a convenience feature to make it easy to compile subsections into a section.
sectionType	section	Type of LaTeX section: usually one of section, subsection or subsubsection. No section statement is written if sectionTitle is empty.
strictBorders	true	Enables the flushing of all document elements before each fragment

19.275 geneExpression (GeneExpressionAnalysis)

Performes the whole analysis of the gene expression data.

Parameter name	Value
arrayType	affy_hg_u133a
fcLimit	2.0
joinProbes	true
pLimit	0.05

19.276 geneExpression-allLogratio (SampleCombiner)

Combine expression data from several samples into one by taking means, medians or log ratios. Combining is done using a sample group table. Each row of the table specifies how one sample group is created from a list of source groups. If the expression input file contains log ratios, both median and log ratio combining are possible and the result are log ratios. If the input are channel values, the groups may be either median groups (result type: channel values) or log ratio groups (result type: log ratio), but not both.

Authors: Kari Nousiainen (Kari.Nousiainen@Helsinki.FI), Kristian Ovaska (Kristian.Ovaska@Helsinki.FI)
Version: 1.0
Category: Filter

Input name	Source	Description
expr	geneExpression-allMeans .expr 19.277	Expression data, either log ratios or channel expressions.
groups	geneExpression-sampleGroups .groups 19.303	Sample group specification.

Parameter name	Value	Description
geometricMean	true	Indicates whether the means will be calculated using geometric(true) or arithmetic mean (false).
groupIDs	tumor_vs_normal	Comma-separated list of sample group IDs. If given, combine only these groups. If not given (empty), combine all sample groups.

Continued on next page...

Parameter name	Value	Description
includeOriginal	false	If true, put input groups into the result even if they are not present in 'groups'.
thresholdRatio	0	Allows to omit those genes that have missing values in some samples. Only genes that have non-missing values in at least the given number of columns are included in the result. The actual threshold T is ceiling(thresholdRatio*n), where n is the number of samples that are used in combining. Only genes that have non-missing values in at least T columns are included. Other genes have the combined value set to NA. Note: this parameter specifies the ratio instead of integer threshold since the number of samples may be different in each sample group.

19.277 geneExpression-allMeans (SampleCombiner)

Combine expression data from several samples into one by taking means, medians or log ratios. Combining is done using a sample group table. Each row of the table specifies how one sample group is created from a list of source groups. If the expression input file contains log ratios, both median and log ratio combining are possible and the result are log ratios. If the input are channel values, the groups may be either median groups (result type: channel values) or log ratio groups (result type: log ratio), but not both.

Authors: Kari Nousiainen (Kari.Nousiainen@Helsinki.FI), Kristian Ovaska (Kristian.Ovaska@Helsinki.FI)
Version: 1.0
Category: Filter

Input name	Source	Description
expr	geneExpression-probeFilter .csv 19.298	Expression data, either log ratios or channel expressions.
groups	geneExpression-sampleGroups .groups 19.303	Sample group specification.

Parameter name	Value	Description
geometricMean	true	Indicates whether the means will be calculated using geometric(true) or arithmetic mean (false).
groupIDs	tumor_solid,normal_tissue	Comma-separated list of sample group IDs. If given, combine only these groups. If not given (empty), combine all sample groups.
includeOriginal	false	If true, put input groups into the result even if they are not present in 'groups'.
thresholdRatio	0	Allows to omit those genes that have missing values in some samples. Only genes that have non-missing values in at least the given number of columns are included in the result. The actual threshold T is ceiling(thresholdRatio*n), where n is the number of samples that are used in combining. Only genes that have non-missing values in at least T columns are included. Other genes have the combined value set to NA. Note: this parameter specifies the ratio instead of integer threshold since the number of samples may be different in each sample group.

19.278 geneExpression-allProbes (IDConvert)

Converts identifiers from a column in a CSV file to another type of identifiers. The conversion is done using an annotation table on the basis of the column named *conversionColumn*.

Author: Kristian Ovaska (kristian.ovaska@helsinki.fi)
Version: 1.0
Category: convert

Input name	Source	Description
csv	geneExpression-allProbesDup .table 19.279	Source CSV file. Contains the column that is to be converted. The column is named by the parameter 'sourceColumn'.
conversionTable	geneExpression-allProbesDup .table 19.279	Conversion table. Must contain the column named by the parameter 'conversionColumn'.

Parameter name	Value	Description
collapseNumeric	median	Method for collapsing numeric columns with duplicate rows (when unique=true). Legal values are "median" (take median of non-NA values), "mean", "max", "min", "first" (take the first row), "majority" (take the value that is present on the largest number of rows), "consensus" (require that all rows have the same value) and "indicator".<br>Here, "majority" and "consensus" are mostly useful for discrete (integer) values). For "consensus", it is an error if duplicate rows contain distinct values. "indicator" assumes that data are divided into negative (-1), neutral (0) and positive (1) items, such as discretized expression values. The result is (a) 1 if there is a 1 and no -1 value; (b) -1 if there is a -1 and not 1 value; (c) 0 if there is a 0 and no -1 or 1; or (d) NA otherwise.
collapseString	consensus	Method for collapsing non-numeric columns with duplicate rows (when unique=true). Legal values are "first" (take the first row), "majority" (take the value that is present on the largest number of rows), "longest" (take the longest value), "shortest" (take the shortest value) and "consensus" (require that all rows have the same value). For "consensus", it is an error if duplicate rows contain distinct values.
conversionColumn	GeneID	Column in 'conversionTable' that is used to fetch new values for the source column.
keyColumn		Column in 'conversionTable' that is used to match ID values from the source CSV. If the parameter is empty, the first column is used.
originalWhen Missing	true	Determines how values are converted when the item is not found in the conversion table. If true, the original (unconverted) ID is inserted to the output. If false, NA is inserted.
sourceColumn	GeneID	Column in 'csv' that is going to be converted. If empty, the first column is used.
split	false	If true, split values in the source column by commas before ID conversion. For example, if the source column contains the string "a,b,c", it is first split into "a", "b" and "c"; then, individual IDs are converted; finally, the converted IDs are joined by commas into a string of format "A,B,C".
splitConverted	false	If true, split converted values by commas after ID conversion. A row with comma separated values in the target column will be split to otherwise identical rows but so that the rows have a single ID value in the target column. This is done before possible collapsing of duplicate rows.
targetColumn		Target column name in 'converted'. If empty, the column name is the same as the original column (sourceColumn).
unique	true	If true, rows with identical identifiers (sourceColumn) are collapsed into one row so that sourceColumn is unique. Collapsing may result in error if duplicate rows contain conflicting values. See parameters collapseNumeric and collapseString for methods to collapse numeric and non-numeric columns, respectively.
varMeasure		Method for calculating the variation of numeric duplicate values. If the variation is above the threshold given in <code>varThreshold</code> , duplicate values are collapsed to NA. Possible values are 'MAD' and 'variance'. 'MAD' stands for the median absolute deviation and 'variance' for the population variance. If no values is given, no threshold for combining numeric values is used.
varThreshold	2	Maximum allowed variability in the duplicate values. If <code>varMeasure</code> is given duplicate values with variance above the threshold are collapsed to NA

19.279 geneExpression-allProbesDup (TableQuery)

Execute an SQL query on input relations and create a result table. TableQuery uses HSQLDB (Hyperthreaded Structured Query Language Database [25]) for executing the query. Consequently, the syntax of the query is defined by HSQLDB.

Author:	Kristian Ovaska (Kristian.Ovaska@Helsinki.FI)
Version:	1.2
Categories:	convert, Filter

Input name	Source	Description
table1	geneExpression-probeSelect .table 19.300	CSV table 1. The table is referred to as 'table1' in the SQL query.
table2	geneExpression-allLogratio .expr 19.276	CSV table 2. The table is referred to as 'table2' in the SQL query.
table3	geneExpression-tTest .pvalues 19.316	CSV table 3. The table is referred to as 'table3' in the SQL query.
query	geneExpression-geneStatistics Query.in 19.291	SQL query. Either this file or the query parameter must be provided, but not both.

Parameter name	Value	Description
numIndices	2	Comma-separated list of index counts for input tables. All indices are single-column indices, running from column 1 to N, where N is retrieved from this parameter. If empty, the default number (1) is used. For example, ",2,,2" sets two indices for table2 and table4 and one index for the rest.
query		SQL query. Either this parameter or the query input must be provided, but not both.

19.280 geneExpression-degAnnot (REvaluate)

This component produces some outputs by executing an R [20] script with the given input data. The exact functionality of this component depends on the given script.

The component is also capable of making branch decisions on Anduril workflow. Two alternative options (*altCase* and *defCase*) are provided.

Authors: Marko Laakso (Marko.Laakso@Helsinki.FI), Erkka Valo (erkka.valo@tkk.fi)
Version: 2.2
Categories: External, Latex, Network Control

Input name	Source	Description
script	geneExpression-degAnnotCols .in 19.281	Data transformation
table1	geneExpression-degAnnotK.bioA nnotation 19.282	Data matrix 1 (data.frame/file table1)

Parameter name	Value	Description
asConnection	false	If true, assign file connections (class file) to the inputs to variables of the same name. This is useful e.g. if you want to read large data files row by row.
param1		Value of this parameter will be assigned to param1 variable
param10		Value of this parameter will be assigned to param10 variable
param2		Value of this parameter will be assigned to param2 variable
param3		Value of this parameter will be assigned to param3 variable
param4		Value of this parameter will be assigned to param4 variable
param5		Value of this parameter will be assigned to param5 variable
param6		Value of this parameter will be assigned to param6 variable
param7		Value of this parameter will be assigned to param7 variable
param8		Value of this parameter will be assigned to param8 variable
param9		Value of this parameter will be assigned to param9 variable

19.281 geneExpression-degAnnotCols (INPUT)

Switch the order of annotation columns.

Category: Internal

Parameter name	Value	Description
path	/gpfs/homes/mlaakso/asserSVN/ andurilCaseStudy/r/ DEGAnnotColumns.r	Path (filename) of the input resource.

19.282 geneExpression-degAnnotK (KorvasieniAnnotator)

Korvasieni is an Ensembl [12] based converter for database identifiers and it can be used to convert genome location, gene, transcript, and translation identifiers across multiple biological databases. The selected `keyColumn` may contain entries with comma separated lists of values in which case `isListKey` parameter may be used to obtain each value separately and to process them as they were given in consecutive lines.

Author: Marko Laakso (Marko.Laakso@Helsinki.FI)
Version: 1.7
Categories: Annotation, GO

Input name	Source	Description
sourceKeys	geneExpression-degProbes .ids 19.283	A list of source database keys.
connection	ensembl.in 19.273	Database connection can be defined using this file. The definition of parameters: database.url, database.user, database.password, database.timeout, database.recycle, and database.driver can be found from the documentation of Korvasieni.

Parameter name	Value	Description
echoColumns	.GeneId	A comma separated list of column names for the columns that will be copied to the output. An asterisk (*) can be used to denote all columns execpt the keyColumn.
inputDB	.GeneId	Type of input keys. This must be a database supported by Korvasieni. If the parameter is omitted, the component tries to derive the database from the type of geneID. If this is not possible, an error is returned. You may define three columns in form of chromosome:start-end in case the inputDB is .DNARegion. This format provides a comfortable compatibility with DNARegion datatype.
inputType	Gene	Ensembl object type for the input keys (Any, Gene, Transcript, Translation)
isListKey	false	Enables the automatic value splits for the comma separated key column
keyColumn		Name of the key column withing sourceKeys file or an empty string for the first column
maxHits	100000	Maximum number of target identifiers for a single source identifier
rename		Comma separated list of column renaming rules (oldname=newname)
skipLevel	never	Skip result rows if the source identifier is unknown or target identifiers are not available. Possible values are: never (no filtering), source (skip if the source ID is unknown), target (skip if no target IDs are found), any (skip if any of the target IDs is missing).
targetDB	.GeneName,.GeneDesc,GO,_Uniprot/SWISSPROT	Comma-separated list of annotation types. Possible values are all databases supported by Korvasieni.
unique	false	This flag can be turned on in order to eliminate duplicate annotations.

19.283 geneExpression-degProbes (CSV2IDList)

Extracts one column from the given CSV file and prints it out without duplicates. The selected column may contain entries with comma separated lists of values in which case *isList* parameter may be used to obtain each value separately.

Output is defined as:

$$ids = \bigcup_{i=1}^{9} \pi_{columnIn_i} \sigma_{regexp_i}(table_i). \quad (1)$$

Author: Marko Laakso (Marko.Laakso@Helsinki.FI)
Version: 1.3
Category: convert

Input name	Source	Description
table1	geneExpression-degSet .result 19.285	The mandatory input relation

Parameter name	Value	Description
acceptMissing	false	Files with missing columnIn are accepted as empty if this is true.
columnIn	Members,Members	A comma separated list of column names for the IDs of interest in each file. Empty values refer to the first column of the file.
columnOut	.GeneId	Name of the only column of the output list. Empty input refers to the name of the input column.
constants		A comma separated list of values that are always included into the output
isList	true	True if the seleted column contains a comma separated list of values to be splitted

Continued on next page...

Parameter name	Value	Description
quotation	false	Indicator that can be used to disable quotation of the output values
regexp1	ID=over|under	Regular expression for the row filtering in table1. A row is included in the result if this parameter is empty or if values in the given columns match given regular expressions. The parameter has a format COLNAME1=EXPRESSION,COLNAME2=EXPRESSION2 where COLNAMEs are column names in "csv" and EXPRESSIONs are regular expressions using Java syntax. For example, "col=a|b" includes rows where the column col has a value of "a" or "b".
regexp2		Regular expression for the row filtering in table2.
regexp3		Regular expression for the row filtering in table3.
regexp4		Regular expression for the row filtering in table4.
regexp5		Regular expression for the row filtering in table5.
regexp6		Regular expression for the row filtering in table6.
regexp7		Regular expression for the row filtering in table7.
regexp8		Regular expression for the row filtering in table8.
regexp9		Regular expression for the row filtering in table9.

19.284 geneExpression-degReport (DEGReport)

Generates a LaTeX report on differentially expressed genes (DEGs). The report has two parts: a summary section that lists the number of over- and underexpressed genes in each sample group; and a gene list section that lists all the gene names.

Authors: Kristian Ovaska (Kristian.Ovaska@Helsinki.FI), Marko Laakso (Marko.Laakso@Helsinki.FI)
Version: 1.1.1
Categories: DEG, Latex

Input name	Source	Description
deg	geneExpression-degSet .result 19.285	Sets of differentially expressed genes. If expression values are given (expr), there must be an annotation column SampleGroup that binds the set to an expression column.
geneAnnotation	geneExpression-degAnnot .table 19.280	Annotation for genes. Must contain the column named by geneColumn.
expr	geneExpression-logratio .expr 19.294	Expression values. Used to sort gene lists.

Parameter name	Value	Description
columns	8	Number of columns in gene list table.
geneColumn	.GeneName	Column name in geneAnnotation that contains gene names. If empty, gene names are not printed.
sectionTitle	Summary of differentially expressed genes	Title for a new latex section. If none is given, a new section is not created.
sectionType	subsection	Type of LaTeX section: usually one of section, subsection or subsubsection. No section statement is written if sectionTitle is empty.
setPattern		R regular expression for matching set names. If empty, all sets are included.

19.285 geneExpression-degSet (SetTransformer)

SetTransformer transform ID lists into new lists by using flexible rules. The rules are read from a *transformation* file. Please, check JEP 2 documentation for the syntax rules.

Author: Kristian Ovaska (kristian.ovaska@helsinki.fi)
Version: 0.6
Category: Convert

Input name	Source	Description
transformation	geneExpression-deg Transformation.in 19.288	Set transformations, one per CSV row. The columns Target (target set ID) and Definition (transformation expression) must be present. The column IterationSet may be present if iterated transformations are used. IterationSet should contain NA for non-iterated transformations. Any other columns are interpreted as annotation columns and are copied to the output. For iterated transformations, the wildcard * in annotations is replaced with the current set ID.
set1	geneExpression-foldChange .deg 19.290	Input sets 1. NA values in Members are interpreted as empty sets.
set2	geneExpression-tTest.idlist 19.316	Input sets 2.

Parameter name	Value	Description
includeAnnotation	*	Comma-separated list of column names in the transformation input that should be used as annotation columns. The wildcard * includes all columns. The special columns Target, Definition and IterationSet are excluded automatically.
includeOriginal	false	If true, the original sets from input files are included in the output as well. If false, only sets defined in transformations are included.

19.286 geneExpression-degStat (TableQuery)

Execute an SQL query on input relations and create a result table. TableQuery uses HSQLDB (Hyperthreaded Structured Query Language Database [25]) for executing the query. Consequently, the syntax of the query is defined by HSQLDB.

Author: Kristian Ovaska (Kristian.Ovaska@Helsinki.FI)
Version: 1.2
Categories: convert, Filter

Input name	Source	Description
table1	geneExpression-degAnnot .table 19.280	CSV table 1. The table is referred to as 'table1' in the SQL query.
table2	geneExpression-logratio .expr 19.294	CSV table 2. The table is referred to as 'table2' in the SQL query.
table3	geneExpression-tTest .pvalues 19.316	CSV table 3. The table is referred to as 'table3' in the SQL query.
query	geneExpression-degStatSQL. in 19.287	SQL query. Either this file or the query parameter must be provided, but not both.

Parameter name	Value	Description
numIndices		Comma-separated list of index counts for input tables. All indices are single-column indices, running from column 1 to N, where N is retrieved from this parameter. If empty, the default number (1) is used. For example, ",2,,2" sets two indices for table2 and table4 and one index for the rest.
query		SQL query. Either this parameter or the query input must be provided, but not both.

19.287 geneExpression-degStatSQL (INPUT)

This query combines DEGs to their fold changes and p-values.

Category: Internal

Parameter name	Value	Description
path	/gpfs/homes/mlaakso/asserSVN/ anduril CaseStudy/sql/ DEGStatistics.sql	Path (filename) of the input resource.

19.288 geneExpression-degTransformation (INPUT)

Selection of differentially expressed genes by their fold change and t-test calls.

Category: Internal

Parameter name	Value	Description
path	/gpfs/homes/mlaakso/asserSVN/ andurilCaseStudy/csv/ FoldChangeAndTTest.csv	Path (filename) of the input resource.

19.289 geneExpression-filterSamplesRScript (INPUT)

R Script for filtering out samples without clinical annotation.

Category: Internal

Parameter name	Value	Description
path	/gpfs/homes/mlaakso/asserSVN/ andurilCaseStudy/r/filterSamples. R	Path (filename) of the input resource.

19.290 geneExpression-foldChange (FoldChange)

Select differentially expressed genes using the fold change criteria. This filter does not compute the fold changes itself but takes them as input.

A gene with a log ratio intensity l is considered to be **under** expressed if and only if $l \leq -\log_2(threshold)$ and **over** expressed if and only if $l \geq \log_2(threshold)$.

Author: Kristian Ovaska (Kristian.Ovaska@Helsinki.FI)
Version: 1.1
Category: DEG

Input name	Source	Description
logratio	geneExpression-logratio .expr 19.294	Log ratio data.

Parameter name	Value	Description
nameDeg	%s_fcDeg	Name of the sets for differentially expressed genes. The pattern %s is expanded to the current sample name in logratio.
nameOver	%s_fcOver	Name of the sets for underexpressed genes. The pattern %s is expanded to the current sample name in logratio.
nameStable	%s_fcStable	Name of the sets for stably expressed genes. The pattern %s is expanded to the current sample name in logratio.
nameUnder	%s_fcUnder	Name of the sets for underexpressed genes. The pattern %s is expanded to the current sample name in logratio.
stableThreshold	0	Fold change threshold (in linear scale) for stable genes. Genes with fold change smaller than the threshold in both directions are considered stably expressed. If the parameter has the value zero, no list of stably expressed genes is included in the results.
threshold	2.0	Fold change threshold (in linear scale). Genes with fold change greater than or equal to the threshold in either direction are considered differentially expressed.

19.291 geneExpression-geneStatisticsQuery (INPUT)

(Technical documentation) Imports an external input file or directory to the pipeline. This is the only way external files are imported. File path is given with the parameter "path".

Category: Internal

Parameter name	Value	Description
path	/gpfs/homes/mlaakso/asserSVN/andurilCaseStudy/sql/GeneStatistics.sql	Path (filename) of the input resource.

19.292 geneExpression-groupMeans (SampleCombiner)

Combine expression data from several samples into one by taking means, medians or log ratios. Combining is done using a sample group table. Each row of the table specifies how one sample group is created from a list of source groups. If the expression input file contains log ratios, both median and log ratio combining are possible and the result are log ratios. If the input are channel values, the groups may be either median groups (result type: channel values) or log ratio groups (result type: log ratio), but not both.

Authors: Kari Nousiainen (Kari.Nousiainen@Helsinki.FI), Kristian Ovaska (Kristian.Ovaska@Helsinki.FI)
Version: 1.0
Category: Filter

Input name	Source	Description
expr	geneExpression-varFilter .matrix 19.318	Expression data, either log ratios or channel expressions.
groups	geneExpression-sampleGroups .groups 19.303	Sample group specification.

Parameter name	Value	Description
geometricMean	true	Indicates whether the means will be calculated using geometric(true) or arithmetic mean (false).
groupIDs	tumor_solid,normal_tissue	Comma-separated list of sample group IDs. If given, combine only these groups. If not given (empty), combine all sample groups.
includeOriginal	false	If true, put input groups into the result even if they are not present in 'groups'.
thresholdRatio	0	Allows to omit those genes that have missing values in some samples. Only genes that have non-missing values in at least the given number of columns are included in the result. The actual threshold T is ceiling(thresholdRatio*n), where n is the number of samples that are used in combining. Only genes that have non-missing values in at least T columns are included. Other genes have the combined value set to NA. Note: this parameter specifies the ratio instead of integer threshold since the number of samples may be different in each sample group.

19.293 geneExpression-heatmap (GOClustering)

This Anduril component uses Gene ontology (GO) [1] annotations to cluster a set of genes. Each cluster consists of genes that share similar GO annotations.

The GO clustering of the genes can be visualized together with expression data (optional). Samples of the expression data are clustered based on their expression profiles and a heatmap is formed by clustering the y-axis of genes using their GO annotations. Component is based on the method descibed in [17].

Authors:	Ping Chen (ping.chen@cs.helsinki.fi), Kristian Ovaska (Kristian.Ovaska@Helsinki.FI), Marko Laakso (Marko.Laakso@Helsinki.FI), Erkka Valo (Erkka.Valo@Helsinki.FI)
Version:	1.1.2
Categories:	Clustering, GO

Input name	Source	Description
goAnnotations	geneExpression-degAnnot .table 19.280	GO annotations for the genes
expr	geneExpression-varFilter .matrix 19.318	Gene expressions

Parameter name	Value	Description
ColumnWidth	15	Control the column width of the GO MEMBER table.
asBitmap	true	Produce heatmap in Portable network graphics (PNG) format instead of Portable document format (PDF). PNG bitmaps are more suitable if the heatmap contains large number of samples and genes.
columnMargin	0	Margin for column names in heatmap
cutLimit	0.5	Clustering cut off height for the hierachical clustering of the genes. This limit is between 0 and 1, where 0 refers to more specific groups.
decimal	3	Control the output of long decimal.
drawHeatmap	true	If true, draw a heatmap.
dropUnknown	true	Genes with no GO annotations are excluded if this flag is true
geneColumn		Name of the gene name column in the annotation file. The value is used for creating the report. The given column is used for listing all the members in the clusters to the report. If the value is empty, cluster member lists are not put into the report.
goColumn	GO	Name of the GO annotation column in the annotation file
grayScale	true	If true, heatmap is in grayscale. If false, heatmap is colorful.
heatmapScale	row	Determines how the values for the heatmap are scaled. Legal values are "row", "column" and "none". This corresponds to the scale parameter of the R function heatmap.
organism	9606	NCBI taxonomy ID for the organism whose gene set is used for GO probabilities. This is used if the input similarityTable is not given. Supported organisms: Homo sapiens: 9606, Saccharomyces cerevisiae: 4932, Caenorhabditis elegans: 6239, Drosophila melanogaster: 7227, Mus musculus: 10090, Rattus norvegicus: 10116.
pvalues	false	If true, compute p-values for information contents of the clusters using a permutation test. The p-values are shown in their own table in the report. The permutation test may be slow.
rowMargin	5	Margin for row names in heatmap
sectionType	subsection	Type of LaTeX section: usually one of section, subsection or subsubsection. No section statement is written if <code>sectionTitle</code> is an empty string.
showClusterNames	true	If true, show cluster names G1, ..., Gn on the right side of the heat map. If false, show gene names.
title	GO clustering	Title for the subsection and for the heatmap image.
xLab	Samples	Label for the x-axis of the heatmap
yLab	GO.groups	Label for the y-axis of the heatmap

19.294 geneExpression-logratio (SampleCombiner)

Combine expression data from several samples into one by taking means, medians or log ratios. Combining is done using a sample group table. Each row of the table specifies how one sample group is created from a list of source groups. If the expression input file contains log ratios, both median and log ratio combining are possible and the result are log ratios. If the input are channel values, the groups may be either median groups (result type: channel values) or log ratio groups (result type: log ratio), but not both.

Authors:	Kari Nousiainen (Kari.Nousiainen@Helsinki.FI), Kristian Ovaska (Kristian.Ovaska@Helsinki.FI)
Version:	1.0
Category:	Filter

Input name	Source	Description
expr	geneExpression-groupMeans .expr 19.292	Expression data, either log ratios or channel expressions.
groups	geneExpression-sampleGroups .groups 19.303	Sample group specification.

Continued on next page...

Input name	Source	Description

Parameter name	Value	Description
geometricMean	true	Indicates whether the means will be calculated using geometric(true) or arithmetic mean (false).
groupIDs	tumor_vs_normal	Comma-separated list of sample group IDs. If given, combine only these groups. If not given (empty), combine all sample groups.
includeOriginal	false	If true, put input groups into the result even if they are not present in 'groups'.
thresholdRatio	0	Allows to omit those genes that have missing values in some samples. Only genes that have non-missing values in at least the given number of columns are included in the result. The actual threshold T is ceiling(thresholdRatio*n), where n is the number of samples that are used in combining. Only genes that have non-missing values in at least T columns are included. Other genes have the combined value set to NA. Note: this parameter specifies the ratio instead of integer threshold since the number of samples may be different in each sample group.

19.295 geneExpression-metadata (INPUT)

(Technical documentation) Imports an external input file or directory to the pipeline. This is the only way external files are imported. File path is given with the parameter "path".

Category: Internal

Parameter name	Value	Description
path	/gpfs/homes/mlaakso/asserSVN/andurilCaseStudy/data/patient-metadata.csv	Path (filename) of the input resource.

19.296 geneExpression-probeAnnot (BiomartAnnotator)

BiomartAnnotator fetches user specified attributes given a set of filter values from the defined BioMart database.

Authors: Erkka Valo (Erkka.Valo@Helsinki.FI), Viljami Aittomaki (Viljami.Aittomaki@Helsinki.FI), Kristian Ovaska (kristian.ovaska@helsinki.fi), Marko Laakso (Marko.Laakso@Helsinki.FI)
Version: 1.1
Category: Annotation

Input name	Source	Description
filter	rawGeneExpression.in 19.439	A list of filter values

Parameter name	Value	Description
attributes	ensembl_gene_id	A comma separated list of attributes to fetch. See biomaRt documentation on how to list available attributes for a given mart and a dataset in R.
batch	true	If true, enable batch mode where all filter values are fetched with one query. This is significantly faster than non-batch mode, but in some instances there may be several result rows for one filter value. If false, filter values are fetched individually.
dataset	hsapiens_gene_ensembl	Dataset to get annotations from. Different BioMart databases (marts) have their own datasets. See biomaRt documentation on how to list available datasets for a mart in R.
filterColumns		Names of the filter column within filter file or an empty string for the first column(s).
filterTypes	affy_hg_u133a	Types of the filter values, as a comma-separated list. See biomaRt documentation on how to list available filters for a given mart and a dataset in R.

Continued on next page...

Parameter name	Value	Description
idAttribute		For batch=true mode, this is the name of the ID attribute that produces values that correspond to filter IDs. If empty, the value of filterType is used. Often, the name of the filter and the corresponding attribute are identical, in which case the default (empty) value can be used. This parameter is not used for batch=false mode.
mart	ensembl	BioMart database to use. See biomaRt documentation on how to list available BioMart databases (marts).
martHost	www.biomart.org	Mart hosting server
martPath	/biomart/martservice	Mart web service URL within the server

19.297 geneExpression-probeAnnotExp (ExpandCollapse)

Converts between the two possible representations of a relations with multivalued columns. By default this component splits rows with multivalued (comma separated lists) columns into multiple rows each representing a single value. The input may contain several columns with comma separated values and all permutations of column values are shown as individual single-valued rows. The alternative mode recovers the original comma separated values from the expanded relations. This is performed by joining values of the their list columns into comma separated lists.

Author: Marko Laakso (Marko.Laakso@Helsinki.FI)
Version: 1.3
Category: convert

Input name	Source	Description
relation	geneExpression-probeAnnot .annotations 19.296	The mandatory input relation

Parameter name	Value	Description
delim	,	Value delimiter between the list column values. Special characters can be encoded as specified in fi.helsinki.ltdk.csbl.asser.ArgumentEncoding.
expand	true	Action mode that is expand (true) or collapse (false)
listCols	ensembl_gene_id	A comma separated list of column names of input (expand) or output (collapse) columns that may contain delim separated values. The asterisk refers to every column of the input relation.

19.298 geneExpression-probeFilter (CSVFilter)

Filters columns and/or rows from CSV files using flexible criteria. Default settings do not filter out anything. The *negate* parameter reverses all inclusion criteria.

Columns are filtered based on column names (see *includeColumns*); it is also possible to rename columns. Columns can be reordered using *includeColumns*.

A row is included in output if all conditions are satisfied. Rows having too many missing values are filtered using the *nonMissing* parameter. The parameters *regexp*, *lowBound* and *highBound* filter rows based on cell contents. If the *auxiliary* input is given, the result contains those rows from the input whose values match those from the auxiliary file.

Authors: Kristian Ovaska (kristian.ovaska@helsinki.fi), Marko Laakso (Marko.Laakso@Helsinki.FI)
Version: 1.1
Category: Filter

Input name	Source	Description
csv	geneExpression-sampleFilter .table 19.302	CSV file to be filtered.
auxiliary	geneExpression-probeSelect .table 19.300	If given, contains one column (see "matchColumn") whose values are matched to a column in the "csv" input (see "idColumn").

Parameter name	Value	Description
highBound		A row is included if the value in given numeric columns are at most as large as given numeric bounds. The parameter has a format COLNAME1=HIGH1,COLNAME2=HIGH2.
idColumn		Column name in "csv" that contains ID values. If the parameter is empty, the first column is used.
includeColumns	*	Comma-separated list of column names that should be included in the result file. The special value * includes all columns. The order of column names is significant: the output columns are in the order given. See also includeColumns input.
lowBound		A row is included if the value in given numeric columns are at least as large as given numeric bounds. The parameter has a format COLNAME1=LOW1,COLNAME2=LOW2. For example, "col=5" includes rows where the column col has a value of at least 5.
matchColumn	probe	Column name in "auxiliary" containing values that must match the ID column in "csv" (specified using "idColumn"). If empty, the first column of "auxiliary" is used.
negate	false	If true, all inclusion criteria are negated so that rows or columns are excluded instead of included if they match the criteria. For example, includeColumns=* then excludes all columns (which would be an error since at least one column must be included).
nonMissing	0	Include those rows that have non-missing (non-NA) values in at least this many columns. Only those columns that are part of the output are counted.
regexp		Row filtering based on regular expression. A row is included in the result if values in the given columns match given regular expressions. The parameter has a format COLNAME1=EXPRESSION,COLNAME2=EXPRESSION2 where COLNAMEs are column names in "csv" and EXPRESSIONs are regular expressions using Java syntax. For example, "col=a|b" includes rows where the column col has a value of "a" or "b". Commas have to be escaped with "\\" if they are used in column names or regular expressions.
rename		Comma-separated list of column renaming rules (OLDNAME=NEWNAME). Parameters that refer to column names refer to old names; renaming is done last.

19.299 geneExpression-probeJoin (IDConvert)

Converts identifiers from a column in a CSV file to another type of identifiers. The conversion is done using an annotation table on the basis of the column named *conversionColumn*.

Author: Kristian Ovaska (kristian.ovaska@helsinki.fi)
Version: 1.0
Category: convert

Input name	Source	Description
csv	geneExpression-probeFilter .csv 19.298	Source CSV file. Contains the column that is to be converted. The column is named by the parameter 'sourceColumn'.
conversionTable	geneExpression-probeSelect .table 19.300	Conversion table. Must contain the column named by the parameter 'conversionColumn'.

Parameter name	Value	Description
collapseNumeric	median	Method for collapsing numeric columns with duplicate rows (when unique=true). Legal values are "median" (take median of non-NA values), "mean", "max", "min", "first" (take the first row), "majority" (take the value that is present on the largest number of rows), "consensus" (require that all rows have the same value) and "indicator".<br>Here, "majority" and "consensus" are mostly useful for discrete (integer) values). For "consensus", it is an error if duplicate rows contain distinct values. "indicator" assumes that data are divided into negative (-1), neutral (0) and positive (1) items, such as discretized expression values. The result is (a) 1 if there is a 1 and no -1 value; (b) -1 if there is a -1 and not 1 value; (c) 0 if there is a 0 and no -1 or 1; or (d) NA otherwise.
collapseString	consensus	Method for collapsing non-numeric columns with duplicate rows (when unique=true). Legal values are "first" (take the first row), "majority" (take the value that is present on the largest number of rows), "longest" (take the longest value), "shortest" (take the shortest value) and "consensus" (require that all rows have the same value). For "consensus", it is an error if duplicate rows contain distinct values.
conversionColumn	.GeneId	Column in 'conversionTable' that is used to fetch new values for the source column.

Continued on next page...

Parameter name	Value	Description
keyColumn		Column in 'conversionTable' that is used to match ID values from the source CSV. If the parameter is empty, the first column is used.
originalWhen Missing	true	Determines how values are converted when the item is not found in the conversion table. If true, the original (unconverted) ID is inserted to the output. If false, NA is inserted.
sourceColumn		Column in 'csv' that is going to be converted. If empty, the first column is used.
split	false	If true, split values in the source column by commas before ID conversion. For example, if the source column contains the string "a,b,c", it is first split into "a", "b" and "c"; then, individual IDs are converted; finally, the converted IDs are joined by commas into a string of format "A,B,C".
splitConverted	false	If true, split converted values by commas after ID conversion. A row with comma separated values in the target column will be split to otherwise identical rows but so that the rows have a single ID value in the target column. This is done before possible collapsing of duplicate rows.
targetColumn		Target column name in 'converted'. If empty, the column name is the same as the original column (sourceColumn).
unique	true	If true, rows with identical identifiers (sourceColumn) are collapsed into one row so that sourceColumn is unique. Collapsing may result in error if duplicate rows contain conflicting values. See parameters collapseNumeric and collapseString for methods to collapse numeric and non-numeric columns, respectively.
varMeasure		Method for calculating the variation of numeric duplicate values. If the variation is above the threshold given in <code>varThreshold</code> , duplicate values are collapsed to NA. Possible values are 'MAD' and 'variance'. 'MAD' stands for the median absolute deviation and 'variance' for the population variance. If no values is given, no threshold for combining numeric values is used.
varThreshold	2	Maximum allowed variability in the duplicate values. If <code>varMeasure</code> is given duplicate values with variance above the threshold are collapsed to NA

19.300 geneExpression-probeSelect (TableQuery)

Execute an SQL query on input relations and create a result table. TableQuery uses HSQLDB (Hyper-threaded Structured Query Language Database [25]) for executing the query. Consequently, the syntax of the query is defined by HSQLDB.

Author: Kristian Ovaska (Kristian.Ovaska@Helsinki.FI)
Version: 1.2
Categories: convert, Filter

Input name	Source	Description
table1	geneExpression-probeAnnotExp .relation 19.297	CSV table 1. The table is referred to as 'table1' in the SQL query.
query	geneExpression-uniqProbesSQL. in 19.317	SQL query. Either this file or the query parameter must be provided, but not both.

Parameter name	Value	Description
numIndices		Comma-separated list of index counts for input tables. All indices are single-column indices, running from column 1 to N, where N is retrieved from this parameter. If empty, the default number (1) is used. For example, ",2,,2" sets two indices for table2 and table4 and one index for the rest.
query		SQL query. Either this parameter or the query input must be provided, but not both.

19.301 geneExpression-report (LatexCombiner)

LatexCombiner joins the given subdocuments together into one LaTeX file.

Authors: Kristian Ovaska (Kristian.Ovaska@Helsinki.FI), Marko Laakso (Marko.Laakso@Helsinki.FI)
Version: 1.3
Category: Latex

Input name	Source	Description
latex1	geneExpression-degReport .summary 19.284	LaTeX fragment 1.
latex2	geneExpression-degReport .genelist 19.284	LaTeX fragment 2.
latex3	geneExpression-heatmap .report 19.293	LaTeX fragment 3.
latex4	geneExpression-survival-km Report.document 19.307	LaTeX fragment 4.

Parameter name	Value	Description
pagebreak	false	Determines if the result document should start with a page break.
sectionTitle	Gene expression analysis	If non-empty, a declaration of a new section with the given name is inserted to the beginning of the combined document. This is a convenience feature to make it easy to compile subsections into a section.
sectionType	section	Type of LaTeX section: usually one of section, subsection or subsubsection. No section statement is written if sectionTitle is empty.
strictBorders	true	Enables the flushing of all document elements before each fragment

19.302 geneExpression-sampleFilter (REvaluate)

This component produces some outputs by executing an R [20] script with the given input data. The exact functionality of this component depends on the given script.

The component is also capable of making branch decisions on Anduril workflow. Two alternative options (*altCase* and *defCase*) are provided.

Authors: Marko Laakso (Marko.Laakso@Helsinki.FI), Erkka Valo (erkka.valo@tkk.fi)
Version: 2.2
Categories: External, Latex, Network Control

Input name	Source	Description
script	geneExpression-filterSamplesRS cript.in 19.289	Data transformation
table1	rawGeneExpression.in 19.439	Data matrix 1 (data.frame/file table1)
table2	geneExpression-samplesWithout ClinicalAnnotation.in 19.304	Data matrix 2 (data.frame/file table2)

Parameter name	Value	Description
asConnection	false	If true, assign file connections (class file) to the inputs to variables of the same name. This is useful e.g. if you want to read large data files row by row.
param1		Value of this parameter will be assigned to param1 variable
param10		Value of this parameter will be assigned to param10 variable
param2		Value of this parameter will be assigned to param2 variable
param3		Value of this parameter will be assigned to param3 variable
param4		Value of this parameter will be assigned to param4 variable
param5		Value of this parameter will be assigned to param5 variable
param6		Value of this parameter will be assigned to param6 variable
param7		Value of this parameter will be assigned to param7 variable
param8		Value of this parameter will be assigned to param8 variable
param9		Value of this parameter will be assigned to param9 variable

19.303 geneExpression-sampleGroups (SampleGroupCreator)

Creates sample group tables based on sample names read from data files. These tables define relationship between samples. For example, which samples are biological or technical replicates. The list of sample names is read from data files (either column names or one column along rows) and sample groups are defined using regular expressions. Also, constant groups where the set of member samples does not depend on data can be created. The type of each definition pattern is given with *patternTypes*, which is one of *re*, *relist* or *verbatim* for each pattern.

Author:	Kristian Ovaska (kristian.ovaska@helsinki.fi)
Version:	1.2.1
Category:	preprocessing

Input name	Source	Description
data1	geneExpression-varFilter .matrix 19.318	Data file 1 for reading sample names. Either column names or values on one column contain sample names.

Parameter name	Value	Description
columns		Defines which columns in input files contain sample names, or whether column names should be used (default). This parameter is a comma-separated list of at most three values which name columns in inputs data1 to data3. If an entry is empty, the column names of the corresponding input file are used instead.
definition1	tumor_solid,mean,Solid tumor samples	Definition of group 1. If empty, the group is omitted. Format: NAME,TYPE or NAME,TYPE,DESCRIPTION.
definition2	normal_tissue,mean,Normal tissue samples	Definition of group 2. If empty, the group is omitted.
definition3	tumor_vs_normal,ratio,fold change of tumor median vs. normal median	Definition of group 3. If empty, the group is omitted.
definition4		Definition of group 4. If empty, the group is omitted.
definition5		Definition of group 5. If empty, the group is omitted.
definition6		Definition of group 6. If empty, the group is omitted.
definition7		Definition of group 7. If empty, the group is omitted.
definition8		Definition of group 8. If empty, the group is omitted.
definition9		Definition of group 9. If empty, the group is omitted.
pattern1	TCGA_[0-9]{2}_[0-9]{4}_01	Pattern for group 1. If empty, the group is omitted. Format depends on the pattern type.
pattern2	TCGA_[0-9]{2}_[0-9]{4}_11	Pattern for group 2. If empty, the group is omitted.
pattern3	tumor_solid,normal_tissue	Pattern for group 3. If empty, the group is omitted.
pattern4		Pattern for group 4. If empty, the group is omitted.
pattern5		Pattern for group 5. If empty, the group is omitted.
pattern6		Pattern for group 6. If empty, the group is omitted.
pattern7		Pattern for group 7. If empty, the group is omitted.
pattern8		Pattern for group 8. If empty, the group is omitted.
pattern9		Pattern for group 9. If empty, the group is omitted.
patternTypes	re,re,verbatim	Comma-separated list of pattern types for each pattern. Each item is one of "re" (default), "relist" or "verbatim". The types are explained above. An empty value is interpreted as "re". For example, ",re,relist,,verbatim" specifies that pattern3 has the type "relist" and pattern5 the type "verbatim"; all others (including pattern6 and above) have the type "re".

19.304 geneExpression-samplesWithoutClinicalAnnotation (INPUT)

List of samples without clinical annotation.

Category: Internal

Parameter name	Value	Description
path	/gpfs/homes/mlaakso/asserSVN/andurilCaseStudy/csv/samplesWithoutClinicalAnnotation.csv	Path (filename) of the input resource.

19.305 geneExpression-survival (TranscriptSurvival)

No description

Parameter name	Value
annotCol	.GeneName,.GeneDesc,Uniprot/SWISSPROT
fcThreshold	2.0
minCount	20
pLimit	0.05
title	Survival analysis for DEGs

Continued on next page...

Parameter name	Value

19.306 geneExpression-survival-km (KaplanMeier)

Produces a Kaplan-Meier plot representing survival estimates based on the given data. Log-rank statistics are calculated in order the address the similarity of the curves.

Authors: Marko Laakso (Marko.Laakso@Helsinki.FI), Riku Louhimo (Riku.Louhimo@Helsinki.FI), Kristian Ovaska (kristian.ovaska@helsinki.fi)
Version: 1.7
Categories: Survival, Latex

Input name	Source	Description
survival	geneExpression-survival-survival TableConv.csv 19.314	Survival time and event indicators together with the sample group assignments
annotation	geneExpression-degAnnot .table 19.280	Additional annotations for the groupCol names. These annotations are listed as column name value pairs in figure caption.

Parameter name	Value	Description
PLimit	0.05	Ignore results with a p-value greater than this limit
annotCol	.GeneName,.GeneDesc,Uniprot /SWISSPROT	A comma separated list of column names of the annotation file that will be shown in the report. An asterisk (*) may be used for all attributes except the first one that is considered as a key.
confInt	0.95	Coverage of the confidence intervals [0,1] or a negative number to disable them
eventSymbol	DECEASED	Value of statusCol column indicating an event. Other values are considered as censorings.
excludeCol	SampleID,PatientID	A comma separated list of column names that should not be used as groupCols even if an asterisk is used. This is especially useful to filter out samples identifiers and other irrelevant columns.
groupCol	*	A comma separated list of column names of sample group identifiers or an empty string for a single curve. An asterisk (*) may be used to plot all attributes except those defined for the other purposes or listed in excludeCol.
minCount	20	Individual groups with less that this many members will be left out
statusCol	VitalStatus	Column name for the event indicator
timeCol	SurvivalEndTime	Column name for the survival time
timeOutLimit	36	Cut survival curves after this limit. A negative value can be used to maximize the length of the period.
title	Kaplan-Meier survival estimates	Figure title
xLabel	survival time	Label for the horizontal time axis

19.307 geneExpression-survival-kmReport (LatexCombiner)

LatexCombiner joins the given subdocuments together into one LaTeX file.

Authors: Kristian Ovaska (Kristian.Ovaska@Helsinki.FI), Marko Laakso (Marko.Laakso@Helsinki.FI)
Version: 1.3
Category: Latex

Input name	Source	Description
latex1	geneExpression-survival-km .report 19.306	LaTeX fragment 1.

Parameter name	Value	Description
pagebreak	true	Determines if the result document should start with a page break.
sectionTitle	Survival analysis for DEGs	If non-empty, a declaration of a new section with the given name is inserted to the beginning of the combined document. This is a convenience feature to make it easy to compile subsections into a section.
sectionType	section	Type of LaTeX section: usually one of section, subsection or subsubsection. No section statement is written if sectionTitle is empty.
strictBorders	true	Enables the flushing of all document elements before each fragment

Continued on next page...

Parameter name	Value	Description

19.308 geneExpression-survival-sampleExpression (SampleExpression)

Create a $\{-1, 0, 1\}$ matrix that indicates whether a given gene/probe is differentially expressed in individual samples. The decision whether a gene is differentially expressed is done:

a) using fold change thresholding or
b) by selecting P largest and P smallest log ratios, where P is a proportion.

The expression input can be either log ratios or channel values, which are handled differently. Log ratios are directly used for fold change thresholding. Channel values are converted to log ratios by dividing them with reference samples. In this case, the expression matrix is assumed to contain both the target and reference samples. Reference samples are converted to a single column by taking a median. The columns that contain reference values are defined in the groups input. If the groups input is not given, values are assumed to be log ratios.

Author: Kristian Ovaska (kristian.ovaska@helsinki.fi)
Version: 1.0
Category: DEG

Input name	Source	Description
expr	geneExpression-varFilter .matrix 19.318	Expression matrix. The values are assumed to be log ratios if the "groups" input is not given; otherwise, they are assumed to be channel values.
groups	geneExpression-sampleGroups .groups 19.303	Sample groups that define reference (control) columns in the expression input. The reference group name is given with the referenceGroup parameter.

Parameter name	Value	Description
referenceGroup	normal_tissue	Group name in the groups input that contains reference samples. Used to compute log ratios from channel values.
threshold	2.0	Threshold that determines whether a gene is under- or overexpressed. Interpretation of the value depends on thresholdType.
thresholdType	fold-change	Defines how differential expression is decided. Legal values are "fold-change" and "top-most". For "fold-change", the threshold parameter is a linear fold change threshold. A gene is overexpressed if its log ratio is over the threshold and underexpressed if the log ratio is under 1/threshold. For "top-most", threshold is a fraction between 0 and 1 that defines how many genes with highest and lowest expression are selected. For example, if threshold is 0.1, the top 10% genes are overexpressed and the bottom 10% are underexpressed.

19.309 geneExpression-survival-sampleExpressionTrans (MatrixTranspose)

Produces a transposed version of the input matrix.

Author: Kristian Ovaska (kristian.ovaska@helsinki.fi)
Version: 1.0
Category: convert

Input name	Source	Description
matrix	geneExpression-survival-sample Expression.indicator 19.308	Input matrix.

Parameter name	Value	Description
rowName	SampleID	Name of the first column in the output.

19.310 geneExpression-survival-sampleNames (ExpandCollapse)

Converts between the two possible representations of a relations with multivalued columns. By default this component splits rows with multivalued (comma separated lists) columns into multiple rows each representing a single value. The input may contain several columns with comma separated values and all permutations of column values are shown as individual single-valued rows. The alternative mode recovers the original comma separated values from the expanded relations. This is performed by joining values of the their list columns into comma separated lists.

Author:	Marko Laakso (Marko.Laakso@Helsinki.FI)
Version:	1.3
Category:	convert

Input name	Source	Description
relation	geneExpression-sampleGroups .groups 19.303	The mandatory input relation

Parameter name	Value	Description
delim	,	Value delimiter between the list column values. Special characters can be encoded as specified in fi.helsinki.ltdk.csbl.asser.ArgumentEncoding.
expand	true	Action mode that is expand (true) or collapse (false)
listCols	*	A comma separated list of column names of input (expand) or output (collapse) columns that may contain delim separated values. The asterisk refers to every column of the input relation.

19.311 geneExpression-survival-survival (TableQuery)

Execute an SQL query on input relations and create a result table. TableQuery uses HSQLDB (Hyperthreaded Structured Query Language Database [25]) for executing the query. Consequently, the syntax of the query is defined by HSQLDB.

Author:	Kristian Ovaska (Kristian.Ovaska@Helsinki.FI)
Version:	1.2
Categories:	convert, Filter

Input name	Source	Description
table1	geneExpression-survival-sample Names.relation 19.310	CSV table 1. The table is referred to as 'table1' in the SQL query.
table2	geneExpression-metadata .in 19.295	CSV table 2. The table is referred to as 'table2' in the SQL query.
query	geneExpression-survival-survival Query.in 19.312	SQL query. Either this file or the query parameter must be provided, but not both.

Parameter name	Value	Description
numIndices		Comma-separated list of index counts for input tables. All indices are single-column indices, running from column 1 to N, where N is retrieved from this parameter. If empty, the default number (1) is used. For example, ",2,,2" sets two indices for table2 and table4 and one index for the rest.
query		SQL query. Either this parameter or the query input must be provided, but not both.

19.312 geneExpression-survival-survivalQuery (INPUT)

Generates a table of samples and their survival times.

Category: Internal

Parameter name	Value	Description
path	/gpfs/homes/mlaakso/asserSVN/ andurilCaseStudy/sql/ exonSurvival.sql	Path (filename) of the input resource.

19.313 geneExpression-survival-survivalTable (CSVJoin)

Join rows from two or more CSV files, optionally using one column as a matching key. If a key column is not used, the result contains all rows and all columns of the input files. Missing values (`NA`) may be introduced when a column is not present in all input files. Each column is present once and duplicate rows are removed.

If a key column is used, the rows in each input file are matched using values from the key column. The result file has one row for each key value. In the result, the first column is the key column; its name is obtained from the first CSV input (csv1). If the intersection parameter is true, a key is included in the result if the key value is present in all inputs. If intersection is false, a key is included if it is present in at least one input (key union). Union semantics may introduce missing values in the result. If several input files have the same column, the value is obtained from the first file. However, if the first file contains a missing value and the second file contains a non-missing value, the non-missing value is used instead.

Author: Kristian Ovaska (kristian.ovaska@helsinki.fi)
Version: 1.0
Category: convert

Input name	Source	Description
csv1	geneExpression-survival-survival .table 19.311	CSV file 1.
csv2	geneExpression-survival-sample ExpressionTrans.matrix 19.309	CSV file 2.

Parameter name	Value	Description
intersection	true	Defines how keys are handled; only used when useKeys=true. If intersection is true, the result contains a key if the key is present in all input files. If false, the result contains a key if the key is present in at least one input file.
keyColumnNames		Comma-separated list of key column names; only used when useKeys=true. The first name refers to csv1, the second to csv2, etc. An empty value refers to the first column. Empty values may be omitted from the list, so all these are equivalent: "col1" ; "col1," ; "col1,," ; etc.
useKeys	true	If true, use one column from each CSV file as a matching key column.

19.314 geneExpression-survival-survivalTableConv (IDConvert)

Converts identifiers from a column in a CSV file to another type of identifiers. The conversion is done using an annotation table on the basis of the column named *conversionColumn*.

Author: Kristian Ovaska (kristian.ovaska@helsinki.fi)
Version: 1.0
Category: convert

Input name	Source	Description
csv	geneExpression-survival-survival Table.csv 19.313	Source CSV file. Contains the column that is to be converted. The column is named by the parameter 'sourceColumn'.
conversionTable	geneExpression-survival-survival .table 19.311	Conversion table. Must contain the column named by the parameter 'conversionColumn'.

Parameter name	Value	Description
collapseNumeric	indicator	Method for collapsing numeric columns with duplicate rows (when unique=true). Legal values are "median" (take median of non-NA values), "mean", "max", "min", "first" (take the first row), "majority" (take the value that is present on the largest number of rows), "consensus" (require that all rows have the same value) and "indicator".<br>Here, "majority" and "consensus" are mostly useful for discrete (integer) values). For "consensus", it is an error if duplicate rows contain distinct values. "indicator" assumes that data are divided into negative (-1), neutral (0) and positive (1) items, such as discretized expression values. The result is (a) 1 if there is a 1 and no -1 value; (b) -1 if there is a -1 and not 1 value; (c) 0 if there is a 0 and no -1 or 1; or (d) NA otherwise.
collapseString	consensus	Method for collapsing non-numeric columns with duplicate rows (when unique=true). Legal values are "first" (take the first row), "majority" (take the value that is present on the largest number of rows), "longest" (take the longest value), "shortest" (take the shortest value) and "consensus" (require that all rows have the same value). For "consensus", it is an error if duplicate rows contain distinct values.
conversionColumn	PatientID	Column in 'conversionTable' that is used to fetch new values for the source column.
keyColumn		Column in 'conversionTable' that is used to match ID values from the source CSV. If the parameter is empty, the first column is used.
originalWhen Missing	true	Determines how values are converted when the item is not found in the conversion table. If true, the original (unconverted) ID is inserted to the output. If false, NA is inserted.
sourceColumn	SampleID	Column in 'csv' that is going to be converted. If empty, the first column is used.
split	false	If true, split values in the source column by commas before ID conversion. For example, if the source column contains the string "a,b,c", it is first split into "a", "b" and "c"; then, individual IDs are converted; finally, the converted IDs are joined by commas into a string of format "A,B,C".
splitConverted	false	If true, split converted values by commas after ID conversion. A row with comma separated values in the target column will be split to otherwise identical rows but so that the rows have a single ID value in the target column. This is done before possible collapsing of duplicate rows.
targetColumn		Target column name in 'converted'. If empty, the column name is the same as the original column (sourceColumn).
unique	true	If true, rows with identical identifiers (sourceColumn) are collapsed into one row so that sourceColumn is unique. Collapsing may result in error if duplicate rows contain conflicting values. See parameters collapseNumeric and collapseString for methods to collapse numeric and non-numeric columns, respectively.
varMeasure		Method for calculating the variation of numeric duplicate values. If the variation is above the threshold given in <code>varThreshold</code> , duplicate values are collapsed to NA. Possible values are 'MAD' and 'variance'. 'MAD' stands for the median absolute deviation and 'variance' for the population variance. If no values is given, no threshold for combining numeric values is used.
varThreshold	2	Maximum allowed variability in the duplicate values. If <code>varMeasure</code> is given duplicate values with variance above the threshold are collapsed to NA

19.315 geneExpression-survivalQuery (INPUT)

(Technical documentation) Imports an external input file or directory to the pipeline. This is the only way external files are imported. File path is given with the parameter "path".

Category: Internal

Parameter name	Value	Description
path	/gpfs/homes/mlaakso/asserSVN/ andurilCaseStudy/sql/ exonSurvival.sql	Path (filename) of the input resource.

19.316 geneExpression-tTest (StatisticalTest)

Compute p-values using statistical tests, optionally with correction for multiple hypotheses. In the current version, only Student's t-test is supported. The t-test can be either paired or non-paired. Two-sided and one-sided are supported. If the data contain missing values, only non-missing values are used.

A test is computed for each row in the input matrix. The columns can be selected using a SampleGroupTable (groups) or, if not present, by giving column names using the parameters targetGroup and

referenceGroup.

If multiple hypothesis correction is enabled (using the correction parameter), corrected p-values are also written to the pvalues output. Identifiers of those rows whose p-value is less than given threshold are written to the idlist output. Identifiers are taken from the first column of the input matrix. If multiple hypothesis correction is enabled, threshold comparison is done using corrected p-values; otherwise, raw p-values are used. The component implements several multiple hypothesis correction methods, some of which produce modified p-values and some produce q-values:

1. Bonferroni correction,
2. false discovery rate (fdr) [4], and
3. Robust estimation of the false discovery rate [19].

Author:	Kristian Ovaska (kristian.ovaska@helsinki.fi)
Version:	1.1
Category:	Analysis

Input name	Source	Description
matrix	geneExpression-varFilter .matrix 19.318	Input matrix, containing values to be tested.
groups	geneExpression-sampleGroups .groups 19.303	If given, must contain the groups named by the parameters targetColumns and referenceColumns. Both groups must have at least two members. The members must be present in the input matrices.

Parameter name	Value	Description
byRow	true	If true, there is one test for each row. If false, there are tests for various column combinations.
contingencyRows	2	For categorical variable independency tests (chi-squared, Fisher), this gives the number of rows in contingency tables. The number of columns is deduced from data. For example, if contingencyRows=3 and target vector has length 12, the table has 4 columns.
correctedColumn	PValueCorrected	Output column name in "pvalues" for corrected p-values. Only used if multiple hypothesis correction has been enabled.
correction	fdr	Type of multiple hypothesis correction. One of "none", "fdr" (Benjamini-Hochberg, 1995), "robustfdr" (Pounds-Cheng 2006), "BY" (FDR, Benjamini-Yekutieli, 2001), "holm", "bonferroni". The value "none" disables correction.
mean	0	Expected mean of the reference distribution (mu) when the reference group is not supplied. Used for t-test and wilcoxon.
outputSet	tumor_vs_normal_tTest	The name of the output set in the idlist output.
paired	false	Indicates whether the test is paired so that i'th element of target vector corresponds to i'th element of reference vector. Ignored for F-test.
prefixColumn		If non-empty, include a column in the pvalues output that contains the outputSet on every row. This parameter gives the name of that column. The column is the first column in pvalues. This prefix column is useful when several separate statistical tests are made and the results are combined into one file. If prefixColumn is empty, do not include the prefix column.
pvalueColumn	PValue	Output column name in "pvalues" for raw p-values.
referenceColumns	normal_tissue	Comma-separated list of reference columns, or, if the groups input is present, the name of a sample group. The special value * means all columns of matrix2 if matrix2 is supplied; otherwise, all columns of matrix are used. May be empty if references are not used.
sided	twosided	For two-sided tests, one of "twosided", "greater" (target greater than reference) or "less" (target smaller than reference).
targetColumns	tumor_solid	Comma-separated list of target columns, or, if the groups input is present, the name of a sample group. The special value * means all columns of matrix.
test	t-test	Type of the statistical test. One of "t-test", "wilcoxon", "chi-squared", "fisher", "F-test", "shapiro" (Shapiro-Wilk), "ks" (Kolmogorov-Smirnov), "cor-pearson", "cor-spearman". Variants of the tests are set with other parameters (sided, paired).
threshold	0.05	P-value threshold for inclusion in the idlist output. Must be between 0 and 1 inclusive. If multiple hypothesis correction has been enabled, the threshold is for corrected p-values.

19.317 geneExpression-uniqProbesSQL (INPUT)

Skip those probes that do not match genes or a mapped to multiple genes.

Category: Internal

Parameter name	Value	Description
path	/gpfs/homes/mlaakso/asserSVN/ andurilCaseStudy/sql/ UniqProbes.sql	Path (filename) of the input resource.

19.318 geneExpression-varFilter (VariationFilter)

VariationFilter is an Anduril component that can filter out those rows from a matrix where the standard deviation is below a threshold. Also rows with less than two non-NA values are filtered as the standard deviation can not be computed. The filter can be used to, for example, remove rows from an expression matrix where the variation between samples is low.

Author: Kristian Ovaska (kristian.ovaska@helsinki.fi)
Version: 1.1
Category: Filter

Input name	Source	Description
matrix	geneExpression-probeJoin .csv 19.299	Input matrix.

Parameter name	Value	Description
columns	*	Names of columns that are included in standard deviation computation. However, all columns are always present in the output matrix. The special value * means all columns.
filterSmall Variation	true	If true, rows with standard deviation BELOW the threshold are filtered out (normal operation). If false, rows with standard deviation ABOVE the threshold are filtered out.
fraction	true	If true, the standard deviation threshold is a fraction of the mean of each row. Otherwise, the threshold is the nominal standard deviation.
threshold	0.1	Standard deviation threshold as a percentage value of the mean of each row (if fraction=true) or as the absolute standard deviation (if fraction=false). For example, assume that fraction=true, the threshold is 0.1 and two rows have means 5 and 3; and standard deviations 0.2 and 0.4. The first row is removed since 0.2 is less than 0.1*5. The second row is kept since 0.4 is greater than 0.1*3.

19.319 geneListFreqs (CSV2IDList)

Extracts one column from the given CSV file and prints it out without duplicates. The selected column may contain entries with comma separated lists of values in which case *isList* parameter may be used to obtain each value separately.

Output is defined as:

$$ids = \bigcup_{i=1}^{9} \pi_{columnIn_i} \sigma_{regexp_i}(table_i). \quad (2)$$

Author: Marko Laakso (Marko.Laakso@Helsinki.FI)
Version: 1.3
Category: convert

Input name	Source	Description
table1	transcriptExpressionResults-all Probes.table 19.889	The mandatory input relation

Parameter name	Value	Description
acceptMissing	false	Files with missing columnIn are accepted as empty if this is true.
columnIn	GeneID	A comma separated list of column names for the IDs of interest in each file. Empty values refer to the first column of the file.
columnOut		Name of the only column of the output list. Empty input refers to the name of the input column.
constants		A comma separated list of values that are always included into the output
isList	false	True if the seleted column contains a comma separated list of values to be splitted
quotation	false	Indicator that can be used to disable quotation of the output values
regexp1		Regular expression for the row filtering in table1. A row is included in the result if this parameter is empty or if values in the given columns match given regular expressions. The parameter has a format COLNAME1=EXPRESSION,COLNAME2=EXPRESSION2 where COLNAMEs are column names in "csv" and EXPRESSIONs are regular expressions using Java syntax. For example, "col=a|b" includes rows where the column col has a value of "a" or "b".
regexp2		Regular expression for the row filtering in table2.
regexp3		Regular expression for the row filtering in table3.
regexp4		Regular expression for the row filtering in table4.
regexp5		Regular expression for the row filtering in table5.
regexp6		Regular expression for the row filtering in table6.
regexp7		Regular expression for the row filtering in table7.
regexp8		Regular expression for the row filtering in table8.
regexp9		Regular expression for the row filtering in table9.

19.320 genotypeDB (INPUT)

Settings for SNPHelistin database (see Section 19.937)

Category: Internal

Parameter name	Value	Description
path	/gpfs/homes/mlaakso/asserSVN/ andurilCaseStudy/csv/snpstorage. properties	Path (filename) of the input resource.

19.321 mainTemplate (LatexTemplate)

This component generates the standard layout configuration for Anduril output documents. The component produces a header and a footer fragment that can be used to build LaTeX documents. The header fragment takes care of the page geometry and other technical details such as library imports and function declarations. Reference list is generated by the footer.

Author: Marko Laakso (Marko.Laakso@Helsinki.FI)
Version: 1.4
Category: Latex

Input name	Source	Description
abstract	abstract.in 19.42	Document abstract including its Latex definition. You may use template entities within this document. The default abstract has been defined in abstractTemplate.tex.
bibtex1	rConfig.citations 19.438	An additional bibliography

Parameter name	Value	Description
authors	Kristian Ovaska,Marko Laakso, Riku Louhimo,Erkka Valo,Viljami Aittom\"aki,Sampsa Hautaniemi	A comma-separated list of document authors on the title page. If authors is empty but title is non-empty, a title page with no author name is printed.
baselineskip	1.2	Baselineskip factor that indicates the line spacing
bibstyle	abbrv	BibTeX reference formatting style
printTOC	true	If true, print Table Of Contents.
title	Analysis of Glioblastoma Samples	Document title on the title page. If empty, no title page is printed. Also see author.
usepackage		Comma-separated list of LaTeX packages that are used in the document. Each package generates a \usepackage{X} line in the header.

19.322 medianExonExpression (MedianExonExpressionAnalysis)

Find differentially expressed genes based on median exon expression.

Parameter name	Value
fcThreshold	2.0
pValueThreshold	0.05

19.323 medianExonExpression-allEntrez (KorvasieniAnnotator)

Korvasieni is an Ensembl [12] based converter for database identifiers and it can be used to convert genome location, gene, transcript, and translation identifiers across multiple biological databases. The selected `keyColumn` may contain entries with comma separated lists of values in which case `isListKey` parameter may be used to obtain each value separately and to process them as they were given in consecutive lines.

Author:	Marko Laakso (Marko.Laakso@Helsinki.FI)
Version:	1.7
Categories:	Annotation, GO

Input name	Source	Description
sourceKeys	medianExonExpression-logratio .expr 19.351	A list of source database keys.
connection	ensembl.in 19.273	Database connection can be defined using this file. The definition of parameters: database.url, database.user, database.password, database.timeout, database.recycle, and database.driver can be found from the documentation of Korvasieni.

Parameter name	Value	Description
echoColumns		A comma separated list of column names for the columns that will be copied to the output. An asterisk (*) can be used to denote all columns execpt the keyColumn.
inputDB	.GeneId	Type of input keys. This must be a database supported by Korvasieni. If the parameter is omitted, the component tries to derive the database from the type of geneID. If this is not possible, an error is returned. You may define three columns in form of chromosome:start-end in case the inputDB is .DNARegion. This format provides a comfortable compatibility with DNARegion datatype.
inputType	Any	Ensembl object type for the input keys (Any, Gene, Transcript, Translation)
isListKey	false	Enables the automatic value splits for the comma separated key column
keyColumn		Name of the key column withing sourceKeys file or an empty string for the first column
maxHits	1	Maximum number of target identifiers for a single source identifier
rename		Comma separated list of column renaming rules (oldname=newname)
skipLevel	never	Skip result rows if the source identifier is unknown or target identifiers are not available. Possible values are: never (no filtering), source (skip if the source ID is unknown), target (skip if no target IDs are found), any (skip if any of the target IDs is missing).
targetDB	_EntrezGene	Comma-separated list of annotation types. Possible values are all databases supported by Korvasieni.
unique	false	This flag can be turned on in order to eliminate duplicate annotations.

19.324 medianExonExpression-allProbes (TableQuery)

Execute an SQL query on input relations and create a result table. TableQuery uses HSQLDB (Hyper-threaded Structured Query Language Database [25]) for executing the query. Consequently, the syntax of the query is defined by HSQLDB.

Author:	Kristian Ovaska (Kristian.Ovaska@Helsinki.FI)
Version:	1.2
Categories:	convert, Filter

Input name	Source	Description
table1	medianExonExpression-logratio .expr 19.351	CSV table 1. The table is referred to as 'table1' in the SQL query.
table2	medianExonExpression-detTTest. pvalues 19.332	CSV table 2. The table is referred to as 'table2' in the SQL query.
query	medianExonExpression-transcript StatisticsQuery.in 19.367	SQL query. Either this file or the query parameter must be provided, but not both.

Parameter name	Value	Description
numIndices		Comma-separated list of index counts for input tables. All indices are single-column indices, running from column 1 to N, where N is retrieved from this parameter. If empty, the default number (1) is used. For example, ",2,,2" sets two indices for table2 and table4 and one index for the rest.
query		SQL query. Either this parameter or the query input must be provided, but not both.

19.325 medianExonExpression-cumDistReport (Plot2D)

Create scatter or line plots having a dot for each supplied (Y, X) coordinate pair. Coordinates for the Y axis must always be provided; values for the X axis are optional. If X coordinates are missing, integers 1 to N are used.

Authors:	Kristian Ovaska (kristian.ovaska@helsinki.fi), Marko Laakso (Marko.Laakso@Helsinki.FI), Sirkku Karinen (sirkku.karinen@Helsinki.FI)
Version:	1.0
Categories:	Latex, Plot

Input name	Source	Description
y	medianExonExpression-tExpr .table 19.366	Coordinates on the Y axis. Columns from this file are selected for plotting using "yColumns".

Parameter name	Value	Description
caption	Distribution of transcript expression values (log2) for the samples.	LaTeX caption of each figure. The caption may contain one or two %s placeholders, which are replaced with the current column names in "y" and "x" (if defined). Column names are not used when multiple plots are created to the same image (imageType = 'single').
colorFunction		Custom function for colors. Example c('red', 'blue', 'green') or rainbow(3) or rainbow(%d), in which %d is replaced with the number of different labels or columns in the plot. Default colors start from black and continue by rainbow()-function until each label or column is assigned with own color.
commonScale	true	If true, all plots have the same limits for X and Y axes. This makes it easier to compare plots. Only relevant when multiple columns have been selected.
dpCm	60	Dots per centimeter. Used for png-images.
drawX	true	If false, do not print X axis ticks and numbers.
height	11	Height of each plot in cm.
imageType	single	Plot multiple images = 'multiple', single image = 'single' or a pairs plot = 'pairs'
labelColumn		Columns in input "label" that is used. If not defined, uses the first column.
legendPosition	off	Legend position 'bottomleft' or 'topright' etc (Can not be used with the pairs plot)
nInf	-2e100	Substitution value for infinitely small values

Continued on next page...

Parameter name	Value	Description
pInf	2e100	Substitution value for infinitely great values
plotType	l	Plot type p=points, l=lines, h=bars
pngImage	true	Switch image format between png and pdf
sectionTitle	Ordered transcript expression values	Title of the LaTeX section or an empty string to omit the section
sectionType	subsection	Type of LaTeX section: usually one of section, subsection or subsubsection. No section statement is written if sectionTitle is empty.
sort	true	If true, sort values before plotting. This creates a cumulative-like distribution plot. (Can not be used with pairs plot)
title		Image title. The caption may contain one or two %s placeholders, which are replaced with the current column names in "y" and "x" (if defined). Column names are not used when multiple plots are created to the same image (imageType = 'single').
width	14	Width of each plot in cm.
xColumns	*	Columns in input "x" that are plotted. The special value * includes all columns except non-numeric ones. There must be as many selected columns in X as there are in Y. Note that the files "y" and "x" may still contain a different number of columns.
xHigh	-0.000001	High bound of the X axis. The special value -0.000001 means that the bound is computed from the data.
xLabel	transcripts	X axis label. The label may contain one %s placeholder, which is replaced with the current column name in "x", or if not defined, in "y".
xLow	-0.000001	Low bound of the X axis. The special value -0.000001 means that the bound is computed from the data.
xTransformation		Numeric R expression that is used to transform X values. The X values are available as a numeric matrix (or vector) under the name "x". This is analogous to "yTransformation".
yColumns	*	Columns in input "y" that are plotted. The special value * includes all columns except non-numeric ones.
yHigh	-0.000001	High bound of the Y axis. The special value -0.000001 means that the bound is computed from the data.
yLabel	intensity	X axis label. The label may contain one %s placeholder, which is replaced with the current column name in "y".
yLow	-0.000001	Low bound of the Y axis. The special value -0.000001 means that the bound is computed from the data.
yTransformation		Numeric R expression that is used to transform Y values. The Y values are available as a numeric matrix (or vector) under the name "y". Only those columns are present that are selected using "yColumns". For example, "-log10(y)" transforms Y values using a negative base 10 logarithm (defined only for positive values). The expression may be any legal R expression that takes a numeric matrix (or vector) and produces a matrix (or vector) of the same size.

19.326 medianExonExpression-deeTableQuery (INPUT)

SQL query to compile the differentially expressed transcript table.

Category: Internal

Parameter name	Value	Description
path	/gpfs/homes/mlaakso/asserSVN/andurilCaseStudy/sql/deeTableQuery.sql	Path (filename) of the input resource.

19.327 medianExonExpression-det (SetTransformer)

SetTransformer transform ID lists into new lists by using flexible rules. The rules are read from a *transformation* file. Please, check JEP 2 documentation for the syntax rules.

Author: Kristian Ovaska (kristian.ovaska@helsinki.fi)
Version: 0.6
Category: Convert

Input name	Source	Description
transformation	medianExonExpression -intersectionTransformation .in 19.339	Set transformations, one per CSV row. The columns Target (target set ID) and Definition (transformation expression) must be present. The column IterationSet may be present if iterated transformations are used. IterationSet should contain NA for non-iterated transformations. Any other columns are interpreted as annotation columns and are copied to the output. For iterated transformations, the wildcard * in annotations is replaced with the current set ID.
set1	medianExonExpression-detFold Change.deg 19.329	Input sets 1. NA values in Members are interpreted as empty sets.
set2	medianExonExpression-detTTest. idlist 19.332	Input sets 2.

Parameter name	Value	Description
includeAnnotation	*	Comma-separated list of column names in the transformation input that should be used as annotation columns. The wildcard * includes all columns. The special columns Target, Definition and IterationSet are excluded automatically.
includeOriginal	true	If true, the original sets from input files are included in the output as well. If false, only sets defined in transformations are included.

19.328 medianExonExpression-detAnnot (KorvasieniAnnotator)

Korvasieni is an Ensembl [12] based converter for database identifiers and it can be used to convert genome location, gene, transcript, and translation identifiers across multiple biological databases. The selected `keyColumn` may contain entries with comma separated lists of values in which case `isListKey` parameter may be used to obtain each value separately and to process them as they were given in consecutive lines.

Author:	Marko Laakso (Marko.Laakso@Helsinki.FI)
Version:	1.7
Categories:	Annotation, GO

Input name	Source	Description
sourceKeys	medianExonExpression-detIDList .ids 19.330	A list of source database keys.
connection	ensembl.in 19.273	Database connection can be defined using this file. The definition of parameters: database.url, database.user, database.password, database.timeout, database.recycle, and database.driver can be found from the documentation of Korvasieni.

Parameter name	Value	Description
echoColumns		A comma separated list of column names for the columns that will be copied to the output. An asterisk (*) can be used to denote all columns execpt the keyColumn.
inputDB	.GeneId	Type of input keys. This must be a database supported by Korvasieni. If the parameter is omitted, the component tries to derive the database from the type of geneID. If this is not possible, an error is returned. You may define three columns in form of chromosome:start-end in case the inputDB is .DNARegion. This format provides a comfortable compatibility with DNARegion datatype.
inputType	Gene	Ensembl object type for the input keys (Any, Gene, Transcript, Translation)
isListKey	false	Enables the automatic value splits for the comma separated key column
keyColumn		Name of the key column withing sourceKeys file or an empty string for the first column
maxHits	100000	Maximum number of target identifiers for a single source identifier
rename		Comma separated list of column renaming rules (oldname=newname)
skipLevel	never	Skip result rows if the source identifier is unknown or target identifiers are not available. Possible values are: never (no filtering), source (skip if the source ID is unknown), target (skip if no target IDs are found), any (skip if any of the target IDs is missing).
targetDB	.GeneId,.GeneName,.GeneDesc, GO	Comma-separated list of annotation types. Possible values are all databases supported by Korvasieni.
unique	false	This flag can be turned on in order to eliminate duplicate annotations.

19.329 medianExonExpression-detFoldChange (FoldChange)

Select differentially expressed genes using the fold change criteria. This filter does not compute the fold changes itself but takes them as input.

A gene with a log ratio intensity l is considered to be **under** expressed if and only if $l \leq -\log_2(threshold)$ and **over** expressed if and only if $l \geq \log_2(threshold)$.

Author: Kristian Ovaska (Kristian.Ovaska@Helsinki.FI)
Version: 1.1
Category: DEG

Input name	Source	Description
logratio	medianExonExpression-logratio .expr 19.351	Log ratio data.

Parameter name	Value	Description
nameDeg	%s_fcDeg	Name of the sets for differentially expressed genes. The pattern %s is expanded to the current sample name in logratio.
nameOver	%s_fcOver	Name of the sets for underexpressed genes. The pattern %s is expanded to the current sample name in logratio.
nameStable	%s_fcStable	Name of the sets for stably expressed genes. The pattern %s is expanded to the current sample name in logratio.
nameUnder	%s_fcUnder	Name of the sets for underexpressed genes. The pattern %s is expanded to the current sample name in logratio.
stableThreshold	0	Fold change threshold (in linear scale) for stable genes. Genes with fold change smaller than the threshold in both directions are considered stably expressed. If the parameter has the value zero, no list of stably expressed genes is included in the results.
threshold	2.0	Fold change threshold (in linear scale). Genes with fold change greater than or equal to the threshold in either direction are considered differentially expressed.

19.330 medianExonExpression-detIDList (CSV2IDList)

Extracts one column from the given CSV file and prints it out without duplicates. The selected column may contain entries with comma separated lists of values in which case *isList* parameter may be used to obtain each value separately.

Output is defined as:

$$ids = \bigcup_{i=1}^{9} \pi_{columnIn_i} \sigma_{regexp_i}(table_i). \quad (3)$$

Author: Marko Laakso (Marko.Laakso@Helsinki.FI)
Version: 1.3
Category: convert

Input name	Source	Description
table1	medianExonExpression-det .result 19.327	The mandatory input relation

Parameter name	Value	Description
acceptMissing	false	Files with missing columnIn are accepted as empty if this is true.
columnIn	Members	A comma separated list of column names for the IDs of interest in each file. Empty values refer to the first column of the file.
columnOut	GeneID	Name of the only column of the output list. Empty input refers to the name of the input column.
constants		A comma separated list of values that are always included into the output
isList	true	True if the seleted column contains a comma separated list of values to be splitted
quotation	false	Indicator that can be used to disable quotation of the output values

Continued on next page...

Parameter name	Value	Description
regexp1	ID=over|under	Regular expression for the row filtering in table1. A row is included in the result if this parameter is empty or if values in the given columns match given regular expressions. The parameter has a format COLNAME1=EXPRESSION,COLNAME2=EXPRESSION2 where COLNAMEs are column names in "csv" and EXPRESSIONs are regular expressions using Java syntax. For example, "col=a|b" includes rows where the column col has a value of "a" or "b".
regexp2		Regular expression for the row filtering in table2.
regexp3		Regular expression for the row filtering in table3.
regexp4		Regular expression for the row filtering in table4.
regexp5		Regular expression for the row filtering in table5.
regexp6		Regular expression for the row filtering in table6.
regexp7		Regular expression for the row filtering in table7.
regexp8		Regular expression for the row filtering in table8.
regexp9		Regular expression for the row filtering in table9.

19.331 medianExonExpression-detReport (DEGReport)

Generates a LaTeX report on differentially expressed genes (DEGs). The report has two parts: a summary section that lists the number of over- and underexpressed genes in each sample group; and a gene list section that lists all the gene names.

Authors:	Kristian Ovaska (Kristian.Ovaska@Helsinki.FI), Marko Laakso (Marko.Laakso@Helsinki.FI)
Version:	1.1.1
Categories:	DEG, Latex

Input name	Source	Description
deg	medianExonExpression-det .result 19.327	Sets of differentially expressed genes. If expression values are given (expr), there must be an annotation column SampleGroup that binds the set to an expression column.
geneAnnotation	medianExonExpression-logratio .expr 19.351	Annotation for genes. Must contain the column named by geneColumn.

Parameter name	Value	Description
columns	4	Number of columns in gene list table.
geneColumn	GeneID	Column name in geneAnnotation that contains gene names. If empty, gene names are not printed.
sectionTitle	Summary of differentially expressed genes: fc > 2.0 and corrected p-value < 0.05	Title for a new latex section. If none is given, a new section is not created.
sectionType	section	Type of LaTeX section: usually one of section, subsection or subsubsection. No section statement is written if sectionTitle is empty.
setPattern	over|under	R regular expression for matching set names. If empty, all sets are included.

19.332 medianExonExpression-detTTest (StatisticalTest)

Compute p-values using statistical tests, optionally with correction for multiple hypotheses. In the current version, only Student's t-test is supported. The t-test can be either paired or non-paired. Two-sided and one-sided are supported. If the data contain missing values, only non-missing values are used.

A test is computed for each row in the input matrix. The columns can be selected using a SampleGroupTable (groups) or, if not present, by giving column names using the parameters targetGroup and referenceGroup.

If multiple hypothesis correction is enabled (using the correction parameter), corrected p-values are also written to the pvalues output. Identifiers of those rows whose p-value is less than given threshold are written to the idlist output. Identifiers are taken from the first column of the input matrix. If multiple hypothesis correction is enabled, threshold comparison is done using corrected p-values; otherwise, raw p-values are used. The component implements several multiple hypothesis correction methods, some of which produce modified p-values and some produce q-values:

1. Bonferroni correction,
2. false discovery rate (fdr) [4], and
3. Robust estimation of the false discovery rate [19].

Author: Kristian Ovaska (kristian.ovaska@helsinki.fi)
Version: 1.1
Category: Analysis

Input name	Source	Description
matrix	medianExonExpression-tExpr .table 19.366	Input matrix, containing values to be tested.
groups	medianExonExpression-sample Groups.groups 19.357	If given, must contain the groups named by the parameters targetColumns and referenceColumns. Both groups must have at least two members. The members must be present in the input matrices.

Parameter name	Value	Description
byRow	true	If true, there is one test for each row. If false, there are tests for various column combinations.
contingencyRows	2	For categorical variable independency tests (chi-squared, Fisher), this gives the number of rows in contingency tables. The number of columns is deduced from data. For example, if contingencyRows=3 and target vector has length 12, the table has 4 columns.
correctedColumn	PValueCorrected	Output column name in "pvalues" for corrected p-values. Only used if multiple hypothesis correction has been enabled.
correction	fdr	Type of multiple hypothesis correction. One of "none", "fdr" (Benjamini-Hochberg, 1995), "robustfdr" (Pounds-Cheng 2006), "BY" (FDR, Benjamini-Yekutieli, 2001), "holm", "bonferroni". The value "none" disables correction.
mean	0	Expected mean of the reference distribution (mu) when the reference group is not supplied. Used for t-test and wilcoxon.
outputSet	tumor_vs_normal_tTest	The name of the output set in the idlist output.
paired	false	Indicates whether the test is paired so that i'th element of target vector corresponds to i'th element of reference vector. Ignored for F-test.
prefixColumn		If non-empty, include a column in the pvalues output that contains the outputSet on every row. This parameter gives the name of that column. The column is the first column in pvalues. This prefix column is useful when several separate statistical tests are made and the results are combined into one file. If prefixColumn is empty, do not include the prefix column.
pvalueColumn	PValue	Output column name in "pvalues" for raw p-values.
referenceColumns	normal_tissue	Comma-separated list of reference columns, or, if the groups input is present, the name of a sample group. The special value * means all columns of matrix2 if matrix2 is supplied; otherwise, all columns of matrix are used. May be empty if references are not used.
sided	twosided	For two-sided tests, one of "twosided", "greater" (target greater than reference) or "less" (target smaller than reference).
targetColumns	tumor_solid	Comma-separated list of target columns, or, if the groups input is present, the name of a sample group. The special value * means all columns of matrix.
test	t-test	Type of the statistical test. One of "t-test", "wilcoxon", "chi-squared", "fisher", "F-test", "shapiro" (Shapiro-Wilk), "ks" (Kolmogorov-Smirnov), "cor-pearson", "cor-spearman". Variants of the tests are set with other parameters (sided, paired).
threshold	0.05	P-value threshold for inclusion in the idlist output. Must be between 0 and 1 inclusive. If multiple hypothesis correction has been enabled, the threshold is for corrected p-values.

19.333 medianExonExpression-detTable (TableQuery)

Execute an SQL query on input relations and create a result table. TableQuery uses HSQLDB (Hyperthreaded Structured Query Language Database [25]) for executing the query. Consequently, the syntax of the query is defined by HSQLDB.

Author: Kristian Ovaska (Kristian.Ovaska@Helsinki.FI)
Version: 1.2
Categories: convert, Filter

Input name	Source	Description
table1	medianExonExpression-detIDList .ids 19.330	CSV table 1. The table is referred to as 'table1' in the SQL query.
table2	medianExonExpression-detTTest. pvalues 19.332	CSV table 2. The table is referred to as 'table2' in the SQL query.
table3	medianExonExpression-logratio .expr 19.351	CSV table 3. The table is referred to as 'table3' in the SQL query.
table4	medianExonExpression-detAnnot .bioAnnotation 19.328	CSV table 4. The table is referred to as 'table4' in the SQL query.
query	medianExonExpression-deeTable Query.in 19.326	SQL query. Either this file or the query parameter must be provided, but not both.

Parameter name	Value	Description
numIndices		Comma-separated list of index counts for input tables. All indices are single-column indices, running from column 1 to N, where N is retrieved from this parameter. If empty, the default number (1) is used. For example, ",2,,2" sets two indices for table2 and table4 and one index for the rest.
query		SQL query. Either this parameter or the query input must be provided, but not both.

19.334 medianExonExpression-detVennDiagram (VennDiagram)

VennDiagram draws Venn diagrams showing the intersections of the given sets of identifiers.

Authors: Erkka Valo (erkka.valo@helsinki.fi), Kristian Ovaska (kristian.ovaska@helsinki.fi)
Version: 1.1.1
Category: Plot

Input name	Source	Description
sets	medianExonExpression-det .result 19.327	Sets containing identifiers.

Parameter name	Value	Description
cex	0.4	A numerical value giving the amount by which the text in the plot should be scaled relative to the default values.
names1	T-test,over,under	Names of the sets to be used in the first Venn diagram. Names are given in a comma-separated list and mapped to sets in <code>sets1</code> positionally. If non are given, the set ID is used as set name.
names2		Names of the sets for the second Venn diagram.
names3		Names of the sets for the third Venn diagram.
names4		Names of the sets for the forth Venn diagram.
sectionTitle		Title for a new latex section. If non is given, a new section is not created.
sectionType	section	Type of LaTeX section: usually one of section, subsection or subsubsection. No section statement is written if sectionTitle is empty.
sets1	tumor_vs_normal_tTest,tumor_vs_normal_fcOver,tumor_vs_normal_fcUnder	A comma-separated list of minimum of two and maximum of three sets identified by the set IDs for which the Venn diagram is drawn.
sets2		Sets for the second Venn diagram.
sets3		Sets for the third Venn diagram.
sets4		Sets for the fourth Venn diagram.

19.335 medianExonExpression-exonSPIArefs (INPUT)

(Technical documentation) Imports an external input file or directory to the pipeline. This is the only way external files are imported. File path is given with the parameter "path".

Category: Internal

Parameter name	Value	Description
path	/gpfs/homes/mlaakso/asserSVN/andurilCaseStudy/csv/exonSPIArefs.csv	Path (filename) of the input resource.

Continued on next page...

Parameter name	Value	Description

19.336 medianExonExpression-filterSamplesRScript (INPUT)

R Script for filtering out samples without clinical annotation.

Category: Internal

Parameter name	Value	Description
path	/gpfs/homes/mlaakso/asserSVN/andurilCaseStudy/r/filterSamples.R	Path (filename) of the input resource.

19.337 medianExonExpression-goClusters (GOClustering)

This Anduril component uses Gene ontology (GO) [1] annotations to cluster a set of genes. Each cluster consists of genes that share similar GO annotations.

The GO clustering of the genes can be visualized together with expression data (optional). Samples of the expression data are clustered based on their expression profiles and a heatmap is formed by clustering the y-axis of genes using their GO annotations. Component is based on the method descibed in [17].

Authors: Ping Chen (ping.chen@cs.helsinki.fi), Kristian Ovaska (Kristian.Ovaska@Helsinki.FI), Marko Laakso (Marko.Laakso@Helsinki.FI), Erkka Valo (Erkka.Valo@Helsinki.FI)

Version: 1.1.2

Categories: Clustering, GO

Input name	Source	Description
goAnnotations	medianExonExpression-detAnnot .bioAnnotation 19.328	GO annotations for the genes
expr	medianExonExpression-tExpr .table 19.366	Gene expressions

Parameter name	Value	Description
ColumnWidth	15	Control the column width of the GO MEMBER table.
asBitmap	true	Produce heatmap in Portable network graphics (PNG) format instead of Portable document format (PDF). PNG bitmaps are more suitable if the heatmap contains large number of samples and genes.
columnMargin	0	Margin for column names in heatmap
cutLimit	0.5	Clustering cut off height for the hierachical clustering of the genes. This limit is between 0 and 1, where 0 refers to more specific groups.
decimal	3	Control the output of long decimal.
drawHeatmap	true	If true, draw a heatmap.
dropUnknown	true	Genes with no GO annotations are excluded if this flag is true
geneColumn		Name of the gene name column in the annotation file. The value is used for creating the report. The given column is used for listing all the members in the clusters to the report. If the value is empty, cluster member lists are not put into the report.
goColumn	GO	Name of the GO annotation column in the annotation file
grayScale	false	If true, heatmap is in grayscale. If false, heatmap is colorful.
heatmapScale	row	Determines how the values for the heatmap are scaled. Legal values are ”row”, ”column” and ”none”. This corresponds to the scale parameter of the R function heatmap.
organism	9606	NCBI taxonomy ID for the organism whose gene set is used for GO probabilities. This is used if the input similarityTable is not given. Supported organisms: Homo sapiens: 9606, Saccharomyces cerevisiae: 4932, Caenorhabditis elegans: 6239, Drosophila melanogaster: 7227, Mus musculus: 10090, Rattus norvegicus: 10116.
pvalues	false	If true, compute p-values for information contents of the clusters using a permutation test. The p-values are shown in their own table in the report. The permutation test may be slow.
rowMargin	5	Margin for row names in heatmap

Continued on next page...

Parameter name	Value	Description
sectionType	subsection	Type of LaTeX section: usually one of section, subsection or subsubsection. No section statement is written if <code>sectionTitle</code> is an empty string.
showClusterNames	true	If true, show cluster names G1, ..., Gn on the right side of the heat map. If false, show gene names.
title	GO clustering	Title for the subsection and for the heatmap image.
xLab	Samples	Label for the x-axis of the heatmap
yLab	GO.groups	Label for the y-axis of the heatmap

19.338 medianExonExpression-groupMeans (SampleCombiner)

Combine expression data from several samples into one by taking means, medians or log ratios. Combining is done using a sample group table. Each row of the table specifies how one sample group is created from a list of source groups. If the expression input file contains log ratios, both median and log ratio combining are possible and the result are log ratios. If the input are channel values, the groups may be either median groups (result type: channel values) or log ratio groups (result type: log ratio), but not both.

Authors: Kari Nousiainen (Kari.Nousiainen@Helsinki.FI), Kristian Ovaska (Kristian.Ovaska@Helsinki.FI)
Version: 1.0
Category: Filter

Input name	Source	Description
expr	medianExonExpression-tExpr .table 19.366	Expression data, either log ratios or channel expressions.
groups	medianExonExpression-sample Groups.groups 19.357	Sample group specification.

Parameter name	Value	Description
geometricMean	true	Indicates whether the means will be calculated using geometric(true) or arithmetic mean (false).
groupIDs	tumor_solid,normal_tissue	Comma-separated list of sample group IDs. If given, combine only these groups. If not given (empty), combine all sample groups.
includeOriginal	false	If true, put input groups into the result even if they are not present in 'groups'.
thresholdRatio	0	Allows to omit those genes that have missing values in some samples. Only genes that have non-missing values in at least the given number of columns are included in the result. The actual threshold T is ceiling(thresholdRatio*n), where n is the number of samples that are used in combining. Only genes that have non-missing values in at least T columns are included. Other genes have the combined value set to NA. Note: this parameter specifies the ratio instead of integer threshold since the number of samples may be different in each sample group.

19.339 medianExonExpression-intersectionTransformation (INPUT)

Intersection transformation of differentially expressed transcripts selected by fold change and differentialyy expressed transcripts selected by t-test.

Category: Internal

Parameter name	Value	Description
path	/gpfs/homes/mlaakso/asserSVN/ andurilCaseStudy/csv/ FoldChangeAndTTest.csv	Path (filename) of the input resource.

19.340 medianExonExpression-km (TranscriptSurvival)

Survival analysis based on transcript expression.

Parameter name	Value
annotCol	.GeneName,.GeneDesc
fcThreshold	2.0
minCount	20
pLimit	0.05
title	Survival analysis for gene expression

19.341 medianExonExpression-km-km (KaplanMeier)

Produces a Kaplan-Meier plot representing survival estimates based on the given data. Log-rank statistics are calculated in order the address the similarity of the curves.

Authors: Marko Laakso (Marko.Laakso@Helsinki.FI), Riku Louhimo (Riku.Louhimo@Helsinki.FI), Kristian Ovaska (kristian.ovaska@helsinki.fi)
Version: 1.7
Categories: Survival, Latex

Input name	Source	Description
survival	medianExonExpression-km -survivalTableConv.csv 19.350	Survival time and event indicators together with the sample group assignments
annotation	medianExonExpression-detAnnot .bioAnnotation 19.328	Additional annotations for the groupCol names. These annotations are listed as column name value pairs in figure caption.

Parameter name	Value	Description
PLimit	0.05	Ignore results with a p-value greater than this limit
annotCol	.GeneName,.GeneDesc	A comma separated list of column names of the annotation file that will be shown in the report. An asterisk (*) may be used for all attributes except the first one that is considered as a key.
confInt	0.95	Coverage of the confidence intervals [0,1] or a negative number to disable them
eventSymbol	DECEASED	Value of statusCol column indicating an event. Other values are considered as censorings.
excludeCol	SampleID,PatientID	A comma separated list of column names that should not be used as groupCols even if an asterisk is used. This is especially useful to filter out samples identifiers and other irrelevant columns.
groupCol	*	A comma separated list of column names of sample group identifiers or an empty string for a single curve. An asterisk (*) may be used to plot all attributes except those defined for the other purposes or listed in excludeCol.
minCount	20	Individual groups with less that this many members will be left out
statusCol	VitalStatus	Column name for the event indicator
timeCol	SurvivalEndTime	Column name for the survival time
timeOutLimit	36	Cut survival curves after this limit. A negative value can be used to maximize the length of the period.
title	Kaplan-Meier survival estimates	Figure title
xLabel	survival time	Label for the horizontal time axis

19.342 medianExonExpression-km-kmReport (LatexCombiner)

LatexCombiner joins the given subdocuments together into one LaTeX file.

Authors: Kristian Ovaska (Kristian.Ovaska@Helsinki.FI), Marko Laakso (Marko.Laakso@Helsinki.FI)
Version: 1.3
Category: Latex

Input name	Source	Description
latex1	medianExonExpression-km-km .report 19.341	LaTeX fragment 1.

Parameter name	Value	Description
pagebreak	true	Determines if the result document should start with a page break.

Continued on next page...

Parameter name	Value	Description
sectionTitle	Survival analysis for gene expression	If non-empty, a declaration of a new section with the given name is inserted to the beginning of the combined document. This is a convenience feature to make it easy to compile subsections into a section.
sectionType	section	Type of LaTeX section: usually one of section, subsection or subsubsection. No section statement is written if sectionTitle is empty.
strictBorders	true	Enables the flushing of all document elements before each fragment

19.343 medianExonExpression-km-sampleExpression (SampleExpression)

Create a $\{-1, 0, 1\}$ matrix that indicates whether a given gene/probe is differentially expressed in individual samples. The decision whether a gene is differentially expressed is done:

a) using fold change thresholding or
b) by selecting P largest and P smallest log ratios, where P is a proportion.

The expression input can be either log ratios or channel values, which are handled differently. Log ratios are directly used for fold change thresholding. Channel values are converted to log ratios by dividing them with reference samples. In this case, the expression matrix is assumed to contain both the target and reference samples. Reference samples are converted to a single column by taking a median. The columns that contain reference values are defined in the groups input. If the groups input is not given, values are assumed to be log ratios.

Author: Kristian Ovaska (kristian.ovaska@helsinki.fi)
Version: 1.0
Category: DEG

Input name	Source	Description
expr	medianExonExpression-tExpr .table 19.366	Expression matrix. The values are assumed to be log ratios if the "groups" input is not given; otherwise, they are assumed to be channel values.
groups	medianExonExpression-sample Groups.groups 19.357	Sample groups that define reference (control) columns in the expression input. The reference group name is given with the referenceGroup parameter.

Parameter name	Value	Description
referenceGroup	normal_tissue	Group name in the groups input that contains reference samples. Used to compute log ratios from channel values.
threshold	2.0	Threshold that determines whether a gene is under- or overexpressed. Interpretation of the value depends on thresholdType.
thresholdType	fold-change	Defines how differential expression is decided. Legal values are "fold-change" and "top-most". For "fold-change", the threshold parameter is a linear fold change threshold. A gene is overexpressed if its log ratio is over the threshold and underexpressed if the log ratio is under 1/threshold. For "top-most", threshold is a fraction between 0 and 1 that defines how many genes with highest and lowest expression are selected. For example, if threshold is 0.1, the top 10% genes are overexpressed and the bottom 10% are underexpressed.

19.344 medianExonExpression-km-sampleExpressionFilt (CSVFilter)

Filters columns and/or rows from CSV files using flexible criteria. Default settings do not filter out anything. The *negate* parameter reverses all inclusion criteria.

Columns are filtered based on column names (see *includeColumns*); it is also possible to rename columns. Columns can be reordered using *includeColumns*.

A row is included in output if all conditions are satisfied. Rows having too many missing values are filtered using the *nonMissing* parameter. The parameters *regexp*, *lowBound* and *highBound* filter rows based on cell contents. If the *auxiliary* input is given, the result contains those rows from the input whose values match those from the auxiliary file.

Authors: Kristian Ovaska (kristian.ovaska@helsinki.fi), Marko Laakso (Marko.Laakso@Helsinki.FI)
Version: 1.1
Category: Filter

Input name	Source	Description
csv	medianExonExpression-km -sampleExpression .indicator 19.343	CSV file to be filtered.
auxiliary	medianExonExpression-detIDList .ids 19.330	If given, contains one column (see "matchColumn") whose values are matched to a column in the "csv" input (see "idColumn").

Parameter name	Value	Description
highBound		A row is included if the value in given numeric columns are at most as large as given numeric bounds. The parameter has a format COLNAME1=HIGH1,COLNAME2=HIGH2.
idColumn		Column name in "csv" that contains ID values. If the parameter is empty, the first column is used.
includeColumns	*	Comma-separated list of column names that should be included in the result file. The special value * includes all columns. The order of column names is significant: the output columns are in the order given. See also includeColumns input.
lowBound		A row is included if the value in given numeric columns are at least as large as given numeric bounds. The parameter has a format COLNAME1=LOW1,COLNAME2=LOW2. For example, "col=5" includes rows where the column col has a value of at least 5.
matchColumn		Column name in "auxiliary" containing values that must match the ID column in "csv" (specified using "idColumn"). If empty, the first column of "auxiliary" is used.
negate	false	If true, all inclusion criteria are negated so that rows or columns are excluded instead of included if they match the criteria. For example, includeColumns=* then excludes all columns (which would be an error since at least one column must be included).
nonMissing	0	Include those rows that have non-missing (non-NA) values in at least this many columns. Only those columns that are part of the output are counted.
regexp		Row filtering based on regular expression. A row is included in the result if values in the given columns match given regular expressions. The parameter has a format COLNAME1=EXPRESSION,COLNAME2=EXPRESSION2 where COLNAMEs are column names in "csv" and EXPRESSIONs are regular expressions using Java syntax. For example, "col=a|b" includes rows where the column col has a value of "a" or "b". Commas have to be escaped with "\\" if they are used in column names or regular expressions.
rename		Comma-separated list of column renaming rules (OLDNAME=NEWNAME). Parameters that refer to column names refer to old names; renaming is done last.

19.345 medianExonExpression-km-sampleExpressionTrans (MatrixTranspose)

Produces a transposed version of the input matrix.

Author: Kristian Ovaska (kristian.ovaska@helsinki.fi)
Version: 1.0
Category: convert

Input name	Source	Description
matrix	medianExonExpression-km -sampleExpressionFilt.csv 19.344	Input matrix.

Parameter name	Value	Description
rowName	SampleID	Name of the first column in the output.

19.346 medianExonExpression-km-sampleNames (ExpandCollapse)

Converts between the two possible representations of a relations with multivalued columns. By default this component splits rows with multivalued (comma separated lists) columns into multiple rows each representing a single value. The input may contain several columns with comma separated values and all permutations of column values are shown as individual single-valued rows. The alternative mode recovers the original comma separated values from the expanded relations. This is performed by joining values of the their list columns into comma separated lists.

Author: Marko Laakso (Marko.Laakso@Helsinki.FI)
Version: 1.3
Category: convert

Input name	Source	Description
relation	medianExonExpression-sample Groups.groups 19.357	The mandatory input relation

Parameter name	Value	Description
delim	,	Value delimiter between the list column values. Special characters can be encoded as specified in fi.helsinki.ltdk.csbl.asser.ArgumentEncoding.
expand	true	Action mode that is expand (true) or collapse (false)
listCols	*	A comma separated list of column names of input (expand) or output (collapse) columns that may contain delim separated values. The asterisk refers to every column of the input relation.

19.347 medianExonExpression-km-survival (TableQuery)

Execute an SQL query on input relations and create a result table. TableQuery uses HSQLDB (Hyperthreaded Structured Query Language Database [25]) for executing the query. Consequently, the syntax of the query is defined by HSQLDB.

Author: Kristian Ovaska (Kristian.Ovaska@Helsinki.FI)
Version: 1.2
Categories: convert, Filter

Input name	Source	Description
table1	medianExonExpression-km -sampleNames.relation 19.346	CSV table 1. The table is referred to as ’table1’ in the SQL query.
table2	medianExonExpression-metadata .in 19.353	CSV table 2. The table is referred to as ’table2’ in the SQL query.
query	medianExonExpression-km -survivalQuery.in 19.348	SQL query. Either this file or the query parameter must be provided, but not both.

Parameter name	Value	Description
numIndices		Comma-separated list of index counts for input tables. All indices are single-column indices, running from column 1 to N, where N is retrieved from this parameter. If empty, the default number (1) is used. For example, ”,2,,2” sets two indices for table2 and table4 and one index for the rest.
query		SQL query. Either this parameter or the query input must be provided, but not both.

19.348 medianExonExpression-km-survivalQuery (INPUT)

Generates a table of samples and their survival times.

Category: Internal

Parameter name	Value	Description
path	/gpfs/homes/mlaakso/asserSVN/andurilCaseStudy/sql/exonSurvival.sql	Path (filename) of the input resource.

19.349 medianExonExpression-km-survivalTable (CSVJoin)

Join rows from two or more CSV files, optionally using one column as a matching key. If a key column is not used, the result contains all rows and all columns of the input files. Missing values (NA) may be introduced when a column is not present in all input files. Each column is present once and duplicate rows are removed.

If a key column is used, the rows in each input file are matched using values from the key column. The result file has one row for each key value. In the result, the first column is the key column; its name is obtained from the first CSV input (csv1). If the intersection parameter is true, a key is included in the result if the key value is present in all inputs. If intersection is false, a key is included if it is present in at least one input (key union). Union semantics may introduce missing values in the result. If several input files have the same column, the value is obtained from the first file. However, if the first file contains a missing value and the second file contains a non-missing value, the non-missing value is used instead.

Author: Kristian Ovaska (kristian.ovaska@helsinki.fi)
Version: 1.0
Category: convert

Input name	Source	Description
csv1	medianExonExpression-km -survival.table 19.347	CSV file 1.
csv2	medianExonExpression-km -sampleExpressionTrans .matrix 19.345	CSV file 2.

Parameter name	Value	Description
intersection	true	Defines how keys are handled; only used when useKeys=true. If intersection is true, the result contains a key if the key is present in all input files. If false, the result contains a key if the key is present in at least one input file.
keyColumnNames		Comma-separated list of key column names; only used when useKeys=true. The first name refers to csv1, the second to csv2, etc. An empty value refers to the first column. Empty values may be omitted from the list, so all these are equivalent: "col1" ; "col1," ; "col1,," ; etc.
useKeys	true	If true, use one column from each CSV file as a matching key column.

19.350 medianExonExpression-km-survivalTableConv (IDConvert)

Converts identifiers from a column in a CSV file to another type of identifiers. The conversion is done using an annotation table on the basis of the column named *conversionColumn*.

Author: Kristian Ovaska (kristian.ovaska@helsinki.fi)
Version: 1.0
Category: convert

Input name	Source	Description
csv	medianExonExpression-km -survivalTable.csv 19.349	Source CSV file. Contains the column that is to be converted. The column is named by the parameter 'sourceColumn'.
conversionTable	medianExonExpression-km -survival.table 19.347	Conversion table. Must contain the column named by the parameter 'conversionColumn'.

Parameter name	Value	Description
collapseNumeric	indicator	Method for collapsing numeric columns with duplicate rows (when unique=true). Legal values are "median" (take median of non-NA values), "mean", "max", "min", "first" (take the first row), "majority" (take the value that is present on the largest number of rows), "consensus" (require that all rows have the same value) and "indicator".<br>Here, "majority" and "consensus" are mostly useful for discrete (integer) values). For "consensus", it is an error if duplicate rows contain distinct values. "indicator" assumes that data are divided into negative (-1), neutral (0) and positive (1) items, such as discretized expression values. The result is (a) 1 if there is a 1 and no -1 value; (b) -1 if there is a -1 and not 1 value; (c) 0 if there is a 0 and no -1 or 1; or (d) NA otherwise.
collapseString	consensus	Method for collapsing non-numeric columns with duplicate rows (when unique=true). Legal values are "first" (take the first row), "majority" (take the value that is present on the largest number of rows), "longest" (take the longest value), "shortest" (take the shortest value) and "consensus" (require that all rows have the same value). For "consensus", it is an error if duplicate rows contain distinct values.
conversionColumn	PatientID	Column in 'conversionTable' that is used to fetch new values for the source column.
keyColumn		Column in 'conversionTable' that is used to match ID values from the source CSV. If the parameter is empty, the first column is used.
originalWhen Missing	true	Determines how values are converted when the item is not found in the conversion table. If true, the original (unconverted) ID is inserted to the output. If false, NA is inserted.
sourceColumn	SampleID	Column in 'csv' that is going to be converted. If empty, the first column is used.
split	false	If true, split values in the source column by commas before ID conversion. For example, if the source column contains the string "a,b,c", it is first split into "a", "b" and "c"; then, individual IDs are converted; finally, the converted IDs are joined by commas into a string of format "A,B,C".
splitConverted	false	If true, split converted values by commas after ID conversion. A row with comma separated values in the target column will be split to otherwise identical rows but so that the rows have a single ID value in the target column. This is done before possible collapsing of duplicate rows.
targetColumn		Target column name in 'converted'. If empty, the column name is the same as the original column (sourceColumn).
unique	true	If true, rows with identical identifiers (sourceColumn) are collapsed into one row so that sourceColumn is unique. Collapsing may result in error if duplicate rows contain conflicting values. See parameters collapseNumeric and collapseString for methods to collapse numeric and non-numeric columns, respectively.
varMeasure		Method for calculating the variation of numeric duplicate values. If the variation is above the threshold given in <code>varThreshold</code> , duplicate values are collapsed to NA. Possible values are 'MAD' and 'variance'. 'MAD' stands for the median absolute deviation and 'variance' for the population variance. If no values is given, no threshold for combining numeric values is used.
varThreshold	2	Maximum allowed variability in the duplicate values. If <code>varMeasure</code> is given duplicate values with variance above the threshold are collapsed to NA

19.351 medianExonExpression-logratio (SampleCombiner)

Combine expression data from several samples into one by taking means, medians or log ratios. Combining is done using a sample group table. Each row of the table specifies how one sample group is created from a list of source groups. If the expression input file contains log ratios, both median and log ratio combining are possible and the result are log ratios. If the input are channel values, the groups may be either median groups (result type: channel values) or log ratio groups (result type: log ratio), but not both.

Authors: Kari Nousiainen (Kari.Nousiainen@Helsinki.FI), Kristian Ovaska (Kristian.Ovaska@Helsinki.FI)
Version: 1.0
Category: Filter

Input name	Source	Description
expr	medianExonExpression-group Means.expr 19.338	Expression data, either log ratios or channel expressions.
groups	medianExonExpression-sample Groups.groups 19.357	Sample group specification.

Parameter name	Value	Description
geometricMean	true	Indicates whether the means will be calculated using geometric(true) or arithmetic mean (false).
groupIDs	tumor_vs_normal	Comma-separated list of sample group IDs. If given, combine only these groups. If not given (empty), combine all sample groups.
includeOriginal	false	If true, put input groups into the result even if they are not present in 'groups'.
thresholdRatio	0	Allows to omit those genes that have missing values in some samples. Only genes that have non-missing values in at least the given number of columns are included in the result. The actual threshold T is ceiling(thresholdRatio*n), where n is the number of samples that are used in combining. Only genes that have non-missing values in at least T columns are included. Other genes have the combined value set to NA. Note: this parameter specifies the ratio instead of integer threshold since the number of samples may be different in each sample group.

19.352 medianExonExpression-mdsReport (MDSPlot)

MDSPlot plots multidimensional scaling on expression data.

This method is used in quality control to visualize similarity within classes and between classes. It works by comparing similarities in the expression values and then assigning them to a 2- or 3-dimensional space.

Author: Kari Nousiainen (Kari.Nousiainen@Helsinki.FI)
Version: 1.0
Categories: Quality Control, Latex

Input name	Source	Description
expr	medianExonExpression-tExpr .table 19.366	Expression data. Can be either channel data or log ratios.
groups	medianExonExpression-sample Groups.groups 19.357	Sample group table maps samples to sample groups. The information is used in plotting to label the samples within the same group with the same color. Note that, a sample may include in many groups. However, the component use only groups whose type is either median or sample.

Parameter name	Value	Description
atomic	false	This parameters shows whether the samples groups are atomic or contain many samples.
caption	Multidimensional Scaling	Label of the figure.
cex	0.2	A numerical value giving the amount by which the size of the sample labels in the plot should be scaled relative to the default value.
dimensions	2	Number of dimensions the data is to be scaled into.
legendPosition	topright	Position of the legend box. One of "bottomright", "bottom", "bottomleft", "left", "topleft", "top", "topright", "right", "center" or "off". If "off", the legend is not shown.
pagebreak	false	Tells if the result document should start with a page break.
plotNames	true	If true sample names are plotted in the MDS plot, else circles are used.
sectionTitle	Multidimensional scaling of the samples	Title of the LaTeX section.
sectionType	subsection	Type of LaTeX section: usually one of section, subsection or subsubsection. If empty, no section statement is written.
width	16	Width of diagram in cm.

19.353 medianExonExpression-metadata (INPUT)

Patient metadata for survival analysis.

Category: Internal

Parameter name	Value	Description
path	/gpfs/homes/mlaakso/asserSVN/ andurilCaseStudy/data/patient-metadata.csv	Path (filename) of the input resource.

19.354 medianExonExpression-qcReport (LatexCombiner)

LatexCombiner joins the given subdocuments together into one LaTeX file.

Authors:	Kristian Ovaska (Kristian.Ovaska@Helsinki.FI), Marko Laakso (Marko.Laakso@Helsinki.FI)
Version:	1.3
Category:	Latex

Input name	Source	Description
latex1	medianExonExpression-cumDist Report.plot 19.325	LaTeX fragment 1.
latex2	medianExonExpression-mds Report.report 19.352	LaTeX fragment 2.

Parameter name	Value	Description
pagebreak	false	Determines if the result document should start with a page break.
sectionTitle		If non-empty, a declaration of a new section with the given name is inserted to the beginning of the combined document. This is a convenience feature to make it easy to compile subsections into a section.
sectionType	section	Type of LaTeX section: usually one of section, subsection or subsubsection. No section statement is written if sectionTitle is empty.
strictBorders	true	Enables the flushing of all document elements before each fragment

19.355 medianExonExpression-reference (CSV2IDList)

Extracts one column from the given CSV file and prints it out without duplicates. The selected column may contain entries with comma separated lists of values in which case *isList* parameter may be used to obtain each value separately.

Output is defined as:

$$ids = \bigcup_{i=1}^{9} \pi_{columnIn_i} \sigma_{regexp_i}(table_i). \qquad (4)$$

Author:	Marko Laakso (Marko.Laakso@Helsinki.FI)
Version:	1.3
Category:	convert

Input name	Source	Description
table1	medianExonExpression-allEntrez .bioAnnotation 19.323	The mandatory input relation

Parameter name	Value	Description
acceptMissing	false	Files with missing columnIn are accepted as empty if this is true.
columnIn	EntrezGene	A comma separated list of column names for the IDs of interest in each file. Empty values refer to the first column of the file.
columnOut		Name of the only column of the output list. Empty input refers to the name of the input column.
constants		A comma separated list of values that are always included into the output
isList	false	True if the seleted column contains a comma separated list of values to be splitted
quotation	false	Indicator that can be used to disable quotation of the output values
regexp1		Regular expression for the row filtering in table1. A row is included in the result if this parameter is empty or if values in the given columns match given regular expressions. The parameter has a format COLNAME1=EXPRESSION,COLNAME2=EXPRESSION2 where COLNAMEs are column names in "csv" and EXPRESSIONs are regular expressions using Java syntax. For example, "col=a|b" includes rows where the column col has a value of "a" or "b".
regexp2		Regular expression for the row filtering in table2.
regexp3		Regular expression for the row filtering in table3.
regexp4		Regular expression for the row filtering in table4.

Continued on next page...

Parameter name	Value	Description
regexp5		Regular expression for the row filtering in table5.
regexp6		Regular expression for the row filtering in table6.
regexp7		Regular expression for the row filtering in table7.
regexp8		Regular expression for the row filtering in table8.
regexp9		Regular expression for the row filtering in table9.

19.356 medianExonExpression-report (LatexCombiner)

LatexCombiner joins the given subdocuments together into one LaTeX file.

Authors: Kristian Ovaska (Kristian.Ovaska@Helsinki.FI), Marko Laakso (Marko.Laakso@Helsinki.FI)
Version: 1.3
Category: Latex

Input name	Source	Description
latex1	medianExonExpression-detReport .summary 19.331	LaTeX fragment 1.
latex2	medianExonExpression-detVenn Diagram.report 19.334	LaTeX fragment 2.
latex3	medianExonExpression-qcReport .document 19.354	LaTeX fragment 3.
latex4	medianExonExpression-goClusters .report 19.337	LaTeX fragment 4.
latex5	medianExonExpression-km-km Report.document 19.342	LaTeX fragment 5.
latex6	medianExonExpression-spia Report.report 19.360	LaTeX fragment 6.

Parameter name	Value	Description
pagebreak	false	Determines if the result document should start with a page break.
sectionTitle		If non-empty, a declaration of a new section with the given name is inserted to the beginning of the combined document. This is a convenience feature to make it easy to compile subsections into a section.
sectionType	section	Type of LaTeX section: usually one of section, subsection or subsubsection. No section statement is written if sectionTitle is empty.
strictBorders	true	Enables the flushing of all document elements before each fragment

19.357 medianExonExpression-sampleGroups (SampleGroupCreator)

Creates sample group tables based on sample names read from data files. These tables define relationship between samples. For example, which samples are biological or technical replicates. The list of sample names is read from data files (either column names or one column along rows) and sample groups are defined using regular expressions. Also, constant groups where the set of member samples does not depend on data can be created. The type of each definition pattern is given with *patternTypes*, which is one of *re*, *relist* or *verbatim* for each pattern.

Author: Kristian Ovaska (kristian.ovaska@helsinki.fi)
Version: 1.2.1
Category: preprocessing

Input name	Source	Description
data1	medianExonExpression-tExpr .table 19.366	Data file 1 for reading sample names. Either column names or values on one column contain sample names.

Parameter name	Value	Description
columns		Defines which columns in input files contain sample names, or whether column names should be used (default). This parameter is a comma-separated list of at most three values which name columns in inputs data1 to data3. If an entry is empty, the column names of the corresponding input file are used instead.
definition1	tumor_solid,mean,Solid tumor samples	Definition of group 1. If empty, the group is omitted. Format: NAME,TYPE or NAME,TYPE,DESCRIPTION.
definition2	normal_tissue,mean,Normal tissue samples	Definition of group 2. If empty, the group is omitted.
definition3	tumor_vs_normal,ratio,fold change of tumor median vs. normal median	Definition of group 3. If empty, the group is omitted.
definition4		Definition of group 4. If empty, the group is omitted.
definition5		Definition of group 5. If empty, the group is omitted.
definition6		Definition of group 6. If empty, the group is omitted.
definition7		Definition of group 7. If empty, the group is omitted.
definition8		Definition of group 8. If empty, the group is omitted.
definition9		Definition of group 9. If empty, the group is omitted.
pattern1	TCGA_[0-9]{2}_[0-9]{4}_01	Pattern for group 1. If empty, the group is omitted. Format depends on the pattern type.
pattern2	TCGA_[0-9]{2}_[0-9]{4}_11	Pattern for group 2. If empty, the group is omitted.
pattern3	tumor_solid,normal_tissue	Pattern for group 3. If empty, the group is omitted.
pattern4		Pattern for group 4. If empty, the group is omitted.
pattern5		Pattern for group 5. If empty, the group is omitted.
pattern6		Pattern for group 6. If empty, the group is omitted.
pattern7		Pattern for group 7. If empty, the group is omitted.
pattern8		Pattern for group 8. If empty, the group is omitted.
pattern9		Pattern for group 9. If empty, the group is omitted.
patternTypes	re,re,verbatim	Comma-separated list of pattern types for each pattern. Each item is one of "re" (default), "relist" or "verbatim". The types are explained above. An empty value is interpreted as "re". For example, ",re,relist,,verbatim" specifies that pattern3 has the type "relist" and pattern5 the type "verbatim"; all others (including pattern6 and above) have the type "re".

19.358 medianExonExpression-samplesWithoutClinicalAnnotation (INPUT)

List of samples without clinical annotation.

Category: Internal

Parameter name	Value	Description
path	/gpfs/homes/mlaakso/asserSVN/andurilCaseStudy/csv/samplesWithoutClinicalAnnotation.csv	Path (filename) of the input resource.

19.359 medianExonExpression-spia (SPIA)

SPIA implements the Signaling Pathway Impact Analysis (SPIA) algorithm described in [24] and [6]. SPIA uses the information from a set of differentially expressed genes and their fold changes, as well as pathways topology in order to assess the significance of the pathways in the condition under the study. The current version of SPIA includes pathway data for 61 KEGG human signaling pathways.

Table 113: Columns of the pathways output.

	column	description
1	Name	Name of the pathway
2	ID	KEGG ID of the pathway (eg. hsa04612)
3	pSize	Number of genes in the pathway
4	NDE	Number of differentially expressed genes in the pathway
5	tA	Observed total perturbation accumulation in the pathway
6	pNDE	Probability of observing at least NDE differentially expressed genes on the pathway by chance
7	pPERT	Probability of of observing a total perturbation accumulation more extreme than tA by chance
8	pG	Global p-value obtained by combining pNDE and pPERT
9	pGFdr	False Discovery Rate adjusted global p-value
10	pFWER	Bonferroni adjusted global p-value
11	Status	Direction in which pathway is perturbed (activated or inhibited)

Author: Viljami Aittomaki (viljami.aittomaki@helsinki.fi)
Version: 1.0.1
Category: Pathway

Input name	Source	Description
deg	medianExonExpression-surv LogratioEntrezFilt.csv 19.365	An expression matrix containing log2 fold-changes for differentially expressed genes (DEG's). Gene ID's must be Entrez Gene ID's! Genes with fold-change 'NA' are removed.
reference	medianExonExpression-reference .ids 19.355	An IDList containing the Entrez ID's in the reference set. If the data was obtained from a microarray experiment, this list will contain all genes present on the specific array used for the experiment. This vector must contain all names of the DEG's in the deg input.

Parameter name	Value	Description
foldChangeColumn		Name of the column in deg that has wanted input fold-change values. If an empty string defaults to the second column in deg (first numerical column).
nboot	1000	Number of bootstrap samples used in computing pPERT. Should be larger than 100. A recommended value is 2000.
organism	hsa	KEGG organism code corresponding to input genes (e.g., hsa=homo sapiens).
verbose	false	If true, report progress (number of pathways already analyzed reported to stdout).

19.360 medianExonExpression-spiaReport (CSV2Latex)

This component converts a tabular CSV file into a printable LaTeX table.

Author: Marko Laakso (Marko.Laakso@Helsinki.FI)
Version: 2.1
Categories: convert, Latex

Input name	Source	Description
tabledata	medianExonExpression-spia .pathways 19.359	Table content
refs	medianExonExpression-exonSPIA refs.in 19.335	Reference rules for the hyperlinks

Parameter name	Value	Description
attach	false	Include the original data as an attachment
caption	SPIA analysis ~\cite{Tarca2008}. Name is the KEGG name for the pathway, and ID is the KEGG internal identication for the pathway. pSize is total number of genes in the pathway according to KEGG, where as NDE is the number of diffentially expressed genes on the pathway. pNDE is the p-value to observe NDE number of genes on the pathway by chance. tA is a perturbation factor defined by SPIA algorithm and pPERT is the probability of observing a more intense perturbation than tA by chance. pG is the combination of pPERT and pNDE. pGFdr is the false discovery rate and pGFWER the Bonferoni corrected p-value.	Caption text for the table.
colFormat	center	LaTeX tabular format for the columns. Default produces a centered layout for all columns.
columns	Name,ID,pSize,NDE,tA,pNDE, pPERT,pG,pGFdr,pGFWER, Status	Comma separated list of column selections for the output. The empty default will use all columns.
countRows	false	Include a row count to the table caption.

Continued on next page. . .

Parameter name	Value	Description
dropMissing	true	This flag can be turned off in order to generate links with missing texts. Link text are substituted with target identifiers.
listCols		Comma separated list of column names. Columns of this list may contain several values separated with commas and the delimiters will be replaced with spaces.
numberFormat	tA=#0.0000,pNDE=#0.0000, pG=#0.0000,pGFdr=#0.0000, pGFWER=#0.0000	A comma separated list of decimal formats for the columns. Each entry consists of the column name and the Java DecimalFormal pattern separated with equal sign. For example, rounding to three decimals can be done like: myColumn=#0.000
pageBreak	true	Use clear page after the table.
rename		Comma separated list of column renaming rules (oldname=newname). New names are used in table header but they do not affect the other behaviour of this component.
ruler	{}	Latex command for the row separating rulers
section	Signalling pathway impact analysis for differentially expressed genes evaluated from median exon expression with survival p $<$ 0.01	Section title for the table container or an empty string if no section should be generated.
sectionType	section	Type of LaTeX section: usually one of: section, subsection, or subsubsection. No section statement is written if section title is empty.
skipEmpty	false	This flag can be used to replace empty tables with a simple LaTeX comment.

19.361 medianExonExpression-survGenes (CSVTransformer)

Transform CSV files using R [20] expressions. This allows to apply arithmetic functions to numeric columns and to combine columns from different CSV files. The R expressions are evaluated and are expected to return R matrices, data frames or vectors that are concatenated to a final result. Concatenations is done on columns, so each transformation creates additional columns to the output. Transformations should create items having the same number of rows. However, the expression may yield a single string or number that is duplicated to fit the number of rows.

Author:	Kristian Ovaska (kristian.ovaska@helsinki.fi)
Version:	1.0
Category:	preprocessing

Input name	Source	Description
csv1	medianExonExpression-km-km .statistics 19.341	Input file 1.

Parameter name	Value	Description
columnNames		R expression that evaluates to the column names of the result CSV file. The evaluated vector must have the same number of items as there are columns in the output. If empty, column names are taken from the input CSV files; depending on the transforms, some column names may be automatically generated.
transform1	csv1[csv1$pValue < 0.01, 'group']	R expression that evaluates to a matrix, data frame, vector or constant. The expression may refer to data frames "csv1" and "csv2" (only if csv2 is given) and matrices "matrix1" and "matrix2" (only if csv2 is given).
transform2		Transformation expression 2. If empty, no transformation is done.
transform3		Transformation expression 3. If empty, no transformation is done.
transform4		Transformation expression 4. If empty, no transformation is done.
transform5		Transformation expression 5. If empty, no transformation is done.
transform6		Transformation expression 6. If empty, no transformation is done.
transform7		Transformation expression 7. If empty, no transformation is done.
transform8		Transformation expression 8. If empty, no transformation is done.
transform9		Transformation expression 9. If empty, no transformation is done.

19.362 medianExonExpression-survGenesEntrez (KorvasieniAnnotator)

Korvasieni is an Ensembl [12] based converter for database identifiers and it can be used to convert genome location, gene, transcript, and translation identifiers across multiple biological databases. The selected `keyColumn` may contain entries with comma separated lists of values in which case `isListKey` parameter

may be used to obtain each value separately and to process them as they were given in consecutive lines.

Author: Marko Laakso (Marko.Laakso@Helsinki.FI)
Version: 1.7
Categories: Annotation, GO

Input name	Source	Description
sourceKeys	medianExonExpression-survGenes .transformed 19.361	A list of source database keys.
connection	ensembl.in 19.273	Database connection can be defined using this file. The definition of parameters: database.url, database.user, database.password, database.timeout, database.recycle, and database.driver can be found from the documentation of Korvasieni.

Parameter name	Value	Description
echoColumns		A comma separated list of column names for the columns that will be copied to the output. An asterisk (*) can be used to denote all columns execpt the keyColumn.
inputDB	.GeneId	Type of input keys. This must be a database supported by Korvasieni. If the parameter is omitted, the component tries to derive the database from the type of geneID. If this is not possible, an error is returned. You may define three columns in form of chromosome:start-end in case the inputDB is .DNARegion. This format provides a comfortable compatibility with DNARegion datatype.
inputType	Any	Ensembl object type for the input keys (Any, Gene, Transcript, Translation)
isListKey	false	Enables the automatic value splits for the comma separated key column
keyColumn		Name of the key column withing sourceKeys file or an empty string for the first column
maxHits	1	Maximum number of target identifiers for a single source identifier
rename		Comma separated list of column renaming rules (oldname=newname)
skipLevel	never	Skip result rows if the source identifier is unknown or target identifiers are not available. Possible values are: never (no filtering), source (skip if the source ID is unknown), target (skip if no target IDs are found), any (skip if any of the target IDs is missing).
targetDB	_EntrezGene	Comma-separated list of annotation types. Possible values are all databases supported by Korvasieni.
unique	false	This flag can be turned on in order to eliminate duplicate annotations.

19.363 medianExonExpression-survLogratio (CSVFilter)

Filters columns and/or rows from CSV files using flexible criteria. Default settings do not filter out anything. The *negate* parameter reverses all inclusion criteria.

Columns are filtered based on column names (see *includeColumns*); it is also possible to rename columns. Columns can be reordered using *includeColumns*.

A row is included in output if all conditions are satisfied. Rows having too many missing values are filtered using the *nonMissing* parameter. The parameters *regexp*, *lowBound* and *highBound* filter rows based on cell contents. If the *auxiliary* input is given, the result contains those rows from the input whose values match those from the auxiliary file.

Authors: Kristian Ovaska (kristian.ovaska@helsinki.fi), Marko Laakso (Marko.Laakso@Helsinki.FI)
Version: 1.1
Category: Filter

Input name	Source	Description
csv	medianExonExpression-logratio .expr 19.351	CSV file to be filtered.
auxiliary	medianExonExpression-survGenes .transformed 19.361	If given, contains one column (see "matchColumn") whose values are matched to a column in the "csv" input (see "idColumn").

Parameter name	Value	Description
highBound		A row is included if the value in given numeric columns are at most as large as given numeric bounds. The parameter has a format COLNAME1=HIGH1,COLNAME2=HIGH2.
idColumn		Column name in "csv" that contains ID values. If the parameter is empty, the first column is used.
includeColumns	*	Comma-separated list of column names that should be included in the result file. The special value * includes all columns. The order of column names is significant: the output columns are in the order given. See also includeColumns input.
lowBound		A row is included if the value in given numeric columns are at least as large as given numeric bounds. The parameter has a format COLNAME1=LOW1,COLNAME2=LOW2. For example, "col=5" includes rows where the column col has a value of at least 5.
matchColumn		Column name in "auxiliary" containing values that must match the ID column in "csv" (specified using "idColumn"). If empty, the first column of "auxiliary" is used.
negate	false	If true, all inclusion criteria are negated so that rows or columns are excluded instead of included if they match the criteria. For example, includeColumns=* then excludes all columns (which would be an error since at least one column must be included).
nonMissing	0	Include those rows that have non-missing (non-NA) values in at least this many columns. Only those columns that are part of the output are counted.
regexp		Row filtering based on regular expression. A row is included in the result if values in the given columns match given regular expressions. The parameter has a format COLNAME1=EXPRESSION,COLNAME2=EXPRESSION2 where COLNAMEs are column names in "csv" and EXPRESSIONs are regular expressions using Java syntax. For example, "col=a|b" includes rows where the column col has a value of "a" or "b". Commas have to be escaped with "\\" if they are used in column names or regular expressions.
rename		Comma-separated list of column renaming rules (OLDNAME=NEWNAME). Parameters that refer to column names refer to old names; renaming is done last.

19.364 medianExonExpression-survLogratioEntrez (IDConvert)

Converts identifiers from a column in a CSV file to another type of identifiers. The conversion is done using an annotation table on the basis of the column named *conversionColumn*.

Author: Kristian Ovaska (kristian.ovaska@helsinki.fi)
Version: 1.0
Category: convert

Input name	Source	Description
csv	medianExonExpression-surv Logratio.csv 19.363	Source CSV file. Contains the column that is to be converted. The column is named by the parameter 'sourceColumn'.
conversionTable	medianExonExpression-survGenes Entrez.bioAnnotation 19.362	Conversion table. Must contain the column named by the parameter 'conversionColumn'.

Parameter name	Value	Description
collapseNumeric	median	Method for collapsing numeric columns with duplicate rows (when unique=true). Legal values are "median" (take median of non-NA values), "mean", "max", "min", "first" (take the first row), "majority" (take the value that is present on the largest number of rows), "consensus" (require that all rows have the same value) and "indicator".<br>Here, "majority" and "consensus" are mostly useful for discrete (integer) values). For "consensus", it is an error if duplicate rows contain distinct values. "indicator" assumes that data are divided into negative (-1), neutral (0) and positive (1) items, such as discretized expression values. The result is (a) 1 if there is a 1 and no -1 value; (b) -1 if there is a -1 and not 1 value; (c) 0 if there is a 0 and no -1 or 1; or (d) NA otherwise.
collapseString	consensus	Method for collapsing non-numeric columns with duplicate rows (when unique=true). Legal values are "first" (take the first row), "majority" (take the value that is present on the largest number of rows), "longest" (take the longest value), "shortest" (take the shortest value) and "consensus" (require that all rows have the same value). For "consensus", it is an error if duplicate rows contain distinct values.
conversionColumn	EntrezGene	Column in 'conversionTable' that is used to fetch new values for the source column.

Continued on next page...

Parameter name	Value	Description
keyColumn		Column in 'conversionTable' that is used to match ID values from the source CSV. If the parameter is empty, the first column is used.
originalWhen Missing	false	Determines how values are converted when the item is not found in the conversion table. If true, the original (unconverted) ID is inserted to the output. If false, NA is inserted.
sourceColumn		Column in 'csv' that is going to be converted. If empty, the first column is used.
split	false	If true, split values in the source column by commas before ID conversion. For example, if the source column contains the string "a,b,c", it is first split into "a", "b" and "c"; then, individual IDs are converted; finally, the converted IDs are joined by commas into a string of format "A,B,C".
splitConverted	false	If true, split converted values by commas after ID conversion. A row with comma separated values in the target column will be split to otherwise identical rows but so that the rows have a single ID value in the target column. This is done before possible collapsing of duplicate rows.
targetColumn		Target column name in 'converted'. If empty, the column name is the same as the original column (sourceColumn).
unique	true	If true, rows with identical identifiers (sourceColumn) are collapsed into one row so that sourceColumn is unique. Collapsing may result in error if duplicate rows contain conflicting values. See parameters collapseNumeric and collapseString for methods to collapse numeric and non-numeric columns, respectively.
varMeasure		Method for calculating the variation of numeric duplicate values. If the variation is above the threshold given in <code>varThreshold</code> , duplicate values are collapsed to NA. Possible values are 'MAD' and 'variance'. 'MAD' stands for the median absolute deviation and 'variance' for the population variance. If no values is given, no threshold for combining numeric values is used.
varThreshold	2	Maximum allowed variability in the duplicate values. If <code>varMeasure</code> is given duplicate values with variance above the threshold are collapsed to NA

19.365 medianExonExpression-survLogratioEntrezFilt (CSVFilter)

Filters columns and/or rows from CSV files using flexible criteria. Default settings do not filter out anything. The *negate* parameter reverses all inclusion criteria.

Columns are filtered based on column names (see *includeColumns*); it is also possible to rename columns. Columns can be reordered using *includeColumns*.

A row is included in output if all conditions are satisfied. Rows having too many missing values are filtered using the *nonMissing* parameter. The parameters *regexp*, *lowBound* and *highBound* filter rows based on cell contents. If the *auxiliary* input is given, the result contains those rows from the input whose values match those from the auxiliary file.

Authors: Kristian Ovaska (kristian.ovaska@helsinki.fi), Marko Laakso (Marko.Laakso@Helsinki.FI)
Version: 1.1
Category: Filter

Input name	Source	Description
csv	medianExonExpression-surv LogratioEntrez.csv 19.364	CSV file to be filtered.

Parameter name	Value	Description
highBound		A row is included if the value in given numeric columns are at most as large as given numeric bounds. The parameter has a format COLNAME1=HIGH1,COLNAME2=HIGH2.
idColumn		Column name in "csv" that contains ID values. If the parameter is empty, the first column is used.
includeColumns	*	Comma-separated list of column names that should be included in the result file. The special value * includes all columns. The order of column names is significant: the output columns are in the order given. See also includeColumns input.
lowBound		A row is included if the value in given numeric columns are at least as large as given numeric bounds. The parameter has a format COLNAME1=LOW1,COLNAME2=LOW2. For example, "col=5" includes rows where the column col has a value of at least 5.
matchColumn		Column name in "auxiliary" containing values that must match the ID column in "csv" (specified using "idColumn"). If empty, the first column of "auxiliary" is used.

Continued on next page...

Parameter name	Value	Description
negate	false	If true, all inclusion criteria are negated so that rows or columns are excluded instead of included if they match the criteria. For example, includeColumns=* then excludes all columns (which would be an error since at least one column must be included).
nonMissing	2	Include those rows that have non-missing (non-NA) values in at least this many columns. Only those columns that are part of the output are counted.
regexp		Row filtering based on regular expression. A row is included in the result if values in the given columns match given regular expressions. The parameter has a format COLNAME1=EXPRESSION,COLNAME2=EXPRESSION2 where COLNAMEs are column names in "csv" and EXPRESSIONs are regular expressions using Java syntax. For example, "col=a|b" includes rows where the column col has a value of "a" or "b". Commas have to be escaped with "\\" if they are used in column names or regular expressions.
rename		Comma-separated list of column renaming rules (OLDNAME=NEWNAME). Parameters that refer to column names refer to old names; renaming is done last.

19.366 medianExonExpression-tExpr (REvaluate)

This component produces some outputs by executing an R [20] script with the given input data. The exact functionality of this component depends on the given script.

The component is also capable of making branch decisions on Anduril workflow. Two alternative options (*altCase* and *defCase*) are provided.

Authors: Marko Laakso (Marko.Laakso@Helsinki.FI), Erkka Valo (erkka.valo@tkk.fi)
Version: 2.2
Categories: External, Latex, Network Control

Input name	Source	Description
script	medianExonExpression-filterSamplesRScript.in 19.336	Data transformation
table1	medianExonExpressionMatrix.in 19.368	Data matrix 1 (data.frame/file table1)
table2	medianExonExpression-samplesWithoutClinicalAnnotation.in 19.358	Data matrix 2 (data.frame/file table2)

Parameter name	Value	Description
asConnection	false	If true, assign file connections (class file) to the inputs to variables of the same name. This is useful e.g. if you want to read large data files row by row.
param1		Value of this parameter will be assigned to param1 variable
param10		Value of this parameter will be assigned to param10 variable
param2		Value of this parameter will be assigned to param2 variable
param3		Value of this parameter will be assigned to param3 variable
param4		Value of this parameter will be assigned to param4 variable
param5		Value of this parameter will be assigned to param5 variable
param6		Value of this parameter will be assigned to param6 variable
param7		Value of this parameter will be assigned to param7 variable
param8		Value of this parameter will be assigned to param8 variable
param9		Value of this parameter will be assigned to param9 variable

19.367 medianExonExpression-transcriptStatisticsQuery (INPUT)

Transcript result data processing.

Category: Internal

Parameter name	Value	Description
path	/gpfs/homes/mlaakso/asserSVN/andurilCaseStudy/sql/MedianExonStatistics.sql	Path (filename) of the input resource.

19.368 medianExonExpressionMatrix (INPUT)

Median exon expression gene expression matrix

Category: Internal

Parameter name	Value	Description
path	/gpfs/homes/mlaakso/asserSVN/ andurilCaseStudy/data/exon-normalized/Expression-Exon/ LBL_HuEx-1_0-st-v2/Level_2/ GeneExpression_v56	Path (filename) of the input resource.

19.369 methylation (Methylation)

Performs methylome analysis.

19.370 methylation-annotation002 (INPUT)

(Technical documentation) Imports an external input file or directory to the pipeline. This is the only way external files are imported. File path is given with the parameter "path".

Category: Internal

Parameter name	Value	Description
path	/gpfs/homes/mlaakso/asserSVN/ andurilCaseStudy/data/ methylation/OMA-002-annotation.csv	Path (filename) of the input resource.

19.371 methylation-annotation003 (INPUT)

(Technical documentation) Imports an external input file or directory to the pipeline. This is the only way external files are imported. File path is given with the parameter "path".

Category: Internal

Parameter name	Value	Description
path	/gpfs/homes/mlaakso/asserSVN/ andurilCaseStudy/data/ methylation/OMA-003-annotation-geneids.csv	Path (filename) of the input resource.

19.372 methylation-beta002raw (INPUT)

(Technical documentation) Imports an external input file or directory to the pipeline. This is the only way external files are imported. File path is given with the parameter "path".

Category: Internal

Parameter name	Value	Description
path	/gpfs/homes/mlaakso/asserSVN/anduril CaseStudy/data/methylation/DNA_Methylation/JHU_USC__IlluminaDNAMethylation_OMA002_CPI/Level_2/jhu-usc.edu__IlluminaDNAMethylation_OMA002_CPI__beta-value	Path (filename) of the input resource.

19.373 methylation-beta003raw (INPUT)

(Technical documentation) Imports an external input file or directory to the pipeline. This is the only way external files are imported. File path is given with the parameter "path".

Category: Internal

Parameter name	Value	Description
path	/gpfs/homes/mlaakso/asserSVN/anduril CaseStudy/data/methylation/DNA_Methylation/JHU_USC__IlluminaDNAMethylation_OMA003_CPI/Level_2/jhu-usc.edu__IlluminaDNAMethylation_OMA003_CPI__beta-value	Path (filename) of the input resource.

19.374 methylation-combined (CSVJoin)

Join rows from two or more CSV files, optionally using one column as a matching key. If a key column is not used, the result contains all rows and all columns of the input files. Missing values (`NA`) may be introduced when a column is not present in all input files. Each column is present once and duplicate rows are removed.

If a key column is used, the rows in each input file are matched using values from the key column. The result file has one row for each key value. In the result, the first column is the key column; its name is obtained from the first CSV input (csv1). If the intersection parameter is true, a key is included in the result if the key value is present in all inputs. If intersection is false, a key is included if it is present in at least one input (key union). Union semantics may introduce missing values in the result. If several input files have the same column, the value is obtained from the first file. However, if the first file contains a missing value and the second file contains a non-missing value, the non-missing value is used instead.

Author: Kristian Ovaska (kristian.ovaska@helsinki.fi)
Version: 1.0
Category: convert

Input name	Source	Description
csv1	methylation-results002-betaResults.transformed 19.380	CSV file 1.
csv2	methylation-results003-betaResults.transformed 19.388	CSV file 2.

Parameter name	Value	Description
intersection	false	Defines how keys are handled; only used when useKeys=true. If intersection is true, the result contains a key if the key is present in all input files. If false, the result contains a key if the key is present in at least one input file.
keyColumnNames		Comma-separated list of key column names; only used when useKeys=true. The first name refers to csv1, the second to csv2, etc. An empty value refers to the first column. Empty values may be omitted from the list, so all these are equivalent: "col1" ; "col1," ; "col1,," ; etc.

Continued on next page...

Parameter name	Value	Description
useKeys	true	If true, use one column from each CSV file as a matching key column.

19.375 methylation-results002 (MethylationAnalysis)

No description

19.376 methylation-results002-beta (SampleCombiner)

Combine expression data from several samples into one by taking means, medians or log ratios. Combining is done using a sample group table. Each row of the table specifies how one sample group is created from a list of source groups. If the expression input file contains log ratios, both median and log ratio combining are possible and the result are log ratios. If the input are channel values, the groups may be either median groups (result type: channel values) or log ratio groups (result type: log ratio), but not both.

Authors: Kari Nousiainen (Kari.Nousiainen@Helsinki.FI), Kristian Ovaska (Kristian.Ovaska@Helsinki.FI)
Version: 1.0
Category: Filter

Input name	Source	Description
expr	methylation-results002-betaFilt .file 19.377	Expression data, either log ratios or channel expressions.
groups	methylation-results002-groups .groups 19.382	Sample group specification.

Parameter name	Value	Description
geometricMean	true	Indicates whether the means will be calculated using geometric(true) or arithmetic mean (false).
groupIDs		Comma-separated list of sample group IDs. If given, combine only these groups. If not given (empty), combine all sample groups.
includeOriginal	false	If true, put input groups into the result even if they are not present in 'groups'.
thresholdRatio	0	Allows to omit those genes that have missing values in some samples. Only genes that have non-missing values in at least the given number of columns are included in the result. The actual threshold T is ceiling(thresholdRatio*n), where n is the number of samples that are used in combining. Only genes that have non-missing values in at least T columns are included. Other genes have the combined value set to NA. Note: this parameter specifies the ratio instead of integer threshold since the number of samples may be different in each sample group.

19.377 methylation-results002-betaFilt (SearchReplace)

Replaces the occurences of the given regular expressions with the corresponding regular expressions. This component is used for transforming given patterns of the input file to a changed form specified by the regular expressions .

Author: Marko Laakso (Marko.Laakso@Helsinki.FI)
Version: 1.0
Category: convert

Input name	Source	Description
file	methylation-results002-betaFilt1 .csv 19.378	Source file to be modified

Parameter name	Value	Description
key00	N/A	Search key 0

Continued on next page...

Parameter name	Value	Description
key01		Search key 1
key02		Search key 2
key03		Search key 3
key04		Search key 4
key05		Search key 5
key06		Search key 6
key07		Search key 7
key08		Search key 8
key09		Search key 9
value00	NA	Replacement 0
value01		Replacement 1
value02		Replacement 2
value03		Replacement 3
value04		Replacement 4
value05		Replacement 5
value06		Replacement 6
value07		Replacement 7
value08		Replacement 8
value09		Replacement 9

19.378 methylation-results002-betaFilt1 (CSVFilter)

Filters columns and/or rows from CSV files using flexible criteria. Default settings do not filter out anything. The *negate* parameter reverses all inclusion criteria.

Columns are filtered based on column names (see *includeColumns*); it is also possible to rename columns. Columns can be reordered using *includeColumns*.

A row is included in output if all conditions are satisfied. Rows having too many missing values are filtered using the *nonMissing* parameter. The parameters *regexp*, *lowBound* and *highBound* filter rows based on cell contents. If the *auxiliary* input is given, the result contains those rows from the input whose values match those from the auxiliary file.

Authors: Kristian Ovaska (kristian.ovaska@helsinki.fi), Marko Laakso (Marko.Laakso@Helsinki.FI)
Version: 1.1
Category: Filter

Input name	Source	Description
csv	methylation-beta002raw.in 19.372	CSV file to be filtered.

Parameter name	Value	Description
highBound		A row is included if the value in given numeric columns are at most as large as given numeric bounds. The parameter has a format COLNAME1=HIGH1,COLNAME2=HIGH2.
idColumn		Column name in "csv" that contains ID values. If the parameter is empty, the first column is used.
includeColumns		Comma-separated list of column names that should be included in the result file. The special value * includes all columns. The order of column names is significant: the output columns are in the order given. See also includeColumns input.
lowBound		A row is included if the value in given numeric columns are at least as large as given numeric bounds. The parameter has a format COLNAME1=LOW1,COLNAME2=LOW2. For example, "col=5" includes rows where the column col has a value of at least 5.
matchColumn		Column name in "auxiliary" containing values that must match the ID column in "csv" (specified using "idColumn"). If empty, the first column of "auxiliary" is used.
negate	true	If true, all inclusion criteria are negated so that rows or columns are excluded instead of included if they match the criteria. For example, includeColumns=* then excludes all columns (which would be an error since at least one column must be included).
nonMissing	0	Include those rows that have non-missing (non-NA) values in at least this many columns. Only those columns that are part of the output are counted.

Continued on next page...

Parameter name	Value	Description
regexp	Hybridization REF=<br>CompositeElement REF	Row filtering based on regular expression. A row is included in the result if values in the given columns match given regular expressions. The parameter has a format COLNAME1=EXPRESSION,COLNAME2=EXPRESSION2 where COLNAMEs are column names in "csv" and EXPRESSIONs are regular expressions using Java syntax. For example, "col=a|b" includes rows where the column col has a value of "a" or "b". Commas have to be escaped with "\\" if they are used in column names or regular expressions.
rename		Comma-separated list of column renaming rules (OLDNAME=NEWNAME). Parameters that refer to column names refer to old names; renaming is done last.

19.379 methylation-results002-betaNames (CSVTransformer)

Transform CSV files using R [20] expressions. This allows to apply arithmetic functions to numeric columns and to combine columns from different CSV files. The R expressions are evaluated and are expected to return R matrices, data frames or vectors that are concatenated to a final result. Concatenations is done on columns, so each transformation creates additional columns to the output. Transformations should create items having the same number of rows. However, the expression may yield a single string or number that is duplicated to fit the number of rows.

Author: Kristian Ovaska (kristian.ovaska@helsinki.fi)
Version: 1.0
Category: preprocessing

Input name	Source	Description
csv1	methylation-results002-beta<br>.expr 19.376	Input file 1.

Parameter name	Value	Description
columnNames	c('GeneName', colnames(csv1)[2:<br>ncol(csv1)])	R expression that evaluates to the column names of the result CSV file. The evaluated vector must have the same number of items as there are columns in the output. If empty, column names are taken from the input CSV files; depending on the transforms, some column names may be automatically generated.
transform1	sapply(strsplit(csv1[,1], '_', fixed=<br>TRUE), function(x) x[1])	R expression that evaluates to a matrix, data frame, vector or constant. The expression may refer to data frames "csv1" and "csv2" (only if csv2 is given) and matrices "matrix1" and "matrix2" (only if csv2 is given).
transform2	csv1[,2:ncol(csv1)]	Transformation expression 2. If empty, no transformation is done.
transform3		Transformation expression 3. If empty, no transformation is done.
transform4		Transformation expression 4. If empty, no transformation is done.
transform5		Transformation expression 5. If empty, no transformation is done.
transform6		Transformation expression 6. If empty, no transformation is done.
transform7		Transformation expression 7. If empty, no transformation is done.
transform8		Transformation expression 8. If empty, no transformation is done.
transform9		Transformation expression 9. If empty, no transformation is done.

19.380 methylation-results002-betaResults (CSVTransformer)

Transform CSV files using R [20] expressions. This allows to apply arithmetic functions to numeric columns and to combine columns from different CSV files. The R expressions are evaluated and are expected to return R matrices, data frames or vectors that are concatenated to a final result. Concatenations is done on columns, so each transformation creates additional columns to the output. Transformations should create items having the same number of rows. However, the expression may yield a single string or number that is duplicated to fit the number of rows.

Author: Kristian Ovaska (kristian.ovaska@helsinki.fi)
Version: 1.0
Category: preprocessing

Input name	Source	Description
csv1	methylation-results002-beta Unique.csv 19.381	Input file 1.

Parameter name	Value	Description
columnNames	c('GeneID', 'Beta', 'BetaSD')	R expression that evaluates to the column names of the result CSV file. The evaluated vector must have the same number of items as there are columns in the output. If empty, column names are taken from the input CSV files; depending on the transforms, some column names may be automatically generated.
transform1	csv1[,1]	R expression that evaluates to a matrix, data frame, vector or constant. The expression may refer to data frames "csv1" and "csv2" (only if csv2 is given) and matrices "matrix1" and "matrix2" (only if csv2 is given).
transform2	sapply(1:nrow(matrix1), function(i) median(matrix1[i,], na.rm= TRUE))	Transformation expression 2. If empty, no transformation is done.
transform3	sapply(1:nrow(matrix1), function(i) sd(matrix1[i,], na.rm=TRUE))	Transformation expression 3. If empty, no transformation is done.
transform4		Transformation expression 4. If empty, no transformation is done.
transform5		Transformation expression 5. If empty, no transformation is done.
transform6		Transformation expression 6. If empty, no transformation is done.
transform7		Transformation expression 7. If empty, no transformation is done.
transform8		Transformation expression 8. If empty, no transformation is done.
transform9		Transformation expression 9. If empty, no transformation is done.

19.381 methylation-results002-betaUnique (IDConvert)

Converts identifiers from a column in a CSV file to another type of identifiers. The conversion is done using an annotation table on the basis of the column named *conversionColumn*.

Author: Kristian Ovaska (kristian.ovaska@helsinki.fi)
Version: 1.0
Category: convert

Input name	Source	Description
csv	methylation-results002-beta Names.transformed 19.379	Source CSV file. Contains the column that is to be converted. The column is named by the parameter 'sourceColumn'.
conversionTable	methylation-annotation002 .in 19.370	Conversion table. Must contain the column named by the parameter 'conversionColumn'.

Parameter name	Value	Description
collapseNumeric	median	Method for collapsing numeric columns with duplicate rows (when unique=true). Legal values are "median" (take median of non-NA values), "mean", "max", "min", "first" (take the first row), "majority" (take the value that is present on the largest number of rows), "consensus" (require that all rows have the same value) and "indicator".<br>Here, "majority" and "consensus" are mostly useful for discrete (integer) values). For "consensus", it is an error if duplicate rows contain distinct values. "indicator" assumes that data are divided into negative (-1), neutral (0) and positive (1) items, such as discretized expression values. The result is (a) 1 if there is a 1 and no -1 value; (b) -1 if there is a -1 and not 1 value; (c) 0 if there is a 0 and no -1 or 1; or (d) NA otherwise.
collapseString	consensus	Method for collapsing non-numeric columns with duplicate rows (when unique=true). Legal values are "first" (take the first row), "majority" (take the value that is present on the largest number of rows), "longest" (take the longest value), "shortest" (take the shortest value) and "consensus" (require that all rows have the same value). For "consensus", it is an error if duplicate rows contain distinct values.
conversionColumn	GeneID	Column in 'conversionTable' that is used to fetch new values for the source column.
keyColumn		Column in 'conversionTable' that is used to match ID values from the source CSV. If the parameter is empty, the first column is used.
originalWhen Missing	true	Determines how values are converted when the item is not found in the conversion table. If true, the original (unconverted) ID is inserted to the output. If false, NA is inserted.
sourceColumn	GeneName	Column in 'csv' that is going to be converted. If empty, the first column is used.

Continued on next page...

Parameter name	Value	Description
split	false	If true, split values in the source column by commas before ID conversion. For example, if the source column contains the string "a,b,c", it is first split into "a", "b" and "c"; then, individual IDs are converted; finally, the converted IDs are joined by commas into a string of format "A,B,C".
splitConverted	false	If true, split converted values by commas after ID conversion. A row with comma separated values in the target column will be split to otherwise identical rows but so that the rows have a single ID value in the target column. This is done before possible collapsing of duplicate rows.
targetColumn		Target column name in 'converted'. If empty, the column name is the same as the original column (sourceColumn).
unique	true	If true, rows with identical identifiers (sourceColumn) are collapsed into one row so that sourceColumn is unique. Collapsing may result in error if duplicate rows contain conflicting values. See parameters collapseNumeric and collapseString for methods to collapse numeric and non-numeric columns, respectively.
varMeasure		Method for calculating the variation of numeric duplicate values. If the variation is above the threshold given in <code>varThreshold</code> , duplicate values are collapsed to NA. Possible values are 'MAD' and 'variance'. 'MAD' stands for the median absolute deviation and 'variance' for the population variance. If no values is given, no threshold for combining numeric values is used.
varThreshold	2	Maximum allowed variability in the duplicate values. If <code>varMeasure</code> is given duplicate values with variance above the threshold are collapsed to NA

19.382 methylation-results002-groups (SampleGroupCreator)

Creates sample group tables based on sample names read from data files. These tables define relationship between samples. For example, which samples are biological or technical replicates. The list of sample names is read from data files (either column names or one column along rows) and sample groups are defined using regular expressions. Also, constant groups where the set of member samples does not depend on data can be created. The type of each definition pattern is given with *patternTypes*, which is one of *re*, *relist* or *verbatim* for each pattern.

Author:	Kristian Ovaska (kristian.ovaska@helsinki.fi)
Version:	1.2.1
Category:	preprocessing

Input name	Source	Description
data1	methylation-results002-betaFilt .file 19.377	Data file 1 for reading sample names. Either column names or values on one column contain sample names.

Parameter name	Value	Description
columns		Defines which columns in input files contain sample names, or whether column names should be used (default). This parameter is a comma-separated list of at most three values which name columns in inputs data1 to data3. If an entry is empty, the column names of the corresponding input file are used instead.
definition1	$1,median	Definition of group 1. If empty, the group is omitted. Format: NAME,TYPE or NAME,TYPE,DESCRIPTION.
definition2		Definition of group 2. If empty, the group is omitted.
definition3		Definition of group 3. If empty, the group is omitted.
definition4		Definition of group 4. If empty, the group is omitted.
definition5		Definition of group 5. If empty, the group is omitted.
definition6		Definition of group 6. If empty, the group is omitted.
definition7		Definition of group 7. If empty, the group is omitted.
definition8		Definition of group 8. If empty, the group is omitted.
definition9		Definition of group 9. If empty, the group is omitted.
pattern1	(TCGA-[0-9]+-[0-9]+)-01.*	Pattern for group 1. If empty, the group is omitted. Format depends on the pattern type.
pattern2		Pattern for group 2. If empty, the group is omitted.
pattern3		Pattern for group 3. If empty, the group is omitted.
pattern4		Pattern for group 4. If empty, the group is omitted.
pattern5		Pattern for group 5. If empty, the group is omitted.
pattern6		Pattern for group 6. If empty, the group is omitted.
pattern7		Pattern for group 7. If empty, the group is omitted.
pattern8		Pattern for group 8. If empty, the group is omitted.
pattern9		Pattern for group 9. If empty, the group is omitted.

Continued on next page...

Parameter name	Value	Description
patternTypes		Comma-separated list of pattern types for each pattern. Each item is one of "re" (default), "relist" or "verbatim". The types are explained above. An empty value is interpreted as "re". For example, ",re,relist,,verbatim" specifies that pattern3 has the type "relist" and pattern5 the type "verbatim"; all others (including pattern6 and above) have the type "re".

19.383 methylation-results003 (MethylationAnalysis)

No description

19.384 methylation-results003-beta (SampleCombiner)

Combine expression data from several samples into one by taking means, medians or log ratios. Combining is done using a sample group table. Each row of the table specifies how one sample group is created from a list of source groups. If the expression input file contains log ratios, both median and log ratio combining are possible and the result are log ratios. If the input are channel values, the groups may be either median groups (result type: channel values) or log ratio groups (result type: log ratio), but not both.

Authors: Kari Nousiainen (Kari.Nousiainen@Helsinki.FI), Kristian Ovaska (Kristian.Ovaska@Helsinki.FI)
Version: 1.0
Category: Filter

Input name	Source	Description
expr	methylation-results003-betaFilt .file 19.385	Expression data, either log ratios or channel expressions.
groups	methylation-results003-groups .groups 19.390	Sample group specification.

Parameter name	Value	Description
geometricMean	true	Indicates whether the means will be calculated using geometric(true) or arithmetic mean (false).
groupIDs		Comma-separated list of sample group IDs. If given, combine only these groups. If not given (empty), combine all sample groups.
includeOriginal	false	If true, put input groups into the result even if they are not present in 'groups'.
thresholdRatio	0	Allows to omit those genes that have missing values in some samples. Only genes that have non-missing values in at least the given number of columns are included in the result. The actual threshold T is ceiling(thresholdRatio*n), where n is the number of samples that are used in combining. Only genes that have non-missing values in at least T columns are included. Other genes have the combined value set to NA. Note: this parameter specifies the ratio instead of integer threshold since the number of samples may be different in each sample group.

19.385 methylation-results003-betaFilt (SearchReplace)

Replaces the occurences of the given regular expressions with the corresponding regular expressions. This component is used for transforming given patterns of the input file to a changed form specified by the regular expressions .

Author: Marko Laakso (Marko.Laakso@Helsinki.FI)
Version: 1.0
Category: convert

Input name	Source	Description
file	methylation-results003-betaFilt1 .csv 19.386	Source file to be modified

Parameter name	Value	Description
key00	N/A	Search key 0
key01		Search key 1
key02		Search key 2
key03		Search key 3
key04		Search key 4
key05		Search key 5
key06		Search key 6
key07		Search key 7
key08		Search key 8
key09		Search key 9
value00	NA	Replacement 0
value01		Replacement 1
value02		Replacement 2
value03		Replacement 3
value04		Replacement 4
value05		Replacement 5
value06		Replacement 6
value07		Replacement 7
value08		Replacement 8
value09		Replacement 9

19.386 methylation-results003-betaFilt1 (CSVFilter)

Filters columns and/or rows from CSV files using flexible criteria. Default settings do not filter out anything. The *negate* parameter reverses all inclusion criteria.

Columns are filtered based on column names (see *includeColumns*); it is also possible to rename columns. Columns can be reordered using *includeColumns*.

A row is included in output if all conditions are satisfied. Rows having too many missing values are filtered using the *nonMissing* parameter. The parameters *regexp*, *lowBound* and *highBound* filter rows based on cell contents. If the *auxiliary* input is given, the result contains those rows from the input whose values match those from the auxiliary file.

Authors: Kristian Ovaska (kristian.ovaska@helsinki.fi), Marko Laakso (Marko.Laakso@Helsinki.FI)
Version: 1.1
Category: Filter

Input name	Source	Description
csv	methylation-beta003raw.in 19.373	CSV file to be filtered.

Parameter name	Value	Description
highBound		A row is included if the value in given numeric columns are at most as large as given numeric bounds. The parameter has a format COLNAME1=HIGH1,COLNAME2=HIGH2.
idColumn		Column name in "csv" that contains ID values. If the parameter is empty, the first column is used.
includeColumns		Comma-separated list of column names that should be included in the result file. The special value * includes all columns. The order of column names is significant: the output columns are in the order given. See also includeColumns input.
lowBound		A row is included if the value in given numeric columns are at least as large as given numeric bounds. The parameter has a format COLNAME1=LOW1,COLNAME2=LOW2. For example, "col=5" includes rows where the column col has a value of at least 5.
matchColumn		Column name in "auxiliary" containing values that must match the ID column in "csv" (specified using "idColumn"). If empty, the first column of "auxiliary" is used.
negate	true	If true, all inclusion criteria are negated so that rows or columns are excluded instead of included if they match the criteria. For example, includeColumns=* then excludes all columns (which would be an error since at least one column must be included).
nonMissing	0	Include those rows that have non-missing (non-NA) values in at least this many columns. Only those columns that are part of the output are counted.

Continued on next page...

Parameter name	Value	Description
regexp	Hybridization REF=<br>CompositeElement REF	Row filtering based on regular expression. A row is included in the result if values in the given columns match given regular expressions. The parameter has a format COLNAME1=EXPRESSION,COLNAME2=EXPRESSION2 where COLNAMEs are column names in "csv" and EXPRESSIONs are regular expressions using Java syntax. For example, "col=a|b" includes rows where the column col has a value of "a" or "b". Commas have to be escaped with "\\" if they are used in column names or regular expressions.
rename		Comma-separated list of column renaming rules (OLDNAME=NEWNAME). Parameters that refer to column names refer to old names; renaming is done last.

19.387 methylation-results003-betaNames (CSVTransformer)

Transform CSV files using R [20] expressions. This allows to apply arithmetic functions to numeric columns and to combine columns from different CSV files. The R expressions are evaluated and are expected to return R matrices, data frames or vectors that are concatenated to a final result. Concatenations is done on columns, so each transformation creates additional columns to the output. Transformations should create items having the same number of rows. However, the expression may yield a single string or number that is duplicated to fit the number of rows.

Author: Kristian Ovaska (kristian.ovaska@helsinki.fi)
Version: 1.0
Category: preprocessing

Input name	Source	Description
csv1	methylation-results003-beta<br>.expr 19.384	Input file 1.

Parameter name	Value	Description
columnNames	c('GeneName', colnames(csv1)[2:<br>ncol(csv1)])	R expression that evaluates to the column names of the result CSV file. The evaluated vector must have the same number of items as there are columns in the output. If empty, column names are taken from the input CSV files; depending on the transforms, some column names may be automatically generated.
transform1	sapply(strsplit(csv1[,1], '_', fixed=<br>TRUE), function(x) x[1])	R expression that evaluates to a matrix, data frame, vector or constant. The expression may refer to data frames "csv1" and "csv2" (only if csv2 is given) and matrices "matrix1" and "matrix2" (only if csv2 is given).
transform2	csv1[,2:ncol(csv1)]	Transformation expression 2. If empty, no transformation is done.
transform3		Transformation expression 3. If empty, no transformation is done.
transform4		Transformation expression 4. If empty, no transformation is done.
transform5		Transformation expression 5. If empty, no transformation is done.
transform6		Transformation expression 6. If empty, no transformation is done.
transform7		Transformation expression 7. If empty, no transformation is done.
transform8		Transformation expression 8. If empty, no transformation is done.
transform9		Transformation expression 9. If empty, no transformation is done.

19.388 methylation-results003-betaResults (CSVTransformer)

Transform CSV files using R [20] expressions. This allows to apply arithmetic functions to numeric columns and to combine columns from different CSV files. The R expressions are evaluated and are expected to return R matrices, data frames or vectors that are concatenated to a final result. Concatenations is done on columns, so each transformation creates additional columns to the output. Transformations should create items having the same number of rows. However, the expression may yield a single string or number that is duplicated to fit the number of rows.

Author: Kristian Ovaska (kristian.ovaska@helsinki.fi)
Version: 1.0
Category: preprocessing

Input name	Source	Description
csv1	methylation-results003-betaUnique.csv 19.389	Input file 1.

Parameter name	Value	Description
columnNames	c('GeneID', 'Beta', 'BetaSD')	R expression that evaluates to the column names of the result CSV file. The evaluated vector must have the same number of items as there are columns in the output. If empty, column names are taken from the input CSV files; depending on the transforms, some column names may be automatically generated.
transform1	csv1[,1]	R expression that evaluates to a matrix, data frame, vector or constant. The expression may refer to data frames "csv1" and "csv2" (only if csv2 is given) and matrices "matrix1" and "matrix2" (only if csv2 is given).
transform2	sapply(1:nrow(matrix1), function(i) median(matrix1[i,], na.rm=TRUE))	Transformation expression 2. If empty, no transformation is done.
transform3	sapply(1:nrow(matrix1), function(i) sd(matrix1[i,], na.rm=TRUE))	Transformation expression 3. If empty, no transformation is done.
transform4		Transformation expression 4. If empty, no transformation is done.
transform5		Transformation expression 5. If empty, no transformation is done.
transform6		Transformation expression 6. If empty, no transformation is done.
transform7		Transformation expression 7. If empty, no transformation is done.
transform8		Transformation expression 8. If empty, no transformation is done.
transform9		Transformation expression 9. If empty, no transformation is done.

19.389 methylation-results003-betaUnique (IDConvert)

Converts identifiers from a column in a CSV file to another type of identifiers. The conversion is done using an annotation table on the basis of the column named *conversionColumn*.

Author: Kristian Ovaska (kristian.ovaska@helsinki.fi)
Version: 1.0
Category: convert

Input name	Source	Description
csv	methylation-results003-betaNames.transformed 19.387	Source CSV file. Contains the column that is to be converted. The column is named by the parameter 'sourceColumn'.
conversionTable	methylation-annotation003.in 19.371	Conversion table. Must contain the column named by the parameter 'conversionColumn'.

Parameter name	Value	Description
collapseNumeric	median	Method for collapsing numeric columns with duplicate rows (when unique=true). Legal values are "median" (take median of non-NA values), "mean", "max", "min", "first" (take the first row), "majority" (take the value that is present on the largest number of rows), "consensus" (require that all rows have the same value) and "indicator".<br>Here, "majority" and "consensus" are mostly useful for discrete (integer) values). For "consensus", it is an error if duplicate rows contain distinct values. "indicator" assumes that data are divided into negative (-1), neutral (0) and positive (1) items, such as discretized expression values. The result is (a) 1 if there is a 1 and no -1 value; (b) -1 if there is a -1 and not 1 value; (c) 0 if there is a 0 and no -1 or 1; or (d) NA otherwise.
collapseString	consensus	Method for collapsing non-numeric columns with duplicate rows (when unique=true). Legal values are "first" (take the first row), "majority" (take the value that is present on the largest number of rows), "longest" (take the longest value), "shortest" (take the shortest value) and "consensus" (require that all rows have the same value). For "consensus", it is an error if duplicate rows contain distinct values.
conversionColumn	GeneID	Column in 'conversionTable' that is used to fetch new values for the source column.
keyColumn		Column in 'conversionTable' that is used to match ID values from the source CSV. If the parameter is empty, the first column is used.
originalWhenMissing	true	Determines how values are converted when the item is not found in the conversion table. If true, the original (unconverted) ID is inserted to the output. If false, NA is inserted.
sourceColumn	GeneName	Column in 'csv' that is going to be converted. If empty, the first column is used.

Continued on next page...

Parameter name	Value	Description
split	false	If true, split values in the source column by commas before ID conversion. For example, if the source column contains the string "a,b,c", it is first split into "a", "b" and "c"; then, individual IDs are converted; finally, the converted IDs are joined by commas into a string of format "A,B,C".
splitConverted	false	If true, split converted values by commas after ID conversion. A row with comma separated values in the target column will be split to otherwise identical rows but so that the rows have a single ID value in the target column. This is done before possible collapsing of duplicate rows.
targetColumn		Target column name in 'converted'. If empty, the column name is the same as the original column (sourceColumn).
unique	true	If true, rows with identical identifiers (sourceColumn) are collapsed into one row so that sourceColumn is unique. Collapsing may result in error if duplicate rows contain conflicting values. See parameters collapseNumeric and collapseString for methods to collapse numeric and non-numeric columns, respectively.
varMeasure		Method for calculating the variation of numeric duplicate values. If the variation is above the threshold given in <code>varThreshold</code> , duplicate values are collapsed to NA. Possible values are 'MAD' and 'variance'. 'MAD' stands for the median absolute deviation and 'variance' for the population variance. If no values is given, no threshold for combining numeric values is used.
varThreshold	2	Maximum allowed variability in the duplicate values. If <code>varMeasure</code> is given duplicate values with variance above the threshold are collapsed to NA

19.390 methylation-results003-groups (SampleGroupCreator)

Creates sample group tables based on sample names read from data files. These tables define relationship between samples. For example, which samples are biological or technical replicates. The list of sample names is read from data files (either column names or one column along rows) and sample groups are defined using regular expressions. Also, constant groups where the set of member samples does not depend on data can be created. The type of each definition pattern is given with *patternTypes*, which is one of *re*, *relist* or *verbatim* for each pattern.

Author: Kristian Ovaska (kristian.ovaska@helsinki.fi)
Version: 1.2.1
Category: preprocessing

Input name	Source	Description
data1	methylation-results003-betaFilt .file 19.385	Data file 1 for reading sample names. Either column names or values on one column contain sample names.

Parameter name	Value	Description
columns		Defines which columns in input files contain sample names, or whether column names should be used (default). This parameter is a comma-separated list of at most three values which name columns in inputs data1 to data3. If an entry is empty, the column names of the corresponding input file are used instead.
definition1	$1,median	Definition of group 1. If empty, the group is omitted. Format: NAME,TYPE or NAME,TYPE,DESCRIPTION.
definition2		Definition of group 2. If empty, the group is omitted.
definition3		Definition of group 3. If empty, the group is omitted.
definition4		Definition of group 4. If empty, the group is omitted.
definition5		Definition of group 5. If empty, the group is omitted.
definition6		Definition of group 6. If empty, the group is omitted.
definition7		Definition of group 7. If empty, the group is omitted.
definition8		Definition of group 8. If empty, the group is omitted.
definition9		Definition of group 9. If empty, the group is omitted.
pattern1	(TCGA-[0-9]+-[0-9]+)-01.*	Pattern for group 1. If empty, the group is omitted. Format depends on the pattern type.
pattern2		Pattern for group 2. If empty, the group is omitted.
pattern3		Pattern for group 3. If empty, the group is omitted.
pattern4		Pattern for group 4. If empty, the group is omitted.
pattern5		Pattern for group 5. If empty, the group is omitted.
pattern6		Pattern for group 6. If empty, the group is omitted.
pattern7		Pattern for group 7. If empty, the group is omitted.
pattern8		Pattern for group 8. If empty, the group is omitted.
pattern9		Pattern for group 9. If empty, the group is omitted.

Continued on next page...

Parameter name	Value	Description
patternTypes		Comma-separated list of pattern types for each pattern. Each item is one of "re" (default), "relist" or "verbatim". The types are explained above. An empty value is interpreted as "re". For example, ",re,relist,,verbatim" specifies that pattern3 has the type "relist" and pattern5 the type "verbatim"; all others (including pattern6 and above) have the type "re".

19.391 miRNAExprNormalized (INPUT)

(Technical documentation) Imports an external input file or directory to the pipeline. This is the only way external files are imported. File path is given with the parameter "path".

Category: Internal

Parameter name	Value	Description
path	/gpfs/homes/mlaakso/asserSVN/anduril CaseStudy/data/mirna/Expression-miRNA/UNC__H-miRNA_8x15K/Level_2/unc.edu__H-miRNA_8x15K__Quantile_Normalized_Log2	Path (filename) of the input resource.

19.392 miRNANames (INPUT)

(Technical documentation) Imports an external input file or directory to the pipeline. This is the only way external files are imported. File path is given with the parameter "path".

Category: Internal

Parameter name	Value	Description
path	/gpfs/homes/mlaakso/asserSVN/andurilCaseStudy/data/mirna/miRBase_Sequences_rel9.1.csv	Path (filename) of the input resource.

19.393 miRNAProbeAnnot (INPUT)

(Technical documentation) Imports an external input file or directory to the pipeline. This is the only way external files are imported. File path is given with the parameter "path".

Category: Internal

Parameter name	Value	Description
path	/gpfs/homes/mlaakso/asserSVN/andurilCaseStudy/data/mirna/agilent_G4470A_probes_CIBEX.csv	Path (filename) of the input resource.

19.394 miRNAResults (MiRNAAnalysis)

Find differentially expressed microRNAs.

Parameter name	Value
fcThreshold	2.0
miRNATargetThreshold	1.0E-5
pValueThreshold	0.05
survivalThreshold	0.1

Continued on next page...

Parameter name	Value
varFilterThreshold	0

19.395 miRNAResults-collapsedMiRNANames (ExpandCollapse)

Converts between the two possible representations of a relations with multivalued columns. By default this component splits rows with multivalued (comma separated lists) columns into multiple rows each representing a single value. The input may contain several columns with comma separated values and all permutations of column values are shown as individual single-valued rows. The alternative mode recovers the original comma separated values from the expanded relations. This is performed by joining values of the their list columns into comma separated lists.

Author: Marko Laakso (Marko.Laakso@Helsinki.FI)
Version: 1.3
Category: convert

Input name	Source	Description
relation	miRNANames.in 19.392	The mandatory input relation

Parameter name	Value	Description
delim	,	Value delimiter between the list column values. Special characters can be encoded as specified in fi.helsinki.ltdk.csbl.asser.ArgumentEncoding.
expand	false	Action mode that is expand (true) or collapse (false)
listCols	stemLoopId,stemLoopName, stemLoopDescription	A comma separated list of column names of input (expand) or output (collapse) columns that may contain delim separated values. The asterisk refers to every column of the input relation.

19.396 miRNAResults-collapsedMiRNATargets (ExpandCollapse)

Converts between the two possible representations of a relations with multivalued columns. By default this component splits rows with multivalued (comma separated lists) columns into multiple rows each representing a single value. The input may contain several columns with comma separated values and all permutations of column values are shown as individual single-valued rows. The alternative mode recovers the original comma separated values from the expanded relations. This is performed by joining values of the their list columns into comma separated lists.

Author: Marko Laakso (Marko.Laakso@Helsinki.FI)
Version: 1.3
Category: convert

Input name	Source	Description
relation	miRNAResults-filteredMiRNA Targets.csv 19.405	The mandatory input relation

Parameter name	Value	Description
delim	,	Value delimiter between the list column values. Special characters can be encoded as specified in fi.helsinki.ltdk.csbl.asser.ArgumentEncoding.
expand	false	Action mode that is expand (true) or collapse (false)
listCols	targetGeneName, targetTranscriptId	A comma separated list of column names of input (expand) or output (collapse) columns that may contain delim separated values. The asterisk refers to every column of the input relation.

19.397 miRNAResults-cumDistReport (Plot2D)

Cumulative distribution for the samples.

Authors:	Kristian Ovaska (kristian.ovaska@helsinki.fi), Marko Laakso (Marko.Laakso@Helsinki.FI), Sirkku Karinen (sirkku.karinen@Helsinki.FI)
Version:	1.0
Categories:	Latex, Plot

Input name	Source	Description
y	miRNAResults-mExpr.matrix 19.410	Coordinates on the Y axis. Columns from this file are selected for plotting using "yColumns".

Parameter name	Value	Description
caption	Cumulative distributions of miRNA expression values for the samples.	LaTeX caption of each figure. The caption may contain one or two %s placeholders, which are replaced with the current column names in "y" and "x" (if defined). Column names are not used when multiple plots are created to the same image (imageType = 'single').
colorFunction		Custom function for colors. Example c('red', 'blue', 'green') or rainbow(3) or rainbow(%d), in which %d is replaced with the number of different labels or columns in the plot. Default colors start from black and continue by rainbow()-function until each label or column is assigned with own color.
commonScale	true	If true, all plots have the same limits for X and Y axes. This makes it easier to compare plots. Only relevant when multiple columns have been selected.
dpCm	60	Dots per centimeter. Used for png-images.
drawX	true	If false, do not print X axis ticks and numbers.
height	11	Height of each plot in cm.
imageType	single	Plot multiple images = 'multiple', single image = 'single' or a pairs plot = 'pairs'
labelColumn		Columns in input "label" that is used. If not defined, uses the first column.
legendPosition	off	Legend position 'bottomleft' or 'topright' etc (Can not be used with the pairs plot)
nInf	-2e100	Substitution value for infinitely small values
pInf	2e100	Substitution value for infinitely great values
plotType	l	Plot type p=points, l=lines, h=bars
pngImage	true	Switch image format between png and pdf
sectionTitle	Ordered miRNA expression values	Title of the LaTeX section or an empty string to omit the section
sectionType	subsubsection	Type of LaTeX section: usually one of section, subsection or subsubsection. No section statement is written if sectionTitle is empty.
sort	true	If true, sort values before plotting. This creates a cumulative-like distribution plot. (Can not be used with pairs plot)
title		Image title. The caption may contain one or two %s placeholders, which are replaced with the current column names in "y" and "x" (if defined). Column names are not used when multiple plots are created to the same image (imageType = 'single').
width	14	Width of each plot in cm.
xColumns	*	Columns in input "x" that are plotted. The special value * includes all columns except non-numeric ones. There must be as many selected columns in X as there are in Y. Note that the files "y" and "x" may still contain a different number of columns.
xHigh	-0.000001	High bound of the X axis. The special value -0.000001 means that the bound is computed from the data.
xLabel	transcripts	X axis label. The label may contain one %s placeholder, which is replaced with the current column name in "x", or if not defined, in "y".
xLow	-0.000001	Low bound of the X axis. The special value -0.000001 means that the bound is computed from the data.
xTransformation		Numeric R expression that is used to transform X values. The X values are available as a numeric matrix (or vector) under the name "x". This is analogous to "yTransformation".
yColumns	*	Columns in input "y" that are plotted. The special value * includes all columns except non-numeric ones.
yHigh	-0.000001	High bound of the Y axis. The special value -0.000001 means that the bound is computed from the data.
yLabel	intensity	X axis label. The label may contain one %s placeholder, which is replaced with the current column name in "y".
yLow	-0.000001	Low bound of the Y axis. The special value -0.000001 means that the bound is computed from the data.
yTransformation		Numeric R expression that is used to transform Y values. The Y values are available as a numeric matrix (or vector) under the name "y". Only those columns are present that are selected using "yColumns". For example, "-log10(y)" transforms Y values using a negative base 10 logarithm (defined only for positive values). The expression may be any legal R expression that takes a numeric matrix (or vector) and produces a matrix (or vector) of the same size.

Continued on next page...

Parameter name	Value	Description

19.398 miRNAResults-demFoldChange (FoldChange)

Calculate differentially expressed genes based on fold change.

Author: Kristian Ovaska (Kristian.Ovaska@Helsinki.FI)
Version: 1.1
Category: DEG

Input name	Source	Description
logratio	miRNAResults-logratio.expr 19.409	Log ratio data.

Parameter name	Value	Description
nameDeg	%s_fcDeg	Name of the sets for differentially expressed genes. The pattern %s is expanded to the current sample name in logratio.
nameOver	%s_fcOver	Name of the sets for underexpressed genes. The pattern %s is expanded to the current sample name in logratio.
nameStable	%s_fcStable	Name of the sets for stably expressed genes. The pattern %s is expanded to the current sample name in logratio.
nameUnder	%s_fcUnder	Name of the sets for underexpressed genes. The pattern %s is expanded to the current sample name in logratio.
stableThreshold	0	Fold change threshold (in linear scale) for stable genes. Genes with fold change smaller than the threshold in both directions are considered stably expressed. If the parameter has the value zero, no list of stably expressed genes is included in the results.
threshold	2.0	Fold change threshold (in linear scale). Genes with fold change greater than or equal to the threshold in either direction are considered differentially expressed.

19.399 miRNAResults-demReport (DEGReport)

Report of differentially expressed miRNAs.

Authors: Kristian Ovaska (Kristian.Ovaska@Helsinki.FI), Marko Laakso (Marko.Laakso@Helsinki.FI)
Version: 1.1.1
Categories: DEG, Latex

Input name	Source	Description
deg	miRNAResults-demSet.result 19.400	Sets of differentially expressed genes. If expression values are given (expr), there must be an annotation column SampleGroup that binds the set to an expression column.
geneAnnotation	miRNAResults-miRNAAnnot.csv 19.412	Annotation for genes. Must contain the column named by geneColumn.
expr	miRNAResults-logratio.expr 19.409	Expression values. Used to sort gene lists.

Parameter name	Value	Description
columns	8	Number of columns in gene list table.
geneColumn	miRNAName	Column name in geneAnnotation that contains gene names. If empty, gene names are not printed.
sectionTitle	Summary of differentially expressed miRNAs	Title for a new latex section. If none is given, a new section is not created.
sectionType	section	Type of LaTeX section: usually one of section, subsection or subsubsection. No section statement is written if sectionTitle is empty.
setPattern	over|under	R regular expression for matching set names. If empty, all sets are included.

19.400 miRNAResults-demSet (SetTransformer)

Take differentially expressed miRNAs based on fold change and t-test.

Author: Kristian Ovaska (kristian.ovaska@helsinki.fi)
Version: 0.6
Category: Convert

Input name	Source	Description
transformation	miRNAResults-demTransformation.in 19.401	Set transformations, one per CSV row. The columns Target (target set ID) and Definition (transformation expression) must be present. The column IterationSet may be present if iterated transformations are used. IterationSet should contain NA for non-iterated transformations. Any other columns are interpreted as annotation columns and are copied to the output. For iterated transformations, the wildcard * in annotations is replaced with the current set ID.
set1	miRNAResults-demFoldChange.deg 19.398	Input sets 1. NA values in Members are interpreted as empty sets.
set2	miRNAResults-tTest.idlist 19.435	Input sets 2.

Parameter name	Value	Description
includeAnnotation	*	Comma-separated list of column names in the transformation input that should be used as annotation columns. The wildcard * includes all columns. The special columns Target, Definition and IterationSet are excluded automatically.
includeOriginal	true	If true, the original sets from input files are included in the output as well. If false, only sets defined in transformations are included.

19.401 miRNAResults-demTransformation (INPUT)

Selection of differentially expressed genes by their fold change and t-test calls.

Category: Internal

Parameter name	Value	Description
path	/gpfs/homes/mlaakso/asserSVN/andurilCaseStudy/csv/FoldChangeAndTTest.csv	Path (filename) of the input resource.

19.402 miRNAResults-demVennDiagram (VennDiagram)

Venn diagram of over, under and statistically significant sets.

Authors: Erkka Valo (erkka.valo@helsinki.fi), Kristian Ovaska (kristian.ovaska@helsinki.fi)
Version: 1.1.1
Category: Plot

Input name	Source	Description
sets	miRNAResults-demSet.result 19.400	Sets containing identifiers.

Parameter name	Value	Description
cex	0.7	A numerical value giving the amount by which the text in the plot should be scaled relative to the default values.
names1	p<0.05,overExpressed, underExpressed	Names of the sets to be used in the first Venn diagram. Names are given in a comma-separated list and mapped to sets in <code>sets1</code> positionally. If non are given, the set ID is used as set name.
names2		Names of the sets for the second Venn diagram.
names3		Names of the sets for the third Venn diagram.

Continued on next page...

Parameter name	Value	Description
names4		Names of the sets for the forth Venn diagram.
sectionTitle	Venn diagram of differentially expressed and statistically significant miRNAs	Title for a new latex section. If non is given, a new section is not created.
sectionType	subsection	Type of LaTeX section: usually one of section, subsection or subsubsection. No section statement is written if sectionTitle is empty.
sets1	tumor_vs_normal_tTest,tumor_vs_normal_fcOver,tumor_vs_normal_fcUnder	A comma-separated list of minimum of two and maximum of three sets identified by the set IDs for which the Venn diagram is drawn.
sets2		Sets for the second Venn diagram.
sets3		Sets for the third Venn diagram.
sets4		Sets for the fourth Venn diagram.

19.403 miRNAResults-dems (CSV2IDList)

Extracts one column from the given CSV file and prints it out without duplicates. The selected column may contain entries with comma separated lists of values in which case *isList* parameter may be used to obtain each value separately.

Output is defined as:

$$ids = \bigcup_{i=1}^{9} \pi_{columnIn_i} \sigma_{regexp_i}(table_i). \quad (5)$$

Author: Marko Laakso (Marko.Laakso@Helsinki.FI)
Version: 1.3
Category: convert

Input name	Source	Description
table1	miRNAResults-demSet.result 19.400	The mandatory input relation

Parameter name	Value	Description
acceptMissing	false	Files with missing columnIn are accepted as empty if this is true.
columnIn	Members	A comma separated list of column names for the IDs of interest in each file. Empty values refer to the first column of the file.
columnOut	miRNAName	Name of the only column of the output list. Empty input refers to the name of the input column.
constants		A comma separated list of values that are always included into the output
isList	true	True if the seleted column contains a comma separated list of values to be splitted
quotation	false	Indicator that can be used to disable quotation of the output values
regexp1	ID=over|under	Regular expression for the row filtering in table1. A row is included in the result if this parameter is empty or if values in the given columns match given regular expressions. The parameter has a format COLNAME1=EXPRESSION,COLNAME2=EXPRESSION2 where COLNAMEs are column names in "csv" and EXPRESSIONs are regular expressions using Java syntax. For example, "col=a|b" includes rows where the column col has a value of "a" or "b".
regexp2		Regular expression for the row filtering in table2.
regexp3		Regular expression for the row filtering in table3.
regexp4		Regular expression for the row filtering in table4.
regexp5		Regular expression for the row filtering in table5.
regexp6		Regular expression for the row filtering in table6.
regexp7		Regular expression for the row filtering in table7.
regexp8		Regular expression for the row filtering in table8.
regexp9		Regular expression for the row filtering in table9.

19.404 miRNAResults-filterSamplesRScript (INPUT)

R Script for filtering out samples without clinical annotation.

Category: Internal

Parameter name	Value	Description
path	/gpfs/homes/mlaakso/asserSVN/anduril CaseStudy/r/filterSamples.R	Path (filename) of the input resource.

19.405 miRNAResults-filteredMiRNATargets (CSVFilter)

Filters columns and/or rows from CSV files using flexible criteria. Default settings do not filter out anything. The *negate* parameter reverses all inclusion criteria.

Columns are filtered based on column names (see *includeColumns*); it is also possible to rename columns. Columns can be reordered using *includeColumns*.

A row is included in output if all conditions are satisfied. Rows having too many missing values are filtered using the *nonMissing* parameter. The parameters *regexp*, *lowBound* and *highBound* filter rows based on cell contents. If the *auxiliary* input is given, the result contains those rows from the input whose values match those from the auxiliary file.

Authors: Kristian Ovaska (kristian.ovaska@helsinki.fi), Marko Laakso (Marko.Laakso@Helsinki.FI)
Version: 1.1
Category: Filter

Input name	Source	Description
csv	miRNATargets.in 19.436	CSV file to be filtered.

Parameter name	Value	Description
highBound	PVALUE_OG=1.0E-5	A row is included if the value in given numeric columns are at most as large as given numeric bounds. The parameter has a format COLNAME1=HIGH1,COLNAME2=HIGH2.
idColumn		Column name in "csv" that contains ID values. If the parameter is empty, the first column is used.
includeColumns	SEQ,EXTERNAL_NAME, TRANSCRIPT_ID	Comma-separated list of column names that should be included in the result file. The special value * includes all columns. The order of column names is significant: the output columns are in the order given. See also includeColumns input.
lowBound		A row is included if the value in given numeric columns are at least as large as given numeric bounds. The parameter has a format COLNAME1=LOW1,COLNAME2=LOW2. For example, "col=5" includes rows where the column col has a value of at least 5.
matchColumn		Column name in "auxiliary" containing values that must match the ID column in "csv" (specified using "idColumn"). If empty, the first column of "auxiliary" is used.
negate	false	If true, all inclusion criteria are negated so that rows or columns are excluded instead of included if they match the criteria. For example, includeColumns=* then excludes all columns (which would be an error since at least one column must be included).
nonMissing	0	Include those rows that have non-missing (non-NA) values in at least this many columns. Only those columns that are part of the output are counted.
regexp		Row filtering based on regular expression. A row is included in the result if values in the given columns match given regular expressions. The parameter has a format COLNAME1=EXPRESSION,COLNAME2=EXPRESSION2 where COLNAMEs are column names in "csv" and EXPRESSIONs are regular expressions using Java syntax. For example, "col=a|b" includes rows where the column col has a value of "a" or "b". Commas have to be escaped with "\\" if they are used in column names or regular expressions.
rename	SEQ=miRNAName,EXTERNAL_NAME=targetGeneName, TRANSCRIPT_ID= targetTranscriptId	Comma-separated list of column renaming rules (OLDNAME=NEWNAME). Parameters that refer to column names refer to old names; renaming is done last.

19.406 miRNAResults-groupMeans (SampleCombiner)

Compute medians of categories and logratios between.

Authors: Kari Nousiainen (Kari.Nousiainen@Helsinki.FI), Kristian Ovaska (Kristian.Ovaska@Helsinki.FI)
Version: 1.0
Category: Filter

Input name	Source	Description
expr	miRNAResults-mExpr.matrix 19.410	Expression data, either log ratios or channel expressions.
groups	miRNAResults-sampleGroups.groups 19.427	Sample group specification.

Parameter name	Value	Description
geometricMean	true	Indicates whether the means will be calculated using geometric(true) or arithmetic mean (false).
groupIDs	tumor_solid,normal_tissue	Comma-separated list of sample group IDs. If given, combine only these groups. If not given (empty), combine all sample groups.
includeOriginal	false	If true, put input groups into the result even if they are not present in 'groups'.
thresholdRatio	0	Allows to omit those genes that have missing values in some samples. Only genes that have non-missing values in at least the given number of columns are included in the result. The actual threshold T is ceiling(thresholdRatio*n), where n is the number of samples that are used in combining. Only genes that have non-missing values in at least T columns are included. Other genes have the combined value set to NA. Note: this parameter specifies the ratio instead of integer threshold since the number of samples may be different in each sample group.

19.407 miRNAResults-km (KaplanMeier)

Produces a Kaplan-Meier plot representing survival estimates based on the given data. Log-rank statistics are calculated in order the address the similarity of the curves.

Authors: Marko Laakso (Marko.Laakso@Helsinki.FI), Riku Louhimo (Riku.Louhimo@Helsinki.FI), Kristian Ovaska (kristian.ovaska@helsinki.fi)
Version: 1.7
Categories: Survival, Latex

Input name	Source	Description
survival	miRNAResults-survivalTableConv.csv 19.434	Survival time and event indicators together with the sample group assignments
annotation	miRNAResults-survivalAnnot.csv 19.431	Additional annotations for the groupCol names. These annotations are listed as column name value pairs in figure caption.

Parameter name	Value	Description
PLimit	0.1	Ignore results with a p-value greater than this limit
annotCol	*	A comma separated list of column names of the annotation file that will be shown in the report. An asterisk (*) may be used for all attributes except the first one that is considered as a key.
confInt	0.95	Coverage of the confidence intervals [0,1] or a negative number to disable them
eventSymbol	DECEASED	Value of statusCol column indicating an event. Other values are considered as censorings.
excludeCol	SampleID,PatientID,VitalStatus, SurvivalEndTime, AgeAtProcedure	A comma separated list of column names that should not be used as groupCols even if an asterisk is used. This is especially useful to filter out samples identifiers and other irrelevant columns.
groupCol	*	A comma separated list of column names of sample group identifiers or an empty string for a single curve. An asterisk (*) may be used to plot all attributes except those defined for the other purposes or listed in excludeCol.
minCount	20	Individual groups with less that this many members will be left out
statusCol	VitalStatus	Column name for the event indicator
timeCol	SurvivalEndTime	Column name for the survival time

Continued on next page...

Parameter name	Value	Description
timeOutLimit	36	Cut survival curves after this limit. A negative value can be used to maximize the length of the period.
title	Kaplan-Meier survival estimates	Figure title
xLabel	survival time	Label for the horizontal time axis

19.408 miRNAResults-kmReport (LatexCombiner)

MiRNA expression survival analysis report.

Authors: Kristian Ovaska (Kristian.Ovaska@Helsinki.FI), Marko Laakso (Marko.Laakso@Helsinki.FI)
Version: 1.3
Category: Latex

Input name	Source	Description
latex1	miRNAResults-km.report 19.407	LaTeX fragment 1.

Parameter name	Value	Description
pagebreak	false	Determines if the result document should start with a page break.
sectionTitle	Survival analysis for miRNA expression	If non-empty, a declaration of a new section with the given name is inserted to the beginning of the combined document. This is a convenience feature to make it easy to compile subsections into a section.
sectionType	section	Type of LaTeX section: usually one of section, subsection or subsubsection. No section statement is written if sectionTitle is empty.
strictBorders	true	Enables the flushing of all document elements before each fragment

19.409 miRNAResults-logratio (SampleCombiner)

Combine expression data from several samples into one by taking means, medians or log ratios. Combining is done using a sample group table. Each row of the table specifies how one sample group is created from a list of source groups. If the expression input file contains log ratios, both median and log ratio combining are possible and the result are log ratios. If the input are channel values, the groups may be either median groups (result type: channel values) or log ratio groups (result type: log ratio), but not both.

Authors: Kari Nousiainen (Kari.Nousiainen@Helsinki.FI), Kristian Ovaska (Kristian.Ovaska@Helsinki.FI)
Version: 1.0
Category: Filter

Input name	Source	Description
expr	miRNAResults-groupMeans. expr 19.406	Expression data, either log ratios or channel expressions.
groups	miRNAResults-sampleGroups. groups 19.427	Sample group specification.

Parameter name	Value	Description
geometricMean	true	Indicates whether the means will be calculated using geometric(true) or arithmetic mean (false).
groupIDs	tumor_vs_normal	Comma-separated list of sample group IDs. If given, combine only these groups. If not given (empty), combine all sample groups.
includeOriginal	false	If true, put input groups into the result even if they are not present in 'groups'.
thresholdRatio	0	Allows to omit those genes that have missing values in some samples. Only genes that have non-missing values in at least the given number of columns are included in the result. The actual threshold T is ceiling(thresholdRatio*n), where n is the number of samples that are used in combining. Only genes that have non-missing values in at least T columns are included. Other genes have the combined value set to NA. Note: this parameter specifies the ratio instead of integer threshold since the number of samples may be different in each sample group.

19.410 miRNAResults-mExpr (VariationFilter)

VariationFilter is an Anduril component that can filter out those rows from a matrix where the standard deviation is below a threshold. Also rows with less than two non-NA values are filtered as the standard deviation can not be computed. The filter can be used to, for example, remove rows from an expression matrix where the variation between samples is low.

Author: Kristian Ovaska (kristian.ovaska@helsinki.fi)
Version: 1.1
Category: Filter

Input name	Source	Description
matrix	miRNAResults-probeJoin.<br>csv 19.420	Input matrix.

Parameter name	Value	Description
columns	*	Names of columns that are included in standard deviation computation. However, all columns are always present in the output matrix. The special value * means all columns.
filterSmall<br>Variation	true	If true, rows with standard deviation BELOW the threshold are filtered out (normal operation). If false, rows with standard deviation ABOVE the threshold are filtered out.
fraction	true	If true, the standard deviation threshold is a fraction of the mean of each row. Otherwise, the threshold is the nominal standard deviation.
threshold	0	Standard deviation threshold as a percentage value of the mean of each row (if fraction=true) or as the absolute standard deviation (if fraction=false). For example, assume that fraction=true, the threshold is 0.1 and two rows have means 5 and 3; and standard deviations 0.2 and 0.4. The first row is removed since 0.2 is less than 0.1*5. The second row is kept since 0.4 is greater than 0.1*3.

19.411 miRNAResults-mds (MDSPlot)

MDS plot of samples.

Author: Kari Nousiainen (Kari.Nousiainen@Helsinki.FI)
Version: 1.0
Categories: Quality Control, Latex

Input name	Source	Description
expr	miRNAResults-mExpr.<br>matrix 19.410	Expression data. Can be either channel data or log ratios.
groups	miRNAResults-sampleGroups.<br>groups 19.427	Sample group table maps samples to sample groups. The information is used in plotting to label the samples within the same group with the same color. Note that, a sample may include in many groups. Howerver, the component use only groups whose type is either median or sample.

Parameter name	Value	Description
atomic	false	This parameters shows whether the samples groups are atomic or contain many samples.
caption	Multidimensional Scaling	Label of the figure.
cex	0.2	A numerical value giving the amount by which the size of the sample labels in the plot should be scaled relative to the default value.
dimensions	2	Number of dimensions the data is to be scaled into.
legendPosition	topright	Position of the legend box. One of "bottomright", "bottom", "bottomleft", "left", "topleft", "top", "topright", "right", "center" or "off". If "off", the legend is not shown.
pagebreak	false	Tells if the result document should start with a page break.
plotNames	true	If true sample names are plotted in the MDS plot, else circles are used.
sectionTitle	Multidimensional scaling of the samples	Title of the LaTeX section.
sectionType	subsubsection	Type of LaTeX section: usually one of section, subsection or subsubsection. If empty, no section statement is written.

Continued on next page...

Parameter name	Value	Description
width	16	Width of diagram in cm.

19.412 miRNAResults-miRNAAnnot (CSVJoin)

Join rows from two or more CSV files, optionally using one column as a matching key. If a key column is not used, the result contains all rows and all columns of the input files. Missing values (`NA`) may be introduced when a column is not present in all input files. Each column is present once and duplicate rows are removed.

If a key column is used, the rows in each input file are matched using values from the key column. The result file has one row for each key value. In the result, the first column is the key column; its name is obtained from the first CSV input (csv1). If the intersection parameter is true, a key is included in the result if the key value is present in all inputs. If intersection is false, a key is included if it is present in at least one input (key union). Union semantics may introduce missing values in the result. If several input files have the same column, the value is obtained from the first file. However, if the first file contains a missing value and the second file contains a non-missing value, the non-missing value is used instead.

Author: Kristian Ovaska (kristian.ovaska@helsinki.fi)
Version: 1.0
Category: convert

Input name	Source	Description
csv1	miRNAResults-collapsedMiRNA Names.relation 19.395	CSV file 1.
csv2	miRNAResults-collapsedMiRNA Targets.relation 19.396	CSV file 2.

Parameter name	Value	Description
intersection	false	Defines how keys are handled; only used when useKeys=true. If intersection is true, the result contains a key if the key is present in all input files. If false, the result contains a key if the key is present in at least one input file.
keyColumnNames	miRNAName,miRNAName	Comma-separated list of key column names; only used when useKeys=true. The first name refers to csv1, the second to csv2, etc. An empty value refers to the first column. Empty values may be omitted from the list, so all these are equivalent: "col1" ; "col1," ; "col1,," ; etc.
useKeys	true	If true, use one column from each CSV file as a matching key column.

19.413 miRNAResults-miRNAStat (TableQuery)

Execute an SQL query on input relations and create a result table. TableQuery uses HSQLDB (Hyper-threaded Structured Query Language Database [25]) for executing the query. Consequently, the syntax of the query is defined by HSQLDB.

Author: Kristian Ovaska (Kristian.Ovaska@Helsinki.FI)
Version: 1.2
Categories: convert, Filter

Input name	Source	Description
table1	miRNAResults-miRNAAnnot .csv 19.412	CSV table 1. The table is referred to as 'table1' in the SQL query.
table2	miRNAResults-logratio. expr 19.409	CSV table 2. The table is referred to as 'table2' in the SQL query.
table3	miRNAResults-tTest. pvalues 19.435	CSV table 3. The table is referred to as 'table3' in the SQL query.
query	miRNAResults-miRNAStatQuery .in 19.418	SQL query. Either this file or the query parameter must be provided, but not both.

Parameter name	Value	Description
numIndices		Comma-separated list of index counts for input tables. All indices are single-column indices, running from column 1 to N, where N is retrieved from this parameter. If empty, the default number (1) is used. For example, ",2,,2" sets two indices for table2 and table4 and one index for the rest.
query		SQL query. Either this parameter or the query input must be provided, but not both.

19.414 miRNAResults-miRNAStatBand (KorvasieniAnnotator)

Korvasieni is an Ensembl [12] based converter for database identifiers and it can be used to convert genome location, gene, transcript, and translation identifiers across multiple biological databases. The selected `keyColumn` may contain entries with comma separated lists of values in which case `isListKey` parameter may be used to obtain each value separately and to process them as they were given in consecutive lines.

Author: Marko Laakso (Marko.Laakso@Helsinki.FI)
Version: 1.7
Categories: Annotation, GO

Input name	Source	Description
sourceKeys	miRNAResults-miRNAStat .table 19.413	A list of source database keys.
connection	ensembl.in 19.273	Database connection can be defined using this file. The definition of parameters: database.url, database.user, database.password, database.timeout, database.recycle, and database.driver can be found from the documentation of Korvasieni.

Parameter name	Value	Description
echoColumns	*	A comma separated list of column names for the columns that will be copied to the output. An asterisk (*) can be used to denote all columns execpt the keyColumn.
inputDB	.GeneName	Type of input keys. This must be a database supported by Korvasieni. If the parameter is omitted, the component tries to derive the database from the type of geneID. If this is not possible, an error is returned. You may define three columns in form of chromosome:start-end in case the inputDB is .DNARegion. This format provides a comfortable compatibility with DNARegion datatype.
inputType	Any	Ensembl object type for the input keys (Any, Gene, Transcript, Translation)
isListKey	false	Enables the automatic value splits for the comma separated key column
keyColumn	miRNAName	Name of the key column withing sourceKeys file or an empty string for the first column
maxHits	100000	Maximum number of target identifiers for a single source identifier
rename	.DNABand=DNABand	Comma separated list of column renaming rules (oldname=newname)
skipLevel	never	Skip result rows if the source identifier is unknown or target identifiers are not available. Possible values are: never (no filtering), source (skip if the source ID is unknown), target (skip if no target IDs are found), any (skip if any of the target IDs is missing).
targetDB	.DNABand	Comma-separated list of annotation types. Possible values are all databases supported by Korvasieni.
unique	false	This flag can be turned on in order to eliminate duplicate annotations.

19.415 miRNAResults-miRNAStatConverted (IDConvert)

Converts identifiers from a column in a CSV file to another type of identifiers. The conversion is done using an annotation table on the basis of the column named *conversionColumn*.

Author: Kristian Ovaska (kristian.ovaska@helsinki.fi)
Version: 1.0
Category: convert

Input name	Source	Description
csv	miRNAResults-miRNAStatBand .bioAnnotation 19.414	Source CSV file. Contains the column that is to be converted. The column is named by the parameter 'sourceColumn'.

Continued on next page...

Input name	Source	Description
conversionTable	miRNAResults-miRNAStatGenes .bioAnnotation 19.417	Conversion table. Must contain the column named by the parameter 'conversionColumn'.

Parameter name	Value	Description
collapseNumeric	median	Method for collapsing numeric columns with duplicate rows (when unique=true). Legal values are "median" (take median of non-NA values), "mean", "max", "min", "first" (take the first row), "majority" (take the value that is present on the largest number of rows), "consensus" (require that all rows have the same value) and "indicator".<br>Here, "majority" and "consensus" are mostly useful for discrete (integer) values). For "consensus", it is an error if duplicate rows contain distinct values. "indicator" assumes that data are divided into negative (-1), neutral (0) and positive (1) items, such as discretized expression values. The result is (a) 1 if there is a 1 and no -1 value; (b) -1 if there is a -1 and not 1 value; (c) 0 if there is a 0 and no -1 or 1; or (d) NA otherwise.
collapseString	consensus	Method for collapsing non-numeric columns with duplicate rows (when unique=true). Legal values are "first" (take the first row), "majority" (take the value that is present on the largest number of rows), "longest" (take the longest value), "shortest" (take the shortest value) and "consensus" (require that all rows have the same value). For "consensus", it is an error if duplicate rows contain distinct values.
conversionColumn	.GeneId	Column in 'conversionTable' that is used to fetch new values for the source column.
keyColumn		Column in 'conversionTable' that is used to match ID values from the source CSV. If the parameter is empty, the first column is used.
originalWhen Missing	false	Determines how values are converted when the item is not found in the conversion table. If true, the original (unconverted) ID is inserted to the output. If false, NA is inserted.
sourceColumn	targetTranscriptId	Column in 'csv' that is going to be converted. If empty, the first column is used.
split	true	If true, split values in the source column by commas before ID conversion. For example, if the source column contains the string "a,b,c", it is first split into "a", "b" and "c"; then, individual IDs are converted; finally, the converted IDs are joined by commas into a string of format "A,B,C".
splitConverted	false	If true, split converted values by commas after ID conversion. A row with comma separated values in the target column will be split to otherwise identical rows but so that the rows have a single ID value in the target column. This is done before possible collapsing of duplicate rows.
targetColumn	GeneID	Target column name in 'converted'. If empty, the column name is the same as the original column (sourceColumn).
unique	false	If true, rows with identical identifiers (sourceColumn) are collapsed into one row so that sourceColumn is unique. Collapsing may result in error if duplicate rows contain conflicting values. See parameters collapseNumeric and collapseString for methods to collapse numeric and non-numeric columns, respectively.
varMeasure		Method for calculating the variation of numeric duplicate values. If the variation is above the threshold given in <code>varThreshold</code> , duplicate values are collapsed to NA. Possible values are 'MAD' and 'variance'. 'MAD' stands for the median absolute deviation and 'variance' for the population variance. If no values is given, no threshold for combining numeric values is used.
varThreshold	2	Maximum allowed variability in the duplicate values. If <code>varMeasure</code> is given duplicate values with variance above the threshold are collapsed to NA

19.416 miRNAResults-miRNAStatFiltered (CSVFilter)

Filters columns and/or rows from CSV files using flexible criteria. Default settings do not filter out anything. The *negate* parameter reverses all inclusion criteria.

Columns are filtered based on column names (see *includeColumns*); it is also possible to rename columns. Columns can be reordered using *includeColumns*.

A row is included in output if all conditions are satisfied. Rows having too many missing values are filtered using the *nonMissing* parameter. The parameters *regexp*, *lowBound* and *highBound* filter rows based on cell contents. If the *auxiliary* input is given, the result contains those rows from the input whose values match those from the auxiliary file.

Authors: Kristian Ovaska (kristian.ovaska@helsinki.fi), Marko Laakso (Marko.Laakso@Helsinki.FI)
Version: 1.1
Category: Filter

Input name	Source	Description
csv	miRNAResults-miRNAStat Converted.csv 19.415	CSV file to be filtered.

Parameter name	Value	Description
highBound		A row is included if the value in given numeric columns are at most as large as given numeric bounds. The parameter has a format COLNAME1=HIGH1,COLNAME2=HIGH2.
idColumn		Column name in "csv" that contains ID values. If the parameter is empty, the first column is used.
includeColumns	miRNAName,miRNAId, FoldChange,PValueCorrected, stemLoopName,GeneID, DNABand	Comma-separated list of column names that should be included in the result file. The special value * includes all columns. The order of column names is significant: the output columns are in the order given. See also includeColumns input.
lowBound		A row is included if the value in given numeric columns are at least as large as given numeric bounds. The parameter has a format COLNAME1=LOW1,COLNAME2=LOW2. For example, "col=5" includes rows where the column col has a value of at least 5.
matchColumn		Column name in "auxiliary" containing values that must match the ID column in "csv" (specified using "idColumn"). If empty, the first column of "auxiliary" is used.
negate	false	If true, all inclusion criteria are negated so that rows or columns are excluded instead of included if they match the criteria. For example, includeColumns=* then excludes all columns (which would be an error since at least one column must be included).
nonMissing	0	Include those rows that have non-missing (non-NA) values in at least this many columns. Only those columns that are part of the output are counted.
regexp		Row filtering based on regular expression. A row is included in the result if values in the given columns match given regular expressions. The parameter has a format COLNAME1=EXPRESSION,COLNAME2=EXPRESSION2 where COLNAMEs are column names in "csv" and EXPRESSIONs are regular expressions using Java syntax. For example, "col=a|b" includes rows where the column col has a value of "a" or "b". Commas have to be escaped with "\\" if they are used in column names or regular expressions.
rename	PValueCorrected=PValue, GeneID=TargetGenes	Comma-separated list of column renaming rules (OLDNAME=NEWNAME). Parameters that refer to column names refer to old names; renaming is done last.

19.417 miRNAResults-miRNAStatGenes (KorvasieniAnnotator)

Korvasieni is an Ensembl [12] based converter for database identifiers and it can be used to convert genome location, gene, transcript, and translation identifiers across multiple biological databases. The selected `keyColumn` may contain entries with comma separated lists of values in which case `isListKey` parameter may be used to obtain each value separately and to process them as they were given in consecutive lines.

Author:	Marko Laakso (Marko.Laakso@Helsinki.FI)
Version:	1.7
Categories:	Annotation, GO

Input name	Source	Description
sourceKeys	miRNAResults-miRNAStat .table 19.413	A list of source database keys.
connection	ensembl.in 19.273	Database connection can be defined using this file. The definition of parameters: database.url, database.user, database.password, database.timeout, database.recycle, and database.driver can be found from the documentation of Korvasieni.

Parameter name	Value	Description
echoColumns		A comma separated list of column names for the columns that will be copied to the output. An asterisk (*) can be used to denote all columns execpt the keyColumn.
inputDB	.TranscriptId	Type of input keys. This must be a database supported by Korvasieni. If the parameter is omitted, the component tries to derive the database from the type of geneID. If this is not possible, an error is returned. You may define three columns in form of chromosome:start-end in case the inputDB is .DNARegion. This format provides a comfortable compatibility with DNARegion datatype.

Continued on next page...

Parameter name	Value	Description
inputType	Any	Ensembl object type for the input keys (Any, Gene, Transcript, Translation)
isListKey	true	Enables the automatic value splits for the comma separated key column
keyColumn	targetTranscriptId	Name of the key column withing sourceKeys file or an empty string for the first column
maxHits	100000	Maximum number of target identifiers for a single source identifier
rename		Comma separated list of column renaming rules (oldname=newname)
skipLevel	never	Skip result rows if the source identifier is unknown or target identifiers are not available. Possible values are: never (no filtering), source (skip if the source ID is unknown), target (skip if no target IDs are found), any (skip if any of the target IDs is missing).
targetDB	.GeneId	Comma-separated list of annotation types. Possible values are all databases supported by Korvasieni.
unique	true	This flag can be turned on in order to eliminate duplicate annotations.

19.418 miRNAResults-miRNAStatQuery (INPUT)

This query combines miRNA fold changes and p-values with annotations.

Category: Internal

Parameter name	Value	Description
path	/gpfs/homes/mlaakso/asserSVN/andurilCaseStudy/sql/miRNAStatistics.sql	Path (filename) of the input resource.

19.419 miRNAResults-patientMetadata (INPUT)

Survival data

Category: Internal

Parameter name	Value	Description
path	/gpfs/homes/mlaakso/asserSVN/andurilCaseStudy/data/patient-metadata.csv	Path (filename) of the input resource.

19.420 miRNAResults-probeJoin (IDConvert)

Converts identifiers from a column in a CSV file to another type of identifiers. The conversion is done using an annotation table on the basis of the column named *conversionColumn*.

Author: Kristian Ovaska (kristian.ovaska@helsinki.fi)
Version: 1.0
Category: convert

Input name	Source	Description
csv	miRNAResults-sampleFilter.table 19.426	Source CSV file. Contains the column that is to be converted. The column is named by the parameter 'sourceColumn'.
conversionTable	miRNAProbeAnnot.in 19.393	Conversion table. Must contain the column named by the parameter 'conversionColumn'.

Parameter name	Value	Description
collapseNumeric	median	Method for collapsing numeric columns with duplicate rows (when unique=true). Legal values are "median" (take median of non-NA values), "mean", "max", "min", "first" (take the first row), "majority" (take the value that is present on the largest number of rows), "consensus" (require that all rows have the same value) and "indicator".<br>Here, "majority" and "consensus" are mostly useful for discrete (integer) values). For "consensus", it is an error if duplicate rows contain distinct values. "indicator" assumes that data are divided into negative (-1), neutral (0) and positive (1) items, such as discretized expression values. The result is (a) 1 if there is a 1 and no -1 value; (b) -1 if there is a -1 and not 1 value; (c) 0 if there is a 0 and no -1 or 1; or (d) NA otherwise.
collapseString	consensus	Method for collapsing non-numeric columns with duplicate rows (when unique=true). Legal values are "first" (take the first row), "majority" (take the value that is present on the largest number of rows), "longest" (take the longest value), "shortest" (take the shortest value) and "consensus" (require that all rows have the same value). For "consensus", it is an error if duplicate rows contain distinct values.
conversionColumn	miRNAName	Column in 'conversionTable' that is used to fetch new values for the source column.
keyColumn		Column in 'conversionTable' that is used to match ID values from the source CSV. If the parameter is empty, the first column is used.
originalWhen Missing	true	Determines how values are converted when the item is not found in the conversion table. If true, the original (unconverted) ID is inserted to the output. If false, NA is inserted.
sourceColumn	AgilentProbe	Column in 'csv' that is going to be converted. If empty, the first column is used.
split	false	If true, split values in the source column by commas before ID conversion. For example, if the source column contains the string "a,b,c", it is first split into "a", "b" and "c"; then, individual IDs are converted; finally, the converted IDs are joined by commas into a string of format "A,B,C".
splitConverted	false	If true, split converted values by commas after ID conversion. A row with comma separated values in the target column will be split to otherwise identical rows but so that the rows have a single ID value in the target column. This is done before possible collapsing of duplicate rows.
targetColumn	miRNAName	Target column name in 'converted'. If empty, the column name is the same as the original column (sourceColumn).
unique	true	If true, rows with identical identifiers (sourceColumn) are collapsed into one row so that sourceColumn is unique. Collapsing may result in error if duplicate rows contain conflicting values. See parameters collapseNumeric and collapseString for methods to collapse numeric and non-numeric columns, respectively.
varMeasure		Method for calculating the variation of numeric duplicate values. If the variation is above the threshold given in <code>varThreshold</code> , duplicate values are collapsed to NA. Possible values are 'MAD' and 'variance'. 'MAD' stands for the median absolute deviation and 'variance' for the population variance. If no values is given, no threshold for combining numeric values is used.
varThreshold	2	Maximum allowed variability in the duplicate values. If <code>varMeasure</code> is given duplicate values with variance above the threshold are collapsed to NA

19.421 miRNAResults-qcReport (LatexCombiner)

Compile quality control report.

Authors: Kristian Ovaska (Kristian.Ovaska@Helsinki.FI), Marko Laakso (Marko.Laakso@Helsinki.FI)
Version: 1.3
Category: Latex

Input name	Source	Description
latex1	miRNAResults-cumDistReport. plot 19.397	LaTeX fragment 1.
latex2	miRNAResults-mds.report 19.411	LaTeX fragment 2.

Parameter name	Value	Description
pagebreak	false	Determines if the result document should start with a page break.
sectionTitle	Expression quality control	If non-empty, a declaration of a new section with the given name is inserted to the beginning of the combined document. This is a convenience feature to make it easy to compile subsections into a section.
sectionType	subsection	Type of LaTeX section: usually one of section, subsection or subsubsection. No section statement is written if sectionTitle is empty.
strictBorders	true	Enables the flushing of all document elements before each fragment

Continued on next page. . .

Parameter name	Value	Description

19.422 miRNAResults-report (LatexCombiner)

Compile report of miRNA analysis.

Authors: Kristian Ovaska (Kristian.Ovaska@Helsinki.FI), Marko Laakso (Marko.Laakso@Helsinki.FI)
Version: 1.3
Category: Latex

Input name	Source	Description
latex1	miRNAResults-demReport. summary 19.399	LaTeX fragment 1.
latex2	miRNAResults-demVennDiagram. report 19.402	LaTeX fragment 2.
latex3	miRNAResults-qcReport. document 19.421	LaTeX fragment 3.
latex4	miRNAResults-kmReport. document 19.408	LaTeX fragment 4.
latex5	miRNAResults-demReport. genelist 19.399	LaTeX fragment 5.

Parameter name	Value	Description
pagebreak	false	Determines if the result document should start with a page break.
sectionTitle		If non-empty, a declaration of a new section with the given name is inserted to the beginning of the combined document. This is a convenience feature to make it easy to compile subsections into a section.
sectionType	section	Type of LaTeX section: usually one of section, subsection or subsubsection. No section statement is written if sectionTitle is empty.
strictBorders	true	Enables the flushing of all document elements before each fragment

19.423 miRNAResults-sampleExpression (SampleExpression)

Create a $\{-1, 0, 1\}$ matrix that indicates whether a given gene/probe is differentially expressed in individual samples. The decision whether a gene is differentially expressed is done:

a) using fold change thresholding or
b) by selecting P largest and P smallest log ratios, where P is a proportion.

The expression input can be either log ratios or channel values, which are handled differently. Log ratios are directly used for fold change thresholding. Channel values are converted to log ratios by dividing them with reference samples. In this case, the expression matrix is assumed to contain both the target and reference samples. Reference samples are converted to a single column by taking a median. The columns that contain reference values are defined in the groups input. If the groups input is not given, values are assumed to be log ratios.

Author: Kristian Ovaska (kristian.ovaska@helsinki.fi)
Version: 1.0
Category: DEG

Input name	Source	Description
expr	miRNAResults-mExpr. matrix 19.410	Expression matrix. The values are assumed to be log ratios if the "groups" input is not given; otherwise, they are assumed to be channel values.
groups	miRNAResults-sampleGroups. groups 19.427	Sample groups that define reference (control) columns in the expression input. The reference group name is given with the referenceGroup parameter.

Parameter name	Value	Description
referenceGroup	normal_tissue	Group name in the groups input that contains reference samples. Used to compute log ratios from channel values.
threshold	2.0	Threshold that determines whether a gene is under- or overexpressed. Interpretation of the value depends on thresholdType.
thresholdType	fold-change	Defines how differential expression is decided. Legal values are "fold-change" and "top-most". For "fold-change", the threshold parameter is a linear fold change threshold. A gene is overexpressed if its log ratio is over the threshold and underexpressed if the log ratio is under 1/threshold. For "top-most", threshold is a fraction between 0 and 1 that defines how many genes with highest and lowest expression are selected. For example, if threshold is 0.1, the top 10% genes are overexpressed and the bottom 10% are underexpressed.

19.424 miRNAResults-sampleExpressionFilt (CSVFilter)

Filters columns and/or rows from CSV files using flexible criteria. Default settings do not filter out anything. The *negate* parameter reverses all inclusion criteria.

Columns are filtered based on column names (see *includeColumns*); it is also possible to rename columns. Columns can be reordered using *includeColumns*.

A row is included in output if all conditions are satisfied. Rows having too many missing values are filtered using the *nonMissing* parameter. The parameters *regexp*, *lowBound* and *highBound* filter rows based on cell contents. If the *auxiliary* input is given, the result contains those rows from the input whose values match those from the auxiliary file.

Authors: Kristian Ovaska (kristian.ovaska@helsinki.fi), Marko Laakso (Marko.Laakso@Helsinki.FI)
Version: 1.1
Category: Filter

Input name	Source	Description
csv	miRNAResults-sampleExpression.indicator 19.423	CSV file to be filtered.
auxiliary	miRNAResults-dems.ids 19.403	If given, contains one column (see "matchColumn") whose values are matched to a column in the "csv" input (see "idColumn").

Parameter name	Value	Description
highBound		A row is included if the value in given numeric columns are at most as large as given numeric bounds. The parameter has a format COLNAME1=HIGH1,COLNAME2=HIGH2.
idColumn		Column name in "csv" that contains ID values. If the parameter is empty, the first column is used.
includeColumns	*	Comma-separated list of column names that should be included in the result file. The special value * includes all columns. The order of column names is significant: the output columns are in the order given. See also includeColumns input.
lowBound		A row is included if the value in given numeric columns are at least as large as given numeric bounds. The parameter has a format COLNAME1=LOW1,COLNAME2=LOW2. For example, "col=5" includes rows where the column col has a value of at least 5.
matchColumn		Column name in "auxiliary" containing values that must match the ID column in "csv" (specified using "idColumn"). If empty, the first column of "auxiliary" is used.
negate	false	If true, all inclusion criteria are negated so that rows or columns are excluded instead of included if they match the criteria. For example, includeColumns=* then excludes all columns (which would be an error since at least one column must be included).
nonMissing	0	Include those rows that have non-missing (non-NA) values in at least this many columns. Only those columns that are part of the output are counted.
regexp		Row filtering based on regular expression. A row is included in the result if values in the given columns match given regular expressions. The parameter has a format COLNAME1=EXPRESSION,COLNAME2=EXPRESSION2 where COLNAMEs are column names in "csv" and EXPRESSIONs are regular expressions using Java syntax. For example, "col=a|b" includes rows where the column col has a value of "a" or "b". Commas have to be escaped with "\\" if they are used in column names or regular expressions.

Continued on next page...

Parameter name	Value	Description
rename		Comma-separated list of column renaming rules (OLDNAME=NEWNAME). Parameters that refer to column names refer to old names; renaming is done last.

19.425 miRNAResults-sampleExpressionTrans (MatrixTranspose)

Produces a transposed version of the input matrix.

Author: Kristian Ovaska (kristian.ovaska@helsinki.fi)
Version: 1.0
Category: convert

Input name	Source	Description
matrix	miRNAResults-sampleExpressionFilt.csv 19.424	Input matrix.

Parameter name	Value	Description
rowName	SampleID	Name of the first column in the output.

19.426 miRNAResults-sampleFilter (REvaluate)

Filter samples without clinical annotation.

Authors: Marko Laakso (Marko.Laakso@Helsinki.FI), Erkka Valo (erkka.valo@tkk.fi)
Version: 2.2
Categories: External, Latex, Network Control

Input name	Source	Description
script	miRNAResults-filterSamplesRScript.in 19.404	Data transformation
table1	miRNAExprNormalized.in 19.391	Data matrix 1 (data.frame/file table1)
table2	miRNAResults-samplesWithoutClinicalAnnotation.in 19.429	Data matrix 2 (data.frame/file table2)

Parameter name	Value	Description
asConnection	false	If true, assign file connections (class file) to the inputs to variables of the same name. This is useful e.g. if you want to read large data files row by row.
param1		Value of this parameter will be assigned to param1 variable
param10		Value of this parameter will be assigned to param10 variable
param2		Value of this parameter will be assigned to param2 variable
param3		Value of this parameter will be assigned to param3 variable
param4		Value of this parameter will be assigned to param4 variable
param5		Value of this parameter will be assigned to param5 variable
param6		Value of this parameter will be assigned to param6 variable
param7		Value of this parameter will be assigned to param7 variable
param8		Value of this parameter will be assigned to param8 variable
param9		Value of this parameter will be assigned to param9 variable

19.427 miRNAResults-sampleGroups (SampleGroupCreator)

Map sample names into their clinical categories.

Author: Kristian Ovaska (kristian.ovaska@helsinki.fi)
Version: 1.2.1
Category: preprocessing

Input name	Source	Description
data1	miRNAResults-mExpr.matrix 19.410	Data file 1 for reading sample names. Either column names or values on one column contain sample names.

Parameter name	Value	Description
columns		Defines which columns in input files contain sample names, or whether column names should be used (default). This parameter is a comma-separated list of at most three values which name columns in inputs data1 to data3. If an entry is empty, the column names of the corresponding input file are used instead.
definition1	tumor_solid,mean,Solid tumor samples	Definition of group 1. If empty, the group is omitted. Format: NAME,TYPE or NAME,TYPE,DESCRIPTION.
definition2	normal_tissue,mean,Normal tissue samples	Definition of group 2. If empty, the group is omitted.
definition3	tumor_vs_normal,ratio,fold change of tumor median vs. normal median	Definition of group 3. If empty, the group is omitted.
definition4		Definition of group 4. If empty, the group is omitted.
definition5		Definition of group 5. If empty, the group is omitted.
definition6		Definition of group 6. If empty, the group is omitted.
definition7		Definition of group 7. If empty, the group is omitted.
definition8		Definition of group 8. If empty, the group is omitted.
definition9		Definition of group 9. If empty, the group is omitted.
pattern1	TCGA_[0-9]{2}_[0-9]{4}_01	Pattern for group 1. If empty, the group is omitted. Format depends on the pattern type.
pattern2	TCGA_[0-9]{2}_[0-9]{4}_11	Pattern for group 2. If empty, the group is omitted.
pattern3	tumor_solid,normal_tissue	Pattern for group 3. If empty, the group is omitted.
pattern4		Pattern for group 4. If empty, the group is omitted.
pattern5		Pattern for group 5. If empty, the group is omitted.
pattern6		Pattern for group 6. If empty, the group is omitted.
pattern7		Pattern for group 7. If empty, the group is omitted.
pattern8		Pattern for group 8. If empty, the group is omitted.
pattern9		Pattern for group 9. If empty, the group is omitted.
patternTypes	re,re,verbatim	Comma-separated list of pattern types for each pattern. Each item is one of "re" (default), "relist" or "verbatim". The types are explained above. An empty value is interpreted as "re". For example, ",re,relist,,verbatim" specifies that pattern3 has the type "relist" and pattern5 the type "verbatim"; all others (including pattern6 and above) have the type "re".

19.428 miRNAResults-sampleNames (ExpandCollapse)

Converts between the two possible representations of a relations with multivalued columns. By default this component splits rows with multivalued (comma separated lists) columns into multiple rows each representing a single value. The input may contain several columns with comma separated values and all permutations of column values are shown as individual single-valued rows. The alternative mode recovers the original comma separated values from the expanded relations. This is performed by joining values of the their list columns into comma separated lists.

Author: Marko Laakso (Marko.Laakso@Helsinki.FI)
Version: 1.3
Category: convert

Input name	Source	Description
relation	miRNAResults-sampleGroups.groups 19.427	The mandatory input relation

Parameter name	Value	Description
delim	,	Value delimiter between the list column values. Special characters can be encoded as specified in fi.helsinki.ltdk.csbl.asser.ArgumentEncoding.

Continued on next page...

Parameter name	Value	Description
expand	true	Action mode that is expand (true) or collapse (false)
listCols	*	A comma separated list of column names of input (expand) or output (collapse) columns that may contain delim separated values. The asterisk refers to every column of the input relation.

19.429 miRNAResults-samplesWithoutClinicalAnnotation (INPUT)

List of samples without clinical annotation.

Category: Internal

Parameter name	Value	Description
path	/gpfs/homes/mlaakso/asserSVN/ andurilCaseStudy/csv/ samplesWithoutClinicalAnnotation. csv	Path (filename) of the input resource.

19.430 miRNAResults-survival (TableQuery)

Execute an SQL query on input relations and create a result table. TableQuery uses HSQLDB (Hyperthreaded Structured Query Language Database [25]) for executing the query. Consequently, the syntax of the query is defined by HSQLDB.

Author: Kristian Ovaska (Kristian.Ovaska@Helsinki.FI)
Version: 1.2
Categories: convert, Filter

Input name	Source	Description
table1	miRNAResults-sampleNames. relation 19.428	CSV table 1. The table is referred to as 'table1' in the SQL query.
table2	miRNAResults-patientMetadata. in 19.419	CSV table 2. The table is referred to as 'table2' in the SQL query.
query	miRNAResults-survivalQuery. in 19.432	SQL query. Either this file or the query parameter must be provided, but not both.

Parameter name	Value	Description
numIndices		Comma-separated list of index counts for input tables. All indices are single-column indices, running from column 1 to N, where N is retrieved from this parameter. If empty, the default number (1) is used. For example, ",2,,2" sets two indices for table2 and table4 and one index for the rest.
query		SQL query. Either this parameter or the query input must be provided, but not both.

19.431 miRNAResults-survivalAnnot (CSVFilter)

Filters columns and/or rows from CSV files using flexible criteria. Default settings do not filter out anything. The *negate* parameter reverses all inclusion criteria.

Columns are filtered based on column names (see *includeColumns*); it is also possible to rename columns. Columns can be reordered using *includeColumns*.

A row is included in output if all conditions are satisfied. Rows having too many missing values are filtered using the *nonMissing* parameter. The parameters *regexp*, *lowBound* and *highBound* filter rows based on cell contents. If the *auxiliary* input is given, the result contains those rows from the input whose values match those from the auxiliary file.

Authors: Kristian Ovaska (kristian.ovaska@helsinki.fi), Marko Laakso (Marko.Laakso@Helsinki.FI)
Version: 1.1
Category: Filter

Input name	Source	Description
csv	miRNAResults-miRNAAnnot .csv 19.412	CSV file to be filtered.

Parameter name	Value	Description
highBound		A row is included if the value in given numeric columns are at most as large as given numeric bounds. The parameter has a format COLNAME1=HIGH1,COLNAME2=HIGH2.
idColumn		Column name in "csv" that contains ID values. If the parameter is empty, the first column is used.
includeColumns	miRNAName,targetGeneName	Comma-separated list of column names that should be included in the result file. The special value * includes all columns. The order of column names is significant: the output columns are in the order given. See also includeColumns input.
lowBound		A row is included if the value in given numeric columns are at least as large as given numeric bounds. The parameter has a format COLNAME1=LOW1,COLNAME2=LOW2. For example, "col=5" includes rows where the column col has a value of at least 5.
matchColumn		Column name in "auxiliary" containing values that must match the ID column in "csv" (specified using "idColumn"). If empty, the first column of "auxiliary" is used.
negate	false	If true, all inclusion criteria are negated so that rows or columns are excluded instead of included if they match the criteria. For example, includeColumns=* then excludes all columns (which would be an error since at least one column must be included).
nonMissing	0	Include those rows that have non-missing (non-NA) values in at least this many columns. Only those columns that are part of the output are counted.
regexp		Row filtering based on regular expression. A row is included in the result if values in the given columns match given regular expressions. The parameter has a format COLNAME1=EXPRESSION,COLNAME2=EXPRESSION2 where COLNAMEs are column names in "csv" and EXPRESSIONs are regular expressions using Java syntax. For example, "col=a|b" includes rows where the column col has a value of "a" or "b". Commas have to be escaped with "\\" if they are used in column names or regular expressions.
rename		Comma-separated list of column renaming rules (OLDNAME=NEWNAME). Parameters that refer to column names refer to old names; renaming is done last.

19.432 miRNAResults-survivalQuery (INPUT)

SQL query to survival data preprocessing.

Category: Internal

Parameter name	Value	Description
path	/gpfs/homes/mlaakso/asserSVN/ anduril CaseStudy/sql/ exonSurvival.sql	Path (filename) of the input resource.

19.433 miRNAResults-survivalTable (CSVJoin)

Join rows from two or more CSV files, optionally using one column as a matching key. If a key column is not used, the result contains all rows and all columns of the input files. Missing values (NA) may be introduced when a column is not present in all input files. Each column is present once and duplicate rows are removed.

If a key column is used, the rows in each input file are matched using values from the key column. The result file has one row for each key value. In the result, the first column is the key column; its name is

obtained from the first CSV input (csv1). If the intersection parameter is true, a key is included in the result if the key value is present in all inputs. If intersection is false, a key is included if it is present in at least one input (key union). Union semantics may introduce missing values in the result. If several input files have the same column, the value is obtained from the first file. However, if the first file contains a missing value and the second file contains a non-missing value, the non-missing value is used instead.

Author: Kristian Ovaska (kristian.ovaska@helsinki.fi)
Version: 1.0
Category: convert

Input name	Source	Description
csv1	miRNAResults-survival.table 19.430	CSV file 1.
csv2	miRNAResults-sampleExpressionTrans.matrix 19.425	CSV file 2.

Parameter name	Value	Description
intersection	true	Defines how keys are handled; only used when useKeys=true. If intersection is true, the result contains a key if the key is present in all input files. If false, the result contains a key if the key is present in at least one input file.
keyColumnNames		Comma-separated list of key column names; only used when useKeys=true. The first name refers to csv1, the second to csv2, etc. An empty value refers to the first column. Empty values may be omitted from the list, so all these are equivalent: "col1" ; "col1," ; "col1,," ; etc.
useKeys	true	If true, use one column from each CSV file as a matching key column.

19.434 miRNAResults-survivalTableConv (IDConvert)

Converts identifiers from a column in a CSV file to another type of identifiers. The conversion is done using an annotation table on the basis of the column named *conversionColumn*.

Author: Kristian Ovaska (kristian.ovaska@helsinki.fi)
Version: 1.0
Category: convert

Input name	Source	Description
csv	miRNAResults-survivalTable.csv 19.433	Source CSV file. Contains the column that is to be converted. The column is named by the parameter 'sourceColumn'.
conversionTable	miRNAResults-survival.table 19.430	Conversion table. Must contain the column named by the parameter 'conversionColumn'.

Parameter name	Value	Description
collapseNumeric	indicator	Method for collapsing numeric columns with duplicate rows (when unique=true). Legal values are "median" (take median of non-NA values), "mean", "max", "min", "first" (take the first row), "majority" (take the value that is present on the largest number of rows), "consensus" (require that all rows have the same value) and "indicator".<br>Here, "majority" and "consensus" are mostly useful for discrete (integer) values). For "consensus", it is an error if duplicate rows contain distinct values. "indicator" assumes that data are divided into negative (-1), neutral (0) and positive (1) items, such as discretized expression values. The result is (a) 1 if there is a 1 and no -1 value; (b) -1 if there is a -1 and not 1 value; (c) 0 if there is a 0 and no -1 or 1; or (d) NA otherwise.
collapseString	consensus	Method for collapsing non-numeric columns with duplicate rows (when unique=true). Legal values are "first" (take the first row), "majority" (take the value that is present on the largest number of rows), "longest" (take the longest value), "shortest" (take the shortest value) and "consensus" (require that all rows have the same value). For "consensus", it is an error if duplicate rows contain distinct values.
conversionColumn	PatientID	Column in 'conversionTable' that is used to fetch new values for the source column.

Continued on next page...

Parameter name	Value	Description
keyColumn		Column in 'conversionTable' that is used to match ID values from the source CSV. If the parameter is empty, the first column is used.
originalWhen Missing	true	Determines how values are converted when the item is not found in the conversion table. If true, the original (unconverted) ID is inserted to the output. If false, NA is inserted.
sourceColumn	SampleID	Column in 'csv' that is going to be converted. If empty, the first column is used.
split	false	If true, split values in the source column by commas before ID conversion. For example, if the source column contains the string "a,b,c", it is first split into "a", "b" and "c"; then, individual IDs are converted; finally, the converted IDs are joined by commas into a string of format "A,B,C".
splitConverted	false	If true, split converted values by commas after ID conversion. A row with comma separated values in the target column will be split to otherwise identical rows but so that the rows have a single ID value in the target column. This is done before possible collapsing of duplicate rows.
targetColumn		Target column name in 'converted'. If empty, the column name is the same as the original column (sourceColumn).
unique	true	If true, rows with identical identifiers (sourceColumn) are collapsed into one row so that sourceColumn is unique. Collapsing may result in error if duplicate rows contain conflicting values. See parameters collapseNumeric and collapseString for methods to collapse numeric and non-numeric columns, respectively.
varMeasure		Method for calculating the variation of numeric duplicate values. If the variation is above the threshold given in <code>varThreshold</code> , duplicate values are collapsed to NA. Possible values are 'MAD' and 'variance'. 'MAD' stands for the median absolute deviation and 'variance' for the population variance. If no values is given, no threshold for combining numeric values is used.
varThreshold	2	Maximum allowed variability in the duplicate values. If <code>varMeasure</code> is given duplicate values with variance above the threshold are collapsed to NA

19.435 miRNAResults-tTest (StatisticalTest)

Calculate differentially expressed genes based on t-test.

Author: Kristian Ovaska (kristian.ovaska@helsinki.fi)
Version: 1.1
Category: Analysis

Input name	Source	Description
matrix	miRNAResults-mExpr. matrix 19.410	Input matrix, containing values to be tested.
groups	miRNAResults-sampleGroups. groups 19.427	If given, must contain the groups named by the parameters targetColumns and referenceColumns. Both groups must have at least two members. The members must be present in the input matrices.

Parameter name	Value	Description
byRow	true	If true, there is one test for each row. If false, there are tests for various column combinations.
contingencyRows	2	For categorical variable independency tests (chi-squared, Fisher), this gives the number of rows in contingency tables. The number of columns is deduced from data. For example, if contingencyRows=3 and target vector has length 12, the table has 4 columns.
correctedColumn	PValueCorrected	Output column name in "pvalues" for corrected p-values. Only used if multiple hypothesis correction has been enabled.
correction	fdr	Type of multiple hypothesis correction. One of "none", "fdr" (Benjamini-Hochberg, 1995), "robustfdr" (Pounds-Cheng 2006), "BY" (FDR, Benjamini-Yekutieli, 2001), "holm", "bonferroni". The value "none" disables correction.
mean	0	Expected mean of the reference distribution (mu) when the reference group is not supplied. Used for t-test and wilcoxon.
outputSet	tumor_vs_normal_tTest	The name of the output set in the idlist output.
paired	false	Indicates whether the test is paired so that i'th element of target vector corresponds to i'th element of reference vector. Ignored for F-test.
prefixColumn		If non-empty, include a column in the pvalues output that contains the outputSet on every row. This parameter gives the name of that column. The column is the first column in pvalues. This prefix column is useful when several separate statistical tests are made and the results are combined into one file. If prefixColumn is empty, do not include the prefix column.
pvalueColumn	PValue	Output column name in "pvalues" for raw p-values.

Continued on next page...

Parameter name	Value	Description
referenceColumns	normal_tissue	Comma-separated list of reference columns, or, if the groups input is present, the name of a sample group. The special value * means all columns of matrix2 if matrix2 is supplied; otherwise, all columns of matrix are used. May be empty if references are not used.
sided	twosided	For two-sided tests, one of "twosided", "greater" (target greater than reference) or "less" (target smaller than reference).
targetColumns	tumor_solid	Comma-separated list of target columns, or, if the groups input is present, the name of a sample group. The special value * means all columns of matrix.
test	t-test	Type of the statistical test. One of "t-test", "wilcoxon", "chi-squared", "fisher", "F-test", "shapiro" (Shapiro-Wilk), "ks" (Kolmogorov-Smirnov), "cor-pearson", "cor-spearman". Variants of the tests are set with other parameters (sided, paired).
threshold	0.05	P-value threshold for inclusion in the idlist output. Must be between 0 and 1 inclusive. If multiple hypothesis correction has been enabled, the threshold is for corrected p-values.

19.436 miRNATargets (INPUT)

(Technical documentation) Imports an external input file or directory to the pipeline. This is the only way external files are imported. File path is given with the parameter "path".

Category: Internal

Parameter name	Value	Description
path	/gpfs/homes/mlaakso/asserSVN/anduril CaseStudy/data/mirna/miRBase_Targets_v4_hsa.csv	Path (filename) of the input resource.

19.437 propertiesDoc (Properties2Latex)

Prepares a LaTeX-representation for the given set of properties files. The document consists of tables of names and values of the properties.

Author: Marko Laakso (Marko.Laakso@Helsinki.FI)
Version: 1.2
Categories: convert, Latex

Input name	Source	Description
props1	ensembl.in 19.273	A set of properties
props2	snpdb.in 19.832	A set of properties
props3	genotypeDB.in 19.320	A set of properties
props4	geneExpression-probeAnnot .databases 19.296	A set of properties

Parameter name	Value	Description
hide	database.password	A comma separated list of property names that should remain invisible. You may use this paramerter to hide passwords and other confidentian settings.
keyWidth	5	Column width for the property names (centimeters)
merge	false	Merge all input properties into a single set
section	System configurations	Section title for the table container or an empty string if no section should be generated
sectionType	subsection	Type of LaTeX section: usually one of section, subsection or subsubsection. No section statement is written if sectionTitle is empty.
valueWidth	13	Column width for the property values (centimeters)

19.438 rConfig (RConfigurationReport)

Generates a short description of the requested R packages and their revisions as available in the active set up.

Author: Marko Laakso (Marko.Laakso@Helsinki.FI)
Version: 1.0
Category: Latex

Parameter name	Value	Description
packages	base,csbl.go,DNAcopy,GTS	A comma separated list of R package names to be included into the document
sectionType	subsection	Type of LaTeX section: usually one of section, subsection or subsubsection.

19.439 rawGeneExpression (INPUT)

Pre-normalized matrix of gene expression data

Category: Internal

Parameter name	Value	Description
path	/gpfs/homes/mlaakso/asserSVN/andurilCaseStudy/data/expression/Expression-Genes/BI__HT_HG-U133A/Level_2/broad.mit.edu__HT_HG-U133A__probeset_rma	Path (filename) of the input resource.

19.440 snpSurvival (SNPSurvival)

SNP survival analysis

19.441 snpSurvival-combinedSurvival (CSVListJoin)

Combines the content of the input files into a single CSV file. The columns of the output file will form a union of all column names of the input files plus an additional column for the names of the source files.

Author: Marko Laakso (Marko.Laakso@Helsinki.FI)
Version: 2.1
Category: convert

Input name	Source	Description
file1	snpSurvival-combinedSurvivalpre.join 19.443	An optional input file
file2	snpSurvival-survivalSet8-survival.statistics 19.793	An optional input file
file3	snpSurvival-survivalSet9-survival.statistics 19.827	An optional input file
file4	snpSurvival-survivalSet10-survival.statistics 19.555	An optional input file

Parameter name	Value	Description
acceptExt		File extension that selects the files of interest. Add a dot (like .csv or .motif) into this parameter if the extension has it. Empty value refers to all files.
fileCol		Column name for the source file names. The whole column will be omitted if this string is empty.
stripExt	true	This flag can be used to remove file extensions from the source file names.

19.442 snpSurvival-combinedSurvivalpost (SearchReplace)

Replaces the occurences of the given regular expressions with the corresponding regular expressions. This component is used for transforming given patterns of the input file to a changed form specified by the regular expressions .

Author: Marko Laakso (Marko.Laakso@Helsinki.FI)
Version: 1.0
Category: convert

Input name	Source	Description
file	snpSurvival-combinedSurvival .join 19.441	Source file to be modified

Parameter name	Value	Description
key00	A\.	Search key 0
key01		Search key 1
key02		Search key 2
key03		Search key 3
key04		Search key 4
key05		Search key 5
key06		Search key 6
key07		Search key 7
key08		Search key 8
key09		Search key 9
value00	A-	Replacement 0
value01		Replacement 1
value02		Replacement 2
value03		Replacement 3
value04		Replacement 4
value05		Replacement 5
value06		Replacement 6
value07		Replacement 7
value08		Replacement 8
value09		Replacement 9

19.443 snpSurvival-combinedSurvivalpre (CSVListJoin)

Combines the content of the input files into a single CSV file. The columns of the output file will form a union of all column names of the input files plus an additional column for the names of the source files.

Author: Marko Laakso (Marko.Laakso@Helsinki.FI)
Version: 2.1
Category: convert

Input name	Source	Description
file1	snpSurvival-survivalSet1-survival .statistics 19.521	An optional input file
file2	snpSurvival-survivalSet2-survival .statistics 19.589	An optional input file
file3	snpSurvival-survivalSet3-survival .statistics 19.623	An optional input file
file4	snpSurvival-survivalSet4-survival .statistics 19.657	An optional input file
file5	snpSurvival-survivalSet5-survival .statistics 19.691	An optional input file
file6	snpSurvival-survivalSet6-survival .statistics 19.725	An optional input file
file7	snpSurvival-survivalSet7-survival .statistics 19.759	An optional input file

Parameter name	Value	Description
acceptExt		File extension that selects the files of interest. Add a dot (like .csv or .motif) into this parameter if the extension has it. Empty value refers to all files.
fileCol		Column name for the source file names. The whole column will be omitted if this string is empty.
stripExt	true	This flag can be used to remove file extensions from the source file names.

19.444 snpSurvival-finalMarkerSet (CSV2IDList)

Extracts one column from the given CSV file and prints it out without duplicates. The selected column may contain entries with comma separated lists of values in which case *isList* parameter may be used to obtain each value separately.

Output is defined as:

$$ids = \bigcup_{i=1}^{9} \pi_{columnIn_i} \sigma_{regexp_i}(table_i). \quad (6)$$

Author: Marko Laakso (Marko.Laakso@Helsinki.FI)
Version: 1.3
Category: convert

Input name	Source	Description
table1	snpSurvival-finalMarkerSetPre-rsNumbers.table 19.447	The mandatory input relation

Parameter name	Value	Description
acceptMissing	false	Files with missing columnIn are accepted as empty if this is true.
columnIn	marker	A comma separated list of column names for the IDs of interest in each file. Empty values refer to the first column of the file.
columnOut		Name of the only column of the output list. Empty input refers to the name of the input column.
constants		A comma separated list of values that are always included into the output
isList	false	True if the seleted column contains a comma separated list of values to be splitted
quotation	false	Indicator that can be used to disable quotation of the output values
regexp1		Regular expression for the row filtering in table1. A row is included in the result if this parameter is empty or if values in the given columns match given regular expressions. The parameter has a format COLNAME1=EXPRESSION,COLNAME2=EXPRESSION2 where COLNAMEs are column names in "csv" and EXPRESSIONs are regular expressions using Java syntax. For example, "col=a|b" includes rows where the column col has a value of "a" or "b".
regexp2		Regular expression for the row filtering in table2.
regexp3		Regular expression for the row filtering in table3.
regexp4		Regular expression for the row filtering in table4.
regexp5		Regular expression for the row filtering in table5.
regexp6		Regular expression for the row filtering in table6.
regexp7		Regular expression for the row filtering in table7.
regexp8		Regular expression for the row filtering in table8.
regexp9		Regular expression for the row filtering in table9.

19.445 snpSurvival-finalMarkerSetPre (AffySNPFetch)

Fetch rsnumbers based on AffySNP-ids

Author: Riku Louhimo (Riku.Louhimo@Helsinki.FI)
Version: 1.0
Categories: Annotation, SNP, Affy

Parameter name	Value	Description
columnIn	marker	Column name of the CSV file containing the IDs of interest.

Continued on next page...

Parameter name	Value	Description

19.446 snpSurvival-finalMarkerSetPre-markers (CSV2IDList)

Extracts one column from the given CSV file and prints it out without duplicates. The selected column may contain entries with comma separated lists of values in which case *isList* parameter may be used to obtain each value separately.

Output is defined as:

$$ids = \bigcup_{i=1}^{9} \pi_{columnIn_i} \sigma_{regexp_i}(table_i). \tag{7}$$

Author: Marko Laakso (Marko.Laakso@Helsinki.FI)
Version: 1.3
Category: convert

Input name	Source	Description
table1	snpSurvival-combinedSurvivalpost .file 19.442	The mandatory input relation

Parameter name	Value	Description
acceptMissing	false	Files with missing columnIn are accepted as empty if this is true.
columnIn	marker	A comma separated list of column names for the IDs of interest in each file. Empty values refer to the first column of the file.
columnOut	markers	Name of the only column of the output list. Empty input refers to the name of the input column.
constants		A comma separated list of values that are always included into the output
isList	false	True if the seleted column contains a comma separated list of values to be splitted
quotation	true	Indicator that can be used to disable quotation of the output values
regexp1		Regular expression for the row filtering in table1. A row is included in the result if this parameter is empty or if values in the given columns match given regular expressions. The parameter has a format COLNAME1=EXPRESSION,COLNAME2=EXPRESSION2 where COLNAMEs are column names in "csv" and EXPRESSIONs are regular expressions using Java syntax. For example, "col=a|b" includes rows where the column col has a value of "a" or "b".
regexp2		Regular expression for the row filtering in table2.
regexp3		Regular expression for the row filtering in table3.
regexp4		Regular expression for the row filtering in table4.
regexp5		Regular expression for the row filtering in table5.
regexp6		Regular expression for the row filtering in table6.
regexp7		Regular expression for the row filtering in table7.
regexp8		Regular expression for the row filtering in table8.
regexp9		Regular expression for the row filtering in table9.

19.447 snpSurvival-finalMarkerSetPre-rsNumbers (SQLSelect)

The given SQL statement is executed against a Java database connectivity (JDBC) database and the result set is written to the output CSV.

Author: Marko Laakso (Marko.Laakso@Helsinki.FI)
Version: 1.0
Category: Data Import

Input name	Source	Description
queryParams	snpSurvival-finalMarkerSetPre -markers.ids 19.446	Each row of this file represents parameters of one SQL select. Parameters should have corresponding question marks within the actual statements. Results of all queries are written to the same output.

Continued on next page...

Input name	Source	Description
connection	snpdb.in 19.832	Database connection can be defined using this file. The definition of parameters: database.url, database.timeout, database.recycle, and database.driver can be found from the documentation of Korvasieni.

Parameter name	Value	Description
defaultQuery	SELECT DISTINCT variation.name AS "marker", variation_synonym.name AS "AffyID" FROM variation_synonym, variation WHERE variation_synonym.name = ? AND variation.variation_id = variation_synonym.variation_id GROUP BY "marker"	SQL select that will be used if query input has not been defined

19.448 snpSurvival-markers1 (INPUT)

(Technical documentation) Imports an external input file or directory to the pipeline. This is the only way external files are imported. File path is given with the parameter "path".

Category: Internal

Parameter name	Value	Description
path	/opt/sandbox/snpDb/markers/markers1.lst	Path (filename) of the input resource.

19.449 snpSurvival-markers10 (INPUT)

(Technical documentation) Imports an external input file or directory to the pipeline. This is the only way external files are imported. File path is given with the parameter "path".

Category: Internal

Parameter name	Value	Description
path	/opt/sandbox/snpDb/markers/markers10.lst	Path (filename) of the input resource.

19.450 snpSurvival-markers2 (INPUT)

(Technical documentation) Imports an external input file or directory to the pipeline. This is the only way external files are imported. File path is given with the parameter "path".

Category: Internal

Parameter name	Value	Description
path	/opt/sandbox/snpDb/markers/markers2.lst	Path (filename) of the input resource.

19.451 snpSurvival-markers3 (INPUT)

(Technical documentation) Imports an external input file or directory to the pipeline. This is the only way external files are imported. File path is given with the parameter "path".

Category: Internal

Parameter name	Value	Description
path	/opt/sandbox/snpDb/markers/ markers3.lst	Path (filename) of the input resource.

19.452 snpSurvival-markers4 (INPUT)

(Technical documentation) Imports an external input file or directory to the pipeline. This is the only way external files are imported. File path is given with the parameter "path".

Category: Internal

Parameter name	Value	Description
path	/opt/sandbox/snpDb/markers/ markers4.lst	Path (filename) of the input resource.

19.453 snpSurvival-markers5 (INPUT)

(Technical documentation) Imports an external input file or directory to the pipeline. This is the only way external files are imported. File path is given with the parameter "path".

Category: Internal

Parameter name	Value	Description
path	/opt/sandbox/snpDb/markers/ markers5.lst	Path (filename) of the input resource.

19.454 snpSurvival-markers6 (INPUT)

(Technical documentation) Imports an external input file or directory to the pipeline. This is the only way external files are imported. File path is given with the parameter "path".

Category: Internal

Parameter name	Value	Description
path	/opt/sandbox/snpDb/markers/ markers6.lst	Path (filename) of the input resource.

19.455 snpSurvival-markers7 (INPUT)

(Technical documentation) Imports an external input file or directory to the pipeline. This is the only way external files are imported. File path is given with the parameter "path".

Category: Internal

Parameter name	Value	Description
path	/opt/sandbox/snpDb/markers/ markers7.lst	Path (filename) of the input resource.

19.456 snpSurvival-markers8 (INPUT)

(Technical documentation) Imports an external input file or directory to the pipeline. This is the only way external files are imported. File path is given with the parameter "path".

Category: Internal

Parameter name	Value	Description
path	/opt/sandbox/snpDb/markers/ markers8.lst	Path (filename) of the input resource.

19.457 snpSurvival-markers9 (INPUT)

(Technical documentation) Imports an external input file or directory to the pipeline. This is the only way external files are imported. File path is given with the parameter "path".

Category: Internal

Parameter name	Value	Description
path	/opt/sandbox/snpDb/markers/ markers9.lst	Path (filename) of the input resource.

19.458 snpSurvival-survivalAll (SurvivalAnalysis)

Redo analysis with rsIDs and more strict p-value.

Parameter name	Value
pvalue	1.0E-4

19.459 snpSurvival-survivalAll-annotSQL (INPUT)

This SQL is used to generate marker annotation report.

Category: Internal

Parameter name	Value	Description
path	/gpfs/homes/mlaakso/asserSVN/ andurilCaseStudy/sql/ SNPgeneTable.sql	Path (filename) of the input resource.

19.460 snpSurvival-survivalAll-caseGenotypes (GetGenotypes)

Load and preprocess the genotype calls for the cancer patient samples.

Parameter name	Value
skipFrq	0.1

19.461 snpSurvival-survivalAll-caseGenotypes-freqCheck (AlleleCounter)

AlleleCounter calculates genotype frequencies for the biallelic markers of the given SNPMatrix. A Pearson's χ^2-test [18] is performed for each marker in order to test if the Hardy-Weinberg equilibrium [9] holds for the population sample.

Author: Marko Laakso (Marko.Laakso@Helsinki.FI)
Version: 1.2
Category: SNP

Input name	Source	Description
genotypes	snpSurvival-survivalAll-case Genotypes-genotypes .genotypes 19.463	SNPHelistin genotype output.

Parameter name	Value	Description
dels	false	Accept homozygous deletions as a possible genotype.
naLimit	0.1	Skip markers where one minus call rate exceeds this limit.
skip	0.1	Drop markers with a relative minor allele frequency lower than this limit. Use negative values to inactivate this limit.

19.462 snpSurvival-survivalAll-caseGenotypes-ftMarkers (CSV2IDList)

Extracts one column from the given CSV file and prints it out without duplicates. The selected column may contain entries with comma separated lists of values in which case *isList* parameter may be used to obtain each value separately.

Output is defined as:

$$ids = \bigcup_{i=1}^{9} \pi_{columnIn_i} \sigma_{regexp_i}(table_i). \tag{8}$$

Author: Marko Laakso (Marko.Laakso@Helsinki.FI)
Version: 1.3
Category: convert

Input name	Source	Description
table1	snpSurvival-survivalAll-case Genotypes-freqCheck .frequencies 19.461	The mandatory input relation

Parameter name	Value	Description
acceptMissing	false	Files with missing columnIn are accepted as empty if this is true.
columnIn	marker	A comma separated list of column names for the IDs of interest in each file. Empty values refer to the first column of the file.
columnOut	marker	Name of the only column of the output list. Empty input refers to the name of the input column.
constants		A comma separated list of values that are always included into the output
isList	false	True if the seleted column contains a comma separated list of values to be splitted
quotation	false	Indicator that can be used to disable quotation of the output values
regexp1		Regular expression for the row filtering in table1. A row is included in the result if this parameter is empty or if values in the given columns match given regular expressions. The parameter has a format COLNAME1=EXPRESSION,COLNAME2=EXPRESSION2 where COLNAMEs are column names in "csv" and EXPRESSIONs are regular expressions using Java syntax. For example, "col=a|b" includes rows where the column col has a value of "a" or "b".
regexp2		Regular expression for the row filtering in table2.
regexp3		Regular expression for the row filtering in table3.
regexp4		Regular expression for the row filtering in table4.
regexp5		Regular expression for the row filtering in table5.
regexp6		Regular expression for the row filtering in table6.
regexp7		Regular expression for the row filtering in table7.
regexp8		Regular expression for the row filtering in table8.
regexp9		Regular expression for the row filtering in table9.

19.463 snpSurvival-survivalAll-caseGenotypes-genotypes (SNPHelistinReader)

This component can be used to read SNP genotype calls for a set of samples and markers of interest. The genotype data is retrieved from a SNPHelistin database that is defined by the input properties.

Author: Marko Laakso (Marko.Laakso@Helsinki.FI)
Version: 1.2
Categories: Data Import, SNP

Input name	Source	Description
sampleNames	snpSurvival-survivalAll-caseNames.ids 19.465	List of sample names of interest.
markerNames	snpSurvival-finalMarkerSet.ids 19.444	List of SNP identifiers of interest.
config	genotypeDB.in 19.320	Connection parameters for SNPHelistin.

Parameter name	Value	Description
errorSymbol	ERR	Symbol for failed measurements. You may for example use 'NA' value in order consider them equal to missing values.
skipUnknown	true	Ignore unknown samples and do not process them. If the parameter is false then the program fails for these samples.

19.464 snpSurvival-survivalAll-caseGenotypes-gtypes (SNPHelistinReader)

This component can be used to read SNP genotype calls for a set of samples and markers of interest. The genotype data is retrieved from a SNPHelistin database that is defined by the input properties.

Author: Marko Laakso (Marko.Laakso@Helsinki.FI)
Version: 1.2
Categories: Data Import, SNP

Input name	Source	Description
sampleNames	snpSurvival-survivalAll-caseNames.ids 19.465	List of sample names of interest.
markerNames	snpSurvival-survivalAll-caseGenotypes-ftMarkers.ids 19.462	List of SNP identifiers of interest.
config	genotypeDB.in 19.320	Connection parameters for SNPHelistin.

Parameter name	Value	Description
errorSymbol	ERR	Symbol for failed measurements. You may for example use 'NA' value in order consider them equal to missing values.
skipUnknown	true	Ignore unknown samples and do not process them. If the parameter is false then the program fails for these samples.

19.465 snpSurvival-survivalAll-caseNames (CSV2IDList)

Extracts one column from the given CSV file and prints it out without duplicates. The selected column may contain entries with comma separated lists of values in which case *isList* parameter may be used to obtain each value separately.

Output is defined as:

$$ids = \bigcup_{i=1}^{9} \pi_{columnIn_i} \sigma_{regexp_i}(table_i). \tag{9}$$

Author: Marko Laakso (Marko.Laakso@Helsinki.FI)
Version: 1.3
Category: convert

Input name	Source	Description
table1	snpSurvival-survivalAll-survival Clinical.table 19.488	The mandatory input relation

Parameter name	Value	Description
acceptMissing	false	Files with missing columnIn are accepted as empty if this is true.
columnIn		A comma separated list of column names for the IDs of interest in each file. Empty values refer to the first column of the file.
columnOut	SampleId	Name of the only column of the output list. Empty input refers to the name of the input column.
constants		A comma separated list of values that are always included into the output
isList	false	True if the seleted column contains a comma separated list of values to be splitted
quotation	false	Indicator that can be used to disable quotation of the output values
regexp1		Regular expression for the row filtering in table1. A row is included in the result if this parameter is empty or if values in the given columns match given regular expressions. The parameter has a format COLNAME1=EXPRESSION,COLNAME2=EXPRESSION2 where COLNAMEs are column names in "csv" and EXPRESSIONs are regular expressions using Java syntax. For example, "col=a|b" includes rows where the column col has a value of "a" or "b".
regexp2		Regular expression for the row filtering in table2.
regexp3		Regular expression for the row filtering in table3.
regexp4		Regular expression for the row filtering in table4.
regexp5		Regular expression for the row filtering in table5.
regexp6		Regular expression for the row filtering in table6.
regexp7		Regular expression for the row filtering in table7.
regexp8		Regular expression for the row filtering in table8.
regexp9		Regular expression for the row filtering in table9.

19.466 snpSurvival-survivalAll-clinicalData (INPUT)

Clinical parameters for the case samples
Category: Internal

Parameter name	Value	Description
path	/gpfs/homes/mlaakso/asserSVN/ andurilCaseStudy/data/snp/ patient-metadata-caucas.csv	Path (filename) of the input resource.

19.467 snpSurvival-survivalAll-clinicalSelect (SourceCode2Latex)

This component converts a source code listing into a LaTeXfragment that may be included into some technical documents.
Author: Marko Laakso (Marko.Laakso@Helsinki.FI)
Version: 1.0
Categories: convert, Latex

Input name	Source	Description
source	snpSurvival-survivalAll-survival ParamsQuery.file 19.489	Source code

Parameter name	Value	Description
caption	This SQL query has been used to select clinical parameters for the survival calculations.	Caption text for the listing
language	SQL	Language defintion for the syntax highlighting. Possible values can be found from the description of listings package.

19.468 snpSurvival-survivalAll-clinicalTypes (INPUT)

Column types for the clinical data

Category: Internal

Parameter name	Value	Description
path	/gpfs/homes/mlaakso/asserSVN/anduriICaseStudy/csv/SNPclinicalTypes.csv	Path (filename) of the input resource.

19.469 snpSurvival-survivalAll-geneRank (QueryReport)

(Technical documentation) Executes an SQL query and prints it with the result table.

Author: Marko Laakso (Marko.Laakso@Helsinki.FI)
Version: 1.1
Category: Latex

Input name	Source	Description
table1	snpSurvival-survivalAll-survSNPAnnot.fullData 19.481	1. data table that is referred as 'table1' in the query

Parameter name	Value	Description
colFormat	lcrp{11cm}	LaTeX tabular formatting for the result columns
listCols		Comma separated list of column names. Columns of this list may contain several values separated with commas and the delimiters will be replaced with spaces.
resTableRef	the previous table	Textual reference from the SQL representation to the result table
section	Gene rank	Subsection title for the output document
showCols	gene,markers,p-value,description	Comma separated list of column selections for the output. The empty default will use all columns.
showQuery	false	This flag tells if the SQL shall be shown on the report
tableCaption	A list of survival affecting genes and the marker counts. P-value is the lowest value for the corresponding markers.	Description for the result table

19.470 snpSurvival-survivalAll-geneRank-eqData (TableQuery)

Execute an SQL query on input relations and create a result table. TableQuery uses HSQLDB (Hyper-threaded Structured Query Language Database [25]) for executing the query. Consequently, the syntax of the query is defined by HSQLDB.

Author: Kristian Ovaska (Kristian.Ovaska@Helsinki.FI)
Version: 1.2
Categories: convert, Filter

Input name	Source	Description
table1	snpSurvival-survivalAll-survSNPAnnot-annotData.table 19.482	CSV table 1. The table is referred to as 'table1' in the SQL query.

Continued on next page...

Input name	Source	Description
table2	snpSurvival-survivalAll-survival .statistics 19.487	CSV table 2. The table is referred to as 'table2' in the SQL query.
query	snpSurvival-survivalAll-geneRank Query.in 19.472	SQL query. Either this file or the query parameter must be provided, but not both.

Parameter name	Value	Description
numIndices		Comma-separated list of index counts for input tables. All indices are single-column indices, running from column 1 to N, where N is retrieved from this parameter. If empty, the default number (1) is used. For example, ",2,,2" sets two indices for table2 and table4 and one index for the rest.
query		SQL query. Either this parameter or the query input must be provided, but not both.

19.471 snpSurvival-survivalAll-geneRank-eqTable (CSV2Latex)

This component converts a tabular CSV file into a printable LaTeX table.

Author: Marko Laakso (Marko.Laakso@Helsinki.FI)
Version: 2.1
Categories: convert, Latex

Input name	Source	Description
tabledata	snpSurvival-survivalAll-geneRank -eqData.table 19.470	Table content
refs	snpSurvival-survivalAll-refsGene Rank.in 19.475	Reference rules for the hyperlinks

Parameter name	Value	Description
attach	false	Include the original data as an attachment
caption	A list of survival affecting genes and the marker counts. P-value is the lowest value for the corresponding markers.	Caption text for the table.
colFormat	lcrp{11cm}	LaTeX tabular format for the columns. Default produces a centered layout for all columns.
columns	gene,markers,p-value,description	Comma separated list of column selections for the output. The empty default will use all columns.
countRows	false	Include a row count to the table caption.
dropMissing	true	This flag can be turned off in order to generate links with missing texts. Link text are substituted with target identifiers.
listCols		Comma separated list of column names. Columns of this list may contain several values separated with commas and the delimiters will be replaced with spaces.
numberFormat		A comma separated list of decimal formats for the columns. Each entry consists of the column name and the Java DecimalFormal pattern separated with equal sign. For example, rounding to three decimals can be done like: myColumn=#0.000
pageBreak	false	Use clear page after the table.
rename		Comma separated list of column renaming rules (oldname=newname). New names are used in table header but they do not affect the other behaviour of this component.
ruler	{}	Latex command for the row separating rulers
section	Gene rank	Section title for the table container or an empty string if no section should be generated.
sectionType	subsection	Type of LaTeX section: usually one of: section, subsection, or subsubsection. No section statement is written if section title is empty.
skipEmpty	false	This flag can be used to replace empty tables with a simple LaTeX comment.

19.472 snpSurvival-survivalAll-geneRankQuery (INPUT)

Generates a list of genes and the marker counts for them.

Category: Internal

Parameter name	Value	Description
path	/gpfs/homes/mlaakso/asserSVN/anduriCaseStudy/sql/SNPinterestingGenes.sql	Path (filename) of the input resource.

19.473 snpSurvival-survivalAll-missingDistC (CSV2Latex)

This component converts a tabular CSV file into a printable LaTeX table.

Author:	Marko Laakso (Marko.Laakso@Helsinki.FI)
Version:	2.1
Categories:	convert, Latex

Input name	Source	Description
tabledata	snpSurvival-survivalAll-caseGenotypes-freqCheck.naDist 19.461	Table content

Parameter name	Value	Description
attach	false	Include the original data as an attachment
caption	Distribution of the missing values in case samples	Caption text for the table.
colFormat	p{3cm}r	LaTeX tabular format for the columns. Default produces a centered layout for all columns.
columns		Comma separated list of column selections for the output. The empty default will use all columns.
countRows	false	Include a row count to the table caption.
dropMissing	true	This flag can be turned off in order to generate links with missing texts. Link text are substituted with target identifiers.
listCols		Comma separated list of column names. Columns of this list may contain several values separated with commas and the delimiters will be replaced with spaces.
numberFormat		A comma separated list of decimal formats for the columns. Each entry consists of the column name and the Java DecimalFormal pattern separated with equal sign. For example, rounding to three decimals can be done like: myColumn=#0.000
pageBreak	false	Use clear page after the table.
rename		Comma separated list of column renaming rules (oldname=newname). New names are used in table header but they do not affect the other behaviour of this component.
ruler	{}	Latex command for the row separating rulers
section		Section title for the table container or an empty string if no section should be generated.
sectionType	subsection	Type of LaTeX section: usually one of: section, subsection, or subsubsection. No section statement is written if section title is empty.
skipEmpty	false	This flag can be used to replace empty tables with a simple LaTeX comment.

19.474 snpSurvival-survivalAll-propertiesDoc (Properties2Latex)

Prepares a LaTeX-representation for the given set of properties files. The document consists of tables of names and values of the properties.

Author:	Marko Laakso (Marko.Laakso@Helsinki.FI)
Version:	1.2
Categories:	convert, Latex

Input name	Source	Description
props1	genotypeDB.in 19.320	A set of properties

Parameter name	Value	Description
hide		A comma separated list of property names that should remain invisible. You may use this paramerter to hide passwords and other confidentian settings.

Continued on next page...

Parameter name	Value	Description
keyWidth	5	Column width for the property names (centimeters)
merge	false	Merge all input properties into a single set
section	SNP database configuration	Section title for the table container or an empty string if no section should be generated
sectionType	subsection	Type of LaTeX section: usually one of section, subsection or subsubsection. No section statement is written if sectionTitle is empty.
valueWidth	13	Column width for the property values (centimeters)

19.475 snpSurvival-survivalAll-refsGeneRank (INPUT)

Hyperlink rules for the gene rank table

Category: Internal

Parameter name	Value	Description
path	/gpfs/homes/mlaakso/asserSVN/andurilCaseStudy/csv/SNPrefsGeneRank.csv	Path (filename) of the input resource.

19.476 snpSurvival-survivalAll-refsHits (INPUT)

Hyperlink rules for the marker annotations

Category: Internal

Parameter name	Value	Description
path	/gpfs/homes/mlaakso/asserSVN/andurilCaseStudy/csv/SNPrefsHits.csv	Path (filename) of the input resource.

19.477 snpSurvival-survivalAll-reportBody (LatexCombiner)

LatexCombiner joins the given subdocuments together into one LaTeX file.

Authors: Kristian Ovaska (Kristian.Ovaska@Helsinki.FI), Marko Laakso (Marko.Laakso@Helsinki.FI)
Version: 1.3
Category: Latex

Input name	Source	Description
latex1	snpSurvival-survivalAll-survivalReport.document 19.491	LaTeX fragment 1.
latex2	snpSurvival-survivalAll-propertiesDoc.report 19.474	LaTeX fragment 2.
latex3	snpSurvival-survivalAll-missingDistC.report 19.473	LaTeX fragment 3.

Parameter name	Value	Description
pagebreak	false	Determines if the result document should start with a page break.
sectionTitle		If non-empty, a declaration of a new section with the given name is inserted to the beginning of the combined document. This is a convenience feature to make it easy to compile subsections into a section.
sectionType	section	Type of LaTeX section: usually one of section, subsection or subsubsection. No section statement is written if sectionTitle is empty.
strictBorders	true	Enables the flushing of all document elements before each fragment

19.478 snpSurvival-survivalAll-reportTemplate (LatexTemplate)

This component generates the standard layout configuration for Anduril output documents. The component produces a header and a footer fragment that can be used to build LaTeX documents. The header fragment takes care of the page geometry and other technical details such as library imports and function declarations. Reference list is generated by the footer.

Author: Marko Laakso (Marko.Laakso@Helsinki.FI)
Version: 1.4
Category: Latex

Input name	Source	Description
abstract	abstract.in 19.42	Document abstract including its Latex definition. You may use template entities within this document. The default abstract has been defined in abstractTemplate.tex.

Parameter name	Value	Description
authors	Marko Laakso, Riku Louhimo, Sampsa Hautaniemi	A comma-separated list of document authors on the title page. If authors is empty but title is non-empty, a title page with no author name is printed.
baselineskip	1.2	Baselineskip factor that indicates the line spacing
bibstyle	abbrv	BibTeX reference formatting style
printTOC	true	If true, print Table Of Contents.
title	Anduril Case Study: Glioblastoma SNP survival analysis	Document title on the title page. If empty, no title page is printed. Also see author.
usepackage		Comma-separated list of LaTeX packages that are used in the document. Each package generates a \usepackage{X} line in the header.

19.479 snpSurvival-survivalAll-snpAnnotations (INPUT)

Annotations for survival component.

Category: Internal

Parameter name	Value	Description
path	/gpfs/homes/mlaakso/asserSVN/andurilCaseStudy/csv/SNPAnnotation.csv	Path (filename) of the input resource.

19.480 snpSurvival-survivalAll-summaryReport (LatexPDF)

This component is a LaTeX compiler that generates a portable document format (PDF) file from the given Anduril document.

Author: Kristian Ovaska (Kristian.Ovaska@Helsinki.FI)
Version: 1.0
Category: Latex

Input name	Source	Description
document	snpSurvival-survivalAll-reportBody.document 19.477	Body of the LaTeX document.
header	snpSurvival-survivalAll-reportTemplate.header 19.478	LaTeX header that is written to the start of the document. If missing, a default header is used.
footer	snpSurvival-survivalAll-reportTemplate.footer 19.478	LaTeX footer that is written to the end of the document. If missing, a default header is used.

Parameter name	Value	Description
bibtexExec	bibtex	Executable command for BibTeX.
latexExec	pdflatex	Executable command for pdflatex.
useRefs	false	This flag can be used to activate BibTeX compiler

19.481 snpSurvival-survivalAll-survSNPAnnot (AnnotateSNPs)

Biological annotations for the survival affecting genes

Parameter name	Value
caption	Gene annotations and the loci of the survival affecting markers

19.482 snpSurvival-survivalAll-survSNPAnnot-annotData (TableQuery)

Execute an SQL query on input relations and create a result table. TableQuery uses HSQLDB (Hyperthreaded Structured Query Language Database [25]) for executing the query. Consequently, the syntax of the query is defined by HSQLDB.

Author: Kristian Ovaska (Kristian.Ovaska@Helsinki.FI)
Version: 1.2
Categories: convert, Filter

Input name	Source	Description
table1	snpSurvival-survivalAll-survSNPAnnot-snpRel.relation 19.486	CSV table 1. The table is referred to as 'table1' in the SQL query.
table2	snpSurvival-survivalAll-survSNPAnnot-genes.bioAnnotation 19.484	CSV table 2. The table is referred to as 'table2' in the SQL query.
table3	snpSurvival-survivalAll-survival.statistics 19.487	CSV table 3. The table is referred to as 'table3' in the SQL query.
query	snpSurvival-survivalAll-annotSQL.in 19.459	SQL query. Either this file or the query parameter must be provided, but not both.

Parameter name	Value	Description
numIndices		Comma-separated list of index counts for input tables. All indices are single-column indices, running from column 1 to N, where N is retrieved from this parameter. If empty, the default number (1) is used. For example, ",2,,2" sets two indices for table2 and table4 and one index for the rest.
query		SQL query. Either this parameter or the query input must be provided, but not both.

19.483 snpSurvival-survivalAll-survSNPAnnot-annotTable (CSV2Latex)

This component converts a tabular CSV file into a printable LaTeX table.

Author: Marko Laakso (Marko.Laakso@Helsinki.FI)
Version: 2.1
Categories: convert, Latex

Input name	Source	Description
tabledata	snpSurvival-survivalAll-survSNPAnnot-annotData.table 19.482	Table content
refs	snpSurvival-survivalAll-refsHits.in 19.476	Reference rules for the hyperlinks

Parameter name	Value	Description
attach	false	Include the original data as an attachment

Continued on next page...

Parameter name	Value	Description
caption	Gene annotations and the loci of the survival affecting markers	Caption text for the table.
colFormat	l@{}lr@{:}lp{1cm}p{1.5cm}p{10 .5cm}	LaTeX tabular format for the columns. Default produces a centered layout for all columns.
columns	hit,marker,chr,locus,band,gene, gene description	Comma separated list of column selections for the output. The empty default will use all columns.
countRows	false	Include a row count to the table caption.
dropMissing	true	This flag can be turned off in order to generate links with missing texts. Link text are substituted with target identifiers.
listCols	band	Comma separated list of column names. Columns of this list may contain several values separated with commas and the delimiters will be replaced with spaces.
numberFormat		A comma separated list of decimal formats for the columns. Each entry consists of the column name and the Java DecimalFormal pattern separated with equal sign. For example, rounding to three decimals can be done like: myColumn=#0.000
pageBreak	false	Use clear page after the table.
rename		Comma separated list of column renaming rules (oldname=newname). New names are used in table header but they do not affect the other behaviour of this component.
ruler	{}	Latex command for the row separating rulers
section		Section title for the table container or an empty string if no section should be generated.
sectionType	subsection	Type of LaTeX section: usually one of: section, subsection, or subsubsection. No section statement is written if section title is empty.
skipEmpty	false	This flag can be used to replace empty tables with a simple LaTeX comment.

19.484 snpSurvival-survivalAll-survSNPAnnot-genes (KorvasieniAnnotator)

Korvasieni is an Ensembl [12] based converter for database identifiers and it can be used to convert genome location, gene, transcript, and translation identifiers across multiple biological databases. The selected `keyColumn` may contain entries with comma separated lists of values in which case `isListKey` parameter may be used to obtain each value separately and to process them as they were given in consecutive lines.

Author:	Marko Laakso (Marko.Laakso@Helsinki.FI)
Version:	1.7
Categories:	Annotation, GO

Input name	Source	Description
sourceKeys	snpSurvival-survivalAll-survSNPAnnot-snpLoci.annotations 19.485	A list of source database keys.
connection	ensembl.in 19.273	Database connection can be defined using this file. The definition of parameters: database.url, database.user, database.password, database.timeout, database.recycle, and database.driver can be found from the documentation of Korvasieni.

Parameter name	Value	Description
echoColumns	refsnp_id	A comma separated list of column names for the columns that will be copied to the output. An asterisk (*) can be used to denote all columns execpt the keyColumn.
inputDB	.GeneId	Type of input keys. This must be a database supported by Korvasieni. If the parameter is omitted, the component tries to derive the database from the type of geneID. If this is not possible, an error is returned. You may define three columns in form of chromosome:start-end in case the inputDB is .DNARegion. This format provides a comfortable compatibility with DNARegion datatype.
inputType	Gene	Ensembl object type for the input keys (Any, Gene, Transcript, Translation)
isListKey	true	Enables the automatic value splits for the comma separated key column
keyColumn	ensembl_gene_stable_id	Name of the key column withing sourceKeys file or an empty string for the first column
maxHits	100000	Maximum number of target identifiers for a single source identifier
rename	UniProt/SwissProt=protein	Comma separated list of column renaming rules (oldname=newname)
skipLevel	never	Skip result rows if the source identifier is unknown or target identifiers are not available. Possible values are: never (no filtering), source (skip if the source ID is unknown), target (skip if no target IDs are found), any (skip if any of the target IDs is missing).
targetDB	.GeneName,.GeneDesc,. DNABand,GO,UniProt/SwissProt	Comma-separated list of annotation types. Possible values are all databases supported by Korvasieni.
unique	true	This flag can be turned on in order to eliminate duplicate annotations.

19.485 snpSurvival-survivalAll-survSNPAnnot-snpLoci (RefSNPAnnotator)

RefSNPAnnotator fetches annotations for given refSNP ID's (also known as rsID's) from BioMart databases. The provided annotations for each rsID include alleles for the SNP, name of the chromosome where the SNP is located, chromosome location of the SNP, the strand of the chromosome, Ensembl gene ID for genes that overlap the SNP's location and validation status of the SNP. The component is implemented using the biomaRt package for Bioconductor [7].

Authors:	Viljami Aittomaki (Viljami.Aittomaki@Helsinki.FI), Marko Laakso (Marko.Laakso@Helsinki.FI)
Version:	1.0.1
Categories:	SNP, Annotation

Input name	Source	Description
rsIDs	snpSurvival-survivalAll-survival .statistics 19.487	List of rsID's for fetching annotations.

Parameter name	Value	Description
dataset	hsapiens_snp	Dataset to get annotations from. Different species have their own datasets. Example values include "hsapiens_snp" and "mmusculus_snp". See biomaRt documentation on how to list available datasets in R.
keyColumn	marker	Name of the key column withing sourceKeys file or an empty string for the first column.

19.486 snpSurvival-survivalAll-survSNPAnnot-snpRel (ExpandCollapse)

Converts between the two possible representations of a relations with multivalued columns. By default this component splits rows with multivalued (comma separated lists) columns into multiple rows each representing a single value. The input may contain several columns with comma separated values and all permutations of column values are shown as individual single-valued rows. The alternative mode recovers the original comma separated values from the expanded relations. This is performed by joining values of the their list columns into comma separated lists.

Author:	Marko Laakso (Marko.Laakso@Helsinki.FI)
Version:	1.3
Category:	convert

Input name	Source	Description
relation	snpSurvival-survivalAll-survSNPA nnot-snpLoci.annotations 19.485	The mandatory input relation

Parameter name	Value	Description
delim	,	Value delimiter between the list column values. Special characters can be encoded as specified in fi.helsinki.ltdk.csbl.asser.ArgumentEncoding.
expand	true	Action mode that is expand (true) or collapse (false)
listCols	ensembl_gene_stable_id	A comma separated list of column names of input (expand) or output (collapse) columns that may contain delim separated values. The asterisk refers to every column of the input relation.

19.487 snpSurvival-survivalAll-survival (SNPKaplanMeier)

This component is used to estimate survival differences between various genotypes. The differences between the survival of different genotypes are measured using G-ρ family of tests [10].

Author: Marko Laakso (Marko.Laakso@Helsinki.FI)
Version: 1.5
Categories: SNP, Latex, Survival

Input name	Source	Description
genotypes	snpSurvival-survivalAll-caseGenotypes-gtypes.genotypes 19.464	Genotype calls
survival	snpSurvival-survivalAll-survivalClinical.table 19.488	Survival time and event indicator
annotations	snpSurvival-survivalAll-snpAnnotations.in 19.479	Additional information about the markers. This data will be shown on the report of interesting markers.

Parameter name	Value	Description
PLimit	1.0E-4	Ignore results with a p-value greater than this limit.
confInt	0.95	Coverage of the confidence intervals [0,1]
minCount	15	Combine rare (aa) homozygotes to heterozygotes (Aa) if their number is below this limit. This parameter is used together with minFreq so that the actual limit is max(minFreq*n, minCount).
minFreq	0.1	Combine rare (aa) homozygotes to heterozygotes (Aa) if their frequency is below this limit. See also minCount.
naLimit	0.1	Skip markers where one minus call rate exceeds this limit.
nullCalls	–,ERR	A comma separated list of genotype calls that will be considered as missing values
sampleCol	sample	Column name for the sample name
showCI	true	Draw confidence intervals to the graph
skipMissingGT	true	Simply skip those samples that lack the genotype information.
statusCol	hasEvent	Column name for the event indicator
survRatePoint	0.5	Sampling height for the distances between the survival expectations. Negative values may be used to suppress these measurements.
timeCol	endTime	Column name for the survival time
timeOutLimit	36	Cut survival curves after this limit. A negative value can be used to maximize the length of the period.
timeStartCol	startTime	Column name for the start time that is reduced from the survival time
title	Genotype Specific Survival Estimates	Section title

19.488 snpSurvival-survivalAll-survivalClinical (TableQuery)

Execute an SQL query on input relations and create a result table. TableQuery uses HSQLDB (Hyper-threaded Structured Query Language Database [25]) for executing the query. Consequently, the syntax of the query is defined by HSQLDB.

Author: Kristian Ovaska (Kristian.Ovaska@Helsinki.FI)
Version: 1.2
Categories: convert, Filter

Input name	Source	Description
table1	snpSurvival-survivalAll-clinicalData.in 19.466	CSV table 1. The table is referred to as 'table1' in the SQL query.
query	snpSurvival-survivalAll-survivalParamsQuery.file 19.489	SQL query. Either this file or the query parameter must be provided, but not both.
columnTypes	snpSurvival-survivalAll-clinicalTypes.in 19.468	Contains SQL types for individual columns. If the file is not provided, the type is inferred from the contents of the columns. This can be used to force the use of VARCHAR for values that are also valid numerics. The file contains the columns Table (refers to one of table1 to table15), Column (refers to a column name in the table), Type (contains an SQL type). A row with Table='result', Column=X and Type='STRING' forces the use string values for result column X.

Parameter name	Value	Description
numIndices		Comma-separated list of index counts for input tables. All indices are single-column indices, running from column 1 to N, where N is retrieved from this parameter. If empty, the default number (1) is used. For example, ",2,,2" sets two indices for table2 and table4 and one index for the rest.
query		SQL query. Either this parameter or the query input must be provided, but not both.

19.489 snpSurvival-survivalAll-survivalParamsQuery (SearchReplace)

Replaces the occurences of the given regular expressions with the corresponding regular expressions. This component is used for transforming given patterns of the input file to a changed form specified by the regular expressions .

Author: Marko Laakso (Marko.Laakso@Helsinki.FI)
Version: 1.0
Category: convert

Input name	Source	Description
file	snpSurvival-survivalAll-survival ParamsTemplate.in 19.490	Source file to be modified

Parameter name	Value	Description
key00	REP.cond00	Search key 0
key01		Search key 1
key02		Search key 2
key03		Search key 3
key04		Search key 4
key05		Search key 5
key06		Search key 6
key07		Search key 7
key08		Search key 8
key09		Search key 9
value00	– No additional restrictions	Replacement 0
value01		Replacement 1
value02		Replacement 2
value03		Replacement 3
value04		Replacement 4
value05		Replacement 5
value06		Replacement 6
value07		Replacement 7
value08		Replacement 8
value09		Replacement 9

19.490 snpSurvival-survivalAll-survivalParamsTemplate (INPUT)

Select relevant parameters for the survival analysis

Category: Internal

Parameter name	Value	Description
path	/gpfs/homes/mlaakso/asserSVN/ andurilCaseStudy/sql/ SNPsurvivalParams.sql	Path (filename) of the input resource.

19.491 snpSurvival-survivalAll-survivalReport (LatexCombiner)

LatexCombiner joins the given subdocuments together into one LaTeX file.

Authors:	Kristian Ovaska (Kristian.Ovaska@Helsinki.FI), Marko Laakso (Marko.Laakso@Helsinki.FI)
Version:	1.3
Category:	Latex

Input name	Source	Description
latex1	snpSurvival-survivalAll-survival .report 19.487	LaTeX fragment 1.
latex2	snpSurvival-survivalAll-clinical Select.report 19.467	LaTeX fragment 2.
latex3	snpSurvival-survivalAll-survSNPA nnot-annotTable.report 19.483	LaTeX fragment 3.
latex4	snpSurvival-survivalAll-geneRank -eqTable.report 19.471	LaTeX fragment 4.

Parameter name	Value	Description
pagebreak	true	Determines if the result document should start with a page break.
sectionTitle		If non-empty, a declaration of a new section with the given name is inserted to the beginning of the combined document. This is a convenience feature to make it easy to compile subsections into a section.
sectionType	section	Type of LaTeX section: usually one of section, subsection or subsubsection. No section statement is written if sectionTitle is empty.
strictBorders	true	Enables the flushing of all document elements before each fragment

19.492 snpSurvival-survivalSet1 (SurvivalAnalysis)

No description

Parameter name	Value
pvalue	0.0010

19.493 snpSurvival-survivalSet1-annotSQL (INPUT)

This SQL is used to generate marker annotation report.

Category: Internal

Parameter name	Value	Description
path	/gpfs/homes/mlaakso/asserSVN/ andurilCaseStudy/sql/ SNPgeneTable.sql	Path (filename) of the input resource.

19.494 snpSurvival-survivalSet1-caseGenotypes (GetGenotypes)

Load and preprocess the genotype calls for the cancer patient samples.

Parameter name	Value
skipFrq	0.1

19.495 snpSurvival-survivalSet1-caseGenotypes-freqCheck (AlleleCounter)

AlleleCounter calculates genotype frequencies for the biallelic markers of the given SNPMatrix. A Pearson's χ^2-test [18] is performed for each marker in order to test if the Hardy-Weinberg equilibrium [9] holds for the population sample.

Author: Marko Laakso (Marko.Laakso@Helsinki.FI)
Version: 1.2
Category: SNP

Input name	Source	Description
genotypes	snpSurvival-survivalSet1-caseGenotypes-genotypes.genotypes 19.497	SNPHelistin genotype output.

Parameter name	Value	Description
dels	false	Accept homozygous deletions as a possible genotype.
naLimit	0.1	Skip markers where one minus call rate exceeds this limit.
skip	0.1	Drop markers with a relative minor allele frequency lower than this limit. Use negative values to inactivate this limit.

19.496 snpSurvival-survivalSet1-caseGenotypes-ftMarkers (CSV2IDList)

Extracts one column from the given CSV file and prints it out without duplicates. The selected column may contain entries with comma separated lists of values in which case *isList* parameter may be used to obtain each value separately.

Output is defined as:

$$ids = \bigcup_{i=1}^{9} \pi_{columnIn_i} \sigma_{regexp_i}(table_i). \tag{10}$$

Author: Marko Laakso (Marko.Laakso@Helsinki.FI)
Version: 1.3
Category: convert

Input name	Source	Description
table1	snpSurvival-survivalSet1-caseGenotypes-freqCheck.frequencies 19.495	The mandatory input relation

Parameter name	Value	Description
acceptMissing	false	Files with missing columnIn are accepted as empty if this is true.
columnIn	marker	A comma separated list of column names for the IDs of interest in each file. Empty values refer to the first column of the file.
columnOut	marker	Name of the only column of the output list. Empty input refers to the name of the input column.
constants		A comma separated list of values that are always included into the output
isList	false	True if the seleted column contains a comma separated list of values to be splitted
quotation	false	Indicator that can be used to disable quotation of the output values
regexp1		Regular expression for the row filtering in table1. A row is included in the result if this parameter is empty or if values in the given columns match given regular expressions. The parameter has a format COLNAME1=EXPRESSION,COLNAME2=EXPRESSION2 where COLNAMEs are column names in "csv" and EXPRESSIONs are regular expressions using Java syntax. For example, "col=a|b" includes rows where the column col has a value of "a" or "b".
regexp2		Regular expression for the row filtering in table2.
regexp3		Regular expression for the row filtering in table3.
regexp4		Regular expression for the row filtering in table4.
regexp5		Regular expression for the row filtering in table5.
regexp6		Regular expression for the row filtering in table6.
regexp7		Regular expression for the row filtering in table7.
regexp8		Regular expression for the row filtering in table8.
regexp9		Regular expression for the row filtering in table9.

19.497 snpSurvival-survivalSet1-caseGenotypes-genotypes (SNPHelistinReader)

This component can be used to read SNP genotype calls for a set of samples and markers of interest. The genotype data is retrieved from a SNPHelistin database that is defined by the input properties.

Author:	Marko Laakso (Marko.Laakso@Helsinki.FI)
Version:	1.2
Categories:	Data Import, SNP

Input name	Source	Description
sampleNames	snpSurvival-survivalSet1-caseNames.ids 19.499	List of sample names of interest.
markerNames	snpSurvival-markers1.in 19.448	List of SNP identifiers of interest.
config	genotypeDB.in 19.320	Connection parameters for SNPHelistin.

Parameter name	Value	Description
errorSymbol	ERR	Symbol for failed measurements. You may for example use 'NA' value in order consider them equal to missing values.
skipUnknown	true	Ignore unknown samples and do not process them. If the parameter is false then the program fails for these samples.

19.498 snpSurvival-survivalSet1-caseGenotypes-gtypes (SNPHelistinReader)

This component can be used to read SNP genotype calls for a set of samples and markers of interest. The genotype data is retrieved from a SNPHelistin database that is defined by the input properties.

Author:	Marko Laakso (Marko.Laakso@Helsinki.FI)
Version:	1.2
Categories:	Data Import, SNP

Input name	Source	Description
sampleNames	snpSurvival-survivalSet1-caseNames.ids 19.499	List of sample names of interest.
markerNames	snpSurvival-survivalSet1-caseGenotypes-ftMarkers.ids 19.496	List of SNP identifiers of interest.
config	genotypeDB.in 19.320	Connection parameters for SNPHelistin.

Parameter name	Value	Description
errorSymbol	ERR	Symbol for failed measurements. You may for example use 'NA' value in order consider them equal to missing values.
skipUnknown	true	Ignore unknown samples and do not process them. If the parameter is false then the program fails for these samples.

19.499 snpSurvival-survivalSet1-caseNames (CSV2IDList)

Extracts one column from the given CSV file and prints it out without duplicates. The selected column may contain entries with comma separated lists of values in which case *isList* parameter may be used to obtain each value separately.

Output is defined as:

$$ids = \bigcup_{i=1}^{9} \pi_{columnIn_i} \sigma_{regexp_i}(table_i). \quad (11)$$

Author: Marko Laakso (Marko.Laakso@Helsinki.FI)
Version: 1.3
Category: convert

Input name	Source	Description
table1	snpSurvival-survivalSet1-survival Clinical.table 19.522	The mandatory input relation

Parameter name	Value	Description
acceptMissing	false	Files with missing columnIn are accepted as empty if this is true.
columnIn		A comma separated list of column names for the IDs of interest in each file. Empty values refer to the first column of the file.
columnOut	SampleId	Name of the only column of the output list. Empty input refers to the name of the input column.
constants		A comma separated list of values that are always included into the output
isList	false	True if the seleted column contains a comma separated list of values to be splitted
quotation	false	Indicator that can be used to disable quotation of the output values
regexp1		Regular expression for the row filtering in table1. A row is included in the result if this parameter is empty or if values in the given columns match given regular expressions. The parameter has a format COLNAME1=EXPRESSION,COLNAME2=EXPRESSION2 where COLNAMEs are column names in "csv" and EXPRESSIONs are regular expressions using Java syntax. For example, "col=a|b" includes rows where the column col has a value of "a" or "b".
regexp2		Regular expression for the row filtering in table2.
regexp3		Regular expression for the row filtering in table3.
regexp4		Regular expression for the row filtering in table4.
regexp5		Regular expression for the row filtering in table5.
regexp6		Regular expression for the row filtering in table6.
regexp7		Regular expression for the row filtering in table7.
regexp8		Regular expression for the row filtering in table8.
regexp9		Regular expression for the row filtering in table9.

19.500 snpSurvival-survivalSet1-clinicalData (INPUT)

Clinical parameters for the case samples
Category: Internal

Parameter name	Value	Description
path	/gpfs/homes/mlaakso/asserSVN/anduril CaseStudy/data/snp/patient-metadata-caucas.csv	Path (filename) of the input resource.

19.501 snpSurvival-survivalSet1-clinicalSelect (SourceCode2Latex)

This component converts a source code listing into a LaTeXfragment that may be included into some technical documents.
Author: Marko Laakso (Marko.Laakso@Helsinki.FI)
Version: 1.0
Categories: convert, Latex

Input name	Source	Description
source	snpSurvival-survivalSet1-survival ParamsQuery.file 19.523	Source code

Parameter name	Value	Description
caption	This SQL query has been used to select clinical parameters for the survival calculations.	Caption text for the listing
language	SQL	Language defintion for the syntax highlighting. Possible values can be found from the description of listings package.

19.502 snpSurvival-survivalSet1-clinicalTypes (INPUT)

Column types for the clinical data

Category: Internal

Parameter name	Value	Description
path	/gpfs/homes/mlaakso/asserSVN/andurilCaseStudy/csv/SNPclinicalTypes.csv	Path (filename) of the input resource.

19.503 snpSurvival-survivalSet1-geneRank (QueryReport)

(Technical documentation) Executes an SQL query and prints it with the result table.

Author: Marko Laakso (Marko.Laakso@Helsinki.FI)
Version: 1.1
Category: Latex

Input name	Source	Description
table1	snpSurvival-survivalSet1-survSNPAnnot.fullData 19.515	1. data table that is referred as 'table1' in the query

Parameter name	Value	Description
colFormat	lcrp{11cm}	LaTeX tabular formatting for the result columns
listCols		Comma separated list of column names. Columns of this list may contain several values separated with commas and the delimiters will be replaced with spaces.
resTableRef	the previous table	Textual reference from the SQL representation to the result table
section	Gene rank	Subsection title for the output document
showCols	gene,markers,p-value,description	Comma separated list of column selections for the output. The empty default will use all columns.
showQuery	false	This flag tells if the SQL shall be shown on the report
tableCaption	A list of survival affecting genes and the marker counts. P-value is the lowest value for the corresponding markers.	Description for the result table

19.504 snpSurvival-survivalSet1-geneRank-eqData (TableQuery)

Execute an SQL query on input relations and create a result table. TableQuery uses HSQLDB (Hyper-threaded Structured Query Language Database [25]) for executing the query. Consequently, the syntax of the query is defined by HSQLDB.

Author: Kristian Ovaska (Kristian.Ovaska@Helsinki.FI)
Version: 1.2
Categories: convert, Filter

Input name	Source	Description
table1	snpSurvival-survivalSet1-survSNPAnnot-annotData.table 19.516	CSV table 1. The table is referred to as 'table1' in the SQL query.

Continued on next page...

Input name	Source	Description
table2	snpSurvival-survivalSet1-survival .statistics 19.521	CSV table 2. The table is referred to as 'table2' in the SQL query.
query	snpSurvival-survivalSet1-gene RankQuery.in 19.506	SQL query. Either this file or the query parameter must be provided, but not both.

Parameter name	Value	Description
numIndices		Comma-separated list of index counts for input tables. All indices are single-column indices, running from column 1 to N, where N is retrieved from this parameter. If empty, the default number (1) is used. For example, ",2,,2" sets two indices for table2 and table4 and one index for the rest.
query		SQL query. Either this parameter or the query input must be provided, but not both.

19.505 snpSurvival-survivalSet1-geneRank-eqTable (CSV2Latex)

This component converts a tabular CSV file into a printable LaTeX table.

Author: Marko Laakso (Marko.Laakso@Helsinki.FI)
Version: 2.1
Categories: convert, Latex

Input name	Source	Description
tabledata	snpSurvival-survivalSet1-gene Rank-eqData.table 19.504	Table content
refs	snpSurvival-survivalSet1-refsGene Rank.in 19.509	Reference rules for the hyperlinks

Parameter name	Value	Description
attach	false	Include the original data as an attachment
caption	A list of survival affecting genes and the marker counts. P-value is the lowest value for the corresponding markers.	Caption text for the table.
colFormat	lcrp{11cm}	LaTeX tabular format for the columns. Default produces a centered layout for all columns.
columns	gene,markers,p-value,description	Comma separated list of column selections for the output. The empty default will use all columns.
countRows	false	Include a row count to the table caption.
dropMissing	true	This flag can be turned off in order to generate links with missing texts. Link text are substituted with target identifiers.
listCols		Comma separated list of column names. Columns of this list may contain several values separated with commas and the delimiters will be replaced with spaces.
numberFormat		A comma separated list of decimal formats for the columns. Each entry consists of the column name and the Java DecimalFormal pattern separated with equal sign. For example, rounding to three decimals can be done like: myColumn=#0.000
pageBreak	false	Use clear page after the table.
rename		Comma separated list of column renaming rules (oldname=newname). New names are used in table header but they do not affect the other behaviour of this component.
ruler	{}	Latex command for the row separating rulers
section	Gene rank	Section title for the table container or an empty string if no section should be generated.
sectionType	subsection	Type of LaTeX section: usually one of: section, subsection, or subsubsection. No section statement is written if section title is empty.
skipEmpty	false	This flag can be used to replace empty tables with a simple LaTeX comment.

19.506 snpSurvival-survivalSet1-geneRankQuery (INPUT)

Generates a list of genes and the marker counts for them.

Category: Internal

Parameter name	Value	Description
path	/gpfs/homes/mlaakso/asserSVN/ andurilCaseStudy/sql/ SNPinterestingGenes.sql	Path (filename) of the input resource.

19.507 snpSurvival-survivalSet1-missingDistC (CSV2Latex)

This component converts a tabular CSV file into a printable LaTeX table.

Author: Marko Laakso (Marko.Laakso@Helsinki.FI)
Version: 2.1
Categories: convert, Latex

Input name	Source	Description
tabledata	snpSurvival-survivalSet1-case Genotypes-freqCheck.na Dist 19.495	Table content

Parameter name	Value	Description
attach	false	Include the original data as an attachment
caption	Distribution of the missing values in case samples	Caption text for the table.
colFormat	p{3cm}r	LaTeX tabular format for the columns. Default produces a centered layout for all columns.
columns		Comma separated list of column selections for the output. The empty default will use all columns.
countRows	false	Include a row count to the table caption.
dropMissing	true	This flag can be turned off in order to generate links with missing texts. Link text are substituted with target identifiers.
listCols		Comma separated list of column names. Columns of this list may contain several values separated with commas and the delimiters will be replaced with spaces.
numberFormat		A comma separated list of decimal formats for the columns. Each entry consists of the column name and the Java DecimalFormal pattern separated with equal sign. For example, rounding to three decimals can be done like: myColumn=#0.000
pageBreak	false	Use clear page after the table.
rename		Comma separated list of column renaming rules (oldname=newname). New names are used in table header but they do not affect the other behaviour of this component.
ruler	{}	Latex command for the row separating rulers
section		Section title for the table container or an empty string if no section should be generated.
sectionType	subsection	Type of LaTeX section: usually one of: section, subsection, or subsubsection. No section statement is written if section title is empty.
skipEmpty	false	This flag can be used to replace empty tables with a simple LaTeX comment.

19.508 snpSurvival-survivalSet1-propertiesDoc (Properties2Latex)

Prepares a LaTeX-representation for the given set of properties files. The document consists of tables of names and values of the properties.

Author: Marko Laakso (Marko.Laakso@Helsinki.FI)
Version: 1.2
Categories: convert, Latex

Input name	Source	Description
props1	genotypeDB.in 19.320	A set of properties

Parameter name	Value	Description
hide		A comma separated list of property names that should remain invisible. You may use this paramerter to hide passwords and other confidentian settings.

Continued on next page...

Parameter name	Value	Description
keyWidth	5	Column width for the property names (centimeters)
merge	false	Merge all input properties into a single set
section	SNP database configuration	Section title for the table container or an empty string if no section should be generated
sectionType	subsection	Type of LaTeX section: usually one of section, subsection or subsubsection. No section statement is written if sectionTitle is empty.
valueWidth	13	Column width for the property values (centimeters)

19.509 snpSurvival-survivalSet1-refsGeneRank (INPUT)

Hyperlink rules for the gene rank table

Category: Internal

Parameter name	Value	Description
path	/gpfs/homes/mlaakso/asserSVN/andurilCaseStudy/csv/SNPrefsGeneRank.csv	Path (filename) of the input resource.

19.510 snpSurvival-survivalSet1-refsHits (INPUT)

Hyperlink rules for the marker annotations

Category: Internal

Parameter name	Value	Description
path	/gpfs/homes/mlaakso/asserSVN/andurilCaseStudy/csv/SNPrefsHits.csv	Path (filename) of the input resource.

19.511 snpSurvival-survivalSet1-reportBody (LatexCombiner)

LatexCombiner joins the given subdocuments together into one LaTeX file.

Authors: Kristian Ovaska (Kristian.Ovaska@Helsinki.FI), Marko Laakso (Marko.Laakso@Helsinki.FI)

Version: 1.3

Category: Latex

Input name	Source	Description
latex1	snpSurvival-survivalSet1-survivalReport.document 19.525	LaTeX fragment 1.
latex2	snpSurvival-survivalSet1-propertiesDoc.report 19.508	LaTeX fragment 2.
latex3	snpSurvival-survivalSet1-missingDistC.report 19.507	LaTeX fragment 3.

Parameter name	Value	Description
pagebreak	false	Determines if the result document should start with a page break.
sectionTitle		If non-empty, a declaration of a new section with the given name is inserted to the beginning of the combined document. This is a convenience feature to make it easy to compile subsections into a section.
sectionType	section	Type of LaTeX section: usually one of section, subsection or subsubsection. No section statement is written if sectionTitle is empty.
strictBorders	true	Enables the flushing of all document elements before each fragment

19.512 snpSurvival-survivalSet1-reportTemplate (LatexTemplate)

This component generates the standard layout configuration for Anduril output documents. The component produces a header and a footer fragment that can be used to build LaTeX documents. The header fragment takes care of the page geometry and other technical details such as library imports and function declarations. Reference list is generated by the footer.

Author: Marko Laakso (Marko.Laakso@Helsinki.FI)
Version: 1.4
Category: Latex

Input name	Source	Description
abstract	abstract.in 19.42	Document abstract including its Latex definition. You may use template entities within this document. The default abstract has been defined in abstractTemplate.tex.

Parameter name	Value	Description
authors	Marko Laakso, Riku Louhimo, Sampsa Hautaniemi	A comma-separated list of document authors on the title page. If authors is empty but title is non-empty, a title page with no author name is printed.
baselineskip	1.2	Baselineskip factor that indicates the line spacing
bibstyle	abbrv	BibTeX reference formatting style
printTOC	true	If true, print Table Of Contents.
title	Anduril Case Study: Glioblastoma SNP survival analysis	Document title on the title page. If empty, no title page is printed. Also see author.
usepackage		Comma-separated list of LaTeX packages that are used in the document. Each package generates a \usepackage{X} line in the header.

19.513 snpSurvival-survivalSet1-snpAnnotations (INPUT)

Annotations for survival component.

Category: Internal

Parameter name	Value	Description
path	/gpfs/homes/mlaakso/asserSVN/andurilCaseStudy/csv/SNPAnnotation.csv	Path (filename) of the input resource.

19.514 snpSurvival-survivalSet1-summaryReport (LatexPDF)

This component is a LaTeX compiler that generates a portable document format (PDF) file from the given Anduril document.

Author: Kristian Ovaska (Kristian.Ovaska@Helsinki.FI)
Version: 1.0
Category: Latex

Input name	Source	Description
document	snpSurvival-survivalSet1-reportBody.document 19.511	Body of the LaTeX document.
header	snpSurvival-survivalSet1-reportTemplate.header 19.512	LaTeX header that is written to the start of the document. If missing, a default header is used.
footer	snpSurvival-survivalSet1-reportTemplate.footer 19.512	LaTeX footer that is written to the end of the document. If missing, a default header is used.

Parameter name	Value	Description
bibtexExec	bibtex	Executable command for BibTeX.
latexExec	pdflatex	Executable command for pdflatex.
useRefs	false	This flag can be used to activate BibTeX compiler

19.515 snpSurvival-survivalSet1-survSNPAnnot (AnnotateSNPs)

Biological annotations for the survival affecting genes

Parameter name	Value
caption	Gene annotations and the loci of the survival affecting markers

19.516 snpSurvival-survivalSet1-survSNPAnnot-annotData (TableQuery)

Execute an SQL query on input relations and create a result table. TableQuery uses HSQLDB (Hyperthreaded Structured Query Language Database [25]) for executing the query. Consequently, the syntax of the query is defined by HSQLDB.

Author: Kristian Ovaska (Kristian.Ovaska@Helsinki.FI)
Version: 1.2
Categories: convert, Filter

Input name	Source	Description
table1	snpSurvival-survivalSet1-survSNPAnnot-snpRel.relation 19.520	CSV table 1. The table is referred to as 'table1' in the SQL query.
table2	snpSurvival-survivalSet1-survSNPAnnot-genes.bioAnnotation 19.518	CSV table 2. The table is referred to as 'table2' in the SQL query.
table3	snpSurvival-survivalSet1-survival.statistics 19.521	CSV table 3. The table is referred to as 'table3' in the SQL query.
query	snpSurvival-survivalSet1-annotSQL.in 19.493	SQL query. Either this file or the query parameter must be provided, but not both.

Parameter name	Value	Description
numIndices		Comma-separated list of index counts for input tables. All indices are single-column indices, running from column 1 to N, where N is retrieved from this parameter. If empty, the default number (1) is used. For example, ",2,,2" sets two indices for table2 and table4 and one index for the rest.
query		SQL query. Either this parameter or the query input must be provided, but not both.

19.517 snpSurvival-survivalSet1-survSNPAnnot-annotTable (CSV2Latex)

This component converts a tabular CSV file into a printable LaTeX table.

Author: Marko Laakso (Marko.Laakso@Helsinki.FI)
Version: 2.1
Categories: convert, Latex

Input name	Source	Description
tabledata	snpSurvival-survivalSet1-survSNPAnnot-annotData.table 19.516	Table content
refs	snpSurvival-survivalSet1-refsHits.in 19.510	Reference rules for the hyperlinks

Continued on next page...

Parameter name	Value	Description

Parameter name	Value	Description
attach	false	Include the original data as an attachment
caption	Gene annotations and the loci of the survival affecting markers	Caption text for the table.
colFormat	l@{}lr@{:}lp{1cm}p{1.5cm}p{10 .5cm}	LaTeX tabular format for the columns. Default produces a centered layout for all columns.
columns	hit,marker,chr,locus,band,gene, gene description	Comma separated list of column selections for the output. The empty default will use all columns.
countRows	false	Include a row count to the table caption.
dropMissing	true	This flag can be turned off in order to generate links with missing texts. Link text are substituted with target identifiers.
listCols	band	Comma separated list of column names. Columns of this list may contain several values separated with commas and the delimiters will be replaced with spaces.
numberFormat		A comma separated list of decimal formats for the columns. Each entry consists of the column name and the Java DecimalFormal pattern separated with equal sign. For example, rounding to three decimals can be done like: myColumn=#0.000
pageBreak	false	Use clear page after the table.
rename		Comma separated list of column renaming rules (oldname=newname). New names are used in table header but they do not affect the other behaviour of this component.
ruler	{}	Latex command for the row separating rulers
section		Section title for the table container or an empty string if no section should be generated.
sectionType	subsection	Type of LaTeX section: usually one of: section, subsection, or subsubsection. No section statement is written if section title is empty.
skipEmpty	false	This flag can be used to replace empty tables with a simple LaTeX comment.

19.518 snpSurvival-survivalSet1-survSNPAnnot-genes (KorvasieniAnnotator)

Korvasieni is an Ensembl [12] based converter for database identifiers and it can be used to convert genome location, gene, transcript, and translation identifiers across multiple biological databases. The selected `keyColumn` may contain entries with comma separated lists of values in which case `isListKey` parameter may be used to obtain each value separately and to process them as they were given in consecutive lines.

Author: Marko Laakso (Marko.Laakso@Helsinki.FI)
Version: 1.7
Categories: Annotation, GO

Input name	Source	Description
sourceKeys	snpSurvival-survivalSet1-survSNPAnnot-snpLoci. annotations 19.519	A list of source database keys.
connection	ensembl.in 19.273	Database connection can be defined using this file. The definition of parameters: database.url, database.user, database.password, database.timeout, database.recycle, and database.driver can be found from the documentation of Korvasieni.

Parameter name	Value	Description
echoColumns	refsnp_id	A comma separated list of column names for the columns that will be copied to the output. An asterisk (*) can be used to denote all columns execpt the keyColumn.
inputDB	.GeneId	Type of input keys. This must be a database supported by Korvasieni. If the parameter is omitted, the component tries to derive the database from the type of geneID. If this is not possible, an error is returned. You may define three columns in form of chromosome:start-end in case the inputDB is .DNARegion. This format provides a comfortable compatibility with DNARegion datatype.
inputType	Gene	Ensembl object type for the input keys (Any, Gene, Transcript, Translation)
isListKey	true	Enables the automatic value splits for the comma separated key column
keyColumn	ensembl_gene_stable_id	Name of the key column withing sourceKeys file or an empty string for the first column
maxHits	100000	Maximum number of target identifiers for a single source identifier
rename	UniProt/SwissProt=protein	Comma separated list of column renaming rules (oldname=newname)
skipLevel	never	Skip result rows if the source identifier is unknown or target identifiers are not available. Possible values are: never (no filtering), source (skip if the source ID is unknown), target (skip if no target IDs are found), any (skip if any of the target IDs is missing).

Continued on next page...

Parameter name	Value	Description
targetDB	.GeneName,.GeneDesc,.DNABand,GO,UniProt/SwissProt	Comma-separated list of annotation types. Possible values are all databases supported by Korvasieni.
unique	true	This flag can be turned on in order to eliminate duplicate annotations.

19.519 snpSurvival-survivalSet1-survSNPAnnot-snpLoci (RefSNPAnnotator)

RefSNPAnnotator fetches annotations for given refSNP ID's (also known as rsID's) from BioMart databases.

The provided annotations for each rsID include alleles for the SNP, name of the chromosome where the SNP is located, chromosome location of the SNP, the strand of the chromosome, Ensembl gene ID for genes that overlap the SNP's location and validation status of the SNP. The component is implemented using the biomaRt package for Bioconductor [7].

Authors: Viljami Aittomaki (Viljami.Aittomaki@Helsinki.FI), Marko Laakso (Marko.Laakso@Helsinki.FI)
Version: 1.0.1
Categories: SNP, Annotation

Input name	Source	Description
rsIDs	snpSurvival-survivalSet1-survival.statistics 19.521	List of rsID's for fetching annotations.

Parameter name	Value	Description
dataset	hsapiens_snp	Dataset to get annotations from. Different species have their own datasets. Example values include "hsapiens_snp" and "mmusculus_snp". See biomaRt documentation on how to list available datasets in R.
keyColumn	marker	Name of the key column withing sourceKeys file or an empty string for the first column.

19.520 snpSurvival-survivalSet1-survSNPAnnot-snpRel (ExpandCollapse)

Converts between the two possible representations of a relations with multivalued columns. By default this component splits rows with multivalued (comma separated lists) columns into multiple rows each representing a single value. The input may contain several columns with comma separated values and all permutations of column values are shown as individual single-valued rows. The alternative mode recovers the original comma separated values from the expanded relations. This is performed by joining values of the their list columns into comma separated lists.

Author: Marko Laakso (Marko.Laakso@Helsinki.FI)
Version: 1.3
Category: convert

Input name	Source	Description
relation	snpSurvival-survivalSet1-survSNPAnnot-snpLoci.annotations 19.519	The mandatory input relation

Parameter name	Value	Description
delim	,	Value delimiter between the list column values. Special characters can be encoded as specified in fi.helsinki.ltdk.csbl.asser.ArgumentEncoding.
expand	true	Action mode that is expand (true) or collapse (false)
listCols	ensembl_gene_stable_id	A comma separated list of column names of input (expand) or output (collapse) columns that may contain delim separated values. The asterisk refers to every column of the input relation.

19.521 snpSurvival-survivalSet1-survival (SNPKaplanMeier)

This component is used to estimate survival differences between various genotypes. The differences between the survival of different genotypes are measured using G-ρ family of tests [10].

Author:	Marko Laakso (Marko.Laakso@Helsinki.FI)
Version:	1.5
Categories:	SNP, Latex, Survival

Input name	Source	Description
genotypes	snpSurvival-survivalSet1-case Genotypes-gtypes .genotypes 19.498	Genotype calls
survival	snpSurvival-survivalSet1-survival Clinical.table 19.522	Survival time and event indicator
annotations	snpSurvival-survivalSet1-snp Annotations.in 19.513	Additional information about the markers. This data will be shown on the report of interesting markers.

Parameter name	Value	Description
PLimit	0.0010	Ignore results with a p-value greater than this limit.
confInt	0.95	Coverage of the confidence intervals [0,1]
minCount	15	Combine rare (aa) homozygotes to heterozygotes (Aa) if their number is below this limit. This parameter is used together with minFreq so that the actual limit is max(minFreq*n, minCount).
minFreq	0.1	Combine rare (aa) homozygotes to heterozygotes (Aa) if their frequency is below this limit. See also minCount.
naLimit	0.1	Skip markers where one minus call rate exceeds this limit.
nullCalls	–,ERR	A comma separated list of genotype calls that will be considered as missing values
sampleCol	sample	Column name for the sample name
showCI	true	Draw confidence intervals to the graph
skipMissingGT	true	Simply skip those samples that lack the genotype information.
statusCol	hasEvent	Column name for the event indicator
survRatePoint	0.5	Sampling height for the distances between the survival expectations. Negative values may be used to suppress these measurements.
timeCol	endTime	Column name for the survival time
timeOutLimit	36	Cut survival curves after this limit. A negative value can be used to maximize the length of the period.
timeStartCol	startTime	Column name for the start time that is reduced from the survival time
title	Genotype Specific Survival Estimates	Section title

19.522 snpSurvival-survivalSet1-survivalClinical (TableQuery)

Execute an SQL query on input relations and create a result table. TableQuery uses HSQLDB (Hyper-threaded Structured Query Language Database [25]) for executing the query. Consequently, the syntax of the query is defined by HSQLDB.

Author:	Kristian Ovaska (Kristian.Ovaska@Helsinki.FI)
Version:	1.2
Categories:	convert, Filter

Input name	Source	Description
table1	snpSurvival-survivalSet1-clinical Data.in 19.500	CSV table 1. The table is referred to as 'table1' in the SQL query.
query	snpSurvival-survivalSet1-survival ParamsQuery.file 19.523	SQL query. Either this file or the query parameter must be provided, but not both.
columnTypes	snpSurvival-survivalSet1-clinical Types.in 19.502	Contains SQL types for individual columns. If the file is not provided, the type is inferred from the contents of the columns. This can be used to force the use of VARCHAR for values that are also valid numerics. The file contains the columns Table (refers to one of table1 to table15), Column (refers to a column name in the table), Type (contains an SQL type). A row with Table='result', Column=X and Type='STRING' forces the use string values for result column X.

Parameter name	Value	Description
numIndices		Comma-separated list of index counts for input tables. All indices are single-column indices, running from column 1 to N, where N is retrieved from this parameter. If empty, the default number (1) is used. For example, ",2,,2" sets two indices for table2 and table4 and one index for the rest.
query		SQL query. Either this parameter or the query input must be provided, but not both.

19.523 snpSurvival-survivalSet1-survivalParamsQuery (SearchReplace)

Replaces the occurences of the given regular expressions with the corresponding regular expressions. This component is used for transforming given patterns of the input file to a changed form specified by the regular expressions .

Author: Marko Laakso (Marko.Laakso@Helsinki.FI)
Version: 1.0
Category: convert

Input name	Source	Description
file	snpSurvival-survivalSet1-survivalParamsTemplate.in 19.524	Source file to be modified

Parameter name	Value	Description
key00	REP.cond00	Search key 0
key01		Search key 1
key02		Search key 2
key03		Search key 3
key04		Search key 4
key05		Search key 5
key06		Search key 6
key07		Search key 7
key08		Search key 8
key09		Search key 9
value00	– No additional restrictions	Replacement 0
value01		Replacement 1
value02		Replacement 2
value03		Replacement 3
value04		Replacement 4
value05		Replacement 5
value06		Replacement 6
value07		Replacement 7
value08		Replacement 8
value09		Replacement 9

19.524 snpSurvival-survivalSet1-survivalParamsTemplate (INPUT)

Select relevant parameters for the survival analysis

Category: Internal

Parameter name	Value	Description
path	/gpfs/homes/mlaakso/asserSVN/andurilCaseStudy/sql/SNPsurvivalParams.sql	Path (filename) of the input resource.

19.525 snpSurvival-survivalSet1-survivalReport (LatexCombiner)

LatexCombiner joins the given subdocuments together into one LaTeX file.

Authors:	Kristian Ovaska (Kristian.Ovaska@Helsinki.FI), Marko Laakso (Marko.Laakso@Helsinki.FI)
Version:	1.3
Category:	Latex

Input name	Source	Description
latex1	snpSurvival-survivalSet1-survival .report 19.521	LaTeX fragment 1.
latex2	snpSurvival-survivalSet1-clinical Select.report 19.501	LaTeX fragment 2.
latex3	snpSurvival-survivalSet1-survSN PAnnot-annotTable.report 19.517	LaTeX fragment 3.
latex4	snpSurvival-survivalSet1-gene Rank-eqTable.report 19.505	LaTeX fragment 4.

Parameter name	Value	Description
pagebreak	true	Determines if the result document should start with a page break.
sectionTitle		If non-empty, a declaration of a new section with the given name is inserted to the beginning of the combined document. This is a convenience feature to make it easy to compile subsections into a section.
sectionType	section	Type of LaTeX section: usually one of section, subsection or subsubsection. No section statement is written if sectionTitle is empty.
strictBorders	true	Enables the flushing of all document elements before each fragment

19.526 snpSurvival-survivalSet10 (SurvivalAnalysis)

No description

Parameter name	Value
pvalue	0.0010

19.527 snpSurvival-survivalSet10-annotSQL (INPUT)

This SQL is used to generate marker annotation report.

Category: Internal

Parameter name	Value	Description
path	/gpfs/homes/mlaakso/asserSVN/ anduril CaseStudy/sql/ SNPgeneTable.sql	Path (filename) of the input resource.

19.528 snpSurvival-survivalSet10-caseGenotypes (GetGenotypes)

Load and preprocess the genotype calls for the cancer patient samples.

Parameter name	Value
skipFrq	0.1

19.529 snpSurvival-survivalSet10-caseGenotypes-freqCheck (AlleleCounter)

AlleleCounter calculates genotype frequencies for the biallelic markers of the given SNPMatrix. A Pearson's χ^2-test [18] is performed for each marker in order to test if the Hardy-Weinberg equilibrium [9] holds for the population sample.

Author:	Marko Laakso (Marko.Laakso@Helsinki.FI)
Version:	1.2
Category:	SNP

Input name	Source	Description
genotypes	snpSurvival-survivalSet10-caseGenotypes-genotypes.genotypes 19.531	SNPHelistin genotype output.

Parameter name	Value	Description
dels	false	Accept homozygous deletions as a possible genotype.
naLimit	0.1	Skip markers where one minus call rate exceeds this limit.
skip	0.1	Drop markers with a relative minor allele frequency lower than this limit. Use negative values to inactivate this limit.

19.530 snpSurvival-survivalSet10-caseGenotypes-ftMarkers (CSV2IDList)

Extracts one column from the given CSV file and prints it out without duplicates. The selected column may contain entries with comma separated lists of values in which case *isList* parameter may be used to obtain each value separately.

Output is defined as:

$$ids = \bigcup_{i=1}^{9} \pi_{columnIn_i} \sigma_{regexp_i}(table_i). \qquad (12)$$

Author:	Marko Laakso (Marko.Laakso@Helsinki.FI)
Version:	1.3
Category:	convert

Input name	Source	Description
table1	snpSurvival-survivalSet10-caseGenotypes-freqCheck.frequencies 19.529	The mandatory input relation

Parameter name	Value	Description
acceptMissing	false	Files with missing columnIn are accepted as empty if this is true.
columnIn	marker	A comma separated list of column names for the IDs of interest in each file. Empty values refer to the first column of the file.
columnOut	marker	Name of the only column of the output list. Empty input refers to the name of the input column.
constants		A comma separated list of values that are always included into the output
isList	false	True if the seleted column contains a comma separated list of values to be splitted
quotation	false	Indicator that can be used to disable quotation of the output values
regexp1		Regular expression for the row filtering in table1. A row is included in the result if this parameter is empty or if values in the given columns match given regular expressions. The parameter has a format COLNAME1=EXPRESSION,COLNAME2=EXPRESSION2 where COLNAMEs are column names in "csv" and EXPRESSIONs are regular expressions using Java syntax. For example, "col=a|b" includes rows where the column col has a value of "a" or "b".
regexp2		Regular expression for the row filtering in table2.
regexp3		Regular expression for the row filtering in table3.
regexp4		Regular expression for the row filtering in table4.
regexp5		Regular expression for the row filtering in table5.
regexp6		Regular expression for the row filtering in table6.
regexp7		Regular expression for the row filtering in table7.
regexp8		Regular expression for the row filtering in table8.
regexp9		Regular expression for the row filtering in table9.

19.531 snpSurvival-survivalSet10-caseGenotypes-genotypes (SNPHelistinReader)

This component can be used to read SNP genotype calls for a set of samples and markers of interest. The genotype data is retrieved from a SNPHelistin database that is defined by the input properties.

Author:	Marko Laakso (Marko.Laakso@Helsinki.FI)
Version:	1.2
Categories:	Data Import, SNP

Input name	Source	Description
sampleNames	snpSurvival-survivalSet10-caseNames.ids 19.533	List of sample names of interest.
markerNames	snpSurvival-markers10.in 19.449	List of SNP identifiers of interest.
config	genotypeDB.in 19.320	Connection parameters for SNPHelistin.

Parameter name	Value	Description
errorSymbol	ERR	Symbol for failed measurements. You may for example use 'NA' value in order consider them equal to missing values.
skipUnknown	true	Ignore unknown samples and do not process them. If the parameter is false then the program fails for these samples.

19.532 snpSurvival-survivalSet10-caseGenotypes-gtypes (SNPHelistinReader)

This component can be used to read SNP genotype calls for a set of samples and markers of interest. The genotype data is retrieved from a SNPHelistin database that is defined by the input properties.

Author:	Marko Laakso (Marko.Laakso@Helsinki.FI)
Version:	1.2
Categories:	Data Import, SNP

Input name	Source	Description
sampleNames	snpSurvival-survivalSet10-caseNames.ids 19.533	List of sample names of interest.
markerNames	snpSurvival-survivalSet10-caseGenotypes-ftMarkers.ids 19.530	List of SNP identifiers of interest.
config	genotypeDB.in 19.320	Connection parameters for SNPHelistin.

Parameter name	Value	Description
errorSymbol	ERR	Symbol for failed measurements. You may for example use 'NA' value in order consider them equal to missing values.
skipUnknown	true	Ignore unknown samples and do not process them. If the parameter is false then the program fails for these samples.

19.533 snpSurvival-survivalSet10-caseNames (CSV2IDList)

Extracts one column from the given CSV file and prints it out without duplicates. The selected column may contain entries with comma separated lists of values in which case *isList* parameter may be used to obtain each value separately.

Output is defined as:

$$ids = \bigcup_{i=1}^{9} \pi_{columnIn_i} \sigma_{regexp_i}(table_i). \tag{13}$$

Author: Marko Laakso (Marko.Laakso@Helsinki.FI)
Version: 1.3
Category: convert

Input name	Source	Description
table1	snpSurvival-survivalSet10-survival Clinical.table 19.556	The mandatory input relation

Parameter name	Value	Description
acceptMissing	false	Files with missing columnIn are accepted as empty if this is true.
columnIn		A comma separated list of column names for the IDs of interest in each file. Empty values refer to the first column of the file.
columnOut	SampleId	Name of the only column of the output list. Empty input refers to the name of the input column.
constants		A comma separated list of values that are always included into the output
isList	false	True if the seleted column contains a comma separated list of values to be splitted
quotation	false	Indicator that can be used to disable quotation of the output values
regexp1		Regular expression for the row filtering in table1. A row is included in the result if this parameter is empty or if values in the given columns match given regular expressions. The parameter has a format COLNAME1=EXPRESSION,COLNAME2=EXPRESSION2 where COLNAMEs are column names in "csv" and EXPRESSIONs are regular expressions using Java syntax. For example, "col=a|b" includes rows where the column col has a value of "a" or "b".
regexp2		Regular expression for the row filtering in table2.
regexp3		Regular expression for the row filtering in table3.
regexp4		Regular expression for the row filtering in table4.
regexp5		Regular expression for the row filtering in table5.
regexp6		Regular expression for the row filtering in table6.
regexp7		Regular expression for the row filtering in table7.
regexp8		Regular expression for the row filtering in table8.
regexp9		Regular expression for the row filtering in table9.

19.534 snpSurvival-survivalSet10-clinicalData (INPUT)

Clinical parameters for the case samples
Category: Internal

Parameter name	Value	Description
path	/gpfs/homes/mlaakso/asserSVN/ andurilCaseStudy/data/snp/ patient-metadata-caucas.csv	Path (filename) of the input resource.

19.535 snpSurvival-survivalSet10-clinicalSelect (SourceCode2Latex)

This component converts a source code listing into a LaTeXfragment that may be included into some technical documents.
Author: Marko Laakso (Marko.Laakso@Helsinki.FI)
Version: 1.0
Categories: convert, Latex

Input name	Source	Description
source	snpSurvival-survivalSet10-survival ParamsQuery.file 19.557	Source code

Parameter name	Value	Description
caption	This SQL query has been used to select clinical parameters for the survival calculations.	Caption text for the listing
language	SQL	Language defintion for the syntax highlighting. Possible values can be found from the description of listings package.

19.536 snpSurvival-survivalSet10-clinicalTypes (INPUT)

Column types for the clinical data

Category: Internal

Parameter name	Value	Description
path	/gpfs/homes/mlaakso/asserSVN/ andurilCaseStudy/csv/ SNPclinicalTypes.csv	Path (filename) of the input resource.

19.537 snpSurvival-survivalSet10-geneRank (QueryReport)

(Technical documentation) Executes an SQL query and prints it with the result table.

Author: Marko Laakso (Marko.Laakso@Helsinki.FI)
Version: 1.1
Category: Latex

Input name	Source	Description
table1	snpSurvival-survivalSet10-survSN PAnnot.fullData 19.549	1. data table that is referred as 'table1' in the query

Parameter name	Value	Description
colFormat	lcrp{11cm}	LaTeX tabular formatting for the result columns
listCols		Comma separated list of column names. Columns of this list may contain several values separated with commas and the delimiters will be replaced with spaces.
resTableRef	the previous table	Textual reference from the SQL representation to the result table
section	Gene rank	Subsection title for the output document
showCols	gene,markers,p-value,description	Comma separated list of column selections for the output. The empty default will use all columns.
showQuery	false	This flag tells if the SQL shall be shown on the report
tableCaption	A list of survival affecting genes and the marker counts. P-value is the lowest value for the corresponding markers.	Description for the result table

19.538 snpSurvival-survivalSet10-geneRank-eqData (TableQuery)

Execute an SQL query on input relations and create a result table. TableQuery uses HSQLDB (Hyper-threaded Structured Query Language Database [25]) for executing the query. Consequently, the syntax of the query is defined by HSQLDB.

Author: Kristian Ovaska (Kristian.Ovaska@Helsinki.FI)
Version: 1.2
Categories: convert, Filter

Input name	Source	Description
table1	snpSurvival-survivalSet10-survSN PAnnot-annotData.table 19.550	CSV table 1. The table is referred to as 'table1' in the SQL query.

Continued on next page...

Input name	Source	Description
table2	snpSurvival-survivalSet10-survival .statistics 19.555	CSV table 2. The table is referred to as 'table2' in the SQL query.
query	snpSurvival-survivalSet10-gene RankQuery.in 19.540	SQL query. Either this file or the query parameter must be provided, but not both.

Parameter name	Value	Description
numIndices		Comma-separated list of index counts for input tables. All indices are single-column indices, running from column 1 to N, where N is retrieved from this parameter. If empty, the default number (1) is used. For example, ",2,,2" sets two indices for table2 and table4 and one index for the rest.
query		SQL query. Either this parameter or the query input must be provided, but not both.

19.539 snpSurvival-survivalSet10-geneRank-eqTable (CSV2Latex)

This component converts a tabular CSV file into a printable LaTeX table.

Author: Marko Laakso (Marko.Laakso@Helsinki.FI)
Version: 2.1
Categories: convert, Latex

Input name	Source	Description
tabledata	snpSurvival-survivalSet10-gene Rank-eqData.table 19.538	Table content
refs	snpSurvival-survivalSet10-refs GeneRank.in 19.543	Reference rules for the hyperlinks

Parameter name	Value	Description
attach	false	Include the original data as an attachment
caption	A list of survival affecting genes and the marker counts. P-value is the lowest value for the corresponding markers.	Caption text for the table.
colFormat	lcrp{11cm}	LaTeX tabular format for the columns. Default produces a centered layout for all columns.
columns	gene,markers,p-value,description	Comma separated list of column selections for the output. The empty default will use all columns.
countRows	false	Include a row count to the table caption.
dropMissing	true	This flag can be turned off in order to generate links with missing texts. Link text are substituted with target identifiers.
listCols		Comma separated list of column names. Columns of this list may contain several values separated with commas and the delimiters will be replaced with spaces.
numberFormat		A comma separated list of decimal formats for the columns. Each entry consists of the column name and the Java DecimalFormal pattern separated with equal sign. For example, rounding to three decimals can be done like: myColumn=#0.000
pageBreak	false	Use clear page after the table.
rename		Comma separated list of column renaming rules (oldname=newname). New names are used in table header but they do not affect the other behaviour of this component.
ruler	{}	Latex command for the row separating rulers
section	Gene rank	Section title for the table container or an empty string if no section should be generated.
sectionType	subsection	Type of LaTeX section: usually one of: section, subsection, or subsubsection. No section statement is written if section title is empty.
skipEmpty	false	This flag can be used to replace empty tables with a simple LaTeX comment.

19.540 snpSurvival-survivalSet10-geneRankQuery (INPUT)

Generates a list of genes and the marker counts for them.

Category: Internal

Parameter name	Value	Description
path	/gpfs/homes/mlaakso/asserSVN/ andurilCaseStudy/sql/ SNPinterestingGenes.sql	Path (filename) of the input resource.

19.541 snpSurvival-survivalSet10-missingDistC (CSV2Latex)

This component converts a tabular CSV file into a printable LaTeX table.

Author:	Marko Laakso (Marko.Laakso@Helsinki.FI)
Version:	2.1
Categories:	convert, Latex

Input name	Source	Description
tabledata	snpSurvival-survivalSet10-case Genotypes-freqCheck.na Dist 19.529	Table content

Parameter name	Value	Description
attach	false	Include the original data as an attachment
caption	Distribution of the missing values in case samples	Caption text for the table.
colFormat	p{3cm}r	LaTeX tabular format for the columns. Default produces a centered layout for all columns.
columns		Comma separated list of column selections for the output. The empty default will use all columns.
countRows	false	Include a row count to the table caption.
dropMissing	true	This flag can be turned off in order to generate links with missing texts. Link text are substituted with target identifiers.
listCols		Comma separated list of column names. Columns of this list may contain several values separated with commas and the delimiters will be replaced with spaces.
numberFormat		A comma separated list of decimal formats for the columns. Each entry consists of the column name and the Java DecimalFormal pattern separated with equal sign. For example, rounding to three decimals can be done like: myColumn=#0.000
pageBreak	false	Use clear page after the table.
rename		Comma separated list of column renaming rules (oldname=newname). New names are used in table header but they do not affect the other behaviour of this component.
ruler	{}	Latex command for the row separating rulers
section		Section title for the table container or an empty string if no section should be generated.
sectionType	subsection	Type of LaTeX section: usually one of: section, subsection, or subsubsection. No section statement is written if section title is empty.
skipEmpty	false	This flag can be used to replace empty tables with a simple LaTeX comment.

19.542 snpSurvival-survivalSet10-propertiesDoc (Properties2Latex)

Prepares a LaTeX-representation for the given set of properties files. The document consists of tables of names and values of the properties.

Author:	Marko Laakso (Marko.Laakso@Helsinki.FI)
Version:	1.2
Categories:	convert, Latex

Input name	Source	Description
props1	genotypeDB.in 19.320	A set of properties

Parameter name	Value	Description
hide		A comma separated list of property names that should remain invisible. You may use this paramerter to hide passwords and other confidentian settings.

Continued on next page...

Parameter name	Value	Description
keyWidth	5	Column width for the property names (centimeters)
merge	false	Merge all input properties into a single set
section	SNP database configuration	Section title for the table container or an empty string if no section should be generated
sectionType	subsection	Type of LaTeX section: usually one of section, subsection or subsubsection. No section statement is written if sectionTitle is empty.
valueWidth	13	Column width for the property values (centimeters)

19.543 snpSurvival-survivalSet10-refsGeneRank (INPUT)

Hyperlink rules for the gene rank table

Category: Internal

Parameter name	Value	Description
path	/gpfs/homes/mlaakso/asserSVN/andurilCaseStudy/csv/SNPrefsGeneRank.csv	Path (filename) of the input resource.

19.544 snpSurvival-survivalSet10-refsHits (INPUT)

Hyperlink rules for the marker annotations

Category: Internal

Parameter name	Value	Description
path	/gpfs/homes/mlaakso/asserSVN/andurilCaseStudy/csv/SNPrefsHits.csv	Path (filename) of the input resource.

19.545 snpSurvival-survivalSet10-reportBody (LatexCombiner)

LatexCombiner joins the given subdocuments together into one LaTeX file.

Authors: Kristian Ovaska (Kristian.Ovaska@Helsinki.FI), Marko Laakso (Marko.Laakso@Helsinki.FI)
Version: 1.3
Category: Latex

Input name	Source	Description
latex1	snpSurvival-survivalSet10-survivalReport.document 19.559	LaTeX fragment 1.
latex2	snpSurvival-survivalSet10-propertiesDoc.report 19.542	LaTeX fragment 2.
latex3	snpSurvival-survivalSet10-missingDistC.report 19.541	LaTeX fragment 3.

Parameter name	Value	Description
pagebreak	false	Determines if the result document should start with a page break.
sectionTitle		If non-empty, a declaration of a new section with the given name is inserted to the beginning of the combined document. This is a convenience feature to make it easy to compile subsections into a section.
sectionType	section	Type of LaTeX section: usually one of section, subsection or subsubsection. No section statement is written if sectionTitle is empty.
strictBorders	true	Enables the flushing of all document elements before each fragment

19.546 snpSurvival-survivalSet10-reportTemplate (LatexTemplate)

This component generates the standard layout configuration for Anduril output documents. The component produces a header and a footer fragment that can be used to build LaTeX documents. The header fragment takes care of the page geometry and other technical details such as library imports and function declarations. Reference list is generated by the footer.

Author: Marko Laakso (Marko.Laakso@Helsinki.FI)
Version: 1.4
Category: Latex

Input name	Source	Description
abstract	abstract.in 19.42	Document abstract including its Latex definition. You may use template entities within this document. The default abstract has been defined in abstractTemplate.tex.

Parameter name	Value	Description
authors	Marko Laakso, Riku Louhimo, Sampsa Hautaniemi	A comma-separated list of document authors on the title page. If authors is empty but title is non-empty, a title page with no author name is printed.
baselineskip	1.2	Baselineskip factor that indicates the line spacing
bibstyle	abbrv	BibTeX reference formatting style
printTOC	true	If true, print Table Of Contents.
title	Anduril Case Study: Glioblastoma SNP survival analysis	Document title on the title page. If empty, no title page is printed. Also see author.
usepackage		Comma-separated list of LaTeX packages that are used in the document. Each package generates a \usepackage{X} line in the header.

19.547 snpSurvival-survivalSet10-snpAnnotations (INPUT)

Annotations for survival component.

Category: Internal

Parameter name	Value	Description
path	/gpfs/homes/mlaakso/asserSVN/andurilCaseStudy/csv/SNPAnnotation.csv	Path (filename) of the input resource.

19.548 snpSurvival-survivalSet10-summaryReport (LatexPDF)

This component is a LaTeX compiler that generates a portable document format (PDF) file from the given Anduril document.

Author: Kristian Ovaska (Kristian.Ovaska@Helsinki.FI)
Version: 1.0
Category: Latex

Input name	Source	Description
document	snpSurvival-survivalSet10-reportBody.document 19.545	Body of the LaTeX document.
header	snpSurvival-survivalSet10-reportTemplate.header 19.546	LaTeX header that is written to the start of the document. If missing, a default header is used.
footer	snpSurvival-survivalSet10-reportTemplate.footer 19.546	LaTeX footer that is written to the end of the document. If missing, a default header is used.

Parameter name	Value	Description
bibtexExec	bibtex	Executable command for BibTeX.
latexExec	pdflatex	Executable command for pdflatex.
useRefs	false	This flag can be used to activate BibTeX compiler

19.549 snpSurvival-survivalSet10-survSNPAnnot (AnnotateSNPs)

Biological annotations for the survival affecting genes

Parameter name	Value
caption	Gene annotations and the loci of the survival affecting markers

19.550 snpSurvival-survivalSet10-survSNPAnnot-annotData (TableQuery)

Execute an SQL query on input relations and create a result table. TableQuery uses HSQLDB (Hyperthreaded Structured Query Language Database [25]) for executing the query. Consequently, the syntax of the query is defined by HSQLDB.

Author: Kristian Ovaska (Kristian.Ovaska@Helsinki.FI)
Version: 1.2
Categories: convert, Filter

Input name	Source	Description
table1	snpSurvival-survivalSet10-survSNPAnnot-snpRel.relation 19.554	CSV table 1. The table is referred to as 'table1' in the SQL query.
table2	snpSurvival-survivalSet10-survSNPAnnot-genes.bioAnnotation 19.552	CSV table 2. The table is referred to as 'table2' in the SQL query.
table3	snpSurvival-survivalSet10-survival.statistics 19.555	CSV table 3. The table is referred to as 'table3' in the SQL query.
query	snpSurvival-survivalSet10-annotSQL.in 19.527	SQL query. Either this file or the query parameter must be provided, but not both.

Parameter name	Value	Description
numIndices		Comma-separated list of index counts for input tables. All indices are single-column indices, running from column 1 to N, where N is retrieved from this parameter. If empty, the default number (1) is used. For example, ",2,,2" sets two indices for table2 and table4 and one index for the rest.
query		SQL query. Either this parameter or the query input must be provided, but not both.

19.551 snpSurvival-survivalSet10-survSNPAnnot-annotTable (CSV2Latex)

This component converts a tabular CSV file into a printable LaTeX table.

Author: Marko Laakso (Marko.Laakso@Helsinki.FI)
Version: 2.1
Categories: convert, Latex

Input name	Source	Description
tabledata	snpSurvival-survivalSet10-survSNPAnnot-annotData.table 19.550	Table content
refs	snpSurvival-survivalSet10-refsHits.in 19.544	Reference rules for the hyperlinks

Continued on next page...

Parameter name	Value	Description

Parameter name	Value	Description
attach	false	Include the original data as an attachment
caption	Gene annotations and the loci of the survival affecting markers	Caption text for the table.
colFormat	l@{}lr@{:}lp{1cm}p{1.5cm}p{10.5cm}	LaTeX tabular format for the columns. Default produces a centered layout for all columns.
columns	hit,marker,chr,locus,band,gene, gene description	Comma separated list of column selections for the output. The empty default will use all columns.
countRows	false	Include a row count to the table caption.
dropMissing	true	This flag can be turned off in order to generate links with missing texts. Link text are substituted with target identifiers.
listCols	band	Comma separated list of column names. Columns of this list may contain several values separated with commas and the delimiters will be replaced with spaces.
numberFormat		A comma separated list of decimal formats for the columns. Each entry consists of the column name and the Java DecimalFormal pattern separated with equal sign. For example, rounding to three decimals can be done like: myColumn=#0.000
pageBreak	false	Use clear page after the table.
rename		Comma separated list of column renaming rules (oldname=newname). New names are used in table header but they do not affect the other behaviour of this component.
ruler	{}	Latex command for the row separating rulers
section		Section title for the table container or an empty string if no section should be generated.
sectionType	subsection	Type of LaTeX section: usually one of: section, subsection, or subsubsection. No section statement is written if section title is empty.
skipEmpty	false	This flag can be used to replace empty tables with a simple LaTeX comment.

19.552 snpSurvival-survivalSet10-survSNPAnnot-genes (KorvasieniAnnotator)

Korvasieni is an Ensembl [12] based converter for database identifiers and it can be used to convert genome location, gene, transcript, and translation identifiers across multiple biological databases. The selected `keyColumn` may contain entries with comma separated lists of values in which case `isListKey` parameter may be used to obtain each value separately and to process them as they were given in consecutive lines.

Author: Marko Laakso (Marko.Laakso@Helsinki.FI)
Version: 1.7
Categories: Annotation, GO

Input name	Source	Description
sourceKeys	snpSurvival-survivalSet10-survSNPAnnot-snpLoci. annotations 19.553	A list of source database keys.
connection	ensembl.in 19.273	Database connection can be defined using this file. The definition of parameters: database.url, database.user, database.password, database.timeout, database.recycle, and database.driver can be found from the documentation of Korvasieni.

Parameter name	Value	Description
echoColumns	refsnp_id	A comma separated list of column names for the columns that will be copied to the output. An asterisk (*) can be used to denote all columns execpt the keyColumn.
inputDB	.GeneId	Type of input keys. This must be a database supported by Korvasieni. If the parameter is omitted, the component tries to derive the database from the type of geneID. If this is not possible, an error is returned. You may define three columns in form of chromosome:start-end in case the inputDB is .DNARegion. This format provides a comfortable compatibility with DNARegion datatype.
inputType	Gene	Ensembl object type for the input keys (Any, Gene, Transcript, Translation)
isListKey	true	Enables the automatic value splits for the comma separated key column
keyColumn	ensembl_gene_stable_id	Name of the key column withing sourceKeys file or an empty string for the first column
maxHits	100000	Maximum number of target identifiers for a single source identifier
rename	UniProt/SwissProt=protein	Comma separated list of column renaming rules (oldname=newname)
skipLevel	never	Skip result rows if the source identifier is unknown or target identifiers are not available. Possible values are: never (no filtering), source (skip if the source ID is unknown), target (skip if no target IDs are found), any (skip if any of the target IDs is missing).

Continued on next page...

Parameter name	Value	Description
targetDB	.GeneName,.GeneDesc,.<br>DNABand,GO,UniProt/SwissProt	Comma-separated list of annotation types. Possible values are all databases supported by Korvasieni.
unique	true	This flag can be turned on in order to eliminate duplicate annotations.

19.553 snpSurvival-survivalSet10-survSNPAnnot-snpLoci (RefSNPAnnotator)

RefSNPAnnotator fetches annotations for given refSNP ID's (also known as rsID's) from BioMart databases.

The provided annotations for each rsID include alleles for the SNP, name of the chromosome where the SNP is located, chromosome location of the SNP, the strand of the chromosome, Ensembl gene ID for genes that overlap the SNP's location and validation status of the SNP. The component is implemented using the biomaRt package for Bioconductor [7].

Authors: Viljami Aittomaki (Viljami.Aittomaki@Helsinki.FI), Marko Laakso (Marko.Laakso@Helsinki.FI)
Version: 1.0.1
Categories: SNP, Annotation

Input name	Source	Description
rsIDs	snpSurvival-survivalSet10-survival<br>.statistics 19.555	List of rsID's for fetching annotations.

Parameter name	Value	Description
dataset	hsapiens_snp	Dataset to get annotations from. Different species have their own datasets. Example values include "hsapiens_snp" and "mmusculus_snp". See biomaRt documentation on how to list available datasets in R.
keyColumn	marker	Name of the key column withing sourceKeys file or an empty string for the first column.

19.554 snpSurvival-survivalSet10-survSNPAnnot-snpRel (ExpandCollapse)

Converts between the two possible representations of a relations with multivalued columns. By default this component splits rows with multivalued (comma separated lists) columns into multiple rows each representing a single value. The input may contain several columns with comma separated values and all permutations of column values are shown as individual single-valued rows. The alternative mode recovers the original comma separated values from the expanded relations. This is performed by joining values of the their list columns into comma separated lists.

Author: Marko Laakso (Marko.Laakso@Helsinki.FI)
Version: 1.3
Category: convert

Input name	Source	Description
relation	snpSurvival-survivalSet10-survSN<br>PAnnot-snpLoci.<br>annotations 19.553	The mandatory input relation

Parameter name	Value	Description
delim	,	Value delimiter between the list column values. Special characters can be encoded as specified in fi.helsinki.ltdk.csbl.asser.ArgumentEncoding.
expand	true	Action mode that is expand (true) or collapse (false)
listCols	ensembl_gene_stable_id	A comma separated list of column names of input (expand) or output (collapse) columns that may contain delim separated values. The asterisk refers to every column of the input relation.

19.555 snpSurvival-survivalSet10-survival (SNPKaplanMeier)

This component is used to estimate survival differences between various genotypes. The differences between the survival of different genotypes are measured using G-ρ family of tests [10].

Author:	Marko Laakso (Marko.Laakso@Helsinki.FI)
Version:	1.5
Categories:	SNP, Latex, Survival

Input name	Source	Description
genotypes	snpSurvival-survivalSet10-caseGenotypes-gtypes.genotypes 19.532	Genotype calls
survival	snpSurvival-survivalSet10-survivalClinical.table 19.556	Survival time and event indicator
annotations	snpSurvival-survivalSet10-snpAnnotations.in 19.547	Additional information about the markers. This data will be shown on the report of interesting markers.

Parameter name	Value	Description
PLimit	0.0010	Ignore results with a p-value greater than this limit.
confInt	0.95	Coverage of the confidence intervals [0,1]
minCount	15	Combine rare (aa) homozygotes to heterozygotes (Aa) if their number is below this limit. This parameter is used together with minFreq so that the actual limit is max(minFreq*n, minCount).
minFreq	0.1	Combine rare (aa) homozygotes to heterozygotes (Aa) if their frequency is below this limit. See also minCount.
naLimit	0.1	Skip markers where one minus call rate exceeds this limit.
nullCalls	–,ERR	A comma separated list of genotype calls that will be considered as missing values
sampleCol	sample	Column name for the sample name
showCI	true	Draw confidence intervals to the graph
skipMissingGT	true	Simply skip those samples that lack the genotype information.
statusCol	hasEvent	Column name for the event indicator
survRatePoint	0.5	Sampling height for the distances between the survival expectations. Negative values may be used to suppress these measurements.
timeCol	endTime	Column name for the survival time
timeOutLimit	36	Cut survival curves after this limit. A negative value can be used to maximize the length of the period.
timeStartCol	startTime	Column name for the start time that is reduced from the survival time
title	Genotype Specific Survival Estimates	Section title

19.556 snpSurvival-survivalSet10-survivalClinical (TableQuery)

Execute an SQL query on input relations and create a result table. TableQuery uses HSQLDB (Hyperthreaded Structured Query Language Database [25]) for executing the query. Consequently, the syntax of the query is defined by HSQLDB.

Author:	Kristian Ovaska (Kristian.Ovaska@Helsinki.FI)
Version:	1.2
Categories:	convert, Filter

Input name	Source	Description
table1	snpSurvival-survivalSet10-clinicalData.in 19.534	CSV table 1. The table is referred to as 'table1' in the SQL query.
query	snpSurvival-survivalSet10-survivalParamsQuery.file 19.557	SQL query. Either this file or the query parameter must be provided, but not both.
columnTypes	snpSurvival-survivalSet10-clinicalTypes.in 19.536	Contains SQL types for individual columns. If the file is not provided, the type is inferred from the contents of the columns. This can be used to force the use of VARCHAR for values that are also valid numerics. The file contains the columns Table (refers to one of table1 to table15), Column (refers to a column name in the table), Type (contains an SQL type). A row with Table='result', Column=X and Type='STRING' forces the use string values for result column X.

Parameter name	Value	Description
numIndices		Comma-separated list of index counts for input tables. All indices are single-column indices, running from column 1 to N, where N is retrieved from this parameter. If empty, the default number (1) is used. For example, ",2,,2" sets two indices for table2 and table4 and one index for the rest.
query		SQL query. Either this parameter or the query input must be provided, but not both.

19.557 snpSurvival-survivalSet10-survivalParamsQuery (SearchReplace)

Replaces the occurences of the given regular expressions with the corresponding regular expressions. This component is used for transforming given patterns of the input file to a changed form specified by the regular expressions .

Author: Marko Laakso (Marko.Laakso@Helsinki.FI)
Version: 1.0
Category: convert

Input name	Source	Description
file	snpSurvival-survivalSet10-survivalParamsTemplate.in 19.558	Source file to be modified

Parameter name	Value	Description
key00	REP.cond00	Search key 0
key01		Search key 1
key02		Search key 2
key03		Search key 3
key04		Search key 4
key05		Search key 5
key06		Search key 6
key07		Search key 7
key08		Search key 8
key09		Search key 9
value00	– No additional restrictions	Replacement 0
value01		Replacement 1
value02		Replacement 2
value03		Replacement 3
value04		Replacement 4
value05		Replacement 5
value06		Replacement 6
value07		Replacement 7
value08		Replacement 8
value09		Replacement 9

19.558 snpSurvival-survivalSet10-survivalParamsTemplate (INPUT)

Select relevant parameters for the survival analysis

Category: Internal

Parameter name	Value	Description
path	/gpfs/homes/mlaakso/asserSVN/andurilCaseStudy/sql/SNPsurvivalParams.sql	Path (filename) of the input resource.

19.559 snpSurvival-survivalSet10-survivalReport (LatexCombiner)

LatexCombiner joins the given subdocuments together into one LaTeX file.

Authors:	Kristian Ovaska (Kristian.Ovaska@Helsinki.FI), Marko Laakso (Marko.Laakso@Helsinki.FI)
Version:	1.3
Category:	Latex

Input name	Source	Description
latex1	snpSurvival-survivalSet10-survival .report 19.555	LaTeX fragment 1.
latex2	snpSurvival-survivalSet10-clinical Select.report 19.535	LaTeX fragment 2.
latex3	snpSurvival-survivalSet10-survSN PAnnot-annotTable.report 19.551	LaTeX fragment 3.
latex4	snpSurvival-survivalSet10-gene Rank-eqTable.report 19.539	LaTeX fragment 4.

Parameter name	Value	Description
pagebreak	true	Determines if the result document should start with a page break.
sectionTitle		If non-empty, a declaration of a new section with the given name is inserted to the beginning of the combined document. This is a convenience feature to make it easy to compile subsections into a section.
sectionType	section	Type of LaTeX section: usually one of section, subsection or subsubsection. No section statement is written if sectionTitle is empty.
strictBorders	true	Enables the flushing of all document elements before each fragment

19.560 snpSurvival-survivalSet2 (SurvivalAnalysis)

No description

Parameter name	Value
pvalue	0.0010

19.561 snpSurvival-survivalSet2-annotSQL (INPUT)

This SQL is used to generate marker annotation report.

Category: Internal

Parameter name	Value	Description
path	/gpfs/homes/mlaakso/asserSVN/andurilCaseStudy/sql/SNPgeneTable.sql	Path (filename) of the input resource.

19.562 snpSurvival-survivalSet2-caseGenotypes (GetGenotypes)

Load and preprocess the genotype calls for the cancer patient samples.

Parameter name	Value
skipFrq	0.1

19.563 snpSurvival-survivalSet2-caseGenotypes-freqCheck (AlleleCounter)

AlleleCounter calculates genotype frequencies for the biallelic markers of the given SNPMatrix. A Pearson's χ^2-test [18] is performed for each marker in order to test if the Hardy-Weinberg equilibrium [9] holds for the population sample.

Author: Marko Laakso (Marko.Laakso@Helsinki.FI)
Version: 1.2
Category: SNP

Input name	Source	Description
genotypes	snpSurvival-survivalSet2-caseGenotypes-genotypes.genotypes 19.565	SNPHelistin genotype output.

Parameter name	Value	Description
dels	false	Accept homozygous deletions as a possible genotype.
naLimit	0.1	Skip markers where one minus call rate exceeds this limit.
skip	0.1	Drop markers with a relative minor allele frequency lower than this limit. Use negative values to inactivate this limit.

19.564 snpSurvival-survivalSet2-caseGenotypes-ftMarkers (CSV2IDList)

Extracts one column from the given CSV file and prints it out without duplicates. The selected column may contain entries with comma separated lists of values in which case *isList* parameter may be used to obtain each value separately.

Output is defined as:

$$ids = \bigcup_{i=1}^{9} \pi_{columnIn_i} \sigma_{regexp_i}(table_i). \qquad (14)$$

Author: Marko Laakso (Marko.Laakso@Helsinki.FI)
Version: 1.3
Category: convert

Input name	Source	Description
table1	snpSurvival-survivalSet2-caseGenotypes-freqCheck.frequencies 19.563	The mandatory input relation

Parameter name	Value	Description
acceptMissing	false	Files with missing columnIn are accepted as empty if this is true.
columnIn	marker	A comma separated list of column names for the IDs of interest in each file. Empty values refer to the first column of the file.
columnOut	marker	Name of the only column of the output list. Empty input refers to the name of the input column.
constants		A comma separated list of values that are always included into the output
isList	false	True if the seleted column contains a comma separated list of values to be splitted
quotation	false	Indicator that can be used to disable quotation of the output values
regexp1		Regular expression for the row filtering in table1. A row is included in the result if this parameter is empty or if values in the given columns match given regular expressions. The parameter has a format COLNAME1=EXPRESSION,COLNAME2=EXPRESSION2 where COLNAMEs are column names in "csv" and EXPRESSIONs are regular expressions using Java syntax. For example, "col=a|b" includes rows where the column col has a value of "a" or "b".
regexp2		Regular expression for the row filtering in table2.
regexp3		Regular expression for the row filtering in table3.
regexp4		Regular expression for the row filtering in table4.
regexp5		Regular expression for the row filtering in table5.
regexp6		Regular expression for the row filtering in table6.
regexp7		Regular expression for the row filtering in table7.
regexp8		Regular expression for the row filtering in table8.
regexp9		Regular expression for the row filtering in table9.

19.565 snpSurvival-survivalSet2-caseGenotypes-genotypes (SNPHelistinReader)

This component can be used to read SNP genotype calls for a set of samples and markers of interest. The genotype data is retrieved from a SNPHelistin database that is defined by the input properties.

Author:	Marko Laakso (Marko.Laakso@Helsinki.FI)
Version:	1.2
Categories:	Data Import, SNP

Input name	Source	Description
sampleNames	snpSurvival-survivalSet2-caseNames.ids 19.567	List of sample names of interest.
markerNames	snpSurvival-markers2.in 19.450	List of SNP identifiers of interest.
config	genotypeDB.in 19.320	Connection parameters for SNPHelistin.

Parameter name	Value	Description
errorSymbol	ERR	Symbol for failed measurements. You may for example use 'NA' value in order consider them equal to missing values.
skipUnknown	true	Ignore unknown samples and do not process them. If the parameter is false then the program fails for these samples.

19.566 snpSurvival-survivalSet2-caseGenotypes-gtypes (SNPHelistinReader)

This component can be used to read SNP genotype calls for a set of samples and markers of interest. The genotype data is retrieved from a SNPHelistin database that is defined by the input properties.

Author:	Marko Laakso (Marko.Laakso@Helsinki.FI)
Version:	1.2
Categories:	Data Import, SNP

Input name	Source	Description
sampleNames	snpSurvival-survivalSet2-caseNames.ids 19.567	List of sample names of interest.
markerNames	snpSurvival-survivalSet2-caseGenotypes-ftMarkers.ids 19.564	List of SNP identifiers of interest.
config	genotypeDB.in 19.320	Connection parameters for SNPHelistin.

Parameter name	Value	Description
errorSymbol	ERR	Symbol for failed measurements. You may for example use 'NA' value in order consider them equal to missing values.
skipUnknown	true	Ignore unknown samples and do not process them. If the parameter is false then the program fails for these samples.

19.567 snpSurvival-survivalSet2-caseNames (CSV2IDList)

Extracts one column from the given CSV file and prints it out without duplicates. The selected column may contain entries with comma separated lists of values in which case *isList* parameter may be used to obtain each value separately.

Output is defined as:

$$ids = \bigcup_{i=1}^{9} \pi_{columnIn_i} \sigma_{regexp_i}(table_i). \quad (15)$$

Author: Marko Laakso (Marko.Laakso@Helsinki.FI)
Version: 1.3
Category: convert

Input name	Source	Description
table1	snpSurvival-survivalSet2-survival Clinical.table 19.590	The mandatory input relation

Parameter name	Value	Description
acceptMissing	false	Files with missing columnIn are accepted as empty if this is true.
columnIn		A comma separated list of column names for the IDs of interest in each file. Empty values refer to the first column of the file.
columnOut	SampleId	Name of the only column of the output list. Empty input refers to the name of the input column.
constants		A comma separated list of values that are always included into the output
isList	false	True if the seleted column contains a comma separated list of values to be splitted
quotation	false	Indicator that can be used to disable quotation of the output values
regexp1		Regular expression for the row filtering in table1. A row is included in the result if this parameter is empty or if values in the given columns match given regular expressions. The parameter has a format COLNAME1=EXPRESSION,COLNAME2=EXPRESSION2 where COLNAMEs are column names in "csv" and EXPRESSIONs are regular expressions using Java syntax. For example, "col=a|b" includes rows where the column col has a value of "a" or "b".
regexp2		Regular expression for the row filtering in table2.
regexp3		Regular expression for the row filtering in table3.
regexp4		Regular expression for the row filtering in table4.
regexp5		Regular expression for the row filtering in table5.
regexp6		Regular expression for the row filtering in table6.
regexp7		Regular expression for the row filtering in table7.
regexp8		Regular expression for the row filtering in table8.
regexp9		Regular expression for the row filtering in table9.

19.568 snpSurvival-survivalSet2-clinicalData (INPUT)

Clinical parameters for the case samples
Category: Internal

Parameter name	Value	Description
path	/gpfs/homes/mlaakso/asserSVN/ andurilCaseStudy/data/snp/ patient-metadata-caucas.csv	Path (filename) of the input resource.

19.569 snpSurvival-survivalSet2-clinicalSelect (SourceCode2Latex)

This component converts a source code listing into a LaTeXfragment that may be included into some technical documents.
Author: Marko Laakso (Marko.Laakso@Helsinki.FI)
Version: 1.0
Categories: convert, Latex

Input name	Source	Description
source	snpSurvival-survivalSet2-survival ParamsQuery.file 19.591	Source code

Parameter name	Value	Description
caption	This SQL query has been used to select clinical parameters for the survival calculations.	Caption text for the listing
language	SQL	Language defintion for the syntax highlighting. Possible values can be found from the description of listings package.

19.570 snpSurvival-survivalSet2-clinicalTypes (INPUT)

Column types for the clinical data

Category: Internal

Parameter name	Value	Description
path	/gpfs/homes/mlaakso/asserSVN/ andurilCaseStudy/csv/ SNPclinicalTypes.csv	Path (filename) of the input resource.

19.571 snpSurvival-survivalSet2-geneRank (QueryReport)

(Technical documentation) Executes an SQL query and prints it with the result table.

Author: Marko Laakso (Marko.Laakso@Helsinki.FI)
Version: 1.1
Category: Latex

Input name	Source	Description
table1	snpSurvival-survivalSet2-survSNPAnnot.fullData 19.583	1. data table that is referred as 'table1' in the query

Parameter name	Value	Description
colFormat	lcrp{11cm}	LaTeX tabular formatting for the result columns
listCols		Comma separated list of column names. Columns of this list may contain several values separated with commas and the delimiters will be replaced with spaces.
resTableRef	the previous table	Textual reference from the SQL representation to the result table
section	Gene rank	Subsection title for the output document
showCols	gene,markers,p-value,description	Comma separated list of column selections for the output. The empty default will use all columns.
showQuery	false	This flag tells if the SQL shall be shown on the report
tableCaption	A list of survival affecting genes and the marker counts. P-value is the lowest value for the corresponding markers.	Description for the result table

19.572 snpSurvival-survivalSet2-geneRank-eqData (TableQuery)

Execute an SQL query on input relations and create a result table. TableQuery uses HSQLDB (Hyper-threaded Structured Query Language Database [25]) for executing the query. Consequently, the syntax of the query is defined by HSQLDB.

Author: Kristian Ovaska (Kristian.Ovaska@Helsinki.FI)
Version: 1.2
Categories: convert, Filter

Input name	Source	Description
table1	snpSurvival-survivalSet2-survSNPAnnot-annotData.table 19.584	CSV table 1. The table is referred to as 'table1' in the SQL query.

Continued on next page...

Input name	Source	Description
table2	snpSurvival-survivalSet2-survival .statistics 19.589	CSV table 2. The table is referred to as 'table2' in the SQL query.
query	snpSurvival-survivalSet2-gene RankQuery.in 19.574	SQL query. Either this file or the query parameter must be provided, but not both.

Parameter name	Value	Description
numIndices		Comma-separated list of index counts for input tables. All indices are single-column indices, running from column 1 to N, where N is retrieved from this parameter. If empty, the default number (1) is used. For example, ",2,,2" sets two indices for table2 and table4 and one index for the rest.
query		SQL query. Either this parameter or the query input must be provided, but not both.

19.573 snpSurvival-survivalSet2-geneRank-eqTable (CSV2Latex)

This component converts a tabular CSV file into a printable LaTeX table.

Author:	Marko Laakso (Marko.Laakso@Helsinki.FI)
Version:	2.1
Categories:	convert, Latex

Input name	Source	Description
tabledata	snpSurvival-survivalSet2-gene Rank-eqData.table 19.572	Table content
refs	snpSurvival-survivalSet2-refsGene Rank.in 19.577	Reference rules for the hyperlinks

Parameter name	Value	Description
attach	false	Include the original data as an attachment
caption	A list of survival affecting genes and the marker counts. P-value is the lowest value for the corresponding markers.	Caption text for the table.
colFormat	lcrp{11cm}	LaTeX tabular format for the columns. Default produces a centered layout for all columns.
columns	gene,markers,p-value,description	Comma separated list of column selections for the output. The empty default will use all columns.
countRows	false	Include a row count to the table caption.
dropMissing	true	This flag can be turned off in order to generate links with missing texts. Link text are substituted with target identifiers.
listCols		Comma separated list of column names. Columns of this list may contain several values separated with commas and the delimiters will be replaced with spaces.
numberFormat		A comma separated list of decimal formats for the columns. Each entry consists of the column name and the Java DecimalFormal pattern separated with equal sign. For example, rounding to three decimals can be done like: myColumn=#0.000
pageBreak	false	Use clear page after the table.
rename		Comma separated list of column renaming rules (oldname=newname). New names are used in table header but they do not affect the other behaviour of this component.
ruler	{}	Latex command for the row separating rulers
section	Gene rank	Section title for the table container or an empty string if no section should be generated.
sectionType	subsection	Type of LaTeX section: usually one of: section, subsection, or subsubsection. No section statement is written if section title is empty.
skipEmpty	false	This flag can be used to replace empty tables with a simple LaTeX comment.

19.574 snpSurvival-survivalSet2-geneRankQuery (INPUT)

Generates a list of genes and the marker counts for them.

Category:	Internal

Parameter name	Value	Description
path	/gpfs/homes/mlaakso/asserSVN/ andurilCaseStudy/sql/ SNPinterestingGenes.sql	Path (filename) of the input resource.

19.575 snpSurvival-survivalSet2-missingDistC (CSV2Latex)

This component converts a tabular CSV file into a printable LaTeX table.

Author: Marko Laakso (Marko.Laakso@Helsinki.FI)
Version: 2.1
Categories: convert, Latex

Input name	Source	Description
tabledata	snpSurvival-survivalSet2-case Genotypes-freqCheck.na Dist 19.563	Table content

Parameter name	Value	Description
attach	false	Include the original data as an attachment
caption	Distribution of the missing values in case samples	Caption text for the table.
colFormat	p{3cm}r	LaTeX tabular format for the columns. Default produces a centered layout for all columns.
columns		Comma separated list of column selections for the output. The empty default will use all columns.
countRows	false	Include a row count to the table caption.
dropMissing	true	This flag can be turned off in order to generate links with missing texts. Link text are substituted with target identifiers.
listCols		Comma separated list of column names. Columns of this list may contain several values separated with commas and the delimiters will be replaced with spaces.
numberFormat		A comma separated list of decimal formats for the columns. Each entry consists of the column name and the Java DecimalFormal pattern separated with equal sign. For example, rounding to three decimals can be done like: myColumn=#0.000
pageBreak	false	Use clear page after the table.
rename		Comma separated list of column renaming rules (oldname=newname). New names are used in table header but they do not affect the other behaviour of this component.
ruler	{}	Latex command for the row separating rulers
section		Section title for the table container or an empty string if no section should be generated.
sectionType	subsection	Type of LaTeX section: usually one of: section, subsection, or subsubsection. No section statement is written if section title is empty.
skipEmpty	false	This flag can be used to replace empty tables with a simple LaTeX comment.

19.576 snpSurvival-survivalSet2-propertiesDoc (Properties2Latex)

Prepares a LaTeX-representation for the given set of properties files. The document consists of tables of names and values of the properties.

Author: Marko Laakso (Marko.Laakso@Helsinki.FI)
Version: 1.2
Categories: convert, Latex

Input name	Source	Description
props1	genotypeDB.in 19.320	A set of properties

Parameter name	Value	Description
hide		A comma separated list of property names that should remain invisible. You may use this paramerter to hide passwords and other confidentian settings.

Continued on next page...

Parameter name	Value	Description
keyWidth	5	Column width for the property names (centimeters)
merge	false	Merge all input properties into a single set
section	SNP database configuration	Section title for the table container or an empty string if no section should be generated
sectionType	subsection	Type of LaTeX section: usually one of section, subsection or subsubsection. No section statement is written if sectionTitle is empty.
valueWidth	13	Column width for the property values (centimeters)

19.577 snpSurvival-survivalSet2-refsGeneRank (INPUT)

Hyperlink rules for the gene rank table

Category: Internal

Parameter name	Value	Description
path	/gpfs/homes/mlaakso/asserSVN/andurilCaseStudy/csv/SNPrefsGeneRank.csv	Path (filename) of the input resource.

19.578 snpSurvival-survivalSet2-refsHits (INPUT)

Hyperlink rules for the marker annotations

Category: Internal

Parameter name	Value	Description
path	/gpfs/homes/mlaakso/asserSVN/andurilCaseStudy/csv/SNPrefsHits.csv	Path (filename) of the input resource.

19.579 snpSurvival-survivalSet2-reportBody (LatexCombiner)

LatexCombiner joins the given subdocuments together into one LaTeX file.

Authors: Kristian Ovaska (Kristian.Ovaska@Helsinki.FI), Marko Laakso (Marko.Laakso@Helsinki.FI)
Version: 1.3
Category: Latex

Input name	Source	Description
latex1	snpSurvival-survivalSet2-survivalReport.document 19.593	LaTeX fragment 1.
latex2	snpSurvival-survivalSet2-propertiesDoc.report 19.576	LaTeX fragment 2.
latex3	snpSurvival-survivalSet2-missingDistC.report 19.575	LaTeX fragment 3.

Parameter name	Value	Description
pagebreak	false	Determines if the result document should start with a page break.
sectionTitle		If non-empty, a declaration of a new section with the given name is inserted to the beginning of the combined document. This is a convenience feature to make it easy to compile subsections into a section.
sectionType	section	Type of LaTeX section: usually one of section, subsection or subsubsection. No section statement is written if sectionTitle is empty.
strictBorders	true	Enables the flushing of all document elements before each fragment

19.580 snpSurvival-survivalSet2-reportTemplate (LatexTemplate)

This component generates the standard layout configuration for Anduril output documents. The component produces a header and a footer fragment that can be used to build LaTeX documents. The header fragment takes care of the page geometry and other technical details such as library imports and function declarations. Reference list is generated by the footer.

Author: Marko Laakso (Marko.Laakso@Helsinki.FI)
Version: 1.4
Category: Latex

Input name	Source	Description
abstract	abstract.in 19.42	Document abstract including its Latex definition. You may use template entities within this document. The default abstract has been defined in abstractTemplate.tex.

Parameter name	Value	Description
authors	Marko Laakso, Riku Louhimo, Sampsa Hautaniemi	A comma-separated list of document authors on the title page. If authors is empty but title is non-empty, a title page with no author name is printed.
baselineskip	1.2	Baselineskip factor that indicates the line spacing
bibstyle	abbrv	BibTeX reference formatting style
printTOC	true	If true, print Table Of Contents.
title	Anduril Case Study: Glioblastoma SNP survival analysis	Document title on the title page. If empty, no title page is printed. Also see author.
usepackage		Comma-separated list of LaTeX packages that are used in the document. Each package generates a \usepackage{X} line in the header.

19.581 snpSurvival-survivalSet2-snpAnnotations (INPUT)

Annotations for survival component.

Category: Internal

Parameter name	Value	Description
path	/gpfs/homes/mlaakso/asserSVN/andurilCaseStudy/csv/SNPAnnotation.csv	Path (filename) of the input resource.

19.582 snpSurvival-survivalSet2-summaryReport (LatexPDF)

This component is a LaTeX compiler that generates a portable document format (PDF) file from the given Anduril document.

Author: Kristian Ovaska (Kristian.Ovaska@Helsinki.FI)
Version: 1.0
Category: Latex

Input name	Source	Description
document	snpSurvival-survivalSet2-reportBody.document 19.579	Body of the LaTeX document.
header	snpSurvival-survivalSet2-reportTemplate.header 19.580	LaTeX header that is written to the start of the document. If missing, a default header is used.
footer	snpSurvival-survivalSet2-reportTemplate.footer 19.580	LaTeX footer that is written to the end of the document. If missing, a default header is used.

Parameter name	Value	Description
bibtexExec	bibtex	Executable command for BibTeX.
latexExec	pdflatex	Executable command for pdflatex.
useRefs	false	This flag can be used to activate BibTeX compiler

19.583 snpSurvival-survivalSet2-survSNPAnnot (AnnotateSNPs)

Biological annotations for the survival affecting genes

Parameter name	Value
caption	Gene annotations and the loci of the survival affecting markers

19.584 snpSurvival-survivalSet2-survSNPAnnot-annotData (TableQuery)

Execute an SQL query on input relations and create a result table. TableQuery uses HSQLDB (Hyperthreaded Structured Query Language Database [25]) for executing the query. Consequently, the syntax of the query is defined by HSQLDB.

Author: Kristian Ovaska (Kristian.Ovaska@Helsinki.FI)
Version: 1.2
Categories: convert, Filter

Input name	Source	Description
table1	snpSurvival-survivalSet2-survSNPAnnot-snpRel.relation 19.588	CSV table 1. The table is referred to as 'table1' in the SQL query.
table2	snpSurvival-survivalSet2-survSNPAnnot-genes.bioAnnotation 19.586	CSV table 2. The table is referred to as 'table2' in the SQL query.
table3	snpSurvival-survivalSet2-survival.statistics 19.589	CSV table 3. The table is referred to as 'table3' in the SQL query.
query	snpSurvival-survivalSet2-annotSQL.in 19.561	SQL query. Either this file or the query parameter must be provided, but not both.

Parameter name	Value	Description
numIndices		Comma-separated list of index counts for input tables. All indices are single-column indices, running from column 1 to N, where N is retrieved from this parameter. If empty, the default number (1) is used. For example, ",2,,2" sets two indices for table2 and table4 and one index for the rest.
query		SQL query. Either this parameter or the query input must be provided, but not both.

19.585 snpSurvival-survivalSet2-survSNPAnnot-annotTable (CSV2Latex)

This component converts a tabular CSV file into a printable LaTeX table.

Author: Marko Laakso (Marko.Laakso@Helsinki.FI)
Version: 2.1
Categories: convert, Latex

Input name	Source	Description
tabledata	snpSurvival-survivalSet2-survSNPAnnot-annotData.table 19.584	Table content
refs	snpSurvival-survivalSet2-refsHits.in 19.578	Reference rules for the hyperlinks

Continued on next page...

Parameter name Value Description

Parameter name	Value	Description
attach	false	Include the original data as an attachment
caption	Gene annotations and the loci of the survival affecting markers	Caption text for the table.
colFormat	l@{}lr@{:}lp{1cm}p{1.5cm}p{10.5cm}	LaTeX tabular format for the columns. Default produces a centered layout for all columns.
columns	hit,marker,chr,locus,band,gene, gene description	Comma separated list of column selections for the output. The empty default will use all columns.
countRows	false	Include a row count to the table caption.
dropMissing	true	This flag can be turned off in order to generate links with missing texts. Link text are substituted with target identifiers.
listCols	band	Comma separated list of column names. Columns of this list may contain several values separated with commas and the delimiters will be replaced with spaces.
numberFormat		A comma separated list of decimal formats for the columns. Each entry consists of the column name and the Java DecimalFormal pattern separated with equal sign. For example, rounding to three decimals can be done like: myColumn=#0.000
pageBreak	false	Use clear page after the table.
rename		Comma separated list of column renaming rules (oldname=newname). New names are used in table header but they do not affect the other behaviour of this component.
ruler	{}	Latex command for the row separating rulers
section		Section title for the table container or an empty string if no section should be generated.
sectionType	subsection	Type of LaTeX section: usually one of: section, subsection, or subsubsection. No section statement is written if section title is empty.
skipEmpty	false	This flag can be used to replace empty tables with a simple LaTeX comment.

19.586 snpSurvival-survivalSet2-survSNPAnnot-genes (KorvasieniAnnotator)

Korvasieni is an Ensembl [12] based converter for database identifiers and it can be used to convert genome location, gene, transcript, and translation identifiers across multiple biological databases. The selected `keyColumn` may contain entries with comma separated lists of values in which case `isListKey` parameter may be used to obtain each value separately and to process them as they were given in consecutive lines.

Author: Marko Laakso (Marko.Laakso@Helsinki.FI)
Version: 1.7
Categories: Annotation, GO

Input name	Source	Description
sourceKeys	snpSurvival-survivalSet2-survSNPAnnot-snpLoci. annotations 19.587	A list of source database keys.
connection	ensembl.in 19.273	Database connection can be defined using this file. The definition of parameters: database.url, database.user, database.password, database.timeout, database.recycle, and database.driver can be found from the documentation of Korvasieni.

Parameter name	Value	Description
echoColumns	refsnp_id	A comma separated list of column names for the columns that will be copied to the output. An asterisk (*) can be used to denote all columns execpt the keyColumn.
inputDB	.GeneId	Type of input keys. This must be a database supported by Korvasieni. If the parameter is omitted, the component tries to derive the database from the type of geneID. If this is not possible, an error is returned. You may define three columns in form of chromosome:start-end in case the inputDB is .DNARegion. This format provides a comfortable compatibility with DNARegion datatype.
inputType	Gene	Ensembl object type for the input keys (Any, Gene, Transcript, Translation)
isListKey	true	Enables the automatic value splits for the comma separated key column
keyColumn	ensembl_gene_stable_id	Name of the key column withing sourceKeys file or an empty string for the first column
maxHits	100000	Maximum number of target identifiers for a single source identifier
rename	UniProt/SwissProt=protein	Comma separated list of column renaming rules (oldname=newname)
skipLevel	never	Skip result rows if the source identifier is unknown or target identifiers are not available. Possible values are: never (no filtering), source (skip if the source ID is unknown), target (skip if no target IDs are found), any (skip if any of the target IDs is missing).

Continued on next page...

Parameter name	Value	Description
targetDB	.GeneName,.GeneDesc,.DNABand,GO,UniProt/SwissProt	Comma-separated list of annotation types. Possible values are all databases supported by Korvasieni.
unique	true	This flag can be turned on in order to eliminate duplicate annotations.

19.587 snpSurvival-survivalSet2-survSNPAnnot-snpLoci (RefSNPAnnotator)

RefSNPAnnotator fetches annotations for given refSNP ID's (also known as rsID's) from BioMart databases.

The provided annotations for each rsID include alleles for the SNP, name of the chromosome where the SNP is located, chromosome location of the SNP, the strand of the chromosome, Ensembl gene ID for genes that overlap the SNP's location and validation status of the SNP. The component is implemented using the biomaRt package for Bioconductor [7].

Authors: Viljami Aittomaki (Viljami.Aittomaki@Helsinki.FI), Marko Laakso (Marko.Laakso@Helsinki.FI)
Version: 1.0.1
Categories: SNP, Annotation

Input name	Source	Description
rsIDs	snpSurvival-survivalSet2-survival.statistics 19.589	List of rsID's for fetching annotations.

Parameter name	Value	Description
dataset	hsapiens_snp	Dataset to get annotations from. Different species have their own datasets. Example values include "hsapiens_snp" and "mmusculus_snp". See biomaRt documentation on how to list available datasets in R.
keyColumn	marker	Name of the key column withing sourceKeys file or an empty string for the first column.

19.588 snpSurvival-survivalSet2-survSNPAnnot-snpRel (ExpandCollapse)

Converts between the two possible representations of a relations with multivalued columns. By default this component splits rows with multivalued (comma separated lists) columns into multiple rows each representing a single value. The input may contain several columns with comma separated values and all permutations of column values are shown as individual single-valued rows. The alternative mode recovers the original comma separated values from the expanded relations. This is performed by joining values of the their list columns into comma separated lists.

Author: Marko Laakso (Marko.Laakso@Helsinki.FI)
Version: 1.3
Category: convert

Input name	Source	Description
relation	snpSurvival-survivalSet2-survSNPAnnot-snpLoci.annotations 19.587	The mandatory input relation

Parameter name	Value	Description
delim	,	Value delimiter between the list column values. Special characters can be encoded as specified in fi.helsinki.ltdk.csbl.asser.ArgumentEncoding.
expand	true	Action mode that is expand (true) or collapse (false)
listCols	ensembl_gene_stable_id	A comma separated list of column names of input (expand) or output (collapse) columns that may contain delim separated values. The asterisk refers to every column of the input relation.

19.589 snpSurvival-survivalSet2-survival (SNPKaplanMeier)

This component is used to estimate survival differences between various genotypes. The differences between the survival of different genotypes are measured using G-ρ family of tests [10].

Author: Marko Laakso (Marko.Laakso@Helsinki.FI)
Version: 1.5
Categories: SNP, Latex, Survival

Input name	Source	Description
genotypes	snpSurvival-survivalSet2-caseGenotypes-gtypes.genotypes 19.566	Genotype calls
survival	snpSurvival-survivalSet2-survivalClinical.table 19.590	Survival time and event indicator
annotations	snpSurvival-survivalSet2-snpAnnotations.in 19.581	Additional information about the markers. This data will be shown on the report of interesting markers.

Parameter name	Value	Description
PLimit	0.0010	Ignore results with a p-value greater than this limit.
confInt	0.95	Coverage of the confidence intervals [0,1]
minCount	15	Combine rare (aa) homozygotes to heterozygotes (Aa) if their number is below this limit. This parameter is used together with minFreq so that the actual limit is max(minFreq*n, minCount).
minFreq	0.1	Combine rare (aa) homozygotes to heterozygotes (Aa) if their frequency is below this limit. See also minCount.
naLimit	0.1	Skip markers where one minus call rate exceeds this limit.
nullCalls	–,ERR	A comma separated list of genotype calls that will be considered as missing values
sampleCol	sample	Column name for the sample name
showCI	true	Draw confidence intervals to the graph
skipMissingGT	true	Simply skip those samples that lack the genotype information.
statusCol	hasEvent	Column name for the event indicator
survRatePoint	0.5	Sampling height for the distances between the survival expectations. Negative values may be used to suppress these measurements.
timeCol	endTime	Column name for the survival time
timeOutLimit	36	Cut survival curves after this limit. A negative value can be used to maximize the length of the period.
timeStartCol	startTime	Column name for the start time that is reduced from the survival time
title	Genotype Specific Survival Estimates	Section title

19.590 snpSurvival-survivalSet2-survivalClinical (TableQuery)

Execute an SQL query on input relations and create a result table. TableQuery uses HSQLDB (Hyper-threaded Structured Query Language Database [25]) for executing the query. Consequently, the syntax of the query is defined by HSQLDB.

Author: Kristian Ovaska (Kristian.Ovaska@Helsinki.FI)
Version: 1.2
Categories: convert, Filter

Input name	Source	Description
table1	snpSurvival-survivalSet2-clinicalData.in 19.568	CSV table 1. The table is referred to as 'table1' in the SQL query.
query	snpSurvival-survivalSet2-survivalParamsQuery.file 19.591	SQL query. Either this file or the query parameter must be provided, but not both.
columnTypes	snpSurvival-survivalSet2-clinicalTypes.in 19.570	Contains SQL types for individual columns. If the file is not provided, the type is inferred from the contents of the columns. This can be used to force the use of VARCHAR for values that are also valid numerics. The file contains the columns Table (refers to one of table1 to table15), Column (refers to a column name in the table), Type (contains an SQL type). A row with Table='result', Column=X and Type='STRING' forces the use string values for result column X.

Parameter name	Value	Description
numIndices		Comma-separated list of index counts for input tables. All indices are single-column indices, running from column 1 to N, where N is retrieved from this parameter. If empty, the default number (1) is used. For example, ",2,,2" sets two indices for table2 and table4 and one index for the rest.
query		SQL query. Either this parameter or the query input must be provided, but not both.

19.591 snpSurvival-survivalSet2-survivalParamsQuery (SearchReplace)

Replaces the occurences of the given regular expressions with the corresponding regular expressions. This component is used for transforming given patterns of the input file to a changed form specified by the regular expressions .

Author: Marko Laakso (Marko.Laakso@Helsinki.FI)
Version: 1.0
Category: convert

Input name	Source	Description
file	snpSurvival-survivalSet2-survivalParamsTemplate.in 19.592	Source file to be modified

Parameter name	Value	Description
key00	REP.cond00	Search key 0
key01		Search key 1
key02		Search key 2
key03		Search key 3
key04		Search key 4
key05		Search key 5
key06		Search key 6
key07		Search key 7
key08		Search key 8
key09		Search key 9
value00	– No additional restrictions	Replacement 0
value01		Replacement 1
value02		Replacement 2
value03		Replacement 3
value04		Replacement 4
value05		Replacement 5
value06		Replacement 6
value07		Replacement 7
value08		Replacement 8
value09		Replacement 9

19.592 snpSurvival-survivalSet2-survivalParamsTemplate (INPUT)

Select relevant parameters for the survival analysis

Category: Internal

Parameter name	Value	Description
path	/gpfs/homes/mlaakso/asserSVN/andurilCaseStudy/sql/SNPsurvivalParams.sql	Path (filename) of the input resource.

19.593 snpSurvival-survivalSet2-survivalReport (LatexCombiner)

LatexCombiner joins the given subdocuments together into one LaTeX file.

Authors:	Kristian Ovaska (Kristian.Ovaska@Helsinki.FI), Marko Laakso (Marko.Laakso@Helsinki.FI)
Version:	1.3
Category:	Latex

Input name	Source	Description
latex1	snpSurvival-survivalSet2-survival.report 19.589	LaTeX fragment 1.
latex2	snpSurvival-survivalSet2-clinicalSelect.report 19.569	LaTeX fragment 2.
latex3	snpSurvival-survivalSet2-survSNPAnnot-annotTable.report 19.585	LaTeX fragment 3.
latex4	snpSurvival-survivalSet2-geneRank-eqTable.report 19.573	LaTeX fragment 4.

Parameter name	Value	Description
pagebreak	true	Determines if the result document should start with a page break.
sectionTitle		If non-empty, a declaration of a new section with the given name is inserted to the beginning of the combined document. This is a convenience feature to make it easy to compile subsections into a section.
sectionType	section	Type of LaTeX section: usually one of section, subsection or subsubsection. No section statement is written if sectionTitle is empty.
strictBorders	true	Enables the flushing of all document elements before each fragment

19.594 snpSurvival-survivalSet3 (SurvivalAnalysis)

No description

Parameter name	Value
pvalue	0.0010

19.595 snpSurvival-survivalSet3-annotSQL (INPUT)

This SQL is used to generate marker annotation report.

Category: Internal

Parameter name	Value	Description
path	/gpfs/homes/mlaakso/asserSVN/andurilCaseStudy/sql/SNPgeneTable.sql	Path (filename) of the input resource.

19.596 snpSurvival-survivalSet3-caseGenotypes (GetGenotypes)

Load and preprocess the genotype calls for the cancer patient samples.

Parameter name	Value
skipFrq	0.1

19.597 snpSurvival-survivalSet3-caseGenotypes-freqCheck (AlleleCounter)

AlleleCounter calculates genotype frequencies for the biallelic markers of the given SNPMatrix. A Pearson's χ^2-test [18] is performed for each marker in order to test if the Hardy-Weinberg equilibrium [9] holds for the population sample.

Author:	Marko Laakso (Marko.Laakso@Helsinki.FI)
Version:	1.2
Category:	SNP

Input name	Source	Description
genotypes	snpSurvival-survivalSet3-caseGenotypes-genotypes.genotypes 19.599	SNPHelistin genotype output.

Parameter name	Value	Description
dels	false	Accept homozygous deletions as a possible genotype.
naLimit	0.1	Skip markers where one minus call rate exceeds this limit.
skip	0.1	Drop markers with a relative minor allele frequency lower than this limit. Use negative values to inactivate this limit.

19.598 snpSurvival-survivalSet3-caseGenotypes-ftMarkers (CSV2IDList)

Extracts one column from the given CSV file and prints it out without duplicates. The selected column may contain entries with comma separated lists of values in which case *isList* parameter may be used to obtain each value separately.

Output is defined as:

$$ids = \bigcup_{i=1}^{9} \pi_{columnIn_i} \sigma_{regexp_i}(table_i). \quad (16)$$

Author:	Marko Laakso (Marko.Laakso@Helsinki.FI)
Version:	1.3
Category:	convert

Input name	Source	Description
table1	snpSurvival-survivalSet3-caseGenotypes-freqCheck.frequencies 19.597	The mandatory input relation

Parameter name	Value	Description
acceptMissing	false	Files with missing columnIn are accepted as empty if this is true.
columnIn	marker	A comma separated list of column names for the IDs of interest in each file. Empty values refer to the first column of the file.
columnOut	marker	Name of the only column of the output list. Empty input refers to the name of the input column.
constants		A comma separated list of values that are always included into the output
isList	false	True if the seleted column contains a comma separated list of values to be splitted
quotation	false	Indicator that can be used to disable quotation of the output values
regexp1		Regular expression for the row filtering in table1. A row is included in the result if this parameter is empty or if values in the given columns match given regular expressions. The parameter has a format COLNAME1=EXPRESSION,COLNAME2=EXPRESSION2 where COLNAMEs are column names in ”csv” and EXPRESSIONs are regular expressions using Java syntax. For example, ”col=a|b” includes rows where the column col has a value of ”a” or ”b”.
regexp2		Regular expression for the row filtering in table2.
regexp3		Regular expression for the row filtering in table3.
regexp4		Regular expression for the row filtering in table4.
regexp5		Regular expression for the row filtering in table5.
regexp6		Regular expression for the row filtering in table6.
regexp7		Regular expression for the row filtering in table7.
regexp8		Regular expression for the row filtering in table8.
regexp9		Regular expression for the row filtering in table9.

19.599 snpSurvival-survivalSet3-caseGenotypes-genotypes (SNPHelistinReader)

This component can be used to read SNP genotype calls for a set of samples and markers of interest. The genotype data is retrieved from a SNPHelistin database that is defined by the input properties.

Author:	Marko Laakso (Marko.Laakso@Helsinki.FI)
Version:	1.2
Categories:	Data Import, SNP

Input name	Source	Description
sampleNames	snpSurvival-survivalSet3-caseNames.ids 19.601	List of sample names of interest.
markerNames	snpSurvival-markers3.in 19.451	List of SNP identifiers of interest.
config	genotypeDB.in 19.320	Connection parameters for SNPHelistin.

Parameter name	Value	Description
errorSymbol	ERR	Symbol for failed measurements. You may for example use 'NA' value in order consider them equal to missing values.
skipUnknown	true	Ignore unknown samples and do not process them. If the parameter is false then the program fails for these samples.

19.600 snpSurvival-survivalSet3-caseGenotypes-gtypes (SNPHelistinReader)

This component can be used to read SNP genotype calls for a set of samples and markers of interest. The genotype data is retrieved from a SNPHelistin database that is defined by the input properties.

Author:	Marko Laakso (Marko.Laakso@Helsinki.FI)
Version:	1.2
Categories:	Data Import, SNP

Input name	Source	Description
sampleNames	snpSurvival-survivalSet3-caseNames.ids 19.601	List of sample names of interest.
markerNames	snpSurvival-survivalSet3-caseGenotypes-ftMarkers.ids 19.598	List of SNP identifiers of interest.
config	genotypeDB.in 19.320	Connection parameters for SNPHelistin.

Parameter name	Value	Description
errorSymbol	ERR	Symbol for failed measurements. You may for example use 'NA' value in order consider them equal to missing values.
skipUnknown	true	Ignore unknown samples and do not process them. If the parameter is false then the program fails for these samples.

19.601 snpSurvival-survivalSet3-caseNames (CSV2IDList)

Extracts one column from the given CSV file and prints it out without duplicates. The selected column may contain entries with comma separated lists of values in which case *isList* parameter may be used to obtain each value separately.

Output is defined as:

$$ids = \bigcup_{i=1}^{9} \pi_{columnIn_i} \sigma_{regexp_i}(table_i). \tag{17}$$

Author: Marko Laakso (Marko.Laakso@Helsinki.FI)
Version: 1.3
Category: convert

Input name	Source	Description
table1	snpSurvival-survivalSet3-survival Clinical.table 19.624	The mandatory input relation

Parameter name	Value	Description
acceptMissing	false	Files with missing columnIn are accepted as empty if this is true.
columnIn		A comma separated list of column names for the IDs of interest in each file. Empty values refer to the first column of the file.
columnOut	SampleId	Name of the only column of the output list. Empty input refers to the name of the input column.
constants		A comma separated list of values that are always included into the output
isList	false	True if the seleted column contains a comma separated list of values to be splitted
quotation	false	Indicator that can be used to disable quotation of the output values
regexp1		Regular expression for the row filtering in table1. A row is included in the result if this parameter is empty or if values in the given columns match given regular expressions. The parameter has a format COLNAME1=EXPRESSION,COLNAME2=EXPRESSION2 where COLNAMEs are column names in "csv" and EXPRESSIONs are regular expressions using Java syntax. For example, "col=a|b" includes rows where the column col has a value of "a" or "b".
regexp2		Regular expression for the row filtering in table2.
regexp3		Regular expression for the row filtering in table3.
regexp4		Regular expression for the row filtering in table4.
regexp5		Regular expression for the row filtering in table5.
regexp6		Regular expression for the row filtering in table6.
regexp7		Regular expression for the row filtering in table7.
regexp8		Regular expression for the row filtering in table8.
regexp9		Regular expression for the row filtering in table9.

19.602 snpSurvival-survivalSet3-clinicalData (INPUT)

Clinical parameters for the case samples
Category: Internal

Parameter name	Value	Description
path	/gpfs/homes/mlaakso/asserSVN/ andurilCaseStudy/data/snp/ patient-metadata-caucas.csv	Path (filename) of the input resource.

19.603 snpSurvival-survivalSet3-clinicalSelect (SourceCode2Latex)

This component converts a source code listing into a LaTeXfragment that may be included into some technical documents.
Author: Marko Laakso (Marko.Laakso@Helsinki.FI)
Version: 1.0
Categories: convert, Latex

Input name	Source	Description
source	snpSurvival-survivalSet3-survival ParamsQuery.file 19.625	Source code

Parameter name	Value	Description
caption	This SQL query has been used to select clinical parameters for the survival calculations.	Caption text for the listing
language	SQL	Language defintion for the syntax highlighting. Possible values can be found from the description of listings package.

19.604 snpSurvival-survivalSet3-clinicalTypes (INPUT)

Column types for the clinical data

Category: Internal

Parameter name	Value	Description
path	/gpfs/homes/mlaakso/asserSVN/ andurilCaseStudy/csv/ SNPclinicalTypes.csv	Path (filename) of the input resource.

19.605 snpSurvival-survivalSet3-geneRank (QueryReport)

(Technical documentation) Executes an SQL query and prints it with the result table.

Author: Marko Laakso (Marko.Laakso@Helsinki.FI)
Version: 1.1
Category: Latex

Input name	Source	Description
table1	snpSurvival-survivalSet3-survSNPAnnot.fullData 19.617	1. data table that is referred as 'table1' in the query

Parameter name	Value	Description
colFormat	lcrp{11cm}	LaTeX tabular formatting for the result columns
listCols		Comma separated list of column names. Columns of this list may contain several values separated with commas and the delimiters will be replaced with spaces.
resTableRef	the previous table	Textual reference from the SQL representation to the result table
section	Gene rank	Subsection title for the output document
showCols	gene,markers,p-value,description	Comma separated list of column selections for the output. The empty default will use all columns.
showQuery	false	This flag tells if the SQL shall be shown on the report
tableCaption	A list of survival affecting genes and the marker counts. P-value is the lowest value for the corresponding markers.	Description for the result table

19.606 snpSurvival-survivalSet3-geneRank-eqData (TableQuery)

Execute an SQL query on input relations and create a result table. TableQuery uses HSQLDB (Hyperthreaded Structured Query Language Database [25]) for executing the query. Consequently, the syntax of the query is defined by HSQLDB.

Author: Kristian Ovaska (Kristian.Ovaska@Helsinki.FI)
Version: 1.2
Categories: convert, Filter

Input name	Source	Description
table1	snpSurvival-survivalSet3-survSNPAnnot-annotData.table 19.618	CSV table 1. The table is referred to as 'table1' in the SQL query.

Continued on next page...

Input name	Source	Description
table2	snpSurvival-survivalSet3-survival .statistics 19.623	CSV table 2. The table is referred to as 'table2' in the SQL query.
query	snpSurvival-survivalSet3-gene RankQuery.in 19.608	SQL query. Either this file or the query parameter must be provided, but not both.

Parameter name	Value	Description
numIndices		Comma-separated list of index counts for input tables. All indices are single-column indices, running from column 1 to N, where N is retrieved from this parameter. If empty, the default number (1) is used. For example, ",2,,2" sets two indices for table2 and table4 and one index for the rest.
query		SQL query. Either this parameter or the query input must be provided, but not both.

19.607 snpSurvival-survivalSet3-geneRank-eqTable (CSV2Latex)

This component converts a tabular CSV file into a printable LaTeX table.

Author: Marko Laakso (Marko.Laakso@Helsinki.FI)
Version: 2.1
Categories: convert, Latex

Input name	Source	Description
tabledata	snpSurvival-survivalSet3-gene Rank-eqData.table 19.606	Table content
refs	snpSurvival-survivalSet3-refsGene Rank.in 19.611	Reference rules for the hyperlinks

Parameter name	Value	Description
attach	false	Include the original data as an attachment
caption	A list of survival affecting genes and the marker counts. P-value is the lowest value for the corresponding markers.	Caption text for the table.
colFormat	lcrp{11cm}	LaTeX tabular format for the columns. Default produces a centered layout for all columns.
columns	gene,markers,p-value,description	Comma separated list of column selections for the output. The empty default will use all columns.
countRows	false	Include a row count to the table caption.
dropMissing	true	This flag can be turned off in order to generate links with missing texts. Link text are substituted with target identifiers.
listCols		Comma separated list of column names. Columns of this list may contain several values separated with commas and the delimiters will be replaced with spaces.
numberFormat		A comma separated list of decimal formats for the columns. Each entry consists of the column name and the Java DecimalFormal pattern separated with equal sign. For example, rounding to three decimals can be done like: myColumn=#0.000
pageBreak	false	Use clear page after the table.
rename		Comma separated list of column renaming rules (oldname=newname). New names are used in table header but they do not affect the other behaviour of this component.
ruler	{}	Latex command for the row separating rulers
section	Gene rank	Section title for the table container or an empty string if no section should be generated.
sectionType	subsection	Type of LaTeX section: usually one of: section, subsection, or subsubsection. No section statement is written if section title is empty.
skipEmpty	false	This flag can be used to replace empty tables with a simple LaTeX comment.

19.608 snpSurvival-survivalSet3-geneRankQuery (INPUT)

Generates a list of genes and the marker counts for them.

Category: Internal

Parameter name	Value	Description
path	/gpfs/homes/mlaakso/asserSVN/anduriICaseStudy/sql/SNPinterestingGenes.sql	Path (filename) of the input resource.

19.609 snpSurvival-survivalSet3-missingDistC (CSV2Latex)

This component converts a tabular CSV file into a printable LATEX table.

Author:	Marko Laakso (Marko.Laakso@Helsinki.FI)
Version:	2.1
Categories:	convert, Latex

Input name	Source	Description
tabledata	snpSurvival-survivalSet3-caseGenotypes-freqCheck.naDist 19.597	Table content

Parameter name	Value	Description
attach	false	Include the original data as an attachment
caption	Distribution of the missing values in case samples	Caption text for the table.
colFormat	p{3cm}r	LaTeX tabular format for the columns. Default produces a centered layout for all columns.
columns		Comma separated list of column selections for the output. The empty default will use all columns.
countRows	false	Include a row count to the table caption.
dropMissing	true	This flag can be turned off in order to generate links with missing texts. Link text are substituted with target identifiers.
listCols		Comma separated list of column names. Columns of this list may contain several values separated with commas and the delimiters will be replaced with spaces.
numberFormat		A comma separated list of decimal formats for the columns. Each entry consists of the column name and the Java DecimalFormal pattern separated with equal sign. For example, rounding to three decimals can be done like: myColumn=#0.000
pageBreak	false	Use clear page after the table.
rename		Comma separated list of column renaming rules (oldname=newname). New names are used in table header but they do not affect the other behaviour of this component.
ruler	{}	Latex command for the row separating rulers
section		Section title for the table container or an empty string if no section should be generated.
sectionType	subsection	Type of LaTeX section: usually one of: section, subsection, or subsubsection. No section statement is written if section title is empty.
skipEmpty	false	This flag can be used to replace empty tables with a simple LaTeX comment.

19.610 snpSurvival-survivalSet3-propertiesDoc (Properties2Latex)

Prepares a LATEX-representation for the given set of properties files. The document consists of tables of names and values of the properties.

Author:	Marko Laakso (Marko.Laakso@Helsinki.FI)
Version:	1.2
Categories:	convert, Latex

Input name	Source	Description
props1	genotypeDB.in 19.320	A set of properties

Parameter name	Value	Description
hide		A comma separated list of property names that should remain invisible. You may use this paramerter to hide passwords and other confidentian settings.

Continued on next page...

Parameter name	Value	Description
keyWidth	5	Column width for the property names (centimeters)
merge	false	Merge all input properties into a single set
section	SNP database configuration	Section title for the table container or an empty string if no section should be generated
sectionType	subsection	Type of LaTeX section: usually one of section, subsection or subsubsection. No section statement is written if sectionTitle is empty.
valueWidth	13	Column width for the property values (centimeters)

19.611 snpSurvival-survivalSet3-refsGeneRank (INPUT)

Hyperlink rules for the gene rank table

Category: Internal

Parameter name	Value	Description
path	/gpfs/homes/mlaakso/asserSVN/anduril CaseStudy/csv/ SNPrefsGeneRank.csv	Path (filename) of the input resource.

19.612 snpSurvival-survivalSet3-refsHits (INPUT)

Hyperlink rules for the marker annotations

Category: Internal

Parameter name	Value	Description
path	/gpfs/homes/mlaakso/asserSVN/ andurilCaseStudy/csv/ SNPrefsHits.csv	Path (filename) of the input resource.

19.613 snpSurvival-survivalSet3-reportBody (LatexCombiner)

LatexCombiner joins the given subdocuments together into one LaTeX file.

Authors: Kristian Ovaska (Kristian.Ovaska@Helsinki.FI), Marko Laakso (Marko.Laakso@Helsinki.FI)
Version: 1.3
Category: Latex

Input name	Source	Description
latex1	snpSurvival-survivalSet3-survival Report.document 19.627	LaTeX fragment 1.
latex2	snpSurvival-survivalSet3 -propertiesDoc.report 19.610	LaTeX fragment 2.
latex3	snpSurvival-survivalSet3-missing DistC.report 19.609	LaTeX fragment 3.

Parameter name	Value	Description
pagebreak	false	Determines if the result document should start with a page break.
sectionTitle		If non-empty, a declaration of a new section with the given name is inserted to the beginning of the combined document. This is a convenience feature to make it easy to compile subsections into a section.
sectionType	section	Type of LaTeX section: usually one of section, subsection or subsubsection. No section statement is written if sectionTitle is empty.
strictBorders	true	Enables the flushing of all document elements before each fragment

19.614 snpSurvival-survivalSet3-reportTemplate (LatexTemplate)

This component generates the standard layout configuration for Anduril output documents. The component produces a header and a footer fragment that can be used to build LaTeX documents. The header fragment takes care of the page geometry and other technical details such as library imports and function declarations. Reference list is generated by the footer.

Author: Marko Laakso (Marko.Laakso@Helsinki.FI)
Version: 1.4
Category: Latex

Input name	Source	Description
abstract	abstract.in 19.42	Document abstract including its Latex definition. You may use template entities within this document. The default abstract has been defined in abstractTemplate.tex.

Parameter name	Value	Description
authors	Marko Laakso, Riku Louhimo, Sampsa Hautaniemi	A comma-separated list of document authors on the title page. If authors is empty but title is non-empty, a title page with no author name is printed.
baselineskip	1.2	Baselineskip factor that indicates the line spacing
bibstyle	abbrv	BibTeX reference formatting style
printTOC	true	If true, print Table Of Contents.
title	Anduril Case Study: Glioblastoma SNP survival analysis	Document title on the title page. If empty, no title page is printed. Also see author.
usepackage		Comma-separated list of LaTeX packages that are used in the document. Each package generates a \usepackage{X} line in the header.

19.615 snpSurvival-survivalSet3-snpAnnotations (INPUT)

Annotations for survival component.

Category: Internal

Parameter name	Value	Description
path	/gpfs/homes/mlaakso/asserSVN/andurilCaseStudy/csv/SNPAnnotation.csv	Path (filename) of the input resource.

19.616 snpSurvival-survivalSet3-summaryReport (LatexPDF)

This component is a LaTeX compiler that generates a portable document format (PDF) file from the given Anduril document.

Author: Kristian Ovaska (Kristian.Ovaska@Helsinki.FI)
Version: 1.0
Category: Latex

Input name	Source	Description
document	snpSurvival-survivalSet3-reportBody.document 19.613	Body of the LaTeX document.
header	snpSurvival-survivalSet3-reportTemplate.header 19.614	LaTeX header that is written to the start of the document. If missing, a default header is used.
footer	snpSurvival-survivalSet3-reportTemplate.footer 19.614	LaTeX footer that is written to the end of the document. If missing, a default header is used.

Parameter name	Value	Description
bibtexExec	bibtex	Executable command for BibTeX.
latexExec	pdflatex	Executable command for pdflatex.
useRefs	false	This flag can be used to activate BibTeX compiler

19.617 snpSurvival-survivalSet3-survSNPAnnot (AnnotateSNPs)

Biological annotations for the survival affecting genes

Parameter name	Value
caption	Gene annotations and the loci of the survival affecting markers

19.618 snpSurvival-survivalSet3-survSNPAnnot-annotData (TableQuery)

Execute an SQL query on input relations and create a result table. TableQuery uses HSQLDB (Hyper-threaded Structured Query Language Database [25]) for executing the query. Consequently, the syntax of the query is defined by HSQLDB.

Author: Kristian Ovaska (Kristian.Ovaska@Helsinki.FI)
Version: 1.2
Categories: convert, Filter

Input name	Source	Description
table1	snpSurvival-survivalSet3-survSNPAnnot-snpRel.relation 19.622	CSV table 1. The table is referred to as 'table1' in the SQL query.
table2	snpSurvival-survivalSet3-survSNPAnnot-genes.bioAnnotation 19.620	CSV table 2. The table is referred to as 'table2' in the SQL query.
table3	snpSurvival-survivalSet3-survival.statistics 19.623	CSV table 3. The table is referred to as 'table3' in the SQL query.
query	snpSurvival-survivalSet3-annotSQL.in 19.595	SQL query. Either this file or the query parameter must be provided, but not both.

Parameter name	Value	Description
numIndices		Comma-separated list of index counts for input tables. All indices are single-column indices, running from column 1 to N, where N is retrieved from this parameter. If empty, the default number (1) is used. For example, ",2,,2" sets two indices for table2 and table4 and one index for the rest.
query		SQL query. Either this parameter or the query input must be provided, but not both.

19.619 snpSurvival-survivalSet3-survSNPAnnot-annotTable (CSV2Latex)

This component converts a tabular CSV file into a printable LaTeX table.

Author: Marko Laakso (Marko.Laakso@Helsinki.FI)
Version: 2.1
Categories: convert, Latex

Input name	Source	Description
tabledata	snpSurvival-survivalSet3-survSNPAnnot-annotData.table 19.618	Table content
refs	snpSurvival-survivalSet3-refsHits.in 19.612	Reference rules for the hyperlinks

Continued on next page...

Parameter name	Value	Description

Parameter name	Value	Description
attach	false	Include the original data as an attachment
caption	Gene annotations and the loci of the survival affecting markers	Caption text for the table.
colFormat	l@{}lr@{:}lp{1cm}p{1.5cm}p{10 .5cm}	LaTeX tabular format for the columns. Default produces a centered layout for all columns.
columns	hit,marker,chr,locus,band,gene, gene description	Comma separated list of column selections for the output. The empty default will use all columns.
countRows	false	Include a row count to the table caption.
dropMissing	true	This flag can be turned off in order to generate links with missing texts. Link text are substituted with target identifiers.
listCols	band	Comma separated list of column names. Columns of this list may contain several values separated with commas and the delimiters will be replaced with spaces.
numberFormat		A comma separated list of decimal formats for the columns. Each entry consists of the column name and the Java DecimalFormal pattern separated with equal sign. For example, rounding to three decimals can be done like: myColumn=#0.000
pageBreak	false	Use clear page after the table.
rename		Comma separated list of column renaming rules (oldname=newname). New names are used in table header but they do not affect the other behaviour of this component.
ruler	{}	Latex command for the row separating rulers
section		Section title for the table container or an empty string if no section should be generated.
sectionType	subsection	Type of LaTeX section: usually one of: section, subsection, or subsubsection. No section statement is written if section title is empty.
skipEmpty	false	This flag can be used to replace empty tables with a simple LaTeX comment.

19.620 snpSurvival-survivalSet3-survSNPAnnot-genes (KorvasieniAnnotator)

Korvasieni is an Ensembl [12] based converter for database identifiers and it can be used to convert genome location, gene, transcript, and translation identifiers across multiple biological databases. The selected `keyColumn` may contain entries with comma separated lists of values in which case `isListKey` parameter may be used to obtain each value separately and to process them as they were given in consecutive lines.

Author: Marko Laakso (Marko.Laakso@Helsinki.FI)
Version: 1.7
Categories: Annotation, GO

Input name	Source	Description
sourceKeys	snpSurvival-survivalSet3-survSNPAnnot-snpLoci. annotations 19.621	A list of source database keys.
connection	ensembl.in 19.273	Database connection can be defined using this file. The definition of parameters: database.url, database.user, database.password, database.timeout, database.recycle, and database.driver can be found from the documentation of Korvasieni.

Parameter name	Value	Description
echoColumns	refsnp_id	A comma separated list of column names for the columns that will be copied to the output. An asterisk (*) can be used to denote all columns execpt the keyColumn.
inputDB	.GeneId	Type of input keys. This must be a database supported by Korvasieni. If the parameter is omitted, the component tries to derive the database from the type of geneID. If this is not possible, an error is returned. You may define three columns in form of chromosome:start-end in case the inputDB is .DNARegion. This format provides a comfortable compatibility with DNARegion datatype.
inputType	Gene	Ensembl object type for the input keys (Any, Gene, Transcript, Translation)
isListKey	true	Enables the automatic value splits for the comma separated key column
keyColumn	ensembl_gene_stable_id	Name of the key column withing sourceKeys file or an empty string for the first column
maxHits	100000	Maximum number of target identifiers for a single source identifier
rename	UniProt/SwissProt=protein	Comma separated list of column renaming rules (oldname=newname)
skipLevel	never	Skip result rows if the source identifier is unknown or target identifiers are not available. Possible values are: never (no filtering), source (skip if the source ID is unknown), target (skip if no target IDs are found), any (skip if any of the target IDs is missing).

Continued on next page...

Parameter name	Value	Description
targetDB	.GeneName,.GeneDesc,.DNABand,GO,UniProt/SwissProt	Comma-separated list of annotation types. Possible values are all databases supported by Korvasieni.
unique	true	This flag can be turned on in order to eliminate duplicate annotations.

19.621 snpSurvival-survivalSet3-survSNPAnnot-snpLoci (RefSNPAnnotator)

RefSNPAnnotator fetches annotations for given refSNP ID's (also known as rsID's) from BioMart databases.

The provided annotations for each rsID include alleles for the SNP, name of the chromosome where the SNP is located, chromosome location of the SNP, the strand of the chromosome, Ensembl gene ID for genes that overlap the SNP's location and validation status of the SNP. The component is implemented using the biomaRt package for Bioconductor [7].

Authors: Viljami Aittomaki (Viljami.Aittomaki@Helsinki.FI), Marko Laakso (Marko.Laakso@Helsinki.FI)
Version: 1.0.1
Categories: SNP, Annotation

Input name	Source	Description
rsIDs	snpSurvival-survivalSet3-survival.statistics 19.623	List of rsID's for fetching annotations.

Parameter name	Value	Description
dataset	hsapiens_snp	Dataset to get annotations from. Different species have their own datasets. Example values include "hsapiens_snp" and "mmusculus_snp". See biomaRt documentation on how to list available datasets in R.
keyColumn	marker	Name of the key column withing sourceKeys file or an empty string for the first column.

19.622 snpSurvival-survivalSet3-survSNPAnnot-snpRel (ExpandCollapse)

Converts between the two possible representations of a relations with multivalued columns. By default this component splits rows with multivalued (comma separated lists) columns into multiple rows each representing a single value. The input may contain several columns with comma separated values and all permutations of column values are shown as individual single-valued rows. The alternative mode recovers the original comma separated values from the expanded relations. This is performed by joining values of the their list columns into comma separated lists.

Author: Marko Laakso (Marko.Laakso@Helsinki.FI)
Version: 1.3
Category: convert

Input name	Source	Description
relation	snpSurvival-survivalSet3-survSNPAnnot-snpLoci.annotations 19.621	The mandatory input relation

Parameter name	Value	Description
delim	,	Value delimiter between the list column values. Special characters can be encoded as specified in fi.helsinki.ltdk.csbl.asser.ArgumentEncoding.
expand	true	Action mode that is expand (true) or collapse (false)
listCols	ensembl_gene_stable_id	A comma separated list of column names of input (expand) or output (collapse) columns that may contain delim separated values. The asterisk refers to every column of the input relation.

19.623 snpSurvival-survivalSet3-survival (SNPKaplanMeier)

This component is used to estimate survival differences between various genotypes. The differences between the survival of different genotypes are measured using G-ρ family of tests [10].

Author: Marko Laakso (Marko.Laakso@Helsinki.FI)
Version: 1.5
Categories: SNP, Latex, Survival

Input name	Source	Description
genotypes	snpSurvival-survivalSet3-caseGenotypes-gtypes.genotypes 19.600	Genotype calls
survival	snpSurvival-survivalSet3-survivalClinical.table 19.624	Survival time and event indicator
annotations	snpSurvival-survivalSet3-snpAnnotations.in 19.615	Additional information about the markers. This data will be shown on the report of interesting markers.

Parameter name	Value	Description
PLimit	0.0010	Ignore results with a p-value greater than this limit.
confInt	0.95	Coverage of the confidence intervals [0,1]
minCount	15	Combine rare (aa) homozygotes to heterozygotes (Aa) if their number is below this limit. This parameter is used together with minFreq so that the actual limit is max(minFreq*n, minCount).
minFreq	0.1	Combine rare (aa) homozygotes to heterozygotes (Aa) if their frequency is below this limit. See also minCount.
naLimit	0.1	Skip markers where one minus call rate exceeds this limit.
nullCalls	–,ERR	A comma separated list of genotype calls that will be considered as missing values
sampleCol	sample	Column name for the sample name
showCI	true	Draw confidence intervals to the graph
skipMissingGT	true	Simply skip those samples that lack the genotype information.
statusCol	hasEvent	Column name for the event indicator
survRatePoint	0.5	Sampling height for the distances between the survival expectations. Negative values may be used to suppress these measurements.
timeCol	endTime	Column name for the survival time
timeOutLimit	36	Cut survival curves after this limit. A negative value can be used to maximize the length of the period.
timeStartCol	startTime	Column name for the start time that is reduced from the survival time
title	Genotype Specific Survival Estimates	Section title

19.624 snpSurvival-survivalSet3-survivalClinical (TableQuery)

Execute an SQL query on input relations and create a result table. TableQuery uses HSQLDB (Hyperthreaded Structured Query Language Database [25]) for executing the query. Consequently, the syntax of the query is defined by HSQLDB.

Author: Kristian Ovaska (Kristian.Ovaska@Helsinki.FI)
Version: 1.2
Categories: convert, Filter

Input name	Source	Description
table1	snpSurvival-survivalSet3-clinicalData.in 19.602	CSV table 1. The table is referred to as 'table1' in the SQL query.
query	snpSurvival-survivalSet3-survivalParamsQuery.file 19.625	SQL query. Either this file or the query parameter must be provided, but not both.
columnTypes	snpSurvival-survivalSet3-clinicalTypes.in 19.604	Contains SQL types for individual columns. If the file is not provided, the type is inferred from the contents of the columns. This can be used to force the use of VARCHAR for values that are also valid numerics. The file contains the columns Table (refers to one of table1 to table15), Column (refers to a column name in the table), Type (contains an SQL type). A row with Table='result', Column=X and Type='STRING' forces the use string values for result column X.

Parameter name	Value	Description
numIndices		Comma-separated list of index counts for input tables. All indices are single-column indices, running from column 1 to N, where N is retrieved from this parameter. If empty, the default number (1) is used. For example, ",2,,2" sets two indices for table2 and table4 and one index for the rest.
query		SQL query. Either this parameter or the query input must be provided, but not both.

19.625 snpSurvival-survivalSet3-survivalParamsQuery (SearchReplace)

Replaces the occurences of the given regular expressions with the corresponding regular expressions. This component is used for transforming given patterns of the input file to a changed form specified by the regular expressions .

Author: Marko Laakso (Marko.Laakso@Helsinki.FI)
Version: 1.0
Category: convert

Input name	Source	Description
file	snpSurvival-survivalSet3-survivalParamsTemplate.in 19.626	Source file to be modified

Parameter name	Value	Description
key00	REP.cond00	Search key 0
key01		Search key 1
key02		Search key 2
key03		Search key 3
key04		Search key 4
key05		Search key 5
key06		Search key 6
key07		Search key 7
key08		Search key 8
key09		Search key 9
value00	– No additional restrictions	Replacement 0
value01		Replacement 1
value02		Replacement 2
value03		Replacement 3
value04		Replacement 4
value05		Replacement 5
value06		Replacement 6
value07		Replacement 7
value08		Replacement 8
value09		Replacement 9

19.626 snpSurvival-survivalSet3-survivalParamsTemplate (INPUT)

Select relevant parameters for the survival analysis

Category: Internal

Parameter name	Value	Description
path	/gpfs/homes/mlaakso/asserSVN/andurilCaseStudy/sql/SNPsurvivalParams.sql	Path (filename) of the input resource.

19.627 snpSurvival-survivalSet3-survivalReport (LatexCombiner)

LatexCombiner joins the given subdocuments together into one LaTeX file.

Authors:	Kristian Ovaska (Kristian.Ovaska@Helsinki.FI), Marko Laakso (Marko.Laakso@Helsinki.FI)
Version:	1.3
Category:	Latex

Input name	Source	Description
latex1	snpSurvival-survivalSet3-survival .report 19.623	LaTeX fragment 1.
latex2	snpSurvival-survivalSet3-clinical Select.report 19.603	LaTeX fragment 2.
latex3	snpSurvival-survivalSet3-survSN PAnnot-annotTable.report 19.619	LaTeX fragment 3.
latex4	snpSurvival-survivalSet3-gene Rank-eqTable.report 19.607	LaTeX fragment 4.

Parameter name	Value	Description
pagebreak	true	Determines if the result document should start with a page break.
sectionTitle		If non-empty, a declaration of a new section with the given name is inserted to the beginning of the combined document. This is a convenience feature to make it easy to compile subsections into a section.
sectionType	section	Type of LaTeX section: usually one of section, subsection or subsubsection. No section statement is written if sectionTitle is empty.
strictBorders	true	Enables the flushing of all document elements before each fragment

19.628 snpSurvival-survivalSet4 (SurvivalAnalysis)

No description

Parameter name	Value
pvalue	0.0010

19.629 snpSurvival-survivalSet4-annotSQL (INPUT)

This SQL is used to generate marker annotation report.

Category: Internal

Parameter name	Value	Description
path	/gpfs/homes/mlaakso/asserSVN/ andurilCaseStudy/sql/ SNPgeneTable.sql	Path (filename) of the input resource.

19.630 snpSurvival-survivalSet4-caseGenotypes (GetGenotypes)

Load and preprocess the genotype calls for the cancer patient samples.

Parameter name	Value
skipFrq	0.1

19.631 snpSurvival-survivalSet4-caseGenotypes-freqCheck (AlleleCounter)

AlleleCounter calculates genotype frequencies for the biallelic markers of the given SNPMatrix. A Pearson's χ^2-test [18] is performed for each marker in order to test if the Hardy-Weinberg equilibrium [9] holds for the population sample.

Author:	Marko Laakso (Marko.Laakso@Helsinki.FI)
Version:	1.2
Category:	SNP

Input name	Source	Description
genotypes	snpSurvival-survivalSet4-case Genotypes-genotypes .genotypes 19.633	SNPHelistin genotype output.

Parameter name	Value	Description
dels	false	Accept homozygous deletions as a possible genotype.
naLimit	0.1	Skip markers where one minus call rate exceeds this limit.
skip	0.1	Drop markers with a relative minor allele frequency lower than this limit. Use negative values to inactivate this limit.

19.632 snpSurvival-survivalSet4-caseGenotypes-ftMarkers (CSV2IDList)

Extracts one column from the given CSV file and prints it out without duplicates. The selected column may contain entries with comma separated lists of values in which case *isList* parameter may be used to obtain each value separately.

Output is defined as:

$$ids = \bigcup_{i=1}^{9} \pi_{columnIn_i} \sigma_{regexp_i}(table_i). \quad (18)$$

Author:	Marko Laakso (Marko.Laakso@Helsinki.FI)
Version:	1.3
Category:	convert

Input name	Source	Description
table1	snpSurvival-survivalSet4-case Genotypes-freqCheck .frequencies 19.631	The mandatory input relation

Parameter name	Value	Description
acceptMissing	false	Files with missing columnIn are accepted as empty if this is true.
columnIn	marker	A comma separated list of column names for the IDs of interest in each file. Empty values refer to the first column of the file.
columnOut	marker	Name of the only column of the output list. Empty input refers to the name of the input column.
constants		A comma separated list of values that are always included into the output
isList	false	True if the seleted column contains a comma separated list of values to be splitted
quotation	false	Indicator that can be used to disable quotation of the output values
regexp1		Regular expression for the row filtering in table1. A row is included in the result if this parameter is empty or if values in the given columns match given regular expressions. The parameter has a format COLNAME1=EXPRESSION,COLNAME2=EXPRESSION2 where COLNAMEs are column names in "csv" and EXPRESSIONs are regular expressions using Java syntax. For example, "col=a|b" includes rows where the column col has a value of "a" or "b".
regexp2		Regular expression for the row filtering in table2.
regexp3		Regular expression for the row filtering in table3.
regexp4		Regular expression for the row filtering in table4.
regexp5		Regular expression for the row filtering in table5.
regexp6		Regular expression for the row filtering in table6.
regexp7		Regular expression for the row filtering in table7.
regexp8		Regular expression for the row filtering in table8.
regexp9		Regular expression for the row filtering in table9.

19.633 snpSurvival-survivalSet4-caseGenotypes-genotypes (SNPHelistinReader)

This component can be used to read SNP genotype calls for a set of samples and markers of interest. The genotype data is retrieved from a SNPHelistin database that is defined by the input properties.

Author:	Marko Laakso (Marko.Laakso@Helsinki.FI)
Version:	1.2
Categories:	Data Import, SNP

Input name	Source	Description
sampleNames	snpSurvival-survivalSet4-caseNames.ids 19.635	List of sample names of interest.
markerNames	snpSurvival-markers4.in 19.452	List of SNP identifiers of interest.
config	genotypeDB.in 19.320	Connection parameters for SNPHelistin.

Parameter name	Value	Description
errorSymbol	ERR	Symbol for failed measurements. You may for example use 'NA' value in order consider them equal to missing values.
skipUnknown	true	Ignore unknown samples and do not process them. If the parameter is false then the program fails for these samples.

19.634 snpSurvival-survivalSet4-caseGenotypes-gtypes (SNPHelistinReader)

This component can be used to read SNP genotype calls for a set of samples and markers of interest. The genotype data is retrieved from a SNPHelistin database that is defined by the input properties.

Author:	Marko Laakso (Marko.Laakso@Helsinki.FI)
Version:	1.2
Categories:	Data Import, SNP

Input name	Source	Description
sampleNames	snpSurvival-survivalSet4-caseNames.ids 19.635	List of sample names of interest.
markerNames	snpSurvival-survivalSet4-caseGenotypes-ftMarkers.ids 19.632	List of SNP identifiers of interest.
config	genotypeDB.in 19.320	Connection parameters for SNPHelistin.

Parameter name	Value	Description
errorSymbol	ERR	Symbol for failed measurements. You may for example use 'NA' value in order consider them equal to missing values.
skipUnknown	true	Ignore unknown samples and do not process them. If the parameter is false then the program fails for these samples.

19.635 snpSurvival-survivalSet4-caseNames (CSV2IDList)

Extracts one column from the given CSV file and prints it out without duplicates. The selected column may contain entries with comma separated lists of values in which case *isList* parameter may be used to obtain each value separately.

Output is defined as:

$$ids = \bigcup_{i=1}^{9} \pi_{columnIn_i} \sigma_{regexp_i}(table_i). \quad (19)$$

Author: Marko Laakso (Marko.Laakso@Helsinki.FI)
Version: 1.3
Category: convert

Input name	Source	Description
table1	snpSurvival-survivalSet4-survival Clinical.table 19.658	The mandatory input relation

Parameter name	Value	Description
acceptMissing	false	Files with missing columnIn are accepted as empty if this is true.
columnIn		A comma separated list of column names for the IDs of interest in each file. Empty values refer to the first column of the file.
columnOut	SampleId	Name of the only column of the output list. Empty input refers to the name of the input column.
constants		A comma separated list of values that are always included into the output
isList	false	True if the seleted column contains a comma separated list of values to be splitted
quotation	false	Indicator that can be used to disable quotation of the output values
regexp1		Regular expression for the row filtering in table1. A row is included in the result if this parameter is empty or if values in the given columns match given regular expressions. The parameter has a format COLNAME1=EXPRESSION,COLNAME2=EXPRESSION2 where COLNAMEs are column names in "csv" and EXPRESSIONs are regular expressions using Java syntax. For example, "col=a|b" includes rows where the column col has a value of "a" or "b".
regexp2		Regular expression for the row filtering in table2.
regexp3		Regular expression for the row filtering in table3.
regexp4		Regular expression for the row filtering in table4.
regexp5		Regular expression for the row filtering in table5.
regexp6		Regular expression for the row filtering in table6.
regexp7		Regular expression for the row filtering in table7.
regexp8		Regular expression for the row filtering in table8.
regexp9		Regular expression for the row filtering in table9.

19.636 snpSurvival-survivalSet4-clinicalData (INPUT)

Clinical parameters for the case samples
Category: Internal

Parameter name	Value	Description
path	/gpfs/homes/mlaakso/asserSVN/ andurilCaseStudy/data/snp/ patient-metadata-caucas.csv	Path (filename) of the input resource.

19.637 snpSurvival-survivalSet4-clinicalSelect (SourceCode2Latex)

This component converts a source code listing into a LaTeXfragment that may be included into some technical documents.
Author: Marko Laakso (Marko.Laakso@Helsinki.FI)
Version: 1.0
Categories: convert, Latex

Input name	Source	Description
source	snpSurvival-survivalSet4-survival ParamsQuery.file 19.659	Source code

Parameter name	Value	Description
caption	This SQL query has been used to select clinical parameters for the survival calculations.	Caption text for the listing
language	SQL	Language defintion for the syntax highlighting. Possible values can be found from the description of listings package.

19.638 snpSurvival-survivalSet4-clinicalTypes (INPUT)

Column types for the clinical data

Category: Internal

Parameter name	Value	Description
path	/gpfs/homes/mlaakso/asserSVN/andurilCaseStudy/csv/SNPclinicalTypes.csv	Path (filename) of the input resource.

19.639 snpSurvival-survivalSet4-geneRank (QueryReport)

(Technical documentation) Executes an SQL query and prints it with the result table.

Author: Marko Laakso (Marko.Laakso@Helsinki.FI)
Version: 1.1
Category: Latex

Input name	Source	Description
table1	snpSurvival-survivalSet4-survSNPAnnot.fullData 19.651	1. data table that is referred as 'table1' in the query

Parameter name	Value	Description
colFormat	lcrp{11cm}	LaTeX tabular formatting for the result columns
listCols		Comma separated list of column names. Columns of this list may contain several values separated with commas and the delimiters will be replaced with spaces.
resTableRef	the previous table	Textual reference from the SQL representation to the result table
section	Gene rank	Subsection title for the output document
showCols	gene,markers,p-value,description	Comma separated list of column selections for the output. The empty default will use all columns.
showQuery	false	This flag tells if the SQL shall be shown on the report
tableCaption	A list of survival affecting genes and the marker counts. P-value is the lowest value for the corresponding markers.	Description for the result table

19.640 snpSurvival-survivalSet4-geneRank-eqData (TableQuery)

Execute an SQL query on input relations and create a result table. TableQuery uses HSQLDB (Hyperthreaded Structured Query Language Database [25]) for executing the query. Consequently, the syntax of the query is defined by HSQLDB.

Author: Kristian Ovaska (Kristian.Ovaska@Helsinki.FI)
Version: 1.2
Categories: convert, Filter

Input name	Source	Description
table1	snpSurvival-survivalSet4-survSNPAnnot-annotData.table 19.652	CSV table 1. The table is referred to as 'table1' in the SQL query.

Continued on next page...

Input name	Source	Description
table2	snpSurvival-survivalSet4-survival .statistics 19.657	CSV table 2. The table is referred to as 'table2' in the SQL query.
query	snpSurvival-survivalSet4-gene RankQuery.in 19.642	SQL query. Either this file or the query parameter must be provided, but not both.

Parameter name	Value	Description
numIndices		Comma-separated list of index counts for input tables. All indices are single-column indices, running from column 1 to N, where N is retrieved from this parameter. If empty, the default number (1) is used. For example, ",2,,2" sets two indices for table2 and table4 and one index for the rest.
query		SQL query. Either this parameter or the query input must be provided, but not both.

19.641 snpSurvival-survivalSet4-geneRank-eqTable (CSV2Latex)

This component converts a tabular CSV file into a printable LaTeX table.

Author:	Marko Laakso (Marko.Laakso@Helsinki.FI)
Version:	2.1
Categories:	convert, Latex

Input name	Source	Description
tabledata	snpSurvival-survivalSet4-gene Rank-eqData.table 19.640	Table content
refs	snpSurvival-survivalSet4-refsGene Rank.in 19.645	Reference rules for the hyperlinks

Parameter name	Value	Description
attach	false	Include the original data as an attachment
caption	A list of survival affecting genes and the marker counts. P-value is the lowest value for the corresponding markers.	Caption text for the table.
colFormat	lcrp{11cm}	LaTeX tabular format for the columns. Default produces a centered layout for all columns.
columns	gene,markers,p-value,description	Comma separated list of column selections for the output. The empty default will use all columns.
countRows	false	Include a row count to the table caption.
dropMissing	true	This flag can be turned off in order to generate links with missing texts. Link text are substituted with target identifiers.
listCols		Comma separated list of column names. Columns of this list may contain several values separated with commas and the delimiters will be replaced with spaces.
numberFormat		A comma separated list of decimal formats for the columns. Each entry consists of the column name and the Java DecimalFormal pattern separated with equal sign. For example, rounding to three decimals can be done like: myColumn=#0.000
pageBreak	false	Use clear page after the table.
rename		Comma separated list of column renaming rules (oldname=newname). New names are used in table header but they do not affect the other behaviour of this component.
ruler	{}	Latex command for the row separating rulers
section	Gene rank	Section title for the table container or an empty string if no section should be generated.
sectionType	subsection	Type of LaTeX section: usually one of: section, subsection, or subsubsection. No section statement is written if section title is empty.
skipEmpty	false	This flag can be used to replace empty tables with a simple LaTeX comment.

19.642 snpSurvival-survivalSet4-geneRankQuery (INPUT)

Generates a list of genes and the marker counts for them.

Category:	Internal

Parameter name	Value	Description
path	/gpfs/homes/mlaakso/asserSVN/andurilCaseStudy/sql/SNPinterestingGenes.sql	Path (filename) of the input resource.

19.643 snpSurvival-survivalSet4-missingDistC (CSV2Latex)

This component converts a tabular CSV file into a printable LATEX table.

Author: Marko Laakso (Marko.Laakso@Helsinki.FI)
Version: 2.1
Categories: convert, Latex

Input name	Source	Description
tabledata	snpSurvival-survivalSet4-caseGenotypes-freqCheck.naDist 19.631	Table content

Parameter name	Value	Description
attach	false	Include the original data as an attachment
caption	Distribution of the missing values in case samples	Caption text for the table.
colFormat	p{3cm}r	LaTeX tabular format for the columns. Default produces a centered layout for all columns.
columns		Comma separated list of column selections for the output. The empty default will use all columns.
countRows	false	Include a row count to the table caption.
dropMissing	true	This flag can be turned off in order to generate links with missing texts. Link text are substituted with target identifiers.
listCols		Comma separated list of column names. Columns of this list may contain several values separated with commas and the delimiters will be replaced with spaces.
numberFormat		A comma separated list of decimal formats for the columns. Each entry consists of the column name and the Java DecimalFormal pattern separated with equal sign. For example, rounding to three decimals can be done like: myColumn=#0.000
pageBreak	false	Use clear page after the table.
rename		Comma separated list of column renaming rules (oldname=newname). New names are used in table header but they do not affect the other behaviour of this component.
ruler	{}	Latex command for the row separating rulers
section		Section title for the table container or an empty string if no section should be generated.
sectionType	subsection	Type of LaTeX section: usually one of: section, subsection, or subsubsection. No section statement is written if section title is empty.
skipEmpty	false	This flag can be used to replace empty tables with a simple LaTeX comment.

19.644 snpSurvival-survivalSet4-propertiesDoc (Properties2Latex)

Prepares a LATEX-representation for the given set of properties files. The document consists of tables of names and values of the properties.

Author: Marko Laakso (Marko.Laakso@Helsinki.FI)
Version: 1.2
Categories: convert, Latex

Input name	Source	Description
props1	genotypeDB.in 19.320	A set of properties

Parameter name	Value	Description
hide		A comma separated list of property names that should remain invisible. You may use this paramerter to hide passwords and other confidentian settings.

Continued on next page...

Parameter name	Value	Description
keyWidth	5	Column width for the property names (centimeters)
merge	false	Merge all input properties into a single set
section	SNP database configuration	Section title for the table container or an empty string if no section should be generated
sectionType	subsection	Type of LaTeX section: usually one of section, subsection or subsubsection. No section statement is written if sectionTitle is empty.
valueWidth	13	Column width for the property values (centimeters)

19.645 snpSurvival-survivalSet4-refsGeneRank (INPUT)

Hyperlink rules for the gene rank table

Category: Internal

Parameter name	Value	Description
path	/gpfs/homes/mlaakso/asserSVN/andurilCaseStudy/csv/SNPrefsGeneRank.csv	Path (filename) of the input resource.

19.646 snpSurvival-survivalSet4-refsHits (INPUT)

Hyperlink rules for the marker annotations

Category: Internal

Parameter name	Value	Description
path	/gpfs/homes/mlaakso/asserSVN/andurilCaseStudy/csv/SNPrefsHits.csv	Path (filename) of the input resource.

19.647 snpSurvival-survivalSet4-reportBody (LatexCombiner)

LatexCombiner joins the given subdocuments together into one LaTeX file.

Authors: Kristian Ovaska (Kristian.Ovaska@Helsinki.FI), Marko Laakso (Marko.Laakso@Helsinki.FI)
Version: 1.3
Category: Latex

Input name	Source	Description
latex1	snpSurvival-survivalSet4-survivalReport.document 19.661	LaTeX fragment 1.
latex2	snpSurvival-survivalSet4-propertiesDoc.report 19.644	LaTeX fragment 2.
latex3	snpSurvival-survivalSet4-missingDistC.report 19.643	LaTeX fragment 3.

Parameter name	Value	Description
pagebreak	false	Determines if the result document should start with a page break.
sectionTitle		If non-empty, a declaration of a new section with the given name is inserted to the beginning of the combined document. This is a convenience feature to make it easy to compile subsections into a section.
sectionType	section	Type of LaTeX section: usually one of section, subsection or subsubsection. No section statement is written if sectionTitle is empty.
strictBorders	true	Enables the flushing of all document elements before each fragment

19.648 snpSurvival-survivalSet4-reportTemplate (LatexTemplate)

This component generates the standard layout configuration for Anduril output documents. The component produces a header and a footer fragment that can be used to build LATEX documents. The header fragment takes care of the page geometry and other technical details such as library imports and function declarations. Reference list is generated by the footer.

Author: Marko Laakso (Marko.Laakso@Helsinki.FI)
Version: 1.4
Category: Latex

Input name	Source	Description
abstract	abstract.in 19.42	Document abstract including its Latex definition. You may use template entities within this document. The default abstract has been defined in abstractTemplate.tex.

Parameter name	Value	Description
authors	Marko Laakso, Riku Louhimo, Sampsa Hautaniemi	A comma-separated list of document authors on the title page. If authors is empty but title is non-empty, a title page with no author name is printed.
baselineskip	1.2	Baselineskip factor that indicates the line spacing
bibstyle	abbrv	BibTeX reference formatting style
printTOC	true	If true, print Table Of Contents.
title	Anduril Case Study: Glioblastoma SNP survival analysis	Document title on the title page. If empty, no title page is printed. Also see author.
usepackage		Comma-separated list of LaTeX packages that are used in the document. Each package generates a \usepackage{X} line in the header.

19.649 snpSurvival-survivalSet4-snpAnnotations (INPUT)

Annotations for survival component.

Category: Internal

Parameter name	Value	Description
path	/gpfs/homes/mlaakso/asserSVN/andurilCaseStudy/csv/SNPAnnotation.csv	Path (filename) of the input resource.

19.650 snpSurvival-survivalSet4-summaryReport (LatexPDF)

This component is a LATEX compiler that generates a portable document format (PDF) file from the given Anduril document.

Author: Kristian Ovaska (Kristian.Ovaska@Helsinki.FI)
Version: 1.0
Category: Latex

Input name	Source	Description
document	snpSurvival-survivalSet4-reportBody.document 19.647	Body of the LaTeX document.
header	snpSurvival-survivalSet4-reportTemplate.header 19.648	LaTeX header that is written to the start of the document. If missing, a default header is used.
footer	snpSurvival-survivalSet4-reportTemplate.footer 19.648	LaTeX footer that is written to the end of the document. If missing, a default header is used.

Parameter name	Value	Description
bibtexExec	bibtex	Executable command for BibTeX.
latexExec	pdflatex	Executable command for pdflatex.
useRefs	false	This flag can be used to activate BibTeX compiler

19.651 snpSurvival-survivalSet4-survSNPAnnot (AnnotateSNPs)

Biological annotations for the survival affecting genes

Parameter name	Value
caption	Gene annotations and the loci of the survival affecting markers

19.652 snpSurvival-survivalSet4-survSNPAnnot-annotData (TableQuery)

Execute an SQL query on input relations and create a result table. TableQuery uses HSQLDB (Hyper-threaded Structured Query Language Database [25]) for executing the query. Consequently, the syntax of the query is defined by HSQLDB.

Author: Kristian Ovaska (Kristian.Ovaska@Helsinki.FI)
Version: 1.2
Categories: convert, Filter

Input name	Source	Description
table1	snpSurvival-survivalSet4-survSNPAnnot-snpRel.relation 19.656	CSV table 1. The table is referred to as 'table1' in the SQL query.
table2	snpSurvival-survivalSet4-survSNPAnnot-genes.bioAnnotation 19.654	CSV table 2. The table is referred to as 'table2' in the SQL query.
table3	snpSurvival-survivalSet4-survival.statistics 19.657	CSV table 3. The table is referred to as 'table3' in the SQL query.
query	snpSurvival-survivalSet4-annotSQL.in 19.629	SQL query. Either this file or the query parameter must be provided, but not both.

Parameter name	Value	Description
numIndices		Comma-separated list of index counts for input tables. All indices are single-column indices, running from column 1 to N, where N is retrieved from this parameter. If empty, the default number (1) is used. For example, ",2,,2" sets two indices for table2 and table4 and one index for the rest.
query		SQL query. Either this parameter or the query input must be provided, but not both.

19.653 snpSurvival-survivalSet4-survSNPAnnot-annotTable (CSV2Latex)

This component converts a tabular CSV file into a printable LaTeX table.

Author: Marko Laakso (Marko.Laakso@Helsinki.FI)
Version: 2.1
Categories: convert, Latex

Input name	Source	Description
tabledata	snpSurvival-survivalSet4-survSNPAnnot-annotData.table 19.652	Table content
refs	snpSurvival-survivalSet4-refsHits.in 19.646	Reference rules for the hyperlinks

Continued on next page...

Parameter name Value Description

Parameter name	Value	Description
attach	false	Include the original data as an attachment
caption	Gene annotations and the loci of the survival affecting markers	Caption text for the table.
colFormat	l@{}lr@{:}lp{1cm}p{1.5cm}p{10.5cm}	LaTeX tabular format for the columns. Default produces a centered layout for all columns.
columns	hit,marker,chr,locus,band,gene, gene description	Comma separated list of column selections for the output. The empty default will use all columns.
countRows	false	Include a row count to the table caption.
dropMissing	true	This flag can be turned off in order to generate links with missing texts. Link text are substituted with target identifiers.
listCols	band	Comma separated list of column names. Columns of this list may contain several values separated with commas and the delimiters will be replaced with spaces.
numberFormat		A comma separated list of decimal formats for the columns. Each entry consists of the column name and the Java DecimalFormal pattern separated with equal sign. For example, rounding to three decimals can be done like: myColumn=#0.000
pageBreak	false	Use clear page after the table.
rename		Comma separated list of column renaming rules (oldname=newname). New names are used in table header but they do not affect the other behaviour of this component.
ruler	{}	Latex command for the row separating rulers
section		Section title for the table container or an empty string if no section should be generated.
sectionType	subsection	Type of LaTeX section: usually one of: section, subsection, or subsubsection. No section statement is written if section title is empty.
skipEmpty	false	This flag can be used to replace empty tables with a simple LaTeX comment.

19.654 snpSurvival-survivalSet4-survSNPAnnot-genes (KorvasieniAnnotator)

Korvasieni is an Ensembl [12] based converter for database identifiers and it can be used to convert genome location, gene, transcript, and translation identifiers across multiple biological databases. The selected `keyColumn` may contain entries with comma separated lists of values in which case `isListKey` parameter may be used to obtain each value separately and to process them as they were given in consecutive lines.

Author: Marko Laakso (Marko.Laakso@Helsinki.FI)
Version: 1.7
Categories: Annotation, GO

Input name	Source	Description
sourceKeys	snpSurvival-survivalSet4-survSNPAnnot-snpLoci. annotations 19.655	A list of source database keys.
connection	ensembl.in 19.273	Database connection can be defined using this file. The definition of parameters: database.url, database.user, database.password, database.timeout, database.recycle, and database.driver can be found from the documentation of Korvasieni.

Parameter name	Value	Description
echoColumns	refsnp_id	A comma separated list of column names for the columns that will be copied to the output. An asterisk (*) can be used to denote all columns execpt the keyColumn.
inputDB	.GeneId	Type of input keys. This must be a database supported by Korvasieni. If the parameter is omitted, the component tries to derive the database from the type of geneID. If this is not possible, an error is returned. You may define three columns in form of chromosome:start-end in case the inputDB is .DNARegion. This format provides a comfortable compatibility with DNARegion datatype.
inputType	Gene	Ensembl object type for the input keys (Any, Gene, Transcript, Translation)
isListKey	true	Enables the automatic value splits for the comma separated key column
keyColumn	ensembl_gene_stable_id	Name of the key column withing sourceKeys file or an empty string for the first column
maxHits	100000	Maximum number of target identifiers for a single source identifier
rename	UniProt/SwissProt=protein	Comma separated list of column renaming rules (oldname=newname)
skipLevel	never	Skip result rows if the source identifier is unknown or target identifiers are not available. Possible values are: never (no filtering), source (skip if the source ID is unknown), target (skip if no target IDs are found), any (skip if any of the target IDs is missing).

Continued on next page...

Parameter name	Value	Description
targetDB	.GeneName,.GeneDesc,. DNABand,GO,UniProt/SwissProt	Comma-separated list of annotation types. Possible values are all databases supported by Korvasieni.
unique	true	This flag can be turned on in order to eliminate duplicate annotations.

19.655 snpSurvival-survivalSet4-survSNPAnnot-snpLoci (RefSNPAnnotator)

RefSNPAnnotator fetches annotations for given refSNP ID's (also known as rsID's) from BioMart databases.

The provided annotations for each rsID include alleles for the SNP, name of the chromosome where the SNP is located, chromosome location of the SNP, the strand of the chromosome, Ensembl gene ID for genes that overlap the SNP's location and validation status of the SNP. The component is implemented using the biomaRt package for Bioconductor [7].

Authors: Viljami Aittomaki (Viljami.Aittomaki@Helsinki.FI), Marko Laakso (Marko.Laakso@Helsinki.FI)
Version: 1.0.1
Categories: SNP, Annotation

Input name	Source	Description
rsIDs	snpSurvival-survivalSet4-survival .statistics 19.657	List of rsID's for fetching annotations.

Parameter name	Value	Description
dataset	hsapiens_snp	Dataset to get annotations from. Different species have their own datasets. Example values include "hsapiens_snp" and "mmusculus_snp". See biomaRt documentation on how to list available datasets in R.
keyColumn	marker	Name of the key column withing sourceKeys file or an empty string for the first column.

19.656 snpSurvival-survivalSet4-survSNPAnnot-snpRel (ExpandCollapse)

Converts between the two possible representations of a relations with multivalued columns. By default this component splits rows with multivalued (comma separated lists) columns into multiple rows each representing a single value. The input may contain several columns with comma separated values and all permutations of column values are shown as individual single-valued rows. The alternative mode recovers the original comma separated values from the expanded relations. This is performed by joining values of the their list columns into comma separated lists.

Author: Marko Laakso (Marko.Laakso@Helsinki.FI)
Version: 1.3
Category: convert

Input name	Source	Description
relation	snpSurvival-survivalSet4-survSN PAnnot-snpLoci. annotations 19.655	The mandatory input relation

Parameter name	Value	Description
delim	,	Value delimiter between the list column values. Special characters can be encoded as specified in fi.helsinki.ltdk.csbl.asser.ArgumentEncoding.
expand	true	Action mode that is expand (true) or collapse (false)
listCols	ensembl_gene_stable_id	A comma separated list of column names of input (expand) or output (collapse) columns that may contain delim separated values. The asterisk refers to every column of the input relation.

19.657 snpSurvival-survivalSet4-survival (SNPKaplanMeier)

This component is used to estimate survival differences between various genotypes. The differences between the survival of different genotypes are measured using G-ρ family of tests [10].

Author:	Marko Laakso (Marko.Laakso@Helsinki.FI)
Version:	1.5
Categories:	SNP, Latex, Survival

Input name	Source	Description
genotypes	snpSurvival-survivalSet4-caseGenotypes-gtypes.genotypes 19.634	Genotype calls
survival	snpSurvival-survivalSet4-survivalClinical.table 19.658	Survival time and event indicator
annotations	snpSurvival-survivalSet4-snpAnnotations.in 19.649	Additional information about the markers. This data will be shown on the report of interesting markers.

Parameter name	Value	Description
PLimit	0.0010	Ignore results with a p-value greater than this limit.
confInt	0.95	Coverage of the confidence intervals [0,1]
minCount	15	Combine rare (aa) homozygotes to heterozygotes (Aa) if their number is below this limit. This parameter is used together with minFreq so that the actual limit is max(minFreq*n, minCount).
minFreq	0.1	Combine rare (aa) homozygotes to heterozygotes (Aa) if their frequency is below this limit. See also minCount.
naLimit	0.1	Skip markers where one minus call rate exceeds this limit.
nullCalls	–,ERR	A comma separated list of genotype calls that will be considered as missing values
sampleCol	sample	Column name for the sample name
showCI	true	Draw confidence intervals to the graph
skipMissingGT	true	Simply skip those samples that lack the genotype information.
statusCol	hasEvent	Column name for the event indicator
survRatePoint	0.5	Sampling height for the distances between the survival expectations. Negative values may be used to suppress these measurements.
timeCol	endTime	Column name for the survival time
timeOutLimit	36	Cut survival curves after this limit. A negative value can be used to maximize the length of the period.
timeStartCol	startTime	Column name for the start time that is reduced from the survival time
title	Genotype Specific Survival Estimates	Section title

19.658 snpSurvival-survivalSet4-survivalClinical (TableQuery)

Execute an SQL query on input relations and create a result table. TableQuery uses HSQLDB (Hyper-threaded Structured Query Language Database [25]) for executing the query. Consequently, the syntax of the query is defined by HSQLDB.

Author:	Kristian Ovaska (Kristian.Ovaska@Helsinki.FI)
Version:	1.2
Categories:	convert, Filter

Input name	Source	Description
table1	snpSurvival-survivalSet4-clinicalData.in 19.636	CSV table 1. The table is referred to as 'table1' in the SQL query.
query	snpSurvival-survivalSet4-survivalParamsQuery.file 19.659	SQL query. Either this file or the query parameter must be provided, but not both.
columnTypes	snpSurvival-survivalSet4-clinicalTypes.in 19.638	Contains SQL types for individual columns. If the file is not provided, the type is inferred from the contents of the columns. This can be used to force the use of VARCHAR for values that are also valid numerics. The file contains the columns Table (refers to one of table1 to table15), Column (refers to a column name in the table), Type (contains an SQL type). A row with Table='result', Column=X and Type='STRING' forces the use string values for result column X.

Parameter name	Value	Description
numIndices		Comma-separated list of index counts for input tables. All indices are single-column indices, running from column 1 to N, where N is retrieved from this parameter. If empty, the default number (1) is used. For example, ",2,,2" sets two indices for table2 and table4 and one index for the rest.
query		SQL query. Either this parameter or the query input must be provided, but not both.

19.659 snpSurvival-survivalSet4-survivalParamsQuery (SearchReplace)

Replaces the occurences of the given regular expressions with the corresponding regular expressions. This component is used for transforming given patterns of the input file to a changed form specified by the regular expressions .

Author: Marko Laakso (Marko.Laakso@Helsinki.FI)
Version: 1.0
Category: convert

Input name	Source	Description
file	snpSurvival-survivalSet4-survival ParamsTemplate.in 19.660	Source file to be modified

Parameter name	Value	Description
key00	REP.cond00	Search key 0
key01		Search key 1
key02		Search key 2
key03		Search key 3
key04		Search key 4
key05		Search key 5
key06		Search key 6
key07		Search key 7
key08		Search key 8
key09		Search key 9
value00	– No additional restrictions	Replacement 0
value01		Replacement 1
value02		Replacement 2
value03		Replacement 3
value04		Replacement 4
value05		Replacement 5
value06		Replacement 6
value07		Replacement 7
value08		Replacement 8
value09		Replacement 9

19.660 snpSurvival-survivalSet4-survivalParamsTemplate (INPUT)

Select relevant parameters for the survival analysis

Category: Internal

Parameter name	Value	Description
path	/gpfs/homes/mlaakso/asserSVN/ anduril CaseStudy/sql/ SNPsurvivalParams.sql	Path (filename) of the input resource.

19.661 snpSurvival-survivalSet4-survivalReport (LatexCombiner)

LatexCombiner joins the given subdocuments together into one LaTeX file.

Authors:	Kristian Ovaska (Kristian.Ovaska@Helsinki.FI), Marko Laakso (Marko.Laakso@Helsinki.FI)
Version:	1.3
Category:	Latex

Input name	Source	Description
latex1	snpSurvival-survivalSet4-survival .report 19.657	LaTeX fragment 1.
latex2	snpSurvival-survivalSet4-clinical Select.report 19.637	LaTeX fragment 2.
latex3	snpSurvival-survivalSet4-survSN PAnnot-annotTable.report 19.653	LaTeX fragment 3.
latex4	snpSurvival-survivalSet4-gene Rank-eqTable.report 19.641	LaTeX fragment 4.

Parameter name	Value	Description
pagebreak	true	Determines if the result document should start with a page break.
sectionTitle		If non-empty, a declaration of a new section with the given name is inserted to the beginning of the combined document. This is a convenience feature to make it easy to compile subsections into a section.
sectionType	section	Type of LaTeX section: usually one of section, subsection or subsubsection. No section statement is written if sectionTitle is empty.
strictBorders	true	Enables the flushing of all document elements before each fragment

19.662 snpSurvival-survivalSet5 (SurvivalAnalysis)

No description

Parameter name	Value
pvalue	0.0010

19.663 snpSurvival-survivalSet5-annotSQL (INPUT)

This SQL is used to generate marker annotation report.

Category: Internal

Parameter name	Value	Description
path	/gpfs/homes/mlaakso/asserSVN/ anduril CaseStudy/sql/ SNPgeneTable.sql	Path (filename) of the input resource.

19.664 snpSurvival-survivalSet5-caseGenotypes (GetGenotypes)

Load and preprocess the genotype calls for the cancer patient samples.

Parameter name	Value
skipFrq	0.1

19.665 snpSurvival-survivalSet5-caseGenotypes-freqCheck (AlleleCounter)

AlleleCounter calculates genotype frequencies for the biallelic markers of the given SNPMatrix. A Pearson's χ^2-test [18] is performed for each marker in order to test if the Hardy-Weinberg equilibrium [9] holds for the population sample.

Author:	Marko Laakso (Marko.Laakso@Helsinki.FI)
Version:	1.2
Category:	SNP

Input name	Source	Description
genotypes	snpSurvival-survivalSet5-caseGenotypes-genotypes.genotypes 19.667	SNPHelistin genotype output.

Parameter name	Value	Description
dels	false	Accept homozygous deletions as a possible genotype.
naLimit	0.1	Skip markers where one minus call rate exceeds this limit.
skip	0.1	Drop markers with a relative minor allele frequency lower than this limit. Use negative values to inactivate this limit.

19.666 snpSurvival-survivalSet5-caseGenotypes-ftMarkers (CSV2IDList)

Extracts one column from the given CSV file and prints it out without duplicates. The selected column may contain entries with comma separated lists of values in which case *isList* parameter may be used to obtain each value separately.

Output is defined as:

$$ids = \bigcup_{i=1}^{9} \pi_{columnIn_i} \sigma_{regexp_i}(table_i). \tag{20}$$

Author:	Marko Laakso (Marko.Laakso@Helsinki.FI)
Version:	1.3
Category:	convert

Input name	Source	Description
table1	snpSurvival-survivalSet5-caseGenotypes-freqCheck.frequencies 19.665	The mandatory input relation

Parameter name	Value	Description
acceptMissing	false	Files with missing columnIn are accepted as empty if this is true.
columnIn	marker	A comma separated list of column names for the IDs of interest in each file. Empty values refer to the first column of the file.
columnOut	marker	Name of the only column of the output list. Empty input refers to the name of the input column.
constants		A comma separated list of values that are always included into the output
isList	false	True if the seleted column contains a comma separated list of values to be splitted
quotation	false	Indicator that can be used to disable quotation of the output values
regexp1		Regular expression for the row filtering in table1. A row is included in the result if this parameter is empty or if values in the given columns match given regular expressions. The parameter has a format COLNAME1=EXPRESSION,COLNAME2=EXPRESSION2 where COLNAMEs are column names in "csv" and EXPRESSIONs are regular expressions using Java syntax. For example, "col=a|b" includes rows where the column col has a value of "a" or "b".
regexp2		Regular expression for the row filtering in table2.
regexp3		Regular expression for the row filtering in table3.
regexp4		Regular expression for the row filtering in table4.
regexp5		Regular expression for the row filtering in table5.
regexp6		Regular expression for the row filtering in table6.
regexp7		Regular expression for the row filtering in table7.
regexp8		Regular expression for the row filtering in table8.
regexp9		Regular expression for the row filtering in table9.

19.667 snpSurvival-survivalSet5-caseGenotypes-genotypes (SNPHelistinReader)

This component can be used to read SNP genotype calls for a set of samples and markers of interest. The genotype data is retrieved from a SNPHelistin database that is defined by the input properties.

Author:	Marko Laakso (Marko.Laakso@Helsinki.FI)
Version:	1.2
Categories:	Data Import, SNP

Input name	Source	Description
sampleNames	snpSurvival-survivalSet5-caseNames.ids 19.669	List of sample names of interest.
markerNames	snpSurvival-markers5.in 19.453	List of SNP identifiers of interest.
config	genotypeDB.in 19.320	Connection parameters for SNPHelistin.

Parameter name	Value	Description
errorSymbol	ERR	Symbol for failed measurements. You may for example use 'NA' value in order consider them equal to missing values.
skipUnknown	true	Ignore unknown samples and do not process them. If the parameter is false then the program fails for these samples.

19.668 snpSurvival-survivalSet5-caseGenotypes-gtypes (SNPHelistinReader)

This component can be used to read SNP genotype calls for a set of samples and markers of interest. The genotype data is retrieved from a SNPHelistin database that is defined by the input properties.

Author:	Marko Laakso (Marko.Laakso@Helsinki.FI)
Version:	1.2
Categories:	Data Import, SNP

Input name	Source	Description
sampleNames	snpSurvival-survivalSet5-caseNames.ids 19.669	List of sample names of interest.
markerNames	snpSurvival-survivalSet5-caseGenotypes-ftMarkers.ids 19.666	List of SNP identifiers of interest.
config	genotypeDB.in 19.320	Connection parameters for SNPHelistin.

Parameter name	Value	Description
errorSymbol	ERR	Symbol for failed measurements. You may for example use 'NA' value in order consider them equal to missing values.
skipUnknown	true	Ignore unknown samples and do not process them. If the parameter is false then the program fails for these samples.

19.669 snpSurvival-survivalSet5-caseNames (CSV2IDList)

Extracts one column from the given CSV file and prints it out without duplicates. The selected column may contain entries with comma separated lists of values in which case *isList* parameter may be used to obtain each value separately.

Output is defined as:

$$ids = \bigcup_{i=1}^{9} \pi_{columnIn_i} \sigma_{regexp_i}(table_i). \tag{21}$$

Author: Marko Laakso (Marko.Laakso@Helsinki.FI)
Version: 1.3
Category: convert

Input name	Source	Description
table1	snpSurvival-survivalSet5-survival Clinical.table 19.692	The mandatory input relation

Parameter name	Value	Description
acceptMissing	false	Files with missing columnIn are accepted as empty if this is true.
columnIn		A comma separated list of column names for the IDs of interest in each file. Empty values refer to the first column of the file.
columnOut	SampleId	Name of the only column of the output list. Empty input refers to the name of the input column.
constants		A comma separated list of values that are always included into the output
isList	false	True if the seleted column contains a comma separated list of values to be splitted
quotation	false	Indicator that can be used to disable quotation of the output values
regexp1		Regular expression for the row filtering in table1. A row is included in the result if this parameter is empty or if values in the given columns match given regular expressions. The parameter has a format COLNAME1=EXPRESSION,COLNAME2=EXPRESSION2 where COLNAMEs are column names in "csv" and EXPRESSIONs are regular expressions using Java syntax. For example, "col=a|b" includes rows where the column col has a value of "a" or "b".
regexp2		Regular expression for the row filtering in table2.
regexp3		Regular expression for the row filtering in table3.
regexp4		Regular expression for the row filtering in table4.
regexp5		Regular expression for the row filtering in table5.
regexp6		Regular expression for the row filtering in table6.
regexp7		Regular expression for the row filtering in table7.
regexp8		Regular expression for the row filtering in table8.
regexp9		Regular expression for the row filtering in table9.

19.670 snpSurvival-survivalSet5-clinicalData (INPUT)

Clinical parameters for the case samples
Category: Internal

Parameter name	Value	Description
path	/gpfs/homes/mlaakso/asserSVN/ andurilCaseStudy/data/snp/ patient-metadata-caucas.csv	Path (filename) of the input resource.

19.671 snpSurvival-survivalSet5-clinicalSelect (SourceCode2Latex)

This component converts a source code listing into a LaTeXfragment that may be included into some technical documents.

Author: Marko Laakso (Marko.Laakso@Helsinki.FI)
Version: 1.0
Categories: convert, Latex

Input name	Source	Description
source	snpSurvival-survivalSet5-survival ParamsQuery.file 19.693	Source code

Parameter name	Value	Description
caption	This SQL query has been used to select clinical parameters for the survival calculations.	Caption text for the listing
language	SQL	Language defintion for the syntax highlighting. Possible values can be found from the description of listings package.

19.672 snpSurvival-survivalSet5-clinicalTypes (INPUT)

Column types for the clinical data

Category: Internal

Parameter name	Value	Description
path	/gpfs/homes/mlaakso/asserSVN/ andurilCaseStudy/csv/ SNPclinicalTypes.csv	Path (filename) of the input resource.

19.673 snpSurvival-survivalSet5-geneRank (QueryReport)

(Technical documentation) Executes an SQL query and prints it with the result table.

Author: Marko Laakso (Marko.Laakso@Helsinki.FI)
Version: 1.1
Category: Latex

Input name	Source	Description
table1	snpSurvival-survivalSet5-survSNPAnnot.fullData 19.685	1. data table that is referred as 'table1' in the query

Parameter name	Value	Description
colFormat	lcrp{11cm}	LaTeX tabular formatting for the result columns
listCols		Comma separated list of column names. Columns of this list may contain several values separated with commas and the delimiters will be replaced with spaces.
resTableRef	the previous table	Textual reference from the SQL representation to the result table
section	Gene rank	Subsection title for the output document
showCols	gene,markers,p-value,description	Comma separated list of column selections for the output. The empty default will use all columns.
showQuery	false	This flag tells if the SQL shall be shown on the report
tableCaption	A list of survival affecting genes and the marker counts. P-value is the lowest value for the corresponding markers.	Description for the result table

19.674 snpSurvival-survivalSet5-geneRank-eqData (TableQuery)

Execute an SQL query on input relations and create a result table. TableQuery uses HSQLDB (Hyperthreaded Structured Query Language Database [25]) for executing the query. Consequently, the syntax of the query is defined by HSQLDB.

Author: Kristian Ovaska (Kristian.Ovaska@Helsinki.FI)
Version: 1.2
Categories: convert, Filter

Input name	Source	Description
table1	snpSurvival-survivalSet5-survSNPAnnot-annotData.table 19.686	CSV table 1. The table is referred to as 'table1' in the SQL query.

Continued on next page...

Input name	Source	Description
table2	snpSurvival-survivalSet5-survival .statistics 19.691	CSV table 2. The table is referred to as 'table2' in the SQL query.
query	snpSurvival-survivalSet5-gene RankQuery.in 19.676	SQL query. Either this file or the query parameter must be provided, but not both.

Parameter name	Value	Description
numIndices		Comma-separated list of index counts for input tables. All indices are single-column indices, running from column 1 to N, where N is retrieved from this parameter. If empty, the default number (1) is used. For example, ",2,,2" sets two indices for table2 and table4 and one index for the rest.
query		SQL query. Either this parameter or the query input must be provided, but not both.

19.675 snpSurvival-survivalSet5-geneRank-eqTable (CSV2Latex)

This component converts a tabular CSV file into a printable LaTeX table.

Author: Marko Laakso (Marko.Laakso@Helsinki.FI)
Version: 2.1
Categories: convert, Latex

Input name	Source	Description
tabledata	snpSurvival-survivalSet5-gene Rank-eqData.table 19.674	Table content
refs	snpSurvival-survivalSet5-refsGene Rank.in 19.679	Reference rules for the hyperlinks

Parameter name	Value	Description
attach	false	Include the original data as an attachment
caption	A list of survival affecting genes and the marker counts. P-value is the lowest value for the corresponding markers.	Caption text for the table.
colFormat	lcrp{11cm}	LaTeX tabular format for the columns. Default produces a centered layout for all columns.
columns	gene,markers,p-value,description	Comma separated list of column selections for the output. The empty default will use all columns.
countRows	false	Include a row count to the table caption.
dropMissing	true	This flag can be turned off in order to generate links with missing texts. Link text are substituted with target identifiers.
listCols		Comma separated list of column names. Columns of this list may contain several values separated with commas and the delimiters will be replaced with spaces.
numberFormat		A comma separated list of decimal formats for the columns. Each entry consists of the column name and the Java DecimalFormal pattern separated with equal sign. For example, rounding to three decimals can be done like: myColumn=#0.000
pageBreak	false	Use clear page after the table.
rename		Comma separated list of column renaming rules (oldname=newname). New names are used in table header but they do not affect the other behaviour of this component.
ruler	{}	Latex command for the row separating rulers
section	Gene rank	Section title for the table container or an empty string if no section should be generated.
sectionType	subsection	Type of LaTeX section: usually one of: section, subsection, or subsubsection. No section statement is written if section title is empty.
skipEmpty	false	This flag can be used to replace empty tables with a simple LaTeX comment.

19.676 snpSurvival-survivalSet5-geneRankQuery (INPUT)

Generates a list of genes and the marker counts for them.

Category: Internal

Parameter name	Value	Description
path	/gpfs/homes/mlaakso/asserSVN/andurilCaseStudy/sql/SNPinterestingGenes.sql	Path (filename) of the input resource.

19.677 snpSurvival-survivalSet5-missingDistC (CSV2Latex)

This component converts a tabular CSV file into a printable LaTeX table.

Author: Marko Laakso (Marko.Laakso@Helsinki.FI)
Version: 2.1
Categories: convert, Latex

Input name	Source	Description
tabledata	snpSurvival-survivalSet5-caseGenotypes-freqCheck.naDist 19.665	Table content

Parameter name	Value	Description
attach	false	Include the original data as an attachment
caption	Distribution of the missing values in case samples	Caption text for the table.
colFormat	p{3cm}r	LaTeX tabular format for the columns. Default produces a centered layout for all columns.
columns		Comma separated list of column selections for the output. The empty default will use all columns.
countRows	false	Include a row count to the table caption.
dropMissing	true	This flag can be turned off in order to generate links with missing texts. Link text are substituted with target identifiers.
listCols		Comma separated list of column names. Columns of this list may contain several values separated with commas and the delimiters will be replaced with spaces.
numberFormat		A comma separated list of decimal formats for the columns. Each entry consists of the column name and the Java DecimalFormal pattern separated with equal sign. For example, rounding to three decimals can be done like: myColumn=#0.000
pageBreak	false	Use clear page after the table.
rename		Comma separated list of column renaming rules (oldname=newname). New names are used in table header but they do not affect the other behaviour of this component.
ruler	{}	Latex command for the row separating rulers
section		Section title for the table container or an empty string if no section should be generated.
sectionType	subsection	Type of LaTeX section: usually one of: section, subsection, or subsubsection. No section statement is written if section title is empty.
skipEmpty	false	This flag can be used to replace empty tables with a simple LaTeX comment.

19.678 snpSurvival-survivalSet5-propertiesDoc (Properties2Latex)

Prepares a LaTeX-representation for the given set of properties files. The document consists of tables of names and values of the properties.

Author: Marko Laakso (Marko.Laakso@Helsinki.FI)
Version: 1.2
Categories: convert, Latex

Input name	Source	Description
props1	genotypeDB.in 19.320	A set of properties

Parameter name	Value	Description
hide		A comma separated list of property names that should remain invisible. You may use this paramerter to hide passwords and other confidentian settings.

Continued on next page...

Parameter name	Value	Description
keyWidth	5	Column width for the property names (centimeters)
merge	false	Merge all input properties into a single set
section	SNP database configuration	Section title for the table container or an empty string if no section should be generated
sectionType	subsection	Type of LaTeX section: usually one of section, subsection or subsubsection. No section statement is written if sectionTitle is empty.
valueWidth	13	Column width for the property values (centimeters)

19.679 snpSurvival-survivalSet5-refsGeneRank (INPUT)

Hyperlink rules for the gene rank table

Category: Internal

Parameter name	Value	Description
path	/gpfs/homes/mlaakso/asserSVN/andurilCaseStudy/csv/SNPrefsGeneRank.csv	Path (filename) of the input resource.

19.680 snpSurvival-survivalSet5-refsHits (INPUT)

Hyperlink rules for the marker annotations

Category: Internal

Parameter name	Value	Description
path	/gpfs/homes/mlaakso/asserSVN/andurilCaseStudy/csv/SNPrefsHits.csv	Path (filename) of the input resource.

19.681 snpSurvival-survivalSet5-reportBody (LatexCombiner)

LatexCombiner joins the given subdocuments together into one LaTeX file.

Authors: Kristian Ovaska (Kristian.Ovaska@Helsinki.FI), Marko Laakso (Marko.Laakso@Helsinki.FI)
Version: 1.3
Category: Latex

Input name	Source	Description
latex1	snpSurvival-survivalSet5-survivalReport.document 19.695	LaTeX fragment 1.
latex2	snpSurvival-survivalSet5-propertiesDoc.report 19.678	LaTeX fragment 2.
latex3	snpSurvival-survivalSet5-missingDistC.report 19.677	LaTeX fragment 3.

Parameter name	Value	Description
pagebreak	false	Determines if the result document should start with a page break.
sectionTitle		If non-empty, a declaration of a new section with the given name is inserted to the beginning of the combined document. This is a convenience feature to make it easy to compile subsections into a section.
sectionType	section	Type of LaTeX section: usually one of section, subsection or subsubsection. No section statement is written if sectionTitle is empty.
strictBorders	true	Enables the flushing of all document elements before each fragment

19.682 snpSurvival-survivalSet5-reportTemplate (LatexTemplate)

This component generates the standard layout configuration for Anduril output documents. The component produces a header and a footer fragment that can be used to build LATEX documents. The header fragment takes care of the page geometry and other technical details such as library imports and function declarations. Reference list is generated by the footer.

Author: Marko Laakso (Marko.Laakso@Helsinki.FI)
Version: 1.4
Category: Latex

Input name	Source	Description
abstract	abstract.in 19.42	Document abstract including its Latex definition. You may use template entities within this document. The default abstract has been defined in abstractTemplate.tex.

Parameter name	Value	Description
authors	Marko Laakso, Riku Louhimo, Sampsa Hautaniemi	A comma-separated list of document authors on the title page. If authors is empty but title is non-empty, a title page with no author name is printed.
baselineskip	1.2	Baselineskip factor that indicates the line spacing
bibstyle	abbrv	BibTeX reference formatting style
printTOC	true	If true, print Table Of Contents.
title	Anduril Case Study: Glioblastoma SNP survival analysis	Document title on the title page. If empty, no title page is printed. Also see author.
usepackage		Comma-separated list of LaTeX packages that are used in the document. Each package generates a \usepackage{X} line in the header.

19.683 snpSurvival-survivalSet5-snpAnnotations (INPUT)

Annotations for survival component.

Category: Internal

Parameter name	Value	Description
path	/gpfs/homes/mlaakso/asserSVN/andurilCaseStudy/csv/SNPAnnotation.csv	Path (filename) of the input resource.

19.684 snpSurvival-survivalSet5-summaryReport (LatexPDF)

This component is a LATEX compiler that generates a portable document format (PDF) file from the given Anduril document.

Author: Kristian Ovaska (Kristian.Ovaska@Helsinki.FI)
Version: 1.0
Category: Latex

Input name	Source	Description
document	snpSurvival-survivalSet5-reportBody.document 19.681	Body of the LaTeX document.
header	snpSurvival-survivalSet5-reportTemplate.header 19.682	LaTeX header that is written to the start of the document. If missing, a default header is used.
footer	snpSurvival-survivalSet5-reportTemplate.footer 19.682	LaTeX footer that is written to the end of the document. If missing, a default header is used.

Parameter name	Value	Description
bibtexExec	bibtex	Executable command for BibTeX.
latexExec	pdflatex	Executable command for pdflatex.
useRefs	false	This flag can be used to activate BibTeX compiler

19.685 snpSurvival-survivalSet5-survSNPAnnot (AnnotateSNPs)

Biological annotations for the survival affecting genes

Parameter name	Value
caption	Gene annotations and the loci of the survival affecting markers

19.686 snpSurvival-survivalSet5-survSNPAnnot-annotData (TableQuery)

Execute an SQL query on input relations and create a result table. TableQuery uses HSQLDB (Hyperthreaded Structured Query Language Database [25]) for executing the query. Consequently, the syntax of the query is defined by HSQLDB.

Author: Kristian Ovaska (Kristian.Ovaska@Helsinki.FI)
Version: 1.2
Categories: convert, Filter

Input name	Source	Description
table1	snpSurvival-survivalSet5-survSNPAnnot-snpRel.relation 19.690	CSV table 1. The table is referred to as 'table1' in the SQL query.
table2	snpSurvival-survivalSet5-survSNPAnnot-genes.bioAnnotation 19.688	CSV table 2. The table is referred to as 'table2' in the SQL query.
table3	snpSurvival-survivalSet5-survival.statistics 19.691	CSV table 3. The table is referred to as 'table3' in the SQL query.
query	snpSurvival-survivalSet5-annotSQL.in 19.663	SQL query. Either this file or the query parameter must be provided, but not both.

Parameter name	Value	Description
numIndices		Comma-separated list of index counts for input tables. All indices are single-column indices, running from column 1 to N, where N is retrieved from this parameter. If empty, the default number (1) is used. For example, ",2,,2" sets two indices for table2 and table4 and one index for the rest.
query		SQL query. Either this parameter or the query input must be provided, but not both.

19.687 snpSurvival-survivalSet5-survSNPAnnot-annotTable (CSV2Latex)

This component converts a tabular CSV file into a printable LaTeX table.

Author: Marko Laakso (Marko.Laakso@Helsinki.FI)
Version: 2.1
Categories: convert, Latex

Input name	Source	Description
tabledata	snpSurvival-survivalSet5-survSNPAnnot-annotData.table 19.686	Table content
refs	snpSurvival-survivalSet5-refsHits.in 19.680	Reference rules for the hyperlinks

Continued on next page...

Parameter name	Value	Description

Parameter name Value Description

Parameter name	Value	Description
attach	false	Include the original data as an attachment
caption	Gene annotations and the loci of the survival affecting markers	Caption text for the table.
colFormat	l@{}lr@{:}lp{1cm}p{1.5cm}p{10 .5cm}	LaTeX tabular format for the columns. Default produces a centered layout for all columns.
columns	hit,marker,chr,locus,band,gene, gene description	Comma separated list of column selections for the output. The empty default will use all columns.
countRows	false	Include a row count to the table caption.
dropMissing	true	This flag can be turned off in order to generate links with missing texts. Link text are substituted with target identifiers.
listCols	band	Comma separated list of column names. Columns of this list may contain several values separated with commas and the delimiters will be replaced with spaces.
numberFormat		A comma separated list of decimal formats for the columns. Each entry consists of the column name and the Java DecimalFormal pattern separated with equal sign. For example, rounding to three decimals can be done like: myColumn=#0.000
pageBreak	false	Use clear page after the table.
rename		Comma separated list of column renaming rules (oldname=newname). New names are used in table header but they do not affect the other behaviour of this component.
ruler	{}	Latex command for the row separating rulers
section		Section title for the table container or an empty string if no section should be generated.
sectionType	subsection	Type of LaTeX section: usually one of: section, subsection, or subsubsection. No section statement is written if section title is empty.
skipEmpty	false	This flag can be used to replace empty tables with a simple LaTeX comment.

19.688 snpSurvival-survivalSet5-survSNPAnnot-genes (KorvasieniAnnotator)

Korvasieni is an Ensembl [12] based converter for database identifiers and it can be used to convert genome location, gene, transcript, and translation identifiers across multiple biological databases. The selected `keyColumn` may contain entries with comma separated lists of values in which case `isListKey` parameter may be used to obtain each value separately and to process them as they were given in consecutive lines.

Author: Marko Laakso (Marko.Laakso@Helsinki.FI)
Version: 1.7
Categories: Annotation, GO

Input name	Source	Description
sourceKeys	snpSurvival-survivalSet5-survSNPAnnot-snpLoci. annotations 19.689	A list of source database keys.
connection	ensembl.in 19.273	Database connection can be defined using this file. The definition of parameters: database.url, database.user, database.password, database.timeout, database.recycle, and database.driver can be found from the documentation of Korvasieni.

Parameter name	Value	Description
echoColumns	refsnp_id	A comma separated list of column names for the columns that will be copied to the output. An asterisk (*) can be used to denote all columns execpt the keyColumn.
inputDB	.GeneId	Type of input keys. This must be a database supported by Korvasieni. If the parameter is omitted, the component tries to derive the database from the type of geneID. If this is not possible, an error is returned. You may define three columns in form of chromosome:start-end in case the inputDB is .DNARegion. This format provides a comfortable compatibility with DNARegion datatype.
inputType	Gene	Ensembl object type for the input keys (Any, Gene, Transcript, Translation)
isListKey	true	Enables the automatic value splits for the comma separated key column
keyColumn	ensembl_gene_stable_id	Name of the key column withing sourceKeys file or an empty string for the first column
maxHits	100000	Maximum number of target identifiers for a single source identifier
rename	UniProt/SwissProt=protein	Comma separated list of column renaming rules (oldname=newname)
skipLevel	never	Skip result rows if the source identifier is unknown or target identifiers are not available. Possible values are: never (no filtering), source (skip if the source ID is unknown), target (skip if no target IDs are found), any (skip if any of the target IDs is missing).

Continued on next page...

Parameter name	Value	Description
targetDB	.GeneName,.GeneDesc,.<br>DNABand,GO,UniProt/SwissProt	Comma-separated list of annotation types. Possible values are all databases supported by Korvasieni.
unique	true	This flag can be turned on in order to eliminate duplicate annotations.

19.689 snpSurvival-survivalSet5-survSNPAnnot-snpLoci (RefSNPAnnotator)

RefSNPAnnotator fetches annotations for given refSNP ID's (also known as rsID's) from BioMart databases.

The provided annotations for each rsID include alleles for the SNP, name of the chromosome where the SNP is located, chromosome location of the SNP, the strand of the chromosome, Ensembl gene ID for genes that overlap the SNP's location and validation status of the SNP. The component is implemented using the biomaRt package for Bioconductor [7].

Authors: Viljami Aittomaki (Viljami.Aittomaki@Helsinki.FI), Marko Laakso (Marko.Laakso@Helsinki.FI)
Version: 1.0.1
Categories: SNP, Annotation

Input name	Source	Description
rsIDs	snpSurvival-survivalSet5-survival<br>.statistics 19.691	List of rsID's for fetching annotations.

Parameter name	Value	Description
dataset	hsapiens_snp	Dataset to get annotations from. Different species have their own datasets. Example values include "hsapiens_snp" and "mmusculus_snp". See biomaRt documentation on how to list available datasets in R.
keyColumn	marker	Name of the key column withing sourceKeys file or an empty string for the first column.

19.690 snpSurvival-survivalSet5-survSNPAnnot-snpRel (ExpandCollapse)

Converts between the two possible representations of a relations with multivalued columns. By default this component splits rows with multivalued (comma separated lists) columns into multiple rows each representing a single value. The input may contain several columns with comma separated values and all permutations of column values are shown as individual single-valued rows. The alternative mode recovers the original comma separated values from the expanded relations. This is performed by joining values of the their list columns into comma separated lists.

Author: Marko Laakso (Marko.Laakso@Helsinki.FI)
Version: 1.3
Category: convert

Input name	Source	Description
relation	snpSurvival-survivalSet5-survSN<br>PAnnot-snpLoci.<br>annotations 19.689	The mandatory input relation

Parameter name	Value	Description
delim	,	Value delimiter between the list column values. Special characters can be encoded as specified in fi.helsinki.ltdk.csbl.asser.ArgumentEncoding.
expand	true	Action mode that is expand (true) or collapse (false)
listCols	ensembl_gene_stable_id	A comma separated list of column names of input (expand) or output (collapse) columns that may contain delim separated values. The asterisk refers to every column of the input relation.

19.691 snpSurvival-survivalSet5-survival (SNPKaplanMeier)

This component is used to estimate survival differences between various genotypes. The differences between the survival of different genotypes are measured using G-ρ family of tests [10].

Author: Marko Laakso (Marko.Laakso@Helsinki.FI)
Version: 1.5
Categories: SNP, Latex, Survival

Input name	Source	Description
genotypes	snpSurvival-survivalSet5-caseGenotypes-gtypes.genotypes 19.668	Genotype calls
survival	snpSurvival-survivalSet5-survivalClinical.table 19.692	Survival time and event indicator
annotations	snpSurvival-survivalSet5-snpAnnotations.in 19.683	Additional information about the markers. This data will be shown on the report of interesting markers.

Parameter name	Value	Description
PLimit	0.0010	Ignore results with a p-value greater than this limit.
confInt	0.95	Coverage of the confidence intervals [0,1]
minCount	15	Combine rare (aa) homozygotes to heterozygotes (Aa) if their number is below this limit. This parameter is used together with minFreq so that the actual limit is max(minFreq*n, minCount).
minFreq	0.1	Combine rare (aa) homozygotes to heterozygotes (Aa) if their frequency is below this limit. See also minCount.
naLimit	0.1	Skip markers where one minus call rate exceeds this limit.
nullCalls	–,ERR	A comma separated list of genotype calls that will be considered as missing values
sampleCol	sample	Column name for the sample name
showCI	true	Draw confidence intervals to the graph
skipMissingGT	true	Simply skip those samples that lack the genotype information.
statusCol	hasEvent	Column name for the event indicator
survRatePoint	0.5	Sampling height for the distances between the survival expectations. Negative values may be used to suppress these measurements.
timeCol	endTime	Column name for the survival time
timeOutLimit	36	Cut survival curves after this limit. A negative value can be used to maximize the length of the period.
timeStartCol	startTime	Column name for the start time that is reduced from the survival time
title	Genotype Specific Survival Estimates	Section title

19.692 snpSurvival-survivalSet5-survivalClinical (TableQuery)

Execute an SQL query on input relations and create a result table. TableQuery uses HSQLDB (Hyperthreaded Structured Query Language Database [25]) for executing the query. Consequently, the syntax of the query is defined by HSQLDB.

Author: Kristian Ovaska (Kristian.Ovaska@Helsinki.FI)
Version: 1.2
Categories: convert, Filter

Input name	Source	Description
table1	snpSurvival-survivalSet5-clinicalData.in 19.670	CSV table 1. The table is referred to as 'table1' in the SQL query.
query	snpSurvival-survivalSet5-survivalParamsQuery.file 19.693	SQL query. Either this file or the query parameter must be provided, but not both.
columnTypes	snpSurvival-survivalSet5-clinicalTypes.in 19.672	Contains SQL types for individual columns. If the file is not provided, the type is inferred from the contents of the columns. This can be used to force the use of VARCHAR for values that are also valid numerics. The file contains the columns Table (refers to one of table1 to table15), Column (refers to a column name in the table), Type (contains an SQL type). A row with Table='result', Column=X and Type='STRING' forces the use string values for result column X.

Parameter name	Value	Description
numIndices		Comma-separated list of index counts for input tables. All indices are single-column indices, running from column 1 to N, where N is retrieved from this parameter. If empty, the default number (1) is used. For example, ",2,,2" sets two indices for table2 and table4 and one index for the rest.
query		SQL query. Either this parameter or the query input must be provided, but not both.

19.693 snpSurvival-survivalSet5-survivalParamsQuery (SearchReplace)

Replaces the occurences of the given regular expressions with the corresponding regular expressions. This component is used for transforming given patterns of the input file to a changed form specified by the regular expressions .

Author: Marko Laakso (Marko.Laakso@Helsinki.FI)
Version: 1.0
Category: convert

Input name	Source	Description
file	snpSurvival-survivalSet5-survivalParamsTemplate.in 19.694	Source file to be modified

Parameter name	Value	Description
key00	REP.cond00	Search key 0
key01		Search key 1
key02		Search key 2
key03		Search key 3
key04		Search key 4
key05		Search key 5
key06		Search key 6
key07		Search key 7
key08		Search key 8
key09		Search key 9
value00	– No additional restrictions	Replacement 0
value01		Replacement 1
value02		Replacement 2
value03		Replacement 3
value04		Replacement 4
value05		Replacement 5
value06		Replacement 6
value07		Replacement 7
value08		Replacement 8
value09		Replacement 9

19.694 snpSurvival-survivalSet5-survivalParamsTemplate (INPUT)

Select relevant parameters for the survival analysis

Category: Internal

Parameter name	Value	Description
path	/gpfs/homes/mlaakso/asserSVN/anduriICaseStudy/sql/SNPsurvivalParams.sql	Path (filename) of the input resource.

19.695 snpSurvival-survivalSet5-survivalReport (LatexCombiner)

LatexCombiner joins the given subdocuments together into one LaTeX file.

Authors:	Kristian Ovaska (Kristian.Ovaska@Helsinki.FI), Marko Laakso (Marko.Laakso@Helsinki.FI)
Version:	1.3
Category:	Latex

Input name	Source	Description
latex1	snpSurvival-survivalSet5-survival .report 19.691	LaTeX fragment 1.
latex2	snpSurvival-survivalSet5-clinical Select.report 19.671	LaTeX fragment 2.
latex3	snpSurvival-survivalSet5-survSN PAnnot-annotTable.report 19.687	LaTeX fragment 3.
latex4	snpSurvival-survivalSet5-gene Rank-eqTable.report 19.675	LaTeX fragment 4.

Parameter name	Value	Description
pagebreak	true	Determines if the result document should start with a page break.
sectionTitle		If non-empty, a declaration of a new section with the given name is inserted to the beginning of the combined document. This is a convenience feature to make it easy to compile subsections into a section.
sectionType	section	Type of LaTeX section: usually one of section, subsection or subsubsection. No section statement is written if sectionTitle is empty.
strictBorders	true	Enables the flushing of all document elements before each fragment

19.696 snpSurvival-survivalSet6 (SurvivalAnalysis)

No description

Parameter name	Value
pvalue	0.0010

19.697 snpSurvival-survivalSet6-annotSQL (INPUT)

This SQL is used to generate marker annotation report.

Category: Internal

Parameter name	Value	Description
path	/gpfs/homes/mlaakso/asserSVN/ andurilCaseStudy/sql/ SNPgeneTable.sql	Path (filename) of the input resource.

19.698 snpSurvival-survivalSet6-caseGenotypes (GetGenotypes)

Load and preprocess the genotype calls for the cancer patient samples.

Parameter name	Value
skipFrq	0.1

19.699 snpSurvival-survivalSet6-caseGenotypes-freqCheck (AlleleCounter)

AlleleCounter calculates genotype frequencies for the biallelic markers of the given SNPMatrix. A Pearson's χ^2-test [18] is performed for each marker in order to test if the Hardy-Weinberg equilibrium [9] holds for the population sample.

Author: Marko Laakso (Marko.Laakso@Helsinki.FI)
Version: 1.2
Category: SNP

Input name	Source	Description
genotypes	snpSurvival-survivalSet6-case Genotypes-genotypes .genotypes 19.701	SNPHelistin genotype output.

Parameter name	Value	Description
dels	false	Accept homozygous deletions as a possible genotype.
naLimit	0.1	Skip markers where one minus call rate exceeds this limit.
skip	0.1	Drop markers with a relative minor allele frequency lower than this limit. Use negative values to inactivate this limit.

19.700 snpSurvival-survivalSet6-caseGenotypes-ftMarkers (CSV2IDList)

Extracts one column from the given CSV file and prints it out without duplicates. The selected column may contain entries with comma separated lists of values in which case *isList* parameter may be used to obtain each value separately.

Output is defined as:

$$ids = \bigcup_{i=1}^{9} \pi_{columnIn_i} \sigma_{regexp_i}(table_i). \quad (22)$$

Author: Marko Laakso (Marko.Laakso@Helsinki.FI)
Version: 1.3
Category: convert

Input name	Source	Description
table1	snpSurvival-survivalSet6-case Genotypes-freqCheck .frequencies 19.699	The mandatory input relation

Parameter name	Value	Description
acceptMissing	false	Files with missing columnIn are accepted as empty if this is true.
columnIn	marker	A comma separated list of column names for the IDs of interest in each file. Empty values refer to the first column of the file.
columnOut	marker	Name of the only column of the output list. Empty input refers to the name of the input column.
constants		A comma separated list of values that are always included into the output
isList	false	True if the seleted column contains a comma separated list of values to be splitted
quotation	false	Indicator that can be used to disable quotation of the output values
regexp1		Regular expression for the row filtering in table1. A row is included in the result if this parameter is empty or if values in the given columns match given regular expressions. The parameter has a format COLNAME1=EXPRESSION,COLNAME2=EXPRESSION2 where COLNAMEs are column names in "csv" and EXPRESSIONs are regular expressions using Java syntax. For example, "col=a|b" includes rows where the column col has a value of "a" or "b".
regexp2		Regular expression for the row filtering in table2.
regexp3		Regular expression for the row filtering in table3.
regexp4		Regular expression for the row filtering in table4.
regexp5		Regular expression for the row filtering in table5.
regexp6		Regular expression for the row filtering in table6.
regexp7		Regular expression for the row filtering in table7.
regexp8		Regular expression for the row filtering in table8.
regexp9		Regular expression for the row filtering in table9.

19.701 snpSurvival-survivalSet6-caseGenotypes-genotypes (SNPHelistinReader)

This component can be used to read SNP genotype calls for a set of samples and markers of interest. The genotype data is retrieved from a SNPHelistin database that is defined by the input properties.

Author:	Marko Laakso (Marko.Laakso@Helsinki.FI)
Version:	1.2
Categories:	Data Import, SNP

Input name	Source	Description
sampleNames	snpSurvival-survivalSet6-caseNames.ids 19.703	List of sample names of interest.
markerNames	snpSurvival-markers6.in 19.454	List of SNP identifiers of interest.
config	genotypeDB.in 19.320	Connection parameters for SNPHelistin.

Parameter name	Value	Description
errorSymbol	ERR	Symbol for failed measurements. You may for example use 'NA' value in order consider them equal to missing values.
skipUnknown	true	Ignore unknown samples and do not process them. If the parameter is false then the program fails for these samples.

19.702 snpSurvival-survivalSet6-caseGenotypes-gtypes (SNPHelistinReader)

This component can be used to read SNP genotype calls for a set of samples and markers of interest. The genotype data is retrieved from a SNPHelistin database that is defined by the input properties.

Author:	Marko Laakso (Marko.Laakso@Helsinki.FI)
Version:	1.2
Categories:	Data Import, SNP

Input name	Source	Description
sampleNames	snpSurvival-survivalSet6-caseNames.ids 19.703	List of sample names of interest.
markerNames	snpSurvival-survivalSet6-caseGenotypes-ftMarkers.ids 19.700	List of SNP identifiers of interest.
config	genotypeDB.in 19.320	Connection parameters for SNPHelistin.

Parameter name	Value	Description
errorSymbol	ERR	Symbol for failed measurements. You may for example use 'NA' value in order consider them equal to missing values.
skipUnknown	true	Ignore unknown samples and do not process them. If the parameter is false then the program fails for these samples.

19.703 snpSurvival-survivalSet6-caseNames (CSV2IDList)

Extracts one column from the given CSV file and prints it out without duplicates. The selected column may contain entries with comma separated lists of values in which case *isList* parameter may be used to obtain each value separately.

Output is defined as:

$$ids = \bigcup_{i=1}^{9} \pi_{columnIn_i} \sigma_{regexp_i}(table_i). \tag{23}$$

Author: Marko Laakso (Marko.Laakso@Helsinki.FI)
Version: 1.3
Category: convert

Input name	Source	Description
table1	snpSurvival-survivalSet6-survival Clinical.table 19.726	The mandatory input relation

Parameter name	Value	Description
acceptMissing	false	Files with missing columnIn are accepted as empty if this is true.
columnIn		A comma separated list of column names for the IDs of interest in each file. Empty values refer to the first column of the file.
columnOut	SampleId	Name of the only column of the output list. Empty input refers to the name of the input column.
constants		A comma separated list of values that are always included into the output
isList	false	True if the seleted column contains a comma separated list of values to be splitted
quotation	false	Indicator that can be used to disable quotation of the output values
regexp1		Regular expression for the row filtering in table1. A row is included in the result if this parameter is empty or if values in the given columns match given regular expressions. The parameter has a format COLNAME1=EXPRESSION,COLNAME2=EXPRESSION2 where COLNAMEs are column names in "csv" and EXPRESSIONs are regular expressions using Java syntax. For example, "col=a|b" includes rows where the column col has a value of "a" or "b".
regexp2		Regular expression for the row filtering in table2.
regexp3		Regular expression for the row filtering in table3.
regexp4		Regular expression for the row filtering in table4.
regexp5		Regular expression for the row filtering in table5.
regexp6		Regular expression for the row filtering in table6.
regexp7		Regular expression for the row filtering in table7.
regexp8		Regular expression for the row filtering in table8.
regexp9		Regular expression for the row filtering in table9.

19.704 snpSurvival-survivalSet6-clinicalData (INPUT)

Clinical parameters for the case samples

Category: Internal

Parameter name	Value	Description
path	/gpfs/homes/mlaakso/asserSVN/anduril CaseStudy/data/snp/patient-metadata-caucas.csv	Path (filename) of the input resource.

19.705 snpSurvival-survivalSet6-clinicalSelect (SourceCode2Latex)

This component converts a source code listing into a LaTeXfragment that may be included into some technical documents.

Author: Marko Laakso (Marko.Laakso@Helsinki.FI)
Version: 1.0
Categories: convert, Latex

Input name	Source	Description
source	snpSurvival-survivalSet6-survival ParamsQuery.file 19.727	Source code

Parameter name	Value	Description
caption	This SQL query has been used to select clinical parameters for the survival calculations.	Caption text for the listing
language	SQL	Language defintion for the syntax highlighting. Possible values can be found from the description of listings package.

19.706 snpSurvival-survivalSet6-clinicalTypes (INPUT)

Column types for the clinical data

Category: Internal

Parameter name	Value	Description
path	/gpfs/homes/mlaakso/asserSVN/andurilCaseStudy/csv/SNPclinicalTypes.csv	Path (filename) of the input resource.

19.707 snpSurvival-survivalSet6-geneRank (QueryReport)

(Technical documentation) Executes an SQL query and prints it with the result table.

Author: Marko Laakso (Marko.Laakso@Helsinki.FI)
Version: 1.1
Category: Latex

Input name	Source	Description
table1	snpSurvival-survivalSet6-survSNPAnnot.fullData 19.719	1. data table that is referred as 'table1' in the query

Parameter name	Value	Description
colFormat	lcrp{11cm}	LaTeX tabular formatting for the result columns
listCols		Comma separated list of column names. Columns of this list may contain several values separated with commas and the delimiters will be replaced with spaces.
resTableRef	the previous table	Textual reference from the SQL representation to the result table
section	Gene rank	Subsection title for the output document
showCols	gene,markers,p-value,description	Comma separated list of column selections for the output. The empty default will use all columns.
showQuery	false	This flag tells if the SQL shall be shown on the report
tableCaption	A list of survival affecting genes and the marker counts. P-value is the lowest value for the corresponding markers.	Description for the result table

19.708 snpSurvival-survivalSet6-geneRank-eqData (TableQuery)

Execute an SQL query on input relations and create a result table. TableQuery uses HSQLDB (Hyper-threaded Structured Query Language Database [25]) for executing the query. Consequently, the syntax of the query is defined by HSQLDB.

Author: Kristian Ovaska (Kristian.Ovaska@Helsinki.FI)
Version: 1.2
Categories: convert, Filter

Input name	Source	Description
table1	snpSurvival-survivalSet6-survSNPAnnot-annotData.table 19.720	CSV table 1. The table is referred to as 'table1' in the SQL query.

Continued on next page...

Input name	Source	Description
table2	snpSurvival-survivalSet6-survival .statistics 19.725	CSV table 2. The table is referred to as 'table2' in the SQL query.
query	snpSurvival-survivalSet6-gene RankQuery.in 19.710	SQL query. Either this file or the query parameter must be provided, but not both.

Parameter name	Value	Description
numIndices		Comma-separated list of index counts for input tables. All indices are single-column indices, running from column 1 to N, where N is retrieved from this parameter. If empty, the default number (1) is used. For example, ",2,,2" sets two indices for table2 and table4 and one index for the rest.
query		SQL query. Either this parameter or the query input must be provided, but not both.

19.709 snpSurvival-survivalSet6-geneRank-eqTable (CSV2Latex)

This component converts a tabular CSV file into a printable LaTeX table.

Author:	Marko Laakso (Marko.Laakso@Helsinki.FI)
Version:	2.1
Categories:	convert, Latex

Input name	Source	Description
tabledata	snpSurvival-survivalSet6-gene Rank-eqData.table 19.708	Table content
refs	snpSurvival-survivalSet6-refsGene Rank.in 19.713	Reference rules for the hyperlinks

Parameter name	Value	Description
attach	false	Include the original data as an attachment
caption	A list of survival affecting genes and the marker counts. P-value is the lowest value for the corresponding markers.	Caption text for the table.
colFormat	lcrp{11cm}	LaTeX tabular format for the columns. Default produces a centered layout for all columns.
columns	gene,markers,p-value,description	Comma separated list of column selections for the output. The empty default will use all columns.
countRows	false	Include a row count to the table caption.
dropMissing	true	This flag can be turned off in order to generate links with missing texts. Link text are substituted with target identifiers.
listCols		Comma separated list of column names. Columns of this list may contain several values separated with commas and the delimiters will be replaced with spaces.
numberFormat		A comma separated list of decimal formats for the columns. Each entry consists of the column name and the Java DecimalFormal pattern separated with equal sign. For example, rounding to three decimals can be done like: myColumn=#0.000
pageBreak	false	Use clear page after the table.
rename		Comma separated list of column renaming rules (oldname=newname). New names are used in table header but they do not affect the other behaviour of this component.
ruler	{}	Latex command for the row separating rulers
section	Gene rank	Section title for the table container or an empty string if no section should be generated.
sectionType	subsection	Type of LaTeX section: usually one of: section, subsection, or subsubsection. No section statement is written if section title is empty.
skipEmpty	false	This flag can be used to replace empty tables with a simple LaTeX comment.

19.710 snpSurvival-survivalSet6-geneRankQuery (INPUT)

Generates a list of genes and the marker counts for them.

Category:	Internal

Parameter name	Value	Description
path	/gpfs/homes/mlaakso/asserSVN/ andurilCaseStudy/sql/ SNPinterestingGenes.sql	Path (filename) of the input resource.

19.711 snpSurvival-survivalSet6-missingDistC (CSV2Latex)

This component converts a tabular CSV file into a printable LATEX table.

Author: Marko Laakso (Marko.Laakso@Helsinki.FI)
Version: 2.1
Categories: convert, Latex

Input name	Source	Description
tabledata	snpSurvival-survivalSet6-case Genotypes-freqCheck.na Dist 19.699	Table content

Parameter name	Value	Description
attach	false	Include the original data as an attachment
caption	Distribution of the missing values in case samples	Caption text for the table.
colFormat	p{3cm}r	LaTeX tabular format for the columns. Default produces a centered layout for all columns.
columns		Comma separated list of column selections for the output. The empty default will use all columns.
countRows	false	Include a row count to the table caption.
dropMissing	true	This flag can be turned off in order to generate links with missing texts. Link text are substituted with target identifiers.
listCols		Comma separated list of column names. Columns of this list may contain several values separated with commas and the delimiters will be replaced with spaces.
numberFormat		A comma separated list of decimal formats for the columns. Each entry consists of the column name and the Java DecimalFormal pattern separated with equal sign. For example, rounding to three decimals can be done like: myColumn=#0.000
pageBreak	false	Use clear page after the table.
rename		Comma separated list of column renaming rules (oldname=newname). New names are used in table header but they do not affect the other behaviour of this component.
ruler	{}	Latex command for the row separating rulers
section		Section title for the table container or an empty string if no section should be generated.
sectionType	subsection	Type of LaTeX section: usually one of: section, subsection, or subsubsection. No section statement is written if section title is empty.
skipEmpty	false	This flag can be used to replace empty tables with a simple LaTeX comment.

19.712 snpSurvival-survivalSet6-propertiesDoc (Properties2Latex)

Prepares a LATEX-representation for the given set of properties files. The document consists of tables of names and values of the properties.

Author: Marko Laakso (Marko.Laakso@Helsinki.FI)
Version: 1.2
Categories: convert, Latex

Input name	Source	Description
props1	genotypeDB.in 19.320	A set of properties

Parameter name	Value	Description
hide		A comma separated list of property names that should remain invisible. You may use this paramerter to hide passwords and other confidentian settings.

Continued on next page...

Parameter name	Value	Description
keyWidth	5	Column width for the property names (centimeters)
merge	false	Merge all input properties into a single set
section	SNP database configuration	Section title for the table container or an empty string if no section should be generated
sectionType	subsection	Type of LaTeX section: usually one of section, subsection or subsubsection. No section statement is written if sectionTitle is empty.
valueWidth	13	Column width for the property values (centimeters)

19.713 snpSurvival-survivalSet6-refsGeneRank (INPUT)

Hyperlink rules for the gene rank table

Category: Internal

Parameter name	Value	Description
path	/gpfs/homes/mlaakso/asserSVN/andurilCaseStudy/csv/SNPrefsGeneRank.csv	Path (filename) of the input resource.

19.714 snpSurvival-survivalSet6-refsHits (INPUT)

Hyperlink rules for the marker annotations

Category: Internal

Parameter name	Value	Description
path	/gpfs/homes/mlaakso/asserSVN/andurilCaseStudy/csv/SNPrefsHits.csv	Path (filename) of the input resource.

19.715 snpSurvival-survivalSet6-reportBody (LatexCombiner)

LatexCombiner joins the given subdocuments together into one LaTeX file.

Authors: Kristian Ovaska (Kristian.Ovaska@Helsinki.FI), Marko Laakso (Marko.Laakso@Helsinki.FI)
Version: 1.3
Category: Latex

Input name	Source	Description
latex1	snpSurvival-survivalSet6-survivalReport.document 19.729	LaTeX fragment 1.
latex2	snpSurvival-survivalSet6-propertiesDoc.report 19.712	LaTeX fragment 2.
latex3	snpSurvival-survivalSet6-missingDistC.report 19.711	LaTeX fragment 3.

Parameter name	Value	Description
pagebreak	false	Determines if the result document should start with a page break.
sectionTitle		If non-empty, a declaration of a new section with the given name is inserted to the beginning of the combined document. This is a convenience feature to make it easy to compile subsections into a section.
sectionType	section	Type of LaTeX section: usually one of section, subsection or subsubsection. No section statement is written if sectionTitle is empty.
strictBorders	true	Enables the flushing of all document elements before each fragment

19.716 snpSurvival-survivalSet6-reportTemplate (LatexTemplate)

This component generates the standard layout configuration for Anduril output documents. The component produces a header and a footer fragment that can be used to build LATEX documents. The header fragment takes care of the page geometry and other technical details such as library imports and function declarations. Reference list is generated by the footer.

Author: Marko Laakso (Marko.Laakso@Helsinki.FI)
Version: 1.4
Category: Latex

Input name	Source	Description
abstract	abstract.in 19.42	Document abstract including its Latex definition. You may use template entities within this document. The default abstract has been defined in abstractTemplate.tex.

Parameter name	Value	Description
authors	Marko Laakso, Riku Louhimo, Sampsa Hautaniemi	A comma-separated list of document authors on the title page. If authors is empty but title is non-empty, a title page with no author name is printed.
baselineskip	1.2	Baselineskip factor that indicates the line spacing
bibstyle	abbrv	BibTeX reference formatting style
printTOC	true	If true, print Table Of Contents.
title	Anduril Case Study: Glioblastoma SNP survival analysis	Document title on the title page. If empty, no title page is printed. Also see author.
usepackage		Comma-separated list of LaTeX packages that are used in the document. Each package generates a \usepackage{X} line in the header.

19.717 snpSurvival-survivalSet6-snpAnnotations (INPUT)

Annotations for survival component.

Category: Internal

Parameter name	Value	Description
path	/gpfs/homes/mlaakso/asserSVN/andurilCaseStudy/csv/SNPAnnotation.csv	Path (filename) of the input resource.

19.718 snpSurvival-survivalSet6-summaryReport (LatexPDF)

This component is a LATEX compiler that generates a portable document format (PDF) file from the given Anduril document.

Author: Kristian Ovaska (Kristian.Ovaska@Helsinki.FI)
Version: 1.0
Category: Latex

Input name	Source	Description
document	snpSurvival-survivalSet6-reportBody.document 19.715	Body of the LaTeX document.
header	snpSurvival-survivalSet6-reportTemplate.header 19.716	LaTeX header that is written to the start of the document. If missing, a default header is used.
footer	snpSurvival-survivalSet6-reportTemplate.footer 19.716	LaTeX footer that is written to the end of the document. If missing, a default header is used.

Parameter name	Value	Description
bibtexExec	bibtex	Executable command for BibTeX.
latexExec	pdflatex	Executable command for pdflatex.
useRefs	false	This flag can be used to activate BibTeX compiler

19.719 snpSurvival-survivalSet6-survSNPAnnot (AnnotateSNPs)

Biological annotations for the survival affecting genes

Parameter name	Value
caption	Gene annotations and the loci of the survival affecting markers

19.720 snpSurvival-survivalSet6-survSNPAnnot-annotData (TableQuery)

Execute an SQL query on input relations and create a result table. TableQuery uses HSQLDB (Hyperthreaded Structured Query Language Database [25]) for executing the query. Consequently, the syntax of the query is defined by HSQLDB.

Author: Kristian Ovaska (Kristian.Ovaska@Helsinki.FI)
Version: 1.2
Categories: convert, Filter

Input name	Source	Description
table1	snpSurvival-survivalSet6-survSNPAnnot-snpRel.relation 19.724	CSV table 1. The table is referred to as 'table1' in the SQL query.
table2	snpSurvival-survivalSet6-survSNPAnnot-genes.bioAnnotation 19.722	CSV table 2. The table is referred to as 'table2' in the SQL query.
table3	snpSurvival-survivalSet6-survival.statistics 19.725	CSV table 3. The table is referred to as 'table3' in the SQL query.
query	snpSurvival-survivalSet6-annotSQL.in 19.697	SQL query. Either this file or the query parameter must be provided, but not both.

Parameter name	Value	Description
numIndices		Comma-separated list of index counts for input tables. All indices are single-column indices, running from column 1 to N, where N is retrieved from this parameter. If empty, the default number (1) is used. For example, ",2,,2" sets two indices for table2 and table4 and one index for the rest.
query		SQL query. Either this parameter or the query input must be provided, but not both.

19.721 snpSurvival-survivalSet6-survSNPAnnot-annotTable (CSV2Latex)

This component converts a tabular CSV file into a printable LaTeX table.

Author: Marko Laakso (Marko.Laakso@Helsinki.FI)
Version: 2.1
Categories: convert, Latex

Input name	Source	Description
tabledata	snpSurvival-survivalSet6-survSNPAnnot-annotData.table 19.720	Table content
refs	snpSurvival-survivalSet6-refsHits.in 19.714	Reference rules for the hyperlinks

Continued on next page...

Parameter name Value Description

Parameter name	Value	Description
attach	false	Include the original data as an attachment
caption	Gene annotations and the loci of the survival affecting markers	Caption text for the table.
colFormat	l@{}lr@{:}lp{1cm}p{1.5cm}p{10 .5cm}	LaTeX tabular format for the columns. Default produces a centered layout for all columns.
columns	hit,marker,chr,locus,band,gene, gene description	Comma separated list of column selections for the output. The empty default will use all columns.
countRows	false	Include a row count to the table caption.
dropMissing	true	This flag can be turned off in order to generate links with missing texts. Link text are substituted with target identifiers.
listCols	band	Comma separated list of column names. Columns of this list may contain several values separated with commas and the delimiters will be replaced with spaces.
numberFormat		A comma separated list of decimal formats for the columns. Each entry consists of the column name and the Java DecimalFormal pattern separated with equal sign. For example, rounding to three decimals can be done like: myColumn=#0.000
pageBreak	false	Use clear page after the table.
rename		Comma separated list of column renaming rules (oldname=newname). New names are used in table header but they do not affect the other behaviour of this component.
ruler	{}	Latex command for the row separating rulers
section		Section title for the table container or an empty string if no section should be generated.
sectionType	subsection	Type of LaTeX section: usually one of: section, subsection, or subsubsection. No section statement is written if section title is empty.
skipEmpty	false	This flag can be used to replace empty tables with a simple LaTeX comment.

19.722 snpSurvival-survivalSet6-survSNPAnnot-genes (KorvasieniAnnotator)

Korvasieni is an Ensembl [12] based converter for database identifiers and it can be used to convert genome location, gene, transcript, and translation identifiers across multiple biological databases. The selected `keyColumn` may contain entries with comma separated lists of values in which case `isListKey` parameter may be used to obtain each value separately and to process them as they were given in consecutive lines.

Author: Marko Laakso (Marko.Laakso@Helsinki.FI)
Version: 1.7
Categories: Annotation, GO

Input name	Source	Description
sourceKeys	snpSurvival-survivalSet6-survSNPAnnot-snpLoci. annotations 19.723	A list of source database keys.
connection	ensembl.in 19.273	Database connection can be defined using this file. The definition of parameters: database.url, database.user, database.password, database.timeout, database.recycle, and database.driver can be found from the documentation of Korvasieni.

Parameter name	Value	Description
echoColumns	refsnp_id	A comma separated list of column names for the columns that will be copied to the output. An asterisk (*) can be used to denote all columns execpt the keyColumn.
inputDB	.GeneId	Type of input keys. This must be a database supported by Korvasieni. If the parameter is omitted, the component tries to derive the database from the type of geneID. If this is not possible, an error is returned. You may define three columns in form of chromosome:start-end in case the inputDB is .DNARegion. This format provides a comfortable compatibility with DNARegion datatype.
inputType	Gene	Ensembl object type for the input keys (Any, Gene, Transcript, Translation)
isListKey	true	Enables the automatic value splits for the comma separated key column
keyColumn	ensembl_gene_stable_id	Name of the key column withing sourceKeys file or an empty string for the first column
maxHits	100000	Maximum number of target identifiers for a single source identifier
rename	UniProt/SwissProt=protein	Comma separated list of column renaming rules (oldname=newname)
skipLevel	never	Skip result rows if the source identifier is unknown or target identifiers are not available. Possible values are: never (no filtering), source (skip if the source ID is unknown), target (skip if no target IDs are found), any (skip if any of the target IDs is missing).

Continued on next page...

Parameter name	Value	Description
targetDB	.GeneName,.GeneDesc,. DNABand,GO,UniProt/SwissProt	Comma-separated list of annotation types. Possible values are all databases supported by Korvasieni.
unique	true	This flag can be turned on in order to eliminate duplicate annotations.

19.723 snpSurvival-survivalSet6-survSNPAnnot-snpLoci (RefSNPAnnotator)

RefSNPAnnotator fetches annotations for given refSNP ID's (also known as rsID's) from BioMart databases.

The provided annotations for each rsID include alleles for the SNP, name of the chromosome where the SNP is located, chromosome location of the SNP, the strand of the chromosome, Ensembl gene ID for genes that overlap the SNP's location and validation status of the SNP. The component is implemented using the biomaRt package for Bioconductor [7].

Authors: Viljami Aittomaki (Viljami.Aittomaki@Helsinki.FI), Marko Laakso (Marko.Laakso@Helsinki.FI)
Version: 1.0.1
Categories: SNP, Annotation

Input name	Source	Description
rsIDs	snpSurvival-survivalSet6-survival .statistics 19.725	List of rsID's for fetching annotations.

Parameter name	Value	Description
dataset	hsapiens_snp	Dataset to get annotations from. Different species have their own datasets. Example values include "hsapiens_snp" and "mmusculus_snp". See biomaRt documentation on how to list available datasets in R.
keyColumn	marker	Name of the key column withing sourceKeys file or an empty string for the first column.

19.724 snpSurvival-survivalSet6-survSNPAnnot-snpRel (ExpandCollapse)

Converts between the two possible representations of a relations with multivalued columns. By default this component splits rows with multivalued (comma separated lists) columns into multiple rows each representing a single value. The input may contain several columns with comma separated values and all permutations of column values are shown as individual single-valued rows. The alternative mode recovers the original comma separated values from the expanded relations. This is performed by joining values of the their list columns into comma separated lists.

Author: Marko Laakso (Marko.Laakso@Helsinki.FI)
Version: 1.3
Category: convert

Input name	Source	Description
relation	snpSurvival-survivalSet6-survSNPAnnot-snpLoci. annotations 19.723	The mandatory input relation

Parameter name	Value	Description
delim	,	Value delimiter between the list column values. Special characters can be encoded as specified in fi.helsinki.ltdk.csbl.asser.ArgumentEncoding.
expand	true	Action mode that is expand (true) or collapse (false)
listCols	ensembl_gene_stable_id	A comma separated list of column names of input (expand) or output (collapse) columns that may contain delim separated values. The asterisk refers to every column of the input relation.

19.725 snpSurvival-survivalSet6-survival (SNPKaplanMeier)

This component is used to estimate survival differences between various genotypes. The differences between the survival of different genotypes are measured using G-ρ family of tests [10].

Author: Marko Laakso (Marko.Laakso@Helsinki.FI)
Version: 1.5
Categories: SNP, Latex, Survival

Input name	Source	Description
genotypes	snpSurvival-survivalSet6-caseGenotypes-gtypes.genotypes 19.702	Genotype calls
survival	snpSurvival-survivalSet6-survivalClinical.table 19.726	Survival time and event indicator
annotations	snpSurvival-survivalSet6-snpAnnotations.in 19.717	Additional information about the markers. This data will be shown on the report of interesting markers.

Parameter name	Value	Description
PLimit	0.0010	Ignore results with a p-value greater than this limit.
confInt	0.95	Coverage of the confidence intervals [0,1]
minCount	15	Combine rare (aa) homozygotes to heterozygotes (Aa) if their number is below this limit. This parameter is used together with minFreq so that the actual limit is max(minFreq*n, minCount).
minFreq	0.1	Combine rare (aa) homozygotes to heterozygotes (Aa) if their frequency is below this limit. See also minCount.
naLimit	0.1	Skip markers where one minus call rate exceeds this limit.
nullCalls	–,ERR	A comma separated list of genotype calls that will be considered as missing values
sampleCol	sample	Column name for the sample name
showCI	true	Draw confidence intervals to the graph
skipMissingGT	true	Simply skip those samples that lack the genotype information.
statusCol	hasEvent	Column name for the event indicator
survRatePoint	0.5	Sampling height for the distances between the survival expectations. Negative values may be used to suppress these measurements.
timeCol	endTime	Column name for the survival time
timeOutLimit	36	Cut survival curves after this limit. A negative value can be used to maximize the length of the period.
timeStartCol	startTime	Column name for the start time that is reduced from the survival time
title	Genotype Specific Survival Estimates	Section title

19.726 snpSurvival-survivalSet6-survivalClinical (TableQuery)

Execute an SQL query on input relations and create a result table. TableQuery uses HSQLDB (Hyperthreaded Structured Query Language Database [25]) for executing the query. Consequently, the syntax of the query is defined by HSQLDB.

Author: Kristian Ovaska (Kristian.Ovaska@Helsinki.FI)
Version: 1.2
Categories: convert, Filter

Input name	Source	Description
table1	snpSurvival-survivalSet6-clinicalData.in 19.704	CSV table 1. The table is referred to as 'table1' in the SQL query.
query	snpSurvival-survivalSet6-survivalParamsQuery.file 19.727	SQL query. Either this file or the query parameter must be provided, but not both.
columnTypes	snpSurvival-survivalSet6-clinicalTypes.in 19.706	Contains SQL types for individual columns. If the file is not provided, the type is inferred from the contents of the columns. This can be used to force the use of VARCHAR for values that are also valid numerics. The file contains the columns Table (refers to one of table1 to table15), Column (refers to a column name in the table), Type (contains an SQL type). A row with Table='result', Column=X and Type='STRING' forces the use string values for result column X.

Parameter name	Value	Description
numIndices		Comma-separated list of index counts for input tables. All indices are single-column indices, running from column 1 to N, where N is retrieved from this parameter. If empty, the default number (1) is used. For example, ",2,,2" sets two indices for table2 and table4 and one index for the rest.
query		SQL query. Either this parameter or the query input must be provided, but not both.

19.727 snpSurvival-survivalSet6-survivalParamsQuery (SearchReplace)

Replaces the occurences of the given regular expressions with the corresponding regular expressions. This component is used for transforming given patterns of the input file to a changed form specified by the regular expressions .

Author: Marko Laakso (Marko.Laakso@Helsinki.FI)
Version: 1.0
Category: convert

Input name	Source	Description
file	snpSurvival-survivalSet6-survival ParamsTemplate.in 19.728	Source file to be modified

Parameter name	Value	Description
key00	REP.cond00	Search key 0
key01		Search key 1
key02		Search key 2
key03		Search key 3
key04		Search key 4
key05		Search key 5
key06		Search key 6
key07		Search key 7
key08		Search key 8
key09		Search key 9
value00	– No additional restrictions	Replacement 0
value01		Replacement 1
value02		Replacement 2
value03		Replacement 3
value04		Replacement 4
value05		Replacement 5
value06		Replacement 6
value07		Replacement 7
value08		Replacement 8
value09		Replacement 9

19.728 snpSurvival-survivalSet6-survivalParamsTemplate (INPUT)

Select relevant parameters for the survival analysis

Category: Internal

Parameter name	Value	Description
path	/gpfs/homes/mlaakso/asserSVN/ andurilCaseStudy/sql/ SNPsurvivalParams.sql	Path (filename) of the input resource.

19.729 snpSurvival-survivalSet6-survivalReport (LatexCombiner)

LatexCombiner joins the given subdocuments together into one LaTeX file.

Authors:	Kristian Ovaska (Kristian.Ovaska@Helsinki.FI), Marko Laakso (Marko.Laakso@Helsinki.FI)
Version:	1.3
Category:	Latex

Input name	Source	Description
latex1	snpSurvival-survivalSet6-survival .report 19.725	LaTeX fragment 1.
latex2	snpSurvival-survivalSet6-clinical Select.report 19.705	LaTeX fragment 2.
latex3	snpSurvival-survivalSet6-survSN PAnnot-annotTable.report 19.721	LaTeX fragment 3.
latex4	snpSurvival-survivalSet6-gene Rank-eqTable.report 19.709	LaTeX fragment 4.

Parameter name	Value	Description
pagebreak	true	Determines if the result document should start with a page break.
sectionTitle		If non-empty, a declaration of a new section with the given name is inserted to the beginning of the combined document. This is a convenience feature to make it easy to compile subsections into a section.
sectionType	section	Type of LaTeX section: usually one of section, subsection or subsubsection. No section statement is written if sectionTitle is empty.
strictBorders	true	Enables the flushing of all document elements before each fragment

19.730 snpSurvival-survivalSet7 (SurvivalAnalysis)

No description

Parameter name	Value
pvalue	0.0010

19.731 snpSurvival-survivalSet7-annotSQL (INPUT)

This SQL is used to generate marker annotation report.

Category: Internal

Parameter name	Value	Description
path	/gpfs/homes/mlaakso/asserSVN/ andurilCaseStudy/sql/ SNPgeneTable.sql	Path (filename) of the input resource.

19.732 snpSurvival-survivalSet7-caseGenotypes (GetGenotypes)

Load and preprocess the genotype calls for the cancer patient samples.

Parameter name	Value
skipFrq	0.1

19.733 snpSurvival-survivalSet7-caseGenotypes-freqCheck (AlleleCounter)

AlleleCounter calculates genotype frequencies for the biallelic markers of the given SNPMatrix. A Pearson's χ^2-test [18] is performed for each marker in order to test if the Hardy-Weinberg equilibrium [9] holds for the population sample.

Author:	Marko Laakso (Marko.Laakso@Helsinki.FI)
Version:	1.2
Category:	SNP

Input name	Source	Description
genotypes	snpSurvival-survivalSet7-case Genotypes-genotypes .genotypes 19.735	SNPHelistin genotype output.

Parameter name	Value	Description
dels	false	Accept homozygous deletions as a possible genotype.
naLimit	0.1	Skip markers where one minus call rate exceeds this limit.
skip	0.1	Drop markers with a relative minor allele frequency lower than this limit. Use negative values to inactivate this limit.

19.734 snpSurvival-survivalSet7-caseGenotypes-ftMarkers (CSV2IDList)

Extracts one column from the given CSV file and prints it out without duplicates. The selected column may contain entries with comma separated lists of values in which case *isList* parameter may be used to obtain each value separately.

Output is defined as:

$$ids = \bigcup_{i=1}^{9} \pi_{columnIn_i} \sigma_{regexp_i}(table_i). \quad (24)$$

Author:	Marko Laakso (Marko.Laakso@Helsinki.FI)
Version:	1.3
Category:	convert

Input name	Source	Description
table1	snpSurvival-survivalSet7-case Genotypes-freqCheck .frequencies 19.733	The mandatory input relation

Parameter name	Value	Description
acceptMissing	false	Files with missing columnIn are accepted as empty if this is true.
columnIn	marker	A comma separated list of column names for the IDs of interest in each file. Empty values refer to the first column of the file.
columnOut	marker	Name of the only column of the output list. Empty input refers to the name of the input column.
constants		A comma separated list of values that are always included into the output
isList	false	True if the seleted column contains a comma separated list of values to be splitted
quotation	false	Indicator that can be used to disable quotation of the output values
regexp1		Regular expression for the row filtering in table1. A row is included in the result if this parameter is empty or if values in the given columns match given regular expressions. The parameter has a format COLNAME1=EXPRESSION,COLNAME2=EXPRESSION2 where COLNAMEs are column names in "csv" and EXPRESSIONs are regular expressions using Java syntax. For example, "col=a|b" includes rows where the column col has a value of "a" or "b".
regexp2		Regular expression for the row filtering in table2.
regexp3		Regular expression for the row filtering in table3.
regexp4		Regular expression for the row filtering in table4.
regexp5		Regular expression for the row filtering in table5.
regexp6		Regular expression for the row filtering in table6.
regexp7		Regular expression for the row filtering in table7.
regexp8		Regular expression for the row filtering in table8.
regexp9		Regular expression for the row filtering in table9.

19.735 snpSurvival-survivalSet7-caseGenotypes-genotypes (SNPHelistinReader)

This component can be used to read SNP genotype calls for a set of samples and markers of interest. The genotype data is retrieved from a SNPHelistin database that is defined by the input properties.

Author: Marko Laakso (Marko.Laakso@Helsinki.FI)
Version: 1.2
Categories: Data Import, SNP

Input name	Source	Description
sampleNames	snpSurvival-survivalSet7-caseNames.ids 19.737	List of sample names of interest.
markerNames	snpSurvival-markers7.in 19.455	List of SNP identifiers of interest.
config	genotypeDB.in 19.320	Connection parameters for SNPHelistin.

Parameter name	Value	Description
errorSymbol	ERR	Symbol for failed measurements. You may for example use 'NA' value in order consider them equal to missing values.
skipUnknown	true	Ignore unknown samples and do not process them. If the parameter is false then the program fails for these samples.

19.736 snpSurvival-survivalSet7-caseGenotypes-gtypes (SNPHelistinReader)

This component can be used to read SNP genotype calls for a set of samples and markers of interest. The genotype data is retrieved from a SNPHelistin database that is defined by the input properties.

Author: Marko Laakso (Marko.Laakso@Helsinki.FI)
Version: 1.2
Categories: Data Import, SNP

Input name	Source	Description
sampleNames	snpSurvival-survivalSet7-caseNames.ids 19.737	List of sample names of interest.
markerNames	snpSurvival-survivalSet7-caseGenotypes-ftMarkers.ids 19.734	List of SNP identifiers of interest.
config	genotypeDB.in 19.320	Connection parameters for SNPHelistin.

Parameter name	Value	Description
errorSymbol	ERR	Symbol for failed measurements. You may for example use 'NA' value in order consider them equal to missing values.
skipUnknown	true	Ignore unknown samples and do not process them. If the parameter is false then the program fails for these samples.

19.737 snpSurvival-survivalSet7-caseNames (CSV2IDList)

Extracts one column from the given CSV file and prints it out without duplicates. The selected column may contain entries with comma separated lists of values in which case *isList* parameter may be used to obtain each value separately.

Output is defined as:

$$ids = \bigcup_{i=1}^{9} \pi_{columnIn_i} \sigma_{regexp_i}(table_i). \quad (25)$$

Author: Marko Laakso (Marko.Laakso@Helsinki.FI)
Version: 1.3
Category: convert

Input name	Source	Description
table1	snpSurvival-survivalSet7-survival Clinical.table 19.760	The mandatory input relation

Parameter name	Value	Description
acceptMissing	false	Files with missing columnIn are accepted as empty if this is true.
columnIn		A comma separated list of column names for the IDs of interest in each file. Empty values refer to the first column of the file.
columnOut	SampleId	Name of the only column of the output list. Empty input refers to the name of the input column.
constants		A comma separated list of values that are always included into the output
isList	false	True if the seleted column contains a comma separated list of values to be splitted
quotation	false	Indicator that can be used to disable quotation of the output values
regexp1		Regular expression for the row filtering in table1. A row is included in the result if this parameter is empty or if values in the given columns match given regular expressions. The parameter has a format COLNAME1=EXPRESSION,COLNAME2=EXPRESSION2 where COLNAMEs are column names in "csv" and EXPRESSIONs are regular expressions using Java syntax. For example, "col=a|b" includes rows where the column col has a value of "a" or "b".
regexp2		Regular expression for the row filtering in table2.
regexp3		Regular expression for the row filtering in table3.
regexp4		Regular expression for the row filtering in table4.
regexp5		Regular expression for the row filtering in table5.
regexp6		Regular expression for the row filtering in table6.
regexp7		Regular expression for the row filtering in table7.
regexp8		Regular expression for the row filtering in table8.
regexp9		Regular expression for the row filtering in table9.

19.738 snpSurvival-survivalSet7-clinicalData (INPUT)

Clinical parameters for the case samples
Category: Internal

Parameter name	Value	Description
path	/gpfs/homes/mlaakso/asserSVN/ andurilCaseStudy/data/snp/ patient-metadata-caucas.csv	Path (filename) of the input resource.

19.739 snpSurvival-survivalSet7-clinicalSelect (SourceCode2Latex)

This component converts a source code listing into a LaTeXfragment that may be included into some technical documents.
Author: Marko Laakso (Marko.Laakso@Helsinki.FI)
Version: 1.0
Categories: convert, Latex

Input name	Source	Description
source	snpSurvival-survivalSet7-survival ParamsQuery.file 19.761	Source code

Parameter name	Value	Description
caption	This SQL query has been used to select clinical parameters for the survival calculations.	Caption text for the listing
language	SQL	Language defintion for the syntax highlighting. Possible values can be found from the description of listings package.

19.740 snpSurvival-survivalSet7-clinicalTypes (INPUT)

Column types for the clinical data

Category: Internal

Parameter name	Value	Description
path	/gpfs/homes/mlaakso/asserSVN/andurilCaseStudy/csv/SNPclinicalTypes.csv	Path (filename) of the input resource.

19.741 snpSurvival-survivalSet7-geneRank (QueryReport)

(Technical documentation) Executes an SQL query and prints it with the result table.

Author: Marko Laakso (Marko.Laakso@Helsinki.FI)
Version: 1.1
Category: Latex

Input name	Source	Description
table1	snpSurvival-survivalSet7-survSNPAnnot.fullData 19.753	1. data table that is referred as 'table1' in the query

Parameter name	Value	Description
colFormat	lcrp{11cm}	LaTeX tabular formatting for the result columns
listCols		Comma separated list of column names. Columns of this list may contain several values separated with commas and the delimiters will be replaced with spaces.
resTableRef	the previous table	Textual reference from the SQL representation to the result table
section	Gene rank	Subsection title for the output document
showCols	gene,markers,p-value,description	Comma separated list of column selections for the output. The empty default will use all columns.
showQuery	false	This flag tells if the SQL shall be shown on the report
tableCaption	A list of survival affecting genes and the marker counts. P-value is the lowest value for the corresponding markers.	Description for the result table

19.742 snpSurvival-survivalSet7-geneRank-eqData (TableQuery)

Execute an SQL query on input relations and create a result table. TableQuery uses HSQLDB (Hyper-threaded Structured Query Language Database [25]) for executing the query. Consequently, the syntax of the query is defined by HSQLDB.

Author: Kristian Ovaska (Kristian.Ovaska@Helsinki.FI)
Version: 1.2
Categories: convert, Filter

Input name	Source	Description
table1	snpSurvival-survivalSet7-survSNPAnnot-annotData.table 19.754	CSV table 1. The table is referred to as 'table1' in the SQL query.

Continued on next page...

Input name	Source	Description
table2	snpSurvival-survivalSet7-survival .statistics 19.759	CSV table 2. The table is referred to as 'table2' in the SQL query.
query	snpSurvival-survivalSet7-gene RankQuery.in 19.744	SQL query. Either this file or the query parameter must be provided, but not both.

Parameter name	Value	Description
numIndices		Comma-separated list of index counts for input tables. All indices are single-column indices, running from column 1 to N, where N is retrieved from this parameter. If empty, the default number (1) is used. For example, ",2,,2" sets two indices for table2 and table4 and one index for the rest.
query		SQL query. Either this parameter or the query input must be provided, but not both.

19.743 snpSurvival-survivalSet7-geneRank-eqTable (CSV2Latex)

This component converts a tabular CSV file into a printable LaTeX table.

Author: Marko Laakso (Marko.Laakso@Helsinki.FI)
Version: 2.1
Categories: convert, Latex

Input name	Source	Description
tabledata	snpSurvival-survivalSet7-gene Rank-eqData.table 19.742	Table content
refs	snpSurvival-survivalSet7-refsGene Rank.in 19.747	Reference rules for the hyperlinks

Parameter name	Value	Description
attach	false	Include the original data as an attachment
caption	A list of survival affecting genes and the marker counts. P-value is the lowest value for the corresponding markers.	Caption text for the table.
colFormat	lcrp{11cm}	LaTeX tabular format for the columns. Default produces a centered layout for all columns.
columns	gene,markers,p-value,description	Comma separated list of column selections for the output. The empty default will use all columns.
countRows	false	Include a row count to the table caption.
dropMissing	true	This flag can be turned off in order to generate links with missing texts. Link text are substituted with target identifiers.
listCols		Comma separated list of column names. Columns of this list may contain several values separated with commas and the delimiters will be replaced with spaces.
numberFormat		A comma separated list of decimal formats for the columns. Each entry consists of the column name and the Java DecimalFormal pattern separated with equal sign. For example, rounding to three decimals can be done like: myColumn=#0.000
pageBreak	false	Use clear page after the table.
rename		Comma separated list of column renaming rules (oldname=newname). New names are used in table header but they do not affect the other behaviour of this component.
ruler	{}	Latex command for the row separating rulers
section	Gene rank	Section title for the table container or an empty string if no section should be generated.
sectionType	subsection	Type of LaTeX section: usually one of: section, subsection, or subsubsection. No section statement is written if section title is empty.
skipEmpty	false	This flag can be used to replace empty tables with a simple LaTeX comment.

19.744 snpSurvival-survivalSet7-geneRankQuery (INPUT)

Generates a list of genes and the marker counts for them.

Category: Internal

Parameter name	Value	Description
path	/gpfs/homes/mlaakso/asserSVN/andurilCaseStudy/sql/SNPinterestingGenes.sql	Path (filename) of the input resource.

19.745 snpSurvival-survivalSet7-missingDistC (CSV2Latex)

This component converts a tabular CSV file into a printable LaTeX table.

Author: Marko Laakso (Marko.Laakso@Helsinki.FI)
Version: 2.1
Categories: convert, Latex

Input name	Source	Description
tabledata	snpSurvival-survivalSet7-caseGenotypes-freqCheck.naDist 19.733	Table content

Parameter name	Value	Description
attach	false	Include the original data as an attachment
caption	Distribution of the missing values in case samples	Caption text for the table.
colFormat	p{3cm}r	LaTeX tabular format for the columns. Default produces a centered layout for all columns.
columns		Comma separated list of column selections for the output. The empty default will use all columns.
countRows	false	Include a row count to the table caption.
dropMissing	true	This flag can be turned off in order to generate links with missing texts. Link text are substituted with target identifiers.
listCols		Comma separated list of column names. Columns of this list may contain several values separated with commas and the delimiters will be replaced with spaces.
numberFormat		A comma separated list of decimal formats for the columns. Each entry consists of the column name and the Java DecimalFormal pattern separated with equal sign. For example, rounding to three decimals can be done like: myColumn=#0.000
pageBreak	false	Use clear page after the table.
rename		Comma separated list of column renaming rules (oldname=newname). New names are used in table header but they do not affect the other behaviour of this component.
ruler	{}	Latex command for the row separating rulers
section		Section title for the table container or an empty string if no section should be generated.
sectionType	subsection	Type of LaTeX section: usually one of: section, subsection, or subsubsection. No section statement is written if section title is empty.
skipEmpty	false	This flag can be used to replace empty tables with a simple LaTeX comment.

19.746 snpSurvival-survivalSet7-propertiesDoc (Properties2Latex)

Prepares a LaTeX-representation for the given set of properties files. The document consists of tables of names and values of the properties.

Author: Marko Laakso (Marko.Laakso@Helsinki.FI)
Version: 1.2
Categories: convert, Latex

Input name	Source	Description
props1	genotypeDB.in 19.320	A set of properties

Parameter name	Value	Description
hide		A comma separated list of property names that should remain invisible. You may use this paramerter to hide passwords and other confidentian settings.

Continued on next page...

Parameter name	Value	Description
keyWidth	5	Column width for the property names (centimeters)
merge	false	Merge all input properties into a single set
section	SNP database configuration	Section title for the table container or an empty string if no section should be generated
sectionType	subsection	Type of LaTeX section: usually one of section, subsection or subsubsection. No section statement is written if sectionTitle is empty.
valueWidth	13	Column width for the property values (centimeters)

19.747 snpSurvival-survivalSet7-refsGeneRank (INPUT)

Hyperlink rules for the gene rank table

Category: Internal

Parameter name	Value	Description
path	/gpfs/homes/mlaakso/asserSVN/andurilCaseStudy/csv/SNPrefsGeneRank.csv	Path (filename) of the input resource.

19.748 snpSurvival-survivalSet7-refsHits (INPUT)

Hyperlink rules for the marker annotations

Category: Internal

Parameter name	Value	Description
path	/gpfs/homes/mlaakso/asserSVN/andurilCaseStudy/csv/SNPrefsHits.csv	Path (filename) of the input resource.

19.749 snpSurvival-survivalSet7-reportBody (LatexCombiner)

LatexCombiner joins the given subdocuments together into one LaTeX file.

Authors: Kristian Ovaska (Kristian.Ovaska@Helsinki.FI), Marko Laakso (Marko.Laakso@Helsinki.FI)
Version: 1.3
Category: Latex

Input name	Source	Description
latex1	snpSurvival-survivalSet7-survivalReport.document 19.763	LaTeX fragment 1.
latex2	snpSurvival-survivalSet7-propertiesDoc.report 19.746	LaTeX fragment 2.
latex3	snpSurvival-survivalSet7-missingDistC.report 19.745	LaTeX fragment 3.

Parameter name	Value	Description
pagebreak	false	Determines if the result document should start with a page break.
sectionTitle		If non-empty, a declaration of a new section with the given name is inserted to the beginning of the combined document. This is a convenience feature to make it easy to compile subsections into a section.
sectionType	section	Type of LaTeX section: usually one of section, subsection or subsubsection. No section statement is written if sectionTitle is empty.
strictBorders	true	Enables the flushing of all document elements before each fragment

19.750 snpSurvival-survivalSet7-reportTemplate (LatexTemplate)

This component generates the standard layout configuration for Anduril output documents. The component produces a header and a footer fragment that can be used to build LaTeX documents. The header fragment takes care of the page geometry and other technical details such as library imports and function declarations. Reference list is generated by the footer.

Author: Marko Laakso (Marko.Laakso@Helsinki.FI)
Version: 1.4
Category: Latex

Input name	Source	Description
abstract	abstract.in 19.42	Document abstract including its Latex definition. You may use template entities within this document. The default abstract has been defined in abstractTemplate.tex.

Parameter name	Value	Description
authors	Marko Laakso, Riku Louhimo, Sampsa Hautaniemi	A comma-separated list of document authors on the title page. If authors is empty but title is non-empty, a title page with no author name is printed.
baselineskip	1.2	Baselineskip factor that indicates the line spacing
bibstyle	abbrv	BibTeX reference formatting style
printTOC	true	If true, print Table Of Contents.
title	Anduril Case Study: Glioblastoma SNP survival analysis	Document title on the title page. If empty, no title page is printed. Also see author.
usepackage		Comma-separated list of LaTeX packages that are used in the document. Each package generates a \usepackage{X} line in the header.

19.751 snpSurvival-survivalSet7-snpAnnotations (INPUT)

Annotations for survival component.

Category: Internal

Parameter name	Value	Description
path	/gpfs/homes/mlaakso/asserSVN/andurilCaseStudy/csv/SNPAnnotation.csv	Path (filename) of the input resource.

19.752 snpSurvival-survivalSet7-summaryReport (LatexPDF)

This component is a LaTeX compiler that generates a portable document format (PDF) file from the given Anduril document.

Author: Kristian Ovaska (Kristian.Ovaska@Helsinki.FI)
Version: 1.0
Category: Latex

Input name	Source	Description
document	snpSurvival-survivalSet7-reportBody.document 19.749	Body of the LaTeX document.
header	snpSurvival-survivalSet7-reportTemplate.header 19.750	LaTeX header that is written to the start of the document. If missing, a default header is used.
footer	snpSurvival-survivalSet7-reportTemplate.footer 19.750	LaTeX footer that is written to the end of the document. If missing, a default header is used.

Parameter name	Value	Description
bibtexExec	bibtex	Executable command for BibTeX.
latexExec	pdflatex	Executable command for pdflatex.
useRefs	false	This flag can be used to activate BibTeX compiler

19.753 snpSurvival-survivalSet7-survSNPAnnot (AnnotateSNPs)

Biological annotations for the survival affecting genes

Parameter name	Value
caption	Gene annotations and the loci of the survival affecting markers

19.754 snpSurvival-survivalSet7-survSNPAnnot-annotData (TableQuery)

Execute an SQL query on input relations and create a result table. TableQuery uses HSQLDB (Hyperthreaded Structured Query Language Database [25]) for executing the query. Consequently, the syntax of the query is defined by HSQLDB.

Author: Kristian Ovaska (Kristian.Ovaska@Helsinki.FI)
Version: 1.2
Categories: convert, Filter

Input name	Source	Description
table1	snpSurvival-survivalSet7-survSNPAnnot-snpRel.relation 19.758	CSV table 1. The table is referred to as 'table1' in the SQL query.
table2	snpSurvival-survivalSet7-survSNPAnnot-genes.bioAnnotation 19.756	CSV table 2. The table is referred to as 'table2' in the SQL query.
table3	snpSurvival-survivalSet7-survival.statistics 19.759	CSV table 3. The table is referred to as 'table3' in the SQL query.
query	snpSurvival-survivalSet7-annotSQL.in 19.731	SQL query. Either this file or the query parameter must be provided, but not both.

Parameter name	Value	Description
numIndices		Comma-separated list of index counts for input tables. All indices are single-column indices, running from column 1 to N, where N is retrieved from this parameter. If empty, the default number (1) is used. For example, ",2,,2" sets two indices for table2 and table4 and one index for the rest.
query		SQL query. Either this parameter or the query input must be provided, but not both.

19.755 snpSurvival-survivalSet7-survSNPAnnot-annotTable (CSV2Latex)

This component converts a tabular CSV file into a printable LaTeX table.

Author: Marko Laakso (Marko.Laakso@Helsinki.FI)
Version: 2.1
Categories: convert, Latex

Input name	Source	Description
tabledata	snpSurvival-survivalSet7-survSNPAnnot-annotData.table 19.754	Table content
refs	snpSurvival-survivalSet7-refsHits.in 19.748	Reference rules for the hyperlinks

Continued on next page...

Parameter name Value Description

Parameter name	Value	Description
attach	false	Include the original data as an attachment
caption	Gene annotations and the loci of the survival affecting markers	Caption text for the table.
colFormat	l@{}lr@{:}lp{1cm}p{1.5cm}p{10 .5cm}	LaTeX tabular format for the columns. Default produces a centered layout for all columns.
columns	hit,marker,chr,locus,band,gene, gene description	Comma separated list of column selections for the output. The empty default will use all columns.
countRows	false	Include a row count to the table caption.
dropMissing	true	This flag can be turned off in order to generate links with missing texts. Link text are substituted with target identifiers.
listCols	band	Comma separated list of column names. Columns of this list may contain several values separated with commas and the delimiters will be replaced with spaces.
numberFormat		A comma separated list of decimal formats for the columns. Each entry consists of the column name and the Java DecimalFormal pattern separated with equal sign. For example, rounding to three decimals can be done like: myColumn=#0.000
pageBreak	false	Use clear page after the table.
rename		Comma separated list of column renaming rules (oldname=newname). New names are used in table header but they do not affect the other behaviour of this component.
ruler	{}	Latex command for the row separating rulers
section		Section title for the table container or an empty string if no section should be generated.
sectionType	subsection	Type of LaTeX section: usually one of: section, subsection, or subsubsection. No section statement is written if section title is empty.
skipEmpty	false	This flag can be used to replace empty tables with a simple LaTeX comment.

19.756 snpSurvival-survivalSet7-survSNPAnnot-genes (KorvasieniAnnotator)

Korvasieni is an Ensembl [12] based converter for database identifiers and it can be used to convert genome location, gene, transcript, and translation identifiers across multiple biological databases. The selected `keyColumn` may contain entries with comma separated lists of values in which case `isListKey` parameter may be used to obtain each value separately and to process them as they were given in consecutive lines.

Author: Marko Laakso (Marko.Laakso@Helsinki.FI)
Version: 1.7
Categories: Annotation, GO

Input name	Source	Description
sourceKeys	snpSurvival-survivalSet7-survSNPAnnot-snpLoci. annotations 19.757	A list of source database keys.
connection	ensembl.in 19.273	Database connection can be defined using this file. The definition of parameters: database.url, database.user, database.password, database.timeout, database.recycle, and database.driver can be found from the documentation of Korvasieni.

Parameter name	Value	Description
echoColumns	refsnp_id	A comma separated list of column names for the columns that will be copied to the output. An asterisk (*) can be used to denote all columns execpt the keyColumn.
inputDB	.GeneId	Type of input keys. This must be a database supported by Korvasieni. If the parameter is omitted, the component tries to derive the database from the type of geneID. If this is not possible, an error is returned. You may define three columns in form of chromosome:start-end in case the inputDB is .DNARegion. This format provides a comfortable compatibility with DNARegion datatype.
inputType	Gene	Ensembl object type for the input keys (Any, Gene, Transcript, Translation)
isListKey	true	Enables the automatic value splits for the comma separated key column
keyColumn	ensembl_gene_stable_id	Name of the key column withing sourceKeys file or an empty string for the first column
maxHits	100000	Maximum number of target identifiers for a single source identifier
rename	UniProt/SwissProt=protein	Comma separated list of column renaming rules (oldname=newname)
skipLevel	never	Skip result rows if the source identifier is unknown or target identifiers are not available. Possible values are: never (no filtering), source (skip if the source ID is unknown), target (skip if no target IDs are found), any (skip if any of the target IDs is missing).

Continued on next page...

Parameter name	Value	Description
targetDB	.GeneName,.GeneDesc,. DNABand,GO,UniProt/SwissProt	Comma-separated list of annotation types. Possible values are all databases supported by Korvasieni.
unique	true	This flag can be turned on in order to eliminate duplicate annotations.

19.757 snpSurvival-survivalSet7-survSNPAnnot-snpLoci (RefSNPAnnotator)

RefSNPAnnotator fetches annotations for given refSNP ID's (also known as rsID's) from BioMart databases.

The provided annotations for each rsID include alleles for the SNP, name of the chromosome where the SNP is located, chromosome location of the SNP, the strand of the chromosome, Ensembl gene ID for genes that overlap the SNP's location and validation status of the SNP. The component is implemented using the biomaRt package for Bioconductor [7].

Authors: Viljami Aittomaki (Viljami.Aittomaki@Helsinki.FI), Marko Laakso (Marko.Laakso@Helsinki.FI)
Version: 1.0.1
Categories: SNP, Annotation

Input name	Source	Description
rsIDs	snpSurvival-survivalSet7-survival .statistics 19.759	List of rsID's for fetching annotations.

Parameter name	Value	Description
dataset	hsapiens_snp	Dataset to get annotations from. Different species have their own datasets. Example values include "hsapiens_snp" and "mmusculus_snp". See biomaRt documentation on how to list available datasets in R.
keyColumn	marker	Name of the key column withing sourceKeys file or an empty string for the first column.

19.758 snpSurvival-survivalSet7-survSNPAnnot-snpRel (ExpandCollapse)

Converts between the two possible representations of a relations with multivalued columns. By default this component splits rows with multivalued (comma separated lists) columns into multiple rows each representing a single value. The input may contain several columns with comma separated values and all permutations of column values are shown as individual single-valued rows. The alternative mode recovers the original comma separated values from the expanded relations. This is performed by joining values of the their list columns into comma separated lists.

Author: Marko Laakso (Marko.Laakso@Helsinki.FI)
Version: 1.3
Category: convert

Input name	Source	Description
relation	snpSurvival-survivalSet7-survSN PAnnot-snpLoci. annotations 19.757	The mandatory input relation

Parameter name	Value	Description
delim	,	Value delimiter between the list column values. Special characters can be encoded as specified in fi.helsinki.ltdk.csbl.asser.ArgumentEncoding.
expand	true	Action mode that is expand (true) or collapse (false)
listCols	ensembl_gene_stable_id	A comma separated list of column names of input (expand) or output (collapse) columns that may contain delim separated values. The asterisk refers to every column of the input relation.

19.759 snpSurvival-survivalSet7-survival (SNPKaplanMeier)

This component is used to estimate survival differences between various genotypes. The differences between the survival of different genotypes are measured using G-ρ family of tests [10].

Author:	Marko Laakso (Marko.Laakso@Helsinki.FI)
Version:	1.5
Categories:	SNP, Latex, Survival

Input name	Source	Description
genotypes	snpSurvival-survivalSet7-caseGenotypes-gtypes.genotypes 19.736	Genotype calls
survival	snpSurvival-survivalSet7-survivalClinical.table 19.760	Survival time and event indicator
annotations	snpSurvival-survivalSet7-snpAnnotations.in 19.751	Additional information about the markers. This data will be shown on the report of interesting markers.

Parameter name	Value	Description
PLimit	0.0010	Ignore results with a p-value greater than this limit.
confInt	0.95	Coverage of the confidence intervals [0,1]
minCount	15	Combine rare (aa) homozygotes to heterozygotes (Aa) if their number is below this limit. This parameter is used together with minFreq so that the actual limit is max(minFreq*n, minCount).
minFreq	0.1	Combine rare (aa) homozygotes to heterozygotes (Aa) if their frequency is below this limit. See also minCount.
naLimit	0.1	Skip markers where one minus call rate exceeds this limit.
nullCalls	–,ERR	A comma separated list of genotype calls that will be considered as missing values
sampleCol	sample	Column name for the sample name
showCI	true	Draw confidence intervals to the graph
skipMissingGT	true	Simply skip those samples that lack the genotype information.
statusCol	hasEvent	Column name for the event indicator
survRatePoint	0.5	Sampling height for the distances between the survival expectations. Negative values may be used to suppress these measurements.
timeCol	endTime	Column name for the survival time
timeOutLimit	36	Cut survival curves after this limit. A negative value can be used to maximize the length of the period.
timeStartCol	startTime	Column name for the start time that is reduced from the survival time
title	Genotype Specific Survival Estimates	Section title

19.760 snpSurvival-survivalSet7-survivalClinical (TableQuery)

Execute an SQL query on input relations and create a result table. TableQuery uses HSQLDB (Hyperthreaded Structured Query Language Database [25]) for executing the query. Consequently, the syntax of the query is defined by HSQLDB.

Author:	Kristian Ovaska (Kristian.Ovaska@Helsinki.FI)
Version:	1.2
Categories:	convert, Filter

Input name	Source	Description
table1	snpSurvival-survivalSet7-clinicalData.in 19.738	CSV table 1. The table is referred to as 'table1' in the SQL query.
query	snpSurvival-survivalSet7-survivalParamsQuery.file 19.761	SQL query. Either this file or the query parameter must be provided, but not both.
columnTypes	snpSurvival-survivalSet7-clinicalTypes.in 19.740	Contains SQL types for individual columns. If the file is not provided, the type is inferred from the contents of the columns. This can be used to force the use of VARCHAR for values that are also valid numerics. The file contains the columns Table (refers to one of table1 to table15), Column (refers to a column name in the table), Type (contains an SQL type). A row with Table='result', Column=X and Type='STRING' forces the use string values for result column X.

Parameter name	Value	Description
numIndices		Comma-separated list of index counts for input tables. All indices are single-column indices, running from column 1 to N, where N is retrieved from this parameter. If empty, the default number (1) is used. For example, ",2,,2" sets two indices for table2 and table4 and one index for the rest.
query		SQL query. Either this parameter or the query input must be provided, but not both.

19.761 snpSurvival-survivalSet7-survivalParamsQuery (SearchReplace)

Replaces the occurences of the given regular expressions with the corresponding regular expressions. This component is used for transforming given patterns of the input file to a changed form specified by the regular expressions .

Author: Marko Laakso (Marko.Laakso@Helsinki.FI)
Version: 1.0
Category: convert

Input name	Source	Description
file	snpSurvival-survivalSet7-survivalParamsTemplate.in 19.762	Source file to be modified

Parameter name	Value	Description
key00	REP.cond00	Search key 0
key01		Search key 1
key02		Search key 2
key03		Search key 3
key04		Search key 4
key05		Search key 5
key06		Search key 6
key07		Search key 7
key08		Search key 8
key09		Search key 9
value00	– No additional restrictions	Replacement 0
value01		Replacement 1
value02		Replacement 2
value03		Replacement 3
value04		Replacement 4
value05		Replacement 5
value06		Replacement 6
value07		Replacement 7
value08		Replacement 8
value09		Replacement 9

19.762 snpSurvival-survivalSet7-survivalParamsTemplate (INPUT)

Select relevant parameters for the survival analysis

Category: Internal

Parameter name	Value	Description
path	/gpfs/homes/mlaakso/asserSVN/andurilCaseStudy/sql/SNPsurvivalParams.sql	Path (filename) of the input resource.

19.763 snpSurvival-survivalSet7-survivalReport (LatexCombiner)

LatexCombiner joins the given subdocuments together into one LaTeX file.

Authors:	Kristian Ovaska (Kristian.Ovaska@Helsinki.FI), Marko Laakso (Marko.Laakso@Helsinki.FI)
Version:	1.3
Category:	Latex

Input name	Source	Description
latex1	snpSurvival-survivalSet7-survival.report 19.759	LaTeX fragment 1.
latex2	snpSurvival-survivalSet7-clinicalSelect.report 19.739	LaTeX fragment 2.
latex3	snpSurvival-survivalSet7-survSNPAnnot-annotTable.report 19.755	LaTeX fragment 3.
latex4	snpSurvival-survivalSet7-geneRank-eqTable.report 19.743	LaTeX fragment 4.

Parameter name	Value	Description
pagebreak	true	Determines if the result document should start with a page break.
sectionTitle		If non-empty, a declaration of a new section with the given name is inserted to the beginning of the combined document. This is a convenience feature to make it easy to compile subsections into a section.
sectionType	section	Type of LaTeX section: usually one of section, subsection or subsubsection. No section statement is written if sectionTitle is empty.
strictBorders	true	Enables the flushing of all document elements before each fragment

19.764 snpSurvival-survivalSet8 (SurvivalAnalysis)

No description

Parameter name	Value
pvalue	0.0010

19.765 snpSurvival-survivalSet8-annotSQL (INPUT)

This SQL is used to generate marker annotation report.

Category: Internal

Parameter name	Value	Description
path	/gpfs/homes/mlaakso/asserSVN/andurilCaseStudy/sql/SNPgeneTable.sql	Path (filename) of the input resource.

19.766 snpSurvival-survivalSet8-caseGenotypes (GetGenotypes)

Load and preprocess the genotype calls for the cancer patient samples.

Parameter name	Value
skipFrq	0.1

19.767 snpSurvival-survivalSet8-caseGenotypes-freqCheck (AlleleCounter)

AlleleCounter calculates genotype frequencies for the biallelic markers of the given SNPMatrix. A Pearson's χ^2-test [18] is performed for each marker in order to test if the Hardy-Weinberg equilibrium [9] holds for the population sample.

Author: Marko Laakso (Marko.Laakso@Helsinki.FI)
Version: 1.2
Category: SNP

Input name	Source	Description
genotypes	snpSurvival-survivalSet8-caseGenotypes-genotypes .genotypes 19.769	SNPHelistin genotype output.

Parameter name	Value	Description
dels	false	Accept homozygous deletions as a possible genotype.
naLimit	0.1	Skip markers where one minus call rate exceeds this limit.
skip	0.1	Drop markers with a relative minor allele frequency lower than this limit. Use negative values to inactivate this limit.

19.768 snpSurvival-survivalSet8-caseGenotypes-ftMarkers (CSV2IDList)

Extracts one column from the given CSV file and prints it out without duplicates. The selected column may contain entries with comma separated lists of values in which case *isList* parameter may be used to obtain each value separately.

Output is defined as:

$$ids = \bigcup_{i=1}^{9} \pi_{columnIn_i} \sigma_{regexp_i}(table_i). \quad (26)$$

Author: Marko Laakso (Marko.Laakso@Helsinki.FI)
Version: 1.3
Category: convert

Input name	Source	Description
table1	snpSurvival-survivalSet8-caseGenotypes-freqCheck .frequencies 19.767	The mandatory input relation

Parameter name	Value	Description
acceptMissing	false	Files with missing columnIn are accepted as empty if this is true.
columnIn	marker	A comma separated list of column names for the IDs of interest in each file. Empty values refer to the first column of the file.
columnOut	marker	Name of the only column of the output list. Empty input refers to the name of the input column.
constants		A comma separated list of values that are always included into the output
isList	false	True if the seleted column contains a comma separated list of values to be splitted
quotation	false	Indicator that can be used to disable quotation of the output values
regexp1		Regular expression for the row filtering in table1. A row is included in the result if this parameter is empty or if values in the given columns match given regular expressions. The parameter has a format COLNAME1=EXPRESSION,COLNAME2=EXPRESSION2 where COLNAMEs are column names in "csv" and EXPRESSIONs are regular expressions using Java syntax. For example, "col=a|b" includes rows where the column col has a value of "a" or "b".
regexp2		Regular expression for the row filtering in table2.
regexp3		Regular expression for the row filtering in table3.
regexp4		Regular expression for the row filtering in table4.
regexp5		Regular expression for the row filtering in table5.
regexp6		Regular expression for the row filtering in table6.
regexp7		Regular expression for the row filtering in table7.
regexp8		Regular expression for the row filtering in table8.
regexp9		Regular expression for the row filtering in table9.

19.769 snpSurvival-survivalSet8-caseGenotypes-genotypes (SNPHelistinReader)

This component can be used to read SNP genotype calls for a set of samples and markers of interest. The genotype data is retrieved from a SNPHelistin database that is defined by the input properties.

Author:	Marko Laakso (Marko.Laakso@Helsinki.FI)
Version:	1.2
Categories:	Data Import, SNP

Input name	Source	Description
sampleNames	snpSurvival-survivalSet8-caseNames.ids 19.771	List of sample names of interest.
markerNames	snpSurvival-markers8.in 19.456	List of SNP identifiers of interest.
config	genotypeDB.in 19.320	Connection parameters for SNPHelistin.

Parameter name	Value	Description
errorSymbol	ERR	Symbol for failed measurements. You may for example use 'NA' value in order consider them equal to missing values.
skipUnknown	true	Ignore unknown samples and do not process them. If the parameter is false then the program fails for these samples.

19.770 snpSurvival-survivalSet8-caseGenotypes-gtypes (SNPHelistinReader)

This component can be used to read SNP genotype calls for a set of samples and markers of interest. The genotype data is retrieved from a SNPHelistin database that is defined by the input properties.

Author:	Marko Laakso (Marko.Laakso@Helsinki.FI)
Version:	1.2
Categories:	Data Import, SNP

Input name	Source	Description
sampleNames	snpSurvival-survivalSet8-caseNames.ids 19.771	List of sample names of interest.
markerNames	snpSurvival-survivalSet8-caseGenotypes-ftMarkers.ids 19.768	List of SNP identifiers of interest.
config	genotypeDB.in 19.320	Connection parameters for SNPHelistin.

Parameter name	Value	Description
errorSymbol	ERR	Symbol for failed measurements. You may for example use 'NA' value in order consider them equal to missing values.
skipUnknown	true	Ignore unknown samples and do not process them. If the parameter is false then the program fails for these samples.

19.771 snpSurvival-survivalSet8-caseNames (CSV2IDList)

Extracts one column from the given CSV file and prints it out without duplicates. The selected column may contain entries with comma separated lists of values in which case *isList* parameter may be used to obtain each value separately.

Output is defined as:

$$ids = \bigcup_{i=1}^{9} \pi_{columnIn_i} \sigma_{regexp_i}(table_i). \tag{27}$$

Author: Marko Laakso (Marko.Laakso@Helsinki.FI)
Version: 1.3
Category: convert

Input name	Source	Description
table1	snpSurvival-survivalSet8-survival Clinical.table 19.794	The mandatory input relation

Parameter name	Value	Description
acceptMissing	false	Files with missing columnIn are accepted as empty if this is true.
columnIn		A comma separated list of column names for the IDs of interest in each file. Empty values refer to the first column of the file.
columnOut	SampleId	Name of the only column of the output list. Empty input refers to the name of the input column.
constants		A comma separated list of values that are always included into the output
isList	false	True if the seleted column contains a comma separated list of values to be splitted
quotation	false	Indicator that can be used to disable quotation of the output values
regexp1		Regular expression for the row filtering in table1. A row is included in the result if this parameter is empty or if values in the given columns match given regular expressions. The parameter has a format COLNAME1=EXPRESSION,COLNAME2=EXPRESSION2 where COLNAMEs are column names in "csv" and EXPRESSIONs are regular expressions using Java syntax. For example, "col=a|b" includes rows where the column col has a value of "a" or "b".
regexp2		Regular expression for the row filtering in table2.
regexp3		Regular expression for the row filtering in table3.
regexp4		Regular expression for the row filtering in table4.
regexp5		Regular expression for the row filtering in table5.
regexp6		Regular expression for the row filtering in table6.
regexp7		Regular expression for the row filtering in table7.
regexp8		Regular expression for the row filtering in table8.
regexp9		Regular expression for the row filtering in table9.

19.772 snpSurvival-survivalSet8-clinicalData (INPUT)

Clinical parameters for the case samples
Category: Internal

Parameter name	Value	Description
path	/gpfs/homes/mlaakso/asserSVN/ andurilCaseStudy/data/snp/ patient-metadata-caucas.csv	Path (filename) of the input resource.

19.773 snpSurvival-survivalSet8-clinicalSelect (SourceCode2Latex)

This component converts a source code listing into a LaTeXfragment that may be included into some technical documents.
Author: Marko Laakso (Marko.Laakso@Helsinki.FI)
Version: 1.0
Categories: convert, Latex

Input name	Source	Description
source	snpSurvival-survivalSet8-survival ParamsQuery.file 19.795	Source code

Parameter name	Value	Description
caption	This SQL query has been used to select clinical parameters for the survival calculations.	Caption text for the listing
language	SQL	Language defintion for the syntax highlighting. Possible values can be found from the description of listings package.

19.774 snpSurvival-survivalSet8-clinicalTypes (INPUT)

Column types for the clinical data

Category: Internal

Parameter name	Value	Description
path	/gpfs/homes/mlaakso/asserSVN/andurilCaseStudy/csv/SNPclinicalTypes.csv	Path (filename) of the input resource.

19.775 snpSurvival-survivalSet8-geneRank (QueryReport)

(Technical documentation) Executes an SQL query and prints it with the result table.

Author: Marko Laakso (Marko.Laakso@Helsinki.FI)
Version: 1.1
Category: Latex

Input name	Source	Description
table1	snpSurvival-survivalSet8-survSNPAnnot.fullData 19.787	1. data table that is referred as 'table1' in the query

Parameter name	Value	Description
colFormat	lcrp{11cm}	LaTeX tabular formatting for the result columns
listCols		Comma separated list of column names. Columns of this list may contain several values separated with commas and the delimiters will be replaced with spaces.
resTableRef	the previous table	Textual reference from the SQL representation to the result table
section	Gene rank	Subsection title for the output document
showCols	gene,markers,p-value,description	Comma separated list of column selections for the output. The empty default will use all columns.
showQuery	false	This flag tells if the SQL shall be shown on the report
tableCaption	A list of survival affecting genes and the marker counts. P-value is the lowest value for the corresponding markers.	Description for the result table

19.776 snpSurvival-survivalSet8-geneRank-eqData (TableQuery)

Execute an SQL query on input relations and create a result table. TableQuery uses HSQLDB (Hyper-threaded Structured Query Language Database [25]) for executing the query. Consequently, the syntax of the query is defined by HSQLDB.

Author: Kristian Ovaska (Kristian.Ovaska@Helsinki.FI)
Version: 1.2
Categories: convert, Filter

Input name	Source	Description
table1	snpSurvival-survivalSet8-survSNPAnnot-annotData.table 19.788	CSV table 1. The table is referred to as 'table1' in the SQL query.

Continued on next page...

Input name	Source	Description
table2	snpSurvival-survivalSet8-survival .statistics 19.793	CSV table 2. The table is referred to as 'table2' in the SQL query.
query	snpSurvival-survivalSet8-gene RankQuery.in 19.778	SQL query. Either this file or the query parameter must be provided, but not both.

Parameter name	Value	Description
numIndices		Comma-separated list of index counts for input tables. All indices are single-column indices, running from column 1 to N, where N is retrieved from this parameter. If empty, the default number (1) is used. For example, ",2,,2" sets two indices for table2 and table4 and one index for the rest.
query		SQL query. Either this parameter or the query input must be provided, but not both.

19.777 snpSurvival-survivalSet8-geneRank-eqTable (CSV2Latex)

This component converts a tabular CSV file into a printable LATEX table.

Author:	Marko Laakso (Marko.Laakso@Helsinki.FI)
Version:	2.1
Categories:	convert, Latex

Input name	Source	Description
tabledata	snpSurvival-survivalSet8-gene Rank-eqData.table 19.776	Table content
refs	snpSurvival-survivalSet8-refsGene Rank.in 19.781	Reference rules for the hyperlinks

Parameter name	Value	Description
attach	false	Include the original data as an attachment
caption	A list of survival affecting genes and the marker counts. P-value is the lowest value for the corresponding markers.	Caption text for the table.
colFormat	lcrp{11cm}	LaTeX tabular format for the columns. Default produces a centered layout for all columns.
columns	gene,markers,p-value,description	Comma separated list of column selections for the output. The empty default will use all columns.
countRows	false	Include a row count to the table caption.
dropMissing	true	This flag can be turned off in order to generate links with missing texts. Link text are substituted with target identifiers.
listCols		Comma separated list of column names. Columns of this list may contain several values separated with commas and the delimiters will be replaced with spaces.
numberFormat		A comma separated list of decimal formats for the columns. Each entry consists of the column name and the Java DecimalFormal pattern separated with equal sign. For example, rounding to three decimals can be done like: myColumn=#0.000
pageBreak	false	Use clear page after the table.
rename		Comma separated list of column renaming rules (oldname=newname). New names are used in table header but they do not affect the other behaviour of this component.
ruler	{}	Latex command for the row separating rulers
section	Gene rank	Section title for the table container or an empty string if no section should be generated.
sectionType	subsection	Type of LaTeX section: usually one of: section, subsection, or subsubsection. No section statement is written if section title is empty.
skipEmpty	false	This flag can be used to replace empty tables with a simple LaTeX comment.

19.778 snpSurvival-survivalSet8-geneRankQuery (INPUT)

Generates a list of genes and the marker counts for them.

Category:	Internal

Parameter name	Value	Description
path	/gpfs/homes/mlaakso/asserSVN/andurilCaseStudy/sql/SNPinterestingGenes.sql	Path (filename) of the input resource.

19.779 snpSurvival-survivalSet8-missingDistC (CSV2Latex)

This component converts a tabular CSV file into a printable LaTeX table.

Author: Marko Laakso (Marko.Laakso@Helsinki.FI)
Version: 2.1
Categories: convert, Latex

Input name	Source	Description
tabledata	snpSurvival-survivalSet8-caseGenotypes-freqCheck.naDist 19.767	Table content

Parameter name	Value	Description
attach	false	Include the original data as an attachment
caption	Distribution of the missing values in case samples	Caption text for the table.
colFormat	p{3cm}r	LaTeX tabular format for the columns. Default produces a centered layout for all columns.
columns		Comma separated list of column selections for the output. The empty default will use all columns.
countRows	false	Include a row count to the table caption.
dropMissing	true	This flag can be turned off in order to generate links with missing texts. Link text are substituted with target identifiers.
listCols		Comma separated list of column names. Columns of this list may contain several values separated with commas and the delimiters will be replaced with spaces.
numberFormat		A comma separated list of decimal formats for the columns. Each entry consists of the column name and the Java DecimalFormal pattern separated with equal sign. For example, rounding to three decimals can be done like: myColumn=#0.000
pageBreak	false	Use clear page after the table.
rename		Comma separated list of column renaming rules (oldname=newname). New names are used in table header but they do not affect the other behaviour of this component.
ruler	{}	Latex command for the row separating rulers
section		Section title for the table container or an empty string if no section should be generated.
sectionType	subsection	Type of LaTeX section: usually one of: section, subsection, or subsubsection. No section statement is written if section title is empty.
skipEmpty	false	This flag can be used to replace empty tables with a simple LaTeX comment.

19.780 snpSurvival-survivalSet8-propertiesDoc (Properties2Latex)

Prepares a LaTeX-representation for the given set of properties files. The document consists of tables of names and values of the properties.

Author: Marko Laakso (Marko.Laakso@Helsinki.FI)
Version: 1.2
Categories: convert, Latex

Input name	Source	Description
props1	genotypeDB.in 19.320	A set of properties

Parameter name	Value	Description
hide		A comma separated list of property names that should remain invisible. You may use this paramerter to hide passwords and other confidentian settings.

Continued on next page...

Parameter name	Value	Description
keyWidth	5	Column width for the property names (centimeters)
merge	false	Merge all input properties into a single set
section	SNP database configuration	Section title for the table container or an empty string if no section should be generated
sectionType	subsection	Type of LaTeX section: usually one of section, subsection or subsubsection. No section statement is written if sectionTitle is empty.
valueWidth	13	Column width for the property values (centimeters)

19.781 snpSurvival-survivalSet8-refsGeneRank (INPUT)

Hyperlink rules for the gene rank table

Category: Internal

Parameter name	Value	Description
path	/gpfs/homes/mlaakso/asserSVN/andurilCaseStudy/csv/SNPrefsGeneRank.csv	Path (filename) of the input resource.

19.782 snpSurvival-survivalSet8-refsHits (INPUT)

Hyperlink rules for the marker annotations

Category: Internal

Parameter name	Value	Description
path	/gpfs/homes/mlaakso/asserSVN/andurilCaseStudy/csv/SNPrefsHits.csv	Path (filename) of the input resource.

19.783 snpSurvival-survivalSet8-reportBody (LatexCombiner)

LatexCombiner joins the given subdocuments together into one LaTeX file.

Authors: Kristian Ovaska (Kristian.Ovaska@Helsinki.FI), Marko Laakso (Marko.Laakso@Helsinki.FI)
Version: 1.3
Category: Latex

Input name	Source	Description
latex1	snpSurvival-survivalSet8-survivalReport.document 19.797	LaTeX fragment 1.
latex2	snpSurvival-survivalSet8-propertiesDoc.report 19.780	LaTeX fragment 2.
latex3	snpSurvival-survivalSet8-missingDistC.report 19.779	LaTeX fragment 3.

Parameter name	Value	Description
pagebreak	false	Determines if the result document should start with a page break.
sectionTitle		If non-empty, a declaration of a new section with the given name is inserted to the beginning of the combined document. This is a convenience feature to make it easy to compile subsections into a section.
sectionType	section	Type of LaTeX section: usually one of section, subsection or subsubsection. No section statement is written if sectionTitle is empty.
strictBorders	true	Enables the flushing of all document elements before each fragment

19.784 snpSurvival-survivalSet8-reportTemplate (LatexTemplate)

This component generates the standard layout configuration for Anduril output documents. The component produces a header and a footer fragment that can be used to build LaTeX documents. The header fragment takes care of the page geometry and other technical details such as library imports and function declarations. Reference list is generated by the footer.

Author: Marko Laakso (Marko.Laakso@Helsinki.FI)
Version: 1.4
Category: Latex

Input name	Source	Description
abstract	abstract.in 19.42	Document abstract including its Latex definition. You may use template entities within this document. The default abstract has been defined in abstractTemplate.tex.

Parameter name	Value	Description
authors	Marko Laakso, Riku Louhimo, Sampsa Hautaniemi	A comma-separated list of document authors on the title page. If authors is empty but title is non-empty, a title page with no author name is printed.
baselineskip	1.2	Baselineskip factor that indicates the line spacing
bibstyle	abbrv	BibTeX reference formatting style
printTOC	true	If true, print Table Of Contents.
title	Anduril Case Study: Glioblastoma SNP survival analysis	Document title on the title page. If empty, no title page is printed. Also see author.
usepackage		Comma-separated list of LaTeX packages that are used in the document. Each package generates a \usepackage{X} line in the header.

19.785 snpSurvival-survivalSet8-snpAnnotations (INPUT)

Annotations for survival component.

Category: Internal

Parameter name	Value	Description
path	/gpfs/homes/mlaakso/asserSVN/andurilCaseStudy/csv/SNPAnnotation.csv	Path (filename) of the input resource.

19.786 snpSurvival-survivalSet8-summaryReport (LatexPDF)

This component is a LaTeX compiler that generates a portable document format (PDF) file from the given Anduril document.

Author: Kristian Ovaska (Kristian.Ovaska@Helsinki.FI)
Version: 1.0
Category: Latex

Input name	Source	Description
document	snpSurvival-survivalSet8-reportBody.document 19.783	Body of the LaTeX document.
header	snpSurvival-survivalSet8-reportTemplate.header 19.784	LaTeX header that is written to the start of the document. If missing, a default header is used.
footer	snpSurvival-survivalSet8-reportTemplate.footer 19.784	LaTeX footer that is written to the end of the document. If missing, a default header is used.

Parameter name	Value	Description
bibtexExec	bibtex	Executable command for BibTeX.
latexExec	pdflatex	Executable command for pdflatex.
useRefs	false	This flag can be used to activate BibTeX compiler

19.787 snpSurvival-survivalSet8-survSNPAnnot (AnnotateSNPs)

Biological annotations for the survival affecting genes

Parameter name	Value
caption	Gene annotations and the loci of the survival affecting markers

19.788 snpSurvival-survivalSet8-survSNPAnnot-annotData (TableQuery)

Execute an SQL query on input relations and create a result table. TableQuery uses HSQLDB (Hyperthreaded Structured Query Language Database [25]) for executing the query. Consequently, the syntax of the query is defined by HSQLDB.

Author: Kristian Ovaska (Kristian.Ovaska@Helsinki.FI)
Version: 1.2
Categories: convert, Filter

Input name	Source	Description
table1	snpSurvival-survivalSet8-survSNPAnnot-snpRel.relation 19.792	CSV table 1. The table is referred to as 'table1' in the SQL query.
table2	snpSurvival-survivalSet8-survSNPAnnot-genes.bioAnnotation 19.790	CSV table 2. The table is referred to as 'table2' in the SQL query.
table3	snpSurvival-survivalSet8-survival.statistics 19.793	CSV table 3. The table is referred to as 'table3' in the SQL query.
query	snpSurvival-survivalSet8-annotSQL.in 19.765	SQL query. Either this file or the query parameter must be provided, but not both.

Parameter name	Value	Description
numIndices		Comma-separated list of index counts for input tables. All indices are single-column indices, running from column 1 to N, where N is retrieved from this parameter. If empty, the default number (1) is used. For example, ",2,,2" sets two indices for table2 and table4 and one index for the rest.
query		SQL query. Either this parameter or the query input must be provided, but not both.

19.789 snpSurvival-survivalSet8-survSNPAnnot-annotTable (CSV2Latex)

This component converts a tabular CSV file into a printable LaTeX table.

Author: Marko Laakso (Marko.Laakso@Helsinki.FI)
Version: 2.1
Categories: convert, Latex

Input name	Source	Description
tabledata	snpSurvival-survivalSet8-survSNPAnnot-annotData.table 19.788	Table content
refs	snpSurvival-survivalSet8-refsHits.in 19.782	Reference rules for the hyperlinks

Continued on next page...

Parameter name Value Description

Parameter name	Value	Description
attach	false	Include the original data as an attachment
caption	Gene annotations and the loci of the survival affecting markers	Caption text for the table.
colFormat	l@{}lr@{:}lp{1cm}p{1.5cm}p{10 .5cm}	LaTeX tabular format for the columns. Default produces a centered layout for all columns.
columns	hit,marker,chr,locus,band,gene, gene description	Comma separated list of column selections for the output. The empty default will use all columns.
countRows	false	Include a row count to the table caption.
dropMissing	true	This flag can be turned off in order to generate links with missing texts. Link text are substituted with target identifiers.
listCols	band	Comma separated list of column names. Columns of this list may contain several values separated with commas and the delimiters will be replaced with spaces.
numberFormat		A comma separated list of decimal formats for the columns. Each entry consists of the column name and the Java DecimalFormal pattern separated with equal sign. For example, rounding to three decimals can be done like: myColumn=#0.000
pageBreak	false	Use clear page after the table.
rename		Comma separated list of column renaming rules (oldname=newname). New names are used in table header but they do not affect the other behaviour of this component.
ruler	{}	Latex command for the row separating rulers
section		Section title for the table container or an empty string if no section should be generated.
sectionType	subsection	Type of LaTeX section: usually one of: section, subsection, or subsubsection. No section statement is written if section title is empty.
skipEmpty	false	This flag can be used to replace empty tables with a simple LaTeX comment.

19.790 snpSurvival-survivalSet8-survSNPAnnot-genes (KorvasieniAnnotator)

Korvasieni is an Ensembl [12] based converter for database identifiers and it can be used to convert genome location, gene, transcript, and translation identifiers across multiple biological databases. The selected `keyColumn` may contain entries with comma separated lists of values in which case `isListKey` parameter may be used to obtain each value separately and to process them as they were given in consecutive lines.

Author: Marko Laakso (Marko.Laakso@Helsinki.FI)
Version: 1.7
Categories: Annotation, GO

Input name	Source	Description
sourceKeys	snpSurvival-survivalSet8-survSNPAnnot-snpLoci. annotations 19.791	A list of source database keys.
connection	ensembl.in 19.273	Database connection can be defined using this file. The definition of parameters: database.url, database.user, database.password, database.timeout, database.recycle, and database.driver can be found from the documentation of Korvasieni.

Parameter name	Value	Description
echoColumns	refsnp_id	A comma separated list of column names for the columns that will be copied to the output. An asterisk (*) can be used to denote all columns execpt the keyColumn.
inputDB	.GeneId	Type of input keys. This must be a database supported by Korvasieni. If the parameter is omitted, the component tries to derive the database from the type of geneID. If this is not possible, an error is returned. You may define three columns in form of chromosome:start-end in case the inputDB is .DNARegion. This format provides a comfortable compatibility with DNARegion datatype.
inputType	Gene	Ensembl object type for the input keys (Any, Gene, Transcript, Translation)
isListKey	true	Enables the automatic value splits for the comma separated key column
keyColumn	ensembl_gene_stable_id	Name of the key column withing sourceKeys file or an empty string for the first column
maxHits	100000	Maximum number of target identifiers for a single source identifier
rename	UniProt/SwissProt=protein	Comma separated list of column renaming rules (oldname=newname)
skipLevel	never	Skip result rows if the source identifier is unknown or target identifiers are not available. Possible values are: never (no filtering), source (skip if the source ID is unknown), target (skip if no target IDs are found), any (skip if any of the target IDs is missing).

Continued on next page...

Parameter name	Value	Description
targetDB	.GeneName,.GeneDesc,.DNABand,GO,UniProt/SwissProt	Comma-separated list of annotation types. Possible values are all databases supported by Korvasieni.
unique	true	This flag can be turned on in order to eliminate duplicate annotations.

19.791 snpSurvival-survivalSet8-survSNPAnnot-snpLoci (RefSNPAnnotator)

RefSNPAnnotator fetches annotations for given refSNP ID's (also known as rsID's) from BioMart databases.

The provided annotations for each rsID include alleles for the SNP, name of the chromosome where the SNP is located, chromosome location of the SNP, the strand of the chromosome, Ensembl gene ID for genes that overlap the SNP's location and validation status of the SNP. The component is implemented using the biomaRt package for Bioconductor [7].

Authors: Viljami Aittomaki (Viljami.Aittomaki@Helsinki.FI), Marko Laakso (Marko.Laakso@Helsinki.FI)
Version: 1.0.1
Categories: SNP, Annotation

Input name	Source	Description
rsIDs	snpSurvival-survivalSet8-survival.statistics 19.793	List of rsID's for fetching annotations.

Parameter name	Value	Description
dataset	hsapiens_snp	Dataset to get annotations from. Different species have their own datasets. Example values include "hsapiens_snp" and "mmusculus_snp". See biomaRt documentation on how to list available datasets in R.
keyColumn	marker	Name of the key column withing sourceKeys file or an empty string for the first column.

19.792 snpSurvival-survivalSet8-survSNPAnnot-snpRel (ExpandCollapse)

Converts between the two possible representations of a relations with multivalued columns. By default this component splits rows with multivalued (comma separated lists) columns into multiple rows each representing a single value. The input may contain several columns with comma separated values and all permutations of column values are shown as individual single-valued rows. The alternative mode recovers the original comma separated values from the expanded relations. This is performed by joining values of the their list columns into comma separated lists.

Author: Marko Laakso (Marko.Laakso@Helsinki.FI)
Version: 1.3
Category: convert

Input name	Source	Description
relation	snpSurvival-survivalSet8-survSNPAnnot-snpLoci.annotations 19.791	The mandatory input relation

Parameter name	Value	Description
delim	,	Value delimiter between the list column values. Special characters can be encoded as specified in fi.helsinki.ltdk.csbl.asser.ArgumentEncoding.
expand	true	Action mode that is expand (true) or collapse (false)
listCols	ensembl_gene_stable_id	A comma separated list of column names of input (expand) or output (collapse) columns that may contain delim separated values. The asterisk refers to every column of the input relation.

19.793 snpSurvival-survivalSet8-survival (SNPKaplanMeier)

This component is used to estimate survival differences between various genotypes. The differences between the survival of different genotypes are measured using G-ρ family of tests [10].

Author: Marko Laakso (Marko.Laakso@Helsinki.FI)
Version: 1.5
Categories: SNP, Latex, Survival

Input name	Source	Description
genotypes	snpSurvival-survivalSet8-case Genotypes-gtypes .genotypes 19.770	Genotype calls
survival	snpSurvival-survivalSet8-survival Clinical.table 19.794	Survival time and event indicator
annotations	snpSurvival-survivalSet8-snp Annotations.in 19.785	Additional information about the markers. This data will be shown on the report of interesting markers.

Parameter name	Value	Description
PLimit	0.0010	Ignore results with a p-value greater than this limit.
confInt	0.95	Coverage of the confidence intervals [0,1]
minCount	15	Combine rare (aa) homozygotes to heterozygotes (Aa) if their number is below this limit. This parameter is used together with minFreq so that the actual limit is max(minFreq*n, minCount).
minFreq	0.1	Combine rare (aa) homozygotes to heterozygotes (Aa) if their frequency is below this limit. See also minCount.
naLimit	0.1	Skip markers where one minus call rate exceeds this limit.
nullCalls	–,ERR	A comma separated list of genotype calls that will be considered as missing values
sampleCol	sample	Column name for the sample name
showCI	true	Draw confidence intervals to the graph
skipMissingGT	true	Simply skip those samples that lack the genotype information.
statusCol	hasEvent	Column name for the event indicator
survRatePoint	0.5	Sampling height for the distances between the survival expectations. Negative values may be used to suppress these measurements.
timeCol	endTime	Column name for the survival time
timeOutLimit	36	Cut survival curves after this limit. A negative value can be used to maximize the length of the period.
timeStartCol	startTime	Column name for the start time that is reduced from the survival time
title	Genotype Specific Survival Estimates	Section title

19.794 snpSurvival-survivalSet8-survivalClinical (TableQuery)

Execute an SQL query on input relations and create a result table. TableQuery uses HSQLDB (Hyperthreaded Structured Query Language Database [25]) for executing the query. Consequently, the syntax of the query is defined by HSQLDB.

Author: Kristian Ovaska (Kristian.Ovaska@Helsinki.FI)
Version: 1.2
Categories: convert, Filter

Input name	Source	Description
table1	snpSurvival-survivalSet8-clinical Data.in 19.772	CSV table 1. The table is referred to as 'table1' in the SQL query.
query	snpSurvival-survivalSet8-survival ParamsQuery.file 19.795	SQL query. Either this file or the query parameter must be provided, but not both.
columnTypes	snpSurvival-survivalSet8-clinical Types.in 19.774	Contains SQL types for individual columns. If the file is not provided, the type is inferred from the contents of the columns. This can be used to force the use of VARCHAR for values that are also valid numerics. The file contains the columns Table (refers to one of table1 to table15), Column (refers to a column name in the table), Type (contains an SQL type). A row with Table='result', Column=X and Type='STRING' forces the use string values for result column X.

Parameter name	Value	Description
numIndices		Comma-separated list of index counts for input tables. All indices are single-column indices, running from column 1 to N, where N is retrieved from this parameter. If empty, the default number (1) is used. For example, ",2,,2" sets two indices for table2 and table4 and one index for the rest.
query		SQL query. Either this parameter or the query input must be provided, but not both.

19.795 snpSurvival-survivalSet8-survivalParamsQuery (SearchReplace)

Replaces the occurences of the given regular expressions with the corresponding regular expressions. This component is used for transforming given patterns of the input file to a changed form specified by the regular expressions .

Author: Marko Laakso (Marko.Laakso@Helsinki.FI)
Version: 1.0
Category: convert

Input name	Source	Description
file	snpSurvival-survivalSet8-survival ParamsTemplate.in 19.796	Source file to be modified

Parameter name	Value	Description
key00	REP.cond00	Search key 0
key01		Search key 1
key02		Search key 2
key03		Search key 3
key04		Search key 4
key05		Search key 5
key06		Search key 6
key07		Search key 7
key08		Search key 8
key09		Search key 9
value00	– No additional restrictions	Replacement 0
value01		Replacement 1
value02		Replacement 2
value03		Replacement 3
value04		Replacement 4
value05		Replacement 5
value06		Replacement 6
value07		Replacement 7
value08		Replacement 8
value09		Replacement 9

19.796 snpSurvival-survivalSet8-survivalParamsTemplate (INPUT)

Select relevant parameters for the survival analysis

Category: Internal

Parameter name	Value	Description
path	/gpfs/homes/mlaakso/asserSVN/ andurilCaseStudy/sql/ SNPsurvivalParams.sql	Path (filename) of the input resource.

19.797 snpSurvival-survivalSet8-survivalReport (LatexCombiner)

LatexCombiner joins the given subdocuments together into one LaTeX file.

Authors:	Kristian Ovaska (Kristian.Ovaska@Helsinki.FI), Marko Laakso (Marko.Laakso@Helsinki.FI)
Version:	1.3
Category:	Latex

Input name	Source	Description
latex1	snpSurvival-survivalSet8-survival .report 19.793	LaTeX fragment 1.
latex2	snpSurvival-survivalSet8-clinical Select.report 19.773	LaTeX fragment 2.
latex3	snpSurvival-survivalSet8-survSN PAnnot-annotTable.report 19.789	LaTeX fragment 3.
latex4	snpSurvival-survivalSet8-gene Rank-eqTable.report 19.777	LaTeX fragment 4.

Parameter name	Value	Description
pagebreak	true	Determines if the result document should start with a page break.
sectionTitle		If non-empty, a declaration of a new section with the given name is inserted to the beginning of the combined document. This is a convenience feature to make it easy to compile subsections into a section.
sectionType	section	Type of LaTeX section: usually one of section, subsection or subsubsection. No section statement is written if sectionTitle is empty.
strictBorders	true	Enables the flushing of all document elements before each fragment

19.798 snpSurvival-survivalSet9 (SurvivalAnalysis)

No description

Parameter name	Value
pvalue	0.0010

19.799 snpSurvival-survivalSet9-annotSQL (INPUT)

This SQL is used to generate marker annotation report.

Category: Internal

Parameter name	Value	Description
path	/gpfs/homes/mlaakso/asserSVN/ anduril CaseStudy/sql/ SNPgeneTable.sql	Path (filename) of the input resource.

19.800 snpSurvival-survivalSet9-caseGenotypes (GetGenotypes)

Load and preprocess the genotype calls for the cancer patient samples.

Parameter name	Value
skipFrq	0.1

19.801 snpSurvival-survivalSet9-caseGenotypes-freqCheck (AlleleCounter)

AlleleCounter calculates genotype frequencies for the biallelic markers of the given SNPMatrix. A Pearson's χ^2-test [18] is performed for each marker in order to test if the Hardy-Weinberg equilibrium [9] holds for the population sample.

Author:	Marko Laakso (Marko.Laakso@Helsinki.FI)
Version:	1.2
Category:	SNP

Input name	Source	Description
genotypes	snpSurvival-survivalSet9-case Genotypes-genotypes .genotypes 19.803	SNPHelistin genotype output.

Parameter name	Value	Description
dels	false	Accept homozygous deletions as a possible genotype.
naLimit	0.1	Skip markers where one minus call rate exceeds this limit.
skip	0.1	Drop markers with a relative minor allele frequency lower than this limit. Use negative values to inactivate this limit.

19.802 snpSurvival-survivalSet9-caseGenotypes-ftMarkers (CSV2IDList)

Extracts one column from the given CSV file and prints it out without duplicates. The selected column may contain entries with comma separated lists of values in which case *isList* parameter may be used to obtain each value separately.

Output is defined as:

$$ids = \bigcup_{i=1}^{9} \pi_{columnIn_i} \sigma_{regexp_i}(table_i). \quad (28)$$

Author:	Marko Laakso (Marko.Laakso@Helsinki.FI)
Version:	1.3
Category:	convert

Input name	Source	Description
table1	snpSurvival-survivalSet9-case Genotypes-freqCheck .frequencies 19.801	The mandatory input relation

Parameter name	Value	Description
acceptMissing	false	Files with missing columnIn are accepted as empty if this is true.
columnIn	marker	A comma separated list of column names for the IDs of interest in each file. Empty values refer to the first column of the file.
columnOut	marker	Name of the only column of the output list. Empty input refers to the name of the input column.
constants		A comma separated list of values that are always included into the output
isList	false	True if the seleted column contains a comma separated list of values to be splitted
quotation	false	Indicator that can be used to disable quotation of the output values
regexp1		Regular expression for the row filtering in table1. A row is included in the result if this parameter is empty or if values in the given columns match given regular expressions. The parameter has a format COLNAME1=EXPRESSION,COLNAME2=EXPRESSION2 where COLNAMEs are column names in "csv" and EXPRESSIONs are regular expressions using Java syntax. For example, "col=a|b" includes rows where the column col has a value of "a" or "b".
regexp2		Regular expression for the row filtering in table2.
regexp3		Regular expression for the row filtering in table3.
regexp4		Regular expression for the row filtering in table4.
regexp5		Regular expression for the row filtering in table5.
regexp6		Regular expression for the row filtering in table6.
regexp7		Regular expression for the row filtering in table7.
regexp8		Regular expression for the row filtering in table8.
regexp9		Regular expression for the row filtering in table9.

19.803 snpSurvival-survivalSet9-caseGenotypes-genotypes (SNPHelistinReader)

This component can be used to read SNP genotype calls for a set of samples and markers of interest. The genotype data is retrieved from a SNPHelistin database that is defined by the input properties.

Author:	Marko Laakso (Marko.Laakso@Helsinki.FI)
Version:	1.2
Categories:	Data Import, SNP

Input name	Source	Description
sampleNames	snpSurvival-survivalSet9-caseNames.ids 19.805	List of sample names of interest.
markerNames	snpSurvival-markers9.in 19.457	List of SNP identifiers of interest.
config	genotypeDB.in 19.320	Connection parameters for SNPHelistin.

Parameter name	Value	Description
errorSymbol	ERR	Symbol for failed measurements. You may for example use 'NA' value in order consider them equal to missing values.
skipUnknown	true	Ignore unknown samples and do not process them. If the parameter is false then the program fails for these samples.

19.804 snpSurvival-survivalSet9-caseGenotypes-gtypes (SNPHelistinReader)

This component can be used to read SNP genotype calls for a set of samples and markers of interest. The genotype data is retrieved from a SNPHelistin database that is defined by the input properties.

Author:	Marko Laakso (Marko.Laakso@Helsinki.FI)
Version:	1.2
Categories:	Data Import, SNP

Input name	Source	Description
sampleNames	snpSurvival-survivalSet9-caseNames.ids 19.805	List of sample names of interest.
markerNames	snpSurvival-survivalSet9-caseGenotypes-ftMarkers.ids 19.802	List of SNP identifiers of interest.
config	genotypeDB.in 19.320	Connection parameters for SNPHelistin.

Parameter name	Value	Description
errorSymbol	ERR	Symbol for failed measurements. You may for example use 'NA' value in order consider them equal to missing values.
skipUnknown	true	Ignore unknown samples and do not process them. If the parameter is false then the program fails for these samples.

19.805 snpSurvival-survivalSet9-caseNames (CSV2IDList)

Extracts one column from the given CSV file and prints it out without duplicates. The selected column may contain entries with comma separated lists of values in which case *isList* parameter may be used to obtain each value separately.

Output is defined as:

$$ids = \bigcup_{i=1}^{9} \pi_{columnIn_i} \sigma_{regexp_i}(table_i). \quad (29)$$

Author: Marko Laakso (Marko.Laakso@Helsinki.FI)
Version: 1.3
Category: convert

Input name	Source	Description
table1	snpSurvival-survivalSet9-survival Clinical.table 19.828	The mandatory input relation

Parameter name	Value	Description
acceptMissing	false	Files with missing columnIn are accepted as empty if this is true.
columnIn		A comma separated list of column names for the IDs of interest in each file. Empty values refer to the first column of the file.
columnOut	SampleId	Name of the only column of the output list. Empty input refers to the name of the input column.
constants		A comma separated list of values that are always included into the output
isList	false	True if the seleted column contains a comma separated list of values to be splitted
quotation	false	Indicator that can be used to disable quotation of the output values
regexp1		Regular expression for the row filtering in table1. A row is included in the result if this parameter is empty or if values in the given columns match given regular expressions. The parameter has a format COLNAME1=EXPRESSION,COLNAME2=EXPRESSION2 where COLNAMEs are column names in "csv" and EXPRESSIONs are regular expressions using Java syntax. For example, "col=a|b" includes rows where the column col has a value of "a" or "b".
regexp2		Regular expression for the row filtering in table2.
regexp3		Regular expression for the row filtering in table3.
regexp4		Regular expression for the row filtering in table4.
regexp5		Regular expression for the row filtering in table5.
regexp6		Regular expression for the row filtering in table6.
regexp7		Regular expression for the row filtering in table7.
regexp8		Regular expression for the row filtering in table8.
regexp9		Regular expression for the row filtering in table9.

19.806 snpSurvival-survivalSet9-clinicalData (INPUT)

Clinical parameters for the case samples
Category: Internal

Parameter name	Value	Description
path	/gpfs/homes/mlaakso/asserSVN/ andurilCaseStudy/data/snp/ patient-metadata-caucas.csv	Path (filename) of the input resource.

19.807 snpSurvival-survivalSet9-clinicalSelect (SourceCode2Latex)

This component converts a source code listing into a LaTeXfragment that may be included into some technical documents.
Author: Marko Laakso (Marko.Laakso@Helsinki.FI)
Version: 1.0
Categories: convert, Latex

Input name	Source	Description
source	snpSurvival-survivalSet9-survival ParamsQuery.file 19.829	Source code

Parameter name	Value	Description
caption	This SQL query has been used to select clinical parameters for the survival calculations.	Caption text for the listing
language	SQL	Language defintion for the syntax highlighting. Possible values can be found from the description of listings package.

19.808 snpSurvival-survivalSet9-clinicalTypes (INPUT)

Column types for the clinical data

Category: Internal

Parameter name	Value	Description
path	/gpfs/homes/mlaakso/asserSVN/andurilCaseStudy/csv/SNPclinicalTypes.csv	Path (filename) of the input resource.

19.809 snpSurvival-survivalSet9-geneRank (QueryReport)

(Technical documentation) Executes an SQL query and prints it with the result table.

Author: Marko Laakso (Marko.Laakso@Helsinki.FI)
Version: 1.1
Category: Latex

Input name	Source	Description
table1	snpSurvival-survivalSet9-survSNPAnnot.fullData 19.821	1. data table that is referred as 'table1' in the query

Parameter name	Value	Description
colFormat	lcrp{11cm}	LaTeX tabular formatting for the result columns
listCols		Comma separated list of column names. Columns of this list may contain several values separated with commas and the delimiters will be replaced with spaces.
resTableRef	the previous table	Textual reference from the SQL representation to the result table
section	Gene rank	Subsection title for the output document
showCols	gene,markers,p-value,description	Comma separated list of column selections for the output. The empty default will use all columns.
showQuery	false	This flag tells if the SQL shall be shown on the report
tableCaption	A list of survival affecting genes and the marker counts. P-value is the lowest value for the corresponding markers.	Description for the result table

19.810 snpSurvival-survivalSet9-geneRank-eqData (TableQuery)

Execute an SQL query on input relations and create a result table. TableQuery uses HSQLDB (Hyper-threaded Structured Query Language Database [25]) for executing the query. Consequently, the syntax of the query is defined by HSQLDB.

Author: Kristian Ovaska (Kristian.Ovaska@Helsinki.FI)
Version: 1.2
Categories: convert, Filter

Input name	Source	Description
table1	snpSurvival-survivalSet9-survSNPAnnot-annotData.table 19.822	CSV table 1. The table is referred to as 'table1' in the SQL query.

Continued on next page...

Input name	Source	Description
table2	snpSurvival-survivalSet9-survival .statistics 19.827	CSV table 2. The table is referred to as 'table2' in the SQL query.
query	snpSurvival-survivalSet9-gene RankQuery.in 19.812	SQL query. Either this file or the query parameter must be provided, but not both.

Parameter name	Value	Description
numIndices		Comma-separated list of index counts for input tables. All indices are single-column indices, running from column 1 to N, where N is retrieved from this parameter. If empty, the default number (1) is used. For example, ",2,,2" sets two indices for table2 and table4 and one index for the rest.
query		SQL query. Either this parameter or the query input must be provided, but not both.

19.811 snpSurvival-survivalSet9-geneRank-eqTable (CSV2Latex)

This component converts a tabular CSV file into a printable LaTeX table.

Author: Marko Laakso (Marko.Laakso@Helsinki.FI)
Version: 2.1
Categories: convert, Latex

Input name	Source	Description
tabledata	snpSurvival-survivalSet9-gene Rank-eqData.table 19.810	Table content
refs	snpSurvival-survivalSet9-refsGene Rank.in 19.815	Reference rules for the hyperlinks

Parameter name	Value	Description
attach	false	Include the original data as an attachment
caption	A list of survival affecting genes and the marker counts. P-value is the lowest value for the corresponding markers.	Caption text for the table.
colFormat	lcrp{11cm}	LaTeX tabular format for the columns. Default produces a centered layout for all columns.
columns	gene,markers,p-value,description	Comma separated list of column selections for the output. The empty default will use all columns.
countRows	false	Include a row count to the table caption.
dropMissing	true	This flag can be turned off in order to generate links with missing texts. Link text are substituted with target identifiers.
listCols		Comma separated list of column names. Columns of this list may contain several values separated with commas and the delimiters will be replaced with spaces.
numberFormat		A comma separated list of decimal formats for the columns. Each entry consists of the column name and the Java DecimalFormal pattern separated with equal sign. For example, rounding to three decimals can be done like: myColumn=#0.000
pageBreak	false	Use clear page after the table.
rename		Comma separated list of column renaming rules (oldname=newname). New names are used in table header but they do not affect the other behaviour of this component.
ruler	{}	Latex command for the row separating rulers
section	Gene rank	Section title for the table container or an empty string if no section should be generated.
sectionType	subsection	Type of LaTeX section: usually one of: section, subsection, or subsubsection. No section statement is written if section title is empty.
skipEmpty	false	This flag can be used to replace empty tables with a simple LaTeX comment.

19.812 snpSurvival-survivalSet9-geneRankQuery (INPUT)

Generates a list of genes and the marker counts for them.

Category: Internal

Parameter name	Value	Description
path	/gpfs/homes/mlaakso/asserSVN/andurilCaseStudy/sql/SNPinterestingGenes.sql	Path (filename) of the input resource.

19.813 snpSurvival-survivalSet9-missingDistC (CSV2Latex)

This component converts a tabular CSV file into a printable LaTeX table.

Author: Marko Laakso (Marko.Laakso@Helsinki.FI)
Version: 2.1
Categories: convert, Latex

Input name	Source	Description
tabledata	snpSurvival-survivalSet9-caseGenotypes-freqCheck.naDist 19.801	Table content

Parameter name	Value	Description
attach	false	Include the original data as an attachment
caption	Distribution of the missing values in case samples	Caption text for the table.
colFormat	p{3cm}r	LaTeX tabular format for the columns. Default produces a centered layout for all columns.
columns		Comma separated list of column selections for the output. The empty default will use all columns.
countRows	false	Include a row count to the table caption.
dropMissing	true	This flag can be turned off in order to generate links with missing texts. Link text are substituted with target identifiers.
listCols		Comma separated list of column names. Columns of this list may contain several values separated with commas and the delimiters will be replaced with spaces.
numberFormat		A comma separated list of decimal formats for the columns. Each entry consists of the column name and the Java DecimalFormal pattern separated with equal sign. For example, rounding to three decimals can be done like: myColumn=#0.000
pageBreak	false	Use clear page after the table.
rename		Comma separated list of column renaming rules (oldname=newname). New names are used in table header but they do not affect the other behaviour of this component.
ruler	{}	Latex command for the row separating rulers
section		Section title for the table container or an empty string if no section should be generated.
sectionType	subsection	Type of LaTeX section: usually one of: section, subsection, or subsubsection. No section statement is written if section title is empty.
skipEmpty	false	This flag can be used to replace empty tables with a simple LaTeX comment.

19.814 snpSurvival-survivalSet9-propertiesDoc (Properties2Latex)

Prepares a LaTeX-representation for the given set of properties files. The document consists of tables of names and values of the properties.

Author: Marko Laakso (Marko.Laakso@Helsinki.FI)
Version: 1.2
Categories: convert, Latex

Input name	Source	Description
props1	genotypeDB.in 19.320	A set of properties

Parameter name	Value	Description
hide		A comma separated list of property names that should remain invisible. You may use this paramerter to hide passwords and other confidentian settings.

Continued on next page...

Parameter name	Value	Description
keyWidth	5	Column width for the property names (centimeters)
merge	false	Merge all input properties into a single set
section	SNP database configuration	Section title for the table container or an empty string if no section should be generated
sectionType	subsection	Type of LaTeX section: usually one of section, subsection or subsubsection. No section statement is written if sectionTitle is empty.
valueWidth	13	Column width for the property values (centimeters)

19.815 snpSurvival-survivalSet9-refsGeneRank (INPUT)

Hyperlink rules for the gene rank table

Category: Internal

Parameter name	Value	Description
path	/gpfs/homes/mlaakso/asserSVN/andurilCaseStudy/csv/SNPrefsGeneRank.csv	Path (filename) of the input resource.

19.816 snpSurvival-survivalSet9-refsHits (INPUT)

Hyperlink rules for the marker annotations

Category: Internal

Parameter name	Value	Description
path	/gpfs/homes/mlaakso/asserSVN/andurilCaseStudy/csv/SNPrefsHits.csv	Path (filename) of the input resource.

19.817 snpSurvival-survivalSet9-reportBody (LatexCombiner)

LatexCombiner joins the given subdocuments together into one LaTeX file.

Authors: Kristian Ovaska (Kristian.Ovaska@Helsinki.FI), Marko Laakso (Marko.Laakso@Helsinki.FI)
Version: 1.3
Category: Latex

Input name	Source	Description
latex1	snpSurvival-survivalSet9-survivalReport.document 19.831	LaTeX fragment 1.
latex2	snpSurvival-survivalSet9-propertiesDoc.report 19.814	LaTeX fragment 2.
latex3	snpSurvival-survivalSet9-missingDistC.report 19.813	LaTeX fragment 3.

Parameter name	Value	Description
pagebreak	false	Determines if the result document should start with a page break.
sectionTitle		If non-empty, a declaration of a new section with the given name is inserted to the beginning of the combined document. This is a convenience feature to make it easy to compile subsections into a section.
sectionType	section	Type of LaTeX section: usually one of section, subsection or subsubsection. No section statement is written if sectionTitle is empty.
strictBorders	true	Enables the flushing of all document elements before each fragment

19.818 snpSurvival-survivalSet9-reportTemplate (LatexTemplate)

This component generates the standard layout configuration for Anduril output documents. The component produces a header and a footer fragment that can be used to build LaTeX documents. The header fragment takes care of the page geometry and other technical details such as library imports and function declarations. Reference list is generated by the footer.

Author: Marko Laakso (Marko.Laakso@Helsinki.FI)
Version: 1.4
Category: Latex

Input name	Source	Description
abstract	abstract.in 19.42	Document abstract including its Latex definition. You may use template entities within this document. The default abstract has been defined in abstractTemplate.tex.

Parameter name	Value	Description
authors	Marko Laakso, Riku Louhimo, Sampsa Hautaniemi	A comma-separated list of document authors on the title page. If authors is empty but title is non-empty, a title page with no author name is printed.
baselineskip	1.2	Baselineskip factor that indicates the line spacing
bibstyle	abbrv	BibTeX reference formatting style
printTOC	true	If true, print Table Of Contents.
title	Anduril Case Study: Glioblastoma SNP survival analysis	Document title on the title page. If empty, no title page is printed. Also see author.
usepackage		Comma-separated list of LaTeX packages that are used in the document. Each package generates a \usepackage{X} line in the header.

19.819 snpSurvival-survivalSet9-snpAnnotations (INPUT)

Annotations for survival component.

Category: Internal

Parameter name	Value	Description
path	/gpfs/homes/mlaakso/asserSVN/anduril CaseStudy/csv/SNPAnnotation.csv	Path (filename) of the input resource.

19.820 snpSurvival-survivalSet9-summaryReport (LatexPDF)

This component is a LaTeX compiler that generates a portable document format (PDF) file from the given Anduril document.

Author: Kristian Ovaska (Kristian.Ovaska@Helsinki.FI)
Version: 1.0
Category: Latex

Input name	Source	Description
document	snpSurvival-survivalSet9-report Body.document 19.817	Body of the LaTeX document.
header	snpSurvival-survivalSet9-report Template.header 19.818	LaTeX header that is written to the start of the document. If missing, a default header is used.
footer	snpSurvival-survivalSet9-report Template.footer 19.818	LaTeX footer that is written to the end of the document. If missing, a default header is used.

Parameter name	Value	Description
bibtexExec	bibtex	Executable command for BibTeX.
latexExec	pdflatex	Executable command for pdflatex.
useRefs	false	This flag can be used to activate BibTeX compiler

19.821 snpSurvival-survivalSet9-survSNPAnnot (AnnotateSNPs)

Biological annotations for the survival affecting genes

Parameter name	Value
caption	Gene annotations and the loci of the survival affecting markers

19.822 snpSurvival-survivalSet9-survSNPAnnot-annotData (TableQuery)

Execute an SQL query on input relations and create a result table. TableQuery uses HSQLDB (Hyperthreaded Structured Query Language Database [25]) for executing the query. Consequently, the syntax of the query is defined by HSQLDB.

Author: Kristian Ovaska (Kristian.Ovaska@Helsinki.FI)
Version: 1.2
Categories: convert, Filter

Input name	Source	Description
table1	snpSurvival-survivalSet9-survSNPAnnot-snpRel.relation 19.826	CSV table 1. The table is referred to as 'table1' in the SQL query.
table2	snpSurvival-survivalSet9-survSNPAnnot-genes.bioAnnotation 19.824	CSV table 2. The table is referred to as 'table2' in the SQL query.
table3	snpSurvival-survivalSet9-survival.statistics 19.827	CSV table 3. The table is referred to as 'table3' in the SQL query.
query	snpSurvival-survivalSet9-annotSQL.in 19.799	SQL query. Either this file or the query parameter must be provided, but not both.

Parameter name	Value	Description
numIndices		Comma-separated list of index counts for input tables. All indices are single-column indices, running from column 1 to N, where N is retrieved from this parameter. If empty, the default number (1) is used. For example, ",2,,2" sets two indices for table2 and table4 and one index for the rest.
query		SQL query. Either this parameter or the query input must be provided, but not both.

19.823 snpSurvival-survivalSet9-survSNPAnnot-annotTable (CSV2Latex)

This component converts a tabular CSV file into a printable LaTeX table.

Author: Marko Laakso (Marko.Laakso@Helsinki.FI)
Version: 2.1
Categories: convert, Latex

Input name	Source	Description
tabledata	snpSurvival-survivalSet9-survSNPAnnot-annotData.table 19.822	Table content
refs	snpSurvival-survivalSet9-refsHits.in 19.816	Reference rules for the hyperlinks

Continued on next page...

Parameter name Value Description

Parameter name	Value	Description
attach	false	Include the original data as an attachment
caption	Gene annotations and the loci of the survival affecting markers	Caption text for the table.
colFormat	l@{}lr@{:}lp{1cm}p{1.5cm}p{10 .5cm}	LaTeX tabular format for the columns. Default produces a centered layout for all columns.
columns	hit,marker,chr,locus,band,gene, gene description	Comma separated list of column selections for the output. The empty default will use all columns.
countRows	false	Include a row count to the table caption.
dropMissing	true	This flag can be turned off in order to generate links with missing texts. Link text are substituted with target identifiers.
listCols	band	Comma separated list of column names. Columns of this list may contain several values separated with commas and the delimiters will be replaced with spaces.
numberFormat		A comma separated list of decimal formats for the columns. Each entry consists of the column name and the Java DecimalFormal pattern separated with equal sign. For example, rounding to three decimals can be done like: myColumn=#0.000
pageBreak	false	Use clear page after the table.
rename		Comma separated list of column renaming rules (oldname=newname). New names are used in table header but they do not affect the other behaviour of this component.
ruler	{}	Latex command for the row separating rulers
section		Section title for the table container or an empty string if no section should be generated.
sectionType	subsection	Type of LaTeX section: usually one of: section, subsection, or subsubsection. No section statement is written if section title is empty.
skipEmpty	false	This flag can be used to replace empty tables with a simple LaTeX comment.

19.824 snpSurvival-survivalSet9-survSNPAnnot-genes (KorvasieniAnnotator)

Korvasieni is an Ensembl [12] based converter for database identifiers and it can be used to convert genome location, gene, transcript, and translation identifiers across multiple biological databases. The selected `keyColumn` may contain entries with comma separated lists of values in which case `isListKey` parameter may be used to obtain each value separately and to process them as they were given in consecutive lines.

Author: Marko Laakso (Marko.Laakso@Helsinki.FI)
Version: 1.7
Categories: Annotation, GO

Input name	Source	Description
sourceKeys	snpSurvival-survivalSet9-survSNPAnnot-snpLoci. annotations 19.825	A list of source database keys.
connection	ensembl.in 19.273	Database connection can be defined using this file. The definition of parameters: database.url, database.user, database.password, database.timeout, database.recycle, and database.driver can be found from the documentation of Korvasieni.

Parameter name	Value	Description
echoColumns	refsnp_id	A comma separated list of column names for the columns that will be copied to the output. An asterisk (*) can be used to denote all columns execpt the keyColumn.
inputDB	.GeneId	Type of input keys. This must be a database supported by Korvasieni. If the parameter is omitted, the component tries to derive the database from the type of geneID. If this is not possible, an error is returned. You may define three columns in form of chromosome:start-end in case the inputDB is .DNARegion. This format provides a comfortable compatibility with DNARegion datatype.
inputType	Gene	Ensembl object type for the input keys (Any, Gene, Transcript, Translation)
isListKey	true	Enables the automatic value splits for the comma separated key column
keyColumn	ensembl_gene_stable_id	Name of the key column withing sourceKeys file or an empty string for the first column
maxHits	100000	Maximum number of target identifiers for a single source identifier
rename	UniProt/SwissProt=protein	Comma separated list of column renaming rules (oldname=newname)
skipLevel	never	Skip result rows if the source identifier is unknown or target identifiers are not available. Possible values are: never (no filtering), source (skip if the source ID is unknown), target (skip if no target IDs are found), any (skip if any of the target IDs is missing).

Continued on next page. . .

Parameter name	Value	Description
targetDB	.GeneName,.GeneDesc,.DNABand,GO,UniProt/SwissProt	Comma-separated list of annotation types. Possible values are all databases supported by Korvasieni.
unique	true	This flag can be turned on in order to eliminate duplicate annotations.

19.825 snpSurvival-survivalSet9-survSNPAnnot-snpLoci (RefSNPAnnotator)

RefSNPAnnotator fetches annotations for given refSNP ID's (also known as rsID's) from BioMart databases.

The provided annotations for each rsID include alleles for the SNP, name of the chromosome where the SNP is located, chromosome location of the SNP, the strand of the chromosome, Ensembl gene ID for genes that overlap the SNP's location and validation status of the SNP. The component is implemented using the biomaRt package for Bioconductor [7].

Authors: Viljami Aittomaki (Viljami.Aittomaki@Helsinki.FI), Marko Laakso (Marko.Laakso@Helsinki.FI)
Version: 1.0.1
Categories: SNP, Annotation

Input name	Source	Description
rsIDs	snpSurvival-survivalSet9-survival.statistics 19.827	List of rsID's for fetching annotations.

Parameter name	Value	Description
dataset	hsapiens_snp	Dataset to get annotations from. Different species have their own datasets. Example values include "hsapiens_snp" and "mmusculus_snp". See biomaRt documentation on how to list available datasets in R.
keyColumn	marker	Name of the key column withing sourceKeys file or an empty string for the first column.

19.826 snpSurvival-survivalSet9-survSNPAnnot-snpRel (ExpandCollapse)

Converts between the two possible representations of a relations with multivalued columns. By default this component splits rows with multivalued (comma separated lists) columns into multiple rows each representing a single value. The input may contain several columns with comma separated values and all permutations of column values are shown as individual single-valued rows. The alternative mode recovers the original comma separated values from the expanded relations. This is performed by joining values of the their list columns into comma separated lists.

Author: Marko Laakso (Marko.Laakso@Helsinki.FI)
Version: 1.3
Category: convert

Input name	Source	Description
relation	snpSurvival-survivalSet9-survSNPAnnot-snpLoci.annotations 19.825	The mandatory input relation

Parameter name	Value	Description
delim	,	Value delimiter between the list column values. Special characters can be encoded as specified in fi.helsinki.ltdk.csbl.asser.ArgumentEncoding.
expand	true	Action mode that is expand (true) or collapse (false)
listCols	ensembl_gene_stable_id	A comma separated list of column names of input (expand) or output (collapse) columns that may contain delim separated values. The asterisk refers to every column of the input relation.

19.827 snpSurvival-survivalSet9-survival (SNPKaplanMeier)

This component is used to estimate survival differences between various genotypes. The differences between the survival of different genotypes are measured using G-ρ family of tests [10].

Author: Marko Laakso (Marko.Laakso@Helsinki.FI)
Version: 1.5
Categories: SNP, Latex, Survival

Input name	Source	Description
genotypes	snpSurvival-survivalSet9-caseGenotypes-gtypes.genotypes 19.804	Genotype calls
survival	snpSurvival-survivalSet9-survivalClinical.table 19.828	Survival time and event indicator
annotations	snpSurvival-survivalSet9-snpAnnotations.in 19.819	Additional information about the markers. This data will be shown on the report of interesting markers.

Parameter name	Value	Description
PLimit	0.0010	Ignore results with a p-value greater than this limit.
confInt	0.95	Coverage of the confidence intervals [0,1]
minCount	15	Combine rare (aa) homozygotes to heterozygotes (Aa) if their number is below this limit. This parameter is used together with minFreq so that the actual limit is max(minFreq*n, minCount).
minFreq	0.1	Combine rare (aa) homozygotes to heterozygotes (Aa) if their frequency is below this limit. See also minCount.
naLimit	0.1	Skip markers where one minus call rate exceeds this limit.
nullCalls	–,ERR	A comma separated list of genotype calls that will be considered as missing values
sampleCol	sample	Column name for the sample name
showCI	true	Draw confidence intervals to the graph
skipMissingGT	true	Simply skip those samples that lack the genotype information.
statusCol	hasEvent	Column name for the event indicator
survRatePoint	0.5	Sampling height for the distances between the survival expectations. Negative values may be used to suppress these measurements.
timeCol	endTime	Column name for the survival time
timeOutLimit	36	Cut survival curves after this limit. A negative value can be used to maximize the length of the period.
timeStartCol	startTime	Column name for the start time that is reduced from the survival time
title	Genotype Specific Survival Estimates	Section title

19.828 snpSurvival-survivalSet9-survivalClinical (TableQuery)

Execute an SQL query on input relations and create a result table. TableQuery uses HSQLDB (Hyperthreaded Structured Query Language Database [25]) for executing the query. Consequently, the syntax of the query is defined by HSQLDB.

Author: Kristian Ovaska (Kristian.Ovaska@Helsinki.FI)
Version: 1.2
Categories: convert, Filter

Input name	Source	Description
table1	snpSurvival-survivalSet9-clinicalData.in 19.806	CSV table 1. The table is referred to as 'table1' in the SQL query.
query	snpSurvival-survivalSet9-survivalParamsQuery.file 19.829	SQL query. Either this file or the query parameter must be provided, but not both.
columnTypes	snpSurvival-survivalSet9-clinicalTypes.in 19.808	Contains SQL types for individual columns. If the file is not provided, the type is inferred from the contents of the columns. This can be used to force the use of VARCHAR for values that are also valid numerics. The file contains the columns Table (refers to one of table1 to table15), Column (refers to a column name in the table), Type (contains an SQL type). A row with Table='result', Column=X and Type='STRING' forces the use string values for result column X.

Parameter name	Value	Description
numIndices		Comma-separated list of index counts for input tables. All indices are single-column indices, running from column 1 to N, where N is retrieved from this parameter. If empty, the default number (1) is used. For example, ",2,,2" sets two indices for table2 and table4 and one index for the rest.
query		SQL query. Either this parameter or the query input must be provided, but not both.

19.829 snpSurvival-survivalSet9-survivalParamsQuery (SearchReplace)

Replaces the occurences of the given regular expressions with the corresponding regular expressions. This component is used for transforming given patterns of the input file to a changed form specified by the regular expressions .

Author: Marko Laakso (Marko.Laakso@Helsinki.FI)
Version: 1.0
Category: convert

Input name	Source	Description
file	snpSurvival-survivalSet9-survival ParamsTemplate.in 19.830	Source file to be modified

Parameter name	Value	Description
key00	REP.cond00	Search key 0
key01		Search key 1
key02		Search key 2
key03		Search key 3
key04		Search key 4
key05		Search key 5
key06		Search key 6
key07		Search key 7
key08		Search key 8
key09		Search key 9
value00	– No additional restrictions	Replacement 0
value01		Replacement 1
value02		Replacement 2
value03		Replacement 3
value04		Replacement 4
value05		Replacement 5
value06		Replacement 6
value07		Replacement 7
value08		Replacement 8
value09		Replacement 9

19.830 snpSurvival-survivalSet9-survivalParamsTemplate (INPUT)

Select relevant parameters for the survival analysis

Category: Internal

Parameter name	Value	Description
path	/gpfs/homes/mlaakso/asserSVN/ andurilCaseStudy/sql/ SNPsurvivalParams.sql	Path (filename) of the input resource.

19.831 snpSurvival-survivalSet9-survivalReport (LatexCombiner)

LatexCombiner joins the given subdocuments together into one LaTeX file.

Authors: Kristian Ovaska (Kristian.Ovaska@Helsinki.FI), Marko Laakso (Marko.Laakso@Helsinki.FI)
Version: 1.3
Category: Latex

Input name	Source	Description
latex1	snpSurvival-survivalSet9-survival .report 19.827	LaTeX fragment 1.
latex2	snpSurvival-survivalSet9-clinical Select.report 19.807	LaTeX fragment 2.
latex3	snpSurvival-survivalSet9-survSN PAnnot-annotTable.report 19.823	LaTeX fragment 3.
latex4	snpSurvival-survivalSet9-gene Rank-eqTable.report 19.811	LaTeX fragment 4.

Parameter name	Value	Description
pagebreak	true	Determines if the result document should start with a page break.
sectionTitle		If non-empty, a declaration of a new section with the given name is inserted to the beginning of the combined document. This is a convenience feature to make it easy to compile subsections into a section.
sectionType	section	Type of LaTeX section: usually one of section, subsection or subsubsection. No section statement is written if sectionTitle is empty.
strictBorders	true	Enables the flushing of all document elements before each fragment

19.832 snpdb (INPUT)

Database configuration for Ensembl variation database.

Category: Internal

Parameter name	Value	Description
path	/gpfs/homes/mlaakso/asserSVN/ anduril CaseStudy/ensemblSNP. properties	Path (filename) of the input resource.

19.833 summaryDoc (LatexCombiner)

LatexCombiner joins the given subdocuments together into one LaTeX file.

Authors: Kristian Ovaska (Kristian.Ovaska@Helsinki.FI), Marko Laakso (Marko.Laakso@Helsinki.FI)
Version: 1.3
Category: Latex

Input name	Source	Description
latex1	expressionSummary .document 19.274	LaTeX fragment 1.
latex2	arrayCGHsummary .document 19.269	LaTeX fragment 2.
latex3	miRNAResults-report. document 19.422	LaTeX fragment 3.
latex4	snpSurvival-survivalAll-report Body.document 19.477	LaTeX fragment 4.
latex5	summaryTable-geneInfo-report .document 19.857	LaTeX fragment 5.
latex6	cfgDoc.document 19.271	LaTeX fragment 6.

Parameter name	Value	Description
pagebreak	false	Determines if the result document should start with a page break.

Continued on next page...

Parameter name	Value	Description
sectionTitle		If non-empty, a declaration of a new section with the given name is inserted to the beginning of the combined document. This is a convenience feature to make it easy to compile subsections into a section.
sectionType	section	Type of LaTeX section: usually one of section, subsection or subsubsection. No section statement is written if sectionTitle is empty.
strictBorders	true	Enables the flushing of all document elements before each fragment

19.834 summaryReport (LatexPDF)

This component is a LaTeX compiler that generates a portable document format (PDF) file from the given Anduril document.

Author: Kristian Ovaska (Kristian.Ovaska@Helsinki.FI)
Version: 1.0
Category: Latex

Input name	Source	Description
document	summaryDoc.document 19.833	Body of the LaTeX document.
header	mainTemplate.header 19.321	LaTeX header that is written to the start of the document. If missing, a default header is used.
footer	mainTemplate.footer 19.321	LaTeX footer that is written to the end of the document. If missing, a default header is used.

Parameter name	Value	Description
bibtexExec	bibtex	Executable command for BibTeX.
latexExec	pdflatex	Executable command for pdflatex.
useRefs	true	This flag can be used to activate BibTeX compiler

19.835 summaryTable (SummaryTable)

This function creates a result spread sheet and website from all analysis results.

Input name	Source
exprProbes	geneExpression.allProbes 19.275
transcriptProbes	transcriptExpressionResults.allProbes 19.887
exonProbes	medianExonExpression.allProbes 19.322
miRNAProbes	miRNAResults.dems 19.394
miRNAgain	ACGHmiRNAgainFreqs.geneFreqs 19.17
miRNAloss	ACGHmiRNAlossFreqs.geneFreqs 19.28
acghExonCorrLoss	arrayCGHexonIntLoss.exons 19.246
acghExonCorr Gain	arrayCGHexonIntGain.exons 19.223
acghGain	ACGHabberGainFreqs.geneFreqs 19.1
acghLoss	ACGHabberLossFreqs.geneFreqs 19.9
kaplanMeier	transcriptExpressionResults.kaplanMeier 19.887
kaplanMeier Statistics	transcriptExpressionResults.kaplanMeierStatistics 19.887
exonKaplanMeier	medianExonExpression.kaplanMeier 19.322
exonKaplanMeier Stat	medianExonExpression.kaplanMeierStatistics 19.322
miRNAKaplanM eier	miRNAResults.kaplanMeier 19.394
miRNAKaplanM eierStat	miRNAResults.kaplanMeierStatistics 19.394
survBloodGenes	snpSurvival.annotResults 19.440
snpSurvImages	snpSurvival.kmReport 19.440
snpSurv Annotation	snpSurvival.annotFull 19.440
methylation	methylation.betaTable 19.369

19.836 summaryTable-acghExonCorrGainConv (CSVFilter)

Filters columns and/or rows from CSV files using flexible criteria. Default settings do not filter out anything. The *negate* parameter reverses all inclusion criteria.

Columns are filtered based on column names (see *includeColumns*); it is also possible to rename columns. Columns can be reordered using *includeColumns*.

A row is included in output if all conditions are satisfied. Rows having too many missing values are filtered using the *nonMissing* parameter. The parameters *regexp*, *lowBound* and *highBound* filter rows based on cell contents. If the *auxiliary* input is given, the result contains those rows from the input whose values match those from the auxiliary file.

Authors: Kristian Ovaska (kristian.ovaska@helsinki.fi), Marko Laakso (Marko.Laakso@Helsinki.FI)
Version: 1.1
Category: Filter

Input name	Source	Description
csv	arrayCGHexonIntGain-integrated ExonACGH.bioAnnotation 19.235	CSV file to be filtered.

Parameter name	Value	Description
highBound		A row is included if the value in given numeric columns are at most as large as given numeric bounds. The parameter has a format COLNAME1=HIGH1,COLNAME2=HIGH2.
idColumn		Column name in "csv" that contains ID values. If the parameter is empty, the first column is used.
includeColumns	Gene,weight,alpha	Comma-separated list of column names that should be included in the result file. The special value * includes all columns. The order of column names is significant: the output columns are in the order given. See also includeColumns input.
lowBound		A row is included if the value in given numeric columns are at least as large as given numeric bounds. The parameter has a format COLNAME1=LOW1,COLNAME2=LOW2. For example, "col=5" includes rows where the column col has a value of at least 5.
matchColumn		Column name in "auxiliary" containing values that must match the ID column in "csv" (specified using "idColumn"). If empty, the first column of "auxiliary" is used.
negate	false	If true, all inclusion criteria are negated so that rows or columns are excluded instead of included if they match the criteria. For example, includeColumns=* then excludes all columns (which would be an error since at least one column must be included).
nonMissing	0	Include those rows that have non-missing (non-NA) values in at least this many columns. Only those columns that are part of the output are counted.
regexp		Row filtering based on regular expression. A row is included in the result if values in the given columns match given regular expressions. The parameter has a format COLNAME1=EXPRESSION,COLNAME2=EXPRESSION2 where COLNAMEs are column names in "csv" and EXPRESSIONs are regular expressions using Java syntax. For example, "col=a|b" includes rows where the column col has a value of "a" or "b". Commas have to be escaped with "\\" if they are used in column names or regular expressions.
rename	weight=GainWeight,alpha= GainAlpha	Comma-separated list of column renaming rules (OLDNAME=NEWNAME). Parameters that refer to column names refer to old names; renaming is done last.

19.837 summaryTable-acghExonCorrLossConv (CSVFilter)

Filters columns and/or rows from CSV files using flexible criteria. Default settings do not filter out anything. The *negate* parameter reverses all inclusion criteria.

Columns are filtered based on column names (see *includeColumns*); it is also possible to rename columns. Columns can be reordered using *includeColumns*.

A row is included in output if all conditions are satisfied. Rows having too many missing values are filtered using the *nonMissing* parameter. The parameters *regexp*, *lowBound* and *highBound* filter rows based on

cell contents. If the *auxiliary* input is given, the result contains those rows from the input whose values match those from the auxiliary file.

Authors: Kristian Ovaska (kristian.ovaska@helsinki.fi), Marko Laakso (Marko.Laakso@Helsinki.FI)
Version: 1.1
Category: Filter

Input name	Source	Description
csv	arrayCGHexonIntLoss-integrated ExonACGH.bioAnnotation 19.258	CSV file to be filtered.

Parameter name	Value	Description
highBound		A row is included if the value in given numeric columns are at most as large as given numeric bounds. The parameter has a format COLNAME1=HIGH1,COLNAME2=HIGH2.
idColumn		Column name in "csv" that contains ID values. If the parameter is empty, the first column is used.
includeColumns	Gene,weight,alpha	Comma-separated list of column names that should be included in the result file. The special value * includes all columns. The order of column names is significant: the output columns are in the order given. See also includeColumns input.
lowBound		A row is included if the value in given numeric columns are at least as large as given numeric bounds. The parameter has a format COLNAME1=LOW1,COLNAME2=LOW2. For example, "col=5" includes rows where the column col has a value of at least 5.
matchColumn		Column name in "auxiliary" containing values that must match the ID column in "csv" (specified using "idColumn"). If empty, the first column of "auxiliary" is used.
negate	false	If true, all inclusion criteria are negated so that rows or columns are excluded instead of included if they match the criteria. For example, includeColumns=* then excludes all columns (which would be an error since at least one column must be included).
nonMissing	0	Include those rows that have non-missing (non-NA) values in at least this many columns. Only those columns that are part of the output are counted.
regexp		Row filtering based on regular expression. A row is included in the result if values in the given columns match given regular expressions. The parameter has a format COLNAME1=EXPRESSION,COLNAME2=EXPRESSION2 where COLNAMEs are column names in "csv" and EXPRESSIONs are regular expressions using Java syntax. For example, "col=a|b" includes rows where the column col has a value of "a" or "b". Commas have to be escaped with "\\" if they are used in column names or regular expressions.
rename	weight=LossWeight,alpha= LossAlpha	Comma-separated list of column renaming rules (OLDNAME=NEWNAME). Parameters that refer to column names refer to old names; renaming is done last.

19.838 summaryTable-acghExonCorrSummary (CSVJoin)

Join rows from two or more CSV files, optionally using one column as a matching key. If a key column is not used, the result contains all rows and all columns of the input files. Missing values (`NA`) may be introduced when a column is not present in all input files. Each column is present once and duplicate rows are removed.

If a key column is used, the rows in each input file are matched using values from the key column. The result file has one row for each key value. In the result, the first column is the key column; its name is obtained from the first CSV input (csv1). If the intersection parameter is true, a key is included in the result if the key value is present in all inputs. If intersection is false, a key is included if it is present in at least one input (key union). Union semantics may introduce missing values in the result. If several input files have the same column, the value is obtained from the first file. However, if the first file contains a missing value and the second file contains a non-missing value, the non-missing value is used instead.

Author: Kristian Ovaska (kristian.ovaska@helsinki.fi)
Version: 1.0
Category: convert

Input name	Source	Description
csv1	summaryTable-acghExonCorrLoss Conv.csv 19.837	CSV file 1.
csv2	summaryTable-acghExonCorr GainConv.csv 19.836	CSV file 2.

Parameter name	Value	Description
intersection	false	Defines how keys are handled; only used when useKeys=true. If intersection is true, the result contains a key if the key is present in all input files. If false, the result contains a key if the key is present in at least one input file.
keyColumnNames		Comma-separated list of key column names; only used when useKeys=true. The first name refers to csv1, the second to csv2, etc. An empty value refers to the first column. Empty values may be omitted from the list, so all these are equivalent: "col1" ; "col1," ; "col1,," ; etc.
useKeys	true	If true, use one column from each CSV file as a matching key column.

19.839 summaryTable-acghGainConv (CSVFilter)

Filters columns and/or rows from CSV files using flexible criteria. Default settings do not filter out anything. The *negate* parameter reverses all inclusion criteria.

Columns are filtered based on column names (see *includeColumns*); it is also possible to rename columns. Columns can be reordered using *includeColumns*.

A row is included in output if all conditions are satisfied. Rows having too many missing values are filtered using the *nonMissing* parameter. The parameters *regexp*, *lowBound* and *highBound* filter rows based on cell contents. If the *auxiliary* input is given, the result contains those rows from the input whose values match those from the auxiliary file.

Authors: Kristian Ovaska (kristian.ovaska@helsinki.fi), Marko Laakso (Marko.Laakso@Helsinki.FI)
Version: 1.1
Category: Filter

Input name	Source	Description
csv	ACGHabberGainFreqs-geneFreqs .table 19.5	CSV file to be filtered.

Parameter name	Value	Description
highBound		A row is included if the value in given numeric columns are at most as large as given numeric bounds. The parameter has a format COLNAME1=HIGH1,COLNAME2=HIGH2.
idColumn		Column name in "csv" that contains ID values. If the parameter is empty, the first column is used.
includeColumns	*	Comma-separated list of column names that should be included in the result file. The special value * includes all columns. The order of column names is significant: the output columns are in the order given. See also includeColumns input.
lowBound		A row is included if the value in given numeric columns are at least as large as given numeric bounds. The parameter has a format COLNAME1=LOW1,COLNAME2=LOW2. For example, "col=5" includes rows where the column col has a value of at least 5.
matchColumn		Column name in "auxiliary" containing values that must match the ID column in "csv" (specified using "idColumn"). If empty, the first column of "auxiliary" is used.
negate	false	If true, all inclusion criteria are negated so that rows or columns are excluded instead of included if they match the criteria. For example, includeColumns=* then excludes all columns (which would be an error since at least one column must be included).
nonMissing	0	Include those rows that have non-missing (non-NA) values in at least this many columns. Only those columns that are part of the output are counted.

Continued on next page...

Parameter name	Value	Description
regexp		Row filtering based on regular expression. A row is included in the result if values in the given columns match given regular expressions. The parameter has a format COLNAME1=EXPRESSION,COLNAME2=EXPRESSION2 where COLNAMEs are column names in "csv" and EXPRESSIONs are regular expressions using Java syntax. For example, "col=a|b" includes rows where the column col has a value of "a" or "b". Commas have to be escaped with "\\" if they are used in column names or regular expressions.
rename	freq=GainFreq	Comma-separated list of column renaming rules (OLDNAME=NEWNAME). Parameters that refer to column names refer to old names; renaming is done last.

19.840 summaryTable-acghLossConv (CSVFilter)

Filters columns and/or rows from CSV files using flexible criteria. Default settings do not filter out anything. The *negate* parameter reverses all inclusion criteria.

Columns are filtered based on column names (see *includeColumns*); it is also possible to rename columns. Columns can be reordered using *includeColumns*.

A row is included in output if all conditions are satisfied. Rows having too many missing values are filtered using the *nonMissing* parameter. The parameters *regexp*, *lowBound* and *highBound* filter rows based on cell contents. If the *auxiliary* input is given, the result contains those rows from the input whose values match those from the auxiliary file.

Authors: Kristian Ovaska (kristian.ovaska@helsinki.fi), Marko Laakso (Marko.Laakso@Helsinki.FI)
Version: 1.1
Category: Filter

Input name	Source	Description
csv	ACGHabberLossFreqs-geneFreqs .table 19.13	CSV file to be filtered.

Parameter name	Value	Description
highBound		A row is included if the value in given numeric columns are at most as large as given numeric bounds. The parameter has a format COLNAME1=HIGH1,COLNAME2=HIGH2.
idColumn		Column name in "csv" that contains ID values. If the parameter is empty, the first column is used.
includeColumns	*	Comma-separated list of column names that should be included in the result file. The special value * includes all columns. The order of column names is significant: the output columns are in the order given. See also includeColumns input.
lowBound		A row is included if the value in given numeric columns are at least as large as given numeric bounds. The parameter has a format COLNAME1=LOW1,COLNAME2=LOW2. For example, "col=5" includes rows where the column col has a value of at least 5.
matchColumn		Column name in "auxiliary" containing values that must match the ID column in "csv" (specified using "idColumn"). If empty, the first column of "auxiliary" is used.
negate	false	If true, all inclusion criteria are negated so that rows or columns are excluded instead of included if they match the criteria. For example, includeColumns=* then excludes all columns (which would be an error since at least one column must be included).
nonMissing	0	Include those rows that have non-missing (non-NA) values in at least this many columns. Only those columns that are part of the output are counted.
regexp		Row filtering based on regular expression. A row is included in the result if values in the given columns match given regular expressions. The parameter has a format COLNAME1=EXPRESSION,COLNAME2=EXPRESSION2 where COLNAMEs are column names in "csv" and EXPRESSIONs are regular expressions using Java syntax. For example, "col=a|b" includes rows where the column col has a value of "a" or "b". Commas have to be escaped with "\\" if they are used in column names or regular expressions.
rename	freq=LossFreq	Comma-separated list of column renaming rules (OLDNAME=NEWNAME). Parameters that refer to column names refer to old names; renaming is done last.

19.841 summaryTable-acghSummary (CSVJoin)

Join rows from two or more CSV files, optionally using one column as a matching key. If a key column is not used, the result contains all rows and all columns of the input files. Missing values (`NA`) may be introduced when a column is not present in all input files. Each column is present once and duplicate rows are removed.

If a key column is used, the rows in each input file are matched using values from the key column. The result file has one row for each key value. In the result, the first column is the key column; its name is obtained from the first CSV input (csv1). If the intersection parameter is true, a key is included in the result if the key value is present in all inputs. If intersection is false, a key is included if it is present in at least one input (key union). Union semantics may introduce missing values in the result. If several input files have the same column, the value is obtained from the first file. However, if the first file contains a missing value and the second file contains a non-missing value, the non-missing value is used instead.

Author: Kristian Ovaska (kristian.ovaska@helsinki.fi)
Version: 1.0
Category: convert

Input name	Source	Description
csv1	summaryTable-acghGainConv .csv 19.839	CSV file 1.
csv2	summaryTable-acghLossConv .csv 19.840	CSV file 2.

Parameter name	Value	Description
intersection	false	Defines how keys are handled; only used when useKeys=true. If intersection is true, the result contains a key if the key is present in all input files. If false, the result contains a key if the key is present in at least one input file.
keyColumnNames		Comma-separated list of key column names; only used when useKeys=true. The first name refers to csv1, the second to csv2, etc. An empty value refers to the first column. Empty values may be omitted from the list, so all these are equivalent: "col1" ; "col1," ; "col1,," ; etc.
useKeys	true	If true, use one column from each CSV file as a matching key column.

19.842 summaryTable-annotation (KorvasieniAnnotator)

Korvasieni is an Ensembl [12] based converter for database identifiers and it can be used to convert genome location, gene, transcript, and translation identifiers across multiple biological databases. The selected `keyColumn` may contain entries with comma separated lists of values in which case `isListKey` parameter may be used to obtain each value separately and to process them as they were given in consecutive lines.

Author: Marko Laakso (Marko.Laakso@Helsinki.FI)
Version: 1.7
Categories: Annotation, GO

Input name	Source	Description
sourceKeys	summaryTable-genes.ids 19.860	A list of source database keys.
connection	ensembl.in 19.273	Database connection can be defined using this file. The definition of parameters: database.url, database.user, database.password, database.timeout, database.recycle, and database.driver can be found from the documentation of Korvasieni.

Parameter name	Value	Description
echoColumns		A comma separated list of column names for the columns that will be copied to the output. An asterisk (*) can be used to denote all columns execpt the keyColumn.

Continued on next page...

Parameter name	Value	Description
inputDB	.GeneId	Type of input keys. This must be a database supported by Korvasieni. If the parameter is omitted, the component tries to derive the database from the type of geneID. If this is not possible, an error is returned. You may define three columns in form of chromosome:start-end in case the inputDB is .DNARegion. This format provides a comfortable compatibility with DNARegion datatype.
inputType	Gene	Ensembl object type for the input keys (Any, Gene, Transcript, Translation)
isListKey	false	Enables the automatic value splits for the comma separated key column
keyColumn		Name of the key column withing sourceKeys file or an empty string for the first column
maxHits	100000	Maximum number of target identifiers for a single source identifier
rename		Comma separated list of column renaming rules (oldname=newname)
skipLevel	never	Skip result rows if the source identifier is unknown or target identifiers are not available. Possible values are: never (no filtering), source (skip if the source ID is unknown), target (skip if no target IDs are found), any (skip if any of the target IDs is missing).
targetDB	.GeneName,_Uniprot /SWISSPROT,.GeneDesc, .DNABand,_EntrezGene	Comma-separated list of annotation types. Possible values are all databases supported by Korvasieni.
unique	false	This flag can be turned on in order to eliminate duplicate annotations.

19.843 summaryTable-correlation (INPUT)

(Technical documentation) Imports an external input file or directory to the pipeline. This is the only way external files are imported. File path is given with the parameter "path".

Category: Internal

Parameter name	Value	Description
path	/gpfs/homes/mlaakso/asserSVN/ andurilCaseStudy/data/results/ correlation/summary/ allcorrelations.csv	Path (filename) of the input resource.

19.844 summaryTable-excel (CSV2Excel)

Represents the content of the given CSV files as a Microsoft Excel spreadsheet. Each *csv* input forms its own sheet. Visual formatting is governed by the optional *style* input.

Authors: Jianmin Wu (jianmin.wu@helsinki.fi), Kristian Ovaska (kristian.ovaska@helsinki.fi)
Version: 1.3
Categories: convert, Excel

Input name	Source	Description
csv	summaryTable-summaryTable .csv 19.877	CSV file corresponding to sheet 1.
style	summaryTable-style.in 19.875	Specifies visual formatting rules for the result spreadsheet.

Parameter name	Value	Description
enableFormulas	false	If true, cell values starting with "=" are treated as formulas and are evaluated by the spreadsheet application at runtime. If false, such strings are treated as string literals.
frozenColumns	1	Number of left-hand columns that are frozen, i.e., always remain visible on the spreadsheet when the columns are scrolled.
frozenRows	1	Number of top rows that are frozen, i.e., always remain visible on the spreadsheet when the rows are scrolled.
missingValue	NA	The string to be inserted into the Excel file for missing values (NAs) in the input.
sheetNames		Comma separated list of sheet names for the result spreadsheet. If a name is empty, a default name is used.

19.845 summaryTable-exonAnnotation (CSVJoin)

Join rows from two or more CSV files, optionally using one column as a matching key. If a key column is not used, the result contains all rows and all columns of the input files. Missing values (`NA`) may be introduced when a column is not present in all input files. Each column is present once and duplicate rows are removed.

If a key column is used, the rows in each input file are matched using values from the key column. The result file has one row for each key value. In the result, the first column is the key column; its name is obtained from the first CSV input (csv1). If the intersection parameter is true, a key is included in the result if the key value is present in all inputs. If intersection is false, a key is included if it is present in at least one input (key union). Union semantics may introduce missing values in the result. If several input files have the same column, the value is obtained from the first file. However, if the first file contains a missing value and the second file contains a non-missing value, the non-missing value is used instead.

Author: Kristian Ovaska (kristian.ovaska@helsinki.fi)
Version: 1.0
Category: convert

Input name	Source	Description
csv1	transcriptExpressionResults-all Probes.table 19.889	CSV file 1.
csv2	transcriptExpressionResults-km -km.statistics 19.907	CSV file 2.

Parameter name	Value	Description
intersection	false	Defines how keys are handled; only used when useKeys=true. If intersection is true, the result contains a key if the key is present in all input files. If false, the result contains a key if the key is present in at least one input file.
keyColumnNames	TranscriptID,group	Comma-separated list of key column names; only used when useKeys=true. The first name refers to csv1, the second to csv2, etc. An empty value refers to the first column. Empty values may be omitted from the list, so all these are equivalent: "col1" ; "col1," ; "col1,," ; etc.
useKeys	true	If true, use one column from each CSV file as a matching key column.

19.846 summaryTable-exonAnnotationFilt (CSVFilter)

Filters columns and/or rows from CSV files using flexible criteria. Default settings do not filter out anything. The *negate* parameter reverses all inclusion criteria.

Columns are filtered based on column names (see *includeColumns*); it is also possible to rename columns. Columns can be reordered using *includeColumns*.

A row is included in output if all conditions are satisfied. Rows having too many missing values are filtered using the *nonMissing* parameter. The parameters *regexp*, *lowBound* and *highBound* filter rows based on cell contents. If the *auxiliary* input is given, the result contains those rows from the input whose values match those from the auxiliary file.

Authors: Kristian Ovaska (kristian.ovaska@helsinki.fi), Marko Laakso (Marko.Laakso@Helsinki.FI)
Version: 1.1
Category: Filter

Input name	Source	Description
csv	summaryTable-exonAnnotation .csv 19.845	CSV file to be filtered.

Parameter name	Value	Description
highBound		A row is included if the value in given numeric columns are at most as large as given numeric bounds. The parameter has a format COLNAME1=HIGH1,COLNAME2=HIGH2.
idColumn		Column name in "csv" that contains ID values. If the parameter is empty, the first column is used.
includeColumns	TranscriptID,GeneID,FoldChange, PValue,pValue	Comma-separated list of column names that should be included in the result file. The special value * includes all columns. The order of column names is significant: the output columns are in the order given. See also includeColumns input.
lowBound		A row is included if the value in given numeric columns are at least as large as given numeric bounds. The parameter has a format COLNAME1=LOW1,COLNAME2=LOW2. For example, "col=5" includes rows where the column col has a value of at least 5.
matchColumn		Column name in "auxiliary" containing values that must match the ID column in "csv" (specified using "idColumn"). If empty, the first column of "auxiliary" is used.
negate	false	If true, all inclusion criteria are negated so that rows or columns are excluded instead of included if they match the criteria. For example, includeColumns=* then excludes all columns (which would be an error since at least one column must be included).
nonMissing	0	Include those rows that have non-missing (non-NA) values in at least this many columns. Only those columns that are part of the output are counted.
regexp		Row filtering based on regular expression. A row is included in the result if values in the given columns match given regular expressions. The parameter has a format COLNAME1=EXPRESSION,COLNAME2=EXPRESSION2 where COLNAMEs are column names in "csv" and EXPRESSIONs are regular expressions using Java syntax. For example, "col=a|b" includes rows where the column col has a value of "a" or "b". Commas have to be escaped with "\\" if they are used in column names or regular expressions.
rename	pValue=SurvivalPValue	Comma-separated list of column renaming rules (OLDNAME=NEWNAME). Parameters that refer to column names refer to old names; renaming is done last.

19.847 summaryTable-geneAlias (INPUT)

(Technical documentation) Imports an external input file or directory to the pipeline. This is the only way external files are imported. File path is given with the parameter "path".

Category: Internal

Parameter name	Value	Description
path	/gpfs/homes/mlaakso/asserSVN/andurilCaseStudy/csv/gene-alias.csv	Path (filename) of the input resource.

19.848 summaryTable-geneInfo (GeneSetInfo)

No description

19.849 summaryTable-geneInfo-annotation (KorvasieniAnnotator)

Korvasieni is an Ensembl [12] based converter for database identifiers and it can be used to convert genome location, gene, transcript, and translation identifiers across multiple biological databases. The selected `keyColumn` may contain entries with comma separated lists of values in which case `isListKey` parameter may be used to obtain each value separately and to process them as they were given in consecutive lines.

Author: Marko Laakso (Marko.Laakso@Helsinki.FI)
Version: 1.7
Categories: Annotation, GO

Continued on next page...

Input name	Source	Description

Input name	Source	Description
sourceKeys	summaryTable-geneSet .transformed 19.859	A list of source database keys.
connection	ensembl.in 19.273	Database connection can be defined using this file. The definition of parameters: database.url, database.user, database.password, database.timeout, database.recycle, and database.driver can be found from the documentation of Korvasieni.

Parameter name	Value	Description
echoColumns		A comma separated list of column names for the columns that will be copied to the output. An asterisk (*) can be used to denote all columns execpt the keyColumn.
inputDB	.GeneId	Type of input keys. This must be a database supported by Korvasieni. If the parameter is omitted, the component tries to derive the database from the type of geneID. If this is not possible, an error is returned. You may define three columns in form of chromosome:start-end in case the inputDB is .DNARegion. This format provides a comfortable compatibility with DNARegion datatype.
inputType	Any	Ensembl object type for the input keys (Any, Gene, Transcript, Translation)
isListKey	false	Enables the automatic value splits for the comma separated key column
keyColumn		Name of the key column withing sourceKeys file or an empty string for the first column
maxHits	100000	Maximum number of target identifiers for a single source identifier
rename	Uniprot/SWISSPROT=Uniprot	Comma separated list of column renaming rules (oldname=newname)
skipLevel	never	Skip result rows if the source identifier is unknown or target identifiers are not available. Possible values are: never (no filtering), source (skip if the source ID is unknown), target (skip if no target IDs are found), any (skip if any of the target IDs is missing).
targetDB	GO,␣EntrezGene,␣Uniprot/ SWISSPROT	Comma-separated list of annotation types. Possible values are all databases supported by Korvasieni.
unique	false	This flag can be turned on in order to eliminate duplicate annotations.

19.850 summaryTable-geneInfo-goEnrichment (GOEnrichment)

GOEnrichment computes enriched GO terms [1] in a set of genes or proteins. Enrichment analysis is done using Fisher's Exact Test. Fisher's test compares the observed frequency of each present GO term to the frequency in a reference gene/protein set. A GO term is present if some input gene/protein is annotated with the GO term or its descendants.

The component also computes adjusted p-values using FDR [4]. However, note that multiple comparison correction might not work well with GO enrichment analysis since a large number of statistical tests are done and no effort is done to reduce the number of tests.

Visualization of enriched GO terms is created in GraphML format. There is one network for each GO ontology. Nodes can be colorized according to the p-value. Colors have a base 10 logarithmic scale, i.e. p-values 1, 0.1 and 0.01 are equally distant from each other. Nodes contain a URL hyperlink to a description of the GO term in the geneontology.org site by default.

Authors: Kristian Ovaska (kristian.ovaska@helsinki.fi), Marko Laakso (Marko.Laakso@Helsinki.FI)
Version: 1.0.1
Category: GO

Input name	Source	Description
goAnnotations	summaryTable-geneInfo -annotation.bioAnnotation 19.849	GO annotations for genes or proteins. GO terms are searched using a regular expression, so the format is very flexible. Each row is considered as a distinct gene or protein.

Parameter name	Value	Description
colorEnd	#ff0000	When colorizing GO graphs, this is the color of a node with a minimally low p-value. The threshold depends on the colorMinP parameter. All nodes with p-value less than the threshold also get this color.

Continued on next page...

Parameter name	Value	Description
colorMiddle		When colorizing GO graphs, this is a color between the two extreme colors. This allows to create color slides between three colors. If the value is empty, a color slide with two colors is used.
colorMinP	0.0001	When colorizing GO graphs, all nodes with p-value below this get the color given with color colorEnd. If the value is 0, the node with the smallest p-value gets the color colorEnd, i.e. the color range is scaled using the p-values present in the data.
colorStart	#ffffff	When colorizing GO graphs, this is the color of a node with p-value 1. Setting this to empty disables node coloring.
filterFDR	false	If true, use FDR-corrected p-values for filtering (column: pvalueCorrected). Otherwise, use raw p-values (colum: pvalue).
filterParents	true	if true, then a GO term is excluded from the result if a child of the term has occurred higher in the list (with a lower p-value).
includeGraph Attributes	true	If true, frequency and p-value of each GO term is included in the graph.
maxEdgeWidth	10	Maximum edge line width in the graphs, in points. Edge widths are computed based on the frequency of the target node so that nodes with a large number of annotations have wide in-coming edges. Setting this to 1 gives the same width for all edges.
maxPriori	1.00	Maximum value of the priori probability that can be accepted for a GO term
organism	9606	NCBI taxonomy ID for the organism whose gene set is used for GO probabilities. This is used if the input enrichmentTable is not given. Supported organisms: Homo sapiens: 9606, Saccharomyces cerevisiae: 4932, Caenorhabditis elegans: 6239, Drosophila melanogaster: 7227, Mus musculus: 10090, Rattus norvegicus: 10116.
threshold	0.2	P-value threshold for filtering GO terms.
urlPattern	http://amigo.geneontology.org/ cgi-bin/amigo/term-details.cgi? term=%s	A printf-like pattern for creating a URL for a GO term. The pattern must contain one %s string that is expanded with the GO term in question, e.g. GO:0005575. If the value is empty, no hyperlinks are created in graphs.

19.851 summaryTable-geneInfo-goHeatmap (GOClustering)

This Anduril component uses Gene ontology (GO) [1] annotations to cluster a set of genes. Each cluster consists of genes that share similar GO annotations.

The GO clustering of the genes can be visualized together with expression data (optional). Samples of the expression data are clustered based on their expression profiles and a heatmap is formed by clustering the y-axis of genes using their GO annotations. Component is based on the method descibed in [17].

Authors:	Ping Chen (ping.chen@cs.helsinki.fi), Kristian Ovaska (Kristian.Ovaska@Helsinki.FI), Marko Laakso (Marko.Laakso@Helsinki.FI), Erkka Valo (Erkka.Valo@Helsinki.FI)
Version:	1.1.2
Categories:	Clustering, GO

Input name	Source	Description
goAnnotations	summaryTable-geneInfo -annotation.bioAnnotation 19.849	GO annotations for the genes

Parameter name	Value	Description
ColumnWidth	15	Control the column width of the GO MEMBER table.
asBitmap	false	Produce heatmap in Portable network graphics (PNG) format instead of Portable document format (PDF). PNG bitmaps are more suitable if the heatmap contains large number of samples and genes.
columnMargin	5	Margin for column names in heatmap
cutLimit	0.5	Clustering cut off height for the hierachical clustering of the genes. This limit is between 0 and 1, where 0 refers to more specific groups.
decimal	3	Control the output of long decimal.
drawHeatmap	true	If true, draw a heatmap.
dropUnknown	false	Genes with no GO annotations are excluded if this flag is true
geneColumn		Name of the gene name column in the annotation file. The value is used for creating the report. The given column is used for listing all the members in the clusters to the report. If the value is empty, cluster member lists are not put into the report.
goColumn	GO	Name of the GO annotation column in the annotation file
grayScale	true	If true, heatmap is in grayscale. If false, heatmap is colorful.
heatmapScale	row	Determines how the values for the heatmap are scaled. Legal values are ”row”, ”column” and ”none”. This corresponds to the scale parameter of the R function heatmap.

Continued on next page...

Parameter name	Value	Description
organism	9606	NCBI taxonomy ID for the organism whose gene set is used for GO probabilities. This is used if the input similarityTable is not given. Supported organisms: Homo sapiens: 9606, Saccharomyces cerevisiae: 4932, Caenorhabditis elegans: 6239, Drosophila melanogaster: 7227, Mus musculus: 10090, Rattus norvegicus: 10116.
pvalues	false	If true, compute p-values for information contents of the clusters using a permutation test. The p-values are shown in their own table in the report. The permutation test may be slow.
rowMargin	5	Margin for row names in heatmap
sectionType	subsection	Type of LaTeX section: usually one of section, subsection or subsubsection. No section statement is written if <code>sectionTitle</code> is an empty string.
showClusterNames	true	If true, show cluster names G1, ..., Gn on the right side of the heat map. If false, show gene names.
title	GO clustering	Title for the subsection and for the heatmap image.
xLab	Samples	Label for the x-axis of the heatmap
yLab	GO.groups	Label for the y-axis of the heatmap

19.852 summaryTable-geneInfo-graphBP (GraphVisualizer)

GraphVisualizer creates a visualization of a graph using Graphviz. There are several layout options (see the parameter layout) and make aspects of the nodes and edges can be customized. In addition to the parameters mentioned here, nodes and edges can have many other GraphML attributes that are used to set rendering options for individual nodes and edges. For the full list, see Graphviz documentation [2].

Author:	Kristian Ovaska (kristian.ovaska@helsinki.fi)
Version:	1.0
Categories:	Graph, Plot

Input name	Source	Description
graph	summaryTable-geneInfo-go Enrichment.graphBP 19.850	Input graph

Parameter name	Value	Description
arrowhead		Type of arrow heads (the target end of the arrow). See Graphviz documentation for the format. If empty, the default type is used. Or, if the edge attribute "arrowhead" is defined in the GraphML file, each edge gets its type from this attribute.
arrowtail		Type of arrow tails (the source end of the arrow). See Graphviz documentation for the format. If empty, the default type is used. Or, if the edge attribute "arrowtail" is defined in the GraphML file, each edge gets its type from this attribute.
bgcolor		Background color for the canvas. See Graphviz documentation for the format. If empty, the default color is used.
circo	circo	Graphviz/circo execution command. Only used if the layout parameter specifies this program.
dot	dot	Graphviz/dot execution command. Only used if the layout parameter specifies this program.
edgecolor		Color for drawing edges and arrows, but not text. See Graphviz documentation for the format. If empty, the default color (black) is used. Or, if the edge attribute "color" is defined in the GraphML file, each edge gets its color from this attribute.
fdp	fdp	Graphviz/fdp execution command. Only used if the layout parameter specifies this program.
fillcolor		Color for filling the background of nodes. See Graphviz documentation for the format. If empty, no filling is done. Or, if the node attribute "fillcolor" is defined in the GraphML file, each node gets its color from this attribute.
fontcolor		Color for text. See Graphviz documentation for the format. If empty, the default color (black) is used. Or, if the node/edge attribute "fontcolor" is defined in the GraphML file, each node/edge gets its color from this attribute.
fontsize	0	Font size of text, in points. A typical value is 14. If zero, the default size is used. Or, if the node/edge attribute "fontsize" is defined in the GraphML file, each node/edge gets its font size from this attribute.
height	0	Minimum height of nodes in INCHES. Depending on layout type, this might also be the final height. If zero, the default height is used. Or, if the node attribute "height" is defined in the GraphML file, each node gets its height from this attribute.

Continued on next page...

Parameter name	Value	Description
layout	hierarchical	Determines how the visualization is layed out. Valid choices are "hierarchical" (layout done using dot), "spring" (neato: Kamada-Kawai algorithm), "spring2" (fdp: Fruchterman-Reingold algorithm), "radial" (twopi) and "circular" (circo) .
margin		Margin around the label of nodes. If given, this is a pair x,y of margin space in inches. If the value is empty, the default margin is used; in Graphviz 2.18 it is "0.11,0.055". Or, if the node attribute "margin" is defined in the GraphML file, each node gets its margin from this attribute.
minSize	0	Minimum number of vertices to render the graph. The whole image will be skipped if there are too few vertices available.
neato	neato	Graphviz/neato execution command. Only used if the layout parameter specifies this program.
nodecolor		Color for drawing the boundaries of nodes, but not text. See Graphviz documentation for the format. If empty, the default color (black) is used. Or, if the node attribute "color" is defined in the GraphML file, each node gets its color from this attribute.
overlap		Determines how overlapping nodes are handled. This corresponds to the "overlap" graph attribute in Graphviz. If the value is "true", overlapping nodes are allowed. The values "false", "scale", "ortho", "compress" and "vpsc" remove overlaps using different methods. See Graphviz documentation for details. If the value is empty, default handling is done.
ps2pdf	ps2pdf	PS2PDF execution command.
rankdir		For hierarchical layouts, the layout direction. One of TB (top-to-bottom, default), BT, LR (left-to-right), RL.
reportCaption		Caption of the figure in the Latex report.
reportHeight	11	Height of the figure in the Latex report in cm.
reportWidth	14	Width of the figure in the Latex report in cm.
shape		Shape of the nodes. Some legal values include box, polygon, ellipse, circle, point, triangle, plaintext, diamond, none, note, box3d, component; for the rest, see Graphviz documentation. If the value is empty, the default shape (ellipse) is used. Or, if the node attribute "shape" is defined in the GraphML file, each node gets its shape from this attribute.
simplify	false	If true, simplify the graph by removing self-loop edges and multiple edges between two vertices.
size	8,8	Maximum width and height of the image, in INCHES.
splines	true	Determines if edges are drawn as straight lines or curves (splines). This corresponds to the "splines" graph attribute in Graphviz. If "true", splines are enabled. If "false", straight lines are used. If the value is empty, default settings are used.
titleAttribute	label,id	Name of the vertex attribute that is used as the title of the vertex in the visualization. The attribute may contain multiple values that are separated by commas. Each attribute is tried in the order given and the first that is defined is used. For example, if the value is "label,id", then label is used if it is defined, and id is used otherwise. The id attribute is always present.
twopi	twopi	Graphviz/twopi execution command. Only used if the layout parameter specifies this program.
width	0	Minimum width of nodes in INCHES. Depending on layout type, this might also be the final width. If zero, the default width is used. Or, if the node attribute "width" is defined in the GraphML file, each node gets its width from this attribute.

19.853 summaryTable-geneInfo-graphCC (GraphVisualizer)

GraphVisualizer creates a visualization of a graph using Graphviz. There are several layout options (see the parameter layout) and make aspects of the nodes and edges can be customized. In addition to the parameters mentioned here, nodes and edges can have many other GraphML attributes that are used to set rendering options for individual nodes and edges. For the full list, see Graphviz documentation [2].

Author:	Kristian Ovaska (kristian.ovaska@helsinki.fi)
Version:	1.0
Categories:	Graph, Plot

Input name	Source	Description
graph	summaryTable-geneInfo-go Enrichment.graphCC 19.850	Input graph

Parameter name	Value	Description
arrowhead		Type of arrow heads (the target end of the arrow). See Graphviz documentation for the format. If empty, the default type is used. Or, if the edge attribute "arrowhead" is defined in the GraphML file, each edge gets its type from this attribute.
arrowtail		Type of arrow tails (the source end of the arrow). See Graphviz documentation for the format. If empty, the default type is used. Or, if the edge attribute "arrowtail" is defined in the GraphML file, each edge gets its type from this attribute.
bgcolor		Background color for the canvas. See Graphviz documentation for the format. If empty, the default color is used.
circo	circo	Graphviz/circo execution command. Only used if the layout parameter specifies this program.
dot	dot	Graphviz/dot execution command. Only used if the layout parameter specifies this program.
edgecolor		Color for drawing edges and arrows, but not text. See Graphviz documentation for the format. If empty, the default color (black) is used. Or, if the edge attribute "color" is defined in the GraphML file, each edge gets its color from this attribute.
fdp	fdp	Graphviz/fdp execution command. Only used if the layout parameter specifies this program.
fillcolor		Color for filling the background of nodes. See Graphviz documentation for the format. If empty, no filling is done. Or, if the node attribute "fillcolor" is defined in the GraphML file, each node gets its color from this attribute.
fontcolor		Color for text. See Graphviz documentation for the format. If empty, the default color (black) is used. Or, if the node/edge attribute "fontcolor" is defined in the GraphML file, each node/edge gets its color from this attribute.
fontsize	0	Font size of text, in points. A typical value is 14. If zero, the default size is used. Or, if the node/edge attribute "fontsize" is defined in the GraphML file, each node/edge gets its font size from this attribute.
height	0	Minimum height of nodes in INCHES. Depending on layout type, this might also be the final height. If zero, the default height is used. Or, if the node attribute "height" is defined in the GraphML file, each node gets its height from this attribute.
layout	hierarchical	Determines how the visualization is layed out. Valid choices are "hierarchical" (layout done using dot), "spring" (neato: Kamada-Kawai algorithm), "spring2" (fdp: Fruchterman-Reingold algorithm), "radial" (twopi) and "circular" (circo) .
margin		Margin around the label of nodes. If given, this is a pair x,y of margin space in inches. If the value is empty, the default margin is used; in Graphviz 2.18 it is "0.11,0.055". Or, if the node attribute "margin" is defined in the GraphML file, each node gets its margin from this attribute.
minSize	0	Minimum number of vertices to render the graph. The whole image will be skipped if there are too few vertices available.
neato	neato	Graphviz/neato execution command. Only used if the layout parameter specifies this program.
nodecolor		Color for drawing the boundaries of nodes, but not text. See Graphviz documentation for the format. If empty, the default color (black) is used. Or, if the node attribute "color" is defined in the GraphML file, each node gets its color from this attribute.
overlap		Determines how overlapping nodes are handled. This corresponds to the "overlap" graph attribute in Graphviz. If the value is "true", overlapping nodes are allowed. The values "false", "scale", "ortho", "compress" and "vpsc" remove overlaps using different methods. See Graphviz documentation for details. If the value is empty, default handling is done.
ps2pdf	ps2pdf	PS2PDF execution command.
rankdir		For hierarchical layouts, the layout direction. One of TB (top-to-bottom, default), BT, LR (left-to-right), RL.
reportCaption		Caption of the figure in the Latex report.
reportHeight	11	Height of the figure in the Latex report in cm.
reportWidth	14	Width of the figure in the Latex report in cm.
shape		Shape of the nodes. Some legal values include box, polygon, ellipse, circle, point, triangle, plaintext, diamond, none, note, box3d, component; for the rest, see Graphviz documentation. If the value is empty, the default shape (ellipse) is used. Or, if the node attribute "shape" is defined in the GraphML file, each node gets its shape from this attribute.
simplify	false	If true, simplify the graph by removing self-loop edges and multiple edges between two vertices.
size	8,8	Maximum width and height of the image, in INCHES.
splines	true	Determines if edges are drawn as straight lines or curves (splines). This corresponds to the "splines" graph attribute in Graphviz. If "true", splines are enabled. If "false", straight lines are used. If the value is empty, default settings are used.
titleAttribute	label,id	Name of the vertex attribute that is used as the title of the vertex in the visualization. The attribute may contain multiple values that are separated by commas. Each attribute is tried in the order given and the first that is defined is used. For example, if the value is "label,id", then label is used if it is defined, and id is used otherwise. The id attribute is always present.
twopi	twopi	Graphviz/twopi execution command. Only used if the layout parameter specifies this program.

Continued on next page...

Parameter name	Value	Description
width	0	Minimum width of nodes in INCHES. Depending on layout type, this might also be the final width. If zero, the default width is used. Or, if the node attribute "width" is defined in the GraphML file, each node gets its width from this attribute.

19.854 summaryTable-geneInfo-graphMF (GraphVisualizer)

GraphVisualizer creates a visualization of a graph using Graphviz. There are several layout options (see the parameter layout) and make aspects of the nodes and edges can be customized. In addition to the parameters mentioned here, nodes and edges can have many other GraphML attributes that are used to set rendering options for individual nodes and edges. For the full list, see Graphviz documentation [2].

Author:	Kristian Ovaska (kristian.ovaska@helsinki.fi)
Version:	1.0
Categories:	Graph, Plot

Input name	Source	Description
graph	summaryTable-geneInfo-go Enrichment.graphMF 19.850	Input graph

Parameter name	Value	Description
arrowhead		Type of arrow heads (the target end of the arrow). See Graphviz documentation for the format. If empty, the default type is used. Or, if the edge attribute "arrowhead" is defined in the GraphML file, each edge gets its type from this attribute.
arrowtail		Type of arrow tails (the source end of the arrow). See Graphviz documentation for the format. If empty, the default type is used. Or, if the edge attribute "arrowtail" is defined in the GraphML file, each edge gets its type from this attribute.
bgcolor		Background color for the canvas. See Graphviz documentation for the format. If empty, the default color is used.
circo	circo	Graphviz/circo execution command. Only used if the layout parameter specifies this program.
dot	dot	Graphviz/dot execution command. Only used if the layout parameter specifies this program.
edgecolor		Color for drawing edges and arrows, but not text. See Graphviz documentation for the format. If empty, the default color (black) is used. Or, if the edge attribute "color" is defined in the GraphML file, each edge gets its color from this attribute.
fdp	fdp	Graphviz/fdp execution command. Only used if the layout parameter specifies this program.
fillcolor		Color for filling the background of nodes. See Graphviz documentation for the format. If empty, no filling is done. Or, if the node attribute "fillcolor" is defined in the GraphML file, each node gets its color from this attribute.
fontcolor		Color for text. See Graphviz documentation for the format. If empty, the default color (black) is used. Or, if the node/edge attribute "fontcolor" is defined in the GraphML file, each node/edge gets its color from this attribute.
fontsize	0	Font size of text, in points. A typical value is 14. If zero, the default size is used. Or, if the node/edge attribute "fontsize" is defined in the GraphML file, each node/edge gets its font size from this attribute.
height	0	Minimum height of nodes in INCHES. Depending on layout type, this might also be the final height. If zero, the default height is used. Or, if the node attribute "height" is defined in the GraphML file, each node gets its height from this attribute.
layout	hierarchical	Determines how the visualization is layed out. Valid choices are "hierarchical" (layout done using dot), "spring" (neato: Kamada-Kawai algorithm), "spring2" (fdp: Fruchterman-Reingold algorithm), "radial" (twopi) and "circular" (circo) .
margin		Margin around the label of nodes. If given, this is a pair x,y of margin space in inches. If the value is empty, the default margin is used; in Graphviz 2.18 it is "0.11,0.055". Or, if the node attribute "margin" is defined in the GraphML file, each node gets its margin from this attribute.
minSize	0	Minimum number of vertices to render the graph. The whole image will be skipped if there are too few vertices available.
neato	neato	Graphviz/neato execution command. Only used if the layout parameter specifies this program.

Continued on next page...

Parameter name	Value	Description
nodecolor		Color for drawing the boundaries of nodes, but not text. See Graphviz documentation for the format. If empty, the default color (black) is used. Or, if the node attribute "color" is defined in the GraphML file, each node gets its color from this attribute.
overlap		Determines how overlapping nodes are handled. This corresponds to the "overlap" graph attribute in Graphviz. If the value is "true", overlapping nodes are allowed. The values "false", "scale", "ortho", "compress" and "vpsc" remove overlaps using different methods. See Graphviz documentation for details. If the value is empty, default handling is done.
ps2pdf	ps2pdf	PS2PDF execution command.
rankdir		For hierarchical layouts, the layout direction. One of TB (top-to-bottom, default), BT, LR (left-to-right), RL.
reportCaption		Caption of the figure in the Latex report.
reportHeight	11	Height of the figure in the Latex report in cm.
reportWidth	14	Width of the figure in the Latex report in cm.
shape		Shape of the nodes. Some legal values include box, polygon, ellipse, circle, point, triangle, plaintext, diamond, none, note, box3d, component; for the rest, see Graphviz documentation. If the value is empty, the default shape (ellipse) is used. Or, if the node attribute "shape" is defined in the GraphML file, each node gets its shape from this attribute.
simplify	false	If true, simplify the graph by removing self-loop edges and multiple edges between two vertices.
size	8,8	Maximum width and height of the image, in INCHES.
splines	true	Determines if edges are drawn as straight lines or curves (splines). This corresponds to the "splines" graph attribute in Graphviz. If "true", splines are enabled. If "false", straight lines are used. If the value is empty, default settings are used.
titleAttribute	label,id	Name of the vertex attribute that is used as the title of the vertex in the visualization. The attribute may contain multiple values that are separated by commas. Each attribute is tried in the order given and the first that is defined is used. For example, if the value is "label,id", then label is used if it is defined, and id is used otherwise. The id attribute is always present.
twopi	twopi	Graphviz/twopi execution command. Only used if the layout parameter specifies this program.
width	0	Minimum width of nodes in INCHES. Depending on layout type, this might also be the final width. If zero, the default width is used. Or, if the node attribute "width" is defined in the GraphML file, each node gets its width from this attribute.

19.855 summaryTable-geneInfo-kegg (KEGGPathway)

Maps UniprotIDs to KEGG [13] pathways. For list of UniprotIDs finds KEGG pathways and for list of pathways finds all UniprotIDs in the pathways.

Authors: Sirkku Karinen (Sirkku.Karinen@Helsinki.FI), Kristian Ovaska (kristian.ovaska@helsinki.fi)
Version: 1.0
Categories: Annotation, Pathway

Input name	Source	Description
query	summaryTable-geneInfo-uniprot Expanded.relation 19.858	Must contain the column named by the parameter "column" that contains query Uniprot IDs, pathway names or pathway IDs.

Parameter name	Value	Description
cacheURL	ftp://ftp.genome.jp/pub/kegg/genes/organisms/hsa/hsa_xrefall.list	If non-empty, this points to a cache table (xrefall) that contains Uniprot and pathway mappings for all genes of the species in question. For human, the URL is "ftp://ftp.genome.jp/pub/kegg/genes/organisms/hsa/hsa_xrefall.list"; for other organisms, hsa needs to be replaced with the organism code. The cache should only be used when several (over ~20) KEGG/Uniprot mappings need to be done.
column	Uniprot	Column name in the input CSV file that contains query values. If empty, the first column is used.
pathway Descriptions	true	If true, query pathway descriptions. If false, do not query and output NA to the pathway description field.
pathwaySizes	true	If false and type=uniprot, pathway sizes (number of genes) are not queried. If pathwaySizes is true or type is not uniprot, sizes are queried.
proteinNames	true	If true, fetch protein names and write them to the "proteins" output. If false, names are not fetched and the Name column of proteins is not present. Fetching protein names may be slow when there are a large number of proteins.
species	homo sapiens	Species name for the query.

Continued on next page...

Parameter name	Value	Description
type	uniprot	Gives the query mode. One of "uniprot" (get pathways for Uniprot IDs), "pid" (get proteins for pathway IDs) or "pname" (get proteins for pathway names).

19.856 summaryTable-geneInfo-keggReport (CSV2Latex)

This component converts a tabular CSV file into a printable LaTeX table.

Author: Marko Laakso (Marko.Laakso@Helsinki.FI)
Version: 2.1
Categories: convert, Latex

Input name	Source	Description
tabledata	summaryTable-geneInfo-kegg .pathways 19.855	Table content

Parameter name	Value	Description
attach	false	Include the original data as an attachment
caption	KEGG pathways	Caption text for the table.
colFormat	center	LaTeX tabular format for the columns. Default produces a centered layout for all columns.
columns	Pathway,Name,QueryProteins	Comma separated list of column selections for the output. The empty default will use all columns.
countRows	false	Include a row count to the table caption.
dropMissing	true	This flag can be turned off in order to generate links with missing texts. Link text are substituted with target identifiers.
listCols		Comma separated list of column names. Columns of this list may contain several values separated with commas and the delimiters will be replaced with spaces.
numberFormat		A comma separated list of decimal formats for the columns. Each entry consists of the column name and the Java DecimalFormal pattern separated with equal sign. For example, rounding to three decimals can be done like: myColumn=#0.000
pageBreak	false	Use clear page after the table.
rename		Comma separated list of column renaming rules (oldname=newname). New names are used in table header but they do not affect the other behaviour of this component.
ruler	{}	Latex command for the row separating rulers
section		Section title for the table container or an empty string if no section should be generated.
sectionType	subsection	Type of LaTeX section: usually one of: section, subsection, or subsubsection. No section statement is written if section title is empty.
skipEmpty	false	This flag can be used to replace empty tables with a simple LaTeX comment.

19.857 summaryTable-geneInfo-report (LatexCombiner)

LatexCombiner joins the given subdocuments together into one LaTeX file.

Authors: Kristian Ovaska (Kristian.Ovaska@Helsinki.FI), Marko Laakso (Marko.Laakso@Helsinki.FI)
Version: 1.3
Category: Latex

Input name	Source	Description
latex1	summaryTable-geneInfo-go Heatmap.report 19.851	LaTeX fragment 1.
latex2	summaryTable-geneInfo-graphBP .figure 19.852	LaTeX fragment 2.
latex3	summaryTable-geneInfo-graphCC .figure 19.853	LaTeX fragment 3.
latex4	summaryTable-geneInfo-graphMF .figure 19.854	LaTeX fragment 4.
latex5	summaryTable-geneInfo-kegg Report.report 19.856	LaTeX fragment 5.

Parameter name	Value	Description
pagebreak	false	Determines if the result document should start with a page break.
sectionTitle	Gene set information	If non-empty, a declaration of a new section with the given name is inserted to the beginning of the combined document. This is a convenience feature to make it easy to compile subsections into a section.
sectionType	section	Type of LaTeX section: usually one of section, subsection or subsubsection. No section statement is written if sectionTitle is empty.
strictBorders	true	Enables the flushing of all document elements before each fragment

19.858 summaryTable-geneInfo-uniprotExpanded (ExpandCollapse)

Converts between the two possible representations of a relations with multivalued columns. By default this component splits rows with multivalued (comma separated lists) columns into multiple rows each representing a single value. The input may contain several columns with comma separated values and all permutations of column values are shown as individual single-valued rows. The alternative mode recovers the original comma separated values from the expanded relations. This is performed by joining values of the their list columns into comma separated lists.

Author: Marko Laakso (Marko.Laakso@Helsinki.FI)
Version: 1.3
Category: convert

Input name	Source	Description
relation	summaryTable-geneInfo -annotation.bioAnnotation 19.849	The mandatory input relation

Parameter name	Value	Description
delim	,	Value delimiter between the list column values. Special characters can be encoded as specified in fi.helsinki.ltdk.csbl.asser.ArgumentEncoding.
expand	true	Action mode that is expand (true) or collapse (false)
listCols	Uniprot	A comma separated list of column names of input (expand) or output (collapse) columns that may contain delim separated values. The asterisk refers to every column of the input relation.

19.859 summaryTable-geneSet (CSVTransformer)

Transform CSV files using R [20] expressions. This allows to apply arithmetic functions to numeric columns and to combine columns from different CSV files. The R expressions are evaluated and are expected to return R matrices, data frames or vectors that are concatenated to a final result. Concatenations is done on columns, so each transformation creates additional columns to the output. Transformations should create items having the same number of rows. However, the expression may yield a single string or number that is duplicated to fit the number of rows.

Author: Kristian Ovaska (kristian.ovaska@helsinki.fi)
Version: 1.0
Category: preprocessing

Input name	Source	Description
csv1	summaryTable-summaryTable .csv 19.877	Input file 1.

Parameter name	Value	Description
columnNames		R expression that evaluates to the column names of the result CSV file. The evaluated vector must have the same number of items as there are columns in the output. If empty, column names are taken from the input CSV files; depending on the transforms, some column names may be automatically generated.

Continued on next page...

Parameter name	Value	Description
transform1	subset(csv1, csv1$'TranscriptExpression: Survival' < 0.002 & csv1$'TranscriptExpression:Max' < 1)['GeneID']	R expression that evaluates to a matrix, data frame, vector or constant. The expression may refer to data frames "csv1" and "csv2" (only if csv2 is given) and matrices "matrix1" and "matrix2" (only if csv2 is given).
transform2		Transformation expression 2. If empty, no transformation is done.
transform3		Transformation expression 3. If empty, no transformation is done.
transform4		Transformation expression 4. If empty, no transformation is done.
transform5		Transformation expression 5. If empty, no transformation is done.
transform6		Transformation expression 6. If empty, no transformation is done.
transform7		Transformation expression 7. If empty, no transformation is done.
transform8		Transformation expression 8. If empty, no transformation is done.
transform9		Transformation expression 9. If empty, no transformation is done.

19.860 summaryTable-genes (CSV2IDList)

Extracts one column from the given CSV file and prints it out without duplicates. The selected column may contain entries with comma separated lists of values in which case *isList* parameter may be used to obtain each value separately.

Output is defined as:

$$ids = \bigcup_{i=1}^{9} \pi_{columnIn_i} \sigma_{regexp_i}(table_i). \quad (30)$$

Author: Marko Laakso (Marko.Laakso@Helsinki.FI)
Version: 1.3
Category: convert

Input name	Source	Description
table1	geneExpression-allProbes .csv 19.278	The mandatory input relation
table2	transcriptExpressionResults-all Probes.table 19.889	The second input relation
table3	medianExonExpression-allProbes .table 19.324	The third input relation

Parameter name	Value	Description
acceptMissing	false	Files with missing columnIn are accepted as empty if this is true.
columnIn	GeneID	A comma separated list of column names for the IDs of interest in each file. Empty values refer to the first column of the file.
columnOut		Name of the only column of the output list. Empty input refers to the name of the input column.
constants		A comma separated list of values that are always included into the output
isList	false	True if the seleted column contains a comma separated list of values to be splitted
quotation	false	Indicator that can be used to disable quotation of the output values
regexp1		Regular expression for the row filtering in table1. A row is included in the result if this parameter is empty or if values in the given columns match given regular expressions. The parameter has a format COLNAME1=EXPRESSION,COLNAME2=EXPRESSION2 where COLNAMEs are column names in "csv" and EXPRESSIONs are regular expressions using Java syntax. For example, "col=a|b" includes rows where the column col has a value of "a" or "b".
regexp2		Regular expression for the row filtering in table2.
regexp3		Regular expression for the row filtering in table3.
regexp4		Regular expression for the row filtering in table4.
regexp5		Regular expression for the row filtering in table5.
regexp6		Regular expression for the row filtering in table6.
regexp7		Regular expression for the row filtering in table7.
regexp8		Regular expression for the row filtering in table8.
regexp9		Regular expression for the row filtering in table9.

19.861 summaryTable-imageMapping (TableQuery)

Execute an SQL query on input relations and create a result table. TableQuery uses HSQLDB (Hyper-threaded Structured Query Language Database [25]) for executing the query. Consequently, the syntax of the query is defined by HSQLDB.

Author: Kristian Ovaska (Kristian.Ovaska@Helsinki.FI)
Version: 1.2
Categories: convert, Filter

Input name	Source	Description
table1	transcriptExpressionResults-km-km.statistics 19.907	CSV table 1. The table is referred to as 'table1' in the SQL query.
table2	snpSurvival-survivalAll-survSNPAnnot-annotData.table 19.482	CSV table 2. The table is referred to as 'table2' in the SQL query.
table3	summaryTable-miRNAstat.file 19.864	CSV table 3. The table is referred to as 'table3' in the SQL query.
table4	medianExonExpression-km-km.statistics 19.341	CSV table 4. The table is referred to as 'table4' in the SQL query.
query	summaryTable-imageMappingQuery.in 19.862	SQL query. Either this file or the query parameter must be provided, but not both.

Parameter name	Value	Description
numIndices		Comma-separated list of index counts for input tables. All indices are single-column indices, running from column 1 to N, where N is retrieved from this parameter. If empty, the default number (1) is used. For example, ",2,,2" sets two indices for table2 and table4 and one index for the rest.
query		SQL query. Either this parameter or the query input must be provided, but not both.

19.862 summaryTable-imageMappingQuery (INPUT)

(Technical documentation) Imports an external input file or directory to the pipeline. This is the only way external files are imported. File path is given with the parameter "path".

Category: Internal

Parameter name	Value	Description
path	/gpfs/homes/mlaakso/asserSVN/andurilCaseStudy/sql/imageMapping.sql	Path (filename) of the input resource.

19.863 summaryTable-images (LatexCombiner)

LatexCombiner joins the given subdocuments together into one LaTeX file.

Authors: Kristian Ovaska (Kristian.Ovaska@Helsinki.FI), Marko Laakso (Marko.Laakso@Helsinki.FI)
Version: 1.3
Category: Latex

Input name	Source	Description
latex1	transcriptExpressionResults-km-kmReport.document 19.908	LaTeX fragment 1.
latex2	snpSurvival-survivalAll-survival.report 19.487	LaTeX fragment 2.
latex3	miRNAResults-km.report 19.407	LaTeX fragment 3.
latex4	medianExonExpression-km-kmReport.document 19.342	LaTeX fragment 4.

Parameter name	Value	Description
pagebreak	false	Determines if the result document should start with a page break.
sectionTitle		If non-empty, a declaration of a new section with the given name is inserted to the beginning of the combined document. This is a convenience feature to make it easy to compile subsections into a section.
sectionType	section	Type of LaTeX section: usually one of section, subsection or subsubsection. No section statement is written if sectionTitle is empty.
strictBorders	true	Enables the flushing of all document elements before each fragment

19.864 summaryTable-miRNAstat (SearchReplace)

Replaces the occurences of the given regular expressions with the corresponding regular expressions. This component is used for transforming given patterns of the input file to a changed form specified by the regular expressions .

Author: Marko Laakso (Marko.Laakso@Helsinki.FI)
Version: 1.0
Category: convert

Input name	Source	Description
file	miRNAResults-km.<br>statistics 19.407	Source file to be modified

Parameter name	Value	Description
key00	hsa.miR.	Search key 0
key01		Search key 1
key02		Search key 2
key03		Search key 3
key04		Search key 4
key05		Search key 5
key06		Search key 6
key07		Search key 7
key08		Search key 8
key09		Search key 9
value00	hsa-miR-	Replacement 0
value01		Replacement 1
value02		Replacement 2
value03		Replacement 3
value04		Replacement 4
value05		Replacement 5
value06		Replacement 6
value07		Replacement 7
value08		Replacement 8
value09		Replacement 9

19.865 summaryTable-miRNAtable (TableQuery)

Execute an SQL query on input relations and create a result table. TableQuery uses HSQLDB (Hyperthreaded Structured Query Language Database [25]) for executing the query. Consequently, the syntax of the query is defined by HSQLDB.

Author: Kristian Ovaska (Kristian.Ovaska@Helsinki.FI)
Version: 1.2
Categories: convert, Filter

Input name	Source	Description
table1	miRNAResults-miRNAStat<br>Filtered.csv 19.416	CSV table 1. The table is referred to as 'table1' in the SQL query.
table2	ACGHmiRNAgainFreqs-gene<br>Freqs.table 19.23	CSV table 2. The table is referred to as 'table2' in the SQL query.
table3	ACGHmiRNAlossFreqs-geneFreqs<br>.table 19.34	CSV table 3. The table is referred to as 'table3' in the SQL query.

Continued on next page...

Input name	Source	Description
table4	summaryTable-miRNAstat .file 19.864	CSV table 4. The table is referred to as 'table4' in the SQL query.

Parameter name	Value	Description
numIndices		Comma-separated list of index counts for input tables. All indices are single-column indices, running from column 1 to N, where N is retrieved from this parameter. If empty, the default number (1) is used. For example, ",2,,2" sets two indices for table2 and table4 and one index for the rest.
query	SELECT table1."miRNAName", table1."miRNAId", table1. "FoldChange", table1."PValue", table4."pValue" AS "SurvivalPValue", table2."freq" AS "CGH:Gain", table3."freq" AS "CGH:Loss", table1. "TargetGenes", table1. "stemLoopName", COALESCE(table1."DNABand", table2. "DNABand", table3. "DNABand") AS "DNABand" FROM table1 LEFT OUTER JOIN table2 ON (table1. "miRNAName" = table2. "miRNAName") LEFT OUTER JOIN table3 ON (table1. "miRNAName" = table3. "miRNAName") LEFT OUTER JOIN table4 ON (table1. "miRNAName" = table4. "group")	SQL query. Either this parameter or the query input must be provided, but not both.

19.866 summaryTable-pathways (KEGGPathway)

Maps UniprotIDs to KEGG [13] pathways. For list of UniprotIDs finds KEGG pathways and for list of pathways finds all UniprotIDs in the pathways.

Authors: Sirkku Karinen (Sirkku.Karinen@Helsinki.FI), Kristian Ovaska (kristian.ovaska@helsinki.fi)
Version: 1.0
Categories: Annotation, Pathway

Input name	Source	Description
query	summaryTable-uniprotExp .relation 19.881	Must contain the column named by the parameter "column" that contains query Uniprot IDs, pathway names or pathway IDs.

Parameter name	Value	Description
cacheURL	ftp://ftp.genome.jp/pub/kegg/ genes/organisms/hsa/hsa_xrefall. list	If non-empty, this points to a cache table (xrefall) that contains Uniprot and pathway mappings for all genes of the species in question. For human, the URL is "ftp://ftp.genome.jp/pub/kegg/genes/organisms/hsa/hsa_xrefall.list"; for other organisms, hsa needs to be replaced with the organism code. The cache should only be used when several (over ~20) KEGG/Uniprot mappings need to be done.
column	Uniprot/SWISSPROT	Column name in the input CSV file that contains query values. If empty, the first column is used.
pathway Descriptions	true	If true, query pathway descriptions. If false, do not query and output NA to the pathway description field.
pathwaySizes	false	If false and type=uniprot, pathway sizes (number of genes) are not queried. If pathwaySizes is true or type is not uniprot, sizes are queried.
proteinNames	false	If true, fetch protein names and write them to the "proteins" output. If false, names are not fetched and the Name column of proteins is not present. Fetching protein names may be slow when there are a large number of proteins.
species	homo sapiens	Species name for the query.
type	uniprot	Gives the query mode. One of "uniprot" (get pathways for Uniprot IDs), "pid" (get proteins for pathway IDs) or "pname" (get proteins for pathway names).

19.867 summaryTable-pathwaysEnsembl (CSVFilter)

Filters columns and/or rows from CSV files using flexible criteria. Default settings do not filter out anything. The *negate* parameter reverses all inclusion criteria.

Columns are filtered based on column names (see *includeColumns*); it is also possible to rename columns. Columns can be reordered using *includeColumns*.

A row is included in output if all conditions are satisfied. Rows having too many missing values are filtered using the *nonMissing* parameter. The parameters *regexp*, *lowBound* and *highBound* filter rows based on cell contents. If the *auxiliary* input is given, the result contains those rows from the input whose values match those from the auxiliary file.

Authors: Kristian Ovaska (kristian.ovaska@helsinki.fi), Marko Laakso (Marko.Laakso@Helsinki.FI)
Version: 1.1
Category: Filter

Input name	Source	Description
csv	summaryTable-pathwaysEnsembl Pre.csv 19.868	CSV file to be filtered.

Parameter name	Value	Description
highBound		A row is included if the value in given numeric columns are at most as large as given numeric bounds. The parameter has a format COLNAME1=HIGH1,COLNAME2=HIGH2.
idColumn		Column name in "csv" that contains ID values. If the parameter is empty, the first column is used.
includeColumns	Pathway,Name,QueryProteins, Description	Comma-separated list of column names that should be included in the result file. The special value * includes all columns. The order of column names is significant: the output columns are in the order given. See also includeColumns input.
lowBound		A row is included if the value in given numeric columns are at least as large as given numeric bounds. The parameter has a format COLNAME1=LOW1,COLNAME2=LOW2. For example, "col=5" includes rows where the column col has a value of at least 5.
matchColumn		Column name in "auxiliary" containing values that must match the ID column in "csv" (specified using "idColumn"). If empty, the first column of "auxiliary" is used.
negate	false	If true, all inclusion criteria are negated so that rows or columns are excluded instead of included if they match the criteria. For example, includeColumns=* then excludes all columns (which would be an error since at least one column must be included).
nonMissing	0	Include those rows that have non-missing (non-NA) values in at least this many columns. Only those columns that are part of the output are counted.
regexp		Row filtering based on regular expression. A row is included in the result if values in the given columns match given regular expressions. The parameter has a format COLNAME1=EXPRESSION,COLNAME2=EXPRESSION2 where COLNAMEs are column names in "csv" and EXPRESSIONs are regular expressions using Java syntax. For example, "col=a|b" includes rows where the column col has a value of "a" or "b". Commas have to be escaped with "\\" if they are used in column names or regular expressions.
rename	QueryProteins=Members	Comma-separated list of column renaming rules (OLDNAME=NEWNAME). Parameters that refer to column names refer to old names; renaming is done last.

19.868 summaryTable-pathwaysEnsemblPre (IDConvert)

Converts identifiers from a column in a CSV file to another type of identifiers. The conversion is done using an annotation table on the basis of the column named *conversionColumn*.

Author: Kristian Ovaska (kristian.ovaska@helsinki.fi)
Version: 1.0
Category: convert

Input name	Source	Description
csv	summaryTable-pathways .pathways 19.866	Source CSV file. Contains the column that is to be converted. The column is named by the parameter 'sourceColumn'.
conversionTable	summaryTable-uniprotExp .relation 19.881	Conversion table. Must contain the column named by the parameter 'conversionColumn'.

Parameter name	Value	Description
collapseNumeric	median	Method for collapsing numeric columns with duplicate rows (when unique=true). Legal values are "median" (take median of non-NA values), "mean", "max", "min", "first" (take the first row), "majority" (take the value that is present on the largest number of rows), "consensus" (require that all rows have the same value) and "indicator".<br>Here, "majority" and "consensus" are mostly useful for discrete (integer) values). For "consensus", it is an error if duplicate rows contain distinct values. "indicator" assumes that data are divided into negative (-1), neutral (0) and positive (1) items, such as discretized expression values. The result is (a) 1 if there is a 1 and no -1 value; (b) -1 if there is a -1 and not 1 value; (c) 0 if there is a 0 and no -1 or 1; or (d) NA otherwise.
collapseString	consensus	Method for collapsing non-numeric columns with duplicate rows (when unique=true). Legal values are "first" (take the first row), "majority" (take the value that is present on the largest number of rows), "longest" (take the longest value), "shortest" (take the shortest value) and "consensus" (require that all rows have the same value). For "consensus", it is an error if duplicate rows contain distinct values.
conversionColumn	GeneID	Column in 'conversionTable' that is used to fetch new values for the source column.
keyColumn	Uniprot/SWISSPROT	Column in 'conversionTable' that is used to match ID values from the source CSV. If the parameter is empty, the first column is used.
originalWhen Missing	true	Determines how values are converted when the item is not found in the conversion table. If true, the original (unconverted) ID is inserted to the output. If false, NA is inserted.
sourceColumn	QueryProteins	Column in 'csv' that is going to be converted. If empty, the first column is used.
split	true	If true, split values in the source column by commas before ID conversion. For example, if the source column contains the string "a,b,c", it is first split into "a", "b" and "c"; then, individual IDs are converted; finally, the converted IDs are joined by commas into a string of format "A,B,C".
splitConverted	false	If true, split converted values by commas after ID conversion. A row with comma separated values in the target column will be split to otherwise identical rows but so that the rows have a single ID value in the target column. This is done before possible collapsing of duplicate rows.
targetColumn		Target column name in 'converted'. If empty, the column name is the same as the original column (sourceColumn).
unique	true	If true, rows with identical identifiers (sourceColumn) are collapsed into one row so that sourceColumn is unique. Collapsing may result in error if duplicate rows contain conflicting values. See parameters collapseNumeric and collapseString for methods to collapse numeric and non-numeric columns, respectively.
varMeasure		Method for calculating the variation of numeric duplicate values. If the variation is above the threshold given in <code>varThreshold</code> , duplicate values are collapsed to NA. Possible values are 'MAD' and 'variance'. 'MAD' stands for the median absolute deviation and 'variance' for the population variance. If no values is given, no threshold for combining numeric values is used.
varThreshold	2	Maximum allowed variability in the duplicate values. If <code>varMeasure</code> is given duplicate values with variance above the threshold are collapsed to NA

19.869 summaryTable-refs (INPUT)

(Technical documentation) Imports an external input file or directory to the pipeline. This is the only way external files are imported. File path is given with the parameter "path".

Category: Internal

Parameter name	Value	Description
path	csv/summary-refs.csv	Path (filename) of the input resource.

19.870 summaryTable-sequenced (INPUT)

(Technical documentation) Imports an external input file or directory to the pipeline. This is the only way external files are imported. File path is given with the parameter "path".

Category: Internal

Parameter name	Value	Description
path	/gpfs/homes/mlaakso/asserSVN/andurilCaseStudy/csv/sequenced.csv	Path (filename) of the input resource.

19.871 summaryTable-site (HTMLReport)

(Technical documentation) Visualizes CSV files and their relationships using statically generated HTML files. The primary input are a set of tables that may represent genes, proteins, SNPs, samples, etc. Each table has a number of attributes (CSV columns) and a number of records (CSV rows). Tables may refer to each other, e.g. an attribute for a protein may name the gene that encodes the protein. References between tables are specified with the "mapping" input. Effectively, the set of tables and mappings between them (foreign keys) constitute a relational database. Records are identified by a single column (primary key) that can be named in the "mapping" input.

For each table, a summary HTML page is generated that shows all records of the table. For each record, a detail page is generated that shows all attributes of the record as well as mappings to other tables. For example, the detail page of a gene may show all proteins encoded by the gene. Mapping to other tables can either be in the form of simple links or as inline records that show a sub-record for each related record. The HTML pages have links to each other when applicable; also, external links can be configured using the "refs" input. Numeric fields can be colorized using the ColorRange column of the "mapping" input and color* parameters. Column names with format PREFIX:NAME are grouped together in the summary page; all neighboring columns with the same prefix belong to the same group. The prefix is displayed as a column group label.

Images (e.g., the 3D structure of a protein) can be attached to records using the "images" and "imageMapping" inputs.

Numeric matrices (e.g., expression values) can be attached to records using the "matrixN" and "matrixMapping" records. Column and/or row names are matched to key values of a table and the correspoding row/column values are printed on the details page of the record. For example, if the column names of matrix1 refer to key values of table1, the row matching a given key is printed on the record details page of the key. If the matrix dimensions are over a specified threshold (matrixThreshold), only distribution statistics are printed.

Author: Kristian Ovaska (kristian.ovaska@helsinki.fi)
Version: 1.0
Category: HTML

Input name	Source	Description
table1	summaryTable-summaryTable.csv 19.877	Input table 1
table2	summaryTable-exonAnnotationFilt.csv 19.846	Input table 2
table3	summaryTable-miRNAtable.table 19.865	Input table 3
table4	summaryTable-pathwaysEnsembl.csv 19.867	Input table 4

Continued on next page...

Input name	Source	Description
mapping	summaryTable-siteMapping .in 19.873	Specifies various properties of tables (tableN), such as key columns, foreign key columns and colorized columns. The following columns may be present; any of them may also be missing.<br>”Table” gives the table ID in question (tableN); if missing, table1 is used.<br>”KeyColumn” gives the column name in the table that contains key values; if missing, the first column is used.<br>”ViewColumn” gives the column name that contains displayable values, such as human-readable gene names; if missing, the key column is used.<br>”ForeignKeys” specifies connections to other tables. It has the format ”MyColumn1=tableM,MyColumn2=tableN”, where MyColumn* are column names in the current table and table* are foreign table IDs. For example, values in MyColumn1 match the key column values of tableM.<br>”SummaryColumns” is a comma-separated list of column names that are included in the summary page; the special value * (default) includes all columns.<br>”IgnoreColumns” is a comma-separated list of column names that are ignored completely (default: none).<br>”SortColumns” is a comma-separated list of column names that can be used to sort the summary page (default: none).<br>”InlineTables” is a comma-separated list of tables (tableN) whose associated records should be printed inline on the same page as column from the primary table.<br>”InlineColumns” is a list of column names that are printed when the record is an inline part of another record page.<br>”AliasColumns” is a list of column names that contain aliases for the record. Aliases can be used in the search box on the index page. KeyColumn and ViewColumn are automatically included.<br>”ColorRange” specifies numeric columns that are colorized. It has the format ”MyColumn1=5 12,MyColumn2=-2 10.5”, meaning that the range of colors is 5 to 12 for MyColumn1 and -2 to 10.5 for MyColumn2. If the keyword ”log” is included in the range specification (e.g., ”5 12 log”), base-2 logarithm is taken before color computation; however, the printed value is the original.
images	summaryTable-images .document 19.863	Directory containing images and other downloadable files (e.g., PDFs) that are shown on the details page of a record.
imageMapping	summaryTable-imageMapping .table 19.861	Specifies how images in the ”images” input are mapped to records. The following columns may be present.<br>”Table” gives the table ID in question (default: table1).<br>”ImageFile” gives the file name in the ”images” folder.<br>”Target” is an ID that matches the primary key of the table; together with ”ImageFile”, this binds an image to a specific record.<br>”Label” gives a human readable label for the image; if missing, an empty label is used.
refs	summaryTable-siteRefs.in 19.874	Formatting rules for external URL references. Contains three columns: ”Table” (table ID), ”Column” (name of the column in the given table) and ”URL” (URL pattern containing an ID tag that is replaced with cell contents). The specified column in the specified table contains HTML links to the external resource.
labels	summaryTable-siteLabels .in 19.872	Contains human-readable labels for table columns, table query boxes and other places. The following columns must be present. ”Table” gives the table ID (tableN), ”Column” gives the column name and ”Label” gives the label for the column. When the column is the special value ”_QUERY”, the entry gives the query box label on the main page. When the table is ”_GLOBAL” and the column is ”_DESCRIPTION”, the entry gives a global description of the site that is shown on the main page. Labels for color slides can be configured using columns ”LowLabel”, ”MiddleLabel” and ”HighLabel”.

Parameter name	Value	Description
colorEnd	#ff6666	For color slides, this is the ending color (high limit).
colorMiddle	#ffffff	For color slides, this is the middle color.
colorStart	#66ff66	For color slides, this is the starting color (low limit).
digits	3	Number of significant digits for printed numeric values.
includeSummaries	*	Comma-separated list of table IDs whose summaries are printed. The special value * prints summaries of all tables.
matrixLabels		Comma-separated list of human-readable labels for matrices. If empty values are present, default labels (matrixN) are used. Even if custom labels are given, matrices are always referred to using matrixN in configuration files.
matrixThreshold	10	The maximum number of matrix values that are printed on the details page of a record. If the matrix dimension is larger than this, statistics on the values are printed.
missingValue	-	Gives the string that is used in HTML pages for missing values (NA).
omitMissing	false	If true, omit missing (NA) attributes in the record details page; the attribute is still printed in the summary page. If false, always print missing values.
recordsPerPage	100	For the summary page, this gives the maximum number of records (rows) per page. If more records are present, they are split into multiple pages.
tableLabels	Gene,Splice variant,miRNA, KEGG pathway	Comma-separated list of human-readable labels for tables. For example, ”Gene,Protein,SNP” would indicate that table1 represents genes, table2 proteins etc. If empty values are present, default labels (tableN) are used. Even if custom labels are given, tables are always referred to using tableN in ”mapping” and other configuration files.

19.872 summaryTable-siteLabels (INPUT)

(Technical documentation) Imports an external input file or directory to the pipeline. This is the only way external files are imported. File path is given with the parameter "path".

Category: Internal

Parameter name	Value	Description
path	/gpfs/homes/mlaakso/asserSVN/ andurilCaseStudy/csv/ summarysite-labels.csv	Path (filename) of the input resource.

19.873 summaryTable-siteMapping (INPUT)

(Technical documentation) Imports an external input file or directory to the pipeline. This is the only way external files are imported. File path is given with the parameter "path".

Category: Internal

Parameter name	Value	Description
path	/gpfs/homes/mlaakso/asserSVN/ andurilCaseStudy/csv/ summarysite-mapping.csv	Path (filename) of the input resource.

19.874 summaryTable-siteRefs (INPUT)

(Technical documentation) Imports an external input file or directory to the pipeline. This is the only way external files are imported. File path is given with the parameter "path".

Category: Internal

Parameter name	Value	Description
path	/gpfs/homes/mlaakso/asserSVN/ andurilCaseStudy/csv/ summarysite-refs.csv	Path (filename) of the input resource.

19.875 summaryTable-style (INPUT)

(Technical documentation) Imports an external input file or directory to the pipeline. This is the only way external files are imported. File path is given with the parameter "path".

Category: Internal

Parameter name	Value	Description
path	/gpfs/homes/mlaakso/asserSVN/ andurilCaseStudy/csv/summary-style.csv	Path (filename) of the input resource.

19.876 summaryTable-summaryQuery (INPUT)

(Technical documentation) Imports an external input file or directory to the pipeline. This is the only way external files are imported. File path is given with the parameter "path".

Category: Internal

Parameter name	Value	Description
path	/gpfs/homes/mlaakso/asserSVN/ andurilCaseStudy/sql/summary. sql	Path (filename) of the input resource.

19.877 summaryTable-summaryTable (IDConvert)

Converts identifiers from a column in a CSV file to another type of identifiers. The conversion is done using an annotation table on the basis of the column named *conversionColumn*.

Author: Kristian Ovaska (kristian.ovaska@helsinki.fi)
Version: 1.0
Category: convert

Input name	Source	Description
csv	summaryTable-summaryTablePre .table 19.878	Source CSV file. Contains the column that is to be converted. The column is named by the parameter 'sourceColumn'.
conversionTable	summaryTable-summaryTablePre .table 19.878	Conversion table. Must contain the column named by the parameter 'conversionColumn'.

Parameter name	Value	Description
collapseNumeric	consensus	Method for collapsing numeric columns with duplicate rows (when unique=true). Legal values are "median" (take median of non-NA values), "mean", "max", "min", "first" (take the first row), "majority" (take the value that is present on the largest number of rows), "consensus" (require that all rows have the same value) and "indicator".<br>Here, "majority" and "consensus" are mostly useful for discrete (integer) values). For "consensus", it is an error if duplicate rows contain distinct values. "indicator" assumes that data are divided into negative (-1), neutral (0) and positive (1) items, such as discretized expression values. The result is (a) 1 if there is a 1 and no -1 value; (b) -1 if there is a -1 and not 1 value; (c) 0 if there is a 0 and no -1 or 1; or (d) NA otherwise.
collapseString	consensus	Method for collapsing non-numeric columns with duplicate rows (when unique=true). Legal values are "first" (take the first row), "majority" (take the value that is present on the largest number of rows), "longest" (take the longest value), "shortest" (take the shortest value) and "consensus" (require that all rows have the same value). For "consensus", it is an error if duplicate rows contain distinct values.
conversionColumn	GeneID	Column in 'conversionTable' that is used to fetch new values for the source column.
keyColumn	GeneID	Column in 'conversionTable' that is used to match ID values from the source CSV. If the parameter is empty, the first column is used.
originalWhen Missing	true	Determines how values are converted when the item is not found in the conversion table. If true, the original (unconverted) ID is inserted to the output. If false, NA is inserted.
sourceColumn	GeneID	Column in 'csv' that is going to be converted. If empty, the first column is used.
split	false	If true, split values in the source column by commas before ID conversion. For example, if the source column contains the string "a,b,c", it is first split into "a", "b" and "c"; then, individual IDs are converted; finally, the converted IDs are joined by commas into a string of format "A,B,C".
splitConverted	false	If true, split converted values by commas after ID conversion. A row with comma separated values in the target column will be split to otherwise identical rows but so that the rows have a single ID value in the target column. This is done before possible collapsing of duplicate rows.
targetColumn		Target column name in 'converted'. If empty, the column name is the same as the original column (sourceColumn).
unique	true	If true, rows with identical identifiers (sourceColumn) are collapsed into one row so that sourceColumn is unique. Collapsing may result in error if duplicate rows contain conflicting values. See parameters collapseNumeric and collapseString for methods to collapse numeric and non-numeric columns, respectively.
varMeasure		Method for calculating the variation of numeric duplicate values. If the variation is above the threshold given in <code>varThreshold</code> , duplicate values are collapsed to NA. Possible values are 'MAD' and 'variance'. 'MAD' stands for the median absolute deviation and 'variance' for the population variance. If no values is given, no threshold for combining numeric values is used.

Continued on next page...

Parameter name	Value	Description
varThreshold	2	Maximum allowed variability in the duplicate values. If <code>varMeasure</code> is given duplicate values with variance above the threshold are collapsed to NA

19.878 summaryTable-summaryTablePre (TableQuery)

Execute an SQL query on input relations and create a result table. TableQuery uses HSQLDB (Hyper-threaded Structured Query Language Database [25]) for executing the query. Consequently, the syntax of the query is defined by HSQLDB.

Author:	Kristian Ovaska (Kristian.Ovaska@Helsinki.FI)
Version:	1.2
Categories:	convert, Filter

Input name	Source	Description
table1	summaryTable-summaryTable Pre2.table 19.879	CSV table 1. The table is referred to as 'table1' in the SQL query.
table2	medianExonExpression-km-km .statistics 19.341	CSV table 2. The table is referred to as 'table2' in the SQL query.

Parameter name	Value	Description
numIndices		Comma-separated list of index counts for input tables. All indices are single-column indices, running from column 1 to N, where N is retrieved from this parameter. If empty, the default number (1) is used. For example, ",2,,2" sets two indices for table2 and table4 and one index for the rest.
query	SELECT T1.*, T2."pValue" AS "MedianExonExpression: Survival" FROM table1 T1 LEFT OUTER JOIN table2 T2 ON T1. "GeneID" = T2."group"	SQL query. Either this parameter or the query input must be provided, but not both.

19.879 summaryTable-summaryTablePre2 (TableQuery)

Execute an SQL query on input relations and create a result table. TableQuery uses HSQLDB (Hyper-threaded Structured Query Language Database [25]) for executing the query. Consequently, the syntax of the query is defined by HSQLDB.

Author:	Kristian Ovaska (Kristian.Ovaska@Helsinki.FI)
Version:	1.2
Categories:	convert, Filter

Input name	Source	Description
table1	summaryTable-annotation.bio Annotation 19.842	CSV table 1. The table is referred to as 'table1' in the SQL query.
table2	geneExpression-allProbes .csv 19.278	CSV table 2. The table is referred to as 'table2' in the SQL query.
table3	transcriptExpressionResults-all Probes.table 19.889	CSV table 3. The table is referred to as 'table3' in the SQL query.
table4	snpSurvival-survivalAll-geneRank -eqData.table 19.470	CSV table 4. The table is referred to as 'table4' in the SQL query.
table5	summaryTable-correlation .in 19.843	CSV table 5. The table is referred to as 'table5' in the SQL query.
table6	summaryTable-sequenced .in 19.870	CSV table 6. The table is referred to as 'table6' in the SQL query.
table7	summaryTable-acghSummary .csv 19.841	CSV table 7. The table is referred to as 'table7' in the SQL query.
table8	summaryTable-vttHits.in 19.882	CSV table 8. The table is referred to as 'table8' in the SQL query.
table9	summaryTable-vttHits2.in 19.883	CSV table 9. The table is referred to as 'table9' in the SQL query.
table10	transcriptExpressionResults-km -km.statistics 19.907	CSV table 10. The table is referred to as 'table10' in the SQL query.

Continued on next page...

Input name	Source	Description
table11	summaryTable-acghExonCorr Summary.csv 19.838	CSV table 11. The table is referred to as 'table11' in the SQL query.
table12	methylation-combined.csv 19.374	CSV table 12. The table is referred to as 'table12' in the SQL query.
table13	summaryTable-vttScreen .in 19.884	CSV table 13. The table is referred to as 'table13' in the SQL query.
table14	summaryTable-geneAlias .in 19.847	CSV table 14. The table is referred to as 'tabl148' in the SQL query.
table15	medianExonExpression-allProbes .table 19.324	CSV table 15. The table is referred to as 'table15' in the SQL query.
query	summaryTable-summaryQuery .in 19.876	SQL query. Either this file or the query parameter must be provided, but not both.

Parameter name	Value	Description
numIndices		Comma-separated list of index counts for input tables. All indices are single-column indices, running from column 1 to N, where N is retrieved from this parameter. If empty, the default number (1) is used. For example, ",2,,2" sets two indices for table2 and table4 and one index for the rest.
query		SQL query. Either this parameter or the query input must be provided, but not both.

19.880 summaryTable-uniprot (CSVFilter)

Filters columns and/or rows from CSV files using flexible criteria. Default settings do not filter out anything. The *negate* parameter reverses all inclusion criteria.

Columns are filtered based on column names (see *includeColumns*); it is also possible to rename columns. Columns can be reordered using *includeColumns*.

A row is included in output if all conditions are satisfied. Rows having too many missing values are filtered using the *nonMissing* parameter. The parameters *regexp*, *lowBound* and *highBound* filter rows based on cell contents. If the *auxiliary* input is given, the result contains those rows from the input whose values match those from the auxiliary file.

Authors:	Kristian Ovaska (kristian.ovaska@helsinki.fi), Marko Laakso (Marko.Laakso@Helsinki.FI)
Version:	1.1
Category:	Filter

Input name	Source	Description
csv	summaryTable-annotation.bio Annotation 19.842	CSV file to be filtered.

Parameter name	Value	Description
highBound		A row is included if the value in given numeric columns are at most as large as given numeric bounds. The parameter has a format COLNAME1=HIGH1,COLNAME2=HIGH2.
idColumn		Column name in "csv" that contains ID values. If the parameter is empty, the first column is used.
includeColumns	GeneID,Uniprot/SWISSPROT	Comma-separated list of column names that should be included in the result file. The special value * includes all columns. The order of column names is significant: the output columns are in the order given. See also includeColumns input.
lowBound		A row is included if the value in given numeric columns are at least as large as given numeric bounds. The parameter has a format COLNAME1=LOW1,COLNAME2=LOW2. For example, "col=5" includes rows where the column col has a value of at least 5.
matchColumn		Column name in "auxiliary" containing values that must match the ID column in "csv" (specified using "idColumn"). If empty, the first column of "auxiliary" is used.
negate	false	If true, all inclusion criteria are negated so that rows or columns are excluded instead of included if they match the criteria. For example, includeColumns=* then excludes all columns (which would be an error since at least one column must be included).
nonMissing	0	Include those rows that have non-missing (non-NA) values in at least this many columns. Only those columns that are part of the output are counted.

Continued on next page...

Parameter name	Value	Description
regexp		Row filtering based on regular expression. A row is included in the result if values in the given columns match given regular expressions. The parameter has a format COLNAME1=EXPRESSION,COLNAME2=EXPRESSION2 where COLNAMEs are column names in "csv" and EXPRESSIONs are regular expressions using Java syntax. For example, "col=a|b" includes rows where the column col has a value of "a" or "b". Commas have to be escaped with "\\" if they are used in column names or regular expressions.
rename		Comma-separated list of column renaming rules (OLDNAME=NEWNAME). Parameters that refer to column names refer to old names; renaming is done last.

19.881 summaryTable-uniprotExp (ExpandCollapse)

Converts between the two possible representations of a relations with multivalued columns. By default this component splits rows with multivalued (comma separated lists) columns into multiple rows each representing a single value. The input may contain several columns with comma separated values and all permutations of column values are shown as individual single-valued rows. The alternative mode recovers the original comma separated values from the expanded relations. This is performed by joining values of the their list columns into comma separated lists.

Author: Marko Laakso (Marko.Laakso@Helsinki.FI)
Version: 1.3
Category: convert

Input name	Source	Description
relation	summaryTable-uniprot.csv 19.880	The mandatory input relation

Parameter name	Value	Description
delim	,	Value delimiter between the list column values. Special characters can be encoded as specified in fi.helsinki.ltdk.csbl.asser.ArgumentEncoding.
expand	true	Action mode that is expand (true) or collapse (false)
listCols	Uniprot/SWISSPROT	A comma separated list of column names of input (expand) or output (collapse) columns that may contain delim separated values. The asterisk refers to every column of the input relation.

19.882 summaryTable-vttHits (INPUT)

(Technical documentation) Imports an external input file or directory to the pipeline. This is the only way external files are imported. File path is given with the parameter "path".

Category: Internal

Parameter name	Value	Description
path	/gpfs/homes/mlaakso/asserSVN/andurilCaseStudy/csv/vtt-hits.csv	Path (filename) of the input resource.

19.883 summaryTable-vttHits2 (INPUT)

(Technical documentation) Imports an external input file or directory to the pipeline. This is the only way external files are imported. File path is given with the parameter "path".

Category: Internal

Parameter name	Value	Description
path	/gpfs/homes/mlaakso/asserSVN/andurilCaseStudy/csv/vtt-hits2.csv	Path (filename) of the input resource.

19.884 summaryTable-vttScreen (INPUT)

(Technical documentation) Imports an external input file or directory to the pipeline. This is the only way external files are imported. File path is given with the parameter "path".

Category: Internal

Parameter name	Value	Description
path	/gpfs/homes/mlaakso/asserSVN/andurilCaseStudy/csv/vtt-screen.csv	Path (filename) of the input resource.

19.885 transcriptAnnotation (INPUT)

Annotation file for transcript expression matrix

Category: Internal

Parameter name	Value	Description
path	/gpfs/homes/mlaakso/asserSVN/andurilCaseStudy/data/exon-normalized/Expression-Exon/LBL_HuEx-1_0-st-v2/Level_2/TranscriptExprAnnotation_v56	Path (filename) of the input resource.

19.886 transcriptExpression (INPUT)

Transcript expression matrix

Category: Internal

Parameter name	Value	Description
path	/gpfs/homes/mlaakso/asserSVN/andurilCaseStudy/data/exon-normalized/Expression-Exon/LBL_HuEx-1_0-st-v2/Level_2/TranscriptExpression_v56	Path (filename) of the input resource.

19.887 transcriptExpressionResults (TranscriptExpressionAnalysis)

Find differentially expressed transcripts.

Parameter name	Value
fcThreshold	2.0
pValueThreshold	0.05

19.888 transcriptExpressionResults-allEntrez (KorvasieniAnnotator)

Korvasieni is an Ensembl [12] based converter for database identifiers and it can be used to convert genome location, gene, transcript, and translation identifiers across multiple biological databases. The selected `keyColumn` may contain entries with comma separated lists of values in which case `isListKey` parameter may be used to obtain each value separately and to process them as they were given in consecutive lines.

Author:	Marko Laakso (Marko.Laakso@Helsinki.FI)
Version:	1.7
Categories:	Annotation, GO

Input name	Source	Description
sourceKeys	transcriptExpressionResults -logratio.expr 19.917	A list of source database keys.
connection	ensembl.in 19.273	Database connection can be defined using this file. The definition of parameters: database.url, database.user, database.password, database.timeout, database.recycle, and database.driver can be found from the documentation of Korvasieni.

Parameter name	Value	Description
echoColumns		A comma separated list of column names for the columns that will be copied to the output. An asterisk (*) can be used to denote all columns execpt the keyColumn.
inputDB	.TranscriptId	Type of input keys. This must be a database supported by Korvasieni. If the parameter is omitted, the component tries to derive the database from the type of geneID. If this is not possible, an error is returned. You may define three columns in form of chromosome:start-end in case the inputDB is .DNARegion. This format provides a comfortable compatibility with DNARegion datatype.
inputType	Any	Ensembl object type for the input keys (Any, Gene, Transcript, Translation)
isListKey	false	Enables the automatic value splits for the comma separated key column
keyColumn		Name of the key column withing sourceKeys file or an empty string for the first column
maxHits	1	Maximum number of target identifiers for a single source identifier
rename		Comma separated list of column renaming rules (oldname=newname)
skipLevel	never	Skip result rows if the source identifier is unknown or target identifiers are not available. Possible values are: never (no filtering), source (skip if the source ID is unknown), target (skip if no target IDs are found), any (skip if any of the target IDs is missing).
targetDB	_EntrezGene	Comma-separated list of annotation types. Possible values are all databases supported by Korvasieni.
unique	false	This flag can be turned on in order to eliminate duplicate annotations.

19.889 transcriptExpressionResults-allProbes (TableQuery)

Execute an SQL query on input relations and create a result table. TableQuery uses HSQLDB (Hyper-threaded Structured Query Language Database [25]) for executing the query. Consequently, the syntax of the query is defined by HSQLDB.

Author:	Kristian Ovaska (Kristian.Ovaska@Helsinki.FI)
Version:	1.2
Categories:	convert, Filter

Input name	Source	Description
table1	transcriptExpressionResults-gene Mapping.csv 19.902	CSV table 1. The table is referred to as 'table1' in the SQL query.
table2	transcriptExpressionResults -logratio.expr 19.917	CSV table 2. The table is referred to as 'table2' in the SQL query.
table3	transcriptExpressionResults-det TTest.pvalues 19.896	CSV table 3. The table is referred to as 'table3' in the SQL query.
query	transcriptExpressionResults -transcriptStatisticsQuery .in 19.935	SQL query. Either this file or the query parameter must be provided, but not both.

Parameter name	Value	Description
numIndices		Comma-separated list of index counts for input tables. All indices are single-column indices, running from column 1 to N, where N is retrieved from this parameter. If empty, the default number (1) is used. For example, ",2,,2" sets two indices for table2 and table4 and one index for the rest.
query		SQL query. Either this parameter or the query input must be provided, but not both.

19.890 transcriptExpressionResults-cumDistReport (Plot2D)

Create scatter or line plots having a dot for each supplied (Y, X) coordinate pair. Coordinates for the Y axis must always be provided; values for the X axis are optional. If X coordinates are missing, integers 1 to N are used.

Authors:	Kristian Ovaska (kristian.ovaska@helsinki.fi), Marko Laakso (Marko.Laakso@Helsinki.FI), Sirkku Karinen (sirkku.karinen@Helsinki.FI)
Version:	1.0
Categories:	Latex, Plot

Input name	Source	Description
y	transcriptExpressionResults-tExpr.table 19.932	Coordinates on the Y axis. Columns from this file are selected for plotting using "yColumns".

Parameter name	Value	Description
caption	Distribution of transcript expression values (log2) for the samples.	LaTeX caption of each figure. The caption may contain one or two %s placeholders, which are replaced with the current column names in "y" and "x" (if defined). Column names are not used when multiple plots are created to the same image (imageType = 'single').
colorFunction		Custom function for colors. Example c('red', 'blue', 'green') or rainbow(3) or rainbow(%d), in which %d is replaced with the number of different labels or columns in the plot. Default colors start from black and continue by rainbow()-function until each label or column is assigned with own color.
commonScale	true	If true, all plots have the same limits for X and Y axes. This makes it easier to compare plots. Only relevant when multiple columns have been selected.
dpCm	60	Dots per centimeter. Used for png-images.
drawX	true	If false, do not print X axis ticks and numbers.
height	11	Height of each plot in cm.
imageType	single	Plot multiple images = 'multiple', single image = 'single' or a pairs plot = 'pairs'
labelColumn		Columns in input "label" that is used. If not defined, uses the first column.
legendPosition	off	Legend position 'bottomleft' or 'topright' etc (Can not be used with the pairs plot)
nInf	-2e100	Substitution value for infinitely small values
pInf	2e100	Substitution value for infinitely great values
plotType	l	Plot type p=points, l=lines, h=bars
pngImage	true	Switch image format between png and pdf
sectionTitle	Ordered transcript expression values	Title of the LaTeX section or an empty string to omit the section
sectionType	subsection	Type of LaTeX section: usually one of section, subsection or subsubsection. No section statement is written if sectionTitle is empty.
sort	true	If true, sort values before plotting. This creates a cumulative-like distribution plot. (Can not be used with pairs plot)
title		Image title. The caption may contain one or two %s placeholders, which are replaced with the current column names in "y" and "x" (if defined). Column names are not used when multiple plots are created to the same image (imageType = 'single').
width	14	Width of each plot in cm.
xColumns	*	Columns in input "x" that are plotted. The special value * includes all columns except non-numeric ones. There must be as many selected columns in X as there are in Y. Note that the files "y" and "x" may still contain a different number of columns.
xHigh	-0.000001	High bound of the X axis. The special value -0.000001 means that the bound is computed from the data.
xLabel	transcripts	X axis label. The label may contain one %s placeholder, which is replaced with the current column name in "x", or if not defined, in "y".
xLow	-0.000001	Low bound of the X axis. The special value -0.000001 means that the bound is computed from the data.

Continued on next page...

Parameter name	Value	Description
xTransformation		Numeric R expression that is used to transform X values. The X values are available as a numeric matrix (or vector) under the name "x". This is analogous to "yTransformation".
yColumns	*	Columns in input "y" that are plotted. The special value * includes all columns except non-numeric ones.
yHigh	-0.000001	High bound of the Y axis. The special value -0.000001 means that the bound is computed from the data.
yLabel	intensity	X axis label. The label may contain one %s placeholder, which is replaced with the current column name in "y".
yLow	-0.000001	Low bound of the Y axis. The special value -0.000001 means that the bound is computed from the data.
yTransformation		Numeric R expression that is used to transform Y values. The Y values are available as a numeric matrix (or vector) under the name "y". Only those columns are present that are selected using "yColumns". For example, "-log10(y)" transforms Y values using a negative base 10 logarithm (defined only for positive values). The expression may be any legal R expression that takes a numeric matrix (or vector) and produces a matrix (or vector) of the same size.

19.891 transcriptExpressionResults-det (SetTransformer)

SetTransformer transform ID lists into new lists by using flexible rules. The rules are read from a *transformation* file. Please, check JEP 2 documentation for the syntax rules.

Author: Kristian Ovaska (kristian.ovaska@helsinki.fi)
Version: 0.6
Category: Convert

Input name	Source	Description
transformation	transcriptExpressionResults -intersectionTransformation .in 19.905	Set transformations, one per CSV row. The columns Target (target set ID) and Definition (transformation expression) must be present. The column IterationSet may be present if iterated transformations are used. IterationSet should contain NA for non-iterated transformations. Any other columns are interpreted as annotation columns and are copied to the output. For iterated transformations, the wildcard * in annotations is replaced with the current set ID.
set1	transcriptExpressionResults-det FoldChange.deg 19.893	Input sets 1. NA values in Members are interpreted as empty sets.
set2	transcriptExpressionResults-det TTest.idlist 19.896	Input sets 2.

Parameter name	Value	Description
includeAnnotation	*	Comma-separated list of column names in the transformation input that should be used as annotation columns. The wildcard * includes all columns. The special columns Target, Definition and IterationSet are excluded automatically.
includeOriginal	true	If true, the original sets from input files are included in the output as well. If false, only sets defined in transformations are included.

19.892 transcriptExpressionResults-detAnnot (KorvasieniAnnotator)

Korvasieni is an Ensembl [12] based converter for database identifiers and it can be used to convert genome location, gene, transcript, and translation identifiers across multiple biological databases. The selected `keyColumn` may contain entries with comma separated lists of values in which case `isListKey` parameter may be used to obtain each value separately and to process them as they were given in consecutive lines.

Author: Marko Laakso (Marko.Laakso@Helsinki.FI)
Version: 1.7
Categories: Annotation, GO

Input name	Source	Description
sourceKeys	transcriptExpressionResults-detID List.ids 19.894	A list of source database keys.

Continued on next page...

Input name	Source	Description
connection	ensembl.in 19.273	Database connection can be defined using this file. The definition of parameters: database.url, database.user, database.password, database.timeout, database.recycle, and database.driver can be found from the documentation of Korvasieni.

Parameter name	Value	Description
echoColumns		A comma separated list of column names for the columns that will be copied to the output. An asterisk (*) can be used to denote all columns execpt the keyColumn.
inputDB	.TranscriptId	Type of input keys. This must be a database supported by Korvasieni. If the parameter is omitted, the component tries to derive the database from the type of geneID. If this is not possible, an error is returned. You may define three columns in form of chromosome:start-end in case the inputDB is .DNARegion. This format provides a comfortable compatibility with DNARegion datatype.
inputType	Transcript	Ensembl object type for the input keys (Any, Gene, Transcript, Translation)
isListKey	false	Enables the automatic value splits for the comma separated key column
keyColumn		Name of the key column withing sourceKeys file or an empty string for the first column
maxHits	100000	Maximum number of target identifiers for a single source identifier
rename		Comma separated list of column renaming rules (oldname=newname)
skipLevel	never	Skip result rows if the source identifier is unknown or target identifiers are not available. Possible values are: never (no filtering), source (skip if the source ID is unknown), target (skip if no target IDs are found), any (skip if any of the target IDs is missing).
targetDB	.GeneId,.GeneName,.GeneDesc, GO	Comma-separated list of annotation types. Possible values are all databases supported by Korvasieni.
unique	false	This flag can be turned on in order to eliminate duplicate annotations.

19.893 transcriptExpressionResults-detFoldChange (FoldChange)

Select differentially expressed genes using the fold change criteria. This filter does not compute the fold changes itself but takes them as input.

A gene with a log ratio intensity l is considered to be **under** expressed if and only if $l \leq -\log_2(threshold)$ and **over** expressed if and only if $l \geq \log_2(threshold)$.

Author: Kristian Ovaska (Kristian.Ovaska@Helsinki.FI)
Version: 1.1
Category: DEG

Input name	Source	Description
logratio	transcriptExpressionResults -logratio.expr 19.917	Log ratio data.

Parameter name	Value	Description
nameDeg	%s_fcDeg	Name of the sets for differentially expressed genes. The pattern %s is expanded to the current sample name in logratio.
nameOver	%s_fcOver	Name of the sets for underexpressed genes. The pattern %s is expanded to the current sample name in logratio.
nameStable	%s_fcStable	Name of the sets for stably expressed genes. The pattern %s is expanded to the current sample name in logratio.
nameUnder	%s_fcUnder	Name of the sets for underexpressed genes. The pattern %s is expanded to the current sample name in logratio.
stableThreshold	0	Fold change threshold (in linear scale) for stable genes. Genes with fold change smaller than the threshold in both directions are considered stably expressed. If the parameter has the value zero, no list of stably expressed genes is included in the results.
threshold	2.0	Fold change threshold (in linear scale). Genes with fold change greater than or equal to the threshold in either direction are considered differentially expressed.

19.894 transcriptExpressionResults-detIDList (CSV2IDList)

Extracts one column from the given CSV file and prints it out without duplicates. The selected column may contain entries with comma separated lists of values in which case *isList* parameter may be used to obtain each value separately.

Output is defined as:

$$ids = \bigcup_{i=1}^{9} \pi_{columnIn_i} \sigma_{regexp_i}(table_i). \qquad (31)$$

Author: Marko Laakso (Marko.Laakso@Helsinki.FI)
Version: 1.3
Category: convert

Input name	Source	Description
table1	transcriptExpressionResults-det .result 19.891	The mandatory input relation

Parameter name	Value	Description
acceptMissing	false	Files with missing columnIn are accepted as empty if this is true.
columnIn	Members	A comma separated list of column names for the IDs of interest in each file. Empty values refer to the first column of the file.
columnOut	.TranscriptId	Name of the only column of the output list. Empty input refers to the name of the input column.
constants		A comma separated list of values that are always included into the output
isList	true	True if the seleted column contains a comma separated list of values to be splitted
quotation	false	Indicator that can be used to disable quotation of the output values
regexp1	ID=over|under	Regular expression for the row filtering in table1. A row is included in the result if this parameter is empty or if values in the given columns match given regular expressions. The parameter has a format COLNAME1=EXPRESSION,COLNAME2=EXPRESSION2 where COLNAMEs are column names in "csv" and EXPRESSIONs are regular expressions using Java syntax. For example, "col=a|b" includes rows where the column col has a value of "a" or "b".
regexp2		Regular expression for the row filtering in table2.
regexp3		Regular expression for the row filtering in table3.
regexp4		Regular expression for the row filtering in table4.
regexp5		Regular expression for the row filtering in table5.
regexp6		Regular expression for the row filtering in table6.
regexp7		Regular expression for the row filtering in table7.
regexp8		Regular expression for the row filtering in table8.
regexp9		Regular expression for the row filtering in table9.

19.895 transcriptExpressionResults-detReport (DEGReport)

Generates a LaTeX report on differentially expressed genes (DEGs). The report has two parts: a summary section that lists the number of over- and underexpressed genes in each sample group; and a gene list section that lists all the gene names.

Authors: Kristian Ovaska (Kristian.Ovaska@Helsinki.FI), Marko Laakso (Marko.Laakso@Helsinki.FI)
Version: 1.1.1
Categories: DEG, Latex

Input name	Source	Description
deg	transcriptExpressionResults-det .result 19.891	Sets of differentially expressed genes. If expression values are given (expr), there must be an annotation column SampleGroup that binds the set to an expression column.
geneAnnotation	transcriptExpressionResults -logratio.expr 19.917	Annotation for genes. Must contain the column named by geneColumn.

Parameter name	Value	Description
columns	4	Number of columns in gene list table.
geneColumn	Transcript	Column name in geneAnnotation that contains gene names. If empty, gene names are not printed.
sectionTitle	Summary of differentially expressed transcripts: fc > 2.0 and corrected p-value < 0.05	Title for a new latex section. If none is given, a new section is not created.
sectionType	section	Type of LaTeX section: usually one of section, subsection or subsubsection. No section statement is written if sectionTitle is empty.
setPattern	over|under	R regular expression for matching set names. If empty, all sets are included.

19.896 transcriptExpressionResults-detTTest (StatisticalTest)

Compute p-values using statistical tests, optionally with correction for multiple hypotheses. In the current version, only Student's t-test is supported. The t-test can be either paired or non-paired. Two-sided and one-sided are supported. If the data contain missing values, only non-missing values are used.

A test is computed for each row in the input matrix. The columns can be selected using a SampleGroupTable (groups) or, if not present, by giving column names using the parameters targetGroup and referenceGroup.

If multiple hypothesis correction is enabled (using the correction parameter), corrected p-values are also written to the pvalues output. Identifiers of those rows whose p-value is less than given threshold are written to the idlist output. Identifiers are taken from the first column of the input matrix. If multiple hypothesis correction is enabled, threshold comparison is done using corrected p-values; otherwise, raw p-values are used. The component implements several multiple hypothesis correction methods, some of which produce modified p-values and some produce q-values:

1. Bonferroni correction,
2. false discovery rate (fdr) [4], and
3. Robust estimation of the false discovery rate [19].

Author: Kristian Ovaska (kristian.ovaska@helsinki.fi)
Version: 1.1
Category: Analysis

Input name	Source	Description
matrix	transcriptExpressionResults-tExpr.table 19.932	Input matrix, containing values to be tested.
groups	transcriptExpressionResults-sampleGroups.groups 19.923	If given, must contain the groups named by the parameters targetColumns and referenceColumns. Both groups must have at least two members. The members must be present in the input matrices.

Parameter name	Value	Description
byRow	true	If true, there is one test for each row. If false, there are tests for various column combinations.
contingencyRows	2	For categorical variable independency tests (chi-squared, Fisher), this gives the number of rows in contingency tables. The number of columns is deduced from data. For example, if contingencyRows=3 and target vector has length 12, the table has 4 columns.
correctedColumn	PValueCorrected	Output column name in "pvalues" for corrected p-values. Only used if multiple hypothesis correction has been enabled.
correction	fdr	Type of multiple hypothesis correction. One of "none", "fdr" (Benjamini-Hochberg, 1995), "robustfdr" (Pounds-Cheng 2006), "BY" (FDR, Benjamini-Yekutieli, 2001), "holm", "bonferroni". The value "none" disables correction.
mean	0	Expected mean of the reference distribution (mu) when the reference group is not supplied. Used for t-test and wilcoxon.
outputSet	tumor_vs_normal_tTest	The name of the output set in the idlist output.
paired	false	Indicates whether the test is paired so that i'th element of target vector corresponds to i'th element of reference vector. Ignored for F-test.

Continued on next page...

Parameter name	Value	Description
prefixColumn		If non-empty, include a column in the pvalues output that contains the outputSet on every row. This parameter gives the name of that column. The column is the first column in pvalues. This prefix column is useful when several separate statistical tests are made and the results are combined into one file. If prefixColumn is empty, do not include the prefix column.
pvalueColumn	PValue	Output column name in "pvalues" for raw p-values.
referenceColumns	normal_tissue	Comma-separated list of reference columns, or, if the groups input is present, the name of a sample group. The special value * means all columns of matrix2 if matrix2 is supplied; otherwise, all columns of matrix are used. May be empty if references are not used.
sided	twosided	For two-sided tests, one of "twosided", "greater" (target greater than reference) or "less" (target smaller than reference).
targetColumns	tumor_solid	Comma-separated list of target columns, or, if the groups input is present, the name of a sample group. The special value * means all columns of matrix.
test	t-test	Type of the statistical test. One of "t-test", "wilcoxon", "chi-squared", "fisher", "F-test", "shapiro" (Shapiro-Wilk), "ks" (Kolmogorov-Smirnov), "cor-pearson", "cor-spearman". Variants of the tests are set with other parameters (sided, paired).
threshold	0.05	P-value threshold for inclusion in the idlist output. Must be between 0 and 1 inclusive. If multiple hypothesis correction has been enabled, the threshold is for corrected p-values.

19.897 transcriptExpressionResults-detTable (TableQuery)

Execute an SQL query on input relations and create a result table. TableQuery uses HSQLDB (Hyperthreaded Structured Query Language Database [25]) for executing the query. Consequently, the syntax of the query is defined by HSQLDB.

Author: Kristian Ovaska (Kristian.Ovaska@Helsinki.FI)
Version: 1.2
Categories: convert, Filter

Input name	Source	Description
table1	transcriptExpressionResults-detIDList.ids 19.894	CSV table 1. The table is referred to as 'table1' in the SQL query.
table2	transcriptExpressionResults-detTTest.pvalues 19.896	CSV table 2. The table is referred to as 'table2' in the SQL query.
table3	transcriptExpressionResults-logratio.expr 19.917	CSV table 3. The table is referred to as 'table3' in the SQL query.
table4	transcriptExpressionResults-detAnnot.bioAnnotation 19.892	CSV table 4. The table is referred to as 'table4' in the SQL query.
query	transcriptExpressionResults-detTableQuery.in 19.898	SQL query. Either this file or the query parameter must be provided, but not both.

Parameter name	Value	Description
numIndices		Comma-separated list of index counts for input tables. All indices are single-column indices, running from column 1 to N, where N is retrieved from this parameter. If empty, the default number (1) is used. For example, ",2,,2" sets two indices for table2 and table4 and one index for the rest.
query		SQL query. Either this parameter or the query input must be provided, but not both.

19.898 transcriptExpressionResults-detTableQuery (INPUT)

SQL query to compile the differentially expressed transcript table.

Category: Internal

Parameter name	Value	Description
path	/gpfs/homes/mlaakso/asserSVN/andurilCaseStudy/sql/detTableQuery.sql	Path (filename) of the input resource.

19.899 transcriptExpressionResults-detVennDiagram (VennDiagram)

VennDiagram draws Venn diagrams showing the intersections of the given sets of identifiers.

Authors: Erkka Valo (erkka.valo@helsinki.fi), Kristian Ovaska (kristian.ovaska@helsinki.fi)
Version: 1.1.1
Category: Plot

Input name	Source	Description
sets	transcriptExpressionResults-det .result 19.891	Sets containing identifiers.

Parameter name	Value	Description
cex	0.4	A numerical value giving the amount by which the text in the plot should be scaled relative to the default values.
names1	T-test,over,under	Names of the sets to be used in the first Venn diagram. Names are given in a comma-separated list and mapped to sets in <code>sets1</code> positionally. If non are given, the set ID is used as set name.
names2		Names of the sets for the second Venn diagram.
names3		Names of the sets for the third Venn diagram.
names4		Names of the sets for the forth Venn diagram.
sectionTitle		Title for a new latex section. If non is given, a new section is not created.
sectionType	section	Type of LaTeX section: usually one of section, subsection or subsubsection. No section statement is written if sectionTitle is empty.
sets1	tumor_vs_normal_tTest,tumor_vs_normal_fcOver,tumor_vs_normal_fcUnder	A comma-separated list of minimum of two and maximum of three sets identified by the set IDs for which the Venn diagram is drawn.
sets2		Sets for the second Venn diagram.
sets3		Sets for the third Venn diagram.
sets4		Sets for the fourth Venn diagram.

19.900 transcriptExpressionResults-exonSPIArefs (INPUT)

(Technical documentation) Imports an external input file or directory to the pipeline. This is the only way external files are imported. File path is given with the parameter "path".

Category: Internal

Parameter name	Value	Description
path	/gpfs/homes/mlaakso/asserSVN/andurilCaseStudy/csv/exonSPIArefs.csv	Path (filename) of the input resource.

19.901 transcriptExpressionResults-filterSamplesRScript (INPUT)

R Script for filtering out samples without clinical annotation.

Category: Internal

Parameter name	Value	Description
path	/gpfs/homes/mlaakso/asserSVN/andurilCaseStudy/r/filterSamples.R	Path (filename) of the input resource.

19.902 transcriptExpressionResults-geneMapping (CSVFilter)

Filters columns and/or rows from CSV files using flexible criteria. Default settings do not filter out anything. The *negate* parameter reverses all inclusion criteria.

Columns are filtered based on column names (see *includeColumns*); it is also possible to rename columns. Columns can be reordered using *includeColumns*.

A row is included in output if all conditions are satisfied. Rows having too many missing values are filtered using the *nonMissing* parameter. The parameters *regexp*, *lowBound* and *highBound* filter rows based on cell contents. If the *auxiliary* input is given, the result contains those rows from the input whose values match those from the auxiliary file.

Authors: Kristian Ovaska (kristian.ovaska@helsinki.fi), Marko Laakso (Marko.Laakso@Helsinki.FI)
Version: 1.1
Category: Filter

Input name	Source	Description
csv	transcriptExpressionResults -transcriptAnnotation.in 19.934	CSV file to be filtered.

Parameter name	Value	Description
highBound		A row is included if the value in given numeric columns are at most as large as given numeric bounds. The parameter has a format COLNAME1=HIGH1,COLNAME2=HIGH2.
idColumn		Column name in "csv" that contains ID values. If the parameter is empty, the first column is used.
includeColumns	Transcript,Gene	Comma-separated list of column names that should be included in the result file. The special value * includes all columns. The order of column names is significant: the output columns are in the order given. See also includeColumns input.
lowBound		A row is included if the value in given numeric columns are at least as large as given numeric bounds. The parameter has a format COLNAME1=LOW1,COLNAME2=LOW2. For example, "col=5" includes rows where the column col has a value of at least 5.
matchColumn		Column name in "auxiliary" containing values that must match the ID column in "csv" (specified using "idColumn"). If empty, the first column of "auxiliary" is used.
negate	false	If true, all inclusion criteria are negated so that rows or columns are excluded instead of included if they match the criteria. For example, includeColumns=* then excludes all columns (which would be an error since at least one column must be included).
nonMissing	0	Include those rows that have non-missing (non-NA) values in at least this many columns. Only those columns that are part of the output are counted.
regexp		Row filtering based on regular expression. A row is included in the result if values in the given columns match given regular expressions. The parameter has a format COLNAME1=EXPRESSION,COLNAME2=EXPRESSION2 where COLNAMEs are column names in "csv" and EXPRESSIONs are regular expressions using Java syntax. For example, "col=a|b" includes rows where the column col has a value of "a" or "b". Commas have to be escaped with "\\" if they are used in column names or regular expressions.
rename		Comma-separated list of column renaming rules (OLDNAME=NEWNAME). Parameters that refer to column names refer to old names; renaming is done last.

19.903 transcriptExpressionResults-goClusters (GOClustering)

This Anduril component uses Gene ontology (GO) [1] annotations to cluster a set of genes. Each cluster consists of genes that share similar GO annotations.

The GO clustering of the genes can be visualized together with expression data (optional). Samples of the expression data are clustered based on their expression profiles and a heatmap is formed by clustering the y-axis of genes using their GO annotations. Component is based on the method descibed in [17].

Authors: Ping Chen (ping.chen@cs.helsinki.fi), Kristian Ovaska (Kristian.Ovaska@Helsinki.FI), Marko Laakso (Marko.Laakso@Helsinki.FI), Erkka Valo (Erkka.Valo@Helsinki.FI)
Version: 1.1.2
Categories: Clustering, GO

Input name	Source	Description
goAnnotations	transcriptExpressionResults-det Annot.bioAnnotation 19.892	GO annotations for the genes
expr	transcriptExpressionResults-t Expr.table 19.932	Gene expressions

Parameter name	Value	Description
ColumnWidth	15	Control the column width of the GO MEMBER table.
asBitmap	true	Produce heatmap in Portable network graphics (PNG) format instead of Portable document format (PDF). PNG bitmaps are more suitable if the heatmap contains large number of samples and genes.
columnMargin	0	Margin for column names in heatmap
cutLimit	0.5	Clustering cut off height for the hierachical clustering of the genes. This limit is between 0 and 1, where 0 refers to more specific groups.
decimal	3	Control the output of long decimal.
drawHeatmap	true	If true, draw a heatmap.
dropUnknown	true	Genes with no GO annotations are excluded if this flag is true
geneColumn		Name of the gene name column in the annotation file. The value is used for creating the report. The given column is used for listing all the members in the clusters to the report. If the value is empty, cluster member lists are not put into the report.
goColumn	GO	Name of the GO annotation column in the annotation file
grayScale	false	If true, heatmap is in grayscale. If false, heatmap is colorful.
heatmapScale	row	Determines how the values for the heatmap are scaled. Legal values are "row", "column" and "none". This corresponds to the scale parameter of the R function heatmap.
organism	9606	NCBI taxonomy ID for the organism whose gene set is used for GO probabilities. This is used if the input similarityTable is not given. Supported organisms: Homo sapiens: 9606, Saccharomyces cerevisiae: 4932, Caenorhabditis elegans: 6239, Drosophila melanogaster: 7227, Mus musculus: 10090, Rattus norvegicus: 10116.
pvalues	false	If true, compute p-values for information contents of the clusters using a permutation test. The p-values are shown in their own table in the report. The permutation test may be slow.
rowMargin	5	Margin for row names in heatmap
sectionType	subsection	Type of LaTeX section: usually one of section, subsection or subsubsection. No section statement is written if <code>sectionTitle</code> is an empty string.
showClusterNames	true	If true, show cluster names G1, ..., Gn on the right side of the heat map. If false, show gene names.
title	GO clustering	Title for the subsection and for the heatmap image.
xLab	Samples	Label for the x-axis of the heatmap
yLab	GO.groups	Label for the y-axis of the heatmap

19.904 transcriptExpressionResults-groupMeans (SampleCombiner)

Combine expression data from several samples into one by taking means, medians or log ratios. Combining is done using a sample group table. Each row of the table specifies how one sample group is created from a list of source groups. If the expression input file contains log ratios, both median and log ratio combining are possible and the result are log ratios. If the input are channel values, the groups may be either median groups (result type: channel values) or log ratio groups (result type: log ratio), but not both.

Authors: Kari Nousiainen (Kari.Nousiainen@Helsinki.FI), Kristian Ovaska (Kristian.Ovaska@Helsinki.FI)
Version: 1.0
Category: Filter

Input name	Source	Description
expr	transcriptExpressionResults-t Expr.table 19.932	Expression data, either log ratios or channel expressions.
groups	transcriptExpressionResults -sampleGroups.groups 19.923	Sample group specification.

Parameter name	Value	Description
geometricMean	true	Indicates whether the means will be calculated using geometric(true) or arithmetic mean (false).
groupIDs	tumor_solid,normal_tissue	Comma-separated list of sample group IDs. If given, combine only these groups. If not given (empty), combine all sample groups.

Continued on next page...

Parameter name	Value	Description
includeOriginal	false	If true, put input groups into the result even if they are not present in 'groups'.
thresholdRatio	0	Allows to omit those genes that have missing values in some samples. Only genes that have non-missing values in at least the given number of columns are included in the result. The actual threshold T is ceiling(thresholdRatio*n), where n is the number of samples that are used in combining. Only genes that have non-missing values in at least T columns are included. Other genes have the combined value set to NA. Note: this parameter specifies the ratio instead of integer threshold since the number of samples may be different in each sample group.

19.905 transcriptExpressionResults-intersectionTransformation (INPUT)

Intersection transformation of differentially expressed transcripts selected by fold change and differentialyy expressed transcripts selected by t-test.

Category: Internal

Parameter name	Value	Description
path	/gpfs/homes/mlaakso/asserSVN/ andurilCaseStudy/csv/ FoldChangeAndTTest.csv	Path (filename) of the input resource.

19.906 transcriptExpressionResults-km (TranscriptSurvival)

Survival analysis based on transcript expression.

Parameter name	Value
annotCol	.GeneId,.GeneName,.GeneDesc
fcThreshold	2.0
minCount	20
pLimit	0.05
title	Survival analysis for transcript expression

19.907 transcriptExpressionResults-km-km (KaplanMeier)

Produces a Kaplan-Meier plot representing survival estimates based on the given data. Log-rank statistics are calculated in order the address the similarity of the curves.

Authors: Marko Laakso (Marko.Laakso@Helsinki.FI), Riku Louhimo (Riku.Louhimo@Helsinki.FI), Kristian Ovaska (kristian.ovaska@helsinki.fi)
Version: 1.7
Categories: Survival, Latex

Input name	Source	Description
survival	transcriptExpressionResults-km -survivalTableConv.csv 19.916	Survival time and event indicators together with the sample group assignments
annotation	transcriptExpressionResults-det Annot.bioAnnotation 19.892	Additional annotations for the groupCol names. These annotations are listed as column name value pairs in figure caption.

Parameter name	Value	Description
PLimit	0.05	Ignore results with a p-value greater than this limit
annotCol	.GeneId,.GeneName,.GeneDesc	A comma separated list of column names of the annotation file that will be shown in the report. An asterisk (*) may be used for all attributes except the first one that is considered as a key.
confInt	0.95	Coverage of the confidence intervals [0,1] or a negative number to disable them
eventSymbol	DECEASED	Value of statusCol column indicating an event. Other values are considered as censorings.

Continued on next page...

Parameter name	Value	Description
excludeCol	SampleID,PatientID	A comma separated list of column names that should not be used as groupCols even if an asterisk is used. This is especially useful to filter out samples identifiers and other irrelevant columns.
groupCol	*	A comma separated list of column names of sample group identifiers or an empty string for a single curve. An asterisk (*) may be used to plot all attributes except those defined for the other purposes or listed in excludeCol.
minCount	20	Individual groups with less that this many members will be left out
statusCol	VitalStatus	Column name for the event indicator
timeCol	SurvivalEndTime	Column name for the survival time
timeOutLimit	36	Cut survival curves after this limit. A negative value can be used to maximize the length of the period.
title	Kaplan-Meier survival estimates	Figure title
xLabel	survival time	Label for the horizontal time axis

19.908 transcriptExpressionResults-km-kmReport (LatexCombiner)

LatexCombiner joins the given subdocuments together into one LaTeX file.

Authors: Kristian Ovaska (Kristian.Ovaska@Helsinki.FI), Marko Laakso (Marko.Laakso@Helsinki.FI)
Version: 1.3
Category: Latex

Input name	Source	Description
latex1	transcriptExpressionResults-km-km.report 19.907	LaTeX fragment 1.

Parameter name	Value	Description
pagebreak	true	Determines if the result document should start with a page break.
sectionTitle	Survival analysis for transcript expression	If non-empty, a declaration of a new section with the given name is inserted to the beginning of the combined document. This is a convenience feature to make it easy to compile subsections into a section.
sectionType	section	Type of LaTeX section: usually one of section, subsection or subsubsection. No section statement is written if sectionTitle is empty.
strictBorders	true	Enables the flushing of all document elements before each fragment

19.909 transcriptExpressionResults-km-sampleExpression (SampleExpression)

Create a $\{-1, 0, 1\}$ matrix that indicates whether a given gene/probe is differentially expressed in individual samples. The decision whether a gene is differentially expressed is done:

a) using fold change thresholding or
b) by selecting P largest and P smallest log ratios, where P is a proportion.

The expression input can be either log ratios or channel values, which are handled differently. Log ratios are directly used for fold change thresholding. Channel values are converted to log ratios by dividing them with reference samples. In this case, the expression matrix is assumed to contain both the target and reference samples. Reference samples are converted to a single column by taking a median. The columns that contain reference values are defined in the groups input. If the groups input is not given, values are assumed to be log ratios.

Author: Kristian Ovaska (kristian.ovaska@helsinki.fi)
Version: 1.0
Category: DEG

Input name	Source	Description
expr	transcriptExpressionResults-tExpr.table 19.932	Expression matrix. The values are assumed to be log ratios if the "groups" input is not given; otherwise, they are assumed to be channel values.

Continued on next page...

Input name	Source	Description
groups	transcriptExpressionResults -sampleGroups.groups 19.923	Sample groups that define reference (control) columns in the expression input. The reference group name is given with the referenceGroup parameter.

Parameter name	Value	Description
referenceGroup	normal_tissue	Group name in the groups input that contains reference samples. Used to compute log ratios from channel values.
threshold	2.0	Threshold that determines whether a gene is under- or overexpressed. Interpretation of the value depends on thresholdType.
thresholdType	fold-change	Defines how differential expression is decided. Legal values are "fold-change" and "top-most". For "fold-change", the threshold parameter is a linear fold change threshold. A gene is overexpressed if its log ratio is over the threshold and underexpressed if the log ratio is under 1/threshold. For "top-most", threshold is a fraction between 0 and 1 that defines how many genes with highest and lowest expression are selected. For example, if threshold is 0.1, the top 10% genes are overexpressed and the bottom 10% are underexpressed.

19.910 transcriptExpressionResults-km-sampleExpressionFilt (CSVFilter)

Filters columns and/or rows from CSV files using flexible criteria. Default settings do not filter out anything. The *negate* parameter reverses all inclusion criteria.

Columns are filtered based on column names (see *includeColumns*); it is also possible to rename columns. Columns can be reordered using *includeColumns*.

A row is included in output if all conditions are satisfied. Rows having too many missing values are filtered using the *nonMissing* parameter. The parameters *regexp*, *lowBound* and *highBound* filter rows based on cell contents. If the *auxiliary* input is given, the result contains those rows from the input whose values match those from the auxiliary file.

Authors: Kristian Ovaska (kristian.ovaska@helsinki.fi), Marko Laakso (Marko.Laakso@Helsinki.FI)
Version: 1.1
Category: Filter

Input name	Source	Description
csv	transcriptExpressionResults-km -sampleExpression .indicator 19.909	CSV file to be filtered.
auxiliary	transcriptExpressionResults-detID List.ids 19.894	If given, contains one column (see "matchColumn") whose values are matched to a column in the "csv" input (see "idColumn").

Parameter name	Value	Description
highBound		A row is included if the value in given numeric columns are at most as large as given numeric bounds. The parameter has a format COLNAME1=HIGH1,COLNAME2=HIGH2.
idColumn		Column name in "csv" that contains ID values. If the parameter is empty, the first column is used.
includeColumns	*	Comma-separated list of column names that should be included in the result file. The special value * includes all columns. The order of column names is significant: the output columns are in the order given. See also includeColumns input.
lowBound		A row is included if the value in given numeric columns are at least as large as given numeric bounds. The parameter has a format COLNAME1=LOW1,COLNAME2=LOW2. For example, "col=5" includes rows where the column col has a value of at least 5.
matchColumn		Column name in "auxiliary" containing values that must match the ID column in "csv" (specified using "idColumn"). If empty, the first column of "auxiliary" is used.
negate	false	If true, all inclusion criteria are negated so that rows or columns are excluded instead of included if they match the criteria. For example, includeColumns=* then excludes all columns (which would be an error since at least one column must be included).
nonMissing	0	Include those rows that have non-missing (non-NA) values in at least this many columns. Only those columns that are part of the output are counted.

Continued on next page...

Parameter name	Value	Description
regexp		Row filtering based on regular expression. A row is included in the result if values in the given columns match given regular expressions. The parameter has a format COLNAME1=EXPRESSION,COLNAME2=EXPRESSION2 where COLNAMEs are column names in "csv" and EXPRESSIONs are regular expressions using Java syntax. For example, "col=a|b" includes rows where the column col has a value of "a" or "b". Commas have to be escaped with "\\" if they are used in column names or regular expressions.
rename		Comma-separated list of column renaming rules (OLDNAME=NEWNAME). Parameters that refer to column names refer to old names; renaming is done last.

19.911 transcriptExpressionResults-km-sampleExpressionTrans (MatrixTranspose)

Produces a transposed version of the input matrix.

Author: Kristian Ovaska (kristian.ovaska@helsinki.fi)
Version: 1.0
Category: convert

Input name	Source	Description
matrix	transcriptExpressionResults-km -sampleExpressionFilt.csv 19.910	Input matrix.

Parameter name	Value	Description
rowName	SampleID	Name of the first column in the output.

19.912 transcriptExpressionResults-km-sampleNames (ExpandCollapse)

Converts between the two possible representations of a relations with multivalued columns. By default this component splits rows with multivalued (comma separated lists) columns into multiple rows each representing a single value. The input may contain several columns with comma separated values and all permutations of column values are shown as individual single-valued rows. The alternative mode recovers the original comma separated values from the expanded relations. This is performed by joining values of the their list columns into comma separated lists.

Author: Marko Laakso (Marko.Laakso@Helsinki.FI)
Version: 1.3
Category: convert

Input name	Source	Description
relation	transcriptExpressionResults -sampleGroups.groups 19.923	The mandatory input relation

Parameter name	Value	Description
delim	,	Value delimiter between the list column values. Special characters can be encoded as specified in fi.helsinki.ltdk.csbl.asser.ArgumentEncoding.
expand	true	Action mode that is expand (true) or collapse (false)
listCols	*	A comma separated list of column names of input (expand) or output (collapse) columns that may contain delim separated values. The asterisk refers to every column of the input relation.

19.913 transcriptExpressionResults-km-survival (TableQuery)

Execute an SQL query on input relations and create a result table. TableQuery uses HSQLDB (Hyperthreaded Structured Query Language Database [25]) for executing the query. Consequently, the syntax of the query is defined by HSQLDB.

Author: Kristian Ovaska (Kristian.Ovaska@Helsinki.FI)
Version: 1.2
Categories: convert, Filter

Input name	Source	Description
table1	transcriptExpressionResults-km -sampleNames.relation 19.912	CSV table 1. The table is referred to as 'table1' in the SQL query.
table2	transcriptExpressionResults -metadata.in 19.919	CSV table 2. The table is referred to as 'table2' in the SQL query.
query	transcriptExpressionResults-km -survivalQuery.in 19.914	SQL query. Either this file or the query parameter must be provided, but not both.

Parameter name	Value	Description
numIndices		Comma-separated list of index counts for input tables. All indices are single-column indices, running from column 1 to N, where N is retrieved from this parameter. If empty, the default number (1) is used. For example, ",2,,2" sets two indices for table2 and table4 and one index for the rest.
query		SQL query. Either this parameter or the query input must be provided, but not both.

19.914 transcriptExpressionResults-km-survivalQuery (INPUT)

Generates a table of samples and their survival times.

Category: Internal

Parameter name	Value	Description
path	/gpfs/homes/mlaakso/asserSVN/ andurilCaseStudy/sql/ exonSurvival.sql	Path (filename) of the input resource.

19.915 transcriptExpressionResults-km-survivalTable (CSVJoin)

Join rows from two or more CSV files, optionally using one column as a matching key. If a key column is not used, the result contains all rows and all columns of the input files. Missing values (NA) may be introduced when a column is not present in all input files. Each column is present once and duplicate rows are removed.

If a key column is used, the rows in each input file are matched using values from the key column. The result file has one row for each key value. In the result, the first column is the key column; its name is obtained from the first CSV input (csv1). If the intersection parameter is true, a key is included in the result if the key value is present in all inputs. If intersection is false, a key is included if it is present in at least one input (key union). Union semantics may introduce missing values in the result. If several input files have the same column, the value is obtained from the first file. However, if the first file contains a missing value and the second file contains a non-missing value, the non-missing value is used instead.

Author: Kristian Ovaska (kristian.ovaska@helsinki.fi)
Version: 1.0
Category: convert

Input name	Source	Description
csv1	transcriptExpressionResults-km -survival.table 19.913	CSV file 1.
csv2	transcriptExpressionResults-km -sampleExpressionTrans .matrix 19.911	CSV file 2.

Parameter name	Value	Description
intersection	true	Defines how keys are handled; only used when useKeys=true. If intersection is true, the result contains a key if the key is present in all input files. If false, the result contains a key if the key is present in at least one input file.
keyColumnNames		Comma-separated list of key column names; only used when useKeys=true. The first name refers to csv1, the second to csv2, etc. An empty value refers to the first column. Empty values may be omitted from the list, so all these are equivalent: "col1" ; "col1," ; "col1,," ; etc.
useKeys	true	If true, use one column from each CSV file as a matching key column.

19.916 transcriptExpressionResults-km-survivalTableConv (IDConvert)

Converts identifiers from a column in a CSV file to another type of identifiers. The conversion is done using an annotation table on the basis of the column named *conversionColumn*.

Author: Kristian Ovaska (kristian.ovaska@helsinki.fi)
Version: 1.0
Category: convert

Input name	Source	Description
csv	transcriptExpressionResults-km -survivalTable.csv 19.915	Source CSV file. Contains the column that is to be converted. The column is named by the parameter 'sourceColumn'.
conversionTable	transcriptExpressionResults-km -survival.table 19.913	Conversion table. Must contain the column named by the parameter 'conversionColumn'.

Parameter name	Value	Description
collapseNumeric	indicator	Method for collapsing numeric columns with duplicate rows (when unique=true). Legal values are "median" (take median of non-NA values), "mean", "max", "min", "first" (take the first row), "majority" (take the value that is present on the largest number of rows), "consensus" (require that all rows have the same value) and "indicator".<br>Here, "majority" and "consensus" are mostly useful for discrete (integer) values). For "consensus", it is an error if duplicate rows contain distinct values. "indicator" assumes that data are divided into negative (-1), neutral (0) and positive (1) items, such as discretized expression values. The result is (a) 1 if there is a 1 and no -1 value; (b) -1 if there is a -1 and not 1 value; (c) 0 if there is a 0 and no -1 or 1; or (d) NA otherwise.
collapseString	consensus	Method for collapsing non-numeric columns with duplicate rows (when unique=true). Legal values are "first" (take the first row), "majority" (take the value that is present on the largest number of rows), "longest" (take the longest value), "shortest" (take the shortest value) and "consensus" (require that all rows have the same value). For "consensus", it is an error if duplicate rows contain distinct values.
conversionColumn	PatientID	Column in 'conversionTable' that is used to fetch new values for the source column.
keyColumn		Column in 'conversionTable' that is used to match ID values from the source CSV. If the parameter is empty, the first column is used.
originalWhen Missing	true	Determines how values are converted when the item is not found in the conversion table. If true, the original (unconverted) ID is inserted to the output. If false, NA is inserted.
sourceColumn	SampleID	Column in 'csv' that is going to be converted. If empty, the first column is used.
split	false	If true, split values in the source column by commas before ID conversion. For example, if the source column contains the string "a,b,c", it is first split into "a", "b" and "c"; then, individual IDs are converted; finally, the converted IDs are joined by commas into a string of format "A,B,C".
splitConverted	false	If true, split converted values by commas after ID conversion. A row with comma separated values in the target column will be split to otherwise identical rows but so that the rows have a single ID value in the target column. This is done before possible collapsing of duplicate rows.

Continued on next page...

Parameter name	Value	Description
targetColumn		Target column name in 'converted'. If empty, the column name is the same as the original column (sourceColumn).
unique	true	If true, rows with identical identifiers (sourceColumn) are collapsed into one row so that sourceColumn is unique. Collapsing may result in error if duplicate rows contain conflicting values. See parameters collapseNumeric and collapseString for methods to collapse numeric and non-numeric columns, respectively.
varMeasure		Method for calculating the variation of numeric duplicate values. If the variation is above the threshold given in <code>varThreshold</code> , duplicate values are collapsed to NA. Possible values are 'MAD' and 'variance'. 'MAD' stands for the median absolute deviation and 'variance' for the population variance. If no values is given, no threshold for combining numeric values is used.
varThreshold	2	Maximum allowed variability in the duplicate values. If <code>varMeasure</code> is given duplicate values with variance above the threshold are collapsed to NA

19.917 transcriptExpressionResults-logratio (SampleCombiner)

Combine expression data from several samples into one by taking means, medians or log ratios. Combining is done using a sample group table. Each row of the table specifies how one sample group is created from a list of source groups. If the expression input file contains log ratios, both median and log ratio combining are possible and the result are log ratios. If the input are channel values, the groups may be either median groups (result type: channel values) or log ratio groups (result type: log ratio), but not both.

Authors: Kari Nousiainen (Kari.Nousiainen@Helsinki.FI), Kristian Ovaska (Kristian.Ovaska@Helsinki.FI)
Version: 1.0
Category: Filter

Input name	Source	Description
expr	transcriptExpressionResults-groupMeans.expr 19.904	Expression data, either log ratios or channel expressions.
groups	transcriptExpressionResults-sampleGroups.groups 19.923	Sample group specification.

Parameter name	Value	Description
geometricMean	true	Indicates whether the means will be calculated using geometric(true) or arithmetic mean (false).
groupIDs	tumor_vs_normal	Comma-separated list of sample group IDs. If given, combine only these groups. If not given (empty), combine all sample groups.
includeOriginal	false	If true, put input groups into the result even if they are not present in 'groups'.
thresholdRatio	0	Allows to omit those genes that have missing values in some samples. Only genes that have non-missing values in at least the given number of columns are included in the result. The actual threshold T is ceiling(thresholdRatio*n), where n is the number of samples that are used in combining. Only genes that have non-missing values in at least T columns are included. Other genes have the combined value set to NA. Note: this parameter specifies the ratio instead of integer threshold since the number of samples may be different in each sample group.

19.918 transcriptExpressionResults-mdsReport (MDSPlot)

MDSPlot plots multidimensional scaling on expression data.

This method is used in quality control to visualize similarity within classes and between classes. It works by comparing similarities in the expression values and then assigning them to a 2- or 3-dimensional space.

Author: Kari Nousiainen (Kari.Nousiainen@Helsinki.FI)
Version: 1.0
Categories: Quality Control, Latex

Input name	Source	Description
expr	transcriptExpressionResults-tExpr.table 19.932	Expression data. Can be either channel data or log ratios.
groups	transcriptExpressionResults-sampleGroups.groups 19.923	Sample group table maps samples to sample groups. The information is used in plotting to label the samples within the same group with the same color. Note that, a sample may include in many groups. However, the component use only groups whose type is either median or sample.

Parameter name	Value	Description
atomic	false	This parameters shows whether the samples groups are atomic or contain many samples.
caption	Multidimensional Scaling	Label of the figure.
cex	0.2	A numerical value giving the amount by which the size of the sample labels in the plot should be scaled relative to the default value.
dimensions	2	Number of dimensions the data is to be scaled into.
legendPosition	topright	Position of the legend box. One of "bottomright", "bottom", "bottomleft", "left", "topleft", "top", "topright", "right", "center" or "off". If "off", the legend is not shown.
pagebreak	false	Tells if the result document should start with a page break.
plotNames	true	If true sample names are plotted in the MDS plot, else circles are used.
sectionTitle	Multidimensional scaling of the samples	Title of the LaTeX section.
sectionType	subsection	Type of LaTeX section: usually one of section, subsection or subsubsection. If empty, no section statement is written.
width	16	Width of diagram in cm.

19.919 transcriptExpressionResults-metadata (INPUT)

Patient metadata for survival analysis.

Category: Internal

Parameter name	Value	Description
path	/gpfs/homes/mlaakso/asserSVN/andurilCaseStudy/data/patient-metadata.csv	Path (filename) of the input resource.

19.920 transcriptExpressionResults-qcReport (LatexCombiner)

LatexCombiner joins the given subdocuments together into one LaTeX file.

Authors: Kristian Ovaska (Kristian.Ovaska@Helsinki.FI), Marko Laakso (Marko.Laakso@Helsinki.FI)
Version: 1.3
Category: Latex

Input name	Source	Description
latex1	transcriptExpressionResults-cumDistReport.plot 19.890	LaTeX fragment 1.
latex2	transcriptExpressionResults-mdsReport.report 19.918	LaTeX fragment 2.

Parameter name	Value	Description
pagebreak	false	Determines if the result document should start with a page break.
sectionTitle		If non-empty, a declaration of a new section with the given name is inserted to the beginning of the combined document. This is a convenience feature to make it easy to compile subsections into a section.
sectionType	section	Type of LaTeX section: usually one of section, subsection or subsubsection. No section statement is written if sectionTitle is empty.
strictBorders	true	Enables the flushing of all document elements before each fragment

19.921 transcriptExpressionResults-reference (CSV2IDList)

Extracts one column from the given CSV file and prints it out without duplicates. The selected column may contain entries with comma separated lists of values in which case *isList* parameter may be used to obtain each value separately.

Output is defined as:

$$ids = \bigcup_{i=1}^{9} \pi_{columnIn_i} \sigma_{regexp_i}(table_i). \quad (32)$$

Author: Marko Laakso (Marko.Laakso@Helsinki.FI)
Version: 1.3
Category: convert

Input name	Source	Description
table1	transcriptExpressionResults-all Entrez.bioAnnotation 19.888	The mandatory input relation

Parameter name	Value	Description
acceptMissing	false	Files with missing columnIn are accepted as empty if this is true.
columnIn	EntrezGene	A comma separated list of column names for the IDs of interest in each file. Empty values refer to the first column of the file.
columnOut		Name of the only column of the output list. Empty input refers to the name of the input column.
constants		A comma separated list of values that are always included into the output
isList	false	True if the seleted column contains a comma separated list of values to be splitted
quotation	false	Indicator that can be used to disable quotation of the output values
regexp1		Regular expression for the row filtering in table1. A row is included in the result if this parameter is empty or if values in the given columns match given regular expressions. The parameter has a format COLNAME1=EXPRESSION,COLNAME2=EXPRESSION2 where COLNAMEs are column names in "csv" and EXPRESSIONs are regular expressions using Java syntax. For example, "col=a|b" includes rows where the column col has a value of "a" or "b".
regexp2		Regular expression for the row filtering in table2.
regexp3		Regular expression for the row filtering in table3.
regexp4		Regular expression for the row filtering in table4.
regexp5		Regular expression for the row filtering in table5.
regexp6		Regular expression for the row filtering in table6.
regexp7		Regular expression for the row filtering in table7.
regexp8		Regular expression for the row filtering in table8.
regexp9		Regular expression for the row filtering in table9.

19.922 transcriptExpressionResults-report (LatexCombiner)

LatexCombiner joins the given subdocuments together into one LaTeX file.

Authors: Kristian Ovaska (Kristian.Ovaska@Helsinki.FI), Marko Laakso (Marko.Laakso@Helsinki.FI)
Version: 1.3
Category: Latex

Input name	Source	Description
latex1	transcriptExpressionResults-det Report.summary 19.895	LaTeX fragment 1.
latex2	transcriptExpressionResults-det VennDiagram.report 19.899	LaTeX fragment 2.
latex3	transcriptExpressionResults-qc Report.document 19.920	LaTeX fragment 3.
latex4	transcriptExpressionResults-go Clusters.report 19.903	LaTeX fragment 4.
latex5	transcriptExpressionResults-km -kmReport.document 19.908	LaTeX fragment 5.

Continued on next page...

Input name	Source	Description
latex6	transcriptExpressionResults-spia Report.report 19.926	LaTeX fragment 6.

Parameter name	Value	Description
pagebreak	false	Determines if the result document should start with a page break.
sectionTitle		If non-empty, a declaration of a new section with the given name is inserted to the beginning of the combined document. This is a convenience feature to make it easy to compile subsections into a section.
sectionType	section	Type of LaTeX section: usually one of section, subsection or subsubsection. No section statement is written if sectionTitle is empty.
strictBorders	true	Enables the flushing of all document elements before each fragment

19.923 transcriptExpressionResults-sampleGroups (SampleGroupCreator)

Creates sample group tables based on sample names read from data files. These tables define relationship between samples. For example, which samples are biological or technical replicates. The list of sample names is read from data files (either column names or one column along rows) and sample groups are defined using regular expressions. Also, constant groups where the set of member samples does not depend on data can be created. The type of each definition pattern is given with *patternTypes*, which is one of *re*, *relist* or *verbatim* for each pattern.

Author:	Kristian Ovaska (kristian.ovaska@helsinki.fi)
Version:	1.2.1
Category:	preprocessing

Input name	Source	Description
data1	transcriptExpressionResults-t Expr.table 19.932	Data file 1 for reading sample names. Either column names or values on one column contain sample names.

Parameter name	Value	Description
columns		Defines which columns in input files contain sample names, or whether column names should be used (default). This parameter is a comma-separated list of at most three values which name columns in inputs data1 to data3. If an entry is empty, the column names of the corresponding input file are used instead.
definition1	tumor_solid,mean,Solid tumor samples	Definition of group 1. If empty, the group is omitted. Format: NAME,TYPE or NAME,TYPE,DESCRIPTION.
definition2	normal_tissue,mean,Normal tissue samples	Definition of group 2. If empty, the group is omitted.
definition3	tumor_vs_normal,ratio,fold change of tumor median vs. normal median	Definition of group 3. If empty, the group is omitted.
definition4		Definition of group 4. If empty, the group is omitted.
definition5		Definition of group 5. If empty, the group is omitted.
definition6		Definition of group 6. If empty, the group is omitted.
definition7		Definition of group 7. If empty, the group is omitted.
definition8		Definition of group 8. If empty, the group is omitted.
definition9		Definition of group 9. If empty, the group is omitted.
pattern1	TCGA_[0-9]{2}_[0-9]{4}_01	Pattern for group 1. If empty, the group is omitted. Format depends on the pattern type.
pattern2	TCGA_[0-9]{2}_[0-9]{4}_11	Pattern for group 2. If empty, the group is omitted.
pattern3	tumor_solid,normal_tissue	Pattern for group 3. If empty, the group is omitted.
pattern4		Pattern for group 4. If empty, the group is omitted.
pattern5		Pattern for group 5. If empty, the group is omitted.
pattern6		Pattern for group 6. If empty, the group is omitted.
pattern7		Pattern for group 7. If empty, the group is omitted.
pattern8		Pattern for group 8. If empty, the group is omitted.
pattern9		Pattern for group 9. If empty, the group is omitted.
patternTypes	re,re,verbatim	Comma-separated list of pattern types for each pattern. Each item is one of "re" (default), "relist" or "verbatim". The types are explained above. An empty value is interpreted as "re". For example, ",re,relist,,verbatim" specifies that pattern3 has the type "relist" and pattern5 the type "verbatim"; all others (including pattern6 and above) have the type "re".

19.924 transcriptExpressionResults-samplesWithoutClinicalAnnotation (INPUT)

List of samples without clinical annotation.

Category: Internal

Parameter name	Value	Description
path	/gpfs/homes/mlaakso/asserSVN/andurilCaseStudy/csv/samplesWithoutClinicalAnnotation.csv	Path (filename) of the input resource.

19.925 transcriptExpressionResults-spia (SPIA)

SPIA implements the Signaling Pathway Impact Analysis (SPIA) algorithm described in [24] and [6]. SPIA uses the information from a set of differentially expressed genes and their fold changes, as well as pathways topology in order to assess the significance of the pathways in the condition under the study. The current version of SPIA includes pathway data for 61 KEGG human signaling pathways.

Table 114: Columns of the pathways output.

	column	description
1	Name	Name of the pathway
2	ID	KEGG ID of the pathway (eg. hsa04612)
3	pSize	Number of genes in the pathway
4	NDE	Number of differentially expressed genes in the pathway
5	tA	Observed total perturbation accumulation in the pathway
6	pNDE	Probability of observing at least NDE differentially expressed genes on the pathway by chance
7	pPERT	Probability of of observing a total perturbation accumulation more extreme than tA by chance
8	pG	Global p-value obtained by combining pNDE and pPERT
9	pGFdr	False Discovery Rate adjusted global p-value
10	pFWER	Bonferroni adjusted global p-value
11	Status	Direction in which pathway is perturbed (activated or inhibited)

Author: Viljami Aittomaki (viljami.aittomaki@helsinki.fi)
Version: 1.0.1
Category: Pathway

Input name	Source	Description
deg	transcriptExpressionResults-survLogratioEntrezFilt.csv 19.931	An expression matrix containing log2 fold-changes for differentially expressed genes (DEG's). Gene ID's must be Entrez Gene ID's! Genes with fold-change 'NA' are removed.
reference	transcriptExpressionResults-reference.ids 19.921	An IDList containing the Entrez ID's in the reference set. If the data was obtained from a microarray experiment, this list will contain all genes present on the specific array used for the experiment. This vector must contain all names of the DEG's in the deg input.

Parameter name	Value	Description
foldChangeColumn		Name of the column in deg that has wanted input fold-change values. If an empty string defaults to the second column in deg (first numerical column).
nboot	1000	Number of bootstrap samples used in computing pPERT. Should be larger than 100. A recommended value is 2000.
organism	hsa	KEGG organism code corresponding to input genes (e.g., hsa=homo sapiens).
verbose	false	If true, report progress (number of pathways already analyzed reported to stdout).

19.926 transcriptExpressionResults-spiaReport (CSV2Latex)

This component converts a tabular CSV file into a printable LaTeX table.

Author: Marko Laakso (Marko.Laakso@Helsinki.FI)
Version: 2.1
Categories: convert, Latex

Input name	Source	Description
tabledata	transcriptExpressionResults-spia .pathways 19.925	Table content
refs	transcriptExpressionResults-exon SPIArefs.in 19.900	Reference rules for the hyperlinks

Parameter name	Value	Description
attach	false	Include the original data as an attachment
caption	SPIA analysis ~\cite{Tarca2008}. Name is the KEGG name for the pathway, and ID is the KEGG internal identication for the pathway. pSize is total number of genes in the pathway according to KEGG, where as NDE is the number of diffentially expressed genes on the pathway. pNDE is the p-value to observe NDE number of genes on the pathway by chance. tA is a perturbation factor defined by SPIA algorithm and pPERT is the probability of observing a more intense perturbation than tA by chance. pG is the combination of pPERT and pNDE. pGFdr is the false discovery rate and pGFWER the Bonferoni corrected p-value.	Caption text for the table.
colFormat	center	LaTeX tabular format for the columns. Default produces a centered layout for all columns.
columns	Name,ID,pSize,NDE,tA,pNDE, pPERT,pG,pGFdr,pGFWER, Status	Comma separated list of column selections for the output. The empty default will use all columns.
countRows	false	Include a row count to the table caption.
dropMissing	true	This flag can be turned off in order to generate links with missing texts. Link text are substituted with target identifiers.
listCols		Comma separated list of column names. Columns of this list may contain several values separated with commas and the delimiters will be replaced with spaces.
numberFormat	tA=#0.0000,pNDE=#0.0000, pG=#0.0000,pGFdr=#0.0000, pGFWER=#0.0000	A comma separated list of decimal formats for the columns. Each entry consists of the column name and the Java DecimalFormal pattern separated with equal sign. For example, rounding to three decimals can be done like: myColumn=#0.000
pageBreak	true	Use clear page after the table.
rename		Comma separated list of column renaming rules (oldname=newname). New names are used in table header but they do not affect the other behaviour of this component.
ruler	{}	Latex command for the row separating rulers
section	Signalling pathway impact analysis for differentially expressed transcripts with survival p $<$ 0.01	Section title for the table container or an empty string if no section should be generated.
sectionType	section	Type of LaTeX section: usually one of: section, subsection, or subsubsection. No section statement is written if section title is empty.
skipEmpty	false	This flag can be used to replace empty tables with a simple LaTeX comment.

19.927 transcriptExpressionResults-survGenes (CSVTransformer)

Transform CSV files using R [20] expressions. This allows to apply arithmetic functions to numeric columns and to combine columns from different CSV files. The R expressions are evaluated and are expected to return R matrices, data frames or vectors that are concatenated to a final result. Concatenations is done on columns, so each transformation creates additional columns to the output. Transformations should create items having the same number of rows. However, the expression may yield a single string or number that is duplicated to fit the number of rows.

Author: Kristian Ovaska (kristian.ovaska@helsinki.fi)
Version: 1.0
Category: preprocessing

Input name	Source	Description
csv1	transcriptExpressionResults-km -km.statistics 19.907	Input file 1.

Parameter name	Value	Description
columnNames		R expression that evaluates to the column names of the result CSV file. The evaluated vector must have the same number of items as there are columns in the output. If empty, column names are taken from the input CSV files; depending on the transforms, some column names may be automatically generated.
transform1	csv1[csv1$pValue < 0.01, 'group']	R expression that evaluates to a matrix, data frame, vector or constant. The expression may refer to data frames "csv1" and "csv2" (only if csv2 is given) and matrices "matrix1" and "matrix2" (only if csv2 is given).
transform2		Transformation expression 2. If empty, no transformation is done.
transform3		Transformation expression 3. If empty, no transformation is done.
transform4		Transformation expression 4. If empty, no transformation is done.
transform5		Transformation expression 5. If empty, no transformation is done.
transform6		Transformation expression 6. If empty, no transformation is done.
transform7		Transformation expression 7. If empty, no transformation is done.
transform8		Transformation expression 8. If empty, no transformation is done.
transform9		Transformation expression 9. If empty, no transformation is done.

19.928 transcriptExpressionResults-survGenesEntrez (KorvasieniAnnotator)

Korvasieni is an Ensembl [12] based converter for database identifiers and it can be used to convert genome location, gene, transcript, and translation identifiers across multiple biological databases. The selected `keyColumn` may contain entries with comma separated lists of values in which case `isListKey` parameter may be used to obtain each value separately and to process them as they were given in consecutive lines.

Author:	Marko Laakso (Marko.Laakso@Helsinki.FI)
Version:	1.7
Categories:	Annotation, GO

Input name	Source	Description
sourceKeys	transcriptExpressionResults-surv Genes.transformed 19.927	A list of source database keys.
connection	ensembl.in 19.273	Database connection can be defined using this file. The definition of parameters: database.url, database.user, database.password, database.timeout, database.recycle, and database.driver can be found from the documentation of Korvasieni.

Parameter name	Value	Description
echoColumns		A comma separated list of column names for the columns that will be copied to the output. An asterisk (*) can be used to denote all columns execpt the keyColumn.
inputDB	.TranscriptId	Type of input keys. This must be a database supported by Korvasieni. If the parameter is omitted, the component tries to derive the database from the type of geneID. If this is not possible, an error is returned. You may define three columns in form of chromosome:start-end in case the inputDB is .DNARegion. This format provides a comfortable compatibility with DNARegion datatype.
inputType	Any	Ensembl object type for the input keys (Any, Gene, Transcript, Translation)
isListKey	false	Enables the automatic value splits for the comma separated key column
keyColumn		Name of the key column withing sourceKeys file or an empty string for the first column
maxHits	1	Maximum number of target identifiers for a single source identifier
rename		Comma separated list of column renaming rules (oldname=newname)
skipLevel	never	Skip result rows if the source identifier is unknown or target identifiers are not available. Possible values are: never (no filtering), source (skip if the source ID is unknown), target (skip if no target IDs are found), any (skip if any of the target IDs is missing).
targetDB	_EntrezGene	Comma-separated list of annotation types. Possible values are all databases supported by Korvasieni.
unique	false	This flag can be turned on in order to eliminate duplicate annotations.

19.929 transcriptExpressionResults-survLogratio (CSVFilter)

Filters columns and/or rows from CSV files using flexible criteria. Default settings do not filter out anything. The *negate* parameter reverses all inclusion criteria.

Columns are filtered based on column names (see *includeColumns*); it is also possible to rename columns. Columns can be reordered using *includeColumns*.

A row is included in output if all conditions are satisfied. Rows having too many missing values are filtered using the *nonMissing* parameter. The parameters *regexp*, *lowBound* and *highBound* filter rows based on cell contents. If the *auxiliary* input is given, the result contains those rows from the input whose values match those from the auxiliary file.

Authors: Kristian Ovaska (kristian.ovaska@helsinki.fi), Marko Laakso (Marko.Laakso@Helsinki.FI)
Version: 1.1
Category: Filter

Input name	Source	Description
csv	transcriptExpressionResults -logratio.expr 19.917	CSV file to be filtered.
auxiliary	transcriptExpressionResults-surv Genes.transformed 19.927	If given, contains one column (see "matchColumn") whose values are matched to a column in the "csv" input (see "idColumn").

Parameter name	Value	Description
highBound		A row is included if the value in given numeric columns are at most as large as given numeric bounds. The parameter has a format COLNAME1=HIGH1,COLNAME2=HIGH2.
idColumn		Column name in "csv" that contains ID values. If the parameter is empty, the first column is used.
includeColumns	*	Comma-separated list of column names that should be included in the result file. The special value * includes all columns. The order of column names is significant: the output columns are in the order given. See also includeColumns input.
lowBound		A row is included if the value in given numeric columns are at least as large as given numeric bounds. The parameter has a format COLNAME1=LOW1,COLNAME2=LOW2. For example, "col=5" includes rows where the column col has a value of at least 5.
matchColumn		Column name in "auxiliary" that must match the ID column in "csv" (specified using "idColumn"). If empty, the first column of "auxiliary" is used.
negate	false	If true, all inclusion criteria are negated so that rows or columns are excluded instead of included if they match the criteria. For example, includeColumns=* then excludes all columns (which would be an error since at least one column must be included).
nonMissing	0	Include those rows that have non-missing (non-NA) values in at least this many columns. Only those columns that are part of the output are counted.
regexp		Row filtering based on regular expression. A row is included in the result if values in the given columns match given regular expressions. The parameter has a format COLNAME1=EXPRESSION,COLNAME2=EXPRESSION2 where COLNAMEs are column names in "csv" and EXPRESSIONs are regular expressions using Java syntax. For example, "col=a|b" includes rows where the column col has a value of "a" or "b". Commas have to be escaped with "\\" if they are used in column names or regular expressions.
rename		Comma-separated list of column renaming rules (OLDNAME=NEWNAME). Parameters that refer to column names refer to old names; renaming is done last.

19.930 transcriptExpressionResults-survLogratioEntrez (IDConvert)

Converts identifiers from a column in a CSV file to another type of identifiers. The conversion is done using an annotation table on the basis of the column named *conversionColumn*.

Author: Kristian Ovaska (kristian.ovaska@helsinki.fi)
Version: 1.0
Category: convert

Input name	Source	Description
csv	transcriptExpressionResults-survLogratio.csv 19.929	Source CSV file. Contains the column that is to be converted. The column is named by the parameter 'sourceColumn'.
conversionTable	transcriptExpressionResults-survGenesEntrez.bioAnnotation 19.928	Conversion table. Must contain the column named by the parameter 'conversionColumn'.

Parameter name	Value	Description
collapseNumeric	median	Method for collapsing numeric columns with duplicate rows (when unique=true). Legal values are "median" (take median of non-NA values), "mean", "max", "min", "first" (take the first row), "majority" (take the value that is present on the largest number of rows), "consensus" (require that all rows have the same value) and "indicator".<br>Here, "majority" and "consensus" are mostly useful for discrete (integer) values). For "consensus", it is an error if duplicate rows contain distinct values. "indicator" assumes that data are divided into negative (-1), neutral (0) and positive (1) items, such as discretized expression values. The result is (a) 1 if there is a 1 and no -1 value; (b) -1 if there is a -1 and not 1 value; (c) 0 if there is a 0 and no -1 or 1; or (d) NA otherwise.
collapseString	consensus	Method for collapsing non-numeric columns with duplicate rows (when unique=true). Legal values are "first" (take the first row), "majority" (take the value that is present on the largest number of rows), "longest" (take the longest value), "shortest" (take the shortest value) and "consensus" (require that all rows have the same value). For "consensus", it is an error if duplicate rows contain distinct values.
conversionColumn	EntrezGene	Column in 'conversionTable' that is used to fetch new values for the source column.
keyColumn		Column in 'conversionTable' that is used to match ID values from the source CSV. If the parameter is empty, the first column is used.
originalWhenMissing	false	Determines how values are converted when the item is not found in the conversion table. If true, the original (unconverted) ID is inserted to the output. If false, NA is inserted.
sourceColumn		Column in 'csv' that is going to be converted. If empty, the first column is used.
split	false	If true, split values in the source column by commas before ID conversion. For example, if the source column contains the string "a,b,c", it is first split into "a", "b" and "c"; then, individual IDs are converted; finally, the converted IDs are joined by commas into a string of format "A,B,C".
splitConverted	false	If true, split converted values by commas after ID conversion. A row with comma separated values in the target column will be split to otherwise identical rows but so that the rows have a single ID value in the target column. This is done before possible collapsing of duplicate rows.
targetColumn		Target column name in 'converted'. If empty, the column name is the same as the original column (sourceColumn).
unique	true	If true, rows with identical identifiers (sourceColumn) are collapsed into one row so that sourceColumn is unique. Collapsing may result in error if duplicate rows contain conflicting values. See parameters collapseNumeric and collapseString for methods to collapse numeric and non-numeric columns, respectively.
varMeasure		Method for calculating the variation of numeric duplicate values. If the variation is above the threshold given in <code>varThreshold</code> , duplicate values are collapsed to NA. Possible values are 'MAD' and 'variance'. 'MAD' stands for the median absolute deviation and 'variance' for the population variance. If no values is given, no threshold for combining numeric values is used.
varThreshold	2	Maximum allowed variability in the duplicate values. If <code>varMeasure</code> is given duplicate values with variance above the threshold are collapsed to NA

19.931 transcriptExpressionResults-survLogratioEntrezFilt (CSVFilter)

Filters columns and/or rows from CSV files using flexible criteria. Default settings do not filter out anything. The *negate* parameter reverses all inclusion criteria.

Columns are filtered based on column names (see *includeColumns*); it is also possible to rename columns. Columns can be reordered using *includeColumns*.

A row is included in output if all conditions are satisfied. Rows having too many missing values are filtered using the *nonMissing* parameter. The parameters *regexp*, *lowBound* and *highBound* filter rows based on cell contents. If the *auxiliary* input is given, the result contains those rows from the input whose values match those from the auxiliary file.

Authors: Kristian Ovaska (kristian.ovaska@helsinki.fi), Marko Laakso (Marko.Laakso@Helsinki.FI)
Version: 1.1
Category: Filter

Input name	Source	Description
csv	transcriptExpressionResults-survLogratioEntrez.csv 19.930	CSV file to be filtered.

Parameter name	Value	Description
highBound		A row is included if the value in given numeric columns are at most as large as given numeric bounds. The parameter has a format COLNAME1=HIGH1,COLNAME2=HIGH2.
idColumn		Column name in "csv" that contains ID values. If the parameter is empty, the first column is used.
includeColumns	*	Comma-separated list of column names that should be included in the result file. The special value * includes all columns. The order of column names is significant: the output columns are in the order given. See also includeColumns input.
lowBound		A row is included if the value in given numeric columns are at least as large as given numeric bounds. The parameter has a format COLNAME1=LOW1,COLNAME2=LOW2. For example, "col=5" includes rows where the column col has a value of at least 5.
matchColumn		Column name in "auxiliary" containing values that must match the ID column in "csv" (specified using "idColumn"). If empty, the first column of "auxiliary" is used.
negate	false	If true, all inclusion criteria are negated so that rows or columns are excluded instead of included if they match the criteria. For example, includeColumns=* then excludes all columns (which would be an error since at least one column must be included).
nonMissing	2	Include those rows that have non-missing (non-NA) values in at least this many columns. Only those columns that are part of the output are counted.
regexp		Row filtering based on regular expression. A row is included in the result if values in the given columns match given regular expressions. The parameter has a format COLNAME1=EXPRESSION,COLNAME2=EXPRESSION2 where COLNAMEs are column names in "csv" and EXPRESSIONs are regular expressions using Java syntax. For example, "col=a|b" includes rows where the column col has a value of "a" or "b". Commas have to be escaped with "\\" if they are used in column names or regular expressions.
rename		Comma-separated list of column renaming rules (OLDNAME=NEWNAME). Parameters that refer to column names refer to old names; renaming is done last.

19.932 transcriptExpressionResults-tExpr (REvaluate)

This component produces some outputs by executing an R [20] script with the given input data. The exact functionality of this component depends on the given script.

The component is also capable of making branch decisions on Anduril workflow. Two alternative options (*altCase* and *defCase*) are provided.

Authors: Marko Laakso (Marko.Laakso@Helsinki.FI), Erkka Valo (erkka.valo@tkk.fi)
Version: 2.2
Categories: External, Latex, Network Control

Input name	Source	Description
script	transcriptExpressionResults-filterSamplesRScript.in 19.901	Data transformation
table1	transcriptExpressionResults-tExprTemp.csv 19.933	Data matrix 1 (data.frame/file table1)
table2	transcriptExpressionResults-samplesWithoutClinicalAnnotation.in 19.924	Data matrix 2 (data.frame/file table2)

Parameter name	Value	Description
asConnection	false	If true, assign file connections (class file) to the inputs to variables of the same name. This is useful e.g. if you want to read large data files row by row.
param1		Value of this parameter will be assigned to param1 variable
param10		Value of this parameter will be assigned to param10 variable
param2		Value of this parameter will be assigned to param2 variable
param3		Value of this parameter will be assigned to param3 variable
param4		Value of this parameter will be assigned to param4 variable
param5		Value of this parameter will be assigned to param5 variable
param6		Value of this parameter will be assigned to param6 variable
param7		Value of this parameter will be assigned to param7 variable
param8		Value of this parameter will be assigned to param8 variable
param9		Value of this parameter will be assigned to param9 variable

19.933 transcriptExpressionResults-tExprTemp (IDConvert)

Converts identifiers from a column in a CSV file to another type of identifiers. The conversion is done using an annotation table on the basis of the column named *conversionColumn*.

Author: Kristian Ovaska (kristian.ovaska@helsinki.fi)
Version: 1.0
Category: convert

Input name	Source	Description
csv	transcriptExpression.in 19.886	Source CSV file. Contains the column that is to be converted. The column is named by the parameter 'sourceColumn'.
conversionTable	transcriptAnnotation.in 19.885	Conversion table. Must contain the column named by the parameter 'conversionColumn'.

Parameter name	Value	Description
collapseNumeric	median	Method for collapsing numeric columns with duplicate rows (when unique=true). Legal values are "median" (take median of non-NA values), "mean", "max", "min", "first" (take the first row), "majority" (take the value that is present on the largest number of rows), "consensus" (require that all rows have the same value) and "indicator".<br>Here, "majority" and "consensus" are mostly useful for discrete (integer) values). For "consensus", it is an error if duplicate rows contain distinct values. "indicator" assumes that data are divided into negative (-1), neutral (0) and positive (1) items, such as discretized expression values. The result is (a) 1 if there is a 1 and no -1 value; (b) -1 if there is a -1 and not 1 value; (c) 0 if there is a 0 and no -1 or 1; or (d) NA otherwise.
collapseString	consensus	Method for collapsing non-numeric columns with duplicate rows (when unique=true). Legal values are "first" (take the first row), "majority" (take the value that is present on the largest number of rows), "longest" (take the longest value), "shortest" (take the shortest value) and "consensus" (require that all rows have the same value). For "consensus", it is an error if duplicate rows contain distinct values.
conversionColumn	Transcript	Column in 'conversionTable' that is used to fetch new values for the source column.
keyColumn	Index	Column in 'conversionTable' that is used to match ID values from the source CSV. If the parameter is empty, the first column is used.
originalWhen Missing	true	Determines how values are converted when the item is not found in the conversion table. If true, the original (unconverted) ID is inserted to the output. If false, NA is inserted.
sourceColumn	Index	Column in 'csv' that is going to be converted. If empty, the first column is used.
split	false	If true, split values in the source column by commas before ID conversion. For example, if the source column contains the string "a,b,c", it is first split into "a", "b" and "c"; then, individual IDs are converted; finally, the converted IDs are joined by commas into a string of format "A,B,C".
splitConverted	false	If true, split converted values by commas after ID conversion. A row with comma separated values in the target column will be split to otherwise identical rows but so that the rows have a single ID value in the target column. This is done before possible collapsing of duplicate rows.
targetColumn	.TranscriptId	Target column name in 'converted'. If empty, the column name is the same as the original column (sourceColumn).
unique	false	If true, rows with identical identifiers (sourceColumn) are collapsed into one row so that sourceColumn is unique. Collapsing may result in error if duplicate rows contain conflicting values. See parameters collapseNumeric and collapseString for methods to collapse numeric and non-numeric columns, respectively.

Continued on next page...

Parameter name	Value	Description
varMeasure		Method for calculating the variation of numeric duplicate values. If the variation is above the threshold given in <code>varThreshold</code> , duplicate values are collapsed to NA. Possible values are 'MAD' and 'variance'. 'MAD' stands for the median absolute deviation and 'variance' for the population variance. If no values is given, no threshold for combining numeric values is used.
varThreshold	2	Maximum allowed variability in the duplicate values. If <code>varMeasure</code> is given duplicate values with variance above the threshold are collapsed to NA

19.934 transcriptExpressionResults-transcriptAnnotation (INPUT)

Transcript expression annotations.

Category: Internal

Parameter name	Value	Description
path	/gpfs/homes/mlaakso/asserSVN/andurilCaseStudy/data/exon-normalized/Expression-Exon/LBL_HuEx-1_0-st-v2/Level_2/TranscriptExprAnnotation_v56	Path (filename) of the input resource.

19.935 transcriptExpressionResults-transcriptStatisticsQuery (INPUT)

Transcript result data processing.

Category: Internal

Parameter name	Value	Description
path	/gpfs/homes/mlaakso/asserSVN/andurilCaseStudy/sql/TranscriptStatistics.sql	Path (filename) of the input resource.

19.936 R configuration

base [21]

Version	2.11.0
Priority	base
Title	The R Base Package
Author	R Development Core Team and contributors worldwide
Maintainer	R Core Team <R-core@r-project.org>
Description	Base R functions
License	Part of R 2.11.0
Built	R 2.11.0; ; 2010-04-22 22:22:16 UTC; unix

csbl.go [16]

Version	1.3.4
Date	2010-04-19
Title	Semantic similarity based on Gene Ontology
Author	Kristian Ovaska <kristian.ovaska@helsinki.fi>, Marko Laakso <Marko.Laakso@Helsinki.FI>
Maintainer	Kristian Ovaska <kristian.ovaska@helsinki.fi>
Depends	methods, cluster, grDevices, Biobase, GO.db, annotate, RUnit
Description	Compute semantic similarity for genes and proteins using Gene Ontology annotations.
License	GPL
Packaged	2010-04-19 15:07:03 UTC; kristian
Built	R 2.10.1; x86_64-pc-linux-gnu; 2010-04-19 15:26:32 UTC; unix

DNAcopy [22]

Title	DNA copy number data analysis
Version	1.22.1
Author	Venkatraman E. Seshan, Adam Olshen
Description	Segments DNA copy number data using circular binary segmentation to detect regions with abnormal copy number
Maintainer	Venkatraman E. Seshan <seshanv@mskcc.org>
biocViews	Microarray, CopyNumberVariants
LazyData	yes
License	GPL (>= 2)
Packaged	2010-05-15 20:14:55 UTC; biocbuild
Built	R 2.11.0; x86_64-pc-linux-gnu; 2010-05-19 07:26:07 UTC; unix

GTS [5]

Type	Package
Title	A package for ranking chromosomal segments
Version	1.1.0
Date	2008-02-01
Author	Cameron Brennan
Maintainer	Cameron Brennan<brennanc2@mskcc.org>
Description	The package processes array-CGH or SNP-derived chromosomal copy number data and provides two complementary indices for each point in the genome - ARI and AFI - that can be used to rank chromosomal regions
biocViews	Microarray
ZipData	no
LazyLoad	Yes
License	The Artistic License, Version 2.0
Packaged	Wed Jun 11 08:16:07 2008; brennan
Built	R 2.10.0; ; 2009-11-27 06:20:12 UTC; unix

19.937 System configurations

The following table shows the properties of **ensembl** component.

property	value
`database.driver`	com.mysql.jdbc.Driver
`database.recycle`	true
`database.timeout`	20
`database.url`	jdbc:mysql://deebee.giu.fi:3306/homo_sapiens_core_58_37c
`database.user`	anonymous

The following table shows the properties of **snpdb** component.

property	value
database.driver	com.mysql.jdbc.Driver
database.recycle	true
database.timeout	20
database.url	jdbc:mysql://ensembldb.ensembl.org:5306/homo_sapiens_variation_57_37b
database.user	anonymous

The following table shows the properties of **genotypeDB** component.

property	value
dir.genotypes	/opt/sandbox/snpDb/repository
dir.suffix.length	5
file.input	
file.markernames	/opt/sandbox/snpDb/catenated.lst
file.output	output.snpr
file.samplenames	/opt/sandbox/snpDb/samples.lst
version	2.22

The following table shows the properties of **geneExpression-probeAnnot** component.

property	value
dataset	Homo sapiens genes (GRCh37)
version	ENSEMBL GENES 58 (SANGER UK)

References

[1] M. Ashburner, C. A. Ball, J. A. Blake, D. Botstein, H. Butler, J. M. Cherry, A. P. Davis, K. Dolinski, S. S. Dwight, and J. T. Eppig. Gene ontology: tool for the unification of biology. The Gene Ontology Consortium. *Nat Genet*, 25(1):25–29, 2000.

[2] AT&T Research. Graphviz documentation, 2008. http://www.graphviz.org/Documentation.php. [13.10.2008].

[3] M. Benelli, G. Marseglia, G. Nannetti, R. Paravidino, F. Zara, F. Bricarelli, F. Torricelli, and A. Magi. A very fast and accurate method for calling aberrations in array-cgh data. *Biostatistics*, 0(0):0–0, 2010.

[4] Y. Benjamini and Y. Hochberg. Controlling the false discovery rate: a practical and powerful approach to multiple testing. *Journal of the Royal Statistical Society, Series B*, 57(1):289–300, 1995.

[5] C. Brennan. *GTS: A package for ranking chromosomal segments*, 2008. R package version 1.1.0.

[6] S. Draghici, P. Khatri, A. Tarca, K. Amin, A. Done, C. Voichita, C. Georgescu, and R. Romero. A systems biology approach for pathway level analysis. *Genome Research*, 17(10):1537, 2007.

[7] S. Durinck, Y. Moreau, A. Kasprzyk, S. Davis, B. De Moor, A. Brazma, and W. Huber. BioMart and Bioconductor: a powerful link between biological databases and microarray data analysis, 2005.

[8] P. Flicek, B. Aken, K. Beal, B. Ballester, M. Caccamo, Y. Chen, L. Clarke, G. Coates, F. Cunningham, T. Cutts, et al. Ensembl 2008. *Nucleic acids research*, 36(Database issue):D707, 2008.

[9] G. H. Hardy. Mendelian proportions in a mixed population, 1908.

[10] D. P. Harrington and T. R. Fleming. A class of rank test procedures for censored survival data. *Biometrika*, 69(3):553–566, 1982.

[11] S. Hautaniemi, M. Ringnr, P. Kauraniemi, R. Autio, H. Edgren, O. Yli-Harja, J. Astola, A. Kallioniemi, and O.-P. Kallioniemi. A strategy for identifying putative causes of gene expression variation in human cancers. *Journal of the Franklin Institute*, 341(1-2):77–88, 2004.

[12] T. J. P. Hubbard, B. L. Aken, S. Ayling, B. Ballester, K. Beal, E. Bragin, S. Brent, Y. Chen, P. Clapham, L. Clarke, et al. Ensembl 2009. *Nucleic acids research*, 37(Database issue):D690, 2009.

[13] M. Kanehisa, S. Goto, M. Furumichi, M. Tanabe, and M. Hirakawa. KEGG for representation and analysis of molecular networks involving diseases and drugs. *Nucleic acids research*, 2009.

[14] R. McLendon, A. Friedman, D. Bigner, E. Van Meir, D. Brat, G. Mastrogianakis, J. Olson, T. Mikkelsen, N. Lehman, K. Aldape, et al. Comprehensive genomic characterization defines human glioblastoma genes and core pathways. *Nature*, 455(7216):1061–1068, 2008.

[15] A. B. Olshen, E. S. Venkatraman, R. Lucito, and M. Wigler. Circular binary segmentation for the analysis of array-based dna copy number data. *Biostatistics*, 5(4):557–572, 2004.

[16] K. Ovaska and M. Laakso. *csbl.go: Semantic similarity based on Gene Ontology*, 2010. R package version 1.3.4.

[17] K. Ovaska, M. Laakso, and S. Hautaniemi. Fast Gene Ontology based clustering for microarray experiments. *BioData Mining*, 1(11), 2008.

[18] R. L. Plackett. Karl Pearson and the Chi-Squared Test. *International Statistical Review/Revue Internationale de Statistique*, 51(1):59–72, 1983.

[19] S. Pounds and C. Cheng. Robust estimation of the false discovery rate. *Bioinformatics*, 22(16):1979, 2006.

[20] R Development Core Team. *R: A Language and Environment for Statistical Computing.* R Foundation for Statistical Computing, Vienna, Austria, 2007. ISBN 3-900051-07-0.

[21] R Development Core Team. *R: A Language and Environment for Statistical Computing*. R Foundation for Statistical Computing, Vienna, Austria, 2010. ISBN 3-900051-07-0.

[22] V. E. Seshan and A. Olshen. *DNAcopy: DNA copy number data analysis*. R package version 1.22.1.

[23] G. K. Smyth. Limma: linear models for microarray data. In R. Gentleman, V. Carey, S. Dudoit, R. Irizarry, and W. Huber, editors, *Bioinformatics and Computational Biology Solutions using R and Bioconductor*, pages 397–420. Springer, New York, 2005.

[24] A. L. Tarca, S. Draghici, P. Khatri, S. S. Hassan, P. Mittal, J. Kim, C. J. Kim, J. P. Kusanovic, and R. Romero. A Novel Signaling Pathway Impact Analysis (SPIA). *Bioinformatics*, page btn577, 2008.

[25] The hsqldb Development Group. HSQLDB, 2008. http://hsqldb.org/. [22.7.2008].

[26] M. A. van de Wiel, K. I. Kim, S. J. Vosse, W. N. van Wieringen, S. M. Wilting, and B. Ylstra. CGHcall: an algorithm for calling aberrations for multiple array CGH tumor profiles. *Bioinformatics*, 23(7):892–894, 2007.

[27] R. Wiedemeyer, C. Brennan, T. P. Heffernan, Y. Xiao, J. Mahoney, A. Protopopov, H. Zheng, G. Bignell, F. Furnari, W. K. Cavenee, W. C. Hahn, K. Ichimura, V. P. Collins, G. C. Chu, M. R. Stratton, K. L. Ligon, A. Futreal, and L. Chin. Feedback Circuit among INK4 Tumor Suppressors Constrains Human Glioblastoma Development. *Cancer Cell*, 13(4):355–364, 2008.
